# Differential SAGE analysis in Arabidopsis uncovers increased transcriptome complexity in response to low temperature

**DOI:** 10.1186/1471-2164-9-434

**Published:** 2008-09-22

**Authors:** Stephen J Robinson, Isobel AP Parkin

**Affiliations:** 1 Agriculture and Agri-Food Canada, Saskatoon Research Centre, 107 Science Place, Saskatoon, SK, S7N 0X2, Canada

## Abstract

**Background:**

Abiotic stress, including low temperature, limits the productivity and geographical distribution of plants, which has led to significant interest in understanding the complex processes that allow plants to adapt to such stresses. The wide range of physiological, biochemical and molecular changes that occur in plants exposed to low temperature require a robust global approach to studying the response. We have employed Serial Analysis of Gene Expression (SAGE) to uncover changes in the transcriptome of *Arabidopsis thaliana *over a time course of low temperature stress.

**Results:**

Five SAGE libraries were generated from *A. thaliana *leaf tissue collected at time points ranging from 30 minutes to one week of low temperature treatment (4°C). Over 240,000 high quality SAGE tags, corresponding to 16,629 annotated genes, provided a comprehensive survey of changes in the transcriptome in response to low temperature, from perception of the stress to acquisition of freezing tolerance. Interpretation of these data was facilitated by representing the SAGE data by gene identifier, allowing more robust statistical analysis, cross-platform comparisons and the identification of genes sharing common expression profiles. Simultaneous statistical calculations across all five libraries identified 920 low temperature responsive genes, only 24% of which overlapped with previous global expression analysis performed using microarrays, although similar functional categories were affected. Clustering of the differentially regulated genes facilitated the identification of novel loci correlated with the development of freezing tolerance. Analysis of their promoter sequences revealed subsets of genes that were independent of CBF and ABA regulation and could provide a mechanism for elucidating complementary signalling pathways. The SAGE data emphasised the complexity of the plant response, with alternate pre-mRNA processing events increasing at low temperatures and antisense transcription being repressed.

**Conclusion:**

Alternate transcript processing appears to play an important role in enhancing the plasticity of the stress induced transcriptome. Novel genes and cis-acting sequences have been identified as compelling targets to allow manipulation of the plant's ability to protect against low temperature stress. The analyses performed provide a contextual framework for the interpretation of quantitative sequence tag based transcriptome analysis which will prevail with the application of next generation sequencing technology.

## Background

Abiotic stresses, including temperature, are a major constraint on the distribution of plant species throughout the world and the impact of these stresses has been estimated to reduce yield potential by 69% [1]. Temperate plants have the ability to increase their freezing tolerance in response to a period of low, non-freezing temperatures through a physiological adaptation known as cold acclimation [2]. In the non-acclimated state, exposure to freezing temperatures causes significant damage to most plant species. However, once acclimated, freezing tolerance is significantly increased and the ability to resist this stress varies both within and between species, for example *Brassica napus*, wheat and rye are able to withstand temperatures of -16°C, -19°C and -29°C respectively [3-5]. *Arabidopsis thaliana *(hereafter referred to as Arabidopsis) has been used as a model to study freezing tolerance and is able exhibit a modest increase of 5°C in freezing tolerance from -3°C to -8°C after exposure to acclimating conditions for one week [6,7]. Cold acclimation is a quantitative trait with a multitude of metabolic, molecular and physiological changes occurring in response to low temperature exposure. An understanding of the molecular components controlling this response has been advanced by the isolation of mutants with a differential response to temperature stress [8-10] and through the identification of genes whose expression is correlated with the development of freezing tolerance [11-14]. However, the individual effects of the majority of these genes are marginal perhaps reflecting redundancy among stress response networks. Therefore, to identify key regulatory proteins within these networks, a comprehensive strategy which may be achieved through the use of genomics technologies is required.

Serial analysis of gene expression (SAGE) has been used to profile the transcriptomes of at least twenty species [15] since the technique was developed by Velculescu et al. ([16]. SAGE is a sequence based technology developed to generate a transcript expression profile in a high throughput, accurate and non-biased manner. Briefly, the method allows the capture of a 14–21 bp cDNA fragment from a defined position within each mRNA molecule. The captured SAGE tags are amplified and concatenated together before being cloned and sequenced, with each sequence read allowing information for approximately 40–60 transcripts to be obtained. The frequency of each tag occurring within a library directly represents the abundance of that mRNA species within the tissue sampled ([16]. SAGE is a powerful technology that has the capacity to detect small differences in gene expression and the reproducibility of SAGE has been proven by comparisons between tag abundance profiles generated from the same mRNA pool [17]. One of the advantages of using SAGE is the ability to reveal the expression of novel genes as data capture is independent from prior knowledge of DNA sequence. The specificity of tag based technologies allows the detection of post transcriptional regulation including the identification of alternate transcript and antisense products [18].

In Arabidopsis, effective tag to gene mapping strategies have been assisted by the completion of the genome sequence where approximately 30,000 gene models have been identified [19]. In general, SAGE data are referenced to the identified tags rather than to the gene from which they are derived. It is commonly observed that multiple SAGE tags uniquely match to a single gene, which together provide the overall expression level of the locus [18]. Therefore, representation of SAGE data based on the annotated gene identifiers would facilitate evaluating gene expression levels, reduce the number of statistical comparisons and simplify comparisons of expression data across expression profiling platforms. In addition, this representation method enables the visualisation of putative alternate transcript processing within the SAGE data. The available analysis tools are adequate for the majority of SAGE experiments conducted to date that have assayed differences in tag frequency between two experimental conditions [20]. However, to determine differences among multiple libraries, which capture the progression of a physiological phenomenon, requires a statistical test with greater power and precision.

This study applied SAGE technology to assess gene expression changes that occur in Arabidopsis leaf tissue exposed to low temperature over a period of one week. Custom Perl scripts were developed to simultaneously analyse multiple libraries and to optimise the presentation of the generated SAGE data. These data demonstrate the changes in the Arabidopsis transcriptome that occur in response to low temperature exposure. Time points were selected to discover gene expression changes correlated with the acclimation process from early signalling events through to the acquisition of freezing tolerance. In addition to identifying a set of novel low temperature regulated loci, the analysis uncovered a disproportionate amount of post transcriptional regulation in response to low temperature, where an increase in alternate transcript processing and a decrease in antisense transcription was observed.

## Results

### Determination of freezing tolerance in Arabidopsis

**Figure 1:**
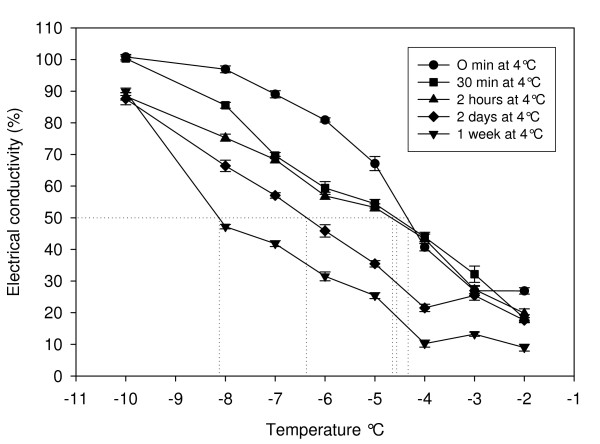
**Freezing tolerance(LT_50_)of Arabidopsis plants exposed to cold acclimating conditions for various lengths of time.**

The degree of freezing tolerance in Arabidopsis was assayed at five time points, 0 minutes, 30 minutes, 2 hours, 2 days and 1 week of exposure to 4°C. As anticipated, the level of freezing tolerance was positively correlated with the period of time under acclimating conditions. Freezing tolerance was measured to be -4.2°C with non-acclimated material under these growth conditions. There was no detectable increase in freezing tolerance prior to 48 hours exposure to low temperature and after 1 week the freezing tolerance increased to -8.3°C (Figure 1). These results are in agreement with previous assessments of freezing tolerance for Arabidopsis [6,10].

### Experimental SAGE library analysis

SAGE libraries were developed from tissue collected at each of the five selected time points, sequencing of these libraries resulted in a total of 242,066 high quality SAGE tags (Table 1). This total comprised 38,179 distinct tags where 20,741 (54%) were observed only once (singleton tags). In common with previous analyses, singleton tags were the most abundant class in each library contributing between 59%–63% of the unique tags [21,22]. Ninety percent (34,146) of the distinct experimental tags could be matched to the Arabidopsis genome sequence. However, due to the short tag length (14 bp) and the non-random nature of genome sequence a number of tags were assigned to multiple locations, complicating the interpretation of these expression data. Thus, all further analyses were restricted to the most informative tags that had been unambiguously assigned to a single location. This resulted in 26,456 (69%) tags matched to 16,629 annotated genes, including 74 genes on the chloroplast and 5 genes on the mitochondrial genome. An additional 1,942 (5%) tags were matched to the pseudochromosome sequences and a further 67 tags and 37 tags were assigned to intergenic regions of the plastid and mitochondria genomes respectively. In comparison, 15,184 (40%) of all unique tags were matched to 13,186 genes using the available Arabidopsis Unigene and full length cDNA sequences, underlining the advantages of a fully sequenced and annotated genome (Additional file 1).

**Table 1:** Summary of SAGE tag abundance of the five Arabidopsis libraries

SAGE library Time treated at 4°C	Total Tags	Reliable tags^a^	Unique tags	Singletons	Genes matched
0 mins	62,292	49,794	13,512	8,208	8,899
30 mins	86,742	65,416	18,056	10,686	11,256
120 mins	68,814	53,154	15,980	10,120	9,476
2 days	69,052	62,032	12,814	8,019	8,239
1 week	43,002	32,648	11,293	7,231	7,407
Combined	329,902	263,044	38,179	20,741	19,630

a. Tags remaining after the removal of duplicate ditags, ditags that fall outside of the 24–28 bp length and ditags possessing a low sequence quality read

### Interpretation of SAGE data

**Figure 2:**
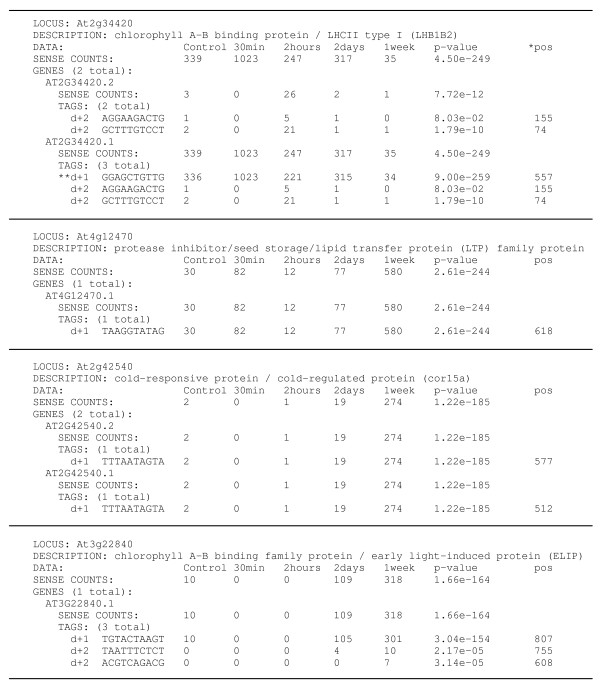
**SAGE tag output referenced according to Arabidopsis gene identifiers**. The tag counts and statistical comparisons are displayed for each locus, gene model and individual tag. To ease comparison across libraries, the tag counts presented were normalized based on 50,000 tags per library. * pos – indicates the position of the SAGE tag within the processed mRNA transcript. ** Codes indicate the dataset matched by the tag, [dvp] [+-] [123] where d = defined UTR sequence, v = virtual UTR sequence and p = pseudogene; + = sense orientation and - = antisense orientation; 1 = canonical (3' most) tag position, 2 = non canonical tag position and 3 = intron.

The SAGE output was restructured to exploit the advantages of utilising a model organism by referencing the results to annotated gene identifiers rather than individual tag sequences (Figure 2). The abundance and relative position of the assigned tags at each locus is indicated along with the overall expression level as determined by summing the individual tag counts. Statistical analysis was performed on both tag and locus counts. SAGE is able to provide evidence for transcription from regions beyond the annotated Arabidopsis gene identifiers (AGI). These include tags matching intergenic regions, which could represent as yet unannotated genes, transposable elements or small RNAs and tags that remain unassigned to the available genome sequence. This study detected 6,079 such orphan tags which were included in the statistical analysis to reveal novel low temperature responsive transcripts. A summary of all these SAGE data are provided as additional data (Additional file 2).

### Low temperature regulation of gene expression detected through SAGE

This is the first report where differences among multiple SAGE libraries are determined simultaneously as previous SAGE analyses have been restricted to pair-wise library comparisons. Statistical analysis was performed using the 2xt chi-squared test for homogeneity among the five SAGE libraries on both discrete SAGE tags and the identified AGI loci.

The SAGE tag analysis revealed 956 tags exhibiting differential expression (p < 0.01), including 874 tags assigned to AGI loci, 54 tags assigned to pseudochromosome sequence, 8 tags assigned to plastid sequence, 5 tags matched to mitochondrial sequence and 15 tags that were not matched to the available genome sequence. Analysis of the total detected expression for each AGI locus as represented by the sum of the individual tag counts revealed 920 genes exhibiting a significant difference in gene expression (p < 0.01). A summary of the expression of 25 genes exhibiting the greatest change in transcript levels in response to low temperature are presented in Table 2 and all differentially expressed genes are available in Additional file 3. It is possible to account for inflation in the frequency of type I errors due to multiple comparisons through the application of the Benjamini-Hochberg [23] or Bonferroni correction [24]. This provides greater confidence for classifying 440 or 300 genes as low temperature responsive, respectively (Additional file 3). However, the application of such corrections assumes independence of loci which may not be appropriate for gene expression data and might increase the occurrence of type 2 errors to unacceptable levels.

**Table 2:** Expression profiles of the 25 Arabidopsis genes most significantly differentially regulated.

**AGI**	**Control**	**30 min**	**2 hours**	**2 days**	**1 week**	**p-value**	**Annotation**
At2g34420	339	1023	247	317	35	4.50e-249	chlorophyll A-B binding protein/LHCII type I (LHB1B2)
At4g12470	30	82	12	77	580	2.61e-244	protease inhibitor/seed storage/lipid transfer protein (LTP) family protein
At2g42540	2	0	1	19	274	1.22e-185	cold-regulated protein (cor15a)
At3g22840	10	0	0	109	318	1.66e-164	chlorophyll A-B binding family protein/early light-induced protein (ELIP)
At4g12480	8	16	6	12	276	1.41e-156	protease inhibitor/seed storage/lipid transfer protein (LTP) family protein
At5g13930	6	4	7	256	84	6.82e-130	chalcone synthase
AtCg00480	51	362	78	49	19	4.97e-126	chloroplast-encoded gene for beta subunit of ATP synthase
At3g50450	55	244	24	48	41	3.48e-75	hypersensitive response protein (HR1)
At3g16770	27	178	15	15	10	3.02e-74	ERF/AP2 transcription factor RAP2.3
At1g30380	413	123	398	327	49	8.89e-69	PSI reaction center subunit psaK
At4g14690	2	0	4	131	87	5.05e-67	chlorophyll A-B binding family protein/early light-induced protein
At3g51600	97	42	22	42	262	8.67e-66	nonspecific lipid transfer protein 5
At5g14740	330	61	197	107	44	1.29e-64	carbonic anhydrase 2
At5g66570	476	118	341	239	134	4.80e-63	oxygen-evolving enhancer protein 1
At3g50990	453	260	581	449	82	3.95e-62	Similar to peroxidase ATP6a
At1g20620	124	280	71	49	56	1.75e-58	catalase 3 (SEN2)
At1g15820	205	43	272	160	25	1.90e-55	chlorophyll A-B binding protein, chloroplast (LHCB6)
At2g05100	207	102	46	16	25	1.86e-48	chlorophyll A-B binding protein/LHCII type II (LHCB2.1) (LHCB2.3)
At5g02960	80	27	70	80	253	2.47e-48	40S ribosomal protein S23 (RPS23B)
At1g31330	24	134	22	20	6	4.83e-46	PSI reaction center subunit III family protein
At3g09390	85	73	162	276	305	1.35e-45	metallothionein protein
At2g42530	3	0	11	45	107	5.28e-45	cold-regulated protein (cor15b)
At4g38970	333	58	229	178	199	1.18e-42	fructose-bisphosphate aldolase
At1g29910	591	404	559	494	131	2.66e-41	chlorophyll A-B binding protein 2
At3g47470	144	49	248	125	43	2.87e-40	chlorophyll A-B binding protein 4

For each Arabidopsis gene the observed SAGE tag abundance for each library is shown. The total tags were normalised to 50,000 per library to facilitate comparative expression analysis.

### Assessment of known low temperature responsive genes

The fidelity of the SAGE data was confirmed by studying the expression of 30 previously identified low temperature induced genes including the *LTI*/*COR*/*ERD *genes [11,14] and the *CBF/DREB1 *transcription factor gene family (hereafter referred to as *CBF*) [25] (Table 3). The anticipated increase in expression level was observed for 28 of the selected genes (p < 0.01). Interestingly, SAGE was able to discriminate between transcripts derived from duplicate members of the *CBF *gene family due to unique tags present in the 3' UTR sequence. Each member of this gene family exhibited a similar expression profile upon exposure to low temperature with maximum expression observed after 2 hours. However, subtle differences were observed, with *CBF2 *expression being detected prior to *CBF1 *or *CBF3 *expression and the majority of the transcripts were derived from *CBF3*. *CBF3 *was the only homologue transcribed after prolonged exposure to low temperature. Additionally, no significant difference was detected in the expression of ten abiotic stress related genes previously demonstrated to be unaffected by low temperature, which included the *DREB2 *family, the *SOS *genes and drought inducible genes (data not shown).

**Table 3:** Expression profiles of previously characterised cold-regulated genes.

AGI	Gene	Control	30 min	2 hour	2 day	1 week	p-value
At1g09070	SRC2	5	19	31	8	7	1.91E-05
At1g13260	RAV1	1	3	16	4	1	5.63E-05
At1g20440	COR47	7	11	35	62	38	9.09E-12
At1g20450	ERD10	2	15	20	49	23	3.04E-09
At1g20620	Catalase	124	280	71	49	56	1.75E-58
At2g15970	WCOR413-like	32	23	46	99	122	1.38E-20
At2g31360	Δ9 desaturase	1	12	14	5	1	3.30E-03
At2g36530	LOS2	26	14	38	77	103	3.05E-18
At2g42530	COR15b	3	0	11	45	107	5.28E-45
At2g42540	COR15a	2	0	1	19	274	1.22E-185
At3g02480	ABA responsive	1	0	1	0	17	1.91E-10
At3g22840	ELIP	10	0	0	109	318	1.66E-164
At3g30775	ERD5	2	18	3	3	4	1.03E-05
At3g50970	XERO2	1	0	3	13	22	9.59E-09
At3g55120	CHI	2	0	0	19	44	4.56E-21
At4g02380	LEA 3	6	26	11	22	44	6.91E-06
At4g12470	pEARLI 1	30	82	12	77	580	2.61E-244
At4g25470	CBF2	0	4	14	1	0	1.56E-05
At4g25480	CBF3	0	0	59	5	3	1.47E-33
At4g25490	CBF1	0	0	4	0	0	5.58E-03
At4g30650	LTI6A	1	0	2	27	56	1.48E-26
At4g39090	RD19a	3	45	4	4	5	7.00E-19
At5g01490	FAD2	8	22	12	1	5	1.05E-04
At5g13930	CHS	6	4	7	256	84	6.82E-130
At5g15960	KIN1	0	0	0	0	0	na
At5g15970	KIN2	0	0	0	0	0	na
At5g52310	COR78	3	0	0	45	10	4.41E-24
At5g52310	LTI65	3	0	0	34	10	4.41E-24
At5g58070	Lipocallin	2	1	8	20	11	5.23E-05
At5g66400	RAB18	8	0	3	5	39	1.20E-14

For each Arabidopsis gene the observed SAGE tag abundance for each library is shown. The total tags were normalised to 50,000 per library to facilitate comparative expression analysis

### SAGE profiles during the development of freezing tolerance

**Figure 3:**
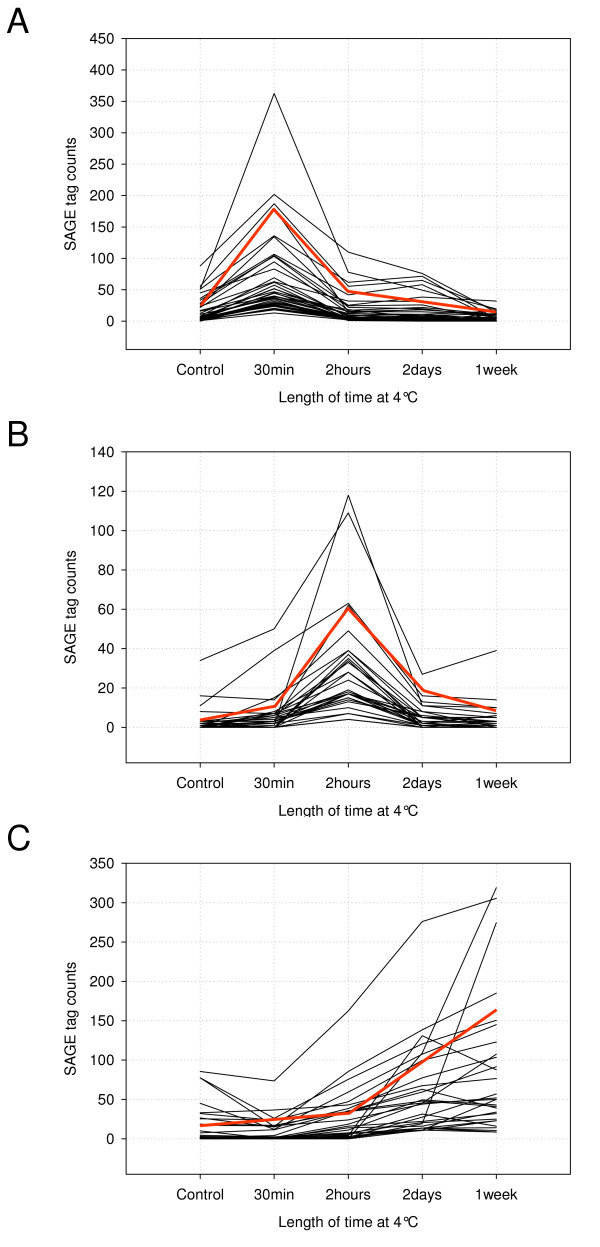
**Genes clustered according to expression profiles**. Genes exhibiting a transient increase in expression after (A) 30 minutes and (B) 2 hours of low temperature treatment. C) Genes mimicking *COR *gene expression, which are positively correlated with an increase in freezing tolerance. The red line represents the desired profile and black lines indicate the expression pattern of genes assigned to that profile, highlighting the variation observed for each profile.

The generation of SAGE data from multiple libraries under cold acclimating conditions allowed gene expression profiles to be explored and genes with similar profiles to be clustered. The profiles of the 920 low temperature responsive genes were clustered using a post-hoc pair-wise analysis for the chi-square test (Figure 3) [26].

Genes involved in the perception of low temperature and those activating cold specific regulons would be expected to exhibit changes in expression at early time points. A comparison of the control library to each low temperature treated library identified those genes only exhibiting a significant difference (p < 0.01) at a single time point. This analysis identified 126 and 47 genes that were up-regulated and 28 and 5 genes which were repressed at the 30 and 120 minute time points, respectively. As anticipated, the major functional class represented among the up-regulated genes were transcriptional activators (25%) which included two members of the well characterised CBF gene family. The third copy of *CBF*, *CBF2 *was significantly up-regulated at both of these time points and nineteen additional genes were found to share a similar profile.

CBF has been demonstrated to control the expression of a suite of low temperature responsive genes exemplified by COR genes whose expression is positively correlated with the development of freezing tolerance [11]. A cluster of 63 genes were identified that were significantly induced to high levels in response to low temperature after 2 days and remained up-regulated after one week, this profile was in common with that exhibited by the majority of *COR *genes. However, it was noted that not all *COR *genes would be observed in this cluster since there are subtle differences among the expression profiles of the previously classified *COR *genes (Table 3). This cluster was enriched for ribosomal proteins (11%, χ^2^_1 _= 72; p < 0.001) and genes involved in oxidative stress protection (8%, χ^2^_1 _= 32; p < 0.001) in addition to previously annotated cold regulated genes (11%, χ^2^_1 _= 17.72; p < 0.001).

**Figure 4:**
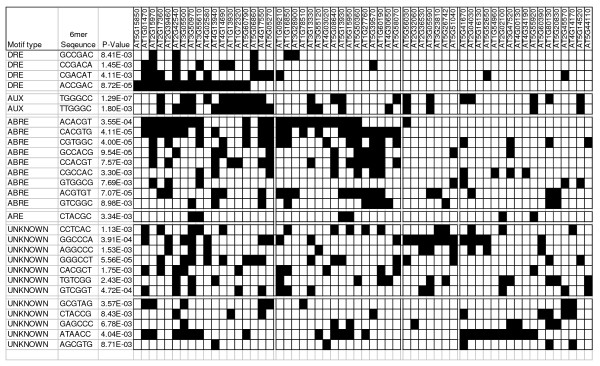
**Promoter sequence analysis for *COR*-like genes**. Distribution of 6 mer promoter elements over-represented among the 63 genes clustered according to the *COR*-gene like profile, described in Figure 3C. Shaded box indicates presence of a cis-element. DRE – CRT/DRE; AUX – auxin responsive; ABRE – ABA responsive; ARE – antioxidant responsive.

An analysis of the promoter sequences (within 1500 bp 5' of the ATG) for the 63 genes in the *COR *gene-like profile (Figure 3C) revealed that 24 possessed the CRT/DRE cis-acting element (CCGAC) recognised by CBF (Figure 4). No correlation was found between the number of CRT/DRE elements in each promoter and the magnitude of gene expression. The promoter sequences of the remaining 39 genes did not possess this element. A comprehensive analysis of 6 mer motifs found to be significantly over-represented within the promoter elements of this cluster identified two additional known cis-acting regulatory elements, 'AUX' and 'ABRE' that are found in the promoters of auxin and absisic acid (ABA) regulated genes respectively [27]. It has been shown that *COR *gene expression responds to the application of ABA, which is reflected by the presence of the ABRE sequence in a high proportion (83%) of those genes whose promoters also contained the CRT/DRE element. However, there were a number of predicted ABA responsive genes that were independent of the CBF regulon (Figure 4). In addition, two further groups of genes appeared to be independent of either CBF or ABA regulation and were characterised by the preponderance of unknown motifs 'GGCCCA' and 'ATAACC'. A similar promoter analysis of the 126 genes found to be up-regulated at 30 minutes only (Figure 3A) identified 193 6 mers which were significantly more abundant in the selected genes compared to the random set (Additional file 4). The majority of the elements were uninformative due to sequence ambiguity. However, for those which could be assigned to a known motif, they were predominantly annotated as light responsive or under circadian control, which may be expected for this timepoint.

### Identification of novel transcripts through SAGE

Alternate transcript processing can be detected due to the precision of SAGE tag assignment [18]. The SAGE method captures a tag from the 3' most anchoring enzyme (*Nla*III) recognition site within each transcript, which will bias the detection of processing events towards those occurring at the 3' end. Although potential differential pre-mRNA processing products have been well described, the information provided by the tag to gene matching is insufficient to distinguish among certain classes [28-30]. SAGE cannot identify mutually exclusive exons and absent exons (exon skip) can only be inferred if the canonical SAGE tag site resides in an internal exon. Thus, using SAGE it is possible to define three general classes of alternate transcript processing; retained introns, modified exon structure (alternative acceptor/donor splice sites) and polymorphic UTR sequences.

Analysis of the SAGE data has revealed that 4,982 (32%) Arabidopsis loci may be subject to differential processing events, this frequency is in accordance with previous estimates based on EST analysis [31]. However, incomplete digestion by the anchoring enzyme can generate SAGE tag artefacts. These potentially misleading tags were eliminated from subsequent analysis as described in Robinson et al. (2004), resulting in a more conservative estimate, with alternative transcript processing events affecting 1,275 loci (8%) (Additional file 5). To further corroborate this phenomenon, these data were compared with loci encoding multiple Unigene and/or full length cDNA sequences possessing unique canonical tags (Additional file 1). For the 993 loci in common between these data, tags matched specific alternative canonical sites in 402 instances among the full length cDNA/EST data. A breakdown of the different classes of pre-mRNA processing events are summarised in Table 4. Similar to previous analyses based on EST sequences the most prevalent type of processing event was intron retention (55%) [29,31]. The vast majority (41%) of the retained intron events were identified in the 3'-UTR sequences.

**Table 4:** Distribution of the different types and frequency of alternative transcription events observed in the SAGE data.

	**No. of Loci**		**No. of Events**	
	All alternative transcripts	Low temperature regulated (p < 001)	All alternative transcripts	Low temperature regulated (p < 001)

Modified Exon Order				
Hits in same exon^a^	105	23	268	67
Hits in different exon^a^	173	26	453	78
Single non-canonical hit^b^	547	67	547	67
Total	755	96	1223	188
				
Retained Introns				
Within coding regions	233	29	298	38
3' UTR introns	768	95	1128	161
5' UTR introns	67	8	88	7
Total	1068	132	1514	206
				
Polymorphic UTRs				
3' UTRs				
Multiple SAGE tags	296	58	682	138
Single non-canonical hits^b^	237	4	237	4
5' UTRs				
Multiple SAGE tags	6	0	16	4
Single non-canonical hits^b^	42	3	42	3

a. There will be some overlap between the alternate transcripts identified in these categories.

b. Due to the addition of virtual UTR sequence for a proportion of the loci it is possible that these categories may be overestimated.

Alternate transcript processing was observed for 138 (15%) of the low temperature responsive genes (p < 0.01), which indicates a two-fold increase of this phenomenon at low temperature. Forty-four of these loci (32%) were functionally assigned to the photosynthetic light harvesting process, electron transport or RuBisCO activase. As anticipated, the majority of loci involved in photosynthesis displayed a decrease in expression after one week at low temperature [32]. However, for 13 of these loci an alternate truncated transcript was observed, which was transiently induced (for example, At2g34420 in Figure 2). Potential alternate transcripts were detected for four low temperature induced chloroplast encoded genes and in each instance tags derived from all possible *Nla*III sites were observed. Chloroplast transcripts are subject to poly-adenylation dependant degradation suggesting that these tags represent different cleavage events rather than the products of alternate transcript processing [33]. In light of this, the relative abundance of organelle encoded transcripts cannot be assessed through expression analysis techniques that capture RNA molecules by poly-T priming.

The 1,952 (5%) tags that were unambiguously matched to intergenic sequence could indicate the presence of non-coding RNAs. Since SAGE tags are captured from polyadenylated transcripts, only a subset of these molecules can be assayed including microRNAs. Presently, 86 microRNA families have been identified in Arabidopsis and 5 of the intergenic SAGE tags were mapped to these microRNA loci [34]. In addition, we tentatively matched 14 SAGE tags to available small RNA databases (Additional file 2). However, these tags were found at levels insufficient to detect any significant expression response.

The orientation of each SAGE tag is known, which facilitates the identification of antisense transcripts [18,35]. The level of antisense expression detected among the SAGE data was estimated to be 21% where tags were matched unambiguously in the antisense orientation to 3,556 genes (Additional file 6). This level is comparable to that previously detected in Arabidopsis [36]. Differential antisense expression (p < 0.01) was detected for 50 genes subsequent to low temperature exposure, 13 of these were in common with the 920 low temperature responsive genes described above. These data indicate that the degree of antisense transcription has been reduced four-fold in response to low temperature.

### Evolutionary functional conservation

**Figure 5:**
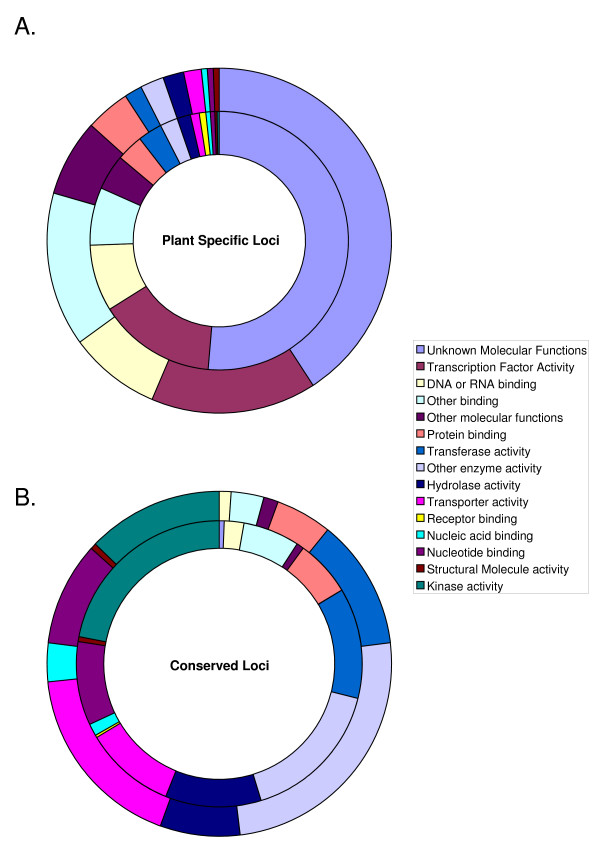
**Frequency of genes distributed according to GO-slim functional categories**. Genes identified under control (inner ring) and low temperature (outer ring) growth conditions and identified as either A) plant specific or those sharing a B) conserved evolution.

The evolutionary origin of the low temperature responsive genes was inferred based on the classifications described by Gutierrez et al. [37] who determined that at least 14% (3,848) of Arabidopsis proteins are plant specific and 9% (2,436) are evolutionarily conserved with the Eukaryota, Bacteria and Archea domains. The SAGE analysis identified 162 plant specific genes and 127 conserved genes that were differentially regulated by low temperature (p < 0.01). Functional characterisation of these subsets revealed that low temperature treatment induced changes in the frequency distribution among several Gene Ontology (GO) slim categories (Figure 5).

Among the plant specific genes the greatest disparity was observed in the 'Other binding' category (χ^2^_1 _= 26; p < 0.001) where the majority of observed genes encoded for lipid binding proteins (Figure 5). Interestingly, although the proportion of genes annotated with 'Transcription factor activity' was similar, there was bias toward the members of two specific gene families, namely *ERF/AP2 *and *AUX/IAA*.

The percentage of genes identified as possessing 'Transporter activity' was significantly increased (χ^2^_1 _= 22; p < 0.001) within the conserved genes subset, whereas genes annotated with 'Kinase activity' were under represented (χ^2^_1 _= 15; p < 0.001) among these data (Figure 5). The majority of the low temperature responsive genes contributing to the 'Transporter activity' category were functionally characterized as either water channel proteins or hexose transporters. In addition, the heat shock protein gene family was over represented within the 'Nucleotide binding' category, although no overall proportional change was observed.

## Discussion

Five SAGE libraries were constructed from tissue harvested throughout the adaptive cold acclimation process exhibiting incremental levels of freezing tolerance, which was positively correlated with exposure to low temperature (Figure 1). Analysing these libraries allows changes in gene expression induced by the initial perception of low temperature, the activation of signalling networks and the perturbation of metabolic pathways to be observed. In total, 242,066 high sequence quality tags were generated to provide a comprehensive assessment of the Arabidopsis leaf transcriptome using SAGE.

The biological interpretation and simultaneous statistical analysis of these complex data necessitated the development of new representation and analysis tools, which was achieved using custom Perl scripts [18,20]. The interpretation of the SAGE data was enhanced by summarising the output according to the accepted gene models. This reduced the complexity of the transcript population, facilitated the identification of differential transcript processing, allowed clustering of similar expression profiles and simplified comparisons with expression data generated using alternate technology platforms. A total of 16,629 annotated Arabidopsis genes were detected with further evidence for 2,150 putative transcriptional units.

### Differential gene expression in response to low temperature

In total, SAGE was able to detect 920 genes responding to low temperature (p < 0.01). Additional low temperature responsive tags were matched to 68 pseudochromosome locations and 15 tags remained unassigned. This suggests that the expression of 6% of the annotated genes detected were affected by low temperature regulation. A set of 30 diagnostic marker genes, known to be regulated by low temperature, were assessed to validate the experimental design. SAGE was able to detect only 28 of these genes due to the lack of an anchoring enzyme site in the *KIN1 *and *KIN2 *genes. In each case, the anticipated expression profile was observed (Table 3). It was determined that SAGE would be unable to detect approximately 2% of the Arabidopsis transcripts due to the absence of the *Nla*III anchoring enzyme site [18].

The effects of low temperature on the Arabidopsis transcriptome have been studied previously using microarray platforms [38-43]. The two most recent analyses which used a comparable duration of low temperature exposure designated 514 [38] and 5,924 genes (p < 0.01) [42] as cold responsive. By comparison to these studies 40% of the differentially regulated loci as determined by SAGE were also classified as low temperature regulated and only 71 of these loci were identified by all three analyses. It is perhaps not surprising that this discrepancy exists, since many factors influence the classification of genes as being responsive to low temperature [44]. Variations in environmental conditions will affect differential gene expression increasing the number of experimental parameters and making direct comparisons difficult to interpret. Technically, SAGE is able to characterise the expression of genes absent from the microarray, as such the profiles of an additional 2,040 genes were determined. Furthermore, SAGE has the ability to detect differences among tag frequencies over a large dynamic range, dependent on the number of tags obtained in each library. This is highlighted by the lack of correspondence between cold responsive genes that are highly expressed based on SAGE analysis, where the signal intensity level detected by microarrays would be saturating and thus prevent identification of differential gene expression [45]. Conversely, a bias exists in SAGE for genes of low abundance where the small number of tags is an impediment to the statistical differentiation of low temperature response. Due to these factors, functional annotation of the identified cold responsive genes was used to compare the data generated from different platforms. This analysis revealed that near identical processes were affected by low temperature, although the response of 'Structural proteins' appeared to be under estimated in the microarray data which could be a function of their relatively high expression levels. The use of complementary technologies allows the complex transcriptional changes taking place during cold acclimation to be fully realised.

### Defining expression profiles

Patterns of co-regulation were assayed among the genes identified as low temperature responsive. This analysis refines the data to assist with candidate gene selection, defines gene regulons to facilitate the capture of regulatory proteins and associates genes of unknown function with well characterised genes. Since the discovery of the COR genes the majority of low temperature research has focused on factors controlling the regulation of these genes, exemplified by the identification of the transcriptional activators CBF and ICE [46,47]. The SAGE data can be mined to find genes with analogous profiles by defining expression patterns representative of the *COR *and *CBF *genes.

*COR *gene-like expression was observed for 63 genes whose transcripts accumulate after 2 days and remain induced after 7 days of low temperature treatment (Figure 3C). Among these genes were the complement of *COR *genes and almost 30% of the genes identified were annotated as stress responsive. The CBF binding element was found in the promoter region of 24 of the 63 *COR*-like genes of which four were common to the previously designated CBF regulon [41]. The remaining 39 genes are likely to be under alternate transcriptional control acting in parallel to and independent of CBF, which appeared to be reflected by the presence of known and new motifs within the regulatory regions of these genes. Motifs which characterise ABA responsive genes were prevalent among genes putatively activated by CBF. A distinct group of genes appeared to respond only to ABA and a number of genes appeared to be independent of either CBF or ABA. The uncharacterised motifs that were significantly over-represented within this category of genes could be used to further dissect low temperature signalling pathways.

A similar approach was applied to identify genes sharing an expression profile comparable to the *CBF *transcriptional activators. The individual members of the *CBF *gene family exhibited overlapping expression profiles, which were compared to the 920 low temperature responsive genes (Table 3). This resulted in the identification of 47, 19 and 13 genes that mimicked the pattern of *CBF1*, *CBF2 *and *CBF3*, respectively. The predominant functional categories observed for these genes were 'Response to stress' (23%) and 'Transcription factor activity' (22%). The novel low temperature inducible transcription factors may allow the detection of further pathways controlling the acquisition of freezing tolerance.

The largest observed cluster of genes represented a rapid transient response observed after 30 minutes of low temperature exposure, where 126 genes were up-regulated and 28 were repressed. The selection of the early time point was designed to capture the immediate transcriptional changes resulting from the plant's perception of the temperature reduction but not to be influenced by the consequent physiological adjustment to the stress. This was indicated by the equivalent distribution of the 126 up-regulated genes across the functional categories compared to those observed from non-stressed tissue, suggesting no significant disruption of cellular homeostasis. Among these data, 16 genes were induced only at this time point and annotated with transcription factor activity and warrant further investigation. A significant proportion of the down-regulated genes were functionally annotated as chloroplast proteins (21%, two-fold increase over non-stressed tissue). The reduction in expression level could be the result of cold induced photoinhibition where the reduction in chlorophyll content, photosystem antenna size and non-photochemical quenching reduce the deleterious effects of reactive oxygen species [48].

### Evolutionary conservation of low temperature induced genes

Sensing and responding to a changing environment is fundamental to all species. Cold adaptation is found in a diverse range of poikilotherm species and different strategies are utilized to survive the environmental stress. The similarities among evolutionarily distant species could illuminate the cellular processes of low temperature perception and signal transduction. Although many adaptations influencing freezing tolerance may be shared among these species, the perception of the temperature change and adaptation to the stress is likely unique in photoautotrophic species as it probably involves perturbations in primary metabolism [49], adjustments to redox homeostasis in the cytosol, chloroplast and mitochondria in conjunction with changes in cell wall-membrane-cytoskeleton conformations [50-52].

Among the low temperature regulated proteins that were found to be conserved across all domains (Eukaryota, Bacteria and Archea) there was a preponderance of membrane transporters, consisting of aquaporins, hexose transporters and a subfamily of the ABC transporters, the soluble GCN type proteins. Although the frequency of genes annotated with 'Kinase activity' were under represented in the low temperature data, 80% of those found were conserved across all three domains. Similarly, the genes which fall under the GO slim classifications 'Other enzyme activity' and 'Nucleotide binding' were over represented among the low temperature responsive genes. On closer examination, these categories largely contained proteins involved in photorespiration, reactive oxygen species metabolism and molecular chaperones. Together, the expression of these proteins, which are essential to protect and restore cellular homeostasis, suggest a conservation of the metabolic response to low temperature stress across a diverse range of organisms.

In contrast, elucidating those responses specific to plants could identify key requirements of the low temperature stress tolerance strategy. This was exemplified by the identification of the COR genes and CBF, among the identified plant-specific proteins. Other plant specific genes regulated by low temperature included those encoding lipid transfer proteins (LTP), additional AP2 binding proteins and auxin regulated proteins. The LTP genes exhibited two distinct expression profiles, where transcript accumulation occurs after 30 minutes or 7 days of treatment. The latter expression pattern is reminiscent of some *COR *genes, indeed the family shares physical properties with COR proteins and one such protein was found to be involved in thylakoid membrane stabilisation [53,54]. It could be inferred from their plant specific origin that their role is to specifically protect plastid or thylakoid membranes. The uncharacterised AP2 binding proteins may act to control CBF independent signalling pathways and represent new targets for manipulating the plant's response to low temperature stress.

### Detection of low temperature induced novel transcripts

The use of SAGE analysis allowed the level of antisense transcription and alternative transcript processing to be estimated throughout the low temperature treatment. Opposing responses to low temperature were observed for antisense and alternative transcript processing. The frequency of antisense transcripts detected was reduced four-fold. It has been proposed that antisense molecules are processed in a similar manner to siRNAs in order to become active [55], thus the reduction in antisense SAGE tags could result from an increase in siRNA production. However, it has been demonstrated that penetrance of RNA mediated gene silencing in tobacco was impaired upon lowering growth temperatures due to a reduction in DICER activity [56]. Therefore, it appears that both siRNA production and antisense transcription decrease in response to low temperatures. This phenomenon may impact the use of RNA interference as a mechanism for controlling gene expression under adverse environmental conditions.

In contrast, the frequency of alternative transcript processing events doubled in response to low temperature, but the relative levels of the different classes of alternative transcripts were unaffected. These data add to the emerging body of evidence indicating that alternate transcript processing is required for plants to adapt to unfavourable conditions. The level of alternate transcript processing was assessed using available EST sequence data for Arabidopsis and it was found that genes with retained introns were biased towards those annotated as being involved in photosynthesis and stress responses [29]. Precise transcript processing is essential for normal plant development, and its relevance to abiotic stress tolerance was exemplified by the identification of the *STA1 *Arabidopsis mutant, since plants possessing defective *sta1 *alleles exhibited mis-splicing of *COR15 *transcripts under cold stress [57]. It has also been observed that critical components of the splicesome, the serine/arginine rich proteins that are involved in the regulation of pre-mRNA splicing, are themselves subject to alternate processing in response to different stress treatments [58]. This might point to a mechanism for generating the increased transcriptome complexity that was observed among the SAGE data in response to low temperature.

The majority of the identified alternate transcript processing events were found to affect genes involved in photosynthesis, light harvesting and electron transport. Notably, the data revealed that 30% of these genes produced a truncated transcript which was transiently induced after two hours of low temperature treatment. The impact of low temperature on photosynthesis leads to an energy imbalance caused by a reduction in the rates of metabolic and photosynthetic enzymes relative to the temperature-independent reaction rates of electron transfer to light harvesting complexes [50]. Adjustments are necessary to prevent oxidative damage, these are known to include alterations in antennal size, heat dissipation through non-photochemical quenching and the diversion of energy from Photosystem II (PSII) to Photosystem I (PSI) [59]. Alterations in antennal size and number could result from truncated transcripts. In mammalian systems, short isoforms of intracellular receptors have been shown to interfere with the formation of functional protein complexes [60]. The alternatively processed photosynthetic transcripts which have lost the conserved chlorophyll binding domain may still function as a structural component of the antennae, but would effectively reduce the efficiency of energy capture, limiting production of reactive oxygen species. This appears to be a short term response to low temperature that acts in concert with an overall reduction in the level of full length transcript observed after one week. This would correlate with the observation that there is no sustained repression of photosynthetic capacity in herbaceous plants after long periods of low temperature treatment [48].

## Conclusion

This study has utilised SAGE to identify differences in gene expression due to low temperature exposure among multiple libraries. It provides a methodology to visualise, interpret and maximise the amount of information which can be obtained from sequence tag data in high-throughput gene expression analysis. Such analyses will become more prevalent with the use of next generation sequencing technologies, which will facilitate the adoption of digital expression analysis. This study, while providing a global view of the low temperature response in Arabidopsis, has identified novel stress regulated genes and potential cis-acting regulatory elements, which will provide avenues for functional characterisation of the plant's response.

## Methods

### Plant Materials and SAGE library construction

The *A. thaliana *ecotype Columbia (Col-4) was used throughout this study. The plant material for SAGE analysis was grown and harvested as described in Robinson et al. [18]. Control seedlings were grown for 14 days at 22°C and 125 μE light with a 16 hr photoperiod. Low temperature treated plants were grown as described for control plants but were exposed to 4°C for 30 minutes, 2 hours, 2 days or 7 days prior to tissue harvest and RNA extraction. Tissue harvest was synchronised to the virtual dawn except where the duration of low temperature exposure was a fraction of 24 hours. The SAGE libraries were generated and the SAGE tags were sequenced and extracted as described in Robinson et al. [18]. For each library, all extracted SAGE tags were submitted to the NCBI Gene Expression Omnibus (GEO) database (Accession No. GSE11461; ).

### Determination of freezing tolerance

The plant material for the freezing tolerance assays was sown in soil and grown at 22°C under 125 μE light with a 16 hr photoperiod until the 6 leaf stage. Low temperature treatment of these plants was performed at 4°C with the light intensity remaining at 125 μE. The plants were sampled at 0 time, 30 minutes, 2 hours, 2 days and 1 week after transfer to the cold acclimating conditions. The degree of freezing tolerance exhibited in these plants was determined by assaying ion leakage by measuring changes in the level of electrical conductivity as described by Sharma et al. [5].

### SAGE tag analysis

The SAGE tag to gene assignment was performed using the cSAGE algorithm [18] with the modification that tags found within 250 bp of an annotated gene's co-ordinates were matched to that gene [20]. This was performed as these tags are likely derived from improperly annotated UTR sequences rather than evidence of novel transcriptional units. Custom Perl scripts were used to organise and count the number of tags matched to each Arabidopsis gene identifier (AGI), in both sense and antisense orientation [20]. Where indicated to facilitate comparison among libraries the tag values have been normalised to 50,000 tags per library based on the number of tags matching to AGIs. All statistical analyses were performed using 2xt contingency tables and employed the chi-square homogeneity test to detect differences in tag frequencies among the libraries where the null hypothesis states that each sample is from the same distribution i.e. *H*_0_: π_1 _= π_2 _π_3 _= π_4 _= π_5 _[61]. Genes were clustered based on expression profiles determined through the use of a post-hoc chi-square test statistic in pair-wise analyses and was achieved using custom Perl scripts, which are available upon request [26].

### Promoter sequence analysis

Custom Perl scripts were developed to analyse the putative promoter sequences from a subset of selected genes. The frequency of individual sequence motifs present within a query set of promoter gene sequences was compared to the frequency observed within the promoters of 6,000 randomly selected genes. The confidence of these frequency distributions was assigned using the binomial distribution. The promoter sequences were defined as the 1500 bp sequence immediately 5' of the start codon of the specified AGI except where there was insufficient sequence between AGI codes, when the promoter sequence was truncated at the boundary of the upstream annotated gene. Promoter sequences were analysed for the presence of sequence motifs (n-mers) of a user-defined length. The frequency of each motif was calculated from both strands and palindromic sequences were only counted once.

### Additional Database resources

Arabidopsis Unigene (build #67) and full length cDNA sequences were obtained from the National Center for Biotechnology Information . The microRNA sequences and small RNA data was obtained from  and , respectively. The gene ontology (GO) slim plant database from The Arabidopsis Information Resource (TAIR; ) was used for functional classification . The plant specific database used for evolutionary comparisons was obtained from . The PLACE database was used to identify known cis-acting regulatory elements (Place: ).

## Authors' contributions

SJR carried out the molecular work, developed targeted Perl scripts, analysed the data, and wrote the manuscript. IAPP conceived of the study, participated in its design and coordination, the analysis of results and helped to write the manuscript. Both authors have read and approved the final manuscript.

## Supplementary Material

Additional file 1 **SAGE tag matches to available Arabidopsis Unigene and full length cDNA sequences.** Click here for file

Additional file 2 **A list of all observed SAGE tags, their abundance in each library and their assigned location in the Arabidopsis genome.** Click here for file

Additional file 3 **SAGE data represented by AGI for those genes whose expression is differentially regulated by low temperature (p < 0.01).** Click here for file

Additional file 4 **Distribution of 6 mer promoter elements over-represented among the regulatory regions of 126 genes significantly up-regulated after 30 minutes exposure to low temperature.** Click here for file

Additional file 5 **SAGE tags suggesting evidence of alternative transcript processing events.** Click here for file

Additional file 6 **Differentially expressed SAGE tags uniquely matching Arabidopsis loci in the antisense orientation.** Click here for file
