# Supplementary material for: Differential SAGE analysis in Arabidopsis uncovers increased transcriptome complexity in response to low temperature
Source: BMC Genomics. 2008 Sep 22;9:434. doi: 10.1186/1471-2164-9-434 (PMC2568001; doi:10.1186/1471-2164-9-434)
Supplement: Additional file 1 — SAGE tag matches to available Arabidopsis Unigene and full length cDNA sequences. [file 1471-2164-9-434-S1.pdf]

Additional file 1: SAGE tag matches to canonical tags in all available Arabidopsis Unigene and full length cDNA sequences and equivalent match in TAIR annotated gene identifiers.

at5g61520	CON	MIN	HOUR	DAY	WEEK	UNIGENE	FLCDNA	TAIR
ATCCAAGATT	0	2	1	0	1	gnl|UG|At#S11815660	gi|22135847|gb|AY128303.1	At5g61520.1

at4g32520	CON	MIN	HOUR	DAY	WEEK	UNIGENE	FLCDNA	TAIR
GCATCTATTA	0	0	0	1	0	gnl|UG|At#S11722187	gi|23308474|gb|BT000641.1	At4g32520.1

at1g73490	CON	MIN	HOUR	DAY	WEEK	UNIGENE	FLCDNA	TAIR
TTCTCTATTG	2	2	0	0	4	gnl|UG|At#S11822140	gi|25083033|gb|BT002025.1	At1g73490.1

at4g27680	CON	MIN	HOUR	DAY	WEEK	UNIGENE	FLCDNA	TAIR
GCAGCGGGAT	0	0	0	4	1	no match found	gi|23197835|gb|BT000126.1	non-canonical match

at1g13930	CON	MIN	HOUR	DAY	WEEK	UNIGENE	FLCDNA	TAIR
AATCTAATCC	57	51	67	87	69	gnl|UG|At#S20296748	gi|15010677|gb|AY045640.1	At1g13930.1
GCAAGTGCCA	1	6	6	2	1	no match found	gi|16323297|gb|AY058230.1	non-canonical match

at1g58440	CON	MIN	HOUR	DAY	WEEK	UNIGENE	FLCDNA	TAIR
CGTCATCCTT	0	0	2	1	0	gnl|UG|At#S11731736	gi|23198061|gb|BT000239.1	At1g58440.1

at4g29930	CON	MIN	HOUR	DAY	WEEK	UNIGENE	FLCDNA	TAIR
TTCAGAAAAA	0	0	0	1	2	no match found	gi|110736371|dbj|AK228201.1|	non-canonical match

at5g58020	CON	MIN	HOUR	DAY	WEEK	UNIGENE	FLCDNA	TAIR
CGGATGTCAA	0	1	3	1	0	no match found	gi|34365742|gb|BT010560.1	non-canonical match

at3g11020	CON	MIN	HOUR	DAY	WEEK	UNIGENE	FLCDNA	TAIR
AACTGTTGCT	0	0	2	1	1	gnl|UG|At#S11824443	gi|26449819|dbj|AK117363.1|	At3g11020.1

at1g12320	CON	MIN	HOUR	DAY	WEEK	UNIGENE	FLCDNA	TAIR
CTTGTCTTAT	1	1	0	0	0	gnl|UG|At#S11708521	gi|17380647|gb|AY063798.1	At1g12320.1

at5g14640	CON	MIN	HOUR	DAY	WEEK	UNIGENE	FLCDNA	TAIR
TATCTTTGTT	2	0	0	2	4	no match found	gi|18086449|gb|AY065043.1	multiple canonical match

at1g73350	CON	MIN	HOUR	DAY	WEEK	UNIGENE	FLCDNA	TAIR
ATTTTGAGCA	0	0	1	0	0	gnl|UG|At#S18894515	gi|17529353|gb|AY065463.1	non-canonical match

at5g37740	CON	MIN	HOUR	DAY	WEEK	UNIGENE	FLCDNA	TAIR
AGGAGTAAGT	1	0	0	0	0	gnl|UG|At#S18911577	gi|23397075|gb|BT000677.1	At5g37740.1

at5g46190	CON	MIN	HOUR	DAY	WEEK	UNIGENE	FLCDNA	TAIR
TTTAGATATG	1	0	0	0	1	gnl|UG|At#S11719107	gi|21404492|gb|AY085782.1	At5g46190.1

at3g01330	CON	MIN	HOUR	DAY	WEEK	UNIGENE	FLCDNA	TAIR
AGAGCACATA	0	0	1	1	0	gnl|UG|At#S11740269	gi|20260249|gb|AY093024.1	At3g01330.1

at2g03470	CON	MIN	HOUR	DAY	WEEK	UNIGENE	FLCDNA	TAIR
AACCTTTTTT	2	2	4	1	2	gnl|UG|At#S11825090	gi|27754609|gb|BT002937.1	At2g03470.1

at3g07510	CON	MIN	HOUR	DAY	WEEK	UNIGENE	FLCDNA	TAIR
GCTTTGTTTT	2	4	2	2	2	gnl|UG|At#S11738488	gi|13877548|gb|AF370475.1	At3g07510.1

at2g35340	CON	MIN	HOUR	DAY	WEEK	UNIGENE	FLCDNA	TAIR
AGCCTAAACA	1	0	0	0	0	no match found	no match found	At2g35340.1
CGACAGGTAA	0	2	0	0	0	gnl|UG|At#S34115257	no match found	multiple non-canonical match

at5g14700	CON	MIN	HOUR	DAY	WEEK	UNIGENE	FLCDNA	TAIR
AGAGTTAGCT	1	0	0	0	0	gnl|UG|At#S11706052	gi|21404418|gb|AY085708.1	At5g14700.1
TGGAAAAAAC	0	0	1	0	0	gnl|UG|At#S18320782	no match found	non-canonical match

at3g03680	CON	MIN	HOUR	DAY	WEEK	UNIGENE	FLCDNA	TAIR
TAATCTTCTG	1	2	1	0	0	gnl|UG|At#S11739603	no match found	At3g03680.1

at3g20700	CON	MIN	HOUR	DAY	WEEK	UNIGENE	FLCDNA	TAIR
GTGTGATCAA	1	1	0	0	0	no match found	no match found	At3g20700.1

at3g55960	CON	MIN	HOUR	DAY	WEEK	UNIGENE	FLCDNA	TAIR
CCTGAATGGT	2	0	2	1	0	gnl|UG|At#S20831441	gi|20466679|gb|AY099806.1	At3g55960.1

at2g40920	CON	MIN	HOUR	DAY	WEEK	UNIGENE	FLCDNA	TAIR
CTAAAAAAAA	16	18	16	13	11	gnl|UG|At#S34115549	gi|110741293|dbj|AK230399.1|	multiple non-canonical match
CTCAAAAAAA	2	9	13	6	4	no match found	gi|110741388|dbj|AK230447.1|	multiple non-canonical match

at3g06100	CON	MIN	HOUR	DAY	WEEK	UNIGENE	FLCDNA	TAIR
ACAGCTCCGG	0	0	2	0	0	gnl|UG|At#S11738901	gi|21406544|gb|AY087797.1	At3g06100.1

at1g77885	CON	MIN	HOUR	DAY	WEEK	UNIGENE	FLCDNA	TAIR
GCGATTGAGT	0	0	1	0	0	gnl|UG|At#S18942241	no match found	At1g77885.1

at4g17970	CON	MIN	HOUR	DAY	WEEK	UNIGENE	FLCDNA	TAIR
TCAACAATAT	3	1	0	0	0	gnl|UG|At#S11724685	no match found	At4g17970.1

at5g66410	CON	MIN	HOUR	DAY	WEEK	UNIGENE	FLCDNA	TAIR
TGGACACAAA	1	2	0	0	0	no match found	no match found	At5g66410.1

at1g21500	CON	MIN	HOUR	DAY	WEEK	UNIGENE	FLCDNA	TAIR
GTGGCACATT	0	10	5	1	0	gnl|UG|At#S11740911	gi|13877530|gb|AF370466.1	At1g21500.1

at5g19180	CON	MIN	HOUR	DAY	WEEK	UNIGENE	FLCDNA	TAIR
AAAACCGACA	0	2	2	3	0	gnl|UG|At#S11721524	gi|110740875|dbj|AK226388.1|	At5g19180.1

at1g68870	CON	MIN	HOUR	DAY	WEEK	UNIGENE	FLCDNA	TAIR
CAGCTGCAAA	1	0	0	0	0	no match found	gi|50198842|gb|BT015000.1	multiple non-canonical match

at2g38040	CON	MIN	HOUR	DAY	WEEK	UNIGENE	FLCDNA	TAIR
ATTTCTGGAT	5	4	6	4	2	gnl|UG|At#S11733092	gi|15294259|gb|AF410321.1	At2g38040.1

at5g16120	CON	MIN	HOUR	DAY	WEEK	UNIGENE	FLCDNA	TAIR
ATTCTCCGTG	3	3	1	0	0	gnl|UG|At#S43849312	gi|13430613|gb|AF360219.1	At5g16120.1

at5g42100	CON	MIN	HOUR	DAY	WEEK	UNIGENE	FLCDNA	TAIR
TCCATTTCCT	3	3	3	1	0	gnl|UG|At#S11719519	gi|15451219|gb|AY054690.1	At5g42100.2

at1g76350	CON	MIN	HOUR	DAY	WEEK	UNIGENE	FLCDNA	TAIR
TCTTCCAATA	0	1	1	2	0	gnl|UG|At#S11727761	no match found	At1g76350.1

at1g30070	CON	MIN	HOUR	DAY	WEEK	UNIGENE	FLCDNA	TAIR
TGATCAGTAT	0	0	0	0	1	gnl|UG|At#S11739003	gi|28973032|gb|BT005421.1	At1g30070.1

at1g22170	CON	MIN	HOUR	DAY	WEEK	UNIGENE	FLCDNA	TAIR
AAGCGGGGAA	0	1	0	0	0	gnl|UG|At#S11740843	gi|56381946|gb|BT020337.1	At1g22170.1

at5g11410	CON	MIN	HOUR	DAY	WEEK	UNIGENE	FLCDNA	TAIR
TATGGACTAA	1	0	1	0	0	gnl|UG|At#S11723402	no match found	At5g11410.1

at1g06060	CON	MIN	HOUR	DAY	WEEK	UNIGENE	FLCDNA	TAIR
GCCACAGGCG	2	4	1	1	0	gnl|UG|At#S11742421	gi|21404226|gb|AY085516.1	non-canonical match

at1g76780	CON	MIN	HOUR	DAY	WEEK	UNIGENE	FLCDNA	TAIR
AAGTATACGC	0	0	1	0	0	no match found	no match found	At1g76780.1
at3g14140	CON	MIN	HOUR	DAY	WEEK	UNIGENE	FLCDNA	TAIR
GTTTCTGGTT	0	1	0	0	0	no match found	no match found	At3g14140.1
at4g19600	CON	MIN	HOUR	DAY	WEEK	UNIGENE	FLCDNA	TAIR
GCGAGATTCT	0	1	0	0	0	no match found	gi|117168064|gb|BT029301.1	non-canonical match
ATGCGAAATG	2	2	2	0	0	gnl|UG|At#S11724400	gi|26450104|dbj|AK117509.1|	At4g19600.1
at5g02570	CON	MIN	HOUR	DAY	WEEK	UNIGENE	FLCDNA	TAIR
CCGTCTCTGA	0	0	1	0	0	gnl|UG|At#S11725518	no match found	non-canonical match
at1g06570	CON	MIN	HOUR	DAY	WEEK	UNIGENE	FLCDNA	TAIR
ATGAAAGATG	7	4	3	1	0	gnl|UG|At#S11742372	gi|16226617|gb|AF428446.1	At1g06570.1
at2g44920	CON	MIN	HOUR	DAY	WEEK	UNIGENE	FLCDNA	TAIR
CAGTTTGATT	1	1	1	1	0	no match found	no match found	At2g44920.2
CTCCGTCTTT	9	4	6	1	2	gnl|UG|At#S11731427	no match found	At2g44920.1
at2g43160	CON	MIN	HOUR	DAY	WEEK	UNIGENE	FLCDNA	TAIR
CCTGGCAATA	0	0	0	1	0	no match found	gi|25090232|gb|BT002247.1	non-canonical match
ATGCACTTGA	1	4	2	4	0	gnl|UG|At#S24443371	gi|110740367|dbj|AK230277.1|	At2g43160.2
at4g13261	CON	MIN	HOUR	DAY	WEEK	UNIGENE	FLCDNA	TAIR
AAATCAATAA	0	0	0	1	0	no match found	no match found	At4g13261.1
at5g43350	CON	MIN	HOUR	DAY	WEEK	UNIGENE	FLCDNA	TAIR
ACGAGAAATA	0	1	0	0	1	no match found	gi|17979403|gb|AY070432.1	At5g43350.1
at1g20340	CON	MIN	HOUR	DAY	WEEK	UNIGENE	FLCDNA	TAIR
AATTTGTTTG	0	0	1	0	0	gnl|UG|At#S20714210	no match found	multiple non-canonical match
GCAATAGAAG	31	120	35	39	2	gnl|UG|At#S11741024	gi|12247994|gb|AF334383.1	At1g20340.1
at5g57080	CON	MIN	HOUR	DAY	WEEK	UNIGENE	FLCDNA	TAIR
GATAAAGTTT	0	3	1	0	0	gnl|UG|At#S35250733	no match found	non-canonical match
at1g07890	CON	MIN	HOUR	DAY	WEEK	UNIGENE	FLCDNA	TAIR
GAGCCAACAG	0	0	1	0	0	gnl|UG|At#S11627533	no match found	non-canonical match
AAGCTTTCTG	0	0	1	0	1	no match found	gi|20334803|gb|AY094002.1	multiple non-canonical match
at5g04830	CON	MIN	HOUR	DAY	WEEK	UNIGENE	FLCDNA	TAIR
TTATAGTGTT	4	0	4	2	4	gnl|UG|At#S15461397	gi|13507538|gb|AF360335.1	At5g04830.1
at2g04830	CON	MIN	HOUR	DAY	WEEK	UNIGENE	FLCDNA	TAIR
TATCGAATTG	0	1	0	0	0	gnl|UG|At#S14959947	gi|45680120|gb|BT011810.1	At2g04830.1
at1g27770	CON	MIN	HOUR	DAY	WEEK	UNIGENE	FLCDNA	TAIR
CCTGTTGCTG	0	2	0	1	0	gnl|UG|At#S11832384	no match found	At1g27770.1
at3g54110	CON	MIN	HOUR	DAY	WEEK	UNIGENE	FLCDNA	TAIR
TTTTTGACCC	2	1	0	2	0	gnl|UG|At#S11728665	gi|62319574|dbj|AK221112.1|	At3g54110.1
at5g37800	CON	MIN	HOUR	DAY	WEEK	UNIGENE	FLCDNA	TAIR
GAAATGGAGA	0	0	1	0	0	gnl|UG|At#S11719955	no match found	multiple non-canonical match
TAATTAAGTG	1	0	0	0	0	no match found	no match found	At5g37800.1
at4g39950	CON	MIN	HOUR	DAY	WEEK	UNIGENE	FLCDNA	TAIR
GCGGAGATGG	0	2	1	0	1	gnl|UG|At#S11720931	gi|15028134|gb|AY046017.1	At4g39950.1

at3g48610	CON	MIN	HOUR	DAY	WEEK	UNIGENE	FLCDNA	TAIR
AGTATGTCAA	0	1	1	1	2	gnl|UG|At#S11729714	no match found	At3g48610.1
at3g20430	CON	MIN	HOUR	DAY	WEEK	UNIGENE	FLCDNA	TAIR
AGAGGATCAG	0	1	0	0	0	gnl|UG|At#S11743574	gi|18175896|gb|AY072125.1	At3g20430.1
at3g04480	CON	MIN	HOUR	DAY	WEEK	UNIGENE	FLCDNA	TAIR
ATATTATTGG	0	1	0	0	0	gnl|UG|At#S11739372	gi|110742422|dbj|AK227077.1|	At3g04480.1
at4g16570	CON	MIN	HOUR	DAY	WEEK	UNIGENE	FLCDNA	TAIR
GAATTGCATT	0	0	1	0	0	no match found	gi|23308404|gb|BT000603.1	At1g04580.1
at5g20580	CON	MIN	HOUR	DAY	WEEK	UNIGENE	FLCDNA	TAIR
AAGCATATAC	0	0	0	0	1	gnl|UG|At#S18941596	gi|110738353|dbj|AK229238.1|	At5g20580.1
at3g11490	CON	MIN	HOUR	DAY	WEEK	UNIGENE	FLCDNA	TAIR
TCGAAAGCGA	1	0	0	1	0	gnl|UG|At#S11737438	no match found	non-canonical match
at5g01050	CON	MIN	HOUR	DAY	WEEK	UNIGENE	FLCDNA	TAIR
GATGCACATT	0	1	0	0	0	no match found	no match found	At5g01050.1
at2g44730	CON	MIN	HOUR	DAY	WEEK	UNIGENE	FLCDNA	TAIR
ACGATGCGTC	1	2	0	0	0	gnl|UG|At#S11731468	no match found	At2g44730.1
at4g11330	CON	MIN	HOUR	DAY	WEEK	UNIGENE	FLCDNA	TAIR
TTGTTATCTT	0	1	0	0	0	no match found	gi|51970863|dbj|AK176361.1|	multiple canonical match
at2g38950	CON	MIN	HOUR	DAY	WEEK	UNIGENE	FLCDNA	TAIR
CGATAACGAT	0	0	1	0	0	no match found	gi|24111436|gb|BT001088.1	non-canonical match
AAGCAACTTG	0	0	2	2	1	gnl|UG|At#S11732862	gi|22022586|gb|AY127026.1	At2g38950.1
at5g14790	CON	MIN	HOUR	DAY	WEEK	UNIGENE	FLCDNA	TAIR
ATCTACCAAA	1	0	0	0	0	no match found	gi|24899826|gb|BT001241.1	non-canonical match
AGTCTTTTGG	2	1	1	2	1	gnl|UG|At#S11722643	gi|21407649|gb|AY088875.1	At5g14790.1
at4g35000	CON	MIN	HOUR	DAY	WEEK	UNIGENE	FLCDNA	TAIR
GACCCAAGAG	1	6	4	2	0	no match found	gi|21404872|gb|AY086162.1	At4g35000.1
at5g03970	CON	MIN	HOUR	DAY	WEEK	UNIGENE	FLCDNA	TAIR
GGTGTCATTA	1	1	0	1	1	gnl|UG|At#S11707618	gi|15215621|gb|AY050339.1	At5g03970.1
at3g16857	CON	MIN	HOUR	DAY	WEEK	UNIGENE	FLCDNA	TAIR
TTAGGGGTTA	2	0	0	0	1	gnl|UG|At#S11735801	gi|15810170|gb|AY056099.1	multiple canonical match
at3g16260	CON	MIN	HOUR	DAY	WEEK	UNIGENE	FLCDNA	TAIR
TGCTTCCAAA	1	0	0	2	0	gnl|UG|At#S11824502	gi|26449702|dbj|AK117304.1|	At3g16260.1
at5g03610	CON	MIN	HOUR	DAY	WEEK	UNIGENE	FLCDNA	TAIR
ACAACTCAAA	0	1	0	0	0	gnl|UG|At#S11725252	gi|21403823|gb|AY085113.1	At5g03610.1
at1g45230	CON	MIN	HOUR	DAY	WEEK	UNIGENE	FLCDNA	TAIR
AGAGAACAAT	0	0	0	1	0	gnl|UG|At#S11816401	gi|48310650|gb|BT014877.1	At1g45230.1
at5g18520	CON	MIN	HOUR	DAY	WEEK	UNIGENE	FLCDNA	TAIR
TTTCATATGT	1	3	0	0	0	gnl|UG|At#S24442758	gi|51969513|dbj|AK175686.1|	At5g18520.1
at4g13000	CON	MIN	HOUR	DAY	WEEK	UNIGENE	FLCDNA	TAIR
AAATATTTAC	0	0	0	0	1	gnl|UG|At#S11823973	gi|28950940|gb|BT005330.1	At4g13000.1
at2g21330	CON	MIN	HOUR	DAY	WEEK	UNIGENE	FLCDNA	TAIR
GTTAATATCT	37	5	25	13	20	gnl|UG|At#S28282295	gi|72196816|gb|DQ108657.1	At2g21330.1

GGGAGGCAAG	12	14	11	7	7	no match found	gi|23297092|gb|AY142633.1	non-canonical match
at5g44070	CON	MIN	HOUR	DAY	WEEK	UNIGENE	FLCDNA	TAIR
GCTTCCCTCC	1	3	3	0	0	gnl|UG|At#S11719321	gi|19310768|gb|AY079384.1	At5g44070.1
at1g23360	CON	MIN	HOUR	DAY	WEEK	UNIGENE	FLCDNA	TAIR
AATCTTTCTC	1	2	0	0	2	gnl|UG|At#S11807236	gi|20259386|gb|AY090980.1	At1g23360.2
GGGAATTTGG	0	1	0	0	0	no match found	gi|48310574|gb|BT014858.1	non-canonical match
at2g02230	CON	MIN	HOUR	DAY	WEEK	UNIGENE	FLCDNA	TAIR
ATAAAAAAAA	28	30	55	37	26	gnl|UG|At#S34117190	no match found	non-canonical match
at2g01220	CON	MIN	HOUR	DAY	WEEK	UNIGENE	FLCDNA	TAIR
GGAAACGTTT	0	0	2	0	0	gnl|UG|At#S21736067	gi|26451898|dbj|AK118433.1|	At2g01220.2
at5g64880	CON	MIN	HOUR	DAY	WEEK	UNIGENE	FLCDNA	TAIR
TACTTTGTTT	1	2	0	0	0	gnl|UG|At#S34117234	gi|110738040|dbj|AK229074.1|	At5g64880.1
at5g04230	CON	MIN	HOUR	DAY	WEEK	UNIGENE	FLCDNA	TAIR
TCCTCTTCGA	0	2	0	1	0	gnl|UG|At#S11725089	no match found	At5g04230.1
at5g05540	CON	MIN	HOUR	DAY	WEEK	UNIGENE	FLCDNA	TAIR
AAGTTAAGCC	0	3	0	0	0	no match found	gi|26451906|dbj|AK118437.1|	At5g05540.1
at3g49160	CON	MIN	HOUR	DAY	WEEK	UNIGENE	FLCDNA	TAIR
GAAGGATTAA	2	0	0	1	0	gnl|UG|At#S11743522	gi|18176181|gb|AY072177.1	At3g49160.1
at3g66658	CON	MIN	HOUR	DAY	WEEK	UNIGENE	FLCDNA	TAIR
TAAGCCTCAT	0	1	2	0	1	gnl|UG|At#S18942169	gi|110735944|dbj|AK227981.1|	At3g66658.1
at1g12130	CON	MIN	HOUR	DAY	WEEK	UNIGENE	FLCDNA	TAIR
TCATTAAATT	0	0	0	1	0	no match found	no match found	At1g12130.1
at3g22540	CON	MIN	HOUR	DAY	WEEK	UNIGENE	FLCDNA	TAIR
TCGCTGATGG	0	1	0	0	0	no match found	gi|52218801|gb|BT015694.1	At3g22540.1
at3g18290	CON	MIN	HOUR	DAY	WEEK	UNIGENE	FLCDNA	TAIR
TCCAATCTGT	2	2	4	0	0	gnl|UG|At#S11735358	gi|20453198|gb|AY094471.1	At3g18290.1
at3g59765	CON	MIN	HOUR	DAY	WEEK	UNIGENE	FLCDNA	TAIR
TAGCCAAACG	0	0	1	0	0	gnl|UG|At#S35283479	no match found	At3g18290.1
at4g36670	CON	MIN	HOUR	DAY	WEEK	UNIGENE	FLCDNA	TAIR
AAAACGACGT	0	0	1	0	0	no match found	gi|145651781|gb|BT030462.1	multiple non-canonical match
ATGAATATAA	0	7	0	0	1	gnl|UG|At#S11721494	gi|26451235|dbj|AK118092.1|	At4g36670.1
at5g59030	CON	MIN	HOUR	DAY	WEEK	UNIGENE	FLCDNA	TAIR
TTGTTCGGAA	0	1	0	1	0	no match found	gi|21689854|gb|AY123038.1	non-canonical match
TGTGTGTGAA	12	13	9	5	1	no match found	gi|20258854|gb|AY091090.1	At5g59030.1
at3g28910	CON	MIN	HOUR	DAY	WEEK	UNIGENE	FLCDNA	TAIR
GTTCTAATTT	0	2	0	1	0	gnl|UG|At#S11732000	gi|21386950|gb|AY114560.1	At3g28910.1
at5g42460	CON	MIN	HOUR	DAY	WEEK	UNIGENE	FLCDNA	TAIR
GCCTCTCTAC	1	1	0	0	1	no match found	gi|110743147|dbj|AK227463.1|	multiple canonical match
at1g77800	CON	MIN	HOUR	DAY	WEEK	UNIGENE	FLCDNA	TAIR
TAGAAACATT	0	0	0	1	0	gnl|UG|At#S11727270	no match found	At1g77800.1
at5g56980	CON	MIN	HOUR	DAY	WEEK	UNIGENE	FLCDNA	TAIR
ATACAGATTA	1	0	2	0	0	gnl|UG|At#S11703372	gi|14326478|gb|AF385692.1	At5g56980.1

CGGAAGCGCC	0	7	1	1	0	no match found	gi|21405873|gb|AY087149.1	non-canonical match
at1g04500	CON	MIN	HOUR	DAY	WEEK	UNIGENE	FLCDNA	TAIR
AATAAAATTG	7	1	3	7	2	no match found	gi|26451418|dbj|AK118186.1|	multiple canonical match
at5g09810	CON	MIN	HOUR	DAY	WEEK	UNIGENE	FLCDNA	TAIR
AAGATCAAGG	7	19	7	4	1	gnl|UG|At#S11723781	gi|17065251|gb|AY062702.1	At5g09810.1
at4g13270	CON	MIN	HOUR	DAY	WEEK	UNIGENE	FLCDNA	TAIR
TAATTCGAAG	2	3	4	3	1	no match found	gi|62867634|gb|BT021984.1	At4g13270.1
TTTTGAGTTC	2	0	0	0	1	no match found	gi|110743778|dbj|AK227741.1|	non-canonical match
at2g11000	CON	MIN	HOUR	DAY	WEEK	UNIGENE	FLCDNA	TAIR
CTTTTTGCTC	2	3	2	2	2	gnl|UG|At#S11739337	gi|110739149|dbj|AK229647.1|	At2g11000.1
at3g23900	CON	MIN	HOUR	DAY	WEEK	UNIGENE	FLCDNA	TAIR
AAGAGTTTGT	3	4	0	0	0	gnl|UG|At#S11733628	no match found	At3g23900.1
at1g22300	CON	MIN	HOUR	DAY	WEEK	UNIGENE	FLCDNA	TAIR
AAAGTCTGAA	0	3	2	0	0	gnl|UG|At#S18942522	no match found	At1g22300.3
CCAATTGGCT	2	0	0	0	0	no match found	gi|20148690|gb|AY081674.1	multiple non-canonical match
ATTTGGGTTT	12	10	13	10	8	gnl|UG|At#S20716286	gi|16604431|gb|AY058834.1	At1g22300.2
at3g02900	CON	MIN	HOUR	DAY	WEEK	UNIGENE	FLCDNA	TAIR
ATTGAATGTA	4	0	2	1	2	gnl|UG|At#S11739808	gi|27311636|gb|BT002424.1	At3g02900.1
at5g23900	CON	MIN	HOUR	DAY	WEEK	UNIGENE	FLCDNA	TAIR
CTGGTGCCAG	3	12	3	3	1	gnl|UG|At#S11720789	gi|14190434|gb|AF378895.1	At5g23900.1
at4g09580	CON	MIN	HOUR	DAY	WEEK	UNIGENE	FLCDNA	TAIR
ATTATGTAAA	0	0	2	1	1	no match found	gi|20465629|gb|AY096649.1	At4g09580.1
at2g15970	CON	MIN	HOUR	DAY	WEEK	UNIGENE	FLCDNA	TAIR
TAAGAGTGAT	19	23	26	59	45	gnl|UG|At#S11675315	gi|20147352|gb|AY093767.1	non-canonical match
AGGTGTTAGT	9	4	16	13	23	no match found	no match found	At2g15970.1
ATCGGATCCG	0	0	1	0	1	no match found	gi|23505784|gb|AY143813.1	non-canonical match
at5g11880	CON	MIN	HOUR	DAY	WEEK	UNIGENE	FLCDNA	TAIR
CTGAGACATT	1	5	2	4	2	no match found	no match found	At5g11880.1
TAAAATTGTC	0	1	0	0	1	gnl|UG|At#S11723277	gi|62319460|dbj|AK221054.1|	At5g11880.1
at1g03440	CON	MIN	HOUR	DAY	WEEK	UNIGENE	FLCDNA	TAIR
TTTTACTCCG	0	3	0	0	0	gnl|UG|At#S18895249	gi|110736866|dbj|AK228464.1|	At1g03440.1
at1g20330	CON	MIN	HOUR	DAY	WEEK	UNIGENE	FLCDNA	TAIR
AGATGTTGTT	4	2	1	2	0	gnl|UG|At#S18925168	gi|23397215|gb|BT000750.1	At1g20330.1
at5g35360	CON	MIN	HOUR	DAY	WEEK	UNIGENE	FLCDNA	TAIR
AAGAGGAGCT	7	13	2	2	1	gnl|UG|At#S28281719	gi|21404678|gb|AY085968.1	multiple non-canonical match
GTTTTGGACT	1	2	3	0	1	no match found	gi|28393663|gb|BT004236.1	At5g41790.1
at5g38810	CON	MIN	HOUR	DAY	WEEK	UNIGENE	FLCDNA	TAIR
TGATATGAAT	0	1	0	0	0	no match found	no match found	At5g38810.1
at4g00530	CON	MIN	HOUR	DAY	WEEK	UNIGENE	FLCDNA	TAIR
TTAGCTAGAA	1	1	0	0	0	gnl|UG|At#S35212006	no match found	non-canonical match
at2g17265	CON	MIN	HOUR	DAY	WEEK	UNIGENE	FLCDNA	TAIR
TTTGGAACAG	0	1	1	0	1	gnl|UG|At#S11815650	gi|22135867|gb|AY128313.1	At2g17265.1
at1g09130	CON	MIN	HOUR	DAY	WEEK	UNIGENE	FLCDNA	TAIR

CGATTAGGAG	13	9	12	15	9	gnl|UG|At#S11742126	gi|51971624|dbj|AK176714.1|	At1g09130.1
at2g39990	CON	MIN	HOUR	DAY	WEEK	UNIGENE	FLCDNA	TAIR
GTTGATGCTG	0	2	0	4	3	gnl|UG|At#S11732605	gi|20259172|gb|AY091363.1	At2g39990.1
at3g18620	CON	MIN	HOUR	DAY	WEEK	UNIGENE	FLCDNA	TAIR
AGAAGAAGAA	0	0	0	1	0	no match found	gi|21281222|gb|AY114003.1	multiple non-canonical match
CCTTTGAACT	0	0	2	3	0	gnl|UG|At#S11744289	gi|19424024|gb|AY080783.1	At3g18620.1
at5g48320	CON	MIN	HOUR	DAY	WEEK	UNIGENE	FLCDNA	TAIR
TAACTTGGCT	1	0	1	0	0	gnl|UG|At#S18909062	no match found	multiple canonical match
at5g56850	CON	MIN	HOUR	DAY	WEEK	UNIGENE	FLCDNA	TAIR
AGGAGCTTGT	0	0	0	1	0	gnl|UG|At#S11718027	no match found	At5g56850.1
at1g05140	CON	MIN	HOUR	DAY	WEEK	UNIGENE	FLCDNA	TAIR
CTTGTATTAT	0	0	1	1	2	gnl|UG|At#S11742515	gi|15010609|gb|AY045606.1	At1g05140.1
at1g28090	CON	MIN	HOUR	DAY	WEEK	UNIGENE	FLCDNA	TAIR
AGACACGTCG	0	1	0	0	0	no match found	no match found	At1g28090.1
TGGAACATTC	1	0	2	1	0	gnl|UG|At#S18942482	no match found	At1g28090.2
at3g21820	CON	MIN	HOUR	DAY	WEEK	UNIGENE	FLCDNA	TAIR
TTTGAGCTGA	1	0	0	0	0	gnl|UG|At#S24442452	gi|62320768|dbj|AK221711.1|	At3g21820.1
at5g38530	CON	MIN	HOUR	DAY	WEEK	UNIGENE	FLCDNA	TAIR
AACAAAATTA	0	5	3	4	2	no match found	gi|19699004|gb|AY081349.1	multiple canonical match
at1g13260	CON	MIN	HOUR	DAY	WEEK	UNIGENE	FLCDNA	TAIR
AGATGATGAA	1	4	15	3	1	gnl|UG|At#S11741711	no match found	At1g13260.1
AGTTGTTTTT	2	16	9	2	4	no match found	gi|17380761|gb|AY063855.1	multiple non-canonical match
at1g19870	CON	MIN	HOUR	DAY	WEEK	UNIGENE	FLCDNA	TAIR
CAGCCAACAC	0	1	1	0	0	gnl|UG|At#S11741067	gi|20856688|gb|AY102110.1	At1g19870.1
AACCGGTCAT	0	1	0	0	0	gnl|UG|At#S30649523	no match found	multiple non-canonical match
at2g38280	CON	MIN	HOUR	DAY	WEEK	UNIGENE	FLCDNA	TAIR
TCACCACTGA	0	1	0	0	0	gnl|UG|At#S15460650	gi|15810524|gb|AY056301.1	At2g38280.1
at2g39460	CON	MIN	HOUR	DAY	WEEK	UNIGENE	FLCDNA	TAIR
TTGCAGTTTA	22	26	31	43	51	no match found	gi|21404922|gb|AY086212.1	At2g39460.1
at5g35430	CON	MIN	HOUR	DAY	WEEK	UNIGENE	FLCDNA	TAIR
TGAGCTTTGT	0	0	0	0	1	gnl|UG|At#S11720160	gi|51970075|dbj|AK175967.1|	At5g35430.1
at1g08820	CON	MIN	HOUR	DAY	WEEK	UNIGENE	FLCDNA	TAIR
AGCTGGCGGA	0	0	0	1	0	gnl|UG|At#S11742152	gi|18377966|gb|AY074512.1	At1g08820.1
at3g04560	CON	MIN	HOUR	DAY	WEEK	UNIGENE	FLCDNA	TAIR
ATGACTCCGT	0	0	0	0	1	no match found	no match found	At3g04560.1
AACTAGAAAA	0	3	0	0	0	gnl|UG|At#S18922795	no match found	non-canonical match
at3g53120	CON	MIN	HOUR	DAY	WEEK	UNIGENE	FLCDNA	TAIR
TTATACCTTT	1	0	0	0	0	no match found	gi|48310465|gb|BT014843.1	multiple non-canonical match
GTACTCGCCT	0	1	0	0	0	gnl|UG|At#S11728850	no match found	At3g53120.1
at5g56630	CON	MIN	HOUR	DAY	WEEK	UNIGENE	FLCDNA	TAIR
GTGCAATGGC	0	2	2	1	0	gnl|UG|At#S11702044	gi|13430589|gb|AF360207.1	At5g56630.1
at5g26860	CON	MIN	HOUR	DAY	WEEK	UNIGENE	FLCDNA	TAIR
ATCACATCTT	1	1	1	0	0	gnl|UG|At#S11700050	gi|21436458|gb|AY117355.1	At5g26860.1

at1g30110	CON	MIN	HOUR	DAY	WEEK	UNIGENE	FLCDNA	TAIR
GGGGTCATTG	1	0	0	0	0	gnl|UG|At#S11738988	gi|110739316|dbj|AK229734.1|	At1g30110.1
GTCAAGCTCA	0	1	0	0	0	no match found	gi|98960928|gb|BT025530.1	non-canonical match

at2g17880	CON	MIN	HOUR	DAY	WEEK	UNIGENE	FLCDNA	TAIR
AAGATCCACG	0	7	0	1	0	gnl|UG|At#S11738022	gi|110742616|dbj|AK227181.1|	At2g17880.1

at1g69780	CON	MIN	HOUR	DAY	WEEK	UNIGENE	FLCDNA	TAIR
GCTTGATCAG	0	0	0	1	0	no match found	gi|22137259|gb|AY133645.1	non-canonical match
TTTTCAAAAA	0	3	1	3	2	gnl|UG|At#S11702682	gi|15982928|gb|AY057572.1	At1g69780.1

at5g56360	CON	MIN	HOUR	DAY	WEEK	UNIGENE	FLCDNA	TAIR
TCATATACCA	0	2	0	0	0	no match found	gi|15081667|gb|AY048226.1	non-canonical match
AAGAAGAGCT	4	2	1	0	1	no match found	no match found	At5g56360.1

at2g34100	CON	MIN	HOUR	DAY	WEEK	UNIGENE	FLCDNA	TAIR
ACGATGAAGA	0	1	0	0	0	gnl|UG|At#S15114814	no match found	At2g34100.1

at2g30070	CON	MIN	HOUR	DAY	WEEK	UNIGENE	FLCDNA	TAIR
AGCACGAATT	0	1	0	0	0	gnl|UG|At#S24442776	gi|62320121|dbj|AK221387.1|	At2g30070.1

at5g43850	CON	MIN	HOUR	DAY	WEEK	UNIGENE	FLCDNA	TAIR
GATTTGCTGG	1	2	0	0	0	gnl|UG|At#S11719343	gi|30102911|gb|BT006566.1	At5g43850.1

at1g21695	CON	MIN	HOUR	DAY	WEEK	UNIGENE	FLCDNA	TAIR
TAGATTTGTA	0	0	0	0	1	gnl|UG|At#S11816482	no match found	At1g21695.1

at5g20600	CON	MIN	HOUR	DAY	WEEK	UNIGENE	FLCDNA	TAIR
AAAATGGAGT	1	1	2	0	1	gnl|UG|At#S34117211	gi|110738084|dbj|AK229097.1|	At5g20600.1

at5g03230	CON	MIN	HOUR	DAY	WEEK	UNIGENE	FLCDNA	TAIR
CGATACCTGA	3	3	0	0	0	gnl|UG|At#S11702533	gi|23296535|gb|AY142539.1	At5g03230.1

at2g37950	CON	MIN	HOUR	DAY	WEEK	UNIGENE	FLCDNA	TAIR
TTTCCTCTTC	0	1	0	0	0	gnl|UG|At#S11733109	gi|54261716|gb|BT015932.1	At2g37950.1

at4g22020	CON	MIN	HOUR	DAY	WEEK	UNIGENE	FLCDNA	TAIR
GTAGTGGTTT	0	0	1	0	0	no match found	no match found	At4g22020.1

at1g58400	CON	MIN	HOUR	DAY	WEEK	UNIGENE	FLCDNA	TAIR
AGAGAAGAAT	1	0	0	0	0	no match found	no match found	At1g58400.1

at1g28400	CON	MIN	HOUR	DAY	WEEK	UNIGENE	FLCDNA	TAIR
GAGAAAGGTA	0	3	2	0	0	gnl|UG|At#S11739480	gi|19347892|gb|AY080623.1	At1g28400.1

at5g59480	CON	MIN	HOUR	DAY	WEEK	UNIGENE	FLCDNA	TAIR
AAATCTTTTT	0	1	0	0	0	gnl|UG|At#S15446799	no match found	non-canonical match
GCAAATCCTG	2	1	0	2	0	no match found	gi|110741501|dbj|AK226585.1|	At5g59480.2

at5g39950	CON	MIN	HOUR	DAY	WEEK	UNIGENE	FLCDNA	TAIR
ATTGATTGAA	7	4	5	4	5	no match found	gi|15081685|gb|AY048235.1	At5g39950.1

at5g20120	CON	MIN	HOUR	DAY	WEEK	UNIGENE	FLCDNA	TAIR
TCTTGTTTCG	0	1	0	0	0	gnl|UG|At#S11721296	gi|110743896|dbj|AK227801.1|	At5g20120.1

at5g03545	CON	MIN	HOUR	DAY	WEEK	UNIGENE	FLCDNA	TAIR
TTTGTGTTGA	1	22	2	4	4	gnl|UG|At#S11832383	gi|14532787|gb|AY040018.1	At5g03545.1

at1g75140	CON	MIN	HOUR	DAY	WEEK	UNIGENE	FLCDNA	TAIR
GATCTTCGGA	0	2	1	0	0	gnl|UG|At#S11728014	gi|26450825|dbj|AK117881.1|	At1g75140.1

at1g16900	CON	MIN	HOUR	DAY	WEEK	UNIGENE	FLCDNA	TAIR
TGGCAGGAGA	0	0	0	1	1	gnl|UG|At#S11741354	gi|22655223|gb|AY140061.1	At1g16900.1
at2g03270	CON	MIN	HOUR	DAY	WEEK	UNIGENE	FLCDNA	TAIR
TGATAATGTA	0	2	0	0	0	gnl|UG|At#S11740492	gi|15027926|gb|AY045820.1	At2g03270.1
at2g40260	CON	MIN	HOUR	DAY	WEEK	UNIGENE	FLCDNA	TAIR
ATGATCATCA	1	1	1	0	0	gnl|UG|At#S11732531	no match found	multiple non-canonical match
at5g09430	CON	MIN	HOUR	DAY	WEEK	UNIGENE	FLCDNA	TAIR
CTGTGAATTT	0	0	1	0	0	gnl|UG|At#S11723884	no match found	multiple non-canonical match
at1g11940	CON	MIN	HOUR	DAY	WEEK	UNIGENE	FLCDNA	TAIR
CTTTCTCTTT	0	1	0	0	0	no match found	gi|28827513|gb|BT005068.1	non-canonical match
at2g13430	CON	MIN	HOUR	DAY	WEEK	UNIGENE	FLCDNA	TAIR
GTTTCGATTA	0	4	0	1	1	no match found	gi|15028178|gb|AY045912.1	At5g66052.1
AAGAAGCAAA	5	1	9	3	2	gnl|UG|At#S11739080	no match found	At2g13430.1
at5g67190	CON	MIN	HOUR	DAY	WEEK	UNIGENE	FLCDNA	TAIR
GAAGGCGGCG	0	1	0	0	0	no match found	no match found	At5g67190.1
at4g14040	CON	MIN	HOUR	DAY	WEEK	UNIGENE	FLCDNA	TAIR
GACCAGATTT	0	1	6	1	1	no match found	gi|17473651|gb|AY065108.1	non-canonical match
GAGACGTATT	1	0	2	1	0	gnl|UG|At#S11725382	no match found	At4g14040.1
at1g51140	CON	MIN	HOUR	DAY	WEEK	UNIGENE	FLCDNA	TAIR
AACAAAGCTA	0	1	1	0	0	gnl|UG|At#S11734092	gi|14334499|gb|AY034941.1	At1g51140.1
at4g14070	CON	MIN	HOUR	DAY	WEEK	UNIGENE	FLCDNA	TAIR
GTCGTGGCTA	0	2	4	1	0	gnl|UG|At#S11725377	gi|62321025|dbj|AK221841.1|	At4g14070.1
at2g18400	CON	MIN	HOUR	DAY	WEEK	UNIGENE	FLCDNA	TAIR
GAAGCCAAGT	0	2	1	1	0	gnl|UG|At#S11737879	gi|14994246|gb|AY044317.1	At2g18400.1
at5g11170	CON	MIN	HOUR	DAY	WEEK	UNIGENE	FLCDNA	TAIR
CCTTCTTAAA	0	1	0	0	0	no match found	gi|62319894|dbj|AK221273.1|	non-canonical match
at3g09720	CON	MIN	HOUR	DAY	WEEK	UNIGENE	FLCDNA	TAIR
TCTTTGAAGA	0	1	1	0	1	no match found	gi|29028777|gb|BT005833.1	multiple non-canonical match
TCGGATTCAA	1	0	1	1	2	gnl|UG|At#S11737965	gi|110742884|dbj|AK227326.1|	At3g09720.1
at3g47940	CON	MIN	HOUR	DAY	WEEK	UNIGENE	FLCDNA	TAIR
AGGAAGTCAT	1	0	0	0	0	gnl|UG|At#S11729814	gi|23197961|gb|BT000189.1	At3g47940.1
at5g14240	CON	MIN	HOUR	DAY	WEEK	UNIGENE	FLCDNA	TAIR
TAAGTTTAGA	0	1	0	0	0	gnl|UG|At#S11722774	no match found	non-canonical match
TGCAACTCGA	0	1	0	0	0	no match found	no match found	At5g14240.1
TTGTTTTTTG	1	0	0	2	1	no match found	gi|21406415|gb|AY087677.1	multiple non-canonical match
at1g70840	CON	MIN	HOUR	DAY	WEEK	UNIGENE	FLCDNA	TAIR
TTTGGTTTTA	0	0	1	0	0	gnl|UG|At#S11744296	gi|19424012|gb|AY080814.1	At1g70840.1
at5g59900	CON	MIN	HOUR	DAY	WEEK	UNIGENE	FLCDNA	TAIR
CAGAAAGCTG	0	1	0	0	0	gnl|UG|At#S11717712	no match found	At5g59900.1
at3g49530	CON	MIN	HOUR	DAY	WEEK	UNIGENE	FLCDNA	TAIR
AGCAATGATG	0	1	0	0	0	no match found	gi|24030449|gb|BT000978.1	non-canonical match
TAGTTAGGCT	0	1	2	1	1	gnl|UG|At#S11760105	gi|16604578|gb|AY059734.1	At3g49530.1

at4g00413	CON	MIN	HOUR	DAY	WEEK	UNIGENE	FLCDNA	TAIR
ATTATGCAAT	0	0	1	0	1	no match found	no match found	At4g00413.1
at3g44560	CON	MIN	HOUR	DAY	WEEK	UNIGENE	FLCDNA	TAIR
AAAATATGTG	0	0	1	0	0	gnl|UG|At#S35290212	no match found	non-canonical match
at1g17145	CON	MIN	HOUR	DAY	WEEK	UNIGENE	FLCDNA	TAIR
TGCCAATAAG	4	2	1	0	1	gnl|UG|At#S11741330	gi|15451137|gb|AY054649.1	At1g17145.1
at1g67920	CON	MIN	HOUR	DAY	WEEK	UNIGENE	FLCDNA	TAIR
ATATGTATGT	0	0	0	1	0	no match found	gi|21403828|gb|AY085118.1	At1g67920.1
at4g35090	CON	MIN	HOUR	DAY	WEEK	UNIGENE	FLCDNA	TAIR
AAATCCGCAG	2	1	3	3	1	no match found	gi|21280988|gb|AY113854.1	non-canonical match
TGGATCTTCT	89	23	106	108	40	no match found	gi|15451165|gb|AY054663.1	multiple canonical match
at5g41150	CON	MIN	HOUR	DAY	WEEK	UNIGENE	FLCDNA	TAIR
GTTACACTCT	1	0	0	0	0	gnl|UG|At#S11719614	gi|22655253|gb|AY140076.1	non-canonical match
at1g76990	CON	MIN	HOUR	DAY	WEEK	UNIGENE	FLCDNA	TAIR
CTTCTCAGGA	0	0	2	0	0	no match found	gi|21280958|gb|AY113891.1	non-canonical match
TCTATAAATG	5	5	3	1	2	gnl|UG|At#S15435741	gi|21406746|gb|AY087972.1	At1g76990.3
at5g23440	CON	MIN	HOUR	DAY	WEEK	UNIGENE	FLCDNA	TAIR
TAAATCGAGT	1	1	0	0	0	gnl|UG|At#S11720833	gi|45752723|gb|BT012165.1	At5g23440.1
at2g42790	CON	MIN	HOUR	DAY	WEEK	UNIGENE	FLCDNA	TAIR
AGACCACAAC	0	2	1	0	0	gnl|UG|At#S24442899	gi|62319876|dbj|AK221264.1|	At2g42790.1
at4g37210	CON	MIN	HOUR	DAY	WEEK	UNIGENE	FLCDNA	TAIR
GTATCAGCTA	0	0	1	0	0	no match found	gi|17065595|gb|AY062946.1	non-canonical match
TTTTGTAATG	2	0	0	2	0	gnl|UG|At#S11721402	gi|13877852|gb|AF370189.1	At4g37210.1
at2g41000	CON	MIN	HOUR	DAY	WEEK	UNIGENE	FLCDNA	TAIR
AAGTGTAATA	1	0	0	0	1	gnl|UG|At#S11732339	gi|110743344|dbj|AK227566.1|	At2g41000.1
at3g15356	CON	MIN	HOUR	DAY	WEEK	UNIGENE	FLCDNA	TAIR
GGACGTGCCG	7	7	10	1	0	gnl|UG|At#S11816215	no match found	At3g15356.1
at4g15460	CON	MIN	HOUR	DAY	WEEK	UNIGENE	FLCDNA	TAIR
ATCATATTTT	0	0	1	0	0	gnl|UG|At#S11725124	no match found	multiple non-canonical match
at1g51085	CON	MIN	HOUR	DAY	WEEK	UNIGENE	FLCDNA	TAIR
AAGGCAAGAA	0	1	0	1	1	no match found	no match found	At1g51085.1
at1g03260	CON	MIN	HOUR	DAY	WEEK	UNIGENE	FLCDNA	TAIR
AGGTGTCAGT	0	0	0	2	0	gnl|UG|At#S11742788	gi|44681459|gb|BT011664.1	At1g03260.1
at2g41560	CON	MIN	HOUR	DAY	WEEK	UNIGENE	FLCDNA	TAIR
ATTGTAGCCG	2	5	2	2	0	gnl|UG|At#S11732198	gi|110741168|dbj|AK230336.1|	At2g41560.1
at3g59780	CON	MIN	HOUR	DAY	WEEK	UNIGENE	FLCDNA	TAIR
AATCGAAACC	2	5	2	2	1	no match found	no match found	At3g59780.1
at2g14120	CON	MIN	HOUR	DAY	WEEK	UNIGENE	FLCDNA	TAIR
ACTCATTCAT	1	1	0	0	0	no match found	gi|20466603|gb|AY099768.1	At1g75360.1
AGACAATGTA	0	0	2	1	0	gnl|UG|At#S11831694	no match found	At2g14120.2
TACGTTCTTG	0	0	1	0	0	no match found	gi|26450374|dbj|AK117648.1|	At2g14120.2
at1g14370	CON	MIN	HOUR	DAY	WEEK	UNIGENE	FLCDNA	TAIR
ACTCCTGGTG	0	3	1	0	0	gnl|UG|At#S11741599	gi|16649048|gb|AY059894.1	non-canonical match

at1g66430	CON	MIN	HOUR	DAY	WEEK	UNIGENE	FLCDNA	TAIR
AGGCTCTACT	0	3	1	0	0	gnl|UG|At#S11729842	gi|24030221|gb|BT000889.1	At1g66430.1
at1g45474	CON	MIN	HOUR	DAY	WEEK	UNIGENE	FLCDNA	TAIR
GCATAAGACC	12	3	4	5	5	gnl|UG|At#S11700840	gi|17063198|gb|AY062097.1	At1g45474.2
at1g16340	CON	MIN	HOUR	DAY	WEEK	UNIGENE	FLCDNA	TAIR
ATGATCCTTT	1	0	2	0	0	gnl|UG|At#S21737268	no match found	multiple canonical match
at5g18200	CON	MIN	HOUR	DAY	WEEK	UNIGENE	FLCDNA	TAIR
TACTGTAGTT	1	0	2	1	0	gnl|UG|At#S11710316	gi|17529045|gb|AY065257.1	At5g18200.1
at3g16670	CON	MIN	HOUR	DAY	WEEK	UNIGENE	FLCDNA	TAIR
TATAGTGCTG	9	10	3	5	3	gnl|UG|At#S11735861	gi|20856563|gb|AY102103.1	At3g16670.1
at1g20100	CON	MIN	HOUR	DAY	WEEK	UNIGENE	FLCDNA	TAIR
TATGTTAGTT	1	2	3	2	1	gnl|UG|At#S35297934	no match found	non-canonical match
AGCTTGCAGA	1	1	1	0	3	gnl|UG|At#S11807232	gi|12083281|gb|AF332437.1	multiple non-canonical match
at5g17800	CON	MIN	HOUR	DAY	WEEK	UNIGENE	FLCDNA	TAIR
TTTGTTTAAC	0	1	0	0	0	gnl|UG|At#S11721870	gi|51970527|dbj|AK176193.1|	At5g17800.1
at2g36800	CON	MIN	HOUR	DAY	WEEK	UNIGENE	FLCDNA	TAIR
GCCGCTATTC	0	3	2	3	0	gnl|UG|At#S11733397	gi|28059217|gb|BT003373.1	At2g36800.1
at3g03100	CON	MIN	HOUR	DAY	WEEK	UNIGENE	FLCDNA	TAIR
GGTACCCACC	1	6	0	4	4	gnl|UG|At#S17007986	gi|62318844|dbj|AK220742.1|	At3g03100.1
at1g28130	CON	MIN	HOUR	DAY	WEEK	UNIGENE	FLCDNA	TAIR
GCTTACTTCG	1	0	0	0	0	gnl|UG|At#S11739583	gi|21360518|gb|AY113067.1	At1g28130.2
at5g15740	CON	MIN	HOUR	DAY	WEEK	UNIGENE	FLCDNA	TAIR
GCCTTGCCAG	1	1	0	2	2	gnl|UG|At#S11722402	gi|62319149|dbj|AK220896.1|	At5g15740.1
at5g35690	CON	MIN	HOUR	DAY	WEEK	UNIGENE	FLCDNA	TAIR
CTATTTACAG	1	4	1	2	5	gnl|UG|At#S11709884	gi|17979188|gb|AY070096.1	At5g35690.1
at3g57280	CON	MIN	HOUR	DAY	WEEK	UNIGENE	FLCDNA	TAIR
TTGTGTTTCT	0	1	0	0	0	gnl|UG|At#S11728089	gi|15450538|gb|AY052733.1	At3g57280.1
CTTCCACCAA	0	0	1	0	0	gnl|UG|At#S11654183	no match found	non-canonical match
at1g28390	CON	MIN	HOUR	DAY	WEEK	UNIGENE	FLCDNA	TAIR
TGTTTGTCTT	0	2	0	0	0	gnl|UG|At#S11739486	gi|19715602|gb|AY075610.1	multiple canonical match
at5g09410	CON	MIN	HOUR	DAY	WEEK	UNIGENE	FLCDNA	TAIR
TGAGAGGACA	0	2	0	0	0	gnl|UG|At#S43849351	gi|110737391|dbj|AK228740.1|	At5g09410.1
at1g17860	CON	MIN	HOUR	DAY	WEEK	UNIGENE	FLCDNA	TAIR
TCAAACTTAA	0	2	0	1	0	gnl|UG|At#S11741259	gi|20453292|gb|AY097369.1	At1g17860.1
at2g03630	CON	MIN	HOUR	DAY	WEEK	UNIGENE	FLCDNA	TAIR
AGAAGATACA	0	0	0	0	1	no match found	no match found	At2g03630.1
at3g54430	CON	MIN	HOUR	DAY	WEEK	UNIGENE	FLCDNA	TAIR
TCTTTCTCAA	0	1	0	0	0	gnl|UG|At#S11706920	gi|15450614|gb|AY052675.1	At3g54430.1
AGCAAAGAAC	0	0	1	0	0	gnl|UG|At#S21736187	no match found	At3g54430.1
at4g26710	CON	MIN	HOUR	DAY	WEEK	UNIGENE	FLCDNA	TAIR
GCTTTTGTTG	1	8	0	2	0	no match found	gi|21403242|gb|AY084532.1	At4g26710.2

at3g45310	CON	MIN	HOUR	DAY	WEEK	UNIGENE	FLCDNA	TAIR
TTCATCGTAT	0	1	0	0	0	gnl|UG|At#S11730324	gi|20147206|gb|AY091771.1	At3g45310.1
at4g34430	CON	MIN	HOUR	DAY	WEEK	UNIGENE	FLCDNA	TAIR
GCCTTTCCAC	0	2	0	0	0	gnl|UG|At#S18941730	gi|20465380|gb|AY096454.1	At4g34430.1
at2g02040	CON	MIN	HOUR	DAY	WEEK	UNIGENE	FLCDNA	TAIR
GCGGTTTACT	2	3	2	3	1	gnl|UG|At#S11740680	gi|23506066|gb|AY143954.1	At2g02040.1
at5g13090	CON	MIN	HOUR	DAY	WEEK	UNIGENE	FLCDNA	TAIR
ACTACTTTGG	4	3	2	0	0	gnl|UG|At#S11811763	gi|21405197|gb|AY086487.1	At5g13090.1
at1g28370	CON	MIN	HOUR	DAY	WEEK	UNIGENE	FLCDNA	TAIR
GGGACGTTAC	0	2	2	0	0	gnl|UG|At#S11739492	gi|13430639|gb|AF360232.1	At1g28370.1
at1g10150	CON	MIN	HOUR	DAY	WEEK	UNIGENE	FLCDNA	TAIR
GATCTTATAT	1	1	0	0	1	gnl|UG|At#S11742019	no match found	At1g10150.1
at5g48620	CON	MIN	HOUR	DAY	WEEK	UNIGENE	FLCDNA	TAIR
ATTGCGAAAA	0	0	1	0	0	no match found	gi|29029047|gb|BT005968.1	non-canonical match
GAACTTGCTT	6	8	13	4	0	gnl|UG|At#S11718857	gi|72196956|gb|DQ108679.1	At5g48620.1
at3g57340	CON	MIN	HOUR	DAY	WEEK	UNIGENE	FLCDNA	TAIR
ATGAGGAGAT	0	5	2	2	2	gnl|UG|At#S18901978	gi|27311872|gb|BT002542.1	At3g57340.2
at2g47390	CON	MIN	HOUR	DAY	WEEK	UNIGENE	FLCDNA	TAIR
CGAGTGCACT	1	4	1	2	2	gnl|UG|At#S15460250	gi|18086369|gb|AY064997.1	non-canonical match
CTGAGAAGGA	0	2	0	0	0	no match found	gi|20466188|gb|AY099560.1	non-canonical match
at5g47860	CON	MIN	HOUR	DAY	WEEK	UNIGENE	FLCDNA	TAIR
AAGAGTTTGG	5	3	8	5	4	no match found	gi|16648765|gb|AY058159.1	At5g47860.1
at1g72220	CON	MIN	HOUR	DAY	WEEK	UNIGENE	FLCDNA	TAIR
TTTCAACAAA	0	1	0	0	0	no match found	gi|25141210|gb|BT002303.1	At5g18300.1
at1g63660	CON	MIN	HOUR	DAY	WEEK	UNIGENE	FLCDNA	TAIR
TAAAATAGAT	5	0	0	1	1	gnl|UG|At#S18942325	gi|28393248|gb|BT004017.1	At1g63660.2
at2g21560	CON	MIN	HOUR	DAY	WEEK	UNIGENE	FLCDNA	TAIR
TCAGAGAACA	1	0	1	0	0	gnl|UG|At#S34117121	gi|110738255|dbj|AK229187.1|	At2g21560.1
at1g20840	CON	MIN	HOUR	DAY	WEEK	UNIGENE	FLCDNA	TAIR
CCTTTGGAAG	0	3	0	1	0	gnl|UG|At#S11816488	no match found	At1g20840.1
at2g16900	CON	MIN	HOUR	DAY	WEEK	UNIGENE	FLCDNA	TAIR
TGTTTCGGTT	2	0	0	1	0	gnl|UG|At#S11738256	gi|14532491|gb|AY039870.1	multiple canonical match
at5g12130	CON	MIN	HOUR	DAY	WEEK	UNIGENE	FLCDNA	TAIR
TGCCGCTTGG	0	0	2	0	0	gnl|UG|At#S11723214	gi|21405111|gb|AY086401.1	At5g12130.1
at4g28706	CON	MIN	HOUR	DAY	WEEK	UNIGENE	FLCDNA	TAIR
GTGGTATGCA	3	0	2	1	0	gnl|UG|At#S15460589	gi|17473610|gb|AY065095.1	At4g28706.1
at5g50280	CON	MIN	HOUR	DAY	WEEK	UNIGENE	FLCDNA	TAIR
ACTTACAATA	0	1	0	0	0	gnl|UG|At#S11718688	no match found	non-canonical match
at5g51640	CON	MIN	HOUR	DAY	WEEK	UNIGENE	FLCDNA	TAIR
GAACGAGATG	2	3	0	0	0	gnl|UG|At#S11744292	gi|19424020|gb|AY080801.1	At5g51640.1
at3g11560	CON	MIN	HOUR	DAY	WEEK	UNIGENE	FLCDNA	TAIR
ATGGAGAAAA	0	0	1	0	0	no match found	gi|62320287|dbj|AK221470.1|	multiple non-canonical match

TCTTTAAAGA	0	0	0	0	1	gnl|UG|At#S11737416	no match found	At3g11560.3
TGTCCGGTAC	3	3	5	1	2	no match found	gi|21405359|gb|AY086649.1	At3g11560.2
at3g04720	CON	MIN	HOUR	DAY	WEEK	UNIGENE	FLCDNA	TAIR
CTTGTTTCGG	4	36	5	3	2	no match found	gi|23197675|gb|BT000046.1	At3g04720.1
GAGTCCTGAA	0	1	0	0	0	gnl|UG|At#S35219294	no match found	multiple non-canonical match
at1g22360	CON	MIN	HOUR	DAY	WEEK	UNIGENE	FLCDNA	TAIR
GTTCTTCTAA	1	3	1	0	0	gnl|UG|At#S11740824	gi|17065005|gb|AY062579.1	At1g22360.1
at1g74810	CON	MIN	HOUR	DAY	WEEK	UNIGENE	FLCDNA	TAIR
TTGATTGTTT	0	1	0	0	0	no match found	no match found	At1g74810.1
at3g43980	CON	MIN	HOUR	DAY	WEEK	UNIGENE	FLCDNA	TAIR
TTGGTGTTGA	5	2	3	4	4	gnl|UG|At#S11701749	gi|11908081|gb|AF326888.1	At3g43980.1
at3g43540	CON	MIN	HOUR	DAY	WEEK	UNIGENE	FLCDNA	TAIR
GGCGACGTCT	3	3	6	3	1	gnl|UG|At#S18942032	gi|14532795|gb|AY040022.1	At3g43540.2
at2g38000	CON	MIN	HOUR	DAY	WEEK	UNIGENE	FLCDNA	TAIR
GGAGAGTCTC	0	1	0	0	0	no match found	no match found	At2g38000.1
at3g07660	CON	MIN	HOUR	DAY	WEEK	UNIGENE	FLCDNA	TAIR
AAGGAGATTT	0	3	1	0	1	gnl|UG|At#S11738444	gi|110742275|dbj|AK226998.1|	At3g07660.1
at5g04780	CON	MIN	HOUR	DAY	WEEK	UNIGENE	FLCDNA	TAIR
AAGGCTGCAT	0	1	0	0	0	gnl|UG|At#S11724937	no match found	non-canonical match
at3g54460	CON	MIN	HOUR	DAY	WEEK	UNIGENE	FLCDNA	TAIR
CTTTGGTTGC	0	1	0	0	0	no match found	gi|14517379|gb|AY039524.1	non-canonical match
ATTTGGTAGA	0	1	0	0	0	gnl|UG|At#S11816066	gi|28393844|gb|BT004336.1	At3g54460.1
at5g62580	CON	MIN	HOUR	DAY	WEEK	UNIGENE	FLCDNA	TAIR
GTGAGTTTGC	1	1	1	0	0	gnl|UG|At#S11717442	no match found	At5g62580.1
at5g18660	CON	MIN	HOUR	DAY	WEEK	UNIGENE	FLCDNA	TAIR
GCGGGTCAAG	3	6	2	2	2	gnl|UG|At#S11721660	gi|26449403|dbj|AK117151.1|	At5g18660.1
at1g06320	CON	MIN	HOUR	DAY	WEEK	UNIGENE	FLCDNA	TAIR
TTTTGTGCGA	0	3	1	1	0	gnl|UG|At#S35280534	no match found	At1g26550.1
GTTTCCGAAA	0	0	1	0	0	gnl|UG|At#S11742395	no match found	non-canonical match
at3g17640	CON	MIN	HOUR	DAY	WEEK	UNIGENE	FLCDNA	TAIR
ATGGATATGG	0	0	0	1	0	no match found	no match found	At3g17640.1
at2g46170	CON	MIN	HOUR	DAY	WEEK	UNIGENE	FLCDNA	TAIR
AAACGTGAGA	0	1	1	2	2	gnl|UG|At#S11731152	gi|15450758|gb|AY053421.1	At2g46170.1
at5g66675	CON	MIN	HOUR	DAY	WEEK	UNIGENE	FLCDNA	TAIR
TAGATAAGTT	1	2	0	0	0	gnl|UG|At#S34117920	gi|28950990|gb|BT005355.1	At5g66675.1
at4g32272	CON	MIN	HOUR	DAY	WEEK	UNIGENE	FLCDNA	TAIR
AGTGGCCTAG	0	0	1	0	0	gnl|UG|At#S38433407	gi|19715587|gb|AY075601.1	non-canonical match
at5g20660	CON	MIN	HOUR	DAY	WEEK	UNIGENE	FLCDNA	TAIR
GATGAAATAG	1	1	1	0	0	no match found	gi|110741499|dbj|AK226584.1|	multiple canonical match
at1g77930	CON	MIN	HOUR	DAY	WEEK	UNIGENE	FLCDNA	TAIR
GCGGTTGCCG	0	0	1	0	0	no match found	gi|107738376|gb|BT025796.1	non-canonical match
TAGATAATCA	2	3	1	0	2	no match found	gi|21407909|gb|AY089135.1	At1g77930.1

at3g12510	CON	MIN	HOUR	DAY	WEEK	UNIGENE	FLCDNA	TAIR
TTCTATCTTT	0	1	0	0	2	gnl|UG|At#S21736771	gi|51969949|dbj|AK175904.1|	At3g12510.1
at4g05390	CON	MIN	HOUR	DAY	WEEK	UNIGENE	FLCDNA	TAIR
AATCTTTCCT	1	3	1	0	2	gnl|UG|At#S11726251	gi|22530947|gb|AY136312.1	At4g05390.1
at1g79560	CON	MIN	HOUR	DAY	WEEK	UNIGENE	FLCDNA	TAIR
TAATGCCAGC	1	3	0	0	1	gnl|UG|At#S11726672	gi|14334985|gb|AY035166.1	non-canonical match
GAGATTGCAA	0	1	0	0	0	gnl|UG|At#S35311425	no match found	non-canonical match
at3g05710	CON	MIN	HOUR	DAY	WEEK	UNIGENE	FLCDNA	TAIR
CTGGTACTCT	0	1	0	0	0	no match found	gi|22136415|gb|AY128886.1	non-canonical match
ATTTTAAGAA	0	2	0	0	0	gnl|UG|At#S11739008	gi|20466513|gb|AY099723.1	At3g05710.2
at5g37360	CON	MIN	HOUR	DAY	WEEK	UNIGENE	FLCDNA	TAIR
TACAGTGCCA	7	4	3	1	0	gnl|UG|At#S11719999	gi|16604481|gb|AY058859.1	At5g37360.1
GCAAATCAGT	0	0	1	0	0	no match found	gi|21655280|gb|AY103300.1	non-canonical match
at3g12000	CON	MIN	HOUR	DAY	WEEK	UNIGENE	FLCDNA	TAIR
AAAATTGGAT	1	0	0	0	1	gnl|UG|At#S11737291	no match found	At3g12000.1
at3g22900	CON	MIN	HOUR	DAY	WEEK	UNIGENE	FLCDNA	TAIR
TTATGTAAAA	1	1	0	0	0	gnl|UG|At#S11733941	gi|21404347|gb|AY085637.1	At3g22900.1
at2g47440	CON	MIN	HOUR	DAY	WEEK	UNIGENE	FLCDNA	TAIR
TCAGCCACTG	6	21	5	9	1	gnl|UG|At#S11730845	gi|25084072|gb|BT002157.1	At2g47440.1
at1g02130	CON	MIN	HOUR	DAY	WEEK	UNIGENE	FLCDNA	TAIR
ATGAGGTCCT	3	2	5	2	0	gnl|UG|At#S11742903	no match found	At1g02130.1
GCAATGTCTG	2	0	1	0	1	no match found	gi|16974456|gb|AY061905.1	non-canonical match
at1g22910	CON	MIN	HOUR	DAY	WEEK	UNIGENE	FLCDNA	TAIR
TATCAGCTTA	2	1	0	1	0	gnl|UG|At#S11703051	gi|13605870|gb|AF367334.1	At1g22910.2
at1g22370	CON	MIN	HOUR	DAY	WEEK	UNIGENE	FLCDNA	TAIR
GACGGAGACA	0	0	0	1	0	gnl|UG|At#S22317525	gi|14532545|gb|AY039897.1	At1g22370.1
at1g22160	CON	MIN	HOUR	DAY	WEEK	UNIGENE	FLCDNA	TAIR
TTAATCATCC	4	4	0	1	0	gnl|UG|At#S11740844	gi|21405028|gb|AY086318.1	At1g22160.1
at1g56660	CON	MIN	HOUR	DAY	WEEK	UNIGENE	FLCDNA	TAIR
ATGCAACAGA	0	2	3	0	1	no match found	gi|16648922|gb|AY059831.1	non-canonical match
TCTCTTGGTA	2	0	0	0	1	gnl|UG|At#S11732045	no match found	At1g56660.1
AGGATGTATC	0	2	1	0	1	no match found	gi|110738560|dbj|AK229343.1|	non-canonical match
at1g62300	CON	MIN	HOUR	DAY	WEEK	UNIGENE	FLCDNA	TAIR
TAATAGGCCA	0	3	1	1	0	gnl|UG|At#S26538503	gi|15810266|gb|AY056172.1	At1g62300.1
at4g28610	CON	MIN	HOUR	DAY	WEEK	UNIGENE	FLCDNA	TAIR
AGCAGCTCGA	0	8	1	1	1	gnl|UG|At#S11722860	gi|19698886|gb|AY081290.1	At4g28610.1
at1g72310	CON	MIN	HOUR	DAY	WEEK	UNIGENE	FLCDNA	TAIR
ATGCTTTGTC	1	0	0	0	0	gnl|UG|At#S11701008	gi|33589685|gb|BT010140.1	At1g72310.1
at5g09250	CON	MIN	HOUR	DAY	WEEK	UNIGENE	FLCDNA	TAIR
TTCTGCTAGT	2	0	1	3	0	gnl|UG|At#S34118229	gi|110736128|dbj|AK228077.1|	At5g09250.1
at3g06440	CON	MIN	HOUR	DAY	WEEK	UNIGENE	FLCDNA	TAIR
ATATAGCGAA	0	1	0	0	0	no match found	gi|25090103|gb|BT002218.1	non-canonical match
TACGTATGTA	0	2	0	0	1	gnl|UG|At#S11738816	gi|13605628|gb|AF361640.1	At3g06440.1

atcg00570	CON	MIN	HOUR	DAY	WEEK	UNIGENE	FLCDNA	TAIR
GACTAGCTGT	0	1	0	0	0	no match found	no match found	AtCg00570
at5g23210	CON	MIN	HOUR	DAY	WEEK	UNIGENE	FLCDNA	TAIR
TTTTCTCGTG	1	0	0	0	2	gnl|UG|At#S15460483	no match found	At5g23210.2
TGCATCAGAG	0	3	0	1	0	no match found	gi|110737894|dbj|AK228998.1|	At5g23210.1
at3g21480	CON	MIN	HOUR	DAY	WEEK	UNIGENE	FLCDNA	TAIR
TTTGGCTTAA	0	1	0	0	0	gnl|UG|At#S11734387	no match found	At3g21480.1
at3g48680	CON	MIN	HOUR	DAY	WEEK	UNIGENE	FLCDNA	TAIR
CACTATCGAA	0	5	0	0	0	gnl|UG|At#S11729701	gi|15293166|gb|AY051017.1	At3g48680.1
at1g31500	CON	MIN	HOUR	DAY	WEEK	UNIGENE	FLCDNA	TAIR
AAGCAGTGAA	0	0	2	2	1	gnl|UG|At#S14829888	gi|110737062|dbj|AK228566.1|	At1g31500.1
at1g19835	CON	MIN	HOUR	DAY	WEEK	UNIGENE	FLCDNA	TAIR
TGTCCTCAAA	1	0	2	0	0	gnl|UG|At#S11741071	gi|62321571|dbj|AK222122.1|	At1g19835.1
at2g36960	CON	MIN	HOUR	DAY	WEEK	UNIGENE	FLCDNA	TAIR
TTTATTTGGC	0	2	0	1	1	no match found	gi|17381223|gb|AY064068.1	non-canonical match
at4g33440	CON	MIN	HOUR	DAY	WEEK	UNIGENE	FLCDNA	TAIR
GTCACTTACG	0	0	0	0	1	gnl|UG|At#S11825241	gi|27754319|gb|BT002785.1	At4g33440.1
at4g13010	CON	MIN	HOUR	DAY	WEEK	UNIGENE	FLCDNA	TAIR
AAAAGAAAAT	8	2	5	10	5	gnl|UG|At#S11725563	gi|15028000|gb|AY045857.1	multiple canonical match
CTACTGGGAA	3	3	2	4	0	no match found	gi|21407953|gb|AY089179.1	non-canonical match
at3g25140	CON	MIN	HOUR	DAY	WEEK	UNIGENE	FLCDNA	TAIR
GCTTGACATA	1	0	0	0	0	gnl|UG|At#S11733235	gi|20466216|gb|AY099574.1	At3g25140.1
at5g22390	CON	MIN	HOUR	DAY	WEEK	UNIGENE	FLCDNA	TAIR
TCGACGAGGA	0	1	2	1	0	gnl|UG|At#S11721002	gi|28827353|gb|BT004988.1	At5g22390.1
at3g47550	CON	MIN	HOUR	DAY	WEEK	UNIGENE	FLCDNA	TAIR
ATACACGGTG	0	3	0	0	0	gnl|UG|At#S15460136	gi|110741113|dbj|AK226509.1|	non-canonical match
at2g21110	CON	MIN	HOUR	DAY	WEEK	UNIGENE	FLCDNA	TAIR
AAAACAAAGT	9	2	3	2	2	no match found	no match found	At2g21110.1
at1g74090	CON	MIN	HOUR	DAY	WEEK	UNIGENE	FLCDNA	TAIR
GTAAATGAAT	1	4	0	1	0	gnl|UG|At#S11728222	gi|26450014|dbj|AK117463.1|	At1g74090.1
at1g56600	CON	MIN	HOUR	DAY	WEEK	UNIGENE	FLCDNA	TAIR
AGAATACAAA	2	1	1	1	3	gnl|UG|At#S11732067	gi|15215761|gb|AY050410.1	At1g56600.1
at1g03240	CON	MIN	HOUR	DAY	WEEK	UNIGENE	FLCDNA	TAIR
AAACAAAAGA	7	2	7	1	1	gnl|UG|At#S11742790	no match found	multiple canonical match
at5g37020	CON	MIN	HOUR	DAY	WEEK	UNIGENE	FLCDNA	TAIR
CTACAACGCG	0	0	1	1	3	gnl|UG|At#S34116234	no match found	At5g37020.1
AACAACAACA	0	1	1	0	2	no match found	gi|110739979|dbj|AK230074.1|	At5g46915.1
at1g41803	CON	MIN	HOUR	DAY	WEEK	UNIGENE	FLCDNA	TAIR
TGTCGCAACT	0	0	1	0	0	no match found	no match found	At1g41803.1
at4g16280	CON	MIN	HOUR	DAY	WEEK	UNIGENE	FLCDNA	TAIR
TTGTGTTACT	0	0	0	0	1	gnl|UG|At#S11832489	no match found	At4g16280.2
at3g11810	CON	MIN	HOUR	DAY	WEEK	UNIGENE	FLCDNA	TAIR

GTGAGCTTGT	2	1	1	0	0	no match found	no match found	At3g11810.1
at3g63130	CON	MIN	HOUR	DAY	WEEK	UNIGENE	FLCDNA	TAIR
TTTAAGGACT	0	0	0	0	1	gnl|UG|At#S11703451	no match found	At3g63130.1
at2g40940	CON	MIN	HOUR	DAY	WEEK	UNIGENE	FLCDNA	TAIR
ACCGGACGGT	2	2	2	3	0	gnl|UG|At#S11732355	gi|15450903|gb|AY054532.1	At2g40940.1
at5g65670	CON	MIN	HOUR	DAY	WEEK	UNIGENE	FLCDNA	TAIR
ACAAAGCTTT	4	10	9	0	4	gnl|UG|At#S15435782	gi|17979423|gb|AY070451.1	At5g65670.2
at4g16420	CON	MIN	HOUR	DAY	WEEK	UNIGENE	FLCDNA	TAIR
AGATATTCAA	0	1	0	0	2	gnl|UG|At#S11724961	gi|23505980|gb|AY143911.1	multiple canonical match
at1g67840	CON	MIN	HOUR	DAY	WEEK	UNIGENE	FLCDNA	TAIR
TCTACATATG	2	3	1	0	1	gnl|UG|At#S18942305	gi|15146205|gb|AY049244.1	At1g67840.1
GAACTTTGTG	1	0	1	0	0	no match found	gi|21360444|gb|AY113030.1	multiple non-canonical match
at1g21060	CON	MIN	HOUR	DAY	WEEK	UNIGENE	FLCDNA	TAIR
ATTCTACTAT	2	0	0	0	0	no match found	no match found	At1g21060.1
at4g13345	CON	MIN	HOUR	DAY	WEEK	UNIGENE	FLCDNA	TAIR
AAGCGTGTAC	0	1	0	0	0	gnl|UG|At#S11725497	no match found	At4g13345.2
AGAGATCACC	0	1	0	1	0	no match found	gi|16604676|gb|AY059783.1	non-canonical match
at1g37080	CON	MIN	HOUR	DAY	WEEK	UNIGENE	FLCDNA	TAIR
GAGAGACTGA	0	0	0	0	1	gnl|UG|At#S22484632	no match found	non-canonical match
at1g74900	CON	MIN	HOUR	DAY	WEEK	UNIGENE	FLCDNA	TAIR
TGAATTAGTT	0	0	0	1	0	gnl|UG|At#S11728055	gi|51971676|dbj|AK176740.1|	At1g74900.1
at5g19220	CON	MIN	HOUR	DAY	WEEK	UNIGENE	FLCDNA	TAIR
GTAGACGTGC	0	2	0	0	0	gnl|UG|At#S35307455	no match found	non-canonical match
at1g14830	CON	MIN	HOUR	DAY	WEEK	UNIGENE	FLCDNA	TAIR
TTTTCTTGGG	2	2	0	0	2	gnl|UG|At#S11700000	gi|21403801|gb|AY085091.1	At1g14830.1
at3g03120	CON	MIN	HOUR	DAY	WEEK	UNIGENE	FLCDNA	TAIR
AGATTATTAA	0	0	0	0	1	gnl|UG|At#S11739751	gi|21403721|gb|AY085011.1	At3g03120.1
at3g12650	CON	MIN	HOUR	DAY	WEEK	UNIGENE	FLCDNA	TAIR
AGAGCTTGAA	0	3	5	4	0	gnl|UG|At#S11705261	gi|16323108|gb|AY057658.1	At3g12650.1
at3g61240	CON	MIN	HOUR	DAY	WEEK	UNIGENE	FLCDNA	TAIR
TAAAGACTCG	0	1	0	1	2	no match found	gi|21539434|gb|AY120712.1	At3g61240.2
at1g20850	CON	MIN	HOUR	DAY	WEEK	UNIGENE	FLCDNA	TAIR
AAGAAATTAA	0	0	2	1	0	gnl|UG|At#S34114266	gi|110743794|dbj|AK227749.1|	At1g20850.1
GACTATGCCT	1	2	0	0	0	no match found	gi|28466958|gb|BT004822.1	multiple non-canonical match
at5g59870	CON	MIN	HOUR	DAY	WEEK	UNIGENE	FLCDNA	TAIR
GCCGCCGTTC	1	0	3	2	1	gnl|UG|At#S17007357	gi|21404658|gb|AY085948.1	At5g59870.1
at1g25260	CON	MIN	HOUR	DAY	WEEK	UNIGENE	FLCDNA	TAIR
GTTTGCATTC	1	1	1	5	1	no match found	gi|15028332|gb|AY045969.1	non-canonical match
TTTTGTAGAA	1	0	0	1	4	gnl|UG|At#S11740404	no match found	At1g25260.1
at5g53930	CON	MIN	HOUR	DAY	WEEK	UNIGENE	FLCDNA	TAIR
AAGATAGTCT	0	1	0	0	0	gnl|UG|At#S11718323	gi|26453017|dbj|AK119009.1|	At5g53930.1
at5g04710	CON	MIN	HOUR	DAY	WEEK	UNIGENE	FLCDNA	TAIR

GTGAGAAATG	2	1	0	1	0	gnl|UG|At#S11724960	gi|62319550|dbj|AK221100.1|	At5g04710.1
at5g23400	CON	MIN	HOUR	DAY	WEEK	UNIGENE	FLCDNA	TAIR
CGAGTTTCAG	0	0	1	0	1	gnl|UG|At#S11720838	gi|28059015|gb|BT003360.1	At5g23400.1
at5g65925	CON	MIN	HOUR	DAY	WEEK	UNIGENE	FLCDNA	TAIR
CTCCGCCGGT	0	3	0	0	0	no match found	gi|38454065|gb|BT010670.1	At5g65925.1
at5g65620	CON	MIN	HOUR	DAY	WEEK	UNIGENE	FLCDNA	TAIR
CTTACGAAGC	0	4	1	0	1	gnl|UG|At#S11717135	gi|23296999|gb|AY142682.1	At5g65620.1
at1g47210	CON	MIN	HOUR	DAY	WEEK	UNIGENE	FLCDNA	TAIR
CCTGTTTCAC	1	1	0	0	1	no match found	no match found	At1g47210.1
ATCTTATTTT	1	0	0	0	0	gnl|UG|At#S11709622	gi|18086354|gb|AY064987.1	At1g47210.2
at3g26760	CON	MIN	HOUR	DAY	WEEK	UNIGENE	FLCDNA	TAIR
ATCCCCGCTG	0	1	0	0	0	gnl|UG|At#S18906424	gi|110737654|dbj|AK228876.1|	At3g26760.1
at5g51180	CON	MIN	HOUR	DAY	WEEK	UNIGENE	FLCDNA	TAIR
TATTCACAAA	2	1	1	1	0	gnl|UG|At#S15459102	gi|15292882|gb|AY050875.1	multiple canonical match
at5g61490	CON	MIN	HOUR	DAY	WEEK	UNIGENE	FLCDNA	TAIR
AGACCATACT	0	0	1	0	0	no match found	no match found	At5g61490.1
at4g39140	CON	MIN	HOUR	DAY	WEEK	UNIGENE	FLCDNA	TAIR
TAAAGTTACA	0	1	0	0	0	gnl|UG|At#S11708991	gi|17065051|gb|AY062602.1	At4g39140.1
at1g44414	CON	MIN	HOUR	DAY	WEEK	UNIGENE	FLCDNA	TAIR
TACTTGGATG	0	0	1	0	0	no match found	no match found	At1g44414.1
at5g08670	CON	MIN	HOUR	DAY	WEEK	UNIGENE	FLCDNA	TAIR
AGAGCTGGCA	14	20	27	20	16	gnl|UG|At#S11710114	gi|15809908|gb|AY054222.1	At5g08670.1
at1g80150	CON	MIN	HOUR	DAY	WEEK	UNIGENE	FLCDNA	TAIR
TGCTGAAGAA	2	2	4	3	0	gnl|UG|At#S11726456	no match found	multiple non-canonical match
at3g54350	CON	MIN	HOUR	DAY	WEEK	UNIGENE	FLCDNA	TAIR
CATAGTTGAC	2	1	1	5	1	gnl|UG|At#S15459253	gi|17063177|gb|AY062111.1	At3g54350.2
at1g75580	CON	MIN	HOUR	DAY	WEEK	UNIGENE	FLCDNA	TAIR
GTGGTGTGTG	1	0	1	1	2	gnl|UG|At#S18896942	no match found	At1g75580.1
at3g21760	CON	MIN	HOUR	DAY	WEEK	UNIGENE	FLCDNA	TAIR
TTGGCAGAGA	4	3	5	3	3	gnl|UG|At#S11734293	gi|22655189|gb|AY140044.1	At3g21760.1
at1g68720	CON	MIN	HOUR	DAY	WEEK	UNIGENE	FLCDNA	TAIR
TTTTGCTTTA	4	4	2	0	0	gnl|UG|At#S11823938	gi|26450840|dbj|AK117889.1|	At1g68720.1
at1g50010	CON	MIN	HOUR	DAY	WEEK	UNIGENE	FLCDNA	TAIR
GCTTGCTGTT	2	7	3	2	2	no match found	no match found	At1g50010.1
at5g63800	CON	MIN	HOUR	DAY	WEEK	UNIGENE	FLCDNA	TAIR
TCGAGTATGG	2	2	1	0	0	no match found	gi|20260007|gb|AY093352.1	non-canonical match
TTCGAACTGT	1	1	0	0	0	gnl|UG|At#S24442779	no match found	non-canonical match
TGGGTGTGTA	4	2	6	1	2	no match found	gi|62320115|dbj|AK221384.1|	pseudo chromosome match
TAAAGAAACT	1	0	0	1	0	gnl|UG|At#S34116522	gi|16649044|gb|AY059892.1	At5g63800.1
at4g03260	CON	MIN	HOUR	DAY	WEEK	UNIGENE	FLCDNA	TAIR
GTATACATTC	6	2	1	1	6	gnl|UG|At#S11726863	no match found	At4g03260.1
at5g45310	CON	MIN	HOUR	DAY	WEEK	UNIGENE	FLCDNA	TAIR

GTGGCTCACT	2	1	1	0	0	gnl|UG|At#S11719196	gi|14030678|gb|AF375430.1	At5g45310.1
at3g49280	CON	MIN	HOUR	DAY	WEEK	UNIGENE	FLCDNA	TAIR
AAGGAGTTAG	1	0	0	0	0	gnl|UG|At#S35284085	no match found	multiple non-canonical match
at5g01075	CON	MIN	HOUR	DAY	WEEK	UNIGENE	FLCDNA	TAIR
GAACTCCTCT	0	2	1	0	0	gnl|UG|At#S14273705	gi|111074275|gb|BT026404.1	At5g01075.1
at3g01450	CON	MIN	HOUR	DAY	WEEK	UNIGENE	FLCDNA	TAIR
ACAGTCACAA	0	0	0	1	0	no match found	gi|21403759|gb|AY085049.1	At3g01450.1
at3g54140	CON	MIN	HOUR	DAY	WEEK	UNIGENE	FLCDNA	TAIR
AGAAGTCTCT	1	8	2	1	3	gnl|UG|At#S11728660	no match found	At3g54140.1
at2g38290	CON	MIN	HOUR	DAY	WEEK	UNIGENE	FLCDNA	TAIR
GTGCTAGAGG	1	2	4	0	2	gnl|UG|At#S18942755	gi|110740542|dbj|AK226212.1|	At2g38290.1
at2g35795	CON	MIN	HOUR	DAY	WEEK	UNIGENE	FLCDNA	TAIR
GCAAGCATTC	1	4	1	0	0	gnl|UG|At#S11733625	gi|114050698|gb|BT028952.1	At2g35795.1
at5g66070	CON	MIN	HOUR	DAY	WEEK	UNIGENE	FLCDNA	TAIR
AGGTATAGGC	0	3	0	0	0	gnl|UG|At#S28281530	gi|46518386|gb|BT012531.1	pseudo chromosome match
at3g47900	CON	MIN	HOUR	DAY	WEEK	UNIGENE	FLCDNA	TAIR
TAGTTTTCTT	0	2	1	2	0	no match found	no match found	At3g47900.1
at4g31350	CON	MIN	HOUR	DAY	WEEK	UNIGENE	FLCDNA	TAIR
TTTTGGTTTT	0	0	0	0	1	gnl|UG|At#S11722387	no match found	At1g73885.1
TTCAGTTTCT	0	1	0	0	0	no match found	no match found	At4g31350.1
at2g45990	CON	MIN	HOUR	DAY	WEEK	UNIGENE	FLCDNA	TAIR
TTTAGGTAAG	3	1	1	3	4	no match found	gi|15292712|gb|AY050790.1	At2g45990.1
at1g74520	CON	MIN	HOUR	DAY	WEEK	UNIGENE	FLCDNA	TAIR
AATATGAAAG	0	0	0	1	0	no match found	gi|51870352|gb|BT015568.1	At1g64710.1
TCTTTGTTGT	1	2	1	2	5	no match found	no match found	At1g74520.1
at3g53270	CON	MIN	HOUR	DAY	WEEK	UNIGENE	FLCDNA	TAIR
TTTGATTAGG	0	0	1	2	1	gnl|UG|At#S18319680	gi|21404725|gb|AY086015.1	At3g53270.3
ATGAAGATGA	0	1	1	1	0	no match found	gi|21280870|gb|AY113949.1	multiple canonical match
TGTTCGATGA	0	0	0	1	0	no match found	gi|26450522|dbj|AK117724.1|	multiple non-canonical match
at4g00400	CON	MIN	HOUR	DAY	WEEK	UNIGENE	FLCDNA	TAIR
TGTGTAATTG	5	1	3	4	2	gnl|UG|At#S11727576	gi|110737643|dbj|AK228870.1|	multiple canonical match
at1g47860	CON	MIN	HOUR	DAY	WEEK	UNIGENE	FLCDNA	TAIR
GCCTAGTCAA	1	1	0	0	0	gnl|UG|At#S34117429	gi|110737660|dbj|AK228879.1|	non-canonical match
at3g01430	CON	MIN	HOUR	DAY	WEEK	UNIGENE	FLCDNA	TAIR
CCCTGCCTTG	0	2	0	0	0	gnl|UG|At#S11740238	gi|28973290|gb|BT005550.1	At3g01430.1
at1g74650	CON	MIN	HOUR	DAY	WEEK	UNIGENE	FLCDNA	TAIR
GAAGCTACAC	0	2	0	0	0	no match found	gi|119360018|gb|BT029481.1	At1g74650.1
at3g08860	CON	MIN	HOUR	DAY	WEEK	UNIGENE	FLCDNA	TAIR
TCCAAGATGT	0	1	1	0	0	gnl|UG|At#S11738239	gi|110740901|dbj|AK226401.1|	At3g08860.1
at3g59800	CON	MIN	HOUR	DAY	WEEK	UNIGENE	FLCDNA	TAIR
CGGCTCGTTT	0	1	1	2	1	no match found	gi|63003845|gb|BT022040.1	At3g59800.1
at1g12640	CON	MIN	HOUR	DAY	WEEK	UNIGENE	FLCDNA	TAIR

GTGTTAAGCT	0	0	1	0	2	gnl|UG|At#S11741774	gi|22136167|gb|AY128762.1	At1g12640.1
at5g07130	CON	MIN	HOUR	DAY	WEEK	UNIGENE	FLCDNA	TAIR
TCATTCATTT	0	2	0	1	0	gnl|UG|At#S11724344	no match found	At5g07130.1
at3g51510	CON	MIN	HOUR	DAY	WEEK	UNIGENE	FLCDNA	TAIR
GTGATGAATG	0	2	0	1	0	no match found	gi|20259288|gb|AY091441.1	non-canonical match
TTCCAACGAG	8	2	6	4	5	gnl|UG|At#S11729153	gi|15027916|gb|AY045815.1	At3g51510.1
at5g54160	CON	MIN	HOUR	DAY	WEEK	UNIGENE	FLCDNA	TAIR
TCGATTGCAT	5	11	7	3	4	gnl|UG|At#S11718299	gi|21406021|gb|AY087297.1	At5g54160.1
at1g61140	CON	MIN	HOUR	DAY	WEEK	UNIGENE	FLCDNA	TAIR
AGAACGAGCT	1	1	2	1	0	gnl|UG|At#S43850406	no match found	At1g61140.1
at5g60660	CON	MIN	HOUR	DAY	WEEK	UNIGENE	FLCDNA	TAIR
GAATTTCCCA	0	0	0	1	0	gnl|UG|At#S11717636	gi|21405969|gb|AY087245.1	At5g60660.1
at1g07150	CON	MIN	HOUR	DAY	WEEK	UNIGENE	FLCDNA	TAIR
TATCTTTAGG	1	0	1	2	0	no match found	gi|110738596|dbj|AK229361.1|	non-canonical match
at2g31390	CON	MIN	HOUR	DAY	WEEK	UNIGENE	FLCDNA	TAIR
TGAACGCTGT	2	3	2	1	1	gnl|UG|At#S11743043	gi|14423527|gb|AF387001.1	non-canonical match
at3g32375	CON	MIN	HOUR	DAY	WEEK	UNIGENE	FLCDNA	TAIR
TCAAACAAAA	0	1	1	0	1	no match found	no match found	At3g32375.1
at2g28630	CON	MIN	HOUR	DAY	WEEK	UNIGENE	FLCDNA	TAIR
TAAAACGTTT	0	4	0	0	0	no match found	gi|20259582|gb|AY091195.1	multiple non-canonical match
at1g75210	CON	MIN	HOUR	DAY	WEEK	UNIGENE	FLCDNA	TAIR
TTATCTTTTG	3	1	1	1	4	gnl|UG|At#S11727999	gi|20260153|gb|AY092976.1	At1g75210.1
at1g63460	CON	MIN	HOUR	DAY	WEEK	UNIGENE	FLCDNA	TAIR
ACATTGACTT	2	2	3	2	2	gnl|UG|At#S34114404	gi|21405715|gb|AY086991.1	At1g63460.1
at1g53570	CON	MIN	HOUR	DAY	WEEK	UNIGENE	FLCDNA	TAIR
AAAATGAATA	0	0	0	0	3	no match found	gi|16930436|gb|AF419572.1	multiple non-canonical match
at2g27730	CON	MIN	HOUR	DAY	WEEK	UNIGENE	FLCDNA	TAIR
GCAACAAGAA	0	5	0	1	1	gnl|UG|At#S11752132	gi|14334933|gb|AY035140.1	At2g27730.1
at5g03890	CON	MIN	HOUR	DAY	WEEK	UNIGENE	FLCDNA	TAIR
TCTCTTCAAA	1	0	0	0	0	no match found	no match found	At5g03890.1
at2g45510	CON	MIN	HOUR	DAY	WEEK	UNIGENE	FLCDNA	TAIR
TAATAAATAA	0	0	0	1	0	gnl|UG|At#S11731298	no match found	At2g45510.1
at1g54320	CON	MIN	HOUR	DAY	WEEK	UNIGENE	FLCDNA	TAIR
TACACATACA	0	2	0	2	3	gnl|UG|At#S11732884	gi|15450728|gb|AY053406.1	multiple canonical match
at5g56190	CON	MIN	HOUR	DAY	WEEK	UNIGENE	FLCDNA	TAIR
GCCGAGCCAG	0	3	0	0	0	no match found	gi|21403204|gb|AY084494.1	non-canonical match
AACTTGGGAA	1	1	2	0	0	gnl|UG|At#S11718093	gi|19423889|gb|AY080763.1	At5g56190.1
at3g09570	CON	MIN	HOUR	DAY	WEEK	UNIGENE	FLCDNA	TAIR
TTGATGTTCT	2	2	0	0	0	gnl|UG|At#S18919050	gi|30387528|gb|BT006586.1	At3g09570.1
at1g09070	CON	MIN	HOUR	DAY	WEEK	UNIGENE	FLCDNA	TAIR
GGTGGTTTCG	0	0	3	0	0	no match found	gi|22655397|gb|AY142027.1	non-canonical match
TCGATCTGCG	0	1	0	0	0	gnl|UG|At#S18920837	no match found	non-canonical match

TGAAGAACGT	5	22	23	6	4	gnl|UG|At#S11699560	gi|15010557|gb|AY045580.1	At1g09070.1
at2g40950	CON	MIN	HOUR	DAY	WEEK	UNIGENE	FLCDNA	TAIR
TTTAGTTCAG	0	1	1	0	0	gnl|UG|At#S11732353	gi|22530913|gb|AY136295.1	At2g40950.1
at2g25100	CON	MIN	HOUR	DAY	WEEK	UNIGENE	FLCDNA	TAIR
CGAGAAAAGA	1	0	1	0	0	gnl|UG|At#S11736247	gi|28973384|gb|BT005597.1	At2g25100.1
at1g20910	CON	MIN	HOUR	DAY	WEEK	UNIGENE	FLCDNA	TAIR
CAACAAACAT	0	1	0	0	1	gnl|UG|At#S11740966	no match found	At1g20910.1
at3g22380	CON	MIN	HOUR	DAY	WEEK	UNIGENE	FLCDNA	TAIR
AATCAGTATG	4	5	1	0	6	gnl|UG|At#S11734107	no match found	At3g22380.1
AAAGGAGGTG	3	6	4	0	1	no match found	gi|14194102|gb|AF367257.1	multiple non-canonical match
at5g65310	CON	MIN	HOUR	DAY	WEEK	UNIGENE	FLCDNA	TAIR
CACGTGTGGC	1	10	7	2	2	gnl|UG|At#S20834112	gi|18377679|gb|AY074293.1	At5g65310.1
at1g66880	CON	MIN	HOUR	DAY	WEEK	UNIGENE	FLCDNA	TAIR
GCTGTATCTA	0	0	0	0	1	gnl|UG|At#S11729740	gi|110741351|dbj|AK230428.1|	At1g66880.1
at2g28290	CON	MIN	HOUR	DAY	WEEK	UNIGENE	FLCDNA	TAIR
GTTGTGGTTG	0	1	1	0	0	no match found	gi|21407264|gb|AY088490.1	At2g28290.2
at1g75550	CON	MIN	HOUR	DAY	WEEK	UNIGENE	FLCDNA	TAIR
AACTAAAAGT	1	1	1	0	1	no match found	no match found	At1g75550.1
at4g11280	CON	MIN	HOUR	DAY	WEEK	UNIGENE	FLCDNA	TAIR
TAATTTTAAC	1	3	1	0	1	gnl|UG|At#S11705476	no match found	At4g11280.1
at5g28150	CON	MIN	HOUR	DAY	WEEK	UNIGENE	FLCDNA	TAIR
ATTGGTTTCT	0	1	1	0	0	gnl|UG|At#S11720396	gi|19699076|gb|AY090242.1	At5g28150.1
at5g62470	CON	MIN	HOUR	DAY	WEEK	UNIGENE	FLCDNA	TAIR
CGGTTTTATG	0	1	3	1	0	gnl|UG|At#S11717454	gi|110737853|dbj|AK228977.1|	At5g62470.2
GTCTAATATC	0	1	0	0	0	no match found	gi|90093291|gb|BT024888.1	non-canonical match
at3g27010	CON	MIN	HOUR	DAY	WEEK	UNIGENE	FLCDNA	TAIR
GAGAAGAAAA	3	1	0	2	4	gnl|UG|At#S11749146	gi|26451402|dbj|AK118178.1|	At3g27010.1
at3g50070	CON	MIN	HOUR	DAY	WEEK	UNIGENE	FLCDNA	TAIR
TTTTACATAT	1	0	0	0	0	no match found	no match found	At3g50070.1
ACTCTACTCA	0	0	0	0	1	no match found	gi|17380631|gb|AY063729.1	non-canonical match
at2g33740	CON	MIN	HOUR	DAY	WEEK	UNIGENE	FLCDNA	TAIR
TCAATGCAAA	2	1	1	1	0	no match found	gi|51971806|dbj|AK176805.1|	At2g33740.2
at3g43425	CON	MIN	HOUR	DAY	WEEK	UNIGENE	FLCDNA	TAIR
TCACTTATGT	0	0	0	0	1	no match found	no match found	At3g43425.1
at5g26600	CON	MIN	HOUR	DAY	WEEK	UNIGENE	FLCDNA	TAIR
AAACTATTTG	0	0	1	0	0	gnl|UG|At#S18941574	gi|110738604|dbj|AK229365.1|	At5g26600.2
at4g17340	CON	MIN	HOUR	DAY	WEEK	UNIGENE	FLCDNA	TAIR
AACCCGGCCA	6	7	5	5	0	gnl|UG|At#S11724795	gi|21407703|gb|AY088929.1	At4g17340.1
at4g02070	CON	MIN	HOUR	DAY	WEEK	UNIGENE	FLCDNA	TAIR
GCCAATACAT	0	0	0	0	1	gnl|UG|At#S11727163	no match found	multiple canonical match
at1g05420	CON	MIN	HOUR	DAY	WEEK	UNIGENE	FLCDNA	TAIR
TTATTTATTT	0	1	1	0	0	gnl|UG|At#S22667349	no match found	non-canonical match

<table>
<tr><td>at3g51220</td><td>CON</td><td>MIN</td><td>HOUR</td><td>DAY</td><td>WEEK</td><td>UNIGENE</td><td>FLCDNA</td><td>TAIR</td></tr>
<tr><td>CGTGCCTTAC</td><td>1</td><td>0</td><td>0</td><td>0</td><td>0</td><td>gnl|UG|At#S11729209</td><td>no match found</td><td>At3g51220.1</td></tr>
<tr><td>at3g55630</td><td>CON</td><td>MIN</td><td>HOUR</td><td>DAY</td><td>WEEK</td><td>UNIGENE</td><td>FLCDNA</td><td>TAIR</td></tr>
<tr><td>ATAGAAACTT</td><td>0</td><td>0</td><td>0</td><td>1</td><td>0</td><td>gnl|UG|At#S18901922</td><td>no match found</td><td>non-canonical match</td></tr>
<tr><td>ATTTAACAAA</td><td>3</td><td>0</td><td>2</td><td>3</td><td>1</td><td>no match found</td><td>gi|62321495|dbj|AK222083.1|</td><td>At3g55630.2</td></tr>
<tr><td>at3g02020</td><td>CON</td><td>MIN</td><td>HOUR</td><td>DAY</td><td>WEEK</td><td>UNIGENE</td><td>FLCDNA</td><td>TAIR</td></tr>
<tr><td>TTGGACATCA</td><td>0</td><td>1</td><td>0</td><td>0</td><td>0</td><td>gnl|UG|At#S11740074</td><td>gi|21407140|gb|AY088366.1</td><td>At3g02020.1</td></tr>
<tr><td>at4g01670</td><td>CON</td><td>MIN</td><td>HOUR</td><td>DAY</td><td>WEEK</td><td>UNIGENE</td><td>FLCDNA</td><td>TAIR</td></tr>
<tr><td>CATTTTCGAG</td><td>0</td><td>0</td><td>1</td><td>0</td><td>0</td><td>gnl|UG|At#S11727269</td><td>gi|26451635|dbj|AK118297.1|</td><td>non-canonical match</td></tr>
<tr><td>GAGGAGCTAA</td><td>0</td><td>1</td><td>0</td><td>0</td><td>0</td><td>gnl|UG|At#S30643174</td><td>gi|115311432|gb|BT028988.1</td><td>non-canonical match</td></tr>
<tr><td>at5g56930</td><td>CON</td><td>MIN</td><td>HOUR</td><td>DAY</td><td>WEEK</td><td>UNIGENE</td><td>FLCDNA</td><td>TAIR</td></tr>
<tr><td>TAGATGAAAC</td><td>0</td><td>0</td><td>1</td><td>0</td><td>0</td><td>gnl|UG|At#S11718020</td><td>no match found</td><td>At5g56930.1</td></tr>
<tr><td>at5g40660</td><td>CON</td><td>MIN</td><td>HOUR</td><td>DAY</td><td>WEEK</td><td>UNIGENE</td><td>FLCDNA</td><td>TAIR</td></tr>
<tr><td>ACATTGATGT</td><td>0</td><td>0</td><td>1</td><td>1</td><td>0</td><td>gnl|UG|At#S11719663</td><td>gi|21405953|gb|AY087229.1</td><td>At5g40660.1</td></tr>
<tr><td>at5g22160</td><td>CON</td><td>MIN</td><td>HOUR</td><td>DAY</td><td>WEEK</td><td>UNIGENE</td><td>FLCDNA</td><td>TAIR</td></tr>
<tr><td>AGTTTTTATG</td><td>1</td><td>0</td><td>0</td><td>0</td><td>0</td><td>no match found</td><td>no match found</td><td>At5g22160.1</td></tr>
<tr><td>at5g45350</td><td>CON</td><td>MIN</td><td>HOUR</td><td>DAY</td><td>WEEK</td><td>UNIGENE</td><td>FLCDNA</td><td>TAIR</td></tr>
<tr><td>GAATGTTTGG</td><td>3</td><td>5</td><td>4</td><td>4</td><td>0</td><td>no match found</td><td>gi|16974402|gb|AY060514.1</td><td>multiple non-canonical match</td></tr>
<tr><td>at5g20980</td><td>CON</td><td>MIN</td><td>HOUR</td><td>DAY</td><td>WEEK</td><td>UNIGENE</td><td>FLCDNA</td><td>TAIR</td></tr>
<tr><td>CAACAAGTCT</td><td>1</td><td>1</td><td>0</td><td>1</td><td>0</td><td>no match found</td><td>gi|110738300|dbj|AK229210.1|</td><td>At5g20980.1</td></tr>
<tr><td>at4g36190</td><td>CON</td><td>MIN</td><td>HOUR</td><td>DAY</td><td>WEEK</td><td>UNIGENE</td><td>FLCDNA</td><td>TAIR</td></tr>
<tr><td>CAGAGTCACA</td><td>0</td><td>1</td><td>3</td><td>0</td><td>1</td><td>gnl|UG|At#S11824688</td><td>gi|26449328|dbj|AK117113.1|</td><td>At4g36190.1</td></tr>
<tr><td>at4g26610</td><td>CON</td><td>MIN</td><td>HOUR</td><td>DAY</td><td>WEEK</td><td>UNIGENE</td><td>FLCDNA</td><td>TAIR</td></tr>
<tr><td>GGAGTGCGGT</td><td>0</td><td>2</td><td>1</td><td>1</td><td>0</td><td>gnl|UG|At#S34115591</td><td>gi|20258942|gb|AY091248.1</td><td>At4g26610.1</td></tr>
<tr><td>at5g65590</td><td>CON</td><td>MIN</td><td>HOUR</td><td>DAY</td><td>WEEK</td><td>UNIGENE</td><td>FLCDNA</td><td>TAIR</td></tr>
<tr><td>ATGTCCTCGG</td><td>0</td><td>0</td><td>0</td><td>1</td><td>0</td><td>gnl|UG|At#S18910783</td><td>gi|52421290|gb|BT015717.1</td><td>At5g65590.1</td></tr>
<tr><td>at4g34820</td><td>CON</td><td>MIN</td><td>HOUR</td><td>DAY</td><td>WEEK</td><td>UNIGENE</td><td>FLCDNA</td><td>TAIR</td></tr>
<tr><td>GTAAAGCAGT</td><td>9</td><td>4</td><td>10</td><td>6</td><td>7</td><td>no match found</td><td>no match found</td><td>At4g34820.1</td></tr>
<tr><td>at5g62400</td><td>CON</td><td>MIN</td><td>HOUR</td><td>DAY</td><td>WEEK</td><td>UNIGENE</td><td>FLCDNA</td><td>TAIR</td></tr>
<tr><td>ACTTTGTTCC</td><td>0</td><td>1</td><td>0</td><td>0</td><td>0</td><td>no match found</td><td>no match found</td><td>At5g62400.1</td></tr>
<tr><td>at5g50380</td><td>CON</td><td>MIN</td><td>HOUR</td><td>DAY</td><td>WEEK</td><td>UNIGENE</td><td>FLCDNA</td><td>TAIR</td></tr>
<tr><td>GGTTTTATAT</td><td>1</td><td>4</td><td>1</td><td>1</td><td>0</td><td>gnl|UG|At#S11718675</td><td>gi|110737816|dbj|AK228958.1|</td><td>At5g50380.1</td></tr>
<tr><td>at4g36720</td><td>CON</td><td>MIN</td><td>HOUR</td><td>DAY</td><td>WEEK</td><td>UNIGENE</td><td>FLCDNA</td><td>TAIR</td></tr>
<tr><td>TGAAGTTTGC</td><td>1</td><td>0</td><td>0</td><td>0</td><td>0</td><td>gnl|UG|At#S11721485</td><td>gi|45752771|gb|BT012190.1</td><td>non-canonical match</td></tr>
<tr><td>at3g13845</td><td>CON</td><td>MIN</td><td>HOUR</td><td>DAY</td><td>WEEK</td><td>UNIGENE</td><td>FLCDNA</td><td>TAIR</td></tr>
<tr><td>TAATTCTTCC</td><td>1</td><td>1</td><td>0</td><td>0</td><td>0</td><td>gnl|UG|At#S11736694</td><td>gi|26453121|dbj|AK119061.1|</td><td>At3g13845.1</td></tr>
<tr><td>at3g50240</td><td>CON</td><td>MIN</td><td>HOUR</td><td>DAY</td><td>WEEK</td><td>UNIGENE</td><td>FLCDNA</td><td>TAIR</td></tr>
<tr><td>ATGATGAGGA</td><td>0</td><td>3</td><td>2</td><td>0</td><td>1</td><td>gnl|UG|At#S11700307</td><td>no match found</td><td>At3g50240.1</td></tr>
<tr><td>at4g25730</td><td>CON</td><td>MIN</td><td>HOUR</td><td>DAY</td><td>WEEK</td><td>UNIGENE</td><td>FLCDNA</td><td>TAIR</td></tr>
<tr><td>GAAGTTCCTA</td><td>0</td><td>1</td><td>4</td><td>1</td><td>1</td><td>gnl|UG|At#S11708237</td><td>gi|17381233|gb|AY064128.1</td><td>At4g25730.1</td></tr>
<tr><td>at4g16340</td><td>CON</td><td>MIN</td><td>HOUR</td><td>DAY</td><td>WEEK</td><td>UNIGENE</td><td>FLCDNA</td><td>TAIR</td></tr>
</table>

GCGGTTTGTA	1	0	0	0	1	no match found	gi|110737384|dbj|AK228736.1|	non-canonical match
at5g66640	CON	MIN	HOUR	DAY	WEEK	UNIGENE	FLCDNA	TAIR
AATCAGATCA	0	1	0	0	0	gnl|UG|At#S11717033	no match found	At5g66640.1
at4g17360	CON	MIN	HOUR	DAY	WEEK	UNIGENE	FLCDNA	TAIR
TCTCTCACGT	2	2	1	0	0	gnl|UG|At#S11724791	no match found	At4g17360.1
at2g33500	CON	MIN	HOUR	DAY	WEEK	UNIGENE	FLCDNA	TAIR
AACGCTCCGC	1	0	0	0	0	gnl|UG|At#S15460825	gi|25083441|gb|BT002067.1	At2g33500.2
at2g37580	CON	MIN	HOUR	DAY	WEEK	UNIGENE	FLCDNA	TAIR
GTGTTCACCG	0	0	2	0	0	gnl|UG|At#S26539684	gi|21403468|gb|AY084758.1	At2g37580.1
GGCCGTTGAA	0	0	1	0	0	no match found	gi|72197598|gb|DQ108758.1	At2g37580.1
at1g11340	CON	MIN	HOUR	DAY	WEEK	UNIGENE	FLCDNA	TAIR
TTGGGTCATA	0	0	0	0	1	gnl|UG|At#S11741901	no match found	At1g11340.1
at1g60440	CON	MIN	HOUR	DAY	WEEK	UNIGENE	FLCDNA	TAIR
TATGACCAAT	1	0	0	0	1	gnl|UG|At#S11731349	no match found	non-canonical match
at3g27820	CON	MIN	HOUR	DAY	WEEK	UNIGENE	FLCDNA	TAIR
ATAAAGAAGC	3	4	6	1	0	gnl|UG|At#S11732370	gi|14532711|gb|AY039980.1	At3g27820.1
at2g45300	CON	MIN	HOUR	DAY	WEEK	UNIGENE	FLCDNA	TAIR
ACCCTTGCCG	0	2	0	2	0	gnl|UG|At#S11731347	gi|63003817|gb|BT022026.1	At2g45300.1
at3g22400	CON	MIN	HOUR	DAY	WEEK	UNIGENE	FLCDNA	TAIR
CAACAATTAT	0	0	1	0	0	gnl|UG|At#S11734104	no match found	At3g22400.1
at1g32120	CON	MIN	HOUR	DAY	WEEK	UNIGENE	FLCDNA	TAIR
TTCTTCATCC	0	0	0	1	0	gnl|UG|At#S11738236	no match found	At1g32120.1
at4g00370	CON	MIN	HOUR	DAY	WEEK	UNIGENE	FLCDNA	TAIR
TTAAAGAAAA	9	4	4	2	7	gnl|UG|At#S11727584	gi|26451813|dbj|AK118390.1|	multiple non-canonical match
GGATGATGTG	0	1	0	0	0	no match found	gi|32306494|gb|BT009663.1	non-canonical match
at3g55440	CON	MIN	HOUR	DAY	WEEK	UNIGENE	FLCDNA	TAIR
CTGTCAACCA	0	0	0	0	1	gnl|UG|At#S11728432	no match found	At3g55440.1
GATGTTGTGG	2	24	8	8	5	no match found	gi|14030670|gb|AF375426.1	non-canonical match
at5g57110	CON	MIN	HOUR	DAY	WEEK	UNIGENE	FLCDNA	TAIR
AACACGCCAC	0	1	0	0	0	gnl|UG|At#S11718002	gi|17978916|gb|AY069869.1	At5g57110.1
at5g10760	CON	MIN	HOUR	DAY	WEEK	UNIGENE	FLCDNA	TAIR
TAGATTTGCT	1	0	1	0	2	no match found	gi|110740987|dbj|AK226446.1|	non-canonical match
TTACGATTTC	0	0	0	1	0	no match found	gi|22136985|gb|AY133788.1	non-canonical match
CTTCAACATT	0	0	0	2	0	gnl|UG|At#S11743531	gi|18176135|gb|AY072168.1	At5g10760.1
at5g22510	CON	MIN	HOUR	DAY	WEEK	UNIGENE	FLCDNA	TAIR
GACCTTCAAC	6	1	9	1	1	gnl|UG|At#S11720966	gi|15912342|gb|AY056449.1	multiple canonical match
at2g29710	CON	MIN	HOUR	DAY	WEEK	UNIGENE	FLCDNA	TAIR
ACGTGATAGG	0	0	1	0	0	gnl|UG|At#S11735115	gi|21405428|gb|AY086718.1	At2g29710.1
at1g33420	CON	MIN	HOUR	DAY	WEEK	UNIGENE	FLCDNA	TAIR
GGATTAAGAC	0	1	2	0	0	gnl|UG|At#S11703603	gi|14334761|gb|AY035054.1	At1g33420.1
at2g36350	CON	MIN	HOUR	DAY	WEEK	UNIGENE	FLCDNA	TAIR
GTGCTGCAGT	0	1	0	1	0	gnl|UG|At#S11733505	no match found	At2g36350.1

at1g30730	CON	MIN	HOUR	DAY	WEEK	UNIGENE	FLCDNA	TAIR
TATTAAAGTT	1	0	0	0	1	gnl|UG|At#S11738760	gi|38566629|gb|BT010838.1	non-canonical match
at3g05727	CON	MIN	HOUR	DAY	WEEK	UNIGENE	FLCDNA	TAIR
TTGTATCCTG	15	16	8	4	10	gnl|UG|At#S24442398	gi|62320875|dbj|AK221765.1|	pseudo chromosome match
at5g09450	CON	MIN	HOUR	DAY	WEEK	UNIGENE	FLCDNA	TAIR
AGACACATAG	0	1	0	0	1	gnl|UG|At#S11723880	gi|14596092|gb|AY042834.1	At5g09450.1
at4g14360	CON	MIN	HOUR	DAY	WEEK	UNIGENE	FLCDNA	TAIR
CTTGGGATAT	1	2	0	0	1	no match found	gi|20259232|gb|AY091393.1	At4g14360.1
at5g11720	CON	MIN	HOUR	DAY	WEEK	UNIGENE	FLCDNA	TAIR
ATTTTTATAA	0	0	0	1	0	gnl|UG|At#S11723316	no match found	non-canonical match
at5g60700	CON	MIN	HOUR	DAY	WEEK	UNIGENE	FLCDNA	TAIR
AAATTCTGAA	0	0	0	1	0	gnl|UG|At#S11717632	gi|26449675|dbj|AK117290.1|	At5g60700.1
at4g27990	CON	MIN	HOUR	DAY	WEEK	UNIGENE	FLCDNA	TAIR
GGATCGTCAG	2	4	3	4	0	gnl|UG|At#S34115628	gi|110741137|dbj|AK226522.1|	At4g27990.1
at1g53450	CON	MIN	HOUR	DAY	WEEK	UNIGENE	FLCDNA	TAIR
TTGAAACCTG	0	0	2	0	0	gnl|UG|At#S11733202	gi|23308272|gb|BT000537.1	At1g53450.2
at2g18750	CON	MIN	HOUR	DAY	WEEK	UNIGENE	FLCDNA	TAIR
AGATGGGGCT	0	0	1	0	0	no match found	gi|23505858|gb|AY143850.1	non-canonical match
AACCCAGAAA	2	3	5	7	2	no match found	no match found	At2g18750.1
at5g05380	CON	MIN	HOUR	DAY	WEEK	UNIGENE	FLCDNA	TAIR
GTTAGAACTC	0	1	0	1	0	gnl|UG|At#S11724790	gi|51971466|dbj|AK176635.1|	non-canonical match
at5g05580	CON	MIN	HOUR	DAY	WEEK	UNIGENE	FLCDNA	TAIR
AGGACATACT	2	0	2	1	1	gnl|UG|At#S11724742	gi|20259911|gb|AY093304.1	non-canonical match
at4g26660	CON	MIN	HOUR	DAY	WEEK	UNIGENE	FLCDNA	TAIR
TATTGATTGA	0	1	0	0	0	gnl|UG|At#S34116768	gi|110738944|dbj|AK229540.1|	At4g26660.1
at3g23550	CON	MIN	HOUR	DAY	WEEK	UNIGENE	FLCDNA	TAIR
CCAAGGGTTT	1	1	1	1	0	gnl|UG|At#S11733737	gi|20465840|gb|AY096384.1	At3g23550.1
at3g46090	CON	MIN	HOUR	DAY	WEEK	UNIGENE	FLCDNA	TAIR
AGGAGACATA	0	0	0	1	0	no match found	gi|89274160|gb|BT024870.1	At3g46080.1
at5g04480	CON	MIN	HOUR	DAY	WEEK	UNIGENE	FLCDNA	TAIR
GTTTTTGTAT	1	1	0	0	1	gnl|UG|At#S11706976	gi|15450502|gb|AY052353.1	At5g04480.1
at5g13950	CON	MIN	HOUR	DAY	WEEK	UNIGENE	FLCDNA	TAIR
GGTTTCTTAG	3	5	4	5	2	gnl|UG|At#S11722848	gi|26449481|dbj|AK117191.1|	At5g13950.1
at2g18120	CON	MIN	HOUR	DAY	WEEK	UNIGENE	FLCDNA	TAIR
CGCAGTACAC	0	0	0	0	1	no match found	no match found	At2g18120.1
at3g23080	CON	MIN	HOUR	DAY	WEEK	UNIGENE	FLCDNA	TAIR
GATTTGGTGG	1	1	0	0	0	no match found	gi|23297763|gb|AY150505.1	non-canonical match
CAGAGTAGTT	2	4	4	1	0	gnl|UG|At#S11706099	gi|15810256|gb|AY056167.1	At3g23080.1
at3g47080	CON	MIN	HOUR	DAY	WEEK	UNIGENE	FLCDNA	TAIR
TGTAGATCCA	2	0	3	0	1	gnl|UG|At#S11729980	gi|14532663|gb|AY039956.1	At3g47080.1
at4g16170	CON	MIN	HOUR	DAY	WEEK	UNIGENE	FLCDNA	TAIR
AGATGCTGTA	1	1	0	1	0	gnl|UG|At#S11724999	no match found	At4g16170.1

at1g51160	CON	MIN	HOUR	DAY	WEEK	UNIGENE	FLCDNA	TAIR
GTTTACTCAA	2	4	5	3	2	no match found	no match found	At1g51160.1
at3g10380	CON	MIN	HOUR	DAY	WEEK	UNIGENE	FLCDNA	TAIR
ATTTTCATAA	1	1	0	0	0	gnl|UG|At#S11737774	gi|26452108|dbj|AK118541.1|	At3g10380.1
atmg00090	CON	MIN	HOUR	DAY	WEEK	UNIGENE	FLCDNA	TAIR
CAATACTTTT	0	0	0	1	0	no match found	no match found	AtMg00090
at5g65840	CON	MIN	HOUR	DAY	WEEK	UNIGENE	FLCDNA	TAIR
GAATGTCTTT	0	1	0	1	0	no match found	gi|23198249|gb|BT000333.1	non-canonical match
TAGATTTTTG	4	1	2	3	0	gnl|UG|At#S11717114	gi|20466749|gb|AY099841.1	At5g65840.1
at3g09020	CON	MIN	HOUR	DAY	WEEK	UNIGENE	FLCDNA	TAIR
ACTTTTCTTT	0	0	1	0	0	gnl|UG|At#S11738194	no match found	At3g09020.1
at5g01270	CON	MIN	HOUR	DAY	WEEK	UNIGENE	FLCDNA	TAIR
TGGATTAGTT	0	1	0	2	0	gnl|UG|At#S11726355	gi|62319789|dbj|AK221220.1|	At5g01270.1
at5g47340	CON	MIN	HOUR	DAY	WEEK	UNIGENE	FLCDNA	TAIR
AGAAACTTCC	0	0	1	0	0	no match found	no match found	At5g47340.1
at3g54250	CON	MIN	HOUR	DAY	WEEK	UNIGENE	FLCDNA	TAIR
AATGACACTT	0	1	0	0	0	gnl|UG|At#S11728640	gi|21406392|gb|AY087654.1	At3g54250.1
at1g04680	CON	MIN	HOUR	DAY	WEEK	UNIGENE	FLCDNA	TAIR
AAAAAAGAGA	0	3	0	1	2	no match found	gi|15809865|gb|AY054200.1	At3g28530.1
ATTAGTACTT	0	1	0	0	0	gnl|UG|At#S11742559	no match found	At1g04680.1
at1g15020	CON	MIN	HOUR	DAY	WEEK	UNIGENE	FLCDNA	TAIR
GTTTAACATT	0	0	1	0	0	gnl|UG|At#S11741532	gi|24899780|gb|BT001218.1	At1g15020.2
at4g32260	CON	MIN	HOUR	DAY	WEEK	UNIGENE	FLCDNA	TAIR
GATCAAAGAG	17	31	8	9	2	gnl|UG|At#S11722232	gi|18377521|gb|AY072512.1	At4g32260.1
at1g20490	CON	MIN	HOUR	DAY	WEEK	UNIGENE	FLCDNA	TAIR
TAGTTTGTTT	0	1	0	1	0	gnl|UG|At#S11741010	no match found	At1g20490.1
at1g67550	CON	MIN	HOUR	DAY	WEEK	UNIGENE	FLCDNA	TAIR
TGCGCCAGCC	0	1	0	1	0	no match found	no match found	At1g67550.1
at4g20310	CON	MIN	HOUR	DAY	WEEK	UNIGENE	FLCDNA	TAIR
TTTGCTCTCA	0	0	0	0	1	gnl|UG|At#S18913925	no match found	At4g20310.1
at5g09460	CON	MIN	HOUR	DAY	WEEK	UNIGENE	FLCDNA	TAIR
CTCTCTCAAA	0	0	0	1	0	no match found	gi|23197601|gb|BT000009.1	At5g09460.1
AAAATGGCGA	0	1	0	0	0	gnl|UG|At#S30626392	no match found	multiple non-canonical match
TAATAAACTC	0	1	0	1	0	gnl|UG|At#S11723876	gi|22135919|gb|AY128339.1	multiple non-canonical match
at3g48590	CON	MIN	HOUR	DAY	WEEK	UNIGENE	FLCDNA	TAIR
AAAGCCGATG	0	2	0	0	0	no match found	gi|21404830|gb|AY086120.1	multiple canonical match
at5g41120	CON	MIN	HOUR	DAY	WEEK	UNIGENE	FLCDNA	TAIR
GTTTCTCTTC	1	0	0	1	0	gnl|UG|At#S11709910	gi|17979136|gb|AY070069.1	At5g41120.1
at5g01100	CON	MIN	HOUR	DAY	WEEK	UNIGENE	FLCDNA	TAIR
AGTCCAAAGT	0	0	0	1	0	gnl|UG|At#S11726445	gi|110736419|dbj|AK228227.1|	At5g01100.1
at2g38640	CON	MIN	HOUR	DAY	WEEK	UNIGENE	FLCDNA	TAIR
AATAGAGAAC	3	1	6	1	2	gnl|UG|At#S34115279	gi|110741792|dbj|AK226738.1|	At2g38640.1

at3g55320	CON	MIN	HOUR	DAY	WEEK	UNIGENE	FLCDNA	TAIR
ATTCTTTAGC	0	0	1	0	0	no match found	gi|28372881|gb|BT003695.1	non-canonical match
GATGATGTAT	1	0	1	0	2	gnl|UG|At#S11728455	gi|26451844|dbj|AK118406.1|	At3g55320.1
at5g41340	CON	MIN	HOUR	DAY	WEEK	UNIGENE	FLCDNA	TAIR
AATTATCTCT	0	1	2	0	1	gnl|UG|At#S18910002	gi|17529149|gb|AY065360.1	At5g41340.1
at3g10110	CON	MIN	HOUR	DAY	WEEK	UNIGENE	FLCDNA	TAIR
ACACTGTAAA	0	0	0	0	1	gnl|UG|At#S11737858	gi|124301047|gb|BT030038.1	multiple canonical match
at4g40045	CON	MIN	HOUR	DAY	WEEK	UNIGENE	FLCDNA	TAIR
GAGATTTCGA	0	1	1	1	0	no match found	gi|21404918|gb|AY086208.1	non-canonical match
AATGTACATT	0	0	0	0	1	gnl|UG|At#S11720915	gi|15450921|gb|AY054541.1	At4g40045.1
at1g77510	CON	MIN	HOUR	DAY	WEEK	UNIGENE	FLCDNA	TAIR
AGTTTTGTTT	2	2	3	0	0	gnl|UG|At#S11727377	gi|62319512|dbj|AK221081.1|	multiple canonical match
at4g23690	CON	MIN	HOUR	DAY	WEEK	UNIGENE	FLCDNA	TAIR
GCTCGTGGGA	1	0	0	0	0	gnl|UG|At#S11723701	gi|27311666|gb|BT002439.1	At4g23690.1
at2g32000	CON	MIN	HOUR	DAY	WEEK	UNIGENE	FLCDNA	TAIR
GGAGGTCCTT	0	1	0	0	1	gnl|UG|At#S24442989	gi|51969987|dbj|AK175923.1|	At2g32000.1
at5g08280	CON	MIN	HOUR	DAY	WEEK	UNIGENE	FLCDNA	TAIR
GTGAAGATGG	0	0	1	0	0	no match found	gi|21689852|gb|AY123037.1	At5g36296.1
ACCTTTTGTT	26	17	11	12	15	gnl|UG|At#S11724042	gi|17979401|gb|AY070431.1	multiple non-canonical match
at5g07475	CON	MIN	HOUR	DAY	WEEK	UNIGENE	FLCDNA	TAIR
TATATGTGTA	0	1	0	0	0	gnl|UG|At#S34118204	gi|110736178|dbj|AK228102.1|	At5g07475.1
at5g58120	CON	MIN	HOUR	DAY	WEEK	UNIGENE	FLCDNA	TAIR
CTTGTTGTTG	3	1	1	1	1	gnl|UG|At#S11717898	gi|18087638|gb|AF462863.1	non-canonical match
at1g12470	CON	MIN	HOUR	DAY	WEEK	UNIGENE	FLCDNA	TAIR
CTTCTGGAGA	2	1	2	1	0	gnl|UG|At#S11741789	no match found	non-canonical match
ATTTTACATC	1	0	0	0	0	no match found	no match found	At1g12470.1
at1g56200	CON	MIN	HOUR	DAY	WEEK	UNIGENE	FLCDNA	TAIR
GCTATGGCGG	0	10	0	0	0	gnl|UG|At#S11732210	gi|21403870|gb|AY085160.1	At1g56200.1
at3g44970	CON	MIN	HOUR	DAY	WEEK	UNIGENE	FLCDNA	TAIR
TAATAAATAC	1	0	0	0	1	no match found	no match found	At3g44970.1
at5g38160	CON	MIN	HOUR	DAY	WEEK	UNIGENE	FLCDNA	TAIR
AAACTTGTTT	0	0	0	1	0	gnl|UG|At#S11823710	gi|26451309|dbj|AK118130.1|	At5g38160.1
at4g38480	CON	MIN	HOUR	DAY	WEEK	UNIGENE	FLCDNA	TAIR
TATTTATCTA	0	1	0	0	3	gnl|UG|At#S11813292	no match found	At4g38480.1
at2g20870	CON	MIN	HOUR	DAY	WEEK	UNIGENE	FLCDNA	TAIR
TTTTTCAGTT	0	1	0	0	0	gnl|UG|At#S30487865	gi|21405147|gb|AY086437.1	At2g20870.1
at5g05200	CON	MIN	HOUR	DAY	WEEK	UNIGENE	FLCDNA	TAIR
ACTATATACA	5	1	0	0	1	no match found	gi|14334951|gb|AY035149.1	non-canonical match
GTTTCTACTT	1	2	0	0	0	gnl|UG|At#S11724832	no match found	multiple non-canonical match
CTGCAGGATC	0	2	0	0	0	no match found	gi|20259937|gb|AY093317.1	multiple non-canonical match
at5g16910	CON	MIN	HOUR	DAY	WEEK	UNIGENE	FLCDNA	TAIR
AATGCTTTGC	1	0	0	0	0	gnl|UG|At#S11722110	no match found	At5g16910.1

at1g10030	CON	MIN	HOUR	DAY	WEEK	UNIGENE	FLCDNA	TAIR
GATTATATGG	0	1	0	2	2	gnl|UG|At#S11742031	gi|110743770|dbj|AK227737.1|	At1g10030.1

at5g01600	CON	MIN	HOUR	DAY	WEEK	UNIGENE	FLCDNA	TAIR
CTGAAAAAGG	5	29	9	16	7	gnl|UG|At#S11725758	gi|12642861|gb|AF339691.1	At5g01600.1

at2g34400	CON	MIN	HOUR	DAY	WEEK	UNIGENE	FLCDNA	TAIR
TCATAAGAGA	0	0	1	0	0	gnl|UG|At#S11733963	no match found	At2g34400.1

at2g26730	CON	MIN	HOUR	DAY	WEEK	UNIGENE	FLCDNA	TAIR
CTCCTGAAGT	0	0	0	0	1	gnl|UG|At#S11735850	gi|15292872|gb|AY050870.1	At2g26730.1

at1g20450	CON	MIN	HOUR	DAY	WEEK	UNIGENE	FLCDNA	TAIR
CCAGCACCAC	1	18	16	30	11	no match found	gi|23397310|gb|BT000799.1	non-canonical match
AAAAGTTTTT	1	1	2	5	2	gnl|UG|At#S15460710	no match found	At1g20450.2

at5g38460	CON	MIN	HOUR	DAY	WEEK	UNIGENE	FLCDNA	TAIR
ATTCTCTGTT	1	3	2	0	1	gnl|UG|At#S11703615	gi|15810650|gb|AY056364.1	At5g38460.1

at4g33585	CON	MIN	HOUR	DAY	WEEK	UNIGENE	FLCDNA	TAIR
CCTCCGATCT	0	0	0	0	1	gnl|UG|At#S38433397	no match found	non-canonical match

at3g43520	CON	MIN	HOUR	DAY	WEEK	UNIGENE	FLCDNA	TAIR
CGCAGCCTCC	3	7	6	1	0	gnl|UG|At#S11730661	gi|23308264|gb|BT000533.1	At3g43520.1

at3g48440	CON	MIN	HOUR	DAY	WEEK	UNIGENE	FLCDNA	TAIR
TCTCAATCTT	0	0	0	1	0	no match found	no match found	At3g48440.1

at2g39920	CON	MIN	HOUR	DAY	WEEK	UNIGENE	FLCDNA	TAIR
TATCCAAGAG	0	3	0	0	1	gnl|UG|At#S11732623	gi|21406014|gb|AY087290.1	At2g39920.1

at5g25560	CON	MIN	HOUR	DAY	WEEK	UNIGENE	FLCDNA	TAIR
TGGTGGCTCA	1	3	0	0	0	gnl|UG|At#S11709759	gi|17979450|gb|AY070720.1	At5g25560.1

at3g06910	CON	MIN	HOUR	DAY	WEEK	UNIGENE	FLCDNA	TAIR
TAGATAGTAT	0	0	0	0	2	gnl|UG|At#S11738659	gi|26450143|dbj|AK117529.1|	At3g06910.1

at2g43570	CON	MIN	HOUR	DAY	WEEK	UNIGENE	FLCDNA	TAIR
TGCACAAGGA	1	0	0	0	0	gnl|UG|At#S11731752	gi|23397324|gb|AY099810.2	At2g43570.1

at3g29075	CON	MIN	HOUR	DAY	WEEK	UNIGENE	FLCDNA	TAIR
ACTAAAAAAG	0	1	1	2	0	gnl|UG|At#S11731942	gi|45773797|gb|BT012216.1	At3g29075.1

at4g37730	CON	MIN	HOUR	DAY	WEEK	UNIGENE	FLCDNA	TAIR
ACTGAACAAA	0	1	0	0	0	gnl|UG|At#S11721314	no match found	At4g37730.1

at4g06614	CON	MIN	HOUR	DAY	WEEK	UNIGENE	FLCDNA	TAIR
TATTATTTTT	1	1	0	0	0	no match found	no match found	At4g06614.1

at1g10870	CON	MIN	HOUR	DAY	WEEK	UNIGENE	FLCDNA	TAIR
ATGAAACAGC	1	1	0	1	0	gnl|UG|At#S11741946	gi|110742045|dbj|AK226875.1|	At1g10870.1

at5g48440	CON	MIN	HOUR	DAY	WEEK	UNIGENE	FLCDNA	TAIR
GTACTAGGAA	0	1	0	0	0	no match found	gi|51969053|dbj|AK175456.1|	At5g48440.2

at1g27290	CON	MIN	HOUR	DAY	WEEK	UNIGENE	FLCDNA	TAIR
ATCATTTCTT	9	5	4	1	0	no match found	gi|14423427|gb|AF386951.1	At1g27290.1

at1g26390	CON	MIN	HOUR	DAY	WEEK	UNIGENE	FLCDNA	TAIR
TGTTATATAG	1	0	0	0	0	gnl|UG|At#S11740188	gi|15293132|gb|AY051000.1	At1g26390.1

at5g48360	CON	MIN	HOUR	DAY	WEEK	UNIGENE	FLCDNA	TAIR
ATTGTGAGAG	2	1	0	0	0	gnl|UG|At#S11823396	gi|26451947|dbj|AK118458.1|	At5g48360.1
at5g14030	CON	MIN	HOUR	DAY	WEEK	UNIGENE	FLCDNA	TAIR
GTGGTTTGTT	1	1	2	5	2	no match found	gi|14517445|gb|AY039558.1	At5g14030.1
TTGATAATCT	0	0	1	0	0	gnl|UG|At#S11722827	no match found	multiple non-canonical match
at2g45950	CON	MIN	HOUR	DAY	WEEK	UNIGENE	FLCDNA	TAIR
ACACGATTGA	0	0	0	0	1	gnl|UG|At#S38433821	gi|13877584|gb|AF370493.1	At2g45950.1
at1g79970	CON	MIN	HOUR	DAY	WEEK	UNIGENE	FLCDNA	TAIR
TTACGAAGAG	2	5	2	2	0	gnl|UG|At#S11726532	gi|21403256|gb|AY084546.1	At1g79970.2
at4g01370	CON	MIN	HOUR	DAY	WEEK	UNIGENE	FLCDNA	TAIR
ATATCAACGA	2	5	5	0	2	gnl|UG|At#S11727342	gi|14532813|gb|AY040031.1	At4g01370.1
at5g13200	CON	MIN	HOUR	DAY	WEEK	UNIGENE	FLCDNA	TAIR
GGTTTTGTGA	0	0	0	2	0	no match found	gi|38454153|gb|BT010714.1	At5g13200.1
at1g04510	CON	MIN	HOUR	DAY	WEEK	UNIGENE	FLCDNA	TAIR
AAGAGATATC	0	2	2	1	1	gnl|UG|At#S11742577	gi|62319610|dbj|AK221130.1|	At1g04510.1
at1g18660	CON	MIN	HOUR	DAY	WEEK	UNIGENE	FLCDNA	TAIR
ATTAAAGGAA	1	3	0	1	1	gnl|UG|At#S18942547	gi|25082693|gb|BT001981.1	At1g18660.3
at1g06720	CON	MIN	HOUR	DAY	WEEK	UNIGENE	FLCDNA	TAIR
GTATTCTTAA	0	2	0	0	0	gnl|UG|At#S11742359	gi|20148442|gb|AY081550.1	At1g06720.1
at5g42880	CON	MIN	HOUR	DAY	WEEK	UNIGENE	FLCDNA	TAIR
TTCTTGTCAA	2	1	1	0	0	no match found	no match found	At5g42880.1
at1g48120	CON	MIN	HOUR	DAY	WEEK	UNIGENE	FLCDNA	TAIR
GCTTTGGATG	0	0	0	1	0	gnl|UG|At#S11735176	gi|17381245|gb|AY064134.1	At1g48120.1
at1g48570	CON	MIN	HOUR	DAY	WEEK	UNIGENE	FLCDNA	TAIR
ATTCATCGTT	0	0	1	0	0	gnl|UG|At#S11735001	no match found	non-canonical match
at3g47830	CON	MIN	HOUR	DAY	WEEK	UNIGENE	FLCDNA	TAIR
GTAAGATCTG	0	0	0	0	1	gnl|UG|At#S11729836	no match found	multiple canonical match
at4g28040	CON	MIN	HOUR	DAY	WEEK	UNIGENE	FLCDNA	TAIR
GTGTAAGTGG	0	5	4	2	1	gnl|UG|At#S28281948	gi|13899060|gb|AF370525.1	non-canonical match
at4g13450	CON	MIN	HOUR	DAY	WEEK	UNIGENE	FLCDNA	TAIR
TTGTGACAAA	0	1	0	0	0	gnl|UG|At#S17865537	gi|38603971|gb|BT010951.1	At4g13450.1
at3g12530	CON	MIN	HOUR	DAY	WEEK	UNIGENE	FLCDNA	TAIR
TATCGGTATC	0	1	0	0	0	gnl|UG|At#S11737137	no match found	At3g12530.1
at1g28290	CON	MIN	HOUR	DAY	WEEK	UNIGENE	FLCDNA	TAIR
TTTAAAAAGA	4	8	1	1	4	gnl|UG|At#S11647401	gi|110738349|dbj|AK229236.1|	At1g28290.1
at2g13610	CON	MIN	HOUR	DAY	WEEK	UNIGENE	FLCDNA	TAIR
TGTTTAATTA	0	1	0	1	3	gnl|UG|At#S11704194	gi|62319010|dbj|AK220826.1|	At2g13610.1
CTATGTTTTG	0	0	1	0	0	no match found	gi|22136941|gb|AY133881.1	non-canonical match
at3g04110	CON	MIN	HOUR	DAY	WEEK	UNIGENE	FLCDNA	TAIR
TAATAATTTA	0	0	0	0	2	gnl|UG|At#S11700764	gi|26450249|dbj|AK117584.1|	At3g04110.1
at5g03345	CON	MIN	HOUR	DAY	WEEK	UNIGENE	FLCDNA	TAIR
AAGTTTTGAA	2	2	5	1	1	gnl|UG|At#S11725326	gi|21404901|gb|AY086191.1	At5g03345.1

<table>
<tr><td>at4g26860</td><td>CON</td><td>MIN</td><td>HOUR</td><td>DAY</td><td>WEEK</td><td>UNIGENE</td><td>FLCDNA</td><td>TAIR</td></tr>
<tr><td>TATAATGTTA</td><td>2</td><td>0</td><td>0</td><td>0</td><td>3</td><td>gnl|UG|At#S11723149</td><td>gi|21403463|gb|AY084753.1</td><td>At4g26860.1</td></tr>
<tr><td>at3g27750</td><td>CON</td><td>MIN</td><td>HOUR</td><td>DAY</td><td>WEEK</td><td>UNIGENE</td><td>FLCDNA</td><td>TAIR</td></tr>
<tr><td>GGAGGCTGAT</td><td>2</td><td>0</td><td>2</td><td>0</td><td>1</td><td>gnl|UG|At#S11732389</td><td>gi|14030622|gb|AF375402.1</td><td>At3g27750.1</td></tr>
<tr><td>at3g44940</td><td>CON</td><td>MIN</td><td>HOUR</td><td>DAY</td><td>WEEK</td><td>UNIGENE</td><td>FLCDNA</td><td>TAIR</td></tr>
<tr><td>AATGTAAACT</td><td>0</td><td>1</td><td>0</td><td>0</td><td>0</td><td>gnl|UG|At#S11730390</td><td>gi|27754370|gb|BT002815.1</td><td>At3g44940.1</td></tr>
<tr><td>at5g19070</td><td>CON</td><td>MIN</td><td>HOUR</td><td>DAY</td><td>WEEK</td><td>UNIGENE</td><td>FLCDNA</td><td>TAIR</td></tr>
<tr><td>AACGAAGCGG</td><td>0</td><td>1</td><td>0</td><td>0</td><td>1</td><td>gnl|UG|At#S11721554</td><td>gi|50201958|gb|BT014970.1</td><td>At5g19070.1</td></tr>
<tr><td>at3g06590</td><td>CON</td><td>MIN</td><td>HOUR</td><td>DAY</td><td>WEEK</td><td>UNIGENE</td><td>FLCDNA</td><td>TAIR</td></tr>
<tr><td>AACTCTTTAG</td><td>1</td><td>2</td><td>1</td><td>2</td><td>0</td><td>no match found</td><td>gi|21407518|gb|AY088744.1</td><td>At3g06590.1</td></tr>
<tr><td>at5g43190</td><td>CON</td><td>MIN</td><td>HOUR</td><td>DAY</td><td>WEEK</td><td>UNIGENE</td><td>FLCDNA</td><td>TAIR</td></tr>
<tr><td>GCATTGGGTT</td><td>2</td><td>2</td><td>1</td><td>0</td><td>0</td><td>gnl|UG|At#S11719409</td><td>gi|27765055|gb|BT003084.1</td><td>At5g43190.1</td></tr>
<tr><td>at3g08505</td><td>CON</td><td>MIN</td><td>HOUR</td><td>DAY</td><td>WEEK</td><td>UNIGENE</td><td>FLCDNA</td><td>TAIR</td></tr>
<tr><td>CTCAGAGAGG</td><td>1</td><td>0</td><td>1</td><td>0</td><td>3</td><td>gnl|UG|At#S18942156</td><td>gi|17979276|gb|AY070366.1</td><td>At3g08505.2</td></tr>
<tr><td>at5g14680</td><td>CON</td><td>MIN</td><td>HOUR</td><td>DAY</td><td>WEEK</td><td>UNIGENE</td><td>FLCDNA</td><td>TAIR</td></tr>
<tr><td>TCTGCTGAAT</td><td>1</td><td>3</td><td>1</td><td>0</td><td>1</td><td>gnl|UG|At#S18909804</td><td>gi|45476562|gb|BT011776.1</td><td>non-canonical match</td></tr>
<tr><td>at3g56460</td><td>CON</td><td>MIN</td><td>HOUR</td><td>DAY</td><td>WEEK</td><td>UNIGENE</td><td>FLCDNA</td><td>TAIR</td></tr>
<tr><td>TAAGCTGAAC</td><td>4</td><td>6</td><td>4</td><td>4</td><td>3</td><td>gnl|UG|At#S11807240</td><td>gi|62320239|dbj|AK221446.1|</td><td>non-canonical match</td></tr>
<tr><td>at2g16980</td><td>CON</td><td>MIN</td><td>HOUR</td><td>DAY</td><td>WEEK</td><td>UNIGENE</td><td>FLCDNA</td><td>TAIR</td></tr>
<tr><td>TTTGTGATGC</td><td>0</td><td>2</td><td>0</td><td>0</td><td>0</td><td>gnl|UG|At#S11738237</td><td>gi|110736471|dbj|AK228256.1|</td><td>At2g16980.2</td></tr>
<tr><td>at1g65250</td><td>CON</td><td>MIN</td><td>HOUR</td><td>DAY</td><td>WEEK</td><td>UNIGENE</td><td>FLCDNA</td><td>TAIR</td></tr>
<tr><td>ACGGTAAACT</td><td>0</td><td>0</td><td>0</td><td>1</td><td>0</td><td>no match found</td><td>no match found</td><td>At1g65250.1</td></tr>
<tr><td>at4g10040</td><td>CON</td><td>MIN</td><td>HOUR</td><td>DAY</td><td>WEEK</td><td>UNIGENE</td><td>FLCDNA</td><td>TAIR</td></tr>
<tr><td>AGATTTTCCT</td><td>3</td><td>2</td><td>2</td><td>12</td><td>6</td><td>no match found</td><td>gi|15028282|gb|AY045944.1</td><td>multiple canonical match</td></tr>
<tr><td>AACTGCTTTT</td><td>0</td><td>0</td><td>0</td><td>0</td><td>2</td><td>gnl|UG|At#S11675519</td><td>gi|21405780|gb|AY087056.1</td><td>non-canonical match</td></tr>
<tr><td>at5g24760</td><td>CON</td><td>MIN</td><td>HOUR</td><td>DAY</td><td>WEEK</td><td>UNIGENE</td><td>FLCDNA</td><td>TAIR</td></tr>
<tr><td>ATGAATCAAA</td><td>0</td><td>1</td><td>0</td><td>0</td><td>1</td><td>gnl|UG|At#S11608035</td><td>gi|19310537|gb|AY079163.1</td><td>At5g24760.2</td></tr>
<tr><td>TCACAGATTC</td><td>1</td><td>0</td><td>0</td><td>0</td><td>0</td><td>no match found</td><td>gi|21404233|gb|AY085523.1</td><td>multiple non-canonical match</td></tr>
<tr><td>at4g19160</td><td>CON</td><td>MIN</td><td>HOUR</td><td>DAY</td><td>WEEK</td><td>UNIGENE</td><td>FLCDNA</td><td>TAIR</td></tr>
<tr><td>GAGCTTTTGT</td><td>6</td><td>11</td><td>4</td><td>5</td><td>0</td><td>gnl|UG|At#S11707420</td><td>gi|15292760|gb|AY050814.1</td><td>At4g19160.1</td></tr>
<tr><td>at4g32930</td><td>CON</td><td>MIN</td><td>HOUR</td><td>DAY</td><td>WEEK</td><td>UNIGENE</td><td>FLCDNA</td><td>TAIR</td></tr>
<tr><td>GGGATTATCA</td><td>0</td><td>0</td><td>0</td><td>2</td><td>4</td><td>no match found</td><td>gi|27754521|gb|BT002892.1</td><td>At1g41850.1</td></tr>
<tr><td>at4g26450</td><td>CON</td><td>MIN</td><td>HOUR</td><td>DAY</td><td>WEEK</td><td>UNIGENE</td><td>FLCDNA</td><td>TAIR</td></tr>
<tr><td>ACTTTTGATT</td><td>1</td><td>0</td><td>1</td><td>1</td><td>0</td><td>no match found</td><td>gi|26451719|dbj|AK118341.1|</td><td>non-canonical match</td></tr>
<tr><td>at5g11090</td><td>CON</td><td>MIN</td><td>HOUR</td><td>DAY</td><td>WEEK</td><td>UNIGENE</td><td>FLCDNA</td><td>TAIR</td></tr>
<tr><td>GCGAAAGCTG</td><td>1</td><td>12</td><td>3</td><td>0</td><td>0</td><td>gnl|UG|At#S11723476</td><td>gi|21405946|gb|AY087222.1</td><td>At5g11090.1</td></tr>
<tr><td>at1g60660</td><td>CON</td><td>MIN</td><td>HOUR</td><td>DAY</td><td>WEEK</td><td>UNIGENE</td><td>FLCDNA</td><td>TAIR</td></tr>
<tr><td>GTGAAGCTAT</td><td>1</td><td>0</td><td>1</td><td>2</td><td>0</td><td>gnl|UG|At#S30644023</td><td>no match found</td><td>pseudo chromosome match</td></tr>
<tr><td>at5g60570</td><td>CON</td><td>MIN</td><td>HOUR</td><td>DAY</td><td>WEEK</td><td>UNIGENE</td><td>FLCDNA</td><td>TAIR</td></tr>
<tr><td>GCGAGGGCAT</td><td>0</td><td>4</td><td>2</td><td>0</td><td>0</td><td>gnl|UG|At#S11717645</td><td>gi|119935920|gb|BT029765.1</td><td>At5g60570.1</td></tr>
<tr><td>at4g37090</td><td>CON</td><td>MIN</td><td>HOUR</td><td>DAY</td><td>WEEK</td><td>UNIGENE</td><td>FLCDNA</td><td>TAIR</td></tr>
</table>

TATCAGATAT	0	1	0	1	0	gnl|UG|At#S11721423	gi|21403179|gb|AY084469.1	At4g37090.1
at5g26740	CON	MIN	HOUR	DAY	WEEK	UNIGENE	FLCDNA	TAIR
CTCCTAATAG	2	1	0	0	0	gnl|UG|At#S18941576	gi|15292726|gb|AY050797.1	At5g26740.1
at1g09590	CON	MIN	HOUR	DAY	WEEK	UNIGENE	FLCDNA	TAIR
GTCGAAGGAA	2	2	5	2	5	gnl|UG|At#S35267093	no match found	At1g09590.1
at3g52110	CON	MIN	HOUR	DAY	WEEK	UNIGENE	FLCDNA	TAIR
ACTTGGAATT	0	1	0	0	0	gnl|UG|At#S11729039	gi|55733772|gb|BT020217.1	At3g52110.1
at1g59760	CON	MIN	HOUR	DAY	WEEK	UNIGENE	FLCDNA	TAIR
GAGATTGCTC	0	1	1	0	0	gnl|UG|At#S21735839	gi|51971868|dbj|AK176836.1|	At1g59760.1
at1g61730	CON	MIN	HOUR	DAY	WEEK	UNIGENE	FLCDNA	TAIR
GACAAGAACA	0	0	0	1	0	gnl|UG|At#S11730890	no match found	At1g61730.1
at3g60220	CON	MIN	HOUR	DAY	WEEK	UNIGENE	FLCDNA	TAIR
CCGGAGAAGA	0	1	0	0	1	gnl|UG|At#S18904759	gi|114050660|gb|BT028933.1	At3g60220.1
at3g51540	CON	MIN	HOUR	DAY	WEEK	UNIGENE	FLCDNA	TAIR
TTTAATGTTT	3	6	2	5	3	no match found	no match found	At3g51540.1
at5g42900	CON	MIN	HOUR	DAY	WEEK	UNIGENE	FLCDNA	TAIR
TGAGTGGATT	0	2	11	5	4	gnl|UG|At#S28281689	gi|21407590|gb|AY088816.1	At5g42900.2
at3g60630	CON	MIN	HOUR	DAY	WEEK	UNIGENE	FLCDNA	TAIR
GTCTCCGGTT	1	1	1	0	0	gnl|UG|At#S11727295	gi|17381211|gb|AY064062.1	At3g60630.1
at1g29700	CON	MIN	HOUR	DAY	WEEK	UNIGENE	FLCDNA	TAIR
TTCCAGTTGC	1	1	2	0	3	gnl|UG|At#S11739117	no match found	At1g29700.1
TGAAATGAAA	1	0	2	2	1	no match found	gi|18377529|gb|AY072516.1	multiple non-canonical match
at1g08520	CON	MIN	HOUR	DAY	WEEK	UNIGENE	FLCDNA	TAIR
AAATCCTCCC	0	1	0	0	0	no match found	gi|27311600|gb|BT002406.1	non-canonical match
AAATTATAAG	14	11	1	6	12	gnl|UG|At#S11707696	gi|110741162|dbj|AK226535.1|	At1g08520.1
at1g02060	CON	MIN	HOUR	DAY	WEEK	UNIGENE	FLCDNA	TAIR
CAACACGGTC	1	0	0	0	0	no match found	no match found	At1g02060.1
at1g76540	CON	MIN	HOUR	DAY	WEEK	UNIGENE	FLCDNA	TAIR
AATACCCACA	0	0	0	1	0	gnl|UG|At#S11727719	gi|21403710|gb|AY085000.1	At1g76540.1
at5g62350	CON	MIN	HOUR	DAY	WEEK	UNIGENE	FLCDNA	TAIR
AAACCAGCAA	13	30	15	16	3	gnl|UG|At#S11717466	gi|18377555|gb|AY072529.1	At5g62350.1
at1g30870	CON	MIN	HOUR	DAY	WEEK	UNIGENE	FLCDNA	TAIR
AGAAGGATCT	1	0	0	0	0	gnl|UG|At#S11825137	gi|21407761|gb|AY088987.1	At1g30870.1
at1g64510	CON	MIN	HOUR	DAY	WEEK	UNIGENE	FLCDNA	TAIR
AACATTGTGT	14	10	5	2	3	gnl|UG|At#S11730233	gi|21386992|gb|AY114581.1	At1g64510.1
at3g16030	CON	MIN	HOUR	DAY	WEEK	UNIGENE	FLCDNA	TAIR
ATATACGGTG	1	0	0	0	0	gnl|UG|At#S11736036	no match found	non-canonical match
at4g36530	CON	MIN	HOUR	DAY	WEEK	UNIGENE	FLCDNA	TAIR
ATGAAGTCCC	0	1	5	3	1	gnl|UG|At#S11721518	gi|30984537|gb|BT008719.1	At4g36530.2
at5g08290	CON	MIN	HOUR	DAY	WEEK	UNIGENE	FLCDNA	TAIR
ATCGATCTTG	0	1	4	0	0	no match found	gi|12642839|gb|AF339680.1	non-canonical match
ATTCAGTGAA	14	16	10	14	8	gnl|UG|At#S11701710	gi|21403765|gb|AY085055.1	At5g08290.1

at3g20362	CON	MIN	HOUR	DAY	WEEK	UNIGENE	FLCDNA	TAIR
GATTATCCAT	0	2	0	0	0	no match found	no match found	At3g20362.1
at1g80720	CON	MIN	HOUR	DAY	WEEK	UNIGENE	FLCDNA	TAIR
GGTACTCATT	1	0	0	4	0	gnl|UG|At#S11726292	gi|21404933|gb|AY086223.1	At1g80720.1
at3g54860	CON	MIN	HOUR	DAY	WEEK	UNIGENE	FLCDNA	TAIR
GAAAAACTAG	0	2	1	1	0	gnl|UG|At#S11743187	gi|18377629|gb|AY074268.1	At3g54860.1
at5g08370	CON	MIN	HOUR	DAY	WEEK	UNIGENE	FLCDNA	TAIR
CCTGCAAAAT	1	2	0	0	0	gnl|UG|At#S11724019	gi|19699068|gb|AY090238.1	non-canonical match
at3g21060	CON	MIN	HOUR	DAY	WEEK	UNIGENE	FLCDNA	TAIR
GAATGATATT	0	1	2	1	0	gnl|UG|At#S14273749	gi|28393596|gb|BT004200.1	non-canonical match
at2g43950	CON	MIN	HOUR	DAY	WEEK	UNIGENE	FLCDNA	TAIR
CTTCAAGTTC	1	3	3	2	2	gnl|UG|At#S15460405	gi|15028228|gb|AY045937.1	At2g43950.1
at4g11360	CON	MIN	HOUR	DAY	WEEK	UNIGENE	FLCDNA	TAIR
GTCGATGCTC	0	2	0	2	0	no match found	no match found	At4g11360.1
at5g07070	CON	MIN	HOUR	DAY	WEEK	UNIGENE	FLCDNA	TAIR
GAAGCTGGAG	0	1	0	0	0	no match found	gi|115311416|gb|BT028980.1	multiple non-canonical match
AGTACAGTAA	0	0	0	0	1	gnl|UG|At#S11701520	no match found	At5g07070.1
at1g75900	CON	MIN	HOUR	DAY	WEEK	UNIGENE	FLCDNA	TAIR
TGTTTTGGGA	0	0	1	1	3	no match found	gi|26449363|dbj|AK117131.1|	non-canonical match
at2g32880	CON	MIN	HOUR	DAY	WEEK	UNIGENE	FLCDNA	TAIR
CTTGTGTTTG	1	0	1	2	0	no match found	no match found	At2g32880.1
at1g05340	CON	MIN	HOUR	DAY	WEEK	UNIGENE	FLCDNA	TAIR
TGTTGCTGTT	0	1	0	1	0	gnl|UG|At#S18321340	no match found	At1g05340.1
at4g31390	CON	MIN	HOUR	DAY	WEEK	UNIGENE	FLCDNA	TAIR
GAGCAACTCA	0	0	0	0	1	no match found	gi|30725521|gb|BT008424.1	multiple non-canonical match
TAATTATATA	0	0	0	1	7	gnl|UG|At#S11722382	gi|20260427|gb|AY093113.1	At4g31390.1
at1g07950	CON	MIN	HOUR	DAY	WEEK	UNIGENE	FLCDNA	TAIR
GACAACGTTT	0	0	1	0	0	gnl|UG|At#S11742239	gi|21405306|gb|AY086596.1	At1g07950.1
at1g80550	CON	MIN	HOUR	DAY	WEEK	UNIGENE	FLCDNA	TAIR
AAGCAATAGA	0	1	0	0	0	no match found	gi|18491212|gb|AY074541.1	multiple non-canonical match
at5g23040	CON	MIN	HOUR	DAY	WEEK	UNIGENE	FLCDNA	TAIR
GACACTCGAG	5	11	6	8	3	gnl|UG|At#S11720874	gi|21928167|gb|AY125520.1	At5g23040.1
at1g67910	CON	MIN	HOUR	DAY	WEEK	UNIGENE	FLCDNA	TAIR
ATCCGATAAC	0	1	0	0	0	gnl|UG|At#S34117456	gi|48309995|gb|BT014751.1	At1g67910.1
ACCAAGACCA	0	0	1	0	0	gnl|UG|At#S22484549	gi|56121921|gb|BT020248.1	non-canonical match
at5g50375	CON	MIN	HOUR	DAY	WEEK	UNIGENE	FLCDNA	TAIR
TTTCTAAGGC	1	0	0	0	0	gnl|UG|At#S11718676	gi|62320904|dbj|AK221780.1|	At5g50375.1
at4g23860	CON	MIN	HOUR	DAY	WEEK	UNIGENE	FLCDNA	TAIR
AAGTTTGTAG	3	1	2	3	1	gnl|UG|At#S11723672	no match found	At4g23860.1
TTAACTTTTT	0	0	0	1	0	no match found	gi|20148674|gb|AY081666.1	non-canonical match
at4g02130	CON	MIN	HOUR	DAY	WEEK	UNIGENE	FLCDNA	TAIR
GACTTGGTGG	0	1	0	0	0	no match found	gi|24030375|gb|BT000950.1	At4g02130.2

TGGATGAGAT	0	0	2	2	1	gnl|UG|At#S28282108	no match found	At4g02130.1
at3g63120	CON	MIN	HOUR	DAY	WEEK	UNIGENE	FLCDNA	TAIR
ATTGATTGTT	5	4	1	3	1	no match found	no match found	At3g63120.1
at5g16760	CON	MIN	HOUR	DAY	WEEK	UNIGENE	FLCDNA	TAIR
TTCAAATGCA	1	2	2	3	1	gnl|UG|At#S11722148	gi|18176068|gb|AY072156.1	At5g16760.1
TCTGAGAAAT	0	1	0	0	0	no match found	gi|20465296|gb|AY096412.1	non-canonical match
at1g12710	CON	MIN	HOUR	DAY	WEEK	UNIGENE	FLCDNA	TAIR
GTTGGGACAT	0	0	0	1	0	gnl|UG|At#S11741767	gi|26450400|dbj|AK117662.1|	At1g12710.1
at2g35800	CON	MIN	HOUR	DAY	WEEK	UNIGENE	FLCDNA	TAIR
GGAGACAATA	0	4	0	0	0	gnl|UG|At#S11708358	gi|20466022|gb|AY096712.1	At2g35800.1
at5g60920	CON	MIN	HOUR	DAY	WEEK	UNIGENE	FLCDNA	TAIR
CCTCCTCCAG	5	7	6	4	2	no match found	gi|20453071|gb|AY094402.1	multiple canonical match
at4g30660	CON	MIN	HOUR	DAY	WEEK	UNIGENE	FLCDNA	TAIR
GGCTACTTCT	0	1	1	0	0	gnl|UG|At#S37211669	no match found	pseudo chromosome match
at1g21780	CON	MIN	HOUR	DAY	WEEK	UNIGENE	FLCDNA	TAIR
GCTCTACTAA	0	1	1	0	0	gnl|UG|At#S38434310	gi|21406286|gb|AY087548.1	At1g21780.1
at4g27710	CON	MIN	HOUR	DAY	WEEK	UNIGENE	FLCDNA	TAIR
TGGTGGTTGT	4	3	2	1	0	gnl|UG|At#S11723006	no match found	At4g27710.1
at4g25490	CON	MIN	HOUR	DAY	WEEK	UNIGENE	FLCDNA	TAIR
CTTTTACCGC	0	0	4	0	0	gnl|UG|At#S11723392	no match found	At4g25490.1
at3g54400	CON	MIN	HOUR	DAY	WEEK	UNIGENE	FLCDNA	TAIR
GCTGCAGCTC	1	4	3	7	3	no match found	gi|17979256|gb|AY070479.1	non-canonical match
AATTTGCGGA	1	4	4	10	2	gnl|UG|At#S11728612	no match found	multiple canonical match
at4g11600	CON	MIN	HOUR	DAY	WEEK	UNIGENE	FLCDNA	TAIR
AAACTGTGTC	0	1	4	0	0	gnl|UG|At#S11725800	no match found	At4g11600.1
GTTTCGAGAT	0	4	5	17	4	no match found	gi|21407421|gb|AY088647.1	non-canonical match
at5g39830	CON	MIN	HOUR	DAY	WEEK	UNIGENE	FLCDNA	TAIR
AAAATATTAT	1	2	0	1	2	gnl|UG|At#S11719748	gi|15912206|gb|AY056381.1	At5g39830.2
at1g25510	CON	MIN	HOUR	DAY	WEEK	UNIGENE	FLCDNA	TAIR
ATTCCGGTTG	0	1	0	2	0	gnl|UG|At#S11740335	gi|110736959|dbj|AK228512.1|	At1g25510.1
at2g41780	CON	MIN	HOUR	DAY	WEEK	UNIGENE	FLCDNA	TAIR
ATCAGCAGCT	0	0	0	0	1	gnl|UG|At#S11732149	gi|27765043|gb|BT003078.1	At2g41780.1
at5g07470	CON	MIN	HOUR	DAY	WEEK	UNIGENE	FLCDNA	TAIR
ACTGAAATCT	2	4	2	3	6	gnl|UG|At#S18913104	gi|14994244|gb|AY044316.1	At5g07470.1
at2g35820	CON	MIN	HOUR	DAY	WEEK	UNIGENE	FLCDNA	TAIR
TTAATCCCAT	4	1	2	0	0	gnl|UG|At#S11733619	gi|21404926|gb|AY086216.1	At2g35820.1
at4g20270	CON	MIN	HOUR	DAY	WEEK	UNIGENE	FLCDNA	TAIR
AGAGAGGTTG	3	2	0	1	0	gnl|UG|At#S11724287	no match found	At4g20270.1
at1g70480	CON	MIN	HOUR	DAY	WEEK	UNIGENE	FLCDNA	TAIR
ACCATTACAA	0	0	0	0	1	no match found	gi|21405023|gb|AY086313.1	non-canonical match
ATTCTGGGAT	0	1	0	0	1	no match found	gi|15451125|gb|AY054643.1	non-canonical match
at4g36290	CON	MIN	HOUR	DAY	WEEK	UNIGENE	FLCDNA	TAIR

TGCATATATG	0	0	0	0	2	gnl|UG|At#S11721549	gi|62321598|dbj|AK222136.1|	At4g36290.1
at1g80120	CON	MIN	HOUR	DAY	WEEK	UNIGENE	FLCDNA	TAIR
GGCTGTTGTA	0	0	1	0	0	gnl|UG|At#S14273907	gi|28393295|gb|BT004042.1	At1g80120.1
at2g47270	CON	MIN	HOUR	DAY	WEEK	UNIGENE	FLCDNA	TAIR
ATCCGACCAA	0	3	0	0	0	gnl|UG|At#S11730885	gi|89111835|gb|BT024807.1	At2g47270.1
at4g17790	CON	MIN	HOUR	DAY	WEEK	UNIGENE	FLCDNA	TAIR
TAGAATGTTA	3	2	5	1	3	gnl|UG|At#S11724712	gi|51968513|dbj|AK175186.1|	At4g17790.1
at5g46520	CON	MIN	HOUR	DAY	WEEK	UNIGENE	FLCDNA	TAIR
TTTGTGAGGC	1	1	0	0	0	gnl|UG|At#S34115235	gi|110741876|dbj|AK226782.1|	non-canonical match
TGAATGTTTT	9	8	10	8	5	no match found	no match found	At5g46520.1
at1g16710	CON	MIN	HOUR	DAY	WEEK	UNIGENE	FLCDNA	TAIR
AAACAGCCAT	2	1	0	0	0	gnl|UG|At#S11741373	no match found	At1g16710.1
at1g49780	CON	MIN	HOUR	DAY	WEEK	UNIGENE	FLCDNA	TAIR
AGTCTTTTTC	0	0	2	0	0	gnl|UG|At#S11734582	gi|26451729|dbj|AK118346.1|	multiple canonical match
at1g48745	CON	MIN	HOUR	DAY	WEEK	UNIGENE	FLCDNA	TAIR
AATTTCCCAA	0	0	0	1	0	gnl|UG|At#S37211367	gi|72198208|gb|DQ108838.1	multiple non-canonical match
at4g18750	CON	MIN	HOUR	DAY	WEEK	UNIGENE	FLCDNA	TAIR
TATTGTAAAC	0	0	0	0	1	gnl|UG|At#S11724544	gi|62320405|dbj|AK221529.1|	non-canonical match
at5g54340	CON	MIN	HOUR	DAY	WEEK	UNIGENE	FLCDNA	TAIR
ATTCGTTTTC	0	0	0	1	0	gnl|UG|At#S11718281	no match found	non-canonical match
at4g30480	CON	MIN	HOUR	DAY	WEEK	UNIGENE	FLCDNA	TAIR
AAAAGATGCA	1	0	1	2	2	no match found	gi|21403760|gb|AY085050.1	At4g30480.2
at3g62910	CON	MIN	HOUR	DAY	WEEK	UNIGENE	FLCDNA	TAIR
GAAGAACTCT	2	5	3	4	3	gnl|UG|At#S11726564	no match found	At3g62910.1
at4g13615	CON	MIN	HOUR	DAY	WEEK	UNIGENE	FLCDNA	TAIR
ACTCGTGGAA	5	14	1	2	1	gnl|UG|At#S30650424	gi|26451117|dbj|AK118031.1|	At4g13615.1
at2g20370	CON	MIN	HOUR	DAY	WEEK	UNIGENE	FLCDNA	TAIR
GAAGAGTGAG	0	1	0	1	0	gnl|UG|At#S34115778	gi|110740844|dbj|AK226372.1|	At2g20370.1
at3g04630	CON	MIN	HOUR	DAY	WEEK	UNIGENE	FLCDNA	TAIR
AAAAACTTTA	4	1	11	6	4	no match found	gi|26449744|dbj|AK117325.1|	multiple canonical match
at2g45340	CON	MIN	HOUR	DAY	WEEK	UNIGENE	FLCDNA	TAIR
ATGTAACAAT	1	1	0	1	3	gnl|UG|At#S11731335	no match found	At2g45340.1
at1g22780	CON	MIN	HOUR	DAY	WEEK	UNIGENE	FLCDNA	TAIR
AAGTTGAGAG	7	6	1	7	5	gnl|UG|At#S11740785	gi|21406152|gb|AY087428.1	At1g22780.1
at4g28440	CON	MIN	HOUR	DAY	WEEK	UNIGENE	FLCDNA	TAIR
ATCTTGCTCT	1	3	1	3	2	gnl|UG|At#S11707332	gi|15912308|gb|AY056432.1	At4g28440.1
at4g10400	CON	MIN	HOUR	DAY	WEEK	UNIGENE	FLCDNA	TAIR
CGAACTCAAC	0	0	0	1	1	gnl|UG|At#S18908258	gi|119935956|gb|BT029774.1	At4g10400.1
ACAATATGGG	1	0	0	0	0	no match found	gi|45680300|gb|BT011990.1	multiple non-canonical match
at1g07320	CON	MIN	HOUR	DAY	WEEK	UNIGENE	FLCDNA	TAIR
AGTAACGTTG	8	7	5	4	4	gnl|UG|At#S11742302	gi|21403791|gb|AY085081.1	At1g07320.2

at3g62290	CON	MIN	HOUR	DAY	WEEK	UNIGENE	FLCDNA	TAIR
TGCTACCTCC	16	60	27	21	3	no match found	gi|21404092|gb|AY085382.1	At3g62290.1

at5g60170	CON	MIN	HOUR	DAY	WEEK	UNIGENE	FLCDNA	TAIR
AAGAGAGGTT	0	2	1	0	0	gnl|UG|At#S11717685	no match found	At5g60170.1

at2g47350	CON	MIN	HOUR	DAY	WEEK	UNIGENE	FLCDNA	TAIR
ACTAGTCGTC	0	1	1	0	0	gnl|UG|At#S34117080	gi|145651797|gb|BT030470.1	At2g47350.1

at5g40190	CON	MIN	HOUR	DAY	WEEK	UNIGENE	FLCDNA	TAIR
GAACGTCTTC	1	2	0	0	0	gnl|UG|At#S21737564	gi|34098924|gb|BT010402.1	At5g40190.1

at5g48340	CON	MIN	HOUR	DAY	WEEK	UNIGENE	FLCDNA	TAIR
AAGGTGTTGA	2	0	0	0	0	gnl|UG|At#S11718886	gi|17528939|gb|AY065204.1	At5g48340.1

at1g66160	CON	MIN	HOUR	DAY	WEEK	UNIGENE	FLCDNA	TAIR
TTAAGAAACC	1	3	0	0	0	gnl|UG|At#S11729894	gi|14334443|gb|AY034913.1	At1g66160.1

at5g27400	CON	MIN	HOUR	DAY	WEEK	UNIGENE	FLCDNA	TAIR
TCTCCCTCAT	1	2	0	0	0	no match found	gi|110743030|dbj|AK227404.1|	non-canonical match
TGTTTGCGGA	1	0	0	0	0	gnl|UG|At#S11720472	no match found	At5g27400.1

at4g00860	CON	MIN	HOUR	DAY	WEEK	UNIGENE	FLCDNA	TAIR
AACCCTATCA	5	6	2	2	4	gnl|UG|At#S11727468	gi|21387038|gb|AY114604.1	At4g00860.1

at2g20430	CON	MIN	HOUR	DAY	WEEK	UNIGENE	FLCDNA	TAIR
TCGGAATATA	0	1	0	0	0	gnl|UG|At#S11737363	no match found	At2g20430.1

at5g18970	CON	MIN	HOUR	DAY	WEEK	UNIGENE	FLCDNA	TAIR
TAAAATGATT	2	0	0	0	0	no match found	gi|21403131|gb|AY084421.1	non-canonical match

at5g60790	CON	MIN	HOUR	DAY	WEEK	UNIGENE	FLCDNA	TAIR
TGCACCTTCT	1	4	4	8	8	gnl|UG|At#S11717623	gi|22655233|gb|AY140066.1	At5g60790.1

at1g05270	CON	MIN	HOUR	DAY	WEEK	UNIGENE	FLCDNA	TAIR
GCATCCTCTT	0	1	3	1	2	gnl|UG|At#S11742501	gi|28950926|gb|BT005323.1	At1g05270.1

at5g12350	CON	MIN	HOUR	DAY	WEEK	UNIGENE	FLCDNA	TAIR
GTTGACAAAG	0	1	0	0	0	no match found	gi|110737153|dbj|AK228615.1|	At5g12350.1

at2g05510	CON	MIN	HOUR	DAY	WEEK	UNIGENE	FLCDNA	TAIR
GAGGAGGAGG	0	0	0	1	0	no match found	gi|30017216|gb|BT006193.1	At5g15540.1

at4g21192	CON	MIN	HOUR	DAY	WEEK	UNIGENE	FLCDNA	TAIR
TGCGTGTGTC	4	5	6	2	0	gnl|UG|At#S11801789	gi|110743949|dbj|AK227829.1|	pseudo chromosome match

at5g08210	CON	MIN	HOUR	DAY	WEEK	UNIGENE	FLCDNA	TAIR
TCTATTTGAC	0	0	0	1	0	no match found	no match found	At5g08210.1

at1g30473	CON	MIN	HOUR	DAY	WEEK	UNIGENE	FLCDNA	TAIR
AACAAAAGAA	0	1	0	0	0	gnl|UG|At#S28282563	no match found	pseudo chromosome match

at3g48330	CON	MIN	HOUR	DAY	WEEK	UNIGENE	FLCDNA	TAIR
TTGGTGATGG	0	0	0	1	0	gnl|UG|At#S15460127	gi|117168206|gb|BT029372.1	At3g48330.2

at4g25970	CON	MIN	HOUR	DAY	WEEK	UNIGENE	FLCDNA	TAIR
CAAATAAGGT	0	0	2	0	0	gnl|UG|At#S34115690	gi|110741015|dbj|AK226460.1|	At4g25970.1
TGAAGAAAGG	0	0	1	0	2	no match found	gi|62319256|dbj|AK220951.1|	multiple non-canonical match

at2g47115	CON	MIN	HOUR	DAY	WEEK	UNIGENE	FLCDNA	TAIR
AAAGTCCCAG	0	0	0	1	0	gnl|UG|At#S11730926	no match found	At2g47115.1

at4g17020	CON	MIN	HOUR	DAY	WEEK	UNIGENE	FLCDNA	TAIR
CTTCGATTCT	1	0	0	1	0	no match found	gi|110736961|dbj|AK228513.1|	non-canonical match
CGCACCCCAA	0	0	0	1	0	gnl|UG|At#S11724849	no match found	non-canonical match

at3g22845	CON	MIN	HOUR	DAY	WEEK	UNIGENE	FLCDNA	TAIR
ACTTAGCCAA	0	3	0	1	0	gnl|UG|At#S11733962	gi|17979491|gb|AY070742.1	At3g22845.1

at1g54490	CON	MIN	HOUR	DAY	WEEK	UNIGENE	FLCDNA	TAIR
AAATCCTCTA	0	1	1	0	0	gnl|UG|At#S11732817	gi|20259664|gb|AY091411.1	At1g54490.1

at4g13395	CON	MIN	HOUR	DAY	WEEK	UNIGENE	FLCDNA	TAIR
AACCGGACGG	1	2	0	0	0	gnl|UG|At#S22665747	no match found	At1g54490.1

at1g55830	CON	MIN	HOUR	DAY	WEEK	UNIGENE	FLCDNA	TAIR
GAAAATCTTT	0	0	0	0	1	no match found	no match found	At1g55830.1

at5g07580	CON	MIN	HOUR	DAY	WEEK	UNIGENE	FLCDNA	TAIR
AAGAGCTTCA	0	1	0	1	0	no match found	gi|16974422|gb|AY060524.1	At5g07580.1

at5g06000	CON	MIN	HOUR	DAY	WEEK	UNIGENE	FLCDNA	TAIR
TTGGGGTCTC	0	0	0	0	1	gnl|UG|At#S11724628	no match found	At5g06000.1

at3g45980	CON	MIN	HOUR	DAY	WEEK	UNIGENE	FLCDNA	TAIR
AGAAAAAAAA	10	6	12	12	8	gnl|UG|At#S20294557	no match found	non-canonical match

at1g70270	CON	MIN	HOUR	DAY	WEEK	UNIGENE	FLCDNA	TAIR
CAACCACCCG	0	1	0	0	1	gnl|UG|At#S30629823	gi|26452253|dbj|AK118616.1|	At1g70270.1

at2g33150	CON	MIN	HOUR	DAY	WEEK	UNIGENE	FLCDNA	TAIR
GGTATCGGTC	1	0	0	0	0	no match found	gi|17380613|gb|AY063720.1	non-canonical match
TGTTTAGTAT	6	4	0	1	7	gnl|UG|At#S11734248	no match found	At2g33150.1
CACCAAAACC	0	0	2	0	0	no match found	gi|13194829|gb|AF349530.1	non-canonical match

at1g33800	CON	MIN	HOUR	DAY	WEEK	UNIGENE	FLCDNA	TAIR
ATTATTTGTG	1	0	0	0	1	gnl|UG|At#S11737662	gi|21403374|gb|AY084664.1	At1g33800.1

at1g58110	CON	MIN	HOUR	DAY	WEEK	UNIGENE	FLCDNA	TAIR
TAAAGTTGTT	1	2	3	1	0	gnl|UG|At#S18892712	gi|28393179|gb|BT003980.1	At1g58110.1

at2g43900	CON	MIN	HOUR	DAY	WEEK	UNIGENE	FLCDNA	TAIR
CTCACTTGTT	0	2	1	2	0	gnl|UG|At#S11731655	gi|19347903|gb|AY080628.1	At2g43900.1

at4g30150	CON	MIN	HOUR	DAY	WEEK	UNIGENE	FLCDNA	TAIR
TGTTTTTGCC	1	1	1	2	2	gnl|UG|At#S11722591	no match found	At4g30150.1

at4g37610	CON	MIN	HOUR	DAY	WEEK	UNIGENE	FLCDNA	TAIR
TACATAGGTT	4	5	0	1	0	gnl|UG|At#S11721335	gi|21403664|gb|AY084954.1	At4g37610.1

at1g20696	CON	MIN	HOUR	DAY	WEEK	UNIGENE	FLCDNA	TAIR
AAAGCCTACA	2	7	1	1	0	gnl|UG|At#S11740988	gi|20453324|gb|AY097385.1	At1g20696.1

at3g60210	CON	MIN	HOUR	DAY	WEEK	UNIGENE	FLCDNA	TAIR
AAAGAGCTCA	3	6	3	3	4	gnl|UG|At#S11727421	gi|21406199|gb|AY087462.1	At3g60210.1

at2g22670	CON	MIN	HOUR	DAY	WEEK	UNIGENE	FLCDNA	TAIR
AAGGGTTCTG	4	13	7	3	0	gnl|UG|At#S11736818	gi|20465376|gb|AY096452.1	At2g22670.2

at3g14172	CON	MIN	HOUR	DAY	WEEK	UNIGENE	FLCDNA	TAIR
TCATCGATGT	3	3	0	0	0	gnl|UG|At#S43849923	no match found	At2g22670.2

<table>
<tr><td>at1g03870</td><td>CON</td><td>MIN</td><td>HOUR</td><td>DAY</td><td>WEEK</td><td>UNIGENE</td><td>FLCDNA</td><td>TAIR</td></tr>
<tr><td>GTTTTGTTGC</td><td>3</td><td>15</td><td>5</td><td>2</td><td>1</td><td>gnl|UG|At#S11810151</td><td>gi|110742298|dbj|AK227010.1|</td><td>At1g03870.1</td></tr>
<tr><td>at4g23180</td><td>CON</td><td>MIN</td><td>HOUR</td><td>DAY</td><td>WEEK</td><td>UNIGENE</td><td>FLCDNA</td><td>TAIR</td></tr>
<tr><td>ACTTGGTCTC</td><td>0</td><td>1</td><td>0</td><td>1</td><td>0</td><td>gnl|UG|At#S11821736</td><td>gi|26450600|dbj|AK117765.1|</td><td>At4g23180.1</td></tr>
<tr><td>at4g27090</td><td>CON</td><td>MIN</td><td>HOUR</td><td>DAY</td><td>WEEK</td><td>UNIGENE</td><td>FLCDNA</td><td>TAIR</td></tr>
<tr><td>TTGGCCAAGA</td><td>32</td><td>20</td><td>30</td><td>21</td><td>24</td><td>no match found</td><td>gi|14190382|gb|AF378869.1</td><td>At4g27090.1</td></tr>
<tr><td>at1g20050</td><td>CON</td><td>MIN</td><td>HOUR</td><td>DAY</td><td>WEEK</td><td>UNIGENE</td><td>FLCDNA</td><td>TAIR</td></tr>
<tr><td>AGTCTCCTTT</td><td>3</td><td>6</td><td>0</td><td>2</td><td>3</td><td>gnl|UG|At#S11741050</td><td>gi|21404797|gb|AY086087.1</td><td>At1g20050.1</td></tr>
<tr><td>at1g18510</td><td>CON</td><td>MIN</td><td>HOUR</td><td>DAY</td><td>WEEK</td><td>UNIGENE</td><td>FLCDNA</td><td>TAIR</td></tr>
<tr><td>CAATAATAAA</td><td>0</td><td>0</td><td>1</td><td>0</td><td>0</td><td>gnl|UG|At#S22484645</td><td>no match found</td><td>At1g18510.1</td></tr>
<tr><td>at2g28550</td><td>CON</td><td>MIN</td><td>HOUR</td><td>DAY</td><td>WEEK</td><td>UNIGENE</td><td>FLCDNA</td><td>TAIR</td></tr>
<tr><td>CCCGAGTTAT</td><td>1</td><td>5</td><td>1</td><td>1</td><td>2</td><td>gnl|UG|At#S11735405</td><td>gi|13272406|gb|AF325074.1</td><td>At2g28550.2</td></tr>
<tr><td>at1g67280</td><td>CON</td><td>MIN</td><td>HOUR</td><td>DAY</td><td>WEEK</td><td>UNIGENE</td><td>FLCDNA</td><td>TAIR</td></tr>
<tr><td>ATGGGATATG</td><td>0</td><td>9</td><td>0</td><td>2</td><td>0</td><td>gnl|UG|At#S11729658</td><td>gi|16930395|gb|AF419551.1</td><td>At1g67280.1</td></tr>
<tr><td>at5g41020</td><td>CON</td><td>MIN</td><td>HOUR</td><td>DAY</td><td>WEEK</td><td>UNIGENE</td><td>FLCDNA</td><td>TAIR</td></tr>
<tr><td>ATGGTGTGAA</td><td>0</td><td>3</td><td>2</td><td>1</td><td>3</td><td>no match found</td><td>gi|15010603|gb|AY045603.1</td><td>non-canonical match</td></tr>
<tr><td>at1g48030</td><td>CON</td><td>MIN</td><td>HOUR</td><td>DAY</td><td>WEEK</td><td>UNIGENE</td><td>FLCDNA</td><td>TAIR</td></tr>
<tr><td>GCCACCTATG</td><td>4</td><td>1</td><td>4</td><td>7</td><td>0</td><td>gnl|UG|At#S11701679</td><td>gi|110742757|dbj|AK227252.1|</td><td>At1g48030.1</td></tr>
<tr><td>at1g09660</td><td>CON</td><td>MIN</td><td>HOUR</td><td>DAY</td><td>WEEK</td><td>UNIGENE</td><td>FLCDNA</td><td>TAIR</td></tr>
<tr><td>AGTTTGCTAA</td><td>0</td><td>0</td><td>1</td><td>2</td><td>1</td><td>gnl|UG|At#S18942606</td><td>gi|51968881|dbj|AK175370.1|</td><td>At1g09660.1</td></tr>
<tr><td>at3g21465</td><td>CON</td><td>MIN</td><td>HOUR</td><td>DAY</td><td>WEEK</td><td>UNIGENE</td><td>FLCDNA</td><td>TAIR</td></tr>
<tr><td>CTACATTAGC</td><td>0</td><td>1</td><td>0</td><td>0</td><td>1</td><td>gnl|UG|At#S28282936</td><td>gi|63147385|gb|BT022105.1</td><td>At3g21465.1</td></tr>
<tr><td>at1g06310</td><td>CON</td><td>MIN</td><td>HOUR</td><td>DAY</td><td>WEEK</td><td>UNIGENE</td><td>FLCDNA</td><td>TAIR</td></tr>
<tr><td>AACAGTCCAC</td><td>0</td><td>2</td><td>2</td><td>0</td><td>0</td><td>no match found</td><td>no match found</td><td>At1g06310.1</td></tr>
<tr><td>at5g20420</td><td>CON</td><td>MIN</td><td>HOUR</td><td>DAY</td><td>WEEK</td><td>UNIGENE</td><td>FLCDNA</td><td>TAIR</td></tr>
<tr><td>GGACATCAAA</td><td>0</td><td>0</td><td>1</td><td>0</td><td>0</td><td>no match found</td><td>no match found</td><td>At5g20420.1</td></tr>
<tr><td>at5g50480</td><td>CON</td><td>MIN</td><td>HOUR</td><td>DAY</td><td>WEEK</td><td>UNIGENE</td><td>FLCDNA</td><td>TAIR</td></tr>
<tr><td>TAAACAATGT</td><td>1</td><td>0</td><td>0</td><td>0</td><td>0</td><td>gnl|UG|At#S11718665</td><td>gi|48310163|gb|BT014783.1</td><td>At5g50480.1</td></tr>
<tr><td>at5g51960</td><td>CON</td><td>MIN</td><td>HOUR</td><td>DAY</td><td>WEEK</td><td>UNIGENE</td><td>FLCDNA</td><td>TAIR</td></tr>
<tr><td>CATTCATTCC</td><td>1</td><td>4</td><td>0</td><td>1</td><td>1</td><td>gnl|UG|At#S21737148</td><td>gi|51969195|dbj|AK175527.1|</td><td>non-canonical match</td></tr>
<tr><td>at3g47150</td><td>CON</td><td>MIN</td><td>HOUR</td><td>DAY</td><td>WEEK</td><td>UNIGENE</td><td>FLCDNA</td><td>TAIR</td></tr>
<tr><td>GCAATCATAA</td><td>0</td><td>0</td><td>0</td><td>1</td><td>0</td><td>no match found</td><td>no match found</td><td>At3g47150.1</td></tr>
<tr><td>at2g46230</td><td>CON</td><td>MIN</td><td>HOUR</td><td>DAY</td><td>WEEK</td><td>UNIGENE</td><td>FLCDNA</td><td>TAIR</td></tr>
<tr><td>TATGTGACTA</td><td>0</td><td>1</td><td>0</td><td>0</td><td>0</td><td>no match found</td><td>gi|16323291|gb|AY058227.1</td><td>non-canonical match</td></tr>
<tr><td>AAGCCCTGAA</td><td>1</td><td>1</td><td>0</td><td>1</td><td>1</td><td>no match found</td><td>gi|13605903|gb|AF367351.1</td><td>multiple non-canonical match</td></tr>
<tr><td>at4g09850</td><td>CON</td><td>MIN</td><td>HOUR</td><td>DAY</td><td>WEEK</td><td>UNIGENE</td><td>FLCDNA</td><td>TAIR</td></tr>
<tr><td>TAAACAAAAA</td><td>0</td><td>3</td><td>0</td><td>1</td><td>0</td><td>gnl|UG|At#S32419178</td><td>no match found</td><td>At4g09850.1</td></tr>
<tr><td>at5g38640</td><td>CON</td><td>MIN</td><td>HOUR</td><td>DAY</td><td>WEEK</td><td>UNIGENE</td><td>FLCDNA</td><td>TAIR</td></tr>
<tr><td>GAGGTTGATA</td><td>0</td><td>0</td><td>0</td><td>2</td><td>1</td><td>no match found</td><td>gi|17064893|gb|AY062523.1</td><td>At5g38640.1</td></tr>
<tr><td>at3g25660</td><td>CON</td><td>MIN</td><td>HOUR</td><td>DAY</td><td>WEEK</td><td>UNIGENE</td><td>FLCDNA</td><td>TAIR</td></tr>
<tr><td>CTTGGTTTAA</td><td>2</td><td>2</td><td>1</td><td>1</td><td>0</td><td>no match found</td><td>gi|15010725|gb|AY045664.1</td><td>non-canonical match</td></tr>
<tr><td>GGCCACATAC</td><td>0</td><td>0</td><td>1</td><td>2</td><td>1</td><td>gnl|UG|At#S11733096</td><td>no match found</td><td>At3g25660.1</td></tr>
</table>

<table>
<tr><td>at4g14480</td><td>CON</td><td>MIN</td><td>HOUR</td><td>DAY</td><td>WEEK</td><td>UNIGENE</td><td>FLCDNA</td><td>TAIR</td></tr>
<tr><td>ACATTACGAT</td><td>1</td><td>0</td><td>0</td><td>0</td><td>0</td><td>no match found</td><td>no match found</td><td>At4g14480.1</td></tr>
<tr><td>at1g69295</td><td>CON</td><td>MIN</td><td>HOUR</td><td>DAY</td><td>WEEK</td><td>UNIGENE</td><td>FLCDNA</td><td>TAIR</td></tr>
<tr><td>GTTGATAGAG</td><td>2</td><td>1</td><td>3</td><td>0</td><td>4</td><td>gnl|UG|At#S11729245</td><td>gi|51970461|dbj|AK176160.1|</td><td>At1g69295.1</td></tr>
<tr><td>at1g51340</td><td>CON</td><td>MIN</td><td>HOUR</td><td>DAY</td><td>WEEK</td><td>UNIGENE</td><td>FLCDNA</td><td>TAIR</td></tr>
<tr><td>TTTTAACTGT</td><td>2</td><td>1</td><td>0</td><td>0</td><td>0</td><td>gnl|UG|At#S18942406</td><td>gi|16604696|gb|AY059793.1</td><td>At1g51340.1</td></tr>
<tr><td>at3g63240</td><td>CON</td><td>MIN</td><td>HOUR</td><td>DAY</td><td>WEEK</td><td>UNIGENE</td><td>FLCDNA</td><td>TAIR</td></tr>
<tr><td>GAAAGGTCAG</td><td>0</td><td>1</td><td>0</td><td>0</td><td>0</td><td>gnl|UG|At#S11726457</td><td>gi|26450975|dbj|AK117957.1|</td><td>At3g63240.1</td></tr>
<tr><td>at1g70420</td><td>CON</td><td>MIN</td><td>HOUR</td><td>DAY</td><td>WEEK</td><td>UNIGENE</td><td>FLCDNA</td><td>TAIR</td></tr>
<tr><td>TTGGGTTCTT</td><td>0</td><td>4</td><td>17</td><td>2</td><td>1</td><td>gnl|UG|At#S11729005</td><td>gi|15010575|gb|AY045589.1</td><td>At1g70420.1</td></tr>
<tr><td>at1g65320</td><td>CON</td><td>MIN</td><td>HOUR</td><td>DAY</td><td>WEEK</td><td>UNIGENE</td><td>FLCDNA</td><td>TAIR</td></tr>
<tr><td>AAGCAAAATT</td><td>0</td><td>1</td><td>0</td><td>0</td><td>2</td><td>gnl|UG|At#S11730063</td><td>gi|26449326|dbj|AK117112.1|</td><td>At1g65320.1</td></tr>
<tr><td>at5g15650</td><td>CON</td><td>MIN</td><td>HOUR</td><td>DAY</td><td>WEEK</td><td>UNIGENE</td><td>FLCDNA</td><td>TAIR</td></tr>
<tr><td>GATTGAAGCT</td><td>1</td><td>2</td><td>0</td><td>0</td><td>3</td><td>gnl|UG|At#S11722425</td><td>gi|21406213|gb|AY087476.1</td><td>At5g15650.1</td></tr>
<tr><td>at3g04770</td><td>CON</td><td>MIN</td><td>HOUR</td><td>DAY</td><td>WEEK</td><td>UNIGENE</td><td>FLCDNA</td><td>TAIR</td></tr>
<tr><td>AGATTAGGTG</td><td>0</td><td>2</td><td>0</td><td>0</td><td>0</td><td>gnl|UG|At#S15461554</td><td>gi|21406147|gb|AY087423.1</td><td>At3g04770.2</td></tr>
<tr><td>at3g01400</td><td>CON</td><td>MIN</td><td>HOUR</td><td>DAY</td><td>WEEK</td><td>UNIGENE</td><td>FLCDNA</td><td>TAIR</td></tr>
<tr><td>GTGGATAAAT</td><td>0</td><td>1</td><td>0</td><td>0</td><td>1</td><td>gnl|UG|At#S11740249</td><td>gi|21928048|gb|AY125543.1</td><td>At3g01400.1</td></tr>
<tr><td>at5g27290</td><td>CON</td><td>MIN</td><td>HOUR</td><td>DAY</td><td>WEEK</td><td>UNIGENE</td><td>FLCDNA</td><td>TAIR</td></tr>
<tr><td>CTCTCATCTA</td><td>2</td><td>2</td><td>2</td><td>1</td><td>1</td><td>no match found</td><td>gi|27754445|gb|BT002853.1</td><td>multiple non-canonical match</td></tr>
<tr><td>at5g64670</td><td>CON</td><td>MIN</td><td>HOUR</td><td>DAY</td><td>WEEK</td><td>UNIGENE</td><td>FLCDNA</td><td>TAIR</td></tr>
<tr><td>AATTGATTAC</td><td>0</td><td>1</td><td>0</td><td>0</td><td>0</td><td>gnl|UG|At#S11717232</td><td>gi|19698998|gb|AY081346.1</td><td>At5g64670.1</td></tr>
<tr><td>at3g51230</td><td>CON</td><td>MIN</td><td>HOUR</td><td>DAY</td><td>WEEK</td><td>UNIGENE</td><td>FLCDNA</td><td>TAIR</td></tr>
<tr><td>AGAAAACAAA</td><td>1</td><td>1</td><td>0</td><td>0</td><td>0</td><td>no match found</td><td>no match found</td><td>At3g51230.1</td></tr>
<tr><td>at4g12980</td><td>CON</td><td>MIN</td><td>HOUR</td><td>DAY</td><td>WEEK</td><td>UNIGENE</td><td>FLCDNA</td><td>TAIR</td></tr>
<tr><td>CCAATTCTCT</td><td>1</td><td>1</td><td>1</td><td>0</td><td>0</td><td>gnl|UG|At#S11725568</td><td>gi|21407014|gb|AY088240.1</td><td>At4g12980.1</td></tr>
<tr><td>at3g33235</td><td>CON</td><td>MIN</td><td>HOUR</td><td>DAY</td><td>WEEK</td><td>UNIGENE</td><td>FLCDNA</td><td>TAIR</td></tr>
<tr><td>CCTATCTCTT</td><td>0</td><td>2</td><td>0</td><td>0</td><td>0</td><td>no match found</td><td>no match found</td><td>At3g33235.1</td></tr>
<tr><td>at1g75040</td><td>CON</td><td>MIN</td><td>HOUR</td><td>DAY</td><td>WEEK</td><td>UNIGENE</td><td>FLCDNA</td><td>TAIR</td></tr>
<tr><td>GATCAGAACA</td><td>0</td><td>1</td><td>0</td><td>0</td><td>0</td><td>gnl|UG|At#S11728030</td><td>gi|16323451|gb|AY059114.1</td><td>At1g75040.1</td></tr>
<tr><td>at2g42410</td><td>CON</td><td>MIN</td><td>HOUR</td><td>DAY</td><td>WEEK</td><td>UNIGENE</td><td>FLCDNA</td><td>TAIR</td></tr>
<tr><td>GGGTTGGAGC</td><td>0</td><td>0</td><td>1</td><td>0</td><td>0</td><td>gnl|UG|At#S11732009</td><td>no match found</td><td>multiple non-canonical match</td></tr>
<tr><td>at5g25620</td><td>CON</td><td>MIN</td><td>HOUR</td><td>DAY</td><td>WEEK</td><td>UNIGENE</td><td>FLCDNA</td><td>TAIR</td></tr>
<tr><td>CTTATAACCC</td><td>0</td><td>2</td><td>0</td><td>0</td><td>0</td><td>gnl|UG|At#S11710344</td><td>gi|17528989|gb|AY065229.1</td><td>At5g25620.1</td></tr>
<tr><td>at1g33700</td><td>CON</td><td>MIN</td><td>HOUR</td><td>DAY</td><td>WEEK</td><td>UNIGENE</td><td>FLCDNA</td><td>TAIR</td></tr>
<tr><td>AGGCCTTTAG</td><td>0</td><td>0</td><td>0</td><td>0</td><td>1</td><td>no match found</td><td>gi|28393604|gb|BT004204.1</td><td>non-canonical match</td></tr>
<tr><td>at5g63650</td><td>CON</td><td>MIN</td><td>HOUR</td><td>DAY</td><td>WEEK</td><td>UNIGENE</td><td>FLCDNA</td><td>TAIR</td></tr>
<tr><td>TTAAAGAGGC</td><td>0</td><td>2</td><td>0</td><td>1</td><td>0</td><td>gnl|UG|At#S11717335</td><td>gi|110743750|dbj|AK227726.1|</td><td>At5g63650.1</td></tr>
<tr><td>at3g56260</td><td>CON</td><td>MIN</td><td>HOUR</td><td>DAY</td><td>WEEK</td><td>UNIGENE</td><td>FLCDNA</td><td>TAIR</td></tr>
<tr><td>AATAAAGCTT</td><td>0</td><td>0</td><td>0</td><td>0</td><td>2</td><td>no match found</td><td>gi|44681331|gb|BT011600.1</td><td>non-canonical match</td></tr>
<tr><td>TCTAAGTAGA</td><td>0</td><td>0</td><td>0</td><td>1</td><td>0</td><td>gnl|UG|At#S18903454</td><td>no match found</td><td>At3g56260.1</td></tr>
</table>

at3g11540	CON	MIN	HOUR	DAY	WEEK	UNIGENE	FLCDNA	TAIR
TGTTGGATTT	1	0	0	1	1	gnl|UG|At#S11737424	gi|62319733|dbj|AK221192.1|	At3g11540.1
ATTCTTGGGA	0	1	0	0	0	no match found	no match found	At3g11540.2
at1g45170	CON	MIN	HOUR	DAY	WEEK	UNIGENE	FLCDNA	TAIR
AAGAATTTGC	0	0	1	1	0	gnl|UG|At#S11735612	gi|26451974|dbj|AK118473.1|	At1g45170.1
GAACCTGGAA	1	0	1	0	0	no match found	gi|45680298|gb|BT011988.1	non-canonical match
at1g07210	CON	MIN	HOUR	DAY	WEEK	UNIGENE	FLCDNA	TAIR
GCTCTAGTTT	2	4	2	1	0	gnl|UG|At#S11742313	gi|62319699|dbj|AK221175.1|	non-canonical match
GAGAACAGAG	0	0	1	0	0	no match found	gi|12083223|gb|AF332408.1	multiple non-canonical match
at1g68690	CON	MIN	HOUR	DAY	WEEK	UNIGENE	FLCDNA	TAIR
CCATAGAAAC	0	0	1	0	0	no match found	gi|21280864|gb|AY113877.1	multiple non-canonical match
AAATTACTTT	1	0	0	0	0	gnl|UG|At#S11703624	gi|14334805|gb|AY035076.1	At1g68690.1
at5g59400	CON	MIN	HOUR	DAY	WEEK	UNIGENE	FLCDNA	TAIR
TCTGTGAGTG	0	1	0	0	0	gnl|UG|At#S11704863	gi|15081619|gb|AY048202.1	At5g59400.1
at1g23820	CON	MIN	HOUR	DAY	WEEK	UNIGENE	FLCDNA	TAIR
TTTCTGTAAC	3	3	0	0	2	no match found	gi|20260023|gb|AY093360.1	non-canonical match
TTCTTATGCT	2	2	0	0	2	gnl|UG|At#S18942506	gi|17065033|gb|AY062593.1	At1g23820.2
at5g46210	CON	MIN	HOUR	DAY	WEEK	UNIGENE	FLCDNA	TAIR
AACTAAATGT	0	2	1	2	1	gnl|UG|At#S11719105	gi|20268718|gb|AY091113.1	At5g46210.1
at2g42740	CON	MIN	HOUR	DAY	WEEK	UNIGENE	FLCDNA	TAIR
CTATGTAACC	0	1	0	0	0	gnl|UG|At#S30643550	gi|18491254|gb|AY074636.1	At2g42740.1
at1g05830	CON	MIN	HOUR	DAY	WEEK	UNIGENE	FLCDNA	TAIR
GTTCTGGCTA	0	2	3	0	1	gnl|UG|At#S34115457	gi|110741465|dbj|AK226560.1|	At1g05830.1
at2g40090	CON	MIN	HOUR	DAY	WEEK	UNIGENE	FLCDNA	TAIR
GAAGCAAGTC	0	1	0	0	0	gnl|UG|At#S11812732	gi|51969457|dbj|AK175658.1|	At2g40090.1
at4g26160	CON	MIN	HOUR	DAY	WEEK	UNIGENE	FLCDNA	TAIR
TGGTGCATCA	1	1	1	1	1	gnl|UG|At#S11723284	gi|21404086|gb|AY085376.1	At4g26160.1
at5g23760	CON	MIN	HOUR	DAY	WEEK	UNIGENE	FLCDNA	TAIR
ACTGATGATA	1	4	1	0	0	gnl|UG|At#S11720803	gi|88011057|gb|BT024557.1	At5g23760.1
at1g13390	CON	MIN	HOUR	DAY	WEEK	UNIGENE	FLCDNA	TAIR
GTGGCTTCTC	0	0	1	1	0	gnl|UG|At#S15461112	gi|124300953|gb|BT029991.1	At1g13390.2
at5g54810	CON	MIN	HOUR	DAY	WEEK	UNIGENE	FLCDNA	TAIR
GGACGGGCTG	1	8	2	3	0	gnl|UG|At#S11718233	gi|14194116|gb|AF367264.1	At5g54810.1
at2g26110	CON	MIN	HOUR	DAY	WEEK	UNIGENE	FLCDNA	TAIR
ACCCAAGGTG	2	2	0	0	0	gnl|UG|At#S11736004	no match found	At2g26110.1
TCGCTAGGAC	2	3	0	0	0	no match found	gi|51971692|dbj|AK176748.1|	non-canonical match
at3g20630	CON	MIN	HOUR	DAY	WEEK	UNIGENE	FLCDNA	TAIR
TGGATTAGTA	4	1	0	3	1	gnl|UG|At#S11734655	gi|22655293|gb|AY140096.1	At3g20630.1
at4g29480	CON	MIN	HOUR	DAY	WEEK	UNIGENE	FLCDNA	TAIR
ATTCTCTCTT	4	2	2	1	1	gnl|UG|At#S18906186	gi|21407278|gb|AY088504.1	At4g29480.1
at4g13560	CON	MIN	HOUR	DAY	WEEK	UNIGENE	FLCDNA	TAIR
TAATCCGAAA	0	0	0	2	1	gnl|UG|At#S11725461	gi|26451721|dbj|AK118342.1|	At4g13560.1

<table>
<tr><td>at4g31700</td><td>CON</td><td>MIN</td><td>HOUR</td><td>DAY</td><td>WEEK</td><td>UNIGENE</td><td>FLCDNA</td><td>TAIR</td></tr>
<tr><td>ATTTATGCTT</td><td>24</td><td>6</td><td>13</td><td>8</td><td>46</td><td>gnl|UG|At#S11722332</td><td>gi|15292738|gb|AY050803.1</td><td>At4g31700.1</td></tr>
<tr><td>at1g06080</td><td>CON</td><td>MIN</td><td>HOUR</td><td>DAY</td><td>WEEK</td><td>UNIGENE</td><td>FLCDNA</td><td>TAIR</td></tr>
<tr><td>TTTGGGGAAG</td><td>0</td><td>1</td><td>2</td><td>0</td><td>0</td><td>gnl|UG|At#S11742419</td><td>gi|12083273|gb|AF332433.1</td><td>At1g06080.1</td></tr>
<tr><td>at1g80270</td><td>CON</td><td>MIN</td><td>HOUR</td><td>DAY</td><td>WEEK</td><td>UNIGENE</td><td>FLCDNA</td><td>TAIR</td></tr>
<tr><td>GCTGCACAGT</td><td>0</td><td>0</td><td>0</td><td>1</td><td>1</td><td>no match found</td><td>gi|20259917|gb|AY093307.1</td><td>multiple non-canonical match</td></tr>
<tr><td>at1g80560</td><td>CON</td><td>MIN</td><td>HOUR</td><td>DAY</td><td>WEEK</td><td>UNIGENE</td><td>FLCDNA</td><td>TAIR</td></tr>
<tr><td>GTTCTGCACC</td><td>0</td><td>0</td><td>2</td><td>0</td><td>0</td><td>gnl|UG|At#S11726308</td><td>gi|21387084|gb|AY114627.1</td><td>At1g80560.1</td></tr>
<tr><td>at5g24660</td><td>CON</td><td>MIN</td><td>HOUR</td><td>DAY</td><td>WEEK</td><td>UNIGENE</td><td>FLCDNA</td><td>TAIR</td></tr>
<tr><td>AACGAGCTCT</td><td>1</td><td>3</td><td>2</td><td>3</td><td>4</td><td>gnl|UG|At#S11720709</td><td>gi|26452226|dbj|AK118602.1|</td><td>At5g24660.1</td></tr>
<tr><td>at3g52360</td><td>CON</td><td>MIN</td><td>HOUR</td><td>DAY</td><td>WEEK</td><td>UNIGENE</td><td>FLCDNA</td><td>TAIR</td></tr>
<tr><td>GACAAGTTCG</td><td>0</td><td>1</td><td>3</td><td>1</td><td>0</td><td>gnl|UG|At#S11708499</td><td>gi|21405798|gb|AY087074.1</td><td>At3g52360.1</td></tr>
<tr><td>at4g26120</td><td>CON</td><td>MIN</td><td>HOUR</td><td>DAY</td><td>WEEK</td><td>UNIGENE</td><td>FLCDNA</td><td>TAIR</td></tr>
<tr><td>TGTTTTAAAA</td><td>1</td><td>0</td><td>1</td><td>0</td><td>0</td><td>no match found</td><td>no match found</td><td>At4g26120.1</td></tr>
<tr><td>at3g50520</td><td>CON</td><td>MIN</td><td>HOUR</td><td>DAY</td><td>WEEK</td><td>UNIGENE</td><td>FLCDNA</td><td>TAIR</td></tr>
<tr><td>GAGGTGTGAT</td><td>0</td><td>0</td><td>0</td><td>2</td><td>0</td><td>no match found</td><td>gi|17104694|gb|AY063062.1</td><td>At3g50520.1</td></tr>
<tr><td>at1g45150</td><td>CON</td><td>MIN</td><td>HOUR</td><td>DAY</td><td>WEEK</td><td>UNIGENE</td><td>FLCDNA</td><td>TAIR</td></tr>
<tr><td>TAGACCATCT</td><td>1</td><td>0</td><td>1</td><td>0</td><td>0</td><td>gnl|UG|At#S11735623</td><td>gi|30725313|gb|BT008320.1</td><td>At1g45150.1</td></tr>
<tr><td>at1g56510</td><td>CON</td><td>MIN</td><td>HOUR</td><td>DAY</td><td>WEEK</td><td>UNIGENE</td><td>FLCDNA</td><td>TAIR</td></tr>
<tr><td>TAATTACTTT</td><td>0</td><td>1</td><td>0</td><td>0</td><td>1</td><td>gnl|UG|At#S34115246</td><td>gi|110741854|dbj|AK226771.1|</td><td>multiple non-canonical match</td></tr>
<tr><td>at3g09440</td><td>CON</td><td>MIN</td><td>HOUR</td><td>DAY</td><td>WEEK</td><td>UNIGENE</td><td>FLCDNA</td><td>TAIR</td></tr>
<tr><td>AGGAACACAA</td><td>2</td><td>1</td><td>0</td><td>17</td><td>0</td><td>gnl|UG|At#S11738053</td><td>no match found</td><td>At3g09440.1</td></tr>
<tr><td>at4g39770</td><td>CON</td><td>MIN</td><td>HOUR</td><td>DAY</td><td>WEEK</td><td>UNIGENE</td><td>FLCDNA</td><td>TAIR</td></tr>
<tr><td>ATGAGAGCTA</td><td>1</td><td>1</td><td>0</td><td>1</td><td>0</td><td>gnl|UG|At#S11720963</td><td>gi|28973300|gb|BT005555.1</td><td>At4g39770.1</td></tr>
<tr><td>at3g50500</td><td>CON</td><td>MIN</td><td>HOUR</td><td>DAY</td><td>WEEK</td><td>UNIGENE</td><td>FLCDNA</td><td>TAIR</td></tr>
<tr><td>GATGACTTTG</td><td>3</td><td>7</td><td>1</td><td>3</td><td>4</td><td>gnl|UG|At#S11705532</td><td>gi|94442422|gb|BT025246.1</td><td>At3g50500.1</td></tr>
<tr><td>at1g30570</td><td>CON</td><td>MIN</td><td>HOUR</td><td>DAY</td><td>WEEK</td><td>UNIGENE</td><td>FLCDNA</td><td>TAIR</td></tr>
<tr><td>AAGCTTGGCT</td><td>0</td><td>1</td><td>0</td><td>1</td><td>0</td><td>gnl|UG|At#S11738819</td><td>no match found</td><td>non-canonical match</td></tr>
<tr><td>at2g32550</td><td>CON</td><td>MIN</td><td>HOUR</td><td>DAY</td><td>WEEK</td><td>UNIGENE</td><td>FLCDNA</td><td>TAIR</td></tr>
<tr><td>GTCAGACCTC</td><td>0</td><td>0</td><td>0</td><td>1</td><td>0</td><td>gnl|UG|At#S11814904</td><td>no match found</td><td>At2g32550.1</td></tr>
<tr><td>at3g50630</td><td>CON</td><td>MIN</td><td>HOUR</td><td>DAY</td><td>WEEK</td><td>UNIGENE</td><td>FLCDNA</td><td>TAIR</td></tr>
<tr><td>TAATTCAACT</td><td>2</td><td>0</td><td>1</td><td>0</td><td>0</td><td>gnl|UG|At#S11729318</td><td>gi|51971652|dbj|AK176728.1|</td><td>At3g50630.1</td></tr>
<tr><td>at2g19160</td><td>CON</td><td>MIN</td><td>HOUR</td><td>DAY</td><td>WEEK</td><td>UNIGENE</td><td>FLCDNA</td><td>TAIR</td></tr>
<tr><td>GAACCCCTAG</td><td>0</td><td>0</td><td>2</td><td>0</td><td>0</td><td>gnl|UG|At#S15461404</td><td>gi|17065505|gb|AY062829.1</td><td>non-canonical match</td></tr>
<tr><td>at1g53190</td><td>CON</td><td>MIN</td><td>HOUR</td><td>DAY</td><td>WEEK</td><td>UNIGENE</td><td>FLCDNA</td><td>TAIR</td></tr>
<tr><td>GAGAAGAGAA</td><td>3</td><td>1</td><td>2</td><td>3</td><td>2</td><td>no match found</td><td>gi|20466833|gb|AY099883.1</td><td>multiple canonical match</td></tr>
<tr><td>at5g42990</td><td>CON</td><td>MIN</td><td>HOUR</td><td>DAY</td><td>WEEK</td><td>UNIGENE</td><td>FLCDNA</td><td>TAIR</td></tr>
<tr><td>GCCTCCCACT</td><td>4</td><td>1</td><td>2</td><td>5</td><td>1</td><td>no match found</td><td>gi|16648721|gb|AY058137.1</td><td>non-canonical match</td></tr>
<tr><td>GAGAGTTTAG</td><td>1</td><td>2</td><td>4</td><td>2</td><td>3</td><td>gnl|UG|At#S11719429</td><td>no match found</td><td>At5g42990.1</td></tr>
<tr><td>at5g14000</td><td>CON</td><td>MIN</td><td>HOUR</td><td>DAY</td><td>WEEK</td><td>UNIGENE</td><td>FLCDNA</td><td>TAIR</td></tr>
<tr><td>GTTCTAGGGG</td><td>0</td><td>1</td><td>0</td><td>0</td><td>0</td><td>gnl|UG|At#S11722833</td><td>gi|21402926|gb|AY084216.1</td><td>At5g14000.1</td></tr>
</table>

at3g53350	CON	MIN	HOUR	DAY	WEEK	UNIGENE	FLCDNA	TAIR
GAAGCTGAGC	0	0	0	2	0	gnl|UG|At#S11709971	gi|17978992|gb|AY069906.1	At3g53350.3
at2g41630	CON	MIN	HOUR	DAY	WEEK	UNIGENE	FLCDNA	TAIR
CGACAGGAGT	1	2	4	1	0	gnl|UG|At#S11732180	gi|21405357|gb|AY086647.1	At2g41630.1
at1g66730	CON	MIN	HOUR	DAY	WEEK	UNIGENE	FLCDNA	TAIR
CTCAAACCAG	0	1	0	0	0	gnl|UG|At#S11729779	no match found	At1g66730.1
at4g00840	CON	MIN	HOUR	DAY	WEEK	UNIGENE	FLCDNA	TAIR
AGAAAAATGG	0	1	0	0	0	gnl|UG|At#S11727471	gi|56461761|gb|BT020391.1	non-canonical match
at2g16360	CON	MIN	HOUR	DAY	WEEK	UNIGENE	FLCDNA	TAIR
GCTGCAATAC	0	0	1	0	0	no match found	no match found	At2g16360.1
at1g06380	CON	MIN	HOUR	DAY	WEEK	UNIGENE	FLCDNA	TAIR
AGTGGGTCTT	0	0	0	0	2	gnl|UG|At#S11742390	no match found	At1g06380.1
at2g23750	CON	MIN	HOUR	DAY	WEEK	UNIGENE	FLCDNA	TAIR
CCATTGTAAA	1	0	0	0	0	no match found	no match found	At2g23750.1
at5g08535	CON	MIN	HOUR	DAY	WEEK	UNIGENE	FLCDNA	TAIR
GCTGGAAGGA	0	1	0	0	0	no match found	gi|21404421|gb|AY085711.1	At5g08535.2
at4g23490	CON	MIN	HOUR	DAY	WEEK	UNIGENE	FLCDNA	TAIR
TCGAGCACGA	0	1	0	0	0	gnl|UG|At#S11723735	gi|110740267|dbj|AK230225.1|	At4g23490.1
at3g50370	CON	MIN	HOUR	DAY	WEEK	UNIGENE	FLCDNA	TAIR
GTTGGTAAAA	1	2	2	3	1	gnl|UG|At#S11729372	gi|62321681|dbj|AK222179.1|	multiple canonical match
at1g23040	CON	MIN	HOUR	DAY	WEEK	UNIGENE	FLCDNA	TAIR
ATCGGAGAAA	2	1	2	2	0	no match found	gi|21406256|gb|AY087519.1	At1g23040.1
at5g51050	CON	MIN	HOUR	DAY	WEEK	UNIGENE	FLCDNA	TAIR
AGGGTCCACG	0	1	0	0	0	gnl|UG|At#S11718611	no match found	non-canonical match
at2g17290	CON	MIN	HOUR	DAY	WEEK	UNIGENE	FLCDNA	TAIR
GAATCAACAT	0	1	0	0	0	gnl|UG|At#S11738159	gi|22655116|gb|AY140007.1	At2g17290.1
at2g39480	CON	MIN	HOUR	DAY	WEEK	UNIGENE	FLCDNA	TAIR
CATCTGATCA	0	0	1	0	0	gnl|UG|At#S11732726	gi|20453063|gb|AY094398.1	At2g39480.1
at3g15760	CON	MIN	HOUR	DAY	WEEK	UNIGENE	FLCDNA	TAIR
TAAGTAGTCA	2	0	0	0	0	no match found	gi|21407374|gb|AY088600.1	At3g15760.1
at4g02540	CON	MIN	HOUR	DAY	WEEK	UNIGENE	FLCDNA	TAIR
AAGCCCGGTT	0	3	0	1	0	gnl|UG|At#S34117311	gi|110737892|dbj|AK228997.1|	At4g02540.1
at4g00810	CON	MIN	HOUR	DAY	WEEK	UNIGENE	FLCDNA	TAIR
AGAGTTATCT	9	7	4	13	20	gnl|UG|At#S20842397	gi|21405346|gb|AY086636.1	At4g00810.1
AATGTTGGTG	0	0	1	0	0	no match found	gi|15777870|gb|AY055096.1	multiple non-canonical match
at5g25120	CON	MIN	HOUR	DAY	WEEK	UNIGENE	FLCDNA	TAIR
TCAACATATG	1	0	1	0	1	gnl|UG|At#S21736052	gi|51971442|dbj|AK176623.1|	multiple non-canonical match
at4g20320	CON	MIN	HOUR	DAY	WEEK	UNIGENE	FLCDNA	TAIR
GATTTCTTCA	1	0	0	1	0	no match found	gi|110736295|dbj|AK228162.1|	multiple non-canonical match
at1g70290	CON	MIN	HOUR	DAY	WEEK	UNIGENE	FLCDNA	TAIR
CACGGTTGGA	4	6	4	0	0	gnl|UG|At#S11729037	gi|110742590|dbj|AK227167.1|	At1g70290.1

at2g25650	CON	MIN	HOUR	DAY	WEEK	UNIGENE	FLCDNA	TAIR
TCCATTGTTG	0	1	2	2	0	gnl|UG|At#S11736111	gi|21402933|gb|AY084223.1	At2g25650.1
ATGACGATGA	1	0	0	0	0	no match found	gi|20465306|gb|AY096417.1	multiple non-canonical match
at4g26555	CON	MIN	HOUR	DAY	WEEK	UNIGENE	FLCDNA	TAIR
CGAACGAGCC	1	1	1	2	1	gnl|UG|At#S11723218	gi|16612239|gb|AF439821.1	At4g26555.1
at1g75540	CON	MIN	HOUR	DAY	WEEK	UNIGENE	FLCDNA	TAIR
TACAAAATCC	2	0	0	0	0	gnl|UG|At#S11727932	gi|110743286|dbj|AK227534.1|	At1g75540.1
AAACAACAAG	0	0	1	0	1	no match found	gi|28416600|gb|BT004585.1	non-canonical match
at1g61260	CON	MIN	HOUR	DAY	WEEK	UNIGENE	FLCDNA	TAIR
ACCGTGATGA	0	1	0	0	0	gnl|UG|At#S34114505	gi|110743238|dbj|AK227510.1|	At1g61260.1
at1g09250	CON	MIN	HOUR	DAY	WEEK	UNIGENE	FLCDNA	TAIR
TATTTTATTT	3	3	1	2	0	gnl|UG|At#S11742113	gi|110743365|dbj|AK227577.1|	At1g09250.1
at5g20040	CON	MIN	HOUR	DAY	WEEK	UNIGENE	FLCDNA	TAIR
TTCTTGAGTA	1	1	0	0	0	gnl|UG|At#S15460640	gi|13430591|gb|AF360208.1	At5g20040.1
CGTGGAAAGC	0	0	1	0	0	no match found	gi|14532863|gb|AY040056.1	At5g20040.2
at1g68920	CON	MIN	HOUR	DAY	WEEK	UNIGENE	FLCDNA	TAIR
ATATTTCGTC	1	2	0	0	0	gnl|UG|At#S11729312	gi|22135840|gb|AY128299.1	At1g68920.1
at1g45332	CON	MIN	HOUR	DAY	WEEK	UNIGENE	FLCDNA	TAIR
ATTGTTGGAC	0	1	0	1	0	no match found	gi|110742890|dbj|AK227329.1|	multiple non-canonical match
ACACAAGGCA	0	1	1	0	0	gnl|UG|At#S11735597	no match found	At1g45332.1
at4g34990	CON	MIN	HOUR	DAY	WEEK	UNIGENE	FLCDNA	TAIR
ATGAGAGGAA	0	0	0	2	0	gnl|UG|At#S11721764	gi|90186243|gb|BT024907.1	At4g34990.1
at2g04240	CON	MIN	HOUR	DAY	WEEK	UNIGENE	FLCDNA	TAIR
TGTATGGAGC	6	3	3	1	1	gnl|UG|At#S11821331	gi|23397317|gb|BT000806.1	multiple canonical match
at1g10370	CON	MIN	HOUR	DAY	WEEK	UNIGENE	FLCDNA	TAIR
CCCGAGACTG	0	0	6	7	2	gnl|UG|At#S11707079	gi|71143087|gb|BT023743.1	At1g10370.1
at4g34220	CON	MIN	HOUR	DAY	WEEK	UNIGENE	FLCDNA	TAIR
AAAGAATTGG	2	2	1	2	0	gnl|UG|At#S11721897	gi|14334871|gb|AY035109.1	At4g34220.1
at3g43870	CON	MIN	HOUR	DAY	WEEK	UNIGENE	FLCDNA	TAIR
TCTGTTGGTT	1	1	1	0	0	no match found	no match found	At3g43870.1
at3g44600	CON	MIN	HOUR	DAY	WEEK	UNIGENE	FLCDNA	TAIR
ACAGGCCGTT	0	0	1	0	2	no match found	gi|30725437|gb|BT008382.1	non-canonical match
at1g29310	CON	MIN	HOUR	DAY	WEEK	UNIGENE	FLCDNA	TAIR
AGAGCCATTG	0	0	0	1	0	no match found	gi|21406232|gb|AY087495.1	multiple non-canonical match
GGAGCCATTG	0	0	0	1	2	gnl|UG|At#S11739258	gi|20260295|gb|AY093047.1	At1g29310.1
at1g35170	CON	MIN	HOUR	DAY	WEEK	UNIGENE	FLCDNA	TAIR
ATCTACGATC	0	2	0	0	0	gnl|UG|At#S11737225	no match found	multiple canonical match
at4g14990	CON	MIN	HOUR	DAY	WEEK	UNIGENE	FLCDNA	TAIR
TCTCAAATGT	3	2	0	1	0	gnl|UG|At#S11725204	gi|110740058|dbj|AK230115.1|	At4g14990.1
at1g28250	CON	MIN	HOUR	DAY	WEEK	UNIGENE	FLCDNA	TAIR
TAAAAAGAGG	6	0	2	0	1	gnl|UG|At#S11739538	no match found	At1g28250.1
at1g04130	CON	MIN	HOUR	DAY	WEEK	UNIGENE	FLCDNA	TAIR
ACGTGTTGAA	3	1	1	0	0	gnl|UG|At#S11742654	gi|17978864|gb|AY066037.1	At1g04130.1

at2g31040	CON	MIN	HOUR	DAY	WEEK	UNIGENE	FLCDNA	TAIR
CATTTGGAGT	2	5	1	5	0	gnl|UG|At#S11734778	gi|15215860|gb|AY050461.1	At2g31040.1
at2g20760	CON	MIN	HOUR	DAY	WEEK	UNIGENE	FLCDNA	TAIR
TAAGAAAGTG	0	0	1	0	1	gnl|UG|At#S11737273	no match found	At2g20760.1
ATGCCGCCTC	0	6	3	1	2	no match found	gi|108385428|gb|BT025885.1	non-canonical match
at3g15360	CON	MIN	HOUR	DAY	WEEK	UNIGENE	FLCDNA	TAIR
GTGTGGACCT	16	37	9	12	0	no match found	gi|16974518|gb|AY060538.1	multiple non-canonical match
CCATTGTTCT	1	0	0	0	0	gnl|UG|At#S11736230	gi|14030704|gb|AF375443.1	At3g15360.1
at1g70090	CON	MIN	HOUR	DAY	WEEK	UNIGENE	FLCDNA	TAIR
AAATAAACCC	0	0	0	0	1	gnl|UG|At#S11729080	no match found	At1g70090.1
GGTGAGACTT	1	1	1	0	0	gnl|UG|At#S43850355	gi|13878002|gb|AF370264.1	non-canonical match
at3g24080	CON	MIN	HOUR	DAY	WEEK	UNIGENE	FLCDNA	TAIR
TTGGGTATTG	2	1	0	1	0	gnl|UG|At#S43849859	gi|110736404|dbj|AK228219.1|	At3g24080.1
at5g13440	CON	MIN	HOUR	DAY	WEEK	UNIGENE	FLCDNA	TAIR
GTCTGGATGA	2	8	8	7	4	gnl|UG|At#S11722990	gi|20148362|gb|AY081510.1	At5g13440.1
at3g63080	CON	MIN	HOUR	DAY	WEEK	UNIGENE	FLCDNA	TAIR
CAGTCAAGTT	3	2	3	1	0	gnl|UG|At#S11726514	gi|21403826|gb|AY085116.1	At3g63080.1
at3g01770	CON	MIN	HOUR	DAY	WEEK	UNIGENE	FLCDNA	TAIR
AGTTGTTGTA	1	2	0	0	3	gnl|UG|At#S11740148	gi|22136455|gb|AY128906.1	At3g01770.1
GGTCAGGGCC	0	2	0	0	0	no match found	gi|23270368|gb|AY045863.2	non-canonical match
at1g06360	CON	MIN	HOUR	DAY	WEEK	UNIGENE	FLCDNA	TAIR
AGAGATTTAT	1	1	1	1	1	gnl|UG|At#S18894051	no match found	At1g06360.1
at2g35710	CON	MIN	HOUR	DAY	WEEK	UNIGENE	FLCDNA	TAIR
GGGACGCCAA	0	1	0	0	0	gnl|UG|At#S30642393	gi|21407710|gb|AY088936.1	At2g35710.3
at1g68000	CON	MIN	HOUR	DAY	WEEK	UNIGENE	FLCDNA	TAIR
GTCACAGATA	0	2	1	0	0	gnl|UG|At#S11729507	gi|22655195|gb|AY140047.1	At1g68000.1
at3g25560	CON	MIN	HOUR	DAY	WEEK	UNIGENE	FLCDNA	TAIR
ATTAGGAGGA	0	0	0	1	0	gnl|UG|At#S18942051	gi|19715614|gb|AY075617.1	At3g25560.2
at4g24690	CON	MIN	HOUR	DAY	WEEK	UNIGENE	FLCDNA	TAIR
CTCAAGGAGC	0	0	1	0	0	no match found	gi|30725523|gb|BT008425.1	non-canonical match
GATGGTTTCT	4	10	7	16	8	gnl|UG|At#S11723526	gi|22655263|gb|AY140081.1	At4g24690.1
at4g23500	CON	MIN	HOUR	DAY	WEEK	UNIGENE	FLCDNA	TAIR
TAATAAGATC	1	0	0	0	0	gnl|UG|At#S11723731	gi|62320483|dbj|AK221568.1|	At4g23500.1
at3g47930	CON	MIN	HOUR	DAY	WEEK	UNIGENE	FLCDNA	TAIR
GTGGAAAAGC	0	0	1	0	2	gnl|UG|At#S11699722	gi|26450909|dbj|AK117924.1|	At3g47930.1
at2g04040	CON	MIN	HOUR	DAY	WEEK	UNIGENE	FLCDNA	TAIR
TTATGTGAGA	0	2	0	0	2	gnl|UG|At#S11740382	gi|111074209|gb|BT026371.1	At2g04040.1
at2g44350	CON	MIN	HOUR	DAY	WEEK	UNIGENE	FLCDNA	TAIR
AATTAAAGCT	6	2	3	3	3	gnl|UG|At#S15460389	no match found	non-canonical match
CTTGGTGCAA	5	1	7	5	6	no match found	gi|21404357|gb|AY085647.1	non-canonical match
at1g73260	CON	MIN	HOUR	DAY	WEEK	UNIGENE	FLCDNA	TAIR
CCTATTTATT	1	3	0	0	0	gnl|UG|At#S34116002	gi|110740416|dbj|AK230302.1|	At1g73260.1

at3g53960	CON	MIN	HOUR	DAY	WEEK	UNIGENE	FLCDNA	TAIR
AGATACACTT	1	0	0	0	0	gnl|UG|At#S11702897	gi|13605522|gb|AF361587.1	At3g53960.1

at3g10820	CON	MIN	HOUR	DAY	WEEK	UNIGENE	FLCDNA	TAIR
TTTCATTATG	0	0	1	0	0	no match found	no match found	At3g10820.1

at4g26230	CON	MIN	HOUR	DAY	WEEK	UNIGENE	FLCDNA	TAIR
ATAATATCTG	0	1	0	0	3	gnl|UG|At#S11723271	no match found	At4g26230.1

at1g60110	CON	MIN	HOUR	DAY	WEEK	UNIGENE	FLCDNA	TAIR
TTCTTTAATT	0	1	0	0	0	gnl|UG|At#S18896537	no match found	multiple non-canonical match

at1g64280	CON	MIN	HOUR	DAY	WEEK	UNIGENE	FLCDNA	TAIR
ATGACTGTAA	0	2	1	0	0	no match found	gi|21406957|gb|AY088183.1	non-canonical match

at5g22330	CON	MIN	HOUR	DAY	WEEK	UNIGENE	FLCDNA	TAIR
GTCGTGAGAT	0	0	1	1	0	gnl|UG|At#S11721015	no match found	At5g22330.1
AGAAGAACAC	1	0	0	0	1	no match found	gi|21403316|gb|AY084606.1	multiple non-canonical match

at1g74100	CON	MIN	HOUR	DAY	WEEK	UNIGENE	FLCDNA	TAIR
ATAACTGAAT	2	6	6	2	3	gnl|UG|At#S11728219	gi|14596198|gb|AY042887.1	At1g74100.1

at5g20680	CON	MIN	HOUR	DAY	WEEK	UNIGENE	FLCDNA	TAIR
TTAATGGCGT	0	1	0	0	0	no match found	gi|15810384|gb|AY056231.1	non-canonical match

at5g65750	CON	MIN	HOUR	DAY	WEEK	UNIGENE	FLCDNA	TAIR
TTAAGGAGCA	0	0	1	0	0	no match found	gi|20465596|gb|AY096631.1	non-canonical match
AGAGAATCTT	4	3	2	3	0	gnl|UG|At#S11717123	gi|110740036|dbj|AK230103.1|	At5g65750.1

at5g29000	CON	MIN	HOUR	DAY	WEEK	UNIGENE	FLCDNA	TAIR
TTTATTGGGA	0	2	0	0	0	no match found	gi|15028280|gb|AY045943.1	non-canonical match

at5g15050	CON	MIN	HOUR	DAY	WEEK	UNIGENE	FLCDNA	TAIR
GGACAATCCT	0	1	0	0	0	gnl|UG|At#S11722579	gi|16209673|gb|AY057600.1	At5g15050.1

at1g55910	CON	MIN	HOUR	DAY	WEEK	UNIGENE	FLCDNA	TAIR
TAGCAATCCT	1	0	2	2	0	gnl|UG|At#S11743288	gi|15294273|gb|AF410328.1	At1g55910.1
TTGATGAGCC	0	0	1	0	0	no match found	gi|20147286|gb|AY093733.1	non-canonical match

at4g32620	CON	MIN	HOUR	DAY	WEEK	UNIGENE	FLCDNA	TAIR
TACCCATCGG	2	8	1	7	2	gnl|UG|At#S34115241	gi|110741864|dbj|AK226776.1|	pseudo chromosome match

at1g73060	CON	MIN	HOUR	DAY	WEEK	UNIGENE	FLCDNA	TAIR
GATTCACACT	5	2	6	4	2	no match found	gi|25082945|gb|BT002009.1	multiple non-canonical match

at4g16143	CON	MIN	HOUR	DAY	WEEK	UNIGENE	FLCDNA	TAIR
GAATAAGCTT	2	2	3	3	1	gnl|UG|At#S11725006	no match found	At4g16143.1

at4g36970	CON	MIN	HOUR	DAY	WEEK	UNIGENE	FLCDNA	TAIR
ACTTGCTTTG	1	4	1	2	2	gnl|UG|At#S11721442	gi|23297308|gb|AY150393.1	At4g36970.1

at3g59380	CON	MIN	HOUR	DAY	WEEK	UNIGENE	FLCDNA	TAIR
TATCAAAACA	2	4	0	1	2	no match found	gi|110736119|dbj|AK228072.1|	At3g59380.1

at1g80010	CON	MIN	HOUR	DAY	WEEK	UNIGENE	FLCDNA	TAIR
TTTTGGAGTA	0	1	0	0	0	gnl|UG|At#S11726519	no match found	At1g80010.1

at1g55205	CON	MIN	HOUR	DAY	WEEK	UNIGENE	FLCDNA	TAIR
GTTATCACTT	0	0	0	1	0	gnl|UG|At#S38434162	gi|72198487|gb|DQ108873.1	multiple non-canonical match

at1g78680	CON	MIN	HOUR	DAY	WEEK	UNIGENE	FLCDNA	TAIR

CGTTGATGAC	1	7	2	1	0	gnl|UG|At#S18895743	gi|17979072|gb|AY070047.1	At1g78680.1
at4g14020	CON	MIN	HOUR	DAY	WEEK	UNIGENE	FLCDNA	TAIR
CAACGTGTGT	4	5	3	0	1	gnl|UG|At#S11813120	gi|21403878|gb|AY085168.1	At4g14020.1
ACACGGCGGC	1	2	0	0	0	no match found	gi|28466806|gb|BT004746.1	multiple non-canonical match
at2g23680	CON	MIN	HOUR	DAY	WEEK	UNIGENE	FLCDNA	TAIR
AATCGCTAGA	0	2	0	0	0	gnl|UG|At#S43850173	gi|17380855|gb|AY063884.1	At2g23680.1
at1g18470	CON	MIN	HOUR	DAY	WEEK	UNIGENE	FLCDNA	TAIR
ATCAAGAGTA	4	4	1	0	0	gnl|UG|At#S14829884	gi|28973677|gb|BT005750.1	At1g18470.1
at3g53030	CON	MIN	HOUR	DAY	WEEK	UNIGENE	FLCDNA	TAIR
AACAAAGTGC	0	2	0	0	0	gnl|UG|At#S11699896	gi|15010661|gb|AY045632.1	At3g53030.1
at4g35750	CON	MIN	HOUR	DAY	WEEK	UNIGENE	FLCDNA	TAIR
AGGTGTATCT	3	66	3	2	0	gnl|UG|At#S11721638	gi|17473777|gb|AY065148.1	At4g35750.1
at5g48160	CON	MIN	HOUR	DAY	WEEK	UNIGENE	FLCDNA	TAIR
TTCCAGCTTA	1	4	1	0	0	gnl|UG|At#S28281657	gi|110742792|dbj|AK227275.1|	At5g48160.1
at3g53490	CON	MIN	HOUR	DAY	WEEK	UNIGENE	FLCDNA	TAIR
GATGCTCACA	1	0	0	1	0	gnl|UG|At#S21736804	gi|51969883|dbj|AK175871.1|	multiple non-canonical match
ATTCTTACTA	7	0	1	2	9	no match found	no match found	At3g53490.1
at4g10450	CON	MIN	HOUR	DAY	WEEK	UNIGENE	FLCDNA	TAIR
TTTGGTCTTT	1	0	3	0	6	gnl|UG|At#S11725920	no match found	At4g10450.1
at5g61000	CON	MIN	HOUR	DAY	WEEK	UNIGENE	FLCDNA	TAIR
TCTTCCGGGT	0	1	0	0	0	gnl|UG|At#S14273999	gi|28393123|gb|BT003950.1	At5g61000.1
at3g01160	CON	MIN	HOUR	DAY	WEEK	UNIGENE	FLCDNA	TAIR
GAATTCAAAC	0	1	2	0	0	gnl|UG|At#S11740319	gi|15529201|gb|AY052225.1	At3g01160.1
at3g13790	CON	MIN	HOUR	DAY	WEEK	UNIGENE	FLCDNA	TAIR
TAAACACATT	0	0	0	0	5	no match found	gi|19310844|gb|AY079422.1	At3g13790.1
at5g64780	CON	MIN	HOUR	DAY	WEEK	UNIGENE	FLCDNA	TAIR
TAATACACAA	2	0	1	0	1	gnl|UG|At#S11743023	gi|21404014|gb|AY085304.1	At5g64780.1
at5g46020	CON	MIN	HOUR	DAY	WEEK	UNIGENE	FLCDNA	TAIR
AGGTTGCAAG	1	9	2	4	3	gnl|UG|At#S11719125	gi|17473781|gb|AY065150.1	At5g46020.1
at5g35630	CON	MIN	HOUR	DAY	WEEK	UNIGENE	FLCDNA	TAIR
AATCTGATGA	56	29	27	36	11	no match found	gi|19698810|gb|AY081252.1	multiple non-canonical match
TCCAATGGTT	12	2	7	3	3	gnl|UG|At#S11720140	no match found	At5g35630.1
at3g54470	CON	MIN	HOUR	DAY	WEEK	UNIGENE	FLCDNA	TAIR
TCTCGTTGAT	1	2	2	2	3	gnl|UG|At#S11816065	gi|22655297|gb|AY140098.1	At3g54470.1
at1g15740	CON	MIN	HOUR	DAY	WEEK	UNIGENE	FLCDNA	TAIR
GCTCTTCTAC	2	6	2	0	0	gnl|UG|At#S11741463	gi|13507546|gb|AF360339.1	non-canonical match
at1g52690	CON	MIN	HOUR	DAY	WEEK	UNIGENE	FLCDNA	TAIR
TAATGGTTTT	3	0	0	4	21	gnl|UG|At#S11821396	gi|23397194|gb|BT000739.1	At1g52690.1
at5g49730	CON	MIN	HOUR	DAY	WEEK	UNIGENE	FLCDNA	TAIR
AATCTCCATA	1	0	1	0	0	gnl|UG|At#S11806614	gi|21281011|gb|AY114002.1	At5g49730.1
at4g33985	CON	MIN	HOUR	DAY	WEEK	UNIGENE	FLCDNA	TAIR
ATGATTAAGG	0	0	1	0	0	gnl|UG|At#S34118206	gi|110736174|dbj|AK228100.1|	At4g33985.1

at5g27930	CON	MIN	HOUR	DAY	WEEK	UNIGENE	FLCDNA	TAIR
ACGATTTTAA	0	1	0	0	1	no match found	gi|26451904|dbj|AK118436.1|	non-canonical match
at1g54340	CON	MIN	HOUR	DAY	WEEK	UNIGENE	FLCDNA	TAIR
AATAAAAGGA	0	0	0	0	1	gnl|UG|At#S34114260	gi|110743806|dbj|AK227755.1|	At1g54340.1
GGAGTTAAAA	0	1	0	0	0	no match found	gi|109134148|gb|BT025983.1	multiple non-canonical match
at4g24830	CON	MIN	HOUR	DAY	WEEK	UNIGENE	FLCDNA	TAIR
TCTTACTTGG	3	4	0	1	2	gnl|UG|At#S11723501	gi|17529035|gb|AY065252.1	At4g24830.1
at5g05400	CON	MIN	HOUR	DAY	WEEK	UNIGENE	FLCDNA	TAIR
GCTGGTTTTT	2	2	1	4	4	gnl|UG|At#S11724785	no match found	multiple non-canonical match
at1g74780	CON	MIN	HOUR	DAY	WEEK	UNIGENE	FLCDNA	TAIR
GTAGCTTATG	0	0	0	1	1	gnl|UG|At#S11728081	gi|110739082|dbj|AK229613.1|	At1g74780.1
at1g76480	CON	MIN	HOUR	DAY	WEEK	UNIGENE	FLCDNA	TAIR
GGAAAGATTC	0	1	0	0	0	gnl|UG|At#S11727731	no match found	multiple non-canonical match
at1g59359	CON	MIN	HOUR	DAY	WEEK	UNIGENE	FLCDNA	TAIR
TTGGTTTGGG	0	0	2	0	0	no match found	gi|22137287|gb|AY133659.1	multiple non-canonical match
at3g61840	CON	MIN	HOUR	DAY	WEEK	UNIGENE	FLCDNA	TAIR
GAAGAACAAG	0	0	3	0	0	gnl|UG|At#S11726899	no match found	multiple canonical match
at5g35410	CON	MIN	HOUR	DAY	WEEK	UNIGENE	FLCDNA	TAIR
GTAGACGTAA	1	0	0	0	0	no match found	gi|51870208|gb|BT015438.1	At5g35410.1
at1g11260	CON	MIN	HOUR	DAY	WEEK	UNIGENE	FLCDNA	TAIR
AAGAAATTAT	14	35	23	7	3	gnl|UG|At#S20800005	gi|16604672|gb|AY059781.1	At1g11260.1
TTTGATTTAT	13	14	6	2	3	no match found	gi|22136869|gb|AY133845.1	non-canonical match
at1g62830	CON	MIN	HOUR	DAY	WEEK	UNIGENE	FLCDNA	TAIR
TCATTGTTGA	0	4	0	0	0	gnl|UG|At#S11730583	gi|62321361|dbj|AK222014.1|	At1g62830.1
at5g20090	CON	MIN	HOUR	DAY	WEEK	UNIGENE	FLCDNA	TAIR
CATCAAACGA	0	1	1	1	0	gnl|UG|At#S43849292	gi|19310836|gb|AY079418.1	non-canonical match
at2g03620	CON	MIN	HOUR	DAY	WEEK	UNIGENE	FLCDNA	TAIR
TAGTAGCTTC	2	1	0	0	1	gnl|UG|At#S43850287	gi|17979300|gb|AY070380.1	At2g03620.1
at4g07507	CON	MIN	HOUR	DAY	WEEK	UNIGENE	FLCDNA	TAIR
AACTTGTTTT	0	1	1	0	1	gnl|UG|At#S18907461	gi|110743154|dbj|AK227467.1|	non-canonical match
at2g39710	CON	MIN	HOUR	DAY	WEEK	UNIGENE	FLCDNA	TAIR
AGAATATCTT	0	0	1	1	0	gnl|UG|At#S11732670	gi|13877758|gb|AF370142.1	At2g39710.1
at5g54490	CON	MIN	HOUR	DAY	WEEK	UNIGENE	FLCDNA	TAIR
TTTAGACTTA	0	1	1	0	0	gnl|UG|At#S22667651	gi|88011163|gb|BT024572.1	At5g54490.1
at2g17110	CON	MIN	HOUR	DAY	WEEK	UNIGENE	FLCDNA	TAIR
CAAGTTACTG	0	2	3	2	1	gnl|UG|At#S11738205	gi|15724285|gb|AF412083.1	At2g17110.1
at1g69980	CON	MIN	HOUR	DAY	WEEK	UNIGENE	FLCDNA	TAIR
TTTGGCTTTG	2	0	2	2	0	gnl|UG|At#S11729103	gi|21403461|gb|AY084751.1	At1g69980.1
at5g17790	CON	MIN	HOUR	DAY	WEEK	UNIGENE	FLCDNA	TAIR
TAGCATAACA	2	0	0	0	0	gnl|UG|At#S11721873	gi|18700081|gb|AY075645.1	At5g17790.1
at5g50130	CON	MIN	HOUR	DAY	WEEK	UNIGENE	FLCDNA	TAIR

TATTTTGAAC	0	0	0	1	0	gnl|UG|At#S11704979	gi|15146201|gb|AY049242.1	At5g50130.1
CCCTTCCTAA	0	1	0	1	0	no match found	gi|19699125|gb|AY090268.1	At5g50130.2

at5g45300	CON	MIN	HOUR	DAY	WEEK	UNIGENE	FLCDNA	TAIR
GTCTTAGGAT	3	0	1	0	0	gnl|UG|At#S11824662	gi|26449381|dbj|AK117140.1|	At5g45300.1
GCGACATAAG	0	0	0	0	1	no match found	gi|30102743|gb|BT006482.1	non-canonical match

at3g13410	CON	MIN	HOUR	DAY	WEEK	UNIGENE	FLCDNA	TAIR
TCTGCAAAGG	1	8	1	0	0	gnl|UG|At#S43850429	gi|14334707|gb|AY035027.1	At3g13410.1

at5g09630	CON	MIN	HOUR	DAY	WEEK	UNIGENE	FLCDNA	TAIR
CAGGCAATTA	0	1	0	0	0	gnl|UG|At#S35175008	gi|21406002|gb|AY087278.1	At5g09630.1

at4g16480	CON	MIN	HOUR	DAY	WEEK	UNIGENE	FLCDNA	TAIR
CTCTCGGCTC	1	1	0	0	0	gnl|UG|At#S11724949	gi|28973604|gb|BT005707.1	At4g16480.1

at4g02630	CON	MIN	HOUR	DAY	WEEK	UNIGENE	FLCDNA	TAIR
GTGGTGGTTT	0	0	1	0	0	gnl|UG|At#S11727033	gi|28393612|gb|BT004208.1	At4g02630.1

at4g38640	CON	MIN	HOUR	DAY	WEEK	UNIGENE	FLCDNA	TAIR
TTCTTGACGA	0	1	1	0	0	gnl|UG|At#S11721163	no match found	At4g38640.1

at1g49840	CON	MIN	HOUR	DAY	WEEK	UNIGENE	FLCDNA	TAIR
ATTGCTCACT	0	0	0	0	1	gnl|UG|At#S34117778	no match found	At1g49840.1

at4g09500	CON	MIN	HOUR	DAY	WEEK	UNIGENE	FLCDNA	TAIR
CCTTAGCAAC	0	1	0	0	0	gnl|UG|At#S18941888	gi|51870316|gb|BT015542.1	At4g09500.1

at2g25355	CON	MIN	HOUR	DAY	WEEK	UNIGENE	FLCDNA	TAIR
GTAGAGAAAT	1	1	0	0	0	gnl|UG|At#S11736182	gi|23308266|gb|BT000534.1	At2g25355.1

at2g38480	CON	MIN	HOUR	DAY	WEEK	UNIGENE	FLCDNA	TAIR
GCGTAACCAA	0	0	1	1	0	no match found	no match found	At2g38480.1

at1g57990	CON	MIN	HOUR	DAY	WEEK	UNIGENE	FLCDNA	TAIR
TTGTATAGAT	5	13	7	0	1	gnl|UG|At#S11708307	gi|17381085|gb|AY063999.1	At1g57990.1

at1g72010	CON	MIN	HOUR	DAY	WEEK	UNIGENE	FLCDNA	TAIR
CTTGAATGAC	0	1	1	0	0	gnl|UG|At#S11728685	no match found	At1g72010.1

at4g04180	CON	MIN	HOUR	DAY	WEEK	UNIGENE	FLCDNA	TAIR
GGCTTCAAAG	0	0	1	0	0	gnl|UG|At#S14273729	gi|28393635|gb|BT004220.1	At4g04180.1

at2g04870	CON	MIN	HOUR	DAY	WEEK	UNIGENE	FLCDNA	TAIR
AATCCAAAAG	0	0	1	0	0	no match found	no match found	At2g04870.1

at4g01090	CON	MIN	HOUR	DAY	WEEK	UNIGENE	FLCDNA	TAIR
AAAACTCCCC	0	2	1	0	0	gnl|UG|At#S11727414	gi|17979013|gb|AY069918.1	At4g01090.1

at5g49760	CON	MIN	HOUR	DAY	WEEK	UNIGENE	FLCDNA	TAIR
ATTCTGTTTG	4	1	0	2	4	no match found	gi|26449347|dbj|AK117123.1|	At5g49760.1

at3g29575	CON	MIN	HOUR	DAY	WEEK	UNIGENE	FLCDNA	TAIR
CTGGTGGTGG	0	0	0	1	0	no match found	gi|22137321|gb|AY133676.1	multiple non-canonical match
TTACAAATCC	2	0	0	2	0	gnl|UG|At#S11731801	gi|14326573|gb|AF385741.1	At3g29575.1

at3g26840	CON	MIN	HOUR	DAY	WEEK	UNIGENE	FLCDNA	TAIR
AACCTACCTA	1	0	0	0	0	gnl|UG|At#S11701982	gi|13430465|gb|AF360145.1	At3g26840.1

at3g52950	CON	MIN	HOUR	DAY	WEEK	UNIGENE	FLCDNA	TAIR
TTTTGCGAAT	1	1	4	1	2	gnl|UG|At#S11728882	gi|22136009|gb|AY128384.1	At3g52950.1

at3g53750	CON	MIN	HOUR	DAY	WEEK	UNIGENE	FLCDNA	TAIR
AAGATCAAAG	3	0	1	0	0	gnl|UG|At#S11728737	no match found	At3g53750.1
at1g18380	CON	MIN	HOUR	DAY	WEEK	UNIGENE	FLCDNA	TAIR
GAAGACAAAG	0	2	0	0	0	gnl|UG|At#S22484646	gi|20258803|gb|AY090931.1	multiple non-canonical match
at5g27430	CON	MIN	HOUR	DAY	WEEK	UNIGENE	FLCDNA	TAIR
AAGTTAAAAA	1	2	0	2	2	gnl|UG|At#S11720469	gi|28393473|gb|BT004137.1	At5g27430.1
at1g01040	CON	MIN	HOUR	DAY	WEEK	UNIGENE	FLCDNA	TAIR
CAAAGAGATT	0	2	1	1	0	gnl|UG|At#S11743010	gi|62320293|dbj|AK221473.1|	At1g01040.1
at5g08080	CON	MIN	HOUR	DAY	WEEK	UNIGENE	FLCDNA	TAIR
ATGCTGTCAG	0	6	2	0	0	gnl|UG|At#S28281834	gi|21436144|gb|AY117244.1	At5g08080.1
at1g66750	CON	MIN	HOUR	DAY	WEEK	UNIGENE	FLCDNA	TAIR
TATCAACTAT	2	1	1	1	2	no match found	gi|22531033|gb|AY136355.1	At1g66750.1
at3g06700	CON	MIN	HOUR	DAY	WEEK	UNIGENE	FLCDNA	TAIR
TTTACGCAAT	21	18	19	28	41	gnl|UG|At#S20705142	gi|21405807|gb|AY087083.1	At3g06700.1
at3g07400	CON	MIN	HOUR	DAY	WEEK	UNIGENE	FLCDNA	TAIR
TTTTCGCCAT	1	1	0	0	0	gnl|UG|At#S11738512	no match found	At3g07400.1
at1g11330	CON	MIN	HOUR	DAY	WEEK	UNIGENE	FLCDNA	TAIR
AGCCTCGCCG	0	1	0	1	1	gnl|UG|At#S11741902	gi|110739550|dbj|AK229855.1|	At1g11330.1
at2g40880	CON	MIN	HOUR	DAY	WEEK	UNIGENE	FLCDNA	TAIR
GATGAACTTC	5	8	4	5	2	gnl|UG|At#S11732371	gi|21403479|gb|AY084769.1	At2g40880.1
at1g10020	CON	MIN	HOUR	DAY	WEEK	UNIGENE	FLCDNA	TAIR
AGATTGTTGC	2	0	0	0	0	gnl|UG|At#S11742032	gi|110738095|dbj|AK229103.1|	At1g10020.1
AGTCACTGAG	0	2	0	0	0	no match found	gi|40823448|gb|BT011247.1	non-canonical match
at1g28200	CON	MIN	HOUR	DAY	WEEK	UNIGENE	FLCDNA	TAIR
GTCCGTGAAG	0	1	1	1	2	no match found	gi|21405718|gb|AY086994.1	non-canonical match
ATTTGTATTC	0	0	0	1	0	gnl|UG|At#S11739559	no match found	At1g28200.1
at4g02405	CON	MIN	HOUR	DAY	WEEK	UNIGENE	FLCDNA	TAIR
GAGACAAGAA	1	0	0	0	0	gnl|UG|At#S11704396	gi|14596176|gb|AY042876.1	non-canonical match
at3g30766	CON	MIN	HOUR	DAY	WEEK	UNIGENE	FLCDNA	TAIR
TTTGGGATGC	0	0	0	1	0	no match found	no match found	At3g30766.1
at2g17840	CON	MIN	HOUR	DAY	WEEK	UNIGENE	FLCDNA	TAIR
TAATGTCTCT	2	6	5	3	3	no match found	gi|16226783|gb|AF428331.1	non-canonical match
at5g13540	CON	MIN	HOUR	DAY	WEEK	UNIGENE	FLCDNA	TAIR
TACGGACAGA	1	1	1	2	3	no match found	no match found	At5g13540.2
TTGTGGGAGT	1	0	0	0	0	gnl|UG|At#S11722963	no match found	At5g13540.1
at5g03170	CON	MIN	HOUR	DAY	WEEK	UNIGENE	FLCDNA	TAIR
TTTGGATCAA	0	0	2	0	0	gnl|UG|At#S11702020	gi|14532749|gb|AY039999.1	At5g03170.1
at5g10490	CON	MIN	HOUR	DAY	WEEK	UNIGENE	FLCDNA	TAIR
GAAACAGATG	0	2	2	1	0	gnl|UG|At#S11723619	gi|62321005|dbj|AK221831.1|	At5g10490.1
at5g23840	CON	MIN	HOUR	DAY	WEEK	UNIGENE	FLCDNA	TAIR
TCTTTTCTTT	0	3	0	0	0	gnl|UG|At#S14273659	gi|21407017|gb|AY088243.1	At5g23840.2

at5g07980	CON	MIN	HOUR	DAY	WEEK	UNIGENE	FLCDNA	TAIR
TAGCTCCAGT	0	1	0	0	0	gnl|UG|At#S11724124	no match found	multiple non-canonical match

at5g64570	CON	MIN	HOUR	DAY	WEEK	UNIGENE	FLCDNA	TAIR
TCGGAGATTT	12	44	16	3	0	gnl|UG|At#S34115437	gi|19423932|gb|AY080756.1	At5g64570.1
AGACCCGATA	0	0	1	1	1	gnl|UG|At#S18907095	no match found	non-canonical match

at2g22990	CON	MIN	HOUR	DAY	WEEK	UNIGENE	FLCDNA	TAIR
AGTTTCTTCA	0	0	1	0	0	no match found	gi|23505918|gb|AY143880.1	multiple non-canonical match

at1g34640	CON	MIN	HOUR	DAY	WEEK	UNIGENE	FLCDNA	TAIR
TAAGACTACC	1	3	0	2	0	gnl|UG|At#S11737346	no match found	non-canonical match

at3g58610	CON	MIN	HOUR	DAY	WEEK	UNIGENE	FLCDNA	TAIR
GTGCCATTGA	9	16	11	11	3	gnl|UG|At#S11727839	gi|23463054|gb|BT000822.1	At3g58610.1

at2g30910	CON	MIN	HOUR	DAY	WEEK	UNIGENE	FLCDNA	TAIR
GAACAAGAAC	0	1	0	0	0	no match found	gi|28416662|gb|BT004616.1	multiple non-canonical match
ATTTGATTCA	1	0	2	0	0	gnl|UG|At#S11734811	gi|110743302|dbj|AK227543.1|	At2g30910.1

at2g36360	CON	MIN	HOUR	DAY	WEEK	UNIGENE	FLCDNA	TAIR
TATCTCTTTG	1	1	0	0	1	no match found	no match found	At2g36360.1

at1g17190	CON	MIN	HOUR	DAY	WEEK	UNIGENE	FLCDNA	TAIR
TGAGGTTTGT	1	0	1	0	1	no match found	no match found	At1g17190.1

at1g08135	CON	MIN	HOUR	DAY	WEEK	UNIGENE	FLCDNA	TAIR
AAGACCAAAG	50	36	55	45	36	gnl|UG|At#S15461411	no match found	At1g02560.1

at1g10480	CON	MIN	HOUR	DAY	WEEK	UNIGENE	FLCDNA	TAIR
ATTCAGTTCC	0	1	0	0	0	no match found	gi|89111901|gb|BT024840.1	multiple non-canonical match

at3g07760	CON	MIN	HOUR	DAY	WEEK	UNIGENE	FLCDNA	TAIR
CACTAGGAAG	4	3	0	4	0	no match found	gi|15451021|gb|AY054591.1	At3g07760.1
GTGGGCACCG	1	0	0	0	0	no match found	gi|47679039|gb|BT014708.1	non-canonical match

at3g48750	CON	MIN	HOUR	DAY	WEEK	UNIGENE	FLCDNA	TAIR
CCTTAGAAAG	3	4	3	2	1	gnl|UG|At#S11729688	gi|21403863|gb|AY085153.1	At3g48750.1

at5g27970	CON	MIN	HOUR	DAY	WEEK	UNIGENE	FLCDNA	TAIR
CTTACAAAAA	0	0	1	0	0	gnl|UG|At#S11720414	gi|17065077|gb|AY062615.1	At5g27970.1

at4g26970	CON	MIN	HOUR	DAY	WEEK	UNIGENE	FLCDNA	TAIR
AACGCTACAC	1	3	1	0	0	gnl|UG|At#S11723132	gi|15215803|gb|AY050431.1	non-canonical match

at2g40680	CON	MIN	HOUR	DAY	WEEK	UNIGENE	FLCDNA	TAIR
ATTTTTTTGA	1	0	0	0	0	gnl|UG|At#S35247895	no match found	multiple non-canonical match

at2g22125	CON	MIN	HOUR	DAY	WEEK	UNIGENE	FLCDNA	TAIR
CAAGAACAAA	0	3	1	2	0	gnl|UG|At#S11736941	gi|110742711|dbj|AK227230.1|	At2g22125.1

at5g66380	CON	MIN	HOUR	DAY	WEEK	UNIGENE	FLCDNA	TAIR
TCATCAGAGA	0	3	0	0	0	gnl|UG|At#S18909358	gi|33589683|gb|BT010139.1	At1g58040.1

at3g05510	CON	MIN	HOUR	DAY	WEEK	UNIGENE	FLCDNA	TAIR
AACTCTTATC	1	0	0	0	1	gnl|UG|At#S11739078	gi|110736431|dbj|AK228236.1|	At3g05510.2

at2g35860	CON	MIN	HOUR	DAY	WEEK	UNIGENE	FLCDNA	TAIR
TTCTATTTGT	4	1	0	0	0	gnl|UG|At#S15460735	gi|20260579|gb|AY093189.1	At2g35860.1

at5g56260	CON	MIN	HOUR	DAY	WEEK	UNIGENE	FLCDNA	TAIR

<table>
<tr><td>GTGAGAAGAA</td><td>0</td><td>1</td><td>0</td><td>2</td><td>0</td><td>no match found</td><td>gi|38603849|gb|BT010892.1</td><td>multiple non-canonical match</td></tr>
<tr><td>at5g58450</td><td>CON</td><td>MIN</td><td>HOUR</td><td>DAY</td><td>WEEK</td><td>UNIGENE</td><td>FLCDNA</td><td>TAIR</td></tr>
<tr><td>ACAGGGACAG</td><td>1</td><td>0</td><td>0</td><td>1</td><td>0</td><td>gnl|UG|At#S11717864</td><td>gi|28392920|gb|BT003846.1</td><td>At5g58450.1</td></tr>
<tr><td>at1g14870</td><td>CON</td><td>MIN</td><td>HOUR</td><td>DAY</td><td>WEEK</td><td>UNIGENE</td><td>FLCDNA</td><td>TAIR</td></tr>
<tr><td>ACCCGCTAAG</td><td>1</td><td>5</td><td>2</td><td>1</td><td>2</td><td>gnl|UG|At#S11741547</td><td>no match found</td><td>At1g14870.1</td></tr>
<tr><td>at1g73940</td><td>CON</td><td>MIN</td><td>HOUR</td><td>DAY</td><td>WEEK</td><td>UNIGENE</td><td>FLCDNA</td><td>TAIR</td></tr>
<tr><td>GTCCTCTCTC</td><td>0</td><td>2</td><td>0</td><td>0</td><td>0</td><td>gnl|UG|At#S30525519</td><td>gi|16323092|gb|AY057650.1</td><td>At1g73940.1</td></tr>
<tr><td>at4g35570</td><td>CON</td><td>MIN</td><td>HOUR</td><td>DAY</td><td>WEEK</td><td>UNIGENE</td><td>FLCDNA</td><td>TAIR</td></tr>
<tr><td>GAACTGGCTA</td><td>1</td><td>5</td><td>4</td><td>0</td><td>2</td><td>no match found</td><td>gi|27754658|gb|BT002963.1</td><td>At4g35570.1</td></tr>
<tr><td>at1g20870</td><td>CON</td><td>MIN</td><td>HOUR</td><td>DAY</td><td>WEEK</td><td>UNIGENE</td><td>FLCDNA</td><td>TAIR</td></tr>
<tr><td>ATTTTGCTAA</td><td>1</td><td>0</td><td>1</td><td>1</td><td>0</td><td>gnl|UG|At#S11740970</td><td>no match found</td><td>At1g20870.1</td></tr>
<tr><td>at5g61150</td><td>CON</td><td>MIN</td><td>HOUR</td><td>DAY</td><td>WEEK</td><td>UNIGENE</td><td>FLCDNA</td><td>TAIR</td></tr>
<tr><td>TAATAATGGA</td><td>1</td><td>1</td><td>0</td><td>1</td><td>2</td><td>gnl|UG|At#S15443007</td><td>gi|25083386|gb|BT002058.1</td><td>At5g61150.1</td></tr>
<tr><td>at4g06568</td><td>CON</td><td>MIN</td><td>HOUR</td><td>DAY</td><td>WEEK</td><td>UNIGENE</td><td>FLCDNA</td><td>TAIR</td></tr>
<tr><td>GAGGAGACTC</td><td>0</td><td>0</td><td>0</td><td>0</td><td>1</td><td>no match found</td><td>no match found</td><td>At4g06568.1</td></tr>
<tr><td>at4g18250</td><td>CON</td><td>MIN</td><td>HOUR</td><td>DAY</td><td>WEEK</td><td>UNIGENE</td><td>FLCDNA</td><td>TAIR</td></tr>
<tr><td>TGTAAGTATC</td><td>0</td><td>1</td><td>0</td><td>0</td><td>0</td><td>no match found</td><td>no match found</td><td>At4g18250.1</td></tr>
<tr><td>at4g36945</td><td>CON</td><td>MIN</td><td>HOUR</td><td>DAY</td><td>WEEK</td><td>UNIGENE</td><td>FLCDNA</td><td>TAIR</td></tr>
<tr><td>TGGTTGTGAC</td><td>0</td><td>1</td><td>0</td><td>0</td><td>0</td><td>gnl|UG|At#S38433383</td><td>gi|62320760|dbj|AK221707.1|</td><td>At4g36950.1</td></tr>
<tr><td>at3g46440</td><td>CON</td><td>MIN</td><td>HOUR</td><td>DAY</td><td>WEEK</td><td>UNIGENE</td><td>FLCDNA</td><td>TAIR</td></tr>
<tr><td>GTGCAAGCGG</td><td>0</td><td>3</td><td>3</td><td>1</td><td>2</td><td>gnl|UG|At#S28282856</td><td>gi|110737126|dbj|AK228600.1|</td><td>At3g46440.1</td></tr>
<tr><td>at3g05690</td><td>CON</td><td>MIN</td><td>HOUR</td><td>DAY</td><td>WEEK</td><td>UNIGENE</td><td>FLCDNA</td><td>TAIR</td></tr>
<tr><td>GTAATGCCTA</td><td>0</td><td>0</td><td>0</td><td>1</td><td>0</td><td>gnl|UG|At#S11739012</td><td>gi|109946478|gb|BT026062.1</td><td>At3g05690.1</td></tr>
<tr><td>at1g47420</td><td>CON</td><td>MIN</td><td>HOUR</td><td>DAY</td><td>WEEK</td><td>UNIGENE</td><td>FLCDNA</td><td>TAIR</td></tr>
<tr><td>CCTGAAATGG</td><td>0</td><td>2</td><td>0</td><td>1</td><td>0</td><td>no match found</td><td>gi|17104550|gb|AY062990.1</td><td>non-canonical match</td></tr>
<tr><td>AATTTGTGTT</td><td>13</td><td>18</td><td>7</td><td>10</td><td>5</td><td>gnl|UG|At#S11735448</td><td>gi|21403091|gb|AY084381.1</td><td>At1g47420.1</td></tr>
<tr><td>at2g17550</td><td>CON</td><td>MIN</td><td>HOUR</td><td>DAY</td><td>WEEK</td><td>UNIGENE</td><td>FLCDNA</td><td>TAIR</td></tr>
<tr><td>GTAACTGTAT</td><td>2</td><td>0</td><td>0</td><td>0</td><td>0</td><td>gnl|UG|At#S34115578</td><td>gi|110741236|dbj|AK230370.1|</td><td>At2g17550.1</td></tr>
<tr><td>at4g16150</td><td>CON</td><td>MIN</td><td>HOUR</td><td>DAY</td><td>WEEK</td><td>UNIGENE</td><td>FLCDNA</td><td>TAIR</td></tr>
<tr><td>AAGAAGCTCA</td><td>0</td><td>3</td><td>1</td><td>1</td><td>0</td><td>no match found</td><td>gi|22135833|gb|AY128295.1</td><td>At3g16940.1</td></tr>
<tr><td>at3g56160</td><td>CON</td><td>MIN</td><td>HOUR</td><td>DAY</td><td>WEEK</td><td>UNIGENE</td><td>FLCDNA</td><td>TAIR</td></tr>
<tr><td>ATTGATTCAG</td><td>0</td><td>1</td><td>1</td><td>0</td><td>0</td><td>gnl|UG|At#S11728303</td><td>no match found</td><td>At3g56160.1</td></tr>
<tr><td>at4g00590</td><td>CON</td><td>MIN</td><td>HOUR</td><td>DAY</td><td>WEEK</td><td>UNIGENE</td><td>FLCDNA</td><td>TAIR</td></tr>
<tr><td>GGACACACCA</td><td>0</td><td>0</td><td>1</td><td>0</td><td>0</td><td>gnl|UG|At#S18907418</td><td>gi|19423929|gb|AY080861.1</td><td>At4g00590.1</td></tr>
<tr><td>at5g27650</td><td>CON</td><td>MIN</td><td>HOUR</td><td>DAY</td><td>WEEK</td><td>UNIGENE</td><td>FLCDNA</td><td>TAIR</td></tr>
<tr><td>TATTACAATT</td><td>0</td><td>0</td><td>1</td><td>0</td><td>0</td><td>gnl|UG|At#S11720447</td><td>no match found</td><td>At5g27650.1</td></tr>
<tr><td>at5g35210</td><td>CON</td><td>MIN</td><td>HOUR</td><td>DAY</td><td>WEEK</td><td>UNIGENE</td><td>FLCDNA</td><td>TAIR</td></tr>
<tr><td>TATTGGTGAT</td><td>3</td><td>0</td><td>0</td><td>0</td><td>0</td><td>no match found</td><td>gi|62320813|dbj|AK221734.1|</td><td>non-canonical match</td></tr>
<tr><td>TAGGTACACT</td><td>1</td><td>1</td><td>0</td><td>0</td><td>1</td><td>gnl|UG|At#S11720182</td><td>gi|30793981|gb|BT008619.1</td><td>At5g35210.1</td></tr>
<tr><td>at2g14880</td><td>CON</td><td>MIN</td><td>HOUR</td><td>DAY</td><td>WEEK</td><td>UNIGENE</td><td>FLCDNA</td><td>TAIR</td></tr>
<tr><td>AAAAGAGAAG</td><td>4</td><td>0</td><td>1</td><td>1</td><td>2</td><td>no match found</td><td>gi|21406909|gb|AY088135.1</td><td>multiple non-canonical match</td></tr>
<tr><td>TACATACTCC</td><td>0</td><td>0</td><td>0</td><td>1</td><td>0</td><td>gnl|UG|At#S11738743</td><td>no match found</td><td>At2g14880.1</td></tr>
</table>

TTTTTCCGTT	3	0	3	3	2	no match found	gi|19698858|gb|AY081276.1	non-canonical match
at2g26975	CON	MIN	HOUR	DAY	WEEK	UNIGENE	FLCDNA	TAIR
CTCTTCGGAA	2	3	4	2	3	gnl|UG|At#S15461106	gi|26452465|dbj|AK118724.1|	At2g26975.1
at1g65910	CON	MIN	HOUR	DAY	WEEK	UNIGENE	FLCDNA	TAIR
ATGGAAAATG	0	0	1	0	0	gnl|UG|At#S18897726	no match found	At1g65910.1
at5g01980	CON	MIN	HOUR	DAY	WEEK	UNIGENE	FLCDNA	TAIR
CTCATTGTAT	0	1	0	0	0	no match found	gi|51970977|dbj|AK176418.1|	At5g01980.1
at1g60950	CON	MIN	HOUR	DAY	WEEK	UNIGENE	FLCDNA	TAIR
GCTACATACA	2	0	3	0	1	gnl|UG|At#S11687700	no match found	non-canonical match
TCTTTATAAT	0	0	0	1	0	gnl|UG|At#S30653240	no match found	non-canonical match
at2g26250	CON	MIN	HOUR	DAY	WEEK	UNIGENE	FLCDNA	TAIR
GAGGCCAAGG	4	17	9	5	3	gnl|UG|At#S11821333	gi|23397315|gb|BT000804.1	At2g26250.1
at5g24810	CON	MIN	HOUR	DAY	WEEK	UNIGENE	FLCDNA	TAIR
CTTCAGTTTC	5	1	2	3	2	gnl|UG|At#S11704918	gi|15081730|gb|AY048258.1	At5g24810.1
at1g12240	CON	MIN	HOUR	DAY	WEEK	UNIGENE	FLCDNA	TAIR
TAATATATTA	1	1	0	0	0	gnl|UG|At#S11741812	no match found	At1g12240.1
TATAACATCA	0	1	0	0	0	no match found	gi|26451949|dbj|AK118459.1|	non-canonical match
at2g40760	CON	MIN	HOUR	DAY	WEEK	UNIGENE	FLCDNA	TAIR
GAAGCTCCAG	0	1	0	0	0	gnl|UG|At#S11732402	gi|62321638|dbj|AK222157.1|	At2g40760.1
at1g35450	CON	MIN	HOUR	DAY	WEEK	UNIGENE	FLCDNA	TAIR
TTCTTTTCAA	0	0	0	1	0	no match found	no match found	At1g35450.1
at5g27000	CON	MIN	HOUR	DAY	WEEK	UNIGENE	FLCDNA	TAIR
AACACACCAG	1	0	0	0	0	gnl|UG|At#S11700826	no match found	At5g27000.1
at5g67480	CON	MIN	HOUR	DAY	WEEK	UNIGENE	FLCDNA	TAIR
CAACGTGTAA	0	6	3	0	1	gnl|UG|At#S18907766	gi|17386119|gb|AF446873.1	At5g67480.1
at4g09810	CON	MIN	HOUR	DAY	WEEK	UNIGENE	FLCDNA	TAIR
ATCATTGCAG	1	0	0	0	0	gnl|UG|At#S34115114	gi|110742097|dbj|AK226903.1|	At4g09810.1
at2g28950	CON	MIN	HOUR	DAY	WEEK	UNIGENE	FLCDNA	TAIR
GGAAAGAACT	1	3	1	0	0	no match found	gi|21404480|gb|AY085770.1	multiple non-canonical match
GGTTTGTGTT	4	2	3	1	0	gnl|UG|At#S11735297	gi|16648831|gb|AY058193.1	At2g28950.1
at3g12950	CON	MIN	HOUR	DAY	WEEK	UNIGENE	FLCDNA	TAIR
GCAATCGAAC	0	0	1	0	1	gnl|UG|At#S11737012	gi|45773813|gb|BT012224.1	At3g12950.1
at2g01600	CON	MIN	HOUR	DAY	WEEK	UNIGENE	FLCDNA	TAIR
ATTGGTTTGT	6	5	5	4	1	gnl|UG|At#S11742644	gi|20465611|gb|AY096639.1	At2g01600.1
at1g65590	CON	MIN	HOUR	DAY	WEEK	UNIGENE	FLCDNA	TAIR
TATAAAAGGC	3	2	0	1	0	gnl|UG|At#S11730007	gi|22135810|gb|AY128283.1	At1g65590.1
TGGGGTGAAC	0	0	1	0	0	no match found	gi|23463072|gb|BT000831.1	non-canonical match
at3g62840	CON	MIN	HOUR	DAY	WEEK	UNIGENE	FLCDNA	TAIR
AATAGTAGTA	1	0	0	0	1	gnl|UG|At#S11726589	no match found	non-canonical match
at1g04620	CON	MIN	HOUR	DAY	WEEK	UNIGENE	FLCDNA	TAIR
TAAGTTTCCA	0	1	4	1	2	no match found	gi|25083090|gb|BT002029.1	At1g04620.1
at1g33260	CON	MIN	HOUR	DAY	WEEK	UNIGENE	FLCDNA	TAIR

<table>
<tr><td>TGATTTGCAT</td><td>0</td><td>1</td><td>0</td><td>0</td><td>1</td><td>gnl|UG|At#S34114486</td><td>gi|110743276|dbj|AK227529.1|</td><td>At1g33260.1</td></tr>
<tr><td>at5g24590</td><td>CON</td><td>MIN</td><td>HOUR</td><td>DAY</td><td>WEEK</td><td>UNIGENE</td><td>FLCDNA</td><td>TAIR</td></tr>
<tr><td>TACGACGACT</td><td>1</td><td>5</td><td>7</td><td>0</td><td>0</td><td>gnl|UG|At#S11701416</td><td>gi|25082961|gb|BT002012.1</td><td>At5g24590.2</td></tr>
<tr><td>at1g68580</td><td>CON</td><td>MIN</td><td>HOUR</td><td>DAY</td><td>WEEK</td><td>UNIGENE</td><td>FLCDNA</td><td>TAIR</td></tr>
<tr><td>TAACCTATGT</td><td>2</td><td>2</td><td>0</td><td>0</td><td>1</td><td>gnl|UG|At#S11729381</td><td>gi|22135895|gb|AY128327.1</td><td>At1g68580.2</td></tr>
<tr><td>at5g16830</td><td>CON</td><td>MIN</td><td>HOUR</td><td>DAY</td><td>WEEK</td><td>UNIGENE</td><td>FLCDNA</td><td>TAIR</td></tr>
<tr><td>CCTACTTATT</td><td>0</td><td>1</td><td>0</td><td>0</td><td>0</td><td>no match found</td><td>gi|90962973|gb|BT025036.1</td><td>non-canonical match</td></tr>
<tr><td>ATCAAAAGAA</td><td>5</td><td>2</td><td>3</td><td>5</td><td>1</td><td>gnl|UG|At#S11722131</td><td>gi|15724188|gb|AF411797.1</td><td>At5g16830.1</td></tr>
<tr><td>at5g06270</td><td>CON</td><td>MIN</td><td>HOUR</td><td>DAY</td><td>WEEK</td><td>UNIGENE</td><td>FLCDNA</td><td>TAIR</td></tr>
<tr><td>AAAACGCCAC</td><td>1</td><td>0</td><td>0</td><td>0</td><td>0</td><td>no match found</td><td>gi|28827635|gb|BT005129.1</td><td>non-canonical match</td></tr>
<tr><td>TGTCATTGTC</td><td>1</td><td>0</td><td>0</td><td>1</td><td>0</td><td>no match found</td><td>gi|21403036|gb|AY084326.1</td><td>At5g06270.1</td></tr>
<tr><td>at1g06690</td><td>CON</td><td>MIN</td><td>HOUR</td><td>DAY</td><td>WEEK</td><td>UNIGENE</td><td>FLCDNA</td><td>TAIR</td></tr>
<tr><td>GCGATTTGGT</td><td>0</td><td>0</td><td>0</td><td>0</td><td>1</td><td>gnl|UG|At#S11742362</td><td>gi|15215593|gb|AY050325.1</td><td>At1g06690.1</td></tr>
<tr><td>at1g78260</td><td>CON</td><td>MIN</td><td>HOUR</td><td>DAY</td><td>WEEK</td><td>UNIGENE</td><td>FLCDNA</td><td>TAIR</td></tr>
<tr><td>CCTTCTCCAT</td><td>0</td><td>1</td><td>0</td><td>0</td><td>0</td><td>no match found</td><td>gi|124300975|gb|BT030002.1</td><td>non-canonical match</td></tr>
<tr><td>at1g21560</td><td>CON</td><td>MIN</td><td>HOUR</td><td>DAY</td><td>WEEK</td><td>UNIGENE</td><td>FLCDNA</td><td>TAIR</td></tr>
<tr><td>ACTCAAGACT</td><td>0</td><td>0</td><td>0</td><td>1</td><td>1</td><td>no match found</td><td>gi|51969117|dbj|AK175488.1|</td><td>non-canonical match</td></tr>
<tr><td>at5g63030</td><td>CON</td><td>MIN</td><td>HOUR</td><td>DAY</td><td>WEEK</td><td>UNIGENE</td><td>FLCDNA</td><td>TAIR</td></tr>
<tr><td>TTGGTGTTTC</td><td>9</td><td>7</td><td>13</td><td>3</td><td>3</td><td>gnl|UG|At#S11717398</td><td>gi|21407599|gb|AY088825.1</td><td>multiple canonical match</td></tr>
<tr><td>at2g21150</td><td>CON</td><td>MIN</td><td>HOUR</td><td>DAY</td><td>WEEK</td><td>UNIGENE</td><td>FLCDNA</td><td>TAIR</td></tr>
<tr><td>GCATTTTTAA</td><td>0</td><td>1</td><td>1</td><td>2</td><td>0</td><td>gnl|UG|At#S11744291</td><td>gi|19424022|gb|AY080816.1</td><td>At2g21150.1</td></tr>
<tr><td>at2g44050</td><td>CON</td><td>MIN</td><td>HOUR</td><td>DAY</td><td>WEEK</td><td>UNIGENE</td><td>FLCDNA</td><td>TAIR</td></tr>
<tr><td>ATCACGTATG</td><td>1</td><td>0</td><td>1</td><td>2</td><td>0</td><td>gnl|UG|At#S11731620</td><td>gi|15292958|gb|AY050913.1</td><td>At2g44050.1</td></tr>
<tr><td>at3g62410</td><td>CON</td><td>MIN</td><td>HOUR</td><td>DAY</td><td>WEEK</td><td>UNIGENE</td><td>FLCDNA</td><td>TAIR</td></tr>
<tr><td>TCTTTGAATC</td><td>16</td><td>10</td><td>18</td><td>5</td><td>7</td><td>gnl|UG|At#S20525059</td><td>gi|20465621|gb|AY096645.1</td><td>At3g62410.1</td></tr>
<tr><td>at5g37510</td><td>CON</td><td>MIN</td><td>HOUR</td><td>DAY</td><td>WEEK</td><td>UNIGENE</td><td>FLCDNA</td><td>TAIR</td></tr>
<tr><td>GAGACAAAGC</td><td>1</td><td>0</td><td>0</td><td>0</td><td>0</td><td>no match found</td><td>gi|24111440|gb|BT001090.1</td><td>multiple non-canonical match</td></tr>
<tr><td>GCGATATATA</td><td>2</td><td>1</td><td>1</td><td>2</td><td>1</td><td>gnl|UG|At#S15460120</td><td>gi|15810150|gb|AY056140.1</td><td>At5g37510.2</td></tr>
<tr><td>at1g26630</td><td>CON</td><td>MIN</td><td>HOUR</td><td>DAY</td><td>WEEK</td><td>UNIGENE</td><td>FLCDNA</td><td>TAIR</td></tr>
<tr><td>GGAGAGGAGC</td><td>17</td><td>41</td><td>32</td><td>31</td><td>11</td><td>no match found</td><td>gi|21403537|gb|AY084827.1</td><td>non-canonical match</td></tr>
<tr><td>ATATTCATTG</td><td>6</td><td>0</td><td>2</td><td>5</td><td>11</td><td>gnl|UG|At#S38434287</td><td>no match found</td><td>At1g26630.1</td></tr>
<tr><td>at5g16290</td><td>CON</td><td>MIN</td><td>HOUR</td><td>DAY</td><td>WEEK</td><td>UNIGENE</td><td>FLCDNA</td><td>TAIR</td></tr>
<tr><td>GCGCTTCGAG</td><td>1</td><td>2</td><td>5</td><td>4</td><td>1</td><td>gnl|UG|At#S15460818</td><td>gi|63003743|gb|BT021989.1</td><td>At5g16290.2</td></tr>
<tr><td>at4g15510</td><td>CON</td><td>MIN</td><td>HOUR</td><td>DAY</td><td>WEEK</td><td>UNIGENE</td><td>FLCDNA</td><td>TAIR</td></tr>
<tr><td>GATTCGTTTA</td><td>1</td><td>2</td><td>4</td><td>0</td><td>4</td><td>gnl|UG|At#S18941857</td><td>gi|21403724|gb|AY085014.1</td><td>At4g15510.1</td></tr>
<tr><td>at3g05900</td><td>CON</td><td>MIN</td><td>HOUR</td><td>DAY</td><td>WEEK</td><td>UNIGENE</td><td>FLCDNA</td><td>TAIR</td></tr>
<tr><td>TAGTCGAAGA</td><td>1</td><td>5</td><td>2</td><td>0</td><td>0</td><td>gnl|UG|At#S11738954</td><td>gi|110741192|dbj|AK230348.1|</td><td>At3g05900.1</td></tr>
<tr><td>at1g27650</td><td>CON</td><td>MIN</td><td>HOUR</td><td>DAY</td><td>WEEK</td><td>UNIGENE</td><td>FLCDNA</td><td>TAIR</td></tr>
<tr><td>GAAGTGGAAA</td><td>0</td><td>1</td><td>0</td><td>1</td><td>0</td><td>gnl|UG|At#S11739761</td><td>gi|12744990|gb|AF344324.1</td><td>At1g27650.1</td></tr>
<tr><td>at1g22885</td><td>CON</td><td>MIN</td><td>HOUR</td><td>DAY</td><td>WEEK</td><td>UNIGENE</td><td>FLCDNA</td><td>TAIR</td></tr>
<tr><td>TATTTATCAT</td><td>2</td><td>0</td><td>0</td><td>0</td><td>0</td><td>gnl|UG|At#S30648569</td><td>gi|21404403|gb|AY085693.1</td><td>At1g22885.1</td></tr>
<tr><td>at1g04040</td><td>CON</td><td>MIN</td><td>HOUR</td><td>DAY</td><td>WEEK</td><td>UNIGENE</td><td>FLCDNA</td><td>TAIR</td></tr>
</table>

AAGAAATTGT	1	5	2	1	0	gnl|UG|At#S11742675	gi|13926197|gb|AF370572.1	At1g04040.1
at3g59540	CON	MIN	HOUR	DAY	WEEK	UNIGENE	FLCDNA	TAIR
GTTTTATATA	6	4	2	2	25	gnl|UG|At#S17006805	gi|13605719|gb|AF361841.1	At3g59540.1
at1g17490	CON	MIN	HOUR	DAY	WEEK	UNIGENE	FLCDNA	TAIR
GAAGAAGTGA	1	3	0	2	0	gnl|UG|At#S30642891	gi|21403867|gb|AY085157.1	At1g17490.1
at3g49870	CON	MIN	HOUR	DAY	WEEK	UNIGENE	FLCDNA	TAIR
CAAGAACTCT	2	10	2	0	1	gnl|UG|At#S11729468	gi|21389676|gb|AY114718.1	At3g49870.1
at4g37740	CON	MIN	HOUR	DAY	WEEK	UNIGENE	FLCDNA	TAIR
GGGAAACTCC	0	2	0	0	0	gnl|UG|At#S11721312	gi|51970885|dbj|AK176372.1|	At4g37740.1
at3g47300	CON	MIN	HOUR	DAY	WEEK	UNIGENE	FLCDNA	TAIR
TGTTAATATA	1	0	0	0	1	gnl|UG|At#S21736313	gi|51970865|dbj|AK176362.1|	At3g47300.1
at2g26990	CON	MIN	HOUR	DAY	WEEK	UNIGENE	FLCDNA	TAIR
GAAGATCTGC	1	0	2	1	1	gnl|UG|At#S11735786	gi|15215815|gb|AY050437.1	At2g26990.1
at2g22240	CON	MIN	HOUR	DAY	WEEK	UNIGENE	FLCDNA	TAIR
GAGTACAAGT	1	0	0	0	0	gnl|UG|At#S11736920	gi|15809869|gb|AY054202.1	multiple non-canonical match
at3g44990	CON	MIN	HOUR	DAY	WEEK	UNIGENE	FLCDNA	TAIR
AATAATTGGA	3	1	3	0	2	gnl|UG|At#S11706103	gi|15810248|gb|AY056163.1	At3g44990.1
ATCCGAAAAG	1	1	1	0	0	no match found	gi|30023801|gb|BT006326.1	non-canonical match
at2g29650	CON	MIN	HOUR	DAY	WEEK	UNIGENE	FLCDNA	TAIR
GCTTGTAGTC	1	1	0	1	0	gnl|UG|At#S15460980	gi|21387196|gb|AY114683.1	At2g29650.1
at3g20770	CON	MIN	HOUR	DAY	WEEK	UNIGENE	FLCDNA	TAIR
TAATATTTTT	1	5	1	1	0	gnl|UG|At#S11734609	gi|34333302|gb|AY069875.2	At3g20770.1
at4g38710	CON	MIN	HOUR	DAY	WEEK	UNIGENE	FLCDNA	TAIR
GAGAAGCCTA	1	1	0	1	1	gnl|UG|At#S11721150	gi|20259813|gb|AY093255.1	At4g38710.1
at1g48460	CON	MIN	HOUR	DAY	WEEK	UNIGENE	FLCDNA	TAIR
TGAAATGAGA	1	0	0	3	2	gnl|UG|At#S11735048	no match found	At1g48460.1
at2g40640	CON	MIN	HOUR	DAY	WEEK	UNIGENE	FLCDNA	TAIR
CTTTCCGCCA	0	2	0	1	0	no match found	gi|51971678|dbj|AK176741.1|	At2g40640.1
at1g67330	CON	MIN	HOUR	DAY	WEEK	UNIGENE	FLCDNA	TAIR
ACCGACCAGC	1	0	2	2	0	gnl|UG|At#S11729647	gi|45752703|gb|BT012155.1	At1g67330.1
TTTGGTTTGC	0	0	1	0	0	no match found	gi|72198516|gb|DQ108876.1	At1g67330.1
at3g50260	CON	MIN	HOUR	DAY	WEEK	UNIGENE	FLCDNA	TAIR
GACGCCGGAG	0	1	0	0	0	no match found	gi|15010555|gb|AY045579.1	At3g50260.1
at1g60430	CON	MIN	HOUR	DAY	WEEK	UNIGENE	FLCDNA	TAIR
AGAATCGCCA	0	2	1	1	0	gnl|UG|At#S38434141	gi|21403324|gb|AY084614.1	At1g60430.1
at1g28680	CON	MIN	HOUR	DAY	WEEK	UNIGENE	FLCDNA	TAIR
GATAAGTTCC	0	1	0	0	1	gnl|UG|At#S11739388	gi|110740648|dbj|AK226268.1|	At1g28680.1
at3g54300	CON	MIN	HOUR	DAY	WEEK	UNIGENE	FLCDNA	TAIR
GAAGAACTTA	0	0	0	2	0	no match found	gi|44681343|gb|BT011606.1	non-canonical match
CTGTGATGCT	3	2	4	3	3	gnl|UG|At#S11728630	gi|110738121|dbj|AK229117.1|	At3g54300.1
at1g80410	CON	MIN	HOUR	DAY	WEEK	UNIGENE	FLCDNA	TAIR
CCAAATGTGT	3	4	2	4	1	gnl|UG|At#S11726330	gi|17381117|gb|AY064015.1	At1g80410.1

at3g61200	CON	MIN	HOUR	DAY	WEEK	UNIGENE	FLCDNA	TAIR
CTCAGTGATC	3	6	5	3	1	gnl|UG|At#S11727115	gi|21407835|gb|AY089061.1	At3g61200.1

at5g61190	CON	MIN	HOUR	DAY	WEEK	UNIGENE	FLCDNA	TAIR
TTTAAGTTGT	0	0	0	0	1	gnl|UG|At#S11717583	no match found	At5g61190.1

at2g13370	CON	MIN	HOUR	DAY	WEEK	UNIGENE	FLCDNA	TAIR
AGTCGAGCGC	0	1	1	0	0	no match found	gi|62319946|dbj|AK221299.1|	non-canonical match
AAGAAGATCT	1	2	0	0	0	gnl|UG|At#S11739088	no match found	At2g13370.1

at2g19640	CON	MIN	HOUR	DAY	WEEK	UNIGENE	FLCDNA	TAIR
ATGCTTCGAG	0	0	1	0	0	gnl|UG|At#S14273939	gi|14334523|gb|AY034953.1	At2g19640.2

at1g30450	CON	MIN	HOUR	DAY	WEEK	UNIGENE	FLCDNA	TAIR
ATGGTCAAAA	1	1	0	1	1	no match found	gi|110741531|dbj|AK226602.1|	multiple canonical match
AGCATCAAAA	1	0	1	2	0	no match found	no match found	At1g30450.3

at5g46180	CON	MIN	HOUR	DAY	WEEK	UNIGENE	FLCDNA	TAIR
ATATCTCAAT	0	0	1	0	0	gnl|UG|At#S11719108	no match found	At5g46180.1
GTGATACTTG	0	2	0	0	0	no match found	gi|66792619|gb|BT023421.1	non-canonical match

at3g57420	CON	MIN	HOUR	DAY	WEEK	UNIGENE	FLCDNA	TAIR
TAATTCGTGA	1	4	0	3	5	gnl|UG|At#S11728067	gi|110739067|dbj|AK229605.1|	At3g57420.1

at5g02800	CON	MIN	HOUR	DAY	WEEK	UNIGENE	FLCDNA	TAIR
GGCAAGGCCT	0	3	0	0	0	gnl|UG|At#S11725463	gi|110736287|dbj|AK228158.1|	At5g02800.1

at1g54290	CON	MIN	HOUR	DAY	WEEK	UNIGENE	FLCDNA	TAIR
GTTTTTGAGC	2	2	1	1	0	gnl|UG|At#S11732890	gi|13926321|gb|AF372910.1	At1g54290.1

at4g15790	CON	MIN	HOUR	DAY	WEEK	UNIGENE	FLCDNA	TAIR
ACACAATGGT	2	0	0	2	1	gnl|UG|At#S11743583	gi|18175845|gb|AY072116.1	At4g15790.1

at1g74450	CON	MIN	HOUR	DAY	WEEK	UNIGENE	FLCDNA	TAIR
GAGACAAATT	0	1	1	2	0	gnl|UG|At#S11728148	gi|21403143|gb|AY084433.1	At1g74450.1

at3g17205	CON	MIN	HOUR	DAY	WEEK	UNIGENE	FLCDNA	TAIR
TTACACAGAA	1	3	2	2	0	gnl|UG|At#S11735685	gi|20260605|gb|AY093202.1	At3g17205.1

at3g55646	CON	MIN	HOUR	DAY	WEEK	UNIGENE	FLCDNA	TAIR
TTGATTCATT	0	2	0	0	0	gnl|UG|At#S37211572	no match found	pseudo chromosome match

at5g38360	CON	MIN	HOUR	DAY	WEEK	UNIGENE	FLCDNA	TAIR
GGAGAAAGGT	0	0	0	1	0	gnl|UG|At#S34115216	gi|56461753|gb|BT020387.1	At5g38360.1

at5g61530	CON	MIN	HOUR	DAY	WEEK	UNIGENE	FLCDNA	TAIR
GGACTTGCTC	0	1	0	0	1	gnl|UG|At#S11717549	gi|15028202|gb|AY045924.1	At5g61530.2

at2g22370	CON	MIN	HOUR	DAY	WEEK	UNIGENE	FLCDNA	TAIR
CTATAGATGA	0	1	0	0	0	gnl|UG|At#S11736893	gi|110736252|dbj|AK228140.1|	At2g22370.1

at1g22620	CON	MIN	HOUR	DAY	WEEK	UNIGENE	FLCDNA	TAIR
TTTGTGAATG	1	1	0	0	1	gnl|UG|At#S11740801	gi|19424034|gb|AY080802.1	multiple non-canonical match

atcg01090	CON	MIN	HOUR	DAY	WEEK	UNIGENE	FLCDNA	TAIR
ATTACCTTAT	1	4	1	0	0	no match found	no match found	AtCg01090

at1g31360	CON	MIN	HOUR	DAY	WEEK	UNIGENE	FLCDNA	TAIR
TAAAAGCAGA	0	0	1	0	0	gnl|UG|At#S11738530	no match found	At1g31360.1

at4g04260	CON	MIN	HOUR	DAY	WEEK	UNIGENE	FLCDNA	TAIR
GAGCTAAAGA	0	0	0	0	1	gnl|UG|At#S11726588	no match found	multiple non-canonical match
at3g03760	CON	MIN	HOUR	DAY	WEEK	UNIGENE	FLCDNA	TAIR
AGGATGAGGA	1	1	0	0	0	no match found	gi|30793926|gb|BT008589.1	multiple non-canonical match
at3g01690	CON	MIN	HOUR	DAY	WEEK	UNIGENE	FLCDNA	TAIR
TGGATGTCGC	7	24	24	14	1	gnl|UG|At#S11740163	gi|27311638|gb|BT002425.1	At3g01690.1
AAAAGACTCG	0	0	0	1	0	no match found	gi|31711863|gb|BT008849.1	non-canonical match
at4g29920	CON	MIN	HOUR	DAY	WEEK	UNIGENE	FLCDNA	TAIR
ATCGATGTTA	0	0	0	1	0	no match found	no match found	At4g29920.1
at1g55930	CON	MIN	HOUR	DAY	WEEK	UNIGENE	FLCDNA	TAIR
TATAGAAAGA	0	0	0	0	2	gnl|UG|At#S15177175	gi|29824373|gb|BT006163.1	At1g55930.1
at1g08290	CON	MIN	HOUR	DAY	WEEK	UNIGENE	FLCDNA	TAIR
TATTTGTATT	1	0	0	0	0	gnl|UG|At#S11742206	gi|26452713|dbj|AK118851.1|	At1g08290.1
at4g37880	CON	MIN	HOUR	DAY	WEEK	UNIGENE	FLCDNA	TAIR
AAGTTTTTTA	1	1	0	2	1	gnl|UG|At#S11721289	gi|110742899|dbj|AK227334.1|	At4g37880.1
at5g17670	CON	MIN	HOUR	DAY	WEEK	UNIGENE	FLCDNA	TAIR
GAAGAATACG	2	1	0	0	1	gnl|UG|At#S18913223	gi|22136267|gb|AY128812.1	At5g17670.1
at4g32605	CON	MIN	HOUR	DAY	WEEK	UNIGENE	FLCDNA	TAIR
AAAACCCATC	0	0	0	0	1	gnl|UG|At#S38433405	gi|23197725|gb|BT000071.1	At4g32610.1
at1g19000	CON	MIN	HOUR	DAY	WEEK	UNIGENE	FLCDNA	TAIR
TGGCCCATCT	25	15	24	7	3	gnl|UG|At#S11704683	gi|21406879|gb|AY088105.1	At1g19000.1
GAGCAAGATC	1	1	2	0	0	no match found	gi|19310830|gb|AY079415.1	non-canonical match
at2g20142	CON	MIN	HOUR	DAY	WEEK	UNIGENE	FLCDNA	TAIR
TTTGCGCTAG	0	0	1	0	0	gnl|UG|At#S11702894	gi|13605516|gb|AF361584.1	At2g20142.1
at5g63790	CON	MIN	HOUR	DAY	WEEK	UNIGENE	FLCDNA	TAIR
AGCTTATTTC	2	0	15	3	2	gnl|UG|At#S11717321	no match found	At5g63790.1
TTTAGTTCCA	0	3	1	2	2	gnl|UG|At#S20707782	gi|24030238|gb|BT000896.1	non-canonical match
at2g26890	CON	MIN	HOUR	DAY	WEEK	UNIGENE	FLCDNA	TAIR
ACTTGTTCCT	0	1	0	0	0	gnl|UG|At#S11735810	no match found	At2g26890.1
at3g56170	CON	MIN	HOUR	DAY	WEEK	UNIGENE	FLCDNA	TAIR
GGAGTGGAGA	0	3	3	0	0	gnl|UG|At#S11699721	no match found	At3g56170.1
at4g20820	CON	MIN	HOUR	DAY	WEEK	UNIGENE	FLCDNA	TAIR
CTCAGCTAAT	0	1	0	1	0	gnl|UG|At#S18902504	no match found	At4g20820.1
at4g25020	CON	MIN	HOUR	DAY	WEEK	UNIGENE	FLCDNA	TAIR
TTAAACTTGA	0	1	0	0	0	gnl|UG|At#S11723474	gi|115311462|gb|BT029003.1	At4g25020.1
at3g13910	CON	MIN	HOUR	DAY	WEEK	UNIGENE	FLCDNA	TAIR
GCGAATTATT	0	3	0	0	0	no match found	gi|17528999|gb|AY065234.1	non-canonical match
at1g10770	CON	MIN	HOUR	DAY	WEEK	UNIGENE	FLCDNA	TAIR
TGTGGACGGG	0	1	0	0	0	gnl|UG|At#S35292767	gi|26451314|dbj|AK118133.1|	At1g10770.1
at5g19540	CON	MIN	HOUR	DAY	WEEK	UNIGENE	FLCDNA	TAIR
TACAAACAGT	1	0	1	0	0	no match found	gi|22136823|gb|AY133822.1	non-canonical match
GCTCTGATGT	11	9	12	12	9	gnl|UG|At#S11721440	gi|21403589|gb|AY084879.1	multiple canonical match

at1g50430 CON MIN HOUR DAY WEEK UNIGENE FLCDNA TAIR
TACCTTGTGA 0 1 2 0 2 gnl|UG|At#S11734345 gi|20466245|gb|AY099589.1 At1g50430.1

at1g29320 CON MIN HOUR DAY WEEK UNIGENE FLCDNA TAIR
GACCAGGAAG 0 0 0 0 1 gnl|UG|At#S11739255 gi|22022567|gb|AY127016.1 At1g29320.1

at3g26100 CON MIN HOUR DAY WEEK UNIGENE FLCDNA TAIR
CAAACGTGCT 0 3 0 1 0 gnl|UG|At#S18942049 gi|16323433|gb|AY059105.1 At3g26100.2

at2g39700 CON MIN HOUR DAY WEEK UNIGENE FLCDNA TAIR
GTTGTATTAT 1 1 2 2 1 gnl|UG|At#S11732672 gi|17065213|gb|AY062683.1 At2g39700.1

at3g12590 CON MIN HOUR DAY WEEK UNIGENE FLCDNA TAIR
AACCTAGGCT 0 1 0 1 0 gnl|UG|At#S11737113 gi|28973650|gb|BT005734.1 At3g12590.1

at5g09850 CON MIN HOUR DAY WEEK UNIGENE FLCDNA TAIR
ACATTCCTAA 1 1 0 0 0 gnl|UG|At#S11723774 gi|20147240|gb|AY093710.1 At5g09850.1

at4g29380 CON MIN HOUR DAY WEEK UNIGENE FLCDNA TAIR
AGCTCCAAGT 0 2 0 0 0 gnl|UG|At#S11722723 no match found At4g29380.1

at4g22550 CON MIN HOUR DAY WEEK UNIGENE FLCDNA TAIR
ACTGGTCCAA 0 0 0 1 0 no match found gi|28973658|gb|BT005739.1 non-canonical match
AATCAAAGAT 2 0 0 1 0 gnl|UG|At#S11723902 no match found At4g22550.1

at1g49950 CON MIN HOUR DAY WEEK UNIGENE FLCDNA TAIR
GCAGAGGCTG 0 1 0 0 0 no match found gi|17065319|gb|AY062736.1 At1g49950.1
CTTATCGCCA 1 0 1 0 1 gnl|UG|At#S18942408 no match found At1g49950.2

at4g29790 CON MIN HOUR DAY WEEK UNIGENE FLCDNA TAIR
ATGGTATAAG 0 1 1 0 0 gnl|UG|At#S11706141 gi|15810172|gb|AY056100.1 At4g29790.1

at5g01510 CON MIN HOUR DAY WEEK UNIGENE FLCDNA TAIR
TTCGGTACAG 1 0 0 1 0 gnl|UG|At#S24442658 gi|62320357|dbj|AK221505.1| At5g01510.1

at2g30880 CON MIN HOUR DAY WEEK UNIGENE FLCDNA TAIR
AAGAACAGAG 1 0 0 1 0 gnl|UG|At#S18942820 gi|17979457|gb|AY070724.1 non-canonical match

at1g36380 CON MIN HOUR DAY WEEK UNIGENE FLCDNA TAIR
AATCTGGTGA 0 2 1 0 0 no match found gi|21404884|gb|AY086174.1 At1g36380.1

at5g60960 CON MIN HOUR DAY WEEK UNIGENE FLCDNA TAIR
AGCAAATTGT 2 1 2 1 4 no match found gi|21405160|gb|AY086450.1 non-canonical match

at2g31720 CON MIN HOUR DAY WEEK UNIGENE FLCDNA TAIR
TGGTATAACT 0 0 0 1 0 no match found no match found At2g31720.1

at5g16410 CON MIN HOUR DAY WEEK UNIGENE FLCDNA TAIR
TGTTTAATCC 0 0 0 1 0 gnl|UG|At#S18910224 gi|53850536|gb|BT015909.1 At5g16410.1

at2g43260 CON MIN HOUR DAY WEEK UNIGENE FLCDNA TAIR
GATACATACA 0 1 0 0 0 gnl|UG|At#S11731823 gi|26449900|dbj|AK117405.1| At2g43260.1

at1g70560 CON MIN HOUR DAY WEEK UNIGENE FLCDNA TAIR
AAGCTCATTA 0 1 0 0 0 gnl|UG|At#S11728973 gi|28950958|gb|BT005339.1 At1g70560.1

at5g12260 CON MIN HOUR DAY WEEK UNIGENE FLCDNA TAIR
GTGTCAAACG 0 1 0 0 0 gnl|UG|At#S11723179 gi|21703126|gb|AY123994.1 At5g12260.1

at4g32000 CON MIN HOUR DAY WEEK UNIGENE FLCDNA TAIR
CGGATGAAGA 0 1 0 0 0 gnl|UG|At#S11722275 no match found multiple non-canonical match

at1g05380	CON	MIN	HOUR	DAY	WEEK	UNIGENE	FLCDNA	TAIR
TAAAGACTAT	0	0	0	1	1	gnl|UG|At#S43850693	gi|14334621|gb|AY034984.1	At1g05380.1
at2g35840	CON	MIN	HOUR	DAY	WEEK	UNIGENE	FLCDNA	TAIR
AGATCCAATA	9	1	7	2	8	no match found	gi|15450787|gb|AY054474.1	At2g35840.2
at3g09520	CON	MIN	HOUR	DAY	WEEK	UNIGENE	FLCDNA	TAIR
TTTCTCATTT	0	0	0	1	0	no match found	no match found	At3g09520.1
at3g53990	CON	MIN	HOUR	DAY	WEEK	UNIGENE	FLCDNA	TAIR
GGAAGCAGAG	10	27	33	21	7	no match found	gi|21405425|gb|AY086715.1	multiple non-canonical match
TGGTGGGCTC	3	4	10	6	4	gnl|UG|At#S18941984	no match found	At3g53990.2
at3g49180	CON	MIN	HOUR	DAY	WEEK	UNIGENE	FLCDNA	TAIR
CAAAAGGTTC	0	1	0	0	0	gnl|UG|At#S11729606	gi|21406020|gb|AY087296.1	At3g49180.1
at2g23290	CON	MIN	HOUR	DAY	WEEK	UNIGENE	FLCDNA	TAIR
ATTTGAGATT	1	4	0	0	1	gnl|UG|At#S11736663	gi|21406781|gb|AY088007.1	At2g23290.1
at2g34430	CON	MIN	HOUR	DAY	WEEK	UNIGENE	FLCDNA	TAIR
GGCCTTCGCT	53	89	41	30	5	gnl|UG|At#S11701719	gi|11908033|gb|AF326864.1	At2g34430.1
at1g66330	CON	MIN	HOUR	DAY	WEEK	UNIGENE	FLCDNA	TAIR
TAATTAATTA	2	2	2	0	1	gnl|UG|At#S11710391	no match found	At1g66330.2
at5g65350	CON	MIN	HOUR	DAY	WEEK	UNIGENE	FLCDNA	TAIR
GGTTTTTCGA	0	0	1	0	0	gnl|UG|At#S11824645	gi|26449415|dbj|AK117157.1|	At5g65350.1
at2g02910	CON	MIN	HOUR	DAY	WEEK	UNIGENE	FLCDNA	TAIR
GAAGAGGAAC	1	0	2	0	0	gnl|UG|At#S11740547	gi|19699299|gb|AY090356.1	At2g02910.1
at5g42500	CON	MIN	HOUR	DAY	WEEK	UNIGENE	FLCDNA	TAIR
AAGAACATAA	0	14	2	0	0	gnl|UG|At#S11719479	gi|30725557|gb|BT008442.1	At5g42500.1
at3g43430	CON	MIN	HOUR	DAY	WEEK	UNIGENE	FLCDNA	TAIR
TGTGCCGTGT	0	0	0	1	0	no match found	gi|21407864|gb|AY089090.1	At3g43430.1
at1g79390	CON	MIN	HOUR	DAY	WEEK	UNIGENE	FLCDNA	TAIR
ATGGGTAAAG	1	2	0	0	0	gnl|UG|At#S11726729	gi|11908091|gb|AF326893.1	non-canonical match
GTACACGCTG	0	1	0	0	0	no match found	no match found	At1g79390.1
at2g46990	CON	MIN	HOUR	DAY	WEEK	UNIGENE	FLCDNA	TAIR
TGCAATGAGA	0	2	0	0	0	no match found	gi|26449565|dbj|AK117233.1|	At2g46990.1
at1g68590	CON	MIN	HOUR	DAY	WEEK	UNIGENE	FLCDNA	TAIR
GATCTCTCAG	10	12	8	6	2	gnl|UG|At#S11729378	gi|21404855|gb|AY086145.1	At1g68590.1
at3g01345	CON	MIN	HOUR	DAY	WEEK	UNIGENE	FLCDNA	TAIR
AAGCTTCTAT	2	0	0	0	3	gnl|UG|At#S34115112	gi|110742100|dbj|AK226905.1|	At3g01345.1
at3g22680	CON	MIN	HOUR	DAY	WEEK	UNIGENE	FLCDNA	TAIR
GCTCTTCTTT	0	0	2	1	0	gnl|UG|At#S11734012	gi|45680408|gb|BT012098.1	At3g22680.1
at5g54500	CON	MIN	HOUR	DAY	WEEK	UNIGENE	FLCDNA	TAIR
ATATTATTTC	3	10	3	3	8	no match found	gi|21539480|gb|AY120735.1	At5g54500.1
TTGAGAAATT	0	10	3	2	0	no match found	gi|25084251|gb|BT002194.1	non-canonical match
at3g46550	CON	MIN	HOUR	DAY	WEEK	UNIGENE	FLCDNA	TAIR
TAAATTTGGA	0	0	0	1	0	gnl|UG|At#S42395155	no match found	multiple non-canonical match

at5g16150	CON	MIN	HOUR	DAY	WEEK	UNIGENE	FLCDNA	TAIR
CACTGGATAT	0	1	0	0	0	no match found	gi|21436466|gb|AY117359.1	non-canonical match
ACCACAAAAA	0	2	1	1	0	no match found	gi|62318984|dbj|AK220813.1|	non-canonical match
at4g20860	CON	MIN	HOUR	DAY	WEEK	UNIGENE	FLCDNA	TAIR
ACTCCGTATG	0	3	3	1	0	gnl|UG|At#S11724199	gi|22137201|gb|AY133616.1	At4g20860.1
at1g11390	CON	MIN	HOUR	DAY	WEEK	UNIGENE	FLCDNA	TAIR
TTGCCTGAGA	1	0	1	0	1	gnl|UG|At#S11741896	no match found	At1g11390.1
at2g16380	CON	MIN	HOUR	DAY	WEEK	UNIGENE	FLCDNA	TAIR
AGTTATCTGA	4	2	0	0	0	gnl|UG|At#S11738383	gi|16612282|gb|AF439836.1	At2g16380.1
at1g29680	CON	MIN	HOUR	DAY	WEEK	UNIGENE	FLCDNA	TAIR
TGATCTGTCC	0	1	0	0	0	no match found	no match found	At1g29680.1
at3g12345	CON	MIN	HOUR	DAY	WEEK	UNIGENE	FLCDNA	TAIR
GATATAAGAT	12	6	8	6	3	gnl|UG|At#S28282997	gi|51968785|dbj|AK175322.1|	At3g12340.2
at1g14860	CON	MIN	HOUR	DAY	WEEK	UNIGENE	FLCDNA	TAIR
ATGTATCCGC	0	0	1	0	0	gnl|UG|At#S11824355	gi|26449994|dbj|AK117453.1|	At1g14860.1
at4g33110	CON	MIN	HOUR	DAY	WEEK	UNIGENE	FLCDNA	TAIR
AACTGGATTC	2	1	2	3	1	no match found	gi|22531139|gb|AY136408.1	multiple canonical match
GAAGTGACTT	0	2	0	0	0	no match found	gi|26451687|dbj|AK118325.1|	non-canonical match
at1g77500	CON	MIN	HOUR	DAY	WEEK	UNIGENE	FLCDNA	TAIR
AAATTGTTAG	0	1	0	0	0	gnl|UG|At#S11727380	gi|19424000|gb|AY080860.1	At1g77500.1
at3g14860	CON	MIN	HOUR	DAY	WEEK	UNIGENE	FLCDNA	TAIR
AGGAGCAGCA	1	0	2	2	0	no match found	gi|25083515|gb|BT002079.1	non-canonical match
ACTGAAAATC	0	1	1	2	2	gnl|UG|At#S20704909	gi|20260313|gb|AY093056.1	At3g14860.2
at2g15820	CON	MIN	HOUR	DAY	WEEK	UNIGENE	FLCDNA	TAIR
TGTTAGAGAA	0	1	0	0	1	no match found	gi|110739798|dbj|AK229981.1|	non-canonical match
at5g36296	CON	MIN	HOUR	DAY	WEEK	UNIGENE	FLCDNA	TAIR
GTGAAGATGG	0	0	1	0	0	no match found	no match found	At5g36296.1
at5g14410	CON	MIN	HOUR	DAY	WEEK	UNIGENE	FLCDNA	TAIR
TGGTTTATGT	1	2	1	2	1	gnl|UG|At#S30642698	gi|51968937|dbj|AK175398.1|	At5g14410.1
at1g01140	CON	MIN	HOUR	DAY	WEEK	UNIGENE	FLCDNA	TAIR
TATAATGAAT	2	4	0	0	0	gnl|UG|At#S15461676	gi|20259787|gb|AY093242.1	At1g01140.1
at5g20230	CON	MIN	HOUR	DAY	WEEK	UNIGENE	FLCDNA	TAIR
TATTTGATTA	1	6	3	3	1	no match found	gi|15450626|gb|AY052681.1	At5g20230.1
ACCGTTCCTC	0	0	2	0	0	no match found	gi|23296683|gb|AY142577.1	non-canonical match
at2g44620	CON	MIN	HOUR	DAY	WEEK	UNIGENE	FLCDNA	TAIR
GTTTCTCCTT	9	8	3	2	4	gnl|UG|At#S11731492	gi|15450374|gb|AY052288.1	At2g44620.1
at4g37800	CON	MIN	HOUR	DAY	WEEK	UNIGENE	FLCDNA	TAIR
GAACCATCTC	2	0	1	1	0	no match found	gi|22136495|gb|AY128926.1	non-canonical match
at5g65630	CON	MIN	HOUR	DAY	WEEK	UNIGENE	FLCDNA	TAIR
GCTTCGGTAG	0	3	0	0	0	gnl|UG|At#S15644895	gi|30793994|gb|BT008626.1	At5g65630.1
at2g35680	CON	MIN	HOUR	DAY	WEEK	UNIGENE	FLCDNA	TAIR
GGAGGCATAA	2	5	4	1	0	no match found	gi|30793896|gb|BT008574.1	non-canonical match
AATTAGTCTC	6	7	4	1	2	gnl|UG|At#S20795516	gi|17528969|gb|AY065219.1	At2g35680.1

at5g53010	CON	MIN	HOUR	DAY	WEEK	UNIGENE	FLCDNA	TAIR
GTCAGACGGG	0	1	0	0	0	gnl|UG|At#S11718415	gi|19310414|gb|AY078942.1	At5g53010.1

at5g39240	CON	MIN	HOUR	DAY	WEEK	UNIGENE	FLCDNA	TAIR
AAAAATTTGT	0	0	1	0	0	no match found	no match found	At5g39240.1

at1g61563	CON	MIN	HOUR	DAY	WEEK	UNIGENE	FLCDNA	TAIR
TTTCTCATTG	6	3	1	0	3	no match found	gi|110740123|dbj|AK230149.1|	At1g61563.1

at5g18580	CON	MIN	HOUR	DAY	WEEK	UNIGENE	FLCDNA	TAIR
TGTGTGTATC	9	0	1	0	4	gnl|UG|At#S11702151	gi|13430807|gb|AF360316.1	non-canonical match

at5g56510	CON	MIN	HOUR	DAY	WEEK	UNIGENE	FLCDNA	TAIR
CTCTTTTGGT	0	1	0	0	0	gnl|UG|At#S11718061	no match found	non-canonical match

at5g02460	CON	MIN	HOUR	DAY	WEEK	UNIGENE	FLCDNA	TAIR
CGTGTAATGT	0	0	1	1	0	gnl|UG|At#S11725544	no match found	At5g02460.1

at1g03780	CON	MIN	HOUR	DAY	WEEK	UNIGENE	FLCDNA	TAIR
AATGCTTGAA	0	0	1	1	0	gnl|UG|At#S18942652	gi|62320543|dbj|AK221598.1|	At1g03780.1

at3g01140	CON	MIN	HOUR	DAY	WEEK	UNIGENE	FLCDNA	TAIR
TAGCAAACCT	1	2	0	0	0	gnl|UG|At#S11740325	no match found	At3g01140.1
GATTGGCATT	1	0	0	0	0	no match found	gi|110737011|dbj|AK228539.1|	At3g01140.1

at5g64180	CON	MIN	HOUR	DAY	WEEK	UNIGENE	FLCDNA	TAIR
GGCCTGACCC	1	0	3	0	1	gnl|UG|At#S11717281	no match found	non-canonical match
CTCGGTCTGA	0	2	0	0	0	gnl|UG|At#S11753399	gi|21280974|gb|AY114022.1	At5g64180.1

at2g13970	CON	MIN	HOUR	DAY	WEEK	UNIGENE	FLCDNA	TAIR
TGTTCCTTTT	0	1	0	0	0	no match found	no match found	At2g13970.1

at1g77020	CON	MIN	HOUR	DAY	WEEK	UNIGENE	FLCDNA	TAIR
TGAAATGTGA	0	0	0	1	0	gnl|UG|At#S11727571	no match found	At1g77020.1

at2g40300	CON	MIN	HOUR	DAY	WEEK	UNIGENE	FLCDNA	TAIR
ATCACATAAC	0	4	2	3	4	gnl|UG|At#S11732523	gi|17065437|gb|AY062795.1	At2g40300.1

at5g54940	CON	MIN	HOUR	DAY	WEEK	UNIGENE	FLCDNA	TAIR
GTTGAGCTAG	2	22	14	2	1	gnl|UG|At#S35286494	gi|14326563|gb|AF385736.1	At5g54940.1

at5g19620	CON	MIN	HOUR	DAY	WEEK	UNIGENE	FLCDNA	TAIR
TAGATGATTT	5	0	0	1	1	gnl|UG|At#S11721420	no match found	non-canonical match
GAGAGAGATG	1	0	0	0	0	no match found	gi|13430585|gb|AF360205.1	multiple non-canonical match

at4g14965	CON	MIN	HOUR	DAY	WEEK	UNIGENE	FLCDNA	TAIR
GAGAAACAGA	0	3	0	0	0	gnl|UG|At#S34116851	gi|21403063|gb|AY084353.1	At4g14965.1

at1g49990	CON	MIN	HOUR	DAY	WEEK	UNIGENE	FLCDNA	TAIR
ATTTCATTTT	5	13	5	5	1	no match found	no match found	At1g49990.1

at5g11340	CON	MIN	HOUR	DAY	WEEK	UNIGENE	FLCDNA	TAIR
TGCAGACAAA	1	0	0	1	0	no match found	gi|28416616|gb|BT004593.1	At5g11340.1

at2g35210	CON	MIN	HOUR	DAY	WEEK	UNIGENE	FLCDNA	TAIR
GCAGAGGAGA	1	0	0	0	0	gnl|UG|At#S11733767	gi|110738610|dbj|AK229368.1|	At2g35210.2

at2g02560	CON	MIN	HOUR	DAY	WEEK	UNIGENE	FLCDNA	TAIR
ATCGTAATGA	0	0	1	0	0	gnl|UG|At#S11740601	gi|20466781|gb|AY099857.1	At2g02560.1

at4g33666	CON	MIN	HOUR	DAY	WEEK	UNIGENE	FLCDNA	TAIR
CTCCCTCTTC	3	0	1	1	1	gnl|UG|At#S11792415	gi|15028390|gb|AY045998.1	At4g33666.1
at1g25098	CON	MIN	HOUR	DAY	WEEK	UNIGENE	FLCDNA	TAIR
TAGTGTGCAA	0	6	0	0	0	gnl|UG|At#S18900598	gi|110738510|dbj|AK229318.1|	pseudo chromosome match
at3g30737	CON	MIN	HOUR	DAY	WEEK	UNIGENE	FLCDNA	TAIR
GAAAACTCTA	1	0	0	0	1	no match found	no match found	At3g30737.1
at2g30200	CON	MIN	HOUR	DAY	WEEK	UNIGENE	FLCDNA	TAIR
TATTTGAGTT	4	0	3	2	0	gnl|UG|At#S18942825	gi|20258829|gb|AY091077.1	At2g30200.1
at4g03690	CON	MIN	HOUR	DAY	WEEK	UNIGENE	FLCDNA	TAIR
ATATACAACT	0	0	1	0	0	no match found	no match found	At4g03690.1
at1g74280	CON	MIN	HOUR	DAY	WEEK	UNIGENE	FLCDNA	TAIR
AGGTTCCGAG	0	1	1	0	0	no match found	gi|21403577|gb|AY084867.1	At1g74290.1
at5g18500	CON	MIN	HOUR	DAY	WEEK	UNIGENE	FLCDNA	TAIR
ACCTAAAGCC	2	1	0	0	0	gnl|UG|At#S28281789	gi|21404227|gb|AY085517.1	At5g18500.1
at4g37280	CON	MIN	HOUR	DAY	WEEK	UNIGENE	FLCDNA	TAIR
TAAATTTCTT	0	1	0	0	1	gnl|UG|At#S11721391	gi|14335027|gb|AY037193.1	At4g37280.1
at4g29870	CON	MIN	HOUR	DAY	WEEK	UNIGENE	FLCDNA	TAIR
TTCGTACTCG	0	2	0	0	0	no match found	gi|21405887|gb|AY087163.1	At4g29870.1
at2g30010	CON	MIN	HOUR	DAY	WEEK	UNIGENE	FLCDNA	TAIR
TCTCATTGAT	1	3	0	1	0	gnl|UG|At#S11735037	gi|110742042|dbj|AK226872.1|	At2g30010.1
at5g58330	CON	MIN	HOUR	DAY	WEEK	UNIGENE	FLCDNA	TAIR
CATCTGTATG	29	7	13	12	15	no match found	gi|110740829|dbj|AK226364.1|	multiple canonical match
CCGGTCGAAG	1	0	0	0	0	no match found	gi|23297667|gb|AY150479.1	non-canonical match
at2g23790	CON	MIN	HOUR	DAY	WEEK	UNIGENE	FLCDNA	TAIR
TTTCCAAGAT	0	4	0	0	0	gnl|UG|At#S11807233	gi|20259392|gb|AY090983.1	At2g23790.1
at3g27690	CON	MIN	HOUR	DAY	WEEK	UNIGENE	FLCDNA	TAIR
CGTCGTACCG	12	40	29	5	3	gnl|UG|At#S11732408	gi|13899124|gb|AF370557.1	At3g27690.1
at5g12120	CON	MIN	HOUR	DAY	WEEK	UNIGENE	FLCDNA	TAIR
AATGGAAGGA	0	5	0	0	0	no match found	gi|20260155|gb|AY092977.1	non-canonical match
at3g12570	CON	MIN	HOUR	DAY	WEEK	UNIGENE	FLCDNA	TAIR
TATCATTGGA	0	5	0	0	0	gnl|UG|At#S15461223	gi|21407751|gb|AY088977.1	At3g12570.1
at2g21380	CON	MIN	HOUR	DAY	WEEK	UNIGENE	FLCDNA	TAIR
CTAACAGAAA	1	0	3	2	1	no match found	gi|14532683|gb|AY039966.1	non-canonical match
at5g18960	CON	MIN	HOUR	DAY	WEEK	UNIGENE	FLCDNA	TAIR
TTTAGAGTAT	0	1	0	0	1	no match found	no match found	At5g18960.1
at4g17565	CON	MIN	HOUR	DAY	WEEK	UNIGENE	FLCDNA	TAIR
CTCTTCTCTG	1	0	2	0	0	gnl|UG|At#S11724752	no match found	multiple non-canonical match
at1g16850	CON	MIN	HOUR	DAY	WEEK	UNIGENE	FLCDNA	TAIR
AATACAGAAT	0	0	0	8	28	gnl|UG|At#S11741359	gi|13358195|gb|AF325003.2	At1g16850.1
at2g25570	CON	MIN	HOUR	DAY	WEEK	UNIGENE	FLCDNA	TAIR
AAGCGATCAT	1	1	1	1	0	no match found	gi|51971682|dbj|AK176743.1|	At2g25570.1

at1g71695	CON	MIN	HOUR	DAY	WEEK	UNIGENE	FLCDNA	TAIR
CACTTGTGGT	17	12	15	9	3	gnl|UG|At#S11640576	gi|23397148|gb|BT000715.1	At1g71695.1
at3g15500	CON	MIN	HOUR	DAY	WEEK	UNIGENE	FLCDNA	TAIR
AGATTGACAA	0	3	0	1	0	gnl|UG|At#S11736196	gi|21403218|gb|AY084508.1	At3g15500.1
at1g13880	CON	MIN	HOUR	DAY	WEEK	UNIGENE	FLCDNA	TAIR
TCGTGTATAG	1	0	0	0	0	gnl|UG|At#S34114203	gi|110743916|dbj|AK227812.1|	At1g13880.1
at3g09085	CON	MIN	HOUR	DAY	WEEK	UNIGENE	FLCDNA	TAIR
GAGAGAGAGA	1	1	0	0	0	no match found	gi|30102819|gb|BT006520.1	At1g30975.1
GAATCGTTGT	1	1	1	1	0	gnl|UG|At#S11738167	gi|21404663|gb|AY085953.1	At3g09085.1
at5g41460	CON	MIN	HOUR	DAY	WEEK	UNIGENE	FLCDNA	TAIR
GTAAATTTTG	0	0	0	0	1	gnl|UG|At#S11719584	gi|53828536|gb|BT015815.1	At5g41460.1
at3g27020	CON	MIN	HOUR	DAY	WEEK	UNIGENE	FLCDNA	TAIR
TTTGTTGGGA	1	2	1	0	0	gnl|UG|At#S11732624	gi|21539460|gb|AY120725.1	At3g27020.1
at1g67070	CON	MIN	HOUR	DAY	WEEK	UNIGENE	FLCDNA	TAIR
TTATCCTAAT	0	0	1	2	0	gnl|UG|At#S21736291	gi|51970909|dbj|AK176384.1|	non-canonical match
at5g59360	CON	MIN	HOUR	DAY	WEEK	UNIGENE	FLCDNA	TAIR
TTGTTTTTCT	0	0	0	0	1	gnl|UG|At#S11717768	no match found	At3g12860.1
at4g24960	CON	MIN	HOUR	DAY	WEEK	UNIGENE	FLCDNA	TAIR
TCCCTGTCTA	0	0	1	0	0	gnl|UG|At#S43849498	gi|21405560|gb|AY086850.1	At4g24960.1
at1g54030	CON	MIN	HOUR	DAY	WEEK	UNIGENE	FLCDNA	TAIR
AGACCGAGAC	1	2	2	1	1	gnl|UG|At#S11732984	gi|33589731|gb|BT010163.1	At1g54030.1
at4g25480	CON	MIN	HOUR	DAY	WEEK	UNIGENE	FLCDNA	TAIR
AAGTCGACGG	0	1	53	4	2	gnl|UG|At#S11699072	gi|88193805|gb|BT024594.1	At4g25480.1
at3g15770	CON	MIN	HOUR	DAY	WEEK	UNIGENE	FLCDNA	TAIR
GTCTTGTGCT	1	3	1	1	0	gnl|UG|At#S11824603	gi|26449499|dbj|AK117200.1|	At3g15770.1
at5g04590	CON	MIN	HOUR	DAY	WEEK	UNIGENE	FLCDNA	TAIR
TAATGTAATG	8	11	0	3	4	gnl|UG|At#S11724992	gi|110739586|dbj|AK229873.1|	At5g04590.1
at5g63810	CON	MIN	HOUR	DAY	WEEK	UNIGENE	FLCDNA	TAIR
AAGATAAAGT	0	3	1	2	0	no match found	gi|20260437|gb|AY093118.1	At5g63810.1
at1g04770	CON	MIN	HOUR	DAY	WEEK	UNIGENE	FLCDNA	TAIR
TCTCATCAAG	0	1	0	1	0	gnl|UG|At#S11742552	gi|110742852|dbj|AK227309.1|	At1g04770.1
at5g53830	CON	MIN	HOUR	DAY	WEEK	UNIGENE	FLCDNA	TAIR
ATATTAAATT	0	1	0	0	0	gnl|UG|At#S11718333	gi|13926341|gb|AF372918.1	At5g53830.1
at1g54850	CON	MIN	HOUR	DAY	WEEK	UNIGENE	FLCDNA	TAIR
TCGAGTAACA	0	2	0	0	0	gnl|UG|At#S11825060	no match found	At1g54850.1
at1g51680	CON	MIN	HOUR	DAY	WEEK	UNIGENE	FLCDNA	TAIR
TAACTTGTGC	12	3	13	7	3	gnl|UG|At#S38434187	gi|20466561|gb|AY099747.1	At1g51680.1
at3g63230	CON	MIN	HOUR	DAY	WEEK	UNIGENE	FLCDNA	TAIR
CTTTTATATA	0	0	0	0	1	gnl|UG|At#S11726464	no match found	At3g63230.1
at5g09540	CON	MIN	HOUR	DAY	WEEK	UNIGENE	FLCDNA	TAIR
CGAAGCTGAC	0	1	1	0	0	gnl|UG|At#S11723852	gi|124300979|gb|BT030004.1	multiple non-canonical match

at5g57180	CON	MIN	HOUR	DAY	WEEK	UNIGENE	FLCDNA	TAIR
TAGGTTTTTT	0	5	1	1	0	gnl|UG|At#S11703085	gi|13937150|gb|AF372929.1	At5g57180.1
GAATCGAAGC	1	4	4	2	0	no match found	gi|18700253|gb|AY078036.1	At5g57180.2

at1g24470	CON	MIN	HOUR	DAY	WEEK	UNIGENE	FLCDNA	TAIR
TGGAATATAA	0	1	0	0	0	gnl|UG|At#S11740515	gi|124301103|gb|BT030066.1	multiple non-canonical match

at2g46930	CON	MIN	HOUR	DAY	WEEK	UNIGENE	FLCDNA	TAIR
TCACAATCTC	1	1	2	0	0	gnl|UG|At#S11730970	gi|15450606|gb|AY052671.1	At2g46930.1

at4g36810	CON	MIN	HOUR	DAY	WEEK	UNIGENE	FLCDNA	TAIR
TGTCTGTAAA	1	0	0	0	0	no match found	gi|110742523|dbj|AK227130.1|	At2g46930.1
TGTCTGTAAT	1	1	0	0	1	gnl|UG|At#S11721468	no match found	At4g36810.1

at1g15940	CON	MIN	HOUR	DAY	WEEK	UNIGENE	FLCDNA	TAIR
AATGAAGGAT	0	0	0	2	0	gnl|UG|At#S11741443	gi|110738086|dbj|AK229098.1|	At1g15940.1

at1g73600	CON	MIN	HOUR	DAY	WEEK	UNIGENE	FLCDNA	TAIR
AAAGTCCTGA	2	2	2	1	0	gnl|UG|At#S11728333	gi|22531067|gb|AY136372.1	At1g73600.1

at1g53380	CON	MIN	HOUR	DAY	WEEK	UNIGENE	FLCDNA	TAIR
ATCGCCGTTT	0	1	0	1	0	gnl|UG|At#S38434173	gi|48958494|gb|BT014949.1	At1g53380.1

at1g43900	CON	MIN	HOUR	DAY	WEEK	UNIGENE	FLCDNA	TAIR
GCGAGTTGGT	1	0	2	0	1	gnl|UG|At#S11708333	gi|17381033|gb|AY063973.1	non-canonical match

at5g17020	CON	MIN	HOUR	DAY	WEEK	UNIGENE	FLCDNA	TAIR
ACTGCAGCAG	0	1	1	1	0	gnl|UG|At#S11722080	gi|20465600|gb|AY096633.1	At5g17020.1

at2g02050	CON	MIN	HOUR	DAY	WEEK	UNIGENE	FLCDNA	TAIR
GAAATGTGAA	0	2	2	0	2	gnl|UG|At#S35247169	gi|15529287|gb|AY052268.1	At2g02050.1

at2g38130	CON	MIN	HOUR	DAY	WEEK	UNIGENE	FLCDNA	TAIR
CTAAGACTAT	2	0	0	0	0	no match found	gi|110737912|dbj|AK229007.1|	non-canonical match

at5g46470	CON	MIN	HOUR	DAY	WEEK	UNIGENE	FLCDNA	TAIR
GATTCTGAAA	0	0	0	1	1	gnl|UG|At#S35259803	no match found	non-canonical match

at1g21270	CON	MIN	HOUR	DAY	WEEK	UNIGENE	FLCDNA	TAIR
TTCATATTTT	1	2	3	4	0	no match found	gi|62318960|dbj|AK220801.1|	At1g21240.1
TATATCTTTT	0	1	0	0	0	no match found	gi|17064909|gb|AY062531.1	multiple non-canonical match

at1g04750	CON	MIN	HOUR	DAY	WEEK	UNIGENE	FLCDNA	TAIR
GGTTTAAGTG	2	2	1	0	3	gnl|UG|At#S11742554	gi|51970859|dbj|AK176359.1|	At1g04750.1

at3g62370	CON	MIN	HOUR	DAY	WEEK	UNIGENE	FLCDNA	TAIR
GAAAACAATC	0	1	0	0	0	gnl|UG|At#S11744369	gi|22136955|gb|AY133773.1	At3g62370.1

at2g25010	CON	MIN	HOUR	DAY	WEEK	UNIGENE	FLCDNA	TAIR
AATATGATTG	0	1	0	0	0	gnl|UG|At#S34116751	gi|110738978|dbj|AK229557.1|	At2g25010.1

at3g58830	CON	MIN	HOUR	DAY	WEEK	UNIGENE	FLCDNA	TAIR
ATAAAAAATG	0	1	2	1	3	no match found	gi|110735772|dbj|AK227892.1|	At3g58830.1
GTGGGTGATC	0	0	0	1	0	no match found	gi|28466952|gb|BT004819.1	non-canonical match

at4g26200	CON	MIN	HOUR	DAY	WEEK	UNIGENE	FLCDNA	TAIR
TTCATCAAGG	0	1	0	0	0	gnl|UG|At#S18905858	no match found	At4g26200.1

at3g07195	CON	MIN	HOUR	DAY	WEEK	UNIGENE	FLCDNA	TAIR
GTGCCGCAGG	0	0	2	0	0	gnl|UG|At#S24442419	gi|45752765|gb|BT012187.1	non-canonical match

at2g43410	CON	MIN	HOUR	DAY	WEEK	UNIGENE	FLCDNA	TAIR
TGGAGGAAGC	0	0	0	0	1	no match found	gi|62318593|dbj|AK220617.1|	non-canonical match

at2g40095	CON	MIN	HOUR	DAY	WEEK	UNIGENE	FLCDNA	TAIR
TAAATAAAAC	0	1	2	0	1	gnl|UG|At#S15460587	gi|26449646|dbj|AK117275.1|	At2g40095.1

at5g64760	CON	MIN	HOUR	DAY	WEEK	UNIGENE	FLCDNA	TAIR
AGAGGATGAA	0	0	1	0	0	no match found	no match found	At5g64760.1
TGGTCTTTTT	0	0	1	0	0	no match found	gi|17063180|gb|AY062113.1	non-canonical match

at1g42440	CON	MIN	HOUR	DAY	WEEK	UNIGENE	FLCDNA	TAIR
AACTTGTACA	0	0	0	0	2	gnl|UG|At#S11630310	gi|13605689|gb|AF361826.1	At1g42440.1

at4g02410	CON	MIN	HOUR	DAY	WEEK	UNIGENE	FLCDNA	TAIR
TTGATATATA	0	1	2	0	0	gnl|UG|At#S11727078	gi|20453207|gb|AY094476.1	At4g02410.1

at3g19520	CON	MIN	HOUR	DAY	WEEK	UNIGENE	FLCDNA	TAIR
TTGAACGCAA	0	1	1	1	0	no match found	gi|28827631|gb|BT005127.1	At3g19520.1

at1g18000	CON	MIN	HOUR	DAY	WEEK	UNIGENE	FLCDNA	TAIR
GGAATAAATA	1	0	0	0	0	no match found	gi|62321546|dbj|AK222109.1|	pseudo chromosome match
TTCGCCGGCG	0	2	0	0	0	gnl|UG|At#S11741246	gi|21403166|gb|AY084456.1	multiple canonical match

at1g11870	CON	MIN	HOUR	DAY	WEEK	UNIGENE	FLCDNA	TAIR
GGAGGCATTG	1	0	0	0	1	gnl|UG|At#S11806639	gi|20268722|gb|AY091115.1	At1g11870.3

at4g37670	CON	MIN	HOUR	DAY	WEEK	UNIGENE	FLCDNA	TAIR
AAGAAGCTAA	0	0	1	0	0	gnl|UG|At#S11721323	gi|21405370|gb|AY086660.1	At4g37670.2

at3g01100	CON	MIN	HOUR	DAY	WEEK	UNIGENE	FLCDNA	TAIR
AAAGAACACA	2	0	0	0	0	gnl|UG|At#S28283063	gi|27311784|gb|BT002498.1	At3g01100.1

at5g23320	CON	MIN	HOUR	DAY	WEEK	UNIGENE	FLCDNA	TAIR
GTGTGTATAG	0	1	0	0	0	gnl|UG|At#S11720846	no match found	non-canonical match
AGTTTCAACT	0	1	0	0	0	gnl|UG|At#S18913980	no match found	non-canonical match

at1g48050	CON	MIN	HOUR	DAY	WEEK	UNIGENE	FLCDNA	TAIR
TCAAAGAAGA	1	0	0	0	0	gnl|UG|At#S11701503	no match found	At1g48050.1

at4g12250	CON	MIN	HOUR	DAY	WEEK	UNIGENE	FLCDNA	TAIR
TCGGAGGAAT	0	3	3	3	2	gnl|UG|At#S11725689	gi|15293118|gb|AY050993.1	non-canonical match

at2g16860	CON	MIN	HOUR	DAY	WEEK	UNIGENE	FLCDNA	TAIR
TTGGAAACAA	2	0	0	1	0	gnl|UG|At#S11738266	gi|20466269|gb|AY099601.1	At2g16860.1

at3g49890	CON	MIN	HOUR	DAY	WEEK	UNIGENE	FLCDNA	TAIR
GAAGTCTGCA	1	3	5	0	1	gnl|UG|At#S11729464	gi|21406223|gb|AY087486.1	At3g49890.1

at3g27190	CON	MIN	HOUR	DAY	WEEK	UNIGENE	FLCDNA	TAIR
TCCTGCTATT	0	1	0	0	0	no match found	gi|38564267|gb|BT010743.1	At3g27190.1
ACAACTTTTA	0	1	1	0	1	gnl|UG|At#S11732573	gi|51969225|dbj|AK175542.1|	pseudo chromosome match

at4g27040	CON	MIN	HOUR	DAY	WEEK	UNIGENE	FLCDNA	TAIR
TATCACTCTG	3	2	1	1	1	gnl|UG|At#S43849481	no match found	non-canonical match

at5g59610	CON	MIN	HOUR	DAY	WEEK	UNIGENE	FLCDNA	TAIR
TGGTGAGAAA	3	0	1	1	1	gnl|UG|At#S11717742	gi|51971227|dbj|AK176543.1|	non-canonical match

at3g19660	CON	MIN	HOUR	DAY	WEEK	UNIGENE	FLCDNA	TAIR
TTTGTGATAA	0	1	1	0	0	gnl|UG|At#S34114204	gi|110743914|dbj|AK227811.1|	At3g19660.1

at4g29210	CON	MIN	HOUR	DAY	WEEK	UNIGENE	FLCDNA	TAIR
AGCCTCAAAG	0	1	1	1	0	gnl|UG|At#S18941776	gi|110737077|dbj|AK228574.1|	non-canonical match

at4g29830	CON	MIN	HOUR	DAY	WEEK	UNIGENE	FLCDNA	TAIR
AACAATTCGG	2	0	0	0	0	gnl|UG|At#S18907448	gi|21406036|gb|AY087312.1	At4g29830.1

at3g18310	CON	MIN	HOUR	DAY	WEEK	UNIGENE	FLCDNA	TAIR
TAACCAAACT	0	0	0	1	0	no match found	no match found	At3g18310.1

at1g09940	CON	MIN	HOUR	DAY	WEEK	UNIGENE	FLCDNA	TAIR
CCCTTGATGG	0	1	1	1	2	gnl|UG|At#S34117657	gi|110737220|dbj|AK228651.1|	At1g09940.1

at3g03330	CON	MIN	HOUR	DAY	WEEK	UNIGENE	FLCDNA	TAIR
GACAAGGTTG	0	1	2	1	0	no match found	gi|23308298|gb|BT000550.1	At3g03330.1

at1g12050	CON	MIN	HOUR	DAY	WEEK	UNIGENE	FLCDNA	TAIR
CACAGGGAAA	1	4	0	2	0	gnl|UG|At#S11741831	gi|20334819|gb|AY094010.1	At1g12050.1

at1g50380	CON	MIN	HOUR	DAY	WEEK	UNIGENE	FLCDNA	TAIR
ATTCCTGCTT	0	2	1	1	0	gnl|UG|At#S11734365	gi|15081691|gb|AY048238.1	At1g50380.1

at3g53930	CON	MIN	HOUR	DAY	WEEK	UNIGENE	FLCDNA	TAIR
AAAGGGGTGA	0	1	0	0	0	no match found	gi|29029001|gb|BT005945.1	multiple non-canonical match

at2g01870	CON	MIN	HOUR	DAY	WEEK	UNIGENE	FLCDNA	TAIR
GATGGACCAG	5	2	5	0	2	gnl|UG|At#S11742603	gi|51968649|dbj|AK175254.1|	At2g01870.1

at3g22945	CON	MIN	HOUR	DAY	WEEK	UNIGENE	FLCDNA	TAIR
TCGTGTTCGG	0	0	1	0	0	no match found	no match found	At3g22945.1

at1g29470	CON	MIN	HOUR	DAY	WEEK	UNIGENE	FLCDNA	TAIR
TATTAGTCGA	8	3	1	5	2	gnl|UG|At#S11739204	gi|110741447|dbj|AK226547.1|	At1g29470.1

at2g34790	CON	MIN	HOUR	DAY	WEEK	UNIGENE	FLCDNA	TAIR
AACAGAGTGT	1	0	0	0	0	gnl|UG|At#S14273927	gi|28827751|gb|BT005187.1	non-canonical match

at2g03120	CON	MIN	HOUR	DAY	WEEK	UNIGENE	FLCDNA	TAIR
ATGAATGAGG	2	0	0	1	3	gnl|UG|At#S11740516	gi|17473841|gb|AY065169.1	At2g03120.1

at1g28120	CON	MIN	HOUR	DAY	WEEK	UNIGENE	FLCDNA	TAIR
ATAATATCCT	0	1	1	0	2	gnl|UG|At#S11739588	gi|22531069|gb|AY136373.1	At1g28120.1

at3g18160	CON	MIN	HOUR	DAY	WEEK	UNIGENE	FLCDNA	TAIR
CCGGAGATTT	1	0	0	0	0	gnl|UG|At#S18942079	gi|21404723|gb|AY086013.1	At3g18160.1

at5g22440	CON	MIN	HOUR	DAY	WEEK	UNIGENE	FLCDNA	TAIR
AGCCTTCTTC	3	1	4	1	5	gnl|UG|At#S18909866	gi|14335147|gb|AY037253.1	At5g22440.1
GAGGAGAAGC	0	1	0	0	1	no match found	gi|18655358|gb|AY077657.1	multiple non-canonical match

at5g15570	CON	MIN	HOUR	DAY	WEEK	UNIGENE	FLCDNA	TAIR
ACTTGTTTTA	0	0	1	1	0	no match found	no match found	At5g15570.1

at4g30067	CON	MIN	HOUR	DAY	WEEK	UNIGENE	FLCDNA	TAIR
TATTTGTACT	1	1	0	0	0	gnl|UG|At#S28281930	no match found	non-canonical match

at1g55190	CON	MIN	HOUR	DAY	WEEK	UNIGENE	FLCDNA	TAIR
ATTAGCACTA	0	1	0	0	0	gnl|UG|At#S14273860	gi|28393383|gb|BT004089.1	At1g55190.1
TTCTTCTACT	1	2	0	1	0	no match found	gi|21404877|gb|AY086167.1	non-canonical match

at3g03780	CON	MIN	HOUR	DAY	WEEK	UNIGENE	FLCDNA	TAIR
GTTGACGCGG	7	3	16	16	5	gnl|UG|At#S11739576	gi|45680417|gb|BT012107.1	At3g03780.2

at1g75680	CON	MIN	HOUR	DAY	WEEK	UNIGENE	FLCDNA	TAIR
CTGGGGAAGA	3	6	3	3	1	gnl|UG|At#S11727907	gi|14532627|gb|AY039938.1	At1g75680.1
at1g01990	CON	MIN	HOUR	DAY	WEEK	UNIGENE	FLCDNA	TAIR
TTCACGTCAT	0	0	2	0	0	gnl|UG|At#S11742917	gi|110743336|dbj|AK227562.1|	At1g01990.1
at3g10220	CON	MIN	HOUR	DAY	WEEK	UNIGENE	FLCDNA	TAIR
GTGAAAGGAA	0	1	0	0	0	gnl|UG|At#S11737822	gi|51971604|dbj|AK176704.1|	non-canonical match
at3g11930	CON	MIN	HOUR	DAY	WEEK	UNIGENE	FLCDNA	TAIR
TTTGGGTTAT	30	31	12	18	24	no match found	gi|13926249|gb|AF372882.1	At3g11930.1
at5g64550	CON	MIN	HOUR	DAY	WEEK	UNIGENE	FLCDNA	TAIR
GGTCTCTGTT	0	0	2	2	0	gnl|UG|At#S11807257	gi|20259337|gb|AY090946.1	At5g64550.1
at3g25500	CON	MIN	HOUR	DAY	WEEK	UNIGENE	FLCDNA	TAIR
TTGTTGTAAA	1	2	0	1	0	gnl|UG|At#S11733149	gi|110741801|dbj|AK226743.1|	At3g25500.1
at5g03120	CON	MIN	HOUR	DAY	WEEK	UNIGENE	FLCDNA	TAIR
CACCGTTCTC	2	0	0	0	0	gnl|UG|At#S18912449	gi|18377539|gb|AY072521.1	At5g03120.1
TCCGGTCTTT	0	0	1	0	0	no match found	gi|21406773|gb|AY087999.1	non-canonical match
at4g03430	CON	MIN	HOUR	DAY	WEEK	UNIGENE	FLCDNA	TAIR
TAGAATTCTC	2	4	4	6	1	gnl|UG|At#S11708009	gi|62319431|dbj|AK221039.1|	At4g03430.1
CCAGGCCATT	0	0	1	0	0	no match found	gi|110741725|dbj|AK226702.1|	non-canonical match
at2g01410	CON	MIN	HOUR	DAY	WEEK	UNIGENE	FLCDNA	TAIR
TAGTGTTGAA	4	4	0	2	3	gnl|UG|At#S18900542	gi|26449450|dbj|AK117175.1|	At2g01410.1
at2g47240	CON	MIN	HOUR	DAY	WEEK	UNIGENE	FLCDNA	TAIR
AAAGATGGAT	0	1	0	0	0	gnl|UG|At#S18901154	gi|22137265|gb|AY133648.1	At2g47240.1
at2g27530	CON	MIN	HOUR	DAY	WEEK	UNIGENE	FLCDNA	TAIR
GGACCACCAC	9	10	13	18	21	gnl|UG|At#S11735663	no match found	multiple canonical match
at5g44578	CON	MIN	HOUR	DAY	WEEK	UNIGENE	FLCDNA	TAIR
TTTCCTCTCC	0	1	0	0	0	no match found	gi|110741794|dbj|AK226739.1|	non-canonical match
at2g47320	CON	MIN	HOUR	DAY	WEEK	UNIGENE	FLCDNA	TAIR
TAAAAGCTTT	1	3	0	0	1	gnl|UG|At#S11730873	gi|15215840|gb|AY050450.1	At2g47320.1
at1g02920	CON	MIN	HOUR	DAY	WEEK	UNIGENE	FLCDNA	TAIR
TCAGTGCTTG	1	8	4	2	0	gnl|UG|At#S11742823	gi|20259865|gb|AY093281.1	At1g02920.1
at3g09925	CON	MIN	HOUR	DAY	WEEK	UNIGENE	FLCDNA	TAIR
CACTGGTCTG	0	2	0	0	0	no match found	no match found	At3g09925.1
at1g01960	CON	MIN	HOUR	DAY	WEEK	UNIGENE	FLCDNA	TAIR
GGAAGAGATT	0	1	0	0	2	gnl|UG|At#S11742920	no match found	At1g01960.1
at1g23290	CON	MIN	HOUR	DAY	WEEK	UNIGENE	FLCDNA	TAIR
TTATTGAACT	27	15	13	22	45	gnl|UG|At#S35295169	gi|21404966|gb|AY086256.1	At1g23290.1
at2g30230	CON	MIN	HOUR	DAY	WEEK	UNIGENE	FLCDNA	TAIR
TGGATAAGAT	0	3	0	0	0	no match found	no match found	At2g30230.1
at3g24140	CON	MIN	HOUR	DAY	WEEK	UNIGENE	FLCDNA	TAIR
CAAATACCAA	0	0	1	0	0	no match found	gi|114213506|gb|BT028961.1	non-canonical match
CAATTTAAAT	0	0	0	1	0	gnl|UG|At#S24442839	gi|62319996|dbj|AK221324.1|	At3g24140.1

at2g18410	CON	MIN	HOUR	DAY	WEEK	UNIGENE	FLCDNA	TAIR
TTGATCAATC	2	3	2	2	2	no match found	no match found	At2g18410.1

at3g16520	CON	MIN	HOUR	DAY	WEEK	UNIGENE	FLCDNA	TAIR
CCGATACATC	4	2	2	1	0	no match found	no match found	At3g16520.3
TCTTTGGGGG	10	9	9	9	1	gnl|UG|At#S23590357	gi|21406985|gb|AY088211.1	non-canonical match
GAGCCCAAAA	0	0	1	0	0	gnl|UG|At#S15461004	no match found	At3g16520.2

at3g20100	CON	MIN	HOUR	DAY	WEEK	UNIGENE	FLCDNA	TAIR
TTTGATGTTG	4	2	2	2	0	gnl|UG|At#S11734821	gi|15215771|gb|AY050415.1	At3g20100.1

at3g06170	CON	MIN	HOUR	DAY	WEEK	UNIGENE	FLCDNA	TAIR
TATATAGTCC	1	2	1	0	1	gnl|UG|At#S11738884	gi|71143053|gb|BT023726.1	At3g06170.1

at5g03620	CON	MIN	HOUR	DAY	WEEK	UNIGENE	FLCDNA	TAIR
TTGTGAGAAG	0	0	2	0	0	gnl|UG|At#S11725251	no match found	At5g03620.1

at4g34620	CON	MIN	HOUR	DAY	WEEK	UNIGENE	FLCDNA	TAIR
CCTTTTTACA	4	3	2	1	5	no match found	gi|62318818|dbj|AK220729.1|	At4g34620.1
TTTCACCCAT	41	45	12	19	15	gnl|UG|At#S20834617	gi|21405680|gb|AY086956.1	multiple non-canonical match

at2g23470	CON	MIN	HOUR	DAY	WEEK	UNIGENE	FLCDNA	TAIR
TTACATACGT	0	0	0	0	1	gnl|UG|At#S11736618	gi|51970901|dbj|AK176380.1|	non-canonical match
GAAACAACAA	0	1	0	0	0	no match found	no match found	At2g23470.1

at5g18770	CON	MIN	HOUR	DAY	WEEK	UNIGENE	FLCDNA	TAIR
TCGTTCGTTT	0	1	1	1	0	gnl|UG|At#S11721631	gi|15983814|gb|AY056813.1	At5g18770.1

at5g08720	CON	MIN	HOUR	DAY	WEEK	UNIGENE	FLCDNA	TAIR
GAAGAGTTGA	0	0	1	0	0	no match found	gi|29029029|gb|BT005959.1	At3g45690.1
TATTTATGTA	1	0	0	0	1	gnl|UG|At#S11823956	gi|26450802|dbj|AK117869.1|	At5g08720.1

at1g44170	CON	MIN	HOUR	DAY	WEEK	UNIGENE	FLCDNA	TAIR
CTCTTTCTCG	1	0	0	0	0	no match found	no match found	At1g44170.1
GACTTAGTTG	0	2	0	4	0	gnl|UG|At#S15459283	gi|18175878|gb|AY072122.1	At1g44170.2

at5g18240	CON	MIN	HOUR	DAY	WEEK	UNIGENE	FLCDNA	TAIR
AACACAGAGG	1	0	0	1	0	no match found	gi|14596180|gb|AY042878.1	non-canonical match

at2g47850	CON	MIN	HOUR	DAY	WEEK	UNIGENE	FLCDNA	TAIR
TGATGTGAAT	0	2	0	1	1	gnl|UG|At#S11730754	gi|28393413|gb|BT004106.1	At2g47850.1

at1g27380	CON	MIN	HOUR	DAY	WEEK	UNIGENE	FLCDNA	TAIR
ACCACTTTCG	0	1	0	0	0	gnl|UG|At#S11675356	gi|26449872|dbj|AK117390.1|	At1g27380.1

at2g25000	CON	MIN	HOUR	DAY	WEEK	UNIGENE	FLCDNA	TAIR
TATATTTCTG	1	0	0	0	1	gnl|UG|At#S11736267	gi|21405701|gb|AY086977.1	At2g25000.1

at4g01040	CON	MIN	HOUR	DAY	WEEK	UNIGENE	FLCDNA	TAIR
TTATGTTTTC	2	0	1	0	0	gnl|UG|At#S11727426	gi|25083712|gb|BT002097.1	At4g01040.1

at4g30780	CON	MIN	HOUR	DAY	WEEK	UNIGENE	FLCDNA	TAIR
TTTTGATGAT	3	1	2	0	1	gnl|UG|At#S11722486	no match found	At4g30780.1

at1g76710	CON	MIN	HOUR	DAY	WEEK	UNIGENE	FLCDNA	TAIR
AAAGAGATAG	0	0	0	0	1	gnl|UG|At#S18942248	gi|25054843|gb|BT001913.1	At1g76710.1

at2g35190	CON	MIN	HOUR	DAY	WEEK	UNIGENE	FLCDNA	TAIR
TTCTGGTAAT	1	1	0	0	0	gnl|UG|At#S11733770	gi|16612242|gb|AF439822.1	At2g35190.1

at5g47580	CON	MIN	HOUR	DAY	WEEK	UNIGENE	FLCDNA	TAIR

AATCTCTTTG	0	0	1	1	1	no match found	gi|16974604|gb|AY060580.1	multiple canonical match
at3g57780	CON	MIN	HOUR	DAY	WEEK	UNIGENE	FLCDNA	TAIR
CTTATGGACC	0	0	1	0	0	gnl|UG|At#S11727993	no match found	At3g57780.1
at2g17790	CON	MIN	HOUR	DAY	WEEK	UNIGENE	FLCDNA	TAIR
TTGGTCAAAA	0	0	0	1	0	no match found	gi|30793854|gb|BT008553.1	non-canonical match
AAAAAAGTAT	3	1	0	0	1	gnl|UG|At#S11738039	gi|110739390|dbj|AK229772.1|	At2g17790.1
GTCTGTAACT	1	1	0	0	1	no match found	gi|30794061|gb|BT008664.1	non-canonical match
at1g10930	CON	MIN	HOUR	DAY	WEEK	UNIGENE	FLCDNA	TAIR
GAGCTTGATT	1	0	0	1	0	gnl|UG|At#S11699946	gi|21539532|gb|AY120761.1	At1g10930.1
at5g17050	CON	MIN	HOUR	DAY	WEEK	UNIGENE	FLCDNA	TAIR
GGCACCGCAA	0	0	0	6	4	gnl|UG|At#S11722073	gi|18252198|gb|AY072325.1	At5g17050.1
at1g23850	CON	MIN	HOUR	DAY	WEEK	UNIGENE	FLCDNA	TAIR
GCTTGGCCAA	0	1	1	0	0	no match found	gi|51968985|dbj|AK175422.1|	non-canonical match
TACACTTATA	0	0	1	0	0	gnl|UG|At#S11740663	no match found	At1g23850.1
at1g01440	CON	MIN	HOUR	DAY	WEEK	UNIGENE	FLCDNA	TAIR
AGGGTTCCAA	0	0	0	0	1	gnl|UG|At#S11742970	gi|37202011|gb|BT010599.1	multiple non-canonical match
at5g11890	CON	MIN	HOUR	DAY	WEEK	UNIGENE	FLCDNA	TAIR
TGAAGGATTT	0	3	1	0	1	gnl|UG|At#S11723275	gi|21403271|gb|AY084561.1	At5g11890.1
at5g02760	CON	MIN	HOUR	DAY	WEEK	UNIGENE	FLCDNA	TAIR
CAATATCATC	4	5	1	1	1	gnl|UG|At#S11725474	no match found	non-canonical match
at1g14270	CON	MIN	HOUR	DAY	WEEK	UNIGENE	FLCDNA	TAIR
ACAGTAATGT	0	0	0	1	1	gnl|UG|At#S18942583	gi|20259410|gb|AY091004.1	At1g14270.2
CTCTTTGTGC	1	2	2	0	1	no match found	gi|21406249|gb|AY087512.1	non-canonical match
at5g64850	CON	MIN	HOUR	DAY	WEEK	UNIGENE	FLCDNA	TAIR
TGTTTCTTCG	6	1	6	4	2	gnl|UG|At#S11717212	gi|21405820|gb|AY087096.1	At5g64850.1
at4g11160	CON	MIN	HOUR	DAY	WEEK	UNIGENE	FLCDNA	TAIR
GAACCAGTCA	1	1	1	0	2	gnl|UG|At#S11725848	gi|51969725|dbj|AK175792.1|	At4g11160.1
at5g48900	CON	MIN	HOUR	DAY	WEEK	UNIGENE	FLCDNA	TAIR
GCAATAGCAC	2	1	2	2	0	gnl|UG|At#S11707437	gi|21404809|gb|AY086099.1	At5g48900.1
at5g40730	CON	MIN	HOUR	DAY	WEEK	UNIGENE	FLCDNA	TAIR
CTCCGGCTCC	0	1	1	1	0	gnl|UG|At#S17007827	gi|21404372|gb|AY085662.1	At5g40730.1
at1g26910	CON	MIN	HOUR	DAY	WEEK	UNIGENE	FLCDNA	TAIR
GATAAGTTTT	2	0	0	0	0	gnl|UG|At#S11740017	gi|21405988|gb|AY087264.1	At1g26910.1
at1g56310	CON	MIN	HOUR	DAY	WEEK	UNIGENE	FLCDNA	TAIR
GAGAGCCCAA	2	0	0	1	0	no match found	no match found	At1g56310.1
at2g31510	CON	MIN	HOUR	DAY	WEEK	UNIGENE	FLCDNA	TAIR
TGTCTGGTTT	2	1	0	0	0	gnl|UG|At#S11734660	no match found	At2g31510.1
at5g10110	CON	MIN	HOUR	DAY	WEEK	UNIGENE	FLCDNA	TAIR
GGCGATATCC	0	2	0	1	0	no match found	gi|20465742|gb|AY096706.1	non-canonical match
at1g10090	CON	MIN	HOUR	DAY	WEEK	UNIGENE	FLCDNA	TAIR
CAACCTGTAG	0	2	0	0	0	gnl|UG|At#S11742025	gi|15146263|gb|AY049273.1	non-canonical match
at5g10530	CON	MIN	HOUR	DAY	WEEK	UNIGENE	FLCDNA	TAIR

GTCGTTGAGC	0	0	0	2	0	no match found	no match found	At5g10530.1
at3g10870	CON	MIN	HOUR	DAY	WEEK	UNIGENE	FLCDNA	TAIR
AAACCGGAGC	0	1	1	0	0	gnl|UG|At#S11708314	no match found	At3g10870.1
at5g06280	CON	MIN	HOUR	DAY	WEEK	UNIGENE	FLCDNA	TAIR
AATTGAATTG	1	2	2	2	1	no match found	no match found	At5g06280.3
ACGACTCTAA	0	0	1	0	0	no match found	gi|21407154|gb|AY088380.1	non-canonical match
at2g34520	CON	MIN	HOUR	DAY	WEEK	UNIGENE	FLCDNA	TAIR
CGAACAATCT	0	2	0	0	0	gnl|UG|At#S15460786	gi|20260273|gb|AY093036.1	At2g34520.1
at5g17380	CON	MIN	HOUR	DAY	WEEK	UNIGENE	FLCDNA	TAIR
GAAGTCGAGA	1	2	0	3	0	gnl|UG|At#S11721982	gi|20466615|gb|AY099774.1	At5g17380.1
at1g23030	CON	MIN	HOUR	DAY	WEEK	UNIGENE	FLCDNA	TAIR
CCAATAATAA	0	4	1	0	0	gnl|UG|At#S11740759	gi|62318740|dbj|AK220690.1|	At1g23030.1
at5g60600	CON	MIN	HOUR	DAY	WEEK	UNIGENE	FLCDNA	TAIR
GTCGTTGGGT	5	9	13	5	2	gnl|UG|At#S43849079	gi|34365568|gb|BT010473.1	At5g60600.2
AATCTTGTGA	4	8	4	3	0	gnl|UG|At#S35307234	no match found	At5g22580.1
at1g24260	CON	MIN	HOUR	DAY	WEEK	UNIGENE	FLCDNA	TAIR
TGTGTGTGTA	1	4	3	0	0	gnl|UG|At#S11740566	no match found	At1g24260.1
at4g05080	CON	MIN	HOUR	DAY	WEEK	UNIGENE	FLCDNA	TAIR
ATTTACAAAA	0	0	0	0	1	no match found	no match found	At4g05080.1
at2g32240	CON	MIN	HOUR	DAY	WEEK	UNIGENE	FLCDNA	TAIR
ACAGTGAAGA	2	2	1	1	0	gnl|UG|At#S11734477	gi|22136177|gb|AY128767.1	At2g32240.1
GGGAAACTGA	0	3	0	0	0	gnl|UG|At#S34115823	gi|110740759|dbj|AK226327.1|	multiple non-canonical match
at5g52470	CON	MIN	HOUR	DAY	WEEK	UNIGENE	FLCDNA	TAIR
CCGAAGAAAC	1	7	3	4	1	gnl|UG|At#S34116069	gi|22654992|gb|AY139769.1	At5g52470.1
at1g24060	CON	MIN	HOUR	DAY	WEEK	UNIGENE	FLCDNA	TAIR
TGGTAAGAGA	1	0	0	0	0	gnl|UG|At#S32419582	no match found	non-canonical match
at4g00200	CON	MIN	HOUR	DAY	WEEK	UNIGENE	FLCDNA	TAIR
CCGAGAAACT	0	1	0	0	0	gnl|UG|At#S34117677	gi|110737182|dbj|AK228631.1|	At4g00200.1
at3g57950	CON	MIN	HOUR	DAY	WEEK	UNIGENE	FLCDNA	TAIR
TTGTAAAATC	0	0	0	1	0	gnl|UG|At#S22666183	no match found	non-canonical match
at5g43320	CON	MIN	HOUR	DAY	WEEK	UNIGENE	FLCDNA	TAIR
TCCGGGGATT	0	1	0	1	0	gnl|UG|At#S11719396	gi|13430689|gb|AF360257.1	At5g43320.1
at5g11700	CON	MIN	HOUR	DAY	WEEK	UNIGENE	FLCDNA	TAIR
TAATCTAAAG	3	2	0	1	1	gnl|UG|At#S11723321	gi|110739163|dbj|AK229654.1|	At5g11700.1
at1g11840	CON	MIN	HOUR	DAY	WEEK	UNIGENE	FLCDNA	TAIR
ATGGGGTATG	0	6	3	1	1	gnl|UG|At#S15461200	gi|18377844|gb|AY074569.1	At1g11840.3
at3g59930	CON	MIN	HOUR	DAY	WEEK	UNIGENE	FLCDNA	TAIR
TTGTGTTCAC	1	0	0	0	0	gnl|UG|At#S11727524	gi|14030664|gb|AF375423.1	At3g59930.1
at2g29530	CON	MIN	HOUR	DAY	WEEK	UNIGENE	FLCDNA	TAIR
TGAACCCATT	3	0	0	6	2	gnl|UG|At#S11735157	gi|110736521|dbj|AK228282.1|	At2g29530.1
at2g45220	CON	MIN	HOUR	DAY	WEEK	UNIGENE	FLCDNA	TAIR
GCCAGGGTTT	0	0	0	1	0	gnl|UG|At#S11731365	gi|13605695|gb|AF361829.1	At2g45220.1

at2g11890	CON	MIN	HOUR	DAY	WEEK	UNIGENE	FLCDNA	TAIR
TTACAACATA	0	1	0	0	0	gnl|UG|At#S34116662	gi|110739147|dbj|AK229646.1|	non-canonical match
AGCCTTGGTA	2	2	0	3	2	no match found	gi|15010775|gb|AY045689.1	At2g11890.1

at1g35365	CON	MIN	HOUR	DAY	WEEK	UNIGENE	FLCDNA	TAIR
TATTTGAAAC	1	0	0	0	0	gnl|UG|At#S35289900	no match found	non-canonical match

at2g39800	CON	MIN	HOUR	DAY	WEEK	UNIGENE	FLCDNA	TAIR
CTCGTGGTCC	3	2	6	2	1	gnl|UG|At#S11732651	gi|23297450|gb|AY150430.1	At2g39800.1

at1g24600	CON	MIN	HOUR	DAY	WEEK	UNIGENE	FLCDNA	TAIR
GGTCCACGTC	0	0	1	0	0	no match found	gi|94807651|gb|BT025307.1	At1g24600.1

at1g27090	CON	MIN	HOUR	DAY	WEEK	UNIGENE	FLCDNA	TAIR
GACAGCAAGA	2	3	0	1	0	gnl|UG|At#S11739963	gi|20259226|gb|AY091390.1	At1g27090.1

at3g28540	CON	MIN	HOUR	DAY	WEEK	UNIGENE	FLCDNA	TAIR
TCTCCAGCTG	0	1	2	1	0	gnl|UG|At#S11732128	no match found	At3g28540.1

at1g26560	CON	MIN	HOUR	DAY	WEEK	UNIGENE	FLCDNA	TAIR
ATTACCTTTC	0	0	0	1	0	gnl|UG|At#S11704760	gi|21403753|gb|AY085043.1	At1g26560.1

at1g04440	CON	MIN	HOUR	DAY	WEEK	UNIGENE	FLCDNA	TAIR
GCTTTTCTTC	8	9	8	10	3	no match found	gi|27754652|gb|BT002960.1	At1g04440.1

at5g19680	CON	MIN	HOUR	DAY	WEEK	UNIGENE	FLCDNA	TAIR
GATATTTATT	1	1	0	0	0	gnl|UG|At#S11721405	gi|21403229|gb|AY084519.1	At5g19680.1

at5g60750	CON	MIN	HOUR	DAY	WEEK	UNIGENE	FLCDNA	TAIR
GAATTGATGC	2	1	0	4	1	gnl|UG|At#S11717627	gi|20465442|gb|AY096531.1	At5g60750.1

at4g17460	CON	MIN	HOUR	DAY	WEEK	UNIGENE	FLCDNA	TAIR
GGTCAACCTT	1	5	1	1	0	no match found	gi|110737100|dbj|AK228587.1|	At4g17460.1
TCTTCTTGAT	1	2	1	1	0	gnl|UG|At#S11699334	no match found	non-canonical match

at4g18100	CON	MIN	HOUR	DAY	WEEK	UNIGENE	FLCDNA	TAIR
AGTTTTGGTT	45	18	36	63	82	gnl|UG|At#S20862528	gi|21405446|gb|AY086736.1	At4g18100.1

at1g31540	CON	MIN	HOUR	DAY	WEEK	UNIGENE	FLCDNA	TAIR
CTAAACTGCA	0	1	0	0	0	no match found	gi|34098812|gb|BT010346.1	At1g31540.1
TGAATGTTTT	9	8	10	8	5	gnl|UG|At#S43850530	no match found	At5g46520.1

at5g41070	CON	MIN	HOUR	DAY	WEEK	UNIGENE	FLCDNA	TAIR
ATAGTTAGCT	0	0	1	1	1	gnl|UG|At#S18911716	gi|26450681|dbj|AK117808.1|	At5g41070.1

at3g52870	CON	MIN	HOUR	DAY	WEEK	UNIGENE	FLCDNA	TAIR
GATGATTGTG	0	1	5	3	6	gnl|UG|At#S11728896	gi|21928169|gb|AY125521.1	At3g52870.1
GATCGGAAGG	0	0	1	0	0	no match found	gi|24111366|gb|BT001053.1	non-canonical match

at1g69800	CON	MIN	HOUR	DAY	WEEK	UNIGENE	FLCDNA	TAIR
AGAGACATTA	0	1	0	0	1	gnl|UG|At#S11729141	gi|15450969|gb|AY054565.1	At1g69800.1

at3g28100	CON	MIN	HOUR	DAY	WEEK	UNIGENE	FLCDNA	TAIR
AAGTAAAGAC	1	1	0	0	0	no match found	no match found	At3g28100.1

at3g08610	CON	MIN	HOUR	DAY	WEEK	UNIGENE	FLCDNA	TAIR
ATACACTGAA	21	10	12	7	6	gnl|UG|At#S35313237	gi|21407533|gb|AY088759.1	At3g08610.1

at5g54280	CON	MIN	HOUR	DAY	WEEK	UNIGENE	FLCDNA	TAIR
TATTTTTGAG	3	2	1	2	1	gnl|UG|At#S34116837	gi|110738811|dbj|AK229471.1|	non-canonical match

at1g09840	CON	MIN	HOUR	DAY	WEEK	UNIGENE	FLCDNA	TAIR
TAAATTACTA	1	0	0	0	0	gnl|UG|At#S18942605	no match found	At1g09840.1
AATACAAACA	0	0	0	1	2	no match found	gi|23197847|gb|BT000132.1	multiple non-canonical match
at1g16300	CON	MIN	HOUR	DAY	WEEK	UNIGENE	FLCDNA	TAIR
GCGTTAGTTG	0	2	0	0	0	gnl|UG|At#S11825160	gi|59958311|gb|BT021096.1	At1g16300.1
at1g13380	CON	MIN	HOUR	DAY	WEEK	UNIGENE	FLCDNA	TAIR
TATTTTACCA	1	3	1	1	0	gnl|UG|At#S11741700	gi|21407065|gb|AY088291.1	At1g13380.1
at1g31812	CON	MIN	HOUR	DAY	WEEK	UNIGENE	FLCDNA	TAIR
AATGACTATA	4	2	2	0	1	gnl|UG|At#S18894113	gi|12642927|gb|AF339724.1	non-canonical match
ATGCAAACTA	35	35	47	33	18	no match found	gi|21407098|gb|AY088324.1	At1g31812.1
GTGTGATCTC	33	16	28	14	8	no match found	gi|11908115|gb|AF326905.1	non-canonical match
at5g01920	CON	MIN	HOUR	DAY	WEEK	UNIGENE	FLCDNA	TAIR
GCACGGGCAT	0	1	2	2	0	no match found	gi|26450335|dbj|AK117628.1|	non-canonical match
at3g24820	CON	MIN	HOUR	DAY	WEEK	UNIGENE	FLCDNA	TAIR
TGAATTTAGT	0	1	0	0	0	gnl|UG|At#S11733308	gi|21405971|gb|AY087247.1	At3g24820.1
at1g17980	CON	MIN	HOUR	DAY	WEEK	UNIGENE	FLCDNA	TAIR
TAAAACTCGT	2	1	2	0	0	gnl|UG|At#S18942551	gi|110743113|dbj|AK227446.1|	At1g17980.2
at4g35060	CON	MIN	HOUR	DAY	WEEK	UNIGENE	FLCDNA	TAIR
GTGATGAATT	2	0	6	4	2	gnl|UG|At#S11721754	gi|21405349|gb|AY086639.1	At4g35060.1
at5g63000	CON	MIN	HOUR	DAY	WEEK	UNIGENE	FLCDNA	TAIR
AAGAAAGCAA	0	0	1	0	0	gnl|UG|At#S14273946	no match found	At5g63000.1
at4g39160	CON	MIN	HOUR	DAY	WEEK	UNIGENE	FLCDNA	TAIR
TAAACTATAG	4	0	0	0	1	gnl|UG|At#S18908693	gi|66792659|gb|BT023441.1	non-canonical match
at4g35910	CON	MIN	HOUR	DAY	WEEK	UNIGENE	FLCDNA	TAIR
GGAAGTTTAC	1	0	1	2	1	gnl|UG|At#S11721611	gi|51970015|dbj|AK175937.1|	non-canonical match
at4g26570	CON	MIN	HOUR	DAY	WEEK	UNIGENE	FLCDNA	TAIR
TCTATTTGTT	4	3	1	0	0	gnl|UG|At#S15460669	gi|18253012|gb|AY072441.1	At4g26570.2
at5g04320	CON	MIN	HOUR	DAY	WEEK	UNIGENE	FLCDNA	TAIR
CTCTTGTATG	1	1	1	0	0	gnl|UG|At#S11725066	gi|110743583|dbj|AK227641.1|	At5g04320.1
CCGCAGAGAA	0	0	1	0	0	no match found	gi|119935824|gb|BT029735.1	multiple non-canonical match
at4g17270	CON	MIN	HOUR	DAY	WEEK	UNIGENE	FLCDNA	TAIR
TACACTCCTT	1	1	2	0	1	gnl|UG|At#S11724803	gi|14190518|gb|AF380659.1	non-canonical match
at5g42650	CON	MIN	HOUR	DAY	WEEK	UNIGENE	FLCDNA	TAIR
TGTTGTGGTC	0	0	0	1	0	no match found	gi|17065503|gb|AY062828.1	non-canonical match
TTCGTTTTTT	6	7	8	3	2	gnl|UG|At#S11719465	gi|17473593|gb|AY065089.1	At5g42650.1
at5g61660	CON	MIN	HOUR	DAY	WEEK	UNIGENE	FLCDNA	TAIR
TCTCTTAAGT	4	5	0	1	4	gnl|UG|At#S11717535	gi|20466347|gb|AY099640.1	At5g61660.1
at1g05560	CON	MIN	HOUR	DAY	WEEK	UNIGENE	FLCDNA	TAIR
GAGGCTTTTG	8	0	1	3	0	no match found	gi|18700283|gb|AY078051.1	At1g45760.1
TTTGATTGAA	4	0	5	3	2	gnl|UG|At#S11742472	no match found	At1g05560.1
at3g06760	CON	MIN	HOUR	DAY	WEEK	UNIGENE	FLCDNA	TAIR
AGACTTGAGG	1	2	2	2	2	gnl|UG|At#S18905144	gi|18377874|gb|AY074585.1	At3g06760.1

at5g52890	CON	MIN	HOUR	DAY	WEEK	UNIGENE	FLCDNA	TAIR
AGTGCATCAG	0	1	0	0	0	gnl|UG|At#S11718428	gi|57222215|gb|BT020514.1	At5g52890.1
at3g26730	CON	MIN	HOUR	DAY	WEEK	UNIGENE	FLCDNA	TAIR
TGACTGATAA	3	5	4	4	2	gnl|UG|At#S11743130	gi|18377742|gb|AY074325.1	At3g26730.1
at1g76940	CON	MIN	HOUR	DAY	WEEK	UNIGENE	FLCDNA	TAIR
TTATCAAGTT	0	0	0	0	1	gnl|UG|At#S11727594	gi|26450150|dbj|AK117533.1|	At1g76940.1
at4g17170	CON	MIN	HOUR	DAY	WEEK	UNIGENE	FLCDNA	TAIR
GAGGCCTCTG	4	0	1	2	0	no match found	gi|30023651|gb|BT006251.1	At4g17170.1
at1g52590	CON	MIN	HOUR	DAY	WEEK	UNIGENE	FLCDNA	TAIR
TGAGCTCTAA	1	0	2	1	2	no match found	gi|51870256|gb|BT015486.1	non-canonical match
TACTCGTCAA	5	0	0	4	0	gnl|UG|At#S11733517	gi|21407013|gb|AY088239.1	At1g52590.1
at2g32950	CON	MIN	HOUR	DAY	WEEK	UNIGENE	FLCDNA	TAIR
GACGATGCAG	1	1	3	0	2	gnl|UG|At#S34115148	gi|110742038|dbj|AK226869.1|	At2g32950.1
at2g44950	CON	MIN	HOUR	DAY	WEEK	UNIGENE	FLCDNA	TAIR
GTTTCCGCAA	1	1	0	5	0	gnl|UG|At#S11731420	no match found	multiple canonical match
at5g46690	CON	MIN	HOUR	DAY	WEEK	UNIGENE	FLCDNA	TAIR
CTAAGTATCA	1	1	1	0	0	no match found	gi|105830422|gb|BT025663.1	At5g46690.1
at5g22950	CON	MIN	HOUR	DAY	WEEK	UNIGENE	FLCDNA	TAIR
TAAACATTAT	2	2	0	1	3	gnl|UG|At#S11706697	gi|62319020|dbj|AK220831.1|	At5g22950.1
CTTCAAAGTT	2	2	1	1	0	no match found	gi|22136127|gb|AY128742.1	multiple non-canonical match
at1g29050	CON	MIN	HOUR	DAY	WEEK	UNIGENE	FLCDNA	TAIR
GAACCAACTT	0	2	0	0	0	gnl|UG|At#S11739350	gi|18377637|gb|AY074272.1	At1g29050.1
CACTTTGTTT	2	6	3	2	7	gnl|UG|At#S18320859	no match found	At2g20820.1
at1g54060	CON	MIN	HOUR	DAY	WEEK	UNIGENE	FLCDNA	TAIR
GGCAAATTAA	4	1	1	0	0	gnl|UG|At#S11732974	gi|21403482|gb|AY084772.1	At1g54060.1
at2g01590	CON	MIN	HOUR	DAY	WEEK	UNIGENE	FLCDNA	TAIR
GAGAAGTACC	0	0	0	0	1	gnl|UG|At#S11742645	gi|14334561|gb|AY035185.1	At2g01590.1
at3g11530	CON	MIN	HOUR	DAY	WEEK	UNIGENE	FLCDNA	TAIR
TGTTTTCACC	0	0	0	1	0	no match found	gi|20466134|gb|AY098979.1	At3g11530.1
at5g52545	CON	MIN	HOUR	DAY	WEEK	UNIGENE	FLCDNA	TAIR
ATCACTCCAA	0	0	1	0	0	gnl|UG|At#S11831824	gi|20259367|gb|AY090967.1	At5g52545.1
at3g28940	CON	MIN	HOUR	DAY	WEEK	UNIGENE	FLCDNA	TAIR
GATAAATTCA	0	5	2	1	1	no match found	gi|20857127|gb|AY102134.1	At3g28940.1
at3g13040	CON	MIN	HOUR	DAY	WEEK	UNIGENE	FLCDNA	TAIR
TCCAAAACCA	1	0	1	2	0	gnl|UG|At#S11736984	gi|21403448|gb|AY084738.1	At3g13040.1
at5g19290	CON	MIN	HOUR	DAY	WEEK	UNIGENE	FLCDNA	TAIR
GTTTACAGTT	4	4	5	8	2	no match found	gi|17380667|gb|AY063808.1	At5g19290.1
at3g16220	CON	MIN	HOUR	DAY	WEEK	UNIGENE	FLCDNA	TAIR
ATTTGCATTA	2	1	2	1	0	no match found	no match found	At3g16220.1
at3g54085	CON	MIN	HOUR	DAY	WEEK	UNIGENE	FLCDNA	TAIR
CCAAATCAAA	0	0	1	1	0	no match found	gi|26450604|dbj|AK117767.1|	non-canonical match
at2g41250	CON	MIN	HOUR	DAY	WEEK	UNIGENE	FLCDNA	TAIR

ATCGTTCTTA	7	2	4	5	2	gnl|UG|At#S11709898	gi|17979160|gb|AY070081.1	At2g41250.1
TTCAGACTTC	0	0	1	1	0	no match found	gi|110742651|dbj|AK227199.1|	non-canonical match
at1g68300	CON	MIN	HOUR	DAY	WEEK	UNIGENE	FLCDNA	TAIR
AAAGAGTTCG	2	3	0	1	2	gnl|UG|At#S11729444	gi|21405441|gb|AY086731.1	At1g68300.1
at1g01540	CON	MIN	HOUR	DAY	WEEK	UNIGENE	FLCDNA	TAIR
GAAGCCGCGA	1	0	1	0	0	no match found	gi|12083265|gb|AF332429.1	non-canonical match
TCACTTATTA	0	2	0	1	3	gnl|UG|At#S11742960	no match found	At1g01540.1
at1g30360	CON	MIN	HOUR	DAY	WEEK	UNIGENE	FLCDNA	TAIR
GTTGTTGTAA	7	8	13	14	9	gnl|UG|At#S11738902	gi|62321289|dbj|AK221977.1|	multiple canonical match
at1g67210	CON	MIN	HOUR	DAY	WEEK	UNIGENE	FLCDNA	TAIR
TCCATCAATG	0	0	0	1	1	gnl|UG|At#S11729670	gi|21404978|gb|AY086268.1	At1g67210.1
at3g13610	CON	MIN	HOUR	DAY	WEEK	UNIGENE	FLCDNA	TAIR
AATTGTGATC	0	0	0	1	0	no match found	no match found	At3g13610.1
at1g23180	CON	MIN	HOUR	DAY	WEEK	UNIGENE	FLCDNA	TAIR
TAAAATTTTG	1	0	1	0	1	gnl|UG|At#S34115041	gi|25083230|gb|BT002042.1	At1g23180.1
at4g28280	CON	MIN	HOUR	DAY	WEEK	UNIGENE	FLCDNA	TAIR
CATCGATCCC	1	0	0	0	0	gnl|UG|At#S11722916	gi|44681401|gb|BT011635.1	non-canonical match
at5g59590	CON	MIN	HOUR	DAY	WEEK	UNIGENE	FLCDNA	TAIR
TGAAGATGAT	0	0	0	1	2	no match found	gi|28973178|gb|BT005494.1	At1g16550.1
at3g50825	CON	MIN	HOUR	DAY	WEEK	UNIGENE	FLCDNA	TAIR
GTTGCGTAAG	2	4	6	2	1	gnl|UG|At#S18902856	gi|110741470|dbj|AK226563.1|	At1g16550.1
at3g53260	CON	MIN	HOUR	DAY	WEEK	UNIGENE	FLCDNA	TAIR
TTCAATCAGC	2	1	0	6	1	gnl|UG|At#S11728826	gi|22137159|gb|AY133595.1	At3g53260.1
atcg00340	CON	MIN	HOUR	DAY	WEEK	UNIGENE	FLCDNA	TAIR
AACGCACACC	16	7	18	12	7	no match found	no match found	AtCg00340
at5g13290	CON	MIN	HOUR	DAY	WEEK	UNIGENE	FLCDNA	TAIR
TATCAAAACC	1	1	0	0	0	gnl|UG|At#S11723033	gi|110737170|dbj|AK228624.1|	At5g13290.1
at3g27770	CON	MIN	HOUR	DAY	WEEK	UNIGENE	FLCDNA	TAIR
GCTCCAGTGT	1	4	7	2	0	gnl|UG|At#S11732388	gi|51971634|dbj|AK176719.1|	non-canonical match
at4g01120	CON	MIN	HOUR	DAY	WEEK	UNIGENE	FLCDNA	TAIR
TCTCTGAGGT	1	0	0	0	1	gnl|UG|At#S11727404	gi|62319868|dbj|AK221260.1|	At4g01120.1
at5g06570	CON	MIN	HOUR	DAY	WEEK	UNIGENE	FLCDNA	TAIR
GTTTCTACTC	0	0	1	0	0	no match found	gi|26452183|dbj|AK118580.1|	At5g06570.1
at5g41990	CON	MIN	HOUR	DAY	WEEK	UNIGENE	FLCDNA	TAIR
GAAGCTGGTC	0	3	0	1	0	gnl|UG|At#S11719530	gi|15983508|gb|AF424629.1	non-canonical match
at2g23670	CON	MIN	HOUR	DAY	WEEK	UNIGENE	FLCDNA	TAIR
CGCGGTTCTT	1	0	0	2	0	no match found	gi|72196942|gb|DQ108677.1	non-canonical match
TTAGCCCACG	2	8	1	3	0	gnl|UG|At#S11736570	gi|16648701|gb|AY058127.1	At2g23670.1
at1g24267	CON	MIN	HOUR	DAY	WEEK	UNIGENE	FLCDNA	TAIR
CTGCGTCTAT	1	1	0	0	2	gnl|UG|At#S28282592	gi|26450523|dbj|AK117725.1|	At1g24267.1
at3g45780	CON	MIN	HOUR	DAY	WEEK	UNIGENE	FLCDNA	TAIR
GCTGAACCAA	1	6	5	2	1	no match found	gi|14532875|gb|AY040062.1	At3g45780.1

at3g52490	CON	MIN	HOUR	DAY	WEEK	UNIGENE	FLCDNA	TAIR
TGATGAGATT	0	0	1	0	0	no match found	gi|51536535|gb|BT015383.1	non-canonical match
at5g59290	CON	MIN	HOUR	DAY	WEEK	UNIGENE	FLCDNA	TAIR
TGTTATTCAT	1	2	0	0	0	no match found	gi|21407217|gb|AY088443.1	multiple non-canonical match
at2g19050	CON	MIN	HOUR	DAY	WEEK	UNIGENE	FLCDNA	TAIR
TTAGGGTTTA	0	0	0	0	1	gnl|UG|At#S11737713	no match found	At2g19050.1
at3g27240	CON	MIN	HOUR	DAY	WEEK	UNIGENE	FLCDNA	TAIR
ATTTTGAGAT	12	8	12	16	18	no match found	no match found	At3g27240.1
at3g61960	CON	MIN	HOUR	DAY	WEEK	UNIGENE	FLCDNA	TAIR
GAAACAATAT	0	1	0	0	0	gnl|UG|At#S11726860	gi|14334751|gb|AY035049.1	At3g61960.1
at5g67340	CON	MIN	HOUR	DAY	WEEK	UNIGENE	FLCDNA	TAIR
ATACACATCT	0	0	0	1	0	gnl|UG|At#S11716961	gi|110738843|dbj|AK229487.1|	At5g67340.1
at1g62500	CON	MIN	HOUR	DAY	WEEK	UNIGENE	FLCDNA	TAIR
GTGGAGGAGG	0	2	0	1	0	no match found	gi|15028318|gb|AY045962.1	At5g38870.1
at5g47780	CON	MIN	HOUR	DAY	WEEK	UNIGENE	FLCDNA	TAIR
AAATTGTATA	2	4	1	0	3	gnl|UG|At#S11706064	gi|15810326|gb|AY056202.1	At5g47780.1
at1g49650	CON	MIN	HOUR	DAY	WEEK	UNIGENE	FLCDNA	TAIR
AAGAGATTTG	2	3	2	1	3	no match found	gi|115311458|gb|BT029001.1	multiple canonical match
at1g20020	CON	MIN	HOUR	DAY	WEEK	UNIGENE	FLCDNA	TAIR
TGTGGACTCA	5	26	9	15	5	gnl|UG|At#S11741052	gi|21387156|gb|AY114663.1	At1g20020.1
at5g65390	CON	MIN	HOUR	DAY	WEEK	UNIGENE	FLCDNA	TAIR
GTTTGTTGTC	5	8	1	0	1	no match found	gi|15215665|gb|AY050361.1	multiple non-canonical match
CCTCGCTCAA	0	1	1	0	2	no match found	gi|20334897|gb|AY094049.1	non-canonical match
at1g52130	CON	MIN	HOUR	DAY	WEEK	UNIGENE	FLCDNA	TAIR
TTTTTAATCC	0	1	0	0	0	no match found	no match found	At1g52130.1
at3g59090	CON	MIN	HOUR	DAY	WEEK	UNIGENE	FLCDNA	TAIR
TGTTGGGGGG	1	3	3	1	2	gnl|UG|At#S28282785	gi|15724161|gb|AF411783.1	At3g59090.1
at4g39100	CON	MIN	HOUR	DAY	WEEK	UNIGENE	FLCDNA	TAIR
GAGCCAAGGA	0	6	0	1	0	gnl|UG|At#S11721082	gi|19310792|gb|AY079396.1	At4g39100.1
at5g27240	CON	MIN	HOUR	DAY	WEEK	UNIGENE	FLCDNA	TAIR
TTTGATAGAT	1	1	3	1	0	no match found	no match found	At5g27240.1
at3g52150	CON	MIN	HOUR	DAY	WEEK	UNIGENE	FLCDNA	TAIR
CTACATCCAA	1	0	0	1	1	gnl|UG|At#S11729033	no match found	pseudo chromosome match
at1g03970	CON	MIN	HOUR	DAY	WEEK	UNIGENE	FLCDNA	TAIR
GAGGTTCTGA	1	5	2	0	0	gnl|UG|At#S34117777	gi|110736995|dbj|AK228531.1|	multiple canonical match
at3g15630	CON	MIN	HOUR	DAY	WEEK	UNIGENE	FLCDNA	TAIR
TCATCGGAAC	1	15	0	1	1	gnl|UG|At#S11736154	gi|15529233|gb|AY052241.1	At3g15630.1
at3g56880	CON	MIN	HOUR	DAY	WEEK	UNIGENE	FLCDNA	TAIR
GAAAGTTATG	0	9	14	3	0	gnl|UG|At#S11728166	gi|21406690|gb|AY087916.1	At3g56880.1
at4g38740	CON	MIN	HOUR	DAY	WEEK	UNIGENE	FLCDNA	TAIR
TGCCAGGGAG	0	0	1	0	0	no match found	gi|28059459|gb|BT003397.1	non-canonical match

GGAACATATA	9	9	5	3	3	gnl|UG|At#S11721145	gi|20260655|gb|AY093227.1	At4g38740.1
at3g60140	CON	MIN	HOUR	DAY	WEEK	UNIGENE	FLCDNA	TAIR
GTAATATTTT	0	0	1	0	0	gnl|UG|At#S11703038	gi|13605842|gb|AF367320.1	At3g60140.1
at5g14710	CON	MIN	HOUR	DAY	WEEK	UNIGENE	FLCDNA	TAIR
GAGTTGTGGA	0	5	1	3	2	gnl|UG|At#S11722662	gi|26450349|dbj|AK117635.1|	At5g14710.1
at4g38500	CON	MIN	HOUR	DAY	WEEK	UNIGENE	FLCDNA	TAIR
TTTTCCAGAC	0	0	0	0	1	no match found	gi|21689814|gb|AY123018.1	At5g01130.1
TTCCAGAACT	0	2	0	0	1	gnl|UG|At#S11704570	gi|15010783|gb|AY045693.1	non-canonical match
at3g14010	CON	MIN	HOUR	DAY	WEEK	UNIGENE	FLCDNA	TAIR
CCATATCATA	1	2	0	2	0	gnl|UG|At#S11704755	gi|15028198|gb|AY045922.1	At3g14010.1
at4g28240	CON	MIN	HOUR	DAY	WEEK	UNIGENE	FLCDNA	TAIR
AACTCCGCTG	7	15	1	10	1	gnl|UG|At#S11722922	gi|22530931|gb|AY136304.1	At4g28240.1
at4g23610	CON	MIN	HOUR	DAY	WEEK	UNIGENE	FLCDNA	TAIR
AAGATGACAA	0	0	0	0	1	gnl|UG|At#S11703077	gi|13937134|gb|AF372921.1	At4g23610.1
at4g35170	CON	MIN	HOUR	DAY	WEEK	UNIGENE	FLCDNA	TAIR
AAGATGAAGA	1	0	0	0	0	no match found	no match found	At4g35170.1
at2g17390	CON	MIN	HOUR	DAY	WEEK	UNIGENE	FLCDNA	TAIR
TAATAGTGTC	4	2	3	1	0	no match found	no match found	At2g17390.1
at2g20835	CON	MIN	HOUR	DAY	WEEK	UNIGENE	FLCDNA	TAIR
AGAACCTCCA	0	1	0	0	0	no match found	gi|26450583|dbj|AK117756.1|	At2g20835.1
at5g44200	CON	MIN	HOUR	DAY	WEEK	UNIGENE	FLCDNA	TAIR
AGACCGGAGA	0	4	2	1	0	gnl|UG|At#S11719308	gi|28466940|gb|BT004813.1	At5g44200.1
at4g33010	CON	MIN	HOUR	DAY	WEEK	UNIGENE	FLCDNA	TAIR
GAAAAAGCCG	27	15	38	24	6	gnl|UG|At#S11722104	gi|16604475|gb|AY058856.1	At4g33010.1
at1g55480	CON	MIN	HOUR	DAY	WEEK	UNIGENE	FLCDNA	TAIR
TTGCCAAACG	0	0	0	1	0	gnl|UG|At#S11732482	gi|14532603|gb|AY039926.1	At1g55480.1
at5g44580	CON	MIN	HOUR	DAY	WEEK	UNIGENE	FLCDNA	TAIR
GTGGGGGTCG	8	26	10	19	4	gnl|UG|At#S18912305	gi|72198282|gb|DQ108847.1	At5g44580.1
at4g32020	CON	MIN	HOUR	DAY	WEEK	UNIGENE	FLCDNA	TAIR
AGATTAAATA	0	0	2	2	1	gnl|UG|At#S11722272	no match found	At4g32020.1
ACCTCGCCGC	1	0	4	0	0	no match found	gi|21928042|gb|AY125540.1	non-canonical match
TAACCGTTTG	19	29	79	23	32	no match found	gi|14326505|gb|AF385706.1	non-canonical match
at5g35840	CON	MIN	HOUR	DAY	WEEK	UNIGENE	FLCDNA	TAIR
GAGAGAGGAA	2	2	0	1	0	gnl|UG|At#S11720119	no match found	At5g35840.1
AAGTGAAGGA	0	1	1	0	0	no match found	gi|18086444|gb|AY065040.1	multiple non-canonical match
at3g59840	CON	MIN	HOUR	DAY	WEEK	UNIGENE	FLCDNA	TAIR
GTTCACGGGA	0	3	1	2	0	gnl|UG|At#S11727553	gi|17473783|gb|AY065151.1	At3g59840.1
at3g05240	CON	MIN	HOUR	DAY	WEEK	UNIGENE	FLCDNA	TAIR
AAGAGCAAAA	0	0	2	0	0	gnl|UG|At#S11739155	no match found	non-canonical match
at4g02390	CON	MIN	HOUR	DAY	WEEK	UNIGENE	FLCDNA	TAIR
CTCCAAGGGG	1	0	0	0	0	gnl|UG|At#S11727081	no match found	At4g02390.1
at1g75720	CON	MIN	HOUR	DAY	WEEK	UNIGENE	FLCDNA	TAIR

ATCTTCACAA	0	0	0	0	1	no match found	no match found	At1g75720.1
at4g14365	CON	MIN	HOUR	DAY	WEEK	UNIGENE	FLCDNA	TAIR
ATCAATGTTA	1	1	0	1	2	gnl|UG|At#S11725323	gi|13358233|gb|AF325044.2	At4g14365.1
at3g10572	CON	MIN	HOUR	DAY	WEEK	UNIGENE	FLCDNA	TAIR
ATTAAGAACC	1	0	1	2	3	gnl|UG|At#S15461315	gi|17065215|gb|AY062684.1	At3g10572.1
at5g66160	CON	MIN	HOUR	DAY	WEEK	UNIGENE	FLCDNA	TAIR
CTCAACACAC	0	4	0	0	0	gnl|UG|At#S24442028	no match found	multiple canonical match
TGTACATTTG	1	0	2	2	0	gnl|UG|At#S20835834	gi|14334831|gb|AY035089.1	non-canonical match
at1g16930	CON	MIN	HOUR	DAY	WEEK	UNIGENE	FLCDNA	TAIR
TTGGTGGATT	0	3	0	0	0	no match found	no match found	At1g16930.1
at3g53860	CON	MIN	HOUR	DAY	WEEK	UNIGENE	FLCDNA	TAIR
TTATAGTAGC	0	1	0	0	1	no match found	gi|110738376|dbj|AK229250.1|	multiple canonical match
at1g32210	CON	MIN	HOUR	DAY	WEEK	UNIGENE	FLCDNA	TAIR
CTTTAATGTA	0	2	3	1	0	gnl|UG|At#S11738193	gi|110740705|dbj|AK226298.1|	At1g32210.1
at3g04090	CON	MIN	HOUR	DAY	WEEK	UNIGENE	FLCDNA	TAIR
GGACCATATC	1	3	0	1	1	no match found	gi|110740960|dbj|AK226432.1|	multiple non-canonical match
at3g55560	CON	MIN	HOUR	DAY	WEEK	UNIGENE	FLCDNA	TAIR
TTCTTGAGAT	2	0	2	1	0	gnl|UG|At#S11728411	no match found	At3g55560.1
at4g29530	CON	MIN	HOUR	DAY	WEEK	UNIGENE	FLCDNA	TAIR
GTAATGGAAA	1	0	0	0	0	gnl|UG|At#S15177183	gi|29824358|gb|BT006155.1	At4g29530.1
at3g28705	CON	MIN	HOUR	DAY	WEEK	UNIGENE	FLCDNA	TAIR
GATAAACGAT	0	1	0	0	0	no match found	no match found	At3g28705.1
at1g31180	CON	MIN	HOUR	DAY	WEEK	UNIGENE	FLCDNA	TAIR
TATTAGAAAC	6	1	3	1	2	gnl|UG|At#S18895173	gi|21406068|gb|AY087344.1	At1g31180.1
at4g29500	CON	MIN	HOUR	DAY	WEEK	UNIGENE	FLCDNA	TAIR
TAGTTTATTT	0	1	2	0	1	no match found	no match found	At4g29500.1
at1g33970	CON	MIN	HOUR	DAY	WEEK	UNIGENE	FLCDNA	TAIR
TCAAGTTTTT	2	7	3	1	0	gnl|UG|At#S28282544	no match found	non-canonical match
at3g48990	CON	MIN	HOUR	DAY	WEEK	UNIGENE	FLCDNA	TAIR
GTGGTAAAGG	30	15	38	28	26	gnl|UG|At#S11729641	gi|17065365|gb|AY062759.1	At3g48990.1
at1g66180	CON	MIN	HOUR	DAY	WEEK	UNIGENE	FLCDNA	TAIR
TGTTTCGATG	3	6	6	3	0	gnl|UG|At#S11729891	gi|15293194|gb|AY051031.1	At1g66180.1
at4g23740	CON	MIN	HOUR	DAY	WEEK	UNIGENE	FLCDNA	TAIR
GGAACATCAA	0	1	0	0	1	gnl|UG|At#S11723693	gi|26451765|dbj|AK118364.1|	At4g23740.1
at4g27830	CON	MIN	HOUR	DAY	WEEK	UNIGENE	FLCDNA	TAIR
GGGTCTTGAA	2	1	3	2	0	gnl|UG|At#S11722988	gi|15982821|gb|AY057518.1	At4g27830.1
at1g14570	CON	MIN	HOUR	DAY	WEEK	UNIGENE	FLCDNA	TAIR
ATCTCTGCTA	2	1	1	1	1	no match found	gi|17978788|gb|AY064683.1	At1g14570.1
at5g06150	CON	MIN	HOUR	DAY	WEEK	UNIGENE	FLCDNA	TAIR
CTTGCTGCTT	0	0	0	0	1	gnl|UG|At#S11724586	no match found	At5g06150.1
at1g06770	CON	MIN	HOUR	DAY	WEEK	UNIGENE	FLCDNA	TAIR

TAACATTGTT	0	0	2	0	1	gnl|UG|At#S18942631	no match found	At1g06770.2
TAAAGAGTCC	0	2	0	1	0	no match found	gi|15809891|gb|AY054213.1	non-canonical match
at1g77380	CON	MIN	HOUR	DAY	WEEK	UNIGENE	FLCDNA	TAIR
AACTAGCACC	0	1	0	0	0	gnl|UG|At#S11727430	no match found	At1g77380.1
at5g23590	CON	MIN	HOUR	DAY	WEEK	UNIGENE	FLCDNA	TAIR
ATTAAGAATT	1	0	1	0	0	no match found	gi|22135987|gb|AY128373.1	At5g23590.1
at1g22400	CON	MIN	HOUR	DAY	WEEK	UNIGENE	FLCDNA	TAIR
TTGTAATATA	0	0	1	0	1	gnl|UG|At#S35299959	no match found	non-canonical match
at3g51840	CON	MIN	HOUR	DAY	WEEK	UNIGENE	FLCDNA	TAIR
GATTTCATCA	2	4	3	1	0	gnl|UG|At#S11729090	no match found	At3g51840.1
at1g25230	CON	MIN	HOUR	DAY	WEEK	UNIGENE	FLCDNA	TAIR
TCAGTTAAGA	0	0	1	0	0	no match found	gi|22655337|gb|AY141997.1	non-canonical match
AAAATCAGTT	4	7	0	2	0	gnl|UG|At#S11740411	gi|17978996|gb|AY069908.1	multiple canonical match
at2g46380	CON	MIN	HOUR	DAY	WEEK	UNIGENE	FLCDNA	TAIR
ATCACCACAT	0	1	0	0	0	no match found	no match found	At2g46380.1
at3g10070	CON	MIN	HOUR	DAY	WEEK	UNIGENE	FLCDNA	TAIR
GGATTGAATT	0	0	0	2	0	gnl|UG|At#S24442473	gi|62320726|dbj|AK221690.1|	multiple canonical match
at5g02040	CON	MIN	HOUR	DAY	WEEK	UNIGENE	FLCDNA	TAIR
ATCGTGTTCT	1	1	1	1	0	gnl|UG|At#S11725648	gi|15028344|gb|AY045975.1	At5g02040.2
at1g66270	CON	MIN	HOUR	DAY	WEEK	UNIGENE	FLCDNA	TAIR
AGTCGTGGTT	0	0	1	0	0	gnl|UG|At#S11729874	gi|15010793|gb|AY045698.1	At1g66270.2
at1g60190	CON	MIN	HOUR	DAY	WEEK	UNIGENE	FLCDNA	TAIR
GGGTCGCTTT	0	0	0	5	3	gnl|UG|At#S11731435	no match found	At1g60190.1
at1g17470	CON	MIN	HOUR	DAY	WEEK	UNIGENE	FLCDNA	TAIR
AATACAAGAT	1	0	0	0	0	gnl|UG|At#S11741297	no match found	At1g17470.1
at4g24190	CON	MIN	HOUR	DAY	WEEK	UNIGENE	FLCDNA	TAIR
ATGATGAGAA	0	0	1	0	0	no match found	gi|18252918|gb|AY072394.1	multiple non-canonical match
TACCAGACAG	8	5	6	0	0	gnl|UG|At#S11723613	gi|14532541|gb|AY039895.1	At4g24190.2
at1g77490	CON	MIN	HOUR	DAY	WEEK	UNIGENE	FLCDNA	TAIR
CCAAGCTTAG	3	4	1	1	0	gnl|UG|At#S11727385	gi|21404264|gb|AY085554.1	At1g77490.1
at4g32530	CON	MIN	HOUR	DAY	WEEK	UNIGENE	FLCDNA	TAIR
TTTGTATTCT	5	4	1	5	5	gnl|UG|At#S43849447	gi|14596150|gb|AY042863.1	At4g32530.1
GCTTCTTGAT	1	2	1	2	2	no match found	gi|62318832|dbj|AK220736.1|	multiple non-canonical match
at5g42330	CON	MIN	HOUR	DAY	WEEK	UNIGENE	FLCDNA	TAIR
GATGTTTTGT	1	0	0	0	0	gnl|UG|At#S11719496	gi|51970797|dbj|AK176328.1|	At5g42330.1
at1g26800	CON	MIN	HOUR	DAY	WEEK	UNIGENE	FLCDNA	TAIR
GGTCGTGTCC	1	0	0	1	0	no match found	gi|15450674|gb|AY052705.1	non-canonical match
at5g17990	CON	MIN	HOUR	DAY	WEEK	UNIGENE	FLCDNA	TAIR
TGGTCTAGAC	0	0	1	0	0	no match found	gi|20259899|gb|AY093298.1	non-canonical match
TAAACACTAC	10	0	1	0	6	gnl|UG|At#S11721825	gi|15450851|gb|AY054506.1	At5g17990.1
at1g04900	CON	MIN	HOUR	DAY	WEEK	UNIGENE	FLCDNA	TAIR
ATACTCAATA	1	1	1	0	1	gnl|UG|At#S11809097	gi|21407664|gb|AY088890.1	non-canonical match

at2g43140	CON	MIN	HOUR	DAY	WEEK	UNIGENE	FLCDNA	TAIR
CCGCAAACAA	0	0	1	0	0	gnl|UG|At#S11731849	no match found	multiple non-canonical match
at1g14850	CON	MIN	HOUR	DAY	WEEK	UNIGENE	FLCDNA	TAIR
GGCTAGACTC	0	1	0	0	0	gnl|UG|At#S11741549	no match found	At1g14850.1
at3g45900	CON	MIN	HOUR	DAY	WEEK	UNIGENE	FLCDNA	TAIR
GAAGATTGAT	0	0	0	1	0	no match found	gi|48310009|gb|BT014753.1	multiple canonical match
at5g63300	CON	MIN	HOUR	DAY	WEEK	UNIGENE	FLCDNA	TAIR
ACAAGTGATC	0	1	0	0	0	gnl|UG|At#S21737549	gi|51968393|dbj|AK175126.1|	non-canonical match
TAGCTAGAGC	0	0	0	0	1	no match found	no match found	At5g63300.1
at4g05100	CON	MIN	HOUR	DAY	WEEK	UNIGENE	FLCDNA	TAIR
CAATCATTGT	0	0	0	0	1	gnl|UG|At#S11726365	gi|14423389|gb|AF386932.1	At4g05100.1
at1g78730	CON	MIN	HOUR	DAY	WEEK	UNIGENE	FLCDNA	TAIR
TGAACCTTAA	1	0	1	0	0	gnl|UG|At#S34114722	gi|110742822|dbj|AK227293.1|	At1g78730.1
at1g58070	CON	MIN	HOUR	DAY	WEEK	UNIGENE	FLCDNA	TAIR
ACGAAATCAG	1	0	0	0	0	no match found	gi|29029101|gb|BT005995.1	non-canonical match
at5g15980	CON	MIN	HOUR	DAY	WEEK	UNIGENE	FLCDNA	TAIR
GATTGTTCCG	2	1	0	0	1	gnl|UG|At#S11722341	no match found	At5g15980.1
TATCACTCAC	0	2	0	3	2	no match found	gi|20856901|gb|AY102122.1	non-canonical match
at5g42310	CON	MIN	HOUR	DAY	WEEK	UNIGENE	FLCDNA	TAIR
AGACACAACA	0	0	1	0	0	gnl|UG|At#S11719498	gi|21539516|gb|AY120753.1	multiple non-canonical match
at2g23520	CON	MIN	HOUR	DAY	WEEK	UNIGENE	FLCDNA	TAIR
TTTTGGCCAA	0	0	0	1	0	gnl|UG|At#S11736604	no match found	multiple non-canonical match
AGAAAAGTTA	0	0	0	1	0	no match found	no match found	At2g23520.1
at1g22070	CON	MIN	HOUR	DAY	WEEK	UNIGENE	FLCDNA	TAIR
TAATGTCTTT	2	2	0	1	0	gnl|UG|At#S34114981	gi|110742347|dbj|AK227036.1|	At1g22070.1
GAGAATCTTC	0	1	0	0	0	no match found	gi|109946422|gb|BT026034.1	At1g66000.1
at5g02930	CON	MIN	HOUR	DAY	WEEK	UNIGENE	FLCDNA	TAIR
TGGAATATCA	0	0	0	0	1	no match found	no match found	At5g02930.1
at3g23600	CON	MIN	HOUR	DAY	WEEK	UNIGENE	FLCDNA	TAIR
GCTGTGATAA	0	7	0	0	0	gnl|UG|At#S28282927	gi|17381243|gb|AY064133.1	At3g23600.1
at2g05185	CON	MIN	HOUR	DAY	WEEK	UNIGENE	FLCDNA	TAIR
TTATGGTTTA	1	1	0	0	0	no match found	no match found	At2g05185.1
at1g10130	CON	MIN	HOUR	DAY	WEEK	UNIGENE	FLCDNA	TAIR
AGATTCAGGT	0	1	0	0	0	no match found	gi|110738279|dbj|AK229199.1|	non-canonical match
at5g28050	CON	MIN	HOUR	DAY	WEEK	UNIGENE	FLCDNA	TAIR
ATGGTAATTA	2	12	4	1	4	gnl|UG|At#S18910189	gi|21407910|gb|AY089136.1	At5g28050.1
at1g67500	CON	MIN	HOUR	DAY	WEEK	UNIGENE	FLCDNA	TAIR
GCTGAGTGGT	1	2	1	2	0	gnl|UG|At#S11729613	no match found	At1g67500.1
at1g15480	CON	MIN	HOUR	DAY	WEEK	UNIGENE	FLCDNA	TAIR
TTGGTAGAAA	0	0	1	1	1	gnl|UG|At#S11741488	no match found	At1g15480.1
at2g17972	CON	MIN	HOUR	DAY	WEEK	UNIGENE	FLCDNA	TAIR
GGAAGAGATG	2	0	2	1	0	no match found	gi|26451251|dbj|AK118100.1|	multiple non-canonical match

at5g42130	CON	MIN	HOUR	DAY	WEEK	UNIGENE	FLCDNA	TAIR
GGACCTCGAG	1	1	3	0	1	gnl|UG|At#S11719516	no match found	At5g42130.1

at1g45110	CON	MIN	HOUR	DAY	WEEK	UNIGENE	FLCDNA	TAIR
TTGTAAAACT	0	0	1	0	0	gnl|UG|At#S11735637	gi|110738767|dbj|AK229448.1|	At1g45110.1

at1g14610	CON	MIN	HOUR	DAY	WEEK	UNIGENE	FLCDNA	TAIR
CAGCACCACC	1	5	1	0	0	no match found	gi|38564251|gb|BT010735.1	At2g13200.1
AACAACTTAA	4	1	2	2	0	gnl|UG|At#S11741575	gi|17065233|gb|AY062693.1	At1g14610.1

at1g47570	CON	MIN	HOUR	DAY	WEEK	UNIGENE	FLCDNA	TAIR
TATGTCGCCC	0	2	0	1	0	gnl|UG|At#S38434197	gi|40823233|gb|BT011232.1	At1g47570.1

at2g05210	CON	MIN	HOUR	DAY	WEEK	UNIGENE	FLCDNA	TAIR
ATATTAACCA	1	0	0	1	0	gnl|UG|At#S43850276	gi|62320289|dbj|AK221471.1|	At2g05210.1

at1g60525	CON	MIN	HOUR	DAY	WEEK	UNIGENE	FLCDNA	TAIR
TTGGTGGTGA	1	0	1	1	0	gnl|UG|At#S18893734	no match found	multiple non-canonical match

at2g41430	CON	MIN	HOUR	DAY	WEEK	UNIGENE	FLCDNA	TAIR
ATCTAGAGTT	0	0	1	0	0	no match found	gi|21405801|gb|AY087077.1	At2g41430.1
GTATTAGATC	1	2	0	0	0	gnl|UG|At#S18942731	no match found	At2g41430.4

at5g43280	CON	MIN	HOUR	DAY	WEEK	UNIGENE	FLCDNA	TAIR
AGAATATGAT	2	1	1	0	0	gnl|UG|At#S20534449	gi|18252832|gb|AY072351.1	At5g43280.1

at4g21570	CON	MIN	HOUR	DAY	WEEK	UNIGENE	FLCDNA	TAIR
ATTGGGGAAA	4	3	3	10	6	gnl|UG|At#S11724077	gi|20260133|gb|AY092966.1	At4g21570.1

at1g45688	CON	MIN	HOUR	DAY	WEEK	UNIGENE	FLCDNA	TAIR
TGATCGGAGA	0	6	3	2	0	gnl|UG|At#S18942424	gi|21407371|gb|AY088597.1	At1g45688.1

at3g59210	CON	MIN	HOUR	DAY	WEEK	UNIGENE	FLCDNA	TAIR
GCTACAAGTA	0	1	0	0	0	gnl|UG|At#S11822860	gi|26453043|dbj|AK119022.1|	At3g59210.1
GTTATTTTGA	5	2	7	3	0	gnl|UG|At#S37211362	no match found	multiple non-canonical match

at4g24440	CON	MIN	HOUR	DAY	WEEK	UNIGENE	FLCDNA	TAIR
TGATTCCAAG	1	1	0	0	0	gnl|UG|At#S11699368	gi|18176270|gb|AY072192.1	At4g24440.2

at3g07160	CON	MIN	HOUR	DAY	WEEK	UNIGENE	FLCDNA	TAIR
CGTATTGGCT	0	1	0	0	1	gnl|UG|At#S11738576	gi|23198275|gb|BT000346.1	At3g07160.1

at3g10270	CON	MIN	HOUR	DAY	WEEK	UNIGENE	FLCDNA	TAIR
CGACGAAGTT	0	0	0	0	1	no match found	no match found	At3g10270.1
TTAGGGAAGG	0	1	0	0	0	gnl|UG|At#S11737810	no match found	multiple non-canonical match

at3g19180	CON	MIN	HOUR	DAY	WEEK	UNIGENE	FLCDNA	TAIR
GGATCATTTA	0	0	0	0	1	gnl|UG|At#S28282951	gi|18377659|gb|AY074283.1	At3g19180.1

at1g64400	CON	MIN	HOUR	DAY	WEEK	UNIGENE	FLCDNA	TAIR
GAAAGAGATC	0	1	1	1	0	gnl|UG|At#S11730256	gi|14532621|gb|AY039935.1	At1g64400.1

at5g07910	CON	MIN	HOUR	DAY	WEEK	UNIGENE	FLCDNA	TAIR
GATCAGTTTC	0	1	0	0	0	no match found	gi|22136191|gb|AY128774.1	At5g07910.1

at4g01590	CON	MIN	HOUR	DAY	WEEK	UNIGENE	FLCDNA	TAIR
TTCAAGCGTA	2	4	1	0	1	gnl|UG|At#S28282112	gi|27754412|gb|BT002836.1	At4g01590.1

at4g27110	CON	MIN	HOUR	DAY	WEEK	UNIGENE	FLCDNA	TAIR
TTTGTTTATA	0	2	0	0	0	no match found	no match found	At4g27110.1

at3g12300	CON	MIN	HOUR	DAY	WEEK	UNIGENE	FLCDNA	TAIR
ATCTTGGGTC	2	0	0	0	0	gnl|UG|At#S11737198	gi|15982827|gb|AY057521.1	At3g12300.1
at2g28600	CON	MIN	HOUR	DAY	WEEK	UNIGENE	FLCDNA	TAIR
ATAAGTCTTT	0	1	0	0	0	gnl|UG|At#S11807179	gi|29824178|gb|BT006065.1	At2g28600.1
at5g51810	CON	MIN	HOUR	DAY	WEEK	UNIGENE	FLCDNA	TAIR
GTGAAATGCA	1	0	0	0	0	gnl|UG|At#S11832515	no match found	At5g51810.1
at2g26210	CON	MIN	HOUR	DAY	WEEK	UNIGENE	FLCDNA	TAIR
TCTTCTTCCG	2	3	3	3	0	gnl|UG|At#S15461135	gi|17644148|gb|AY065296.1	At2g26210.1
at5g52010	CON	MIN	HOUR	DAY	WEEK	UNIGENE	FLCDNA	TAIR
GTGAATTGAG	0	0	1	0	1	no match found	gi|14517513|gb|AY039592.1	non-canonical match
at2g34490	CON	MIN	HOUR	DAY	WEEK	UNIGENE	FLCDNA	TAIR
AGTGTGATTA	0	0	1	1	0	no match found	gi|21404287|gb|AY085577.1	non-canonical match
TATTTTATGT	1	0	1	0	2	gnl|UG|At#S11733945	gi|18650636|gb|AY074867.1	At2g34490.1
at3g16950	CON	MIN	HOUR	DAY	WEEK	UNIGENE	FLCDNA	TAIR
GTTTCAATTA	1	3	0	0	0	gnl|UG|At#S11735770	gi|15215695|gb|AY050376.1	At3g16950.1
at4g12110	CON	MIN	HOUR	DAY	WEEK	UNIGENE	FLCDNA	TAIR
TGGGTCATTG	1	3	2	3	1	gnl|UG|At#S18337916	gi|21928126|gb|AY125499.1	At4g12110.1
at2g30350	CON	MIN	HOUR	DAY	WEEK	UNIGENE	FLCDNA	TAIR
GAACTATAGG	0	0	0	0	1	gnl|UG|At#S15460960	gi|51968919|dbj|AK175389.1|	At2g30350.2
at4g31460	CON	MIN	HOUR	DAY	WEEK	UNIGENE	FLCDNA	TAIR
GGCAGAGCCA	0	0	1	0	0	no match found	gi|15724233|gb|AF412057.1	At4g31460.1
at2g20740	CON	MIN	HOUR	DAY	WEEK	UNIGENE	FLCDNA	TAIR
TGTGCATTTA	2	2	0	1	0	gnl|UG|At#S20720587	gi|18087598|gb|AF462842.1	At2g20740.3
TATGGAGCCA	0	0	0	1	0	no match found	gi|20453254|gb|AY097350.1	non-canonical match
at5g38840	CON	MIN	HOUR	DAY	WEEK	UNIGENE	FLCDNA	TAIR
TAAACGAAGA	2	3	1	1	1	gnl|UG|At#S11719849	gi|110741019|dbj|AK226462.1|	At5g38840.1
at2g37160	CON	MIN	HOUR	DAY	WEEK	UNIGENE	FLCDNA	TAIR
CGCGATGTCC	0	1	0	0	0	gnl|UG|At#S11823614	gi|26451504|dbj|AK118231.1|	At2g37160.1
at1g26830	CON	MIN	HOUR	DAY	WEEK	UNIGENE	FLCDNA	TAIR
AAGTCCAGGA	0	1	2	2	1	gnl|UG|At#S34115556	gi|110741279|dbj|AK230392.1|	At1g26830.1
at4g27550	CON	MIN	HOUR	DAY	WEEK	UNIGENE	FLCDNA	TAIR
CCGTTGGTGT	0	1	0	0	0	gnl|UG|At#S11723035	no match found	non-canonical match
at3g01820	CON	MIN	HOUR	DAY	WEEK	UNIGENE	FLCDNA	TAIR
GCAGGGCTTG	0	1	0	4	1	gnl|UG|At#S11740131	gi|21928120|gb|AY125496.1	At3g01820.1
at3g02470	CON	MIN	HOUR	DAY	WEEK	UNIGENE	FLCDNA	TAIR
TGACCCCTGA	9	49	20	24	8	gnl|UG|At#S11739947	gi|16226704|gb|AF428468.1	At3g02470.1
at2g31970	CON	MIN	HOUR	DAY	WEEK	UNIGENE	FLCDNA	TAIR
CTGAAAAATA	1	0	0	0	0	gnl|UG|At#S11734536	gi|22654996|gb|AY139771.1	At2g31970.1
at5g47420	CON	MIN	HOUR	DAY	WEEK	UNIGENE	FLCDNA	TAIR
AGAGAGAATC	1	0	0	0	0	gnl|UG|At#S18912026	gi|19424008|gb|AY080805.1	At5g47420.1
at5g26820	CON	MIN	HOUR	DAY	WEEK	UNIGENE	FLCDNA	TAIR
TCTTACGGCG	0	1	0	0	0	gnl|UG|At#S11720530	gi|17065059|gb|AY062606.1	At5g26820.1

at5g06700	CON	MIN	HOUR	DAY	WEEK	UNIGENE	FLCDNA	TAIR
CGATAGCGAA	1	6	3	3	2	gnl|UG|At#S11724447	gi|110738759|dbj|AK229444.1|	At5g06700.1
at5g62960	CON	MIN	HOUR	DAY	WEEK	UNIGENE	FLCDNA	TAIR
CTATGGAGCC	1	0	0	0	0	gnl|UG|At#S11717405	gi|38604057|gb|BT010994.1	At5g62960.1
at3g27650	CON	MIN	HOUR	DAY	WEEK	UNIGENE	FLCDNA	TAIR
GGAGAGGTCA	0	2	0	0	0	gnl|UG|At#S11732422	gi|21407652|gb|AY088878.1	At3g27650.1
at3g50310	CON	MIN	HOUR	DAY	WEEK	UNIGENE	FLCDNA	TAIR
TCGCTTTTGA	0	1	0	0	0	gnl|UG|At#S11729386	gi|21405194|gb|AY086484.1	At3g50310.1
at4g19490	CON	MIN	HOUR	DAY	WEEK	UNIGENE	FLCDNA	TAIR
TCGAGCTAAT	1	0	0	0	1	gnl|UG|At#S15460933	gi|110742432|dbj|AK227083.1|	At4g19490.1
at4g25150	CON	MIN	HOUR	DAY	WEEK	UNIGENE	FLCDNA	TAIR
TAATCACTTG	1	0	0	0	0	gnl|UG|At#S11723450	gi|18389259|gb|AY074377.1	At4g25150.1
at3g21580	CON	MIN	HOUR	DAY	WEEK	UNIGENE	FLCDNA	TAIR
ATTGTTAGAG	0	1	0	0	0	gnl|UG|At#S30651334	gi|119360058|gb|BT029502.1	At3g21580.1
at5g53940	CON	MIN	HOUR	DAY	WEEK	UNIGENE	FLCDNA	TAIR
TTTATAGAGA	0	1	1	0	2	no match found	gi|51970423|dbj|AK176141.1|	multiple canonical match
at2g39770	CON	MIN	HOUR	DAY	WEEK	UNIGENE	FLCDNA	TAIR
TGAGCGATGA	1	7	4	1	4	gnl|UG|At#S11732659	gi|16226304|gb|AF428297.1	At2g39770.1
at1g54830	CON	MIN	HOUR	DAY	WEEK	UNIGENE	FLCDNA	TAIR
GATTTATCTA	1	1	3	1	1	gnl|UG|At#S18942376	gi|21406254|gb|AY087517.1	multiple non-canonical match
at5g59240	CON	MIN	HOUR	DAY	WEEK	UNIGENE	FLCDNA	TAIR
TTTATGGATT	0	0	0	1	0	gnl|UG|At#S34118379	gi|110735840|dbj|AK227927.1|	At5g59240.1
at5g58900	CON	MIN	HOUR	DAY	WEEK	UNIGENE	FLCDNA	TAIR
GAGGAAACTA	1	0	0	0	0	gnl|UG|At#S11717815	gi|28973136|gb|BT005473.1	At5g58900.1
at5g61900	CON	MIN	HOUR	DAY	WEEK	UNIGENE	FLCDNA	TAIR
AATGATCTCG	1	1	0	1	0	gnl|UG|At#S18941435	gi|21404049|gb|AY085339.1	At5g61900.1
at5g20840	CON	MIN	HOUR	DAY	WEEK	UNIGENE	FLCDNA	TAIR
TATATGAGGC	1	2	0	1	0	gnl|UG|At#S11721122	gi|19424023|gb|AY080794.1	At5g20840.1
at1g22950	CON	MIN	HOUR	DAY	WEEK	UNIGENE	FLCDNA	TAIR
TGCCGAGTAA	0	1	0	1	0	gnl|UG|At#S11740768	no match found	At1g22950.1
at3g12980	CON	MIN	HOUR	DAY	WEEK	UNIGENE	FLCDNA	TAIR
TGTATAAGGG	0	1	0	2	1	gnl|UG|At#S11736998	no match found	At3g12980.1
at4g18710	CON	MIN	HOUR	DAY	WEEK	UNIGENE	FLCDNA	TAIR
TATCAGATCT	1	4	1	0	0	no match found	gi|109946416|gb|BT026031.1	non-canonical match
GTTTTGCTTT	0	2	2	3	1	gnl|UG|At#S11724551	no match found	At4g18710.1
at3g19553	CON	MIN	HOUR	DAY	WEEK	UNIGENE	FLCDNA	TAIR
TATTGCTTAT	1	2	2	1	2	gnl|UG|At#S11734988	no match found	At3g19553.1
at1g63740	CON	MIN	HOUR	DAY	WEEK	UNIGENE	FLCDNA	TAIR
AAATTTCTCA	0	0	0	1	0	gnl|UG|At#S11730389	gi|20466557|gb|AY099745.1	At1g63740.1
at3g16720	CON	MIN	HOUR	DAY	WEEK	UNIGENE	FLCDNA	TAIR
GATCTGGATC	0	4	2	0	0	gnl|UG|At#S11735846	gi|21407006|gb|AY088232.1	At3g16720.1

at3g26030	CON	MIN	HOUR	DAY	WEEK	UNIGENE	FLCDNA	TAIR
TAAACATCTT	9	1	1	4	3	gnl|UG|At#S20714701	gi|20259475|gb|AY091037.1	At3g26030.1
at1g65290	CON	MIN	HOUR	DAY	WEEK	UNIGENE	FLCDNA	TAIR
AATTACGAGG	12	10	13	2	11	gnl|UG|At#S11730072	gi|21403946|gb|AY085236.1	At1g65290.1
at1g29418	CON	MIN	HOUR	DAY	WEEK	UNIGENE	FLCDNA	TAIR
AATTCTTATG	14	11	4	6	0	gnl|UG|At#S18323115	no match found	non-canonical match
at2g04790	CON	MIN	HOUR	DAY	WEEK	UNIGENE	FLCDNA	TAIR
ATTGCCGACA	2	1	1	0	0	gnl|UG|At#S28282360	gi|48596994|gb|BT014909.1	At2g04790.1
at1g30960	CON	MIN	HOUR	DAY	WEEK	UNIGENE	FLCDNA	TAIR
TTGTCAAAGA	1	0	0	0	1	gnl|UG|At#S11738670	no match found	pseudo chromosome match
at3g44110	CON	MIN	HOUR	DAY	WEEK	UNIGENE	FLCDNA	TAIR
GGTTTGGGCT	1	5	6	17	3	no match found	gi|110741023|dbj|AK226464.1|	multiple non-canonical match
CAGAACAAAT	0	0	1	4	1	gnl|UG|At#S11730545	no match found	At3g44110.2
at1g08700	CON	MIN	HOUR	DAY	WEEK	UNIGENE	FLCDNA	TAIR
TTGGGTGTTG	1	1	2	1	0	no match found	gi|21406982|gb|AY088208.1	non-canonical match
TATTTCACCT	1	1	1	0	0	gnl|UG|At#S11742165	no match found	At1g08700.1
at3g09360	CON	MIN	HOUR	DAY	WEEK	UNIGENE	FLCDNA	TAIR
AAGACAGATT	0	0	0	1	0	gnl|UG|At#S34115329	gi|110741697|dbj|AK226688.1|	At3g09360.1
at4g29890	CON	MIN	HOUR	DAY	WEEK	UNIGENE	FLCDNA	TAIR
ATAAATAGGT	0	1	0	0	0	gnl|UG|At#S18905502	gi|19699339|gb|AY090377.1	At4g29890.1
at1g51460	CON	MIN	HOUR	DAY	WEEK	UNIGENE	FLCDNA	TAIR
CTCTCTCTTC	0	1	0	0	0	gnl|UG|At#S11733972	gi|26451908|dbj|AK118438.1|	At1g51460.1
at3g53600	CON	MIN	HOUR	DAY	WEEK	UNIGENE	FLCDNA	TAIR
CTGATTTCTG	0	0	1	0	0	no match found	no match found	At3g53600.1
at1g22590	CON	MIN	HOUR	DAY	WEEK	UNIGENE	FLCDNA	TAIR
GTTTTTAATT	0	2	2	0	1	no match found	gi|26450735|dbj|AK117835.1|	At1g22590.1
at4g26510	CON	MIN	HOUR	DAY	WEEK	UNIGENE	FLCDNA	TAIR
TGGGCCAATG	0	0	1	0	0	gnl|UG|At#S11723224	no match found	At4g26510.2
at4g14600	CON	MIN	HOUR	DAY	WEEK	UNIGENE	FLCDNA	TAIR
CACGTGGTTC	0	1	0	0	0	gnl|UG|At#S11743038	gi|21404665|gb|AY085955.1	At4g14600.1
at1g04210	CON	MIN	HOUR	DAY	WEEK	UNIGENE	FLCDNA	TAIR
AATCTCATCA	0	2	0	2	0	no match found	no match found	At1g04210.1
AAATGATTCT	0	0	0	0	1	gnl|UG|At#S11742627	gi|20466531|gb|AY099732.1	non-canonical match
at3g04760	CON	MIN	HOUR	DAY	WEEK	UNIGENE	FLCDNA	TAIR
GTACGCAAAG	1	0	0	0	0	gnl|UG|At#S11706051	gi|15810358|gb|AY056218.1	At3g04760.1
at1g23960	CON	MIN	HOUR	DAY	WEEK	UNIGENE	FLCDNA	TAIR
GATAGTACAG	1	2	1	0	0	gnl|UG|At#S18894353	gi|45680404|gb|BT012094.1	At1g23960.1
at5g08260	CON	MIN	HOUR	DAY	WEEK	UNIGENE	FLCDNA	TAIR
ATGGCAAGTT	0	3	1	1	8	gnl|UG|At#S11724048	gi|17065641|gb|AY062969.1	At5g08260.1
at3g18990	CON	MIN	HOUR	DAY	WEEK	UNIGENE	FLCDNA	TAIR
TATCTTCCTT	0	2	0	0	0	no match found	gi|89000958|gb|BT024731.1	multiple canonical match

at3g56490	CON	MIN	HOUR	DAY	WEEK	UNIGENE	FLCDNA	TAIR
TTCATCTCAT	1	0	2	0	0	no match found	gi|26983873|gb|BT002356.1	multiple non-canonical match
ACTTTTATGA	5	3	2	3	5	no match found	gi|21405584|gb|AY086874.1	multiple non-canonical match

at1g51740	CON	MIN	HOUR	DAY	WEEK	UNIGENE	FLCDNA	TAIR
TTCTGCAGCA	0	1	0	0	0	gnl|UG|At#S11733872	gi|21405279|gb|AY086569.1	At1g51740.1

at2g42350	CON	MIN	HOUR	DAY	WEEK	UNIGENE	FLCDNA	TAIR
AACCATAATA	0	1	0	0	0	gnl|UG|At#S11732020	gi|38566659|gb|BT010853.1	At2g42350.1

at1g49480	CON	MIN	HOUR	DAY	WEEK	UNIGENE	FLCDNA	TAIR
TACTTGCCAT	0	1	0	0	0	gnl|UG|At#S11734687	gi|51972153|gb|BT015682.1	At1g49480.1

at1g12040	CON	MIN	HOUR	DAY	WEEK	UNIGENE	FLCDNA	TAIR
TAAAGAAGGC	0	1	0	0	0	gnl|UG|At#S11702727	no match found	At1g12040.1

at2g23090	CON	MIN	HOUR	DAY	WEEK	UNIGENE	FLCDNA	TAIR
GCTCGTGCCA	7	24	6	6	5	gnl|UG|At#S20525310	gi|15450483|gb|AY052343.1	At2g23090.1

at4g37760	CON	MIN	HOUR	DAY	WEEK	UNIGENE	FLCDNA	TAIR
CGCCATCCTC	0	2	1	0	0	no match found	gi|18086576|gb|AY072019.1	non-canonical match

at3g18970	CON	MIN	HOUR	DAY	WEEK	UNIGENE	FLCDNA	TAIR
CATTGGATTC	0	1	0	0	1	gnl|UG|At#S11735158	no match found	At3g18970.1

at5g01230	CON	MIN	HOUR	DAY	WEEK	UNIGENE	FLCDNA	TAIR
TGGTGACTTA	0	0	1	0	0	gnl|UG|At#S11707384	gi|21406726|gb|AY087952.1	At5g01230.2

at3g29030	CON	MIN	HOUR	DAY	WEEK	UNIGENE	FLCDNA	TAIR
CGACCCTCCC	0	1	0	0	0	gnl|UG|At#S26538919	gi|110737468|dbj|AK228779.1|	At3g29030.1

at1g24460	CON	MIN	HOUR	DAY	WEEK	UNIGENE	FLCDNA	TAIR
TTTTGTGTCG	1	0	1	1	0	no match found	no match found	At1g24460.1

at2g44200	CON	MIN	HOUR	DAY	WEEK	UNIGENE	FLCDNA	TAIR
ATAAGCACTT	0	2	0	1	0	no match found	gi|21360458|gb|AY113037.1	non-canonical match

at4g10480	CON	MIN	HOUR	DAY	WEEK	UNIGENE	FLCDNA	TAIR
AGCTCTCAGC	2	11	1	4	5	gnl|UG|At#S11725917	gi|20465564|gb|AY096615.1	At4g10480.1

at2g31060	CON	MIN	HOUR	DAY	WEEK	UNIGENE	FLCDNA	TAIR
ACCCAAGTTA	1	1	0	2	1	gnl|UG|At#S28282229	gi|62319234|dbj|AK220940.1|	At4g10480.1

at4g38170	CON	MIN	HOUR	DAY	WEEK	UNIGENE	FLCDNA	TAIR
AGCAATATGT	0	0	0	1	0	gnl|UG|At#S11721240	no match found	At4g38170.1

at1g02475	CON	MIN	HOUR	DAY	WEEK	UNIGENE	FLCDNA	TAIR
GAGTGGCAAG	0	0	3	1	1	gnl|UG|At#S11742867	gi|13878058|gb|AF370292.1	At1g02475.1

at2g46200	CON	MIN	HOUR	DAY	WEEK	UNIGENE	FLCDNA	TAIR
CAACACCCTT	0	1	0	0	0	gnl|UG|At#S18942693	gi|18176103|gb|AY072162.1	At2g46200.1

at4g13180	CON	MIN	HOUR	DAY	WEEK	UNIGENE	FLCDNA	TAIR
GGCCGTGAAA	0	1	0	0	0	gnl|UG|At#S11725534	gi|17933306|gb|AF446363.1	At4g13180.1

at4g13900	CON	MIN	HOUR	DAY	WEEK	UNIGENE	FLCDNA	TAIR
AGAATTGTTT	5	1	1	3	1	no match found	gi|62320421|dbj|AK221537.1|	multiple non-canonical match

at5g64905	CON	MIN	HOUR	DAY	WEEK	UNIGENE	FLCDNA	TAIR
ACCATTGAAA	0	0	1	0	0	gnl|UG|At#S30614154	gi|21406289|gb|AY087551.1	At5g64905.1

at1g26230	CON	MIN	HOUR	DAY	WEEK	UNIGENE	FLCDNA	TAIR
AGAAGTCACA	1	1	0	0	0	gnl|UG|At#S24442526	gi|62320621|dbj|AK221637.1|	pseudo chromosome match
at4g37790	CON	MIN	HOUR	DAY	WEEK	UNIGENE	FLCDNA	TAIR
TTAGTTACTT	0	2	0	0	1	gnl|UG|At#S11704695	gi|21406301|gb|AY087563.1	At4g37790.1
at1g68910	CON	MIN	HOUR	DAY	WEEK	UNIGENE	FLCDNA	TAIR
GAGACCTTAA	0	1	0	0	0	gnl|UG|At#S38434087	no match found	At1g68910.1
at5g02820	CON	MIN	HOUR	DAY	WEEK	UNIGENE	FLCDNA	TAIR
TGAATTATTA	0	0	0	1	1	gnl|UG|At#S11700068	no match found	At5g02820.1
TCATATGATA	1	0	0	0	0	no match found	gi|114213516|gb|BT028966.1	multiple non-canonical match
at2g28250	CON	MIN	HOUR	DAY	WEEK	UNIGENE	FLCDNA	TAIR
CAAAAATAAC	0	1	0	0	0	gnl|UG|At#S11710170	gi|28394006|gb|BT004417.1	At2g28250.1
at2g12462	CON	MIN	HOUR	DAY	WEEK	UNIGENE	FLCDNA	TAIR
TATTAATTTT	0	0	1	0	0	gnl|UG|At#S37211619	no match found	non-canonical match
at3g57190	CON	MIN	HOUR	DAY	WEEK	UNIGENE	FLCDNA	TAIR
AGAAGATCAA	1	1	0	1	0	no match found	gi|30725573|gb|BT008450.1	multiple non-canonical match
at1g26690	CON	MIN	HOUR	DAY	WEEK	UNIGENE	FLCDNA	TAIR
AGTTTTGTAC	0	3	0	2	1	gnl|UG|At#S11740093	gi|21403475|gb|AY084765.1	At1g26690.1
at2g40350	CON	MIN	HOUR	DAY	WEEK	UNIGENE	FLCDNA	TAIR
GGGTAAATGG	0	1	0	1	0	gnl|UG|At#S11732506	no match found	At2g40350.1
at5g22000	CON	MIN	HOUR	DAY	WEEK	UNIGENE	FLCDNA	TAIR
TGCGACTGGT	3	9	6	2	1	gnl|UG|At#S15460574	gi|26451709|dbj|AK118336.1|	At5g22000.3
at3g59770	CON	MIN	HOUR	DAY	WEEK	UNIGENE	FLCDNA	TAIR
AGCGTAAGAA	1	0	0	0	0	gnl|UG|At#S28282781	gi|110742407|dbj|AK227068.1|	At3g59770.1
at3g25800	CON	MIN	HOUR	DAY	WEEK	UNIGENE	FLCDNA	TAIR
CTCTAAGCTC	1	0	1	0	0	no match found	gi|27311764|gb|BT002488.1	At3g25800.1
at5g24530	CON	MIN	HOUR	DAY	WEEK	UNIGENE	FLCDNA	TAIR
GGCATATTTG	0	1	7	6	1	no match found	gi|14423475|gb|AF386975.1	At5g24530.1
AGCCCGGCCA	0	0	0	1	0	no match found	gi|20148252|gb|AY081455.1	non-canonical match
at5g25220	CON	MIN	HOUR	DAY	WEEK	UNIGENE	FLCDNA	TAIR
AACTCAAACA	1	2	1	1	2	no match found	gi|21539534|gb|AY120762.1	multiple non-canonical match
AGTTTTTTTT	0	0	1	2	2	gnl|UG|At#S11720661	no match found	At5g25220.1
at2g29540	CON	MIN	HOUR	DAY	WEEK	UNIGENE	FLCDNA	TAIR
GACATTGAAA	0	0	0	0	1	gnl|UG|At#S11700525	gi|28827295|gb|BT004959.1	At2g29540.1
at1g81020	CON	MIN	HOUR	DAY	WEEK	UNIGENE	FLCDNA	TAIR
TGTTTCTTAA	2	1	0	0	0	no match found	no match found	At1g81020.1
at1g71090	CON	MIN	HOUR	DAY	WEEK	UNIGENE	FLCDNA	TAIR
ATGCTAGTAG	0	1	0	0	0	no match found	gi|19310750|gb|AY079375.1	non-canonical match
AACCGTGCAC	2	1	2	0	0	gnl|UG|At#S11704844	gi|15028380|gb|AY045993.1	At1g71090.1
at5g21060	CON	MIN	HOUR	DAY	WEEK	UNIGENE	FLCDNA	TAIR
TCTGTGGATG	0	0	0	0	1	no match found	gi|28973500|gb|BT005655.1	At5g21060.1
at3g02910	CON	MIN	HOUR	DAY	WEEK	UNIGENE	FLCDNA	TAIR
GTCTTTAGAT	0	0	0	1	0	gnl|UG|At#S11739807	no match found	At3g02910.1

at1g15175	CON	MIN	HOUR	DAY	WEEK	UNIGENE	FLCDNA	TAIR
TCTGTATTGA	0	2	0	0	0	gnl|UG|At#S26539667	gi|72197616|gb|DQ108760.1	At3g02910.1

at2g26430	CON	MIN	HOUR	DAY	WEEK	UNIGENE	FLCDNA	TAIR
CTAAGATCAC	4	2	3	2	0	gnl|UG|At#S38433923	gi|20258845|gb|AY091085.1	At2g26430.1

at5g55570	CON	MIN	HOUR	DAY	WEEK	UNIGENE	FLCDNA	TAIR
GAGAAGATTC	0	0	0	0	1	no match found	no match found	At5g55570.1

at5g39960	CON	MIN	HOUR	DAY	WEEK	UNIGENE	FLCDNA	TAIR
GAAAGACACG	0	1	0	0	0	gnl|UG|At#S11719735	no match found	non-canonical match

at3g29580	CON	MIN	HOUR	DAY	WEEK	UNIGENE	FLCDNA	TAIR
AAGAGAAAGA	1	5	1	3	1	no match found	no match found	At3g29580.1

at5g07100	CON	MIN	HOUR	DAY	WEEK	UNIGENE	FLCDNA	TAIR
CGCCTATACA	0	0	0	1	0	gnl|UG|At#S18941666	no match found	At5g07100.1

at3g63170	CON	MIN	HOUR	DAY	WEEK	UNIGENE	FLCDNA	TAIR
TCGGGAGTGT	1	2	1	0	1	gnl|UG|At#S11726481	gi|17473779|gb|AY065149.1	non-canonical match

at1g25490	CON	MIN	HOUR	DAY	WEEK	UNIGENE	FLCDNA	TAIR
CTCTAAGTTT	2	1	0	2	0	gnl|UG|At#S11740344	gi|62321444|dbj|AK222057.1|	At1g25490.1

at1g09820	CON	MIN	HOUR	DAY	WEEK	UNIGENE	FLCDNA	TAIR
ACACCACCAT	2	1	0	0	0	gnl|UG|At#S11742052	no match found	At1g09820.1

at3g23050	CON	MIN	HOUR	DAY	WEEK	UNIGENE	FLCDNA	TAIR
GGAAATGTTT	4	39	6	4	2	no match found	gi|18377419|gb|AY072461.1	multiple non-canonical match
TGTTTTGATC	1	3	4	5	0	gnl|UG|At#S20803856	gi|21406503|gb|AY087765.1	At3g23050.1

at3g46070	CON	MIN	HOUR	DAY	WEEK	UNIGENE	FLCDNA	TAIR
ATAAGTTTCT	1	0	0	0	1	no match found	no match found	At3g46070.1

at2g38010	CON	MIN	HOUR	DAY	WEEK	UNIGENE	FLCDNA	TAIR
TCGTAATCGC	0	1	0	0	0	gnl|UG|At#S18942761	no match found	At2g38010.1

at3g05680	CON	MIN	HOUR	DAY	WEEK	UNIGENE	FLCDNA	TAIR
TACATTAGCT	0	2	0	0	0	gnl|UG|At#S11739015	gi|28393003|gb|BT003888.1	At3g05680.1

at5g37340	CON	MIN	HOUR	DAY	WEEK	UNIGENE	FLCDNA	TAIR
GGAGCAAAAC	1	0	0	0	0	gnl|UG|At#S18941556	gi|17979122|gb|AY070062.1	At5g37340.2

at5g52250	CON	MIN	HOUR	DAY	WEEK	UNIGENE	FLCDNA	TAIR
ATTAATACAA	0	0	1	0	2	gnl|UG|At#S11718491	gi|110736197|dbj|AK228112.1|	At5g52250.1

at1g79790	CON	MIN	HOUR	DAY	WEEK	UNIGENE	FLCDNA	TAIR
GACGTTTTGC	0	0	1	0	0	no match found	gi|110735804|dbj|AK227909.1|	At1g79790.1

at1g18720	CON	MIN	HOUR	DAY	WEEK	UNIGENE	FLCDNA	TAIR
TATTACAATC	0	1	0	0	2	no match found	no match found	At1g18720.1
GATTGATGTA	3	2	3	1	1	gnl|UG|At#S18893710	gi|15809883|gb|AY054209.1	non-canonical match

at4g36240	CON	MIN	HOUR	DAY	WEEK	UNIGENE	FLCDNA	TAIR
AAGTTAAGCT	0	1	0	0	2	no match found	gi|18252998|gb|AY072434.1	non-canonical match
ATCATTAAAA	1	0	0	0	0	gnl|UG|At#S11721557	no match found	At4g36240.1

at3g61930	CON	MIN	HOUR	DAY	WEEK	UNIGENE	FLCDNA	TAIR
GTGTATCGGA	0	0	1	0	0	gnl|UG|At#S11726865	gi|26451344|dbj|AK118148.1|	At3g61930.1

at3g09922	CON	MIN	HOUR	DAY	WEEK	UNIGENE	FLCDNA	TAIR

TAAATTTGCT	0	3	1	0	0	no match found	gi|26452915|dbj|AK118956.1|	At3g09922.1

at1g15080	CON	MIN	HOUR	DAY	WEEK	UNIGENE	FLCDNA	TAIR
GAAATATCAA	2	0	1	1	2	gnl|UG|At#S14274037	gi|21406411|gb|AY087673.1	At1g15080.1

at3g27260	CON	MIN	HOUR	DAY	WEEK	UNIGENE	FLCDNA	TAIR
AAGAGTCCCT	1	1	2	3	0	gnl|UG|At#S11732553	gi|15724175|gb|AF411790.1	At3g27260.1
AGGGAAATAT	0	1	0	0	0	no match found	gi|20259927|gb|AY093312.1	non-canonical match

at3g24150	CON	MIN	HOUR	DAY	WEEK	UNIGENE	FLCDNA	TAIR
GATTGATCTT	2	1	0	0	0	gnl|UG|At#S34117481	gi|110737561|dbj|AK228827.1|	At3g24150.1

at1g18490	CON	MIN	HOUR	DAY	WEEK	UNIGENE	FLCDNA	TAIR
CGTCCCGGAT	0	1	1	1	0	no match found	gi|18377499|gb|AY072501.1	non-canonical match

at1g72630	CON	MIN	HOUR	DAY	WEEK	UNIGENE	FLCDNA	TAIR
TTTTTGTTGT	0	1	0	1	1	no match found	gi|14532583|gb|AY039916.1	non-canonical match

at1g07610	CON	MIN	HOUR	DAY	WEEK	UNIGENE	FLCDNA	TAIR
GTCTCAAACC	3	5	1	2	1	gnl|UG|At#S20705126	gi|14030618|gb|AF375400.1	At1g07610.1

at5g17210	CON	MIN	HOUR	DAY	WEEK	UNIGENE	FLCDNA	TAIR
AGAAGACAGT	0	1	0	0	0	gnl|UG|At#S18941618	gi|14334547|gb|AY035178.1	At5g17210.2

at3g16100	CON	MIN	HOUR	DAY	WEEK	UNIGENE	FLCDNA	TAIR
TGTTTTAAGG	0	3	1	2	0	gnl|UG|At#S11736019	gi|21403401|gb|AY084691.1	At3g16100.1

at3g16040	CON	MIN	HOUR	DAY	WEEK	UNIGENE	FLCDNA	TAIR
TTGCGATGTT	1	0	0	0	0	gnl|UG|At#S11736035	gi|21405411|gb|AY086701.1	At3g16040.1

at3g52750	CON	MIN	HOUR	DAY	WEEK	UNIGENE	FLCDNA	TAIR
ATATTGTTGA	0	0	0	1	0	gnl|UG|At#S11705939	gi|15810584|gb|AY056331.1	At3g52750.1

at3g57510	CON	MIN	HOUR	DAY	WEEK	UNIGENE	FLCDNA	TAIR
AATAAAGTAA	0	0	0	0	1	gnl|UG|At#S11728049	gi|26450945|dbj|AK117942.1|	At3g57510.1

at1g21370	CON	MIN	HOUR	DAY	WEEK	UNIGENE	FLCDNA	TAIR
TGTTGTCGAA	0	2	0	1	0	gnl|UG|At#S14273754	gi|28393586|gb|BT004195.1	non-canonical match

at3g62770	CON	MIN	HOUR	DAY	WEEK	UNIGENE	FLCDNA	TAIR
GATGGGAGCT	1	3	1	0	0	no match found	gi|16323361|gb|AY059169.1	At3g62770.2

at1g74510	CON	MIN	HOUR	DAY	WEEK	UNIGENE	FLCDNA	TAIR
TTGTCTTGTC	0	0	2	0	1	gnl|UG|At#S15435755	no match found	At1g74510.1
ATTGGGGTCA	1	1	0	1	0	no match found	gi|110736699|dbj|AK228375.1|	non-canonical match

at3g02430	CON	MIN	HOUR	DAY	WEEK	UNIGENE	FLCDNA	TAIR
TTACTTGGAA	1	0	0	0	0	no match found	no match found	At3g02430.1

at3g26420	CON	MIN	HOUR	DAY	WEEK	UNIGENE	FLCDNA	TAIR
AAAGTGAAAT	2	2	0	0	0	no match found	no match found	At3g26420.1

at1g22190	CON	MIN	HOUR	DAY	WEEK	UNIGENE	FLCDNA	TAIR
CTTCGACCGT	4	2	0	2	2	gnl|UG|At#S11740841	gi|15292782|gb|AY050825.1	At1g22190.1

at4g10360	CON	MIN	HOUR	DAY	WEEK	UNIGENE	FLCDNA	TAIR
TTGTGGTTGT	1	0	2	4	1	no match found	no match found	At4g10360.1
ATGTTACTGT	1	0	0	0	1	gnl|UG|At#S28282071	no match found	non-canonical match

at2g37090	CON	MIN	HOUR	DAY	WEEK	UNIGENE	FLCDNA	TAIR
TATAGACGTA	1	0	0	0	0	no match found	gi|53828582|gb|BT015838.1	non-canonical match

at1g65430	CON	MIN	HOUR	DAY	WEEK	UNIGENE	FLCDNA	TAIR
AGAGGTGAGT	1	3	2	0	2	gnl|UG|At#S11730041	gi|17065463|gb|AY062808.1	At1g65430.1
at1g79930	CON	MIN	HOUR	DAY	WEEK	UNIGENE	FLCDNA	TAIR
GCTGCGGGGA	0	3	1	7	1	gnl|UG|At#S28282375	gi|110741979|dbj|AK226836.1|	At1g79930.1
at5g67350	CON	MIN	HOUR	DAY	WEEK	UNIGENE	FLCDNA	TAIR
GAAGACGAAC	1	0	2	1	0	gnl|UG|At#S14273780	gi|28393536|gb|BT004169.1	At5g67350.1
at3g51670	CON	MIN	HOUR	DAY	WEEK	UNIGENE	FLCDNA	TAIR
TTTAGCTATT	0	0	2	0	0	gnl|UG|At#S11709398	gi|16930482|gb|AF419595.1	At3g51670.1
at4g31780	CON	MIN	HOUR	DAY	WEEK	UNIGENE	FLCDNA	TAIR
ATATGCACGA	7	2	3	6	2	gnl|UG|At#S11806935	gi|31711945|gb|BT008890.1	At4g31780.1
at3g43330	CON	MIN	HOUR	DAY	WEEK	UNIGENE	FLCDNA	TAIR
TACACAATTG	12	9	14	9	6	no match found	no match found	At3g43330.1
at5g04990	CON	MIN	HOUR	DAY	WEEK	UNIGENE	FLCDNA	TAIR
GGCGTGCGCC	1	2	1	1	0	gnl|UG|At#S11724884	gi|15912310|gb|AY056433.1	At5g04990.1
at5g17310	CON	MIN	HOUR	DAY	WEEK	UNIGENE	FLCDNA	TAIR
TGAAAAGACA	2	3	2	1	0	no match found	gi|13430663|gb|AF360244.1	non-canonical match
TAGAATTTTA	0	0	1	3	3	gnl|UG|At#S11702969	gi|13605670|gb|AF361816.1	At5g17310.1
at5g67540	CON	MIN	HOUR	DAY	WEEK	UNIGENE	FLCDNA	TAIR
GCAGATAGGT	0	1	0	0	0	gnl|UG|At#S18941404	gi|20856123|gb|AY101528.1	At5g67540.1
at2g27790	CON	MIN	HOUR	DAY	WEEK	UNIGENE	FLCDNA	TAIR
ACTTATGGAG	0	1	0	0	0	no match found	gi|110741299|dbj|AK230402.1|	multiple non-canonical match
at1g53800	CON	MIN	HOUR	DAY	WEEK	UNIGENE	FLCDNA	TAIR
AGATCTAACG	0	0	1	0	1	gnl|UG|At#S22666648	gi|15724263|gb|AF412072.1	At1g53800.1
at5g10820	CON	MIN	HOUR	DAY	WEEK	UNIGENE	FLCDNA	TAIR
AAACATTTCG	0	1	0	1	0	no match found	no match found	At5g10820.1
at5g11770	CON	MIN	HOUR	DAY	WEEK	UNIGENE	FLCDNA	TAIR
CTGACTATTG	1	3	2	3	6	gnl|UG|At#S11723301	gi|16226315|gb|AF428300.1	At5g11770.1
at4g32180	CON	MIN	HOUR	DAY	WEEK	UNIGENE	FLCDNA	TAIR
AGGGTTCAGT	2	1	1	1	1	gnl|UG|At#S43849454	no match found	At5g11770.1
ATTATCCAGG	0	0	1	0	0	gnl|UG|At#S11831581	no match found	pseudo chromosome match
at5g51200	CON	MIN	HOUR	DAY	WEEK	UNIGENE	FLCDNA	TAIR
ATTTCAAATG	3	3	1	1	3	no match found	no match found	At5g51200.1
at1g72175	CON	MIN	HOUR	DAY	WEEK	UNIGENE	FLCDNA	TAIR
CCGGTTCGTG	0	1	2	0	0	gnl|UG|At#S11728654	gi|13878188|gb|AF370357.1	At1g72175.1
at5g59160	CON	MIN	HOUR	DAY	WEEK	UNIGENE	FLCDNA	TAIR
TTATATTATC	2	4	0	0	0	gnl|UG|At#S11717789	no match found	At5g59160.1
at5g63750	CON	MIN	HOUR	DAY	WEEK	UNIGENE	FLCDNA	TAIR
CAGAGTATGA	0	1	0	0	0	gnl|UG|At#S11717325	no match found	At5g63750.1
at3g52310	CON	MIN	HOUR	DAY	WEEK	UNIGENE	FLCDNA	TAIR
TATCTTACTT	0	0	0	1	0	gnl|UG|At#S11729004	no match found	At3g52310.1
at1g01720	CON	MIN	HOUR	DAY	WEEK	UNIGENE	FLCDNA	TAIR

CAGAAGCCTT	2	3	4	7	4	gnl|UG|At#S18896550	gi|87116659|gb|BT024513.1	At1g01720.1
at2g27140	CON	MIN	HOUR	DAY	WEEK	UNIGENE	FLCDNA	TAIR
ATTCTCAAAG	0	1	1	0	0	gnl|UG|At#S11735755	gi|21405730|gb|AY087006.1	At2g27140.1
at1g50900	CON	MIN	HOUR	DAY	WEEK	UNIGENE	FLCDNA	TAIR
GGTATGCTTG	3	8	2	2	4	gnl|UG|At#S11734186	gi|21404562|gb|AY085852.1	At1g50900.1
at4g27690	CON	MIN	HOUR	DAY	WEEK	UNIGENE	FLCDNA	TAIR
TCGAGACAGA	0	6	3	0	0	gnl|UG|At#S11723009	gi|45680198|gb|BT011888.1	At4g27690.1
at1g73460	CON	MIN	HOUR	DAY	WEEK	UNIGENE	FLCDNA	TAIR
GCCTTGGACT	0	1	0	0	1	no match found	gi|19424094|gb|AY080848.1	At1g73450.1
at3g16780	CON	MIN	HOUR	DAY	WEEK	UNIGENE	FLCDNA	TAIR
AAAGTGAAGG	0	1	0	2	0	no match found	gi|21407847|gb|AY089073.1	At3g16780.1
at4g09840	CON	MIN	HOUR	DAY	WEEK	UNIGENE	FLCDNA	TAIR
AAGCTGGAGA	1	2	3	2	1	no match found	gi|52218805|gb|BT015696.1	multiple non-canonical match
at2g42170	CON	MIN	HOUR	DAY	WEEK	UNIGENE	FLCDNA	TAIR
AAGTGTGATG	0	1	2	0	0	no match found	gi|26452949|dbj|AK118975.1|	At2g42170.1
at3g30460	CON	MIN	HOUR	DAY	WEEK	UNIGENE	FLCDNA	TAIR
TAGTATTTTC	0	0	0	0	2	gnl|UG|At#S21737086	gi|51969319|dbj|AK175589.1|	multiple non-canonical match
at5g36700	CON	MIN	HOUR	DAY	WEEK	UNIGENE	FLCDNA	TAIR
TATACATAAA	2	0	0	0	1	gnl|UG|At#S43849229	no match found	multiple non-canonical match
at1g58370	CON	MIN	HOUR	DAY	WEEK	UNIGENE	FLCDNA	TAIR
TGCTAATGTA	0	0	1	0	0	gnl|UG|At#S11699464	no match found	At1g58370.1
at5g18420	CON	MIN	HOUR	DAY	WEEK	UNIGENE	FLCDNA	TAIR
CAGAATAGGC	0	0	0	0	1	gnl|UG|At#S11721718	gi|21539502|gb|AY120746.1	At5g18420.3
at2g32690	CON	MIN	HOUR	DAY	WEEK	UNIGENE	FLCDNA	TAIR
CATTCGTAAC	10	5	1	4	4	gnl|UG|At#S18899457	no match found	At2g32690.1
at5g60120	CON	MIN	HOUR	DAY	WEEK	UNIGENE	FLCDNA	TAIR
AACCGCCCAC	1	2	0	0	1	gnl|UG|At#S11743035	gi|18389251|gb|AY074373.1	At5g60120.1
at1g30835	CON	MIN	HOUR	DAY	WEEK	UNIGENE	FLCDNA	TAIR
TGTAATTTGT	0	1	1	0	0	gnl|UG|At#S20722524	gi|110737347|dbj|AK228717.1|	multiple non-canonical match
GACCTAACCA	0	0	0	1	0	no match found	gi|110735858|dbj|AK227937.1|	At1g30835.1
at3g29320	CON	MIN	HOUR	DAY	WEEK	UNIGENE	FLCDNA	TAIR
TAATGATGCA	5	8	9	2	3	gnl|UG|At#S11731863	gi|15146188|gb|AY049235.1	At3g29320.1
at4g11100	CON	MIN	HOUR	DAY	WEEK	UNIGENE	FLCDNA	TAIR
AGTATAATAA	0	0	0	0	1	no match found	gi|21407895|gb|AY089121.1	At4g11100.1
at3g10090	CON	MIN	HOUR	DAY	WEEK	UNIGENE	FLCDNA	TAIR
AGGAACGTTA	1	4	2	3	0	no match found	gi|40823560|gb|BT011253.1	At3g10090.1
at3g62860	CON	MIN	HOUR	DAY	WEEK	UNIGENE	FLCDNA	TAIR
ATTTTACCTC	1	0	0	0	0	gnl|UG|At#S11705456	gi|16226352|gb|AF428376.1	At3g62860.1
at5g10810	CON	MIN	HOUR	DAY	WEEK	UNIGENE	FLCDNA	TAIR
GACTATGACT	0	2	1	0	0	gnl|UG|At#S11676418	gi|28827443|gb|BT005033.1	At5g10810.1
at1g68400	CON	MIN	HOUR	DAY	WEEK	UNIGENE	FLCDNA	TAIR

<table>
<tr><td>TGGTGAAGTT</td><td>0</td><td>1</td><td>1</td><td>0</td><td>0</td><td>gnl|UG|At#S11729419</td><td>gi|14190424|gb|AF378890.1</td><td>At1g68400.1</td></tr>
<tr><td>at4g34480</td><td>CON</td><td>MIN</td><td>HOUR</td><td>DAY</td><td>WEEK</td><td>UNIGENE</td><td>FLCDNA</td><td>TAIR</td></tr>
<tr><td>TAAACGCAAA</td><td>0</td><td>0</td><td>0</td><td>0</td><td>1</td><td>gnl|UG|At#S11721852</td><td>no match found</td><td>At4g34480.1</td></tr>
<tr><td>at1g69550</td><td>CON</td><td>MIN</td><td>HOUR</td><td>DAY</td><td>WEEK</td><td>UNIGENE</td><td>FLCDNA</td><td>TAIR</td></tr>
<tr><td>ATTTTAGCCT</td><td>1</td><td>1</td><td>1</td><td>0</td><td>0</td><td>gnl|UG|At#S11729196</td><td>gi|62318996|dbj|AK220819.1|</td><td>At1g69550.1</td></tr>
<tr><td>at2g46090</td><td>CON</td><td>MIN</td><td>HOUR</td><td>DAY</td><td>WEEK</td><td>UNIGENE</td><td>FLCDNA</td><td>TAIR</td></tr>
<tr><td>GGCCATCATA</td><td>0</td><td>0</td><td>2</td><td>0</td><td>0</td><td>gnl|UG|At#S11731169</td><td>gi|18377559|gb|AY072531.1</td><td>At2g46090.1</td></tr>
<tr><td>at1g73450</td><td>CON</td><td>MIN</td><td>HOUR</td><td>DAY</td><td>WEEK</td><td>UNIGENE</td><td>FLCDNA</td><td>TAIR</td></tr>
<tr><td>GCCTTGGACT</td><td>0</td><td>1</td><td>0</td><td>0</td><td>1</td><td>gnl|UG|At#S11728365</td><td>no match found</td><td>At1g73450.1</td></tr>
<tr><td>at2g42610</td><td>CON</td><td>MIN</td><td>HOUR</td><td>DAY</td><td>WEEK</td><td>UNIGENE</td><td>FLCDNA</td><td>TAIR</td></tr>
<tr><td>TATCTCTGGC</td><td>0</td><td>0</td><td>1</td><td>0</td><td>0</td><td>gnl|UG|At#S18898574</td><td>no match found</td><td>non-canonical match</td></tr>
<tr><td>TCCTCTCCAA</td><td>0</td><td>1</td><td>0</td><td>0</td><td>0</td><td>no match found</td><td>gi|26452445|dbj|AK118714.1|</td><td>At2g42610.1</td></tr>
<tr><td>at1g45545</td><td>CON</td><td>MIN</td><td>HOUR</td><td>DAY</td><td>WEEK</td><td>UNIGENE</td><td>FLCDNA</td><td>TAIR</td></tr>
<tr><td>AGCATTTTGA</td><td>0</td><td>0</td><td>2</td><td>0</td><td>0</td><td>no match found</td><td>no match found</td><td>At1g45545.1</td></tr>
<tr><td>at4g14890</td><td>CON</td><td>MIN</td><td>HOUR</td><td>DAY</td><td>WEEK</td><td>UNIGENE</td><td>FLCDNA</td><td>TAIR</td></tr>
<tr><td>GCGACTCTTC</td><td>0</td><td>2</td><td>0</td><td>0</td><td>2</td><td>gnl|UG|At#S11793078</td><td>gi|21404949|gb|AY086239.1</td><td>At4g14890.1</td></tr>
<tr><td>at3g44340</td><td>CON</td><td>MIN</td><td>HOUR</td><td>DAY</td><td>WEEK</td><td>UNIGENE</td><td>FLCDNA</td><td>TAIR</td></tr>
<tr><td>ACCTTGATGC</td><td>0</td><td>1</td><td>1</td><td>0</td><td>1</td><td>gnl|UG|At#S28282868</td><td>no match found</td><td>At3g44340.1</td></tr>
<tr><td>at2g34610</td><td>CON</td><td>MIN</td><td>HOUR</td><td>DAY</td><td>WEEK</td><td>UNIGENE</td><td>FLCDNA</td><td>TAIR</td></tr>
<tr><td>GAGATATTAA</td><td>0</td><td>1</td><td>0</td><td>1</td><td>0</td><td>no match found</td><td>gi|17979356|gb|AY070408.1</td><td>At2g34610.1</td></tr>
<tr><td>TAACTCATTT</td><td>1</td><td>0</td><td>0</td><td>0</td><td>0</td><td>gnl|UG|At#S30627186</td><td>no match found</td><td>pseudo chromosome match</td></tr>
<tr><td>at3g17170</td><td>CON</td><td>MIN</td><td>HOUR</td><td>DAY</td><td>WEEK</td><td>UNIGENE</td><td>FLCDNA</td><td>TAIR</td></tr>
<tr><td>TTTTGGATAA</td><td>1</td><td>0</td><td>4</td><td>1</td><td>3</td><td>gnl|UG|At#S11706514</td><td>gi|18377865|gb|AY074580.1</td><td>At3g17170.1</td></tr>
<tr><td>at5g41940</td><td>CON</td><td>MIN</td><td>HOUR</td><td>DAY</td><td>WEEK</td><td>UNIGENE</td><td>FLCDNA</td><td>TAIR</td></tr>
<tr><td>AAAATGTCTT</td><td>2</td><td>1</td><td>0</td><td>0</td><td>0</td><td>gnl|UG|At#S11719535</td><td>no match found</td><td>At5g41940.1</td></tr>
<tr><td>at3g58100</td><td>CON</td><td>MIN</td><td>HOUR</td><td>DAY</td><td>WEEK</td><td>UNIGENE</td><td>FLCDNA</td><td>TAIR</td></tr>
<tr><td>ATGCTCTCAT</td><td>0</td><td>3</td><td>0</td><td>0</td><td>0</td><td>gnl|UG|At#S11727933</td><td>gi|21404709|gb|AY085999.1</td><td>At3g58100.1</td></tr>
<tr><td>at5g65540</td><td>CON</td><td>MIN</td><td>HOUR</td><td>DAY</td><td>WEEK</td><td>UNIGENE</td><td>FLCDNA</td><td>TAIR</td></tr>
<tr><td>TGTAAAAGAG</td><td>0</td><td>0</td><td>0</td><td>1</td><td>0</td><td>gnl|UG|At#S11717143</td><td>gi|26451237|dbj|AK118093.1|</td><td>non-canonical match</td></tr>
<tr><td>at2g21500</td><td>CON</td><td>MIN</td><td>HOUR</td><td>DAY</td><td>WEEK</td><td>UNIGENE</td><td>FLCDNA</td><td>TAIR</td></tr>
<tr><td>TGGGCACGTC</td><td>0</td><td>1</td><td>0</td><td>0</td><td>0</td><td>gnl|UG|At#S15461312</td><td>gi|20260435|gb|AY093117.1</td><td>At2g21500.1</td></tr>
<tr><td>at1g64355</td><td>CON</td><td>MIN</td><td>HOUR</td><td>DAY</td><td>WEEK</td><td>UNIGENE</td><td>FLCDNA</td><td>TAIR</td></tr>
<tr><td>AGTTCAACGC</td><td>2</td><td>2</td><td>2</td><td>4</td><td>2</td><td>gnl|UG|At#S28282450</td><td>gi|18700138|gb|AY075674.1</td><td>At1g64355.1</td></tr>
<tr><td>GGCTCCAACC</td><td>1</td><td>2</td><td>2</td><td>1</td><td>0</td><td>no match found</td><td>gi|88193799|gb|BT024591.1</td><td>non-canonical match</td></tr>
<tr><td>at1g28510</td><td>CON</td><td>MIN</td><td>HOUR</td><td>DAY</td><td>WEEK</td><td>UNIGENE</td><td>FLCDNA</td><td>TAIR</td></tr>
<tr><td>TACCATCCAT</td><td>0</td><td>0</td><td>1</td><td>0</td><td>0</td><td>gnl|UG|At#S18895905</td><td>gi|21404405|gb|AY085695.1</td><td>non-canonical match</td></tr>
<tr><td>at5g54830</td><td>CON</td><td>MIN</td><td>HOUR</td><td>DAY</td><td>WEEK</td><td>UNIGENE</td><td>FLCDNA</td><td>TAIR</td></tr>
<tr><td>GATTTATGAT</td><td>0</td><td>1</td><td>0</td><td>1</td><td>0</td><td>gnl|UG|At#S34114986</td><td>gi|19699058|gb|AY090233.1</td><td>multiple canonical match</td></tr>
<tr><td>at4g12420</td><td>CON</td><td>MIN</td><td>HOUR</td><td>DAY</td><td>WEEK</td><td>UNIGENE</td><td>FLCDNA</td><td>TAIR</td></tr>
<tr><td>GTCAGCGATA</td><td>0</td><td>5</td><td>1</td><td>1</td><td>0</td><td>gnl|UG|At#S11725661</td><td>gi|110739786|dbj|AK229975.1|</td><td>At4g12420.1</td></tr>
<tr><td>at2g20585</td><td>CON</td><td>MIN</td><td>HOUR</td><td>DAY</td><td>WEEK</td><td>UNIGENE</td><td>FLCDNA</td><td>TAIR</td></tr>
<tr><td>TTGTATTGGT</td><td>1</td><td>0</td><td>0</td><td>0</td><td>0</td><td>gnl|UG|At#S43850194</td><td>no match found</td><td>non-canonical match</td></tr>
</table>

at5g62670	CON	MIN	HOUR	DAY	WEEK	UNIGENE	FLCDNA	TAIR
TAAGTATCAG	4	1	3	3	3	gnl|UG|At#S11717433	gi|21928114|gb|AY125493.1	non-canonical match
at4g20780	CON	MIN	HOUR	DAY	WEEK	UNIGENE	FLCDNA	TAIR
ATGCGTACTG	0	1	2	0	0	gnl|UG|At#S11724213	gi|26450754|dbj|AK117845.1|	At4g20780.1
at5g65207	CON	MIN	HOUR	DAY	WEEK	UNIGENE	FLCDNA	TAIR
GCTTCTTCTC	4	7	6	1	0	no match found	gi|26453217|dbj|AK119111.1|	At5g65207.1
at3g16350	CON	MIN	HOUR	DAY	WEEK	UNIGENE	FLCDNA	TAIR
GACGAGTTGG	0	1	0	0	0	no match found	gi|29028775|gb|BT005832.1	multiple non-canonical match
TTTAAGGCAA	0	0	0	0	1	gnl|UG|At#S11735946	no match found	At3g16350.1
at5g48385	CON	MIN	HOUR	DAY	WEEK	UNIGENE	FLCDNA	TAIR
GGAACTTCTA	0	1	1	0	0	gnl|UG|At#S15459150	gi|110740813|dbj|AK226356.1|	At5g48385.1
at4g27870	CON	MIN	HOUR	DAY	WEEK	UNIGENE	FLCDNA	TAIR
GATGGTCTGA	0	4	1	1	0	gnl|UG|At#S11705863	gi|20453049|gb|AY094391.1	At4g27870.1
at1g77250	CON	MIN	HOUR	DAY	WEEK	UNIGENE	FLCDNA	TAIR
CTTCTAAACG	0	1	0	0	1	gnl|UG|At#S21736811	gi|51969559|dbj|AK175709.1|	At1g77250.1
at1g30630	CON	MIN	HOUR	DAY	WEEK	UNIGENE	FLCDNA	TAIR
GAATCAATTT	1	3	9	6	2	gnl|UG|At#S11738794	gi|21406211|gb|AY087474.1	multiple canonical match
at5g40670	CON	MIN	HOUR	DAY	WEEK	UNIGENE	FLCDNA	TAIR
TTTAAAGATC	0	2	1	0	1	gnl|UG|At#S11719662	gi|15529265|gb|AY052257.1	At5g40670.1
at1g60090	CON	MIN	HOUR	DAY	WEEK	UNIGENE	FLCDNA	TAIR
TGATTGATGC	0	1	1	0	0	no match found	no match found	At1g60090.1
at1g54460	CON	MIN	HOUR	DAY	WEEK	UNIGENE	FLCDNA	TAIR
ATGGAGAGAC	1	2	1	0	0	no match found	gi|15777864|gb|AY055093.1	At1g54460.1
at5g47020	CON	MIN	HOUR	DAY	WEEK	UNIGENE	FLCDNA	TAIR
TAAATCTTTT	1	3	0	2	1	gnl|UG|At#S11719022	no match found	At5g47020.1
at1g10920	CON	MIN	HOUR	DAY	WEEK	UNIGENE	FLCDNA	TAIR
TGAAGAGATT	1	0	0	0	0	no match found	gi|18491219|gb|AY074545.1	non-canonical match
at5g11060	CON	MIN	HOUR	DAY	WEEK	UNIGENE	FLCDNA	TAIR
GAAATTTCTC	3	2	5	4	1	gnl|UG|At#S11823550	gi|26451633|dbj|AK118296.1|	At5g11060.1
at5g44050	CON	MIN	HOUR	DAY	WEEK	UNIGENE	FLCDNA	TAIR
AATTAAATCT	0	0	0	1	0	no match found	no match found	At5g44050.1
at4g27410	CON	MIN	HOUR	DAY	WEEK	UNIGENE	FLCDNA	TAIR
TGAATCGGCA	2	6	1	4	1	no match found	gi|16226942|gb|AF428375.1	At4g27410.1
TCTAGTTTAT	0	0	0	0	1	gnl|UG|At#S11723061	gi|15982728|gb|AY057578.1	At4g27410.2
at2g39681	CON	MIN	HOUR	DAY	WEEK	UNIGENE	FLCDNA	TAIR
GATATGCAAA	0	1	0	0	0	gnl|UG|At#S27318332	gi|72197936|gb|DQ108799.1	non-canonical match
at4g39840	CON	MIN	HOUR	DAY	WEEK	UNIGENE	FLCDNA	TAIR
TTGCAGATTC	1	0	1	1	1	no match found	gi|26449928|dbj|AK117420.1|	non-canonical match
TACCTCGTTC	0	2	1	0	0	gnl|UG|At#S11720948	gi|22136847|gb|AY133834.1	At4g39840.1
at4g34450	CON	MIN	HOUR	DAY	WEEK	UNIGENE	FLCDNA	TAIR
CAGACTTGTG	2	1	1	0	0	gnl|UG|At#S11721858	gi|62321021|dbj|AK221839.1|	At4g34450.1

at1g23200	CON	MIN	HOUR	DAY	WEEK	UNIGENE	FLCDNA	TAIR
TAACAGATGG	1	0	0	0	0	gnl|UG|At#S11740742	gi|110742327|dbj|AK227025.1|	At1g23200.1
at5g02780	CON	MIN	HOUR	DAY	WEEK	UNIGENE	FLCDNA	TAIR
GTTTATTCCG	0	0	1	0	0	gnl|UG|At#S11725469	gi|38454161|gb|BT010718.1	pseudo chromosome match
at2g22450	CON	MIN	HOUR	DAY	WEEK	UNIGENE	FLCDNA	TAIR
TGATCTGATA	0	2	0	0	2	gnl|UG|At#S34117347	gi|110737822|dbj|AK228961.1|	At2g22450.1
at5g48020	CON	MIN	HOUR	DAY	WEEK	UNIGENE	FLCDNA	TAIR
GGTCGACAAA	1	1	3	2	1	gnl|UG|At#S11718919	gi|110743712|dbj|AK227707.1|	At5g48020.1
at2g39750	CON	MIN	HOUR	DAY	WEEK	UNIGENE	FLCDNA	TAIR
CGAGAAGCGT	0	4	0	2	1	gnl|UG|At#S11732663	gi|21700884|gb|AY124857.1	At2g39750.1
at1g18950	CON	MIN	HOUR	DAY	WEEK	UNIGENE	FLCDNA	TAIR
AAGAAAGAAA	3	11	12	2	4	gnl|UG|At#S11741157	no match found	At1g18950.1
at4g18197	CON	MIN	HOUR	DAY	WEEK	UNIGENE	FLCDNA	TAIR
ATAGAATGGA	0	1	0	1	0	gnl|UG|At#S34114378	gi|110743482|dbj|AK227637.1|	non-canonical match
at5g48870	CON	MIN	HOUR	DAY	WEEK	UNIGENE	FLCDNA	TAIR
GTTCTTGAAG	0	1	0	1	1	gnl|UG|At#S20526198	gi|110739169|dbj|AK229657.1|	At5g48870.1
at3g42786	CON	MIN	HOUR	DAY	WEEK	UNIGENE	FLCDNA	TAIR
TGTCCGAAAT	1	0	0	0	0	no match found	no match found	At3g42786.1
at1g60960	CON	MIN	HOUR	DAY	WEEK	UNIGENE	FLCDNA	TAIR
TCTTCTCTTG	1	0	0	0	0	no match found	gi|21404314|gb|AY085604.1	non-canonical match
TATAGTTCTT	0	2	0	0	0	gnl|UG|At#S11731182	gi|18491214|gb|AY074542.1	At1g60960.1
at3g19590	CON	MIN	HOUR	DAY	WEEK	UNIGENE	FLCDNA	TAIR
TAAACATCTA	1	0	1	0	0	gnl|UG|At#S11734977	gi|29824352|gb|BT006152.1	At3g19590.1
at5g64130	CON	MIN	HOUR	DAY	WEEK	UNIGENE	FLCDNA	TAIR
TGCTCCATCT	3	7	1	2	4	gnl|UG|At#S38433095	gi|20453352|gb|AY097399.1	At5g64130.1
at4g09620	CON	MIN	HOUR	DAY	WEEK	UNIGENE	FLCDNA	TAIR
GTGTTAATGG	1	1	0	0	0	no match found	gi|32189298|gb|BT009654.1	At4g09620.1
ATTTAAGGAC	1	2	0	0	1	gnl|UG|At#S11817497	gi|22655265|gb|AY140082.1	pseudo chromosome match
at1g63240	CON	MIN	HOUR	DAY	WEEK	UNIGENE	FLCDNA	TAIR
GGTACTAGTA	0	1	0	0	0	gnl|UG|At#S11730495	gi|21281037|gb|AY114080.1	At1g63240.1
at4g01400	CON	MIN	HOUR	DAY	WEEK	UNIGENE	FLCDNA	TAIR
ACTTGGAGAC	1	1	0	0	0	gnl|UG|At#S11727333	gi|21539536|gb|AY120763.1	At4g01400.1
at4g28030	CON	MIN	HOUR	DAY	WEEK	UNIGENE	FLCDNA	TAIR
TAATTGTAAA	0	2	3	0	0	gnl|UG|At#S35305294	gi|21403432|gb|AY084722.1	At4g28030.1
at5g47820	CON	MIN	HOUR	DAY	WEEK	UNIGENE	FLCDNA	TAIR
AGGAAATAAA	1	2	1	0	0	gnl|UG|At#S11718939	no match found	At5g47820.2
at1g30820	CON	MIN	HOUR	DAY	WEEK	UNIGENE	FLCDNA	TAIR
GAGATCATTG	2	0	1	0	0	gnl|UG|At#S11738726	no match found	At1g30820.1
at5g59780	CON	MIN	HOUR	DAY	WEEK	UNIGENE	FLCDNA	TAIR
GTAGTGGTCG	4	2	1	1	2	gnl|UG|At#S15446004	gi|110737990|dbj|AK229048.1|	At5g59780.3
TACAAAGCAC	0	1	0	0	0	gnl|UG|At#S18913988	no match found	non-canonical match
at3g27170	CON	MIN	HOUR	DAY	WEEK	UNIGENE	FLCDNA	TAIR

TCAGTGGCCA	0	1	0	1	0	gnl|UG|At#S11732579	gi|17064883|gb|AY062518.1	At3g27170.1
at5g24710	CON	MIN	HOUR	DAY	WEEK	UNIGENE	FLCDNA	TAIR
AAAAATCATT	1	1	1	0	0	no match found	no match found	At5g24710.1
at1g09620	CON	MIN	HOUR	DAY	WEEK	UNIGENE	FLCDNA	TAIR
CTTCGCTGCT	2	0	2	3	0	gnl|UG|At#S11706009	gi|15810444|gb|AY056261.1	At1g09620.1
at5g23120	CON	MIN	HOUR	DAY	WEEK	UNIGENE	FLCDNA	TAIR
GAACCGTGAC	7	14	6	5	2	gnl|UG|At#S11720866	gi|15010779|gb|AY045691.1	At5g23120.1
at2g22770	CON	MIN	HOUR	DAY	WEEK	UNIGENE	FLCDNA	TAIR
TTTTGGCTGA	0	0	0	1	0	gnl|UG|At#S15461269	gi|51970637|dbj|AK176248.1|	At2g22770.1
at3g24800	CON	MIN	HOUR	DAY	WEEK	UNIGENE	FLCDNA	TAIR
GATACAGATG	0	0	0	2	0	gnl|UG|At#S11733313	gi|21280980|gb|AY114077.1	At3g24800.1
at1g72910	CON	MIN	HOUR	DAY	WEEK	UNIGENE	FLCDNA	TAIR
AAGGAACTAA	0	1	1	0	0	gnl|UG|At#S11728485	gi|23198071|gb|BT000244.1	At1g72910.1
at4g02360	CON	MIN	HOUR	DAY	WEEK	UNIGENE	FLCDNA	TAIR
TCAAGAATCT	0	1	0	0	0	gnl|UG|At#S11727087	no match found	At4g02360.1
at1g53490	CON	MIN	HOUR	DAY	WEEK	UNIGENE	FLCDNA	TAIR
TAAATCTGCC	0	1	0	0	0	no match found	gi|62320696|dbj|AK221675.1|	At4g02360.1
TCCCATTTGT	0	2	0	0	0	gnl|UG|At#S11733189	no match found	pseudo chromosome match
at1g67360	CON	MIN	HOUR	DAY	WEEK	UNIGENE	FLCDNA	TAIR
CAATGGGTGA	0	2	0	1	0	no match found	gi|20259668|gb|AY091413.1	non-canonical match
at5g55740	CON	MIN	HOUR	DAY	WEEK	UNIGENE	FLCDNA	TAIR
TGTTTGTTGC	0	0	0	1	0	gnl|UG|At#S11718139	no match found	non-canonical match
at5g66050	CON	MIN	HOUR	DAY	WEEK	UNIGENE	FLCDNA	TAIR
TACCTGAGAG	2	0	1	0	0	gnl|UG|At#S18941413	gi|26452558|dbj|AK118771.1|	At5g66050.1
at1g72510	CON	MIN	HOUR	DAY	WEEK	UNIGENE	FLCDNA	TAIR
ATTGTGTTTC	1	3	0	2	0	no match found	gi|48958478|gb|BT014941.1	At2g09970.1
at3g59150	CON	MIN	HOUR	DAY	WEEK	UNIGENE	FLCDNA	TAIR
AGATTGGTAG	0	1	0	1	0	gnl|UG|At#S11727742	gi|20466629|gb|AY099781.1	At3g59150.1
at1g79660	CON	MIN	HOUR	DAY	WEEK	UNIGENE	FLCDNA	TAIR
CTCTTAAGAA	0	2	0	0	0	no match found	gi|15529237|gb|AY052243.1	At1g79660.1
at1g01430	CON	MIN	HOUR	DAY	WEEK	UNIGENE	FLCDNA	TAIR
GAATGATCTA	2	4	1	1	1	gnl|UG|At#S14273648	gi|28393778|gb|BT004301.1	At1g01430.1
at4g15000	CON	MIN	HOUR	DAY	WEEK	UNIGENE	FLCDNA	TAIR
GTGAAAAATG	37	21	30	39	35	gnl|UG|At#S17006705	gi|21404197|gb|AY085487.1	At4g15000.1
at1g11860	CON	MIN	HOUR	DAY	WEEK	UNIGENE	FLCDNA	TAIR
ACTTTGTCTC	19	11	5	4	7	no match found	gi|21928146|gb|AY125509.1	non-canonical match
GTTTTAATGT	0	1	0	0	0	gnl|UG|At#S11741851	no match found	At1g11860.2
at4g02230	CON	MIN	HOUR	DAY	WEEK	UNIGENE	FLCDNA	TAIR
CTCGACGAGA	2	8	5	8	4	gnl|UG|At#S11727116	gi|14423511|gb|AF386993.1	At4g02230.1
at5g50410	CON	MIN	HOUR	DAY	WEEK	UNIGENE	FLCDNA	TAIR
TAGCTGTATC	0	0	0	0	1	gnl|UG|At#S11718672	no match found	At5g50410.1

at3g62530	CON	MIN	HOUR	DAY	WEEK	UNIGENE	FLCDNA	TAIR
GTTCGAGTAG	5	5	6	4	5	gnl|UG|At#S11726685	gi|110739206|dbj|AK229677.1|	At3g62530.1

at1g19110	CON	MIN	HOUR	DAY	WEEK	UNIGENE	FLCDNA	TAIR
TAATTTCTGT	1	1	1	0	2	gnl|UG|At#S11741140	gi|23197959|gb|BT000188.1	At1g19110.1

at1g59620	CON	MIN	HOUR	DAY	WEEK	UNIGENE	FLCDNA	TAIR
TCATTCTAGA	0	1	0	0	0	gnl|UG|At#S34115262	gi|110741824|dbj|AK226755.1|	At1g59620.1

at5g18460	CON	MIN	HOUR	DAY	WEEK	UNIGENE	FLCDNA	TAIR
ATGTCAAGAT	0	1	0	0	0	gnl|UG|At#S11721705	gi|110736744|dbj|AK228398.1|	At5g18460.1

at4g25170	CON	MIN	HOUR	DAY	WEEK	UNIGENE	FLCDNA	TAIR
CTTATGATCA	0	0	1	0	0	no match found	gi|21689834|gb|AY123028.1	non-canonical match
CTGCTTGTTT	4	8	4	4	4	gnl|UG|At#S11723447	gi|17979332|gb|AY070396.1	At4g25170.1

at5g25980	CON	MIN	HOUR	DAY	WEEK	UNIGENE	FLCDNA	TAIR
TCTCTTCTTT	13	10	8	7	4	no match found	gi|110740760|dbj|AK226328.1|	At5g64820.1
GGATGGGGAA	2	1	3	0	3	gnl|UG|At#S11720593	gi|13605679|gb|AF361821.1	At5g25980.1

at2g24290	CON	MIN	HOUR	DAY	WEEK	UNIGENE	FLCDNA	TAIR
TTCTCTATAT	1	0	0	0	1	gnl|UG|At#S11702422	gi|13877800|gb|AF370163.1	At2g24290.1

at5g14040	CON	MIN	HOUR	DAY	WEEK	UNIGENE	FLCDNA	TAIR
ACCGTGACTC	3	7	3	4	2	no match found	gi|16604459|gb|AY058848.1	At5g14040.1

at3g23400	CON	MIN	HOUR	DAY	WEEK	UNIGENE	FLCDNA	TAIR
TGAACATATA	13	3	11	15	23	gnl|UG|At#S18901643	gi|21405644|gb|AY086921.1	At3g23400.1

at5g49930	CON	MIN	HOUR	DAY	WEEK	UNIGENE	FLCDNA	TAIR
TTACAGCCCA	5	3	2	4	1	gnl|UG|At#S11718724	no match found	At5g49930.1

at5g67260	CON	MIN	HOUR	DAY	WEEK	UNIGENE	FLCDNA	TAIR
TGATGAAGTT	0	3	0	1	0	gnl|UG|At#S11716969	gi|21406278|gb|AY087540.1	At5g67260.1

at3g11740	CON	MIN	HOUR	DAY	WEEK	UNIGENE	FLCDNA	TAIR
TTGGGTGATG	0	0	0	0	1	no match found	no match found	At3g11740.1

at1g79880	CON	MIN	HOUR	DAY	WEEK	UNIGENE	FLCDNA	TAIR
GAACAACAGT	0	0	0	0	1	gnl|UG|At#S18942221	gi|110736336|dbj|AK228183.1|	At1g79880.1

at1g15810	CON	MIN	HOUR	DAY	WEEK	UNIGENE	FLCDNA	TAIR
TCCTCTGCGG	0	2	0	0	0	no match found	gi|22137169|gb|AY133600.1	non-canonical match
CATTTGTGGC	0	1	1	0	0	no match found	gi|13605576|gb|AF361614.1	non-canonical match

at1g26730	CON	MIN	HOUR	DAY	WEEK	UNIGENE	FLCDNA	TAIR
AGAATATTGA	0	1	0	0	0	no match found	no match found	At1g26730.1

at1g15670	CON	MIN	HOUR	DAY	WEEK	UNIGENE	FLCDNA	TAIR
TACTATTTGG	0	4	0	0	0	gnl|UG|At#S34118218	gi|110736150|dbj|AK228088.1|	At1g15670.1
ATAAATATGA	0	9	0	0	0	no match found	gi|16323267|gb|AY057738.1	non-canonical match

at2g32030	CON	MIN	HOUR	DAY	WEEK	UNIGENE	FLCDNA	TAIR
TATAATTTGA	0	0	0	1	0	gnl|UG|At#S11734520	no match found	At2g32030.1

at1g79110	CON	MIN	HOUR	DAY	WEEK	UNIGENE	FLCDNA	TAIR
AAACCAAAAA	0	0	1	0	0	gnl|UG|At#S34116269	gi|110739911|dbj|AK230039.1|	non-canonical match

at3g12080	CON	MIN	HOUR	DAY	WEEK	UNIGENE	FLCDNA	TAIR
CATAACAGAG	1	3	0	0	0	gnl|UG|At#S11737269	gi|19310575|gb|AY080655.1	At3g12080.1

at1g15660	CON	MIN	HOUR	DAY	WEEK	UNIGENE	FLCDNA	TAIR
AGACTTTGTC	0	0	1	0	0	gnl|UG|At#S11741471	no match found	At1g15660.1
at2g07460	CON	MIN	HOUR	DAY	WEEK	UNIGENE	FLCDNA	TAIR
AATAAGAAGA	1	2	0	2	1	no match found	no match found	At2g07460.1
at5g57410	CON	MIN	HOUR	DAY	WEEK	UNIGENE	FLCDNA	TAIR
GTCCAGTTAC	0	1	0	1	0	gnl|UG|At#S11717971	gi|58743303|gb|BT020622.1	At5g57410.1
at1g24880	CON	MIN	HOUR	DAY	WEEK	UNIGENE	FLCDNA	TAIR
GACTGAAGAG	0	2	1	0	0	gnl|UG|At#S11823712	no match found	multiple non-canonical match
at3g02540	CON	MIN	HOUR	DAY	WEEK	UNIGENE	FLCDNA	TAIR
TGTTCTTTCT	3	8	3	4	0	gnl|UG|At#S11739925	gi|14517453|gb|AY039562.1	At3g02540.1
at3g06210	CON	MIN	HOUR	DAY	WEEK	UNIGENE	FLCDNA	TAIR
TTCCTCTTGA	0	1	0	1	0	gnl|UG|At#S11738875	gi|20268714|gb|AY091111.1	At3g06210.1
at5g11330	CON	MIN	HOUR	DAY	WEEK	UNIGENE	FLCDNA	TAIR
CCATTCTTTT	1	2	1	1	0	gnl|UG|At#S11723423	gi|28950968|gb|BT005344.1	At5g11330.1
at1g02730	CON	MIN	HOUR	DAY	WEEK	UNIGENE	FLCDNA	TAIR
TTTGTAATCA	3	3	0	0	0	gnl|UG|At#S11742843	gi|110742040|dbj|AK226870.1|	At1g02730.1
at4g25630	CON	MIN	HOUR	DAY	WEEK	UNIGENE	FLCDNA	TAIR
TGTCGTTGGT	1	2	1	6	2	gnl|UG|At#S11723370	gi|23297149|gb|AY142647.1	At4g25630.1
at5g19430	CON	MIN	HOUR	DAY	WEEK	UNIGENE	FLCDNA	TAIR
ATGATTTTGA	0	1	1	0	0	gnl|UG|At#S11702912	gi|13605552|gb|AF361602.1	At5g19430.1
at2g21370	CON	MIN	HOUR	DAY	WEEK	UNIGENE	FLCDNA	TAIR
TGAACACATT	1	1	2	0	0	no match found	gi|51969455|dbj|AK175657.1|	At2g21370.2
CGTGTCGATT	0	1	3	1	1	gnl|UG|At#S18942884	gi|51971762|dbj|AK176783.1|	pseudo chromosome match
at2g05630	CON	MIN	HOUR	DAY	WEEK	UNIGENE	FLCDNA	TAIR
GTGTTAATTT	2	0	0	1	0	gnl|UG|At#S34114676	gi|110742907|dbj|AK227339.1|	At2g05630.1
at1g05805	CON	MIN	HOUR	DAY	WEEK	UNIGENE	FLCDNA	TAIR
CTGGATTTAG	0	3	1	0	1	gnl|UG|At#S11742446	gi|21404816|gb|AY086106.1	At1g05805.1
at4g27450	CON	MIN	HOUR	DAY	WEEK	UNIGENE	FLCDNA	TAIR
AATAAAACTG	9	19	7	8	0	no match found	gi|21406144|gb|AY087420.1	At4g27450.1
at1g19800	CON	MIN	HOUR	DAY	WEEK	UNIGENE	FLCDNA	TAIR
TCTCTTGTCG	0	1	0	1	0	gnl|UG|At#S18925455	gi|21281061|gb|AY114021.1	At1g19800.3
at2g20670	CON	MIN	HOUR	DAY	WEEK	UNIGENE	FLCDNA	TAIR
AGTGTTAATT	3	30	2	4	0	gnl|UG|At#S11737295	gi|15724181|gb|AF411793.1	At2g20670.1
at5g15802	CON	MIN	HOUR	DAY	WEEK	UNIGENE	FLCDNA	TAIR
TGTCGAAAAT	0	2	1	2	0	gnl|UG|At#S15460844	gi|26450076|dbj|AK117495.1|	At5g15802.1
at2g01820	CON	MIN	HOUR	DAY	WEEK	UNIGENE	FLCDNA	TAIR
GCTCACATCG	1	3	2	1	1	gnl|UG|At#S11742612	gi|28393528|gb|BT004165.1	At2g01820.1
at3g50845	CON	MIN	HOUR	DAY	WEEK	UNIGENE	FLCDNA	TAIR
GGTTTATATA	0	0	0	0	1	gnl|UG|At#S38433619	no match found	non-canonical match
at4g02580	CON	MIN	HOUR	DAY	WEEK	UNIGENE	FLCDNA	TAIR
CTAAAGCTAA	0	2	0	0	3	no match found	gi|15450450|gb|AY052326.1	non-canonical match
TGAAATTGTA	2	5	5	8	8	gnl|UG|At#S11727043	no match found	At4g02580.1

at3g05350	CON	MIN	HOUR	DAY	WEEK	UNIGENE	FLCDNA	TAIR
ATTAGTACCA	4	1	1	0	3	gnl|UG|At#S11744145	gi|19310477|gb|AY079018.1	At3g05350.1
at5g19760	CON	MIN	HOUR	DAY	WEEK	UNIGENE	FLCDNA	TAIR
GATCTTCCTA	7	9	4	6	2	gnl|UG|At#S11721382	gi|15810536|gb|AY056307.1	multiple non-canonical match
at5g24210	CON	MIN	HOUR	DAY	WEEK	UNIGENE	FLCDNA	TAIR
TATAAGAATG	0	0	1	0	1	gnl|UG|At#S11710204	gi|110742243|dbj|AK226981.1|	multiple canonical match
at5g16480	CON	MIN	HOUR	DAY	WEEK	UNIGENE	FLCDNA	TAIR
TGTGATGTGA	0	2	0	0	0	gnl|UG|At#S34115622	gi|110741148|dbj|AK226528.1|	At5g16480.1
at5g45650	CON	MIN	HOUR	DAY	WEEK	UNIGENE	FLCDNA	TAIR
TTGTGAGGAG	1	0	0	1	1	no match found	gi|28973548|gb|BT005679.1	At5g45650.1
at4g12780	CON	MIN	HOUR	DAY	WEEK	UNIGENE	FLCDNA	TAIR
GGGCTATGTT	1	2	0	0	0	gnl|UG|At#S11725601	gi|19698974|gb|AY081334.1	At4g12780.1
at3g15060	CON	MIN	HOUR	DAY	WEEK	UNIGENE	FLCDNA	TAIR
CAGATGATTC	1	2	2	1	0	no match found	no match found	At3g15060.1
at1g75730	CON	MIN	HOUR	DAY	WEEK	UNIGENE	FLCDNA	TAIR
ACTGCTGCTG	0	0	1	0	0	gnl|UG|At#S11727894	gi|133778891|gb|BT030373.1	At1g75730.1
at3g29390	CON	MIN	HOUR	DAY	WEEK	UNIGENE	FLCDNA	TAIR
CTTCCTCCAC	1	4	0	0	0	gnl|UG|At#S11731845	gi|17473752|gb|AY065141.1	At3g29390.1
at2g44180	CON	MIN	HOUR	DAY	WEEK	UNIGENE	FLCDNA	TAIR
TTAGCTTATT	0	0	1	0	0	gnl|UG|At#S11731592	no match found	pseudo chromosome match
at4g24940	CON	MIN	HOUR	DAY	WEEK	UNIGENE	FLCDNA	TAIR
TATATCTCAA	0	0	2	0	2	gnl|UG|At#S11723486	gi|18252880|gb|AY072375.1	At4g24940.1
at1g34760	CON	MIN	HOUR	DAY	WEEK	UNIGENE	FLCDNA	TAIR
CAACTTCTCA	1	0	0	0	0	no match found	gi|117958764|gb|BT029457.1	non-canonical match
at1g02170	CON	MIN	HOUR	DAY	WEEK	UNIGENE	FLCDNA	TAIR
CTTCTGACAG	1	4	0	4	0	gnl|UG|At#S11742899	no match found	non-canonical match
at3g17900	CON	MIN	HOUR	DAY	WEEK	UNIGENE	FLCDNA	TAIR
TGTTTGCGTT	1	1	1	0	0	no match found	gi|13605520|gb|AF361586.1	At3g17900.1
at3g23280	CON	MIN	HOUR	DAY	WEEK	UNIGENE	FLCDNA	TAIR
TTTTCTTTTT	2	3	1	0	2	no match found	gi|15010671|gb|AY045637.1	multiple canonical match
at1g70230	CON	MIN	HOUR	DAY	WEEK	UNIGENE	FLCDNA	TAIR
GAGGAGTACA	3	2	0	2	0	gnl|UG|At#S11729051	gi|19310588|gb|AY080707.1	At1g70230.1
ATCGAGATGT	0	0	1	1	0	no match found	gi|21436298|gb|AY117213.1	multiple non-canonical match
at4g00490	CON	MIN	HOUR	DAY	WEEK	UNIGENE	FLCDNA	TAIR
CTACTAAAAA	0	0	0	1	0	gnl|UG|At#S11727555	no match found	non-canonical match
at2g31305	CON	MIN	HOUR	DAY	WEEK	UNIGENE	FLCDNA	TAIR
GAAGAACACA	0	0	0	1	0	gnl|UG|At#S11734711	gi|21405410|gb|AY086700.1	At2g31305.1
at3g18420	CON	MIN	HOUR	DAY	WEEK	UNIGENE	FLCDNA	TAIR
GACAGGTCCG	0	0	1	3	1	gnl|UG|At#S11678561	gi|18655374|gb|AY077665.1	At3g18420.1
at5g19310	CON	MIN	HOUR	DAY	WEEK	UNIGENE	FLCDNA	TAIR
GAAGAGAAGG	1	0	0	0	0	gnl|UG|At#S11721493	no match found	At5g19310.1

at1g26750	CON	MIN	HOUR	DAY	WEEK	UNIGENE	FLCDNA	TAIR
TCTGTAGCTA	1	0	0	0	1	no match found	gi|21405458|gb|AY086748.1	non-canonical match
AGGCAAGGAA	1	0	0	0	0	no match found	gi|18252978|gb|AY072424.1	At1g26750.1
at1g49240	CON	MIN	HOUR	DAY	WEEK	UNIGENE	FLCDNA	TAIR
ACGGGATCAC	7	19	7	5	3	gnl|UG|At#S11734771	gi|21406072|gb|AY087348.1	At1g49240.1
at2g45210	CON	MIN	HOUR	DAY	WEEK	UNIGENE	FLCDNA	TAIR
GGGCCGTCAT	0	1	0	0	1	no match found	gi|28393886|gb|BT004357.1	non-canonical match
GGGACACTTA	0	0	1	0	0	gnl|UG|At#S11825239	gi|27754323|gb|BT002787.1	At2g45210.1
at5g14370	CON	MIN	HOUR	DAY	WEEK	UNIGENE	FLCDNA	TAIR
ATATTGCTAT	0	3	0	0	0	gnl|UG|At#S11707345	gi|15292916|gb|AY050892.1	At5g14370.1
at1g44110	CON	MIN	HOUR	DAY	WEEK	UNIGENE	FLCDNA	TAIR
ATTTTGCTGT	2	1	0	0	0	gnl|UG|At#S21737269	gi|51968953|dbj|AK175406.1|	At1g44110.1
at5g43580	CON	MIN	HOUR	DAY	WEEK	UNIGENE	FLCDNA	TAIR
TGCTTTCTCA	0	1	0	1	0	no match found	gi|38566531|gb|BT010789.1	non-canonical match
at5g15100	CON	MIN	HOUR	DAY	WEEK	UNIGENE	FLCDNA	TAIR
TGGTACAAAA	0	1	0	0	0	gnl|UG|At#S11722567	no match found	multiple non-canonical match
at4g38200	CON	MIN	HOUR	DAY	WEEK	UNIGENE	FLCDNA	TAIR
TATGGGTGCA	0	0	0	1	0	gnl|UG|At#S11721235	gi|110742670|dbj|AK227209.1|	At4g38200.1
at3g43300	CON	MIN	HOUR	DAY	WEEK	UNIGENE	FLCDNA	TAIR
AGAGAGTTCT	4	1	1	1	0	gnl|UG|At#S11730709	gi|110739725|dbj|AK229944.1|	At3g43300.1
at5g16130	CON	MIN	HOUR	DAY	WEEK	UNIGENE	FLCDNA	TAIR
AAGGTCTTTT	1	7	5	7	10	gnl|UG|At#S11722307	gi|90567999|gb|BT024914.1	At5g16130.1
at5g20220	CON	MIN	HOUR	DAY	WEEK	UNIGENE	FLCDNA	TAIR
CCGGAAGCCG	1	0	1	0	0	gnl|UG|At#S38433279	gi|21404173|gb|AY085463.1	At5g20220.1
at5g09330	CON	MIN	HOUR	DAY	WEEK	UNIGENE	FLCDNA	TAIR
AGACATAGAC	0	0	0	0	1	gnl|UG|At#S38433336	gi|56236055|gb|BT020263.1	At5g09330.1
at2g42490	CON	MIN	HOUR	DAY	WEEK	UNIGENE	FLCDNA	TAIR
CGAATTGGAG	3	4	3	2	0	gnl|UG|At#S11731992	gi|62321695|dbj|AK222186.1|	At2g42490.1
at5g26850	CON	MIN	HOUR	DAY	WEEK	UNIGENE	FLCDNA	TAIR
ATGAGACTAC	0	1	0	0	0	gnl|UG|At#S11720527	gi|37201993|gb|BT010590.1	At5g26850.1
at5g65830	CON	MIN	HOUR	DAY	WEEK	UNIGENE	FLCDNA	TAIR
CCTGTTTACT	0	1	0	0	0	no match found	gi|87116643|gb|BT024505.1	non-canonical match
at1g59520	CON	MIN	HOUR	DAY	WEEK	UNIGENE	FLCDNA	TAIR
GAGTAGCACA	1	0	1	0	1	gnl|UG|At#S11731674	gi|110743902|dbj|AK227804.1|	At1g59520.1
at1g22750	CON	MIN	HOUR	DAY	WEEK	UNIGENE	FLCDNA	TAIR
GGTCAATGGC	1	4	6	3	4	gnl|UG|At#S11707387	gi|15292832|gb|AY050850.1	At1g22750.2
GAGCTTCTAT	0	2	0	0	0	no match found	gi|20465788|gb|AY096749.1	multiple non-canonical match
at5g20070	CON	MIN	HOUR	DAY	WEEK	UNIGENE	FLCDNA	TAIR
GGTAAATCAG	0	2	4	3	0	gnl|UG|At#S11721307	gi|15146277|gb|AY049280.1	At5g20070.1
at1g26300	CON	MIN	HOUR	DAY	WEEK	UNIGENE	FLCDNA	TAIR
GAAATGAAGA	0	3	0	1	0	no match found	gi|51970993|dbj|AK176426.1|	multiple canonical match

<table>
<tr><td>at1g20980</td><td>CON</td><td>MIN</td><td>HOUR</td><td>DAY</td><td>WEEK</td><td>UNIGENE</td><td>FLCDNA</td><td>TAIR</td></tr>
<tr><td>CTGCTTCATA</td><td>5</td><td>4</td><td>0</td><td>3</td><td>4</td><td>gnl|UG|At#S11740959</td><td>gi|110742104|dbj|AK226907.1|</td><td>At1g20980.1</td></tr>
<tr><td>at2g38540</td><td>CON</td><td>MIN</td><td>HOUR</td><td>DAY</td><td>WEEK</td><td>UNIGENE</td><td>FLCDNA</td><td>TAIR</td></tr>
<tr><td>TGGAGTCAAT</td><td>17</td><td>41</td><td>3</td><td>10</td><td>16</td><td>no match found</td><td>gi|20147118|gb|AY091677.1</td><td>At2g38540.1</td></tr>
<tr><td>at5g19950</td><td>CON</td><td>MIN</td><td>HOUR</td><td>DAY</td><td>WEEK</td><td>UNIGENE</td><td>FLCDNA</td><td>TAIR</td></tr>
<tr><td>TATCTTATTA</td><td>0</td><td>0</td><td>0</td><td>0</td><td>1</td><td>no match found</td><td>gi|20466595|gb|AY099764.1</td><td>non-canonical match</td></tr>
<tr><td>at5g51980</td><td>CON</td><td>MIN</td><td>HOUR</td><td>DAY</td><td>WEEK</td><td>UNIGENE</td><td>FLCDNA</td><td>TAIR</td></tr>
<tr><td>TTAGGAGTTT</td><td>0</td><td>2</td><td>0</td><td>1</td><td>0</td><td>gnl|UG|At#S11718518</td><td>no match found</td><td>At5g51980.1</td></tr>
<tr><td>at2g33100</td><td>CON</td><td>MIN</td><td>HOUR</td><td>DAY</td><td>WEEK</td><td>UNIGENE</td><td>FLCDNA</td><td>TAIR</td></tr>
<tr><td>GAACAGAAAC</td><td>1</td><td>0</td><td>0</td><td>0</td><td>0</td><td>gnl|UG|At#S11734257</td><td>no match found</td><td>At2g33100.1</td></tr>
<tr><td>at1g19580</td><td>CON</td><td>MIN</td><td>HOUR</td><td>DAY</td><td>WEEK</td><td>UNIGENE</td><td>FLCDNA</td><td>TAIR</td></tr>
<tr><td>AAGAATAAAG</td><td>2</td><td>0</td><td>2</td><td>3</td><td>4</td><td>gnl|UG|At#S11741094</td><td>gi|21403735|gb|AY085025.1</td><td>At1g19580.1</td></tr>
<tr><td>at1g10470</td><td>CON</td><td>MIN</td><td>HOUR</td><td>DAY</td><td>WEEK</td><td>UNIGENE</td><td>FLCDNA</td><td>TAIR</td></tr>
<tr><td>TCGTCGGAGA</td><td>1</td><td>2</td><td>3</td><td>2</td><td>1</td><td>gnl|UG|At#S11741987</td><td>gi|110740749|dbj|AK226322.1|</td><td>At1g10470.1</td></tr>
<tr><td>at4g18230</td><td>CON</td><td>MIN</td><td>HOUR</td><td>DAY</td><td>WEEK</td><td>UNIGENE</td><td>FLCDNA</td><td>TAIR</td></tr>
<tr><td>CAAATTTACC</td><td>0</td><td>1</td><td>1</td><td>0</td><td>0</td><td>gnl|UG|At#S15177156</td><td>gi|30793784|gb|BT008518.1</td><td>At4g18230.1</td></tr>
<tr><td>at3g53530</td><td>CON</td><td>MIN</td><td>HOUR</td><td>DAY</td><td>WEEK</td><td>UNIGENE</td><td>FLCDNA</td><td>TAIR</td></tr>
<tr><td>CTTCTTTATG</td><td>0</td><td>4</td><td>0</td><td>1</td><td>3</td><td>gnl|UG|At#S28282824</td><td>gi|19424069|gb|AY080885.1</td><td>multiple non-canonical match</td></tr>
<tr><td>at1g67250</td><td>CON</td><td>MIN</td><td>HOUR</td><td>DAY</td><td>WEEK</td><td>UNIGENE</td><td>FLCDNA</td><td>TAIR</td></tr>
<tr><td>ACTCATCGTG</td><td>4</td><td>5</td><td>3</td><td>2</td><td>0</td><td>gnl|UG|At#S11729662</td><td>gi|21405491|gb|AY086781.1</td><td>At1g67250.1</td></tr>
<tr><td>at2g22100</td><td>CON</td><td>MIN</td><td>HOUR</td><td>DAY</td><td>WEEK</td><td>UNIGENE</td><td>FLCDNA</td><td>TAIR</td></tr>
<tr><td>TTCTCTGATA</td><td>1</td><td>1</td><td>0</td><td>0</td><td>0</td><td>gnl|UG|At#S11736948</td><td>gi|21407007|gb|AY088233.1</td><td>At2g22100.1</td></tr>
<tr><td>at5g50550</td><td>CON</td><td>MIN</td><td>HOUR</td><td>DAY</td><td>WEEK</td><td>UNIGENE</td><td>FLCDNA</td><td>TAIR</td></tr>
<tr><td>GAGAAGAAGA</td><td>1</td><td>0</td><td>0</td><td>0</td><td>0</td><td>no match found</td><td>gi|119935806|gb|BT029728.1</td><td>multiple non-canonical match</td></tr>
<tr><td>CTTTTATTAG</td><td>1</td><td>0</td><td>0</td><td>0</td><td>2</td><td>gnl|UG|At#S18912275</td><td>gi|62320333|dbj|AK221493.1|</td><td>At5g50550.1</td></tr>
<tr><td>at5g06865</td><td>CON</td><td>MIN</td><td>HOUR</td><td>DAY</td><td>WEEK</td><td>UNIGENE</td><td>FLCDNA</td><td>TAIR</td></tr>
<tr><td>AACCAAACCT</td><td>15</td><td>19</td><td>21</td><td>32</td><td>29</td><td>no match found</td><td>gi|21407071|gb|AY088297.1</td><td>At5g06860.1</td></tr>
<tr><td>TTCCAGTTCG</td><td>0</td><td>0</td><td>0</td><td>0</td><td>1</td><td>no match found</td><td>gi|15081804|gb|AY048295.1</td><td>multiple non-canonical match</td></tr>
<tr><td>GACCGTAATA</td><td>1</td><td>1</td><td>0</td><td>1</td><td>0</td><td>gnl|UG|At#S26540350</td><td>gi|72197046|gb|DQ108691.1</td><td>multiple non-canonical match</td></tr>
<tr><td>at5g48920</td><td>CON</td><td>MIN</td><td>HOUR</td><td>DAY</td><td>WEEK</td><td>UNIGENE</td><td>FLCDNA</td><td>TAIR</td></tr>
<tr><td>CCATTGATAT</td><td>1</td><td>0</td><td>0</td><td>0</td><td>0</td><td>gnl|UG|At#S11718826</td><td>gi|38566633|gb|BT010840.1</td><td>non-canonical match</td></tr>
<tr><td>at5g55610</td><td>CON</td><td>MIN</td><td>HOUR</td><td>DAY</td><td>WEEK</td><td>UNIGENE</td><td>FLCDNA</td><td>TAIR</td></tr>
<tr><td>GGTAGTTCAA</td><td>0</td><td>1</td><td>0</td><td>1</td><td>0</td><td>gnl|UG|At#S18941473</td><td>gi|28393904|gb|BT004366.1</td><td>At5g55610.1</td></tr>
<tr><td>at1g68190</td><td>CON</td><td>MIN</td><td>HOUR</td><td>DAY</td><td>WEEK</td><td>UNIGENE</td><td>FLCDNA</td><td>TAIR</td></tr>
<tr><td>TAACTGTGAA</td><td>1</td><td>2</td><td>0</td><td>1</td><td>0</td><td>gnl|UG|At#S11729465</td><td>gi|26450668|dbj|AK117800.1|</td><td>At1g68190.1</td></tr>
<tr><td>at3g17050</td><td>CON</td><td>MIN</td><td>HOUR</td><td>DAY</td><td>WEEK</td><td>UNIGENE</td><td>FLCDNA</td><td>TAIR</td></tr>
<tr><td>GTGGTCGTTT</td><td>2</td><td>2</td><td>0</td><td>0</td><td>0</td><td>gnl|UG|At#S11699990</td><td>no match found</td><td>At1g68190.1</td></tr>
<tr><td>GTGGCGGTTT</td><td>0</td><td>4</td><td>0</td><td>0</td><td>0</td><td>no match found</td><td>no match found</td><td>At3g17050.1</td></tr>
<tr><td>at5g65410</td><td>CON</td><td>MIN</td><td>HOUR</td><td>DAY</td><td>WEEK</td><td>UNIGENE</td><td>FLCDNA</td><td>TAIR</td></tr>
<tr><td>AATCTTGAAT</td><td>1</td><td>1</td><td>1</td><td>0</td><td>0</td><td>gnl|UG|At#S11717156</td><td>gi|27311558|gb|BT002385.1</td><td>At5g65410.1</td></tr>
<tr><td>at5g43830</td><td>CON</td><td>MIN</td><td>HOUR</td><td>DAY</td><td>WEEK</td><td>UNIGENE</td><td>FLCDNA</td><td>TAIR</td></tr>
<tr><td>TTTGAGGTGG</td><td>16</td><td>27</td><td>12</td><td>4</td><td>3</td><td>gnl|UG|At#S11704671</td><td>gi|62319427|dbj|AK221037.1|</td><td>At5g43830.1</td></tr>
</table>

at1g29980	CON	MIN	HOUR	DAY	WEEK	UNIGENE	FLCDNA	TAIR
GCGCAAGCGA	0	0	1	0	1	gnl|UG|At#S11704528	gi|15010695|gb|AY045649.1	At1g29980.2
at4g24890	CON	MIN	HOUR	DAY	WEEK	UNIGENE	FLCDNA	TAIR
TTGTTGTTGG	0	0	0	0	1	gnl|UG|At#S11723493	gi|23296458|gb|AY142520.1	At4g24890.1
at1g01880	CON	MIN	HOUR	DAY	WEEK	UNIGENE	FLCDNA	TAIR
AGAAGAAACT	0	0	1	0	0	gnl|UG|At#S11742927	no match found	multiple non-canonical match
at4g17300	CON	MIN	HOUR	DAY	WEEK	UNIGENE	FLCDNA	TAIR
CAGGATTTGG	0	1	1	0	1	gnl|UG|At#S11724800	gi|19310457|gb|AY078967.1	At4g17300.1
at2g28510	CON	MIN	HOUR	DAY	WEEK	UNIGENE	FLCDNA	TAIR
CAATGGATCC	0	0	2	0	0	no match found	no match found	At2g28510.1
at3g57170	CON	MIN	HOUR	DAY	WEEK	UNIGENE	FLCDNA	TAIR
GATTCCTTTT	0	1	0	0	0	gnl|UG|At#S11728113	gi|62321115|dbj|AK221887.1|	At3g57170.1
at3g54020	CON	MIN	HOUR	DAY	WEEK	UNIGENE	FLCDNA	TAIR
AGATCTCTGC	0	0	1	0	0	no match found	gi|15081788|gb|AY048287.1	At3g54020.1
ACTAAAAACA	0	0	0	0	1	gnl|UG|At#S11728686	no match found	multiple non-canonical match
at4g17570	CON	MIN	HOUR	DAY	WEEK	UNIGENE	FLCDNA	TAIR
AGTACTACTT	0	0	1	0	0	gnl|UG|At#S11724749	no match found	At4g17570.1
at1g33960	CON	MIN	HOUR	DAY	WEEK	UNIGENE	FLCDNA	TAIR
CTCTTGGACT	0	0	2	0	0	gnl|UG|At#S11737585	gi|51971454|dbj|AK176629.1|	At1g33960.1
at3g51930	CON	MIN	HOUR	DAY	WEEK	UNIGENE	FLCDNA	TAIR
AGGGATTTTT	0	0	0	1	0	gnl|UG|At#S34118005	gi|110736560|dbj|AK228303.1|	At3g51930.1
at3g08980	CON	MIN	HOUR	DAY	WEEK	UNIGENE	FLCDNA	TAIR
TTCCTCAAGA	0	0	0	2	0	gnl|UG|At#S18904962	no match found	At3g08980.1
at5g14780	CON	MIN	HOUR	DAY	WEEK	UNIGENE	FLCDNA	TAIR
TTGGAGAGAT	2	18	3	5	5	gnl|UG|At#S11722646	gi|15810033|gb|AY054285.1	At5g14780.1
at2g18220	CON	MIN	HOUR	DAY	WEEK	UNIGENE	FLCDNA	TAIR
TGTTTATACA	0	0	0	1	0	gnl|UG|At#S11737919	no match found	non-canonical match
at3g25650	CON	MIN	HOUR	DAY	WEEK	UNIGENE	FLCDNA	TAIR
AAGAGCAAAC	0	1	0	1	0	no match found	no match found	At3g25650.1
at3g12670	CON	MIN	HOUR	DAY	WEEK	UNIGENE	FLCDNA	TAIR
GGATCTTTTG	0	0	1	0	0	no match found	gi|30725393|gb|BT008360.1	non-canonical match
GATATGAATG	1	0	0	1	2	gnl|UG|At#S11737092	gi|20260421|gb|AY093110.1	At3g12670.1
at1g76170	CON	MIN	HOUR	DAY	WEEK	UNIGENE	FLCDNA	TAIR
TATGCTTATT	0	0	0	0	1	gnl|UG|At#S11727801	no match found	non-canonical match
at2g42030	CON	MIN	HOUR	DAY	WEEK	UNIGENE	FLCDNA	TAIR
AGAACAGTTC	1	0	0	0	0	gnl|UG|At#S11707824	gi|20465662|gb|AY096666.1	At2g42030.1
at2g38450	CON	MIN	HOUR	DAY	WEEK	UNIGENE	FLCDNA	TAIR
TCCTGATCGA	1	1	1	1	2	gnl|UG|At#S11732987	gi|21404005|gb|AY085295.1	At2g38450.1
at3g60290	CON	MIN	HOUR	DAY	WEEK	UNIGENE	FLCDNA	TAIR
CATCTTGTAT	1	0	0	0	0	gnl|UG|At#S11727397	no match found	multiple non-canonical match
at2g17640	CON	MIN	HOUR	DAY	WEEK	UNIGENE	FLCDNA	TAIR
AGATCTTTGT	2	0	0	0	0	gnl|UG|At#S18898965	gi|51971342|dbj|AK176573.1|	At2g17640.1

<table>
<tr><td>at1g06148</td><td>CON</td><td>MIN</td><td>HOUR</td><td>DAY</td><td>WEEK</td><td>UNIGENE</td><td>FLCDNA</td><td>TAIR</td></tr>
<tr><td>AAGATTATTG</td><td>1</td><td>0</td><td>1</td><td>0</td><td>0</td><td>gnl|UG|At#S37211329</td><td>no match found</td><td>multiple non-canonical match</td></tr>
<tr><td>at2g35500</td><td>CON</td><td>MIN</td><td>HOUR</td><td>DAY</td><td>WEEK</td><td>UNIGENE</td><td>FLCDNA</td><td>TAIR</td></tr>
<tr><td>GCTCACCAAA</td><td>5</td><td>1</td><td>0</td><td>5</td><td>1</td><td>gnl|UG|At#S11733696</td><td>no match found</td><td>non-canonical match</td></tr>
<tr><td>at3g10930</td><td>CON</td><td>MIN</td><td>HOUR</td><td>DAY</td><td>WEEK</td><td>UNIGENE</td><td>FLCDNA</td><td>TAIR</td></tr>
<tr><td>TATTACAGAA</td><td>0</td><td>0</td><td>1</td><td>0</td><td>0</td><td>gnl|UG|At#S11823501</td><td>gi|28973084|gb|BT005447.1</td><td>At3g10930.1</td></tr>
<tr><td>at3g60500</td><td>CON</td><td>MIN</td><td>HOUR</td><td>DAY</td><td>WEEK</td><td>UNIGENE</td><td>FLCDNA</td><td>TAIR</td></tr>
<tr><td>TTGAAAGACT</td><td>0</td><td>1</td><td>0</td><td>0</td><td>0</td><td>no match found</td><td>gi|28973592|gb|BT005701.1</td><td>multiple non-canonical match</td></tr>
<tr><td>ATTAAAAACC</td><td>0</td><td>0</td><td>1</td><td>0</td><td>0</td><td>gnl|UG|At#S18941945</td><td>gi|28393588|gb|BT004196.1</td><td>At3g60500.1</td></tr>
<tr><td>at5g02370</td><td>CON</td><td>MIN</td><td>HOUR</td><td>DAY</td><td>WEEK</td><td>UNIGENE</td><td>FLCDNA</td><td>TAIR</td></tr>
<tr><td>GAGCAATAAT</td><td>0</td><td>0</td><td>1</td><td>0</td><td>0</td><td>no match found</td><td>gi|59958333|gb|BT021107.1</td><td>At5g02370.1</td></tr>
<tr><td>TCTTGCTGTG</td><td>25</td><td>119</td><td>23</td><td>28</td><td>18</td><td>no match found</td><td>gi|13265412|gb|AF324665.2</td><td>At5g02380.1</td></tr>
<tr><td>at3g17365</td><td>CON</td><td>MIN</td><td>HOUR</td><td>DAY</td><td>WEEK</td><td>UNIGENE</td><td>FLCDNA</td><td>TAIR</td></tr>
<tr><td>TTTACTCCGT</td><td>0</td><td>0</td><td>0</td><td>0</td><td>1</td><td>gnl|UG|At#S11735634</td><td>gi|15293078|gb|AY050973.1</td><td>At3g17365.1</td></tr>
<tr><td>at2g33540</td><td>CON</td><td>MIN</td><td>HOUR</td><td>DAY</td><td>WEEK</td><td>UNIGENE</td><td>FLCDNA</td><td>TAIR</td></tr>
<tr><td>ATCGGTCAGA</td><td>0</td><td>0</td><td>0</td><td>2</td><td>0</td><td>gnl|UG|At#S11814883</td><td>no match found</td><td>At2g33540.1</td></tr>
<tr><td>at5g16390</td><td>CON</td><td>MIN</td><td>HOUR</td><td>DAY</td><td>WEEK</td><td>UNIGENE</td><td>FLCDNA</td><td>TAIR</td></tr>
<tr><td>AGAAATGGAG</td><td>3</td><td>9</td><td>8</td><td>3</td><td>1</td><td>gnl|UG|At#S18941623</td><td>gi|21406610|gb|AY087857.1</td><td>At5g16390.1</td></tr>
<tr><td>at1g08480</td><td>CON</td><td>MIN</td><td>HOUR</td><td>DAY</td><td>WEEK</td><td>UNIGENE</td><td>FLCDNA</td><td>TAIR</td></tr>
<tr><td>AATGTTTTGA</td><td>0</td><td>7</td><td>5</td><td>5</td><td>1</td><td>no match found</td><td>gi|15450426|gb|AY052314.1</td><td>At1g08480.1</td></tr>
<tr><td>at1g75990</td><td>CON</td><td>MIN</td><td>HOUR</td><td>DAY</td><td>WEEK</td><td>UNIGENE</td><td>FLCDNA</td><td>TAIR</td></tr>
<tr><td>GCTGAGGAAG</td><td>1</td><td>0</td><td>2</td><td>1</td><td>4</td><td>gnl|UG|At#S11705770</td><td>gi|15982802|gb|AY057508.1</td><td>At1g75990.1</td></tr>
<tr><td>at2g35610</td><td>CON</td><td>MIN</td><td>HOUR</td><td>DAY</td><td>WEEK</td><td>UNIGENE</td><td>FLCDNA</td><td>TAIR</td></tr>
<tr><td>TTATAACCAT</td><td>0</td><td>1</td><td>0</td><td>0</td><td>0</td><td>gnl|UG|At#S11743128</td><td>gi|18377746|gb|AY074327.1</td><td>At2g35610.1</td></tr>
<tr><td>at3g29770</td><td>CON</td><td>MIN</td><td>HOUR</td><td>DAY</td><td>WEEK</td><td>UNIGENE</td><td>FLCDNA</td><td>TAIR</td></tr>
<tr><td>TAATGGGACA</td><td>0</td><td>0</td><td>0</td><td>0</td><td>1</td><td>gnl|UG|At#S11731719</td><td>no match found</td><td>At3g29770.1</td></tr>
<tr><td>at3g55820</td><td>CON</td><td>MIN</td><td>HOUR</td><td>DAY</td><td>WEEK</td><td>UNIGENE</td><td>FLCDNA</td><td>TAIR</td></tr>
<tr><td>GAATCAGGAA</td><td>0</td><td>0</td><td>1</td><td>0</td><td>0</td><td>no match found</td><td>no match found</td><td>At3g55820.1</td></tr>
<tr><td>at5g27470</td><td>CON</td><td>MIN</td><td>HOUR</td><td>DAY</td><td>WEEK</td><td>UNIGENE</td><td>FLCDNA</td><td>TAIR</td></tr>
<tr><td>GAAGAGACAC</td><td>1</td><td>1</td><td>1</td><td>0</td><td>0</td><td>gnl|UG|At#S11720465</td><td>gi|15293240|gb|AY051054.1</td><td>At5g27470.1</td></tr>
<tr><td>at4g03000</td><td>CON</td><td>MIN</td><td>HOUR</td><td>DAY</td><td>WEEK</td><td>UNIGENE</td><td>FLCDNA</td><td>TAIR</td></tr>
<tr><td>GAAGATTGCC</td><td>1</td><td>1</td><td>1</td><td>1</td><td>1</td><td>gnl|UG|At#S18941908</td><td>gi|110738034|dbj|AK229071.1|</td><td>At4g03000.2</td></tr>
<tr><td>TTACTGCATT</td><td>0</td><td>1</td><td>0</td><td>0</td><td>0</td><td>no match found</td><td>gi|110741543|dbj|AK226608.1|</td><td>non-canonical match</td></tr>
<tr><td>at5g02310</td><td>CON</td><td>MIN</td><td>HOUR</td><td>DAY</td><td>WEEK</td><td>UNIGENE</td><td>FLCDNA</td><td>TAIR</td></tr>
<tr><td>TTTGTAAATA</td><td>6</td><td>1</td><td>1</td><td>2</td><td>2</td><td>gnl|UG|At#S11725581</td><td>no match found</td><td>At5g02310.1</td></tr>
<tr><td>at3g52040</td><td>CON</td><td>MIN</td><td>HOUR</td><td>DAY</td><td>WEEK</td><td>UNIGENE</td><td>FLCDNA</td><td>TAIR</td></tr>
<tr><td>GCAAATCCAT</td><td>1</td><td>1</td><td>1</td><td>0</td><td>1</td><td>no match found</td><td>gi|88010972|gb|BT024542.1</td><td>At3g52040.1</td></tr>
<tr><td>at3g62590</td><td>CON</td><td>MIN</td><td>HOUR</td><td>DAY</td><td>WEEK</td><td>UNIGENE</td><td>FLCDNA</td><td>TAIR</td></tr>
<tr><td>GATTATTTTT</td><td>0</td><td>0</td><td>1</td><td>1</td><td>0</td><td>gnl|UG|At#S11706214</td><td>gi|15810023|gb|AY054280.1</td><td>At3g62590.1</td></tr>
<tr><td>at5g60390</td><td>CON</td><td>MIN</td><td>HOUR</td><td>DAY</td><td>WEEK</td><td>UNIGENE</td><td>FLCDNA</td><td>TAIR</td></tr>
<tr><td>AGGCAGACCG</td><td>28</td><td>42</td><td>37</td><td>49</td><td>40</td><td>gnl|UG|At#S11717663</td><td>gi|13430509|gb|AF360167.1</td><td>At5g60390.1</td></tr>
</table>

at1g55260	CON	MIN	HOUR	DAY	WEEK	UNIGENE	FLCDNA	TAIR
TAAATGAGTC	3	1	3	0	3	no match found	gi|21403887|gb|AY085177.1	multiple canonical match
at5g58510	CON	MIN	HOUR	DAY	WEEK	UNIGENE	FLCDNA	TAIR
GTCTTTGAAA	0	2	1	0	1	gnl|UG|At#S11807174	gi|20259511|gb|AY091055.1	At5g58510.1
at2g05220	CON	MIN	HOUR	DAY	WEEK	UNIGENE	FLCDNA	TAIR
ACTCTTGACT	4	10	3	4	2	gnl|UG|At#S28282355	no match found	multiple canonical match
at1g18460	CON	MIN	HOUR	DAY	WEEK	UNIGENE	FLCDNA	TAIR
AACGATATGC	0	0	1	3	0	gnl|UG|At#S11741206	gi|24111438|gb|BT001089.1	At1g18460.1
atcg00490	CON	MIN	HOUR	DAY	WEEK	UNIGENE	FLCDNA	TAIR
TGTACAAGCT	0	1	3	0	0	no match found	no match found	AtCg00490
at2g13100	CON	MIN	HOUR	DAY	WEEK	UNIGENE	FLCDNA	TAIR
ATCTGAATAA	1	0	0	1	1	no match found	gi|46931239|gb|BT012605.1	non-canonical match
CTTATTGTTT	0	2	0	1	0	gnl|UG|At#S11739127	gi|48310445|gb|BT014839.1	At2g13100.1
at5g63870	CON	MIN	HOUR	DAY	WEEK	UNIGENE	FLCDNA	TAIR
AAGGTCCAGA	1	2	0	0	0	gnl|UG|At#S11707339	gi|15292928|gb|AY050898.1	At5g63870.1
at4g12880	CON	MIN	HOUR	DAY	WEEK	UNIGENE	FLCDNA	TAIR
AAGCTAGATG	4	4	2	0	0	gnl|UG|At#S11725585	gi|21406899|gb|AY088125.1	At4g12880.1
at1g02450	CON	MIN	HOUR	DAY	WEEK	UNIGENE	FLCDNA	TAIR
AGCAAGGATG	0	0	0	0	1	no match found	gi|28372815|gb|BT003662.1	non-canonical match
at4g23800	CON	MIN	HOUR	DAY	WEEK	UNIGENE	FLCDNA	TAIR
TTCAAGTCCT	2	7	1	2	0	gnl|UG|At#S11723682	gi|18377798|gb|AY074353.1	At4g23800.1
at4g36250	CON	MIN	HOUR	DAY	WEEK	UNIGENE	FLCDNA	TAIR
GAACAACTTC	2	4	4	2	1	gnl|UG|At#S11721555	gi|133778905|gb|BT030380.1	At4g36250.1
at1g11915	CON	MIN	HOUR	DAY	WEEK	UNIGENE	FLCDNA	TAIR
GTCACACTCA	4	0	0	3	1	gnl|UG|At#S11702220	gi|13507566|gb|AF360349.1	At1g11915.1
at1g32230	CON	MIN	HOUR	DAY	WEEK	UNIGENE	FLCDNA	TAIR
AAGAAGGTGC	2	3	2	5	1	gnl|UG|At#S11738190	gi|16604702|gb|AY059796.1	At1g32230.2
at3g43083	CON	MIN	HOUR	DAY	WEEK	UNIGENE	FLCDNA	TAIR
CTGTTTTTAT	0	0	1	0	0	gnl|UG|At#S28282875	no match found	pseudo chromosome match
at2g24570	CON	MIN	HOUR	DAY	WEEK	UNIGENE	FLCDNA	TAIR
TAAGAAAATG	0	0	0	1	0	gnl|UG|At#S34114411	gi|110743417|dbj|AK227604.1|	At2g24570.1
at1g74920	CON	MIN	HOUR	DAY	WEEK	UNIGENE	FLCDNA	TAIR
GAAATGGAAA	4	9	9	3	3	gnl|UG|At#S11728052	gi|21406119|gb|AY087395.1	At1g74920.1
GGGTGGTGTC	0	0	0	1	0	no match found	gi|31711909|gb|BT008872.1	non-canonical match
at2g28740	CON	MIN	HOUR	DAY	WEEK	UNIGENE	FLCDNA	TAIR
CGAGGAGGAA	1	2	4	2	2	gnl|UG|At#S16347387	gi|23296861|gb|AY142651.1	At2g28740.1
at3g46000	CON	MIN	HOUR	DAY	WEEK	UNIGENE	FLCDNA	TAIR
TACAATACTT	4	5	2	1	6	no match found	gi|13877562|gb|AF370482.1	At3g46000.1
at3g54170	CON	MIN	HOUR	DAY	WEEK	UNIGENE	FLCDNA	TAIR
AAACAGAAAG	3	3	1	1	2	gnl|UG|At#S11728653	gi|17380967|gb|AY063940.1	multiple canonical match
at5g25752	CON	MIN	HOUR	DAY	WEEK	UNIGENE	FLCDNA	TAIR
CTAACTGGAA	0	1	0	0	0	gnl|UG|At#S11817036	gi|110736073|dbj|AK228048.1|	At5g25752.1

at3g01320	CON	MIN	HOUR	DAY	WEEK	UNIGENE	FLCDNA	TAIR
TAGTGTGTGT	1	1	0	0	1	gnl|UG|At#S11740273	no match found	At3g01320.1
at3g06860	CON	MIN	HOUR	DAY	WEEK	UNIGENE	FLCDNA	TAIR
TTCTGGGCTG	1	0	1	0	0	gnl|UG|At#S11708943	no match found	At3g06860.1
at4g38140	CON	MIN	HOUR	DAY	WEEK	UNIGENE	FLCDNA	TAIR
ATGATGACGG	1	0	1	0	0	gnl|UG|At#S26110449	gi|124301119|gb|BT030074.1	non-canonical match
at3g02170	CON	MIN	HOUR	DAY	WEEK	UNIGENE	FLCDNA	TAIR
AACTTGAAGG	1	8	1	0	0	gnl|UG|At#S11706160	gi|23297750|gb|AY150501.1	At3g02170.1
at5g60450	CON	MIN	HOUR	DAY	WEEK	UNIGENE	FLCDNA	TAIR
CCATTGGATT	1	1	2	1	2	gnl|UG|At#S11717657	gi|19424050|gb|AY080832.1	At5g60450.1
at5g48250	CON	MIN	HOUR	DAY	WEEK	UNIGENE	FLCDNA	TAIR
TAGAAGTTTG	0	2	27	5	3	gnl|UG|At#S11718895	gi|14532665|gb|AY039957.1	At5g48250.1
at3g21200	CON	MIN	HOUR	DAY	WEEK	UNIGENE	FLCDNA	TAIR
GAAGACATCT	1	1	0	1	1	gnl|UG|At#S11734467	gi|17065155|gb|AY062654.1	At3g21200.1
at1g16680	CON	MIN	HOUR	DAY	WEEK	UNIGENE	FLCDNA	TAIR
GGAGCTTGAA	0	0	3	0	0	gnl|UG|At#S11741377	no match found	At1g16680.1
at1g42540	CON	MIN	HOUR	DAY	WEEK	UNIGENE	FLCDNA	TAIR
GATGAGAAAG	1	4	1	0	0	gnl|UG|At#S11736291	no match found	non-canonical match
at3g10300	CON	MIN	HOUR	DAY	WEEK	UNIGENE	FLCDNA	TAIR
CTCACTGTTT	0	4	1	1	2	gnl|UG|At#S15461328	gi|17064843|gb|AY062498.1	At3g10300.2
at1g53240	CON	MIN	HOUR	DAY	WEEK	UNIGENE	FLCDNA	TAIR
CTTGAAAGGA	0	0	0	0	1	no match found	gi|12642847|gb|AF339684.1	non-canonical match
AACAAGAGAT	21	10	14	9	27	gnl|UG|At#S11733277	gi|13265429|gb|AF324670.2	At1g53240.1
at3g43410	CON	MIN	HOUR	DAY	WEEK	UNIGENE	FLCDNA	TAIR
TTGTTTTCTT	0	0	0	1	0	no match found	no match found	At3g43410.1
at2g44080	CON	MIN	HOUR	DAY	WEEK	UNIGENE	FLCDNA	TAIR
TTTCTTCTTC	3	8	1	0	0	no match found	gi|19423989|gb|AY080817.1	At2g44080.1
at2g20825	CON	MIN	HOUR	DAY	WEEK	UNIGENE	FLCDNA	TAIR
CGAGGAGGAG	0	0	0	1	0	gnl|UG|At#S14233106	no match found	At2g20825.1
at2g01180	CON	MIN	HOUR	DAY	WEEK	UNIGENE	FLCDNA	TAIR
AAATTGACGT	2	3	0	3	1	no match found	gi|21404579|gb|AY085869.1	non-canonical match
at3g16480	CON	MIN	HOUR	DAY	WEEK	UNIGENE	FLCDNA	TAIR
TCTCCAATTT	3	4	2	2	0	gnl|UG|At#S11735914	no match found	At3g16480.1
at1g18560	CON	MIN	HOUR	DAY	WEEK	UNIGENE	FLCDNA	TAIR
AATCATTGAA	0	0	0	0	1	gnl|UG|At#S11741195	no match found	At1g18560.1
at1g19670	CON	MIN	HOUR	DAY	WEEK	UNIGENE	FLCDNA	TAIR
TGTATTTTTA	1	1	0	0	0	gnl|UG|At#S11741086	gi|23198201|gb|BT000309.1	multiple canonical match
at5g58820	CON	MIN	HOUR	DAY	WEEK	UNIGENE	FLCDNA	TAIR
AAAAATAAAT	0	0	0	1	0	no match found	no match found	At5g58820.1
at1g01070	CON	MIN	HOUR	DAY	WEEK	UNIGENE	FLCDNA	TAIR
TTCTTGTGGG	0	0	0	0	1	gnl|UG|At#S18893489	gi|21407813|gb|AY089039.1	At1g01070.2

at4g33680	CON	MIN	HOUR	DAY	WEEK	UNIGENE	FLCDNA	TAIR
ATTTGAGTTA	8	2	3	5	4	gnl|UG|At#S11721993	gi|15912290|gb|AY056423.1	At4g33680.1

at2g18770	CON	MIN	HOUR	DAY	WEEK	UNIGENE	FLCDNA	TAIR
AGGATTGTGT	2	1	0	0	0	gnl|UG|At#S21989818	gi|21403947|gb|AY085237.1	At2g18770.1

at3g55500	CON	MIN	HOUR	DAY	WEEK	UNIGENE	FLCDNA	TAIR
GTTTGCTTGT	0	0	0	1	0	gnl|UG|At#S21736800	gi|51969891|dbj|AK175875.1|	pseudo chromosome match

at4g30010	CON	MIN	HOUR	DAY	WEEK	UNIGENE	FLCDNA	TAIR
CTAAGGAGCA	0	0	2	0	0	no match found	gi|15809751|gb|AY054143.1	At4g30010.1
AATAATGAAA	8	9	8	3	12	gnl|UG|At#S11722615	gi|14190500|gb|AF380650.1	non-canonical match

at5g55460	CON	MIN	HOUR	DAY	WEEK	UNIGENE	FLCDNA	TAIR
TAACATCAAC	1	0	0	0	0	gnl|UG|At#S11718167	gi|114050638|gb|BT028922.1	At5g55460.1

at5g27620	CON	MIN	HOUR	DAY	WEEK	UNIGENE	FLCDNA	TAIR
ACGAGAGTAA	1	1	0	0	0	gnl|UG|At#S11823159	gi|26452431|dbj|AK118707.1|	At5g27620.1

at3g15351	CON	MIN	HOUR	DAY	WEEK	UNIGENE	FLCDNA	TAIR
CTGTGCATAC	0	1	0	0	0	no match found	gi|58331790|gb|BT020547.1	At3g15351.1

atcg00120	CON	MIN	HOUR	DAY	WEEK	UNIGENE	FLCDNA	TAIR
ATCCCTATAG	0	1	0	1	0	no match found	no match found	AtCg00120

at1g78900	CON	MIN	HOUR	DAY	WEEK	UNIGENE	FLCDNA	TAIR
CCTGTGGCTG	0	0	1	0	0	no match found	gi|30725439|gb|BT008383.1	non-canonical match

at2g44290	CON	MIN	HOUR	DAY	WEEK	UNIGENE	FLCDNA	TAIR
AAGTGTCTTT	1	0	0	0	0	gnl|UG|At#S11731567	gi|26451352|dbj|AK118152.1|	At2g44290.1

at5g05310	CON	MIN	HOUR	DAY	WEEK	UNIGENE	FLCDNA	TAIR
AAGATGAAGG	1	0	0	0	0	gnl|UG|At#S11724807	gi|110738395|dbj|AK229260.1|	At5g05310.1

at2g18910	CON	MIN	HOUR	DAY	WEEK	UNIGENE	FLCDNA	TAIR
TATAAACTCA	0	0	1	0	1	gnl|UG|At#S11737751	gi|21405998|gb|AY087274.1	At2g18910.1

at4g26795	CON	MIN	HOUR	DAY	WEEK	UNIGENE	FLCDNA	TAIR
TTCTGAAATA	0	0	0	0	1	no match found	gi|21405259|gb|AY086549.1	At4g26790.2

at3g26000	CON	MIN	HOUR	DAY	WEEK	UNIGENE	FLCDNA	TAIR
AGAATGCGAG	0	2	1	0	0	gnl|UG|At#S11732985	gi|22655120|gb|AY140009.1	At3g26000.1

at3g09250	CON	MIN	HOUR	DAY	WEEK	UNIGENE	FLCDNA	TAIR
AATCTTGGTC	6	4	2	0	1	no match found	no match found	At3g09250.1
TAGACAATGA	14	9	12	5	1	gnl|UG|At#S11738114	gi|21405928|gb|AY087204.1	non-canonical match
CTTCTCCTGT	0	1	0	0	0	no match found	gi|17380785|gb|AY063867.1	multiple non-canonical match

at1g33400	CON	MIN	HOUR	DAY	WEEK	UNIGENE	FLCDNA	TAIR
TCTTAAGCAA	0	1	0	0	2	gnl|UG|At#S11824150	gi|26450408|dbj|AK117666.1|	At1g33400.1

at3g45230	CON	MIN	HOUR	DAY	WEEK	UNIGENE	FLCDNA	TAIR
CTAATATAAT	0	1	0	0	0	gnl|UG|At#S11730337	no match found	At3g45230.1

at5g50350	CON	MIN	HOUR	DAY	WEEK	UNIGENE	FLCDNA	TAIR
CCTTATCAAG	0	0	0	0	1	gnl|UG|At#S11718679	gi|22531047|gb|AY136362.1	At5g50350.1

at1g68110	CON	MIN	HOUR	DAY	WEEK	UNIGENE	FLCDNA	TAIR
AAGAAGATTT	0	1	0	0	1	gnl|UG|At#S18921897	no match found	multiple non-canonical match

at1g26970	CON	MIN	HOUR	DAY	WEEK	UNIGENE	FLCDNA	TAIR
CATTTTTTTT	1	0	0	0	0	gnl|UG|At#S18893457	gi|56236065|gb|BT020268.1	At1g26970.1
at3g51940	CON	MIN	HOUR	DAY	WEEK	UNIGENE	FLCDNA	TAIR
GTTTTGTGTT	5	3	6	4	5	gnl|UG|At#S11729071	no match found	multiple canonical match
at4g16141	CON	MIN	HOUR	DAY	WEEK	UNIGENE	FLCDNA	TAIR
GCTCTTTCTT	0	2	0	0	0	gnl|UG|At#S18906060	no match found	At4g16141.1
at1g10560	CON	MIN	HOUR	DAY	WEEK	UNIGENE	FLCDNA	TAIR
AGTTCCAGGA	0	2	0	0	0	gnl|UG|At#S11741977	gi|18700156|gb|AY075683.1	At1g10560.1
at5g23300	CON	MIN	HOUR	DAY	WEEK	UNIGENE	FLCDNA	TAIR
AAGCAATTGG	0	1	0	0	0	gnl|UG|At#S34118141	gi|111074515|gb|BT026524.1	multiple canonical match
at1g22990	CON	MIN	HOUR	DAY	WEEK	UNIGENE	FLCDNA	TAIR
AGCCGATGTT	0	1	2	1	0	gnl|UG|At#S11740763	gi|18377445|gb|AY072474.1	At1g22990.1
at4g32370	CON	MIN	HOUR	DAY	WEEK	UNIGENE	FLCDNA	TAIR
TAAACCAAAA	1	0	0	0	0	no match found	no match found	At4g32370.1
at5g18470	CON	MIN	HOUR	DAY	WEEK	UNIGENE	FLCDNA	TAIR
GAACCTGCTG	0	0	1	0	0	gnl|UG|At#S11721703	gi|21407780|gb|AY089006.1	non-canonical match
at1g65230	CON	MIN	HOUR	DAY	WEEK	UNIGENE	FLCDNA	TAIR
AAGAGTCTTG	2	0	2	4	0	gnl|UG|At#S11730083	gi|23198127|gb|BT000272.1	At1g65230.1
at1g64850	CON	MIN	HOUR	DAY	WEEK	UNIGENE	FLCDNA	TAIR
GGACAAGTCT	1	2	0	0	0	gnl|UG|At#S11676725	gi|21405755|gb|AY087031.1	At1g64850.1
at1g75330	CON	MIN	HOUR	DAY	WEEK	UNIGENE	FLCDNA	TAIR
CGGTTGGTTC	1	7	3	5	3	gnl|UG|At#S11727975	gi|13878014|gb|AF370270.1	At1g75330.1
at3g26744	CON	MIN	HOUR	DAY	WEEK	UNIGENE	FLCDNA	TAIR
TAATGCAATT	5	3	3	2	1	gnl|UG|At#S11732718	gi|19310474|gb|AY079016.1	multiple non-canonical match
at1g26820	CON	MIN	HOUR	DAY	WEEK	UNIGENE	FLCDNA	TAIR
GTCGATGCGA	0	0	1	0	0	no match found	gi|17380739|gb|AY063844.1	multiple non-canonical match
GTAAAAAGAT	1	0	0	0	0	no match found	gi|21407595|gb|AY088821.1	non-canonical match
at1g08030	CON	MIN	HOUR	DAY	WEEK	UNIGENE	FLCDNA	TAIR
AATTAGTGTC	1	1	0	0	0	gnl|UG|At#S11742231	no match found	At1g08020.1
at3g22370	CON	MIN	HOUR	DAY	WEEK	UNIGENE	FLCDNA	TAIR
TAAAGATACC	3	0	0	0	2	gnl|UG|At#S11734108	no match found	At3g22370.1
GAAGTCGCGA	0	3	1	0	0	no match found	gi|21407744|gb|AY088970.1	multiple non-canonical match
at2g14410	CON	MIN	HOUR	DAY	WEEK	UNIGENE	FLCDNA	TAIR
AAGATGATTT	0	0	0	1	0	no match found	no match found	At2g14410.1
at1g68450	CON	MIN	HOUR	DAY	WEEK	UNIGENE	FLCDNA	TAIR
AAAGTTAAGG	0	1	0	0	0	gnl|UG|At#S11729410	gi|26450536|dbj|AK117732.1|	At1g68450.1
at5g52547	CON	MIN	HOUR	DAY	WEEK	UNIGENE	FLCDNA	TAIR
CAAGTGTTGT	1	0	0	0	0	no match found	gi|110741265|dbj|AK230385.1|	multiple non-canonical match
at3g13990	CON	MIN	HOUR	DAY	WEEK	UNIGENE	FLCDNA	TAIR
GGACCTGGCC	0	2	0	1	0	gnl|UG|At#S11736654	gi|110742568|dbj|AK227156.1|	non-canonical match
at4g01220	CON	MIN	HOUR	DAY	WEEK	UNIGENE	FLCDNA	TAIR
TGAAGAGGTG	1	0	0	0	1	gnl|UG|At#S15461593	no match found	At4g01220.1

at1g12400	CON	MIN	HOUR	DAY	WEEK	UNIGENE	FLCDNA	TAIR
TAGAGCAGAT	0	1	0	0	0	no match found	gi|40823746|gb|BT011266.1	non-canonical match

at1g27850	CON	MIN	HOUR	DAY	WEEK	UNIGENE	FLCDNA	TAIR
AATTGTAAAG	1	0	3	1	1	gnl|UG|At#S11739685	no match found	At1g27850.1

at3g55730	CON	MIN	HOUR	DAY	WEEK	UNIGENE	FLCDNA	TAIR
GATAAAGAGT	0	0	0	1	0	gnl|UG|At#S11701216	gi|23197767|gb|BT000092.1	At3g55730.1

at5g23430	CON	MIN	HOUR	DAY	WEEK	UNIGENE	FLCDNA	TAIR
AATAGAGGTT	0	1	0	0	0	gnl|UG|At#S15460468	gi|25083344|gb|BT002053.1	At5g23430.2

at1g69210	CON	MIN	HOUR	DAY	WEEK	UNIGENE	FLCDNA	TAIR
GATGAGCTTG	0	0	2	0	0	gnl|UG|At#S43850359	gi|13937140|gb|AF372924.1	multiple non-canonical match

at2g41530	CON	MIN	HOUR	DAY	WEEK	UNIGENE	FLCDNA	TAIR
CTCAAGCCCT	3	7	4	4	2	gnl|UG|At#S11732205	gi|17386095|gb|AF446861.1	At2g41530.1

at2g44770	CON	MIN	HOUR	DAY	WEEK	UNIGENE	FLCDNA	TAIR
GAGTTTAACA	0	2	1	0	0	gnl|UG|At#S11731459	no match found	multiple canonical match

at4g39050	CON	MIN	HOUR	DAY	WEEK	UNIGENE	FLCDNA	TAIR
CGGTTCTGAA	0	4	1	2	0	gnl|UG|At#S11807169	gi|23297816|gb|AY150516.1	At4g39050.1

at1g70770	CON	MIN	HOUR	DAY	WEEK	UNIGENE	FLCDNA	TAIR
TGAATGCAGA	1	0	0	0	0	gnl|UG|At#S11815900	gi|22531183|gb|AY136430.1	At1g70770.1
AATACATCAA	3	2	0	0	0	no match found	gi|30725429|gb|BT008378.1	non-canonical match

at1g03600	CON	MIN	HOUR	DAY	WEEK	UNIGENE	FLCDNA	TAIR
ATAATTGCTT	60	20	48	25	21	no match found	gi|21403261|gb|AY084551.1	At1g03600.1

at1g42470	CON	MIN	HOUR	DAY	WEEK	UNIGENE	FLCDNA	TAIR
TAGAAGGGGA	1	0	0	0	0	gnl|UG|At#S11736304	no match found	At1g42470.1

at5g42660	CON	MIN	HOUR	DAY	WEEK	UNIGENE	FLCDNA	TAIR
TACGCAAGGC	0	0	1	0	0	no match found	gi|21700922|gb|AY124876.1	non-canonical match
TTTTAAACTG	0	1	0	0	0	gnl|UG|At#S11719464	gi|16323219|gb|AY057714.1	At5g42660.1

at5g21080	CON	MIN	HOUR	DAY	WEEK	UNIGENE	FLCDNA	TAIR
TGTTTCAGTT	0	0	0	2	0	no match found	no match found	At5g21080.1

at2g16880	CON	MIN	HOUR	DAY	WEEK	UNIGENE	FLCDNA	TAIR
CAACATTCTC	1	0	0	0	0	gnl|UG|At#S11743620	gi|18175642|gb|AY072079.1	At2g16880.1

at3g08770	CON	MIN	HOUR	DAY	WEEK	UNIGENE	FLCDNA	TAIR
AGATCTCTCT	0	1	0	0	0	no match found	gi|16649162|gb|AY059951.1	multiple canonical match

at3g46150	CON	MIN	HOUR	DAY	WEEK	UNIGENE	FLCDNA	TAIR
GCAATGTTAG	0	1	0	0	0	no match found	no match found	At3g46150.1

at3g20980	CON	MIN	HOUR	DAY	WEEK	UNIGENE	FLCDNA	TAIR
TGGGAAAAGA	0	0	1	0	0	gnl|UG|At#S11734542	no match found	non-canonical match

at4g26670	CON	MIN	HOUR	DAY	WEEK	UNIGENE	FLCDNA	TAIR
TTTTGTGAAA	2	7	3	3	6	gnl|UG|At#S11723198	gi|15294263|gb|AF410323.1	At4g26670.1

at1g06500	CON	MIN	HOUR	DAY	WEEK	UNIGENE	FLCDNA	TAIR
TAGCAGCTTG	0	1	2	0	0	gnl|UG|At#S38434417	gi|21389682|gb|AY114721.1	At1g06500.1

at2g45750	CON	MIN	HOUR	DAY	WEEK	UNIGENE	FLCDNA	TAIR

AAAGAGAGAA	0	0	1	0	0	no match found	no match found	At2g45750.1
at1g32360	CON	MIN	HOUR	DAY	WEEK	UNIGENE	FLCDNA	TAIR
GTACATTTTG	0	2	2	1	0	gnl|UG|At#S11738144	gi|26451082|dbj|AK118013.1|	At1g32360.1
GAGCTGCAGA	0	0	1	0	0	no match found	gi|19547998|gb|AY081710.1	multiple non-canonical match
at1g36160	CON	MIN	HOUR	DAY	WEEK	UNIGENE	FLCDNA	TAIR
TCACTCTTGC	6	6	3	4	5	gnl|UG|At#S11736810	gi|110738845|dbj|AK229488.1|	At1g36160.1
at2g38580	CON	MIN	HOUR	DAY	WEEK	UNIGENE	FLCDNA	TAIR
GATACTGCAT	1	0	0	0	0	gnl|UG|At#S11732950	gi|51968993|dbj|AK175426.1|	At2g38580.1
at4g08730	CON	MIN	HOUR	DAY	WEEK	UNIGENE	FLCDNA	TAIR
GAAAAGCAAA	0	0	1	0	0	no match found	no match found	At4g08730.1
at1g04160	CON	MIN	HOUR	DAY	WEEK	UNIGENE	FLCDNA	TAIR
ACAGAAGAAT	0	1	0	0	0	gnl|UG|At#S11742649	no match found	At1g04160.1
at2g41835	CON	MIN	HOUR	DAY	WEEK	UNIGENE	FLCDNA	TAIR
TCCAGAAGTG	0	1	1	0	0	gnl|UG|At#S11710301	gi|17529075|gb|AY065272.1	At2g41835.1
at1g60670	CON	MIN	HOUR	DAY	WEEK	UNIGENE	FLCDNA	TAIR
TTAAAAGTTG	1	2	0	0	0	gnl|UG|At#S11704698	gi|15028080|gb|AY045897.1	non-canonical match
at3g55140	CON	MIN	HOUR	DAY	WEEK	UNIGENE	FLCDNA	TAIR
GAGTTCCTAA	0	2	1	1	0	gnl|UG|At#S18941972	no match found	At3g55140.1
at1g32400	CON	MIN	HOUR	DAY	WEEK	UNIGENE	FLCDNA	TAIR
TGAGTCCATT	1	6	1	1	1	gnl|UG|At#S28282553	gi|14326468|gb|AF385687.1	At1g32400.2
at5g63350	CON	MIN	HOUR	DAY	WEEK	UNIGENE	FLCDNA	TAIR
GCCCTTCGTA	0	0	1	0	0	gnl|UG|At#S11825206	gi|27754382|gb|BT002821.1	At5g63350.1
at3g29130	CON	MIN	HOUR	DAY	WEEK	UNIGENE	FLCDNA	TAIR
TGTATTACTT	1	0	1	0	0	gnl|UG|At#S11731923	no match found	non-canonical match
at5g40150	CON	MIN	HOUR	DAY	WEEK	UNIGENE	FLCDNA	TAIR
TGTGATTATT	0	3	1	0	3	gnl|UG|At#S11719716	gi|26452284|dbj|AK118632.1|	At5g40150.1
at1g60180	CON	MIN	HOUR	DAY	WEEK	UNIGENE	FLCDNA	TAIR
AGATAGAGAA	0	1	0	0	0	no match found	no match found	At1g60180.1
at2g38310	CON	MIN	HOUR	DAY	WEEK	UNIGENE	FLCDNA	TAIR
CATATCATAA	8	3	1	0	0	no match found	gi|21405870|gb|AY087146.1	At2g38310.1
at1g51800	CON	MIN	HOUR	DAY	WEEK	UNIGENE	FLCDNA	TAIR
AATTTCACAA	1	2	2	0	0	gnl|UG|At#S11823034	gi|39104600|dbj|AK118836.2|	non-canonical match
at2g33250	CON	MIN	HOUR	DAY	WEEK	UNIGENE	FLCDNA	TAIR
CTCAAGTGAA	2	0	5	3	3	gnl|UG|At#S11734225	gi|15010591|gb|AY045597.1	At2g33250.1
at1g05760	CON	MIN	HOUR	DAY	WEEK	UNIGENE	FLCDNA	TAIR
GGACTTATGA	0	0	0	1	0	no match found	no match found	At1g05760.1
at5g46230	CON	MIN	HOUR	DAY	WEEK	UNIGENE	FLCDNA	TAIR
ATACTTTGCT	1	0	0	1	0	gnl|UG|At#S21737104	gi|51969283|dbj|AK175571.1|	At5g46230.1
at4g25160	CON	MIN	HOUR	DAY	WEEK	UNIGENE	FLCDNA	TAIR
GAATGGAGGT	0	1	0	0	0	no match found	no match found	At4g25160.1
at3g54610	CON	MIN	HOUR	DAY	WEEK	UNIGENE	FLCDNA	TAIR

TATACTGGAG	0	0	0	0	1	gnl|UG|At#S11728581	no match found		At3g54610.1
at1g63490	CON	MIN	HOUR	DAY	WEEK	UNIGENE	FLCDNA		TAIR
GTTTCTTGTA	1	0	0	0	0	gnl|UG|At#S11730439	gi|42821104|gb|BT011572.1		At1g63490.1
at4g30810	CON	MIN	HOUR	DAY	WEEK	UNIGENE	FLCDNA		TAIR
TCTGAACTCG	1	0	2	3	0	gnl|UG|At#S11707285	gi|15293048|gb|AY050958.1		At4g30810.1
at3g29310	CON	MIN	HOUR	DAY	WEEK	UNIGENE	FLCDNA		TAIR
TTCTGTGAAC	2	1	1	0	0	gnl|UG|At#S11731867	gi|19310461|gb|AY078969.1		At3g29310.1
at1g32640	CON	MIN	HOUR	DAY	WEEK	UNIGENE	FLCDNA		TAIR
GAAACTGCGT	0	0	4	0	0	no match found	gi|110738293|dbj|AK229206.1|		At3g29310.1
TTTTTTTCCA	12	6	5	3	9	gnl|UG|At#S11698912	no match found		multiple canonical match
at3g53290	CON	MIN	HOUR	DAY	WEEK	UNIGENE	FLCDNA		TAIR
GAAGAAGTTG	0	0	0	1	1	gnl|UG|At#S11728822	no match found		multiple non-canonical match
at3g14660	CON	MIN	HOUR	DAY	WEEK	UNIGENE	FLCDNA		TAIR
GTTTGGACCC	1	2	1	0	1	no match found	gi|27754240|gb|BT002745.1		non-canonical match
ACACAACAAC	0	1	0	0	0	no match found	gi|13605900|gb|AF367349.1		At3g14660.1
TTGGATTACG	0	0	1	1	0	gnl|UG|At#S11743643	no match found		At3g14660.1
at1g29550	CON	MIN	HOUR	DAY	WEEK	UNIGENE	FLCDNA		TAIR
ATTTCGTAAT	0	1	0	1	0	no match found	no match found		At1g29550.1
at5g47930	CON	MIN	HOUR	DAY	WEEK	UNIGENE	FLCDNA		TAIR
GATGTTAAGT	0	0	0	0	1	no match found	gi|19310838|gb|AY079419.1		multiple non-canonical match
TTTACAATTC	1	1	1	2	8	no match found	no match found		At5g47930.1
ATAAAACTAC	13	4	5	11	14	no match found	gi|21407406|gb|AY088632.1		non-canonical match
at1g51590	CON	MIN	HOUR	DAY	WEEK	UNIGENE	FLCDNA		TAIR
AGTGTTGGCA	0	1	0	0	0	gnl|UG|At#S11733920	gi|22136333|gb|AY128845.1		At1g51590.1
at1g01260	CON	MIN	HOUR	DAY	WEEK	UNIGENE	FLCDNA		TAIR
CGTTTGAAGA	0	1	0	0	0	gnl|UG|At#S11742987	gi|21539514|gb|AY120752.1		At1g01260.1
at5g38510	CON	MIN	HOUR	DAY	WEEK	UNIGENE	FLCDNA		TAIR
AGCTTTCTGC	1	1	0	0	0	gnl|UG|At#S11719882	gi|46518448|gb|BT012562.1		At5g38510.1
at5g35800	CON	MIN	HOUR	DAY	WEEK	UNIGENE	FLCDNA		TAIR
AATTGCAAGC	0	1	0	0	0	gnl|UG|At#S35290856	gi|106500278|gb|BT025690.1		At2g07520.1
at5g15490	CON	MIN	HOUR	DAY	WEEK	UNIGENE	FLCDNA		TAIR
AGTTTTTCCA	0	3	5	2	4	gnl|UG|At#S11706066	gi|15810322|gb|AY056200.1		At5g15490.1
at1g09950	CON	MIN	HOUR	DAY	WEEK	UNIGENE	FLCDNA		TAIR
TTTTTTATTT	0	1	0	0	0	no match found	gi|110737844|dbj|AK228972.1|		At1g09950.1
at3g23570	CON	MIN	HOUR	DAY	WEEK	UNIGENE	FLCDNA		TAIR
TTTGTGGCTA	1	1	2	0	2	gnl|UG|At#S11733735	gi|13899072|gb|AF370531.1		At3g23570.1
CTCGCTTGGC	0	0	1	0	0	no match found	gi|24899686|gb|BT001171.1		non-canonical match
at1g23950	CON	MIN	HOUR	DAY	WEEK	UNIGENE	FLCDNA		TAIR
GGCGTCTGCG	2	5	6	1	0	gnl|UG|At#S11740643	gi|13878050|gb|AF370288.1		At1g23950.2
at5g11070	CON	MIN	HOUR	DAY	WEEK	UNIGENE	FLCDNA		TAIR
ATGATCGGAG	0	8	0	0	0	gnl|UG|At#S34115859	gi|110740692|dbj|AK226291.1|		At5g11070.1
at1g34350	CON	MIN	HOUR	DAY	WEEK	UNIGENE	FLCDNA		TAIR
CTTTGTTGTT	0	0	0	0	1	gnl|UG|At#S11737463	gi|21405191|gb|AY086481.1		At1g34350.1

at4g11530	CON	MIN	HOUR	DAY	WEEK	UNIGENE	FLCDNA	TAIR
TTTTGAACCT	0	0	0	1	0	no match found	no match found	At4g11530.1
at5g63420	CON	MIN	HOUR	DAY	WEEK	UNIGENE	FLCDNA	TAIR
GAAAGAACAG	0	1	0	0	0	gnl|UG|At#S14273739	gi|28393616|gb|BT004210.1	At5g63420.1
at1g27410	CON	MIN	HOUR	DAY	WEEK	UNIGENE	FLCDNA	TAIR
AAAGGAATTG	0	2	0	0	0	no match found	gi|17473670|gb|AY065116.1	At1g27410.1
at1g24330	CON	MIN	HOUR	DAY	WEEK	UNIGENE	FLCDNA	TAIR
TAGATAGTCT	1	0	0	0	0	no match found	no match found	At1g24330.1
at1g17440	CON	MIN	HOUR	DAY	WEEK	UNIGENE	FLCDNA	TAIR
AGTCTTATAG	0	2	1	0	1	gnl|UG|At#S11706148	gi|15810158|gb|AY056144.1	At1g17440.1
at5g61600	CON	MIN	HOUR	DAY	WEEK	UNIGENE	FLCDNA	TAIR
ATTTCATTTA	0	11	1	0	0	no match found	no match found	At5g61600.1
GAAACTGATC	2	1	3	2	4	no match found	gi|23297104|gb|AY142636.1	At5g26717.1
at1g64430	CON	MIN	HOUR	DAY	WEEK	UNIGENE	FLCDNA	TAIR
TAAAGCAATT	0	1	0	0	0	gnl|UG|At#S28282449	no match found	non-canonical match
GAACTTGAAA	0	1	1	2	0	no match found	gi|17529265|gb|AY065419.1	At1g64430.1
at2g40110	CON	MIN	HOUR	DAY	WEEK	UNIGENE	FLCDNA	TAIR
TAAATGTAAA	0	3	0	0	0	gnl|UG|At#S11704582	gi|21404327|gb|AY085617.1	At2g40110.1
at5g63220	CON	MIN	HOUR	DAY	WEEK	UNIGENE	FLCDNA	TAIR
AAAGATGCAA	0	1	1	0	0	gnl|UG|At#S11717378	gi|51870413|gb|BT015610.1	At5g63220.1
at4g17310	CON	MIN	HOUR	DAY	WEEK	UNIGENE	FLCDNA	TAIR
TACTTTGATT	1	0	1	0	3	no match found	gi|110737986|dbj|AK229046.1|	At5g34834.1
at5g60550	CON	MIN	HOUR	DAY	WEEK	UNIGENE	FLCDNA	TAIR
TTATTGTCCA	0	1	1	1	0	gnl|UG|At#S11717647	no match found	pseudo chromosome match
at2g20420	CON	MIN	HOUR	DAY	WEEK	UNIGENE	FLCDNA	TAIR
GACATCATTA	0	1	1	0	0	no match found	gi|21406334|gb|AY087596.1	multiple non-canonical match
AATAAAAATC	1	0	1	2	3	gnl|UG|At#S11737366	no match found	At2g20420.1
at5g36940	CON	MIN	HOUR	DAY	WEEK	UNIGENE	FLCDNA	TAIR
AAACCATCAA	0	2	0	1	3	gnl|UG|At#S11720042	gi|26450565|dbj|AK117747.1|	At5g36940.1
at4g12490	CON	MIN	HOUR	DAY	WEEK	UNIGENE	FLCDNA	TAIR
TATGCAAGTT	1	0	2	3	21	gnl|UG|At#S11725649	gi|15450470|gb|AY052336.1	At4g12490.1
at5g44568	CON	MIN	HOUR	DAY	WEEK	UNIGENE	FLCDNA	TAIR
GTATCACGTT	0	0	0	0	1	gnl|UG|At#S22666544	no match found	At4g12490.1
TACACACACA	3	1	0	0	3	no match found	gi|62318760|dbj|AK220700.1|	multiple non-canonical match
at1g24510	CON	MIN	HOUR	DAY	WEEK	UNIGENE	FLCDNA	TAIR
ACTACTCTGT	0	3	1	0	2	gnl|UG|At#S18942499	gi|19715604|gb|AY075611.1	At1g24510.1
at3g21080	CON	MIN	HOUR	DAY	WEEK	UNIGENE	FLCDNA	TAIR
ATTTTTGGAT	0	0	0	1	0	gnl|UG|At#S11734504	gi|30017282|gb|BT006226.1	At3g21080.1
at4g27750	CON	MIN	HOUR	DAY	WEEK	UNIGENE	FLCDNA	TAIR
AAAATAGAGT	0	4	2	0	0	gnl|UG|At#S11723000	gi|28393093|gb|BT003934.1	At4g27750.1
at3g23450	CON	MIN	HOUR	DAY	WEEK	UNIGENE	FLCDNA	TAIR
GTTTACAAGT	1	3	1	4	0	no match found	gi|110743296|dbj|AK227540.1|	At3g23450.1

TAAAACGCTA	6	9	13	9	3	gnl|UG|At#S20761159	no match found	non-canonical match
TTTTTGTGGT	0	0	0	1	0	gnl|UG|At#S11831735	no match found	non-canonical match
at3g48380	CON	MIN	HOUR	DAY	WEEK	UNIGENE	FLCDNA	TAIR
TAACAACAAT	2	2	2	1	3	no match found	gi|110742155|dbj|AK226935.1|	At3g48380.2
at1g70370	CON	MIN	HOUR	DAY	WEEK	UNIGENE	FLCDNA	TAIR
TAAAGAATGC	5	1	3	2	2	gnl|UG|At#S11729015	gi|15912220|gb|AY056388.1	At1g70370.1
at5g50915	CON	MIN	HOUR	DAY	WEEK	UNIGENE	FLCDNA	TAIR
TCGACACAAA	0	0	1	0	1	gnl|UG|At#S15459110	gi|21406340|gb|AY087602.1	At5g50915.2
at3g26935	CON	MIN	HOUR	DAY	WEEK	UNIGENE	FLCDNA	TAIR
GTTTAGCACC	1	1	0	0	0	gnl|UG|At#S15460538	gi|110737301|dbj|AK228693.1|	non-canonical match
at3g14220	CON	MIN	HOUR	DAY	WEEK	UNIGENE	FLCDNA	TAIR
TCGTCTCTCC	2	4	1	2	0	gnl|UG|At#S11736592	gi|28973440|gb|BT005625.1	At3g14220.1
at5g57020	CON	MIN	HOUR	DAY	WEEK	UNIGENE	FLCDNA	TAIR
GTTTACTGAA	4	14	2	2	0	gnl|UG|At#S11701159	gi|15027994|gb|AY045854.1	At5g57020.1
at1g65480	CON	MIN	HOUR	DAY	WEEK	UNIGENE	FLCDNA	TAIR
AAATGAAGTG	0	0	1	0	0	gnl|UG|At#S11699520	gi|62319119|dbj|AK220881.1|	At1g65480.1
at5g15140	CON	MIN	HOUR	DAY	WEEK	UNIGENE	FLCDNA	TAIR
AACAAGAAAC	4	1	3	4	8	gnl|UG|At#S11722555	gi|26452397|dbj|AK118690.1|	multiple canonical match
at4g11800	CON	MIN	HOUR	DAY	WEEK	UNIGENE	FLCDNA	TAIR
GAATGGAATC	0	1	0	1	0	no match found	no match found	At4g11800.1
at3g56710	CON	MIN	HOUR	DAY	WEEK	UNIGENE	FLCDNA	TAIR
TGATAATCTA	2	0	4	0	0	gnl|UG|At#S11704353	gi|14596086|gb|AY042831.1	At3g56710.1
at3g11220	CON	MIN	HOUR	DAY	WEEK	UNIGENE	FLCDNA	TAIR
CTCTCATTCA	0	0	1	0	1	gnl|UG|At#S11708543	gi|17063184|gb|AY062115.1	At3g11220.1
at4g32790	CON	MIN	HOUR	DAY	WEEK	UNIGENE	FLCDNA	TAIR
AAGAGCAGCA	0	1	1	0	0	gnl|UG|At#S11722143	no match found	multiple non-canonical match
at2g35540	CON	MIN	HOUR	DAY	WEEK	UNIGENE	FLCDNA	TAIR
ACTGAAACGT	1	1	0	0	0	gnl|UG|At#S11733687	no match found	non-canonical match
at1g12669	CON	MIN	HOUR	DAY	WEEK	UNIGENE	FLCDNA	TAIR
CTCAGAAAAA	0	0	0	0	1	gnl|UG|At#S37211714	no match found	pseudo chromosome match
at3g17160	CON	MIN	HOUR	DAY	WEEK	UNIGENE	FLCDNA	TAIR
ACCCAAAAAC	0	1	1	1	1	no match found	no match found	At3g17160.1
at1g71040	CON	MIN	HOUR	DAY	WEEK	UNIGENE	FLCDNA	TAIR
AAGACAATAT	3	2	1	1	3	gnl|UG|At#S11728872	gi|17064775|gb|AY062464.1	At1g71040.1
at4g09750	CON	MIN	HOUR	DAY	WEEK	UNIGENE	FLCDNA	TAIR
GCAGCTCTTA	0	0	5	2	2	gnl|UG|At#S34114582	no match found	At4g09750.1
at3g48400	CON	MIN	HOUR	DAY	WEEK	UNIGENE	FLCDNA	TAIR
TCGTTCGTGT	0	1	0	0	0	gnl|UG|At#S11816078	no match found	non-canonical match
at5g57345	CON	MIN	HOUR	DAY	WEEK	UNIGENE	FLCDNA	TAIR
TTTTTTGGAG	5	3	1	9	2	gnl|UG|At#S34114367	gi|98960954|gb|BT025543.1	At5g57345.1
at3g15880	CON	MIN	HOUR	DAY	WEEK	UNIGENE	FLCDNA	TAIR

CAGCCTCCCA	1	2	2	1	0	no match found	gi|14532677|gb|AY039963.1	At3g15880.2
at5g58350	CON	MIN	HOUR	DAY	WEEK	UNIGENE	FLCDNA	TAIR
TGTTTAATAA	1	0	0	0	1	gnl|UG|At#S11717875	gi|14532571|gb|AY039910.1	At5g58350.1
at1g79820	CON	MIN	HOUR	DAY	WEEK	UNIGENE	FLCDNA	TAIR
TAAGGAAATG	0	0	0	1	0	no match found	gi|19347893|gb|AY080624.1	non-canonical match
at2g44525	CON	MIN	HOUR	DAY	WEEK	UNIGENE	FLCDNA	TAIR
GTGCGACTTT	1	1	4	2	2	gnl|UG|At#S11731517	gi|21407389|gb|AY088615.1	At2g44525.1
at1g33265	CON	MIN	HOUR	DAY	WEEK	UNIGENE	FLCDNA	TAIR
AGGTTGTAAC	0	1	1	0	0	gnl|UG|At#S11737834	gi|20466807|gb|AY099870.1	non-canonical match
at3g24010	CON	MIN	HOUR	DAY	WEEK	UNIGENE	FLCDNA	TAIR
TTGACTATAA	2	0	1	2	3	gnl|UG|At#S18902211	gi|28393137|gb|BT003957.1	At3g24010.1
at5g63460	CON	MIN	HOUR	DAY	WEEK	UNIGENE	FLCDNA	TAIR
CTCAAGGAGA	0	2	1	2	0	gnl|UG|At#S21735963	gi|51971874|dbj|AK176839.1|	At5g63460.1
at1g15500	CON	MIN	HOUR	DAY	WEEK	UNIGENE	FLCDNA	TAIR
TCTGAGGAAG	2	10	5	8	4	gnl|UG|At#S20534736	gi|110742044|dbj|AK226874.1|	multiple canonical match
at4g33900	CON	MIN	HOUR	DAY	WEEK	UNIGENE	FLCDNA	TAIR
AGAGAAAAAC	3	0	2	0	0	no match found	no match found	At4g33900.1
at3g04820	CON	MIN	HOUR	DAY	WEEK	UNIGENE	FLCDNA	TAIR
TATTTGTGCG	1	0	0	0	0	gnl|UG|At#S21989795	no match found	At3g04820.1
at5g62300	CON	MIN	HOUR	DAY	WEEK	UNIGENE	FLCDNA	TAIR
GGACAGATTC	3	8	4	10	3	gnl|UG|At#S17007193	gi|23198347|gb|BT000382.1	At5g62300.1
at2g04400	CON	MIN	HOUR	DAY	WEEK	UNIGENE	FLCDNA	TAIR
ATTGTGGTTG	0	0	2	0	0	gnl|UG|At#S11740306	gi|26450092|dbj|AK117503.1|	non-canonical match
at5g59450	CON	MIN	HOUR	DAY	WEEK	UNIGENE	FLCDNA	TAIR
AAGGAGGCTA	0	1	0	0	1	gnl|UG|At#S11703550	gi|17065587|gb|AY062942.1	At5g59450.1
TGTTGTTGGT	0	1	0	0	0	gnl|UG|At#S35255457	no match found	non-canonical match
at1g79600	CON	MIN	HOUR	DAY	WEEK	UNIGENE	FLCDNA	TAIR
TTACAGTAAT	15	2	1	0	6	gnl|UG|At#S11726660	gi|14532489|gb|AY039869.1	At1g79600.1
at1g05510	CON	MIN	HOUR	DAY	WEEK	UNIGENE	FLCDNA	TAIR
TGTATAAGAT	0	0	0	0	1	no match found	gi|17979350|gb|AY070405.1	non-canonical match
at5g13840	CON	MIN	HOUR	DAY	WEEK	UNIGENE	FLCDNA	TAIR
TCAAAGGTTG	0	1	0	0	0	gnl|UG|At#S11722877	gi|20466230|gb|AY099581.1	At5g13840.1
at1g68790	CON	MIN	HOUR	DAY	WEEK	UNIGENE	FLCDNA	TAIR
GGCATTGTAG	0	0	0	3	2	gnl|UG|At#S11729339	no match found	At1g68790.1
ATCGAAGAGG	1	0	0	1	0	no match found	gi|18176291|gb|AY072196.1	non-canonical match
at2g47370	CON	MIN	HOUR	DAY	WEEK	UNIGENE	FLCDNA	TAIR
GACGGCGGCG	0	1	0	0	0	gnl|UG|At#S11730861	gi|170291230|gb|BT031352.1	At2g47370.1
at2g37760	CON	MIN	HOUR	DAY	WEEK	UNIGENE	FLCDNA	TAIR
ATGTTTGGCA	1	2	3	3	0	gnl|UG|At#S38433867	gi|16604706|gb|AY059798.1	At2g37760.1
AGACACACGG	2	0	3	1	0	no match found	gi|21436090|gb|AY117171.1	At2g37760.3
at5g50400	CON	MIN	HOUR	DAY	WEEK	UNIGENE	FLCDNA	TAIR
ATTTCTTCGA	0	1	0	0	0	no match found	no match found	At5g50400.1

at5g04460	CON	MIN	HOUR	DAY	WEEK	UNIGENE	FLCDNA	TAIR
TGTACTTGCT	0	0	0	1	0	gnl|UG|At#S11725028	no match found	At5g04460.1
at2g41800	CON	MIN	HOUR	DAY	WEEK	UNIGENE	FLCDNA	TAIR
ACTTCGGCCA	0	1	0	0	0	gnl|UG|At#S11732143	gi|17979519|gb|AY070757.1	At2g41800.1
at5g38030	CON	MIN	HOUR	DAY	WEEK	UNIGENE	FLCDNA	TAIR
TCGAATCTTT	0	0	1	0	0	no match found	gi|15810017|gb|AY054277.1	multiple non-canonical match
at3g62980	CON	MIN	HOUR	DAY	WEEK	UNIGENE	FLCDNA	TAIR
AGCAATGAAT	1	1	0	0	2	gnl|UG|At#S11726545	gi|62318938|dbj|AK220790.1|	At3g62980.1
at1g05000	CON	MIN	HOUR	DAY	WEEK	UNIGENE	FLCDNA	TAIR
GAGATATTCG	0	0	1	1	0	no match found	gi|28950700|gb|BT005210.1	multiple non-canonical match
at1g45904	CON	MIN	HOUR	DAY	WEEK	UNIGENE	FLCDNA	TAIR
GTGAAAAGGA	0	1	2	3	0	no match found	gi|46359832|gb|BT012390.1	non-canonical match
at1g63010	CON	MIN	HOUR	DAY	WEEK	UNIGENE	FLCDNA	TAIR
CTCAAATATT	0	2	0	2	1	gnl|UG|At#S34116046	gi|14334841|gb|AY035094.1	At1g63010.1
at1g06010	CON	MIN	HOUR	DAY	WEEK	UNIGENE	FLCDNA	TAIR
TTTTAATGGT	1	2	1	1	0	gnl|UG|At#S11742426	gi|110739092|dbj|AK229618.1|	At1g06010.1
at4g37660	CON	MIN	HOUR	DAY	WEEK	UNIGENE	FLCDNA	TAIR
TAATACTTTG	1	0	1	0	1	gnl|UG|At#S11721325	no match found	At4g37660.1
GAGAAACTCA	1	0	0	2	1	no match found	gi|20334823|gb|AY094012.1	multiple non-canonical match
at1g05750	CON	MIN	HOUR	DAY	WEEK	UNIGENE	FLCDNA	TAIR
GGACTTATGA	0	0	0	1	0	no match found	gi|17978784|gb|AY064681.1	At1g05760.1
CTTCCCTACA	0	1	0	0	0	gnl|UG|At#S11702286	no match found	non-canonical match
at1g78955	CON	MIN	HOUR	DAY	WEEK	UNIGENE	FLCDNA	TAIR
TTCTTGTCGG	0	0	0	0	1	no match found	no match found	At1g78955.1
at1g77870	CON	MIN	HOUR	DAY	WEEK	UNIGENE	FLCDNA	TAIR
GATCTTCATT	0	0	0	1	0	gnl|UG|At#S11727244	gi|21406089|gb|AY087365.1	At1g77870.1
at5g58375	CON	MIN	HOUR	DAY	WEEK	UNIGENE	FLCDNA	TAIR
GCAAGTGGTC	3	8	2	1	0	gnl|UG|At#S11717872	gi|21404433|gb|AY085723.1	At5g58375.1
at3g06750	CON	MIN	HOUR	DAY	WEEK	UNIGENE	FLCDNA	TAIR
TAATCCTGTC	1	1	2	0	0	gnl|UG|At#S11704171	gi|21281033|gb|AY113863.1	At3g06750.1
at5g02880	CON	MIN	HOUR	DAY	WEEK	UNIGENE	FLCDNA	TAIR
GTTTTAAAAA	1	0	4	2	1	gnl|UG|At#S11725445	no match found	At5g02880.1
at1g78920	CON	MIN	HOUR	DAY	WEEK	UNIGENE	FLCDNA	TAIR
GATTAATGGT	0	4	1	0	0	no match found	gi|15450809|gb|AY054485.1	At1g78920.1
ACATTTTGCA	0	0	1	1	0	gnl|UG|At#S43850312	no match found	pseudo chromosome match
at5g16110	CON	MIN	HOUR	DAY	WEEK	UNIGENE	FLCDNA	TAIR
GCTTAGTAGA	0	7	0	0	0	no match found	no match found	At5g16110.1
at2g43430	CON	MIN	HOUR	DAY	WEEK	UNIGENE	FLCDNA	TAIR
AGGTTCGGAT	0	0	1	0	0	gnl|UG|At#S18942710	gi|20259002|gb|AY091278.1	At2g43430.1
at1g28760	CON	MIN	HOUR	DAY	WEEK	UNIGENE	FLCDNA	TAIR
CTGACAGAAT	0	1	0	0	0	gnl|UG|At#S11739375	no match found	At1g28760.1

at2g03870	CON	MIN	HOUR	DAY	WEEK	UNIGENE	FLCDNA	TAIR
GACACTTACC	2	0	2	0	3	gnl|UG|At#S15461664	no match found	multiple non-canonical match
at5g64630	CON	MIN	HOUR	DAY	WEEK	UNIGENE	FLCDNA	TAIR
AGATGGTTTT	0	0	0	1	1	gnl|UG|At#S11717236	gi|14030688|gb|AF375435.1	At5g64630.2
at2g32910	CON	MIN	HOUR	DAY	WEEK	UNIGENE	FLCDNA	TAIR
GTTTCTCCAG	0	1	1	0	0	no match found	gi|28393782|gb|BT004303.1	multiple canonical match
at3g52160	CON	MIN	HOUR	DAY	WEEK	UNIGENE	FLCDNA	TAIR
AATAAAATAA	0	1	1	0	1	gnl|UG|At#S11729030	gi|21403423|gb|AY084713.1	At3g52160.1
at4g36270	CON	MIN	HOUR	DAY	WEEK	UNIGENE	FLCDNA	TAIR
AGGTGCGAGG	1	0	2	1	0	gnl|UG|At#S11721552	no match found	multiple non-canonical match
at5g06660	CON	MIN	HOUR	DAY	WEEK	UNIGENE	FLCDNA	TAIR
GCTTTTCTTT	0	1	0	0	0	no match found	gi|16974514|gb|AY060536.1	non-canonical match
at2g28460	CON	MIN	HOUR	DAY	WEEK	UNIGENE	FLCDNA	TAIR
TGTTCTGGAT	0	0	0	1	0	gnl|UG|At#S11735428	gi|110741658|dbj|AK226668.1|	multiple non-canonical match
at5g66730	CON	MIN	HOUR	DAY	WEEK	UNIGENE	FLCDNA	TAIR
ACAGCTAAAT	1	2	3	1	0	gnl|UG|At#S11717024	no match found	At5g66730.1
TTGTACTTTG	2	0	1	0	0	no match found	gi|20466785|gb|AY099859.1	non-canonical match
at4g26600	CON	MIN	HOUR	DAY	WEEK	UNIGENE	FLCDNA	TAIR
TCCAAAAGTT	0	0	0	4	0	gnl|UG|At#S11709789	gi|17979389|gb|AY070425.1	At4g26600.1
at5g11490	CON	MIN	HOUR	DAY	WEEK	UNIGENE	FLCDNA	TAIR
CAGAGTCATT	0	1	0	0	0	gnl|UG|At#S34116635	gi|110739200|dbj|AK229673.1|	At5g11490.1
at5g14540	CON	MIN	HOUR	DAY	WEEK	UNIGENE	FLCDNA	TAIR
TATGTTTGAA	4	2	2	1	1	no match found	gi|22530951|gb|AY136314.1	multiple canonical match
TCAGATTTCC	1	0	4	1	0	no match found	gi|28059780|gb|BT003429.1	non-canonical match
at3g55830	CON	MIN	HOUR	DAY	WEEK	UNIGENE	FLCDNA	TAIR
GCAGTACATT	1	4	2	1	1	gnl|UG|At#S11728358	gi|110736935|dbj|AK228499.1|	At3g55830.1
at4g00560	CON	MIN	HOUR	DAY	WEEK	UNIGENE	FLCDNA	TAIR
ATAGTATTCC	0	0	0	1	1	gnl|UG|At#S18898688	gi|110736654|dbj|AK228352.1|	At4g00560.3
at1g71980	CON	MIN	HOUR	DAY	WEEK	UNIGENE	FLCDNA	TAIR
TCACCGTATA	1	2	0	0	1	gnl|UG|At#S11728694	gi|22136865|gb|AY133843.1	At1g71980.1
at5g15850	CON	MIN	HOUR	DAY	WEEK	UNIGENE	FLCDNA	TAIR
CTTGTAAATT	3	1	3	8	8	gnl|UG|At#S11722373	gi|18389243|gb|AY074369.1	At5g15850.1
at5g33234	CON	MIN	HOUR	DAY	WEEK	UNIGENE	FLCDNA	TAIR
AAAAATATTT	0	1	1	0	0	no match found	gi|110739961|dbj|AK230065.1|	non-canonical match
at3g28180	CON	MIN	HOUR	DAY	WEEK	UNIGENE	FLCDNA	TAIR
GTGTTCAAGA	1	2	2	0	1	gnl|UG|At#S11705984	gi|15810494|gb|AY056286.1	At3g28180.1
at1g49230	CON	MIN	HOUR	DAY	WEEK	UNIGENE	FLCDNA	TAIR
CGAGAAGATT	0	1	1	0	0	gnl|UG|At#S21736440	gi|34365600|gb|BT010489.1	At1g49230.1
at3g06780	CON	MIN	HOUR	DAY	WEEK	UNIGENE	FLCDNA	TAIR
TAAATACCAT	0	0	0	1	0	gnl|UG|At#S11738698	no match found	At3g06780.1
AATGATGATT	0	1	0	0	0	no match found	gi|15450370|gb|AY052286.1	multiple non-canonical match
at4g35780	CON	MIN	HOUR	DAY	WEEK	UNIGENE	FLCDNA	TAIR

TATAATTTTC	1	2	1	0	0	gnl|UG|At#S11721633	gi|20260235|gb|AY093017.1	At4g35780.1
at1g74260	CON	MIN	HOUR	DAY	WEEK	UNIGENE	FLCDNA	TAIR
AGTTACTCTC	7	3	8	6	3	gnl|UG|At#S30654053	gi|62318842|dbj|AK220741.1|	At1g74260.1
at1g22900	CON	MIN	HOUR	DAY	WEEK	UNIGENE	FLCDNA	TAIR
CTCTGAAAAG	1	0	0	0	0	no match found	no match found	At1g22900.1
at3g15310	CON	MIN	HOUR	DAY	WEEK	UNIGENE	FLCDNA	TAIR
CGAAATCAAC	0	0	0	1	0	gnl|UG|At#S11707361	gi|15292884|gb|AY050876.1	At3g15310.1
at3g12450	CON	MIN	HOUR	DAY	WEEK	UNIGENE	FLCDNA	TAIR
AAAACATAGA	1	0	0	0	0	no match found	no match found	At3g12450.1
at5g51400	CON	MIN	HOUR	DAY	WEEK	UNIGENE	FLCDNA	TAIR
ATTTATCATT	1	1	1	0	3	gnl|UG|At#S11718576	gi|15451199|gb|AY054680.1	multiple canonical match
at4g33360	CON	MIN	HOUR	DAY	WEEK	UNIGENE	FLCDNA	TAIR
GCTGCACTGA	0	0	1	0	0	gnl|UG|At#S43849437	gi|13926212|gb|AF370578.1	At4g33360.1
at5g61760	CON	MIN	HOUR	DAY	WEEK	UNIGENE	FLCDNA	TAIR
AACGTTTGTC	0	0	0	0	1	no match found	gi|15724265|gb|AF412073.1	non-canonical match
AAAGTAAGCT	0	1	0	0	1	gnl|UG|At#S11717525	no match found	At5g61760.1
at3g10330	CON	MIN	HOUR	DAY	WEEK	UNIGENE	FLCDNA	TAIR
CTGGAGATTT	1	0	1	0	0	gnl|UG|At#S11737789	gi|21406601|gb|AY087850.1	At3g10330.1
at1g66240	CON	MIN	HOUR	DAY	WEEK	UNIGENE	FLCDNA	TAIR
TGAGGGATGT	4	9	2	1	1	no match found	gi|18655400|gb|AY077678.1	At1g66240.1
at2g20390	CON	MIN	HOUR	DAY	WEEK	UNIGENE	FLCDNA	TAIR
ATTGAGAGTC	1	1	0	0	0	gnl|UG|At#S11737373	gi|20260283|gb|AY093041.1	At2g20390.1
at2g11462	CON	MIN	HOUR	DAY	WEEK	UNIGENE	FLCDNA	TAIR
TCGTTGGAGT	0	1	0	0	0	no match found	no match found	At2g11462.1
at1g17060	CON	MIN	HOUR	DAY	WEEK	UNIGENE	FLCDNA	TAIR
GAGCTCATTT	0	0	0	1	0	gnl|UG|At#S34117226	gi|110738056|dbj|AK229082.1|	At1g17060.1
at1g05205	CON	MIN	HOUR	DAY	WEEK	UNIGENE	FLCDNA	TAIR
GGGTTTACCG	0	0	2	0	1	no match found	gi|21403162|gb|AY084452.1	At1g05205.1
at4g13340	CON	MIN	HOUR	DAY	WEEK	UNIGENE	FLCDNA	TAIR
GTTCACCACA	1	18	3	6	1	gnl|UG|At#S11725499	no match found	At4g13340.1
at1g09760	CON	MIN	HOUR	DAY	WEEK	UNIGENE	FLCDNA	TAIR
CGCAACAGTT	0	0	1	0	0	no match found	gi|23197869|gb|BT000143.1	non-canonical match
GAGAGCGAGG	5	4	4	5	2	gnl|UG|At#S11742060	gi|16649064|gb|AY059902.1	non-canonical match
at3g11110	CON	MIN	HOUR	DAY	WEEK	UNIGENE	FLCDNA	TAIR
AATCTGGAAG	0	1	0	0	0	no match found	gi|62320008|dbj|AK221330.1|	non-canonical match
at1g60610	CON	MIN	HOUR	DAY	WEEK	UNIGENE	FLCDNA	TAIR
TAGGCACTTG	1	0	0	1	0	gnl|UG|At#S11731297	gi|45680301|gb|BT011991.1	At1g60610.2
at1g12660	CON	MIN	HOUR	DAY	WEEK	UNIGENE	FLCDNA	TAIR
TGTGAAAAAA	27	18	41	32	9	no match found	no match found	At1g12660.1
at5g28850	CON	MIN	HOUR	DAY	WEEK	UNIGENE	FLCDNA	TAIR
AATCTTTTAG	0	1	0	0	0	gnl|UG|At#S11706131	gi|15810191|gb|AY056110.1	At5g28850.1
ATCTTCAGTT	1	0	2	1	0	gnl|UG|At#S20808834	no match found	non-canonical match

at2g35810	CON	MIN	HOUR	DAY	WEEK	UNIGENE	FLCDNA	TAIR
AGTGTGCTTC	1	2	1	1	2	no match found	gi|17380999|gb|AY063956.1	At2g35810.1
at3g60110	CON	MIN	HOUR	DAY	WEEK	UNIGENE	FLCDNA	TAIR
TTCACAAAAC	1	0	0	0	0	gnl|UG|At#S11705874	gi|15912230|gb|AY056393.1	At3g60110.1
at5g49890	CON	MIN	HOUR	DAY	WEEK	UNIGENE	FLCDNA	TAIR
TAATTATATT	2	2	0	0	5	gnl|UG|At#S11718728	gi|21539518|gb|AY120754.1	At5g49890.1
at3g13740	CON	MIN	HOUR	DAY	WEEK	UNIGENE	FLCDNA	TAIR
GGTTGATTCT	1	1	3	3	7	gnl|UG|At#S11736735	gi|14423483|gb|AF386979.1	At3g13740.1
at3g53890	CON	MIN	HOUR	DAY	WEEK	UNIGENE	FLCDNA	TAIR
TTTTCGATTT	23	8	19	20	15	gnl|UG|At#S35265557	gi|17473932|gb|AY065200.1	At3g53890.1
at4g14605	CON	MIN	HOUR	DAY	WEEK	UNIGENE	FLCDNA	TAIR
TGAGTCGCAG	1	0	0	3	0	gnl|UG|At#S11725276	gi|110741630|dbj|AK226653.1|	At4g14605.1
at1g23390	CON	MIN	HOUR	DAY	WEEK	UNIGENE	FLCDNA	TAIR
GACGCAATTT	0	1	1	1	0	gnl|UG|At#S11809484	gi|21402980|gb|AY084270.1	At1g23390.1
GCGACGATGG	1	0	0	0	0	gnl|UG|At#S35308888	no match found	non-canonical match
at1g30240	CON	MIN	HOUR	DAY	WEEK	UNIGENE	FLCDNA	TAIR
ATGGTGATGA	0	0	0	1	0	no match found	no match found	At1g30240.1
at5g49910	CON	MIN	HOUR	DAY	WEEK	UNIGENE	FLCDNA	TAIR
ACCAAGATCA	0	0	0	0	1	no match found	gi|19698968|gb|AY081331.1	non-canonical match
ATTGAAGCAA	3	1	2	2	7	gnl|UG|At#S11718726	no match found	non-canonical match
at1g80530	CON	MIN	HOUR	DAY	WEEK	UNIGENE	FLCDNA	TAIR
GCCACTCTGA	0	0	1	0	0	gnl|UG|At#S35283466	no match found	non-canonical match
GGGTTTCAGT	0	1	1	2	0	gnl|UG|At#S11726311	gi|15810606|gb|AY056342.1	At1g80530.1
at1g48090	CON	MIN	HOUR	DAY	WEEK	UNIGENE	FLCDNA	TAIR
TATACGGGTA	4	2	0	1	2	gnl|UG|At#S11735191	gi|21402979|gb|AY084269.1	At1g48090.1
at3g59280	CON	MIN	HOUR	DAY	WEEK	UNIGENE	FLCDNA	TAIR
GTTTCTTTGA	3	1	3	1	3	gnl|UG|At#S11727717	gi|14190486|gb|AF380643.1	At3g59280.1
at2g39500	CON	MIN	HOUR	DAY	WEEK	UNIGENE	FLCDNA	TAIR
ACTTTATTAC	0	1	1	0	0	gnl|UG|At#S30556097	gi|14335015|gb|AY037187.1	At2g39500.1
at3g18130	CON	MIN	HOUR	DAY	WEEK	UNIGENE	FLCDNA	TAIR
AAGTGTGTTT	9	14	10	3	6	gnl|UG|At#S30650459	no match found	multiple non-canonical match
GCCGAGGGAC	0	0	1	3	0	gnl|UG|At#S30557087	gi|15215619|gb|AY050338.1	At3g18130.1
at3g13300	CON	MIN	HOUR	DAY	WEEK	UNIGENE	FLCDNA	TAIR
CTTATGGGTT	2	0	2	2	2	gnl|UG|At#S11736883	gi|22135923|gb|AY128341.1	At3g13300.1
at2g30710	CON	MIN	HOUR	DAY	WEEK	UNIGENE	FLCDNA	TAIR
GTGCTTGCTG	0	0	1	0	1	no match found	gi|22136273|gb|AY128815.1	non-canonical match
at5g34880	CON	MIN	HOUR	DAY	WEEK	UNIGENE	FLCDNA	TAIR
TCTCTTGGGA	0	1	0	0	0	no match found	no match found	At5g34880.1
at3g16310	CON	MIN	HOUR	DAY	WEEK	UNIGENE	FLCDNA	TAIR
CCTTTACCTC	1	3	0	0	0	gnl|UG|At#S11709779	gi|21405661|gb|AY086937.1	At3g16310.1
at4g17220	CON	MIN	HOUR	DAY	WEEK	UNIGENE	FLCDNA	TAIR
TTTCCAGATG	0	1	0	0	0	gnl|UG|At#S11824470	gi|26449765|dbj|AK117336.1|	At4g17220.1

at1g03090	CON	MIN	HOUR	DAY	WEEK	UNIGENE	FLCDNA	TAIR
GCTGGTTTGG	0	10	2	1	0	gnl|UG|At#S15461625	gi|17979455|gb|AY070723.1	At1g03090.2
at1g13280	CON	MIN	HOUR	DAY	WEEK	UNIGENE	FLCDNA	TAIR
TTTGAGCTAA	1	3	0	0	1	gnl|UG|At#S11741709	no match found	At1g13280.1
TTGAGCCGTC	0	0	1	0	0	no match found	gi|22655329|gb|AY141993.1	non-canonical match
at1g18600	CON	MIN	HOUR	DAY	WEEK	UNIGENE	FLCDNA	TAIR
AACAGTAATT	1	3	1	0	0	gnl|UG|At#S11741191	gi|13877606|gb|AF370504.1	At1g18600.1
at3g15580	CON	MIN	HOUR	DAY	WEEK	UNIGENE	FLCDNA	TAIR
TGCTATAGCA	1	6	4	0	2	gnl|UG|At#S11736174	gi|88011042|gb|BT024554.1	At3g15580.1
at3g57800	CON	MIN	HOUR	DAY	WEEK	UNIGENE	FLCDNA	TAIR
AATGTGTATA	0	0	0	1	3	gnl|UG|At#S11727988	gi|22654999|gb|AY139773.1	At3g57800.1
at1g34120	CON	MIN	HOUR	DAY	WEEK	UNIGENE	FLCDNA	TAIR
TATAATAGTG	0	1	0	0	0	gnl|UG|At#S26539385	gi|110742962|dbj|AK227368.1|	At1g34120.1
at4g18600	CON	MIN	HOUR	DAY	WEEK	UNIGENE	FLCDNA	TAIR
AAAAAAAGAA	3	2	5	3	0	no match found	no match found	At4g18600.1
at1g32060	CON	MIN	HOUR	DAY	WEEK	UNIGENE	FLCDNA	TAIR
CTGATTTCCC	0	1	4	0	0	no match found	gi|23197621|gb|BT000019.1	non-canonical match
GCGAAAAGGA	336	202	446	416	98	gnl|UG|At#S11738253	gi|22135951|gb|AY128355.1	multiple canonical match
at2g33560	CON	MIN	HOUR	DAY	WEEK	UNIGENE	FLCDNA	TAIR
TTTTGAAATT	0	1	0	1	0	gnl|UG|At#S11734146	gi|21406980|gb|AY088206.1	multiple canonical match
at5g05510	CON	MIN	HOUR	DAY	WEEK	UNIGENE	FLCDNA	TAIR
TTTGGCGAAC	0	0	1	0	0	gnl|UG|At#S18908490	gi|56381968|gb|BT020348.1	At5g05510.1
at2g15790	CON	MIN	HOUR	DAY	WEEK	UNIGENE	FLCDNA	TAIR
GCAAAGACTG	0	0	1	1	1	no match found	no match found	At2g15790.1
at3g50390	CON	MIN	HOUR	DAY	WEEK	UNIGENE	FLCDNA	TAIR
CTGGTCCGGT	0	0	1	0	0	gnl|UG|At#S11729368	gi|124301031|gb|BT030030.1	At3g50390.1
at1g24450	CON	MIN	HOUR	DAY	WEEK	UNIGENE	FLCDNA	TAIR
TTTTTTCGGA	0	4	1	0	0	gnl|UG|At#S11740521	gi|15010593|gb|AY045598.1	At1g24450.1
at3g49650	CON	MIN	HOUR	DAY	WEEK	UNIGENE	FLCDNA	TAIR
ACTCGATTTT	0	0	1	0	0	gnl|UG|At#S11812806	gi|110737802|dbj|AK228951.1|	At3g49650.1
at1g11680	CON	MIN	HOUR	DAY	WEEK	UNIGENE	FLCDNA	TAIR
TTAATGAGAG	0	3	0	0	0	no match found	gi|15292852|gb|AY050860.1	At1g11680.1
at5g05970	CON	MIN	HOUR	DAY	WEEK	UNIGENE	FLCDNA	TAIR
TCACAACGTT	1	0	0	0	0	gnl|UG|At#S43849370	no match found	multiple non-canonical match
at5g04730	CON	MIN	HOUR	DAY	WEEK	UNIGENE	FLCDNA	TAIR
GTTTAGATGA	0	1	0	1	0	gnl|UG|At#S11724951	no match found	multiple non-canonical match
at4g11175	CON	MIN	HOUR	DAY	WEEK	UNIGENE	FLCDNA	TAIR
ATTAGGTTTA	2	0	1	0	0	gnl|UG|At#S38432437	no match found	At4g11175.1
TAATCGTACA	5	1	5	7	6	no match found	gi|62319974|dbj|AK221313.1|	non-canonical match
at1g75390	CON	MIN	HOUR	DAY	WEEK	UNIGENE	FLCDNA	TAIR
GAGATGGTGA	1	2	1	1	0	gnl|UG|At#S11727963	no match found	At1g75390.1
GCTTCTGCTT	0	0	1	1	1	no match found	gi|28466850|gb|BT004768.1	multiple non-canonical match

at3g14690	CON	MIN	HOUR	DAY	WEEK	UNIGENE	FLCDNA	TAIR
CACAAGCTCT	10	7	6	18	12	gnl|UG|At#S11736442	gi|15292786|gb|AY050827.1	At3g14690.1
at1g71440	CON	MIN	HOUR	DAY	WEEK	UNIGENE	FLCDNA	TAIR
GAATTGAGGA	0	0	2	0	0	no match found	gi|31711941|gb|BT008888.1	non-canonical match
at1g12780	CON	MIN	HOUR	DAY	WEEK	UNIGENE	FLCDNA	TAIR
TATCGAACTG	6	44	15	4	2	gnl|UG|At#S11741760	gi|15809879|gb|AY054207.1	At1g12780.1
GGGTTACCAG	0	1	2	0	0	no match found	gi|12248020|gb|AF334724.1	non-canonical match
at4g39850	CON	MIN	HOUR	DAY	WEEK	UNIGENE	FLCDNA	TAIR
GGAGTCACTT	0	3	0	1	1	gnl|UG|At#S11720947	no match found	At4g39850.1
at5g24770	CON	MIN	HOUR	DAY	WEEK	UNIGENE	FLCDNA	TAIR
AAAATCCTCT	0	1	0	0	0	no match found	gi|23397238|gb|BT000762.1	non-canonical match
TAAATCTAAT	0	0	1	0	0	gnl|UG|At#S28281750	no match found	pseudo chromosome match
at1g08550	CON	MIN	HOUR	DAY	WEEK	UNIGENE	FLCDNA	TAIR
TGGTCCTGAA	0	3	3	1	1	gnl|UG|At#S11742180	gi|13877976|gb|AF370251.1	At1g08550.1
at1g18250	CON	MIN	HOUR	DAY	WEEK	UNIGENE	FLCDNA	TAIR
TTTCGAATTC	4	1	0	2	0	gnl|UG|At#S28282616	no match found	At1g18250.1
at2g44190	CON	MIN	HOUR	DAY	WEEK	UNIGENE	FLCDNA	TAIR
GCATACTCGC	0	1	1	0	0	gnl|UG|At#S11731589	no match found	At2g44190.1
at2g03500	CON	MIN	HOUR	DAY	WEEK	UNIGENE	FLCDNA	TAIR
GTGGCACTCC	0	2	0	0	0	gnl|UG|At#S11740455	gi|16974543|gb|AY060559.1	At2g03500.1
at2g34560	CON	MIN	HOUR	DAY	WEEK	UNIGENE	FLCDNA	TAIR
ATGTGTTGGT	0	3	0	0	0	gnl|UG|At#S18942789	gi|21403568|gb|AY084858.1	At2g34560.2
at4g34120	CON	MIN	HOUR	DAY	WEEK	UNIGENE	FLCDNA	TAIR
ACTCCGAGAC	1	4	0	2	0	gnl|UG|At#S11721915	gi|13430837|gb|AF360331.1	non-canonical match
at1g12450	CON	MIN	HOUR	DAY	WEEK	UNIGENE	FLCDNA	TAIR
ACCACAACAT	0	0	0	0	1	gnl|UG|At#S11741791	gi|62320871|dbj|AK221763.1|	At1g12450.1
at2g34410	CON	MIN	HOUR	DAY	WEEK	UNIGENE	FLCDNA	TAIR
AATCTAAAGC	0	0	1	0	0	gnl|UG|At#S28282203	gi|51536463|gb|BT015347.1	non-canonical match
at3g28020	CON	MIN	HOUR	DAY	WEEK	UNIGENE	FLCDNA	TAIR
CCTTTGAAAA	0	1	0	0	0	gnl|UG|At#S11732298	no match found	non-canonical match
at1g59833	CON	MIN	HOUR	DAY	WEEK	UNIGENE	FLCDNA	TAIR
TAACGAAGAG	0	1	0	0	0	gnl|UG|At#S28282471	no match found	non-canonical match
at2g42890	CON	MIN	HOUR	DAY	WEEK	UNIGENE	FLCDNA	TAIR
ATCAGTATTT	2	1	0	0	0	gnl|UG|At#S11731907	gi|51969501|dbj|AK175680.1|	At2g42890.1
at1g48920	CON	MIN	HOUR	DAY	WEEK	UNIGENE	FLCDNA	TAIR
TGGAGAGATC	2	6	2	5	2	gnl|UG|At#S11734876	gi|28973758|gb|BT005793.1	At1g48920.1
at3g28925	CON	MIN	HOUR	DAY	WEEK	UNIGENE	FLCDNA	TAIR
AATTTCGTCG	0	1	0	0	0	no match found	no match found	At3g28925.1
at1g62305	CON	MIN	HOUR	DAY	WEEK	UNIGENE	FLCDNA	TAIR
TATATTATGA	0	1	0	0	0	no match found	gi|61656150|gb|BT021929.1	At1g42852.1
GATTTTCTTT	1	1	0	1	0	gnl|UG|At#S21132609	gi|58743301|gb|BT020621.1	At1g62305.2

at3g59220	CON	MIN	HOUR	DAY	WEEK	UNIGENE	FLCDNA	TAIR
TTGTTGTGTT	2	0	1	0	0	no match found	no match found	At3g59220.1
at3g01960	CON	MIN	HOUR	DAY	WEEK	UNIGENE	FLCDNA	TAIR
ATGGCAGAGA	0	0	1	1	0	gnl|UG|At#S11740097	gi|51870389|gb|BT015594.1	multiple non-canonical match
at1g55255	CON	MIN	HOUR	DAY	WEEK	UNIGENE	FLCDNA	TAIR
CTTACAACAC	0	1	0	0	0	no match found	gi|115646856|gb|BT029207.1	non-canonical match
at5g15810	CON	MIN	HOUR	DAY	WEEK	UNIGENE	FLCDNA	TAIR
AAGCAGTAAA	0	2	1	0	0	gnl|UG|At#S11722385	gi|19347968|gb|AY080697.1	At5g15810.1
at5g64740	CON	MIN	HOUR	DAY	WEEK	UNIGENE	FLCDNA	TAIR
CAATTGCTGG	0	0	1	1	0	no match found	gi|110738823|dbj|AK229477.1|	non-canonical match
GCGCTCTTCT	2	5	2	2	2	gnl|UG|At#S11717225	gi|23506072|gb|AY143957.1	At5g64740.1
at5g35180	CON	MIN	HOUR	DAY	WEEK	UNIGENE	FLCDNA	TAIR
TGACATATGT	0	1	0	1	0	gnl|UG|At#S43849236	gi|16930704|gb|AF436836.1	At5g35180.1
at5g03190	CON	MIN	HOUR	DAY	WEEK	UNIGENE	FLCDNA	TAIR
CTTCAAACTC	1	0	1	0	1	gnl|UG|At#S11725363	gi|27311560|gb|BT002386.1	At5g03190.1
at2g10232	CON	MIN	HOUR	DAY	WEEK	UNIGENE	FLCDNA	TAIR
TATGTGAAAT	1	0	0	0	1	no match found	no match found	At2g10232.1
at1g42430	CON	MIN	HOUR	DAY	WEEK	UNIGENE	FLCDNA	TAIR
TATCACCTAA	0	1	0	0	0	gnl|UG|At#S34117524	gi|47679073|gb|BT014742.1	At1g42430.1
at5g63660	CON	MIN	HOUR	DAY	WEEK	UNIGENE	FLCDNA	TAIR
TGTGTTTATT	0	0	1	1	0	no match found	no match found	At5g63660.1
GCTTCCATCG	0	1	0	0	0	gnl|UG|At#S11717334	no match found	non-canonical match
at1g16180	CON	MIN	HOUR	DAY	WEEK	UNIGENE	FLCDNA	TAIR
AATCTTCAAT	11	13	7	10	7	gnl|UG|At#S11741420	gi|62319552|dbj|AK221101.1|	At1g16180.1
at5g42050	CON	MIN	HOUR	DAY	WEEK	UNIGENE	FLCDNA	TAIR
GAATCTACGA	0	14	4	2	1	gnl|UG|At#S11719524	gi|21281017|gb|AY114057.1	At5g42050.1
at4g27180	CON	MIN	HOUR	DAY	WEEK	UNIGENE	FLCDNA	TAIR
TTAACGCCAG	0	1	1	1	0	gnl|UG|At#S11723099	no match found	At4g27180.1
at1g54410	CON	MIN	HOUR	DAY	WEEK	UNIGENE	FLCDNA	TAIR
ATGATGGTCA	0	0	0	4	1	gnl|UG|At#S11683358	no match found	multiple non-canonical match
TATCTTATCT	92	77	26	37	190	gnl|UG|At#S11732849	no match found	multiple canonical match
at1g21000	CON	MIN	HOUR	DAY	WEEK	UNIGENE	FLCDNA	TAIR
GCTTTGTCTT	6	2	4	2	1	gnl|UG|At#S18896401	gi|14030626|gb|AF375404.1	At1g21000.1
GGAGAGAGTA	1	3	0	0	0	no match found	gi|13877712|gb|AF370615.1	non-canonical match
at1g19700	CON	MIN	HOUR	DAY	WEEK	UNIGENE	FLCDNA	TAIR
CTTGCAAAGC	0	1	0	0	0	gnl|UG|At#S11706963	gi|15450528|gb|AY052366.1	At1g19700.1
at4g16430	CON	MIN	HOUR	DAY	WEEK	UNIGENE	FLCDNA	TAIR
ATTCCAACGT	2	0	0	1	0	gnl|UG|At#S11724959	gi|16226918|gb|AF428368.1	At4g16430.1
at2g23350	CON	MIN	HOUR	DAY	WEEK	UNIGENE	FLCDNA	TAIR
AATGTAATGC	1	2	0	0	0	gnl|UG|At#S11702054	gi|13430609|gb|AF360217.1	At2g23350.1
at3g54650	CON	MIN	HOUR	DAY	WEEK	UNIGENE	FLCDNA	TAIR
TTTATCATTG	1	2	0	0	0	gnl|UG|At#S11728576	gi|18087569|gb|AF462827.1	At3g54650.1

<table>
<tr><td>at1g78050</td><td>CON</td><td>MIN</td><td>HOUR</td><td>DAY</td><td>WEEK</td><td>UNIGENE</td><td>FLCDNA</td><td>TAIR</td></tr>
<tr><td>TTATTTCAAA</td><td>0</td><td>1</td><td>0</td><td>0</td><td>1</td><td>gnl|UG|At#S11727186</td><td>gi|26451922|dbj|AK118445.1|</td><td>non-canonical match</td></tr>
<tr><td>AAACGAGGAA</td><td>0</td><td>0</td><td>1</td><td>1</td><td>0</td><td>no match found</td><td>gi|28372947|gb|BT003728.1</td><td>non-canonical match</td></tr>
<tr><td>at1g04260</td><td>CON</td><td>MIN</td><td>HOUR</td><td>DAY</td><td>WEEK</td><td>UNIGENE</td><td>FLCDNA</td><td>TAIR</td></tr>
<tr><td>GGGCGATTTC</td><td>0</td><td>2</td><td>0</td><td>2</td><td>0</td><td>gnl|UG|At#S11742615</td><td>gi|17104668|gb|AY063049.1</td><td>At1g04260.1</td></tr>
<tr><td>at3g47850</td><td>CON</td><td>MIN</td><td>HOUR</td><td>DAY</td><td>WEEK</td><td>UNIGENE</td><td>FLCDNA</td><td>TAIR</td></tr>
<tr><td>GAATTAGCCA</td><td>0</td><td>0</td><td>1</td><td>0</td><td>0</td><td>gnl|UG|At#S11729830</td><td>gi|21405096|gb|AY086386.1</td><td>At3g47850.1</td></tr>
<tr><td>at1g62770</td><td>CON</td><td>MIN</td><td>HOUR</td><td>DAY</td><td>WEEK</td><td>UNIGENE</td><td>FLCDNA</td><td>TAIR</td></tr>
<tr><td>GGAGGCAAAG</td><td>1</td><td>3</td><td>0</td><td>0</td><td>0</td><td>gnl|UG|At#S11730595</td><td>gi|21407519|gb|AY088745.1</td><td>At1g62770.1</td></tr>
<tr><td>at5g08510</td><td>CON</td><td>MIN</td><td>HOUR</td><td>DAY</td><td>WEEK</td><td>UNIGENE</td><td>FLCDNA</td><td>TAIR</td></tr>
<tr><td>TCAGGTGCAT</td><td>0</td><td>0</td><td>0</td><td>0</td><td>1</td><td>gnl|UG|At#S11723984</td><td>gi|110741622|dbj|AK226649.1|</td><td>At5g08510.1</td></tr>
<tr><td>at1g44910</td><td>CON</td><td>MIN</td><td>HOUR</td><td>DAY</td><td>WEEK</td><td>UNIGENE</td><td>FLCDNA</td><td>TAIR</td></tr>
<tr><td>GTCTCCTCGT</td><td>0</td><td>1</td><td>3</td><td>1</td><td>1</td><td>no match found</td><td>gi|25083461|gb|BT002071.1</td><td>non-canonical match</td></tr>
<tr><td>at3g16980</td><td>CON</td><td>MIN</td><td>HOUR</td><td>DAY</td><td>WEEK</td><td>UNIGENE</td><td>FLCDNA</td><td>TAIR</td></tr>
<tr><td>TAATCTGTTA</td><td>1</td><td>0</td><td>0</td><td>0</td><td>0</td><td>gnl|UG|At#S11702902</td><td>no match found</td><td>multiple non-canonical match</td></tr>
<tr><td>at5g06160</td><td>CON</td><td>MIN</td><td>HOUR</td><td>DAY</td><td>WEEK</td><td>UNIGENE</td><td>FLCDNA</td><td>TAIR</td></tr>
<tr><td>GCCTTGGCCA</td><td>1</td><td>1</td><td>0</td><td>1</td><td>1</td><td>gnl|UG|At#S18913117</td><td>gi|21281055|gb|AY113862.1</td><td>At5g06160.1</td></tr>
<tr><td>at1g72500</td><td>CON</td><td>MIN</td><td>HOUR</td><td>DAY</td><td>WEEK</td><td>UNIGENE</td><td>FLCDNA</td><td>TAIR</td></tr>
<tr><td>AGAAGGAGTT</td><td>1</td><td>2</td><td>0</td><td>0</td><td>0</td><td>gnl|UG|At#S11728575</td><td>no match found</td><td>At2g21800.1</td></tr>
<tr><td>at4g24230</td><td>CON</td><td>MIN</td><td>HOUR</td><td>DAY</td><td>WEEK</td><td>UNIGENE</td><td>FLCDNA</td><td>TAIR</td></tr>
<tr><td>TGAAGCCTTC</td><td>0</td><td>1</td><td>0</td><td>0</td><td>0</td><td>gnl|UG|At#S11723604</td><td>no match found</td><td>non-canonical match</td></tr>
<tr><td>AGTCAAGAAG</td><td>0</td><td>2</td><td>2</td><td>1</td><td>0</td><td>no match found</td><td>gi|45773893|gb|BT012264.1</td><td>multiple non-canonical match</td></tr>
<tr><td>at3g49620</td><td>CON</td><td>MIN</td><td>HOUR</td><td>DAY</td><td>WEEK</td><td>UNIGENE</td><td>FLCDNA</td><td>TAIR</td></tr>
<tr><td>GATTTCGAGT</td><td>0</td><td>0</td><td>1</td><td>1</td><td>0</td><td>gnl|UG|At#S11729516</td><td>gi|19423909|gb|AY080850.1</td><td>At3g49620.1</td></tr>
<tr><td>at4g33300</td><td>CON</td><td>MIN</td><td>HOUR</td><td>DAY</td><td>WEEK</td><td>UNIGENE</td><td>FLCDNA</td><td>TAIR</td></tr>
<tr><td>TAATCTGCGA</td><td>1</td><td>6</td><td>4</td><td>0</td><td>2</td><td>gnl|UG|At#S28281905</td><td>gi|62321424|dbj|AK222047.1|</td><td>At4g33300.1</td></tr>
<tr><td>at1g70620</td><td>CON</td><td>MIN</td><td>HOUR</td><td>DAY</td><td>WEEK</td><td>UNIGENE</td><td>FLCDNA</td><td>TAIR</td></tr>
<tr><td>ACAAATCCAG</td><td>1</td><td>6</td><td>0</td><td>1</td><td>1</td><td>gnl|UG|At#S15435767</td><td>no match found</td><td>At1g70620.2</td></tr>
<tr><td>at1g43700</td><td>CON</td><td>MIN</td><td>HOUR</td><td>DAY</td><td>WEEK</td><td>UNIGENE</td><td>FLCDNA</td><td>TAIR</td></tr>
<tr><td>GCGACATCGG</td><td>3</td><td>4</td><td>7</td><td>3</td><td>2</td><td>gnl|UG|At#S11736032</td><td>gi|17529333|gb|AY065453.1</td><td>At1g43700.1</td></tr>
<tr><td>GATTTCACCA</td><td>0</td><td>1</td><td>0</td><td>0</td><td>0</td><td>no match found</td><td>gi|21436378|gb|AY117284.1</td><td>multiple non-canonical match</td></tr>
<tr><td>at3g60260</td><td>CON</td><td>MIN</td><td>HOUR</td><td>DAY</td><td>WEEK</td><td>UNIGENE</td><td>FLCDNA</td><td>TAIR</td></tr>
<tr><td>AAATTTGGGT</td><td>0</td><td>1</td><td>0</td><td>0</td><td>0</td><td>gnl|UG|At#S38433566</td><td>gi|17473881|gb|AY065185.1</td><td>At3g60260.1</td></tr>
<tr><td>at3g23690</td><td>CON</td><td>MIN</td><td>HOUR</td><td>DAY</td><td>WEEK</td><td>UNIGENE</td><td>FLCDNA</td><td>TAIR</td></tr>
<tr><td>CCAAGATTCT</td><td>1</td><td>3</td><td>1</td><td>0</td><td>1</td><td>gnl|UG|At#S11733702</td><td>gi|20465452|gb|AY096536.1</td><td>At3g23690.1</td></tr>
<tr><td>at1g14400</td><td>CON</td><td>MIN</td><td>HOUR</td><td>DAY</td><td>WEEK</td><td>UNIGENE</td><td>FLCDNA</td><td>TAIR</td></tr>
<tr><td>CTTTGATGTA</td><td>7</td><td>11</td><td>1</td><td>7</td><td>10</td><td>gnl|UG|At#S11741596</td><td>gi|17979146|gb|AY070074.1</td><td>At1g14400.1</td></tr>
<tr><td>at3g12720</td><td>CON</td><td>MIN</td><td>HOUR</td><td>DAY</td><td>WEEK</td><td>UNIGENE</td><td>FLCDNA</td><td>TAIR</td></tr>
<tr><td>GATGATCTTG</td><td>1</td><td>1</td><td>4</td><td>2</td><td>3</td><td>gnl|UG|At#S11704004</td><td>gi|14517437|gb|AY039554.1</td><td>multiple canonical match</td></tr>
<tr><td>at4g22740</td><td>CON</td><td>MIN</td><td>HOUR</td><td>DAY</td><td>WEEK</td><td>UNIGENE</td><td>FLCDNA</td><td>TAIR</td></tr>
<tr><td>ATTTGAGGGT</td><td>3</td><td>3</td><td>3</td><td>2</td><td>1</td><td>gnl|UG|At#S15460805</td><td>gi|15809949|gb|AY054243.1</td><td>non-canonical match</td></tr>
<tr><td>at1g21610</td><td>CON</td><td>MIN</td><td>HOUR</td><td>DAY</td><td>WEEK</td><td>UNIGENE</td><td>FLCDNA</td><td>TAIR</td></tr>
</table>

GTTTATAAAG	0	2	1	0	0	gnl|UG|At#S11740900	gi|19699099|gb|AY090255.1	At1g21610.2
at5g14120	CON	MIN	HOUR	DAY	WEEK	UNIGENE	FLCDNA	TAIR
AGTAGACTTA	11	14	5	3	0	gnl|UG|At#S11722804	gi|110742036|dbj|AK226867.1|	At5g14120.1
at3g54190	CON	MIN	HOUR	DAY	WEEK	UNIGENE	FLCDNA	TAIR
TGTGGTCCAA	0	0	2	0	0	gnl|UG|At#S11702098	gi|13430701|gb|AF360263.1	At3g54190.1
at5g62900	CON	MIN	HOUR	DAY	WEEK	UNIGENE	FLCDNA	TAIR
AAAAGCAGAG	0	1	1	0	0	gnl|UG|At#S11717411	gi|13877822|gb|AF370174.1	At5g62900.1
at5g44860	CON	MIN	HOUR	DAY	WEEK	UNIGENE	FLCDNA	TAIR
GAGGTGATGA	1	4	1	0	1	gnl|UG|At#S11719241	no match found	At5g44860.1
at2g28470	CON	MIN	HOUR	DAY	WEEK	UNIGENE	FLCDNA	TAIR
TAATTGTTGG	9	2	4	2	2	gnl|UG|At#S15461043	gi|110741448|dbj|AK226548.1|	At2g28470.1
at5g07920	CON	MIN	HOUR	DAY	WEEK	UNIGENE	FLCDNA	TAIR
CTAAAGAGAG	0	1	0	0	1	no match found	gi|28393495|gb|BT004148.1	non-canonical match
at1g19485	CON	MIN	HOUR	DAY	WEEK	UNIGENE	FLCDNA	TAIR
AATAAAGGAA	1	0	0	0	0	gnl|UG|At#S11741104	no match found	At1g19485.1
at3g06300	CON	MIN	HOUR	DAY	WEEK	UNIGENE	FLCDNA	TAIR
TATCGTCTCG	1	5	5	2	2	gnl|UG|At#S11738853	gi|110738389|dbj|AK229257.1|	At3g06300.1
at5g08450	CON	MIN	HOUR	DAY	WEEK	UNIGENE	FLCDNA	TAIR
AGTGAACCCA	4	6	1	6	2	no match found	gi|19424082|gb|AY080766.1	non-canonical match
CTTTGCTTCT	0	1	1	1	0	gnl|UG|At#S11724000	gi|20259546|gb|AY091073.1	At5g08450.2
at1g23140	CON	MIN	HOUR	DAY	WEEK	UNIGENE	FLCDNA	TAIR
ATTCTCCAGC	0	1	0	0	0	gnl|UG|At#S22666582	no match found	At1g23140.1
at3g02190	CON	MIN	HOUR	DAY	WEEK	UNIGENE	FLCDNA	TAIR
ATTATGAATT	2	2	3	4	5	gnl|UG|At#S30504393	gi|21407537|gb|AY088763.1	At3g02190.1
at1g76090	CON	MIN	HOUR	DAY	WEEK	UNIGENE	FLCDNA	TAIR
TTTTGCCCCT	4	9	7	0	1	gnl|UG|At#S11727819	gi|14030612|gb|AF375397.1	multiple canonical match
at4g08310	CON	MIN	HOUR	DAY	WEEK	UNIGENE	FLCDNA	TAIR
AAGCTACACA	0	2	1	0	0	gnl|UG|At#S11806626	gi|23297036|gb|AY142689.1	At4g08310.1
at2g02090	CON	MIN	HOUR	DAY	WEEK	UNIGENE	FLCDNA	TAIR
TGTAACCACT	1	0	1	0	3	gnl|UG|At#S11707745	gi|16648974|gb|AY059857.1	At2g02090.1
at4g15475	CON	MIN	HOUR	DAY	WEEK	UNIGENE	FLCDNA	TAIR
TGCCGAAGCC	2	0	0	0	0	gnl|UG|At#S11725121	no match found	At4g15475.1
at4g30280	CON	MIN	HOUR	DAY	WEEK	UNIGENE	FLCDNA	TAIR
AATAAGTTAC	0	3	2	0	0	gnl|UG|At#S18906625	gi|62318660|dbj|AK220650.1|	At4g30280.1
at2g42160	CON	MIN	HOUR	DAY	WEEK	UNIGENE	FLCDNA	TAIR
ATCACGTTCT	1	2	1	0	0	gnl|UG|At#S11732063	no match found	At4g30280.1
at2g37975	CON	MIN	HOUR	DAY	WEEK	UNIGENE	FLCDNA	TAIR
CTCTTTAACT	0	0	0	3	1	gnl|UG|At#S11733104	gi|62319248|dbj|AK220947.1|	At2g37975.1
at4g18670	CON	MIN	HOUR	DAY	WEEK	UNIGENE	FLCDNA	TAIR
TAACACGGCG	4	5	4	4	1	gnl|UG|At#S11724558	no match found	At4g18670.1
at5g18900	CON	MIN	HOUR	DAY	WEEK	UNIGENE	FLCDNA	TAIR

GTTGGAACTA	0	1	0	0	2	gnl|UG|At#S11721596	gi|20260279|gb|AY093039.1	At5g18900.1
at4g16660	CON	MIN	HOUR	DAY	WEEK	UNIGENE	FLCDNA	TAIR
GATGGAGAAG	2	1	3	0	0	gnl|UG|At#S11724915	gi|110739796|dbj|AK229980.1|	At4g16660.1
at4g39080	CON	MIN	HOUR	DAY	WEEK	UNIGENE	FLCDNA	TAIR
AGCCCTCCGG	1	0	0	0	0	no match found	no match found	At4g39080.1
at1g23280	CON	MIN	HOUR	DAY	WEEK	UNIGENE	FLCDNA	TAIR
ACTCAGATGA	1	1	0	0	0	gnl|UG|At#S11740733	no match found	At1g23280.1
at3g30122	CON	MIN	HOUR	DAY	WEEK	UNIGENE	FLCDNA	TAIR
ATTGTCTCAA	0	1	0	1	1	no match found	no match found	At3g30122.1
at5g17190	CON	MIN	HOUR	DAY	WEEK	UNIGENE	FLCDNA	TAIR
AAGTCTCAGC	1	4	0	6	1	gnl|UG|At#S11722037	gi|21280894|gb|AY114029.1	At5g17190.1
at4g17010	CON	MIN	HOUR	DAY	WEEK	UNIGENE	FLCDNA	TAIR
TTGACACAGA	0	1	0	0	0	gnl|UG|At#S11724851	gi|15010699|gb|AY045651.1	At4g17010.1
at3g50800	CON	MIN	HOUR	DAY	WEEK	UNIGENE	FLCDNA	TAIR
GCTTAATCAT	0	2	0	0	0	no match found	gi|28827349|gb|BT004986.1	At3g50800.1
at5g50115	CON	MIN	HOUR	DAY	WEEK	UNIGENE	FLCDNA	TAIR
AGCTTCTCAA	0	0	0	1	0	gnl|UG|At#S11816845	no match found	multiple non-canonical match
at5g16590	CON	MIN	HOUR	DAY	WEEK	UNIGENE	FLCDNA	TAIR
ATCAGGCTTT	2	0	0	0	0	gnl|UG|At#S11722194	gi|110737671|dbj|AK228885.1|	At5g16590.1
at5g51690	CON	MIN	HOUR	DAY	WEEK	UNIGENE	FLCDNA	TAIR
GGTATGGTTG	0	1	0	0	0	no match found	gi|62318678|dbj|AK220659.1|	multiple non-canonical match
TTGACTTTAT	0	0	0	0	1	no match found	no match found	At5g51690.1
at5g09240	CON	MIN	HOUR	DAY	WEEK	UNIGENE	FLCDNA	TAIR
TGTTGAATCT	2	0	0	0	0	gnl|UG|At#S15461177	gi|26450869|dbj|AK117904.1|	At5g09240.2
at1g43710	CON	MIN	HOUR	DAY	WEEK	UNIGENE	FLCDNA	TAIR
TGGTGGTTAT	1	1	2	3	1	no match found	gi|14532817|gb|AY040033.1	non-canonical match
ATCAACCTTC	0	0	0	2	1	gnl|UG|At#S11736028	no match found	At1g43710.1
at1g58230	CON	MIN	HOUR	DAY	WEEK	UNIGENE	FLCDNA	TAIR
GACGGATTGT	0	0	0	0	1	gnl|UG|At#S11731802	no match found	At1g58230.1
at3g45600	CON	MIN	HOUR	DAY	WEEK	UNIGENE	FLCDNA	TAIR
CTCTGTTATC	2	1	0	0	0	no match found	gi|30023773|gb|BT006312.1	non-canonical match
TTCTGGTTTC	0	0	0	1	0	gnl|UG|At#S11730262	no match found	At3g45600.1
at3g43730	CON	MIN	HOUR	DAY	WEEK	UNIGENE	FLCDNA	TAIR
TTCAAGATGA	1	0	0	0	0	no match found	no match found	At3g43730.1
at1g28320	CON	MIN	HOUR	DAY	WEEK	UNIGENE	FLCDNA	TAIR
TTTGTAAAGC	0	0	1	1	0	gnl|UG|At#S11739514	gi|18086452|gb|AY065045.1	At1g28320.1
at1g02335	CON	MIN	HOUR	DAY	WEEK	UNIGENE	FLCDNA	TAIR
AAAATATAAT	1	1	0	0	1	gnl|UG|At#S21736270	gi|72198168|gb|DQ108833.1	multiple non-canonical match
at3g24506	CON	MIN	HOUR	DAY	WEEK	UNIGENE	FLCDNA	TAIR
TTTAGATCTT	0	2	0	3	2	gnl|UG|At#S11733415	gi|26452532|dbj|AK118758.1|	At3g24506.1
at1g31070	CON	MIN	HOUR	DAY	WEEK	UNIGENE	FLCDNA	TAIR
AGAGAGAGAA	1	1	0	0	0	no match found	gi|15450738|gb|AY053411.1	At1g31070.2

at3g27810	CON	MIN	HOUR	DAY	WEEK	UNIGENE	FLCDNA	TAIR
GTTAAATATT	1	0	0	0	0	gnl|UG|At#S30651889	no match found	multiple non-canonical match
TTATACATAA	1	0	0	0	0	gnl|UG|At#S11732375	gi|26451910|dbj|AK118439.1|	At3g27810.1

at5g13930	CON	MIN	HOUR	DAY	WEEK	UNIGENE	FLCDNA	TAIR
TCGTCTTCTG	0	0	1	0	0	gnl|UG|At#S35295444	no match found	non-canonical match
TCGAGCGCGT	6	5	6	184	46	gnl|UG|At#S11722854	gi|21406516|gb|AY087778.1	At5g13930.1

at4g27120	CON	MIN	HOUR	DAY	WEEK	UNIGENE	FLCDNA	TAIR
GTTGCTTTAG	1	0	0	1	0	gnl|UG|At#S11723108	no match found	pseudo chromosome match

at5g52510	CON	MIN	HOUR	DAY	WEEK	UNIGENE	FLCDNA	TAIR
GCTGGGTTTG	2	2	2	3	1	gnl|UG|At#S11718465	gi|22136379|gb|AY128868.1	At5g52510.1

at1g30590	CON	MIN	HOUR	DAY	WEEK	UNIGENE	FLCDNA	TAIR
TGGTTTTGTA	1	8	1	1	1	gnl|UG|At#S11738811	no match found	At1g30590.1

at3g03470	CON	MIN	HOUR	DAY	WEEK	UNIGENE	FLCDNA	TAIR
AAAAACCCTT	4	4	2	2	1	gnl|UG|At#S11739655	gi|15983413|gb|AF424581.1	At3g03470.1

at3g56480	CON	MIN	HOUR	DAY	WEEK	UNIGENE	FLCDNA	TAIR
CAGCTCTGTG	0	0	1	1	0	gnl|UG|At#S11728242	gi|29028897|gb|BT005893.1	At3g56480.1

at5g66360	CON	MIN	HOUR	DAY	WEEK	UNIGENE	FLCDNA	TAIR
ATATTACATC	0	1	0	0	0	no match found	gi|31711837|gb|BT008836.1	At5g66360.1

at1g01290	CON	MIN	HOUR	DAY	WEEK	UNIGENE	FLCDNA	TAIR
TTCGTGTAGA	0	0	1	1	0	gnl|UG|At#S11742985	gi|105829725|gb|BT025641.1	At1g01290.1

at1g68260	CON	MIN	HOUR	DAY	WEEK	UNIGENE	FLCDNA	TAIR
AGTTTCTAGA	0	1	1	0	0	no match found	gi|16930434|gb|AF419571.1	non-canonical match

at2g40710	CON	MIN	HOUR	DAY	WEEK	UNIGENE	FLCDNA	TAIR
TTCTGGTAGT	0	1	0	0	0	no match found	gi|124300943|gb|BT029986.1	multiple canonical match

at1g13800	CON	MIN	HOUR	DAY	WEEK	UNIGENE	FLCDNA	TAIR
AGTGATCTCC	0	0	0	1	0	no match found	no match found	At1g13800.1

at1g11990	CON	MIN	HOUR	DAY	WEEK	UNIGENE	FLCDNA	TAIR
AATCAATAAT	1	0	0	1	1	gnl|UG|At#S26540539	no match found	multiple non-canonical match

at1g57790	CON	MIN	HOUR	DAY	WEEK	UNIGENE	FLCDNA	TAIR
ATCTTGATTG	0	0	1	2	0	gnl|UG|At#S11731919	gi|30984577|gb|BT008739.1	At1g57790.1

at2g07050	CON	MIN	HOUR	DAY	WEEK	UNIGENE	FLCDNA	TAIR
TTTCCCGCTT	1	3	1	0	0	gnl|UG|At#S11739738	no match found	At2g07050.1
CTTTACTCTT	1	2	0	0	0	no match found	gi|20453055|gb|AY094394.1	non-canonical match

at2g13800	CON	MIN	HOUR	DAY	WEEK	UNIGENE	FLCDNA	TAIR
CTGGCACTGA	0	1	0	1	0	gnl|UG|At#S11739005	gi|20453087|gb|AY094412.1	At2g13800.1

at4g37340	CON	MIN	HOUR	DAY	WEEK	UNIGENE	FLCDNA	TAIR
TGCAAAGCAC	0	0	1	0	0	gnl|UG|At#S21737302	no match found	At4g37340.1

at4g28230	CON	MIN	HOUR	DAY	WEEK	UNIGENE	FLCDNA	TAIR
AAGAAAGATT	0	0	2	0	0	gnl|UG|At#S34118068	gi|110736439|dbj|AK228240.1|	At4g28230.1

at3g49220	CON	MIN	HOUR	DAY	WEEK	UNIGENE	FLCDNA	TAIR
TACACACGAG	2	2	1	0	0	no match found	gi|110741468|dbj|AK226562.1|	At3g49220.1
ACACGTGTAC	0	0	1	0	0	no match found	gi|110738026|dbj|AK229067.1|	non-canonical match

at5g23340	CON	MIN	HOUR	DAY	WEEK	UNIGENE	FLCDNA	TAIR	
TGACAGAAGT	1	1	0	0	0	gnl|UG|At#S11720844	gi|15809999|gb|AY054268.1	non-canonical match	
at3g11590	CON	MIN	HOUR	DAY	WEEK	UNIGENE	FLCDNA	TAIR	
AATTTACACG	2	0	0	2	1	gnl|UG|At#S11737408	no match found	At3g11590.1	
AATTTGAACT	1	0	1	0	0	no match found	gi|15724287|gb|AF412084.1	non-canonical match	
at5g43710	CON	MIN	HOUR	DAY	WEEK	UNIGENE	FLCDNA	TAIR	
CTCAGAAATA	1	0	1	0	0	gnl|UG|At#S11719358	no match found	At5g43710.1	
at2g03310	CON	MIN	HOUR	DAY	WEEK	UNIGENE	FLCDNA	TAIR	
TTTGATTAAA	1	1	2	1	0	gnl|UG|At#S11740483	gi|26453243|dbj|AK119125.1|	At2g03310.1	
AAGAGAAACT	1	1	0	2	1	no match found	gi|28372965|gb|BT003737.1	multiple non-canonical match	
at1g68820	CON	MIN	HOUR	DAY	WEEK	UNIGENE	FLCDNA	TAIR	
AATCTTCTTT	0	3	1	0	3	gnl|UG|At#S11729331	gi|13878072|gb|AF370299.1	At1g68820.1	
at2g38400	CON	MIN	HOUR	DAY	WEEK	UNIGENE	FLCDNA	TAIR	
GCAAAGGGAA	1	1	0	0	0	no match found	gi|15028236|gb|AY045941.1	non-canonical match	
at2g26380	CON	MIN	HOUR	DAY	WEEK	UNIGENE	FLCDNA	TAIR	
GAAACAATCC	0	0	1	0	0	gnl|UG|At#S11735938	no match found	non-canonical match	
at5g24400	CON	MIN	HOUR	DAY	WEEK	UNIGENE	FLCDNA	TAIR	
GAAATAATGA	3	2	3	1	2	no match found	gi|13878084|gb|AF370305.1	At5g24400.1	
at3g56670	CON	MIN	HOUR	DAY	WEEK	UNIGENE	FLCDNA	TAIR	
GAATGAAAAA	1	0	0	0	0	gnl|UG|At#S35283757	no match found	multiple non-canonical match	
at4g02850	CON	MIN	HOUR	DAY	WEEK	UNIGENE	FLCDNA	TAIR	
AAAGGCTCTA	0	0	1	0	1	gnl|UG|At#S11726973	gi|51972139|gb|BT015675.1	At4g02850.1	
at2g17570	CON	MIN	HOUR	DAY	WEEK	UNIGENE	FLCDNA	TAIR	
AAAGCCACAG	0	2	0	0	0	gnl|UG|At#S11738092	gi|18377517|gb|AY072510.1	At2g17570.1	
at2g38940	CON	MIN	HOUR	DAY	WEEK	UNIGENE	FLCDNA	TAIR	
TACTCGTTAA	1	2	0	0	1	no match found	no match found	At2g38940.1	
at3g26470	CON	MIN	HOUR	DAY	WEEK	UNIGENE	FLCDNA	TAIR	
AATCCTCCTG	0	1	0	0	0	no match found	gi|28950764|gb|BT005242.1	non-canonical match	
at1g31010	CON	MIN	HOUR	DAY	WEEK	UNIGENE	FLCDNA	TAIR	
AAAATATGGA	0	1	1	1	0	gnl|UG|At#S11738658	gi|21405290|gb|AY086580.1	At1g31010.1	
at4g08870	CON	MIN	HOUR	DAY	WEEK	UNIGENE	FLCDNA	TAIR	
GTATCGAATA	3	1	4	5	1	gnl|UG|At#S11726073	gi|19424033|gb|AY080768.1	At4g08870.1	
at1g25141	CON	MIN	HOUR	DAY	WEEK	UNIGENE	FLCDNA	TAIR	
TTATCTTTCT	0	2	0	0	0	no match found	gi|110739269|dbj|AK229710.1|	At1g25210.1	
GTTTAAGTTT	6	5	9	12	6	gnl|UG|At#S11740427	gi|62320788|dbj|AK221721.1|	multiple canonical match	
at3g51895	CON	MIN	HOUR	DAY	WEEK	UNIGENE	FLCDNA	TAIR	
CTTCACACGT	1	0	0	1	1	gnl|UG|At#S11729078	gi|20466813|gb|AY099873.1	At3g51895.1	
at3g23480	CON	MIN	HOUR	DAY	WEEK	UNIGENE	FLCDNA	TAIR	
ATTATGCATT	0	1	0	0	1	gnl|UG|At#S11706640	gi|15451181|gb|AY054671.1	At3g23480.1	
at2g05990	CON	MIN	HOUR	DAY	WEEK	UNIGENE	FLCDNA	TAIR	
ATTGAGTATT	0	5	0	0	0	no match found	gi|13265591|gb|AF324719.2	multiple non-canonical match	
TTTCTTGTAA	1	1	3	0	0	gnl|UG|At#S15461647	no match found	At2g05990.1	

at5g38140	CON	MIN	HOUR	DAY	WEEK	UNIGENE	FLCDNA	TAIR
AAGCTGCTCT	0	4	0	1	0	gnl|UG|At#S11719920	gi|61656130|gb|BT021919.1	At5g38140.1
at1g33940	CON	MIN	HOUR	DAY	WEEK	UNIGENE	FLCDNA	TAIR
GAGTTGCAGA	0	0	1	0	0	gnl|UG|At#S35281701	no match found	multiple non-canonical match
at5g24600	CON	MIN	HOUR	DAY	WEEK	UNIGENE	FLCDNA	TAIR
GCTGTTTCTT	0	1	1	1	0	gnl|UG|At#S21736235	gi|50198890|gb|BT015014.1	At5g24600.1
at1g03750	CON	MIN	HOUR	DAY	WEEK	UNIGENE	FLCDNA	TAIR
CTCTACGATT	0	0	1	0	0	no match found	no match found	At1g03750.1
TGTTCTTACT	1	0	0	0	0	gnl|UG|At#S11742742	no match found	non-canonical match
at1g59830	CON	MIN	HOUR	DAY	WEEK	UNIGENE	FLCDNA	TAIR
GCCGCAATTC	1	3	3	0	1	gnl|UG|At#S18942356	gi|21406295|gb|AY087557.1	At1g59830.1
at1g30270	CON	MIN	HOUR	DAY	WEEK	UNIGENE	FLCDNA	TAIR
GAATGTTGTG	1	2	4	0	1	gnl|UG|At#S11738934	gi|15912282|gb|AY056419.1	At1g30270.1
at4g39450	CON	MIN	HOUR	DAY	WEEK	UNIGENE	FLCDNA	TAIR
TGAGTGAGTG	1	0	1	0	0	gnl|UG|At#S11721018	no match found	At4g39450.1
at5g14570	CON	MIN	HOUR	DAY	WEEK	UNIGENE	FLCDNA	TAIR
AACAATAAAA	2	3	4	0	2	no match found	gi|26450057|dbj|AK117485.1|	multiple non-canonical match
TGGGTCTTCT	0	0	0	1	1	gnl|UG|At#S11722698	gi|14334783|gb|AY035065.1	At5g14570.1
at4g26190	CON	MIN	HOUR	DAY	WEEK	UNIGENE	FLCDNA	TAIR
GGAGTTTTAC	19	13	29	25	8	gnl|UG|At#S11723279	gi|62320629|dbj|AK221641.1|	At4g26190.1
AAAAGCGGGC	0	0	1	0	1	no match found	gi|110738749|dbj|AK229439.1|	non-canonical match
at1g53710	CON	MIN	HOUR	DAY	WEEK	UNIGENE	FLCDNA	TAIR
TTTAGTCAAA	1	0	0	0	1	no match found	gi|27754646|gb|BT002956.1	multiple non-canonical match
TTTAGTCTAT	0	0	1	0	0	gnl|UG|At#S11733111	no match found	non-canonical match
at4g29780	CON	MIN	HOUR	DAY	WEEK	UNIGENE	FLCDNA	TAIR
TATTTTCTAT	1	3	1	0	0	gnl|UG|At#S11825105	gi|21405117|gb|AY086407.1	At4g29780.1
at2g17480	CON	MIN	HOUR	DAY	WEEK	UNIGENE	FLCDNA	TAIR
CATTTCGCCT	0	0	2	0	0	gnl|UG|At#S11738110	gi|27754573|gb|BT002918.1	At2g17480.1
at5g46220	CON	MIN	HOUR	DAY	WEEK	UNIGENE	FLCDNA	TAIR
AATCTTGGTC	6	4	2	0	1	no match found	gi|50253509|gb|BT015085.1	At3g09250.1
at4g33467	CON	MIN	HOUR	DAY	WEEK	UNIGENE	FLCDNA	TAIR
CTTGGGACAT	0	0	4	0	1	gnl|UG|At#S37211479	gi|62318591|dbj|AK220616.1|	pseudo chromosome match
GATTTTCCTG	0	0	1	0	0	gnl|UG|At#S24442810	no match found	pseudo chromosome match
at5g65740	CON	MIN	HOUR	DAY	WEEK	UNIGENE	FLCDNA	TAIR
TAGCTTTCAA	4	1	1	0	1	no match found	gi|39104596|dbj|AK118792.2|	At5g65740.1
at2g01210	CON	MIN	HOUR	DAY	WEEK	UNIGENE	FLCDNA	TAIR
AGGCACGTTT	0	1	0	0	0	gnl|UG|At#S11742710	no match found	At2g01210.1
at3g52930	CON	MIN	HOUR	DAY	WEEK	UNIGENE	FLCDNA	TAIR
GGCAGGTAAA	6	12	6	9	3	gnl|UG|At#S11728887	gi|17380609|gb|AY063718.1	At3g52930.1
at1g20900	CON	MIN	HOUR	DAY	WEEK	UNIGENE	FLCDNA	TAIR
GAAGGTTTGA	1	0	0	0	0	gnl|UG|At#S11740967	gi|30102699|gb|BT006460.1	multiple canonical match
at1g64830	CON	MIN	HOUR	DAY	WEEK	UNIGENE	FLCDNA	TAIR

TTGATCTTTT	0	1	2	2	0	no match found	no match found		At1g64830.1
at2g26470	CON	MIN	HOUR	DAY	WEEK	UNIGENE	FLCDNA		TAIR
TTGGCCTACT	1	1	0	0	0	gnl|UG|At#S11735915	gi|26449483|dbj|AK117192.1|		At2g26470.1
at1g76680	CON	MIN	HOUR	DAY	WEEK	UNIGENE	FLCDNA		TAIR
CTTGTCCTCA	2	5	4	1	0	gnl|UG|At#S16343875	gi|18650649|gb|AY074874.1		non-canonical match
at4g33910	CON	MIN	HOUR	DAY	WEEK	UNIGENE	FLCDNA		TAIR
TTCCCATTTG	0	0	1	1	0	gnl|UG|At#S11708240	gi|20465826|gb|AY096377.1		At4g33910.1
at1g54520	CON	MIN	HOUR	DAY	WEEK	UNIGENE	FLCDNA		TAIR
GCGTCTTCTT	1	3	0	0	0	gnl|UG|At#S11732809	gi|11993860|gb|AF327533.1		multiple canonical match
at1g14060	CON	MIN	HOUR	DAY	WEEK	UNIGENE	FLCDNA		TAIR
TTTCTTCTCT	0	0	0	0	1	gnl|UG|At#S11823043	gi|21405671|gb|AY086947.1		At1g14060.1
at1g79850	CON	MIN	HOUR	DAY	WEEK	UNIGENE	FLCDNA		TAIR
AGTGTGTTTT	44	24	41	33	30	gnl|UG|At#S11726577	gi|14423473|gb|AF386974.1		At1g79850.1
GCATAAGCAA	11	7	15	3	11	gnl|UG|At#S35300126	no match found		multiple non-canonical match
AAAACGATGC	3	5	1	1	0	no match found	gi|18377575|gb|AY072539.1		non-canonical match
at2g04160	CON	MIN	HOUR	DAY	WEEK	UNIGENE	FLCDNA		TAIR
AAGGCAACAC	0	3	1	0	0	gnl|UG|At#S11740361	gi|15010691|gb|AY045647.1		non-canonical match
at2g27680	CON	MIN	HOUR	DAY	WEEK	UNIGENE	FLCDNA		TAIR
TAATTACACT	11	4	3	3	16	no match found	gi|14326472|gb|AF385689.1		non-canonical match
TTTGGAGAGA	0	1	0	0	0	no match found	no match found		At2g27680.1
at1g76460	CON	MIN	HOUR	DAY	WEEK	UNIGENE	FLCDNA		TAIR
GTTATACAGC	0	2	0	0	0	no match found	gi|23308242|gb|BT000522.1		non-canonical match
TCCTTTTCTG	1	2	1	0	0	gnl|UG|At#S11727736	gi|14030648|gb|AF375415.1		At1g76460.1
at5g11800	CON	MIN	HOUR	DAY	WEEK	UNIGENE	FLCDNA		TAIR
TAATGTATCA	2	2	0	0	0	gnl|UG|At#S34115440	gi|110741491|dbj|AK226577.1|		At5g11800.1
at1g29750	CON	MIN	HOUR	DAY	WEEK	UNIGENE	FLCDNA		TAIR
GAGAACAATA	0	0	0	1	2	gnl|UG|At#S11807221	gi|23296777|gb|AY142598.1		At1g29750.2
at2g39970	CON	MIN	HOUR	DAY	WEEK	UNIGENE	FLCDNA		TAIR
ATCAAGGAAG	0	2	2	0	0	gnl|UG|At#S11732611	gi|21407085|gb|AY088311.1		At2g39970.1
at1g25400	CON	MIN	HOUR	DAY	WEEK	UNIGENE	FLCDNA		TAIR
TTGTAAGTAG	0	3	2	0	1	gnl|UG|At#S11813936	gi|21403017|gb|AY084307.1		At1g25400.1
at2g34650	CON	MIN	HOUR	DAY	WEEK	UNIGENE	FLCDNA		TAIR
CGCGGAATTT	1	0	0	0	0	gnl|UG|At#S11733902	no match found		At2g34650.1
at5g54510	CON	MIN	HOUR	DAY	WEEK	UNIGENE	FLCDNA		TAIR
GATTATGCGA	4	4	0	1	1	gnl|UG|At#S11718264	gi|59958335|gb|BT021108.1		At5g54510.1
at4g18700	CON	MIN	HOUR	DAY	WEEK	UNIGENE	FLCDNA		TAIR
AGGGGTATGT	2	0	3	0	4	gnl|UG|At#S11724552	gi|16648898|gb|AY059819.1		At4g18700.1
ATCGGAATGT	0	1	0	1	0	no match found	gi|24899684|gb|BT001170.1		multiple non-canonical match
at5g24870	CON	MIN	HOUR	DAY	WEEK	UNIGENE	FLCDNA		TAIR
ATGATGGGAT	0	1	0	0	1	gnl|UG|At#S18941580	gi|22531177|gb|AY136427.1		At5g24870.1
at5g50460	CON	MIN	HOUR	DAY	WEEK	UNIGENE	FLCDNA		TAIR
GACGCCATTG	2	3	1	1	4	no match found	gi|13877812|gb|AF370169.1		At5g50460.1

at3g11900	CON	MIN	HOUR	DAY	WEEK	UNIGENE	FLCDNA	TAIR
AATCTGAAAT	1	1	5	4	1	gnl|UG|At#S34114401	gi|110743437|dbj|AK227614.1|	At3g11900.1
GTACATCCGC	0	0	0	1	0	no match found	gi|30725277|gb|BT008302.1	non-canonical match

at4g32070	CON	MIN	HOUR	DAY	WEEK	UNIGENE	FLCDNA	TAIR
ATATTTTGGA	0	0	2	0	0	gnl|UG|At#S35238281	no match found	At2g25290.1

at3g23000	CON	MIN	HOUR	DAY	WEEK	UNIGENE	FLCDNA	TAIR
AAGAAATCAA	17	14	23	13	13	no match found	gi|57222171|gb|BT020492.1	multiple non-canonical match
GTACTCAATA	1	0	0	0	2	gnl|UG|At#S11733910	no match found	At3g23000.1
AAGTTATGGC	0	1	0	0	0	gnl|UG|At#S18925876	no match found	non-canonical match

at3g16700	CON	MIN	HOUR	DAY	WEEK	UNIGENE	FLCDNA	TAIR
TTTAAGATTC	4	2	4	2	2	no match found	gi|51969473|dbj|AK175666.1|	non-canonical match

at1g29070	CON	MIN	HOUR	DAY	WEEK	UNIGENE	FLCDNA	TAIR
GTTCGTTGAG	31	25	34	38	15	gnl|UG|At#S18896040	gi|17380839|gb|AY063876.1	multiple non-canonical match
TACTACTATA	19	2	2	1	10	no match found	gi|21403380|gb|AY084670.1	At1g29070.1
GTTTCCGTAG	0	0	4	0	1	no match found	gi|20259638|gb|AY091237.1	multiple non-canonical match

at1g48850	CON	MIN	HOUR	DAY	WEEK	UNIGENE	FLCDNA	TAIR
ATTTTTTATA	1	0	1	1	3	no match found	gi|15982823|gb|AY057519.1	non-canonical match

at5g65240	CON	MIN	HOUR	DAY	WEEK	UNIGENE	FLCDNA	TAIR
AATGAAGTTT	0	0	0	0	1	gnl|UG|At#S11707758	gi|16648948|gb|AY059844.1	At5g65240.1

at1g70730	CON	MIN	HOUR	DAY	WEEK	UNIGENE	FLCDNA	TAIR
GGACTTTTGG	0	0	2	0	0	gnl|UG|At#S11728939	gi|27363247|gb|BT002627.1	At1g70730.1

at3g10350	CON	MIN	HOUR	DAY	WEEK	UNIGENE	FLCDNA	TAIR
TTCACCAGAA	1	1	0	0	0	gnl|UG|At#S11737782	gi|110740613|dbj|AK226249.1|	At3g10350.1

at5g23690	CON	MIN	HOUR	DAY	WEEK	UNIGENE	FLCDNA	TAIR
TATTTGCGAG	0	3	0	0	0	gnl|UG|At#S11720810	gi|45680370|gb|BT012060.1	At5g23690.1

at4g37608	CON	MIN	HOUR	DAY	WEEK	UNIGENE	FLCDNA	TAIR
TATAAATATG	0	0	0	0	1	gnl|UG|At#S26538764	gi|72198569|gb|DQ108883.1	multiple non-canonical match

at2g17030	CON	MIN	HOUR	DAY	WEEK	UNIGENE	FLCDNA	TAIR
TTATCTCCAA	0	1	1	2	0	gnl|UG|At#S11738225	no match found	At2g17030.1

at4g22140	CON	MIN	HOUR	DAY	WEEK	UNIGENE	FLCDNA	TAIR
AACAACGCTT	2	2	3	1	1	gnl|UG|At#S28281986	gi|62321729|dbj|AK222203.1|	At2g17030.1

atcg00330	CON	MIN	HOUR	DAY	WEEK	UNIGENE	FLCDNA	TAIR
TTTGTTGCCA	0	2	0	0	0	no match found	no match found	AtCg00330

at2g02880	CON	MIN	HOUR	DAY	WEEK	UNIGENE	FLCDNA	TAIR
CAATTTCTTG	0	0	1	0	0	gnl|UG|At#S11740550	gi|26451249|dbj|AK118099.1|	At2g02880.1

at2g17380	CON	MIN	HOUR	DAY	WEEK	UNIGENE	FLCDNA	TAIR
TGATTGTTTG	0	0	1	1	1	gnl|UG|At#S11738138	gi|110743942|dbj|AK227825.1|	At2g17380.1

at2g29960	CON	MIN	HOUR	DAY	WEEK	UNIGENE	FLCDNA	TAIR
AAAATAGTAC	1	0	1	0	2	gnl|UG|At#S11735050	no match found	pseudo chromosome match

at1g65260	CON	MIN	HOUR	DAY	WEEK	UNIGENE	FLCDNA	TAIR
GTGCTACTAT	0	1	0	0	0	no match found	gi|110738046|dbj|AK229077.1|	non-canonical match

at2g03390	CON	MIN	HOUR	DAY	WEEK	UNIGENE	FLCDNA	TAIR
TAGATGTAGT	2	1	0	1	4	gnl|UG|At#S11740470	gi|51971334|dbj|AK176569.1|	At2g03390.1

at1g50560	CON	MIN	HOUR	DAY	WEEK	UNIGENE	FLCDNA		TAIR
ATGGTTGAAT	0	0	1	0	0	gnl|UG|At#S11709018	gi|21387180|gb|AY114675.1		non-canonical match
at2g29680	CON	MIN	HOUR	DAY	WEEK	UNIGENE	FLCDNA		TAIR
TAAATGCAGG	0	0	1	0	0	no match found	no match found		At2g29680.2
AGAAAGGCTC	1	0	0	0	0	gnl|UG|At#S11709663	no match found		At2g29680.1
at5g66250	CON	MIN	HOUR	DAY	WEEK	UNIGENE	FLCDNA		TAIR
AAATTGCGTT	2	3	1	1	1	gnl|UG|At#S11815714	gi|22022593|gb|AY127030.1		At5g66250.1
TTTGTTGATA	1	0	1	0	0	no match found	gi|21403285|gb|AY084575.1		non-canonical match
at5g61240	CON	MIN	HOUR	DAY	WEEK	UNIGENE	FLCDNA		TAIR
TTCAAGTCAG	0	2	1	0	2	gnl|UG|At#S11717578	gi|22135941|gb|AY128350.1		non-canonical match
at1g10590	CON	MIN	HOUR	DAY	WEEK	UNIGENE	FLCDNA		TAIR
GGATGATCTT	4	7	7	6	4	gnl|UG|At#S11741973	gi|14335099|gb|AY037229.1		multiple canonical match
TACAAGGGTT	0	0	2	0	3	no match found	gi|21281076|gb|AY113861.1		non-canonical match
at1g64140	CON	MIN	HOUR	DAY	WEEK	UNIGENE	FLCDNA		TAIR
TCTTTTGTTT	11	8	4	4	6	gnl|UG|At#S11730310	no match found		At1g64140.1
at3g13860	CON	MIN	HOUR	DAY	WEEK	UNIGENE	FLCDNA		TAIR
TGATCTTGTG	0	3	2	1	2	gnl|UG|At#S11736689	gi|15982716|gb|AY056782.1		At3g13860.1
at1g36340	CON	MIN	HOUR	DAY	WEEK	UNIGENE	FLCDNA		TAIR
CTAATAATTA	0	1	0	0	0	gnl|UG|At#S18895346	gi|51972105|gb|BT015658.1		At1g36340.1
at4g12300	CON	MIN	HOUR	DAY	WEEK	UNIGENE	FLCDNA		TAIR
TCAAAGCCTT	0	0	0	0	1	no match found	gi|28973098|gb|BT005454.1		non-canonical match
at4g22220	CON	MIN	HOUR	DAY	WEEK	UNIGENE	FLCDNA		TAIR
AGAACGTCAT	2	6	2	0	1	gnl|UG|At#S11723962	gi|19548020|gb|AY081721.1		At4g22220.1
at2g45330	CON	MIN	HOUR	DAY	WEEK	UNIGENE	FLCDNA		TAIR
CGAAGATTGA	1	2	0	0	0	no match found	no match found		At2g45330.1
at1g73630	CON	MIN	HOUR	DAY	WEEK	UNIGENE	FLCDNA		TAIR
TTGATACTGA	0	1	1	0	0	gnl|UG|At#S11728327	gi|21407534|gb|AY088760.1		multiple canonical match
at4g32480	CON	MIN	HOUR	DAY	WEEK	UNIGENE	FLCDNA		TAIR
GTATACAAAG	0	10	0	0	0	gnl|UG|At#S11722193	no match found		At4g32480.1
CAGGCTAAAT	0	3	0	0	0	no match found	gi|16974484|gb|AY061919.1		non-canonical match
at5g43670	CON	MIN	HOUR	DAY	WEEK	UNIGENE	FLCDNA		TAIR
GATCAAAAAT	0	1	0	2	0	gnl|UG|At#S11705995	gi|15810472|gb|AY056275.1		At5g43670.1
at3g15930	CON	MIN	HOUR	DAY	WEEK	UNIGENE	FLCDNA		TAIR
CAGATGTTGG	0	1	0	0	0	no match found	no match found		At3g15930.1
at2g07690	CON	MIN	HOUR	DAY	WEEK	UNIGENE	FLCDNA		TAIR
TGTATTTGAT	0	0	2	1	1	gnl|UG|At#S11739504	no match found		At2g07690.1
at1g62880	CON	MIN	HOUR	DAY	WEEK	UNIGENE	FLCDNA		TAIR
TTTGTTAATT	1	1	0	0	0	gnl|UG|At#S35285665	no match found		non-canonical match
at3g57120	CON	MIN	HOUR	DAY	WEEK	UNIGENE	FLCDNA		TAIR
TCGCCGGAAT	0	1	0	0	0	gnl|UG|At#S11728123	gi|21689862|gb|AY122959.1		At3g57120.1
at5g65200	CON	MIN	HOUR	DAY	WEEK	UNIGENE	FLCDNA		TAIR
ATTCGGCTCT	0	1	0	0	0	gnl|UG|At#S21621224	gi|52421296|gb|BT015720.1		non-canonical match

at2g37460	CON	MIN	HOUR	DAY	WEEK	UNIGENE	FLCDNA	TAIR
GGAGAATAAT	0	0	1	0	0	gnl|UG|At#S11733236	gi|14334857|gb|AY035102.1	At2g37460.1
at2g32750	CON	MIN	HOUR	DAY	WEEK	UNIGENE	FLCDNA	TAIR
AAAAAACTGC	1	0	0	0	0	gnl|UG|At#S11734347	no match found	non-canonical match
at2g37620	CON	MIN	HOUR	DAY	WEEK	UNIGENE	FLCDNA	TAIR
GATTTATGGT	3	1	0	1	1	no match found	gi|21407806|gb|AY089032.1	multiple canonical match
TAAAGATATA	1	1	0	0	0	gnl|UG|At#S15460674	no match found	non-canonical match
at3g09200	CON	MIN	HOUR	DAY	WEEK	UNIGENE	FLCDNA	TAIR
ACTTTGATAA	2	0	1	0	0	gnl|UG|At#S11738126	no match found	At3g09200.1
GTCACTTCCT	4	9	5	4	5	no match found	gi|13877924|gb|AF370225.1	multiple non-canonical match
at2g46020	CON	MIN	HOUR	DAY	WEEK	UNIGENE	FLCDNA	TAIR
AGGTGAGATC	0	1	1	0	0	gnl|UG|At#S18942694	no match found	non-canonical match
at3g04870	CON	MIN	HOUR	DAY	WEEK	UNIGENE	FLCDNA	TAIR
AAGAAGAAGC	4	4	4	6	4	gnl|UG|At#S18942188	gi|16649100|gb|AY059920.1	multiple non-canonical match
at2g12200	CON	MIN	HOUR	DAY	WEEK	UNIGENE	FLCDNA	TAIR
ATACATCAAA	0	1	0	0	1	gnl|UG|At#S11739211	no match found	At2g12200.1
at1g07370	CON	MIN	HOUR	DAY	WEEK	UNIGENE	FLCDNA	TAIR
AATTCCTTCA	0	1	0	0	0	gnl|UG|At#S11701152	gi|18087558|gb|AF462821.1	multiple canonical match
at4g04692	CON	MIN	HOUR	DAY	WEEK	UNIGENE	FLCDNA	TAIR
GCTGTCTGTT	6	4	6	1	0	gnl|UG|At#S26538777	gi|72196884|gb|DQ108666.1	multiple canonical match
at3g57150	CON	MIN	HOUR	DAY	WEEK	UNIGENE	FLCDNA	TAIR
ATGATAGCAG	3	4	0	3	4	gnl|UG|At#S11728118	gi|15146211|gb|AY049247.1	At3g57150.1
at3g51240	CON	MIN	HOUR	DAY	WEEK	UNIGENE	FLCDNA	TAIR
GATTACGGTT	0	0	1	6	1	no match found	gi|16604532|gb|AY058886.1	multiple canonical match
CGTCGATATG	0	0	0	0	1	no match found	gi|21406297|gb|AY087559.1	non-canonical match
at2g16490	CON	MIN	HOUR	DAY	WEEK	UNIGENE	FLCDNA	TAIR
GAATCTTCAG	3	4	1	1	0	gnl|UG|At#S18906937	gi|15809936|gb|AY054236.1	non-canonical match
at1g09750	CON	MIN	HOUR	DAY	WEEK	UNIGENE	FLCDNA	TAIR
GCCGGGATAC	0	0	2	0	0	no match found	gi|26983825|gb|BT002332.1	non-canonical match
GCCTGCAGTT	13	17	25	22	4	gnl|UG|At#S11742061	gi|13878056|gb|AF370291.1	At1g09750.1
at1g51760	CON	MIN	HOUR	DAY	WEEK	UNIGENE	FLCDNA	TAIR
TAAAGATTAA	1	1	0	0	2	no match found	gi|14030706|gb|AF375444.1	At1g51760.1
GCCACAAGAT	0	0	1	0	0	no match found	gi|23506080|gb|AY143961.1	multiple non-canonical match
at1g19600	CON	MIN	HOUR	DAY	WEEK	UNIGENE	FLCDNA	TAIR
CAGTGGTGGA	1	4	0	0	0	gnl|UG|At#S11741093	gi|28466956|gb|BT004821.1	At1g19600.1
at5g67300	CON	MIN	HOUR	DAY	WEEK	UNIGENE	FLCDNA	TAIR
ATTCCGATGA	0	26	11	5	1	gnl|UG|At#S20718314	gi|12642875|gb|AF339698.1	At5g67300.1
at2g34250	CON	MIN	HOUR	DAY	WEEK	UNIGENE	FLCDNA	TAIR
GGAGCCATCG	9	14	3	8	3	gnl|UG|At#S28282205	gi|23308212|gb|BT000507.1	At2g34250.1
at1g79620	CON	MIN	HOUR	DAY	WEEK	UNIGENE	FLCDNA	TAIR
TTTCAACACA	1	0	1	1	0	gnl|UG|At#S11726650	gi|26450790|dbj|AK117863.1|	At1g79620.1
at3g01200	CON	MIN	HOUR	DAY	WEEK	UNIGENE	FLCDNA	TAIR

ATGAATCATA	1	0	1	0	0	gnl|UG|At#S11740307	no match found	At3g01200.1
at3g06140	CON	MIN	HOUR	DAY	WEEK	UNIGENE	FLCDNA	TAIR
TATAGATAAC	3	0	0	1	0	gnl|UG|At#S11738893	gi|21406833|gb|AY088059.1	At3g06140.1
at1g69790	CON	MIN	HOUR	DAY	WEEK	UNIGENE	FLCDNA	TAIR
TCTCCTTCAT	2	1	0	0	0	gnl|UG|At#S11729143	gi|57222227|gb|BT020520.1	At1g69790.1
at5g64250	CON	MIN	HOUR	DAY	WEEK	UNIGENE	FLCDNA	TAIR
TGTAATCCAG	1	0	3	1	2	no match found	gi|21404742|gb|AY086032.1	non-canonical match
at2g36305	CON	MIN	HOUR	DAY	WEEK	UNIGENE	FLCDNA	TAIR
TCTTAGAATC	0	2	0	1	0	gnl|UG|At#S15460722	gi|26453263|dbj|AK119135.1|	At2g36305.1
at5g15780	CON	MIN	HOUR	DAY	WEEK	UNIGENE	FLCDNA	TAIR
GAGAAAATAC	0	0	1	0	0	no match found	gi|53828648|gb|BT015871.1	multiple non-canonical match
GAGAGAATAC	3	11	2	1	0	gnl|UG|At#S11722393	gi|27311644|gb|BT002428.1	At5g15780.1
at3g11410	CON	MIN	HOUR	DAY	WEEK	UNIGENE	FLCDNA	TAIR
TGCCTTCGAG	0	0	0	0	1	no match found	gi|20259228|gb|AY091391.1	non-canonical match
at3g05152	CON	MIN	HOUR	DAY	WEEK	UNIGENE	FLCDNA	TAIR
GATGTATGTA	0	0	1	0	0	gnl|UG|At#S18904334	no match found	pseudo chromosome match
at1g01170	CON	MIN	HOUR	DAY	WEEK	UNIGENE	FLCDNA	TAIR
GCCAAGAACC	6	9	6	5	2	gnl|UG|At#S11676355	gi|26451443|dbj|AK118199.1|	At1g01170.1
at4g14230	CON	MIN	HOUR	DAY	WEEK	UNIGENE	FLCDNA	TAIR
TCGATATAAT	3	3	4	1	1	gnl|UG|At#S11725347	gi|110740974|dbj|AK226439.1|	At4g14230.1
at1g45760	CON	MIN	HOUR	DAY	WEEK	UNIGENE	FLCDNA	TAIR
GAGGCTTTTG	8	0	1	3	0	no match found	no match found	At1g45760.1
at3g26612	CON	MIN	HOUR	DAY	WEEK	UNIGENE	FLCDNA	TAIR
GATGTTGTTC	0	0	1	0	0	gnl|UG|At#S26539319	gi|72198089|gb|DQ108822.1	multiple non-canonical match
at5g01810	CON	MIN	HOUR	DAY	WEEK	UNIGENE	FLCDNA	TAIR
AAGGAGAGTA	0	2	2	0	0	gnl|UG|At#S28281867	no match found	At5g01810.1
at3g16080	CON	MIN	HOUR	DAY	WEEK	UNIGENE	FLCDNA	TAIR
TGGATCTCAT	9	7	3	14	20	gnl|UG|At#S11736025	gi|110741049|dbj|AK226477.1|	At3g16080.1
at2g18020	CON	MIN	HOUR	DAY	WEEK	UNIGENE	FLCDNA	TAIR
GAGGAGGTAA	7	12	10	9	13	no match found	gi|22135869|gb|AY128314.1	At2g18020.1
at3g15820	CON	MIN	HOUR	DAY	WEEK	UNIGENE	FLCDNA	TAIR
AGAAGAATGC	0	2	0	0	0	gnl|UG|At#S11736100	gi|13926234|gb|AF372876.1	At3g15820.1
at2g25490	CON	MIN	HOUR	DAY	WEEK	UNIGENE	FLCDNA	TAIR
AAAAAGGCGA	0	7	1	0	0	gnl|UG|At#S11743494	gi|20259112|gb|AY091333.1	At2g25490.1
at5g37660	CON	MIN	HOUR	DAY	WEEK	UNIGENE	FLCDNA	TAIR
TTCTTGGCTA	1	0	0	0	0	no match found	gi|63025161|gb|BT022067.1	At5g37660.1
at3g24720	CON	MIN	HOUR	DAY	WEEK	UNIGENE	FLCDNA	TAIR
TCAATGGAAG	0	0	1	0	0	no match found	no match found	At3g24720.1
at2g23590	CON	MIN	HOUR	DAY	WEEK	UNIGENE	FLCDNA	TAIR
ACACCAGAAG	0	0	1	0	0	gnl|UG|At#S11736584	no match found	At2g23590.1
at1g76160	CON	MIN	HOUR	DAY	WEEK	UNIGENE	FLCDNA	TAIR

GACGGCTATA	0	2	0	0	0	gnl|UG|At#S11727803	gi|22531131|gb|AY136404.1	At1g76160.1
at1g22770	CON	MIN	HOUR	DAY	WEEK	UNIGENE	FLCDNA	TAIR
AACCAAAGCT	1	4	1	1	2	gnl|UG|At#S11740786	no match found	At1g22770.1
at2g27030	CON	MIN	HOUR	DAY	WEEK	UNIGENE	FLCDNA	TAIR
TAATGACAAA	0	2	0	0	0	no match found	gi|26983863|gb|BT002351.1	At2g27030.3
TTATGAACTA	5	6	0	3	4	gnl|UG|At#S11807229	gi|20259399|gb|AY090987.1	At2g27030.2
at1g62390	CON	MIN	HOUR	DAY	WEEK	UNIGENE	FLCDNA	TAIR
CTGTTCGAAA	1	6	2	0	1	gnl|UG|At#S11730669	gi|21281130|gb|AY113996.1	At1g62390.1
at5g62700	CON	MIN	HOUR	DAY	WEEK	UNIGENE	FLCDNA	TAIR
TTTACAATAT	3	0	0	0	0	gnl|UG|At#S35215689	no match found	non-canonical match
at5g51120	CON	MIN	HOUR	DAY	WEEK	UNIGENE	FLCDNA	TAIR
CCAGGAGTTC	2	2	0	3	1	gnl|UG|At#S11813795	gi|21403174|gb|AY084464.1	At5g51120.1
at2g27100	CON	MIN	HOUR	DAY	WEEK	UNIGENE	FLCDNA	TAIR
AGAATTTCTT	1	2	5	5	2	gnl|UG|At#S11735761	gi|16226330|gb|AF428305.1	At2g27100.1
at5g16030	CON	MIN	HOUR	DAY	WEEK	UNIGENE	FLCDNA	TAIR
GGTGAGATCA	5	4	3	4	1	no match found	gi|28059401|gb|BT003392.1	non-canonical match
at1g16700	CON	MIN	HOUR	DAY	WEEK	UNIGENE	FLCDNA	TAIR
AGGAACTTCT	1	2	2	0	3	gnl|UG|At#S11741375	gi|21404158|gb|AY085448.1	At1g16700.1
at1g55750	CON	MIN	HOUR	DAY	WEEK	UNIGENE	FLCDNA	TAIR
AAGCTTATGG	1	0	0	0	0	no match found	no match found	At1g55750.1
at5g28460	CON	MIN	HOUR	DAY	WEEK	UNIGENE	FLCDNA	TAIR
GGTTTGCACT	1	0	0	0	0	no match found	gi|62321165|dbj|AK221912.1|	multiple canonical match
at2g07170	CON	MIN	HOUR	DAY	WEEK	UNIGENE	FLCDNA	TAIR
AAATCATTTT	1	0	0	0	0	gnl|UG|At#S11739715	no match found	multiple non-canonical match
at5g64980	CON	MIN	HOUR	DAY	WEEK	UNIGENE	FLCDNA	TAIR
AAGAAATGTA	0	0	0	1	1	gnl|UG|At#S11717198	gi|26451338|dbj|AK118145.1|	At5g64980.1
at3g21055	CON	MIN	HOUR	DAY	WEEK	UNIGENE	FLCDNA	TAIR
TCTATCTCTC	161	73	63	32	29	no match found	gi|11908023|gb|AF326859.1	At3g21055.1
at1g43670	CON	MIN	HOUR	DAY	WEEK	UNIGENE	FLCDNA	TAIR
AGCGTTCTCC	3	7	6	3	1	gnl|UG|At#S11736043	gi|30984563|gb|BT008732.1	At1g43670.1
at3g54880	CON	MIN	HOUR	DAY	WEEK	UNIGENE	FLCDNA	TAIR
AGAAAAAGAC	0	5	0	2	1	gnl|UG|At#S21735756	gi|51972034|dbj|AK176919.1|	multiple non-canonical match
at5g27700	CON	MIN	HOUR	DAY	WEEK	UNIGENE	FLCDNA	TAIR
TCTTCTAATT	9	13	9	6	10	gnl|UG|At#S30646720	gi|19424103|gb|AY080873.1	At5g27700.1
CCTCTGTTCA	4	14	3	5	1	no match found	gi|117168166|gb|BT029352.1	multiple non-canonical match
at5g33355	CON	MIN	HOUR	DAY	WEEK	UNIGENE	FLCDNA	TAIR
AGCTAATCTA	5	1	4	5	2	no match found	no match found	At5g33355.1
at4g39250	CON	MIN	HOUR	DAY	WEEK	UNIGENE	FLCDNA	TAIR
AGGCTGCAGT	0	0	1	0	0	gnl|UG|At#S11721055	no match found	non-canonical match
at4g15890	CON	MIN	HOUR	DAY	WEEK	UNIGENE	FLCDNA	TAIR
TTTGTCCTTG	1	0	0	0	0	gnl|UG|At#S11725050	no match found	At4g15890.1

at1g79230	CON	MIN	HOUR	DAY	WEEK	UNIGENE	FLCDNA	TAIR
TTTAAGTTGA	5	5	1	0	1	gnl|UG|At#S38434036	gi|21405861|gb|AY087137.1	multiple canonical match

at1g50320	CON	MIN	HOUR	DAY	WEEK	UNIGENE	FLCDNA	TAIR
TTCTCCGGCA	1	1	1	2	0	gnl|UG|At#S11734385	gi|21404915|gb|AY086205.1	At1g50320.1

at3g62130	CON	MIN	HOUR	DAY	WEEK	UNIGENE	FLCDNA	TAIR
TGCGTCTAAT	1	3	2	1	1	gnl|UG|At#S11726810	gi|21405517|gb|AY086807.1	At3g62130.1

at1g20480	CON	MIN	HOUR	DAY	WEEK	UNIGENE	FLCDNA	TAIR
AGGATGTGAT	0	1	0	0	0	no match found	no match found	At1g20480.1

at5g42797	CON	MIN	HOUR	DAY	WEEK	UNIGENE	FLCDNA	TAIR
TTTATATTAA	0	0	1	0	0	gnl|UG|At#S28281690	no match found	multiple non-canonical match

at3g13440	CON	MIN	HOUR	DAY	WEEK	UNIGENE	FLCDNA	TAIR
TTTTGTGTTG	2	1	1	1	0	gnl|UG|At#S11736841	gi|18377857|gb|AY074576.1	At3g13440.1
GGAAGATTCA	0	0	1	0	0	gnl|UG|At#S37211165	no match found	At3g13440.1

at5g55530	CON	MIN	HOUR	DAY	WEEK	UNIGENE	FLCDNA	TAIR
TTGTATCTCC	2	0	1	0	2	no match found	gi|28393795|gb|BT004310.1	non-canonical match
TTTGTATTTT	3	0	2	2	2	gnl|UG|At#S11718160	no match found	multiple canonical match
CAACAGTTTA	0	0	1	0	0	no match found	gi|57222229|gb|BT020521.1	non-canonical match

at1g21065	CON	MIN	HOUR	DAY	WEEK	UNIGENE	FLCDNA	TAIR
GGAACTTGGC	7	3	11	9	2	gnl|UG|At#S11740950	gi|12083271|gb|AF332432.1	At1g21065.1

at3g52840	CON	MIN	HOUR	DAY	WEEK	UNIGENE	FLCDNA	TAIR
TACAGATATA	4	4	1	0	0	gnl|UG|At#S11728901	gi|13605856|gb|AF367327.1	At3g52840.1

at3g27310	CON	MIN	HOUR	DAY	WEEK	UNIGENE	FLCDNA	TAIR
TTACCTTTGA	0	0	2	0	2	gnl|UG|At#S18902788	gi|51968709|dbj|AK175284.1|	At3g27310.1

at5g38410	CON	MIN	HOUR	DAY	WEEK	UNIGENE	FLCDNA	TAIR
CCATTCAATA	0	1	0	0	1	no match found	gi|28973645|gb|BT005730.1	At1g06820.1

at4g02380	CON	MIN	HOUR	DAY	WEEK	UNIGENE	FLCDNA	TAIR
TAATTTTGTC	6	30	10	16	25	gnl|UG|At#S11675984	gi|15294219|gb|AF410301.1	At4g02380.1

at1g24490	CON	MIN	HOUR	DAY	WEEK	UNIGENE	FLCDNA	TAIR
TCTTAAGGCG	0	0	1	1	0	gnl|UG|At#S32321350	no match found	At1g24490.1

at1g49110	CON	MIN	HOUR	DAY	WEEK	UNIGENE	FLCDNA	TAIR
TAAACTTTAA	0	1	0	0	0	no match found	no match found	At1g49110.1

at4g17190	CON	MIN	HOUR	DAY	WEEK	UNIGENE	FLCDNA	TAIR
CTTATAGTAT	1	0	0	0	0	gnl|UG|At#S18941847	gi|18176349|gb|AY072207.1	At4g17190.1

at3g46820	CON	MIN	HOUR	DAY	WEEK	UNIGENE	FLCDNA	TAIR
ATTCTTATTA	0	1	0	0	1	no match found	gi|110742415|dbj|AK227072.1|	multiple canonical match
TCAGAGATAC	0	0	0	1	1	no match found	gi|62318870|dbj|AK220755.1|	non-canonical match

at1g17745	CON	MIN	HOUR	DAY	WEEK	UNIGENE	FLCDNA	TAIR
TTTCTCTTGT	3	1	0	1	16	gnl|UG|At#S11741272	gi|15215739|gb|AY050399.1	multiple canonical match
CACAAAAAAA	2	3	3	2	1	no match found	gi|15028272|gb|AY046051.1	pseudo chromosome match

at3g51700	CON	MIN	HOUR	DAY	WEEK	UNIGENE	FLCDNA	TAIR
GTGTTTCTTA	0	0	0	0	1	gnl|UG|At#S11729116	no match found	At3g51700.1

at2g43540	CON	MIN	HOUR	DAY	WEEK	UNIGENE	FLCDNA	TAIR
CTGTGCAAGA	0	0	1	0	0	no match found	gi|62319507|dbj|AK221078.1|	At2g43540.1

CTTTCGCCGG	0	1	0	1	0	gnl|UG|At#S24442431	gi|21405461|gb|AY086751.1	At2g43540.1
at4g36400	CON	MIN	HOUR	DAY	WEEK	UNIGENE	FLCDNA	TAIR
AGCAGCCAAG	2	1	0	2	0	gnl|UG|At#S11708120	gi|16604325|gb|AY058061.1	At4g36400.2
at2g16800	CON	MIN	HOUR	DAY	WEEK	UNIGENE	FLCDNA	TAIR
TACCAACAAA	4	3	2	0	1	gnl|UG|At#S30644483	gi|21405020|gb|AY086310.1	At2g16800.1
at5g56040	CON	MIN	HOUR	DAY	WEEK	UNIGENE	FLCDNA	TAIR
AGAGGAGAGC	0	0	1	0	0	no match found	gi|110740398|dbj|AK230293.1|	non-canonical match
at4g13920	CON	MIN	HOUR	DAY	WEEK	UNIGENE	FLCDNA	TAIR
AGAATCTTTT	0	0	2	0	0	gnl|UG|At#S11707953	gi|16604662|gb|AY059776.1	At4g13920.1
at3g28920	CON	MIN	HOUR	DAY	WEEK	UNIGENE	FLCDNA	TAIR
TCGTTTTGCA	1	3	3	1	0	gnl|UG|At#S11731997	gi|22136283|gb|AY128820.1	At3g28920.1
at5g25800	CON	MIN	HOUR	DAY	WEEK	UNIGENE	FLCDNA	TAIR
TAACAAAAAG	1	2	0	0	0	gnl|UG|At#S11720611	gi|22135981|gb|AY128370.1	At5g25800.1
at3g46668	CON	MIN	HOUR	DAY	WEEK	UNIGENE	FLCDNA	TAIR
ATGCTCCAAA	0	1	0	1	0	gnl|UG|At#S11831636	gi|26452788|dbj|AK118890.1|	pseudo chromosome match
at2g15320	CON	MIN	HOUR	DAY	WEEK	UNIGENE	FLCDNA	TAIR
AGTTTACTCT	0	1	2	0	2	gnl|UG|At#S11811947	gi|15292804|gb|AY050836.1	At2g15320.1
at5g43720	CON	MIN	HOUR	DAY	WEEK	UNIGENE	FLCDNA	TAIR
GAGCTTTTTT	0	2	1	2	0	gnl|UG|At#S11719357	gi|51969353|dbj|AK175606.1|	At5g43720.1
at1g68780	CON	MIN	HOUR	DAY	WEEK	UNIGENE	FLCDNA	TAIR
TTCCTTTCGG	1	0	1	3	0	gnl|UG|At#S11729341	gi|13605580|gb|AF361616.1	At1g68780.1
at5g53950	CON	MIN	HOUR	DAY	WEEK	UNIGENE	FLCDNA	TAIR
GCTAATTATT	0	0	1	0	0	no match found	no match found	At5g53950.1
at2g01510	CON	MIN	HOUR	DAY	WEEK	UNIGENE	FLCDNA	TAIR
CGAGACAAGA	4	1	2	2	0	gnl|UG|At#S11742661	no match found	At2g01510.1
at2g18050	CON	MIN	HOUR	DAY	WEEK	UNIGENE	FLCDNA	TAIR
TAATATACTC	0	0	0	0	1	gnl|UG|At#S11704601	gi|21403313|gb|AY084603.1	At2g18050.1
at3g10690	CON	MIN	HOUR	DAY	WEEK	UNIGENE	FLCDNA	TAIR
ATCGAAGTTA	1	0	2	5	1	gnl|UG|At#S11737680	no match found	At3g10690.1
at5g48830	CON	MIN	HOUR	DAY	WEEK	UNIGENE	FLCDNA	TAIR
ATAACATTGG	0	2	0	0	0	gnl|UG|At#S34116867	gi|21404715|gb|AY086005.1	At5g48830.1
at5g48490	CON	MIN	HOUR	DAY	WEEK	UNIGENE	FLCDNA	TAIR
TTTAATGGAA	9	1	0	1	5	gnl|UG|At#S30582342	gi|27754510|gb|BT002886.1	At5g48490.1
CTGACTACAC	1	0	1	0	0	no match found	gi|106879148|gb|BT025701.1	non-canonical match
at4g33670	CON	MIN	HOUR	DAY	WEEK	UNIGENE	FLCDNA	TAIR
GCCAAGTGGA	0	8	2	1	0	gnl|UG|At#S11721994	gi|15215697|gb|AY050377.1	multiple non-canonical match
at1g70200	CON	MIN	HOUR	DAY	WEEK	UNIGENE	FLCDNA	TAIR
AGGAACTAGA	0	2	1	0	0	no match found	gi|28827687|gb|BT005155.1	non-canonical match
AATAGGTGAT	5	2	0	0	2	gnl|UG|At#S11729058	gi|28392944|gb|BT003858.1	At1g70200.1
at2g32940	CON	MIN	HOUR	DAY	WEEK	UNIGENE	FLCDNA	TAIR
TTCTTCTGCT	1	0	0	0	0	gnl|UG|At#S11734299	no match found	At2g32940.1

at5g39730	CON	MIN	HOUR	DAY	WEEK	UNIGENE	FLCDNA	TAIR
TGGATTGTAT	3	5	3	4	2	no match found	gi|14334937|gb|AY035142.1	multiple canonical match

at3g18430	CON	MIN	HOUR	DAY	WEEK	UNIGENE	FLCDNA	TAIR
TCTGATGAGC	2	0	1	0	1	gnl|UG|At#S11735315	gi|20259020|gb|AY091287.1	At3g18430.1

at5g65020	CON	MIN	HOUR	DAY	WEEK	UNIGENE	FLCDNA	TAIR
GTTTGTTTTA	1	4	0	4	1	gnl|UG|At#S11709813	gi|21404423|gb|AY085713.1	At5g65020.1

at1g75370	CON	MIN	HOUR	DAY	WEEK	UNIGENE	FLCDNA	TAIR
AGGCTTTAAT	0	3	1	2	0	no match found	gi|26451649|dbj|AK118304.1|	At1g75370.1

at5g51460	CON	MIN	HOUR	DAY	WEEK	UNIGENE	FLCDNA	TAIR
GAAGAGATCA	0	1	0	1	0	no match found	gi|30725607|gb|BT008467.1	multiple non-canonical match
CCGGAGATAC	1	1	1	0	1	gnl|UG|At#S11700451	gi|20260495|gb|AY093147.1	At5g51460.2

at1g04120	CON	MIN	HOUR	DAY	WEEK	UNIGENE	FLCDNA	TAIR
TTTATCAATA	0	3	1	0	4	gnl|UG|At#S11742655	gi|110738795|dbj|AK229463.1|	At1g04120.1

at1g71940	CON	MIN	HOUR	DAY	WEEK	UNIGENE	FLCDNA	TAIR
AAATATTTTG	1	0	1	1	0	gnl|UG|At#S11728701	gi|21406481|gb|AY087743.1	At1g71940.1
GCCTAACATT	0	0	2	1	0	no match found	gi|18700126|gb|AY075668.1	non-canonical match

at4g02440	CON	MIN	HOUR	DAY	WEEK	UNIGENE	FLCDNA	TAIR
TCTCTGGTGC	0	2	2	0	0	gnl|UG|At#S34116475	gi|110739506|dbj|AK229833.1|	At4g02440.1

at4g18920	CON	MIN	HOUR	DAY	WEEK	UNIGENE	FLCDNA	TAIR
AGAATTTTTT	0	0	1	0	0	gnl|UG|At#S11809338	gi|21407415|gb|AY088641.1	At4g18920.1

at3g06350	CON	MIN	HOUR	DAY	WEEK	UNIGENE	FLCDNA	TAIR
GTTTTGGCAA	0	0	0	0	1	no match found	gi|45773759|gb|BT012197.1	non-canonical match
AAAAGTCGTC	1	1	7	3	2	gnl|UG|At#S11738839	gi|62319330|dbj|AK220988.1|	At3g06350.1

at1g44920	CON	MIN	HOUR	DAY	WEEK	UNIGENE	FLCDNA	TAIR
AATATTCGGT	0	3	2	1	1	gnl|UG|At#S11735691	gi|13878106|gb|AF370316.1	At1g44920.1

at3g12930	CON	MIN	HOUR	DAY	WEEK	UNIGENE	FLCDNA	TAIR
TTATGGAACA	3	0	0	4	2	gnl|UG|At#S11810167	gi|21406694|gb|AY087920.1	At3g12930.1
TTTCTAGATT	0	1	0	0	1	gnl|UG|At#S11770578	gi|27311970|gb|BT002591.1	multiple non-canonical match

at3g21670	CON	MIN	HOUR	DAY	WEEK	UNIGENE	FLCDNA	TAIR
AAACATCAGT	2	0	1	2	1	gnl|UG|At#S11734322	gi|21403249|gb|AY084539.1	At3g21670.1

at3g17240	CON	MIN	HOUR	DAY	WEEK	UNIGENE	FLCDNA	TAIR
TAGAAGCCCT	1	1	0	1	1	gnl|UG|At#S15460970	no match found	At3g17240.1

at1g56000	CON	MIN	HOUR	DAY	WEEK	UNIGENE	FLCDNA	TAIR
TACACAGGTA	0	1	0	0	0	gnl|UG|At#S11732281	no match found	non-canonical match
TAAAATGACT	0	1	1	0	0	no match found	gi|17978975|gb|AY069897.1	non-canonical match

at3g49390	CON	MIN	HOUR	DAY	WEEK	UNIGENE	FLCDNA	TAIR
TCGGGTACTG	1	0	0	0	0	gnl|UG|At#S28282843	gi|110737685|dbj|AK228892.1|	At3g49390.1

at3g05250	CON	MIN	HOUR	DAY	WEEK	UNIGENE	FLCDNA	TAIR
TCCTCTCTGT	0	1	0	0	0	gnl|UG|At#S11739152	gi|26450610|dbj|AK117770.1|	At3g05250.1

at4g18975	CON	MIN	HOUR	DAY	WEEK	UNIGENE	FLCDNA	TAIR
TATATGTAGA	0	2	0	0	1	no match found	gi|62320587|dbj|AK221620.1|	non-canonical match
CTTTATAGTG	0	1	0	0	0	gnl|UG|At#S28282014	no match found	At4g18975.1

at3g26090	CON	MIN	HOUR	DAY	WEEK	UNIGENE	FLCDNA	TAIR

TCATTTTCTC	1	1	0	1	3	gnl|UG|At#S11806612	gi|20268775|gb|AY091142.1	At3g26090.1
at3g07560	CON	MIN	HOUR	DAY	WEEK	UNIGENE	FLCDNA	TAIR
GAAACCAGAA	0	0	1	1	0	no match found	gi|20147310|gb|AY093745.1	At5g10970.1
ACAACCAGTT	3	12	5	6	0	gnl|UG|At#S11738475	gi|18700168|gb|AY075689.1	At3g07560.1
at3g56050	CON	MIN	HOUR	DAY	WEEK	UNIGENE	FLCDNA	TAIR
TGACTTTACC	0	1	0	1	0	gnl|UG|At#S11728321	gi|20466709|gb|AY099821.1	At3g56050.1
at3g07540	CON	MIN	HOUR	DAY	WEEK	UNIGENE	FLCDNA	TAIR
TTGATCCTGC	0	1	0	0	0	gnl|UG|At#S11738479	gi|15215733|gb|AY050396.1	At3g07540.1
at5g53040	CON	MIN	HOUR	DAY	WEEK	UNIGENE	FLCDNA	TAIR
GAAGATAAAA	0	0	1	0	0	no match found	no match found	At5g53040.1
at4g10330	CON	MIN	HOUR	DAY	WEEK	UNIGENE	FLCDNA	TAIR
ATGTATATGT	1	0	0	0	0	gnl|UG|At#S11725932	no match found	At4g10330.1
at5g52070	CON	MIN	HOUR	DAY	WEEK	UNIGENE	FLCDNA	TAIR
TCAGATCGAA	0	0	0	1	0	no match found	gi|21436380|gb|AY117285.1	non-canonical match
at4g04330	CON	MIN	HOUR	DAY	WEEK	UNIGENE	FLCDNA	TAIR
TCGAAACCGA	1	9	3	1	3	gnl|UG|At#S11704586	gi|23296671|gb|AY142573.1	At4g04330.1
at1g05575	CON	MIN	HOUR	DAY	WEEK	UNIGENE	FLCDNA	TAIR
GGACCTTCTC	0	4	1	0	0	gnl|UG|At#S30460298	gi|21405438|gb|AY086728.1	At1g05575.1
at1g28395	CON	MIN	HOUR	DAY	WEEK	UNIGENE	FLCDNA	TAIR
GGAAAAAAAA	5	2	5	1	2	no match found	gi|110736255|dbj|AK228142.1|	non-canonical match
GGTTCTTACA	0	0	0	0	1	gnl|UG|At#S43850554	no match found	non-canonical match
at1g12920	CON	MIN	HOUR	DAY	WEEK	UNIGENE	FLCDNA	TAIR
AGGACATTTG	1	4	2	4	1	gnl|UG|At#S11741745	gi|21405333|gb|AY086623.1	At1g12920.1
at5g27280	CON	MIN	HOUR	DAY	WEEK	UNIGENE	FLCDNA	TAIR
AGGTTAAGTA	1	2	0	0	0	no match found	gi|21404984|gb|AY086274.1	non-canonical match
at1g72250	CON	MIN	HOUR	DAY	WEEK	UNIGENE	FLCDNA	TAIR
TAGACACCAA	0	0	1	0	0	gnl|UG|At#S11728633	no match found	At1g72250.1
at1g62970	CON	MIN	HOUR	DAY	WEEK	UNIGENE	FLCDNA	TAIR
TTCTGCTTAG	0	2	0	1	0	gnl|UG|At#S34117639	gi|110737256|dbj|AK228669.1|	At1g62970.1
at5g03500	CON	MIN	HOUR	DAY	WEEK	UNIGENE	FLCDNA	TAIR
ATTCAACAAG	2	0	0	0	0	gnl|UG|At#S38433362	gi|19715580|gb|AY075596.1	non-canonical match
TAATGTTAAA	0	0	1	0	0	no match found	no match found	At5g03500.1
at3g17090	CON	MIN	HOUR	DAY	WEEK	UNIGENE	FLCDNA	TAIR
ACCTCATATC	1	3	1	2	0	gnl|UG|At#S11735725	gi|111074403|gb|BT026468.1	At3g17090.1
at5g39860	CON	MIN	HOUR	DAY	WEEK	UNIGENE	FLCDNA	TAIR
TAATCTTAAA	3	1	1	0	0	gnl|UG|At#S11809850	gi|21407020|gb|AY088246.1	At5g39860.1
at4g16360	CON	MIN	HOUR	DAY	WEEK	UNIGENE	FLCDNA	TAIR
GAGTATGGGT	1	1	0	0	0	gnl|UG|At#S28282031	gi|13430671|gb|AF360248.1	At4g16360.1
at2g36880	CON	MIN	HOUR	DAY	WEEK	UNIGENE	FLCDNA	TAIR
CAAGTTAGAA	24	8	41	54	21	gnl|UG|At#S43850088	no match found	At2g36880.1
GTGGAGGTGC	0	1	4	2	3	no match found	gi|22137171|gb|AY133601.1	non-canonical match
at2g45730	CON	MIN	HOUR	DAY	WEEK	UNIGENE	FLCDNA	TAIR

CAGATGAGTT	0	0	2	0	0	gnl|UG|At#S11731250	gi|17473623|gb|AY065099.1	At2g45730.1
at2g27590	CON	MIN	HOUR	DAY	WEEK	UNIGENE	FLCDNA	TAIR
ATTTCAATAT	1	0	0	1	1	no match found	gi|18175867|gb|AY072120.1	At2g27590.1
at1g79570	CON	MIN	HOUR	DAY	WEEK	UNIGENE	FLCDNA	TAIR
GATGCTGCAT	0	0	1	1	0	no match found	gi|62321388|dbj|AK222029.1|	non-canonical match
CATCCCTTTT	1	0	0	0	0	gnl|UG|At#S34116581	gi|110739303|dbj|AK229727.1|	At1g79570.1
at3g21530	CON	MIN	HOUR	DAY	WEEK	UNIGENE	FLCDNA	TAIR
GAAACTAGGA	2	2	1	0	1	gnl|UG|At#S21735945	gi|51971656|dbj|AK176730.1|	At3g21530.1
at1g77620	CON	MIN	HOUR	DAY	WEEK	UNIGENE	FLCDNA	TAIR
ACATAGTAGT	0	1	0	0	0	gnl|UG|At#S11727343	no match found	At1g77620.1
at1g24100	CON	MIN	HOUR	DAY	WEEK	UNIGENE	FLCDNA	TAIR
AAATGTAAGT	5	3	3	0	0	gnl|UG|At#S34116044	gi|110740341|dbj|AK230264.1|	multiple canonical match
at1g56170	CON	MIN	HOUR	DAY	WEEK	UNIGENE	FLCDNA	TAIR
TTAGTATATC	0	0	0	1	1	no match found	gi|21389648|gb|AY114704.1	multiple non-canonical match
at1g50590	CON	MIN	HOUR	DAY	WEEK	UNIGENE	FLCDNA	TAIR
TTAGAGGCTT	0	0	0	1	0	gnl|UG|At#S18897562	no match found	At1g50590.1
at5g47455	CON	MIN	HOUR	DAY	WEEK	UNIGENE	FLCDNA	TAIR
ACTAGTTTGA	0	0	0	0	1	no match found	gi|21405551|gb|AY086841.1	At5g47455.2
AAGGGAAAAA	1	1	1	1	3	gnl|UG|At#S18941515	gi|26450573|dbj|AK117751.1|	At5g47455.6
at3g24660	CON	MIN	HOUR	DAY	WEEK	UNIGENE	FLCDNA	TAIR
ATGATAAAAA	5	1	2	1	0	gnl|UG|At#S11699007	no match found	non-canonical match
at1g01600	CON	MIN	HOUR	DAY	WEEK	UNIGENE	FLCDNA	TAIR
ATGAAGAAAA	0	0	2	1	1	gnl|UG|At#S11742954	gi|26449890|dbj|AK117400.1|	At1g01600.1
at1g65700	CON	MIN	HOUR	DAY	WEEK	UNIGENE	FLCDNA	TAIR
TGTTGGGTTT	0	2	0	1	1	gnl|UG|At#S11729990	gi|21405739|gb|AY087015.1	At1g65700.1
at5g15330	CON	MIN	HOUR	DAY	WEEK	UNIGENE	FLCDNA	TAIR
ATAATAACAA	0	1	0	0	0	gnl|UG|At#S43850026	no match found	non-canonical match
GCGAGATGGT	0	1	0	0	0	gnl|UG|At#S11707518	gi|15215821|gb|AY050440.1	At5g15330.1
at5g43930	CON	MIN	HOUR	DAY	WEEK	UNIGENE	FLCDNA	TAIR
TTAAAAATAG	1	1	0	1	0	gnl|UG|At#S28281680	gi|15450893|gb|AY054527.1	At5g43930.1
at1g75510	CON	MIN	HOUR	DAY	WEEK	UNIGENE	FLCDNA	TAIR
TGACATAGTC	1	0	0	0	2	gnl|UG|At#S11727940	gi|13265438|gb|AF324673.2	At1g75510.1
at3g58230	CON	MIN	HOUR	DAY	WEEK	UNIGENE	FLCDNA	TAIR
AACAACCTCC	0	0	0	1	0	gnl|UG|At#S11727908	no match found	multiple non-canonical match
at5g17540	CON	MIN	HOUR	DAY	WEEK	UNIGENE	FLCDNA	TAIR
TCTTCTCTGT	0	1	0	0	0	gnl|UG|At#S11721941	no match found	At5g17540.1
at1g19350	CON	MIN	HOUR	DAY	WEEK	UNIGENE	FLCDNA	TAIR
ATTAAGTGGC	6	0	3	4	6	gnl|UG|At#S18942541	gi|13937166|gb|AF372937.1	At1g19350.5
at5g10960	CON	MIN	HOUR	DAY	WEEK	UNIGENE	FLCDNA	TAIR
GTGGATATGA	4	5	3	0	0	gnl|UG|At#S11723511	gi|15292842|gb|AY050855.1	At5g10960.1
at3g60830	CON	MIN	HOUR	DAY	WEEK	UNIGENE	FLCDNA	TAIR
ACAGGATTCG	1	1	0	0	0	gnl|UG|At#S11727235	gi|20466389|gb|AY099661.1	At3g60830.1

at4g13780	CON	MIN	HOUR	DAY	WEEK	UNIGENE	FLCDNA	TAIR
TTCTTGTAAA	5	4	3	1	2	gnl|UG|At#S11725424	gi|17381105|gb|AY064009.1	At4g13780.1
at1g75270	CON	MIN	HOUR	DAY	WEEK	UNIGENE	FLCDNA	TAIR
CTTCTCTAGT	2	2	1	0	2	gnl|UG|At#S11727986	gi|22655140|gb|AY140019.1	At1g75270.1
at1g55530	CON	MIN	HOUR	DAY	WEEK	UNIGENE	FLCDNA	TAIR
GAGCTGAGAA	2	0	2	0	1	no match found	gi|23308202|gb|BT000502.1	non-canonical match
AAATCTCGGT	2	4	0	1	1	gnl|UG|At#S11732466	gi|15983407|gb|AF424578.1	At1g55530.1
at1g07170	CON	MIN	HOUR	DAY	WEEK	UNIGENE	FLCDNA	TAIR
GGTTCAAACT	1	1	0	1	2	gnl|UG|At#S30649176	no match found	pseudo chromosome match
at5g44250	CON	MIN	HOUR	DAY	WEEK	UNIGENE	FLCDNA	TAIR
AGTACCCATT	0	7	0	3	1	gnl|UG|At#S38433181	gi|15450861|gb|AY054511.1	At5g44250.1
at4g19865	CON	MIN	HOUR	DAY	WEEK	UNIGENE	FLCDNA	TAIR
TCAGCTCTTG	0	1	0	0	0	gnl|UG|At#S15460915	no match found	At4g19865.1
at4g23530	CON	MIN	HOUR	DAY	WEEK	UNIGENE	FLCDNA	TAIR
GATGTCAATC	0	1	0	0	0	no match found	gi|21436290|gb|AY117209.1	multiple non-canonical match
TTGCTTTCCT	0	1	2	0	0	gnl|UG|At#S11723727	gi|17380709|gb|AY063829.1	At4g23530.1
at1g52855	CON	MIN	HOUR	DAY	WEEK	UNIGENE	FLCDNA	TAIR
AAGACTTCGA	0	0	0	0	1	no match found	gi|21405518|gb|AY086808.1	At1g52855.1
at2g27360	CON	MIN	HOUR	DAY	WEEK	UNIGENE	FLCDNA	TAIR
CCTCGACTCT	1	2	0	2	0	gnl|UG|At#S11735703	gi|51972006|dbj|AK176905.1|	At2g27360.1
at2g20830	CON	MIN	HOUR	DAY	WEEK	UNIGENE	FLCDNA	TAIR
AGTGACAATT	1	0	3	0	2	gnl|UG|At#S11702090	gi|13430685|gb|AF360255.1	At2g20830.1
TCTTGCTCGA	0	0	1	0	0	no match found	gi|62318615|dbj|AK220628.1|	non-canonical match
at4g01060	CON	MIN	HOUR	DAY	WEEK	UNIGENE	FLCDNA	TAIR
AAAAATTGAA	0	0	1	1	0	no match found	gi|26451140|dbj|AK118043.1|	At4g01060.1
AGTCAAGAAG	0	2	2	1	0	gnl|UG|At#S11688121	no match found	multiple non-canonical match
at1g79000	CON	MIN	HOUR	DAY	WEEK	UNIGENE	FLCDNA	TAIR
CGAGCTTTAA	0	1	1	0	0	gnl|UG|At#S11726847	no match found	At1g79000.1
at5g03940	CON	MIN	HOUR	DAY	WEEK	UNIGENE	FLCDNA	TAIR
ATTATCTAGG	2	1	1	0	0	gnl|UG|At#S11725163	no match found	At5g03940.1
at5g47540	CON	MIN	HOUR	DAY	WEEK	UNIGENE	FLCDNA	TAIR
TCTTTAAGCT	0	0	0	2	0	gnl|UG|At#S18910639	gi|16226300|gb|AF428296.1	non-canonical match
AATGTTGTAA	0	0	0	0	1	no match found	no match found	At5g47540.1
at1g10360	CON	MIN	HOUR	DAY	WEEK	UNIGENE	FLCDNA	TAIR
AGTGTACCAA	3	3	2	3	0	gnl|UG|At#S11807246	gi|20259356|gb|AY090957.1	non-canonical match
at5g57710	CON	MIN	HOUR	DAY	WEEK	UNIGENE	FLCDNA	TAIR
GATCGGGTTT	0	4	1	0	0	gnl|UG|At#S11717941	no match found	At5g57710.1
at3g22220	CON	MIN	HOUR	DAY	WEEK	UNIGENE	FLCDNA	TAIR
GAAGTTCTTG	0	0	1	1	0	gnl|UG|At#S11734158	gi|28393359|gb|BT004077.1	At3g22220.1
at4g24670	CON	MIN	HOUR	DAY	WEEK	UNIGENE	FLCDNA	TAIR
TGATTATGAT	2	0	0	0	1	gnl|UG|At#S18941804	gi|20259316|gb|AY091455.1	At4g24670.1
at1g14670	CON	MIN	HOUR	DAY	WEEK	UNIGENE	FLCDNA	TAIR

GCTTGCATTT	1	4	0	0	0	gnl|UG|At#S11741569	gi|15450754|gb|AY053419.1	At1g14670.1
at3g20920	CON	MIN	HOUR	DAY	WEEK	UNIGENE	FLCDNA	TAIR
CCCCTGATGA	0	2	0	1	0	gnl|UG|At#S11705940	gi|21406915|gb|AY088141.1	At3g20920.1
at4g21620	CON	MIN	HOUR	DAY	WEEK	UNIGENE	FLCDNA	TAIR
AGGATCCTTT	4	9	16	11	9	no match found	gi|16648719|gb|AY058136.1	At4g21620.1
at5g18850	CON	MIN	HOUR	DAY	WEEK	UNIGENE	FLCDNA	TAIR
TTTTTAGGTC	1	3	0	0	0	gnl|UG|At#S18912366	gi|110736390|dbj|AK228211.1|	At5g18850.1
at1g15700	CON	MIN	HOUR	DAY	WEEK	UNIGENE	FLCDNA	TAIR
AAAGAAAAAA	2	1	2	3	1	gnl|UG|At#S11710318	gi|17529041|gb|AY065255.1	At4g04270.1
at1g19715	CON	MIN	HOUR	DAY	WEEK	UNIGENE	FLCDNA	TAIR
CTCCTAAACG	1	3	2	3	0	gnl|UG|At#S11747739	gi|17064841|gb|AY062497.1	At1g19715.1
at2g11240	CON	MIN	HOUR	DAY	WEEK	UNIGENE	FLCDNA	TAIR
ATTAAAAGTT	0	2	0	0	0	no match found	gi|110738188|dbj|AK229152.1|	non-canonical match
at4g22320	CON	MIN	HOUR	DAY	WEEK	UNIGENE	FLCDNA	TAIR
GCTTATTCCT	0	0	0	0	1	gnl|UG|At#S11723945	gi|26450260|dbj|AK117590.1|	At4g22320.1
at1g35680	CON	MIN	HOUR	DAY	WEEK	UNIGENE	FLCDNA	TAIR
CTGTTGTGGA	9	6	11	4	3	gnl|UG|At#S11736977	gi|21436000|gb|AY116944.1	At1g35680.1
at5g49520	CON	MIN	HOUR	DAY	WEEK	UNIGENE	FLCDNA	TAIR
GATTGCTTCA	1	1	0	0	0	no match found	gi|20259612|gb|AY091224.1	multiple non-canonical match
CTATCACTAG	0	1	0	0	0	gnl|UG|At#S11708453	gi|17380791|gb|AY063870.1	At5g49520.1
at3g14000	CON	MIN	HOUR	DAY	WEEK	UNIGENE	FLCDNA	TAIR
ATTCATCTGG	0	0	1	0	0	gnl|UG|At#S18942102	gi|115311460|gb|BT029002.1	At3g14000.1
at4g37590	CON	MIN	HOUR	DAY	WEEK	UNIGENE	FLCDNA	TAIR
TTTCTTAAGG	0	3	1	0	1	gnl|UG|At#S34115592	gi|110741208|dbj|AK230356.1|	At4g37590.1
at1g50150	CON	MIN	HOUR	DAY	WEEK	UNIGENE	FLCDNA	TAIR
GCAGCTTGGG	0	2	0	1	0	gnl|UG|At#S11734449	no match found	At1g50140.1
at1g58030	CON	MIN	HOUR	DAY	WEEK	UNIGENE	FLCDNA	TAIR
AAGGTTCTTT	1	7	1	1	1	gnl|UG|At#S15459087	gi|23306413|gb|BT000457.1	At1g58030.1
at1g19620	CON	MIN	HOUR	DAY	WEEK	UNIGENE	FLCDNA	TAIR
GTCATCGTAG	0	0	0	1	0	gnl|UG|At#S11741091	no match found	At1g19620.1
at4g02520	CON	MIN	HOUR	DAY	WEEK	UNIGENE	FLCDNA	TAIR
GCCATTGGAA	2	23	11	3	0	gnl|UG|At#S11727057	gi|13265466|gb|AF324681.2	At4g02520.1
at1g12380	CON	MIN	HOUR	DAY	WEEK	UNIGENE	FLCDNA	TAIR
CAGCCGTTGA	0	0	1	1	0	gnl|UG|At#S34115463	gi|110741457|dbj|AK226554.1|	At1g12380.1
at5g56000	CON	MIN	HOUR	DAY	WEEK	UNIGENE	FLCDNA	TAIR
GATGAGCTGA	1	0	1	1	1	gnl|UG|At#S34114761	gi|110742759|dbj|AK227254.1|	At5g56000.1
at2g37570	CON	MIN	HOUR	DAY	WEEK	UNIGENE	FLCDNA	TAIR
ATAGAACATT	2	2	0	1	0	gnl|UG|At#S11733210	gi|15451173|gb|AY054667.1	At2g39620.1
at4g11200	CON	MIN	HOUR	DAY	WEEK	UNIGENE	FLCDNA	TAIR
CTTAAAACTT	0	1	0	0	0	no match found	no match found	At4g11200.1
at3g61050	CON	MIN	HOUR	DAY	WEEK	UNIGENE	FLCDNA	TAIR

GAAGAAAGGA	1	0	0	1	0	no match found	gi|28416558|gb|BT004564.1	At1g71190.1
at1g16620	CON	MIN	HOUR	DAY	WEEK	UNIGENE	FLCDNA	TAIR
AACGTTTCAA	0	0	1	0	0	no match found	no match found	At1g16620.1
at5g23860	CON	MIN	HOUR	DAY	WEEK	UNIGENE	FLCDNA	TAIR
AATGATTTGG	2	3	1	0	0	gnl|UG|At#S11720793	gi|15451225|gb|AY054693.1	At5g23860.1
at1g68760	CON	MIN	HOUR	DAY	WEEK	UNIGENE	FLCDNA	TAIR
TGTCCTATCG	3	1	0	1	0	gnl|UG|At#S21735790	gi|51971966|dbj|AK176885.1|	At1g68760.1
at4g25230	CON	MIN	HOUR	DAY	WEEK	UNIGENE	FLCDNA	TAIR
CCACACGTGC	0	2	2	2	0	gnl|UG|At#S11743521	gi|18176186|gb|AY072178.1	At4g25230.2
at2g35830	CON	MIN	HOUR	DAY	WEEK	UNIGENE	FLCDNA	TAIR
ATTTCACCAA	5	4	3	4	0	no match found	gi|21436044|gb|AY116966.1	At2g35830.1
at1g03080	CON	MIN	HOUR	DAY	WEEK	UNIGENE	FLCDNA	TAIR
TGAATGGAAA	5	3	5	2	0	gnl|UG|At#S11742806	no match found	At1g03080.1
at3g11380	CON	MIN	HOUR	DAY	WEEK	UNIGENE	FLCDNA	TAIR
TTCTCGAAAA	1	2	1	1	0	no match found	no match found	At3g11380.1
at1g47240	CON	MIN	HOUR	DAY	WEEK	UNIGENE	FLCDNA	TAIR
TCAAGAAGAT	0	2	0	0	0	no match found	gi|15294257|gb|AF410320.1	At1g47240.1
at4g22590	CON	MIN	HOUR	DAY	WEEK	UNIGENE	FLCDNA	TAIR
ACCACCTGAA	0	0	1	0	0	gnl|UG|At#S11723893	gi|62321205|dbj|AK221933.1|	At4g22590.1
at3g22530	CON	MIN	HOUR	DAY	WEEK	UNIGENE	FLCDNA	TAIR
AGAAGAAGAC	1	1	1	2	1	gnl|UG|At#S11734058	gi|21404035|gb|AY085325.1	At3g22530.1
at5g44490	CON	MIN	HOUR	DAY	WEEK	UNIGENE	FLCDNA	TAIR
TGTCAAATCA	0	0	0	1	0	gnl|UG|At#S11719279	no match found	multiple non-canonical match
at1g14800	CON	MIN	HOUR	DAY	WEEK	UNIGENE	FLCDNA	TAIR
ATGAGTTCAG	0	0	1	2	0	gnl|UG|At#S11741554	no match found	At1g14800.1
at5g01160	CON	MIN	HOUR	DAY	WEEK	UNIGENE	FLCDNA	TAIR
TAATTCGAGT	1	0	1	1	1	gnl|UG|At#S11726410	gi|21403076|gb|AY084366.1	At5g01160.1
GAATCCCGGA	0	0	1	1	3	no match found	gi|89000984|gb|BT024744.1	non-canonical match
at4g35870	CON	MIN	HOUR	DAY	WEEK	UNIGENE	FLCDNA	TAIR
AGATTCTGTG	0	0	0	1	0	no match found	gi|19699092|gb|AY090251.1	At4g35870.1
CCAATAGTAT	0	0	1	0	1	gnl|UG|At#S11808713	gi|20465419|gb|AY096495.1	At4g35880.1
at1g07200	CON	MIN	HOUR	DAY	WEEK	UNIGENE	FLCDNA	TAIR
GAAAGTTTTT	2	0	0	1	0	gnl|UG|At#S34115020	gi|110742274|dbj|AK226997.1|	At1g07200.1
at2g37025	CON	MIN	HOUR	DAY	WEEK	UNIGENE	FLCDNA	TAIR
TGAAGTTTCT	0	0	1	1	2	gnl|UG|At#S34115500	gi|110741389|dbj|AK230448.1|	At2g37025.1
at3g54840	CON	MIN	HOUR	DAY	WEEK	UNIGENE	FLCDNA	TAIR
TCTTCAGACT	2	2	0	1	1	gnl|UG|At#S38433599	gi|27754458|gb|BT002860.1	At3g54840.1
at3g15190	CON	MIN	HOUR	DAY	WEEK	UNIGENE	FLCDNA	TAIR
GGGATAACGG	18	11	23	16	13	gnl|UG|At#S11736292	gi|21405062|gb|AY086352.1	At3g15190.1
at5g44730	CON	MIN	HOUR	DAY	WEEK	UNIGENE	FLCDNA	TAIR
CTTTGTTGGT	1	1	0	0	1	gnl|UG|At#S18910546	gi|106879170|gb|BT025712.1	At5g44730.1

at1g61360	CON	MIN	HOUR	DAY	WEEK	UNIGENE	FLCDNA	TAIR
AATGTTCCAA	0	0	1	1	0	gnl|UG|At#S34117306	gi|110737902|dbj|AK229002.1|	At1g61360.1
at3g10910	CON	MIN	HOUR	DAY	WEEK	UNIGENE	FLCDNA	TAIR
ATCGGTGATT	0	0	1	1	0	gnl|UG|At#S11737609	gi|110735946|dbj|AK227982.1|	At3g10910.1
at4g36680	CON	MIN	HOUR	DAY	WEEK	UNIGENE	FLCDNA	TAIR
ATGAATTCAA	3	1	1	3	2	gnl|UG|At#S11721492	gi|17381285|gb|AY064155.1	At4g36680.1
at4g16162	CON	MIN	HOUR	DAY	WEEK	UNIGENE	FLCDNA	TAIR
CAGTATTTTA	0	1	0	0	0	gnl|UG|At#S37211674	no match found	multiple non-canonical match
at3g17820	CON	MIN	HOUR	DAY	WEEK	UNIGENE	FLCDNA	TAIR
ATTTGATTTC	3	7	1	3	3	no match found	gi|21407086|gb|AY088312.1	At3g17820.1
at1g64620	CON	MIN	HOUR	DAY	WEEK	UNIGENE	FLCDNA	TAIR
TTTTAAAAAG	1	0	0	0	0	gnl|UG|At#S14273902	gi|21403608|gb|AY084898.1	At1g64620.1
at1g78490	CON	MIN	HOUR	DAY	WEEK	UNIGENE	FLCDNA	TAIR
CAGTTTCCAA	0	0	0	1	0	no match found	gi|17473540|gb|AY065073.1	non-canonical match
at2g24030	CON	MIN	HOUR	DAY	WEEK	UNIGENE	FLCDNA	TAIR
GCTTTGCTTG	0	0	1	0	0	no match found	gi|109946626|gb|BT026136.1	non-canonical match
at2g20840	CON	MIN	HOUR	DAY	WEEK	UNIGENE	FLCDNA	TAIR
TACTTCAGGG	0	1	1	0	0	gnl|UG|At#S11737247	gi|46931313|gb|BT012642.1	At2g20840.1
at2g36620	CON	MIN	HOUR	DAY	WEEK	UNIGENE	FLCDNA	TAIR
GTTCTCAAGA	2	4	0	1	5	gnl|UG|At#S11733449	gi|18252830|gb|AY072350.1	At2g36620.1
at3g13320	CON	MIN	HOUR	DAY	WEEK	UNIGENE	FLCDNA	TAIR
TTTGCGGTCC	1	2	0	0	0	gnl|UG|At#S11736877	gi|15983506|gb|AF424628.1	At3g13320.1
at5g12010	CON	MIN	HOUR	DAY	WEEK	UNIGENE	FLCDNA	TAIR
CGTTTAATGA	0	1	0	0	0	gnl|UG|At#S11708107	gi|25141198|gb|BT002297.1	At5g12010.1
at1g14280	CON	MIN	HOUR	DAY	WEEK	UNIGENE	FLCDNA	TAIR
TAAAATGTCT	5	3	2	1	0	gnl|UG|At#S11741609	gi|21407638|gb|AY088864.1	At1g14280.1
at2g33040	CON	MIN	HOUR	DAY	WEEK	UNIGENE	FLCDNA	TAIR
TACTCCGGTT	5	7	11	9	8	gnl|UG|At#S11817200	gi|17065101|gb|AY062627.1	pseudo chromosome match
GACAGCTCAA	0	6	8	3	1	no match found	gi|23198401|gb|BT000409.1	At2g33040.1
at1g48410	CON	MIN	HOUR	DAY	WEEK	UNIGENE	FLCDNA	TAIR
GTGAGAAAGA	0	0	1	0	0	no match found	gi|110735728|dbj|AK227868.1|	non-canonical match
at2g42220	CON	MIN	HOUR	DAY	WEEK	UNIGENE	FLCDNA	TAIR
TTTTCACATT	34	22	13	13	6	no match found	gi|15010629|gb|AY045616.1	At2g42220.1
at4g25840	CON	MIN	HOUR	DAY	WEEK	UNIGENE	FLCDNA	TAIR
AATTTGTAAT	0	1	1	0	0	no match found	gi|21407656|gb|AY088882.1	At4g25840.1
at4g01310	CON	MIN	HOUR	DAY	WEEK	UNIGENE	FLCDNA	TAIR
TTTCAGAAAA	11	13	20	16	9	gnl|UG|At#S11727356	gi|21404953|gb|AY086243.1	At4g01310.1
at4g25360	CON	MIN	HOUR	DAY	WEEK	UNIGENE	FLCDNA	TAIR
GAACGAAATG	0	1	1	1	0	gnl|UG|At#S28281970	gi|17979114|gb|AY070058.1	At4g25360.1
at3g56950	CON	MIN	HOUR	DAY	WEEK	UNIGENE	FLCDNA	TAIR
AACCCTGCAG	0	1	0	0	0	gnl|UG|At#S11728153	gi|21405877|gb|AY087153.1	non-canonical match
AGATAAGCAG	3	5	5	4	1	no match found	no match found	At3g56950.1

at1g27530	CON	MIN	HOUR	DAY	WEEK	UNIGENE	FLCDNA	TAIR
AAACCCAATA	4	11	1	1	0	gnl|UG|At#S11739810	gi|15451103|gb|AY054632.1	At1g27530.1
at5g23520	CON	MIN	HOUR	DAY	WEEK	UNIGENE	FLCDNA	TAIR
AATAATTTGT	1	1	0	0	0	no match found	no match found	At5g23520.1
at4g34350	CON	MIN	HOUR	DAY	WEEK	UNIGENE	FLCDNA	TAIR
TCAAGAGTGT	19	8	8	1	5	gnl|UG|At#S11721874	gi|20148250|gb|AY081454.1	At4g34350.1
at5g62430	CON	MIN	HOUR	DAY	WEEK	UNIGENE	FLCDNA	TAIR
AAGCTTTATA	1	1	1	1	1	gnl|UG|At#S11717458	gi|17933304|gb|AF446362.1	At5g62430.1
at3g63010	CON	MIN	HOUR	DAY	WEEK	UNIGENE	FLCDNA	TAIR
CATTAAAAAA	1	0	3	0	0	gnl|UG|At#S34118124	gi|110736334|dbj|AK228182.1|	multiple non-canonical match
at5g11540	CON	MIN	HOUR	DAY	WEEK	UNIGENE	FLCDNA	TAIR
TCGAATGAGA	0	0	1	0	0	no match found	no match found	At5g11540.1
at5g65230	CON	MIN	HOUR	DAY	WEEK	UNIGENE	FLCDNA	TAIR
TTATTTGTGT	0	1	0	0	0	gnl|UG|At#S11717174	no match found	At5g65230.1
at3g26360	CON	MIN	HOUR	DAY	WEEK	UNIGENE	FLCDNA	TAIR
AGATTAGAAA	0	0	0	0	1	no match found	no match found	At3g26360.1
at1g71390	CON	MIN	HOUR	DAY	WEEK	UNIGENE	FLCDNA	TAIR
AGTGGTTTAC	0	0	0	0	1	gnl|UG|At#S11728791	no match found	non-canonical match
at5g56990	CON	MIN	HOUR	DAY	WEEK	UNIGENE	FLCDNA	TAIR
AGGAAACCTA	0	1	2	0	1	gnl|UG|At#S21989658	no match found	multiple non-canonical match
at4g17486	CON	MIN	HOUR	DAY	WEEK	UNIGENE	FLCDNA	TAIR
GAAGCTGAAA	0	2	2	1	0	gnl|UG|At#S11724770	gi|14194146|gb|AF367279.1	At4g17486.1
at2g44160	CON	MIN	HOUR	DAY	WEEK	UNIGENE	FLCDNA	TAIR
GCTGTGAACA	4	3	1	3	1	gnl|UG|At#S11699575	gi|15215809|gb|AY050434.1	At2g44160.1
at5g19050	CON	MIN	HOUR	DAY	WEEK	UNIGENE	FLCDNA	TAIR
AATTAATTCA	0	0	1	1	2	no match found	gi|18377687|gb|AY074297.1	At5g19050.1
at3g03640	CON	MIN	HOUR	DAY	WEEK	UNIGENE	FLCDNA	TAIR
AGAAACAATC	0	0	2	0	2	gnl|UG|At#S11739614	no match found	At3g03640.1
at1g08800	CON	MIN	HOUR	DAY	WEEK	UNIGENE	FLCDNA	TAIR
CAACTTTGTT	0	1	3	1	0	no match found	gi|26451365|dbj|AK118159.1|	At1g08800.1
at4g33980	CON	MIN	HOUR	DAY	WEEK	UNIGENE	FLCDNA	TAIR
ACGAAACCAA	0	3	4	0	0	gnl|UG|At#S11721938	gi|21407359|gb|AY088585.1	At4g33980.1
at4g15075	CON	MIN	HOUR	DAY	WEEK	UNIGENE	FLCDNA	TAIR
AATGTAATGA	1	0	2	0	2	no match found	no match found	At4g15075.1
at1g23780	CON	MIN	HOUR	DAY	WEEK	UNIGENE	FLCDNA	TAIR
GTTGCTCTCT	2	3	3	5	2	gnl|UG|At#S18922926	gi|15146299|gb|AY049291.1	multiple non-canonical match
at2g42800	CON	MIN	HOUR	DAY	WEEK	UNIGENE	FLCDNA	TAIR
GCGTTGAGCA	0	0	0	1	0	gnl|UG|At#S11731925	gi|66792705|gb|BT023464.1	At2g42800.1
at1g73430	CON	MIN	HOUR	DAY	WEEK	UNIGENE	FLCDNA	TAIR
GCTCAATCGA	1	1	3	3	0	gnl|UG|At#S11728369	gi|22654988|gb|AY139767.1	At1g73430.1

at1g74580	CON	MIN	HOUR	DAY	WEEK	UNIGENE	FLCDNA	TAIR
ATCTAAGTCC	0	0	0	0	1	no match found	no match found	At1g74580.1
at2g28200	CON	MIN	HOUR	DAY	WEEK	UNIGENE	FLCDNA	TAIR
AGGCGGCACA	0	3	0	1	0	gnl|UG|At#S21737182	gi|51969127|dbj|AK175493.1|	At2g28200.1
at3g43270	CON	MIN	HOUR	DAY	WEEK	UNIGENE	FLCDNA	TAIR
TTTGATCGAA	2	1	0	0	0	gnl|UG|At#S11730721	gi|17979140|gb|AY070071.1	At3g43270.1
at3g28370	CON	MIN	HOUR	DAY	WEEK	UNIGENE	FLCDNA	TAIR
TTTTGCCTGA	0	0	0	1	0	no match found	no match found	At3g28370.1
at5g05950	CON	MIN	HOUR	DAY	WEEK	UNIGENE	FLCDNA	TAIR
GTTGATGCAA	0	2	1	1	1	gnl|UG|At#S11724639	gi|110737480|dbj|AK228786.1|	At5g05950.1
at3g57560	CON	MIN	HOUR	DAY	WEEK	UNIGENE	FLCDNA	TAIR
AGATTATGTC	1	1	6	0	0	gnl|UG|At#S11728039	gi|21406841|gb|AY088067.1	At3g57560.1
at1g06890	CON	MIN	HOUR	DAY	WEEK	UNIGENE	FLCDNA	TAIR
CCTGGTTCTA	1	1	1	0	0	gnl|UG|At#S11742344	gi|21436372|gb|AY117281.1	At1g06890.1
at1g79720	CON	MIN	HOUR	DAY	WEEK	UNIGENE	FLCDNA	TAIR
AGTTTGATCG	0	0	1	0	0	no match found	gi|115646740|gb|BT029166.1	non-canonical match
AAAGCCAATC	3	1	2	3	2	gnl|UG|At#S11726620	gi|19699358|gb|AY090445.1	At1g79720.1
at2g22590	CON	MIN	HOUR	DAY	WEEK	UNIGENE	FLCDNA	TAIR
ACTCGATCGG	0	0	0	1	0	gnl|UG|At#S11736838	gi|45680106|gb|BT011796.1	At2g22590.1
at3g16890	CON	MIN	HOUR	DAY	WEEK	UNIGENE	FLCDNA	TAIR
AGTATTGAAT	0	0	0	0	1	no match found	no match found	At3g16890.1
at1g22850	CON	MIN	HOUR	DAY	WEEK	UNIGENE	FLCDNA	TAIR
TATCAACGTT	14	13	13	9	11	gnl|UG|At#S11740779	gi|15215601|gb|AY050329.1	At1g22850.1
at1g59710	CON	MIN	HOUR	DAY	WEEK	UNIGENE	FLCDNA	TAIR
ACTGTTGTCT	0	0	0	1	1	gnl|UG|At#S11731622	gi|15010641|gb|AY045622.1	At1g59710.1
at5g10020	CON	MIN	HOUR	DAY	WEEK	UNIGENE	FLCDNA	TAIR
GATTTCCTTT	0	1	2	0	0	gnl|UG|At#S11723736	gi|17978959|gb|AY070134.1	At5g10020.1
at5g52110	CON	MIN	HOUR	DAY	WEEK	UNIGENE	FLCDNA	TAIR
GATTAAAGGA	0	0	0	0	1	no match found	gi|21407812|gb|AY089038.1	multiple canonical match
at1g72550	CON	MIN	HOUR	DAY	WEEK	UNIGENE	FLCDNA	TAIR
TCGAGAGAAT	3	1	4	3	2	no match found	gi|17065261|gb|AY062707.1	At1g72550.2
at1g07000	CON	MIN	HOUR	DAY	WEEK	UNIGENE	FLCDNA	TAIR
TATTGCTTTT	0	0	0	1	1	gnl|UG|At#S11742335	gi|18700110|gb|AY075660.1	At1g07000.1
at1g19080	CON	MIN	HOUR	DAY	WEEK	UNIGENE	FLCDNA	TAIR
TCAAAATGAT	0	1	2	2	1	gnl|UG|At#S11741143	no match found	At1g19080.1
at5g04950	CON	MIN	HOUR	DAY	WEEK	UNIGENE	FLCDNA	TAIR
TCTTGTGTGT	4	1	0	1	3	gnl|UG|At#S11724897	gi|18252858|gb|AY072364.1	At5g04950.1
at3g61620	CON	MIN	HOUR	DAY	WEEK	UNIGENE	FLCDNA	TAIR
CAGTGGTGTT	0	0	0	2	0	gnl|UG|At#S43849668	gi|26452704|dbj|AK118846.1|	At3g61620.1
at4g36090	CON	MIN	HOUR	DAY	WEEK	UNIGENE	FLCDNA	TAIR
GAGGAGGAAG	1	0	0	0	0	gnl|UG|At#S21736051	gi|51971444|dbj|AK176624.1|	At4g36090.1

at3g22270	CON	MIN	HOUR	DAY	WEEK	UNIGENE	FLCDNA	TAIR
GAGTAGTAGG	2	2	0	1	1	gnl|UG|At#S11734143	gi|110737820|dbj|AK228960.1|	At3g22270.1
at3g09850	CON	MIN	HOUR	DAY	WEEK	UNIGENE	FLCDNA	TAIR
TGGCCTTTGT	3	1	2	3	3	gnl|UG|At#S11737930	gi|15982780|gb|AY057497.1	At3g09850.1
at3g51010	CON	MIN	HOUR	DAY	WEEK	UNIGENE	FLCDNA	TAIR
TGTTTCTTGT	0	1	1	2	2	no match found	gi|21404024|gb|AY085314.1	At5g59740.1
at4g10760	CON	MIN	HOUR	DAY	WEEK	UNIGENE	FLCDNA	TAIR
ATTTTGTAAA	0	1	0	2	2	gnl|UG|At#S34114630	gi|110742995|dbj|AK227385.1|	multiple non-canonical match
at1g27595	CON	MIN	HOUR	DAY	WEEK	UNIGENE	FLCDNA	TAIR
TAATGGATGT	1	1	2	0	1	gnl|UG|At#S34117614	gi|110737303|dbj|AK228694.1|	At1g27595.1
at3g61220	CON	MIN	HOUR	DAY	WEEK	UNIGENE	FLCDNA	TAIR
GGTCAACTAA	0	1	1	0	1	gnl|UG|At#S11727109	gi|15028054|gb|AY045884.1	At3g61220.1
at1g02860	CON	MIN	HOUR	DAY	WEEK	UNIGENE	FLCDNA	TAIR
TGTGCTTGCT	0	1	0	1	0	no match found	gi|21407597|gb|AY088823.1	At1g02860.1
at2g30575	CON	MIN	HOUR	DAY	WEEK	UNIGENE	FLCDNA	TAIR
AGAGTTGATG	0	0	0	1	0	gnl|UG|At#S33259836	no match found	pseudo chromosome match
TACCTTACCA	0	0	0	1	0	gnl|UG|At#S11744651	gi|19698932|gb|AY081313.1	At2g30575.1
at3g18050	CON	MIN	HOUR	DAY	WEEK	UNIGENE	FLCDNA	TAIR
TTCTTTAACA	8	9	1	0	1	gnl|UG|At#S11706628	gi|15451205|gb|AY054683.1	At3g18050.1
at3g15840	CON	MIN	HOUR	DAY	WEEK	UNIGENE	FLCDNA	TAIR
GATACAAATT	7	13	13	7	1	gnl|UG|At#S43849904	gi|13358213|gb|AF325024.2	At3g15840.1
at2g21080	CON	MIN	HOUR	DAY	WEEK	UNIGENE	FLCDNA	TAIR
CGCTTCTGAA	0	2	0	1	0	gnl|UG|At#S11737186	gi|30725677|gb|BT008502.1	At2g21080.1
at3g23830	CON	MIN	HOUR	DAY	WEEK	UNIGENE	FLCDNA	TAIR
TCCTCTAAGC	0	2	0	0	0	no match found	gi|20453302|gb|AY097374.1	non-canonical match
at5g05620	CON	MIN	HOUR	DAY	WEEK	UNIGENE	FLCDNA	TAIR
AGAGTTTGCA	0	1	0	0	0	gnl|UG|At#S11724731	gi|134031919|gb|BT030394.1	multiple non-canonical match
at1g10490	CON	MIN	HOUR	DAY	WEEK	UNIGENE	FLCDNA	TAIR
TATGCTTCTG	1	0	0	0	0	no match found	gi|110741535|dbj|AK226604.1|	non-canonical match
at5g64440	CON	MIN	HOUR	DAY	WEEK	UNIGENE	FLCDNA	TAIR
TTATTTCGAT	0	1	0	0	0	gnl|UG|At#S18913258	gi|23505898|gb|AY143870.1	At5g64440.1
at3g02950	CON	MIN	HOUR	DAY	WEEK	UNIGENE	FLCDNA	TAIR
TTTCAAGCGT	0	0	1	0	0	gnl|UG|At#S11739797	gi|15983443|gb|AF424596.1	At3g02950.1
at2g45120	CON	MIN	HOUR	DAY	WEEK	UNIGENE	FLCDNA	TAIR
TTCTAACGCC	0	1	0	0	0	gnl|UG|At#S11731386	no match found	At2g45120.1
at4g25180	CON	MIN	HOUR	DAY	WEEK	UNIGENE	FLCDNA	TAIR
ATGAAGACCA	0	1	0	0	0	no match found	no match found	At4g25180.1
at1g16040	CON	MIN	HOUR	DAY	WEEK	UNIGENE	FLCDNA	TAIR
GCAGGAATGG	0	1	0	0	0	gnl|UG|At#S21737221	gi|51969049|dbj|AK175454.1|	multiple canonical match
at5g64300	CON	MIN	HOUR	DAY	WEEK	UNIGENE	FLCDNA	TAIR
TATGGCTTGA	2	5	2	1	0	gnl|UG|At#S11820522	gi|119935814|gb|BT029731.1	At5g64300.1

at1g37130	CON	MIN	HOUR	DAY	WEEK	UNIGENE	FLCDNA	TAIR
GCTTGCGGAC	20	34	34	27	1	gnl|UG|At#S34116078	gi|16930702|gb|AF436835.1	At1g37130.1
at2g38500	CON	MIN	HOUR	DAY	WEEK	UNIGENE	FLCDNA	TAIR
TCTCTTTTCT	0	0	1	0	0	gnl|UG|At#S11732972	gi|30017322|gb|BT006246.1	At2g38500.1
at1g50640	CON	MIN	HOUR	DAY	WEEK	UNIGENE	FLCDNA	TAIR
AGCAGCACCG	0	1	1	0	0	gnl|UG|At#S11610532	gi|94442520|gb|BT025295.1	multiple non-canonical match
ATTCTAAATA	5	4	2	4	3	gnl|UG|At#S11734284	gi|110736156|dbj|AK228091.1|	At1g50640.1
at2g03180	CON	MIN	HOUR	DAY	WEEK	UNIGENE	FLCDNA	TAIR
TGGTTATAGA	0	0	0	0	1	gnl|UG|At#S11740507	no match found	non-canonical match
at1g15900	CON	MIN	HOUR	DAY	WEEK	UNIGENE	FLCDNA	TAIR
ATTTTTTTTC	0	0	0	1	0	no match found	no match found	At1g15900.1
at1g68370	CON	MIN	HOUR	DAY	WEEK	UNIGENE	FLCDNA	TAIR
GGAATGGTTC	1	4	2	1	3	gnl|UG|At#S34117300	gi|110737914|dbj|AK229008.1|	At1g68370.1
TATTTCTTAC	0	1	0	0	0	no match found	gi|98960980|gb|BT025556.1	non-canonical match
at5g04170	CON	MIN	HOUR	DAY	WEEK	UNIGENE	FLCDNA	TAIR
TATAAAAGAA	2	3	0	0	3	gnl|UG|At#S11725104	gi|19698990|gb|AY081342.1	At5g04170.1
CTCACCGTCC	2	3	2	1	0	no match found	gi|31711841|gb|BT008838.1	non-canonical match
at1g79090	CON	MIN	HOUR	DAY	WEEK	UNIGENE	FLCDNA	TAIR
CAGAGTTTGC	1	1	1	2	0	gnl|UG|At#S30654024	gi|110738019|dbj|AK229063.1|	At1g79090.1
at1g56490	CON	MIN	HOUR	DAY	WEEK	UNIGENE	FLCDNA	TAIR
AAAAAAAAAT	5	3	4	1	0	no match found	no match found	At1g56490.1
at1g26270	CON	MIN	HOUR	DAY	WEEK	UNIGENE	FLCDNA	TAIR
GTCTTTGCTT	0	5	0	0	0	gnl|UG|At#S11703563	gi|18700158|gb|AY075684.1	At1g26270.1
at5g50310	CON	MIN	HOUR	DAY	WEEK	UNIGENE	FLCDNA	TAIR
AACTATGGCG	0	2	1	0	2	no match found	no match found	At5g50310.1
AACTAAATTA	1	0	0	1	0	gnl|UG|At#S11706454	gi|15724227|gb|AF412054.1	non-canonical match
at5g41380	CON	MIN	HOUR	DAY	WEEK	UNIGENE	FLCDNA	TAIR
TAATTGAAAA	3	0	0	0	0	gnl|UG|At#S22484584	no match found	non-canonical match
at4g21020	CON	MIN	HOUR	DAY	WEEK	UNIGENE	FLCDNA	TAIR
CAAAAGAGAA	1	0	0	0	0	no match found	gi|21436216|gb|AY117321.1	multiple non-canonical match
at1g21110	CON	MIN	HOUR	DAY	WEEK	UNIGENE	FLCDNA	TAIR
CCTCTCTTTG	0	1	7	0	0	gnl|UG|At#S11740945	gi|26452596|dbj|AK118791.1|	At1g21110.1
at1g09540	CON	MIN	HOUR	DAY	WEEK	UNIGENE	FLCDNA	TAIR
GGCGAGCTGT	0	0	0	1	0	gnl|UG|At#S11708366	gi|17380965|gb|AY063939.1	At1g09540.1
at5g11240	CON	MIN	HOUR	DAY	WEEK	UNIGENE	FLCDNA	TAIR
ATCAAGAGAT	0	1	0	1	0	no match found	gi|26451882|dbj|AK118425.1|	multiple canonical match
at3g08943	CON	MIN	HOUR	DAY	WEEK	UNIGENE	FLCDNA	TAIR
ATGCCCTATG	0	1	0	0	0	gnl|UG|At#S34118018	gi|110736537|dbj|AK228290.1|	At3g08943.1
at2g42380	CON	MIN	HOUR	DAY	WEEK	UNIGENE	FLCDNA	TAIR
CATCATCATC	0	1	1	0	0	gnl|UG|At#S15460466	gi|18491296|gb|AY074657.1	At2g42380.1
at3g09240	CON	MIN	HOUR	DAY	WEEK	UNIGENE	FLCDNA	TAIR
GCTCTTCTCT	0	0	0	0	1	gnl|UG|At#S11738115	no match found	multiple non-canonical match

at2g07180	CON	MIN	HOUR	DAY	WEEK	UNIGENE	FLCDNA	TAIR
ACTAATCTTC	0	1	0	0	0	gnl|UG|At#S11739714	gi|19423981|gb|AY080806.1	At2g07180.1
at2g40230	CON	MIN	HOUR	DAY	WEEK	UNIGENE	FLCDNA	TAIR
TTGGATCGGT	1	0	0	0	0	gnl|UG|At#S11732538	gi|21403075|gb|AY084365.1	At2g40230.1
at5g50840	CON	MIN	HOUR	DAY	WEEK	UNIGENE	FLCDNA	TAIR
AAACAGAGAG	2	0	0	2	1	no match found	gi|19698942|gb|AY081318.1	At5g50840.1
at5g25480	CON	MIN	HOUR	DAY	WEEK	UNIGENE	FLCDNA	TAIR
TTGATGGCGA	0	1	0	0	0	gnl|UG|At#S11720633	gi|21403991|gb|AY085281.1	At5g25480.1
at1g06870	CON	MIN	HOUR	DAY	WEEK	UNIGENE	FLCDNA	TAIR
GAATGCACTG	1	0	2	0	0	gnl|UG|At#S11742345	gi|21404199|gb|AY085489.1	At1g06870.1
at4g39390	CON	MIN	HOUR	DAY	WEEK	UNIGENE	FLCDNA	TAIR
AAGACAATTC	0	1	0	0	0	gnl|UG|At#S11721031	gi|23296522|gb|AY142535.1	At4g39390.2
at5g02560	CON	MIN	HOUR	DAY	WEEK	UNIGENE	FLCDNA	TAIR
TTCTATTAGC	0	1	0	0	0	gnl|UG|At#S11725523	gi|21404225|gb|AY085515.1	At5g02560.1
at1g58983	CON	MIN	HOUR	DAY	WEEK	UNIGENE	FLCDNA	TAIR
TATAAATTGT	1	0	1	0	0	gnl|UG|At#S11731710	no match found	At1g58983.1
TCTATTTCTA	15	2	8	6	7	no match found	gi|18086425|gb|AY065030.1	At2g42460.1
at3g23090	CON	MIN	HOUR	DAY	WEEK	UNIGENE	FLCDNA	TAIR
ATGATGAAAC	1	0	0	0	1	gnl|UG|At#S34114377	gi|110743578|dbj|AK227638.1|	At3g23090.1
at5g10970	CON	MIN	HOUR	DAY	WEEK	UNIGENE	FLCDNA	TAIR
GAAACCAGAA	0	0	1	1	0	no match found	no match found	At5g10970.1
at4g24780	CON	MIN	HOUR	DAY	WEEK	UNIGENE	FLCDNA	TAIR
AGGACGCGCC	2	3	3	3	2	gnl|UG|At#S11701977	gi|13430455|gb|AF360140.1	At4g24780.1
at1g23260	CON	MIN	HOUR	DAY	WEEK	UNIGENE	FLCDNA	TAIR
GCTTGTGTTA	0	3	0	0	1	no match found	gi|20259983|gb|AY093340.1	At1g23260.1
at5g10190	CON	MIN	HOUR	DAY	WEEK	UNIGENE	FLCDNA	TAIR
GAACGGTGCA	0	0	0	1	0	gnl|UG|At#S11701999	gi|21281224|gb|AY113893.1	At5g10190.1
at3g29070	CON	MIN	HOUR	DAY	WEEK	UNIGENE	FLCDNA	TAIR
AAGAAATGTT	0	0	0	1	0	gnl|UG|At#S11731946	no match found	multiple non-canonical match
at5g51010	CON	MIN	HOUR	DAY	WEEK	UNIGENE	FLCDNA	TAIR
AATGCAGTAT	5	10	4	0	2	gnl|UG|At#S11718615	gi|19310696|gb|AY079348.1	At5g51010.1
at1g17340	CON	MIN	HOUR	DAY	WEEK	UNIGENE	FLCDNA	TAIR
GTAAAGAGGG	1	0	0	0	0	no match found	gi|110739344|dbj|AK229748.1|	non-canonical match
CGCGGCAATA	3	1	1	4	0	gnl|UG|At#S11741310	no match found	At1g17340.1
at3g01310	CON	MIN	HOUR	DAY	WEEK	UNIGENE	FLCDNA	TAIR
CTTTTTTTCT	0	0	0	0	1	no match found	gi|110743926|dbj|AK227817.1|	multiple non-canonical match
at5g50260	CON	MIN	HOUR	DAY	WEEK	UNIGENE	FLCDNA	TAIR
GGGAGAGGAA	0	1	0	0	0	gnl|UG|At#S11718690	gi|20258849|gb|AY091087.1	At5g50260.1
at5g41950	CON	MIN	HOUR	DAY	WEEK	UNIGENE	FLCDNA	TAIR
GGAGTCTTTA	0	0	0	2	0	no match found	gi|133778835|gb|BT030345.1	non-canonical match
at1g18530	CON	MIN	HOUR	DAY	WEEK	UNIGENE	FLCDNA	TAIR
TGGTTTCATT	1	1	2	2	0	no match found	no match found	At1g18530.1

at3g22425	CON	MIN	HOUR	DAY	WEEK	UNIGENE	FLCDNA	TAIR
AGCCATCGTC	0	2	0	1	1	no match found	gi|26452643|dbj|AK118815.1|	At3g22425.1
at4g26630	CON	MIN	HOUR	DAY	WEEK	UNIGENE	FLCDNA	TAIR
TGACAGGTGA	7	6	11	2	3	gnl|UG|At#S28281960	gi|30725655|gb|BT008491.1	At4g26630.1
GAGATGAGAA	5	1	3	2	1	no match found	gi|110741443|dbj|AK226545.1|	multiple non-canonical match
AAGCTGACAA	3	9	3	4	2	no match found	gi|110739060|dbj|AK229601.1|	non-canonical match
at5g07960	CON	MIN	HOUR	DAY	WEEK	UNIGENE	FLCDNA	TAIR
TCTTAAGAGT	1	1	0	0	0	gnl|UG|At#S30640774	gi|110742985|dbj|AK227380.1|	At5g07960.1
at4g12590	CON	MIN	HOUR	DAY	WEEK	UNIGENE	FLCDNA	TAIR
GGTCAACTTC	1	2	0	1	1	gnl|UG|At#S11725633	gi|27311748|gb|BT002480.1	At4g12590.1
at2g36490	CON	MIN	HOUR	DAY	WEEK	UNIGENE	FLCDNA	TAIR
CATCCAGCCT	0	1	0	1	0	gnl|UG|At#S15660591	no match found	At2g36490.1
at5g24430	CON	MIN	HOUR	DAY	WEEK	UNIGENE	FLCDNA	TAIR
TGAATTGAGT	1	3	1	1	1	gnl|UG|At#S11720733	gi|62321266|dbj|AK221965.1|	At5g24430.1
at1g03190	CON	MIN	HOUR	DAY	WEEK	UNIGENE	FLCDNA	TAIR
GCTTATTAGT	1	0	0	0	1	gnl|UG|At#S11817672	gi|17064789|gb|AY062471.1	At1g03190.1
at5g58140	CON	MIN	HOUR	DAY	WEEK	UNIGENE	FLCDNA	TAIR
AAAAGAGCCG	0	1	0	1	0	no match found	gi|20268687|gb|AY091097.1	At5g58150.1
TAGAGCCCCT	0	1	0	0	0	no match found	gi|31711967|gb|BT008901.1	non-canonical match
at3g52660	CON	MIN	HOUR	DAY	WEEK	UNIGENE	FLCDNA	TAIR
TTACCCGATG	1	0	0	0	0	gnl|UG|At#S21736350	gi|51970791|dbj|AK176325.1|	non-canonical match
TCTTTTTCTG	1	0	0	0	0	no match found	no match found	At3g52660.1
at2g23840	CON	MIN	HOUR	DAY	WEEK	UNIGENE	FLCDNA	TAIR
CCAGAAGAAT	0	1	1	0	1	gnl|UG|At#S11736524	gi|44681451|gb|BT011660.1	At2g23840.1
at5g46250	CON	MIN	HOUR	DAY	WEEK	UNIGENE	FLCDNA	TAIR
AAGTTTGATC	1	11	5	3	2	gnl|UG|At#S15459193	gi|15215747|gb|AY050403.1	At5g46250.1
at5g16690	CON	MIN	HOUR	DAY	WEEK	UNIGENE	FLCDNA	TAIR
CCTTCAAAAA	0	0	1	0	0	gnl|UG|At#S11722168	no match found	multiple non-canonical match
at2g18170	CON	MIN	HOUR	DAY	WEEK	UNIGENE	FLCDNA	TAIR
GAGGAACCAG	1	0	0	2	0	gnl|UG|At#S11737935	gi|62321751|dbj|AK222214.1|	At2g18170.1
at4g37480	CON	MIN	HOUR	DAY	WEEK	UNIGENE	FLCDNA	TAIR
TGTACTAAAG	1	1	0	0	0	no match found	no match found	At4g37480.1
at3g27025	CON	MIN	HOUR	DAY	WEEK	UNIGENE	FLCDNA	TAIR
GAGCTTCTTC	0	1	0	0	0	gnl|UG|At#S35239258	no match found	multiple non-canonical match
at3g15290	CON	MIN	HOUR	DAY	WEEK	UNIGENE	FLCDNA	TAIR
CCTATGATCA	1	2	0	0	0	no match found	gi|24899760|gb|BT001208.1	multiple non-canonical match
TTGTTATCTA	0	0	0	1	1	gnl|UG|At#S11736260	no match found	At3g15290.1
at4g26780	CON	MIN	HOUR	DAY	WEEK	UNIGENE	FLCDNA	TAIR
CTATAATTGA	1	0	0	2	0	gnl|UG|At#S11723180	gi|21406149|gb|AY087425.1	At4g26780.1
at2g36060	CON	MIN	HOUR	DAY	WEEK	UNIGENE	FLCDNA	TAIR
ATACCGGCGT	2	3	3	1	0	no match found	gi|17065367|gb|AY062760.1	At2g36060.1
at2g47170	CON	MIN	HOUR	DAY	WEEK	UNIGENE	FLCDNA	TAIR

TGCCACCTCT	5	5	6	8	4	gnl|UG|At#S11730911	gi|22655407|gb|AY142032.1	At2g47170.1
at4g33090	CON	MIN	HOUR	DAY	WEEK	UNIGENE	FLCDNA	TAIR
TTTTCTATAA	4	4	0	1	4	gnl|UG|At#S11722090	gi|110742476|dbj|AK227105.1|	At4g33090.1
GGGCTCCGGA	0	0	1	0	0	no match found	gi|29028733|gb|BT005811.1	non-canonical match
at3g07890	CON	MIN	HOUR	DAY	WEEK	UNIGENE	FLCDNA	TAIR
TTTTTATAGA	0	0	0	1	0	gnl|UG|At#S11738389	gi|110737641|dbj|AK228869.1|	At3g07890.1
at5g63110	CON	MIN	HOUR	DAY	WEEK	UNIGENE	FLCDNA	TAIR
TCGTGGTGGC	4	3	0	0	0	gnl|UG|At#S11717390	gi|21407088|gb|AY088314.1	At5g63110.1
at1g05520	CON	MIN	HOUR	DAY	WEEK	UNIGENE	FLCDNA	TAIR
ACAAGACTAC	0	3	0	1	1	gnl|UG|At#S11705841	gi|15912296|gb|AY056426.1	At1g05520.1
at4g03210	CON	MIN	HOUR	DAY	WEEK	UNIGENE	FLCDNA	TAIR
TGGTTACTCT	5	15	3	5	0	gnl|UG|At#S28282100	gi|18252836|gb|AY072353.1	At4g03210.1
at4g22490	CON	MIN	HOUR	DAY	WEEK	UNIGENE	FLCDNA	TAIR
TCCCAGAAAC	0	6	1	2	1	gnl|UG|At#S21736027	gi|21405431|gb|AY086721.1	At4g22490.1
at3g23310	CON	MIN	HOUR	DAY	WEEK	UNIGENE	FLCDNA	TAIR
ATCAATTACC	1	1	0	0	0	gnl|UG|At#S11733813	gi|17381025|gb|AY063969.1	At3g23310.1
at5g06350	CON	MIN	HOUR	DAY	WEEK	UNIGENE	FLCDNA	TAIR
TCTCTACTAT	0	1	1	0	0	gnl|UG|At#S18908479	no match found	At5g06350.1
at4g26940	CON	MIN	HOUR	DAY	WEEK	UNIGENE	FLCDNA	TAIR
GGTTGTTTAT	0	1	2	0	3	gnl|UG|At#S11723136	gi|20147375|gb|AY093781.1	At4g26940.2
at5g11020	CON	MIN	HOUR	DAY	WEEK	UNIGENE	FLCDNA	TAIR
TGATTTCTAT	2	0	0	0	0	gnl|UG|At#S15461091	no match found	pseudo chromosome match
at1g33050	CON	MIN	HOUR	DAY	WEEK	UNIGENE	FLCDNA	TAIR
ACTAATTGTG	1	3	2	1	1	no match found	gi|17529299|gb|AY065436.1	At1g33050.1
TCGATTCAAG	0	1	0	0	0	gnl|UG|At#S43850524	no match found	non-canonical match
at1g03220	CON	MIN	HOUR	DAY	WEEK	UNIGENE	FLCDNA	TAIR
CTTCTCCCCT	0	4	0	1	1	gnl|UG|At#S11658081	gi|21403726|gb|AY085016.1	At1g03220.1
at3g20070	CON	MIN	HOUR	DAY	WEEK	UNIGENE	FLCDNA	TAIR
ATAAAACGTA	0	1	0	0	0	no match found	gi|21689808|gb|AY123015.1	non-canonical match
at5g42300	CON	MIN	HOUR	DAY	WEEK	UNIGENE	FLCDNA	TAIR
GGTCTTGAGC	0	0	0	0	1	no match found	gi|18377565|gb|AY072534.1	non-canonical match
TTACATCTAA	18	6	4	9	11	gnl|UG|At#S18910200	gi|13899064|gb|AF370527.1	At5g42300.1
at4g24350	CON	MIN	HOUR	DAY	WEEK	UNIGENE	FLCDNA	TAIR
ATCGTAACAA	0	0	0	0	1	gnl|UG|At#S43849503	gi|51971622|dbj|AK176713.1|	At4g24350.1
at1g24170	CON	MIN	HOUR	DAY	WEEK	UNIGENE	FLCDNA	TAIR
GGTTAGACTT	0	0	2	0	1	gnl|UG|At#S11740585	gi|26453137|dbj|AK119069.1|	At1g24170.1
at1g03910	CON	MIN	HOUR	DAY	WEEK	UNIGENE	FLCDNA	TAIR
CTGGTCCACC	1	0	0	1	0	gnl|UG|At#S11742708	no match found	non-canonical match
at3g06120	CON	MIN	HOUR	DAY	WEEK	UNIGENE	FLCDNA	TAIR
ACATTTTATA	0	1	0	0	0	no match found	no match found	At3g06120.1
at3g44050	CON	MIN	HOUR	DAY	WEEK	UNIGENE	FLCDNA	TAIR
ATAATCGAGA	0	2	0	0	0	gnl|UG|At#S11730559	no match found	At3g44050.1

at1g65800	CON	MIN	HOUR	DAY	WEEK	UNIGENE	FLCDNA	TAIR
GAACGTATAC	2	4	2	1	0	gnl|UG|At#S11704581	gi|15027840|gb|AY045777.1	At1g65800.1
at3g46530	CON	MIN	HOUR	DAY	WEEK	UNIGENE	FLCDNA	TAIR
ATTTTTTGTG	0	2	4	0	0	gnl|UG|At#S11730093	no match found	At3g46530.1
TGAACCAGGC	0	3	3	0	1	no match found	gi|14334999|gb|AY037179.1	non-canonical match
at4g18120	CON	MIN	HOUR	DAY	WEEK	UNIGENE	FLCDNA	TAIR
TGAATTGATG	3	4	3	2	2	gnl|UG|At#S15644881	gi|30794017|gb|BT008640.1	At4g18120.1
at1g13820	CON	MIN	HOUR	DAY	WEEK	UNIGENE	FLCDNA	TAIR
TCGAAAAGCC	0	0	0	2	0	no match found	gi|48310455|gb|BT014841.1	non-canonical match
GTTTGGTTGG	2	2	2	2	1	gnl|UG|At#S11741653	no match found	At1g13820.1
at1g53500	CON	MIN	HOUR	DAY	WEEK	UNIGENE	FLCDNA	TAIR
ACCGTACTAG	0	1	2	2	1	gnl|UG|At#S11733186	gi|15081768|gb|AY048277.1	At1g53500.1
at2g43870	CON	MIN	HOUR	DAY	WEEK	UNIGENE	FLCDNA	TAIR
AGCCTGTGGC	1	0	0	0	0	no match found	no match found	At2g43870.1
at1g31550	CON	MIN	HOUR	DAY	WEEK	UNIGENE	FLCDNA	TAIR
TCTCAGCTCA	1	0	1	0	0	gnl|UG|At#S24442552	gi|62320569|dbj|AK221611.1|	At1g31550.1
at3g12070	CON	MIN	HOUR	DAY	WEEK	UNIGENE	FLCDNA	TAIR
GCACTACTCT	0	0	1	0	0	gnl|UG|At#S11737272	gi|21403303|gb|AY084593.1	At3g12070.1
at1g22310	CON	MIN	HOUR	DAY	WEEK	UNIGENE	FLCDNA	TAIR
GAGAATTTGG	0	0	1	0	0	no match found	gi|22136681|gb|AY133726.1	multiple non-canonical match
at3g48430	CON	MIN	HOUR	DAY	WEEK	UNIGENE	FLCDNA	TAIR
AAGAAGATTA	0	1	0	1	1	gnl|UG|At#S11816077	no match found	multiple canonical match
at1g18680	CON	MIN	HOUR	DAY	WEEK	UNIGENE	FLCDNA	TAIR
TCTTTGTAAA	1	0	0	0	0	no match found	gi|28973584|gb|BT005697.1	non-canonical match
CTCATACATT	0	2	1	0	0	gnl|UG|At#S11741183	gi|26451200|dbj|AK118073.1|	At1g18680.1
at5g14970	CON	MIN	HOUR	DAY	WEEK	UNIGENE	FLCDNA	TAIR
CTATTGGGAC	6	1	2	2	0	gnl|UG|At#S11722599	gi|22655277|gb|AY140088.1	At5g14970.1
at3g14870	CON	MIN	HOUR	DAY	WEEK	UNIGENE	FLCDNA	TAIR
TCAACGCCGT	0	0	1	0	0	gnl|UG|At#S14273615	gi|28973372|gb|BT005591.1	At3g14870.1
at5g52850	CON	MIN	HOUR	DAY	WEEK	UNIGENE	FLCDNA	TAIR
AGTTCGTGTC	0	1	0	0	0	gnl|UG|At#S11718432	no match found	non-canonical match
at4g13930	CON	MIN	HOUR	DAY	WEEK	UNIGENE	FLCDNA	TAIR
TCTGAGATGA	4	4	10	10	6	gnl|UG|At#S11725398	gi|13358183|gb|AF324991.2	At4g13930.1
at4g11990	CON	MIN	HOUR	DAY	WEEK	UNIGENE	FLCDNA	TAIR
TTCACAAAAA	2	0	0	0	0	gnl|UG|At#S11725733	gi|66792669|gb|BT023446.1	At4g11990.1
at3g16910	CON	MIN	HOUR	DAY	WEEK	UNIGENE	FLCDNA	TAIR
ACCAGAATAA	1	2	2	0	1	gnl|UG|At#S11735781	gi|19699127|gb|AY090269.1	At3g16910.1
at1g04410	CON	MIN	HOUR	DAY	WEEK	UNIGENE	FLCDNA	TAIR
GTTATCTTTT	10	8	5	4	3	gnl|UG|At#S11742586	no match found	At1g04410.1
AAAAGAAGAA	10	11	17	10	9	no match found	gi|20148468|gb|AY081563.1	multiple non-canonical match
at3g55600	CON	MIN	HOUR	DAY	WEEK	UNIGENE	FLCDNA	TAIR
ATCTGTGTGT	0	1	1	0	0	gnl|UG|At#S11728404	gi|46931289|gb|BT012630.1	At3g55600.1

at5g27330	CON	MIN	HOUR	DAY	WEEK	UNIGENE	FLCDNA	TAIR
TAGTTGATTC	0	0	1	0	1	gnl|UG|At#S11720479	gi|62318762|dbj|AK220701.1|	At5g27330.1
at5g24390	CON	MIN	HOUR	DAY	WEEK	UNIGENE	FLCDNA	TAIR
TAAGTTTGGT	0	0	0	0	1	gnl|UG|At#S21735986	gi|51971574|dbj|AK176689.1|	non-canonical match
at5g49960	CON	MIN	HOUR	DAY	WEEK	UNIGENE	FLCDNA	TAIR
GTACCTTGAG	0	1	0	0	0	gnl|UG|At#S11718720	no match found	non-canonical match
at2g44280	CON	MIN	HOUR	DAY	WEEK	UNIGENE	FLCDNA	TAIR
TGGGCTTGCT	1	0	0	0	0	gnl|UG|At#S11731569	gi|22135823|gb|AY128290.1	At2g44280.1
at2g29290	CON	MIN	HOUR	DAY	WEEK	UNIGENE	FLCDNA	TAIR
CTTGAGCTAG	8	17	12	5	0	no match found	gi|51968391|dbj|AK175125.1|	multiple canonical match
at1g66340	CON	MIN	HOUR	DAY	WEEK	UNIGENE	FLCDNA	TAIR
TATGACATTG	1	2	0	2	0	no match found	gi|110742821|dbj|AK227292.1|	non-canonical match
at4g27745	CON	MIN	HOUR	DAY	WEEK	UNIGENE	FLCDNA	TAIR
ATGAATTCAT	0	1	0	0	0	gnl|UG|At#S18322103	no match found	At4g27740.1
at5g13100	CON	MIN	HOUR	DAY	WEEK	UNIGENE	FLCDNA	TAIR
TTAGGGAGGA	0	2	1	0	1	gnl|UG|At#S11723083	gi|21407929|gb|AY089155.1	At5g13100.1
AGAAAGTCGC	1	1	0	0	1	no match found	gi|56381998|gb|BT020363.1	non-canonical match
at3g13228	CON	MIN	HOUR	DAY	WEEK	UNIGENE	FLCDNA	TAIR
ACTTTGACGA	1	0	0	1	0	no match found	gi|21403384|gb|AY084674.1	At3g13228.1
at2g18250	CON	MIN	HOUR	DAY	WEEK	UNIGENE	FLCDNA	TAIR
TAATTTAATA	0	0	0	0	2	gnl|UG|At#S21735778	gi|51971990|dbj|AK176897.1|	multiple canonical match
at1g12830	CON	MIN	HOUR	DAY	WEEK	UNIGENE	FLCDNA	TAIR
TCAACGACGA	1	0	0	0	0	gnl|UG|At#S11741755	gi|15215675|gb|AY050366.1	At1g12830.1
at3g06550	CON	MIN	HOUR	DAY	WEEK	UNIGENE	FLCDNA	TAIR
CTCACTACTT	0	0	1	0	0	gnl|UG|At#S38433770	gi|110741437|dbj|AK226542.1|	At3g06547.1
at2g17870	CON	MIN	HOUR	DAY	WEEK	UNIGENE	FLCDNA	TAIR
TGTGGTGGTG	0	1	0	0	0	gnl|UG|At#S11738025	gi|17104540|gb|AY062985.1	At2g17870.1
at1g55160	CON	MIN	HOUR	DAY	WEEK	UNIGENE	FLCDNA	TAIR
TTTTGCTTTT	1	4	2	0	0	no match found	gi|13937162|gb|AF372935.1	At1g55160.1
at2g17990	CON	MIN	HOUR	DAY	WEEK	UNIGENE	FLCDNA	TAIR
CGGTGTGGAA	0	0	1	0	0	gnl|UG|At#S11737990	gi|56121927|gb|BT020251.1	At2g17990.1
at2g43650	CON	MIN	HOUR	DAY	WEEK	UNIGENE	FLCDNA	TAIR
CTTCGTTTTG	0	1	0	3	1	gnl|UG|At#S15460422	gi|20466445|gb|AY099689.1	At2g43650.1
TTGAAGAGAT	0	0	0	1	0	no match found	gi|110741705|dbj|AK226692.1|	At5g60690.1
at5g24420	CON	MIN	HOUR	DAY	WEEK	UNIGENE	FLCDNA	TAIR
AAGCACAACA	1	0	0	0	0	no match found	gi|14190402|gb|AF378879.1	non-canonical match
AATGGTGTTT	1	0	0	0	0	no match found	gi|21403913|gb|AY085203.1	multiple non-canonical match
at1g50140	CON	MIN	HOUR	DAY	WEEK	UNIGENE	FLCDNA	TAIR
GCAGCTTGGG	0	2	0	1	0	no match found	no match found	At1g50140.1
at2g01505	CON	MIN	HOUR	DAY	WEEK	UNIGENE	FLCDNA	TAIR
GAAGCTTGTT	0	1	0	0	0	gnl|UG|At#S18900402	no match found	At2g36320.1

at1g53460	CON	MIN	HOUR	DAY	WEEK	UNIGENE	FLCDNA	TAIR
AGTTAACATT	1	0	0	0	0	gnl|UG|At#S11813835	gi|15450676|gb|AY052706.1	At1g53460.1
at3g07940	CON	MIN	HOUR	DAY	WEEK	UNIGENE	FLCDNA	TAIR
GGAGAGGCAG	0	0	0	1	0	no match found	gi|25083804|gb|BT002109.1	non-canonical match
at4g24340	CON	MIN	HOUR	DAY	WEEK	UNIGENE	FLCDNA	TAIR
ACTTGCGTGT	0	1	0	0	0	gnl|UG|At#S11723587	gi|28950908|gb|BT005314.1	At4g24340.1
at2g17140	CON	MIN	HOUR	DAY	WEEK	UNIGENE	FLCDNA	TAIR
GCTGGACAAA	0	2	1	0	0	no match found	gi|110737728|dbj|AK228914.1|	multiple canonical match
at3g52500	CON	MIN	HOUR	DAY	WEEK	UNIGENE	FLCDNA	TAIR
GAGCGACCCG	1	11	2	1	1	gnl|UG|At#S34116761	gi|13430561|gb|AF360193.1	At3g52500.1
ATGATACAGA	1	0	0	0	0	no match found	gi|110738958|dbj|AK229547.1|	non-canonical match
at5g27200	CON	MIN	HOUR	DAY	WEEK	UNIGENE	FLCDNA	TAIR
ACTTCTAGGA	0	0	0	0	1	no match found	no match found	At5g27200.1
at5g45680	CON	MIN	HOUR	DAY	WEEK	UNIGENE	FLCDNA	TAIR
CCTTATTCCT	3	1	2	0	1	gnl|UG|At#S11719160	gi|18086456|gb|AY065047.1	At5g45680.1
at2g39795	CON	MIN	HOUR	DAY	WEEK	UNIGENE	FLCDNA	TAIR
GAACAGTGAA	1	0	1	0	1	gnl|UG|At#S11732653	gi|21406658|gb|AY087884.1	At2g39795.1
at5g16160	CON	MIN	HOUR	DAY	WEEK	UNIGENE	FLCDNA	TAIR
AAAAGATTTC	0	2	1	0	0	gnl|UG|At#S30642243	gi|51970649|dbj|AK176254.1|	At5g16160.1
at2g31310	CON	MIN	HOUR	DAY	WEEK	UNIGENE	FLCDNA	TAIR
GATCATAATG	0	0	0	1	0	gnl|UG|At#S11734708	no match found	multiple non-canonical match
at1g69460	CON	MIN	HOUR	DAY	WEEK	UNIGENE	FLCDNA	TAIR
AAGAGATGTA	0	3	0	0	0	gnl|UG|At#S11729214	gi|18252234|gb|AY072343.1	At1g69460.1
at5g56140	CON	MIN	HOUR	DAY	WEEK	UNIGENE	FLCDNA	TAIR
ATGATGGTTT	6	3	3	8	3	no match found	no match found	At5g56140.1
ATATGTACAA	0	1	0	0	0	no match found	gi|24030183|gb|BT000873.1	non-canonical match
at2g21870	CON	MIN	HOUR	DAY	WEEK	UNIGENE	FLCDNA	TAIR
GCCAAAGCAC	1	2	5	2	2	no match found	gi|19310624|gb|AY079312.1	multiple non-canonical match
TTTTTTTGAT	15	18	5	8	12	gnl|UG|At#S11737001	gi|21405831|gb|AY087107.1	At2g21870.1
at1g73570	CON	MIN	HOUR	DAY	WEEK	UNIGENE	FLCDNA	TAIR
GAGAAGGTCT	0	2	0	0	0	gnl|UG|At#S11728341	no match found	non-canonical match
at4g29440	CON	MIN	HOUR	DAY	WEEK	UNIGENE	FLCDNA	TAIR
TTTGGCTTTC	0	0	0	1	0	gnl|UG|At#S34116410	gi|110739635|dbj|AK229898.1|	At4g29440.1
at1g29785	CON	MIN	HOUR	DAY	WEEK	UNIGENE	FLCDNA	TAIR
TTTTGGATCG	0	1	1	0	0	no match found	gi|72197818|gb|DQ108784.1	At4g29440.1
at2g45250	CON	MIN	HOUR	DAY	WEEK	UNIGENE	FLCDNA	TAIR
TGGTGGAAAG	3	1	0	0	0	gnl|UG|At#S11731356	gi|110738729|dbj|AK229429.1|	At2g45250.1
at3g16240	CON	MIN	HOUR	DAY	WEEK	UNIGENE	FLCDNA	TAIR
TGAACCCAGC	0	0	1	0	0	gnl|UG|At#S34115021	no match found	non-canonical match
TTTCTGTGTT	91	60	40	20	2	gnl|UG|At#S23657995	no match found	At3g16240.1
at5g66985	CON	MIN	HOUR	DAY	WEEK	UNIGENE	FLCDNA	TAIR
ATGAATTGAA	0	1	0	0	0	gnl|UG|At#S30650636	gi|21406997|gb|AY088223.1	multiple canonical match

<table>
<tr><td>at1g12210</td><td>CON</td><td>MIN</td><td>HOUR</td><td>DAY</td><td>WEEK</td><td>UNIGENE</td><td>FLCDNA</td><td>TAIR</td></tr>
<tr><td>GAAAAAAAAT</td><td>0</td><td>0</td><td>1</td><td>0</td><td>0</td><td>gnl|UG|At#S11741815</td><td>no match found</td><td>multiple non-canonical match</td></tr>
<tr><td>ACCCAAAAAA</td><td>0</td><td>5</td><td>2</td><td>4</td><td>1</td><td>no match found</td><td>no match found</td><td>At1g12210.1</td></tr>
<tr><td>at2g43810</td><td>CON</td><td>MIN</td><td>HOUR</td><td>DAY</td><td>WEEK</td><td>UNIGENE</td><td>FLCDNA</td><td>TAIR</td></tr>
<tr><td>TCTTTTTCTT</td><td>3</td><td>1</td><td>0</td><td>1</td><td>2</td><td>no match found</td><td>gi|110735707|dbj|AK227857.1|</td><td>At2g43810.1</td></tr>
<tr><td>at2g19900</td><td>CON</td><td>MIN</td><td>HOUR</td><td>DAY</td><td>WEEK</td><td>UNIGENE</td><td>FLCDNA</td><td>TAIR</td></tr>
<tr><td>CCAAGATCTC</td><td>0</td><td>0</td><td>0</td><td>0</td><td>1</td><td>gnl|UG|At#S11737494</td><td>gi|17065315|gb|AY062734.1</td><td>At2g19900.1</td></tr>
<tr><td>at4g04955</td><td>CON</td><td>MIN</td><td>HOUR</td><td>DAY</td><td>WEEK</td><td>UNIGENE</td><td>FLCDNA</td><td>TAIR</td></tr>
<tr><td>TATATATTGT</td><td>0</td><td>0</td><td>0</td><td>0</td><td>1</td><td>gnl|UG|At#S11726395</td><td>no match found</td><td>At4g04955.1</td></tr>
<tr><td>CTTCTGATGC</td><td>1</td><td>0</td><td>1</td><td>0</td><td>0</td><td>no match found</td><td>gi|21281138|gb|AY113948.1</td><td>multiple canonical match</td></tr>
<tr><td>at1g73610</td><td>CON</td><td>MIN</td><td>HOUR</td><td>DAY</td><td>WEEK</td><td>UNIGENE</td><td>FLCDNA</td><td>TAIR</td></tr>
<tr><td>TCAAAAAAAA</td><td>21</td><td>32</td><td>61</td><td>30</td><td>28</td><td>no match found</td><td>no match found</td><td>At1g73610.1</td></tr>
<tr><td>at4g15093</td><td>CON</td><td>MIN</td><td>HOUR</td><td>DAY</td><td>WEEK</td><td>UNIGENE</td><td>FLCDNA</td><td>TAIR</td></tr>
<tr><td>TGAATTTCAA</td><td>1</td><td>1</td><td>0</td><td>1</td><td>1</td><td>gnl|UG|At#S11725187</td><td>gi|15293028|gb|AY050948.1</td><td>At4g15093.1</td></tr>
<tr><td>at5g65210</td><td>CON</td><td>MIN</td><td>HOUR</td><td>DAY</td><td>WEEK</td><td>UNIGENE</td><td>FLCDNA</td><td>TAIR</td></tr>
<tr><td>TATTGATAAG</td><td>0</td><td>0</td><td>0</td><td>0</td><td>1</td><td>gnl|UG|At#S38433087</td><td>gi|20466253|gb|AY099593.1</td><td>At5g65210.1</td></tr>
<tr><td>at1g24090</td><td>CON</td><td>MIN</td><td>HOUR</td><td>DAY</td><td>WEEK</td><td>UNIGENE</td><td>FLCDNA</td><td>TAIR</td></tr>
<tr><td>GAGGTGTGAA</td><td>0</td><td>1</td><td>0</td><td>0</td><td>0</td><td>gnl|UG|At#S11740606</td><td>no match found</td><td>At1g24090.1</td></tr>
<tr><td>at5g46790</td><td>CON</td><td>MIN</td><td>HOUR</td><td>DAY</td><td>WEEK</td><td>UNIGENE</td><td>FLCDNA</td><td>TAIR</td></tr>
<tr><td>CCTTCCGATT</td><td>0</td><td>1</td><td>1</td><td>0</td><td>0</td><td>gnl|UG|At#S11719046</td><td>gi|17380841|gb|AY063877.1</td><td>At5g46790.1</td></tr>
<tr><td>at5g16280</td><td>CON</td><td>MIN</td><td>HOUR</td><td>DAY</td><td>WEEK</td><td>UNIGENE</td><td>FLCDNA</td><td>TAIR</td></tr>
<tr><td>AGAATTGTTC</td><td>0</td><td>1</td><td>0</td><td>1</td><td>0</td><td>gnl|UG|At#S11722271</td><td>no match found</td><td>At5g16280.1</td></tr>
<tr><td>at4g35080</td><td>CON</td><td>MIN</td><td>HOUR</td><td>DAY</td><td>WEEK</td><td>UNIGENE</td><td>FLCDNA</td><td>TAIR</td></tr>
<tr><td>TAACCATACT</td><td>3</td><td>2</td><td>0</td><td>2</td><td>2</td><td>gnl|UG|At#S11721751</td><td>gi|15146229|gb|AY049256.1</td><td>At4g35080.1</td></tr>
<tr><td>GTATCAACAG</td><td>0</td><td>0</td><td>0</td><td>1</td><td>0</td><td>no match found</td><td>gi|19699141|gb|AY090276.1</td><td>non-canonical match</td></tr>
<tr><td>at3g12260</td><td>CON</td><td>MIN</td><td>HOUR</td><td>DAY</td><td>WEEK</td><td>UNIGENE</td><td>FLCDNA</td><td>TAIR</td></tr>
<tr><td>GACATTTACA</td><td>0</td><td>5</td><td>0</td><td>1</td><td>1</td><td>gnl|UG|At#S11737208</td><td>gi|15450991|gb|AY054576.1</td><td>At3g12260.1</td></tr>
<tr><td>at4g25130</td><td>CON</td><td>MIN</td><td>HOUR</td><td>DAY</td><td>WEEK</td><td>UNIGENE</td><td>FLCDNA</td><td>TAIR</td></tr>
<tr><td>AAAAATACAA</td><td>5</td><td>3</td><td>2</td><td>2</td><td>4</td><td>gnl|UG|At#S11723454</td><td>gi|21406288|gb|AY087550.1</td><td>At4g25130.1</td></tr>
<tr><td>at1g68530</td><td>CON</td><td>MIN</td><td>HOUR</td><td>DAY</td><td>WEEK</td><td>UNIGENE</td><td>FLCDNA</td><td>TAIR</td></tr>
<tr><td>GTCCGATTGT</td><td>8</td><td>9</td><td>3</td><td>5</td><td>1</td><td>gnl|UG|At#S15439854</td><td>gi|62319437|dbj|AK221042.1|</td><td>At1g68530.1</td></tr>
<tr><td>at2g01640</td><td>CON</td><td>MIN</td><td>HOUR</td><td>DAY</td><td>WEEK</td><td>UNIGENE</td><td>FLCDNA</td><td>TAIR</td></tr>
<tr><td>ATTTTGGAAA</td><td>0</td><td>1</td><td>1</td><td>1</td><td>1</td><td>gnl|UG|At#S11742639</td><td>gi|26451705|dbj|AK118334.1|</td><td>At2g01640.1</td></tr>
<tr><td>at5g66540</td><td>CON</td><td>MIN</td><td>HOUR</td><td>DAY</td><td>WEEK</td><td>UNIGENE</td><td>FLCDNA</td><td>TAIR</td></tr>
<tr><td>TTGAAGAAAG</td><td>0</td><td>0</td><td>2</td><td>0</td><td>0</td><td>gnl|UG|At#S11717043</td><td>gi|14488062|gb|AF389279.1</td><td>At5g66540.1</td></tr>
<tr><td>at1g16170</td><td>CON</td><td>MIN</td><td>HOUR</td><td>DAY</td><td>WEEK</td><td>UNIGENE</td><td>FLCDNA</td><td>TAIR</td></tr>
<tr><td>GTGAAATAAA</td><td>2</td><td>3</td><td>0</td><td>1</td><td>0</td><td>gnl|UG|At#S11741421</td><td>gi|21405894|gb|AY087170.1</td><td>At1g16170.1</td></tr>
<tr><td>at2g32730</td><td>CON</td><td>MIN</td><td>HOUR</td><td>DAY</td><td>WEEK</td><td>UNIGENE</td><td>FLCDNA</td><td>TAIR</td></tr>
<tr><td>TTCAACTACG</td><td>3</td><td>2</td><td>2</td><td>3</td><td>7</td><td>gnl|UG|At#S11734352</td><td>gi|110737044|dbj|AK228556.1|</td><td>At2g32730.1</td></tr>
<tr><td>at3g58820</td><td>CON</td><td>MIN</td><td>HOUR</td><td>DAY</td><td>WEEK</td><td>UNIGENE</td><td>FLCDNA</td><td>TAIR</td></tr>
<tr><td>ATGCATTTGG</td><td>0</td><td>0</td><td>0</td><td>0</td><td>1</td><td>no match found</td><td>no match found</td><td>At3g58820.1</td></tr>
<tr><td>at4g37000</td><td>CON</td><td>MIN</td><td>HOUR</td><td>DAY</td><td>WEEK</td><td>UNIGENE</td><td>FLCDNA</td><td>TAIR</td></tr>
</table>

TTAGTCCAGC	1	1	1	1	2	gnl|UG|At#S11721437	gi|20147382|gb|AY093785.1	At4g37000.1
at2g28056	CON	MIN	HOUR	DAY	WEEK	UNIGENE	FLCDNA	TAIR
ATGATATCTT	0	0	0	0	3	no match found	gi|26452427|dbj|AK118705.1|	At2g28056.1
at5g16715	CON	MIN	HOUR	DAY	WEEK	UNIGENE	FLCDNA	TAIR
GACCATTTAA	0	0	2	0	1	gnl|UG|At#S11722164	no match found	At5g16715.1
at1g04640	CON	MIN	HOUR	DAY	WEEK	UNIGENE	FLCDNA	TAIR
AACAGTTGGT	1	1	0	3	1	gnl|UG|At#S28282717	gi|22137225|gb|AY133628.1	At1g04640.1
at5g20650	CON	MIN	HOUR	DAY	WEEK	UNIGENE	FLCDNA	TAIR
TGCTTGATAA	2	3	5	2	0	gnl|UG|At#S11721165	gi|14517485|gb|AY039578.1	At5g20650.1
at4g03140	CON	MIN	HOUR	DAY	WEEK	UNIGENE	FLCDNA	TAIR
GAAGCTTTCT	0	0	1	1	1	gnl|UG|At#S11726898	gi|62318976|dbj|AK220809.1|	multiple non-canonical match
GATCGACGAT	0	0	1	0	0	gnl|UG|At#S18906191	no match found	non-canonical match
at5g25490	CON	MIN	HOUR	DAY	WEEK	UNIGENE	FLCDNA	TAIR
TCGAGAGTTT	0	0	1	0	0	gnl|UG|At#S11720632	gi|26452847|dbj|AK118921.1|	At5g25490.1
at3g11780	CON	MIN	HOUR	DAY	WEEK	UNIGENE	FLCDNA	TAIR
AATCCCCCCA	3	3	7	4	1	no match found	no match found	At3g11780.1
GAACCACTCT	8	3	12	4	2	no match found	gi|21405128|gb|AY086418.1	non-canonical match
at5g05250	CON	MIN	HOUR	DAY	WEEK	UNIGENE	FLCDNA	TAIR
TAAATAAATG	3	0	0	0	0	gnl|UG|At#S11706203	gi|15810044|gb|AY054291.1	At5g05250.1
at5g26000	CON	MIN	HOUR	DAY	WEEK	UNIGENE	FLCDNA	TAIR
TTTCCTCTTT	77	55	45	30	22	no match found	gi|15010759|gb|AY045681.1	At5g26000.1
at1g47260	CON	MIN	HOUR	DAY	WEEK	UNIGENE	FLCDNA	TAIR
TGACAGATTT	0	0	0	1	0	no match found	no match found	At1g47260.1
AGAAAGTTAA	0	5	0	0	1	no match found	gi|15028352|gb|AY045979.1	non-canonical match
at3g25740	CON	MIN	HOUR	DAY	WEEK	UNIGENE	FLCDNA	TAIR
AATTATATCA	2	1	0	0	0	gnl|UG|At#S24442619	gi|62320435|dbj|AK221544.1|	At3g25740.1
at5g08180	CON	MIN	HOUR	DAY	WEEK	UNIGENE	FLCDNA	TAIR
AATCCTATGG	2	1	0	4	10	gnl|UG|At#S11724068	gi|14190414|gb|AF378885.1	At5g08180.1
TTGTGTCTTG	0	0	0	0	1	no match found	gi|15215892|gb|AY050477.1	multiple non-canonical match
at2g30060	CON	MIN	HOUR	DAY	WEEK	UNIGENE	FLCDNA	TAIR
GATGATTCGC	5	4	4	3	0	gnl|UG|At#S11735021	gi|20260399|gb|AY093099.1	At2g30060.1
at1g74456	CON	MIN	HOUR	DAY	WEEK	UNIGENE	FLCDNA	TAIR
ATGCCTTTTC	3	2	0	2	1	gnl|UG|At#S11831821	gi|20259370|gb|AY090970.1	non-canonical match
at1g11670	CON	MIN	HOUR	DAY	WEEK	UNIGENE	FLCDNA	TAIR
CAAACTATTA	0	0	1	0	0	gnl|UG|At#S11741869	gi|22655138|gb|AY140018.1	At1g11670.1
at3g53410	CON	MIN	HOUR	DAY	WEEK	UNIGENE	FLCDNA	TAIR
CTACTTTGTC	0	0	1	0	0	gnl|UG|At#S11728801	gi|21403226|gb|AY084516.1	At3g53410.1
at3g28530	CON	MIN	HOUR	DAY	WEEK	UNIGENE	FLCDNA	TAIR
AAAAAAGAGA	0	3	0	1	2	no match found	no match found	At3g28530.1
at5g53370	CON	MIN	HOUR	DAY	WEEK	UNIGENE	FLCDNA	TAIR
TTATTACCTC	0	0	1	0	0	gnl|UG|At#S11718379	gi|13507548|gb|AF360340.1	At5g53370.1
at1g45249	CON	MIN	HOUR	DAY	WEEK	UNIGENE	FLCDNA	TAIR

GAAATGCAAA	1	1	1	0	0	no match found	gi|111074353|gb|BT026443.1	At1g45249.2
at1g30440	CON	MIN	HOUR	DAY	WEEK	UNIGENE	FLCDNA	TAIR
TTTCTTTTTT	1	3	1	3	1	gnl|UG|At#S11738871	no match found	multiple canonical match
at3g25640	CON	MIN	HOUR	DAY	WEEK	UNIGENE	FLCDNA	TAIR
ATGAACCCTG	0	1	0	0	0	gnl|UG|At#S11825194	gi|50253583|gb|BT015122.1	At3g25640.1
at3g22980	CON	MIN	HOUR	DAY	WEEK	UNIGENE	FLCDNA	TAIR
TTGGAGAAAT	0	3	5	0	1	gnl|UG|At#S11733914	no match found	At3g22980.1
at5g10600	CON	MIN	HOUR	DAY	WEEK	UNIGENE	FLCDNA	TAIR
CCCAGAAATG	0	1	0	0	0	no match found	gi|110737419|dbj|AK228754.1|	At5g10600.1
at4g02600	CON	MIN	HOUR	DAY	WEEK	UNIGENE	FLCDNA	TAIR
GATATGTTTC	2	2	2	1	2	gnl|UG|At#S11727040	gi|18175952|gb|AY072135.1	multiple canonical match
at2g20690	CON	MIN	HOUR	DAY	WEEK	UNIGENE	FLCDNA	TAIR
GGGAAGTATG	0	1	0	1	0	gnl|UG|At#S11737292	gi|19548076|gb|AY081833.1	At2g20690.1
at1g27190	CON	MIN	HOUR	DAY	WEEK	UNIGENE	FLCDNA	TAIR
GCAGATAAAC	0	3	0	0	0	gnl|UG|At#S11739918	gi|110737780|dbj|AK228940.1|	At1g27190.1
at2g43510	CON	MIN	HOUR	DAY	WEEK	UNIGENE	FLCDNA	TAIR
GTTTGTGTAA	2	5	0	1	1	gnl|UG|At#S11792323	no match found	multiple canonical match
at4g16760	CON	MIN	HOUR	DAY	WEEK	UNIGENE	FLCDNA	TAIR
AAGACAGTCG	2	5	0	2	1	gnl|UG|At#S11724895	gi|24111394|gb|BT001067.1	At4g16760.1
at1g43190	CON	MIN	HOUR	DAY	WEEK	UNIGENE	FLCDNA	TAIR
GGCTTAACAT	0	0	3	0	0	gnl|UG|At#S34117581	gi|110737367|dbj|AK228727.1|	At1g43190.1
TCCAAGAACA	1	1	0	0	0	no match found	gi|50198951|gb|BT015028.1	non-canonical match
at3g56660	CON	MIN	HOUR	DAY	WEEK	UNIGENE	FLCDNA	TAIR
CTGAGCATAA	1	1	0	1	0	no match found	no match found	At3g56660.1
at3g09800	CON	MIN	HOUR	DAY	WEEK	UNIGENE	FLCDNA	TAIR
GACTTCAATT	0	0	0	1	0	no match found	gi|27754541|gb|BT002902.1	At3g09800.1
at2g20750	CON	MIN	HOUR	DAY	WEEK	UNIGENE	FLCDNA	TAIR
TGAACGCAGG	0	0	1	0	0	gnl|UG|At#S11737274	gi|28827425|gb|BT005024.1	At2g20750.1
at2g29995	CON	MIN	HOUR	DAY	WEEK	UNIGENE	FLCDNA	TAIR
ATCCACCCCG	1	0	0	0	0	no match found	gi|88010830|gb|BT024520.1	non-canonical match
ACAGAAAACG	0	0	1	0	0	gnl|UG|At#S35297354	no match found	non-canonical match
TAATTTTATG	1	0	0	1	0	gnl|UG|At#S30651296	no match found	pseudo chromosome match
at1g54010	CON	MIN	HOUR	DAY	WEEK	UNIGENE	FLCDNA	TAIR
TTTCTCTAGT	12	9	1	3	0	gnl|UG|At#S11732992	gi|17064951|gb|AY062552.1	At1g54010.1
at4g02200	CON	MIN	HOUR	DAY	WEEK	UNIGENE	FLCDNA	TAIR
GTTTGGGTTT	0	1	0	0	0	gnl|UG|At#S11727122	no match found	At4g02200.1
GAAGACAATA	0	1	0	1	0	no match found	gi|90568011|gb|BT024920.1	multiple non-canonical match
at5g18180	CON	MIN	HOUR	DAY	WEEK	UNIGENE	FLCDNA	TAIR
CTTGTGAGGG	1	0	0	0	0	gnl|UG|At#S11721779	no match found	multiple non-canonical match
at3g21100	CON	MIN	HOUR	DAY	WEEK	UNIGENE	FLCDNA	TAIR
GGAGATCAAA	0	0	2	1	0	no match found	no match found	At3g21100.1
at5g56600	CON	MIN	HOUR	DAY	WEEK	UNIGENE	FLCDNA	TAIR

GTAATCCAAG	2	3	3	1	1	gnl|UG|At#S43849100	gi|21402995|gb|AY084285.1	At5g56600.1
at1g09340	CON	MIN	HOUR	DAY	WEEK	UNIGENE	FLCDNA	TAIR
ATTCTGAGCA	22	20	25	18	7	gnl|UG|At#S11742104	gi|16226246|gb|AF428282.1	At1g09340.1
at4g39580	CON	MIN	HOUR	DAY	WEEK	UNIGENE	FLCDNA	TAIR
ATGATTTGGA	0	0	1	2	0	no match found	no match found	At4g39580.1
at4g23230	CON	MIN	HOUR	DAY	WEEK	UNIGENE	FLCDNA	TAIR
GTGGTTAACC	1	0	0	0	0	gnl|UG|At#S11723785	gi|17064985|gb|AY062569.1	At4g23230.1
at5g13490	CON	MIN	HOUR	DAY	WEEK	UNIGENE	FLCDNA	TAIR
ATGGGTGGTG	0	0	0	1	0	gnl|UG|At#S11698549	gi|15292846|gb|AY050857.1	At5g13490.1
at5g20510	CON	MIN	HOUR	DAY	WEEK	UNIGENE	FLCDNA	TAIR
ATCAAGCTGC	0	0	0	1	1	no match found	no match found	At5g20510.1
TCCAGATTTG	0	2	1	0	1	gnl|UG|At#S11721199	no match found	non-canonical match
at4g23400	CON	MIN	HOUR	DAY	WEEK	UNIGENE	FLCDNA	TAIR
TGTGATGATC	9	22	17	7	2	no match found	gi|62319898|dbj|AK221275.1|	At4g23400.1
at1g69850	CON	MIN	HOUR	DAY	WEEK	UNIGENE	FLCDNA	TAIR
AACACAAAGA	0	2	0	0	0	gnl|UG|At#S11729134	gi|23397157|gb|BT000720.1	At1g69850.1
at2g33370	CON	MIN	HOUR	DAY	WEEK	UNIGENE	FLCDNA	TAIR
TACTTTGAAG	4	7	1	3	1	gnl|UG|At#S11734195	gi|98961078|gb|BT025605.1	At2g33370.1
at2g11140	CON	MIN	HOUR	DAY	WEEK	UNIGENE	FLCDNA	TAIR
GAAATAGTAG	0	0	1	0	0	gnl|UG|At#S11814371	gi|17065313|gb|AY062733.1	At2g11140.1
at4g20020	CON	MIN	HOUR	DAY	WEEK	UNIGENE	FLCDNA	TAIR
CTGCAGTGAA	0	0	2	2	1	gnl|UG|At#S18941833	gi|19310563|gb|AY080648.1	At4g20020.2
ACTGAACAAG	0	0	0	0	1	no match found	no match found	At4g20020.1
at1g11410	CON	MIN	HOUR	DAY	WEEK	UNIGENE	FLCDNA	TAIR
TTAGGTCATA	0	1	1	0	0	gnl|UG|At#S11741894	no match found	At1g11410.1
at3g50570	CON	MIN	HOUR	DAY	WEEK	UNIGENE	FLCDNA	TAIR
GACATATCTT	1	3	0	2	0	gnl|UG|At#S11823856	gi|26451006|dbj|AK117973.1|	At3g50570.1
at3g05380	CON	MIN	HOUR	DAY	WEEK	UNIGENE	FLCDNA	TAIR
TCCCTTGTAC	0	0	0	0	1	gnl|UG|At#S43849966	gi|19347723|gb|AY080604.1	At3g05380.1
at2g25830	CON	MIN	HOUR	DAY	WEEK	UNIGENE	FLCDNA	TAIR
GAATTGAACA	0	1	0	2	0	gnl|UG|At#S11736069	gi|15810382|gb|AY056230.1	At2g25830.1
at1g79050	CON	MIN	HOUR	DAY	WEEK	UNIGENE	FLCDNA	TAIR
TTCACCTCTT	3	2	1	5	3	gnl|UG|At#S11726829	gi|15292902|gb|AY050885.1	At1g79050.1
at3g49710	CON	MIN	HOUR	DAY	WEEK	UNIGENE	FLCDNA	TAIR
TGGGGATTAT	2	0	0	1	0	gnl|UG|At#S11729498	no match found	non-canonical match
at2g26690	CON	MIN	HOUR	DAY	WEEK	UNIGENE	FLCDNA	TAIR
CAAGATGAAG	2	4	4	1	0	no match found	gi|20466667|gb|AY099800.1	At2g26690.1
at5g66820	CON	MIN	HOUR	DAY	WEEK	UNIGENE	FLCDNA	TAIR
GAGTCGAGAG	1	2	1	1	0	gnl|UG|At#S18913055	no match found	At5g66820.1
at1g78830	CON	MIN	HOUR	DAY	WEEK	UNIGENE	FLCDNA	TAIR
TGAAGCATTA	12	8	11	6	6	gnl|UG|At#S11726918	no match found	At1g78830.1

at5g01410	CON	MIN	HOUR	DAY	WEEK	UNIGENE	FLCDNA	TAIR
GTTGGGATCA	4	3	5	18	2	gnl|UG|At#S11809329	gi|110742647|dbj|AK227197.1|	At5g01410.1
at5g53045	CON	MIN	HOUR	DAY	WEEK	UNIGENE	FLCDNA	TAIR
CTTTGATTGC	0	1	0	0	0	gnl|UG|At#S30457176	gi|26452832|dbj|AK118913.1|	At5g53045.1
at3g28950	CON	MIN	HOUR	DAY	WEEK	UNIGENE	FLCDNA	TAIR
GAGGAGGCTG	3	4	5	3	5	no match found	gi|110735960|dbj|AK227989.1|	multiple canonical match
at5g54890	CON	MIN	HOUR	DAY	WEEK	UNIGENE	FLCDNA	TAIR
TCTCAGAAGT	1	0	0	0	1	gnl|UG|At#S11718224	no match found	At5g54890.1
at5g58640	CON	MIN	HOUR	DAY	WEEK	UNIGENE	FLCDNA	TAIR
TGCCAAAAGT	3	6	1	4	4	gnl|UG|At#S18909935	gi|17933296|gb|AF446358.1	At5g58640.1
at4g39690	CON	MIN	HOUR	DAY	WEEK	UNIGENE	FLCDNA	TAIR
TTATCTGAAT	1	1	2	1	1	no match found	gi|23397146|gb|BT000714.1	non-canonical match
at1g69730	CON	MIN	HOUR	DAY	WEEK	UNIGENE	FLCDNA	TAIR
AGGGCAGAAT	0	2	0	1	0	gnl|UG|At#S11729156	no match found	At1g69730.1
at4g26270	CON	MIN	HOUR	DAY	WEEK	UNIGENE	FLCDNA	TAIR
ATGCTTTAGT	0	1	0	0	0	no match found	gi|15146207|gb|AY049245.1	non-canonical match
at1g65190	CON	MIN	HOUR	DAY	WEEK	UNIGENE	FLCDNA	TAIR
TTCGTATTAA	1	1	0	0	0	gnl|UG|At#S11730091	gi|14334553|gb|AY035181.1	At1g65190.1
at1g47290	CON	MIN	HOUR	DAY	WEEK	UNIGENE	FLCDNA	TAIR
ACTCCTGCCT	1	0	0	0	0	gnl|UG|At#S15459254	gi|26450043|dbj|AK117478.1|	At1g47290.1
at2g22120	CON	MIN	HOUR	DAY	WEEK	UNIGENE	FLCDNA	TAIR
TCCAGCAGCT	0	2	0	0	0	gnl|UG|At#S11736944	gi|28827341|gb|BT004982.1	At2g22120.1
at5g26230	CON	MIN	HOUR	DAY	WEEK	UNIGENE	FLCDNA	TAIR
TCTTAATTTT	0	2	0	1	0	no match found	no match found	At5g26230.1
at3g61630	CON	MIN	HOUR	DAY	WEEK	UNIGENE	FLCDNA	TAIR
GGATGTGGAT	0	1	1	0	0	gnl|UG|At#S11726962	gi|21407712|gb|AY088938.1	At3g61630.1
at5g15320	CON	MIN	HOUR	DAY	WEEK	UNIGENE	FLCDNA	TAIR
AAGCTCAAGG	0	0	1	0	0	no match found	gi|88010804|gb|BT024516.1	non-canonical match
ATTCATCCTT	2	3	0	0	1	no match found	gi|26451597|dbj|AK118278.1|	At5g15320.1
at1g16570	CON	MIN	HOUR	DAY	WEEK	UNIGENE	FLCDNA	TAIR
AGTCTGTGTA	0	0	1	0	1	gnl|UG|At#S43850629	gi|22135999|gb|AY128379.1	At1g16570.1
at5g03740	CON	MIN	HOUR	DAY	WEEK	UNIGENE	FLCDNA	TAIR
TTGCAACTCC	0	0	1	0	0	no match found	gi|22655421|gb|AY142039.1	multiple non-canonical match
ATGACTACTC	0	2	1	0	0	no match found	gi|13926267|gb|AF372889.1	At5g03740.1
at2g04550	CON	MIN	HOUR	DAY	WEEK	UNIGENE	FLCDNA	TAIR
AGATTGGAAG	0	6	1	1	1	gnl|UG|At#S11740272	no match found	At2g04550.2
GAGATTGGTG	1	0	1	0	0	no match found	gi|28393744|gb|BT004283.1	At2g04550.3
at3g10230	CON	MIN	HOUR	DAY	WEEK	UNIGENE	FLCDNA	TAIR
ACAAAGGGGA	0	0	2	0	0	gnl|UG|At#S11707980	gi|16604608|gb|AY059749.1	At3g10230.1
at5g44316	CON	MIN	HOUR	DAY	WEEK	UNIGENE	FLCDNA	TAIR
AGAGAGCATT	1	1	0	1	0	gnl|UG|At#S11816866	no match found	non-canonical match
AACCAACATT	1	1	0	0	0	no match found	no match found	At5g44316.1

at3g54200	CON	MIN	HOUR	DAY	WEEK	UNIGENE	FLCDNA	TAIR
GCTGGCGTCA	1	2	1	0	0	gnl|UG|At#S11728648	gi|110738153|dbj|AK229134.1|	At3g54200.1
at2g15910	CON	MIN	HOUR	DAY	WEEK	UNIGENE	FLCDNA	TAIR
TGTTATTACT	1	0	0	1	0	gnl|UG|At#S11738491	no match found	At2g15910.1
GAATTGATTA	2	7	2	0	0	gnl|UG|At#S35304445	gi|26450810|dbj|AK117873.1|	non-canonical match
at1g08810	CON	MIN	HOUR	DAY	WEEK	UNIGENE	FLCDNA	TAIR
AATAAAATCC	0	0	0	1	0	gnl|UG|At#S11742153	gi|26450025|dbj|AK117469.1|	At1g08810.2
at5g66880	CON	MIN	HOUR	DAY	WEEK	UNIGENE	FLCDNA	TAIR
CACACATATA	1	5	1	0	0	gnl|UG|At#S11717008	gi|20260397|gb|AY093098.1	At5g66880.1
at3g07350	CON	MIN	HOUR	DAY	WEEK	UNIGENE	FLCDNA	TAIR
TGTCGTGCCA	1	5	0	0	1	gnl|UG|At#S18893845	gi|21405146|gb|AY086436.1	At3g07350.1
at5g22460	CON	MIN	HOUR	DAY	WEEK	UNIGENE	FLCDNA	TAIR
AGGTCTTAGG	0	1	2	2	0	gnl|UG|At#S15460548	gi|20465594|gb|AY096630.1	At5g22460.1
at3g07680	CON	MIN	HOUR	DAY	WEEK	UNIGENE	FLCDNA	TAIR
TCTCGTGTCT	2	4	0	0	2	no match found	gi|14532909|gb|AY040079.1	non-canonical match
TTCAAGAACT	1	3	0	2	1	gnl|UG|At#S11738438	no match found	At3g07680.1
at4g33890	CON	MIN	HOUR	DAY	WEEK	UNIGENE	FLCDNA	TAIR
TGTATTTTTC	0	0	0	2	2	gnl|UG|At#S11721956	gi|20260335|gb|AY093067.1	At4g33890.1
at1g08830	CON	MIN	HOUR	DAY	WEEK	UNIGENE	FLCDNA	TAIR
AATAAAAAAG	1	3	3	4	0	gnl|UG|At#S11742151	no match found	At1g08830.1
at1g48550	CON	MIN	HOUR	DAY	WEEK	UNIGENE	FLCDNA	TAIR
TTTCTCATAC	0	0	0	1	0	gnl|UG|At#S11735015	gi|26450433|dbj|AK117679.1|	multiple canonical match
at1g05060	CON	MIN	HOUR	DAY	WEEK	UNIGENE	FLCDNA	TAIR
TGGATAGAGA	0	3	0	1	0	gnl|UG|At#S18893999	gi|21406399|gb|AY087661.1	At1g05060.1
at5g04870	CON	MIN	HOUR	DAY	WEEK	UNIGENE	FLCDNA	TAIR
GGTTCAAGTC	1	0	0	1	0	gnl|UG|At#S11724914	gi|29028975|gb|BT005932.1	At5g04870.1
at5g35480	CON	MIN	HOUR	DAY	WEEK	UNIGENE	FLCDNA	TAIR
TAATTTTATT	0	1	0	0	0	no match found	gi|62320417|dbj|AK221535.1|	multiple canonical match
at3g52290	CON	MIN	HOUR	DAY	WEEK	UNIGENE	FLCDNA	TAIR
GCACCTACAC	0	1	0	0	0	gnl|UG|At#S11825091	gi|27754607|gb|BT002936.1	At3g52290.1
at5g66930	CON	MIN	HOUR	DAY	WEEK	UNIGENE	FLCDNA	TAIR
TTCCTCCCAT	0	1	0	0	1	gnl|UG|At#S18911533	gi|15810344|gb|AY056211.1	At5g66930.1
at4g05020	CON	MIN	HOUR	DAY	WEEK	UNIGENE	FLCDNA	TAIR
GTCTTTTTTT	0	1	0	1	0	gnl|UG|At#S11726384	gi|14532463|gb|AY039856.1	At4g05020.1
at5g13320	CON	MIN	HOUR	DAY	WEEK	UNIGENE	FLCDNA	TAIR
TGTGGTAGCC	0	1	0	0	1	gnl|UG|At#S11723022	no match found	At5g13320.1
at2g25080	CON	MIN	HOUR	DAY	WEEK	UNIGENE	FLCDNA	TAIR
AGTGAGCATA	20	15	17	11	3	gnl|UG|At#S11736251	gi|14334959|gb|AY035153.1	At2g25080.1
at5g47040	CON	MIN	HOUR	DAY	WEEK	UNIGENE	FLCDNA	TAIR
GCGGAACAAC	2	0	1	1	1	gnl|UG|At#S11719020	gi|34098904|gb|BT010392.1	At5g47040.1
at5g20340	CON	MIN	HOUR	DAY	WEEK	UNIGENE	FLCDNA	TAIR
TTTGATGCAA	1	0	0	0	0	gnl|UG|At#S11721243	no match found	At5g20340.1

at1g35220	CON	MIN	HOUR	DAY	WEEK	UNIGENE	FLCDNA	TAIR
AACTACGCAA	0	1	0	0	0	gnl|UG|At#S11737195	gi|26451884|dbj|AK118426.1|	non-canonical match
at1g52960	CON	MIN	HOUR	DAY	WEEK	UNIGENE	FLCDNA	TAIR
GTTTCAGCTT	0	0	0	1	0	gnl|UG|At#S35311172	no match found	multiple non-canonical match
at5g53400	CON	MIN	HOUR	DAY	WEEK	UNIGENE	FLCDNA	TAIR
TCTGAGCATC	1	2	0	0	5	gnl|UG|At#S11718376	gi|21406479|gb|AY087741.1	At5g53400.1
at5g49710	CON	MIN	HOUR	DAY	WEEK	UNIGENE	FLCDNA	TAIR
TATAAAGAAT	0	0	0	0	1	no match found	gi|110738368|dbj|AK229246.1|	At5g49710.3
TGTTTGTAAT	0	0	1	0	0	no match found	gi|21405045|gb|AY086335.1	non-canonical match
at4g30180	CON	MIN	HOUR	DAY	WEEK	UNIGENE	FLCDNA	TAIR
TTTTTTTTGT	0	1	0	0	0	no match found	gi|38454067|gb|BT010671.1	multiple non-canonical match
at3g58730	CON	MIN	HOUR	DAY	WEEK	UNIGENE	FLCDNA	TAIR
TTGTCCTCGA	1	14	0	3	0	no match found	gi|110740939|dbj|AK226421.1|	At3g58730.1
at5g27120	CON	MIN	HOUR	DAY	WEEK	UNIGENE	FLCDNA	TAIR
CATCTGTTGT	1	0	1	1	2	gnl|UG|At#S11720500	gi|25083409|gb|BT002060.1	At5g27120.1
at2g05840	CON	MIN	HOUR	DAY	WEEK	UNIGENE	FLCDNA	TAIR
TAAGGGTGTC	3	4	1	4	6	gnl|UG|At#S11739971	gi|15450622|gb|AY052679.1	At2g05840.1
GTTCTAGGTA	0	0	0	1	0	no match found	gi|23505816|gb|AY143829.1	non-canonical match
at5g57700	CON	MIN	HOUR	DAY	WEEK	UNIGENE	FLCDNA	TAIR
GTGAAGCAGG	1	2	1	0	0	gnl|UG|At#S15450549	gi|21403349|gb|AY084639.1	At5g57700.1
at1g74470	CON	MIN	HOUR	DAY	WEEK	UNIGENE	FLCDNA	TAIR
TAGCTGTTGG	13	31	9	12	1	gnl|UG|At#S11707344	gi|23397034|gb|BT000656.1	At1g74470.1
at2g31820	CON	MIN	HOUR	DAY	WEEK	UNIGENE	FLCDNA	TAIR
GTGGTTGTTT	0	1	0	0	0	no match found	no match found	At2g31820.1
CATAGGATGG	0	0	0	2	1	gnl|UG|At#S11734581	no match found	non-canonical match
at1g56150	CON	MIN	HOUR	DAY	WEEK	UNIGENE	FLCDNA	TAIR
ACCTACACGA	1	3	0	0	0	gnl|UG|At#S11607119	gi|26450660|dbj|AK117796.1|	At1g56150.1
at1g56590	CON	MIN	HOUR	DAY	WEEK	UNIGENE	FLCDNA	TAIR
CCGGTCAAAA	2	1	0	0	0	gnl|UG|At#S11732072	gi|23198149|gb|BT000283.1	At1g56590.1
at3g58170	CON	MIN	HOUR	DAY	WEEK	UNIGENE	FLCDNA	TAIR
ATATTCCCAG	3	1	3	3	0	gnl|UG|At#S11727919	gi|26449795|dbj|AK117351.1|	At3g58170.1
at4g01895	CON	MIN	HOUR	DAY	WEEK	UNIGENE	FLCDNA	TAIR
TTCTTCAATC	0	1	0	0	0	gnl|UG|At#S18322357	gi|109946490|gb|BT026068.1	non-canonical match
at3g56040	CON	MIN	HOUR	DAY	WEEK	UNIGENE	FLCDNA	TAIR
AAAAAAGCTC	0	0	5	2	0	gnl|UG|At#S11728322	no match found	At4g16045.1
AAAATAAAAA	3	1	11	5	5	no match found	gi|14596046|gb|AY042811.1	multiple non-canonical match
at1g17310	CON	MIN	HOUR	DAY	WEEK	UNIGENE	FLCDNA	TAIR
GCCAATCAAT	0	1	0	0	0	no match found	gi|94442498|gb|BT025284.1	non-canonical match
at1g51820	CON	MIN	HOUR	DAY	WEEK	UNIGENE	FLCDNA	TAIR
GAATCAAAGA	0	0	0	0	1	gnl|UG|At#S11733832	no match found	multiple non-canonical match
at5g13450	CON	MIN	HOUR	DAY	WEEK	UNIGENE	FLCDNA	TAIR
TCAATCAGGA	0	0	0	0	1	no match found	gi|15809743|gb|AY054139.1	non-canonical match

CTTGTGATGA	7	9	6	4	10	gnl|UG|At#S11722987	gi|14190494|gb|AF380647.1	At5g13450.1
at4g38540	CON	MIN	HOUR	DAY	WEEK	UNIGENE	FLCDNA	TAIR
ATGATTCTCA	1	3	0	1	0	no match found	no match found	At4g38540.1
at1g75460	CON	MIN	HOUR	DAY	WEEK	UNIGENE	FLCDNA	TAIR
ATGCACACGC	2	4	2	1	0	no match found	gi|15028232|gb|AY045939.1	non-canonical match
at5g58700	CON	MIN	HOUR	DAY	WEEK	UNIGENE	FLCDNA	TAIR
AGAAATATAT	0	0	0	0	1	gnl|UG|At#S11806688	gi|20260635|gb|AY093217.1	At5g58700.1
at5g62880	CON	MIN	HOUR	DAY	WEEK	UNIGENE	FLCDNA	TAIR
TAAGAGATTA	0	0	0	2	1	gnl|UG|At#S11717413	gi|51968569|dbj|AK175214.1|	multiple canonical match
at3g11320	CON	MIN	HOUR	DAY	WEEK	UNIGENE	FLCDNA	TAIR
GAAAAAAATG	1	0	0	0	0	gnl|UG|At#S34118435	gi|53828520|gb|BT015807.1	At3g11320.1
at4g36180	CON	MIN	HOUR	DAY	WEEK	UNIGENE	FLCDNA	TAIR
CTTGAAGGCT	1	2	1	1	1	gnl|UG|At#S11721567	gi|19347727|gb|AY080606.1	At4g36180.1
at5g25415	CON	MIN	HOUR	DAY	WEEK	UNIGENE	FLCDNA	TAIR
TGATGGGTTT	0	0	1	0	0	gnl|UG|At#S11720641	no match found	At5g25415.1
at4g18205	CON	MIN	HOUR	DAY	WEEK	UNIGENE	FLCDNA	TAIR
CAAATGGGAT	1	6	3	0	0	gnl|UG|At#S28282017	gi|18491220|gb|AY074546.1	multiple non-canonical match
at1g20540	CON	MIN	HOUR	DAY	WEEK	UNIGENE	FLCDNA	TAIR
TCTGGGATCT	0	2	0	0	0	no match found	gi|23198163|gb|BT000290.1	non-canonical match
at4g33500	CON	MIN	HOUR	DAY	WEEK	UNIGENE	FLCDNA	TAIR
TGAACCTGGA	1	1	1	1	1	gnl|UG|At#S11722025	gi|110742596|dbj|AK227170.1|	At4g33500.1
at1g55220	CON	MIN	HOUR	DAY	WEEK	UNIGENE	FLCDNA	TAIR
TTTTATCAAA	3	1	2	0	0	no match found	no match found	At1g55220.1
at4g27100	CON	MIN	HOUR	DAY	WEEK	UNIGENE	FLCDNA	TAIR
GTTTTTGTTA	0	0	0	0	1	gnl|UG|At#S28281954	gi|62320589|dbj|AK221621.1|	multiple canonical match
at5g24314	CON	MIN	HOUR	DAY	WEEK	UNIGENE	FLCDNA	TAIR
TTCTAAGCAA	2	1	4	1	0	gnl|UG|At#S11817050	gi|17380655|gb|AY063802.1	At5g24314.1
at5g15920	CON	MIN	HOUR	DAY	WEEK	UNIGENE	FLCDNA	TAIR
AACGGTCCTT	1	0	0	0	0	gnl|UG|At#S11722358	no match found	At5g15920.1
at3g57470	CON	MIN	HOUR	DAY	WEEK	UNIGENE	FLCDNA	TAIR
AATAGTAAAC	0	1	0	1	0	gnl|UG|At#S11703608	gi|14334771|gb|AY035059.1	At3g57470.1
at2g37680	CON	MIN	HOUR	DAY	WEEK	UNIGENE	FLCDNA	TAIR
CGATGCACGT	1	3	0	0	1	gnl|UG|At#S11733180	no match found	non-canonical match
AGACAAAGAA	1	0	2	1	4	no match found	no match found	At2g37680.1
at5g02940	CON	MIN	HOUR	DAY	WEEK	UNIGENE	FLCDNA	TAIR
TTATATTTTC	1	0	1	0	0	gnl|UG|At#S11725431	gi|62318897|dbj|AK220769.1|	At5g02940.1
at3g08510	CON	MIN	HOUR	DAY	WEEK	UNIGENE	FLCDNA	TAIR
ATTAAGAAAG	1	11	1	1	2	gnl|UG|At#S11738338	gi|21403175|gb|AY084465.1	At3g08510.1
at5g13820	CON	MIN	HOUR	DAY	WEEK	UNIGENE	FLCDNA	TAIR
GAAAAAGGGG	0	0	0	1	1	gnl|UG|At#S11722882	no match found	At5g13820.1
at2g43850	CON	MIN	HOUR	DAY	WEEK	UNIGENE	FLCDNA	TAIR

GACAAACTCA	0	1	1	0	1	gnl|UG|At#S11709710	gi|17979550|gb|AY070773.1	At2g43850.1
at4g02710	CON	MIN	HOUR	DAY	WEEK	UNIGENE	FLCDNA	TAIR
CGAGGAGAGG	0	2	0	0	0	gnl|UG|At#S11727011	no match found	non-canonical match
at4g03220	CON	MIN	HOUR	DAY	WEEK	UNIGENE	FLCDNA	TAIR
ATTCTTGTAA	0	0	0	1	0	no match found	no match found	At4g03220.1
at4g27060	CON	MIN	HOUR	DAY	WEEK	UNIGENE	FLCDNA	TAIR
AGCTTGTGAA	0	2	0	0	1	gnl|UG|At#S11723117	gi|110736008|dbj|AK228014.1|	At4g27060.1
at4g15180	CON	MIN	HOUR	DAY	WEEK	UNIGENE	FLCDNA	TAIR
AACAAAGCCA	0	1	0	0	0	gnl|UG|At#S11725171	gi|110741648|dbj|AK226663.1|	At4g15180.1
at3g01930	CON	MIN	HOUR	DAY	WEEK	UNIGENE	FLCDNA	TAIR
TGTATTTGGT	0	1	0	0	0	gnl|UG|At#S11740109	gi|22136035|gb|AY128397.1	At3g01930.1
at1g18900	CON	MIN	HOUR	DAY	WEEK	UNIGENE	FLCDNA	TAIR
TTGAGAGGAT	0	1	1	0	1	gnl|UG|At#S18942546	gi|26450016|dbj|AK117464.1|	At1g18900.2
at5g45340	CON	MIN	HOUR	DAY	WEEK	UNIGENE	FLCDNA	TAIR
CCTTTTGGTA	0	1	1	0	0	no match found	gi|18086489|gb|AY065065.1	non-canonical match
CCTCTATATC	0	1	2	0	0	gnl|UG|At#S11719193	no match found	At5g45340.1
at5g46170	CON	MIN	HOUR	DAY	WEEK	UNIGENE	FLCDNA	TAIR
TAAGAGTTTT	1	2	1	0	2	gnl|UG|At#S11709875	gi|17979206|gb|AY070105.1	At5g46170.1
at1g09850	CON	MIN	HOUR	DAY	WEEK	UNIGENE	FLCDNA	TAIR
TTACAGAAAA	0	4	0	0	1	gnl|UG|At#S11704426	gi|110741820|dbj|AK226753.1|	At1g09850.1
at2g36390	CON	MIN	HOUR	DAY	WEEK	UNIGENE	FLCDNA	TAIR
GTGTATGCAC	1	2	1	0	0	gnl|UG|At#S11759934	gi|110742083|dbj|AK226896.1|	At2g36390.1
at1g55000	CON	MIN	HOUR	DAY	WEEK	UNIGENE	FLCDNA	TAIR
TCCATTCTTG	2	1	1	3	3	gnl|UG|At#S11732648	gi|15983459|gb|AF424604.1	At1g55000.1
at4g11680	CON	MIN	HOUR	DAY	WEEK	UNIGENE	FLCDNA	TAIR
TATAATTGCA	0	1	0	0	0	gnl|UG|At#S11725787	gi|38564281|gb|BT010750.1	At4g11680.1
at5g20490	CON	MIN	HOUR	DAY	WEEK	UNIGENE	FLCDNA	TAIR
CAACAAGTGG	1	1	0	0	0	gnl|UG|At#S11721204	gi|110737321|dbj|AK228703.1|	At5g20490.1
at5g39080	CON	MIN	HOUR	DAY	WEEK	UNIGENE	FLCDNA	TAIR
AAAAAATTGA	1	0	0	0	0	no match found	gi|21406442|gb|AY087704.1	non-canonical match
at2g47250	CON	MIN	HOUR	DAY	WEEK	UNIGENE	FLCDNA	TAIR
CTTGCTGGTT	0	0	1	0	0	gnl|UG|At#S11730889	gi|22135844|gb|AY128301.1	At2g47250.1
at3g12830	CON	MIN	HOUR	DAY	WEEK	UNIGENE	FLCDNA	TAIR
GAATCGCTTC	0	4	1	0	0	no match found	gi|110737863|dbj|AK228982.1|	non-canonical match
at3g57220	CON	MIN	HOUR	DAY	WEEK	UNIGENE	FLCDNA	TAIR
TCCAACAATC	1	1	0	0	1	gnl|UG|At#S21737507	gi|21407705|gb|AY088931.1	At3g57220.1
atcg01060	CON	MIN	HOUR	DAY	WEEK	UNIGENE	FLCDNA	TAIR
AAACAACTCG	1	1	0	0	0	no match found	no match found	AtCg01060
at5g07900	CON	MIN	HOUR	DAY	WEEK	UNIGENE	FLCDNA	TAIR
AATCTTTGTG	2	1	1	1	1	gnl|UG|At#S11724142	gi|21404909|gb|AY086199.1	multiple canonical match
at1g04790	CON	MIN	HOUR	DAY	WEEK	UNIGENE	FLCDNA	TAIR

TCCGGTTTGC	0	0	2	1	0	gnl|UG|At#S11742550	gi|30725481|gb|BT008404.1	At1g04790.1
at1g62110	CON	MIN	HOUR	DAY	WEEK	UNIGENE	FLCDNA	TAIR
GTGACAAGGA	0	1	0	0	1	gnl|UG|At#S11730756	gi|28951040|gb|BT005380.1	At1g62110.1
at1g31330	CON	MIN	HOUR	DAY	WEEK	UNIGENE	FLCDNA	TAIR
AAAGCTTTCT	21	153	19	14	3	no match found	no match found	At1g31330.1
at1g01180	CON	MIN	HOUR	DAY	WEEK	UNIGENE	FLCDNA	TAIR
GGCGGATATA	0	1	0	1	0	gnl|UG|At#S34117936	gi|110736694|dbj|AK228372.1|	non-canonical match
at3g55380	CON	MIN	HOUR	DAY	WEEK	UNIGENE	FLCDNA	TAIR
GATACGAGCT	0	0	0	1	1	gnl|UG|At#S11704685	gi|21436334|gb|AY117262.1	At3g55380.1
at1g31650	CON	MIN	HOUR	DAY	WEEK	UNIGENE	FLCDNA	TAIR
AACAAAGCCG	1	1	1	1	0	gnl|UG|At#S11738415	gi|62321354|dbj|AK222010.1|	At1g31650.1
at3g05290	CON	MIN	HOUR	DAY	WEEK	UNIGENE	FLCDNA	TAIR
GATTCTCATT	0	1	0	0	0	gnl|UG|At#S11739140	no match found	At3g05290.1
at1g66940	CON	MIN	HOUR	DAY	WEEK	UNIGENE	FLCDNA	TAIR
ATTTCGGTTT	1	0	0	0	0	gnl|UG|At#S18942309	no match found	At1g66940.2
CAGTTCGGGC	0	3	0	0	3	no match found	gi|21403221|gb|AY084511.1	At1g66940.1
at5g62190	CON	MIN	HOUR	DAY	WEEK	UNIGENE	FLCDNA	TAIR
GTGAAATACC	0	2	0	0	1	gnl|UG|At#S11717482	gi|14517481|gb|AY039576.1	At5g62190.1
at1g69330	CON	MIN	HOUR	DAY	WEEK	UNIGENE	FLCDNA	TAIR
TGTGTTATCT	0	1	2	0	0	gnl|UG|At#S11729240	gi|19699329|gb|AY090372.1	At1g69330.1
AGACTGGTGC	0	0	0	1	0	no match found	gi|21407204|gb|AY088430.1	non-canonical match
at1g18630	CON	MIN	HOUR	DAY	WEEK	UNIGENE	FLCDNA	TAIR
GGTTTGTTTA	1	0	0	0	0	gnl|UG|At#S34114533	gi|110743183|dbj|AK227482.1|	At1g18630.1
at4g02840	CON	MIN	HOUR	DAY	WEEK	UNIGENE	FLCDNA	TAIR
AATTTGAGAT	4	3	2	0	3	gnl|UG|At#S11726976	gi|26450420|dbj|AK117672.1|	At4g02840.1
AACACTCATT	0	0	0	0	2	no match found	gi|114050552|gb|BT028879.1	non-canonical match
at1g15960	CON	MIN	HOUR	DAY	WEEK	UNIGENE	FLCDNA	TAIR
AAGACTTGAC	0	0	0	1	1	gnl|UG|At#S11743805	gi|18650633|gb|AY074865.1	At1g15960.1
at5g07180	CON	MIN	HOUR	DAY	WEEK	UNIGENE	FLCDNA	TAIR
GGATAGGTGA	1	0	0	0	0	gnl|UG|At#S11724330	no match found	At5g07180.1
at1g68570	CON	MIN	HOUR	DAY	WEEK	UNIGENE	FLCDNA	TAIR
TCCATCGGAC	0	3	0	3	0	gnl|UG|At#S11729383	gi|20147230|gb|AY091784.1	At1g68570.1
at1g52890	CON	MIN	HOUR	DAY	WEEK	UNIGENE	FLCDNA	TAIR
TAAGTTTAAT	0	0	1	1	1	no match found	no match found	At1g52890.1
CATAGAAAAT	0	2	4	0	2	no match found	gi|17529067|gb|AY065268.1	non-canonical match
at2g29700	CON	MIN	HOUR	DAY	WEEK	UNIGENE	FLCDNA	TAIR
TTCTTCATTG	0	1	0	2	0	no match found	gi|15215631|gb|AY050344.1	At2g29700.1
at5g53580	CON	MIN	HOUR	DAY	WEEK	UNIGENE	FLCDNA	TAIR
TTGAAGATAA	0	2	0	0	1	gnl|UG|At#S11718357	gi|46931335|gb|BT012653.1	At5g53580.1
at2g32600	CON	MIN	HOUR	DAY	WEEK	UNIGENE	FLCDNA	TAIR
GATTCTCAAA	0	5	0	4	3	gnl|UG|At#S11734384	gi|15450714|gb|AY052725.1	At2g32600.1
at2g29790	CON	MIN	HOUR	DAY	WEEK	UNIGENE	FLCDNA	TAIR

GATCTGGAAA	0	0	1	0	0	gnl|UG|At#S11714246	gi|46931197|gb|BT012584.1	multiple canonical match
at1g80780	CON	MIN	HOUR	DAY	WEEK	UNIGENE	FLCDNA	TAIR
GACACTGAGT	1	5	0	0	0	gnl|UG|At#S15435715	gi|17979380|gb|AY070420.1	At1g80780.1
at3g55220	CON	MIN	HOUR	DAY	WEEK	UNIGENE	FLCDNA	TAIR
AGAAGCATTG	0	0	0	1	0	no match found	no match found	At3g55220.1
AAACAGTTGC	0	0	1	0	1	no match found	gi|62318968|dbj|AK220805.1|	non-canonical match
at1g31860	CON	MIN	HOUR	DAY	WEEK	UNIGENE	FLCDNA	TAIR
GACTCGACGG	0	1	3	0	1	gnl|UG|At#S11704851	gi|21405224|gb|AY086514.1	At1g31860.1
at1g52730	CON	MIN	HOUR	DAY	WEEK	UNIGENE	FLCDNA	TAIR
ATTTGCTATG	1	0	0	0	1	gnl|UG|At#S20861283	no match found	multiple non-canonical match
GGTGTTAAAA	1	0	3	4	1	gnl|UG|At#S34118336	gi|110735923|dbj|AK227970.1|	At1g52730.1
at4g11980	CON	MIN	HOUR	DAY	WEEK	UNIGENE	FLCDNA	TAIR
ACAACTTGGG	1	1	2	0	0	gnl|UG|At#S11725734	gi|15450340|gb|AY052271.1	At4g11980.1
at5g57640	CON	MIN	HOUR	DAY	WEEK	UNIGENE	FLCDNA	TAIR
TATGATAACC	1	0	0	0	0	gnl|UG|At#S21989657	no match found	non-canonical match
at3g27300	CON	MIN	HOUR	DAY	WEEK	UNIGENE	FLCDNA	TAIR
GCTACATTTG	1	1	0	2	0	gnl|UG|At#S28282896	gi|117168058|gb|BT029298.1	At3g27300.1
at3g11130	CON	MIN	HOUR	DAY	WEEK	UNIGENE	FLCDNA	TAIR
GTGGTATTCG	6	6	5	3	2	gnl|UG|At#S11737545	gi|110738757|dbj|AK229443.1|	At3g11130.1
at1g01900	CON	MIN	HOUR	DAY	WEEK	UNIGENE	FLCDNA	TAIR
ACGACGGCGA	1	0	0	0	0	gnl|UG|At#S11825187	gi|27754420|gb|BT002840.1	At1g01900.1
at5g13420	CON	MIN	HOUR	DAY	WEEK	UNIGENE	FLCDNA	TAIR
CTTTCTAAAG	3	2	4	3	2	gnl|UG|At#S11722994	gi|15810514|gb|AY056296.1	At5g13420.1
at3g51480	CON	MIN	HOUR	DAY	WEEK	UNIGENE	FLCDNA	TAIR
TGTTCTTGCT	0	2	0	0	0	gnl|UG|At#S14273685	gi|28393708|gb|BT004264.1	At3g51480.1
at5g51840	CON	MIN	HOUR	DAY	WEEK	UNIGENE	FLCDNA	TAIR
GACAGAGACC	1	1	0	0	0	no match found	gi|51971125|dbj|AK176492.1|	non-canonical match
at2g36480	CON	MIN	HOUR	DAY	WEEK	UNIGENE	FLCDNA	TAIR
ATGCTAGTTT	0	0	0	0	1	gnl|UG|At#S11733475	gi|110741828|dbj|AK226757.1|	At2g36480.1
at5g57000	CON	MIN	HOUR	DAY	WEEK	UNIGENE	FLCDNA	TAIR
ATAGACTTGC	0	0	3	0	0	gnl|UG|At#S11718013	gi|21407450|gb|AY088676.1	At5g57000.1
at1g55460	CON	MIN	HOUR	DAY	WEEK	UNIGENE	FLCDNA	TAIR
TGTTGAGAGT	0	2	0	1	0	no match found	gi|15293154|gb|AY051011.1	multiple non-canonical match
AATTCGACGG	0	2	0	0	0	no match found	gi|23270364|gb|AY039912.2	non-canonical match
TTATTCGATC	4	1	1	2	1	gnl|UG|At#S11701969	gi|13430439|gb|AF360132.1	At1g55460.1
at1g22430	CON	MIN	HOUR	DAY	WEEK	UNIGENE	FLCDNA	TAIR
CAGCGCCAAT	1	5	0	1	0	gnl|UG|At#S11740818	gi|22136285|gb|AY128821.1	At1g22430.1
at2g21620	CON	MIN	HOUR	DAY	WEEK	UNIGENE	FLCDNA	TAIR
GTAAAGAGTG	1	1	0	4	1	gnl|UG|At#S15461306	gi|20334875|gb|AY094038.1	At2g21620.1
at5g54100	CON	MIN	HOUR	DAY	WEEK	UNIGENE	FLCDNA	TAIR
ATCGCTCAAG	1	0	0	0	1	gnl|UG|At#S11718305	gi|26452346|dbj|AK118664.1|	At5g54100.1
at2g47730	CON	MIN	HOUR	DAY	WEEK	UNIGENE	FLCDNA	TAIR

ACCACTGACC	7	19	15	22	4	gnl|UG|At#S25665942	gi|14532561|gb|AY039905.1	At2g47730.1
at3g16920	CON	MIN	HOUR	DAY	WEEK	UNIGENE	FLCDNA	TAIR
GGCTTGGTTT	2	1	2	2	0	no match found	gi|110740739|dbj|AK226317.1|	multiple non-canonical match
at5g45800	CON	MIN	HOUR	DAY	WEEK	UNIGENE	FLCDNA	TAIR
TGAGTATTAG	3	1	2	0	0	gnl|UG|At#S11719147	gi|62319002|dbj|AK220822.1|	At5g45800.1
at5g64020	CON	MIN	HOUR	DAY	WEEK	UNIGENE	FLCDNA	TAIR
TAAATGGAAC	0	0	0	0	1	gnl|UG|At#S11717297	gi|119935929|gb|BT029767.1	At5g64020.1
at1g76300	CON	MIN	HOUR	DAY	WEEK	UNIGENE	FLCDNA	TAIR
TTTTGTTAAT	1	1	0	0	3	gnl|UG|At#S11727770	gi|14190502|gb|AF380651.1	At1g76300.1
at2g38180	CON	MIN	HOUR	DAY	WEEK	UNIGENE	FLCDNA	TAIR
AGTTAACCCA	0	0	0	1	0	gnl|UG|At#S11733057	gi|59958351|gb|BT021116.1	At2g38180.1
at1g18060	CON	MIN	HOUR	DAY	WEEK	UNIGENE	FLCDNA	TAIR
AAATGAAAGT	10	2	5	4	5	gnl|UG|At#S11741240	gi|110738962|dbj|AK229549.1|	At1g18060.1
GAAGAATCGG	0	0	4	0	1	no match found	gi|15081663|gb|AY048224.1	non-canonical match
at3g17300	CON	MIN	HOUR	DAY	WEEK	UNIGENE	FLCDNA	TAIR
TTGTTTGAGT	0	2	1	0	0	no match found	gi|20260057|gb|AY093377.1	At3g17300.1
at4g22190	CON	MIN	HOUR	DAY	WEEK	UNIGENE	FLCDNA	TAIR
CGGTAGTCAA	0	5	0	0	0	no match found	gi|20466281|gb|AY099607.1	non-canonical match
TAATTAAGAA	7	8	0	3	6	gnl|UG|At#S11723970	no match found	At4g22190.1
at4g24800	CON	MIN	HOUR	DAY	WEEK	UNIGENE	FLCDNA	TAIR
AGGTGGTGAA	3	4	2	3	1	gnl|UG|At#S28281974	gi|17063162|gb|AY062102.1	At4g24800.1
at1g15405	CON	MIN	HOUR	DAY	WEEK	UNIGENE	FLCDNA	TAIR
GCCTGCTGGC	0	1	1	0	0	gnl|UG|At#S20297450	no match found	pseudo chromosome match
at3g22740	CON	MIN	HOUR	DAY	WEEK	UNIGENE	FLCDNA	TAIR
CTTTAATTAT	1	1	1	1	1	gnl|UG|At#S11733993	gi|28950916|gb|BT005318.1	At3g22740.1
at5g26220	CON	MIN	HOUR	DAY	WEEK	UNIGENE	FLCDNA	TAIR
AAGACAGCAA	1	2	1	0	0	gnl|UG|At#S11720570	gi|26450774|dbj|AK117855.1|	At5g26220.1
at4g22260	CON	MIN	HOUR	DAY	WEEK	UNIGENE	FLCDNA	TAIR
AACAATAGAT	1	1	0	0	2	gnl|UG|At#S11700751	gi|13265406|gb|AF324663.2	At4g22260.1
at4g30850	CON	MIN	HOUR	DAY	WEEK	UNIGENE	FLCDNA	TAIR
AAGCTTGAAC	0	0	0	0	1	no match found	gi|72198664|gb|DQ108896.1	At4g22260.1
at2g24580	CON	MIN	HOUR	DAY	WEEK	UNIGENE	FLCDNA	TAIR
GTTTTAAGAT	0	0	1	0	1	gnl|UG|At#S11736361	gi|21436150|gb|AY117247.1	At2g24580.1
at5g11200	CON	MIN	HOUR	DAY	WEEK	UNIGENE	FLCDNA	TAIR
GAGGTCCACA	3	3	4	4	4	gnl|UG|At#S18913145	gi|14190380|gb|AF378868.1	At5g11200.1
at1g70470	CON	MIN	HOUR	DAY	WEEK	UNIGENE	FLCDNA	TAIR
TGTAAGGTGA	1	1	0	0	0	gnl|UG|At#S11728992	gi|38566585|gb|BT010816.1	At1g70470.1
at2g40220	CON	MIN	HOUR	DAY	WEEK	UNIGENE	FLCDNA	TAIR
CAAAATTTTG	2	5	7	4	1	no match found	no match found	At2g40220.1
at4g08230	CON	MIN	HOUR	DAY	WEEK	UNIGENE	FLCDNA	TAIR
TGGCTCGTGC	1	3	2	0	1	gnl|UG|At#S11726137	gi|18252942|gb|AY072406.1	At4g08230.1

at1g26890	CON	MIN	HOUR	DAY	WEEK	UNIGENE	FLCDNA	TAIR
TTATTAACAA	2	0	0	0	0	no match found	no match found	At1g26890.1
at5g39040	CON	MIN	HOUR	DAY	WEEK	UNIGENE	FLCDNA	TAIR
ATGAGCTTCT	0	2	0	3	0	gnl|UG|At#S34116498	no match found	At5g39040.1
at1g50500	CON	MIN	HOUR	DAY	WEEK	UNIGENE	FLCDNA	TAIR
ATTTGTTCCT	1	1	1	0	0	gnl|UG|At#S34117563	gi|110737401|dbj|AK228745.1|	At1g50500.1
at3g63330	CON	MIN	HOUR	DAY	WEEK	UNIGENE	FLCDNA	TAIR
GAAATGCTCT	1	0	0	0	0	gnl|UG|At#S11726419	gi|21689732|gb|AY122955.1	At3g63330.1
at3g17450	CON	MIN	HOUR	DAY	WEEK	UNIGENE	FLCDNA	TAIR
GAATCTGGTA	1	1	1	1	0	gnl|UG|At#S11735613	gi|110737505|dbj|AK228799.1|	At3g17450.1
at4g27280	CON	MIN	HOUR	DAY	WEEK	UNIGENE	FLCDNA	TAIR
GCCGGAAACC	0	9	1	0	0	gnl|UG|At#S11723081	gi|16648776|gb|AY058165.1	At4g27280.1
at1g77610	CON	MIN	HOUR	DAY	WEEK	UNIGENE	FLCDNA	TAIR
GTTGATATTC	0	1	0	0	0	gnl|UG|At#S11727348	gi|13430497|gb|AF360161.1	At1g77610.1
at4g37520	CON	MIN	HOUR	DAY	WEEK	UNIGENE	FLCDNA	TAIR
GGTCTCATAA	2	2	3	0	1	gnl|UG|At#S11721348	gi|17065479|gb|AY062816.1	At4g37520.1
at1g19020	CON	MIN	HOUR	DAY	WEEK	UNIGENE	FLCDNA	TAIR
GATTAATTAA	2	5	4	0	0	gnl|UG|At#S30645513	gi|51970815|dbj|AK176337.1|	At1g19020.1
at5g54970	CON	MIN	HOUR	DAY	WEEK	UNIGENE	FLCDNA	TAIR
AAGGATATCT	0	1	0	1	1	gnl|UG|At#S30644536	no match found	At5g54970.1
at2g36885	CON	MIN	HOUR	DAY	WEEK	UNIGENE	FLCDNA	TAIR
CTGCTTTCGG	0	2	0	1	3	gnl|UG|At#S11709840	gi|17979286|gb|AY070371.1	At2g36885.2
at1g49760	CON	MIN	HOUR	DAY	WEEK	UNIGENE	FLCDNA	TAIR
CTTTCTGTTC	0	2	0	4	1	gnl|UG|At#S11734590	gi|17978684|gb|AY064622.1	At1g49760.1
at5g25210	CON	MIN	HOUR	DAY	WEEK	UNIGENE	FLCDNA	TAIR
AGAAAGTGGT	3	1	4	1	2	no match found	gi|29824284|gb|BT006118.1	multiple non-canonical match
AGCTCGAAAA	1	3	1	0	0	gnl|UG|At#S11720662	gi|21403897|gb|AY085187.1	At5g25210.1
at4g27700	CON	MIN	HOUR	DAY	WEEK	UNIGENE	FLCDNA	TAIR
AAATCCAGCA	15	11	19	7	4	gnl|UG|At#S11723008	gi|21403936|gb|AY085226.1	At4g27700.1
GGGCAAGGAA	2	1	3	1	1	no match found	gi|20334877|gb|AY094039.1	non-canonical match
at4g33640	CON	MIN	HOUR	DAY	WEEK	UNIGENE	FLCDNA	TAIR
TAGTAACAAA	5	3	0	1	4	no match found	gi|110738472|dbj|AK229299.1|	At4g33640.1
at2g46420	CON	MIN	HOUR	DAY	WEEK	UNIGENE	FLCDNA	TAIR
TTCTCAGTCA	0	1	0	0	0	gnl|UG|At#S43850010	gi|15450510|gb|AY052357.1	At2g46420.1
at5g63490	CON	MIN	HOUR	DAY	WEEK	UNIGENE	FLCDNA	TAIR
CAAAATCAAT	1	0	2	1	0	gnl|UG|At#S11717351	gi|110739693|dbj|AK229928.1|	At5g63490.1
at1g33600	CON	MIN	HOUR	DAY	WEEK	UNIGENE	FLCDNA	TAIR
ATTTTTATTT	0	5	2	1	0	gnl|UG|At#S11737722	gi|51969895|dbj|AK175877.1|	At2g02650.1
GGGAAGCTGA	2	3	5	4	0	gnl|UG|At#S18919062	no match found	non-canonical match
at5g09740	CON	MIN	HOUR	DAY	WEEK	UNIGENE	FLCDNA	TAIR
TGATCTGTGC	1	0	1	0	0	gnl|UG|At#S11723799	gi|26449353|dbj|AK117126.1|	At5g09740.1
at2g05170	CON	MIN	HOUR	DAY	WEEK	UNIGENE	FLCDNA	TAIR

TGTATGCATT	0	1	0	0	0	gnl|UG|At#S11740114	gi|27363389|gb|BT002698.1	At2g05170.1
at5g24850	CON	MIN	HOUR	DAY	WEEK	UNIGENE	FLCDNA	TAIR
GTAATGGTCC	0	0	0	2	1	gnl|UG|At#S11817042	gi|20857181|gb|AY102138.1	At5g24850.1
at5g20165	CON	MIN	HOUR	DAY	WEEK	UNIGENE	FLCDNA	TAIR
ATGGGTGTGT	1	2	2	1	0	gnl|UG|At#S30649596	gi|50198834|gb|BT014998.1	At5g20165.1
at5g17290	CON	MIN	HOUR	DAY	WEEK	UNIGENE	FLCDNA	TAIR
AACGGTGATC	0	0	1	0	0	gnl|UG|At#S11722007	gi|28827577|gb|BT005100.1	At5g17290.1
at1g09570	CON	MIN	HOUR	DAY	WEEK	UNIGENE	FLCDNA	TAIR
CTGATGGCTG	0	4	1	0	0	no match found	gi|14517371|gb|AY039520.1	non-canonical match
ATGTATCATT	1	1	1	2	0	gnl|UG|At#S11742080	no match found	At1g09570.1
at5g08000	CON	MIN	HOUR	DAY	WEEK	UNIGENE	FLCDNA	TAIR
GTGTGTGTGC	0	2	0	0	0	gnl|UG|At#S11724117	gi|21405977|gb|AY087253.1	At5g08000.1
at5g52460	CON	MIN	HOUR	DAY	WEEK	UNIGENE	FLCDNA	TAIR
GATATAGAGG	0	1	0	0	0	gnl|UG|At#S11718470	no match found	multiple canonical match
at4g01915	CON	MIN	HOUR	DAY	WEEK	UNIGENE	FLCDNA	TAIR
GTTATAGTTT	0	3	0	0	1	gnl|UG|At#S15982960	no match found	At4g01915.2
at1g71350	CON	MIN	HOUR	DAY	WEEK	UNIGENE	FLCDNA	TAIR
TATTACCTTG	0	1	0	0	0	gnl|UG|At#S11808280	gi|30023809|gb|BT006330.1	At1g71350.1
at3g20083	CON	MIN	HOUR	DAY	WEEK	UNIGENE	FLCDNA	TAIR
AAAGAGGCCA	0	1	0	0	0	gnl|UG|At#S11708290	no match found	multiple canonical match
at5g50200	CON	MIN	HOUR	DAY	WEEK	UNIGENE	FLCDNA	TAIR
AAGTTGCCTA	0	0	0	0	1	gnl|UG|At#S15459120	no match found	At5g50200.1
at1g23840	CON	MIN	HOUR	DAY	WEEK	UNIGENE	FLCDNA	TAIR
ATTGTAACAC	0	1	0	0	0	gnl|UG|At#S11703728	gi|22655413|gb|AY142035.1	At1g23840.1
at2g42910	CON	MIN	HOUR	DAY	WEEK	UNIGENE	FLCDNA	TAIR
GTGTCTTCCC	1	0	0	3	1	gnl|UG|At#S11731904	gi|51970065|dbj|AK175962.1|	At2g42910.1
at5g41190	CON	MIN	HOUR	DAY	WEEK	UNIGENE	FLCDNA	TAIR
TAAACCACGA	0	0	1	3	1	gnl|UG|At#S11719610	gi|51969177|dbj|AK175518.1|	non-canonical match
at4g13250	CON	MIN	HOUR	DAY	WEEK	UNIGENE	FLCDNA	TAIR
TAAACACTTC	8	2	1	0	5	gnl|UG|At#S11725519	gi|16323185|gb|AY057697.1	At4g13250.1
at2g05940	CON	MIN	HOUR	DAY	WEEK	UNIGENE	FLCDNA	TAIR
TATGAGTGCC	1	1	0	0	0	gnl|UG|At#S11739946	gi|15810412|gb|AY056245.1	At2g05940.1
at4g40010	CON	MIN	HOUR	DAY	WEEK	UNIGENE	FLCDNA	TAIR
TATGCCGCTT	0	1	1	0	1	no match found	no match found	At4g40010.1
at5g62200	CON	MIN	HOUR	DAY	WEEK	UNIGENE	FLCDNA	TAIR
GCTCTAAAGC	0	0	0	1	1	gnl|UG|At#S21737544	gi|51968403|dbj|AK175131.1|	At5g62200.1
at5g54760	CON	MIN	HOUR	DAY	WEEK	UNIGENE	FLCDNA	TAIR
GTTTCTAAGC	3	10	4	5	4	no match found	no match found	At5g54760.1
at5g61130	CON	MIN	HOUR	DAY	WEEK	UNIGENE	FLCDNA	TAIR
GTGTGTGTGT	1	4	0	0	0	gnl|UG|At#S14274073	gi|28827767|gb|BT005195.1	At5g61130.1
at1g06950	CON	MIN	HOUR	DAY	WEEK	UNIGENE	FLCDNA	TAIR

GGAGGTCTCA	2	2	2	2	0	gnl|UG|At#S11742340	gi|20466767|gb|AY099850.1	At1g06950.1
at2g36990	CON	MIN	HOUR	DAY	WEEK	UNIGENE	FLCDNA	TAIR
GAGATATTGG	4	2	5	3	8	gnl|UG|At#S11733353	gi|20259556|gb|AY091182.1	At2g36990.1
at5g64120	CON	MIN	HOUR	DAY	WEEK	UNIGENE	FLCDNA	TAIR
GCCAAGCCAC	0	0	1	0	0	no match found	gi|27363237|gb|BT002622.1	non-canonical match
ACGCAAAGTA	4	9	6	1	3	gnl|UG|At#S11698858	gi|21404992|gb|AY086282.1	At5g64120.1
at3g56320	CON	MIN	HOUR	DAY	WEEK	UNIGENE	FLCDNA	TAIR
GACAAACTCT	0	1	0	0	0	no match found	gi|110736146|dbj|AK228086.1|	multiple non-canonical match
at1g77180	CON	MIN	HOUR	DAY	WEEK	UNIGENE	FLCDNA	TAIR
GGGAAGAAGA	2	2	1	1	1	no match found	gi|14423425|gb|AF386950.1	At1g77180.1
at5g36880	CON	MIN	HOUR	DAY	WEEK	UNIGENE	FLCDNA	TAIR
AGGTAAAAGG	0	2	0	0	0	no match found	gi|15028046|gb|AY045880.1	non-canonical match
AATTTGGTTT	6	2	2	3	1	gnl|UG|At#S28281713	no match found	multiple canonical match
at2g33770	CON	MIN	HOUR	DAY	WEEK	UNIGENE	FLCDNA	TAIR
TAGAATATGT	1	0	3	0	0	gnl|UG|At#S11743163	gi|110739705|dbj|AK229934.1|	At2g33770.1
at1g44800	CON	MIN	HOUR	DAY	WEEK	UNIGENE	FLCDNA	TAIR
CATTGGAGGT	0	7	2	0	2	gnl|UG|At#S11735748	gi|21404108|gb|AY085398.1	At1g44800.1
at5g60680	CON	MIN	HOUR	DAY	WEEK	UNIGENE	FLCDNA	TAIR
AAGGAGTAGG	0	0	1	0	0	no match found	gi|20259626|gb|AY091231.1	non-canonical match
TAATATTTCT	9	4	5	2	4	gnl|UG|At#S11717634	gi|21404364|gb|AY085654.1	At5g60680.1
at1g62360	CON	MIN	HOUR	DAY	WEEK	UNIGENE	FLCDNA	TAIR
TTCCGATTGA	0	0	1	3	0	gnl|UG|At#S11730675	gi|19424063|gb|AY080857.1	At1g62360.1
at2g41620	CON	MIN	HOUR	DAY	WEEK	UNIGENE	FLCDNA	TAIR
AGATCCAAGA	0	0	2	2	0	no match found	gi|21281025|gb|AY113905.1	multiple non-canonical match
CAGCTTTTTA	2	4	0	0	1	no match found	gi|14334431|gb|AY034907.1	At2g41620.1
AGAAAAGAGA	1	0	0	0	0	gnl|UG|At#S11732181	no match found	multiple non-canonical match
at1g25682	CON	MIN	HOUR	DAY	WEEK	UNIGENE	FLCDNA	TAIR
CATCTTCCAT	0	6	1	1	0	gnl|UG|At#S11740308	gi|62320730|dbj|AK221692.1|	At1g25682.1
at5g41350	CON	MIN	HOUR	DAY	WEEK	UNIGENE	FLCDNA	TAIR
CATTCTAGAA	0	1	0	1	0	no match found	gi|23505814|gb|AY143828.1	non-canonical match
TCAGTGTTGA	0	1	3	2	0	gnl|UG|At#S11719594	gi|15081800|gb|AY048293.1	At5g41350.1
at2g24150	CON	MIN	HOUR	DAY	WEEK	UNIGENE	FLCDNA	TAIR
GCTATTTCCT	6	1	3	3	1	gnl|UG|At#S11736460	no match found	At2g24150.1
at1g55690	CON	MIN	HOUR	DAY	WEEK	UNIGENE	FLCDNA	TAIR
TTAGTTTCTT	2	1	0	0	1	gnl|UG|At#S11732407	no match found	At1g55690.3
at5g54840	CON	MIN	HOUR	DAY	WEEK	UNIGENE	FLCDNA	TAIR
TTTGATCTAA	0	1	1	1	1	gnl|UG|At#S18941479	gi|124301145|gb|BT030087.1	At5g54840.1
at1g71970	CON	MIN	HOUR	DAY	WEEK	UNIGENE	FLCDNA	TAIR
ATATAGCATA	1	4	1	1	0	gnl|UG|At#S11728697	gi|20856774|gb|AY102115.1	At1g71970.1
GGAACAACTT	1	0	1	0	0	no match found	gi|23505804|gb|AY143823.1	multiple non-canonical match
at3g33151	CON	MIN	HOUR	DAY	WEEK	UNIGENE	FLCDNA	TAIR
GCCAAATGAT	0	1	0	0	0	no match found	no match found	At3g33151.1
at3g14840	CON	MIN	HOUR	DAY	WEEK	UNIGENE	FLCDNA	TAIR

AGAGGCCATT	0	2	1	0	2	gnl|UG|At#S35297036	no match found	multiple non-canonical match
AAACAGAAAA	3	6	4	2	0	gnl|UG|At#S11736396	no match found	At3g14840.2
at5g25270	CON	MIN	HOUR	DAY	WEEK	UNIGENE	FLCDNA	TAIR
CCGTTTATAT	0	3	0	0	0	no match found	gi|26452236|dbj|AK118607.1|	multiple non-canonical match
AGAGTCAAAA	0	0	1	1	0	no match found	no match found	At5g25270.1
at1g29950	CON	MIN	HOUR	DAY	WEEK	UNIGENE	FLCDNA	TAIR
AATACCCCTT	0	1	0	0	0	gnl|UG|At#S11702917	gi|18491144|gb|AY074843.1	At1g29950.1
at4g30930	CON	MIN	HOUR	DAY	WEEK	UNIGENE	FLCDNA	TAIR
CTTGTGGGAG	0	0	0	2	0	gnl|UG|At#S11722462	gi|110736392|dbj|AK228212.1|	At4g30930.1
at2g43920	CON	MIN	HOUR	DAY	WEEK	UNIGENE	FLCDNA	TAIR
TTCCTTATCT	1	2	0	0	0	gnl|UG|At#S43850025	gi|17979490|gb|AY070741.1	At2g43920.1
at3g09920	CON	MIN	HOUR	DAY	WEEK	UNIGENE	FLCDNA	TAIR
CTTATAAATC	0	3	0	1	0	gnl|UG|At#S34116493	gi|21539494|gb|AY120742.1	At3g09920.1
at5g63260	CON	MIN	HOUR	DAY	WEEK	UNIGENE	FLCDNA	TAIR
TCCTGTTTAA	0	1	0	0	1	gnl|UG|At#S34117518	gi|110737488|dbj|AK228790.1|	non-canonical match
at3g28350	CON	MIN	HOUR	DAY	WEEK	UNIGENE	FLCDNA	TAIR
TTTTAGGCTT	0	0	0	0	1	no match found	no match found	At3g28350.1
at5g24150	CON	MIN	HOUR	DAY	WEEK	UNIGENE	FLCDNA	TAIR
AACCCTCGTC	1	0	0	1	0	gnl|UG|At#S11806833	gi|22136197|gb|AY128777.1	At5g24150.1
at2g39280	CON	MIN	HOUR	DAY	WEEK	UNIGENE	FLCDNA	TAIR
GTCAGTCCCT	0	1	0	0	0	gnl|UG|At#S11732781	no match found	non-canonical match
at3g07525	CON	MIN	HOUR	DAY	WEEK	UNIGENE	FLCDNA	TAIR
GTTCTCTGTT	0	2	0	0	0	no match found	gi|23296728|gb|AY142587.1	At3g07525.1
at5g46440	CON	MIN	HOUR	DAY	WEEK	UNIGENE	FLCDNA	TAIR
AACGACGCCG	0	0	0	1	0	gnl|UG|At#S17865564	gi|44681395|gb|BT011632.1	At5g46440.1
at5g14430	CON	MIN	HOUR	DAY	WEEK	UNIGENE	FLCDNA	TAIR
TTCCTCTTTA	6	6	3	0	4	no match found	gi|62321803|dbj|AK222240.1|	At5g14430.1
GAACACCTTC	0	2	0	0	0	no match found	gi|21280806|gb|AY113997.1	non-canonical match
at5g20180	CON	MIN	HOUR	DAY	WEEK	UNIGENE	FLCDNA	TAIR
ACCTGAACAA	4	1	4	4	8	gnl|UG|At#S15460629	gi|21405531|gb|AY086821.1	At5g20180.2
at1g15060	CON	MIN	HOUR	DAY	WEEK	UNIGENE	FLCDNA	TAIR
GATTTATGTG	1	1	0	0	1	gnl|UG|At#S11741527	gi|51970191|dbj|AK176025.1|	At1g15060.1
at1g77170	CON	MIN	HOUR	DAY	WEEK	UNIGENE	FLCDNA	TAIR
TTCGAAAAAT	1	0	0	0	0	gnl|UG|At#S18893301	no match found	At1g77170.1
at5g08120	CON	MIN	HOUR	DAY	WEEK	UNIGENE	FLCDNA	TAIR
TATGTATCTA	1	0	0	0	0	gnl|UG|At#S11724085	gi|13877922|gb|AF370224.1	At5g08120.1
at5g47680	CON	MIN	HOUR	DAY	WEEK	UNIGENE	FLCDNA	TAIR
ATCTTCTGGA	2	1	0	0	0	no match found	gi|31711823|gb|BT008829.1	non-canonical match
CGGTTTGCAA	0	0	1	0	0	gnl|UG|At#S11718953	gi|21404241|gb|AY085531.1	non-canonical match
at5g45850	CON	MIN	HOUR	DAY	WEEK	UNIGENE	FLCDNA	TAIR
ATGTCGGATA	0	0	1	0	0	gnl|UG|At#S11719142	no match found	non-canonical match
at5g67590	CON	MIN	HOUR	DAY	WEEK	UNIGENE	FLCDNA	TAIR

GCTATGTTTA	13	14	6	5	9	gnl|UG|At#S11716936	gi|26452344|dbj|AK118663.1|	At5g67590.1
at4g27430	CON	MIN	HOUR	DAY	WEEK	UNIGENE	FLCDNA	TAIR
TTGAGTTCAG	1	1	3	2	1	no match found	gi|62321193|dbj|AK221927.1|	At4g27430.1
at3g13780	CON	MIN	HOUR	DAY	WEEK	UNIGENE	FLCDNA	TAIR
GTGAAATCGA	0	1	0	0	0	gnl|UG|At#S11736719	gi|29824387|gb|BT006170.1	At3g13780.1
at1g25390	CON	MIN	HOUR	DAY	WEEK	UNIGENE	FLCDNA	TAIR
CGACCCACGA	0	1	2	1	0	gnl|UG|At#S11706548	gi|15529241|gb|AY052245.1	At1g25390.1
at1g80190	CON	MIN	HOUR	DAY	WEEK	UNIGENE	FLCDNA	TAIR
TAACAATTAT	0	0	1	1	0	gnl|UG|At#S21737123	gi|51969245|dbj|AK175552.1|	At1g80190.1
at1g54000	CON	MIN	HOUR	DAY	WEEK	UNIGENE	FLCDNA	TAIR
CGTGAATTTT	0	0	0	1	0	no match found	gi|98961030|gb|BT025581.1	non-canonical match
AGTTAATTTA	0	1	0	0	0	gnl|UG|At#S11732994	gi|21406096|gb|AY087372.1	At1g54000.1
at5g15410	CON	MIN	HOUR	DAY	WEEK	UNIGENE	FLCDNA	TAIR
ATCATCTCGA	3	6	3	2	3	no match found	gi|110742426|dbj|AK227080.1|	At5g15410.2
at5g06290	CON	MIN	HOUR	DAY	WEEK	UNIGENE	FLCDNA	TAIR
TTTGGTGAAA	8	8	12	4	2	gnl|UG|At#S11724553	gi|20148348|gb|AY081503.1	At5g06290.1
at3g13560	CON	MIN	HOUR	DAY	WEEK	UNIGENE	FLCDNA	TAIR
ACCGCCTTTT	0	0	0	1	0	no match found	gi|14334497|gb|AY034940.1	At3g13560.3
at2g21300	CON	MIN	HOUR	DAY	WEEK	UNIGENE	FLCDNA	TAIR
GCCACACTGT	3	0	1	0	0	gnl|UG|At#S11737131	gi|62319878|dbj|AK221265.1|	At2g21300.1
at1g27300	CON	MIN	HOUR	DAY	WEEK	UNIGENE	FLCDNA	TAIR
AAAAAGGGTT	4	1	0	0	2	gnl|UG|At#S11739880	gi|15450913|gb|AY054537.1	non-canonical match
GGAATGGATC	1	0	0	1	0	no match found	gi|17978742|gb|AY064655.1	non-canonical match
GTTCAGTGCC	2	3	1	0	0	no match found	no match found	At1g27300.1
at3g11470	CON	MIN	HOUR	DAY	WEEK	UNIGENE	FLCDNA	TAIR
ATATCTTAGC	0	1	0	0	0	gnl|UG|At#S11737447	gi|20147156|gb|AY091696.1	At3g11470.1
at4g04570	CON	MIN	HOUR	DAY	WEEK	UNIGENE	FLCDNA	TAIR
AGCTCTGTAA	1	0	0	1	2	gnl|UG|At#S11726508	gi|22022568|gb|AY127017.1	At4g04570.1
at1g22860	CON	MIN	HOUR	DAY	WEEK	UNIGENE	FLCDNA	TAIR
GACAAATGTA	1	1	0	1	0	gnl|UG|At#S11740778	gi|110738219|dbj|AK229169.1|	multiple canonical match
at2g47400	CON	MIN	HOUR	DAY	WEEK	UNIGENE	FLCDNA	TAIR
CTAGGGACAA	47	73	26	29	7	gnl|UG|At#S18322588	gi|17065525|gb|AY062839.1	At2g47400.1
at4g15670	CON	MIN	HOUR	DAY	WEEK	UNIGENE	FLCDNA	TAIR
CATTTAAGAA	0	0	0	1	1	gnl|UG|At#S19066830	gi|44681327|gb|BT011598.1	pseudo chromosome match
at1g05840	CON	MIN	HOUR	DAY	WEEK	UNIGENE	FLCDNA	TAIR
ACTCTTTTCC	0	1	0	0	0	gnl|UG|At#S11742442	no match found	At1g05840.1
at5g57130	CON	MIN	HOUR	DAY	WEEK	UNIGENE	FLCDNA	TAIR
GCTACATTTC	1	2	1	0	0	gnl|UG|At#S11718000	gi|28393848|gb|BT004338.1	At5g57130.1
at1g62740	CON	MIN	HOUR	DAY	WEEK	UNIGENE	FLCDNA	TAIR
CGTTTTAAGA	0	1	0	0	1	gnl|UG|At#S11730602	no match found	non-canonical match
AACAAAATCC	1	2	3	4	3	no match found	gi|28973652|gb|BT005735.1	At4g01000.1
TCTATATAGA	1	1	1	0	1	no match found	no match found	At1g62740.1

at2g13840	CON	MIN	HOUR	DAY	WEEK	UNIGENE	FLCDNA	TAIR
TGTCACAGTT	2	1	0	0	2	gnl|UG|At#S11738998	gi|20466106|gb|AY098965.1	At2g13840.1
at3g18860	CON	MIN	HOUR	DAY	WEEK	UNIGENE	FLCDNA	TAIR
TGGAAAACAA	0	1	1	0	0	gnl|UG|At#S11735192	gi|15294239|gb|AF410311.1	At3g18860.1
at5g49080	CON	MIN	HOUR	DAY	WEEK	UNIGENE	FLCDNA	TAIR
AAATTTCTAA	1	0	0	1	4	gnl|UG|At#S11650794	no match found	At5g49080.1
at1g22050	CON	MIN	HOUR	DAY	WEEK	UNIGENE	FLCDNA	TAIR
TGTGTGGTTT	1	0	1	0	0	gnl|UG|At#S11823067	gi|26452617|dbj|AK118802.1|	multiple non-canonical match
at5g55250	CON	MIN	HOUR	DAY	WEEK	UNIGENE	FLCDNA	TAIR
GGGTTTTTAT	0	0	0	1	0	gnl|UG|At#S11718188	gi|62319071|dbj|AK220857.1|	pseudo chromosome match
at1g16670	CON	MIN	HOUR	DAY	WEEK	UNIGENE	FLCDNA	TAIR
GTTTCAAACA	0	0	2	0	0	gnl|UG|At#S11741378	gi|23197887|gb|BT000152.1	At1g16670.1
at1g54230	CON	MIN	HOUR	DAY	WEEK	UNIGENE	FLCDNA	TAIR
ATGATGATGC	0	0	0	0	1	gnl|UG|At#S11732914	no match found	At1g54230.1
at1g77280	CON	MIN	HOUR	DAY	WEEK	UNIGENE	FLCDNA	TAIR
GGAAGCATTG	0	0	0	1	0	gnl|UG|At#S11727483	no match found	non-canonical match
at1g74230	CON	MIN	HOUR	DAY	WEEK	UNIGENE	FLCDNA	TAIR
TAGAGAGACC	3	4	3	4	2	gnl|UG|At#S11728193	gi|25083153|gb|BT002037.1	At1g74230.1
at4g26180	CON	MIN	HOUR	DAY	WEEK	UNIGENE	FLCDNA	TAIR
TCCCTCCGGA	0	0	0	1	0	gnl|UG|At#S11723280	no match found	At4g26180.1
at5g62380	CON	MIN	HOUR	DAY	WEEK	UNIGENE	FLCDNA	TAIR
ATGGAGTAGA	4	1	2	3	2	no match found	no match found	At5g62380.1
at4g23810	CON	MIN	HOUR	DAY	WEEK	UNIGENE	FLCDNA	TAIR
ATTTCAATAC	0	0	1	0	1	gnl|UG|At#S11723681	gi|110735860|dbj|AK227938.1|	At4g23810.1
at3g46130	CON	MIN	HOUR	DAY	WEEK	UNIGENE	FLCDNA	TAIR
GTCGTCAGGT	1	0	0	1	0	gnl|UG|At#S25698260	gi|51971091|dbj|AK176475.1|	At3g46130.1
at1g16260	CON	MIN	HOUR	DAY	WEEK	UNIGENE	FLCDNA	TAIR
CACACATCCA	2	0	0	1	0	gnl|UG|At#S34116480	gi|110739497|dbj|AK229828.1|	non-canonical match
at5g51140	CON	MIN	HOUR	DAY	WEEK	UNIGENE	FLCDNA	TAIR
GAAGGGTATC	0	2	2	2	0	no match found	gi|21403209|gb|AY084499.1	non-canonical match
AATCCAAAAG	0	0	1	0	0	gnl|UG|At#S43849382	no match found	At2g04870.1
at5g15390	CON	MIN	HOUR	DAY	WEEK	UNIGENE	FLCDNA	TAIR
TACAACTCTT	1	1	0	0	1	gnl|UG|At#S34117477	gi|110737569|dbj|AK228831.1|	At5g15390.1
at3g61770	CON	MIN	HOUR	DAY	WEEK	UNIGENE	FLCDNA	TAIR
TTGTAGTATC	2	0	0	0	3	gnl|UG|At#S11726919	gi|21405613|gb|AY086903.1	At3g61770.1
at5g59540	CON	MIN	HOUR	DAY	WEEK	UNIGENE	FLCDNA	TAIR
TACCAAATAT	8	6	3	0	7	gnl|UG|At#S28281584	no match found	At5g59540.1
at3g53230	CON	MIN	HOUR	DAY	WEEK	UNIGENE	FLCDNA	TAIR
ATGAAATAAA	1	0	1	1	0	gnl|UG|At#S11728831	gi|110737509|dbj|AK228801.1|	At3g53230.1
at5g24650	CON	MIN	HOUR	DAY	WEEK	UNIGENE	FLCDNA	TAIR
GGTGGTGTTC	0	2	1	2	0	gnl|UG|At#S11720711	gi|21407101|gb|AY088327.1	At5g24650.1

at4g34750	CON	MIN	HOUR	DAY	WEEK	UNIGENE	FLCDNA	TAIR
TTGGATTGGC	0	0	1	0	0	gnl|UG|At#S35306981	gi|41349925|gb|BT011508.1	At4g34750.1
at5g26800	CON	MIN	HOUR	DAY	WEEK	UNIGENE	FLCDNA	TAIR
GTTATAAACA	4	0	0	0	1	gnl|UG|At#S17008049	gi|48310022|gb|BT014755.1	At5g26800.1
at1g54180	CON	MIN	HOUR	DAY	WEEK	UNIGENE	FLCDNA	TAIR
CGAGGTTATG	0	0	1	0	0	no match found	gi|57222217|gb|BT020515.1	non-canonical match
at4g37830	CON	MIN	HOUR	DAY	WEEK	UNIGENE	FLCDNA	TAIR
ACGATGCTTA	2	12	1	0	0	gnl|UG|At#S11604500	gi|15810104|gb|AY056090.1	At4g37830.1
at4g34710	CON	MIN	HOUR	DAY	WEEK	UNIGENE	FLCDNA	TAIR
ATCCATCGTT	18	13	13	12	12	gnl|UG|At#S11721813	gi|110739385|dbj|AK229769.1|	At4g34710.1
CCGTATCTTG	1	1	0	0	0	no match found	gi|15982890|gb|AY057553.1	non-canonical match
at5g32620	CON	MIN	HOUR	DAY	WEEK	UNIGENE	FLCDNA	TAIR
TGTCTATGAG	2	5	0	0	0	gnl|UG|At#S11831825	gi|20259364|gb|AY090965.1	non-canonical match
atmg00590	CON	MIN	HOUR	DAY	WEEK	UNIGENE	FLCDNA	TAIR
AAACGATCGA	1	0	0	0	0	no match found	no match found	AtMg00590
at2g14660	CON	MIN	HOUR	DAY	WEEK	UNIGENE	FLCDNA	TAIR
ACTTTAGGAG	0	1	0	0	0	gnl|UG|At#S11811312	gi|149944292|gb|BT030609.1	At2g14660.1
at1g12820	CON	MIN	HOUR	DAY	WEEK	UNIGENE	FLCDNA	TAIR
TGTTTTCAAA	1	0	2	3	1	gnl|UG|At#S11741756	gi|110742802|dbj|AK227280.1|	non-canonical match
at1g65440	CON	MIN	HOUR	DAY	WEEK	UNIGENE	FLCDNA	TAIR
GTCCTCCTAA	0	0	2	0	1	no match found	gi|110738993|dbj|AK229565.1|	non-canonical match
GATCATCTGG	0	1	0	0	0	gnl|UG|At#S11730038	no match found	At1g65440.1
at2g14860	CON	MIN	HOUR	DAY	WEEK	UNIGENE	FLCDNA	TAIR
GAAAATTTGT	0	1	0	1	0	no match found	no match found	At2g14860.1
TATATCACGA	0	0	0	1	1	gnl|UG|At#S11738747	no match found	non-canonical match
at5g16510	CON	MIN	HOUR	DAY	WEEK	UNIGENE	FLCDNA	TAIR
TGTTTTTGAG	1	3	2	2	0	gnl|UG|At#S15460808	gi|20268773|gb|AY091141.1	At5g16510.1
at5g19140	CON	MIN	HOUR	DAY	WEEK	UNIGENE	FLCDNA	TAIR
TCTCTTAAAG	29	118	16	16	6	gnl|UG|At#S28281787	gi|14532643|gb|AY039946.1	At5g19140.1
at5g18570	CON	MIN	HOUR	DAY	WEEK	UNIGENE	FLCDNA	TAIR
ATTCAGCCAA	0	1	0	0	0	gnl|UG|At#S11815569	gi|22136031|gb|AY128395.1	At5g18570.1
at5g10770	CON	MIN	HOUR	DAY	WEEK	UNIGENE	FLCDNA	TAIR
ATACAACAAT	1	3	1	1	1	gnl|UG|At#S11723552	gi|110740048|dbj|AK230109.1|	multiple canonical match
at1g77300	CON	MIN	HOUR	DAY	WEEK	UNIGENE	FLCDNA	TAIR
GTTGCGGAAC	0	1	0	1	0	gnl|UG|At#S11727466	gi|62321172|dbj|AK221916.1|	At1g77300.1
at2g05380	CON	MIN	HOUR	DAY	WEEK	UNIGENE	FLCDNA	TAIR
AGTGTACGAT	63	55	41	71	106	gnl|UG|At#S11676738	gi|15146251|gb|AY049267.1	At2g05380.1
at5g22290	CON	MIN	HOUR	DAY	WEEK	UNIGENE	FLCDNA	TAIR
ATAGACAAAC	0	1	2	0	0	gnl|UG|At#S11721024	gi|14326464|gb|AF385685.1	At5g22290.1
at5g55700	CON	MIN	HOUR	DAY	WEEK	UNIGENE	FLCDNA	TAIR
TGACTGGTAG	0	2	0	0	0	gnl|UG|At#S11718143	gi|25054835|gb|BT001909.1	At5g55700.1
at2g31360	CON	MIN	HOUR	DAY	WEEK	UNIGENE	FLCDNA	TAIR

CGTTCGAGTC	1	14	12	4	1	gnl|UG|At#S11734696	gi|19310776|gb|AY079388.1	At2g31360.1
at5g42950	CON	MIN	HOUR	DAY	WEEK	UNIGENE	FLCDNA	TAIR
TTTGTTGTTT	4	1	1	4	2	gnl|UG|At#S11719434	no match found	At5g42950.1
at4g04840	CON	MIN	HOUR	DAY	WEEK	UNIGENE	FLCDNA	TAIR
TCTTAAAAAA	3	2	4	2	1	gnl|UG|At#S35244714	gi|26452764|dbj|AK118878.1|	At4g04840.1
at1g30135	CON	MIN	HOUR	DAY	WEEK	UNIGENE	FLCDNA	TAIR
AAGCTGCAGC	1	4	4	6	1	gnl|UG|At#S30645653	gi|21405846|gb|AY087122.1	non-canonical match
at1g33250	CON	MIN	HOUR	DAY	WEEK	UNIGENE	FLCDNA	TAIR
TTCCACTTTC	0	0	1	0	0	no match found	no match found	At1g33250.1
at1g21510	CON	MIN	HOUR	DAY	WEEK	UNIGENE	FLCDNA	TAIR
TTTTTTTTTA	2	0	4	1	2	no match found	no match found	At1g21510.1
at4g30750	CON	MIN	HOUR	DAY	WEEK	UNIGENE	FLCDNA	TAIR
CACTATTTAT	0	2	0	2	0	gnl|UG|At#S20823704	gi|14335143|gb|AY037251.1	At4g30750.1
at5g01881	CON	MIN	HOUR	DAY	WEEK	UNIGENE	FLCDNA	TAIR
GATCAACAAG	2	1	0	1	0	gnl|UG|At#S35270998	no match found	At5g01880.1
at1g17615	CON	MIN	HOUR	DAY	WEEK	UNIGENE	FLCDNA	TAIR
AGTCAGAAAG	1	0	0	0	0	gnl|UG|At#S11741284	no match found	multiple non-canonical match
at4g03410	CON	MIN	HOUR	DAY	WEEK	UNIGENE	FLCDNA	TAIR
GATTCCAAGA	4	3	2	8	4	no match found	gi|30794096|gb|BT008684.1	non-canonical match
at3g55400	CON	MIN	HOUR	DAY	WEEK	UNIGENE	FLCDNA	TAIR
GTTTCTGTTC	0	2	0	0	0	gnl|UG|At#S38433591	gi|13605909|gb|AF367354.1	At3g55400.1
at5g55600	CON	MIN	HOUR	DAY	WEEK	UNIGENE	FLCDNA	TAIR
TAAAACCAAA	0	0	1	0	0	no match found	gi|110737698|dbj|AK228899.1|	multiple non-canonical match
TAAAACCACT	1	0	0	0	0	gnl|UG|At#S11709504	gi|18087547|gb|AF462815.1	At5g55600.1
at2g01970	CON	MIN	HOUR	DAY	WEEK	UNIGENE	FLCDNA	TAIR
CTTGGAACCG	1	1	0	1	2	gnl|UG|At#S11741672	gi|62321046|dbj|AK221852.1|	At2g01970.1
at1g74560	CON	MIN	HOUR	DAY	WEEK	UNIGENE	FLCDNA	TAIR
ATGAGGTTGC	0	0	1	0	0	no match found	gi|14326532|gb|AF385720.1	non-canonical match
TCCTAAATCT	4	5	3	6	9	gnl|UG|At#S28282398	no match found	At1g74560.1
at1g59900	CON	MIN	HOUR	DAY	WEEK	UNIGENE	FLCDNA	TAIR
AGAAAGTTGG	6	9	3	3	4	gnl|UG|At#S11731539	gi|13430787|gb|AF360306.1	At1g59900.1
at1g34630	CON	MIN	HOUR	DAY	WEEK	UNIGENE	FLCDNA	TAIR
TTCTACAAAG	3	2	0	0	0	gnl|UG|At#S11709626	gi|18086346|gb|AY064983.1	At1g34630.1
at4g29680	CON	MIN	HOUR	DAY	WEEK	UNIGENE	FLCDNA	TAIR
GTCCTATGTT	0	0	1	0	0	gnl|UG|At#S11722672	gi|23198335|gb|BT000376.1	At4g29680.1
at1g15410	CON	MIN	HOUR	DAY	WEEK	UNIGENE	FLCDNA	TAIR
TGTTGGCTTG	2	0	3	2	0	gnl|UG|At#S34116141	gi|18176343|gb|AY072206.1	At1g15410.1
at1g63610	CON	MIN	HOUR	DAY	WEEK	UNIGENE	FLCDNA	TAIR
AATACATTTG	0	0	0	0	1	gnl|UG|At#S18893787	gi|30102763|gb|BT006492.1	At1g63610.2
at2g35370	CON	MIN	HOUR	DAY	WEEK	UNIGENE	FLCDNA	TAIR
CTTGTTTTCT	14	19	5	3	8	gnl|UG|At#S11733731	no match found	multiple canonical match

at2g33120	CON	MIN	HOUR	DAY	WEEK	UNIGENE	FLCDNA	TAIR
AAGATAAAGC	0	0	1	0	0	no match found	gi|21389652|gb|AY114706.1	multiple non-canonical match
TTCGACAATC	6	6	13	4	6	gnl|UG|At#S28282212	gi|21405073|gb|AY086363.1	At2g33120.1

at5g62370	CON	MIN	HOUR	DAY	WEEK	UNIGENE	FLCDNA	TAIR
GCTGCTCTTG	0	0	0	0	1	gnl|UG|At#S11717464	no match found	multiple non-canonical match

at4g14716	CON	MIN	HOUR	DAY	WEEK	UNIGENE	FLCDNA	TAIR
ATCAATGCCT	2	6	0	0	0	gnl|UG|At#S11725253	gi|28973070|gb|BT005440.1	At4g14716.1

at4g15540	CON	MIN	HOUR	DAY	WEEK	UNIGENE	FLCDNA	TAIR
ATACATTGAA	4	14	1	1	1	gnl|UG|At#S11824357	gi|19347831|gb|AY080726.1	At4g15540.1

at5g13410	CON	MIN	HOUR	DAY	WEEK	UNIGENE	FLCDNA	TAIR
CCAGCGCTTA	0	1	0	0	0	gnl|UG|At#S11722996	gi|46931301|gb|BT012636.1	At5g13410.1

at3g43190	CON	MIN	HOUR	DAY	WEEK	UNIGENE	FLCDNA	TAIR
AACAATGTAA	1	0	0	0	0	gnl|UG|At#S11730749	no match found	non-canonical match

at3g10370	CON	MIN	HOUR	DAY	WEEK	UNIGENE	FLCDNA	TAIR
TTTCTCGGAT	2	2	0	1	0	gnl|UG|At#S11737776	no match found	At3g10370.1

at4g07390	CON	MIN	HOUR	DAY	WEEK	UNIGENE	FLCDNA	TAIR
AACTTTGCCG	1	1	1	2	0	gnl|UG|At#S11726218	gi|18252960|gb|AY072415.1	At4g07390.1

at3g17185	CON	MIN	HOUR	DAY	WEEK	UNIGENE	FLCDNA	TAIR
GATGAAATTA	0	1	1	0	0	no match found	gi|109134192|gb|BT026005.1	At3g17185.1

at2g19930	CON	MIN	HOUR	DAY	WEEK	UNIGENE	FLCDNA	TAIR
TCTAATAATA	1	0	0	0	0	gnl|UG|At#S11737487	gi|110738635|dbj|AK229381.1|	At2g19930.1

at5g27640	CON	MIN	HOUR	DAY	WEEK	UNIGENE	FLCDNA	TAIR
TAATGGTACT	3	0	1	1	1	gnl|UG|At#S28281733	gi|62319195|dbj|AK220920.1|	non-canonical match

at1g19880	CON	MIN	HOUR	DAY	WEEK	UNIGENE	FLCDNA	TAIR
CGGTTTTTGC	1	0	0	1	0	gnl|UG|At#S11741066	gi|23197983|gb|BT000200.1	At1g19880.1

at1g27540	CON	MIN	HOUR	DAY	WEEK	UNIGENE	FLCDNA	TAIR
TTGTTGGGAT	0	0	1	0	1	gnl|UG|At#S11739806	gi|26452076|dbj|AK118525.1|	At1g27540.1

at3g51430	CON	MIN	HOUR	DAY	WEEK	UNIGENE	FLCDNA	TAIR
ATCAGAGACT	1	3	1	0	1	gnl|UG|At#S11729169	gi|38564287|gb|BT010753.1	At3g51430.1

at3g27380	CON	MIN	HOUR	DAY	WEEK	UNIGENE	FLCDNA	TAIR
TGCTATGAAC	0	1	0	0	1	no match found	gi|17104676|gb|AY063053.1	At3g27380.1

at1g33330	CON	MIN	HOUR	DAY	WEEK	UNIGENE	FLCDNA	TAIR
GTGGATTATA	1	0	0	2	3	gnl|UG|At#S11737811	no match found	At1g33330.1
AGAGCAGCCA	0	2	0	1	0	no match found	gi|29824306|gb|BT006129.1	non-canonical match

at1g55960	CON	MIN	HOUR	DAY	WEEK	UNIGENE	FLCDNA	TAIR
TTGTAGCCGC	3	1	5	5	0	gnl|UG|At#S11732299	gi|16604620|gb|AY059755.1	At1g55960.1

at4g23930	CON	MIN	HOUR	DAY	WEEK	UNIGENE	FLCDNA	TAIR
TATGTGAAGT	1	0	0	1	0	gnl|UG|At#S11825184	gi|27754426|gb|BT002843.1	At4g23930.2

at1g11185	CON	MIN	HOUR	DAY	WEEK	UNIGENE	FLCDNA	TAIR
GATCCTAAAC	0	1	0	0	0	gnl|UG|At#S37211214	gi|72197374|gb|DQ108731.1	non-canonical match

at5g08230	CON	MIN	HOUR	DAY	WEEK	UNIGENE	FLCDNA	TAIR
TTCTTCACAG	1	1	0	0	0	gnl|UG|At#S11724056	no match found	At5g08230.1

at5g09380	CON	MIN	HOUR	DAY	WEEK	UNIGENE	FLCDNA	TAIR
CAGTTTTGAC	0	1	1	0	0	gnl|UG|At#S11723895	no match found	non-canonical match
at3g50470	CON	MIN	HOUR	DAY	WEEK	UNIGENE	FLCDNA	TAIR
TATATAGATT	0	1	0	0	0	gnl|UG|At#S21736925	gi|51969641|dbj|AK175750.1|	multiple canonical match
at4g19370	CON	MIN	HOUR	DAY	WEEK	UNIGENE	FLCDNA	TAIR
ATGAGCACCA	0	1	0	0	0	gnl|UG|At#S11724437	no match found	non-canonical match
at3g30465	CON	MIN	HOUR	DAY	WEEK	UNIGENE	FLCDNA	TAIR
AGAAGAAAGC	0	1	0	1	0	no match found	no match found	At3g30465.1
at3g19780	CON	MIN	HOUR	DAY	WEEK	UNIGENE	FLCDNA	TAIR
TGGGTAGAAG	1	2	0	1	1	gnl|UG|At#S11734919	gi|26450831|dbj|AK117884.1|	At3g19780.1
at1g18400	CON	MIN	HOUR	DAY	WEEK	UNIGENE	FLCDNA	TAIR
GTCCCTTTGA	1	1	1	0	0	gnl|UG|At#S11741211	gi|28950816|gb|BT005268.1	At1g18400.1
at1g36500	CON	MIN	HOUR	DAY	WEEK	UNIGENE	FLCDNA	TAIR
TATGGTTGAT	1	0	1	0	0	no match found	no match found	At1g36500.1
at2g39960	CON	MIN	HOUR	DAY	WEEK	UNIGENE	FLCDNA	TAIR
GAGGAGAAGA	0	1	0	1	0	gnl|UG|At#S11732614	no match found	At2g39960.1
at4g31550	CON	MIN	HOUR	DAY	WEEK	UNIGENE	FLCDNA	TAIR
AGCACTCAGA	0	14	3	2	0	no match found	gi|23297313|gb|AY150394.1	At4g31550.1
at1g34418	CON	MIN	HOUR	DAY	WEEK	UNIGENE	FLCDNA	TAIR
TGGTTATGAT	1	0	0	0	2	gnl|UG|At#S18923767	gi|20259371|gb|AY090971.1	non-canonical match
at4g37530	CON	MIN	HOUR	DAY	WEEK	UNIGENE	FLCDNA	TAIR
GATCCCAATA	0	0	2	0	0	no match found	gi|23297813|gb|AY150515.1	multiple non-canonical match
ATTAGTCCCA	0	0	2	2	0	gnl|UG|At#S11721346	no match found	At4g37530.1
at2g02800	CON	MIN	HOUR	DAY	WEEK	UNIGENE	FLCDNA	TAIR
GGTACTCACG	1	2	1	0	0	gnl|UG|At#S11740562	gi|26449870|dbj|AK117389.1|	At2g02800.1
at2g40400	CON	MIN	HOUR	DAY	WEEK	UNIGENE	FLCDNA	TAIR
GGTGTGATTC	0	0	1	1	0	gnl|UG|At#S15460572	no match found	At2g40400.2
ATTTCATTTG	0	1	1	0	0	no match found	gi|15294187|gb|AF410285.1	non-canonical match
TTGGTTTGCC	0	0	1	0	0	no match found	no match found	At2g40400.1
at1g55840	CON	MIN	HOUR	DAY	WEEK	UNIGENE	FLCDNA	TAIR
GTTCAGTCCA	1	2	1	1	1	gnl|UG|At#S11732343	gi|19310516|gb|AY079108.1	At1g55840.1
at4g11340	CON	MIN	HOUR	DAY	WEEK	UNIGENE	FLCDNA	TAIR
AGATGAAGGA	0	0	1	0	0	no match found	no match found	At4g11340.1
at4g00550	CON	MIN	HOUR	DAY	WEEK	UNIGENE	FLCDNA	TAIR
TATGATGACA	0	1	0	0	0	no match found	no match found	At4g00550.1
at3g21110	CON	MIN	HOUR	DAY	WEEK	UNIGENE	FLCDNA	TAIR
AAAAAAACTA	0	1	2	3	3	gnl|UG|At#S11734493	gi|19347901|gb|AY080627.1	non-canonical match
at4g18370	CON	MIN	HOUR	DAY	WEEK	UNIGENE	FLCDNA	TAIR
TTATTGCTAA	1	1	0	0	0	no match found	gi|51971740|dbj|AK176772.1|	multiple non-canonical match
at1g55590	CON	MIN	HOUR	DAY	WEEK	UNIGENE	FLCDNA	TAIR
ATTCAGTAGG	1	0	0	0	0	gnl|UG|At#S11732446	gi|20856546|gb|AY102102.1	At1g55590.1

at3g11830 CON MIN HOUR DAY WEEK UNIGENE FLCDNA TAIR
CAAAGCGGTG 1 7 0 2 1 gnl|UG|At#S11737345 gi|20857171|gb|AY102137.1 At3g11830.1

at4g36640 CON MIN HOUR DAY WEEK UNIGENE FLCDNA TAIR
TGTAATACTA 1 1 0 0 0 gnl|UG|At#S11721500 gi|26450473|dbj|AK117699.1| At4g36640.1

at2g44130 CON MIN HOUR DAY WEEK UNIGENE FLCDNA TAIR
CTTCAATCTA 4 3 0 1 0 no match found no match found At2g44130.1

at3g20800 CON MIN HOUR DAY WEEK UNIGENE FLCDNA TAIR
TGCTTTTTAA 1 0 0 0 1 no match found gi|21403744|gb|AY085034.1 multiple non-canonical match
TTGAACGGTA 3 0 3 0 0 gnl|UG|At#S11807503 gi|20258839|gb|AY091082.1 At3g20800.1

at1g23480 CON MIN HOUR DAY WEEK UNIGENE FLCDNA TAIR
ACGTCGAGGT 0 1 0 0 0 gnl|UG|At#S28282596 gi|23197989|gb|BT000203.1 At1g23480.1

at5g09990 CON MIN HOUR DAY WEEK UNIGENE FLCDNA TAIR
ATATATTGAT 1 0 0 0 0 gnl|UG|At#S30644282 gi|21404891|gb|AY086181.1 At5g09990.1

at3g60640 CON MIN HOUR DAY WEEK UNIGENE FLCDNA TAIR
ATTTCGAGAA 0 1 0 0 0 gnl|UG|At#S11727291 gi|21407023|gb|AY088249.1 At3g60640.1

at2g44100 CON MIN HOUR DAY WEEK UNIGENE FLCDNA TAIR
CGTACCTATT 1 1 2 3 1 no match found gi|13605640|gb|AF361801.1 non-canonical match
CTCTTCCGTT 12 9 7 2 6 no match found gi|21405642|gb|AY086920.1 At2g44100.1

at3g15560 CON MIN HOUR DAY WEEK UNIGENE FLCDNA TAIR
TTTTCAAGGC 0 0 1 0 0 gnl|UG|At#S11736178 no match found At3g15560.1

at5g26030 CON MIN HOUR DAY WEEK UNIGENE FLCDNA TAIR
CGTTCCGGAA 0 2 4 5 1 gnl|UG|At#S11720588 gi|19310444|gb|AY078959.1 At5g26030.1

at5g19770 CON MIN HOUR DAY WEEK UNIGENE FLCDNA TAIR
TATGCGAAGA 4 10 4 5 7 gnl|UG|At#S11721380 no match found At5g19770.1

at2g25180 CON MIN HOUR DAY WEEK UNIGENE FLCDNA TAIR
CATATGAGAG 1 2 1 1 1 gnl|UG|At#S11736228 no match found At2g25180.1

at3g05600 CON MIN HOUR DAY WEEK UNIGENE FLCDNA TAIR
TGTTGTTTCG 5 8 6 4 1 gnl|UG|At#S11739046 no match found multiple canonical match

at1g21550 CON MIN HOUR DAY WEEK UNIGENE FLCDNA TAIR
TTTGATGTGT 1 0 0 0 0 no match found gi|13605536|gb|AF361594.1 multiple canonical match

at5g33330 CON MIN HOUR DAY WEEK UNIGENE FLCDNA TAIR
GTTCGATTCA 0 0 0 0 1 no match found no match found At5g33330.1

at4g01070 CON MIN HOUR DAY WEEK UNIGENE FLCDNA TAIR
GCCATTATAC 1 0 1 2 1 gnl|UG|At#S11727419 gi|13430699|gb|AF360262.1 At4g01070.1

at3g20340 CON MIN HOUR DAY WEEK UNIGENE FLCDNA TAIR
ACGCTTCTGT 0 3 2 0 0 gnl|UG|At#S11734745 no match found At3g20340.1
TTTGTATATG 1 0 1 0 0 no match found gi|44681325|gb|BT011597.1 multiple non-canonical match

at2g39200 CON MIN HOUR DAY WEEK UNIGENE FLCDNA TAIR
TCAAGGTTGA 0 0 0 1 0 gnl|UG|At#S11732800 gi|110737700|dbj|AK228900.1| At2g39200.1

at1g68670 CON MIN HOUR DAY WEEK UNIGENE FLCDNA TAIR
AAGGTTGATG 0 9 2 0 0 no match found no match found At1g68670.1

at1g27500 CON MIN HOUR DAY WEEK UNIGENE FLCDNA TAIR

TGGTTGGGGT	0	0	1	0	0	gnl|UG|At#S11739815	gi|29028989|gb|BT005939.1	At1g27500.1
at4g01600	CON	MIN	HOUR	DAY	WEEK	UNIGENE	FLCDNA	TAIR
TAATCAAATT	0	3	0	1	0	gnl|UG|At#S11727283	gi|50897219|gb|BT015153.1	At4g01600.1
at1g12570	CON	MIN	HOUR	DAY	WEEK	UNIGENE	FLCDNA	TAIR
GAGGATGCGT	0	1	0	0	0	gnl|UG|At#S11741780	gi|17978953|gb|AY069890.1	At1g12570.1
at1g11800	CON	MIN	HOUR	DAY	WEEK	UNIGENE	FLCDNA	TAIR
AACTGGTGCG	0	1	0	0	0	gnl|UG|At#S11741856	gi|21404353|gb|AY085643.1	At1g11800.1
at3g15860	CON	MIN	HOUR	DAY	WEEK	UNIGENE	FLCDNA	TAIR
GTGATTGTTG	0	0	1	0	1	no match found	no match found	At3g15860.1
AACATAACAA	0	1	0	0	0	gnl|UG|At#S21989792	no match found	non-canonical match
at5g08010	CON	MIN	HOUR	DAY	WEEK	UNIGENE	FLCDNA	TAIR
GAAGAAATAC	2	0	0	0	0	gnl|UG|At#S11724114	no match found	At5g08010.1
at1g61240	CON	MIN	HOUR	DAY	WEEK	UNIGENE	FLCDNA	TAIR
ATCTTCTTTA	1	0	1	0	0	gnl|UG|At#S43850404	gi|26449862|dbj|AK117385.1|	At1g61240.1
at1g27750	CON	MIN	HOUR	DAY	WEEK	UNIGENE	FLCDNA	TAIR
ACACAATAGA	0	1	0	0	1	gnl|UG|At#S11807205	gi|20259446|gb|AY091022.1	non-canonical match
AGGATTTGCA	0	0	0	1	0	no match found	no match found	At1g27750.1
at1g35186	CON	MIN	HOUR	DAY	WEEK	UNIGENE	FLCDNA	TAIR
TGGTTCCATT	1	0	0	0	0	no match found	no match found	At1g35186.1
at2g06912	CON	MIN	HOUR	DAY	WEEK	UNIGENE	FLCDNA	TAIR
AAAAGAAATT	1	1	4	0	0	no match found	no match found	At2g06912.1
at1g23150	CON	MIN	HOUR	DAY	WEEK	UNIGENE	FLCDNA	TAIR
TTTGGTTTGT	0	0	0	0	1	gnl|UG|At#S11825081	gi|27754626|gb|BT002946.1	At1g23150.1
at4g16155	CON	MIN	HOUR	DAY	WEEK	UNIGENE	FLCDNA	TAIR
AAGAATTTAA	1	1	0	0	0	no match found	gi|21281172|gb|AY113958.1	multiple non-canonical match
GTTTTGACAA	2	3	5	4	0	gnl|UG|At#S11725002	gi|15809855|gb|AY054195.1	At4g16155.1
at2g01190	CON	MIN	HOUR	DAY	WEEK	UNIGENE	FLCDNA	TAIR
TAGTGCTTTA	2	1	3	1	3	gnl|UG|At#S11742715	gi|17979118|gb|AY070060.1	At2g01190.1
at3g25760	CON	MIN	HOUR	DAY	WEEK	UNIGENE	FLCDNA	TAIR
ACAACTCTCA	0	4	0	1	0	gnl|UG|At#S21735898	gi|87116591|gb|BT024479.1	At3g25760.1
at2g24430	CON	MIN	HOUR	DAY	WEEK	UNIGENE	FLCDNA	TAIR
GACTTGGATG	0	0	1	0	0	gnl|UG|At#S15461204	no match found	At2g24430.1
at1g48840	CON	MIN	HOUR	DAY	WEEK	UNIGENE	FLCDNA	TAIR
TTCTTTGTTT	1	0	0	0	0	no match found	no match found	At1g48840.1
TATGTTGTAG	2	0	1	1	3	gnl|UG|At#S11743125	gi|18377752|gb|AY074330.1	non-canonical match
at3g18210	CON	MIN	HOUR	DAY	WEEK	UNIGENE	FLCDNA	TAIR
AAAGAATCTA	3	1	4	0	1	gnl|UG|At#S11735378	gi|14423467|gb|AF386971.1	At3g18210.1
at5g12890	CON	MIN	HOUR	DAY	WEEK	UNIGENE	FLCDNA	TAIR
TCCGGTAATT	7	10	15	11	16	gnl|UG|At#S11723138	no match found	At5g12890.1
at5g17930	CON	MIN	HOUR	DAY	WEEK	UNIGENE	FLCDNA	TAIR
TGATCCTGTT	0	2	1	1	1	gnl|UG|At#S21736436	gi|51970619|dbj|AK176239.1|	non-canonical match
at1g36960	CON	MIN	HOUR	DAY	WEEK	UNIGENE	FLCDNA	TAIR

ACTCTTACTT	0	0	2	0	0	gnl|UG|At#S11816442	no match found	non-canonical match
at4g31040	CON	MIN	HOUR	DAY	WEEK	UNIGENE	FLCDNA	TAIR
TACACAATAA	2	2	1	2	2	gnl|UG|At#S11743872	gi|51968781|dbj|AK175320.1|	At4g31040.1
at2g41730	CON	MIN	HOUR	DAY	WEEK	UNIGENE	FLCDNA	TAIR
ATGAACATTG	0	3	0	1	0	gnl|UG|At#S20814515	gi|30793798|gb|BT008525.1	At2g41730.1
at2g44065	CON	MIN	HOUR	DAY	WEEK	UNIGENE	FLCDNA	TAIR
AAACTAGAAG	0	3	4	0	0	no match found	gi|17380713|gb|AY063831.1	At2g44065.2
at4g38830	CON	MIN	HOUR	DAY	WEEK	UNIGENE	FLCDNA	TAIR
GCTTCTGTTG	0	5	3	1	2	no match found	gi|110736138|dbj|AK228082.1|	At4g38830.1
at1g67480	CON	MIN	HOUR	DAY	WEEK	UNIGENE	FLCDNA	TAIR
TTGAAGTAGG	0	2	1	0	0	gnl|UG|At#S11729618	gi|119935812|gb|BT029730.1	At1g67480.1
at4g00430	CON	MIN	HOUR	DAY	WEEK	UNIGENE	FLCDNA	TAIR
ACTTCGTGAA	0	0	1	1	0	gnl|UG|At#S11727568	gi|20466717|gb|AY099825.1	At4g00430.1
GCTACACCAA	4	7	8	9	1	no match found	gi|21539580|gb|AY120785.1	At4g00430.2
at4g36360	CON	MIN	HOUR	DAY	WEEK	UNIGENE	FLCDNA	TAIR
TGCCGAGAGC	0	3	0	1	0	gnl|UG|At#S11721546	gi|110739913|dbj|AK230040.1|	At4g36360.1
at5g63120	CON	MIN	HOUR	DAY	WEEK	UNIGENE	FLCDNA	TAIR
ATATTTCTAA	0	0	0	0	1	gnl|UG|At#S11717389	gi|16974622|gb|AY060589.1	At5g63120.2
ACAATGCTAA	0	0	0	1	0	no match found	gi|110741549|dbj|AK226611.1|	non-canonical match
at5g63970	CON	MIN	HOUR	DAY	WEEK	UNIGENE	FLCDNA	TAIR
TTTATACTTC	1	0	0	0	0	gnl|UG|At#S11717302	gi|20259325|gb|AY090939.1	At5g63970.1
at2g14220	CON	MIN	HOUR	DAY	WEEK	UNIGENE	FLCDNA	TAIR
TTTCCACAAC	1	0	0	1	0	no match found	no match found	At2g14220.1
at5g18940	CON	MIN	HOUR	DAY	WEEK	UNIGENE	FLCDNA	TAIR
AAACTGCTGC	2	3	3	4	1	gnl|UG|At#S34118468	gi|21407133|gb|AY088359.1	At5g18940.2
at3g22290	CON	MIN	HOUR	DAY	WEEK	UNIGENE	FLCDNA	TAIR
GATATAGTTT	1	1	2	0	1	gnl|UG|At#S11734135	gi|51970733|dbj|AK176296.1|	At3g22290.1
GCCAGCTGTT	1	2	0	2	0	no match found	gi|45680160|gb|BT011850.1	multiple non-canonical match
at2g14680	CON	MIN	HOUR	DAY	WEEK	UNIGENE	FLCDNA	TAIR
GGATGATTCA	3	1	2	1	1	gnl|UG|At#S11823037	gi|39104598|dbj|AK118833.2|	At2g14680.1
at1g33110	CON	MIN	HOUR	DAY	WEEK	UNIGENE	FLCDNA	TAIR
ACTCTGAGAA	1	1	3	4	1	no match found	gi|17065359|gb|AY062756.1	At1g33110.1
at5g08150	CON	MIN	HOUR	DAY	WEEK	UNIGENE	FLCDNA	TAIR
ATTATGATGA	0	1	0	0	0	no match found	gi|44917536|gb|BT011730.1	multiple non-canonical match
at4g37270	CON	MIN	HOUR	DAY	WEEK	UNIGENE	FLCDNA	TAIR
GTCGTGGAAA	1	1	2	0	0	no match found	gi|18086406|gb|AY065018.1	non-canonical match
GACCTTTAAT	1	0	0	0	0	gnl|UG|At#S11721392	no match found	At4g37270.1
at5g42730	CON	MIN	HOUR	DAY	WEEK	UNIGENE	FLCDNA	TAIR
AACTCTGCTT	1	2	0	0	1	no match found	no match found	At5g42730.1
at2g39900	CON	MIN	HOUR	DAY	WEEK	UNIGENE	FLCDNA	TAIR
TTCTTTGTCT	0	2	0	1	0	no match found	gi|20453155|gb|AY094448.1	multiple canonical match
at5g63320	CON	MIN	HOUR	DAY	WEEK	UNIGENE	FLCDNA	TAIR

ATAATGGGGA	0	1	0	0	0	gnl|UG|At#S11717368	no match found	At5g63320.1
at5g39850	CON	MIN	HOUR	DAY	WEEK	UNIGENE	FLCDNA	TAIR
TCACTTTCGA	1	0	1	7	0	gnl|UG|At#S11809981	gi|21406883|gb|AY088109.1	At5g39850.1
at2g04120	CON	MIN	HOUR	DAY	WEEK	UNIGENE	FLCDNA	TAIR
AGCAAAAAAG	0	0	0	1	0	no match found	no match found	At2g04120.1
at5g61010	CON	MIN	HOUR	DAY	WEEK	UNIGENE	FLCDNA	TAIR
CTTAATACAA	3	0	1	0	1	gnl|UG|At#S11717601	no match found	At5g61010.1
CAAATAAAGT	2	2	2	1	0	no match found	gi|15215763|gb|AY050411.1	non-canonical match
GAGCTCCATA	0	1	0	0	0	no match found	gi|16974328|gb|AY059656.1	non-canonical match
at1g14690	CON	MIN	HOUR	DAY	WEEK	UNIGENE	FLCDNA	TAIR
TGTGTATATT	0	0	1	0	0	gnl|UG|At#S11741565	gi|51970741|dbj|AK176300.1|	non-canonical match
CAGAACGCGC	1	1	1	1	0	no match found	gi|30725419|gb|BT008373.1	non-canonical match
at3g02360	CON	MIN	HOUR	DAY	WEEK	UNIGENE	FLCDNA	TAIR
AACCTTATCC	2	4	1	1	0	gnl|UG|At#S15461611	gi|21406065|gb|AY087341.1	At3g02360.2
at5g02710	CON	MIN	HOUR	DAY	WEEK	UNIGENE	FLCDNA	TAIR
TTATTAATAG	1	0	0	0	2	no match found	gi|15450945|gb|AY054553.1	non-canonical match
ATCGGAGATG	0	0	1	0	2	no match found	gi|30102907|gb|BT006564.1	non-canonical match
ATTCTTGACC	1	0	1	1	1	gnl|UG|At#S24443579	gi|62318527|dbj|AK220584.1|	pseudo chromosome match
at4g24730	CON	MIN	HOUR	DAY	WEEK	UNIGENE	FLCDNA	TAIR
GGGTTCACCA	1	0	1	0	0	gnl|UG|At#S18941803	gi|31711725|gb|BT008780.1	At4g24730.2
at2g26530	CON	MIN	HOUR	DAY	WEEK	UNIGENE	FLCDNA	TAIR
CAAATAGGAC	0	5	1	1	0	gnl|UG|At#S11735902	gi|21406011|gb|AY087287.1	At2g26530.1
at5g13620	CON	MIN	HOUR	DAY	WEEK	UNIGENE	FLCDNA	TAIR
GACTTTTTTT	1	0	0	0	0	no match found	no match found	At5g13620.1
at5g67560	CON	MIN	HOUR	DAY	WEEK	UNIGENE	FLCDNA	TAIR
ATATCTTGCA	0	0	2	0	0	no match found	gi|15450887|gb|AY054524.1	non-canonical match
ATGAGATCAC	0	1	0	0	3	no match found	gi|21406224|gb|AY087487.1	At5g67560.1
at1g49980	CON	MIN	HOUR	DAY	WEEK	UNIGENE	FLCDNA	TAIR
GATGCTAATA	0	1	0	0	0	gnl|UG|At#S11734507	gi|62319916|dbj|AK221284.1|	At1g49980.1
at1g77080	CON	MIN	HOUR	DAY	WEEK	UNIGENE	FLCDNA	TAIR
GGAAGAAGAA	0	1	0	0	0	no match found	no match found	At1g77080.2
AACTTTTGTA	0	0	0	1	0	gnl|UG|At#S35310936	no match found	non-canonical match
at2g43330	CON	MIN	HOUR	DAY	WEEK	UNIGENE	FLCDNA	TAIR
ACTTACACAT	2	0	0	0	0	gnl|UG|At#S11697057	gi|17380889|gb|AY063901.1	At2g43330.1
at2g46800	CON	MIN	HOUR	DAY	WEEK	UNIGENE	FLCDNA	TAIR
TCACGATACA	4	6	3	1	2	gnl|UG|At#S15460275	gi|21404743|gb|AY086033.1	At2g46800.2
at2g40970	CON	MIN	HOUR	DAY	WEEK	UNIGENE	FLCDNA	TAIR
GTAGTATGAA	0	1	0	0	0	gnl|UG|At#S11732346	gi|18252892|gb|AY072381.1	At2g40970.1
at3g08955	CON	MIN	HOUR	DAY	WEEK	UNIGENE	FLCDNA	TAIR
ATTCCCTACA	0	2	0	0	0	no match found	gi|50253457|gb|BT015059.1	At3g08955.1
at5g65480	CON	MIN	HOUR	DAY	WEEK	UNIGENE	FLCDNA	TAIR
GAACAGTGGC	18	7	12	9	4	no match found	gi|23397175|gb|BT000729.1	non-canonical match
TGGGTGTTTT	7	2	4	2	0	no match found	gi|16604319|gb|AY058058.1	non-canonical match
TGCTTACTTT	11	4	12	6	2	gnl|UG|At#S11717149	no match found	At5g65480.1

at2g05070	CON	MIN	HOUR	DAY	WEEK	UNIGENE	FLCDNA	TAIR
ACAGGTTCAT	1	0	0	1	0	gnl|UG|At#S11740132	no match found	At2g05070.1
at2g13143	CON	MIN	HOUR	DAY	WEEK	UNIGENE	FLCDNA	TAIR
TCATATATGT	1	1	0	0	0	no match found	no match found	At2g13143.1
at5g23460	CON	MIN	HOUR	DAY	WEEK	UNIGENE	FLCDNA	TAIR
AGTTTAATTT	0	0	1	0	0	gnl|UG|At#S18897099	no match found	multiple non-canonical match
at3g12750	CON	MIN	HOUR	DAY	WEEK	UNIGENE	FLCDNA	TAIR
ATAGGAAGCA	0	2	1	0	0	gnl|UG|At#S34115619	gi|110741154|dbj|AK226531.1|	At3g12750.1
at5g50810	CON	MIN	HOUR	DAY	WEEK	UNIGENE	FLCDNA	TAIR
AAACGCTTTA	1	2	1	3	0	gnl|UG|At#S11718636	gi|21406025|gb|AY087301.1	At5g50810.1
at3g19670	CON	MIN	HOUR	DAY	WEEK	UNIGENE	FLCDNA	TAIR
AGCTCCAGCT	0	2	3	2	0	gnl|UG|At#S11734951	no match found	At3g19670.1
at3g61890	CON	MIN	HOUR	DAY	WEEK	UNIGENE	FLCDNA	TAIR
AGCTGATGAA	2	3	1	1	4	gnl|UG|At#S11726879	gi|21405911|gb|AY087187.1	multiple canonical match
at4g37930	CON	MIN	HOUR	DAY	WEEK	UNIGENE	FLCDNA	TAIR
AAGTACAAGA	0	0	2	0	0	no match found	gi|30102485|gb|BT006353.1	non-canonical match
TAAAACTCTA	169	69	114	103	75	gnl|UG|At#S11721281	gi|17979461|gb|AY070726.1	At4g37930.1
at3g57720	CON	MIN	HOUR	DAY	WEEK	UNIGENE	FLCDNA	TAIR
CGTTGTACTT	0	1	0	1	0	gnl|UG|At#S18903723	no match found	At3g57720.1
at5g02480	CON	MIN	HOUR	DAY	WEEK	UNIGENE	FLCDNA	TAIR
TTTTAAAGTG	5	3	1	0	2	gnl|UG|At#S11725541	gi|15724273|gb|AF412077.1	At5g02480.1
at1g18090	CON	MIN	HOUR	DAY	WEEK	UNIGENE	FLCDNA	TAIR
ACGACAAAGA	0	0	0	0	1	gnl|UG|At#S11741237	gi|15215781|gb|AY050420.1	At1g18090.2
at1g21200	CON	MIN	HOUR	DAY	WEEK	UNIGENE	FLCDNA	TAIR
TGAATTCTCT	0	2	1	0	0	gnl|UG|At#S11704654	gi|15027986|gb|AY045850.1	At1g21200.1
at5g09690	CON	MIN	HOUR	DAY	WEEK	UNIGENE	FLCDNA	TAIR
GTTACATTTT	0	1	0	0	0	gnl|UG|At#S11723813	gi|110736531|dbj|AK228287.1|	At5g09690.3
at5g41260	CON	MIN	HOUR	DAY	WEEK	UNIGENE	FLCDNA	TAIR
CAAGACAGAG	0	2	0	0	0	gnl|UG|At#S11719603	gi|115311488|gb|BT029016.1	At5g41260.1
at3g19980	CON	MIN	HOUR	DAY	WEEK	UNIGENE	FLCDNA	TAIR
GAAAGGGAAG	2	1	0	1	3	gnl|UG|At#S11734859	no match found	At3g19980.1
at5g40450	CON	MIN	HOUR	DAY	WEEK	UNIGENE	FLCDNA	TAIR
AATCTAAAAT	0	5	0	0	1	gnl|UG|At#S43849204	gi|62320449|dbj|AK221551.1|	multiple non-canonical match
TTCAATAAAT	5	16	8	6	1	no match found	gi|26449801|dbj|AK117354.1|	At5g58090.1
at2g47980	CON	MIN	HOUR	DAY	WEEK	UNIGENE	FLCDNA	TAIR
AAATATCTAT	2	0	0	1	0	gnl|UG|At#S11730733	no match found	At2g47980.1
at1g17690	CON	MIN	HOUR	DAY	WEEK	UNIGENE	FLCDNA	TAIR
TTTGCTTTCT	0	0	1	1	1	gnl|UG|At#S11705970	gi|15810522|gb|AY056300.1	At1g17690.1
at3g01840	CON	MIN	HOUR	DAY	WEEK	UNIGENE	FLCDNA	TAIR
GCCCTAAACT	0	1	0	0	0	no match found	no match found	At3g01840.1
at2g37585	CON	MIN	HOUR	DAY	WEEK	UNIGENE	FLCDNA	TAIR

AAAAGTGTAT	0	1	0	0	0	gnl|UG|At#S34118178	gi|110736228|dbj|AK228128.1|	At2g37585.1
TTTTCTCTAA	0	0	0	0	1	gnl|UG|At#S11820517	gi|23296568|gb|AY142550.1	non-canonical match
at3g49910	CON	MIN	HOUR	DAY	WEEK	UNIGENE	FLCDNA	TAIR
CCGATCCGTA	0	0	2	0	0	gnl|UG|At#S30451010	gi|16323425|gb|AY059101.1	non-canonical match
AGAACTTATG	43	19	38	44	65	gnl|UG|At#S11702417	gi|13877790|gb|AF370158.1	At3g49910.1
at5g09270	CON	MIN	HOUR	DAY	WEEK	UNIGENE	FLCDNA	TAIR
TTAAGCGTTG	0	0	0	2	0	no match found	gi|21406575|gb|AY087824.1	At5g09270.1
at5g14660	CON	MIN	HOUR	DAY	WEEK	UNIGENE	FLCDNA	TAIR
GTGTTTAATC	0	0	0	1	0	no match found	gi|20465676|gb|AY096673.1	non-canonical match
TCGTGGTTTA	1	0	0	0	1	no match found	gi|21404127|gb|AY085417.1	At5g14660.2
TGTTGGTAAT	0	0	1	0	0	gnl|UG|At#S11722677	no match found	pseudo chromosome match
at2g25910	CON	MIN	HOUR	DAY	WEEK	UNIGENE	FLCDNA	TAIR
AATGATGCTG	0	1	0	0	0	no match found	gi|18491138|gb|AY074840.1	multiple non-canonical match
GTTCAGTACG	4	9	3	1	1	gnl|UG|At#S28282263	gi|13605560|gb|AF361606.1	At2g25910.1
at1g01200	CON	MIN	HOUR	DAY	WEEK	UNIGENE	FLCDNA	TAIR
TGTCACTCGT	0	0	0	0	1	gnl|UG|At#S34117979	gi|110736610|dbj|AK228329.1|	At1g01200.1
at5g16300	CON	MIN	HOUR	DAY	WEEK	UNIGENE	FLCDNA	TAIR
TAATTAAGTA	3	0	0	0	3	gnl|UG|At#S11722266	no match found	non-canonical match
at2g26650	CON	MIN	HOUR	DAY	WEEK	UNIGENE	FLCDNA	TAIR
GGTTCTTCTC	0	1	0	0	0	no match found	no match found	At2g26650.1
AGAGGAGAGT	0	0	2	0	0	gnl|UG|At#S11735870	gi|30102663|gb|BT006442.1	non-canonical match
at3g47520	CON	MIN	HOUR	DAY	WEEK	UNIGENE	FLCDNA	TAIR
GCATTGAAGC	2	0	6	8	8	gnl|UG|At#S11815682	gi|22135806|gb|AY128281.1	At3g47520.1
CTGGAATCAC	0	1	0	2	0	no match found	gi|23308436|gb|BT000621.1	non-canonical match
at1g69490	CON	MIN	HOUR	DAY	WEEK	UNIGENE	FLCDNA	TAIR
AATGAAAATT	1	4	4	2	4	gnl|UG|At#S11702038	gi|13430577|gb|AF360201.1	At1g69490.1
GGACCCGTCT	1	0	1	1	4	no match found	gi|15293162|gb|AY051015.1	non-canonical match
at1g32490	CON	MIN	HOUR	DAY	WEEK	UNIGENE	FLCDNA	TAIR
GAGCAGTTGT	1	0	0	1	2	gnl|UG|At#S11738097	gi|18377728|gb|AY074318.1	At1g32490.1
at1g53645	CON	MIN	HOUR	DAY	WEEK	UNIGENE	FLCDNA	TAIR
GACAAGCTCG	1	1	3	1	0	gnl|UG|At#S21736495	gi|51970531|dbj|AK176195.1|	At1g53645.1
at5g16840	CON	MIN	HOUR	DAY	WEEK	UNIGENE	FLCDNA	TAIR
GAAGTTGGAA	8	1	9	7	7	gnl|UG|At#S38433292	gi|16648739|gb|AY058146.1	At5g16840.1
at5g23310	CON	MIN	HOUR	DAY	WEEK	UNIGENE	FLCDNA	TAIR
AGTCGGATGG	0	1	1	4	1	gnl|UG|At#S11720847	gi|20259614|gb|AY091225.1	At5g23310.1
at5g48610	CON	MIN	HOUR	DAY	WEEK	UNIGENE	FLCDNA	TAIR
TTGTGTTATA	0	0	0	1	1	gnl|UG|At#S34115281	gi|110741788|dbj|AK226736.1|	multiple canonical match
AGGTTGATGA	0	1	0	0	0	gnl|UG|At#S35292126	no match found	multiple non-canonical match
at4g38510	CON	MIN	HOUR	DAY	WEEK	UNIGENE	FLCDNA	TAIR
GACTCTCCTC	0	3	1	4	0	gnl|UG|At#S28281879	gi|19699257|gb|AY090334.1	multiple non-canonical match
at1g04730	CON	MIN	HOUR	DAY	WEEK	UNIGENE	FLCDNA	TAIR
CTTGATATAT	0	0	0	1	0	no match found	no match found	At1g04730.1
at4g19110	CON	MIN	HOUR	DAY	WEEK	UNIGENE	FLCDNA	TAIR
CCTCTTCTGA	6	5	0	4	0	gnl|UG|At#S43849535	gi|110741343|dbj|AK230424.1|	At4g19110.1

at1g55870	CON	MIN	HOUR	DAY	WEEK	UNIGENE	FLCDNA	TAIR
TTCTCTGACC	0	2	0	0	0	gnl|UG|At#S28103976	gi|110743151|dbj|AK227465.1|	At1g55870.1
at4g16670	CON	MIN	HOUR	DAY	WEEK	UNIGENE	FLCDNA	TAIR
ATGTCTTGTG	0	1	1	0	0	gnl|UG|At#S14274055	gi|28393014|gb|BT003894.1	At4g16670.1
at4g15765	CON	MIN	HOUR	DAY	WEEK	UNIGENE	FLCDNA	TAIR
AGGTTGGTTA	1	3	1	0	0	gnl|UG|At#S24442868	gi|62319938|dbj|AK221295.1|	At4g15765.1
at5g01590	CON	MIN	HOUR	DAY	WEEK	UNIGENE	FLCDNA	TAIR
GGCTTATGAA	0	0	0	0	1	gnl|UG|At#S11725761	no match found	At5g01590.1
GTTTGTTTGT	4	0	1	1	1	no match found	gi|15292666|gb|AY050767.1	non-canonical match
at3g22190	CON	MIN	HOUR	DAY	WEEK	UNIGENE	FLCDNA	TAIR
GGAGAAACAG	1	1	1	1	0	no match found	no match found	At3g22190.1
at1g15950	CON	MIN	HOUR	DAY	WEEK	UNIGENE	FLCDNA	TAIR
TTTGATCTAT	9	5	3	3	9	gnl|UG|At#S11741442	gi|110736783|dbj|AK228419.1|	At1g15950.1
at2g28800	CON	MIN	HOUR	DAY	WEEK	UNIGENE	FLCDNA	TAIR
CCCGCTTCAG	10	6	15	18	12	gnl|UG|At#S43850141	gi|27311660|gb|BT002436.1	At2g28800.1
at3g49990	CON	MIN	HOUR	DAY	WEEK	UNIGENE	FLCDNA	TAIR
GTCAAGAGTC	0	2	1	1	3	gnl|UG|At#S11824485	gi|29028881|gb|BT005885.1	At3g49990.1
at3g13120	CON	MIN	HOUR	DAY	WEEK	UNIGENE	FLCDNA	TAIR
ATTGATATTC	2	9	2	2	3	gnl|UG|At#S11736955	gi|16226586|gb|AF428438.1	At3g13120.1
at1g08710	CON	MIN	HOUR	DAY	WEEK	UNIGENE	FLCDNA	TAIR
AAGTATCATC	3	1	0	2	4	no match found	gi|22136745|gb|AY133758.1	At5g46580.1
AATAAAGATG	1	1	0	0	1	no match found	gi|17529081|gb|AY065275.1	At1g08710.1
at3g47770	CON	MIN	HOUR	DAY	WEEK	UNIGENE	FLCDNA	TAIR
TAACCACAAT	0	1	0	0	0	no match found	no match found	At3g47770.1
at5g17870	CON	MIN	HOUR	DAY	WEEK	UNIGENE	FLCDNA	TAIR
TCTGTGTCAG	5	13	1	1	1	no match found	gi|21405379|gb|AY086669.1	At5g17870.1
at4g18780	CON	MIN	HOUR	DAY	WEEK	UNIGENE	FLCDNA	TAIR
CTGTTTCTAG	0	3	0	0	0	no match found	gi|20466339|gb|AY099636.1	multiple non-canonical match
TTTTTAAAAA	1	3	1	1	1	gnl|UG|At#S11724540	no match found	At4g18780.1
at5g54730	CON	MIN	HOUR	DAY	WEEK	UNIGENE	FLCDNA	TAIR
AACATAGTTA	0	1	4	0	0	gnl|UG|At#S11718241	no match found	At5g54730.1
at2g42690	CON	MIN	HOUR	DAY	WEEK	UNIGENE	FLCDNA	TAIR
CGAGTTCTTG	0	0	1	0	0	no match found	gi|19310712|gb|AY079356.1	non-canonical match
ATAAATCAAC	6	3	5	5	1	gnl|UG|At#S11731950	gi|15293128|gb|AY050998.1	At2g42690.1
at2g07777	CON	MIN	HOUR	DAY	WEEK	UNIGENE	FLCDNA	TAIR
CTCCCCAGGG	2	0	2	3	1	no match found	no match found	At2g07777.1
at5g11950	CON	MIN	HOUR	DAY	WEEK	UNIGENE	FLCDNA	TAIR
AAAATGTTGC	3	2	1	1	5	gnl|UG|At#S11723261	no match found	At5g11950.2
TTGCATCGCA	0	0	1	0	0	no match found	gi|110743603|dbj|AK227652.1|	At5g11950.1
at5g46510	CON	MIN	HOUR	DAY	WEEK	UNIGENE	FLCDNA	TAIR
TCTATATATG	0	0	0	0	2	gnl|UG|At#S11719074	no match found	At5g46510.1
at2g31580	CON	MIN	HOUR	DAY	WEEK	UNIGENE	FLCDNA	TAIR

TATGTCAACC	0	1	1	0	0	gnl|UG|At#S11807182	gi|20259495|gb|AY091047.1	At2g31580.1
at5g08030	CON	MIN	HOUR	DAY	WEEK	UNIGENE	FLCDNA	TAIR
GATGTTTCCA	0	0	2	0	0	gnl|UG|At#S11724109	gi|66792677|gb|BT023450.1	At5g08030.1
at3g49790	CON	MIN	HOUR	DAY	WEEK	UNIGENE	FLCDNA	TAIR
GATGCTGACT	1	3	0	0	1	gnl|UG|At#S34117449	gi|46931271|gb|BT012621.1	At3g49790.1
at4g36210	CON	MIN	HOUR	DAY	WEEK	UNIGENE	FLCDNA	TAIR
TTTGAAATGT	4	3	2	0	2	gnl|UG|At#S34114720	gi|110742825|dbj|AK227295.1|	At4g36210.1
ACTGCTGCAA	0	0	2	0	0	no match found	no match found	At4g36210.2
at3g10570	CON	MIN	HOUR	DAY	WEEK	UNIGENE	FLCDNA	TAIR
GTTAAGCCAA	0	0	1	0	0	gnl|UG|At#S11737717	gi|59958319|gb|BT021100.1	At3g10570.1
at5g62690	CON	MIN	HOUR	DAY	WEEK	UNIGENE	FLCDNA	TAIR
ACGGAGATGA	0	1	0	0	0	gnl|UG|At#S11824377	no match found	non-canonical match
at5g24290	CON	MIN	HOUR	DAY	WEEK	UNIGENE	FLCDNA	TAIR
TTATGGAATT	0	0	1	0	0	gnl|UG|At#S15460426	gi|110736396|dbj|AK228214.1|	At5g24290.2
at5g43970	CON	MIN	HOUR	DAY	WEEK	UNIGENE	FLCDNA	TAIR
GTTAAGTAGT	0	0	0	1	0	gnl|UG|At#S35234681	no match found	non-canonical match
at1g16840	CON	MIN	HOUR	DAY	WEEK	UNIGENE	FLCDNA	TAIR
TTAATGAAAT	0	2	3	1	2	gnl|UG|At#S15460904	no match found	At1g16840.3
at2g04865	CON	MIN	HOUR	DAY	WEEK	UNIGENE	FLCDNA	TAIR
GAACTGTCAG	0	1	0	1	0	no match found	no match found	At2g04865.1
at2g25840	CON	MIN	HOUR	DAY	WEEK	UNIGENE	FLCDNA	TAIR
TCGAGTTAAT	1	1	0	0	0	gnl|UG|At#S18942856	gi|24899786|gb|BT001221.1	At2g25840.2
at5g38200	CON	MIN	HOUR	DAY	WEEK	UNIGENE	FLCDNA	TAIR
CAGAGGATGC	1	0	1	0	0	no match found	gi|16604299|gb|AY058048.1	At5g38200.1
ATTTGCTTTA	0	0	1	0	3	gnl|UG|At#S11719913	no match found	non-canonical match
at2g33400	CON	MIN	HOUR	DAY	WEEK	UNIGENE	FLCDNA	TAIR
TCAAATACCA	0	1	0	0	0	gnl|UG|At#S11744079	no match found	At2g33390.1
TAACTGTCTG	0	0	1	1	0	no match found	gi|19310602|gb|AY080714.1	At2g33390.1
at5g51970	CON	MIN	HOUR	DAY	WEEK	UNIGENE	FLCDNA	TAIR
GCCATTGTGT	0	7	4	0	4	gnl|UG|At#S34115581	gi|21403923|gb|AY085213.1	At5g51970.2
at4g10320	CON	MIN	HOUR	DAY	WEEK	UNIGENE	FLCDNA	TAIR
AGCCGTCCCT	1	2	2	0	2	gnl|UG|At#S11725933	no match found	At4g10320.1
at5g37570	CON	MIN	HOUR	DAY	WEEK	UNIGENE	FLCDNA	TAIR
TAGGTTAACG	0	0	0	1	1	no match found	no match found	At5g37570.1
at2g04360	CON	MIN	HOUR	DAY	WEEK	UNIGENE	FLCDNA	TAIR
ATAAACCACC	1	1	1	1	1	gnl|UG|At#S34117314	gi|110737887|dbj|AK228994.1|	At2g04360.1
at5g37690	CON	MIN	HOUR	DAY	WEEK	UNIGENE	FLCDNA	TAIR
TGTTATAAAT	1	0	0	0	0	gnl|UG|At#S11813207	gi|21403788|gb|AY085078.1	At5g37690.1
at5g22480	CON	MIN	HOUR	DAY	WEEK	UNIGENE	FLCDNA	TAIR
ATTTAATTTT	0	1	0	1	1	gnl|UG|At#S11720977	gi|21404338|gb|AY085628.1	At5g22480.1
at5g23940	CON	MIN	HOUR	DAY	WEEK	UNIGENE	FLCDNA	TAIR
TACTGTTGTC	2	0	1	1	1	gnl|UG|At#S11720785	gi|21407891|gb|AY089117.1	At5g23940.1

GTTTTTTAGT	0	1	0	1	0	gnl|UG|At#S20720356	no match found	non-canonical match
at4g05530	CON	MIN	HOUR	DAY	WEEK	UNIGENE	FLCDNA	TAIR
GCTGCTGCGG	0	0	2	0	0	no match found	gi|21281152|gb|AY113900.1	non-canonical match
TATGTGTATA	1	0	0	0	0	gnl|UG|At#S11726238	no match found	At4g05530.1
at3g22440	CON	MIN	HOUR	DAY	WEEK	UNIGENE	FLCDNA	TAIR
GAGGACTCTA	1	4	2	1	1	gnl|UG|At#S11707462	gi|15292672|gb|AY050770.1	At3g22440.1
at3g05040	CON	MIN	HOUR	DAY	WEEK	UNIGENE	FLCDNA	TAIR
TCATAAGCAA	1	2	0	2	2	gnl|UG|At#S11739212	gi|110740172|dbj|AK230176.1|	non-canonical match
at2g10950	CON	MIN	HOUR	DAY	WEEK	UNIGENE	FLCDNA	TAIR
CACATTTTTA	0	0	0	1	0	gnl|UG|At#S11739340	gi|110736707|dbj|AK228379.1|	At2g10950.1
at5g64820	CON	MIN	HOUR	DAY	WEEK	UNIGENE	FLCDNA	TAIR
TCTCTTCTTT	13	10	8	7	4	no match found	no match found	At5g64820.1
at4g26840	CON	MIN	HOUR	DAY	WEEK	UNIGENE	FLCDNA	TAIR
GAGGATGGTG	2	6	5	2	2	gnl|UG|At#S11723151	gi|18252866|gb|AY072368.1	At4g26840.1
at3g54590	CON	MIN	HOUR	DAY	WEEK	UNIGENE	FLCDNA	TAIR
TTATTCTCCA	0	3	0	0	0	gnl|UG|At#S11728584	no match found	multiple non-canonical match
at4g12900	CON	MIN	HOUR	DAY	WEEK	UNIGENE	FLCDNA	TAIR
GATGACACTC	0	1	0	0	1	gnl|UG|At#S11725582	no match found	At4g12900.1
at1g07130	CON	MIN	HOUR	DAY	WEEK	UNIGENE	FLCDNA	TAIR
AAATGTGTGC	1	0	0	0	0	no match found	gi|21405137|gb|AY086427.1	At3g31960.1
at5g12150	CON	MIN	HOUR	DAY	WEEK	UNIGENE	FLCDNA	TAIR
CTTGTAAAAC	8	3	4	2	2	gnl|UG|At#S43849333	gi|17473858|gb|AY065176.1	At5g12150.1
at2g23910	CON	MIN	HOUR	DAY	WEEK	UNIGENE	FLCDNA	TAIR
AAGAGAGATG	0	1	0	2	2	gnl|UG|At#S11736512	gi|21403953|gb|AY085243.1	At2g23910.1
at5g43940	CON	MIN	HOUR	DAY	WEEK	UNIGENE	FLCDNA	TAIR
AACAAGGAGA	4	7	2	7	0	gnl|UG|At#S11698521	gi|22022511|gb|AY126986.1	multiple non-canonical match
at1g72470	CON	MIN	HOUR	DAY	WEEK	UNIGENE	FLCDNA	TAIR
AGCTGTAATG	1	0	0	0	1	gnl|UG|At#S11728586	gi|110738011|dbj|AK229059.1|	At1g72470.1
at3g15470	CON	MIN	HOUR	DAY	WEEK	UNIGENE	FLCDNA	TAIR
TAAGCTTGAT	0	0	1	0	0	gnl|UG|At#S11736204	no match found	At3g15470.1
at1g55320	CON	MIN	HOUR	DAY	WEEK	UNIGENE	FLCDNA	TAIR
ACTAAACACT	0	0	0	0	2	gnl|UG|At#S38434161	gi|26449359|dbj|AK117129.1|	At1g55320.1
at5g49510	CON	MIN	HOUR	DAY	WEEK	UNIGENE	FLCDNA	TAIR
AACTAGAGAT	1	1	0	0	3	gnl|UG|At#S11702612	gi|13878182|gb|AF370354.1	At5g49510.1
at4g09460	CON	MIN	HOUR	DAY	WEEK	UNIGENE	FLCDNA	TAIR
GTTTCCAAAA	1	2	0	3	0	gnl|UG|At#S11726019	gi|28393540|gb|BT004171.1	At4g09460.1
GGAAGATCTC	1	0	1	0	0	no match found	gi|21405523|gb|AY086813.1	At3g54030.1
at5g38940	CON	MIN	HOUR	DAY	WEEK	UNIGENE	FLCDNA	TAIR
GATCTACAGA	0	1	0	0	0	no match found	gi|66841351|gb|BT023474.1	At5g38940.1
at1g20070	CON	MIN	HOUR	DAY	WEEK	UNIGENE	FLCDNA	TAIR
CCTAGGTTTT	0	0	1	0	0	gnl|UG|At#S11741048	gi|26450343|dbj|AK117632.1|	At1g20070.1

at3g55330	CON	MIN	HOUR	DAY	WEEK	UNIGENE	FLCDNA	TAIR
CTCTGGGTAC	1	7	3	1	1	gnl|UG|At#S11728453	gi|16930398|gb|AF419553.1	At3g55330.1
at3g44280	CON	MIN	HOUR	DAY	WEEK	UNIGENE	FLCDNA	TAIR
AAGTTTCGTT	0	1	0	1	0	no match found	gi|26451430|dbj|AK118192.1|	At3g44280.1
at1g51600	CON	MIN	HOUR	DAY	WEEK	UNIGENE	FLCDNA	TAIR
TTGGCTCTTG	1	1	1	0	0	gnl|UG|At#S11733919	gi|23297317|gb|AY150395.1	At1g51600.2
at1g47640	CON	MIN	HOUR	DAY	WEEK	UNIGENE	FLCDNA	TAIR
GAAACTTTGA	1	1	1	1	0	gnl|UG|At#S11735359	gi|110742812|dbj|AK227286.1|	At1g47640.1
at2g38600	CON	MIN	HOUR	DAY	WEEK	UNIGENE	FLCDNA	TAIR
GGCGATGAAA	0	1	0	0	0	gnl|UG|At#S11732943	no match found	non-canonical match
at2g46680	CON	MIN	HOUR	DAY	WEEK	UNIGENE	FLCDNA	TAIR
CTTTGTAGCT	0	1	0	2	5	gnl|UG|At#S11731033	no match found	At2g46680.1
AACCGAGCCA	0	1	3	1	0	no match found	gi|15027938|gb|AY045826.1	multiple non-canonical match
at2g17730	CON	MIN	HOUR	DAY	WEEK	UNIGENE	FLCDNA	TAIR
TAAATTCCAG	0	2	3	0	0	gnl|UG|At#S11738054	gi|26452209|dbj|AK118593.1|	At2g17730.1
at2g40840	CON	MIN	HOUR	DAY	WEEK	UNIGENE	FLCDNA	TAIR
TTGAAGCTCC	0	6	0	0	1	gnl|UG|At#S11732382	gi|19698936|gb|AY081315.1	At2g40840.1
at3g47610	CON	MIN	HOUR	DAY	WEEK	UNIGENE	FLCDNA	TAIR
TGATTACGTG	2	0	1	0	0	gnl|UG|At#S11729879	no match found	At3g47610.1
TAGAAGATGG	1	1	0	1	0	no match found	gi|21407767|gb|AY088993.1	non-canonical match
at4g17260	CON	MIN	HOUR	DAY	WEEK	UNIGENE	FLCDNA	TAIR
CAGAGTAATC	1	1	1	3	1	gnl|UG|At#S11724805	gi|21406000|gb|AY087276.1	At4g17260.1
at2g46030	CON	MIN	HOUR	DAY	WEEK	UNIGENE	FLCDNA	TAIR
ACTGTTTACA	1	2	0	1	0	gnl|UG|At#S34118063	gi|110736449|dbj|AK228245.1|	non-canonical match
at4g32850	CON	MIN	HOUR	DAY	WEEK	UNIGENE	FLCDNA	TAIR
TCGGAAGACC	1	2	1	1	1	no match found	gi|110740406|dbj|AK230297.1|	At4g32850.2
at5g45820	CON	MIN	HOUR	DAY	WEEK	UNIGENE	FLCDNA	TAIR
GAGAATTCCT	2	3	7	1	0	gnl|UG|At#S24442653	gi|114213524|gb|BT028970.1	non-canonical match
at4g33510	CON	MIN	HOUR	DAY	WEEK	UNIGENE	FLCDNA	TAIR
ATCAAGAAGG	0	5	0	1	0	gnl|UG|At#S11699204	gi|21700886|gb|AY124858.1	At4g33510.1
at3g55020	CON	MIN	HOUR	DAY	WEEK	UNIGENE	FLCDNA	TAIR
TTAATGACGA	0	1	0	0	0	gnl|UG|At#S11706128	gi|15810197|gb|AY056113.1	At3g55020.1
at4g32915	CON	MIN	HOUR	DAY	WEEK	UNIGENE	FLCDNA	TAIR
GCGACCAGAG	0	0	0	1	0	gnl|UG|At#S11722120	no match found	At4g32915.1
at4g25910	CON	MIN	HOUR	DAY	WEEK	UNIGENE	FLCDNA	TAIR
ACTGTTCGTG	1	4	0	0	0	gnl|UG|At#S11723325	gi|88196758|gb|BT024624.1	At4g25910.1
at5g45720	CON	MIN	HOUR	DAY	WEEK	UNIGENE	FLCDNA	TAIR
ACAAAATTTC	0	0	0	0	1	no match found	no match found	At5g45720.1
at3g08690	CON	MIN	HOUR	DAY	WEEK	UNIGENE	FLCDNA	TAIR
TACAAGACAG	1	4	2	2	1	gnl|UG|At#S11738290	gi|21404819|gb|AY086109.1	At3g08690.1
at1g69390	CON	MIN	HOUR	DAY	WEEK	UNIGENE	FLCDNA	TAIR
TATATATATA	0	1	0	0	1	gnl|UG|At#S11729233	gi|21407602|gb|AY088828.1	At1g69390.1

at2g12875	CON	MIN	HOUR	DAY	WEEK	UNIGENE	FLCDNA	TAIR
TGCCACTCCA	0	1	0	0	0	gnl|UG|At#S11817270	no match found	At2g12875.1
at1g05960	CON	MIN	HOUR	DAY	WEEK	UNIGENE	FLCDNA	TAIR
GAAGAGCACA	1	0	0	0	0	gnl|UG|At#S11742430	no match found	At1g05960.1
at3g23290	CON	MIN	HOUR	DAY	WEEK	UNIGENE	FLCDNA	TAIR
CCCTTTCCTA	1	0	0	1	0	gnl|UG|At#S38433684	no match found	non-canonical match
at2g16950	CON	MIN	HOUR	DAY	WEEK	UNIGENE	FLCDNA	TAIR
GTTCCTGTCT	2	1	1	0	0	gnl|UG|At#S11738244	no match found	multiple canonical match
at5g48470	CON	MIN	HOUR	DAY	WEEK	UNIGENE	FLCDNA	TAIR
TGACGTAAAA	0	0	0	0	2	gnl|UG|At#S11718873	no match found	At5g48470.1
at5g22220	CON	MIN	HOUR	DAY	WEEK	UNIGENE	FLCDNA	TAIR
TGGCCAGATG	0	2	0	0	0	no match found	gi|30387576|gb|BT006610.1	At5g22220.1
at1g48420	CON	MIN	HOUR	DAY	WEEK	UNIGENE	FLCDNA	TAIR
AACACGTCAG	0	3	1	0	0	gnl|UG|At#S11735062	gi|20259953|gb|AY093325.1	At1g48420.1
at3g32034	CON	MIN	HOUR	DAY	WEEK	UNIGENE	FLCDNA	TAIR
GAAAGAAAAT	0	2	0	1	4	no match found	gi|110742046|dbj|AK226876.1|	multiple non-canonical match
GTTGCTCAGA	1	1	1	3	1	no match found	gi|110738673|dbj|AK229400.1|	non-canonical match
TGTATTTATT	2	0	0	0	1	no match found	gi|110741723|dbj|AK226701.1|	non-canonical match
at3g52440	CON	MIN	HOUR	DAY	WEEK	UNIGENE	FLCDNA	TAIR
AAAAGAAAAA	7	6	10	10	3	no match found	no match found	At3g52440.1
at3g02920	CON	MIN	HOUR	DAY	WEEK	UNIGENE	FLCDNA	TAIR
TTCAAAAGTC	0	0	0	1	0	gnl|UG|At#S11739805	gi|21403516|gb|AY084806.1	At3g02920.1
at3g10240	CON	MIN	HOUR	DAY	WEEK	UNIGENE	FLCDNA	TAIR
ATCTTCTTCT	0	1	0	0	0	no match found	no match found	At3g10240.1
at1g65410	CON	MIN	HOUR	DAY	WEEK	UNIGENE	FLCDNA	TAIR
GCTGGATCAA	2	2	3	1	1	gnl|UG|At#S11702883	gi|13605494|gb|AF361573.1	At1g65410.1
at2g39020	CON	MIN	HOUR	DAY	WEEK	UNIGENE	FLCDNA	TAIR
TTGTTGACTG	1	0	0	1	1	gnl|UG|At#S11732844	gi|28466876|gb|BT004781.1	At2g39020.1
at4g28400	CON	MIN	HOUR	DAY	WEEK	UNIGENE	FLCDNA	TAIR
TATCGTTGTA	2	3	2	0	0	gnl|UG|At#S11722894	gi|16604584|gb|AY059737.1	At4g28400.1
at3g14440	CON	MIN	HOUR	DAY	WEEK	UNIGENE	FLCDNA	TAIR
AATTAAGGAA	0	0	1	1	1	gnl|UG|At#S11706015	gi|15810432|gb|AY056255.1	At3g14440.1
at5g42030	CON	MIN	HOUR	DAY	WEEK	UNIGENE	FLCDNA	TAIR
GGGAAGAGTG	0	2	1	1	0	gnl|UG|At#S11719526	gi|21403105|gb|AY084395.1	At5g42030.1
at5g01260	CON	MIN	HOUR	DAY	WEEK	UNIGENE	FLCDNA	TAIR
ATAAAAGTTT	0	0	0	1	0	gnl|UG|At#S11705295	gi|16323040|gb|AY057624.1	At5g01260.1
TTTACTGAGG	0	1	0	0	0	no match found	gi|23505872|gb|AY143857.1	At5g01260.2
at1g56145	CON	MIN	HOUR	DAY	WEEK	UNIGENE	FLCDNA	TAIR
GACGACGACA	0	2	0	1	0	gnl|UG|At#S11732229	no match found	At1g56145.1
at5g18920	CON	MIN	HOUR	DAY	WEEK	UNIGENE	FLCDNA	TAIR
AAAGGAGAAT	1	0	1	0	1	gnl|UG|At#S11721591	gi|21404203|gb|AY085493.1	At5g18920.1

at3g27906	CON	MIN	HOUR	DAY	WEEK	UNIGENE	FLCDNA	TAIR
TAAGAAGCAA	0	0	1	0	0	no match found	gi|62319711|dbj|AK221181.1|	pseudo chromosome match
at4g35480	CON	MIN	HOUR	DAY	WEEK	UNIGENE	FLCDNA	TAIR
ATTAAAAGTA	0	0	1	0	0	gnl|UG|At#S11721687	gi|21405806|gb|AY087082.1	At4g35480.1
at4g33560	CON	MIN	HOUR	DAY	WEEK	UNIGENE	FLCDNA	TAIR
CGTCTATGAC	0	2	0	0	1	gnl|UG|At#S35279436	gi|21404411|gb|AY085701.1	At4g33560.1
at1g03900	CON	MIN	HOUR	DAY	WEEK	UNIGENE	FLCDNA	TAIR
AAAAGTACGT	1	7	1	1	0	gnl|UG|At#S21737115	gi|51969261|dbj|AK175560.1|	multiple canonical match
at2g47700	CON	MIN	HOUR	DAY	WEEK	UNIGENE	FLCDNA	TAIR
GGAGAGAGAT	1	1	0	0	1	gnl|UG|At#S15460237	gi|31711855|gb|BT008845.1	At2g47700.1
at4g25650	CON	MIN	HOUR	DAY	WEEK	UNIGENE	FLCDNA	TAIR
GTGTTGTACA	7	3	2	2	0	gnl|UG|At#S15460696	gi|17065309|gb|AY062731.1	At4g25650.1
at1g69526	CON	MIN	HOUR	DAY	WEEK	UNIGENE	FLCDNA	TAIR
TCTATGGAGC	1	0	0	0	0	no match found	no match found	At1g69526.1
at4g30580	CON	MIN	HOUR	DAY	WEEK	UNIGENE	FLCDNA	TAIR
TGGAAGAATT	2	4	0	4	2	gnl|UG|At#S11815870	gi|26451111|dbj|AK118028.1|	At4g30580.1
at5g64270	CON	MIN	HOUR	DAY	WEEK	UNIGENE	FLCDNA	TAIR
GACAGGGATT	0	1	1	2	2	no match found	gi|62320129|dbj|AK221391.1|	non-canonical match
at5g52040	CON	MIN	HOUR	DAY	WEEK	UNIGENE	FLCDNA	TAIR
TAGTATTTAG	4	6	1	0	3	no match found	gi|13877816|gb|AF370171.1	multiple canonical match
AAAATAGTGC	0	0	1	0	0	gnl|UG|At#S15459090	no match found	At5g52040.1
at5g51210	CON	MIN	HOUR	DAY	WEEK	UNIGENE	FLCDNA	TAIR
CAATGTATGA	1	0	0	0	0	gnl|UG|At#S11718595	gi|27754367|gb|BT002813.1	At5g51210.1
at2g48110	CON	MIN	HOUR	DAY	WEEK	UNIGENE	FLCDNA	TAIR
ATTCGCTATG	0	1	3	0	2	gnl|UG|At#S11730708	no match found	At2g48110.1
at1g52740	CON	MIN	HOUR	DAY	WEEK	UNIGENE	FLCDNA	TAIR
GAAGGGTTGG	6	16	8	2	3	gnl|UG|At#S11733460	gi|15450953|gb|AY054557.1	At1g52740.1
at2g42040	CON	MIN	HOUR	DAY	WEEK	UNIGENE	FLCDNA	TAIR
TGTATGTGTA	0	2	0	0	0	gnl|UG|At#S11813536	no match found	multiple non-canonical match
TCTATGTGTA	1	0	1	0	1	no match found	gi|17529347|gb|AY065460.1	At2g42040.1
at3g11620	CON	MIN	HOUR	DAY	WEEK	UNIGENE	FLCDNA	TAIR
GGTTGCGCAG	1	1	0	0	0	gnl|UG|At#S28283004	gi|21403436|gb|AY084726.1	At3g11620.2
at1g07300	CON	MIN	HOUR	DAY	WEEK	UNIGENE	FLCDNA	TAIR
TAAATAATGT	0	2	0	2	0	gnl|UG|At#S11742304	gi|38454075|gb|BT010675.1	multiple non-canonical match
at1g67170	CON	MIN	HOUR	DAY	WEEK	UNIGENE	FLCDNA	TAIR
ACTATGGCTA	1	0	0	0	0	gnl|UG|At#S11729676	gi|110736465|dbj|AK228253.1|	At1g67170.1
at2g33430	CON	MIN	HOUR	DAY	WEEK	UNIGENE	FLCDNA	TAIR
TGAGAGGTAT	0	1	0	0	2	no match found	gi|21407411|gb|AY088637.1	non-canonical match
TGGGAAATGG	3	2	3	0	3	gnl|UG|At#S11734180	gi|17933284|gb|AF446351.1	At2g33430.1
at5g63480	CON	MIN	HOUR	DAY	WEEK	UNIGENE	FLCDNA	TAIR
AAGCCAAAAA	1	0	0	0	0	gnl|UG|At#S11717352	gi|51969383|dbj|AK175621.1|	At5g63480.1
at1g02816	CON	MIN	HOUR	DAY	WEEK	UNIGENE	FLCDNA	TAIR

GTTTCGGATG	0	0	1	0	0	gnl|UG|At#S11742834	no match found	At1g02816.1
at4g25700	CON	MIN	HOUR	DAY	WEEK	UNIGENE	FLCDNA	TAIR
TTTGTCCACG	3	1	0	1	1	gnl|UG|At#S11723359	gi|13877914|gb|AF370220.1	At4g25700.1
at4g34760	CON	MIN	HOUR	DAY	WEEK	UNIGENE	FLCDNA	TAIR
ATCCGATGAT	0	3	0	0	2	gnl|UG|At#S11721804	gi|21403058|gb|AY084348.1	At4g34760.1
at5g66210	CON	MIN	HOUR	DAY	WEEK	UNIGENE	FLCDNA	TAIR
AGTTCAGGAG	0	6	1	1	0	gnl|UG|At#S43849038	gi|110742378|dbj|AK227052.1|	At5g66210.2
at1g07280	CON	MIN	HOUR	DAY	WEEK	UNIGENE	FLCDNA	TAIR
TGGCAAACTT	4	8	6	5	3	gnl|UG|At#S11742306	gi|18086483|gb|AY065061.1	At1g07280.1
at1g04170	CON	MIN	HOUR	DAY	WEEK	UNIGENE	FLCDNA	TAIR
GATGGTGCAC	1	1	2	1	1	gnl|UG|At#S11742646	gi|21406427|gb|AY087689.1	At1g04170.1
at1g72770	CON	MIN	HOUR	DAY	WEEK	UNIGENE	FLCDNA	TAIR
GTACTTGAAT	2	0	2	2	1	gnl|UG|At#S38434069	no match found	non-canonical match
TTGTGTTCTT	0	0	1	0	0	gnl|UG|At#S11786188	no match found	At1g72770.1
at3g59630	CON	MIN	HOUR	DAY	WEEK	UNIGENE	FLCDNA	TAIR
TCCCCTGTCA	0	1	0	0	0	gnl|UG|At#S18901608	gi|53749153|gb|BT015772.1	At3g59630.1
at2g30760	CON	MIN	HOUR	DAY	WEEK	UNIGENE	FLCDNA	TAIR
TCGCAAACAG	8	0	17	5	3	no match found	gi|62320323|dbj|AK221488.1|	At3g59630.1
at4g27840	CON	MIN	HOUR	DAY	WEEK	UNIGENE	FLCDNA	TAIR
AGGTAAAAAG	1	0	0	0	1	gnl|UG|At#S11709973	gi|17978988|gb|AY069904.1	At4g27840.1
at1g13330	CON	MIN	HOUR	DAY	WEEK	UNIGENE	FLCDNA	TAIR
GAACTCAGTA	0	0	0	0	1	gnl|UG|At#S21736065	gi|51971416|dbj|AK176610.1|	At1g13330.1
at5g03200	CON	MIN	HOUR	DAY	WEEK	UNIGENE	FLCDNA	TAIR
CTTAATTAAG	0	0	1	1	1	gnl|UG|At#S11725360	gi|21407658|gb|AY088884.1	At5g03200.1
at1g07230	CON	MIN	HOUR	DAY	WEEK	UNIGENE	FLCDNA	TAIR
TACCCACTTT	2	5	3	1	0	gnl|UG|At#S11814596	gi|21703146|gb|AY124005.1	At1g07230.1
at4g23650	CON	MIN	HOUR	DAY	WEEK	UNIGENE	FLCDNA	TAIR
GCCAGCACTA	0	2	0	1	0	no match found	gi|23397318|gb|BT000807.1	multiple non-canonical match
GGTGATGATA	4	6	1	2	0	no match found	gi|15450436|gb|AY052319.1	At4g23650.1
at3g24870	CON	MIN	HOUR	DAY	WEEK	UNIGENE	FLCDNA	TAIR
CAGGTCTTTT	0	0	1	1	0	gnl|UG|At#S11733292	gi|110742114|dbj|AK226913.1|	At3g24870.1
at5g24340	CON	MIN	HOUR	DAY	WEEK	UNIGENE	FLCDNA	TAIR
TGTTCCAGAG	0	1	1	1	0	gnl|UG|At#S11720742	gi|110743599|dbj|AK227650.1|	At5g24340.1
at2g39290	CON	MIN	HOUR	DAY	WEEK	UNIGENE	FLCDNA	TAIR
GTTATTGACC	0	0	1	0	0	gnl|UG|At#S11700252	gi|89274134|gb|BT024857.1	At2g39290.1
at2g04030	CON	MIN	HOUR	DAY	WEEK	UNIGENE	FLCDNA	TAIR
GCAGGAGAAA	0	2	0	1	0	no match found	gi|110739264|dbj|AK229707.1|	non-canonical match
GGCCTAATGA	8	6	13	24	11	gnl|UG|At#S11740391	gi|15450722|gb|AY053403.1	At2g04030.1
AGAGGTAGGA	0	0	1	0	0	no match found	gi|25090167|gb|BT002234.1	multiple non-canonical match
at4g28811	CON	MIN	HOUR	DAY	WEEK	UNIGENE	FLCDNA	TAIR
CAAATGTTTT	0	0	0	1	0	gnl|UG|At#S38433424	no match found	multiple non-canonical match
at5g64460	CON	MIN	HOUR	DAY	WEEK	UNIGENE	FLCDNA	TAIR

TGATCAGAGG	0	1	0	1	0	gnl|UG|At#S18910175	gi|24030310|gb|BT000924.1	At5g64460.2
at1g34220	CON	MIN	HOUR	DAY	WEEK	UNIGENE	FLCDNA	TAIR
AAGATTATCA	0	0	1	0	0	no match found	no match found	At1g34220.1
ATTCGTGATT	0	4	1	0	1	gnl|UG|At#S18893578	gi|110743176|dbj|AK227478.1|	non-canonical match
at3g46100	CON	MIN	HOUR	DAY	WEEK	UNIGENE	FLCDNA	TAIR
ATGAACCATT	1	3	1	0	0	gnl|UG|At#S11730168	gi|15810388|gb|AY056233.1	At3g46100.1
at4g05120	CON	MIN	HOUR	DAY	WEEK	UNIGENE	FLCDNA	TAIR
TCATTAAGCC	0	1	0	0	0	gnl|UG|At#S21736922	gi|51969647|dbj|AK175753.1|	At4g05120.1
at3g25870	CON	MIN	HOUR	DAY	WEEK	UNIGENE	FLCDNA	TAIR
GATGTATTCG	0	1	0	0	0	gnl|UG|At#S11733021	gi|26451334|dbj|AK118143.1|	At3g25870.1
at5g13740	CON	MIN	HOUR	DAY	WEEK	UNIGENE	FLCDNA	TAIR
ATACTGCATA	0	2	0	1	0	gnl|UG|At#S11722904	gi|22136243|gb|AY128800.1	At5g13740.1
at2g19090	CON	MIN	HOUR	DAY	WEEK	UNIGENE	FLCDNA	TAIR
ATGTGCCTTA	0	0	1	0	0	no match found	no match found	At2g19090.1
at4g19985	CON	MIN	HOUR	DAY	WEEK	UNIGENE	FLCDNA	TAIR
TTTGACTTTT	0	0	0	2	0	gnl|UG|At#S11724336	gi|15451111|gb|AY054636.1	At4g19985.1
TGGGTTTGGA	0	3	0	0	0	no match found	gi|18377479|gb|AY072491.1	multiple non-canonical match
at3g01280	CON	MIN	HOUR	DAY	WEEK	UNIGENE	FLCDNA	TAIR
GTTTGAGTGG	8	8	6	6	7	gnl|UG|At#S11740283	gi|21406753|gb|AY087979.1	At3g01280.1
at1g66130	CON	MIN	HOUR	DAY	WEEK	UNIGENE	FLCDNA	TAIR
CTTTATGGTA	0	0	1	0	1	gnl|UG|At#S11729900	no match found	At1g66130.1
at2g29020	CON	MIN	HOUR	DAY	WEEK	UNIGENE	FLCDNA	TAIR
CTTTTGTTTT	4	6	5	1	0	gnl|UG|At#S11735281	gi|18086566|gb|AY072014.1	At2g29020.1
at5g24980	CON	MIN	HOUR	DAY	WEEK	UNIGENE	FLCDNA	TAIR
TCATACTAAT	1	0	0	1	0	gnl|UG|At#S35302058	no match found	At5g24980.1
at1g73240	CON	MIN	HOUR	DAY	WEEK	UNIGENE	FLCDNA	TAIR
ATCAAAAAGC	1	0	1	2	1	gnl|UG|At#S11728412	gi|20466305|gb|AY099619.1	At1g73240.1
at1g06135	CON	MIN	HOUR	DAY	WEEK	UNIGENE	FLCDNA	TAIR
GTTGAGTACT	0	0	2	0	0	gnl|UG|At#S30644573	gi|21405061|gb|AY086351.1	At1g06135.1
at3g42410	CON	MIN	HOUR	DAY	WEEK	UNIGENE	FLCDNA	TAIR
TAGCTAATGA	0	1	0	0	0	no match found	no match found	At3g42410.1
at1g14650	CON	MIN	HOUR	DAY	WEEK	UNIGENE	FLCDNA	TAIR
TCGCTTGCAC	0	5	1	4	1	no match found	gi|19698892|gb|AY081293.1	At1g14650.1
TTCACTTTTT	0	0	0	2	0	no match found	gi|20148646|gb|AY081652.1	multiple non-canonical match
ACTCTTTTAA	33	21	25	26	25	no match found	gi|110740976|dbj|AK226440.1|	At4g30960.1
at3g16500	CON	MIN	HOUR	DAY	WEEK	UNIGENE	FLCDNA	TAIR
TCTGCAAAAC	0	1	0	0	0	gnl|UG|At#S11735908	no match found	At3g16500.1
at4g03490	CON	MIN	HOUR	DAY	WEEK	UNIGENE	FLCDNA	TAIR
GCTCTACTTC	0	0	1	0	0	gnl|UG|At#S11726797	no match found	multiple non-canonical match
at5g53170	CON	MIN	HOUR	DAY	WEEK	UNIGENE	FLCDNA	TAIR
TTACAAAATT	6	9	2	5	0	gnl|UG|At#S11718399	gi|20258847|gb|AY091086.1	At5g53170.1
at2g25760	CON	MIN	HOUR	DAY	WEEK	UNIGENE	FLCDNA	TAIR

AAGTTCTAAA	2	0	1	1	0	gnl|UG|At#S11709932	gi|17979090|gb|AY070056.1	At2g25760.2
at1g72440	CON	MIN	HOUR	DAY	WEEK	UNIGENE	FLCDNA	TAIR
GACCTGATTG	0	1	1	4	3	gnl|UG|At#S11728591	no match found	At1g72440.1
at5g23790	CON	MIN	HOUR	DAY	WEEK	UNIGENE	FLCDNA	TAIR
AAGGTGTGGA	1	4	1	0	0	gnl|UG|At#S11720800	no match found	At5g23790.1
at2g44690	CON	MIN	HOUR	DAY	WEEK	UNIGENE	FLCDNA	TAIR
TTCTTTGACT	1	0	0	0	0	gnl|UG|At#S11731477	no match found	At2g44690.1
at1g67195	CON	MIN	HOUR	DAY	WEEK	UNIGENE	FLCDNA	TAIR
TAAAAAAAAT	1	0	0	0	0	gnl|UG|At#S37211634	no match found	multiple non-canonical match
at3g04450	CON	MIN	HOUR	DAY	WEEK	UNIGENE	FLCDNA	TAIR
AGTATGATCC	1	1	0	0	1	gnl|UG|At#S11823233	gi|26452280|dbj|AK118630.1|	At3g04450.1
at1g02950	CON	MIN	HOUR	DAY	WEEK	UNIGENE	FLCDNA	TAIR
AACCTTGTAA	0	0	0	1	0	no match found	gi|72197486|gb|DQ108744.1	non-canonical match
at2g34750	CON	MIN	HOUR	DAY	WEEK	UNIGENE	FLCDNA	TAIR
TATGTGCTCT	1	5	1	0	0	no match found	gi|20465616|gb|AY096642.1	At2g34750.1
at3g60240	CON	MIN	HOUR	DAY	WEEK	UNIGENE	FLCDNA	TAIR
CACCTAACCG	2	5	3	3	2	gnl|UG|At#S34115236	gi|110741874|dbj|AK226781.1|	At3g60240.1
at1g59780	CON	MIN	HOUR	DAY	WEEK	UNIGENE	FLCDNA	TAIR
CCCCTTCTTC	0	1	0	0	0	gnl|UG|At#S11731586	no match found	multiple non-canonical match
at5g42610	CON	MIN	HOUR	DAY	WEEK	UNIGENE	FLCDNA	TAIR
TTTCATTTCA	0	0	0	0	1	no match found	no match found	At5g42610.1
GATTTGATTT	3	10	1	3	2	gnl|UG|At#S21989701	no match found	multiple non-canonical match
at1g63780	CON	MIN	HOUR	DAY	WEEK	UNIGENE	FLCDNA	TAIR
AACACCTCTA	1	1	0	1	1	gnl|UG|At#S11730382	gi|21406921|gb|AY088147.1	At1g63780.1
at4g00090	CON	MIN	HOUR	DAY	WEEK	UNIGENE	FLCDNA	TAIR
AGGCGTATTT	1	0	0	2	1	gnl|UG|At#S11727644	gi|15912316|gb|AY056436.1	At4g00090.1
at1g75260	CON	MIN	HOUR	DAY	WEEK	UNIGENE	FLCDNA	TAIR
GATCCACATC	0	0	2	0	0	no match found	no match found	At1g75260.1
at1g06515	CON	MIN	HOUR	DAY	WEEK	UNIGENE	FLCDNA	TAIR
TTTTTGCAAT	3	1	0	1	0	gnl|UG|At#S38434415	gi|72198134|gb|DQ108829.1	multiple non-canonical match
at1g02140	CON	MIN	HOUR	DAY	WEEK	UNIGENE	FLCDNA	TAIR
AACCACAACC	11	5	9	12	5	gnl|UG|At#S11742902	gi|17473797|gb|AY065157.1	At1g02140.1
at3g03160	CON	MIN	HOUR	DAY	WEEK	UNIGENE	FLCDNA	TAIR
ATGAAATCCC	0	0	0	2	2	gnl|UG|At#S11710233	gi|20466042|gb|AY096722.1	At3g03160.1
at3g01420	CON	MIN	HOUR	DAY	WEEK	UNIGENE	FLCDNA	TAIR
TGATTCAATA	0	1	0	0	0	no match found	gi|14595998|gb|AY042787.1	non-canonical match
at1g04690	CON	MIN	HOUR	DAY	WEEK	UNIGENE	FLCDNA	TAIR
ACGTCAATAT	0	0	2	0	5	gnl|UG|At#S11742558	no match found	At1g04690.1
GTGTGCTTCA	2	3	0	1	0	no match found	gi|21407830|gb|AY089056.1	multiple non-canonical match
at3g54670	CON	MIN	HOUR	DAY	WEEK	UNIGENE	FLCDNA	TAIR
AGAGAGCTGG	0	1	0	0	0	no match found	gi|110743856|dbj|AK227781.1|	non-canonical match
CCAAGCCGCA	0	1	0	2	0	gnl|UG|At#S11728572	no match found	At3g54670.1

at5g13160	CON	MIN	HOUR	DAY	WEEK	UNIGENE	FLCDNA	TAIR
GAGAGCAGAA	1	0	1	2	0	no match found	gi|13937146|gb|AF372927.1	non-canonical match
at3g18670	CON	MIN	HOUR	DAY	WEEK	UNIGENE	FLCDNA	TAIR
TGTTTTTAAA	0	0	0	0	1	no match found	no match found	At3g18670.1
at5g63940	CON	MIN	HOUR	DAY	WEEK	UNIGENE	FLCDNA	TAIR
ACCGTCCGCA	0	0	0	2	1	gnl|UG|At#S11717305	gi|20260317|gb|AY093058.1	At5g63940.1
at1g24625	CON	MIN	HOUR	DAY	WEEK	UNIGENE	FLCDNA	TAIR
GGAAGAATGT	0	0	1	0	0	no match found	gi|28973448|gb|BT005629.1	At1g24625.1
at3g19640	CON	MIN	HOUR	DAY	WEEK	UNIGENE	FLCDNA	TAIR
GTCCAAGTAG	1	1	1	0	0	gnl|UG|At#S11734961	gi|30387600|gb|BT006622.1	At3g19640.1
at5g37070	CON	MIN	HOUR	DAY	WEEK	UNIGENE	FLCDNA	TAIR
AAGACAAAGG	0	3	0	0	0	gnl|UG|At#S11720028	gi|51971131|dbj|AK176495.1|	At4g12160.1
at3g04930	CON	MIN	HOUR	DAY	WEEK	UNIGENE	FLCDNA	TAIR
TTTACACGGC	0	1	0	0	0	no match found	gi|18958043|gb|AY079045.1	non-canonical match
at1g14240	CON	MIN	HOUR	DAY	WEEK	UNIGENE	FLCDNA	TAIR
GGATCGACTC	1	0	0	0	0	gnl|UG|At#S18942584	gi|110743020|dbj|AK227399.1|	At1g14240.2
at3g48700	CON	MIN	HOUR	DAY	WEEK	UNIGENE	FLCDNA	TAIR
TTGCTTACAA	1	0	0	0	0	gnl|UG|At#S11822914	gi|26452934|dbj|AK118967.1|	At3g48700.1
at5g54770	CON	MIN	HOUR	DAY	WEEK	UNIGENE	FLCDNA	TAIR
AACACTGCTG	48	168	84	50	27	gnl|UG|At#S11718237	gi|15809896|gb|AY054216.1	At5g54770.1
at5g23630	CON	MIN	HOUR	DAY	WEEK	UNIGENE	FLCDNA	TAIR
AGCATTTTTC	0	1	2	6	1	gnl|UG|At#S11720816	no match found	pseudo chromosome match
GGAGCGGTTA	0	0	0	1	0	no match found	no match found	At5g23630.1
at4g26240	CON	MIN	HOUR	DAY	WEEK	UNIGENE	FLCDNA	TAIR
GCTTGCTCCC	0	1	0	0	0	no match found	gi|22136339|gb|AY128848.1	non-canonical match
TAATACTCTT	1	0	0	1	1	gnl|UG|At#S11723269	gi|21404001|gb|AY085291.1	At4g26240.1
at5g51890	CON	MIN	HOUR	DAY	WEEK	UNIGENE	FLCDNA	TAIR
GTAAAACTTG	0	1	1	0	0	no match found	gi|21689880|gb|AY122968.1	multiple non-canonical match
AGTTGCAGAG	2	0	3	2	1	gnl|UG|At#S11743578	gi|18175872|gb|AY072121.1	At5g51890.1
at5g12860	CON	MIN	HOUR	DAY	WEEK	UNIGENE	FLCDNA	TAIR
GCAGCTATCT	6	10	9	1	2	gnl|UG|At#S11723146	no match found	At5g12860.1
at5g01950	CON	MIN	HOUR	DAY	WEEK	UNIGENE	FLCDNA	TAIR
GAAAAAATAT	0	1	0	0	0	gnl|UG|At#S11725669	gi|110739663|dbj|AK229912.1|	At5g01950.1
at4g19540	CON	MIN	HOUR	DAY	WEEK	UNIGENE	FLCDNA	TAIR
AGTTGCTTCG	0	2	0	0	0	gnl|UG|At#S11813151	gi|21403847|gb|AY085137.1	At4g19540.1
at2g16485	CON	MIN	HOUR	DAY	WEEK	UNIGENE	FLCDNA	TAIR
GAGACTCCTC	0	0	0	1	0	gnl|UG|At#S11738353	no match found	non-canonical match
at4g38220	CON	MIN	HOUR	DAY	WEEK	UNIGENE	FLCDNA	TAIR
ACCACAATGA	1	0	0	0	0	gnl|UG|At#S15460213	gi|18650599|gb|AY074847.1	At4g38220.1
at2g33220	CON	MIN	HOUR	DAY	WEEK	UNIGENE	FLCDNA	TAIR
TACCAAGTTG	0	0	1	0	0	no match found	gi|20259080|gb|AY091317.1	non-canonical match
TTCATATATT	1	0	1	0	2	gnl|UG|At#S11734231	no match found	non-canonical match

TATTGCTGGT	7	7	2	2	3	no match found	gi|17529029|gb|AY065249.1	At2g33220.1
at3g09100	CON	MIN	HOUR	DAY	WEEK	UNIGENE	FLCDNA	TAIR
TCTGGGTTGG	1	1	0	1	0	gnl|UG|At#S18942148	gi|110741477|dbj|AK226567.1|	At3g09100.2
at5g67090	CON	MIN	HOUR	DAY	WEEK	UNIGENE	FLCDNA	TAIR
AATACAAAAC	0	1	0	0	0	no match found	no match found	At5g67090.1
at3g25030	CON	MIN	HOUR	DAY	WEEK	UNIGENE	FLCDNA	TAIR
CAAAGAGAGC	0	0	0	1	0	gnl|UG|At#S34115491	gi|51536563|gb|BT015397.1	At3g25030.1
at3g12420	CON	MIN	HOUR	DAY	WEEK	UNIGENE	FLCDNA	TAIR
TTTAATCGAA	0	0	1	0	0	no match found	no match found	At3g12420.1
at5g61170	CON	MIN	HOUR	DAY	WEEK	UNIGENE	FLCDNA	TAIR
AACATTGTTG	3	7	3	2	3	no match found	gi|34365670|gb|BT010524.1	At5g61170.1
AAATTAGTTA	3	2	1	1	1	gnl|UG|At#S21736686	gi|51970119|dbj|AK175989.1|	pseudo chromosome match
at2g20940	CON	MIN	HOUR	DAY	WEEK	UNIGENE	FLCDNA	TAIR
CGTTTTTGTT	0	0	1	2	0	gnl|UG|At#S11737226	gi|21404133|gb|AY085423.1	At2g20940.1
at2g44860	CON	MIN	HOUR	DAY	WEEK	UNIGENE	FLCDNA	TAIR
CGAATGGTTA	0	3	1	5	2	gnl|UG|At#S11731439	no match found	non-canonical match
GAAGAGTGAT	2	0	2	1	3	no match found	gi|15146219|gb|AY049251.1	multiple non-canonical match
at1g02330	CON	MIN	HOUR	DAY	WEEK	UNIGENE	FLCDNA	TAIR
TTTGCAGACT	1	1	1	0	1	gnl|UG|At#S11742882	gi|13937156|gb|AF372932.1	At1g02330.1
at4g30420	CON	MIN	HOUR	DAY	WEEK	UNIGENE	FLCDNA	TAIR
ATGAATCAAG	0	0	1	0	0	gnl|UG|At#S18907434	no match found	multiple non-canonical match
at2g17720	CON	MIN	HOUR	DAY	WEEK	UNIGENE	FLCDNA	TAIR
CTTGCTTTCT	0	0	2	0	0	gnl|UG|At#S11738055	no match found	multiple canonical match
AATTTTCCGT	3	3	4	2	5	no match found	gi|21406234|gb|AY087497.1	At2g17720.1
at1g61690	CON	MIN	HOUR	DAY	WEEK	UNIGENE	FLCDNA	TAIR
TTACTCGAGT	1	0	0	1	0	gnl|UG|At#S24442887	gi|62319900|dbj|AK221276.1|	At1g61690.1
at2g27820	CON	MIN	HOUR	DAY	WEEK	UNIGENE	FLCDNA	TAIR
GCCGAGTCAC	2	3	1	3	0	gnl|UG|At#S11810386	gi|21406433|gb|AY087695.1	At2g27820.1
at5g22280	CON	MIN	HOUR	DAY	WEEK	UNIGENE	FLCDNA	TAIR
CTAGTCTTTC	0	1	1	0	0	gnl|UG|At#S11721025	gi|17065493|gb|AY062823.1	At5g22280.1
at5g53860	CON	MIN	HOUR	DAY	WEEK	UNIGENE	FLCDNA	TAIR
GCTGTTCTTC	0	1	2	2	1	no match found	gi|20334787|gb|AY093994.1	non-canonical match
CTTTGAACTT	0	2	2	0	3	gnl|UG|At#S11718330	gi|62319185|dbj|AK220915.1|	At5g53860.2
at1g64640	CON	MIN	HOUR	DAY	WEEK	UNIGENE	FLCDNA	TAIR
TGATGGAATT	1	1	1	5	0	gnl|UG|At#S11730204	gi|45773785|gb|BT012210.1	multiple non-canonical match
at2g38750	CON	MIN	HOUR	DAY	WEEK	UNIGENE	FLCDNA	TAIR
AAAATTTGAT	1	1	0	0	0	gnl|UG|At#S11732908	gi|21405918|gb|AY087194.1	multiple canonical match
at5g58740	CON	MIN	HOUR	DAY	WEEK	UNIGENE	FLCDNA	TAIR
TCTCATAAAT	0	8	1	0	6	gnl|UG|At#S18909036	gi|18389273|gb|AY074384.1	At5g58740.1
at2g15400	CON	MIN	HOUR	DAY	WEEK	UNIGENE	FLCDNA	TAIR
ATAGTTTTGT	0	0	0	0	2	no match found	gi|29824192|gb|BT006072.1	multiple non-canonical match
at3g55050	CON	MIN	HOUR	DAY	WEEK	UNIGENE	FLCDNA	TAIR

AGAGCGGAAC	1	1	0	0	0	gnl|UG|At#S34117016	gi|110738458|dbj|AK229292.1|	At3g55050.1
at3g54480	CON	MIN	HOUR	DAY	WEEK	UNIGENE	FLCDNA	TAIR
TATTCCAAGG	0	1	0	1	0	gnl|UG|At#S11701455	gi|29028837|gb|BT005863.1	At3g54480.1
at3g24190	CON	MIN	HOUR	DAY	WEEK	UNIGENE	FLCDNA	TAIR
GCGAGAATAG	6	0	7	15	9	gnl|UG|At#S11733531	gi|15294249|gb|AF410316.1	At3g24190.1
at5g61020	CON	MIN	HOUR	DAY	WEEK	UNIGENE	FLCDNA	TAIR
CATCCTCGAT	0	1	3	2	1	no match found	gi|20465482|gb|AY096551.1	non-canonical match
AGCATTCATC	1	0	0	1	5	gnl|UG|At#S35264278	gi|110742188|dbj|AK226952.1|	At5g61020.1
at3g04470	CON	MIN	HOUR	DAY	WEEK	UNIGENE	FLCDNA	TAIR
TTATTTCTGA	0	5	2	0	1	gnl|UG|At#S11739376	gi|18377984|gb|AY074522.1	At3g04470.1
at1g65370	CON	MIN	HOUR	DAY	WEEK	UNIGENE	FLCDNA	TAIR
CATATCTGAC	1	0	0	2	0	gnl|UG|At#S11730053	gi|15810005|gb|AY054271.1	At1g65370.1
at3g16140	CON	MIN	HOUR	DAY	WEEK	UNIGENE	FLCDNA	TAIR
TTACCTTTCT	103	94	49	24	12	gnl|UG|At#S30651531	gi|21407596|gb|AY088822.1	At3g16140.1
at1g44350	CON	MIN	HOUR	DAY	WEEK	UNIGENE	FLCDNA	TAIR
CCGCCGTGGC	2	0	0	1	0	no match found	gi|110740052|dbj|AK230111.1|	non-canonical match
at3g13870	CON	MIN	HOUR	DAY	WEEK	UNIGENE	FLCDNA	TAIR
AACCTCCTCA	0	1	0	0	0	no match found	gi|22136245|gb|AY128801.1	multiple non-canonical match
ATTTGTTTGC	7	3	8	6	6	gnl|UG|At#S18942103	gi|110740459|dbj|AK230324.1|	At3g13870.1
at1g35580	CON	MIN	HOUR	DAY	WEEK	UNIGENE	FLCDNA	TAIR
AAACCAGTCA	4	1	1	1	0	gnl|UG|At#S15460113	gi|17529025|gb|AY065247.1	At1g35580.1
at3g06145	CON	MIN	HOUR	DAY	WEEK	UNIGENE	FLCDNA	TAIR
CTTCGAATTA	0	0	2	2	0	no match found	gi|62320487|dbj|AK221570.1|	non-canonical match
TATAACTCCG	1	0	1	0	0	gnl|UG|At#S11816252	no match found	At3g06145.1
at2g07520	CON	MIN	HOUR	DAY	WEEK	UNIGENE	FLCDNA	TAIR
AATTGCAAGC	0	1	0	0	0	no match found	no match found	At2g07520.1
at5g38480	CON	MIN	HOUR	DAY	WEEK	UNIGENE	FLCDNA	TAIR
TGATAATCTG	7	9	6	9	5	gnl|UG|At#S11719885	gi|15724305|gb|AF412093.1	At5g38480.1
at5g26667	CON	MIN	HOUR	DAY	WEEK	UNIGENE	FLCDNA	TAIR
CGATTTTATA	1	3	3	2	1	gnl|UG|At#S28281745	no match found	pseudo chromosome match
at1g08230	CON	MIN	HOUR	DAY	WEEK	UNIGENE	FLCDNA	TAIR
CAAAGCAAAG	3	1	1	0	1	gnl|UG|At#S37211287	no match found	non-canonical match
at2g37500	CON	MIN	HOUR	DAY	WEEK	UNIGENE	FLCDNA	TAIR
GGGATGCGAT	2	1	0	1	1	gnl|UG|At#S11733228	gi|21403640|gb|AY084930.1	non-canonical match
at4g01900	CON	MIN	HOUR	DAY	WEEK	UNIGENE	FLCDNA	TAIR
GAGAATCCAG	1	0	0	0	0	no match found	gi|28950698|gb|BT005209.1	multiple non-canonical match
AATTATGCAG	2	2	1	4	0	gnl|UG|At#S11727208	gi|110736347|dbj|AK228189.1|	At4g01900.1
at1g55310	CON	MIN	HOUR	DAY	WEEK	UNIGENE	FLCDNA	TAIR
GTACTGTTAT	1	2	3	1	0	gnl|UG|At#S11732539	gi|15293080|gb|AY050974.1	At1g55310.1
GGGGCCAGTA	0	0	0	0	1	no match found	gi|56744213|gb|BT020420.1	non-canonical match
at2g01090	CON	MIN	HOUR	DAY	WEEK	UNIGENE	FLCDNA	TAIR
CCAGAAGAAG	3	1	0	1	0	gnl|UG|At#S11742728	gi|13430615|gb|AF360220.1	At2g01090.1

at1g70780	CON	MIN	HOUR	DAY	WEEK	UNIGENE	FLCDNA	TAIR
GGATGCAATT	0	0	2	0	0	no match found	gi|23506214|gb|AY149965.1	non-canonical match
at4g30940	CON	MIN	HOUR	DAY	WEEK	UNIGENE	FLCDNA	TAIR
ATCATAGACT	1	0	0	0	0	no match found	gi|16226414|gb|AF428394.1	multiple non-canonical match
at3g42450	CON	MIN	HOUR	DAY	WEEK	UNIGENE	FLCDNA	TAIR
AGGTTTGAAA	1	0	0	0	0	no match found	no match found	At3g42450.1
at1g20110	CON	MIN	HOUR	DAY	WEEK	UNIGENE	FLCDNA	TAIR
CACTGTGCAC	0	0	1	0	0	no match found	gi|23506042|gb|AY143942.1	non-canonical match
TAGTCTGTTG	2	2	4	0	1	gnl|UG|At#S11741044	gi|62319717|dbj|AK221184.1|	At1g20110.1
at2g43360	CON	MIN	HOUR	DAY	WEEK	UNIGENE	FLCDNA	TAIR
TTCAAGACAT	0	0	1	0	0	no match found	gi|90093313|gb|BT024899.1	non-canonical match
GTTTGACGAT	5	3	5	5	4	gnl|UG|At#S11731804	gi|21406926|gb|AY088152.1	At2g43360.1
at4g20070	CON	MIN	HOUR	DAY	WEEK	UNIGENE	FLCDNA	TAIR
AAAAATGTAT	2	2	0	0	4	gnl|UG|At#S11808303	gi|20466244|gb|AY099588.1	At4g20070.1
at5g60800	CON	MIN	HOUR	DAY	WEEK	UNIGENE	FLCDNA	TAIR
CAAATATATC	0	1	0	0	0	gnl|UG|At#S34118321	gi|110735952|dbj|AK227985.1|	At5g60800.1
at5g35530	CON	MIN	HOUR	DAY	WEEK	UNIGENE	FLCDNA	TAIR
TTATTCTAAG	12	8	4	5	1	no match found	gi|21403030|gb|AY084320.1	At5g35530.1
CTTGACTGGG	1	0	3	0	0	no match found	gi|20466074|gb|AY098949.1	multiple non-canonical match
at2g33240	CON	MIN	HOUR	DAY	WEEK	UNIGENE	FLCDNA	TAIR
TCATTAAAAA	0	0	1	1	0	no match found	no match found	At2g33240.1
at1g47520	CON	MIN	HOUR	DAY	WEEK	UNIGENE	FLCDNA	TAIR
ATTGTCACTT	0	1	0	0	0	gnl|UG|At#S21737032	gi|51969427|dbj|AK175643.1|	non-canonical match
at3g25585	CON	MIN	HOUR	DAY	WEEK	UNIGENE	FLCDNA	TAIR
TAGAACCTTT	2	4	6	1	5	gnl|UG|At#S28282909	gi|15810001|gb|AY054269.1	At3g25585.2
AGATCACAAC	1	0	0	0	0	no match found	gi|23296819|gb|AY142609.1	multiple non-canonical match
at2g24640	CON	MIN	HOUR	DAY	WEEK	UNIGENE	FLCDNA	TAIR
GCTCTTGAAA	0	2	1	1	0	gnl|UG|At#S11736349	gi|13430805|gb|AF360315.1	At2g24640.1
at4g20930	CON	MIN	HOUR	DAY	WEEK	UNIGENE	FLCDNA	TAIR
AAACGAAGGA	1	0	0	0	0	gnl|UG|At#S11806408	gi|21405555|gb|AY086845.1	non-canonical match
at2g47000	CON	MIN	HOUR	DAY	WEEK	UNIGENE	FLCDNA	TAIR
GATTCATCAG	0	0	1	0	0	gnl|UG|At#S11730954	no match found	At2g47000.1
at1g06560	CON	MIN	HOUR	DAY	WEEK	UNIGENE	FLCDNA	TAIR
CATCCTACTC	1	0	0	0	0	no match found	no match found	At1g06560.1
at5g36170	CON	MIN	HOUR	DAY	WEEK	UNIGENE	FLCDNA	TAIR
GCTTCTGCTG	2	3	3	0	1	gnl|UG|At#S11720086	gi|21436316|gb|AY117253.1	At5g36170.2
at3g44730	CON	MIN	HOUR	DAY	WEEK	UNIGENE	FLCDNA	TAIR
TCGCAAAAGT	0	1	0	0	1	gnl|UG|At#S11730430	no match found	non-canonical match
at1g61890	CON	MIN	HOUR	DAY	WEEK	UNIGENE	FLCDNA	TAIR
GTCTCCGTTG	0	2	1	1	0	gnl|UG|At#S11704749	gi|13272458|gb|AF325100.1	At1g61890.1
at2g23140	CON	MIN	HOUR	DAY	WEEK	UNIGENE	FLCDNA	TAIR
GAAACGCTGG	1	3	0	0	0	gnl|UG|At#S11736705	no match found	non-canonical match

at1g21600	CON	MIN	HOUR	DAY	WEEK	UNIGENE	FLCDNA	TAIR
GAGAAACATC	1	1	0	1	4	gnl|UG|At#S11740901	gi|21280836|gb|AY113873.1	At1g21600.1
at4g19500	CON	MIN	HOUR	DAY	WEEK	UNIGENE	FLCDNA	TAIR
TCTATTCCTT	1	0	1	0	2	gnl|UG|At#S34116979	gi|110738532|dbj|AK229329.1|	non-canonical match
at2g25800	CON	MIN	HOUR	DAY	WEEK	UNIGENE	FLCDNA	TAIR
GAGCCAAACA	0	1	1	0	0	gnl|UG|At#S34117523	gi|110737478|dbj|AK228785.1|	At2g25800.1
at3g17830	CON	MIN	HOUR	DAY	WEEK	UNIGENE	FLCDNA	TAIR
GCTTTGTAGT	0	0	0	0	1	gnl|UG|At#S18901753	no match found	At2g25800.1
at3g52115	CON	MIN	HOUR	DAY	WEEK	UNIGENE	FLCDNA	TAIR
AAGGTGTGTC	0	1	0	0	0	gnl|UG|At#S41605475	no match found	non-canonical match
at3g51440	CON	MIN	HOUR	DAY	WEEK	UNIGENE	FLCDNA	TAIR
TTTATAAAAA	0	0	1	0	0	no match found	gi|23296633|gb|AY142567.1	non-canonical match
at5g59000	CON	MIN	HOUR	DAY	WEEK	UNIGENE	FLCDNA	TAIR
GTCGCCCTGT	0	0	0	2	0	gnl|UG|At#S11717805	gi|88196734|gb|BT024612.1	At5g59000.1
at5g52580	CON	MIN	HOUR	DAY	WEEK	UNIGENE	FLCDNA	TAIR
TTACCAAAAT	1	0	0	1	2	gnl|UG|At#S11718458	no match found	At5g52590.1
at3g61250	CON	MIN	HOUR	DAY	WEEK	UNIGENE	FLCDNA	TAIR
TTTGAGAAAA	1	2	0	0	0	gnl|UG|At#S11727097	no match found	At3g61250.1
at4g23870	CON	MIN	HOUR	DAY	WEEK	UNIGENE	FLCDNA	TAIR
TAAGGTTCAA	0	3	1	1	0	gnl|UG|At#S11723670	gi|15450915|gb|AY054538.1	At4g23870.1
at5g56480	CON	MIN	HOUR	DAY	WEEK	UNIGENE	FLCDNA	TAIR
TTACAAATTA	0	1	0	0	0	no match found	no match found	At5g56480.1
at1g22740	CON	MIN	HOUR	DAY	WEEK	UNIGENE	FLCDNA	TAIR
TAATGGGTTG	2	1	0	2	2	no match found	no match found	At1g22740.1
at4g23493	CON	MIN	HOUR	DAY	WEEK	UNIGENE	FLCDNA	TAIR
GACGTCTAGC	0	1	1	0	0	gnl|UG|At#S30652946	gi|21405430|gb|AY086720.1	At4g23493.1
at1g07040	CON	MIN	HOUR	DAY	WEEK	UNIGENE	FLCDNA	TAIR
TGTAACGAGT	1	1	0	2	4	gnl|UG|At#S11742330	gi|27311554|gb|BT002383.1	At1g07040.1
at3g12050	CON	MIN	HOUR	DAY	WEEK	UNIGENE	FLCDNA	TAIR
GCAAAGTTCG	0	2	0	1	0	gnl|UG|At#S11737279	gi|21406542|gb|AY087796.1	At3g12050.1
at2g27510	CON	MIN	HOUR	DAY	WEEK	UNIGENE	FLCDNA	TAIR
AATCGTCACC	5	2	2	10	6	gnl|UG|At#S18898828	gi|21405332|gb|AY086622.1	At2g27510.1
ACCAAGGCCG	0	1	0	0	0	no match found	gi|28972976|gb|BT005393.1	non-canonical match
at5g59930	CON	MIN	HOUR	DAY	WEEK	UNIGENE	FLCDNA	TAIR
TTTTCTGATG	0	1	0	0	0	gnl|UG|At#S11717709	no match found	non-canonical match
at3g01790	CON	MIN	HOUR	DAY	WEEK	UNIGENE	FLCDNA	TAIR
GATCTGTTTC	2	1	0	1	0	gnl|UG|At#S15461632	no match found	At3g01790.2
CTTCCAAGTA	1	2	0	0	1	no match found	gi|33589789|gb|BT010192.1	At3g01790.1
at5g39060	CON	MIN	HOUR	DAY	WEEK	UNIGENE	FLCDNA	TAIR
GCTGTTCTCT	1	0	0	0	0	no match found	no match found	At5g39060.1
at1g43245	CON	MIN	HOUR	DAY	WEEK	UNIGENE	FLCDNA	TAIR
GAGTTTCCTC	1	1	1	0	0	gnl|UG|At#S11816427	no match found	At1g43245.1

at1g59600	CON	MIN	HOUR	DAY	WEEK	UNIGENE	FLCDNA	TAIR
GGACAAGATT	1	0	0	0	0	no match found	gi|51968593|dbj|AK175226.1|	At1g59600.1
at4g29350	CON	MIN	HOUR	DAY	WEEK	UNIGENE	FLCDNA	TAIR
GTTGTCCAAG	0	0	3	0	0	no match found	gi|21281043|gb|AY114048.1	non-canonical match
TATTGTGATT	10	37	6	8	10	gnl|UG|At#S11722728	gi|21405286|gb|AY086576.1	At4g29350.1
at2g27350	CON	MIN	HOUR	DAY	WEEK	UNIGENE	FLCDNA	TAIR
ATGTATGCAA	1	0	0	0	0	gnl|UG|At#S18942843	gi|27311746|gb|BT002479.1	At2g27350.2
at4g14560	CON	MIN	HOUR	DAY	WEEK	UNIGENE	FLCDNA	TAIR
AAAGGATCCG	0	3	3	0	0	gnl|UG|At#S11725284	gi|22136657|gb|AY133714.1	At4g14560.1
at5g21170	CON	MIN	HOUR	DAY	WEEK	UNIGENE	FLCDNA	TAIR
TGTTCATAGA	0	0	1	0	0	no match found	gi|89000912|gb|BT024708.1	non-canonical match
TTCTTTGTTC	2	8	2	0	0	no match found	no match found	At5g21170.1
at2g41140	CON	MIN	HOUR	DAY	WEEK	UNIGENE	FLCDNA	TAIR
GTTAAGCTTC	1	0	0	3	0	gnl|UG|At#S11700917	no match found	At2g41140.1
at4g01810	CON	MIN	HOUR	DAY	WEEK	UNIGENE	FLCDNA	TAIR
GCACAGATGT	0	3	2	0	0	no match found	gi|14334823|gb|AY035085.1	non-canonical match
at2g17695	CON	MIN	HOUR	DAY	WEEK	UNIGENE	FLCDNA	TAIR
TCAATGCTTC	5	2	1	3	0	no match found	gi|26451699|dbj|AK118331.1|	At2g17695.1
at1g03410	CON	MIN	HOUR	DAY	WEEK	UNIGENE	FLCDNA	TAIR
AAGCCGAATC	0	0	0	1	0	gnl|UG|At#S11742773	gi|110741631|dbj|AK226654.1|	At1g03410.1
at4g38100	CON	MIN	HOUR	DAY	WEEK	UNIGENE	FLCDNA	TAIR
GATATCTAGC	0	1	0	1	0	gnl|UG|At#S11721252	gi|21404781|gb|AY086071.1	At4g38100.1
at5g04430	CON	MIN	HOUR	DAY	WEEK	UNIGENE	FLCDNA	TAIR
GAAATCACTC	0	3	0	1	0	no match found	gi|23507774|gb|BT000854.1	non-canonical match
at1g67780	CON	MIN	HOUR	DAY	WEEK	UNIGENE	FLCDNA	TAIR
AACACGAACT	0	0	1	0	0	no match found	no match found	At1g67780.1
at1g12350	CON	MIN	HOUR	DAY	WEEK	UNIGENE	FLCDNA	TAIR
TTGGGAATAT	1	0	1	0	1	no match found	no match found	At1g12350.1
at3g48070	CON	MIN	HOUR	DAY	WEEK	UNIGENE	FLCDNA	TAIR
TGGTGGTGGT	2	1	2	1	1	gnl|UG|At#S11729791	gi|62320900|dbj|AK221778.1|	At3g48070.1
at4g35420	CON	MIN	HOUR	DAY	WEEK	UNIGENE	FLCDNA	TAIR
AAATCAAAGA	0	2	0	0	0	gnl|UG|At#S11721695	gi|110742938|dbj|AK227355.1|	At4g35420.1
at5g49220	CON	MIN	HOUR	DAY	WEEK	UNIGENE	FLCDNA	TAIR
GATTTGGCTC	0	0	0	0	1	gnl|UG|At#S11718796	gi|17381185|gb|AY064049.1	At5g49220.1
at1g20090	CON	MIN	HOUR	DAY	WEEK	UNIGENE	FLCDNA	TAIR
CTCATTTCTT	0	3	0	0	0	gnl|UG|At#S11701991	gi|15810538|gb|AY056308.1	At1g20090.1
at5g52380	CON	MIN	HOUR	DAY	WEEK	UNIGENE	FLCDNA	TAIR
GAATCTACCC	0	2	0	0	0	gnl|UG|At#S11718478	gi|90962969|gb|BT025034.1	At5g52380.1
at2g19385	CON	MIN	HOUR	DAY	WEEK	UNIGENE	FLCDNA	TAIR
TCTCCGACTG	1	1	2	0	0	gnl|UG|At#S11737628	gi|21403389|gb|AY084679.1	At2g19385.1
at5g12080	CON	MIN	HOUR	DAY	WEEK	UNIGENE	FLCDNA	TAIR

AACAAGCTGA	1	0	0	0	0	gnl|UG|At#S15461040	gi|25090178|gb|BT002236.1	At5g12080.2
at4g21600	CON	MIN	HOUR	DAY	WEEK	UNIGENE	FLCDNA	TAIR
GTTCAAACTT	0	1	0	0	0	gnl|UG|At#S11810094	gi|21406770|gb|AY087996.1	At4g21600.1
at1g05590	CON	MIN	HOUR	DAY	WEEK	UNIGENE	FLCDNA	TAIR
CTCTTGATCA	1	1	1	1	1	gnl|UG|At#S34117189	gi|110738125|dbj|AK229119.1|	At1g05590.1
at2g46980	CON	MIN	HOUR	DAY	WEEK	UNIGENE	FLCDNA	TAIR
AGTTCAGGCG	0	1	0	0	0	gnl|UG|At#S34117852	gi|110736850|dbj|AK228456.1|	multiple canonical match
at1g05490	CON	MIN	HOUR	DAY	WEEK	UNIGENE	FLCDNA	TAIR
TCAAAAAAAA	21	32	61	30	28	gnl|UG|At#S34115799	gi|110740803|dbj|AK226351.1|	At1g73610.1
at1g11545	CON	MIN	HOUR	DAY	WEEK	UNIGENE	FLCDNA	TAIR
CGTCTCTACC	0	1	0	0	0	gnl|UG|At#S11741880	gi|110736798|dbj|AK228427.1|	At1g11545.1
at2g45880	CON	MIN	HOUR	DAY	WEEK	UNIGENE	FLCDNA	TAIR
GGGAAGCTGT	0	0	1	0	0	gnl|UG|At#S34114692	gi|110742878|dbj|AK227323.1|	At2g45880.1
at3g17020	CON	MIN	HOUR	DAY	WEEK	UNIGENE	FLCDNA	TAIR
CCCTGTGACC	3	29	7	3	5	gnl|UG|At#S11735745	gi|15215898|gb|AY050480.1	At3g17020.1
at5g32470	CON	MIN	HOUR	DAY	WEEK	UNIGENE	FLCDNA	TAIR
GCTTAAAAGT	0	0	3	1	0	gnl|UG|At#S34117548	gi|110737431|dbj|AK228760.1|	non-canonical match
at4g23050	CON	MIN	HOUR	DAY	WEEK	UNIGENE	FLCDNA	TAIR
GTTAAAGGAT	1	0	0	0	0	gnl|UG|At#S11706013	gi|17065375|gb|AY062764.1	At4g23050.1
at1g65220	CON	MIN	HOUR	DAY	WEEK	UNIGENE	FLCDNA	TAIR
GAGATGGAAC	0	0	2	1	0	gnl|UG|At#S11730085	gi|29824394|gb|BT006174.1	At1g65220.1
GAGAGAAGAG	1	0	2	2	2	gnl|UG|At#S43849932	no match found	non-canonical match
at2g44910	CON	MIN	HOUR	DAY	WEEK	UNIGENE	FLCDNA	TAIR
TGCCCTTCTT	0	1	0	0	0	gnl|UG|At#S26112092	no match found	non-canonical match
at5g18310	CON	MIN	HOUR	DAY	WEEK	UNIGENE	FLCDNA	TAIR
AATCGAAAAG	1	0	0	0	0	no match found	gi|24030403|gb|BT000961.1	non-canonical match
AATGAATAAT	2	9	2	0	3	gnl|UG|At#S11743839	gi|19347949|gb|AY080687.1	At5g18310.2
at3g05460	CON	MIN	HOUR	DAY	WEEK	UNIGENE	FLCDNA	TAIR
ATCTTGTGAA	0	1	0	0	0	no match found	gi|21407044|gb|AY088270.1	At3g05460.1
at4g18380	CON	MIN	HOUR	DAY	WEEK	UNIGENE	FLCDNA	TAIR
TAGCAAGTTT	0	1	1	1	0	gnl|UG|At#S11724615	gi|110738384|dbj|AK229254.1|	At4g18380.1
at5g36710	CON	MIN	HOUR	DAY	WEEK	UNIGENE	FLCDNA	TAIR
GTAGCCACAG	0	1	0	0	0	no match found	no match found	At5g36710.1
at3g14060	CON	MIN	HOUR	DAY	WEEK	UNIGENE	FLCDNA	TAIR
TCCGGCTACG	0	1	0	0	0	gnl|UG|At#S34115213	gi|110741919|dbj|AK226804.1|	At3g14060.1
at5g47520	CON	MIN	HOUR	DAY	WEEK	UNIGENE	FLCDNA	TAIR
GAGACATCGG	0	0	0	0	1	no match found	gi|28466932|gb|BT004809.1	non-canonical match
ATAATGAATG	0	0	0	0	1	gnl|UG|At#S24442003	gi|62321644|dbj|AK222160.1|	multiple canonical match
at2g26140	CON	MIN	HOUR	DAY	WEEK	UNIGENE	FLCDNA	TAIR
TATTTGTGTA	0	1	2	3	1	gnl|UG|At#S11735997	gi|62319042|dbj|AK220842.1|	At2g26140.1
at1g61520	CON	MIN	HOUR	DAY	WEEK	UNIGENE	FLCDNA	TAIR
TGTTTTTATG	333	373	253	142	54	gnl|UG|At#S28282463	gi|21405149|gb|AY086439.1	At1g61520.1

at2g03720	CON	MIN	HOUR	DAY	WEEK	UNIGENE	FLCDNA	TAIR
AACTAGTTCA	0	0	1	0	0	no match found	gi|56381932|gb|BT020330.1	non-canonical match
at5g01890	CON	MIN	HOUR	DAY	WEEK	UNIGENE	FLCDNA	TAIR
ACTTAGAGTG	2	0	0	0	1	gnl|UG|At#S11725685	gi|28416470|gb|BT004520.1	At5g01890.1
at4g31340	CON	MIN	HOUR	DAY	WEEK	UNIGENE	FLCDNA	TAIR
GTCGTCGTAA	1	3	5	0	0	gnl|UG|At#S28281924	gi|62321514|dbj|AK222093.1|	At4g31340.1
at3g49640	CON	MIN	HOUR	DAY	WEEK	UNIGENE	FLCDNA	TAIR
GAAATGGATA	0	2	1	0	1	gnl|UG|At#S18902815	no match found	non-canonical match
TTTTATGATA	0	1	0	0	1	gnl|UG|At#S18902043	gi|26452530|dbj|AK118757.1|	multiple non-canonical match
at5g24930	CON	MIN	HOUR	DAY	WEEK	UNIGENE	FLCDNA	TAIR
GTGGTATCTT	10	11	12	6	2	gnl|UG|At#S11720690	gi|15450658|gb|AY052697.1	At5g24930.1
at1g03230	CON	MIN	HOUR	DAY	WEEK	UNIGENE	FLCDNA	TAIR
CTTCTCTCCT	0	1	0	0	0	gnl|UG|At#S11742791	gi|12083229|gb|AF332411.1	At1g03230.1
at2g36400	CON	MIN	HOUR	DAY	WEEK	UNIGENE	FLCDNA	TAIR
GGCGGACCAT	0	2	0	0	0	no match found	gi|111074411|gb|BT026472.1	non-canonical match
TCTAAATAGG	1	1	1	0	0	gnl|UG|At#S11812776	no match found	At2g36400.1
at1g55730	CON	MIN	HOUR	DAY	WEEK	UNIGENE	FLCDNA	TAIR
TACATTTGAG	0	0	0	0	1	gnl|UG|At#S11732390	gi|22531155|gb|AY136416.1	At1g55730.1
at1g59970	CON	MIN	HOUR	DAY	WEEK	UNIGENE	FLCDNA	TAIR
GAAATGAGAG	0	1	1	0	1	gnl|UG|At#S21735966	gi|51971614|dbj|AK176709.1|	At1g59970.1
at4g30840	CON	MIN	HOUR	DAY	WEEK	UNIGENE	FLCDNA	TAIR
TGGGACGCGT	0	0	0	1	0	gnl|UG|At#S11722476	gi|90093295|gb|BT024890.1	At4g30840.1
at5g57685	CON	MIN	HOUR	DAY	WEEK	UNIGENE	FLCDNA	TAIR
GGAGGAGGAC	1	0	0	0	0	no match found	gi|46931211|gb|BT012591.1	multiple non-canonical match
at2g02570	CON	MIN	HOUR	DAY	WEEK	UNIGENE	FLCDNA	TAIR
CTTTCAAATC	0	0	0	2	5	no match found	gi|27754611|gb|BT002938.1	multiple canonical match
at2g35010	CON	MIN	HOUR	DAY	WEEK	UNIGENE	FLCDNA	TAIR
GAACAGCTCT	2	0	1	0	0	gnl|UG|At#S38433880	gi|107738215|gb|BT025773.1	At2g35010.1
at1g17350	CON	MIN	HOUR	DAY	WEEK	UNIGENE	FLCDNA	TAIR
GAGAGGAAAT	0	2	0	0	0	no match found	gi|117168230|gb|BT029384.1	multiple non-canonical match
TCATAAAATA	0	0	1	1	1	gnl|UG|At#S11824125	gi|26450457|dbj|AK117691.1|	At1g17350.1
at2g39760	CON	MIN	HOUR	DAY	WEEK	UNIGENE	FLCDNA	TAIR
ATACAACATC	5	0	4	1	3	no match found	gi|22135871|gb|AY128315.1	multiple canonical match
at3g55000	CON	MIN	HOUR	DAY	WEEK	UNIGENE	FLCDNA	TAIR
AAACAGTAAA	2	3	0	2	1	gnl|UG|At#S11728516	gi|21403006|gb|AY084296.1	At3g55000.1
at3g48510	CON	MIN	HOUR	DAY	WEEK	UNIGENE	FLCDNA	TAIR
CAGAATGAAA	1	0	1	1	0	no match found	gi|108385249|gb|BT025864.1	multiple non-canonical match
at1g08840	CON	MIN	HOUR	DAY	WEEK	UNIGENE	FLCDNA	TAIR
AAATAATAAA	0	2	0	1	0	no match found	no match found	At1g08840.1
at5g03730	CON	MIN	HOUR	DAY	WEEK	UNIGENE	FLCDNA	TAIR
ATATGCACAT	0	0	0	0	1	no match found	gi|110742597|dbj|AK227171.1|	At5g03730.2
ACTATTGTCA	1	2	1	0	1	gnl|UG|At#S11725226	gi|19715616|gb|AY075618.1	At5g03730.1

at5g04760	CON	MIN	HOUR	DAY	WEEK	UNIGENE	FLCDNA	TAIR
GACTGGACTG	0	1	1	0	0	gnl|UG|At#S11724943	gi|15293084|gb|AY050976.1	At5g04760.1
at5g45810	CON	MIN	HOUR	DAY	WEEK	UNIGENE	FLCDNA	TAIR
ATCGGAATGT	0	1	0	1	0	gnl|UG|At#S11702703	no match found	multiple non-canonical match
at5g05830	CON	MIN	HOUR	DAY	WEEK	UNIGENE	FLCDNA	TAIR
GCTCTTCCAC	0	2	1	1	0	gnl|UG|At#S34114498	gi|30102533|gb|BT006377.1	At5g05830.1
at4g08350	CON	MIN	HOUR	DAY	WEEK	UNIGENE	FLCDNA	TAIR
CCCGATATTT	0	4	0	2	0	gnl|UG|At#S11726125	no match found	non-canonical match
at5g04970	CON	MIN	HOUR	DAY	WEEK	UNIGENE	FLCDNA	TAIR
AACTTCACCG	0	1	0	0	0	gnl|UG|At#S11724890	no match found	At5g04970.1
at3g29034	CON	MIN	HOUR	DAY	WEEK	UNIGENE	FLCDNA	TAIR
CAATCTCTGG	0	0	1	0	2	gnl|UG|At#S15460420	gi|28416776|gb|BT004673.1	At3g29034.1
at1g14900	CON	MIN	HOUR	DAY	WEEK	UNIGENE	FLCDNA	TAIR
GTGAAGAACA	1	2	0	1	0	gnl|UG|At#S11741544	gi|28466926|gb|BT004806.1	At1g14900.1
at1g41830	CON	MIN	HOUR	DAY	WEEK	UNIGENE	FLCDNA	TAIR
TTCTTTTAAT	2	1	1	0	0	gnl|UG|At#S11736424	gi|16648888|gb|AY059814.1	At1g41830.1
GACAGCCATA	0	1	0	0	0	no match found	gi|22136093|gb|AY128725.1	non-canonical match
at1g49040	CON	MIN	HOUR	DAY	WEEK	UNIGENE	FLCDNA	TAIR
CTGGGCCAGT	0	1	0	0	0	gnl|UG|At#S15459226	no match found	At1g49040.1
at3g05480	CON	MIN	HOUR	DAY	WEEK	UNIGENE	FLCDNA	TAIR
CTGAAGGATC	0	0	1	0	0	gnl|UG|At#S19551140	gi|115646871|gb|BT029213.1	At3g05480.1
at5g63910	CON	MIN	HOUR	DAY	WEEK	UNIGENE	FLCDNA	TAIR
ACAAAGGAAT	1	3	0	0	0	gnl|UG|At#S11717308	gi|19698816|gb|AY081255.1	At5g63910.1
at5g63130	CON	MIN	HOUR	DAY	WEEK	UNIGENE	FLCDNA	TAIR
ATAATGACGA	1	1	3	0	1	gnl|UG|At#S11717388	gi|26449809|dbj|AK117358.1|	At5g63130.1
at5g46420	CON	MIN	HOUR	DAY	WEEK	UNIGENE	FLCDNA	TAIR
TTCAGAATGT	4	3	7	3	2	gnl|UG|At#S11707452	gi|15292692|gb|AY050780.1	At5g46420.1
at5g37720	CON	MIN	HOUR	DAY	WEEK	UNIGENE	FLCDNA	TAIR
AACACCTCTT	1	0	0	0	0	no match found	gi|37201989|gb|BT010588.1	multiple non-canonical match
CTCTTCGTTG	3	3	1	2	1	gnl|UG|At#S34118375	gi|110735848|dbj|AK227931.1|	At5g37720.1
at4g29950	CON	MIN	HOUR	DAY	WEEK	UNIGENE	FLCDNA	TAIR
ACTGCTCTGA	3	4	4	3	1	no match found	gi|18377649|gb|AY074278.1	non-canonical match
TGTAAGATGT	0	0	1	1	1	gnl|UG|At#S11722624	no match found	At4g29950.1
at2g45440	CON	MIN	HOUR	DAY	WEEK	UNIGENE	FLCDNA	TAIR
ATTCCAGATG	0	2	0	0	0	gnl|UG|At#S11731314	gi|21407844|gb|AY089070.1	At2g45440.1
at5g14610	CON	MIN	HOUR	DAY	WEEK	UNIGENE	FLCDNA	TAIR
AGGCGATGAT	0	1	1	0	0	gnl|UG|At#S11722689	gi|110737545|dbj|AK228819.1|	At5g14610.1
at1g27480	CON	MIN	HOUR	DAY	WEEK	UNIGENE	FLCDNA	TAIR
GGGTGGGACG	1	1	0	0	0	gnl|UG|At#S11739823	gi|21406157|gb|AY087433.1	At1g27480.1
at2g30950	CON	MIN	HOUR	DAY	WEEK	UNIGENE	FLCDNA	TAIR
GATAAGCTCG	11	14	15	22	12	gnl|UG|At#S15460934	gi|62319660|dbj|AK221155.1|	At2g30950.1

at5g52750	CON	MIN	HOUR	DAY	WEEK	UNIGENE	FLCDNA	TAIR
CCTCCAATGA	0	2	0	0	0	gnl|UG|At#S11718442	gi|21404615|gb|AY085905.1	At5g52750.1
at1g79550	CON	MIN	HOUR	DAY	WEEK	UNIGENE	FLCDNA	TAIR
GGTGTGTTTG	5	8	3	5	3	no match found	gi|62319056|dbj|AK220849.1|	non-canonical match
at1g49720	CON	MIN	HOUR	DAY	WEEK	UNIGENE	FLCDNA	TAIR
CAGCAGCAGC	0	1	0	1	0	gnl|UG|At#S15644874	gi|30793898|gb|BT008575.1	At1g49720.1
at4g21705	CON	MIN	HOUR	DAY	WEEK	UNIGENE	FLCDNA	TAIR
CTAAAAGTTT	0	0	0	0	1	gnl|UG|At#S14273990	gi|28827499|gb|BT005061.1	At4g21705.1
at1g69545	CON	MIN	HOUR	DAY	WEEK	UNIGENE	FLCDNA	TAIR
TCTCTACAGT	1	0	1	0	0	gnl|UG|At#S34115265	gi|110741818|dbj|AK226752.1|	At4g21705.1
ACTTGTGTCG	0	0	0	1	0	no match found	no match found	At1g69545.1
at5g65270	CON	MIN	HOUR	DAY	WEEK	UNIGENE	FLCDNA	TAIR
TGTTGTAACT	0	0	0	1	0	gnl|UG|At#S11717170	gi|19310618|gb|AY079309.1	At5g65270.1
at1g70580	CON	MIN	HOUR	DAY	WEEK	UNIGENE	FLCDNA	TAIR
CTTTAGGACA	0	0	1	0	0	gnl|UG|At#S18942284	gi|14334913|gb|AY035130.1	At1g70580.1
at5g62680	CON	MIN	HOUR	DAY	WEEK	UNIGENE	FLCDNA	TAIR
GATTTGTCAT	2	2	1	1	2	gnl|UG|At#S11717432	no match found	pseudo chromosome match
at1g02420	CON	MIN	HOUR	DAY	WEEK	UNIGENE	FLCDNA	TAIR
CTTGTTTCCA	0	1	0	0	0	no match found	no match found	At1g02420.1
at4g39955	CON	MIN	HOUR	DAY	WEEK	UNIGENE	FLCDNA	TAIR
TTAGCAAACT	0	2	0	0	0	no match found	gi|116325915|gb|BT029227.1	non-canonical match
TTTTTAGCTT	1	1	0	0	0	gnl|UG|At#S11666007	no match found	multiple non-canonical match
at3g03810	CON	MIN	HOUR	DAY	WEEK	UNIGENE	FLCDNA	TAIR
ACCTTCTCGC	0	0	0	0	1	gnl|UG|At#S11739566	gi|110736065|dbj|AK228044.1|	At3g03810.1
at3g61670	CON	MIN	HOUR	DAY	WEEK	UNIGENE	FLCDNA	TAIR
AAAGAGGTTT	0	2	0	0	0	no match found	gi|110742112|dbj|AK226912.1|	At3g61670.1
at1g18880	CON	MIN	HOUR	DAY	WEEK	UNIGENE	FLCDNA	TAIR
GACAAGACTT	0	2	1	0	0	gnl|UG|At#S11741164	gi|21436190|gb|AY117308.1	At1g18880.1
at5g66400	CON	MIN	HOUR	DAY	WEEK	UNIGENE	FLCDNA	TAIR
GGATCCGGAT	0	0	3	1	0	no match found	gi|25090136|gb|BT002226.1	multiple non-canonical match
AGTGTGTAAT	7	1	3	4	22	gnl|UG|At#S28281526	gi|16226664|gb|AF428458.1	At5g66400.1
at2g44870	CON	MIN	HOUR	DAY	WEEK	UNIGENE	FLCDNA	TAIR
TTAGACAAAT	6	3	6	8	9	gnl|UG|At#S11731437	gi|21407880|gb|AY089106.1	At2g44870.1
at4g21110	CON	MIN	HOUR	DAY	WEEK	UNIGENE	FLCDNA	TAIR
TGTATGCCGT	2	3	3	3	1	gnl|UG|At#S11724156	gi|23308280|gb|BT000541.1	At4g21110.1
at1g29960	CON	MIN	HOUR	DAY	WEEK	UNIGENE	FLCDNA	TAIR
GGAAAATCTT	1	0	0	0	0	gnl|UG|At#S11739036	gi|51969681|dbj|AK175770.1|	non-canonical match
at3g59650	CON	MIN	HOUR	DAY	WEEK	UNIGENE	FLCDNA	TAIR
TCACCAAACA	1	1	0	0	0	no match found	gi|27808539|gb|BT003118.1	At3g59650.1
at1g73470	CON	MIN	HOUR	DAY	WEEK	UNIGENE	FLCDNA	TAIR
ATGTGGGGGA	0	1	0	0	0	gnl|UG|At#S18896235	gi|124301177|gb|BT030103.1	At1g73470.2
at2g35910	CON	MIN	HOUR	DAY	WEEK	UNIGENE	FLCDNA	TAIR

TCATCATCGA	0	1	8	0	1	gnl|UG|At#S11807521	gi|20258807|gb|AY090933.1	At2g35910.1
at2g25740	CON	MIN	HOUR	DAY	WEEK	UNIGENE	FLCDNA	TAIR
CGCTGAAACA	0	1	2	0	0	gnl|UG|At#S15461147	gi|18377846|gb|AY074570.1	At2g25740.1
at2g14750	CON	MIN	HOUR	DAY	WEEK	UNIGENE	FLCDNA	TAIR
GCTGTTTTAT	2	6	9	1	2	gnl|UG|At#S11738776	gi|15810037|gb|AY054287.1	At2g14750.1
at5g43440	CON	MIN	HOUR	DAY	WEEK	UNIGENE	FLCDNA	TAIR
CAGCTGATCA	0	1	0	0	0	no match found	gi|23505904|gb|AY143873.1	At5g43440.1
CAGGCAAGCT	0	1	0	0	0	gnl|UG|At#S34115161	gi|110742016|dbj|AK226856.1|	non-canonical match
at3g57050	CON	MIN	HOUR	DAY	WEEK	UNIGENE	FLCDNA	TAIR
CAAGCATACC	0	1	0	1	0	gnl|UG|At#S15459218	gi|21281080|gb|AY114051.1	At3g57050.3
at2g19810	CON	MIN	HOUR	DAY	WEEK	UNIGENE	FLCDNA	TAIR
GGAGGTTTTT	0	4	0	0	1	gnl|UG|At#S11737520	gi|22531113|gb|AY136395.1	At2g19810.1
at3g24093	CON	MIN	HOUR	DAY	WEEK	UNIGENE	FLCDNA	TAIR
TTCAAAAAAA	11	4	20	10	6	gnl|UG|At#S37211579	no match found	non-canonical match
at3g44880	CON	MIN	HOUR	DAY	WEEK	UNIGENE	FLCDNA	TAIR
AACAAGAGAA	1	3	3	2	2	no match found	no match found	At3g44880.1
at3g21190	CON	MIN	HOUR	DAY	WEEK	UNIGENE	FLCDNA	TAIR
TGAACTAAAA	1	1	1	0	0	no match found	gi|28973698|gb|BT005762.1	multiple non-canonical match
at1g48130	CON	MIN	HOUR	DAY	WEEK	UNIGENE	FLCDNA	TAIR
CAAGTATTGT	0	1	0	0	0	no match found	gi|28393057|gb|BT003916.1	At1g48130.1
at5g49720	CON	MIN	HOUR	DAY	WEEK	UNIGENE	FLCDNA	TAIR
GAAACCTTGA	10	21	10	11	5	gnl|UG|At#S11718745	gi|21404875|gb|AY086165.1	At5g49720.1
at3g18110	CON	MIN	HOUR	DAY	WEEK	UNIGENE	FLCDNA	TAIR
TAATATTTAT	2	0	0	0	1	gnl|UG|At#S11735408	no match found	multiple canonical match
at3g15160	CON	MIN	HOUR	DAY	WEEK	UNIGENE	FLCDNA	TAIR
ACTTTGCTTT	0	1	0	0	1	gnl|UG|At#S11736301	gi|23297453|gb|AY150431.1	At3g15160.1
at4g34250	CON	MIN	HOUR	DAY	WEEK	UNIGENE	FLCDNA	TAIR
CGGGTGGTAG	0	2	0	0	0	gnl|UG|At#S11721892	gi|110738399|dbj|AK229262.1|	At4g34250.1
at1g02750	CON	MIN	HOUR	DAY	WEEK	UNIGENE	FLCDNA	TAIR
TTTGATGATG	0	1	2	0	1	gnl|UG|At#S11742841	gi|26452594|dbj|AK118790.1|	At1g02750.1
at2g02080	CON	MIN	HOUR	DAY	WEEK	UNIGENE	FLCDNA	TAIR
GTCTGATACA	0	2	0	0	0	gnl|UG|At#S43850292	gi|26450538|dbj|AK117733.1|	At2g02080.1
at4g32190	CON	MIN	HOUR	DAY	WEEK	UNIGENE	FLCDNA	TAIR
GAAGGTGTAA	1	0	0	0	0	gnl|UG|At#S11722244	gi|13430501|gb|AF360163.1	At4g32190.1
at2g42400	CON	MIN	HOUR	DAY	WEEK	UNIGENE	FLCDNA	TAIR
TGCGTTATAT	0	1	0	1	1	gnl|UG|At#S11732011	gi|18700277|gb|AY078048.1	At2g42400.1
at5g40720	CON	MIN	HOUR	DAY	WEEK	UNIGENE	FLCDNA	TAIR
TCGTATGGCG	0	2	0	0	0	gnl|UG|At#S11719657	gi|15912254|gb|AY056405.1	At5g40720.1
at3g19690	CON	MIN	HOUR	DAY	WEEK	UNIGENE	FLCDNA	TAIR
CAACTATGAT	0	0	1	0	0	no match found	gi|124301043|gb|BT030036.1	multiple non-canonical match
at1g69870	CON	MIN	HOUR	DAY	WEEK	UNIGENE	FLCDNA	TAIR

ATCGTCCGGA	0	4	3	6	2	gnl|UG|At#S11744579	gi|21928024|gb|AY125531.1	At1g69870.1
at2g47890	CON	MIN	HOUR	DAY	WEEK	UNIGENE	FLCDNA	TAIR
AATGATGATG	0	2	3	0	2	gnl|UG|At#S18942677	no match found	At2g47890.2
at5g22050	CON	MIN	HOUR	DAY	WEEK	UNIGENE	FLCDNA	TAIR
TGGTTTATCA	4	0	2	1	0	gnl|UG|At#S11817073	gi|62319556|dbj|AK221103.1|	At5g22050.2
at5g46880	CON	MIN	HOUR	DAY	WEEK	UNIGENE	FLCDNA	TAIR
TCAGGGGCAA	0	0	1	0	0	no match found	no match found	At5g46880.1
at4g28270	CON	MIN	HOUR	DAY	WEEK	UNIGENE	FLCDNA	TAIR
TTTATGTGTC	3	2	0	0	0	gnl|UG|At#S34114705	gi|27764993|gb|BT003053.1	At4g28270.1
at5g56670	CON	MIN	HOUR	DAY	WEEK	UNIGENE	FLCDNA	TAIR
GGAAAGGTTC	3	3	6	6	8	no match found	gi|22135963|gb|AY128361.1	non-canonical match
at2g42670	CON	MIN	HOUR	DAY	WEEK	UNIGENE	FLCDNA	TAIR
GCTTGAGGAA	0	0	1	0	0	gnl|UG|At#S38433844	gi|88196754|gb|BT024622.1	At2g42670.1
at2g45790	CON	MIN	HOUR	DAY	WEEK	UNIGENE	FLCDNA	TAIR
TCTTGAAACA	1	0	0	3	1	no match found	gi|21407704|gb|AY088930.1	non-canonical match
GTCCTCAAAG	1	2	3	2	2	gnl|UG|At#S11731238	gi|15292744|gb|AY050806.1	At2g45790.1
at5g13330	CON	MIN	HOUR	DAY	WEEK	UNIGENE	FLCDNA	TAIR
TGTAACAATT	0	0	1	2	0	gnl|UG|At#S11723020	gi|44681349|gb|BT011609.1	At5g13330.1
AACTCACCTC	0	0	0	1	0	no match found	gi|45773865|gb|BT012250.1	multiple non-canonical match
at3g47833	CON	MIN	HOUR	DAY	WEEK	UNIGENE	FLCDNA	TAIR
GAATCACGAG	1	0	1	1	1	gnl|UG|At#S11729835	no match found	At3g47833.1
at2g47780	CON	MIN	HOUR	DAY	WEEK	UNIGENE	FLCDNA	TAIR
ATTCCTATTG	0	1	0	0	0	gnl|UG|At#S11730770	gi|23306435|gb|BT000468.1	At2g47780.1
at3g56900	CON	MIN	HOUR	DAY	WEEK	UNIGENE	FLCDNA	TAIR
GCTAGAGTTC	0	0	0	1	0	no match found	no match found	At3g56900.1
at5g40870	CON	MIN	HOUR	DAY	WEEK	UNIGENE	FLCDNA	TAIR
ATTTTGACAA	0	0	0	1	0	gnl|UG|At#S14961127	no match found	At5g40870.1
at3g21175	CON	MIN	HOUR	DAY	WEEK	UNIGENE	FLCDNA	TAIR
CTGATGGTGG	1	0	0	0	0	gnl|UG|At#S11734473	gi|14596058|gb|AY042817.1	At3g21175.2
at5g46430	CON	MIN	HOUR	DAY	WEEK	UNIGENE	FLCDNA	TAIR
ATGCACAACA	4	5	3	7	1	gnl|UG|At#S23687403	gi|14596168|gb|AY042872.1	At5g46430.2
at4g18480	CON	MIN	HOUR	DAY	WEEK	UNIGENE	FLCDNA	TAIR
CACAAGTAGG	0	0	3	0	0	gnl|UG|At#S11724595	gi|20260585|gb|AY093192.1	At4g18480.1
at2g27480	CON	MIN	HOUR	DAY	WEEK	UNIGENE	FLCDNA	TAIR
TTGATGGTCA	0	0	0	0	1	gnl|UG|At#S34117188	gi|110738126|dbj|AK229120.1|	At2g27480.1
at3g10080	CON	MIN	HOUR	DAY	WEEK	UNIGENE	FLCDNA	TAIR
TCTAATGAAC	0	0	0	0	1	no match found	gi|21404590|gb|AY085880.1	non-canonical match
at4g15610	CON	MIN	HOUR	DAY	WEEK	UNIGENE	FLCDNA	TAIR
TTGGAGGAGC	1	2	0	2	0	no match found	gi|21405304|gb|AY086594.1	At4g15610.1
at1g18710	CON	MIN	HOUR	DAY	WEEK	UNIGENE	FLCDNA	TAIR
TTGTTAGCAT	1	0	0	0	0	gnl|UG|At#S11710411	gi|17473831|gb|AY065166.1	At1g18710.1

at1g77090	CON	MIN	HOUR	DAY	WEEK	UNIGENE	FLCDNA	TAIR
TACTGATAAC	0	1	0	0	0	gnl|UG|At#S11727549	gi|13926194|gb|AF370571.1	At1g77090.1
at4g10280	CON	MIN	HOUR	DAY	WEEK	UNIGENE	FLCDNA	TAIR
GAAGGGAAAG	0	1	0	1	0	no match found	gi|15529211|gb|AY052230.1	multiple non-canonical match
at4g00100	CON	MIN	HOUR	DAY	WEEK	UNIGENE	FLCDNA	TAIR
GCCTTGCTCC	6	15	6	8	7	gnl|UG|At#S11675282	gi|30102865|gb|BT006543.1	At4g00100.1
at2g37130	CON	MIN	HOUR	DAY	WEEK	UNIGENE	FLCDNA	TAIR
AGCAGTTCTC	2	19	5	2	0	gnl|UG|At#S11733321	gi|20148518|gb|AY081588.1	At2g37130.1
at2g36670	CON	MIN	HOUR	DAY	WEEK	UNIGENE	FLCDNA	TAIR
AATGATGAAA	1	0	2	0	0	gnl|UG|At#S11733436	gi|110737615|dbj|AK228855.1|	At2g36670.2
at5g28500	CON	MIN	HOUR	DAY	WEEK	UNIGENE	FLCDNA	TAIR
GAGAGAGTGA	3	4	3	0	0	gnl|UG|At#S11720361	gi|53749183|gb|BT015787.1	At5g28500.1
at1g54380	CON	MIN	HOUR	DAY	WEEK	UNIGENE	FLCDNA	TAIR
AGCTTTAATG	2	0	1	0	0	gnl|UG|At#S11701978	gi|13430457|gb|AF360141.1	At1g54380.1
at1g12390	CON	MIN	HOUR	DAY	WEEK	UNIGENE	FLCDNA	TAIR
TTCAGATTAA	0	1	1	0	1	no match found	gi|110743210|dbj|AK227496.1|	At1g12390.1
at4g33480	CON	MIN	HOUR	DAY	WEEK	UNIGENE	FLCDNA	TAIR
TAATCTAAAT	0	2	0	1	1	no match found	gi|26450035|dbj|AK117474.1|	non-canonical match
CACCTACTTG	0	1	0	0	1	gnl|UG|At#S11722028	no match found	At4g33480.1
at1g78180	CON	MIN	HOUR	DAY	WEEK	UNIGENE	FLCDNA	TAIR
TACGGTGCTA	1	1	0	0	0	gnl|UG|At#S21736546	gi|51970335|dbj|AK176097.1|	non-canonical match
GTGAAGATAA	0	0	1	0	0	gnl|UG|At#S30516874	no match found	multiple non-canonical match
at2g29400	CON	MIN	HOUR	DAY	WEEK	UNIGENE	FLCDNA	TAIR
TAGCTGTTGA	0	4	2	0	2	gnl|UG|At#S11735190	gi|19424067|gb|AY080871.1	At2g29400.1
at5g50790	CON	MIN	HOUR	DAY	WEEK	UNIGENE	FLCDNA	TAIR
TTAAGATAGG	0	1	0	0	0	gnl|UG|At#S11718638	gi|21403382|gb|AY084672.1	At5g50790.1
at2g22360	CON	MIN	HOUR	DAY	WEEK	UNIGENE	FLCDNA	TAIR
ACTTGCTCGT	0	3	1	1	0	gnl|UG|At#S11736894	gi|110738884|dbj|AK229509.1|	At2g22360.1
at1g19150	CON	MIN	HOUR	DAY	WEEK	UNIGENE	FLCDNA	TAIR
TAAAATATAT	4	1	0	0	0	no match found	gi|21405171|gb|AY086461.1	At1g19150.1
GCTCATCTGG	0	0	2	0	0	no match found	gi|56381916|gb|BT020322.1	non-canonical match
at5g63290	CON	MIN	HOUR	DAY	WEEK	UNIGENE	FLCDNA	TAIR
TGAGGTACCT	0	0	0	0	1	gnl|UG|At#S11717371	gi|115311486|gb|BT029015.1	At5g63290.1
at5g44820	CON	MIN	HOUR	DAY	WEEK	UNIGENE	FLCDNA	TAIR
ATCTGAATCT	1	1	1	0	0	gnl|UG|At#S11719245	gi|109946406|gb|BT026026.1	At5g44820.1
at1g07990	CON	MIN	HOUR	DAY	WEEK	UNIGENE	FLCDNA	TAIR
ATTCCAAACG	0	0	0	1	0	no match found	gi|28416500|gb|BT004535.1	non-canonical match
TATCTTTTTC	1	1	0	1	1	gnl|UG|At#S11742235	gi|110738741|dbj|AK229435.1|	At1g07990.1
at5g55290	CON	MIN	HOUR	DAY	WEEK	UNIGENE	FLCDNA	TAIR
CGCAAAGTCA	4	6	1	8	1	gnl|UG|At#S18908316	gi|13926254|gb|AF372884.1	At5g55290.1
at4g22820	CON	MIN	HOUR	DAY	WEEK	UNIGENE	FLCDNA	TAIR
ATTGCAGCTT	1	2	0	2	1	gnl|UG|At#S18941818	gi|25082837|gb|BT001995.1	At4g22820.1

at3g12965	CON	MIN	HOUR	DAY	WEEK	UNIGENE	FLCDNA	TAIR
AGAGCCAAGT	4	4	1	10	4	no match found	gi|110736012|dbj|AK228017.1|	non-canonical match
at2g34900	CON	MIN	HOUR	DAY	WEEK	UNIGENE	FLCDNA	TAIR
CCAATAAGCA	0	0	1	0	0	no match found	gi|111074405|gb|BT026469.1	At2g34900.1
TTGCTTCAAA	1	2	0	0	0	gnl|UG|At#S11733841	gi|110741363|dbj|AK230434.1|	At2g34900.2
at4g13840	CON	MIN	HOUR	DAY	WEEK	UNIGENE	FLCDNA	TAIR
AGAAATCAAA	4	8	2	7	3	gnl|UG|At#S11725414	no match found	At4g13840.1
at5g47660	CON	MIN	HOUR	DAY	WEEK	UNIGENE	FLCDNA	TAIR
AACAAGTACT	1	0	0	0	0	gnl|UG|At#S11718955	gi|26452348|dbj|AK118665.1|	non-canonical match
at4g10080	CON	MIN	HOUR	DAY	WEEK	UNIGENE	FLCDNA	TAIR
TGTTTATGTC	7	4	4	0	1	no match found	gi|26449948|dbj|AK117430.1|	At4g10080.1
at3g57740	CON	MIN	HOUR	DAY	WEEK	UNIGENE	FLCDNA	TAIR
GATAAGATTA	0	0	0	0	1	no match found	no match found	At3g57740.1
at4g19870	CON	MIN	HOUR	DAY	WEEK	UNIGENE	FLCDNA	TAIR
AATTCTTTGT	0	1	0	0	1	no match found	no match found	At4g19870.2
at3g17910	CON	MIN	HOUR	DAY	WEEK	UNIGENE	FLCDNA	TAIR
GCATACAAGA	0	0	1	0	0	no match found	gi|89000976|gb|BT024740.1	At3g17910.1
at3g48500	CON	MIN	HOUR	DAY	WEEK	UNIGENE	FLCDNA	TAIR
TCTTACGATG	1	1	1	0	2	gnl|UG|At#S38433627	gi|22135911|gb|AY128335.1	At3g48500.1
at1g53870	CON	MIN	HOUR	DAY	WEEK	UNIGENE	FLCDNA	TAIR
ACCGTGATTA	0	1	0	0	0	gnl|UG|At#S18942385	gi|51971618|dbj|AK176711.1|	multiple non-canonical match
TTTTAAAGCT	0	1	0	0	1	no match found	gi|21403198|gb|AY084488.1	multiple canonical match
at2g46340	CON	MIN	HOUR	DAY	WEEK	UNIGENE	FLCDNA	TAIR
AAGCTACTAA	1	2	1	1	1	gnl|UG|At#S11731108	gi|110742625|dbj|AK227186.1|	At2g46340.1
at3g27060	CON	MIN	HOUR	DAY	WEEK	UNIGENE	FLCDNA	TAIR
TCTTCTCTCT	8	2	1	0	0	gnl|UG|At#S11732612	gi|21436296|gb|AY117212.1	At3g27060.1
at3g01440	CON	MIN	HOUR	DAY	WEEK	UNIGENE	FLCDNA	TAIR
GTTGATAACT	3	0	2	0	1	gnl|UG|At#S11740237	gi|21403894|gb|AY085184.1	At3g01440.1
at4g35980	CON	MIN	HOUR	DAY	WEEK	UNIGENE	FLCDNA	TAIR
ATAGTGTCAA	1	2	0	0	0	gnl|UG|At#S11721601	gi|21407384|gb|AY088610.1	At4g35980.1
at1g03055	CON	MIN	HOUR	DAY	WEEK	UNIGENE	FLCDNA	TAIR
CTTTGAATTC	1	0	0	0	1	gnl|UG|At#S18942658	gi|14488101|gb|AF389299.1	At1g03055.1
at2g32150	CON	MIN	HOUR	DAY	WEEK	UNIGENE	FLCDNA	TAIR
TGGACACCAA	7	12	11	2	2	gnl|UG|At#S11734496	gi|15982855|gb|AY057535.1	At2g32150.1
CGGTGAAGGT	0	1	0	0	0	no match found	gi|20334755|gb|AY093978.1	non-canonical match
at5g07730	CON	MIN	HOUR	DAY	WEEK	UNIGENE	FLCDNA	TAIR
GATCATCTTC	1	0	1	0	0	gnl|UG|At#S11706712	no match found	At5g07730.1
at4g28703	CON	MIN	HOUR	DAY	WEEK	UNIGENE	FLCDNA	TAIR
AGATAGATTT	0	1	0	0	0	gnl|UG|At#S11680800	no match found	non-canonical match
GCCCAAGTGG	0	3	1	0	0	no match found	gi|21406559|gb|AY087808.1	At4g28703.1
at1g10830	CON	MIN	HOUR	DAY	WEEK	UNIGENE	FLCDNA	TAIR
CTTTAGGCTT	2	1	0	0	1	gnl|UG|At#S18922920	gi|21404878|gb|AY086168.1	At1g10830.1

at2g07698	CON	MIN	HOUR	DAY	WEEK	UNIGENE	FLCDNA	TAIR
GGGGAATATT	0	1	0	0	0	gnl|UG|At#S11739571	no match found	At2g07698.1

at2g27420	CON	MIN	HOUR	DAY	WEEK	UNIGENE	FLCDNA	TAIR
AAGATTGATT	1	0	0	0	0	no match found	gi|20465848|gb|AY096388.1	multiple non-canonical match
TTTTCGTGCA	1	0	0	1	1	gnl|UG|At#S11708273	gi|17381153|gb|AY064033.1	At2g27420.1

at1g56345	CON	MIN	HOUR	DAY	WEEK	UNIGENE	FLCDNA	TAIR
AGCTTCACGC	0	1	0	0	0	gnl|UG|At#S34118082	gi|33589805|gb|BT010200.1	At1g56345.1

at1g18730	CON	MIN	HOUR	DAY	WEEK	UNIGENE	FLCDNA	TAIR
CAACATTATA	9	0	3	2	5	gnl|UG|At#S43850619	gi|19310577|gb|AY080701.1	multiple canonical match

at3g28340	CON	MIN	HOUR	DAY	WEEK	UNIGENE	FLCDNA	TAIR
GGTTAGGCTT	0	0	1	0	0	no match found	gi|44917576|gb|BT011750.1	non-canonical match

at2g38550	CON	MIN	HOUR	DAY	WEEK	UNIGENE	FLCDNA	TAIR
GTATCGGGAA	0	3	0	0	0	gnl|UG|At#S11732962	gi|22136953|gb|AY133772.1	At2g38550.1

at3g27740	CON	MIN	HOUR	DAY	WEEK	UNIGENE	FLCDNA	TAIR
GCACTTAAAT	7	5	3	10	6	gnl|UG|At#S11732392	gi|15028108|gb|AY046004.1	At3g27740.1

at5g17780	CON	MIN	HOUR	DAY	WEEK	UNIGENE	FLCDNA	TAIR
TTTGAGTCCG	1	1	2	0	2	gnl|UG|At#S43849305	no match found	At5g17780.1

at5g65570	CON	MIN	HOUR	DAY	WEEK	UNIGENE	FLCDNA	TAIR
GCGTAAGCAG	0	0	0	0	1	no match found	no match found	At5g65570.1

at1g10720	CON	MIN	HOUR	DAY	WEEK	UNIGENE	FLCDNA	TAIR
TACAGAAGAT	1	4	2	0	1	gnl|UG|At#S11741960	gi|21405541|gb|AY086831.1	At1g10720.1
AAACATCATC	1	0	0	0	0	no match found	gi|21436186|gb|AY117306.1	multiple non-canonical match

at1g19050	CON	MIN	HOUR	DAY	WEEK	UNIGENE	FLCDNA	TAIR
TTCAAAACGA	0	5	5	2	0	gnl|UG|At#S11741146	gi|88193787|gb|BT024585.1	multiple canonical match

at1g27370	CON	MIN	HOUR	DAY	WEEK	UNIGENE	FLCDNA	TAIR
TCTACGACTA	0	2	0	0	0	gnl|UG|At#S11739864	gi|18650610|gb|AY074853.1	At1g27370.1

at2g25900	CON	MIN	HOUR	DAY	WEEK	UNIGENE	FLCDNA	TAIR
GAGTTTTCGA	2	19	2	5	0	gnl|UG|At#S11736051	gi|14030624|gb|AF375403.1	At2g25900.1

at3g10700	CON	MIN	HOUR	DAY	WEEK	UNIGENE	FLCDNA	TAIR
CGAGTTATCA	0	1	0	0	0	gnl|UG|At#S11743596	gi|20465754|gb|AY096732.1	At3g10700.1

at4g28050	CON	MIN	HOUR	DAY	WEEK	UNIGENE	FLCDNA	TAIR
GAAAAAGGTG	0	2	0	0	0	gnl|UG|At#S11722953	gi|17065395|gb|AY062774.1	multiple non-canonical match
AGAAGAGGTT	0	1	1	0	0	no match found	no match found	At4g28050.1

at1g34300	CON	MIN	HOUR	DAY	WEEK	UNIGENE	FLCDNA	TAIR
CATCTATGTT	0	2	0	0	1	gnl|UG|At#S11744590	gi|24111428|gb|BT001084.1	At1g34300.1

at1g12620	CON	MIN	HOUR	DAY	WEEK	UNIGENE	FLCDNA	TAIR
AAAGCAAGCT	0	0	1	0	0	gnl|UG|At#S11702888	gi|13605504|gb|AF361578.1	At1g12620.1

at3g07860	CON	MIN	HOUR	DAY	WEEK	UNIGENE	FLCDNA	TAIR
GCAGGATATC	0	0	0	0	2	gnl|UG|At#S18903655	no match found	non-canonical match

at4g09000	CON	MIN	HOUR	DAY	WEEK	UNIGENE	FLCDNA	TAIR
GAGAAAGTCG	6	18	7	2	2	no match found	gi|15724253|gb|AF412067.1	non-canonical match

at4g39630	CON	MIN	HOUR	DAY	WEEK	UNIGENE	FLCDNA	TAIR

CTAGGGTAAT	0	0	0	0	1	gnl|UG|At#S11720988	gi|21404792|gb|AY086082.1	At4g39630.1
at4g02350	CON	MIN	HOUR	DAY	WEEK	UNIGENE	FLCDNA	TAIR
GCTGCAATCA	0	0	0	0	1	gnl|UG|At#S11727088	no match found	At4g02350.1
at1g06240	CON	MIN	HOUR	DAY	WEEK	UNIGENE	FLCDNA	TAIR
GAATTGCTCT	0	1	1	1	1	no match found	no match found	At1g06240.1
at5g61450	CON	MIN	HOUR	DAY	WEEK	UNIGENE	FLCDNA	TAIR
CAAAAGAAGT	0	1	0	0	0	no match found	gi|24899652|gb|BT001154.1	non-canonical match
AACTAATACT	0	1	1	0	1	gnl|UG|At#S11703898	gi|14423543|gb|AF387009.1	At5g61450.1
at5g14260	CON	MIN	HOUR	DAY	WEEK	UNIGENE	FLCDNA	TAIR
AATGAACCTT	6	5	4	6	2	gnl|UG|At#S15460929	gi|18377717|gb|AY074312.1	At5g14260.1
CCCAGCACCA	0	0	1	0	0	gnl|UG|At#S37062927	gi|23297670|gb|AY150480.1	non-canonical match
at2g34840	CON	MIN	HOUR	DAY	WEEK	UNIGENE	FLCDNA	TAIR
GTTCAAGCTA	0	1	0	1	0	gnl|UG|At#S43850103	gi|19715633|gb|AY075627.1	At2g34840.1
at5g57930	CON	MIN	HOUR	DAY	WEEK	UNIGENE	FLCDNA	TAIR
ATGAAAGATT	0	1	0	2	0	no match found	gi|14030660|gb|AF375421.1	multiple non-canonical match
TTCCTGATGT	1	1	1	2	0	gnl|UG|At#S11717917	gi|21387174|gb|AY114672.1	At5g57930.2
at5g10280	CON	MIN	HOUR	DAY	WEEK	UNIGENE	FLCDNA	TAIR
TTGTAGAATG	0	0	1	0	0	gnl|UG|At#S11700705	gi|27754492|gb|BT002877.1	At5g10280.1
at2g42120	CON	MIN	HOUR	DAY	WEEK	UNIGENE	FLCDNA	TAIR
TCTACTTCGT	0	1	0	0	0	gnl|UG|At#S24442508	gi|62320657|dbj|AK221655.1|	At2g42120.1
at1g27400	CON	MIN	HOUR	DAY	WEEK	UNIGENE	FLCDNA	TAIR
CCACATTGAG	7	6	7	3	9	gnl|UG|At#S11739854	gi|16226208|gb|AF428271.1	At1g27400.1
at1g02305	CON	MIN	HOUR	DAY	WEEK	UNIGENE	FLCDNA	TAIR
GTGTTGTAGC	5	7	1	1	0	gnl|UG|At#S11742885	gi|14532525|gb|AY039887.1	At1g02305.1
at3g10500	CON	MIN	HOUR	DAY	WEEK	UNIGENE	FLCDNA	TAIR
TGTTTCTGGG	1	0	0	1	0	no match found	gi|15293138|gb|AY051003.1	multiple non-canonical match
GTTGACACCC	0	0	1	0	0	gnl|UG|At#S11737742	no match found	At3g10500.1
at4g28370	CON	MIN	HOUR	DAY	WEEK	UNIGENE	FLCDNA	TAIR
TGAACATTTC	1	0	2	0	0	no match found	gi|56236077|gb|BT020274.1	non-canonical match
GTATTGGTTT	0	0	1	0	0	gnl|UG|At#S11722900	gi|20465613|gb|AY096640.1	At4g28370.1
at5g67410	CON	MIN	HOUR	DAY	WEEK	UNIGENE	FLCDNA	TAIR
TCAAAGAACA	0	0	0	0	1	no match found	no match found	At5g67410.1
TTGGAGAAGT	0	0	1	0	0	gnl|UG|At#S11716954	no match found	non-canonical match
at1g18070	CON	MIN	HOUR	DAY	WEEK	UNIGENE	FLCDNA	TAIR
CGGTTGTCGA	0	0	2	1	1	no match found	gi|31711943|gb|BT008889.1	non-canonical match
AATGATTCGG	1	2	7	4	3	gnl|UG|At#S11741239	gi|21539548|gb|AY120769.1	At1g18070.1
at1g07473	CON	MIN	HOUR	DAY	WEEK	UNIGENE	FLCDNA	TAIR
TCTCTCACTC	25	11	7	10	4	no match found	no match found	At1g07473.1
at5g49300	CON	MIN	HOUR	DAY	WEEK	UNIGENE	FLCDNA	TAIR
ATTAGGGTTT	1	0	0	1	0	no match found	no match found	At5g49300.1
at5g62090	CON	MIN	HOUR	DAY	WEEK	UNIGENE	FLCDNA	TAIR
AGAGGCATTT	1	2	2	1	1	gnl|UG|At#S15441005	gi|14532713|gb|AY039981.1	At5g62090.2
at2g40140	CON	MIN	HOUR	DAY	WEEK	UNIGENE	FLCDNA	TAIR

AGCAGCAGAT	0	1	0	1	0	no match found	gi|22136517|gb|AY128937.1	non-canonical match
AGTTCAAATG	1	6	16	3	1	gnl|UG|At#S11732564	gi|20260233|gb|AY093016.1	At2g40140.1
at1g20780	CON	MIN	HOUR	DAY	WEEK	UNIGENE	FLCDNA	TAIR
AAAATGTGAA	0	0	0	0	1	gnl|UG|At#S11740979	gi|16604660|gb|AY059775.1	At1g20780.1
at2g16660	CON	MIN	HOUR	DAY	WEEK	UNIGENE	FLCDNA	TAIR
ATACAATATC	1	0	0	0	1	gnl|UG|At#S11821335	gi|17979282|gb|AY070369.1	At2g16660.1
at4g21090	CON	MIN	HOUR	DAY	WEEK	UNIGENE	FLCDNA	TAIR
TTGTATTCTA	2	1	0	2	1	gnl|UG|At#S15460868	no match found	At4g21090.2
AAAAAGCTAT	0	0	0	0	1	no match found	gi|110743956|dbj|AK227833.1|	non-canonical match
TGATTGTTAT	0	1	1	3	6	no match found	gi|28466922|gb|BT004804.1	At2g36530.1
at4g09980	CON	MIN	HOUR	DAY	WEEK	UNIGENE	FLCDNA	TAIR
TACGGCTTTA	1	1	0	3	0	gnl|UG|At#S11725967	gi|15028132|gb|AY046016.1	At4g09980.1
at4g02190	CON	MIN	HOUR	DAY	WEEK	UNIGENE	FLCDNA	TAIR
AGCCTCAAAA	0	2	0	0	0	no match found	no match found	At4g02190.1
at4g03960	CON	MIN	HOUR	DAY	WEEK	UNIGENE	FLCDNA	TAIR
CTCCAAGAGG	1	3	2	0	0	no match found	gi|15810021|gb|AY054279.1	non-canonical match
TAGAACAAGT	1	0	0	1	0	gnl|UG|At#S11726673	no match found	At4g03960.1
at2g24260	CON	MIN	HOUR	DAY	WEEK	UNIGENE	FLCDNA	TAIR
ACCATACAGT	0	0	0	1	0	gnl|UG|At#S11736433	gi|51970881|dbj|AK176370.1|	At2g24260.1
at4g15236	CON	MIN	HOUR	DAY	WEEK	UNIGENE	FLCDNA	TAIR
GGTTTTGAAC	0	0	0	0	1	no match found	no match found	At4g15236.1
at1g16530	CON	MIN	HOUR	DAY	WEEK	UNIGENE	FLCDNA	TAIR
TTTCTGTTAA	0	0	1	0	0	no match found	no match found	At1g16530.1
at1g25210	CON	MIN	HOUR	DAY	WEEK	UNIGENE	FLCDNA	TAIR
TTATCTTTCT	0	2	0	0	0	no match found	no match found	At1g25210.1
at1g23205	CON	MIN	HOUR	DAY	WEEK	UNIGENE	FLCDNA	TAIR
ATAGAGATAT	1	0	0	0	0	gnl|UG|At#S11740741	gi|62321550|dbj|AK222111.1|	At1g23205.1
at2g35670	CON	MIN	HOUR	DAY	WEEK	UNIGENE	FLCDNA	TAIR
GTAGTACTCA	0	1	0	0	0	no match found	no match found	At2g35670.1
at2g36900	CON	MIN	HOUR	DAY	WEEK	UNIGENE	FLCDNA	TAIR
TCGAGGAATC	0	0	1	0	0	gnl|UG|At#S11733371	gi|21405051|gb|AY086341.1	At2g36900.1
GATCCTGCTC	2	1	1	0	0	gnl|UG|At#S18901130	no match found	At2g36895.1
at5g39680	CON	MIN	HOUR	DAY	WEEK	UNIGENE	FLCDNA	TAIR
TCTGTGACAA	0	0	0	0	1	gnl|UG|At#S11719764	no match found	non-canonical match
at2g17540	CON	MIN	HOUR	DAY	WEEK	UNIGENE	FLCDNA	TAIR
GAAGTGTCAG	2	0	1	1	0	gnl|UG|At#S18942909	gi|110743096|dbj|AK227437.1|	At2g17540.2
at1g31340	CON	MIN	HOUR	DAY	WEEK	UNIGENE	FLCDNA	TAIR
GGAAAAAAGT	3	1	2	5	3	gnl|UG|At#S11738538	gi|28973675|gb|BT005749.1	At1g31340.1
at2g22690	CON	MIN	HOUR	DAY	WEEK	UNIGENE	FLCDNA	TAIR
TCCTTTGCAA	0	0	0	0	1	gnl|UG|At#S28282284	gi|109946418|gb|BT026032.1	At2g22690.1
at3g04940	CON	MIN	HOUR	DAY	WEEK	UNIGENE	FLCDNA	TAIR
AACTTTAAGA	1	1	2	1	2	no match found	gi|22531163|gb|AY136420.1	At3g04940.1
TAGGATTTCA	1	0	0	1	0	gnl|UG|At#S11739243	no match found	non-canonical match

at1g12840	CON	MIN	HOUR	DAY	WEEK	UNIGENE	FLCDNA	TAIR
GTTGATTTAT	7	3	5	6	9	gnl|UG|At#S11741754	gi|16649004|gb|AY059872.1	At1g12840.1
GCTGGTTTAG	6	5	13	6	3	no match found	gi|12248022|gb|AF334725.1	multiple non-canonical match
at5g36890	CON	MIN	HOUR	DAY	WEEK	UNIGENE	FLCDNA	TAIR
AAATGTGTTG	0	0	1	0	0	no match found	gi|51969661|dbj|AK175760.1|	multiple non-canonical match
at5g19480	CON	MIN	HOUR	DAY	WEEK	UNIGENE	FLCDNA	TAIR
TGACTAAAAA	0	1	3	0	1	gnl|UG|At#S21737372	gi|51968747|dbj|AK175303.1|	non-canonical match
at4g30670	CON	MIN	HOUR	DAY	WEEK	UNIGENE	FLCDNA	TAIR
AAGAAAACAA	0	1	3	1	2	no match found	gi|21403028|gb|AY084318.1	multiple non-canonical match
at3g20260	CON	MIN	HOUR	DAY	WEEK	UNIGENE	FLCDNA	TAIR
GGAAGGTGTT	0	0	0	1	0	gnl|UG|At#S11816205	gi|62320337|dbj|AK221495.1|	At3g20260.1
at1g78420	CON	MIN	HOUR	DAY	WEEK	UNIGENE	FLCDNA	TAIR
GGTCTGAATC	1	3	2	0	1	gnl|UG|At#S11727069	gi|16604612|gb|AY059751.1	At1g78420.1
at1g72700	CON	MIN	HOUR	DAY	WEEK	UNIGENE	FLCDNA	TAIR
TAAAGAGTAG	0	1	0	1	1	gnl|UG|At#S11728529	no match found	At1g72700.1
at5g21040	CON	MIN	HOUR	DAY	WEEK	UNIGENE	FLCDNA	TAIR
TTGGCTTTAA	1	1	1	4	0	gnl|UG|At#S11721075	gi|15081722|gb|AY048254.1	At5g21040.1
at5g07830	CON	MIN	HOUR	DAY	WEEK	UNIGENE	FLCDNA	TAIR
ATGTTAATGG	0	3	1	1	1	gnl|UG|At#S11704975	gi|15146194|gb|AY049238.1	multiple non-canonical match
at5g06910	CON	MIN	HOUR	DAY	WEEK	UNIGENE	FLCDNA	TAIR
CTTGATGAGG	0	1	0	0	1	gnl|UG|At#S11724394	gi|30017234|gb|BT006202.1	At5g06910.1
at2g46370	CON	MIN	HOUR	DAY	WEEK	UNIGENE	FLCDNA	TAIR
ACTACGGTAG	0	2	0	0	0	gnl|UG|At#S11731100	gi|23297476|gb|AY150437.1	At2g46370.2
at2g24190	CON	MIN	HOUR	DAY	WEEK	UNIGENE	FLCDNA	TAIR
TAATGAGAAT	2	0	1	0	0	gnl|UG|At#S11736450	no match found	multiple non-canonical match
at1g21090	CON	MIN	HOUR	DAY	WEEK	UNIGENE	FLCDNA	TAIR
TTTCTTTTTC	0	1	0	0	0	gnl|UG|At#S18896155	gi|51970103|dbj|AK175981.1|	At1g21090.1
at2g34860	CON	MIN	HOUR	DAY	WEEK	UNIGENE	FLCDNA	TAIR
TTTTTATAAT	5	2	3	0	10	gnl|UG|At#S38433882	gi|20466395|gb|AY099664.1	At2g34860.1
at1g24610	CON	MIN	HOUR	DAY	WEEK	UNIGENE	FLCDNA	TAIR
AAAGACCTCA	1	0	1	0	0	no match found	gi|18377561|gb|AY072532.1	non-canonical match
at5g22020	CON	MIN	HOUR	DAY	WEEK	UNIGENE	FLCDNA	TAIR
TTTTGGAAGA	0	0	0	3	1	gnl|UG|At#S11817076	gi|62320740|dbj|AK221697.1|	At5g22020.1
at1g61990	CON	MIN	HOUR	DAY	WEEK	UNIGENE	FLCDNA	TAIR
GATACATTGG	1	0	1	0	1	gnl|UG|At#S11730802	no match found	At1g61990.1
at1g11580	CON	MIN	HOUR	DAY	WEEK	UNIGENE	FLCDNA	TAIR
CTCTGTTTCG	2	3	8	1	1	gnl|UG|At#S11741877	gi|14334991|gb|AY037175.1	At1g11580.1
at3g24400	CON	MIN	HOUR	DAY	WEEK	UNIGENE	FLCDNA	TAIR
AACAAGTTTA	0	1	0	0	0	no match found	no match found	At3g24400.1
at2g46470	CON	MIN	HOUR	DAY	WEEK	UNIGENE	FLCDNA	TAIR
CAGGAAGGTC	0	4	0	0	0	gnl|UG|At#S11731077	gi|26450687|dbj|AK117811.1|	At2g46470.1

at4g28790	CON	MIN	HOUR	DAY	WEEK	UNIGENE	FLCDNA	TAIR
TTCACATAAC	0	0	1	0	0	gnl|UG|At#S18941778	no match found	At4g28790.2
at2g44270	CON	MIN	HOUR	DAY	WEEK	UNIGENE	FLCDNA	TAIR
TATGCTTATT	0	0	0	0	1	no match found	gi|30725379|gb|BT008353.1	non-canonical match
at3g28270	CON	MIN	HOUR	DAY	WEEK	UNIGENE	FLCDNA	TAIR
AAGATAATGA	2	0	1	0	0	gnl|UG|At#S11732223	no match found	At3g28270.1
at5g47830	CON	MIN	HOUR	DAY	WEEK	UNIGENE	FLCDNA	TAIR
AGAATCTGCG	0	3	3	0	0	gnl|UG|At#S18941510	gi|26452550|dbj|AK118767.1|	At5g47830.2
at4g02425	CON	MIN	HOUR	DAY	WEEK	UNIGENE	FLCDNA	TAIR
ATGATCCGCA	0	0	1	0	0	gnl|UG|At#S15461565	gi|26450274|dbj|AK117597.1|	At4g02425.1
at1g20120	CON	MIN	HOUR	DAY	WEEK	UNIGENE	FLCDNA	TAIR
GAGCGATAAA	1	0	0	0	0	gnl|UG|At#S11741043	no match found	pseudo chromosome match
at1g22210	CON	MIN	HOUR	DAY	WEEK	UNIGENE	FLCDNA	TAIR
TTTCGTTGAA	2	0	1	1	1	gnl|UG|At#S11740839	no match found	multiple canonical match
at4g01270	CON	MIN	HOUR	DAY	WEEK	UNIGENE	FLCDNA	TAIR
GAGAGAGAAA	0	0	0	0	1	gnl|UG|At#S11727367	no match found	At4g01270.1
at2g18328	CON	MIN	HOUR	DAY	WEEK	UNIGENE	FLCDNA	TAIR
GACAGCTAGA	0	2	0	1	0	gnl|UG|At#S37211245	no match found	multiple non-canonical match
at1g20650	CON	MIN	HOUR	DAY	WEEK	UNIGENE	FLCDNA	TAIR
AAGCTCGAAA	1	2	2	2	0	gnl|UG|At#S11740993	gi|51971073|dbj|AK176466.1|	At1g20650.1
at1g27030	CON	MIN	HOUR	DAY	WEEK	UNIGENE	FLCDNA	TAIR
GGAAATAAAA	0	3	1	1	0	gnl|UG|At#S11739990	gi|14517457|gb|AY039564.1	At1g27030.1
at4g08390	CON	MIN	HOUR	DAY	WEEK	UNIGENE	FLCDNA	TAIR
CGAAGCTCAG	0	0	0	0	1	no match found	gi|21281098|gb|AY114065.1	non-canonical match
TTTTTTCTTG	2	1	2	3	11	no match found	gi|15810560|gb|AY056319.1	At4g08390.2
TTTTTTTCTT	0	0	0	0	1	gnl|UG|At#S11698976	no match found	At3g46280.1
AGCGTATCGT	0	0	0	0	1	gnl|UG|At#S11831130	no match found	non-canonical match
at4g15760	CON	MIN	HOUR	DAY	WEEK	UNIGENE	FLCDNA	TAIR
TTAGGGAAAT	0	1	1	0	0	no match found	gi|51971187|dbj|AK176523.1|	non-canonical match
ACCATACATT	0	0	1	0	1	gnl|UG|At#S11725071	gi|51968447|dbj|AK175153.1|	At4g15760.1
ATCAAAAAAA	10	11	13	8	7	no match found	gi|51968729|dbj|AK175294.1|	multiple non-canonical match
ACCATAAAAA	2	2	2	0	0	no match found	gi|51969073|dbj|AK175466.1|	pseudo chromosome match
at5g27370	CON	MIN	HOUR	DAY	WEEK	UNIGENE	FLCDNA	TAIR
ATTAATACAC	1	0	0	0	0	no match found	no match found	At5g27370.1
at3g49070	CON	MIN	HOUR	DAY	WEEK	UNIGENE	FLCDNA	TAIR
TCAATTTTTA	0	0	0	1	0	no match found	no match found	At3g49070.1
at4g28310	CON	MIN	HOUR	DAY	WEEK	UNIGENE	FLCDNA	TAIR
GGCGAGAGAC	0	1	2	0	0	gnl|UG|At#S11812221	gi|51972008|dbj|AK176906.1|	non-canonical match
at3g60910	CON	MIN	HOUR	DAY	WEEK	UNIGENE	FLCDNA	TAIR
TGTGTTTGTC	3	0	0	1	0	gnl|UG|At#S11743909	gi|19347802|gb|AY080676.1	At3g60910.1
at4g07410	CON	MIN	HOUR	DAY	WEEK	UNIGENE	FLCDNA	TAIR
GATGGATGTT	1	2	1	2	3	gnl|UG|At#S38433531	gi|22136757|gb|AY133764.1	At4g07410.1

at4g32250	CON	MIN	HOUR	DAY	WEEK	UNIGENE	FLCDNA	TAIR
TACATAATGA	2	0	3	0	0	gnl|UG|At#S18905892	gi|20259491|gb|AY091045.1	non-canonical match
at1g62380	CON	MIN	HOUR	DAY	WEEK	UNIGENE	FLCDNA	TAIR
AAGCTTTATG	18	48	6	7	0	gnl|UG|At#S34115597	gi|22136881|gb|AY133851.1	At1g62380.1
at2g39780	CON	MIN	HOUR	DAY	WEEK	UNIGENE	FLCDNA	TAIR
TCTCTGATGT	2	4	1	1	0	gnl|UG|At#S11732657	gi|110742770|dbj|AK227261.1|	At2g39780.1
at2g42580	CON	MIN	HOUR	DAY	WEEK	UNIGENE	FLCDNA	TAIR
GCGTTGGCGA	0	6	2	1	0	gnl|UG|At#S11731973	gi|23506040|gb|AY143941.1	At2g42580.1
at5g57655	CON	MIN	HOUR	DAY	WEEK	UNIGENE	FLCDNA	TAIR
GTTATGATGA	1	15	3	1	0	no match found	gi|21403668|gb|AY084958.1	multiple canonical match
at2g39250	CON	MIN	HOUR	DAY	WEEK	UNIGENE	FLCDNA	TAIR
AGGTGATCAC	0	0	1	0	0	gnl|UG|At#S15460611	gi|17065003|gb|AY062578.1	At2g39250.1
at3g56860	CON	MIN	HOUR	DAY	WEEK	UNIGENE	FLCDNA	TAIR
TATCCATCCA	2	1	0	1	0	no match found	gi|21436450|gb|AY117351.1	multiple non-canonical match
ATTGCAGATC	0	1	0	0	0	no match found	gi|20259481|gb|AY091040.1	non-canonical match
TGGGTCTTTG	2	0	1	3	0	no match found	no match found	At3g56860.2
at4g16880	CON	MIN	HOUR	DAY	WEEK	UNIGENE	FLCDNA	TAIR
TCTCTTTCAG	5	5	4	1	1	gnl|UG|At#S11724875	gi|110739134|dbj|AK229639.1|	At4g16880.1
at2g41610	CON	MIN	HOUR	DAY	WEEK	UNIGENE	FLCDNA	TAIR
AAATTTGAAA	0	0	0	1	0	gnl|UG|At#S15114831	no match found	At2g41610.1
at2g39720	CON	MIN	HOUR	DAY	WEEK	UNIGENE	FLCDNA	TAIR
AGCTACCGGC	0	0	1	0	0	gnl|UG|At#S18899706	no match found	non-canonical match
at2g46630	CON	MIN	HOUR	DAY	WEEK	UNIGENE	FLCDNA	TAIR
TCAGTGATTA	1	1	0	0	1	no match found	no match found	At2g46630.1
at3g46180	CON	MIN	HOUR	DAY	WEEK	UNIGENE	FLCDNA	TAIR
CTCTCCTGCA	0	0	0	0	1	no match found	gi|45752697|gb|BT012152.1	multiple non-canonical match
ACGTAGGACG	1	0	0	3	0	gnl|UG|At#S11730151	gi|110743334|dbj|AK227561.1|	At3g46180.1
at4g14930	CON	MIN	HOUR	DAY	WEEK	UNIGENE	FLCDNA	TAIR
AGAGAAAATA	2	7	2	1	1	no match found	gi|27311790|gb|BT002501.1	multiple non-canonical match
at3g03530	CON	MIN	HOUR	DAY	WEEK	UNIGENE	FLCDNA	TAIR
ATTGAAAGAG	0	1	0	0	0	gnl|UG|At#S11739639	gi|14335155|gb|AY037257.1	At3g03530.1
at2g16740	CON	MIN	HOUR	DAY	WEEK	UNIGENE	FLCDNA	TAIR
GCTCGAAGCT	1	4	2	2	1	no match found	gi|21405497|gb|AY086787.1	non-canonical match
at5g42060	CON	MIN	HOUR	DAY	WEEK	UNIGENE	FLCDNA	TAIR
TGTCTAAGCA	0	0	2	0	0	gnl|UG|At#S11790798	gi|21386924|gb|AY114547.1	At5g42060.1
at1g01930	CON	MIN	HOUR	DAY	WEEK	UNIGENE	FLCDNA	TAIR
AAGCCTTGTT	1	1	2	2	2	gnl|UG|At#S11742923	gi|62321054|dbj|AK221856.1|	At1g01930.1
ATGCTAAAGT	0	1	0	0	0	no match found	gi|29824278|gb|BT006115.1	non-canonical match
at4g27950	CON	MIN	HOUR	DAY	WEEK	UNIGENE	FLCDNA	TAIR
TCGGTTTCGA	0	0	1	0	0	gnl|UG|At#S11722969	no match found	At4g27950.1
at1g73230	CON	MIN	HOUR	DAY	WEEK	UNIGENE	FLCDNA	TAIR
GGTTGTGAGT	3	6	5	7	7	gnl|UG|At#S11728413	gi|21403551|gb|AY084841.1	At1g73230.1

at1g47270	CON	MIN	HOUR	DAY	WEEK	UNIGENE	FLCDNA	TAIR
AACATACCAG	0	0	1	0	0	gnl|UG|At#S11735500	gi|110737760|dbj|AK228930.1|	At1g47270.1
at1g62200	CON	MIN	HOUR	DAY	WEEK	UNIGENE	FLCDNA	TAIR
GTATACTCTG	1	0	0	0	1	gnl|UG|At#S11708065	gi|16604435|gb|AY058836.1	At1g62200.1
at1g51805	CON	MIN	HOUR	DAY	WEEK	UNIGENE	FLCDNA	TAIR
TGTGAATAAC	3	4	10	3	5	gnl|UG|At#S11733840	gi|17380979|gb|AY063946.1	At1g51805.1
at5g61810	CON	MIN	HOUR	DAY	WEEK	UNIGENE	FLCDNA	TAIR
GGCCAAGAGT	1	1	0	0	0	gnl|UG|At#S11706050	gi|15810360|gb|AY056219.1	non-canonical match
at1g17020	CON	MIN	HOUR	DAY	WEEK	UNIGENE	FLCDNA	TAIR
AAAGAGTATA	0	0	0	0	1	no match found	gi|15081818|gb|AY048302.1	non-canonical match
at2g21210	CON	MIN	HOUR	DAY	WEEK	UNIGENE	FLCDNA	TAIR
ATTCCTTCTA	33	10	4	5	2	gnl|UG|At#S11737151	gi|51970087|dbj|AK175973.1|	At2g21210.1
at5g20590	CON	MIN	HOUR	DAY	WEEK	UNIGENE	FLCDNA	TAIR
TAGAGGTGTG	0	1	1	2	1	gnl|UG|At#S11721179	gi|21404957|gb|AY086247.1	non-canonical match
at1g43560	CON	MIN	HOUR	DAY	WEEK	UNIGENE	FLCDNA	TAIR
TCAGCTTATG	1	4	0	0	2	gnl|UG|At#S11736089	gi|22135796|gb|AY128276.1	At1g43560.1
at5g63840	CON	MIN	HOUR	DAY	WEEK	UNIGENE	FLCDNA	TAIR
GCCGTTAGAG	0	2	2	2	1	gnl|UG|At#S11717316	gi|110742140|dbj|AK226927.1|	At5g63840.1
at1g72645	CON	MIN	HOUR	DAY	WEEK	UNIGENE	FLCDNA	TAIR
TACTAAGTAA	2	0	0	0	2	gnl|UG|At#S18923704	gi|19347743|gb|AY080613.1	At1g72645.1
at3g10770	CON	MIN	HOUR	DAY	WEEK	UNIGENE	FLCDNA	TAIR
TATAAACCGG	0	3	1	1	0	gnl|UG|At#S11737656	gi|110740805|dbj|AK226352.1|	At3g10770.2
at1g77990	CON	MIN	HOUR	DAY	WEEK	UNIGENE	FLCDNA	TAIR
TCTTGCGTGA	0	0	0	0	1	gnl|UG|At#S11727205	gi|18377974|gb|AY074516.1	At1g77990.1
GTGGCTCTGG	0	1	0	0	0	no match found	gi|110741894|dbj|AK226791.1|	non-canonical match
at5g19150	CON	MIN	HOUR	DAY	WEEK	UNIGENE	FLCDNA	TAIR
AAGTAGAGTT	0	1	2	1	0	gnl|UG|At#S11721531	gi|15081665|gb|AY048225.1	At5g19150.1
at1g02380	CON	MIN	HOUR	DAY	WEEK	UNIGENE	FLCDNA	TAIR
ATAATAACGG	0	1	0	0	0	gnl|UG|At#S11742877	gi|26450695|dbj|AK117815.1|	At1g02380.1
at5g42820	CON	MIN	HOUR	DAY	WEEK	UNIGENE	FLCDNA	TAIR
AACTCCTTAG	2	2	1	2	2	no match found	gi|23198021|gb|BT000219.1	At5g42820.1
at3g03940	CON	MIN	HOUR	DAY	WEEK	UNIGENE	FLCDNA	TAIR
TTTGTTATAA	5	1	0	0	3	gnl|UG|At#S11739532	gi|22135891|gb|AY128325.1	multiple non-canonical match
GAGTATACTG	0	4	0	1	1	no match found	no match found	At3g03940.1
at3g20740	CON	MIN	HOUR	DAY	WEEK	UNIGENE	FLCDNA	TAIR
TTGCTACTCC	0	0	2	0	0	gnl|UG|At#S11700825	no match found	At3g20740.1
at1g04190	CON	MIN	HOUR	DAY	WEEK	UNIGENE	FLCDNA	TAIR
AGATTCTCCC	1	1	1	0	1	gnl|UG|At#S11744311	gi|19423983|gb|AY080790.1	At1g04190.1
at1g06250	CON	MIN	HOUR	DAY	WEEK	UNIGENE	FLCDNA	TAIR
AGCTAGATTA	1	0	0	0	0	gnl|UG|At#S18892765	no match found	non-canonical match
at2g47770	CON	MIN	HOUR	DAY	WEEK	UNIGENE	FLCDNA	TAIR
ATATACTTGA	0	0	0	0	3	gnl|UG|At#S11730772	gi|16226869|gb|AF428356.1	At2g47770.1

at5g05090	CON	MIN	HOUR	DAY	WEEK	UNIGENE	FLCDNA	TAIR
CAGCTTATGA	0	2	0	0	0	gnl|UG|At#S11724860	gi|20260577|gb|AY093188.1	At5g05090.1
at5g58990	CON	MIN	HOUR	DAY	WEEK	UNIGENE	FLCDNA	TAIR
GAAAAGCTTG	0	0	0	1	0	no match found	gi|20148678|gb|AY081668.1	multiple non-canonical match
TAATAAATGA	1	0	0	0	0	no match found	gi|15450823|gb|AY054492.1	At5g58990.1
at1g63260	CON	MIN	HOUR	DAY	WEEK	UNIGENE	FLCDNA	TAIR
TTGGTGAATG	1	1	0	2	1	gnl|UG|At#S11730491	gi|42600562|gb|BT011569.1	At1g63260.1
AAAACCGAGT	0	1	0	0	0	no match found	gi|45773871|gb|BT012253.1	non-canonical match
at3g26380	CON	MIN	HOUR	DAY	WEEK	UNIGENE	FLCDNA	TAIR
GTCCTGCTCT	0	5	0	0	1	gnl|UG|At#S11732848	gi|110742015|dbj|AK226855.1|	At3g26380.1
at1g68440	CON	MIN	HOUR	DAY	WEEK	UNIGENE	FLCDNA	TAIR
AGAATGTTGT	0	6	1	2	0	gnl|UG|At#S11729412	gi|21700822|gb|AY124826.1	At1g68440.1
at4g36195	CON	MIN	HOUR	DAY	WEEK	UNIGENE	FLCDNA	TAIR
TAAAGAGTCA	1	2	1	2	1	gnl|UG|At#S11703510	gi|51970331|dbj|AK176095.1|	At4g36195.1
at3g62940	CON	MIN	HOUR	DAY	WEEK	UNIGENE	FLCDNA	TAIR
CTTTTGGGCT	0	1	0	1	0	gnl|UG|At#S11726555	no match found	At3g62940.1
at5g64450	CON	MIN	HOUR	DAY	WEEK	UNIGENE	FLCDNA	TAIR
GAAAGAAAAA	1	0	3	2	1	no match found	no match found	At5g64450.1
TTGACAATAA	0	0	0	0	1	gnl|UG|At#S19189588	gi|45680333|gb|BT012023.1	At1g44190.1
at2g23940	CON	MIN	HOUR	DAY	WEEK	UNIGENE	FLCDNA	TAIR
TCTGCGTGTA	1	0	1	0	1	gnl|UG|At#S11736506	gi|26450204|dbj|AK117560.1|	multiple canonical match
at2g43980	CON	MIN	HOUR	DAY	WEEK	UNIGENE	FLCDNA	TAIR
TCCAAGAGAA	2	0	0	1	1	gnl|UG|At#S18898553	gi|20465988|gb|AY096566.1	At2g43980.1
at3g26520	CON	MIN	HOUR	DAY	WEEK	UNIGENE	FLCDNA	TAIR
AACCCAGCCG	44	155	44	51	2	no match found	gi|16226820|gb|AF428341.1	non-canonical match
GAAATTTTAA	1	2	1	1	0	gnl|UG|At#S11732807	no match found	multiple canonical match
CTTTCGGTCT	0	0	11	0	0	gnl|UG|At#S23733728	no match found	non-canonical match
at1g79640	CON	MIN	HOUR	DAY	WEEK	UNIGENE	FLCDNA	TAIR
GTTACATAAG	4	0	0	0	1	no match found	no match found	At1g79640.1
at3g48150	CON	MIN	HOUR	DAY	WEEK	UNIGENE	FLCDNA	TAIR
TACACTATAA	2	0	0	0	1	gnl|UG|At#S11729775	gi|15982908|gb|AY057562.1	At3g48150.1
at5g50900	CON	MIN	HOUR	DAY	WEEK	UNIGENE	FLCDNA	TAIR
TTGATTGATG	3	0	2	0	0	gnl|UG|At#S11704228	gi|14532769|gb|AY040009.1	At5g50900.1
at5g01960	CON	MIN	HOUR	DAY	WEEK	UNIGENE	FLCDNA	TAIR
TTTTGCGAAG	0	2	0	0	1	no match found	gi|20466489|gb|AY099711.1	non-canonical match
at5g11430	CON	MIN	HOUR	DAY	WEEK	UNIGENE	FLCDNA	TAIR
ATACTGCTGC	0	0	1	0	0	gnl|UG|At#S11723397	no match found	At5g11430.1
at2g45630	CON	MIN	HOUR	DAY	WEEK	UNIGENE	FLCDNA	TAIR
TTGACTTAGT	0	0	1	0	0	no match found	gi|18700247|gb|AY078033.1	non-canonical match
GCAGTTGTAT	1	0	0	0	1	gnl|UG|At#S21736008	gi|62320835|dbj|AK221745.1|	non-canonical match
GATATAGAGG	0	1	0	0	0	no match found	gi|15724345|gb|AF412113.1	multiple canonical match
at2g47930	CON	MIN	HOUR	DAY	WEEK	UNIGENE	FLCDNA	TAIR
TAATATTAAT	0	3	1	0	1	gnl|UG|At#S11703366	gi|14326466|gb|AF385686.1	At2g47930.1

at2g20880	CON	MIN	HOUR	DAY	WEEK	UNIGENE	FLCDNA	TAIR
GTTCAATGCA	0	0	0	1	0	gnl|UG|At#S11737238	gi|20148660|gb|AY081659.1	At2g20880.1
at5g42240	CON	MIN	HOUR	DAY	WEEK	UNIGENE	FLCDNA	TAIR
TAAAAGTTTG	2	1	0	3	1	gnl|UG|At#S11719505	gi|13605556|gb|AF361604.1	At5g42240.1
at1g17460	CON	MIN	HOUR	DAY	WEEK	UNIGENE	FLCDNA	TAIR
ATTTAAACAG	1	0	0	0	0	gnl|UG|At#S11743920	gi|19347779|gb|AY080665.1	At1g17460.1
at1g67785	CON	MIN	HOUR	DAY	WEEK	UNIGENE	FLCDNA	TAIR
CTTTTGGGTG	9	5	0	2	0	gnl|UG|At#S30458997	gi|21403155|gb|AY084445.1	At1g67785.1
at5g02790	CON	MIN	HOUR	DAY	WEEK	UNIGENE	FLCDNA	TAIR
TAACTGTAAG	5	7	6	3	1	gnl|UG|At#S11725465	gi|25084107|gb|BT002166.1	At5g02790.1
at5g10910	CON	MIN	HOUR	DAY	WEEK	UNIGENE	FLCDNA	TAIR
CATCCAACTT	0	1	0	0	0	gnl|UG|At#S11723521	gi|28416478|gb|BT004524.1	At5g10910.1
at3g14680	CON	MIN	HOUR	DAY	WEEK	UNIGENE	FLCDNA	TAIR
CAAGCTTTTC	0	0	0	0	1	gnl|UG|At#S11736444	no match found	At3g14680.1
at5g38560	CON	MIN	HOUR	DAY	WEEK	UNIGENE	FLCDNA	TAIR
ATATAATGTA	2	0	0	0	0	gnl|UG|At#S11719877	gi|18700152|gb|AY075681.1	At5g38560.1
at4g27600	CON	MIN	HOUR	DAY	WEEK	UNIGENE	FLCDNA	TAIR
CCTTCCGTCT	0	0	1	0	0	no match found	gi|16974332|gb|AY059658.1	non-canonical match
TAGTAGTGCC	9	2	4	5	3	no match found	gi|15450506|gb|AY052355.1	non-canonical match
at5g20050	CON	MIN	HOUR	DAY	WEEK	UNIGENE	FLCDNA	TAIR
ATTGTCGATC	2	6	1	0	0	gnl|UG|At#S11703755	gi|27363357|gb|BT002682.1	At5g20050.1
at1g27340	CON	MIN	HOUR	DAY	WEEK	UNIGENE	FLCDNA	TAIR
GTCTCTGGGA	0	1	0	1	0	gnl|UG|At#S11739870	gi|21404181|gb|AY085471.1	At1g27340.1
at1g75310	CON	MIN	HOUR	DAY	WEEK	UNIGENE	FLCDNA	TAIR
GAACAAATTT	1	1	1	1	0	gnl|UG|At#S11727977	no match found	non-canonical match
at5g15750	CON	MIN	HOUR	DAY	WEEK	UNIGENE	FLCDNA	TAIR
CTTGCTTAGC	4	3	0	0	4	gnl|UG|At#S11722401	gi|51968507|dbj|AK175183.1|	At5g15750.1
at3g02940	CON	MIN	HOUR	DAY	WEEK	UNIGENE	FLCDNA	TAIR
ACTCAAAAAA	1	0	2	2	0	gnl|UG|At#S34118040	gi|110736495|dbj|AK228268.1|	pseudo chromosome match
at4g28025	CON	MIN	HOUR	DAY	WEEK	UNIGENE	FLCDNA	TAIR
CAAGTTAAGT	9	15	9	10	3	gnl|UG|At#S11722958	gi|21405834|gb|AY087110.1	At4g28025.1
at5g26940	CON	MIN	HOUR	DAY	WEEK	UNIGENE	FLCDNA	TAIR
GCCAAGAACA	1	0	0	1	0	no match found	gi|51968683|dbj|AK175271.1|	At5g26940.4
at3g03730	CON	MIN	HOUR	DAY	WEEK	UNIGENE	FLCDNA	TAIR
AGTTACTTAG	0	0	0	0	2	gnl|UG|At#S11739587	no match found	non-canonical match
at5g51070	CON	MIN	HOUR	DAY	WEEK	UNIGENE	FLCDNA	TAIR
GTTCAATAGG	1	3	2	1	2	no match found	gi|14335169|gb|AY037264.1	non-canonical match
AAGAAGAGTT	0	1	3	0	1	gnl|UG|At#S11718609	gi|14334877|gb|AY035112.1	At5g51070.1
at1g30900	CON	MIN	HOUR	DAY	WEEK	UNIGENE	FLCDNA	TAIR
CCATTAGAGA	0	0	0	0	1	gnl|UG|At#S11738700	no match found	non-canonical match
at2g43640	CON	MIN	HOUR	DAY	WEEK	UNIGENE	FLCDNA	TAIR

ACTGCTTTGA	0	2	1	1	0	gnl|UG|At#S11731733	gi|27765063|gb|BT003088.1	At2g43640.1
at2g46580	CON	MIN	HOUR	DAY	WEEK	UNIGENE	FLCDNA	TAIR
GCTACTGGAA	1	0	0	1	0	gnl|UG|At#S11731055	gi|21405122|gb|AY086412.1	At2g46580.1
at3g44630	CON	MIN	HOUR	DAY	WEEK	UNIGENE	FLCDNA	TAIR
CTTGTTAAGG	0	1	0	0	1	no match found	gi|28973752|gb|BT005790.1	multiple non-canonical match
at5g44160	CON	MIN	HOUR	DAY	WEEK	UNIGENE	FLCDNA	TAIR
ATCATCAAGA	0	1	0	0	0	gnl|UG|At#S11719312	gi|27363363|gb|BT002685.1	At5g44160.1
at2g03150	CON	MIN	HOUR	DAY	WEEK	UNIGENE	FLCDNA	TAIR
TTGCCATACC	0	1	1	2	0	no match found	gi|17064957|gb|AY062555.1	non-canonical match
AAGACAAGGA	1	0	0	0	0	gnl|UG|At#S11740511	no match found	At2g03150.1
at5g59200	CON	MIN	HOUR	DAY	WEEK	UNIGENE	FLCDNA	TAIR
AGTTTCTTGT	0	1	0	0	0	gnl|UG|At#S11717785	no match found	multiple non-canonical match
at2g46330	CON	MIN	HOUR	DAY	WEEK	UNIGENE	FLCDNA	TAIR
TTCTCGATTA	17	35	12	8	5	gnl|UG|At#S35311452	gi|15294169|gb|AF410276.1	At2g46330.2
at5g35460	CON	MIN	HOUR	DAY	WEEK	UNIGENE	FLCDNA	TAIR
TTTTGTTCTA	2	1	0	0	3	gnl|UG|At#S18911408	gi|14335067|gb|AY037213.1	At5g35460.1
at4g30993	CON	MIN	HOUR	DAY	WEEK	UNIGENE	FLCDNA	TAIR
ACCAAATATG	2	3	2	1	0	no match found	gi|21406453|gb|AY087715.1	non-canonical match
GCTGAAGAGT	2	1	0	1	0	gnl|UG|At#S15460481	gi|62320861|dbj|AK221758.1|	At4g30993.2
at5g50420	CON	MIN	HOUR	DAY	WEEK	UNIGENE	FLCDNA	TAIR
TCCAGCGTAT	1	1	0	0	0	gnl|UG|At#S11718671	gi|21407335|gb|AY088561.1	At5g50420.1
at2g28130	CON	MIN	HOUR	DAY	WEEK	UNIGENE	FLCDNA	TAIR
TGGGCTGTGT	0	1	1	0	0	gnl|UG|At#S34117340	gi|110737836|dbj|AK228968.1|	At2g28130.1
at1g76810	CON	MIN	HOUR	DAY	WEEK	UNIGENE	FLCDNA	TAIR
CTGGCAAATT	2	8	5	4	2	gnl|UG|At#S11727654	gi|62321276|dbj|AK221970.1|	At1g76810.1
at1g17110	CON	MIN	HOUR	DAY	WEEK	UNIGENE	FLCDNA	TAIR
ATTATTACGA	0	1	1	0	1	gnl|UG|At#S11741334	no match found	At1g17110.1
at4g31510	CON	MIN	HOUR	DAY	WEEK	UNIGENE	FLCDNA	TAIR
ATCAGGCCGT	2	3	0	0	0	gnl|UG|At#S11722363	no match found	At4g31510.1
at5g16660	CON	MIN	HOUR	DAY	WEEK	UNIGENE	FLCDNA	TAIR
TCTACATATT	0	1	0	0	0	gnl|UG|At#S11722173	no match found	At5g16660.1
at1g08210	CON	MIN	HOUR	DAY	WEEK	UNIGENE	FLCDNA	TAIR
TCCTTTGATA	0	1	0	0	0	no match found	gi|11993876|gb|AF329505.1	At1g08210.1
at2g21350	CON	MIN	HOUR	DAY	WEEK	UNIGENE	FLCDNA	TAIR
TTCTTAAAGA	0	2	0	0	0	no match found	no match found	At2g21350.1
at1g31580	CON	MIN	HOUR	DAY	WEEK	UNIGENE	FLCDNA	TAIR
TGTTTTTCAT	40	10	29	8	27	gnl|UG|At#S11738440	no match found	multiple canonical match
at5g08710	CON	MIN	HOUR	DAY	WEEK	UNIGENE	FLCDNA	TAIR
GTAGTGATGT	0	0	0	1	0	gnl|UG|At#S11822991	gi|28973186|gb|BT005498.1	At5g08710.1
at3g53430	CON	MIN	HOUR	DAY	WEEK	UNIGENE	FLCDNA	TAIR
CCGCCGAAGT	2	18	2	5	3	gnl|UG|At#S11728798	gi|110741063|dbj|AK226484.1|	At3g53430.1

at3g43600	CON	MIN	HOUR	DAY	WEEK	UNIGENE	FLCDNA	TAIR
AAAGAAGGTA	0	0	1	1	1	gnl|UG|At#S11730646	no match found	At3g43600.1
at5g54170	CON	MIN	HOUR	DAY	WEEK	UNIGENE	FLCDNA	TAIR
GCTCAAATCA	0	1	0	0	0	no match found	gi|21406422|gb|AY087684.1	non-canonical match
at4g33700	CON	MIN	HOUR	DAY	WEEK	UNIGENE	FLCDNA	TAIR
GCTGTTGTTG	0	0	0	0	1	no match found	gi|20856057|gb|AY101524.1	multiple non-canonical match
AACAACAACT	0	1	1	1	3	gnl|UG|At#S11721990	gi|17381275|gb|AY064150.1	At4g33700.1
at3g52140	CON	MIN	HOUR	DAY	WEEK	UNIGENE	FLCDNA	TAIR
TCTGGCTTTT	1	2	0	5	3	gnl|UG|At#S11729034	no match found	At3g52140.1
at3g59420	CON	MIN	HOUR	DAY	WEEK	UNIGENE	FLCDNA	TAIR
GATAACATTC	0	0	0	1	0	gnl|UG|At#S11807665	no match found	At3g59420.1
at5g01670	CON	MIN	HOUR	DAY	WEEK	UNIGENE	FLCDNA	TAIR
AAGACTAAAC	1	0	0	0	0	gnl|UG|At#S18911338	gi|26451076|dbj|AK118010.1|	At5g01670.2
at4g17840	CON	MIN	HOUR	DAY	WEEK	UNIGENE	FLCDNA	TAIR
GTATATACTC	0	1	0	1	0	gnl|UG|At#S11724706	gi|110739173|dbj|AK229659.1|	At4g17840.1
at2g26080	CON	MIN	HOUR	DAY	WEEK	UNIGENE	FLCDNA	TAIR
GCTCCGCTCC	5	6	8	10	2	gnl|UG|At#S11736010	gi|110742033|dbj|AK226865.1|	At2g26080.1
at5g19600	CON	MIN	HOUR	DAY	WEEK	UNIGENE	FLCDNA	TAIR
AAATGGTTTT	1	0	0	0	0	gnl|UG|At#S34115232	gi|110741882|dbj|AK226785.1|	At5g19600.1
at1g14320	CON	MIN	HOUR	DAY	WEEK	UNIGENE	FLCDNA	TAIR
GACCTTTGGC	3	6	7	3	4	no match found	gi|21281240|gb|AY113989.1	non-canonical match
GCTTTAGCTT	0	1	1	1	2	gnl|UG|At#S11741605	gi|15028018|gb|AY045866.1	At1g14320.1
at1g20795	CON	MIN	HOUR	DAY	WEEK	UNIGENE	FLCDNA	TAIR
AGACCAATCT	0	0	1	0	0	no match found	no match found	At1g20795.1
at5g40770	CON	MIN	HOUR	DAY	WEEK	UNIGENE	FLCDNA	TAIR
TTTATCTAAT	1	2	1	0	5	no match found	gi|21406379|gb|AY087641.1	multiple non-canonical match
CGTGAGAAAA	0	0	1	0	2	gnl|UG|At#S23627098	no match found	non-canonical match
CTCTTTGCCC	1	2	2	0	2	no match found	gi|21387092|gb|AY114631.1	non-canonical match
at2g38270	CON	MIN	HOUR	DAY	WEEK	UNIGENE	FLCDNA	TAIR
GGCTAAGGGA	0	0	1	0	1	no match found	gi|28827325|gb|BT004974.1	non-canonical match
CTTGGATTCT	7	5	9	9	11	gnl|UG|At#S11733031	gi|21404983|gb|AY086273.1	At2g38270.1
at5g52210	CON	MIN	HOUR	DAY	WEEK	UNIGENE	FLCDNA	TAIR
ATGAGTAGGA	0	1	0	1	0	gnl|UG|At#S11700945	gi|110743284|dbj|AK227533.1|	At5g52210.1
at2g38770	CON	MIN	HOUR	DAY	WEEK	UNIGENE	FLCDNA	TAIR
AGGAAAAAAA	2	1	4	2	1	gnl|UG|At#S15460639	gi|20466795|gb|AY099864.1	At2g38770.1
at5g63580	CON	MIN	HOUR	DAY	WEEK	UNIGENE	FLCDNA	TAIR
CGGTGGTGAA	0	1	0	0	0	no match found	gi|170291236|gb|BT031358.1	non-canonical match
CACTCAAAAT	0	1	3	0	0	gnl|UG|At#S11717342	gi|27754527|gb|BT002895.1	At5g63580.1
at1g71710	CON	MIN	HOUR	DAY	WEEK	UNIGENE	FLCDNA	TAIR
TCGTTATACT	1	1	1	0	1	gnl|UG|At#S11728751	gi|15081806|gb|AY048296.1	At1g71710.1
at3g47870	CON	MIN	HOUR	DAY	WEEK	UNIGENE	FLCDNA	TAIR
GCTGAGTGAA	0	1	0	0	1	no match found	no match found	At3g47870.1
at5g30510	CON	MIN	HOUR	DAY	WEEK	UNIGENE	FLCDNA	TAIR

TGTACTTTGT	37	23	29	16	23	gnl|UG|At#S15460177	gi|62319676|dbj|AK221163.1|	At5g30510.1
CTTCGCTTCC	1	1	3	0	0	no match found	gi|23296538|gb|AY142540.1	non-canonical match
at3g15570	CON	MIN	HOUR	DAY	WEEK	UNIGENE	FLCDNA	TAIR
GAGGAGCAAA	0	2	2	0	1	gnl|UG|At#S21737131	gi|51969229|dbj|AK175544.1|	At3g15570.1
at3g24110	CON	MIN	HOUR	DAY	WEEK	UNIGENE	FLCDNA	TAIR
AGTTGGGTTG	0	1	0	0	1	gnl|UG|At#S11733556	no match found	At3g24110.1
at1g66100	CON	MIN	HOUR	DAY	WEEK	UNIGENE	FLCDNA	TAIR
TTAAGATAAT	11	3	11	1	0	gnl|UG|At#S11676426	gi|14190504|gb|AF380652.1	At1g66100.1
at1g45050	CON	MIN	HOUR	DAY	WEEK	UNIGENE	FLCDNA	TAIR
TCCACAAAAA	1	2	1	3	2	no match found	gi|51971710|dbj|AK176757.1|	At1g45050.1
at5g02270	CON	MIN	HOUR	DAY	WEEK	UNIGENE	FLCDNA	TAIR
GAGACTGCAC	0	1	1	0	1	gnl|UG|At#S11725591	gi|21404842|gb|AY086132.1	At5g02270.1
at1g80490	CON	MIN	HOUR	DAY	WEEK	UNIGENE	FLCDNA	TAIR
TTTGCAGTGG	1	2	0	1	0	gnl|UG|At#S15435717	gi|110742051|dbj|AK226879.1|	At1g80490.1
at5g45170	CON	MIN	HOUR	DAY	WEEK	UNIGENE	FLCDNA	TAIR
CGAAGCAGTT	1	0	1	0	0	gnl|UG|At#S24442036	gi|44917580|gb|BT011752.1	At5g45170.1
at4g02195	CON	MIN	HOUR	DAY	WEEK	UNIGENE	FLCDNA	TAIR
ATTGTTCTTT	0	1	0	0	0	gnl|UG|At#S34116989	gi|110738512|dbj|AK229319.1|	At4g02195.1
at3g52220	CON	MIN	HOUR	DAY	WEEK	UNIGENE	FLCDNA	TAIR
AAAAATGTGG	9	5	6	5	2	gnl|UG|At#S11729021	gi|13899104|gb|AF370547.1	At3g52220.1
at2g02070	CON	MIN	HOUR	DAY	WEEK	UNIGENE	FLCDNA	TAIR
TCGTCATCAA	2	5	4	6	0	gnl|UG|At#S11706092	gi|15810270|gb|AY056174.1	At2g02070.1
at3g06320	CON	MIN	HOUR	DAY	WEEK	UNIGENE	FLCDNA	TAIR
TCCTCTTTAC	0	1	0	0	1	no match found	gi|109134210|gb|BT026014.1	multiple non-canonical match
at3g52120	CON	MIN	HOUR	DAY	WEEK	UNIGENE	FLCDNA	TAIR
GGTGATTATA	0	1	2	0	0	gnl|UG|At#S43849724	gi|14335159|gb|AY037259.1	At3g52120.1
at4g37190	CON	MIN	HOUR	DAY	WEEK	UNIGENE	FLCDNA	TAIR
GGATCAGATG	0	0	0	1	0	gnl|UG|At#S11721406	gi|45680375|gb|BT012065.1	multiple non-canonical match
at1g43910	CON	MIN	HOUR	DAY	WEEK	UNIGENE	FLCDNA	TAIR
TTGATGTATG	1	1	1	0	0	gnl|UG|At#S11735933	gi|39104582|dbj|AK118165.2|	non-canonical match
at3g27660	CON	MIN	HOUR	DAY	WEEK	UNIGENE	FLCDNA	TAIR
AGCCGGGTAA	0	0	0	1	0	gnl|UG|At#S11732418	gi|21404596|gb|AY085886.1	At3g27660.1
at5g58200	CON	MIN	HOUR	DAY	WEEK	UNIGENE	FLCDNA	TAIR
TAAATGGAAG	1	0	0	0	0	gnl|UG|At#S11717890	gi|107738308|gb|BT025785.1	At5g58200.1
at5g25900	CON	MIN	HOUR	DAY	WEEK	UNIGENE	FLCDNA	TAIR
GCAGGCATTG	1	0	0	0	0	gnl|UG|At#S11720601	gi|16323134|gb|AY057671.1	At5g25900.1
at1g10450	CON	MIN	HOUR	DAY	WEEK	UNIGENE	FLCDNA	TAIR
ATTCACTACG	1	2	0	1	0	gnl|UG|At#S11741989	no match found	At1g10450.1
at4g13640	CON	MIN	HOUR	DAY	WEEK	UNIGENE	FLCDNA	TAIR
ATGCTGGATG	0	4	3	2	1	gnl|UG|At#S11744654	gi|21387090|gb|AY114630.1	non-canonical match
at2g43100	CON	MIN	HOUR	DAY	WEEK	UNIGENE	FLCDNA	TAIR

ATGGTGTTCT	2	4	1	0	1	no match found	gi|21436288|gb|AY117208.1	multiple non-canonical match
TTCCAAGATA	5	1	1	3	3	gnl|UG|At#S11731859	gi|16974632|gb|AY060594.1	At2g43100.1
at5g62030	CON	MIN	HOUR	DAY	WEEK	UNIGENE	FLCDNA	TAIR
AAGATTGGAA	0	1	2	0	1	gnl|UG|At#S11807166	gi|20259527|gb|AY091063.1	At5g62030.1
at1g53700	CON	MIN	HOUR	DAY	WEEK	UNIGENE	FLCDNA	TAIR
CTGGTCACGT	0	0	0	1	0	gnl|UG|At#S11733115	gi|134031903|gb|BT030386.1	At1g53700.1
at1g68070	CON	MIN	HOUR	DAY	WEEK	UNIGENE	FLCDNA	TAIR
TCCACTATGC	0	1	1	0	0	no match found	gi|90093275|gb|BT024880.1	non-canonical match
at3g07790	CON	MIN	HOUR	DAY	WEEK	UNIGENE	FLCDNA	TAIR
GGGTGAGATT	0	1	3	0	1	gnl|UG|At#S11738414	no match found	At3g07790.1
at2g20320	CON	MIN	HOUR	DAY	WEEK	UNIGENE	FLCDNA	TAIR
GAGTCTCTTT	0	1	1	0	0	gnl|UG|At#S11737390	no match found	non-canonical match
at5g20150	CON	MIN	HOUR	DAY	WEEK	UNIGENE	FLCDNA	TAIR
TTTCTGGATA	5	15	6	7	10	gnl|UG|At#S11810856	gi|19715594|gb|AY075605.1	At5g20150.1
at5g49440	CON	MIN	HOUR	DAY	WEEK	UNIGENE	FLCDNA	TAIR
GTGTTGTCGG	4	24	1	4	0	no match found	gi|26451575|dbj|AK118267.1|	non-canonical match
at5g24120	CON	MIN	HOUR	DAY	WEEK	UNIGENE	FLCDNA	TAIR
TAAAGTTTGA	14	3	32	45	14	gnl|UG|At#S11720767	no match found	At5g24120.1
CTTGGTTGTT	1	1	1	4	1	no match found	gi|62320237|dbj|AK221445.1|	non-canonical match
at4g23540	CON	MIN	HOUR	DAY	WEEK	UNIGENE	FLCDNA	TAIR
AATCTAATCT	1	1	0	1	0	no match found	no match found	At4g23540.1
at3g27050	CON	MIN	HOUR	DAY	WEEK	UNIGENE	FLCDNA	TAIR
TCATCTCCAT	3	0	0	1	1	no match found	gi|13937132|gb|AF372920.1	At3g27050.1
TGAAGCCATT	1	1	0	3	1	gnl|UG|At#S11732615	no match found	non-canonical match
at2g38530	CON	MIN	HOUR	DAY	WEEK	UNIGENE	FLCDNA	TAIR
CAAAGTCAAT	3	15	0	3	2	gnl|UG|At#S20800355	gi|16649114|gb|AY059927.1	At2g38530.1
at2g22000	CON	MIN	HOUR	DAY	WEEK	UNIGENE	FLCDNA	TAIR
TTTACCTTCT	0	0	1	0	1	gnl|UG|At#S11736969	no match found	non-canonical match
at5g43010	CON	MIN	HOUR	DAY	WEEK	UNIGENE	FLCDNA	TAIR
AAGGCGGTGA	3	5	2	2	2	no match found	gi|18700261|gb|AY078040.1	At5g43010.1
at1g55300	CON	MIN	HOUR	DAY	WEEK	UNIGENE	FLCDNA	TAIR
ATTTGATGAT	2	0	1	1	0	gnl|UG|At#S28282486	gi|38638687|gb|BT011002.1	At1g55300.1
at1g73220	CON	MIN	HOUR	DAY	WEEK	UNIGENE	FLCDNA	TAIR
ATTACTTGAT	0	1	0	0	0	gnl|UG|At#S11728415	gi|21406706|gb|AY087932.1	At1g73220.1
at4g08850	CON	MIN	HOUR	DAY	WEEK	UNIGENE	FLCDNA	TAIR
GAATTTTAGT	2	7	17	4	5	gnl|UG|At#S15461370	gi|18086326|gb|AY064972.1	At4g08850.1
ATCGGTCTCC	0	1	1	0	0	gnl|UG|At#S35277386	no match found	At4g08850.2
at3g59900	CON	MIN	HOUR	DAY	WEEK	UNIGENE	FLCDNA	TAIR
TAAATCTGAA	1	5	0	2	0	gnl|UG|At#S21736035	gi|51971476|dbj|AK176640.1|	At3g59900.1
at1g04520	CON	MIN	HOUR	DAY	WEEK	UNIGENE	FLCDNA	TAIR
AAAATTGTTA	2	0	1	0	0	gnl|UG|At#S11742576	no match found	At1g04520.1
at5g66010	CON	MIN	HOUR	DAY	WEEK	UNIGENE	FLCDNA	TAIR

TTTGGGAGCG	0	1	0	0	0	gnl|UG|At#S11717097	no match found	At5g66010.1

at1g78560	CON	MIN	HOUR	DAY	WEEK	UNIGENE	FLCDNA	TAIR
AAAATCATAT	1	1	0	0	1	no match found	no match found	At1g78560.1
AAGTCCTCAC	1	1	1	1	0	no match found	gi|23197631|gb|BT000024.1	non-canonical match

at1g70750	CON	MIN	HOUR	DAY	WEEK	UNIGENE	FLCDNA	TAIR
TTGCTTGTGA	0	0	0	0	1	gnl|UG|At#S34117392	gi|110737732|dbj|AK228916.1|	At1g70750.1

at5g01660	CON	MIN	HOUR	DAY	WEEK	UNIGENE	FLCDNA	TAIR
AGAGACAGAG	0	0	1	0	1	no match found	no match found	At5g01660.1

at1g53640	CON	MIN	HOUR	DAY	WEEK	UNIGENE	FLCDNA	TAIR
AAATTCGATG	1	0	0	0	0	no match found	no match found	At1g53640.1

at5g15190	CON	MIN	HOUR	DAY	WEEK	UNIGENE	FLCDNA	TAIR
TGTATATAAA	0	0	0	0	1	gnl|UG|At#S11822803	gi|26453157|dbj|AK119079.1|	At5g15190.2

at1g79030	CON	MIN	HOUR	DAY	WEEK	UNIGENE	FLCDNA	TAIR
GGATCTAGAT	0	0	0	0	1	gnl|UG|At#S11726836	no match found	At1g79030.1

at2g20900	CON	MIN	HOUR	DAY	WEEK	UNIGENE	FLCDNA	TAIR
ACTGCCGGTC	1	1	0	0	0	gnl|UG|At#S18942891	gi|21404299|gb|AY085589.1	At2g20900.3

at3g13460	CON	MIN	HOUR	DAY	WEEK	UNIGENE	FLCDNA	TAIR
GAAGGAGCTT	3	2	4	5	1	no match found	gi|110742753|dbj|AK227250.1|	non-canonical match
TTTTAAGATT	6	1	7	4	3	gnl|UG|At#S20757719	gi|110740176|dbj|AK230178.1|	At3g13460.1

at5g47120	CON	MIN	HOUR	DAY	WEEK	UNIGENE	FLCDNA	TAIR
TTTCCACTAA	1	1	1	6	2	gnl|UG|At#S11719012	gi|20268759|gb|AY091134.1	At5g47120.1

at1g20080	CON	MIN	HOUR	DAY	WEEK	UNIGENE	FLCDNA	TAIR
ATTTGGTTAA	1	0	0	2	0	no match found	no match found	At1g20080.1

at5g64930	CON	MIN	HOUR	DAY	WEEK	UNIGENE	FLCDNA	TAIR
GTGGGTCTGA	1	1	1	1	2	gnl|UG|At#S11717203	gi|110737976|dbj|AK229041.1|	At5g64930.1

at1g17640	CON	MIN	HOUR	DAY	WEEK	UNIGENE	FLCDNA	TAIR
GAAACGGAAA	0	1	1	0	1	gnl|UG|At#S11741281	no match found	At1g17640.1

at3g15120	CON	MIN	HOUR	DAY	WEEK	UNIGENE	FLCDNA	TAIR
TTTTTGCAAA	0	0	2	0	0	gnl|UG|At#S11736312	no match found	multiple non-canonical match

at5g42280	CON	MIN	HOUR	DAY	WEEK	UNIGENE	FLCDNA	TAIR
AAGCCCCACT	0	1	0	0	0	gnl|UG|At#S34115905	gi|110740606|dbj|AK226245.1|	non-canonical match
AACTCTTCTC	6	3	7	0	4	no match found	no match found	At5g42280.1

at4g21210	CON	MIN	HOUR	DAY	WEEK	UNIGENE	FLCDNA	TAIR
ACCATATTAT	3	0	1	2	3	gnl|UG|At#S11724139	no match found	At4g21210.1

at1g27050	CON	MIN	HOUR	DAY	WEEK	UNIGENE	FLCDNA	TAIR
GGAGAAGCAG	0	1	5	0	0	gnl|UG|At#S11739980	gi|18087566|gb|AF462825.1	At1g27050.1

at1g72230	CON	MIN	HOUR	DAY	WEEK	UNIGENE	FLCDNA	TAIR
ATTTTTCCTA	1	1	0	2	0	gnl|UG|At#S11728638	gi|15529179|gb|AY052214.1	At1g72230.1

at2g34660	CON	MIN	HOUR	DAY	WEEK	UNIGENE	FLCDNA	TAIR
TTAAACTAGG	2	0	4	4	6	gnl|UG|At#S11733899	gi|110740125|dbj|AK230151.1|	At2g34660.1

at4g28320	CON	MIN	HOUR	DAY	WEEK	UNIGENE	FLCDNA	TAIR
TAAACTGTCT	0	0	0	0	1	gnl|UG|At#S34117576	gi|110737377|dbj|AK228732.1|	At4g28320.1

at1g80950	CON	MIN	HOUR	DAY	WEEK	UNIGENE	FLCDNA	TAIR
GGATACCATA	0	2	0	0	0	gnl|UG|At#S11726269	gi|28058881|gb|BT003346.1	At1g80950.1
at2g09800	CON	MIN	HOUR	DAY	WEEK	UNIGENE	FLCDNA	TAIR
AGCTAAGGAG	0	1	0	0	0	gnl|UG|At#S18901274	no match found	pseudo chromosome match
at5g43680	CON	MIN	HOUR	DAY	WEEK	UNIGENE	FLCDNA	TAIR
GTTTTCTCAA	0	0	1	0	0	gnl|UG|At#S11704462	gi|15010561|gb|AY045582.1	At5g43680.1
at3g19370	CON	MIN	HOUR	DAY	WEEK	UNIGENE	FLCDNA	TAIR
GAAATCTAAT	0	1	0	0	1	gnl|UG|At#S11735036	gi|110737806|dbj|AK228953.1|	At3g19370.1
at3g50550	CON	MIN	HOUR	DAY	WEEK	UNIGENE	FLCDNA	TAIR
CTCTTAATAC	2	0	1	0	0	gnl|UG|At#S35280401	gi|21407509|gb|AY088735.1	At3g50550.1
at3g22480	CON	MIN	HOUR	DAY	WEEK	UNIGENE	FLCDNA	TAIR
TTTTGAATGT	3	1	1	1	2	no match found	gi|62320961|dbj|AK221809.1|	At3g22480.1
ATAGGAGGCG	0	1	0	0	0	gnl|UG|At#S18925537	gi|21405345|gb|AY086635.1	non-canonical match
at4g39800	CON	MIN	HOUR	DAY	WEEK	UNIGENE	FLCDNA	TAIR
AATCTAAGAC	2	1	2	0	2	no match found	gi|13937199|gb|AF372954.1	non-canonical match
GTTTTGGTTC	18	5	18	29	20	gnl|UG|At#S11703594	no match found	At4g39800.1
at1g20920	CON	MIN	HOUR	DAY	WEEK	UNIGENE	FLCDNA	TAIR
GACTTGCGAC	0	3	0	0	0	gnl|UG|At#S24442821	gi|25083095|gb|BT002030.1	pseudo chromosome match
at1g04610	CON	MIN	HOUR	DAY	WEEK	UNIGENE	FLCDNA	TAIR
ATATTGCTAA	0	1	1	0	0	gnl|UG|At#S34117542	gi|40823346|gb|BT011241.1	At1g04610.1
at3g09990	CON	MIN	HOUR	DAY	WEEK	UNIGENE	FLCDNA	TAIR
CGATTTTAAA	0	1	0	0	0	no match found	no match found	At3g09990.1
at3g17950	CON	MIN	HOUR	DAY	WEEK	UNIGENE	FLCDNA	TAIR
TTGAATGTAG	0	1	0	0	0	gnl|UG|At#S21126608	no match found	non-canonical match
at3g17740	CON	MIN	HOUR	DAY	WEEK	UNIGENE	FLCDNA	TAIR
GTCGAAGAAA	0	0	2	0	0	gnl|UG|At#S11735519	gi|26449767|dbj|AK117337.1|	multiple canonical match
at4g26130	CON	MIN	HOUR	DAY	WEEK	UNIGENE	FLCDNA	TAIR
ACCCAACCCG	1	7	3	0	0	gnl|UG|At#S11723289	gi|18377836|gb|AY074565.1	At4g26130.1
at5g26710	CON	MIN	HOUR	DAY	WEEK	UNIGENE	FLCDNA	TAIR
GCTTCCTGAT	3	2	2	1	1	no match found	gi|110739667|dbj|AK229914.1|	multiple non-canonical match
at5g62140	CON	MIN	HOUR	DAY	WEEK	UNIGENE	FLCDNA	TAIR
GCTTCAACAA	0	0	2	1	0	no match found	gi|62320179|dbj|AK221416.1|	At5g62140.1
at4g26500	CON	MIN	HOUR	DAY	WEEK	UNIGENE	FLCDNA	TAIR
ATTCACCATT	0	0	1	0	0	gnl|UG|At#S11723226	gi|28393577|gb|BT004190.1	At4g26500.1
at3g54830	CON	MIN	HOUR	DAY	WEEK	UNIGENE	FLCDNA	TAIR
TCACTGATTG	0	2	0	0	0	gnl|UG|At#S11728544	no match found	non-canonical match
at3g22200	CON	MIN	HOUR	DAY	WEEK	UNIGENE	FLCDNA	TAIR
GATTTACTTA	0	2	0	1	2	gnl|UG|At#S11734162	gi|23296653|gb|AY142571.1	At3g22200.1
at4g36280	CON	MIN	HOUR	DAY	WEEK	UNIGENE	FLCDNA	TAIR
TGCATATTTG	0	0	0	1	0	gnl|UG|At#S11825072	gi|27754644|gb|BT002955.1	At4g36280.1
at4g03820	CON	MIN	HOUR	DAY	WEEK	UNIGENE	FLCDNA	TAIR

AAGACAGAAT	0	0	0	0	1	gnl|UG|At#S18941905	gi|26449460|dbj|AK117180.1|	At4g03820.2
at2g20770	CON	MIN	HOUR	DAY	WEEK	UNIGENE	FLCDNA	TAIR
AAGTGAACAC	0	0	1	0	0	gnl|UG|At#S11708371	gi|17380955|gb|AY063934.1	non-canonical match
at2g44610	CON	MIN	HOUR	DAY	WEEK	UNIGENE	FLCDNA	TAIR
ATTCGATCAG	1	3	2	6	1	gnl|UG|At#S11731494	gi|21406678|gb|AY087904.1	At2g44610.1
at3g45260	CON	MIN	HOUR	DAY	WEEK	UNIGENE	FLCDNA	TAIR
TTTTAATTTT	2	5	2	0	0	gnl|UG|At#S11730332	gi|16226321|gb|AF428302.1	At3g45260.1
at3g18230	CON	MIN	HOUR	DAY	WEEK	UNIGENE	FLCDNA	TAIR
TAACGTCCTG	1	0	0	1	1	gnl|UG|At#S11735372	gi|110738032|dbj|AK229070.1|	At3g18230.1
at3g15620	CON	MIN	HOUR	DAY	WEEK	UNIGENE	FLCDNA	TAIR
TAACACTTCT	0	0	2	0	0	gnl|UG|At#S28282973	gi|26449887|dbj|AK117398.1|	At3g15620.1
at4g05070	CON	MIN	HOUR	DAY	WEEK	UNIGENE	FLCDNA	TAIR
TATAAAATGA	8	17	10	5	4	no match found	gi|18175931|gb|AY072131.1	At4g05070.1
at5g57887	CON	MIN	HOUR	DAY	WEEK	UNIGENE	FLCDNA	TAIR
TATTTGTACA	2	6	1	1	1	gnl|UG|At#S30641665	gi|21407103|gb|AY088329.1	non-canonical match
TCGATCACGA	0	0	1	0	0	no match found	gi|28973492|gb|BT005651.1	At5g57880.1
at3g62270	CON	MIN	HOUR	DAY	WEEK	UNIGENE	FLCDNA	TAIR
AAACATCAGC	1	0	3	2	3	gnl|UG|At#S11726763	no match found	At3g62270.1
at2g38590	CON	MIN	HOUR	DAY	WEEK	UNIGENE	FLCDNA	TAIR
TTGAGACTTG	0	0	0	0	1	gnl|UG|At#S11732946	no match found	multiple canonical match
at1g65350	CON	MIN	HOUR	DAY	WEEK	UNIGENE	FLCDNA	TAIR
GGTAATGTCC	0	0	0	1	0	no match found	no match found	At1g65350.1
at1g27840	CON	MIN	HOUR	DAY	WEEK	UNIGENE	FLCDNA	TAIR
TGCGTTGTTC	1	0	2	0	0	gnl|UG|At#S11739688	gi|18377623|gb|AY074265.1	At1g27840.1
at3g18940	CON	MIN	HOUR	DAY	WEEK	UNIGENE	FLCDNA	TAIR
GAAGAGTATG	0	1	0	0	1	gnl|UG|At#S18902187	gi|21407297|gb|AY088523.1	At3g18940.1
at2g41090	CON	MIN	HOUR	DAY	WEEK	UNIGENE	FLCDNA	TAIR
CAATTTATTA	3	20	4	8	20	gnl|UG|At#S11732318	gi|15028258|gb|AY046044.1	At2g41090.1
at1g52780	CON	MIN	HOUR	DAY	WEEK	UNIGENE	FLCDNA	TAIR
CAAGTATATA	4	2	1	0	1	gnl|UG|At#S11733442	gi|110738200|dbj|AK229158.1|	At1g52780.1
at2g07671	CON	MIN	HOUR	DAY	WEEK	UNIGENE	FLCDNA	TAIR
CTCAGTAGTT	2	1	0	0	0	gnl|UG|At#S35308968	no match found	non-canonical match
at5g41800	CON	MIN	HOUR	DAY	WEEK	UNIGENE	FLCDNA	TAIR
AATTTCTCGA	1	2	5	1	0	gnl|UG|At#S11719549	gi|20466437|gb|AY099685.1	At5g41800.1
at5g65220	CON	MIN	HOUR	DAY	WEEK	UNIGENE	FLCDNA	TAIR
AATCCCTTAA	33	19	33	21	24	no match found	gi|21404373|gb|AY085663.1	At5g65220.1
GTGTCAGAAT	2	8	0	0	0	no match found	gi|19310702|gb|AY079351.1	At4g29580.1
at3g57530	CON	MIN	HOUR	DAY	WEEK	UNIGENE	FLCDNA	TAIR
CCATTCCGCA	0	3	2	0	1	gnl|UG|At#S34115802	gi|44681391|gb|BT011630.1	At3g57530.1
at3g57910	CON	MIN	HOUR	DAY	WEEK	UNIGENE	FLCDNA	TAIR
TGGAACTCTG	0	0	0	1	0	gnl|UG|At#S11727968	gi|62320599|dbj|AK221626.1|	non-canonical match

at4g07600	CON	MIN	HOUR	DAY	WEEK	UNIGENE	FLCDNA	TAIR
GAGTATATGT	0	0	0	1	0	no match found	no match found	At4g07600.1
at4g23910	CON	MIN	HOUR	DAY	WEEK	UNIGENE	FLCDNA	TAIR
AAAGAATTTT	1	5	1	1	2	no match found	gi|110743730|dbj|AK227716.1|	multiple non-canonical match
at5g01460	CON	MIN	HOUR	DAY	WEEK	UNIGENE	FLCDNA	TAIR
GCCTATTGAA	3	2	2	0	0	gnl|UG|At#S11725791	gi|18176295|gb|AY072197.1	At5g01460.1
at1g54680	CON	MIN	HOUR	DAY	WEEK	UNIGENE	FLCDNA	TAIR
TCTATGTTAC	0	3	0	0	1	no match found	no match found	At1g54680.1
at2g32040	CON	MIN	HOUR	DAY	WEEK	UNIGENE	FLCDNA	TAIR
TAACCAAAAT	1	1	1	2	1	no match found	gi|19698918|gb|AY081306.1	non-canonical match
at2g21385	CON	MIN	HOUR	DAY	WEEK	UNIGENE	FLCDNA	TAIR
TAATAATTTT	1	3	0	3	0	gnl|UG|At#S11737115	gi|15027870|gb|AY045792.1	At2g21385.1
at3g12390	CON	MIN	HOUR	DAY	WEEK	UNIGENE	FLCDNA	TAIR
AAGTGATTTT	10	8	4	11	10	gnl|UG|At#S11737178	gi|15081679|gb|AY048232.1	At3g12390.1
at3g21550	CON	MIN	HOUR	DAY	WEEK	UNIGENE	FLCDNA	TAIR
TGTTTATTCG	2	2	0	1	1	gnl|UG|At#S35278751	gi|15529148|gb|AY052198.1	At3g21550.1
at1g66045	CON	MIN	HOUR	DAY	WEEK	UNIGENE	FLCDNA	TAIR
GCCGATGTCT	0	0	1	0	0	gnl|UG|At#S11816323	no match found	At1g66045.1
at2g39620	CON	MIN	HOUR	DAY	WEEK	UNIGENE	FLCDNA	TAIR
ATAGAACATT	2	2	0	1	0	no match found	no match found	At2g39620.1
at1g58360	CON	MIN	HOUR	DAY	WEEK	UNIGENE	FLCDNA	TAIR
AGTGAGTTTG	2	1	2	0	1	gnl|UG|At#S35287910	gi|17978952|gb|AY069889.1	non-canonical match
AATCTCTCTG	5	1	4	1	10	gnl|UG|At#S11731764	no match found	At1g58360.1
at5g64370	CON	MIN	HOUR	DAY	WEEK	UNIGENE	FLCDNA	TAIR
GATCTCAATC	0	1	1	0	0	gnl|UG|At#S11717262	gi|30725679|gb|BT008503.1	At5g64370.1
at3g13350	CON	MIN	HOUR	DAY	WEEK	UNIGENE	FLCDNA	TAIR
AATCAGGAGC	1	0	0	1	0	gnl|UG|At#S11736872	gi|21406508|gb|AY087770.1	At3g13350.1
at1g07250	CON	MIN	HOUR	DAY	WEEK	UNIGENE	FLCDNA	TAIR
CTAAGTTTTT	4	2	4	3	2	gnl|UG|At#S11742309	gi|14532789|gb|AY040019.1	At1g07250.1
at5g23540	CON	MIN	HOUR	DAY	WEEK	UNIGENE	FLCDNA	TAIR
TCATCCAACA	1	7	1	2	0	gnl|UG|At#S11720823	gi|20259104|gb|AY091329.1	At5g23540.1
at1g73830	CON	MIN	HOUR	DAY	WEEK	UNIGENE	FLCDNA	TAIR
CAGAAGGCAA	2	5	0	1	0	gnl|UG|At#S11728280	gi|29824158|gb|BT006055.1	At1g73830.1
at2g35060	CON	MIN	HOUR	DAY	WEEK	UNIGENE	FLCDNA	TAIR
TTCCGTTGCG	1	2	0	0	0	gnl|UG|At#S28282197	gi|25084026|gb|BT002147.1	At2g35060.1
atcg01110	CON	MIN	HOUR	DAY	WEEK	UNIGENE	FLCDNA	TAIR
GGATCTTCGT	0	0	0	1	0	no match found	no match found	AtCg01110
at3g58370	CON	MIN	HOUR	DAY	WEEK	UNIGENE	FLCDNA	TAIR
GATTATACAA	12	1	4	6	4	no match found	no match found	At3g58370.1
at3g62550	CON	MIN	HOUR	DAY	WEEK	UNIGENE	FLCDNA	TAIR
ACTATGGCTT	1	8	12	1	1	gnl|UG|At#S11703693	gi|14334945|gb|AY035146.1	At3g62550.1

at5g12390	CON	MIN	HOUR	DAY	WEEK	UNIGENE	FLCDNA	TAIR
TTTCCCTTAT	0	1	0	0	0	no match found	gi|26451524|dbj|AK118241.1|	non-canonical match
at1g47740	CON	MIN	HOUR	DAY	WEEK	UNIGENE	FLCDNA	TAIR
TATTGCATCA	5	1	1	2	2	gnl|UG|At#S11735328	gi|21406839|gb|AY088065.1	multiple non-canonical match
at5g13280	CON	MIN	HOUR	DAY	WEEK	UNIGENE	FLCDNA	TAIR
TTCTTTATAC	2	0	0	0	0	gnl|UG|At#S11698542	gi|23308184|gb|BT000493.1	At5g13280.1
at4g29030	CON	MIN	HOUR	DAY	WEEK	UNIGENE	FLCDNA	TAIR
GCTTGGTCCA	4	5	5	5	1	gnl|UG|At#S11722785	gi|21404934|gb|AY086224.1	At4g29030.1
at1g26550	CON	MIN	HOUR	DAY	WEEK	UNIGENE	FLCDNA	TAIR
TTTTGTGCGA	0	3	1	1	0	no match found	gi|21403996|gb|AY085286.1	At1g26550.1
at1g05870	CON	MIN	HOUR	DAY	WEEK	UNIGENE	FLCDNA	TAIR
TATGTGTACT	0	0	0	0	2	no match found	gi|26451182|dbj|AK118064.1|	At1g05870.1
TTAACGACTC	1	2	2	1	3	gnl|UG|At#S20854293	no match found	non-canonical match
AAGAATCGAG	0	1	0	0	0	gnl|UG|At#S18942640	no match found	At1g05870.2
at2g47520	CON	MIN	HOUR	DAY	WEEK	UNIGENE	FLCDNA	TAIR
AGATTCTATC	0	1	0	0	0	no match found	gi|18491272|gb|AY074645.1	multiple canonical match
at4g17085	CON	MIN	HOUR	DAY	WEEK	UNIGENE	FLCDNA	TAIR
AAAATTGATC	2	2	1	0	0	no match found	gi|110736817|dbj|AK228438.1|	non-canonical match
at5g58190	CON	MIN	HOUR	DAY	WEEK	UNIGENE	FLCDNA	TAIR
GCAACAAAAA	0	1	2	0	0	gnl|UG|At#S11717891	gi|18086574|gb|AY072018.1	At5g58190.1
at3g57600	CON	MIN	HOUR	DAY	WEEK	UNIGENE	FLCDNA	TAIR
TTCATTTTTT	0	0	0	1	1	no match found	gi|21405967|gb|AY087243.1	At3g57600.1
at3g11120	CON	MIN	HOUR	DAY	WEEK	UNIGENE	FLCDNA	TAIR
AGGGCTAAGT	12	5	10	19	22	gnl|UG|At#S16349040	gi|28393754|gb|BT004288.1	At3g11120.1
at5g40810	CON	MIN	HOUR	DAY	WEEK	UNIGENE	FLCDNA	TAIR
ATTTTGTAAC	0	2	0	0	0	gnl|UG|At#S11719648	no match found	At5g40810.1
at5g22120	CON	MIN	HOUR	DAY	WEEK	UNIGENE	FLCDNA	TAIR
GGATTGAAGC	0	2	1	0	2	gnl|UG|At#S11817067	gi|51968895|dbj|AK175377.1|	At5g22120.1
at5g42960	CON	MIN	HOUR	DAY	WEEK	UNIGENE	FLCDNA	TAIR
GAACCTTGAA	2	3	1	2	2	gnl|UG|At#S11719432	gi|15451133|gb|AY054647.1	At5g42960.1
at5g55840	CON	MIN	HOUR	DAY	WEEK	UNIGENE	FLCDNA	TAIR
TTTAGATCAT	0	0	0	2	0	no match found	no match found	At5g55840.1
at5g22320	CON	MIN	HOUR	DAY	WEEK	UNIGENE	FLCDNA	TAIR
GAGGTTGAGA	0	1	0	2	0	gnl|UG|At#S11721019	gi|15912298|gb|AY056427.1	At5g22320.1
at5g15950	CON	MIN	HOUR	DAY	WEEK	UNIGENE	FLCDNA	TAIR
GACTTGAGCC	0	0	0	1	2	no match found	gi|17978850|gb|AY066030.1	non-canonical match
ACACCTTTGG	0	0	5	6	15	gnl|UG|At#S28281799	no match found	pseudo chromosome match
ATTTTAGTGT	1	1	1	0	15	no match found	no match found	At5g15950.1
at3g45100	CON	MIN	HOUR	DAY	WEEK	UNIGENE	FLCDNA	TAIR
GTAATTGGTA	0	0	1	0	1	no match found	no match found	At3g45100.1
at5g44005	CON	MIN	HOUR	DAY	WEEK	UNIGENE	FLCDNA	TAIR
TTGTGGAAAA	0	0	0	0	1	gnl|UG|At#S18941531	gi|27764977|gb|BT003045.1	At5g44005.1

at3g11770	CON	MIN	HOUR	DAY	WEEK	UNIGENE	FLCDNA	TAIR
CCCGGCCTTG	4	2	6	7	2	gnl|UG|At#S11737361	gi|110736242|dbj|AK228135.1|	At3g11770.1
at1g25380	CON	MIN	HOUR	DAY	WEEK	UNIGENE	FLCDNA	TAIR
GCAAAGATGG	0	1	0	0	0	no match found	gi|30984591|gb|BT008746.1	multiple non-canonical match
CGGTGATTGA	3	2	3	4	1	gnl|UG|At#S11740376	gi|20260665|gb|AY093232.1	At1g25380.1
at5g42490	CON	MIN	HOUR	DAY	WEEK	UNIGENE	FLCDNA	TAIR
GAACCAAAGA	0	1	0	0	0	gnl|UG|At#S11719480	no match found	multiple non-canonical match
at5g44575	CON	MIN	HOUR	DAY	WEEK	UNIGENE	FLCDNA	TAIR
GTTTTTAGCA	2	0	2	0	1	gnl|UG|At#S35312289	gi|26452487|dbj|AK118735.1|	At5g44575.1
at4g38240	CON	MIN	HOUR	DAY	WEEK	UNIGENE	FLCDNA	TAIR
CAGCCTCCTT	1	1	0	0	0	no match found	gi|23198251|gb|BT000334.1	multiple non-canonical match
at5g03415	CON	MIN	HOUR	DAY	WEEK	UNIGENE	FLCDNA	TAIR
AAGCCGGAGA	1	2	0	1	0	gnl|UG|At#S15461503	gi|21404728|gb|AY086018.1	At5g03415.1
at3g54010	CON	MIN	HOUR	DAY	WEEK	UNIGENE	FLCDNA	TAIR
AGAGAGATAT	2	0	0	0	1	gnl|UG|At#S15459256	gi|15810452|gb|AY056265.1	At3g54010.2
at1g11630	CON	MIN	HOUR	DAY	WEEK	UNIGENE	FLCDNA	TAIR
AAGTGGCTTG	0	1	0	0	0	no match found	gi|19548050|gb|AY081736.1	non-canonical match
at4g16563	CON	MIN	HOUR	DAY	WEEK	UNIGENE	FLCDNA	TAIR
TTTTGGGGAT	1	1	1	0	3	gnl|UG|At#S11724934	gi|15809799|gb|AY054167.1	At4g16563.1
at3g53580	CON	MIN	HOUR	DAY	WEEK	UNIGENE	FLCDNA	TAIR
GATTTTGTTT	5	3	4	3	3	gnl|UG|At#S11728771	gi|22022529|gb|AY126996.1	At3g53580.1
at3g66654	CON	MIN	HOUR	DAY	WEEK	UNIGENE	FLCDNA	TAIR
AAATGAAAAT	0	1	0	0	0	gnl|UG|At#S18942170	gi|28973050|gb|BT005430.1	multiple canonical match
at2g40860	CON	MIN	HOUR	DAY	WEEK	UNIGENE	FLCDNA	TAIR
TGTGTTATCA	2	1	0	0	0	no match found	gi|15810224|gb|AY056151.1	At2g40860.1
at1g48040	CON	MIN	HOUR	DAY	WEEK	UNIGENE	FLCDNA	TAIR
GTGACCCGAG	0	1	0	0	0	gnl|UG|At#S21735999	gi|51970161|dbj|AK176010.1|	At1g48040.1
at2g42310	CON	MIN	HOUR	DAY	WEEK	UNIGENE	FLCDNA	TAIR
AAATAAGTGT	4	4	4	2	4	gnl|UG|At#S11732033	gi|18176257|gb|AY072190.1	At2g42310.1
at1g69040	CON	MIN	HOUR	DAY	WEEK	UNIGENE	FLCDNA	TAIR
ACTAAGTGTT	2	1	0	0	0	gnl|UG|At#S18942299	gi|26452307|dbj|AK118644.1|	At1g69040.1
at4g25000	CON	MIN	HOUR	DAY	WEEK	UNIGENE	FLCDNA	TAIR
AAGATTGGAC	0	1	0	0	0	gnl|UG|At#S11710340	gi|21436162|gb|AY117294.1	At4g25000.1
at2g42760	CON	MIN	HOUR	DAY	WEEK	UNIGENE	FLCDNA	TAIR
TGAATTAAAA	3	0	0	1	0	gnl|UG|At#S11731933	no match found	multiple canonical match
CTGTGGCCTC	0	0	1	0	0	no match found	gi|107738371|gb|BT025795.1	non-canonical match
at1g49430	CON	MIN	HOUR	DAY	WEEK	UNIGENE	FLCDNA	TAIR
TGATTCTTGT	0	3	2	1	2	gnl|UG|At#S11734695	gi|20453101|gb|AY094420.1	At1g49430.1
at2g47450	CON	MIN	HOUR	DAY	WEEK	UNIGENE	FLCDNA	TAIR
GCGGCTGGTT	0	5	1	0	0	no match found	gi|25084230|gb|BT002191.1	non-canonical match
ATGCTGGCTG	1	1	1	0	2	gnl|UG|At#S20829323	no match found	non-canonical match
TAAACCCAAT	3	1	0	0	1	gnl|UG|At#S11730843	gi|15982849|gb|AY057532.1	At2g47450.1

at3g59810	CON	MIN	HOUR	DAY	WEEK	UNIGENE	FLCDNA	TAIR
TGACCCCAGA	0	1	2	2	0	gnl|UG|At#S11825177	gi|21405140|gb|AY086430.1	At3g59810.1
at1g03150	CON	MIN	HOUR	DAY	WEEK	UNIGENE	FLCDNA	TAIR
TGACAGCCGT	0	1	0	0	0	gnl|UG|At#S11742799	gi|14423551|gb|AF387013.1	At1g03150.1
at3g06810	CON	MIN	HOUR	DAY	WEEK	UNIGENE	FLCDNA	TAIR
GAGCAGCCGG	0	1	2	0	0	gnl|UG|At#S11738689	gi|51970185|dbj|AK176022.1|	At3g06810.1
at3g52430	CON	MIN	HOUR	DAY	WEEK	UNIGENE	FLCDNA	TAIR
TTTATGCAAG	1	1	3	1	1	no match found	gi|110741462|dbj|AK226558.1|	At3g52430.1
at3g62360	CON	MIN	HOUR	DAY	WEEK	UNIGENE	FLCDNA	TAIR
TCGAAGGAAA	1	1	0	0	0	no match found	gi|62320465|dbj|AK221559.1|	multiple non-canonical match
ACTATTTCCA	1	0	1	3	1	gnl|UG|At#S11726738	no match found	At3g62360.1
at3g27320	CON	MIN	HOUR	DAY	WEEK	UNIGENE	FLCDNA	TAIR
AAGCGGAAAG	0	1	0	0	0	no match found	gi|51971958|dbj|AK176881.1|	At3g27320.1
at1g13080	CON	MIN	HOUR	DAY	WEEK	UNIGENE	FLCDNA	TAIR
GAAAGTGTAT	0	0	5	0	0	gnl|UG|At#S11741730	gi|17381189|gb|AY064051.1	multiple canonical match
at5g66100	CON	MIN	HOUR	DAY	WEEK	UNIGENE	FLCDNA	TAIR
ACTCTATCAG	2	1	0	0	1	gnl|UG|At#S18911068	gi|23506194|gb|AY149955.1	At5g66100.1
at4g23300	CON	MIN	HOUR	DAY	WEEK	UNIGENE	FLCDNA	TAIR
TCTGAAAATA	0	0	0	1	1	gnl|UG|At#S11723771	gi|51971336|dbj|AK176570.1|	At4g23300.1
at3g12700	CON	MIN	HOUR	DAY	WEEK	UNIGENE	FLCDNA	TAIR
TTTGTTAGTG	2	1	0	0	2	gnl|UG|At#S11737082	no match found	At3g12700.1
at2g30150	CON	MIN	HOUR	DAY	WEEK	UNIGENE	FLCDNA	TAIR
CGGCTATAGG	0	1	0	1	0	gnl|UG|At#S18894401	gi|23197783|gb|BT000100.1	multiple non-canonical match
at3g58740	CON	MIN	HOUR	DAY	WEEK	UNIGENE	FLCDNA	TAIR
TTTTTAAGAA	0	1	0	0	0	no match found	gi|20466289|gb|AY099611.1	At3g58740.1
at5g13070	CON	MIN	HOUR	DAY	WEEK	UNIGENE	FLCDNA	TAIR
GTTATAATAA	1	1	1	0	1	gnl|UG|At#S11723090	no match found	At5g13070.1
at5g01090	CON	MIN	HOUR	DAY	WEEK	UNIGENE	FLCDNA	TAIR
TAGAAGAATC	6	2	3	0	1	gnl|UG|At#S11726450	gi|110736880|dbj|AK228471.1|	At5g01090.1
at1g63970	CON	MIN	HOUR	DAY	WEEK	UNIGENE	FLCDNA	TAIR
AAGAAATAGA	4	4	3	3	0	gnl|UG|At#S18894462	gi|13877782|gb|AF370154.1	At1g63970.2
at1g63880	CON	MIN	HOUR	DAY	WEEK	UNIGENE	FLCDNA	TAIR
GGAAAGGCTG	0	2	0	0	0	gnl|UG|At#S18895109	gi|39104606|dbj|AK119065.2|	At1g63880.1
at5g02010	CON	MIN	HOUR	DAY	WEEK	UNIGENE	FLCDNA	TAIR
AAGGAGTCGT	0	2	1	0	0	gnl|UG|At#S11725656	gi|55733764|gb|BT020213.1	At5g02010.1
at3g19450	CON	MIN	HOUR	DAY	WEEK	UNIGENE	FLCDNA	TAIR
TGACTGTGAT	4	0	4	7	4	no match found	gi|14596106|gb|AY042841.1	multiple non-canonical match
AACGAAAAAT	4	5	8	10	8	gnl|UG|At#S11735017	gi|21406994|gb|AY088220.1	At3g19450.1
at4g21050	CON	MIN	HOUR	DAY	WEEK	UNIGENE	FLCDNA	TAIR
GTTTATGTTG	5	2	1	2	2	no match found	no match found	At4g21050.1
at3g61190	CON	MIN	HOUR	DAY	WEEK	UNIGENE	FLCDNA	TAIR
GTGTGTTTGT	1	1	0	0	1	gnl|UG|At#S11727119	gi|26449567|dbj|AK117234.1|	At3g61190.1

at1g11090	CON	MIN	HOUR	DAY	WEEK	UNIGENE	FLCDNA	TAIR
GAAAAATGTT	0	0	2	1	1	gnl|UG|At#S11741926	gi|26450506|dbj|AK117716.1|	At1g11090.1
at1g11310	CON	MIN	HOUR	DAY	WEEK	UNIGENE	FLCDNA	TAIR
ATATGTTATT	1	3	1	0	4	gnl|UG|At#S43850658	gi|62319528|dbj|AK221089.1|	At1g11310.1
at3g17180	CON	MIN	HOUR	DAY	WEEK	UNIGENE	FLCDNA	TAIR
CATAAATAAT	1	0	0	1	0	no match found	no match found	At3g17180.1
at5g57910	CON	MIN	HOUR	DAY	WEEK	UNIGENE	FLCDNA	TAIR
GCAAGATCCT	0	1	0	0	0	gnl|UG|At#S18910058	gi|26453249|dbj|AK119128.1|	At5g57910.1
at4g33320	CON	MIN	HOUR	DAY	WEEK	UNIGENE	FLCDNA	TAIR
GATCCACTAG	0	0	0	1	0	gnl|UG|At#S11722054	no match found	At4g33320.1
at2g22830	CON	MIN	HOUR	DAY	WEEK	UNIGENE	FLCDNA	TAIR
TGGGTGTTTA	1	3	0	0	0	gnl|UG|At#S11736773	no match found	At2g22830.1
at2g44480	CON	MIN	HOUR	DAY	WEEK	UNIGENE	FLCDNA	TAIR
CAACTTTAGC	0	1	0	0	0	no match found	no match found	At2g44480.1
at5g15080	CON	MIN	HOUR	DAY	WEEK	UNIGENE	FLCDNA	TAIR
GTTCTTCGCC	1	1	1	0	0	gnl|UG|At#S11722572	gi|27311942|gb|BT002577.1	At5g15080.1
at5g48485	CON	MIN	HOUR	DAY	WEEK	UNIGENE	FLCDNA	TAIR
GCTCGGTTCT	10	9	8	10	2	gnl|UG|At#S30642984	gi|30102799|gb|BT006510.1	At5g48485.1
at4g38160	CON	MIN	HOUR	DAY	WEEK	UNIGENE	FLCDNA	TAIR
AGAAGTTTGG	2	2	4	11	3	no match found	gi|45680169|gb|BT011859.1	At4g38160.1
at2g43910	CON	MIN	HOUR	DAY	WEEK	UNIGENE	FLCDNA	TAIR
TATCCGATTA	1	11	4	0	1	gnl|UG|At#S18901111	gi|21405070|gb|AY086360.1	At2g43910.1
at1g15140	CON	MIN	HOUR	DAY	WEEK	UNIGENE	FLCDNA	TAIR
TTTATCCTGC	0	1	0	0	0	gnl|UG|At#S18942574	gi|15451091|gb|AY054626.1	At1g15140.2
at5g18590	CON	MIN	HOUR	DAY	WEEK	UNIGENE	FLCDNA	TAIR
TGTGTTGTGG	4	2	7	2	2	gnl|UG|At#S11721674	gi|110740536|dbj|AK226209.1|	non-canonical match
at2g39410	CON	MIN	HOUR	DAY	WEEK	UNIGENE	FLCDNA	TAIR
GAAAATCTGA	1	0	0	0	0	gnl|UG|At#S15460607	gi|21403728|gb|AY085018.1	At2g39410.2
at5g52300	CON	MIN	HOUR	DAY	WEEK	UNIGENE	FLCDNA	TAIR
GTGAGGCGGT	1	0	1	0	1	gnl|UG|At#S11718486	gi|19698870|gb|AY081282.1	At5g52300.1
at5g44000	CON	MIN	HOUR	DAY	WEEK	UNIGENE	FLCDNA	TAIR
GGAATTCAAG	0	1	0	0	0	gnl|UG|At#S21735988	gi|51971570|dbj|AK176687.1|	At5g44000.1
at4g07935	CON	MIN	HOUR	DAY	WEEK	UNIGENE	FLCDNA	TAIR
TAGACGGATC	0	0	0	1	0	no match found	no match found	At4g07935.1
at5g11550	CON	MIN	HOUR	DAY	WEEK	UNIGENE	FLCDNA	TAIR
CGAGGTCTCG	0	3	0	0	0	gnl|UG|At#S11723363	gi|27754692|gb|BT002981.1	At5g11550.1
at1g11925	CON	MIN	HOUR	DAY	WEEK	UNIGENE	FLCDNA	TAIR
TAACAATCCA	0	0	1	0	1	gnl|UG|At#S11816509	no match found	At1g11925.1
at3g24495	CON	MIN	HOUR	DAY	WEEK	UNIGENE	FLCDNA	TAIR
AGATCAAATC	0	0	0	1	0	gnl|UG|At#S11701029	no match found	multiple canonical match

at2g41160	CON	MIN	HOUR	DAY	WEEK	UNIGENE	FLCDNA	TAIR
GCTTCGTTAT	1	0	1	0	0	gnl|UG|At#S15460540	gi|22136995|gb|AY133793.1	At2g41160.1

at3g09540	CON	MIN	HOUR	DAY	WEEK	UNIGENE	FLCDNA	TAIR
AACCACAACA	0	0	1	2	0	gnl|UG|At#S11738021	gi|119360158|gb|BT029552.1	At3g09540.1

at3g22620	CON	MIN	HOUR	DAY	WEEK	UNIGENE	FLCDNA	TAIR
CCTAGAGTCC	0	0	0	1	0	gnl|UG|At#S11734034	gi|14334663|gb|AY035005.1	non-canonical match

at3g16710	CON	MIN	HOUR	DAY	WEEK	UNIGENE	FLCDNA	TAIR
AAGCTGATTC	0	1	0	0	0	gnl|UG|At#S11735849	no match found	non-canonical match
TTGCATATTA	0	3	0	0	0	no match found	no match found	At3g16710.1

at1g56010	CON	MIN	HOUR	DAY	WEEK	UNIGENE	FLCDNA	TAIR
TAAAATACCA	2	3	1	0	0	no match found	gi|110736645|dbj|AK228347.1|	non-canonical match

at2g20190	CON	MIN	HOUR	DAY	WEEK	UNIGENE	FLCDNA	TAIR
TCTCAAAGGA	0	2	0	1	0	gnl|UG|At#S11807202	gi|20259451|gb|AY091025.1	At2g20190.1

at1g13810	CON	MIN	HOUR	DAY	WEEK	UNIGENE	FLCDNA	TAIR
AAGATTGCAA	0	1	1	1	0	gnl|UG|At#S11741654	no match found	At1g13810.1

at1g21140	CON	MIN	HOUR	DAY	WEEK	UNIGENE	FLCDNA	TAIR
CAAGCGGCTG	0	0	1	0	0	no match found	gi|28827637|gb|BT005130.1	non-canonical match
CAACACAATT	0	1	0	0	0	gnl|UG|At#S14274120	gi|28392888|gb|BT003829.1	At1g21140.1

at5g22210	CON	MIN	HOUR	DAY	WEEK	UNIGENE	FLCDNA	TAIR
GATTCAAACC	1	2	2	1	1	no match found	gi|51970445|dbj|AK176152.1|	At5g22210.1
TTTCTAAAAA	0	3	4	2	4	gnl|UG|At#S11676811	no match found	multiple non-canonical match

at5g67050	CON	MIN	HOUR	DAY	WEEK	UNIGENE	FLCDNA	TAIR
AAAGCTCTAG	0	0	1	0	0	gnl|UG|At#S18909516	no match found	At5g67050.1

at3g52240	CON	MIN	HOUR	DAY	WEEK	UNIGENE	FLCDNA	TAIR
GTTACCCATA	0	1	0	0	0	gnl|UG|At#S11824098	gi|26450512|dbj|AK117719.1|	At3g52240.1

at3g17310	CON	MIN	HOUR	DAY	WEEK	UNIGENE	FLCDNA	TAIR
CGGTCAATGT	1	2	1	0	1	gnl|UG|At#S11735652	gi|15292896|gb|AY050882.1	At3g17310.1

at2g28540	CON	MIN	HOUR	DAY	WEEK	UNIGENE	FLCDNA	TAIR
TATGGAAGCG	1	2	0	1	0	gnl|UG|At#S28282244	gi|110737439|dbj|AK228764.1|	At2g28540.1

at1g23380	CON	MIN	HOUR	DAY	WEEK	UNIGENE	FLCDNA	TAIR
AGGTAGCAGA	0	1	0	0	0	no match found	gi|28393912|gb|BT004370.1	non-canonical match
TAGTAGAAGG	0	1	0	0	0	gnl|UG|At#S11825097	gi|27754595|gb|BT002930.1	At1g23380.2

at3g48560	CON	MIN	HOUR	DAY	WEEK	UNIGENE	FLCDNA	TAIR
TGTTGCCGAT	10	29	15	14	1	gnl|UG|At#S11729722	gi|14596062|gb|AY042819.1	At3g48560.1

at3g02550	CON	MIN	HOUR	DAY	WEEK	UNIGENE	FLCDNA	TAIR
TGGTACCTGT	0	8	0	0	0	gnl|UG|At#S11739924	gi|19699325|gb|AY090370.1	At3g02550.1

at4g35550	CON	MIN	HOUR	DAY	WEEK	UNIGENE	FLCDNA	TAIR
GATTGTTGAA	0	0	0	2	0	gnl|UG|At#S11721675	no match found	multiple non-canonical match

at4g39210	CON	MIN	HOUR	DAY	WEEK	UNIGENE	FLCDNA	TAIR
TAAATTAGAA	0	0	0	0	5	gnl|UG|At#S11707740	gi|16648984|gb|AY059862.1	multiple canonical match

at2g23400	CON	MIN	HOUR	DAY	WEEK	UNIGENE	FLCDNA	TAIR
AATCAAAAAA	6	5	5	10	8	no match found	no match found	At2g23400.1

<table>
<tr><td>at5g43920</td><td>CON</td><td>MIN</td><td>HOUR</td><td>DAY</td><td>WEEK</td><td>UNIGENE</td><td>FLCDNA</td><td>TAIR</td></tr>
<tr><td>TGGGTTCCCC</td><td>2</td><td>1</td><td>2</td><td>0</td><td>0</td><td>gnl|UG|At#S11706017</td><td>gi|15810428|gb|AY056253.1</td><td>At5g43920.1</td></tr>
<tr><td>at1g55545</td><td>CON</td><td>MIN</td><td>HOUR</td><td>DAY</td><td>WEEK</td><td>UNIGENE</td><td>FLCDNA</td><td>TAIR</td></tr>
<tr><td>TGGGGATTTC</td><td>0</td><td>0</td><td>0</td><td>1</td><td>0</td><td>no match found</td><td>no match found</td><td>At1g55545.1</td></tr>
<tr><td>at3g49920</td><td>CON</td><td>MIN</td><td>HOUR</td><td>DAY</td><td>WEEK</td><td>UNIGENE</td><td>FLCDNA</td><td>TAIR</td></tr>
<tr><td>CTGCTAATGT</td><td>0</td><td>1</td><td>1</td><td>1</td><td>1</td><td>gnl|UG|At#S11729457</td><td>no match found</td><td>multiple non-canonical match</td></tr>
<tr><td>at5g05570</td><td>CON</td><td>MIN</td><td>HOUR</td><td>DAY</td><td>WEEK</td><td>UNIGENE</td><td>FLCDNA</td><td>TAIR</td></tr>
<tr><td>TTTATTTATC</td><td>0</td><td>0</td><td>0</td><td>0</td><td>1</td><td>gnl|UG|At#S11724745</td><td>no match found</td><td>multiple canonical match</td></tr>
<tr><td>at4g12570</td><td>CON</td><td>MIN</td><td>HOUR</td><td>DAY</td><td>WEEK</td><td>UNIGENE</td><td>FLCDNA</td><td>TAIR</td></tr>
<tr><td>CCTCTGTTTA</td><td>0</td><td>1</td><td>2</td><td>0</td><td>0</td><td>gnl|UG|At#S11725636</td><td>gi|110738332|dbj|AK229227.1|</td><td>At4g12570.1</td></tr>
<tr><td>at3g11945</td><td>CON</td><td>MIN</td><td>HOUR</td><td>DAY</td><td>WEEK</td><td>UNIGENE</td><td>FLCDNA</td><td>TAIR</td></tr>
<tr><td>TACATACATC</td><td>2</td><td>1</td><td>0</td><td>0</td><td>2</td><td>gnl|UG|At#S38433748</td><td>no match found</td><td>At3g11950.1</td></tr>
<tr><td>at2g27610</td><td>CON</td><td>MIN</td><td>HOUR</td><td>DAY</td><td>WEEK</td><td>UNIGENE</td><td>FLCDNA</td><td>TAIR</td></tr>
<tr><td>TGGAGACTTT</td><td>0</td><td>0</td><td>0</td><td>0</td><td>1</td><td>gnl|UG|At#S11735646</td><td>no match found</td><td>non-canonical match</td></tr>
<tr><td>at3g57350</td><td>CON</td><td>MIN</td><td>HOUR</td><td>DAY</td><td>WEEK</td><td>UNIGENE</td><td>FLCDNA</td><td>TAIR</td></tr>
<tr><td>TCACAATGAG</td><td>0</td><td>0</td><td>0</td><td>0</td><td>2</td><td>no match found</td><td>no match found</td><td>At3g57350.1</td></tr>
<tr><td>GTTAGCAAAA</td><td>0</td><td>0</td><td>1</td><td>0</td><td>0</td><td>gnl|UG|At#S11728077</td><td>no match found</td><td>non-canonical match</td></tr>
<tr><td>at2g28670</td><td>CON</td><td>MIN</td><td>HOUR</td><td>DAY</td><td>WEEK</td><td>UNIGENE</td><td>FLCDNA</td><td>TAIR</td></tr>
<tr><td>AGCTCGGATC</td><td>1</td><td>1</td><td>0</td><td>0</td><td>0</td><td>gnl|UG|At#S43850142</td><td>gi|53749145|gb|BT015768.1</td><td>At2g28670.1</td></tr>
<tr><td>at5g62460</td><td>CON</td><td>MIN</td><td>HOUR</td><td>DAY</td><td>WEEK</td><td>UNIGENE</td><td>FLCDNA</td><td>TAIR</td></tr>
<tr><td>GCTTGGGCTA</td><td>0</td><td>3</td><td>0</td><td>0</td><td>0</td><td>gnl|UG|At#S11717455</td><td>gi|22655447|gb|AY142052.1</td><td>At5g62460.1</td></tr>
<tr><td>at4g31400</td><td>CON</td><td>MIN</td><td>HOUR</td><td>DAY</td><td>WEEK</td><td>UNIGENE</td><td>FLCDNA</td><td>TAIR</td></tr>
<tr><td>ATCCATATGT</td><td>1</td><td>0</td><td>0</td><td>0</td><td>0</td><td>no match found</td><td>no match found</td><td>At4g31400.1</td></tr>
<tr><td>TGGCTATGCC</td><td>0</td><td>0</td><td>0</td><td>0</td><td>1</td><td>no match found</td><td>gi|72197296|gb|DQ108721.1</td><td>non-canonical match</td></tr>
<tr><td>at4g25740</td><td>CON</td><td>MIN</td><td>HOUR</td><td>DAY</td><td>WEEK</td><td>UNIGENE</td><td>FLCDNA</td><td>TAIR</td></tr>
<tr><td>TTTCATAAAA</td><td>0</td><td>0</td><td>0</td><td>1</td><td>2</td><td>no match found</td><td>gi|14334535|gb|AY035172.1</td><td>multiple non-canonical match</td></tr>
<tr><td>CAGAGTTTCA</td><td>3</td><td>2</td><td>4</td><td>2</td><td>1</td><td>no match found</td><td>gi|21281206|gb|AY113926.1</td><td>non-canonical match</td></tr>
<tr><td>GAATTAGGTT</td><td>1</td><td>1</td><td>0</td><td>0</td><td>1</td><td>gnl|UG|At#S11723352</td><td>no match found</td><td>non-canonical match</td></tr>
<tr><td>at3g14100</td><td>CON</td><td>MIN</td><td>HOUR</td><td>DAY</td><td>WEEK</td><td>UNIGENE</td><td>FLCDNA</td><td>TAIR</td></tr>
<tr><td>GATTGTAAGA</td><td>11</td><td>7</td><td>3</td><td>2</td><td>5</td><td>gnl|UG|At#S11736626</td><td>gi|21406430|gb|AY087692.1</td><td>non-canonical match</td></tr>
<tr><td>at2g37450</td><td>CON</td><td>MIN</td><td>HOUR</td><td>DAY</td><td>WEEK</td><td>UNIGENE</td><td>FLCDNA</td><td>TAIR</td></tr>
<tr><td>GATGAGCCAT</td><td>2</td><td>0</td><td>1</td><td>0</td><td>0</td><td>no match found</td><td>gi|14994262|gb|AY044325.1</td><td>multiple non-canonical match</td></tr>
<tr><td>TAACATTTTC</td><td>3</td><td>1</td><td>4</td><td>2</td><td>0</td><td>gnl|UG|At#S34118108</td><td>gi|110736365|dbj|AK228198.1|</td><td>At2g37450.1</td></tr>
<tr><td>at4g32270</td><td>CON</td><td>MIN</td><td>HOUR</td><td>DAY</td><td>WEEK</td><td>UNIGENE</td><td>FLCDNA</td><td>TAIR</td></tr>
<tr><td>TTAATGGTGA</td><td>0</td><td>0</td><td>0</td><td>0</td><td>1</td><td>gnl|UG|At#S11722231</td><td>gi|26450558|dbj|AK117743.1|</td><td>At1g12770.1</td></tr>
<tr><td>at2g38800</td><td>CON</td><td>MIN</td><td>HOUR</td><td>DAY</td><td>WEEK</td><td>UNIGENE</td><td>FLCDNA</td><td>TAIR</td></tr>
<tr><td>GTAGAGAACC</td><td>1</td><td>3</td><td>3</td><td>2</td><td>1</td><td>gnl|UG|At#S11732896</td><td>no match found</td><td>At2g38800.1</td></tr>
<tr><td>at2g23420</td><td>CON</td><td>MIN</td><td>HOUR</td><td>DAY</td><td>WEEK</td><td>UNIGENE</td><td>FLCDNA</td><td>TAIR</td></tr>
<tr><td>CGACCTGATC</td><td>2</td><td>3</td><td>1</td><td>1</td><td>1</td><td>gnl|UG|At#S11736628</td><td>gi|25054895|gb|BT001932.1</td><td>At2g23420.1</td></tr>
<tr><td>at3g59890</td><td>CON</td><td>MIN</td><td>HOUR</td><td>DAY</td><td>WEEK</td><td>UNIGENE</td><td>FLCDNA</td><td>TAIR</td></tr>
<tr><td>CCAAGTTAAA</td><td>1</td><td>0</td><td>0</td><td>1</td><td>0</td><td>gnl|UG|At#S18902413</td><td>gi|21406242|gb|AY087505.1</td><td>At3g59890.2</td></tr>
<tr><td>at4g39470</td><td>CON</td><td>MIN</td><td>HOUR</td><td>DAY</td><td>WEEK</td><td>UNIGENE</td><td>FLCDNA</td><td>TAIR</td></tr>
<tr><td>CTAGTGATTT</td><td>1</td><td>0</td><td>3</td><td>0</td><td>0</td><td>gnl|UG|At#S11721016</td><td>gi|15215655|gb|AY050356.1</td><td>multiple canonical match</td></tr>
</table>

at3g10150	CON	MIN	HOUR	DAY	WEEK	UNIGENE	FLCDNA	TAIR
GACTAGACTG	0	1	0	0	0	gnl|UG|At#S11825070	gi|50198969|gb|BT015036.1	non-canonical match
ATGGAGAATC	0	0	0	0	1	no match found	no match found	At3g10150.1
at4g14570	CON	MIN	HOUR	DAY	WEEK	UNIGENE	FLCDNA	TAIR
GCATCAATGG	0	0	0	1	0	gnl|UG|At#S11725282	no match found	At4g14570.1
at5g40000	CON	MIN	HOUR	DAY	WEEK	UNIGENE	FLCDNA	TAIR
ATCTCAGCTA	1	0	0	0	0	gnl|UG|At#S11719731	no match found	At5g40000.1
at2g37920	CON	MIN	HOUR	DAY	WEEK	UNIGENE	FLCDNA	TAIR
GGAACCGATT	0	3	1	0	0	no match found	gi|20260589|gb|AY093194.1	At2g37920.1
at1g24147	CON	MIN	HOUR	DAY	WEEK	UNIGENE	FLCDNA	TAIR
TGGATGAAGC	0	0	1	0	0	gnl|UG|At#S37211437	no match found	pseudo chromosome match
TTTTCACATA	1	2	0	0	0	gnl|UG|At#S11792033	no match found	multiple non-canonical match
at5g24200	CON	MIN	HOUR	DAY	WEEK	UNIGENE	FLCDNA	TAIR
TCACATCAAA	0	0	0	1	1	gnl|UG|At#S11720758	no match found	non-canonical match
at2g22900	CON	MIN	HOUR	DAY	WEEK	UNIGENE	FLCDNA	TAIR
AAGTGAAAGG	1	0	2	1	0	gnl|UG|At#S35310011	gi|14334701|gb|AY035024.1	At2g22900.1
at3g11340	CON	MIN	HOUR	DAY	WEEK	UNIGENE	FLCDNA	TAIR
TATAAAAAAA	3	3	3	2	5	no match found	gi|26452975|dbj|AK118988.1|	non-canonical match
at4g09510	CON	MIN	HOUR	DAY	WEEK	UNIGENE	FLCDNA	TAIR
GACTTGCTGA	0	2	3	0	2	gnl|UG|At#S11726014	gi|21407784|gb|AY089010.1	At4g09510.1
at5g58960	CON	MIN	HOUR	DAY	WEEK	UNIGENE	FLCDNA	TAIR
GAGAGTGTCG	1	1	0	0	0	gnl|UG|At#S11717809	gi|19715644|gb|AY075634.1	At5g58960.1
at5g41180	CON	MIN	HOUR	DAY	WEEK	UNIGENE	FLCDNA	TAIR
CCTTGGATAA	0	1	0	0	0	no match found	no match found	At5g41180.1
at3g01120	CON	MIN	HOUR	DAY	WEEK	UNIGENE	FLCDNA	TAIR
TCCTACTGGG	9	14	9	4	9	no match found	gi|20453136|gb|AY094438.1	non-canonical match
at3g60800	CON	MIN	HOUR	DAY	WEEK	UNIGENE	FLCDNA	TAIR
ATTTACCCTA	1	3	3	1	3	gnl|UG|At#S11709832	gi|110738423|dbj|AK229274.1|	At3g60800.1
at3g25920	CON	MIN	HOUR	DAY	WEEK	UNIGENE	FLCDNA	TAIR
AAGCTCACCT	2	20	11	6	4	gnl|UG|At#S11733006	gi|62318706|dbj|AK220673.1|	non-canonical match
at2g05310	CON	MIN	HOUR	DAY	WEEK	UNIGENE	FLCDNA	TAIR
TTTCTTATAC	25	6	2	0	6	no match found	no match found	At2g05310.1
at1g03030	CON	MIN	HOUR	DAY	WEEK	UNIGENE	FLCDNA	TAIR
GAGTTGGGGA	0	1	0	0	0	gnl|UG|At#S11742812	gi|63147411|gb|BT022118.1	At1g03030.1
at5g48430	CON	MIN	HOUR	DAY	WEEK	UNIGENE	FLCDNA	TAIR
TCATTATATT	0	0	0	1	1	no match found	no match found	At5g48430.1
TCAAGTGTGG	0	1	1	0	0	gnl|UG|At#S24442676	gi|62320321|dbj|AK221487.1|	multiple non-canonical match
at1g06540	CON	MIN	HOUR	DAY	WEEK	UNIGENE	FLCDNA	TAIR
GTGGAAGAAC	0	0	0	0	1	gnl|UG|At#S11742375	gi|20258789|gb|AY090922.1	multiple canonical match
at1g64270	CON	MIN	HOUR	DAY	WEEK	UNIGENE	FLCDNA	TAIR
GGTTATTGTT	0	0	1	0	0	gnl|UG|At#S11709412	gi|16930454|gb|AF419581.1	non-canonical match

<table>
<tr><td>at4g34660</td><td>CON</td><td>MIN</td><td>HOUR</td><td>DAY</td><td>WEEK</td><td>UNIGENE</td><td>FLCDNA</td><td>TAIR</td></tr>
<tr><td>GGTTACTTCT</td><td>0</td><td>0</td><td>0</td><td>0</td><td>1</td><td>no match found</td><td>gi|20465386|gb|AY096478.1</td><td>non-canonical match</td></tr>
<tr><td>TTCTGAATCT</td><td>3</td><td>0</td><td>1</td><td>0</td><td>0</td><td>gnl|UG|At#S11721821</td><td>gi|21405882|gb|AY087158.1</td><td>At4g34660.1</td></tr>
<tr><td>at4g14700</td><td>CON</td><td>MIN</td><td>HOUR</td><td>DAY</td><td>WEEK</td><td>UNIGENE</td><td>FLCDNA</td><td>TAIR</td></tr>
<tr><td>GTTGGCCAAT</td><td>0</td><td>0</td><td>0</td><td>1</td><td>0</td><td>gnl|UG|At#S11704302</td><td>no match found</td><td>At4g14700.1</td></tr>
<tr><td>at1g08410</td><td>CON</td><td>MIN</td><td>HOUR</td><td>DAY</td><td>WEEK</td><td>UNIGENE</td><td>FLCDNA</td><td>TAIR</td></tr>
<tr><td>CTGCAGAGCT</td><td>0</td><td>0</td><td>1</td><td>0</td><td>0</td><td>no match found</td><td>gi|66792665|gb|BT023444.1</td><td>non-canonical match</td></tr>
<tr><td>at4g14819</td><td>CON</td><td>MIN</td><td>HOUR</td><td>DAY</td><td>WEEK</td><td>UNIGENE</td><td>FLCDNA</td><td>TAIR</td></tr>
<tr><td>TAGTCATCTT</td><td>1</td><td>0</td><td>0</td><td>0</td><td>0</td><td>gnl|UG|At#S37211678</td><td>no match found</td><td>non-canonical match</td></tr>
<tr><td>at2g41120</td><td>CON</td><td>MIN</td><td>HOUR</td><td>DAY</td><td>WEEK</td><td>UNIGENE</td><td>FLCDNA</td><td>TAIR</td></tr>
<tr><td>AATGAAATTT</td><td>1</td><td>1</td><td>2</td><td>3</td><td>0</td><td>gnl|UG|At#S11732309</td><td>gi|110738995|dbj|AK229566.1|</td><td>multiple non-canonical match</td></tr>
<tr><td>at5g48330</td><td>CON</td><td>MIN</td><td>HOUR</td><td>DAY</td><td>WEEK</td><td>UNIGENE</td><td>FLCDNA</td><td>TAIR</td></tr>
<tr><td>TATTTACGTG</td><td>1</td><td>1</td><td>1</td><td>1</td><td>0</td><td>gnl|UG|At#S11718887</td><td>gi|26450137|dbj|AK117526.1|</td><td>At5g48330.1</td></tr>
<tr><td>at3g17470</td><td>CON</td><td>MIN</td><td>HOUR</td><td>DAY</td><td>WEEK</td><td>UNIGENE</td><td>FLCDNA</td><td>TAIR</td></tr>
<tr><td>ATCTTCCACA</td><td>2</td><td>1</td><td>1</td><td>0</td><td>0</td><td>gnl|UG|At#S11735602</td><td>no match found</td><td>At3g17470.1</td></tr>
<tr><td>TCGCCTGCAA</td><td>0</td><td>0</td><td>0</td><td>1</td><td>0</td><td>gnl|UG|At#S43849737</td><td>no match found</td><td>non-canonical match</td></tr>
<tr><td>at1g48750</td><td>CON</td><td>MIN</td><td>HOUR</td><td>DAY</td><td>WEEK</td><td>UNIGENE</td><td>FLCDNA</td><td>TAIR</td></tr>
<tr><td>CTTGAATGTT</td><td>9</td><td>3</td><td>8</td><td>4</td><td>13</td><td>gnl|UG|At#S17007056</td><td>no match found</td><td>At1g48750.1</td></tr>
<tr><td>at3g44160</td><td>CON</td><td>MIN</td><td>HOUR</td><td>DAY</td><td>WEEK</td><td>UNIGENE</td><td>FLCDNA</td><td>TAIR</td></tr>
<tr><td>TAATATTACA</td><td>1</td><td>0</td><td>0</td><td>0</td><td>0</td><td>gnl|UG|At#S11730536</td><td>gi|56121917|gb|BT020246.1</td><td>At3g44160.1</td></tr>
<tr><td>at3g19340</td><td>CON</td><td>MIN</td><td>HOUR</td><td>DAY</td><td>WEEK</td><td>UNIGENE</td><td>FLCDNA</td><td>TAIR</td></tr>
<tr><td>GGGCTGAAGC</td><td>2</td><td>0</td><td>1</td><td>1</td><td>0</td><td>gnl|UG|At#S11735047</td><td>gi|20260587|gb|AY093193.1</td><td>At3g19340.1</td></tr>
<tr><td>at2g25240</td><td>CON</td><td>MIN</td><td>HOUR</td><td>DAY</td><td>WEEK</td><td>UNIGENE</td><td>FLCDNA</td><td>TAIR</td></tr>
<tr><td>GTTTGATGGA</td><td>0</td><td>1</td><td>0</td><td>0</td><td>0</td><td>gnl|UG|At#S11736214</td><td>gi|56121911|gb|BT020243.1</td><td>At2g25240.1</td></tr>
<tr><td>at1g02080</td><td>CON</td><td>MIN</td><td>HOUR</td><td>DAY</td><td>WEEK</td><td>UNIGENE</td><td>FLCDNA</td><td>TAIR</td></tr>
<tr><td>TTTTCTAATT</td><td>2</td><td>3</td><td>1</td><td>0</td><td>0</td><td>gnl|UG|At#S11742908</td><td>gi|27754386|gb|BT002823.1</td><td>At1g02080.1</td></tr>
<tr><td>at5g18150</td><td>CON</td><td>MIN</td><td>HOUR</td><td>DAY</td><td>WEEK</td><td>UNIGENE</td><td>FLCDNA</td><td>TAIR</td></tr>
<tr><td>TCTAGCTAAA</td><td>0</td><td>1</td><td>0</td><td>0</td><td>0</td><td>no match found</td><td>no match found</td><td>At5g18150.1</td></tr>
<tr><td>GAAGACGGTG</td><td>0</td><td>0</td><td>1</td><td>2</td><td>0</td><td>gnl|UG|At#S30454935</td><td>gi|28973484|gb|BT005647.1</td><td>non-canonical match</td></tr>
<tr><td>TACTTGAATT</td><td>0</td><td>0</td><td>1</td><td>0</td><td>0</td><td>gnl|UG|At#S43849321</td><td>no match found</td><td>pseudo chromosome match</td></tr>
<tr><td>at1g63980</td><td>CON</td><td>MIN</td><td>HOUR</td><td>DAY</td><td>WEEK</td><td>UNIGENE</td><td>FLCDNA</td><td>TAIR</td></tr>
<tr><td>AAGCTGAAAC</td><td>0</td><td>2</td><td>4</td><td>1</td><td>0</td><td>no match found</td><td>no match found</td><td>At1g63980.1</td></tr>
<tr><td>at5g50565</td><td>CON</td><td>MIN</td><td>HOUR</td><td>DAY</td><td>WEEK</td><td>UNIGENE</td><td>FLCDNA</td><td>TAIR</td></tr>
<tr><td>TAAGATCTGC</td><td>0</td><td>1</td><td>0</td><td>0</td><td>2</td><td>gnl|UG|At#S24442403</td><td>gi|62321741|dbj|AK222209.1|</td><td>multiple canonical match</td></tr>
<tr><td>at3g55770</td><td>CON</td><td>MIN</td><td>HOUR</td><td>DAY</td><td>WEEK</td><td>UNIGENE</td><td>FLCDNA</td><td>TAIR</td></tr>
<tr><td>GAGGTTGCCC</td><td>2</td><td>13</td><td>3</td><td>5</td><td>1</td><td>gnl|UG|At#S18903591</td><td>gi|21403071|gb|AY084361.1</td><td>At3g55770.1</td></tr>
<tr><td>at1g49710</td><td>CON</td><td>MIN</td><td>HOUR</td><td>DAY</td><td>WEEK</td><td>UNIGENE</td><td>FLCDNA</td><td>TAIR</td></tr>
<tr><td>AGATTGGTGA</td><td>0</td><td>1</td><td>1</td><td>0</td><td>0</td><td>gnl|UG|At#S11734601</td><td>gi|27311928|gb|BT002570.1</td><td>At1g49710.1</td></tr>
<tr><td>at5g44720</td><td>CON</td><td>MIN</td><td>HOUR</td><td>DAY</td><td>WEEK</td><td>UNIGENE</td><td>FLCDNA</td><td>TAIR</td></tr>
<tr><td>AGGATTTCGT</td><td>1</td><td>0</td><td>1</td><td>0</td><td>0</td><td>gnl|UG|At#S11719256</td><td>gi|21406778|gb|AY088004.1</td><td>At5g44720.1</td></tr>
<tr><td>TTAATTCCAA</td><td>2</td><td>2</td><td>4</td><td>3</td><td>2</td><td>gnl|UG|At#S20756797</td><td>gi|19423896|gb|AY080879.1</td><td>non-canonical match</td></tr>
<tr><td>at2g38970</td><td>CON</td><td>MIN</td><td>HOUR</td><td>DAY</td><td>WEEK</td><td>UNIGENE</td><td>FLCDNA</td><td>TAIR</td></tr>
<tr><td>GCAAAGAGCA</td><td>0</td><td>1</td><td>0</td><td>0</td><td>0</td><td>gnl|UG|At#S15460625</td><td>gi|18700172|gb|AY075691.1</td><td>At2g38970.1</td></tr>
</table>

at1g80070	CON	MIN	HOUR	DAY	WEEK	UNIGENE	FLCDNA	TAIR
GAGGAGGCTG	3	4	5	3	5	gnl|UG|At#S11726500	no match found	multiple canonical match
at2g25170	CON	MIN	HOUR	DAY	WEEK	UNIGENE	FLCDNA	TAIR
AAATTATAGT	2	2	1	0	2	gnl|UG|At#S11736229	gi|110738691|dbj|AK229409.1|	At2g25170.1
at3g04610	CON	MIN	HOUR	DAY	WEEK	UNIGENE	FLCDNA	TAIR
GCTGAAGCTG	1	1	0	2	0	gnl|UG|At#S11709859	gi|17979248|gb|AY070475.1	At3g04610.1
at5g59250	CON	MIN	HOUR	DAY	WEEK	UNIGENE	FLCDNA	TAIR
GGTAGCTGTT	0	1	0	0	0	no match found	gi|56381948|gb|BT020338.1	non-canonical match
TATGAGGATG	3	2	1	0	1	gnl|UG|At#S34115936	gi|51536477|gb|BT015354.1	multiple non-canonical match
at1g02700	CON	MIN	HOUR	DAY	WEEK	UNIGENE	FLCDNA	TAIR
GCCGAGAGAC	0	2	0	0	0	gnl|UG|At#S11705942	gi|20259576|gb|AY091192.1	At1g02700.1
at2g22230	CON	MIN	HOUR	DAY	WEEK	UNIGENE	FLCDNA	TAIR
TGGACTCTTC	1	1	2	1	1	gnl|UG|At#S11736923	gi|21928165|gb|AY125519.1	non-canonical match
at5g63680	CON	MIN	HOUR	DAY	WEEK	UNIGENE	FLCDNA	TAIR
GTGATGCTGT	0	2	0	0	1	gnl|UG|At#S34116670	gi|110739132|dbj|AK229638.1|	At5g63680.1
at1g74270	CON	MIN	HOUR	DAY	WEEK	UNIGENE	FLCDNA	TAIR
TATCCGAGTA	2	4	1	0	2	no match found	gi|17104786|gb|AY063108.1	non-canonical match
GATCCGGTTT	7	10	11	8	5	gnl|UG|At#S11728184	gi|21404666|gb|AY085956.1	At1g74270.1
at1g04570	CON	MIN	HOUR	DAY	WEEK	UNIGENE	FLCDNA	TAIR
CACCTTGTGC	0	0	1	0	1	gnl|UG|At#S11742571	gi|29028943|gb|BT005916.1	At1g04570.1
at2g05100	CON	MIN	HOUR	DAY	WEEK	UNIGENE	FLCDNA	TAIR
TTCTCTATGT	179	117	42	12	14	no match found	no match found	At2g05100.1
at2g40800	CON	MIN	HOUR	DAY	WEEK	UNIGENE	FLCDNA	TAIR
AATCTTTTCC	0	1	0	0	1	gnl|UG|At#S11732391	gi|17473708|gb|AY065132.1	At2g40800.1
at4g30360	CON	MIN	HOUR	DAY	WEEK	UNIGENE	FLCDNA	TAIR
ATGATTGACT	0	0	0	0	2	gnl|UG|At#S11722557	gi|21703137|gb|AY124000.1	At4g30360.1
at3g02450	CON	MIN	HOUR	DAY	WEEK	UNIGENE	FLCDNA	TAIR
GATGCCATCT	3	0	1	0	1	no match found	gi|17065469|gb|AY062811.1	At3g02450.1
at2g39570	CON	MIN	HOUR	DAY	WEEK	UNIGENE	FLCDNA	TAIR
AACGTTATGC	2	7	0	0	0	no match found	gi|15010737|gb|AY045670.1	non-canonical match
TAGTGGCCTT	2	2	3	1	1	gnl|UG|At#S11732702	no match found	At2g39570.1
at2g06510	CON	MIN	HOUR	DAY	WEEK	UNIGENE	FLCDNA	TAIR
CTTGATTTGC	1	0	0	0	1	gnl|UG|At#S11739832	gi|29029003|gb|BT005946.1	At2g06510.1
at5g24610	CON	MIN	HOUR	DAY	WEEK	UNIGENE	FLCDNA	TAIR
TTGATCTCCG	0	3	0	0	0	gnl|UG|At#S21736310	gi|21403598|gb|AY084888.1	At5g24610.1
at4g33100	CON	MIN	HOUR	DAY	WEEK	UNIGENE	FLCDNA	TAIR
TTTTTCGAGA	1	0	1	0	0	gnl|UG|At#S35271463	gi|51969897|dbj|AK175878.1|	At4g33100.1
at1g04970	CON	MIN	HOUR	DAY	WEEK	UNIGENE	FLCDNA	TAIR
TCTTTGAAAT	0	2	0	1	0	gnl|UG|At#S14273673	gi|21406473|gb|AY087735.1	At1g04970.1
at1g74850	CON	MIN	HOUR	DAY	WEEK	UNIGENE	FLCDNA	TAIR
ATTTTGCAGC	2	1	3	2	0	gnl|UG|At#S11728066	no match found	At1g74850.1

at4g04720	CON	MIN	HOUR	DAY	WEEK	UNIGENE	FLCDNA	TAIR
TCTTAGCTCC	1	3	2	1	1	gnl|UG|At#S11726470	gi|15292914|gb|AY050891.1	At4g04720.1
at2g30140	CON	MIN	HOUR	DAY	WEEK	UNIGENE	FLCDNA	TAIR
CTCACTAAAT	3	0	1	1	3	gnl|UG|At#S11734998	gi|110740801|dbj|AK226350.1|	At2g30140.1
at4g20260	CON	MIN	HOUR	DAY	WEEK	UNIGENE	FLCDNA	TAIR
TGAATTTGTA	17	33	37	21	15	gnl|UG|At#S30652228	gi|20260369|gb|AY093084.1	At4g20260.1
at1g58160	CON	MIN	HOUR	DAY	WEEK	UNIGENE	FLCDNA	TAIR
GATCTTTTGA	1	0	0	0	0	gnl|UG|At#S11594719	no match found	multiple non-canonical match
at5g53480	CON	MIN	HOUR	DAY	WEEK	UNIGENE	FLCDNA	TAIR
GAACATCTCA	1	0	1	0	1	no match found	gi|21404684|gb|AY085974.1	multiple non-canonical match
at1g18800	CON	MIN	HOUR	DAY	WEEK	UNIGENE	FLCDNA	TAIR
TCTGTTGTGT	0	0	0	3	3	gnl|UG|At#S11741171	gi|26451104|dbj|AK118024.1|	At1g18800.1
TCTTCTCTAT	1	0	0	0	0	no match found	gi|21405471|gb|AY086761.1	non-canonical match
at5g66630	CON	MIN	HOUR	DAY	WEEK	UNIGENE	FLCDNA	TAIR
TTCTTCTTCA	0	1	0	0	0	gnl|UG|At#S30637461	no match found	multiple non-canonical match
CTTGGCTTAG	0	1	0	0	0	no match found	gi|27808629|gb|BT003163.1	multiple non-canonical match
at3g16840	CON	MIN	HOUR	DAY	WEEK	UNIGENE	FLCDNA	TAIR
GTTACAGGCG	0	4	0	2	0	no match found	gi|15010735|gb|AY045669.1	non-canonical match
CTGAATCAAT	0	0	1	0	1	gnl|UG|At#S11735809	gi|15450789|gb|AY054475.1	At3g16840.1
at4g36770	CON	MIN	HOUR	DAY	WEEK	UNIGENE	FLCDNA	TAIR
AACTTTGAAT	0	1	0	0	0	no match found	no match found	At4g36770.1
at3g02870	CON	MIN	HOUR	DAY	WEEK	UNIGENE	FLCDNA	TAIR
AAGTCATCTG	1	2	0	1	2	no match found	gi|21404258|gb|AY085548.1	non-canonical match
at2g32810	CON	MIN	HOUR	DAY	WEEK	UNIGENE	FLCDNA	TAIR
AGTGACCTCA	3	0	1	1	0	gnl|UG|At#S11703984	gi|25090388|gb|BT002279.1	non-canonical match
at5g23140	CON	MIN	HOUR	DAY	WEEK	UNIGENE	FLCDNA	TAIR
TTTAGTGTTT	0	1	0	0	0	no match found	gi|26452072|dbj|AK118523.1|	non-canonical match
at2g15000	CON	MIN	HOUR	DAY	WEEK	UNIGENE	FLCDNA	TAIR
CGTGAATCAG	1	0	0	3	0	gnl|UG|At#S38433995	gi|110736915|dbj|AK228489.1|	At2g15000.1
at3g54740	CON	MIN	HOUR	DAY	WEEK	UNIGENE	FLCDNA	TAIR
TCTAGATTTC	0	3	0	0	0	gnl|UG|At#S38433601	gi|51970113|dbj|AK175986.1|	At3g54740.1
at5g11450	CON	MIN	HOUR	DAY	WEEK	UNIGENE	FLCDNA	TAIR
AAATTTCTTT	0	3	1	2	1	gnl|UG|At#S11723390	gi|18252954|gb|AY072412.1	At5g11450.1
at2g16050	CON	MIN	HOUR	DAY	WEEK	UNIGENE	FLCDNA	TAIR
TGAAAGTTGA	2	2	2	2	1	gnl|UG|At#S11738457	no match found	pseudo chromosome match
at4g08040	CON	MIN	HOUR	DAY	WEEK	UNIGENE	FLCDNA	TAIR
TGGGATACAA	0	1	0	0	0	gnl|UG|At#S11701542	gi|12083217|gb|AF332405.1	At4g08040.1
at3g01150	CON	MIN	HOUR	DAY	WEEK	UNIGENE	FLCDNA	TAIR
TTTAGGTTGT	3	0	1	0	0	gnl|UG|At#S11806687	gi|20260637|gb|AY093218.1	At3g01150.1
at2g29910	CON	MIN	HOUR	DAY	WEEK	UNIGENE	FLCDNA	TAIR
TGTATCAAAT	0	1	0	2	0	gnl|UG|At#S15460971	no match found	At2g29910.2
at4g30490	CON	MIN	HOUR	DAY	WEEK	UNIGENE	FLCDNA	TAIR

AAAAGGAAAT	2	2	0	2	1	no match found	gi|20466287|gb|AY099610.1	non-canonical match
at3g54320	CON	MIN	HOUR	DAY	WEEK	UNIGENE	FLCDNA	TAIR
TTCGATGATG	0	1	0	0	0	gnl|UG|At#S28282819	no match found	At3g54320.1
at2g39950	CON	MIN	HOUR	DAY	WEEK	UNIGENE	FLCDNA	TAIR
GAAGCAGAGG	0	5	4	0	0	gnl|UG|At#S34115795	gi|110740811|dbj|AK226355.1|	At2g39950.1
at3g20510	CON	MIN	HOUR	DAY	WEEK	UNIGENE	FLCDNA	TAIR
CAGATGATAC	0	0	1	0	1	gnl|UG|At#S11734691	gi|51971330|dbj|AK176567.1|	non-canonical match
at3g14990	CON	MIN	HOUR	DAY	WEEK	UNIGENE	FLCDNA	TAIR
GTCAAGAAAC	1	7	0	1	0	gnl|UG|At#S28282978	gi|22136579|gb|AY129490.1	At3g14990.2
at1g17520	CON	MIN	HOUR	DAY	WEEK	UNIGENE	FLCDNA	TAIR
TAGCGAGACC	0	2	2	1	1	gnl|UG|At#S11741292	gi|26452092|dbj|AK118533.1|	At1g17520.1
at3g02930	CON	MIN	HOUR	DAY	WEEK	UNIGENE	FLCDNA	TAIR
AAACTTGAGG	0	1	0	0	0	gnl|UG|At#S11739801	no match found	At3g02930.1
at5g56590	CON	MIN	HOUR	DAY	WEEK	UNIGENE	FLCDNA	TAIR
ATCTCGTTGT	1	1	1	0	0	gnl|UG|At#S11718053	gi|22137243|gb|AY133637.1	At5g56590.1
at3g11880	CON	MIN	HOUR	DAY	WEEK	UNIGENE	FLCDNA	TAIR
GTCGTGTAAC	0	0	1	0	0	gnl|UG|At#S11737330	gi|16226859|gb|AF428353.1	At3g11880.1
at3g30837	CON	MIN	HOUR	DAY	WEEK	UNIGENE	FLCDNA	TAIR
AAAGTTCTCA	1	1	1	2	0	no match found	no match found	At3g30837.1
at4g22290	CON	MIN	HOUR	DAY	WEEK	UNIGENE	FLCDNA	TAIR
CAGAGAAATA	0	1	0	0	0	gnl|UG|At#S11723950	gi|22655309|gb|AY140104.1	non-canonical match
at3g13710	CON	MIN	HOUR	DAY	WEEK	UNIGENE	FLCDNA	TAIR
AAGATGAATC	0	0	0	1	0	gnl|UG|At#S11736740	no match found	At3g13710.1
at2g07771	CON	MIN	HOUR	DAY	WEEK	UNIGENE	FLCDNA	TAIR
GCTGAATGGT	0	0	0	1	0	no match found	gi|48310002|gb|BT014752.1	multiple canonical match
at2g29900	CON	MIN	HOUR	DAY	WEEK	UNIGENE	FLCDNA	TAIR
TTAGGTGTTG	2	1	1	2	0	gnl|UG|At#S11735065	gi|114050622|gb|BT028914.1	At2g29900.1
at4g23260	CON	MIN	HOUR	DAY	WEEK	UNIGENE	FLCDNA	TAIR
GATCCACTAG	0	0	0	1	0	no match found	gi|20466601|gb|AY099767.1	At4g33320.1
at3g02820	CON	MIN	HOUR	DAY	WEEK	UNIGENE	FLCDNA	TAIR
GATAAAAGCA	0	1	0	0	0	gnl|UG|At#S11739838	gi|106879190|gb|BT025722.1	At3g02820.1
at3g10410	CON	MIN	HOUR	DAY	WEEK	UNIGENE	FLCDNA	TAIR
AAAACAAAAG	2	2	2	1	3	gnl|UG|At#S11737764	gi|20147198|gb|AY091767.1	multiple canonical match
at2g45030	CON	MIN	HOUR	DAY	WEEK	UNIGENE	FLCDNA	TAIR
ACACAAGGAA	0	0	1	1	0	gnl|UG|At#S11731403	gi|62321174|dbj|AK221917.1|	At2g45030.1
at3g30775	CON	MIN	HOUR	DAY	WEEK	UNIGENE	FLCDNA	TAIR
AGGGACTATG	2	11	3	2	2	gnl|UG|At#S11731451	gi|16226334|gb|AF428306.1	At3g30775.1
at5g06440	CON	MIN	HOUR	DAY	WEEK	UNIGENE	FLCDNA	TAIR
TATAAAGTTT	2	2	1	1	1	gnl|UG|At#S11724514	gi|20260311|gb|AY093055.1	At5g06440.2
at2g35390	CON	MIN	HOUR	DAY	WEEK	UNIGENE	FLCDNA	TAIR
TTCTAACCGG	5	4	6	0	1	gnl|UG|At#S11733725	gi|51971798|dbj|AK176801.1|	At2g35390.2

at5g20560	CON	MIN	HOUR	DAY	WEEK	UNIGENE	FLCDNA	TAIR
TTGCAAGTGG	1	0	1	0	0	gnl|UG|At#S11721185	no match found	multiple non-canonical match
at3g58050	CON	MIN	HOUR	DAY	WEEK	UNIGENE	FLCDNA	TAIR
CGGCAACTCC	0	3	0	1	0	gnl|UG|At#S11727941	no match found	At3g58050.1
at5g38490	CON	MIN	HOUR	DAY	WEEK	UNIGENE	FLCDNA	TAIR
GAAGTGATCC	0	0	1	0	0	no match found	no match found	At5g38490.1
at1g78190	CON	MIN	HOUR	DAY	WEEK	UNIGENE	FLCDNA	TAIR
TTTGCCAAAA	2	0	0	1	0	gnl|UG|At#S11744880	gi|30725331|gb|BT008329.1	non-canonical match
at1g17760	CON	MIN	HOUR	DAY	WEEK	UNIGENE	FLCDNA	TAIR
GATCAGCCAG	0	1	1	0	0	gnl|UG|At#S11710351	gi|26983801|gb|BT002320.1	At1g17760.1
at1g49570	CON	MIN	HOUR	DAY	WEEK	UNIGENE	FLCDNA	TAIR
CGCTGACATA	0	1	0	0	0	gnl|UG|At#S11698579	gi|30017314|gb|BT006242.1	At1g49570.1
at4g21215	CON	MIN	HOUR	DAY	WEEK	UNIGENE	FLCDNA	TAIR
CCACAGTCTC	0	0	2	2	2	gnl|UG|At#S11706466	gi|15724203|gb|AF412042.1	At4g21215.1
at4g20870	CON	MIN	HOUR	DAY	WEEK	UNIGENE	FLCDNA	TAIR
GTCAACCTAA	1	2	1	0	0	gnl|UG|At#S11724196	gi|14994242|gb|AY044315.1	At4g20870.1
at3g16660	CON	MIN	HOUR	DAY	WEEK	UNIGENE	FLCDNA	TAIR
AATAAGGTCT	1	2	0	0	0	gnl|UG|At#S11735865	gi|26449714|dbj|AK117310.1|	At3g16660.1
at1g53200	CON	MIN	HOUR	DAY	WEEK	UNIGENE	FLCDNA	TAIR
GACTCTTCGG	0	2	0	0	0	gnl|UG|At#S11733281	no match found	At1g53200.2
at4g36515	CON	MIN	HOUR	DAY	WEEK	UNIGENE	FLCDNA	TAIR
ATCATCGTTT	0	0	0	1	0	no match found	gi|62321667|dbj|AK222172.1|	non-canonical match
at1g78070	CON	MIN	HOUR	DAY	WEEK	UNIGENE	FLCDNA	TAIR
TTTGGTCAAC	0	1	1	0	2	no match found	gi|62320748|dbj|AK221701.1|	multiple non-canonical match
at2g02160	CON	MIN	HOUR	DAY	WEEK	UNIGENE	FLCDNA	TAIR
GGCGAGAAGA	0	5	2	4	0	gnl|UG|At#S16346982	gi|24030233|gb|BT000894.1	At2g02160.1
at2g44710	CON	MIN	HOUR	DAY	WEEK	UNIGENE	FLCDNA	TAIR
CAAAGTTGTG	0	0	1	0	0	gnl|UG|At#S15460377	gi|20147225|gb|AY091781.1	At2g44710.1
at1g60080	CON	MIN	HOUR	DAY	WEEK	UNIGENE	FLCDNA	TAIR
GACACTCTTG	0	0	0	1	0	gnl|UG|At#S11731470	gi|110735925|dbj|AK227971.1|	At1g60080.1
at1g10410	CON	MIN	HOUR	DAY	WEEK	UNIGENE	FLCDNA	TAIR
AACTATCATC	0	2	2	0	3	gnl|UG|At#S11741992	gi|20453051|gb|AY094392.1	At1g10410.1
at5g47070	CON	MIN	HOUR	DAY	WEEK	UNIGENE	FLCDNA	TAIR
ATCGTTGACC	0	1	1	0	0	gnl|UG|At#S11824256	gi|26450196|dbj|AK117556.1|	multiple canonical match
at3g15590	CON	MIN	HOUR	DAY	WEEK	UNIGENE	FLCDNA	TAIR
AAAATGAAAA	48	213	50	47	10	no match found	gi|20856657|gb|AY102108.1	At3g15590.1
at1g50470	CON	MIN	HOUR	DAY	WEEK	UNIGENE	FLCDNA	TAIR
GTTGTTTGTT	0	0	1	0	0	gnl|UG|At#S11734331	no match found	non-canonical match
at1g80940	CON	MIN	HOUR	DAY	WEEK	UNIGENE	FLCDNA	TAIR
ATACAAGAAG	3	5	0	2	0	gnl|UG|At#S11726270	gi|20259216|gb|AY091385.1	At1g80940.2

at3g47820	CON	MIN	HOUR	DAY	WEEK	UNIGENE	FLCDNA	TAIR
TAATCGGAGC	0	0	1	0	0	gnl|UG|At#S11729837	gi|20259438|gb|AY091018.1	At3g47820.1
at1g36640	CON	MIN	HOUR	DAY	WEEK	UNIGENE	FLCDNA	TAIR
TAATAATAGT	0	0	1	0	0	gnl|UG|At#S11691360	no match found	At1g36640.1
at2g42730	CON	MIN	HOUR	DAY	WEEK	UNIGENE	FLCDNA	TAIR
GAAATGAGGA	1	0	0	0	0	no match found	gi|110736976|dbj|AK228521.1|	non-canonical match
at4g11970	CON	MIN	HOUR	DAY	WEEK	UNIGENE	FLCDNA	TAIR
GTACCAAGTT	0	0	0	1	0	no match found	no match found	At4g11970.2
TAAACTTTTG	2	0	1	1	2	gnl|UG|At#S11725735	no match found	non-canonical match
at2g01680	CON	MIN	HOUR	DAY	WEEK	UNIGENE	FLCDNA	TAIR
TATCCGATTT	2	0	1	2	0	gnl|UG|At#S11742635	gi|110740934|dbj|AK226418.1|	multiple canonical match
at4g30996	CON	MIN	HOUR	DAY	WEEK	UNIGENE	FLCDNA	TAIR
AGATGTCTGG	1	1	0	0	0	no match found	gi|21281114|gb|AY114012.1	At4g30996.1
at3g18320	CON	MIN	HOUR	DAY	WEEK	UNIGENE	FLCDNA	TAIR
GCCTAAGTTT	0	1	0	0	0	no match found	gi|51870220|gb|BT015450.1	multiple non-canonical match
at1g14920	CON	MIN	HOUR	DAY	WEEK	UNIGENE	FLCDNA	TAIR
TTGGGTTGGC	0	2	1	1	0	no match found	gi|22655347|gb|AY142002.1	non-canonical match
at3g61860	CON	MIN	HOUR	DAY	WEEK	UNIGENE	FLCDNA	TAIR
AAATCTGGAT	0	2	0	0	0	no match found	gi|16612267|gb|AF439831.1	At3g61860.1
GAATGCCTCT	0	1	0	0	0	gnl|UG|At#S18903176	no match found	non-canonical match
at3g50770	CON	MIN	HOUR	DAY	WEEK	UNIGENE	FLCDNA	TAIR
AGTTTCGTCA	0	0	0	0	1	gnl|UG|At#S11832540	gi|45680293|gb|BT011983.1	At3g50770.1
at5g42220	CON	MIN	HOUR	DAY	WEEK	UNIGENE	FLCDNA	TAIR
GCTCAAATGA	1	0	0	0	1	gnl|UG|At#S11817640	no match found	At5g42220.1
at3g57920	CON	MIN	HOUR	DAY	WEEK	UNIGENE	FLCDNA	TAIR
GAACCCGAGA	1	0	0	0	0	gnl|UG|At#S11727966	gi|21405094|gb|AY086384.1	At3g57920.1
at1g08250	CON	MIN	HOUR	DAY	WEEK	UNIGENE	FLCDNA	TAIR
AAAATGTAAA	0	1	0	1	2	gnl|UG|At#S11742210	gi|15810502|gb|AY056290.1	At1g08250.1
at2g47220	CON	MIN	HOUR	DAY	WEEK	UNIGENE	FLCDNA	TAIR
GATTCACGGT	0	1	0	0	0	gnl|UG|At#S34116773	gi|15293072|gb|AY050970.1	At2g47220.1
at1g27520	CON	MIN	HOUR	DAY	WEEK	UNIGENE	FLCDNA	TAIR
GATATATAGT	0	0	0	0	2	gnl|UG|At#S11807237	gi|20259384|gb|AY090979.1	At1g27520.1
at4g08500	CON	MIN	HOUR	DAY	WEEK	UNIGENE	FLCDNA	TAIR
TAATGTATAA	0	1	0	0	1	gnl|UG|At#S11726110	gi|17064765|gb|AY062459.1	At4g08500.1
at3g03440	CON	MIN	HOUR	DAY	WEEK	UNIGENE	FLCDNA	TAIR
CATTACTTAT	1	1	0	3	3	gnl|UG|At#S11743845	gi|19347935|gb|AY080643.1	At3g03440.1
at5g16220	CON	MIN	HOUR	DAY	WEEK	UNIGENE	FLCDNA	TAIR
CTTCTGCTTT	1	2	0	1	1	gnl|UG|At#S11722286	gi|17064963|gb|AY062558.1	At5g16220.1
at5g35730	CON	MIN	HOUR	DAY	WEEK	UNIGENE	FLCDNA	TAIR
AATTGAACCA	0	1	1	1	1	gnl|UG|At#S11720131	gi|21404774|gb|AY086064.1	At5g35730.1
at3g20250	CON	MIN	HOUR	DAY	WEEK	UNIGENE	FLCDNA	TAIR
TCCTGGAGAG	1	0	0	0	0	gnl|UG|At#S34117898	gi|110736765|dbj|AK228410.1|	non-canonical match

at2g33585	CON	MIN	HOUR	DAY	WEEK	UNIGENE	FLCDNA	TAIR
TATAGACACT	2	0	0	0	1	gnl|UG|At#S15460820	gi|26452981|dbj|AK118991.1|	non-canonical match
at4g06540	CON	MIN	HOUR	DAY	WEEK	UNIGENE	FLCDNA	TAIR
GATGTTGATA	1	1	0	0	0	no match found	no match found	At4g06540.1
at5g15530	CON	MIN	HOUR	DAY	WEEK	UNIGENE	FLCDNA	TAIR
GAACTACTGG	0	1	2	1	1	no match found	gi|114050598|gb|BT028902.1	non-canonical match
at4g17550	CON	MIN	HOUR	DAY	WEEK	UNIGENE	FLCDNA	TAIR
GTGGTGTTTC	0	0	0	13	3	gnl|UG|At#S11724755	no match found	At4g17550.1
at5g43860	CON	MIN	HOUR	DAY	WEEK	UNIGENE	FLCDNA	TAIR
TAAACATAAG	1	0	0	1	3	no match found	no match found	At5g43860.1
at4g35950	CON	MIN	HOUR	DAY	WEEK	UNIGENE	FLCDNA	TAIR
GAAGTCAAGA	0	1	0	0	0	gnl|UG|At#S18906481	gi|21406060|gb|AY087336.1	At4g35950.1
at3g50410	CON	MIN	HOUR	DAY	WEEK	UNIGENE	FLCDNA	TAIR
GATCCGGTTC	0	0	1	0	0	gnl|UG|At#S11729362	no match found	At3g50410.1
at5g17760	CON	MIN	HOUR	DAY	WEEK	UNIGENE	FLCDNA	TAIR
GGACATTGCT	0	0	0	0	2	gnl|UG|At#S11721882	gi|15810570|gb|AY056324.1	At5g17760.1
at3g27570	CON	MIN	HOUR	DAY	WEEK	UNIGENE	FLCDNA	TAIR
AAGCTAAACT	0	0	0	1	0	no match found	gi|19699187|gb|AY090299.1	multiple non-canonical match
GAGAGGTAAT	2	0	2	2	1	gnl|UG|At#S11732448	gi|110736943|dbj|AK228503.1|	At3g27570.1
at2g21250	CON	MIN	HOUR	DAY	WEEK	UNIGENE	FLCDNA	TAIR
TTGTTAAACA	5	5	6	7	7	gnl|UG|At#S18942886	gi|21405947|gb|AY087223.1	At2g21250.1
at5g49980	CON	MIN	HOUR	DAY	WEEK	UNIGENE	FLCDNA	TAIR
ATTGTTGATG	0	0	2	0	1	gnl|UG|At#S11718718	gi|62319765|dbj|AK221208.1|	At5g49980.1
at3g27280	CON	MIN	HOUR	DAY	WEEK	UNIGENE	FLCDNA	TAIR
GTAAATAAAT	0	0	1	2	0	no match found	no match found	At3g27280.1
CTCTTTAATC	0	1	0	0	0	gnl|UG|At#S18942042	gi|21406017|gb|AY087293.1	At3g27280.2
at2g01620	CON	MIN	HOUR	DAY	WEEK	UNIGENE	FLCDNA	TAIR
AGATTGCAAT	0	1	1	0	0	gnl|UG|At#S11742641	gi|28827727|gb|BT005175.1	At2g01620.1
at5g42800	CON	MIN	HOUR	DAY	WEEK	UNIGENE	FLCDNA	TAIR
CTAGCACAAC	0	0	0	0	1	gnl|UG|At#S11719449	gi|62320591|dbj|AK221622.1|	At5g42800.1
at3g50750	CON	MIN	HOUR	DAY	WEEK	UNIGENE	FLCDNA	TAIR
ATTTTTCAAT	0	0	2	1	0	gnl|UG|At#S11729298	gi|27311692|gb|BT002452.1	At3g50750.1
at1g72020	CON	MIN	HOUR	DAY	WEEK	UNIGENE	FLCDNA	TAIR
AAGTACCAAG	3	11	2	2	2	gnl|UG|At#S11728684	gi|18253028|gb|AY072449.1	At1g72020.1
at2g46260	CON	MIN	HOUR	DAY	WEEK	UNIGENE	FLCDNA	TAIR
GACTTCGTTC	1	1	1	1	1	gnl|UG|At#S11704718	gi|62321197|dbj|AK221929.1|	At2g46260.1
at5g23470	CON	MIN	HOUR	DAY	WEEK	UNIGENE	FLCDNA	TAIR
ACAAGAAAAA	1	4	5	7	16	gnl|UG|At#S11720830	no match found	multiple non-canonical match
at1g31130	CON	MIN	HOUR	DAY	WEEK	UNIGENE	FLCDNA	TAIR
GTGGTGGGAA	0	2	1	1	0	gnl|UG|At#S11629818	gi|13272450|gb|AF325096.1	multiple non-canonical match
ATTTGAGTTT	3	4	7	9	0	no match found	gi|19698822|gb|AY081258.1	non-canonical match

at4g32570	CON	MIN	HOUR	DAY	WEEK	UNIGENE	FLCDNA	TAIR
ATTAAAGTCT	0	3	1	1	1	gnl|UG|At#S11722180	gi|21405141|gb|AY086431.1	non-canonical match
at2g16090	CON	MIN	HOUR	DAY	WEEK	UNIGENE	FLCDNA	TAIR
GATATGAATC	0	1	0	0	0	gnl|UG|At#S11709587	gi|18086413|gb|AY065022.1	At2g16090.1
at1g69740	CON	MIN	HOUR	DAY	WEEK	UNIGENE	FLCDNA	TAIR
ATCTGAGTTT	7	7	3	2	3	gnl|UG|At#S11729154	no match found	At1g69740.1
TCATACACAG	0	3	0	0	1	no match found	gi|16323331|gb|AY059154.1	multiple non-canonical match
TTGCGGCACA	1	3	0	4	1	gnl|UG|At#S34116188	no match found	At3g53610.2
at4g22830	CON	MIN	HOUR	DAY	WEEK	UNIGENE	FLCDNA	TAIR
TGCATTCAAC	1	0	1	0	1	gnl|UG|At#S11723849	gi|26452655|dbj|AK118821.1|	At4g22830.1
ATGGCTAAAC	1	1	0	1	0	no match found	gi|28973426|gb|BT005618.1	multiple non-canonical match
at1g53580	CON	MIN	HOUR	DAY	WEEK	UNIGENE	FLCDNA	TAIR
AGCTTTTTCA	0	0	0	1	0	gnl|UG|At#S11733160	no match found	multiple non-canonical match
TCACTGGTAC	0	3	0	0	0	no match found	gi|15450394|gb|AY052298.1	At1g53580.2
at3g22070	CON	MIN	HOUR	DAY	WEEK	UNIGENE	FLCDNA	TAIR
CCAAGCTAGT	1	0	1	0	0	no match found	gi|28827399|gb|BT005011.1	non-canonical match
TTGAATACTT	0	1	1	0	0	gnl|UG|At#S11824308	gi|26450090|dbj|AK117502.1|	At3g22070.1
at3g49010	CON	MIN	HOUR	DAY	WEEK	UNIGENE	FLCDNA	TAIR
CCGGAGCTAG	19	23	24	23	21	no match found	gi|16226380|gb|AF428384.1	At3g49010.2
at1g80750	CON	MIN	HOUR	DAY	WEEK	UNIGENE	FLCDNA	TAIR
AGTTCAGGGC	0	4	1	0	0	gnl|UG|At#S11726289	gi|27765019|gb|BT003066.1	At1g80750.1
at1g10600	CON	MIN	HOUR	DAY	WEEK	UNIGENE	FLCDNA	TAIR
AAACAAGAGT	1	0	0	0	0	no match found	no match found	At1g10600.1
TCCTTAGATT	2	3	0	0	0	gnl|UG|At#S38434379	gi|51969057|dbj|AK175458.1|	non-canonical match
at5g10695	CON	MIN	HOUR	DAY	WEEK	UNIGENE	FLCDNA	TAIR
ATTATGAAAA	0	3	2	3	1	gnl|UG|At#S11723568	gi|19347740|gb|AY080612.1	At5g10695.1
at5g40580	CON	MIN	HOUR	DAY	WEEK	UNIGENE	FLCDNA	TAIR
TTTACGTTTT	0	0	0	0	1	no match found	gi|21406488|gb|AY087750.1	non-canonical match
TTTACATTTT	11	4	5	5	7	gnl|UG|At#S15459296	no match found	multiple canonical match
at2g05590	CON	MIN	HOUR	DAY	WEEK	UNIGENE	FLCDNA	TAIR
CGTCTCAATA	1	0	0	0	0	gnl|UG|At#S15461650	gi|13569551|gb|AF345342.1	At2g05590.2
at1g80000	CON	MIN	HOUR	DAY	WEEK	UNIGENE	FLCDNA	TAIR
GAGAGACCTG	0	1	0	0	0	gnl|UG|At#S18942219	gi|28416530|gb|BT004550.1	At1g80000.2
at1g18390	CON	MIN	HOUR	DAY	WEEK	UNIGENE	FLCDNA	TAIR
TATGTCGCAC	0	1	0	0	0	gnl|UG|At#S11741212	no match found	At1g18390.1
at5g41410	CON	MIN	HOUR	DAY	WEEK	UNIGENE	FLCDNA	TAIR
ATATTGTTTG	0	0	1	1	0	gnl|UG|At#S11719588	gi|15146192|gb|AY049237.1	At5g41410.1
at2g14900	CON	MIN	HOUR	DAY	WEEK	UNIGENE	FLCDNA	TAIR
AATGTGCTTG	0	3	0	0	0	gnl|UG|At#S11738737	gi|28394018|gb|BT004423.1	At2g14900.1
at5g27670	CON	MIN	HOUR	DAY	WEEK	UNIGENE	FLCDNA	TAIR
GGAAGATGAT	4	5	8	2	3	gnl|UG|At#S20298065	gi|14326515|gb|AF385711.1	At5g27670.1
at1g60260	CON	MIN	HOUR	DAY	WEEK	UNIGENE	FLCDNA	TAIR
GGCGCATATG	0	3	0	0	0	no match found	gi|19423881|gb|AY080772.1	non-canonical match
AGAGCCACTT	1	4	1	1	0	gnl|UG|At#S11744486	gi|19699288|gb|AY090350.1	At1g60260.1

at1g61780	CON	MIN	HOUR	DAY	WEEK	UNIGENE	FLCDNA	TAIR
TATTTTTTTG	1	0	1	0	0	gnl|UG|At#S30488502	gi|21407593|gb|AY088819.1	At1g61780.1

at1g73840	CON	MIN	HOUR	DAY	WEEK	UNIGENE	FLCDNA	TAIR
TTAGAACTTT	2	3	1	0	3	gnl|UG|At#S11709790	gi|17979387|gb|AY070424.1	At1g73840.1
TATCATTCAG	1	0	0	0	0	no match found	gi|20465538|gb|AY096602.1	non-canonical match

at5g64830	CON	MIN	HOUR	DAY	WEEK	UNIGENE	FLCDNA	TAIR
GAATTATCTT	0	2	0	0	0	gnl|UG|At#S28281545	gi|15724301|gb|AF412091.1	At5g64830.1

at4g39795	CON	MIN	HOUR	DAY	WEEK	UNIGENE	FLCDNA	TAIR
TAAAATTAAT	0	1	0	0	0	gnl|UG|At#S11693091	no match found	At4g39795.1

at5g04190	CON	MIN	HOUR	DAY	WEEK	UNIGENE	FLCDNA	TAIR
GGCTCCACCA	3	0	0	0	0	gnl|UG|At#S11725099	gi|51315393|gb|BT015323.1	At5g04190.1

at3g13224	CON	MIN	HOUR	DAY	WEEK	UNIGENE	FLCDNA	TAIR
TAGCGCCAAC	0	0	0	1	0	no match found	gi|19347907|gb|AY080630.1	non-canonical match
TGTAAGTAGG	0	1	0	0	0	no match found	gi|62320243|dbj|AK221448.1|	non-canonical match

at5g04150	CON	MIN	HOUR	DAY	WEEK	UNIGENE	FLCDNA	TAIR
TTGCTTAGGT	0	0	0	1	0	gnl|UG|At#S26110025	no match found	non-canonical match
TGAATGATAA	0	0	0	1	0	no match found	no match found	At5g04150.1

at2g29480	CON	MIN	HOUR	DAY	WEEK	UNIGENE	FLCDNA	TAIR
GTTGCGGGAA	0	0	1	0	0	gnl|UG|At#S11735168	gi|20453169|gb|AY094455.1	non-canonical match

at5g07350	CON	MIN	HOUR	DAY	WEEK	UNIGENE	FLCDNA	TAIR
CTTACAGTTT	1	0	0	3	1	gnl|UG|At#S11709634	gi|18086331|gb|AY064975.1	At5g07350.1

at1g71950	CON	MIN	HOUR	DAY	WEEK	UNIGENE	FLCDNA	TAIR
GCTGATGAAT	0	3	0	0	0	no match found	gi|21406435|gb|AY087697.1	multiple non-canonical match

at5g15550	CON	MIN	HOUR	DAY	WEEK	UNIGENE	FLCDNA	TAIR
ATCCTGGAAT	1	1	2	1	0	gnl|UG|At#S11722451	gi|17381109|gb|AY064011.1	At5g15550.1

at2g27760	CON	MIN	HOUR	DAY	WEEK	UNIGENE	FLCDNA	TAIR
AAAAGGTTAT	0	1	0	0	2	gnl|UG|At#S11744355	gi|62320645|dbj|AK221649.1|	At2g27760.1

at2g27460	CON	MIN	HOUR	DAY	WEEK	UNIGENE	FLCDNA	TAIR
AAGTGAAAGC	0	0	0	0	1	gnl|UG|At#S11735678	gi|15450829|gb|AY054495.1	non-canonical match

at5g43750	CON	MIN	HOUR	DAY	WEEK	UNIGENE	FLCDNA	TAIR
ACTTTTGATG	10	5	2	5	7	gnl|UG|At#S14273793	gi|28393511|gb|BT004156.1	At5g43750.1

at5g65495	CON	MIN	HOUR	DAY	WEEK	UNIGENE	FLCDNA	TAIR
ACGGAGAAGA	0	1	0	0	0	gnl|UG|At#S30642146	gi|26452271|dbj|AK118625.1|	At5g65495.1

at2g33170	CON	MIN	HOUR	DAY	WEEK	UNIGENE	FLCDNA	TAIR
CCACTATCAA	0	2	1	2	0	gnl|UG|At#S11734242	no match found	At2g33170.1

at3g15353	CON	MIN	HOUR	DAY	WEEK	UNIGENE	FLCDNA	TAIR
GACGTTGGTG	0	0	4	0	0	no match found	gi|20259218|gb|AY091386.1	non-canonical match
TCTCTCTTAT	54	64	21	9	13	no match found	gi|18377772|gb|AY074340.1	At3g15353.1

at1g67400	CON	MIN	HOUR	DAY	WEEK	UNIGENE	FLCDNA	TAIR
GAATTCAACG	0	3	0	0	0	no match found	gi|51968573|dbj|AK175216.1|	At1g67400.1

at5g19130	CON	MIN	HOUR	DAY	WEEK	UNIGENE	FLCDNA	TAIR
GTAATTAGCA	1	1	1	1	0	gnl|UG|At#S11721535	gi|27311614|gb|BT002413.1	non-canonical match

at2g31880	CON	MIN	HOUR	DAY	WEEK	UNIGENE	FLCDNA	TAIR
TTGTCCCAGA	1	3	4	4	2	gnl|UG|At#S11734562	gi|16648754|gb|AY058153.1	At2g31880.1
at2g40475	CON	MIN	HOUR	DAY	WEEK	UNIGENE	FLCDNA	TAIR
TTTTGATTAT	6	1	0	0	6	gnl|UG|At#S11732473	no match found	At2g40475.1
at1g64750	CON	MIN	HOUR	DAY	WEEK	UNIGENE	FLCDNA	TAIR
GATTAGTTAC	0	0	0	1	1	no match found	no match found	At1g64750.1
at4g37260	CON	MIN	HOUR	DAY	WEEK	UNIGENE	FLCDNA	TAIR
TATAGAGCCA	6	40	12	1	2	gnl|UG|At#S11708393	no match found	multiple canonical match
at5g15160	CON	MIN	HOUR	DAY	WEEK	UNIGENE	FLCDNA	TAIR
CAATTCCATT	0	2	0	0	0	no match found	gi|26452782|dbj|AK118887.1|	At5g15160.1
at1g06650	CON	MIN	HOUR	DAY	WEEK	UNIGENE	FLCDNA	TAIR
ACAATATATG	2	1	1	0	3	gnl|UG|At#S11742366	gi|13878118|gb|AF370322.1	At1g06650.2
at3g23255	CON	MIN	HOUR	DAY	WEEK	UNIGENE	FLCDNA	TAIR
GTGATGGATC	0	0	0	1	0	no match found	gi|110736760|dbj|AK228407.1|	multiple non-canonical match
at4g33410	CON	MIN	HOUR	DAY	WEEK	UNIGENE	FLCDNA	TAIR
AAATAGAGAT	0	2	0	2	2	gnl|UG|At#S18907755	gi|16648808|gb|AY058181.1	At4g33410.1
at4g14090	CON	MIN	HOUR	DAY	WEEK	UNIGENE	FLCDNA	TAIR
GAAGCGCTTA	0	0	0	1	0	gnl|UG|At#S34115479	gi|110741429|dbj|AK226538.1|	At4g14090.1
at3g49060	CON	MIN	HOUR	DAY	WEEK	UNIGENE	FLCDNA	TAIR
TCACAGTAAT	0	0	0	0	1	gnl|UG|At#S21736252	gi|51970987|dbj|AK176423.1|	At3g49060.1
at1g79270	CON	MIN	HOUR	DAY	WEEK	UNIGENE	FLCDNA	TAIR
TAGTGTTTTT	1	1	3	4	2	gnl|UG|At#S11726765	no match found	multiple non-canonical match
at1g31800	CON	MIN	HOUR	DAY	WEEK	UNIGENE	FLCDNA	TAIR
TTTGCTTCCT	0	0	0	1	1	no match found	gi|22655377|gb|AY142017.1	multiple non-canonical match
ACCAATGCTT	8	2	8	6	3	gnl|UG|At#S11738364	gi|62319773|dbj|AK221212.1|	non-canonical match
at2g05500	CON	MIN	HOUR	DAY	WEEK	UNIGENE	FLCDNA	TAIR
CAATTTAAAA	0	0	0	1	0	no match found	no match found	At2g05500.1
at3g03770	CON	MIN	HOUR	DAY	WEEK	UNIGENE	FLCDNA	TAIR
AGAGGTAGAG	1	0	2	0	1	gnl|UG|At#S11822649	gi|27311538|gb|BT002375.1	At3g03770.1
at4g27340	CON	MIN	HOUR	DAY	WEEK	UNIGENE	FLCDNA	TAIR
GCTGAATATA	0	0	2	1	2	gnl|UG|At#S34115013	gi|110742287|dbj|AK227004.1|	At4g27340.1
at2g38050	CON	MIN	HOUR	DAY	WEEK	UNIGENE	FLCDNA	TAIR
TTCCAGAAGC	0	0	2	0	0	no match found	no match found	At2g38050.1
at1g14810	CON	MIN	HOUR	DAY	WEEK	UNIGENE	FLCDNA	TAIR
CAGAGAGTGT	1	3	3	2	2	no match found	gi|17979523|gb|AY070759.1	non-canonical match
CACACGCTTG	0	0	1	0	1	gnl|UG|At#S11741553	no match found	At1g14810.1
at4g16060	CON	MIN	HOUR	DAY	WEEK	UNIGENE	FLCDNA	TAIR
GAAGAAGAAC	3	4	2	0	0	gnl|UG|At#S11725020	gi|28950778|gb|BT005249.1	At4g16060.1
at5g08139	CON	MIN	HOUR	DAY	WEEK	UNIGENE	FLCDNA	TAIR
TACTTTGAAT	1	0	0	0	1	gnl|UG|At#S15461222	gi|62319842|dbj|AK221247.1|	At5g08139.1
at2g23610	CON	MIN	HOUR	DAY	WEEK	UNIGENE	FLCDNA	TAIR

GCCAAGAACT	0	5	1	1	1	gnl|UG|At#S11736579	gi|50198957|gb|BT015031.1	At2g23610.1
at5g07250	CON	MIN	HOUR	DAY	WEEK	UNIGENE	FLCDNA	TAIR
TGGGTGGATT	0	2	0	0	0	gnl|UG|At#S11724312	gi|20466142|gb|AY098983.1	non-canonical match
at1g52220	CON	MIN	HOUR	DAY	WEEK	UNIGENE	FLCDNA	TAIR
CATTTGAAAC	34	17	26	21	4	gnl|UG|At#S11733669	gi|15294155|gb|AF410269.1	At1g52220.1
at1g08890	CON	MIN	HOUR	DAY	WEEK	UNIGENE	FLCDNA	TAIR
TATTATCCGA	0	0	0	1	0	gnl|UG|At#S11742147	gi|110742684|dbj|AK227216.1|	At1g08890.1
at3g27430	CON	MIN	HOUR	DAY	WEEK	UNIGENE	FLCDNA	TAIR
TTTAAGGATC	6	0	0	3	3	gnl|UG|At#S11732501	gi|28058827|gb|BT003339.1	At3g27430.2
GAACCAAACC	0	0	1	1	0	no match found	gi|30023761|gb|BT006306.1	multiple non-canonical match
at1g15920	CON	MIN	HOUR	DAY	WEEK	UNIGENE	FLCDNA	TAIR
TTCGCCTTGG	0	4	0	0	0	no match found	gi|21281270|gb|AY114040.1	At1g15920.1
TATTTAACAT	0	0	0	0	1	gnl|UG|At#S18942568	no match found	At1g15920.2
at2g10410	CON	MIN	HOUR	DAY	WEEK	UNIGENE	FLCDNA	TAIR
GACGCTAGCG	0	4	0	2	3	gnl|UG|At#S11832190	gi|15215848|gb|AY050454.1	At2g10410.1
at4g26080	CON	MIN	HOUR	DAY	WEEK	UNIGENE	FLCDNA	TAIR
TCGAGATCCA	1	2	0	1	0	gnl|UG|At#S11723297	gi|23297059|gb|AY142623.1	At4g26080.1
at5g52810	CON	MIN	HOUR	DAY	WEEK	UNIGENE	FLCDNA	TAIR
AAATGAAGGA	0	0	0	0	1	gnl|UG|At#S11718436	gi|21403381|gb|AY084671.1	At5g52810.1
at2g32540	CON	MIN	HOUR	DAY	WEEK	UNIGENE	FLCDNA	TAIR
ACATTTTCTC	6	0	3	0	0	no match found	no match found	At2g32540.1
at1g08650	CON	MIN	HOUR	DAY	WEEK	UNIGENE	FLCDNA	TAIR
TGGGGTGAGT	0	5	4	6	4	gnl|UG|At#S11710155	gi|17644150|gb|AY065297.1	At1g08650.1
at4g14440	CON	MIN	HOUR	DAY	WEEK	UNIGENE	FLCDNA	TAIR
ATAGTGCGGA	1	2	2	2	0	gnl|UG|At#S11725309	gi|30023699|gb|BT006275.1	At4g14440.1
at2g01950	CON	MIN	HOUR	DAY	WEEK	UNIGENE	FLCDNA	TAIR
ATGTATCAAG	0	0	1	0	0	gnl|UG|At#S11743142	gi|18377719|gb|AY074313.1	At2g01950.1
at2g25820	CON	MIN	HOUR	DAY	WEEK	UNIGENE	FLCDNA	TAIR
TCTCAAAGAA	0	0	0	0	1	no match found	gi|13878156|gb|AF370341.1	At2g25820.1
at3g03890	CON	MIN	HOUR	DAY	WEEK	UNIGENE	FLCDNA	TAIR
AAAAAAAGTT	2	4	10	4	4	gnl|UG|At#S11739547	gi|21404512|gb|AY085802.1	At3g03890.2
at1g79670	CON	MIN	HOUR	DAY	WEEK	UNIGENE	FLCDNA	TAIR
GTGATGATGG	0	2	2	0	0	no match found	gi|19310446|gb|AY078961.1	At1g79670.2
at4g12160	CON	MIN	HOUR	DAY	WEEK	UNIGENE	FLCDNA	TAIR
AAGACAAAGG	0	3	0	0	0	no match found	no match found	At4g12160.1
at3g50820	CON	MIN	HOUR	DAY	WEEK	UNIGENE	FLCDNA	TAIR
ACCCGTCTCA	11	33	11	10	1	gnl|UG|At#S11729285	gi|51870320|gb|BT015545.1	At3g50820.1
at3g05230	CON	MIN	HOUR	DAY	WEEK	UNIGENE	FLCDNA	TAIR
CCCAAGACCG	2	1	0	0	0	gnl|UG|At#S11739159	gi|110743607|dbj|AK227654.1|	At3g05230.1
at4g25225	CON	MIN	HOUR	DAY	WEEK	UNIGENE	FLCDNA	TAIR
GGAACAGTGT	0	0	1	1	0	gnl|UG|At#S20295316	no match found	At3g05230.1

at3g05170	CON	MIN	HOUR	DAY	WEEK	UNIGENE	FLCDNA	TAIR
AGAAGAAACG	0	2	0	0	0	gnl|UG|At#S11739173	no match found	At3g05170.1
at2g45150	CON	MIN	HOUR	DAY	WEEK	UNIGENE	FLCDNA	TAIR
GTAAGATCCG	0	1	1	0	0	gnl|UG|At#S18942698	gi|15292688|gb|AY050778.1	At2g45150.1
at5g16200	CON	MIN	HOUR	DAY	WEEK	UNIGENE	FLCDNA	TAIR
GCGACCAGTC	2	0	0	1	1	gnl|UG|At#S21138589	gi|51972087|gb|BT015649.1	At5g16200.1
at1g30260	CON	MIN	HOUR	DAY	WEEK	UNIGENE	FLCDNA	TAIR
CATATGTTTG	3	1	0	0	1	gnl|UG|At#S11738935	gi|16323183|gb|AY057696.1	At1g30260.1
CACTTCCGGT	1	0	1	1	0	no match found	gi|21436006|gb|AY116947.1	non-canonical match
at5g08540	CON	MIN	HOUR	DAY	WEEK	UNIGENE	FLCDNA	TAIR
ACACAGCTCG	3	5	0	4	2	gnl|UG|At#S11723977	gi|21405114|gb|AY086404.1	non-canonical match
at2g01060	CON	MIN	HOUR	DAY	WEEK	UNIGENE	FLCDNA	TAIR
AAGAATGAAT	2	4	1	2	4	gnl|UG|At#S18943004	gi|13899110|gb|AF370550.1	At2g01060.2
at3g20320	CON	MIN	HOUR	DAY	WEEK	UNIGENE	FLCDNA	TAIR
GCTGAAGATG	1	0	2	0	0	gnl|UG|At#S18942066	gi|21404432|gb|AY085722.1	At3g20320.2
at2g44410	CON	MIN	HOUR	DAY	WEEK	UNIGENE	FLCDNA	TAIR
GAGAAAACAA	0	0	0	1	0	gnl|UG|At#S11731543	gi|45773899|gb|BT012267.1	At2g44410.1
at2g39640	CON	MIN	HOUR	DAY	WEEK	UNIGENE	FLCDNA	TAIR
CGTCGTACCA	0	0	0	1	0	gnl|UG|At#S11732687	no match found	At2g39640.1
at2g01890	CON	MIN	HOUR	DAY	WEEK	UNIGENE	FLCDNA	TAIR
GAGACATATC	0	0	2	1	0	no match found	gi|17529295|gb|AY065434.1	At2g01890.1
at3g24160	CON	MIN	HOUR	DAY	WEEK	UNIGENE	FLCDNA	TAIR
CCTTTGACGA	4	2	5	1	2	no match found	gi|15450742|gb|AY053413.1	non-canonical match
AATCTGAATA	1	2	2	0	1	no match found	no match found	At3g24160.1
at3g61210	CON	MIN	HOUR	DAY	WEEK	UNIGENE	FLCDNA	TAIR
TCATCAATGA	1	1	4	1	1	gnl|UG|At#S11823339	gi|39104590|dbj|AK118519.2|	At3g61210.1
at1g09830	CON	MIN	HOUR	DAY	WEEK	UNIGENE	FLCDNA	TAIR
ATATTGGTTG	0	1	0	1	0	gnl|UG|At#S11699426	gi|15292772|gb|AY050820.1	At1g09830.1
at5g59730	CON	MIN	HOUR	DAY	WEEK	UNIGENE	FLCDNA	TAIR
AAGAGAAGGT	1	4	1	1	0	gnl|UG|At#S11717730	gi|15010739|gb|AY045671.1	At5g59730.1
at3g45640	CON	MIN	HOUR	DAY	WEEK	UNIGENE	FLCDNA	TAIR
TTAACCCAAT	0	0	1	0	0	no match found	gi|23197597|gb|BT000007.1	multiple non-canonical match
TTATTTACTG	6	8	2	1	2	no match found	gi|14423447|gb|AF386961.1	At3g45640.1
at3g12520	CON	MIN	HOUR	DAY	WEEK	UNIGENE	FLCDNA	TAIR
ACGCAGTCCA	1	1	0	0	0	gnl|UG|At#S11737141	gi|26450309|dbj|AK117615.1|	At3g12520.1
at4g00525	CON	MIN	HOUR	DAY	WEEK	UNIGENE	FLCDNA	TAIR
ATATTTGATT	0	1	0	0	0	gnl|UG|At#S11816781	no match found	At4g00525.1
at4g31080	CON	MIN	HOUR	DAY	WEEK	UNIGENE	FLCDNA	TAIR
GAGGAAACTC	0	0	1	0	0	no match found	no match found	At4g31080.1
ATGGCAGTTG	0	1	0	0	0	no match found	gi|30725689|gb|BT008508.1	non-canonical match
TGTGTTTGTG	1	2	2	1	0	gnl|UG|At#S11722433	gi|51971081|dbj|AK176470.1|	multiple canonical match
at2g46590	CON	MIN	HOUR	DAY	WEEK	UNIGENE	FLCDNA	TAIR
GGTTACTTTA	1	0	0	0	0	gnl|UG|At#S11831503	gi|28058738|gb|BT003328.1	At2g46590.1

at1g35340	CON	MIN	HOUR	DAY	WEEK	UNIGENE	FLCDNA	TAIR
AGCGAGGTTC	0	4	0	1	0	no match found	gi|51968885|dbj|AK175372.1|	At1g35340.1
at1g77440	CON	MIN	HOUR	DAY	WEEK	UNIGENE	FLCDNA	TAIR
TCTACGTTGT	0	2	0	0	1	gnl|UG|At#S11700414	gi|14326551|gb|AF385730.1	At1g77440.1
at1g72150	CON	MIN	HOUR	DAY	WEEK	UNIGENE	FLCDNA	TAIR
ATTTCTTACC	6	11	9	1	3	gnl|UG|At#S11728659	no match found	At1g72150.1
at3g19510	CON	MIN	HOUR	DAY	WEEK	UNIGENE	FLCDNA	TAIR
AAGGTTTTAG	0	0	1	1	0	gnl|UG|At#S11735007	gi|26449312|dbj|AK117105.1|	At3g19510.1
at1g50410	CON	MIN	HOUR	DAY	WEEK	UNIGENE	FLCDNA	TAIR
AGCTGCATTC	0	0	0	1	1	no match found	no match found	At1g50410.1
at3g16190	CON	MIN	HOUR	DAY	WEEK	UNIGENE	FLCDNA	TAIR
TAATAAAGAA	2	6	2	0	5	no match found	gi|16648697|gb|AY058125.1	multiple canonical match
at2g21410	CON	MIN	HOUR	DAY	WEEK	UNIGENE	FLCDNA	TAIR
GACACGAAGA	0	3	0	1	0	gnl|UG|At#S11737107	gi|20466447|gb|AY099690.1	At2g21410.1
at5g59350	CON	MIN	HOUR	DAY	WEEK	UNIGENE	FLCDNA	TAIR
AGAGATGCTG	1	0	0	0	0	gnl|UG|At#S14829869	gi|21404088|gb|AY085378.1	At5g59350.1
at5g46920	CON	MIN	HOUR	DAY	WEEK	UNIGENE	FLCDNA	TAIR
TAAGAAACCA	0	1	0	0	1	gnl|UG|At#S11719032	gi|13926343|gb|AF372919.1	At5g46920.1
at1g20440	CON	MIN	HOUR	DAY	WEEK	UNIGENE	FLCDNA	TAIR
CCAAGACCAC	6	13	28	43	21	gnl|UG|At#S34115588	gi|21389638|gb|AY114699.1	non-canonical match
at5g01740	CON	MIN	HOUR	DAY	WEEK	UNIGENE	FLCDNA	TAIR
GGGCGTTGTA	0	4	0	0	0	gnl|UG|At#S11725723	gi|48310613|gb|BT014870.1	At5g01740.1
at3g45970	CON	MIN	HOUR	DAY	WEEK	UNIGENE	FLCDNA	TAIR
ATTCTACATT	1	0	0	0	2	gnl|UG|At#S11730192	gi|21405478|gb|AY086768.1	At3g45970.1
ACAAGAAGCC	0	2	0	0	1	no match found	gi|15450562|gb|AY052745.1	non-canonical match
at2g27830	CON	MIN	HOUR	DAY	WEEK	UNIGENE	FLCDNA	TAIR
AAGAACGGTA	0	3	0	1	0	no match found	gi|21406013|gb|AY087289.1	multiple canonical match
at1g14150	CON	MIN	HOUR	DAY	WEEK	UNIGENE	FLCDNA	TAIR
AAATCTCTGG	7	3	7	4	0	gnl|UG|At#S11741623	gi|21407099|gb|AY088325.1	At1g14150.1
at5g07340	CON	MIN	HOUR	DAY	WEEK	UNIGENE	FLCDNA	TAIR
AATCTGAATG	2	2	0	0	1	gnl|UG|At#S11822166	gi|25082879|gb|BT001999.1	At5g07340.1
at3g56220	CON	MIN	HOUR	DAY	WEEK	UNIGENE	FLCDNA	TAIR
ATTCAAAAAG	0	0	1	0	0	gnl|UG|At#S11728293	gi|110736047|dbj|AK228035.1|	At3g56220.1
at1g66000	CON	MIN	HOUR	DAY	WEEK	UNIGENE	FLCDNA	TAIR
GAGAATCTTC	0	1	0	0	0	gnl|UG|At#S11729927	no match found	At1g66000.1
at2g22870	CON	MIN	HOUR	DAY	WEEK	UNIGENE	FLCDNA	TAIR
AATTGCCGAA	0	1	0	0	0	gnl|UG|At#S11736764	gi|21406001|gb|AY087277.1	non-canonical match
at1g64870	CON	MIN	HOUR	DAY	WEEK	UNIGENE	FLCDNA	TAIR
CCAGAGGACG	1	6	1	3	1	gnl|UG|At#S11730159	no match found	At1g64870.1
at1g22650	CON	MIN	HOUR	DAY	WEEK	UNIGENE	FLCDNA	TAIR
GTCTATCGCC	0	2	0	2	0	gnl|UG|At#S11740798	gi|21407162|gb|AY088388.1	At1g22650.1

at4g25370	CON	MIN	HOUR	DAY	WEEK	UNIGENE	FLCDNA	TAIR
TACTTTTTCA	2	4	1	0	0	gnl|UG|At#S11723411	gi|21405534|gb|AY086824.1	At4g25370.1
at5g23660	CON	MIN	HOUR	DAY	WEEK	UNIGENE	FLCDNA	TAIR
TAACTGTAAC	0	3	0	0	0	gnl|UG|At#S11705810	gi|15982722|gb|AY057575.1	At5g23660.1
at3g46080	CON	MIN	HOUR	DAY	WEEK	UNIGENE	FLCDNA	TAIR
AGGAGACATA	0	0	0	1	0	gnl|UG|At#S22665713	no match found	At3g46080.1
at2g45740	CON	MIN	HOUR	DAY	WEEK	UNIGENE	FLCDNA	TAIR
GTCTTATCAG	0	4	1	1	0	gnl|UG|At#S11703748	gi|21403653|gb|AY084943.1	At2g45740.2
at1g13460	CON	MIN	HOUR	DAY	WEEK	UNIGENE	FLCDNA	TAIR
TTTCTAAACG	0	0	0	0	1	gnl|UG|At#S18942590	gi|21403779|gb|AY085069.1	At1g13460.2
at1g51980	CON	MIN	HOUR	DAY	WEEK	UNIGENE	FLCDNA	TAIR
CACTGCATTC	1	5	2	1	0	gnl|UG|At#S11733758	no match found	At1g51980.1
at1g06800	CON	MIN	HOUR	DAY	WEEK	UNIGENE	FLCDNA	TAIR
CTCCTGACAT	0	0	1	0	0	gnl|UG|At#S15461513	gi|20466265|gb|AY099599.1	At1g06800.1
at1g79360	CON	MIN	HOUR	DAY	WEEK	UNIGENE	FLCDNA	TAIR
TGAAATTAAT	0	1	0	0	0	gnl|UG|At#S34114646	gi|110742964|dbj|AK227369.1|	At1g79360.1
at3g30180	CON	MIN	HOUR	DAY	WEEK	UNIGENE	FLCDNA	TAIR
GAGATGGATG	1	1	1	0	0	gnl|UG|At#S11731671	gi|15450574|gb|AY052655.1	multiple canonical match
at5g63200	CON	MIN	HOUR	DAY	WEEK	UNIGENE	FLCDNA	TAIR
TCAAGGCTCT	1	2	3	0	0	gnl|UG|At#S21736206	gi|27764941|gb|BT003027.1	At5g63200.1
at3g25220	CON	MIN	HOUR	DAY	WEEK	UNIGENE	FLCDNA	TAIR
AAAGCTGTTG	0	1	0	1	0	gnl|UG|At#S11733205	gi|22136163|gb|AY128760.1	At3g25220.1
at1g21070	CON	MIN	HOUR	DAY	WEEK	UNIGENE	FLCDNA	TAIR
AAATCAAAAT	1	0	0	0	0	gnl|UG|At#S11740949	no match found	At1g21070.1
TACTCTTTTC	1	0	0	0	0	no match found	gi|26450365|dbj|AK117643.1|	multiple non-canonical match
at1g56070	CON	MIN	HOUR	DAY	WEEK	UNIGENE	FLCDNA	TAIR
GTTGAGATCC	9	13	11	11	12	no match found	gi|62321133|dbj|AK221896.1|	multiple non-canonical match
at2g01660	CON	MIN	HOUR	DAY	WEEK	UNIGENE	FLCDNA	TAIR
GCAAGCGGTG	0	1	0	0	0	no match found	gi|17065481|gb|AY062817.1	At2g01660.1
at2g06025	CON	MIN	HOUR	DAY	WEEK	UNIGENE	FLCDNA	TAIR
TACAATTGTG	1	2	1	0	0	no match found	gi|62318764|dbj|AK220702.1|	non-canonical match
at1g05070	CON	MIN	HOUR	DAY	WEEK	UNIGENE	FLCDNA	TAIR
CGAAGAAGCT	0	4	4	0	2	gnl|UG|At#S18893542	gi|21403903|gb|AY085193.1	At1g05070.1
at2g45140	CON	MIN	HOUR	DAY	WEEK	UNIGENE	FLCDNA	TAIR
CACCTTTGGT	1	2	0	0	0	no match found	gi|21407173|gb|AY088399.1	non-canonical match
at3g12760	CON	MIN	HOUR	DAY	WEEK	UNIGENE	FLCDNA	TAIR
GCCTTACCTG	2	4	2	4	1	gnl|UG|At#S11707311	gi|15292984|gb|AY050926.1	At3g12760.1
at2g35100	CON	MIN	HOUR	DAY	WEEK	UNIGENE	FLCDNA	TAIR
TCATAAAAGC	2	2	0	2	0	gnl|UG|At#S15460763	gi|50253447|gb|BT015054.1	At2g35100.1
at3g49980	CON	MIN	HOUR	DAY	WEEK	UNIGENE	FLCDNA	TAIR
GTTGAGGCGG	0	1	0	0	0	gnl|UG|At#S11729446	no match found	multiple non-canonical match

at2g43060	CON	MIN	HOUR	DAY	WEEK	UNIGENE	FLCDNA	TAIR
CAGTGTCTCG	2	8	0	0	0	gnl|UG|At#S11709134	gi|20259861|gb|AY093279.1	At2g43060.1
at4g16830	CON	MIN	HOUR	DAY	WEEK	UNIGENE	FLCDNA	TAIR
ATAACGTCCA	1	2	0	0	0	no match found	gi|20465928|gb|AY096500.1	non-canonical match
TTTTGAGACG	7	7	4	6	1	gnl|UG|At#S38433486	gi|21403538|gb|AY084828.1	At4g16830.1
at5g10180	CON	MIN	HOUR	DAY	WEEK	UNIGENE	FLCDNA	TAIR
CAAACTATAA	4	3	3	1	0	gnl|UG|At#S11723692	gi|17064939|gb|AY062546.1	At5g10180.1
at5g12000	CON	MIN	HOUR	DAY	WEEK	UNIGENE	FLCDNA	TAIR
AAACATTGTC	0	1	0	0	0	no match found	no match found	At5g12000.1
at3g03190	CON	MIN	HOUR	DAY	WEEK	UNIGENE	FLCDNA	TAIR
GAATTGGCTG	1	0	0	0	0	gnl|UG|At#S11739732	gi|20466619|gb|AY099776.1	At3g03190.1
at3g07690	CON	MIN	HOUR	DAY	WEEK	UNIGENE	FLCDNA	TAIR
GCTTCTTGGT	0	1	0	0	0	gnl|UG|At#S11738435	no match found	At3g07690.1
at3g28850	CON	MIN	HOUR	DAY	WEEK	UNIGENE	FLCDNA	TAIR
TCCTGATTGT	1	0	1	0	0	gnl|UG|At#S11732021	no match found	At3g28850.1
at3g52260	CON	MIN	HOUR	DAY	WEEK	UNIGENE	FLCDNA	TAIR
CTTGTTCATC	1	0	0	1	0	gnl|UG|At#S18902471	gi|17978939|gb|AY069882.1	At3g52260.1
at5g37380	CON	MIN	HOUR	DAY	WEEK	UNIGENE	FLCDNA	TAIR
GCTCTTAAGT	1	2	6	1	0	gnl|UG|At#S15460123	gi|15450366|gb|AY052284.1	At5g37380.1
GAACCAAAGA	0	1	0	0	0	no match found	gi|27363369|gb|BT002688.1	multiple non-canonical match
at5g15680	CON	MIN	HOUR	DAY	WEEK	UNIGENE	FLCDNA	TAIR
TAAGAAGGAA	4	4	2	0	0	gnl|UG|At#S11722417	gi|26451355|dbj|AK118154.1|	non-canonical match
at5g19840	CON	MIN	HOUR	DAY	WEEK	UNIGENE	FLCDNA	TAIR
AAAGTCTTGT	0	0	3	0	1	gnl|UG|At#S11824580	gi|26449545|dbj|AK117223.1|	At5g19840.1
at5g64580	CON	MIN	HOUR	DAY	WEEK	UNIGENE	FLCDNA	TAIR
TAAAACATAG	0	1	1	0	4	gnl|UG|At#S11717241	gi|19347915|gb|AY080633.1	At5g64580.1
at4g36690	CON	MIN	HOUR	DAY	WEEK	UNIGENE	FLCDNA	TAIR
AGACAGGAAG	0	3	4	3	0	gnl|UG|At#S11744082	gi|24030413|gb|BT000965.1	At4g36690.3
at3g59070	CON	MIN	HOUR	DAY	WEEK	UNIGENE	FLCDNA	TAIR
AGTTATAAAT	1	0	0	0	0	no match found	no match found	At3g59070.1
at4g09640	CON	MIN	HOUR	DAY	WEEK	UNIGENE	FLCDNA	TAIR
TGTCACTTGT	0	0	1	0	0	gnl|UG|At#S14829917	gi|110736746|dbj|AK228399.1|	At4g09640.1
at3g50010	CON	MIN	HOUR	DAY	WEEK	UNIGENE	FLCDNA	TAIR
TTTACTTACT	0	0	0	0	1	no match found	no match found	At3g50010.1
at5g49460	CON	MIN	HOUR	DAY	WEEK	UNIGENE	FLCDNA	TAIR
GGAAGATGTG	2	0	1	0	0	no match found	gi|62318794|dbj|AK220717.1|	non-canonical match
TCTATATCTT	0	1	0	0	0	gnl|UG|At#S11718772	no match found	At5g49460.1
TATGTTTGTT	8	2	2	0	1	no match found	gi|14334787|gb|AY035067.1	non-canonical match
at4g23130	CON	MIN	HOUR	DAY	WEEK	UNIGENE	FLCDNA	TAIR
AACAAGCAGG	0	0	1	0	0	gnl|UG|At#S11702688	gi|23296341|gb|AY142496.1	At4g23130.2
at3g22630	CON	MIN	HOUR	DAY	WEEK	UNIGENE	FLCDNA	TAIR
TCTGTAGAAG	0	1	0	0	2	gnl|UG|At#S11734027	gi|15529249|gb|AY052249.1	At3g22630.1

at1g07840	CON	MIN	HOUR	DAY	WEEK	UNIGENE	FLCDNA	TAIR
CTCTTGCAGT	0	1	0	0	2	gnl|UG|At#S18942621	gi|15810572|gb|AY056325.1	At1g07840.1
AACTAACCGA	0	1	0	0	0	no match found	gi|20259572|gb|AY091190.1	non-canonical match

at4g14500	CON	MIN	HOUR	DAY	WEEK	UNIGENE	FLCDNA	TAIR
GATTCTGCAG	1	0	0	0	0	no match found	gi|31376366|gb|BT008748.1	multiple non-canonical match
TAGAGTAGTA	5	2	1	1	0	gnl|UG|At#S11815971	gi|22531041|gb|AY136359.1	At4g14500.1

at5g52570	CON	MIN	HOUR	DAY	WEEK	UNIGENE	FLCDNA	TAIR
TTCGTTCACG	1	0	0	0	0	gnl|UG|At#S11718459	gi|18389293|gb|AY074394.1	non-canonical match

at4g04950	CON	MIN	HOUR	DAY	WEEK	UNIGENE	FLCDNA	TAIR
AAAGGTTCAC	1	3	1	1	1	gnl|UG|At#S11726396	gi|16648848|gb|AY058202.1	At4g04950.1

at3g54920	CON	MIN	HOUR	DAY	WEEK	UNIGENE	FLCDNA	TAIR
TAGTGAATAA	1	1	2	0	1	gnl|UG|At#S11728528	gi|18377754|gb|AY074331.1	At3g54920.1

at5g53900	CON	MIN	HOUR	DAY	WEEK	UNIGENE	FLCDNA	TAIR
AAACTCTCTG	2	0	0	1	0	gnl|UG|At#S11743433	gi|21387110|gb|AY114640.1	At5g53900.2

at5g17350	CON	MIN	HOUR	DAY	WEEK	UNIGENE	FLCDNA	TAIR
TTTTGCTATT	0	1	0	0	0	no match found	gi|110737523|dbj|AK228808.1|	non-canonical match

at1g21760	CON	MIN	HOUR	DAY	WEEK	UNIGENE	FLCDNA	TAIR
ACTTTACCGT	0	1	0	0	1	no match found	no match found	At1g21760.1
AACTTTAAGG	0	3	0	1	0	gnl|UG|At#S11740885	gi|21403410|gb|AY084700.1	non-canonical match

at4g35680	CON	MIN	HOUR	DAY	WEEK	UNIGENE	FLCDNA	TAIR
AGGAGGATGG	0	0	1	0	0	gnl|UG|At#S11721651	gi|110736821|dbj|AK228440.1|	At4g35680.1

at3g10670	CON	MIN	HOUR	DAY	WEEK	UNIGENE	FLCDNA	TAIR
ACAAACTACT	2	3	3	3	1	gnl|UG|At#S11737688	gi|21403720|gb|AY085010.1	At3g10670.1

at2g01610	CON	MIN	HOUR	DAY	WEEK	UNIGENE	FLCDNA	TAIR
TTTTACATTT	0	1	1	0	1	gnl|UG|At#S11742643	gi|51969329|dbj|AK175594.1|	multiple non-canonical match

at4g34640	CON	MIN	HOUR	DAY	WEEK	UNIGENE	FLCDNA	TAIR
GATTCTATTT	2	5	5	3	1	gnl|UG|At#S11699155	gi|20466803|gb|AY099868.1	At4g34640.1

at1g08180	CON	MIN	HOUR	DAY	WEEK	UNIGENE	FLCDNA	TAIR
TCCGTAAGAA	0	0	0	0	1	no match found	gi|28466808|gb|BT004747.1	non-canonical match

at2g45590	CON	MIN	HOUR	DAY	WEEK	UNIGENE	FLCDNA	TAIR
ATTATAGTTT	1	2	0	0	0	gnl|UG|At#S11731281	no match found	At2g45590.1
GGCTAAGCAA	0	1	0	1	0	no match found	gi|27754325|gb|BT002788.1	non-canonical match

at3g52890	CON	MIN	HOUR	DAY	WEEK	UNIGENE	FLCDNA	TAIR
GCGCTGCAGT	1	2	1	1	0	gnl|UG|At#S11728893	gi|25054837|gb|BT001910.1	At3g52890.1
CATTTGTTGA	0	2	0	0	0	no match found	gi|18175716|gb|AY072093.1	non-canonical match

at3g23100	CON	MIN	HOUR	DAY	WEEK	UNIGENE	FLCDNA	TAIR
ATTGTTCATC	1	0	0	0	0	gnl|UG|At#S35293086	no match found	At1g68230.1

at3g21350	CON	MIN	HOUR	DAY	WEEK	UNIGENE	FLCDNA	TAIR
ATTACTTCTC	2	3	4	3	0	no match found	gi|17979204|gb|AY070104.1	At3g21350.1

at4g31450	CON	MIN	HOUR	DAY	WEEK	UNIGENE	FLCDNA	TAIR
TGAAGTGTGT	2	0	1	1	0	no match found	gi|110741107|dbj|AK226506.1|	non-canonical match
ATCTTTTAAT	0	0	1	0	2	gnl|UG|At#S11722374	gi|21406955|gb|AY088181.1	At4g31450.1

at1g67750	CON	MIN	HOUR	DAY	WEEK	UNIGENE	FLCDNA	TAIR
TTGTCCCTGG	0	1	1	0	0	gnl|UG|At#S11729559	gi|18087516|gb|AF462797.1	At1g67750.1
TCTTTAAAGA	0	0	0	0	1	no match found	gi|20856265|gb|AY101535.1	At3g11560.3

at1g53040	CON	MIN	HOUR	DAY	WEEK	UNIGENE	FLCDNA	TAIR
TGATAGAGAG	1	1	0	0	0	gnl|UG|At#S11733345	gi|110738522|dbj|AK229324.1|	At1g53040.1

at3g55605	CON	MIN	HOUR	DAY	WEEK	UNIGENE	FLCDNA	TAIR
ATGAAGAAAG	1	0	1	1	0	gnl|UG|At#S34114220	gi|21405127|gb|AY086417.1	At3g55605.1

at1g73970	CON	MIN	HOUR	DAY	WEEK	UNIGENE	FLCDNA	TAIR
GAATTATGTT	2	0	0	0	1	gnl|UG|At#S14829903	gi|28973646|gb|BT005731.1	At1g73970.1

at2g37070	CON	MIN	HOUR	DAY	WEEK	UNIGENE	FLCDNA	TAIR
TATAGCTTTG	0	0	0	1	0	gnl|UG|At#S11733334	no match found	At2g37070.1

at5g44910	CON	MIN	HOUR	DAY	WEEK	UNIGENE	FLCDNA	TAIR
AGAGACTTGA	1	0	0	0	0	no match found	no match found	At5g44910.1

at1g28330	CON	MIN	HOUR	DAY	WEEK	UNIGENE	FLCDNA	TAIR
CGGGTGATGT	2	39	1	0	0	gnl|UG|At#S15460294	gi|14335095|gb|AY037227.1	At1g28330.3

at1g57760	CON	MIN	HOUR	DAY	WEEK	UNIGENE	FLCDNA	TAIR
TTCTTTGATT	1	1	0	2	0	no match found	no match found	At1g57760.1

at3g27230	CON	MIN	HOUR	DAY	WEEK	UNIGENE	FLCDNA	TAIR
CTTCTAGCGG	0	1	0	1	0	no match found	gi|21405247|gb|AY086537.1	At3g27230.1

at1g10190	CON	MIN	HOUR	DAY	WEEK	UNIGENE	FLCDNA	TAIR
TTACAATAAT	1	0	0	0	0	gnl|UG|At#S11743558	gi|21689892|gb|AY122974.1	At1g10190.1

at3g27700	CON	MIN	HOUR	DAY	WEEK	UNIGENE	FLCDNA	TAIR
AAACACAGAA	1	0	0	1	0	gnl|UG|At#S15460480	gi|23397274|gb|BT000780.1	At3g27700.1

at1g78460	CON	MIN	HOUR	DAY	WEEK	UNIGENE	FLCDNA	TAIR
TTAATGACGC	3	15	1	1	0	gnl|UG|At#S11705258	gi|16323114|gb|AY057661.1	At1g78460.1

at2g26740	CON	MIN	HOUR	DAY	WEEK	UNIGENE	FLCDNA	TAIR
TGATTGTTTA	9	5	3	1	0	gnl|UG|At#S11735847	no match found	At2g26740.1
TATGGTTGTA	4	10	2	1	0	no match found	gi|11935192|gb|AF327422.1	non-canonical match

at1g02840	CON	MIN	HOUR	DAY	WEEK	UNIGENE	FLCDNA	TAIR
AAGTATGCGC	0	4	0	2	0	no match found	gi|21404630|gb|AY085920.1	At1g02840.3
TTTGGATTTT	1	2	7	0	0	gnl|UG|At#S15461636	no match found	multiple canonical match

at2g14095	CON	MIN	HOUR	DAY	WEEK	UNIGENE	FLCDNA	TAIR
AGTCGCCTTC	0	0	0	1	0	gnl|UG|At#S11738932	gi|21407942|gb|AY089168.1	At2g14095.1

at5g35890	CON	MIN	HOUR	DAY	WEEK	UNIGENE	FLCDNA	TAIR
ATTATCAAAA	0	0	0	0	1	gnl|UG|At#S35283251	no match found	non-canonical match
TGTTTTAACA	2	0	1	0	1	no match found	no match found	At5g35890.1

at2g25110	CON	MIN	HOUR	DAY	WEEK	UNIGENE	FLCDNA	TAIR
ACAAGAAGTA	2	3	1	0	0	gnl|UG|At#S11736246	gi|16209644|gb|AY057588.1	At2g25110.1

at3g02800	CON	MIN	HOUR	DAY	WEEK	UNIGENE	FLCDNA	TAIR
CTTATGTTGC	0	1	0	0	0	gnl|UG|At#S18902322	no match found	non-canonical match

at5g56030	CON	MIN	HOUR	DAY	WEEK	UNIGENE	FLCDNA	TAIR
GTTGATGCGA	5	4	4	21	7	no match found	gi|23270384|gb|AY064070.2	non-canonical match
GATGAGTTGA	3	3	4	12	5	gnl|UG|At#S11718110	gi|27311858|gb|BT002535.1	At5g56030.1

at4g00755	CON	MIN	HOUR	DAY	WEEK	UNIGENE	FLCDNA	TAIR
GATGCAGAGG	0	2	0	0	0	no match found	gi|28393061|gb|BT003918.1	At4g00755.1

at2g35110	CON	MIN	HOUR	DAY	WEEK	UNIGENE	FLCDNA	TAIR
TCTTTATCTT	1	1	2	0	6	no match found	no match found	At2g35110.1

at5g43130	CON	MIN	HOUR	DAY	WEEK	UNIGENE	FLCDNA	TAIR
GCCATTTCAA	4	1	0	1	2	gnl|UG|At#S18912742	gi|110738777|dbj|AK229454.1|	At5g43130.1

at3g26450	CON	MIN	HOUR	DAY	WEEK	UNIGENE	FLCDNA	TAIR
GTTTCATTTA	0	1	0	0	0	gnl|UG|At#S11805477	no match found	non-canonical match
CAACTCCTCA	14	10	16	4	0	gnl|UG|At#S18919197	gi|21407420|gb|AY088646.1	At3g26450.1
TCCTATCTTT	2	0	0	0	0	gnl|UG|At#S35255674	no match found	non-canonical match

at5g15560	CON	MIN	HOUR	DAY	WEEK	UNIGENE	FLCDNA	TAIR
TCAAAATTTA	0	1	0	0	0	no match found	no match found	At5g15560.1

at5g11000	CON	MIN	HOUR	DAY	WEEK	UNIGENE	FLCDNA	TAIR
TTCAGATCTC	0	1	1	0	1	gnl|UG|At#S11723500	gi|21436002|gb|AY116945.1	At5g11000.1

at1g27730	CON	MIN	HOUR	DAY	WEEK	UNIGENE	FLCDNA	TAIR
AGCCCTATGC	1	12	7	2	1	gnl|UG|At#S11698982	gi|17104582|gb|AY063006.1	At1g27730.1

at3g07770	CON	MIN	HOUR	DAY	WEEK	UNIGENE	FLCDNA	TAIR
AAAGGGAGAA	0	0	1	1	0	gnl|UG|At#S34118081	gi|110736415|dbj|AK228225.1|	At3g07770.1

at1g18740	CON	MIN	HOUR	DAY	WEEK	UNIGENE	FLCDNA	TAIR
AAGCTGGACT	0	2	1	1	0	no match found	gi|17978920|gb|AY069871.1	pseudo chromosome match
GTCAGCTTCT	1	0	0	0	0	no match found	gi|22136561|gb|AY129481.1	non-canonical match

at3g19390	CON	MIN	HOUR	DAY	WEEK	UNIGENE	FLCDNA	TAIR
CTAGTGGGCA	1	1	0	0	0	no match found	gi|17065297|gb|AY062725.1	At3g19390.1
ATGTAAGATA	0	0	1	0	0	gnl|UG|At#S11735034	no match found	multiple non-canonical match

at2g39340	CON	MIN	HOUR	DAY	WEEK	UNIGENE	FLCDNA	TAIR
TAATGAAACC	0	1	1	0	0	no match found	no match found	At2g39340.1
ATGAAATATT	1	1	0	0	0	gnl|UG|At#S11709613	gi|18086368|gb|AY064996.1	non-canonical match

at1g06930	CON	MIN	HOUR	DAY	WEEK	UNIGENE	FLCDNA	TAIR
AGCACTTCAA	0	0	1	0	0	no match found	no match found	At1g06930.1

at2g28450	CON	MIN	HOUR	DAY	WEEK	UNIGENE	FLCDNA	TAIR
TAGCTGATCA	1	0	0	0	0	gnl|UG|At#S11735432	gi|22135821|gb|AY128289.1	At2g28450.1

at5g59080	CON	MIN	HOUR	DAY	WEEK	UNIGENE	FLCDNA	TAIR
TCATCTAAGC	1	0	2	0	0	gnl|UG|At#S11717797	gi|17380959|gb|AY063936.1	At5g59080.1

at3g03110	CON	MIN	HOUR	DAY	WEEK	UNIGENE	FLCDNA	TAIR
GCCGATTCTT	0	2	0	0	0	gnl|UG|At#S11739753	gi|14194144|gb|AF367278.1	At3g03110.1

at1g58460	CON	MIN	HOUR	DAY	WEEK	UNIGENE	FLCDNA	TAIR
TTATTTTGAT	0	0	1	0	0	no match found	no match found	At1g58460.1

at2g34960	CON	MIN	HOUR	DAY	WEEK	UNIGENE	FLCDNA	TAIR
AACTGCTTCG	1	0	0	1	0	gnl|UG|At#S11733829	gi|19699364|gb|AY090448.1	At2g34960.1

at1g60930	CON	MIN	HOUR	DAY	WEEK	UNIGENE	FLCDNA	TAIR
CAATTCAAAT	0	1	1	1	0	gnl|UG|At#S11699947	no match found	pseudo chromosome match

at4g19660	CON	MIN	HOUR	DAY	WEEK	UNIGENE	FLCDNA	TAIR

GTTTAGGTTT	1	2	2	2	0	no match found	no match found	At4g19660.1
at5g50180	CON	MIN	HOUR	DAY	WEEK	UNIGENE	FLCDNA	TAIR
TTCCCTAAAT	0	0	0	0	1	gnl|UG|At#S11718698	no match found	At5g50180.1
at4g34280	CON	MIN	HOUR	DAY	WEEK	UNIGENE	FLCDNA	TAIR
TATTGTTGTA	1	0	0	0	0	gnl|UG|At#S11721886	gi|26449712|dbj|AK117309.1|	At4g34280.1
at4g10750	CON	MIN	HOUR	DAY	WEEK	UNIGENE	FLCDNA	TAIR
AATCCAACAA	1	2	1	0	3	gnl|UG|At#S11725891	gi|26451477|dbj|AK118216.1|	At4g10750.1
ATAAAGATGT	1	0	0	0	0	no match found	gi|29824140|gb|BT006046.1	non-canonical match
at1g54610	CON	MIN	HOUR	DAY	WEEK	UNIGENE	FLCDNA	TAIR
TATTTGTAAC	1	0	2	2	6	gnl|UG|At#S43850440	gi|13877618|gb|AF370510.1	At1g54610.1
at2g40930	CON	MIN	HOUR	DAY	WEEK	UNIGENE	FLCDNA	TAIR
ACTTAGATCT	0	1	0	0	0	gnl|UG|At#S11702616	gi|23198183|gb|BT000300.1	At2g40930.1
at2g38240	CON	MIN	HOUR	DAY	WEEK	UNIGENE	FLCDNA	TAIR
GCTCCTTCGC	1	1	1	2	0	no match found	no match found	At2g38240.1
CAGGCTTTAG	0	0	0	1	0	gnl|UG|At#S11733035	gi|17065133|gb|AY062643.1	non-canonical match
at4g10180	CON	MIN	HOUR	DAY	WEEK	UNIGENE	FLCDNA	TAIR
TTGTAATCTA	0	0	1	0	2	gnl|UG|At#S11725947	no match found	At4g10180.1
at2g25060	CON	MIN	HOUR	DAY	WEEK	UNIGENE	FLCDNA	TAIR
TTGTGTTGTT	3	3	2	1	1	gnl|UG|At#S11736255	gi|51969611|dbj|AK175735.1|	At2g25060.1
at1g09815	CON	MIN	HOUR	DAY	WEEK	UNIGENE	FLCDNA	TAIR
TAGTGTCCTC	0	1	1	2	1	gnl|UG|At#S35319594	gi|21407207|gb|AY088433.1	At1g09815.1
at3g01910	CON	MIN	HOUR	DAY	WEEK	UNIGENE	FLCDNA	TAIR
TGGTGGATTA	3	1	4	2	2	gnl|UG|At#S11817386	gi|13430669|gb|AF360247.1	At3g01910.1
GGTGTTGTTT	0	2	2	1	0	no match found	gi|22136905|gb|AY133863.1	non-canonical match
at5g57270	CON	MIN	HOUR	DAY	WEEK	UNIGENE	FLCDNA	TAIR
GTACTCTACT	1	0	1	0	0	gnl|UG|At#S18909565	gi|20260013|gb|AY093355.1	At5g57270.1
at5g45250	CON	MIN	HOUR	DAY	WEEK	UNIGENE	FLCDNA	TAIR
TAAAAAGTAC	1	0	0	0	0	gnl|UG|At#S11811127	no match found	pseudo chromosome match
CACACGGTTA	1	1	1	1	0	gnl|UG|At#S11719202	no match found	non-canonical match
at2g42710	CON	MIN	HOUR	DAY	WEEK	UNIGENE	FLCDNA	TAIR
TGCCACTTGG	1	0	0	0	0	gnl|UG|At#S18900509	gi|26451658|dbj|AK118309.1|	At2g42710.1
at5g24300	CON	MIN	HOUR	DAY	WEEK	UNIGENE	FLCDNA	TAIR
TGTGTCTTAC	6	2	2	4	2	gnl|UG|At#S11720748	gi|110742055|dbj|AK226881.1|	At5g24300.1
at2g44430	CON	MIN	HOUR	DAY	WEEK	UNIGENE	FLCDNA	TAIR
TTTGCCCCTT	0	1	0	0	1	gnl|UG|At#S11731538	gi|21539424|gb|AY120707.1	At2g44430.1
at3g44010	CON	MIN	HOUR	DAY	WEEK	UNIGENE	FLCDNA	TAIR
TCTTATTTTG	26	13	19	16	39	gnl|UG|At#S35307634	gi|27754281|gb|BT002766.1	At3g44010.1
AAGGCGTCGA	3	1	3	1	6	no match found	gi|72198261|gb|DQ108844.1	non-canonical match
at4g31120	CON	MIN	HOUR	DAY	WEEK	UNIGENE	FLCDNA	TAIR
TTCAGCTGGT	0	0	0	1	0	gnl|UG|At#S11722426	gi|26452315|dbj|AK118648.1|	At4g31120.1
at3g23580	CON	MIN	HOUR	DAY	WEEK	UNIGENE	FLCDNA	TAIR
AGTCAATACA	1	0	1	0	1	gnl|UG|At#S11733732	gi|23505944|gb|AY143893.1	At3g23580.1

at2g07751	CON	MIN	HOUR	DAY	WEEK	UNIGENE	FLCDNA	TAIR
ATATATTTGC	1	0	1	0	0	no match found	no match found	At2g07751.1
ATGGCCTTTT	1	0	0	0	0	gnl|UG|At#S11817310	no match found	multiple canonical match

at5g05440	CON	MIN	HOUR	DAY	WEEK	UNIGENE	FLCDNA	TAIR
GTGGTCTCTG	0	13	2	0	0	no match found	gi|16974394|gb|AY060510.1	non-canonical match
GTATCATTCT	0	1	1	0	0	gnl|UG|At#S11706542	gi|15529253|gb|AY052251.1	At5g05440.1

at5g66490	CON	MIN	HOUR	DAY	WEEK	UNIGENE	FLCDNA	TAIR
TAATTTAAGT	1	2	2	2	1	no match found	no match found	At5g66490.1
GTCAATCCCC	1	2	0	1	0	no match found	gi|26450202|dbj|AK117559.1|	non-canonical match

at2g27430	CON	MIN	HOUR	DAY	WEEK	UNIGENE	FLCDNA	TAIR
GAGATGATAA	0	0	1	0	0	gnl|UG|At#S11825315	no match found	At2g27430.1

at2g32390	CON	MIN	HOUR	DAY	WEEK	UNIGENE	FLCDNA	TAIR
TCGACAGCGA	0	1	0	1	0	gnl|UG|At#S11734438	no match found	At2g32390.1

at3g62990	CON	MIN	HOUR	DAY	WEEK	UNIGENE	FLCDNA	TAIR
AACGTGGACA	0	1	1	0	0	gnl|UG|At#S18904407	no match found	At2g32390.1

at1g65270	CON	MIN	HOUR	DAY	WEEK	UNIGENE	FLCDNA	TAIR
ACATATCAAG	3	1	0	2	5	gnl|UG|At#S18942313	gi|21407240|gb|AY088466.1	At1g65270.3
GCTGAAGAAC	0	0	2	0	0	no match found	gi|27311648|gb|BT002430.1	multiple non-canonical match

at3g45590	CON	MIN	HOUR	DAY	WEEK	UNIGENE	FLCDNA	TAIR
TTAACAAGTC	0	0	1	0	0	gnl|UG|At#S11699823	gi|62320331|dbj|AK221492.1|	non-canonical match
CTCTGGTCGT	0	0	1	0	0	no match found	no match found	At3g45590.1

at2g05635	CON	MIN	HOUR	DAY	WEEK	UNIGENE	FLCDNA	TAIR
ATTTGTTGGT	0	1	0	1	0	no match found	no match found	At2g05635.1

at3g09810	CON	MIN	HOUR	DAY	WEEK	UNIGENE	FLCDNA	TAIR
GGATAATCTT	1	0	0	0	0	gnl|UG|At#S15461353	gi|51970679|dbj|AK176269.1|	At3g09810.1

at3g49780	CON	MIN	HOUR	DAY	WEEK	UNIGENE	FLCDNA	TAIR
GCTCTCCTTC	1	1	0	0	0	no match found	gi|21406780|gb|AY088006.1	At3g49780.1
TATCTCAACA	0	3	1	0	1	gnl|UG|At#S11822180	gi|25082735|gb|BT001985.1	pseudo chromosome match

at4g19390	CON	MIN	HOUR	DAY	WEEK	UNIGENE	FLCDNA	TAIR
TAATAGTGAT	0	1	0	0	0	gnl|UG|At#S11724433	gi|14190356|gb|AF378856.1	At4g19390.1

at2g40060	CON	MIN	HOUR	DAY	WEEK	UNIGENE	FLCDNA	TAIR
TGTGTTATTA	8	4	6	3	2	gnl|UG|At#S11732585	gi|20260191|gb|AY092995.1	At2g40060.1

at4g16590	CON	MIN	HOUR	DAY	WEEK	UNIGENE	FLCDNA	TAIR
TTGTATAAGT	0	0	0	0	1	gnl|UG|At#S11724928	gi|62321079|dbj|AK221869.1|	multiple canonical match

at4g23890	CON	MIN	HOUR	DAY	WEEK	UNIGENE	FLCDNA	TAIR
TATTGTGGAA	3	2	1	1	0	no match found	gi|23197587|gb|BT000002.1	multiple non-canonical match
TGATATAAAA	7	17	1	7	3	gnl|UG|At#S11723666	gi|22135939|gb|AY128349.1	At4g23890.1

at1g52540	CON	MIN	HOUR	DAY	WEEK	UNIGENE	FLCDNA	TAIR
TGTGCTCAGA	0	1	0	1	1	gnl|UG|At#S11733535	gi|21404806|gb|AY086096.1	At1g52540.1

at5g26110	CON	MIN	HOUR	DAY	WEEK	UNIGENE	FLCDNA	TAIR
CGGGAATGTG	0	0	1	0	0	gnl|UG|At#S11720580	gi|21407819|gb|AY089045.1	At5g26110.1

at1g73250	CON	MIN	HOUR	DAY	WEEK	UNIGENE	FLCDNA	TAIR
TTAACATTGG	0	0	1	0	0	gnl|UG|At#S11728410	gi|119360016|gb|BT029480.1	At1g73250.1

at2g41760	CON	MIN	HOUR	DAY	WEEK	UNIGENE	FLCDNA	TAIR
TTTTCAGTTG	0	1	0	2	0	no match found	gi|14335091|gb|AY037225.1	multiple non-canonical match
ATTGTTCTTG	0	1	0	0	0	no match found	no match found	At2g41760.1
at3g13190	CON	MIN	HOUR	DAY	WEEK	UNIGENE	FLCDNA	TAIR
ATTAATGATG	1	1	0	0	0	gnl|UG|At#S24442513	gi|23197915|gb|BT000166.1	multiple canonical match
at5g53300	CON	MIN	HOUR	DAY	WEEK	UNIGENE	FLCDNA	TAIR
GGCTAAATGG	42	86	51	49	17	gnl|UG|At#S11675360	gi|21280892|gb|AY113937.1	At5g53300.1
at2g45540	CON	MIN	HOUR	DAY	WEEK	UNIGENE	FLCDNA	TAIR
TTTGTTCGTC	0	1	0	0	0	gnl|UG|At#S11731293	gi|110741668|dbj|AK226673.1|	At2g45540.1
at3g05160	CON	MIN	HOUR	DAY	WEEK	UNIGENE	FLCDNA	TAIR
TTTCAGAAGA	0	3	0	0	0	no match found	gi|16604315|gb|AY058056.1	multiple canonical match
at2g37100	CON	MIN	HOUR	DAY	WEEK	UNIGENE	FLCDNA	TAIR
ATCCAAAAAA	0	1	1	3	0	no match found	gi|72198358|gb|DQ108857.1	multiple non-canonical match
at2g14520	CON	MIN	HOUR	DAY	WEEK	UNIGENE	FLCDNA	TAIR
TAATAACACT	0	1	0	0	1	no match found	gi|19423999|gb|AY080819.1	multiple canonical match
at3g05800	CON	MIN	HOUR	DAY	WEEK	UNIGENE	FLCDNA	TAIR
ACAGCTCTAT	0	1	7	1	1	gnl|UG|At#S21735822	gi|51970371|dbj|AK176115.1|	At3g05800.1
at2g05810	CON	MIN	HOUR	DAY	WEEK	UNIGENE	FLCDNA	TAIR
TATTTGTGTG	10	3	3	0	4	gnl|UG|At#S11739977	no match found	multiple canonical match
GAGGAGTGGT	0	0	0	2	0	no match found	gi|62321007|dbj|AK221832.1|	multiple canonical match
at4g37240	CON	MIN	HOUR	DAY	WEEK	UNIGENE	FLCDNA	TAIR
AGAGGTGGTG	0	1	0	0	0	gnl|UG|At#S11721397	gi|21404320|gb|AY085610.1	At4g37240.1
at2g17410	CON	MIN	HOUR	DAY	WEEK	UNIGENE	FLCDNA	TAIR
AGAAGAGCCT	2	2	0	1	1	gnl|UG|At#S21736293	gi|110740375|dbj|AK230281.1|	At2g17410.1
at3g01590	CON	MIN	HOUR	DAY	WEEK	UNIGENE	FLCDNA	TAIR
ATCTCATTAT	0	1	0	0	0	no match found	gi|15810276|gb|AY056177.1	At3g01590.2
at5g63470	CON	MIN	HOUR	DAY	WEEK	UNIGENE	FLCDNA	TAIR
GCTTGTAGTA	0	1	0	0	1	gnl|UG|At#S11717353	no match found	At3g01590.2
GTAATCTCGA	3	5	0	0	0	no match found	gi|23198019|gb|BT000218.1	At5g63470.1
at3g28917	CON	MIN	HOUR	DAY	WEEK	UNIGENE	FLCDNA	TAIR
TTATTGTATT	1	0	0	0	0	no match found	gi|21405595|gb|AY086885.1	At3g28917.1
at4g35040	CON	MIN	HOUR	DAY	WEEK	UNIGENE	FLCDNA	TAIR
GCTAATCAGA	0	0	0	1	1	gnl|UG|At#S11820870	gi|18252902|gb|AY072386.1	At4g35040.1
at1g14430	CON	MIN	HOUR	DAY	WEEK	UNIGENE	FLCDNA	TAIR
TTTGTTATTT	0	0	0	1	0	gnl|UG|At#S11741593	no match found	At1g14430.1
at1g58270	CON	MIN	HOUR	DAY	WEEK	UNIGENE	FLCDNA	TAIR
GCCCAATGCA	0	0	1	2	0	gnl|UG|At#S11731793	gi|16930452|gb|AF419580.1	At1g58270.1
at3g25880	CON	MIN	HOUR	DAY	WEEK	UNIGENE	FLCDNA	TAIR
TATACTATGA	0	1	0	0	0	no match found	gi|45680185|gb|BT011875.1	multiple non-canonical match
at1g42550	CON	MIN	HOUR	DAY	WEEK	UNIGENE	FLCDNA	TAIR
GTGGATGATG	2	7	3	4	0	gnl|UG|At#S11736287	gi|18252230|gb|AY072341.1	At1g42550.1
at2g03730	CON	MIN	HOUR	DAY	WEEK	UNIGENE	FLCDNA	TAIR

CTAGCATTGA	2	1	0	1	0	gnl|UG|At#S28282714	gi|15292816|gb|AY050842.1	At2g03730.1
at2g13290	CON	MIN	HOUR	DAY	WEEK	UNIGENE	FLCDNA	TAIR
CTTCCTGAAA	0	1	0	0	0	no match found	gi|28393637|gb|BT004221.1	non-canonical match
at1g54170	CON	MIN	HOUR	DAY	WEEK	UNIGENE	FLCDNA	TAIR
AATGGTGGTG	1	2	0	2	0	gnl|UG|At#S11732936	gi|22531119|gb|AY136398.1	At1g54170.1
at3g55740	CON	MIN	HOUR	DAY	WEEK	UNIGENE	FLCDNA	TAIR
TTTTTGCTGA	1	3	0	0	1	gnl|UG|At#S11728377	gi|19698890|gb|AY081292.1	multiple canonical match
at1g71260	CON	MIN	HOUR	DAY	WEEK	UNIGENE	FLCDNA	TAIR
GGTCTTCTTG	0	2	6	0	2	gnl|UG|At#S11728821	gi|110740243|dbj|AK230212.1|	At1g71260.1
at2g45290	CON	MIN	HOUR	DAY	WEEK	UNIGENE	FLCDNA	TAIR
CCCAATATTA	0	1	0	0	0	gnl|UG|At#S11731348	gi|110741476|dbj|AK226566.1|	At2g45290.1
at1g62660	CON	MIN	HOUR	DAY	WEEK	UNIGENE	FLCDNA	TAIR
TGAGTGAAGA	5	3	5	1	2	gnl|UG|At#S20756761	gi|17064809|gb|AY062481.1	multiple non-canonical match
TATTACCTCA	0	0	1	0	0	no match found	gi|23308216|gb|BT000509.1	non-canonical match
at5g04240	CON	MIN	HOUR	DAY	WEEK	UNIGENE	FLCDNA	TAIR
TAACGATTGG	0	0	2	0	0	gnl|UG|At#S11725086	gi|62321458|dbj|AK222064.1|	pseudo chromosome match
at1g50920	CON	MIN	HOUR	DAY	WEEK	UNIGENE	FLCDNA	TAIR
AAGTTGTGCC	4	6	1	1	1	gnl|UG|At#S11734179	gi|20465635|gb|AY096652.1	At1g50920.1
at4g03030	CON	MIN	HOUR	DAY	WEEK	UNIGENE	FLCDNA	TAIR
TTGTGTGAGC	3	5	2	2	0	gnl|UG|At#S11726927	no match found	At4g03030.1
at5g20300	CON	MIN	HOUR	DAY	WEEK	UNIGENE	FLCDNA	TAIR
AACAATAGGA	0	1	0	1	1	gnl|UG|At#S28281775	gi|19423980|gb|AY080845.1	At5g20300.1
at3g16250	CON	MIN	HOUR	DAY	WEEK	UNIGENE	FLCDNA	TAIR
GATCCACCGG	0	0	0	1	0	no match found	no match found	At3g16250.1
AGTGGAACAT	3	5	7	1	2	gnl|UG|At#S11735973	gi|14596158|gb|AY042867.1	non-canonical match
at1g78200	CON	MIN	HOUR	DAY	WEEK	UNIGENE	FLCDNA	TAIR
AAAAGAATAG	1	1	0	2	0	gnl|UG|At#S18942238	gi|15081702|gb|AY048244.1	At1g78200.1
at1g63390	CON	MIN	HOUR	DAY	WEEK	UNIGENE	FLCDNA	TAIR
GTTGCTCCGG	0	0	0	1	0	gnl|UG|At#S11730460	no match found	At1g63390.1
at3g10420	CON	MIN	HOUR	DAY	WEEK	UNIGENE	FLCDNA	TAIR
TCTGACTTCA	0	4	1	2	2	gnl|UG|At#S34115953	gi|110740512|dbj|AK226197.1|	At3g10420.1
at1g69440	CON	MIN	HOUR	DAY	WEEK	UNIGENE	FLCDNA	TAIR
TTTTACTGCT	0	1	1	0	0	gnl|UG|At#S11729220	no match found	At1g69440.1
at1g20760	CON	MIN	HOUR	DAY	WEEK	UNIGENE	FLCDNA	TAIR
GATACTCGTT	0	2	1	0	1	gnl|UG|At#S11740981	gi|110742186|dbj|AK226951.1|	At1g20760.1
at1g70460	CON	MIN	HOUR	DAY	WEEK	UNIGENE	FLCDNA	TAIR
CTTGAGCTCT	0	1	0	0	0	no match found	no match found	At1g70460.1
at4g06642	CON	MIN	HOUR	DAY	WEEK	UNIGENE	FLCDNA	TAIR
GAACAGAAGA	0	0	1	0	0	no match found	no match found	At4g06642.1
at1g06150	CON	MIN	HOUR	DAY	WEEK	UNIGENE	FLCDNA	TAIR
AACACTTCTC	1	0	1	0	4	gnl|UG|At#S11742412	no match found	non-canonical match

at2g31400	CON	MIN	HOUR	DAY	WEEK	UNIGENE	FLCDNA	TAIR
ACCACATAAC	1	5	1	2	3	gnl|UG|At#S11734685	no match found	At2g31400.1
at5g01010	CON	MIN	HOUR	DAY	WEEK	UNIGENE	FLCDNA	TAIR
AACACTCATC	0	1	4	0	1	gnl|UG|At#S21736617	gi|51970049|dbj|AK175954.1|	At5g01010.1
at5g52870	CON	MIN	HOUR	DAY	WEEK	UNIGENE	FLCDNA	TAIR
GCTCGAGAGA	0	3	1	1	0	gnl|UG|At#S11704706	gi|20258914|gb|AY091212.1	At5g52870.1
at2g46080	CON	MIN	HOUR	DAY	WEEK	UNIGENE	FLCDNA	TAIR
TTGTCTTCCG	0	3	2	1	0	gnl|UG|At#S11731170	gi|18252158|gb|AY072305.1	At2g46080.1
at1g64350	CON	MIN	HOUR	DAY	WEEK	UNIGENE	FLCDNA	TAIR
TAGTCTTTGT	1	1	1	0	5	gnl|UG|At#S11702480	gi|21406226|gb|AY087489.1	At1g64350.1
at4g35580	CON	MIN	HOUR	DAY	WEEK	UNIGENE	FLCDNA	TAIR
TTTGTGTGTT	2	2	1	0	1	gnl|UG|At#S43849424	gi|15912330|gb|AY056443.1	At4g35580.1
at3g62660	CON	MIN	HOUR	DAY	WEEK	UNIGENE	FLCDNA	TAIR
GGCTTGGTGG	3	6	4	3	0	gnl|UG|At#S11726645	gi|23397212|gb|BT000748.1	At3g62660.1
at1g49320	CON	MIN	HOUR	DAY	WEEK	UNIGENE	FLCDNA	TAIR
TAGCTTTTAA	1	1	1	0	1	gnl|UG|At#S11734733	gi|30102635|gb|BT006428.1	At1g49320.1
at3g01510	CON	MIN	HOUR	DAY	WEEK	UNIGENE	FLCDNA	TAIR
AAAAAATGTC	0	0	0	1	0	gnl|UG|At#S11740217	no match found	At3g01510.1
at5g08550	CON	MIN	HOUR	DAY	WEEK	UNIGENE	FLCDNA	TAIR
GTTTTTTGCA	0	0	0	2	0	no match found	no match found	At5g08550.1
ACCACGCCAG	2	0	0	2	1	gnl|UG|At#S11723973	no match found	non-canonical match
at3g26618	CON	MIN	HOUR	DAY	WEEK	UNIGENE	FLCDNA	TAIR
ATGCAGAGAC	0	1	0	1	0	gnl|UG|At#S11707606	gi|21435996|gb|AY116942.1	At3g26618.1
at5g50530	CON	MIN	HOUR	DAY	WEEK	UNIGENE	FLCDNA	TAIR
GGCAGCCGCT	0	0	1	0	0	no match found	no match found	At5g50530.1
at3g09620	CON	MIN	HOUR	DAY	WEEK	UNIGENE	FLCDNA	TAIR
AGGTTTTTGA	1	0	0	0	0	no match found	no match found	At3g09620.1
at1g21360	CON	MIN	HOUR	DAY	WEEK	UNIGENE	FLCDNA	TAIR
GAAGCGATCG	0	3	0	0	0	no match found	gi|45773919|gb|BT012277.1	At1g21360.1
at5g13590	CON	MIN	HOUR	DAY	WEEK	UNIGENE	FLCDNA	TAIR
TTTCTGAAGC	1	0	2	0	1	gnl|UG|At#S34117609	no match found	multiple non-canonical match
at5g15580	CON	MIN	HOUR	DAY	WEEK	UNIGENE	FLCDNA	TAIR
AACTGGAAGG	1	0	0	0	0	no match found	no match found	At5g15580.1
at4g35783	CON	MIN	HOUR	DAY	WEEK	UNIGENE	FLCDNA	TAIR
GTGAAGGAGA	0	0	2	0	0	gnl|UG|At#S17479695	no match found	At5g15580.1
at5g10570	CON	MIN	HOUR	DAY	WEEK	UNIGENE	FLCDNA	TAIR
GTTACTTCGG	0	1	0	0	0	gnl|UG|At#S11806570	no match found	At5g10570.1
at1g27910	CON	MIN	HOUR	DAY	WEEK	UNIGENE	FLCDNA	TAIR
TCTTTATTAC	1	1	1	0	0	gnl|UG|At#S11739663	gi|110739991|dbj|AK230080.1|	At1g27910.1
at5g43260	CON	MIN	HOUR	DAY	WEEK	UNIGENE	FLCDNA	TAIR
GACCAAAAGC	0	1	6	5	2	gnl|UG|At#S11719402	gi|14334627|gb|AY034987.1	At5g43260.1
ACTGATTTGA	1	0	1	0	0	no match found	gi|110741337|dbj|AK230421.1|	pseudo chromosome match

at1g29520	CON	MIN	HOUR	DAY	WEEK	UNIGENE	FLCDNA	TAIR
GAATAAAATC	0	0	1	0	0	gnl|UG|At#S11743131	gi|18377740|gb|AY074324.1	At1g29520.1
at1g76510	CON	MIN	HOUR	DAY	WEEK	UNIGENE	FLCDNA	TAIR
GTCGACTGTT	0	0	1	1	0	gnl|UG|At#S11727725	gi|110738614|dbj|AK229370.1|	At1g76510.1
at2g15690	CON	MIN	HOUR	DAY	WEEK	UNIGENE	FLCDNA	TAIR
AGGAATTATC	0	1	1	5	0	no match found	gi|14335135|gb|AY037247.1	multiple non-canonical match
at4g01490	CON	MIN	HOUR	DAY	WEEK	UNIGENE	FLCDNA	TAIR
CATCGACTTT	1	0	0	0	0	no match found	no match found	At4g01490.1
at1g05940	CON	MIN	HOUR	DAY	WEEK	UNIGENE	FLCDNA	TAIR
TTGGATTATC	0	1	0	2	0	gnl|UG|At#S11702156	gi|21280890|gb|AY113890.1	At1g05940.1
at1g15980	CON	MIN	HOUR	DAY	WEEK	UNIGENE	FLCDNA	TAIR
TATTGGGTTG	3	6	5	1	0	gnl|UG|At#S11741439	gi|16323197|gb|AY057703.1	At1g15980.1
at1g67700	CON	MIN	HOUR	DAY	WEEK	UNIGENE	FLCDNA	TAIR
AGAGCTGCAC	2	10	9	3	3	gnl|UG|At#S15442151	gi|15215605|gb|AY050331.1	At1g67700.2
at2g39450	CON	MIN	HOUR	DAY	WEEK	UNIGENE	FLCDNA	TAIR
CCTCTGCAAG	0	1	0	2	0	gnl|UG|At#S11815881	gi|30725627|gb|BT008477.1	At2g39450.1
at5g01340	CON	MIN	HOUR	DAY	WEEK	UNIGENE	FLCDNA	TAIR
CCATTAGAAC	0	0	0	1	1	gnl|UG|At#S11726327	gi|21406440|gb|AY087702.1	At5g01340.1
at4g26700	CON	MIN	HOUR	DAY	WEEK	UNIGENE	FLCDNA	TAIR
CTTCCAAACC	1	1	0	0	0	gnl|UG|At#S28281958	no match found	At4g26700.1
at1g69230	CON	MIN	HOUR	DAY	WEEK	UNIGENE	FLCDNA	TAIR
CGTGCAGAAG	0	1	0	0	0	gnl|UG|At#S18942296	gi|21405740|gb|AY087016.1	At1g69230.2
at2g17525	CON	MIN	HOUR	DAY	WEEK	UNIGENE	FLCDNA	TAIR
GCTGAAAGAG	0	0	3	0	0	gnl|UG|At#S11817248	no match found	At2g17525.1
at2g13410	CON	MIN	HOUR	DAY	WEEK	UNIGENE	FLCDNA	TAIR
CCAAACTTTT	1	0	1	0	0	no match found	no match found	At2g13410.1
at4g38960	CON	MIN	HOUR	DAY	WEEK	UNIGENE	FLCDNA	TAIR
AATGTAAGCT	0	0	0	1	1	no match found	no match found	At4g38960.1
at2g31960	CON	MIN	HOUR	DAY	WEEK	UNIGENE	FLCDNA	TAIR
TATATAGTTT	0	2	0	0	0	no match found	gi|26449799|dbj|AK117353.1|	non-canonical match
at2g41110	CON	MIN	HOUR	DAY	WEEK	UNIGENE	FLCDNA	TAIR
TTATTGACAG	4	4	2	1	7	gnl|UG|At#S17007813	no match found	At2g41110.1
at3g08940	CON	MIN	HOUR	DAY	WEEK	UNIGENE	FLCDNA	TAIR
TAATGATGTA	96	74	31	32	7	gnl|UG|At#S15461391	gi|17473744|gb|AY065140.1	At3g08940.2
TAATGATTAA	0	1	0	0	0	gnl|UG|At#S20845636	no match found	multiple non-canonical match
GGTACCCGGG	0	0	2	0	0	gnl|UG|At#S11611176	no match found	non-canonical match
at1g75360	CON	MIN	HOUR	DAY	WEEK	UNIGENE	FLCDNA	TAIR
ACTCATTCAT	1	1	0	0	0	no match found	no match found	At1g75360.1
at5g05740	CON	MIN	HOUR	DAY	WEEK	UNIGENE	FLCDNA	TAIR
GAGCAGTTTA	0	1	2	2	6	gnl|UG|At#S18941676	gi|18650612|gb|AY074854.1	At5g05740.1
at3g07340	CON	MIN	HOUR	DAY	WEEK	UNIGENE	FLCDNA	TAIR

TTGTTATCCG	0	1	0	0	1	gnl|UG|At#S21736012	gi|51968879|dbj|AK175369.1|	At3g07340.1
at2g31200	CON	MIN	HOUR	DAY	WEEK	UNIGENE	FLCDNA	TAIR
TTCTATGTCA	5	6	3	4	2	no match found	gi|13926244|gb|AF372880.1	non-canonical match
ATGAGAGAGT	0	0	1	0	0	gnl|UG|At#S11734741	no match found	At2g31200.1
at5g26920	CON	MIN	HOUR	DAY	WEEK	UNIGENE	FLCDNA	TAIR
ACCTCAAGCT	0	1	2	1	0	gnl|UG|At#S11720520	no match found	At5g26920.1
at3g09550	CON	MIN	HOUR	DAY	WEEK	UNIGENE	FLCDNA	TAIR
CGAATCCGTC	0	0	1	0	0	gnl|UG|At#S34115338	gi|46518452|gb|BT012564.1	At3g09550.1
at3g02040	CON	MIN	HOUR	DAY	WEEK	UNIGENE	FLCDNA	TAIR
AGGTGAAAGC	3	18	6	5	4	gnl|UG|At#S11740069	gi|28058727|gb|BT003327.1	At3g02040.1
TGGAGGAGAT	1	1	1	0	0	no match found	gi|21280828|gb|AY114073.1	non-canonical match
at1g73080	CON	MIN	HOUR	DAY	WEEK	UNIGENE	FLCDNA	TAIR
TATTCCAGTG	0	0	2	0	0	gnl|UG|At#S11824422	gi|26449860|dbj|AK117384.1|	multiple canonical match
GACTCAGATT	0	2	0	0	0	gnl|UG|At#S11824625	gi|22655011|gb|AY139779.1	At1g73080.1
at4g30790	CON	MIN	HOUR	DAY	WEEK	UNIGENE	FLCDNA	TAIR
AGATAGCAGC	0	0	0	1	0	gnl|UG|At#S11722485	no match found	At4g30790.1
at5g20160	CON	MIN	HOUR	DAY	WEEK	UNIGENE	FLCDNA	TAIR
GCTGCAGATG	0	4	1	6	2	gnl|UG|At#S15460632	gi|20334835|gb|AY094018.1	multiple canonical match
at4g28220	CON	MIN	HOUR	DAY	WEEK	UNIGENE	FLCDNA	TAIR
AAGCTGACAT	1	0	1	1	0	gnl|UG|At#S11722925	gi|21404756|gb|AY086046.1	multiple non-canonical match
GAAGAGTTAG	0	0	0	0	1	no match found	gi|95147319|gb|BT025339.1	multiple non-canonical match
at1g53840	CON	MIN	HOUR	DAY	WEEK	UNIGENE	FLCDNA	TAIR
ATCTGTAATT	4	5	8	0	3	gnl|UG|At#S11733051	gi|21406878|gb|AY088104.1	At1g53840.1
at1g05620	CON	MIN	HOUR	DAY	WEEK	UNIGENE	FLCDNA	TAIR
ATCCTGCGAC	0	1	0	1	0	gnl|UG|At#S18896974	gi|21406687|gb|AY087913.1	At1g05620.1
at2g45720	CON	MIN	HOUR	DAY	WEEK	UNIGENE	FLCDNA	TAIR
GAGCTGTTGG	2	1	0	0	1	gnl|UG|At#S28282138	gi|53850494|gb|BT015888.1	At2g45720.1
at4g02370	CON	MIN	HOUR	DAY	WEEK	UNIGENE	FLCDNA	TAIR
ATTGAGAGAA	0	1	1	2	0	no match found	gi|13430841|gb|AF360333.1	At4g02370.1
at2g30260	CON	MIN	HOUR	DAY	WEEK	UNIGENE	FLCDNA	TAIR
ATGCTACAGC	0	1	1	0	0	no match found	gi|27765023|gb|BT003068.1	At2g30260.1
at5g16060	CON	MIN	HOUR	DAY	WEEK	UNIGENE	FLCDNA	TAIR
TCGTAAGCAA	1	0	1	0	0	no match found	gi|28416800|gb|BT004685.1	At5g16060.1
at4g36800	CON	MIN	HOUR	DAY	WEEK	UNIGENE	FLCDNA	TAIR
TTTGATCTTT	6	4	8	3	6	gnl|UG|At#S11721470	no match found	multiple canonical match
at2g15960	CON	MIN	HOUR	DAY	WEEK	UNIGENE	FLCDNA	TAIR
TTTTTAAGTT	9	78	13	5	2	gnl|UG|At#S16347517	gi|110741170|dbj|AK230337.1|	multiple non-canonical match
TAGTATGTCT	0	3	1	0	0	no match found	gi|15028290|gb|AY045948.1	At2g15960.1
GACCGTTTTG	0	0	1	0	0	no match found	gi|19310616|gb|AY079308.1	multiple non-canonical match
at1g24350	CON	MIN	HOUR	DAY	WEEK	UNIGENE	FLCDNA	TAIR
CATAGACACA	0	2	2	0	0	no match found	gi|26449728|dbj|AK117317.1|	multiple non-canonical match
at3g26170	CON	MIN	HOUR	DAY	WEEK	UNIGENE	FLCDNA	TAIR
TTCGGATCTG	0	1	0	0	0	gnl|UG|At#S11732921	no match found	pseudo chromosome match

at5g47690	CON	MIN	HOUR	DAY	WEEK	UNIGENE	FLCDNA	TAIR
TATTGTAGCC	1	4	0	4	3	gnl|UG|At#S43849156	gi|110738714|dbj|AK229421.1|	At5g47690.1
at3g09390	CON	MIN	HOUR	DAY	WEEK	UNIGENE	FLCDNA	TAIR
TGATGAGTTT	74	84	146	199	170	gnl|UG|At#S18322200	gi|14335167|gb|AY037263.1	At3g09390.1
at5g51080	CON	MIN	HOUR	DAY	WEEK	UNIGENE	FLCDNA	TAIR
GCTGCTCGTC	0	1	0	0	0	gnl|UG|At#S11718608	gi|29028871|gb|BT005880.1	At5g51080.1
at2g06950	CON	MIN	HOUR	DAY	WEEK	UNIGENE	FLCDNA	TAIR
TCAGCTTGGG	2	1	3	5	3	no match found	gi|13878086|gb|AF370306.1	non-canonical match
at3g18000	CON	MIN	HOUR	DAY	WEEK	UNIGENE	FLCDNA	TAIR
ACGTTCAAGC	0	0	0	2	2	gnl|UG|At#S11735444	gi|20260387|gb|AY093093.1	At3g18000.1
at1g31730	CON	MIN	HOUR	DAY	WEEK	UNIGENE	FLCDNA	TAIR
CTCTTCGATT	1	3	0	0	0	gnl|UG|At#S11738378	gi|22531061|gb|AY136369.1	At1g31730.1
at3g22142	CON	MIN	HOUR	DAY	WEEK	UNIGENE	FLCDNA	TAIR
GCAAAGCCAC	0	0	0	1	1	gnl|UG|At#S38433687	no match found	At3g22140.1
at4g01000	CON	MIN	HOUR	DAY	WEEK	UNIGENE	FLCDNA	TAIR
GAGGTTTTGG	2	4	4	2	2	no match found	gi|16209706|gb|AY057615.1	multiple non-canonical match
AACAAAATCC	1	2	3	4	3	gnl|UG|At#S11727438	no match found	At4g01000.1
at4g08953	CON	MIN	HOUR	DAY	WEEK	UNIGENE	FLCDNA	TAIR
ATCAACCAAT	0	1	1	0	0	no match found	no match found	At4g08953.1
at2g41230	CON	MIN	HOUR	DAY	WEEK	UNIGENE	FLCDNA	TAIR
TGATAGATCT	2	2	0	0	2	no match found	gi|20268788|gb|AY091149.1	non-canonical match
at4g13300	CON	MIN	HOUR	DAY	WEEK	UNIGENE	FLCDNA	TAIR
GTTAATGAAT	1	0	0	0	0	no match found	no match found	At4g13300.1
at5g55380	CON	MIN	HOUR	DAY	WEEK	UNIGENE	FLCDNA	TAIR
CTATTAAATT	1	0	1	0	1	gnl|UG|At#S11718175	gi|19699362|gb|AY090447.1	At5g55380.1
at5g01240	CON	MIN	HOUR	DAY	WEEK	UNIGENE	FLCDNA	TAIR
CTCACGTACC	0	2	1	0	0	gnl|UG|At#S11726366	no match found	At5g01240.2
at5g59140	CON	MIN	HOUR	DAY	WEEK	UNIGENE	FLCDNA	TAIR
TACTTCTTTA	1	2	0	1	0	gnl|UG|At#S11717791	gi|15028384|gb|AY045995.1	At5g59140.1
at2g21130	CON	MIN	HOUR	DAY	WEEK	UNIGENE	FLCDNA	TAIR
TGGTGTTTGG	1	5	1	1	1	gnl|UG|At#S11737171	no match found	At2g21130.1
at2g39130	CON	MIN	HOUR	DAY	WEEK	UNIGENE	FLCDNA	TAIR
TCATTGATTG	2	1	0	0	0	gnl|UG|At#S15460615	gi|15292734|gb|AY050801.1	At2g39130.1
at3g45820	CON	MIN	HOUR	DAY	WEEK	UNIGENE	FLCDNA	TAIR
TACTTGTTAT	0	1	0	0	0	gnl|UG|At#S37211321	gi|110735672|dbj|AK227839.1|	non-canonical match
at3g09430	CON	MIN	HOUR	DAY	WEEK	UNIGENE	FLCDNA	TAIR
GAGGAAGATT	1	0	0	0	0	gnl|UG|At#S11738056	no match found	non-canonical match
at4g13160	CON	MIN	HOUR	DAY	WEEK	UNIGENE	FLCDNA	TAIR
ATTCAGTATC	1	1	0	0	0	gnl|UG|At#S34117305	gi|45752691|gb|BT012149.1	At4g13160.1
at3g10850	CON	MIN	HOUR	DAY	WEEK	UNIGENE	FLCDNA	TAIR
GGCCCGTCAA	0	4	1	1	0	gnl|UG|At#S11737629	gi|15450456|gb|AY052329.1	At3g10850.1

at1g17280	CON	MIN	HOUR	DAY	WEEK	UNIGENE	FLCDNA	TAIR
GTTTGTGCTT	0	3	0	1	0	gnl|UG|At#S11741315	gi|21403386|gb|AY084676.1	At1g17280.1
at4g24400	CON	MIN	HOUR	DAY	WEEK	UNIGENE	FLCDNA	TAIR
GTAGACATTC	0	1	0	0	0	gnl|UG|At#S11723579	gi|24030395|gb|BT000958.1	At4g24400.1
at4g36870	CON	MIN	HOUR	DAY	WEEK	UNIGENE	FLCDNA	TAIR
CCTGACAAAG	0	1	2	2	0	gnl|UG|At#S11707500	gi|15215856|gb|AY050459.1	At4g36870.1
at2g18790	CON	MIN	HOUR	DAY	WEEK	UNIGENE	FLCDNA	TAIR
ATGCTGATGA	1	0	1	1	0	gnl|UG|At#S34116705	gi|110739064|dbj|AK229603.1|	At2g18790.1
at4g10925	CON	MIN	HOUR	DAY	WEEK	UNIGENE	FLCDNA	TAIR
TACTGAATGT	2	2	1	0	1	gnl|UG|At#S15461292	gi|21403366|gb|AY084656.1	At4g10925.1
at2g26570	CON	MIN	HOUR	DAY	WEEK	UNIGENE	FLCDNA	TAIR
AACAAAAGAG	1	2	1	0	1	gnl|UG|At#S34117533	gi|110737460|dbj|AK228775.1|	At2g26570.1
at3g23910	CON	MIN	HOUR	DAY	WEEK	UNIGENE	FLCDNA	TAIR
GATGCAATTG	1	0	2	0	0	gnl|UG|At#S11698709	gi|20259509|gb|AY091054.1	At3g23910.1
at5g16400	CON	MIN	HOUR	DAY	WEEK	UNIGENE	FLCDNA	TAIR
TAAACTATAA	7	1	1	1	0	gnl|UG|At#S11722241	no match found	At5g16400.1
at1g35190	CON	MIN	HOUR	DAY	WEEK	UNIGENE	FLCDNA	TAIR
AATGCTCTTT	0	2	0	0	0	gnl|UG|At#S11737210	gi|21407838|gb|AY089064.1	At1g35190.1
at1g25540	CON	MIN	HOUR	DAY	WEEK	UNIGENE	FLCDNA	TAIR
TCCTATTCTG	0	1	0	2	4	gnl|UG|At#S11740323	no match found	At1g25540.1
at5g62390	CON	MIN	HOUR	DAY	WEEK	UNIGENE	FLCDNA	TAIR
GTGCTATCGT	0	2	2	1	0	no match found	gi|20260357|gb|AY093078.1	non-canonical match
AATTAGAAAT	1	0	1	1	1	gnl|UG|At#S11717462	no match found	At5g62390.1
at2g16440	CON	MIN	HOUR	DAY	WEEK	UNIGENE	FLCDNA	TAIR
AGGAAAGCTT	0	0	0	1	0	gnl|UG|At#S34115222	gi|110741902|dbj|AK226795.1|	At2g16440.1
at5g51420	CON	MIN	HOUR	DAY	WEEK	UNIGENE	FLCDNA	TAIR
TTCTCTTTTG	0	0	1	0	1	no match found	no match found	At5g51420.1
at5g25754	CON	MIN	HOUR	DAY	WEEK	UNIGENE	FLCDNA	TAIR
GATCGTGTCA	1	5	5	1	3	gnl|UG|At#S11817034	no match found	At5g25757.1
TGGTCAAATG	4	4	2	3	8	gnl|UG|At#S11767350	gi|23397252|gb|BT000769.1	At5g25754.1
at5g34835	CON	MIN	HOUR	DAY	WEEK	UNIGENE	FLCDNA	TAIR
AACACCATCA	1	0	0	0	0	no match found	no match found	At5g34835.1
at3g52090	CON	MIN	HOUR	DAY	WEEK	UNIGENE	FLCDNA	TAIR
CAACTTCATC	1	0	2	2	1	gnl|UG|At#S11729043	gi|29029079|gb|BT005984.1	At3g52090.1
at1g52410	CON	MIN	HOUR	DAY	WEEK	UNIGENE	FLCDNA	TAIR
GCGCGGTGAG	0	6	1	1	0	gnl|UG|At#S15459166	gi|15081708|gb|AY048247.1	At1g52410.1
at3g09180	CON	MIN	HOUR	DAY	WEEK	UNIGENE	FLCDNA	TAIR
TGAATGTTGT	1	1	0	2	1	gnl|UG|At#S11738132	gi|21403573|gb|AY084863.1	multiple canonical match
CTGCTACAGC	0	0	1	0	0	no match found	gi|56382014|gb|BT020371.1	non-canonical match
at5g06320	CON	MIN	HOUR	DAY	WEEK	UNIGENE	FLCDNA	TAIR
TAAATAAATA	1	15	3	3	6	gnl|UG|At#S11724545	gi|13878138|gb|AF370332.1	non-canonical match

at3g54690	CON	MIN	HOUR	DAY	WEEK	UNIGENE	FLCDNA	TAIR
TATTGTCCAT	1	0	1	1	3	gnl|UG|At#S18904154	gi|21407901|gb|AY089127.1	At3g54690.1
at3g07200	CON	MIN	HOUR	DAY	WEEK	UNIGENE	FLCDNA	TAIR
TAGGAAAAAA	1	0	0	0	0	no match found	gi|28372917|gb|BT003713.1	non-canonical match
at4g35230	CON	MIN	HOUR	DAY	WEEK	UNIGENE	FLCDNA	TAIR
TTGTAAGTTT	6	9	4	7	2	gnl|UG|At#S11721727	gi|16612248|gb|AF439824.1	At4g35230.1
AACACAGACG	0	5	1	1	0	no match found	gi|22655469|gb|AY142063.1	non-canonical match
at5g15870	CON	MIN	HOUR	DAY	WEEK	UNIGENE	FLCDNA	TAIR
GCGGTGGCGG	1	3	2	3	0	gnl|UG|At#S11722368	no match found	multiple non-canonical match
at1g31430	CON	MIN	HOUR	DAY	WEEK	UNIGENE	FLCDNA	TAIR
AGTTTATTGT	0	1	0	0	0	gnl|UG|At#S11738493	no match found	At1g31430.1
at3g53280	CON	MIN	HOUR	DAY	WEEK	UNIGENE	FLCDNA	TAIR
TTATCTTTCA	4	5	0	0	1	gnl|UG|At#S18901921	gi|22655215|gb|AY140057.1	At3g53280.1
at1g14170	CON	MIN	HOUR	DAY	WEEK	UNIGENE	FLCDNA	TAIR
TTTCTTAAAT	0	0	0	1	1	gnl|UG|At#S43850644	gi|110743443|dbj|AK227617.1|	At1g14170.1
at1g09580	CON	MIN	HOUR	DAY	WEEK	UNIGENE	FLCDNA	TAIR
GATTGTTTAT	0	0	0	1	2	gnl|UG|At#S11742078	no match found	At1g09580.1
at1g49670	CON	MIN	HOUR	DAY	WEEK	UNIGENE	FLCDNA	TAIR
AGTGGAGCTT	0	2	2	1	0	gnl|UG|At#S11734612	gi|18252160|gb|AY072306.1	At1g49670.1
at5g24655	CON	MIN	HOUR	DAY	WEEK	UNIGENE	FLCDNA	TAIR
GGAAAAGGAG	0	0	1	0	0	no match found	gi|56236103|gb|BT020287.1	non-canonical match
at5g13430	CON	MIN	HOUR	DAY	WEEK	UNIGENE	FLCDNA	TAIR
CCTATTTGTC	0	1	0	1	1	gnl|UG|At#S11722993	no match found	At5g13430.1
TCTGCAAGTA	0	0	1	0	0	no match found	gi|17978904|gb|AY066055.1	multiple non-canonical match
TTAGAGCAAG	3	6	4	7	7	no match found	gi|62319497|dbj|AK221073.1|	non-canonical match
at3g28210	CON	MIN	HOUR	DAY	WEEK	UNIGENE	FLCDNA	TAIR
AGTAATATTT	0	0	0	0	1	gnl|UG|At#S11732242	no match found	At3g28210.1
at5g05370	CON	MIN	HOUR	DAY	WEEK	UNIGENE	FLCDNA	TAIR
GAGATATCTT	4	1	1	2	2	gnl|UG|At#S20706368	gi|110741472|dbj|AK226564.1|	At5g05370.1
at2g34570	CON	MIN	HOUR	DAY	WEEK	UNIGENE	FLCDNA	TAIR
AAGAAAAAGA	2	3	2	1	4	gnl|UG|At#S11814901	no match found	At2g34570.1
at5g52550	CON	MIN	HOUR	DAY	WEEK	UNIGENE	FLCDNA	TAIR
GGAATATCAG	1	2	6	2	2	no match found	no match found	At5g52550.1
at3g48780	CON	MIN	HOUR	DAY	WEEK	UNIGENE	FLCDNA	TAIR
TGATTGTTGT	1	5	3	0	1	no match found	gi|110739189|dbj|AK229667.1|	At3g48780.1
at5g27830	CON	MIN	HOUR	DAY	WEEK	UNIGENE	FLCDNA	TAIR
AATTTAACTG	1	1	1	0	0	no match found	gi|14532675|gb|AY039962.1	non-canonical match
TGAAATTTTG	1	0	3	1	0	gnl|UG|At#S11720429	no match found	At5g27830.1
at2g27450	CON	MIN	HOUR	DAY	WEEK	UNIGENE	FLCDNA	TAIR
GACCTAGTCA	0	3	0	1	1	gnl|UG|At#S11743586	gi|18175829|gb|AY072113.1	At2g27450.1
at2g40100	CON	MIN	HOUR	DAY	WEEK	UNIGENE	FLCDNA	TAIR
GCAAGACGCC	0	0	1	0	0	no match found	gi|20465760|gb|AY096735.1	non-canonical match
TTACGCTATA	2	1	3	0	3	gnl|UG|At#S11732577	gi|17979324|gb|AY070392.1	At2g40100.1

at5g49210	CON	MIN	HOUR	DAY	WEEK	UNIGENE	FLCDNA	TAIR
AGATTGAGAT	10	9	16	9	6	gnl|UG|At#S15459138	no match found	multiple canonical match
at3g49400	CON	MIN	HOUR	DAY	WEEK	UNIGENE	FLCDNA	TAIR
GAAGATCTCG	0	1	0	0	0	gnl|UG|At#S11729564	gi|14517467|gb|AY039569.1	At3g49400.1
at5g42620	CON	MIN	HOUR	DAY	WEEK	UNIGENE	FLCDNA	TAIR
GTTTAGCAGT	1	3	3	1	1	gnl|UG|At#S11719468	gi|13430719|gb|AF360272.1	At5g42620.1
at2g46810	CON	MIN	HOUR	DAY	WEEK	UNIGENE	FLCDNA	TAIR
GAACTTGACT	1	0	1	1	1	gnl|UG|At#S11730999	no match found	multiple canonical match
at3g19380	CON	MIN	HOUR	DAY	WEEK	UNIGENE	FLCDNA	TAIR
GCCTGACTAC	2	2	1	0	0	no match found	gi|21405213|gb|AY086503.1	non-canonical match
AATTAGTTTT	0	0	1	0	0	gnl|UG|At#S11735035	no match found	At3g19380.1
at3g02790	CON	MIN	HOUR	DAY	WEEK	UNIGENE	FLCDNA	TAIR
TTAAATCAAC	4	0	0	0	0	gnl|UG|At#S11796032	gi|13605546|gb|AF361599.1	At3g02790.1
at5g59850	CON	MIN	HOUR	DAY	WEEK	UNIGENE	FLCDNA	TAIR
CAGAAACACG	2	4	1	3	3	gnl|UG|At#S11717717	gi|21406314|gb|AY087576.1	At5g59850.1
at3g60600	CON	MIN	HOUR	DAY	WEEK	UNIGENE	FLCDNA	TAIR
TACGTTCTTT	1	4	4	2	2	gnl|UG|At#S28282773	gi|21404632|gb|AY085922.1	At3g60600.1
at2g38390	CON	MIN	HOUR	DAY	WEEK	UNIGENE	FLCDNA	TAIR
TTGGGGATAT	1	0	0	0	1	gnl|UG|At#S11733000	no match found	At2g38390.1
at2g22720	CON	MIN	HOUR	DAY	WEEK	UNIGENE	FLCDNA	TAIR
GAAATTGTAC	2	3	4	2	0	gnl|UG|At#S18942877	gi|16323050|gb|AY057629.1	At2g22720.1
at1g62820	CON	MIN	HOUR	DAY	WEEK	UNIGENE	FLCDNA	TAIR
CTATTCGACA	0	1	1	0	0	gnl|UG|At#S11710325	gi|17529027|gb|AY065248.1	At1g62820.1
at1g19910	CON	MIN	HOUR	DAY	WEEK	UNIGENE	FLCDNA	TAIR
GGTGAAATTT	16	18	19	26	10	gnl|UG|At#S18896506	no match found	At1g19910.1
at1g70890	CON	MIN	HOUR	DAY	WEEK	UNIGENE	FLCDNA	TAIR
ATGGAAAGTT	1	12	1	1	0	gnl|UG|At#S11728908	gi|30725555|gb|BT008441.1	At1g70890.1
at2g28150	CON	MIN	HOUR	DAY	WEEK	UNIGENE	FLCDNA	TAIR
ATGCTTGCAG	0	1	0	0	0	gnl|UG|At#S11735504	gi|29824308|gb|BT006130.1	At2g28150.1
at4g29580	CON	MIN	HOUR	DAY	WEEK	UNIGENE	FLCDNA	TAIR
GTGTCAGAAT	2	8	0	0	0	no match found	no match found	At4g29580.1
at5g25820	CON	MIN	HOUR	DAY	WEEK	UNIGENE	FLCDNA	TAIR
ATTCTTCATT	0	0	0	0	1	gnl|UG|At#S11720609	gi|110738110|dbj|AK229111.1|	multiple canonical match
at1g09320	CON	MIN	HOUR	DAY	WEEK	UNIGENE	FLCDNA	TAIR
ACATAGTGCG	0	0	1	2	0	gnl|UG|At#S11742106	gi|51971127|dbj|AK176493.1|	At1g09320.1
at2g25950	CON	MIN	HOUR	DAY	WEEK	UNIGENE	FLCDNA	TAIR
ATATCTTTAA	0	1	0	0	0	no match found	gi|110742983|dbj|AK227379.1|	non-canonical match
at5g38610	CON	MIN	HOUR	DAY	WEEK	UNIGENE	FLCDNA	TAIR
ATACAACAGC	0	0	1	0	0	no match found	no match found	At5g38610.1
AGTAACTAAC	0	1	0	0	0	gnl|UG|At#S11719872	no match found	multiple non-canonical match
at5g10730	CON	MIN	HOUR	DAY	WEEK	UNIGENE	FLCDNA	TAIR

GAATACAACG	0	0	1	0	0	gnl|UG|At#S11723560	gi|16323237|gb|AY057723.1	At5g10730.1
at3g04050	CON	MIN	HOUR	DAY	WEEK	UNIGENE	FLCDNA	TAIR
GGCTTATTTT	0	1	0	0	1	no match found	no match found	At3g04050.1
at1g14020	CON	MIN	HOUR	DAY	WEEK	UNIGENE	FLCDNA	TAIR
TCAAACACTT	2	1	0	0	0	gnl|UG|At#S18921219	gi|110739823|dbj|AK229994.1|	At1g14020.1
at3g56910	CON	MIN	HOUR	DAY	WEEK	UNIGENE	FLCDNA	TAIR
GAAGAATTGT	7	23	6	8	2	gnl|UG|At#S11675412	no match found	multiple canonical match
at1g19380	CON	MIN	HOUR	DAY	WEEK	UNIGENE	FLCDNA	TAIR
AGGATCACAT	0	0	1	0	0	no match found	gi|45752639|gb|BT012123.1	multiple canonical match
AATTGCTTCG	1	13	9	1	2	gnl|UG|At#S18896870	gi|110737808|dbj|AK228954.1|	At1g19380.1
at5g54780	CON	MIN	HOUR	DAY	WEEK	UNIGENE	FLCDNA	TAIR
AAGAATGGAG	0	1	0	0	0	gnl|UG|At#S34115087	gi|110742145|dbj|AK226930.1|	multiple canonical match
at5g23370	CON	MIN	HOUR	DAY	WEEK	UNIGENE	FLCDNA	TAIR
AGCAAGAGAT	0	1	0	0	0	gnl|UG|At#S11720841	no match found	At5g23370.1
at2g27860	CON	MIN	HOUR	DAY	WEEK	UNIGENE	FLCDNA	TAIR
TATGTCGGAG	18	28	21	13	7	no match found	gi|21405540|gb|AY086830.1	non-canonical match
ACCATCATTA	0	2	1	1	0	no match found	gi|24111292|gb|BT001016.1	non-canonical match
at5g25780	CON	MIN	HOUR	DAY	WEEK	UNIGENE	FLCDNA	TAIR
AAGAGGAGAA	2	2	0	3	0	gnl|UG|At#S11720613	no match found	At5g25780.1
at3g02340	CON	MIN	HOUR	DAY	WEEK	UNIGENE	FLCDNA	TAIR
TTAAACTTTG	1	0	0	0	0	gnl|UG|At#S11739983	no match found	At3g02340.1
at3g14610	CON	MIN	HOUR	DAY	WEEK	UNIGENE	FLCDNA	TAIR
GATATGTCAT	1	0	0	0	0	gnl|UG|At#S11736467	gi|18252154|gb|AY072303.1	At3g14610.1
at4g34530	CON	MIN	HOUR	DAY	WEEK	UNIGENE	FLCDNA	TAIR
TGCAGAATCT	1	4	4	0	1	no match found	no match found	At4g34530.1
at4g39540	CON	MIN	HOUR	DAY	WEEK	UNIGENE	FLCDNA	TAIR
AATTTGTGCC	2	1	1	2	1	no match found	gi|26450650|dbj|AK117791.1|	At4g39540.2
at4g11630	CON	MIN	HOUR	DAY	WEEK	UNIGENE	FLCDNA	TAIR
TTATAGTTTT	0	0	0	0	1	gnl|UG|At#S11725795	gi|62320251|dbj|AK221452.1|	At4g11630.1
at1g54360	CON	MIN	HOUR	DAY	WEEK	UNIGENE	FLCDNA	TAIR
GTAATTGTAT	0	2	0	0	0	gnl|UG|At#S28282492	gi|28059030|gb|BT003362.1	At1g54360.2
at5g03520	CON	MIN	HOUR	DAY	WEEK	UNIGENE	FLCDNA	TAIR
TCAATCGCAA	0	0	1	0	0	no match found	gi|15810624|gb|AY056351.1	non-canonical match
TTGTTACCTA	2	2	1	1	1	gnl|UG|At#S11725280	gi|14334901|gb|AY035124.1	At5g03520.1
at1g62170	CON	MIN	HOUR	DAY	WEEK	UNIGENE	FLCDNA	TAIR
AGTGAATCAA	0	0	1	0	0	no match found	no match found	At1g62170.1
at1g13780	CON	MIN	HOUR	DAY	WEEK	UNIGENE	FLCDNA	TAIR
AAACACTGAT	1	1	0	0	0	gnl|UG|At#S24442568	gi|62320537|dbj|AK221595.1|	At1g13780.1
at3g53800	CON	MIN	HOUR	DAY	WEEK	UNIGENE	FLCDNA	TAIR
GACTTCAAGT	0	4	1	1	0	gnl|UG|At#S11728726	gi|26449489|dbj|AK117195.1|	At3g53800.1
at5g24735	CON	MIN	HOUR	DAY	WEEK	UNIGENE	FLCDNA	TAIR
GCAAGACTCC	0	6	0	3	0	gnl|UG|At#S30470211	gi|72198537|gb|DQ108879.1	At3g53800.1

```
GAAAATCATT    4     4     1     4     0     gnl|UG|At#S34115423   no match found               At1g32050.1

at1g71830     CON   MIN   HOUR  DAY   WEEK  UNIGENE               FLCDNA                       TAIR
TTACTTGACT    0     2     0     0     0     gnl|UG|At#S11728725   gi|15081615|gb|AY048200.1    At1g71830.1

at3g19240     CON   MIN   HOUR  DAY   WEEK  UNIGENE               FLCDNA                       TAIR
CACGATAACT    2     1     0     3     0     gnl|UG|At#S11735074   no match found               At3g19240.1

at3g04840     CON   MIN   HOUR  DAY   WEEK  UNIGENE               FLCDNA                       TAIR
GATGTTCACG    16    6     15    12    14    no match found        no match found               At3g04840.1

at1g55630     CON   MIN   HOUR  DAY   WEEK  UNIGENE               FLCDNA                       TAIR
GTAGAGAAAG    0     1     0     0     0     no match found        gi|30793840|gb|BT008546.1    multiple non-canonical match

at5g05170     CON   MIN   HOUR  DAY   WEEK  UNIGENE               FLCDNA                       TAIR
GGGACCACTC    2     4     2     2     2     gnl|UG|At#S11700449   gi|26983831|gb|BT002335.1    At5g05170.1

at3g33072     CON   MIN   HOUR  DAY   WEEK  UNIGENE               FLCDNA                       TAIR
AAGAAGAGAG    0     0     1     0     0     no match found        no match found               At3g33072.1

at3g46770     CON   MIN   HOUR  DAY   WEEK  UNIGENE               FLCDNA                       TAIR
AGCACTTCTT    0     0     0     1     0     no match found        no match found               At3g46770.1

at3g22520     CON   MIN   HOUR  DAY   WEEK  UNIGENE               FLCDNA                       TAIR
TAATGTTGTA    0     0     0     1     0     gnl|UG|At#S11708303   gi|17381093|gb|AY064003.1    At3g22520.1

at3g07020     CON   MIN   HOUR  DAY   WEEK  UNIGENE               FLCDNA                       TAIR
CTTGATTGCT    1     1     0     0     1     gnl|UG|At#S11738621   no match found               At3g07020.1
CTCGACGATA    0     2     0     0     0     no match found        gi|15982778|gb|AY057496.1    multiple non-canonical match

at5g47720     CON   MIN   HOUR  DAY   WEEK  UNIGENE               FLCDNA                       TAIR
TGTGTGTCAC    0     2     0     0     0     gnl|UG|At#S28281660   gi|21407514|gb|AY088740.1    At5g47720.2

at5g45480     CON   MIN   HOUR  DAY   WEEK  UNIGENE               FLCDNA                       TAIR
TCCTTCTTCT    1     1     1     0     0     gnl|UG|At#S11719179   no match found               At5g45480.1

at5g26990     CON   MIN   HOUR  DAY   WEEK  UNIGENE               FLCDNA                       TAIR
ATAATGAGTA    0     0     0     0     1     gnl|UG|At#S11720513   gi|110736607|dbj|AK228327.1| At5g26990.1

at1g67850     CON   MIN   HOUR  DAY   WEEK  UNIGENE               FLCDNA                       TAIR
GCAAGGGGTG    0     1     0     1     0     no match found        gi|25090151|gb|BT002230.1    multiple canonical match

at5g01180     CON   MIN   HOUR  DAY   WEEK  UNIGENE               FLCDNA                       TAIR
CGCTTTGATG    0     1     1     0     0     gnl|UG|At#S11726401   gi|110736811|dbj|AK228435.1| At5g01180.1

at4g21860     CON   MIN   HOUR  DAY   WEEK  UNIGENE               FLCDNA                       TAIR
GCAGATTTTT    1     0     1     1     2     gnl|UG|At#S11724032   no match found               At5g01180.1
TGCTGCTTGT    12    15    7     4     0     no match found        gi|21404365|gb|AY085655.1    At4g21860.1

at5g10350     CON   MIN   HOUR  DAY   WEEK  UNIGENE               FLCDNA                       TAIR
TACGGATGAG    1     6     2     1     2     no match found        gi|28393012|gb|BT003893.1    At5g10350.1

at1g69990     CON   MIN   HOUR  DAY   WEEK  UNIGENE               FLCDNA                       TAIR
GTTTCTTCTC    0     3     1     0     0     gnl|UG|At#S11729101   no match found               non-canonical match

at5g57350     CON   MIN   HOUR  DAY   WEEK  UNIGENE               FLCDNA                       TAIR
TGTTTTTGTT    3     3     6     4     1     gnl|UG|At#S11717977   gi|18176051|gb|AY072153.1    At5g57350.1

at3g01050     CON   MIN   HOUR  DAY   WEEK  UNIGENE               FLCDNA                       TAIR
TAAGAGAGTA    1     1     0     0     2     gnl|UG|At#S18904203   gi|38454047|gb|BT010661.1    pseudo chromosome match
```

at4g12340	CON	MIN	HOUR	DAY	WEEK	UNIGENE	FLCDNA	TAIR
AGATAAAACT	2	3	3	4	2	gnl|UG|At#S11725674	gi|16648694|gb|AY058123.1	At4g12340.1
at3g11440	CON	MIN	HOUR	DAY	WEEK	UNIGENE	FLCDNA	TAIR
ACTTGGTGGT	0	1	0	0	0	gnl|UG|At#S11737456	gi|28393103|gb|BT003940.1	At3g11440.1
at4g33040	CON	MIN	HOUR	DAY	WEEK	UNIGENE	FLCDNA	TAIR
TAGAAAATGA	0	0	4	0	0	gnl|UG|At#S11722099	gi|18377673|gb|AY074290.1	At4g33040.1
at2g28890	CON	MIN	HOUR	DAY	WEEK	UNIGENE	FLCDNA	TAIR
AACTGCTTGA	1	1	2	0	0	gnl|UG|At#S11735311	gi|20465372|gb|AY096450.1	At2g28890.1
at5g23090	CON	MIN	HOUR	DAY	WEEK	UNIGENE	FLCDNA	TAIR
GTGGGTCAGG	0	0	1	0	0	gnl|UG|At#S15460488	no match found	At5g23090.2
TTCTCAAGGC	1	0	0	0	0	no match found	gi|21436032|gb|AY116960.1	At5g23090.3
at2g30460	CON	MIN	HOUR	DAY	WEEK	UNIGENE	FLCDNA	TAIR
GTTTATTCTA	0	0	0	0	2	gnl|UG|At#S21736401	gi|62319791|dbj|AK221221.1|	non-canonical match
at5g52140	CON	MIN	HOUR	DAY	WEEK	UNIGENE	FLCDNA	TAIR
AAGCAAATAT	1	0	1	0	0	no match found	no match found	At5g52140.1
at5g06560	CON	MIN	HOUR	DAY	WEEK	UNIGENE	FLCDNA	TAIR
GGATTGCAAA	0	0	0	1	0	no match found	gi|15293220|gb|AY051044.1	non-canonical match
at1g62045	CON	MIN	HOUR	DAY	WEEK	UNIGENE	FLCDNA	TAIR
TAGGCAACGC	1	0	1	2	1	no match found	no match found	At1g62045.1
at2g47630	CON	MIN	HOUR	DAY	WEEK	UNIGENE	FLCDNA	TAIR
AACCTAGAAG	1	0	0	0	0	gnl|UG|At#S18900108	gi|16323213|gb|AY057711.1	multiple non-canonical match
at5g02960	CON	MIN	HOUR	DAY	WEEK	UNIGENE	FLCDNA	TAIR
ATCGTTTAAT	69	31	63	58	141	gnl|UG|At#S11725426	gi|14532717|gb|AY039983.1	At5g02960.1
at2g24350	CON	MIN	HOUR	DAY	WEEK	UNIGENE	FLCDNA	TAIR
AGACAGAAAC	0	0	0	1	0	gnl|UG|At#S11736409	no match found	non-canonical match
at2g33290	CON	MIN	HOUR	DAY	WEEK	UNIGENE	FLCDNA	TAIR
GTCGGTGAGT	0	1	0	1	0	gnl|UG|At#S11734215	no match found	At2g33290.1
at5g67450	CON	MIN	HOUR	DAY	WEEK	UNIGENE	FLCDNA	TAIR
CTCGCTCGTG	0	2	0	0	0	gnl|UG|At#S11716950	gi|27765015|gb|BT003064.1	At5g67450.1
at3g55450	CON	MIN	HOUR	DAY	WEEK	UNIGENE	FLCDNA	TAIR
TCTTCTTCTT	0	1	0	0	2	no match found	gi|28058889|gb|BT003347.1	At3g55450.1
at1g13670	CON	MIN	HOUR	DAY	WEEK	UNIGENE	FLCDNA	TAIR
AAGCGAGGTT	0	2	2	2	0	gnl|UG|At#S21737250	gi|51968991|dbj|AK175425.1|	pseudo chromosome match
at1g12770	CON	MIN	HOUR	DAY	WEEK	UNIGENE	FLCDNA	TAIR
TTAATGGTGA	0	0	0	0	1	no match found	no match found	At1g12770.1
AACCACTCAA	1	1	1	2	0	gnl|UG|At#S11741761	gi|51969315|dbj|AK175587.1|	non-canonical match
at5g47550	CON	MIN	HOUR	DAY	WEEK	UNIGENE	FLCDNA	TAIR
TAACCGCTCT	3	5	0	4	1	no match found	gi|18252838|gb|AY072354.1	At5g47550.1
at5g07740	CON	MIN	HOUR	DAY	WEEK	UNIGENE	FLCDNA	TAIR
ATTGACTCCA	0	2	1	1	0	gnl|UG|At#S20295581	no match found	At5g07740.1
at5g21326	CON	MIN	HOUR	DAY	WEEK	UNIGENE	FLCDNA	TAIR
TCACAGAAAA	0	2	0	0	0	no match found	gi|48310655|gb|BT014878.1	multiple non-canonical match

TGCAGTAAGT	4	6	4	5	2	gnl|UG|At#S15460578	gi|21928176|gb|AY125525.1	At5g21326.1
at2g44850	CON	MIN	HOUR	DAY	WEEK	UNIGENE	FLCDNA	TAIR
GCTTCGTTTT	0	0	0	0	2	no match found	no match found	At2g44850.1
at5g10370	CON	MIN	HOUR	DAY	WEEK	UNIGENE	FLCDNA	TAIR
GTGGAATCGG	1	0	2	0	0	gnl|UG|At#S11723644	gi|26451969|dbj|AK118469.1|	At5g10370.1
at3g60770	CON	MIN	HOUR	DAY	WEEK	UNIGENE	FLCDNA	TAIR
TCCTTCAAGA	9	16	11	13	18	no match found	gi|14190472|gb|AF380636.1	At3g60770.1
at1g67230	CON	MIN	HOUR	DAY	WEEK	UNIGENE	FLCDNA	TAIR
ATATTGAAAT	3	2	0	1	0	no match found	gi|110741401|dbj|AK230454.1|	multiple non-canonical match
GAGGAGAAAG	4	3	5	2	0	no match found	gi|17933293|gb|AF446356.1	multiple non-canonical match
AAGCTACGGC	0	1	0	2	1	gnl|UG|At#S11729667	gi|26449389|dbj|AK117144.1|	At1g67230.1
at1g31480	CON	MIN	HOUR	DAY	WEEK	UNIGENE	FLCDNA	TAIR
GTTTTGCCAC	1	1	1	1	2	gnl|UG|At#S11738473	no match found	At1g31480.1
at5g06260	CON	MIN	HOUR	DAY	WEEK	UNIGENE	FLCDNA	TAIR
GCAGGCAATT	1	1	3	1	2	gnl|UG|At#S11724560	gi|15292792|gb|AY050830.1	At5g06260.1
at3g52820	CON	MIN	HOUR	DAY	WEEK	UNIGENE	FLCDNA	TAIR
TTGGCCTTCA	0	2	0	0	0	gnl|UG|At#S11728905	gi|26452117|dbj|AK118546.1|	At3g52820.1
at4g35860	CON	MIN	HOUR	DAY	WEEK	UNIGENE	FLCDNA	TAIR
GCCTGCTATT	1	2	0	0	0	gnl|UG|At#S38433387	gi|21436444|gb|AY117348.1	At4g35860.1
at3g45740	CON	MIN	HOUR	DAY	WEEK	UNIGENE	FLCDNA	TAIR
AGTTATATCA	1	0	0	0	0	gnl|UG|At#S11730236	gi|110738249|dbj|AK229184.1|	At3g45740.1
at3g62460	CON	MIN	HOUR	DAY	WEEK	UNIGENE	FLCDNA	TAIR
CTAATTGTTG	0	1	0	1	0	gnl|UG|At#S11726705	gi|18650644|gb|AY074871.1	At3g62460.1
at5g50430	CON	MIN	HOUR	DAY	WEEK	UNIGENE	FLCDNA	TAIR
TTGTTGCTCG	0	0	1	0	0	no match found	gi|22655423|gb|AY142040.1	multiple non-canonical match
ACTGCAAATC	2	1	0	3	1	gnl|UG|At#S11718670	gi|14190508|gb|AF380654.1	At5g50430.1
at3g11250	CON	MIN	HOUR	DAY	WEEK	UNIGENE	FLCDNA	TAIR
GGTAAGAACA	0	1	0	0	0	gnl|UG|At#S11737516	gi|15810222|gb|AY056150.1	At3g11250.1
at1g60490	CON	MIN	HOUR	DAY	WEEK	UNIGENE	FLCDNA	TAIR
TTAACTCCTC	0	0	0	0	1	gnl|UG|At#S11731331	gi|19347827|gb|AY080724.1	At1g60490.1
at4g05150	CON	MIN	HOUR	DAY	WEEK	UNIGENE	FLCDNA	TAIR
TACCAATGGG	0	4	2	0	3	gnl|UG|At#S11645341	gi|110740815|dbj|AK226357.1|	At4g05150.1
at3g04740	CON	MIN	HOUR	DAY	WEEK	UNIGENE	FLCDNA	TAIR
TTGTGTAGCC	6	3	1	0	0	gnl|UG|At#S11806593	no match found	At3g04740.1
at1g60600	CON	MIN	HOUR	DAY	WEEK	UNIGENE	FLCDNA	TAIR
GCCAAGTATT	0	0	2	1	0	no match found	gi|110742780|dbj|AK227267.1|	At1g60600.1
GTGAGCGATT	1	2	1	1	1	gnl|UG|At#S43850413	no match found	pseudo chromosome match
at5g14720	CON	MIN	HOUR	DAY	WEEK	UNIGENE	FLCDNA	TAIR
CTACTACAAC	0	1	0	0	0	gnl|UG|At#S11722660	gi|17529279|gb|AY065426.1	At5g14720.1
at4g00630	CON	MIN	HOUR	DAY	WEEK	UNIGENE	FLCDNA	TAIR
GTCTCAATCT	0	1	0	0	0	gnl|UG|At#S11727515	no match found	non-canonical match
at5g67400	CON	MIN	HOUR	DAY	WEEK	UNIGENE	FLCDNA	TAIR

TTATGAGTTT	0	1	1	0	0	gnl|UG|At#S11698837	no match found	At5g67400.1
GACCCAACCA	0	0	1	0	0	no match found	gi|126352291|gb|BT030328.1	non-canonical match
at2g17200	CON	MIN	HOUR	DAY	WEEK	UNIGENE	FLCDNA	TAIR
AACTGATGTC	3	4	1	3	0	gnl|UG|At#S11738182	gi|23296305|gb|AY142486.1	At2g17200.1
at5g18860	CON	MIN	HOUR	DAY	WEEK	UNIGENE	FLCDNA	TAIR
ATTAGTTCTA	0	0	0	1	0	gnl|UG|At#S11744172	gi|19310426|gb|AY078950.1	At5g18860.1
TTCTTAGGGG	0	2	0	0	0	no match found	gi|22137231|gb|AY133631.1	multiple non-canonical match
at1g18580	CON	MIN	HOUR	DAY	WEEK	UNIGENE	FLCDNA	TAIR
AAGCCTTGGC	1	2	0	0	1	gnl|UG|At#S11709146	gi|15293066|gb|AY050967.1	At1g18580.1
at1g06030	CON	MIN	HOUR	DAY	WEEK	UNIGENE	FLCDNA	TAIR
TTGACGCTGT	0	1	0	0	0	no match found	gi|20258777|gb|AY090916.1	At1g06030.1
at5g27520	CON	MIN	HOUR	DAY	WEEK	UNIGENE	FLCDNA	TAIR
TACAAAAAAA	0	3	1	2	4	no match found	gi|17380871|gb|AY063892.1	non-canonical match
at2g17230	CON	MIN	HOUR	DAY	WEEK	UNIGENE	FLCDNA	TAIR
AGTTAGCTGA	0	9	1	0	0	gnl|UG|At#S11708118	gi|19699195|gb|AY090303.1	At2g17230.1
at4g32390	CON	MIN	HOUR	DAY	WEEK	UNIGENE	FLCDNA	TAIR
GCTTTTTGCT	1	1	0	0	0	gnl|UG|At#S11722211	no match found	multiple non-canonical match
at5g15940	CON	MIN	HOUR	DAY	WEEK	UNIGENE	FLCDNA	TAIR
AATATGAGAA	0	0	0	1	0	gnl|UG|At#S11722352	no match found	At5g15940.1
at4g39910	CON	MIN	HOUR	DAY	WEEK	UNIGENE	FLCDNA	TAIR
GTTACATCCT	0	1	0	1	0	no match found	gi|16604538|gb|AY058889.1	non-canonical match
TACAGTTTAT	3	0	1	2	0	gnl|UG|At#S11720938	no match found	At4g39910.1
at1g07350	CON	MIN	HOUR	DAY	WEEK	UNIGENE	FLCDNA	TAIR
TCTTTCTACT	2	0	1	0	0	no match found	gi|15292732|gb|AY050800.1	At1g07350.2
at2g43320	CON	MIN	HOUR	DAY	WEEK	UNIGENE	FLCDNA	TAIR
GATGGATGTA	0	0	1	0	0	gnl|UG|At#S11731813	gi|22136567|gb|AY129484.1	At2g43320.1
at4g28070	CON	MIN	HOUR	DAY	WEEK	UNIGENE	FLCDNA	TAIR
GTATGATGAT	0	3	1	0	0	gnl|UG|At#S22220276	no match found	non-canonical match
at2g15570	CON	MIN	HOUR	DAY	WEEK	UNIGENE	FLCDNA	TAIR
AAGAAGGTAA	1	1	1	1	0	no match found	gi|51969097|dbj|AK175478.1|	multiple canonical match
at5g40390	CON	MIN	HOUR	DAY	WEEK	UNIGENE	FLCDNA	TAIR
CTAAACACAA	1	0	0	0	0	gnl|UG|At#S11719691	gi|20148632|gb|AY081645.1	At5g40390.1
at3g55110	CON	MIN	HOUR	DAY	WEEK	UNIGENE	FLCDNA	TAIR
GGAATATTGT	2	1	0	1	1	gnl|UG|At#S11728493	gi|62319522|dbj|AK221086.1|	At3g55110.1
at1g50660	CON	MIN	HOUR	DAY	WEEK	UNIGENE	FLCDNA	TAIR
TCCTTAAACA	1	2	0	0	0	gnl|UG|At#S11734276	gi|20453097|gb|AY094417.1	At1g50660.1
at5g63780	CON	MIN	HOUR	DAY	WEEK	UNIGENE	FLCDNA	TAIR
CTTCATTGAA	6	3	5	3	4	gnl|UG|At#S11717322	gi|27311830|gb|BT002521.1	non-canonical match
GACGTGTCAT	0	0	0	1	0	no match found	no match found	At5g63780.1
at1g79010	CON	MIN	HOUR	DAY	WEEK	UNIGENE	FLCDNA	TAIR
AGTTCCGGTT	11	3	3	9	13	gnl|UG|At#S11726843	gi|15081696|gb|AY048241.1	At1g79010.1
ACAAAATGCA	0	3	0	1	0	no match found	gi|18252264|gb|AY072622.1	non-canonical match

at4g00040	CON	MIN	HOUR	DAY	WEEK	UNIGENE	FLCDNA	TAIR
AGGAACCTCT	0	0	0	1	0	no match found	gi|30725599|gb|BT008463.1	non-canonical match
TAATGGTTGC	3	1	0	1	1	gnl|UG|At#S11822172	gi|25082808|gb|BT001993.1	non-canonical match
at3g57580	CON	MIN	HOUR	DAY	WEEK	UNIGENE	FLCDNA	TAIR
TCATCTATGC	0	0	0	0	1	gnl|UG|At#S11728034	gi|62321050|dbj|AK221854.1|	At3g57580.1
at1g78020	CON	MIN	HOUR	DAY	WEEK	UNIGENE	FLCDNA	TAIR
GTCACACCGA	2	11	1	5	0	gnl|UG|At#S11809742	gi|17380851|gb|AY063882.1	At1g78020.1
at1g43170	CON	MIN	HOUR	DAY	WEEK	UNIGENE	FLCDNA	TAIR
GTCGCTTCCA	0	0	3	0	0	no match found	gi|15450558|gb|AY052743.1	non-canonical match
at4g40042	CON	MIN	HOUR	DAY	WEEK	UNIGENE	FLCDNA	TAIR
TATCTGTAGT	1	0	0	0	1	gnl|UG|At#S11816543	no match found	pseudo chromosome match
at3g03460	CON	MIN	HOUR	DAY	WEEK	UNIGENE	FLCDNA	TAIR
ACTCAGATTT	0	0	1	0	0	gnl|UG|At#S11739658	no match found	non-canonical match
at1g10060	CON	MIN	HOUR	DAY	WEEK	UNIGENE	FLCDNA	TAIR
TTCGCCACCA	0	0	1	0	0	gnl|UG|At#S43850669	gi|22136199|gb|AY128778.1	At1g10060.2
at1g09490	CON	MIN	HOUR	DAY	WEEK	UNIGENE	FLCDNA	TAIR
TCGGTAGAGC	0	1	0	0	0	gnl|UG|At#S18898151	gi|21689622|gb|AY122900.1	At1g09490.1
at2g37250	CON	MIN	HOUR	DAY	WEEK	UNIGENE	FLCDNA	TAIR
ATTGTTTGAG	4	0	2	3	8	no match found	gi|15450418|gb|AY052310.1	At2g37250.1
at2g16390	CON	MIN	HOUR	DAY	WEEK	UNIGENE	FLCDNA	TAIR
CAACAATTGT	0	0	1	0	0	no match found	no match found	At2g16390.1
at2g27600	CON	MIN	HOUR	DAY	WEEK	UNIGENE	FLCDNA	TAIR
AGCAAACTCC	0	0	2	2	1	gnl|UG|At#S11735647	no match found	non-canonical match
atcg00720	CON	MIN	HOUR	DAY	WEEK	UNIGENE	FLCDNA	TAIR
TCAACATATT	0	2	0	1	0	no match found	no match found	AtCg00720
at5g17300	CON	MIN	HOUR	DAY	WEEK	UNIGENE	FLCDNA	TAIR
AGATTCATCT	1	0	2	2	0	gnl|UG|At#S11722006	gi|15215872|gb|AY050467.1	At5g17300.1
at4g01480	CON	MIN	HOUR	DAY	WEEK	UNIGENE	FLCDNA	TAIR
CAGGAGCCTG	0	2	0	0	0	gnl|UG|At#S11727313	gi|15450871|gb|AY054516.1	At4g01480.1
at3g20050	CON	MIN	HOUR	DAY	WEEK	UNIGENE	FLCDNA	TAIR
ATCAAACTGG	2	1	1	0	0	no match found	gi|59958325|gb|BT021103.1	multiple non-canonical match
TAATGAAGTT	6	3	3	5	4	gnl|UG|At#S11734832	gi|25083338|gb|BT002052.1	At3g20050.1
at5g41770	CON	MIN	HOUR	DAY	WEEK	UNIGENE	FLCDNA	TAIR
GAGAGTAGCT	0	1	1	0	0	gnl|UG|At#S11719552	gi|25083214|gb|BT002040.1	At5g41770.1
at3g43573	CON	MIN	HOUR	DAY	WEEK	UNIGENE	FLCDNA	TAIR
ATCCAAGAAA	0	0	1	1	0	no match found	no match found	At3g43573.1
at5g02260	CON	MIN	HOUR	DAY	WEEK	UNIGENE	FLCDNA	TAIR
GTTTCTTCGA	0	0	1	0	2	gnl|UG|At#S11725595	gi|21404795|gb|AY086085.1	At5g02260.1
at2g01250	CON	MIN	HOUR	DAY	WEEK	UNIGENE	FLCDNA	TAIR
GTATCATTTG	3	13	3	4	5	gnl|UG|At#S11742701	gi|19310656|gb|AY079328.1	At2g01250.1
at3g27460	CON	MIN	HOUR	DAY	WEEK	UNIGENE	FLCDNA	TAIR
GGTATGTATA	0	1	0	0	1	gnl|UG|At#S11743940	gi|19347734|gb|AY080609.1	At3g27460.1

at1g42980	CON	MIN	HOUR	DAY	WEEK	UNIGENE	FLCDNA	TAIR
ATTTTCTTTG	0	0	0	0	1	gnl|UG|At#S11736209	no match found	At1g42980.1
at1g20300	CON	MIN	HOUR	DAY	WEEK	UNIGENE	FLCDNA	TAIR
TGTATTATTT	0	1	0	0	0	gnl|UG|At#S11741028	no match found	At1g20300.1
TACTCTTTTT	0	1	1	0	1	no match found	gi|15450346|gb|AY052274.1	non-canonical match
AAGAGCTGGT	0	1	0	0	0	no match found	gi|17386103|gb|AF446865.1	non-canonical match
at1g10820	CON	MIN	HOUR	DAY	WEEK	UNIGENE	FLCDNA	TAIR
GTTTAAGTCG	0	2	0	2	0	gnl|UG|At#S18942599	gi|26452007|dbj|AK118490.1|	At1g10820.2
at3g12730	CON	MIN	HOUR	DAY	WEEK	UNIGENE	FLCDNA	TAIR
GACTCTACGT	0	0	0	1	0	gnl|UG|At#S11737077	gi|17979532|gb|AY070764.1	At3g12730.1
at3g57570	CON	MIN	HOUR	DAY	WEEK	UNIGENE	FLCDNA	TAIR
CATAACGCCG	0	2	0	1	1	gnl|UG|At#S11728037	no match found	At3g57570.1
at1g76980	CON	MIN	HOUR	DAY	WEEK	UNIGENE	FLCDNA	TAIR
AACATCGTCT	0	0	1	0	0	no match found	no match found	At1g76980.1
TGAATCGTGT	0	1	0	0	0	gnl|UG|At#S17865650	gi|38454133|gb|BT010704.1	multiple non-canonical match
at3g16510	CON	MIN	HOUR	DAY	WEEK	UNIGENE	FLCDNA	TAIR
GTTCTTCGTC	0	1	0	0	0	gnl|UG|At#S11735904	gi|40823213|gb|BT011231.1	At3g16510.1
at3g08990	CON	MIN	HOUR	DAY	WEEK	UNIGENE	FLCDNA	TAIR
AACCAGAACG	0	1	0	0	0	gnl|UG|At#S11738200	gi|106879156|gb|BT025705.1	At3g08990.1
at5g20410	CON	MIN	HOUR	DAY	WEEK	UNIGENE	FLCDNA	TAIR
ATGTCTTTGC	0	2	1	1	0	gnl|UG|At#S11721226	gi|13937144|gb|AF372926.1	At5g20410.1
at1g09730	CON	MIN	HOUR	DAY	WEEK	UNIGENE	FLCDNA	TAIR
ATCTTGAAAT	0	0	0	0	1	gnl|UG|At#S11742063	gi|24111338|gb|BT001039.1	At1g09730.1
at5g46620	CON	MIN	HOUR	DAY	WEEK	UNIGENE	FLCDNA	TAIR
GAGCTGTGAG	0	1	0	1	0	no match found	gi|114050608|gb|BT028907.1	At5g46620.1
at4g22350	CON	MIN	HOUR	DAY	WEEK	UNIGENE	FLCDNA	TAIR
CAGATATATG	0	2	0	0	0	no match found	no match found	At4g22350.1
at3g26910	CON	MIN	HOUR	DAY	WEEK	UNIGENE	FLCDNA	TAIR
CAGGAGTAGG	1	1	0	0	0	gnl|UG|At#S11732673	gi|23197973|gb|BT000195.1	At3g26910.1
at5g06710	CON	MIN	HOUR	DAY	WEEK	UNIGENE	FLCDNA	TAIR
TTTTATTGAA	1	1	0	0	1	gnl|UG|At#S18941669	gi|110743068|dbj|AK227423.1|	At5g06710.2
at2g21470	CON	MIN	HOUR	DAY	WEEK	UNIGENE	FLCDNA	TAIR
ATGGTGAGCA	0	0	1	1	0	gnl|UG|At#S18942883	gi|26450534|dbj|AK117731.1|	At2g21470.1
at3g24550	CON	MIN	HOUR	DAY	WEEK	UNIGENE	FLCDNA	TAIR
ATAAAGTTTA	2	3	0	0	0	gnl|UG|At#S11733399	gi|22136227|gb|AY128792.1	At3g24550.1
at3g09580	CON	MIN	HOUR	DAY	WEEK	UNIGENE	FLCDNA	TAIR
TCTTGTATGT	3	0	0	0	0	gnl|UG|At#S11738006	gi|17381087|gb|AY064000.1	At3g09580.1
at1g28070	CON	MIN	HOUR	DAY	WEEK	UNIGENE	FLCDNA	TAIR
GTACCTAAAC	0	1	0	0	0	gnl|UG|At#S24443553	gi|44681469|gb|BT011669.1	non-canonical match
at5g06870	CON	MIN	HOUR	DAY	WEEK	UNIGENE	FLCDNA	TAIR
AACCAATGTT	6	2	6	2	2	gnl|UG|At#S11724401	gi|21406177|gb|AY087447.1	At5g06870.1

<table>
<tr><td>at1g34050</td><td>CON</td><td>MIN</td><td>HOUR</td><td>DAY</td><td>WEEK</td><td>UNIGENE</td><td>FLCDNA</td><td>TAIR</td></tr>
<tr><td>AAAGTTATGT</td><td>0</td><td>0</td><td>1</td><td>0</td><td>0</td><td>no match found</td><td>no match found</td><td>At1g34050.1</td></tr>
<tr><td>TCGAAGGAAA</td><td>1</td><td>1</td><td>0</td><td>0</td><td>0</td><td>gnl|UG|At#S11737560</td><td>no match found</td><td>multiple non-canonical match</td></tr>
<tr><td>at5g65260</td><td>CON</td><td>MIN</td><td>HOUR</td><td>DAY</td><td>WEEK</td><td>UNIGENE</td><td>FLCDNA</td><td>TAIR</td></tr>
<tr><td>GTTAAGCATA</td><td>2</td><td>1</td><td>0</td><td>0</td><td>0</td><td>gnl|UG|At#S11717171</td><td>gi|14423493|gb|AF386984.1</td><td>At5g65260.1</td></tr>
<tr><td>at2g27190</td><td>CON</td><td>MIN</td><td>HOUR</td><td>DAY</td><td>WEEK</td><td>UNIGENE</td><td>FLCDNA</td><td>TAIR</td></tr>
<tr><td>GCTTGATGCA</td><td>3</td><td>3</td><td>2</td><td>2</td><td>0</td><td>gnl|UG|At#S11735740</td><td>gi|21406056|gb|AY087332.1</td><td>At2g27190.1</td></tr>
<tr><td>at3g19895</td><td>CON</td><td>MIN</td><td>HOUR</td><td>DAY</td><td>WEEK</td><td>UNIGENE</td><td>FLCDNA</td><td>TAIR</td></tr>
<tr><td>AGAAGGCGTA</td><td>0</td><td>1</td><td>0</td><td>0</td><td>0</td><td>gnl|UG|At#S11807786</td><td>gi|20453174|gb|AY094458.1</td><td>At3g19895.1</td></tr>
<tr><td>at5g07820</td><td>CON</td><td>MIN</td><td>HOUR</td><td>DAY</td><td>WEEK</td><td>UNIGENE</td><td>FLCDNA</td><td>TAIR</td></tr>
<tr><td>AATCTTTATA</td><td>0</td><td>2</td><td>0</td><td>0</td><td>0</td><td>gnl|UG|At#S34117387</td><td>gi|110737742|dbj|AK228921.1|</td><td>At5g07820.1</td></tr>
<tr><td>at4g26210</td><td>CON</td><td>MIN</td><td>HOUR</td><td>DAY</td><td>WEEK</td><td>UNIGENE</td><td>FLCDNA</td><td>TAIR</td></tr>
<tr><td>CGTTTCAGAT</td><td>0</td><td>1</td><td>2</td><td>1</td><td>0</td><td>gnl|UG|At#S18921944</td><td>gi|21407288|gb|AY088514.1</td><td>At4g26210.2</td></tr>
<tr><td>at3g16620</td><td>CON</td><td>MIN</td><td>HOUR</td><td>DAY</td><td>WEEK</td><td>UNIGENE</td><td>FLCDNA</td><td>TAIR</td></tr>
<tr><td>TTTTTCTTTT</td><td>3</td><td>4</td><td>0</td><td>0</td><td>2</td><td>gnl|UG|At#S34117175</td><td>no match found</td><td>multiple canonical match</td></tr>
<tr><td>at1g21750</td><td>CON</td><td>MIN</td><td>HOUR</td><td>DAY</td><td>WEEK</td><td>UNIGENE</td><td>FLCDNA</td><td>TAIR</td></tr>
<tr><td>GTGTGGACAC</td><td>1</td><td>2</td><td>0</td><td>0</td><td>3</td><td>gnl|UG|At#S11740886</td><td>gi|17104688|gb|AY063059.1</td><td>At1g21750.1</td></tr>
<tr><td>at4g23640</td><td>CON</td><td>MIN</td><td>HOUR</td><td>DAY</td><td>WEEK</td><td>UNIGENE</td><td>FLCDNA</td><td>TAIR</td></tr>
<tr><td>AACTATTACT</td><td>0</td><td>1</td><td>0</td><td>1</td><td>0</td><td>gnl|UG|At#S11723709</td><td>gi|110741515|dbj|AK226593.1|</td><td>At4g23640.1</td></tr>
<tr><td>at2g39840</td><td>CON</td><td>MIN</td><td>HOUR</td><td>DAY</td><td>WEEK</td><td>UNIGENE</td><td>FLCDNA</td><td>TAIR</td></tr>
<tr><td>CATATGGTCG</td><td>1</td><td>0</td><td>0</td><td>1</td><td>0</td><td>gnl|UG|At#S11732638</td><td>gi|110737953|dbj|AK229028.1|</td><td>At2g39840.1</td></tr>
<tr><td>at2g30470</td><td>CON</td><td>MIN</td><td>HOUR</td><td>DAY</td><td>WEEK</td><td>UNIGENE</td><td>FLCDNA</td><td>TAIR</td></tr>
<tr><td>GAACTGGCTG</td><td>0</td><td>2</td><td>0</td><td>1</td><td>0</td><td>gnl|UG|At#S15460952</td><td>gi|17064831|gb|AY062492.1</td><td>At2g30470.1</td></tr>
<tr><td>at3g21330</td><td>CON</td><td>MIN</td><td>HOUR</td><td>DAY</td><td>WEEK</td><td>UNIGENE</td><td>FLCDNA</td><td>TAIR</td></tr>
<tr><td>GCGTCTTGAT</td><td>0</td><td>0</td><td>0</td><td>0</td><td>1</td><td>gnl|UG|At#S14273670</td><td>gi|28393736|gb|BT004279.1</td><td>At3g21330.1</td></tr>
<tr><td>at1g11950</td><td>CON</td><td>MIN</td><td>HOUR</td><td>DAY</td><td>WEEK</td><td>UNIGENE</td><td>FLCDNA</td><td>TAIR</td></tr>
<tr><td>GCTATTGTAG</td><td>0</td><td>0</td><td>1</td><td>0</td><td>0</td><td>gnl|UG|At#S11741841</td><td>no match found</td><td>At1g11950.1</td></tr>
<tr><td>at3g07320</td><td>CON</td><td>MIN</td><td>HOUR</td><td>DAY</td><td>WEEK</td><td>UNIGENE</td><td>FLCDNA</td><td>TAIR</td></tr>
<tr><td>AAATAATTAA</td><td>0</td><td>1</td><td>0</td><td>0</td><td>0</td><td>gnl|UG|At#S11816248</td><td>gi|21406233|gb|AY087496.1</td><td>At3g07320.1</td></tr>
<tr><td>at1g12440</td><td>CON</td><td>MIN</td><td>HOUR</td><td>DAY</td><td>WEEK</td><td>UNIGENE</td><td>FLCDNA</td><td>TAIR</td></tr>
<tr><td>AACCTCTGCT</td><td>0</td><td>1</td><td>0</td><td>0</td><td>0</td><td>no match found</td><td>gi|38566601|gb|BT010824.1</td><td>non-canonical match</td></tr>
<tr><td>TTTTGCAACA</td><td>5</td><td>0</td><td>3</td><td>1</td><td>3</td><td>gnl|UG|At#S15461164</td><td>gi|110740564|dbj|AK226223.1|</td><td>At1g12440.1</td></tr>
<tr><td>at3g47348</td><td>CON</td><td>MIN</td><td>HOUR</td><td>DAY</td><td>WEEK</td><td>UNIGENE</td><td>FLCDNA</td><td>TAIR</td></tr>
<tr><td>GTTCTGTAAA</td><td>1</td><td>0</td><td>0</td><td>2</td><td>0</td><td>no match found</td><td>gi|26451682|dbj|AK118322.1|</td><td>non-canonical match</td></tr>
<tr><td>at2g32410</td><td>CON</td><td>MIN</td><td>HOUR</td><td>DAY</td><td>WEEK</td><td>UNIGENE</td><td>FLCDNA</td><td>TAIR</td></tr>
<tr><td>TATATTGATT</td><td>0</td><td>2</td><td>0</td><td>0</td><td>0</td><td>gnl|UG|At#S28282216</td><td>no match found</td><td>At2g32410.1</td></tr>
<tr><td>at3g03290</td><td>CON</td><td>MIN</td><td>HOUR</td><td>DAY</td><td>WEEK</td><td>UNIGENE</td><td>FLCDNA</td><td>TAIR</td></tr>
<tr><td>AATGAATGCA</td><td>1</td><td>0</td><td>0</td><td>0</td><td>0</td><td>no match found</td><td>no match found</td><td>At3g03290.1</td></tr>
<tr><td>at3g14790</td><td>CON</td><td>MIN</td><td>HOUR</td><td>DAY</td><td>WEEK</td><td>UNIGENE</td><td>FLCDNA</td><td>TAIR</td></tr>
<tr><td>TAGCTGTCTT</td><td>4</td><td>1</td><td>1</td><td>4</td><td>2</td><td>gnl|UG|At#S11736413</td><td>gi|19310442|gb|AY078958.1</td><td>At3g14790.1</td></tr>
<tr><td>at3g08960</td><td>CON</td><td>MIN</td><td>HOUR</td><td>DAY</td><td>WEEK</td><td>UNIGENE</td><td>FLCDNA</td><td>TAIR</td></tr>
<tr><td>TGCTTGTGTA</td><td>0</td><td>1</td><td>0</td><td>0</td><td>0</td><td>no match found</td><td>no match found</td><td>At3g08960.1</td></tr>
</table>

at4g13220	CON	MIN	HOUR	DAY	WEEK	UNIGENE	FLCDNA	TAIR
AGACAGAACT	7	4	7	6	5	gnl|UG|At#S11725524	gi|26453225|dbj|AK119115.1|	At4g13220.1
at5g48910	CON	MIN	HOUR	DAY	WEEK	UNIGENE	FLCDNA	TAIR
CTCGTGTATG	0	0	1	0	0	gnl|UG|At#S11705952	gi|15810558|gb|AY056318.1	At5g48910.1
at5g13190	CON	MIN	HOUR	DAY	WEEK	UNIGENE	FLCDNA	TAIR
AATGAACCGT	5	2	6	1	2	no match found	gi|21407076|gb|AY088302.1	At5g13190.1
at1g51560	CON	MIN	HOUR	DAY	WEEK	UNIGENE	FLCDNA	TAIR
CAAAATCTCA	0	1	1	1	0	gnl|UG|At#S11733933	gi|18377804|gb|AY074356.1	At1g51560.1
at4g24390	CON	MIN	HOUR	DAY	WEEK	UNIGENE	FLCDNA	TAIR
ATTTGTTTTG	0	3	0	1	0	no match found	no match found	At4g24390.2
at2g30380	CON	MIN	HOUR	DAY	WEEK	UNIGENE	FLCDNA	TAIR
CCGATTAAAA	1	0	0	0	0	no match found	no match found	At2g30380.1
at2g18100	CON	MIN	HOUR	DAY	WEEK	UNIGENE	FLCDNA	TAIR
GCATTGCAAA	0	1	0	0	0	no match found	gi|28416556|gb|BT004563.1	multiple non-canonical match
at5g52120	CON	MIN	HOUR	DAY	WEEK	UNIGENE	FLCDNA	TAIR
CGGGTGACTT	0	0	1	0	0	gnl|UG|At#S11718504	gi|18175687|gb|AY072088.1	At5g52120.1
at5g67150	CON	MIN	HOUR	DAY	WEEK	UNIGENE	FLCDNA	TAIR
TATGTGTTGT	1	0	1	0	0	gnl|UG|At#S11716980	gi|21436436|gb|AY117344.1	At5g67150.1
at5g13480	CON	MIN	HOUR	DAY	WEEK	UNIGENE	FLCDNA	TAIR
AATCCTCAGA	0	2	0	0	0	gnl|UG|At#S18909025	gi|45773815|gb|BT012225.1	multiple non-canonical match
at5g48790	CON	MIN	HOUR	DAY	WEEK	UNIGENE	FLCDNA	TAIR
CTTGTCAAGC	1	3	0	3	1	gnl|UG|At#S11718839	gi|14326507|gb|AF385707.1	At5g48790.1
at5g44785	CON	MIN	HOUR	DAY	WEEK	UNIGENE	FLCDNA	TAIR
GCCTAAGCTT	1	2	1	2	0	gnl|UG|At#S18941527	gi|28973012|gb|BT005411.1	At5g44785.1
at2g36570	CON	MIN	HOUR	DAY	WEEK	UNIGENE	FLCDNA	TAIR
TTTTGTGTTT	1	0	0	1	0	gnl|UG|At#S11733457	gi|110735701|dbj|AK227854.1|	At2g36570.1
at1g03060	CON	MIN	HOUR	DAY	WEEK	UNIGENE	FLCDNA	TAIR
TTTTGCTTCA	0	1	2	0	0	gnl|UG|At#S11742808	no match found	At1g03060.1
at4g21105	CON	MIN	HOUR	DAY	WEEK	UNIGENE	FLCDNA	TAIR
TCGAATGGAA	5	14	17	5	2	gnl|UG|At#S11724157	gi|17386155|gb|AF446891.1	At4g21105.1
at5g59613	CON	MIN	HOUR	DAY	WEEK	UNIGENE	FLCDNA	TAIR
AAAGAGAGGA	6	14	12	4	6	gnl|UG|At#S11816799	gi|11762191|gb|AF325022.1	At5g59613.1
at5g22030	CON	MIN	HOUR	DAY	WEEK	UNIGENE	FLCDNA	TAIR
TTGCGGGGAA	0	0	1	0	0	gnl|UG|At#S11721041	no match found	At5g22030.1
at3g48320	CON	MIN	HOUR	DAY	WEEK	UNIGENE	FLCDNA	TAIR
AGCTACTTAA	1	0	0	0	0	gnl|UG|At#S34115460	gi|110741461|dbj|AK226557.1|	At3g48320.1
at4g04540	CON	MIN	HOUR	DAY	WEEK	UNIGENE	FLCDNA	TAIR
GAAAAGATGG	0	0	1	1	0	gnl|UG|At#S11726517	no match found	multiple non-canonical match
at3g24630	CON	MIN	HOUR	DAY	WEEK	UNIGENE	FLCDNA	TAIR
AGCCAGAAAC	0	1	0	0	0	gnl|UG|At#S11733372	no match found	multiple non-canonical match

at2g14255	CON	MIN	HOUR	DAY	WEEK	UNIGENE	FLCDNA	TAIR
CATAAACCCT	1	0	1	2	1	gnl|UG|At#S18942936	no match found	At2g14255.1

at1g34340	CON	MIN	HOUR	DAY	WEEK	UNIGENE	FLCDNA	TAIR
GATTGCCTCC	0	1	0	0	0	no match found	gi|30102821|gb|BT006521.1	non-canonical match
GTTACAAGTT	0	0	1	0	0	gnl|UG|At#S11822100	gi|25083435|gb|BT002066.1	non-canonical match

at1g12020	CON	MIN	HOUR	DAY	WEEK	UNIGENE	FLCDNA	TAIR
TGACAACATT	0	1	0	0	1	gnl|UG|At#S11741834	no match found	At1g12020.1

at1g03350	CON	MIN	HOUR	DAY	WEEK	UNIGENE	FLCDNA	TAIR
CGATTGATGA	0	1	0	0	1	gnl|UG|At#S11742779	gi|15293190|gb|AY051029.1	At1g03350.1

at1g19440	CON	MIN	HOUR	DAY	WEEK	UNIGENE	FLCDNA	TAIR
GTTGTTTTTT	0	0	0	1	1	no match found	gi|21407786|gb|AY089012.1	non-canonical match
TTTTCAGATC	2	0	0	1	1	gnl|UG|At#S11741109	gi|110740670|dbj|AK226279.1|	At1g19440.1

at5g44770	CON	MIN	HOUR	DAY	WEEK	UNIGENE	FLCDNA	TAIR
TATCATTTGC	1	0	0	0	0	no match found	no match found	At5g44770.1

at5g11110	CON	MIN	HOUR	DAY	WEEK	UNIGENE	FLCDNA	TAIR
GTGGTGTTCG	1	1	0	0	1	gnl|UG|At#S11723471	gi|17978914|gb|AY069868.1	At5g11110.1

at5g19630	CON	MIN	HOUR	DAY	WEEK	UNIGENE	FLCDNA	TAIR
AAAAGATAGA	10	0	2	2	6	gnl|UG|At#S11721419	gi|28827701|gb|BT005162.1	At5g19630.1

at5g22920	CON	MIN	HOUR	DAY	WEEK	UNIGENE	FLCDNA	TAIR
AGGAGATTAT	2	19	3	4	0	gnl|UG|At#S11720886	gi|15450520|gb|AY052362.1	At5g22920.1

at5g22360	CON	MIN	HOUR	DAY	WEEK	UNIGENE	FLCDNA	TAIR
GTCCTCCTTG	3	3	0	1	0	gnl|UG|At#S11705220	gi|110743435|dbj|AK227613.1|	At5g22360.1

at2g38320	CON	MIN	HOUR	DAY	WEEK	UNIGENE	FLCDNA	TAIR
TTTTTCTTAA	0	0	0	1	0	no match found	gi|13878094|gb|AF370310.1	non-canonical match

at2g47090	CON	MIN	HOUR	DAY	WEEK	UNIGENE	FLCDNA	TAIR
GTTCCATCGA	0	1	0	0	0	gnl|UG|At#S11730928	gi|20334759|gb|AY093980.1	At2g47090.1

at3g53520	CON	MIN	HOUR	DAY	WEEK	UNIGENE	FLCDNA	TAIR
GTTAGTGACT	2	4	1	0	1	gnl|UG|At#S15459278	gi|17473548|gb|AY065075.1	At3g53520.2

at1g46768	CON	MIN	HOUR	DAY	WEEK	UNIGENE	FLCDNA	TAIR
CCTTCTTCCT	0	1	1	2	1	no match found	no match found	At1g46768.1
TAGTTTGTTT	0	1	0	1	0	no match found	gi|21405548|gb|AY086838.1	At1g20490.1

at3g51860	CON	MIN	HOUR	DAY	WEEK	UNIGENE	FLCDNA	TAIR
AACAATTTGG	0	1	2	1	1	no match found	gi|56382020|gb|BT020374.1	non-canonical match
AATGAGGGAG	0	2	0	0	2	gnl|UG|At#S11729086	gi|16604311|gb|AY058054.1	At3g51860.1

at5g18490	CON	MIN	HOUR	DAY	WEEK	UNIGENE	FLCDNA	TAIR
TTGGTGATTG	1	2	1	2	0	gnl|UG|At#S11706798	gi|21405892|gb|AY087168.1	At5g18490.1

at5g53880	CON	MIN	HOUR	DAY	WEEK	UNIGENE	FLCDNA	TAIR
TTATTTTTAT	7	3	1	2	6	gnl|UG|At#S11718328	gi|23198025|gb|BT000221.1	At5g53880.1

at3g02370	CON	MIN	HOUR	DAY	WEEK	UNIGENE	FLCDNA	TAIR
GAGTTCCTTG	0	1	0	0	0	gnl|UG|At#S11739975	gi|45680353|gb|BT012043.1	multiple non-canonical match

at1g48350	CON	MIN	HOUR	DAY	WEEK	UNIGENE	FLCDNA	TAIR
ATTGAAATTT	0	1	0	0	0	gnl|UG|At#S43849896	no match found	multiple non-canonical match
GACGTATTGA	13	10	9	16	4	gnl|UG|At#S11735088	gi|12484208|gb|AF336922.1	At1g48350.1

at2g25050	CON	MIN	HOUR	DAY	WEEK	UNIGENE	FLCDNA	TAIR
ACAAAACACA	1	0	0	1	0	no match found	gi|72197155|gb|DQ108704.1	At5g08310.1

at2g42300	CON	MIN	HOUR	DAY	WEEK	UNIGENE	FLCDNA	TAIR
GCCTTTCTAT	0	1	1	2	1	gnl|UG|At#S21736656	gi|17529249|gb|AY065411.1	At2g42300.1

at5g09620	CON	MIN	HOUR	DAY	WEEK	UNIGENE	FLCDNA	TAIR
GCTGCACCAC	1	1	0	1	1	no match found	gi|40823502|gb|BT011250.1	At5g09620.1

at2g17150	CON	MIN	HOUR	DAY	WEEK	UNIGENE	FLCDNA	TAIR
ATAAAGGCAA	1	0	0	0	0	no match found	gi|62320800|dbj|AK221727.1|	multiple non-canonical match
GGAATCCTGT	0	0	0	1	0	gnl|UG|At#S11807260	gi|20259332|gb|AY090943.1	At2g17150.1

at3g48860	CON	MIN	HOUR	DAY	WEEK	UNIGENE	FLCDNA	TAIR
TAAGTTTCAC	2	2	1	0	0	no match found	gi|20260533|gb|AY093166.1	At3g48860.2

at3g22160	CON	MIN	HOUR	DAY	WEEK	UNIGENE	FLCDNA	TAIR
CTTGCATTTT	0	2	2	0	0	gnl|UG|At#S11734172	no match found	At3g22160.1
AACAACCCTA	0	0	1	0	0	no match found	gi|21407354|gb|AY088580.1	non-canonical match
GCACGGAAGT	0	1	0	0	0	no match found	gi|26449919|dbj|AK117415.1|	non-canonical match

at5g17260	CON	MIN	HOUR	DAY	WEEK	UNIGENE	FLCDNA	TAIR
GAAATGAAGA	0	3	0	1	0	gnl|UG|At#S11722017	no match found	multiple canonical match

at3g44670	CON	MIN	HOUR	DAY	WEEK	UNIGENE	FLCDNA	TAIR
GGTTTGATTT	1	2	1	2	0	gnl|UG|At#S38433649	gi|19347775|gb|AY080663.1	At3g44670.1

at4g06746	CON	MIN	HOUR	DAY	WEEK	UNIGENE	FLCDNA	TAIR
AATAAGTTGC	0	0	0	2	1	no match found	gi|88900403|gb|BT024689.1	At4g06746.1

at2g22980	CON	MIN	HOUR	DAY	WEEK	UNIGENE	FLCDNA	TAIR
TTCAAAAGAT	1	2	2	0	0	gnl|UG|At#S11806840	gi|15294269|gb|AF410326.1	At2g22980.1

at3g49840	CON	MIN	HOUR	DAY	WEEK	UNIGENE	FLCDNA	TAIR
TGTGTAGTCA	1	0	0	0	0	gnl|UG|At#S11729475	no match found	non-canonical match
CTACCTCGTT	0	1	0	0	0	gnl|UG|At#S18918802	gi|110741809|dbj|AK226747.1|	non-canonical match

at1g07240	CON	MIN	HOUR	DAY	WEEK	UNIGENE	FLCDNA	TAIR
TAATTTTATC	0	2	0	0	0	gnl|UG|At#S11742310	gi|20259789|gb|AY093243.1	At1g07240.1
TGTTAAACTA	0	1	1	0	0	no match found	gi|110742255|dbj|AK226987.1|	non-canonical match

at1g69880	CON	MIN	HOUR	DAY	WEEK	UNIGENE	FLCDNA	TAIR
GTTGTGGGTG	0	0	1	1	0	no match found	gi|28372831|gb|BT003670.1	multiple non-canonical match
TAATATAAAA	0	3	1	0	0	gnl|UG|At#S11729126	gi|26450715|dbj|AK117825.1|	At1g69880.1

at1g15910	CON	MIN	HOUR	DAY	WEEK	UNIGENE	FLCDNA	TAIR
ATCTCTTTTG	0	1	1	0	0	gnl|UG|At#S11741446	no match found	multiple canonical match

at1g48160	CON	MIN	HOUR	DAY	WEEK	UNIGENE	FLCDNA	TAIR
CAAGTAGGGA	0	1	0	0	0	gnl|UG|At#S11707905	gi|21407120|gb|AY088346.1	At1g48160.1

at1g60970	CON	MIN	HOUR	DAY	WEEK	UNIGENE	FLCDNA	TAIR
GTGAATGTGT	1	0	0	0	0	gnl|UG|At#S11731181	gi|15450726|gb|AY053405.1	At1g60970.1

at4g39730	CON	MIN	HOUR	DAY	WEEK	UNIGENE	FLCDNA	TAIR
GTTGGTACGT	2	8	3	12	12	gnl|UG|At#S20298048	gi|20147334|gb|AY093757.1	At4g39730.1

at3g52580	CON	MIN	HOUR	DAY	WEEK	UNIGENE	FLCDNA	TAIR
AAAATTGGTC	1	3	1	1	1	gnl|UG|At#S11728956	gi|21700836|gb|AY124833.1	At3g52580.1

at4g32160	CON	MIN	HOUR	DAY	WEEK	UNIGENE	FLCDNA	TAIR
GATACTGAAA	0	1	0	0	0	gnl|UG|At#S11707984	gi|16604600|gb|AY059745.1	At4g32160.1
at1g35515	CON	MIN	HOUR	DAY	WEEK	UNIGENE	FLCDNA	TAIR
GATGTCTGGC	1	3	0	0	0	gnl|UG|At#S21736319	gi|51970853|dbj|AK176356.1|	non-canonical match
at4g14905	CON	MIN	HOUR	DAY	WEEK	UNIGENE	FLCDNA	TAIR
TGCTTACTTT	11	4	12	6	2	no match found	gi|110740532|dbj|AK226207.1|	At5g65480.1
TCGGTATATA	1	1	0	0	2	gnl|UG|At#S18941862	gi|26451032|dbj|AK117987.1|	At4g14905.2
at4g26910	CON	MIN	HOUR	DAY	WEEK	UNIGENE	FLCDNA	TAIR
CTTTCTTTGT	1	0	0	0	0	gnl|UG|At#S11723141	no match found	At4g26910.1
CACTCGATTG	0	3	1	1	0	no match found	gi|21402958|gb|AY084248.1	non-canonical match
at1g72416	CON	MIN	HOUR	DAY	WEEK	UNIGENE	FLCDNA	TAIR
TTGTAAAAGT	0	5	1	1	1	gnl|UG|At#S37211312	no match found	pseudo chromosome match
at3g57020	CON	MIN	HOUR	DAY	WEEK	UNIGENE	FLCDNA	TAIR
TTGTATCTAC	0	0	2	1	7	gnl|UG|At#S18903159	gi|21407987|gb|AY089213.1	At3g57020.1
at1g06740	CON	MIN	HOUR	DAY	WEEK	UNIGENE	FLCDNA	TAIR
AACTTGATCA	0	0	1	0	0	gnl|UG|At#S24442885	gi|62319904|dbj|AK221278.1|	At1g06740.1
at1g80460	CON	MIN	HOUR	DAY	WEEK	UNIGENE	FLCDNA	TAIR
AGAGACATCT	2	0	1	1	0	gnl|UG|At#S11726318	no match found	At1g80460.1
at2g35640	CON	MIN	HOUR	DAY	WEEK	UNIGENE	FLCDNA	TAIR
GAGTAATTAA	0	0	1	1	0	no match found	no match found	At2g35640.1
at2g46060	CON	MIN	HOUR	DAY	WEEK	UNIGENE	FLCDNA	TAIR
AAAAGATCAC	0	3	1	0	1	no match found	gi|20259485|gb|AY091042.1	At2g46060.2
at3g52210	CON	MIN	HOUR	DAY	WEEK	UNIGENE	FLCDNA	TAIR
TCTTTCTTCC	1	0	0	0	0	no match found	no match found	At3g52210.1
TGTTTTAACA	2	0	1	0	1	gnl|UG|At#S11729022	gi|51971450|dbj|AK176627.1|	At5g35890.1
at1g79510	CON	MIN	HOUR	DAY	WEEK	UNIGENE	FLCDNA	TAIR
AGCTTGTACA	2	2	3	4	3	gnl|UG|At#S11702562	gi|21404879|gb|AY086169.1	At1g79510.2
at5g03700	CON	MIN	HOUR	DAY	WEEK	UNIGENE	FLCDNA	TAIR
GCTTTGGTTT	0	0	0	0	1	gnl|UG|At#S11725235	no match found	multiple non-canonical match
at1g26110	CON	MIN	HOUR	DAY	WEEK	UNIGENE	FLCDNA	TAIR
GGCTGCCAAT	0	3	1	0	0	gnl|UG|At#S11740287	gi|18700275|gb|AY078047.1	At1g26110.1
at5g07300	CON	MIN	HOUR	DAY	WEEK	UNIGENE	FLCDNA	TAIR
AATGCCTACA	0	1	0	0	0	gnl|UG|At#S11724300	gi|33589665|gb|BT010130.1	At5g07300.1
at1g69930	CON	MIN	HOUR	DAY	WEEK	UNIGENE	FLCDNA	TAIR
GAATTAATAA	0	1	0	0	0	gnl|UG|At#S11729115	gi|26453279|dbj|AK119143.1|	At1g69930.1
at2g25200	CON	MIN	HOUR	DAY	WEEK	UNIGENE	FLCDNA	TAIR
GGATGTTTTC	0	0	0	1	0	gnl|UG|At#S21737357	gi|51968777|dbj|AK175318.1|	At2g25200.1
at4g38950	CON	MIN	HOUR	DAY	WEEK	UNIGENE	FLCDNA	TAIR
TCTTGTATAT	0	0	0	0	1	gnl|UG|At#S11721107	gi|110740202|dbj|AK230191.1|	At4g38950.1
at5g63440	CON	MIN	HOUR	DAY	WEEK	UNIGENE	FLCDNA	TAIR
CTTATGCGAC	2	1	0	0	1	gnl|UG|At#S15437859	gi|26452403|dbj|AK118693.1|	At5g63440.1
at5g01820	CON	MIN	HOUR	DAY	WEEK	UNIGENE	FLCDNA	TAIR

GGGGATGAAG	2	2	0	3	1	gnl|UG|At#S11725702	gi|13430553|gb|AF360189.1	At5g01820.1
at2g34925	CON	MIN	HOUR	DAY	WEEK	UNIGENE	FLCDNA	TAIR
GAGTCCCAAG	0	0	1	0	0	gnl|UG|At#S38433881	no match found	At5g01820.1
at3g03520	CON	MIN	HOUR	DAY	WEEK	UNIGENE	FLCDNA	TAIR
AGATCGTTGA	0	1	1	0	0	gnl|UG|At#S11739640	gi|23297653|gb|AY150477.1	At3g03520.1
at1g60990	CON	MIN	HOUR	DAY	WEEK	UNIGENE	FLCDNA	TAIR
AGTTTTGCTG	2	0	2	1	0	gnl|UG|At#S38434136	no match found	At3g03520.1
ACATATGATG	0	1	0	0	0	no match found	gi|51969109|dbj|AK175484.1|	non-canonical match
at2g19270	CON	MIN	HOUR	DAY	WEEK	UNIGENE	FLCDNA	TAIR
AAGCACAAGG	0	2	1	1	1	gnl|UG|At#S11737655	gi|22135887|gb|AY128323.1	At2g19270.1
at1g71380	CON	MIN	HOUR	DAY	WEEK	UNIGENE	FLCDNA	TAIR
GTTGGATTCT	1	0	16	1	1	gnl|UG|At#S11728794	no match found	At1g71380.1
at3g14910	CON	MIN	HOUR	DAY	WEEK	UNIGENE	FLCDNA	TAIR
CTCCACGCAA	0	0	1	1	0	gnl|UG|At#S24639683	gi|115646782|gb|BT029181.1	At3g14910.1
at5g25590	CON	MIN	HOUR	DAY	WEEK	UNIGENE	FLCDNA	TAIR
TCACAGAGAG	0	1	1	0	1	gnl|UG|At#S11807176	gi|20259507|gb|AY091053.1	At5g25590.1
at3g26580	CON	MIN	HOUR	DAY	WEEK	UNIGENE	FLCDNA	TAIR
TATAAATGTG	5	6	3	0	1	gnl|UG|At#S11732782	gi|22655243|gb|AY140071.1	At3g26580.1
at3g17570	CON	MIN	HOUR	DAY	WEEK	UNIGENE	FLCDNA	TAIR
GAGGAGATTT	0	0	0	1	0	gnl|UG|At#S11735570	no match found	At3g17570.1
at2g23070	CON	MIN	HOUR	DAY	WEEK	UNIGENE	FLCDNA	TAIR
GACAAAGTTC	0	4	0	0	0	gnl|UG|At#S11736720	gi|20148298|gb|AY081478.1	At2g23070.1
at3g48710	CON	MIN	HOUR	DAY	WEEK	UNIGENE	FLCDNA	TAIR
TAGTATCAGG	1	0	0	1	1	gnl|UG|At#S34116174	gi|110739699|dbj|AK229931.1|	At3g48710.1
at3g45638	CON	MIN	HOUR	DAY	WEEK	UNIGENE	FLCDNA	TAIR
GCTCCATCAG	0	1	0	0	0	gnl|UG|At#S37211565	gi|62320886|dbj|AK221771.1|	At3g48710.1
at3g20000	CON	MIN	HOUR	DAY	WEEK	UNIGENE	FLCDNA	TAIR
TACAACTACT	0	3	0	0	1	gnl|UG|At#S11734853	gi|21436304|gb|AY117216.1	At3g20000.1
at3g60350	CON	MIN	HOUR	DAY	WEEK	UNIGENE	FLCDNA	TAIR
CTGCAAAGAA	1	1	1	1	0	gnl|UG|At#S11727378	no match found	non-canonical match
at2g32980	CON	MIN	HOUR	DAY	WEEK	UNIGENE	FLCDNA	TAIR
TCTGCGATGA	2	0	1	0	1	no match found	gi|16612287|gb|AF439838.1	multiple non-canonical match
TGACTATCAT	0	1	0	0	2	gnl|UG|At#S11734291	no match found	At2g32980.1
at1g12860	CON	MIN	HOUR	DAY	WEEK	UNIGENE	FLCDNA	TAIR
TGTTTGGGGA	2	4	2	2	1	no match found	gi|26451078|dbj|AK118011.1|	multiple non-canonical match
at3g61320	CON	MIN	HOUR	DAY	WEEK	UNIGENE	FLCDNA	TAIR
TGATGGCTTA	0	1	2	0	0	gnl|UG|At#S18919088	gi|16604548|gb|AY058894.1	At3g61320.1
at5g14850	CON	MIN	HOUR	DAY	WEEK	UNIGENE	FLCDNA	TAIR
ATCCAACATT	0	1	0	0	0	no match found	gi|15215851|gb|AY050456.1	multiple canonical match
at1g48600	CON	MIN	HOUR	DAY	WEEK	UNIGENE	FLCDNA	TAIR
GGCTAATTGA	1	0	0	0	1	gnl|UG|At#S43850476	no match found	pseudo chromosome match

at2g30695	CON	MIN	HOUR	DAY	WEEK	UNIGENE	FLCDNA	TAIR
TTTTTGAGAA	1	1	1	0	0	no match found	gi|20334857|gb|AY094029.1	At2g30695.1
TGTTTTTATT	0	2	0	0	1	gnl|UG|At#S18898719	no match found	non-canonical match
at1g33680	CON	MIN	HOUR	DAY	WEEK	UNIGENE	FLCDNA	TAIR
ACTCATTCTC	4	4	4	2	3	gnl|UG|At#S34116253	gi|110739540|dbj|AK229850.1|	multiple non-canonical match
at2g20260	CON	MIN	HOUR	DAY	WEEK	UNIGENE	FLCDNA	TAIR
GTTTATCTCT	37	56	29	18	9	no match found	gi|14596070|gb|AY042823.1	At2g20260.1
at1g31870	CON	MIN	HOUR	DAY	WEEK	UNIGENE	FLCDNA	TAIR
TGAATCAGAT	0	1	0	0	0	gnl|UG|At#S28282556	no match found	At1g31870.1
at1g14980	CON	MIN	HOUR	DAY	WEEK	UNIGENE	FLCDNA	TAIR
GATAACCCAA	10	4	5	8	7	gnl|UG|At#S11741536	gi|21405418|gb|AY086708.1	At1g14980.1
at1g09810	CON	MIN	HOUR	DAY	WEEK	UNIGENE	FLCDNA	TAIR
AATTGCTTTA	0	1	1	0	0	gnl|UG|At#S11742054	gi|62320738|dbj|AK221696.1|	At1g09810.1
at1g18570	CON	MIN	HOUR	DAY	WEEK	UNIGENE	FLCDNA	TAIR
AATGTAGTTC	1	8	1	0	0	gnl|UG|At#S11741194	no match found	At1g18570.1
TCAATATGAT	0	1	0	0	0	no match found	gi|26449755|dbj|AK117331.1|	non-canonical match
at1g21525	CON	MIN	HOUR	DAY	WEEK	UNIGENE	FLCDNA	TAIR
ATCGTTTATG	0	0	2	1	1	gnl|UG|At#S11810946	gi|21405838|gb|AY087114.1	At1g21525.1
at2g04020	CON	MIN	HOUR	DAY	WEEK	UNIGENE	FLCDNA	TAIR
ACTTCTTTTG	1	0	0	0	0	no match found	no match found	At2g04020.1
at2g29890	CON	MIN	HOUR	DAY	WEEK	UNIGENE	FLCDNA	TAIR
ACAAACAGCA	1	0	0	0	0	gnl|UG|At#S28282237	gi|26451416|dbj|AK118185.1|	At2g29890.1
at1g03660	CON	MIN	HOUR	DAY	WEEK	UNIGENE	FLCDNA	TAIR
TCTTTAGCTC	1	0	0	0	0	gnl|UG|At#S11742751	no match found	At1g03660.1
at4g33070	CON	MIN	HOUR	DAY	WEEK	UNIGENE	FLCDNA	TAIR
GAATAAAGTA	1	0	0	3	9	gnl|UG|At#S11722093	gi|17979050|gb|AY070036.1	At4g33070.1
at5g04740	CON	MIN	HOUR	DAY	WEEK	UNIGENE	FLCDNA	TAIR
TGAAAGAAGA	0	1	0	2	0	gnl|UG|At#S18910659	gi|21389644|gb|AY114702.1	At5g04740.1
at1g19850	CON	MIN	HOUR	DAY	WEEK	UNIGENE	FLCDNA	TAIR
CTGATTTGTA	2	0	1	0	0	gnl|UG|At#S11741069	gi|25083307|gb|BT002050.1	At1g19850.1
at3g04490	CON	MIN	HOUR	DAY	WEEK	UNIGENE	FLCDNA	TAIR
AAGGAATATT	0	0	0	1	0	gnl|UG|At#S11739369	no match found	At3g04490.1
at5g58730	CON	MIN	HOUR	DAY	WEEK	UNIGENE	FLCDNA	TAIR
CGTAGACTCT	0	1	1	1	0	gnl|UG|At#S11717835	gi|16604508|gb|AY058873.1	At5g58730.1
at1g48610	CON	MIN	HOUR	DAY	WEEK	UNIGENE	FLCDNA	TAIR
GCGAAAACAG	0	0	1	0	0	gnl|UG|At#S18942411	gi|15081762|gb|AY048274.1	At1g48610.2
at4g31210	CON	MIN	HOUR	DAY	WEEK	UNIGENE	FLCDNA	TAIR
CTAATAAACA	0	2	0	0	1	gnl|UG|At#S11722413	no match found	At4g31210.1
at5g35970	CON	MIN	HOUR	DAY	WEEK	UNIGENE	FLCDNA	TAIR
TATATTTTTA	12	6	7	7	12	gnl|UG|At#S11720106	no match found	At5g60930.1
at4g22380	CON	MIN	HOUR	DAY	WEEK	UNIGENE	FLCDNA	TAIR
GTTCTTTAAC	2	2	1	7	2	gnl|UG|At#S11723932	no match found	At4g22380.1

at1g70120	CON	MIN	HOUR	DAY	WEEK	UNIGENE	FLCDNA	TAIR
CTTGGTCTAG	0	1	0	0	1	no match found	no match found	At1g70120.1
at1g65500	CON	MIN	HOUR	DAY	WEEK	UNIGENE	FLCDNA	TAIR
TGCTCCTTTC	1	2	1	0	4	gnl|UG|At#S11730027	gi|21405152|gb|AY086442.1	At1g65500.1
at1g34095	CON	MIN	HOUR	DAY	WEEK	UNIGENE	FLCDNA	TAIR
GCACGTGGTG	0	0	1	0	0	gnl|UG|At#S11816452	no match found	multiple non-canonical match
at2g27310	CON	MIN	HOUR	DAY	WEEK	UNIGENE	FLCDNA	TAIR
CGGGTCAGCG	0	1	0	0	0	gnl|UG|At#S11735713	gi|13877666|gb|AF370592.1	At2g27310.1
at3g55470	CON	MIN	HOUR	DAY	WEEK	UNIGENE	FLCDNA	TAIR
TGTATGAAAT	2	2	0	1	1	gnl|UG|At#S11728428	gi|26449379|dbj|AK117139.1|	non-canonical match
TGAAAGAGCT	0	2	0	0	0	no match found	gi|107738043|gb|BT025730.1	non-canonical match
at5g03240	CON	MIN	HOUR	DAY	WEEK	UNIGENE	FLCDNA	TAIR
TTAAACAATT	4	1	2	4	1	gnl|UG|At#S28281863	gi|27311628|gb|BT002420.1	At5g03240.1
at1g49550	CON	MIN	HOUR	DAY	WEEK	UNIGENE	FLCDNA	TAIR
GCTTGTGATG	0	0	0	0	1	no match found	no match found	At1g49550.1
at4g09647	CON	MIN	HOUR	DAY	WEEK	UNIGENE	FLCDNA	TAIR
TAAAAAAAAG	0	1	0	2	0	gnl|UG|At#S28282076	no match found	non-canonical match
at1g10960	CON	MIN	HOUR	DAY	WEEK	UNIGENE	FLCDNA	TAIR
CCGTGCCGGT	13	16	25	22	0	gnl|UG|At#S11811993	gi|22135780|gb|AY127948.1	At1g10960.1
at5g45110	CON	MIN	HOUR	DAY	WEEK	UNIGENE	FLCDNA	TAIR
AAACTGCACC	1	0	0	0	0	gnl|UG|At#S11817641	gi|22654982|gb|AY139763.1	At5g45110.1
at1g63840	CON	MIN	HOUR	DAY	WEEK	UNIGENE	FLCDNA	TAIR
GCCCGACCCA	0	1	2	0	0	gnl|UG|At#S11730371	gi|33589711|gb|BT010153.1	At1g63840.1
at4g15420	CON	MIN	HOUR	DAY	WEEK	UNIGENE	FLCDNA	TAIR
GCAAGAGTAG	1	2	1	0	1	gnl|UG|At#S11725130	gi|23506012|gb|AY143927.1	At4g15420.1
at1g71760	CON	MIN	HOUR	DAY	WEEK	UNIGENE	FLCDNA	TAIR
TGATCAGAAT	0	1	0	0	0	gnl|UG|At#S21989838	no match found	At1g71760.1
at5g28040	CON	MIN	HOUR	DAY	WEEK	UNIGENE	FLCDNA	TAIR
CTCTCGGTGT	0	1	0	0	0	no match found	gi|28950992|gb|BT005356.1	multiple non-canonical match
at4g37920	CON	MIN	HOUR	DAY	WEEK	UNIGENE	FLCDNA	TAIR
AACACACTCT	2	0	0	0	2	gnl|UG|At#S11721282	gi|27754706|gb|BT002988.1	multiple non-canonical match
TAGGGTCCAG	0	0	0	0	1	no match found	no match found	At4g37920.1
at1g80930	CON	MIN	HOUR	DAY	WEEK	UNIGENE	FLCDNA	TAIR
AAGACTGTTT	1	2	0	2	1	gnl|UG|At#S11726271	gi|22654970|gb|AY139757.1	At1g80930.1
at5g25050	CON	MIN	HOUR	DAY	WEEK	UNIGENE	FLCDNA	TAIR
AGACTTTTGC	0	2	0	0	0	gnl|UG|At#S11720678	gi|110737282|dbj|AK228683.1|	At5g25050.1
at4g04710	CON	MIN	HOUR	DAY	WEEK	UNIGENE	FLCDNA	TAIR
CTTTGTATCA	0	3	0	0	0	no match found	no match found	At4g04710.1
at2g44175	CON	MIN	HOUR	DAY	WEEK	UNIGENE	FLCDNA	TAIR
CGACTTGAAC	0	0	1	0	0	gnl|UG|At#S15460394	no match found	non-canonical match
at2g39470	CON	MIN	HOUR	DAY	WEEK	UNIGENE	FLCDNA	TAIR

<table>
<tr><td>AAGTAGGTCC</td><td>0</td><td>3</td><td>1</td><td>1</td><td>0</td><td>gnl|UG|At#S18901236</td><td>gi|14532687|gb|AY039968.1</td><td>non-canonical match</td></tr>
<tr><td>at3g03860</td><td>CON</td><td>MIN</td><td>HOUR</td><td>DAY</td><td>WEEK</td><td>UNIGENE</td><td>FLCDNA</td><td>TAIR</td></tr>
<tr><td>TCAGTATTGG</td><td>0</td><td>0</td><td>1</td><td>0</td><td>0</td><td>no match found</td><td>gi|20259148|gb|AY091351.1</td><td>non-canonical match</td></tr>
<tr><td>TAAATCCAGG</td><td>2</td><td>3</td><td>4</td><td>3</td><td>1</td><td>gnl|UG|At#S18901867</td><td>gi|16604552|gb|AY059721.1</td><td>At3g03860.1</td></tr>
<tr><td>at1g44900</td><td>CON</td><td>MIN</td><td>HOUR</td><td>DAY</td><td>WEEK</td><td>UNIGENE</td><td>FLCDNA</td><td>TAIR</td></tr>
<tr><td>AAAAAAATGT</td><td>0</td><td>1</td><td>1</td><td>0</td><td>0</td><td>gnl|UG|At#S11735696</td><td>gi|110743289|dbj|AK227536.1|</td><td>At1g44900.1</td></tr>
<tr><td>at3g17660</td><td>CON</td><td>MIN</td><td>HOUR</td><td>DAY</td><td>WEEK</td><td>UNIGENE</td><td>FLCDNA</td><td>TAIR</td></tr>
<tr><td>TGGGCTTTAA</td><td>0</td><td>1</td><td>0</td><td>0</td><td>0</td><td>gnl|UG|At#S11735546</td><td>gi|110737198|dbj|AK228639.1|</td><td>pseudo chromosome match</td></tr>
<tr><td>at2g36630</td><td>CON</td><td>MIN</td><td>HOUR</td><td>DAY</td><td>WEEK</td><td>UNIGENE</td><td>FLCDNA</td><td>TAIR</td></tr>
<tr><td>GGATTCTTAG</td><td>3</td><td>1</td><td>3</td><td>0</td><td>1</td><td>gnl|UG|At#S18898033</td><td>gi|22137235|gb|AY133633.1</td><td>At2g36630.1</td></tr>
<tr><td>at1g79690</td><td>CON</td><td>MIN</td><td>HOUR</td><td>DAY</td><td>WEEK</td><td>UNIGENE</td><td>FLCDNA</td><td>TAIR</td></tr>
<tr><td>AGATACTAAC</td><td>0</td><td>1</td><td>0</td><td>1</td><td>0</td><td>gnl|UG|At#S11726629</td><td>gi|21539558|gb|AY120774.1</td><td>non-canonical match</td></tr>
<tr><td>at1g08930</td><td>CON</td><td>MIN</td><td>HOUR</td><td>DAY</td><td>WEEK</td><td>UNIGENE</td><td>FLCDNA</td><td>TAIR</td></tr>
<tr><td>GATTATAATG</td><td>1</td><td>2</td><td>1</td><td>0</td><td>1</td><td>gnl|UG|At#S11742143</td><td>gi|110740843|dbj|AK226371.1|</td><td>non-canonical match</td></tr>
<tr><td>at3g21220</td><td>CON</td><td>MIN</td><td>HOUR</td><td>DAY</td><td>WEEK</td><td>UNIGENE</td><td>FLCDNA</td><td>TAIR</td></tr>
<tr><td>GTCGTTACGA</td><td>0</td><td>1</td><td>0</td><td>0</td><td>0</td><td>gnl|UG|At#S11734462</td><td>gi|19698850|gb|AY081272.1</td><td>At3g21220.1</td></tr>
<tr><td>at3g53470</td><td>CON</td><td>MIN</td><td>HOUR</td><td>DAY</td><td>WEEK</td><td>UNIGENE</td><td>FLCDNA</td><td>TAIR</td></tr>
<tr><td>CTCTGGACAA</td><td>3</td><td>4</td><td>1</td><td>1</td><td>1</td><td>gnl|UG|At#S11638304</td><td>gi|28416886|gb|BT004728.1</td><td>At3g53470.2</td></tr>
<tr><td>at4g32240</td><td>CON</td><td>MIN</td><td>HOUR</td><td>DAY</td><td>WEEK</td><td>UNIGENE</td><td>FLCDNA</td><td>TAIR</td></tr>
<tr><td>TCATAATCTT</td><td>1</td><td>0</td><td>1</td><td>0</td><td>0</td><td>gnl|UG|At#S11722236</td><td>gi|21405683|gb|AY086959.1</td><td>At4g32240.1</td></tr>
<tr><td>at2g20530</td><td>CON</td><td>MIN</td><td>HOUR</td><td>DAY</td><td>WEEK</td><td>UNIGENE</td><td>FLCDNA</td><td>TAIR</td></tr>
<tr><td>GTTCGAAAGG</td><td>1</td><td>3</td><td>2</td><td>2</td><td>0</td><td>gnl|UG|At#S38433962</td><td>gi|18252886|gb|AY072378.1</td><td>At2g20530.1</td></tr>
<tr><td>at1g62180</td><td>CON</td><td>MIN</td><td>HOUR</td><td>DAY</td><td>WEEK</td><td>UNIGENE</td><td>FLCDNA</td><td>TAIR</td></tr>
<tr><td>TCGTTTGTGA</td><td>0</td><td>1</td><td>0</td><td>0</td><td>1</td><td>no match found</td><td>gi|14532761|gb|AY040005.1</td><td>non-canonical match</td></tr>
<tr><td>ACTTTGTATA</td><td>3</td><td>0</td><td>0</td><td>0</td><td>9</td><td>gnl|UG|At#S11702029</td><td>gi|21407439|gb|AY088665.1</td><td>At1g62180.1</td></tr>
<tr><td>at1g65490</td><td>CON</td><td>MIN</td><td>HOUR</td><td>DAY</td><td>WEEK</td><td>UNIGENE</td><td>FLCDNA</td><td>TAIR</td></tr>
<tr><td>CAGACGTACT</td><td>0</td><td>1</td><td>0</td><td>1</td><td>0</td><td>gnl|UG|At#S11789034</td><td>no match found</td><td>pseudo chromosome match</td></tr>
<tr><td>CAGACGTAGT</td><td>1</td><td>3</td><td>4</td><td>2</td><td>0</td><td>no match found</td><td>gi|26451810|dbj|AK118388.1|</td><td>At1g65490.1</td></tr>
<tr><td>at2g18193</td><td>CON</td><td>MIN</td><td>HOUR</td><td>DAY</td><td>WEEK</td><td>UNIGENE</td><td>FLCDNA</td><td>TAIR</td></tr>
<tr><td>GTAAATCAAA</td><td>0</td><td>0</td><td>0</td><td>1</td><td>0</td><td>gnl|UG|At#S21736095</td><td>gi|51968373|dbj|AK175116.1|</td><td>At2g18193.1</td></tr>
<tr><td>at5g56710</td><td>CON</td><td>MIN</td><td>HOUR</td><td>DAY</td><td>WEEK</td><td>UNIGENE</td><td>FLCDNA</td><td>TAIR</td></tr>
<tr><td>ATTGACTCTT</td><td>6</td><td>1</td><td>3</td><td>1</td><td>0</td><td>no match found</td><td>gi|21404107|gb|AY085397.1</td><td>At5g56710.1</td></tr>
<tr><td>at2g23130</td><td>CON</td><td>MIN</td><td>HOUR</td><td>DAY</td><td>WEEK</td><td>UNIGENE</td><td>FLCDNA</td><td>TAIR</td></tr>
<tr><td>TATTACAGCT</td><td>3</td><td>3</td><td>1</td><td>0</td><td>4</td><td>no match found</td><td>gi|30793948|gb|BT008601.1</td><td>non-canonical match</td></tr>
<tr><td>CTATTTTCTG</td><td>3</td><td>5</td><td>9</td><td>1</td><td>2</td><td>gnl|UG|At#S11825101</td><td>gi|27754588|gb|BT002926.1</td><td>At2g23130.1</td></tr>
<tr><td>at2g23950</td><td>CON</td><td>MIN</td><td>HOUR</td><td>DAY</td><td>WEEK</td><td>UNIGENE</td><td>FLCDNA</td><td>TAIR</td></tr>
<tr><td>TAAATGGTTA</td><td>0</td><td>0</td><td>0</td><td>0</td><td>1</td><td>gnl|UG|At#S11736502</td><td>gi|62318664|dbj|AK220652.1|</td><td>At2g23950.1</td></tr>
<tr><td>at2g33350</td><td>CON</td><td>MIN</td><td>HOUR</td><td>DAY</td><td>WEEK</td><td>UNIGENE</td><td>FLCDNA</td><td>TAIR</td></tr>
<tr><td>CCCCCAACAA</td><td>1</td><td>1</td><td>0</td><td>0</td><td>0</td><td>gnl|UG|At#S21126626</td><td>no match found</td><td>multiple non-canonical match</td></tr>
<tr><td>at3g26370</td><td>CON</td><td>MIN</td><td>HOUR</td><td>DAY</td><td>WEEK</td><td>UNIGENE</td><td>FLCDNA</td><td>TAIR</td></tr>
<tr><td>TGTAAAGCTT</td><td>0</td><td>1</td><td>2</td><td>0</td><td>0</td><td>gnl|UG|At#S11732851</td><td>gi|110741759|dbj|AK226720.1|</td><td>At3g26370.1</td></tr>
<tr><td>at2g47380</td><td>CON</td><td>MIN</td><td>HOUR</td><td>DAY</td><td>WEEK</td><td>UNIGENE</td><td>FLCDNA</td><td>TAIR</td></tr>
</table>

CGACATTGAA	3	6	1	2	1	no match found	gi|17979510|gb|AY070752.1	At2g47380.1
at5g37350	CON	MIN	HOUR	DAY	WEEK	UNIGENE	FLCDNA	TAIR
CTGTACCAAA	0	2	0	0	1	no match found	gi|17065285|gb|AY062719.1	non-canonical match
ATAGGTTTGC	1	1	0	1	1	gnl|UG|At#S11720000	gi|19423895|gb|AY080757.1	At5g37350.2
at1g63730	CON	MIN	HOUR	DAY	WEEK	UNIGENE	FLCDNA	TAIR
GCTACCTGGA	0	0	1	0	0	gnl|UG|At#S11730391	no match found	non-canonical match
at5g49990	CON	MIN	HOUR	DAY	WEEK	UNIGENE	FLCDNA	TAIR
GTCAATGTGC	0	3	0	1	0	gnl|UG|At#S11718717	gi|110739351|dbj|AK229752.1|	At5g49990.1
at5g24313	CON	MIN	HOUR	DAY	WEEK	UNIGENE	FLCDNA	TAIR
TTTTGAAAGA	0	0	1	0	0	gnl|UG|At#S11801516	gi|110743274|dbj|AK227528.1|	At5g24313.1
at5g57950	CON	MIN	HOUR	DAY	WEEK	UNIGENE	FLCDNA	TAIR
GATGTGGATG	0	1	0	0	0	gnl|UG|At#S11717915	gi|21406514|gb|AY087776.1	At5g57950.1
at4g00580	CON	MIN	HOUR	DAY	WEEK	UNIGENE	FLCDNA	TAIR
TTGAATTTGT	1	0	0	0	0	no match found	no match found	At4g00580.1
at1g13350	CON	MIN	HOUR	DAY	WEEK	UNIGENE	FLCDNA	TAIR
TACATTGAAT	0	0	0	0	1	no match found	no match found	At1g13350.1
at5g51290	CON	MIN	HOUR	DAY	WEEK	UNIGENE	FLCDNA	TAIR
GCAAACATAT	0	1	2	0	0	no match found	gi|14334905|gb|AY035126.1	At5g51290.1
at1g08200	CON	MIN	HOUR	DAY	WEEK	UNIGENE	FLCDNA	TAIR
GTGGTTGATT	16	9	7	12	6	gnl|UG|At#S11742214	gi|14596184|gb|AY042880.1	At1g08200.1
at2g23930	CON	MIN	HOUR	DAY	WEEK	UNIGENE	FLCDNA	TAIR
AATACTCTTT	2	2	4	3	13	gnl|UG|At#S11790735	gi|25083130|gb|BT002035.1	At2g23930.1
at4g21810	CON	MIN	HOUR	DAY	WEEK	UNIGENE	FLCDNA	TAIR
CGCCTTTTGA	2	6	5	3	0	gnl|UG|At#S18906172	gi|17473758|gb|AY065142.1	At4g21810.1
at4g26410	CON	MIN	HOUR	DAY	WEEK	UNIGENE	FLCDNA	TAIR
GGTATGTCGG	1	0	2	0	1	gnl|UG|At#S11723241	gi|28394052|gb|BT004440.1	At4g26410.1
at5g43450	CON	MIN	HOUR	DAY	WEEK	UNIGENE	FLCDNA	TAIR
CTTAAAACAG	0	1	0	3	1	gnl|UG|At#S11719383	gi|14596164|gb|AY042870.1	At5g43450.1
at1g75080	CON	MIN	HOUR	DAY	WEEK	UNIGENE	FLCDNA	TAIR
AATTTGGTTT	6	2	2	3	1	no match found	gi|18086460|gb|AY065049.1	multiple canonical match
at1g28010	CON	MIN	HOUR	DAY	WEEK	UNIGENE	FLCDNA	TAIR
AAATATTGGA	1	2	1	0	0	gnl|UG|At#S11739624	no match found	At1g28010.1
at5g38990	CON	MIN	HOUR	DAY	WEEK	UNIGENE	FLCDNA	TAIR
TCTCCACCGT	1	1	0	0	0	no match found	gi|23308460|gb|BT000634.1	At5g38990.1
at4g14970	CON	MIN	HOUR	DAY	WEEK	UNIGENE	FLCDNA	TAIR
CAAGAGGATG	1	0	0	0	0	gnl|UG|At#S11725206	no match found	non-canonical match
at4g39060	CON	MIN	HOUR	DAY	WEEK	UNIGENE	FLCDNA	TAIR
ATTACAAACC	2	1	0	2	1	no match found	no match found	At4g39060.1
at5g05450	CON	MIN	HOUR	DAY	WEEK	UNIGENE	FLCDNA	TAIR
TTATGTCCTT	1	1	1	1	0	gnl|UG|At#S11724772	no match found	At5g05450.1
GGATATGGCC	0	2	1	0	0	no match found	gi|110736441|dbj|AK228241.1|	non-canonical match

at3g50950	CON	MIN	HOUR	DAY	WEEK	UNIGENE	FLCDNA	TAIR
GACTGGGAAG	0	6	1	1	0	gnl|UG|At#S11729260	gi|110742312|dbj|AK227017.1|	At3g50950.1

at5g56870	CON	MIN	HOUR	DAY	WEEK	UNIGENE	FLCDNA	TAIR
AAAAGCTTCT	0	2	1	0	0	no match found	gi|17978798|gb|AY064690.1	multiple non-canonical match

at5g45390	CON	MIN	HOUR	DAY	WEEK	UNIGENE	FLCDNA	TAIR
TCTCCAATTG	1	4	2	2	0	no match found	gi|14596088|gb|AY042832.1	multiple non-canonical match
AACTTTTACA	0	1	0	0	1	gnl|UG|At#S11719188	no match found	At5g45390.1

at1g10865	CON	MIN	HOUR	DAY	WEEK	UNIGENE	FLCDNA	TAIR
TCAATGAAAA	6	0	7	3	5	no match found	gi|72198608|gb|DQ108888.1	non-canonical match

at4g28550	CON	MIN	HOUR	DAY	WEEK	UNIGENE	FLCDNA	TAIR
ATGTTTTCAT	0	1	0	0	0	gnl|UG|At#S11722871	no match found	At4g28550.1

at3g58990	CON	MIN	HOUR	DAY	WEEK	UNIGENE	FLCDNA	TAIR
TCCTCTCGAA	0	2	2	0	1	gnl|UG|At#S11727771	no match found	At3g58990.1
ATTCCTTCTG	2	1	2	0	0	no match found	gi|17529161|gb|AY065366.1	non-canonical match

at4g31480	CON	MIN	HOUR	DAY	WEEK	UNIGENE	FLCDNA	TAIR
AAATGTCTCA	2	5	0	0	1	gnl|UG|At#S11722369	gi|62320683|dbj|AK221668.1|	At4g31480.1

at5g45410	CON	MIN	HOUR	DAY	WEEK	UNIGENE	FLCDNA	TAIR
GCTTTGACCA	1	4	1	1	1	gnl|UG|At#S11816860	gi|21407312|gb|AY088538.1	At5g45410.1

at1g24190	CON	MIN	HOUR	DAY	WEEK	UNIGENE	FLCDNA	TAIR
ACTGCACTCT	0	1	0	0	0	gnl|UG|At#S11740581	gi|110743451|dbj|AK227621.1|	At1g24190.1

at4g24550	CON	MIN	HOUR	DAY	WEEK	UNIGENE	FLCDNA	TAIR
ACGTTCACGA	0	2	2	0	1	gnl|UG|At#S11723549	gi|18176153|gb|AY072171.1	At4g24550.1

at5g03150	CON	MIN	HOUR	DAY	WEEK	UNIGENE	FLCDNA	TAIR
GATATGTATT	1	0	0	0	0	gnl|UG|At#S11725373	gi|110737691|dbj|AK228895.1|	At5g03150.1

at1g21050	CON	MIN	HOUR	DAY	WEEK	UNIGENE	FLCDNA	TAIR
TTCGTTTTTA	4	2	1	2	0	gnl|UG|At#S11740952	gi|26452694|dbj|AK118841.1|	At1g21050.1

at5g46760	CON	MIN	HOUR	DAY	WEEK	UNIGENE	FLCDNA	TAIR
TTGTTAAATG	0	0	0	1	0	no match found	no match found	At5g46760.1

at5g07270	CON	MIN	HOUR	DAY	WEEK	UNIGENE	FLCDNA	TAIR
GAACAGAGAG	0	1	0	0	0	gnl|UG|At#S11724306	gi|117168056|gb|BT029297.1	At5g07270.1

at3g54826	CON	MIN	HOUR	DAY	WEEK	UNIGENE	FLCDNA	TAIR
GATTCTCTTA	0	3	0	1	0	gnl|UG|At#S18941977	gi|110743000|dbj|AK227388.1|	At3g54826.1

at1g11200	CON	MIN	HOUR	DAY	WEEK	UNIGENE	FLCDNA	TAIR
TGCGTCAAAG	0	0	3	0	0	gnl|UG|At#S11741916	gi|21406851|gb|AY088077.1	At1g11200.1

at2g20570	CON	MIN	HOUR	DAY	WEEK	UNIGENE	FLCDNA	TAIR
TGACTAATAA	4	3	0	2	2	gnl|UG|At#S11737327	gi|15451039|gb|AY054600.1	At2g20570.1

at5g16610	CON	MIN	HOUR	DAY	WEEK	UNIGENE	FLCDNA	TAIR
GAAGATGTTA	0	0	2	2	0	gnl|UG|At#S18941621	gi|23463074|gb|BT000832.1	At5g16610.1

at2g15080	CON	MIN	HOUR	DAY	WEEK	UNIGENE	FLCDNA	TAIR
GCGCCAGAGC	0	0	0	1	0	gnl|UG|At#S15461548	no match found	At2g15080.2

at3g18820	CON	MIN	HOUR	DAY	WEEK	UNIGENE	FLCDNA	TAIR
CTAGCTAAAT	2	5	4	2	11	gnl|UG|At#S11735203	gi|21402976|gb|AY084266.1	At3g18820.1

AAATCATTTG	0	6	0	1	1	no match found	gi|16323367|gb|AY059072.1	non-canonical match
at2g37060	CON	MIN	HOUR	DAY	WEEK	UNIGENE	FLCDNA	TAIR
GAACCTCTCA	0	0	0	1	0	gnl|UG|At#S28282181	gi|17979252|gb|AY070477.1	At2g37060.2
at4g18890	CON	MIN	HOUR	DAY	WEEK	UNIGENE	FLCDNA	TAIR
GAGAATGTGT	1	0	0	0	0	gnl|UG|At#S11724520	gi|21407153|gb|AY088379.1	At4g18890.1
at1g21880	CON	MIN	HOUR	DAY	WEEK	UNIGENE	FLCDNA	TAIR
CTGACCAGGT	0	1	1	0	0	gnl|UG|At#S11740874	gi|15982869|gb|AY057542.1	At1g21880.2
at5g63380	CON	MIN	HOUR	DAY	WEEK	UNIGENE	FLCDNA	TAIR
AAAGGTTATG	0	0	0	1	0	gnl|UG|At#S11717362	gi|34098908|gb|BT010394.1	At5g63380.1
at1g25570	CON	MIN	HOUR	DAY	WEEK	UNIGENE	FLCDNA	TAIR
ATTATGACTT	1	0	0	0	0	gnl|UG|At#S11740313	no match found	At1g25570.1
at1g08390	CON	MIN	HOUR	DAY	WEEK	UNIGENE	FLCDNA	TAIR
GGTATGTACT	0	0	1	0	0	gnl|UG|At#S11742196	gi|110737423|dbj|AK228756.1|	At1g08390.1
at5g14480	CON	MIN	HOUR	DAY	WEEK	UNIGENE	FLCDNA	TAIR
GCTGGAAACA	1	2	0	0	0	no match found	gi|53850466|gb|BT015874.1	multiple canonical match
at2g25605	CON	MIN	HOUR	DAY	WEEK	UNIGENE	FLCDNA	TAIR
ACGGAGATGT	0	0	1	0	0	no match found	gi|45752741|gb|BT012174.1	non-canonical match
ATTAGATTTA	0	0	2	0	1	gnl|UG|At#S24442380	gi|62320910|dbj|AK221783.1|	At2g25605.1
at3g62970	CON	MIN	HOUR	DAY	WEEK	UNIGENE	FLCDNA	TAIR
TAAACTGAAA	6	1	3	4	1	gnl|UG|At#S34114185	gi|110743951|dbj|AK227830.1|	At3g62970.1
at5g65380	CON	MIN	HOUR	DAY	WEEK	UNIGENE	FLCDNA	TAIR
CCTTGTAATA	5	2	0	0	4	gnl|UG|At#S11717159	gi|14030730|gb|AF375456.1	At5g65380.1
at5g67440	CON	MIN	HOUR	DAY	WEEK	UNIGENE	FLCDNA	TAIR
TTCTTGAAGC	0	2	0	0	0	no match found	gi|15451227|gb|AY054694.1	multiple canonical match
at3g01300	CON	MIN	HOUR	DAY	WEEK	UNIGENE	FLCDNA	TAIR
TTTCTTCGTC	1	0	1	0	1	gnl|UG|At#S11740278	gi|16209632|gb|AY057584.1	At3g01300.1
at2g47650	CON	MIN	HOUR	DAY	WEEK	UNIGENE	FLCDNA	TAIR
CTCGAGCTCG	2	2	0	0	0	gnl|UG|At#S11730799	gi|63003797|gb|BT022016.1	At2g47650.1
at5g03110	CON	MIN	HOUR	DAY	WEEK	UNIGENE	FLCDNA	TAIR
GGTCAAGTCC	0	0	0	1	0	gnl|UG|At#S11725384	gi|38603961|gb|BT010948.1	At5g03110.1
at3g62250	CON	MIN	HOUR	DAY	WEEK	UNIGENE	FLCDNA	TAIR
GATTCTATGT	41	12	24	30	49	gnl|UG|At#S11593942	no match found	At3g62250.1
at5g42010	CON	MIN	HOUR	DAY	WEEK	UNIGENE	FLCDNA	TAIR
TAGATTTTGA	0	0	0	1	0	gnl|UG|At#S34115452	gi|110741474|dbj|AK226565.1|	At5g42010.1
at3g28480	CON	MIN	HOUR	DAY	WEEK	UNIGENE	FLCDNA	TAIR
TGCAAGTTAT	1	1	1	2	1	gnl|UG|At#S11732145	gi|21407382|gb|AY088608.1	At3g28480.1
at3g48195	CON	MIN	HOUR	DAY	WEEK	UNIGENE	FLCDNA	TAIR
GCAGTACTGC	0	1	0	0	0	gnl|UG|At#S11816089	gi|110739353|dbj|AK229753.1|	At3g48195.1
at2g43110	CON	MIN	HOUR	DAY	WEEK	UNIGENE	FLCDNA	TAIR
TATGGTCCAA	0	1	0	0	1	gnl|UG|At#S11822922	gi|114050604|gb|BT028905.1	At2g43110.1
at1g77120	CON	MIN	HOUR	DAY	WEEK	UNIGENE	FLCDNA	TAIR

GGTGCTTGAA	0	3	4	23	9	gnl|UG|At#S11727536	gi|15010621|gb|AY045612.1	At1g77120.1
at2g21790	CON	MIN	HOUR	DAY	WEEK	UNIGENE	FLCDNA	TAIR
TCTCTTGGAC	0	1	1	1	1	gnl|UG|At#S11737017	gi|13358239|gb|AF325052.2	At2g21790.1
at4g39660	CON	MIN	HOUR	DAY	WEEK	UNIGENE	FLCDNA	TAIR
GGAACGTTTT	0	0	0	0	1	no match found	gi|15809991|gb|AY054264.1	At4g39660.1
at5g05750	CON	MIN	HOUR	DAY	WEEK	UNIGENE	FLCDNA	TAIR
ATGAAGAGGT	0	2	1	0	0	gnl|UG|At#S11724691	gi|15810414|gb|AY056246.1	At5g05750.1
at2g13950	CON	MIN	HOUR	DAY	WEEK	UNIGENE	FLCDNA	TAIR
TTGATTGTTT	0	1	0	0	0	gnl|UG|At#S11738969	no match found	At1g74810.1
at1g03160	CON	MIN	HOUR	DAY	WEEK	UNIGENE	FLCDNA	TAIR
TATCACGAGA	2	0	1	0	0	gnl|UG|At#S38434432	gi|51970735|dbj|AK176297.1|	pseudo chromosome match
at1g25520	CON	MIN	HOUR	DAY	WEEK	UNIGENE	FLCDNA	TAIR
AGCTCGGTTT	0	2	0	1	0	gnl|UG|At#S11740331	gi|27754431|gb|BT002846.1	At1g25520.1
at1g36936	CON	MIN	HOUR	DAY	WEEK	UNIGENE	FLCDNA	TAIR
CTTCACAAAA	1	0	0	0	1	no match found	no match found	At1g36936.1
at1g22610	CON	MIN	HOUR	DAY	WEEK	UNIGENE	FLCDNA	TAIR
CTGCGTCATC	4	4	6	4	5	gnl|UG|At#S34114371	gi|110743588|dbj|AK227644.1|	At1g22610.1
at2g13560	CON	MIN	HOUR	DAY	WEEK	UNIGENE	FLCDNA	TAIR
TGGAATCCAG	4	3	5	3	5	gnl|UG|At#S11739061	gi|20268708|gb|AY091108.1	At2g13560.1
at3g57760	CON	MIN	HOUR	DAY	WEEK	UNIGENE	FLCDNA	TAIR
ATTCAAGTGA	1	0	0	1	0	gnl|UG|At#S28282798	gi|13937221|gb|AF372966.1	At3g57760.1
at5g24105	CON	MIN	HOUR	DAY	WEEK	UNIGENE	FLCDNA	TAIR
CTCAATCCGC	0	0	2	0	0	gnl|UG|At#S18912589	gi|62319354|dbj|AK221000.1|	At5g24105.1
at1g60590	CON	MIN	HOUR	DAY	WEEK	UNIGENE	FLCDNA	TAIR
ATATATGTTT	1	0	0	0	0	gnl|UG|At#S11731306	gi|16974584|gb|AY060568.1	At1g60590.1
at3g49800	CON	MIN	HOUR	DAY	WEEK	UNIGENE	FLCDNA	TAIR
GTTTGTTTTG	3	0	1	3	0	no match found	gi|15983353|gb|AF424551.1	multiple non-canonical match
GTGGCTCAGA	2	1	3	1	0	gnl|UG|At#S11729481	no match found	At3g49800.1
at1g53760	CON	MIN	HOUR	DAY	WEEK	UNIGENE	FLCDNA	TAIR
AGTGGAACAA	1	1	0	1	0	gnl|UG|At#S34115607	no match found	At1g53760.1
at1g78030	CON	MIN	HOUR	DAY	WEEK	UNIGENE	FLCDNA	TAIR
AACCCTAATT	1	0	0	2	0	no match found	gi|62319960|dbj|AK221306.1|	non-canonical match
GATCGATGTC	0	0	0	1	1	gnl|UG|At#S24442857	no match found	non-canonical match
at1g53050	CON	MIN	HOUR	DAY	WEEK	UNIGENE	FLCDNA	TAIR
ACCGACATAT	1	0	2	1	0	gnl|UG|At#S14273787	gi|28393522|gb|BT004162.1	At1g53050.1
at1g17710	CON	MIN	HOUR	DAY	WEEK	UNIGENE	FLCDNA	TAIR
CACGTTAAGT	0	5	5	1	0	gnl|UG|At#S38434328	no match found	non-canonical match
at2g45060	CON	MIN	HOUR	DAY	WEEK	UNIGENE	FLCDNA	TAIR
AAGGTGTATT	1	1	0	0	0	gnl|UG|At#S11731397	gi|15010567|gb|AY045585.1	At2g45060.1
at3g04060	CON	MIN	HOUR	DAY	WEEK	UNIGENE	FLCDNA	TAIR
TACATAAGAT	0	1	0	0	0	gnl|UG|At#S11739498	gi|13605594|gb|AF361623.1	At3g04060.1

at4g39260	CON	MIN	HOUR	DAY	WEEK	UNIGENE	FLCDNA	TAIR
AGGGATGCGA	3	39	6	5	5	gnl|UG|At#S18895991	gi|14334647|gb|AY034997.1	At4g39260.4
at5g67130	CON	MIN	HOUR	DAY	WEEK	UNIGENE	FLCDNA	TAIR
AATTGCCTTA	1	2	3	0	1	gnl|UG|At#S11716982	gi|15451187|gb|AY054674.1	non-canonical match
at1g17890	CON	MIN	HOUR	DAY	WEEK	UNIGENE	FLCDNA	TAIR
GATTTCATAC	1	0	1	2	2	gnl|UG|At#S18942553	gi|21407805|gb|AY089031.1	At1g17890.3
at1g62050	CON	MIN	HOUR	DAY	WEEK	UNIGENE	FLCDNA	TAIR
AGTAGTAGTA	0	1	0	0	0	no match found	gi|14334425|gb|AY034904.1	non-canonical match
at4g19190	CON	MIN	HOUR	DAY	WEEK	UNIGENE	FLCDNA	TAIR
ACTCTTTTTA	1	0	0	0	0	gnl|UG|At#S11724468	gi|51970223|dbj|AK176041.1|	At4g19190.1
at1g78230	CON	MIN	HOUR	DAY	WEEK	UNIGENE	FLCDNA	TAIR
TAAAACCGAA	0	0	3	2	1	gnl|UG|At#S11727124	gi|17528979|gb|AY065224.1	At1g78230.1
at1g09795	CON	MIN	HOUR	DAY	WEEK	UNIGENE	FLCDNA	TAIR
AGGAGACTCC	0	1	1	0	1	gnl|UG|At#S11699535	gi|26452033|dbj|AK118503.1|	At1g09795.1
at4g21560	CON	MIN	HOUR	DAY	WEEK	UNIGENE	FLCDNA	TAIR
AAGTGTAATG	1	0	1	0	0	no match found	gi|22531272|gb|AY136475.1	multiple non-canonical match
at2g43620	CON	MIN	HOUR	DAY	WEEK	UNIGENE	FLCDNA	TAIR
TGGTTCTGGA	0	0	1	4	0	gnl|UG|At#S11731739	no match found	At2g43620.1
at4g29140	CON	MIN	HOUR	DAY	WEEK	UNIGENE	FLCDNA	TAIR
ATCGTTTTGT	0	0	0	1	0	gnl|UG|At#S34114524	no match found	multiple non-canonical match
CCGGTGGCAG	0	1	0	2	0	gnl|UG|At#S11722766	no match found	At4g29140.1
at1g12140	CON	MIN	HOUR	DAY	WEEK	UNIGENE	FLCDNA	TAIR
AATAATTCCT	0	1	1	0	2	no match found	no match found	At1g12140.1
at1g71480	CON	MIN	HOUR	DAY	WEEK	UNIGENE	FLCDNA	TAIR
TGTATCCAAC	3	1	12	5	2	no match found	gi|51969761|dbj|AK175810.1|	At1g71480.1
at2g47060	CON	MIN	HOUR	DAY	WEEK	UNIGENE	FLCDNA	TAIR
GGCTACACCT	0	2	0	0	0	no match found	gi|19424107|gb|AY080876.1	At2g47060.2
at3g27250	CON	MIN	HOUR	DAY	WEEK	UNIGENE	FLCDNA	TAIR
CGGGTAAGAT	0	0	1	0	0	gnl|UG|At#S24442591	gi|62320491|dbj|AK221572.1|	pseudo chromosome match
at1g21680	CON	MIN	HOUR	DAY	WEEK	UNIGENE	FLCDNA	TAIR
GGCCCCTCGG	1	15	4	6	0	gnl|UG|At#S11740893	gi|15028242|gb|AY046036.1	At1g21680.1
at2g45690	CON	MIN	HOUR	DAY	WEEK	UNIGENE	FLCDNA	TAIR
AGAGATCCAT	0	0	0	1	0	gnl|UG|At#S21737261	gi|51968969|dbj|AK175414.1|	At2g45690.1
at3g03790	CON	MIN	HOUR	DAY	WEEK	UNIGENE	FLCDNA	TAIR
TGAAACACTT	1	1	0	0	0	no match found	gi|110741852|dbj|AK226770.1|	non-canonical match
AAGCTCTTTA	2	1	5	1	0	gnl|UG|At#S18942197	gi|26449495|dbj|AK117198.1|	At3g03790.1
at3g09090	CON	MIN	HOUR	DAY	WEEK	UNIGENE	FLCDNA	TAIR
GTCTTGGCTG	0	1	0	0	0	gnl|UG|At#S43849944	gi|110737093|dbj|AK228583.1|	non-canonical match
at3g12775	CON	MIN	HOUR	DAY	WEEK	UNIGENE	FLCDNA	TAIR
ACTCCTGCTA	1	0	1	0	0	no match found	no match found	At3g12775.1
at1g29690	CON	MIN	HOUR	DAY	WEEK	UNIGENE	FLCDNA	TAIR
TTTCGGTTGC	0	0	1	0	0	no match found	gi|18650617|gb|AY074857.1	non-canonical match

at4g33565	CON	MIN	HOUR	DAY	WEEK	UNIGENE	FLCDNA	TAIR
GTGAGTCACA	1	1	1	0	0	gnl|UG|At#S11722013	gi|94442458|gb|BT025264.1	At4g33565.1
at4g34150	CON	MIN	HOUR	DAY	WEEK	UNIGENE	FLCDNA	TAIR
TTTAACCAGA	5	9	11	4	3	gnl|UG|At#S18907694	gi|15724315|gb|AF412098.1	At4g34150.1
at3g53880	CON	MIN	HOUR	DAY	WEEK	UNIGENE	FLCDNA	TAIR
AGACGTTGAG	1	0	0	0	0	gnl|UG|At#S18903635	gi|21403575|gb|AY084865.1	At3g53880.1
at5g47000	CON	MIN	HOUR	DAY	WEEK	UNIGENE	FLCDNA	TAIR
AAAATTCAAA	0	0	0	1	1	gnl|UG|At#S11806772	gi|21406882|gb|AY088108.1	At5g47000.1
at3g15510	CON	MIN	HOUR	DAY	WEEK	UNIGENE	FLCDNA	TAIR
CATTATATAA	0	2	0	0	0	no match found	gi|28393363|gb|BT004079.1	multiple canonical match
at3g46940	CON	MIN	HOUR	DAY	WEEK	UNIGENE	FLCDNA	TAIR
GCGATTCATC	1	0	0	1	0	gnl|UG|At#S11702592	gi|17104548|gb|AY062989.1	multiple non-canonical match
at2g35155	CON	MIN	HOUR	DAY	WEEK	UNIGENE	FLCDNA	TAIR
GCTTTAAAGA	0	0	1	0	0	gnl|UG|At#S11733776	gi|16604658|gb|AY059774.1	At2g35155.1
at5g24270	CON	MIN	HOUR	DAY	WEEK	UNIGENE	FLCDNA	TAIR
CAAGGAAAGA	0	1	0	1	0	gnl|UG|At#S11708313	gi|20465716|gb|AY096693.1	non-canonical match
GTTTATTATT	2	1	0	0	1	no match found	no match found	At5g24270.1
at1g30230	CON	MIN	HOUR	DAY	WEEK	UNIGENE	FLCDNA	TAIR
TGGTCTGGTT	28	31	51	39	22	gnl|UG|At#S11738946	gi|110740550|dbj|AK226216.1|	At1g30230.1
at2g22710	CON	MIN	HOUR	DAY	WEEK	UNIGENE	FLCDNA	TAIR
TTCCACTTGA	0	1	0	0	0	gnl|UG|At#S11831663	gi|26451657|dbj|AK118308.1|	At2g22710.1
at4g30710	CON	MIN	HOUR	DAY	WEEK	UNIGENE	FLCDNA	TAIR
TAAGCTCTGA	1	1	0	1	0	gnl|UG|At#S11722498	gi|110742693|dbj|AK227221.1|	At4g30710.1
at1g79975	CON	MIN	HOUR	DAY	WEEK	UNIGENE	FLCDNA	TAIR
TTGCTTCTTT	1	2	0	0	1	gnl|UG|At#S11726530	gi|13877732|gb|AF370129.1	At1g79975.1
at1g48380	CON	MIN	HOUR	DAY	WEEK	UNIGENE	FLCDNA	TAIR
CAGTTGGATA	1	0	0	3	0	gnl|UG|At#S43850479	gi|16323205|gb|AY057707.1	At1g48380.1
at1g50600	CON	MIN	HOUR	DAY	WEEK	UNIGENE	FLCDNA	TAIR
ATCCGAAGGA	2	2	2	1	2	gnl|UG|At#S11734298	gi|14334475|gb|AY034929.1	At1g50600.1
at5g64280	CON	MIN	HOUR	DAY	WEEK	UNIGENE	FLCDNA	TAIR
CTCGGCGCTA	0	0	1	1	0	gnl|UG|At#S11717271	gi|62321448|dbj|AK222059.1|	At5g64280.1
at4g30110	CON	MIN	HOUR	DAY	WEEK	UNIGENE	FLCDNA	TAIR
TGATTCATCA	1	0	1	1	0	no match found	no match found	At4g30110.1
at3g55240	CON	MIN	HOUR	DAY	WEEK	UNIGENE	FLCDNA	TAIR
CAAACATCAC	1	0	0	0	0	no match found	gi|24030410|gb|BT000964.1	non-canonical match
GATCGATGGT	2	3	3	0	1	gnl|UG|At#S34114908	gi|19310582|gb|AY080704.1	At3g55240.1
at3g52990	CON	MIN	HOUR	DAY	WEEK	UNIGENE	FLCDNA	TAIR
ACAGAGTAGT	1	3	1	1	3	gnl|UG|At#S38433611	gi|14194098|gb|AF367255.1	At3g52990.1
at3g61690	CON	MIN	HOUR	DAY	WEEK	UNIGENE	FLCDNA	TAIR
ATCATCGGTT	1	0	0	1	0	gnl|UG|At#S11726944	gi|110739041|dbj|AK229591.1|	At3g61690.1
at5g01020	CON	MIN	HOUR	DAY	WEEK	UNIGENE	FLCDNA	TAIR

CAGAGTTCGG	2	10	4	2	0	gnl|UG|At#S11726495	gi|26450965|dbj|AK117952.1|	At5g01020.1
at1g62330	CON	MIN	HOUR	DAY	WEEK	UNIGENE	FLCDNA	TAIR
TATTGGTTAG	1	0	0	0	1	no match found	gi|51971800|dbj|AK176802.1|	multiple non-canonical match
GCATTTGTAG	0	2	0	0	1	gnl|UG|At#S11730680	no match found	At1g62330.1
at5g58550	CON	MIN	HOUR	DAY	WEEK	UNIGENE	FLCDNA	TAIR
GCCACAACAC	0	0	1	0	0	gnl|UG|At#S11717854	gi|110737397|dbj|AK228743.1|	At5g58550.1
at1g61610	CON	MIN	HOUR	DAY	WEEK	UNIGENE	FLCDNA	TAIR
AATTGGAGAA	0	0	1	0	0	no match found	no match found	At1g61610.1
at3g18480	CON	MIN	HOUR	DAY	WEEK	UNIGENE	FLCDNA	TAIR
GAGCAGAGGA	0	1	0	0	1	gnl|UG|At#S11706044	gi|15810372|gb|AY056225.1	At3g18480.1
at4g03560	CON	MIN	HOUR	DAY	WEEK	UNIGENE	FLCDNA	TAIR
AAAACATATG	12	6	6	2	3	no match found	gi|110741497|dbj|AK226582.1|	At4g03560.1
at4g33770	CON	MIN	HOUR	DAY	WEEK	UNIGENE	FLCDNA	TAIR
GGAGCAAAAA	0	0	1	0	0	gnl|UG|At#S11721976	gi|116325931|gb|BT029235.1	At4g33770.1
at1g09575	CON	MIN	HOUR	DAY	WEEK	UNIGENE	FLCDNA	TAIR
TCCTCCGGTG	0	1	0	0	0	gnl|UG|At#S11706116	gi|15810220|gb|AY056149.1	At1g09575.1
at5g25360	CON	MIN	HOUR	DAY	WEEK	UNIGENE	FLCDNA	TAIR
TCTTCTATTA	0	2	0	1	0	gnl|UG|At#S11720647	gi|110736785|dbj|AK228420.1|	At5g25360.1
at3g18800	CON	MIN	HOUR	DAY	WEEK	UNIGENE	FLCDNA	TAIR
ATCTCTTTCT	2	2	0	0	0	gnl|UG|At#S11735210	gi|21405434|gb|AY086724.1	At3g18800.1
at1g18750	CON	MIN	HOUR	DAY	WEEK	UNIGENE	FLCDNA	TAIR
TTTTTGTATG	0	1	0	0	0	gnl|UG|At#S18893210	no match found	multiple non-canonical match
AACCATTGAT	0	1	0	1	1	no match found	no match found	At1g18750.1
at5g42350	CON	MIN	HOUR	DAY	WEEK	UNIGENE	FLCDNA	TAIR
TATTGAAAAG	0	0	1	1	0	gnl|UG|At#S11719494	no match found	At5g42350.1
at1g21160	CON	MIN	HOUR	DAY	WEEK	UNIGENE	FLCDNA	TAIR
CCAGTTGATT	0	0	0	1	0	gnl|UG|At#S11740940	gi|110740628|dbj|AK226257.1|	At1g21160.1
at3g44260	CON	MIN	HOUR	DAY	WEEK	UNIGENE	FLCDNA	TAIR
CTGGGGTTTT	1	12	0	0	0	gnl|UG|At#S11730518	gi|15292828|gb|AY050848.1	At3g44260.1
at2g06555	CON	MIN	HOUR	DAY	WEEK	UNIGENE	FLCDNA	TAIR
ATGATGATAC	1	0	0	1	0	no match found	no match found	At2g06555.1
at3g48640	CON	MIN	HOUR	DAY	WEEK	UNIGENE	FLCDNA	TAIR
ATGTTTCGAC	0	0	1	0	0	no match found	no match found	At3g48640.1
TATTTTGCTT	0	0	0	1	0	no match found	gi|62320060|dbj|AK221356.1|	non-canonical match
CAAAGAGAAA	0	1	0	0	0	gnl|UG|At#S16341712	no match found	multiple non-canonical match
at3g28930	CON	MIN	HOUR	DAY	WEEK	UNIGENE	FLCDNA	TAIR
GAGTGGAAGA	0	2	0	0	0	gnl|UG|At#S11731994	gi|16323241|gb|AY057725.1	At3g28930.1
GAGGAAGAAG	0	2	2	0	1	no match found	gi|21407850|gb|AY089076.1	At2g06210.1
at3g46800	CON	MIN	HOUR	DAY	WEEK	UNIGENE	FLCDNA	TAIR
AATTATATAT	0	1	0	0	1	no match found	no match found	At3g46800.1
at3g22840	CON	MIN	HOUR	DAY	WEEK	UNIGENE	FLCDNA	TAIR
TGTACTAAGT	9	0	0	76	168	gnl|UG|At#S11733965	gi|21404027|gb|AY085317.1	At3g22840.1
ACGTCAGACG	0	0	0	0	4	no match found	gi|20453400|gb|AY097423.1	non-canonical match

at1g80900	CON	MIN	HOUR	DAY	WEEK	UNIGENE	FLCDNA	TAIR
CCTCTGTGAA	0	1	0	1	0	gnl|UG|At#S11726274	gi|62319465|dbj|AK221057.1|	At1g80900.1
at2g13790	CON	MIN	HOUR	DAY	WEEK	UNIGENE	FLCDNA	TAIR
CACTTTTGAA	1	1	3	1	0	gnl|UG|At#S34115444	gi|110741485|dbj|AK226573.1|	At2g13790.1
at3g01210	CON	MIN	HOUR	DAY	WEEK	UNIGENE	FLCDNA	TAIR
AATCTAACAA	0	1	0	0	0	gnl|UG|At#S11740303	gi|28372925|gb|BT003717.1	At3g01210.1
at2g35620	CON	MIN	HOUR	DAY	WEEK	UNIGENE	FLCDNA	TAIR
AGATTAAAAT	3	1	1	1	0	gnl|UG|At#S11733662	gi|110740018|dbj|AK230094.1|	At2g35620.1
at1g56230	CON	MIN	HOUR	DAY	WEEK	UNIGENE	FLCDNA	TAIR
TAATATTCGG	1	1	0	0	1	gnl|UG|At#S34115367	gi|46518488|gb|BT012582.1	At1g56230.1
at3g10160	CON	MIN	HOUR	DAY	WEEK	UNIGENE	FLCDNA	TAIR
AGAAGTGAGA	4	1	0	2	0	no match found	no match found	At3g10160.1
at5g49780	CON	MIN	HOUR	DAY	WEEK	UNIGENE	FLCDNA	TAIR
ACGAATCAAT	0	1	0	0	0	no match found	no match found	At5g49780.1
at5g52540	CON	MIN	HOUR	DAY	WEEK	UNIGENE	FLCDNA	TAIR
TGAAGATGGT	0	0	2	0	1	gnl|UG|At#S11718462	gi|17064941|gb|AY062547.1	At5g52540.1
at5g13650	CON	MIN	HOUR	DAY	WEEK	UNIGENE	FLCDNA	TAIR
CCCTTTGATC	6	15	14	12	24	gnl|UG|At#S11722931	gi|16649146|gb|AY059943.1	At5g13650.1
at3g59530	CON	MIN	HOUR	DAY	WEEK	UNIGENE	FLCDNA	TAIR
TGGAAGGAAA	0	1	0	0	0	gnl|UG|At#S14273740	gi|28393614|gb|BT004209.1	At3g59530.2
at3g61650	CON	MIN	HOUR	DAY	WEEK	UNIGENE	FLCDNA	TAIR
TTGTGTTGAT	1	0	0	0	3	no match found	no match found	At3g61650.1
at5g54250	CON	MIN	HOUR	DAY	WEEK	UNIGENE	FLCDNA	TAIR
TGGATATTTT	2	4	8	3	0	no match found	no match found	At5g54250.1
at4g06676	CON	MIN	HOUR	DAY	WEEK	UNIGENE	FLCDNA	TAIR
AAGAATACAA	0	3	2	0	0	gnl|UG|At#S21989758	no match found	non-canonical match
at2g04880	CON	MIN	HOUR	DAY	WEEK	UNIGENE	FLCDNA	TAIR
GGGATGTATA	1	1	3	0	1	gnl|UG|At#S11740181	no match found	non-canonical match
at2g35020	CON	MIN	HOUR	DAY	WEEK	UNIGENE	FLCDNA	TAIR
TGAAATCTCC	0	2	0	1	0	gnl|UG|At#S11733812	gi|18087510|gb|AF462794.1	At2g35020.1
at1g51850	CON	MIN	HOUR	DAY	WEEK	UNIGENE	FLCDNA	TAIR
TAATGAAATT	0	0	0	0	1	gnl|UG|At#S11733819	no match found	At1g51850.1
at1g50490	CON	MIN	HOUR	DAY	WEEK	UNIGENE	FLCDNA	TAIR
ATTTCTGCAA	0	1	0	2	0	no match found	gi|110742989|dbj|AK227382.1|	multiple non-canonical match
CATATGGGAA	0	0	0	0	1	no match found	no match found	At1g50490.1
at4g32650	CON	MIN	HOUR	DAY	WEEK	UNIGENE	FLCDNA	TAIR
TTGAACAAAT	2	2	0	1	0	gnl|UG|At#S11722167	gi|15529217|gb|AY052233.1	At4g32650.1
at1g76490	CON	MIN	HOUR	DAY	WEEK	UNIGENE	FLCDNA	TAIR
GATAATTCGT	13	26	27	14	3	gnl|UG|At#S11727729	gi|23397125|gb|BT000703.1	At1g76490.1
at2g36300	CON	MIN	HOUR	DAY	WEEK	UNIGENE	FLCDNA	TAIR
CTTGCTGGTA	0	3	0	0	0	gnl|UG|At#S11733515	gi|107738349|gb|BT025791.1	At2g36300.1

<table>
<tr><td>at2g03820</td><td>CON</td><td>MIN</td><td>HOUR</td><td>DAY</td><td>WEEK</td><td>UNIGENE</td><td>FLCDNA</td><td>TAIR</td></tr>
<tr><td>GCTGCTGAAT</td><td>4</td><td>2</td><td>1</td><td>7</td><td>7</td><td>gnl|UG|At#S11706030</td><td>gi|15810402|gb|AY056240.1</td><td>At2g03820.1</td></tr>
<tr><td>at3g14600</td><td>CON</td><td>MIN</td><td>HOUR</td><td>DAY</td><td>WEEK</td><td>UNIGENE</td><td>FLCDNA</td><td>TAIR</td></tr>
<tr><td>ATTCTCATCA</td><td>4</td><td>8</td><td>9</td><td>2</td><td>10</td><td>gnl|UG|At#S11736471</td><td>gi|21407125|gb|AY088351.1</td><td>At3g14600.1</td></tr>
<tr><td>at1g71810</td><td>CON</td><td>MIN</td><td>HOUR</td><td>DAY</td><td>WEEK</td><td>UNIGENE</td><td>FLCDNA</td><td>TAIR</td></tr>
<tr><td>TATCAGCTTC</td><td>1</td><td>1</td><td>0</td><td>0</td><td>0</td><td>gnl|UG|At#S11728728</td><td>gi|14532543|gb|AY039896.1</td><td>At1g71810.1</td></tr>
<tr><td>at3g54720</td><td>CON</td><td>MIN</td><td>HOUR</td><td>DAY</td><td>WEEK</td><td>UNIGENE</td><td>FLCDNA</td><td>TAIR</td></tr>
<tr><td>AGAAAATCTG</td><td>1</td><td>4</td><td>0</td><td>2</td><td>0</td><td>gnl|UG|At#S11728564</td><td>gi|21407719|gb|AY088945.1</td><td>At3g54720.1</td></tr>
<tr><td>at3g03600</td><td>CON</td><td>MIN</td><td>HOUR</td><td>DAY</td><td>WEEK</td><td>UNIGENE</td><td>FLCDNA</td><td>TAIR</td></tr>
<tr><td>GATGATTTTG</td><td>2</td><td>1</td><td>4</td><td>2</td><td>2</td><td>gnl|UG|At#S34114679</td><td>gi|110742902|dbj|AK227336.1|</td><td>At3g03600.1</td></tr>
<tr><td>at3g56130</td><td>CON</td><td>MIN</td><td>HOUR</td><td>DAY</td><td>WEEK</td><td>UNIGENE</td><td>FLCDNA</td><td>TAIR</td></tr>
<tr><td>TGTGCCACGG</td><td>0</td><td>7</td><td>0</td><td>1</td><td>0</td><td>no match found</td><td>gi|14190386|gb|AF378871.1</td><td>At3g56130.1</td></tr>
<tr><td>at5g64730</td><td>CON</td><td>MIN</td><td>HOUR</td><td>DAY</td><td>WEEK</td><td>UNIGENE</td><td>FLCDNA</td><td>TAIR</td></tr>
<tr><td>TGATTGGAGG</td><td>0</td><td>2</td><td>0</td><td>0</td><td>1</td><td>gnl|UG|At#S11717226</td><td>gi|21281168|gb|AY113956.1</td><td>At5g64730.1</td></tr>
<tr><td>at3g54770</td><td>CON</td><td>MIN</td><td>HOUR</td><td>DAY</td><td>WEEK</td><td>UNIGENE</td><td>FLCDNA</td><td>TAIR</td></tr>
<tr><td>TATGGTGGAG</td><td>0</td><td>1</td><td>0</td><td>0</td><td>0</td><td>gnl|UG|At#S34116021</td><td>gi|110740387|dbj|AK230287.1|</td><td>At3g54770.1</td></tr>
<tr><td>at2g38090</td><td>CON</td><td>MIN</td><td>HOUR</td><td>DAY</td><td>WEEK</td><td>UNIGENE</td><td>FLCDNA</td><td>TAIR</td></tr>
<tr><td>GCAAAACTTC</td><td>0</td><td>0</td><td>0</td><td>1</td><td>0</td><td>gnl|UG|At#S11733081</td><td>gi|29824292|gb|BT006122.1</td><td>At2g38090.1</td></tr>
<tr><td>at1g19170</td><td>CON</td><td>MIN</td><td>HOUR</td><td>DAY</td><td>WEEK</td><td>UNIGENE</td><td>FLCDNA</td><td>TAIR</td></tr>
<tr><td>TCCGTTGGGT</td><td>0</td><td>0</td><td>2</td><td>2</td><td>0</td><td>gnl|UG|At#S11741134</td><td>gi|19698860|gb|AY081277.1</td><td>At1g19170.1</td></tr>
<tr><td>at1g17210</td><td>CON</td><td>MIN</td><td>HOUR</td><td>DAY</td><td>WEEK</td><td>UNIGENE</td><td>FLCDNA</td><td>TAIR</td></tr>
<tr><td>GGAAAAAGTA</td><td>0</td><td>4</td><td>0</td><td>1</td><td>0</td><td>gnl|UG|At#S11741323</td><td>gi|110738069|dbj|AK229089.1|</td><td>At1g17210.1</td></tr>
<tr><td>at2g39730</td><td>CON</td><td>MIN</td><td>HOUR</td><td>DAY</td><td>WEEK</td><td>UNIGENE</td><td>FLCDNA</td><td>TAIR</td></tr>
<tr><td>CTTGTGATGG</td><td>161</td><td>152</td><td>137</td><td>69</td><td>19</td><td>gnl|UG|At#S15460597</td><td>gi|15810187|gb|AY056108.1</td><td>At2g39730.1</td></tr>
<tr><td>at1g61370</td><td>CON</td><td>MIN</td><td>HOUR</td><td>DAY</td><td>WEEK</td><td>UNIGENE</td><td>FLCDNA</td><td>TAIR</td></tr>
<tr><td>ATTAATCACA</td><td>1</td><td>0</td><td>0</td><td>0</td><td>0</td><td>no match found</td><td>no match found</td><td>At1g61370.1</td></tr>
<tr><td>at1g31220</td><td>CON</td><td>MIN</td><td>HOUR</td><td>DAY</td><td>WEEK</td><td>UNIGENE</td><td>FLCDNA</td><td>TAIR</td></tr>
<tr><td>TTTCTGATAC</td><td>1</td><td>0</td><td>0</td><td>0</td><td>0</td><td>gnl|UG|At#S14274072</td><td>gi|28392981|gb|BT003877.1</td><td>At1g31220.1</td></tr>
<tr><td>at3g52610</td><td>CON</td><td>MIN</td><td>HOUR</td><td>DAY</td><td>WEEK</td><td>UNIGENE</td><td>FLCDNA</td><td>TAIR</td></tr>
<tr><td>CAGGGATCCT</td><td>0</td><td>0</td><td>1</td><td>0</td><td>0</td><td>no match found</td><td>no match found</td><td>At3g52610.1</td></tr>
<tr><td>at4g38580</td><td>CON</td><td>MIN</td><td>HOUR</td><td>DAY</td><td>WEEK</td><td>UNIGENE</td><td>FLCDNA</td><td>TAIR</td></tr>
<tr><td>TGATGTCATA</td><td>0</td><td>2</td><td>0</td><td>2</td><td>1</td><td>gnl|UG|At#S11721172</td><td>gi|21402998|gb|AY084288.1</td><td>At4g38580.1</td></tr>
<tr><td>at1g33811</td><td>CON</td><td>MIN</td><td>HOUR</td><td>DAY</td><td>WEEK</td><td>UNIGENE</td><td>FLCDNA</td><td>TAIR</td></tr>
<tr><td>TAATATGGCT</td><td>5</td><td>1</td><td>1</td><td>0</td><td>2</td><td>gnl|UG|At#S11737659</td><td>gi|20466731|gb|AY099832.1</td><td>At1g33811.1</td></tr>
<tr><td>at4g30760</td><td>CON</td><td>MIN</td><td>HOUR</td><td>DAY</td><td>WEEK</td><td>UNIGENE</td><td>FLCDNA</td><td>TAIR</td></tr>
<tr><td>CTTATGCTAA</td><td>0</td><td>4</td><td>0</td><td>0</td><td>0</td><td>gnl|UG|At#S11722490</td><td>gi|21404807|gb|AY086097.1</td><td>At4g30760.2</td></tr>
<tr><td>at3g06390</td><td>CON</td><td>MIN</td><td>HOUR</td><td>DAY</td><td>WEEK</td><td>UNIGENE</td><td>FLCDNA</td><td>TAIR</td></tr>
<tr><td>CGTTTCTATA</td><td>0</td><td>1</td><td>0</td><td>0</td><td>0</td><td>gnl|UG|At#S14273996</td><td>gi|28393129|gb|BT003953.1</td><td>At3g06390.1</td></tr>
<tr><td>at3g60810</td><td>CON</td><td>MIN</td><td>HOUR</td><td>DAY</td><td>WEEK</td><td>UNIGENE</td><td>FLCDNA</td><td>TAIR</td></tr>
<tr><td>TGGAATATGA</td><td>0</td><td>3</td><td>0</td><td>0</td><td>0</td><td>gnl|UG|At#S11727239</td><td>gi|117168212|gb|BT029375.1</td><td>non-canonical match</td></tr>
</table>

at4g31270	CON	MIN	HOUR	DAY	WEEK	UNIGENE	FLCDNA	TAIR
AATCGTAATA	0	0	1	0	0	gnl|UG|At#S11722400	gi|26452366|dbj|AK118674.1|	At4g31270.1
at4g18590	CON	MIN	HOUR	DAY	WEEK	UNIGENE	FLCDNA	TAIR
TTAGTTTCAC	2	3	1	3	1	gnl|UG|At#S11724576	no match found	At4g18590.1
at5g45260	CON	MIN	HOUR	DAY	WEEK	UNIGENE	FLCDNA	TAIR
GTCAACATTC	1	0	0	0	1	no match found	gi|110741007|dbj|AK226456.1|	At5g45270.1
at1g23060	CON	MIN	HOUR	DAY	WEEK	UNIGENE	FLCDNA	TAIR
TCAAGCTCCA	0	2	0	0	0	gnl|UG|At#S11705720	gi|19699223|gb|AY090317.1	At1g23060.1
at2g11910	CON	MIN	HOUR	DAY	WEEK	UNIGENE	FLCDNA	TAIR
AGTGTTTGTG	1	5	7	5	3	gnl|UG|At#S11739240	gi|21436270|gb|AY117199.1	At2g11910.2
at5g05965	CON	MIN	HOUR	DAY	WEEK	UNIGENE	FLCDNA	TAIR
ATCAATCATC	1	1	0	0	0	gnl|UG|At#S21989722	no match found	multiple canonical match
at4g14340	CON	MIN	HOUR	DAY	WEEK	UNIGENE	FLCDNA	TAIR
TCCTCGAATT	0	1	0	1	0	no match found	gi|15450524|gb|AY052364.1	non-canonical match
TGTACTTCAA	0	2	1	0	0	gnl|UG|At#S11725329	no match found	At4g14340.1
at2g28370	CON	MIN	HOUR	DAY	WEEK	UNIGENE	FLCDNA	TAIR
GTGTGTTTTT	3	2	0	0	1	gnl|UG|At#S11735446	gi|14030700|gb|AF375441.1	At2g28370.1
at1g24310	CON	MIN	HOUR	DAY	WEEK	UNIGENE	FLCDNA	TAIR
TACGTAGTAG	1	0	0	0	1	gnl|UG|At#S11740551	gi|26450406|dbj|AK117665.1|	At1g24310.1
GTGGCTGAAG	1	0	0	0	0	no match found	gi|28827331|gb|BT004977.1	non-canonical match
at1g13750	CON	MIN	HOUR	DAY	WEEK	UNIGENE	FLCDNA	TAIR
AGAAGAGTAA	0	1	0	0	0	gnl|UG|At#S11741660	gi|20466208|gb|AY099570.1	non-canonical match
at3g23430	CON	MIN	HOUR	DAY	WEEK	UNIGENE	FLCDNA	TAIR
GCTAAGAGAC	0	1	0	0	0	gnl|UG|At#S11806592	no match found	At3g23430.1
at3g26890	CON	MIN	HOUR	DAY	WEEK	UNIGENE	FLCDNA	TAIR
GTTACACAAG	2	2	1	1	0	gnl|UG|At#S28282899	gi|15293102|gb|AY050985.1	At3g26890.2
at4g09420	CON	MIN	HOUR	DAY	WEEK	UNIGENE	FLCDNA	TAIR
ATGTATGATA	0	0	1	0	0	gnl|UG|At#S18908714	no match found	At4g09420.1
at2g33710	CON	MIN	HOUR	DAY	WEEK	UNIGENE	FLCDNA	TAIR
ATGAGGGTGT	0	0	1	0	0	no match found	gi|23198055|gb|BT000236.1	non-canonical match
at2g43820	CON	MIN	HOUR	DAY	WEEK	UNIGENE	FLCDNA	TAIR
GTGGCAATGC	2	2	2	3	1	gnl|UG|At#S11731682	gi|21406064|gb|AY087340.1	At2g43820.1
at1g57870	CON	MIN	HOUR	DAY	WEEK	UNIGENE	FLCDNA	TAIR
GCATTACACT	0	2	0	0	1	gnl|UG|At#S11731896	gi|31711887|gb|BT008861.1	At1g57870.1
at1g64820	CON	MIN	HOUR	DAY	WEEK	UNIGENE	FLCDNA	TAIR
GTCACTCGTA	0	1	0	0	0	no match found	no match found	At1g64820.1
at5g63610	CON	MIN	HOUR	DAY	WEEK	UNIGENE	FLCDNA	TAIR
TGGAATGTTG	0	2	1	1	1	gnl|UG|At#S11717339	gi|110737226|dbj|AK228654.1|	At5g63610.1
at5g49100	CON	MIN	HOUR	DAY	WEEK	UNIGENE	FLCDNA	TAIR
GTGGTGGAGG	0	0	1	1	0	no match found	gi|18377645|gb|AY074276.1	At5g49100.1
at5g64500	CON	MIN	HOUR	DAY	WEEK	UNIGENE	FLCDNA	TAIR
AGACAAAAAA	2	3	5	4	2	gnl|UG|At#S11717249	no match found	At5g64500.1

at2g40620	CON	MIN	HOUR	DAY	WEEK	UNIGENE	FLCDNA	TAIR
AAGACCATCT	0	1	1	0	0	gnl|UG|At#S11732433	gi|20465782|gb|AY096746.1	At2g40620.1

at1g64450	CON	MIN	HOUR	DAY	WEEK	UNIGENE	FLCDNA	TAIR
TTTAAGTTTG	0	0	0	1	0	gnl|UG|At#S11730246	gi|110735736|dbj|AK227872.1|	At1g64450.1

at5g62410	CON	MIN	HOUR	DAY	WEEK	UNIGENE	FLCDNA	TAIR
GCAAAACTAG	0	1	0	0	0	gnl|UG|At#S11703360	no match found	At5g62410.1

at2g17033	CON	MIN	HOUR	DAY	WEEK	UNIGENE	FLCDNA	TAIR
GTATGCACTT	1	0	2	0	0	no match found	gi|21280878|gb|AY113883.1	multiple non-canonical match
AGAGTGTTGA	0	1	0	0	0	no match found	gi|110738239|dbj|AK229179.1|	non-canonical match
AAGTTTCTAG	1	0	0	0	0	no match found	gi|26452936|dbj|AK118968.1|	At2g17033.2

at3g56840	CON	MIN	HOUR	DAY	WEEK	UNIGENE	FLCDNA	TAIR
GAGTTCCTGG	1	2	0	1	0	gnl|UG|At#S11728174	no match found	At3g56840.1

at2g25270	CON	MIN	HOUR	DAY	WEEK	UNIGENE	FLCDNA	TAIR
TCTGAACATA	0	1	0	0	0	gnl|UG|At#S18921454	gi|26452741|dbj|AK118866.1|	multiple non-canonical match

at3g05280	CON	MIN	HOUR	DAY	WEEK	UNIGENE	FLCDNA	TAIR
ATTTACTTTG	6	0	1	0	0	gnl|UG|At#S11739143	gi|26452920|dbj|AK118959.1|	At3g05280.1
TCCGCAACAT	1	1	0	0	0	no match found	gi|28973138|gb|BT005474.1	non-canonical match

at2g31260	CON	MIN	HOUR	DAY	WEEK	UNIGENE	FLCDNA	TAIR
CAATTCTTTA	1	0	1	0	2	gnl|UG|At#S15460918	gi|19715617|gb|AY075619.1	At2g31260.1

at4g20330	CON	MIN	HOUR	DAY	WEEK	UNIGENE	FLCDNA	TAIR
TATTCTCACG	2	2	5	1	1	gnl|UG|At#S11724278	gi|14326522|gb|AF385715.1	At4g20330.1

at1g28440	CON	MIN	HOUR	DAY	WEEK	UNIGENE	FLCDNA	TAIR
AGGCGTGTGG	0	2	0	1	2	gnl|UG|At#S11739464	gi|20260671|gb|AY093235.1	At1g28440.1

at1g79350	CON	MIN	HOUR	DAY	WEEK	UNIGENE	FLCDNA	TAIR
ACAAATTGGC	0	2	4	4	3	gnl|UG|At#S34117410	gi|110737696|dbj|AK228898.1|	At1g79350.1

at2g38630	CON	MIN	HOUR	DAY	WEEK	UNIGENE	FLCDNA	TAIR
AGATATGTAA	0	0	0	0	1	gnl|UG|At#S11732935	gi|28393696|gb|BT004257.1	At2g38630.1

at4g32690	CON	MIN	HOUR	DAY	WEEK	UNIGENE	FLCDNA	TAIR
CAAAATGCTC	0	1	1	0	1	gnl|UG|At#S11722159	gi|89001032|gb|BT024768.1	At4g32690.1

at4g09730	CON	MIN	HOUR	DAY	WEEK	UNIGENE	FLCDNA	TAIR
TTTGATTTCC	0	2	0	0	2	gnl|UG|At#S21736909	gi|51969011|dbj|AK175435.1|	At4g09730.1

at1g72320	CON	MIN	HOUR	DAY	WEEK	UNIGENE	FLCDNA	TAIR
AAACTTTTTG	0	0	1	5	3	gnl|UG|At#S15435761	no match found	At1g72320.2
CAAAGGATAA	0	1	0	1	0	no match found	gi|15293278|gb|AY051073.1	At1g72320.1

at5g15030	CON	MIN	HOUR	DAY	WEEK	UNIGENE	FLCDNA	TAIR
TGAATTTTAT	0	0	1	1	1	gnl|UG|At#S11722584	no match found	At5g15030.1

at3g26780	CON	MIN	HOUR	DAY	WEEK	UNIGENE	FLCDNA	TAIR
AATCTGCAGA	0	1	2	0	0	no match found	gi|26449921|dbj|AK117416.1|	multiple non-canonical match
AGGAAAAAAG	6	3	2	5	4	gnl|UG|At#S11732706	no match found	non-canonical match

at1g50450	CON	MIN	HOUR	DAY	WEEK	UNIGENE	FLCDNA	TAIR
TTCATCAGAA	6	2	10	5	14	no match found	gi|14517415|gb|AY039543.1	At1g50450.1

at5g47190	CON	MIN	HOUR	DAY	WEEK	UNIGENE	FLCDNA	TAIR

TAATTTACAG	15	16	4	4	12	gnl|UG|At#S11743862	gi|21405694|gb|AY086970.1	At5g47190.1
at1g75780	CON	MIN	HOUR	DAY	WEEK	UNIGENE	FLCDNA	TAIR
TATCAAGTTG	0	1	1	1	0	gnl|UG|At#S11727887	gi|26449622|dbj|AK117262.1|	At1g75780.1
at2g27080	CON	MIN	HOUR	DAY	WEEK	UNIGENE	FLCDNA	TAIR
ACGTGGATCT	0	2	1	0	0	no match found	gi|13877664|gb|AF370591.1	At2g27080.2
at5g19550	CON	MIN	HOUR	DAY	WEEK	UNIGENE	FLCDNA	TAIR
CTGCAGTGAC	2	0	1	2	3	no match found	gi|22135927|gb|AY128343.1	non-canonical match
TCTTTTGACT	1	3	0	3	9	gnl|UG|At#S11721438	no match found	At5g19550.1
at4g10060	CON	MIN	HOUR	DAY	WEEK	UNIGENE	FLCDNA	TAIR
AATGATGAAT	0	0	0	0	1	gnl|UG|At#S11807783	gi|20453180|gb|AY094461.1	At4g10060.1
at5g67320	CON	MIN	HOUR	DAY	WEEK	UNIGENE	FLCDNA	TAIR
ACCTCCGAGA	0	2	0	0	0	gnl|UG|At#S11716963	gi|16323187|gb|AY057698.1	At5g67320.1
AAGGAGATCG	2	3	2	0	0	no match found	gi|62320313|dbj|AK221483.1|	multiple canonical match
at5g58950	CON	MIN	HOUR	DAY	WEEK	UNIGENE	FLCDNA	TAIR
AAAGCTCTGA	2	3	2	0	1	gnl|UG|At#S11717810	gi|17063197|gb|AY062096.1	At5g58950.1
at3g15640	CON	MIN	HOUR	DAY	WEEK	UNIGENE	FLCDNA	TAIR
TGGAGAAGAA	0	0	0	1	0	gnl|UG|At#S11736148	gi|14194104|gb|AF367258.1	At3g15640.1
at4g35470	CON	MIN	HOUR	DAY	WEEK	UNIGENE	FLCDNA	TAIR
TTTGTCTATC	1	0	0	0	0	no match found	gi|21407477|gb|AY088703.1	non-canonical match
TCTCTCCACA	1	0	0	2	0	no match found	gi|22136103|gb|AY128730.1	non-canonical match
AGTTGGCTTT	1	4	1	0	0	no match found	gi|18252196|gb|AY072324.1	non-canonical match
at3g55850	CON	MIN	HOUR	DAY	WEEK	UNIGENE	FLCDNA	TAIR
ATGATGCAGT	1	0	1	0	0	gnl|UG|At#S18941966	gi|16209665|gb|AY057597.1	At3g55850.1
at3g43720	CON	MIN	HOUR	DAY	WEEK	UNIGENE	FLCDNA	TAIR
GCGGCACAAC	4	3	3	4	1	no match found	gi|15028170|gb|AY045908.1	At3g43720.1
at4g04940	CON	MIN	HOUR	DAY	WEEK	UNIGENE	FLCDNA	TAIR
TCAACGTTAT	0	1	0	0	0	gnl|UG|At#S11726399	gi|16604678|gb|AY059784.1	At4g04940.1
at4g18040	CON	MIN	HOUR	DAY	WEEK	UNIGENE	FLCDNA	TAIR
ACTTGACAGT	0	3	8	6	6	gnl|UG|At#S11724674	gi|21405206|gb|AY086496.1	At4g18040.1
at5g36910	CON	MIN	HOUR	DAY	WEEK	UNIGENE	FLCDNA	TAIR
TTCTTCTGTC	0	0	0	1	0	no match found	gi|149944362|gb|BT030644.1	multiple non-canonical match
AGGGTTTTTG	7	0	10	7	2	gnl|UG|At#S11720045	no match found	At5g36910.1
at2g15765	CON	MIN	HOUR	DAY	WEEK	UNIGENE	FLCDNA	TAIR
TTTGAGAGAG	31	20	27	33	32	no match found	no match found	At2g15765.1
at4g00780	CON	MIN	HOUR	DAY	WEEK	UNIGENE	FLCDNA	TAIR
CCTCTGGGGA	4	7	6	0	0	gnl|UG|At#S11727479	gi|17529101|gb|AY065285.1	At4g00780.1
at2g33850	CON	MIN	HOUR	DAY	WEEK	UNIGENE	FLCDNA	TAIR
AAACAATAAT	1	1	0	1	0	gnl|UG|At#S11734080	gi|15451031|gb|AY054596.1	At2g33850.1
at2g27840	CON	MIN	HOUR	DAY	WEEK	UNIGENE	FLCDNA	TAIR
CCTCTCAGGT	0	0	1	0	0	gnl|UG|At#S11805169	gi|110739094|dbj|AK229619.1|	At2g27840.1
at5g55310	CON	MIN	HOUR	DAY	WEEK	UNIGENE	FLCDNA	TAIR
TTCGTTGAGG	0	3	0	0	1	gnl|UG|At#S11718182	gi|110741972|dbj|AK226832.1|	pseudo chromosome match

at2g37970	CON	MIN	HOUR	DAY	WEEK	UNIGENE	FLCDNA	TAIR
ATTCCTGTTG	4	2	10	2	2	gnl|UG|At#S11733106	gi|20148420|gb|AY081539.1	At2g37970.1
at2g44745	CON	MIN	HOUR	DAY	WEEK	UNIGENE	FLCDNA	TAIR
TGTTTTTCAA	4	4	5	4	6	no match found	gi|21406935|gb|AY088161.1	At2g44745.1
at5g61560	CON	MIN	HOUR	DAY	WEEK	UNIGENE	FLCDNA	TAIR
AATCGTTGTG	1	0	0	0	0	gnl|UG|At#S34117561	gi|110737405|dbj|AK228747.1|	At5g61560.1
at1g46480	CON	MIN	HOUR	DAY	WEEK	UNIGENE	FLCDNA	TAIR
AAGGTGAGGC	0	4	1	0	0	gnl|UG|At#S11735555	gi|38454109|gb|BT010692.1	At1g46480.1
at1g63110	CON	MIN	HOUR	DAY	WEEK	UNIGENE	FLCDNA	TAIR
TATACAAGGT	0	0	0	0	1	gnl|UG|At#S18942331	no match found	multiple non-canonical match
at1g48970	CON	MIN	HOUR	DAY	WEEK	UNIGENE	FLCDNA	TAIR
GAAAAATTGT	0	0	0	1	0	no match found	gi|62318752|dbj|AK220696.1|	multiple non-canonical match
at5g27140	CON	MIN	HOUR	DAY	WEEK	UNIGENE	FLCDNA	TAIR
CGAAGAAGAA	1	2	0	0	0	gnl|UG|At#S11720498	no match found	multiple non-canonical match
at1g23860	CON	MIN	HOUR	DAY	WEEK	UNIGENE	FLCDNA	TAIR
CTGTTTTCCA	2	2	0	1	0	no match found	gi|51969659|dbj|AK175759.1|	multiple non-canonical match
at5g28067	CON	MIN	HOUR	DAY	WEEK	UNIGENE	FLCDNA	TAIR
AATGGAACGA	0	0	1	0	0	no match found	no match found	At5g28067.1
at3g33545	CON	MIN	HOUR	DAY	WEEK	UNIGENE	FLCDNA	TAIR
AGAAATTGGT	1	4	1	1	2	no match found	no match found	At3g33545.1
at2g31865	CON	MIN	HOUR	DAY	WEEK	UNIGENE	FLCDNA	TAIR
ATATAAAGTC	0	0	0	0	1	gnl|UG|At#S18942811	gi|18252208|gb|AY072330.1	At2g31865.1
at5g67070	CON	MIN	HOUR	DAY	WEEK	UNIGENE	FLCDNA	TAIR
GTTTATTATT	2	1	0	0	1	no match found	gi|21407027|gb|AY088253.1	At5g24270.1
TTCCAATTTC	2	0	2	1	1	gnl|UG|At#S11716988	no match found	At5g67070.1
at2g17000	CON	MIN	HOUR	DAY	WEEK	UNIGENE	FLCDNA	TAIR
TTTTTGAAAG	0	0	1	0	0	no match found	no match found	At2g17000.1
at3g13110	CON	MIN	HOUR	DAY	WEEK	UNIGENE	FLCDNA	TAIR
ACAAGATTCC	3	6	4	4	4	no match found	gi|111074409|gb|BT026471.1	non-canonical match
ATTTCTGTGA	0	1	6	2	4	gnl|UG|At#S11736958	gi|110740577|dbj|AK226230.1|	At3g13110.1
at5g40250	CON	MIN	HOUR	DAY	WEEK	UNIGENE	FLCDNA	TAIR
CTCAACAAAA	0	1	0	1	1	gnl|UG|At#S21736362	gi|51970767|dbj|AK176313.1|	non-canonical match
CTGGTTCCTG	0	0	0	1	0	no match found	no match found	At5g40250.1
at4g14950	CON	MIN	HOUR	DAY	WEEK	UNIGENE	FLCDNA	TAIR
CTCCAACATC	1	0	0	0	1	gnl|UG|At#S18941861	gi|16604570|gb|AY059730.1	At4g14950.2
at4g19700	CON	MIN	HOUR	DAY	WEEK	UNIGENE	FLCDNA	TAIR
TCTTCTTGAC	0	1	0	1	1	gnl|UG|At#S11724384	gi|17065053|gb|AY062603.1	At4g19700.1
at3g07550	CON	MIN	HOUR	DAY	WEEK	UNIGENE	FLCDNA	TAIR
ATTCTTTTAA	0	0	0	0	1	gnl|UG|At#S11738477	gi|26452862|dbj|AK118929.1|	At3g07550.1
at5g22080	CON	MIN	HOUR	DAY	WEEK	UNIGENE	FLCDNA	TAIR
AAGAACAGTG	4	3	2	0	0	gnl|UG|At#S11817071	gi|26453259|dbj|AK119133.1|	At5g22080.1
at3g16410	CON	MIN	HOUR	DAY	WEEK	UNIGENE	FLCDNA	TAIR

GTGGTAAAGC	0	0	1	0	0	gnl|UG|At#S34118167	no match found	multiple canonical match
at4g28850	CON	MIN	HOUR	DAY	WEEK	UNIGENE	FLCDNA	TAIR
ATGAAGAAGA	1	1	0	0	2	no match found	gi|110740299|dbj|AK230242.1|	multiple non-canonical match
at1g20880	CON	MIN	HOUR	DAY	WEEK	UNIGENE	FLCDNA	TAIR
TTCTACAGCT	0	2	1	0	0	gnl|UG|At#S11740969	gi|23505938|gb|AY143890.1	At1g20880.1
at3g13720	CON	MIN	HOUR	DAY	WEEK	UNIGENE	FLCDNA	TAIR
AGACGACGTC	0	1	0	0	0	no match found	gi|110742661|dbj|AK227204.1|	At1g20880.1
at4g02990	CON	MIN	HOUR	DAY	WEEK	UNIGENE	FLCDNA	TAIR
AGAGGAGAAT	0	0	1	0	1	no match found	gi|14190446|gb|AF378901.1	non-canonical match
TTGTTGAAAT	2	1	4	3	3	gnl|UG|At#S11726936	no match found	At4g02990.1
at2g03760	CON	MIN	HOUR	DAY	WEEK	UNIGENE	FLCDNA	TAIR
TTCTTGTTTT	1	1	0	3	0	gnl|UG|At#S11740418	gi|14030734|gb|AF375458.1	At2g03760.1
at2g27050	CON	MIN	HOUR	DAY	WEEK	UNIGENE	FLCDNA	TAIR
CTACAAGTTT	1	1	1	0	1	gnl|UG|At#S11735772	no match found	At2g27050.1
at2g20610	CON	MIN	HOUR	DAY	WEEK	UNIGENE	FLCDNA	TAIR
CCAAGAAGAC	4	2	5	1	0	no match found	gi|20259032|gb|AY091293.1	non-canonical match
CTCTTCGACA	0	1	1	0	0	gnl|UG|At#S23740556	no match found	multiple non-canonical match
AAAGAGAATT	1	0	1	1	3	gnl|UG|At#S18942900	gi|15293106|gb|AY050987.1	At2g20610.1
at2g07696	CON	MIN	HOUR	DAY	WEEK	UNIGENE	FLCDNA	TAIR
AATGAAGAAG	1	0	0	0	0	no match found	no match found	At2g07696.1
GACTGGCTTC	1	0	0	0	0	gnl|UG|At#S17854093	gi|40824097|gb|BT011309.1	non-canonical match
at3g53940	CON	MIN	HOUR	DAY	WEEK	UNIGENE	FLCDNA	TAIR
ACATTCGAGG	0	0	1	1	0	gnl|UG|At#S11728698	gi|20259827|gb|AY093262.1	At3g53940.1
at1g14770	CON	MIN	HOUR	DAY	WEEK	UNIGENE	FLCDNA	TAIR
TGTTGATGAA	0	0	1	1	1	gnl|UG|At#S18942577	gi|26452751|dbj|AK118871.1|	At1g14770.1
at1g63800	CON	MIN	HOUR	DAY	WEEK	UNIGENE	FLCDNA	TAIR
TATATATGAA	0	1	0	0	0	no match found	gi|110741373|dbj|AK230439.1|	non-canonical match
TTGTGCACCT	1	3	0	0	0	gnl|UG|At#S11743108	gi|18377786|gb|AY074347.1	At1g63800.1
at1g78370	CON	MIN	HOUR	DAY	WEEK	UNIGENE	FLCDNA	TAIR
GAGAAAGAGA	4	20	13	5	3	gnl|UG|At#S11727083	gi|23197919|gb|BT000168.1	At1g78370.1
at1g65140	CON	MIN	HOUR	DAY	WEEK	UNIGENE	FLCDNA	TAIR
GAAATCTATA	2	2	0	1	0	gnl|UG|At#S11730101	no match found	multiple non-canonical match
at3g02120	CON	MIN	HOUR	DAY	WEEK	UNIGENE	FLCDNA	TAIR
TATCTAATGT	2	1	0	0	0	gnl|UG|At#S11740044	gi|21407115|gb|AY088341.1	At3g02120.1
at3g18690	CON	MIN	HOUR	DAY	WEEK	UNIGENE	FLCDNA	TAIR
TATAAAAAGA	0	1	0	0	0	gnl|UG|At#S11735241	gi|21403040|gb|AY084330.1	At3g18690.1
at5g14840	CON	MIN	HOUR	DAY	WEEK	UNIGENE	FLCDNA	TAIR
CTTTCATTAT	0	0	0	1	0	gnl|UG|At#S11705356	gi|20466467|gb|AY099700.1	non-canonical match
at5g23420	CON	MIN	HOUR	DAY	WEEK	UNIGENE	FLCDNA	TAIR
AGTGACTTCC	0	1	0	0	0	gnl|UG|At#S11720835	gi|26452393|dbj|AK118688.1|	At5g23420.1
at1g75440	CON	MIN	HOUR	DAY	WEEK	UNIGENE	FLCDNA	TAIR
CTCTCAAGCT	1	4	0	1	0	no match found	no match found	At1g75440.1

<table>
<tr><td>at2g05260</td><td>CON</td><td>MIN</td><td>HOUR</td><td>DAY</td><td>WEEK</td><td>UNIGENE</td><td>FLCDNA</td><td>TAIR</td></tr>
<tr><td>TACAAATAGC</td><td>0</td><td>1</td><td>0</td><td>0</td><td>0</td><td>gnl|UG|At#S34114434</td><td>gi|110743373|dbj|AK227581.1|</td><td>At2g05260.1</td></tr>
<tr><td>at3g20650</td><td>CON</td><td>MIN</td><td>HOUR</td><td>DAY</td><td>WEEK</td><td>UNIGENE</td><td>FLCDNA</td><td>TAIR</td></tr>
<tr><td>ATTTGTATTG</td><td>1</td><td>0</td><td>0</td><td>0</td><td>0</td><td>no match found</td><td>no match found</td><td>At3g20650.2</td></tr>
<tr><td>AGAAGGCTTG</td><td>0</td><td>0</td><td>1</td><td>1</td><td>0</td><td>no match found</td><td>gi|28973780|gb|BT005804.1</td><td>non-canonical match</td></tr>
<tr><td>CCTTTTTATT</td><td>0</td><td>0</td><td>0</td><td>1</td><td>0</td><td>no match found</td><td>no match found</td><td>At3g20650.1</td></tr>
<tr><td>at3g05560</td><td>CON</td><td>MIN</td><td>HOUR</td><td>DAY</td><td>WEEK</td><td>UNIGENE</td><td>FLCDNA</td><td>TAIR</td></tr>
<tr><td>GAGATTGCTT</td><td>0</td><td>0</td><td>0</td><td>0</td><td>1</td><td>no match found</td><td>gi|30102825|gb|BT006523.1</td><td>multiple non-canonical match</td></tr>
<tr><td>TTTCTGTTTT</td><td>17</td><td>11</td><td>7</td><td>7</td><td>18</td><td>gnl|UG|At#S18904247</td><td>gi|27311726|gb|BT002469.1</td><td>At3g05560.2</td></tr>
<tr><td>at3g29350</td><td>CON</td><td>MIN</td><td>HOUR</td><td>DAY</td><td>WEEK</td><td>UNIGENE</td><td>FLCDNA</td><td>TAIR</td></tr>
<tr><td>GCTAGAGCTT</td><td>1</td><td>1</td><td>0</td><td>0</td><td>0</td><td>no match found</td><td>gi|20453360|gb|AY097403.1</td><td>multiple canonical match</td></tr>
<tr><td>at1g48620</td><td>CON</td><td>MIN</td><td>HOUR</td><td>DAY</td><td>WEEK</td><td>UNIGENE</td><td>FLCDNA</td><td>TAIR</td></tr>
<tr><td>CTTCCCATCT</td><td>4</td><td>4</td><td>6</td><td>3</td><td>4</td><td>gnl|UG|At#S20804434</td><td>gi|66792627|gb|BT023425.1</td><td>At1g48620.1</td></tr>
<tr><td>at3g55120</td><td>CON</td><td>MIN</td><td>HOUR</td><td>DAY</td><td>WEEK</td><td>UNIGENE</td><td>FLCDNA</td><td>TAIR</td></tr>
<tr><td>CATTTACATT</td><td>2</td><td>0</td><td>0</td><td>14</td><td>25</td><td>gnl|UG|At#S14273684</td><td>gi|28393710|gb|BT004265.1</td><td>At3g55120.1</td></tr>
<tr><td>at3g58970</td><td>CON</td><td>MIN</td><td>HOUR</td><td>DAY</td><td>WEEK</td><td>UNIGENE</td><td>FLCDNA</td><td>TAIR</td></tr>
<tr><td>TAGATGGAAA</td><td>1</td><td>1</td><td>1</td><td>3</td><td>1</td><td>no match found</td><td>gi|16209701|gb|AY057613.1</td><td>At3g58970.1</td></tr>
<tr><td>at4g09800</td><td>CON</td><td>MIN</td><td>HOUR</td><td>DAY</td><td>WEEK</td><td>UNIGENE</td><td>FLCDNA</td><td>TAIR</td></tr>
<tr><td>AAGCTGAGAG</td><td>10</td><td>18</td><td>3</td><td>5</td><td>11</td><td>gnl|UG|At#S11725985</td><td>gi|21405044|gb|AY086334.1</td><td>At4g09800.1</td></tr>
<tr><td>at4g25620</td><td>CON</td><td>MIN</td><td>HOUR</td><td>DAY</td><td>WEEK</td><td>UNIGENE</td><td>FLCDNA</td><td>TAIR</td></tr>
<tr><td>AAAAAGCAAG</td><td>0</td><td>0</td><td>1</td><td>0</td><td>0</td><td>no match found</td><td>gi|28951010|gb|BT005365.1</td><td>non-canonical match</td></tr>
<tr><td>GGAAGCAAAG</td><td>3</td><td>2</td><td>3</td><td>0</td><td>2</td><td>gnl|UG|At#S11723371</td><td>gi|26449761|dbj|AK117334.1|</td><td>At4g25620.1</td></tr>
<tr><td>at3g55410</td><td>CON</td><td>MIN</td><td>HOUR</td><td>DAY</td><td>WEEK</td><td>UNIGENE</td><td>FLCDNA</td><td>TAIR</td></tr>
<tr><td>TCAAAGAGCA</td><td>0</td><td>1</td><td>1</td><td>1</td><td>0</td><td>gnl|UG|At#S11728437</td><td>gi|110739608|dbj|AK229884.1|</td><td>At3g55410.1</td></tr>
<tr><td>at3g17650</td><td>CON</td><td>MIN</td><td>HOUR</td><td>DAY</td><td>WEEK</td><td>UNIGENE</td><td>FLCDNA</td><td>TAIR</td></tr>
<tr><td>TGAAAGTGTA</td><td>3</td><td>2</td><td>1</td><td>3</td><td>1</td><td>gnl|UG|At#S11705775</td><td>gi|15982792|gb|AY057503.1</td><td>At3g17650.1</td></tr>
<tr><td>at1g16800</td><td>CON</td><td>MIN</td><td>HOUR</td><td>DAY</td><td>WEEK</td><td>UNIGENE</td><td>FLCDNA</td><td>TAIR</td></tr>
<tr><td>AAAAGAAAGA</td><td>0</td><td>4</td><td>0</td><td>0</td><td>0</td><td>gnl|UG|At#S11741364</td><td>no match found</td><td>multiple non-canonical match</td></tr>
<tr><td>CCATTATGAT</td><td>0</td><td>1</td><td>0</td><td>0</td><td>0</td><td>no match found</td><td>no match found</td><td>At1g16800.1</td></tr>
<tr><td>at5g63890</td><td>CON</td><td>MIN</td><td>HOUR</td><td>DAY</td><td>WEEK</td><td>UNIGENE</td><td>FLCDNA</td><td>TAIR</td></tr>
<tr><td>ACTGTACAAT</td><td>1</td><td>0</td><td>0</td><td>0</td><td>0</td><td>gnl|UG|At#S11717311</td><td>gi|21406761|gb|AY087987.1</td><td>At5g63890.1</td></tr>
<tr><td>at1g80040</td><td>CON</td><td>MIN</td><td>HOUR</td><td>DAY</td><td>WEEK</td><td>UNIGENE</td><td>FLCDNA</td><td>TAIR</td></tr>
<tr><td>GCAACTCAAT</td><td>0</td><td>2</td><td>2</td><td>2</td><td>2</td><td>gnl|UG|At#S18942218</td><td>gi|18176073|gb|AY072157.1</td><td>At1g80040.2</td></tr>
<tr><td>at1g08680</td><td>CON</td><td>MIN</td><td>HOUR</td><td>DAY</td><td>WEEK</td><td>UNIGENE</td><td>FLCDNA</td><td>TAIR</td></tr>
<tr><td>ATTAATAATA</td><td>0</td><td>0</td><td>0</td><td>0</td><td>1</td><td>no match found</td><td>gi|19698912|gb|AY081303.1</td><td>non-canonical match</td></tr>
<tr><td>at3g15210</td><td>CON</td><td>MIN</td><td>HOUR</td><td>DAY</td><td>WEEK</td><td>UNIGENE</td><td>FLCDNA</td><td>TAIR</td></tr>
<tr><td>TTGTTTCTCT</td><td>0</td><td>8</td><td>2</td><td>0</td><td>4</td><td>no match found</td><td>no match found</td><td>At3g15210.1</td></tr>
<tr><td>GATCGTTATG</td><td>2</td><td>1</td><td>4</td><td>0</td><td>3</td><td>no match found</td><td>gi|22655161|gb|AY140030.1</td><td>non-canonical match</td></tr>
<tr><td>CGTTTCTTCT</td><td>0</td><td>1</td><td>0</td><td>1</td><td>1</td><td>no match found</td><td>gi|21404942|gb|AY086232.1</td><td>non-canonical match</td></tr>
<tr><td>at5g47790</td><td>CON</td><td>MIN</td><td>HOUR</td><td>DAY</td><td>WEEK</td><td>UNIGENE</td><td>FLCDNA</td><td>TAIR</td></tr>
<tr><td>GGCTTCTGTG</td><td>0</td><td>1</td><td>0</td><td>0</td><td>0</td><td>no match found</td><td>gi|33589741|gb|BT010168.1</td><td>multiple non-canonical match</td></tr>
<tr><td>at1g09900</td><td>CON</td><td>MIN</td><td>HOUR</td><td>DAY</td><td>WEEK</td><td>UNIGENE</td><td>FLCDNA</td><td>TAIR</td></tr>
<tr><td>AAGAAAAGCT</td><td>1</td><td>1</td><td>0</td><td>1</td><td>0</td><td>gnl|UG|At#S11742044</td><td>no match found</td><td>multiple non-canonical match</td></tr>
</table>

at1g67865	CON	MIN	HOUR	DAY	WEEK	UNIGENE		FLCDNA	TAIR
TTTCAGTATC	5	7	1	2	1	gnl|UG|At#S35265483		gi|15529158|gb|AY052203.1	At1g67865.1
at4g02860	CON	MIN	HOUR	DAY	WEEK	UNIGENE		FLCDNA	TAIR
TCTTGGTCTG	0	0	2	1	1	no match found		gi|51972157|gb|BT015684.1	non-canonical match
at2g20515	CON	MIN	HOUR	DAY	WEEK	UNIGENE		FLCDNA	TAIR
GTTTCGGAGT	1	0	0	0	0	gnl|UG|At#S11737341		gi|21405066|gb|AY086356.1	At2g20515.1
at5g60490	CON	MIN	HOUR	DAY	WEEK	UNIGENE		FLCDNA	TAIR
TGGGTGATTT	1	1	0	0	0	gnl|UG|At#S11717653		gi|21404167|gb|AY085457.1	At5g60490.1
at3g09970	CON	MIN	HOUR	DAY	WEEK	UNIGENE		FLCDNA	TAIR
GAAAACTTCA	0	0	3	0	0	no match found		gi|23297515|gb|AY150444.1	non-canonical match
CTATAATCCA	2	0	0	0	0	gnl|UG|At#S11737901		gi|19424084|gb|AY080859.1	At3g09970.1
at1g16820	CON	MIN	HOUR	DAY	WEEK	UNIGENE		FLCDNA	TAIR
TTAGCCAGAT	0	0	1	0	0	no match found		no match found	At1g16820.1
at3g29090	CON	MIN	HOUR	DAY	WEEK	UNIGENE		FLCDNA	TAIR
AAGAACCTAT	0	0	1	0	1	gnl|UG|At#S11731938		gi|21403128|gb|AY084418.1	multiple canonical match
at3g01740	CON	MIN	HOUR	DAY	WEEK	UNIGENE		FLCDNA	TAIR
ATTCTCTTTG	0	1	0	0	0	gnl|UG|At#S11740155		no match found	At3g01740.1
at1g35510	CON	MIN	HOUR	DAY	WEEK	UNIGENE		FLCDNA	TAIR
AGTCCTGTAT	3	1	0	0	2	gnl|UG|At#S11737094		gi|15293042|gb|AY050955.1	At1g35510.1
at1g51370	CON	MIN	HOUR	DAY	WEEK	UNIGENE		FLCDNA	TAIR
GTGATTCCTT	0	0	0	1	0	gnl|UG|At#S34117414		gi|110737689|dbj|AK228894.1|	At1g51370.1
at5g08780	CON	MIN	HOUR	DAY	WEEK	UNIGENE		FLCDNA	TAIR
AAAGAACAAG	0	0	1	0	0	gnl|UG|At#S11817111		no match found	multiple non-canonical match
at3g17510	CON	MIN	HOUR	DAY	WEEK	UNIGENE		FLCDNA	TAIR
TTCATATAAT	1	1	1	1	1	no match found		gi|20260555|gb|AY093177.1	non-canonical match
TGTTTAGTTT	6	3	11	5	2	gnl|UG|At#S11735591		no match found	At3g17510.2
at1g13170	CON	MIN	HOUR	DAY	WEEK	UNIGENE		FLCDNA	TAIR
TTTGTTTATA	0	2	0	0	0	gnl|UG|At#S11709505		gi|18087545|gb|AF462814.1	At4g27110.1
at5g02610	CON	MIN	HOUR	DAY	WEEK	UNIGENE		FLCDNA	TAIR
AGCTTAGAGA	0	1	3	0	0	gnl|UG|At#S11725511		gi|21407773|gb|AY088999.1	At5g02610.1
at5g67370	CON	MIN	HOUR	DAY	WEEK	UNIGENE		FLCDNA	TAIR
AATTATCCAC	3	1	4	3	5	gnl|UG|At#S11701964		gi|13430429|gb|AF360127.1	At5g67370.1
at5g25610	CON	MIN	HOUR	DAY	WEEK	UNIGENE		FLCDNA	TAIR
TTGTGTGGTT	21	4	12	9	10	gnl|UG|At#S11720620		gi|16974545|gb|AY060560.1	At5g25610.1
at5g23450	CON	MIN	HOUR	DAY	WEEK	UNIGENE		FLCDNA	TAIR
GAGAAGTGAT	0	0	0	0	1	no match found		gi|28416518|gb|BT004544.1	non-canonical match
AGAGAGTATA	3	2	2	1	1	gnl|UG|At#S28281758		gi|22654974|gb|AY139759.1	multiple canonical match
at4g17610	CON	MIN	HOUR	DAY	WEEK	UNIGENE		FLCDNA	TAIR
TCAGTGGTGC	0	3	0	0	1	gnl|UG|At#S11724741		gi|110740265|dbj|AK230224.1|	non-canonical match
at1g09060	CON	MIN	HOUR	DAY	WEEK	UNIGENE		FLCDNA	TAIR
CTTCATATAG	3	3	1	0	1	gnl|UG|At#S34118287		gi|110736016|dbj|AK228019.1|	At1g09060.1
at5g19690	CON	MIN	HOUR	DAY	WEEK	UNIGENE		FLCDNA	TAIR

TTTATTGCGG	3	6	7	6	6	gnl|UG|At#S11706075	gi|15810304|gb|AY056191.1	At5g19690.1
at2g01940	CON	MIN	HOUR	DAY	WEEK	UNIGENE	FLCDNA	TAIR
TCGTCAGCGA	0	1	1	0	0	gnl|UG|At#S18920315	gi|110741727|dbj|AK226703.1|	At2g01940.1
at3g18140	CON	MIN	HOUR	DAY	WEEK	UNIGENE	FLCDNA	TAIR
ACGGCTAGAT	0	5	3	0	0	gnl|UG|At#S11704607	gi|21406200|gb|AY087463.1	At3g18140.1
at2g37940	CON	MIN	HOUR	DAY	WEEK	UNIGENE	FLCDNA	TAIR
GATATTGTTA	2	2	3	0	2	gnl|UG|At#S11733112	gi|16604321|gb|AY058059.1	At2g37940.1
at1g33470	CON	MIN	HOUR	DAY	WEEK	UNIGENE	FLCDNA	TAIR
GTTAGGAATT	1	3	0	1	0	gnl|UG|At#S11737766	gi|14532531|gb|AY039890.1	At1g33470.1
TCTCCTACTA	0	0	1	0	0	gnl|UG|At#S20835239	no match found	pseudo chromosome match
at3g50530	CON	MIN	HOUR	DAY	WEEK	UNIGENE	FLCDNA	TAIR
GTGTGTCTAG	0	2	1	0	1	gnl|UG|At#S11729340	gi|110736730|dbj|AK228391.1|	At3g50530.1
at4g04770	CON	MIN	HOUR	DAY	WEEK	UNIGENE	FLCDNA	TAIR
TGAATCTTGT	11	13	10	14	15	gnl|UG|At#S11726449	gi|15912264|gb|AY056410.1	At4g04770.1
at3g55260	CON	MIN	HOUR	DAY	WEEK	UNIGENE	FLCDNA	TAIR
ACAACATCTG	4	1	1	2	1	gnl|UG|At#S34114755	gi|21403511|gb|AY084801.1	At3g55260.1
at4g28710	CON	MIN	HOUR	DAY	WEEK	UNIGENE	FLCDNA	TAIR
TTTTGGTTTG	1	1	0	2	0	no match found	gi|62319884|dbj|AK221268.1|	multiple canonical match
at2g47590	CON	MIN	HOUR	DAY	WEEK	UNIGENE	FLCDNA	TAIR
TTCGATGAGC	0	4	1	0	1	gnl|UG|At#S11730810	gi|115646758|gb|BT029173.1	At2g47590.1
at5g63630	CON	MIN	HOUR	DAY	WEEK	UNIGENE	FLCDNA	TAIR
TCGAGATGAG	1	0	0	0	0	gnl|UG|At#S11717337	no match found	non-canonical match
at1g52040	CON	MIN	HOUR	DAY	WEEK	UNIGENE	FLCDNA	TAIR
TGTCTTATCA	1	1	0	0	0	gnl|UG|At#S11733733	gi|110740204|dbj|AK230192.1|	At1g52040.1
at3g19680	CON	MIN	HOUR	DAY	WEEK	UNIGENE	FLCDNA	TAIR
AGTTGTCTTC	4	15	3	0	1	no match found	no match found	At3g19680.1
at1g32690	CON	MIN	HOUR	DAY	WEEK	UNIGENE	FLCDNA	TAIR
TTTTTTTTAA	0	3	0	0	0	gnl|UG|At#S11738019	gi|38566627|gb|BT010837.1	At1g32690.1
atcg00180	CON	MIN	HOUR	DAY	WEEK	UNIGENE	FLCDNA	TAIR
AGATTTATGC	0	0	0	1	0	no match found	no match found	AtCg00180
at4g27595	CON	MIN	HOUR	DAY	WEEK	UNIGENE	FLCDNA	TAIR
ACTTTGCTGA	0	1	0	0	0	gnl|UG|At#S11816579	no match found	non-canonical match
at4g14310	CON	MIN	HOUR	DAY	WEEK	UNIGENE	FLCDNA	TAIR
TGGTGTTTTG	2	0	1	0	0	gnl|UG|At#S15461163	no match found	At4g14310.2
at1g05200	CON	MIN	HOUR	DAY	WEEK	UNIGENE	FLCDNA	TAIR
GCGAAATTAC	3	3	0	0	1	gnl|UG|At#S11823638	gi|26451457|dbj|AK118206.1|	At1g05200.1
at1g12890	CON	MIN	HOUR	DAY	WEEK	UNIGENE	FLCDNA	TAIR
TAACAGTAAA	17	1	16	6	7	no match found	no match found	At1g12890.1
at3g07090	CON	MIN	HOUR	DAY	WEEK	UNIGENE	FLCDNA	TAIR
CAAAAGTACG	1	1	0	4	2	gnl|UG|At#S11738599	gi|23296880|gb|AY142656.1	At3g07090.1
at2g01850	CON	MIN	HOUR	DAY	WEEK	UNIGENE	FLCDNA	TAIR

ACATATTCAT	4	4	0	0	0	gnl|UG|At#S11742607	gi|21404545|gb|AY085835.1	At2g01850.1
at2g30050	CON	MIN	HOUR	DAY	WEEK	UNIGENE	FLCDNA	TAIR
ATATATAGTT	0	0	1	0	0	gnl|UG|At#S11735023	no match found	At2g30050.1
GAAGATGGAT	0	1	2	0	0	no match found	gi|21406291|gb|AY087553.1	multiple non-canonical match
at1g77210	CON	MIN	HOUR	DAY	WEEK	UNIGENE	FLCDNA	TAIR
GTTGTAGTAG	0	7	0	0	0	gnl|UG|At#S11727508	gi|29028885|gb|BT005887.1	At1g77210.1
at4g15802	CON	MIN	HOUR	DAY	WEEK	UNIGENE	FLCDNA	TAIR
GGAGGCAGAA	0	0	1	0	0	no match found	gi|28416758|gb|BT004664.1	non-canonical match
TCCGAAGGAA	7	7	5	7	1	gnl|UG|At#S15461094	gi|26453170|dbj|AK119086.1|	At4g15802.1
at2g02990	CON	MIN	HOUR	DAY	WEEK	UNIGENE	FLCDNA	TAIR
GAGATTGGTT	0	1	2	0	1	gnl|UG|At#S11740536	gi|21405457|gb|AY086747.1	At2g02990.1
at1g27752	CON	MIN	HOUR	DAY	WEEK	UNIGENE	FLCDNA	TAIR
AGGATTTGCA	0	0	0	1	0	gnl|UG|At#S34115568	gi|110741256|dbj|AK230380.1|	At1g27750.1
at2g29580	CON	MIN	HOUR	DAY	WEEK	UNIGENE	FLCDNA	TAIR
ATTACCCACA	0	0	1	0	0	gnl|UG|At#S11705317	gi|27363235|gb|BT002621.1	At2g29580.1
at2g36340	CON	MIN	HOUR	DAY	WEEK	UNIGENE	FLCDNA	TAIR
ATGTATCCAC	0	1	0	0	0	gnl|UG|At#S11733508	no match found	non-canonical match
CCACTAATTT	0	1	0	0	0	no match found	no match found	At2g36340.1
at1g70680	CON	MIN	HOUR	DAY	WEEK	UNIGENE	FLCDNA	TAIR
CTCACACACA	0	0	1	0	0	gnl|UG|At#S11728948	gi|110737207|dbj|AK228644.1|	At1g70680.1
at3g22590	CON	MIN	HOUR	DAY	WEEK	UNIGENE	FLCDNA	TAIR
TTGAGATATT	0	0	0	1	0	gnl|UG|At#S11710190	gi|17529301|gb|AY065437.1	At3g22590.1
at4g34170	CON	MIN	HOUR	DAY	WEEK	UNIGENE	FLCDNA	TAIR
TCGGCGAATG	0	0	1	0	0	gnl|UG|At#S11721905	no match found	non-canonical match
at1g78270	CON	MIN	HOUR	DAY	WEEK	UNIGENE	FLCDNA	TAIR
TCACACGATT	2	3	0	0	0	gnl|UG|At#S11727113	gi|23198067|gb|BT000242.1	At1g78270.1
at5g47230	CON	MIN	HOUR	DAY	WEEK	UNIGENE	FLCDNA	TAIR
CGATGAAAAA	2	4	9	3	1	gnl|UG|At#S11719001	gi|26450220|dbj|AK117568.1|	At5g47230.1
at4g28300	CON	MIN	HOUR	DAY	WEEK	UNIGENE	FLCDNA	TAIR
GCTCCCCTCT	2	5	2	3	1	gnl|UG|At#S18925512	gi|17380877|gb|AY063895.1	At4g28300.1
at4g12040	CON	MIN	HOUR	DAY	WEEK	UNIGENE	FLCDNA	TAIR
AATCAAAATG	3	7	8	2	4	gnl|UG|At#S11702885	gi|13605498|gb|AF361575.1	At4g12040.2
at3g18930	CON	MIN	HOUR	DAY	WEEK	UNIGENE	FLCDNA	TAIR
GAACAAACGT	0	1	0	0	0	gnl|UG|At#S18942075	gi|21689662|gb|AY122920.1	At3g18930.2
at2g04032	CON	MIN	HOUR	DAY	WEEK	UNIGENE	FLCDNA	TAIR
TCCTCGGTTA	0	0	0	1	0	gnl|UG|At#S24442330	gi|50253555|gb|BT015108.1	At2g03900.1
at4g10840	CON	MIN	HOUR	DAY	WEEK	UNIGENE	FLCDNA	TAIR
TATAGTGTTT	7	2	2	2	1	no match found	gi|21405199|gb|AY086489.1	multiple canonical match
at1g27000	CON	MIN	HOUR	DAY	WEEK	UNIGENE	FLCDNA	TAIR
TGTAGTGTCC	2	0	1	0	0	no match found	gi|15982835|gb|AY057525.1	non-canonical match
CTAAACTTGT	0	1	0	0	0	no match found	gi|15810656|gb|AY056367.1	non-canonical match
at5g63770	CON	MIN	HOUR	DAY	WEEK	UNIGENE	FLCDNA	TAIR

CCGCAGCCAT	1	0	1	0	0	no match found	gi|31711749|gb|BT008792.1	non-canonical match
at3g50930	CON	MIN	HOUR	DAY	WEEK	UNIGENE	FLCDNA	TAIR
AGGAGGTTGA	0	2	3	0	0	gnl|UG|At#S11729265	gi|20856862|gb|AY102119.1	At3g50930.1
at1g18270	CON	MIN	HOUR	DAY	WEEK	UNIGENE	FLCDNA	TAIR
AAAGCTGTGA	1	3	1	0	2	gnl|UG|At#S43850623	gi|22136929|gb|AY133875.1	At1g18270.1
at4g15340	CON	MIN	HOUR	DAY	WEEK	UNIGENE	FLCDNA	TAIR
GTGCGGAAAC	0	1	0	0	0	gnl|UG|At#S11725144	no match found	At4g15340.1
at3g61830	CON	MIN	HOUR	DAY	WEEK	UNIGENE	FLCDNA	TAIR
TCAATTTTTC	1	0	0	1	0	gnl|UG|At#S11726901	gi|16604602|gb|AY059746.1	At3g61830.1
at4g32950	CON	MIN	HOUR	DAY	WEEK	UNIGENE	FLCDNA	TAIR
ACACGACAGC	0	1	0	0	1	no match found	no match found	At4g32950.1
at4g27010	CON	MIN	HOUR	DAY	WEEK	UNIGENE	FLCDNA	TAIR
TCTATTTAAA	2	0	0	0	0	no match found	no match found	At4g27010.1
at5g12980	CON	MIN	HOUR	DAY	WEEK	UNIGENE	FLCDNA	TAIR
CGATGCACTT	0	0	0	0	1	gnl|UG|At#S18913204	gi|110739141|dbj|AK229643.1|	At5g12980.1
at5g51560	CON	MIN	HOUR	DAY	WEEK	UNIGENE	FLCDNA	TAIR
AGCTAGGTAA	1	3	1	0	0	gnl|UG|At#S11718560	gi|21404111|gb|AY085401.1	At5g51560.1
at4g10030	CON	MIN	HOUR	DAY	WEEK	UNIGENE	FLCDNA	TAIR
TCTTGGAAGA	0	3	0	1	0	gnl|UG|At#S11725962	gi|21407220|gb|AY088446.1	At4g10030.1
at4g26650	CON	MIN	HOUR	DAY	WEEK	UNIGENE	FLCDNA	TAIR
GGAACTTTTG	1	0	2	4	1	gnl|UG|At#S23626812	no match found	multiple non-canonical match
GATGATAAAA	0	0	2	2	0	no match found	gi|16930502|gb|AF419605.1	non-canonical match
at2g44970	CON	MIN	HOUR	DAY	WEEK	UNIGENE	FLCDNA	TAIR
CCTGGATGGT	0	0	1	0	0	gnl|UG|At#S15460369	gi|18377627|gb|AY074267.1	At2g44970.1
at1g34210	CON	MIN	HOUR	DAY	WEEK	UNIGENE	FLCDNA	TAIR
CTATGGAGTT	0	3	1	1	0	gnl|UG|At#S11737509	gi|110739279|dbj|AK229715.1|	non-canonical match
at2g26830	CON	MIN	HOUR	DAY	WEEK	UNIGENE	FLCDNA	TAIR
CTGAATGATG	0	0	0	1	0	gnl|UG|At#S18898578	gi|21406419|gb|AY087681.1	At2g26830.1
at2g45310	CON	MIN	HOUR	DAY	WEEK	UNIGENE	FLCDNA	TAIR
CTAATATTAG	0	0	0	1	0	gnl|UG|At#S11731344	gi|28973494|gb|BT005652.1	At2g45310.1
at5g08660	CON	MIN	HOUR	DAY	WEEK	UNIGENE	FLCDNA	TAIR
TTTGGTTGTG	0	1	0	0	0	gnl|UG|At#S11817117	no match found	At5g08660.1
at3g14830	CON	MIN	HOUR	DAY	WEEK	UNIGENE	FLCDNA	TAIR
TACATATATA	1	0	0	0	0	gnl|UG|At#S28282981	gi|20466192|gb|AY099562.1	multiple canonical match
AGATTTAGTT	0	0	1	0	0	no match found	gi|45680405|gb|BT012095.1	non-canonical match
at3g03880	CON	MIN	HOUR	DAY	WEEK	UNIGENE	FLCDNA	TAIR
GAGACTGATT	0	1	1	0	0	gnl|UG|At#S11825139	gi|28393970|gb|BT004399.1	At3g03880.1
at3g53570	CON	MIN	HOUR	DAY	WEEK	UNIGENE	FLCDNA	TAIR
TGGATCACTC	1	1	0	0	0	no match found	gi|23270394|gb|AY034936.2	At3g53570.3
TATAGAAATG	2	2	0	0	0	gnl|UG|At#S15459276	no match found	At3g53570.2
at1g52720	CON	MIN	HOUR	DAY	WEEK	UNIGENE	FLCDNA	TAIR
TGTTCCCTAG	1	0	0	0	0	gnl|UG|At#S11733467	gi|21404680|gb|AY085970.1	At1g52720.1

at3g23620	CON	MIN	HOUR	DAY	WEEK	UNIGENE	FLCDNA	TAIR
GACTTGGTTA	0	1	0	0	1	no match found	gi|21689700|gb|AY122939.1	non-canonical match
at5g57630	CON	MIN	HOUR	DAY	WEEK	UNIGENE	FLCDNA	TAIR
GAGTTTTGCC	0	0	3	0	0	gnl|UG|At#S18912781	gi|22136499|gb|AY128928.1	At5g57630.1
at2g41040	CON	MIN	HOUR	DAY	WEEK	UNIGENE	FLCDNA	TAIR
TAACTAAATC	0	0	2	4	1	gnl|UG|At#S11732329	gi|110737846|dbj|AK228973.1|	At2g41040.1
at1g79870	CON	MIN	HOUR	DAY	WEEK	UNIGENE	FLCDNA	TAIR
AAAAGGGTAA	5	3	3	2	2	gnl|UG|At#S11726569	gi|17978982|gb|AY069901.1	At1g79870.1
at5g14080	CON	MIN	HOUR	DAY	WEEK	UNIGENE	FLCDNA	TAIR
TCGTGTTGCT	0	1	1	0	0	gnl|UG|At#S11722815	no match found	At5g14080.1
at5g13000	CON	MIN	HOUR	DAY	WEEK	UNIGENE	FLCDNA	TAIR
GATGATTAGG	5	4	2	3	1	gnl|UG|At#S11723106	no match found	At5g13000.1
at3g55510	CON	MIN	HOUR	DAY	WEEK	UNIGENE	FLCDNA	TAIR
AATTATTTTC	0	0	1	1	2	gnl|UG|At#S34115237	gi|110741872|dbj|AK226780.1|	At3g55510.1
at1g16730	CON	MIN	HOUR	DAY	WEEK	UNIGENE	FLCDNA	TAIR
ACTATCTGAG	1	0	1	0	1	gnl|UG|At#S11741371	gi|51970027|dbj|AK175943.1|	non-canonical match
TGTTCTTCTC	1	0	0	0	0	no match found	no match found	At1g16730.1
at5g46710	CON	MIN	HOUR	DAY	WEEK	UNIGENE	FLCDNA	TAIR
GTCTGTTATC	0	5	0	0	0	gnl|UG|At#S11719054	gi|21406964|gb|AY088190.1	At5g46710.1
at1g35670	CON	MIN	HOUR	DAY	WEEK	UNIGENE	FLCDNA	TAIR
AGCAACTTTC	1	6	3	1	5	gnl|UG|At#S11736982	gi|15293094|gb|AY050981.1	At1g35670.1
at4g32330	CON	MIN	HOUR	DAY	WEEK	UNIGENE	FLCDNA	TAIR
TGGATTTGGT	1	5	5	3	3	gnl|UG|At#S11703501	gi|14334555|gb|AY035182.1	At4g32330.2
at2g30790	CON	MIN	HOUR	DAY	WEEK	UNIGENE	FLCDNA	TAIR
ATCACTCCTA	0	1	0	1	0	gnl|UG|At#S21736741	gi|51969783|dbj|AK175821.1|	non-canonical match
at4g39200	CON	MIN	HOUR	DAY	WEEK	UNIGENE	FLCDNA	TAIR
GTGCTGTTTG	23	30	16	9	15	no match found	gi|21404285|gb|AY085575.1	At4g39200.1
at1g35780	CON	MIN	HOUR	DAY	WEEK	UNIGENE	FLCDNA	TAIR
TCGACGGCGG	0	2	0	0	0	no match found	gi|16226791|gb|AF428333.1	non-canonical match
at3g57880	CON	MIN	HOUR	DAY	WEEK	UNIGENE	FLCDNA	TAIR
ATGACTCTTT	3	1	0	0	2	gnl|UG|At#S34116452	gi|14030690|gb|AF375436.1	At3g57880.1
at1g52905	CON	MIN	HOUR	DAY	WEEK	UNIGENE	FLCDNA	TAIR
TCTAAATCTG	0	2	0	0	0	no match found	gi|21405492|gb|AY086782.1	At1g52905.1
at4g34100	CON	MIN	HOUR	DAY	WEEK	UNIGENE	FLCDNA	TAIR
TAAGCCTTAA	0	0	0	0	2	no match found	no match found	At4g34100.1
TCTGGTTTAG	4	4	3	1	1	gnl|UG|At#S11721918	gi|26451661|dbj|AK118311.1|	non-canonical match
at1g22570	CON	MIN	HOUR	DAY	WEEK	UNIGENE	FLCDNA	TAIR
AAAGTTAATC	0	0	2	0	0	no match found	no match found	At1g22570.1
TGGATTACTT	0	0	1	0	2	gnl|UG|At#S11740805	no match found	non-canonical match
at1g35160	CON	MIN	HOUR	DAY	WEEK	UNIGENE	FLCDNA	TAIR
CAGGACGAAA	4	4	3	1	3	no match found	gi|110735756|dbj|AK227883.1|	At1g35160.1
AGCTTCTTGG	0	1	0	0	0	gnl|UG|At#S18896260	no match found	non-canonical match

at5g48630	CON	MIN	HOUR	DAY	WEEK	UNIGENE	FLCDNA	TAIR
AACATTGTGA	1	2	0	0	2	gnl|UG|At#S11718856	gi|87116579|gb|BT024473.1	At5g48630.1
at1g73740	CON	MIN	HOUR	DAY	WEEK	UNIGENE	FLCDNA	TAIR
GAAGATATTC	0	0	0	1	0	gnl|UG|At#S11728298	gi|16648912|gb|AY059826.1	At1g73740.1
at1g13020	CON	MIN	HOUR	DAY	WEEK	UNIGENE	FLCDNA	TAIR
GCACAGGCGA	1	2	1	0	2	gnl|UG|At#S11741736	gi|110739185|dbj|AK229665.1|	At1g13020.1
at3g08880	CON	MIN	HOUR	DAY	WEEK	UNIGENE	FLCDNA	TAIR
TAACAGTATC	1	0	0	0	0	gnl|UG|At#S21735940	gi|51969185|dbj|AK175522.1|	pseudo chromosome match
at3g52570	CON	MIN	HOUR	DAY	WEEK	UNIGENE	FLCDNA	TAIR
GACCTTTAAC	0	0	0	1	0	gnl|UG|At#S11743075	gi|20453340|gb|AY097393.1	At3g52570.1
at1g01010	CON	MIN	HOUR	DAY	WEEK	UNIGENE	FLCDNA	TAIR
ATCATTGGTG	0	0	0	0	1	gnl|UG|At#S11743013	gi|110742029|dbj|AK226863.1|	At1g01010.1
at1g03370	CON	MIN	HOUR	DAY	WEEK	UNIGENE	FLCDNA	TAIR
TGAAGACGAG	0	1	0	1	0	gnl|UG|At#S11742777	gi|21539552|gb|AY120771.1	At1g03370.1
at3g21250	CON	MIN	HOUR	DAY	WEEK	UNIGENE	FLCDNA	TAIR
AACTTTGAGA	0	0	6	4	1	gnl|UG|At#S11817674	no match found	At3g21250.1
at5g17880	CON	MIN	HOUR	DAY	WEEK	UNIGENE	FLCDNA	TAIR
TCTTTATTGG	0	1	0	1	0	gnl|UG|At#S11721850	no match found	At5g17880.1
at3g07570	CON	MIN	HOUR	DAY	WEEK	UNIGENE	FLCDNA	TAIR
TTATTGACTA	1	0	0	0	0	gnl|UG|At#S11738469	gi|110736321|dbj|AK228175.1|	At3g07570.1
at4g14110	CON	MIN	HOUR	DAY	WEEK	UNIGENE	FLCDNA	TAIR
ATTTAGCTCT	1	0	0	0	0	gnl|UG|At#S11725368	gi|26452096|dbj|AK118535.1|	At4g14110.1
at5g37072	CON	MIN	HOUR	DAY	WEEK	UNIGENE	FLCDNA	TAIR
GATGTTGTGT	0	0	1	0	0	gnl|UG|At#S30624005	no match found	multiple non-canonical match
at3g58700	CON	MIN	HOUR	DAY	WEEK	UNIGENE	FLCDNA	TAIR
AAATGGTTCC	6	2	4	1	4	no match found	gi|21403942|gb|AY085232.1	At3g58700.1
at1g76850	CON	MIN	HOUR	DAY	WEEK	UNIGENE	FLCDNA	TAIR
CTTTGCAGAG	0	5	0	0	0	gnl|UG|At#S11744375	gi|110741240|dbj|AK230372.1|	At1g76850.1
at3g20540	CON	MIN	HOUR	DAY	WEEK	UNIGENE	FLCDNA	TAIR
TCTGAACCCT	0	2	0	0	0	gnl|UG|At#S11818422	gi|110741932|dbj|AK226811.1|	At3g20540.1
at1g15780	CON	MIN	HOUR	DAY	WEEK	UNIGENE	FLCDNA	TAIR
TCAAAACTAA	2	1	2	1	0	gnl|UG|At#S11741459	gi|62321228|dbj|AK221945.1|	At1g15780.1
at3g28130	CON	MIN	HOUR	DAY	WEEK	UNIGENE	FLCDNA	TAIR
TTACGGACAA	0	1	0	0	0	gnl|UG|At#S11705013	gi|15146273|gb|AY049278.1	At3g28130.1
GTCCACGAGT	1	2	0	0	0	no match found	gi|22136585|gb|AY129493.1	non-canonical match
at3g22320	CON	MIN	HOUR	DAY	WEEK	UNIGENE	FLCDNA	TAIR
TTCTTGTTCC	1	1	0	0	0	gnl|UG|At#S11734125	gi|15724321|gb|AF412101.1	At3g22320.1
at3g17250	CON	MIN	HOUR	DAY	WEEK	UNIGENE	FLCDNA	TAIR
GTGGATCTGA	0	1	0	0	0	gnl|UG|At#S34117448	gi|110737625|dbj|AK228860.1|	At3g17250.1
at4g39970	CON	MIN	HOUR	DAY	WEEK	UNIGENE	FLCDNA	TAIR
TGTGATCACG	1	4	0	0	0	gnl|UG|At#S18906188	gi|22022551|gb|AY127008.1	At4g39970.1

at4g28600	CON	MIN	HOUR	DAY	WEEK	UNIGENE	FLCDNA	TAIR
TCTCTCGAGA	0	1	0	0	0	gnl|UG|At#S11722861	gi|56381952|gb|BT020340.1	At4g28600.1
at5g20740	CON	MIN	HOUR	DAY	WEEK	UNIGENE	FLCDNA	TAIR
TGTTTTAAGT	0	0	2	0	0	no match found	gi|28393055|gb|BT003915.1	non-canonical match
at5g24030	CON	MIN	HOUR	DAY	WEEK	UNIGENE	FLCDNA	TAIR
TATACTTGTT	1	1	0	0	1	gnl|UG|At#S11720776	gi|51536497|gb|BT015364.1	At5g24030.1
at5g41270	CON	MIN	HOUR	DAY	WEEK	UNIGENE	FLCDNA	TAIR
CTTTCTTCAC	2	0	0	0	0	gnl|UG|At#S34118409	gi|28466942|gb|BT004814.1	At5g41270.1
at2g04520	CON	MIN	HOUR	DAY	WEEK	UNIGENE	FLCDNA	TAIR
TCTGATGAGG	2	5	2	1	0	gnl|UG|At#S11740280	gi|14190444|gb|AF378900.1	At2g04520.1
at2g11190	CON	MIN	HOUR	DAY	WEEK	UNIGENE	FLCDNA	TAIR
GTCGAAGCCA	0	1	1	1	0	no match found	no match found	At2g11190.1
at3g52190	CON	MIN	HOUR	DAY	WEEK	UNIGENE	FLCDNA	TAIR
GCATCAGTGA	1	1	0	1	0	gnl|UG|At#S18902625	gi|21407763|gb|AY088989.1	At3g52190.1
at1g71010	CON	MIN	HOUR	DAY	WEEK	UNIGENE	FLCDNA	TAIR
GACCTCTTGA	2	1	2	2	0	gnl|UG|At#S11728878	gi|17473835|gb|AY065168.1	At1g71010.1
at3g01890	CON	MIN	HOUR	DAY	WEEK	UNIGENE	FLCDNA	TAIR
AGTTCACTAC	0	0	0	0	1	gnl|UG|At#S11816268	gi|26453079|dbj|AK119040.1|	At3g01890.1
at3g54510	CON	MIN	HOUR	DAY	WEEK	UNIGENE	FLCDNA	TAIR
GTTTTGTTTT	0	1	0	0	0	gnl|UG|At#S11825100	gi|27754590|gb|BT002927.1	multiple canonical match
at1g68030	CON	MIN	HOUR	DAY	WEEK	UNIGENE	FLCDNA	TAIR
ACAACAACAA	3	2	1	0	0	no match found	no match found	At1g68030.1
at1g06400	CON	MIN	HOUR	DAY	WEEK	UNIGENE	FLCDNA	TAIR
ATTGGTAATT	4	2	3	1	1	no match found	gi|17644168|gb|AY065306.1	At1g06400.1
GAGACTTCAG	0	1	0	0	0	gnl|UG|At#S18895155	gi|12083263|gb|AF332428.1	non-canonical match
at1g29370	CON	MIN	HOUR	DAY	WEEK	UNIGENE	FLCDNA	TAIR
CAACTCTGAT	4	10	5	9	4	gnl|UG|At#S11739237	gi|17380759|gb|AY063854.1	At1g29370.1
at3g09000	CON	MIN	HOUR	DAY	WEEK	UNIGENE	FLCDNA	TAIR
ACTGGAAACC	0	3	1	0	0	gnl|UG|At#S11821388	gi|56381990|gb|BT020359.1	At3g09000.1
at1g49590	CON	MIN	HOUR	DAY	WEEK	UNIGENE	FLCDNA	TAIR
ACAGTTTATT	0	0	1	0	0	gnl|UG|At#S18896442	gi|15010771|gb|AY045687.1	At1g49590.1
at4g16920	CON	MIN	HOUR	DAY	WEEK	UNIGENE	FLCDNA	TAIR
ATAACTGAAG	3	5	3	4	2	no match found	no match found	At4g16920.1
GCGATGTATC	1	1	0	0	0	gnl|UG|At#S34117948	no match found	pseudo chromosome match
at3g53950	CON	MIN	HOUR	DAY	WEEK	UNIGENE	FLCDNA	TAIR
GAAAAACTTA	1	0	1	0	1	gnl|UG|At#S11728696	gi|26449361|dbj|AK117130.1|	At3g53950.1
at5g13240	CON	MIN	HOUR	DAY	WEEK	UNIGENE	FLCDNA	TAIR
TGCAAATACT	3	3	2	1	1	gnl|UG|At#S11743841	gi|19347945|gb|AY080685.1	At5g13240.1
at3g17920	CON	MIN	HOUR	DAY	WEEK	UNIGENE	FLCDNA	TAIR
ACTTGAGACT	0	0	0	0	2	no match found	no match found	At3g17920.1
at4g24015	CON	MIN	HOUR	DAY	WEEK	UNIGENE	FLCDNA	TAIR

TTGTTTTGTT	0	1	0	1	0	gnl|UG|At#S18941808	gi|110736596|dbj|AK228321.1|	At4g24015.1
at5g02810	CON	MIN	HOUR	DAY	WEEK	UNIGENE	FLCDNA	TAIR
TTTTAACATT	2	5	2	0	2	gnl|UG|At#S11725462	gi|14532637|gb|AY039943.1	At5g02810.1
at3g46560	CON	MIN	HOUR	DAY	WEEK	UNIGENE	FLCDNA	TAIR
ATCGATCAGC	0	0	1	3	0	gnl|UG|At#S18901821	gi|15028234|gb|AY045940.1	At3g46560.1
at1g01730	CON	MIN	HOUR	DAY	WEEK	UNIGENE	FLCDNA	TAIR
AAGCCTTAAT	0	1	2	3	2	gnl|UG|At#S11742940	gi|21407879|gb|AY089105.1	At1g01730.1
at3g56590	CON	MIN	HOUR	DAY	WEEK	UNIGENE	FLCDNA	TAIR
AAGCAGCTTC	0	0	1	1	0	gnl|UG|At#S34115190	gi|110741963|dbj|AK226827.1|	non-canonical match
at4g19820	CON	MIN	HOUR	DAY	WEEK	UNIGENE	FLCDNA	TAIR
GGATGCTAAG	0	1	0	0	0	no match found	no match found	At4g19820.1
at2g39930	CON	MIN	HOUR	DAY	WEEK	UNIGENE	FLCDNA	TAIR
GTCTTGCTCC	0	1	0	0	0	gnl|UG|At#S11732620	gi|34098816|gb|BT010348.1	non-canonical match
AAACAAAAAA	60	10	70	52	37	no match found	no match found	At2g39930.1
at4g02280	CON	MIN	HOUR	DAY	WEEK	UNIGENE	FLCDNA	TAIR
TTGAAGATTA	1	0	0	1	0	gnl|UG|At#S11727105	gi|15982720|gb|AY056784.1	At4g02280.1
at5g06370	CON	MIN	HOUR	DAY	WEEK	UNIGENE	FLCDNA	TAIR
CCTTAACTGT	0	1	0	0	0	gnl|UG|At#S11724533	gi|21407747|gb|AY088973.1	At5g06370.1
at1g49410	CON	MIN	HOUR	DAY	WEEK	UNIGENE	FLCDNA	TAIR
AGACATTTAG	1	1	3	4	3	gnl|UG|At#S18895338	gi|14335019|gb|AY037189.1	pseudo chromosome match
at1g10100	CON	MIN	HOUR	DAY	WEEK	UNIGENE	FLCDNA	TAIR
TCCTTGAATC	1	0	0	0	0	no match found	no match found	At1g10100.1
at4g34860	CON	MIN	HOUR	DAY	WEEK	UNIGENE	FLCDNA	TAIR
TTGGTATGGT	1	0	0	0	0	no match found	gi|27363383|gb|BT002695.1	non-canonical match
AGAGCTCTTC	0	0	1	0	0	no match found	no match found	At4g34860.1
at5g37370	CON	MIN	HOUR	DAY	WEEK	UNIGENE	FLCDNA	TAIR
TTGTTTAACT	0	3	0	0	0	gnl|UG|At#S11806689	gi|20260633|gb|AY093216.1	multiple canonical match
atcg00480	CON	MIN	HOUR	DAY	WEEK	UNIGENE	FLCDNA	TAIR
GAAATGAAAG	44	413	70	36	11	no match found	no match found	AtCg00480
at2g27775	CON	MIN	HOUR	DAY	WEEK	UNIGENE	FLCDNA	TAIR
AGATCCATCA	1	0	0	0	0	no match found	gi|169881289|gb|BT031350.1	non-canonical match
at1g72880	CON	MIN	HOUR	DAY	WEEK	UNIGENE	FLCDNA	TAIR
ATTTGGCCAA	1	2	0	1	0	gnl|UG|At#S11728492	gi|110742850|dbj|AK227308.1|	At1g72880.2
at1g42990	CON	MIN	HOUR	DAY	WEEK	UNIGENE	FLCDNA	TAIR
ATGTCGAAGC	1	4	2	0	0	gnl|UG|At#S11704817	gi|22136789|gb|AY133805.1	At1g42990.1
at1g26670	CON	MIN	HOUR	DAY	WEEK	UNIGENE	FLCDNA	TAIR
GTGTGGATGA	2	5	1	1	0	gnl|UG|At#S11740099	gi|26451850|dbj|AK118409.1|	At1g26670.1
at2g32100	CON	MIN	HOUR	DAY	WEEK	UNIGENE	FLCDNA	TAIR
GAAGAGTAAC	2	0	3	1	0	gnl|UG|At#S11734505	no match found	At2g32100.1
at1g66900	CON	MIN	HOUR	DAY	WEEK	UNIGENE	FLCDNA	TAIR
CCAGAGAAAG	2	1	4	3	1	gnl|UG|At#S11709942	gi|17979070|gb|AY070046.1	At1g66900.1
GTACTGCGGA	0	1	1	0	0	no match found	gi|51870369|gb|BT015578.1	non-canonical match

at2g19880	CON	MIN	HOUR	DAY	WEEK	UNIGENE	FLCDNA		TAIR
GGGTTTTGTT	0	3	0	0	0	gnl|UG|At#S11737501	gi|15983421|gb|AF424585.1		At2g19880.1
at1g30600	CON	MIN	HOUR	DAY	WEEK	UNIGENE	FLCDNA		TAIR
CAGAGGTTTT	0	0	1	0	0	gnl|UG|At#S11743557	gi|20465292|gb|AY096410.1		At1g30600.1
at3g20410	CON	MIN	HOUR	DAY	WEEK	UNIGENE	FLCDNA		TAIR
GATGCTGCTG	1	3	0	0	0	no match found	no match found		At3g20410.1
at5g16870	CON	MIN	HOUR	DAY	WEEK	UNIGENE	FLCDNA		TAIR
TAGCATTTCC	1	2	0	0	0	no match found	gi|51968873|dbj|AK175366.1|		At3g20410.1
AACTCCACCT	0	0	1	0	0	no match found	gi|21405787|gb|AY087063.1		At5g16870.1
at1g07140	CON	MIN	HOUR	DAY	WEEK	UNIGENE	FLCDNA		TAIR
GATCCTTTTT	3	2	4	0	0	no match found	gi|15294205|gb|AF410294.1		multiple non-canonical match
TTTTGTCGAG	0	0	1	0	0	no match found	gi|25084244|gb|BT002193.1		non-canonical match
TGTGTGGCAC	0	10	1	0	1	no match found	gi|20453282|gb|AY097364.1		non-canonical match
TGATTTTTTA	1	1	5	5	2	no match found	gi|13877580|gb|AF370491.1		non-canonical match
ATTTATATAA	0	1	0	0	0	gnl|UG|At#S11742320	no match found		multiple canonical match
at2g24100	CON	MIN	HOUR	DAY	WEEK	UNIGENE	FLCDNA		TAIR
CCCGAAGGAG	0	0	1	2	1	gnl|UG|At#S11736468	gi|31376398|gb|BT008764.1		At2g24100.1
at1g78590	CON	MIN	HOUR	DAY	WEEK	UNIGENE	FLCDNA		TAIR
GACGTTAACT	0	1	4	0	0	gnl|UG|At#S11726997	gi|63025183|gb|BT022078.1		At1g78590.1
at1g66190	CON	MIN	HOUR	DAY	WEEK	UNIGENE	FLCDNA		TAIR
AAAAAGGTGA	0	1	0	0	0	gnl|UG|At#S21989847	no match found		multiple non-canonical match
at4g28180	CON	MIN	HOUR	DAY	WEEK	UNIGENE	FLCDNA		TAIR
GAATGCTTCA	0	1	0	0	1	no match found	gi|34146807|gb|BT010411.1		multiple non-canonical match
at5g03360	CON	MIN	HOUR	DAY	WEEK	UNIGENE	FLCDNA		TAIR
AAACCTTATT	0	1	0	0	0	no match found	gi|26450156|dbj|AK117536.1|		non-canonical match
at5g37475	CON	MIN	HOUR	DAY	WEEK	UNIGENE	FLCDNA		TAIR
TGAGAAAATG	0	0	1	1	2	gnl|UG|At#S15460122	gi|24899744|gb|BT001200.1		At5g37475.1
at5g35540	CON	MIN	HOUR	DAY	WEEK	UNIGENE	FLCDNA		TAIR
ATTATTACTT	0	0	1	1	0	gnl|UG|At#S11720149	no match found		multiple non-canonical match
at3g56690	CON	MIN	HOUR	DAY	WEEK	UNIGENE	FLCDNA		TAIR
AATGTAAAGA	0	3	0	1	1	no match found	no match found		At3g56690.1
at4g40060	CON	MIN	HOUR	DAY	WEEK	UNIGENE	FLCDNA		TAIR
TAATTAACGA	4	4	0	1	0	gnl|UG|At#S11720912	gi|15450981|gb|AY054571.1		At4g40060.1
at1g72210	CON	MIN	HOUR	DAY	WEEK	UNIGENE	FLCDNA		TAIR
TGTGATCTTT	1	0	1	0	0	gnl|UG|At#S11728645	gi|28392969|gb|BT003871.1		At1g72210.1
at4g35770	CON	MIN	HOUR	DAY	WEEK	UNIGENE	FLCDNA		TAIR
TACAGGGCTT	0	3	1	0	0	gnl|UG|At#S11715382	no match found		non-canonical match
TACAGAGTCG	5	56	2	5	0	no match found	gi|18958010|gb|AY078973.1		At4g35770.1
CCTACACACG	0	1	0	0	0	gnl|UG|At#S38433390	no match found		multiple non-canonical match
at1g63360	CON	MIN	HOUR	DAY	WEEK	UNIGENE	FLCDNA		TAIR
CCAATTGATT	1	2	1	2	1	gnl|UG|At#S11730467	no match found		non-canonical match
at3g23980	CON	MIN	HOUR	DAY	WEEK	UNIGENE	FLCDNA		TAIR
AACTTGAAGA	0	0	1	0	3	gnl|UG|At#S11733596	gi|110741487|dbj|AK226574.1|		At3g23980.1

at3g22330	CON	MIN	HOUR	DAY	WEEK	UNIGENE	FLCDNA	TAIR
AGGGATCGTG	0	4	0	1	0	gnl|UG|At#S11734122	gi|17064851|gb|AY062502.1	At3g22330.1
at5g55640	CON	MIN	HOUR	DAY	WEEK	UNIGENE	FLCDNA	TAIR
AATCTCAGTT	1	1	0	0	0	gnl|UG|At#S11718149	gi|13899122|gb|AF370556.1	At5g55640.1
at1g51540	CON	MIN	HOUR	DAY	WEEK	UNIGENE	FLCDNA	TAIR
GAATTTTGTA	0	0	0	0	1	gnl|UG|At#S11733939	gi|110743234|dbj|AK227508.1|	non-canonical match
at5g39500	CON	MIN	HOUR	DAY	WEEK	UNIGENE	FLCDNA	TAIR
TCAACTGAAT	0	1	0	0	0	gnl|UG|At#S11719782	no match found	At5g39500.1
at1g79940	CON	MIN	HOUR	DAY	WEEK	UNIGENE	FLCDNA	TAIR
GATCGGGTGT	0	4	1	0	1	gnl|UG|At#S43850308	gi|110743726|dbj|AK227714.1|	At1g79940.1
at3g07600	CON	MIN	HOUR	DAY	WEEK	UNIGENE	FLCDNA	TAIR
TAAAGCCAAA	0	0	1	1	0	gnl|UG|At#S11738460	no match found	multiple canonical match
at4g18290	CON	MIN	HOUR	DAY	WEEK	UNIGENE	FLCDNA	TAIR
AAGCTGCAAA	0	0	1	1	0	gnl|UG|At#S11724631	no match found	At4g18290.1
at1g19730	CON	MIN	HOUR	DAY	WEEK	UNIGENE	FLCDNA	TAIR
TCAAGTGCCA	0	0	0	0	1	no match found	gi|28416850|gb|BT004710.1	non-canonical match
CTTTTTTTAT	0	1	0	0	1	no match found	gi|21407472|gb|AY088698.1	At1g19730.1
at2g46710	CON	MIN	HOUR	DAY	WEEK	UNIGENE	FLCDNA	TAIR
TTTTTGAGAG	2	2	0	0	0	no match found	gi|26449716|dbj|AK117311.1|	At2g46710.1
at3g05910	CON	MIN	HOUR	DAY	WEEK	UNIGENE	FLCDNA	TAIR
GAAAATGTAC	0	3	1	1	0	gnl|UG|At#S11738953	gi|20465792|gb|AY096751.1	At3g05910.1
at2g37040	CON	MIN	HOUR	DAY	WEEK	UNIGENE	FLCDNA	TAIR
TCTGAATAAT	7	0	5	23	11	gnl|UG|At#S11733341	gi|62321195|dbj|AK221928.1|	At2g37040.1
CTTTGATCAA	0	0	0	0	1	no match found	gi|19310726|gb|AY079363.1	multiple non-canonical match
at3g60860	CON	MIN	HOUR	DAY	WEEK	UNIGENE	FLCDNA	TAIR
TTAATACTTG	2	2	1	1	0	gnl|UG|At#S11727222	no match found	At3g60860.1
at4g38680	CON	MIN	HOUR	DAY	WEEK	UNIGENE	FLCDNA	TAIR
GCGAGAGACT	1	4	2	2	0	gnl|UG|At#S11721156	gi|14326486|gb|AF385696.1	At4g38680.1
at4g01650	CON	MIN	HOUR	DAY	WEEK	UNIGENE	FLCDNA	TAIR
ATCTTTGAGT	1	0	0	0	0	no match found	gi|110743082|dbj|AK227430.1|	multiple canonical match
at5g60640	CON	MIN	HOUR	DAY	WEEK	UNIGENE	FLCDNA	TAIR
AGTTTGGACT	11	10	11	7	6	gnl|UG|At#S11717638	gi|25082812|gb|BT001994.1	At5g60640.1
at5g62720	CON	MIN	HOUR	DAY	WEEK	UNIGENE	FLCDNA	TAIR
TACGATCGAA	1	9	6	1	1	gnl|UG|At#S18941429	gi|26449513|dbj|AK117207.1|	At5g62720.1
at3g16370	CON	MIN	HOUR	DAY	WEEK	UNIGENE	FLCDNA	TAIR
CTCCAATGCT	11	16	9	7	3	gnl|UG|At#S11735943	gi|18700185|gb|AY075698.1	At3g16370.1
at2g35290	CON	MIN	HOUR	DAY	WEEK	UNIGENE	FLCDNA	TAIR
TCGACTCGAT	0	1	0	0	0	gnl|UG|At#S11733752	gi|26452265|dbj|AK118622.1|	At2g35290.1
at1g69360	CON	MIN	HOUR	DAY	WEEK	UNIGENE	FLCDNA	TAIR
ATTGGTTATC	0	1	1	1	1	gnl|UG|At#S11707942	gi|16604686|gb|AY059788.1	At1g69360.1
at5g59880	CON	MIN	HOUR	DAY	WEEK	UNIGENE	FLCDNA	TAIR

GTCTCCGGAC	4	5	3	7	2	gnl|UG|At#S15445668	no match found	At5g59880.1
at5g07630	CON	MIN	HOUR	DAY	WEEK	UNIGENE	FLCDNA	TAIR
AATGAAAAGG	1	0	0	0	0	no match found	no match found	At5g07630.1
at5g61500	CON	MIN	HOUR	DAY	WEEK	UNIGENE	FLCDNA	TAIR
GATGGCTGGA	4	5	1	2	1	gnl|UG|At#S11717552	gi|110740690|dbj|AK226290.1|	At5g61500.1
at1g77590	CON	MIN	HOUR	DAY	WEEK	UNIGENE	FLCDNA	TAIR
TCTATACAAC	0	1	0	0	0	gnl|UG|At#S11727353	no match found	At1g77590.1
GACGCCAGAG	0	1	5	2	0	no match found	gi|110740111|dbj|AK230143.1|	multiple non-canonical match
at3g50080	CON	MIN	HOUR	DAY	WEEK	UNIGENE	FLCDNA	TAIR
GCATAAGATT	0	0	1	0	0	gnl|UG|At#S11729428	no match found	At3g50080.1
at4g30920	CON	MIN	HOUR	DAY	WEEK	UNIGENE	FLCDNA	TAIR
AAATTCGATA	1	1	0	0	0	gnl|UG|At#S11722463	gi|27363297|gb|BT002652.1	At4g30920.1
at5g40210	CON	MIN	HOUR	DAY	WEEK	UNIGENE	FLCDNA	TAIR
AGTGATTTCT	0	0	1	2	0	gnl|UG|At#S11719709	gi|15724297|gb|AF412089.1	At5g40210.1
at3g19630	CON	MIN	HOUR	DAY	WEEK	UNIGENE	FLCDNA	TAIR
GAGGTTTAAC	0	1	0	0	0	gnl|UG|At#S11734965	no match found	At3g19630.1
at1g77390	CON	MIN	HOUR	DAY	WEEK	UNIGENE	FLCDNA	TAIR
TAAAACAAAA	0	1	0	0	1	no match found	no match found	At1g77390.1
CGTTAAGAAT	0	1	0	0	0	gnl|UG|At#S11727424	no match found	non-canonical match
at4g16146	CON	MIN	HOUR	DAY	WEEK	UNIGENE	FLCDNA	TAIR
TTTCCATTAA	1	2	1	1	1	gnl|UG|At#S11725005	gi|26453127|dbj|AK119064.1|	At4g16146.1
TTTCCATAAA	1	1	1	0	0	no match found	gi|21405713|gb|AY086989.1	multiple non-canonical match
at1g69370	CON	MIN	HOUR	DAY	WEEK	UNIGENE	FLCDNA	TAIR
CCCCTCACAA	0	1	0	0	0	gnl|UG|At#S11729235	gi|28950892|gb|BT005306.1	At1g69370.1
at3g09670	CON	MIN	HOUR	DAY	WEEK	UNIGENE	FLCDNA	TAIR
AGGATAAGAA	0	2	0	0	0	gnl|UG|At#S38433758	gi|110739281|dbj|AK229716.1|	At3g09670.1
at3g62810	CON	MIN	HOUR	DAY	WEEK	UNIGENE	FLCDNA	TAIR
GCTAATATTG	0	0	1	0	0	gnl|UG|At#S11726598	no match found	pseudo chromosome match
ATCGTCCAGG	0	0	1	0	1	no match found	gi|28466830|gb|BT004758.1	At3g62810.1
at5g11760	CON	MIN	HOUR	DAY	WEEK	UNIGENE	FLCDNA	TAIR
TTTGATGAGA	0	1	0	3	1	gnl|UG|At#S11723304	no match found	At5g11760.1
at1g21130	CON	MIN	HOUR	DAY	WEEK	UNIGENE	FLCDNA	TAIR
GTACTAGAAC	0	0	1	0	0	no match found	gi|124301153|gb|BT030091.1	non-canonical match
at5g53420	CON	MIN	HOUR	DAY	WEEK	UNIGENE	FLCDNA	TAIR
AAGAATAAGG	1	2	0	0	1	gnl|UG|At#S18911187	gi|20466573|gb|AY099753.1	At5g53420.1
at5g34940	CON	MIN	HOUR	DAY	WEEK	UNIGENE	FLCDNA	TAIR
TGCTTAAGGT	0	1	0	0	0	gnl|UG|At#S11720209	gi|110738425|dbj|AK229275.1|	At5g34940.2
at2g47910	CON	MIN	HOUR	DAY	WEEK	UNIGENE	FLCDNA	TAIR
GCCAGGATGC	1	1	2	3	2	gnl|UG|At#S18942676	gi|12642887|gb|AF339704.1	At2g47910.2
at2g20790	CON	MIN	HOUR	DAY	WEEK	UNIGENE	FLCDNA	TAIR
TGATAATAAA	0	1	0	0	0	gnl|UG|At#S15461343	no match found	At2g20790.1
at1g38131	CON	MIN	HOUR	DAY	WEEK	UNIGENE	FLCDNA	TAIR

GAAATGAGGT	0	1	0	0	0	gnl|UG|At#S11816437	gi|27311576|gb|BT002394.1	multiple canonical match
at3g13510	CON	MIN	HOUR	DAY	WEEK	UNIGENE	FLCDNA	TAIR
TAACTAGATG	1	0	0	0	0	gnl|UG|At#S11736817	gi|21406444|gb|AY087706.1	At3g13510.1
at2g46110	CON	MIN	HOUR	DAY	WEEK	UNIGENE	FLCDNA	TAIR
GAGCCTTCAA	0	1	1	0	0	no match found	gi|28466954|gb|BT004820.1	non-canonical match
GTTTCTCTGT	0	0	0	0	14	gnl|UG|At#S34118457	gi|110735691|dbj|AK227849.1|	At2g46110.1
at3g05100	CON	MIN	HOUR	DAY	WEEK	UNIGENE	FLCDNA	TAIR
TATTGTTAAT	1	0	0	0	0	no match found	gi|17979284|gb|AY070370.1	At3g05100.1
at4g10260	CON	MIN	HOUR	DAY	WEEK	UNIGENE	FLCDNA	TAIR
AAATCTAAGT	0	0	0	0	1	gnl|UG|At#S18906476	no match found	non-canonical match
at1g69935	CON	MIN	HOUR	DAY	WEEK	UNIGENE	FLCDNA	TAIR
ATGCAAAAAA	7	11	13	5	1	gnl|UG|At#S34114523	gi|110743202|dbj|AK227492.1|	At1g69935.1
at4g37430	CON	MIN	HOUR	DAY	WEEK	UNIGENE	FLCDNA	TAIR
TGTAGGCCTA	1	1	0	0	0	gnl|UG|At#S11721368	gi|18491104|gb|AY074823.1	At4g37430.1
AGGTGGAGAT	0	0	1	0	0	no match found	gi|62320145|dbj|AK221399.1|	At4g37430.1
at3g11200	CON	MIN	HOUR	DAY	WEEK	UNIGENE	FLCDNA	TAIR
TTTTAATCAA	1	4	0	0	2	no match found	gi|16604429|gb|AY058833.1	non-canonical match
GGGTTTGTAT	1	0	0	0	0	gnl|UG|At#S18902208	gi|21403848|gb|AY085138.1	At3g11200.1
at1g77420	CON	MIN	HOUR	DAY	WEEK	UNIGENE	FLCDNA	TAIR
CAGTCAGCCA	1	1	1	0	0	gnl|UG|At#S11727406	gi|21403083|gb|AY084373.1	At1g77420.1
at3g09630	CON	MIN	HOUR	DAY	WEEK	UNIGENE	FLCDNA	TAIR
TGGAGAGGCT	3	6	5	1	6	gnl|UG|At#S28283013	gi|21406737|gb|AY087963.1	At3g09630.1
at3g55200	CON	MIN	HOUR	DAY	WEEK	UNIGENE	FLCDNA	TAIR
GCTTACAGAT	0	4	1	0	0	no match found	no match found	At3g55200.1
at5g25955	CON	MIN	HOUR	DAY	WEEK	UNIGENE	FLCDNA	TAIR
GTCTTGTGGT	1	0	0	0	0	no match found	no match found	At5g25955.1
at5g13560	CON	MIN	HOUR	DAY	WEEK	UNIGENE	FLCDNA	TAIR
TGGTTTTGCG	1	2	1	3	1	no match found	gi|15982808|gb|AY057511.1	non-canonical match
at3g18960	CON	MIN	HOUR	DAY	WEEK	UNIGENE	FLCDNA	TAIR
TGCTTAAAGA	0	1	0	0	0	gnl|UG|At#S11735164	gi|30793830|gb|BT008541.1	At3g18960.1
at2g18990	CON	MIN	HOUR	DAY	WEEK	UNIGENE	FLCDNA	TAIR
ATCTTCACTC	2	0	1	0	0	no match found	gi|26453306|dbj|AK119157.1|	At2g18990.1
at4g18060	CON	MIN	HOUR	DAY	WEEK	UNIGENE	FLCDNA	TAIR
CAACGGAAGT	0	2	0	1	0	gnl|UG|At#S11724671	gi|25090268|gb|BT002255.1	non-canonical match
at3g55005	CON	MIN	HOUR	DAY	WEEK	UNIGENE	FLCDNA	TAIR
TATGGTTTTA	0	0	2	2	1	gnl|UG|At#S11728515	gi|18700181|gb|AY075696.1	At3g55005.1
at2g25310	CON	MIN	HOUR	DAY	WEEK	UNIGENE	FLCDNA	TAIR
TTTCATCTAT	1	0	0	0	1	no match found	gi|28393809|gb|BT004317.1	At2g25310.1
GTCGTTGTTG	1	1	0	0	1	no match found	gi|29824320|gb|BT006136.1	non-canonical match
at5g14800	CON	MIN	HOUR	DAY	WEEK	UNIGENE	FLCDNA	TAIR
AACTAGAGAA	3	3	0	1	2	no match found	gi|21406267|gb|AY087530.1	At5g14800.1
at5g64610	CON	MIN	HOUR	DAY	WEEK	UNIGENE	FLCDNA	TAIR

ACAGCGATTA	0	1	0	0	0	gnl|UG|At#S11808209	gi|23198137|gb|BT000277.1	At5g64610.1
at4g24620	CON	MIN	HOUR	DAY	WEEK	UNIGENE	FLCDNA	TAIR
TCTATGATTT	0	1	0	1	0	no match found	gi|19423954|gb|AY080758.1	non-canonical match
at1g34030	CON	MIN	HOUR	DAY	WEEK	UNIGENE	FLCDNA	TAIR
AAGCTCAGGG	3	7	3	3	3	gnl|UG|At#S11737568	gi|21405510|gb|AY086800.1	At1g34030.1
at3g13550	CON	MIN	HOUR	DAY	WEEK	UNIGENE	FLCDNA	TAIR
ACGAGGTAGC	1	1	1	2	1	gnl|UG|At#S11736808	gi|110738727|dbj|AK229428.1|	At3g13550.1
at2g21960	CON	MIN	HOUR	DAY	WEEK	UNIGENE	FLCDNA	TAIR
AAGAAGAGCA	2	0	2	1	1	no match found	no match found	At2g21960.1
at4g31115	CON	MIN	HOUR	DAY	WEEK	UNIGENE	FLCDNA	TAIR
GAACATCCAG	0	0	0	1	0	gnl|UG|At#S43849462	gi|19310419|gb|AY078946.1	At4g31115.1
at4g10120	CON	MIN	HOUR	DAY	WEEK	UNIGENE	FLCDNA	TAIR
TCGAAGACTG	2	5	1	0	0	gnl|UG|At#S28282072	gi|62319201|dbj|AK220923.1|	At4g10120.1
at5g52970	CON	MIN	HOUR	DAY	WEEK	UNIGENE	FLCDNA	TAIR
GCGATGCTTT	1	1	0	0	0	gnl|UG|At#S11718420	gi|15081647|gb|AY048216.1	multiple canonical match
at3g19960	CON	MIN	HOUR	DAY	WEEK	UNIGENE	FLCDNA	TAIR
CTGATCCAAT	0	0	0	1	0	gnl|UG|At#S11734865	gi|25054926|gb|BT001941.1	At3g19960.1
at5g03905	CON	MIN	HOUR	DAY	WEEK	UNIGENE	FLCDNA	TAIR
TATAATGTCA	0	0	2	0	0	gnl|UG|At#S11725175	no match found	At5g03905.1
at4g33750	CON	MIN	HOUR	DAY	WEEK	UNIGENE	FLCDNA	TAIR
AAACTTGACT	2	3	3	0	0	gnl|UG|At#S21736225	gi|51971041|dbj|AK176450.1|	pseudo chromosome match
at3g26710	CON	MIN	HOUR	DAY	WEEK	UNIGENE	FLCDNA	TAIR
GTTTATGTCA	2	7	1	1	1	gnl|UG|At#S11732733	gi|22655151|gb|AY140025.1	At3g26710.1
at5g05190	CON	MIN	HOUR	DAY	WEEK	UNIGENE	FLCDNA	TAIR
ATATGTTTGT	1	3	0	0	1	gnl|UG|At#S11724833	gi|20466819|gb|AY099876.1	non-canonical match
at1g76730	CON	MIN	HOUR	DAY	WEEK	UNIGENE	FLCDNA	TAIR
ATTGTCAGCT	0	2	0	1	0	gnl|UG|At#S18895047	gi|26453209|dbj|AK119107.1|	multiple canonical match
at2g41910	CON	MIN	HOUR	DAY	WEEK	UNIGENE	FLCDNA	TAIR
TGAAGAATGA	0	0	1	1	0	gnl|UG|At#S11732120	no match found	non-canonical match
at1g56290	CON	MIN	HOUR	DAY	WEEK	UNIGENE	FLCDNA	TAIR
TTTTTTTTCA	1	0	0	0	0	gnl|UG|At#S11706061	gi|15810332|gb|AY056205.1	At1g56290.1
at3g42600	CON	MIN	HOUR	DAY	WEEK	UNIGENE	FLCDNA	TAIR
TGTATCTCTA	1	0	0	0	0	no match found	no match found	At3g42600.1
at1g55680	CON	MIN	HOUR	DAY	WEEK	UNIGENE	FLCDNA	TAIR
TGTATGATGT	0	1	0	0	0	no match found	gi|17979110|gb|AY070028.1	non-canonical match
at3g47070	CON	MIN	HOUR	DAY	WEEK	UNIGENE	FLCDNA	TAIR
CCTCTGTTTT	47	9	51	41	27	no match found	gi|21405276|gb|AY086566.1	non-canonical match
ATCTAGTTAT	9	4	10	6	3	gnl|UG|At#S11729982	no match found	At3g47070.1
at3g13857	CON	MIN	HOUR	DAY	WEEK	UNIGENE	FLCDNA	TAIR
GCCCCTGCGC	5	1	5	36	18	gnl|UG|At#S15644818	gi|110738916|dbj|AK229525.1|	multiple canonical match
at3g48420	CON	MIN	HOUR	DAY	WEEK	UNIGENE	FLCDNA	TAIR

CTTGCTTGGA	0	0	1	0	0	no match found	gi|17104702|gb|AY063066.1	non-canonical match
TAAAACCATA	12	4	3	0	7	gnl|UG|At#S18905178	gi|21405352|gb|AY086642.1	At3g48420.1
at3g22170	CON	MIN	HOUR	DAY	WEEK	UNIGENE	FLCDNA	TAIR
AGCCAAAGCT	5	3	3	0	2	gnl|UG|At#S43849873	gi|110739305|dbj|AK229728.1|	At3g22170.1
at3g55280	CON	MIN	HOUR	DAY	WEEK	UNIGENE	FLCDNA	TAIR
AAAAAGATTG	2	0	1	3	4	gnl|UG|At#S11728463	gi|23308154|gb|BT000478.1	At3g55280.1
at2g33380	CON	MIN	HOUR	DAY	WEEK	UNIGENE	FLCDNA	TAIR
GGAGTGATTC	0	0	0	1	0	gnl|UG|At#S11708342	gi|20259945|gb|AY093321.1	At2g33380.1
at4g37200	CON	MIN	HOUR	DAY	WEEK	UNIGENE	FLCDNA	TAIR
CTTCTGGCAG	4	11	11	3	2	gnl|UG|At#S11721403	gi|18700132|gb|AY075671.1	At4g37200.1
at3g57160	CON	MIN	HOUR	DAY	WEEK	UNIGENE	FLCDNA	TAIR
TTGCACATTA	0	0	0	0	1	gnl|UG|At#S11811727	gi|21405236|gb|AY086526.1	At3g57160.1
at3g60840	CON	MIN	HOUR	DAY	WEEK	UNIGENE	FLCDNA	TAIR
AGAAGGCAAC	0	1	0	0	0	gnl|UG|At#S11727232	no match found	non-canonical match
at5g20700	CON	MIN	HOUR	DAY	WEEK	UNIGENE	FLCDNA	TAIR
GCGAAAAATA	1	5	3	2	4	gnl|UG|At#S11721153	no match found	At5g20700.1
at4g03390	CON	MIN	HOUR	DAY	WEEK	UNIGENE	FLCDNA	TAIR
GTTCGGGTGA	3	2	2	1	3	gnl|UG|At#S11726824	gi|110736191|dbj|AK228109.1|	At4g03390.1
at5g10300	CON	MIN	HOUR	DAY	WEEK	UNIGENE	FLCDNA	TAIR
GTGATGCTCT	0	1	1	0	0	gnl|UG|At#S11723662	gi|16648678|gb|AY058115.1	At5g10300.1
at3g12740	CON	MIN	HOUR	DAY	WEEK	UNIGENE	FLCDNA	TAIR
CTCATTACCT	1	0	0	0	1	gnl|UG|At#S11737074	gi|15028094|gb|AY045904.1	non-canonical match
TATCTTGTGA	0	3	1	0	0	no match found	gi|20258910|gb|AY091210.1	non-canonical match
at2g34710	CON	MIN	HOUR	DAY	WEEK	UNIGENE	FLCDNA	TAIR
TTCGTGAATT	0	0	1	1	0	gnl|UG|At#S11733885	gi|20466649|gb|AY099791.1	At2g34710.1
at2g38700	CON	MIN	HOUR	DAY	WEEK	UNIGENE	FLCDNA	TAIR
AAGAGTTACG	0	2	0	1	0	gnl|UG|At#S11699461	gi|31711703|gb|BT008769.1	At2g38700.1
at5g42720	CON	MIN	HOUR	DAY	WEEK	UNIGENE	FLCDNA	TAIR
GCTCCATTGT	0	0	1	0	1	gnl|UG|At#S11719458	gi|23297708|gb|AY150489.1	At5g42720.1
at1g23080	CON	MIN	HOUR	DAY	WEEK	UNIGENE	FLCDNA	TAIR
GCATTGCAAC	4	4	6	3	1	gnl|UG|At#S11706973	gi|15450508|gb|AY052356.1	At1g23080.1
at1g02660	CON	MIN	HOUR	DAY	WEEK	UNIGENE	FLCDNA	TAIR
ATATGAACTC	0	3	0	0	0	gnl|UG|At#S11704906	gi|22655023|gb|AY139797.1	At1g02660.1
at3g49130	CON	MIN	HOUR	DAY	WEEK	UNIGENE	FLCDNA	TAIR
GAACTGTTCT	0	0	0	0	1	no match found	no match found	At3g49130.1
at2g20890	CON	MIN	HOUR	DAY	WEEK	UNIGENE	FLCDNA	TAIR
GAAGGATATC	1	1	2	1	0	no match found	gi|20148534|gb|AY081596.1	non-canonical match
TAGTATTTTA	4	5	1	3	6	gnl|UG|At#S11737237	gi|21406118|gb|AY087394.1	At2g20890.1
at1g71840	CON	MIN	HOUR	DAY	WEEK	UNIGENE	FLCDNA	TAIR
TTGCTATCTG	1	1	0	1	0	gnl|UG|At#S11743889	gi|19347843|gb|AY080732.1	At1g71840.1
at1g24290	CON	MIN	HOUR	DAY	WEEK	UNIGENE	FLCDNA	TAIR
TTGTTCAAGT	0	0	0	0	1	gnl|UG|At#S11740556	gi|26451435|dbj|AK118195.1|	At1g24290.1

at2g33255	CON	MIN	HOUR	DAY	WEEK	UNIGENE	FLCDNA	TAIR
TGTGTGAGGG	4	0	1	2	0	gnl|UG|At#S15460839	gi|16648737|gb|AY058145.1	pseudo chromosome match
TGGGAAACGA	0	0	0	0	1	no match found	gi|20147252|gb|AY093716.1	At2g33255.1

at3g18760	CON	MIN	HOUR	DAY	WEEK	UNIGENE	FLCDNA	TAIR
TGTTTTATTG	0	0	0	0	1	gnl|UG|At#S11735220	gi|26451218|dbj|AK118083.1|	At3g18760.1

at5g54130	CON	MIN	HOUR	DAY	WEEK	UNIGENE	FLCDNA	TAIR
TACAAAATAA	1	0	1	2	3	gnl|UG|At#S38433133	gi|51970737|dbj|AK176298.1|	At5g54130.1

at2g44760	CON	MIN	HOUR	DAY	WEEK	UNIGENE	FLCDNA	TAIR
AATATAATAG	1	0	0	0	2	no match found	no match found	At2g44760.1

at3g54030	CON	MIN	HOUR	DAY	WEEK	UNIGENE	FLCDNA	TAIR
GGAAGATCTC	1	0	1	0	0	gnl|UG|At#S11728683	no match found	At3g54030.1

at4g19880	CON	MIN	HOUR	DAY	WEEK	UNIGENE	FLCDNA	TAIR
TGGCGGATTA	1	3	10	1	0	gnl|UG|At#S11724350	no match found	pseudo chromosome match
AGTTGAGTTC	2	6	6	2	2	no match found	gi|13878018|gb|AF370272.1	At4g19880.1

at5g45490	CON	MIN	HOUR	DAY	WEEK	UNIGENE	FLCDNA	TAIR
GACAAACCGA	1	3	1	1	0	gnl|UG|At#S11719178	gi|18086407|gb|AY065019.1	At5g45490.1

at3g03960	CON	MIN	HOUR	DAY	WEEK	UNIGENE	FLCDNA	TAIR
CACTGTACTT	0	2	1	0	0	gnl|UG|At#S11739530	gi|13877578|gb|AF370490.1	At3g03960.1

at2g15535	CON	MIN	HOUR	DAY	WEEK	UNIGENE	FLCDNA	TAIR
CATATTATTA	0	1	0	0	0	gnl|UG|At#S30642264	gi|21407442|gb|AY088668.1	At2g15535.1

at4g28010	CON	MIN	HOUR	DAY	WEEK	UNIGENE	FLCDNA	TAIR
TACAAATGAT	0	0	1	0	1	gnl|UG|At#S11815688	gi|22135794|gb|AY128275.1	At4g28010.1

at5g26290	CON	MIN	HOUR	DAY	WEEK	UNIGENE	FLCDNA	TAIR
TTTCACTTTA	0	0	0	1	0	gnl|UG|At#S11704003	gi|14517435|gb|AY039553.1	non-canonical match

at3g60520	CON	MIN	HOUR	DAY	WEEK	UNIGENE	FLCDNA	TAIR
GATTAGTAAG	1	0	4	1	0	gnl|UG|At#S11727327	gi|110735834|dbj|AK227924.1|	At3g60520.1

at3g01470	CON	MIN	HOUR	DAY	WEEK	UNIGENE	FLCDNA	TAIR
TTGACGATAA	9	10	7	4	5	gnl|UG|At#S11740229	gi|16648821|gb|AY058188.1	At3g01470.1

at2g32050	CON	MIN	HOUR	DAY	WEEK	UNIGENE	FLCDNA	TAIR
TAAAAGCCTG	0	0	0	1	0	no match found	no match found	At2g32050.1

at2g46830	CON	MIN	HOUR	DAY	WEEK	UNIGENE	FLCDNA	TAIR
AGACTCTATT	5	0	3	8	7	gnl|UG|At#S11707282	gi|24429605|gb|BT001096.1	At2g46830.2

at5g56050	CON	MIN	HOUR	DAY	WEEK	UNIGENE	FLCDNA	TAIR
AATACTTTTT	0	2	0	0	0	no match found	no match found	At5g56050.1

at3g52070	CON	MIN	HOUR	DAY	WEEK	UNIGENE	FLCDNA	TAIR
GTAGTAGCTA	3	5	1	0	1	no match found	gi|21403514|gb|AY084804.1	At3g52070.1

at3g24570	CON	MIN	HOUR	DAY	WEEK	UNIGENE	FLCDNA	TAIR
GGTCGAGCAA	1	4	0	0	0	gnl|UG|At#S34117324	gi|41349919|gb|BT011505.1	non-canonical match

at1g78300	CON	MIN	HOUR	DAY	WEEK	UNIGENE	FLCDNA	TAIR
CAGCTTCTTC	1	3	3	4	3	gnl|UG|At#S11727103	gi|18655378|gb|AY077667.1	At1g78300.1

at2g23310	CON	MIN	HOUR	DAY	WEEK	UNIGENE	FLCDNA	TAIR

GTGGAAAACT	2	1	4	0	4	no match found	gi|17978977|gb|AY069898.1	At2g23310.1
at4g22130	CON	MIN	HOUR	DAY	WEEK	UNIGENE	FLCDNA	TAIR
TTTCAATCAT	0	0	1	0	0	gnl|UG|At#S11723981	no match found	At4g22130.1
at2g44750	CON	MIN	HOUR	DAY	WEEK	UNIGENE	FLCDNA	TAIR
ATGCATCGAA	2	3	1	0	1	gnl|UG|At#S15460375	gi|20148328|gb|AY081493.1	At2g44750.1
at2g41550	CON	MIN	HOUR	DAY	WEEK	UNIGENE	FLCDNA	TAIR
GACGGAAAAA	0	0	1	0	0	gnl|UG|At#S11732200	gi|26452552|dbj|AK118768.1|	non-canonical match
at2g14530	CON	MIN	HOUR	DAY	WEEK	UNIGENE	FLCDNA	TAIR
CACTTTTCAT	0	1	2	0	1	gnl|UG|At#S11743796	gi|18650651|gb|AY074875.1	At2g14530.1
at5g11960	CON	MIN	HOUR	DAY	WEEK	UNIGENE	FLCDNA	TAIR
TTACTCTAAT	1	0	0	0	0	no match found	no match found	At5g11960.1
at2g23600	CON	MIN	HOUR	DAY	WEEK	UNIGENE	FLCDNA	TAIR
AAGCACCGTC	3	1	2	1	0	gnl|UG|At#S11736582	gi|15810084|gb|AY056068.1	At2g23600.1
at4g21700	CON	MIN	HOUR	DAY	WEEK	UNIGENE	FLCDNA	TAIR
GACTTCTTGT	0	1	0	2	0	no match found	no match found	At4g21700.1
at5g04810	CON	MIN	HOUR	DAY	WEEK	UNIGENE	FLCDNA	TAIR
GCTTCTTAGA	4	1	2	2	0	gnl|UG|At#S11724927	gi|110739198|dbj|AK229672.1|	At5g04810.1
at5g58600	CON	MIN	HOUR	DAY	WEEK	UNIGENE	FLCDNA	TAIR
GAACCAGTTG	0	0	0	2	2	no match found	gi|28827569|gb|BT005096.1	multiple non-canonical match
at2g26060	CON	MIN	HOUR	DAY	WEEK	UNIGENE	FLCDNA	TAIR
ATGTTTCATA	2	2	0	0	4	gnl|UG|At#S28282260	gi|28059423|gb|BT003394.1	At2g26060.1
at2g27402	CON	MIN	HOUR	DAY	WEEK	UNIGENE	FLCDNA	TAIR
TAAGAAGACA	0	3	2	0	0	gnl|UG|At#S35243420	gi|109134188|gb|BT026003.1	At2g27402.1
at1g30520	CON	MIN	HOUR	DAY	WEEK	UNIGENE	FLCDNA	TAIR
ACTAGCTCTC	3	1	1	0	1	gnl|UG|At#S11738836	gi|34365546|gb|BT010462.1	At1g30520.1
at4g21940	CON	MIN	HOUR	DAY	WEEK	UNIGENE	FLCDNA	TAIR
AAAGAGTATG	0	1	0	0	0	no match found	gi|17104578|gb|AY063004.1	multiple non-canonical match
at5g27630	CON	MIN	HOUR	DAY	WEEK	UNIGENE	FLCDNA	TAIR
AAACTTTTTT	0	0	0	1	0	gnl|UG|At#S11720449	gi|20260513|gb|AY093156.1	multiple non-canonical match
at2g40510	CON	MIN	HOUR	DAY	WEEK	UNIGENE	FLCDNA	TAIR
TGGATCTTGA	20	11	18	12	23	gnl|UG|At#S11732457	gi|20466413|gb|AY099673.1	At2g40510.1
TCGTTAGAGT	0	0	1	0	2	no match found	gi|111074311|gb|BT026422.1	non-canonical match
at2g28105	CON	MIN	HOUR	DAY	WEEK	UNIGENE	FLCDNA	TAIR
AGAGTCGCTT	0	1	0	0	0	no match found	no match found	At2g28105.1
at1g04710	CON	MIN	HOUR	DAY	WEEK	UNIGENE	FLCDNA	TAIR
AGGGAAAACT	0	1	2	3	0	gnl|UG|At#S11742556	gi|20466549|gb|AY099741.1	At1g04710.1
at1g72090	CON	MIN	HOUR	DAY	WEEK	UNIGENE	FLCDNA	TAIR
TAGAGTTTTG	0	1	0	0	0	no match found	gi|27363417|gb|BT002712.1	non-canonical match
TAGAATACTA	1	0	0	0	0	gnl|UG|At#S11728673	gi|14194136|gb|AF367274.1	At1g72090.1
at2g19610	CON	MIN	HOUR	DAY	WEEK	UNIGENE	FLCDNA	TAIR
TTTTCTGAAA	3	0	2	3	1	gnl|UG|At#S11737574	no match found	At2g19610.1

at4g04910	CON	MIN	HOUR	DAY	WEEK	UNIGENE	FLCDNA	TAIR
TTATTTTGCT	3	0	0	1	0	no match found	gi|20856712|gb|AY102111.1	non-canonical match

at3g49900	CON	MIN	HOUR	DAY	WEEK	UNIGENE	FLCDNA	TAIR
GTAGCTGCTG	0	0	1	0	0	gnl|UG|At#S11729462	no match found	non-canonical match

at3g52640	CON	MIN	HOUR	DAY	WEEK	UNIGENE	FLCDNA	TAIR
TGTACACAGT	1	0	1	0	1	no match found	gi|30725507|gb|BT008417.1	non-canonical match
TTAAATTCTT	0	0	0	1	0	gnl|UG|At#S28282828	gi|27311552|gb|BT002382.1	At3g52640.2

at3g51910	CON	MIN	HOUR	DAY	WEEK	UNIGENE	FLCDNA	TAIR
GAAAACAGAG	0	0	1	0	0	gnl|UG|At#S11729076	no match found	multiple non-canonical match

at3g16160	CON	MIN	HOUR	DAY	WEEK	UNIGENE	FLCDNA	TAIR
AATCCATCCT	1	0	1	0	0	no match found	no match found	At3g16160.1

at5g19010	CON	MIN	HOUR	DAY	WEEK	UNIGENE	FLCDNA	TAIR
AACTCAATCT	7	3	1	4	3	gnl|UG|At#S11721570	gi|17064905|gb|AY062529.1	At5g19010.1

at1g32220	CON	MIN	HOUR	DAY	WEEK	UNIGENE	FLCDNA	TAIR
AATAGCTCTC	8	3	8	7	7	gnl|UG|At#S11738191	gi|22530967|gb|AY136322.1	At1g32220.1

at5g40490	CON	MIN	HOUR	DAY	WEEK	UNIGENE	FLCDNA	TAIR
TGACATCTTT	0	0	1	1	0	gnl|UG|At#S11719681	gi|22531254|gb|AY136466.1	At5g40490.1

at1g08160	CON	MIN	HOUR	DAY	WEEK	UNIGENE	FLCDNA	TAIR
CGTATCTCCA	0	2	0	0	0	gnl|UG|At#S11710238	gi|17529201|gb|AY065386.1	At1g08160.1

at3g21680	CON	MIN	HOUR	DAY	WEEK	UNIGENE	FLCDNA	TAIR
GCTAGTTATG	0	0	1	0	0	gnl|UG|At#S17007115	no match found	multiple non-canonical match

at1g61590	CON	MIN	HOUR	DAY	WEEK	UNIGENE	FLCDNA	TAIR
TGTGATGTGG	1	0	0	0	0	gnl|UG|At#S11708223	gi|17381260|gb|AY064142.1	At1g61590.1

at4g39670	CON	MIN	HOUR	DAY	WEEK	UNIGENE	FLCDNA	TAIR
GAAGCATCGC	0	0	0	1	0	gnl|UG|At#S11815757	gi|23308250|gb|BT000526.1	At4g39670.1

at1g20970	CON	MIN	HOUR	DAY	WEEK	UNIGENE	FLCDNA	TAIR
AGGCAGTTCC	1	7	1	1	2	gnl|UG|At#S11740960	no match found	non-canonical match

at1g22890	CON	MIN	HOUR	DAY	WEEK	UNIGENE	FLCDNA	TAIR
TTGGAATTTT	4	7	7	5	0	no match found	gi|28393813|gb|BT004319.1	At1g22890.1
GCCCCTCCAA	0	2	0	0	0	no match found	gi|117168238|gb|BT029388.1	non-canonical match

at2g43945	CON	MIN	HOUR	DAY	WEEK	UNIGENE	FLCDNA	TAIR
TCAAGAAGCC	5	2	4	1	1	gnl|UG|At#S15460406	gi|28393676|gb|BT004247.1	At2g43945.1

at1g14520	CON	MIN	HOUR	DAY	WEEK	UNIGENE	FLCDNA	TAIR
TGTAAATGTA	0	0	0	0	1	no match found	gi|51969807|dbj|AK175833.1|	At1g14520.1

at4g24460	CON	MIN	HOUR	DAY	WEEK	UNIGENE	FLCDNA	TAIR
ATGCTCTCAG	0	1	0	0	0	gnl|UG|At#S14829863	gi|28973716|gb|BT005771.1	At4g24460.1

at5g09320	CON	MIN	HOUR	DAY	WEEK	UNIGENE	FLCDNA	TAIR
TCTATGGAGA	0	0	1	0	3	gnl|UG|At#S11723909	gi|21403069|gb|AY084359.1	At5g09320.1

at5g53220	CON	MIN	HOUR	DAY	WEEK	UNIGENE	FLCDNA	TAIR
ACTTGGAGAA	0	1	0	0	0	gnl|UG|At#S21989671	no match found	non-canonical match

at5g22500	CON	MIN	HOUR	DAY	WEEK	UNIGENE	FLCDNA	TAIR
CATATGATAT	0	0	0	1	0	gnl|UG|At#S11720972	no match found	multiple non-canonical match

TGCTTAAACA	0	2	0	1	0	no match found	gi|14334737|gb|AY035042.1	non-canonical match
at3g04730	CON	MIN	HOUR	DAY	WEEK	UNIGENE	FLCDNA	TAIR
CAAACGTATA	9	32	13	1	1	gnl|UG|At#S11739300	gi|14030658|gb|AF375420.1	At3g04730.1
at2g43030	CON	MIN	HOUR	DAY	WEEK	UNIGENE	FLCDNA	TAIR
ATCAAAGGTG	11	17	10	9	3	gnl|UG|At#S11731877	gi|21403997|gb|AY085287.1	multiple canonical match
at1g34440	CON	MIN	HOUR	DAY	WEEK	UNIGENE	FLCDNA	TAIR
AGAGAGATGG	0	0	1	0	0	no match found	no match found	At1g34440.1
at5g59910	CON	MIN	HOUR	DAY	WEEK	UNIGENE	FLCDNA	TAIR
AATTGGGTTC	0	0	2	0	0	gnl|UG|At#S35300214	no match found	pseudo chromosome match
at4g33650	CON	MIN	HOUR	DAY	WEEK	UNIGENE	FLCDNA	TAIR
ATCGGTCCAC	1	0	1	0	0	no match found	no match found	At4g33650.1
ATCCAAATAT	1	3	0	0	0	gnl|UG|At#S11744212	gi|14334427|gb|AY034905.1	At1g66710.1
at2g42810	CON	MIN	HOUR	DAY	WEEK	UNIGENE	FLCDNA	TAIR
ATAATTAAAG	0	4	0	0	1	gnl|UG|At#S28282147	gi|19347798|gb|AY080674.1	At2g42810.1
ACGGTAAACT	0	0	0	1	0	no match found	gi|33589765|gb|BT010180.1	At1g65250.1
at2g47990	CON	MIN	HOUR	DAY	WEEK	UNIGENE	FLCDNA	TAIR
GTAATGTCTA	1	1	0	0	3	gnl|UG|At#S11730731	gi|26451322|dbj|AK118137.1|	At2g47990.1
at4g31490	CON	MIN	HOUR	DAY	WEEK	UNIGENE	FLCDNA	TAIR
GGTTTGTTCC	0	2	2	2	2	gnl|UG|At#S11722367	no match found	At4g31490.1
GAGAGAAGAG	1	0	2	2	2	no match found	gi|110737664|dbj|AK228881.1|	non-canonical match
at1g49600	CON	MIN	HOUR	DAY	WEEK	UNIGENE	FLCDNA	TAIR
TACGCTACTG	2	1	0	2	1	gnl|UG|At#S11734638	no match found	At1g49600.1
at5g03030	CON	MIN	HOUR	DAY	WEEK	UNIGENE	FLCDNA	TAIR
TGTCTGATAC	0	1	1	2	3	no match found	no match found	At5g03030.1
at4g30680	CON	MIN	HOUR	DAY	WEEK	UNIGENE	FLCDNA	TAIR
TTTTGATCAG	0	1	0	0	0	no match found	gi|16612266|gb|AF439830.1	non-canonical match
at1g72070	CON	MIN	HOUR	DAY	WEEK	UNIGENE	FLCDNA	TAIR
TCCTTCCTCC	1	1	2	1	0	no match found	gi|51969031|dbj|AK175445.1|	At1g72060.1
at2g16365	CON	MIN	HOUR	DAY	WEEK	UNIGENE	FLCDNA	TAIR
GTTTGGAATG	3	1	11	1	0	no match found	gi|30387552|gb|BT006598.1	At2g16365.2
at2g23150	CON	MIN	HOUR	DAY	WEEK	UNIGENE	FLCDNA	TAIR
AGTTCTTAGC	2	0	1	0	1	gnl|UG|At#S11736702	gi|16648850|gb|AY058203.1	At2g23150.1
at2g36080	CON	MIN	HOUR	DAY	WEEK	UNIGENE	FLCDNA	TAIR
CGATGTTCTT	0	0	1	0	0	no match found	no match found	At2g36080.2
GACATAAGTT	0	2	1	3	0	gnl|UG|At#S15460726	gi|26450254|dbj|AK117587.1|	At2g36080.1
at2g21070	CON	MIN	HOUR	DAY	WEEK	UNIGENE	FLCDNA	TAIR
GATATGAAAA	0	0	0	1	0	no match found	gi|53850468|gb|BT015875.1	non-canonical match
at4g19040	CON	MIN	HOUR	DAY	WEEK	UNIGENE	FLCDNA	TAIR
ACAAATTAGT	0	0	0	0	1	gnl|UG|At#S43849536	no match found	At3g59750.1
at4g15360	CON	MIN	HOUR	DAY	WEEK	UNIGENE	FLCDNA	TAIR
GAAGAGACTT	1	0	0	1	0	no match found	no match found	At4g15360.1
at1g60160	CON	MIN	HOUR	DAY	WEEK	UNIGENE	FLCDNA	TAIR

AATTAGGGTT	3	4	1	0	0	gnl|UG|At#S11731444	no match found	non-canonical match
at3g24040	CON	MIN	HOUR	DAY	WEEK	UNIGENE	FLCDNA	TAIR
AACTTCTGGG	1	2	0	0	0	gnl|UG|At#S11733579	gi|46518432|gb|BT012554.1	At3g24040.1
at1g12530	CON	MIN	HOUR	DAY	WEEK	UNIGENE	FLCDNA	TAIR
TAGTGATGAT	1	0	1	0	1	gnl|UG|At#S22484655	no match found	At1g12530.1
at5g08300	CON	MIN	HOUR	DAY	WEEK	UNIGENE	FLCDNA	TAIR
CCGGAGCCAT	3	6	5	3	4	gnl|UG|At#S11724038	gi|18252214|gb|AY072333.1	At5g08300.1
at3g07565	CON	MIN	HOUR	DAY	WEEK	UNIGENE	FLCDNA	TAIR
AGTAGGGTCG	1	2	2	0	0	gnl|UG|At#S28283019	gi|19347921|gb|AY080636.1	At3g07565.1
at1g68185	CON	MIN	HOUR	DAY	WEEK	UNIGENE	FLCDNA	TAIR
GGAAGGGATC	0	0	0	0	1	gnl|UG|At#S11729467	gi|21405328|gb|AY086618.1	At1g68185.1
at3g27880	CON	MIN	HOUR	DAY	WEEK	UNIGENE	FLCDNA	TAIR
AGAAGTTTTA	0	1	1	2	0	no match found	gi|21404176|gb|AY085466.1	non-canonical match
at4g14713	CON	MIN	HOUR	DAY	WEEK	UNIGENE	FLCDNA	TAIR
CACTTTGCAG	0	1	2	0	0	gnl|UG|At#S18941864	gi|18086339|gb|AY064979.1	At4g14713.1
at5g48760	CON	MIN	HOUR	DAY	WEEK	UNIGENE	FLCDNA	TAIR
TTTTGAGCCA	0	3	1	5	2	gnl|UG|At#S42369531	gi|21406965|gb|AY088191.1	At5g48760.1
GACCTATTCA	0	0	1	0	0	no match found	gi|19699304|gb|AY090359.1	non-canonical match
at2g38790	CON	MIN	HOUR	DAY	WEEK	UNIGENE	FLCDNA	TAIR
GATGATCAAA	0	0	1	1	0	no match found	gi|27754464|gb|BT002863.1	multiple canonical match
at2g03380	CON	MIN	HOUR	DAY	WEEK	UNIGENE	FLCDNA	TAIR
TATGGTTGAT	1	0	1	0	0	gnl|UG|At#S11740472	no match found	At1g36500.1
at5g03630	CON	MIN	HOUR	DAY	WEEK	UNIGENE	FLCDNA	TAIR
GCAATTCTAC	1	3	2	3	0	gnl|UG|At#S11725250	gi|22655343|gb|AY142000.1	At5g03630.1
at1g65845	CON	MIN	HOUR	DAY	WEEK	UNIGENE	FLCDNA	TAIR
CAGCAGTTCC	3	7	6	2	0	gnl|UG|At#S11611428	no match found	At1g65845.1
at1g69270	CON	MIN	HOUR	DAY	WEEK	UNIGENE	FLCDNA	TAIR
AGAACGGGTT	0	4	1	0	0	gnl|UG|At#S11729251	gi|20466783|gb|AY099858.1	non-canonical match
at1g20380	CON	MIN	HOUR	DAY	WEEK	UNIGENE	FLCDNA	TAIR
GATCGACTAG	0	1	0	1	0	gnl|UG|At#S11741020	no match found	At1g20380.1
at5g03160	CON	MIN	HOUR	DAY	WEEK	UNIGENE	FLCDNA	TAIR
TAACTTAAGA	0	1	1	2	2	gnl|UG|At#S11725369	gi|110737081|dbj|AK228576.1|	non-canonical match
at1g40230	CON	MIN	HOUR	DAY	WEEK	UNIGENE	FLCDNA	TAIR
GCACAGAGAG	0	0	0	1	0	no match found	no match found	At1g40230.1
at1g74240	CON	MIN	HOUR	DAY	WEEK	UNIGENE	FLCDNA	TAIR
AAGGTTAGTG	1	0	1	0	0	gnl|UG|At#S11824184	gi|26450339|dbj|AK117630.1|	At1g74240.1
at1g19860	CON	MIN	HOUR	DAY	WEEK	UNIGENE	FLCDNA	TAIR
CTCTCTGCCT	3	2	0	0	2	gnl|UG|At#S11741068	gi|62320505|dbj|AK221579.1|	At1g19860.1
at1g20470	CON	MIN	HOUR	DAY	WEEK	UNIGENE	FLCDNA	TAIR
ATCAGAGCAA	1	0	0	0	0	gnl|UG|At#S11804437	gi|26450200|dbj|AK117558.1|	At1g20470.1
at3g13520	CON	MIN	HOUR	DAY	WEEK	UNIGENE	FLCDNA	TAIR

AGCTTTTTGT	12	8	10	9	9	no match found	gi|21404749|gb|AY086039.1	At3g13520.1
at5g04260	CON	MIN	HOUR	DAY	WEEK	UNIGENE	FLCDNA	TAIR
TTGATGTCAA	0	2	0	0	0	gnl|UG|At#S18910845	gi|21436390|gb|AY117290.1	At5g04260.1
at4g27310	CON	MIN	HOUR	DAY	WEEK	UNIGENE	FLCDNA	TAIR
CGTCGCTCTT	4	6	8	2	0	gnl|UG|At#S11709758	gi|17979451|gb|AY070721.1	At4g27310.1
at5g55660	CON	MIN	HOUR	DAY	WEEK	UNIGENE	FLCDNA	TAIR
CAATTTCGTT	0	0	1	1	2	gnl|UG|At#S11718147	no match found	At5g55660.1
at1g25450	CON	MIN	HOUR	DAY	WEEK	UNIGENE	FLCDNA	TAIR
TTCTAATAAC	0	0	0	0	1	gnl|UG|At#S11740360	gi|28393606|gb|BT004205.1	At1g25450.1
at2g14610	CON	MIN	HOUR	DAY	WEEK	UNIGENE	FLCDNA	TAIR
CATACACACG	3	0	1	2	2	gnl|UG|At#S11738810	gi|21436246|gb|AY117187.1	At2g14610.1
at5g13960	CON	MIN	HOUR	DAY	WEEK	UNIGENE	FLCDNA	TAIR
GACCGGATGG	0	1	1	0	0	gnl|UG|At#S11744262	gi|26983787|gb|BT002313.1	At5g13960.1
at3g13360	CON	MIN	HOUR	DAY	WEEK	UNIGENE	FLCDNA	TAIR
TAAGCTCGGT	0	1	2	0	3	gnl|UG|At#S11736869	gi|15010791|gb|AY045697.1	At3g13360.1
at1g52230	CON	MIN	HOUR	DAY	WEEK	UNIGENE	FLCDNA	TAIR
GCGTCTTTTG	17	36	19	15	1	no match found	gi|14326517|gb|AF385712.1	At1g52230.1
at5g55000	CON	MIN	HOUR	DAY	WEEK	UNIGENE	FLCDNA	TAIR
TGAATCTGCG	1	0	1	1	0	no match found	gi|124301157|gb|BT030093.1	At5g55000.2
at4g01240	CON	MIN	HOUR	DAY	WEEK	UNIGENE	FLCDNA	TAIR
GAAGCGTTTA	0	0	0	1	0	gnl|UG|At#S11727376	no match found	At4g01240.1
at1g49310	CON	MIN	HOUR	DAY	WEEK	UNIGENE	FLCDNA	TAIR
AAGCAACAGC	0	0	0	1	0	gnl|UG|At#S34114674	gi|110742911|dbj|AK227341.1|	At1g49310.1
at5g46580	CON	MIN	HOUR	DAY	WEEK	UNIGENE	FLCDNA	TAIR
AAGTATCATC	3	1	0	2	4	gnl|UG|At#S11719067	no match found	At5g46580.1
at1g50510	CON	MIN	HOUR	DAY	WEEK	UNIGENE	FLCDNA	TAIR
TGGTACCCTT	0	1	0	1	0	gnl|UG|At#S11734327	no match found	At1g50510.1
ATTCAGGACA	0	0	0	0	1	no match found	gi|27754327|gb|BT002789.1	non-canonical match
at3g20910	CON	MIN	HOUR	DAY	WEEK	UNIGENE	FLCDNA	TAIR
AAAGAGTATT	1	0	0	0	0	gnl|UG|At#S18903612	gi|21406434|gb|AY087696.1	At3g20910.1
at1g56580	CON	MIN	HOUR	DAY	WEEK	UNIGENE	FLCDNA	TAIR
AATTACTTTC	9	5	1	14	7	gnl|UG|At#S30641572	gi|16648668|gb|AY058110.1	At1g56580.1
at1g32450	CON	MIN	HOUR	DAY	WEEK	UNIGENE	FLCDNA	TAIR
AGTGATGATG	0	2	0	0	0	gnl|UG|At#S11738113	no match found	At1g32450.1
at5g14330	CON	MIN	HOUR	DAY	WEEK	UNIGENE	FLCDNA	TAIR
TATTCCTTCT	0	1	0	0	0	gnl|UG|At#S11722757	no match found	At5g14330.1
at4g00710	CON	MIN	HOUR	DAY	WEEK	UNIGENE	FLCDNA	TAIR
TCGCATCATA	0	0	1	1	0	no match found	gi|17064837|gb|AY062495.1	non-canonical match
TATGAACTCT	4	0	2	1	0	gnl|UG|At#S11727495	no match found	At4g00710.1
at5g27820	CON	MIN	HOUR	DAY	WEEK	UNIGENE	FLCDNA	TAIR
TTACAAGATA	3	0	0	1	1	gnl|UG|At#S11720430	gi|28392880|gb|BT003825.1	At5g27820.1

at5g03400	CON	MIN	HOUR	DAY	WEEK	UNIGENE	FLCDNA	TAIR
TTAATGAAAC	0	0	0	0	1	no match found	no match found	At5g03400.1
at2g17820	CON	MIN	HOUR	DAY	WEEK	UNIGENE	FLCDNA	TAIR
CAATGTCTTC	0	0	1	1	0	gnl|UG|At#S11699825	no match found	At2g17820.1
at5g51300	CON	MIN	HOUR	DAY	WEEK	UNIGENE	FLCDNA	TAIR
AGAATTTTAT	1	2	1	2	3	gnl|UG|At#S15459100	gi|13877738|gb|AF370132.1	At5g51300.1
CGTGTTCTGA	0	2	1	0	1	no match found	gi|23297081|gb|AY142629.1	At5g51300.2
at2g21640	CON	MIN	HOUR	DAY	WEEK	UNIGENE	FLCDNA	TAIR
TATTTGGGTG	1	0	0	0	1	no match found	gi|26452866|dbj|AK118931.1|	At2g21640.1
at1g33410	CON	MIN	HOUR	DAY	WEEK	UNIGENE	FLCDNA	TAIR
CATTACTCAA	0	1	0	0	0	gnl|UG|At#S11737788	no match found	At1g33410.1
at3g61700	CON	MIN	HOUR	DAY	WEEK	UNIGENE	FLCDNA	TAIR
TCGGGTATGG	0	2	0	0	0	gnl|UG|At#S38433561	gi|62320427|dbj|AK221540.1|	At3g61700.1
at2g30100	CON	MIN	HOUR	DAY	WEEK	UNIGENE	FLCDNA	TAIR
CCTATATATT	0	0	0	0	1	gnl|UG|At#S11735006	no match found	At2g30100.1
at3g55690	CON	MIN	HOUR	DAY	WEEK	UNIGENE	FLCDNA	TAIR
AAAAGCCTCT	1	0	0	0	0	gnl|UG|At#S11728387	gi|34146855|gb|BT010435.1	At3g55690.1
at2g17190	CON	MIN	HOUR	DAY	WEEK	UNIGENE	FLCDNA	TAIR
CTAGATATGA	0	1	0	0	0	gnl|UG|At#S11738184	gi|110737154|dbj|AK228616.1|	At2g17190.1
at1g01470	CON	MIN	HOUR	DAY	WEEK	UNIGENE	FLCDNA	TAIR
ACGGCTCTTG	0	2	2	7	3	gnl|UG|At#S11742967	gi|18650613|gb|AY074855.1	At1g01470.1
at5g40480	CON	MIN	HOUR	DAY	WEEK	UNIGENE	FLCDNA	TAIR
AGGAATCTCC	0	1	2	2	1	gnl|UG|At#S11719682	no match found	non-canonical match
at1g67860	CON	MIN	HOUR	DAY	WEEK	UNIGENE	FLCDNA	TAIR
TATTTTTGTT	0	0	0	1	0	gnl|UG|At#S11591774	no match found	At3g58310.1
at1g29930	CON	MIN	HOUR	DAY	WEEK	UNIGENE	FLCDNA	TAIR
GGCCTTTGCC	3	2	0	1	0	gnl|UG|At#S11625712	no match found	non-canonical match
GGCCTTCGCC	646	986	638	736	259	gnl|UG|At#S30639484	gi|15293002|gb|AY050935.1	At1g29930.1
at3g19120	CON	MIN	HOUR	DAY	WEEK	UNIGENE	FLCDNA	TAIR
TAAGAAGCAG	2	2	0	0	1	gnl|UG|At#S11735111	gi|21404300|gb|AY085590.1	At3g19120.1
at3g06020	CON	MIN	HOUR	DAY	WEEK	UNIGENE	FLCDNA	TAIR
AAGATATGTT	1	0	0	0	1	gnl|UG|At#S35217509	no match found	At3g06020.1
at4g14430	CON	MIN	HOUR	DAY	WEEK	UNIGENE	FLCDNA	TAIR
TTCAATGTTG	4	8	6	1	3	no match found	gi|110738733|dbj|AK229431.1|	multiple non-canonical match
at5g46270	CON	MIN	HOUR	DAY	WEEK	UNIGENE	FLCDNA	TAIR
GTCATTAACT	0	1	0	0	0	no match found	no match found	At5g46270.1
at5g27035	CON	MIN	HOUR	DAY	WEEK	UNIGENE	FLCDNA	TAIR
TCTCCATTGG	0	1	0	0	0	no match found	no match found	At5g27035.1
at4g34700	CON	MIN	HOUR	DAY	WEEK	UNIGENE	FLCDNA	TAIR
AAAACTTGTC	7	5	11	3	4	gnl|UG|At#S11721815	gi|21404535|gb|AY085825.1	At4g34700.1
GGCTCCTGGT	1	2	0	1	1	no match found	gi|24111282|gb|BT001011.1	multiple non-canonical match
at5g02290	CON	MIN	HOUR	DAY	WEEK	UNIGENE	FLCDNA	TAIR

TTATCTCACA	1	1	1	0	0	gnl|UG|At#S11725586	no match found	At5g02290.1
at5g23390	CON	MIN	HOUR	DAY	WEEK	UNIGENE	FLCDNA	TAIR
ATCTTGGTCA	1	1	0	0	0	gnl|UG|At#S11720839	gi|19699052|gb|AY090230.1	At5g23390.1
at1g56060	CON	MIN	HOUR	DAY	WEEK	UNIGENE	FLCDNA	TAIR
AGGGTCAGAG	0	0	0	1	0	gnl|UG|At#S17854152	gi|38566503|gb|BT010775.1	At1g56060.1
at5g19875	CON	MIN	HOUR	DAY	WEEK	UNIGENE	FLCDNA	TAIR
TGAACTTAAA	0	0	1	2	1	gnl|UG|At#S11721353	gi|26453289|dbj|AK119148.1|	At5g19875.1
at2g34310	CON	MIN	HOUR	DAY	WEEK	UNIGENE	FLCDNA	TAIR
GTCTGTAGAT	1	0	0	0	0	gnl|UG|At#S11733986	gi|110737026|dbj|AK228547.1|	At2g34310.2
at3g48570	CON	MIN	HOUR	DAY	WEEK	UNIGENE	FLCDNA	TAIR
TATACAAATT	1	1	1	1	3	no match found	gi|21404052|gb|AY085342.1	At3g48570.1
at1g70640	CON	MIN	HOUR	DAY	WEEK	UNIGENE	FLCDNA	TAIR
ATGTTGATGA	0	0	6	1	1	gnl|UG|At#S11728954	no match found	At1g70640.1
at5g41315	CON	MIN	HOUR	DAY	WEEK	UNIGENE	FLCDNA	TAIR
GATCTGTTGA	0	1	0	0	0	gnl|UG|At#S11816884	no match found	multiple non-canonical match
at3g17770	CON	MIN	HOUR	DAY	WEEK	UNIGENE	FLCDNA	TAIR
ATCCCTTCTC	1	0	0	0	1	gnl|UG|At#S11735510	gi|71143067|gb|BT023733.1	At3g17770.1
at1g19450	CON	MIN	HOUR	DAY	WEEK	UNIGENE	FLCDNA	TAIR
GAGCAGTGGG	0	0	0	0	1	no match found	gi|20259851|gb|AY093274.1	non-canonical match
CTTTGTTCAA	2	1	2	1	5	gnl|UG|At#S11741108	gi|16648956|gb|AY059848.1	At1g19450.1
at5g58030	CON	MIN	HOUR	DAY	WEEK	UNIGENE	FLCDNA	TAIR
GGAACATTCA	0	0	0	0	1	no match found	gi|110735662|dbj|AK227834.1|	At5g58030.1
TTTACTGTTT	0	0	0	0	1	gnl|UG|At#S11717907	no match found	multiple non-canonical match
at5g64920	CON	MIN	HOUR	DAY	WEEK	UNIGENE	FLCDNA	TAIR
TTGCATCAAA	2	12	3	4	0	gnl|UG|At#S20832122	gi|19698864|gb|AY081279.1	At5g64920.1
at5g40070	CON	MIN	HOUR	DAY	WEEK	UNIGENE	FLCDNA	TAIR
TCAAGAGAGT	1	0	0	0	0	gnl|UG|At#S35302237	no match found	At5g37415.1
at1g32510	CON	MIN	HOUR	DAY	WEEK	UNIGENE	FLCDNA	TAIR
AGCCATCACA	0	0	1	0	0	no match found	no match found	At1g32510.1
at5g44340	CON	MIN	HOUR	DAY	WEEK	UNIGENE	FLCDNA	TAIR
TGTGTTCGTT	18	21	15	24	16	gnl|UG|At#S11703688	gi|14334935|gb|AY035141.1	At5g44340.1
at5g52910	CON	MIN	HOUR	DAY	WEEK	UNIGENE	FLCDNA	TAIR
TTACATTTAG	0	1	0	0	1	gnl|UG|At#S11718426	gi|110740741|dbj|AK226318.1|	At5g52910.1
at1g04980	CON	MIN	HOUR	DAY	WEEK	UNIGENE	FLCDNA	TAIR
GGAAATGATG	2	2	0	0	0	gnl|UG|At#S11742531	gi|20466693|gb|AY099813.1	At1g04980.1
at3g17440	CON	MIN	HOUR	DAY	WEEK	UNIGENE	FLCDNA	TAIR
TTCTTTTACT	3	0	0	1	0	gnl|UG|At#S18902077	gi|21403355|gb|AY084645.1	At3g17440.1
at3g53920	CON	MIN	HOUR	DAY	WEEK	UNIGENE	FLCDNA	TAIR
ATCTTCTAGA	0	1	0	1	0	gnl|UG|At#S11728704	gi|22135903|gb|AY128331.1	At3g53920.1
at5g59750	CON	MIN	HOUR	DAY	WEEK	UNIGENE	FLCDNA	TAIR
GAGTTAATCG	0	1	1	0	1	gnl|UG|At#S11717728	gi|15215751|gb|AY050405.1	At5g59750.1

at5g44410 CON MIN HOUR DAY WEEK UNIGENE FLCDNA TAIR
GAAGATTAGA 0 0 0 1 0 gnl|UG|At#S11719287 gi|110743375|dbj|AK227582.1| At5g44410.1

at1g09960 CON MIN HOUR DAY WEEK UNIGENE FLCDNA TAIR
AGATTCTCTC 0 0 0 1 0 gnl|UG|At#S11743607 gi|18175711|gb|AY072092.1 At1g09960.1

at4g00290 CON MIN HOUR DAY WEEK UNIGENE FLCDNA TAIR
GGAAAAGAAG 0 0 0 1 0 gnl|UG|At#S18906870 gi|51971718|dbj|AK176761.1| At4g00290.1

at2g07718 CON MIN HOUR DAY WEEK UNIGENE FLCDNA TAIR
AAACGATCGA 1 0 0 0 0 gnl|UG|At#S11739546 no match found AtMg00590

at3g10540 CON MIN HOUR DAY WEEK UNIGENE FLCDNA TAIR
CCTCGGGCCG 0 1 1 1 1 gnl|UG|At#S34114199 gi|66792633|gb|BT023428.1 At3g10540.1

at2g41020 CON MIN HOUR DAY WEEK UNIGENE FLCDNA TAIR
TGAATTTTTT 1 4 1 1 0 gnl|UG|At#S18942735 gi|21406186|gb|AY087452.1 At2g41020.1

at1g13730 CON MIN HOUR DAY WEEK UNIGENE FLCDNA TAIR
GACGCTACAC 0 1 0 0 0 gnl|UG|At#S11741662 gi|16648784|gb|AY058169.1 At1g13730.1

at5g45900 CON MIN HOUR DAY WEEK UNIGENE FLCDNA TAIR
GTGCTGGCCC 0 0 0 3 0 gnl|UG|At#S11719137 gi|23297571|gb|AY150456.1 At5g45900.1

at5g61430 CON MIN HOUR DAY WEEK UNIGENE FLCDNA TAIR
TCCATTAATT 0 0 1 0 0 gnl|UG|At#S11717559 gi|21403812|gb|AY085102.1 At5g61430.1

at3g48100 CON MIN HOUR DAY WEEK UNIGENE FLCDNA TAIR
AGAGATATGT 1 1 3 1 0 no match found gi|18252910|gb|AY072390.1 non-canonical match

at5g17280 CON MIN HOUR DAY WEEK UNIGENE FLCDNA TAIR
GCGACGACCG 0 3 2 1 0 gnl|UG|At#S35277899 gi|17979261|gb|AY070482.1 At5g17280.1

at1g03430 CON MIN HOUR DAY WEEK UNIGENE FLCDNA TAIR
TCCATATGTA 1 0 0 0 0 gnl|UG|At#S21737314 gi|51968863|dbj|AK175361.1| At1g03430.1

at3g18773 CON MIN HOUR DAY WEEK UNIGENE FLCDNA TAIR
CCAAAAAGTC 0 0 1 0 0 gnl|UG|At#S15460898 gi|94442514|gb|BT025292.1 At3g18773.1

at5g42140 CON MIN HOUR DAY WEEK UNIGENE FLCDNA TAIR
ATTGTTTAAA 0 0 0 0 1 gnl|UG|At#S11719515 no match found At5g42140.1

at2g40420 CON MIN HOUR DAY WEEK UNIGENE FLCDNA TAIR
TCTATTGTAT 0 0 0 0 1 gnl|UG|At#S11732489 gi|110737319|dbj|AK228702.1| At2g40420.1

at4g28860 CON MIN HOUR DAY WEEK UNIGENE FLCDNA TAIR
AAGATTATGT 2 2 1 2 0 gnl|UG|At#S11722816 gi|51971352|dbj|AK176578.1| At4g28860.1

at1g06410 CON MIN HOUR DAY WEEK UNIGENE FLCDNA TAIR
CTTGAATCTC 2 1 0 0 0 gnl|UG|At#S11742387 gi|110737794|dbj|AK228947.1| At1g06410.1

at4g00230 CON MIN HOUR DAY WEEK UNIGENE FLCDNA TAIR
GACGACATCT 1 0 1 0 0 gnl|UG|At#S11701276 gi|20258809|gb|AY090934.1 At4g00230.1

at4g20400 CON MIN HOUR DAY WEEK UNIGENE FLCDNA TAIR
TCTTTCCTTT 2 0 1 0 1 gnl|UG|At#S11724266 gi|27311760|gb|BT002486.1 At4g20400.1

at5g08415 CON MIN HOUR DAY WEEK UNIGENE FLCDNA TAIR
CAAAGATCAG 1 0 0 1 0 gnl|UG|At#S11724010 gi|98960966|gb|BT025549.1 At5g08415.1

at1g64780 CON MIN HOUR DAY WEEK UNIGENE FLCDNA TAIR

ATCAGTATGT	11	0	3	2	1	gnl|UG|At#S11730178	gi|17064989|gb|AY062571.1	At1g64780.1
TTGGACGCAT	0	0	1	0	0	no match found	gi|20260051|gb|AY093374.1	non-canonical match
at1g77480	CON	MIN	HOUR	DAY	WEEK	UNIGENE	FLCDNA	TAIR
GCTGGACCAG	0	1	1	0	0	gnl|UG|At#S11727389	gi|24899794|gb|BT001225.1	At1g77480.2
at2g22840	CON	MIN	HOUR	DAY	WEEK	UNIGENE	FLCDNA	TAIR
GGAGGTCCAC	0	0	3	1	1	gnl|UG|At#S11812778	no match found	At2g22840.1
at3g10400	CON	MIN	HOUR	DAY	WEEK	UNIGENE	FLCDNA	TAIR
TTTGATGACT	0	1	0	0	0	gnl|UG|At#S34117619	gi|110737294|dbj|AK228689.1|	At3g10400.1
at4g03290	CON	MIN	HOUR	DAY	WEEK	UNIGENE	FLCDNA	TAIR
ATCATAGACT	1	0	0	0	0	gnl|UG|At#S20824919	no match found	multiple non-canonical match
at5g57330	CON	MIN	HOUR	DAY	WEEK	UNIGENE	FLCDNA	TAIR
CCTGGTCTAG	1	7	4	5	2	gnl|UG|At#S11717980	gi|20260439|gb|AY093119.1	At5g57330.1
at5g67230	CON	MIN	HOUR	DAY	WEEK	UNIGENE	FLCDNA	TAIR
ACCGAGACTT	0	0	1	0	0	no match found	no match found	At5g67230.1
GTGGAGCCAC	1	0	0	0	0	gnl|UG|At#S11709903	gi|17979150|gb|AY070076.1	non-canonical match
at1g25550	CON	MIN	HOUR	DAY	WEEK	UNIGENE	FLCDNA	TAIR
TTGCTACGCC	0	5	0	2	0	gnl|UG|At#S11740320	gi|12642893|gb|AF339707.1	At1g25550.1
at5g58920	CON	MIN	HOUR	DAY	WEEK	UNIGENE	FLCDNA	TAIR
TTTTGGCTTA	2	0	0	1	1	gnl|UG|At#S11717813	gi|21405762|gb|AY087038.1	At5g58920.1
at3g56430	CON	MIN	HOUR	DAY	WEEK	UNIGENE	FLCDNA	TAIR
TTTGGAATCG	0	1	0	0	0	gnl|UG|At#S11728252	gi|114213520|gb|BT028968.1	At3g56430.1
at3g25100	CON	MIN	HOUR	DAY	WEEK	UNIGENE	FLCDNA	TAIR
TAACTTGTTT	0	0	0	0	1	gnl|UG|At#S11733247	no match found	At3g25100.1
at5g36790	CON	MIN	HOUR	DAY	WEEK	UNIGENE	FLCDNA	TAIR
GTTGGTGATA	4	6	5	1	1	no match found	gi|20453152|gb|AY094446.1	At5g36790.1
at4g17960	CON	MIN	HOUR	DAY	WEEK	UNIGENE	FLCDNA	TAIR
AGTTTTTGGA	0	2	0	0	0	no match found	gi|21403876|gb|AY085166.1	pseudo chromosome match
at1g67365	CON	MIN	HOUR	DAY	WEEK	UNIGENE	FLCDNA	TAIR
TAGAGCATTA	0	0	0	0	1	gnl|UG|At#S18897321	no match found	pseudo chromosome match
TTTTCTGTCG	0	0	1	0	0	no match found	gi|110737007|dbj|AK228537.1|	pseudo chromosome match
at3g06730	CON	MIN	HOUR	DAY	WEEK	UNIGENE	FLCDNA	TAIR
CAGGTTCGAG	0	6	1	1	0	no match found	gi|13878104|gb|AF370315.1	non-canonical match
at1g80840	CON	MIN	HOUR	DAY	WEEK	UNIGENE	FLCDNA	TAIR
TAATCGTTTT	2	8	6	0	1	gnl|UG|At#S11806602	gi|21407169|gb|AY088395.1	At1g80840.1
at5g04420	CON	MIN	HOUR	DAY	WEEK	UNIGENE	FLCDNA	TAIR
AGGCTAAAAC	0	2	0	0	0	no match found	gi|63003777|gb|BT022006.1	non-canonical match
TTTAGACCAA	0	1	0	0	1	gnl|UG|At#S28281854	gi|15724205|gb|AF412043.1	multiple non-canonical match
at4g10170	CON	MIN	HOUR	DAY	WEEK	UNIGENE	FLCDNA	TAIR
TGATTGAGCT	0	2	0	0	0	gnl|UG|At#S34114518	gi|110743212|dbj|AK227497.1|	At4g10170.1
at1g02940	CON	MIN	HOUR	DAY	WEEK	UNIGENE	FLCDNA	TAIR
TGATGTGAAG	0	0	2	0	0	gnl|UG|At#S11742821	no match found	multiple non-canonical match
at3g63430	CON	MIN	HOUR	DAY	WEEK	UNIGENE	FLCDNA	TAIR

CGAGAGTGAT	0	1	0	0	0	gnl|UG|At#S11726375	no match found	At3g63430.1
at2g39350	CON	MIN	HOUR	DAY	WEEK	UNIGENE	FLCDNA	TAIR
TCATTGGTGA	0	1	1	0	0	gnl|UG|At#S11732761	gi|26449769|dbj|AK117338.1|	multiple non-canonical match
at1g19640	CON	MIN	HOUR	DAY	WEEK	UNIGENE	FLCDNA	TAIR
ATTTGGTAGT	0	0	1	0	0	no match found	gi|110736897|dbj|AK228480.1|	non-canonical match
at4g19450	CON	MIN	HOUR	DAY	WEEK	UNIGENE	FLCDNA	TAIR
GTTTCACCGG	1	2	2	1	1	gnl|UG|At#S11724426	gi|24030180|gb|BT000872.1	At4g19450.1
at3g15430	CON	MIN	HOUR	DAY	WEEK	UNIGENE	FLCDNA	TAIR
TTTGTATGAA	1	1	0	0	0	no match found	gi|14334415|gb|AY034899.1	non-canonical match
AGTTAATTAA	0	0	1	0	0	gnl|UG|At#S18942088	no match found	At3g15430.1
at1g75030	CON	MIN	HOUR	DAY	WEEK	UNIGENE	FLCDNA	TAIR
TTTAAGAACG	0	0	0	1	0	gnl|UG|At#S11700194	gi|124301077|gb|BT030053.1	At1g75030.1
at2g32720	CON	MIN	HOUR	DAY	WEEK	UNIGENE	FLCDNA	TAIR
ACTGTTGGAT	1	1	0	0	0	gnl|UG|At#S11734355	gi|110743052|dbj|AK227415.1|	At2g32720.1
at3g14620	CON	MIN	HOUR	DAY	WEEK	UNIGENE	FLCDNA	TAIR
AACACGTGAT	5	0	1	0	0	gnl|UG|At#S11736464	gi|15529168|gb|AY052208.1	At3g14620.1
at1g15260	CON	MIN	HOUR	DAY	WEEK	UNIGENE	FLCDNA	TAIR
TATTACGAGT	1	3	2	0	0	gnl|UG|At#S11741509	gi|21405716|gb|AY086992.1	At1g15260.1
at2g37195	CON	MIN	HOUR	DAY	WEEK	UNIGENE	FLCDNA	TAIR
GAAATTAACT	0	0	1	0	0	gnl|UG|At#S34114325	gi|110743679|dbj|AK227690.1|	At2g37195.1
at4g35530	CON	MIN	HOUR	DAY	WEEK	UNIGENE	FLCDNA	TAIR
ATGTATCTAG	0	1	0	0	0	gnl|UG|At#S11721679	gi|18253010|gb|AY072440.1	At4g35530.1
at4g32940	CON	MIN	HOUR	DAY	WEEK	UNIGENE	FLCDNA	TAIR
TACAGAAGCT	7	9	7	2	5	gnl|UG|At#S34116150	gi|15983488|gb|AF424619.1	non-canonical match
GATGGTTTAC	1	6	2	3	4	no match found	no match found	At4g32940.1
AGGTCTTTTG	0	1	0	0	0	no match found	gi|22137031|gb|AY133531.1	non-canonical match
at2g26170	CON	MIN	HOUR	DAY	WEEK	UNIGENE	FLCDNA	TAIR
GGTTTGGTTA	0	2	0	0	0	gnl|UG|At#S15461138	gi|15810029|gb|AY054283.1	At3g55940.1
at1g08920	CON	MIN	HOUR	DAY	WEEK	UNIGENE	FLCDNA	TAIR
CTAGAGTGGA	0	2	1	0	0	gnl|UG|At#S15461373	gi|22137063|gb|AY133547.1	At1g08920.1
at3g50690	CON	MIN	HOUR	DAY	WEEK	UNIGENE	FLCDNA	TAIR
ATGCTGTGGG	2	3	1	0	0	gnl|UG|At#S11729308	no match found	At3g50690.1
at1g64770	CON	MIN	HOUR	DAY	WEEK	UNIGENE	FLCDNA	TAIR
TTAATGACTA	10	2	2	2	8	gnl|UG|At#S11730180	gi|21404638|gb|AY085928.1	At1g64770.1
at1g22810	CON	MIN	HOUR	DAY	WEEK	UNIGENE	FLCDNA	TAIR
GGCGAAAGCA	1	0	1	0	0	no match found	no match found	At1g22810.1
at2g38465	CON	MIN	HOUR	DAY	WEEK	UNIGENE	FLCDNA	TAIR
CCAGGAAGGG	0	0	0	1	2	gnl|UG|At#S16345907	gi|21403704|gb|AY084994.1	At2g38465.1
at2g25150	CON	MIN	HOUR	DAY	WEEK	UNIGENE	FLCDNA	TAIR
AAGTTGCCAC	1	0	0	0	0	no match found	gi|32441257|gb|BT009686.1	non-canonical match
at3g56110	CON	MIN	HOUR	DAY	WEEK	UNIGENE	FLCDNA	TAIR
GCGAGACCTG	0	2	0	0	0	gnl|UG|At#S11728311	gi|15450977|gb|AY054569.1	At3g56110.1

at3g11950	CON	MIN	HOUR	DAY	WEEK	UNIGENE	FLCDNA	TAIR
TACATACATC	2	1	0	0	2	no match found	no match found	At3g11950.1
GAGTGTATGT	0	1	0	1	0	gnl|UG|At#S34115399	gi|110741562|dbj|AK226618.1|	non-canonical match
at5g59320	CON	MIN	HOUR	DAY	WEEK	UNIGENE	FLCDNA	TAIR
TATTTGTTTT	4	0	3	7	6	no match found	gi|21407330|gb|AY088556.1	At5g59320.1
at1g69680	CON	MIN	HOUR	DAY	WEEK	UNIGENE	FLCDNA	TAIR
ATCTTATTTC	0	2	0	0	0	gnl|UG|At#S11729166	gi|14335133|gb|AY037246.1	At1g69680.1
at4g25190	CON	MIN	HOUR	DAY	WEEK	UNIGENE	FLCDNA	TAIR
TGTTAATGTG	0	0	1	0	0	gnl|UG|At#S43849493	no match found	multiple non-canonical match
AAATCTAAAA	1	0	0	1	0	no match found	no match found	At4g25190.1
at4g35360	CON	MIN	HOUR	DAY	WEEK	UNIGENE	FLCDNA	TAIR
GGCCGTGGAA	0	0	1	2	0	gnl|UG|At#S11721706	gi|17063178|gb|AY062112.1	At4g35360.1
at1g75100	CON	MIN	HOUR	DAY	WEEK	UNIGENE	FLCDNA	TAIR
GGACCACTTC	1	2	1	2	0	gnl|UG|At#S11728022	gi|21655286|gb|AY103303.1	At1g75100.1
at5g58320	CON	MIN	HOUR	DAY	WEEK	UNIGENE	FLCDNA	TAIR
AGATCAGGGA	1	1	0	0	0	gnl|UG|At#S34114317	gi|110743695|dbj|AK227698.1|	At5g58320.2
TAAATAAGAA	0	0	1	1	0	no match found	no match found	At5g58320.1
at2g38740	CON	MIN	HOUR	DAY	WEEK	UNIGENE	FLCDNA	TAIR
CACAATGCTA	2	0	1	0	3	gnl|UG|At#S18901174	gi|15912256|gb|AY056406.1	multiple non-canonical match
at3g44520	CON	MIN	HOUR	DAY	WEEK	UNIGENE	FLCDNA	TAIR
TTGCAAAAAA	10	1	5	8	4	no match found	no match found	At3g44520.1
at2g22510	CON	MIN	HOUR	DAY	WEEK	UNIGENE	FLCDNA	TAIR
ACCACGATAC	1	0	0	0	0	gnl|UG|At#S11736853	no match found	At2g22510.1
at5g58800	CON	MIN	HOUR	DAY	WEEK	UNIGENE	FLCDNA	TAIR
GCAAGTACTT	0	2	0	1	0	gnl|UG|At#S28281589	gi|17381197|gb|AY064055.1	At5g58800.1
at2g46880	CON	MIN	HOUR	DAY	WEEK	UNIGENE	FLCDNA	TAIR
GGTACGGACA	1	0	0	0	0	no match found	no match found	At2g46880.2
at5g15210	CON	MIN	HOUR	DAY	WEEK	UNIGENE	FLCDNA	TAIR
CTTCTCGCAC	0	1	0	0	0	gnl|UG|At#S11722539	gi|45773755|gb|BT012195.1	At5g15210.1
at1g31230	CON	MIN	HOUR	DAY	WEEK	UNIGENE	FLCDNA	TAIR
CTAGCATATG	3	0	0	0	1	gnl|UG|At#S11738581	gi|26452574|dbj|AK118779.1|	non-canonical match
at1g63270	CON	MIN	HOUR	DAY	WEEK	UNIGENE	FLCDNA	TAIR
ACGGTGGTGC	0	1	0	0	0	gnl|UG|At#S11730488	no match found	non-canonical match
AGCTGTTTTT	0	0	0	1	0	no match found	no match found	At1g63270.1
at5g66055	CON	MIN	HOUR	DAY	WEEK	UNIGENE	FLCDNA	TAIR
TGGAGAGTTT	0	0	0	1	0	no match found	gi|24111370|gb|BT001055.1	At5g66055.2
at5g43700	CON	MIN	HOUR	DAY	WEEK	UNIGENE	FLCDNA	TAIR
AAAGGATCTG	2	6	1	0	0	gnl|UG|At#S11719359	gi|29028817|gb|BT005853.1	At5g43700.1
at1g54090	CON	MIN	HOUR	DAY	WEEK	UNIGENE	FLCDNA	TAIR
TGGTGGCAGT	1	0	2	1	0	gnl|UG|At#S11806708	gi|20260593|gb|AY093196.1	At1g54090.1
at3g06960	CON	MIN	HOUR	DAY	WEEK	UNIGENE	FLCDNA	TAIR
GTTGAGCTTC	0	1	2	1	0	gnl|UG|At#S18942167	gi|14194138|gb|AF367275.1	At3g06960.2

at3g22780	CON	MIN	HOUR	DAY	WEEK	UNIGENE	FLCDNA	TAIR
GAAAGCAAAC	0	0	0	1	0	gnl|UG|At#S11701219	gi|23297129|gb|AY142642.1	multiple non-canonical match
at4g27940	CON	MIN	HOUR	DAY	WEEK	UNIGENE	FLCDNA	TAIR
GATCTGTGGA	0	0	1	0	1	gnl|UG|At#S11722971	gi|16226566|gb|AF428433.1	At4g27940.1
at4g15415	CON	MIN	HOUR	DAY	WEEK	UNIGENE	FLCDNA	TAIR
GGTTCAATTG	1	1	0	1	1	gnl|UG|At#S15461116	gi|20147342|gb|AY093761.1	At4g15415.1
at3g54640	CON	MIN	HOUR	DAY	WEEK	UNIGENE	FLCDNA	TAIR
TGAAACAGAT	0	3	0	2	2	gnl|UG|At#S11728577	gi|108385366|gb|BT025875.1	At3g54640.1
at5g29562	CON	MIN	HOUR	DAY	WEEK	UNIGENE	FLCDNA	TAIR
GGAAGATGAA	0	0	1	0	0	no match found	no match found	At5g29562.1
at4g17730	CON	MIN	HOUR	DAY	WEEK	UNIGENE	FLCDNA	TAIR
GACAAGTGCT	2	3	0	0	0	gnl|UG|At#S11724722	gi|23397134|gb|BT000708.1	At4g17730.1
at2g31230	CON	MIN	HOUR	DAY	WEEK	UNIGENE	FLCDNA	TAIR
TTGATCTTGT	1	1	0	0	1	no match found	gi|17979407|gb|AY070443.1	At2g31230.1
AACAAATAAA	1	0	0	0	0	gnl|UG|At#S26111501	no match found	non-canonical match
at4g14030	CON	MIN	HOUR	DAY	WEEK	UNIGENE	FLCDNA	TAIR
GAGAAAGGCT	3	20	15	5	0	no match found	no match found	At4g14030.1
at4g13980	CON	MIN	HOUR	DAY	WEEK	UNIGENE	FLCDNA	TAIR
TCATTTAAAT	0	1	0	0	0	gnl|UG|At#S11725391	gi|62321095|dbj|AK221877.1|	At4g13980.1
at1g72330	CON	MIN	HOUR	DAY	WEEK	UNIGENE	FLCDNA	TAIR
TCATATGAAG	0	2	0	0	2	gnl|UG|At#S11728617	gi|14334825|gb|AY035086.1	At1g72330.1
at5g11710	CON	MIN	HOUR	DAY	WEEK	UNIGENE	FLCDNA	TAIR
GGTGCAGGAT	0	1	1	0	0	no match found	gi|20465544|gb|AY096605.1	At2g07715.1
GGACAATCTG	1	1	4	2	1	gnl|UG|At#S11723319	gi|18377701|gb|AY074304.1	At5g11710.1
at5g51720	CON	MIN	HOUR	DAY	WEEK	UNIGENE	FLCDNA	TAIR
TGAAGCACAA	2	4	5	4	1	gnl|UG|At#S30640788	gi|16323223|gb|AY057716.1	At5g51720.1
at1g69160	CON	MIN	HOUR	DAY	WEEK	UNIGENE	FLCDNA	TAIR
GTTTTTTTTG	6	2	2	1	2	gnl|UG|At#S11729273	gi|20260557|gb|AY093178.1	At1g69160.1
AGAAGCACGA	0	1	0	0	0	no match found	gi|28059381|gb|BT003390.1	non-canonical match
at3g52560	CON	MIN	HOUR	DAY	WEEK	UNIGENE	FLCDNA	TAIR
TGTTCAATCT	5	6	3	4	5	gnl|UG|At#S38433614	gi|14596116|gb|AY042846.1	At3g52560.1
at1g56045	CON	MIN	HOUR	DAY	WEEK	UNIGENE	FLCDNA	TAIR
AGGAGATTAA	14	16	18	21	15	no match found	gi|51971578|dbj|AK176691.1|	At1g56045.1
at5g65710	CON	MIN	HOUR	DAY	WEEK	UNIGENE	FLCDNA	TAIR
AGGAAAGTAG	1	0	0	0	0	gnl|UG|At#S11717127	no match found	non-canonical match
at2g46505	CON	MIN	HOUR	DAY	WEEK	UNIGENE	FLCDNA	TAIR
AAGGAATGGA	1	7	0	2	3	gnl|UG|At#S11731071	gi|15450444|gb|AY052323.1	At2g46505.1
at3g51500	CON	MIN	HOUR	DAY	WEEK	UNIGENE	FLCDNA	TAIR
CGATTTCACA	0	0	0	0	1	gnl|UG|At#S34114739	gi|110742794|dbj|AK227276.1|	At3g51500.1
at3g60590	CON	MIN	HOUR	DAY	WEEK	UNIGENE	FLCDNA	TAIR
TCAGAAAGAA	0	3	0	0	0	no match found	no match found	At3g60590.3
TCAAAGATTT	1	3	0	0	0	gnl|UG|At#S15459165	gi|27311682|gb|BT002447.1	At3g60590.1

at3g53630	CON	MIN	HOUR	DAY	WEEK	UNIGENE	FLCDNA	TAIR
TTTGTTTATT	2	2	1	2	3	gnl|UG|At#S11728758	gi|18377876|gb|AY074586.1	At3g53630.1

at1g01830	CON	MIN	HOUR	DAY	WEEK	UNIGENE	FLCDNA	TAIR
TACTCAAGTC	0	0	0	0	1	no match found	gi|20258992|gb|AY091273.1	At1g01830.1

at3g58500	CON	MIN	HOUR	DAY	WEEK	UNIGENE	FLCDNA	TAIR
TGTTTGGCTG	5	6	3	8	3	gnl|UG|At#S11706047	gi|15810366|gb|AY056222.1	At3g58500.1
GCCTCAATTC	0	2	0	0	0	no match found	gi|21360430|gb|AY113023.1	non-canonical match

at1g61180	CON	MIN	HOUR	DAY	WEEK	UNIGENE	FLCDNA	TAIR
AGATATTGTC	1	0	0	0	0	gnl|UG|At#S20795789	no match found	multiple non-canonical match
GATGTTTCAA	1	0	1	1	0	no match found	gi|15450875|gb|AY054518.1	At1g61180.1

at4g36750	CON	MIN	HOUR	DAY	WEEK	UNIGENE	FLCDNA	TAIR
GCTGCAATAG	0	1	0	0	0	no match found	gi|23505772|gb|AY143807.1	non-canonical match
TTTCTTTATA	0	1	1	0	0	gnl|UG|At#S11721478	gi|15529267|gb|AY052258.1	At4g36750.1

at4g15810	CON	MIN	HOUR	DAY	WEEK	UNIGENE	FLCDNA	TAIR
TCTGTTTTAG	0	0	0	0	1	gnl|UG|At#S11725063	no match found	non-canonical match

at1g19270	CON	MIN	HOUR	DAY	WEEK	UNIGENE	FLCDNA	TAIR
CGTGGATGAG	0	0	0	1	0	gnl|UG|At#S11741124	gi|110742546|dbj|AK227143.1|	At1g19270.1

at5g20060	CON	MIN	HOUR	DAY	WEEK	UNIGENE	FLCDNA	TAIR
GTTGACATCC	2	3	1	0	2	gnl|UG|At#S18941597	gi|23306355|gb|BT000428.1	At5g20060.1

at5g35490	CON	MIN	HOUR	DAY	WEEK	UNIGENE	FLCDNA	TAIR
CCTATTTTGG	1	0	0	0	0	gnl|UG|At#S11720154	no match found	At5g35490.1

at4g32175	CON	MIN	HOUR	DAY	WEEK	UNIGENE	FLCDNA	TAIR
ATGCTCTTTA	0	1	0	0	1	gnl|UG|At#S24442391	gi|62320888|dbj|AK221772.1|	multiple non-canonical match

at3g19130	CON	MIN	HOUR	DAY	WEEK	UNIGENE	FLCDNA	TAIR
GTTTCACATA	0	3	0	1	1	gnl|UG|At#S11735108	gi|110741039|dbj|AK226472.1|	At3g19130.1

at1g03400	CON	MIN	HOUR	DAY	WEEK	UNIGENE	FLCDNA	TAIR
ATATAATACG	2	0	0	0	1	gnl|UG|At#S11742774	gi|15146295|gb|AY049289.1	At1g03400.1

at5g66520	CON	MIN	HOUR	DAY	WEEK	UNIGENE	FLCDNA	TAIR
AGAAGGAAAC	0	0	1	1	0	gnl|UG|At#S11717045	no match found	multiple non-canonical match

at3g30530	CON	MIN	HOUR	DAY	WEEK	UNIGENE	FLCDNA	TAIR
AGCCTAAGCA	0	1	0	0	0	gnl|UG|At#S26111679	no match found	non-canonical match

at2g21650	CON	MIN	HOUR	DAY	WEEK	UNIGENE	FLCDNA	TAIR
AAGCTGCAGT	1	0	0	0	1	no match found	gi|30793856|gb|BT008554.1	multiple non-canonical match

at2g39870	CON	MIN	HOUR	DAY	WEEK	UNIGENE	FLCDNA	TAIR
TTGGCGAGAA	1	5	2	1	0	gnl|UG|At#S11732636	gi|18086440|gb|AY065038.1	At2g39870.1

at1g05410	CON	MIN	HOUR	DAY	WEEK	UNIGENE	FLCDNA	TAIR
AATCGAAACC	2	5	2	2	1	no match found	gi|18086399|gb|AY065014.1	At3g59780.1
CTCAGAAAGA	1	0	0	2	1	no match found	gi|18491166|gb|AY074502.1	At1g05410.1

at1g68060	CON	MIN	HOUR	DAY	WEEK	UNIGENE	FLCDNA	TAIR
AAAAGGATCA	0	2	0	1	1	gnl|UG|At#S11729494	gi|20465646|gb|AY096658.1	At1g68060.1

at3g15355	CON	MIN	HOUR	DAY	WEEK	UNIGENE	FLCDNA	TAIR
AGCTCTCTGA	0	1	0	0	0	no match found	gi|16323333|gb|AY059155.1	multiple non-canonical match

GATCCTCTTT	0	0	1	1	0	gnl|UG|At#S11702959	gi|13605650|gb|AF361806.1	At3g15355.1
at3g05060	CON	MIN	HOUR	DAY	WEEK	UNIGENE	FLCDNA	TAIR
GAGGTAGTTT	0	3	0	1	1	no match found	gi|16974594|gb|AY060573.1	multiple non-canonical match
at4g03320	CON	MIN	HOUR	DAY	WEEK	UNIGENE	FLCDNA	TAIR
ATTTGAAAGT	0	0	1	0	0	no match found	gi|13605614|gb|AF361633.1	multiple non-canonical match
TGTAACACAG	0	0	1	0	0	no match found	gi|21403253|gb|AY084543.1	non-canonical match
at1g72680	CON	MIN	HOUR	DAY	WEEK	UNIGENE	FLCDNA	TAIR
TCTCTCTTGA	0	1	1	1	0	gnl|UG|At#S11728534	gi|31711845|gb|BT008840.1	At1g72680.1
at1g34020	CON	MIN	HOUR	DAY	WEEK	UNIGENE	FLCDNA	TAIR
AAGACGATCC	0	0	1	1	0	gnl|UG|At#S11737570	gi|21407309|gb|AY088535.1	At1g34020.1
at4g32420	CON	MIN	HOUR	DAY	WEEK	UNIGENE	FLCDNA	TAIR
TCAGAAGGAG	1	1	0	0	0	gnl|UG|At#S11722206	gi|20259448|gb|AY091023.1	At4g32420.1
at4g39960	CON	MIN	HOUR	DAY	WEEK	UNIGENE	FLCDNA	TAIR
CTCAGGAGAT	1	1	1	0	3	gnl|UG|At#S11720929	gi|15450580|gb|AY052658.1	At4g39960.1
at4g37040	CON	MIN	HOUR	DAY	WEEK	UNIGENE	FLCDNA	TAIR
ATCTTGCAGA	0	0	1	0	0	no match found	gi|26451667|dbj|AK118314.1|	multiple canonical match
at1g22882	CON	MIN	HOUR	DAY	WEEK	UNIGENE	FLCDNA	TAIR
TTACCAATGG	1	0	0	0	2	gnl|UG|At#S11816480	gi|26452062|dbj|AK118518.1|	At1g22882.1
at4g25720	CON	MIN	HOUR	DAY	WEEK	UNIGENE	FLCDNA	TAIR
GGACAGTGAC	2	0	0	1	1	gnl|UG|At#S35294068	gi|17529255|gb|AY065414.1	At4g25720.1
at2g19480	CON	MIN	HOUR	DAY	WEEK	UNIGENE	FLCDNA	TAIR
GTCCGGTTTA	11	13	13	16	10	gnl|UG|At#S11737607	gi|20466609|gb|AY099771.1	At2g19480.1
at4g38545	CON	MIN	HOUR	DAY	WEEK	UNIGENE	FLCDNA	TAIR
ATGATTCTCA	1	3	0	1	0	no match found	gi|14335041|gb|AY037200.1	At4g38540.1
GTGCGTTGAT	1	0	0	0	0	gnl|UG|At#S18910551	gi|72198641|gb|DQ108893.1	At4g38540.1
at1g17050	CON	MIN	HOUR	DAY	WEEK	UNIGENE	FLCDNA	TAIR
CTTATTATTG	0	0	0	4	0	gnl|UG|At#S11741340	no match found	multiple non-canonical match
at5g50920	CON	MIN	HOUR	DAY	WEEK	UNIGENE	FLCDNA	TAIR
CTTTTTAAGG	45	25	40	30	26	no match found	gi|62319203|dbj|AK220924.1|	non-canonical match
CAAAGAGGAG	10	0	13	8	6	gnl|UG|At#S11718624	no match found	At5g50920.1
at3g04920	CON	MIN	HOUR	DAY	WEEK	UNIGENE	FLCDNA	TAIR
ACCAACAGAC	1	1	1	0	0	no match found	gi|20334887|gb|AY094044.1	non-canonical match
TTTACTGAAA	8	1	3	1	5	gnl|UG|At#S11739248	gi|15215671|gb|AY050364.1	At3g04920.1
at4g25500	CON	MIN	HOUR	DAY	WEEK	UNIGENE	FLCDNA	TAIR
AGACCTTCCA	0	1	3	1	0	gnl|UG|At#S38433439	gi|23270365|gb|AY046008.2	At4g25500.2
at4g37450	CON	MIN	HOUR	DAY	WEEK	UNIGENE	FLCDNA	TAIR
GGCCGTTCTC	2	6	1	3	0	no match found	gi|20334855|gb|AY094028.1	At4g37450.1
at5g02410	CON	MIN	HOUR	DAY	WEEK	UNIGENE	FLCDNA	TAIR
ATGTTTTCTG	0	0	1	1	0	gnl|UG|At#S11725557	gi|20466148|gb|AY099540.1	At5g02410.1
at3g46310	CON	MIN	HOUR	DAY	WEEK	UNIGENE	FLCDNA	TAIR
GAAATGTTTG	0	0	1	0	1	gnl|UG|At#S11730130	no match found	non-canonical match
at5g21100	CON	MIN	HOUR	DAY	WEEK	UNIGENE	FLCDNA	TAIR

ATTTCTGGGT	0	1	0	0	0	gnl|UG|At#S11721060	gi|28059565|gb|BT003407.1	At5g21100.1
at1g04950	CON	MIN	HOUR	DAY	WEEK	UNIGENE	FLCDNA	TAIR
AAATAATTTC	3	2	0	0	1	no match found	gi|15293056|gb|AY050962.1	non-canonical match
TATGCTCTCT	0	0	0	0	2	gnl|UG|At#S28282710	no match found	At1g04950.1
at5g20250	CON	MIN	HOUR	DAY	WEEK	UNIGENE	FLCDNA	TAIR
TACAATTCGG	7	41	5	3	0	gnl|UG|At#S28281777	gi|19699066|gb|AY090237.1	At5g20250.2
at4g16860	CON	MIN	HOUR	DAY	WEEK	UNIGENE	FLCDNA	TAIR
TTTGTAATTA	1	2	0	0	2	gnl|UG|At#S11724877	gi|21539520|gb|AY120755.1	At4g16860.1
CGAGGACAAA	0	2	1	0	0	no match found	gi|26453194|dbj|AK119099.1|	multiple non-canonical match
at3g09890	CON	MIN	HOUR	DAY	WEEK	UNIGENE	FLCDNA	TAIR
AAATTATTAT	0	0	0	2	0	gnl|UG|At#S43849940	gi|110743882|dbj|AK227794.1|	At3g09890.1
at5g56170	CON	MIN	HOUR	DAY	WEEK	UNIGENE	FLCDNA	TAIR
TCCTGTGAAC	3	0	2	3	0	gnl|UG|At#S11718096	gi|23306373|gb|BT000437.1	At5g56170.1
at2g05250	CON	MIN	HOUR	DAY	WEEK	UNIGENE	FLCDNA	TAIR
TTAACATCTT	0	1	0	0	0	gnl|UG|At#S11740102	gi|15983798|gb|AY056805.1	multiple canonical match
at1g06040	CON	MIN	HOUR	DAY	WEEK	UNIGENE	FLCDNA	TAIR
TTCATTCATA	1	0	0	0	0	no match found	gi|19310754|gb|AY079377.1	non-canonical match
TGTGGAGTTA	26	9	31	23	10	no match found	gi|15027874|gb|AY045794.1	multiple non-canonical match
GTCTATGCTT	12	3	19	18	17	gnl|UG|At#S15461535	gi|21407932|gb|AY089158.1	At1g06040.2
at5g12020	CON	MIN	HOUR	DAY	WEEK	UNIGENE	FLCDNA	TAIR
ACGGTGTGTT	0	0	1	0	0	gnl|UG|At#S11823018	gi|26452717|dbj|AK118853.1|	At5g12020.1
at5g08380	CON	MIN	HOUR	DAY	WEEK	UNIGENE	FLCDNA	TAIR
TGTATAGAGA	0	1	1	0	5	gnl|UG|At#S11724016	gi|20260601|gb|AY093200.1	multiple canonical match
at5g20920	CON	MIN	HOUR	DAY	WEEK	UNIGENE	FLCDNA	TAIR
TTATGCAATA	1	0	0	0	0	no match found	gi|23197695|gb|BT000056.1	multiple non-canonical match
GACTTCTTTT	3	4	7	8	6	gnl|UG|At#S38433277	no match found	At5g20920.2
at3g57660	CON	MIN	HOUR	DAY	WEEK	UNIGENE	FLCDNA	TAIR
GAGACAATCC	0	0	0	0	1	gnl|UG|At#S11728017	no match found	non-canonical match
at1g48520	CON	MIN	HOUR	DAY	WEEK	UNIGENE	FLCDNA	TAIR
CAAGATGTGC	2	0	0	1	0	no match found	gi|18377820|gb|AY074557.1	non-canonical match
at1g69580	CON	MIN	HOUR	DAY	WEEK	UNIGENE	FLCDNA	TAIR
GAGGCGTGTA	0	0	1	0	0	gnl|UG|At#S38434086	gi|119360086|gb|BT029516.1	non-canonical match
at2g14280	CON	MIN	HOUR	DAY	WEEK	UNIGENE	FLCDNA	TAIR
CAATTAAAAC	0	1	0	0	0	no match found	no match found	At2g14280.1
at3g09450	CON	MIN	HOUR	DAY	WEEK	UNIGENE	FLCDNA	TAIR
CTACTGAAGG	1	0	1	2	7	gnl|UG|At#S11738050	no match found	non-canonical match
TTGAGTCTGA	0	1	0	0	0	no match found	no match found	At3g09450.1
at5g66005	CON	MIN	HOUR	DAY	WEEK	UNIGENE	FLCDNA	TAIR
TTTTGTTGTC	0	1	0	0	0	gnl|UG|At#S18941414	gi|62321771|dbj|AK222224.1|	At5g66005.2
at1g74290	CON	MIN	HOUR	DAY	WEEK	UNIGENE	FLCDNA	TAIR
AGGTTCCGAG	0	1	1	0	0	gnl|UG|At#S11728180	no match found	At1g74290.1
at4g38430	CON	MIN	HOUR	DAY	WEEK	UNIGENE	FLCDNA	TAIR
GTCGTATGCA	4	0	1	2	1	gnl|UG|At#S11721196	gi|15912194|gb|AY056375.1	At4g38430.1

at4g39940	CON	MIN	HOUR	DAY	WEEK	UNIGENE	FLCDNA	TAIR
TGTGCGAGTC	1	9	1	1	0	gnl|UG|At#S11700471	gi|18087562|gb|AF462823.1	At4g39940.1
at3g24927	CON	MIN	HOUR	DAY	WEEK	UNIGENE	FLCDNA	TAIR
GGGCGTTTCA	1	1	0	4	0	gnl|UG|At#S18904341	gi|19423922|gb|AY080780.1	At3g24927.1
at5g61670	CON	MIN	HOUR	DAY	WEEK	UNIGENE	FLCDNA	TAIR
ATCCTCGTAT	1	0	2	0	0	no match found	gi|23506190|gb|AY149953.1	non-canonical match
at4g11890	CON	MIN	HOUR	DAY	WEEK	UNIGENE	FLCDNA	TAIR
AAGTGATGAG	1	0	0	0	0	gnl|UG|At#S11725749	gi|30017316|gb|BT006243.1	At4g11890.1
at1g11592	CON	MIN	HOUR	DAY	WEEK	UNIGENE	FLCDNA	TAIR
AAAGTCCTCT	1	1	0	0	0	gnl|UG|At#S18913449	gi|72197105|gb|DQ108698.1	At4g11890.1
at1g10690	CON	MIN	HOUR	DAY	WEEK	UNIGENE	FLCDNA	TAIR
TTTATGTATA	1	1	0	0	0	no match found	no match found	At1g10690.1
at2g27250	CON	MIN	HOUR	DAY	WEEK	UNIGENE	FLCDNA	TAIR
ATTTTCCAAA	0	0	1	0	0	gnl|UG|At#S14233018	no match found	multiple non-canonical match
at4g17510	CON	MIN	HOUR	DAY	WEEK	UNIGENE	FLCDNA	TAIR
GTTTAGAGAA	2	0	3	1	2	gnl|UG|At#S11724763	gi|26452815|dbj|AK118904.1|	At4g17510.1
ACTCCTATGG	0	1	0	0	0	no match found	gi|28973166|gb|BT005488.1	non-canonical match
at1g79830	CON	MIN	HOUR	DAY	WEEK	UNIGENE	FLCDNA	TAIR
TATTGTCTGT	3	1	1	0	0	no match found	gi|62318820|dbj|AK220730.1|	At1g79830.1
CTGGTGAACA	0	1	0	0	0	gnl|UG|At#S34115290	gi|110741772|dbj|AK226727.1|	non-canonical match
at1g06450	CON	MIN	HOUR	DAY	WEEK	UNIGENE	FLCDNA	TAIR
GCTTCACTTT	0	2	2	1	2	no match found	gi|26451266|dbj|AK118108.1|	At4g08170.1
at5g23010	CON	MIN	HOUR	DAY	WEEK	UNIGENE	FLCDNA	TAIR
AGAGCGGCAT	1	15	7	5	0	gnl|UG|At#S11709649	gi|15809871|gb|AY054203.1	At5g23010.1
at3g30390	CON	MIN	HOUR	DAY	WEEK	UNIGENE	FLCDNA	TAIR
AGTGTTGTTT	19	23	20	10	10	no match found	gi|110739794|dbj|AK229979.1|	non-canonical match
ACTCTTAGCT	2	0	2	0	2	gnl|UG|At#S28282881	gi|16226266|gb|AF428287.1	At3g30390.1
at4g03600	CON	MIN	HOUR	DAY	WEEK	UNIGENE	FLCDNA	TAIR
GAACCTCCTG	0	1	0	0	0	gnl|UG|At#S11726769	gi|21406584|gb|AY087833.1	At4g03600.1
at3g57550	CON	MIN	HOUR	DAY	WEEK	UNIGENE	FLCDNA	TAIR
ATCTCTTATG	1	2	1	1	0	gnl|UG|At#S11701209	gi|27311720|gb|BT002466.1	At3g57550.1
at1g04920	CON	MIN	HOUR	DAY	WEEK	UNIGENE	FLCDNA	TAIR
TAGATTAATT	0	0	1	0	0	gnl|UG|At#S11744173	gi|19310424|gb|AY078949.1	non-canonical match
at3g58120	CON	MIN	HOUR	DAY	WEEK	UNIGENE	FLCDNA	TAIR
GCGAAAAGAT	1	0	1	0	0	gnl|UG|At#S11727930	gi|20260307|gb|AY093053.1	At3g58120.1
at2g31680	CON	MIN	HOUR	DAY	WEEK	UNIGENE	FLCDNA	TAIR
AAAAACAGAG	0	0	1	0	0	gnl|UG|At#S34114310	gi|21404204|gb|AY085494.1	At2g31680.1
at1g58060	CON	MIN	HOUR	DAY	WEEK	UNIGENE	FLCDNA	TAIR
TATAAGACAC	0	0	0	0	2	gnl|UG|At#S11731865	gi|110743198|dbj|AK227490.1|	At1g58060.1
at5g52340	CON	MIN	HOUR	DAY	WEEK	UNIGENE	FLCDNA	TAIR
GAGTGAGCCA	0	0	0	2	0	gnl|UG|At#S11718482	no match found	multiple non-canonical match

at3g46610 CON MIN HOUR DAY WEEK UNIGENE FLCDNA TAIR
TGAAGGCTCA 0 1 0 2 0 gnl|UG|At#S11730075 no match found At3g46610.1

at3g12380 CON MIN HOUR DAY WEEK UNIGENE FLCDNA TAIR
GCGAGGAGCA 0 2 0 1 0 gnl|UG|At#S11737181 gi|15450489|gb|AY052346.1 At3g12380.1

at4g31420 CON MIN HOUR DAY WEEK UNIGENE FLCDNA TAIR
TACTTTTGAG 2 1 3 6 2 no match found gi|62319189|dbj|AK220917.1| multiple canonical match

at5g28300 CON MIN HOUR DAY WEEK UNIGENE FLCDNA TAIR
TAGATGGAGA 1 2 3 1 0 gnl|UG|At#S11720381 gi|30725451|gb|BT008389.1 At5g28300.1

at1g17420 CON MIN HOUR DAY WEEK UNIGENE FLCDNA TAIR
ATATATAATA 0 1 0 0 0 gnl|UG|At#S11747710 gi|19715629|gb|AY075625.1 At1g17420.1

at2g34590 CON MIN HOUR DAY WEEK UNIGENE FLCDNA TAIR
ATTACTTAGA 0 6 0 0 0 gnl|UG|At#S11701006 gi|21406536|gb|AY087792.1 At2g34590.1

at2g18970 CON MIN HOUR DAY WEEK UNIGENE FLCDNA TAIR
TGATATTTGA 1 0 0 0 0 gnl|UG|At#S11737732 no match found At2g18970.1

at5g16710 CON MIN HOUR DAY WEEK UNIGENE FLCDNA TAIR
GAGGATGTTT 0 0 0 0 1 no match found gi|51969651|dbj|AK175755.1| multiple non-canonical match
GAGAATGTTT 4 6 5 2 2 gnl|UG|At#S11722165 gi|26452228|dbj|AK118603.1| At5g16710.1

at1g30690 CON MIN HOUR DAY WEEK UNIGENE FLCDNA TAIR
TAGTCTCTGG 4 1 6 0 2 gnl|UG|At#S28282561 gi|14334977|gb|AY035162.1 At1g30690.1

at2g28850 CON MIN HOUR DAY WEEK UNIGENE FLCDNA TAIR
TTTCTCTTAA 0 0 0 1 0 no match found no match found At2g28850.1

at5g37850 CON MIN HOUR DAY WEEK UNIGENE FLCDNA TAIR
ACTGCTCTTC 0 1 0 1 0 gnl|UG|At#S38433210 gi|21403129|gb|AY084419.1 At5g37850.1

at5g47890 CON MIN HOUR DAY WEEK UNIGENE FLCDNA TAIR
GAGAGGAAGC 3 4 1 1 0 gnl|UG|At#S17007776 gi|17644164|gb|AY065304.1 At5g47890.1

at1g14460 CON MIN HOUR DAY WEEK UNIGENE FLCDNA TAIR
GAAATCTCCT 1 1 0 0 1 gnl|UG|At#S35282430 no match found multiple non-canonical match
TGCTAGGCCA 0 2 0 1 0 gnl|UG|At#S11741590 no match found At1g14460.1

at3g26483 CON MIN HOUR DAY WEEK UNIGENE FLCDNA TAIR
GAGGAAGAGA 0 0 0 0 1 gnl|UG|At#S34116381 no match found multiple non-canonical match

at1g69340 CON MIN HOUR DAY WEEK UNIGENE FLCDNA TAIR
CTTATCTTCT 1 2 0 1 1 no match found gi|13937138|gb|AF372923.1 multiple canonical match

at5g65760 CON MIN HOUR DAY WEEK UNIGENE FLCDNA TAIR
TAACTATGTA 2 3 1 0 0 gnl|UG|At#S11717122 gi|110736176|dbj|AK228101.1| At5g65760.1

at2g35733 CON MIN HOUR DAY WEEK UNIGENE FLCDNA TAIR
TGAAAGAGAA 0 0 0 0 1 no match found no match found At2g35733.1

at4g26830 CON MIN HOUR DAY WEEK UNIGENE FLCDNA TAIR
GTGCGTTTCG 0 1 0 0 0 gnl|UG|At#S11723157 gi|110740156|dbj|AK230168.1| At4g26830.1

at4g20360 CON MIN HOUR DAY WEEK UNIGENE FLCDNA TAIR
AACGACAAAG 26 32 25 23 16 gnl|UG|At#S20871215 gi|24030502|gb|BT000998.1 At4g20360.1
GCTCTCGCTT 0 0 4 0 0 gnl|UG|At#S35259386 no match found non-canonical match

at5g43745 CON MIN HOUR DAY WEEK UNIGENE FLCDNA TAIR

AAAGAGGGGG	0	0	0	2	0	no match found	gi|20259817|gb|AY093257.1	non-canonical match
TCTCCAACTC	0	0	1	1	0	gnl|UG|At#S11719354	gi|15450497|gb|AY052350.1	At5g43745.1
at2g07775	CON	MIN	HOUR	DAY	WEEK	UNIGENE	FLCDNA	TAIR
CTTCCTTCCT	1	3	0	0	0	no match found	no match found	At2g07775.1
AGCCTGATGA	0	0	1	0	0	gnl|UG|At#S35237285	no match found	non-canonical match
at1g74970	CON	MIN	HOUR	DAY	WEEK	UNIGENE	FLCDNA	TAIR
GAGGCGGTCT	0	1	1	1	0	no match found	gi|20259210|gb|AY091382.1	non-canonical match
TTAATGTTTA	17	7	6	6	11	gnl|UG|At#S11728044	gi|51969143|dbj|AK175501.1|	At1g74970.1
at3g26740	CON	MIN	HOUR	DAY	WEEK	UNIGENE	FLCDNA	TAIR
CGAAGTAATC	0	0	3	0	0	no match found	gi|16974526|gb|AY060542.1	non-canonical match
TGAATATGTG	38	79	27	6	6	gnl|UG|At#S11703229	gi|21406859|gb|AY088085.1	multiple canonical match
at4g32150	CON	MIN	HOUR	DAY	WEEK	UNIGENE	FLCDNA	TAIR
TTCTATAAAT	3	6	1	3	4	gnl|UG|At#S11722250	gi|110740909|dbj|AK226405.1|	At4g32150.1
at5g43980	CON	MIN	HOUR	DAY	WEEK	UNIGENE	FLCDNA	TAIR
TCCAATTGTA	0	0	1	0	0	gnl|UG|At#S11823817	gi|26451088|dbj|AK118016.1|	At5g43980.1
at5g14140	CON	MIN	HOUR	DAY	WEEK	UNIGENE	FLCDNA	TAIR
GTCTTGTAGT	1	0	0	0	0	no match found	gi|110736662|dbj|AK228356.1|	non-canonical match
at2g41460	CON	MIN	HOUR	DAY	WEEK	UNIGENE	FLCDNA	TAIR
TAAAAATATG	2	0	2	1	1	no match found	no match found	At2g41460.1
at1g31160	CON	MIN	HOUR	DAY	WEEK	UNIGENE	FLCDNA	TAIR
TCAACCCAAA	2	1	1	0	0	gnl|UG|At#S11738604	gi|28827591|gb|BT005107.1	At1g31160.1
at1g65020	CON	MIN	HOUR	DAY	WEEK	UNIGENE	FLCDNA	TAIR
ACTGAAGTAA	2	0	3	0	1	gnl|UG|At#S18895284	gi|110736719|dbj|AK228385.1|	non-canonical match
at4g21510	CON	MIN	HOUR	DAY	WEEK	UNIGENE	FLCDNA	TAIR
TATTTTAGTC	1	0	1	0	0	gnl|UG|At#S11724089	gi|21407904|gb|AY089130.1	At4g21510.1
at1g13690	CON	MIN	HOUR	DAY	WEEK	UNIGENE	FLCDNA	TAIR
TATCTAAACC	0	2	0	1	0	gnl|UG|At#S11741665	no match found	At1g13690.1
at1g72890	CON	MIN	HOUR	DAY	WEEK	UNIGENE	FLCDNA	TAIR
TGTTTTTGAC	0	1	1	0	0	gnl|UG|At#S11728489	no match found	multiple non-canonical match
at3g04460	CON	MIN	HOUR	DAY	WEEK	UNIGENE	FLCDNA	TAIR
CAAGTTAGAG	0	0	1	1	0	gnl|UG|At#S11739377	gi|26450379|dbj|AK117651.1|	At3g04460.1
at4g24530	CON	MIN	HOUR	DAY	WEEK	UNIGENE	FLCDNA	TAIR
TGTAAAGTAG	0	1	0	0	0	gnl|UG|At#S34114202	gi|110743918|dbj|AK227813.1|	At4g24530.1
at4g32350	CON	MIN	HOUR	DAY	WEEK	UNIGENE	FLCDNA	TAIR
TATGTGGTGC	4	1	1	0	0	gnl|UG|At#S11743842	gi|19347943|gb|AY080684.1	At4g32350.1
at2g29570	CON	MIN	HOUR	DAY	WEEK	UNIGENE	FLCDNA	TAIR
AACTCCTTCA	1	1	0	0	0	gnl|UG|At#S11735145	gi|21407884|gb|AY089110.1	At2g29570.1
at1g69030	CON	MIN	HOUR	DAY	WEEK	UNIGENE	FLCDNA	TAIR
GAGGAACTTA	0	0	1	0	0	gnl|UG|At#S11729292	no match found	At1g69030.1
at1g07010	CON	MIN	HOUR	DAY	WEEK	UNIGENE	FLCDNA	TAIR
TTCAAGATTC	1	2	2	0	1	gnl|UG|At#S43850686	gi|22531179|gb|AY136428.1	At1g07010.1
AACTTCAGGT	0	1	0	2	0	no match found	gi|30725625|gb|BT008476.1	non-canonical match

at5g53000	CON	MIN	HOUR	DAY	WEEK	UNIGENE	FLCDNA	TAIR
GTACAATGAT	2	3	1	0	1	gnl|UG|At#S11700923	gi|28393349|gb|BT004072.1	At5g53000.1
at1g55350	CON	MIN	HOUR	DAY	WEEK	UNIGENE	FLCDNA	TAIR
GCTCACCGGC	0	1	1	0	1	gnl|UG|At#S11732518	gi|18377970|gb|AY074514.1	At1g55350.4
at5g27850	CON	MIN	HOUR	DAY	WEEK	UNIGENE	FLCDNA	TAIR
AGTATTAAAA	1	0	0	0	0	gnl|UG|At#S11706150	no match found	At5g27850.1
AAGGTTACTG	5	14	11	4	7	no match found	gi|21689770|gb|AY122996.1	multiple non-canonical match
at1g11170	CON	MIN	HOUR	DAY	WEEK	UNIGENE	FLCDNA	TAIR
TCCATTATGT	0	1	0	0	0	gnl|UG|At#S18942597	gi|18700104|gb|AY075657.1	At1g11170.2
at1g52140	CON	MIN	HOUR	DAY	WEEK	UNIGENE	FLCDNA	TAIR
AGGAAAAAGA	1	3	1	2	1	gnl|UG|At#S11733689	gi|28973120|gb|BT005465.1	At1g52140.1
at1g34150	CON	MIN	HOUR	DAY	WEEK	UNIGENE	FLCDNA	TAIR
TGTAGTGTAA	0	0	1	0	2	gnl|UG|At#S11737527	gi|13430549|gb|AF360187.1	At1g34150.1
at1g64720	CON	MIN	HOUR	DAY	WEEK	UNIGENE	FLCDNA	TAIR
TAATAGCAAA	98	127	77	43	12	gnl|UG|At#S11730187	gi|62321028|dbj|AK221843.1|	At1g64720.1
TAAGTAGACA	1	0	0	0	0	no match found	gi|23197607|gb|BT000012.1	non-canonical match
at3g11420	CON	MIN	HOUR	DAY	WEEK	UNIGENE	FLCDNA	TAIR
GATGGTGCCG	0	1	3	0	0	no match found	gi|34098872|gb|BT010376.1	non-canonical match
AACGTAACTA	0	1	1	1	0	gnl|UG|At#S11737466	no match found	At3g11420.1
at1g06960	CON	MIN	HOUR	DAY	WEEK	UNIGENE	FLCDNA	TAIR
CAGGCTCTTC	0	0	0	1	0	gnl|UG|At#S11743880	gi|23296312|gb|AY142487.1	At1g06960.1
at1g28685	CON	MIN	HOUR	DAY	WEEK	UNIGENE	FLCDNA	TAIR
CTTCTTGTCT	0	0	1	0	0	gnl|UG|At#S24442425	gi|62320821|dbj|AK221738.1|	non-canonical match
at3g02140	CON	MIN	HOUR	DAY	WEEK	UNIGENE	FLCDNA	TAIR
TACATTTTAT	1	1	0	1	0	gnl|UG|At#S11740039	no match found	At3g02140.1
at5g52530	CON	MIN	HOUR	DAY	WEEK	UNIGENE	FLCDNA	TAIR
GCGTCATCTG	3	4	4	7	5	gnl|UG|At#S15459088	gi|17381191|gb|AY064052.1	At5g52530.1
ATAACAGATA	0	1	0	0	0	no match found	gi|21689754|gb|AY122988.1	non-canonical match
at4g31740	CON	MIN	HOUR	DAY	WEEK	UNIGENE	FLCDNA	TAIR
ATCTATTGGG	1	0	0	0	0	no match found	no match found	At4g31740.1
at1g15215	CON	MIN	HOUR	DAY	WEEK	UNIGENE	FLCDNA	TAIR
ATCACGCGAG	0	0	1	0	0	no match found	gi|28827771|gb|BT005197.1	non-canonical match
at1g47410	CON	MIN	HOUR	DAY	WEEK	UNIGENE	FLCDNA	TAIR
TGAGTTTATT	0	0	1	1	0	no match found	no match found	At1g47410.1
GTCTTGGAGG	1	0	0	0	0	no match found	gi|52218787|gb|BT015687.1	non-canonical match
at5g66030	CON	MIN	HOUR	DAY	WEEK	UNIGENE	FLCDNA	TAIR
CAGAAAACCG	0	2	1	3	0	gnl|UG|At#S15435780	no match found	At5g66030.2
at2g12490	CON	MIN	HOUR	DAY	WEEK	UNIGENE	FLCDNA	TAIR
ATATTCGACA	0	1	0	0	0	no match found	no match found	At2g12490.1
at4g00180	CON	MIN	HOUR	DAY	WEEK	UNIGENE	FLCDNA	TAIR
GCTGACCATC	0	1	2	2	0	gnl|UG|At#S18906183	gi|14335013|gb|AY037186.1	At4g00180.1
at2g36485	CON	MIN	HOUR	DAY	WEEK	UNIGENE	FLCDNA	TAIR
CCCACTGTTT	0	1	0	0	0	gnl|UG|At#S21736568	gi|15450487|gb|AY052345.1	At2g36485.1

at2g40150	CON	MIN	HOUR	DAY	WEEK	UNIGENE	FLCDNA	TAIR
GAATGAGTTT	0	2	1	0	1	gnl|UG|At#S11702321	gi|28059702|gb|BT003422.1	At2g40150.1
at3g22550	CON	MIN	HOUR	DAY	WEEK	UNIGENE	FLCDNA	TAIR
TACAGGGGAG	0	1	0	0	0	no match found	gi|20466495|gb|AY099714.1	non-canonical match
at4g22760	CON	MIN	HOUR	DAY	WEEK	UNIGENE	FLCDNA	TAIR
AGGAGAACAA	1	1	0	0	0	no match found	gi|62320700|dbj|AK221677.1|	multiple non-canonical match
at1g58200	CON	MIN	HOUR	DAY	WEEK	UNIGENE	FLCDNA	TAIR
CTGATTGAGC	1	5	0	0	0	gnl|UG|At#S18942360	gi|27363433|gb|BT002720.1	At1g58200.2
at3g19850	CON	MIN	HOUR	DAY	WEEK	UNIGENE	FLCDNA	TAIR
CAAAATGTAT	0	0	1	1	0	no match found	gi|62321793|dbj|AK222235.1|	non-canonical match
at2g28320	CON	MIN	HOUR	DAY	WEEK	UNIGENE	FLCDNA	TAIR
TTAAGCATTG	1	0	1	0	1	gnl|UG|At#S34115614	gi|110741164|dbj|AK226536.1|	At2g28320.1
AAAGACCAAG	0	0	1	0	0	no match found	gi|22655301|gb|AY140100.1	multiple non-canonical match
at1g06470	CON	MIN	HOUR	DAY	WEEK	UNIGENE	FLCDNA	TAIR
TGGACCTTCC	0	1	2	2	0	gnl|UG|At#S18942634	gi|23306365|gb|BT000433.1	At1g06470.2
at1g44000	CON	MIN	HOUR	DAY	WEEK	UNIGENE	FLCDNA	TAIR
GAGATTCAGT	1	1	2	3	1	gnl|UG|At#S11704672	gi|15028026|gb|AY045870.1	At1g44000.1
at1g44190	CON	MIN	HOUR	DAY	WEEK	UNIGENE	FLCDNA	TAIR
TTGACAATAA	0	0	0	0	1	no match found	no match found	At1g44190.1
at3g43230	CON	MIN	HOUR	DAY	WEEK	UNIGENE	FLCDNA	TAIR
AATGTAAAGT	0	1	1	1	0	no match found	gi|21403126|gb|AY084416.1	non-canonical match
at5g25880	CON	MIN	HOUR	DAY	WEEK	UNIGENE	FLCDNA	TAIR
TACAGTCCTG	0	1	1	2	1	gnl|UG|At#S11720603	no match found	At5g25880.1
at4g39480	CON	MIN	HOUR	DAY	WEEK	UNIGENE	FLCDNA	TAIR
CATCCTGAAA	0	2	0	0	0	gnl|UG|At#S11721014	gi|110736832|dbj|AK228446.1|	non-canonical match
at5g35200	CON	MIN	HOUR	DAY	WEEK	UNIGENE	FLCDNA	TAIR
AGAAAGCAGG	2	3	2	1	0	gnl|UG|At#S11720183	gi|23198333|gb|BT000375.1	At5g35200.1
at3g57610	CON	MIN	HOUR	DAY	WEEK	UNIGENE	FLCDNA	TAIR
TGGAGTATGA	0	2	3	2	1	gnl|UG|At#S11728029	gi|21403843|gb|AY085133.1	At3g57610.1
at2g18160	CON	MIN	HOUR	DAY	WEEK	UNIGENE	FLCDNA	TAIR
ATTTTGTCTT	1	0	1	1	0	no match found	no match found	At2g18160.1
AAACGTTGTA	0	1	0	0	0	no match found	gi|110740302|dbj|AK230244.1|	pseudo chromosome match
TATTGGTGTG	2	3	4	0	2	gnl|UG|At#S11701327	gi|23397119|gb|BT000700.1	non-canonical match
AAGATCCAAG	2	4	2	0	0	no match found	gi|22137059|gb|AY133545.1	non-canonical match
at2g32230	CON	MIN	HOUR	DAY	WEEK	UNIGENE	FLCDNA	TAIR
TACCAATGAG	2	1	1	2	0	gnl|UG|At#S11807170	gi|51536605|gb|BT015418.1	At2g32230.1
at5g53920	CON	MIN	HOUR	DAY	WEEK	UNIGENE	FLCDNA	TAIR
ATATGAAACT	0	0	0	0	1	gnl|UG|At#S11718324	no match found	At5g53920.1
at5g58710	CON	MIN	HOUR	DAY	WEEK	UNIGENE	FLCDNA	TAIR
TGGTATTCGG	5	9	3	6	1	gnl|UG|At#S11717837	gi|20334833|gb|AY094017.1	At5g58710.1
at1g06900	CON	MIN	HOUR	DAY	WEEK	UNIGENE	FLCDNA	TAIR
TTTGATTTCT	13	9	13	6	9	gnl|UG|At#S34117004	no match found	multiple canonical match

at2g02180	CON	MIN	HOUR	DAY	WEEK	UNIGENE	FLCDNA	TAIR
TGGTAAAAAA	7	4	4	2	1	no match found	gi|21403758|gb|AY085048.1	non-canonical match
at3g42790	CON	MIN	HOUR	DAY	WEEK	UNIGENE	FLCDNA	TAIR
CAGCAACAAA	2	2	1	1	1	gnl|UG|At#S11730884	gi|17065549|gb|AY062851.1	At3g42790.1
at5g36210	CON	MIN	HOUR	DAY	WEEK	UNIGENE	FLCDNA	TAIR
CTTGGACTAA	2	2	4	2	5	gnl|UG|At#S11720082	no match found	At5g36210.1
at5g13230	CON	MIN	HOUR	DAY	WEEK	UNIGENE	FLCDNA	TAIR
TCGGCAAAGA	0	0	0	1	0	no match found	no match found	At5g13230.1
at4g32050	CON	MIN	HOUR	DAY	WEEK	UNIGENE	FLCDNA	TAIR
GTTTCTCTAT	0	0	1	3	1	gnl|UG|At#S11722267	no match found	non-canonical match
at5g57770	CON	MIN	HOUR	DAY	WEEK	UNIGENE	FLCDNA	TAIR
CTCACTCTCT	1	0	0	0	0	gnl|UG|At#S18913012	no match found	At5g57770.1
at3g25670	CON	MIN	HOUR	DAY	WEEK	UNIGENE	FLCDNA	TAIR
TGGGATTTGC	0	0	0	1	0	gnl|UG|At#S11733091	no match found	multiple non-canonical match
at1g07470	CON	MIN	HOUR	DAY	WEEK	UNIGENE	FLCDNA	TAIR
ATTATTTATG	2	2	0	1	1	no match found	gi|15983369|gb|AF424559.1	non-canonical match
AGTTTTATAT	1	0	0	0	0	gnl|UG|At#S11742287	no match found	non-canonical match
at3g46060	CON	MIN	HOUR	DAY	WEEK	UNIGENE	FLCDNA	TAIR
CTGTGGAACT	3	6	1	8	0	gnl|UG|At#S11703680	gi|14596014|gb|AY042795.1	At3g46060.1
at5g61990	CON	MIN	HOUR	DAY	WEEK	UNIGENE	FLCDNA	TAIR
GGAGCAATTT	1	0	0	0	0	gnl|UG|At#S15644832	gi|30794105|gb|BT008689.1	At5g61990.1
at1g77570	CON	MIN	HOUR	DAY	WEEK	UNIGENE	FLCDNA	TAIR
AAGATTTTAT	0	0	1	0	0	no match found	gi|51969425|dbj|AK175642.1|	At1g77570.1
at4g05620	CON	MIN	HOUR	DAY	WEEK	UNIGENE	FLCDNA	TAIR
AAACCTAATC	0	1	0	0	0	gnl|UG|At#S11726229	no match found	At4g12030.1
at5g05000	CON	MIN	HOUR	DAY	WEEK	UNIGENE	FLCDNA	TAIR
TTGACAAGAA	0	1	1	3	1	gnl|UG|At#S18941680	gi|21407221|gb|AY088447.1	At5g05000.1
at1g71190	CON	MIN	HOUR	DAY	WEEK	UNIGENE	FLCDNA	TAIR
GAAGAAAGGA	1	0	0	1	0	gnl|UG|At#S11728842	no match found	At1g71190.1
at3g06980	CON	MIN	HOUR	DAY	WEEK	UNIGENE	FLCDNA	TAIR
GAGTCTTGCA	0	0	2	0	2	gnl|UG|At#S11708140	gi|27311828|gb|BT002520.1	At3g06980.1
ATGTCCCAAA	0	0	0	1	1	no match found	gi|21360512|gb|AY113064.1	non-canonical match
at3g12480	CON	MIN	HOUR	DAY	WEEK	UNIGENE	FLCDNA	TAIR
CACTCTATGA	0	4	2	2	0	gnl|UG|At#S11737150	gi|18087596|gb|AF462841.1	At3g12480.1
at1g08770	CON	MIN	HOUR	DAY	WEEK	UNIGENE	FLCDNA	TAIR
GCGCATCTAA	0	0	0	1	0	gnl|UG|At#S11742157	no match found	At1g08770.1
at1g20140	CON	MIN	HOUR	DAY	WEEK	UNIGENE	FLCDNA	TAIR
ATTGAGGACG	0	1	0	0	0	gnl|UG|At#S11741041	gi|20466136|gb|AY098980.1	At1g20140.1
at4g30290	CON	MIN	HOUR	DAY	WEEK	UNIGENE	FLCDNA	TAIR
GTTTACAACT	1	0	0	0	1	no match found	gi|15215689|gb|AY050373.1	multiple non-canonical match
at1g61960	CON	MIN	HOUR	DAY	WEEK	UNIGENE	FLCDNA	TAIR

AGAAGTGAAA	0	0	1	1	0	gnl|UG|At#S11730814	gi|26450010|dbj|AK117461.1|	At1g61960.1
at1g14720	CON	MIN	HOUR	DAY	WEEK	UNIGENE	FLCDNA	TAIR
ACTTATTCGT	0	2	0	0	1	gnl|UG|At#S11741562	gi|14326520|gb|AF385714.1	At1g14720.1
at1g01080	CON	MIN	HOUR	DAY	WEEK	UNIGENE	FLCDNA	TAIR
ATCGTAAGAC	2	0	1	1	1	gnl|UG|At#S28282752	gi|21404848|gb|AY086138.1	At1g01080.1
at4g28640	CON	MIN	HOUR	DAY	WEEK	UNIGENE	FLCDNA	TAIR
GAAACACCGG	0	1	1	0	0	gnl|UG|At#S11722855	gi|12083203|gb|AF332397.1	At4g28640.1
at5g10150	CON	MIN	HOUR	DAY	WEEK	UNIGENE	FLCDNA	TAIR
TTGTTTTTTT	1	0	0	0	0	gnl|UG|At#S11723700	no match found	At5g10150.1
TCGGAGAATC	0	3	1	1	0	no match found	gi|50198937|gb|BT015021.1	non-canonical match
at4g16330	CON	MIN	HOUR	DAY	WEEK	UNIGENE	FLCDNA	TAIR
ATCCCTCCAA	1	3	0	0	0	gnl|UG|At#S11724974	gi|20260149|gb|AY092974.1	At4g16330.1
at1g09980	CON	MIN	HOUR	DAY	WEEK	UNIGENE	FLCDNA	TAIR
CGCTGTGATG	3	0	0	1	0	gnl|UG|At#S11742036	gi|21689820|gb|AY123021.1	At1g09980.1
at5g10740	CON	MIN	HOUR	DAY	WEEK	UNIGENE	FLCDNA	TAIR
ATAGTTTATT	0	0	1	2	4	gnl|UG|At#S11817108	gi|26450182|dbj|AK117549.1|	At5g10740.1
at1g13990	CON	MIN	HOUR	DAY	WEEK	UNIGENE	FLCDNA	TAIR
AAAACAAGAA	1	1	0	0	0	no match found	gi|22137313|gb|AY133672.1	At1g13990.1
at5g25460	CON	MIN	HOUR	DAY	WEEK	UNIGENE	FLCDNA	TAIR
AGGAGCGATG	13	27	16	17	3	gnl|UG|At#S11720636	gi|62320879|dbj|AK221767.1|	At5g25460.1
at2g21240	CON	MIN	HOUR	DAY	WEEK	UNIGENE	FLCDNA	TAIR
TGTTCGTGCA	1	1	1	0	0	gnl|UG|At#S11737143	gi|109946572|gb|BT026109.1	At2g21240.2
at3g19790	CON	MIN	HOUR	DAY	WEEK	UNIGENE	FLCDNA	TAIR
ATGGGTTTTA	1	0	0	1	1	gnl|UG|At#S19719637	gi|46518360|gb|BT012518.1	non-canonical match
at1g29660	CON	MIN	HOUR	DAY	WEEK	UNIGENE	FLCDNA	TAIR
TTTACCGGGA	3	10	8	5	3	no match found	no match found	At1g29660.1
at3g14130	CON	MIN	HOUR	DAY	WEEK	UNIGENE	FLCDNA	TAIR
TTAGGACAGA	1	0	0	0	0	gnl|UG|At#S11736616	no match found	At3g14130.1
at4g10070	CON	MIN	HOUR	DAY	WEEK	UNIGENE	FLCDNA	TAIR
CTTCAAAAGA	1	0	0	0	0	no match found	no match found	At4g10070.1
at1g72340	CON	MIN	HOUR	DAY	WEEK	UNIGENE	FLCDNA	TAIR
GCACCGGCGT	2	1	2	2	0	no match found	gi|17065009|gb|AY062581.1	non-canonical match
at2g30870	CON	MIN	HOUR	DAY	WEEK	UNIGENE	FLCDNA	TAIR
TTAGCGCTTG	2	22	4	2	1	no match found	gi|22136037|gb|AY128398.1	non-canonical match
TATATTCCTC	0	0	0	0	1	gnl|UG|At#S11734822	no match found	multiple non-canonical match
at4g00850	CON	MIN	HOUR	DAY	WEEK	UNIGENE	FLCDNA	TAIR
TACAATGTTA	0	0	1	0	0	gnl|UG|At#S11727470	gi|14190376|gb|AF378866.1	At4g00850.1
GCGGTGGTGA	0	0	0	1	0	no match found	gi|15450536|gb|AY052732.1	multiple non-canonical match
at2g29950	CON	MIN	HOUR	DAY	WEEK	UNIGENE	FLCDNA	TAIR
ATGAATTGAT	1	0	1	0	0	gnl|UG|At#S21132600	no match found	non-canonical match
TATTTGTTTT	4	0	3	7	6	gnl|UG|At#S35218709	no match found	At5g59320.1
at5g64050	CON	MIN	HOUR	DAY	WEEK	UNIGENE	FLCDNA	TAIR

GAGGAACGGT	1	0	0	1	2	gnl|UG|At#S11717294	gi|110739975|dbj|AK230072.1|	At5g64050.1
at3g07580	CON	MIN	HOUR	DAY	WEEK	UNIGENE	FLCDNA	TAIR
ACTGAAGTGC	0	0	1	0	0	gnl|UG|At#S21736363	gi|51970765|dbj|AK176312.1|	At3g07580.1
at1g48320	CON	MIN	HOUR	DAY	WEEK	UNIGENE	FLCDNA	TAIR
CTCAAAATGG	1	4	0	1	1	gnl|UG|At#S11735095	gi|110738946|dbj|AK229541.1|	At1g48320.1
at5g01970	CON	MIN	HOUR	DAY	WEEK	UNIGENE	FLCDNA	TAIR
AAAATTCTGT	0	1	0	1	0	gnl|UG|At#S11725665	gi|23198057|gb|BT000237.1	At5g01970.1
at1g04580	CON	MIN	HOUR	DAY	WEEK	UNIGENE	FLCDNA	TAIR
GAATTGCATT	0	0	1	0	0	gnl|UG|At#S11742570	no match found	At1g04580.1
at4g19810	CON	MIN	HOUR	DAY	WEEK	UNIGENE	FLCDNA	TAIR
GGATGCTACG	0	1	0	0	0	gnl|UG|At#S11724363	gi|119360132|gb|BT029539.1	At4g19810.1
at5g41590	CON	MIN	HOUR	DAY	WEEK	UNIGENE	FLCDNA	TAIR
ATCTTCTTTG	0	0	1	0	0	gnl|UG|At#S11719571	no match found	At5g41590.1
at3g56410	CON	MIN	HOUR	DAY	WEEK	UNIGENE	FLCDNA	TAIR
GTTTGGTGAT	1	0	0	0	0	no match found	gi|110737855|dbj|AK228978.1|	pseudo chromosome match
CGTTATACCA	0	1	0	0	0	gnl|UG|At#S34117330	gi|110737734|dbj|AK228917.1|	At3g56410.1
at5g65950	CON	MIN	HOUR	DAY	WEEK	UNIGENE	FLCDNA	TAIR
ACTTGGGTTT	1	0	2	1	0	gnl|UG|At#S11717103	gi|20466411|gb|AY099672.1	At5g65950.1
at5g03650	CON	MIN	HOUR	DAY	WEEK	UNIGENE	FLCDNA	TAIR
GTCTATGCAC	5	3	1	3	0	gnl|UG|At#S11725245	gi|26450530|dbj|AK117729.1|	At5g03650.1
at2g02860	CON	MIN	HOUR	DAY	WEEK	UNIGENE	FLCDNA	TAIR
GGATCAATTG	0	0	0	0	1	gnl|UG|At#S11740553	gi|110742222|dbj|AK226970.1|	At2g02860.1
at5g13980	CON	MIN	HOUR	DAY	WEEK	UNIGENE	FLCDNA	TAIR
GAATTTACTT	0	5	3	1	1	gnl|UG|At#S28281809	gi|23198185|gb|BT000301.1	At5g13980.1
at5g16750	CON	MIN	HOUR	DAY	WEEK	UNIGENE	FLCDNA	TAIR
AATTATAAAT	1	0	0	0	1	gnl|UG|At#S11722151	gi|15810484|gb|AY056281.1	At5g16750.1
ATTCAACCGC	0	1	0	0	0	no match found	gi|21539572|gb|AY120781.1	multiple non-canonical match
at1g22060	CON	MIN	HOUR	DAY	WEEK	UNIGENE	FLCDNA	TAIR
TATATAAAGT	0	4	0	0	0	gnl|UG|At#S11740854	no match found	At1g22060.1
at3g14430	CON	MIN	HOUR	DAY	WEEK	UNIGENE	FLCDNA	TAIR
TTTCTGTAGG	1	0	0	0	1	gnl|UG|At#S11689013	gi|21405732|gb|AY087008.1	At3g14430.1
at5g27690	CON	MIN	HOUR	DAY	WEEK	UNIGENE	FLCDNA	TAIR
GGCCAATTAA	0	1	0	0	0	gnl|UG|At#S14274076	gi|28973190|gb|BT005500.1	At5g27690.1
at5g27840	CON	MIN	HOUR	DAY	WEEK	UNIGENE	FLCDNA	TAIR
AGGATGCTAA	0	3	0	1	0	no match found	gi|14596132|gb|AY042854.1	At5g27840.1
at1g23550	CON	MIN	HOUR	DAY	WEEK	UNIGENE	FLCDNA	TAIR
AGAGAAGTTC	0	0	1	0	0	gnl|UG|At#S11740706	gi|62321284|dbj|AK221974.1|	At5g27840.1
atcg00160	CON	MIN	HOUR	DAY	WEEK	UNIGENE	FLCDNA	TAIR
TTAACAAATT	5	1	1	1	3	no match found	no match found	AtCg00160
at3g56940	CON	MIN	HOUR	DAY	WEEK	UNIGENE	FLCDNA	TAIR
CCTCCTGTTG	102	99	95	57	21	gnl|UG|At#S11728155	no match found	At3g56940.1

at3g06530	CON	MIN	HOUR	DAY	WEEK	UNIGENE	FLCDNA	TAIR
CATCGGTCAA	0	1	0	0	0	gnl|UG|At#S11738785	gi|62320951|dbj|AK221804.1|	non-canonical match
at3g61130	CON	MIN	HOUR	DAY	WEEK	UNIGENE	FLCDNA	TAIR
AATAAAATCA	1	2	0	2	0	gnl|UG|At#S11727136	gi|14517361|gb|AY039515.1	At3g61130.1
at5g07320	CON	MIN	HOUR	DAY	WEEK	UNIGENE	FLCDNA	TAIR
AACTCACATC	0	0	2	0	1	gnl|UG|At#S21737447	gi|51968597|dbj|AK175228.1|	At5g07320.1
at4g15090	CON	MIN	HOUR	DAY	WEEK	UNIGENE	FLCDNA	TAIR
GAGCTGAGTT	1	0	1	1	0	gnl|UG|At#S11725188	gi|19310413|gb|AY078941.1	At4g15090.1
at1g16020	CON	MIN	HOUR	DAY	WEEK	UNIGENE	FLCDNA	TAIR
GCTTTGGAGA	0	1	0	0	1	gnl|UG|At#S11741435	gi|45680343|gb|BT012033.1	At1g16020.1
at1g09330	CON	MIN	HOUR	DAY	WEEK	UNIGENE	FLCDNA	TAIR
GTTGGTTTTT	1	0	3	3	0	gnl|UG|At#S11742105	gi|21404089|gb|AY085379.1	At1g09330.1
at1g67960	CON	MIN	HOUR	DAY	WEEK	UNIGENE	FLCDNA	TAIR
GATCATTTGG	0	1	1	1	0	gnl|UG|At#S11708304	gi|17381091|gb|AY064002.1	At1g67960.1
at2g33300	CON	MIN	HOUR	DAY	WEEK	UNIGENE	FLCDNA	TAIR
AGAAGCAGAA	0	0	0	0	1	gnl|UG|At#S43850112	no match found	At1g67960.1
at2g38660	CON	MIN	HOUR	DAY	WEEK	UNIGENE	FLCDNA	TAIR
GTTTATCCAA	0	0	0	0	1	no match found	gi|28393824|gb|BT004325.1	At1g67960.1
GTTTATCCTA	0	1	1	0	0	gnl|UG|At#S18897999	no match found	At2g38660.1
at1g52400	CON	MIN	HOUR	DAY	WEEK	UNIGENE	FLCDNA	TAIR
TTGGAGGTTC	1	9	0	2	1	gnl|UG|At#S28282505	gi|23397308|gb|BT000797.1	At1g52400.1
at4g32680	CON	MIN	HOUR	DAY	WEEK	UNIGENE	FLCDNA	TAIR
TTTCAGATAT	1	0	1	1	0	gnl|UG|At#S11722161	gi|13877552|gb|AF370477.1	At4g32680.1
at4g08960	CON	MIN	HOUR	DAY	WEEK	UNIGENE	FLCDNA	TAIR
CCATCTTCTT	0	2	0	0	0	gnl|UG|At#S11704490	gi|21406300|gb|AY087562.1	At4g08960.1
at3g60180	CON	MIN	HOUR	DAY	WEEK	UNIGENE	FLCDNA	TAIR
TTCTCATCAT	3	2	0	0	1	gnl|UG|At#S43849676	gi|18252846|gb|AY072358.1	multiple canonical match
at2g24820	CON	MIN	HOUR	DAY	WEEK	UNIGENE	FLCDNA	TAIR
AAATAAATTT	4	3	14	2	3	gnl|UG|At#S11736306	gi|21407857|gb|AY089083.1	non-canonical match
at1g22020	CON	MIN	HOUR	DAY	WEEK	UNIGENE	FLCDNA	TAIR
GTAATCCATA	0	1	0	0	0	gnl|UG|At#S11740858	gi|21928156|gb|AY125514.1	At1g22020.1
at2g38995	CON	MIN	HOUR	DAY	WEEK	UNIGENE	FLCDNA	TAIR
GAGAAAGGAG	1	0	0	0	0	gnl|UG|At#S15114817	no match found	At2g38995.1
at5g44610	CON	MIN	HOUR	DAY	WEEK	UNIGENE	FLCDNA	TAIR
TGAATTTCCT	0	1	0	0	1	gnl|UG|At#S11831991	gi|21407866|gb|AY089092.1	At5g44610.1
at3g48600	CON	MIN	HOUR	DAY	WEEK	UNIGENE	FLCDNA	TAIR
GAGAAAGTGG	0	1	0	0	0	gnl|UG|At#S18902915	no match found	At3g48600.1
at3g58770	CON	MIN	HOUR	DAY	WEEK	UNIGENE	FLCDNA	TAIR
TATCAATTTG	1	0	0	0	0	no match found	no match found	At3g58770.1
atcg00950	CON	MIN	HOUR	DAY	WEEK	UNIGENE	FLCDNA	TAIR
CTATACTCCC	20	49	5	9	9	no match found	gi|20466066|gb|AY096798.1	pseudo chromosome match

at2g27810	CON	MIN	HOUR	DAY	WEEK	UNIGENE	FLCDNA	TAIR
TTGTTGTAAG	5	0	0	0	0	gnl|UG|At#S11776903	gi|110742099|dbj|AK226904.1|	At2g27810.2
at3g11325	CON	MIN	HOUR	DAY	WEEK	UNIGENE	FLCDNA	TAIR
TTCAGAGAAT	0	1	0	1	0	gnl|UG|At#S11816234	no match found	multiple non-canonical match
at4g16935	CON	MIN	HOUR	DAY	WEEK	UNIGENE	FLCDNA	TAIR
ATTTGCCTTT	0	4	1	0	0	no match found	no match found	At4g16935.1
at1g76965	CON	MIN	HOUR	DAY	WEEK	UNIGENE	FLCDNA	TAIR
TATAACGAAA	1	0	0	0	0	no match found	no match found	At1g76965.1
at2g45600	CON	MIN	HOUR	DAY	WEEK	UNIGENE	FLCDNA	TAIR
TTTTACGAAC	2	0	1	0	2	gnl|UG|At#S11810486	gi|21406328|gb|AY087590.1	At2g45600.1
at5g35170	CON	MIN	HOUR	DAY	WEEK	UNIGENE	FLCDNA	TAIR
AATGGCCGAG	0	0	2	1	0	no match found	no match found	At5g35170.1
TTCATCTTAG	1	2	0	1	0	gnl|UG|At#S28281721	gi|110740592|dbj|AK226238.1|	non-canonical match
at5g07010	CON	MIN	HOUR	DAY	WEEK	UNIGENE	FLCDNA	TAIR
TGTGAAGCTA	0	1	0	0	0	no match found	no match found	At5g07010.1
at1g73770	CON	MIN	HOUR	DAY	WEEK	UNIGENE	FLCDNA	TAIR
TTGGCTGTAA	0	1	0	0	0	gnl|UG|At#S35225389	no match found	pseudo chromosome match
at4g37130	CON	MIN	HOUR	DAY	WEEK	UNIGENE	FLCDNA	TAIR
GCTTGAGTTC	1	2	0	1	1	gnl|UG|At#S11721415	gi|20260345|gb|AY093072.1	At4g37130.1
at4g34980	CON	MIN	HOUR	DAY	WEEK	UNIGENE	FLCDNA	TAIR
ATCGTAAACC	3	2	4	1	1	gnl|UG|At#S11743033	gi|18389255|gb|AY074375.1	At4g34980.1
TGAACGATAA	0	1	0	0	0	gnl|UG|At#S35303397	no match found	At4g34980.1
at1g63530	CON	MIN	HOUR	DAY	WEEK	UNIGENE	FLCDNA	TAIR
TTCTTCCTTT	0	0	0	1	0	gnl|UG|At#S21099298	no match found	At1g63530.1
at3g57480	CON	MIN	HOUR	DAY	WEEK	UNIGENE	FLCDNA	TAIR
GGTTTCTTGA	0	1	0	0	0	gnl|UG|At#S11728056	gi|30017276|gb|BT006223.1	At3g57480.1
at4g28770	CON	MIN	HOUR	DAY	WEEK	UNIGENE	FLCDNA	TAIR
GCGATTGGGA	1	0	1	1	0	gnl|UG|At#S11722832	gi|29029075|gb|BT005982.1	non-canonical match
GGTGTGTTTT	1	0	0	0	0	gnl|UG|At#S43849597	no match found	non-canonical match
at2g44740	CON	MIN	HOUR	DAY	WEEK	UNIGENE	FLCDNA	TAIR
GTCGCTGCTA	0	1	0	0	0	gnl|UG|At#S11731465	gi|66841349|gb|BT023473.1	At2g44740.1
at5g54660	CON	MIN	HOUR	DAY	WEEK	UNIGENE	FLCDNA	TAIR
TCGCCTTGAC	3	0	0	0	0	gnl|UG|At#S18911050	gi|21407808|gb|AY089034.1	At5g54660.1
at2g36307	CON	MIN	HOUR	DAY	WEEK	UNIGENE	FLCDNA	TAIR
GTGGATTCTG	0	0	0	1	0	no match found	gi|62321378|dbj|AK222023.1|	At5g54660.1
at4g30935	CON	MIN	HOUR	DAY	WEEK	UNIGENE	FLCDNA	TAIR
AAGATTACCA	1	3	1	0	1	gnl|UG|At#S11722461	gi|28393377|gb|BT004086.1	At4g30935.1
at3g15920	CON	MIN	HOUR	DAY	WEEK	UNIGENE	FLCDNA	TAIR
TTTGGCAAAA	0	1	0	1	0	gnl|UG|At#S21736675	gi|51970141|dbj|AK176000.1|	non-canonical match
at3g10610	CON	MIN	HOUR	DAY	WEEK	UNIGENE	FLCDNA	TAIR
TAATTCATCT	2	0	0	2	4	gnl|UG|At#S11737705	no match found	At3g10610.1
at4g26640	CON	MIN	HOUR	DAY	WEEK	UNIGENE	FLCDNA	TAIR

GGGAGAGTAC	0	0	2	0	0	gnl|UG|At#S15460663	gi|15028070|gb|AY045892.1	At4g26640.1
at3g04120	CON	MIN	HOUR	DAY	WEEK	UNIGENE	FLCDNA	TAIR
TGAGGTGATG	67	30	68	87	83	gnl|UG|At#S35285007	gi|22655269|gb|AY140084.1	At3g04120.1
at3g14370	CON	MIN	HOUR	DAY	WEEK	UNIGENE	FLCDNA	TAIR
CTAGTCACGT	0	3	0	0	0	gnl|UG|At#S11736546	gi|116325945|gb|BT029242.1	At3g14370.1
at5g67500	CON	MIN	HOUR	DAY	WEEK	UNIGENE	FLCDNA	TAIR
GCACACTTGG	4	6	3	4	2	gnl|UG|At#S11716945	gi|107738406|gb|BT025802.1	At5g67500.1
at5g11650	CON	MIN	HOUR	DAY	WEEK	UNIGENE	FLCDNA	TAIR
GGTTTAATCT	0	1	2	1	2	gnl|UG|At#S34117045	gi|21404177|gb|AY085467.1	At5g11650.1
at1g02300	CON	MIN	HOUR	DAY	WEEK	UNIGENE	FLCDNA	TAIR
TTAAGAAAAA	0	0	2	0	0	gnl|UG|At#S24442627	gi|62320419|dbj|AK221536.1|	At1g22335.1
at5g62540	CON	MIN	HOUR	DAY	WEEK	UNIGENE	FLCDNA	TAIR
GATGTTTAAA	1	1	2	2	1	gnl|UG|At#S11717447	gi|21404077|gb|AY085367.1	At5g62540.1
at2g45135	CON	MIN	HOUR	DAY	WEEK	UNIGENE	FLCDNA	TAIR
GGGGCACTTG	0	2	0	0	0	gnl|UG|At#S15460363	no match found	non-canonical match
at1g66380	CON	MIN	HOUR	DAY	WEEK	UNIGENE	FLCDNA	TAIR
TAACAAAGAT	0	0	0	0	1	no match found	no match found	At1g66380.1
at5g44140	CON	MIN	HOUR	DAY	WEEK	UNIGENE	FLCDNA	TAIR
TGAATCTTTT	0	2	0	0	0	no match found	no match found	At5g44140.1
at4g17620	CON	MIN	HOUR	DAY	WEEK	UNIGENE	FLCDNA	TAIR
TTCACCTTCT	0	1	0	0	0	gnl|UG|At#S11724738	gi|110742073|dbj|AK226891.1|	At4g17620.1
at3g63200	CON	MIN	HOUR	DAY	WEEK	UNIGENE	FLCDNA	TAIR
TCATCATCAC	0	1	0	0	0	no match found	gi|24111290|gb|BT001015.1	At3g63200.1
at3g50230	CON	MIN	HOUR	DAY	WEEK	UNIGENE	FLCDNA	TAIR
GAAACCTTAA	5	9	19	9	12	gnl|UG|At#S11729400	no match found	multiple non-canonical match
at2g32415	CON	MIN	HOUR	DAY	WEEK	UNIGENE	FLCDNA	TAIR
GAGAAGATGG	0	0	0	1	0	gnl|UG|At#S11807228	gi|20259401|gb|AY090988.1	At2g32415.1
at5g10710	CON	MIN	HOUR	DAY	WEEK	UNIGENE	FLCDNA	TAIR
CCGAATGAAA	2	0	0	2	0	gnl|UG|At#S11702116	gi|13430737|gb|AF360281.1	At5g10710.2
at2g42080	CON	MIN	HOUR	DAY	WEEK	UNIGENE	FLCDNA	TAIR
CGTATCGAAC	0	0	0	1	0	gnl|UG|At#S11732083	gi|25083941|gb|BT002128.1	At2g42080.1
at2g01320	CON	MIN	HOUR	DAY	WEEK	UNIGENE	FLCDNA	TAIR
ACAGTTTTCA	1	0	1	0	0	gnl|UG|At#S15461674	gi|13605838|gb|AF367318.1	At2g01320.4
at3g07630	CON	MIN	HOUR	DAY	WEEK	UNIGENE	FLCDNA	TAIR
CGAAGAGTTT	0	2	0	0	0	gnl|UG|At#S11738451	gi|21280852|gb|AY113967.1	At3g07630.1
at4g35290	CON	MIN	HOUR	DAY	WEEK	UNIGENE	FLCDNA	TAIR
AAAGATGAAG	0	0	0	2	0	gnl|UG|At#S18941726	gi|16604666|gb|AY059778.1	At4g35290.2
at2g19560	CON	MIN	HOUR	DAY	WEEK	UNIGENE	FLCDNA	TAIR
GTTGAGGAGT	1	0	0	0	0	gnl|UG|At#S11737588	no match found	At2g19560.1
at4g33220	CON	MIN	HOUR	DAY	WEEK	UNIGENE	FLCDNA	TAIR
TTCTTCTAAG	5	3	3	0	2	gnl|UG|At#S11722071	gi|14190428|gb|AF378892.1	At4g33220.1

at4g32700	CON	MIN	HOUR	DAY	WEEK	UNIGENE	FLCDNA	TAIR
GTTCCAAAAG	1	0	3	2	0	gnl|UG|At#S24430076	no match found	pseudo chromosome match
at1g62870	CON	MIN	HOUR	DAY	WEEK	UNIGENE	FLCDNA	TAIR
CAACTTCTTG	1	0	0	0	0	gnl|UG|At#S34115294	gi|110741765|dbj|AK226723.1|	non-canonical match
at2g15830	CON	MIN	HOUR	DAY	WEEK	UNIGENE	FLCDNA	TAIR
GTCTCTACTC	0	1	0	2	1	gnl|UG|At#S11738507	gi|57222203|gb|BT020508.1	At2g15830.1
at2g05790	CON	MIN	HOUR	DAY	WEEK	UNIGENE	FLCDNA	TAIR
GGTCAAACGC	0	2	0	0	0	gnl|UG|At#S11739978	no match found	At2g05790.1
at2g43350	CON	MIN	HOUR	DAY	WEEK	UNIGENE	FLCDNA	TAIR
GAAAAGAAGA	1	4	2	1	2	no match found	gi|21405754|gb|AY087030.1	multiple canonical match
at4g33050	CON	MIN	HOUR	DAY	WEEK	UNIGENE	FLCDNA	TAIR
GGAATCCCAA	0	6	3	3	1	gnl|UG|At#S11722097	no match found	At4g33050.3
at3g62170	CON	MIN	HOUR	DAY	WEEK	UNIGENE	FLCDNA	TAIR
TTGTATCCTT	0	0	1	0	0	gnl|UG|At#S11726798	gi|20260675|gb|AY093237.1	At3g62170.1
at5g19460	CON	MIN	HOUR	DAY	WEEK	UNIGENE	FLCDNA	TAIR
GAATTGAAGG	0	0	2	0	1	gnl|UG|At#S11721460	gi|18377776|gb|AY074342.1	At5g19460.1
at4g20830	CON	MIN	HOUR	DAY	WEEK	UNIGENE	FLCDNA	TAIR
GGGCGAATAG	0	0	1	3	0	no match found	gi|22137035|gb|AY133533.1	non-canonical match
GAGTTTGGAC	1	0	1	1	0	gnl|UG|At#S11708997	gi|17065037|gb|AY062595.1	At4g20840.1
GAACTCTACA	2	8	6	6	4	no match found	gi|15983492|gb|AF424621.1	At4g20830.2
at5g19855	CON	MIN	HOUR	DAY	WEEK	UNIGENE	FLCDNA	TAIR
TACTATATGA	3	1	1	0	1	no match found	gi|21406943|gb|AY088169.1	At5g19855.1
at5g48990	CON	MIN	HOUR	DAY	WEEK	UNIGENE	FLCDNA	TAIR
GGAAGCGGTA	0	1	2	0	0	gnl|UG|At#S11743626	gi|18175610|gb|AY072073.1	At5g48990.1
at1g21326	CON	MIN	HOUR	DAY	WEEK	UNIGENE	FLCDNA	TAIR
CTGGGATTTT	0	1	0	0	0	gnl|UG|At#S11816484	no match found	multiple non-canonical match
at1g18990	CON	MIN	HOUR	DAY	WEEK	UNIGENE	FLCDNA	TAIR
AATCTGTTTT	0	1	0	0	1	gnl|UG|At#S11825095	gi|27754599|gb|BT002932.1	At1g18990.1
at5g36290	CON	MIN	HOUR	DAY	WEEK	UNIGENE	FLCDNA	TAIR
CTGGCTTCAA	1	4	0	1	1	gnl|UG|At#S18913253	gi|32362302|gb|BT009678.1	At5g36290.1
at2g23780	CON	MIN	HOUR	DAY	WEEK	UNIGENE	FLCDNA	TAIR
ATGCTACGCT	1	3	0	0	0	gnl|UG|At#S11736543	gi|30794129|gb|BT008701.1	At2g23780.1
at3g19280	CON	MIN	HOUR	DAY	WEEK	UNIGENE	FLCDNA	TAIR
CTTTGAGTGT	0	1	0	1	0	gnl|UG|At#S11735064	no match found	At3g19280.1
at2g07723	CON	MIN	HOUR	DAY	WEEK	UNIGENE	FLCDNA	TAIR
TTCCCAGAAA	0	0	1	0	0	gnl|UG|At#S35294260	no match found	multiple canonical match
at1g03610	CON	MIN	HOUR	DAY	WEEK	UNIGENE	FLCDNA	TAIR
ATGTTTGCTT	1	1	2	0	0	no match found	gi|21403679|gb|AY084969.1	non-canonical match
at1g71430	CON	MIN	HOUR	DAY	WEEK	UNIGENE	FLCDNA	TAIR
TTGTCTCTTC	0	1	0	0	1	gnl|UG|At#S11728783	gi|26450703|dbj|AK117819.1|	At1g71430.1
at1g23980	CON	MIN	HOUR	DAY	WEEK	UNIGENE	FLCDNA	TAIR

AGATTTTAAG	0	0	3	1	0	gnl|UG|At#S11740636	gi|26453198|dbj|AK119101.1|	At1g23980.1
at2g19710	CON	MIN	HOUR	DAY	WEEK	UNIGENE	FLCDNA	TAIR
TTCACCCCAA	0	1	0	1	0	gnl|UG|At#S11737547	gi|34222083|gb|BT010458.1	At2g19710.1
at3g53400	CON	MIN	HOUR	DAY	WEEK	UNIGENE	FLCDNA	TAIR
TATAGAGCAA	1	2	0	0	1	gnl|UG|At#S11728803	gi|17528947|gb|AY065208.1	At3g53400.1
at3g26670	CON	MIN	HOUR	DAY	WEEK	UNIGENE	FLCDNA	TAIR
CACATTCTTC	1	0	1	1	1	gnl|UG|At#S15460555	gi|62319469|dbj|AK221059.1|	At3g26670.1
at2g30090	CON	MIN	HOUR	DAY	WEEK	UNIGENE	FLCDNA	TAIR
AACCCTACAT	0	0	0	0	1	no match found	no match found	At2g30090.1
at2g34070	CON	MIN	HOUR	DAY	WEEK	UNIGENE	FLCDNA	TAIR
TATATTACAT	1	0	0	0	1	gnl|UG|At#S18899784	gi|21405214|gb|AY086504.1	At2g34070.1
at5g54900	CON	MIN	HOUR	DAY	WEEK	UNIGENE	FLCDNA	TAIR
CACTTTCGGT	0	3	0	0	0	gnl|UG|At#S11718223	gi|119360144|gb|BT029545.1	At5g54900.1
at5g25350	CON	MIN	HOUR	DAY	WEEK	UNIGENE	FLCDNA	TAIR
CATACAAAAA	2	15	2	0	2	gnl|UG|At#S34118448	gi|110735709|dbj|AK227858.1|	At5g25350.1
at1g15430	CON	MIN	HOUR	DAY	WEEK	UNIGENE	FLCDNA	TAIR
AAGATTCTGA	1	0	2	1	0	gnl|UG|At#S18942571	gi|13430773|gb|AF360299.1	At1g15430.2
at5g48570	CON	MIN	HOUR	DAY	WEEK	UNIGENE	FLCDNA	TAIR
AGTCTTTGAA	0	0	1	1	0	gnl|UG|At#S34118277	gi|110736035|dbj|AK228029.1|	At5g48570.1
at5g67290	CON	MIN	HOUR	DAY	WEEK	UNIGENE	FLCDNA	TAIR
GTGGTGAAGC	1	0	1	0	0	gnl|UG|At#S11716966	gi|21403150|gb|AY084440.1	At5g67290.1
at1g02270	CON	MIN	HOUR	DAY	WEEK	UNIGENE	FLCDNA	TAIR
CTTGTCTCAC	0	0	1	1	2	no match found	gi|17380605|gb|AY063716.1	non-canonical match
at3g28960	CON	MIN	HOUR	DAY	WEEK	UNIGENE	FLCDNA	TAIR
GCGACCGTCG	0	1	0	0	0	gnl|UG|At#S11731985	no match found	non-canonical match
at5g05480	CON	MIN	HOUR	DAY	WEEK	UNIGENE	FLCDNA	TAIR
TTCAAGGTTG	0	1	0	2	0	no match found	gi|24111304|gb|BT001022.1	multiple non-canonical match
TTTGGGGTCA	2	2	1	2	0	gnl|UG|At#S11724764	gi|13605890|gb|AF367344.1	non-canonical match
at3g24050	CON	MIN	HOUR	DAY	WEEK	UNIGENE	FLCDNA	TAIR
GAATGGAAGA	4	1	7	7	4	gnl|UG|At#S11733573	gi|21406335|gb|AY087597.1	At3g24050.1
at4g25440	CON	MIN	HOUR	DAY	WEEK	UNIGENE	FLCDNA	TAIR
TTTCAGTGTA	0	1	1	0	0	no match found	no match found	At4g25440.1
at4g38280	CON	MIN	HOUR	DAY	WEEK	UNIGENE	FLCDNA	TAIR
TGGTGGATGC	0	0	1	0	0	no match found	no match found	At4g38280.1
at5g52090	CON	MIN	HOUR	DAY	WEEK	UNIGENE	FLCDNA	TAIR
GAAGAAGAAA	2	2	2	2	0	gnl|UG|At#S11718507	no match found	multiple non-canonical match
at1g15400	CON	MIN	HOUR	DAY	WEEK	UNIGENE	FLCDNA	TAIR
CGGTTGTTGC	0	2	0	0	0	gnl|UG|At#S11741495	gi|12484218|gb|AF336927.1	At1g15400.1
AGGAAAAAAT	3	3	1	1	0	no match found	gi|21403881|gb|AY085171.1	At1g15400.2
at2g31570	CON	MIN	HOUR	DAY	WEEK	UNIGENE	FLCDNA	TAIR
TGACTCTCGT	19	13	31	19	16	gnl|UG|At#S11734645	no match found	At2g31570.1

at5g64680	CON	MIN	HOUR	DAY	WEEK	UNIGENE	FLCDNA	TAIR
TTCTCTGGCT	0	1	0	1	1	gnl|UG|At#S11717231	no match found	At5g64680.1
at4g30260	CON	MIN	HOUR	DAY	WEEK	UNIGENE	FLCDNA	TAIR
TATGTCTCTG	1	0	1	0	0	gnl|UG|At#S11722573	gi|20260559|gb|AY093179.1	At4g30260.1
at1g25320	CON	MIN	HOUR	DAY	WEEK	UNIGENE	FLCDNA	TAIR
GATCTCTGAA	1	1	0	0	0	gnl|UG|At#S11705826	gi|34365564|gb|BT010471.1	At1g25320.1
at2g37750	CON	MIN	HOUR	DAY	WEEK	UNIGENE	FLCDNA	TAIR
GTAAAAAAGA	1	0	0	0	0	gnl|UG|At#S11710276	gi|17529125|gb|AY065348.1	non-canonical match
at4g38260	CON	MIN	HOUR	DAY	WEEK	UNIGENE	FLCDNA	TAIR
TAAACACAAA	0	1	0	3	1	no match found	gi|15451201|gb|AY054681.1	non-canonical match
GAAGGAACAC	0	0	1	0	0	gnl|UG|At#S19189591	gi|45680330|gb|BT012020.1	non-canonical match
at3g51390	CON	MIN	HOUR	DAY	WEEK	UNIGENE	FLCDNA	TAIR
TTTCCTCTAA	2	3	0	1	0	gnl|UG|At#S11729176	gi|21406313|gb|AY087575.1	At3g51390.1
at3g09880	CON	MIN	HOUR	DAY	WEEK	UNIGENE	FLCDNA	TAIR
GGTTCAAAAT	1	0	1	3	1	gnl|UG|At#S11737921	no match found	At3g09880.1
at2g09970	CON	MIN	HOUR	DAY	WEEK	UNIGENE	FLCDNA	TAIR
ATTGTGTTTC	1	3	0	2	0	no match found	no match found	At2g09970.1
at3g24503	CON	MIN	HOUR	DAY	WEEK	UNIGENE	FLCDNA	TAIR
GACGCTCTCG	0	3	7	7	7	gnl|UG|At#S11733416	gi|62319809|dbj|AK221230.1|	At3g24503.1
at4g35335	CON	MIN	HOUR	DAY	WEEK	UNIGENE	FLCDNA	TAIR
AAACCTTCTA	1	1	0	1	0	no match found	gi|51971023|dbj|AK176441.1|	pseudo chromosome match
at2g07140	CON	MIN	HOUR	DAY	WEEK	UNIGENE	FLCDNA	TAIR
GCCTAAGTTT	0	1	0	0	0	gnl|UG|At#S11739718	no match found	multiple non-canonical match
at1g03510	CON	MIN	HOUR	DAY	WEEK	UNIGENE	FLCDNA	TAIR
AAATTTTTGT	0	0	1	0	0	no match found	no match found	At1g03510.1
at1g64580	CON	MIN	HOUR	DAY	WEEK	UNIGENE	FLCDNA	TAIR
AGTAAAGCGG	0	0	0	1	0	no match found	gi|110741327|dbj|AK230416.1|	pseudo chromosome match
at4g35785	CON	MIN	HOUR	DAY	WEEK	UNIGENE	FLCDNA	TAIR
GAAGAAGCCC	3	1	0	1	0	gnl|UG|At#S18905576	gi|28393646|gb|BT004226.1	At4g35785.1
at2g25250	CON	MIN	HOUR	DAY	WEEK	UNIGENE	FLCDNA	TAIR
GGGATTGAAA	3	3	1	1	0	gnl|UG|At#S11736210	gi|110736279|dbj|AK228154.1|	At2g25250.1
at1g70700	CON	MIN	HOUR	DAY	WEEK	UNIGENE	FLCDNA	TAIR
TCTGTCAAGA	6	13	48	4	4	gnl|UG|At#S38434079	gi|110740150|dbj|AK230165.1|	multiple canonical match
at2g05060	CON	MIN	HOUR	DAY	WEEK	UNIGENE	FLCDNA	TAIR
GCAAGACGAC	15	7	15	10	1	no match found	no match found	At2g05060.1
at5g54980	CON	MIN	HOUR	DAY	WEEK	UNIGENE	FLCDNA	TAIR
TCTTTGTTCT	0	2	0	1	0	gnl|UG|At#S11825066	gi|27754654|gb|BT002961.1	At5g54980.1
at2g42820	CON	MIN	HOUR	DAY	WEEK	UNIGENE	FLCDNA	TAIR
TATGTACATA	1	0	0	0	0	gnl|UG|At#S11731922	gi|51968935|dbj|AK175397.1|	At2g42820.1
at4g18170	CON	MIN	HOUR	DAY	WEEK	UNIGENE	FLCDNA	TAIR
ATATGTTTAG	0	0	0	0	1	gnl|UG|At#S18907479	gi|21435998|gb|AY116943.1	non-canonical match
AAAGAATGAG	0	2	2	0	0	no match found	gi|17979527|gb|AY070761.1	At4g18170.1

at3g17130	CON	MIN	HOUR	DAY	WEEK	UNIGENE	FLCDNA	TAIR
CTCACGATCC	1	0	0	2	0	gnl|UG|At#S18911503	no match found	At3g17130.1
at1g65960	CON	MIN	HOUR	DAY	WEEK	UNIGENE	FLCDNA	TAIR
CTTGTTGATT	17	6	12	8	14	no match found	no match found	At1g65960.1
AGCTAGATAC	3	12	1	2	4	no match found	gi|16226293|gb|AF428294.1	non-canonical match
at5g54470	CON	MIN	HOUR	DAY	WEEK	UNIGENE	FLCDNA	TAIR
CGTTGTCTTC	0	0	1	0	1	gnl|UG|At#S24442644	gi|62320385|dbj|AK221519.1|	At5g54470.1
at4g19020	CON	MIN	HOUR	DAY	WEEK	UNIGENE	FLCDNA	TAIR
AGGCTTCGTC	1	1	0	1	0	gnl|UG|At#S11724498	no match found	At4g19020.1
at1g48490	CON	MIN	HOUR	DAY	WEEK	UNIGENE	FLCDNA	TAIR
GTCTCTTAGG	0	4	2	2	0	gnl|UG|At#S28282525	gi|14326577|gb|AF385743.1	At1g48490.1
at5g11680	CON	MIN	HOUR	DAY	WEEK	UNIGENE	FLCDNA	TAIR
CGTACGTGGA	1	1	1	0	1	gnl|UG|At#S11723328	gi|17065569|gb|AY062861.1	At5g11680.1
at5g47435	CON	MIN	HOUR	DAY	WEEK	UNIGENE	FLCDNA	TAIR
GTCTCTTACC	1	2	0	1	0	no match found	gi|20148260|gb|AY081459.1	At5g47435.1
at4g15410	CON	MIN	HOUR	DAY	WEEK	UNIGENE	FLCDNA	TAIR
GGGTTCCCTC	0	2	0	2	0	gnl|UG|At#S18894591	gi|15292664|gb|AY050766.1	At4g15410.1
at3g47340	CON	MIN	HOUR	DAY	WEEK	UNIGENE	FLCDNA	TAIR
CCGATGATGA	8	45	5	1	0	gnl|UG|At#S15460140	gi|20465518|gb|AY096592.1	multiple canonical match
at1g77890	CON	MIN	HOUR	DAY	WEEK	UNIGENE	FLCDNA	TAIR
TGCACTTGTG	0	1	0	0	0	no match found	gi|51970759|dbj|AK176309.1|	non-canonical match
at3g58620	CON	MIN	HOUR	DAY	WEEK	UNIGENE	FLCDNA	TAIR
TTGGTTTAAA	0	2	0	0	0	no match found	gi|28827389|gb|BT005006.1	non-canonical match
at4g30190	CON	MIN	HOUR	DAY	WEEK	UNIGENE	FLCDNA	TAIR
AGCCTATGTA	9	6	2	6	16	gnl|UG|At#S11821352	gi|23397276|gb|BT000781.1	At4g30190.1
TGGAATCAGT	0	0	1	0	0	no match found	gi|25055008|gb|BT001969.1	multiple non-canonical match
at1g71240	CON	MIN	HOUR	DAY	WEEK	UNIGENE	FLCDNA	TAIR
TTTTAACTAG	0	0	1	0	1	gnl|UG|At#S34116841	gi|110738803|dbj|AK229467.1|	At1g71240.1
at5g55507	CON	MIN	HOUR	DAY	WEEK	UNIGENE	FLCDNA	TAIR
GATTATGGTC	0	1	0	0	0	gnl|UG|At#S14960002	no match found	At5g55507.1
at2g44820	CON	MIN	HOUR	DAY	WEEK	UNIGENE	FLCDNA	TAIR
TGTTTTCAGA	1	0	0	1	0	gnl|UG|At#S34117682	gi|110737173|dbj|AK228626.1|	multiple non-canonical match
GAAGGATAAG	0	1	0	1	0	no match found	gi|19310390|gb|AY078929.1	non-canonical match
at1g27930	CON	MIN	HOUR	DAY	WEEK	UNIGENE	FLCDNA	TAIR
GGGCATTATA	1	0	2	0	1	gnl|UG|At#S11739657	no match found	At1g27930.1
TGGGCTTCTT	0	3	1	1	0	no match found	gi|15215712|gb|AY050385.1	multiple non-canonical match
at4g11270	CON	MIN	HOUR	DAY	WEEK	UNIGENE	FLCDNA	TAIR
TTCTAGGTTC	1	0	0	0	2	gnl|UG|At#S11725836	no match found	At4g11270.1
at5g55300	CON	MIN	HOUR	DAY	WEEK	UNIGENE	FLCDNA	TAIR
TCAAGCTGTT	1	1	2	1	0	gnl|UG|At#S11718183	gi|20258814|gb|AY090993.1	At5g55300.1
at1g76010	CON	MIN	HOUR	DAY	WEEK	UNIGENE	FLCDNA	TAIR
AACACCGTGG	0	6	1	0	0	no match found	gi|21700864|gb|AY124847.1	non-canonical match

<table>
<tr><td>TTTGCCATAA</td><td>8</td><td>13</td><td>8</td><td>4</td><td>1</td><td>gnl|UG|At#S11727840</td><td>gi|15809881|gb|AY054208.1</td><td>At1g76010.1</td></tr>
<tr><td>at4g18880</td><td>CON</td><td>MIN</td><td>HOUR</td><td>DAY</td><td>WEEK</td><td>UNIGENE</td><td>FLCDNA</td><td>TAIR</td></tr>
<tr><td>GAGAAGCGTC</td><td>0</td><td>3</td><td>3</td><td>0</td><td>1</td><td>gnl|UG|At#S11815775</td><td>gi|24111358|gb|BT001049.1</td><td>At4g18880.1</td></tr>
<tr><td>at2g30110</td><td>CON</td><td>MIN</td><td>HOUR</td><td>DAY</td><td>WEEK</td><td>UNIGENE</td><td>FLCDNA</td><td>TAIR</td></tr>
<tr><td>TTCTATCAAA</td><td>6</td><td>4</td><td>5</td><td>3</td><td>2</td><td>gnl|UG|At#S11735004</td><td>gi|62318770|dbj|AK220705.1|</td><td>At2g30110.1</td></tr>
<tr><td>GCTTGGACAG</td><td>0</td><td>0</td><td>0</td><td>1</td><td>1</td><td>no match found</td><td>gi|30102505|gb|BT006363.1</td><td>multiple non-canonical match</td></tr>
<tr><td>at4g37470</td><td>CON</td><td>MIN</td><td>HOUR</td><td>DAY</td><td>WEEK</td><td>UNIGENE</td><td>FLCDNA</td><td>TAIR</td></tr>
<tr><td>AGACAGATCT</td><td>2</td><td>4</td><td>3</td><td>3</td><td>2</td><td>no match found</td><td>gi|20259140|gb|AY091347.1</td><td>multiple non-canonical match</td></tr>
<tr><td>at5g56750</td><td>CON</td><td>MIN</td><td>HOUR</td><td>DAY</td><td>WEEK</td><td>UNIGENE</td><td>FLCDNA</td><td>TAIR</td></tr>
<tr><td>GGATTAAAGC</td><td>0</td><td>2</td><td>0</td><td>0</td><td>0</td><td>gnl|UG|At#S11718037</td><td>gi|19699253|gb|AY090332.1</td><td>At5g56750.1</td></tr>
<tr><td>at1g02150</td><td>CON</td><td>MIN</td><td>HOUR</td><td>DAY</td><td>WEEK</td><td>UNIGENE</td><td>FLCDNA</td><td>TAIR</td></tr>
<tr><td>AGACCGATGC</td><td>1</td><td>1</td><td>2</td><td>0</td><td>0</td><td>no match found</td><td>gi|29028735|gb|BT005812.1</td><td>non-canonical match</td></tr>
<tr><td>AATGTAAGAG</td><td>3</td><td>2</td><td>4</td><td>2</td><td>4</td><td>gnl|UG|At#S11742901</td><td>gi|20453084|gb|AY094410.1</td><td>At1g02150.1</td></tr>
<tr><td>at1g73440</td><td>CON</td><td>MIN</td><td>HOUR</td><td>DAY</td><td>WEEK</td><td>UNIGENE</td><td>FLCDNA</td><td>TAIR</td></tr>
<tr><td>TTGAAAGGTT</td><td>1</td><td>2</td><td>1</td><td>0</td><td>0</td><td>no match found</td><td>gi|13358218|gb|AF325029.2</td><td>At1g73440.1</td></tr>
<tr><td>at2g25480</td><td>CON</td><td>MIN</td><td>HOUR</td><td>DAY</td><td>WEEK</td><td>UNIGENE</td><td>FLCDNA</td><td>TAIR</td></tr>
<tr><td>ATGTTTGGTC</td><td>1</td><td>0</td><td>1</td><td>2</td><td>1</td><td>gnl|UG|At#S34114247</td><td>gi|110743830|dbj|AK227768.1|</td><td>At2g25480.1</td></tr>
<tr><td>at1g64520</td><td>CON</td><td>MIN</td><td>HOUR</td><td>DAY</td><td>WEEK</td><td>UNIGENE</td><td>FLCDNA</td><td>TAIR</td></tr>
<tr><td>TAATGACCGA</td><td>6</td><td>3</td><td>5</td><td>2</td><td>6</td><td>gnl|UG|At#S11730230</td><td>gi|14532465|gb|AY039857.1</td><td>At1g64520.1</td></tr>
<tr><td>at1g75230</td><td>CON</td><td>MIN</td><td>HOUR</td><td>DAY</td><td>WEEK</td><td>UNIGENE</td><td>FLCDNA</td><td>TAIR</td></tr>
<tr><td>TCCTCTGCTT</td><td>0</td><td>1</td><td>0</td><td>0</td><td>0</td><td>no match found</td><td>no match found</td><td>At1g75230.2</td></tr>
<tr><td>at3g13230</td><td>CON</td><td>MIN</td><td>HOUR</td><td>DAY</td><td>WEEK</td><td>UNIGENE</td><td>FLCDNA</td><td>TAIR</td></tr>
<tr><td>CTTGGTTTTG</td><td>0</td><td>1</td><td>0</td><td>2</td><td>0</td><td>gnl|UG|At#S15644854</td><td>gi|30794067|gb|BT008667.1</td><td>At3g13230.1</td></tr>
<tr><td>at5g40520</td><td>CON</td><td>MIN</td><td>HOUR</td><td>DAY</td><td>WEEK</td><td>UNIGENE</td><td>FLCDNA</td><td>TAIR</td></tr>
<tr><td>GTGAGCAGAA</td><td>0</td><td>1</td><td>0</td><td>0</td><td>0</td><td>gnl|UG|At#S11719678</td><td>gi|51968741|dbj|AK175300.1|</td><td>At5g40520.1</td></tr>
<tr><td>at2g39910</td><td>CON</td><td>MIN</td><td>HOUR</td><td>DAY</td><td>WEEK</td><td>UNIGENE</td><td>FLCDNA</td><td>TAIR</td></tr>
<tr><td>AATGAGATGC</td><td>0</td><td>1</td><td>0</td><td>0</td><td>0</td><td>gnl|UG|At#S24442020</td><td>gi|62321612|dbj|AK222143.1|</td><td>At2g39910.1</td></tr>
<tr><td>at4g35250</td><td>CON</td><td>MIN</td><td>HOUR</td><td>DAY</td><td>WEEK</td><td>UNIGENE</td><td>FLCDNA</td><td>TAIR</td></tr>
<tr><td>GACAACACAA</td><td>2</td><td>2</td><td>1</td><td>2</td><td>0</td><td>gnl|UG|At#S11721722</td><td>gi|18087582|gb|AF462834.1</td><td>At4g35250.1</td></tr>
<tr><td>at4g21390</td><td>CON</td><td>MIN</td><td>HOUR</td><td>DAY</td><td>WEEK</td><td>UNIGENE</td><td>FLCDNA</td><td>TAIR</td></tr>
<tr><td>GCTTCAGTTT</td><td>0</td><td>1</td><td>0</td><td>0</td><td>0</td><td>no match found</td><td>no match found</td><td>At4g21390.1</td></tr>
<tr><td>at5g65730</td><td>CON</td><td>MIN</td><td>HOUR</td><td>DAY</td><td>WEEK</td><td>UNIGENE</td><td>FLCDNA</td><td>TAIR</td></tr>
<tr><td>TTTCGTCTTG</td><td>4</td><td>5</td><td>1</td><td>2</td><td>2</td><td>no match found</td><td>no match found</td><td>At5g65730.1</td></tr>
<tr><td>GCAAAGGAGA</td><td>0</td><td>1</td><td>0</td><td>0</td><td>0</td><td>no match found</td><td>gi|20334765|gb|AY093983.1</td><td>multiple non-canonical match</td></tr>
<tr><td>at1g29170</td><td>CON</td><td>MIN</td><td>HOUR</td><td>DAY</td><td>WEEK</td><td>UNIGENE</td><td>FLCDNA</td><td>TAIR</td></tr>
<tr><td>GAGTGATACG</td><td>1</td><td>0</td><td>0</td><td>0</td><td>1</td><td>gnl|UG|At#S43850550</td><td>no match found</td><td>non-canonical match</td></tr>
<tr><td>at2g19330</td><td>CON</td><td>MIN</td><td>HOUR</td><td>DAY</td><td>WEEK</td><td>UNIGENE</td><td>FLCDNA</td><td>TAIR</td></tr>
<tr><td>AGGCAGTTGA</td><td>0</td><td>1</td><td>0</td><td>0</td><td>0</td><td>no match found</td><td>gi|17979058|gb|AY070040.1</td><td>At2g19340.1</td></tr>
<tr><td>at4g01020</td><td>CON</td><td>MIN</td><td>HOUR</td><td>DAY</td><td>WEEK</td><td>UNIGENE</td><td>FLCDNA</td><td>TAIR</td></tr>
<tr><td>TATCTATATC</td><td>1</td><td>0</td><td>0</td><td>0</td><td>0</td><td>gnl|UG|At#S11727434</td><td>no match found</td><td>At4g01020.1</td></tr>
<tr><td>at1g80310</td><td>CON</td><td>MIN</td><td>HOUR</td><td>DAY</td><td>WEEK</td><td>UNIGENE</td><td>FLCDNA</td><td>TAIR</td></tr>
<tr><td>CTGGTCTGCG</td><td>1</td><td>1</td><td>1</td><td>0</td><td>0</td><td>gnl|UG|At#S11726390</td><td>gi|17381199|gb|AY064056.1</td><td>At1g80310.1</td></tr>
</table>

at1g23400	CON	MIN	HOUR	DAY	WEEK	UNIGENE	FLCDNA	TAIR
TGGAGAGGAC	1	6	0	0	0	gnl|UG|At#S11740721	gi|21387128|gb|AY114649.1	At1g23400.1
at1g09500	CON	MIN	HOUR	DAY	WEEK	UNIGENE	FLCDNA	TAIR
TTATTTAAAA	0	0	2	1	1	gnl|UG|At#S15461337	no match found	At1g09500.2
at5g05220	CON	MIN	HOUR	DAY	WEEK	UNIGENE	FLCDNA	TAIR
GGGGCTGATG	0	0	0	2	1	gnl|UG|At#S11706904	gi|15450646|gb|AY052691.1	At5g05220.1
at1g14000	CON	MIN	HOUR	DAY	WEEK	UNIGENE	FLCDNA	TAIR
AACCAGAGAC	2	2	0	0	0	no match found	gi|111074449|gb|BT026491.1	multiple non-canonical match
GATTGGATTG	5	4	8	2	4	gnl|UG|At#S11741637	gi|62319095|dbj|AK220869.1|	At1g14000.1
at3g51050	CON	MIN	HOUR	DAY	WEEK	UNIGENE	FLCDNA	TAIR
CACTTATCAC	0	1	1	0	1	gnl|UG|At#S11729237	gi|18087607|gb|AF462847.1	At3g51050.1
at1g56280	CON	MIN	HOUR	DAY	WEEK	UNIGENE	FLCDNA	TAIR
GAGGAACTAA	1	22	9	6	1	no match found	gi|62319443|dbj|AK221045.1|	multiple canonical match
at4g37300	CON	MIN	HOUR	DAY	WEEK	UNIGENE	FLCDNA	TAIR
GTAAAGTTCT	5	9	0	0	1	no match found	gi|17065601|gb|AY062949.1	non-canonical match
at5g53350	CON	MIN	HOUR	DAY	WEEK	UNIGENE	FLCDNA	TAIR
GACTCTATGT	1	4	0	0	1	gnl|UG|At#S11718381	gi|23296602|gb|AY142561.1	At5g53350.1
at1g69640	CON	MIN	HOUR	DAY	WEEK	UNIGENE	FLCDNA	TAIR
CCTTACTCGC	0	0	0	0	1	no match found	gi|23505994|gb|AY143918.1	non-canonical match
TTTTTTTTAT	1	0	0	1	3	gnl|UG|At#S11729175	gi|17979534|gb|AY070765.1	multiple canonical match
at5g55260	CON	MIN	HOUR	DAY	WEEK	UNIGENE	FLCDNA	TAIR
TGTTGCCAAA	1	0	1	0	0	no match found	no match found	At5g55260.1
at1g12940	CON	MIN	HOUR	DAY	WEEK	UNIGENE	FLCDNA	TAIR
GAATGAACAA	0	0	1	0	0	gnl|UG|At#S11741743	no match found	At1g12940.1
at2g24250	CON	MIN	HOUR	DAY	WEEK	UNIGENE	FLCDNA	TAIR
ATCTCTAGCA	1	1	0	0	0	gnl|UG|At#S11750418	gi|14335043|gb|AY037201.1	At2g24250.1
at2g01720	CON	MIN	HOUR	DAY	WEEK	UNIGENE	FLCDNA	TAIR
CTTGTATCGG	1	3	0	2	3	gnl|UG|At#S11742630	gi|110740743|dbj|AK226319.1|	At2g01720.1
at4g10600	CON	MIN	HOUR	DAY	WEEK	UNIGENE	FLCDNA	TAIR
TACAAGGAGT	0	0	1	0	0	gnl|UG|At#S11824133	gi|26450441|dbj|AK117683.1|	At4g10600.1
at3g59940	CON	MIN	HOUR	DAY	WEEK	UNIGENE	FLCDNA	TAIR
GTCGACAATC	2	6	2	1	0	gnl|UG|At#S11727521	gi|20258926|gb|AY091240.1	At3g59940.1
at2g31650	CON	MIN	HOUR	DAY	WEEK	UNIGENE	FLCDNA	TAIR
CAAAAATATG	0	1	0	0	0	gnl|UG|At#S15460905	gi|27754616|gb|BT002941.1	At2g31650.1
at4g33470	CON	MIN	HOUR	DAY	WEEK	UNIGENE	FLCDNA	TAIR
TCCTGGATCC	1	1	0	0	0	no match found	gi|15529219|gb|AY052234.1	At4g33470.1
at3g12630	CON	MIN	HOUR	DAY	WEEK	UNIGENE	FLCDNA	TAIR
TCTTTCTGGT	4	2	7	1	2	gnl|UG|At#S11737104	no match found	At3g12630.1
CAACTATGAT	0	0	1	0	0	gnl|UG|At#S35277419	no match found	multiple non-canonical match
ATTGTAGCTA	0	6	0	0	0	no match found	gi|32815926|gb|BT009714.1	non-canonical match
GATTTGATTT	3	10	1	3	2	no match found	gi|21403465|gb|AY084755.1	multiple non-canonical match
at2g29990	CON	MIN	HOUR	DAY	WEEK	UNIGENE	FLCDNA	TAIR

GCTACTATCG	0	0	0	1	0	gnl|UG|At#S11735040	no match found	non-canonical match
at2g04660	CON	MIN	HOUR	DAY	WEEK	UNIGENE	FLCDNA	TAIR
TGTTAGACTA	0	0	0	1	2	gnl|UG|At#S11823401	gi|26451937|dbj|AK118453.1|	At2g04660.1
at4g38370	CON	MIN	HOUR	DAY	WEEK	UNIGENE	FLCDNA	TAIR
CTCATTTATG	0	1	0	0	2	gnl|UG|At#S11721207	gi|110741101|dbj|AK226503.1|	non-canonical match
at4g23790	CON	MIN	HOUR	DAY	WEEK	UNIGENE	FLCDNA	TAIR
TAAAATTATG	0	0	0	1	2	gnl|UG|At#S18907673	no match found	At4g23790.1
at5g59070	CON	MIN	HOUR	DAY	WEEK	UNIGENE	FLCDNA	TAIR
GTTTCAGGAA	0	1	0	0	1	gnl|UG|At#S14273961	gi|28393194|gb|BT003988.1	At5g59070.1
at1g73885	CON	MIN	HOUR	DAY	WEEK	UNIGENE	FLCDNA	TAIR
TTTTGGTTTT	0	0	0	0	1	no match found	no match found	At1g73885.1
at5g16380	CON	MIN	HOUR	DAY	WEEK	UNIGENE	FLCDNA	TAIR
AATCTTTTTG	0	0	1	0	0	no match found	gi|17381065|gb|AY063989.1	At5g16380.1
at3g08730	CON	MIN	HOUR	DAY	WEEK	UNIGENE	FLCDNA	TAIR
TGTCAGAAGG	3	0	0	0	1	no match found	gi|110739899|dbj|AK230033.1|	At5g16380.1
AACATTAAAG	5	1	1	3	4	gnl|UG|At#S11738278	gi|21403645|gb|AY084935.1	At3g08730.1
at2g37410	CON	MIN	HOUR	DAY	WEEK	UNIGENE	FLCDNA	TAIR
CTATGTTCTC	7	7	7	2	4	gnl|UG|At#S11733248	gi|13430691|gb|AF360258.1	At2g37410.1
GTTTGGAGGG	0	0	1	0	0	no match found	gi|14532895|gb|AY040072.1	non-canonical match
at5g23060	CON	MIN	HOUR	DAY	WEEK	UNIGENE	FLCDNA	TAIR
GATACGATTT	5	7	7	2	2	gnl|UG|At#S11720872	gi|19310662|gb|AY079331.1	At5g23060.1
at2g04780	CON	MIN	HOUR	DAY	WEEK	UNIGENE	FLCDNA	TAIR
GTTATTTCCG	3	3	4	1	1	gnl|UG|At#S15461657	gi|21403251|gb|AY084541.1	At2g04780.1
at5g37400	CON	MIN	HOUR	DAY	WEEK	UNIGENE	FLCDNA	TAIR
CTCAATACTT	1	0	0	0	0	gnl|UG|At#S11719995	gi|110739839|dbj|AK230002.1|	At5g37400.1
at3g12180	CON	MIN	HOUR	DAY	WEEK	UNIGENE	FLCDNA	TAIR
GAGGGTTTCA	0	1	0	1	1	gnl|UG|At#S11737231	gi|110735779|dbj|AK227896.1|	At3g12180.1
GTTGTCTTTA	0	1	0	1	0	no match found	gi|30017218|gb|BT006194.1	non-canonical match
at1g78440	CON	MIN	HOUR	DAY	WEEK	UNIGENE	FLCDNA	TAIR
CAAGAGTGTG	0	0	0	0	1	gnl|UG|At#S11699419	gi|13430551|gb|AF360188.1	At1g78440.1
at1g53330	CON	MIN	HOUR	DAY	WEEK	UNIGENE	FLCDNA	TAIR
AGTTCATTAT	1	0	0	0	0	no match found	no match found	At1g53330.1
at3g49730	CON	MIN	HOUR	DAY	WEEK	UNIGENE	FLCDNA	TAIR
TCCTAAATCA	0	1	0	0	0	gnl|UG|At#S11729495	gi|110743593|dbj|AK227647.1|	At3g49730.1
at1g76150	CON	MIN	HOUR	DAY	WEEK	UNIGENE	FLCDNA	TAIR
CCGGATGAAG	2	0	2	0	1	no match found	gi|17979530|gb|AY070763.1	At1g76150.1
at3g29180	CON	MIN	HOUR	DAY	WEEK	UNIGENE	FLCDNA	TAIR
AGCATTGTAC	0	2	0	0	1	no match found	gi|17978712|gb|AY064637.1	At3g29180.1
at4g16720	CON	MIN	HOUR	DAY	WEEK	UNIGENE	FLCDNA	TAIR
GAAGAAAAAC	2	0	1	0	0	no match found	gi|23505794|gb|AY143818.1	multiple non-canonical match
TTGTTGCACT	18	18	19	17	26	gnl|UG|At#S11724902	gi|21405026|gb|AY086316.1	At4g16720.1
at5g64640	CON	MIN	HOUR	DAY	WEEK	UNIGENE	FLCDNA	TAIR

AGGTTTTAGG	0	1	0	1	0	gnl|UG|At#S11717235	gi|22135881|gb|AY128320.1	pseudo chromosome match
at1g55760	CON	MIN	HOUR	DAY	WEEK	UNIGENE	FLCDNA	TAIR
ATTTGATTGA	1	1	0	1	1	gnl|UG|At#S11732377	gi|51969391|dbj|AK175625.1|	At1g55760.1
at3g09260	CON	MIN	HOUR	DAY	WEEK	UNIGENE	FLCDNA	TAIR
ATTTGCCAGA	33	60	40	22	1	no match found	gi|22655221|gb|AY140060.1	At3g09260.1
at1g73090	CON	MIN	HOUR	DAY	WEEK	UNIGENE	FLCDNA	TAIR
GCAAGAAGGT	0	0	0	0	1	gnl|UG|At#S11728447	gi|51971644|dbj|AK176724.1|	At1g73090.1
at1g55440	CON	MIN	HOUR	DAY	WEEK	UNIGENE	FLCDNA	TAIR
GCGGATGTGA	0	0	0	1	0	no match found	no match found	At1g55440.1
at4g32610	CON	MIN	HOUR	DAY	WEEK	UNIGENE	FLCDNA	TAIR
ACAAGAAGAA	4	2	1	0	1	no match found	gi|110743170|dbj|AK227475.1|	multiple non-canonical match
AAAACCCATC	0	0	0	0	1	no match found	no match found	At4g32610.1
GTTATACTTG	0	0	1	0	1	gnl|UG|At#S11722174	no match found	non-canonical match
at1g02780	CON	MIN	HOUR	DAY	WEEK	UNIGENE	FLCDNA	TAIR
CTAGGAGAGA	23	37	17	14	14	gnl|UG|At#S11742839	gi|21405592|gb|AY086882.1	At1g02780.1
at5g07670	CON	MIN	HOUR	DAY	WEEK	UNIGENE	FLCDNA	TAIR
AATACTTAAC	1	0	0	0	0	gnl|UG|At#S11709949	gi|20465474|gb|AY096547.1	At5g07670.1
at1g14960	CON	MIN	HOUR	DAY	WEEK	UNIGENE	FLCDNA	TAIR
GTGTTGTATG	0	0	0	1	0	no match found	gi|14596068|gb|AY042822.1	multiple canonical match
at1g26220	CON	MIN	HOUR	DAY	WEEK	UNIGENE	FLCDNA	TAIR
TTTCTCGGAG	2	1	1	0	0	gnl|UG|At#S35310273	gi|26450705|dbj|AK117820.1|	At1g26220.1
at1g35490	CON	MIN	HOUR	DAY	WEEK	UNIGENE	FLCDNA	TAIR
TTATGTAATA	0	0	0	0	1	gnl|UG|At#S11737102	gi|21406043|gb|AY087319.1	At1g35490.1
at3g18510	CON	MIN	HOUR	DAY	WEEK	UNIGENE	FLCDNA	TAIR
ATGGTGGACC	0	0	0	1	2	gnl|UG|At#S11823860	gi|26450998|dbj|AK117969.1|	At3g18510.1
at3g60320	CON	MIN	HOUR	DAY	WEEK	UNIGENE	FLCDNA	TAIR
GAGTCTCTTC	0	5	2	2	1	gnl|UG|At#S11727388	gi|16604628|gb|AY059759.1	At3g60320.1
at1g48240	CON	MIN	HOUR	DAY	WEEK	UNIGENE	FLCDNA	TAIR
TTGTATAATT	0	10	0	1	1	gnl|UG|At#S11735128	gi|26449738|dbj|AK117322.1|	At1g48240.1
at1g76400	CON	MIN	HOUR	DAY	WEEK	UNIGENE	FLCDNA	TAIR
ATAAGCTTGA	0	1	1	0	1	gnl|UG|At#S11727749	gi|22136201|gb|AY128779.1	multiple canonical match
at5g22875	CON	MIN	HOUR	DAY	WEEK	UNIGENE	FLCDNA	TAIR
TAACACCAGT	4	0	3	0	1	no match found	gi|21405634|gb|AY086916.1	At5g22875.1
at4g32590	CON	MIN	HOUR	DAY	WEEK	UNIGENE	FLCDNA	TAIR
TCAAACAATA	2	2	2	0	1	no match found	gi|22136785|gb|AY133803.1	At4g32590.2
at2g45710	CON	MIN	HOUR	DAY	WEEK	UNIGENE	FLCDNA	TAIR
ACAATCCATA	1	2	2	0	3	gnl|UG|At#S11731253	gi|110736238|dbj|AK228133.1|	At2g45710.1
at3g05220	CON	MIN	HOUR	DAY	WEEK	UNIGENE	FLCDNA	TAIR
TATGCTTGTC	3	9	5	0	2	gnl|UG|At#S34115076	gi|110742166|dbj|AK226941.1|	At3g05220.2
at1g54115	CON	MIN	HOUR	DAY	WEEK	UNIGENE	FLCDNA	TAIR
TTACGTAACA	0	0	0	0	1	no match found	no match found	At1g54115.1
TTGTTAGAGA	3	0	0	0	0	gnl|UG|At#S11732953	no match found	non-canonical match

at5g07890	CON	MIN	HOUR	DAY	WEEK	UNIGENE	FLCDNA	TAIR
GGTTTTCTGT	0	1	2	0	1	gnl|UG|At#S28281837	no match found	At5g07890.1
at2g17280	CON	MIN	HOUR	DAY	WEEK	UNIGENE	FLCDNA	TAIR
AATTTATTGT	0	1	0	0	3	gnl|UG|At#S38433980	gi|26450628|dbj|AK117779.1|	At2g17280.1
at2g41705	CON	MIN	HOUR	DAY	WEEK	UNIGENE	FLCDNA	TAIR
GAGAGCCTAT	3	0	0	2	0	gnl|UG|At#S11732165	gi|26453265|dbj|AK119136.1|	At2g41705.1
at2g46700	CON	MIN	HOUR	DAY	WEEK	UNIGENE	FLCDNA	TAIR
TGCATAAGGT	0	1	0	0	0	gnl|UG|At#S11731027	gi|18087554|gb|AF462819.1	At2g46700.1
at3g51250	CON	MIN	HOUR	DAY	WEEK	UNIGENE	FLCDNA	TAIR
GGTTGCTTTC	1	0	2	1	0	gnl|UG|At#S11729200	gi|15215687|gb|AY050372.1	At3g51250.1
at4g03250	CON	MIN	HOUR	DAY	WEEK	UNIGENE	FLCDNA	TAIR
AGAAAAAATC	0	0	1	0	0	no match found	no match found	At4g03250.1
at1g01710	CON	MIN	HOUR	DAY	WEEK	UNIGENE	FLCDNA	TAIR
AGACCCGTGG	1	0	1	0	0	no match found	gi|28950988|gb|BT005354.1	non-canonical match
at3g05165	CON	MIN	HOUR	DAY	WEEK	UNIGENE	FLCDNA	TAIR
GACTAGTGGG	1	4	2	2	0	gnl|UG|At#S11704868	gi|62319580|dbj|AK221115.1|	At3g05165.2
at5g13750	CON	MIN	HOUR	DAY	WEEK	UNIGENE	FLCDNA	TAIR
ATGTTTCGCG	0	0	4	2	0	gnl|UG|At#S15460958	gi|21405468|gb|AY086758.1	At5g13750.2
ACAGCGATGT	0	3	2	0	0	no match found	gi|17065581|gb|AY062867.1	At5g13750.1
at3g57940	CON	MIN	HOUR	DAY	WEEK	UNIGENE	FLCDNA	TAIR
ATAAGCTATG	0	0	1	1	1	gnl|UG|At#S34116951	gi|110738588|dbj|AK229357.1|	At3g57940.1
at3g11397	CON	MIN	HOUR	DAY	WEEK	UNIGENE	FLCDNA	TAIR
TGCACAAACA	1	0	2	2	1	gnl|UG|At#S11709519	gi|18087518|gb|AF462798.1	At3g11397.1
at3g16690	CON	MIN	HOUR	DAY	WEEK	UNIGENE	FLCDNA	TAIR
TATGGTTCTC	0	1	0	0	0	gnl|UG|At#S11735855	gi|62321642|dbj|AK222159.1|	At3g16690.1
at5g48590	CON	MIN	HOUR	DAY	WEEK	UNIGENE	FLCDNA	TAIR
AAGCAAAAAG	0	2	0	0	1	gnl|UG|At#S14274077	gi|21407967|gb|AY089193.1	multiple canonical match
at3g55880	CON	MIN	HOUR	DAY	WEEK	UNIGENE	FLCDNA	TAIR
TGAGACTACA	1	1	0	0	3	gnl|UG|At#S18904663	gi|22530949|gb|AY136313.1	At3g55880.2
at5g27680	CON	MIN	HOUR	DAY	WEEK	UNIGENE	FLCDNA	TAIR
GAGCTTAGAA	0	0	0	1	0	gnl|UG|At#S11707975	gi|16604618|gb|AY059754.1	At5g27680.1
GAGAAGCAAA	0	1	0	0	0	no match found	gi|22136825|gb|AY133823.1	multiple non-canonical match
at4g30610	CON	MIN	HOUR	DAY	WEEK	UNIGENE	FLCDNA	TAIR
TTGCCCATAT	1	0	0	0	0	gnl|UG|At#S11722514	gi|26983829|gb|BT002334.1	At4g30610.1
at1g03380	CON	MIN	HOUR	DAY	WEEK	UNIGENE	FLCDNA	TAIR
TGGAGATCAA	0	0	3	2	0	gnl|UG|At#S11822616	gi|27311608|gb|BT002410.1	At1g03380.1
at4g34910	CON	MIN	HOUR	DAY	WEEK	UNIGENE	FLCDNA	TAIR
TAGCATCAGG	0	0	2	2	0	no match found	gi|27754372|gb|BT002816.1	multiple non-canonical match
at4g04630	CON	MIN	HOUR	DAY	WEEK	UNIGENE	FLCDNA	TAIR
TTTTGGTGAA	0	0	3	0	0	gnl|UG|At#S11726493	no match found	At4g04630.1
at2g05520	CON	MIN	HOUR	DAY	WEEK	UNIGENE	FLCDNA	TAIR

TAATAGTGTC	4	2	3	1	0	no match found	gi|23296557|gb|AY142547.1	At2g17390.1
GTGCGTTTGT	128	162	172	178	154	no match found	gi|20148476|gb|AY081567.1	At2g05520.1
at1g31200	CON	MIN	HOUR	DAY	WEEK	UNIGENE	FLCDNA	TAIR
TCTGCTAAAA	1	0	1	0	2	gnl|UG|At#S35289199	no match found	non-canonical match
at3g11060	CON	MIN	HOUR	DAY	WEEK	UNIGENE	FLCDNA	TAIR
TTTTGTGAAT	0	1	0	0	0	gnl|UG|At#S35267487	no match found	non-canonical match
at3g25540	CON	MIN	HOUR	DAY	WEEK	UNIGENE	FLCDNA	TAIR
AATCTGAACC	1	0	2	0	0	gnl|UG|At#S11733139	gi|13430479|gb|AF360152.1	At3g25540.1
at4g00730	CON	MIN	HOUR	DAY	WEEK	UNIGENE	FLCDNA	TAIR
CACCGTTCAG	0	6	0	5	0	gnl|UG|At#S43849639	gi|17063159|gb|AY062100.1	At4g00730.1
at4g09010	CON	MIN	HOUR	DAY	WEEK	UNIGENE	FLCDNA	TAIR
AGAGGAAACT	32	22	44	30	2	gnl|UG|At#S11726059	gi|13899078|gb|AF370534.1	At4g09010.1
at3g59990	CON	MIN	HOUR	DAY	WEEK	UNIGENE	FLCDNA	TAIR
AAGAACTTTG	0	2	2	1	0	no match found	gi|23197709|gb|BT000063.1	multiple non-canonical match
AATTTGCTCA	4	1	1	2	2	no match found	gi|17473818|gb|AY065161.1	multiple canonical match
at5g64490	CON	MIN	HOUR	DAY	WEEK	UNIGENE	FLCDNA	TAIR
AAAGTTTGGA	0	1	0	0	0	gnl|UG|At#S14273777	gi|28393542|gb|BT004172.1	At5g64490.1
at3g49210	CON	MIN	HOUR	DAY	WEEK	UNIGENE	FLCDNA	TAIR
AACCAGATGA	0	0	2	0	0	gnl|UG|At#S34117087	gi|110738320|dbj|AK229221.1|	At3g49210.1
at1g79950	CON	MIN	HOUR	DAY	WEEK	UNIGENE	FLCDNA	TAIR
CAAGCATTGA	0	1	0	0	0	no match found	gi|110741965|dbj|AK226828.1|	multiple non-canonical match
at1g17170	CON	MIN	HOUR	DAY	WEEK	UNIGENE	FLCDNA	TAIR
CCAAATTCTG	0	0	1	3	2	no match found	gi|45752759|gb|BT012184.1	At1g17170.1
at3g16570	CON	MIN	HOUR	DAY	WEEK	UNIGENE	FLCDNA	TAIR
TAAATGTTTC	1	5	5	3	0	gnl|UG|At#S35283633	gi|21403298|gb|AY084588.1	At3g16570.1
at1g26530	CON	MIN	HOUR	DAY	WEEK	UNIGENE	FLCDNA	TAIR
CGTGTAATTT	0	1	0	0	0	no match found	no match found	At1g26530.1
at4g19050	CON	MIN	HOUR	DAY	WEEK	UNIGENE	FLCDNA	TAIR
GTAATAAGTT	1	0	1	0	0	no match found	gi|26449775|dbj|AK117341.1|	At4g19050.1
at5g13720	CON	MIN	HOUR	DAY	WEEK	UNIGENE	FLCDNA	TAIR
AACCCATATT	3	4	1	4	3	gnl|UG|At#S11722909	gi|20465417|gb|AY096494.1	At5g13720.1
at5g67420	CON	MIN	HOUR	DAY	WEEK	UNIGENE	FLCDNA	TAIR
TCCTTTATCT	0	1	0	0	0	gnl|UG|At#S11716953	gi|87116587|gb|BT024477.1	At5g67420.1
at4g40080	CON	MIN	HOUR	DAY	WEEK	UNIGENE	FLCDNA	TAIR
TAGATGATTA	0	2	0	0	0	gnl|UG|At#S11720909	gi|21407430|gb|AY088656.1	At4g40080.1
at3g23230	CON	MIN	HOUR	DAY	WEEK	UNIGENE	FLCDNA	TAIR
GATTCAAATA	0	0	1	0	0	no match found	no match found	At3g23230.1
at4g26100	CON	MIN	HOUR	DAY	WEEK	UNIGENE	FLCDNA	TAIR
ATCCCGAGCT	0	0	0	0	1	no match found	gi|20259044|gb|AY091299.1	multiple non-canonical match
CTTGTAATTT	0	0	1	0	0	no match found	no match found	At4g26100.3
AAATTTGGTC	1	2	1	0	0	no match found	gi|15292700|gb|AY050784.1	non-canonical match
at2g17020	CON	MIN	HOUR	DAY	WEEK	UNIGENE	FLCDNA	TAIR

GCTCTTGCAT	0	1	0	0	0	gnl|UG|At#S11738228	gi|21403837|gb|AY085127.1	At2g17020.1
at1g58250	CON	MIN	HOUR	DAY	WEEK	UNIGENE	FLCDNA	TAIR
GTTTAAGCGT	0	1	0	1	0	no match found	gi|110739030|dbj|AK229585.1|	non-canonical match
at3g28040	CON	MIN	HOUR	DAY	WEEK	UNIGENE	FLCDNA	TAIR
GATAGTTTCT	4	4	4	3	1	gnl|UG|At#S11732291	no match found	multiple canonical match
at3g24090	CON	MIN	HOUR	DAY	WEEK	UNIGENE	FLCDNA	TAIR
GACCTTTAGC	0	0	0	1	0	gnl|UG|At#S11733562	no match found	non-canonical match
at1g28490	CON	MIN	HOUR	DAY	WEEK	UNIGENE	FLCDNA	TAIR
CAAGTGAAGT	0	0	1	0	0	no match found	gi|23297415|gb|AY150420.1	non-canonical match
TTTGTGGTGA	2	1	1	1	2	no match found	gi|18175839|gb|AY072115.1	At1g28490.1
at3g06460	CON	MIN	HOUR	DAY	WEEK	UNIGENE	FLCDNA	TAIR
GTTTGAAATT	0	0	1	0	0	gnl|UG|At#S11738809	gi|18252966|gb|AY072418.1	At3g06460.1
at2g39980	CON	MIN	HOUR	DAY	WEEK	UNIGENE	FLCDNA	TAIR
CTCTCTTGCC	0	0	1	0	0	no match found	no match found	At2g39980.1
at1g31810	CON	MIN	HOUR	DAY	WEEK	UNIGENE	FLCDNA	TAIR
TCACACTTCT	0	0	1	0	0	gnl|UG|At#S35281188	no match found	non-canonical match
AACAAACATC	1	1	0	0	0	gnl|UG|At#S11738358	no match found	At1g31810.1
at1g68310	CON	MIN	HOUR	DAY	WEEK	UNIGENE	FLCDNA	TAIR
TCTCTGTTTT	2	0	0	2	0	gnl|UG|At#S14829847	gi|110736709|dbj|AK228380.1|	At1g68310.1
at4g24590	CON	MIN	HOUR	DAY	WEEK	UNIGENE	FLCDNA	TAIR
CAATTTTAAT	4	2	0	3	4	gnl|UG|At#S11723542	gi|26449864|dbj|AK117386.1|	At4g24590.1
at5g57150	CON	MIN	HOUR	DAY	WEEK	UNIGENE	FLCDNA	TAIR
CAGTTTTTGC	0	0	1	0	0	gnl|UG|At#S11815950	gi|22531083|gb|AY136380.1	At5g57150.2
at5g03080	CON	MIN	HOUR	DAY	WEEK	UNIGENE	FLCDNA	TAIR
TATTCTAGGG	0	1	0	0	0	gnl|UG|At#S11725396	gi|46402485|gb|BT012429.1	At5g03080.1
at3g11580	CON	MIN	HOUR	DAY	WEEK	UNIGENE	FLCDNA	TAIR
AATATATCAT	0	1	0	0	0	gnl|UG|At#S11737411	gi|51968703|dbj|AK175281.1|	At3g11580.1
at2g47810	CON	MIN	HOUR	DAY	WEEK	UNIGENE	FLCDNA	TAIR
TATGTGGTGT	1	0	0	0	0	gnl|UG|At#S14273981	gi|28393158|gb|BT003968.1	At2g47810.1
at1g02280	CON	MIN	HOUR	DAY	WEEK	UNIGENE	FLCDNA	TAIR
TAGACAAGAA	0	2	0	0	0	no match found	gi|105830153|gb|BT025654.1	multiple non-canonical match
CTTTCTGGTT	5	3	7	11	15	no match found	no match found	At1g02280.1
at5g42090	CON	MIN	HOUR	DAY	WEEK	UNIGENE	FLCDNA	TAIR
GTTGGGATGT	0	4	2	0	0	gnl|UG|At#S11719520	gi|20259829|gb|AY093263.1	At5g42090.1
at4g17830	CON	MIN	HOUR	DAY	WEEK	UNIGENE	FLCDNA	TAIR
TGCCAAGGCT	1	2	1	2	1	gnl|UG|At#S11724707	gi|17979096|gb|AY070021.1	At4g17830.1
at1g29790	CON	MIN	HOUR	DAY	WEEK	UNIGENE	FLCDNA	TAIR
ATAAAGTTCA	0	1	5	1	4	gnl|UG|At#S11739085	gi|20260609|gb|AY093204.1	At1g29790.1
GTGAAGTTTT	0	1	0	0	0	no match found	gi|30725595|gb|BT008461.1	multiple non-canonical match
at5g10630	CON	MIN	HOUR	DAY	WEEK	UNIGENE	FLCDNA	TAIR
CAAAATTCGA	2	1	5	7	0	gnl|UG|At#S11723581	gi|62321614|dbj|AK222144.1|	At5g10630.1
at5g54790	CON	MIN	HOUR	DAY	WEEK	UNIGENE	FLCDNA	TAIR

TGAGTTGTAT	1	0	0	0	0	gnl|UG|At#S26540431	no match found	non-canonical match
at2g13540	CON	MIN	HOUR	DAY	WEEK	UNIGENE	FLCDNA	TAIR
GAGAAGTTGG	1	2	0	1	1	no match found	gi|23308492|gb|BT000650.1	At2g13540.1
at3g03320	CON	MIN	HOUR	DAY	WEEK	UNIGENE	FLCDNA	TAIR
AGAATTAGTT	0	0	0	1	0	no match found	gi|32189306|gb|BT009658.1	At3g03320.1
at3g30834	CON	MIN	HOUR	DAY	WEEK	UNIGENE	FLCDNA	TAIR
AATGTGCTGG	1	0	0	0	0	no match found	no match found	At3g30834.1
at3g07880	CON	MIN	HOUR	DAY	WEEK	UNIGENE	FLCDNA	TAIR
TTTGCTCGAG	0	1	4	0	1	no match found	gi|28973346|gb|BT005578.1	non-canonical match
TGTTTGATGA	3	2	1	1	0	gnl|UG|At#S11738390	no match found	At3g07880.1
at5g56550	CON	MIN	HOUR	DAY	WEEK	UNIGENE	FLCDNA	TAIR
TCACACCTCC	1	0	0	0	0	no match found	no match found	At5g56550.1
ACAACAACAA	3	2	1	0	0	gnl|UG|At#S11718057	gi|51971388|dbj|AK176596.1|	At1g68030.1
at5g25920	CON	MIN	HOUR	DAY	WEEK	UNIGENE	FLCDNA	TAIR
GACAAGAAAA	1	1	2	2	3	gnl|UG|At#S11720599	no match found	non-canonical match
at4g21910	CON	MIN	HOUR	DAY	WEEK	UNIGENE	FLCDNA	TAIR
GTTATTATGC	0	1	1	1	1	gnl|UG|At#S11724021	gi|21404428|gb|AY085718.1	At4g21910.2
at2g29450	CON	MIN	HOUR	DAY	WEEK	UNIGENE	FLCDNA	TAIR
CAATGTTTGT	8	3	7	1	5	gnl|UG|At#S11735178	gi|17065199|gb|AY062676.1	At2g29450.1
at3g25860	CON	MIN	HOUR	DAY	WEEK	UNIGENE	FLCDNA	TAIR
AATACAACTC	1	1	1	1	0	gnl|UG|At#S11733023	gi|15028224|gb|AY045935.1	At3g25860.1
at5g13470	CON	MIN	HOUR	DAY	WEEK	UNIGENE	FLCDNA	TAIR
CTTCCGTTGT	0	0	0	1	0	gnl|UG|At#S11722981	gi|34365684|gb|BT010531.1	At5g13470.1
at1g62910	CON	MIN	HOUR	DAY	WEEK	UNIGENE	FLCDNA	TAIR
GATTTTTTCA	0	1	0	0	0	no match found	no match found	At1g62910.1
at1g29150	CON	MIN	HOUR	DAY	WEEK	UNIGENE	FLCDNA	TAIR
TCCTGAGAGA	1	2	2	2	1	no match found	gi|15810544|gb|AY056311.1	non-canonical match
GTTTCTTACC	8	3	4	5	6	gnl|UG|At#S11739315	no match found	At1g29150.1
at3g56300	CON	MIN	HOUR	DAY	WEEK	UNIGENE	FLCDNA	TAIR
ACAGCCATCG	0	0	0	1	0	gnl|UG|At#S11728276	no match found	At3g56300.1
at3g16460	CON	MIN	HOUR	DAY	WEEK	UNIGENE	FLCDNA	TAIR
TCGCGCCAAT	5	7	5	2	0	gnl|UG|At#S18942087	gi|14334869|gb|AY035108.1	At3g16460.2
at5g45270	CON	MIN	HOUR	DAY	WEEK	UNIGENE	FLCDNA	TAIR
GTCAACATTC	1	0	0	0	1	no match found	no match found	At5g45270.1
at5g45360	CON	MIN	HOUR	DAY	WEEK	UNIGENE	FLCDNA	TAIR
GTGTGTACTA	0	1	1	0	0	gnl|UG|At#S11719191	gi|27754484|gb|BT002873.1	At5g45360.1
at3g16170	CON	MIN	HOUR	DAY	WEEK	UNIGENE	FLCDNA	TAIR
TACTTTGACT	1	2	2	2	2	gnl|UG|At#S11736000	gi|23397256|gb|BT000771.1	At3g16170.1
at1g29030	CON	MIN	HOUR	DAY	WEEK	UNIGENE	FLCDNA	TAIR
ACACTCTTTT	4	2	0	0	0	no match found	gi|28973374|gb|BT005592.1	multiple canonical match
at3g09680	CON	MIN	HOUR	DAY	WEEK	UNIGENE	FLCDNA	TAIR
TCTATGTACT	0	0	0	0	1	gnl|UG|At#S11737975	gi|21403194|gb|AY084484.1	At3g09680.1

at1g03520	CON	MIN	HOUR	DAY	WEEK	UNIGENE	FLCDNA	TAIR
TCCGTGAAGA	0	0	0	1	0	gnl|UG|At#S11742762	gi|15292806|gb|AY050837.1	At1g03520.1
at5g34850	CON	MIN	HOUR	DAY	WEEK	UNIGENE	FLCDNA	TAIR
GGTGTGAATT	3	1	7	3	1	gnl|UG|At#S20816059	gi|15292756|gb|AY050812.1	non-canonical match
CTTTGTATCA	0	3	0	0	0	no match found	gi|20259672|gb|AY091415.1	At4g04710.1
GCTGTTTTTG	14	28	15	27	7	gnl|UG|At#S34116212	no match found	At1g07890.1
at1g78320	CON	MIN	HOUR	DAY	WEEK	UNIGENE	FLCDNA	TAIR
TTTTTTCTCA	0	0	0	0	1	gnl|UG|At#S11727099	gi|21404523|gb|AY085813.1	multiple canonical match
at4g02700	CON	MIN	HOUR	DAY	WEEK	UNIGENE	FLCDNA	TAIR
CTCCACACGG	0	1	0	0	0	gnl|UG|At#S11727013	gi|110738059|dbj|AK229084.1|	At4g02700.1
at5g08650	CON	MIN	HOUR	DAY	WEEK	UNIGENE	FLCDNA	TAIR
GCCGTTCTCA	5	4	12	4	2	gnl|UG|At#S11723938	gi|22136081|gb|AY128719.1	At5g08650.1
at1g50940	CON	MIN	HOUR	DAY	WEEK	UNIGENE	FLCDNA	TAIR
GCATTTGGTG	0	3	0	0	0	gnl|UG|At#S11734168	gi|26450116|dbj|AK117515.1|	At1g50940.1
at1g11430	CON	MIN	HOUR	DAY	WEEK	UNIGENE	FLCDNA	TAIR
TATGCATTCA	1	2	1	2	1	gnl|UG|At#S11741892	gi|26450102|dbj|AK117508.1|	At1g11430.1
at5g08420	CON	MIN	HOUR	DAY	WEEK	UNIGENE	FLCDNA	TAIR
AGAGATAAAA	1	0	1	0	0	no match found	gi|21405726|gb|AY087002.1	non-canonical match
AGTGATAAAA	0	7	3	0	1	gnl|UG|At#S11702540	gi|23296613|gb|AY142563.1	At5g08420.1
at5g39510	CON	MIN	HOUR	DAY	WEEK	UNIGENE	FLCDNA	TAIR
GTTAAACATT	0	0	1	0	1	gnl|UG|At#S11719781	no match found	non-canonical match
ACGAGGAGGA	4	2	1	0	1	no match found	gi|20147178|gb|AY091707.1	At5g39510.1
at2g37640	CON	MIN	HOUR	DAY	WEEK	UNIGENE	FLCDNA	TAIR
GAACGTTGCT	0	5	1	1	0	gnl|UG|At#S11733190	gi|21689618|gb|AY122898.1	At2g37640.1
at1g74310	CON	MIN	HOUR	DAY	WEEK	UNIGENE	FLCDNA	TAIR
CTCTGTTTTG	1	0	1	1	0	gnl|UG|At#S34117751	no match found	multiple non-canonical match
at1g32470	CON	MIN	HOUR	DAY	WEEK	UNIGENE	FLCDNA	TAIR
CTTTAGTTTC	22	34	22	23	13	gnl|UG|At#S11738105	gi|14326571|gb|AF385740.1	At1g32470.1
CGCAGGACCA	0	0	1	0	0	no match found	gi|12083337|gb|AF332465.1	non-canonical match
at5g48740	CON	MIN	HOUR	DAY	WEEK	UNIGENE	FLCDNA	TAIR
AAAAAAGCTG	0	1	0	0	0	gnl|UG|At#S14273727	gi|28393639|gb|BT004222.1	non-canonical match
at3g17120	CON	MIN	HOUR	DAY	WEEK	UNIGENE	FLCDNA	TAIR
TACCGATAAA	0	0	0	0	1	gnl|UG|At#S18942085	gi|18087520|gb|AF462799.1	At3g17120.2
at2g46000	CON	MIN	HOUR	DAY	WEEK	UNIGENE	FLCDNA	TAIR
TTTGGTTTTT	0	1	0	1	1	gnl|UG|At#S11731190	gi|21404646|gb|AY085936.1	At2g46000.1
at2g01440	CON	MIN	HOUR	DAY	WEEK	UNIGENE	FLCDNA	TAIR
TATGACTTGG	1	0	0	0	0	gnl|UG|At#S11742673	no match found	At2g01440.1
at2g35050	CON	MIN	HOUR	DAY	WEEK	UNIGENE	FLCDNA	TAIR
TCCTCCTCTG	1	7	1	2	0	gnl|UG|At#S11733803	no match found	At2g35050.1
at1g19300	CON	MIN	HOUR	DAY	WEEK	UNIGENE	FLCDNA	TAIR
GGCTAGGCTT	0	1	1	1	0	gnl|UG|At#S23591632	gi|21403684|gb|AY084974.1	At1g19300.1
at2g21950	CON	MIN	HOUR	DAY	WEEK	UNIGENE	FLCDNA	TAIR

AGAAAGCTAC	4	1	2	1	1	gnl|UG|At#S11806617	gi|20268765|gb|AY091137.1	At2g21950.1

at5g05080	CON	MIN	HOUR	DAY	WEEK	UNIGENE	FLCDNA	TAIR
CTAAACCAAA	0	4	1	0	0	no match found	gi|16226746|gb|AF428320.1	non-canonical match
GTTTTGCTGA	1	0	2	0	0	gnl|UG|At#S11724863	gi|21406880|gb|AY088106.1	At5g05080.1

at1g30610	CON	MIN	HOUR	DAY	WEEK	UNIGENE	FLCDNA	TAIR
GTCCAGAGTC	0	0	1	0	0	gnl|UG|At#S11738802	no match found	non-canonical match

at3g27925	CON	MIN	HOUR	DAY	WEEK	UNIGENE	FLCDNA	TAIR
TTATTCGTGG	0	1	1	0	0	no match found	gi|21360530|gb|AY113073.1	non-canonical match
CGAAAGGTAA	14	5	7	11	2	gnl|UG|At#S11732332	gi|14517499|gb|AY039585.1	At3g27925.1

at1g54120	CON	MIN	HOUR	DAY	WEEK	UNIGENE	FLCDNA	TAIR
ACCGAGCACG	1	0	0	0	1	gnl|UG|At#S11791608	gi|21407546|gb|AY088772.1	At1g54120.1

at3g01860	CON	MIN	HOUR	DAY	WEEK	UNIGENE	FLCDNA	TAIR
CCCCTATTGA	2	3	2	0	3	gnl|UG|At#S11740124	gi|28827301|gb|BT004962.1	At3g01860.2

at2g23390	CON	MIN	HOUR	DAY	WEEK	UNIGENE	FLCDNA	TAIR
TTCGTTTTCT	1	2	3	2	0	gnl|UG|At#S34114966	gi|110742376|dbj|AK227051.1|	At2g23390.1

at4g40000	CON	MIN	HOUR	DAY	WEEK	UNIGENE	FLCDNA	TAIR
ACCAAGCAAA	0	0	0	0	1	gnl|UG|At#S11720923	no match found	At4g40000.1

at4g28470	CON	MIN	HOUR	DAY	WEEK	UNIGENE	FLCDNA	TAIR
AAATCCATTA	1	0	0	0	1	gnl|UG|At#S11722883	gi|17978938|gb|AY069881.1	non-canonical match

at4g30410	CON	MIN	HOUR	DAY	WEEK	UNIGENE	FLCDNA	TAIR
ATGTCGATCG	1	0	1	0	0	gnl|UG|At#S34114327	gi|110743675|dbj|AK227688.1|	At4g30410.1

at1g56700	CON	MIN	HOUR	DAY	WEEK	UNIGENE	FLCDNA	TAIR
GACCGATTGT	10	8	5	2	1	gnl|UG|At#S11732028	gi|110743236|dbj|AK227509.1|	At1g56700.1

at3g51920	CON	MIN	HOUR	DAY	WEEK	UNIGENE	FLCDNA	TAIR
ATTTAAGAGT	8	12	13	1	3	gnl|UG|At#S11729075	gi|14190470|gb|AF380635.1	At3g51920.1

at4g27350	CON	MIN	HOUR	DAY	WEEK	UNIGENE	FLCDNA	TAIR
TGGATTATTG	0	0	0	2	0	gnl|UG|At#S11701838	gi|21406204|gb|AY087467.1	At4g27350.1

at3g15020	CON	MIN	HOUR	DAY	WEEK	UNIGENE	FLCDNA	TAIR
CTTAAAGGGA	0	0	0	0	1	gnl|UG|At#S11753001	gi|20147388|gb|AY093788.1	At3g15020.1

at5g12430	CON	MIN	HOUR	DAY	WEEK	UNIGENE	FLCDNA	TAIR
TGAAGAAACA	0	0	1	0	0	gnl|UG|At#S11723158	no match found	At5g12430.1

at1g05680	CON	MIN	HOUR	DAY	WEEK	UNIGENE	FLCDNA	TAIR
CAGGATGTGT	2	0	1	4	0	gnl|UG|At#S11742459	gi|63003803|gb|BT022019.1	At1g05680.1

at4g26620	CON	MIN	HOUR	DAY	WEEK	UNIGENE	FLCDNA	TAIR
GTTAGAGAGC	0	1	0	0	0	gnl|UG|At#S11723205	gi|27363399|gb|BT002703.1	At4g26620.1

at5g16180	CON	MIN	HOUR	DAY	WEEK	UNIGENE	FLCDNA	TAIR
GAGCGAGCTC	0	0	1	0	0	gnl|UG|At#S11722296	no match found	non-canonical match

at5g47630	CON	MIN	HOUR	DAY	WEEK	UNIGENE	FLCDNA	TAIR
ATGCTTACAG	2	0	0	0	0	gnl|UG|At#S38433161	gi|17380989|gb|AY063951.1	non-canonical match

at3g01680	CON	MIN	HOUR	DAY	WEEK	UNIGENE	FLCDNA	TAIR
TTCGATTGAA	0	1	1	1	0	gnl|UG|At#S11740169	gi|110736166|dbj|AK228096.1|	At3g01680.1

at5g13180	CON	MIN	HOUR	DAY	WEEK	UNIGENE	FLCDNA	TAIR
CTTCAAGTGG	9	12	16	4	3	gnl|UG|At#S11723060	gi|14326559|gb|AF385734.1	At5g13180.1
at2g38350	CON	MIN	HOUR	DAY	WEEK	UNIGENE	FLCDNA	TAIR
AACAAGAACC	0	1	0	0	0	gnl|UG|At#S18251578	no match found	At2g38350.1
at1g73860	CON	MIN	HOUR	DAY	WEEK	UNIGENE	FLCDNA	TAIR
TTAATTTGTG	1	0	0	0	0	gnl|UG|At#S34116727	gi|110739023|dbj|AK229581.1|	At1g73860.1
at3g06940	CON	MIN	HOUR	DAY	WEEK	UNIGENE	FLCDNA	TAIR
CAAAGCTACT	1	2	0	1	0	gnl|UG|At#S11708395	gi|17380907|gb|AY063910.1	At3g06940.1
at4g35240	CON	MIN	HOUR	DAY	WEEK	UNIGENE	FLCDNA	TAIR
GTGGAGAAAG	2	0	1	0	0	no match found	no match found	At4g35240.1
at2g21320	CON	MIN	HOUR	DAY	WEEK	UNIGENE	FLCDNA	TAIR
TATCTTTACA	2	1	0	1	0	gnl|UG|At#S18900180	no match found	pseudo chromosome match
AATAGTGATC	1	0	1	2	5	no match found	gi|16974588|gb|AY060570.1	At2g21320.1
at3g01370	CON	MIN	HOUR	DAY	WEEK	UNIGENE	FLCDNA	TAIR
TGTCTCCTGC	1	1	0	2	1	no match found	gi|37202001|gb|BT010594.1	non-canonical match
TATCGGTGAA	5	2	3	2	1	gnl|UG|At#S11740255	no match found	At3g01370.1
at1g05030	CON	MIN	HOUR	DAY	WEEK	UNIGENE	FLCDNA	TAIR
GGTTTCAGCT	0	1	0	1	0	gnl|UG|At#S34115215	gi|55733726|gb|BT020194.1	At1g05030.1
at3g57690	CON	MIN	HOUR	DAY	WEEK	UNIGENE	FLCDNA	TAIR
GCTGCTGAGG	0	0	0	1	0	gnl|UG|At#S30653804	gi|114050692|gb|BT028949.1	At3g57690.1
at2g32220	CON	MIN	HOUR	DAY	WEEK	UNIGENE	FLCDNA	TAIR
AATTGTTTTT	0	0	0	1	1	gnl|UG|At#S35315143	gi|62867610|gb|BT021972.1	At2g32220.1
at1g04310	CON	MIN	HOUR	DAY	WEEK	UNIGENE	FLCDNA	TAIR
GAAGTTAGAT	0	1	0	0	0	gnl|UG|At#S34117157	gi|110738187|dbj|AK229151.1|	At1g04310.1
at1g63100	CON	MIN	HOUR	DAY	WEEK	UNIGENE	FLCDNA	TAIR
TGGGTTTTCG	0	0	0	1	0	gnl|UG|At#S11730526	no match found	multiple non-canonical match
at1g19330	CON	MIN	HOUR	DAY	WEEK	UNIGENE	FLCDNA	TAIR
TCTCAGCAAA	0	1	0	1	0	no match found	gi|21406775|gb|AY088001.1	multiple canonical match
at3g22231	CON	MIN	HOUR	DAY	WEEK	UNIGENE	FLCDNA	TAIR
ATGAGACAGA	1	1	1	0	3	gnl|UG|At#S18901463	gi|14335055|gb|AY037207.1	At3g22231.1
at3g06240	CON	MIN	HOUR	DAY	WEEK	UNIGENE	FLCDNA	TAIR
TAATTAACAA	2	0	2	0	0	gnl|UG|At#S11738867	gi|21403133|gb|AY084423.1	At3g06240.1
at3g22960	CON	MIN	HOUR	DAY	WEEK	UNIGENE	FLCDNA	TAIR
CTGCAATCAA	5	11	7	4	2	gnl|UG|At#S11733921	gi|15810314|gb|AY056196.1	At3g22960.1
at2g43710	CON	MIN	HOUR	DAY	WEEK	UNIGENE	FLCDNA	TAIR
ATGAGAAAGA	3	0	5	2	0	no match found	gi|20334827|gb|AY094014.1	multiple non-canonical match
ATGAATAATT	8	2	4	8	8	gnl|UG|At#S11731716	no match found	At2g43710.1
at2g42280	CON	MIN	HOUR	DAY	WEEK	UNIGENE	FLCDNA	TAIR
TCTGAATATT	2	1	2	0	1	gnl|UG|At#S11732040	gi|51536447|gb|BT015339.1	At2g42280.2
at5g54090	CON	MIN	HOUR	DAY	WEEK	UNIGENE	FLCDNA	TAIR
AAAGCCATCG	0	1	0	0	0	no match found	no match found	At5g54090.1
AATAGAATGC	0	0	0	1	0	no match found	gi|22655251|gb|AY140075.1	At5g54080.2
AAGAAACATT	1	1	0	0	1	gnl|UG|At#S11718307	gi|51968459|dbj|AK175159.1|	At4g06556.1

at1g04870	CON	MIN	HOUR	DAY	WEEK	UNIGENE	FLCDNA	TAIR
TATGAACCCC	0	2	0	3	3	gnl|UG|At#S11742542	gi|16930706|gb|AF436837.1	At1g04870.1
at5g25540	CON	MIN	HOUR	DAY	WEEK	UNIGENE	FLCDNA	TAIR
ATATATTGTT	0	0	0	1	1	gnl|UG|At#S11720627	no match found	At5g25540.1
at3g04250	CON	MIN	HOUR	DAY	WEEK	UNIGENE	FLCDNA	TAIR
ACTTGTACTT	0	1	0	0	0	no match found	no match found	At3g04250.1
at2g34460	CON	MIN	HOUR	DAY	WEEK	UNIGENE	FLCDNA	TAIR
CTCAAACCTT	6	2	17	11	16	no match found	gi|15912294|gb|AY056425.1	non-canonical match
at2g17900	CON	MIN	HOUR	DAY	WEEK	UNIGENE	FLCDNA	TAIR
AATCACTTTC	0	0	1	1	1	gnl|UG|At#S15461489	no match found	At2g17900.1
at2g22740	CON	MIN	HOUR	DAY	WEEK	UNIGENE	FLCDNA	TAIR
AAGACTCTAG	0	0	1	0	0	gnl|UG|At#S11710250	gi|27754252|gb|BT002751.1	At2g22740.2
at3g15810	CON	MIN	HOUR	DAY	WEEK	UNIGENE	FLCDNA	TAIR
GACGCGACCG	0	4	3	0	0	gnl|UG|At#S11736102	gi|20259608|gb|AY091222.1	At3g15810.1
at2g47920	CON	MIN	HOUR	DAY	WEEK	UNIGENE	FLCDNA	TAIR
CTGTTCTGAT	1	1	0	0	0	gnl|UG|At#S11710483	gi|17473608|gb|AY065094.1	At2g47920.1
at2g37930	CON	MIN	HOUR	DAY	WEEK	UNIGENE	FLCDNA	TAIR
GCATAATTAA	1	0	0	0	0	gnl|UG|At#S11733113	gi|26451004|dbj|AK117972.1|	At2g37930.1
at3g46930	CON	MIN	HOUR	DAY	WEEK	UNIGENE	FLCDNA	TAIR
AAAGAGCTGA	1	1	0	0	0	gnl|UG|At#S11730013	no match found	At3g46930.1
at1g75800	CON	MIN	HOUR	DAY	WEEK	UNIGENE	FLCDNA	TAIR
GGAGAATTCT	0	4	1	1	1	gnl|UG|At#S11727882	gi|18377859|gb|AY074577.1	At1g75800.1
at4g11790	CON	MIN	HOUR	DAY	WEEK	UNIGENE	FLCDNA	TAIR
AAGTCAAATG	0	0	1	0	0	gnl|UG|At#S11725768	gi|15982859|gb|AY057537.1	At4g11790.1
at5g11970	CON	MIN	HOUR	DAY	WEEK	UNIGENE	FLCDNA	TAIR
GAAGATTTCG	0	3	0	0	0	gnl|UG|At#S11709822	gi|17979322|gb|AY070391.1	At5g11970.1
at5g47990	CON	MIN	HOUR	DAY	WEEK	UNIGENE	FLCDNA	TAIR
TTTCCTGATT	0	0	1	0	0	gnl|UG|At#S11718922	no match found	At5g47990.1
at3g60450	CON	MIN	HOUR	DAY	WEEK	UNIGENE	FLCDNA	TAIR
ATCCTGTCAG	0	2	0	0	0	gnl|UG|At#S11727347	gi|21404284|gb|AY085574.1	At3g60450.1
at3g18830	CON	MIN	HOUR	DAY	WEEK	UNIGENE	FLCDNA	TAIR
GAGAAGGAGT	0	4	0	1	1	gnl|UG|At#S11735200	gi|17473875|gb|AY065183.1	At3g18830.1
at5g16250	CON	MIN	HOUR	DAY	WEEK	UNIGENE	FLCDNA	TAIR
GCCGTTCCAA	3	4	1	2	0	gnl|UG|At#S11722278	gi|110743062|dbj|AK227420.1|	At5g16250.1
at5g41450	CON	MIN	HOUR	DAY	WEEK	UNIGENE	FLCDNA	TAIR
ATAAATTAAT	0	1	0	0	0	no match found	no match found	At5g41450.1
at1g18890	CON	MIN	HOUR	DAY	WEEK	UNIGENE	FLCDNA	TAIR
ACGCTCTCAC	1	1	1	1	0	no match found	gi|28416562|gb|BT004566.1	non-canonical match
at1g66020	CON	MIN	HOUR	DAY	WEEK	UNIGENE	FLCDNA	TAIR
ATGTTTTTGA	77	87	36	39	4	gnl|UG|At#S11824300	no match found	At1g66020.1

at1g10180	CON	MIN	HOUR	DAY	WEEK	UNIGENE	FLCDNA	TAIR
GCTGACCTAG	0	2	0	3	2	gnl|UG|At#S11742016	gi|62321414|dbj|AK222042.1|	At1g10180.1
at3g26540	CON	MIN	HOUR	DAY	WEEK	UNIGENE	FLCDNA	TAIR
TATATAGAGG	0	0	0	0	1	no match found	no match found	At3g26540.1
at2g20660	CON	MIN	HOUR	DAY	WEEK	UNIGENE	FLCDNA	TAIR
TCCAAAATCC	0	1	1	0	0	no match found	no match found	At2g20660.1
at4g23620	CON	MIN	HOUR	DAY	WEEK	UNIGENE	FLCDNA	TAIR
AAACAATCCG	0	0	0	0	1	gnl|UG|At#S11723713	no match found	multiple non-canonical match
at1g63080	CON	MIN	HOUR	DAY	WEEK	UNIGENE	FLCDNA	TAIR
TAAACCAAAA	1	0	0	0	0	gnl|UG|At#S34115293	gi|110741767|dbj|AK226724.1|	At4g32370.1
at4g19420	CON	MIN	HOUR	DAY	WEEK	UNIGENE	FLCDNA	TAIR
GTTCTGGCAA	2	7	3	1	0	gnl|UG|At#S11724429	gi|50198790|gb|BT014978.1	At4g19420.1
at5g07790	CON	MIN	HOUR	DAY	WEEK	UNIGENE	FLCDNA	TAIR
TTTTTTTGGA	0	0	0	0	1	gnl|UG|At#S11743021	gi|18389279|gb|AY074387.1	multiple canonical match
at1g19400	CON	MIN	HOUR	DAY	WEEK	UNIGENE	FLCDNA	TAIR
GTTTAATGCA	2	8	3	5	0	gnl|UG|At#S15460758	gi|62321295|dbj|AK221980.1|	At1g19400.1
at4g07932	CON	MIN	HOUR	DAY	WEEK	UNIGENE	FLCDNA	TAIR
TGCTTTGATC	0	1	0	0	0	no match found	no match found	At4g07932.1
at4g00670	CON	MIN	HOUR	DAY	WEEK	UNIGENE	FLCDNA	TAIR
AAACCACAGC	0	0	1	0	0	no match found	no match found	At4g00670.1
at5g10780	CON	MIN	HOUR	DAY	WEEK	UNIGENE	FLCDNA	TAIR
CATCAGATTG	0	7	0	3	0	gnl|UG|At#S11723551	gi|14335059|gb|AY037209.1	At5g10780.1
at1g17820	CON	MIN	HOUR	DAY	WEEK	UNIGENE	FLCDNA	TAIR
AGAAGGAAGA	0	0	0	0	1	gnl|UG|At#S11741263	gi|22135975|gb|AY128367.1	multiple non-canonical match
at3g04260	CON	MIN	HOUR	DAY	WEEK	UNIGENE	FLCDNA	TAIR
TAGTTCCGAG	6	3	2	4	0	gnl|UG|At#S11739438	no match found	At3g04260.1
at1g42852	CON	MIN	HOUR	DAY	WEEK	UNIGENE	FLCDNA	TAIR
TATATTATGA	0	1	0	0	0	no match found	no match found	At1g42852.1
at4g24030	CON	MIN	HOUR	DAY	WEEK	UNIGENE	FLCDNA	TAIR
TCTCAGAATT	0	0	0	1	0	no match found	gi|110737702|dbj|AK228901.1|	pseudo chromosome match
GCTTCTGGAT	0	0	0	1	0	gnl|UG|At#S43849509	no match found	At4g24030.1
at1g26210	CON	MIN	HOUR	DAY	WEEK	UNIGENE	FLCDNA	TAIR
TGTAGGTAGT	0	0	2	1	0	no match found	no match found	At1g26210.1
at1g74710	CON	MIN	HOUR	DAY	WEEK	UNIGENE	FLCDNA	TAIR
AATGTTCATA	0	0	2	0	2	gnl|UG|At#S18942263	gi|13605886|gb|AF367342.1	At1g74710.1
at1g77410	CON	MIN	HOUR	DAY	WEEK	UNIGENE	FLCDNA	TAIR
AGAGTCCAAT	0	1	1	0	0	gnl|UG|At#S11727411	gi|29029059|gb|BT005974.1	At1g77410.1
at5g22040	CON	MIN	HOUR	DAY	WEEK	UNIGENE	FLCDNA	TAIR
GGGATGATGG	0	2	2	1	0	gnl|UG|At#S11817074	gi|58531329|gb|BT020568.1	At5g22040.1
at5g06780	CON	MIN	HOUR	DAY	WEEK	UNIGENE	FLCDNA	TAIR
AGCTTCCAAT	0	3	0	0	1	gnl|UG|At#S11705396	gi|16226578|gb|AF428436.1	At5g06780.1

```
at5g65010    CON   MIN   HOUR   DAY   WEEK   UNIGENE              FLCDNA                         TAIR
GATCTCCAGT   11    3     17     14    5      gnl|UG|At#S11717195  gi|13605882|gb|AF367340.1      At5g65010.2

at3g15010    CON   MIN   HOUR   DAY   WEEK   UNIGENE              FLCDNA                         TAIR
GCGGAATGTA   0     0     1      0     0      no match found       gi|20259620|gb|AY091228.1      At3g15010.1

at5g52190    CON   MIN   HOUR   DAY   WEEK   UNIGENE              FLCDNA                         TAIR
CAACGGATGT   0     0     1      0     0      no match found       gi|19347959|gb|AY080691.1      non-canonical match
TGCTGTTGTT   1     0     1      1     0      gnl|UG|At#S11718497  gi|21404109|gb|AY085399.1      At5g52190.1

at3g23730    CON   MIN   HOUR   DAY   WEEK   UNIGENE              FLCDNA                         TAIR
CAGCTTAAAC   0     2     0      0     0      gnl|UG|At#S21736870  gi|51969749|dbj|AK175804.1|    At3g23730.1

at1g08510    CON   MIN   HOUR   DAY   WEEK   UNIGENE              FLCDNA                         TAIR
TGCCAGCGGA   5     7     12     9     2      gnl|UG|At#S11698510  gi|15451197|gb|AY054679.1      At1g08510.1
TCCTCCTCCT   0     2     0      0     3      no match found       gi|16648914|gb|AY059827.1      non-canonical match
ACTCTGGAGT   0     2     0      0     0      no match found       gi|30725683|gb|BT008505.1      non-canonical match

at1g71780    CON   MIN   HOUR   DAY   WEEK   UNIGENE              FLCDNA                         TAIR
TGTAATGCGA   2     0     1      0     2      gnl|UG|At#S11728735  gi|21405225|gb|AY086515.1      At1g71780.1

at5g03310    CON   MIN   HOUR   DAY   WEEK   UNIGENE              FLCDNA                         TAIR
ACTTGTCTTG   0     0     0      1     0      no match found       no match found                 At5g03310.1

at1g13110    CON   MIN   HOUR   DAY   WEEK   UNIGENE              FLCDNA                         TAIR
AAACAATTAG   4     16    5      5     4      gnl|UG|At#S11741727  gi|18087622|gb|AF462855.1      At1g13110.1

at5g48180    CON   MIN   HOUR   DAY   WEEK   UNIGENE              FLCDNA                         TAIR
GTGGGAATTC   1     8     3      3     3      gnl|UG|At#S18318877  gi|20148580|gb|AY081619.1      At5g48180.1

at2g36750    CON   MIN   HOUR   DAY   WEEK   UNIGENE              FLCDNA                         TAIR
AGATGGGGAG   0     0     0      3     1      gnl|UG|At#S11733414  gi|111074233|gb|BT026383.1     At2g36750.1

at1g78895    CON   MIN   HOUR   DAY   WEEK   UNIGENE              FLCDNA                         TAIR
TACTTGCCGA   1     0     2      3     2      gnl|UG|At#S34117352  gi|110737812|dbj|AK228956.1|   At1g78895.1

at2g36290    CON   MIN   HOUR   DAY   WEEK   UNIGENE              FLCDNA                         TAIR
AGAATCTCAA   2     3     1      6     4      gnl|UG|At#S11733516  gi|21407948|gb|AY089174.1      multiple canonical match

at3g02280    CON   MIN   HOUR   DAY   WEEK   UNIGENE              FLCDNA                         TAIR
CTCTCTGAAG   1     0     0      0     0      gnl|UG|At#S11740002  gi|38564259|gb|BT010739.1      At3g02280.1

at5g43220    CON   MIN   HOUR   DAY   WEEK   UNIGENE              FLCDNA                         TAIR
AACAAGAATA   1     0     0      0     0      no match found       no match found                 At5g43220.1

at5g47730    CON   MIN   HOUR   DAY   WEEK   UNIGENE              FLCDNA                         TAIR
CAAATGTAAA   1     0     0      0     0      gnl|UG|At#S38433157  gi|14532535|gb|AY039892.1      At5g47730.1

at3g62000    CON   MIN   HOUR   DAY   WEEK   UNIGENE              FLCDNA                         TAIR
TACTTTTGTC   0     0     0      0     2      gnl|UG|At#S11709852  gi|17979263|gb|AY070483.1      At3g62000.1

at2g06210    CON   MIN   HOUR   DAY   WEEK   UNIGENE              FLCDNA                         TAIR
GAGGAAGAAG   0     2     2      0     1      gnl|UG|At#S18942957  no match found                 At2g06210.1

at2g16640    CON   MIN   HOUR   DAY   WEEK   UNIGENE              FLCDNA                         TAIR
TTAGAGTGAG   0     1     0      0     1      gnl|UG|At#S11655209  gi|20466387|gb|AY099660.1      At2g16640.1

at5g50020    CON   MIN   HOUR   DAY   WEEK   UNIGENE              FLCDNA                         TAIR
GAAACTGGGG   0     0     2      1     0      gnl|UG|At#S11709938  gi|17979078|gb|AY070050.1      At5g50020.1

at5g22620    CON   MIN   HOUR   DAY   WEEK   UNIGENE              FLCDNA                         TAIR
```

GTGGTGGTTG	0	1	0	2	0	no match found	gi|17064807|gb|AY062480.1	At5g22620.1
ATAACAGAAT	0	0	1	2	0	gnl|UG|At#S35269944	no match found	multiple non-canonical match
at4g35270	CON	MIN	HOUR	DAY	WEEK	UNIGENE	FLCDNA	TAIR
CTTATATGTA	2	0	0	0	1	gnl|UG|At#S11721719	no match found	At4g35270.1
at3g14230	CON	MIN	HOUR	DAY	WEEK	UNIGENE	FLCDNA	TAIR
AGCCTCTCTT	2	16	3	1	1	gnl|UG|At#S11736587	gi|21404290|gb|AY085580.1	At3g14230.1
at4g36790	CON	MIN	HOUR	DAY	WEEK	UNIGENE	FLCDNA	TAIR
TTGTGATTCT	0	1	1	0	0	gnl|UG|At#S11721471	gi|17380885|gb|AY063899.1	At4g36790.1
at5g52840	CON	MIN	HOUR	DAY	WEEK	UNIGENE	FLCDNA	TAIR
TTCCTCAGCA	5	5	0	3	2	gnl|UG|At#S11718433	gi|18958051|gb|AY079117.1	At5g52840.1
at4g22910	CON	MIN	HOUR	DAY	WEEK	UNIGENE	FLCDNA	TAIR
ATAATAATGC	1	0	0	0	0	gnl|UG|At#S11723837	gi|20466238|gb|AY099585.1	At4g22910.1
at2g18960	CON	MIN	HOUR	DAY	WEEK	UNIGENE	FLCDNA	TAIR
ACTCATCTTT	7	21	5	5	7	gnl|UG|At#S11737735	gi|62321203|dbj|AK221932.1|	At2g18960.1
at1g77580	CON	MIN	HOUR	DAY	WEEK	UNIGENE	FLCDNA	TAIR
ACAAGAAATC	1	0	0	1	0	gnl|UG|At#S11825106	gi|27754579|gb|BT002921.1	At1g77580.2
at5g35732	CON	MIN	HOUR	DAY	WEEK	UNIGENE	FLCDNA	TAIR
CGGTTCCCGT	0	2	0	0	1	gnl|UG|At#S30623986	gi|48958496|gb|BT014950.1	At5g35732.1
at4g35440	CON	MIN	HOUR	DAY	WEEK	UNIGENE	FLCDNA	TAIR
GAGCGTGGTT	0	3	0	0	0	gnl|UG|At#S11721694	gi|26451751|dbj|AK118357.1|	multiple canonical match
at5g49230	CON	MIN	HOUR	DAY	WEEK	UNIGENE	FLCDNA	TAIR
CTGAGTATGT	2	1	0	0	2	gnl|UG|At#S11718795	gi|13937215|gb|AF372963.1	At5g49230.1
at1g16190	CON	MIN	HOUR	DAY	WEEK	UNIGENE	FLCDNA	TAIR
CATCTCCTTC	0	0	0	0	1	no match found	no match found	At1g16190.1
at5g47380	CON	MIN	HOUR	DAY	WEEK	UNIGENE	FLCDNA	TAIR
CATACTTAGC	0	0	0	0	1	gnl|UG|At#S34116898	gi|110738693|dbj|AK229410.1|	non-canonical match
at3g02300	CON	MIN	HOUR	DAY	WEEK	UNIGENE	FLCDNA	TAIR
AAAGAAGTTG	0	1	0	0	0	gnl|UG|At#S34117000	gi|110738490|dbj|AK229308.1|	At3g02300.1
at4g00030	CON	MIN	HOUR	DAY	WEEK	UNIGENE	FLCDNA	TAIR
ACGGTTATCG	0	1	0	1	0	no match found	gi|18377822|gb|AY074558.1	At4g00030.1
at5g46550	CON	MIN	HOUR	DAY	WEEK	UNIGENE	FLCDNA	TAIR
AAGAAAAAGG	0	0	0	0	1	gnl|UG|At#S11719070	gi|26451382|dbj|AK118168.1|	At5g46550.1
at1g26250	CON	MIN	HOUR	DAY	WEEK	UNIGENE	FLCDNA	TAIR
ATGAAGCGAG	0	0	1	0	0	gnl|UG|At#S11740240	no match found	At1g26250.1
at1g64130	CON	MIN	HOUR	DAY	WEEK	UNIGENE	FLCDNA	TAIR
AGTTTTATTA	0	1	0	0	1	no match found	no match found	At1g64130.1
at3g48350	CON	MIN	HOUR	DAY	WEEK	UNIGENE	FLCDNA	TAIR
GGTTTCAGTT	18	20	23	24	12	gnl|UG|At#S11729753	no match found	multiple canonical match
at1g27600	CON	MIN	HOUR	DAY	WEEK	UNIGENE	FLCDNA	TAIR
TGCTGTGCTC	0	0	1	0	0	gnl|UG|At#S11739781	gi|21404968|gb|AY086258.1	At1g27600.1
at4g35110	CON	MIN	HOUR	DAY	WEEK	UNIGENE	FLCDNA	TAIR

ATGGTTTAGT	0	1	0	0	0	gnl|UG|At#S11721745	gi|23397132|gb|BT000707.1	At4g35110.1
at1g09415	CON	MIN	HOUR	DAY	WEEK	UNIGENE	FLCDNA	TAIR
GAATTCTAGT	5	2	3	0	0	gnl|UG|At#S11742096	gi|18377469|gb|AY072486.1	At1g09415.1
AATAAAGCAG	0	1	2	1	0	no match found	gi|51969979|dbj|AK175919.1|	non-canonical match
at3g47370	CON	MIN	HOUR	DAY	WEEK	UNIGENE	FLCDNA	TAIR
GCGTATGAAC	0	5	1	0	1	gnl|UG|At#S35259671	gi|110743827|dbj|AK227766.1|	At3g47370.1
at5g47030	CON	MIN	HOUR	DAY	WEEK	UNIGENE	FLCDNA	TAIR
AAGGCACTGA	3	12	4	3	0	gnl|UG|At#S11719021	gi|13430525|gb|AF360175.1	At5g47030.1
at5g32800	CON	MIN	HOUR	DAY	WEEK	UNIGENE	FLCDNA	TAIR
GGAACAAGTT	0	2	0	0	1	no match found	no match found	At5g32800.1
at5g65050	CON	MIN	HOUR	DAY	WEEK	UNIGENE	FLCDNA	TAIR
CGAAAGAATT	2	1	1	3	0	gnl|UG|At#S14872064	gi|26449998|dbj|AK117455.1|	At5g65050.1
at1g12650	CON	MIN	HOUR	DAY	WEEK	UNIGENE	FLCDNA	TAIR
AGAGACTCTA	1	1	0	2	0	gnl|UG|At#S11741773	no match found	non-canonical match
at5g63040	CON	MIN	HOUR	DAY	WEEK	UNIGENE	FLCDNA	TAIR
TGGTTATCAT	0	0	1	0	0	gnl|UG|At#S18941428	gi|89000982|gb|BT024743.1	At5g63040.1
at5g05410	CON	MIN	HOUR	DAY	WEEK	UNIGENE	FLCDNA	TAIR
TTTGATGTCG	0	0	2	1	0	gnl|UG|At#S11708334	gi|17381031|gb|AY063972.1	At5g05410.1
at3g25520	CON	MIN	HOUR	DAY	WEEK	UNIGENE	FLCDNA	TAIR
CCTCTTGTAG	16	14	4	9	14	gnl|UG|At#S11733143	gi|27311642|gb|BT002427.1	At3g25520.1
at1g51200	CON	MIN	HOUR	DAY	WEEK	UNIGENE	FLCDNA	TAIR
AAGCTTATTT	24	29	22	19	14	gnl|UG|At#S11734073	gi|25082725|gb|BT001984.1	At1g51200.1
TTAAAAAGGT	3	2	1	1	3	no match found	gi|15912334|gb|AY056445.1	multiple non-canonical match
at3g09410	CON	MIN	HOUR	DAY	WEEK	UNIGENE	FLCDNA	TAIR
AACTTGCCTT	0	1	0	0	0	gnl|UG|At#S18942144	gi|110743350|dbj|AK227569.1|	At3g09410.3
at2g30960	CON	MIN	HOUR	DAY	WEEK	UNIGENE	FLCDNA	TAIR
ATTCCATAAC	1	0	0	1	0	gnl|UG|At#S11734798	gi|110738799|dbj|AK229465.1|	non-canonical match
at4g01280	CON	MIN	HOUR	DAY	WEEK	UNIGENE	FLCDNA	TAIR
AATCGACAAA	0	1	0	0	0	gnl|UG|At#S11727364	gi|89000918|gb|BT024711.1	At4g01280.1
at4g25690	CON	MIN	HOUR	DAY	WEEK	UNIGENE	FLCDNA	TAIR
GTAATATCTG	1	0	1	0	0	gnl|UG|At#S11709574	gi|53850544|gb|BT015913.1	At4g25690.2
at4g37750	CON	MIN	HOUR	DAY	WEEK	UNIGENE	FLCDNA	TAIR
TGAAACAGAA	2	1	1	0	0	no match found	gi|19310586|gb|AY080706.1	At4g37750.1
at5g62630	CON	MIN	HOUR	DAY	WEEK	UNIGENE	FLCDNA	TAIR
AAGGATCTGC	1	0	1	0	0	gnl|UG|At#S11717437	gi|20148432|gb|AY081545.1	At5g62630.1
at3g47250	CON	MIN	HOUR	DAY	WEEK	UNIGENE	FLCDNA	TAIR
ACGCAAGCCG	7	13	12	10	4	gnl|UG|At#S11729949	gi|110740817|dbj|AK226358.1|	At3g47250.2
at2g40470	CON	MIN	HOUR	DAY	WEEK	UNIGENE	FLCDNA	TAIR
GGAGCCATCT	0	0	1	0	0	gnl|UG|At#S11708148	gi|94442506|gb|BT025288.1	At2g40470.1
at3g08947	CON	MIN	HOUR	DAY	WEEK	UNIGENE	FLCDNA	TAIR
TGAATAGTGG	0	0	1	0	0	gnl|UG|At#S34116500	gi|110739458|dbj|AK229808.1|	non-canonical match

at2g20960	CON	MIN	HOUR	DAY	WEEK	UNIGENE	FLCDNA	TAIR
CTTGCCTCGG	0	3	4	3	0	gnl|UG|At#S11737224	gi|16648886|gb|AY059813.1	At2g20960.1

at2g24330	CON	MIN	HOUR	DAY	WEEK	UNIGENE	FLCDNA	TAIR
GTGAAAATCA	1	1	2	3	2	gnl|UG|At#S11736414	gi|26452965|dbj|AK118983.1|	At2g24330.1

at3g22260	CON	MIN	HOUR	DAY	WEEK	UNIGENE	FLCDNA	TAIR
TATATTGACT	1	0	0	0	1	gnl|UG|At#S18942059	no match found	At3g22260.2

at3g43590	CON	MIN	HOUR	DAY	WEEK	UNIGENE	FLCDNA	TAIR
AAATCTATTA	0	1	0	0	0	gnl|UG|At#S11730648	gi|20466715|gb|AY099824.1	At3g43590.1
GAAGAGCTAA	0	0	1	0	0	no match found	gi|25084295|gb|BT002203.1	multiple non-canonical match

at5g52980	CON	MIN	HOUR	DAY	WEEK	UNIGENE	FLCDNA	TAIR
TTCACTGGAT	1	0	1	0	1	gnl|UG|At#S34114591	gi|110743070|dbj|AK227424.1|	At5g52980.1

at3g03305	CON	MIN	HOUR	DAY	WEEK	UNIGENE	FLCDNA	TAIR
AGATTTGAAT	1	1	1	1	0	gnl|UG|At#S11739700	gi|110741513|dbj|AK226592.1|	At3g03305.1

at3g23150	CON	MIN	HOUR	DAY	WEEK	UNIGENE	FLCDNA	TAIR
AGGATCAGGA	0	1	0	0	0	gnl|UG|At#S11733860	gi|110737918|dbj|AK229010.1|	At3g23150.1

at1g57980	CON	MIN	HOUR	DAY	WEEK	UNIGENE	FLCDNA	TAIR
TCAACTGTAT	1	0	0	0	0	gnl|UG|At#S11731882	gi|26452463|dbj|AK118723.1|	At1g57980.1

at4g12130	CON	MIN	HOUR	DAY	WEEK	UNIGENE	FLCDNA	TAIR
GTGGCCAGCT	1	0	0	0	0	gnl|UG|At#S11725710	gi|22655069|gb|AY139983.1	non-canonical match

at4g22880	CON	MIN	HOUR	DAY	WEEK	UNIGENE	FLCDNA	TAIR
AATTTGATGT	0	0	0	0	5	gnl|UG|At#S11723841	gi|110740932|dbj|AK226417.1|	At4g22880.1

at2g47790	CON	MIN	HOUR	DAY	WEEK	UNIGENE	FLCDNA	TAIR
GACGACGTCA	0	1	0	0	0	gnl|UG|At#S11730768	gi|15983389|gb|AF424569.1	multiple non-canonical match

at1g23440	CON	MIN	HOUR	DAY	WEEK	UNIGENE	FLCDNA	TAIR
CTCGACGGAG	3	6	1	4	0	no match found	gi|51969519|dbj|AK175689.1|	At1g23440.1

at1g09230	CON	MIN	HOUR	DAY	WEEK	UNIGENE	FLCDNA	TAIR
TAACTCTTTG	1	2	3	1	1	gnl|UG|At#S11807509	gi|20258827|gb|AY091076.1	non-canonical match
AGACTGTTGG	0	2	0	0	0	no match found	gi|21689716|gb|AY122947.1	non-canonical match

at5g47140	CON	MIN	HOUR	DAY	WEEK	UNIGENE	FLCDNA	TAIR
ATATGAAAGA	0	1	0	1	0	gnl|UG|At#S11719010	gi|56236047|gb|BT020259.1	At5g47140.1

at1g17010	CON	MIN	HOUR	DAY	WEEK	UNIGENE	FLCDNA	TAIR
GAAGAAAAAA	18	1	10	15	8	gnl|UG|At#S11741344	gi|26451336|dbj|AK118144.1|	At1g17010.1

at3g51450	CON	MIN	HOUR	DAY	WEEK	UNIGENE	FLCDNA	TAIR
TGACAAGATT	0	0	1	0	0	gnl|UG|At#S11729164	gi|51969269|dbj|AK175564.1|	At3g51450.1

at5g39050	CON	MIN	HOUR	DAY	WEEK	UNIGENE	FLCDNA	TAIR
AAATGGATGT	1	2	2	2	0	no match found	gi|25090340|gb|BT002269.1	At5g39050.1

at5g43160	CON	MIN	HOUR	DAY	WEEK	UNIGENE	FLCDNA	TAIR
AGACAGAAAG	0	0	2	0	1	gnl|UG|At#S18908487	no match found	non-canonical match

at2g30270	CON	MIN	HOUR	DAY	WEEK	UNIGENE	FLCDNA	TAIR
AATATTGAGA	0	0	0	0	3	gnl|UG|At#S11734971	gi|26452564|dbj|AK118774.1|	At2g30270.1

at1g74670	CON	MIN	HOUR	DAY	WEEK	UNIGENE	FLCDNA	TAIR
TTCTTCTGCC	4	4	3	0	0	gnl|UG|At#S11671862	gi|44681329|gb|BT011599.1	At1g74670.1

```
at4g35900    CON   MIN   HOUR   DAY   WEEK   UNIGENE               FLCDNA                          TAIR
AAGAAGTGAA   0     2     0      0     0      gnl|UG|At#S11721613   no match found                  At4g35900.1

at1g34130    CON   MIN   HOUR   DAY   WEEK   UNIGENE               FLCDNA                          TAIR
TACAAGTTAT   0     0     0      0     1      gnl|UG|At#S11737533   gi|110739025|dbj|AK229582.1|    At1g34130.1

at3g01390    CON   MIN   HOUR   DAY   WEEK   UNIGENE               FLCDNA                          TAIR
TGACCACAGT   9     8     5      4     6      gnl|UG|At#S15461643   gi|21389700|gb|AY114730.1       At3g01390.1

at5g10380    CON   MIN   HOUR   DAY   WEEK   UNIGENE               FLCDNA                          TAIR
CCTTTCATCT   0     3     0      0     0      gnl|UG|At#S11723642   gi|51971141|dbj|AK176500.1|     At5g10380.1

at4g06576    CON   MIN   HOUR   DAY   WEEK   UNIGENE               FLCDNA                          TAIR
ATTACAGAGA   0     0     2      0     0      no match found        no match found                  At4g06576.1

at3g32027    CON   MIN   HOUR   DAY   WEEK   UNIGENE               FLCDNA                          TAIR
GAGAGTGTGG   0     1     0      0     0      no match found        no match found                  At3g32027.1

at3g17580    CON   MIN   HOUR   DAY   WEEK   UNIGENE               FLCDNA                          TAIR
TCATAAATTA   0     1     0      0     0      no match found        no match found                  At3g17580.1

at1g32750    CON   MIN   HOUR   DAY   WEEK   UNIGENE               FLCDNA                          TAIR
AGTAGTCTTG   1     1     2      0     0      gnl|UG|At#S11738003   no match found                  At1g32750.1

at1g09240    CON   MIN   HOUR   DAY   WEEK   UNIGENE               FLCDNA                          TAIR
ATGATCGAGG   1     2     2      0     2      no match found        gi|21406358|gb|AY087620.1       non-canonical match
CTCTTAACAT   1     0     0      0     1      gnl|UG|At#S11742114   no match found                  At1g09240.1

at4g06744    CON   MIN   HOUR   DAY   WEEK   UNIGENE               FLCDNA                          TAIR
GTGGCTCCCT   0     0     0      1     1      gnl|UG|At#S15461439   no match found                  At4g06744.1

at3g29375    CON   MIN   HOUR   DAY   WEEK   UNIGENE               FLCDNA                          TAIR
AGATCAAAAC   0     0     0      1     0      no match found        no match found                  At3g29375.1

at5g10690    CON   MIN   HOUR   DAY   WEEK   UNIGENE               FLCDNA                          TAIR
TGTGAGTACA   1     2     0      0     0      gnl|UG|At#S11723569   gi|18176045|gb|AY072152.1       At5g10690.1

at5g52480    CON   MIN   HOUR   DAY   WEEK   UNIGENE               FLCDNA                          TAIR
GTCACGCTCA   0     0     0      1     0      no match found        no match found                  At5g52480.1

at4g29010    CON   MIN   HOUR   DAY   WEEK   UNIGENE               FLCDNA                          TAIR
CCGCGTCCTA   0     9     4      1     0      gnl|UG|At#S11722789   gi|16648890|gb|AY059815.1       At4g29010.1

at2g20210    CON   MIN   HOUR   DAY   WEEK   UNIGENE               FLCDNA                          TAIR
AAATAGCATC   0     0     0      0     1      gnl|UG|At#S11737423   gi|110737873|dbj|AK228987.1|    At2g20210.1

at1g11700    CON   MIN   HOUR   DAY   WEEK   UNIGENE               FLCDNA                          TAIR
AGTACTTGGC   0     2     1      2     0      gnl|UG|At#S34114334   gi|110743661|dbj|AK227681.1|    At1g11700.1

at1g53910    CON   MIN   HOUR   DAY   WEEK   UNIGENE               FLCDNA                          TAIR
AGCCTCTATC   2     3     1      2     3      gnl|UG|At#S11733033   gi|14335161|gb|AY037260.1       At1g53910.1

at2g02930    CON   MIN   HOUR   DAY   WEEK   UNIGENE               FLCDNA                          TAIR
TCCATTGGAA   0     1     0      0     0      gnl|UG|At#S34117082   gi|110738330|dbj|AK229226.1|    At2g02930.1

at5g53320    CON   MIN   HOUR   DAY   WEEK   UNIGENE               FLCDNA                          TAIR
ATTTCAGCTA   46    23    42     37    13     no match found        no match found                  At5g53320.1
AAAGAATCCA   0     0     1      0     0      gnl|UG|At#S34114866   gi|110742560|dbj|AK227151.1|    multiple non-canonical match

at3g53980    CON   MIN   HOUR   DAY   WEEK   UNIGENE               FLCDNA                          TAIR
```

TACCTTATAA	0	0	1	0	1	gnl|UG|At#S17006729	gi|21407124|gb|AY088350.1	At3g53980.1
at2g25620	CON	MIN	HOUR	DAY	WEEK	UNIGENE	FLCDNA	TAIR
AGGCGGCATA	0	1	1	5	2	no match found	gi|11908107|gb|AF326901.1	At2g25620.1
at4g34790	CON	MIN	HOUR	DAY	WEEK	UNIGENE	FLCDNA	TAIR
GGTTTAATGA	0	2	0	0	0	gnl|UG|At#S11721799	gi|110739436|dbj|AK229797.1|	non-canonical match
at3g23750	CON	MIN	HOUR	DAY	WEEK	UNIGENE	FLCDNA	TAIR
GAGACTTCTC	1	2	1	0	2	gnl|UG|At#S11733685	gi|110738813|dbj|AK229472.1|	At3g23750.1
at1g15930	CON	MIN	HOUR	DAY	WEEK	UNIGENE	FLCDNA	TAIR
ATCAAGTTTT	30	18	35	46	20	gnl|UG|At#S11741444	gi|14190452|gb|AF380626.1	At1g15930.2
at2g06820	CON	MIN	HOUR	DAY	WEEK	UNIGENE	FLCDNA	TAIR
AGAAGAAAAA	0	0	1	0	1	gnl|UG|At#S14233065	no match found	multiple non-canonical match
at1g28600	CON	MIN	HOUR	DAY	WEEK	UNIGENE	FLCDNA	TAIR
GAAAGACCCT	1	0	0	1	1	gnl|UG|At#S18895246	gi|22137319|gb|AY133675.1	At1g28600.1
at3g04880	CON	MIN	HOUR	DAY	WEEK	UNIGENE	FLCDNA	TAIR
ACCTTGTAGT	0	1	0	0	0	gnl|UG|At#S11739261	gi|23505882|gb|AY143862.1	At3g04880.1
at2g21270	CON	MIN	HOUR	DAY	WEEK	UNIGENE	FLCDNA	TAIR
TTGTGTACTA	1	2	1	0	3	no match found	gi|110741144|dbj|AK226526.1|	At2g21270.1
CTCTGTAGCT	0	0	0	1	0	gnl|UG|At#S28282297	no match found	non-canonical match
at2g46520	CON	MIN	HOUR	DAY	WEEK	UNIGENE	FLCDNA	TAIR
TTTCATAAAC	0	0	0	1	0	gnl|UG|At#S11731067	no match found	At2g46520.1
TTTCACAAAA	0	1	0	0	1	no match found	gi|62318913|dbj|AK220777.1|	At2g11390.1
at3g04980	CON	MIN	HOUR	DAY	WEEK	UNIGENE	FLCDNA	TAIR
TAAAAGTCTA	1	0	1	0	0	no match found	no match found	At3g04980.1
at4g19400	CON	MIN	HOUR	DAY	WEEK	UNIGENE	FLCDNA	TAIR
TACAGTCGTT	0	1	0	0	0	gnl|UG|At#S34117372	gi|110737772|dbj|AK228936.1|	At4g19400.1
AGAACTGGTT	0	1	1	0	0	no match found	gi|21403560|gb|AY084850.1	multiple non-canonical match
at1g54040	CON	MIN	HOUR	DAY	WEEK	UNIGENE	FLCDNA	TAIR
GTTGTGTGAG	0	0	1	1	0	gnl|UG|At#S30641717	gi|18491228|gb|AY074550.1	At1g54040.2
at1g06640	CON	MIN	HOUR	DAY	WEEK	UNIGENE	FLCDNA	TAIR
TTGGAGATGT	6	8	2	2	1	gnl|UG|At#S18942632	gi|110740891|dbj|AK226396.1|	At1g06640.2
at2g19240	CON	MIN	HOUR	DAY	WEEK	UNIGENE	FLCDNA	TAIR
AAAGAAATTG	10	7	9	13	11	no match found	no match found	At2g19240.1
at3g63400	CON	MIN	HOUR	DAY	WEEK	UNIGENE	FLCDNA	TAIR
ATGCTGCTGA	2	6	2	1	1	gnl|UG|At#S11708331	gi|24030285|gb|BT000915.1	At3g63400.2
at4g31140	CON	MIN	HOUR	DAY	WEEK	UNIGENE	FLCDNA	TAIR
ATGCCTATAA	1	1	0	0	0	gnl|UG|At#S11722422	gi|110738669|dbj|AK229398.1|	At4g31140.1
at5g45290	CON	MIN	HOUR	DAY	WEEK	UNIGENE	FLCDNA	TAIR
ATCCATCATC	0	1	1	2	2	no match found	gi|58652065|gb|BT020585.1	multiple non-canonical match
at5g25930	CON	MIN	HOUR	DAY	WEEK	UNIGENE	FLCDNA	TAIR
AAAGAGGTCT	1	0	0	0	0	gnl|UG|At#S14273891	gi|28393325|gb|BT004058.1	At5g25930.1
at5g08130	CON	MIN	HOUR	DAY	WEEK	UNIGENE	FLCDNA	TAIR
AAGTTCGTGA	1	3	1	2	1	no match found	gi|119935974|gb|BT029780.1	non-canonical match

AACGATTTTG	0	1	1	0	0	gnl|UG|At#S23655042	no match found	At5g08130.1
at1g30650	CON	MIN	HOUR	DAY	WEEK	UNIGENE	FLCDNA	TAIR
ACAGACCGTA	0	0	1	0	0	gnl|UG|At#S11738787	gi|21404192|gb|AY085482.1	At1g30650.1
at4g13510	CON	MIN	HOUR	DAY	WEEK	UNIGENE	FLCDNA	TAIR
GGGACACTTT	0	0	2	0	0	gnl|UG|At#S18909795	no match found	non-canonical match
GGGTTGTGAT	4	3	12	10	11	gnl|UG|At#S11725470	gi|14335079|gb|AY037219.1	At4g13510.1
at4g13500	CON	MIN	HOUR	DAY	WEEK	UNIGENE	FLCDNA	TAIR
CCAAATTAGG	1	0	0	1	0	no match found	gi|109946534|gb|BT026090.1	non-canonical match
TCTTCATTAC	0	0	1	0	0	no match found	no match found	At4g13500.1
at2g46550	CON	MIN	HOUR	DAY	WEEK	UNIGENE	FLCDNA	TAIR
CCTCTTCCTC	0	2	0	0	0	no match found	gi|25083762|gb|BT002105.1	non-canonical match
TAATATCTGC	0	0	1	0	0	gnl|UG|At#S11731062	gi|22655071|gb|AY139984.1	At2g46550.1
at3g50060	CON	MIN	HOUR	DAY	WEEK	UNIGENE	FLCDNA	TAIR
GCGGAAATGC	0	3	3	1	0	gnl|UG|At#S11729431	gi|15983427|gb|AF424588.1	At3g50060.1
at5g11150	CON	MIN	HOUR	DAY	WEEK	UNIGENE	FLCDNA	TAIR
GGCCAAGTCT	1	0	0	1	1	no match found	gi|21689690|gb|AY122934.1	At5g11150.1
at1g67050	CON	MIN	HOUR	DAY	WEEK	UNIGENE	FLCDNA	TAIR
TTTAAATCTA	1	2	1	0	0	gnl|UG|At#S11729703	gi|20260171|gb|AY092985.1	At1g67050.1
CCAGTATTAT	0	0	0	1	1	no match found	gi|21403729|gb|AY085019.1	non-canonical match
at1g32130	CON	MIN	HOUR	DAY	WEEK	UNIGENE	FLCDNA	TAIR
AGAAATCTCG	0	5	1	1	2	gnl|UG|At#S11738232	no match found	At1g32130.1
at2g19430	CON	MIN	HOUR	DAY	WEEK	UNIGENE	FLCDNA	TAIR
TACAGGATGT	0	0	0	0	1	gnl|UG|At#S18900517	gi|20466503|gb|AY099718.1	At2g19430.1
at1g21590	CON	MIN	HOUR	DAY	WEEK	UNIGENE	FLCDNA	TAIR
TAATTTGTAT	4	2	0	0	2	gnl|UG|At#S11740902	gi|110740060|dbj|AK230116.1|	At1g21590.1
at2g19680	CON	MIN	HOUR	DAY	WEEK	UNIGENE	FLCDNA	TAIR
GGAATTCGTA	2	0	0	1	0	gnl|UG|At#S11737552	gi|13899094|gb|AF370542.1	At2g19680.1
at2g45850	CON	MIN	HOUR	DAY	WEEK	UNIGENE	FLCDNA	TAIR
GGCCTTCATA	1	0	1	0	3	gnl|UG|At#S11731222	gi|17065245|gb|AY062699.1	multiple non-canonical match
atcg01130	CON	MIN	HOUR	DAY	WEEK	UNIGENE	FLCDNA	TAIR
AAATGGGTTG	1	22	3	3	1	no match found	no match found	AtCg01130
TATACGCGAT	1	0	0	1	0	no match found	no match found	AtCg01130
at1g78800	CON	MIN	HOUR	DAY	WEEK	UNIGENE	FLCDNA	TAIR
TCGTTGAATC	0	0	1	1	1	gnl|UG|At#S18893825	gi|23308430|gb|BT000618.1	At1g78800.1
at1g52310	CON	MIN	HOUR	DAY	WEEK	UNIGENE	FLCDNA	TAIR
GAGAAAGTAA	0	2	1	2	1	gnl|UG|At#S11708843	gi|62319230|dbj|AK220938.1|	At1g52310.1
at2g18196	CON	MIN	HOUR	DAY	WEEK	UNIGENE	FLCDNA	TAIR
GTATCAAAGT	0	0	0	1	0	gnl|UG|At#S15461452	no match found	At2g18196.1
at5g39530	CON	MIN	HOUR	DAY	WEEK	UNIGENE	FLCDNA	TAIR
TATAGTTCTC	8	7	1	2	0	gnl|UG|At#S11719779	gi|26452528|dbj|AK118756.1|	At5g39530.1
at2g34680	CON	MIN	HOUR	DAY	WEEK	UNIGENE	FLCDNA	TAIR
AAACCTTAAA	1	1	0	0	1	gnl|UG|At#S11733894	gi|110737896|dbj|AK228999.1|	At2g34680.1

at4g25470	CON	MIN	HOUR	DAY	WEEK	UNIGENE	FLCDNA	TAIR
GCCGAAGGGA	0	5	13	1	0	gnl|UG|At#S11699074	gi|115311438|gb|BT028991.1	At4g25470.1
at1g79200	CON	MIN	HOUR	DAY	WEEK	UNIGENE	FLCDNA	TAIR
GCTGAAAGAA	1	1	0	0	0	gnl|UG|At#S11726778	gi|26452152|dbj|AK118564.1|	At1g79200.1
at1g62540	CON	MIN	HOUR	DAY	WEEK	UNIGENE	FLCDNA	TAIR
ATGCCCTGAT	0	2	3	0	0	gnl|UG|At#S11730638	gi|13878100|gb|AF370313.1	At1g62540.1
at4g28060	CON	MIN	HOUR	DAY	WEEK	UNIGENE	FLCDNA	TAIR
TCAGAATTTT	0	0	2	1	1	no match found	no match found	At4g28060.1
AAATCTATAA	2	6	0	1	3	no match found	gi|17979512|gb|AY070753.1	non-canonical match
at4g31750	CON	MIN	HOUR	DAY	WEEK	UNIGENE	FLCDNA	TAIR
GTATCGTACC	0	1	0	0	0	gnl|UG|At#S11743927	gi|22136751|gb|AY133761.1	At4g31750.1
at5g20270	CON	MIN	HOUR	DAY	WEEK	UNIGENE	FLCDNA	TAIR
ATTTTCAGAA	3	2	2	3	1	gnl|UG|At#S11721262	no match found	At5g20270.1
TGTTCGTTTT	0	0	1	1	1	no match found	gi|21405087|gb|AY086377.1	multiple non-canonical match
at3g12203	CON	MIN	HOUR	DAY	WEEK	UNIGENE	FLCDNA	TAIR
TTCAAACTTT	0	0	0	1	0	no match found	no match found	At3g12203.1
at1g67810	CON	MIN	HOUR	DAY	WEEK	UNIGENE	FLCDNA	TAIR
ATTATTCCAT	0	1	0	0	1	gnl|UG|At#S17007816	gi|117958778|gb|BT029461.1	At1g67810.1
at5g25190	CON	MIN	HOUR	DAY	WEEK	UNIGENE	FLCDNA	TAIR
GCTTCTCTTC	0	2	0	0	0	gnl|UG|At#S11720664	no match found	At5g25190.1
at1g62730	CON	MIN	HOUR	DAY	WEEK	UNIGENE	FLCDNA	TAIR
GAGAGCAATG	0	1	1	0	0	no match found	no match found	At1g62730.1
at2g20820	CON	MIN	HOUR	DAY	WEEK	UNIGENE	FLCDNA	TAIR
GATCTACCAA	1	0	0	0	0	no match found	gi|12642909|gb|AF339715.1	non-canonical match
at1g63940	CON	MIN	HOUR	DAY	WEEK	UNIGENE	FLCDNA	TAIR
ATCTATGGGT	1	2	0	3	2	gnl|UG|At#S15450438	no match found	At1g63940.2
GAGTTTTTTT	1	6	2	2	5	no match found	gi|23397056|gb|BT000667.1	multiple non-canonical match
at5g61880	CON	MIN	HOUR	DAY	WEEK	UNIGENE	FLCDNA	TAIR
TCTATTGAAT	2	1	1	0	0	gnl|UG|At#S15441569	gi|14517497|gb|AY039584.1	At5g61880.1
at5g42920	CON	MIN	HOUR	DAY	WEEK	UNIGENE	FLCDNA	TAIR
TAACATTTTT	0	1	0	1	1	gnl|UG|At#S11704148	gi|14532601|gb|AY039925.1	At5g42920.2
at3g62920	CON	MIN	HOUR	DAY	WEEK	UNIGENE	FLCDNA	TAIR
GCTGATGATA	0	2	1	0	2	gnl|UG|At#S11726561	gi|26452614|dbj|AK118800.1|	At3g62920.1
at5g11030	CON	MIN	HOUR	DAY	WEEK	UNIGENE	FLCDNA	TAIR
GCCAAGAATC	1	0	1	0	0	gnl|UG|At#S11723494	gi|28416704|gb|BT004637.1	At5g11030.1
at3g51550	CON	MIN	HOUR	DAY	WEEK	UNIGENE	FLCDNA	TAIR
AATCGCGTCA	8	15	17	9	8	gnl|UG|At#S11729146	gi|14334759|gb|AY035053.1	At3g51550.1
at5g53430	CON	MIN	HOUR	DAY	WEEK	UNIGENE	FLCDNA	TAIR
AATTAGAGAG	0	1	0	0	0	gnl|UG|At#S11824582	gi|26449541|dbj|AK117221.1|	At5g53430.1
at1g04110	CON	MIN	HOUR	DAY	WEEK	UNIGENE	FLCDNA	TAIR
TGAATCCGCA	1	0	0	0	0	gnl|UG|At#S11742658	no match found	non-canonical match
at3g11700	CON	MIN	HOUR	DAY	WEEK	UNIGENE	FLCDNA	TAIR

TAGTATGCTT	11	11	6	0	5	gnl|UG|At#S11706069	gi|22136767|gb|AY133794.1	At3g11700.1
at1g51700	CON	MIN	HOUR	DAY	WEEK	UNIGENE	FLCDNA	TAIR
GGTCCGGGTC	0	1	1	1	3	no match found	gi|107738210|gb|BT025772.1	non-canonical match
at2g13960	CON	MIN	HOUR	DAY	WEEK	UNIGENE	FLCDNA	TAIR
AACTTGCAAT	0	0	0	1	0	gnl|UG|At#S11738966	no match found	At2g13960.1
at1g78660	CON	MIN	HOUR	DAY	WEEK	UNIGENE	FLCDNA	TAIR
ATTTCCCTTG	2	4	5	1	2	no match found	gi|16323195|gb|AY057702.1	multiple canonical match
at2g24765	CON	MIN	HOUR	DAY	WEEK	UNIGENE	FLCDNA	TAIR
TTCACTTAAT	0	0	1	0	0	no match found	gi|26450542|dbj|AK117735.1|	At2g24765.1
TTCACTTAAA	1	4	1	1	0	no match found	gi|17380731|gb|AY063840.1	At2g24765.1
at5g17610	CON	MIN	HOUR	DAY	WEEK	UNIGENE	FLCDNA	TAIR
TAAGGCTTCT	0	3	1	2	1	gnl|UG|At#S11721924	gi|51970569|dbj|AK176214.1|	At5g17610.1
at2g24860	CON	MIN	HOUR	DAY	WEEK	UNIGENE	FLCDNA	TAIR
TGTTAAGTAT	6	1	1	1	1	no match found	gi|51970709|dbj|AK176284.1|	non-canonical match
TGCATCTTCC	0	0	0	0	1	gnl|UG|At#S11736295	no match found	At2g24860.1
at4g38360	CON	MIN	HOUR	DAY	WEEK	UNIGENE	FLCDNA	TAIR
ATTTCTTATT	0	2	0	0	2	gnl|UG|At#S11704713	gi|15028110|gb|AY046005.1	At4g38360.1
at3g28670	CON	MIN	HOUR	DAY	WEEK	UNIGENE	FLCDNA	TAIR
CTTTACCAGA	1	4	0	1	1	gnl|UG|At#S11732086	gi|34365728|gb|BT010553.1	At3g28670.1
at4g10470	CON	MIN	HOUR	DAY	WEEK	UNIGENE	FLCDNA	TAIR
TCAATTCTAG	0	1	0	0	1	gnl|UG|At#S11725918	gi|16226644|gb|AF428453.1	At4g10470.1
at5g44565	CON	MIN	HOUR	DAY	WEEK	UNIGENE	FLCDNA	TAIR
TAAGGATTTT	0	0	0	2	5	no match found	no match found	At5g44565.1
at3g53710	CON	MIN	HOUR	DAY	WEEK	UNIGENE	FLCDNA	TAIR
ATGCCAAAGA	0	0	0	0	1	no match found	gi|134031917|gb|BT030393.1	At3g53710.1
TGTTAAGGAG	2	4	1	3	3	gnl|UG|At#S11728743	gi|23296550|gb|AY142544.1	multiple canonical match
at2g43240	CON	MIN	HOUR	DAY	WEEK	UNIGENE	FLCDNA	TAIR
TAATTTTAAA	1	1	0	0	0	gnl|UG|At#S11731828	gi|110738592|dbj|AK229359.1|	non-canonical match
GTGTTATAAA	1	0	1	1	2	no match found	no match found	At2g43240.1
at5g05060	CON	MIN	HOUR	DAY	WEEK	UNIGENE	FLCDNA	TAIR
GCTATCTCCT	2	0	0	0	0	no match found	gi|45680216|gb|BT011906.1	At5g05060.1
at3g12360	CON	MIN	HOUR	DAY	WEEK	UNIGENE	FLCDNA	TAIR
ACTTATTACG	1	2	1	0	0	gnl|UG|At#S11812504	gi|21404195|gb|AY085485.1	At3g12360.1
at3g21140	CON	MIN	HOUR	DAY	WEEK	UNIGENE	FLCDNA	TAIR
GTGTTGAGAC	0	0	1	0	1	gnl|UG|At#S11734483	gi|25083911|gb|BT002122.1	At3g21140.1
at3g04790	CON	MIN	HOUR	DAY	WEEK	UNIGENE	FLCDNA	TAIR
GTCTGTTTCT	9	1	9	4	4	gnl|UG|At#S30647780	gi|21407156|gb|AY088382.1	At3g04790.1
at4g02715	CON	MIN	HOUR	DAY	WEEK	UNIGENE	FLCDNA	TAIR
CCTAATCTGC	2	3	1	2	0	gnl|UG|At#S11815856	gi|26451579|dbj|AK118269.1|	At3g04790.1
TCGCGTAAAA	0	0	0	0	1	no match found	no match found	At4g02715.1
at1g72900	CON	MIN	HOUR	DAY	WEEK	UNIGENE	FLCDNA	TAIR
AACGAACTTA	0	1	0	0	0	gnl|UG|At#S11728487	gi|23198013|gb|BT000215.1	At1g72900.1

at2g22970	CON	MIN	HOUR	DAY	WEEK	UNIGENE	FLCDNA	TAIR
CTATAGTTAC	0	1	0	0	0	gnl|UG|At#S11821386	gi|14517521|gb|AY039596.1	At2g22970.1
at3g62400	CON	MIN	HOUR	DAY	WEEK	UNIGENE	FLCDNA	TAIR
CCACATTGAA	1	5	3	2	1	gnl|UG|At#S14273665	gi|21407770|gb|AY088996.1	At3g62400.2
at1g65080	CON	MIN	HOUR	DAY	WEEK	UNIGENE	FLCDNA	TAIR
GGGCAGGCGT	0	1	0	0	0	gnl|UG|At#S11730113	gi|22655124|gb|AY140011.1	At1g65080.1
at5g04410	CON	MIN	HOUR	DAY	WEEK	UNIGENE	FLCDNA	TAIR
TTACATTGTG	0	0	2	2	0	gnl|UG|At#S11725042	gi|20466285|gb|AY099609.1	multiple non-canonical match
TTTCCCCCAC	1	0	0	0	0	no match found	no match found	At5g04410.1
GTGTCTGCTC	1	3	4	1	1	no match found	gi|22136361|gb|AY128859.1	non-canonical match
at5g38980	CON	MIN	HOUR	DAY	WEEK	UNIGENE	FLCDNA	TAIR
TAACATACTC	13	5	7	4	5	gnl|UG|At#S11716102	gi|26450980|dbj|AK117960.1|	At5g38980.1
at1g05820	CON	MIN	HOUR	DAY	WEEK	UNIGENE	FLCDNA	TAIR
TAGTCTCGAG	1	1	0	0	0	gnl|UG|At#S43850691	no match found	At1g05820.1
at2g35240	CON	MIN	HOUR	DAY	WEEK	UNIGENE	FLCDNA	TAIR
CGTTGACTCT	1	1	0	1	0	gnl|UG|At#S11733762	gi|18253008|gb|AY072439.1	At2g35240.1
at1g55130	CON	MIN	HOUR	DAY	WEEK	UNIGENE	FLCDNA	TAIR
TTTGTGGTTC	0	1	0	0	0	gnl|UG|At#S11732603	gi|27754303|gb|BT002777.1	At1g55130.1
at5g09970	CON	MIN	HOUR	DAY	WEEK	UNIGENE	FLCDNA	TAIR
GGGCTAGCTA	0	0	1	0	0	gnl|UG|At#S34115016	gi|110742281|dbj|AK227001.1|	At5g09970.1
at3g47810	CON	MIN	HOUR	DAY	WEEK	UNIGENE	FLCDNA	TAIR
CTTCAGAGAC	1	3	0	0	0	gnl|UG|At#S18942012	gi|28393866|gb|BT004347.1	non-canonical match
at5g22140	CON	MIN	HOUR	DAY	WEEK	UNIGENE	FLCDNA	TAIR
TTGTTTGTGT	2	0	2	1	0	gnl|UG|At#S11817065	gi|26450664|dbj|AK117798.1|	At5g22140.1
at2g11290	CON	MIN	HOUR	DAY	WEEK	UNIGENE	FLCDNA	TAIR
GCATTTCTCA	0	1	8	1	0	no match found	no match found	At2g11290.1
at5g42540	CON	MIN	HOUR	DAY	WEEK	UNIGENE	FLCDNA	TAIR
ATGGAAGAGG	1	2	0	4	2	gnl|UG|At#S11719475	gi|62319143|dbj|AK220893.1|	At5g42540.1
at5g45750	CON	MIN	HOUR	DAY	WEEK	UNIGENE	FLCDNA	TAIR
GAAACCTCGG	0	4	0	0	0	gnl|UG|At#S11719153	gi|13877912|gb|AF370219.1	At5g45750.1
at2g15050	CON	MIN	HOUR	DAY	WEEK	UNIGENE	FLCDNA	TAIR
ATCTCTACAA	3	0	0	0	0	gnl|UG|At#S35265955	no match found	non-canonical match
TCGTTGCATT	2	2	0	2	0	no match found	gi|88011109|gb|BT024565.1	At2g15050.2
TGGCATAAGT	4	2	0	2	0	no match found	gi|62318670|dbj|AK220655.1|	At2g15050.1
at1g07970	CON	MIN	HOUR	DAY	WEEK	UNIGENE	FLCDNA	TAIR
TGTGCTTGCA	0	0	0	1	0	gnl|UG|At#S34117636	gi|110737262|dbj|AK228672.1|	At1g07970.1
at1g50370	CON	MIN	HOUR	DAY	WEEK	UNIGENE	FLCDNA	TAIR
ATTGCCATTG	3	0	5	0	0	gnl|UG|At#S11734367	gi|21406146|gb|AY087422.1	At1g50370.1
at1g67660	CON	MIN	HOUR	DAY	WEEK	UNIGENE	FLCDNA	TAIR
TCGAGTTCTT	0	1	1	1	0	gnl|UG|At#S11822886	gi|26452991|dbj|AK118996.1|	At1g67660.1
at3g44680	CON	MIN	HOUR	DAY	WEEK	UNIGENE	FLCDNA	TAIR
CTGAATGTGT	0	1	0	0	0	no match found	gi|47679043|gb|BT014712.1	multiple non-canonical match
TTTTCGTCTT	1	0	0	1	0	gnl|UG|At#S11730475	gi|25082913|gb|BT002003.1	At3g44680.1

at1g18485	CON	MIN	HOUR	DAY	WEEK	UNIGENE	FLCDNA	TAIR
GAAAGGGAAA	0	1	0	0	0	gnl|UG|At#S11741203	no match found	multiple canonical match
at3g09860	CON	MIN	HOUR	DAY	WEEK	UNIGENE	FLCDNA	TAIR
ACAAGTGGAT	1	11	3	0	0	gnl|UG|At#S11737928	gi|48310048|gb|BT014760.1	At3g09860.1
at5g09770	CON	MIN	HOUR	DAY	WEEK	UNIGENE	FLCDNA	TAIR
GGAAAGATCT	0	3	0	0	0	gnl|UG|At#S11723792	gi|110736201|dbj|AK228114.1|	At5g09770.1
at2g41945	CON	MIN	HOUR	DAY	WEEK	UNIGENE	FLCDNA	TAIR
CACAGATAAA	0	0	0	3	0	no match found	gi|48958502|gb|BT014953.1	non-canonical match
GTATTTGATT	0	0	0	3	2	no match found	gi|110737099|dbj|AK228586.1|	At2g41945.1
at5g52590	CON	MIN	HOUR	DAY	WEEK	UNIGENE	FLCDNA	TAIR
TTACCAAAAT	1	0	0	1	2	no match found	gi|26450725|dbj|AK117830.1|	At5g52590.1
at5g02770	CON	MIN	HOUR	DAY	WEEK	UNIGENE	FLCDNA	TAIR
TAAGACATCA	0	0	2	0	0	gnl|UG|At#S11709812	gi|62320776|dbj|AK221715.1|	At5g02770.1
at3g54090	CON	MIN	HOUR	DAY	WEEK	UNIGENE	FLCDNA	TAIR
ACAAGCTGAA	0	0	1	0	0	no match found	gi|22136277|gb|AY128817.1	non-canonical match
at1g30090	CON	MIN	HOUR	DAY	WEEK	UNIGENE	FLCDNA	TAIR
GACTGGATTG	1	2	2	3	2	gnl|UG|At#S11705504	gi|16209716|gb|AY057619.1	At1g30090.1
at5g05790	CON	MIN	HOUR	DAY	WEEK	UNIGENE	FLCDNA	TAIR
CCCAAAAGTA	1	0	0	0	0	gnl|UG|At#S11724679	gi|109946462|gb|BT026054.1	At5g05790.1
at3g61580	CON	MIN	HOUR	DAY	WEEK	UNIGENE	FLCDNA	TAIR
GCTCTTTTTT	0	0	2	0	0	gnl|UG|At#S11726978	no match found	At3g61580.1
GAAGAGATGT	18	10	21	19	14	no match found	gi|23306383|gb|BT000442.1	non-canonical match
GCTAAATGAT	1	1	3	0	0	no match found	gi|21406069|gb|AY087345.1	non-canonical match
at1g05385	CON	MIN	HOUR	DAY	WEEK	UNIGENE	FLCDNA	TAIR
TTATACATAT	3	0	0	0	4	gnl|UG|At#S11702500	gi|13877958|gb|AF370242.1	At1g05385.1
at2g02650	CON	MIN	HOUR	DAY	WEEK	UNIGENE	FLCDNA	TAIR
ATTTTTATTT	0	5	2	1	0	no match found	no match found	At2g02650.1
at2g23170	CON	MIN	HOUR	DAY	WEEK	UNIGENE	FLCDNA	TAIR
GACTATGCCA	0	2	0	0	0	gnl|UG|At#S11736697	gi|23463056|gb|BT000823.1	At2g23170.1
at1g14120	CON	MIN	HOUR	DAY	WEEK	UNIGENE	FLCDNA	TAIR
CTGGCGAATC	0	1	2	0	0	no match found	gi|19347940|gb|AY080683.1	At1g14120.1
at4g29820	CON	MIN	HOUR	DAY	WEEK	UNIGENE	FLCDNA	TAIR
AAACCGAGAA	0	1	0	1	2	no match found	gi|15081673|gb|AY048229.1	At4g29820.1
AGTAAAACAA	0	0	1	0	1	no match found	gi|21404694|gb|AY085984.1	multiple non-canonical match
at3g06430	CON	MIN	HOUR	DAY	WEEK	UNIGENE	FLCDNA	TAIR
GTCGAGGCGT	1	3	1	2	1	gnl|UG|At#S11738817	gi|20466299|gb|AY099616.1	At3g06430.1
at5g47430	CON	MIN	HOUR	DAY	WEEK	UNIGENE	FLCDNA	TAIR
GCACAAGGAT	1	0	0	0	0	no match found	gi|110741715|dbj|AK226697.1|	multiple non-canonical match
ATCTTTATAA	0	0	0	0	2	no match found	gi|72198598|gb|DQ108887.1	At1g23020.1
at1g17970	CON	MIN	HOUR	DAY	WEEK	UNIGENE	FLCDNA	TAIR
TCCATTGCGT	0	0	2	0	0	gnl|UG|At#S11741248	gi|16226278|gb|AF428290.1	At1g17970.1
at3g28200	CON	MIN	HOUR	DAY	WEEK	UNIGENE	FLCDNA	TAIR

GATTATATTC	3	2	1	0	0	gnl|UG|At#S11703522	gi|23297262|gb|AY150382.1	At3g28200.1
at2g31850	CON	MIN	HOUR	DAY	WEEK	UNIGENE	FLCDNA	TAIR
AGTTTGCAAC	0	1	0	0	0	no match found	no match found	At2g31850.1
at5g66650	CON	MIN	HOUR	DAY	WEEK	UNIGENE	FLCDNA	TAIR
CGGGGGTAAC	0	1	2	0	0	gnl|UG|At#S11717032	no match found	At5g66650.1
at2g40410	CON	MIN	HOUR	DAY	WEEK	UNIGENE	FLCDNA	TAIR
GGACTGGAGA	1	1	2	1	0	gnl|UG|At#S18900201	gi|20260255|gb|AY093027.1	At2g40410.1
at1g77450	CON	MIN	HOUR	DAY	WEEK	UNIGENE	FLCDNA	TAIR
GCTTTGTACG	0	0	1	0	1	gnl|UG|At#S11727400	gi|26452969|dbj|AK118985.1|	At1g77450.1
at2g03240	CON	MIN	HOUR	DAY	WEEK	UNIGENE	FLCDNA	TAIR
GGCATCAACA	1	0	2	0	0	gnl|UG|At#S11740497	gi|110742014|dbj|AK226854.1|	At2g03240.1
at3g16730	CON	MIN	HOUR	DAY	WEEK	UNIGENE	FLCDNA	TAIR
TTCTGTCTCA	0	0	0	1	0	gnl|UG|At#S34115200	gi|110741944|dbj|AK226817.1|	multiple non-canonical match
GTACAAAAAA	0	2	0	0	0	no match found	no match found	At3g16730.1
at4g12000	CON	MIN	HOUR	DAY	WEEK	UNIGENE	FLCDNA	TAIR
ATGTGAAAGA	1	0	1	1	0	gnl|UG|At#S11725731	gi|110740782|dbj|AK226340.1|	At4g12000.1
at2g46735	CON	MIN	HOUR	DAY	WEEK	UNIGENE	FLCDNA	TAIR
TAAATTTTCC	2	0	1	2	0	no match found	gi|21403064|gb|AY084354.1	non-canonical match
at5g25100	CON	MIN	HOUR	DAY	WEEK	UNIGENE	FLCDNA	TAIR
CTCATCGCCT	3	3	2	0	0	gnl|UG|At#S11720673	gi|13430445|gb|AF360135.1	multiple canonical match
at5g45140	CON	MIN	HOUR	DAY	WEEK	UNIGENE	FLCDNA	TAIR
CAAGCTACTA	0	0	0	1	0	gnl|UG|At#S11719213	gi|21703115|gb|AY123987.1	At5g45140.1
at3g54710	CON	MIN	HOUR	DAY	WEEK	UNIGENE	FLCDNA	TAIR
ACATTTCACT	0	1	0	1	0	gnl|UG|At#S11728565	gi|115646792|gb|BT029185.1	At3g54710.1
at2g31150	CON	MIN	HOUR	DAY	WEEK	UNIGENE	FLCDNA	TAIR
AGTCAGATTA	2	0	0	0	0	gnl|UG|At#S15460925	no match found	At2g31150.1
at3g05660	CON	MIN	HOUR	DAY	WEEK	UNIGENE	FLCDNA	TAIR
GTTTAACCAC	0	0	0	1	1	gnl|UG|At#S11739022	no match found	At2g31150.1
at2g45695	CON	MIN	HOUR	DAY	WEEK	UNIGENE	FLCDNA	TAIR
AAAGGCGATA	0	1	0	0	0	gnl|UG|At#S43849674	no match found	non-canonical match
GTGGATAAAA	0	0	1	2	0	gnl|UG|At#S37211181	gi|62318674|dbj|AK220657.1|	multiple non-canonical match
at5g56660	CON	MIN	HOUR	DAY	WEEK	UNIGENE	FLCDNA	TAIR
AAGAACTCTA	2	1	0	0	0	gnl|UG|At#S11743615	gi|45680364|gb|BT012054.1	At5g56660.1
at4g16800	CON	MIN	HOUR	DAY	WEEK	UNIGENE	FLCDNA	TAIR
AGAAAGCAAT	0	2	1	0	0	gnl|UG|At#S11724889	gi|22136625|gb|AY133698.1	At4g16800.1
at5g63010	CON	MIN	HOUR	DAY	WEEK	UNIGENE	FLCDNA	TAIR
TTGTTGTACT	1	0	0	0	0	gnl|UG|At#S11717400	gi|26450620|dbj|AK117775.1|	At5g63010.1
at4g39170	CON	MIN	HOUR	DAY	WEEK	UNIGENE	FLCDNA	TAIR
GCTTTTGTGA	0	1	0	0	0	gnl|UG|At#S11721069	gi|14335005|gb|AY037182.1	At4g39170.1
at3g48880	CON	MIN	HOUR	DAY	WEEK	UNIGENE	FLCDNA	TAIR
GATCACAAGA	1	3	0	1	0	gnl|UG|At#S11729663	gi|15028346|gb|AY045976.1	At3g48880.1

at1g70590	CON	MIN	HOUR	DAY	WEEK	UNIGENE	FLCDNA	TAIR
TCCATAACTT	0	0	0	2	0	no match found	gi|14335049|gb|AY037204.1	At1g70590.1
at2g25260	CON	MIN	HOUR	DAY	WEEK	UNIGENE	FLCDNA	TAIR
AAGGGGAAGA	0	0	1	0	0	gnl|UG|At#S11736205	gi|71143049|gb|BT023724.1	At2g25260.1
at3g06820	CON	MIN	HOUR	DAY	WEEK	UNIGENE	FLCDNA	TAIR
CCCGTCTTCT	1	2	2	2	0	gnl|UG|At#S11738685	gi|19715646|gb|AY075635.1	At3g06820.1
at5g06210	CON	MIN	HOUR	DAY	WEEK	UNIGENE	FLCDNA	TAIR
TGTAAGAGAT	1	1	0	0	0	gnl|UG|At#S11724571	gi|20260001|gb|AY093349.1	At5g06210.1
at4g14800	CON	MIN	HOUR	DAY	WEEK	UNIGENE	FLCDNA	TAIR
TGTTGAACTT	2	1	1	4	5	gnl|UG|At#S11725234	gi|110735664|dbj|AK227835.1|	At4g14800.1
TCTGTTGAAG	1	0	0	0	0	no match found	gi|88193793|gb|BT024588.1	multiple non-canonical match
at4g17100	CON	MIN	HOUR	DAY	WEEK	UNIGENE	FLCDNA	TAIR
TCTTGTCCTT	4	2	2	2	1	gnl|UG|At#S18896200	gi|20260611|gb|AY093205.1	At4g17100.1
at4g37310	CON	MIN	HOUR	DAY	WEEK	UNIGENE	FLCDNA	TAIR
CATATATGAT	0	0	1	1	1	no match found	gi|110742804|dbj|AK227281.1|	multiple non-canonical match
at1g22710	CON	MIN	HOUR	DAY	WEEK	UNIGENE	FLCDNA	TAIR
ACGGTTGTGG	4	14	4	2	1	gnl|UG|At#S11740792	gi|20147212|gb|AY091774.1	At1g22710.1
at1g04280	CON	MIN	HOUR	DAY	WEEK	UNIGENE	FLCDNA	TAIR
GGAGCCATTC	2	2	3	1	2	gnl|UG|At#S11742610	gi|110742283|dbj|AK227002.1|	At1g04280.1
at4g24275	CON	MIN	HOUR	DAY	WEEK	UNIGENE	FLCDNA	TAIR
AAACGTTTAA	3	1	1	0	0	gnl|UG|At#S30651580	gi|110739267|dbj|AK229709.1|	At4g24275.1
AAGGAGATGA	0	1	0	0	0	no match found	gi|109946538|gb|BT026092.1	non-canonical match
at1g31460	CON	MIN	HOUR	DAY	WEEK	UNIGENE	FLCDNA	TAIR
CATCAGCTAG	0	1	0	0	1	gnl|UG|At#S11738483	gi|21407754|gb|AY088980.1	At1g31460.1
at4g12600	CON	MIN	HOUR	DAY	WEEK	UNIGENE	FLCDNA	TAIR
TGATGTAACC	3	4	2	0	0	gnl|UG|At#S11725631	gi|15777868|gb|AY055095.1	At4g12600.1
at1g47580	CON	MIN	HOUR	DAY	WEEK	UNIGENE	FLCDNA	TAIR
AGTTTTTGCT	0	0	0	1	0	gnl|UG|At#S11735384	no match found	At1g47580.1
at2g35520	CON	MIN	HOUR	DAY	WEEK	UNIGENE	FLCDNA	TAIR
AGATTTTTGA	5	1	3	2	0	gnl|UG|At#S11705006	gi|21406733|gb|AY087959.1	At2g35520.2
at4g18490	CON	MIN	HOUR	DAY	WEEK	UNIGENE	FLCDNA	TAIR
AGGAAGCAAA	0	0	1	0	0	gnl|UG|At#S11724593	no match found	non-canonical match
at2g45070	CON	MIN	HOUR	DAY	WEEK	UNIGENE	FLCDNA	TAIR
TTATGGGCAA	9	7	3	3	5	gnl|UG|At#S18899180	gi|24030257|gb|BT000904.1	At2g45070.1
at4g14520	CON	MIN	HOUR	DAY	WEEK	UNIGENE	FLCDNA	TAIR
TAACCCAAAC	1	2	1	0	0	gnl|UG|At#S34115620	gi|110741152|dbj|AK226530.1|	At4g14520.1
at5g08060	CON	MIN	HOUR	DAY	WEEK	UNIGENE	FLCDNA	TAIR
GCAGATCACC	1	4	2	2	4	gnl|UG|At#S11724102	gi|17065637|gb|AY062967.1	At5g08060.1
at3g13882	CON	MIN	HOUR	DAY	WEEK	UNIGENE	FLCDNA	TAIR
TGTTGTTGAT	1	1	1	0	2	gnl|UG|At#S17007164	gi|13877910|gb|AF370218.1	non-canonical match
at1g66070	CON	MIN	HOUR	DAY	WEEK	UNIGENE	FLCDNA	TAIR
TTTTTGGTTC	0	1	0	0	2	gnl|UG|At#S18894232	gi|21407816|gb|AY089042.1	At1g66070.1

at5g42080	CON	MIN	HOUR	DAY	WEEK	UNIGENE	FLCDNA	TAIR
GAGAGACGCA	0	0	1	0	0	no match found	gi|24111386|gb|BT001063.1	non-canonical match
CAGTATCGTT	10	6	4	6	5	gnl|UG|At#S15459255	gi|20453080|gb|AY094408.1	At5g42080.1
at5g19440	CON	MIN	HOUR	DAY	WEEK	UNIGENE	FLCDNA	TAIR
TCAATGTTCT	4	4	2	5	10	gnl|UG|At#S11721465	gi|21407222|gb|AY088448.1	At5g19440.1
at5g23850	CON	MIN	HOUR	DAY	WEEK	UNIGENE	FLCDNA	TAIR
TAATAGCTTT	0	1	0	0	0	gnl|UG|At#S24442708	gi|62320257|dbj|AK221455.1|	At5g23850.1
at1g32190	CON	MIN	HOUR	DAY	WEEK	UNIGENE	FLCDNA	TAIR
GAAAGAGACT	0	2	3	0	2	gnl|UG|At#S11738202	gi|28393310|gb|BT004050.1	At1g32190.1
at5g45610	CON	MIN	HOUR	DAY	WEEK	UNIGENE	FLCDNA	TAIR
GATTTAGCCC	0	0	1	0	0	gnl|UG|At#S18909236	no match found	At5g45610.1
at2g14260	CON	MIN	HOUR	DAY	WEEK	UNIGENE	FLCDNA	TAIR
GCCAGAGGCA	0	1	3	0	0	no match found	gi|21387210|gb|AY114690.1	non-canonical match
GACGTTTATA	2	0	2	2	0	gnl|UG|At#S11738898	gi|15450388|gb|AY052295.1	At2g14260.2
at3g28660	CON	MIN	HOUR	DAY	WEEK	UNIGENE	FLCDNA	TAIR
GTGTTTGGGC	0	1	0	0	0	gnl|UG|At#S11807165	gi|20259530|gb|AY091065.1	At3g28660.1
at3g47630	CON	MIN	HOUR	DAY	WEEK	UNIGENE	FLCDNA	TAIR
AAAGTTTAGA	0	2	1	0	1	gnl|UG|At#S11729875	gi|110738627|dbj|AK229377.1|	At3g47630.1
at2g26030	CON	MIN	HOUR	DAY	WEEK	UNIGENE	FLCDNA	TAIR
TTGCAGTTGT	0	1	0	0	0	gnl|UG|At#S11821798	gi|51971320|dbj|AK176562.1|	At2g26030.2
at3g54750	CON	MIN	HOUR	DAY	WEEK	UNIGENE	FLCDNA	TAIR
GTTGAGATTA	0	0	0	0	1	gnl|UG|At#S15459250	gi|26451475|dbj|AK118215.1|	At3g54750.1
at4g20150	CON	MIN	HOUR	DAY	WEEK	UNIGENE	FLCDNA	TAIR
TGGTGGGTAT	0	4	4	3	1	gnl|UG|At#S11724308	gi|15028286|gb|AY045946.1	At4g20150.1
at5g07840	CON	MIN	HOUR	DAY	WEEK	UNIGENE	FLCDNA	TAIR
TTGTATTGAA	2	0	0	0	0	gnl|UG|At#S34114984	gi|110742341|dbj|AK227033.1|	At5g07840.1
at5g50570	CON	MIN	HOUR	DAY	WEEK	UNIGENE	FLCDNA	TAIR
ACTCTGATTG	0	1	0	0	0	gnl|UG|At#S15459119	no match found	multiple canonical match
at5g54870	CON	MIN	HOUR	DAY	WEEK	UNIGENE	FLCDNA	TAIR
TAAACTGTGT	7	2	1	2	1	gnl|UG|At#S11718226	gi|14596222|gb|AY042899.1	multiple canonical match
at3g05780	CON	MIN	HOUR	DAY	WEEK	UNIGENE	FLCDNA	TAIR
ATCATAAGAT	1	0	0	0	0	no match found	no match found	At3g05780.1
at5g61060	CON	MIN	HOUR	DAY	WEEK	UNIGENE	FLCDNA	TAIR
TGTTTGTACC	1	2	0	0	0	gnl|UG|At#S11717596	gi|23197729|gb|BT000073.1	At5g61060.1
at1g58350	CON	MIN	HOUR	DAY	WEEK	UNIGENE	FLCDNA	TAIR
ACCGTTTATG	0	1	0	1	0	gnl|UG|At#S43850417	gi|110741888|dbj|AK226788.1|	At1g58350.1
at4g00720	CON	MIN	HOUR	DAY	WEEK	UNIGENE	FLCDNA	TAIR
AAGCTTCTTC	4	6	2	2	0	no match found	gi|110740516|dbj|AK226199.1|	multiple non-canonical match
TTCCTCTCTA	1	2	0	1	1	gnl|UG|At#S11727491	no match found	At4g00720.1
at3g46970	CON	MIN	HOUR	DAY	WEEK	UNIGENE	FLCDNA	TAIR
GACGCTCAGG	0	9	1	2	4	gnl|UG|At#S11705595	gi|15983802|gb|AY056807.1	At3g46970.1

at1g03280	CON	MIN	HOUR	DAY	WEEK	UNIGENE	FLCDNA	TAIR
GATGATCAAG	0	0	0	1	0	no match found	gi|20465666|gb|AY096668.1	multiple non-canonical match

at1g49540	CON	MIN	HOUR	DAY	WEEK	UNIGENE	FLCDNA	TAIR
TTTAGCAAAA	0	1	2	1	3	gnl|UG|At#S34115359	gi|110741638|dbj|AK226658.1|	multiple non-canonical match

at1g04430	CON	MIN	HOUR	DAY	WEEK	UNIGENE	FLCDNA	TAIR
GGTTCTTTCG	0	4	0	0	0	gnl|UG|At#S11742584	gi|21403167|gb|AY084457.1	At1g04430.1

at3g12340	CON	MIN	HOUR	DAY	WEEK	UNIGENE	FLCDNA	TAIR
TTAAAAAAAT	0	0	2	1	1	gnl|UG|At#S37211167	no match found	At3g12340.1
GATATAAGAT	12	6	8	6	3	no match found	no match found	At3g12340.2

at3g46880	CON	MIN	HOUR	DAY	WEEK	UNIGENE	FLCDNA	TAIR
AAAGCTTCTC	0	1	0	0	0	no match found	no match found	At3g46880.1

at1g17910	CON	MIN	HOUR	DAY	WEEK	UNIGENE	FLCDNA	TAIR
GTGCGTTCTT	0	0	0	2	0	no match found	no match found	At1g17910.1

at1g29260	CON	MIN	HOUR	DAY	WEEK	UNIGENE	FLCDNA	TAIR
GATATGCGGT	1	2	0	0	0	gnl|UG|At#S11739282	gi|89274146|gb|BT024863.1	At1g29260.1

at3g63090	CON	MIN	HOUR	DAY	WEEK	UNIGENE	FLCDNA	TAIR
TGAAGAGAGA	1	0	0	0	0	gnl|UG|At#S11726510	gi|23198213|gb|BT000315.1	At3g63090.1

at5g07020	CON	MIN	HOUR	DAY	WEEK	UNIGENE	FLCDNA	TAIR
TCGAATGTCT	1	6	5	2	0	gnl|UG|At#S11724370	gi|15293030|gb|AY050949.1	At5g07020.1

at3g07050	CON	MIN	HOUR	DAY	WEEK	UNIGENE	FLCDNA	TAIR
TTGAATACAA	2	1	0	2	2	gnl|UG|At#S11738610	gi|15451213|gb|AY054687.1	multiple canonical match

at5g58250	CON	MIN	HOUR	DAY	WEEK	UNIGENE	FLCDNA	TAIR
CTTGACGAAG	0	9	3	1	1	gnl|UG|At#S11717885	gi|45773779|gb|BT012207.1	At5g58250.1

at5g54600	CON	MIN	HOUR	DAY	WEEK	UNIGENE	FLCDNA	TAIR
AAGAGCCGAG	10	25	8	11	4	no match found	gi|21405520|gb|AY086810.1	non-canonical match
ATTTAACATA	3	1	1	0	3	gnl|UG|At#S15455700	gi|25082790|gb|BT001990.1	At5g54600.1

at1g80860	CON	MIN	HOUR	DAY	WEEK	UNIGENE	FLCDNA	TAIR
GATAATGCAG	2	0	2	2	2	no match found	gi|21406929|gb|AY088155.1	At1g80860.2

at2g03350	CON	MIN	HOUR	DAY	WEEK	UNIGENE	FLCDNA	TAIR
AATTTATTTG	2	1	1	1	0	gnl|UG|At#S11740477	gi|21404387|gb|AY085677.1	At2g03350.1

at1g19770	CON	MIN	HOUR	DAY	WEEK	UNIGENE	FLCDNA	TAIR
GATGATGAGT	2	6	5	1	0	gnl|UG|At#S11741076	no match found	At1g19770.1

at1g25440	CON	MIN	HOUR	DAY	WEEK	UNIGENE	FLCDNA	TAIR
TTATATCTCA	4	4	0	0	2	gnl|UG|At#S11740364	gi|110740968|dbj|AK226436.1|	At1g25440.1

at1g73950	CON	MIN	HOUR	DAY	WEEK	UNIGENE	FLCDNA	TAIR
TAGACACCGT	0	2	1	0	0	gnl|UG|At#S11728256	gi|17529335|gb|AY065454.1	At1g73950.1

at4g03080	CON	MIN	HOUR	DAY	WEEK	UNIGENE	FLCDNA	TAIR
TGTAATATAA	2	1	0	2	1	gnl|UG|At#S11726911	gi|22022521|gb|AY126992.1	multiple non-canonical match

at2g04650	CON	MIN	HOUR	DAY	WEEK	UNIGENE	FLCDNA	TAIR
AATTGAGTTC	0	0	0	1	1	gnl|UG|At#S11740251	gi|23197829|gb|BT000123.1	At2g04650.1

at1g66410	CON	MIN	HOUR	DAY	WEEK	UNIGENE	FLCDNA	TAIR
TGATGACGAA	5	20	4	2	8	no match found	gi|21280868|gb|AY113902.1	non-canonical match

TTTAGATTTC	3	5	2	11	12	gnl|UG|At#S11729846	gi|21407250|gb|AY088476.1	At1g66410.1
at2g22795	CON	MIN	HOUR	DAY	WEEK	UNIGENE	FLCDNA	TAIR
AATCGGATTC	0	2	1	1	0	gnl|UG|At#S15461268	no match found	At2g22795.1
at1g23730	CON	MIN	HOUR	DAY	WEEK	UNIGENE	FLCDNA	TAIR
GCCATTGAAG	0	2	0	0	0	gnl|UG|At#S11740687	gi|21386932|gb|AY114551.1	multiple non-canonical match
at5g07460	CON	MIN	HOUR	DAY	WEEK	UNIGENE	FLCDNA	TAIR
GAAACGCTCA	0	3	1	0	1	gnl|UG|At#S11724259	gi|46402479|gb|BT012426.1	At5g07460.1
at2g17630	CON	MIN	HOUR	DAY	WEEK	UNIGENE	FLCDNA	TAIR
CTTGATTCTT	1	0	0	1	1	gnl|UG|At#S34117346	gi|110737824|dbj|AK228962.1|	At2g17630.1
at1g11910	CON	MIN	HOUR	DAY	WEEK	UNIGENE	FLCDNA	TAIR
GGCAAATACC	4	14	6	8	6	gnl|UG|At#S11741846	gi|21407431|gb|AY088657.1	At1g11910.1
at5g09820	CON	MIN	HOUR	DAY	WEEK	UNIGENE	FLCDNA	TAIR
GATCTTCTTT	0	1	1	1	0	gnl|UG|At#S28281825	gi|110737315|dbj|AK228700.1|	At5g09820.1
at1g75350	CON	MIN	HOUR	DAY	WEEK	UNIGENE	FLCDNA	TAIR
TGTTCCTTCT	1	14	2	1	1	no match found	gi|16323459|gb|AY059118.1	At1g75350.1
at3g61560	CON	MIN	HOUR	DAY	WEEK	UNIGENE	FLCDNA	TAIR
GTGTTTGATG	0	0	0	0	1	gnl|UG|At#S11726985	gi|50198811|gb|BT014988.1	At3g61560.1
at3g05420	CON	MIN	HOUR	DAY	WEEK	UNIGENE	FLCDNA	TAIR
GACAATGTTA	1	1	0	0	0	gnl|UG|At#S11739101	gi|110736069|dbj|AK228046.1|	At3g05420.2
at1g32860	CON	MIN	HOUR	DAY	WEEK	UNIGENE	FLCDNA	TAIR
TTACTGGCGA	0	0	0	2	1	gnl|UG|At#S11737958	gi|23197679|gb|BT000048.1	At1g32860.1
at4g39900	CON	MIN	HOUR	DAY	WEEK	UNIGENE	FLCDNA	TAIR
TAAACTGTAG	4	2	1	0	0	gnl|UG|At#S11720939	gi|21403680|gb|AY084970.1	At4g39900.1
at1g70490	CON	MIN	HOUR	DAY	WEEK	UNIGENE	FLCDNA	TAIR
ATGAGGGAGA	4	10	7	3	1	gnl|UG|At#S18942286	gi|15810604|gb|AY056341.1	At1g70490.3
at1g64670	CON	MIN	HOUR	DAY	WEEK	UNIGENE	FLCDNA	TAIR
TTTCTTTTGT	2	1	1	0	0	gnl|UG|At#S11730198	gi|21403300|gb|AY084590.1	At1g64670.1
at1g08600	CON	MIN	HOUR	DAY	WEEK	UNIGENE	FLCDNA	TAIR
TTGAAATTTG	2	1	1	0	2	gnl|UG|At#S11742175	no match found	At1g08600.1
at5g22630	CON	MIN	HOUR	DAY	WEEK	UNIGENE	FLCDNA	TAIR
ATTTGAGTCG	0	1	0	0	0	no match found	gi|26451802|dbj|AK118384.1|	non-canonical match
TGAAGTGGGA	0	2	3	1	0	gnl|UG|At#S11720937	gi|19699062|gb|AY090235.1	At5g22630.1
at1g17720	CON	MIN	HOUR	DAY	WEEK	UNIGENE	FLCDNA	TAIR
ATGTAACCAG	0	2	1	0	0	gnl|UG|At#S11741274	gi|110741433|dbj|AK226540.1|	At1g17720.1
at5g12140	CON	MIN	HOUR	DAY	WEEK	UNIGENE	FLCDNA	TAIR
AGTAAAAGCT	2	3	2	2	4	no match found	gi|15724167|gb|AF411786.1	At5g12140.1
TGTTTGAACT	10	14	15	20	16	no match found	gi|21407106|gb|AY088332.1	non-canonical match
at2g21600	CON	MIN	HOUR	DAY	WEEK	UNIGENE	FLCDNA	TAIR
ATCAAGCACA	0	6	1	1	1	gnl|UG|At#S11814573	gi|16323080|gb|AY057644.1	At2g21600.1
at3g13222	CON	MIN	HOUR	DAY	WEEK	UNIGENE	FLCDNA	TAIR
CAGGCTGGGA	0	3	0	0	0	gnl|UG|At#S11736917	gi|23296733|gb|AY142588.1	At3g13222.1

at3g42052	CON	MIN	HOUR	DAY	WEEK	UNIGENE	FLCDNA	TAIR
TGAACCATTT	0	2	0	1	1	gnl|UG|At#S24442349	gi|62320971|dbj|AK221814.1|	non-canonical match
at4g34680	CON	MIN	HOUR	DAY	WEEK	UNIGENE	FLCDNA	TAIR
TGGAGTCCGG	0	1	1	0	1	gnl|UG|At#S28281897	gi|22136457|gb|AY128907.1	At4g34680.1
at2g30500	CON	MIN	HOUR	DAY	WEEK	UNIGENE	FLCDNA	TAIR
TTGATATAGT	2	0	0	0	1	gnl|UG|At#S34114510	gi|110743228|dbj|AK227505.1|	At2g30500.1
at5g01620	CON	MIN	HOUR	DAY	WEEK	UNIGENE	FLCDNA	TAIR
CGAGGAGGTG	0	1	0	1	0	gnl|UG|At#S11725753	gi|19347853|gb|AY080736.1	At5g01620.1
at4g04340	CON	MIN	HOUR	DAY	WEEK	UNIGENE	FLCDNA	TAIR
ATCGGAAAAT	2	7	4	0	2	gnl|UG|At#S15461520	no match found	At4g04340.1
at3g10060	CON	MIN	HOUR	DAY	WEEK	UNIGENE	FLCDNA	TAIR
ACAAGTCGAC	2	1	0	1	0	gnl|UG|At#S11737871	gi|20453144|gb|AY094442.1	At3g10060.1
at2g40690	CON	MIN	HOUR	DAY	WEEK	UNIGENE	FLCDNA	TAIR
AATGTGAATT	1	0	0	1	0	gnl|UG|At#S11732419	gi|15293068|gb|AY050968.1	At2g40690.1
at4g16120	CON	MIN	HOUR	DAY	WEEK	UNIGENE	FLCDNA	TAIR
GAGGGCCTTC	0	1	0	0	0	gnl|UG|At#S11824542	gi|29028917|gb|BT005903.1	At4g16120.1
at1g57630	CON	MIN	HOUR	DAY	WEEK	UNIGENE	FLCDNA	TAIR
GAGGAAAGCT	0	1	0	0	1	gnl|UG|At#S11731980	no match found	non-canonical match
at5g67280	CON	MIN	HOUR	DAY	WEEK	UNIGENE	FLCDNA	TAIR
TTTGAAAATG	3	3	1	1	1	no match found	gi|110742582|dbj|AK227163.1|	multiple canonical match
at4g23290	CON	MIN	HOUR	DAY	WEEK	UNIGENE	FLCDNA	TAIR
AATATTTTTC	1	1	2	3	0	gnl|UG|At#S11723773	gi|20466232|gb|AY099582.1	At4g23290.1
at3g11450	CON	MIN	HOUR	DAY	WEEK	UNIGENE	FLCDNA	TAIR
AGAATTTTGG	1	2	2	2	0	gnl|UG|At#S11737454	no match found	At3g11450.1
at2g43020	CON	MIN	HOUR	DAY	WEEK	UNIGENE	FLCDNA	TAIR
ATCTCTACGA	0	2	0	0	0	gnl|UG|At#S11731879	gi|18650597|gb|AY074846.1	At2g43020.1
at1g43850	CON	MIN	HOUR	DAY	WEEK	UNIGENE	FLCDNA	TAIR
ATTTTTTGTT	2	2	0	2	0	gnl|UG|At#S11735963	gi|26451595|dbj|AK118277.1|	At1g43850.1
at1g26150	CON	MIN	HOUR	DAY	WEEK	UNIGENE	FLCDNA	TAIR
GCTCCGGAAT	0	3	2	0	0	gnl|UG|At#S11740271	no match found	At1g26150.1
at4g24520	CON	MIN	HOUR	DAY	WEEK	UNIGENE	FLCDNA	TAIR
AATGATTTGT	4	8	5	5	2	gnl|UG|At#S11723556	gi|15451215|gb|AY054688.1	At4g24520.1
at5g21930	CON	MIN	HOUR	DAY	WEEK	UNIGENE	FLCDNA	TAIR
AATCTGAGGT	4	2	6	2	1	no match found	gi|26453111|dbj|AK119056.1|	multiple canonical match
at1g57540	CON	MIN	HOUR	DAY	WEEK	UNIGENE	FLCDNA	TAIR
AATTTAGGTA	1	0	1	0	3	gnl|UG|At#S18895512	gi|26449332|dbj|AK117115.1|	At1g57540.2
at1g08125	CON	MIN	HOUR	DAY	WEEK	UNIGENE	FLCDNA	TAIR
GAGACTCTAT	1	0	0	0	0	gnl|UG|At#S18942615	gi|26452645|dbj|AK118816.1|	At1g08125.1
at3g02720	CON	MIN	HOUR	DAY	WEEK	UNIGENE	FLCDNA	TAIR
TTATGTAAAC	2	0	2	0	0	no match found	gi|24030367|gb|BT000947.1	At3g02720.1
at1g10522	CON	MIN	HOUR	DAY	WEEK	UNIGENE	FLCDNA	TAIR

TAGACAAGGA	1	2	0	0	0	gnl|UG|At#S11741981	gi|21405344|gb|AY086634.1	multiple non-canonical match
at2g35630	CON	MIN	HOUR	DAY	WEEK	UNIGENE	FLCDNA	TAIR
CAACTGGCTT	0	1	0	0	0	gnl|UG|At#S11733661	gi|62320882|dbj|AK221769.1|	At2g35630.1
at1g80020	CON	MIN	HOUR	DAY	WEEK	UNIGENE	FLCDNA	TAIR
TGACCTTGGA	0	4	1	1	0	no match found	no match found	At1g80020.1
at1g48860	CON	MIN	HOUR	DAY	WEEK	UNIGENE	FLCDNA	TAIR
AACAAAATGC	1	2	0	0	0	gnl|UG|At#S11734895	gi|21405427|gb|AY086717.1	At1g48860.2
at1g30490	CON	MIN	HOUR	DAY	WEEK	UNIGENE	FLCDNA	TAIR
GGCAGACCGG	1	3	1	0	0	gnl|UG|At#S18897685	gi|62319445|dbj|AK221046.1|	At1g30490.1
at1g71030	CON	MIN	HOUR	DAY	WEEK	UNIGENE	FLCDNA	TAIR
TGGTGTAACA	6	10	2	0	0	gnl|UG|At#S11728873	gi|13877700|gb|AF370609.1	At1g71030.1
at1g36430	CON	MIN	HOUR	DAY	WEEK	UNIGENE	FLCDNA	TAIR
TCCAGAAGAA	0	0	1	0	0	no match found	no match found	At1g36430.1
at2g02500	CON	MIN	HOUR	DAY	WEEK	UNIGENE	FLCDNA	TAIR
AAAAATGTTT	0	2	0	2	1	gnl|UG|At#S11740611	gi|26451270|dbj|AK118110.1|	At2g02500.1
at4g21610	CON	MIN	HOUR	DAY	WEEK	UNIGENE	FLCDNA	TAIR
GTCTGAGCAG	1	1	1	0	0	gnl|UG|At#S18907503	gi|45476530|gb|BT011760.1	At4g21610.1
at4g38040	CON	MIN	HOUR	DAY	WEEK	UNIGENE	FLCDNA	TAIR
TACACCTCTT	1	2	1	0	0	gnl|UG|At#S11721261	gi|15293110|gb|AY050989.1	At4g38040.1
at3g22110	CON	MIN	HOUR	DAY	WEEK	UNIGENE	FLCDNA	TAIR
TTCACTCACC	0	7	0	2	3	gnl|UG|At#S11734189	gi|21404624|gb|AY085914.1	At3g22110.1
at1g52300	CON	MIN	HOUR	DAY	WEEK	UNIGENE	FLCDNA	TAIR
GATGGATTTC	33	22	55	60	41	gnl|UG|At#S11733623	gi|13877906|gb|AF370216.1	At1g52300.1
at1g31920	CON	MIN	HOUR	DAY	WEEK	UNIGENE	FLCDNA	TAIR
ATCTACGAAA	1	1	2	2	0	gnl|UG|At#S11738304	no match found	At1g31920.1
at1g48300	CON	MIN	HOUR	DAY	WEEK	UNIGENE	FLCDNA	TAIR
TAATTGCGAT	16	1	11	5	9	gnl|UG|At#S11735103	gi|110743457|dbj|AK227624.1|	non-canonical match
GGGAAGTGTC	2	2	4	1	0	no match found	gi|30102609|gb|BT006415.1	non-canonical match
at1g76050	CON	MIN	HOUR	DAY	WEEK	UNIGENE	FLCDNA	TAIR
AATGATAATT	3	2	3	0	4	gnl|UG|At#S18895540	gi|26450632|dbj|AK117781.1|	non-canonical match
at3g29360	CON	MIN	HOUR	DAY	WEEK	UNIGENE	FLCDNA	TAIR
CCTGCCGTTG	2	3	5	5	5	gnl|UG|At#S11731852	gi|15983403|gb|AF424576.1	At3g29360.1
at4g27440	CON	MIN	HOUR	DAY	WEEK	UNIGENE	FLCDNA	TAIR
AAGAAACTGG	0	34	4	2	4	gnl|UG|At#S11723054	no match found	At4g27440.1
at1g55340	CON	MIN	HOUR	DAY	WEEK	UNIGENE	FLCDNA	TAIR
GAAAGCGATT	0	2	3	0	1	gnl|UG|At#S11732522	gi|110741494|dbj|AK226579.1|	At1g55340.1
at3g53100	CON	MIN	HOUR	DAY	WEEK	UNIGENE	FLCDNA	TAIR
CTGCGGAACA	0	1	0	0	0	gnl|UG|At#S11728853	gi|110738050|dbj|AK229079.1|	At3g53100.1
at4g27000	CON	MIN	HOUR	DAY	WEEK	UNIGENE	FLCDNA	TAIR
ATGTGTAAGT	0	0	1	0	0	no match found	gi|20259887|gb|AY093292.1	non-canonical match
ACTAATATGA	13	7	2	2	11	gnl|UG|At#S11723127	gi|15450813|gb|AY054487.1	At4g27000.1

at2g10400	CON	MIN	HOUR	DAY	WEEK	UNIGENE	FLCDNA	TAIR
GTAAATGTAG	0	0	0	0	2	no match found	no match found	At2g10400.1
at5g02870	CON	MIN	HOUR	DAY	WEEK	UNIGENE	FLCDNA	TAIR
AAGAGGCACG	3	11	4	1	2	no match found	gi|22137185|gb|AY133608.1	At5g02870.1
at4g03520	CON	MIN	HOUR	DAY	WEEK	UNIGENE	FLCDNA	TAIR
TCTTATGTCA	28	17	15	10	28	gnl|UG|At#S11726789	gi|15028102|gb|AY046001.1	At4g03520.1
AAATATCACT	2	0	0	0	0	no match found	gi|19310724|gb|AY079362.1	non-canonical match
at3g23070	CON	MIN	HOUR	DAY	WEEK	UNIGENE	FLCDNA	TAIR
GAAAGAAAAT	0	2	0	1	4	gnl|UG|At#S11733889	no match found	multiple non-canonical match
at1g24050	CON	MIN	HOUR	DAY	WEEK	UNIGENE	FLCDNA	TAIR
TTGTTGTCTT	1	1	0	0	0	gnl|UG|At#S11740616	gi|20453159|gb|AY094450.1	At1g24050.1
at4g32830	CON	MIN	HOUR	DAY	WEEK	UNIGENE	FLCDNA	TAIR
TTCCAAATAA	0	2	1	0	0	gnl|UG|At#S11722135	gi|21405666|gb|AY086942.1	At4g32830.1
at4g25960	CON	MIN	HOUR	DAY	WEEK	UNIGENE	FLCDNA	TAIR
ATAGAGGGAA	0	1	0	1	0	gnl|UG|At#S11723317	gi|20466379|gb|AY099656.1	At4g25960.1
at5g61840	CON	MIN	HOUR	DAY	WEEK	UNIGENE	FLCDNA	TAIR
AGAGGAGTGT	0	0	1	0	0	gnl|UG|At#S11717517	gi|15809825|gb|AY054180.1	At5g61840.1
at2g21280	CON	MIN	HOUR	DAY	WEEK	UNIGENE	FLCDNA	TAIR
ATGATACCCT	0	1	1	0	0	gnl|UG|At#S11821376	gi|23397229|gb|BT000757.1	At2g21280.1
at2g38440	CON	MIN	HOUR	DAY	WEEK	UNIGENE	FLCDNA	TAIR
ATGAGTGCGC	0	1	1	1	0	gnl|UG|At#S11732988	no match found	At2g38440.1
at2g43590	CON	MIN	HOUR	DAY	WEEK	UNIGENE	FLCDNA	TAIR
TAATAAGTGA	0	0	0	1	1	gnl|UG|At#S11731747	gi|110736332|dbj|AK228181.1|	At2g43590.1
at4g12290	CON	MIN	HOUR	DAY	WEEK	UNIGENE	FLCDNA	TAIR
ATCTCCCGGT	0	2	0	2	0	no match found	gi|28416506|gb|BT004538.1	non-canonical match
CGGATTATGG	1	3	11	1	3	gnl|UG|At#S11725682	gi|22654994|gb|AY139770.1	At4g12290.1
at1g13980	CON	MIN	HOUR	DAY	WEEK	UNIGENE	FLCDNA	TAIR
TGAATAACAT	1	0	0	0	0	no match found	gi|62319012|dbj|AK220827.1|	non-canonical match
at1g61580	CON	MIN	HOUR	DAY	WEEK	UNIGENE	FLCDNA	TAIR
ACCGAGTATG	0	0	0	0	1	gnl|UG|At#S14274080	gi|28973594|gb|BT005702.1	At1g61580.1
at2g43150	CON	MIN	HOUR	DAY	WEEK	UNIGENE	FLCDNA	TAIR
TTGGCGTTAC	0	7	1	0	0	gnl|UG|At#S11731847	no match found	At2g43150.1
at3g18640	CON	MIN	HOUR	DAY	WEEK	UNIGENE	FLCDNA	TAIR
TTGTCTTAAT	0	0	1	0	2	gnl|UG|At#S21736878	gi|51969735|dbj|AK175797.1|	At3g18640.1
at1g36730	CON	MIN	HOUR	DAY	WEEK	UNIGENE	FLCDNA	TAIR
AAAAGCTTGT	1	0	0	0	0	gnl|UG|At#S11671793	no match found	non-canonical match
ACTTAGACAA	6	2	2	0	5	gnl|UG|At#S11736712	gi|17381209|gb|AY064061.1	At1g36730.1
at5g42070	CON	MIN	HOUR	DAY	WEEK	UNIGENE	FLCDNA	TAIR
GACGAGGTTG	0	2	2	3	0	gnl|UG|At#S11806601	gi|21407627|gb|AY088853.1	At5g42070.1
at1g41795	CON	MIN	HOUR	DAY	WEEK	UNIGENE	FLCDNA	TAIR
GGAAAGTCAA	0	0	1	0	0	no match found	no match found	At1g41795.1
at2g01520	CON	MIN	HOUR	DAY	WEEK	UNIGENE	FLCDNA	TAIR

TATCCTTGTT	0	0	1	0	0	gnl|UG|At#S11742659	gi|15809957|gb|AY054247.1	At2g01520.1
at1g22500	CON	MIN	HOUR	DAY	WEEK	UNIGENE	FLCDNA	TAIR
ACCAGCTTGT	0	0	1	0	0	gnl|UG|At#S11740811	gi|40823266|gb|BT011235.1	At1g22500.1
at3g21290	CON	MIN	HOUR	DAY	WEEK	UNIGENE	FLCDNA	TAIR
GGTATCAAGA	0	1	2	0	0	gnl|UG|At#S11704150	gi|14532605|gb|AY039927.1	At3g21290.1
at1g04020	CON	MIN	HOUR	DAY	WEEK	UNIGENE	FLCDNA	TAIR
GGTCTTGGAG	0	1	0	0	0	gnl|UG|At#S11742681	no match found	At1g04020.2
at1g29910	CON	MIN	HOUR	DAY	WEEK	UNIGENE	FLCDNA	TAIR
AGGAGAAAGA	501	455	479	348	69	gnl|UG|At#S11739048	gi|17473827|gb|AY065165.1	At1g29910.1
at2g14330	CON	MIN	HOUR	DAY	WEEK	UNIGENE	FLCDNA	TAIR
GTATGTGTTC	1	0	0	0	0	no match found	no match found	At2g14330.1
at5g41210	CON	MIN	HOUR	DAY	WEEK	UNIGENE	FLCDNA	TAIR
AGATCCTTTT	1	2	1	0	0	no match found	gi|15451157|gb|AY054659.1	At5g41210.1
AAGGTTTAGA	0	0	0	1	1	gnl|UG|At#S11719608	no match found	non-canonical match
at1g61010	CON	MIN	HOUR	DAY	WEEK	UNIGENE	FLCDNA	TAIR
AATACACGGT	1	0	0	0	0	gnl|UG|At#S15455458	no match found	At1g61010.2
TTTAGTAATA	0	0	0	0	2	no match found	gi|18377653|gb|AY074280.1	At1g61010.1
at4g08240	CON	MIN	HOUR	DAY	WEEK	UNIGENE	FLCDNA	TAIR
CCACAACCAC	0	0	1	0	0	gnl|UG|At#S15461392	gi|28416888|gb|BT004729.1	At4g08240.2
at5g22800	CON	MIN	HOUR	DAY	WEEK	UNIGENE	FLCDNA	TAIR
TATAGATGAT	5	1	0	1	0	gnl|UG|At#S11817055	no match found	At5g22800.1
at2g40080	CON	MIN	HOUR	DAY	WEEK	UNIGENE	FLCDNA	TAIR
TATTCTGATC	0	0	1	0	1	gnl|UG|At#S11732580	gi|21407522|gb|AY088748.1	At2g40080.1
at5g67250	CON	MIN	HOUR	DAY	WEEK	UNIGENE	FLCDNA	TAIR
TGTGCAAGAC	2	2	5	3	1	gnl|UG|At#S11716970	gi|15810226|gb|AY056152.1	At5g67250.1
AAGCAGCTGA	0	1	2	0	0	no match found	gi|31711937|gb|BT008886.1	non-canonical match
at5g48380	CON	MIN	HOUR	DAY	WEEK	UNIGENE	FLCDNA	TAIR
TTTGAAGTTT	4	6	7	5	6	gnl|UG|At#S11718882	gi|13605826|gb|AF367312.1	multiple canonical match
at1g26760	CON	MIN	HOUR	DAY	WEEK	UNIGENE	FLCDNA	TAIR
GAGATGGCGA	0	0	0	1	0	gnl|UG|At#S11705528	gi|16209660|gb|AY057595.1	multiple non-canonical match
at5g40710	CON	MIN	HOUR	DAY	WEEK	UNIGENE	FLCDNA	TAIR
TTCAGGAGGA	0	1	0	0	0	gnl|UG|At#S11719658	gi|90962967|gb|BT025033.1	At5g40710.1
at2g40430	CON	MIN	HOUR	DAY	WEEK	UNIGENE	FLCDNA	TAIR
TTGCACTCTT	0	2	4	1	1	no match found	gi|20259831|gb|AY093264.1	At2g40430.1
at1g74070	CON	MIN	HOUR	DAY	WEEK	UNIGENE	FLCDNA	TAIR
GTGGTTTGGT	0	2	2	2	2	gnl|UG|At#S11728228	no match found	At1g74070.1
at3g02580	CON	MIN	HOUR	DAY	WEEK	UNIGENE	FLCDNA	TAIR
TAAATGTTTG	4	5	1	2	0	gnl|UG|At#S11698928	gi|21407301|gb|AY088527.1	At3g02580.1
TGTCTCTCTC	1	0	1	0	0	no match found	gi|51971638|dbj|AK176721.1|	non-canonical match
at4g15417	CON	MIN	HOUR	DAY	WEEK	UNIGENE	FLCDNA	TAIR
ACTCTTTTGG	1	1	0	0	0	gnl|UG|At#S34116418	no match found	non-canonical match
at5g43460	CON	MIN	HOUR	DAY	WEEK	UNIGENE	FLCDNA	TAIR

ACCTTGGAAT	2	0	5	3	3	gnl|UG|At#S11719382	no match found	At5g43460.1
at1g60420	CON	MIN	HOUR	DAY	WEEK	UNIGENE	FLCDNA	TAIR
CGATAAGTGT	0	0	1	0	0	gnl|UG|At#S11731357	gi|21406120|gb|AY087396.1	non-canonical match
at5g37410	CON	MIN	HOUR	DAY	WEEK	UNIGENE	FLCDNA	TAIR
AGATGGGAAA	0	1	0	0	0	gnl|UG|At#S11719994	no match found	At5g37410.1
at4g17530	CON	MIN	HOUR	DAY	WEEK	UNIGENE	FLCDNA	TAIR
TAAGAAATCT	5	3	9	7	6	gnl|UG|At#S11724759	gi|15529160|gb|AY052204.1	At4g17530.1
ACTGCTGCAA	0	0	2	0	0	no match found	gi|16974364|gb|AY060495.1	At4g36210.2
at1g14880	CON	MIN	HOUR	DAY	WEEK	UNIGENE	FLCDNA	TAIR
AGTATTTCTT	0	2	0	0	3	gnl|UG|At#S11741546	gi|26452732|dbj|AK118861.1|	At1g14880.1
at3g01640	CON	MIN	HOUR	DAY	WEEK	UNIGENE	FLCDNA	TAIR
TGAGCTTTAA	1	1	0	2	0	gnl|UG|At#S11705289	gi|16323052|gb|AY057630.1	At3g01640.1
at4g27080	CON	MIN	HOUR	DAY	WEEK	UNIGENE	FLCDNA	TAIR
AAAAAGATAG	0	1	0	0	1	gnl|UG|At#S11723114	gi|15450831|gb|AY054496.1	At4g27080.1
at1g03860	CON	MIN	HOUR	DAY	WEEK	UNIGENE	FLCDNA	TAIR
AAACCCTAAA	0	1	0	1	0	gnl|UG|At#S18942650	gi|21407952|gb|AY089178.1	At1g03860.2
at3g61550	CON	MIN	HOUR	DAY	WEEK	UNIGENE	FLCDNA	TAIR
TAATTAAGTT	7	3	3	0	0	no match found	gi|16226238|gb|AF428280.1	At3g61550.1
at1g75380	CON	MIN	HOUR	DAY	WEEK	UNIGENE	FLCDNA	TAIR
AGAGAGCTCG	21	41	16	11	1	no match found	gi|15809928|gb|AY054232.1	At1g75380.3
AGAGAGGTAA	1	2	1	0	0	gnl|UG|At#S11727965	gi|21406141|gb|AY087417.1	At1g75380.1
at5g60900	CON	MIN	HOUR	DAY	WEEK	UNIGENE	FLCDNA	TAIR
AGAAATGTTA	0	0	0	0	1	gnl|UG|At#S11807924	gi|20856746|gb|AY102113.1	non-canonical match
at4g07475	CON	MIN	HOUR	DAY	WEEK	UNIGENE	FLCDNA	TAIR
TTTATTGAAT	1	0	0	0	1	no match found	no match found	At4g07475.1
at1g29280	CON	MIN	HOUR	DAY	WEEK	UNIGENE	FLCDNA	TAIR
GGAAGCGTTG	0	2	0	0	0	no match found	no match found	At1g29280.1
at1g22180	CON	MIN	HOUR	DAY	WEEK	UNIGENE	FLCDNA	TAIR
AAATCCTAAA	0	1	0	0	1	gnl|UG|At#S18942524	gi|18253034|gb|AY072452.1	At1g22180.3
at1g07360	CON	MIN	HOUR	DAY	WEEK	UNIGENE	FLCDNA	TAIR
ATGCCTCCAC	0	0	0	0	2	no match found	gi|15450877|gb|AY054519.1	multiple non-canonical match
at2g46400	CON	MIN	HOUR	DAY	WEEK	UNIGENE	FLCDNA	TAIR
ATTTTGGCCT	0	0	0	0	1	gnl|UG|At#S11744334	gi|19423941|gb|AY080812.1	At2g46400.1
AGATTGAGAA	0	0	0	0	1	no match found	gi|45680163|gb|BT011853.1	multiple non-canonical match
at5g40540	CON	MIN	HOUR	DAY	WEEK	UNIGENE	FLCDNA	TAIR
AGAGAATGTG	0	0	1	0	2	gnl|UG|At#S11719676	gi|110738179|dbj|AK229147.1|	At5g40540.1
at4g37820	CON	MIN	HOUR	DAY	WEEK	UNIGENE	FLCDNA	TAIR
TGTATGTATA	3	0	0	0	1	gnl|UG|At#S11721300	gi|26450587|dbj|AK117758.1|	At4g37820.1
at1g71720	CON	MIN	HOUR	DAY	WEEK	UNIGENE	FLCDNA	TAIR
TTGTACAATG	0	0	2	1	1	gnl|UG|At#S24442530	gi|62320613|dbj|AK221633.1|	At1g71720.1
at5g19580	CON	MIN	HOUR	DAY	WEEK	UNIGENE	FLCDNA	TAIR
TAAACCTTTT	0	0	1	0	0	gnl|UG|At#S11721429	no match found	At5g19580.1

at5g15730	CON	MIN	HOUR	DAY	WEEK	UNIGENE	FLCDNA	TAIR
AGTCTCAAAA	1	1	1	1	0	gnl|UG|At#S38433296	gi|62318724|dbj|AK220682.1|	At5g15730.1
at5g63330	CON	MIN	HOUR	DAY	WEEK	UNIGENE	FLCDNA	TAIR
TAGAAATAAT	1	0	0	0	0	gnl|UG|At#S11717367	gi|50253451|gb|BT015056.1	At5g63330.1
at1g13470	CON	MIN	HOUR	DAY	WEEK	UNIGENE	FLCDNA	TAIR
AGAGATTTCT	0	0	0	1	1	gnl|UG|At#S24442581	gi|62320511|dbj|AK221582.1|	At1g13470.1
at3g58750	CON	MIN	HOUR	DAY	WEEK	UNIGENE	FLCDNA	TAIR
TACTTTTGAT	0	1	1	0	0	no match found	no match found	At3g58750.1
TGTTGTTTTC	2	2	0	0	2	no match found	gi|62318808|dbj|AK220724.1|	multiple non-canonical match
at1g69950	CON	MIN	HOUR	DAY	WEEK	UNIGENE	FLCDNA	TAIR
ATCATCATTT	0	0	0	1	0	no match found	no match found	At1g69950.1
at3g17850	CON	MIN	HOUR	DAY	WEEK	UNIGENE	FLCDNA	TAIR
CAGAGTTTGA	4	2	8	2	3	no match found	gi|26449547|dbj|AK117224.1|	multiple canonical match
at3g11800	CON	MIN	HOUR	DAY	WEEK	UNIGENE	FLCDNA	TAIR
AATTAAATAG	2	0	0	0	0	gnl|UG|At#S11704719	gi|15028124|gb|AY046012.1	multiple canonical match
TTCTGGAAGA	0	2	0	0	0	no match found	gi|19310798|gb|AY079399.1	non-canonical match
at2g34600	CON	MIN	HOUR	DAY	WEEK	UNIGENE	FLCDNA	TAIR
TGTGTTTCTT	1	1	0	0	0	gnl|UG|At#S11733915	gi|38454057|gb|BT010666.1	At2g34600.1
at1g33270	CON	MIN	HOUR	DAY	WEEK	UNIGENE	FLCDNA	TAIR
GGCTGAGATG	0	1	1	0	1	no match found	gi|38603807|gb|BT010871.1	non-canonical match
TGCTTCGAGG	1	1	3	2	1	gnl|UG|At#S18942449	gi|110742516|dbj|AK227126.1|	multiple non-canonical match
at2g40020	CON	MIN	HOUR	DAY	WEEK	UNIGENE	FLCDNA	TAIR
CATATTGAGT	0	1	0	0	0	gnl|UG|At#S18942746	gi|28973452|gb|BT005631.1	At2g40020.1
at4g28200	CON	MIN	HOUR	DAY	WEEK	UNIGENE	FLCDNA	TAIR
TGTTTTCTCT	0	0	0	1	0	no match found	gi|30725461|gb|BT008394.1	multiple non-canonical match
TTGAAGTTCA	1	1	1	3	0	no match found	gi|62321470|dbj|AK222070.1|	non-canonical match
at3g12580	CON	MIN	HOUR	DAY	WEEK	UNIGENE	FLCDNA	TAIR
AGGAACACGA	1	1	2	2	0	no match found	gi|16649030|gb|AY059885.1	multiple non-canonical match
at1g16650	CON	MIN	HOUR	DAY	WEEK	UNIGENE	FLCDNA	TAIR
GAATGAAGCT	0	1	0	0	1	gnl|UG|At#S11741379	no match found	At1g16650.1
at5g18640	CON	MIN	HOUR	DAY	WEEK	UNIGENE	FLCDNA	TAIR
ATCACATATG	1	2	0	0	1	gnl|UG|At#S11721663	no match found	At5g18640.1
at5g18700	CON	MIN	HOUR	DAY	WEEK	UNIGENE	FLCDNA	TAIR
TGACAAAGTG	2	0	0	0	0	gnl|UG|At#S11721650	no match found	non-canonical match
at2g28840	CON	MIN	HOUR	DAY	WEEK	UNIGENE	FLCDNA	TAIR
GGGGAGCAAT	0	10	2	4	1	gnl|UG|At#S11735325	gi|13926221|gb|AF370581.1	At2g28840.1
at5g27860	CON	MIN	HOUR	DAY	WEEK	UNIGENE	FLCDNA	TAIR
GATTTGTTTT	1	1	1	0	0	gnl|UG|At#S42407268	gi|110737879|dbj|AK228990.1|	At5g27860.1
at1g01770	CON	MIN	HOUR	DAY	WEEK	UNIGENE	FLCDNA	TAIR
ATTTTTTTTG	0	3	1	1	1	no match found	gi|16604690|gb|AY059790.1	multiple canonical match
GGTTGCACCA	4	1	1	3	0	no match found	gi|26449407|dbj|AK117153.1|	At1g01770.1
at5g10450	CON	MIN	HOUR	DAY	WEEK	UNIGENE	FLCDNA	TAIR

AAAACTATTT	1	2	1	0	0	no match found	gi|15450384|gb|AY052293.1	At5g10450.1
GCCAAACAGG	6	23	10	11	2	no match found	gi|14335137|gb|AY037248.1	multiple non-canonical match
at5g22820	CON	MIN	HOUR	DAY	WEEK	UNIGENE	FLCDNA	TAIR
AAGTCAGCTT	0	0	0	0	1	gnl|UG|At#S11720897	no match found	multiple non-canonical match
at4g36730	CON	MIN	HOUR	DAY	WEEK	UNIGENE	FLCDNA	TAIR
AAGCCGGTAG	0	1	1	0	0	gnl|UG|At#S11721483	gi|17978766|gb|AY064670.1	At4g36730.2
at3g47590	CON	MIN	HOUR	DAY	WEEK	UNIGENE	FLCDNA	TAIR
AAAGTTTGTA	0	4	3	1	1	gnl|UG|At#S11729885	gi|51969609|dbj|AK175734.1|	At3g47590.1
at3g12640	CON	MIN	HOUR	DAY	WEEK	UNIGENE	FLCDNA	TAIR
CTTCTTCAAT	1	0	0	1	1	gnl|UG|At#S34117603	gi|21406003|gb|AY087279.1	At3g12640.1
at4g17490	CON	MIN	HOUR	DAY	WEEK	UNIGENE	FLCDNA	TAIR
CCGCTTTTAT	0	6	3	0	0	gnl|UG|At#S11724768	gi|17473791|gb|AY065155.1	At4g17490.1
at3g15730	CON	MIN	HOUR	DAY	WEEK	UNIGENE	FLCDNA	TAIR
ACCTTCCTGG	0	0	1	0	0	no match found	gi|47679066|gb|BT014735.1	multiple non-canonical match
TCAAGTAATG	4	15	3	8	5	gnl|UG|At#S11736125	gi|110742065|dbj|AK226887.1|	At3g15730.1
at1g27950	CON	MIN	HOUR	DAY	WEEK	UNIGENE	FLCDNA	TAIR
TCACATTAGG	13	7	11	10	16	no match found	gi|62318722|dbj|AK220681.1|	At1g27950.1
at3g54290	CON	MIN	HOUR	DAY	WEEK	UNIGENE	FLCDNA	TAIR
ACATTTGGTG	0	2	0	0	0	gnl|UG|At#S11728634	gi|21407940|gb|AY089166.1	At3g54290.1
at5g20010	CON	MIN	HOUR	DAY	WEEK	UNIGENE	FLCDNA	TAIR
TATTTTTGGA	1	0	0	0	0	no match found	gi|62319117|dbj|AK220880.1|	At3g54290.1
GCACCGTGAT	0	11	1	4	1	gnl|UG|At#S11721321	gi|16226504|gb|AF428417.1	At5g20010.1
at5g27360	CON	MIN	HOUR	DAY	WEEK	UNIGENE	FLCDNA	TAIR
GTCAAGTAGT	0	2	1	1	0	gnl|UG|At#S11720476	no match found	At5g27360.1
at1g55325	CON	MIN	HOUR	DAY	WEEK	UNIGENE	FLCDNA	TAIR
TAGGTATCTC	0	1	0	0	0	gnl|UG|At#S35309386	gi|26451641|dbj|AK118300.1|	At1g55325.1
at5g59050	CON	MIN	HOUR	DAY	WEEK	UNIGENE	FLCDNA	TAIR
CCTATTGTAT	0	1	0	0	0	gnl|UG|At#S11708845	gi|17065345|gb|AY062749.1	At5g59050.2
at3g42150	CON	MIN	HOUR	DAY	WEEK	UNIGENE	FLCDNA	TAIR
TCCTAATCTT	3	3	2	0	0	gnl|UG|At#S24442420	gi|62320831|dbj|AK221743.1|	At3g42150.2
at1g20990	CON	MIN	HOUR	DAY	WEEK	UNIGENE	FLCDNA	TAIR
TTTTGAAAAA	1	0	0	0	0	no match found	no match found	At1g20990.1
at1g78000	CON	MIN	HOUR	DAY	WEEK	UNIGENE	FLCDNA	TAIR
TTAGGACAAG	0	2	0	0	1	gnl|UG|At#S11727203	gi|110743254|dbj|AK227518.1|	At1g78000.2
at1g76320	CON	MIN	HOUR	DAY	WEEK	UNIGENE	FLCDNA	TAIR
CTTGGAAAAT	0	0	1	0	0	gnl|UG|At#S24442757	gi|62320159|dbj|AK221406.1|	multiple non-canonical match
at5g57260	CON	MIN	HOUR	DAY	WEEK	UNIGENE	FLCDNA	TAIR
ATTGTCCAGA	0	1	1	0	0	no match found	no match found	At5g57260.1
at1g10140	CON	MIN	HOUR	DAY	WEEK	UNIGENE	FLCDNA	TAIR
TTGATACAAG	0	3	0	0	0	gnl|UG|At#S34116178	gi|110740086|dbj|AK230130.1|	At1g10140.1
at1g26580	CON	MIN	HOUR	DAY	WEEK	UNIGENE	FLCDNA	TAIR
TGGATTCTTG	0	4	3	2	0	gnl|UG|At#S11740128	gi|44681377|gb|BT011623.1	At1g26580.1

at5g23575	CON	MIN	HOUR	DAY	WEEK	UNIGENE	FLCDNA	TAIR
GAGACAGATG	0	9	1	1	5	no match found	gi|62321715|dbj|AK222196.1|	At5g23575.1
at1g04450	CON	MIN	HOUR	DAY	WEEK	UNIGENE	FLCDNA	TAIR
GTTTTGATCA	0	0	1	0	0	gnl|UG|At#S11742582	no match found	multiple non-canonical match
at2g45270	CON	MIN	HOUR	DAY	WEEK	UNIGENE	FLCDNA	TAIR
GCATTGCTTA	0	0	0	0	1	no match found	gi|21403287|gb|AY084577.1	non-canonical match
at1g34360	CON	MIN	HOUR	DAY	WEEK	UNIGENE	FLCDNA	TAIR
TAAACCCACA	2	2	2	2	2	gnl|UG|At#S11737458	gi|45773941|gb|BT012288.1	At1g34360.1
at5g53530	CON	MIN	HOUR	DAY	WEEK	UNIGENE	FLCDNA	TAIR
TAATATTTCA	0	2	0	0	0	gnl|UG|At#S11718362	gi|45680200|gb|BT011890.1	At5g53530.1
at4g31590	CON	MIN	HOUR	DAY	WEEK	UNIGENE	FLCDNA	TAIR
TCAAATCACT	1	0	0	0	0	gnl|UG|At#S11722349	gi|28058783|gb|BT003334.1	At4g31590.1
at5g13770	CON	MIN	HOUR	DAY	WEEK	UNIGENE	FLCDNA	TAIR
TCATAAGCAT	0	0	0	0	1	no match found	no match found	At5g13770.1
AAAGTTGAAG	1	3	4	3	0	gnl|UG|At#S11722897	gi|51536485|gb|BT015358.1	non-canonical match
at5g35560	CON	MIN	HOUR	DAY	WEEK	UNIGENE	FLCDNA	TAIR
TATACGTAAA	0	1	0	0	0	gnl|UG|At#S11720147	gi|15146184|gb|AY049233.1	At5g35560.1
at2g22880	CON	MIN	HOUR	DAY	WEEK	UNIGENE	FLCDNA	TAIR
TGAAGAAGAA	1	0	2	1	3	no match found	no match found	At2g22880.1
AATTTGTTTC	0	4	1	0	0	gnl|UG|At#S11692800	no match found	non-canonical match
at4g28900	CON	MIN	HOUR	DAY	WEEK	UNIGENE	FLCDNA	TAIR
GCTTGCTACT	0	2	0	1	0	no match found	gi|110737085|dbj|AK228579.1|	non-canonical match
at1g74430	CON	MIN	HOUR	DAY	WEEK	UNIGENE	FLCDNA	TAIR
CCTATGAGCC	1	0	0	0	0	gnl|UG|At#S11823470	gi|26451797|dbj|AK118381.1|	At1g74430.1
at4g13770	CON	MIN	HOUR	DAY	WEEK	UNIGENE	FLCDNA	TAIR
ACTGGTCTTG	5	7	6	1	2	no match found	gi|18700183|gb|AY075697.1	non-canonical match
ATGTCTTTTG	0	0	0	2	0	gnl|UG|At#S11725425	no match found	At4g13770.1
TTACTCGAGC	2	4	1	1	1	gnl|UG|At#S11806471	gi|20147222|gb|AY091779.1	At5g65110.1
at2g40960	CON	MIN	HOUR	DAY	WEEK	UNIGENE	FLCDNA	TAIR
GCTAAGGATT	0	0	0	2	0	gnl|UG|At#S11811412	gi|21404993|gb|AY086283.1	At2g40960.1
at5g60100	CON	MIN	HOUR	DAY	WEEK	UNIGENE	FLCDNA	TAIR
ACAGTCCTAT	0	0	0	0	1	gnl|UG|At#S11717692	no match found	At5g60100.1
at4g38020	CON	MIN	HOUR	DAY	WEEK	UNIGENE	FLCDNA	TAIR
CTTCAAAATC	2	1	1	1	1	gnl|UG|At#S11721265	gi|90093283|gb|BT024884.1	At4g38020.1
at3g21300	CON	MIN	HOUR	DAY	WEEK	UNIGENE	FLCDNA	TAIR
GCTGCTTCAT	0	0	0	1	0	gnl|UG|At#S11744098	gi|19310569|gb|AY080651.1	At3g21300.1
at4g35390	CON	MIN	HOUR	DAY	WEEK	UNIGENE	FLCDNA	TAIR
GTCGGTTTGA	0	0	0	1	0	gnl|UG|At#S11721701	gi|50198776|gb|BT014971.1	At4g35390.1
at3g20040	CON	MIN	HOUR	DAY	WEEK	UNIGENE	FLCDNA	TAIR
CGTTACCCGA	0	1	0	0	1	no match found	no match found	At3g20040.1
at5g59310	CON	MIN	HOUR	DAY	WEEK	UNIGENE	FLCDNA	TAIR
TTTAAGATAT	3	0	3	0	2	no match found	gi|15010685|gb|AY045644.1	At5g59310.1

at5g45380	CON	MIN	HOUR	DAY	WEEK	UNIGENE	FLCDNA	TAIR
ATTAATTTAG	1	0	0	2	0	gnl|UG|At#S11719189	no match found	multiple non-canonical match
at5g45430	CON	MIN	HOUR	DAY	WEEK	UNIGENE	FLCDNA	TAIR
ACGATTATGT	10	2	5	1	4	gnl|UG|At#S11719184	gi|15450859|gb|AY054510.1	At5g45430.1
TTCCTCAGAC	0	0	0	0	1	no match found	gi|24899764|gb|BT001210.1	non-canonical match
at5g09390	CON	MIN	HOUR	DAY	WEEK	UNIGENE	FLCDNA	TAIR
AGTTTATATA	0	2	0	0	1	gnl|UG|At#S11723894	gi|21403925|gb|AY085215.1	At5g09390.1
at1g19680	CON	MIN	HOUR	DAY	WEEK	UNIGENE	FLCDNA	TAIR
GGGAAAATAT	0	1	1	0	0	gnl|UG|At#S11741085	gi|110742809|dbj|AK227284.1|	multiple non-canonical match
at1g02340	CON	MIN	HOUR	DAY	WEEK	UNIGENE	FLCDNA	TAIR
TAACAATTTT	1	3	2	0	0	gnl|UG|At#S11701844	no match found	At1g02340.1
CGATGAGAAG	1	1	0	2	0	no match found	gi|26449594|dbj|AK117248.1|	non-canonical match
at5g01470	CON	MIN	HOUR	DAY	WEEK	UNIGENE	FLCDNA	TAIR
CTCGCCTCTG	1	0	2	0	0	gnl|UG|At#S11811837	gi|21405121|gb|AY086411.1	At5g01470.1
at2g28900	CON	MIN	HOUR	DAY	WEEK	UNIGENE	FLCDNA	TAIR
CGCTTAAGAA	1	6	3	3	5	gnl|UG|At#S11735309	gi|20147376|gb|AY093782.1	At2g28900.1
at2g19870	CON	MIN	HOUR	DAY	WEEK	UNIGENE	FLCDNA	TAIR
TACCGATTTA	1	0	0	0	1	no match found	gi|17065307|gb|AY062730.1	non-canonical match
ACCTGATCTA	0	0	0	0	3	gnl|UG|At#S11807546	no match found	At2g19870.1
at2g33590	CON	MIN	HOUR	DAY	WEEK	UNIGENE	FLCDNA	TAIR
GATTTCTGAG	2	1	2	4	4	gnl|UG|At#S11734139	gi|21403294|gb|AY084584.1	At2g33590.1
at2g01260	CON	MIN	HOUR	DAY	WEEK	UNIGENE	FLCDNA	TAIR
TTGTCTTGCA	0	0	0	1	0	gnl|UG|At#S18942998	gi|110736022|dbj|AK228022.1|	At2g01260.2
at1g60460	CON	MIN	HOUR	DAY	WEEK	UNIGENE	FLCDNA	TAIR
ATCAAATCTG	0	0	0	1	0	gnl|UG|At#S22484623	no match found	multiple non-canonical match
at4g00165	CON	MIN	HOUR	DAY	WEEK	UNIGENE	FLCDNA	TAIR
CCCTCCCCCT	1	1	0	2	0	gnl|UG|At#S11793175	gi|56236105|gb|BT020288.1	At4g00165.1
at1g15340	CON	MIN	HOUR	DAY	WEEK	UNIGENE	FLCDNA	TAIR
AAGGCAGAAG	3	4	1	4	1	gnl|UG|At#S11741501	gi|56382006|gb|BT020367.1	At1g15340.1
at5g60540	CON	MIN	HOUR	DAY	WEEK	UNIGENE	FLCDNA	TAIR
TACTTGTTTC	1	2	3	5	1	gnl|UG|At#S18910108	gi|62319183|dbj|AK220914.1|	At5g60540.1
at1g17780	CON	MIN	HOUR	DAY	WEEK	UNIGENE	FLCDNA	TAIR
TGATGATGAA	2	0	0	0	1	gnl|UG|At#S11817509	gi|22655241|gb|AY140070.1	At1g17780.1
at2g06850	CON	MIN	HOUR	DAY	WEEK	UNIGENE	FLCDNA	TAIR
TTATAATTCA	21	15	4	3	9	gnl|UG|At#S11699156	no match found	At2g06850.1
at1g63020	CON	MIN	HOUR	DAY	WEEK	UNIGENE	FLCDNA	TAIR
CAAATTCCGG	0	0	1	0	0	gnl|UG|At#S11730543	no match found	At1g63020.1
at1g43860	CON	MIN	HOUR	DAY	WEEK	UNIGENE	FLCDNA	TAIR
GACGACTCTA	0	0	2	0	1	no match found	gi|30725591|gb|BT008459.1	At1g43860.1
at2g30800	CON	MIN	HOUR	DAY	WEEK	UNIGENE	FLCDNA	TAIR
TTGCTCTAAT	1	0	0	0	0	gnl|UG|At#S15460936	no match found	At2g30800.1

at5g67385	CON	MIN	HOUR	DAY	WEEK	UNIGENE	FLCDNA	TAIR
CAGCTCAGAA	1	5	1	3	1	gnl|UG|At#S11816783	gi|51536493|gb|BT015362.1	At5g67385.1
at3g57520	CON	MIN	HOUR	DAY	WEEK	UNIGENE	FLCDNA	TAIR
TTGAGATTCT	1	5	0	1	0	gnl|UG|At#S11728045	gi|110740841|dbj|AK226370.1|	At3g57520.3
at1g20930	CON	MIN	HOUR	DAY	WEEK	UNIGENE	FLCDNA	TAIR
AATACCCGCA	0	0	1	0	0	gnl|UG|At#S34116852	gi|21403151|gb|AY084441.1	At1g20930.1
at5g42270	CON	MIN	HOUR	DAY	WEEK	UNIGENE	FLCDNA	TAIR
TTCAGAGACT	39	14	49	73	79	gnl|UG|At#S11719502	gi|22022512|gb|AY126987.1	non-canonical match
at3g19400	CON	MIN	HOUR	DAY	WEEK	UNIGENE	FLCDNA	TAIR
GGGGTTGAAT	2	2	3	0	0	no match found	gi|26452045|dbj|AK118509.1|	non-canonical match
TTCACATTAA	0	2	0	1	0	gnl|UG|At#S11735032	no match found	At3g19400.1
at3g25770	CON	MIN	HOUR	DAY	WEEK	UNIGENE	FLCDNA	TAIR
TAATCAACAA	4	1	2	3	1	no match found	gi|15809727|gb|AY054131.1	At5g19790.1
AAATTAAAAT	3	5	2	2	2	gnl|UG|At#S11733062	gi|14190460|gb|AF380630.1	At3g25770.1
at4g08099	CON	MIN	HOUR	DAY	WEEK	UNIGENE	FLCDNA	TAIR
ATTAGTGTTT	1	0	0	0	0	no match found	no match found	At4g08099.1
at3g53670	CON	MIN	HOUR	DAY	WEEK	UNIGENE	FLCDNA	TAIR
GGCTGAAGAT	0	0	1	0	0	gnl|UG|At#S11728750	gi|14423469|gb|AF386972.1	At3g53670.1
atcg00750	CON	MIN	HOUR	DAY	WEEK	UNIGENE	FLCDNA	TAIR
TTCAAGCAAG	1	5	0	0	0	no match found	no match found	AtCg00750
at1g49580	CON	MIN	HOUR	DAY	WEEK	UNIGENE	FLCDNA	TAIR
ACCTAAGCGA	0	0	1	0	0	gnl|UG|At#S34116892	gi|110738704|dbj|AK229416.1|	At1g49580.1
at3g51830	CON	MIN	HOUR	DAY	WEEK	UNIGENE	FLCDNA	TAIR
GGCCAAAAAA	1	0	3	3	0	gnl|UG|At#S11729092	gi|22136711|gb|AY133741.1	At3g51830.1
at5g14200	CON	MIN	HOUR	DAY	WEEK	UNIGENE	FLCDNA	TAIR
CAACATTGTA	6	7	11	2	0	gnl|UG|At#S11722786	gi|18377878|gb|AY074587.1	At5g14200.1
at4g37110	CON	MIN	HOUR	DAY	WEEK	UNIGENE	FLCDNA	TAIR
TAAGAACTTG	0	0	0	1	0	gnl|UG|At#S34117334	gi|110737848|dbj|AK228974.1|	At4g37110.1
at1g62855	CON	MIN	HOUR	DAY	WEEK	UNIGENE	FLCDNA	TAIR
AAGCCGAAGA	0	5	1	1	0	no match found	no match found	At1g62855.1
at1g07480	CON	MIN	HOUR	DAY	WEEK	UNIGENE	FLCDNA	TAIR
AACTCTTATT	1	0	0	0	0	no match found	gi|17065293|gb|AY062723.1	At1g07480.1
at1g64500	CON	MIN	HOUR	DAY	WEEK	UNIGENE	FLCDNA	TAIR
GAGCAGCAAG	0	0	2	1	0	no match found	gi|15810442|gb|AY056260.1	At1g64500.1
at1g73390	CON	MIN	HOUR	DAY	WEEK	UNIGENE	FLCDNA	TAIR
GTCTGAAGCC	0	0	0	3	0	gnl|UG|At#S43850337	gi|54606849|gb|BT015943.1	At1g73390.1
at1g16810	CON	MIN	HOUR	DAY	WEEK	UNIGENE	FLCDNA	TAIR
AGTGAGCACT	2	6	3	5	1	gnl|UG|At#S11741363	gi|15146197|gb|AY049240.1	At1g16810.1
at2g01470	CON	MIN	HOUR	DAY	WEEK	UNIGENE	FLCDNA	TAIR
TAAACTAACA	4	10	2	1	5	gnl|UG|At#S11742669	gi|18377756|gb|AY074332.1	At2g01470.1
at1g68130	CON	MIN	HOUR	DAY	WEEK	UNIGENE	FLCDNA	TAIR
TCTTCGGCGA	1	2	0	1	0	no match found	no match found	At1g68130.1

at5g18255	CON	MIN	HOUR	DAY	WEEK	UNIGENE		FLCDNA	TAIR
ACCCGATTAA	0	1	1	1	1	no match found		gi|20465922|gb|AY096474.1	At5g18260.1
at3g15940	CON	MIN	HOUR	DAY	WEEK	UNIGENE		FLCDNA	TAIR
CTATTCTTTG	2	0	0	0	0	gnl|UG|At#S11736064		gi|62321119|dbj|AK221889.1|	At3g15940.1
at3g56820	CON	MIN	HOUR	DAY	WEEK	UNIGENE		FLCDNA	TAIR
TCTTTCTAAA	0	1	0	0	1	gnl|UG|At#S11728179		no match found	multiple non-canonical match
at5g51280	CON	MIN	HOUR	DAY	WEEK	UNIGENE		FLCDNA	TAIR
TCAAAATATT	0	0	1	2	0	gnl|UG|At#S34115770		gi|110740860|dbj|AK226380.1|	At5g51280.1
at2g38025	CON	MIN	HOUR	DAY	WEEK	UNIGENE		FLCDNA	TAIR
GAAGAGGAGG	0	1	0	1	0	gnl|UG|At#S15460659		gi|28950786|gb|BT005253.1	At2g38025.1
at5g45060	CON	MIN	HOUR	DAY	WEEK	UNIGENE		FLCDNA	TAIR
GTGGTGTATC	0	1	2	0	0	gnl|UG|At#S11719221		no match found	At5g45060.1
at4g25835	CON	MIN	HOUR	DAY	WEEK	UNIGENE		FLCDNA	TAIR
AGTTACTGTA	0	1	0	1	0	gnl|UG|At#S11723336		gi|19715598|gb|AY075607.1	At4g25835.1
at3g14960	CON	MIN	HOUR	DAY	WEEK	UNIGENE		FLCDNA	TAIR
TCCACCATTG	0	1	0	0	0	gnl|UG|At#S11736356		gi|17979336|gb|AY070398.1	At3g14960.1
atcg00380	CON	MIN	HOUR	DAY	WEEK	UNIGENE		FLCDNA	TAIR
GACATATTTT	1	1	1	3	1	no match found		no match found	AtCg00380
at1g70710	CON	MIN	HOUR	DAY	WEEK	UNIGENE		FLCDNA	TAIR
GTTGGATACG	0	1	0	0	0	gnl|UG|At#S11728942		gi|15081780|gb|AY048283.1	At1g70710.1
at3g19260	CON	MIN	HOUR	DAY	WEEK	UNIGENE		FLCDNA	TAIR
TTTCCACTTC	1	1	1	0	1	gnl|UG|At#S18905249		gi|21403690|gb|AY084980.1	At3g19260.1
at1g06780	CON	MIN	HOUR	DAY	WEEK	UNIGENE		FLCDNA	TAIR
AAGCCGTGGT	1	0	0	3	0	no match found		gi|15028086|gb|AY045900.1	non-canonical match
at4g12010	CON	MIN	HOUR	DAY	WEEK	UNIGENE		FLCDNA	TAIR
CTTTGAATTA	0	0	1	1	0	no match found		gi|51970067|dbj|AK175963.1|	non-canonical match
at1g65310	CON	MIN	HOUR	DAY	WEEK	UNIGENE		FLCDNA	TAIR
AATATGTTAC	1	1	0	0	0	gnl|UG|At#S11730065		no match found	At1g65310.1
at1g80670	CON	MIN	HOUR	DAY	WEEK	UNIGENE		FLCDNA	TAIR
ACATTTTGAA	0	1	1	0	0	gnl|UG|At#S11726297		gi|21406421|gb|AY087683.1	At1g80670.1
at2g32440	CON	MIN	HOUR	DAY	WEEK	UNIGENE		FLCDNA	TAIR
AATCTTCTGG	1	0	1	0	0	gnl|UG|At#S18899900		gi|115646745|gb|BT029168.1	At2g32440.1
at3g01780	CON	MIN	HOUR	DAY	WEEK	UNIGENE		FLCDNA	TAIR
CCAGAGGACG	1	6	1	3	1	no match found		gi|28393647|gb|BT004227.1	At1g64870.1
GCGCTTCAGG	1	4	0	2	1	gnl|UG|At#S11740146		no match found	At3g01780.1
at4g18280	CON	MIN	HOUR	DAY	WEEK	UNIGENE		FLCDNA	TAIR
CGTAAGATGA	2	3	0	3	3	gnl|UG|At#S11724633		gi|14030676|gb|AF375429.1	At4g18280.1
at3g53200	CON	MIN	HOUR	DAY	WEEK	UNIGENE		FLCDNA	TAIR
TTCTCTATGG	0	1	0	0	0	gnl|UG|At#S26111659		gi|28950794|gb|BT005257.1	At3g53200.1
at3g13435	CON	MIN	HOUR	DAY	WEEK	UNIGENE		FLCDNA	TAIR
TAACGATCTT	0	1	0	0	0	gnl|UG|At#S30523531		gi|26449654|dbj|AK117279.1|	At3g13435.1

at3g22750	CON	MIN	HOUR	DAY	WEEK	UNIGENE	FLCDNA	TAIR
GCTCCAGAGG	0	0	0	1	0	gnl|UG|At#S11703553	gi|14334661|gb|AY035004.1	At3g22750.1
at2g44640	CON	MIN	HOUR	DAY	WEEK	UNIGENE	FLCDNA	TAIR
ATCGAGCTCC	1	0	1	1	0	no match found	gi|19699151|gb|AY090281.1	non-canonical match
ACTATAAAAA	4	2	0	3	0	no match found	gi|15146245|gb|AY049264.1	non-canonical match
at2g33840	CON	MIN	HOUR	DAY	WEEK	UNIGENE	FLCDNA	TAIR
GTTTGATGAG	0	1	0	1	0	gnl|UG|At#S15460810	gi|21464572|gb|AY120698.1	At2g33840.1
at2g40316	CON	MIN	HOUR	DAY	WEEK	UNIGENE	FLCDNA	TAIR
GATCCCTGTA	1	2	3	0	0	gnl|UG|At#S18913838	gi|110743338|dbj|AK227563.1|	At2g40316.1
at5g26717	CON	MIN	HOUR	DAY	WEEK	UNIGENE	FLCDNA	TAIR
GAAACTGATC	2	1	3	2	4	no match found	no match found	At5g26717.1
at3g28080	CON	MIN	HOUR	DAY	WEEK	UNIGENE	FLCDNA	TAIR
GGCAATACGT	1	1	0	0	0	gnl|UG|At#S18901737	gi|21436252|gb|AY117190.1	At3g28080.1
at5g01130	CON	MIN	HOUR	DAY	WEEK	UNIGENE	FLCDNA	TAIR
TTTTCCAGAC	0	0	0	0	1	gnl|UG|At#S11726431	no match found	At5g01130.1
at1g70720	CON	MIN	HOUR	DAY	WEEK	UNIGENE	FLCDNA	TAIR
AAACAGATGA	0	0	1	0	0	no match found	gi|107738149|gb|BT025760.1	multiple non-canonical match
AAACAGAGAA	2	0	3	1	1	gnl|UG|At#S30654803	no match found	multiple non-canonical match
at1g80600	CON	MIN	HOUR	DAY	WEEK	UNIGENE	FLCDNA	TAIR
TCTCAAAATT	4	4	3	5	1	gnl|UG|At#S11726304	gi|27311956|gb|BT002584.1	At1g80600.1
at5g46280	CON	MIN	HOUR	DAY	WEEK	UNIGENE	FLCDNA	TAIR
AGGACACACA	0	0	1	0	1	gnl|UG|At#S11698524	gi|28466888|gb|BT004787.1	At5g46280.1
at4g36030	CON	MIN	HOUR	DAY	WEEK	UNIGENE	FLCDNA	TAIR
GATAACGAAA	2	1	0	0	0	no match found	gi|110742182|dbj|AK226949.1|	At5g46280.1
at5g06265	CON	MIN	HOUR	DAY	WEEK	UNIGENE	FLCDNA	TAIR
ACATTTGGTT	0	1	0	0	0	gnl|UG|At#S43849368	gi|21405385|gb|AY086675.1	At5g06265.1
at5g64220	CON	MIN	HOUR	DAY	WEEK	UNIGENE	FLCDNA	TAIR
TCTCTTGCAT	1	1	0	0	0	no match found	gi|38603813|gb|BT010874.1	multiple non-canonical match
GTGATTGGTT	3	0	2	2	2	gnl|UG|At#S28281553	no match found	At5g64220.1
at3g19770	CON	MIN	HOUR	DAY	WEEK	UNIGENE	FLCDNA	TAIR
AAGAATATGG	0	1	0	0	0	gnl|UG|At#S11734920	gi|15027906|gb|AY045810.1	At3g19770.1
at3g06280	CON	MIN	HOUR	DAY	WEEK	UNIGENE	FLCDNA	TAIR
CTCTGTATTT	1	0	0	0	0	gnl|UG|At#S11738858	no match found	non-canonical match
at2g38170	CON	MIN	HOUR	DAY	WEEK	UNIGENE	FLCDNA	TAIR
AAGGGACTGG	1	7	4	6	0	gnl|UG|At#S15460654	gi|62321556|dbj|AK222114.1|	At2g38170.2
at3g22942	CON	MIN	HOUR	DAY	WEEK	UNIGENE	FLCDNA	TAIR
GGATCAATGG	1	0	0	0	0	no match found	gi|88900339|gb|BT024657.1	non-canonical match
TCGTGTTCGG	0	0	1	0	0	gnl|UG|At#S34118002	gi|110736566|dbj|AK228306.1|	At3g22945.1
at5g51450	CON	MIN	HOUR	DAY	WEEK	UNIGENE	FLCDNA	TAIR
TGCCTGATGA	0	0	0	1	0	gnl|UG|At#S11709185	gi|17063160|gb|AY062101.1	At5g51450.1
at2g26710	CON	MIN	HOUR	DAY	WEEK	UNIGENE	FLCDNA	TAIR
CTCTTTTTGT	0	1	1	0	0	gnl|UG|At#S11735854	gi|110739334|dbj|AK229743.1|	At2g26710.1

at1g16320	CON	MIN	HOUR	DAY	WEEK	UNIGENE	FLCDNA	TAIR
CTCATAGTGG	2	2	1	2	2	gnl|UG|At#S11741407	gi|18087578|gb|AF462832.1	At1g16320.1
at1g19340	CON	MIN	HOUR	DAY	WEEK	UNIGENE	FLCDNA	TAIR
ATCGTTTTTC	1	0	0	0	0	gnl|UG|At#S11741118	gi|21403662|gb|AY084952.1	At1g19340.1
at4g26260	CON	MIN	HOUR	DAY	WEEK	UNIGENE	FLCDNA	TAIR
GAGGATTAGT	0	0	1	0	0	gnl|UG|At#S11723266	no match found	non-canonical match
at2g34640	CON	MIN	HOUR	DAY	WEEK	UNIGENE	FLCDNA	TAIR
GACTTTGAAG	4	0	0	0	1	gnl|UG|At#S11733904	gi|39104569|dbj|AK117813.2|	At2g34640.1
at1g67620	CON	MIN	HOUR	DAY	WEEK	UNIGENE	FLCDNA	TAIR
CACTTGATGA	0	1	0	0	0	gnl|UG|At#S21736999	gi|51970209|dbj|AK176034.1|	At1g67620.1
at5g45113	CON	MIN	HOUR	DAY	WEEK	UNIGENE	FLCDNA	TAIR
AAGCATATCT	0	0	0	0	2	gnl|UG|At#S11816861	no match found	At5g45113.1
at1g52500	CON	MIN	HOUR	DAY	WEEK	UNIGENE	FLCDNA	TAIR
CAGTTCAAGT	0	0	1	0	0	gnl|UG|At#S15459163	no match found	At1g52500.1
at4g36960	CON	MIN	HOUR	DAY	WEEK	UNIGENE	FLCDNA	TAIR
AGCTTGGACG	1	2	0	0	0	gnl|UG|At#S11721443	gi|15081786|gb|AY048286.1	At4g36960.1
at4g05050	CON	MIN	HOUR	DAY	WEEK	UNIGENE	FLCDNA	TAIR
TACAGCCTCT	47	34	37	40	28	gnl|UG|At#S20793400	gi|15810025|gb|AY054281.1	At4g05050.1
at3g25690	CON	MIN	HOUR	DAY	WEEK	UNIGENE	FLCDNA	TAIR
AAAGCATTTG	17	12	12	3	3	gnl|UG|At#S11733087	no match found	At3g25690.1
at5g19485	CON	MIN	HOUR	DAY	WEEK	UNIGENE	FLCDNA	TAIR
AAGTCTTGAA	0	3	1	0	1	gnl|UG|At#S37211454	no match found	At3g25690.1
at1g34260	CON	MIN	HOUR	DAY	WEEK	UNIGENE	FLCDNA	TAIR
TGAAAGTATT	0	2	1	1	1	gnl|UG|At#S11737495	no match found	At1g34260.1
at4g17720	CON	MIN	HOUR	DAY	WEEK	UNIGENE	FLCDNA	TAIR
GTCGATTTGG	0	3	10	1	3	gnl|UG|At#S14273910	gi|28393290|gb|BT004039.1	At4g17720.1
at1g34315	CON	MIN	HOUR	DAY	WEEK	UNIGENE	FLCDNA	TAIR
GAGAAAGGTG	0	3	0	0	0	gnl|UG|At#S35309797	no match found	multiple non-canonical match
at3g06540	CON	MIN	HOUR	DAY	WEEK	UNIGENE	FLCDNA	TAIR
GGGAGTGAAG	1	2	0	0	0	gnl|UG|At#S11738781	gi|23463084|gb|BT000837.1	At3g06540.1
at5g20950	CON	MIN	HOUR	DAY	WEEK	UNIGENE	FLCDNA	TAIR
CTGATAACTT	0	1	0	0	0	gnl|UG|At#S15460591	gi|23296984|gb|AY142679.1	At5g20950.1
at5g13680	CON	MIN	HOUR	DAY	WEEK	UNIGENE	FLCDNA	TAIR
AAAATCTCTA	0	1	0	0	0	gnl|UG|At#S11722920	no match found	At5g13680.1
at2g40330	CON	MIN	HOUR	DAY	WEEK	UNIGENE	FLCDNA	TAIR
AGTCGGAGGA	0	2	1	1	0	gnl|UG|At#S11732511	gi|28393740|gb|BT004281.1	At2g40330.1
at2g39090	CON	MIN	HOUR	DAY	WEEK	UNIGENE	FLCDNA	TAIR
TGAAGAGTAG	1	0	0	0	1	gnl|UG|At#S28282168	gi|27311882|gb|BT002547.1	At2g39090.1
at5g51220	CON	MIN	HOUR	DAY	WEEK	UNIGENE	FLCDNA	TAIR
AAAACAATGT	1	1	0	2	1	gnl|UG|At#S34117370	gi|110737776|dbj|AK228938.1|	multiple canonical match

at3g52720	CON	MIN	HOUR	DAY	WEEK	UNIGENE	FLCDNA	TAIR
GTACACCAAG	1	4	0	0	0	gnl|UG|At#S11706842	gi|15450772|gb|AY054466.1	At3g52720.1
at5g53050	CON	MIN	HOUR	DAY	WEEK	UNIGENE	FLCDNA	TAIR
TCAAACATTT	4	3	5	0	0	gnl|UG|At#S28281623	gi|110740735|dbj|AK226315.1|	At5g53050.1
at1g05020	CON	MIN	HOUR	DAY	WEEK	UNIGENE	FLCDNA	TAIR
CAAGAGAGTT	1	2	3	1	0	no match found	gi|28827745|gb|BT005184.1	At1g05020.1
at1g05300	CON	MIN	HOUR	DAY	WEEK	UNIGENE	FLCDNA	TAIR
GCTCATATCT	0	0	0	0	1	no match found	gi|48596980|gb|BT014902.1	At1g05300.2
at1g03360	CON	MIN	HOUR	DAY	WEEK	UNIGENE	FLCDNA	TAIR
ATATGCTTGG	0	0	0	0	1	gnl|UG|At#S11742778	gi|34365688|gb|BT010533.1	At1g03360.1
at4g16442	CON	MIN	HOUR	DAY	WEEK	UNIGENE	FLCDNA	TAIR
AAGGCGGTTG	0	2	1	0	0	gnl|UG|At#S11724957	gi|29824212|gb|BT006082.1	At4g16442.1
at5g14460	CON	MIN	HOUR	DAY	WEEK	UNIGENE	FLCDNA	TAIR
GCTACTGGTC	0	1	0	0	0	gnl|UG|At#S34115343	gi|110741669|dbj|AK226674.1|	non-canonical match
at1g63220	CON	MIN	HOUR	DAY	WEEK	UNIGENE	FLCDNA	TAIR
GATCCTTATG	0	0	0	0	1	no match found	gi|20148350|gb|AY081504.1	At1g63220.1
at2g35000	CON	MIN	HOUR	DAY	WEEK	UNIGENE	FLCDNA	TAIR
ACCAACAAAA	0	0	1	1	0	gnl|UG|At#S11733818	gi|52627086|gb|BT015733.1	At2g35000.1
at3g05675	CON	MIN	HOUR	DAY	WEEK	UNIGENE	FLCDNA	TAIR
TCTTAACTCA	0	0	1	0	1	gnl|UG|At#S11807224	gi|20259408|gb|AY091003.1	At3g05675.1
at5g02380	CON	MIN	HOUR	DAY	WEEK	UNIGENE	FLCDNA	TAIR
TCTTGCTGTG	25	119	23	28	18	no match found	no match found	At5g02380.1
at2g20920	CON	MIN	HOUR	DAY	WEEK	UNIGENE	FLCDNA	TAIR
ATGGTTTTGA	1	5	0	1	1	gnl|UG|At#S11737233	gi|17978971|gb|AY069895.1	At2g20920.1
at4g02030	CON	MIN	HOUR	DAY	WEEK	UNIGENE	FLCDNA	TAIR
ATTTTTGCCA	0	3	0	0	2	gnl|UG|At#S34117480	gi|110737563|dbj|AK228828.1|	At4g02030.1
at3g28740	CON	MIN	HOUR	DAY	WEEK	UNIGENE	FLCDNA	TAIR
AGAGAAAGTG	1	0	11	7	8	gnl|UG|At#S11707388	gi|21280798|gb|AY113869.1	At3g28740.1
at2g43680	CON	MIN	HOUR	DAY	WEEK	UNIGENE	FLCDNA	TAIR
GCAACAACAG	4	13	3	5	1	gnl|UG|At#S18942708	gi|20466711|gb|AY099822.1	At2g43680.1
at1g67340	CON	MIN	HOUR	DAY	WEEK	UNIGENE	FLCDNA	TAIR
GTATAAATCT	0	0	1	0	0	no match found	no match found	At1g67340.1
AGTTTAGGAG	0	1	2	1	0	gnl|UG|At#S18895908	gi|23197875|gb|BT000146.1	non-canonical match
at5g35740	CON	MIN	HOUR	DAY	WEEK	UNIGENE	FLCDNA	TAIR
TACTGTTCTC	1	2	0	1	1	gnl|UG|At#S30642644	gi|21403513|gb|AY084803.1	At5g35740.1
at3g57360	CON	MIN	HOUR	DAY	WEEK	UNIGENE	FLCDNA	TAIR
GAGTTTCTTG	0	0	0	0	1	gnl|UG|At#S34116634	gi|51870295|gb|BT015522.1	At3g57360.1
at1g63050	CON	MIN	HOUR	DAY	WEEK	UNIGENE	FLCDNA	TAIR
GTTTTAAGCT	0	1	0	0	0	gnl|UG|At#S11730537	gi|21689736|gb|AY122979.1	At1g63050.1
at3g20470	CON	MIN	HOUR	DAY	WEEK	UNIGENE	FLCDNA	TAIR
ATTTTGTGTT	11	13	4	2	0	gnl|UG|At#S18901426	no match found	At3g20470.1
GCAATGGGTT	0	1	1	2	0	no match found	gi|15010655|gb|AY045629.1	non-canonical match

at5g40950 CON MIN HOUR DAY WEEK UNIGENE FLCDNA TAIR
CTGGGAAAAA 7 10 1 4 2 gnl|UG|At#S11719634 gi|21436344|gb|AY117267.1 At5g40950.1

at1g04530 CON MIN HOUR DAY WEEK UNIGENE FLCDNA TAIR
TCCGTTATGT 5 6 7 2 3 gnl|UG|At#S11742575 gi|17644166|gb|AY065305.1 At1g04530.1

at3g13235 CON MIN HOUR DAY WEEK UNIGENE FLCDNA TAIR
TCTTTACGTC 2 1 2 3 2 gnl|UG|At#S34115217 gi|110741911|dbj|AK226800.1| At3g13235.1

at2g07733 CON MIN HOUR DAY WEEK UNIGENE FLCDNA TAIR
GAGTTGTATT 0 0 1 0 0 gnl|UG|At#S19117004 gi|44917484|gb|BT011704.1 multiple non-canonical match

at4g30000 CON MIN HOUR DAY WEEK UNIGENE FLCDNA TAIR
GGCTCCAATG 0 1 1 0 0 gnl|UG|At#S11722617 gi|26451955|dbj|AK118462.1| At4g30000.1

at2g35930 CON MIN HOUR DAY WEEK UNIGENE FLCDNA TAIR
ATTCTTGCAT 0 1 1 0 0 gnl|UG|At#S11733594 gi|29028769|gb|BT005829.1 At2g35930.1

at5g09530 CON MIN HOUR DAY WEEK UNIGENE FLCDNA TAIR
TCGTATGGTT 0 1 1 1 0 no match found gi|15451135|gb|AY054648.1 non-canonical match

at3g55460 CON MIN HOUR DAY WEEK UNIGENE FLCDNA TAIR
GACGGTCTCG 2 4 2 3 0 gnl|UG|At#S18904716 gi|20466365|gb|AY099649.1 At3g55460.1

at4g16015 CON MIN HOUR DAY WEEK UNIGENE FLCDNA TAIR
TGATCATTGT 0 0 1 0 0 gnl|UG|At#S43849569 no match found multiple non-canonical match

at2g18730 CON MIN HOUR DAY WEEK UNIGENE FLCDNA TAIR
GAAGCAACCA 1 1 0 0 0 gnl|UG|At#S15461419 gi|18086344|gb|AY064982.1 At2g18730.1

at5g09580 CON MIN HOUR DAY WEEK UNIGENE FLCDNA TAIR
TCTACTCCAG 0 0 0 1 0 gnl|UG|At#S11723842 no match found At5g09580.1

at5g64080 CON MIN HOUR DAY WEEK UNIGENE FLCDNA TAIR
CAAGCTTCAC 0 0 0 1 1 gnl|UG|At#S11717291 gi|20147300|gb|AY093740.1 multiple canonical match

at2g24850 CON MIN HOUR DAY WEEK UNIGENE FLCDNA TAIR
CCATTAACCA 0 0 2 0 0 gnl|UG|At#S11736296 gi|21404034|gb|AY085324.1 At2g24850.1

at4g29360 CON MIN HOUR DAY WEEK UNIGENE FLCDNA TAIR
AATGCACTGT 2 0 0 1 0 gnl|UG|At#S11722727 gi|18175942|gb|AY072133.1 At4g29360.1

at5g49215 CON MIN HOUR DAY WEEK UNIGENE FLCDNA TAIR
TGTAACCAAA 0 1 0 1 0 gnl|UG|At#S11807512 gi|20258820|gb|AY090997.1 multiple canonical match

at4g35850 CON MIN HOUR DAY WEEK UNIGENE FLCDNA TAIR
GCTTCTTCGT 2 0 0 0 0 gnl|UG|At#S11721622 gi|21407084|gb|AY088310.1 At4g35850.1

at1g01120 CON MIN HOUR DAY WEEK UNIGENE FLCDNA TAIR
GAACCTTCTA 0 1 1 2 0 gnl|UG|At#S11743002 gi|18377663|gb|AY074285.1 At1g01120.1

at4g22750 CON MIN HOUR DAY WEEK UNIGENE FLCDNA TAIR
GTGTAAGATA 0 2 0 0 0 gnl|UG|At#S11723865 gi|21406515|gb|AY087777.1 At4g22750.1

at3g20015 CON MIN HOUR DAY WEEK UNIGENE FLCDNA TAIR
TACGGAGACG 0 1 0 0 0 gnl|UG|At#S11734847 gi|14532701|gb|AY039975.1 At3g20015.1

at3g47000 CON MIN HOUR DAY WEEK UNIGENE FLCDNA TAIR
CAAAAACAAT 2 1 2 0 1 gnl|UG|At#S11729997 gi|17065279|gb|AY062716.1 At3g47000.1

at3g12860	CON	MIN	HOUR	DAY	WEEK	UNIGENE	FLCDNA	TAIR
TTGTTTTTCT	0	0	0	0	1	no match found	no match found	At3g12860.1
CAGGAAGTCT	0	0	1	0	0	gnl|UG|At#S11737037	gi|110741585|dbj|AK226630.1|	non-canonical match

at4g16520	CON	MIN	HOUR	DAY	WEEK	UNIGENE	FLCDNA	TAIR
TCTTCTGTGT	2	9	0	1	0	gnl|UG|At#S15461067	gi|20147098|gb|AY091667.1	At4g16520.2

at4g33150	CON	MIN	HOUR	DAY	WEEK	UNIGENE	FLCDNA	TAIR
TGTAGCATAC	9	5	2	3	0	gnl|UG|At#S15460397	gi|62319366|dbj|AK221006.1|	At4g33150.1
GTGCTTTTGC	0	1	1	0	0	no match found	gi|20334813|gb|AY094007.1	non-canonical match

at1g49970	CON	MIN	HOUR	DAY	WEEK	UNIGENE	FLCDNA	TAIR
TGGTAGTAAC	8	4	5	3	9	no match found	gi|15010751|gb|AY045677.1	non-canonical match
TGGATAAAGG	0	0	1	0	0	no match found	gi|23308342|gb|BT000572.1	non-canonical match

at4g00050	CON	MIN	HOUR	DAY	WEEK	UNIGENE	FLCDNA	TAIR
AATTATGATA	1	0	1	0	2	no match found	no match found	At4g00050.1
CTCTACTCAG	1	0	0	1	1	gnl|UG|At#S18907142	gi|109134122|gb|BT025971.1	non-canonical match

at3g56150	CON	MIN	HOUR	DAY	WEEK	UNIGENE	FLCDNA	TAIR
AAGTGCAACA	1	7	0	0	0	no match found	gi|25054976|gb|BT001961.1	At3g56150.1

at5g25440	CON	MIN	HOUR	DAY	WEEK	UNIGENE	FLCDNA	TAIR
CAAGAAGTGC	0	2	2	0	0	gnl|UG|At#S18908471	gi|20466369|gb|AY099651.1	At5g25440.1

at3g58460	CON	MIN	HOUR	DAY	WEEK	UNIGENE	FLCDNA	TAIR
GGCTTTGACA	0	1	0	0	0	gnl|UG|At#S11727863	gi|21406217|gb|AY087480.1	At3g58460.1

at4g00150	CON	MIN	HOUR	DAY	WEEK	UNIGENE	FLCDNA	TAIR
GAGATGTCGC	0	3	1	0	0	gnl|UG|At#S11727631	gi|18087576|gb|AF462831.1	At4g00150.1

at1g23540	CON	MIN	HOUR	DAY	WEEK	UNIGENE	FLCDNA	TAIR
ATTCTTACAA	1	0	1	0	0	no match found	no match found	At1g23540.1

at5g60210	CON	MIN	HOUR	DAY	WEEK	UNIGENE	FLCDNA	TAIR
TTCCAAAGAT	1	0	1	0	0	gnl|UG|At#S11717681	gi|110737621|dbj|AK228858.1|	At5g60210.1

at1g14130	CON	MIN	HOUR	DAY	WEEK	UNIGENE	FLCDNA	TAIR
CAGGACTAGA	0	0	0	2	1	gnl|UG|At#S11741625	gi|28973390|gb|BT005600.1	At1g14130.1

at1g49170	CON	MIN	HOUR	DAY	WEEK	UNIGENE	FLCDNA	TAIR
TAAAGTATTT	0	0	0	0	1	gnl|UG|At#S11734799	no match found	At1g49170.1

at3g10113	CON	MIN	HOUR	DAY	WEEK	UNIGENE	FLCDNA	TAIR
GAAACGGTTA	0	1	0	0	0	gnl|UG|At#S11816242	no match found	multiple non-canonical match

at5g49360	CON	MIN	HOUR	DAY	WEEK	UNIGENE	FLCDNA	TAIR
CATAAATAAT	1	0	0	1	0	no match found	gi|110737297|dbj|AK228691.1|	At3g17180.1
GCAGGGGCAA	6	22	10	4	1	gnl|UG|At#S34115969	gi|110740480|dbj|AK230335.1|	At5g49360.1

at5g52420	CON	MIN	HOUR	DAY	WEEK	UNIGENE	FLCDNA	TAIR
TTTGGCTTCT	0	2	1	1	1	gnl|UG|At#S11718474	gi|15146255|gb|AY049269.1	non-canonical match

at3g07310	CON	MIN	HOUR	DAY	WEEK	UNIGENE	FLCDNA	TAIR
ATCTTCTGGT	3	5	2	2	2	gnl|UG|At#S11738532	gi|14596186|gb|AY042881.1	At3g07310.1

at2g14890	CON	MIN	HOUR	DAY	WEEK	UNIGENE	FLCDNA	TAIR
CCTTATTTCT	2	1	2	1	1	no match found	no match found	At2g14890.1

at5g45500	CON	MIN	HOUR	DAY	WEEK	UNIGENE	FLCDNA	TAIR
TTCCGTAAGG	0	1	0	1	1	gnl|UG|At#S11719177	gi|23308326|gb|BT000564.1	At5g45500.1

at1g12090	CON	MIN	HOUR	DAY	WEEK	UNIGENE	FLCDNA	TAIR
CTGCAGCCTC	19	108	12	14	5	gnl|UG|At#S18893129	gi|16648817|gb|AY058186.1	At1g12090.1
at4g27880	CON	MIN	HOUR	DAY	WEEK	UNIGENE	FLCDNA	TAIR
AATGAATGTT	1	1	2	0	0	gnl|UG|At#S11722980	no match found	multiple canonical match
at4g36370	CON	MIN	HOUR	DAY	WEEK	UNIGENE	FLCDNA	TAIR
ACAAACAAAA	0	0	3	0	1	no match found	no match found	At4g36370.1
at1g18670	CON	MIN	HOUR	DAY	WEEK	UNIGENE	FLCDNA	TAIR
TATAGGCATA	0	0	1	0	1	gnl|UG|At#S11741184	no match found	At4g36370.1
at1g15200	CON	MIN	HOUR	DAY	WEEK	UNIGENE	FLCDNA	TAIR
AGGTTCTCTA	3	0	1	3	1	gnl|UG|At#S11702110	gi|15293206|gb|AY051037.1	At1g15200.1
at1g10840	CON	MIN	HOUR	DAY	WEEK	UNIGENE	FLCDNA	TAIR
GCTCGTAAAT	0	2	3	2	1	gnl|UG|At#S11741948	gi|21406062|gb|AY087338.1	At1g10840.2
at4g34310	CON	MIN	HOUR	DAY	WEEK	UNIGENE	FLCDNA	TAIR
GTTAGGTTAA	0	1	0	0	0	gnl|UG|At#S11721880	no match found	At4g34310.2
at5g58880	CON	MIN	HOUR	DAY	WEEK	UNIGENE	FLCDNA	TAIR
ATGACGAAAA	0	1	0	0	0	no match found	no match found	At5g58880.1
at1g57620	CON	MIN	HOUR	DAY	WEEK	UNIGENE	FLCDNA	TAIR
GATAGTTTCT	4	4	4	3	1	no match found	gi|21404454|gb|AY085744.1	multiple canonical match
at1g47710	CON	MIN	HOUR	DAY	WEEK	UNIGENE	FLCDNA	TAIR
TGATGCATCT	0	0	0	3	0	gnl|UG|At#S11735339	no match found	At1g47710.1
at3g23650	CON	MIN	HOUR	DAY	WEEK	UNIGENE	FLCDNA	TAIR
TTTACAAAAC	0	1	0	0	0	no match found	no match found	At3g23650.1
at1g35350	CON	MIN	HOUR	DAY	WEEK	UNIGENE	FLCDNA	TAIR
GTTCTATTCC	0	1	1	0	0	gnl|UG|At#S11737147	no match found	At1g35350.1
at4g38970	CON	MIN	HOUR	DAY	WEEK	UNIGENE	FLCDNA	TAIR
TTCGTCAAAG	2	0	3	0	0	no match found	gi|53828646|gb|BT015870.1	non-canonical match
TCAAATCATT	286	67	198	128	111	gnl|UG|At#S11721104	gi|110739482|dbj|AK229820.1|	At4g38970.1
at5g23820	CON	MIN	HOUR	DAY	WEEK	UNIGENE	FLCDNA	TAIR
TCTTTTCTTC	9	11	11	6	0	gnl|UG|At#S11720797	gi|15215722|gb|AY050390.1	At5g23820.1
TAACAATGAA	1	1	0	1	0	no match found	gi|20453248|gb|AY097347.1	non-canonical match
at5g57460	CON	MIN	HOUR	DAY	WEEK	UNIGENE	FLCDNA	TAIR
TAAGGTTTTG	2	1	2	2	1	gnl|UG|At#S11717967	gi|44917456|gb|BT011690.1	multiple canonical match
at4g16490	CON	MIN	HOUR	DAY	WEEK	UNIGENE	FLCDNA	TAIR
TATTGATGGT	2	3	0	2	3	gnl|UG|At#S11707372	gi|26452477|dbj|AK118730.1|	At4g16490.1
at1g70300	CON	MIN	HOUR	DAY	WEEK	UNIGENE	FLCDNA	TAIR
CTTACCTCGC	2	2	1	0	0	gnl|UG|At#S11729035	gi|20259959|gb|AY093328.1	At1g70300.1
at5g10470	CON	MIN	HOUR	DAY	WEEK	UNIGENE	FLCDNA	TAIR
TGTATTTATT	2	0	0	0	1	gnl|UG|At#S38433322	no match found	non-canonical match
at3g21295	CON	MIN	HOUR	DAY	WEEK	UNIGENE	FLCDNA	TAIR
TGGCCACATT	0	1	0	0	0	gnl|UG|At#S34118372	gi|110735853|dbj|AK227934.1|	pseudo chromosome match
at1g20190	CON	MIN	HOUR	DAY	WEEK	UNIGENE	FLCDNA	TAIR

TCTCGTAACT	1	2	3	0	0	gnl|UG|At#S11741037	gi|12083279|gb|AF332436.1	At1g20190.1
at5g22060	CON	MIN	HOUR	DAY	WEEK	UNIGENE	FLCDNA	TAIR
CGCATCTTAG	2	3	0	2	5	gnl|UG|At#S11721040	gi|26451806|dbj|AK118386.1|	At5g22060.1
at1g59820	CON	MIN	HOUR	DAY	WEEK	UNIGENE	FLCDNA	TAIR
TACATAAAAT	0	0	0	1	0	gnl|UG|At#S11731566	gi|20147218|gb|AY091777.1	At1g59820.1
at1g62990	CON	MIN	HOUR	DAY	WEEK	UNIGENE	FLCDNA	TAIR
TACTTACTTA	1	0	0	0	0	gnl|UG|At#S11702268	gi|26451689|dbj|AK118326.1|	At1g62990.1
at1g13250	CON	MIN	HOUR	DAY	WEEK	UNIGENE	FLCDNA	TAIR
TGATAAAGAA	1	2	0	0	1	gnl|UG|At#S11741712	gi|21403480|gb|AY084770.1	At1g13250.1
at1g43130	CON	MIN	HOUR	DAY	WEEK	UNIGENE	FLCDNA	TAIR
TGGACAGGAC	0	1	2	0	1	gnl|UG|At#S11736167	gi|21405013|gb|AY086303.1	At1g43130.1
at5g39450	CON	MIN	HOUR	DAY	WEEK	UNIGENE	FLCDNA	TAIR
TTTGAATTTT	0	1	0	2	0	gnl|UG|At#S11806739	gi|31711987|gb|BT008911.1	At5g39450.1
at2g17040	CON	MIN	HOUR	DAY	WEEK	UNIGENE	FLCDNA	TAIR
GCTCCAGAAT	0	0	4	0	0	gnl|UG|At#S11738222	gi|13605646|gb|AF361804.1	At2g17040.1
at3g18980	CON	MIN	HOUR	DAY	WEEK	UNIGENE	FLCDNA	TAIR
TTCTTTGAAT	0	0	0	1	1	no match found	gi|15027964|gb|AY045839.1	non-canonical match
at2g34300	CON	MIN	HOUR	DAY	WEEK	UNIGENE	FLCDNA	TAIR
GTGGCGTCCT	0	1	6	1	1	gnl|UG|At#S11733989	no match found	At2g34300.1
at3g08920	CON	MIN	HOUR	DAY	WEEK	UNIGENE	FLCDNA	TAIR
ATCAATGACG	1	2	1	0	1	gnl|UG|At#S30585693	gi|21405763|gb|AY087039.1	At3g08920.1
at1g09270	CON	MIN	HOUR	DAY	WEEK	UNIGENE	FLCDNA	TAIR
AGATTCAGCT	1	2	3	0	1	gnl|UG|At#S11707349	gi|20259333|gb|AY090944.1	At1g09270.1
GACCTTGTCC	0	0	1	0	0	no match found	gi|14194156|gb|AF367284.1	non-canonical match
at5g50800	CON	MIN	HOUR	DAY	WEEK	UNIGENE	FLCDNA	TAIR
ACCGATCCGA	0	0	0	3	0	gnl|UG|At#S11718637	gi|21406253|gb|AY087516.1	At5g50800.1
at5g56340	CON	MIN	HOUR	DAY	WEEK	UNIGENE	FLCDNA	TAIR
GCCGTTTAGT	0	1	1	1	0	gnl|UG|At#S11718078	gi|20259580|gb|AY091194.1	At5g56340.1
at3g06035	CON	MIN	HOUR	DAY	WEEK	UNIGENE	FLCDNA	TAIR
TACTCTTCCT	2	1	0	0	0	gnl|UG|At#S11738916	gi|110735966|dbj|AK227992.1|	At3g06035.1
at5g06920	CON	MIN	HOUR	DAY	WEEK	UNIGENE	FLCDNA	TAIR
GCCATCTCCA	0	0	1	0	0	gnl|UG|At#S35279059	no match found	non-canonical match
at2g16850	CON	MIN	HOUR	DAY	WEEK	UNIGENE	FLCDNA	TAIR
TTCGTATAAA	0	2	0	0	0	gnl|UG|At#S11738267	gi|21407898|gb|AY089124.1	At2g16850.1
AGACTTCTTA	1	1	1	1	0	no match found	gi|110736168|dbj|AK228097.1|	non-canonical match
at1g15000	CON	MIN	HOUR	DAY	WEEK	UNIGENE	FLCDNA	TAIR
TTCACAAGTT	1	1	0	1	0	gnl|UG|At#S11741534	gi|19715606|gb|AY075612.1	At1g15000.1
at2g41990	CON	MIN	HOUR	DAY	WEEK	UNIGENE	FLCDNA	TAIR
TTGTCTCTTA	0	0	0	1	0	no match found	gi|89000924|gb|BT024714.1	non-canonical match
at2g40935	CON	MIN	HOUR	DAY	WEEK	UNIGENE	FLCDNA	TAIR
GAATCAGCTA	2	1	2	1	2	gnl|UG|At#S15460549	gi|117958398|gb|BT029441.1	At2g40935.1

at3g10760	CON	MIN	HOUR	DAY	WEEK	UNIGENE	FLCDNA	TAIR
AGTCAGCTGA	1	3	1	2	1	gnl|UG|At#S11737657	no match found	At3g10760.1
at1g80260	CON	MIN	HOUR	DAY	WEEK	UNIGENE	FLCDNA	TAIR
CTTTTCTTGT	0	0	3	0	0	gnl|UG|At#S34117388	gi|110737740|dbj|AK228920.1|	At1g80260.1
at4g35300	CON	MIN	HOUR	DAY	WEEK	UNIGENE	FLCDNA	TAIR
TGGGGAGAAA	0	1	2	4	3	gnl|UG|At#S11721714	gi|20453188|gb|AY094465.1	At4g35300.1
at2g26590	CON	MIN	HOUR	DAY	WEEK	UNIGENE	FLCDNA	TAIR
GGATTTTCCT	1	0	2	2	1	gnl|UG|At#S28282257	gi|13926226|gb|AF372873.1	At2g26590.1
at4g28540	CON	MIN	HOUR	DAY	WEEK	UNIGENE	FLCDNA	TAIR
ACAATTTGTT	0	0	2	2	2	gnl|UG|At#S11722872	gi|21403330|gb|AY084620.1	At4g28540.1
at4g00820	CON	MIN	HOUR	DAY	WEEK	UNIGENE	FLCDNA	TAIR
ATTTTTTTCC	0	0	1	0	0	gnl|UG|At#S11727474	gi|110737786|dbj|AK228943.1|	At4g00820.1
at2g23770	CON	MIN	HOUR	DAY	WEEK	UNIGENE	FLCDNA	TAIR
AATTATGAGA	0	0	0	2	0	no match found	no match found	At2g23770.1
at5g25770	CON	MIN	HOUR	DAY	WEEK	UNIGENE	FLCDNA	TAIR
ATCAAAGAAT	0	2	1	0	0	gnl|UG|At#S11720614	gi|22530943|gb|AY136310.1	At5g25770.2
at4g38420	CON	MIN	HOUR	DAY	WEEK	UNIGENE	FLCDNA	TAIR
GTCAAAAGCA	2	3	0	0	0	gnl|UG|At#S11721197	gi|18176391|gb|AY072215.1	At4g38420.1
at5g47770	CON	MIN	HOUR	DAY	WEEK	UNIGENE	FLCDNA	TAIR
ACTATGATCT	5	11	4	3	3	gnl|UG|At#S11718944	gi|13878122|gb|AF370324.1	At5g47770.1
at5g46800	CON	MIN	HOUR	DAY	WEEK	UNIGENE	FLCDNA	TAIR
GCGGGTGGAG	0	3	1	4	1	gnl|UG|At#S11719044	gi|45680159|gb|BT011849.1	At5g46800.1
at4g25800	CON	MIN	HOUR	DAY	WEEK	UNIGENE	FLCDNA	TAIR
AGAAATTTGA	0	0	0	0	1	gnl|UG|At#S34115386	gi|110741587|dbj|AK226631.1|	At4g25800.1
at1g43620	CON	MIN	HOUR	DAY	WEEK	UNIGENE	FLCDNA	TAIR
TGGTGGCGTG	1	1	2	1	2	gnl|UG|At#S11736062	gi|29028779|gb|BT005834.1	At1g43620.1
CGGTGAGAGA	1	0	0	0	0	gnl|UG|At#S35304304	no match found	At1g43620.1
at4g28740	CON	MIN	HOUR	DAY	WEEK	UNIGENE	FLCDNA	TAIR
GCAAGCCTTC	2	0	1	1	0	gnl|UG|At#S11722837	gi|20147174|gb|AY091705.1	At4g28740.1
at5g66090	CON	MIN	HOUR	DAY	WEEK	UNIGENE	FLCDNA	TAIR
GTTGTTGTTG	4	4	5	1	8	gnl|UG|At#S11717088	gi|21403730|gb|AY085020.1	At5g66090.1
at2g03070	CON	MIN	HOUR	DAY	WEEK	UNIGENE	FLCDNA	TAIR
ATGCAGAATG	0	0	1	0	1	no match found	gi|110738570|dbj|AK229348.1|	At2g03070.1
at1g03457	CON	MIN	HOUR	DAY	WEEK	UNIGENE	FLCDNA	TAIR
TATAAATGTT	0	2	0	0	0	gnl|UG|At#S11742768	gi|26449590|dbj|AK117246.1|	At1g03457.1
at2g31280	CON	MIN	HOUR	DAY	WEEK	UNIGENE	FLCDNA	TAIR
ACTTGAAACT	0	0	2	2	1	gnl|UG|At#S28282227	gi|62321209|dbj|AK221935.1|	At2g31280.1
at5g50870	CON	MIN	HOUR	DAY	WEEK	UNIGENE	FLCDNA	TAIR
AGATCTTATC	0	1	0	0	2	gnl|UG|At#S11718630	no match found	pseudo chromosome match
at2g16580	CON	MIN	HOUR	DAY	WEEK	UNIGENE	FLCDNA	TAIR
ATCCGATGAG	0	1	1	0	0	gnl|UG|At#S17007155	no match found	At2g16580.1

at1g32790	CON	MIN	HOUR	DAY	WEEK	UNIGENE	FLCDNA	TAIR
TAAAGATGTT	6	11	4	5	4	gnl|UG|At#S28282549	gi|21407939|gb|AY089165.1	At1g32790.1
GCTCAGCGAG	0	0	1	0	0	no match found	gi|25090127|gb|BT002225.1	non-canonical match
at4g00310	CON	MIN	HOUR	DAY	WEEK	UNIGENE	FLCDNA	TAIR
TGAAATATTT	1	0	0	1	0	gnl|UG|At#S11704978	gi|15146199|gb|AY049241.1	At4g00310.1
at1g50575	CON	MIN	HOUR	DAY	WEEK	UNIGENE	FLCDNA	TAIR
TAGCATATAG	0	0	0	0	1	gnl|UG|At#S11734312	gi|16649026|gb|AY059883.1	At1g50575.1
at5g02970	CON	MIN	HOUR	DAY	WEEK	UNIGENE	FLCDNA	TAIR
CTCAAGCAAC	1	1	1	1	1	gnl|UG|At#S11725423	gi|13605548|gb|AF361600.1	At5g02970.1
at1g73660	CON	MIN	HOUR	DAY	WEEK	UNIGENE	FLCDNA	TAIR
CGTAATTTCC	1	2	3	2	2	gnl|UG|At#S11728319	gi|20466321|gb|AY099627.1	At1g73660.1
at2g38730	CON	MIN	HOUR	DAY	WEEK	UNIGENE	FLCDNA	TAIR
TTGTCTTTGG	0	0	0	1	0	no match found	gi|25083101|gb|BT002031.1	non-canonical match
TTACTTTCAT	1	0	0	0	0	gnl|UG|At#S11732912	no match found	At2g38730.1
at1g29900	CON	MIN	HOUR	DAY	WEEK	UNIGENE	FLCDNA	TAIR
CTGCTGATAT	0	4	3	1	2	no match found	gi|22137065|gb|AY133548.1	non-canonical match
TAACGCTTGG	1	2	0	1	1	gnl|UG|At#S11703331	gi|14194124|gb|AF367268.1	At1g29900.1
at4g04760	CON	MIN	HOUR	DAY	WEEK	UNIGENE	FLCDNA	TAIR
AAACTACCAA	0	2	1	0	1	no match found	no match found	At4g04760.1
GCTTGTTGCT	0	0	0	1	0	gnl|UG|At#S11726452	no match found	non-canonical match
at3g18370	CON	MIN	HOUR	DAY	WEEK	UNIGENE	FLCDNA	TAIR
TCAGAGTCAC	0	0	0	1	0	gnl|UG|At#S11735332	gi|15983786|gb|AY056799.1	At3g18370.1
at5g58110	CON	MIN	HOUR	DAY	WEEK	UNIGENE	FLCDNA	TAIR
CTTGTTGACT	8	10	6	4	7	gnl|UG|At#S11717899	gi|18087564|gb|AF462824.1	At5g58110.1
at1g72560	CON	MIN	HOUR	DAY	WEEK	UNIGENE	FLCDNA	TAIR
AAAATACTTT	0	0	1	0	0	gnl|UG|At#S11728561	no match found	At1g72560.1
at4g23980	CON	MIN	HOUR	DAY	WEEK	UNIGENE	FLCDNA	TAIR
GCTTAAACAA	1	0	3	1	0	no match found	gi|110736485|dbj|AK228263.1|	At4g23980.1
at1g71020	CON	MIN	HOUR	DAY	WEEK	UNIGENE	FLCDNA	TAIR
AATTAATTTA	0	1	0	0	0	gnl|UG|At#S43850349	gi|62319296|dbj|AK220971.1|	At1g71020.1
at5g61800	CON	MIN	HOUR	DAY	WEEK	UNIGENE	FLCDNA	TAIR
GAGCGGTTTG	0	1	0	0	0	gnl|UG|At#S11717521	no match found	At5g61800.1
atmg00170	CON	MIN	HOUR	DAY	WEEK	UNIGENE	FLCDNA	TAIR
CTATCCTTAC	0	1	0	0	0	gnl|UG|At#S35249522	no match found	AtMg00620
at5g43900	CON	MIN	HOUR	DAY	WEEK	UNIGENE	FLCDNA	TAIR
ACAGAGGAAT	0	1	0	0	0	no match found	gi|110738134|dbj|AK229124.1|	non-canonical match
CTCCATTATA	3	1	0	0	0	gnl|UG|At#S18317827	no match found	pseudo chromosome match
at1g48260	CON	MIN	HOUR	DAY	WEEK	UNIGENE	FLCDNA	TAIR
TACCTGGCTC	2	1	1	0	0	gnl|UG|At#S11735119	no match found	At1g48260.1
at1g47970	CON	MIN	HOUR	DAY	WEEK	UNIGENE	FLCDNA	TAIR
ACTGGTGTTA	0	3	0	0	1	gnl|UG|At#S11821402	gi|14423519|gb|AF386997.1	At1g47970.1
at3g05937	CON	MIN	HOUR	DAY	WEEK	UNIGENE	FLCDNA	TAIR
CAGGAAATGA	0	1	2	0	2	gnl|UG|At#S18942178	no match found	At3g05937.1

at4g34140	CON	MIN	HOUR	DAY	WEEK	UNIGENE	FLCDNA	TAIR
GGTTGGAAGG	1	0	0	0	0	gnl|UG|At#S34114415	gi|110743409|dbj|AK227600.1|	At4g34140.1
at5g48150	CON	MIN	HOUR	DAY	WEEK	UNIGENE	FLCDNA	TAIR
TGAAGGAGCT	1	7	0	0	0	gnl|UG|At#S18941509	gi|95147293|gb|BT025326.1	At5g48150.2
at3g02660	CON	MIN	HOUR	DAY	WEEK	UNIGENE	FLCDNA	TAIR
GCGAAGAGGG	0	0	0	1	1	gnl|UG|At#S24442554	gi|117168054|gb|BT029296.1	At3g02660.1
at4g39780	CON	MIN	HOUR	DAY	WEEK	UNIGENE	FLCDNA	TAIR
TATTCTCTCA	1	2	0	0	0	gnl|UG|At#S11720961	no match found	At4g39780.1
TGGAATCTTC	0	0	0	0	1	no match found	gi|106879184|gb|BT025719.1	non-canonical match
at1g47310	CON	MIN	HOUR	DAY	WEEK	UNIGENE	FLCDNA	TAIR
TCATTCACAA	0	1	0	1	0	no match found	gi|30102707|gb|BT006464.1	non-canonical match
at2g26910	CON	MIN	HOUR	DAY	WEEK	UNIGENE	FLCDNA	TAIR
AACCAGTACT	3	0	4	0	0	gnl|UG|At#S11735806	no match found	At2g26910.1
at1g55510	CON	MIN	HOUR	DAY	WEEK	UNIGENE	FLCDNA	TAIR
TCTTTGTTAT	1	0	0	0	0	no match found	no match found	At1g55510.1
at5g10650	CON	MIN	HOUR	DAY	WEEK	UNIGENE	FLCDNA	TAIR
TGAGCTGTGT	0	1	0	0	0	gnl|UG|At#S18909538	gi|53850554|gb|BT015918.1	At5g10650.1
TTTTGGTTTA	1	0	0	0	0	gnl|UG|At#S28281818	no match found	multiple non-canonical match
at1g43775	CON	MIN	HOUR	DAY	WEEK	UNIGENE	FLCDNA	TAIR
AGAAGATAGA	0	0	0	0	1	no match found	no match found	At1g43775.1
at1g04140	CON	MIN	HOUR	DAY	WEEK	UNIGENE	FLCDNA	TAIR
GTTCTCTTTT	1	4	0	0	0	gnl|UG|At#S15461595	no match found	At1g04140.1
at1g13195	CON	MIN	HOUR	DAY	WEEK	UNIGENE	FLCDNA	TAIR
AAGAGAGTGA	0	1	1	0	0	gnl|UG|At#S43850648	gi|87116601|gb|BT024484.1	non-canonical match
at4g05470	CON	MIN	HOUR	DAY	WEEK	UNIGENE	FLCDNA	TAIR
TCTCCTCTTT	0	1	0	0	1	no match found	no match found	At4g05470.1
at3g61470	CON	MIN	HOUR	DAY	WEEK	UNIGENE	FLCDNA	TAIR
TTTGTACAAA	199	188	85	54	37	gnl|UG|At#S11727015	gi|21407757|gb|AY088983.1	At3g61470.1
at3g16630	CON	MIN	HOUR	DAY	WEEK	UNIGENE	FLCDNA	TAIR
TTTGTGAATT	2	1	3	5	1	gnl|UG|At#S34115448	gi|110741479|dbj|AK226569.1|	At3g16630.2
at4g19645	CON	MIN	HOUR	DAY	WEEK	UNIGENE	FLCDNA	TAIR
AACTTGATCT	0	1	0	0	1	gnl|UG|At#S15460926	gi|27311620|gb|BT002416.1	At4g19645.2
at1g68230	CON	MIN	HOUR	DAY	WEEK	UNIGENE	FLCDNA	TAIR
ATTGTTCATC	1	0	0	0	0	no match found	no match found	At1g68230.1
at1g27970	CON	MIN	HOUR	DAY	WEEK	UNIGENE	FLCDNA	TAIR
TATTTGGTTT	14	15	6	6	16	gnl|UG|At#S11739642	gi|21405462|gb|AY086752.1	At1g27970.1
at4g26520	CON	MIN	HOUR	DAY	WEEK	UNIGENE	FLCDNA	TAIR
TGAAATTCAC	1	0	2	2	1	no match found	no match found	At4g26520.1
at1g26940	CON	MIN	HOUR	DAY	WEEK	UNIGENE	FLCDNA	TAIR
GATTGTTTCC	1	3	0	1	0	no match found	no match found	At1g26940.1
at4g24050	CON	MIN	HOUR	DAY	WEEK	UNIGENE	FLCDNA	TAIR

TAGCTAAGAA	1	1	0	0	0	no match found	gi|16612263|gb|AF439829.1	multiple non-canonical match
at2g17240	CON	MIN	HOUR	DAY	WEEK	UNIGENE	FLCDNA	TAIR
GCGTCGCTTT	0	3	0	0	0	no match found	gi|14190506|gb|AF380653.1	At2g17240.1
at4g08950	CON	MIN	HOUR	DAY	WEEK	UNIGENE	FLCDNA	TAIR
TTGTATGACT	1	3	0	0	1	gnl|UG|At#S11726065	gi|20259893|gb|AY093295.1	At4g08950.1
at1g22140	CON	MIN	HOUR	DAY	WEEK	UNIGENE	FLCDNA	TAIR
AGCCTTGGGT	0	0	1	0	0	no match found	gi|20334899|gb|AY094050.1	multiple non-canonical match
TTCCCACTTT	4	2	4	2	0	no match found	gi|21407267|gb|AY088493.1	At1g22140.1
at1g30320	CON	MIN	HOUR	DAY	WEEK	UNIGENE	FLCDNA	TAIR
ATTGGATTCT	1	2	1	0	1	gnl|UG|At#S11738915	gi|26452635|dbj|AK118811.1|	At1g30320.1
AGGCATTGGA	0	0	1	0	0	no match found	gi|29824296|gb|BT006124.1	multiple non-canonical match
at5g47060	CON	MIN	HOUR	DAY	WEEK	UNIGENE	FLCDNA	TAIR
TTTGAGTTAT	1	1	1	0	1	gnl|UG|At#S34116065	gi|110740301|dbj|AK230243.1|	multiple canonical match
at3g15480	CON	MIN	HOUR	DAY	WEEK	UNIGENE	FLCDNA	TAIR
ATTTGTTGTG	11	5	3	1	2	gnl|UG|At#S11736203	gi|21404905|gb|AY086195.1	At3g15480.1
at5g43380	CON	MIN	HOUR	DAY	WEEK	UNIGENE	FLCDNA	TAIR
ATTTCCTTCG	0	2	0	1	0	gnl|UG|At#S15459240	gi|22531250|gb|AY136464.1	At5g43380.1
at3g07460	CON	MIN	HOUR	DAY	WEEK	UNIGENE	FLCDNA	TAIR
GTCAGATGCT	0	0	1	0	0	no match found	gi|23197757|gb|BT000087.1	non-canonical match
TAAGCACAGA	7	8	8	4	4	no match found	gi|21405058|gb|AY086348.1	non-canonical match
at5g01790	CON	MIN	HOUR	DAY	WEEK	UNIGENE	FLCDNA	TAIR
CGAAGATTTT	1	1	6	3	4	gnl|UG|At#S11725709	gi|40824034|gb|BT011294.1	At5g01790.1
at4g23660	CON	MIN	HOUR	DAY	WEEK	UNIGENE	FLCDNA	TAIR
ACCTTCTGGT	2	0	0	0	0	gnl|UG|At#S11723706	no match found	At4g23660.2
at2g32210	CON	MIN	HOUR	DAY	WEEK	UNIGENE	FLCDNA	TAIR
CTTCTGAAGA	1	2	0	0	0	gnl|UG|At#S11743050	gi|21405823|gb|AY087099.1	At2g32210.1
at1g14040	CON	MIN	HOUR	DAY	WEEK	UNIGENE	FLCDNA	TAIR
CATAGACAGA	0	2	0	0	0	gnl|UG|At#S11741633	no match found	At1g14040.1
at1g73550	CON	MIN	HOUR	DAY	WEEK	UNIGENE	FLCDNA	TAIR
ATCCGTCAGT	0	0	1	1	0	gnl|UG|At#S32740785	gi|124301115|gb|BT030072.1	At1g73550.1
at1g02410	CON	MIN	HOUR	DAY	WEEK	UNIGENE	FLCDNA	TAIR
TTTTTTAAGA	2	2	0	0	1	gnl|UG|At#S11742874	gi|26452391|dbj|AK118687.1|	At1g02410.1
at1g16420	CON	MIN	HOUR	DAY	WEEK	UNIGENE	FLCDNA	TAIR
ACCGTTTTGT	0	0	1	0	0	gnl|UG|At#S21737471	gi|51968549|dbj|AK175204.1|	At1g16420.1
at4g20980	CON	MIN	HOUR	DAY	WEEK	UNIGENE	FLCDNA	TAIR
TGGGGGATTG	1	2	0	2	0	gnl|UG|At#S28281998	gi|17528987|gb|AY065228.1	At4g20980.1
at4g31820	CON	MIN	HOUR	DAY	WEEK	UNIGENE	FLCDNA	TAIR
AAGGAACATC	0	1	0	0	0	gnl|UG|At#S11807206	gi|20259444|gb|AY091021.1	At4g31820.1
at1g32550	CON	MIN	HOUR	DAY	WEEK	UNIGENE	FLCDNA	TAIR
TAAACTGTAA	0	1	1	0	0	no match found	gi|21281216|gb|AY114079.1	non-canonical match
at4g01690	CON	MIN	HOUR	DAY	WEEK	UNIGENE	FLCDNA	TAIR
TCACGGTACG	4	5	3	5	2	no match found	gi|14423413|gb|AF386944.1	At4g01690.1

at4g29900	CON	MIN	HOUR	DAY	WEEK	UNIGENE	FLCDNA	TAIR
TGGAGAAACT	0	2	0	0	0	gnl|UG|At#S11722633	no match found	At4g29900.1
at2g25970	CON	MIN	HOUR	DAY	WEEK	UNIGENE	FLCDNA	TAIR
CTCTTATCTT	2	3	0	1	2	gnl|UG|At#S11736040	gi|19310434|gb|AY078954.1	At2g25970.1
at5g40170	CON	MIN	HOUR	DAY	WEEK	UNIGENE	FLCDNA	TAIR
CGTTGAACTG	1	2	3	3	0	gnl|UG|At#S11719713	no match found	At5g40170.1
at2g41950	CON	MIN	HOUR	DAY	WEEK	UNIGENE	FLCDNA	TAIR
ACTCCCTTTT	0	1	0	0	0	gnl|UG|At#S14233015	gi|18491232|gb|AY074625.1	non-canonical match
at5g14590	CON	MIN	HOUR	DAY	WEEK	UNIGENE	FLCDNA	TAIR
GTCCCAAGGT	3	0	0	3	0	gnl|UG|At#S11722693	gi|20466433|gb|AY099683.1	At5g14590.1
at1g07790	CON	MIN	HOUR	DAY	WEEK	UNIGENE	FLCDNA	TAIR
GGGATTATGA	1	2	2	1	1	gnl|UG|At#S11742255	gi|21407410|gb|AY088636.1	At1g07790.1
at4g04780	CON	MIN	HOUR	DAY	WEEK	UNIGENE	FLCDNA	TAIR
AAGAAACCTG	1	1	2	1	1	gnl|UG|At#S11726444	gi|27754567|gb|BT002915.1	At4g04780.1
at3g02530	CON	MIN	HOUR	DAY	WEEK	UNIGENE	FLCDNA	TAIR
ACAAAGGAAA	0	0	1	0	0	no match found	gi|14423531|gb|AF387003.1	multiple non-canonical match
ATGAATGTTT	2	4	0	5	2	gnl|UG|At#S11786940	gi|21387134|gb|AY114652.1	At3g02530.1
at4g29520	CON	MIN	HOUR	DAY	WEEK	UNIGENE	FLCDNA	TAIR
CACAGAAAGT	0	2	0	0	1	gnl|UG|At#S11722699	gi|21403993|gb|AY085283.1	At4g29520.1
at1g30890	CON	MIN	HOUR	DAY	WEEK	UNIGENE	FLCDNA	TAIR
ACGTGAAGAA	2	1	2	1	2	gnl|UG|At#S11738705	gi|14532657|gb|AY039953.1	At1g30890.1
at5g27300	CON	MIN	HOUR	DAY	WEEK	UNIGENE	FLCDNA	TAIR
TCGAAATTTT	0	1	0	0	0	gnl|UG|At#S11817499	gi|22655261|gb|AY140080.1	non-canonical match
at4g16070	CON	MIN	HOUR	DAY	WEEK	UNIGENE	FLCDNA	TAIR
AGCTCCCTCA	0	1	1	0	2	gnl|UG|At#S11709100	gi|17064827|gb|AY062490.1	At4g16070.1
at2g39190	CON	MIN	HOUR	DAY	WEEK	UNIGENE	FLCDNA	TAIR
TCTCTCACCA	2	1	2	2	0	gnl|UG|At#S11732802	gi|15081812|gb|AY048299.1	At2g39190.2
at1g44750	CON	MIN	HOUR	DAY	WEEK	UNIGENE	FLCDNA	TAIR
GGGAAATGGA	1	3	1	0	0	gnl|UG|At#S18942434	gi|28058998|gb|BT003358.1	At1g44750.2
at4g22240	CON	MIN	HOUR	DAY	WEEK	UNIGENE	FLCDNA	TAIR
AGAATTATCC	0	0	1	2	1	no match found	no match found	At4g22240.1
at1g10430	CON	MIN	HOUR	DAY	WEEK	UNIGENE	FLCDNA	TAIR
GAGCAAAACT	0	1	2	0	0	gnl|UG|At#S11741990	gi|20259837|gb|AY093267.1	At1g10430.1
at3g57930	CON	MIN	HOUR	DAY	WEEK	UNIGENE	FLCDNA	TAIR
GAATCAAAAC	1	4	2	2	4	gnl|UG|At#S14273721	gi|28393649|gb|BT004228.1	At3g57930.1
at1g27200	CON	MIN	HOUR	DAY	WEEK	UNIGENE	FLCDNA	TAIR
TAAATTTCGG	3	4	1	1	0	gnl|UG|At#S11739917	no match found	At1g27200.1
TTTGGCCTTG	0	1	0	0	0	no match found	gi|23297604|gb|AY150464.1	non-canonical match
at3g15180	CON	MIN	HOUR	DAY	WEEK	UNIGENE	FLCDNA	TAIR
GTGCCTGGTG	1	0	0	1	0	gnl|UG|At#S11736297	gi|30102759|gb|BT006490.1	At3g15180.1
at1g79960	CON	MIN	HOUR	DAY	WEEK	UNIGENE	FLCDNA	TAIR

GAAATAGAGG	0	1	0	0	0	no match found	no match found	At1g79960.1
at4g05400	CON	MIN	HOUR	DAY	WEEK	UNIGENE	FLCDNA	TAIR
ATTCGAGTTA	0	2	0	0	0	gnl|UG|At#S21737105	gi|51969281|dbj|AK175570.1|	At4g05400.1
at1g09157	CON	MIN	HOUR	DAY	WEEK	UNIGENE	FLCDNA	TAIR
TTTTCCTTGA	0	0	1	0	0	gnl|UG|At#S37211615	no match found	non-canonical match
at2g04025	CON	MIN	HOUR	DAY	WEEK	UNIGENE	FLCDNA	TAIR
AAGATGGGAA	0	0	1	0	0	no match found	gi|52218797|gb|BT015692.1	multiple non-canonical match
at1g64960	CON	MIN	HOUR	DAY	WEEK	UNIGENE	FLCDNA	TAIR
GTATTTATTG	1	0	0	0	1	gnl|UG|At#S11730139	gi|26451482|dbj|AK118219.1|	non-canonical match
at1g38950	CON	MIN	HOUR	DAY	WEEK	UNIGENE	FLCDNA	TAIR
GAAAAGCTTA	1	0	0	0	0	no match found	no match found	At1g38950.1
at2g39810	CON	MIN	HOUR	DAY	WEEK	UNIGENE	FLCDNA	TAIR
CCGGTGAAGG	0	4	1	0	1	gnl|UG|At#S11732647	gi|28393692|gb|BT004255.1	At2g39810.1
at3g19100	CON	MIN	HOUR	DAY	WEEK	UNIGENE	FLCDNA	TAIR
GAAGACAGAT	1	0	4	2	2	gnl|UG|At#S11735117	gi|16604465|gb|AY058851.1	At3g19100.1
CTTATGAGCT	0	0	0	0	1	no match found	gi|18958023|gb|AY079035.1	multiple non-canonical match
at3g24350	CON	MIN	HOUR	DAY	WEEK	UNIGENE	FLCDNA	TAIR
TAAAATTGCT	0	1	0	0	0	no match found	gi|28393776|gb|BT004300.1	At3g24350.1
at5g48880	CON	MIN	HOUR	DAY	WEEK	UNIGENE	FLCDNA	TAIR
GCAGATTTGG	0	1	1	4	1	gnl|UG|At#S11718830	no match found	multiple canonical match
at4g13970	CON	MIN	HOUR	DAY	WEEK	UNIGENE	FLCDNA	TAIR
AAAGCCACTA	0	0	4	0	0	gnl|UG|At#S11725393	gi|110739076|dbj|AK229610.1|	At4g13970.1
at1g74045	CON	MIN	HOUR	DAY	WEEK	UNIGENE	FLCDNA	TAIR
GGGCTTCATT	2	0	2	3	0	no match found	no match found	At1g74045.1
at1g13090	CON	MIN	HOUR	DAY	WEEK	UNIGENE	FLCDNA	TAIR
ATCAACGCTT	4	2	1	0	0	gnl|UG|At#S11741729	gi|22136137|gb|AY128747.1	At1g13090.1
at1g10657	CON	MIN	HOUR	DAY	WEEK	UNIGENE	FLCDNA	TAIR
CTTATTATCT	1	0	0	0	0	gnl|UG|At#S26539156	gi|110736950|dbj|AK228507.1|	pseudo chromosome match
at3g28760	CON	MIN	HOUR	DAY	WEEK	UNIGENE	FLCDNA	TAIR
TATTCTATTT	0	1	0	0	0	gnl|UG|At#S28282885	gi|28973462|gb|BT005636.1	At3g28760.1
at3g58390	CON	MIN	HOUR	DAY	WEEK	UNIGENE	FLCDNA	TAIR
TGTCAGGAGA	2	0	0	1	0	gnl|UG|At#S11723019	no match found	At4g27650.1
at1g32520	CON	MIN	HOUR	DAY	WEEK	UNIGENE	FLCDNA	TAIR
TGTTATCATA	1	0	0	0	0	gnl|UG|At#S21737364	gi|51968763|dbj|AK175311.1|	At1g32520.1
at1g63090	CON	MIN	HOUR	DAY	WEEK	UNIGENE	FLCDNA	TAIR
CTGGAGACTT	0	3	0	1	0	gnl|UG|At#S18895154	gi|27754672|gb|BT002971.1	At1g63090.1
at1g75810	CON	MIN	HOUR	DAY	WEEK	UNIGENE	FLCDNA	TAIR
TGTTAGATAT	1	0	0	0	0	gnl|UG|At#S21736103	gi|21407843|gb|AY089069.1	At1g75810.1
at3g32226	CON	MIN	HOUR	DAY	WEEK	UNIGENE	FLCDNA	TAIR
AAGGTTCTGA	0	1	0	0	0	no match found	no match found	At3g32226.1
at1g01160	CON	MIN	HOUR	DAY	WEEK	UNIGENE	FLCDNA	TAIR

CGATGCAACA	0	0	0	1	0	no match found	gi|20857111|gb|AY102133.1	At1g01160.1
at2g39030	CON	MIN	HOUR	DAY	WEEK	UNIGENE	FLCDNA	TAIR
AGACCCATCG	0	1	1	2	0	no match found	gi|18377431|gb|AY072467.1	non-canonical match
at4g15010	CON	MIN	HOUR	DAY	WEEK	UNIGENE	FLCDNA	TAIR
GCAGTTTCTT	0	1	0	0	0	gnl|UG|At#S18941859	gi|30725619|gb|BT008473.1	At4g15010.3
at3g07780	CON	MIN	HOUR	DAY	WEEK	UNIGENE	FLCDNA	TAIR
GATACAATGC	0	0	0	1	2	no match found	gi|21280842|gb|AY113947.1	multiple non-canonical match
CAACCGAATT	0	2	1	0	0	no match found	gi|23397314|gb|BT000803.1	multiple non-canonical match
ATCGTCTATG	3	2	1	0	1	gnl|UG|At#S11738418	gi|15028084|gb|AY045899.1	multiple canonical match
at5g35510	CON	MIN	HOUR	DAY	WEEK	UNIGENE	FLCDNA	TAIR
CAAATCAGAT	0	0	0	0	1	gnl|UG|At#S11720152	no match found	multiple non-canonical match
at5g04060	CON	MIN	HOUR	DAY	WEEK	UNIGENE	FLCDNA	TAIR
AGCTTCAAGA	0	1	0	1	0	no match found	gi|18086556|gb|AY072008.1	At5g04060.1
TTTTGTTTCG	0	1	0	0	0	gnl|UG|At#S11725135	no match found	non-canonical match
at5g12170	CON	MIN	HOUR	DAY	WEEK	UNIGENE	FLCDNA	TAIR
GGAGGAACAA	0	0	2	0	0	gnl|UG|At#S38433312	gi|51971015|dbj|AK176437.1|	At5g12170.1
at1g09420	CON	MIN	HOUR	DAY	WEEK	UNIGENE	FLCDNA	TAIR
GTGGGGTCCA	1	0	0	0	0	gnl|UG|At#S11706038	no match found	At1g09420.1
at2g29970	CON	MIN	HOUR	DAY	WEEK	UNIGENE	FLCDNA	TAIR
TTCTTGAAGA	0	1	0	0	0	gnl|UG|At#S11735049	gi|14532595|gb|AY039922.1	At2g29970.1
at4g31410	CON	MIN	HOUR	DAY	WEEK	UNIGENE	FLCDNA	TAIR
CTCAGTCAGA	1	1	1	0	0	no match found	gi|20453260|gb|AY097353.1	At4g31410.2
CATCTCTGGA	1	1	2	0	0	gnl|UG|At#S15460464	gi|21404144|gb|AY085434.1	At4g31410.1
at5g54850	CON	MIN	HOUR	DAY	WEEK	UNIGENE	FLCDNA	TAIR
AAACAAACTT	0	0	0	0	1	gnl|UG|At#S11718229	gi|46518370|gb|BT012523.1	At5g54850.1
at1g66150	CON	MIN	HOUR	DAY	WEEK	UNIGENE	FLCDNA	TAIR
GCTGTCTCTT	0	1	1	0	1	no match found	no match found	At1g66150.1
TTTCTTGTGA	7	2	3	1	1	no match found	gi|29824402|gb|BT006178.1	multiple non-canonical match
at4g20840	CON	MIN	HOUR	DAY	WEEK	UNIGENE	FLCDNA	TAIR
GAGTTTGGAC	1	0	1	1	0	no match found	no match found	At4g20840.1
at2g47070	CON	MIN	HOUR	DAY	WEEK	UNIGENE	FLCDNA	TAIR
TTGGAGAAGA	11	2	12	6	5	gnl|UG|At#S15460259	gi|110740351|dbj|AK230269.1|	At2g47070.1
at3g11890	CON	MIN	HOUR	DAY	WEEK	UNIGENE	FLCDNA	TAIR
CAATAGGTTG	2	0	0	0	1	gnl|UG|At#S18942121	gi|17979222|gb|AY070462.1	At3g11890.2
at1g02205	CON	MIN	HOUR	DAY	WEEK	UNIGENE	FLCDNA	TAIR
TTCCAGAATA	1	0	0	0	0	gnl|UG|At#S15461646	gi|110739919|dbj|AK230043.1|	At1g02205.1
AGTGTGGCAC	1	0	0	0	0	gnl|UG|At#S35315170	gi|110742081|dbj|AK226895.1|	non-canonical match
at2g04530	CON	MIN	HOUR	DAY	WEEK	UNIGENE	FLCDNA	TAIR
TAGAGGAAAT	0	1	0	1	2	no match found	gi|20466196|gb|AY099564.1	At2g04530.1
at5g57990	CON	MIN	HOUR	DAY	WEEK	UNIGENE	FLCDNA	TAIR
CTGCTGTATC	1	4	2	2	0	gnl|UG|At#S34117092	gi|110738312|dbj|AK229216.1|	At5g57990.1
at1g32580	CON	MIN	HOUR	DAY	WEEK	UNIGENE	FLCDNA	TAIR
TGTCTGCAAG	4	5	1	4	0	gnl|UG|At#S11738062	gi|17381077|gb|AY063995.1	At1g32580.1

at4g20940	CON	MIN	HOUR	DAY	WEEK	UNIGENE	FLCDNA	TAIR
AGTGAATGAA	3	0	0	1	0	gnl|UG|At#S11724186	no match found	At4g20940.1
at4g32750	CON	MIN	HOUR	DAY	WEEK	UNIGENE	FLCDNA	TAIR
GTCGGTTTTG	0	1	0	1	0	no match found	gi|51968853|dbj|AK175356.1|	At4g32750.1
at3g26680	CON	MIN	HOUR	DAY	WEEK	UNIGENE	FLCDNA	TAIR
TTTCAGAGAA	1	0	0	0	0	gnl|UG|At#S15460552	gi|26449932|dbj|AK117422.1|	At3g26680.1
at5g21274	CON	MIN	HOUR	DAY	WEEK	UNIGENE	FLCDNA	TAIR
ATGGCTAAGT	0	5	0	2	0	gnl|UG|At#S11707608	no match found	multiple canonical match
at4g17280	CON	MIN	HOUR	DAY	WEEK	UNIGENE	FLCDNA	TAIR
TGAATTCATA	0	1	0	2	2	gnl|UG|At#S11724801	no match found	non-canonical match
CTCAAGCAAG	0	0	1	0	0	no match found	no match found	At4g17280.1
at3g47510	CON	MIN	HOUR	DAY	WEEK	UNIGENE	FLCDNA	TAIR
CTTCGAGACT	0	1	0	0	0	gnl|UG|At#S35300892	no match found	At3g47510.1
at1g62680	CON	MIN	HOUR	DAY	WEEK	UNIGENE	FLCDNA	TAIR
ATGTTGTAAT	0	0	0	0	1	gnl|UG|At#S34115384	gi|110741591|dbj|AK226633.1|	pseudo chromosome match
at3g16960	CON	MIN	HOUR	DAY	WEEK	UNIGENE	FLCDNA	TAIR
GCTTCACCAC	2	0	2	0	0	gnl|UG|At#S11735766	no match found	multiple non-canonical match
at1g70940	CON	MIN	HOUR	DAY	WEEK	UNIGENE	FLCDNA	TAIR
GGGCAACGAG	2	5	4	3	2	no match found	gi|110742071|dbj|AK226890.1|	non-canonical match
AGCAAATAAC	6	3	18	7	4	gnl|UG|At#S11728897	no match found	At1g70940.1
at1g60000	CON	MIN	HOUR	DAY	WEEK	UNIGENE	FLCDNA	TAIR
GACTGTTACT	1	0	0	2	2	gnl|UG|At#S11731499	gi|13194797|gb|AF348590.1	At1g60000.1
at5g12090	CON	MIN	HOUR	DAY	WEEK	UNIGENE	FLCDNA	TAIR
TCCGAATATT	0	0	1	1	0	no match found	no match found	At5g12090.1
at4g26150	CON	MIN	HOUR	DAY	WEEK	UNIGENE	FLCDNA	TAIR
AAATTTTGTT	0	0	1	1	0	gnl|UG|At#S11723285	no match found	At4g26150.1
at5g09880	CON	MIN	HOUR	DAY	WEEK	UNIGENE	FLCDNA	TAIR
AATATGAAGC	1	1	0	0	1	gnl|UG|At#S11723769	gi|15451045|gb|AY054603.1	At5g09880.1
at3g11500	CON	MIN	HOUR	DAY	WEEK	UNIGENE	FLCDNA	TAIR
AATCAATCTT	3	6	4	5	2	gnl|UG|At#S11737435	gi|24899750|gb|BT001203.1	At3g11500.1
at1g27670	CON	MIN	HOUR	DAY	WEEK	UNIGENE	FLCDNA	TAIR
CTATAAGGAG	0	2	0	0	0	gnl|UG|At#S11739749	gi|26449581|dbj|AK117241.1|	At1g27670.1
at4g13940	CON	MIN	HOUR	DAY	WEEK	UNIGENE	FLCDNA	TAIR
AAACTTAAAT	25	39	29	50	35	no match found	gi|13358226|gb|AF325037.2	non-canonical match
GTTCGATTCG	1	0	1	1	1	gnl|UG|At#S11725397	no match found	At4g13940.1
at3g59360	CON	MIN	HOUR	DAY	WEEK	UNIGENE	FLCDNA	TAIR
ACACCAACTC	0	2	0	0	1	gnl|UG|At#S15459188	gi|13430657|gb|AF360241.1	At3g59360.2
at5g41400	CON	MIN	HOUR	DAY	WEEK	UNIGENE	FLCDNA	TAIR
GCTGGAACCA	0	0	0	1	0	gnl|UG|At#S11825049	gi|27754688|gb|BT002979.1	At5g41400.1
at5g12840	CON	MIN	HOUR	DAY	WEEK	UNIGENE	FLCDNA	TAIR
CAATGAGAAG	0	1	0	0	0	gnl|UG|At#S11709557	gi|107738402|gb|BT025801.1	At5g12840.1

at5g51620	CON	MIN	HOUR	DAY	WEEK	UNIGENE	FLCDNA	TAIR
CAAACGAGAG	1	2	2	0	0	no match found	no match found	At5g51620.2
at4g32880	CON	MIN	HOUR	DAY	WEEK	UNIGENE	FLCDNA	TAIR
TTTTTCCTTA	1	0	0	0	0	gnl|UG|At#S11722126	gi|20466329|gb|AY099631.1	multiple canonical match
at5g27740	CON	MIN	HOUR	DAY	WEEK	UNIGENE	FLCDNA	TAIR
AATTGCTGAA	0	1	1	0	1	gnl|UG|At#S11720438	gi|20259636|gb|AY091236.1	At5g27740.1
at3g23410	CON	MIN	HOUR	DAY	WEEK	UNIGENE	FLCDNA	TAIR
ACCACTGGTT	0	0	0	0	1	no match found	gi|27363419|gb|BT002713.1	non-canonical match
TTTTGGAAAC	0	1	3	2	1	gnl|UG|At#S11733784	gi|13605691|gb|AF361827.1	At3g23410.1
at5g53470	CON	MIN	HOUR	DAY	WEEK	UNIGENE	FLCDNA	TAIR
GGACCGGTTT	0	0	1	1	0	gnl|UG|At#S11718369	gi|20260069|gb|AY093383.1	At5g53470.1
at1g43160	CON	MIN	HOUR	DAY	WEEK	UNIGENE	FLCDNA	TAIR
GACCGAATAT	0	0	1	0	2	no match found	gi|17065541|gb|AY062847.1	non-canonical match
GGTGTATATA	0	0	0	0	4	gnl|UG|At#S11736156	no match found	At1g43160.1
at4g11470	CON	MIN	HOUR	DAY	WEEK	UNIGENE	FLCDNA	TAIR
TCTGTTCCTT	0	1	0	0	0	gnl|UG|At#S11725816	no match found	non-canonical match
at1g26090	CON	MIN	HOUR	DAY	WEEK	UNIGENE	FLCDNA	TAIR
CCGGGGTTTG	0	1	1	1	0	gnl|UG|At#S18893500	no match found	non-canonical match
at1g03250	CON	MIN	HOUR	DAY	WEEK	UNIGENE	FLCDNA	TAIR
GATTCCTTGA	0	2	0	0	0	gnl|UG|At#S11742789	gi|22022559|gb|AY127012.1	At1g03250.1
at4g33000	CON	MIN	HOUR	DAY	WEEK	UNIGENE	FLCDNA	TAIR
CAACACCGGT	1	2	4	1	2	gnl|UG|At#S43849441	gi|26449966|dbj|AK117439.1|	At4g33000.2
at3g02750	CON	MIN	HOUR	DAY	WEEK	UNIGENE	FLCDNA	TAIR
TAATAATGGC	1	4	2	1	1	gnl|UG|At#S11739859	gi|17064839|gb|AY062496.1	At3g02750.1
at2g03930	CON	MIN	HOUR	DAY	WEEK	UNIGENE	FLCDNA	TAIR
AAACATCAAA	4	2	2	1	2	no match found	no match found	At2g03930.2
at5g62220	CON	MIN	HOUR	DAY	WEEK	UNIGENE	FLCDNA	TAIR
CAGTATCAGT	1	1	0	0	2	gnl|UG|At#S11717479	no match found	At5g62220.1
at1g31930	CON	MIN	HOUR	DAY	WEEK	UNIGENE	FLCDNA	TAIR
AGGAAACTCT	3	3	3	2	0	gnl|UG|At#S11738301	gi|110738986|dbj|AK229561.1|	At1g31930.2
at3g25410	CON	MIN	HOUR	DAY	WEEK	UNIGENE	FLCDNA	TAIR
GTATAAAAGG	6	2	2	4	2	gnl|UG|At#S11744663	gi|19698908|gb|AY081301.1	At3g25410.1
at5g47130	CON	MIN	HOUR	DAY	WEEK	UNIGENE	FLCDNA	TAIR
TGAATTGAAA	0	1	0	0	0	no match found	no match found	At5g47130.1
at2g02020	CON	MIN	HOUR	DAY	WEEK	UNIGENE	FLCDNA	TAIR
AAACGAACCA	0	0	1	1	0	gnl|UG|At#S14274089	gi|28392948|gb|BT003860.1	At2g02020.1
at1g51620	CON	MIN	HOUR	DAY	WEEK	UNIGENE	FLCDNA	TAIR
GTTGCTGCAT	0	0	1	0	0	gnl|UG|At#S11733913	no match found	At1g51620.1
at3g21280	CON	MIN	HOUR	DAY	WEEK	UNIGENE	FLCDNA	TAIR
CTCCTCTTTA	0	1	0	0	0	gnl|UG|At#S11734447	gi|28393201|gb|BT003992.1	At3g21280.1
at3g17590	CON	MIN	HOUR	DAY	WEEK	UNIGENE	FLCDNA	TAIR
CTTAACTTGA	0	1	0	2	0	gnl|UG|At#S11735565	gi|26449425|dbj|AK117162.1|	At3g17590.1

at4g26900	CON	MIN	HOUR	DAY	WEEK	UNIGENE	FLCDNA		TAIR
ACACATTCAA	0	0	0	0	1	no match found	gi|26452023|dbj|AK118498.1|		non-canonical match
at5g64650	CON	MIN	HOUR	DAY	WEEK	UNIGENE	FLCDNA		TAIR
TTGTTTCTCC	1	1	0	1	0	gnl|UG|At#S11717234	gi|21405528|gb|AY086818.1		At5g64650.1
at5g50050	CON	MIN	HOUR	DAY	WEEK	UNIGENE	FLCDNA		TAIR
TCAACTTGAA	0	0	1	0	0	no match found	gi|51972115|gb|BT015663.1		multiple non-canonical match
at2g35490	CON	MIN	HOUR	DAY	WEEK	UNIGENE	FLCDNA		TAIR
TAAAAACCGT	10	3	1	1	7	gnl|UG|At#S11733698	gi|21406566|gb|AY087815.1		At2g35490.1
at1g06130	CON	MIN	HOUR	DAY	WEEK	UNIGENE	FLCDNA		TAIR
GTCATACTCG	4	2	1	1	1	gnl|UG|At#S11742414	gi|21404464|gb|AY085754.1		At1g06130.2
at4g01460	CON	MIN	HOUR	DAY	WEEK	UNIGENE	FLCDNA		TAIR
TGGTCAAATC	1	1	0	0	0	no match found	gi|22136405|gb|AY128881.1		non-canonical match
at5g15200	CON	MIN	HOUR	DAY	WEEK	UNIGENE	FLCDNA		TAIR
GTGAGACTTG	14	30	19	27	17	gnl|UG|At#S11722541	gi|110736815|dbj|AK228437.1|		At5g15200.1
at2g27330	CON	MIN	HOUR	DAY	WEEK	UNIGENE	FLCDNA		TAIR
CAGGTGGTGA	0	0	0	1	0	no match found	gi|106879154|gb|BT025704.1		non-canonical match
at3g26165	CON	MIN	HOUR	DAY	WEEK	UNIGENE	FLCDNA		TAIR
TACTGTGTTT	1	0	0	1	0	gnl|UG|At#S34116612	gi|110739243|dbj|AK229696.1|		non-canonical match
at4g16840	CON	MIN	HOUR	DAY	WEEK	UNIGENE	FLCDNA		TAIR
TTGTCTTGTC	0	0	2	0	1	no match found	gi|28973106|gb|BT005458.1		At1g74510.1
AAATGTATGT	0	0	0	1	0	gnl|UG|At#S11823114	gi|26452522|dbj|AK118753.1|		pseudo chromosome match
at1g54385	CON	MIN	HOUR	DAY	WEEK	UNIGENE	FLCDNA		TAIR
ATTCACCTCT	0	0	0	0	1	gnl|UG|At#S21989855	no match found		multiple non-canonical match
at2g18950	CON	MIN	HOUR	DAY	WEEK	UNIGENE	FLCDNA		TAIR
TCAGAACATC	2	0	0	0	2	gnl|UG|At#S15461409	gi|17380873|gb|AY063893.1		At2g18950.1
at4g32640	CON	MIN	HOUR	DAY	WEEK	UNIGENE	FLCDNA		TAIR
TTCGGAATTT	1	1	0	1	0	gnl|UG|At#S43849445	gi|28973656|gb|BT005737.1		At4g32640.1
at5g16730	CON	MIN	HOUR	DAY	WEEK	UNIGENE	FLCDNA		TAIR
ACACGGAAAC	1	5	1	0	0	no match found	gi|62320247|dbj|AK221450.1|		non-canonical match
TAAAACGTAT	0	0	2	0	0	gnl|UG|At#S11722156	no match found		At5g16730.1
at1g60640	CON	MIN	HOUR	DAY	WEEK	UNIGENE	FLCDNA		TAIR
GAGAAAACGA	1	2	1	0	0	no match found	no match found		At1g60640.1
at1g21280	CON	MIN	HOUR	DAY	WEEK	UNIGENE	FLCDNA		TAIR
TTCCAGAAAC	2	0	0	1	0	gnl|UG|At#S14274050	gi|28827239|gb|BT004931.1		multiple non-canonical match
at5g28640	CON	MIN	HOUR	DAY	WEEK	UNIGENE	FLCDNA		TAIR
TAAATCTGCT	3	0	1	1	0	gnl|UG|At#S11720347	no match found		At5g28640.1
at5g28750	CON	MIN	HOUR	DAY	WEEK	UNIGENE	FLCDNA		TAIR
AAGAGTTTAG	19	10	20	19	10	gnl|UG|At#S30587814	gi|15810474|gb|AY056276.1		At5g28750.1
atmg00580	CON	MIN	HOUR	DAY	WEEK	UNIGENE	FLCDNA		TAIR
GCAAATTTCA	2	3	6	2	1	no match found	no match found		AtMg00580
at3g46280	CON	MIN	HOUR	DAY	WEEK	UNIGENE	FLCDNA		TAIR

TTTTTTTCTT	0	0	0	0	1	no match found	gi|19347807|gb|AY080678.1	At3g46280.1
at4g13850	CON	MIN	HOUR	DAY	WEEK	UNIGENE	FLCDNA	TAIR
GGGAACCGAT	1	5	1	4	7	gnl|UG|At#S11725412	gi|18252852|gb|AY072361.1	At4g13850.2
at3g19150	CON	MIN	HOUR	DAY	WEEK	UNIGENE	FLCDNA	TAIR
AAGAAGACAA	3	5	3	5	1	no match found	gi|110738488|dbj|AK229307.1|	multiple non-canonical match
AATATAGAAA	0	0	1	1	0	gnl|UG|At#S11735101	no match found	At3g19150.1
ACGTCATCGT	1	0	0	0	0	no match found	gi|149944320|gb|BT030623.1	non-canonical match
at1g62790	CON	MIN	HOUR	DAY	WEEK	UNIGENE	FLCDNA	TAIR
TTAGTATATT	0	0	0	0	2	gnl|UG|At#S34116576	gi|110739313|dbj|AK229732.1|	At1g62790.2
at4g01710	CON	MIN	HOUR	DAY	WEEK	UNIGENE	FLCDNA	TAIR
TCTCACATTT	1	1	0	1	2	gnl|UG|At#S11727260	gi|15529134|gb|AY052191.1	At4g01710.1
at3g49850	CON	MIN	HOUR	DAY	WEEK	UNIGENE	FLCDNA	TAIR
TTATGTCAAG	0	0	0	2	1	gnl|UG|At#S11716925	gi|16604534|gb|AY058887.1	At3g49850.1
at2g17710	CON	MIN	HOUR	DAY	WEEK	UNIGENE	FLCDNA	TAIR
TGTTATTGCT	0	0	1	0	0	gnl|UG|At#S11738058	gi|17933280|gb|AF446349.1	At2g17710.1
at1g64460	CON	MIN	HOUR	DAY	WEEK	UNIGENE	FLCDNA	TAIR
AAAGAAGCCT	0	0	1	0	0	gnl|UG|At#S11730244	no match found	At1g64460.1
at4g10140	CON	MIN	HOUR	DAY	WEEK	UNIGENE	FLCDNA	TAIR
TTTCACAATC	0	0	0	1	0	no match found	gi|19347932|gb|AY080641.1	At4g10140.1
TCTTTCTTAT	1	0	0	0	0	gnl|UG|At#S18922715	no match found	non-canonical match
at4g17040	CON	MIN	HOUR	DAY	WEEK	UNIGENE	FLCDNA	TAIR
TTAAAAAAGG	6	8	5	3	5	gnl|UG|At#S18906232	gi|21406229|gb|AY087492.1	At4g17040.1
at5g56280	CON	MIN	HOUR	DAY	WEEK	UNIGENE	FLCDNA	TAIR
TAAAAGATCC	1	1	0	0	0	gnl|UG|At#S11706371	gi|28393280|gb|BT004034.1	At5g56280.1
at2g37860	CON	MIN	HOUR	DAY	WEEK	UNIGENE	FLCDNA	TAIR
ACTGCCAAAC	0	1	0	1	0	no match found	no match found	At2g37860.2
at4g23700	CON	MIN	HOUR	DAY	WEEK	UNIGENE	FLCDNA	TAIR
TGTTTCATAT	0	0	1	0	0	gnl|UG|At#S11723699	no match found	At4g23700.1
at5g44110	CON	MIN	HOUR	DAY	WEEK	UNIGENE	FLCDNA	TAIR
AAAGAATTTC	0	0	1	5	4	gnl|UG|At#S11719317	no match found	At5g44110.1
at1g10585	CON	MIN	HOUR	DAY	WEEK	UNIGENE	FLCDNA	TAIR
TGTTAGTTCT	1	1	1	1	1	gnl|UG|At#S11741974	gi|15293050|gb|AY050959.1	At1g10585.1
at3g44713	CON	MIN	HOUR	DAY	WEEK	UNIGENE	FLCDNA	TAIR
AGAAGTTGGC	0	1	0	1	0	no match found	no match found	At3g44713.1
at3g06850	CON	MIN	HOUR	DAY	WEEK	UNIGENE	FLCDNA	TAIR
TTCGAATAAG	1	7	3	1	0	gnl|UG|At#S11738678	gi|21405151|gb|AY086441.1	At3g06850.2
at1g55490	CON	MIN	HOUR	DAY	WEEK	UNIGENE	FLCDNA	TAIR
ATTCCCTCAA	31	19	20	32	57	gnl|UG|At#S15459112	gi|14423415|gb|AF386945.1	At1g55490.2
at5g02110	CON	MIN	HOUR	DAY	WEEK	UNIGENE	FLCDNA	TAIR
ATTTTGTTTT	20	2	7	7	4	no match found	no match found	At5g02110.1
at1g10270	CON	MIN	HOUR	DAY	WEEK	UNIGENE	FLCDNA	TAIR
AGTGCCAAGA	0	0	1	0	0	gnl|UG|At#S11742007	no match found	At1g10270.1

<table>
<tr><td>at2g34350</td><td>CON</td><td>MIN</td><td>HOUR</td><td>DAY</td><td>WEEK</td><td>UNIGENE</td><td>FLCDNA</td><td>TAIR</td></tr>
<tr><td>ATCATAAAAA</td><td>0</td><td>0</td><td>0</td><td>1</td><td>0</td><td>gnl|UG|At#S34116602</td><td>gi|110739262|dbj|AK229706.1|</td><td>non-canonical match</td></tr>
<tr><td>at1g64490</td><td>CON</td><td>MIN</td><td>HOUR</td><td>DAY</td><td>WEEK</td><td>UNIGENE</td><td>FLCDNA</td><td>TAIR</td></tr>
<tr><td>CTCCATCAAT</td><td>3</td><td>0</td><td>3</td><td>1</td><td>4</td><td>gnl|UG|At#S21736930</td><td>gi|51969631|dbj|AK175745.1|</td><td>At1g64490.1</td></tr>
<tr><td>at1g28420</td><td>CON</td><td>MIN</td><td>HOUR</td><td>DAY</td><td>WEEK</td><td>UNIGENE</td><td>FLCDNA</td><td>TAIR</td></tr>
<tr><td>AAGTTGAGAT</td><td>0</td><td>0</td><td>1</td><td>0</td><td>0</td><td>gnl|UG|At#S11739472</td><td>no match found</td><td>At1g28420.1</td></tr>
<tr><td>at4g14420</td><td>CON</td><td>MIN</td><td>HOUR</td><td>DAY</td><td>WEEK</td><td>UNIGENE</td><td>FLCDNA</td><td>TAIR</td></tr>
<tr><td>TGACAACTCA</td><td>2</td><td>4</td><td>0</td><td>0</td><td>0</td><td>gnl|UG|At#S11725313</td><td>gi|21404409|gb|AY085699.1</td><td>At4g14420.1</td></tr>
<tr><td>at2g38670</td><td>CON</td><td>MIN</td><td>HOUR</td><td>DAY</td><td>WEEK</td><td>UNIGENE</td><td>FLCDNA</td><td>TAIR</td></tr>
<tr><td>AAGCTTATCA</td><td>0</td><td>0</td><td>2</td><td>1</td><td>2</td><td>gnl|UG|At#S11732926</td><td>gi|22136257|gb|AY128807.1</td><td>At2g38670.1</td></tr>
<tr><td>at2g02960</td><td>CON</td><td>MIN</td><td>HOUR</td><td>DAY</td><td>WEEK</td><td>UNIGENE</td><td>FLCDNA</td><td>TAIR</td></tr>
<tr><td>GAGCCATTAC</td><td>1</td><td>0</td><td>0</td><td>1</td><td>0</td><td>gnl|UG|At#S18942982</td><td>gi|17529099|gb|AY065284.1</td><td>At2g02960.1</td></tr>
<tr><td>at5g60300</td><td>CON</td><td>MIN</td><td>HOUR</td><td>DAY</td><td>WEEK</td><td>UNIGENE</td><td>FLCDNA</td><td>TAIR</td></tr>
<tr><td>TTTCTTTGTT</td><td>1</td><td>0</td><td>1</td><td>1</td><td>0</td><td>no match found</td><td>gi|62320754|dbj|AK221704.1|</td><td>multiple canonical match</td></tr>
<tr><td>ATTTGGTCCA</td><td>3</td><td>0</td><td>4</td><td>1</td><td>0</td><td>no match found</td><td>gi|21539486|gb|AY120738.1</td><td>multiple canonical match</td></tr>
<tr><td>at5g35370</td><td>CON</td><td>MIN</td><td>HOUR</td><td>DAY</td><td>WEEK</td><td>UNIGENE</td><td>FLCDNA</td><td>TAIR</td></tr>
<tr><td>GTTGAAGGAC</td><td>2</td><td>1</td><td>1</td><td>0</td><td>0</td><td>gnl|UG|At#S11720166</td><td>gi|110740453|dbj|AK230321.1|</td><td>non-canonical match</td></tr>
<tr><td>at5g16190</td><td>CON</td><td>MIN</td><td>HOUR</td><td>DAY</td><td>WEEK</td><td>UNIGENE</td><td>FLCDNA</td><td>TAIR</td></tr>
<tr><td>CAGGCTCTTG</td><td>0</td><td>2</td><td>0</td><td>0</td><td>0</td><td>gnl|UG|At#S11722294</td><td>no match found</td><td>At5g16190.1</td></tr>
<tr><td>at1g10500</td><td>CON</td><td>MIN</td><td>HOUR</td><td>DAY</td><td>WEEK</td><td>UNIGENE</td><td>FLCDNA</td><td>TAIR</td></tr>
<tr><td>CTCTTTCTTT</td><td>1</td><td>4</td><td>1</td><td>0</td><td>2</td><td>gnl|UG|At#S34115693</td><td>gi|38603853|gb|BT010894.1</td><td>At1g10500.1</td></tr>
<tr><td>at3g52370</td><td>CON</td><td>MIN</td><td>HOUR</td><td>DAY</td><td>WEEK</td><td>UNIGENE</td><td>FLCDNA</td><td>TAIR</td></tr>
<tr><td>TGATAAAAAT</td><td>0</td><td>3</td><td>1</td><td>1</td><td>0</td><td>gnl|UG|At#S11728995</td><td>gi|26450295|dbj|AK117608.1|</td><td>At3g52370.1</td></tr>
<tr><td>at3g18680</td><td>CON</td><td>MIN</td><td>HOUR</td><td>DAY</td><td>WEEK</td><td>UNIGENE</td><td>FLCDNA</td><td>TAIR</td></tr>
<tr><td>AAGCTCATTT</td><td>4</td><td>3</td><td>3</td><td>11</td><td>4</td><td>gnl|UG|At#S11735243</td><td>gi|15292788|gb|AY050828.1</td><td>non-canonical match</td></tr>
<tr><td>at1g47056</td><td>CON</td><td>MIN</td><td>HOUR</td><td>DAY</td><td>WEEK</td><td>UNIGENE</td><td>FLCDNA</td><td>TAIR</td></tr>
<tr><td>GCATCAAGCA</td><td>0</td><td>2</td><td>0</td><td>0</td><td>1</td><td>gnl|UG|At#S11735531</td><td>no match found</td><td>non-canonical match</td></tr>
<tr><td>at5g15540</td><td>CON</td><td>MIN</td><td>HOUR</td><td>DAY</td><td>WEEK</td><td>UNIGENE</td><td>FLCDNA</td><td>TAIR</td></tr>
<tr><td>GAGGAGGAGG</td><td>0</td><td>0</td><td>0</td><td>1</td><td>0</td><td>gnl|UG|At#S11722454</td><td>no match found</td><td>At5g15540.1</td></tr>
<tr><td>at1g76200</td><td>CON</td><td>MIN</td><td>HOUR</td><td>DAY</td><td>WEEK</td><td>UNIGENE</td><td>FLCDNA</td><td>TAIR</td></tr>
<tr><td>AGAAAAAAGG</td><td>0</td><td>0</td><td>0</td><td>1</td><td>0</td><td>no match found</td><td>gi|21404674|gb|AY085964.1</td><td>multiple non-canonical match</td></tr>
<tr><td>ACGAAAAAAG</td><td>7</td><td>7</td><td>15</td><td>5</td><td>3</td><td>gnl|UG|At#S30651935</td><td>gi|110740809|dbj|AK226354.1|</td><td>non-canonical match</td></tr>
<tr><td>TTGTTATTTT</td><td>9</td><td>10</td><td>9</td><td>5</td><td>8</td><td>no match found</td><td>no match found</td><td>At1g76200.1</td></tr>
<tr><td>at1g03495</td><td>CON</td><td>MIN</td><td>HOUR</td><td>DAY</td><td>WEEK</td><td>UNIGENE</td><td>FLCDNA</td><td>TAIR</td></tr>
<tr><td>GATTTTGGAT</td><td>0</td><td>1</td><td>0</td><td>1</td><td>0</td><td>no match found</td><td>gi|62320553|dbj|AK221603.1|</td><td>multiple canonical match</td></tr>
<tr><td>at5g10790</td><td>CON</td><td>MIN</td><td>HOUR</td><td>DAY</td><td>WEEK</td><td>UNIGENE</td><td>FLCDNA</td><td>TAIR</td></tr>
<tr><td>GCTGATGCAT</td><td>0</td><td>0</td><td>1</td><td>1</td><td>1</td><td>gnl|UG|At#S11701488</td><td>no match found</td><td>At5g10790.1</td></tr>
<tr><td>at4g07675</td><td>CON</td><td>MIN</td><td>HOUR</td><td>DAY</td><td>WEEK</td><td>UNIGENE</td><td>FLCDNA</td><td>TAIR</td></tr>
<tr><td>ATGAAGACCA</td><td>0</td><td>1</td><td>0</td><td>0</td><td>0</td><td>gnl|UG|At#S11816696</td><td>no match found</td><td>At4g25180.1</td></tr>
<tr><td>at4g01897</td><td>CON</td><td>MIN</td><td>HOUR</td><td>DAY</td><td>WEEK</td><td>UNIGENE</td><td>FLCDNA</td><td>TAIR</td></tr>
<tr><td>CAGCTTCTTG</td><td>0</td><td>6</td><td>0</td><td>0</td><td>0</td><td>gnl|UG|At#S15461577</td><td>gi|89001076|gb|BT024790.1</td><td>multiple non-canonical match</td></tr>
</table>

at5g18270	CON	MIN	HOUR	DAY	WEEK	UNIGENE	FLCDNA	TAIR
TTAGTTAATA	1	0	0	0	0	gnl|UG|At#S14829921	gi|110736688|dbj|AK228369.1|	At5g18270.2
at3g10140	CON	MIN	HOUR	DAY	WEEK	UNIGENE	FLCDNA	TAIR
TATACTGCAG	0	0	1	1	0	gnl|UG|At#S11737847	gi|22531123|gb|AY136400.1	At3g10140.1
at1g06670	CON	MIN	HOUR	DAY	WEEK	UNIGENE	FLCDNA	TAIR
TAACTGAAAG	0	0	0	1	0	gnl|UG|At#S11699382	no match found	At1g06670.1
at5g58430	CON	MIN	HOUR	DAY	WEEK	UNIGENE	FLCDNA	TAIR
TATTGTGTAT	1	0	1	0	0	no match found	gi|20453215|gb|AY094480.1	non-canonical match
TTAGGGACTT	0	1	5	3	0	gnl|UG|At#S11717866	no match found	At5g58430.1
at4g02100	CON	MIN	HOUR	DAY	WEEK	UNIGENE	FLCDNA	TAIR
GCTGGTCTCA	0	1	0	0	0	gnl|UG|At#S34114462	gi|110743319|dbj|AK227553.1|	At4g02100.1
at5g62570	CON	MIN	HOUR	DAY	WEEK	UNIGENE	FLCDNA	TAIR
TCACAGAAGA	0	2	0	0	0	gnl|UG|At#S20191547	gi|20260395|gb|AY093097.1	At5g62570.1
at1g05430	CON	MIN	HOUR	DAY	WEEK	UNIGENE	FLCDNA	TAIR
TATTTGTCTC	1	1	0	0	2	gnl|UG|At#S14274106	gi|21405516|gb|AY086806.1	multiple canonical match
at2g30550	CON	MIN	HOUR	DAY	WEEK	UNIGENE	FLCDNA	TAIR
TTCATAAAAA	1	0	0	0	0	no match found	no match found	At2g30550.1
CGCTAGTGAA	0	1	0	0	0	no match found	gi|21404731|gb|AY086021.1	non-canonical match
CTTTGGTCAA	1	2	2	0	0	gnl|UG|At#S11734903	gi|20268777|gb|AY091143.1	At2g30550.2
at4g11690	CON	MIN	HOUR	DAY	WEEK	UNIGENE	FLCDNA	TAIR
TAAGCTCAAA	0	0	1	0	0	gnl|UG|At#S11725784	no match found	non-canonical match
at4g29160	CON	MIN	HOUR	DAY	WEEK	UNIGENE	FLCDNA	TAIR
TATGCATTGA	0	4	0	0	2	gnl|UG|At#S18941777	gi|21407293|gb|AY088519.1	At4g29160.1
at5g01540	CON	MIN	HOUR	DAY	WEEK	UNIGENE	FLCDNA	TAIR
ATTTAACTAG	0	0	1	0	0	gnl|UG|At#S11725772	gi|13605542|gb|AF361597.1	At5g01540.1
at3g17810	CON	MIN	HOUR	DAY	WEEK	UNIGENE	FLCDNA	TAIR
CAAATTACGA	1	3	5	4	2	no match found	gi|14334711|gb|AY035029.1	non-canonical match
at4g04885	CON	MIN	HOUR	DAY	WEEK	UNIGENE	FLCDNA	TAIR
TAAATGGAAT	0	1	0	0	0	gnl|UG|At#S11816730	no match found	multiple canonical match
at3g13850	CON	MIN	HOUR	DAY	WEEK	UNIGENE	FLCDNA	TAIR
AAGCTTTCGT	0	0	0	0	1	gnl|UG|At#S11736691	no match found	non-canonical match
at5g04520	CON	MIN	HOUR	DAY	WEEK	UNIGENE	FLCDNA	TAIR
TAACTTTAAC	1	1	1	0	0	no match found	no match found	At5g04520.1
at2g46220	CON	MIN	HOUR	DAY	WEEK	UNIGENE	FLCDNA	TAIR
AATACATTAC	7	5	0	2	1	gnl|UG|At#S11750462	gi|15215834|gb|AY050447.1	non-canonical match
at1g34320	CON	MIN	HOUR	DAY	WEEK	UNIGENE	FLCDNA	TAIR
AAAGCTCTCG	1	0	1	1	0	gnl|UG|At#S34116153	gi|51971087|dbj|AK176473.1|	At1g34320.1
at3g23930	CON	MIN	HOUR	DAY	WEEK	UNIGENE	FLCDNA	TAIR
CAAAGGAAAC	0	1	0	0	0	gnl|UG|At#S11733614	gi|26450467|dbj|AK117696.1|	At3g23930.1
at5g04940	CON	MIN	HOUR	DAY	WEEK	UNIGENE	FLCDNA	TAIR
CTTTTATCCT	1	0	1	0	0	gnl|UG|At#S11702224	no match found	At5g04940.1
at5g13350	CON	MIN	HOUR	DAY	WEEK	UNIGENE	FLCDNA	TAIR

TGTTGAATTT	0	0	0	0	1	no match found	no match found	At5g13350.1
at1g68840	CON	MIN	HOUR	DAY	WEEK	UNIGENE	FLCDNA	TAIR
CTAGGTTGTA	5	9	1	2	0	gnl|UG|At#S11729329	gi|21404618|gb|AY085908.1	At1g68840.1
at3g24770	CON	MIN	HOUR	DAY	WEEK	UNIGENE	FLCDNA	TAIR
TGACTGTGTG	0	0	0	0	1	gnl|UG|At#S11809390	gi|21406206|gb|AY087469.1	At3g24770.1
AGGTTCCTAG	0	1	0	0	1	no match found	gi|28393379|gb|BT004087.1	non-canonical match
at2g07080	CON	MIN	HOUR	DAY	WEEK	UNIGENE	FLCDNA	TAIR
GATAAGCTCA	92	141	77	81	26	no match found	no match found	At2g07080.1
at1g05790	CON	MIN	HOUR	DAY	WEEK	UNIGENE	FLCDNA	TAIR
AACAGGTTTC	4	0	1	0	0	gnl|UG|At#S34117465	gi|110737592|dbj|AK228843.1|	At1g05790.1
at1g73650	CON	MIN	HOUR	DAY	WEEK	UNIGENE	FLCDNA	TAIR
GAAACTCAGG	1	5	0	8	3	gnl|UG|At#S18942266	gi|21403857|gb|AY085147.1	At1g73650.1
at5g25170	CON	MIN	HOUR	DAY	WEEK	UNIGENE	FLCDNA	TAIR
TTTTCTTTGG	1	0	1	0	0	gnl|UG|At#S11720666	no match found	At5g25170.1
at1g49960	CON	MIN	HOUR	DAY	WEEK	UNIGENE	FLCDNA	TAIR
TATAATACAA	1	0	0	0	0	no match found	gi|15983804|gb|AY056808.1	multiple non-canonical match
at2g47330	CON	MIN	HOUR	DAY	WEEK	UNIGENE	FLCDNA	TAIR
ATGATAGTGG	0	2	0	2	2	no match found	gi|16323191|gb|AY057700.1	At2g47330.1
at3g18170	CON	MIN	HOUR	DAY	WEEK	UNIGENE	FLCDNA	TAIR
GTGTTCTTGC	0	0	0	1	0	gnl|UG|At#S34118211	gi|110736164|dbj|AK228095.1|	At3g18170.1
at1g70790	CON	MIN	HOUR	DAY	WEEK	UNIGENE	FLCDNA	TAIR
TCGAATAACT	3	0	1	0	0	gnl|UG|At#S17016099	gi|21404074|gb|AY085364.1	At1g70790.1
at1g16610	CON	MIN	HOUR	DAY	WEEK	UNIGENE	FLCDNA	TAIR
GATGGTGCCC	0	2	0	3	0	gnl|UG|At#S11741382	gi|15027956|gb|AY045835.1	At1g16610.1
at4g29260	CON	MIN	HOUR	DAY	WEEK	UNIGENE	FLCDNA	TAIR
TACTACATTC	2	0	1	0	0	gnl|UG|At#S11722744	gi|51870251|gb|BT015481.1	At4g29260.1
at1g15180	CON	MIN	HOUR	DAY	WEEK	UNIGENE	FLCDNA	TAIR
TTTTCAGCAG	0	1	0	1	0	gnl|UG|At#S43850633	gi|19310610|gb|AY079305.1	At1g15180.1
at5g54430	CON	MIN	HOUR	DAY	WEEK	UNIGENE	FLCDNA	TAIR
AAGGAGTTGT	6	4	9	2	4	gnl|UG|At#S18908483	gi|17979328|gb|AY070394.1	At5g54430.1
at3g09560	CON	MIN	HOUR	DAY	WEEK	UNIGENE	FLCDNA	TAIR
TAGGAGTTAA	0	1	0	1	0	gnl|UG|At#S11738015	gi|110738847|dbj|AK229489.1|	At3g09560.2
at3g48020	CON	MIN	HOUR	DAY	WEEK	UNIGENE	FLCDNA	TAIR
ATCGAGATGT	0	0	1	1	0	gnl|UG|At#S18899801	no match found	multiple non-canonical match
at1g27470	CON	MIN	HOUR	DAY	WEEK	UNIGENE	FLCDNA	TAIR
GATGGAGGTT	0	0	0	1	1	gnl|UG|At#S11709920	gi|23297581|gb|AY150459.1	At1g27470.1
at4g00620	CON	MIN	HOUR	DAY	WEEK	UNIGENE	FLCDNA	TAIR
TAGTTTTTAA	0	1	0	2	2	gnl|UG|At#S11727517	gi|16649148|gb|AY059944.1	At4g00620.1
at1g15390	CON	MIN	HOUR	DAY	WEEK	UNIGENE	FLCDNA	TAIR
AGTGTGATCA	2	1	1	0	0	gnl|UG|At#S11701417	gi|22136569|gb|AY129485.1	At1g15390.1
at3g58160	CON	MIN	HOUR	DAY	WEEK	UNIGENE	FLCDNA	TAIR

AATGAGAAAA	0	0	0	1	0	no match found	no match found	At3g58160.1
at2g28000	CON	MIN	HOUR	DAY	WEEK	UNIGENE	FLCDNA	TAIR
GTGTAATCAA	12	9	6	8	16	gnl|UG|At#S11735547	gi|62321526|dbj|AK222099.1|	At2g28000.1
at3g49360	CON	MIN	HOUR	DAY	WEEK	UNIGENE	FLCDNA	TAIR
GCTGGTGCTC	0	1	0	0	0	gnl|UG|At#S11729573	gi|21403144|gb|AY084434.1	At3g49360.1
at5g23530	CON	MIN	HOUR	DAY	WEEK	UNIGENE	FLCDNA	TAIR
CGTTTTACAT	3	0	4	2	0	no match found	gi|21407550|gb|AY088776.1	multiple canonical match
at1g08450	CON	MIN	HOUR	DAY	WEEK	UNIGENE	FLCDNA	TAIR
AAAACTTGAC	5	3	4	4	0	gnl|UG|At#S11742190	gi|110740184|dbj|AK230182.1|	At1g08450.2
AAAACTTGAA	0	1	0	0	0	no match found	gi|15810562|gb|AY056320.1	non-canonical match
at1g68810	CON	MIN	HOUR	DAY	WEEK	UNIGENE	FLCDNA	TAIR
ATTAAAACAT	0	1	0	0	0	gnl|UG|At#S11729332	gi|18176097|gb|AY072161.1	At1g68810.1
at1g54390	CON	MIN	HOUR	DAY	WEEK	UNIGENE	FLCDNA	TAIR
TACAAAGGTT	1	0	0	2	0	no match found	gi|21404279|gb|AY085569.1	At1g54390.2
at2g16780	CON	MIN	HOUR	DAY	WEEK	UNIGENE	FLCDNA	TAIR
TTGGATTTTG	2	0	1	0	3	gnl|UG|At#S11738285	gi|15983816|gb|AY056814.1	At2g16780.1
at2g31340	CON	MIN	HOUR	DAY	WEEK	UNIGENE	FLCDNA	TAIR
TTGCTGTGTT	0	0	0	0	2	gnl|UG|At#S11744153	gi|19310463|gb|AY078970.1	At2g31340.1
at1g26610	CON	MIN	HOUR	DAY	WEEK	UNIGENE	FLCDNA	TAIR
AGATGCATAC	0	1	0	0	0	gnl|UG|At#S11740118	no match found	At1g26610.1
at2g01860	CON	MIN	HOUR	DAY	WEEK	UNIGENE	FLCDNA	TAIR
AGCTGAAACT	0	3	0	0	1	gnl|UG|At#S11742604	gi|55733738|gb|BT020200.1	non-canonical match
at1g54840	CON	MIN	HOUR	DAY	WEEK	UNIGENE	FLCDNA	TAIR
TGTTTGAAAT	0	0	0	0	1	gnl|UG|At#S11732716	gi|22136529|gb|AY128943.1	multiple non-canonical match
at5g57800	CON	MIN	HOUR	DAY	WEEK	UNIGENE	FLCDNA	TAIR
AAGTATGGTC	1	2	2	0	2	gnl|UG|At#S11817377	gi|25083877|gb|BT002119.1	At5g57800.1
at4g17110	CON	MIN	HOUR	DAY	WEEK	UNIGENE	FLCDNA	TAIR
CAACGAAGTA	2	3	2	1	1	no match found	no match found	At4g17110.1
at5g54080	CON	MIN	HOUR	DAY	WEEK	UNIGENE	FLCDNA	TAIR
TCACTTTAAA	4	1	4	0	0	no match found	no match found	At5g54080.1
AATAGAATGC	0	0	0	1	0	no match found	no match found	At5g54080.2
at1g60710	CON	MIN	HOUR	DAY	WEEK	UNIGENE	FLCDNA	TAIR
GTTAATGCCT	3	6	5	1	2	gnl|UG|At#S11731260	gi|20466377|gb|AY099655.1	At1g60710.1
at1g53260	CON	MIN	HOUR	DAY	WEEK	UNIGENE	FLCDNA	TAIR
GCAAAAAGAC	0	0	1	0	0	no match found	no match found	At1g53260.1
at2g11230	CON	MIN	HOUR	DAY	WEEK	UNIGENE	FLCDNA	TAIR
GCAATAAACT	0	1	1	1	0	no match found	no match found	At2g11230.1
at1g73170	CON	MIN	HOUR	DAY	WEEK	UNIGENE	FLCDNA	TAIR
AAGAGGAAAC	0	1	0	0	0	gnl|UG|At#S38434067	gi|18086458|gb|AY065048.1	At1g73170.1
at1g52870	CON	MIN	HOUR	DAY	WEEK	UNIGENE	FLCDNA	TAIR
GCTCGTTATC	0	7	2	2	0	gnl|UG|At#S11733409	gi|14326544|gb|AF385726.1	At1g52870.1

at3g51120	CON	MIN	HOUR	DAY	WEEK	UNIGENE	FLCDNA	TAIR
ACAAAATTTC	0	0	0	0	1	gnl|UG|At#S20820425	no match found	At5g45720.1
at1g04240	CON	MIN	HOUR	DAY	WEEK	UNIGENE	FLCDNA	TAIR
AAAGGATCAG	0	0	1	0	0	no match found	gi|12083195|gb|AF332393.1	non-canonical match
CTTAATGGTC	1	1	0	0	0	gnl|UG|At#S11742619	gi|17381101|gb|AY064007.1	At1g04240.1
at5g16960	CON	MIN	HOUR	DAY	WEEK	UNIGENE	FLCDNA	TAIR
TGGGATGATC	0	1	0	0	0	gnl|UG|At#S11722098	gi|21407341|gb|AY088567.1	At5g16960.1
at1g08420	CON	MIN	HOUR	DAY	WEEK	UNIGENE	FLCDNA	TAIR
TCATATCTTT	0	2	0	0	0	gnl|UG|At#S11742193	gi|110741369|dbj|AK230437.1|	multiple non-canonical match
at3g18490	CON	MIN	HOUR	DAY	WEEK	UNIGENE	FLCDNA	TAIR
TCAAAGTAGA	23	7	18	10	8	gnl|UG|At#S11735298	gi|23297731|gb|AY150497.1	At3g18490.1
at1g44790	CON	MIN	HOUR	DAY	WEEK	UNIGENE	FLCDNA	TAIR
TCACAGATCT	0	1	0	1	1	gnl|UG|At#S11735753	gi|110736692|dbj|AK228371.1|	At1g44790.1
at2g47490	CON	MIN	HOUR	DAY	WEEK	UNIGENE	FLCDNA	TAIR
TTACTGAAAG	2	0	1	3	2	gnl|UG|At#S11730833	gi|13937205|gb|AF372957.1	multiple canonical match
TTGCCATTCA	1	0	0	0	0	no match found	gi|18491124|gb|AY074833.1	non-canonical match
at1g10630	CON	MIN	HOUR	DAY	WEEK	UNIGENE	FLCDNA	TAIR
CGCAACTAGC	0	0	1	0	2	no match found	gi|50198854|gb|BT015004.1	non-canonical match
TTTTACTGTC	4	1	1	0	3	gnl|UG|At#S11741969	gi|48310129|gb|BT014776.1	At1g10630.1
at1g51940	CON	MIN	HOUR	DAY	WEEK	UNIGENE	FLCDNA	TAIR
AAGCAAGTGG	1	0	1	0	0	gnl|UG|At#S11733775	no match found	At1g51940.1
at3g59020	CON	MIN	HOUR	DAY	WEEK	UNIGENE	FLCDNA	TAIR
TCTTAAGAGA	0	0	1	0	0	no match found	no match found	At3g59020.1
GGCACGACAT	1	1	0	2	0	gnl|UG|At#S37211430	no match found	pseudo chromosome match
at5g57660	CON	MIN	HOUR	DAY	WEEK	UNIGENE	FLCDNA	TAIR
TTTATGCTTC	4	18	4	0	2	gnl|UG|At#S11717946	gi|21407206|gb|AY088432.1	multiple canonical match
at5g38310	CON	MIN	HOUR	DAY	WEEK	UNIGENE	FLCDNA	TAIR
TGAATTTAAA	0	0	0	1	0	no match found	no match found	At5g38310.1
at3g63110	CON	MIN	HOUR	DAY	WEEK	UNIGENE	FLCDNA	TAIR
GAACGCGAGT	1	0	1	1	0	gnl|UG|At#S11726505	gi|21928144|gb|AY125508.1	At3g63110.1
at3g28780	CON	MIN	HOUR	DAY	WEEK	UNIGENE	FLCDNA	TAIR
GATATAAAAT	1	1	1	0	0	gnl|UG|At#S21736967	gi|51969557|dbj|AK175708.1|	At3g28780.1
at1g69010	CON	MIN	HOUR	DAY	WEEK	UNIGENE	FLCDNA	TAIR
TAACCTATCA	0	0	1	0	3	gnl|UG|At#S11729297	gi|19310567|gb|AY080650.1	At1g69010.1
at1g71990	CON	MIN	HOUR	DAY	WEEK	UNIGENE	FLCDNA	TAIR
TGGTTTCATT	1	1	2	2	0	no match found	gi|110737724|dbj|AK228912.1|	At1g18530.1
at5g09870	CON	MIN	HOUR	DAY	WEEK	UNIGENE	FLCDNA	TAIR
GCACTTTTCG	0	1	3	3	2	gnl|UG|At#S11723770	gi|31711829|gb|BT008832.1	At5g09870.1
at5g05520	CON	MIN	HOUR	DAY	WEEK	UNIGENE	FLCDNA	TAIR
GCGGAGTCTT	1	2	1	0	1	gnl|UG|At#S11724757	gi|16648862|gb|AY059801.1	At5g05520.1
at5g55500	CON	MIN	HOUR	DAY	WEEK	UNIGENE	FLCDNA	TAIR
AAGAGCCTTG	0	0	1	0	0	no match found	gi|23505972|gb|AY143907.1	non-canonical match

at2g32295	CON	MIN	HOUR	DAY	WEEK	UNIGENE	FLCDNA	TAIR
AAGATGTGTA	1	0	0	2	0	gnl|UG|At#S15460872	gi|62321285|dbj|AK221975.1|	At2g32295.1
at3g10620	CON	MIN	HOUR	DAY	WEEK	UNIGENE	FLCDNA	TAIR
TCAGCTTTCG	3	1	3	0	0	gnl|UG|At#S11737702	gi|13877848|gb|AF370187.1	non-canonical match
at3g28455	CON	MIN	HOUR	DAY	WEEK	UNIGENE	FLCDNA	TAIR
CGGGCTTCGT	0	0	0	1	0	gnl|UG|At#S11816181	gi|21403486|gb|AY084776.1	At3g28455.1
at1g33590	CON	MIN	HOUR	DAY	WEEK	UNIGENE	FLCDNA	TAIR
CCAGAGCTCC	1	8	1	1	0	gnl|UG|At#S11737727	gi|21403060|gb|AY084350.1	At1g33590.1
at3g04420	CON	MIN	HOUR	DAY	WEEK	UNIGENE	FLCDNA	TAIR
GTTGACTGAT	1	1	1	2	1	gnl|UG|At#S11709213	gi|16974618|gb|AY060587.1	At3g04420.1
at2g47640	CON	MIN	HOUR	DAY	WEEK	UNIGENE	FLCDNA	TAIR
GTTCTTGAAA	1	2	1	1	1	no match found	gi|28466880|gb|BT004783.1	At1g77940.1
TCCAAGCTCA	0	0	0	0	1	gnl|UG|At#S11730800	no match found	multiple non-canonical match
TTCCTTGTTT	5	2	1	1	8	no match found	gi|21405311|gb|AY086601.1	At2g47640.2
at1g74550	CON	MIN	HOUR	DAY	WEEK	UNIGENE	FLCDNA	TAIR
GTTCGTACAA	0	0	0	1	1	gnl|UG|At#S11728124	no match found	At1g74550.1
at5g45960	CON	MIN	HOUR	DAY	WEEK	UNIGENE	FLCDNA	TAIR
CAAGTGGGTT	0	1	0	0	0	gnl|UG|At#S11719131	gi|21403431|gb|AY084721.1	At5g45960.1
at1g54790	CON	MIN	HOUR	DAY	WEEK	UNIGENE	FLCDNA	TAIR
GGAACTACAA	0	1	1	2	0	no match found	no match found	At1g54790.2
at3g23890	CON	MIN	HOUR	DAY	WEEK	UNIGENE	FLCDNA	TAIR
AAGAAAACCA	0	1	1	0	0	gnl|UG|At#S11733631	no match found	At3g23890.1
at3g52450	CON	MIN	HOUR	DAY	WEEK	UNIGENE	FLCDNA	TAIR
AAATTATGAA	0	0	1	0	0	no match found	no match found	At3g52450.1
at2g42620	CON	MIN	HOUR	DAY	WEEK	UNIGENE	FLCDNA	TAIR
AACTTTTTGT	0	3	1	1	2	gnl|UG|At#S11707062	no match found	At2g42620.1
at3g47620	CON	MIN	HOUR	DAY	WEEK	UNIGENE	FLCDNA	TAIR
AGATTAATGA	4	7	1	0	0	gnl|UG|At#S11729877	gi|16604510|gb|AY058874.1	At3g47620.1
at4g12320	CON	MIN	HOUR	DAY	WEEK	UNIGENE	FLCDNA	TAIR
TCAAAGCCGT	0	0	1	0	0	gnl|UG|At#S11807518	gi|51536591|gb|BT015411.1	At4g12320.1
at5g46630	CON	MIN	HOUR	DAY	WEEK	UNIGENE	FLCDNA	TAIR
GCTTCTAGTT	1	4	1	5	1	gnl|UG|At#S18941517	gi|14326519|gb|AF385713.1	At5g46630.2
at2g36070	CON	MIN	HOUR	DAY	WEEK	UNIGENE	FLCDNA	TAIR
TTTCAGGGGA	1	1	2	1	1	no match found	gi|53828526|gb|BT015810.1	At2g36070.1
at3g13150	CON	MIN	HOUR	DAY	WEEK	UNIGENE	FLCDNA	TAIR
GTTATATTGG	0	1	0	0	1	gnl|UG|At#S11736943	gi|66792657|gb|BT023440.1	At3g13150.1
at4g26820	CON	MIN	HOUR	DAY	WEEK	UNIGENE	FLCDNA	TAIR
TTGATGACGA	0	0	1	0	0	gnl|UG|At#S43849993	no match found	non-canonical match
at1g29240	CON	MIN	HOUR	DAY	WEEK	UNIGENE	FLCDNA	TAIR
TGCAGCTTCA	0	1	0	0	0	gnl|UG|At#S11739286	gi|19347725|gb|AY080605.1	At1g29240.1
at1g13635	CON	MIN	HOUR	DAY	WEEK	UNIGENE	FLCDNA	TAIR
GAGGCATATA	1	0	0	0	0	gnl|UG|At#S18897452	no match found	At1g13635.1

at3g13330	CON	MIN	HOUR	DAY	WEEK	UNIGENE	FLCDNA	TAIR
GTTTCGGTTT	2	6	3	14	11	gnl|UG|At#S11736874	gi|19347796|gb|AY080673.1	At3g13330.1
at5g61390	CON	MIN	HOUR	DAY	WEEK	UNIGENE	FLCDNA	TAIR
AAAGAAACAC	0	0	0	1	0	no match found	no match found	At5g61390.1
at5g05720	CON	MIN	HOUR	DAY	WEEK	UNIGENE	FLCDNA	TAIR
TCACCAGTGA	0	0	1	0	0	no match found	no match found	At5g05720.1
at4g22970	CON	MIN	HOUR	DAY	WEEK	UNIGENE	FLCDNA	TAIR
ATGTATTGTC	1	0	0	0	0	gnl|UG|At#S11723826	no match found	At4g22970.1
at5g41520	CON	MIN	HOUR	DAY	WEEK	UNIGENE	FLCDNA	TAIR
ATCATATCAG	0	5	2	0	1	no match found	gi|14030698|gb|AF375440.1	non-canonical match
AATCTTTTTA	3	1	2	2	2	gnl|UG|At#S11719578	no match found	At5g41520.1
at1g26460	CON	MIN	HOUR	DAY	WEEK	UNIGENE	FLCDNA	TAIR
CCCAGTGTTT	1	1	0	0	0	gnl|UG|At#S11740166	gi|19698978|gb|AY081336.1	At1g26460.1
at1g05135	CON	MIN	HOUR	DAY	WEEK	UNIGENE	FLCDNA	TAIR
GAGGAGGCGT	3	33	2	12	0	gnl|UG|At#S34117575	gi|110737379|dbj|AK228733.1|	At1g05135.1
at2g40000	CON	MIN	HOUR	DAY	WEEK	UNIGENE	FLCDNA	TAIR
TTCAGCACCT	0	21	1	0	0	gnl|UG|At#S18904005	gi|21406131|gb|AY087407.1	At2g40000.1
at5g41470	CON	MIN	HOUR	DAY	WEEK	UNIGENE	FLCDNA	TAIR
CTTATTGCCT	0	0	0	1	0	no match found	no match found	At5g41470.1
at1g77350	CON	MIN	HOUR	DAY	WEEK	UNIGENE	FLCDNA	TAIR
TATCGAAACC	0	0	0	2	0	gnl|UG|At#S28282387	no match found	pseudo chromosome match
AATTGTGATA	1	0	0	1	1	no match found	gi|16648790|gb|AY058172.1	At1g77350.1
at3g50760	CON	MIN	HOUR	DAY	WEEK	UNIGENE	FLCDNA	TAIR
GGTAAGGTTA	0	0	2	0	1	gnl|UG|At#S24442665	gi|44917560|gb|BT011742.1	At3g50760.1
at5g44240	CON	MIN	HOUR	DAY	WEEK	UNIGENE	FLCDNA	TAIR
GGTCCTATTT	0	3	0	0	0	gnl|UG|At#S11719304	gi|16930514|gb|AF419611.1	At5g44240.1
at5g24010	CON	MIN	HOUR	DAY	WEEK	UNIGENE	FLCDNA	TAIR
TGCTTCAGCT	0	1	0	0	0	gnl|UG|At#S11720778	gi|19347927|gb|AY080639.1	At5g24010.1
at2g30170	CON	MIN	HOUR	DAY	WEEK	UNIGENE	FLCDNA	TAIR
ATAAGATCTT	7	7	2	1	0	gnl|UG|At#S18898610	gi|13878070|gb|AF370298.1	At2g30170.1
at3g19750	CON	MIN	HOUR	DAY	WEEK	UNIGENE	FLCDNA	TAIR
TCGTAACAAT	1	0	2	0	7	gnl|UG|At#S11734929	no match found	At3g19740.1
at5g27060	CON	MIN	HOUR	DAY	WEEK	UNIGENE	FLCDNA	TAIR
TGATAGTTAA	0	0	0	1	0	no match found	no match found	At5g27060.1
at5g44450	CON	MIN	HOUR	DAY	WEEK	UNIGENE	FLCDNA	TAIR
ACTAAACCTG	0	2	1	0	0	no match found	no match found	At5g44450.1
at5g04930	CON	MIN	HOUR	DAY	WEEK	UNIGENE	FLCDNA	TAIR
GATGTTCTGG	0	1	0	0	0	gnl|UG|At#S34117567	gi|110737393|dbj|AK228741.1|	At5g04930.1
at4g14145	CON	MIN	HOUR	DAY	WEEK	UNIGENE	FLCDNA	TAIR
TTGTCGTGTT	0	6	1	1	0	gnl|UG|At#S11816639	gi|26453207|dbj|AK119106.1|	At4g14145.1
at1g51720	CON	MIN	HOUR	DAY	WEEK	UNIGENE	FLCDNA	TAIR

ACGGACCAAG	0	2	4	3	1	gnl|UG|At#S11733876	gi|28058695|gb|BT003324.1	At1g51720.1
at5g10550	CON	MIN	HOUR	DAY	WEEK	UNIGENE	FLCDNA	TAIR
CCTCCGGTGA	0	0	0	1	0	gnl|UG|At#S11723605	no match found	non-canonical match
at2g21140	CON	MIN	HOUR	DAY	WEEK	UNIGENE	FLCDNA	TAIR
TTTATGTATC	5	3	1	1	5	gnl|UG|At#S11737168	gi|26449759|dbj|AK117333.1|	At2g21140.1
at5g24580	CON	MIN	HOUR	DAY	WEEK	UNIGENE	FLCDNA	TAIR
CACTGCCAAG	0	1	0	0	0	gnl|UG|At#S18941582	gi|18175600|gb|AY072071.1	At5g24580.1
at1g50260	CON	MIN	HOUR	DAY	WEEK	UNIGENE	FLCDNA	TAIR
GAAGGCTCAA	0	1	0	0	1	gnl|UG|At#S11808268	gi|20466317|gb|AY099625.1	At1g50260.1
at1g01020	CON	MIN	HOUR	DAY	WEEK	UNIGENE	FLCDNA	TAIR
GTGTTATGTC	0	0	0	0	1	no match found	no match found	At1g01020.1
GCTCTTAAAG	1	1	1	0	0	gnl|UG|At#S28282754	gi|110736773|dbj|AK228414.1|	At1g01020.1
at4g14740	CON	MIN	HOUR	DAY	WEEK	UNIGENE	FLCDNA	TAIR
TTGGAGGAAC	1	0	2	0	0	gnl|UG|At#S11743181	gi|23297567|gb|AY150455.1	At4g14740.2
at3g19360	CON	MIN	HOUR	DAY	WEEK	UNIGENE	FLCDNA	TAIR
GCCAAGCAGA	0	0	1	0	0	gnl|UG|At#S11735042	gi|19548068|gb|AY081745.1	At3g19360.1
at3g08840	CON	MIN	HOUR	DAY	WEEK	UNIGENE	FLCDNA	TAIR
TTGAAACCGG	1	1	0	0	0	gnl|UG|At#S11823023	gi|26452706|dbj|AK118847.1|	At3g08840.3
at3g54080	CON	MIN	HOUR	DAY	WEEK	UNIGENE	FLCDNA	TAIR
TGTGTAAGCT	1	1	0	0	0	no match found	gi|13877872|gb|AF370199.1	non-canonical match
at5g26980	CON	MIN	HOUR	DAY	WEEK	UNIGENE	FLCDNA	TAIR
GTGGAGTATT	2	0	0	0	1	gnl|UG|At#S18910024	gi|110736331|dbj|AK228180.1|	At5g26980.1
at5g08590	CON	MIN	HOUR	DAY	WEEK	UNIGENE	FLCDNA	TAIR
TCAAAGAGGC	0	0	1	0	0	no match found	gi|90093287|gb|BT024886.1	non-canonical match
AAACAGAAGG	4	3	5	4	2	gnl|UG|At#S11723956	gi|110741508|dbj|AK226589.1|	At5g08590.1
at5g63530	CON	MIN	HOUR	DAY	WEEK	UNIGENE	FLCDNA	TAIR
TACTGTAATG	1	1	1	1	3	no match found	gi|15810596|gb|AY056337.1	non-canonical match
TGGTTTCTTT	0	1	2	1	1	gnl|UG|At#S11717347	gi|13430829|gb|AF360327.1	At5g63530.1
at5g04800	CON	MIN	HOUR	DAY	WEEK	UNIGENE	FLCDNA	TAIR
TGTGTTCTTG	7	4	6	10	14	gnl|UG|At#S18911254	gi|21406619|gb|AY087862.1	At5g04800.1
GACTTTGTTC	0	0	1	0	1	no match found	gi|23507768|gb|BT000851.1	multiple non-canonical match
at5g62650	CON	MIN	HOUR	DAY	WEEK	UNIGENE	FLCDNA	TAIR
CTCTCAATAG	1	0	0	0	0	gnl|UG|At#S11717435	gi|15809801|gb|AY054168.1	At5g62650.1
at3g03070	CON	MIN	HOUR	DAY	WEEK	UNIGENE	FLCDNA	TAIR
CCATTGAGCT	4	4	3	2	0	gnl|UG|At#S22666383	no match found	At3g03070.1
TTGTAACATT	2	1	0	0	0	no match found	gi|21406208|gb|AY087471.1	non-canonical match
at2g44420	CON	MIN	HOUR	DAY	WEEK	UNIGENE	FLCDNA	TAIR
TGATTGATTC	0	0	0	1	0	gnl|UG|At#S11731541	gi|26450108|dbj|AK117511.1|	At2g44420.1
at1g14620	CON	MIN	HOUR	DAY	WEEK	UNIGENE	FLCDNA	TAIR
TCTCTGGTTG	1	1	2	1	1	gnl|UG|At#S11741574	gi|22022539|gb|AY127001.1	At1g14620.1
at1g06840	CON	MIN	HOUR	DAY	WEEK	UNIGENE	FLCDNA	TAIR
AAGCATCATT	1	0	0	0	1	gnl|UG|At#S11742347	no match found	At1g06840.1

at5g11520	CON	MIN	HOUR	DAY	WEEK	UNIGENE	FLCDNA	TAIR
ACACCAAAGC	5	3	3	3	2	gnl|UG|At#S11707467	gi|15292662|gb|AY050765.1	At5g11520.1
at1g26120	CON	MIN	HOUR	DAY	WEEK	UNIGENE	FLCDNA	TAIR
TTGAAGTTGG	0	1	0	0	0	gnl|UG|At#S11709837	gi|20259110|gb|AY091332.1	At1g26120.1
at1g50460	CON	MIN	HOUR	DAY	WEEK	UNIGENE	FLCDNA	TAIR
AATCATCATC	0	1	0	1	0	gnl|UG|At#S11734336	gi|18377721|gb|AY074314.1	At1g50460.1
at4g11380	CON	MIN	HOUR	DAY	WEEK	UNIGENE	FLCDNA	TAIR
TTCTTTATAG	0	1	0	0	0	no match found	gi|34098844|gb|BT010362.1	non-canonical match
TTTGTGTTAC	2	1	1	1	1	no match found	gi|20260511|gb|AY093155.1	non-canonical match
at1g73720	CON	MIN	HOUR	DAY	WEEK	UNIGENE	FLCDNA	TAIR
AGAAGGATGT	1	0	0	0	0	gnl|UG|At#S11728304	gi|20857032|gb|AY102129.1	At1g73720.1
at2g29370	CON	MIN	HOUR	DAY	WEEK	UNIGENE	FLCDNA	TAIR
CCTTCTAATG	1	0	0	0	0	gnl|UG|At#S15460995	gi|19424098|gb|AY080866.1	At2g29370.1
at1g60760	CON	MIN	HOUR	DAY	WEEK	UNIGENE	FLCDNA	TAIR
AACAAACAGT	0	0	0	0	1	no match found	no match found	At1g60760.1
at4g05590	CON	MIN	HOUR	DAY	WEEK	UNIGENE	FLCDNA	TAIR
TCAAGGCTTG	2	1	3	0	0	gnl|UG|At#S28282094	gi|21404938|gb|AY086228.1	At4g05590.1
at4g10130	CON	MIN	HOUR	DAY	WEEK	UNIGENE	FLCDNA	TAIR
GTCTGGTTCG	0	1	2	1	0	gnl|UG|At#S11710231	gi|17529215|gb|AY065394.1	At4g10130.1
at1g21080	CON	MIN	HOUR	DAY	WEEK	UNIGENE	FLCDNA	TAIR
TTATCTCATC	2	1	2	2	5	gnl|UG|At#S11704791	gi|15028270|gb|AY046050.1	At1g21080.1
at3g53330	CON	MIN	HOUR	DAY	WEEK	UNIGENE	FLCDNA	TAIR
AAAGAATCAA	0	1	0	1	0	no match found	no match found	At3g53330.1
at5g39740	CON	MIN	HOUR	DAY	WEEK	UNIGENE	FLCDNA	TAIR
CCTCTAGTAG	13	14	11	11	13	gnl|UG|At#S11719758	gi|19310481|gb|AY079020.1	At5g39740.1
at5g20900	CON	MIN	HOUR	DAY	WEEK	UNIGENE	FLCDNA	TAIR
TCTGGGAGTG	8	11	6	4	4	gnl|UG|At#S18911081	gi|13430543|gb|AF360184.1	At5g20900.1
at2g26870	CON	MIN	HOUR	DAY	WEEK	UNIGENE	FLCDNA	TAIR
GGAGCGAATA	1	0	0	0	0	gnl|UG|At#S11735816	no match found	At2g26870.1
at4g14730	CON	MIN	HOUR	DAY	WEEK	UNIGENE	FLCDNA	TAIR
ATTTCTCCTT	0	0	1	0	0	gnl|UG|At#S18905370	no match found	At4g14730.1
at2g20060	CON	MIN	HOUR	DAY	WEEK	UNIGENE	FLCDNA	TAIR
TAGTATCTGT	0	0	1	0	1	gnl|UG|At#S11737457	gi|21404255|gb|AY085545.1	At2g20060.1
at3g27090	CON	MIN	HOUR	DAY	WEEK	UNIGENE	FLCDNA	TAIR
GTATCTTTGA	0	8	1	0	0	gnl|UG|At#S11732604	gi|21405735|gb|AY087011.1	At3g27090.1
at4g05180	CON	MIN	HOUR	DAY	WEEK	UNIGENE	FLCDNA	TAIR
TTCTGATTCT	6	2	7	3	0	gnl|UG|At#S11726339	no match found	At4g05180.1
at3g06250	CON	MIN	HOUR	DAY	WEEK	UNIGENE	FLCDNA	TAIR
CGAGAGGGTG	0	0	1	1	0	no match found	gi|23297524|gb|AY150446.1	non-canonical match
TAAAATCGTT	1	0	3	0	0	gnl|UG|At#S11702105	gi|13430715|gb|AF360270.1	At3g06250.1
at5g19990	CON	MIN	HOUR	DAY	WEEK	UNIGENE	FLCDNA	TAIR
AAAACATTTT	5	3	1	2	4	gnl|UG|At#S11721327	gi|17473853|gb|AY065174.1	At5g19990.1

TAATTAATAA	0	0	0	0	1	no match found	gi|110741172|dbj|AK230338.1|	At5g43600.1
at1g74730	CON	MIN	HOUR	DAY	WEEK	UNIGENE	FLCDNA	TAIR
TCTTGTCCAA	19	4	14	15	10	gnl|UG|At#S11728092	gi|25084055|gb|BT002152.1	multiple non-canonical match
at4g13660	CON	MIN	HOUR	DAY	WEEK	UNIGENE	FLCDNA	TAIR
GCCTTTTGTT	0	3	0	0	0	no match found	gi|27754502|gb|BT002882.1	At4g13660.1
at1g58180	CON	MIN	HOUR	DAY	WEEK	UNIGENE	FLCDNA	TAIR
GATGTTATTA	4	5	0	0	1	gnl|UG|At#S28282474	no match found	At1g58180.2
GATAAGAGAT	1	0	0	0	0	no match found	gi|21539554|gb|AY120772.1	At1g58180.1
at3g25940	CON	MIN	HOUR	DAY	WEEK	UNIGENE	FLCDNA	TAIR
TAGTGTTCTT	0	1	0	0	4	no match found	no match found	At3g25940.1
at3g07170	CON	MIN	HOUR	DAY	WEEK	UNIGENE	FLCDNA	TAIR
GTGAAACATA	0	1	2	0	3	no match found	gi|15294217|gb|AF410300.1	At3g07170.1
at1g10940	CON	MIN	HOUR	DAY	WEEK	UNIGENE	FLCDNA	TAIR
AAGATAGTGG	1	2	0	1	0	no match found	gi|31711865|gb|BT008850.1	non-canonical match
GTACTATGAA	0	0	2	1	1	no match found	gi|20260461|gb|AY093130.1	At1g10940.1
at3g11510	CON	MIN	HOUR	DAY	WEEK	UNIGENE	FLCDNA	TAIR
AAGATTGGTC	15	10	11	8	9	gnl|UG|At#S11737432	gi|110740919|dbj|AK226410.1|	At3g11510.1
at1g23190	CON	MIN	HOUR	DAY	WEEK	UNIGENE	FLCDNA	TAIR
GATGTCTATA	0	0	0	0	1	no match found	gi|20148520|gb|AY081589.1	non-canonical match
CTACGACCAT	1	0	0	0	0	gnl|UG|At#S11740743	no match found	At1g23190.1
at4g00330	CON	MIN	HOUR	DAY	WEEK	UNIGENE	FLCDNA	TAIR
AAAAAATGCA	1	2	4	2	0	gnl|UG|At#S11727595	gi|22136737|gb|AY133754.1	At4g00330.1
at1g78890	CON	MIN	HOUR	DAY	WEEK	UNIGENE	FLCDNA	TAIR
GTTTAGACTT	3	3	0	1	1	no match found	gi|110738173|dbj|AK229144.1|	At1g78890.1
at1g51100	CON	MIN	HOUR	DAY	WEEK	UNIGENE	FLCDNA	TAIR
CTTTTGGTTT	0	0	1	0	1	gnl|UG|At#S34117431	gi|110737656|dbj|AK228877.1|	non-canonical match
at1g12330	CON	MIN	HOUR	DAY	WEEK	UNIGENE	FLCDNA	TAIR
GAGGAAACCG	1	0	2	0	0	gnl|UG|At#S11741803	gi|62318595|dbj|AK220618.1|	At1g12330.1
at3g42160	CON	MIN	HOUR	DAY	WEEK	UNIGENE	FLCDNA	TAIR
AAGAGAAGCT	0	1	0	0	0	gnl|UG|At#S11731098	no match found	multiple canonical match
at3g48650	CON	MIN	HOUR	DAY	WEEK	UNIGENE	FLCDNA	TAIR
TTTGTTGTGT	2	1	0	1	2	no match found	gi|26451398|dbj|AK118176.1|	multiple canonical match
at3g17700	CON	MIN	HOUR	DAY	WEEK	UNIGENE	FLCDNA	TAIR
TTGTTGGTTC	0	2	0	0	0	gnl|UG|At#S11701221	no match found	At3g17700.1
at4g31440	CON	MIN	HOUR	DAY	WEEK	UNIGENE	FLCDNA	TAIR
TAATAGTTGC	0	2	0	0	0	no match found	no match found	At4g31440.1
at4g20300	CON	MIN	HOUR	DAY	WEEK	UNIGENE	FLCDNA	TAIR
CACAACCACC	0	1	0	0	0	no match found	no match found	At4g20300.1
GAGAATATGG	0	3	1	0	1	gnl|UG|At#S11724283	gi|21404550|gb|AY085840.1	At4g20300.2
at3g13772	CON	MIN	HOUR	DAY	WEEK	UNIGENE	FLCDNA	TAIR
CCTTTTTCTG	3	5	3	0	3	gnl|UG|At#S11736723	gi|110737752|dbj|AK228926.1|	At3g13772.1
at3g22310	CON	MIN	HOUR	DAY	WEEK	UNIGENE	FLCDNA	TAIR

TTTGAAGGAG	2	0	2	1	1	gnl|UG|At#S11806662	gi|20268675|gb|AY091091.1	At3g22310.1
at5g16720	CON	MIN	HOUR	DAY	WEEK	UNIGENE	FLCDNA	TAIR
GAGGACTAAC	0	1	0	0	0	gnl|UG|At#S34116986	gi|110738518|dbj|AK229322.1|	At5g16720.1
at3g07870	CON	MIN	HOUR	DAY	WEEK	UNIGENE	FLCDNA	TAIR
TCTTCGGTAA	3	2	2	0	1	gnl|UG|At#S11738394	no match found	At3g07870.1
CTGGAACATT	1	1	0	0	0	no match found	gi|15983478|gb|AF424614.1	non-canonical match
at4g13520	CON	MIN	HOUR	DAY	WEEK	UNIGENE	FLCDNA	TAIR
GAGATACTCA	6	7	5	2	0	gnl|UG|At#S11725468	gi|13605761|gb|AF361862.1	At4g13520.1
at2g46070	CON	MIN	HOUR	DAY	WEEK	UNIGENE	FLCDNA	TAIR
TTTGAATGAT	1	1	1	1	1	gnl|UG|At#S11731171	no match found	At2g46070.1
at4g17650	CON	MIN	HOUR	DAY	WEEK	UNIGENE	FLCDNA	TAIR
TTTTGTTTTC	0	0	0	2	0	gnl|UG|At#S11724735	gi|13878164|gb|AF370345.1	At4g17650.1
at2g05920	CON	MIN	HOUR	DAY	WEEK	UNIGENE	FLCDNA	TAIR
AGCCATAAGT	6	2	3	2	1	gnl|UG|At#S11739952	gi|14334833|gb|AY035090.1	At2g05920.1
at3g12977	CON	MIN	HOUR	DAY	WEEK	UNIGENE	FLCDNA	TAIR
ATTCGAAACA	0	0	1	0	0	gnl|UG|At#S11832259	no match found	pseudo chromosome match
at4g16980	CON	MIN	HOUR	DAY	WEEK	UNIGENE	FLCDNA	TAIR
CGTTGTTGGA	51	35	36	36	9	gnl|UG|At#S35284464	gi|21405704|gb|AY086980.1	At4g16980.1
at4g38470	CON	MIN	HOUR	DAY	WEEK	UNIGENE	FLCDNA	TAIR
ATTCGACGCA	3	12	3	0	0	gnl|UG|At#S11721191	gi|62319532|dbj|AK221091.1|	At4g38470.1
at3g18240	CON	MIN	HOUR	DAY	WEEK	UNIGENE	FLCDNA	TAIR
AACCTTGGCT	1	0	0	2	2	gnl|UG|At#S11735371	gi|11908119|gb|AF326907.1	At3g18240.1
at1g73910	CON	MIN	HOUR	DAY	WEEK	UNIGENE	FLCDNA	TAIR
GAGCTTCTTA	0	1	0	0	0	no match found	no match found	At1g73910.1
at5g45740	CON	MIN	HOUR	DAY	WEEK	UNIGENE	FLCDNA	TAIR
CGTGAGGAAT	0	0	0	0	2	gnl|UG|At#S11719154	gi|124301037|gb|BT030033.1	At5g45740.1
at4g30220	CON	MIN	HOUR	DAY	WEEK	UNIGENE	FLCDNA	TAIR
AACTTGCAGC	2	6	4	2	1	gnl|UG|At#S30432195	gi|30102543|gb|BT006382.1	At4g30220.1
at4g00990	CON	MIN	HOUR	DAY	WEEK	UNIGENE	FLCDNA	TAIR
ATTACCGAGT	1	0	1	0	0	no match found	gi|18176334|gb|AY072204.1	non-canonical match
at1g32070	CON	MIN	HOUR	DAY	WEEK	UNIGENE	FLCDNA	TAIR
AGTATGATCA	4	2	2	1	1	gnl|UG|At#S18942456	no match found	At1g32070.2
TTTTGGTACC	3	1	0	1	0	no match found	gi|21404893|gb|AY086183.1	At1g32070.1
at4g08685	CON	MIN	HOUR	DAY	WEEK	UNIGENE	FLCDNA	TAIR
AAGCTATACC	5	0	3	1	0	gnl|UG|At#S34117754	gi|21280950|gb|AY114069.1	non-canonical match
at5g15120	CON	MIN	HOUR	DAY	WEEK	UNIGENE	FLCDNA	TAIR
GCTGCAAGAA	0	1	0	0	0	gnl|UG|At#S18911336	gi|30725347|gb|BT008337.1	At5g15120.1
at1g17200	CON	MIN	HOUR	DAY	WEEK	UNIGENE	FLCDNA	TAIR
TTTCAAATGT	4	6	1	4	4	gnl|UG|At#S11741324	gi|21405536|gb|AY086826.1	At1g17200.1
at4g21650	CON	MIN	HOUR	DAY	WEEK	UNIGENE	FLCDNA	TAIR
ATGTTATAAT	0	1	2	0	0	no match found	gi|16649028|gb|AY059884.1	At4g21650.1

at4g01730	CON	MIN	HOUR	DAY	WEEK	UNIGENE	FLCDNA	TAIR
AGATGCAAAA	2	1	1	0	0	no match found	no match found	At4g01730.1
at2g43700	CON	MIN	HOUR	DAY	WEEK	UNIGENE	FLCDNA	TAIR
TTTGTTTGTA	1	0	0	0	0	gnl|UG|At#S18897953	no match found	multiple non-canonical match
at5g51545	CON	MIN	HOUR	DAY	WEEK	UNIGENE	FLCDNA	TAIR
GATATTTGAG	1	1	1	1	2	gnl|UG|At#S28281631	gi|110738466|dbj|AK229296.1|	non-canonical match
at1g40124	CON	MIN	HOUR	DAY	WEEK	UNIGENE	FLCDNA	TAIR
TTGTGTTCAA	0	0	1	0	0	no match found	no match found	At1g40124.1
at5g53850	CON	MIN	HOUR	DAY	WEEK	UNIGENE	FLCDNA	TAIR
GGTTCAAGAC	1	4	0	0	1	gnl|UG|At#S18941480	gi|110738253|dbj|AK229186.1|	At5g53850.1
at3g13200	CON	MIN	HOUR	DAY	WEEK	UNIGENE	FLCDNA	TAIR
AAGTGATTGT	6	7	2	5	1	gnl|UG|At#S11736929	gi|21404944|gb|AY086234.1	At3g13200.1
at5g59920	CON	MIN	HOUR	DAY	WEEK	UNIGENE	FLCDNA	TAIR
TTTATCGAGT	1	0	1	1	2	gnl|UG|At#S11717710	no match found	non-canonical match
at1g06180	CON	MIN	HOUR	DAY	WEEK	UNIGENE	FLCDNA	TAIR
AATAAAAGTC	2	1	2	5	1	gnl|UG|At#S11742409	no match found	At1g06180.1
at4g02770	CON	MIN	HOUR	DAY	WEEK	UNIGENE	FLCDNA	TAIR
TGGTGATGAT	20	5	12	6	1	no match found	gi|20148684|gb|AY081671.1	multiple non-canonical match
AGATCTATTG	4	2	7	0	0	no match found	gi|21281090|gb|AY113939.1	non-canonical match
TTAATTTTTA	204	131	147	128	54	gnl|UG|At#S11726994	gi|13265444|gb|AF324675.2	At4g02770.1
AGAGAAGGTC	1	0	1	0	0	gnl|UG|At#S30579843	no match found	non-canonical match
at5g48060	CON	MIN	HOUR	DAY	WEEK	UNIGENE	FLCDNA	TAIR
TCATAAGGTT	0	1	0	1	0	gnl|UG|At#S11718915	no match found	At5g48060.1
at3g17710	CON	MIN	HOUR	DAY	WEEK	UNIGENE	FLCDNA	TAIR
TGTTGTGGCG	1	0	0	0	0	gnl|UG|At#S11735533	gi|21406736|gb|AY087962.1	At3g17710.1
at4g19530	CON	MIN	HOUR	DAY	WEEK	UNIGENE	FLCDNA	TAIR
AAGCACAATC	2	4	0	2	0	gnl|UG|At#S11724413	no match found	At4g19530.1
at4g39820	CON	MIN	HOUR	DAY	WEEK	UNIGENE	FLCDNA	TAIR
TATTTTGAGT	1	1	1	0	0	gnl|UG|At#S11720952	no match found	At4g39820.1
at5g16800	CON	MIN	HOUR	DAY	WEEK	UNIGENE	FLCDNA	TAIR
GCTGCATTGC	1	0	0	0	0	gnl|UG|At#S11722139	no match found	At5g16800.1
at5g24500	CON	MIN	HOUR	DAY	WEEK	UNIGENE	FLCDNA	TAIR
ATGAACCAGA	0	1	0	0	0	gnl|UG|At#S14829875	gi|110736860|dbj|AK228461.1|	At5g24500.1
at1g74940	CON	MIN	HOUR	DAY	WEEK	UNIGENE	FLCDNA	TAIR
TACAAAGGAG	4	7	2	2	0	no match found	gi|23306419|gb|BT000460.1	At1g74940.1
at2g37790	CON	MIN	HOUR	DAY	WEEK	UNIGENE	FLCDNA	TAIR
AGACGAGTCC	1	3	2	0	0	gnl|UG|At#S11733151	gi|110742936|dbj|AK227354.1|	At2g37790.1
at5g47360	CON	MIN	HOUR	DAY	WEEK	UNIGENE	FLCDNA	TAIR
AGTCGTTTCT	0	1	0	0	1	gnl|UG|At#S11718988	no match found	non-canonical match
at3g25190	CON	MIN	HOUR	DAY	WEEK	UNIGENE	FLCDNA	TAIR
CATCACAACT	1	1	0	0	1	gnl|UG|At#S11703182	gi|14030610|gb|AF375396.1	At3g25190.1
at4g00170	CON	MIN	HOUR	DAY	WEEK	UNIGENE	FLCDNA	TAIR

GGGTTAGCTT	0	1	0	0	0	gnl|UG|At#S11727629	gi|15292934|gb|AY050901.1	At4g00170.1
at3g22237	CON	MIN	HOUR	DAY	WEEK	UNIGENE	FLCDNA	TAIR
GTATCCAAGT	1	1	0	0	0	gnl|UG|At#S17865569	gi|38603907|gb|BT010921.1	At3g22237.1
at3g56630	CON	MIN	HOUR	DAY	WEEK	UNIGENE	FLCDNA	TAIR
ACCATATTTT	3	2	0	1	1	gnl|UG|At#S11728214	no match found	At3g56630.1
at4g22200	CON	MIN	HOUR	DAY	WEEK	UNIGENE	FLCDNA	TAIR
GTGAATCTCC	1	0	0	1	1	gnl|UG|At#S11723969	gi|110739318|dbj|AK229735.1|	At4g22200.1
at3g55610	CON	MIN	HOUR	DAY	WEEK	UNIGENE	FLCDNA	TAIR
TCCACAACCT	0	1	1	2	1	no match found	gi|20147196|gb|AY091766.1	multiple non-canonical match
CAGAATAAGT	6	3	14	16	9	gnl|UG|At#S11728401	gi|62319127|dbj|AK220885.1|	At3g55610.1
at3g54130	CON	MIN	HOUR	DAY	WEEK	UNIGENE	FLCDNA	TAIR
TCGTCTTCTT	0	1	0	0	0	gnl|UG|At#S11728662	gi|38564305|gb|BT010762.1	At3g54130.1
at4g20850	CON	MIN	HOUR	DAY	WEEK	UNIGENE	FLCDNA	TAIR
GCGAGAATCG	0	1	3	2	0	gnl|UG|At#S11724201	gi|20465633|gb|AY096651.1	At4g20850.1
at5g66000	CON	MIN	HOUR	DAY	WEEK	UNIGENE	FLCDNA	TAIR
GAGTTAGATA	0	0	1	0	0	gnl|UG|At#S11704669	gi|15028020|gb|AY045867.1	multiple non-canonical match
at1g45020	CON	MIN	HOUR	DAY	WEEK	UNIGENE	FLCDNA	TAIR
AGCAGATCGC	1	0	0	0	0	no match found	no match found	At1g45020.1
at5g15930	CON	MIN	HOUR	DAY	WEEK	UNIGENE	FLCDNA	TAIR
GATCCTGATA	0	0	1	0	0	gnl|UG|At#S11722354	gi|21406691|gb|AY087917.1	At5g15930.1
at1g08910	CON	MIN	HOUR	DAY	WEEK	UNIGENE	FLCDNA	TAIR
TGAAACATTA	3	0	1	0	1	gnl|UG|At#S11742145	no match found	At1g08910.1
at2g36030	CON	MIN	HOUR	DAY	WEEK	UNIGENE	FLCDNA	TAIR
TGGTGACTGA	0	0	0	1	0	no match found	no match found	At2g36030.1
at5g11910	CON	MIN	HOUR	DAY	WEEK	UNIGENE	FLCDNA	TAIR
AGATCAGTTG	2	3	2	0	2	gnl|UG|At#S11723270	gi|89274136|gb|BT024858.1	At5g11910.1
at3g05000	CON	MIN	HOUR	DAY	WEEK	UNIGENE	FLCDNA	TAIR
TTTGAAATGA	2	1	0	1	1	gnl|UG|At#S11739221	gi|27754553|gb|BT002908.1	At3g05000.1
at3g24320	CON	MIN	HOUR	DAY	WEEK	UNIGENE	FLCDNA	TAIR
CAGTTCGGGT	0	0	1	0	0	gnl|UG|At#S11733476	no match found	At3g24320.1
at1g69890	CON	MIN	HOUR	DAY	WEEK	UNIGENE	FLCDNA	TAIR
TCATTCTAGT	0	1	1	0	0	gnl|UG|At#S11729125	gi|21406115|gb|AY087391.1	At1g69890.1
at3g06950	CON	MIN	HOUR	DAY	WEEK	UNIGENE	FLCDNA	TAIR
GTCTTTGTCT	1	1	0	0	0	gnl|UG|At#S11738643	gi|110737768|dbj|AK228934.1|	At3g06950.1
at1g04590	CON	MIN	HOUR	DAY	WEEK	UNIGENE	FLCDNA	TAIR
GACTGTAAGG	0	3	1	0	0	gnl|UG|At#S11702072	gi|13430645|gb|AF360235.1	At1g04590.2
at4g13050	CON	MIN	HOUR	DAY	WEEK	UNIGENE	FLCDNA	TAIR
TGTTTTTTTG	4	0	1	2	0	gnl|UG|At#S11725556	gi|26452269|dbj|AK118624.1|	At4g13050.1
at4g20365	CON	MIN	HOUR	DAY	WEEK	UNIGENE	FLCDNA	TAIR
ATGTGAAAAT	0	0	1	0	0	no match found	no match found	At4g20365.1
at2g25640	CON	MIN	HOUR	DAY	WEEK	UNIGENE	FLCDNA	TAIR

<table>
<tr><td>TCATCCATAG</td><td>0</td><td>0</td><td>0</td><td>1</td><td>0</td><td>gnl|UG|At#S21737310</td><td>gi|51968871|dbj|AK175365.1|</td><td>multiple non-canonical match</td></tr>
<tr><td>at4g14220</td><td>CON</td><td>MIN</td><td>HOUR</td><td>DAY</td><td>WEEK</td><td>UNIGENE</td><td>FLCDNA</td><td>TAIR</td></tr>
<tr><td>TATCAACCTC</td><td>0</td><td>1</td><td>0</td><td>0</td><td>1</td><td>gnl|UG|At#S11705361</td><td>gi|26450996|dbj|AK117968.1|</td><td>At4g14220.1</td></tr>
<tr><td>at2g19520</td><td>CON</td><td>MIN</td><td>HOUR</td><td>DAY</td><td>WEEK</td><td>UNIGENE</td><td>FLCDNA</td><td>TAIR</td></tr>
<tr><td>ATCCTGTTTC</td><td>2</td><td>0</td><td>2</td><td>1</td><td>3</td><td>gnl|UG|At#S11737599</td><td>gi|16323102|gb|AY057655.1</td><td>At2g19520.1</td></tr>
<tr><td>TTATGACTTG</td><td>0</td><td>0</td><td>1</td><td>0</td><td>0</td><td>no match found</td><td>gi|20148236|gb|AY081447.1</td><td>non-canonical match</td></tr>
<tr><td>at5g52900</td><td>CON</td><td>MIN</td><td>HOUR</td><td>DAY</td><td>WEEK</td><td>UNIGENE</td><td>FLCDNA</td><td>TAIR</td></tr>
<tr><td>TAAGTTAAAT</td><td>4</td><td>1</td><td>0</td><td>2</td><td>0</td><td>no match found</td><td>gi|21404081|gb|AY085371.1</td><td>multiple non-canonical match</td></tr>
<tr><td>TTTACTTTTA</td><td>2</td><td>0</td><td>0</td><td>1</td><td>0</td><td>gnl|UG|At#S34117753</td><td>gi|28973706|gb|BT005766.1</td><td>At5g52900.1</td></tr>
<tr><td>GAAGAGGAGC</td><td>0</td><td>1</td><td>0</td><td>0</td><td>0</td><td>no match found</td><td>gi|29824226|gb|BT006089.1</td><td>non-canonical match</td></tr>
<tr><td>at5g61380</td><td>CON</td><td>MIN</td><td>HOUR</td><td>DAY</td><td>WEEK</td><td>UNIGENE</td><td>FLCDNA</td><td>TAIR</td></tr>
<tr><td>TTCACTCACA</td><td>0</td><td>1</td><td>5</td><td>1</td><td>2</td><td>gnl|UG|At#S11717564</td><td>gi|20453053|gb|AY094393.1</td><td>At5g61380.1</td></tr>
<tr><td>at1g69250</td><td>CON</td><td>MIN</td><td>HOUR</td><td>DAY</td><td>WEEK</td><td>UNIGENE</td><td>FLCDNA</td><td>TAIR</td></tr>
<tr><td>CTTGCAGACC</td><td>1</td><td>2</td><td>0</td><td>0</td><td>0</td><td>gnl|UG|At#S18942295</td><td>gi|30017246|gb|BT006208.1</td><td>At1g69250.2</td></tr>
<tr><td>at5g60460</td><td>CON</td><td>MIN</td><td>HOUR</td><td>DAY</td><td>WEEK</td><td>UNIGENE</td><td>FLCDNA</td><td>TAIR</td></tr>
<tr><td>TAAATCGCTA</td><td>6</td><td>5</td><td>2</td><td>0</td><td>1</td><td>gnl|UG|At#S20294428</td><td>gi|26451289|dbj|AK118120.1|</td><td>At5g60460.1</td></tr>
<tr><td>at5g45130</td><td>CON</td><td>MIN</td><td>HOUR</td><td>DAY</td><td>WEEK</td><td>UNIGENE</td><td>FLCDNA</td><td>TAIR</td></tr>
<tr><td>ATGATACTTA</td><td>2</td><td>2</td><td>1</td><td>2</td><td>3</td><td>gnl|UG|At#S11719214</td><td>no match found</td><td>At5g45130.1</td></tr>
<tr><td>at2g45400</td><td>CON</td><td>MIN</td><td>HOUR</td><td>DAY</td><td>WEEK</td><td>UNIGENE</td><td>FLCDNA</td><td>TAIR</td></tr>
<tr><td>TTCTAGTTAC</td><td>0</td><td>0</td><td>1</td><td>0</td><td>0</td><td>gnl|UG|At#S11731323</td><td>no match found</td><td>At2g45400.1</td></tr>
<tr><td>at1g10650</td><td>CON</td><td>MIN</td><td>HOUR</td><td>DAY</td><td>WEEK</td><td>UNIGENE</td><td>FLCDNA</td><td>TAIR</td></tr>
<tr><td>TCGGCACTTG</td><td>3</td><td>2</td><td>1</td><td>6</td><td>0</td><td>gnl|UG|At#S43850666</td><td>gi|27754498|gb|BT002880.1</td><td>At1g10650.1</td></tr>
<tr><td>at5g15500</td><td>CON</td><td>MIN</td><td>HOUR</td><td>DAY</td><td>WEEK</td><td>UNIGENE</td><td>FLCDNA</td><td>TAIR</td></tr>
<tr><td>GTGGAAAACG</td><td>0</td><td>0</td><td>1</td><td>0</td><td>0</td><td>no match found</td><td>gi|28951038|gb|BT005379.1</td><td>non-canonical match</td></tr>
<tr><td>at2g44790</td><td>CON</td><td>MIN</td><td>HOUR</td><td>DAY</td><td>WEEK</td><td>UNIGENE</td><td>FLCDNA</td><td>TAIR</td></tr>
<tr><td>AAGCTAGCCG</td><td>0</td><td>1</td><td>0</td><td>1</td><td>0</td><td>gnl|UG|At#S30610330</td><td>gi|20147336|gb|AY093758.1</td><td>multiple non-canonical match</td></tr>
<tr><td>TGGAACCACT</td><td>0</td><td>1</td><td>0</td><td>1</td><td>0</td><td>gnl|UG|At#S11731457</td><td>no match found</td><td>At2g44790.1</td></tr>
<tr><td>at2g25280</td><td>CON</td><td>MIN</td><td>HOUR</td><td>DAY</td><td>WEEK</td><td>UNIGENE</td><td>FLCDNA</td><td>TAIR</td></tr>
<tr><td>AACTAAAGAT</td><td>0</td><td>1</td><td>0</td><td>0</td><td>1</td><td>no match found</td><td>gi|21405150|gb|AY086440.1</td><td>multiple non-canonical match</td></tr>
<tr><td>at3g52940</td><td>CON</td><td>MIN</td><td>HOUR</td><td>DAY</td><td>WEEK</td><td>UNIGENE</td><td>FLCDNA</td><td>TAIR</td></tr>
<tr><td>AATCCTTTCA</td><td>0</td><td>1</td><td>0</td><td>1</td><td>0</td><td>gnl|UG|At#S18903072</td><td>gi|110742631|dbj|AK227189.1|</td><td>At3g52940.1</td></tr>
<tr><td>at1g74680</td><td>CON</td><td>MIN</td><td>HOUR</td><td>DAY</td><td>WEEK</td><td>UNIGENE</td><td>FLCDNA</td><td>TAIR</td></tr>
<tr><td>TCTGTTTTCT</td><td>0</td><td>0</td><td>1</td><td>1</td><td>0</td><td>gnl|UG|At#S11728101</td><td>gi|16209708|gb|AY057616.1</td><td>At1g74680.1</td></tr>
<tr><td>at4g15080</td><td>CON</td><td>MIN</td><td>HOUR</td><td>DAY</td><td>WEEK</td><td>UNIGENE</td><td>FLCDNA</td><td>TAIR</td></tr>
<tr><td>TCTGCTATTT</td><td>1</td><td>1</td><td>0</td><td>1</td><td>2</td><td>gnl|UG|At#S11725189</td><td>gi|20466787|gb|AY099860.1</td><td>At4g15080.1</td></tr>
<tr><td>at4g27130</td><td>CON</td><td>MIN</td><td>HOUR</td><td>DAY</td><td>WEEK</td><td>UNIGENE</td><td>FLCDNA</td><td>TAIR</td></tr>
<tr><td>TTACTTCTGT</td><td>4</td><td>12</td><td>2</td><td>3</td><td>4</td><td>gnl|UG|At#S11723107</td><td>gi|14335109|gb|AY037234.1</td><td>At4g27130.1</td></tr>
<tr><td>at5g38700</td><td>CON</td><td>MIN</td><td>HOUR</td><td>DAY</td><td>WEEK</td><td>UNIGENE</td><td>FLCDNA</td><td>TAIR</td></tr>
<tr><td>AGGTTCCTCT</td><td>0</td><td>1</td><td>0</td><td>0</td><td>0</td><td>gnl|UG|At#S24443475</td><td>gi|62318736|dbj|AK220688.1|</td><td>non-canonical match</td></tr>
<tr><td>at5g06410</td><td>CON</td><td>MIN</td><td>HOUR</td><td>DAY</td><td>WEEK</td><td>UNIGENE</td><td>FLCDNA</td><td>TAIR</td></tr>
<tr><td>TTTTAGTTTA</td><td>0</td><td>1</td><td>0</td><td>0</td><td>0</td><td>gnl|UG|At#S11724524</td><td>gi|22136055|gb|AY128407.1</td><td>At5g06410.1</td></tr>
<tr><td>at1g55080</td><td>CON</td><td>MIN</td><td>HOUR</td><td>DAY</td><td>WEEK</td><td>UNIGENE</td><td>FLCDNA</td><td>TAIR</td></tr>
</table>

ACCAGCAACT	0	0	0	0	1	gnl|UG|At#S34114895	gi|30023711|gb|BT006281.1	At1g55080.1
at4g15780	CON	MIN	HOUR	DAY	WEEK	UNIGENE	FLCDNA	TAIR
GATTTAATTG	0	2	1	0	0	gnl|UG|At#S11725068	gi|50198885|gb|BT015012.1	At4g15780.1
at5g60710	CON	MIN	HOUR	DAY	WEEK	UNIGENE	FLCDNA	TAIR
ACTTTCGGAA	0	5	0	0	0	gnl|UG|At#S11717631	gi|21539496|gb|AY120743.1	At5g60710.1
at3g06480	CON	MIN	HOUR	DAY	WEEK	UNIGENE	FLCDNA	TAIR
GAGAATTAGT	1	2	0	1	0	gnl|UG|At#S11738804	no match found	At3g06480.1
at1g69610	CON	MIN	HOUR	DAY	WEEK	UNIGENE	FLCDNA	TAIR
TCGAAACTTA	1	0	0	0	0	gnl|UG|At#S11729184	no match found	At1g69610.1
at4g00060	CON	MIN	HOUR	DAY	WEEK	UNIGENE	FLCDNA	TAIR
CACTTTAGAA	1	1	1	0	0	gnl|UG|At#S11727650	gi|19699088|gb|AY090249.1	At4g00060.1
at1g53035	CON	MIN	HOUR	DAY	WEEK	UNIGENE	FLCDNA	TAIR
GTTTCGTTTG	0	0	0	0	1	gnl|UG|At#S11733349	gi|26453131|dbj|AK119066.1|	At1g53035.1
at2g28230	CON	MIN	HOUR	DAY	WEEK	UNIGENE	FLCDNA	TAIR
TTTATCTATA	1	0	0	0	0	no match found	gi|38454105|gb|BT010690.1	multiple canonical match
at3g53900	CON	MIN	HOUR	DAY	WEEK	UNIGENE	FLCDNA	TAIR
TATATGCTGG	1	1	0	0	0	gnl|UG|At#S11728708	gi|21403466|gb|AY084756.1	At3g53900.1
at1g30680	CON	MIN	HOUR	DAY	WEEK	UNIGENE	FLCDNA	TAIR
GTGTTTCTCT	0	0	2	0	7	gnl|UG|At#S15460206	no match found	At1g30680.1
at1g43580	CON	MIN	HOUR	DAY	WEEK	UNIGENE	FLCDNA	TAIR
TATAGTGGTC	0	1	0	1	0	gnl|UG|At#S11736083	gi|19699333|gb|AY090374.1	At1g43580.1
at2g33980	CON	MIN	HOUR	DAY	WEEK	UNIGENE	FLCDNA	TAIR
TATGAAGTGA	1	1	0	0	0	gnl|UG|At#S34116713	gi|110739049|dbj|AK229595.1|	non-canonical match
CTACAACTTA	1	0	0	0	0	no match found	gi|13430755|gb|AF360290.1	At2g33980.1
at3g23490	CON	MIN	HOUR	DAY	WEEK	UNIGENE	FLCDNA	TAIR
TCGGCGATAG	0	0	1	0	0	no match found	gi|20147236|gb|AY093708.1	non-canonical match
AAGGCCTTAT	1	2	1	1	0	no match found	gi|15294213|gb|AF410298.1	non-canonical match
at4g23730	CON	MIN	HOUR	DAY	WEEK	UNIGENE	FLCDNA	TAIR
TTGTGTGTGG	1	0	0	1	0	gnl|UG|At#S11723694	gi|105830213|gb|BT025656.1	At4g23730.1
at4g19850	CON	MIN	HOUR	DAY	WEEK	UNIGENE	FLCDNA	TAIR
GAATTTCTCA	0	1	0	0	0	no match found	no match found	At4g19850.1
at5g25840	CON	MIN	HOUR	DAY	WEEK	UNIGENE	FLCDNA	TAIR
CAGACGGAGA	2	8	8	5	3	gnl|UG|At#S11720607	gi|21405558|gb|AY086848.1	At5g25840.1
at3g15890	CON	MIN	HOUR	DAY	WEEK	UNIGENE	FLCDNA	TAIR
TTGCAGAGGA	0	1	0	0	0	gnl|UG|At#S11736080	gi|21407135|gb|AY088361.1	At3g15890.1
at5g54650	CON	MIN	HOUR	DAY	WEEK	UNIGENE	FLCDNA	TAIR
TCAGGTTTTA	1	0	0	0	0	gnl|UG|At#S11704324	gi|14596026|gb|AY042801.1	At5g54650.1
at1g07135	CON	MIN	HOUR	DAY	WEEK	UNIGENE	FLCDNA	TAIR
AATCTTCGAG	0	6	4	0	0	gnl|UG|At#S21735843	gi|51968433|dbj|AK175146.1|	At1g07135.1
at4g03060	CON	MIN	HOUR	DAY	WEEK	UNIGENE	FLCDNA	TAIR
TACAATAATG	2	0	0	0	0	no match found	no match found	At4g03060.1

at3g12320	CON	MIN	HOUR	DAY	WEEK	UNIGENE	FLCDNA	TAIR
TTAGATTCAA	2	2	0	0	0	gnl|UG|At#S11737197	gi|15293280|gb|AY051074.1	At3g12320.1
at2g45190	CON	MIN	HOUR	DAY	WEEK	UNIGENE	FLCDNA	TAIR
TGTCTTTTTC	4	1	4	6	3	gnl|UG|At#S34117406	gi|110737704|dbj|AK228902.1|	At2g45190.1
at2g18440	CON	MIN	HOUR	DAY	WEEK	UNIGENE	FLCDNA	TAIR
AAATTTTAAT	0	1	4	2	1	gnl|UG|At#S34114999	gi|51970127|dbj|AK175993.1|	At2g18440.1
GCAGGTCAAA	2	0	0	5	1	no match found	gi|51971910|dbj|AK176857.1|	multiple non-canonical match
at2g07040	CON	MIN	HOUR	DAY	WEEK	UNIGENE	FLCDNA	TAIR
AAAAGTAAAT	1	0	0	0	0	no match found	gi|28827613|gb|BT005118.1	multiple non-canonical match
at3g01060	CON	MIN	HOUR	DAY	WEEK	UNIGENE	FLCDNA	TAIR
TTGGATAAAG	2	1	6	2	7	gnl|UG|At#S11740347	gi|13878026|gb|AF370276.1	At3g01060.3
at3g54360	CON	MIN	HOUR	DAY	WEEK	UNIGENE	FLCDNA	TAIR
AGACTGCTGG	0	0	0	1	0	no match found	gi|21404775|gb|AY086065.1	non-canonical match
ATTGTACTCT	3	1	1	0	1	gnl|UG|At#S11728621	no match found	At3g54360.1
at1g04985	CON	MIN	HOUR	DAY	WEEK	UNIGENE	FLCDNA	TAIR
GGTTTTGTCT	0	2	2	0	1	gnl|UG|At#S18897740	gi|27754676|gb|BT002973.1	At1g04985.1
at5g57625	CON	MIN	HOUR	DAY	WEEK	UNIGENE	FLCDNA	TAIR
GATCGTTGAA	0	2	0	0	0	no match found	gi|30023647|gb|BT006249.1	multiple non-canonical match
at1g69260	CON	MIN	HOUR	DAY	WEEK	UNIGENE	FLCDNA	TAIR
TAGATCATCC	0	1	0	0	1	gnl|UG|At#S11729254	gi|21407608|gb|AY088834.1	At1g69260.1
at5g19350	CON	MIN	HOUR	DAY	WEEK	UNIGENE	FLCDNA	TAIR
GTCAATGATA	11	4	1	3	6	gnl|UG|At#S11721484	gi|18377730|gb|AY074319.1	At5g19350.1
at1g08980	CON	MIN	HOUR	DAY	WEEK	UNIGENE	FLCDNA	TAIR
GATCTCGTCG	0	0	0	0	1	no match found	gi|28416538|gb|BT004554.1	non-canonical match
TCATAAACTT	1	9	1	1	2	gnl|UG|At#S11742139	gi|14335037|gb|AY037198.1	At1g08980.1
at2g45470	CON	MIN	HOUR	DAY	WEEK	UNIGENE	FLCDNA	TAIR
GGATTAGTGA	21	13	24	20	11	gnl|UG|At#S18899783	gi|62319040|dbj|AK220841.1|	At2g45470.1
GCGGCGTCAC	0	0	1	0	0	gnl|UG|At#S35289128	no match found	non-canonical match
at4g36120	CON	MIN	HOUR	DAY	WEEK	UNIGENE	FLCDNA	TAIR
CGTTTTGAGT	0	1	0	0	0	no match found	gi|110738659|dbj|AK229393.1|	non-canonical match
at2g26900	CON	MIN	HOUR	DAY	WEEK	UNIGENE	FLCDNA	TAIR
ATGACAACTT	9	10	7	11	11	gnl|UG|At#S15461109	gi|28393075|gb|BT003925.1	At2g26900.1
at1g09630	CON	MIN	HOUR	DAY	WEEK	UNIGENE	FLCDNA	TAIR
TTGATTGGGA	0	0	0	1	0	gnl|UG|At#S11742073	gi|15010637|gb|AY045620.1	At1g09630.1
at3g12020	CON	MIN	HOUR	DAY	WEEK	UNIGENE	FLCDNA	TAIR
ACAGCCCATC	2	3	0	0	0	gnl|UG|At#S11737286	gi|26450188|dbj|AK117552.1|	non-canonical match
at5g53895	CON	MIN	HOUR	DAY	WEEK	UNIGENE	FLCDNA	TAIR
GATAAAGCCA	0	0	0	1	0	gnl|UG|At#S24443550	gi|44917522|gb|BT011723.1	multiple non-canonical match
at2g31610	CON	MIN	HOUR	DAY	WEEK	UNIGENE	FLCDNA	TAIR
CTCCTAAAGA	0	0	0	1	0	no match found	gi|15450570|gb|AY052749.1	multiple non-canonical match
TTGTTTCTTA	4	5	1	2	8	gnl|UG|At#S18900653	gi|14190418|gb|AF378887.1	multiple non-canonical match
GGATTTTGTT	0	3	0	3	0	no match found	gi|20148376|gb|AY081517.1	multiple non-canonical match
at3g06200	CON	MIN	HOUR	DAY	WEEK	UNIGENE	FLCDNA	TAIR

GATTATGTTA	1	1	0	0	0	no match found	gi|14190398|gb|AF378877.1	At3g06200.1
TTTATTGTAT	0	0	0	0	1	gnl|UG|At#S11738878	no match found	non-canonical match
at5g47480	CON	MIN	HOUR	DAY	WEEK	UNIGENE	FLCDNA	TAIR
AGTGGTAATG	2	0	0	1	0	gnl|UG|At#S11718974	no match found	At5g47480.1
at1g69960	CON	MIN	HOUR	DAY	WEEK	UNIGENE	FLCDNA	TAIR
GACCAGAATT	1	4	0	1	0	gnl|UG|At#S11729107	gi|108385336|gb|BT025871.1	At1g69960.1
at5g49810	CON	MIN	HOUR	DAY	WEEK	UNIGENE	FLCDNA	TAIR
CTAATAAAGG	6	1	2	0	2	gnl|UG|At#S11718736	gi|20453176|gb|AY094459.1	At5g49810.1
at1g21310	CON	MIN	HOUR	DAY	WEEK	UNIGENE	FLCDNA	TAIR
TGCAAGTTTG	6	36	14	32	25	gnl|UG|At#S11740927	no match found	At1g21310.1
at1g69620	CON	MIN	HOUR	DAY	WEEK	UNIGENE	FLCDNA	TAIR
TTGAGTTCAC	9	9	8	7	9	gnl|UG|At#S18895853	no match found	At1g69620.1
at5g56320	CON	MIN	HOUR	DAY	WEEK	UNIGENE	FLCDNA	TAIR
TCAAGAAACT	0	0	2	0	1	gnl|UG|At#S34116034	gi|21406953|gb|AY088179.1	At5g56320.1
at5g40090	CON	MIN	HOUR	DAY	WEEK	UNIGENE	FLCDNA	TAIR
GAACAAGGGA	0	2	0	0	0	no match found	gi|116325927|gb|BT029233.1	multiple non-canonical match
at4g33080	CON	MIN	HOUR	DAY	WEEK	UNIGENE	FLCDNA	TAIR
GAATGTGATT	0	1	0	0	0	no match found	gi|31711929|gb|BT008882.1	At4g33080.1
at2g47460	CON	MIN	HOUR	DAY	WEEK	UNIGENE	FLCDNA	TAIR
GGATGTTTGG	0	0	0	1	0	gnl|UG|At#S11730841	gi|62320881|dbj|AK221768.1|	At2g47460.1
at1g47550	CON	MIN	HOUR	DAY	WEEK	UNIGENE	FLCDNA	TAIR
TAAAAAAACA	2	0	2	2	1	no match found	no match found	At1g47550.1
at2g42590	CON	MIN	HOUR	DAY	WEEK	UNIGENE	FLCDNA	TAIR
CCTGTCTCAG	14	21	11	18	15	gnl|UG|At#S11706328	gi|21404637|gb|AY085927.1	At2g42590.1
at5g11010	CON	MIN	HOUR	DAY	WEEK	UNIGENE	FLCDNA	TAIR
TTTTTTTATA	0	0	0	0	2	gnl|UG|At#S11706433	gi|17979298|gb|AY070379.1	At5g11010.2
at3g06483	CON	MIN	HOUR	DAY	WEEK	UNIGENE	FLCDNA	TAIR
GAAGGATATG	0	0	0	0	1	no match found	gi|16323389|gb|AY059083.1	At3g06483.1
TATTTATGGT	3	1	0	1	2	gnl|UG|At#S11738801	gi|14334687|gb|AY035017.1	pseudo chromosome match
at1g43080	CON	MIN	HOUR	DAY	WEEK	UNIGENE	FLCDNA	TAIR
AAAAAAAGGG	0	2	1	0	0	gnl|UG|At#S11736179	no match found	multiple non-canonical match
at1g16445	CON	MIN	HOUR	DAY	WEEK	UNIGENE	FLCDNA	TAIR
TTCAAGAGAG	1	0	0	0	0	gnl|UG|At#S11807247	gi|20259355|gb|AY090956.1	At1g16445.1
at5g23080	CON	MIN	HOUR	DAY	WEEK	UNIGENE	FLCDNA	TAIR
TCCTTCATTT	0	1	1	0	1	gnl|UG|At#S11720870	gi|26451307|dbj|AK118129.1|	At5g23080.1
GGAAAGCTGC	0	2	1	0	1	no match found	gi|30793924|gb|BT008588.1	non-canonical match
at2g04820	CON	MIN	HOUR	DAY	WEEK	UNIGENE	FLCDNA	TAIR
TAGCTCTTGA	0	0	1	0	0	no match found	no match found	At2g04820.1
at4g12690	CON	MIN	HOUR	DAY	WEEK	UNIGENE	FLCDNA	TAIR
AGATCGTAGT	0	0	1	0	0	gnl|UG|At#S11813193	gi|110738544|dbj|AK229335.1|	At4g12690.1
at4g25810	CON	MIN	HOUR	DAY	WEEK	UNIGENE	FLCDNA	TAIR
AAAAGTAATA	1	1	0	1	0	gnl|UG|At#S11723340	gi|17064791|gb|AY062472.1	At4g25810.1

at4g30140	CON	MIN	HOUR	DAY	WEEK	UNIGENE	FLCDNA	TAIR
GACAGCAAAC	0	4	0	0	0	gnl|UG|At#S11722593	gi|13877600|gb|AF370501.1	At4g30140.1
at5g10800	CON	MIN	HOUR	DAY	WEEK	UNIGENE	FLCDNA	TAIR
TGAGAAGCAT	1	0	0	0	0	gnl|UG|At#S11723547	no match found	pseudo chromosome match
at4g04614	CON	MIN	HOUR	DAY	WEEK	UNIGENE	FLCDNA	TAIR
GACTACGGGA	1	0	1	1	0	gnl|UG|At#S37211687	gi|149944330|gb|BT030628.1	pseudo chromosome match
at4g18390	CON	MIN	HOUR	DAY	WEEK	UNIGENE	FLCDNA	TAIR
TAATTGGTAT	0	0	0	1	0	gnl|UG|At#S11724614	no match found	At4g18390.1
at1g61980	CON	MIN	HOUR	DAY	WEEK	UNIGENE	FLCDNA	TAIR
AGGACAAGCA	0	0	0	1	0	gnl|UG|At#S34114424	gi|119935862|gb|BT029746.1	At1g61980.1
at1g21380	CON	MIN	HOUR	DAY	WEEK	UNIGENE	FLCDNA	TAIR
GATTTTTTTA	0	1	6	4	1	gnl|UG|At#S11740920	gi|14334601|gb|AY034974.1	non-canonical match
at3g27670	CON	MIN	HOUR	DAY	WEEK	UNIGENE	FLCDNA	TAIR
TCTTAATTCC	2	2	0	0	1	gnl|UG|At#S16149923	no match found	At3g27670.1
at1g08790	CON	MIN	HOUR	DAY	WEEK	UNIGENE	FLCDNA	TAIR
CTAAACAAAA	2	1	1	0	1	no match found	no match found	At1g08790.1
at1g14740	CON	MIN	HOUR	DAY	WEEK	UNIGENE	FLCDNA	TAIR
AAGCTGAGAA	0	1	1	1	0	no match found	gi|20148718|gb|AY081688.1	non-canonical match
at4g16740	CON	MIN	HOUR	DAY	WEEK	UNIGENE	FLCDNA	TAIR
GTTTCAGTAT	0	0	5	0	0	no match found	gi|26452938|dbj|AK118969.1|	non-canonical match
at4g15480	CON	MIN	HOUR	DAY	WEEK	UNIGENE	FLCDNA	TAIR
CTTCGTGACT	0	0	0	1	0	no match found	gi|53793642|gb|BT015796.1	non-canonical match
TACAACCAGT	0	0	0	1	0	gnl|UG|At#S11725120	no match found	non-canonical match
at4g23270	CON	MIN	HOUR	DAY	WEEK	UNIGENE	FLCDNA	TAIR
TAAGTTAAAC	3	5	6	3	0	gnl|UG|At#S11823069	gi|26452612|dbj|AK118799.1|	At4g23270.1
at1g03130	CON	MIN	HOUR	DAY	WEEK	UNIGENE	FLCDNA	TAIR
AGATCCATCG	45	52	46	30	1	no match found	gi|23507778|gb|BT000856.1	multiple non-canonical match
TGGTAAGTGA	2	0	3	0	0	gnl|UG|At#S11742801	no match found	At1g03130.1
TAAAATCTTG	130	83	146	70	17	no match found	gi|14517459|gb|AY039565.1	non-canonical match
at1g57720	CON	MIN	HOUR	DAY	WEEK	UNIGENE	FLCDNA	TAIR
GAGCTGTACG	12	10	7	3	5	gnl|UG|At#S11731944	gi|17978698|gb|AY064630.1	At1g57720.1
at5g65000	CON	MIN	HOUR	DAY	WEEK	UNIGENE	FLCDNA	TAIR
CCGGTGGTGT	1	2	0	1	0	gnl|UG|At#S18941417	gi|110742239|dbj|AK226979.1|	At5g65000.2
at1g74200	CON	MIN	HOUR	DAY	WEEK	UNIGENE	FLCDNA	TAIR
GTTTCTTTCT	1	0	1	0	0	no match found	gi|13430727|gb|AF360276.1	At1g74200.1
at1g12120	CON	MIN	HOUR	DAY	WEEK	UNIGENE	FLCDNA	TAIR
TAATCTTGTA	1	1	4	2	1	gnl|UG|At#S11741824	gi|17380909|gb|AY063911.1	At1g12120.1
at2g42200	CON	MIN	HOUR	DAY	WEEK	UNIGENE	FLCDNA	TAIR
AAAATCCAAG	1	0	0	0	0	gnl|UG|At#S11704715	no match found	At2g42200.2
at3g58200	CON	MIN	HOUR	DAY	WEEK	UNIGENE	FLCDNA	TAIR
AAATAAGAAG	0	1	0	0	0	gnl|UG|At#S14273835	gi|28393429|gb|BT004114.1	At3g58200.1

at2g07728	CON	MIN	HOUR	DAY	WEEK	UNIGENE	FLCDNA	TAIR
CAGTACTTTG	0	2	0	0	0	no match found	gi|110739717|dbj|AK229940.1|	pseudo chromosome match
at3g57750	CON	MIN	HOUR	DAY	WEEK	UNIGENE	FLCDNA	TAIR
TGTCTGAGGT	0	1	0	1	0	gnl|UG|At#S11728000	gi|51968379|dbj|AK175119.1|	At3g57750.1
at1g70570	CON	MIN	HOUR	DAY	WEEK	UNIGENE	FLCDNA	TAIR
AAGGCTACGA	0	2	0	0	0	gnl|UG|At#S11702121	gi|15293216|gb|AY051042.1	At1g70570.1
at1g70280	CON	MIN	HOUR	DAY	WEEK	UNIGENE	FLCDNA	TAIR
AAAAGTCTCC	1	4	1	1	0	gnl|UG|At#S15435794	gi|17065223|gb|AY062688.1	At1g70280.1
at2g32900	CON	MIN	HOUR	DAY	WEEK	UNIGENE	FLCDNA	TAIR
AGGTATTTTA	1	3	0	0	0	gnl|UG|At#S11734308	gi|17978941|gb|AY069883.1	At2g32900.1
at5g15020	CON	MIN	HOUR	DAY	WEEK	UNIGENE	FLCDNA	TAIR
GAAGGTGTAC	0	0	0	0	1	gnl|UG|At#S11722587	no match found	At5g15020.1
at5g25810	CON	MIN	HOUR	DAY	WEEK	UNIGENE	FLCDNA	TAIR
GAGACCTCAC	0	1	0	0	0	no match found	gi|90568017|gb|BT024923.1	multiple non-canonical match
at3g24270	CON	MIN	HOUR	DAY	WEEK	UNIGENE	FLCDNA	TAIR
TAGAGATCAC	1	1	0	1	0	no match found	no match found	At3g24270.1
at1g53430	CON	MIN	HOUR	DAY	WEEK	UNIGENE	FLCDNA	TAIR
GCTCCTGAGT	0	2	2	0	1	no match found	no match found	At1g53430.1
at5g49130	CON	MIN	HOUR	DAY	WEEK	UNIGENE	FLCDNA	TAIR
GCGGCCACCG	0	1	1	0	0	no match found	gi|110736941|dbj|AK228502.1|	multiple non-canonical match
at3g51800	CON	MIN	HOUR	DAY	WEEK	UNIGENE	FLCDNA	TAIR
GTCATTTGCA	0	0	1	1	3	no match found	gi|23297075|gb|AY142627.1	At3g51800.1
at4g15110	CON	MIN	HOUR	DAY	WEEK	UNIGENE	FLCDNA	TAIR
CCAAAAATGG	0	2	4	3	0	no match found	no match found	At4g15110.1
at5g10290	CON	MIN	HOUR	DAY	WEEK	UNIGENE	FLCDNA	TAIR
ATGTATCTTT	1	1	2	1	2	gnl|UG|At#S11723664	gi|20260259|gb|AY093029.1	At5g10290.1
at4g24020	CON	MIN	HOUR	DAY	WEEK	UNIGENE	FLCDNA	TAIR
ATGTAACGAC	1	1	0	1	0	gnl|UG|At#S11723645	gi|110736842|dbj|AK228452.1|	At4g24020.1
at2g34390	CON	MIN	HOUR	DAY	WEEK	UNIGENE	FLCDNA	TAIR
CTGATCCAGA	0	1	0	0	0	no match found	no match found	At2g34390.1
at1g04230	CON	MIN	HOUR	DAY	WEEK	UNIGENE	FLCDNA	TAIR
CTGCCACTAG	0	1	0	0	0	no match found	gi|31711801|gb|BT008818.1	non-canonical match
at3g10640	CON	MIN	HOUR	DAY	WEEK	UNIGENE	FLCDNA	TAIR
GAAGAGCTCA	0	2	0	0	0	gnl|UG|At#S18942138	gi|110736411|dbj|AK228223.1|	At3g10640.2
at3g61820	CON	MIN	HOUR	DAY	WEEK	UNIGENE	FLCDNA	TAIR
AAGATCCCTA	5	2	4	1	1	gnl|UG|At#S11751806	gi|14532549|gb|AY039899.1	At3g61820.1
at3g58660	CON	MIN	HOUR	DAY	WEEK	UNIGENE	FLCDNA	TAIR
TTTTTCACTG	0	0	0	0	1	no match found	gi|17979348|gb|AY070404.1	At3g58660.1
at5g13790	CON	MIN	HOUR	DAY	WEEK	UNIGENE	FLCDNA	TAIR
AGAAGGAAGG	1	0	0	0	0	gnl|UG|At#S43849615	no match found	non-canonical match
at4g26480	CON	MIN	HOUR	DAY	WEEK	UNIGENE	FLCDNA	TAIR

AAGAGAGCTA	0	1	0	0	0	gnl|UG|At#S11723229	no match found	non-canonical match
GTTTTGTGTT	5	3	6	4	5	no match found	gi|110740088|dbj|AK230131.1|	multiple canonical match
at5g51170	CON	MIN	HOUR	DAY	WEEK	UNIGENE	FLCDNA	TAIR
TCGATGGAAA	0	3	0	0	0	gnl|UG|At#S11718599	gi|28393838|gb|BT004333.1	At5g51170.1
at2g38330	CON	MIN	HOUR	DAY	WEEK	UNIGENE	FLCDNA	TAIR
GAAAATGCTA	1	0	2	1	0	gnl|UG|At#S11733019	gi|28973232|gb|BT005521.1	non-canonical match
at2g37678	CON	MIN	HOUR	DAY	WEEK	UNIGENE	FLCDNA	TAIR
AGACAAAGAA	1	0	2	1	4	gnl|UG|At#S15644839	gi|30794092|gb|BT008682.1	At2g37680.1
at2g29560	CON	MIN	HOUR	DAY	WEEK	UNIGENE	FLCDNA	TAIR
TGTATATAAT	0	1	0	0	0	gnl|UG|At#S16592710	no match found	multiple non-canonical match
TTTTTTACAA	1	0	0	0	0	gnl|UG|At#S11735147	no match found	At2g29560.1
TGATATTAAT	0	0	0	1	0	no match found	gi|14334909|gb|AY035128.1	non-canonical match
at1g56460	CON	MIN	HOUR	DAY	WEEK	UNIGENE	FLCDNA	TAIR
TAAATACTAA	3	0	0	0	0	gnl|UG|At#S11732116	gi|26450455|dbj|AK117690.1|	multiple canonical match
at2g22300	CON	MIN	HOUR	DAY	WEEK	UNIGENE	FLCDNA	TAIR
ACTCTAATTT	1	6	7	3	1	gnl|UG|At#S15461285	gi|27311706|gb|BT002459.1	non-canonical match
at3g24730	CON	MIN	HOUR	DAY	WEEK	UNIGENE	FLCDNA	TAIR
ATGAAATCCT	0	1	0	0	0	gnl|UG|At#S11733335	gi|51970439|dbj|AK176149.1|	non-canonical match
at4g13700	CON	MIN	HOUR	DAY	WEEK	UNIGENE	FLCDNA	TAIR
TGGACCAGTT	0	1	0	0	0	gnl|UG|At#S11725436	no match found	non-canonical match
at4g29390	CON	MIN	HOUR	DAY	WEEK	UNIGENE	FLCDNA	TAIR
AAGCTTGAAG	3	3	12	6	6	no match found	no match found	At4g29390.1
at4g13990	CON	MIN	HOUR	DAY	WEEK	UNIGENE	FLCDNA	TAIR
TCGATGTTGT	70	92	50	40	8	gnl|UG|At#S11725389	no match found	multiple canonical match
at2g42650	CON	MIN	HOUR	DAY	WEEK	UNIGENE	FLCDNA	TAIR
AGGTTAGATA	1	0	0	0	0	no match found	gi|19310374|gb|AY078921.1	non-canonical match
at3g54630	CON	MIN	HOUR	DAY	WEEK	UNIGENE	FLCDNA	TAIR
GATTCGAAGA	0	0	0	1	0	gnl|UG|At#S11728578	gi|133778859|gb|BT030357.1	At3g54630.1
at3g45830	CON	MIN	HOUR	DAY	WEEK	UNIGENE	FLCDNA	TAIR
TTTTTTATTC	0	1	0	0	0	gnl|UG|At#S11730220	no match found	At3g45830.1
at1g17140	CON	MIN	HOUR	DAY	WEEK	UNIGENE	FLCDNA	TAIR
GATCCACCGG	0	0	0	1	0	no match found	gi|89000950|gb|BT024727.1	At3g16250.1
AACAAATTTC	1	1	1	1	0	gnl|UG|At#S11741331	no match found	At1g17140.1
at1g57770	CON	MIN	HOUR	DAY	WEEK	UNIGENE	FLCDNA	TAIR
TCTTTTTAAA	1	0	2	2	0	gnl|UG|At#S11710295	gi|17529087|gb|AY065278.1	At1g57770.1
TTCTACACGC	0	0	0	0	1	no match found	gi|22136841|gb|AY133831.1	At1g31530.1
at4g02510	CON	MIN	HOUR	DAY	WEEK	UNIGENE	FLCDNA	TAIR
TATCAGTAAT	6	2	5	3	6	gnl|UG|At#S11727060	gi|15724245|gb|AF412063.1	At4g02510.1
at1g04220	CON	MIN	HOUR	DAY	WEEK	UNIGENE	FLCDNA	TAIR
TATAATACTA	0	0	0	1	1	gnl|UG|At#S11742624	gi|62321530|dbj|AK222101.1|	At1g04220.1
at1g23410	CON	MIN	HOUR	DAY	WEEK	UNIGENE	FLCDNA	TAIR
GCGAGTCATT	13	15	18	13	12	gnl|UG|At#S11740720	no match found	multiple canonical match

at1g17080	CON	MIN	HOUR	DAY	WEEK	UNIGENE	FLCDNA	TAIR
ATGTTTGGAA	1	3	3	1	1	gnl|UG|At#S11741337	gi|20453322|gb|AY097384.1	At1g17080.1

at1g29400	CON	MIN	HOUR	DAY	WEEK	UNIGENE	FLCDNA	TAIR
AATACTCGGT	6	7	9	8	3	gnl|UG|At#S15460244	gi|110740424|dbj|AK230306.1|	At1g29400.2

at5g04910	CON	MIN	HOUR	DAY	WEEK	UNIGENE	FLCDNA	TAIR
AGATTACTGA	0	1	1	1	1	gnl|UG|At#S11724907	no match found	non-canonical match

at1g33060	CON	MIN	HOUR	DAY	WEEK	UNIGENE	FLCDNA	TAIR
TTTTTCGTTT	0	1	0	0	1	gnl|UG|At#S18942452	no match found	At1g33060.2

at5g11590	CON	MIN	HOUR	DAY	WEEK	UNIGENE	FLCDNA	TAIR
GGATCTCTAA	0	1	0	0	0	gnl|UG|At#S24244345	no match found	At5g11590.1

at3g49680	CON	MIN	HOUR	DAY	WEEK	UNIGENE	FLCDNA	TAIR
AAATGGACTG	7	3	3	2	0	gnl|UG|At#S11729505	gi|21406357|gb|AY087619.1	At3g49680.1

at3g09900	CON	MIN	HOUR	DAY	WEEK	UNIGENE	FLCDNA	TAIR
GACGAAAGCA	1	0	0	0	0	gnl|UG|At#S34117014	gi|110738462|dbj|AK229294.1|	At3g09900.1

at3g48930	CON	MIN	HOUR	DAY	WEEK	UNIGENE	FLCDNA	TAIR
CTTCCGTGTT	7	8	5	1	8	gnl|UG|At#S11729654	gi|16649154|gb|AY059947.1	At3g48930.1

at3g10730	CON	MIN	HOUR	DAY	WEEK	UNIGENE	FLCDNA	TAIR
CTTGATCTGA	1	1	1	0	0	gnl|UG|At#S11737669	gi|26451213|dbj|AK118080.1|	At3g10730.1

at1g10640	CON	MIN	HOUR	DAY	WEEK	UNIGENE	FLCDNA	TAIR
TATATCATCT	1	0	0	0	1	gnl|UG|At#S11741968	gi|14532455|gb|AY039852.1	At1g10640.1

at5g59060	CON	MIN	HOUR	DAY	WEEK	UNIGENE	FLCDNA	TAIR
AAAAAAGAAA	2	2	2	0	2	gnl|UG|At#S21989655	no match found	non-canonical match

at2g26980	CON	MIN	HOUR	DAY	WEEK	UNIGENE	FLCDNA	TAIR
GACGATATTG	0	0	0	0	1	no match found	gi|20268689|gb|AY091098.1	At2g26980.3

at5g46390	CON	MIN	HOUR	DAY	WEEK	UNIGENE	FLCDNA	TAIR
CTATGAAACT	0	0	0	1	1	gnl|UG|At#S11708827	gi|15912238|gb|AY056397.1	At5g46390.2

at5g55480	CON	MIN	HOUR	DAY	WEEK	UNIGENE	FLCDNA	TAIR
ATCCCAGTTC	0	0	1	1	0	gnl|UG|At#S11718165	gi|53828544|gb|BT015819.1	At5g55480.1

at5g18400	CON	MIN	HOUR	DAY	WEEK	UNIGENE	FLCDNA	TAIR
GGTGTTTCTC	0	1	2	2	1	gnl|UG|At#S18910557	gi|21703113|gb|AY123986.1	At5g18400.1

at2g17787	CON	MIN	HOUR	DAY	WEEK	UNIGENE	FLCDNA	TAIR
GTGTGGCCGA	0	0	1	0	0	no match found	gi|114050648|gb|BT028927.1	non-canonical match
ACCAACATAT	1	0	0	0	0	gnl|UG|At#S18900497	gi|110736053|dbj|AK228038.1|	At2g17787.1

at2g17450	CON	MIN	HOUR	DAY	WEEK	UNIGENE	FLCDNA	TAIR
CTTCTACGGC	2	13	0	1	0	gnl|UG|At#S11738116	gi|13877952|gb|AF370239.1	At2g17450.1

at5g42765	CON	MIN	HOUR	DAY	WEEK	UNIGENE	FLCDNA	TAIR
GCTCGTCTTA	0	2	0	0	0	no match found	gi|21407237|gb|AY088463.1	multiple non-canonical match
ACAAGCGTTC	1	3	2	0	0	gnl|UG|At#S11719453	gi|23306449|gb|BT000475.1	multiple non-canonical match

at5g56500	CON	MIN	HOUR	DAY	WEEK	UNIGENE	FLCDNA	TAIR
AGCAGCAACA	2	0	1	0	0	gnl|UG|At#S28281605	gi|110736631|dbj|AK228340.1|	At5g56500.1
CTTCATCTGT	0	0	1	1	1	no match found	gi|62319197|dbj|AK220921.1|	multiple non-canonical match

at5g05100	CON	MIN	HOUR	DAY	WEEK	UNIGENE	FLCDNA	TAIR

ACGCAATTTT	1	1	2	2	0	gnl|UG|At#S11724859	gi|16649096|gb|AY059918.1	At5g05100.1
at2g33670	CON	MIN	HOUR	DAY	WEEK	UNIGENE	FLCDNA	TAIR
GCGTTGGAAA	0	0	0	0	1	gnl|UG|At#S11734126	no match found	At2g33670.1
at3g51000	CON	MIN	HOUR	DAY	WEEK	UNIGENE	FLCDNA	TAIR
ATGCTCTAGT	3	2	3	2	2	no match found	gi|13937212|gb|AF372961.1	non-canonical match
at1g63520	CON	MIN	HOUR	DAY	WEEK	UNIGENE	FLCDNA	TAIR
ACTCAAAGAA	0	0	1	0	0	gnl|UG|At#S11730435	gi|63147389|gb|BT022107.1	At1g63520.1
at2g27040	CON	MIN	HOUR	DAY	WEEK	UNIGENE	FLCDNA	TAIR
TTCTTCTGTT	1	1	0	0	0	gnl|UG|At#S11703629	gi|15293198|gb|AY051033.1	At2g27040.1
at5g61370	CON	MIN	HOUR	DAY	WEEK	UNIGENE	FLCDNA	TAIR
AGATTCTTTT	0	0	0	0	1	gnl|UG|At#S11717565	gi|17529063|gb|AY065266.1	At5g61370.1
at4g01160	CON	MIN	HOUR	DAY	WEEK	UNIGENE	FLCDNA	TAIR
TCAGATCATT	1	0	0	0	2	no match found	no match found	At4g01160.1
at5g20960	CON	MIN	HOUR	DAY	WEEK	UNIGENE	FLCDNA	TAIR
GCAATTGTGG	0	0	1	0	0	no match found	no match found	At5g20960.2
GCAATGGCAA	1	0	2	1	1	gnl|UG|At#S11699507	no match found	At5g20960.1
at5g58290	CON	MIN	HOUR	DAY	WEEK	UNIGENE	FLCDNA	TAIR
GAATCAAATT	6	6	7	13	7	no match found	gi|17979230|gb|AY070466.1	At5g58290.1
at5g54200	CON	MIN	HOUR	DAY	WEEK	UNIGENE	FLCDNA	TAIR
TCTAACATAC	0	1	0	0	0	gnl|UG|At#S11718295	no match found	At5g54200.1
at1g05500	CON	MIN	HOUR	DAY	WEEK	UNIGENE	FLCDNA	TAIR
TAGCCCAGAA	2	2	3	3	0	gnl|UG|At#S11742478	gi|110737089|dbj|AK228581.1|	non-canonical match
at5g48840	CON	MIN	HOUR	DAY	WEEK	UNIGENE	FLCDNA	TAIR
TTTGTGTGTA	0	1	0	0	0	gnl|UG|At#S11718834	no match found	multiple canonical match
at1g03290	CON	MIN	HOUR	DAY	WEEK	UNIGENE	FLCDNA	TAIR
AACAAAGAGA	1	2	1	0	3	gnl|UG|At#S11742785	no match found	At1g03290.1
at1g02820	CON	MIN	HOUR	DAY	WEEK	UNIGENE	FLCDNA	TAIR
GGTTCCAGAT	1	0	1	7	2	gnl|UG|At#S21735928	gi|21407598|gb|AY088824.1	At1g02820.1
at3g51320	CON	MIN	HOUR	DAY	WEEK	UNIGENE	FLCDNA	TAIR
AGTTTGGTCT	0	0	0	1	0	gnl|UG|At#S34115370	gi|110741619|dbj|AK226647.1|	non-canonical match
at3g20970	CON	MIN	HOUR	DAY	WEEK	UNIGENE	FLCDNA	TAIR
CTGATGCATT	0	0	1	0	1	gnl|UG|At#S11734551	gi|13899084|gb|AF370537.1	At3g20970.1
at4g12990	CON	MIN	HOUR	DAY	WEEK	UNIGENE	FLCDNA	TAIR
TCACAAGAAG	0	1	0	0	0	gnl|UG|At#S18913560	no match found	non-canonical match
at5g23880	CON	MIN	HOUR	DAY	WEEK	UNIGENE	FLCDNA	TAIR
TAATCTCGTA	0	2	0	0	1	gnl|UG|At#S11720791	gi|14334617|gb|AY034982.1	At5g23880.1
at4g13310	CON	MIN	HOUR	DAY	WEEK	UNIGENE	FLCDNA	TAIR
GCTTTTCTTT	0	1	0	0	0	gnl|UG|At#S37211266	no match found	non-canonical match
CAGTACTTTG	0	2	0	0	0	gnl|UG|At#S15982944	no match found	pseudo chromosome match
at5g66800	CON	MIN	HOUR	DAY	WEEK	UNIGENE	FLCDNA	TAIR
TTTCATCGAT	1	0	0	0	0	gnl|UG|At#S11717017	gi|21402993|gb|AY084283.1	At5g66800.1

at3g46430	CON	MIN	HOUR	DAY	WEEK	UNIGENE	FLCDNA	TAIR
CGAACGCACA	7	5	10	9	10	gnl|UG|At#S18904861	gi|17381283|gb|AY064154.1	non-canonical match
at5g52060	CON	MIN	HOUR	DAY	WEEK	UNIGENE	FLCDNA	TAIR
CCGATACAAT	0	1	1	0	1	no match found	gi|110742456|dbj|AK227095.1|	At5g52060.1
TGTGTTTTCT	2	2	1	0	1	gnl|UG|At#S11718510	no match found	non-canonical match
at5g63160	CON	MIN	HOUR	DAY	WEEK	UNIGENE	FLCDNA	TAIR
TCTTCGTTGT	1	13	3	0	0	gnl|UG|At#S11717384	gi|110742798|dbj|AK227278.1|	At5g63160.1
at4g34110	CON	MIN	HOUR	DAY	WEEK	UNIGENE	FLCDNA	TAIR
CCATTGACTA	9	14	7	6	4	gnl|UG|At#S11721916	gi|19347815|gb|AY080718.1	At4g34110.1
at1g26440	CON	MIN	HOUR	DAY	WEEK	UNIGENE	FLCDNA	TAIR
GGTGGACAAG	0	1	0	0	0	gnl|UG|At#S11708115	gi|16604335|gb|AY058066.1	At1g26440.1
at5g16010	CON	MIN	HOUR	DAY	WEEK	UNIGENE	FLCDNA	TAIR
TTTTGCTGGC	0	0	3	0	0	gnl|UG|At#S11702818	gi|14190512|gb|AF380656.1	At5g16010.1
at2g34160	CON	MIN	HOUR	DAY	WEEK	UNIGENE	FLCDNA	TAIR
ACTTCAACTG	1	1	4	1	0	gnl|UG|At#S11734028	gi|21403121|gb|AY084411.1	At2g34160.1
at2g01710	CON	MIN	HOUR	DAY	WEEK	UNIGENE	FLCDNA	TAIR
ATTGTTAAAA	0	3	0	2	0	gnl|UG|At#S11742631	gi|15529207|gb|AY052228.1	At2g01710.1
at1g76260	CON	MIN	HOUR	DAY	WEEK	UNIGENE	FLCDNA	TAIR
GGCTGTCAGG	0	0	0	1	0	gnl|UG|At#S18896416	gi|51970961|dbj|AK176410.1|	non-canonical match
at3g62030	CON	MIN	HOUR	DAY	WEEK	UNIGENE	FLCDNA	TAIR
TCGTGTTTGG	15	15	15	9	8	no match found	gi|16648946|gb|AY059843.1	non-canonical match
GTTGCAACCG	1	1	0	0	0	gnl|UG|At#S11726838	no match found	At3g62030.1
AGTTATAAGT	17	7	20	18	11	no match found	gi|21405609|gb|AY086899.1	non-canonical match
at4g08262	CON	MIN	HOUR	DAY	WEEK	UNIGENE	FLCDNA	TAIR
GAAGTTGCCA	0	0	0	1	1	no match found	no match found	At4g08262.1
at4g14160	CON	MIN	HOUR	DAY	WEEK	UNIGENE	FLCDNA	TAIR
CCCGTTTGTA	2	2	4	5	1	gnl|UG|At#S20707985	gi|18389287|gb|AY074391.1	At4g14160.3
GATCACAGGC	0	0	1	0	0	no match found	gi|21689678|gb|AY122928.1	non-canonical match
at4g19410	CON	MIN	HOUR	DAY	WEEK	UNIGENE	FLCDNA	TAIR
CGAACACCGC	2	10	1	3	3	no match found	gi|21406095|gb|AY087371.1	At4g19410.1
at3g13445	CON	MIN	HOUR	DAY	WEEK	UNIGENE	FLCDNA	TAIR
TATGTTGCTG	2	1	0	0	1	gnl|UG|At#S28282988	gi|21403591|gb|AY084881.1	At3g13445.1
at5g61230	CON	MIN	HOUR	DAY	WEEK	UNIGENE	FLCDNA	TAIR
TAATCTTCAG	10	2	8	2	4	no match found	gi|14517559|gb|AY039615.1	multiple canonical match
at1g67590	CON	MIN	HOUR	DAY	WEEK	UNIGENE	FLCDNA	TAIR
GCTAGGTATA	1	0	0	0	0	gnl|UG|At#S28282432	gi|21402988|gb|AY084278.1	At1g67590.1
at2g21180	CON	MIN	HOUR	DAY	WEEK	UNIGENE	FLCDNA	TAIR
GAGAACCCTG	0	1	0	0	0	gnl|UG|At#S26539357	gi|16974342|gb|AY059663.1	multiple non-canonical match
TTATCCAATA	3	1	0	0	2	gnl|UG|At#S11737161	no match found	At2g21180.1
at5g47640	CON	MIN	HOUR	DAY	WEEK	UNIGENE	FLCDNA	TAIR
TGATAGAACG	1	0	2	2	3	gnl|UG|At#S11718957	gi|14326579|gb|AF385744.1	At5g47640.1
at1g56240	CON	MIN	HOUR	DAY	WEEK	UNIGENE	FLCDNA	TAIR
AAAGTAACTA	0	1	0	0	0	gnl|UG|At#S18896214	gi|52218823|gb|BT015705.1	At1g56240.1

at1g15750	CON	MIN	HOUR	DAY	WEEK	UNIGENE	FLCDNA	TAIR	
TTTGCAGTTG	1	4	0	4	0	gnl|UG|At#S28282630	gi|23296856|gb|AY142619.1	multiple non-canonical match	
at2g43230	CON	MIN	HOUR	DAY	WEEK	UNIGENE	FLCDNA	TAIR	
AGTTTCTCCA	0	0	0	0	1	no match found	no match found	At2g43230.1	
at1g70885	CON	MIN	HOUR	DAY	WEEK	UNIGENE	FLCDNA	TAIR	
TATCAAAAGC	0	0	0	0	2	gnl|UG|At#S11831822	gi|20259369|gb|AY090969.1	At1g70885.1	
at3g27110	CON	MIN	HOUR	DAY	WEEK	UNIGENE	FLCDNA	TAIR	
TATAGAAGGA	0	0	1	0	0	no match found	no match found	At3g27110.2	
TATAGGAACA	0	0	0	0	1	gnl|UG|At#S18903801	gi|15010605|gb|AY045604.1	At3g27110.1	
at3g09740	CON	MIN	HOUR	DAY	WEEK	UNIGENE	FLCDNA	TAIR	
TTGTTCGTGT	9	13	2	1	2	gnl|UG|At#S11737959	gi|19699302|gb|AY090358.1	At3g09740.1	
at3g09700	CON	MIN	HOUR	DAY	WEEK	UNIGENE	FLCDNA	TAIR	
TAGTTCCAAG	0	1	0	0	0	gnl|UG|At#S30641606	gi|21407063|gb|AY088289.1	non-canonical match	
at1g75060	CON	MIN	HOUR	DAY	WEEK	UNIGENE	FLCDNA	TAIR	
TAATTAGGTC	0	1	0	0	0	gnl|UG|At#S11728026	no match found	At1g75060.1	
at3g02630	CON	MIN	HOUR	DAY	WEEK	UNIGENE	FLCDNA	TAIR	
AGACTGCTTA	1	1	0	0	0	gnl|UG|At#S11739898	gi|21406018|gb|AY087294.1	At3g02630.1	
at5g60930	CON	MIN	HOUR	DAY	WEEK	UNIGENE	FLCDNA	TAIR	
TATATTTTTA	12	6	7	7	12	no match found	no match found	At5g60930.1	
TGGATGTTCT	0	0	0	1	0	gnl|UG|At#S11717609	gi|26451781|dbj|AK118373.1|	non-canonical match	
at3g05530	CON	MIN	HOUR	DAY	WEEK	UNIGENE	FLCDNA	TAIR	
AAGACTTCAA	2	3	1	3	0	no match found	gi|17065257|gb|AY062705.1	multiple non-canonical match	
at5g01400	CON	MIN	HOUR	DAY	WEEK	UNIGENE	FLCDNA	TAIR	
TATTGTAGCA	1	0	0	0	0	gnl|UG|At#S11725805	no match found	At5g01400.1	
at1g48110	CON	MIN	HOUR	DAY	WEEK	UNIGENE	FLCDNA	TAIR	
TGCTGTTCAC	1	0	2	0	0	gnl|UG|At#S43850482	gi|110738437|dbj|AK229281.1|	At3g43360.1	
TTTTGACTCG	0	0	0	0	1	no match found	no match found	At1g48110.1	
at3g21500	CON	MIN	HOUR	DAY	WEEK	UNIGENE	FLCDNA	TAIR	
AGAGTGCTCT	0	1	0	0	0	gnl|UG|At#S15460768	gi|51969369|dbj|AK175614.1|	At3g21500.1	
at3g04830	CON	MIN	HOUR	DAY	WEEK	UNIGENE	FLCDNA	TAIR	
GCTGCACCAG	1	1	2	1	1	gnl|UG|At#S15461553	gi|21406597|gb|AY087846.1	At3g04830.2	
at4g12070	CON	MIN	HOUR	DAY	WEEK	UNIGENE	FLCDNA	TAIR	
GATGCTTGTT	0	1	1	0	0	no match found	gi|26451707|dbj|AK118335.1|	multiple non-canonical match	
ATTTAACTTT	0	0	1	0	0	no match found	gi|21406105|gb|AY087381.1	At4g12070.1	
at1g29250	CON	MIN	HOUR	DAY	WEEK	UNIGENE	FLCDNA	TAIR	
ACATCGACTG	1	2	1	3	0	gnl|UG|At#S11739285	gi|16974408|gb|AY060517.1	At1g29250.1	
at1g61770	CON	MIN	HOUR	DAY	WEEK	UNIGENE	FLCDNA	TAIR	
GTCCAGTTTT	1	3	5	3	2	gnl|UG|At#S11730874	gi|19347730|gb|AY080607.1	At1g61770.1	
CAACGATACA	0	0	1	0	1	no match found	gi|26983835|gb|BT002337.1	non-canonical match	
at3g18165	CON	MIN	HOUR	DAY	WEEK	UNIGENE	FLCDNA	TAIR	
CTTGAGACAC	0	0	1	1	1	gnl|UG|At#S11735391	gi|21406886|gb|AY088112.1	At3g18165.1	
at2g23060	CON	MIN	HOUR	DAY	WEEK	UNIGENE	FLCDNA	TAIR	

AATGTGAAAT	1	1	1	0	1	gnl|UG|At#S35252520	no match found	pseudo chromosome match
at3g57140	CON	MIN	HOUR	DAY	WEEK	UNIGENE	FLCDNA	TAIR
TGGTTAAGAA	1	1	0	1	1	no match found	gi|62319950|dbj|AK221301.1|	non-canonical match
at5g06360	CON	MIN	HOUR	DAY	WEEK	UNIGENE	FLCDNA	TAIR
TTGGCTAGTT	4	6	4	0	3	gnl|UG|At#S11724534	gi|21404164|gb|AY085454.1	At5g06360.1
at5g58380	CON	MIN	HOUR	DAY	WEEK	UNIGENE	FLCDNA	TAIR
ACTCCAATCT	0	3	0	0	0	gnl|UG|At#S11814599	gi|21703140|gb|AY124002.1	At5g58380.1
at4g25300	CON	MIN	HOUR	DAY	WEEK	UNIGENE	FLCDNA	TAIR
CTTTTGCAAA	0	1	1	0	0	gnl|UG|At#S18941798	gi|110743936|dbj|AK227822.1|	At4g25300.1
at1g69700	CON	MIN	HOUR	DAY	WEEK	UNIGENE	FLCDNA	TAIR
ATCTTTGATG	2	1	0	1	0	no match found	gi|23296864|gb|AY142652.1	At1g69700.1
at4g31290	CON	MIN	HOUR	DAY	WEEK	UNIGENE	FLCDNA	TAIR
AGGAGGACTA	0	5	3	0	0	gnl|UG|At#S11722397	gi|110743782|dbj|AK227743.1|	At4g31290.1
at3g54220	CON	MIN	HOUR	DAY	WEEK	UNIGENE	FLCDNA	TAIR
TCGTGGAACA	1	1	0	0	0	gnl|UG|At#S11728644	gi|15810552|gb|AY056315.1	At3g54220.1
at2g44630	CON	MIN	HOUR	DAY	WEEK	UNIGENE	FLCDNA	TAIR
GAACTGAAGA	0	0	0	1	0	gnl|UG|At#S11731489	gi|26451651|dbj|AK118305.1|	At2g44630.1
at1g21670	CON	MIN	HOUR	DAY	WEEK	UNIGENE	FLCDNA	TAIR
TGGTTCCCTA	1	4	3	3	4	gnl|UG|At#S11703627	gi|24030457|gb|BT000981.1	At1g21670.1
at1g09390	CON	MIN	HOUR	DAY	WEEK	UNIGENE	FLCDNA	TAIR
AAAGTATTGT	1	0	0	0	0	gnl|UG|At#S11742099	gi|27754508|gb|BT002885.1	non-canonical match
at1g12880	CON	MIN	HOUR	DAY	WEEK	UNIGENE	FLCDNA	TAIR
ATTCTTCAAA	1	0	0	0	0	no match found	gi|20258974|gb|AY091264.1	non-canonical match
at1g02560	CON	MIN	HOUR	DAY	WEEK	UNIGENE	FLCDNA	TAIR
AAGACCAAAG	50	36	55	45	36	no match found	gi|21403104|gb|AY084394.1	At1g02560.1
at1g19940	CON	MIN	HOUR	DAY	WEEK	UNIGENE	FLCDNA	TAIR
TTAGGGAAAA	0	3	0	0	0	no match found	gi|21360510|gb|AY113063.1	non-canonical match
TATATGGTTT	0	1	0	0	1	gnl|UG|At#S11704905	gi|15081704|gb|AY048245.1	At1g19940.1
at4g27270	CON	MIN	HOUR	DAY	WEEK	UNIGENE	FLCDNA	TAIR
TGGAGAAATT	0	0	0	0	1	gnl|UG|At#S24442967	gi|34365650|gb|BT010514.1	At4g27270.1
at5g18190	CON	MIN	HOUR	DAY	WEEK	UNIGENE	FLCDNA	TAIR
TCAAGGAAAA	1	2	0	1	0	gnl|UG|At#S38433287	gi|28392918|gb|BT003845.1	At5g18190.1
at3g27930	CON	MIN	HOUR	DAY	WEEK	UNIGENE	FLCDNA	TAIR
GAAGCAAAGA	1	1	0	0	0	no match found	gi|18087556|gb|AF462820.1	At3g27930.1
at2g36470	CON	MIN	HOUR	DAY	WEEK	UNIGENE	FLCDNA	TAIR
ATGTGGGATT	0	1	0	0	1	gnl|UG|At#S11733477	gi|21403186|gb|AY084476.1	At2g36470.1
at3g16175	CON	MIN	HOUR	DAY	WEEK	UNIGENE	FLCDNA	TAIR
TCGTGGGAGA	0	1	0	0	0	gnl|UG|At#S11706941	gi|15450572|gb|AY052654.1	non-canonical match
at2g04515	CON	MIN	HOUR	DAY	WEEK	UNIGENE	FLCDNA	TAIR
CAACATAATG	1	0	0	0	0	gnl|UG|At#S11817338	gi|62318688|dbj|AK220664.1|	At2g04515.1
at4g14710	CON	MIN	HOUR	DAY	WEEK	UNIGENE	FLCDNA	TAIR

GACTTTTGTG	1	0	0	0	0	no match found	gi|58331818|gb|BT020561.1	multiple non-canonical match
TTGGATATTA	1	1	3	2	4	gnl|UG|At#S38433496	gi|21405464|gb|AY086754.1	At4g14710.1
at5g41360	CON	MIN	HOUR	DAY	WEEK	UNIGENE	FLCDNA	TAIR
GAATACAACT	1	0	1	2	0	gnl|UG|At#S11719593	no match found	At5g41360.1
at3g17280	CON	MIN	HOUR	DAY	WEEK	UNIGENE	FLCDNA	TAIR
GTTTGAAATA	0	0	1	0	0	gnl|UG|At#S11735659	no match found	non-canonical match
at5g13510	CON	MIN	HOUR	DAY	WEEK	UNIGENE	FLCDNA	TAIR
CCCACTCGTG	2	9	9	8	5	gnl|UG|At#S11722970	gi|21405594|gb|AY086884.1	At5g13510.1
at5g03910	CON	MIN	HOUR	DAY	WEEK	UNIGENE	FLCDNA	TAIR
TAATAAACAA	0	1	0	1	2	no match found	no match found	At5g03910.1
TACAGACCAA	2	2	2	0	2	no match found	gi|62321356|dbj|AK222011.1|	non-canonical match
at1g79500	CON	MIN	HOUR	DAY	WEEK	UNIGENE	FLCDNA	TAIR
TTAATGCATC	0	1	0	0	0	gnl|UG|At#S18942230	gi|13937186|gb|AF372947.1	At1g79500.1
at5g24775	CON	MIN	HOUR	DAY	WEEK	UNIGENE	FLCDNA	TAIR
CGTTCCAAAT	0	1	0	0	0	no match found	no match found	At5g24775.1
at3g29590	CON	MIN	HOUR	DAY	WEEK	UNIGENE	FLCDNA	TAIR
ATTGATCGTT	0	0	0	1	1	no match found	no match found	At3g29590.1
at2g42830	CON	MIN	HOUR	DAY	WEEK	UNIGENE	FLCDNA	TAIR
TATTCAATCT	0	0	1	0	0	no match found	gi|26451976|dbj|AK118474.1|	At2g42830.1
at1g13300	CON	MIN	HOUR	DAY	WEEK	UNIGENE	FLCDNA	TAIR
GCCGCACCGC	0	3	0	0	0	gnl|UG|At#S11703053	gi|21700900|gb|AY124865.1	At1g13300.1
at2g40600	CON	MIN	HOUR	DAY	WEEK	UNIGENE	FLCDNA	TAIR
CTATGAGGTT	0	1	0	0	0	gnl|UG|At#S11732439	gi|24030241|gb|BT000897.1	At2g40600.1
at4g25640	CON	MIN	HOUR	DAY	WEEK	UNIGENE	FLCDNA	TAIR
TAACCCAAAT	0	0	4	4	3	gnl|UG|At#S11723368	no match found	At4g25640.1
GTTCTCAACA	0	2	1	0	0	no match found	gi|110740945|dbj|AK226424.1|	non-canonical match
at4g02220	CON	MIN	HOUR	DAY	WEEK	UNIGENE	FLCDNA	TAIR
GAAAGGAGAT	0	1	0	0	0	gnl|UG|At#S11727118	gi|15810626|gb|AY056352.1	At4g02220.1
at5g03300	CON	MIN	HOUR	DAY	WEEK	UNIGENE	FLCDNA	TAIR
GCTGGGAGAC	0	3	2	2	0	no match found	gi|14596134|gb|AY042855.1	multiple non-canonical match
TTTAATCAAT	0	1	0	0	0	gnl|UG|At#S11725337	no match found	non-canonical match
at5g04920	CON	MIN	HOUR	DAY	WEEK	UNIGENE	FLCDNA	TAIR
CGACAAACAA	0	0	1	0	0	gnl|UG|At#S11724904	no match found	non-canonical match
at5g65450	CON	MIN	HOUR	DAY	WEEK	UNIGENE	FLCDNA	TAIR
TAGCACAAAA	0	1	0	0	1	gnl|UG|At#S11701485	no match found	non-canonical match
at4g35790	CON	MIN	HOUR	DAY	WEEK	UNIGENE	FLCDNA	TAIR
GCTCTTCCTG	3	4	2	3	2	gnl|UG|At#S15460276	gi|19310476|gb|AY079017.1	At4g35790.2
at4g34412	CON	MIN	HOUR	DAY	WEEK	UNIGENE	FLCDNA	TAIR
CTTTCTTGTT	0	1	0	2	1	no match found	no match found	At4g34412.1
at5g46290	CON	MIN	HOUR	DAY	WEEK	UNIGENE	FLCDNA	TAIR
CAACTTCCAC	3	5	2	0	0	gnl|UG|At#S11719097	gi|21703100|gb|AY123979.1	At5g46290.1
at1g06220	CON	MIN	HOUR	DAY	WEEK	UNIGENE	FLCDNA	TAIR

AAGGACCTCA	0	2	0	0	0	gnl|UG|At#S11756253	gi|110741073|dbj|AK226489.1|	multiple non-canonical match
GTCAAGACCC	0	0	0	0	1	no match found	gi|62320052|dbj|AK221352.1|	multiple non-canonical match
ACTCGCAAAT	0	1	2	3	1	gnl|UG|At#S15461528	no match found	At1g06220.2
at2g41540	CON	MIN	HOUR	DAY	WEEK	UNIGENE	FLCDNA	TAIR
GCTTTTATAA	0	0	0	1	0	gnl|UG|At#S15460507	gi|17380927|gb|AY063920.1	non-canonical match
at1g03630	CON	MIN	HOUR	DAY	WEEK	UNIGENE	FLCDNA	TAIR
TACAATAAGT	6	0	4	2	6	no match found	gi|21407303|gb|AY088529.1	At1g03630.1
at5g20190	CON	MIN	HOUR	DAY	WEEK	UNIGENE	FLCDNA	TAIR
TCTTAAACTC	2	10	9	6	1	gnl|UG|At#S11721280	no match found	At5g20190.1
at4g29310	CON	MIN	HOUR	DAY	WEEK	UNIGENE	FLCDNA	TAIR
AACTTATGAC	1	0	0	0	0	gnl|UG|At#S11722735	gi|20260629|gb|AY093214.1	multiple canonical match
at1g08050	CON	MIN	HOUR	DAY	WEEK	UNIGENE	FLCDNA	TAIR
GTTTCTACTA	0	0	0	0	1	gnl|UG|At#S11742229	gi|27754462|gb|BT002862.1	At1g08050.1
at1g08490	CON	MIN	HOUR	DAY	WEEK	UNIGENE	FLCDNA	TAIR
AAGAAAAGTT	1	4	2	3	3	no match found	gi|20453111|gb|AY094425.1	At1g08490.1
at3g28700	CON	MIN	HOUR	DAY	WEEK	UNIGENE	FLCDNA	TAIR
GACCTATGAC	0	0	0	0	1	no match found	gi|95147285|gb|BT025322.1	non-canonical match
at3g51310	CON	MIN	HOUR	DAY	WEEK	UNIGENE	FLCDNA	TAIR
AAGAGTAATC	0	2	0	2	0	gnl|UG|At#S34114395	gi|110743449|dbj|AK227620.1|	At3g51310.1
at1g26770	CON	MIN	HOUR	DAY	WEEK	UNIGENE	FLCDNA	TAIR
GCTTCTTCTG	0	0	1	0	1	no match found	gi|16974398|gb|AY060512.1	multiple canonical match
at5g42830	CON	MIN	HOUR	DAY	WEEK	UNIGENE	FLCDNA	TAIR
TCACTTGTTT	1	1	1	0	0	gnl|UG|At#S14273845	gi|28393410|gb|BT004104.1	At5g42830.1
at2g22400	CON	MIN	HOUR	DAY	WEEK	UNIGENE	FLCDNA	TAIR
ATGACTTTTG	3	4	3	6	2	gnl|UG|At#S15461283	gi|20466585|gb|AY099759.1	multiple canonical match
at5g49950	CON	MIN	HOUR	DAY	WEEK	UNIGENE	FLCDNA	TAIR
AGATAAGCCT	2	2	2	0	0	gnl|UG|At#S11718721	gi|13877626|gb|AF370514.1	non-canonical match
at1g75420	CON	MIN	HOUR	DAY	WEEK	UNIGENE	FLCDNA	TAIR
GCCTTTAAGC	0	1	0	0	0	gnl|UG|At#S15644900	gi|110739258|dbj|AK229704.1|	At1g75420.1
at5g14910	CON	MIN	HOUR	DAY	WEEK	UNIGENE	FLCDNA	TAIR
TTTTTCCAGG	2	8	1	1	1	gnl|UG|At#S11722614	gi|15810112|gb|AY056094.1	At5g14910.1
at5g26670	CON	MIN	HOUR	DAY	WEEK	UNIGENE	FLCDNA	TAIR
AATGCAGATT	0	0	0	1	0	gnl|UG|At#S15460309	gi|51536517|gb|BT015374.1	At5g26670.2
at3g16900	CON	MIN	HOUR	DAY	WEEK	UNIGENE	FLCDNA	TAIR
TGTGTTTTGC	1	0	0	1	0	no match found	no match found	At3g16900.1
at2g22475	CON	MIN	HOUR	DAY	WEEK	UNIGENE	FLCDNA	TAIR
TTGATCTCCA	0	0	1	1	0	gnl|UG|At#S18942879	gi|21405906|gb|AY087182.1	At2g22475.2
at2g04270	CON	MIN	HOUR	DAY	WEEK	UNIGENE	FLCDNA	TAIR
GATGAGAAGA	0	1	0	1	0	gnl|UG|At#S11824711	gi|20260627|gb|AY093213.1	multiple non-canonical match
at1g21910	CON	MIN	HOUR	DAY	WEEK	UNIGENE	FLCDNA	TAIR
TGAGGTTTCT	1	3	1	0	0	gnl|UG|At#S11812077	gi|21404635|gb|AY085925.1	At1g21910.1

at4g34740	CON	MIN	HOUR	DAY	WEEK	UNIGENE	FLCDNA	TAIR
CTTCACGGGG	1	0	0	2	3	gnl|UG|At#S11710454	gi|20148564|gb|AY081611.1	At4g34740.1
at4g38810	CON	MIN	HOUR	DAY	WEEK	UNIGENE	FLCDNA	TAIR
GGATGCAATG	4	3	10	3	3	gnl|UG|At#S11721133	gi|15810420|gb|AY056249.1	At4g38810.2
at4g25990	CON	MIN	HOUR	DAY	WEEK	UNIGENE	FLCDNA	TAIR
TACACTATTA	1	0	0	0	2	gnl|UG|At#S15460684	no match found	At4g25990.2
TAAGGTTGGG	0	0	1	0	1	no match found	gi|126352271|gb|BT030317.1	non-canonical match
at2g17510	CON	MIN	HOUR	DAY	WEEK	UNIGENE	FLCDNA	TAIR
GAGGTAGTAG	0	1	1	0	0	gnl|UG|At#S20760421	gi|19699185|gb|AY090298.1	At2g17510.1
at2g33420	CON	MIN	HOUR	DAY	WEEK	UNIGENE	FLCDNA	TAIR
AAACAGAACT	1	0	0	0	0	no match found	gi|26451959|dbj|AK118464.1|	At2g33420.1
at4g03200	CON	MIN	HOUR	DAY	WEEK	UNIGENE	FLCDNA	TAIR
TTTAATAGTT	1	3	0	1	1	gnl|UG|At#S43849622	gi|62318792|dbj|AK220716.1|	At4g03200.1
at4g24630	CON	MIN	HOUR	DAY	WEEK	UNIGENE	FLCDNA	TAIR
AAAGCTGGGG	0	0	0	1	0	gnl|UG|At#S11723535	no match found	non-canonical match
at5g06550	CON	MIN	HOUR	DAY	WEEK	UNIGENE	FLCDNA	TAIR
ACAAATTCAA	0	0	0	1	0	gnl|UG|At#S21735980	gi|51968945|dbj|AK175402.1|	At5g06550.1
at4g14272	CON	MIN	HOUR	DAY	WEEK	UNIGENE	FLCDNA	TAIR
GATATACAGT	1	0	0	0	0	gnl|UG|At#S28282046	no match found	non-canonical match
at2g17340	CON	MIN	HOUR	DAY	WEEK	UNIGENE	FLCDNA	TAIR
GGTCGTGGAA	2	1	3	1	3	gnl|UG|At#S11738149	gi|21405266|gb|AY086556.1	At2g17340.1
at4g20760	CON	MIN	HOUR	DAY	WEEK	UNIGENE	FLCDNA	TAIR
CATTCTACTA	0	1	1	0	1	gnl|UG|At#S11707017	gi|15450422|gb|AY052312.1	At4g20760.1
at2g02790	CON	MIN	HOUR	DAY	WEEK	UNIGENE	FLCDNA	TAIR
TTGTCTTCAG	3	2	0	1	1	no match found	no match found	At2g02790.1
at1g61040	CON	MIN	HOUR	DAY	WEEK	UNIGENE	FLCDNA	TAIR
AAATTAGAAA	2	3	2	2	5	gnl|UG|At#S11731154	gi|20147192|gb|AY091764.1	At1g61040.1
at4g20380	CON	MIN	HOUR	DAY	WEEK	UNIGENE	FLCDNA	TAIR
GAACTTGACC	0	0	0	2	0	gnl|UG|At#S18905541	no match found	At4g20380.2
TCCGTTGATG	0	1	0	1	0	no match found	gi|19423935|gb|AY080824.1	non-canonical match
at1g61600	CON	MIN	HOUR	DAY	WEEK	UNIGENE	FLCDNA	TAIR
ACAAGAAACC	0	0	1	1	0	no match found	gi|19347754|gb|AY080618.1	At4g20490.1
at5g57490	CON	MIN	HOUR	DAY	WEEK	UNIGENE	FLCDNA	TAIR
CTACGCTCAA	0	1	0	0	0	gnl|UG|At#S11717965	gi|14334473|gb|AY034928.1	At5g57490.1
at1g67630	CON	MIN	HOUR	DAY	WEEK	UNIGENE	FLCDNA	TAIR
AGGTACTTCG	0	0	1	0	0	no match found	gi|26452175|dbj|AK118576.1|	non-canonical match
CTACGGTTAG	0	1	0	0	0	gnl|UG|At#S11729585	no match found	At1g67630.1
at1g04940	CON	MIN	HOUR	DAY	WEEK	UNIGENE	FLCDNA	TAIR
AACGCAGCCG	5	6	4	3	4	gnl|UG|At#S11742535	gi|26449431|dbj|AK117165.1|	non-canonical match
CCTGAAGCTC	0	0	0	1	0	no match found	no match found	At1g04940.1
at5g25890	CON	MIN	HOUR	DAY	WEEK	UNIGENE	FLCDNA	TAIR
CCTTCGTTTC	0	0	0	2	0	gnl|UG|At#S11720602	no match found	pseudo chromosome match

at4g27730	CON	MIN	HOUR	DAY	WEEK	UNIGENE	FLCDNA	TAIR
AGACAAAAGC	0	1	0	0	0	no match found	gi|23296483|gb|AY142525.1	At4g27730.1
CTTCAGAAAA	0	0	1	1	0	no match found	gi|62321176|dbj|AK221918.1|	non-canonical match
CTTCAGAAAG	0	0	0	2	2	gnl|UG|At#S11723004	no match found	non-canonical match
at3g05670	CON	MIN	HOUR	DAY	WEEK	UNIGENE	FLCDNA	TAIR
ATGATACAAG	1	1	1	0	1	gnl|UG|At#S11739019	gi|21539512|gb|AY120751.1	At3g05670.1
at2g28930	CON	MIN	HOUR	DAY	WEEK	UNIGENE	FLCDNA	TAIR
AACGAGGTTG	0	0	1	0	0	no match found	gi|119935889|gb|BT029755.1	non-canonical match
GCTCGTTTAC	1	1	1	0	0	gnl|UG|At#S11823276	gi|26452193|dbj|AK118585.1|	non-canonical match
at2g30700	CON	MIN	HOUR	DAY	WEEK	UNIGENE	FLCDNA	TAIR
TATAGTGGCA	1	3	0	1	1	gnl|UG|At#S15460938	no match found	At2g30700.1
at3g61430	CON	MIN	HOUR	DAY	WEEK	UNIGENE	FLCDNA	TAIR
TGGTTGTCAT	0	0	1	0	0	no match found	gi|20453350|gb|AY097398.1	non-canonical match
TTTATTACAG	4	0	0	0	3	no match found	no match found	At3g61430.1
TATGTGTGCA	18	11	10	4	6	no match found	gi|16648674|gb|AY058113.1	non-canonical match
at1g80620	CON	MIN	HOUR	DAY	WEEK	UNIGENE	FLCDNA	TAIR
TCATCTGCGG	0	2	1	1	1	gnl|UG|At#S15435716	gi|18377873|gb|AY074584.1	At1g80620.1
at2g33570	CON	MIN	HOUR	DAY	WEEK	UNIGENE	FLCDNA	TAIR
TATCATTTTT	0	2	0	0	0	gnl|UG|At#S11734144	gi|22655219|gb|AY140059.1	At2g33570.1
at4g35490	CON	MIN	HOUR	DAY	WEEK	UNIGENE	FLCDNA	TAIR
AATGAATTTC	3	5	4	3	3	no match found	gi|21407578|gb|AY088804.1	At4g35490.1
at2g21590	CON	MIN	HOUR	DAY	WEEK	UNIGENE	FLCDNA	TAIR
AACAAAGGCG	0	0	0	2	0	gnl|UG|At#S28282290	gi|17979397|gb|AY070429.1	At2g21590.1
at4g02680	CON	MIN	HOUR	DAY	WEEK	UNIGENE	FLCDNA	TAIR
AACCTTGAAC	0	1	0	2	0	gnl|UG|At#S34117113	gi|110738271|dbj|AK229195.1|	At4g02680.1
at3g24180	CON	MIN	HOUR	DAY	WEEK	UNIGENE	FLCDNA	TAIR
TATAGGCCAA	0	1	1	0	1	gnl|UG|At#S43849796	no match found	non-canonical match
ATGGACAGAG	1	0	0	0	0	gnl|UG|At#S11733532	gi|17529231|gb|AY065402.1	At3g24180.1
at5g13760	CON	MIN	HOUR	DAY	WEEK	UNIGENE	FLCDNA	TAIR
GACACAATCC	0	2	0	0	0	gnl|UG|At#S11817600	gi|17064961|gb|AY062557.1	At5g13760.1
at2g32870	CON	MIN	HOUR	DAY	WEEK	UNIGENE	FLCDNA	TAIR
CTTGTCTATA	2	2	4	1	0	gnl|UG|At#S11734317	gi|27311690|gb|BT002451.1	At2g32870.1
at5g28840	CON	MIN	HOUR	DAY	WEEK	UNIGENE	FLCDNA	TAIR
AATGAGATGG	4	10	2	5	2	gnl|UG|At#S11720327	gi|16323179|gb|AY057694.1	At5g28840.1
at3g25400	CON	MIN	HOUR	DAY	WEEK	UNIGENE	FLCDNA	TAIR
ATCAAGAACA	1	2	0	0	0	gnl|UG|At#S14273628	gi|28393816|gb|BT004321.1	At3g25400.1
at3g01360	CON	MIN	HOUR	DAY	WEEK	UNIGENE	FLCDNA	TAIR
ATTAAAGGTA	3	0	0	0	0	no match found	gi|110738890|dbj|AK229512.1|	non-canonical match
at5g49740	CON	MIN	HOUR	DAY	WEEK	UNIGENE	FLCDNA	TAIR
GTGGTACAAG	2	0	0	0	0	no match found	gi|110742440|dbj|AK227087.1|	multiple non-canonical match
ATAGCAATTT	6	3	4	5	1	gnl|UG|At#S11718743	no match found	At5g49740.1
at1g79170	CON	MIN	HOUR	DAY	WEEK	UNIGENE	FLCDNA	TAIR
AGAACACGCG	1	0	0	0	0	no match found	no match found	At1g79170.1

at3g44370	CON	MIN	HOUR	DAY	WEEK	UNIGENE	FLCDNA	TAIR
TGCTCTGTAT	1	1	0	0	0	no match found	no match found	At3g44370.1
at4g22120	CON	MIN	HOUR	DAY	WEEK	UNIGENE	FLCDNA	TAIR
GTCAAAAGAA	0	0	1	1	0	gnl|UG|At#S24442944	gi|62319787|dbj|AK221219.1|	At4g22120.1
at1g75640	CON	MIN	HOUR	DAY	WEEK	UNIGENE	FLCDNA	TAIR
CTCGAAGGGT	0	0	2	1	0	gnl|UG|At#S34115208	gi|110741928|dbj|AK226809.1|	non-canonical match
at3g62450	CON	MIN	HOUR	DAY	WEEK	UNIGENE	FLCDNA	TAIR
TTACCAAGAT	2	1	0	1	0	gnl|UG|At#S18904806	gi|26453057|dbj|AK119029.1|	non-canonical match
at2g31350	CON	MIN	HOUR	DAY	WEEK	UNIGENE	FLCDNA	TAIR
TTGCAGAGCT	0	5	0	0	0	gnl|UG|At#S18897987	gi|18176313|gb|AY072200.1	At2g31350.2
at2g21170	CON	MIN	HOUR	DAY	WEEK	UNIGENE	FLCDNA	TAIR
TAGCTGTCCG	0	0	1	0	0	no match found	gi|15450568|gb|AY052748.1	non-canonical match
GAACAGATGA	45	28	31	18	11	gnl|UG|At#S18899648	gi|14190440|gb|AF378898.1	At2g21170.1
at4g01540	CON	MIN	HOUR	DAY	WEEK	UNIGENE	FLCDNA	TAIR
GTTATTTCCT	0	1	0	0	0	no match found	no match found	At4g01540.1
AAGATGATCA	2	8	0	1	0	gnl|UG|At#S11727297	no match found	multiple non-canonical match
at3g58690	CON	MIN	HOUR	DAY	WEEK	UNIGENE	FLCDNA	TAIR
GAGTCATAGA	1	1	0	1	5	no match found	gi|26449988|dbj|AK117450.1|	At3g58690.1
at5g23150	CON	MIN	HOUR	DAY	WEEK	UNIGENE	FLCDNA	TAIR
GAGGCCTTTT	1	0	2	0	0	gnl|UG|At#S11720863	no match found	At5g23150.1
at4g33350	CON	MIN	HOUR	DAY	WEEK	UNIGENE	FLCDNA	TAIR
CAGGATTTGA	4	3	5	5	5	gnl|UG|At#S11722049	gi|28973024|gb|BT005417.1	At4g33350.1
at5g18620	CON	MIN	HOUR	DAY	WEEK	UNIGENE	FLCDNA	TAIR
GTCCATAAAC	0	1	0	0	0	gnl|UG|At#S15460701	gi|14334971|gb|AY035159.1	At5g18620.1
at3g23700	CON	MIN	HOUR	DAY	WEEK	UNIGENE	FLCDNA	TAIR
TACTAGAACG	4	0	5	1	2	gnl|UG|At#S11733701	gi|15146283|gb|AY049283.1	At3g23700.1
at4g14940	CON	MIN	HOUR	DAY	WEEK	UNIGENE	FLCDNA	TAIR
GACATTGGAG	0	0	1	0	0	gnl|UG|At#S11725212	no match found	At4g14940.1
at3g23040	CON	MIN	HOUR	DAY	WEEK	UNIGENE	FLCDNA	TAIR
TACTTAGATT	1	0	0	0	0	no match found	no match found	At3g23040.1
at4g26870	CON	MIN	HOUR	DAY	WEEK	UNIGENE	FLCDNA	TAIR
ACCCAGAACT	0	4	1	0	1	gnl|UG|At#S11723148	gi|24030348|gb|BT000939.1	At4g26870.1
at4g12830	CON	MIN	HOUR	DAY	WEEK	UNIGENE	FLCDNA	TAIR
TGCTTTCTAT	0	0	1	0	3	gnl|UG|At#S11725593	no match found	At4g12830.1
TACAAGAAGA	0	0	0	0	1	no match found	gi|19699239|gb|AY090325.1	multiple non-canonical match
at4g25433	CON	MIN	HOUR	DAY	WEEK	UNIGENE	FLCDNA	TAIR
GAATCTATTG	0	1	0	0	0	no match found	no match found	At4g25433.1
TCGTTTTATG	0	0	0	1	2	gnl|UG|At#S34115357	gi|110741642|dbj|AK226660.1|	non-canonical match
at5g62940	CON	MIN	HOUR	DAY	WEEK	UNIGENE	FLCDNA	TAIR
CCAAAGACTT	0	0	1	0	0	gnl|UG|At#S34117316	gi|58531345|gb|BT020576.1	At5g62940.1
at1g32610	CON	MIN	HOUR	DAY	WEEK	UNIGENE	FLCDNA	TAIR
CATCTCCGTT	0	0	1	1	0	gnl|UG|At#S21736088	gi|34146859|gb|BT010437.1	At1g32610.1

at4g11940	CON	MIN	HOUR	DAY	WEEK	UNIGENE	FLCDNA	TAIR
ATTATATATG	1	0	0	0	0	no match found	no match found	At4g11940.1
at4g03280	CON	MIN	HOUR	DAY	WEEK	UNIGENE	FLCDNA	TAIR
GTGGTCTTAA	69	68	47	33	12	gnl|UG|At#S11789802	gi|13926181|gb|AF370566.1	At4g03280.1
at2g02360	CON	MIN	HOUR	DAY	WEEK	UNIGENE	FLCDNA	TAIR
GTATGTTTAT	1	0	0	0	0	gnl|UG|At#S11740629	gi|110742666|dbj|AK227207.1|	At2g02360.1
at2g03050	CON	MIN	HOUR	DAY	WEEK	UNIGENE	FLCDNA	TAIR
AAGGTGTAAT	0	0	0	0	1	gnl|UG|At#S14233111	no match found	At2g03050.1
at5g16000	CON	MIN	HOUR	DAY	WEEK	UNIGENE	FLCDNA	TAIR
CATCTGTTTC	0	0	1	0	0	no match found	gi|18377619|gb|AY074263.1	multiple non-canonical match
TTTGAGAATG	0	2	0	0	0	gnl|UG|At#S11722336	no match found	At5g16000.1
at4g33400	CON	MIN	HOUR	DAY	WEEK	UNIGENE	FLCDNA	TAIR
TGAAATTACA	0	0	0	1	0	no match found	gi|20465422|gb|AY096497.1	non-canonical match
at1g63640	CON	MIN	HOUR	DAY	WEEK	UNIGENE	FLCDNA	TAIR
ACTTCTGAGA	0	1	2	0	0	gnl|UG|At#S18893422	no match found	At1g63640.2
at3g19050	CON	MIN	HOUR	DAY	WEEK	UNIGENE	FLCDNA	TAIR
TAACTGTTAT	1	0	1	1	0	gnl|UG|At#S11735140	no match found	non-canonical match
at5g22310	CON	MIN	HOUR	DAY	WEEK	UNIGENE	FLCDNA	TAIR
GAGAGTGGTA	1	5	0	0	0	gnl|UG|At#S11705607	gi|15983778|gb|AY056795.1	At5g22310.1
at2g11460	CON	MIN	HOUR	DAY	WEEK	UNIGENE	FLCDNA	TAIR
TCTTTTTTCT	0	1	0	0	0	no match found	no match found	At2g11460.1
at3g19420	CON	MIN	HOUR	DAY	WEEK	UNIGENE	FLCDNA	TAIR
ACCGGCAAGG	0	3	1	0	0	gnl|UG|At#S11735025	gi|17979062|gb|AY070042.1	At3g19420.1
at1g65950	CON	MIN	HOUR	DAY	WEEK	UNIGENE	FLCDNA	TAIR
CAGCGTATGA	0	0	1	0	1	gnl|UG|At#S11743949	gi|19347715|gb|AY080591.1	At1g65950.1
at2g42600	CON	MIN	HOUR	DAY	WEEK	UNIGENE	FLCDNA	TAIR
AAGGGTATCG	20	13	19	15	9	gnl|UG|At#S15460459	gi|23270355|gb|AY074346.2	At2g42600.2
at5g47370	CON	MIN	HOUR	DAY	WEEK	UNIGENE	FLCDNA	TAIR
AACTAGTCCT	2	2	0	0	0	gnl|UG|At#S11718987	gi|16226633|gb|AF428450.1	At5g47370.1
at3g48550	CON	MIN	HOUR	DAY	WEEK	UNIGENE	FLCDNA	TAIR
ATCCACCACG	0	1	0	0	0	gnl|UG|At#S11729724	gi|88900423|gb|BT024699.1	At3g48550.1
at1g35180	CON	MIN	HOUR	DAY	WEEK	UNIGENE	FLCDNA	TAIR
TAACATTTGA	0	0	1	1	0	gnl|UG|At#S11737220	gi|110736907|dbj|AK228485.1|	At1g35180.1
at1g62420	CON	MIN	HOUR	DAY	WEEK	UNIGENE	FLCDNA	TAIR
CAAGCTAAGT	0	1	0	0	0	gnl|UG|At#S21737414	gi|26450591|dbj|AK117760.1|	At1g29180.1
at2g05300	CON	MIN	HOUR	DAY	WEEK	UNIGENE	FLCDNA	TAIR
GTTGGTTTGG	0	0	1	0	0	gnl|UG|At#S11743954	gi|19347704|gb|AY080586.1	non-canonical match
at5g53180	CON	MIN	HOUR	DAY	WEEK	UNIGENE	FLCDNA	TAIR
GCACCACCAT	0	1	0	0	0	gnl|UG|At#S34117451	gi|55733736|gb|BT020199.1	At5g53180.1
at5g10160	CON	MIN	HOUR	DAY	WEEK	UNIGENE	FLCDNA	TAIR
GTTGAGGCAA	1	0	1	1	0	gnl|UG|At#S11723697	gi|110743615|dbj|AK227658.1|	non-canonical match

at4g39280	CON	MIN	HOUR	DAY	WEEK	UNIGENE	FLCDNA	TAIR
AAGGTTTAGG	2	1	1	1	0	no match found	gi|25141224|gb|BT002310.1	multiple canonical match

at1g12110	CON	MIN	HOUR	DAY	WEEK	UNIGENE	FLCDNA	TAIR
TCTATGTTGA	7	8	0	3	5	gnl|UG|At#S11741825	gi|25082982|gb|BT002016.1	At1g12110.1

at4g33810	CON	MIN	HOUR	DAY	WEEK	UNIGENE	FLCDNA	TAIR
TCAACCAGTT	0	0	1	0	0	gnl|UG|At#S11721971	no match found	non-canonical match

at5g33370	CON	MIN	HOUR	DAY	WEEK	UNIGENE	FLCDNA	TAIR
ACCTAAGCTT	2	0	1	1	0	gnl|UG|At#S11720233	gi|28827339|gb|BT004981.1	At5g33370.1

at3g53540	CON	MIN	HOUR	DAY	WEEK	UNIGENE	FLCDNA	TAIR
GCAACAACAC	0	2	0	0	1	no match found	gi|23308446|gb|BT000627.1	multiple non-canonical match
TGTCCCTGTA	1	1	2	0	1	gnl|UG|At#S11728778	no match found	At3g53540.1

at1g67090	CON	MIN	HOUR	DAY	WEEK	UNIGENE	FLCDNA	TAIR
CAGGTGTGGC	320	539	226	337	106	no match found	gi|110741031|dbj|AK226468.1|	non-canonical match

at4g14240	CON	MIN	HOUR	DAY	WEEK	UNIGENE	FLCDNA	TAIR
ACAATGTCAT	1	1	1	0	1	gnl|UG|At#S21735907	gi|51970403|dbj|AK176131.1|	At4g14240.1

at4g23060	CON	MIN	HOUR	DAY	WEEK	UNIGENE	FLCDNA	TAIR
GCTTGTACAG	0	3	0	0	0	gnl|UG|At#S11723812	no match found	At4g23060.1

at3g07360	CON	MIN	HOUR	DAY	WEEK	UNIGENE	FLCDNA	TAIR
GAACGATAAC	1	3	2	0	1	gnl|UG|At#S11738525	gi|21407735|gb|AY088961.1	At3g07360.2

at5g21990	CON	MIN	HOUR	DAY	WEEK	UNIGENE	FLCDNA	TAIR
GCTTCTCTTT	2	1	1	0	0	gnl|UG|At#S11817078	gi|26452526|dbj|AK118755.1|	At5g21990.1

at1g52930	CON	MIN	HOUR	DAY	WEEK	UNIGENE	FLCDNA	TAIR
AGCTCTCAAA	2	1	0	1	1	gnl|UG|At#S11702548	gi|13878054|gb|AF370290.1	At1g52930.1

at1g19130	CON	MIN	HOUR	DAY	WEEK	UNIGENE	FLCDNA	TAIR
TCTTGGTCCT	1	1	0	0	0	gnl|UG|At#S17008117	gi|26449698|dbj|AK117302.1|	non-canonical match

at3g55210	CON	MIN	HOUR	DAY	WEEK	UNIGENE	FLCDNA	TAIR
ATATGGAGAA	0	1	0	0	1	no match found	gi|45680236|gb|BT011926.1	At3g55210.1

at5g54290	CON	MIN	HOUR	DAY	WEEK	UNIGENE	FLCDNA	TAIR
TAAAAACTTA	1	2	3	3	3	gnl|UG|At#S11718286	gi|15292990|gb|AY050929.1	At5g54290.1

at1g01520	CON	MIN	HOUR	DAY	WEEK	UNIGENE	FLCDNA	TAIR
GGATATGAAT	0	0	2	3	2	no match found	no match found	At1g01520.1

at5g43100	CON	MIN	HOUR	DAY	WEEK	UNIGENE	FLCDNA	TAIR
AGTTAGACAT	0	1	0	0	0	no match found	no match found	At5g43100.1

at3g13670	CON	MIN	HOUR	DAY	WEEK	UNIGENE	FLCDNA	TAIR
TAACCTCAAT	0	2	0	0	1	gnl|UG|At#S11744090	gi|21436280|gb|AY117204.1	At3g13670.1

at4g13260	CON	MIN	HOUR	DAY	WEEK	UNIGENE	FLCDNA	TAIR
GTCCACATTT	1	0	1	0	0	gnl|UG|At#S18906251	no match found	At4g13260.1

at1g68520	CON	MIN	HOUR	DAY	WEEK	UNIGENE	FLCDNA	TAIR
AAAGGAAGGT	4	9	4	2	1	gnl|UG|At#S11729394	gi|20148424|gb|AY081541.1	At1g68520.1

at5g27590	CON	MIN	HOUR	DAY	WEEK	UNIGENE	FLCDNA	TAIR
TAATTTGCAT	0	0	1	0	0	no match found	no match found	At5g27590.1

at3g49570	CON	MIN	HOUR	DAY	WEEK	UNIGENE	FLCDNA	TAIR
GACCAAATCT	1	2	1	1	4	gnl|UG|At#S11729526	gi|29824294|gb|BT006123.1	At3g49570.1
at1g02000	CON	MIN	HOUR	DAY	WEEK	UNIGENE	FLCDNA	TAIR
ATTCATTCGA	0	1	1	1	0	gnl|UG|At#S11742916	gi|21403464|gb|AY084754.1	At1g02000.1
at5g49650	CON	MIN	HOUR	DAY	WEEK	UNIGENE	FLCDNA	TAIR
GCTGGCTCTG	0	1	3	0	0	gnl|UG|At#S11707394	gi|23296763|gb|AY142596.1	At5g49650.1
at5g18670	CON	MIN	HOUR	DAY	WEEK	UNIGENE	FLCDNA	TAIR
CTTTCACAGT	2	3	2	0	0	gnl|UG|At#S11710001	gi|17978934|gb|AY069879.1	At5g18670.1
at5g66120	CON	MIN	HOUR	DAY	WEEK	UNIGENE	FLCDNA	TAIR
TTCAAGTCCA	1	1	2	1	1	gnl|UG|At#S11717085	gi|17978955|gb|AY069891.1	At5g66120.2
at3g52180	CON	MIN	HOUR	DAY	WEEK	UNIGENE	FLCDNA	TAIR
TTAGTAATAA	0	1	0	0	0	no match found	gi|62320002|dbj|AK221327.1|	pseudo chromosome match
AAGTTAGTAG	1	3	2	1	10	no match found	gi|21403385|gb|AY084675.1	At3g52180.1
at5g19120	CON	MIN	HOUR	DAY	WEEK	UNIGENE	FLCDNA	TAIR
GGAAGAATCT	3	17	2	0	0	no match found	gi|15810068|gb|AY055793.1	At5g19120.1
CATTTGTACA	6	9	2	0	0	gnl|UG|At#S11721538	gi|110742807|dbj|AK227283.1|	pseudo chromosome match
at3g51110	CON	MIN	HOUR	DAY	WEEK	UNIGENE	FLCDNA	TAIR
GAGGCTAGCT	2	1	1	0	0	gnl|UG|At#S11707251	gi|15293116|gb|AY050992.1	At3g51110.1
at1g07750	CON	MIN	HOUR	DAY	WEEK	UNIGENE	FLCDNA	TAIR
TCTTGGTTCT	3	2	0	0	0	gnl|UG|At#S11742258	gi|12248028|gb|AF334728.1	At1g07750.1
at5g08020	CON	MIN	HOUR	DAY	WEEK	UNIGENE	FLCDNA	TAIR
GGTTTGTAAA	0	0	1	0	0	no match found	no match found	At5g08020.1
at5g64110	CON	MIN	HOUR	DAY	WEEK	UNIGENE	FLCDNA	TAIR
ACCAAGATGA	0	4	0	1	0	gnl|UG|At#S11717288	gi|17473851|gb|AY065173.1	At5g64110.1
at5g65370	CON	MIN	HOUR	DAY	WEEK	UNIGENE	FLCDNA	TAIR
CTATAAAAAG	4	1	3	3	1	no match found	no match found	At5g65370.1
at3g09050	CON	MIN	HOUR	DAY	WEEK	UNIGENE	FLCDNA	TAIR
AAAGACCTTG	0	3	0	1	0	gnl|UG|At#S11738181	gi|62320253|dbj|AK221453.1|	At3g09050.1
at5g65205	CON	MIN	HOUR	DAY	WEEK	UNIGENE	FLCDNA	TAIR
TTACATCTTT	0	1	0	0	2	gnl|UG|At#S11717177	gi|15983431|gb|AF424590.1	At5g65205.1
AAAGATTTTC	0	0	0	1	0	no match found	gi|20466144|gb|AY098984.1	multiple non-canonical match
at1g74800	CON	MIN	HOUR	DAY	WEEK	UNIGENE	FLCDNA	TAIR
GAGTAGGGAA	1	0	0	0	0	gnl|UG|At#S11728076	gi|19699370|gb|AY090451.1	At1g74800.1
at1g11960	CON	MIN	HOUR	DAY	WEEK	UNIGENE	FLCDNA	TAIR
CAAGCCGCGG	0	0	1	0	0	gnl|UG|At#S11741840	gi|18176280|gb|AY072194.1	At1g11960.1
at1g01390	CON	MIN	HOUR	DAY	WEEK	UNIGENE	FLCDNA	TAIR
CTCAAGATAA	0	1	1	0	0	gnl|UG|At#S11708925	gi|17065183|gb|AY062668.1	At1g01390.1
at4g20480	CON	MIN	HOUR	DAY	WEEK	UNIGENE	FLCDNA	TAIR
CACAACTGCT	1	2	1	2	0	gnl|UG|At#S11724254	gi|23297297|gb|AY150390.1	At4g20480.1
at1g54570	CON	MIN	HOUR	DAY	WEEK	UNIGENE	FLCDNA	TAIR
ATTCGAACAA	0	1	3	4	3	gnl|UG|At#S11732792	gi|21405201|gb|AY086491.1	At1g54570.1
at5g02590	CON	MIN	HOUR	DAY	WEEK	UNIGENE	FLCDNA	TAIR

TTGTTGATTG	0	0	0	0	2	gnl|UG|At#S11725514	gi|21403930|gb|AY085220.1	At5g02590.1
at4g22745	CON	MIN	HOUR	DAY	WEEK	UNIGENE	FLCDNA	TAIR
GGTCATCGAC	0	0	0	0	1	gnl|UG|At#S11723866	gi|54261714|gb|BT015931.1	At4g22745.1
at1g34460	CON	MIN	HOUR	DAY	WEEK	UNIGENE	FLCDNA	TAIR
GAAAAGAAGT	0	0	2	0	0	gnl|UG|At#S18912696	no match found	multiple non-canonical match
at3g24430	CON	MIN	HOUR	DAY	WEEK	UNIGENE	FLCDNA	TAIR
CCTCCTGGAA	0	0	1	0	0	gnl|UG|At#S11733440	gi|110740871|dbj|AK226386.1|	At3g24430.1
at4g19150	CON	MIN	HOUR	DAY	WEEK	UNIGENE	FLCDNA	TAIR
GGTGCGATTC	0	1	0	0	0	gnl|UG|At#S18906775	gi|26452015|dbj|AK118494.1|	At4g19150.1
at1g11210	CON	MIN	HOUR	DAY	WEEK	UNIGENE	FLCDNA	TAIR
GTCTTTCTTA	0	2	2	0	4	gnl|UG|At#S11741915	gi|15146279|gb|AY049281.1	At1g11210.1
at1g69380	CON	MIN	HOUR	DAY	WEEK	UNIGENE	FLCDNA	TAIR
CTTCTGGATT	1	0	0	0	0	gnl|UG|At#S11813786	gi|21403184|gb|AY084474.1	non-canonical match
at2g31090	CON	MIN	HOUR	DAY	WEEK	UNIGENE	FLCDNA	TAIR
GGAATTTGCA	3	2	3	4	1	gnl|UG|At#S21737335	gi|51968821|dbj|AK175340.1|	pseudo chromosome match
at4g19100	CON	MIN	HOUR	DAY	WEEK	UNIGENE	FLCDNA	TAIR
GGTTCCGTTT	0	2	0	0	0	gnl|UG|At#S18941841	gi|119360096|gb|BT029521.1	non-canonical match
TCACGTGTTT	0	2	0	1	1	no match found	no match found	At4g19100.1
at2g34040	CON	MIN	HOUR	DAY	WEEK	UNIGENE	FLCDNA	TAIR
ACTGTGTTTC	1	4	0	2	0	gnl|UG|At#S11734055	gi|21403929|gb|AY085219.1	At2g34040.1
at5g39520	CON	MIN	HOUR	DAY	WEEK	UNIGENE	FLCDNA	TAIR
TATAGTTCTG	1	2	1	0	0	gnl|UG|At#S11719780	gi|110741123|dbj|AK226515.1|	non-canonical match
at3g20080	CON	MIN	HOUR	DAY	WEEK	UNIGENE	FLCDNA	TAIR
AATCTTTGGT	0	2	2	1	0	no match found	gi|21404066|gb|AY085356.1	At1g19220.1
at5g14740	CON	MIN	HOUR	DAY	WEEK	UNIGENE	FLCDNA	TAIR
ATCAGCATCT	20	15	31	6	1	no match found	gi|16226546|gb|AF428428.1	non-canonical match
TTTACTTTCA	264	55	143	72	24	gnl|UG|At#S18941630	gi|23397026|gb|BT000652.1	At5g14740.2
TGGTGGCATC	1	1	4	0	0	no match found	gi|12642851|gb|AF339686.1	non-canonical match
at4g31560	CON	MIN	HOUR	DAY	WEEK	UNIGENE	FLCDNA	TAIR
TTGTGTTTTG	3	3	3	1	2	gnl|UG|At#S11790189	gi|13926336|gb|AF372916.1	At4g31560.1
at4g29080	CON	MIN	HOUR	DAY	WEEK	UNIGENE	FLCDNA	TAIR
AAGAGCTCTG	1	1	0	1	0	gnl|UG|At#S11722778	gi|15450724|gb|AY053404.1	At4g29080.1
at3g53310	CON	MIN	HOUR	DAY	WEEK	UNIGENE	FLCDNA	TAIR
ACTTCTTATT	1	0	0	0	0	gnl|UG|At#S11728818	gi|21406324|gb|AY087586.1	At3g53310.1
at5g27380	CON	MIN	HOUR	DAY	WEEK	UNIGENE	FLCDNA	TAIR
AAGATCAATA	2	5	4	0	1	no match found	gi|15983423|gb|AF424586.1	At5g27380.1
AATTGAATAA	2	7	0	0	0	no match found	gi|110742643|dbj|AK227195.1|	multiple non-canonical match
at4g24240	CON	MIN	HOUR	DAY	WEEK	UNIGENE	FLCDNA	TAIR
TGAATGTCCT	0	5	1	0	0	gnl|UG|At#S11723602	gi|18176040|gb|AY072151.1	At4g24240.1
at3g06930	CON	MIN	HOUR	DAY	WEEK	UNIGENE	FLCDNA	TAIR
TCTTTCTAAA	0	1	0	0	1	no match found	gi|28393407|gb|BT004102.1	multiple non-canonical match
at1g79730	CON	MIN	HOUR	DAY	WEEK	UNIGENE	FLCDNA	TAIR

AGGTTGAACT	1	0	0	2	0	no match found	gi|20147373|gb|AY093780.1	At1g79730.1
TACACGTCCA	1	2	1	0	3	gnl|UG|At#S11726617	no match found	pseudo chromosome match
at5g67240	CON	MIN	HOUR	DAY	WEEK	UNIGENE	FLCDNA	TAIR
GCTCAAGTTA	1	1	3	0	1	gnl|UG|At#S11744367	gi|25055028|gb|BT001977.1	At5g67240.1
at3g15110	CON	MIN	HOUR	DAY	WEEK	UNIGENE	FLCDNA	TAIR
TAGCCAACTA	5	4	8	2	8	gnl|UG|At#S11736317	gi|21407404|gb|AY088630.1	At3g15110.1
at5g50450	CON	MIN	HOUR	DAY	WEEK	UNIGENE	FLCDNA	TAIR
TTATTTTAAA	2	0	0	0	1	gnl|UG|At#S11718668	gi|20466553|gb|AY099743.1	multiple canonical match
GTGAGCGGTA	0	0	1	0	0	no match found	gi|23198133|gb|BT000275.1	non-canonical match
at4g20720	CON	MIN	HOUR	DAY	WEEK	UNIGENE	FLCDNA	TAIR
TGGATACACA	0	1	0	0	0	gnl|UG|At#S11724223	no match found	multiple canonical match
at1g53670	CON	MIN	HOUR	DAY	WEEK	UNIGENE	FLCDNA	TAIR
TCTTTGACGA	2	3	3	4	1	gnl|UG|At#S11733129	gi|26449722|dbj|AK117314.1|	At1g53670.1
at5g50640	CON	MIN	HOUR	DAY	WEEK	UNIGENE	FLCDNA	TAIR
AATTGTATAT	0	1	0	0	0	gnl|UG|At#S34116030	gi|110740369|dbj|AK230278.1|	multiple non-canonical match
GGCAGCCGCT	0	0	1	0	0	gnl|UG|At#S11718655	no match found	At5g50530.1
at3g02080	CON	MIN	HOUR	DAY	WEEK	UNIGENE	FLCDNA	TAIR
GACAGACATT	3	6	3	5	2	gnl|UG|At#S11740055	gi|13878028|gb|AF370277.1	At3g02080.1
at5g41700	CON	MIN	HOUR	DAY	WEEK	UNIGENE	FLCDNA	TAIR
TCTGTTTCTT	17	37	19	14	7	no match found	gi|15451029|gb|AY054595.1	At5g41700.4
ATGATAAGAC	0	2	0	0	0	gnl|UG|At#S11824428	no match found	At5g41080.2
GGCTAAACAA	0	0	1	0	0	no match found	gi|18377525|gb|AY072514.1	non-canonical match
at2g18040	CON	MIN	HOUR	DAY	WEEK	UNIGENE	FLCDNA	TAIR
GTCTTACTGC	6	0	2	3	2	gnl|UG|At#S11737978	gi|21403769|gb|AY085059.1	At2g18040.1
at5g02840	CON	MIN	HOUR	DAY	WEEK	UNIGENE	FLCDNA	TAIR
ATCGAACGTC	0	1	0	0	0	no match found	no match found	At5g02840.2
AACACTTAAG	0	0	1	0	0	no match found	gi|20148386|gb|AY081522.1	non-canonical match
CCTTTTGTAA	5	4	5	1	0	no match found	gi|14596212|gb|AY042894.1	multiple non-canonical match
at1g79910	CON	MIN	HOUR	DAY	WEEK	UNIGENE	FLCDNA	TAIR
ATGAGAGCAT	1	1	0	0	0	gnl|UG|At#S11726554	gi|22136367|gb|AY128862.1	At1g79910.1
at5g10830	CON	MIN	HOUR	DAY	WEEK	UNIGENE	FLCDNA	TAIR
GTCGGCCATC	0	0	2	0	0	no match found	gi|27808551|gb|BT003124.1	multiple non-canonical match
at4g01250	CON	MIN	HOUR	DAY	WEEK	UNIGENE	FLCDNA	TAIR
GCGAAAGTAC	0	5	0	0	0	no match found	gi|15028172|gb|AY045909.1	At4g01250.1
at5g61970	CON	MIN	HOUR	DAY	WEEK	UNIGENE	FLCDNA	TAIR
AGATTTCTGT	3	2	3	3	1	no match found	gi|15293136|gb|AY051002.1	non-canonical match
at2g29170	CON	MIN	HOUR	DAY	WEEK	UNIGENE	FLCDNA	TAIR
GAAAACGTTG	0	1	1	1	0	gnl|UG|At#S11735247	no match found	non-canonical match
at4g28620	CON	MIN	HOUR	DAY	WEEK	UNIGENE	FLCDNA	TAIR
AATTTCTAGG	0	0	1	0	1	no match found	no match found	At4g28620.1
at5g56150	CON	MIN	HOUR	DAY	WEEK	UNIGENE	FLCDNA	TAIR
GGTCCAGCAG	1	3	2	1	0	no match found	gi|20148364|gb|AY081511.1	non-canonical match
AGCTTTATGC	7	4	1	5	2	gnl|UG|At#S15453242	gi|21402930|gb|AY084220.1	At5g56150.2

at1g29710	CON	MIN	HOUR	DAY	WEEK	UNIGENE	FLCDNA	TAIR
ATATGATGAA	1	0	0	0	0	gnl|UG|At#S11739113	no match found	non-canonical match

at2g39440	CON	MIN	HOUR	DAY	WEEK	UNIGENE	FLCDNA	TAIR
AACGAGAGAT	0	0	0	1	0	gnl|UG|At#S43849234	no match found	pseudo chromosome match
GACCTAAACC	0	1	0	0	0	gnl|UG|At#S43850069	no match found	pseudo chromosome match

at5g51340	CON	MIN	HOUR	DAY	WEEK	UNIGENE	FLCDNA	TAIR
TCGACCGCTT	0	1	0	0	0	gnl|UG|At#S34117255	gi|110737999|dbj|AK229053.1|	At5g51340.1

at3g12490	CON	MIN	HOUR	DAY	WEEK	UNIGENE	FLCDNA	TAIR
ACTAGTCCCT	2	9	2	6	9	gnl|UG|At#S15461229	gi|17473692|gb|AY065127.1	non-canonical match

at1g55475	CON	MIN	HOUR	DAY	WEEK	UNIGENE	FLCDNA	TAIR
GAGATTGTGG	0	0	0	1	0	gnl|UG|At#S11706146	gi|15810162|gb|AY056146.1	At1g55475.1

at4g16530	CON	MIN	HOUR	DAY	WEEK	UNIGENE	FLCDNA	TAIR
CTCAAAGAGT	2	1	1	0	0	gnl|UG|At#S11724940	no match found	At4g16530.1

at5g46240	CON	MIN	HOUR	DAY	WEEK	UNIGENE	FLCDNA	TAIR
TATATCTGCA	1	3	1	0	0	gnl|UG|At#S11699357	gi|51971151|dbj|AK176505.1|	At5g46240.1

at5g51390	CON	MIN	HOUR	DAY	WEEK	UNIGENE	FLCDNA	TAIR
AGAGCTTATA	0	1	0	0	0	gnl|UG|At#S30640908	gi|21403119|gb|AY084409.1	At5g51390.1

at1g24520	CON	MIN	HOUR	DAY	WEEK	UNIGENE	FLCDNA	TAIR
TCGAGGCTCC	0	1	0	0	0	gnl|UG|At#S11740502	gi|114050568|gb|BT028887.1	At1g24520.1

at4g32840	CON	MIN	HOUR	DAY	WEEK	UNIGENE	FLCDNA	TAIR
AACCCTCCTA	1	2	0	0	0	gnl|UG|At#S34114544	gi|21406794|gb|AY088020.1	At4g32840.1

at3g57990	CON	MIN	HOUR	DAY	WEEK	UNIGENE	FLCDNA	TAIR
AGCGCTGAAT	1	2	0	1	0	gnl|UG|At#S11727954	gi|20259404|gb|AY091001.1	At3g57990.1

at3g06400	CON	MIN	HOUR	DAY	WEEK	UNIGENE	FLCDNA	TAIR
AATGTTTGTC	6	0	2	5	6	gnl|UG|At#S11738825	no match found	At3g06400.1

at4g16400	CON	MIN	HOUR	DAY	WEEK	UNIGENE	FLCDNA	TAIR
GCAGAAACAA	0	1	0	1	0	no match found	no match found	At4g16400.1

at5g25265	CON	MIN	HOUR	DAY	WEEK	UNIGENE	FLCDNA	TAIR
GGATATAGAG	1	3	1	0	2	gnl|UG|At#S34116048	gi|30725567|gb|BT008447.1	At5g25265.1

at4g14910	CON	MIN	HOUR	DAY	WEEK	UNIGENE	FLCDNA	TAIR
CTGGATCTTT	0	1	2	0	1	no match found	gi|15081689|gb|AY048237.1	non-canonical match

at1g04300	CON	MIN	HOUR	DAY	WEEK	UNIGENE	FLCDNA	TAIR
AATATACAAT	3	4	2	0	1	gnl|UG|At#S38434430	gi|110738785|dbj|AK229458.1|	At1g04300.1

at5g18230	CON	MIN	HOUR	DAY	WEEK	UNIGENE	FLCDNA	TAIR
TTTGTATATA	1	0	0	0	2	no match found	gi|13877644|gb|AF370523.1	At5g18230.1

at1g10910	CON	MIN	HOUR	DAY	WEEK	UNIGENE	FLCDNA	TAIR
AAATAGATGA	0	3	0	1	2	gnl|UG|At#S34115380	gi|110741599|dbj|AK226637.1|	non-canonical match

at2g35650	CON	MIN	HOUR	DAY	WEEK	UNIGENE	FLCDNA	TAIR
GGCTTGTTCG	0	2	1	0	0	gnl|UG|At#S11708005	gi|21403317|gb|AY084607.1	At2g35650.1

at3g26810	CON	MIN	HOUR	DAY	WEEK	UNIGENE	FLCDNA	TAIR
GCTCAATGTA	1	4	2	0	0	gnl|UG|At#S11732699	gi|17064983|gb|AY062568.1	At3g26810.1

at2g41740	CON	MIN	HOUR	DAY	WEEK	UNIGENE	FLCDNA	TAIR
CTTCTTCACC	0	1	0	0	0	no match found	gi|22136973|gb|AY133782.1	non-canonical match
GGTTGGTTTG	3	3	6	6	1	gnl|UG|At#S11732156	gi|110742057|dbj|AK226882.1|	At2g41740.1
at3g58670	CON	MIN	HOUR	DAY	WEEK	UNIGENE	FLCDNA	TAIR
GCTTGAAGAG	0	2	3	2	1	no match found	gi|23296433|gb|AY142515.1	At3g58670.1
at5g49660	CON	MIN	HOUR	DAY	WEEK	UNIGENE	FLCDNA	TAIR
TATTTAAATA	0	0	0	0	2	gnl|UG|At#S11718751	gi|26450864|dbj|AK117901.1|	At5g49660.1
at3g13674	CON	MIN	HOUR	DAY	WEEK	UNIGENE	FLCDNA	TAIR
CAATTTCTTT	0	1	0	0	0	gnl|UG|At#S11736755	no match found	At3g13674.1
at1g67870	CON	MIN	HOUR	DAY	WEEK	UNIGENE	FLCDNA	TAIR
GGATGCAGCA	0	0	1	0	0	no match found	gi|20466104|gb|AY098964.1	multiple non-canonical match
TTTCTGTATC	14	5	10	1	0	gnl|UG|At#S11729532	gi|15450342|gb|AY052272.1	At1g67870.1
at5g48640	CON	MIN	HOUR	DAY	WEEK	UNIGENE	FLCDNA	TAIR
TAACACTGTT	3	0	0	0	0	gnl|UG|At#S11718855	no match found	At5g48640.1
at1g60940	CON	MIN	HOUR	DAY	WEEK	UNIGENE	FLCDNA	TAIR
GGAGAAGTCC	11	20	17	11	3	gnl|UG|At#S11731188	gi|21407576|gb|AY088802.1	At1g60940.1
at2g07715	CON	MIN	HOUR	DAY	WEEK	UNIGENE	FLCDNA	TAIR
GGTGCAGGAT	0	1	1	0	0	no match found	no match found	At2g07715.1
at4g01110	CON	MIN	HOUR	DAY	WEEK	UNIGENE	FLCDNA	TAIR
TTGTAGCACA	1	2	2	0	0	no match found	no match found	At4g01110.1
CGTGTGATCA	0	1	0	0	0	gnl|UG|At#S37211365	no match found	At4g01110.1
at4g18220	CON	MIN	HOUR	DAY	WEEK	UNIGENE	FLCDNA	TAIR
AAGTATATTT	2	1	0	0	2	no match found	no match found	At4g18220.1
at1g60770	CON	MIN	HOUR	DAY	WEEK	UNIGENE	FLCDNA	TAIR
TTTTTGTGAA	1	1	0	0	0	no match found	gi|27754490|gb|BT002876.1	multiple canonical match
TCAAAGGCAG	0	0	1	0	0	no match found	gi|28393974|gb|BT004401.1	non-canonical match
at3g52605	CON	MIN	HOUR	DAY	WEEK	UNIGENE	FLCDNA	TAIR
CAGGGATCCT	0	0	1	0	0	no match found	gi|23296329|gb|AY142492.1	At3g52610.1
at1g31410	CON	MIN	HOUR	DAY	WEEK	UNIGENE	FLCDNA	TAIR
AAACAGATTA	0	1	0	0	0	no match found	gi|51536593|gb|BT015412.1	non-canonical match
TTACTAGTTA	0	0	1	0	0	no match found	no match found	At1g31410.1
TTGGTTAACA	1	0	0	0	0	no match found	gi|28393764|gb|BT004294.1	multiple non-canonical match
at1g62422	CON	MIN	HOUR	DAY	WEEK	UNIGENE	FLCDNA	TAIR
TAATATAGAT	0	3	0	1	0	gnl|UG|At#S24443122	gi|62319435|dbj|AK221041.1|	non-canonical match
at1g75450	CON	MIN	HOUR	DAY	WEEK	UNIGENE	FLCDNA	TAIR
CTCTCAAGCT	1	4	0	1	0	no match found	gi|17529345|gb|AY065459.1	At1g75440.1
at1g34430	CON	MIN	HOUR	DAY	WEEK	UNIGENE	FLCDNA	TAIR
ATTTTGCTAC	3	0	0	0	0	no match found	gi|22135831|gb|AY128294.1	non-canonical match
GTTGAAGAAG	0	1	0	0	0	gnl|UG|At#S30567557	no match found	non-canonical match
at1g72160	CON	MIN	HOUR	DAY	WEEK	UNIGENE	FLCDNA	TAIR
ACTTTATGAA	3	2	4	2	0	gnl|UG|At#S11728657	gi|15081613|gb|AY048199.1	At1g72160.1
at1g64680	CON	MIN	HOUR	DAY	WEEK	UNIGENE	FLCDNA	TAIR
TTGGTTATAA	2	4	3	0	0	gnl|UG|At#S34115853	gi|21407716|gb|AY088942.1	At1g64680.1

at4g36490	CON	MIN	HOUR	DAY	WEEK	UNIGENE	FLCDNA	TAIR
TACATCAAAT	2	0	0	0	0	no match found	gi|15215779|gb|AY050419.1	multiple canonical match
at3g61460	CON	MIN	HOUR	DAY	WEEK	UNIGENE	FLCDNA	TAIR
ATTTTCACTG	1	1	0	1	0	no match found	gi|21405272|gb|AY086562.1	At3g61460.1
at4g21480	CON	MIN	HOUR	DAY	WEEK	UNIGENE	FLCDNA	TAIR
ATCTTCACGT	1	1	0	0	0	gnl|UG|At#S11706612	no match found	multiple non-canonical match
at3g12145	CON	MIN	HOUR	DAY	WEEK	UNIGENE	FLCDNA	TAIR
TTTAATTTCG	1	0	0	0	1	gnl|UG|At#S18942120	gi|24899688|gb|BT001172.1	At3g12145.1
at3g24560	CON	MIN	HOUR	DAY	WEEK	UNIGENE	FLCDNA	TAIR
TTGCATTCAT	0	0	0	1	0	gnl|UG|At#S18902134	gi|14532773|gb|AY040011.1	non-canonical match
at3g44100	CON	MIN	HOUR	DAY	WEEK	UNIGENE	FLCDNA	TAIR
AAACAAAGAG	1	0	3	1	1	gnl|UG|At#S11730550	gi|21407094|gb|AY088320.1	At3g44100.1
TCCGGTTGCA	2	5	2	2	1	no match found	gi|27765003|gb|BT003058.1	non-canonical match
at1g19960	CON	MIN	HOUR	DAY	WEEK	UNIGENE	FLCDNA	TAIR
TTAACTGTGT	0	0	0	0	3	gnl|UG|At#S35312582	gi|110736570|dbj|AK228308.1|	non-canonical match
at1g50340	CON	MIN	HOUR	DAY	WEEK	UNIGENE	FLCDNA	TAIR
AAATCAATCC	0	0	0	0	1	gnl|UG|At#S11734380	no match found	non-canonical match
at5g37830	CON	MIN	HOUR	DAY	WEEK	UNIGENE	FLCDNA	TAIR
GAACGGGACA	0	0	1	0	0	gnl|UG|At#S11719952	gi|20856447|gb|AY102096.1	At5g37830.1
at5g11480	CON	MIN	HOUR	DAY	WEEK	UNIGENE	FLCDNA	TAIR
GATTATGACC	1	2	2	0	0	gnl|UG|At#S11723382	gi|21405916|gb|AY087192.1	At5g11480.1
at3g20310	CON	MIN	HOUR	DAY	WEEK	UNIGENE	FLCDNA	TAIR
CGATTAAGAG	1	9	0	1	1	no match found	gi|110736858|dbj|AK228460.1|	non-canonical match
GTCAATGGGC	0	3	1	0	1	gnl|UG|At#S11703794	gi|14335149|gb|AY037254.1	At3g20310.1
at4g36410	CON	MIN	HOUR	DAY	WEEK	UNIGENE	FLCDNA	TAIR
ACGATAAAGT	0	4	2	1	0	gnl|UG|At#S11721539	gi|21406850|gb|AY088076.1	At4g36410.1
at2g38420	CON	MIN	HOUR	DAY	WEEK	UNIGENE	FLCDNA	TAIR
AACGTGTTCG	1	0	0	0	0	gnl|UG|At#S11732993	gi|22655257|gb|AY140078.1	At2g38420.1
at5g65490	CON	MIN	HOUR	DAY	WEEK	UNIGENE	FLCDNA	TAIR
TACATTACTA	0	2	1	0	1	gnl|UG|At#S11717148	no match found	non-canonical match
at3g19910	CON	MIN	HOUR	DAY	WEEK	UNIGENE	FLCDNA	TAIR
CAAACATTCT	0	1	1	0	0	gnl|UG|At#S11734883	gi|19310706|gb|AY079353.1	At3g19910.1
at1g54575	CON	MIN	HOUR	DAY	WEEK	UNIGENE	FLCDNA	TAIR
GAAGTTGATA	3	3	9	6	5	gnl|UG|At#S21989853	gi|50253455|gb|BT015058.1	At1g54575.1
at3g08670	CON	MIN	HOUR	DAY	WEEK	UNIGENE	FLCDNA	TAIR
TTTGACAATG	0	1	1	0	0	no match found	gi|34365760|gb|BT010569.1	At3g08670.1
at1g21730	CON	MIN	HOUR	DAY	WEEK	UNIGENE	FLCDNA	TAIR
ATGTAAATAT	2	3	0	0	0	gnl|UG|At#S11740888	no match found	At1g21730.1
at3g01460	CON	MIN	HOUR	DAY	WEEK	UNIGENE	FLCDNA	TAIR
TCATTCGATA	1	2	0	0	0	gnl|UG|At#S11740232	no match found	non-canonical match
at2g42010	CON	MIN	HOUR	DAY	WEEK	UNIGENE	FLCDNA	TAIR
AGAGGACATT	1	0	1	0	0	gnl|UG|At#S11732095	no match found	At2g42010.1

at1g30640	CON	MIN	HOUR	DAY	WEEK	UNIGENE	FLCDNA	TAIR
GCGGAACTGA	0	2	0	1	0	gnl|UG|At#S11738789	gi|26452212|dbj|AK118595.1|	non-canonical match
at2g32070	CON	MIN	HOUR	DAY	WEEK	UNIGENE	FLCDNA	TAIR
GAGGTCTGAA	0	0	2	1	0	gnl|UG|At#S11734510	gi|15293024|gb|AY050946.1	At2g32070.1
at3g19740	CON	MIN	HOUR	DAY	WEEK	UNIGENE	FLCDNA	TAIR
TCGTAACAAT	1	0	2	0	7	no match found	no match found	At3g19740.1
at5g27600	CON	MIN	HOUR	DAY	WEEK	UNIGENE	FLCDNA	TAIR
GTGTAATTTT	3	0	2	1	1	gnl|UG|At#S11720452	gi|26452250|dbj|AK118614.1|	At5g27600.1
at3g56380	CON	MIN	HOUR	DAY	WEEK	UNIGENE	FLCDNA	TAIR
TTTTAGCAGT	0	0	0	1	0	gnl|UG|At#S11701516	no match found	multiple non-canonical match
at5g06310	CON	MIN	HOUR	DAY	WEEK	UNIGENE	FLCDNA	TAIR
GAACCATACA	3	6	9	5	3	gnl|UG|At#S11724547	gi|62320105|dbj|AK221379.1|	At5g06310.1
at5g20570	CON	MIN	HOUR	DAY	WEEK	UNIGENE	FLCDNA	TAIR
TGTCTAATCG	1	6	3	6	5	gnl|UG|At#S17008027	gi|18252990|gb|AY072430.1	At5g20570.1
at1g12310	CON	MIN	HOUR	DAY	WEEK	UNIGENE	FLCDNA	TAIR
TTTGTTTTCA	8	3	4	3	6	gnl|UG|At#S11741805	no match found	multiple canonical match
GCGAAACATC	0	0	1	0	0	no match found	gi|20334851|gb|AY094026.1	non-canonical match
at1g50120	CON	MIN	HOUR	DAY	WEEK	UNIGENE	FLCDNA	TAIR
CAATAATTTG	1	1	0	0	1	gnl|UG|At#S11734454	gi|20268710|gb|AY091109.1	At1g50120.1
at5g10030	CON	MIN	HOUR	DAY	WEEK	UNIGENE	FLCDNA	TAIR
AGACATTGCA	0	1	0	0	0	no match found	gi|110738576|dbj|AK229351.1|	non-canonical match
at3g50970	CON	MIN	HOUR	DAY	WEEK	UNIGENE	FLCDNA	TAIR
AAAATAAAAG	1	0	3	10	12	no match found	gi|15809983|gb|AY054260.1	At3g50970.1
at2g36000	CON	MIN	HOUR	DAY	WEEK	UNIGENE	FLCDNA	TAIR
GAAGCAAGTA	3	1	1	0	0	gnl|UG|At#S15460731	gi|14532527|gb|AY039888.1	At2g36000.1
AAGCTTGTGC	0	2	0	0	0	no match found	gi|21403701|gb|AY084991.1	At2g36000.2
at5g58070	CON	MIN	HOUR	DAY	WEEK	UNIGENE	FLCDNA	TAIR
AATTTGTTTT	0	0	1	0	0	gnl|UG|At#S11717903	no match found	At5g58070.1
GGCCGTTGGT	1	2	7	14	6	no match found	gi|21404395|gb|AY085685.1	non-canonical match
at1g50290	CON	MIN	HOUR	DAY	WEEK	UNIGENE	FLCDNA	TAIR
TTTATGGAGC	0	1	1	0	0	gnl|UG|At#S11705664	gi|21700824|gb|AY124827.1	At1g50290.1
at1g03050	CON	MIN	HOUR	DAY	WEEK	UNIGENE	FLCDNA	TAIR
GTTGTTTCAT	3	0	0	0	0	no match found	no match found	At1g03050.1
at5g50360	CON	MIN	HOUR	DAY	WEEK	UNIGENE	FLCDNA	TAIR
CAAGTTAGAT	0	0	0	3	3	gnl|UG|At#S11702579	gi|13878116|gb|AF370321.1	At5g50360.1
at5g11460	CON	MIN	HOUR	DAY	WEEK	UNIGENE	FLCDNA	TAIR
GTTTTGGTGG	0	0	1	0	0	no match found	no match found	At5g11460.1
at4g38570	CON	MIN	HOUR	DAY	WEEK	UNIGENE	FLCDNA	TAIR
TTGTGTACGA	0	0	1	1	0	gnl|UG|At#S18908169	gi|27311652|gb|BT002432.1	non-canonical match
at2g26260	CON	MIN	HOUR	DAY	WEEK	UNIGENE	FLCDNA	TAIR
ACTAAAAACC	1	0	1	0	1	gnl|UG|At#S24442940	gi|51970495|dbj|AK176177.1|	At2g26260.1

at3g01040	CON	MIN	HOUR	DAY	WEEK	UNIGENE	FLCDNA	TAIR
ATCACAACAG	1	0	1	0	1	gnl|UG|At#S11740352	gi|110742216|dbj|AK226967.1|	At3g01040.1
at3g20270	CON	MIN	HOUR	DAY	WEEK	UNIGENE	FLCDNA	TAIR
TACGTAAGCC	1	0	0	0	3	gnl|UG|At#S11734762	gi|18176285|gb|AY072195.1	At3g20270.1
at1g64860	CON	MIN	HOUR	DAY	WEEK	UNIGENE	FLCDNA	TAIR
GGAAGACATT	4	2	3	2	1	gnl|UG|At#S11699057	gi|20259915|gb|AY093306.1	At1g64860.1
at1g26450	CON	MIN	HOUR	DAY	WEEK	UNIGENE	FLCDNA	TAIR
TTTCTCTAAA	1	1	0	0	1	gnl|UG|At#S34118099	gi|110736383|dbj|AK228207.1|	multiple non-canonical match
TTTCTCTTTG	0	1	1	1	0	no match found	no match found	At1g26450.1
at4g35600	CON	MIN	HOUR	DAY	WEEK	UNIGENE	FLCDNA	TAIR
AAGGAAGTAG	0	0	1	1	0	gnl|UG|At#S11721666	gi|17529233|gb|AY065403.1	At4g35600.1
at1g23490	CON	MIN	HOUR	DAY	WEEK	UNIGENE	FLCDNA	TAIR
TGCCACTTCA	5	8	6	2	5	gnl|UG|At#S11740712	gi|16323245|gb|AY057727.1	At1g23490.1
at5g38520	CON	MIN	HOUR	DAY	WEEK	UNIGENE	FLCDNA	TAIR
GCTGGCTCAA	4	4	3	3	2	no match found	gi|24899772|gb|BT001214.1	non-canonical match
at1g34000	CON	MIN	HOUR	DAY	WEEK	UNIGENE	FLCDNA	TAIR
TGTCTTCATC	3	8	0	4	2	gnl|UG|At#S11737575	gi|21403239|gb|AY084529.1	At1g34000.2
at2g10940	CON	MIN	HOUR	DAY	WEEK	UNIGENE	FLCDNA	TAIR
TCCAATAGAC	5	16	2	1	2	gnl|UG|At#S11675875	gi|17473864|gb|AY065178.1	non-canonical match
ATACTGTCAA	21	15	18	22	9	gnl|UG|At#S15461603	no match found	At2g10940.2
at3g49940	CON	MIN	HOUR	DAY	WEEK	UNIGENE	FLCDNA	TAIR
TATTCAATGG	1	2	0	0	0	gnl|UG|At#S11729454	gi|27311686|gb|BT002449.1	At3g49940.1
at5g09220	CON	MIN	HOUR	DAY	WEEK	UNIGENE	FLCDNA	TAIR
AACAACAGAG	2	5	4	2	2	gnl|UG|At#S11650790	gi|19699270|gb|AY090341.1	At5g09220.1
at2g29740	CON	MIN	HOUR	DAY	WEEK	UNIGENE	FLCDNA	TAIR
GGTTTGGGCC	0	0	0	0	1	no match found	gi|28973168|gb|BT005489.1	non-canonical match
at4g27320	CON	MIN	HOUR	DAY	WEEK	UNIGENE	FLCDNA	TAIR
TATGACTTTC	3	3	4	5	1	gnl|UG|At#S11723075	gi|51971834|dbj|AK176819.1|	At4g27320.1
at1g69060	CON	MIN	HOUR	DAY	WEEK	UNIGENE	FLCDNA	TAIR
AAGGACAAAT	0	0	1	0	1	gnl|UG|At#S15438527	gi|21407675|gb|AY088901.1	At1g69060.1
at1g70740	CON	MIN	HOUR	DAY	WEEK	UNIGENE	FLCDNA	TAIR
AGACGGGTAT	0	1	0	0	0	gnl|UG|At#S11728937	gi|26449976|dbj|AK117444.1|	At1g70740.1
at3g14067	CON	MIN	HOUR	DAY	WEEK	UNIGENE	FLCDNA	TAIR
GGGTGTGAAT	10	11	10	7	5	gnl|UG|At#S34115776	gi|110738007|dbj|AK229057.1|	multiple non-canonical match
GTTATCAAAT	1	0	0	0	1	no match found	no match found	At3g14067.1
TTGTTAATGG	1	0	0	0	0	no match found	gi|44917460|gb|BT011692.1	multiple non-canonical match
AATTCGGGTC	1	6	3	3	0	no match found	gi|45773915|gb|BT012275.1	non-canonical match
at4g04480	CON	MIN	HOUR	DAY	WEEK	UNIGENE	FLCDNA	TAIR
AATCTTTGTC	0	1	1	0	0	no match found	no match found	At4g04480.1
at3g25840	CON	MIN	HOUR	DAY	WEEK	UNIGENE	FLCDNA	TAIR
GGTGGAGTTC	2	1	2	1	0	gnl|UG|At#S11806922	gi|22136221|gb|AY128789.1	multiple canonical match
ACAGAGTTGA	0	1	0	0	0	no match found	gi|62320365|dbj|AK221509.1|	multiple non-canonical match
at2g13975	CON	MIN	HOUR	DAY	WEEK	UNIGENE	FLCDNA	TAIR

AAGACCCGAA	1	0	1	0	0	no match found	gi|110740400|dbj|AK230294.1|	At2g13975.1
at1g79410	CON	MIN	HOUR	DAY	WEEK	UNIGENE	FLCDNA	TAIR
TATTTTTTAT	1	0	0	1	0	gnl|UG|At#S11726719	gi|21928172|gb|AY125523.1	At1g79410.1
at2g30320	CON	MIN	HOUR	DAY	WEEK	UNIGENE	FLCDNA	TAIR
GATGAATTTG	0	1	0	0	0	gnl|UG|At#S11734959	no match found	multiple non-canonical match
at1g22640	CON	MIN	HOUR	DAY	WEEK	UNIGENE	FLCDNA	TAIR
AGAGTGATGC	2	1	0	1	0	no match found	gi|18176477|gb|AY072543.1	non-canonical match
at1g10550	CON	MIN	HOUR	DAY	WEEK	UNIGENE	FLCDNA	TAIR
CCTGCTGAGT	0	0	0	0	1	gnl|UG|At#S11741978	gi|21405512|gb|AY086802.1	multiple canonical match
at5g55910	CON	MIN	HOUR	DAY	WEEK	UNIGENE	FLCDNA	TAIR
GAAGTGCAGT	0	2	1	2	0	gnl|UG|At#S11718122	gi|110742480|dbj|AK227107.1|	At5g55910.1
at5g16050	CON	MIN	HOUR	DAY	WEEK	UNIGENE	FLCDNA	TAIR
ATTTGGTTAT	12	2	6	2	3	no match found	gi|14334531|gb|AY035170.1	multiple non-canonical match
GAATGATTTG	1	0	0	0	0	no match found	gi|16323453|gb|AY059115.1	multiple non-canonical match
TTTTGGTTGT	1	0	0	0	0	gnl|UG|At#S20798378	no match found	non-canonical match
at3g18890	CON	MIN	HOUR	DAY	WEEK	UNIGENE	FLCDNA	TAIR
AAGCCTCCAA	0	1	0	0	0	no match found	gi|30725479|gb|BT008403.1	multiple non-canonical match
CCTTATATAT	3	1	0	0	2	gnl|UG|At#S11735184	gi|25083200|gb|BT002039.1	At3g18890.1
at2g44500	CON	MIN	HOUR	DAY	WEEK	UNIGENE	FLCDNA	TAIR
TTTTTACGAT	0	3	0	0	0	gnl|UG|At#S18942701	gi|62319105|dbj|AK220874.1|	At2g44500.2
at1g78540	CON	MIN	HOUR	DAY	WEEK	UNIGENE	FLCDNA	TAIR
GATGCAAAAC	0	0	0	1	0	gnl|UG|At#S24442631	gi|62320411|dbj|AK221532.1|	multiple non-canonical match
at1g04860	CON	MIN	HOUR	DAY	WEEK	UNIGENE	FLCDNA	TAIR
AAATTTCATT	0	0	2	2	2	gnl|UG|At#S11742543	gi|17064773|gb|AY062463.1	At1g04860.1
at1g06430	CON	MIN	HOUR	DAY	WEEK	UNIGENE	FLCDNA	TAIR
GACAAGATTG	3	3	4	3	1	no match found	gi|28392857|gb|BT003813.1	multiple canonical match
at4g09680	CON	MIN	HOUR	DAY	WEEK	UNIGENE	FLCDNA	TAIR
AGTGTAGACA	2	0	0	0	0	gnl|UG|At#S34115565	gi|110741261|dbj|AK230383.1|	At4g09680.1
at3g49380	CON	MIN	HOUR	DAY	WEEK	UNIGENE	FLCDNA	TAIR
TGGCTGTCGT	1	0	0	0	0	no match found	no match found	At3g49380.1
at1g23530	CON	MIN	HOUR	DAY	WEEK	UNIGENE	FLCDNA	TAIR
CAGTGAGGTT	1	0	0	0	0	gnl|UG|At#S21737234	gi|51969023|dbj|AK175441.1|	At1g23530.1
at5g28927	CON	MIN	HOUR	DAY	WEEK	UNIGENE	FLCDNA	TAIR
AAGTTTTCAA	1	1	0	0	0	no match found	no match found	At5g28927.1
at1g71170	CON	MIN	HOUR	DAY	WEEK	UNIGENE	FLCDNA	TAIR
GAGTCTCACC	2	1	1	1	3	no match found	gi|21407860|gb|AY089086.1	non-canonical match
at5g28450	CON	MIN	HOUR	DAY	WEEK	UNIGENE	FLCDNA	TAIR
ACAAATTTAA	0	0	1	0	0	no match found	no match found	At5g28450.1
AGGTCCATCA	0	0	0	1	0	gnl|UG|At#S18910770	no match found	multiple non-canonical match
at4g36160	CON	MIN	HOUR	DAY	WEEK	UNIGENE	FLCDNA	TAIR
TTTGATAAAA	0	0	1	1	2	gnl|UG|At#S11721571	no match found	At4g36160.1
at1g77630	CON	MIN	HOUR	DAY	WEEK	UNIGENE	FLCDNA	TAIR

AACACTGCTA	0	0	0	0	1	gnl|UG|At#S18896327	gi|51971734|dbj|AK176769.1|	At1g77630.1
at1g22630	CON	MIN	HOUR	DAY	WEEK	UNIGENE	FLCDNA	TAIR
TAAAAATAAA	9	9	3	10	2	gnl|UG|At#S11740800	gi|21406407|gb|AY087669.1	At1g22630.1
at5g08410	CON	MIN	HOUR	DAY	WEEK	UNIGENE	FLCDNA	TAIR
TGGTTCGTGT	0	2	3	1	1	gnl|UG|At#S11724012	gi|28372907|gb|BT003708.1	At5g08410.1
at1g63810	CON	MIN	HOUR	DAY	WEEK	UNIGENE	FLCDNA	TAIR
GAACAAACAC	1	2	0	0	0	gnl|UG|At#S11730378	gi|110741754|dbj|AK226717.1|	At1g63810.1
at2g27020	CON	MIN	HOUR	DAY	WEEK	UNIGENE	FLCDNA	TAIR
AACCATTGGA	12	2	5	3	10	gnl|UG|At#S11735779	gi|14030728|gb|AF375455.1	At2g27020.1
CAAAGAAGGC	0	2	0	0	0	no match found	gi|21700782|gb|AY124806.1	non-canonical match
at1g44960	CON	MIN	HOUR	DAY	WEEK	UNIGENE	FLCDNA	TAIR
GCTTATTTTG	1	0	2	1	1	gnl|UG|At#S11735682	gi|18176197|gb|AY072180.1	At1g44960.1
at5g57750	CON	MIN	HOUR	DAY	WEEK	UNIGENE	FLCDNA	TAIR
TTGGAATTGA	1	1	0	0	0	no match found	no match found	At5g57750.1
at5g26340	CON	MIN	HOUR	DAY	WEEK	UNIGENE	FLCDNA	TAIR
TATATGTCAA	0	3	2	2	1	gnl|UG|At#S11720559	no match found	At5g26340.1
at4g31980	CON	MIN	HOUR	DAY	WEEK	UNIGENE	FLCDNA	TAIR
AATCTCTCTC	1	2	0	0	1	no match found	gi|17529023|gb|AY065246.1	non-canonical match
GAACAGGTGG	0	1	0	0	0	gnl|UG|At#S18905473	no match found	multiple non-canonical match
at2g32560	CON	MIN	HOUR	DAY	WEEK	UNIGENE	FLCDNA	TAIR
GGGAAAGATA	2	0	2	0	0	gnl|UG|At#S18913798	gi|21404277|gb|AY085567.1	At2g32560.1
at3g12920	CON	MIN	HOUR	DAY	WEEK	UNIGENE	FLCDNA	TAIR
TTAATCTTTC	1	3	1	1	1	gnl|UG|At#S11737020	gi|21407481|gb|AY088707.1	At3g12920.1
at3g21790	CON	MIN	HOUR	DAY	WEEK	UNIGENE	FLCDNA	TAIR
TAGCTCTTAA	2	1	4	0	1	gnl|UG|At#S11734282	no match found	non-canonical match
at3g61140	CON	MIN	HOUR	DAY	WEEK	UNIGENE	FLCDNA	TAIR
CCAGATCGGC	0	1	1	0	0	no match found	gi|26450148|dbj|AK117532.1|	non-canonical match
at1g14340	CON	MIN	HOUR	DAY	WEEK	UNIGENE	FLCDNA	TAIR
AAGTGTGATA	0	1	0	1	0	no match found	gi|15292970|gb|AY050919.1	non-canonical match
ATGATGCTGA	0	1	0	0	1	gnl|UG|At#S11741603	no match found	At1g14340.1
at5g55140	CON	MIN	HOUR	DAY	WEEK	UNIGENE	FLCDNA	TAIR
TCTTGAGAGT	1	0	0	0	0	gnl|UG|At#S11718199	gi|21407470|gb|AY088696.1	At5g55140.1
at5g67100	CON	MIN	HOUR	DAY	WEEK	UNIGENE	FLCDNA	TAIR
CAAGCACACA	0	0	1	0	0	gnl|UG|At#S11716985	no match found	multiple non-canonical match
at2g31740	CON	MIN	HOUR	DAY	WEEK	UNIGENE	FLCDNA	TAIR
GATGCTTGCC	0	2	0	1	0	gnl|UG|At#S15460901	gi|62320758|dbj|AK221706.1|	At2g31740.1
at3g15357	CON	MIN	HOUR	DAY	WEEK	UNIGENE	FLCDNA	TAIR
GAAAGCGAGT	0	0	0	1	0	gnl|UG|At#S11736234	gi|89111861|gb|BT024820.1	At3g15357.1
at2g48070	CON	MIN	HOUR	DAY	WEEK	UNIGENE	FLCDNA	TAIR
AAGATTCTTT	3	5	1	0	2	no match found	gi|15027852|gb|AY045783.1	At2g48070.1
CTCTTTTCCC	1	0	0	0	0	gnl|UG|At#S37211589	no match found	At2g48070.2
at4g01830	CON	MIN	HOUR	DAY	WEEK	UNIGENE	FLCDNA	TAIR

TTTGTAGTTA	0	1	0	0	0	no match found	no match found	At4g01830.1
at2g19340	CON	MIN	HOUR	DAY	WEEK	UNIGENE	FLCDNA	TAIR
AGGCAGTTGA	0	1	0	0	0	no match found	no match found	At2g19340.1
TCTCTTTGGA	0	0	0	1	1	no match found	gi|51969711|dbj|AK175785.1|	At2g19340.2
at1g75980	CON	MIN	HOUR	DAY	WEEK	UNIGENE	FLCDNA	TAIR
ATCAATATAT	0	0	0	0	1	no match found	gi|117168224|gb|BT029381.1	multiple non-canonical match
GGCGTTCGGG	0	1	1	0	1	gnl|UG|At#S11727845	no match found	At1g75980.1
at1g58807	CON	MIN	HOUR	DAY	WEEK	UNIGENE	FLCDNA	TAIR
TCACTCCCAC	1	4	4	2	0	gnl|UG|At#S35312938	no match found	non-canonical match
at1g24577	CON	MIN	HOUR	DAY	WEEK	UNIGENE	FLCDNA	TAIR
ACAAAGCCAG	0	1	1	0	0	gnl|UG|At#S37211431	gi|62320056|dbj|AK221354.1|	non-canonical match
at1g74320	CON	MIN	HOUR	DAY	WEEK	UNIGENE	FLCDNA	TAIR
GAGTATGCAA	0	1	0	0	0	gnl|UG|At#S11728175	gi|22655287|gb|AY140093.1	At1g74320.1
at3g28160	CON	MIN	HOUR	DAY	WEEK	UNIGENE	FLCDNA	TAIR
TTACATTTAT	1	1	1	2	0	gnl|UG|At#S34115910	gi|110740596|dbj|AK226240.1|	At3g28160.1
at4g21890	CON	MIN	HOUR	DAY	WEEK	UNIGENE	FLCDNA	TAIR
ATCAATCTGC	0	1	0	0	0	no match found	gi|60543312|gb|BT021119.1	At4g21890.1
at2g40290	CON	MIN	HOUR	DAY	WEEK	UNIGENE	FLCDNA	TAIR
ACTCATTATT	1	0	0	0	0	gnl|UG|At#S18942741	no match found	At2g40290.2
GTTTTATAAA	2	3	2	0	1	no match found	gi|19347785|gb|AY080668.1	non-canonical match
GCTAAGCTAC	0	0	1	1	0	no match found	gi|21436248|gb|AY117188.1	non-canonical match
at1g02010	CON	MIN	HOUR	DAY	WEEK	UNIGENE	FLCDNA	TAIR
ATCGAGGAGC	0	1	0	0	0	gnl|UG|At#S18898157	no match found	At1g02010.1
at1g80170	CON	MIN	HOUR	DAY	WEEK	UNIGENE	FLCDNA	TAIR
TTTTCAGATT	0	0	1	0	0	no match found	gi|23296941|gb|AY142668.1	multiple non-canonical match
at1g18640	CON	MIN	HOUR	DAY	WEEK	UNIGENE	FLCDNA	TAIR
ATTGGAGATG	0	1	1	0	0	gnl|UG|At#S18942548	gi|17529131|gb|AY065351.1	At1g18640.2
at3g29185	CON	MIN	HOUR	DAY	WEEK	UNIGENE	FLCDNA	TAIR
TACTTTTGTT	0	1	0	0	0	gnl|UG|At#S11799226	no match found	multiple non-canonical match
AATCATCGAC	4	4	6	2	3	gnl|UG|At#S15460410	gi|110736911|dbj|AK228487.1|	At3g29185.1
at3g57270	CON	MIN	HOUR	DAY	WEEK	UNIGENE	FLCDNA	TAIR
TGAAGAATGG	0	1	0	1	1	no match found	no match found	At3g57270.1
at3g08680	CON	MIN	HOUR	DAY	WEEK	UNIGENE	FLCDNA	TAIR
AATGGTTGTA	2	0	2	1	0	gnl|UG|At#S18942154	gi|15982756|gb|AY057485.1	At3g08680.2
at5g40850	CON	MIN	HOUR	DAY	WEEK	UNIGENE	FLCDNA	TAIR
GTCTCCCTTC	0	1	1	1	1	no match found	gi|16604389|gb|AY058093.1	non-canonical match
at5g67200	CON	MIN	HOUR	DAY	WEEK	UNIGENE	FLCDNA	TAIR
TGGGGTTGGA	0	0	1	0	0	gnl|UG|At#S11707705	gi|16649054|gb|AY059897.1	At5g67200.1
at5g32440	CON	MIN	HOUR	DAY	WEEK	UNIGENE	FLCDNA	TAIR
ATGCTGAAGC	0	4	1	1	0	gnl|UG|At#S11702399	gi|13877754|gb|AF370140.1	At5g32440.1
at3g17225	CON	MIN	HOUR	DAY	WEEK	UNIGENE	FLCDNA	TAIR
ATTTGTTGGT	0	1	0	1	0	no match found	gi|110741421|dbj|AK230465.1|	At2g05635.1
ATCTTTTTAC	6	2	5	2	1	gnl|UG|At#S34115483	no match found	non-canonical match

at2g35960	CON	MIN	HOUR	DAY	WEEK	UNIGENE	FLCDNA	TAIR
TCATATAAAT	4	0	1	3	1	gnl|UG|At#S11733586	gi|51969899|dbj|AK175879.1|	non-canonical match
TTGATCAATA	0	1	2	1	6	gnl|UG|At#S30643836	gi|107738274|gb|BT025781.1	multiple canonical match
at1g30510	CON	MIN	HOUR	DAY	WEEK	UNIGENE	FLCDNA	TAIR
ATATGATGAT	1	0	0	0	2	gnl|UG|At#S11738842	gi|21406792|gb|AY088018.1	At1g30510.2
at1g44835	CON	MIN	HOUR	DAY	WEEK	UNIGENE	FLCDNA	TAIR
AAAGCTCGAA	0	3	3	4	3	gnl|UG|At#S11735734	gi|110742856|dbj|AK227311.1|	At1g44835.1
TTGAACCGGA	4	3	8	4	1	no match found	gi|28973134|gb|BT005472.1	multiple non-canonical match
at5g62490	CON	MIN	HOUR	DAY	WEEK	UNIGENE	FLCDNA	TAIR
AACGGTGCTG	0	1	0	0	0	no match found	no match found	At5g62490.1
at1g29970	CON	MIN	HOUR	DAY	WEEK	UNIGENE	FLCDNA	TAIR
CTCGGGATGA	1	8	0	0	1	no match found	gi|149944288|gb|BT030607.1	At5g30440.1
TCGAAAAAAA	0	1	0	1	2	no match found	gi|110738496|dbj|AK229311.1|	At5g30440.1
at3g54810	CON	MIN	HOUR	DAY	WEEK	UNIGENE	FLCDNA	TAIR
CGTTGAAGTG	1	6	0	0	0	gnl|UG|At#S11706401	gi|15724333|gb|AF412107.1	At3g54810.2
at1g17220	CON	MIN	HOUR	DAY	WEEK	UNIGENE	FLCDNA	TAIR
GCAAATGTTC	9	3	10	14	11	gnl|UG|At#S11741322	gi|13605888|gb|AF367343.1	At1g17220.1
TCGGTGTTCT	1	0	0	0	0	no match found	gi|24111274|gb|BT001007.1	non-canonical match
at2g42880	CON	MIN	HOUR	DAY	WEEK	UNIGENE	FLCDNA	TAIR
TCATAAAAGA	4	8	4	3	0	gnl|UG|At#S11731909	gi|15724283|gb|AF412082.1	At2g42880.1
at5g13710	CON	MIN	HOUR	DAY	WEEK	UNIGENE	FLCDNA	TAIR
CCCAAAATAT	7	6	5	3	2	gnl|UG|At#S11722912	no match found	At5g13710.1
at1g06510	CON	MIN	HOUR	DAY	WEEK	UNIGENE	FLCDNA	TAIR
ATTATTAATG	0	1	0	0	0	gnl|UG|At#S11742378	gi|27754365|gb|BT002812.1	At1g06510.1
at5g16505	CON	MIN	HOUR	DAY	WEEK	UNIGENE	FLCDNA	TAIR
CCGTCGTTGG	0	2	0	1	0	gnl|UG|At#S11707760	gi|20259875|gb|AY093286.1	At5g16505.1
at1g73820	CON	MIN	HOUR	DAY	WEEK	UNIGENE	FLCDNA	TAIR
TTAGAAACTG	2	0	1	0	0	gnl|UG|At#S11728282	gi|26453039|dbj|AK119020.1|	At1g73820.1
at1g14730	CON	MIN	HOUR	DAY	WEEK	UNIGENE	FLCDNA	TAIR
CAGAGAGCTA	0	1	0	0	0	gnl|UG|At#S21736514	gi|37202069|gb|BT010628.1	At1g14730.1
at3g26050	CON	MIN	HOUR	DAY	WEEK	UNIGENE	FLCDNA	TAIR
GAGAAGCACA	1	0	0	0	0	gnl|UG|At#S14273906	gi|28827265|gb|BT004944.1	At3g26050.1
at4g38060	CON	MIN	HOUR	DAY	WEEK	UNIGENE	FLCDNA	TAIR
TAAAGTCTTG	1	0	0	0	1	gnl|UG|At#S11706302	gi|15809847|gb|AY054191.1	At4g38060.2
GAGCAATGGC	1	0	0	1	0	no match found	gi|17978852|gb|AY066031.1	multiple non-canonical match
at1g12430	CON	MIN	HOUR	DAY	WEEK	UNIGENE	FLCDNA	TAIR
AAGGGAATGC	1	1	0	0	0	gnl|UG|At#S11701295	no match found	At1g12430.1
at1g26420	CON	MIN	HOUR	DAY	WEEK	UNIGENE	FLCDNA	TAIR
AAATCGGTTT	1	0	1	0	0	gnl|UG|At#S11740176	no match found	At1g26420.1
at1g63430	CON	MIN	HOUR	DAY	WEEK	UNIGENE	FLCDNA	TAIR
GAAGCCCGAG	0	1	0	0	0	gnl|UG|At#S11730451	gi|26450925|dbj|AK117932.1|	At1g63430.1
at1g30410	CON	MIN	HOUR	DAY	WEEK	UNIGENE	FLCDNA	TAIR

ATTACCATTT	0	1	0	0	0	gnl|UG|At#S11738879	no match found		At1g30410.1
at1g67530	CON	MIN	HOUR	DAY	WEEK	UNIGENE	FLCDNA		TAIR
GATCAACTCC	0	0	1	0	0	no match found	gi|30102747|gb|BT006484.1		non-canonical match
TACATACAAG	3	0	2	0	0	gnl|UG|At#S11824606	gi|26449493|dbj|AK117197.1|		At1g67530.1
at5g09670	CON	MIN	HOUR	DAY	WEEK	UNIGENE	FLCDNA		TAIR
GTTCCGGAGG	2	0	2	2	1	gnl|UG|At#S11723819	gi|22530997|gb|AY136337.1		At5g09670.2
at2g37110	CON	MIN	HOUR	DAY	WEEK	UNIGENE	FLCDNA		TAIR
ATCCTTGTCT	5	3	3	2	4	gnl|UG|At#S11733326	gi|110740714|dbj|AK226303.1|		At2g37110.1
at5g55060	CON	MIN	HOUR	DAY	WEEK	UNIGENE	FLCDNA		TAIR
GACCGATAAG	0	1	0	0	0	gnl|UG|At#S21737490	gi|51968511|dbj|AK175185.1|		At5g55060.1
at5g41850	CON	MIN	HOUR	DAY	WEEK	UNIGENE	FLCDNA		TAIR
TGATTGATGG	0	1	0	0	0	gnl|UG|At#S11719544	gi|45752763|gb|BT012186.1		non-canonical match
at4g16990	CON	MIN	HOUR	DAY	WEEK	UNIGENE	FLCDNA		TAIR
GCTTTAATAC	4	4	5	1	3	gnl|UG|At#S11822502	gi|51968607|dbj|AK175233.1|		At4g16990.1
at3g01540	CON	MIN	HOUR	DAY	WEEK	UNIGENE	FLCDNA		TAIR
CTTCGGATCT	4	9	4	5	1	gnl|UG|At#S11709454	gi|17065029|gb|AY062591.1		At3g01540.2
at3g13690	CON	MIN	HOUR	DAY	WEEK	UNIGENE	FLCDNA		TAIR
AATATAATTT	0	1	0	0	0	gnl|UG|At#S18921206	no match found		non-canonical match
at1g26208	CON	MIN	HOUR	DAY	WEEK	UNIGENE	FLCDNA		TAIR
GCAACCCTAG	0	0	0	1	0	gnl|UG|At#S11740257	no match found		At1g26210.1
AATTGGATAA	0	0	0	0	1	gnl|UG|At#S11824852	no match found		non-canonical match
at1g49560	CON	MIN	HOUR	DAY	WEEK	UNIGENE	FLCDNA		TAIR
GAAGATGTTG	0	0	0	0	1	gnl|UG|At#S11734656	gi|21405404|gb|AY086694.1		At1g49560.1
at3g50340	CON	MIN	HOUR	DAY	WEEK	UNIGENE	FLCDNA		TAIR
ATTATTATTC	1	1	0	0	0	gnl|UG|At#S11729380	gi|110743321|dbj|AK227554.1|		At3g50340.1
at4g37080	CON	MIN	HOUR	DAY	WEEK	UNIGENE	FLCDNA		TAIR
AGTTCAATTT	1	0	0	2	0	gnl|UG|At#S18941714	gi|19715621|gb|AY075621.1		At4g37080.2
at5g59430	CON	MIN	HOUR	DAY	WEEK	UNIGENE	FLCDNA		TAIR
GCTACTGGAC	1	1	2	1	0	gnl|UG|At#S11717761	gi|22136005|gb|AY128382.1		At5g59430.1
at5g43600	CON	MIN	HOUR	DAY	WEEK	UNIGENE	FLCDNA		TAIR
GCTAATGGAG	1	0	0	0	0	no match found	gi|18377778|gb|AY074343.1		multiple non-canonical match
TAATTAATAA	0	0	0	0	1	no match found	no match found		At5g43600.1
at4g15953	CON	MIN	HOUR	DAY	WEEK	UNIGENE	FLCDNA		TAIR
ATACGCAACT	0	1	0	0	0	gnl|UG|At#S38433489	gi|51971720|dbj|AK176762.1|		pseudo chromosome match
at1g77540	CON	MIN	HOUR	DAY	WEEK	UNIGENE	FLCDNA		TAIR
GAAGCCTCTG	1	5	0	1	0	gnl|UG|At#S30643363	gi|21404047|gb|AY085337.1		At1g77540.1
at1g71400	CON	MIN	HOUR	DAY	WEEK	UNIGENE	FLCDNA		TAIR
AGTGGTTCAC	0	0	1	0	0	gnl|UG|At#S11728789	no match found		At1g71400.1
at4g01290	CON	MIN	HOUR	DAY	WEEK	UNIGENE	FLCDNA		TAIR
AACTCGACCT	6	5	7	1	3	gnl|UG|At#S34116000	gi|110739739|dbj|AK229951.1|		At4g01290.1
at4g31920	CON	MIN	HOUR	DAY	WEEK	UNIGENE	FLCDNA		TAIR
CATACGAGAA	1	0	1	0	0	gnl|UG|At#S11722288	gi|110736269|dbj|AK228149.1|		At4g31920.1

at1g21520	CON	MIN	HOUR	DAY	WEEK	UNIGENE	FLCDNA	TAIR
TGATTGTAGT	2	2	3	2	2	gnl|UG|At#S22668313	no match found	At1g21520.1
ACGGCAAGCG	0	0	1	0	0	no match found	gi|28827363|gb|BT004993.1	multiple non-canonical match
at1g32990	CON	MIN	HOUR	DAY	WEEK	UNIGENE	FLCDNA	TAIR
TATGTGTTTT	18	16	18	18	15	no match found	gi|13358212|gb|AF325023.2	At1g32990.1
GGGATAGACA	0	0	1	0	0	no match found	gi|17104710|gb|AY063070.1	multiple non-canonical match
at3g26950	CON	MIN	HOUR	DAY	WEEK	UNIGENE	FLCDNA	TAIR
TCTGGAACCG	0	1	2	0	0	gnl|UG|At#S21736407	gi|62320092|dbj|AK221372.1|	At3g26950.1
at2g31840	CON	MIN	HOUR	DAY	WEEK	UNIGENE	FLCDNA	TAIR
GCTTTCTTCT	0	0	2	0	0	gnl|UG|At#S15114798	no match found	At2g31840.1
at3g51370	CON	MIN	HOUR	DAY	WEEK	UNIGENE	FLCDNA	TAIR
AGATCCAACC	1	5	1	1	1	gnl|UG|At#S11729178	gi|15292742|gb|AY050805.1	At3g51370.1
at4g12520	CON	MIN	HOUR	DAY	WEEK	UNIGENE	FLCDNA	TAIR
TTTTTCTTTG	0	1	0	0	1	no match found	gi|110743044|dbj|AK227411.1|	multiple canonical match
at3g01500	CON	MIN	HOUR	DAY	WEEK	UNIGENE	FLCDNA	TAIR
CAGATCTTGA	18	2	19	9	1	no match found	gi|16226254|gb|AF428284.1	At3g01500.1
ACCATCCATC	103	67	64	57	5	gnl|UG|At#S15461641	gi|20148658|gb|AY081658.1	At3g01500.3
at5g17460	CON	MIN	HOUR	DAY	WEEK	UNIGENE	FLCDNA	TAIR
ATCACTCACT	0	0	0	1	1	gnl|UG|At#S37211176	no match found	At3g01500.3
AAAGATATTC	0	0	12	13	17	no match found	gi|13358185|gb|AF324993.2	non-canonical match
at4g25830	CON	MIN	HOUR	DAY	WEEK	UNIGENE	FLCDNA	TAIR
GTCCTCCTCT	1	1	0	0	0	gnl|UG|At#S11723337	gi|51970319|dbj|AK176089.1|	At4g25830.1
at3g45620	CON	MIN	HOUR	DAY	WEEK	UNIGENE	FLCDNA	TAIR
TTCTTAGGCT	2	2	0	0	0	gnl|UG|At#S11824110	gi|21405565|gb|AY086855.1	At3g45620.1
at4g17910	CON	MIN	HOUR	DAY	WEEK	UNIGENE	FLCDNA	TAIR
CCTTACGTTA	0	1	0	0	0	gnl|UG|At#S11724694	no match found	non-canonical match
at1g74690	CON	MIN	HOUR	DAY	WEEK	UNIGENE	FLCDNA	TAIR
AAGAATGCAA	1	0	2	0	0	gnl|UG|At#S11728099	gi|20466425|gb|AY099679.1	multiple non-canonical match
at2g11390	CON	MIN	HOUR	DAY	WEEK	UNIGENE	FLCDNA	TAIR
TTTCACAAAA	0	1	0	0	1	no match found	no match found	At2g11390.1
at3g03340	CON	MIN	HOUR	DAY	WEEK	UNIGENE	FLCDNA	TAIR
TATGTTACTC	1	1	0	0	0	gnl|UG|At#S11739693	gi|21407348|gb|AY088574.1	At3g03340.1
at1g78570	CON	MIN	HOUR	DAY	WEEK	UNIGENE	FLCDNA	TAIR
ACTGTGTTGG	0	1	1	5	1	gnl|UG|At#S11727004	gi|20148284|gb|AY081471.1	non-canonical match
at3g58030	CON	MIN	HOUR	DAY	WEEK	UNIGENE	FLCDNA	TAIR
CTCCTGTTGT	1	1	2	1	1	gnl|UG|At#S11727946	gi|19698884|gb|AY081289.1	At3g58030.2
at2g17250	CON	MIN	HOUR	DAY	WEEK	UNIGENE	FLCDNA	TAIR
AAAACGAGTT	0	0	0	0	1	gnl|UG|At#S11709588	gi|18086411|gb|AY065021.1	At2g17250.1
at1g04000	CON	MIN	HOUR	DAY	WEEK	UNIGENE	FLCDNA	TAIR
AACACCAAAA	0	1	0	1	0	gnl|UG|At#S11742686	gi|21407637|gb|AY088863.1	At1g04000.1
at1g36320	CON	MIN	HOUR	DAY	WEEK	UNIGENE	FLCDNA	TAIR
ATGAAAGAAG	0	2	0	0	1	no match found	gi|21655272|gb|AY103296.1	At5g67640.1

TTTCAGTAGT	2	3	0	0	1	gnl|UG|At#S11736783	no match found	At1g36320.1
at3g60250	CON	MIN	HOUR	DAY	WEEK	UNIGENE	FLCDNA	TAIR
TAAAACTCAT	4	1	2	0	1	gnl|UG|At#S11727409	gi|110738340|dbj|AK229231.1|	At3g60250.1
at3g63490	CON	MIN	HOUR	DAY	WEEK	UNIGENE	FLCDNA	TAIR
TCAGGACGCA	24	11	23	19	10	no match found	gi|24899726|gb|BT001191.1	non-canonical match
TGATTTACTA	2	1	0	0	0	gnl|UG|At#S11726353	no match found	non-canonical match
CCAAATCCCA	0	2	2	0	1	no match found	gi|22137007|gb|AY133519.1	non-canonical match
at1g49200	CON	MIN	HOUR	DAY	WEEK	UNIGENE	FLCDNA	TAIR
TCTTTAGCTA	1	2	0	0	0	gnl|UG|At#S11734787	gi|14517431|gb|AY039551.1	At1g49200.1
at5g05530	CON	MIN	HOUR	DAY	WEEK	UNIGENE	FLCDNA	TAIR
TTTTTCATAA	0	1	0	0	0	gnl|UG|At#S11724754	gi|26452928|dbj|AK118964.1|	At5g05530.1
at2g46160	CON	MIN	HOUR	DAY	WEEK	UNIGENE	FLCDNA	TAIR
TAACGTTGAC	1	0	0	1	0	no match found	gi|62320379|dbj|AK221516.1|	pseudo chromosome match
at3g42722	CON	MIN	HOUR	DAY	WEEK	UNIGENE	FLCDNA	TAIR
GTGGAAGGAA	0	0	2	0	0	no match found	no match found	At3g42722.1
at3g28956	CON	MIN	HOUR	DAY	WEEK	UNIGENE	FLCDNA	TAIR
AATTGAAGCA	0	1	0	0	0	gnl|UG|At#S16341565	no match found	At3g28956.1
at5g60820	CON	MIN	HOUR	DAY	WEEK	UNIGENE	FLCDNA	TAIR
GCTTGGGATT	1	0	0	0	0	gnl|UG|At#S11717620	no match found	At5g60820.1
at3g42924	CON	MIN	HOUR	DAY	WEEK	UNIGENE	FLCDNA	TAIR
TTTCTACAAA	2	0	0	1	0	no match found	no match found	At3g42924.1
at1g08360	CON	MIN	HOUR	DAY	WEEK	UNIGENE	FLCDNA	TAIR
GGAGTTGCAG	7	14	13	6	5	gnl|UG|At#S11742199	gi|17473552|gb|AY065077.1	At1g08360.1
at4g04800	CON	MIN	HOUR	DAY	WEEK	UNIGENE	FLCDNA	TAIR
TTTTCAAAGG	1	0	0	0	0	no match found	gi|20453300|gb|AY097373.1	multiple non-canonical match
ACTATTTAAA	0	1	0	0	2	gnl|UG|At#S11726439	gi|21406703|gb|AY087929.1	At4g04800.1
at2g20000	CON	MIN	HOUR	DAY	WEEK	UNIGENE	FLCDNA	TAIR
AGTTGATCTG	1	1	2	0	0	gnl|UG|At#S15461375	gi|17064787|gb|AY062470.1	At2g20000.1
at3g53130	CON	MIN	HOUR	DAY	WEEK	UNIGENE	FLCDNA	TAIR
ATAATTGATT	0	2	2	1	0	no match found	gi|20258841|gb|AY091083.1	non-canonical match
at5g06130	CON	MIN	HOUR	DAY	WEEK	UNIGENE	FLCDNA	TAIR
GGAAAGTATC	7	5	10	12	4	gnl|UG|At#S15461322	gi|18176019|gb|AY072147.1	At5g06130.2
ACCCCCGCTT	0	0	0	0	1	no match found	gi|22136729|gb|AY133750.1	non-canonical match
at1g51630	CON	MIN	HOUR	DAY	WEEK	UNIGENE	FLCDNA	TAIR
CTTTTGCTGA	2	1	0	3	2	gnl|UG|At#S11733908	no match found	At1g51630.1
at3g27990	CON	MIN	HOUR	DAY	WEEK	UNIGENE	FLCDNA	TAIR
GAATGGTATT	0	0	2	0	0	gnl|UG|At#S18902523	gi|110742731|dbj|AK227240.1|	At3g27990.1
at1g69325	CON	MIN	HOUR	DAY	WEEK	UNIGENE	FLCDNA	TAIR
ATGATGTTAT	0	0	1	0	0	no match found	no match found	At1g69325.1
at3g02710	CON	MIN	HOUR	DAY	WEEK	UNIGENE	FLCDNA	TAIR
GGACCAGATA	2	0	0	0	0	gnl|UG|At#S11739878	gi|26452562|dbj|AK118773.1|	At3g02710.1
at1g17100	CON	MIN	HOUR	DAY	WEEK	UNIGENE	FLCDNA	TAIR

GCCACCGGTT	0	2	0	6	0	gnl|UG|At#S11741335	gi|21405686|gb|AY086962.1	non-canonical match
at4g28910	CON	MIN	HOUR	DAY	WEEK	UNIGENE	FLCDNA	TAIR
GGAATGACTG	0	2	0	1	0	gnl|UG|At#S15460586	gi|20466050|gb|AY096726.1	At4g28910.1
at1g73120	CON	MIN	HOUR	DAY	WEEK	UNIGENE	FLCDNA	TAIR
AAGGTACGGG	0	0	1	0	0	gnl|UG|At#S11824523	gi|28416834|gb|BT004702.1	At1g73120.1
at4g01960	CON	MIN	HOUR	DAY	WEEK	UNIGENE	FLCDNA	TAIR
TTTGTCTCTC	1	1	1	0	0	gnl|UG|At#S11727191	gi|21403841|gb|AY085131.1	multiple canonical match
ATAGTGACGG	0	0	1	0	0	gnl|UG|At#S31153424	gi|88196762|gb|BT024626.1	non-canonical match
at3g25185	CON	MIN	HOUR	DAY	WEEK	UNIGENE	FLCDNA	TAIR
GAACAGAGAA	0	0	2	0	0	no match found	no match found	At3g25185.1
at4g34770	CON	MIN	HOUR	DAY	WEEK	UNIGENE	FLCDNA	TAIR
ATAAGGGAGC	0	3	0	0	0	gnl|UG|At#S15644852	gi|30793862|gb|BT008557.1	At4g34770.1
at2g27740	CON	MIN	HOUR	DAY	WEEK	UNIGENE	FLCDNA	TAIR
AATGGCAGTC	1	0	0	0	0	gnl|UG|At#S11735611	gi|48310366|gb|BT014824.1	At2g27740.1
at1g04350	CON	MIN	HOUR	DAY	WEEK	UNIGENE	FLCDNA	TAIR
CATCTATTGG	10	4	3	4	0	gnl|UG|At#S11742597	gi|15292974|gb|AY050921.1	At1g04350.1
at4g34970	CON	MIN	HOUR	DAY	WEEK	UNIGENE	FLCDNA	TAIR
GAGATGAAAT	0	1	0	0	0	gnl|UG|At#S11721767	no match found	multiple non-canonical match
at4g00830	CON	MIN	HOUR	DAY	WEEK	UNIGENE	FLCDNA	TAIR
ATGGTAACCG	0	1	2	0	0	no match found	gi|21360554|gb|AY113171.1	non-canonical match
TGTTATAAAA	0	1	0	0	2	gnl|UG|At#S28282115	gi|110743367|dbj|AK227578.1|	At4g00830.1
at2g46720	CON	MIN	HOUR	DAY	WEEK	UNIGENE	FLCDNA	TAIR
TATCGAGGCA	0	1	0	0	1	no match found	no match found	At2g46720.1
at2g22090	CON	MIN	HOUR	DAY	WEEK	UNIGENE	FLCDNA	TAIR
CAGTTTCTGG	0	0	1	0	0	no match found	gi|51969709|dbj|AK175784.1|	At2g22090.1
at3g47500	CON	MIN	HOUR	DAY	WEEK	UNIGENE	FLCDNA	TAIR
TTTTGGTTCT	2	1	8	2	3	gnl|UG|At#S11729903	no match found	At3g47500.1
at2g18700	CON	MIN	HOUR	DAY	WEEK	UNIGENE	FLCDNA	TAIR
AGCAACAGAA	1	1	0	0	0	no match found	gi|20148416|gb|AY081537.1	multiple non-canonical match
TGAGATGCTT	0	5	4	3	1	gnl|UG|At#S11737804	gi|14596154|gb|AY042865.1	At2g18700.1
at3g07810	CON	MIN	HOUR	DAY	WEEK	UNIGENE	FLCDNA	TAIR
GTTTGGTTTT	1	0	0	1	1	no match found	gi|16930692|gb|AF436830.1	multiple non-canonical match
TAGGGCTTTC	3	2	1	3	1	gnl|UG|At#S11738411	gi|15292696|gb|AY050782.1	At3g07810.1
at3g55970	CON	MIN	HOUR	DAY	WEEK	UNIGENE	FLCDNA	TAIR
TTCAAGTAAA	0	0	0	1	0	no match found	gi|110738291|dbj|AK229205.1|	pseudo chromosome match
at1g56650	CON	MIN	HOUR	DAY	WEEK	UNIGENE	FLCDNA	TAIR
CCTTGGACTT	0	0	1	0	1	gnl|UG|At#S11700697	gi|62320625|dbj|AK221639.1|	At1g56650.1
at4g38495	CON	MIN	HOUR	DAY	WEEK	UNIGENE	FLCDNA	TAIR
TTGAATCCTT	1	0	2	1	0	gnl|UG|At#S11806605	gi|20268790|gb|AY091150.1	At4g38495.1
at4g34131	CON	MIN	HOUR	DAY	WEEK	UNIGENE	FLCDNA	TAIR
GCCTGTAGCA	0	0	0	1	0	gnl|UG|At#S11721914	gi|21387190|gb|AY114680.1	At4g34131.1
at5g02050	CON	MIN	HOUR	DAY	WEEK	UNIGENE	FLCDNA	TAIR

TTTTTAATGG	2	1	0	0	1	gnl|UG|At#S11725645	gi|21404903|gb|AY086193.1	At5g02050.1	
at5g38860	CON	MIN	HOUR	DAY	WEEK	UNIGENE	FLCDNA	TAIR	
ATATCTGTGC	1	2	0	0	0	gnl|UG|At#S18911266	no match found	At5g38860.1	
at2g37420	CON	MIN	HOUR	DAY	WEEK	UNIGENE	FLCDNA	TAIR	
GATAAATCTG	0	0	0	1	0	gnl|UG|At#S34117610	gi|110737311|dbj|AK228698.1|	At2g37420.1	
at1g14290	CON	MIN	HOUR	DAY	WEEK	UNIGENE	FLCDNA	TAIR	
ATGTTCACCA	0	3	0	0	0	gnl|UG|At#S11741608	gi|24797013|gb|BT001128.1	At1g14290.1	
at1g07420	CON	MIN	HOUR	DAY	WEEK	UNIGENE	FLCDNA	TAIR	
TGTCTTAGTT	0	0	3	3	3	gnl|UG|At#S18942629	gi|27754334|gb|BT002793.1	At1g07420.2	
at2g19600	CON	MIN	HOUR	DAY	WEEK	UNIGENE	FLCDNA	TAIR	
TGCTCTGAAA	0	0	1	0	0	gnl|UG|At#S11806635	gi|20268730|gb|AY091119.1	At2g19600.1	
at5g65930	CON	MIN	HOUR	DAY	WEEK	UNIGENE	FLCDNA	TAIR	
AAATTCGTCT	0	1	2	2	3	gnl|UG|At#S11717105	gi|110739743|dbj|AK229953.1|	At5g65930.2	
TCGCAGTTGA	2	1	2	1	2	no match found	gi|14517406|gb|AY039538.1	multiple non-canonical match	
at5g55130	CON	MIN	HOUR	DAY	WEEK	UNIGENE	FLCDNA	TAIR	
TACTCGTTTG	1	3	0	0	1	gnl|UG|At#S14274085	gi|28392956|gb|BT003864.1	At5g55130.1	
at1g49975	CON	MIN	HOUR	DAY	WEEK	UNIGENE	FLCDNA	TAIR	
GCTTTAACAG	0	4	0	0	0	gnl|UG|At#S11734513	gi|88011186|gb|BT024576.1	At1g49975.1	
at2g47880	CON	MIN	HOUR	DAY	WEEK	UNIGENE	FLCDNA	TAIR	
TCCCTTCACC	0	1	0	0	0	gnl|UG|At#S24442720	gi|62320233|dbj|AK221443.1|	At2g47880.1	
at1g66890	CON	MIN	HOUR	DAY	WEEK	UNIGENE	FLCDNA	TAIR	
TCATCTGTTA	0	3	0	0	0	gnl|UG|At#S30641972	gi|109946560|gb|BT026103.1	At1g66890.1	
at3g22990	CON	MIN	HOUR	DAY	WEEK	UNIGENE	FLCDNA	TAIR	
TGTTTTGTTT	0	0	1	1	0	no match found	no match found	At3g22990.1	
TAAAAGTCTT	1	0	0	0	0	no match found	gi|20465316|gb|AY096422.1	non-canonical match	
at5g56420	CON	MIN	HOUR	DAY	WEEK	UNIGENE	FLCDNA	TAIR	
TGCTTCTTAT	0	1	0	0	0	gnl|UG|At#S11705715	gi|15982910|gb|AY057563.1	At5g56420.2	
at1g36370	CON	MIN	HOUR	DAY	WEEK	UNIGENE	FLCDNA	TAIR	
GAAAGTCACA	0	3	1	0	2	gnl|UG|At#S11744091	gi|19310579|gb|AY080702.1	At1g36370.1	
at4g12720	CON	MIN	HOUR	DAY	WEEK	UNIGENE	FLCDNA	TAIR	
TGTGTGTCCA	1	9	6	6	5	gnl|UG|At#S15461227	gi|13877892|gb|AF370209.1	At4g12720.3	
at4g00340	CON	MIN	HOUR	DAY	WEEK	UNIGENE	FLCDNA	TAIR	
GATTATGTCT	0	2	0	0	0	gnl|UG|At#S11727591	gi|26451703|dbj|AK118333.1|	At4g00340.1	
at4g36760	CON	MIN	HOUR	DAY	WEEK	UNIGENE	FLCDNA	TAIR	
TCTTTATTCA	2	1	0	0	3	gnl|UG|At#S11721476	gi|13430673|gb|AF360249.1	At4g36760.2	
at2g47600	CON	MIN	HOUR	DAY	WEEK	UNIGENE	FLCDNA	TAIR	
TCTCAGTTTT	1	1	1	0	2	gnl|UG|At#S11743884	gi|19347855|gb|AY080737.1	At2g47600.1	
at5g04440	CON	MIN	HOUR	DAY	WEEK	UNIGENE	FLCDNA	TAIR	
TTAATGATCC	5	0	1	0	1	gnl|UG|At#S11725033	no match found	At5g04440.1	
GGCTTCGGGA	1	1	0	0	0	no match found	gi|87116597|gb|BT024482.1	non-canonical match	
at1g31910	CON	MIN	HOUR	DAY	WEEK	UNIGENE	FLCDNA	TAIR	
TATTACTTCA	2	1	1	0	2	no match found	gi|110740133|dbj|AK230156.1|	At1g31910.1	

at5g25510	CON	MIN	HOUR	DAY	WEEK	UNIGENE	FLCDNA	TAIR
TCTTGCCACG	0	1	0	0	0	no match found	gi|23296588|gb|AY142557.1	non-canonical match
CTGCAGATTT	4	4	1	5	3	gnl|UG|At#S11707981	gi|16604606|gb|AY059748.1	At5g25510.1
at3g62310	CON	MIN	HOUR	DAY	WEEK	UNIGENE	FLCDNA	TAIR
TCGGCATCTG	0	0	1	0	0	gnl|UG|At#S11726751	gi|21539574|gb|AY120782.1	At3g62310.1
at4g11170	CON	MIN	HOUR	DAY	WEEK	UNIGENE	FLCDNA	TAIR
CAGAAAGAGA	1	0	0	1	0	gnl|UG|At#S11725847	no match found	non-canonical match
at1g32350	CON	MIN	HOUR	DAY	WEEK	UNIGENE	FLCDNA	TAIR
TGTGTGTAAA	0	0	1	0	0	gnl|UG|At#S11738145	gi|21404169|gb|AY085459.1	At1g32350.1
AACTCAAGGA	0	0	1	0	0	no match found	gi|115311484|gb|BT029014.1	non-canonical match
at4g24900	CON	MIN	HOUR	DAY	WEEK	UNIGENE	FLCDNA	TAIR
TTGAGCTATG	0	1	0	0	0	gnl|UG|At#S11723492	gi|22136381|gb|AY128869.1	At4g24900.1
at5g13850	CON	MIN	HOUR	DAY	WEEK	UNIGENE	FLCDNA	TAIR
AGTTTATACT	6	0	0	2	4	gnl|UG|At#S11722874	no match found	At5g13850.1
at2g37035	CON	MIN	HOUR	DAY	WEEK	UNIGENE	FLCDNA	TAIR
AGGTGATCAT	0	2	0	0	1	gnl|UG|At#S15460690	no match found	At2g37035.1
at5g51770	CON	MIN	HOUR	DAY	WEEK	UNIGENE	FLCDNA	TAIR
TCGTGGGCGA	0	1	0	0	0	gnl|UG|At#S11718539	no match found	non-canonical match
at3g12500	CON	MIN	HOUR	DAY	WEEK	UNIGENE	FLCDNA	TAIR
CCGTGATAGC	0	5	1	0	0	gnl|UG|At#S11737148	gi|20148380|gb|AY081519.1	At3g12500.1
at5g01350	CON	MIN	HOUR	DAY	WEEK	UNIGENE	FLCDNA	TAIR
GGTCAGCTCA	3	5	7	4	4	gnl|UG|At#S18910641	gi|15010719|gb|AY045661.1	At5g01350.1
at5g12400	CON	MIN	HOUR	DAY	WEEK	UNIGENE	FLCDNA	TAIR
AAGTTAGTAT	1	2	0	0	0	no match found	no match found	At5g12400.1
at3g10340	CON	MIN	HOUR	DAY	WEEK	UNIGENE	FLCDNA	TAIR
ATTTGTGTTT	0	0	2	0	0	gnl|UG|At#S11737785	gi|23198087|gb|BT000252.1	At3g10340.1
at4g24270	CON	MIN	HOUR	DAY	WEEK	UNIGENE	FLCDNA	TAIR
GGGATTTGGA	0	1	0	0	1	gnl|UG|At#S11723598	gi|18700130|gb|AY075670.1	At4g24270.2
at1g11050	CON	MIN	HOUR	DAY	WEEK	UNIGENE	FLCDNA	TAIR
TAAAATTCTG	0	3	7	0	0	gnl|UG|At#S11741930	gi|20466405|gb|AY099669.1	At1g11050.1
at5g17920	CON	MIN	HOUR	DAY	WEEK	UNIGENE	FLCDNA	TAIR
GTTGATGCGG	27	56	34	34	15	gnl|UG|At#S11721841	gi|13877642|gb|AF370522.1	At5g17920.1
ATGAATCCCT	0	0	0	1	0	gnl|UG|At#S30643240	no match found	non-canonical match
at5g05870	CON	MIN	HOUR	DAY	WEEK	UNIGENE	FLCDNA	TAIR
AAATGGGATT	1	1	0	1	1	gnl|UG|At#S34117997	gi|110736576|dbj|AK228311.1|	At5g05870.1
at2g07340	CON	MIN	HOUR	DAY	WEEK	UNIGENE	FLCDNA	TAIR
TTCTAGTTAA	2	0	1	1	4	gnl|UG|At#S15461638	gi|21406034|gb|AY087310.1	At2g07340.1
at2g43090	CON	MIN	HOUR	DAY	WEEK	UNIGENE	FLCDNA	TAIR
AGAGTCACTT	8	4	11	12	6	no match found	gi|14334969|gb|AY035158.1	multiple non-canonical match
at3g12400	CON	MIN	HOUR	DAY	WEEK	UNIGENE	FLCDNA	TAIR
TTGGGGATCT	3	0	0	1	0	gnl|UG|At#S11705987	gi|15810488|gb|AY056283.1	At3g12400.1

at1g79630	CON	MIN	HOUR	DAY	WEEK	UNIGENE	FLCDNA	TAIR
TTAGCTGTAG	1	0	2	0	0	gnl|UG|At#S11744105	gi|19310555|gb|AY080600.1	At1g79630.2
GAAGTCTCAA	0	0	0	1	0	no match found	gi|21280854|gb|AY114037.1	multiple non-canonical match
at4g04230	CON	MIN	HOUR	DAY	WEEK	UNIGENE	FLCDNA	TAIR
CGCCTAATGA	0	0	0	0	1	no match found	no match found	At4g04230.1
at5g19960	CON	MIN	HOUR	DAY	WEEK	UNIGENE	FLCDNA	TAIR
AACTAAATAA	1	1	1	0	0	gnl|UG|At#S11721333	gi|21404179|gb|AY085469.1	At5g19960.1
at4g39350	CON	MIN	HOUR	DAY	WEEK	UNIGENE	FLCDNA	TAIR
TACTTTTAGT	2	3	2	4	0	gnl|UG|At#S11721037	no match found	At4g39350.1
at4g33230	CON	MIN	HOUR	DAY	WEEK	UNIGENE	FLCDNA	TAIR
ACAAGAAAAC	67	18	77	100	103	no match found	no match found	At4g33230.1
at3g03850	CON	MIN	HOUR	DAY	WEEK	UNIGENE	FLCDNA	TAIR
TGAATAGATA	0	2	0	0	0	gnl|UG|At#S17865570	gi|38603905|gb|BT010920.1	At3g03850.1
at1g11530	CON	MIN	HOUR	DAY	WEEK	UNIGENE	FLCDNA	TAIR
GAATTTCTAT	0	4	1	0	0	gnl|UG|At#S11741882	gi|30794085|gb|BT008678.1	At1g11530.1
at5g26240	CON	MIN	HOUR	DAY	WEEK	UNIGENE	FLCDNA	TAIR
TTGCCACCCA	0	2	0	1	0	no match found	gi|26449821|dbj|AK117364.1|	non-canonical match
at3g30749	CON	MIN	HOUR	DAY	WEEK	UNIGENE	FLCDNA	TAIR
AGTTCATTAT	1	0	0	0	0	gnl|UG|At#S35247712	no match found	At1g53330.1
at4g19860	CON	MIN	HOUR	DAY	WEEK	UNIGENE	FLCDNA	TAIR
CCTCGTGTAC	5	14	5	3	0	gnl|UG|At#S11724353	gi|110742758|dbj|AK227253.1|	At4g19860.1
at1g20830	CON	MIN	HOUR	DAY	WEEK	UNIGENE	FLCDNA	TAIR
AAGCTATGCA	1	3	0	0	0	gnl|UG|At#S11740973	gi|26452944|dbj|AK118972.1|	At1g20830.1
at3g48110	CON	MIN	HOUR	DAY	WEEK	UNIGENE	FLCDNA	TAIR
CAATTGGTGG	1	1	1	0	0	gnl|UG|At#S11729782	gi|22531149|gb|AY136413.1	At3g48110.2
at4g10000	CON	MIN	HOUR	DAY	WEEK	UNIGENE	FLCDNA	TAIR
TCTTTTTTTT	1	1	0	1	0	no match found	no match found	At4g10000.2
GTCAAAATCT	0	1	0	0	0	no match found	gi|21406763|gb|AY087989.1	At4g10000.1
at5g45100	CON	MIN	HOUR	DAY	WEEK	UNIGENE	FLCDNA	TAIR
ACCATTTTTT	0	0	1	0	0	gnl|UG|At#S11719217	gi|16604441|gb|AY058839.1	At5g45100.1
at3g30800	CON	MIN	HOUR	DAY	WEEK	UNIGENE	FLCDNA	TAIR
AATCAGCCAG	0	0	0	1	0	gnl|UG|At#S37211201	no match found	pseudo chromosome match
at5g64070	CON	MIN	HOUR	DAY	WEEK	UNIGENE	FLCDNA	TAIR
ATATTTCATT	1	0	1	2	0	gnl|UG|At#S11717292	no match found	At5g64070.1
ATGTGATATT	0	2	1	0	1	no match found	gi|18087620|gb|AF462854.1	non-canonical match
at1g03960	CON	MIN	HOUR	DAY	WEEK	UNIGENE	FLCDNA	TAIR
AAATTTTTGC	0	1	0	3	0	gnl|UG|At#S18942649	gi|28393300|gb|BT004045.1	At1g03960.2
at2g15930	CON	MIN	HOUR	DAY	WEEK	UNIGENE	FLCDNA	TAIR
TTCTGTTGAT	0	1	0	0	0	no match found	no match found	At2g15930.1
at5g63180	CON	MIN	HOUR	DAY	WEEK	UNIGENE	FLCDNA	TAIR
TGTGTATATA	4	0	1	1	2	gnl|UG|At#S11717382	no match found	At5g63180.1
at4g30160	CON	MIN	HOUR	DAY	WEEK	UNIGENE	FLCDNA	TAIR

GCTTGTTTAA	0	1	1	0	0	gnl|UG|At#S11722590	gi|26449687|dbj|AK117296.1|	At4g30160.1
at5g26780	CON	MIN	HOUR	DAY	WEEK	UNIGENE	FLCDNA	TAIR
GACCAGAGGC	1	0	2	1	1	gnl|UG|At#S11720534	no match found	At5g26780.1
at5g50100	CON	MIN	HOUR	DAY	WEEK	UNIGENE	FLCDNA	TAIR
AATAATTTCA	8	2	7	10	9	gnl|UG|At#S11718706	gi|21406196|gb|AY087459.1	At5g50100.1
at5g08470	CON	MIN	HOUR	DAY	WEEK	UNIGENE	FLCDNA	TAIR
GTAAGTTCTA	1	1	1	2	2	gnl|UG|At#S11723995	gi|20453046|gb|AY094389.1	At5g08470.1
at3g26180	CON	MIN	HOUR	DAY	WEEK	UNIGENE	FLCDNA	TAIR
TTCCGGTTCT	1	0	1	0	0	gnl|UG|At#S11732916	gi|15810462|gb|AY056270.1	At3g26180.2
at5g20380	CON	MIN	HOUR	DAY	WEEK	UNIGENE	FLCDNA	TAIR
GAGAAACTTT	2	0	4	3	2	gnl|UG|At#S11721233	gi|20465259|gb|AY095993.1	At5g20380.1
at3g58560	CON	MIN	HOUR	DAY	WEEK	UNIGENE	FLCDNA	TAIR
TCCCTGGGGT	2	0	1	0	0	gnl|UG|At#S11727848	gi|18087603|gb|AF462845.1	At3g58560.1
at1g33780	CON	MIN	HOUR	DAY	WEEK	UNIGENE	FLCDNA	TAIR
TTGCTGCGTG	2	3	2	0	1	gnl|UG|At#S11737665	gi|20148514|gb|AY081586.1	At1g33780.1
at3g07610	CON	MIN	HOUR	DAY	WEEK	UNIGENE	FLCDNA	TAIR
TCTGATGCGG	0	1	0	0	0	no match found	gi|51971856|dbj|AK176830.1|	non-canonical match
CAGTTGATAA	0	1	0	0	0	gnl|UG|At#S11738458	no match found	At3g07610.1
at4g33690	CON	MIN	HOUR	DAY	WEEK	UNIGENE	FLCDNA	TAIR
CACTCTGTGT	0	0	0	1	0	no match found	no match found	At4g33690.1
at3g58510	CON	MIN	HOUR	DAY	WEEK	UNIGENE	FLCDNA	TAIR
TTGTAAACTT	0	3	0	0	0	no match found	gi|17064739|gb|AY062446.1	multiple canonical match
at4g16650	CON	MIN	HOUR	DAY	WEEK	UNIGENE	FLCDNA	TAIR
TACATTTGTC	0	0	1	0	0	gnl|UG|At#S11724916	gi|20856497|gb|AY102099.1	At4g16650.1
at5g54630	CON	MIN	HOUR	DAY	WEEK	UNIGENE	FLCDNA	TAIR
TTGGGGAAAA	2	3	0	0	1	gnl|UG|At#S11718251	gi|15010657|gb|AY045630.1	At5g54630.1
at2g19800	CON	MIN	HOUR	DAY	WEEK	UNIGENE	FLCDNA	TAIR
TCTTCAATAA	0	6	1	0	0	gnl|UG|At#S11737522	gi|13877656|gb|AF370587.1	At2g19800.1
at5g07150	CON	MIN	HOUR	DAY	WEEK	UNIGENE	FLCDNA	TAIR
TTTATTCCTT	2	1	0	0	4	no match found	no match found	At5g07150.1
at2g45560	CON	MIN	HOUR	DAY	WEEK	UNIGENE	FLCDNA	TAIR
CCGTACCCGT	0	3	3	2	1	gnl|UG|At#S15460347	no match found	At2g45560.1
at5g35610	CON	MIN	HOUR	DAY	WEEK	UNIGENE	FLCDNA	TAIR
TACAAAAAGA	3	2	2	0	0	no match found	no match found	At5g35610.1
at3g54620	CON	MIN	HOUR	DAY	WEEK	UNIGENE	FLCDNA	TAIR
CAGAATCCAT	0	1	3	0	1	no match found	gi|20148330|gb|AY081494.1	multiple non-canonical match
ATGGAAGAAT	2	4	1	1	1	gnl|UG|At#S43849708	no match found	At3g54620.1
at4g09430	CON	MIN	HOUR	DAY	WEEK	UNIGENE	FLCDNA	TAIR
TATACTCACT	1	0	0	0	0	no match found	gi|110735764|dbj|AK227888.1|	multiple non-canonical match
at1g31780	CON	MIN	HOUR	DAY	WEEK	UNIGENE	FLCDNA	TAIR
CTTTATTTGA	0	0	0	1	0	gnl|UG|At#S11738368	gi|110740764|dbj|AK226330.1|	At1g31780.1

at3g62830	CON	MIN	HOUR	DAY	WEEK	UNIGENE	FLCDNA	TAIR
TTCTCACTGT	1	0	0	0	0	no match found	no match found	At3g62830.1
GACCTCACTA	4	3	3	5	7	gnl|UG|At#S11726591	gi|15215720|gb|AY050389.1	non-canonical match
at5g55030	CON	MIN	HOUR	DAY	WEEK	UNIGENE	FLCDNA	TAIR
TGAATCAAAA	0	1	1	1	0	gnl|UG|At#S11718209	gi|18377657|gb|AY074282.1	multiple canonical match
at2g37470	CON	MIN	HOUR	DAY	WEEK	UNIGENE	FLCDNA	TAIR
CGGTTTCTGA	2	2	7	2	0	gnl|UG|At#S30580219	gi|28950692|gb|BT005206.1	At2g37470.1
at1g12000	CON	MIN	HOUR	DAY	WEEK	UNIGENE	FLCDNA	TAIR
GTCTAAATTT	1	1	1	0	0	gnl|UG|At#S11741836	no match found	At1g12000.1
GGACAGTCAC	2	2	0	1	0	no match found	gi|20259823|gb|AY093260.1	non-canonical match
at1g01630	CON	MIN	HOUR	DAY	WEEK	UNIGENE	FLCDNA	TAIR
TAATTTTTCA	3	0	0	0	0	gnl|UG|At#S11742951	no match found	At1g01630.1
GAAGGTCATT	1	2	2	1	0	no match found	gi|21404669|gb|AY085959.1	At2g16170.1
at1g29840	CON	MIN	HOUR	DAY	WEEK	UNIGENE	FLCDNA	TAIR
GCTCGGCTGA	0	0	1	0	0	no match found	gi|45825152|gb|BT012359.1	non-canonical match
CTTCTTAACA	0	1	0	1	0	gnl|UG|At#S24442615	gi|62320443|dbj|AK221548.1|	non-canonical match
at5g37540	CON	MIN	HOUR	DAY	WEEK	UNIGENE	FLCDNA	TAIR
CTTGGTGCAG	0	1	0	0	0	gnl|UG|At#S11719981	gi|15809849|gb|AY054192.1	At5g37540.1
at2g28820	CON	MIN	HOUR	DAY	WEEK	UNIGENE	FLCDNA	TAIR
TAACCAAGCC	1	1	0	1	0	no match found	no match found	At2g28820.1
at3g57500	CON	MIN	HOUR	DAY	WEEK	UNIGENE	FLCDNA	TAIR
TTCCTTTTTT	0	0	2	2	0	gnl|UG|At#S22484603	no match found	At3g57500.1
at2g42770	CON	MIN	HOUR	DAY	WEEK	UNIGENE	FLCDNA	TAIR
GAAAACTATC	2	1	2	0	1	gnl|UG|At#S11731932	gi|21407854|gb|AY089080.1	At2g42770.1
at5g11180	CON	MIN	HOUR	DAY	WEEK	UNIGENE	FLCDNA	TAIR
ATAAATTGAT	0	0	1	0	0	no match found	no match found	At5g11180.1
at5g02600	CON	MIN	HOUR	DAY	WEEK	UNIGENE	FLCDNA	TAIR
TTGTAAAATT	1	0	0	0	0	no match found	gi|62320853|dbj|AK221754.1|	At5g02600.2
at3g14290	CON	MIN	HOUR	DAY	WEEK	UNIGENE	FLCDNA	TAIR
ATGAAAATGG	1	3	1	0	0	gnl|UG|At#S18904102	gi|13877964|gb|AF370245.1	At3g14290.1
at4g02560	CON	MIN	HOUR	DAY	WEEK	UNIGENE	FLCDNA	TAIR
GTTGAATTAG	0	0	1	1	0	gnl|UG|At#S11727045	gi|110741807|dbj|AK226746.1|	At4g02560.1
at1g71960	CON	MIN	HOUR	DAY	WEEK	UNIGENE	FLCDNA	TAIR
TGAAAATGAA	0	1	1	0	1	gnl|UG|At#S11707424	gi|15292752|gb|AY050810.1	At1g71960.1
at3g25165	CON	MIN	HOUR	DAY	WEEK	UNIGENE	FLCDNA	TAIR
TGTAATAGTA	0	0	0	0	1	gnl|UG|At#S11733226	gi|21406681|gb|AY087907.1	At3g25165.1
at4g08930	CON	MIN	HOUR	DAY	WEEK	UNIGENE	FLCDNA	TAIR
GGCTTCCAAG	0	1	2	1	0	gnl|UG|At#S11726067	gi|110743757|dbj|AK227730.1|	At4g08930.1
at1g06050	CON	MIN	HOUR	DAY	WEEK	UNIGENE	FLCDNA	TAIR
TACAGAAGCA	0	1	0	0	0	gnl|UG|At#S11742422	gi|21407696|gb|AY088922.1	At1g06050.1
at2g44360	CON	MIN	HOUR	DAY	WEEK	UNIGENE	FLCDNA	TAIR
AGTGATATTG	2	2	5	1	3	gnl|UG|At#S11731553	gi|26452604|dbj|AK118795.1|	non-canonical match

at3g21240	CON	MIN	HOUR	DAY	WEEK	UNIGENE	FLCDNA	TAIR
AAGGAAGAAG	0	1	0	0	0	gnl|UG|At#S11734456	gi|23198175|gb|BT000296.1	At3g21240.1

at5g25425	CON	MIN	HOUR	DAY	WEEK	UNIGENE	FLCDNA	TAIR
CCTGAAAAAA	0	0	0	1	0	no match found	no match found	At5g25425.1

at4g22670	CON	MIN	HOUR	DAY	WEEK	UNIGENE	FLCDNA	TAIR
GCTGCTCTTC	1	5	1	2	1	gnl|UG|At#S11723878	gi|21406209|gb|AY087472.1	At4g22670.1

at1g08190	CON	MIN	HOUR	DAY	WEEK	UNIGENE	FLCDNA	TAIR
TGTGCCAGAA	8	2	12	6	1	gnl|UG|At#S34115445	gi|62321626|dbj|AK222151.1|	At1g08190.1

at1g30470	CON	MIN	HOUR	DAY	WEEK	UNIGENE	FLCDNA	TAIR
GGACAAAAGA	1	1	0	2	1	gnl|UG|At#S11738857	no match found	At1g30470.1

at3g57390	CON	MIN	HOUR	DAY	WEEK	UNIGENE	FLCDNA	TAIR
GAAATGAAGC	0	0	1	0	0	gnl|UG|At#S18941961	gi|62321763|dbj|AK222220.1|	At3g57390.1

at1g05320	CON	MIN	HOUR	DAY	WEEK	UNIGENE	FLCDNA	TAIR
ACATTGAAGA	2	2	0	0	0	gnl|UG|At#S28282706	gi|62321592|dbj|AK222133.1|	At1g05320.1

at3g10250	CON	MIN	HOUR	DAY	WEEK	UNIGENE	FLCDNA	TAIR
TAATTTTCAG	1	1	2	0	0	gnl|UG|At#S11737814	gi|15809981|gb|AY054259.1	At3g10250.1

at1g02145	CON	MIN	HOUR	DAY	WEEK	UNIGENE	FLCDNA	TAIR
ATGTAATGTA	1	0	0	0	1	gnl|UG|At#S37211202	gi|110743748|dbj|AK227725.1|	At3g10250.1

at5g60730	CON	MIN	HOUR	DAY	WEEK	UNIGENE	FLCDNA	TAIR
GGTGATCTCA	0	0	1	0	0	gnl|UG|At#S11717629	gi|52627092|gb|BT015736.1	At5g60730.1

at4g10090	CON	MIN	HOUR	DAY	WEEK	UNIGENE	FLCDNA	TAIR
GAAAATTAGT	1	0	1	0	1	gnl|UG|At#S34118443	gi|110735718|dbj|AK227863.1|	At4g10090.1

at1g19970	CON	MIN	HOUR	DAY	WEEK	UNIGENE	FLCDNA	TAIR
CAGAACACTA	0	1	0	0	1	gnl|UG|At#S11741057	no match found	At1g19970.1

at1g55500	CON	MIN	HOUR	DAY	WEEK	UNIGENE	FLCDNA	TAIR
ACTCTGTTGA	2	0	1	0	2	gnl|UG|At#S11707533	gi|15215791|gb|AY050425.1	At1g55500.1

at5g23330	CON	MIN	HOUR	DAY	WEEK	UNIGENE	FLCDNA	TAIR
CTCGCTTATA	1	1	0	1	2	gnl|UG|At#S34118213	gi|21404297|gb|AY085587.1	At5g23330.1

at2g27210	CON	MIN	HOUR	DAY	WEEK	UNIGENE	FLCDNA	TAIR
TTGTGTACAG	1	0	0	0	0	gnl|UG|At#S11735735	gi|110738114|dbj|AK229113.1|	At2g27210.1

at5g45620	CON	MIN	HOUR	DAY	WEEK	UNIGENE	FLCDNA	TAIR
TCCTTGACAA	1	1	0	0	2	gnl|UG|At#S18941523	no match found	At5g45620.1
GGCGCAACCG	5	3	6	5	2	gnl|UG|At#S18912263	gi|20260473|gb|AY093136.1	multiple non-canonical match

at1g24530	CON	MIN	HOUR	DAY	WEEK	UNIGENE	FLCDNA	TAIR
ATAAAGCAAT	0	1	0	0	0	gnl|UG|At#S11740501	gi|15028340|gb|AY045973.1	At1g24530.1

at4g12470	CON	MIN	HOUR	DAY	WEEK	UNIGENE	FLCDNA	TAIR
TAAGGTATAG	26	94	11	56	323	gnl|UG|At#S11725652	gi|20260265|gb|AY093032.1	At4g12470.1

at5g26622	CON	MIN	HOUR	DAY	WEEK	UNIGENE	FLCDNA	TAIR
CTCCACAAAG	0	3	0	0	0	gnl|UG|At#S37211658	no match found	At4g12470.1

at3g08820	CON	MIN	HOUR	DAY	WEEK	UNIGENE	FLCDNA	TAIR
AAGTGATGAA	0	0	0	1	0	gnl|UG|At#S11738249	no match found	At3g08820.1

at4g08770	CON	MIN	HOUR	DAY	WEEK	UNIGENE	FLCDNA	TAIR
GATGTGGTAG	0	1	0	0	0	no match found	gi|22531051|gb|AY136364.1	multiple canonical match
at2g20340	CON	MIN	HOUR	DAY	WEEK	UNIGENE	FLCDNA	TAIR
TCGCACACTG	0	0	0	1	0	gnl|UG|At#S11716869	gi|20465924|gb|AY096475.1	At2g20340.1
at1g20560	CON	MIN	HOUR	DAY	WEEK	UNIGENE	FLCDNA	TAIR
TTTGTGTCTA	0	0	1	0	0	gnl|UG|At#S11741003	gi|13937176|gb|AF372942.1	At1g20560.1
at3g48000	CON	MIN	HOUR	DAY	WEEK	UNIGENE	FLCDNA	TAIR
GTTTGTAATT	16	14	21	11	6	gnl|UG|At#S11729802	gi|13194813|gb|AF349522.1	At3g48000.1
CTTATCGCTC	1	0	0	0	0	no match found	gi|30102521|gb|BT006371.1	non-canonical match
at5g20140	CON	MIN	HOUR	DAY	WEEK	UNIGENE	FLCDNA	TAIR
GAACTTCATA	1	0	1	1	0	gnl|UG|At#S11721293	gi|23197727|gb|BT000072.1	At5g20140.1
at1g02110	CON	MIN	HOUR	DAY	WEEK	UNIGENE	FLCDNA	TAIR
TAAATTTGAG	1	1	1	0	1	gnl|UG|At#S24441961	gi|62321727|dbj|AK222202.1|	At1g02110.1
at5g56460	CON	MIN	HOUR	DAY	WEEK	UNIGENE	FLCDNA	TAIR
CTAATTCACA	0	0	1	0	0	gnl|UG|At#S11718066	gi|22135983|gb|AY128371.1	At5g56460.1
at2g17420	CON	MIN	HOUR	DAY	WEEK	UNIGENE	FLCDNA	TAIR
ATAAAATCAC	1	0	1	0	0	gnl|UG|At#S11738124	gi|20466579|gb|AY099756.1	At2g17420.1
at1g32940	CON	MIN	HOUR	DAY	WEEK	UNIGENE	FLCDNA	TAIR
GGTCCAAGAG	0	0	0	1	0	gnl|UG|At#S11743129	gi|18377744|gb|AY074326.1	At1g32940.1
at1g31835	CON	MIN	HOUR	DAY	WEEK	UNIGENE	FLCDNA	TAIR
GCCATTTTCG	1	0	0	0	0	gnl|UG|At#S18893020	gi|21405251|gb|AY086541.1	At1g31835.1
at2g38820	CON	MIN	HOUR	DAY	WEEK	UNIGENE	FLCDNA	TAIR
CATACGTAGA	0	2	0	0	0	gnl|UG|At#S18942753	gi|16649128|gb|AY059934.1	At2g38820.1
at1g30130	CON	MIN	HOUR	DAY	WEEK	UNIGENE	FLCDNA	TAIR
TGACTTGATT	1	0	2	2	1	gnl|UG|At#S11738981	gi|21407549|gb|AY088775.1	At1g30130.1
at5g55620	CON	MIN	HOUR	DAY	WEEK	UNIGENE	FLCDNA	TAIR
TATATCAATC	2	1	3	1	7	no match found	gi|21405495|gb|AY086785.1	non-canonical match
at3g49250	CON	MIN	HOUR	DAY	WEEK	UNIGENE	FLCDNA	TAIR
TAGTTGGAAT	0	1	0	0	0	gnl|UG|At#S11729593	gi|15146293|gb|AY049288.1	At3g49250.1
CACTAATCAG	0	0	1	0	0	no match found	gi|19699161|gb|AY090286.1	non-canonical match
at3g03220	CON	MIN	HOUR	DAY	WEEK	UNIGENE	FLCDNA	TAIR
ATTCTTTTTT	0	1	0	0	0	gnl|UG|At#S11707997	gi|16604574|gb|AY059732.1	At3g03220.1
at2g01570	CON	MIN	HOUR	DAY	WEEK	UNIGENE	FLCDNA	TAIR
TCTGAAGTTT	0	7	1	1	0	gnl|UG|At#S11742648	gi|34365556|gb|BT010467.1	At2g01570.1
at5g65910	CON	MIN	HOUR	DAY	WEEK	UNIGENE	FLCDNA	TAIR
CCTTGCTTCT	0	1	1	0	0	gnl|UG|At#S11703464	gi|21281218|gb|AY113924.1	At5g65910.1
at3g62070	CON	MIN	HOUR	DAY	WEEK	UNIGENE	FLCDNA	TAIR
GATGATGAGA	0	0	1	0	0	no match found	gi|110737464|dbj|AK228777.1|	multiple canonical match
at5g54145	CON	MIN	HOUR	DAY	WEEK	UNIGENE	FLCDNA	TAIR
TGATTTGTGT	6	6	4	3	0	gnl|UG|At#S11791298	no match found	At5g54145.1
at3g25040	CON	MIN	HOUR	DAY	WEEK	UNIGENE	FLCDNA	TAIR
GATCGCCGGG	1	1	0	1	1	gnl|UG|At#S11733270	gi|21404990|gb|AY086280.1	At3g25040.1

at5g59500	CON	MIN	HOUR	DAY	WEEK	UNIGENE	FLCDNA	TAIR
GTTCTCTTAA	0	0	1	0	1	gnl|UG|At#S11717754	gi|19347911|gb|AY080632.1	At5g59500.1
at4g04040	CON	MIN	HOUR	DAY	WEEK	UNIGENE	FLCDNA	TAIR
TGTTAATAAT	4	4	3	2	5	gnl|UG|At#S11726649	no match found	At4g04040.1
at3g14240	CON	MIN	HOUR	DAY	WEEK	UNIGENE	FLCDNA	TAIR
TTCACCCAAC	0	5	0	1	0	gnl|UG|At#S11709368	gi|16930700|gb|AF436834.1	At3g14240.1
at4g35540	CON	MIN	HOUR	DAY	WEEK	UNIGENE	FLCDNA	TAIR
TTTTCAACTC	1	0	1	0	0	no match found	no match found	At4g35540.1
at1g51570	CON	MIN	HOUR	DAY	WEEK	UNIGENE	FLCDNA	TAIR
TTTTTTTCTG	2	5	4	1	4	gnl|UG|At#S11816380	gi|15010787|gb|AY045695.1	At1g51570.1
at4g27760	CON	MIN	HOUR	DAY	WEEK	UNIGENE	FLCDNA	TAIR
TAGTTTGCTT	1	1	1	0	0	gnl|UG|At#S34115221	gi|110741904|dbj|AK226796.1|	At4g27760.1
at5g14420	CON	MIN	HOUR	DAY	WEEK	UNIGENE	FLCDNA	TAIR
GCCTTTGGTT	0	2	0	0	0	gnl|UG|At#S15460917	gi|20466261|gb|AY099597.1	At5g14420.1
at4g15900	CON	MIN	HOUR	DAY	WEEK	UNIGENE	FLCDNA	TAIR
TATTGTTGTT	4	1	1	0	3	no match found	gi|27754712|gb|BT002991.1	At4g15900.1
at2g25430	CON	MIN	HOUR	DAY	WEEK	UNIGENE	FLCDNA	TAIR
TCTGATTTTC	0	2	0	0	0	no match found	gi|30725271|gb|BT008299.1	multiple non-canonical match
TTTCCACTGT	2	2	0	3	2	gnl|UG|At#S11736166	gi|22654976|gb|AY139760.1	At2g25430.1
at4g26720	CON	MIN	HOUR	DAY	WEEK	UNIGENE	FLCDNA	TAIR
TGTGATCTCC	1	0	0	0	0	gnl|UG|At#S11808014	gi|20466827|gb|AY099880.1	At4g26720.1
at3g04680	CON	MIN	HOUR	DAY	WEEK	UNIGENE	FLCDNA	TAIR
TTTTTTGCTT	0	0	0	1	0	gnl|UG|At#S11708728	gi|17065579|gb|AY062866.1	At3g04680.2
at5g10610	CON	MIN	HOUR	DAY	WEEK	UNIGENE	FLCDNA	TAIR
GTCTGAAATG	2	0	1	1	1	gnl|UG|At#S11723589	no match found	At5g10610.1
at2g43390	CON	MIN	HOUR	DAY	WEEK	UNIGENE	FLCDNA	TAIR
TAAATATTTT	0	0	0	1	0	gnl|UG|At#S11817369	no match found	At2g43390.1
at2g46430	CON	MIN	HOUR	DAY	WEEK	UNIGENE	FLCDNA	TAIR
TTAACTTTGT	0	3	0	1	0	gnl|UG|At#S11731088	gi|16648780|gb|AY058167.1	At2g46430.1
at5g10840	CON	MIN	HOUR	DAY	WEEK	UNIGENE	FLCDNA	TAIR
GCGATTAAGG	5	0	0	1	4	gnl|UG|At#S11723538	gi|62319389|dbj|AK221018.1|	At2g46430.1
at2g42110	CON	MIN	HOUR	DAY	WEEK	UNIGENE	FLCDNA	TAIR
GCGTAAAACA	0	1	0	0	0	gnl|UG|At#S11732074	gi|149944370|gb|BT030648.1	At2g42110.1
at4g19185	CON	MIN	HOUR	DAY	WEEK	UNIGENE	FLCDNA	TAIR
GTCTAAAAAC	0	2	0	0	0	gnl|UG|At#S11724469	gi|16974592|gb|AY060572.1	At4g19185.1
at2g21340	CON	MIN	HOUR	DAY	WEEK	UNIGENE	FLCDNA	TAIR
AAAATGTATT	5	2	2	1	0	gnl|UG|At#S11737124	gi|22136237|gb|AY128797.1	At2g21340.1
at3g53180	CON	MIN	HOUR	DAY	WEEK	UNIGENE	FLCDNA	TAIR
CTTTCAAAAG	0	0	0	0	1	gnl|UG|At#S11708915	gi|17065203|gb|AY062678.1	At3g53180.1
AAGGGTCGTA	1	0	0	0	0	no match found	gi|17065449|gb|AY062801.1	non-canonical match
at1g73500	CON	MIN	HOUR	DAY	WEEK	UNIGENE	FLCDNA	TAIR

GATCCGATTT	1	7	0	0	0	gnl|UG|At#S11728355	gi|26452086|dbj|AK118530.1|	At1g73500.1
at4g05460	CON	MIN	HOUR	DAY	WEEK	UNIGENE	FLCDNA	TAIR
ACTTGTTTTA	0	0	1	1	0	gnl|UG|At#S11726244	gi|21406512|gb|AY087774.1	At5g15570.1
at1g79740	CON	MIN	HOUR	DAY	WEEK	UNIGENE	FLCDNA	TAIR
ATGTCGGAGT	1	0	0	0	1	gnl|UG|At#S19208374	gi|45752651|gb|BT012129.1	non-canonical match
TTTCGTTCAT	0	0	1	0	1	no match found	no match found	At1g79740.1
at5g38660	CON	MIN	HOUR	DAY	WEEK	UNIGENE	FLCDNA	TAIR
TATACGGCGA	7	8	22	4	3	gnl|UG|At#S11719867	gi|25082753|gb|BT001987.1	At5g38660.1
at4g37540	CON	MIN	HOUR	DAY	WEEK	UNIGENE	FLCDNA	TAIR
TCTTTCATCT	0	5	0	0	0	gnl|UG|At#S11743555	gi|18176002|gb|AY072144.1	At4g37540.1
at2g41790	CON	MIN	HOUR	DAY	WEEK	UNIGENE	FLCDNA	TAIR
GGTCGTTCAG	1	4	3	0	1	gnl|UG|At#S11732146	gi|110741611|dbj|AK226643.1|	At2g41790.1
at1g17680	CON	MIN	HOUR	DAY	WEEK	UNIGENE	FLCDNA	TAIR
CATTACTTAG	1	0	0	0	0	gnl|UG|At#S24442859	gi|62319956|dbj|AK221304.1|	At1g17680.1
at2g26240	CON	MIN	HOUR	DAY	WEEK	UNIGENE	FLCDNA	TAIR
ACTGGAGCTT	0	6	1	1	0	gnl|UG|At#S11735977	gi|18252992|gb|AY072431.1	At2g26240.1
at5g24800	CON	MIN	HOUR	DAY	WEEK	UNIGENE	FLCDNA	TAIR
TTCGTTAAAT	2	1	4	5	1	gnl|UG|At#S11817047	gi|110742043|dbj|AK226873.1|	non-canonical match
AGCAGACAAA	0	0	0	1	0	no match found	no match found	At5g24800.1
at2g34355	CON	MIN	HOUR	DAY	WEEK	UNIGENE	FLCDNA	TAIR
GCTTCTGTGG	0	1	0	0	0	gnl|UG|At#S15460795	gi|110736937|dbj|AK228500.1|	At2g34355.1
at3g11170	CON	MIN	HOUR	DAY	WEEK	UNIGENE	FLCDNA	TAIR
TGATACATCA	3	0	0	0	1	gnl|UG|At#S11703958	gi|20908083|gb|AY102158.1	At3g11170.1
at3g24840	CON	MIN	HOUR	DAY	WEEK	UNIGENE	FLCDNA	TAIR
CTGGCCAAGA	0	0	0	1	0	gnl|UG|At#S11733303	no match found	At3g24840.1
at3g18500	CON	MIN	HOUR	DAY	WEEK	UNIGENE	FLCDNA	TAIR
GTTCTATATC	1	0	0	0	0	gnl|UG|At#S18903605	gi|20260445|gb|AY093122.1	At3g18500.1
at2g18300	CON	MIN	HOUR	DAY	WEEK	UNIGENE	FLCDNA	TAIR
GTTGACATCA	4	9	5	3	0	gnl|UG|At#S15461434	gi|15724317|gb|AF412099.1	At2g18300.2
at3g54100	CON	MIN	HOUR	DAY	WEEK	UNIGENE	FLCDNA	TAIR
GGAAAACAGT	1	1	0	0	0	gnl|UG|At#S11728667	no match found	At3g54100.1
at2g37690	CON	MIN	HOUR	DAY	WEEK	UNIGENE	FLCDNA	TAIR
TGGATTTTGA	1	1	1	1	1	gnl|UG|At#S14829864	gi|28973714|gb|BT005770.1	At2g37690.1
at5g06810	CON	MIN	HOUR	DAY	WEEK	UNIGENE	FLCDNA	TAIR
TCCTTTGAAA	0	1	2	1	0	gnl|UG|At#S11603472	no match found	At2g37690.1
TCTTAAGTAC	0	1	0	0	0	gnl|UG|At#S11724415	no match found	At5g06810.1
at5g65770	CON	MIN	HOUR	DAY	WEEK	UNIGENE	FLCDNA	TAIR
AACTACCATC	1	1	0	0	0	gnl|UG|At#S11717121	no match found	non-canonical match
at4g01940	CON	MIN	HOUR	DAY	WEEK	UNIGENE	FLCDNA	TAIR
AAGAAAGCCA	1	3	5	3	4	no match found	gi|14517433|gb|AY039552.1	non-canonical match
at2g39080	CON	MIN	HOUR	DAY	WEEK	UNIGENE	FLCDNA	TAIR
TATCCTCTTG	1	5	1	1	1	no match found	gi|51969825|dbj|AK175842.1|	non-canonical match

GTTCATATAT	0	0	0	0	2	gnl|UG|At#S11732830	no match found	multiple non-canonical match
at1g27150	CON	MIN	HOUR	DAY	WEEK	UNIGENE	FLCDNA	TAIR
TTTTTAAATA	0	4	0	0	0	no match found	gi|21403865|gb|AY085155.1	At1g27150.1
at2g43960	CON	MIN	HOUR	DAY	WEEK	UNIGENE	FLCDNA	TAIR
TGATTTCAAT	1	0	0	0	0	gnl|UG|At#S15114787	no match found	At2g43960.1
at4g02120	CON	MIN	HOUR	DAY	WEEK	UNIGENE	FLCDNA	TAIR
CTGCTGCTTG	0	0	0	1	0	gnl|UG|At#S11727148	gi|22655203|gb|AY140051.1	At4g02120.1
at5g41370	CON	MIN	HOUR	DAY	WEEK	UNIGENE	FLCDNA	TAIR
TATGTAAGTA	0	0	0	2	1	gnl|UG|At#S11719592	gi|14517423|gb|AY039547.1	At5g41370.1
GAATACAACT	1	0	1	2	0	no match found	gi|21360400|gb|AY113008.1	At5g41360.1
at1g33170	CON	MIN	HOUR	DAY	WEEK	UNIGENE	FLCDNA	TAIR
AGAGAGGTCC	1	0	0	0	0	gnl|UG|At#S11737852	gi|22655267|gb|AY140083.1	At1g33170.1
at5g35700	CON	MIN	HOUR	DAY	WEEK	UNIGENE	FLCDNA	TAIR
ACGCGTTTGA	0	2	0	1	0	gnl|UG|At#S11720133	gi|15027846|gb|AY045780.1	At5g35700.1
at5g66420	CON	MIN	HOUR	DAY	WEEK	UNIGENE	FLCDNA	TAIR
GTTTTTTTTT	2	0	7	1	3	no match found	gi|14190422|gb|AF378889.1	multiple canonical match
at2g04940	CON	MIN	HOUR	DAY	WEEK	UNIGENE	FLCDNA	TAIR
GCGGTGGGTG	1	1	1	0	0	gnl|UG|At#S11740167	gi|24899824|gb|BT001240.1	At2g04940.1
at5g42760	CON	MIN	HOUR	DAY	WEEK	UNIGENE	FLCDNA	TAIR
CTGCCGTTTG	1	0	0	4	2	gnl|UG|At#S11719454	gi|16648882|gb|AY059811.1	At5g42760.1
at2g27200	CON	MIN	HOUR	DAY	WEEK	UNIGENE	FLCDNA	TAIR
CCATTGAGGA	0	1	0	0	0	gnl|UG|At#S24634123	gi|63003825|gb|BT022030.1	At2g27200.1
at1g31850	CON	MIN	HOUR	DAY	WEEK	UNIGENE	FLCDNA	TAIR
CACTACCATC	2	0	1	2	1	gnl|UG|At#S11660522	no match found	multiple non-canonical match
GACGCAATCA	0	2	1	1	1	gnl|UG|At#S11738331	gi|22135825|gb|AY128291.1	At1g31850.3
at2g38100	CON	MIN	HOUR	DAY	WEEK	UNIGENE	FLCDNA	TAIR
TTCAATCTCT	0	1	0	0	0	gnl|UG|At#S11733078	no match found	multiple non-canonical match
at5g49160	CON	MIN	HOUR	DAY	WEEK	UNIGENE	FLCDNA	TAIR
GGTAAGGTTG	0	4	0	3	0	gnl|UG|At#S11718802	gi|110737924|dbj|AK229013.1|	At5g49160.1
at5g52650	CON	MIN	HOUR	DAY	WEEK	UNIGENE	FLCDNA	TAIR
AAGGAGGAGA	0	0	3	0	0	no match found	gi|21689686|gb|AY122932.1	multiple non-canonical match
CGAGAATATG	0	1	0	1	0	gnl|UG|At#S11718452	no match found	non-canonical match
GAGGAGTTTT	18	12	9	32	21	no match found	gi|21407452|gb|AY088678.1	At5g52650.1
at4g20420	CON	MIN	HOUR	DAY	WEEK	UNIGENE	FLCDNA	TAIR
GAAAAATGAA	0	0	1	1	0	gnl|UG|At#S11724263	gi|26451030|dbj|AK117986.1|	At4g20420.1
at1g22280	CON	MIN	HOUR	DAY	WEEK	UNIGENE	FLCDNA	TAIR
AGCCTCGTAC	0	3	3	0	1	gnl|UG|At#S18942523	gi|21403584|gb|AY084874.1	At1g22280.1
at3g15780	CON	MIN	HOUR	DAY	WEEK	UNIGENE	FLCDNA	TAIR
GAAGAAATTG	0	0	1	0	0	gnl|UG|At#S16342797	no match found	non-canonical match
at1g05210	CON	MIN	HOUR	DAY	WEEK	UNIGENE	FLCDNA	TAIR
CCCAGAAGAA	2	0	1	1	0	gnl|UG|At#S11742507	gi|21405664|gb|AY086940.1	At1g05210.1
at1g15170	CON	MIN	HOUR	DAY	WEEK	UNIGENE	FLCDNA	TAIR

GTTTTCCAAA	1	1	1	2	1	gnl|UG|At#S11741518	no match found		At1g15170.1
at3g47860	CON	MIN	HOUR	DAY	WEEK	UNIGENE	FLCDNA		TAIR
CCAGGTATGG	0	5	0	0	1	gnl|UG|At#S11729828	gi|27754733|gb|BT003002.1		At3g47860.1
at2g47710	CON	MIN	HOUR	DAY	WEEK	UNIGENE	FLCDNA		TAIR
CTCATTGCTC	4	7	2	4	1	no match found	gi|21405552|gb|AY086842.1		At2g47710.1
at2g14620	CON	MIN	HOUR	DAY	WEEK	UNIGENE	FLCDNA		TAIR
ACAAGATTGA	0	1	0	0	0	no match found	gi|20465526|gb|AY096596.1		non-canonical match
TTATACTGTT	0	0	0	1	0	gnl|UG|At#S18318426	no match found		multiple non-canonical match
at1g03850	CON	MIN	HOUR	DAY	WEEK	UNIGENE	FLCDNA		TAIR
GCGTGAATCC	0	1	2	2	1	no match found	gi|21405442|gb|AY086732.1		At1g03850.2
at5g17710	CON	MIN	HOUR	DAY	WEEK	UNIGENE	FLCDNA		TAIR
AGGCAATAAT	1	2	0	0	0	gnl|UG|At#S15460747	gi|17104678|gb|AY063054.1		At5g17710.1
at3g14735	CON	MIN	HOUR	DAY	WEEK	UNIGENE	FLCDNA		TAIR
GCCCTGCGAT	3	5	0	4	1	gnl|UG|At#S17479688	no match found		At5g17710.1
at3g29240	CON	MIN	HOUR	DAY	WEEK	UNIGENE	FLCDNA		TAIR
GTCTTTGGGA	14	5	5	5	0	gnl|UG|At#S18903584	gi|28416520|gb|BT004545.1		At3g29240.1
GCAGGAACGT	0	0	1	0	0	gnl|UG|At#S26539776	no match found		non-canonical match
at4g22160	CON	MIN	HOUR	DAY	WEEK	UNIGENE	FLCDNA		TAIR
TTATTTACTC	0	1	0	0	0	gnl|UG|At#S11723975	gi|21407688|gb|AY088914.1		At4g22160.2
at4g19080	CON	MIN	HOUR	DAY	WEEK	UNIGENE	FLCDNA		TAIR
CAGCTCAACT	0	0	0	1	0	gnl|UG|At#S11724487	no match found		At4g19080.1
at1g70600	CON	MIN	HOUR	DAY	WEEK	UNIGENE	FLCDNA		TAIR
GTTTCTTCAA	5	9	9	8	6	gnl|UG|At#S11603464	no match found		multiple non-canonical match
ATTCATTCGT	2	1	3	2	2	gnl|UG|At#S11728965	no match found		At1g70600.1
at3g49670	CON	MIN	HOUR	DAY	WEEK	UNIGENE	FLCDNA		TAIR
ACGGATTCAA	1	1	1	1	0	no match found	gi|29824274|gb|BT006113.1		non-canonical match
ATAAGTGGTT	1	0	4	3	0	gnl|UG|At#S11729506	gi|110740138|dbj|AK230159.1|		At3g49670.1
at1g21790	CON	MIN	HOUR	DAY	WEEK	UNIGENE	FLCDNA		TAIR
TAAATGTTAT	0	0	1	0	0	gnl|UG|At#S11740882	gi|21406398|gb|AY087660.1		At1g21790.1
at2g02680	CON	MIN	HOUR	DAY	WEEK	UNIGENE	FLCDNA		TAIR
ATCCCGAAAA	1	1	1	1	0	gnl|UG|At#S20838542	no match found		At1g21790.1
at5g38470	CON	MIN	HOUR	DAY	WEEK	UNIGENE	FLCDNA		TAIR
GGAGTTGATG	11	6	14	14	9	gnl|UG|At#S38433207	gi|14335003|gb|AY037181.1		At5g38470.1
at1g53560	CON	MIN	HOUR	DAY	WEEK	UNIGENE	FLCDNA		TAIR
GGAGGAAGAG	4	5	7	2	0	gnl|UG|At#S35281854	gi|20466665|gb|AY099799.1		At1g53560.1
at5g49630	CON	MIN	HOUR	DAY	WEEK	UNIGENE	FLCDNA		TAIR
CACTCTCTTC	1	2	0	1	0	gnl|UG|At#S11718754	gi|110738093|dbj|AK229102.1|		multiple canonical match
at1g73530	CON	MIN	HOUR	DAY	WEEK	UNIGENE	FLCDNA		TAIR
TCAAAATGGA	0	0	1	2	1	gnl|UG|At#S11728350	no match found		At1g73530.1
at1g08630	CON	MIN	HOUR	DAY	WEEK	UNIGENE	FLCDNA		TAIR
GATATGGAGG	0	5	1	0	0	gnl|UG|At#S18926144	gi|21405444|gb|AY086734.1		At1g08630.2
at1g67430	CON	MIN	HOUR	DAY	WEEK	UNIGENE	FLCDNA		TAIR

TCCCTTGTGT	4	4	1	1	5	no match found	gi|110736680|dbj|AK228365.1|	multiple non-canonical match
GTTCTGCAAG	1	1	1	1	2	gnl|UG|At#S11729629	no match found	pseudo chromosome match
at5g51510	CON	MIN	HOUR	DAY	WEEK	UNIGENE	FLCDNA	TAIR
GAGGAACGCA	0	0	0	1	0	gnl|UG|At#S11718565	gi|28973526|gb|BT005668.1	At5g51510.1
at3g05840	CON	MIN	HOUR	DAY	WEEK	UNIGENE	FLCDNA	TAIR
CGAGGAAGCA	2	10	2	4	1	gnl|UG|At#S11738974	gi|21404462|gb|AY085752.1	At3g05840.1
at5g44100	CON	MIN	HOUR	DAY	WEEK	UNIGENE	FLCDNA	TAIR
TAAAAACGGT	1	2	3	3	1	gnl|UG|At#S11719318	gi|18087639|gb|AF462864.1	At5g44100.1
at2g47500	CON	MIN	HOUR	DAY	WEEK	UNIGENE	FLCDNA	TAIR
GCTTCTTATT	0	2	0	1	0	gnl|UG|At#S14273861	gi|28393381|gb|BT004088.1	At2g47500.1
at1g23600	CON	MIN	HOUR	DAY	WEEK	UNIGENE	FLCDNA	TAIR
GTATAAAAAA	0	1	0	1	0	no match found	no match found	At1g23600.1
at1g64585	CON	MIN	HOUR	DAY	WEEK	UNIGENE	FLCDNA	TAIR
CTGCTCTGTT	1	0	0	0	0	gnl|UG|At#S38434113	no match found	non-canonical match
at5g12230	CON	MIN	HOUR	DAY	WEEK	UNIGENE	FLCDNA	TAIR
TATAATAATT	1	4	0	0	1	gnl|UG|At#S34114952	gi|110742402|dbj|AK227065.1|	At5g12230.1
at4g29330	CON	MIN	HOUR	DAY	WEEK	UNIGENE	FLCDNA	TAIR
CCAGATCTTC	0	1	0	0	0	gnl|UG|At#S11708503	gi|17380685|gb|AY063817.1	At4g29330.1
at3g42050	CON	MIN	HOUR	DAY	WEEK	UNIGENE	FLCDNA	TAIR
CAGCGGGGAG	4	13	5	5	3	gnl|UG|At#S11731138	gi|14334993|gb|AY037176.1	At3g42050.1
at5g53440	CON	MIN	HOUR	DAY	WEEK	UNIGENE	FLCDNA	TAIR
AATCTGATGA	56	29	27	36	11	gnl|UG|At#S11718372	no match found	multiple non-canonical match
TTCCTTTTGT	1	0	2	1	2	no match found	no match found	At5g53440.1
at3g30727	CON	MIN	HOUR	DAY	WEEK	UNIGENE	FLCDNA	TAIR
GAAGGTGGCT	0	2	0	0	0	no match found	no match found	At3g30727.1
at3g25990	CON	MIN	HOUR	DAY	WEEK	UNIGENE	FLCDNA	TAIR
TCTGAGAGAG	0	0	0	0	1	no match found	gi|45680261|gb|BT011951.1	non-canonical match
at1g72200	CON	MIN	HOUR	DAY	WEEK	UNIGENE	FLCDNA	TAIR
TCTCCAGGTG	0	1	0	0	0	gnl|UG|At#S14273681	gi|57222161|gb|BT020487.1	At1g72200.1
at3g07280	CON	MIN	HOUR	DAY	WEEK	UNIGENE	FLCDNA	TAIR
TTCTTTTCTG	0	0	0	1	1	gnl|UG|At#S35292127	gi|18700174|gb|AY075692.1	non-canonical match
GATCAGAAAG	0	1	1	0	0	no match found	gi|18377830|gb|AY074562.1	At3g07280.1
at5g42670	CON	MIN	HOUR	DAY	WEEK	UNIGENE	FLCDNA	TAIR
AACTTTGGAA	1	1	2	1	0	gnl|UG|At#S11719463	no match found	multiple non-canonical match
at3g11010	CON	MIN	HOUR	DAY	WEEK	UNIGENE	FLCDNA	TAIR
TGATAGTTAA	0	0	0	1	0	gnl|UG|At#S11737583	no match found	At5g27060.1
AACCCTTTTG	0	0	0	1	1	no match found	gi|26449946|dbj|AK117429.1|	multiple canonical match
at3g56340	CON	MIN	HOUR	DAY	WEEK	UNIGENE	FLCDNA	TAIR
TGAAACCAAT	3	7	4	7	4	gnl|UG|At#S11728269	gi|14532523|gb|AY039886.1	At3g56340.1
at1g71730	CON	MIN	HOUR	DAY	WEEK	UNIGENE	FLCDNA	TAIR
GGACTCAAGA	0	2	2	0	0	no match found	gi|12083315|gb|AF332454.1	At1g71730.1
at2g30530	CON	MIN	HOUR	DAY	WEEK	UNIGENE	FLCDNA	TAIR

AAAACTCGAT	1	0	0	0	0	gnl|UG|At#S11734907	gi|15450620|gb|AY052678.1	At2g30530.1
at3g01760	CON	MIN	HOUR	DAY	WEEK	UNIGENE	FLCDNA	TAIR
TTTCATAAAT	2	0	0	0	2	no match found	no match found	At3g01760.1
at3g18190	CON	MIN	HOUR	DAY	WEEK	UNIGENE	FLCDNA	TAIR
TGCTATTGCA	3	4	1	2	4	gnl|UG|At#S11735382	gi|21407344|gb|AY088570.1	At3g18190.1
at4g12770	CON	MIN	HOUR	DAY	WEEK	UNIGENE	FLCDNA	TAIR
TGGCATTCAT	0	0	0	2	0	gnl|UG|At#S34117460	gi|110737601|dbj|AK228848.1|	At4g12770.1
at2g39000	CON	MIN	HOUR	DAY	WEEK	UNIGENE	FLCDNA	TAIR
ATGAAGCTAG	1	3	0	0	0	gnl|UG|At#S34117436	gi|48310208|gb|BT014792.1	At2g39000.3
at3g17210	CON	MIN	HOUR	DAY	WEEK	UNIGENE	FLCDNA	TAIR
AGTTTATTAA	15	10	8	18	18	no match found	gi|21405406|gb|AY086696.1	At3g17210.1
at5g22250	CON	MIN	HOUR	DAY	WEEK	UNIGENE	FLCDNA	TAIR
TTTTATTCAA	0	1	0	0	0	gnl|UG|At#S11721033	no match found	At5g22250.1
at4g37980	CON	MIN	HOUR	DAY	WEEK	UNIGENE	FLCDNA	TAIR
TACTTTATCA	38	11	13	10	5	gnl|UG|At#S11721271	gi|15912214|gb|AY056385.1	At4g37980.1
at2g28790	CON	MIN	HOUR	DAY	WEEK	UNIGENE	FLCDNA	TAIR
TTCTAGGGTT	0	1	0	0	0	gnl|UG|At#S11735340	gi|26452025|dbj|AK118499.1|	At2g28790.1
ACTGTGCTTC	0	0	1	0	0	no match found	gi|30017274|gb|BT006222.1	non-canonical match
at4g28820	CON	MIN	HOUR	DAY	WEEK	UNIGENE	FLCDNA	TAIR
GGAATAGAAG	1	1	0	0	0	gnl|UG|At#S21736055	gi|92856577|gb|BT025164.1	At4g28820.1
at4g38690	CON	MIN	HOUR	DAY	WEEK	UNIGENE	FLCDNA	TAIR
TGAGTGTGTA	4	1	3	0	0	gnl|UG|At#S18906154	gi|17979228|gb|AY070465.1	At4g38690.1
at1g49190	CON	MIN	HOUR	DAY	WEEK	UNIGENE	FLCDNA	TAIR
GAAAAGAAAC	0	0	1	0	0	gnl|UG|At#S11734792	no match found	multiple non-canonical match
at5g64200	CON	MIN	HOUR	DAY	WEEK	UNIGENE	FLCDNA	TAIR
TGCAAGAATT	2	5	1	2	4	no match found	gi|22022545|gb|AY127004.1	At5g64200.2
AGGAGAAGGA	4	6	3	0	0	no match found	gi|23505860|gb|AY143851.1	multiple non-canonical match
TTGAGATTGT	1	0	1	0	1	gnl|UG|At#S11717279	gi|17380867|gb|AY063890.1	multiple non-canonical match
at5g46050	CON	MIN	HOUR	DAY	WEEK	UNIGENE	FLCDNA	TAIR
AGCAGTTTCT	0	1	2	0	0	no match found	no match found	At5g46050.1
at1g43800	CON	MIN	HOUR	DAY	WEEK	UNIGENE	FLCDNA	TAIR
AGGTTTGCTT	1	3	0	0	0	no match found	gi|28416680|gb|BT004625.1	non-canonical match
at4g16515	CON	MIN	HOUR	DAY	WEEK	UNIGENE	FLCDNA	TAIR
ACCAACTTCT	0	3	4	1	0	gnl|UG|At#S11668198	gi|14190464|gb|AF380632.1	At4g16515.1
at4g20440	CON	MIN	HOUR	DAY	WEEK	UNIGENE	FLCDNA	TAIR
TTGCCTTTCA	6	1	1	0	5	gnl|UG|At#S11724260	gi|21407896|gb|AY089122.1	At4g20440.1
at1g16740	CON	MIN	HOUR	DAY	WEEK	UNIGENE	FLCDNA	TAIR
TACCCCGGTG	2	3	3	8	5	gnl|UG|At#S30640782	gi|21405211|gb|AY086501.1	At1g16740.1
at4g18810	CON	MIN	HOUR	DAY	WEEK	UNIGENE	FLCDNA	TAIR
TTTGTATACT	0	1	1	1	4	gnl|UG|At#S11724535	gi|18252186|gb|AY072319.1	At4g18810.1
at3g50590	CON	MIN	HOUR	DAY	WEEK	UNIGENE	FLCDNA	TAIR
GAAGTTCAAA	0	1	0	0	0	no match found	gi|28973770|gb|BT005799.1	multiple non-canonical match

<table>
<tr><td>TACGAACTCA</td><td>4</td><td>3</td><td>3</td><td>1</td><td>0</td><td>gnl|UG|At#S11729326</td><td>gi|16604640|gb|AY059765.1</td><td>At3g50590.1</td></tr>
<tr><td>at5g13050</td><td>CON</td><td>MIN</td><td>HOUR</td><td>DAY</td><td>WEEK</td><td>UNIGENE</td><td>FLCDNA</td><td>TAIR</td></tr>
<tr><td>TGAGCTCAGG</td><td>0</td><td>5</td><td>0</td><td>0</td><td>0</td><td>gnl|UG|At#S18912722</td><td>gi|21403106|gb|AY084396.1</td><td>At5g13050.1</td></tr>
<tr><td>at1g13320</td><td>CON</td><td>MIN</td><td>HOUR</td><td>DAY</td><td>WEEK</td><td>UNIGENE</td><td>FLCDNA</td><td>TAIR</td></tr>
<tr><td>TTTTCTGTGA</td><td>0</td><td>2</td><td>1</td><td>0</td><td>3</td><td>gnl|UG|At#S28282645</td><td>no match found</td><td>At1g13320.1</td></tr>
<tr><td>at3g06050</td><td>CON</td><td>MIN</td><td>HOUR</td><td>DAY</td><td>WEEK</td><td>UNIGENE</td><td>FLCDNA</td><td>TAIR</td></tr>
<tr><td>TCTCAAGGAT</td><td>5</td><td>1</td><td>4</td><td>3</td><td>1</td><td>gnl|UG|At#S11738912</td><td>gi|21404106|gb|AY085396.1</td><td>At3g06050.1</td></tr>
<tr><td>at4g06547</td><td>CON</td><td>MIN</td><td>HOUR</td><td>DAY</td><td>WEEK</td><td>UNIGENE</td><td>FLCDNA</td><td>TAIR</td></tr>
<tr><td>TATACGTCGA</td><td>0</td><td>0</td><td>0</td><td>1</td><td>0</td><td>no match found</td><td>gi|72197535|gb|DQ108750.1</td><td>non-canonical match</td></tr>
<tr><td>at1g30910</td><td>CON</td><td>MIN</td><td>HOUR</td><td>DAY</td><td>WEEK</td><td>UNIGENE</td><td>FLCDNA</td><td>TAIR</td></tr>
<tr><td>GTTTGGAAAG</td><td>0</td><td>4</td><td>2</td><td>0</td><td>0</td><td>gnl|UG|At#S18896608</td><td>gi|26452770|dbj|AK118881.1|</td><td>At1g30910.1</td></tr>
<tr><td>at1g12930</td><td>CON</td><td>MIN</td><td>HOUR</td><td>DAY</td><td>WEEK</td><td>UNIGENE</td><td>FLCDNA</td><td>TAIR</td></tr>
<tr><td>AAGAATCTTG</td><td>2</td><td>1</td><td>0</td><td>0</td><td>1</td><td>gnl|UG|At#S11741744</td><td>gi|15215745|gb|AY050402.1</td><td>At1g12930.1</td></tr>
<tr><td>at1g05690</td><td>CON</td><td>MIN</td><td>HOUR</td><td>DAY</td><td>WEEK</td><td>UNIGENE</td><td>FLCDNA</td><td>TAIR</td></tr>
<tr><td>CCGAGTCGTG</td><td>0</td><td>0</td><td>1</td><td>0</td><td>0</td><td>gnl|UG|At#S11742458</td><td>no match found</td><td>At1g05690.1</td></tr>
<tr><td>at1g22530</td><td>CON</td><td>MIN</td><td>HOUR</td><td>DAY</td><td>WEEK</td><td>UNIGENE</td><td>FLCDNA</td><td>TAIR</td></tr>
<tr><td>GCTCAAGAAG</td><td>0</td><td>10</td><td>1</td><td>0</td><td>0</td><td>no match found</td><td>gi|23397357|gb|AY034966.2</td><td>multiple non-canonical match</td></tr>
<tr><td>GGAGCTTAGG</td><td>4</td><td>34</td><td>5</td><td>6</td><td>1</td><td>gnl|UG|At#S11740808</td><td>no match found</td><td>At1g22530.1</td></tr>
<tr><td>at3g61760</td><td>CON</td><td>MIN</td><td>HOUR</td><td>DAY</td><td>WEEK</td><td>UNIGENE</td><td>FLCDNA</td><td>TAIR</td></tr>
<tr><td>GTCTGCGCTG</td><td>1</td><td>0</td><td>0</td><td>0</td><td>0</td><td>gnl|UG|At#S11726922</td><td>no match found</td><td>At3g61760.1</td></tr>
<tr><td>at5g32450</td><td>CON</td><td>MIN</td><td>HOUR</td><td>DAY</td><td>WEEK</td><td>UNIGENE</td><td>FLCDNA</td><td>TAIR</td></tr>
<tr><td>GTCGTTGTTT</td><td>0</td><td>5</td><td>2</td><td>0</td><td>0</td><td>gnl|UG|At#S11720270</td><td>no match found</td><td>At5g32450.1</td></tr>
<tr><td>TTTCTCATTA</td><td>3</td><td>4</td><td>2</td><td>0</td><td>0</td><td>no match found</td><td>gi|26450492|dbj|AK117709.1|</td><td>non-canonical match</td></tr>
<tr><td>at1g12460</td><td>CON</td><td>MIN</td><td>HOUR</td><td>DAY</td><td>WEEK</td><td>UNIGENE</td><td>FLCDNA</td><td>TAIR</td></tr>
<tr><td>ATGAAGCTTT</td><td>1</td><td>1</td><td>0</td><td>1</td><td>0</td><td>gnl|UG|At#S11825076</td><td>gi|27754636|gb|BT002951.1</td><td>multiple canonical match</td></tr>
<tr><td>at2g42230</td><td>CON</td><td>MIN</td><td>HOUR</td><td>DAY</td><td>WEEK</td><td>UNIGENE</td><td>FLCDNA</td><td>TAIR</td></tr>
<tr><td>TATTTCAAAG</td><td>1</td><td>0</td><td>0</td><td>0</td><td>1</td><td>gnl|UG|At#S11732052</td><td>gi|53850496|gb|BT015889.1</td><td>At2g42230.1</td></tr>
<tr><td>at1g74440</td><td>CON</td><td>MIN</td><td>HOUR</td><td>DAY</td><td>WEEK</td><td>UNIGENE</td><td>FLCDNA</td><td>TAIR</td></tr>
<tr><td>TAATTTTTTA</td><td>1</td><td>4</td><td>1</td><td>1</td><td>0</td><td>no match found</td><td>no match found</td><td>At1g74440.1</td></tr>
<tr><td>at4g10720</td><td>CON</td><td>MIN</td><td>HOUR</td><td>DAY</td><td>WEEK</td><td>UNIGENE</td><td>FLCDNA</td><td>TAIR</td></tr>
<tr><td>GCCAAAAAAA</td><td>3</td><td>2</td><td>1</td><td>1</td><td>1</td><td>no match found</td><td>gi|62320712|dbj|AK221683.1|</td><td>multiple non-canonical match</td></tr>
<tr><td>at5g14620</td><td>CON</td><td>MIN</td><td>HOUR</td><td>DAY</td><td>WEEK</td><td>UNIGENE</td><td>FLCDNA</td><td>TAIR</td></tr>
<tr><td>AAGGTACATT</td><td>0</td><td>1</td><td>0</td><td>0</td><td>0</td><td>gnl|UG|At#S34117111</td><td>gi|51970417|dbj|AK176138.1|</td><td>At5g14620.1</td></tr>
<tr><td>at5g07990</td><td>CON</td><td>MIN</td><td>HOUR</td><td>DAY</td><td>WEEK</td><td>UNIGENE</td><td>FLCDNA</td><td>TAIR</td></tr>
<tr><td>TTCACAGCTG</td><td>0</td><td>0</td><td>0</td><td>2</td><td>0</td><td>gnl|UG|At#S11724119</td><td>gi|110742562|dbj|AK227152.1|</td><td>At5g07990.1</td></tr>
<tr><td>at3g56980</td><td>CON</td><td>MIN</td><td>HOUR</td><td>DAY</td><td>WEEK</td><td>UNIGENE</td><td>FLCDNA</td><td>TAIR</td></tr>
<tr><td>TCATCTTCAA</td><td>0</td><td>0</td><td>0</td><td>1</td><td>0</td><td>gnl|UG|At#S11806581</td><td>no match found</td><td>At3g56980.1</td></tr>
<tr><td>at5g66180</td><td>CON</td><td>MIN</td><td>HOUR</td><td>DAY</td><td>WEEK</td><td>UNIGENE</td><td>FLCDNA</td><td>TAIR</td></tr>
<tr><td>AACAACAAGT</td><td>1</td><td>2</td><td>2</td><td>0</td><td>1</td><td>gnl|UG|At#S28281528</td><td>gi|51968869|dbj|AK175364.1|</td><td>At5g66180.1</td></tr>
<tr><td>at1g80890</td><td>CON</td><td>MIN</td><td>HOUR</td><td>DAY</td><td>WEEK</td><td>UNIGENE</td><td>FLCDNA</td><td>TAIR</td></tr>
<tr><td>TCTGTTCTAG</td><td>1</td><td>2</td><td>1</td><td>0</td><td>0</td><td>gnl|UG|At#S11726275</td><td>gi|110738948|dbj|AK229542.1|</td><td>non-canonical match</td></tr>
</table>

at1g04400	CON	MIN	HOUR	DAY	WEEK	UNIGENE	FLCDNA	TAIR
AAGAAATCTA	0	0	0	1	0	no match found	gi|30793900|gb|BT008576.1	multiple non-canonical match
TAGATGTGTG	10	10	10	3	6	gnl|UG|At#S11742587	gi|30794032|gb|BT008648.1	At1g04400.1

at1g08760	CON	MIN	HOUR	DAY	WEEK	UNIGENE	FLCDNA	TAIR
TGGAGTCAGC	0	0	1	0	0	gnl|UG|At#S11808260	gi|34098802|gb|BT010341.1	At1g08760.1

at3g26340	CON	MIN	HOUR	DAY	WEEK	UNIGENE	FLCDNA	TAIR
TGATGGAAGA	1	0	0	0	0	gnl|UG|At#S11732861	gi|16612307|gb|AF439846.1	At3g26340.1

at5g11670	CON	MIN	HOUR	DAY	WEEK	UNIGENE	FLCDNA	TAIR
TACAGCCCTG	6	23	12	5	1	gnl|UG|At#S11723331	gi|16226465|gb|AF428407.1	At5g11670.1

at3g30720	CON	MIN	HOUR	DAY	WEEK	UNIGENE	FLCDNA	TAIR
GGTCGGGCTT	0	2	0	0	0	gnl|UG|At#S11731472	no match found	At3g30720.1

at1g75150	CON	MIN	HOUR	DAY	WEEK	UNIGENE	FLCDNA	TAIR
TGGCCACCAA	0	0	1	0	0	gnl|UG|At#S11728011	no match found	non-canonical match

at3g26980	CON	MIN	HOUR	DAY	WEEK	UNIGENE	FLCDNA	TAIR
TACCATAATG	0	1	0	0	0	gnl|UG|At#S11732639	gi|15028294|gb|AY045950.1	At3g26980.1

at3g54960	CON	MIN	HOUR	DAY	WEEK	UNIGENE	FLCDNA	TAIR
AAATCTGATG	1	2	0	0	1	gnl|UG|At#S11709642	gi|23197927|gb|BT000172.1	At3g54960.1

at3g14770	CON	MIN	HOUR	DAY	WEEK	UNIGENE	FLCDNA	TAIR
GTGGGTCGAT	2	0	4	5	0	gnl|UG|At#S11736418	gi|110740117|dbj|AK230146.1|	At3g14770.1

at1g23830	CON	MIN	HOUR	DAY	WEEK	UNIGENE	FLCDNA	TAIR
ATTGTAAAAC	0	0	1	0	1	gnl|UG|At#S11708081	gi|20466076|gb|AY098950.1	At1g23830.1

at5g16920	CON	MIN	HOUR	DAY	WEEK	UNIGENE	FLCDNA	TAIR
TAAAAGAAAA	1	0	0	0	0	gnl|UG|At#S11812401	gi|21404302|gb|AY085592.1	At5g16920.1

at3g58270	CON	MIN	HOUR	DAY	WEEK	UNIGENE	FLCDNA	TAIR
AAATTGAAGA	0	0	0	0	1	gnl|UG|At#S11727902	gi|63003779|gb|BT022007.1	multiple canonical match

at2g38960	CON	MIN	HOUR	DAY	WEEK	UNIGENE	FLCDNA	TAIR
AAACTAAAAA	0	2	2	0	2	gnl|UG|At#S24442650	gi|62320373|dbj|AK221513.1|	multiple non-canonical match

at1g64440	CON	MIN	HOUR	DAY	WEEK	UNIGENE	FLCDNA	TAIR
GGATTATATT	1	0	0	0	1	gnl|UG|At#S11730248	gi|40823150|gb|BT011226.1	At1g64440.1

at1g14890	CON	MIN	HOUR	DAY	WEEK	UNIGENE	FLCDNA	TAIR
GTTTTGATGA	0	4	2	0	0	gnl|UG|At#S11741545	no match found	non-canonical match

at3g24300	CON	MIN	HOUR	DAY	WEEK	UNIGENE	FLCDNA	TAIR
CCTCGGCTAA	0	1	0	1	0	gnl|UG|At#S34114217	gi|110743890|dbj|AK227798.1|	At3g24300.1

at4g04830	CON	MIN	HOUR	DAY	WEEK	UNIGENE	FLCDNA	TAIR
TAGTGAATAT	1	5	1	0	2	no match found	gi|20148560|gb|AY081609.1	At4g04830.1

at2g36830	CON	MIN	HOUR	DAY	WEEK	UNIGENE	FLCDNA	TAIR
GACCAACCAC	18	24	6	6	1	gnl|UG|At#S11733390	gi|13877818|gb|AF370172.1	At2g36830.1

at2g01450	CON	MIN	HOUR	DAY	WEEK	UNIGENE	FLCDNA	TAIR
ATGTCTCTCC	3	11	4	1	0	gnl|UG|At#S34116537	gi|110736491|dbj|AK228266.1|	At2g01450.1

at5g32610	CON	MIN	HOUR	DAY	WEEK	UNIGENE	FLCDNA	TAIR
AGCTTCAAGA	0	1	0	1	0	gnl|UG|At#S37211141	no match found	At5g04060.1

at5g50740	CON	MIN	HOUR	DAY	WEEK	UNIGENE	FLCDNA	TAIR
CCGCGGTTGT	1	0	5	0	0	gnl|UG|At#S11718643	gi|62318563|dbj|AK220602.1|	non-canonical match

at5g20330	CON	MIN	HOUR	DAY	WEEK	UNIGENE	FLCDNA	TAIR
GTTTCTTAAA	1	0	1	0	1	gnl|UG|At#S11721246	gi|28392901|gb|BT003836.1	At5g20330.1

at2g37020	CON	MIN	HOUR	DAY	WEEK	UNIGENE	FLCDNA	TAIR
AAAACGTCAA	3	0	1	1	1	no match found	gi|14596008|gb|AY042792.1	non-canonical match

at4g10430	CON	MIN	HOUR	DAY	WEEK	UNIGENE	FLCDNA	TAIR
CTTTTACAAA	1	1	0	0	0	gnl|UG|At#S11725922	gi|16648994|gb|AY059867.1	At4g10430.1

at5g49750	CON	MIN	HOUR	DAY	WEEK	UNIGENE	FLCDNA	TAIR
AAATTAGAAT	0	1	0	0	0	no match found	no match found	At5g49750.1

at2g15040	CON	MIN	HOUR	DAY	WEEK	UNIGENE	FLCDNA	TAIR
TTTTCAGTAA	0	1	1	0	0	gnl|UG|At#S18900858	no match found	non-canonical match

at4g24130	CON	MIN	HOUR	DAY	WEEK	UNIGENE	FLCDNA	TAIR
GACGAGGAAG	1	0	0	0	0	gnl|UG|At#S18907877	gi|30793804|gb|BT008528.1	At4g24130.1

at3g11940	CON	MIN	HOUR	DAY	WEEK	UNIGENE	FLCDNA	TAIR
GAGATTATCC	10	30	8	8	11	gnl|UG|At#S11737313	no match found	multiple canonical match

at1g71420	CON	MIN	HOUR	DAY	WEEK	UNIGENE	FLCDNA	TAIR
AAATTAGCTT	1	0	0	0	0	gnl|UG|At#S11728784	gi|110741295|dbj|AK230400.1|	non-canonical match

at3g51160	CON	MIN	HOUR	DAY	WEEK	UNIGENE	FLCDNA	TAIR
GATGCTAAGC	2	1	1	0	1	gnl|UG|At#S11729218	gi|21403284|gb|AY084574.1	At3g51160.1

at2g15490	CON	MIN	HOUR	DAY	WEEK	UNIGENE	FLCDNA	TAIR
TTTAATGTTG	0	0	0	0	1	gnl|UG|At#S34114331	gi|110743667|dbj|AK227684.1|	At2g15490.2

at4g08900	CON	MIN	HOUR	DAY	WEEK	UNIGENE	FLCDNA	TAIR
ACAGCAATGG	0	0	1	3	0	gnl|UG|At#S11699742	gi|15450350|gb|AY052276.1	At4g08900.1

at1g19180	CON	MIN	HOUR	DAY	WEEK	UNIGENE	FLCDNA	TAIR
TCGTGGCTCG	0	0	1	0	0	no match found	gi|19548054|gb|AY081738.1	non-canonical match
CTTTTTGATT	0	8	3	5	3	gnl|UG|At#S18942544	gi|14532539|gb|AY039894.1	At1g19180.2

at1g62750	CON	MIN	HOUR	DAY	WEEK	UNIGENE	FLCDNA	TAIR
GATATGAGAA	39	8	31	33	38	gnl|UG|At#S11730601	gi|14532623|gb|AY039936.1	multiple canonical match
AGTGATCCTT	0	0	0	1	0	no match found	gi|62320531|dbj|AK221592.1|	non-canonical match

at3g09190	CON	MIN	HOUR	DAY	WEEK	UNIGENE	FLCDNA	TAIR
TACAATCTCT	1	0	0	0	0	no match found	no match found	At3g09190.1

at5g07030	CON	MIN	HOUR	DAY	WEEK	UNIGENE	FLCDNA	TAIR
CAGCAGCAGA	2	2	0	0	0	gnl|UG|At#S11724367	gi|109134178|gb|BT025998.1	At5g07030.1
GACACGAGCA	0	0	1	0	0	gnl|UG|At#S24442538	no match found	multiple non-canonical match

at4g15680	CON	MIN	HOUR	DAY	WEEK	UNIGENE	FLCDNA	TAIR
TATGTTGTCT	1	2	0	0	0	gnl|UG|At#S11725084	gi|21405868|gb|AY087144.1	At4g15680.1

at3g44330	CON	MIN	HOUR	DAY	WEEK	UNIGENE	FLCDNA	TAIR
AAGAATTCAA	1	1	1	1	2	gnl|UG|At#S11730503	gi|27311778|gb|BT002495.1	At3g44330.1

at4g16680	CON	MIN	HOUR	DAY	WEEK	UNIGENE	FLCDNA	TAIR
TCTGCTTATC	0	1	0	0	0	no match found	gi|110740630|dbj|AK226258.1|	non-canonical match

at4g19710	CON	MIN	HOUR	DAY	WEEK	UNIGENE	FLCDNA	TAIR

TACAATAGCA	1	1	0	0	2	gnl|UG|At#S18941836	gi|110736463|dbj|AK228252.1|	At4g19710.2
at3g17930	CON	MIN	HOUR	DAY	WEEK	UNIGENE	FLCDNA	TAIR
CAGAAACGAT	5	2	2	1	2	gnl|UG|At#S11735470	gi|20147322|gb|AY093751.1	multiple non-canonical match
at4g11860	CON	MIN	HOUR	DAY	WEEK	UNIGENE	FLCDNA	TAIR
TAAATCCTCA	2	3	2	0	0	gnl|UG|At#S34116889	gi|21404308|gb|AY085598.1	At4g11860.1
at5g38830	CON	MIN	HOUR	DAY	WEEK	UNIGENE	FLCDNA	TAIR
GAGATAGTTA	0	3	2	3	0	gnl|UG|At#S11719850	no match found	pseudo chromosome match
at2g21390	CON	MIN	HOUR	DAY	WEEK	UNIGENE	FLCDNA	TAIR
AGAACTTTAT	1	0	3	7	1	gnl|UG|At#S11737112	gi|110737299|dbj|AK228692.1|	At2g21390.1
at5g21950	CON	MIN	HOUR	DAY	WEEK	UNIGENE	FLCDNA	TAIR
TTAATAAAAA	1	0	0	0	0	no match found	no match found	At5g21950.1
at1g51580	CON	MIN	HOUR	DAY	WEEK	UNIGENE	FLCDNA	TAIR
ATAGTAAAAA	3	2	0	1	2	no match found	gi|110735739|dbj|AK227874.1|	multiple canonical match
at5g47620	CON	MIN	HOUR	DAY	WEEK	UNIGENE	FLCDNA	TAIR
GAGATCAGAT	2	5	6	7	3	gnl|UG|At#S11710353	gi|21404538|gb|AY085828.1	At5g47620.2
at5g64770	CON	MIN	HOUR	DAY	WEEK	UNIGENE	FLCDNA	TAIR
GTTTTCTGTT	5	2	12	6	5	no match found	gi|27311820|gb|BT002516.1	At5g64770.1
at5g56210	CON	MIN	HOUR	DAY	WEEK	UNIGENE	FLCDNA	TAIR
ATAGTTTTGT	0	0	0	0	2	gnl|UG|At#S21989661	no match found	multiple non-canonical match
TACAATGAAA	3	5	2	0	0	no match found	no match found	At5g56210.1
at2g35720	CON	MIN	HOUR	DAY	WEEK	UNIGENE	FLCDNA	TAIR
GGTTTTTGCG	0	0	0	1	0	gnl|UG|At#S11733642	no match found	At2g35720.1
at4g33180	CON	MIN	HOUR	DAY	WEEK	UNIGENE	FLCDNA	TAIR
TAAGAACTTT	2	4	0	0	0	no match found	gi|51968981|dbj|AK175420.1|	multiple non-canonical match
at3g09350	CON	MIN	HOUR	DAY	WEEK	UNIGENE	FLCDNA	TAIR
CTAATATTCG	1	0	0	1	0	gnl|UG|At#S38433760	gi|28393836|gb|BT004332.1	At3g09350.1
at3g50380	CON	MIN	HOUR	DAY	WEEK	UNIGENE	FLCDNA	TAIR
TGGAAAGCTG	1	2	0	0	0	gnl|UG|At#S11729370	no match found	At3g50380.1
at2g12685	CON	MIN	HOUR	DAY	WEEK	UNIGENE	FLCDNA	TAIR
TGACTCAATC	0	0	0	1	0	no match found	no match found	At2g12685.1
at1g76880	CON	MIN	HOUR	DAY	WEEK	UNIGENE	FLCDNA	TAIR
GAGATGGGTG	0	2	0	0	0	gnl|UG|At#S26539034	no match found	non-canonical match
AGAATTTAAA	1	2	0	0	1	gnl|UG|At#S11727623	gi|110740127|dbj|AK230152.1|	multiple canonical match
at2g36895	CON	MIN	HOUR	DAY	WEEK	UNIGENE	FLCDNA	TAIR
GATCCTGCTC	2	1	1	0	0	no match found	gi|110741109|dbj|AK226507.1|	At2g36895.1
at5g21482	CON	MIN	HOUR	DAY	WEEK	UNIGENE	FLCDNA	TAIR
TCCAATTATG	1	0	1	1	1	gnl|UG|At#S11806600	gi|20268800|gb|AY091158.1	At5g21482.1
GCTATACTTT	0	0	2	0	0	no match found	gi|27754264|gb|BT002757.1	multiple non-canonical match
at3g23805	CON	MIN	HOUR	DAY	WEEK	UNIGENE	FLCDNA	TAIR
TCTAGATCCT	0	1	0	0	0	gnl|UG|At#S11733666	gi|21403442|gb|AY084732.1	At3g23805.1
at5g06030	CON	MIN	HOUR	DAY	WEEK	UNIGENE	FLCDNA	TAIR
GAAAAACAAA	0	0	1	0	0	no match found	no match found	At5g06030.1

at2g33410	CON	MIN	HOUR	DAY	WEEK	UNIGENE	FLCDNA	TAIR
TCACGTCTTT	1	3	3	0	2	gnl|UG|At#S11734185	gi|22530919|gb|AY136298.1	At2g33410.1
at4g39640	CON	MIN	HOUR	DAY	WEEK	UNIGENE	FLCDNA	TAIR
TCCTATCTCC	1	2	1	0	1	gnl|UG|At#S11720986	gi|23308188|gb|BT000495.1	At4g39640.1
at3g13590	CON	MIN	HOUR	DAY	WEEK	UNIGENE	FLCDNA	TAIR
TCACCGTATT	0	0	1	0	0	gnl|UG|At#S11736795	no match found	non-canonical match
at1g56340	CON	MIN	HOUR	DAY	WEEK	UNIGENE	FLCDNA	TAIR
ACGAGCTCTA	3	3	6	8	3	gnl|UG|At#S11732163	gi|30725695|gb|BT008511.1	At1g56340.1
at5g54540	CON	MIN	HOUR	DAY	WEEK	UNIGENE	FLCDNA	TAIR
GTAGTTTTAA	1	12	4	4	2	gnl|UG|At#S11718261	gi|16648815|gb|AY058185.1	At5g54540.1
at2g39180	CON	MIN	HOUR	DAY	WEEK	UNIGENE	FLCDNA	TAIR
TTGCAGGCTG	0	1	0	0	0	gnl|UG|At#S11732803	gi|110736344|dbj|AK228187.1|	At2g39180.1
at3g05850	CON	MIN	HOUR	DAY	WEEK	UNIGENE	FLCDNA	TAIR
GCTGAGTGTG	0	1	0	0	0	gnl|UG|At#S15644893	gi|110742267|dbj|AK226993.1|	At3g05850.1
at2g19720	CON	MIN	HOUR	DAY	WEEK	UNIGENE	FLCDNA	TAIR
TCATTTGTCT	1	0	1	1	0	gnl|UG|At#S11737544	no match found	non-canonical match
at1g01790	CON	MIN	HOUR	DAY	WEEK	UNIGENE	FLCDNA	TAIR
GTCTTAATCT	0	2	0	0	0	gnl|UG|At#S11742934	no match found	At1g01790.1
at5g13120	CON	MIN	HOUR	DAY	WEEK	UNIGENE	FLCDNA	TAIR
AGATGGAGCT	7	8	7	10	2	gnl|UG|At#S11723078	gi|13605816|gb|AF367307.1	At5g13120.1
at1g73010	CON	MIN	HOUR	DAY	WEEK	UNIGENE	FLCDNA	TAIR
AACCAATATT	1	9	3	0	5	gnl|UG|At#S11728462	gi|133778813|gb|BT030334.1	multiple canonical match
at3g07110	CON	MIN	HOUR	DAY	WEEK	UNIGENE	FLCDNA	TAIR
AGGTTTCTCC	1	3	0	5	2	no match found	gi|21387008|gb|AY114589.1	non-canonical match
CGCCTCCTCC	0	0	0	3	0	gnl|UG|At#S28283022	no match found	At3g07110.1
at1g07180	CON	MIN	HOUR	DAY	WEEK	UNIGENE	FLCDNA	TAIR
TGAAAGACGA	0	1	1	0	1	gnl|UG|At#S11742316	gi|28973318|gb|BT005564.1	At1g07180.1
at3g54820	CON	MIN	HOUR	DAY	WEEK	UNIGENE	FLCDNA	TAIR
GTGGCTCAGT	1	1	2	0	0	gnl|UG|At#S11728545	gi|17104532|gb|AY062981.1	At3g54820.1
at1g01920	CON	MIN	HOUR	DAY	WEEK	UNIGENE	FLCDNA	TAIR
CCCTTCACTT	1	1	2	0	0	gnl|UG|At#S11742924	gi|110742945|dbj|AK227359.1|	At1g01920.1
at5g50330	CON	MIN	HOUR	DAY	WEEK	UNIGENE	FLCDNA	TAIR
AGCAGGATCT	0	2	0	0	0	gnl|UG|At#S34114196	gi|110743930|dbj|AK227819.1|	At5g50330.1
at3g55980	CON	MIN	HOUR	DAY	WEEK	UNIGENE	FLCDNA	TAIR
GGTTAACTCT	0	3	6	2	0	gnl|UG|At#S11643720	gi|62318806|dbj|AK220723.1|	non-canonical match
AAATGGCTAA	0	1	9	0	0	gnl|UG|At#S11705988	gi|15810486|gb|AY056282.1	At3g55980.1
at1g20575	CON	MIN	HOUR	DAY	WEEK	UNIGENE	FLCDNA	TAIR
TCGTTTAGAA	1	1	0	2	0	gnl|UG|At#S21737116	gi|110743121|dbj|AK227450.1|	At1g20575.1
at3g47342	CON	MIN	HOUR	DAY	WEEK	UNIGENE	FLCDNA	TAIR
GTTCTGTAAA	1	0	0	2	0	gnl|UG|At#S18901932	no match found	non-canonical match
at3g48185	CON	MIN	HOUR	DAY	WEEK	UNIGENE	FLCDNA	TAIR

TATGATCACT	0	3	0	0	0	gnl|UG|At#S11708452	gi|20259014|gb|AY091284.1	At3g48185.1
at3g49430	CON	MIN	HOUR	DAY	WEEK	UNIGENE	FLCDNA	TAIR
AAGTATGCAA	0	0	1	0	0	no match found	gi|124301105|gb|BT030067.1	At3g49430.1
at3g61160	CON	MIN	HOUR	DAY	WEEK	UNIGENE	FLCDNA	TAIR
CAAGGAAATG	0	2	0	0	0	gnl|UG|At#S11727126	gi|110738546|dbj|AK229336.1|	At3g61160.2
at4g34730	CON	MIN	HOUR	DAY	WEEK	UNIGENE	FLCDNA	TAIR
GAAAGAGGAA	2	0	0	0	0	no match found	gi|30023649|gb|BT006250.1	non-canonical match
TATGCAGTAA	0	0	3	0	1	gnl|UG|At#S11721810	gi|25082956|gb|BT002011.1	At4g34730.1
at4g38550	CON	MIN	HOUR	DAY	WEEK	UNIGENE	FLCDNA	TAIR
CGTGTGTTTC	0	1	8	2	3	no match found	gi|17064935|gb|AY062544.1	At4g38550.1
AAATGTTTAT	1	2	0	0	0	no match found	gi|13430739|gb|AF360282.1	non-canonical match
at1g04270	CON	MIN	HOUR	DAY	WEEK	UNIGENE	FLCDNA	TAIR
TTACAAGAGG	41	13	20	19	61	gnl|UG|At#S11742614	gi|15081657|gb|AY048221.1	At1g04270.1
ATCATCGTCC	0	3	1	0	0	no match found	gi|20147170|gb|AY091703.1	non-canonical match
at1g54650	CON	MIN	HOUR	DAY	WEEK	UNIGENE	FLCDNA	TAIR
GGAAGTTCCA	0	1	0	0	0	gnl|UG|At#S14273925	gi|56550684|gb|BT020405.1	At1g54650.1
at5g47840	CON	MIN	HOUR	DAY	WEEK	UNIGENE	FLCDNA	TAIR
ACGGGCTGTG	0	2	0	1	0	gnl|UG|At#S11718937	gi|15146327|gb|AY049305.1	At5g47840.1
at1g76660	CON	MIN	HOUR	DAY	WEEK	UNIGENE	FLCDNA	TAIR
GCTTTTTTGT	2	2	3	1	0	gnl|UG|At#S11727690	gi|26449383|dbj|AK117141.1|	At1g76660.1
CGGATGAAGA	0	1	0	0	0	no match found	gi|28951036|gb|BT005378.1	multiple non-canonical match
at1g13755	CON	MIN	HOUR	DAY	WEEK	UNIGENE	FLCDNA	TAIR
CATATATGAT	0	0	1	1	1	gnl|UG|At#S37211794	no match found	multiple non-canonical match
at5g17040	CON	MIN	HOUR	DAY	WEEK	UNIGENE	FLCDNA	TAIR
TTCTGTTTTG	0	0	0	0	1	gnl|UG|At#S11722076	no match found	At5g17040.1
at3g58600	CON	MIN	HOUR	DAY	WEEK	UNIGENE	FLCDNA	TAIR
ACCATATGAA	0	1	0	0	0	gnl|UG|At#S11704838	gi|25054904|gb|BT001934.1	non-canonical match
at5g15520	CON	MIN	HOUR	DAY	WEEK	UNIGENE	FLCDNA	TAIR
AAGAGCTGTT	0	0	1	2	5	gnl|UG|At#S17006707	gi|21405215|gb|AY086505.1	At5g15520.1
at1g25425	CON	MIN	HOUR	DAY	WEEK	UNIGENE	FLCDNA	TAIR
ACCTCCAAGA	1	0	1	0	1	gnl|UG|At#S18893313	no match found	At1g53160.2
at4g36480	CON	MIN	HOUR	DAY	WEEK	UNIGENE	FLCDNA	TAIR
CATTAGCCAC	1	0	0	0	0	no match found	gi|23197845|gb|BT000131.1	non-canonical match
at2g16250	CON	MIN	HOUR	DAY	WEEK	UNIGENE	FLCDNA	TAIR
CCTAAACCCA	0	1	0	0	0	gnl|UG|At#S11738409	no match found	At2g16250.1
at4g31990	CON	MIN	HOUR	DAY	WEEK	UNIGENE	FLCDNA	TAIR
TGTATATGAC	2	0	0	0	1	gnl|UG|At#S11816568	gi|21405990|gb|AY087266.1	At4g31990.2
at2g26580	CON	MIN	HOUR	DAY	WEEK	UNIGENE	FLCDNA	TAIR
GCAGCTGCTC	0	1	1	0	0	no match found	gi|26453179|dbj|AK119091.1|	At2g26580.1
at2g31070	CON	MIN	HOUR	DAY	WEEK	UNIGENE	FLCDNA	TAIR
CATTGAACAA	0	3	0	1	1	gnl|UG|At#S11734770	gi|16649150|gb|AY059945.1	At2g31070.1
at4g31810	CON	MIN	HOUR	DAY	WEEK	UNIGENE	FLCDNA	TAIR

GTGGATTGCT	0	1	1	0	0	gnl|UG|At#S11722311	gi|19424018|gb|AY080787.1	At4g31810.1
at2g46280	CON	MIN	HOUR	DAY	WEEK	UNIGENE	FLCDNA	TAIR
TTTTTATTTA	2	6	2	1	1	gnl|UG|At#S18942692	gi|13877568|gb|AF370485.1	multiple canonical match
at4g35840	CON	MIN	HOUR	DAY	WEEK	UNIGENE	FLCDNA	TAIR
TGGTTAGAGT	3	0	1	2	0	gnl|UG|At#S11823811	gi|26451100|dbj|AK118022.1|	At4g35840.1
at5g13080	CON	MIN	HOUR	DAY	WEEK	UNIGENE	FLCDNA	TAIR
ATCTGATGAT	1	1	0	0	0	no match found	gi|27311640|gb|BT002426.1	At5g13080.1
at5g05470	CON	MIN	HOUR	DAY	WEEK	UNIGENE	FLCDNA	TAIR
GCTAAGCTTA	0	0	1	1	0	no match found	gi|26450112|dbj|AK117513.1|	multiple non-canonical match
GTTAGTAGTA	0	0	0	0	1	gnl|UG|At#S11724765	gi|13358180|gb|AF324988.2	At5g05470.1
at5g16560	CON	MIN	HOUR	DAY	WEEK	UNIGENE	FLCDNA	TAIR
TATGCAGTAT	0	1	0	0	0	gnl|UG|At#S11722201	no match found	At5g16560.1
at4g22340	CON	MIN	HOUR	DAY	WEEK	UNIGENE	FLCDNA	TAIR
CTTTATGAAA	2	1	1	0	1	gnl|UG|At#S28281984	gi|21403280|gb|AY084570.1	At4g22340.2
at3g45890	CON	MIN	HOUR	DAY	WEEK	UNIGENE	FLCDNA	TAIR
GCCAATGGAT	0	2	0	0	0	no match found	gi|30793914|gb|BT008583.1	multiple canonical match
TCTCTCAAGT	1	0	0	0	0	gnl|UG|At#S15644838	gi|30794094|gb|BT008683.1	At3g45890.1
at1g31530	CON	MIN	HOUR	DAY	WEEK	UNIGENE	FLCDNA	TAIR
TTCTACACGC	0	0	0	0	1	gnl|UG|At#S32419557	no match found	At1g31530.1
at5g02100	CON	MIN	HOUR	DAY	WEEK	UNIGENE	FLCDNA	TAIR
GGGTGATCTC	0	3	0	0	0	gnl|UG|At#S11707398	gi|20258866|gb|AY091166.1	At5g02100.1
at1g16470	CON	MIN	HOUR	DAY	WEEK	UNIGENE	FLCDNA	TAIR
GAACTTGACG	3	0	6	2	1	gnl|UG|At#S28282623	gi|12083341|gb|AF332467.1	At1g16470.1
at3g60300	CON	MIN	HOUR	DAY	WEEK	UNIGENE	FLCDNA	TAIR
CATCAACGTC	0	6	1	0	0	gnl|UG|At#S11649921	gi|17065267|gb|AY062710.1	At3g60300.1
at5g28740	CON	MIN	HOUR	DAY	WEEK	UNIGENE	FLCDNA	TAIR
TGGAGATCTC	0	1	1	1	0	gnl|UG|At#S11720337	no match found	At5g28740.1
at1g75860	CON	MIN	HOUR	DAY	WEEK	UNIGENE	FLCDNA	TAIR
TGTATTGCTG	0	1	1	0	1	gnl|UG|At#S11743898	gi|19347825|gb|AY080723.1	non-canonical match
at2g16150	CON	MIN	HOUR	DAY	WEEK	UNIGENE	FLCDNA	TAIR
CGAGAATGCT	0	0	1	0	0	no match found	no match found	At2g16150.1
at3g29784	CON	MIN	HOUR	DAY	WEEK	UNIGENE	FLCDNA	TAIR
AAGAAGATGT	0	0	0	1	0	no match found	no match found	At3g29784.1
at1g16980	CON	MIN	HOUR	DAY	WEEK	UNIGENE	FLCDNA	TAIR
TTGATGAGCT	0	1	0	0	0	no match found	no match found	At1g16980.1
at3g03990	CON	MIN	HOUR	DAY	WEEK	UNIGENE	FLCDNA	TAIR
GGCCAAAAAC	0	1	0	0	0	no match found	gi|110739438|dbj|AK229798.1|	At3g03990.1
GATTTGCTCC	0	4	0	0	0	gnl|UG|At#S11739520	gi|21407127|gb|AY088353.1	At3g03990.1
at1g68945	CON	MIN	HOUR	DAY	WEEK	UNIGENE	FLCDNA	TAIR
AAAGAGAGTG	1	0	0	0	0	gnl|UG|At#S20710442	no match found	At1g68945.1
GCGGATGAGT	12	7	5	12	6	no match found	gi|15028210|gb|AY045928.1	non-canonical match
at1g54350	CON	MIN	HOUR	DAY	WEEK	UNIGENE	FLCDNA	TAIR

<table>
<tr><td>TTTGGTGTCC</td><td>1</td><td>1</td><td>2</td><td>1</td><td>0</td><td>gnl|UG|At#S11732868</td><td>gi|45773817|gb|BT012226.1</td><td>At1g54350.1</td></tr>
<tr><td>at5g56890</td><td>CON</td><td>MIN</td><td>HOUR</td><td>DAY</td><td>WEEK</td><td>UNIGENE</td><td>FLCDNA</td><td>TAIR</td></tr>
<tr><td>GAAACCGGTT</td><td>1</td><td>1</td><td>2</td><td>0</td><td>0</td><td>gnl|UG|At#S11816810</td><td>gi|20453161|gb|AY094451.1</td><td>At5g56890.1</td></tr>
<tr><td>at2g29210</td><td>CON</td><td>MIN</td><td>HOUR</td><td>DAY</td><td>WEEK</td><td>UNIGENE</td><td>FLCDNA</td><td>TAIR</td></tr>
<tr><td>GTTCACAGAT</td><td>1</td><td>1</td><td>0</td><td>0</td><td>0</td><td>no match found</td><td>gi|23308458|gb|BT000633.1</td><td>non-canonical match</td></tr>
<tr><td>TTCCAAACTT</td><td>0</td><td>4</td><td>2</td><td>2</td><td>3</td><td>gnl|UG|At#S11735236</td><td>gi|21928130|gb|AY125501.1</td><td>multiple canonical match</td></tr>
<tr><td>at1g06200</td><td>CON</td><td>MIN</td><td>HOUR</td><td>DAY</td><td>WEEK</td><td>UNIGENE</td><td>FLCDNA</td><td>TAIR</td></tr>
<tr><td>GACGAGGATT</td><td>0</td><td>1</td><td>1</td><td>0</td><td>0</td><td>no match found</td><td>gi|20148504|gb|AY081581.1</td><td>multiple non-canonical match</td></tr>
<tr><td>AGCTTCATCC</td><td>1</td><td>1</td><td>2</td><td>4</td><td>0</td><td>no match found</td><td>gi|17065431|gb|AY062792.1</td><td>non-canonical match</td></tr>
<tr><td>GTTTTAAACC</td><td>0</td><td>0</td><td>1</td><td>3</td><td>0</td><td>gnl|UG|At#S11742407</td><td>no match found</td><td>At1g06200.1</td></tr>
<tr><td>at4g15230</td><td>CON</td><td>MIN</td><td>HOUR</td><td>DAY</td><td>WEEK</td><td>UNIGENE</td><td>FLCDNA</td><td>TAIR</td></tr>
<tr><td>AGCAAACTCA</td><td>1</td><td>0</td><td>3</td><td>0</td><td>1</td><td>no match found</td><td>gi|26449505|dbj|AK117203.1|</td><td>At4g15230.1</td></tr>
<tr><td>at4g04360</td><td>CON</td><td>MIN</td><td>HOUR</td><td>DAY</td><td>WEEK</td><td>UNIGENE</td><td>FLCDNA</td><td>TAIR</td></tr>
<tr><td>TGGGAGCTTC</td><td>0</td><td>0</td><td>1</td><td>0</td><td>1</td><td>gnl|UG|At#S22484600</td><td>no match found</td><td>non-canonical match</td></tr>
<tr><td>at3g56870</td><td>CON</td><td>MIN</td><td>HOUR</td><td>DAY</td><td>WEEK</td><td>UNIGENE</td><td>FLCDNA</td><td>TAIR</td></tr>
<tr><td>AGATATAACG</td><td>0</td><td>0</td><td>0</td><td>1</td><td>0</td><td>gnl|UG|At#S21989774</td><td>no match found</td><td>At3g56870.1</td></tr>
<tr><td>at3g53190</td><td>CON</td><td>MIN</td><td>HOUR</td><td>DAY</td><td>WEEK</td><td>UNIGENE</td><td>FLCDNA</td><td>TAIR</td></tr>
<tr><td>AATTGAATTT</td><td>1</td><td>0</td><td>0</td><td>0</td><td>0</td><td>gnl|UG|At#S24442588</td><td>gi|62320497|dbj|AK221575.1|</td><td>At3g53190.1</td></tr>
<tr><td>at1g64970</td><td>CON</td><td>MIN</td><td>HOUR</td><td>DAY</td><td>WEEK</td><td>UNIGENE</td><td>FLCDNA</td><td>TAIR</td></tr>
<tr><td>CTTCAGCAGT</td><td>0</td><td>0</td><td>0</td><td>1</td><td>3</td><td>gnl|UG|At#S11730137</td><td>no match found</td><td>multiple non-canonical match</td></tr>
<tr><td>at3g28900</td><td>CON</td><td>MIN</td><td>HOUR</td><td>DAY</td><td>WEEK</td><td>UNIGENE</td><td>FLCDNA</td><td>TAIR</td></tr>
<tr><td>AGTTTTTGTT</td><td>6</td><td>3</td><td>5</td><td>12</td><td>10</td><td>gnl|UG|At#S11732004</td><td>gi|15027922|gb|AY045818.1</td><td>At3g28900.1</td></tr>
<tr><td>at2g04100</td><td>CON</td><td>MIN</td><td>HOUR</td><td>DAY</td><td>WEEK</td><td>UNIGENE</td><td>FLCDNA</td><td>TAIR</td></tr>
<tr><td>AGTTGGAACG</td><td>0</td><td>1</td><td>0</td><td>0</td><td>0</td><td>gnl|UG|At#S11740365</td><td>gi|30387604|gb|BT006624.1</td><td>At2g04100.1</td></tr>
<tr><td>at1g02260</td><td>CON</td><td>MIN</td><td>HOUR</td><td>DAY</td><td>WEEK</td><td>UNIGENE</td><td>FLCDNA</td><td>TAIR</td></tr>
<tr><td>GATACACTCT</td><td>0</td><td>0</td><td>0</td><td>0</td><td>1</td><td>gnl|UG|At#S11742890</td><td>gi|66792703|gb|BT023463.1</td><td>At1g02260.1</td></tr>
<tr><td>at4g31540</td><td>CON</td><td>MIN</td><td>HOUR</td><td>DAY</td><td>WEEK</td><td>UNIGENE</td><td>FLCDNA</td><td>TAIR</td></tr>
<tr><td>TTAGGGACCA</td><td>0</td><td>0</td><td>1</td><td>0</td><td>0</td><td>gnl|UG|At#S11722357</td><td>gi|22654980|gb|AY139762.1</td><td>At4g31540.1</td></tr>
<tr><td>at1g80210</td><td>CON</td><td>MIN</td><td>HOUR</td><td>DAY</td><td>WEEK</td><td>UNIGENE</td><td>FLCDNA</td><td>TAIR</td></tr>
<tr><td>GAAGATTAAC</td><td>2</td><td>0</td><td>3</td><td>1</td><td>0</td><td>no match found</td><td>gi|20147354|gb|AY093768.1</td><td>non-canonical match</td></tr>
<tr><td>at1g36050</td><td>CON</td><td>MIN</td><td>HOUR</td><td>DAY</td><td>WEEK</td><td>UNIGENE</td><td>FLCDNA</td><td>TAIR</td></tr>
<tr><td>GTCAAAAAGC</td><td>0</td><td>0</td><td>0</td><td>0</td><td>1</td><td>no match found</td><td>gi|27808597|gb|BT003147.1</td><td>non-canonical match</td></tr>
<tr><td>CTCCAAATGT</td><td>2</td><td>2</td><td>0</td><td>0</td><td>0</td><td>gnl|UG|At#S11736857</td><td>gi|110736189|dbj|AK228108.1|</td><td>At1g36050.1</td></tr>
<tr><td>at2g04740</td><td>CON</td><td>MIN</td><td>HOUR</td><td>DAY</td><td>WEEK</td><td>UNIGENE</td><td>FLCDNA</td><td>TAIR</td></tr>
<tr><td>TTGTAGATAT</td><td>0</td><td>0</td><td>0</td><td>1</td><td>1</td><td>gnl|UG|At#S11740224</td><td>no match found</td><td>At2g04740.1</td></tr>
<tr><td>at5g64480</td><td>CON</td><td>MIN</td><td>HOUR</td><td>DAY</td><td>WEEK</td><td>UNIGENE</td><td>FLCDNA</td><td>TAIR</td></tr>
<tr><td>TTTTTCGATT</td><td>0</td><td>2</td><td>3</td><td>4</td><td>6</td><td>no match found</td><td>gi|51969101|dbj|AK175480.1|</td><td>multiple canonical match</td></tr>
<tr><td>at2g17500</td><td>CON</td><td>MIN</td><td>HOUR</td><td>DAY</td><td>WEEK</td><td>UNIGENE</td><td>FLCDNA</td><td>TAIR</td></tr>
<tr><td>CGTACCTTTC</td><td>2</td><td>0</td><td>1</td><td>0</td><td>1</td><td>gnl|UG|At#S15461505</td><td>gi|21403057|gb|AY084347.1</td><td>At2g17500.2</td></tr>
<tr><td>at3g20790</td><td>CON</td><td>MIN</td><td>HOUR</td><td>DAY</td><td>WEEK</td><td>UNIGENE</td><td>FLCDNA</td><td>TAIR</td></tr>
<tr><td>ATAATACAAA</td><td>2</td><td>1</td><td>2</td><td>0</td><td>0</td><td>gnl|UG|At#S11734603</td><td>gi|20260135|gb|AY092967.1</td><td>non-canonical match</td></tr>
<tr><td>at1g63770</td><td>CON</td><td>MIN</td><td>HOUR</td><td>DAY</td><td>WEEK</td><td>UNIGENE</td><td>FLCDNA</td><td>TAIR</td></tr>
</table>

CCAAGGATGG	3	2	4	1	0	gnl|UG|At#S11776800	gi|25083481|gb|BT002074.1	At1g63770.1
at4g14270	CON	MIN	HOUR	DAY	WEEK	UNIGENE	FLCDNA	TAIR
AATCCGAACT	1	8	2	0	0	gnl|UG|At#S28282047	gi|21405708|gb|AY086984.1	At4g14270.1
at3g19820	CON	MIN	HOUR	DAY	WEEK	UNIGENE	FLCDNA	TAIR
AGTGTTTACT	3	5	1	2	0	gnl|UG|At#S20866803	gi|23198317|gb|BT000367.1	At3g19820.1
at1g50440	CON	MIN	HOUR	DAY	WEEK	UNIGENE	FLCDNA	TAIR
TGATGGAACT	0	0	0	1	0	no match found	gi|107738345|gb|BT025790.1	multiple non-canonical match
TTTATCTGTT	2	0	1	1	3	gnl|UG|At#S15459202	gi|21403773|gb|AY085063.1	At1g50440.2
at2g38840	CON	MIN	HOUR	DAY	WEEK	UNIGENE	FLCDNA	TAIR
CTCGTTGTTT	1	1	0	0	0	gnl|UG|At#S11732887	gi|45752655|gb|BT012131.1	At2g38840.1
at5g19340	CON	MIN	HOUR	DAY	WEEK	UNIGENE	FLCDNA	TAIR
TTCCTGTTTG	0	2	0	0	0	gnl|UG|At#S11721486	gi|45752681|gb|BT012144.1	At5g19340.1
at3g48410	CON	MIN	HOUR	DAY	WEEK	UNIGENE	FLCDNA	TAIR
AAATATCTGG	0	1	0	0	0	gnl|UG|At#S11710291	gi|17529095|gb|AY065282.1	At3g48410.1
at3g63270	CON	MIN	HOUR	DAY	WEEK	UNIGENE	FLCDNA	TAIR
CCAGTTGGAC	0	1	1	2	0	gnl|UG|At#S11726446	gi|13878074|gb|AF370300.1	At3g63270.1
at1g55850	CON	MIN	HOUR	DAY	WEEK	UNIGENE	FLCDNA	TAIR
TTTCTCGTCC	0	1	0	0	0	gnl|UG|At#S11708267	gi|17381167|gb|AY064040.1	At1g55850.1
at1g29810	CON	MIN	HOUR	DAY	WEEK	UNIGENE	FLCDNA	TAIR
CCACACAAGC	1	1	0	1	0	gnl|UG|At#S18893953	gi|26449458|dbj|AK117179.1|	At1g29810.1
at5g56730	CON	MIN	HOUR	DAY	WEEK	UNIGENE	FLCDNA	TAIR
CCGCAGAGAT	0	1	0	0	0	gnl|UG|At#S11718039	no match found	non-canonical match
ACTATCATAG	0	0	1	0	1	no match found	no match found	At5g56730.1
at3g60360	CON	MIN	HOUR	DAY	WEEK	UNIGENE	FLCDNA	TAIR
GTTGTGTAGC	1	0	1	1	0	no match found	gi|21403866|gb|AY085156.1	At3g60360.1
AATCCAAAAT	0	0	1	0	0	no match found	gi|23397167|gb|BT000725.1	multiple non-canonical match
at3g05880	CON	MIN	HOUR	DAY	WEEK	UNIGENE	FLCDNA	TAIR
GTTTCAAGTT	0	1	2	1	0	gnl|UG|At#S35281098	no match found	non-canonical match
at4g17870	CON	MIN	HOUR	DAY	WEEK	UNIGENE	FLCDNA	TAIR
CCTTCGGAGT	0	2	0	0	0	gnl|UG|At#S11724701	gi|14596204|gb|AY042890.1	At4g17870.1
at1g56550	CON	MIN	HOUR	DAY	WEEK	UNIGENE	FLCDNA	TAIR
CTCTTGAGTC	0	1	0	0	0	gnl|UG|At#S11732087	gi|26449883|dbj|AK117396.1|	At1g56550.1
at3g04550	CON	MIN	HOUR	DAY	WEEK	UNIGENE	FLCDNA	TAIR
TAAAAATTTT	1	3	5	8	4	gnl|UG|At#S11703447	gi|17064727|gb|AY062440.1	At3g04550.1
at5g08310	CON	MIN	HOUR	DAY	WEEK	UNIGENE	FLCDNA	TAIR
ACAAAACACA	1	0	0	1	0	gnl|UG|At#S11724034	no match found	At5g08310.1
at4g17470	CON	MIN	HOUR	DAY	WEEK	UNIGENE	FLCDNA	TAIR
TTCCAGAATG	0	1	1	0	0	gnl|UG|At#S11724774	gi|63003765|gb|BT022000.1	At4g17470.1
at1g74910	CON	MIN	HOUR	DAY	WEEK	UNIGENE	FLCDNA	TAIR
GAAAATGCAG	0	1	0	0	0	no match found	gi|21700876|gb|AY124853.1	multiple non-canonical match
TATCATTTAT	2	5	3	1	2	no match found	gi|13937223|gb|AF372967.1	multiple non-canonical match
at3g21310	CON	MIN	HOUR	DAY	WEEK	UNIGENE	FLCDNA	TAIR

GACACAACTC	2	0	0	0	0	gnl|UG|At#S11811702	gi|21405262|gb|AY086552.1	At3g21310.1
at1g27330	CON	MIN	HOUR	DAY	WEEK	UNIGENE	FLCDNA	TAIR
TTATTGAAAG	5	7	6	3	9	gnl|UG|At#S18897368	gi|110743216|dbj|AK227499.1|	At1g27330.1
at5g27460	CON	MIN	HOUR	DAY	WEEK	UNIGENE	FLCDNA	TAIR
CCAAAAGACC	1	2	0	0	0	gnl|UG|At#S11720466	no match found	non-canonical match
GTATAATAAT	0	1	0	0	1	no match found	no match found	At5g27460.1
at2g30440	CON	MIN	HOUR	DAY	WEEK	UNIGENE	FLCDNA	TAIR
ACAAAAGAAA	0	5	1	2	0	no match found	gi|22135949|gb|AY128354.1	multiple non-canonical match
TTTTGGCTAC	1	1	2	2	0	gnl|UG|At#S11734930	no match found	At2g30440.1
at3g50110	CON	MIN	HOUR	DAY	WEEK	UNIGENE	FLCDNA	TAIR
AAGACTCAGA	0	0	0	0	1	gnl|UG|At#S11729423	gi|24030327|gb|BT000931.1	At3g50110.1
at5g22350	CON	MIN	HOUR	DAY	WEEK	UNIGENE	FLCDNA	TAIR
GGGCATCTAG	0	2	0	0	1	gnl|UG|At#S11721012	gi|62321059|dbj|AK221859.1|	At5g22350.1
at4g23680	CON	MIN	HOUR	DAY	WEEK	UNIGENE	FLCDNA	TAIR
ATCATATGTC	6	2	5	4	1	gnl|UG|At#S30651766	gi|13605713|gb|AF361838.1	At4g23680.1
GGAAACCACG	0	0	1	1	0	no match found	gi|22655453|gb|AY142055.1	non-canonical match
at1g08540	CON	MIN	HOUR	DAY	WEEK	UNIGENE	FLCDNA	TAIR
AGGCAGGACT	2	2	1	3	0	no match found	gi|133778837|gb|BT030346.1	At1g08540.1
at5g16360	CON	MIN	HOUR	DAY	WEEK	UNIGENE	FLCDNA	TAIR
AAGATGACTC	0	0	0	1	0	gnl|UG|At#S11651983	gi|27754414|gb|BT002837.1	At5g16360.1
at4g02330	CON	MIN	HOUR	DAY	WEEK	UNIGENE	FLCDNA	TAIR
TGATTAATTC	0	1	1	1	0	gnl|UG|At#S11744279	gi|23297460|gb|AY150433.1	At4g02330.1
at3g55580	CON	MIN	HOUR	DAY	WEEK	UNIGENE	FLCDNA	TAIR
CGGTTGGTGG	0	0	2	0	0	gnl|UG|At#S34115948	gi|110740522|dbj|AK226202.1|	At3g55580.1
at3g03420	CON	MIN	HOUR	DAY	WEEK	UNIGENE	FLCDNA	TAIR
TATTTCTCTC	0	0	0	1	0	gnl|UG|At#S11739672	gi|13877934|gb|AF370230.1	At3g03420.1
at3g02060	CON	MIN	HOUR	DAY	WEEK	UNIGENE	FLCDNA	TAIR
CTTCTTCTTA	0	2	0	0	1	gnl|UG|At#S11740064	gi|13877638|gb|AF370520.1	multiple canonical match
at5g04560	CON	MIN	HOUR	DAY	WEEK	UNIGENE	FLCDNA	TAIR
AGAGGAAGTT	0	0	1	0	0	gnl|UG|At#S11725000	gi|28950994|gb|BT005357.1	At5g04560.1
at1g52320	CON	MIN	HOUR	DAY	WEEK	UNIGENE	FLCDNA	TAIR
TTCGGATATG	0	0	1	0	0	gnl|UG|At#S34117780	gi|62320231|dbj|AK221442.1|	At1g52320.1
at5g20670	CON	MIN	HOUR	DAY	WEEK	UNIGENE	FLCDNA	TAIR
TTCTGTGTTC	0	0	0	0	1	gnl|UG|At#S11721159	no match found	At5g20670.1
at1g56670	CON	MIN	HOUR	DAY	WEEK	UNIGENE	FLCDNA	TAIR
AAAGTTCTTT	0	0	1	0	0	gnl|UG|At#S11732041	no match found	At1g56670.1
at1g64630	CON	MIN	HOUR	DAY	WEEK	UNIGENE	FLCDNA	TAIR
TAAAAATATG	2	0	2	1	1	no match found	gi|19698920|gb|AY081307.1	At2g41460.1
at1g23970	CON	MIN	HOUR	DAY	WEEK	UNIGENE	FLCDNA	TAIR
GCCGTCTGCG	0	1	1	0	0	gnl|UG|At#S11740639	gi|21406881|gb|AY088107.1	At1g23970.2
at3g14410	CON	MIN	HOUR	DAY	WEEK	UNIGENE	FLCDNA	TAIR
GAACTTCCAC	0	0	1	0	0	gnl|UG|At#S11736535	gi|15294189|gb|AF410286.1	At3g14410.1

at3g26210	CON	MIN	HOUR	DAY	WEEK	UNIGENE	FLCDNA	TAIR
ATAGGTAACC	0	1	1	0	0	gnl|UG|At#S11732906	no match found	pseudo chromosome match
at5g27510	CON	MIN	HOUR	DAY	WEEK	UNIGENE	FLCDNA	TAIR
GATAGGGTTT	1	0	0	1	0	gnl|UG|At#S11720461	no match found	multiple non-canonical match
at3g09730	CON	MIN	HOUR	DAY	WEEK	UNIGENE	FLCDNA	TAIR
CTACATCGAA	0	0	1	1	3	no match found	no match found	At3g09730.1
at2g43520	CON	MIN	HOUR	DAY	WEEK	UNIGENE	FLCDNA	TAIR
GTTTGTAACT	2	3	2	2	0	no match found	gi|51969551|dbj|AK175705.1|	multiple canonical match
at5g53770	CON	MIN	HOUR	DAY	WEEK	UNIGENE	FLCDNA	TAIR
GACCTACTGA	2	0	0	1	0	gnl|UG|At#S11718339	gi|14334639|gb|AY034993.1	At5g53770.1
at5g54390	CON	MIN	HOUR	DAY	WEEK	UNIGENE	FLCDNA	TAIR
TCGTTTTCCT	2	3	1	1	1	gnl|UG|At#S11718276	no match found	At5g54390.1
AGAAGATTAT	1	0	0	0	0	no match found	gi|21405198|gb|AY086488.1	multiple non-canonical match
at1g01970	CON	MIN	HOUR	DAY	WEEK	UNIGENE	FLCDNA	TAIR
CTTGAAGCCT	3	1	2	0	1	gnl|UG|At#S34118453	gi|110735699|dbj|AK227853.1|	At1g01970.1
at5g38220	CON	MIN	HOUR	DAY	WEEK	UNIGENE	FLCDNA	TAIR
GTACAAAACT	0	1	3	1	1	gnl|UG|At#S11719911	no match found	At5g38220.1
at3g25150	CON	MIN	HOUR	DAY	WEEK	UNIGENE	FLCDNA	TAIR
CTCAATGGAC	0	3	2	0	0	no match found	gi|17979474|gb|AY070733.1	non-canonical match
at1g65630	CON	MIN	HOUR	DAY	WEEK	UNIGENE	FLCDNA	TAIR
ATTTTTAATG	0	0	0	1	0	no match found	no match found	At1g65630.1
at4g38490	CON	MIN	HOUR	DAY	WEEK	UNIGENE	FLCDNA	TAIR
AGGAAGGCAA	1	2	1	0	1	no match found	gi|15451093|gb|AY054627.1	multiple canonical match
at1g65660	CON	MIN	HOUR	DAY	WEEK	UNIGENE	FLCDNA	TAIR
TAACCTGAAG	1	4	2	2	2	gnl|UG|At#S11729998	gi|27754340|gb|BT002797.1	At1g65660.1
at4g35350	CON	MIN	HOUR	DAY	WEEK	UNIGENE	FLCDNA	TAIR
GTAAGAGTTC	4	1	3	1	0	gnl|UG|At#S18941725	gi|26449880|dbj|AK117394.1|	At4g35350.1
at2g42190	CON	MIN	HOUR	DAY	WEEK	UNIGENE	FLCDNA	TAIR
AAATCAATCT	4	5	2	2	3	no match found	gi|21403663|gb|AY084953.1	multiple canonical match
at5g19080	CON	MIN	HOUR	DAY	WEEK	UNIGENE	FLCDNA	TAIR
TCATACTCTT	1	0	0	0	0	gnl|UG|At#S11721551	gi|21928148|gb|AY125510.1	At5g19080.1
at2g22420	CON	MIN	HOUR	DAY	WEEK	UNIGENE	FLCDNA	TAIR
GTGAAGTTGG	0	0	0	1	0	gnl|UG|At#S11736884	gi|21407737|gb|AY088963.1	At2g22420.1
at3g44750	CON	MIN	HOUR	DAY	WEEK	UNIGENE	FLCDNA	TAIR
CAAGAAGACT	0	1	1	0	1	gnl|UG|At#S11730426	gi|17529219|gb|AY065396.1	At3g44750.1
at1g38423	CON	MIN	HOUR	DAY	WEEK	UNIGENE	FLCDNA	TAIR
TTAAGATTTA	0	1	0	0	0	gnl|UG|At#S11817989	no match found	pseudo chromosome match
at3g10880	CON	MIN	HOUR	DAY	WEEK	UNIGENE	FLCDNA	TAIR
TTTTTTCATT	0	1	0	1	0	gnl|UG|At#S11737618	no match found	non-canonical match
at4g31500	CON	MIN	HOUR	DAY	WEEK	UNIGENE	FLCDNA	TAIR
ACTGGACTCG	4	12	20	4	5	gnl|UG|At#S11722365	gi|14334699|gb|AY035023.1	At4g31500.1

at4g08090	CON	MIN	HOUR	DAY	WEEK	UNIGENE	FLCDNA	TAIR
CCAAAGCTAA	0	0	0	0	1	no match found	no match found	At4g08090.1
at4g25550	CON	MIN	HOUR	DAY	WEEK	UNIGENE	FLCDNA	TAIR
TGTTTCATTC	2	0	2	0	1	gnl|UG|At#S34117832	gi|110736889|dbj|AK228476.1|	At4g25550.1
AGGACTAGCG	1	0	0	0	0	no match found	gi|62318511|dbj|AK220576.1|	non-canonical match
ATTAGTTCGT	1	2	1	0	1	no match found	gi|26451194|dbj|AK118070.1|	non-canonical match
at1g21580	CON	MIN	HOUR	DAY	WEEK	UNIGENE	FLCDNA	TAIR
TAGTCTCTGT	0	1	0	0	1	no match found	gi|110737277|dbj|AK228680.1|	non-canonical match
at5g66680	CON	MIN	HOUR	DAY	WEEK	UNIGENE	FLCDNA	TAIR
ATGAGCCCGT	0	0	0	1	0	gnl|UG|At#S11717029	gi|14334505|gb|AY034944.1	At5g66680.1
at3g47560	CON	MIN	HOUR	DAY	WEEK	UNIGENE	FLCDNA	TAIR
GATCGGGTGA	0	3	0	2	1	gnl|UG|At#S18942014	gi|56381918|gb|BT020323.1	non-canonical match
at3g51660	CON	MIN	HOUR	DAY	WEEK	UNIGENE	FLCDNA	TAIR
TTATTCTTTT	2	1	2	1	2	no match found	gi|21403114|gb|AY084404.1	non-canonical match
at1g21830	CON	MIN	HOUR	DAY	WEEK	UNIGENE	FLCDNA	TAIR
TGGCACCATC	1	2	0	0	0	no match found	gi|110739340|dbj|AK229746.1|	non-canonical match
GATTTAAAAG	1	2	3	2	0	no match found	gi|62321333|dbj|AK221999.1|	At1g21830.1
TGGTCTGAAT	1	1	5	0	2	gnl|UG|At#S11740879	gi|62320857|dbj|AK221756.1|	pseudo chromosome match
at3g32445	CON	MIN	HOUR	DAY	WEEK	UNIGENE	FLCDNA	TAIR
TCGCTTGTTC	1	0	0	0	0	no match found	no match found	At3g32445.1
at4g22000	CON	MIN	HOUR	DAY	WEEK	UNIGENE	FLCDNA	TAIR
ATTACTATAG	3	1	0	0	1	gnl|UG|At#S11709068	gi|21404804|gb|AY086094.1	At4g22000.1
at1g64065	CON	MIN	HOUR	DAY	WEEK	UNIGENE	FLCDNA	TAIR
TACACATCAA	1	0	0	0	0	no match found	no match found	At1g64065.1
ATCCGGTTTA	0	1	0	0	0	gnl|UG|At#S21132608	no match found	non-canonical match
at1g16080	CON	MIN	HOUR	DAY	WEEK	UNIGENE	FLCDNA	TAIR
GTGGAAAGAC	1	2	3	2	0	gnl|UG|At#S11741429	gi|18176354|gb|AY072208.1	At1g16080.1
at5g32482	CON	MIN	HOUR	DAY	WEEK	UNIGENE	FLCDNA	TAIR
TACTCTTACC	12	1	8	2	7	no match found	no match found	At5g32482.1
at2g37480	CON	MIN	HOUR	DAY	WEEK	UNIGENE	FLCDNA	TAIR
TTGAGTTTCG	2	10	5	3	0	gnl|UG|At#S15460676	gi|51971001|dbj|AK176430.1|	At2g37480.1
at1g78240	CON	MIN	HOUR	DAY	WEEK	UNIGENE	FLCDNA	TAIR
TCTACTTATT	0	4	0	0	0	gnl|UG|At#S11701976	gi|13430453|gb|AF360139.1	At1g78240.1
at1g25420	CON	MIN	HOUR	DAY	WEEK	UNIGENE	FLCDNA	TAIR
CCATCATCTA	1	2	0	0	0	no match found	gi|20465253|gb|AY095990.1	At1g25420.3
at1g13770	CON	MIN	HOUR	DAY	WEEK	UNIGENE	FLCDNA	TAIR
TAACCGATAC	1	0	3	0	0	gnl|UG|At#S43850646	gi|28393738|gb|BT004280.1	At1g13770.1
at3g49240	CON	MIN	HOUR	DAY	WEEK	UNIGENE	FLCDNA	TAIR
AGGGCGTTAA	0	1	0	0	1	gnl|UG|At#S11729594	gi|31711737|gb|BT008786.1	At3g49240.1
at3g29000	CON	MIN	HOUR	DAY	WEEK	UNIGENE	FLCDNA	TAIR
GAGACTAGCT	0	3	3	0	0	gnl|UG|At#S11731971	no match found	At3g29000.1
at5g59330	CON	MIN	HOUR	DAY	WEEK	UNIGENE	FLCDNA	TAIR

TTAAAGATAA	0	0	0	0	1	no match found	no match found	At5g59330.1
at5g57860	CON	MIN	HOUR	DAY	WEEK	UNIGENE	FLCDNA	TAIR
AAGAGTTGCT	0	2	0	1	0	gnl|UG|At#S18908901	gi|21407625|gb|AY088851.1	At5g57860.1
at4g09570	CON	MIN	HOUR	DAY	WEEK	UNIGENE	FLCDNA	TAIR
GATAATTTTG	2	1	2	4	3	gnl|UG|At#S11726008	no match found	multiple canonical match
at4g16380	CON	MIN	HOUR	DAY	WEEK	UNIGENE	FLCDNA	TAIR
TTCTTCTTTA	2	3	1	0	1	no match found	gi|17473637|gb|AY065105.1	At4g16380.1
TGAATTTGAC	3	7	5	1	1	gnl|UG|At#S18907118	no match found	multiple non-canonical match
at5g46490	CON	MIN	HOUR	DAY	WEEK	UNIGENE	FLCDNA	TAIR
AGAGTGCCAT	1	1	0	1	0	no match found	gi|16323098|gb|AY057653.1	At5g46490.1
at1g07090	CON	MIN	HOUR	DAY	WEEK	UNIGENE	FLCDNA	TAIR
CCCTCTCAAG	0	2	1	0	0	no match found	gi|21405579|gb|AY086869.1	At1g07090.1
at1g56210	CON	MIN	HOUR	DAY	WEEK	UNIGENE	FLCDNA	TAIR
CACAACGGTT	0	0	0	1	0	gnl|UG|At#S11825144	gi|21403127|gb|AY084417.1	At1g56210.1
at5g47330	CON	MIN	HOUR	DAY	WEEK	UNIGENE	FLCDNA	TAIR
CAATATATGT	0	0	0	0	1	gnl|UG|At#S14273779	gi|28393538|gb|BT004170.1	At5g47330.1
at5g06220	CON	MIN	HOUR	DAY	WEEK	UNIGENE	FLCDNA	TAIR
TATCTGGTAC	0	1	1	1	2	gnl|UG|At#S11724569	gi|110740046|dbj|AK230108.1|	At5g06220.1
at5g19900	CON	MIN	HOUR	DAY	WEEK	UNIGENE	FLCDNA	TAIR
GTAAGCTTGT	1	2	2	0	0	gnl|UG|At#S11721347	no match found	At5g19900.1
at1g03980	CON	MIN	HOUR	DAY	WEEK	UNIGENE	FLCDNA	TAIR
AGCTTCTTGT	0	1	0	0	0	gnl|UG|At#S11742692	no match found	non-canonical match
at1g08880	CON	MIN	HOUR	DAY	WEEK	UNIGENE	FLCDNA	TAIR
TATCAATGGA	3	7	5	5	10	gnl|UG|At#S11742148	gi|21406698|gb|AY087924.1	At1g08880.1
at3g52800	CON	MIN	HOUR	DAY	WEEK	UNIGENE	FLCDNA	TAIR
GATGCAGCTA	1	11	3	0	0	no match found	gi|14596166|gb|AY042871.1	At3g52800.1
at2g18390	CON	MIN	HOUR	DAY	WEEK	UNIGENE	FLCDNA	TAIR
CTTGACTAAT	0	1	0	1	0	gnl|UG|At#S11737880	gi|21407105|gb|AY088331.1	At2g18390.1
at5g42860	CON	MIN	HOUR	DAY	WEEK	UNIGENE	FLCDNA	TAIR
TTACAAGGTA	0	2	0	0	0	gnl|UG|At#S11719443	gi|110743327|dbj|AK227557.1|	At5g42860.1
at4g20880	CON	MIN	HOUR	DAY	WEEK	UNIGENE	FLCDNA	TAIR
ATTCAGACTT	0	2	1	0	0	gnl|UG|At#S11724195	gi|26452094|dbj|AK118534.1|	At4g20880.1
at3g57870	CON	MIN	HOUR	DAY	WEEK	UNIGENE	FLCDNA	TAIR
GTTTTGTGGC	1	4	1	1	0	no match found	gi|14596100|gb|AY042838.1	At3g57870.1
AGTGTTGTCC	0	1	0	0	0	gnl|UG|At#S11727976	no match found	At3g57870.1
at2g44980	CON	MIN	HOUR	DAY	WEEK	UNIGENE	FLCDNA	TAIR
TAACGGTGTC	1	1	2	0	0	gnl|UG|At#S18942700	no match found	At2g44980.1
at5g62910	CON	MIN	HOUR	DAY	WEEK	UNIGENE	FLCDNA	TAIR
TTGTCTTTTG	0	4	0	1	0	gnl|UG|At#S11717410	gi|19347721|gb|AY080603.1	At5g62910.1
at1g48430	CON	MIN	HOUR	DAY	WEEK	UNIGENE	FLCDNA	TAIR
GTACAGCGCC	4	0	1	0	0	gnl|UG|At#S11735058	gi|22136003|gb|AY128381.1	At1g48430.1

at5g57360	CON	MIN	HOUR	DAY	WEEK	UNIGENE	FLCDNA	TAIR
GCACTTGTGT	0	0	3	2	0	gnl|UG|At#S11717976	gi|31711709|gb|BT008772.1	At5g57360.1
at4g05010	CON	MIN	HOUR	DAY	WEEK	UNIGENE	FLCDNA	TAIR
TGAATATAAC	0	2	0	0	0	no match found	no match found	At4g05010.1
TGGATCACGA	0	2	0	0	0	gnl|UG|At#S11726386	no match found	non-canonical match
at5g54670	CON	MIN	HOUR	DAY	WEEK	UNIGENE	FLCDNA	TAIR
AAGACCTTTT	1	1	0	0	0	gnl|UG|At#S34118168	gi|110736248|dbj|AK228138.1|	At5g54670.1
at5g63400	CON	MIN	HOUR	DAY	WEEK	UNIGENE	FLCDNA	TAIR
ATCAAAGACT	5	5	1	3	1	no match found	gi|14532641|gb|AY039945.1	At5g63400.1
at5g05730	CON	MIN	HOUR	DAY	WEEK	UNIGENE	FLCDNA	TAIR
GTCAATAATA	0	1	1	0	2	gnl|UG|At#S11724696	no match found	At5g63400.1
TGTATAAAAC	1	1	1	0	2	no match found	gi|18086565|gb|AY072013.1	non-canonical match
at3g01090	CON	MIN	HOUR	DAY	WEEK	UNIGENE	FLCDNA	TAIR
CCACCAAGAT	0	1	1	1	1	gnl|UG|At#S11806733	gi|20260541|gb|AY093170.1	At3g01090.2
at1g20580	CON	MIN	HOUR	DAY	WEEK	UNIGENE	FLCDNA	TAIR
TAAGCTTTGA	1	0	0	1	0	gnl|UG|At#S11741000	gi|21405507|gb|AY086797.1	At1g20580.1
CTCCAATGTT	0	0	1	0	0	gnl|UG|At#S23789158	gi|20334747|gb|AY093974.1	non-canonical match
at3g44710	CON	MIN	HOUR	DAY	WEEK	UNIGENE	FLCDNA	TAIR
TTTTTTTGTT	3	1	2	0	3	no match found	gi|110743780|dbj|AK227742.1|	At3g44710.1
at1g04420	CON	MIN	HOUR	DAY	WEEK	UNIGENE	FLCDNA	TAIR
TAATGACGTA	7	1	3	2	7	gnl|UG|At#S11710263	gi|17529151|gb|AY065361.1	At1g04420.1
at3g58630	CON	MIN	HOUR	DAY	WEEK	UNIGENE	FLCDNA	TAIR
AGTTTCTCTT	0	2	1	0	1	gnl|UG|At#S11727835	no match found	At3g58630.1
at5g23535	CON	MIN	HOUR	DAY	WEEK	UNIGENE	FLCDNA	TAIR
ACTGAAACGG	1	2	2	1	2	no match found	gi|110735794|dbj|AK227904.1|	At5g23535.1
TTTAGTTATT	0	0	0	1	1	gnl|UG|At#S11817053	no match found	non-canonical match
at3g45690	CON	MIN	HOUR	DAY	WEEK	UNIGENE	FLCDNA	TAIR
GAAGAGTTGA	0	0	1	0	0	gnl|UG|At#S11730245	no match found	At3g45690.1
at2g43280	CON	MIN	HOUR	DAY	WEEK	UNIGENE	FLCDNA	TAIR
GATTTAACTA	1	2	0	0	0	gnl|UG|At#S11825136	gi|27754519|gb|BT002891.1	At3g45690.1
at2g18510	CON	MIN	HOUR	DAY	WEEK	UNIGENE	FLCDNA	TAIR
TCTATGCCTC	0	1	0	2	1	gnl|UG|At#S11737851	gi|14334957|gb|AY035152.1	At2g18510.1
at3g13600	CON	MIN	HOUR	DAY	WEEK	UNIGENE	FLCDNA	TAIR
CAAATGGACA	0	0	0	1	0	gnl|UG|At#S11736792	no match found	non-canonical match
at3g20870	CON	MIN	HOUR	DAY	WEEK	UNIGENE	FLCDNA	TAIR
TGCAATAATT	1	0	0	0	1	gnl|UG|At#S11734578	gi|15809843|gb|AY054189.1	At3g20870.1
at1g05550	CON	MIN	HOUR	DAY	WEEK	UNIGENE	FLCDNA	TAIR
TGCATTTTCC	0	1	0	0	0	gnl|UG|At#S11742473	no match found	non-canonical match
at3g23210	CON	MIN	HOUR	DAY	WEEK	UNIGENE	FLCDNA	TAIR
TTGATTCATC	0	0	2	0	0	gnl|UG|At#S11733842	gi|22136349|gb|AY128853.1	At3g23210.1
at3g61440	CON	MIN	HOUR	DAY	WEEK	UNIGENE	FLCDNA	TAIR
CGAGCTTCGG	23	65	42	34	4	gnl|UG|At#S11727022	gi|20260389|gb|AY093094.1	At3g61440.1

at5g08610	CON	MIN	HOUR	DAY	WEEK	UNIGENE	FLCDNA	TAIR
GGACTTGACA	1	1	0	0	1	gnl|UG|At#S11723949	no match found	At5g08610.1
at5g24160	CON	MIN	HOUR	DAY	WEEK	UNIGENE	FLCDNA	TAIR
AAAATGTTGG	0	1	1	0	0	no match found	no match found	At5g24160.1
TATTATTGGA	1	0	1	0	0	gnl|UG|At#S11720763	no match found	multiple non-canonical match
at4g03380	CON	MIN	HOUR	DAY	WEEK	UNIGENE	FLCDNA	TAIR
CATTGTTTTT	0	1	0	0	0	no match found	no match found	At4g03380.1
at4g30440	CON	MIN	HOUR	DAY	WEEK	UNIGENE	FLCDNA	TAIR
CCAATATTAG	4	15	4	1	2	gnl|UG|At#S11722543	gi|21404215|gb|AY085505.1	At4g30440.1
at4g21710	CON	MIN	HOUR	DAY	WEEK	UNIGENE	FLCDNA	TAIR
TGTGTGAAGT	1	2	0	1	0	gnl|UG|At#S11724055	no match found	At4g21710.1
at3g12680	CON	MIN	HOUR	DAY	WEEK	UNIGENE	FLCDNA	TAIR
CAAGTTTGAC	0	1	0	0	1	gnl|UG|At#S11737089	gi|110738175|dbj|AK229145.1|	At3g12680.1
at4g01320	CON	MIN	HOUR	DAY	WEEK	UNIGENE	FLCDNA	TAIR
TATGGTTTCT	0	0	0	0	1	gnl|UG|At#S11727355	gi|30102489|gb|BT006355.1	At4g01320.1
at4g21160	CON	MIN	HOUR	DAY	WEEK	UNIGENE	FLCDNA	TAIR
CAAAAGAATC	2	0	0	0	0	no match found	no match found	At4g21160.1
CTCTCTGTTC	0	2	0	0	0	no match found	gi|110738685|dbj|AK229406.1|	non-canonical match
at3g63150	CON	MIN	HOUR	DAY	WEEK	UNIGENE	FLCDNA	TAIR
AGCATTCCAG	0	1	1	0	0	gnl|UG|At#S11726490	gi|16648802|gb|AY058178.1	At3g63150.1
at3g09840	CON	MIN	HOUR	DAY	WEEK	UNIGENE	FLCDNA	TAIR
TGTTCTTTTC	0	7	3	1	0	gnl|UG|At#S11737932	gi|17473550|gb|AY065076.1	At3g09840.1
at5g22780	CON	MIN	HOUR	DAY	WEEK	UNIGENE	FLCDNA	TAIR
CTTCAAGAAT	0	1	0	0	0	no match found	gi|14488102|gb|AF389300.1	multiple non-canonical match
at2g40070	CON	MIN	HOUR	DAY	WEEK	UNIGENE	FLCDNA	TAIR
GTAACTTGTC	0	1	1	0	0	gnl|UG|At#S11732581	gi|71143065|gb|BT023732.1	At2g40070.1
at4g29510	CON	MIN	HOUR	DAY	WEEK	UNIGENE	FLCDNA	TAIR
TGCCACAAGC	1	0	0	3	2	gnl|UG|At#S11722700	gi|17381027|gb|AY063970.1	At4g29510.1
at2g06925	CON	MIN	HOUR	DAY	WEEK	UNIGENE	FLCDNA	TAIR
GAAGCTGCTC	2	6	1	1	0	gnl|UG|At#S11739770	gi|25084279|gb|BT002200.1	At2g06925.1
at1g76060	CON	MIN	HOUR	DAY	WEEK	UNIGENE	FLCDNA	TAIR
TCATTGTAGA	2	0	0	1	1	gnl|UG|At#S11825200	gi|27754394|gb|BT002827.1	At1g76060.1
at1g14510	CON	MIN	HOUR	DAY	WEEK	UNIGENE	FLCDNA	TAIR
GGAAATGCGT	0	0	2	1	0	gnl|UG|At#S11741585	gi|110736109|dbj|AK228067.1|	At1g14510.1
at2g25320	CON	MIN	HOUR	DAY	WEEK	UNIGENE	FLCDNA	TAIR
CCCTTCAACA	0	0	0	1	0	gnl|UG|At#S11736190	no match found	At2g25320.1
at5g13260	CON	MIN	HOUR	DAY	WEEK	UNIGENE	FLCDNA	TAIR
CTTTCTTCTC	2	1	1	2	0	gnl|UG|At#S11708270	gi|110742979|dbj|AK227377.1|	At5g13260.1
at5g37710	CON	MIN	HOUR	DAY	WEEK	UNIGENE	FLCDNA	TAIR
CGATTCTATG	0	1	0	0	0	gnl|UG|At#S11703607	gi|14334769|gb|AY035058.1	non-canonical match
at3g26650	CON	MIN	HOUR	DAY	WEEK	UNIGENE	FLCDNA	TAIR
TTTTTCCATT	72	59	60	45	35	gnl|UG|At#S11732754	gi|16648727|gb|AY058140.1	At3g26650.1

CTCTTCCGTT	12	9	7	2	6	gnl|UG|At#S11678064	no match found	At2g44100.1
at2g31110	CON	MIN	HOUR	DAY	WEEK	UNIGENE	FLCDNA	TAIR
GGACAAAGAT	0	1	0	0	0	gnl|UG|At#S38433901	gi|51971986|dbj|AK176895.1|	At2g31110.1
at5g16440	CON	MIN	HOUR	DAY	WEEK	UNIGENE	FLCDNA	TAIR
AAAACCATTC	1	1	0	1	1	gnl|UG|At#S11722229	gi|20147318|gb|AY093749.1	At5g16440.1
at3g50560	CON	MIN	HOUR	DAY	WEEK	UNIGENE	FLCDNA	TAIR
AACTCGATAT	2	0	0	0	0	gnl|UG|At#S11729335	gi|16612319|gb|AF439850.1	At3g50560.1
at3g06410	CON	MIN	HOUR	DAY	WEEK	UNIGENE	FLCDNA	TAIR
AACTAAGCAA	0	1	3	3	2	gnl|UG|At#S34115992	gi|110741371|dbj|AK230438.1|	At3g06410.1
at3g18250	CON	MIN	HOUR	DAY	WEEK	UNIGENE	FLCDNA	TAIR
CAGTGACCGA	0	2	2	1	0	gnl|UG|At#S37211318	no match found	pseudo chromosome match
at1g59560	CON	MIN	HOUR	DAY	WEEK	UNIGENE	FLCDNA	TAIR
AGTTTTTGAC	0	2	0	0	0	gnl|UG|At#S34118230	gi|110736126|dbj|AK228076.1|	At1g59560.1
at1g14580	CON	MIN	HOUR	DAY	WEEK	UNIGENE	FLCDNA	TAIR
GGTGGCTCGG	0	0	0	0	1	no match found	gi|22136761|gb|AY133766.1	non-canonical match
AAAAGTAATT	0	2	0	1	1	gnl|UG|At#S11807187	gi|20259483|gb|AY091041.1	At1g14580.1
at4g30800	CON	MIN	HOUR	DAY	WEEK	UNIGENE	FLCDNA	TAIR
ATTTCGATGA	0	0	2	0	1	gnl|UG|At#S11722483	gi|15028244|gb|AY046037.1	At4g30800.1
at4g29220	CON	MIN	HOUR	DAY	WEEK	UNIGENE	FLCDNA	TAIR
AAATGGTGAT	0	0	1	0	0	no match found	gi|22136017|gb|AY128388.1	non-canonical match
ATAGAGAATG	5	1	1	1	0	gnl|UG|At#S11722751	no match found	multiple non-canonical match
at1g61000	CON	MIN	HOUR	DAY	WEEK	UNIGENE	FLCDNA	TAIR
AATACACGGT	1	0	0	0	0	no match found	gi|22136891|gb|AY133856.1	At1g61010.2
TATTGAAGTA	0	0	0	0	1	gnl|UG|At#S11744323	gi|19423961|gb|AY080858.1	non-canonical match
at1g56450	CON	MIN	HOUR	DAY	WEEK	UNIGENE	FLCDNA	TAIR
CAGACCTGAG	1	4	2	2	3	gnl|UG|At#S11732119	gi|62320869|dbj|AK221762.1|	At1g56450.1
at5g58100	CON	MIN	HOUR	DAY	WEEK	UNIGENE	FLCDNA	TAIR
ACAACAATAC	0	0	2	1	0	gnl|UG|At#S11717900	gi|110737998|dbj|AK229052.1|	At5g58100.1
at3g63420	CON	MIN	HOUR	DAY	WEEK	UNIGENE	FLCDNA	TAIR
GGATCCAAAA	0	1	2	0	2	gnl|UG|At#S11726383	gi|51969771|dbj|AK175815.1|	At3g63420.1
at2g25670	CON	MIN	HOUR	DAY	WEEK	UNIGENE	FLCDNA	TAIR
TATTTCGCCG	1	4	3	2	4	no match found	gi|17529065|gb|AY065267.1	non-canonical match
CCTTGTCACA	0	1	1	1	2	gnl|UG|At#S15461151	gi|21403657|gb|AY084947.1	At2g25670.1
at5g27450	CON	MIN	HOUR	DAY	WEEK	UNIGENE	FLCDNA	TAIR
ATGGGAAATA	3	0	0	0	3	gnl|UG|At#S11720467	gi|20260531|gb|AY093165.1	At5g27450.2
at2g31450	CON	MIN	HOUR	DAY	WEEK	UNIGENE	FLCDNA	TAIR
ATCCGAAGTC	1	1	5	0	0	gnl|UG|At#S38433898	gi|17380753|gb|AY063851.1	At2g31450.1
at5g22400	CON	MIN	HOUR	DAY	WEEK	UNIGENE	FLCDNA	TAIR
TTCTTCCTGT	0	0	1	0	0	gnl|UG|At#S11721000	no match found	At5g22400.1
at1g24400	CON	MIN	HOUR	DAY	WEEK	UNIGENE	FLCDNA	TAIR
TCTAATTTTA	0	1	0	0	0	gnl|UG|At#S11740530	no match found	At1g24400.1
at2g15900	CON	MIN	HOUR	DAY	WEEK	UNIGENE	FLCDNA	TAIR

TGTATAAAAA	0	1	0	0	0	no match found	no match found	At2g15900.1
at3g17970	CON	MIN	HOUR	DAY	WEEK	UNIGENE	FLCDNA	TAIR
AGAAATGCCC	0	1	1	0	1	no match found	no match found	At3g17970.1
at5g22130	CON	MIN	HOUR	DAY	WEEK	UNIGENE	FLCDNA	TAIR
ATCTGCAAAT	0	0	1	0	0	gnl|UG|At#S34115403	gi|63147375|gb|BT022100.1	At5g22130.1
at2g39670	CON	MIN	HOUR	DAY	WEEK	UNIGENE	FLCDNA	TAIR
GCTCAATCAC	3	2	0	1	2	gnl|UG|At#S15460601	gi|23197703|gb|BT000060.1	At2g39670.1
at5g18260	CON	MIN	HOUR	DAY	WEEK	UNIGENE	FLCDNA	TAIR
ACCCGATTAA	0	1	1	1	1	no match found	no match found	At5g18260.1
at5g64350	CON	MIN	HOUR	DAY	WEEK	UNIGENE	FLCDNA	TAIR
GGGGATCCAG	5	12	1	4	5	gnl|UG|At#S11717264	gi|16649116|gb|AY059928.1	At5g64350.1
at2g21580	CON	MIN	HOUR	DAY	WEEK	UNIGENE	FLCDNA	TAIR
GTGTTGTTTG	3	13	5	2	8	gnl|UG|At#S28282291	gi|15081637|gb|AY048211.1	multiple canonical match
at1g01610	CON	MIN	HOUR	DAY	WEEK	UNIGENE	FLCDNA	TAIR
GAGTAATAAT	1	0	0	0	0	no match found	gi|20259891|gb|AY093294.1	multiple non-canonical match
TAGTGTACCA	1	0	1	1	1	gnl|UG|At#S11742953	no match found	At1g01610.1
at5g60770	CON	MIN	HOUR	DAY	WEEK	UNIGENE	FLCDNA	TAIR
TGGCATTTTT	0	0	0	1	0	no match found	no match found	At5g60770.1
at3g05390	CON	MIN	HOUR	DAY	WEEK	UNIGENE	FLCDNA	TAIR
TTCCTGCAGT	1	0	0	0	0	gnl|UG|At#S21126621	no match found	non-canonical match
at3g09375	CON	MIN	HOUR	DAY	WEEK	UNIGENE	FLCDNA	TAIR
AATCAGTTTC	0	0	0	1	0	no match found	no match found	At3g09375.1
AATGAGTTTC	1	3	2	2	0	gnl|UG|At#S11734921	gi|25090063|gb|BT002207.1	At3g19760.1
at4g21920	CON	MIN	HOUR	DAY	WEEK	UNIGENE	FLCDNA	TAIR
ATTTTTTTTT	1	0	0	1	0	no match found	no match found	At4g21920.1
at1g42480	CON	MIN	HOUR	DAY	WEEK	UNIGENE	FLCDNA	TAIR
CAAATGACAT	0	1	0	0	0	gnl|UG|At#S11736303	gi|149944312|gb|BT030619.1	At1g42480.1
at5g01250	CON	MIN	HOUR	DAY	WEEK	UNIGENE	FLCDNA	TAIR
TGGAAACTGG	0	0	1	0	0	gnl|UG|At#S34116890	gi|110738708|dbj|AK229418.1|	At5g01250.1
at4g09830	CON	MIN	HOUR	DAY	WEEK	UNIGENE	FLCDNA	TAIR
TAAATCATTG	6	7	6	4	1	no match found	no match found	At4g09830.1
CAGAATAAGA	0	1	0	0	0	no match found	gi|20259186|gb|AY091370.1	non-canonical match
at2g22955	CON	MIN	HOUR	DAY	WEEK	UNIGENE	FLCDNA	TAIR
ATGTCTTAAA	1	0	0	0	0	gnl|UG|At#S30651099	gi|21403637|gb|AY084927.1	pseudo chromosome match
at5g01500	CON	MIN	HOUR	DAY	WEEK	UNIGENE	FLCDNA	TAIR
CCAAACAGCA	0	1	0	1	0	gnl|UG|At#S11725783	gi|30102451|gb|BT006336.1	At5g01500.1
at2g36145	CON	MIN	HOUR	DAY	WEEK	UNIGENE	FLCDNA	TAIR
ATCACTGGTA	3	0	1	3	5	no match found	gi|18389237|gb|AY074366.1	At2g36145.1
at3g20155	CON	MIN	HOUR	DAY	WEEK	UNIGENE	FLCDNA	TAIR
AGAAGAAGAA	0	0	0	1	0	gnl|UG|At#S11816206	no match found	multiple non-canonical match
at4g24760	CON	MIN	HOUR	DAY	WEEK	UNIGENE	FLCDNA	TAIR
GAAGGGTGTG	0	1	1	0	0	gnl|UG|At#S11723512	gi|21403622|gb|AY084912.1	At4g24760.1

at4g36420	CON	MIN	HOUR	DAY	WEEK	UNIGENE	FLCDNA	TAIR
TTATTTCCAA	2	3	3	7	4	gnl|UG|At#S11702552	gi|13878062|gb|AF370294.1	pseudo chromosome match
at4g15630	CON	MIN	HOUR	DAY	WEEK	UNIGENE	FLCDNA	TAIR
TTATGTGGAA	0	0	0	0	1	no match found	gi|110738514|dbj|AK229320.1|	non-canonical match
TTTCTATTTC	2	0	0	0	1	gnl|UG|At#S11725094	gi|62318776|dbj|AK220708.1|	At4g15630.1
at5g63980	CON	MIN	HOUR	DAY	WEEK	UNIGENE	FLCDNA	TAIR
TCGCTGGTGC	1	1	1	0	0	gnl|UG|At#S11717301	gi|110743141|dbj|AK227460.1|	At5g63980.1
at3g07700	CON	MIN	HOUR	DAY	WEEK	UNIGENE	FLCDNA	TAIR
TATCTTTAGC	2	3	5	4	4	gnl|UG|At#S11738434	gi|19424002|gb|AY080823.1	At3g07700.1
at2g17670	CON	MIN	HOUR	DAY	WEEK	UNIGENE	FLCDNA	TAIR
GATTGTGTAA	0	1	0	0	0	no match found	gi|21404188|gb|AY085478.1	non-canonical match
at4g20340	CON	MIN	HOUR	DAY	WEEK	UNIGENE	FLCDNA	TAIR
GAAAATGGCG	0	1	0	0	0	gnl|UG|At#S11724276	no match found	non-canonical match
at4g18070	CON	MIN	HOUR	DAY	WEEK	UNIGENE	FLCDNA	TAIR
GTTTAGTTGC	0	2	0	1	1	no match found	gi|21406991|gb|AY088217.1	At4g18070.1
at3g56580	CON	MIN	HOUR	DAY	WEEK	UNIGENE	FLCDNA	TAIR
CCCAGTCTGT	1	0	0	0	0	no match found	gi|20465560|gb|AY096613.1	multiple non-canonical match
TTAGCTTGTT	0	0	0	0	2	gnl|UG|At#S11728224	gi|15028360|gb|AY045983.1	At3g56580.2
at4g38870	CON	MIN	HOUR	DAY	WEEK	UNIGENE	FLCDNA	TAIR
ATCTAGATTG	0	1	0	0	0	no match found	no match found	At4g38870.1
at3g49080	CON	MIN	HOUR	DAY	WEEK	UNIGENE	FLCDNA	TAIR
GAATTTGAAA	0	0	0	1	0	no match found	gi|30387522|gb|BT006583.1	non-canonical match
at5g04050	CON	MIN	HOUR	DAY	WEEK	UNIGENE	FLCDNA	TAIR
TAAAGAAAGA	1	5	3	1	1	no match found	gi|20453206|gb|AY094475.1	multiple canonical match
at5g61820	CON	MIN	HOUR	DAY	WEEK	UNIGENE	FLCDNA	TAIR
TCGACTCTAT	10	3	9	10	17	gnl|UG|At#S11673951	gi|17529155|gb|AY065363.1	At5g61820.1
at5g19450	CON	MIN	HOUR	DAY	WEEK	UNIGENE	FLCDNA	TAIR
TTGTCTGGAT	2	0	1	0	3	gnl|UG|At#S15460660	no match found	At5g19450.1
at5g06600	CON	MIN	HOUR	DAY	WEEK	UNIGENE	FLCDNA	TAIR
CAATTTTTAT	2	2	1	1	2	gnl|UG|At#S11782800	gi|13430571|gb|AF360198.1	At5g06600.2
at2g40160	CON	MIN	HOUR	DAY	WEEK	UNIGENE	FLCDNA	TAIR
GAACGAGTTG	0	1	0	0	0	gnl|UG|At#S11705611	gi|21360562|gb|AY113175.1	At2g40160.1
at3g12250	CON	MIN	HOUR	DAY	WEEK	UNIGENE	FLCDNA	TAIR
ACAGAAGATG	0	2	1	0	1	gnl|UG|At#S18942118	gi|51971828|dbj|AK176816.1|	At3g12250.2
at1g24150	CON	MIN	HOUR	DAY	WEEK	UNIGENE	FLCDNA	TAIR
TCGGATAGAT	1	0	0	0	0	gnl|UG|At#S11740590	no match found	At1g24150.1
at3g13880	CON	MIN	HOUR	DAY	WEEK	UNIGENE	FLCDNA	TAIR
AAAATTTAAT	1	1	0	0	0	gnl|UG|At#S11736683	no match found	non-canonical match
at5g01560	CON	MIN	HOUR	DAY	WEEK	UNIGENE	FLCDNA	TAIR
GATTTTGCTC	0	1	1	0	0	no match found	gi|110738681|dbj|AK229404.1|	non-canonical match
at1g02890	CON	MIN	HOUR	DAY	WEEK	UNIGENE	FLCDNA	TAIR

TAAAGAGAAA	2	2	0	2	1	gnl|UG|At#S11742826	no match found	At1g02890.1
at1g53720	CON	MIN	HOUR	DAY	WEEK	UNIGENE	FLCDNA	TAIR
GGCTTCTCAT	0	0	1	0	0	gnl|UG|At#S11733105	no match found	At1g53720.1
at3g14720	CON	MIN	HOUR	DAY	WEEK	UNIGENE	FLCDNA	TAIR
TCTTAGAATT	2	0	0	3	2	gnl|UG|At#S11736431	no match found	At3g14720.1
at5g42470	CON	MIN	HOUR	DAY	WEEK	UNIGENE	FLCDNA	TAIR
GCTGAGCGAC	0	1	1	2	0	gnl|UG|At#S11719482	gi|52627090|gb|BT015735.1	At5g42470.1
at1g15880	CON	MIN	HOUR	DAY	WEEK	UNIGENE	FLCDNA	TAIR
ATGTCAATTT	4	3	1	0	1	gnl|UG|At#S11702619	gi|15294143|gb|AF410263.1	At1g15880.1
at1g27130	CON	MIN	HOUR	DAY	WEEK	UNIGENE	FLCDNA	TAIR
CCAACCGTCG	4	7	4	1	1	no match found	no match found	At1g27130.1
at1g53750	CON	MIN	HOUR	DAY	WEEK	UNIGENE	FLCDNA	TAIR
AACTGCGAAA	1	3	3	5	0	gnl|UG|At#S11733095	gi|17065567|gb|AY062860.1	At1g53750.1
at2g25920	CON	MIN	HOUR	DAY	WEEK	UNIGENE	FLCDNA	TAIR
GCAATCCTCC	1	1	2	3	0	gnl|UG|At#S11707089	gi|15294291|gb|AF410337.1	At2g25920.1
AGACTTTGTG	0	0	1	0	0	no match found	gi|23507800|gb|BT000867.1	multiple non-canonical match
at2g17560	CON	MIN	HOUR	DAY	WEEK	UNIGENE	FLCDNA	TAIR
AAAGGCGGCG	1	1	3	2	1	no match found	gi|13877774|gb|AF370150.1	At2g17560.1
at2g36355	CON	MIN	HOUR	DAY	WEEK	UNIGENE	FLCDNA	TAIR
TATCTCTTTG	1	1	0	0	1	no match found	gi|20260247|gb|AY093023.1	At2g36360.1
AGGCTTTCGA	0	0	1	0	0	gnl|UG|At#S23623350	no match found	At2g36360.1
at5g26751	CON	MIN	HOUR	DAY	WEEK	UNIGENE	FLCDNA	TAIR
GTGGAGAAAG	2	0	1	0	0	gnl|UG|At#S34116266	no match found	At4g35240.1
at5g61030	CON	MIN	HOUR	DAY	WEEK	UNIGENE	FLCDNA	TAIR
GAGAAAAGTG	0	0	1	7	1	gnl|UG|At#S11717599	gi|21928058|gb|AY125548.1	At5g61030.1
at3g23560	CON	MIN	HOUR	DAY	WEEK	UNIGENE	FLCDNA	TAIR
GCTTGAATAT	0	0	1	3	2	gnl|UG|At#S11701955	gi|17064869|gb|AY062511.1	At3g23560.1
at4g35830	CON	MIN	HOUR	DAY	WEEK	UNIGENE	FLCDNA	TAIR
TACACTCCGA	0	0	3	1	0	no match found	gi|17065391|gb|AY062772.1	non-canonical match
CGAGCTCTTT	16	34	18	22	10	gnl|UG|At#S43849421	no match found	At4g35830.1
at5g57015	CON	MIN	HOUR	DAY	WEEK	UNIGENE	FLCDNA	TAIR
GCAAGGACTT	0	1	0	0	0	no match found	gi|26452844|dbj|AK118919.1|	non-canonical match
at5g09590	CON	MIN	HOUR	DAY	WEEK	UNIGENE	FLCDNA	TAIR
GTGGGTTCTA	0	3	2	3	3	no match found	no match found	At5g09590.1
TCTGGTGGTT	0	0	0	1	0	gnl|UG|At#S35308032	no match found	multiple non-canonical match
at2g01100	CON	MIN	HOUR	DAY	WEEK	UNIGENE	FLCDNA	TAIR
GCAGAGATTC	1	0	0	0	0	no match found	gi|21928014|gb|AY125526.1	non-canonical match
TGCAGATGAT	0	1	0	0	0	no match found	gi|21406709|gb|AY087935.1	At2g01100.1
ATTACTACCC	0	0	1	1	0	gnl|UG|At#S18943003	gi|18086560|gb|AY072010.1	At2g01100.2
at1g57660	CON	MIN	HOUR	DAY	WEEK	UNIGENE	FLCDNA	TAIR
TGCAACAGTC	1	0	1	0	1	gnl|UG|At#S35272574	no match found	multiple non-canonical match
GTCGAAGGTA	6	5	3	3	13	gnl|UG|At#S11707397	gi|21405561|gb|AY086851.1	At1g57660.1
at3g57040	CON	MIN	HOUR	DAY	WEEK	UNIGENE	FLCDNA	TAIR

GTTAATAACC	1	0	0	4	1	gnl|UG|At#S11728136	no match found	At3g57040.1
at4g13750	CON	MIN	HOUR	DAY	WEEK	UNIGENE	FLCDNA	TAIR
CCCAACCAGA	0	0	1	0	0	gnl|UG|At#S11725429	gi|110739954|dbj|AK230061.1|	non-canonical match
at4g31790	CON	MIN	HOUR	DAY	WEEK	UNIGENE	FLCDNA	TAIR
GAGATTCTGT	0	0	0	1	1	gnl|UG|At#S11722315	gi|13605812|gb|AF367305.1	At4g31790.1
at1g55900	CON	MIN	HOUR	DAY	WEEK	UNIGENE	FLCDNA	TAIR
AGCTACCGTT	1	2	2	0	2	gnl|UG|At#S11732319	gi|18175980|gb|AY072140.1	At1g55900.1
at4g08980	CON	MIN	HOUR	DAY	WEEK	UNIGENE	FLCDNA	TAIR
AACCAGGCCT	1	2	1	4	0	gnl|UG|At#S15461363	gi|21403295|gb|AY084585.1	At4g08980.3
at4g16745	CON	MIN	HOUR	DAY	WEEK	UNIGENE	FLCDNA	TAIR
TTACCGTTCA	0	0	0	0	1	gnl|UG|At#S11724898	gi|26983907|gb|BT002373.1	At4g16745.1
at5g52200	CON	MIN	HOUR	DAY	WEEK	UNIGENE	FLCDNA	TAIR
TTTCACAGAG	1	1	1	2	2	gnl|UG|At#S11718496	gi|15215637|gb|AY050347.1	At5g52200.1
at1g76890	CON	MIN	HOUR	DAY	WEEK	UNIGENE	FLCDNA	TAIR
AGAGGTCCAA	0	4	2	0	1	gnl|UG|At#S11727619	gi|133778871|gb|BT030363.1	At1g76890.2
at5g13060	CON	MIN	HOUR	DAY	WEEK	UNIGENE	FLCDNA	TAIR
ACTGATATTC	0	0	0	1	1	gnl|UG|At#S11716883	gi|18491178|gb|AY074524.1	At5g13060.1
at4g19840	CON	MIN	HOUR	DAY	WEEK	UNIGENE	FLCDNA	TAIR
CGGCTGGTGT	0	1	1	0	0	no match found	gi|21689634|gb|AY122906.1	non-canonical match
TGTGTATAAT	2	0	2	0	1	gnl|UG|At#S11724358	gi|21404440|gb|AY085730.1	At4g19840.1
at5g66290	CON	MIN	HOUR	DAY	WEEK	UNIGENE	FLCDNA	TAIR
ATGATGCTAT	2	1	1	0	0	no match found	no match found	At5g66290.1
at5g06970	CON	MIN	HOUR	DAY	WEEK	UNIGENE	FLCDNA	TAIR
TGGCTACGCT	0	0	1	0	0	gnl|UG|At#S11724378	gi|27363441|gb|BT002724.1	At5g06970.1
at3g58220	CON	MIN	HOUR	DAY	WEEK	UNIGENE	FLCDNA	TAIR
CGTCTAGTAA	1	1	1	0	0	no match found	gi|15451069|gb|AY054615.1	multiple canonical match
at2g18450	CON	MIN	HOUR	DAY	WEEK	UNIGENE	FLCDNA	TAIR
CACTTCTTCA	0	0	1	0	0	gnl|UG|At#S11818035	no match found	non-canonical match
at2g06010	CON	MIN	HOUR	DAY	WEEK	UNIGENE	FLCDNA	TAIR
TGAAAGCACA	0	1	0	0	1	gnl|UG|At#S11739929	gi|18252842|gb|AY072356.1	At2g06010.1
at2g33440	CON	MIN	HOUR	DAY	WEEK	UNIGENE	FLCDNA	TAIR
TGATGCAGCC	1	0	0	0	1	gnl|UG|At#S15460829	no match found	At2g33440.1
at5g42740	CON	MIN	HOUR	DAY	WEEK	UNIGENE	FLCDNA	TAIR
TCAAACTTTT	0	0	0	1	0	gnl|UG|At#S11719456	gi|13937229|gb|AF372970.1	At5g42740.1
at3g05370	CON	MIN	HOUR	DAY	WEEK	UNIGENE	FLCDNA	TAIR
GAAAAGTTTC	1	1	3	0	0	gnl|UG|At#S11744463	gi|30102497|gb|BT006359.1	multiple canonical match
at1g04250	CON	MIN	HOUR	DAY	WEEK	UNIGENE	FLCDNA	TAIR
AAAGGATCGG	1	1	0	5	0	gnl|UG|At#S11742617	gi|21406008|gb|AY087284.1	At1g04250.1
at5g20350	CON	MIN	HOUR	DAY	WEEK	UNIGENE	FLCDNA	TAIR
TTCATTCTGC	2	1	1	0	0	no match found	gi|110737118|dbj|AK228596.1|	non-canonical match
TTTCTTATCA	10	3	2	3	5	gnl|UG|At#S11721241	no match found	At5g20350.1

at4g18130	CON	MIN	HOUR	DAY	WEEK	UNIGENE	FLCDNA	TAIR
CGCCGTTTCC	0	1	1	0	0	gnl|UG|At#S11724659	no match found	non-canonical match
TCTAGAACAA	0	0	0	0	1	no match found	no match found	At4g18130.1

at3g17609	CON	MIN	HOUR	DAY	WEEK	UNIGENE	FLCDNA	TAIR
TGTTCTAAGC	0	0	2	0	0	no match found	gi|51969021|dbj|AK175440.1|	At3g17609.2

at1g74055	CON	MIN	HOUR	DAY	WEEK	UNIGENE	FLCDNA	TAIR
TGACAGAGAC	1	1	0	0	0	gnl|UG|At#S24443096	no match found	At1g74055.1

at2g02220	CON	MIN	HOUR	DAY	WEEK	UNIGENE	FLCDNA	TAIR
AGAGTCGAGC	0	4	2	0	0	gnl|UG|At#S18897996	gi|110738594|dbj|AK229360.1|	multiple non-canonical match

at5g44750	CON	MIN	HOUR	DAY	WEEK	UNIGENE	FLCDNA	TAIR
AATTGTCTTT	2	0	0	1	0	gnl|UG|At#S43849173	no match found	At5g44750.1
ACACGAGCTA	0	1	0	1	0	no match found	gi|19424046|gb|AY080825.1	non-canonical match

at4g29670	CON	MIN	HOUR	DAY	WEEK	UNIGENE	FLCDNA	TAIR
TTCTCTTGCT	0	3	1	0	0	no match found	gi|18377421|gb|AY072462.1	At4g29670.2
TGAGGTTGCA	1	0	1	1	0	no match found	no match found	At4g29670.1

at2g37240	CON	MIN	HOUR	DAY	WEEK	UNIGENE	FLCDNA	TAIR
GAAGGATACC	0	1	0	2	1	gnl|UG|At#S11733294	gi|30102453|gb|BT006337.1	At2g37240.1

at3g02460	CON	MIN	HOUR	DAY	WEEK	UNIGENE	FLCDNA	TAIR
CTCCAGCTGA	0	0	1	0	0	no match found	gi|110737292|dbj|AK228688.1|	non-canonical match

at5g54910	CON	MIN	HOUR	DAY	WEEK	UNIGENE	FLCDNA	TAIR
ATGGTTCTGG	0	2	0	1	1	gnl|UG|At#S11718222	no match found	At5g54910.1

at5g58870	CON	MIN	HOUR	DAY	WEEK	UNIGENE	FLCDNA	TAIR
TGTTGTTGTA	3	1	4	2	0	gnl|UG|At#S11707746	gi|16648972|gb|AY059856.1	At5g58870.1

at3g55360	CON	MIN	HOUR	DAY	WEEK	UNIGENE	FLCDNA	TAIR
TTTATTCCTT	2	1	0	0	4	no match found	gi|20148508|gb|AY081583.1	At5g07150.1
AGTCAATAAT	4	3	0	2	7	gnl|UG|At#S11728446	gi|21407226|gb|AY088452.1	At3g55360.1

at4g38120	CON	MIN	HOUR	DAY	WEEK	UNIGENE	FLCDNA	TAIR
ACAAATTTTG	1	0	0	2	2	gnl|UG|At#S34117589	gi|110737351|dbj|AK228719.1|	At4g38110.1

at4g32960	CON	MIN	HOUR	DAY	WEEK	UNIGENE	FLCDNA	TAIR
TGAAAAAAAT	1	2	1	1	0	gnl|UG|At#S11752649	no match found	At4g32960.1

at2g20990	CON	MIN	HOUR	DAY	WEEK	UNIGENE	FLCDNA	TAIR
TGAAAGTTGT	1	2	1	4	1	gnl|UG|At#S11737216	gi|15027958|gb|AY045836.1	At2g20990.1

at3g56420	CON	MIN	HOUR	DAY	WEEK	UNIGENE	FLCDNA	TAIR
GCTCTCTTCT	0	1	0	0	1	no match found	no match found	At3g56420.1

at1g50170	CON	MIN	HOUR	DAY	WEEK	UNIGENE	FLCDNA	TAIR
TAAGAACGTT	1	0	1	0	3	gnl|UG|At#S18913375	gi|28393328|gb|BT004060.1	At1g50170.1

at5g67030	CON	MIN	HOUR	DAY	WEEK	UNIGENE	FLCDNA	TAIR
ACAATGTATA	10	3	5	0	17	gnl|UG|At#S11744660	gi|19698914|gb|AY081304.1	At5g67030.2

at5g43430	CON	MIN	HOUR	DAY	WEEK	UNIGENE	FLCDNA	TAIR
TTGTTTGAAG	0	2	1	0	0	gnl|UG|At#S28281685	gi|15010711|gb|AY045657.1	At5g43430.1

at3g43690	CON	MIN	HOUR	DAY	WEEK	UNIGENE	FLCDNA	TAIR
GAAAGAAGAT	1	0	0	0	0	gnl|UG|At#S34117820	gi|110736913|dbj|AK228488.1|	At3g43690.1

at1g01050	CON	MIN	HOUR	DAY	WEEK	UNIGENE	FLCDNA	TAIR
CAGGAACCTG	1	2	0	1	0	gnl|UG|At#S11743009	gi|21403725|gb|AY085015.1	At1g01050.1
at3g55250	CON	MIN	HOUR	DAY	WEEK	UNIGENE	FLCDNA	TAIR
AGCAAGTAAT	2	0	0	3	1	gnl|UG|At#S11728467	gi|22136461|gb|AY128909.1	At3g55250.1
at5g67380	CON	MIN	HOUR	DAY	WEEK	UNIGENE	FLCDNA	TAIR
GCGCACGCTT	0	0	0	1	0	no match found	gi|14423457|gb|AF386966.1	non-canonical match
at1g58602	CON	MIN	HOUR	DAY	WEEK	UNIGENE	FLCDNA	TAIR
GGTGAGTGTT	0	3	3	1	1	no match found	gi|15146241|gb|AY049262.1	multiple non-canonical match
at5g66570	CON	MIN	HOUR	DAY	WEEK	UNIGENE	FLCDNA	TAIR
TCCTTCATCG	122	50	86	31	28	no match found	no match found	At5g66570.1
CCTAAGATCT	289	86	218	141	47	no match found	gi|13926290|gb|AF372898.1	non-canonical match
ACCCGTCTTA	0	0	3	1	0	no match found	gi|51870301|gb|BT015528.1	non-canonical match
TTATTATATT	28	32	11	4	12	no match found	gi|15451005|gb|AY054583.1	multiple canonical match
at5g47510	CON	MIN	HOUR	DAY	WEEK	UNIGENE	FLCDNA	TAIR
AGACTAAAAA	0	1	0	1	0	gnl|UG|At#S11718971	no match found	At5g47510.1
at3g09735	CON	MIN	HOUR	DAY	WEEK	UNIGENE	FLCDNA	TAIR
ATATTTTCTT	10	5	2	11	17	gnl|UG|At#S11737960	gi|26452940|dbj|AK118970.1|	At3g09735.1
at3g48740	CON	MIN	HOUR	DAY	WEEK	UNIGENE	FLCDNA	TAIR
AATAAAAATA	2	9	2	1	1	no match found	gi|17979364|gb|AY070412.1	non-canonical match
at3g52200	CON	MIN	HOUR	DAY	WEEK	UNIGENE	FLCDNA	TAIR
TTTCATTTTT	2	1	1	2	0	gnl|UG|At#S11729024	gi|110737395|dbj|AK228742.1|	At3g52200.1
at1g18500	CON	MIN	HOUR	DAY	WEEK	UNIGENE	FLCDNA	TAIR
TCAAAAGAGT	4	3	8	5	0	gnl|UG|At#S11741201	no match found	At1g18500.1
at4g34200	CON	MIN	HOUR	DAY	WEEK	UNIGENE	FLCDNA	TAIR
AGCGTTGGGA	4	6	4	3	2	gnl|UG|At#S11721901	gi|62321125|dbj|AK221892.1|	At4g34200.1
at2g35040	CON	MIN	HOUR	DAY	WEEK	UNIGENE	FLCDNA	TAIR
TCAAGAGCAA	1	3	0	0	0	gnl|UG|At#S11806632	gi|20268736|gb|AY091122.1	At2g35040.1
at5g21970	CON	MIN	HOUR	DAY	WEEK	UNIGENE	FLCDNA	TAIR
GAAGAGAGAG	0	2	0	0	0	gnl|UG|At#S34114959	no match found	multiple canonical match
at1g70500	CON	MIN	HOUR	DAY	WEEK	UNIGENE	FLCDNA	TAIR
ATGAGCTTTA	0	1	1	0	0	gnl|UG|At#S11728985	no match found	At1g70500.1
at1g08900	CON	MIN	HOUR	DAY	WEEK	UNIGENE	FLCDNA	TAIR
ATACAGTGGA	2	3	0	1	0	gnl|UG|At#S20804345	gi|14423387|gb|AF386931.1	At1g08900.1
at5g18680	CON	MIN	HOUR	DAY	WEEK	UNIGENE	FLCDNA	TAIR
GCCGTGTCAC	0	1	0	1	0	gnl|UG|At#S34118445	gi|38564317|gb|BT010768.1	At5g18680.1
ATTCCTCTGT	0	0	0	0	1	gnl|UG|At#S43849707	no match found	pseudo chromosome match
at4g29750	CON	MIN	HOUR	DAY	WEEK	UNIGENE	FLCDNA	TAIR
ATTTGTAATT	0	1	0	0	1	no match found	no match found	At4g29750.1
TTGCAGACCT	0	0	1	0	0	gnl|UG|At#S11722659	no match found	non-canonical match
at1g53880	CON	MIN	HOUR	DAY	WEEK	UNIGENE	FLCDNA	TAIR
ATGGGAACTT	0	0	0	0	1	gnl|UG|At#S11733037	no match found	multiple canonical match
at1g08660	CON	MIN	HOUR	DAY	WEEK	UNIGENE	FLCDNA	TAIR
AGTGAGACCA	1	3	0	0	0	gnl|UG|At#S11742169	gi|17381246|gb|AY064135.1	At1g08660.1

at4g09760	CON	MIN	HOUR	DAY	WEEK	UNIGENE	FLCDNA	TAIR
ATATAGCTAT	1	0	1	0	1	gnl|UG|At#S18941886	gi|21407286|gb|AY088512.1	At4g09760.3
at2g15695	CON	MIN	HOUR	DAY	WEEK	UNIGENE	FLCDNA	TAIR
AGGGATCTTC	2	3	2	4	0	gnl|UG|At#S11738543	gi|110741489|dbj|AK226576.1|	At2g15695.1
at1g30880	CON	MIN	HOUR	DAY	WEEK	UNIGENE	FLCDNA	TAIR
AGCTTCAGTT	0	1	0	0	0	gnl|UG|At#S11677217	no match found	At2g15695.1
AGTTTCAGTA	8	7	7	10	10	no match found	gi|110743123|dbj|AK227451.1|	At1g30880.1
at3g58290	CON	MIN	HOUR	DAY	WEEK	UNIGENE	FLCDNA	TAIR
GCTTTGCAAA	0	0	1	1	1	no match found	no match found	At3g58290.1
at2g26550	CON	MIN	HOUR	DAY	WEEK	UNIGENE	FLCDNA	TAIR
AGATTACGCA	0	1	0	0	0	gnl|UG|At#S11735897	no match found	non-canonical match
ATTGTTTGAT	1	0	1	0	2	no match found	no match found	At2g26550.1
at1g19650	CON	MIN	HOUR	DAY	WEEK	UNIGENE	FLCDNA	TAIR
AACATTGACA	0	0	0	1	0	gnl|UG|At#S43850612	gi|62321366|dbj|AK222017.1|	At1g19650.1
at5g05610	CON	MIN	HOUR	DAY	WEEK	UNIGENE	FLCDNA	TAIR
TGTTTGTATA	2	1	1	0	1	gnl|UG|At#S11724734	gi|51971808|dbj|AK176806.1|	multiple canonical match
GGAAGTGTGT	0	1	0	0	0	no match found	gi|87116575|gb|BT024471.1	multiple non-canonical match
at1g76955	CON	MIN	HOUR	DAY	WEEK	UNIGENE	FLCDNA	TAIR
CTCGAGTTGT	0	0	1	0	0	no match found	gi|111074269|gb|BT026401.1	non-canonical match
TGGCTGCCTC	1	0	2	0	0	gnl|UG|At#S18895532	no match found	At1g76955.1
at1g79590	CON	MIN	HOUR	DAY	WEEK	UNIGENE	FLCDNA	TAIR
TCTATGCTCT	0	1	0	0	0	gnl|UG|At#S28282379	gi|21404995|gb|AY086285.1	At1g79590.1
at5g64430	CON	MIN	HOUR	DAY	WEEK	UNIGENE	FLCDNA	TAIR
GCACCTAAAG	2	7	2	2	2	gnl|UG|At#S11781521	gi|17064915|gb|AY062534.1	At5g64430.1
at4g38920	CON	MIN	HOUR	DAY	WEEK	UNIGENE	FLCDNA	TAIR
TCTTCTCGAA	5	8	9	11	5	gnl|UG|At#S11707766	gi|15724313|gb|AF412097.1	At4g38920.1
at3g15660	CON	MIN	HOUR	DAY	WEEK	UNIGENE	FLCDNA	TAIR
TACGGTAAAA	3	3	3	7	1	gnl|UG|At#S11736143	gi|110743465|dbj|AK227628.1|	At3g15660.1
at4g28720	CON	MIN	HOUR	DAY	WEEK	UNIGENE	FLCDNA	TAIR
GTCGTGAGAA	0	1	0	0	0	gnl|UG|At#S11722840	gi|116325937|gb|BT029238.1	At4g28720.1
at4g02050	CON	MIN	HOUR	DAY	WEEK	UNIGENE	FLCDNA	TAIR
GGGTCCTCTA	0	4	0	0	2	gnl|UG|At#S11727169	no match found	At4g02050.1
at3g21510	CON	MIN	HOUR	DAY	WEEK	UNIGENE	FLCDNA	TAIR
TCATAGATGT	0	0	1	0	0	no match found	gi|17104734|gb|AY063082.1	non-canonical match
ACTACAATTA	0	2	0	0	0	gnl|UG|At#S11734378	no match found	At3g21510.1
at5g59130	CON	MIN	HOUR	DAY	WEEK	UNIGENE	FLCDNA	TAIR
AACTACAATA	0	0	0	0	1	gnl|UG|At#S14274088	gi|28392950|gb|BT003861.1	multiple non-canonical match
at1g33120	CON	MIN	HOUR	DAY	WEEK	UNIGENE	FLCDNA	TAIR
CGCTTTGATC	11	22	9	6	7	gnl|UG|At#S11737853	no match found	At1g33140.1
at3g04890	CON	MIN	HOUR	DAY	WEEK	UNIGENE	FLCDNA	TAIR
TCGAGAGCTG	0	0	1	1	0	no match found	gi|17104638|gb|AY063034.1	multiple non-canonical match
GCCCAAACAA	0	1	1	3	0	no match found	gi|21403677|gb|AY084967.1	non-canonical match

at2g21970	CON	MIN	HOUR	DAY	WEEK	UNIGENE	FLCDNA	TAIR
TGAAACAATT	1	1	1	1	0	gnl|UG|At#S11701164	gi|14423379|gb|AF386927.1	At2g21970.1
ATTGTATTTG	0	0	0	1	0	no match found	gi|30102887|gb|BT006554.1	non-canonical match

at3g04670	CON	MIN	HOUR	DAY	WEEK	UNIGENE	FLCDNA	TAIR
TGGAGCGATG	2	0	0	0	0	no match found	gi|22137141|gb|AY133586.1	non-canonical match

at5g02530	CON	MIN	HOUR	DAY	WEEK	UNIGENE	FLCDNA	TAIR
TATTCCTTCC	3	1	1	1	0	no match found	gi|22655122|gb|AY140010.1	At5g02530.1

at4g35310	CON	MIN	HOUR	DAY	WEEK	UNIGENE	FLCDNA	TAIR
GAATCAACAA	1	3	4	3	3	gnl|UG|At#S11721713	gi|110742474|dbj|AK227104.1|	At4g35310.1

at5g09660	CON	MIN	HOUR	DAY	WEEK	UNIGENE	FLCDNA	TAIR
CCTATGTCTC	73	38	66	29	15	gnl|UG|At#S43849347	gi|16226936|gb|AF428373.1	At5g09660.1

at1g77460	CON	MIN	HOUR	DAY	WEEK	UNIGENE	FLCDNA	TAIR
GTCCAATAGG	0	1	0	0	0	gnl|UG|At#S11727396	gi|26451198|dbj|AK118072.1|	At1g77460.1

at5g64340	CON	MIN	HOUR	DAY	WEEK	UNIGENE	FLCDNA	TAIR
CTACTGGTAT	0	2	1	0	1	gnl|UG|At#S11709030	gi|17064969|gb|AY062561.1	At5g64340.1

at3g02180	CON	MIN	HOUR	DAY	WEEK	UNIGENE	FLCDNA	TAIR
CAGCTCCTGG	5	15	5	1	0	gnl|UG|At#S18942201	gi|15028042|gb|AY045878.1	At3g02180.1

at1g07390	CON	MIN	HOUR	DAY	WEEK	UNIGENE	FLCDNA	TAIR
ATACCAGTTT	1	0	1	0	0	gnl|UG|At#S11742295	gi|110741870|dbj|AK226779.1|	At1g07390.1

at2g32920	CON	MIN	HOUR	DAY	WEEK	UNIGENE	FLCDNA	TAIR
TACGGATTTT	4	1	3	3	5	gnl|UG|At#S11734302	gi|18252190|gb|AY072321.1	At2g32920.1

at3g23325	CON	MIN	HOUR	DAY	WEEK	UNIGENE	FLCDNA	TAIR
ATCTAAACAT	5	0	0	0	2	gnl|UG|At#S11733810	gi|21407853|gb|AY089079.1	At3g23325.1

at1g67440	CON	MIN	HOUR	DAY	WEEK	UNIGENE	FLCDNA	TAIR
ATGTAAAAGA	0	1	1	0	0	no match found	gi|66792691|gb|BT023457.1	At1g67440.1
ATTTAAAAGA	0	0	0	0	1	gnl|UG|At#S18893698	no match found	non-canonical match

at5g19980	CON	MIN	HOUR	DAY	WEEK	UNIGENE	FLCDNA	TAIR
CCACTCCTGT	0	1	3	0	0	gnl|UG|At#S11721328	gi|89000964|gb|BT024734.1	At5g19980.1

at5g20080	CON	MIN	HOUR	DAY	WEEK	UNIGENE	FLCDNA	TAIR
TAATAAAATG	0	2	1	0	2	gnl|UG|At#S11721306	gi|21406004|gb|AY087280.1	At5g20080.1

at5g01800	CON	MIN	HOUR	DAY	WEEK	UNIGENE	FLCDNA	TAIR
ATGACAACCA	0	0	1	0	0	no match found	gi|19548000|gb|AY081711.1	non-canonical match
TAAATACTGA	3	0	2	0	1	no match found	gi|15912284|gb|AY056420.1	non-canonical match
TTGTGTAGTC	2	0	0	0	0	gnl|UG|At#S11725707	no match found	At5g01800.1

at5g45280	CON	MIN	HOUR	DAY	WEEK	UNIGENE	FLCDNA	TAIR
CTCATTGCCA	0	0	1	2	1	gnl|UG|At#S11706068	gi|23297553|gb|AY150453.1	At5g45280.2

at5g43150	CON	MIN	HOUR	DAY	WEEK	UNIGENE	FLCDNA	TAIR
AGTGTTGTAT	2	0	2	3	4	gnl|UG|At#S11719413	no match found	At5g43150.1
ACGTGAGTTC	0	1	0	0	0	no match found	gi|110736407|dbj|AK228221.1|	non-canonical match

at2g24050	CON	MIN	HOUR	DAY	WEEK	UNIGENE	FLCDNA	TAIR
CCAGAGTTTG	1	1	0	1	1	gnl|UG|At#S11709096	gi|17064835|gb|AY062494.1	At2g24050.1

at4g01840	CON	MIN	HOUR	DAY	WEEK	UNIGENE	FLCDNA	TAIR
GTTTCATCAG	0	1	0	0	0	gnl|UG|At#S11645173	gi|21403567|gb|AY084857.1	At4g01840.1

at5g61580	CON	MIN	HOUR	DAY	WEEK	UNIGENE	FLCDNA	TAIR
AAGTAATTTC	3	2	0	1	0	gnl|UG|At#S11717544	gi|22136389|gb|AY128873.1	At5g61580.1
at1g53170	CON	MIN	HOUR	DAY	WEEK	UNIGENE	FLCDNA	TAIR
GCGACGTATC	1	3	0	0	0	gnl|UG|At#S11733293	gi|26450166|dbj|AK117541.1|	At1g53170.1
at5g15220	CON	MIN	HOUR	DAY	WEEK	UNIGENE	FLCDNA	TAIR
TGATCTCTCG	0	5	3	3	3	gnl|UG|At#S11722538	gi|15081776|gb|AY048281.1	At5g15220.1
at1g50720	CON	MIN	HOUR	DAY	WEEK	UNIGENE	FLCDNA	TAIR
GCTTCATAAG	0	0	1	0	0	no match found	no match found	At1g50720.1
at2g40660	CON	MIN	HOUR	DAY	WEEK	UNIGENE	FLCDNA	TAIR
ACCTCGGCTG	0	1	2	3	0	gnl|UG|At#S11732427	gi|47679076|gb|BT014745.1	At2g40660.1
at1g30400	CON	MIN	HOUR	DAY	WEEK	UNIGENE	FLCDNA	TAIR
ATAAAGAGAT	2	1	1	0	0	no match found	no match found	At1g30400.1
at3g14900	CON	MIN	HOUR	DAY	WEEK	UNIGENE	FLCDNA	TAIR
TATGTAAATG	0	0	0	1	2	gnl|UG|At#S34114837	gi|110742614|dbj|AK227180.1|	At3g14900.1
at1g58380	CON	MIN	HOUR	DAY	WEEK	UNIGENE	FLCDNA	TAIR
AGATAAGTTT	1	3	1	4	0	gnl|UG|At#S11731753	gi|21407517|gb|AY088743.1	At1g58380.1
at1g13220	CON	MIN	HOUR	DAY	WEEK	UNIGENE	FLCDNA	TAIR
CTCATAATAG	1	0	0	0	0	no match found	gi|15010713|gb|AY045658.1	At1g13220.1
at5g59530	CON	MIN	HOUR	DAY	WEEK	UNIGENE	FLCDNA	TAIR
TATCAGAATA	0	1	0	0	0	gnl|UG|At#S11717750	no match found	At5g59530.1
at5g28220	CON	MIN	HOUR	DAY	WEEK	UNIGENE	FLCDNA	TAIR
CTTGCGACGT	1	0	2	1	0	gnl|UG|At#S11720389	gi|28393701|gb|BT004260.1	At5g28220.1
at3g49050	CON	MIN	HOUR	DAY	WEEK	UNIGENE	FLCDNA	TAIR
TTCTCTACTC	1	1	0	0	0	gnl|UG|At#S11729630	gi|30793790|gb|BT008521.1	At3g49050.1
at2g20950	CON	MIN	HOUR	DAY	WEEK	UNIGENE	FLCDNA	TAIR
TCAACGTGTG	1	0	4	1	0	gnl|UG|At#S11743894	gi|30794057|gb|BT008662.1	At2g20950.4
at3g61610	CON	MIN	HOUR	DAY	WEEK	UNIGENE	FLCDNA	TAIR
CTTTGTGTGG	0	1	0	0	0	gnl|UG|At#S11726970	no match found	At3g61610.1
at2g34810	CON	MIN	HOUR	DAY	WEEK	UNIGENE	FLCDNA	TAIR
GGCAGTTGAT	2	0	2	2	1	gnl|UG|At#S11733861	gi|20466739|gb|AY099836.1	At2g34810.1
AATTGGTATA	1	0	0	0	1	no match found	gi|31711959|gb|BT008897.1	non-canonical match
at3g14160	CON	MIN	HOUR	DAY	WEEK	UNIGENE	FLCDNA	TAIR
GATCCAGTAT	1	0	0	2	0	gnl|UG|At#S11736609	gi|13877856|gb|AF370191.1	At3g14160.1
at4g17880	CON	MIN	HOUR	DAY	WEEK	UNIGENE	FLCDNA	TAIR
AGATAAATAA	1	1	0	0	1	gnl|UG|At#S24442656	gi|62320361|dbj|AK221507.1|	At4g17880.1
at2g19940	CON	MIN	HOUR	DAY	WEEK	UNIGENE	FLCDNA	TAIR
CCAATGATCC	0	2	1	0	0	gnl|UG|At#S15461377	gi|19699207|gb|AY090309.1	At2g19940.2
at2g21010	CON	MIN	HOUR	DAY	WEEK	UNIGENE	FLCDNA	TAIR
TAACAACGTT	0	0	1	0	0	no match found	no match found	At2g21010.1
at4g16444	CON	MIN	HOUR	DAY	WEEK	UNIGENE	FLCDNA	TAIR
TAATGGTCGG	2	2	0	0	0	gnl|UG|At#S11724956	gi|98960932|gb|BT025532.1	At4g16444.1

at3g02050	CON	MIN	HOUR	DAY	WEEK	UNIGENE	FLCDNA	TAIR
GGGGGAATAA	2	0	2	3	1	gnl|UG|At#S11708847	gi|28059236|gb|BT003375.1	At3g02050.1
at1g43650	CON	MIN	HOUR	DAY	WEEK	UNIGENE	FLCDNA	TAIR
TAATGTGTAA	1	0	0	0	0	gnl|UG|At#S21736457	gi|51970577|dbj|AK176218.1|	multiple non-canonical match
at2g43550	CON	MIN	HOUR	DAY	WEEK	UNIGENE	FLCDNA	TAIR
TGTTAGATTA	4	2	0	1	0	gnl|UG|At#S30640765	gi|21407970|gb|AY089196.1	At2g43550.1
at3g26080	CON	MIN	HOUR	DAY	WEEK	UNIGENE	FLCDNA	TAIR
TATGTAAAAA	0	0	0	2	5	gnl|UG|At#S11732958	no match found	At3g26080.1
AACTTGTCGA	0	1	0	0	0	no match found	gi|50198816|gb|BT014990.1	non-canonical match
at2g27505	CON	MIN	HOUR	DAY	WEEK	UNIGENE	FLCDNA	TAIR
AACACTAAAA	0	0	0	0	1	no match found	no match found	At2g27505.1
at3g53640	CON	MIN	HOUR	DAY	WEEK	UNIGENE	FLCDNA	TAIR
TACATTGAAT	0	0	0	0	1	gnl|UG|At#S11706829	gi|15450801|gb|AY054481.1	At1g13350.1
at2g29980	CON	MIN	HOUR	DAY	WEEK	UNIGENE	FLCDNA	TAIR
TGTTGGGAAG	1	4	2	0	1	gnl|UG|At#S15460969	gi|20465898|gb|AY096462.1	At2g29980.2
at1g34550	CON	MIN	HOUR	DAY	WEEK	UNIGENE	FLCDNA	TAIR
AAAGGGTTCC	1	1	1	2	0	gnl|UG|At#S11737377	no match found	non-canonical match
at4g32920	CON	MIN	HOUR	DAY	WEEK	UNIGENE	FLCDNA	TAIR
GACGAGAACG	0	0	0	1	1	no match found	gi|25090273|gb|BT002256.1	non-canonical match
GTTTGAGATA	1	0	1	0	0	gnl|UG|At#S18319581	gi|16323058|gb|AY057633.1	At4g32920.1
at2g39530	CON	MIN	HOUR	DAY	WEEK	UNIGENE	FLCDNA	TAIR
CTGTCCGCTG	0	1	0	0	0	gnl|UG|At#S11732714	gi|28973478|gb|BT005644.1	At2g39530.1
at1g06820	CON	MIN	HOUR	DAY	WEEK	UNIGENE	FLCDNA	TAIR
CCATTCAATA	0	1	0	0	1	gnl|UG|At#S18894418	no match found	At1g06820.1
at4g39980	CON	MIN	HOUR	DAY	WEEK	UNIGENE	FLCDNA	TAIR
AAACTTGTCT	2	0	3	3	2	no match found	no match found	At4g39980.1
TCATTGTTCT	1	2	1	2	0	no match found	gi|22655205|gb|AY140052.1	non-canonical match
GAAACACCAT	0	0	2	0	0	no match found	gi|23463052|gb|BT000821.1	multiple non-canonical match
at1g73730	CON	MIN	HOUR	DAY	WEEK	UNIGENE	FLCDNA	TAIR
GCAATAGTCT	3	1	0	2	0	gnl|UG|At#S11728301	gi|17979066|gb|AY070044.1	At1g73730.1
at4g07825	CON	MIN	HOUR	DAY	WEEK	UNIGENE	FLCDNA	TAIR
ATCTCGTTTG	1	2	0	1	0	gnl|UG|At#S11824626	gi|26449454|dbj|AK117177.1|	At4g07825.1
CGGCGGCATC	0	1	0	0	1	no match found	gi|115311498|gb|BT029021.1	non-canonical match
at5g48810	CON	MIN	HOUR	DAY	WEEK	UNIGENE	FLCDNA	TAIR
CTAGATGAGT	3	8	2	1	1	gnl|UG|At#S11718837	gi|17104716|gb|AY063073.1	non-canonical match
at4g19510	CON	MIN	HOUR	DAY	WEEK	UNIGENE	FLCDNA	TAIR
TTTGAAGGAA	0	1	0	0	0	gnl|UG|At#S34117268	gi|110737975|dbj|AK229040.1|	At4g19510.1
at1g21250	CON	MIN	HOUR	DAY	WEEK	UNIGENE	FLCDNA	TAIR
TATTTCTTTT	0	1	0	1	1	no match found	gi|14532585|gb|AY039917.1	non-canonical match
TTTGTAATTC	0	0	0	0	1	gnl|UG|At#S11740932	no match found	At1g21250.1
TTTGTAAAAA	1	1	3	1	2	no match found	gi|62321238|dbj|AK221950.1|	multiple non-canonical match
at2g40205	CON	MIN	HOUR	DAY	WEEK	UNIGENE	FLCDNA	TAIR
AGAGCTAAGT	2	9	1	4	4	no match found	gi|17065407|gb|AY062780.1	non-canonical match

at5g52310	CON	MIN	HOUR	DAY	WEEK	UNIGENE	FLCDNA	TAIR
AGTTGGATCT	3	0	0	33	6	gnl|UG|At#S11718485	gi|15450770|gb|AY054465.1	At5g52310.1
at2g16600	CON	MIN	HOUR	DAY	WEEK	UNIGENE	FLCDNA	TAIR
TTGTGTTTGG	4	22	1	7	10	gnl|UG|At#S11738329	gi|15081645|gb|AY048215.1	At2g16600.1
at4g23750	CON	MIN	HOUR	DAY	WEEK	UNIGENE	FLCDNA	TAIR
GAGGACCATT	0	0	5	0	0	gnl|UG|At#S11705803	gi|50198973|gb|BT015038.1	At4g23750.2
at3g11070	CON	MIN	HOUR	DAY	WEEK	UNIGENE	FLCDNA	TAIR
GATTTCTCAT	0	0	1	0	0	gnl|UG|At#S11737562	gi|20466220|gb|AY099576.1	At3g11070.1
at1g09530	CON	MIN	HOUR	DAY	WEEK	UNIGENE	FLCDNA	TAIR
TCAATGGCGT	0	2	1	0	0	gnl|UG|At#S15461334	gi|26449608|dbj|AK117255.1|	At1g09530.2
at1g78850	CON	MIN	HOUR	DAY	WEEK	UNIGENE	FLCDNA	TAIR
ACAAAATATA	0	5	2	2	1	gnl|UG|At#S11628645	gi|23397231|gb|BT000758.1	At1g78850.1
at1g16515	CON	MIN	HOUR	DAY	WEEK	UNIGENE	FLCDNA	TAIR
TTAAAAAAAA	41	46	52	33	33	gnl|UG|At#S18893775	no match found	non-canonical match
at2g14850	CON	MIN	HOUR	DAY	WEEK	UNIGENE	FLCDNA	TAIR
AAAGCAAAAG	1	2	1	0	0	gnl|UG|At#S11710200	gi|17529281|gb|AY065427.1	At2g14850.1
at3g61800	CON	MIN	HOUR	DAY	WEEK	UNIGENE	FLCDNA	TAIR
AAGTTCTGCG	0	1	0	0	0	gnl|UG|At#S11726912	no match found	At3g61800.1
at1g78380	CON	MIN	HOUR	DAY	WEEK	UNIGENE	FLCDNA	TAIR
GTTTCCAGCA	7	13	11	9	3	gnl|UG|At#S20705106	gi|21405756|gb|AY087032.1	At1g78380.1
at5g40690	CON	MIN	HOUR	DAY	WEEK	UNIGENE	FLCDNA	TAIR
TGGAAGAATA	0	1	1	0	0	gnl|UG|At#S11719660	gi|110737091|dbj|AK228582.1|	multiple canonical match
at2g18290	CON	MIN	HOUR	DAY	WEEK	UNIGENE	FLCDNA	TAIR
AAGAGTCTAT	0	1	0	0	0	gnl|UG|At#S11737900	gi|110738940|dbj|AK229538.1|	At2g18290.1
at5g06460	CON	MIN	HOUR	DAY	WEEK	UNIGENE	FLCDNA	TAIR
GCTGAGCCGG	1	1	3	0	1	no match found	no match found	At5g06460.1
at4g01130	CON	MIN	HOUR	DAY	WEEK	UNIGENE	FLCDNA	TAIR
GCCATTCTCG	0	0	0	1	0	gnl|UG|At#S11727402	gi|119360142|gb|BT029544.1	At4g01130.1
at5g19790	CON	MIN	HOUR	DAY	WEEK	UNIGENE	FLCDNA	TAIR
TAATCAACAA	4	1	2	3	1	no match found	no match found	At5g19790.1
at5g56220	CON	MIN	HOUR	DAY	WEEK	UNIGENE	FLCDNA	TAIR
ATCTCACTTT	0	0	1	0	0	no match found	gi|28973200|gb|BT005505.1	non-canonical match
at1g34470	CON	MIN	HOUR	DAY	WEEK	UNIGENE	FLCDNA	TAIR
TTTCATTTAT	0	1	0	0	0	gnl|UG|At#S34114432	gi|110743377|dbj|AK227583.1|	multiple canonical match
at3g04780	CON	MIN	HOUR	DAY	WEEK	UNIGENE	FLCDNA	TAIR
TGCTTCAGCA	0	2	1	2	0	gnl|UG|At#S11739288	gi|21406999|gb|AY088225.1	At3g04780.1
at5g04360	CON	MIN	HOUR	DAY	WEEK	UNIGENE	FLCDNA	TAIR
GCTTGTCTTT	1	0	0	0	1	gnl|UG|At#S11725055	gi|27311610|gb|BT002411.1	At5g04360.1
at1g80230	CON	MIN	HOUR	DAY	WEEK	UNIGENE	FLCDNA	TAIR
GTGATGACCA	0	1	0	7	0	no match found	gi|110743471|dbj|AK227631.1|	non-canonical match
TGTTTATACG	0	0	3	0	1	gnl|UG|At#S11726426	no match found	At1g80230.1

at2g02400	CON	MIN	HOUR	DAY	WEEK	UNIGENE	FLCDNA	TAIR
AGAAACCCAT	3	1	2	0	0	gnl|UG|At#S11740623	gi|110736833|dbj|AK228447.1|	At2g02400.1
at4g08105	CON	MIN	HOUR	DAY	WEEK	UNIGENE	FLCDNA	TAIR
AGATTTGTGT	8	18	0	0	0	gnl|UG|At#S34116273	no match found	At5g49450.1
at2g38060	CON	MIN	HOUR	DAY	WEEK	UNIGENE	FLCDNA	TAIR
TGTGAACTAT	0	0	1	0	0	no match found	no match found	At2g38060.1
at5g42000	CON	MIN	HOUR	DAY	WEEK	UNIGENE	FLCDNA	TAIR
CACAAGGTCC	1	0	0	0	0	no match found	gi|44681461|gb|BT011665.1	non-canonical match
at5g47180	CON	MIN	HOUR	DAY	WEEK	UNIGENE	FLCDNA	TAIR
AATTGGAAAC	0	1	0	0	1	gnl|UG|At#S18909889	gi|21403984|gb|AY085274.1	At5g47180.2
at5g16270	CON	MIN	HOUR	DAY	WEEK	UNIGENE	FLCDNA	TAIR
AGCACCAAGT	5	2	1	2	1	gnl|UG|At#S11722274	gi|15451167|gb|AY054664.1	At5g16270.1
AGGAGGATGA	0	1	0	0	0	no match found	gi|18377435|gb|AY072469.1	multiple non-canonical match
at3g19620	CON	MIN	HOUR	DAY	WEEK	UNIGENE	FLCDNA	TAIR
ACAGAGAAAT	0	0	0	1	0	gnl|UG|At#S11734967	no match found	multiple non-canonical match
at3g06150	CON	MIN	HOUR	DAY	WEEK	UNIGENE	FLCDNA	TAIR
TTGATTCGAT	0	1	0	0	0	gnl|UG|At#S18901785	no match found	At3g06150.1
at4g13590	CON	MIN	HOUR	DAY	WEEK	UNIGENE	FLCDNA	TAIR
AGAGCAATAA	4	0	0	1	1	gnl|UG|At#S11725455	no match found	non-canonical match
at2g02510	CON	MIN	HOUR	DAY	WEEK	UNIGENE	FLCDNA	TAIR
GTTTCGTTAT	4	3	5	3	11	gnl|UG|At#S17007301	gi|51971684|dbj|AK176744.1|	At2g02510.1
at1g66980	CON	MIN	HOUR	DAY	WEEK	UNIGENE	FLCDNA	TAIR
AATCTTCTAT	1	1	0	0	0	gnl|UG|At#S11729716	gi|38566513|gb|BT010780.1	multiple canonical match
at5g16540	CON	MIN	HOUR	DAY	WEEK	UNIGENE	FLCDNA	TAIR
AAGACAGGAG	1	2	0	0	0	gnl|UG|At#S18941622	gi|21403344|gb|AY084634.1	At5g16540.3
at4g31600	CON	MIN	HOUR	DAY	WEEK	UNIGENE	FLCDNA	TAIR
AAATGTTTGT	0	2	1	1	0	no match found	gi|14596060|gb|AY042818.1	multiple canonical match
at5g44870	CON	MIN	HOUR	DAY	WEEK	UNIGENE	FLCDNA	TAIR
TTTTTATCGG	0	0	1	0	0	gnl|UG|At#S11719240	no match found	non-canonical match
TGAGATAGAA	1	1	0	0	0	no match found	no match found	At5g44870.1
at5g35375	CON	MIN	HOUR	DAY	WEEK	UNIGENE	FLCDNA	TAIR
TGTATTCGAT	0	0	2	5	1	gnl|UG|At#S34115864	gi|110740683|dbj|AK226286.1|	pseudo chromosome match
at1g53785	CON	MIN	HOUR	DAY	WEEK	UNIGENE	FLCDNA	TAIR
TTATAGCATT	0	1	0	0	0	gnl|UG|At#S11824209	gi|26450289|dbj|AK117605.1|	At1g53785.1
at1g26520	CON	MIN	HOUR	DAY	WEEK	UNIGENE	FLCDNA	TAIR
AACAATACTC	0	0	1	0	1	gnl|UG|At#S11740149	gi|62321491|dbj|AK222081.1|	At1g26520.1
at3g54060	CON	MIN	HOUR	DAY	WEEK	UNIGENE	FLCDNA	TAIR
ACTCAAGCCA	0	0	1	1	1	no match found	no match found	At3g54060.1
at5g10920	CON	MIN	HOUR	DAY	WEEK	UNIGENE	FLCDNA	TAIR
TTGCAGCGGT	1	2	4	4	1	gnl|UG|At#S11723518	gi|15028082|gb|AY045898.1	At5g10920.1
at5g03455	CON	MIN	HOUR	DAY	WEEK	UNIGENE	FLCDNA	TAIR

GAGCACAAAC	0	0	1	2	0	gnl|UG|At#S11725297	gi|21405439|gb|AY086729.1	At5g03455.1
ATCTTGGAAC	0	1	1	1	0	no match found	gi|28372807|gb|BT003658.1	non-canonical match
at1g07960	CON	MIN	HOUR	DAY	WEEK	UNIGENE	FLCDNA	TAIR
GTTTTCAGCA	0	0	1	0	1	gnl|UG|At#S18942619	gi|26450502|dbj|AK117714.1|	At1g07960.1
at1g02880	CON	MIN	HOUR	DAY	WEEK	UNIGENE	FLCDNA	TAIR
AAATACATAT	1	0	0	0	0	no match found	gi|16648735|gb|AY058144.1	At1g02880.3
at1g76100	CON	MIN	HOUR	DAY	WEEK	UNIGENE	FLCDNA	TAIR
ATTTATAAAT	15	3	4	5	1	gnl|UG|At#S11727817	gi|110740506|dbj|AK226194.1|	At1g76100.1
at1g76280	CON	MIN	HOUR	DAY	WEEK	UNIGENE	FLCDNA	TAIR
AGCGAAGACC	0	0	0	1	0	no match found	no match found	At1g76280.1
at5g22700	CON	MIN	HOUR	DAY	WEEK	UNIGENE	FLCDNA	TAIR
GATGATGCTT	1	0	0	1	1	gnl|UG|At#S11720918	no match found	At5g22700.1
at3g05400	CON	MIN	HOUR	DAY	WEEK	UNIGENE	FLCDNA	TAIR
GTGCTTGGTG	0	2	0	0	0	gnl|UG|At#S11739105	gi|20259596|gb|AY091216.1	At3g05400.1
at3g23880	CON	MIN	HOUR	DAY	WEEK	UNIGENE	FLCDNA	TAIR
GACGATCATA	0	1	0	0	0	gnl|UG|At#S11824507	gi|30725381|gb|BT008354.1	At3g23880.1
at5g45020	CON	MIN	HOUR	DAY	WEEK	UNIGENE	FLCDNA	TAIR
TTATGTTCTA	0	0	1	1	1	no match found	gi|14030630|gb|AF375406.1	multiple canonical match
at2g16720	CON	MIN	HOUR	DAY	WEEK	UNIGENE	FLCDNA	TAIR
GAGTGTAGTA	0	0	1	0	0	gnl|UG|At#S11738298	gi|23197905|gb|BT000161.1	At2g16720.1
at3g60620	CON	MIN	HOUR	DAY	WEEK	UNIGENE	FLCDNA	TAIR
GAGTTTGATT	1	1	0	0	0	no match found	gi|16323391|gb|AY059084.1	non-canonical match
at1g65580	CON	MIN	HOUR	DAY	WEEK	UNIGENE	FLCDNA	TAIR
GAAGTGACAG	0	2	0	0	0	gnl|UG|At#S14273738	gi|28393618|gb|BT004211.1	At1g65580.1
at2g18030	CON	MIN	HOUR	DAY	WEEK	UNIGENE	FLCDNA	TAIR
GTAGAAGAAG	1	2	0	0	0	gnl|UG|At#S11737981	gi|27765029|gb|BT003071.1	At2g18030.1
at1g45000	CON	MIN	HOUR	DAY	WEEK	UNIGENE	FLCDNA	TAIR
CATCCTGTAT	0	0	1	0	0	gnl|UG|At#S11735660	no match found	At1g45000.1
AAGGCTGTGA	1	4	2	3	1	no match found	gi|21387176|gb|AY114673.1	non-canonical match
at1g70860	CON	MIN	HOUR	DAY	WEEK	UNIGENE	FLCDNA	TAIR
GCAAGTCAAA	0	0	0	1	0	no match found	no match found	At1g70860.1
at5g61040	CON	MIN	HOUR	DAY	WEEK	UNIGENE	FLCDNA	TAIR
GAAGAAACTG	0	0	0	2	1	gnl|UG|At#S34117145	gi|110738209|dbj|AK229163.1|	At5g61040.1
at2g35130	CON	MIN	HOUR	DAY	WEEK	UNIGENE	FLCDNA	TAIR
GACCTCGAGG	0	1	0	0	0	gnl|UG|At#S11733781	no match found	At2g35130.1
at3g08930	CON	MIN	HOUR	DAY	WEEK	UNIGENE	FLCDNA	TAIR
TAATCATATT	0	4	4	3	2	gnl|UG|At#S11703639	gi|14334835|gb|AY035091.1	At3g08930.2
at2g25210	CON	MIN	HOUR	DAY	WEEK	UNIGENE	FLCDNA	TAIR
ATTAAGAAGA	3	9	2	2	3	gnl|UG|At#S11676328	gi|34365572|gb|BT010475.1	At2g25210.1
at3g47740	CON	MIN	HOUR	DAY	WEEK	UNIGENE	FLCDNA	TAIR
ATGATGTTGA	1	0	0	0	0	no match found	no match found	At3g47740.1

at5g19850	CON	MIN	HOUR	DAY	WEEK	UNIGENE	FLCDNA	TAIR
GGAGCCCATC	0	0	1	0	0	no match found	gi|28950928|gb|BT005324.1	non-canonical match
AACAGTCACA	0	0	3	5	4	no match found	gi|26450540|dbj|AK117734.1|	non-canonical match

at4g31150	CON	MIN	HOUR	DAY	WEEK	UNIGENE	FLCDNA	TAIR
CTCTAAGACA	0	0	0	1	0	gnl|UG|At#S18906013	gi|51968463|dbj|AK175161.1|	At4g31150.2

at3g59470	CON	MIN	HOUR	DAY	WEEK	UNIGENE	FLCDNA	TAIR
ATGGATCTCC	0	1	0	0	0	gnl|UG|At#S11708092	gi|16604381|gb|AY058089.1	At3g59470.1

at3g51300	CON	MIN	HOUR	DAY	WEEK	UNIGENE	FLCDNA	TAIR
CTCCATTCTA	0	0	2	0	0	gnl|UG|At#S11729190	gi|17978902|gb|AY066054.1	At3g51300.1

at1g52670	CON	MIN	HOUR	DAY	WEEK	UNIGENE	FLCDNA	TAIR
CCCAAGTCCT	0	1	0	0	0	gnl|UG|At#S11733484	gi|17528957|gb|AY065213.1	At1g52670.1

at4g26980	CON	MIN	HOUR	DAY	WEEK	UNIGENE	FLCDNA	TAIR
CCTCTTTTCT	0	0	1	1	0	gnl|UG|At#S34117258	gi|63003789|gb|BT022012.1	At4g26980.1

at3g45210	CON	MIN	HOUR	DAY	WEEK	UNIGENE	FLCDNA	TAIR
AAGGAATTGG	0	2	1	0	0	gnl|UG|At#S11730340	gi|20334743|gb|AY093972.1	At3g45210.1

at5g06960	CON	MIN	HOUR	DAY	WEEK	UNIGENE	FLCDNA	TAIR
GCGATGGGGA	0	0	1	0	0	no match found	gi|23505886|gb|AY143864.1	multiple non-canonical match

at2g28100	CON	MIN	HOUR	DAY	WEEK	UNIGENE	FLCDNA	TAIR
AGAGTCGCTT	0	1	0	0	0	gnl|UG|At#S21989805	no match found	At2g28105.1
ATTTGTAAGA	0	2	0	1	2	gnl|UG|At#S11735518	gi|26453077|dbj|AK119039.1|	At2g28100.1

at1g49750	CON	MIN	HOUR	DAY	WEEK	UNIGENE	FLCDNA	TAIR
CGGAAACAGA	4	6	3	5	4	no match found	gi|45773903|gb|BT012269.1	At1g49750.1

at4g33630	CON	MIN	HOUR	DAY	WEEK	UNIGENE	FLCDNA	TAIR
AGATCGGTTG	2	2	1	3	0	no match found	gi|16604594|gb|AY059742.1	non-canonical match

at5g43780	CON	MIN	HOUR	DAY	WEEK	UNIGENE	FLCDNA	TAIR
TGTCCCTCTG	0	0	1	1	1	gnl|UG|At#S11719350	gi|21436220|gb|AY117323.1	non-canonical match

at1g63055	CON	MIN	HOUR	DAY	WEEK	UNIGENE	FLCDNA	TAIR
AAAAAATGTT	4	2	0	4	4	no match found	gi|72196913|gb|DQ108670.1	non-canonical match

at5g66200	CON	MIN	HOUR	DAY	WEEK	UNIGENE	FLCDNA	TAIR
GGTAACGCAG	0	2	1	1	0	gnl|UG|At#S11717077	gi|23198097|gb|BT000257.1	At5g66200.1

at1g48210	CON	MIN	HOUR	DAY	WEEK	UNIGENE	FLCDNA	TAIR
TGTATGTCTC	2	0	1	1	6	gnl|UG|At#S11735139	gi|15451195|gb|AY054678.1	At1g48210.1

at3g56230	CON	MIN	HOUR	DAY	WEEK	UNIGENE	FLCDNA	TAIR
GCCCAAAAAA	2	1	2	3	1	no match found	gi|51970739|dbj|AK176299.1|	At1g48210.1

at3g25882	CON	MIN	HOUR	DAY	WEEK	UNIGENE	FLCDNA	TAIR
TTATCCAAAA	0	0	1	0	0	no match found	gi|51968619|dbj|AK175239.1|	non-canonical match

at5g46340	CON	MIN	HOUR	DAY	WEEK	UNIGENE	FLCDNA	TAIR
CTGACAACTG	0	0	1	0	0	gnl|UG|At#S11719091	gi|15983463|gb|AF424606.1	At5g46340.1

at5g60970	CON	MIN	HOUR	DAY	WEEK	UNIGENE	FLCDNA	TAIR
ATACGGATCA	1	0	0	0	0	gnl|UG|At#S11717605	gi|119360034|gb|BT029489.1	At5g60970.1

at4g27657	CON	MIN	HOUR	DAY	WEEK	UNIGENE	FLCDNA	TAIR
ATATCTGTTG	0	0	2	1	1	gnl|UG|At#S30428310	no match found	At4g27657.1

at1g05010	CON	MIN	HOUR	DAY	WEEK	UNIGENE	FLCDNA	TAIR
AAAGCTATGG	0	3	1	0	1	no match found	gi|23297271|gb|AY150384.1	non-canonical match
TTGTTGTATG	47	34	16	4	13	gnl|UG|At#S11742527	gi|13877784|gb|AF370155.1	At1g05010.1
at5g08560	CON	MIN	HOUR	DAY	WEEK	UNIGENE	FLCDNA	TAIR
TTGGCTTCAG	0	4	1	2	0	gnl|UG|At#S11723965	gi|17978716|gb|AY064639.1	At5g08560.1
at4g02260	CON	MIN	HOUR	DAY	WEEK	UNIGENE	FLCDNA	TAIR
TAGAGGTAGA	1	7	6	1	4	gnl|UG|At#S18941911	gi|110742285|dbj|AK227003.1|	At4g02260.2
at1g23220	CON	MIN	HOUR	DAY	WEEK	UNIGENE	FLCDNA	TAIR
GCACTGCATT	0	0	0	1	0	gnl|UG|At#S11740739	gi|20465366|gb|AY096447.1	At1g23220.1
at1g27350	CON	MIN	HOUR	DAY	WEEK	UNIGENE	FLCDNA	TAIR
TAATGAAGAT	0	5	5	1	3	gnl|UG|At#S18897473	gi|27754569|gb|BT002916.1	At1g27350.1
at4g22300	CON	MIN	HOUR	DAY	WEEK	UNIGENE	FLCDNA	TAIR
AAAACCGGTT	1	1	1	1	0	gnl|UG|At#S11723948	gi|26450210|dbj|AK117563.1|	multiple canonical match
ATTCTCCAAT	0	0	1	0	0	gnl|UG|At#S43850618	no match found	pseudo chromosome match
at4g17120	CON	MIN	HOUR	DAY	WEEK	UNIGENE	FLCDNA	TAIR
CAACGAAGTA	2	3	2	1	1	gnl|UG|At#S11724830	no match found	At4g17110.1
at5g06340	CON	MIN	HOUR	DAY	WEEK	UNIGENE	FLCDNA	TAIR
AACTCGTCTA	0	0	0	1	1	no match found	gi|21360538|gb|AY113163.1	non-canonical match
AATCTCAGAC	6	9	7	7	2	gnl|UG|At#S11724539	no match found	At5g06340.1
at1g68585	CON	MIN	HOUR	DAY	WEEK	UNIGENE	FLCDNA	TAIR
CCACTTATTC	1	0	0	0	0	gnl|UG|At#S11729379	gi|114050550|gb|BT028878.1	non-canonical match
at3g06660	CON	MIN	HOUR	DAY	WEEK	UNIGENE	FLCDNA	TAIR
GAAGACGGAA	0	1	0	0	0	gnl|UG|At#S11738734	gi|14334461|gb|AY034922.1	At3g06660.1
at2g25440	CON	MIN	HOUR	DAY	WEEK	UNIGENE	FLCDNA	TAIR
TTTGTCAAAT	1	0	0	0	0	no match found	no match found	At2g25440.1
at3g60090	CON	MIN	HOUR	DAY	WEEK	UNIGENE	FLCDNA	TAIR
AACAAGAACT	0	0	1	0	0	gnl|UG|At#S11727464	no match found	multiple non-canonical match
at4g33060	CON	MIN	HOUR	DAY	WEEK	UNIGENE	FLCDNA	TAIR
TTATATGTAG	3	0	1	0	1	no match found	no match found	At4g33060.1
at5g20520	CON	MIN	HOUR	DAY	WEEK	UNIGENE	FLCDNA	TAIR
GTTATATGAT	6	4	0	0	7	gnl|UG|At#S11721198	gi|21406307|gb|AY087569.1	At5g20520.1
at3g47360	CON	MIN	HOUR	DAY	WEEK	UNIGENE	FLCDNA	TAIR
TGGCATACGA	0	1	0	0	0	gnl|UG|At#S11747727	gi|19715600|gb|AY075608.1	multiple non-canonical match
at1g48830	CON	MIN	HOUR	DAY	WEEK	UNIGENE	FLCDNA	TAIR
AAGGTCTTCT	5	3	0	0	6	gnl|UG|At#S11734906	gi|21405576|gb|AY086866.1	At1g48830.2
at5g41240	CON	MIN	HOUR	DAY	WEEK	UNIGENE	FLCDNA	TAIR
GCGGGAGCTC	0	1	0	0	0	gnl|UG|At#S11719605	gi|30725395|gb|BT008361.1	At5g41240.1
at2g22790	CON	MIN	HOUR	DAY	WEEK	UNIGENE	FLCDNA	TAIR
TGTTCGGTAC	0	0	1	0	0	no match found	no match found	At2g22790.1
GTTTCTCTTT	0	0	0	2	0	gnl|UG|At#S11736789	no match found	multiple non-canonical match
at4g11420	CON	MIN	HOUR	DAY	WEEK	UNIGENE	FLCDNA	TAIR
TTAGACTCGT	7	6	5	2	8	gnl|UG|At#S11725821	gi|110741455|dbj|AK226553.1|	At4g11420.1

at4g13550	CON	MIN	HOUR	DAY	WEEK	UNIGENE	FLCDNA	TAIR
GTATCAAAAG	3	0	1	1	5	gnl|UG|At#S34115250	gi|110741847|dbj|AK226767.1|	multiple non-canonical match
at3g21260	CON	MIN	HOUR	DAY	WEEK	UNIGENE	FLCDNA	TAIR
GATGGATCTC	0	0	2	0	0	gnl|UG|At#S34117990	gi|117168090|gb|BT029314.1	At3g21260.1
at3g52900	CON	MIN	HOUR	DAY	WEEK	UNIGENE	FLCDNA	TAIR
GATCTTACCT	1	1	0	0	0	gnl|UG|At#S11728891	gi|110738966|dbj|AK229551.1|	At3g52900.1
at3g54230	CON	MIN	HOUR	DAY	WEEK	UNIGENE	FLCDNA	TAIR
TCTGACAATA	0	2	0	0	0	gnl|UG|At#S11709913	gi|20465338|gb|AY096433.1	non-canonical match
at4g36860	CON	MIN	HOUR	DAY	WEEK	UNIGENE	FLCDNA	TAIR
GTCTGAGGCG	2	2	3	2	1	gnl|UG|At#S18941715	gi|21387148|gb|AY114659.1	At4g36860.1
at1g72450	CON	MIN	HOUR	DAY	WEEK	UNIGENE	FLCDNA	TAIR
AATCTTGACA	1	4	4	0	0	gnl|UG|At#S11728590	gi|16323401|gb|AY059089.1	At1g72450.1
at3g04590	CON	MIN	HOUR	DAY	WEEK	UNIGENE	FLCDNA	TAIR
AGTCGTCGGA	1	0	0	0	0	gnl|UG|At#S11739344	gi|119935917|gb|BT029764.1	At3g04590.2
at3g01380	CON	MIN	HOUR	DAY	WEEK	UNIGENE	FLCDNA	TAIR
TTTTGGGGTT	1	0	0	0	0	no match found	no match found	At3g01380.1
at5g07220	CON	MIN	HOUR	DAY	WEEK	UNIGENE	FLCDNA	TAIR
ATAAATATGT	1	1	0	0	1	gnl|UG|At#S11697207	gi|16226482|gb|AF428411.1	At5g07220.1
at3g47910	CON	MIN	HOUR	DAY	WEEK	UNIGENE	FLCDNA	TAIR
TACGATGACA	0	0	0	1	0	no match found	gi|119360022|gb|BT029483.1	non-canonical match
at5g39320	CON	MIN	HOUR	DAY	WEEK	UNIGENE	FLCDNA	TAIR
CCTGCCTTTG	0	3	0	1	1	gnl|UG|At#S11719800	gi|110743318|dbj|AK227552.1|	At5g39320.1
at2g15560	CON	MIN	HOUR	DAY	WEEK	UNIGENE	FLCDNA	TAIR
AAGATCCTGC	0	4	0	1	0	gnl|UG|At#S21737356	gi|51970841|dbj|AK176350.1|	At2g15560.1
at1g05630	CON	MIN	HOUR	DAY	WEEK	UNIGENE	FLCDNA	TAIR
TATTCAACTT	0	1	1	0	0	gnl|UG|At#S11742464	no match found	At1g05630.1
at5g28290	CON	MIN	HOUR	DAY	WEEK	UNIGENE	FLCDNA	TAIR
ACGTTTCCAC	2	0	0	1	1	gnl|UG|At#S11720382	gi|19699306|gb|AY090360.1	At5g28290.1
at1g68620	CON	MIN	HOUR	DAY	WEEK	UNIGENE	FLCDNA	TAIR
GAGATGTGCG	0	0	0	1	0	gnl|UG|At#S11729374	gi|14335125|gb|AY037242.1	At1g68620.1
at3g47470	CON	MIN	HOUR	DAY	WEEK	UNIGENE	FLCDNA	TAIR
GCACAACACT	3	1	1	0	0	no match found	gi|51870199|gb|BT015429.1	non-canonical match
TGGCAACAGT	118	56	211	90	24	gnl|UG|At#S34115989	gi|13358203|gb|AF325012.2	At3g47470.1
CCTCGGCCTC	1	1	1	0	0	gnl|UG|At#S30494349	no match found	non-canonical match
at1g62850	CON	MIN	HOUR	DAY	WEEK	UNIGENE	FLCDNA	TAIR
AAGCCGAAGA	0	5	1	1	0	no match found	gi|28393872|gb|BT004350.1	At1g62855.1
TTATTCGAAA	0	1	1	0	0	no match found	gi|51971434|dbj|AK176619.1|	non-canonical match
at2g07709	CON	MIN	HOUR	DAY	WEEK	UNIGENE	FLCDNA	TAIR
CTAGCAGATG	0	0	0	1	0	gnl|UG|At#S35232503	no match found	non-canonical match
at2g38880	CON	MIN	HOUR	DAY	WEEK	UNIGENE	FLCDNA	TAIR
AAGAAAGCGT	1	1	1	1	0	no match found	gi|28973262|gb|BT005536.1	At2g38880.1

at3g59290	CON	MIN	HOUR	DAY	WEEK	UNIGENE	FLCDNA	TAIR
GTAAAAAGAG	1	0	1	0	0	gnl|UG|At#S11727716	no match found	At3g59290.1
at1g10760	CON	MIN	HOUR	DAY	WEEK	UNIGENE	FLCDNA	TAIR
GCTGTTTTGG	0	4	0	0	1	gnl|UG|At#S11741956	gi|110740253|dbj|AK230217.1|	At1g10760.1
at5g54680	CON	MIN	HOUR	DAY	WEEK	UNIGENE	FLCDNA	TAIR
TAAATTTAAG	9	3	7	11	4	gnl|UG|At#S11718246	gi|15451009|gb|AY054585.1	non-canonical match
at4g02620	CON	MIN	HOUR	DAY	WEEK	UNIGENE	FLCDNA	TAIR
ATTCAGTTCT	2	6	0	1	0	gnl|UG|At#S11727034	gi|21402987|gb|AY084277.1	At4g02620.1
at3g02350	CON	MIN	HOUR	DAY	WEEK	UNIGENE	FLCDNA	TAIR
AAACCGTGGC	2	2	1	1	2	gnl|UG|At#S11739982	gi|28416490|gb|BT004530.1	At3g02350.1
at4g29840	CON	MIN	HOUR	DAY	WEEK	UNIGENE	FLCDNA	TAIR
GATGTTCTCA	1	4	1	6	3	gnl|UG|At#S11722644	no match found	multiple non-canonical match
at5g17640	CON	MIN	HOUR	DAY	WEEK	UNIGENE	FLCDNA	TAIR
GCACTTGCTG	1	3	2	1	0	gnl|UG|At#S11702464	gi|13877884|gb|AF370205.1	At5g17640.1
at1g24210	CON	MIN	HOUR	DAY	WEEK	UNIGENE	FLCDNA	TAIR
AAGATTTGAT	0	1	0	0	0	no match found	gi|88900411|gb|BT024693.1	multiple non-canonical match
at5g50250	CON	MIN	HOUR	DAY	WEEK	UNIGENE	FLCDNA	TAIR
GGAAAGTAGT	0	2	0	0	0	gnl|UG|At#S11718691	gi|14532487|gb|AY039868.1	At5g50250.1
at3g13450	CON	MIN	HOUR	DAY	WEEK	UNIGENE	FLCDNA	TAIR
TAATGGATAC	1	3	0	1	0	gnl|UG|At#S11736834	gi|110738413|dbj|AK229269.1|	At3g13450.1
CCAACCAAGA	0	1	1	0	0	no match found	gi|90093293|gb|BT024889.1	multiple non-canonical match
at3g12690	CON	MIN	HOUR	DAY	WEEK	UNIGENE	FLCDNA	TAIR
GAGGTGGTGA	194	261	147	178	48	gnl|UG|At#S18942116	gi|56381936|gb|BT020332.1	multiple canonical match
at1g33290	CON	MIN	HOUR	DAY	WEEK	UNIGENE	FLCDNA	TAIR
CATCCGTCTG	0	5	2	2	1	gnl|UG|At#S11737820	gi|110737928|dbj|AK229015.1|	At1g33290.1
at4g12970	CON	MIN	HOUR	DAY	WEEK	UNIGENE	FLCDNA	TAIR
AAATGATGAA	0	0	1	0	0	no match found	gi|51969893|dbj|AK175876.1|	At4g12970.1
at1g67730	CON	MIN	HOUR	DAY	WEEK	UNIGENE	FLCDNA	TAIR
AAGAAAGAAT	3	3	0	1	2	gnl|UG|At#S11729562	gi|13605725|gb|AF361844.1	At1g67730.1
at4g38110	CON	MIN	HOUR	DAY	WEEK	UNIGENE	FLCDNA	TAIR
ACAAATTTTG	1	0	0	2	2	no match found	no match found	At4g38110.1
at5g47570	CON	MIN	HOUR	DAY	WEEK	UNIGENE	FLCDNA	TAIR
GTTGAGTGGC	3	10	2	5	1	gnl|UG|At#S11718965	gi|18087586|gb|AF462836.1	At5g47570.1
at5g59890	CON	MIN	HOUR	DAY	WEEK	UNIGENE	FLCDNA	TAIR
GTGTCCCGAC	1	1	3	0	1	gnl|UG|At#S15445613	gi|15215858|gb|AY050460.1	At5g59890.1
at4g02610	CON	MIN	HOUR	DAY	WEEK	UNIGENE	FLCDNA	TAIR
TGAAACAGGT	0	3	1	1	0	gnl|UG|At#S11727037	gi|26450412|dbj|AK117668.1|	non-canonical match
at3g14280	CON	MIN	HOUR	DAY	WEEK	UNIGENE	FLCDNA	TAIR
AGGAAGTAAG	3	0	2	1	2	no match found	no match found	At3g14280.1
ATGATCAAAT	0	1	1	0	0	gnl|UG|At#S11736572	gi|30725287|gb|BT008307.1	multiple non-canonical match
at5g57160	CON	MIN	HOUR	DAY	WEEK	UNIGENE	FLCDNA	TAIR
TTTTGTAACT	0	0	1	1	1	gnl|UG|At#S11701284	no match found	At5g57160.1

at5g05780	CON	MIN	HOUR	DAY	WEEK	UNIGENE	FLCDNA	TAIR
AAAAAGCAGA	2	5	0	1	1	gnl|UG|At#S11724682	gi|62321365|dbj|AK222016.1|	multiple canonical match
at3g50430	CON	MIN	HOUR	DAY	WEEK	UNIGENE	FLCDNA	TAIR
GCTAATTCTA	1	0	0	0	0	no match found	no match found	At3g50430.1
at1g49740	CON	MIN	HOUR	DAY	WEEK	UNIGENE	FLCDNA	TAIR
TTACCAAGCC	0	0	0	1	0	no match found	gi|20260217|gb|AY093008.1	non-canonical match
at2g25790	CON	MIN	HOUR	DAY	WEEK	UNIGENE	FLCDNA	TAIR
CACCAATAAC	0	0	0	1	0	gnl|UG|At#S11736076	gi|17063166|gb|AY062104.1	At2g25790.1
at1g65130	CON	MIN	HOUR	DAY	WEEK	UNIGENE	FLCDNA	TAIR
ACCATCTTTG	0	0	0	1	0	no match found	no match found	At1g65130.1
at3g47380	CON	MIN	HOUR	DAY	WEEK	UNIGENE	FLCDNA	TAIR
CATAATCCGA	0	0	0	0	1	gnl|UG|At#S11729925	no match found	At3g47380.1
at1g61100	CON	MIN	HOUR	DAY	WEEK	UNIGENE	FLCDNA	TAIR
GCCTTCAAGG	0	1	3	0	0	no match found	gi|19347712|gb|AY080590.1	multiple non-canonical match
ACTGCATCTC	0	3	6	1	0	gnl|UG|At#S11731127	gi|110737497|dbj|AK228795.1|	At1g61100.1
at4g06634	CON	MIN	HOUR	DAY	WEEK	UNIGENE	FLCDNA	TAIR
GCAAGCAAAA	0	1	0	1	0	gnl|UG|At#S28282092	no match found	At4g06634.1
at1g54370	CON	MIN	HOUR	DAY	WEEK	UNIGENE	FLCDNA	TAIR
TCAATAATCT	5	0	0	0	0	gnl|UG|At#S34115715	gi|110740966|dbj|AK226435.1|	At1g54370.1
at2g38255	CON	MIN	HOUR	DAY	WEEK	UNIGENE	FLCDNA	TAIR
TTAAAGTTAA	1	1	0	0	0	gnl|UG|At#S15460651	no match found	At2g38255.1
at3g16760	CON	MIN	HOUR	DAY	WEEK	UNIGENE	FLCDNA	TAIR
GAGAAATACA	1	0	1	1	1	no match found	gi|50253543|gb|BT015102.1	non-canonical match
at5g51880	CON	MIN	HOUR	DAY	WEEK	UNIGENE	FLCDNA	TAIR
AAGAAGACAT	0	0	0	0	1	no match found	gi|21404827|gb|AY086117.1	non-canonical match
GCTCATTCAT	1	3	0	0	1	gnl|UG|At#S11718528	gi|110735882|dbj|AK227949.1|	At5g51880.1
at4g32460	CON	MIN	HOUR	DAY	WEEK	UNIGENE	FLCDNA	TAIR
CAGTGATGCC	0	4	1	0	0	gnl|UG|At#S11722196	gi|110740604|dbj|AK226244.1|	At4g32460.1
at2g47310	CON	MIN	HOUR	DAY	WEEK	UNIGENE	FLCDNA	TAIR
TTCATAAAAG	0	2	2	0	0	gnl|UG|At#S34116956	gi|110738578|dbj|AK229352.1|	At2g47310.1
at3g49880	CON	MIN	HOUR	DAY	WEEK	UNIGENE	FLCDNA	TAIR
TGTTGGCGGG	0	1	1	0	0	gnl|UG|At#S11708320	gi|17381059|gb|AY063986.1	At3g49880.1
at1g73670	CON	MIN	HOUR	DAY	WEEK	UNIGENE	FLCDNA	TAIR
TAAAAATTTG	1	1	2	4	1	gnl|UG|At#S11703908	gi|14423563|gb|AF387019.1	At1g73670.1
at2g30360	CON	MIN	HOUR	DAY	WEEK	UNIGENE	FLCDNA	TAIR
GACGTCTTAG	0	1	0	0	0	gnl|UG|At#S11822548	gi|16226429|gb|AF428398.1	At2g30360.1
at4g17130	CON	MIN	HOUR	DAY	WEEK	UNIGENE	FLCDNA	TAIR
CTTACTATCT	0	1	0	0	0	no match found	no match found	At4g17130.1
at1g01060	CON	MIN	HOUR	DAY	WEEK	UNIGENE	FLCDNA	TAIR
TCTTGTCTCT	1	0	2	3	1	gnl|UG|At#S15461680	no match found	At1g01060.1
at1g70070	CON	MIN	HOUR	DAY	WEEK	UNIGENE	FLCDNA	TAIR

GACCGTCCAC	0	2	0	1	2	gnl|UG|At#S11729084	no match found	At1g70070.1
at1g62810	CON	MIN	HOUR	DAY	WEEK	UNIGENE	FLCDNA	TAIR
GATATTGATG	1	2	0	0	0	no match found	no match found	At1g62810.1
at3g51100	CON	MIN	HOUR	DAY	WEEK	UNIGENE	FLCDNA	TAIR
AAGAGAGTTG	0	2	0	1	1	no match found	gi|110735878|dbj|AK227947.1|	multiple non-canonical match
at4g23630	CON	MIN	HOUR	DAY	WEEK	UNIGENE	FLCDNA	TAIR
TGGAAGAACA	2	4	3	6	2	gnl|UG|At#S11723711	gi|21406653|gb|AY087881.1	At4g23630.1
at5g06860	CON	MIN	HOUR	DAY	WEEK	UNIGENE	FLCDNA	TAIR
AACCAAACCT	15	19	21	32	29	no match found	no match found	At5g06860.1
at4g23470	CON	MIN	HOUR	DAY	WEEK	UNIGENE	FLCDNA	TAIR
ACTTTTTCCT	6	7	4	3	3	gnl|UG|At#S18941815	gi|51969137|dbj|AK175498.1|	At4g23470.2
at3g14415	CON	MIN	HOUR	DAY	WEEK	UNIGENE	FLCDNA	TAIR
GCACTAAGTG	53	55	53	39	24	gnl|UG|At#S23593209	gi|22531127|gb|AY136402.1	At3g14415.1
at1g60870	CON	MIN	HOUR	DAY	WEEK	UNIGENE	FLCDNA	TAIR
TTATAGGATT	2	6	3	0	2	gnl|UG|At#S11731213	gi|21403985|gb|AY085275.1	At1g60870.1
at5g18650	CON	MIN	HOUR	DAY	WEEK	UNIGENE	FLCDNA	TAIR
TATTGCCTCC	8	15	16	5	4	gnl|UG|At#S11721661	gi|17381207|gb|AY064060.1	At5g18650.1
at3g21650	CON	MIN	HOUR	DAY	WEEK	UNIGENE	FLCDNA	TAIR
GCAACGGTTA	0	5	2	3	0	gnl|UG|At#S11734332	gi|110742348|dbj|AK227037.1|	At3g21650.1
at5g57830	CON	MIN	HOUR	DAY	WEEK	UNIGENE	FLCDNA	TAIR
CGGCGTGGTT	0	0	0	2	0	gnl|UG|At#S11717927	no match found	At5g57830.1
at5g60200	CON	MIN	HOUR	DAY	WEEK	UNIGENE	FLCDNA	TAIR
TTTTTTAATG	0	0	1	1	0	gnl|UG|At#S11717682	gi|21404764|gb|AY086054.1	At5g60200.1
at1g02640	CON	MIN	HOUR	DAY	WEEK	UNIGENE	FLCDNA	TAIR
GGATTCATAT	3	3	0	0	0	gnl|UG|At#S11742852	gi|23506062|gb|AY143952.1	At1g02640.1
at1g18850	CON	MIN	HOUR	DAY	WEEK	UNIGENE	FLCDNA	TAIR
AAGAGCTAAA	1	0	0	0	0	gnl|UG|At#S11706055	gi|21407463|gb|AY088689.1	At1g18850.1
at4g21130	CON	MIN	HOUR	DAY	WEEK	UNIGENE	FLCDNA	TAIR
GAAGGGAAGA	1	0	0	0	0	no match found	no match found	At4g21130.1
at4g02400	CON	MIN	HOUR	DAY	WEEK	UNIGENE	FLCDNA	TAIR
CCTATAGGAC	0	0	0	0	1	gnl|UG|At#S34116077	gi|110740278|dbj|AK230231.1|	non-canonical match
at1g28670	CON	MIN	HOUR	DAY	WEEK	UNIGENE	FLCDNA	TAIR
TCAACATCAT	0	6	0	0	1	no match found	no match found	At1g28670.1
at1g61280	CON	MIN	HOUR	DAY	WEEK	UNIGENE	FLCDNA	TAIR
AAAAAACATT	0	0	2	1	1	no match found	no match found	At1g61280.1
at1g04630	CON	MIN	HOUR	DAY	WEEK	UNIGENE	FLCDNA	TAIR
TTCCTTGCCG	0	2	0	0	0	no match found	gi|25083851|gb|BT002116.1	multiple non-canonical match
at5g64960	CON	MIN	HOUR	DAY	WEEK	UNIGENE	FLCDNA	TAIR
CCGCTCCACT	2	4	1	2	0	no match found	gi|13430443|gb|AF360134.1	non-canonical match
at4g12730	CON	MIN	HOUR	DAY	WEEK	UNIGENE	FLCDNA	TAIR
CCGGTTTACC	2	5	2	2	0	gnl|UG|At#S34115586	gi|16974608|gb|AY060582.1	At4g12730.1

at4g25570	CON	MIN	HOUR	DAY	WEEK	UNIGENE	FLCDNA	TAIR
CCATTGCTCT	2	7	2	1	2	gnl|UG|At#S11723380	gi|21404313|gb|AY085603.1	At4g25570.1
at3g47650	CON	MIN	HOUR	DAY	WEEK	UNIGENE	FLCDNA	TAIR
TTCTCAATGC	0	1	1	0	0	no match found	gi|17065647|gb|AY062972.1	non-canonical match
TATTACTATT	8	10	6	4	29	gnl|UG|At#S11729871	gi|13877966|gb|AF370246.1	At3g47650.1
at5g19390	CON	MIN	HOUR	DAY	WEEK	UNIGENE	FLCDNA	TAIR
AGAAAAAAAG	2	2	2	1	2	gnl|UG|At#S28281783	gi|20147216|gb|AY091776.1	At5g19390.2
at1g06350	CON	MIN	HOUR	DAY	WEEK	UNIGENE	FLCDNA	TAIR
CCATATTTGG	0	1	0	0	0	gnl|UG|At#S11742392	gi|110737136|dbj|AK228606.1|	At1g06350.1
at5g35330	CON	MIN	HOUR	DAY	WEEK	UNIGENE	FLCDNA	TAIR
GATATCTCCA	3	1	0	1	0	no match found	gi|110736705|dbj|AK228378.1|	non-canonical match
at5g07360	CON	MIN	HOUR	DAY	WEEK	UNIGENE	FLCDNA	TAIR
CTTTGTAGTG	2	1	0	0	0	gnl|UG|At#S11724286	no match found	At5g07360.1
at4g23770	CON	MIN	HOUR	DAY	WEEK	UNIGENE	FLCDNA	TAIR
ACCACTTCAT	0	0	1	0	0	gnl|UG|At#S14273664	gi|28973418|gb|BT005614.1	At4g23770.1
at5g57050	CON	MIN	HOUR	DAY	WEEK	UNIGENE	FLCDNA	TAIR
GGAATAGTGT	1	1	2	1	0	gnl|UG|At#S11718008	gi|22531153|gb|AY136415.1	At5g57050.1
at1g78010	CON	MIN	HOUR	DAY	WEEK	UNIGENE	FLCDNA	TAIR
GCTGTGAGTG	1	1	0	0	0	gnl|UG|At#S34117101	gi|110738295|dbj|AK229207.1|	At1g78010.1
at5g08770	CON	MIN	HOUR	DAY	WEEK	UNIGENE	FLCDNA	TAIR
ACCCGAGAAG	0	2	1	0	0	gnl|UG|At#S11817112	no match found	At5g08770.1
at5g64816	CON	MIN	HOUR	DAY	WEEK	UNIGENE	FLCDNA	TAIR
GCGAAGAAGA	0	3	1	0	0	no match found	gi|98961066|gb|BT025599.1	At5g64816.1
at4g37925	CON	MIN	HOUR	DAY	WEEK	UNIGENE	FLCDNA	TAIR
ACATATGCTA	9	1	4	0	0	no match found	gi|110742536|dbj|AK227138.1|	non-canonical match
at4g21380	CON	MIN	HOUR	DAY	WEEK	UNIGENE	FLCDNA	TAIR
TATTCTGTAA	0	1	0	0	0	gnl|UG|At#S11724110	gi|26452797|dbj|AK118895.1|	At4g21380.1
at5g56940	CON	MIN	HOUR	DAY	WEEK	UNIGENE	FLCDNA	TAIR
ATTCATTAGC	0	0	2	2	0	gnl|UG|At#S11718019	gi|14334471|gb|AY034927.1	At5g56940.1
at3g46660	CON	MIN	HOUR	DAY	WEEK	UNIGENE	FLCDNA	TAIR
ATGACTCTAT	0	0	1	0	0	gnl|UG|At#S34115713	gi|110740970|dbj|AK226437.1|	non-canonical match
at4g33940	CON	MIN	HOUR	DAY	WEEK	UNIGENE	FLCDNA	TAIR
AGTACCCTTT	0	2	0	0	0	gnl|UG|At#S11721946	gi|63003761|gb|BT021998.1	At4g33940.1
at1g60550	CON	MIN	HOUR	DAY	WEEK	UNIGENE	FLCDNA	TAIR
CCGGCCTTCA	1	2	0	2	1	gnl|UG|At#S11731317	gi|26450041|dbj|AK117477.1|	At1g60550.1
at2g40770	CON	MIN	HOUR	DAY	WEEK	UNIGENE	FLCDNA	TAIR
ATAAATGCAA	1	0	1	1	0	gnl|UG|At#S11732399	no match found	At2g40770.1
at2g12730	CON	MIN	HOUR	DAY	WEEK	UNIGENE	FLCDNA	TAIR
AACTTGTGGA	0	0	1	0	0	no match found	no match found	At2g12730.1
at5g57880	CON	MIN	HOUR	DAY	WEEK	UNIGENE	FLCDNA	TAIR
TCGATCACGA	0	0	1	0	0	no match found	no match found	At5g57880.1

at1g50520	CON	MIN	HOUR	DAY	WEEK	UNIGENE	FLCDNA	TAIR
CTGTCTTTGT	0	1	0	0	0	gnl|UG|At#S11734321	gi|26452479|dbj|AK118731.1|	At1g50520.1
at5g62270	CON	MIN	HOUR	DAY	WEEK	UNIGENE	FLCDNA	TAIR
ATCTTGTATT	0	0	1	0	0	gnl|UG|At#S11717474	no match found	At5g62270.1
at2g39740	CON	MIN	HOUR	DAY	WEEK	UNIGENE	FLCDNA	TAIR
GTTCAGAGAT	1	2	0	0	1	no match found	gi|110735730|dbj|AK227869.1|	At2g39740.1
GCCTTTGTGA	0	0	0	1	0	gnl|UG|At#S11732664	no match found	non-canonical match
at1g80820	CON	MIN	HOUR	DAY	WEEK	UNIGENE	FLCDNA	TAIR
AAAAGCTTTA	1	0	0	0	0	gnl|UG|At#S11726282	gi|21405872|gb|AY087148.1	At1g80820.1
at1g69570	CON	MIN	HOUR	DAY	WEEK	UNIGENE	FLCDNA	TAIR
AACTGAGAGA	1	1	1	1	1	gnl|UG|At#S11729191	gi|51970833|dbj|AK176346.1|	At1g69570.1
at4g10570	CON	MIN	HOUR	DAY	WEEK	UNIGENE	FLCDNA	TAIR
TCAGGTGATG	1	1	1	2	1	gnl|UG|At#S14273817	gi|28393464|gb|BT004132.1	At4g10570.1
at1g63830	CON	MIN	HOUR	DAY	WEEK	UNIGENE	FLCDNA	TAIR
TATGCAGACA	1	2	2	1	0	gnl|UG|At#S11744253	no match found	At1g63830.1
at3g10405	CON	MIN	HOUR	DAY	WEEK	UNIGENE	FLCDNA	TAIR
AGTTACTGAC	0	2	4	0	3	gnl|UG|At#S11816240	gi|62320686|dbj|AK221670.1|	At3g10405.1
at1g64700	CON	MIN	HOUR	DAY	WEEK	UNIGENE	FLCDNA	TAIR
CTCTTTTCTC	0	2	0	0	0	gnl|UG|At#S11730193	gi|38454063|gb|BT010669.1	At1g64700.1
at3g58790	CON	MIN	HOUR	DAY	WEEK	UNIGENE	FLCDNA	TAIR
AACTGAGACT	2	0	2	1	0	no match found	gi|22136431|gb|AY128894.1	multiple non-canonical match
TTGTAATGAT	0	0	0	1	2	no match found	gi|20466463|gb|AY099698.1	At3g58790.1
at2g24690	CON	MIN	HOUR	DAY	WEEK	UNIGENE	FLCDNA	TAIR
TATTTAAGTT	0	0	0	1	0	gnl|UG|At#S11736336	no match found	non-canonical match
at3g06770	CON	MIN	HOUR	DAY	WEEK	UNIGENE	FLCDNA	TAIR
TGTAAACAAA	0	0	1	0	1	gnl|UG|At#S15461506	gi|22136513|gb|AY128935.1	At3g06770.3
at2g07020	CON	MIN	HOUR	DAY	WEEK	UNIGENE	FLCDNA	TAIR
TGTTTTTATC	1	0	0	0	0	no match found	no match found	At2g07020.1
at5g08070	CON	MIN	HOUR	DAY	WEEK	UNIGENE	FLCDNA	TAIR
TGTTTGATCA	0	1	0	1	0	no match found	gi|124300961|gb|BT029995.1	multiple non-canonical match
at3g14310	CON	MIN	HOUR	DAY	WEEK	UNIGENE	FLCDNA	TAIR
GAAGGAATAT	0	4	0	0	0	no match found	gi|15529255|gb|AY052252.1	multiple non-canonical match
ATGGATGTCA	0	0	0	0	1	no match found	gi|16604544|gb|AY058892.1	non-canonical match
TATTTTCTTG	1	4	0	0	2	gnl|UG|At#S11772147	no match found	At3g14310.1
at1g17500	CON	MIN	HOUR	DAY	WEEK	UNIGENE	FLCDNA	TAIR
GAAGAAAGTA	0	3	0	1	0	gnl|UG|At#S11741294	no match found	At1g17500.1
at5g55860	CON	MIN	HOUR	DAY	WEEK	UNIGENE	FLCDNA	TAIR
ATGAAGTTGA	0	2	1	0	0	gnl|UG|At#S11718127	no match found	At5g55860.1
at4g12390	CON	MIN	HOUR	DAY	WEEK	UNIGENE	FLCDNA	TAIR
GTTTCGACTA	2	0	2	1	0	gnl|UG|At#S11725666	gi|15450853|gb|AY054507.1	At4g12390.1
at5g49820	CON	MIN	HOUR	DAY	WEEK	UNIGENE	FLCDNA	TAIR
AAGAAAAAAA	11	8	17	16	5	no match found	gi|16604642|gb|AY059766.1	multiple canonical match

TGAAGGAGGG	1	0	0	0	0	no match found	gi|23296702|gb|AY142582.1	non-canonical match
ATTGTTACAC	0	0	1	1	0	gnl|UG|At#S11718735	no match found	non-canonical match
at1g77940	CON	MIN	HOUR	DAY	WEEK	UNIGENE	FLCDNA	TAIR
GTTCTTGAAA	1	2	1	1	1	no match found	no match found	At1g77940.1
AGGGTATCAA	2	8	2	0	3	gnl|UG|At#S35288873	gi|21404591|gb|AY085881.1	At5g40855.1
at5g60320	CON	MIN	HOUR	DAY	WEEK	UNIGENE	FLCDNA	TAIR
TTTGTTACTC	0	0	0	0	1	gnl|UG|At#S11717670	no match found	At5g60320.1
at5g10070	CON	MIN	HOUR	DAY	WEEK	UNIGENE	FLCDNA	TAIR
TAGTATAATG	2	0	0	0	2	gnl|UG|At#S34114341	gi|110743647|dbj|AK227674.1|	At5g10070.1
at4g38980	CON	MIN	HOUR	DAY	WEEK	UNIGENE	FLCDNA	TAIR
AGCACATACG	0	1	0	0	0	gnl|UG|At#S11721102	gi|19423913|gb|AY080872.1	At4g38980.1
at5g60270	CON	MIN	HOUR	DAY	WEEK	UNIGENE	FLCDNA	TAIR
AAGCTTAGCT	0	4	0	0	1	gnl|UG|At#S21737161	gi|51969169|dbj|AK175514.1|	non-canonical match
at4g18160	CON	MIN	HOUR	DAY	WEEK	UNIGENE	FLCDNA	TAIR
GTTGTCCGAT	0	1	0	0	0	gnl|UG|At#S11724654	no match found	At4g18160.1
at5g56900	CON	MIN	HOUR	DAY	WEEK	UNIGENE	FLCDNA	TAIR
GTAGAATTTG	1	1	1	2	1	gnl|UG|At#S11743828	gi|19347973|gb|AY080699.1	At5g56900.1
at5g05987	CON	MIN	HOUR	DAY	WEEK	UNIGENE	FLCDNA	TAIR
CAAGCATAAG	0	0	0	1	0	no match found	gi|28973352|gb|BT005581.1	non-canonical match
GTTAAGATGG	0	0	0	2	0	gnl|UG|At#S15461332	gi|26452889|dbj|AK118943.1|	At5g05987.1
at1g09260	CON	MIN	HOUR	DAY	WEEK	UNIGENE	FLCDNA	TAIR
TCTTTCTTGG	0	0	0	0	1	no match found	no match found	At1g09260.1
at5g12240	CON	MIN	HOUR	DAY	WEEK	UNIGENE	FLCDNA	TAIR
GACGACCAAG	0	1	2	0	0	no match found	gi|51969327|dbj|AK175593.1|	At5g12240.1
at3g06720	CON	MIN	HOUR	DAY	WEEK	UNIGENE	FLCDNA	TAIR
GTTATACTCC	1	1	1	1	1	gnl|UG|At#S11738712	gi|21407506|gb|AY088732.1	At3g06720.1
at1g56500	CON	MIN	HOUR	DAY	WEEK	UNIGENE	FLCDNA	TAIR
ACCTTTTCTT	6	3	3	3	0	no match found	gi|26452041|dbj|AK118507.1|	non-canonical match
ACCGTGGTAT	1	0	0	0	0	gnl|UG|At#S11732114	gi|17529225|gb|AY065399.1	At1g56500.1
at1g80500	CON	MIN	HOUR	DAY	WEEK	UNIGENE	FLCDNA	TAIR
AGCTTTATAT	0	0	0	0	1	gnl|UG|At#S18896325	gi|110736373|dbj|AK228202.1|	non-canonical match
at2g26410	CON	MIN	HOUR	DAY	WEEK	UNIGENE	FLCDNA	TAIR
GAGAAACTCT	0	1	0	0	0	gnl|UG|At#S11735930	no match found	At2g26410.1
at5g20935	CON	MIN	HOUR	DAY	WEEK	UNIGENE	FLCDNA	TAIR
TCTCTAAAAT	0	0	2	3	4	gnl|UG|At#S30457658	gi|21407683|gb|AY088909.1	multiple canonical match
at4g37460	CON	MIN	HOUR	DAY	WEEK	UNIGENE	FLCDNA	TAIR
AGCAGTAAGA	1	2	4	1	3	gnl|UG|At#S11824466	gi|26449773|dbj|AK117340.1|	At4g37460.1
GATTAGGCGA	0	1	0	0	0	no match found	gi|29029043|gb|BT005966.1	non-canonical match
at5g14180	CON	MIN	HOUR	DAY	WEEK	UNIGENE	FLCDNA	TAIR
GGTGTTACTG	0	0	1	0	0	no match found	gi|20147186|gb|AY091711.1	non-canonical match
TGCCCATATT	1	2	2	2	0	gnl|UG|At#S11722790	gi|51969555|dbj|AK175707.1|	At5g14180.1
at1g74410	CON	MIN	HOUR	DAY	WEEK	UNIGENE	FLCDNA	TAIR
AATAAGAATA	1	0	1	0	0	no match found	gi|15215807|gb|AY050433.1	At1g74410.1

at5g63570	CON	MIN	HOUR	DAY	WEEK	UNIGENE	FLCDNA	TAIR
TTTTGGGGAC	4	0	4	2	1	gnl|UG|At#S11717343	gi|20856672|gb|AY102109.1	At5g63570.1
at5g43400	CON	MIN	HOUR	DAY	WEEK	UNIGENE	FLCDNA	TAIR
AATGAATCTT	1	0	0	0	0	no match found	gi|18450362|gb|AY048243.2	multiple canonical match
at2g24755	CON	MIN	HOUR	DAY	WEEK	UNIGENE	FLCDNA	TAIR
TGGCTTAAGA	0	0	1	0	0	no match found	gi|62320377|dbj|AK221515.1|	pseudo chromosome match
at3g10050	CON	MIN	HOUR	DAY	WEEK	UNIGENE	FLCDNA	TAIR
GTTGTTCTTG	2	2	0	0	1	gnl|UG|At#S11737875	gi|18086438|gb|AY065037.1	At3g10050.1
at2g01630	CON	MIN	HOUR	DAY	WEEK	UNIGENE	FLCDNA	TAIR
GATGATTGTA	1	0	0	0	0	gnl|UG|At#S38434020	gi|21404210|gb|AY085500.1	At2g01630.1
at1g24590	CON	MIN	HOUR	DAY	WEEK	UNIGENE	FLCDNA	TAIR
ATGATTATTA	1	0	0	0	0	gnl|UG|At#S11740491	gi|109946500|gb|BT026073.1	At1g24590.1
at5g44710	CON	MIN	HOUR	DAY	WEEK	UNIGENE	FLCDNA	TAIR
GAAAACGTGC	3	1	0	0	0	gnl|UG|At#S11719257	gi|28973444|gb|BT005627.1	At5g44710.1
at1g45976	CON	MIN	HOUR	DAY	WEEK	UNIGENE	FLCDNA	TAIR
TGATTGTGAT	3	1	1	0	3	gnl|UG|At#S24442426	no match found	At1g45976.1
at3g50660	CON	MIN	HOUR	DAY	WEEK	UNIGENE	FLCDNA	TAIR
CCGTTTGGAG	0	2	1	0	0	no match found	gi|15724347|gb|AF412114.1	At3g50660.1
at1g23870	CON	MIN	HOUR	DAY	WEEK	UNIGENE	FLCDNA	TAIR
ATGGGGACTG	2	2	4	0	1	gnl|UG|At#S11743489	gi|18176366|gb|AY072210.1	At1g23870.1
at5g07970	CON	MIN	HOUR	DAY	WEEK	UNIGENE	FLCDNA	TAIR
TAGCATTACT	0	1	1	0	0	gnl|UG|At#S11724126	gi|110737307|dbj|AK228696.1|	At5g07970.1
at4g39190	CON	MIN	HOUR	DAY	WEEK	UNIGENE	FLCDNA	TAIR
ATCCCAAGTG	0	1	0	0	0	gnl|UG|At#S21989728	no match found	non-canonical match
at3g25230	CON	MIN	HOUR	DAY	WEEK	UNIGENE	FLCDNA	TAIR
TTTGCGAAAC	0	1	2	2	3	gnl|UG|At#S11733201	no match found	At3g25230.1
at1g14310	CON	MIN	HOUR	DAY	WEEK	UNIGENE	FLCDNA	TAIR
TCGGAGATGA	0	1	1	1	0	gnl|UG|At#S18897143	gi|50897253|gb|BT015170.1	At1g14310.1
at1g14685	CON	MIN	HOUR	DAY	WEEK	UNIGENE	FLCDNA	TAIR
GAACTAACAA	2	1	0	0	1	gnl|UG|At#S18942578	gi|19699199|gb|AY090305.1	At1g14685.2
at2g20850	CON	MIN	HOUR	DAY	WEEK	UNIGENE	FLCDNA	TAIR
ATCCAGAGAG	0	1	0	0	0	gnl|UG|At#S35034674	gi|15810274|gb|AY056176.1	At2g20850.1
at2g47160	CON	MIN	HOUR	DAY	WEEK	UNIGENE	FLCDNA	TAIR
TTCTTAATCC	0	1	0	0	0	gnl|UG|At#S38433815	gi|23397181|gb|BT000732.1	At2g47160.1
at4g33380	CON	MIN	HOUR	DAY	WEEK	UNIGENE	FLCDNA	TAIR
TTTTTGTTCA	4	5	1	2	1	gnl|UG|At#S11708222	gi|17381262|gb|AY064143.1	At4g33380.1
at1g72030	CON	MIN	HOUR	DAY	WEEK	UNIGENE	FLCDNA	TAIR
AACTGAACTG	0	0	1	1	1	gnl|UG|At#S11823776	gi|26451172|dbj|AK118059.1|	non-canonical match
TACATTTTGG	2	3	1	1	4	no match found	gi|28973336|gb|BT005573.1	non-canonical match
at5g35570	CON	MIN	HOUR	DAY	WEEK	UNIGENE	FLCDNA	TAIR
GAGATTTGTT	0	1	0	0	1	gnl|UG|At#S11704007	gi|14517443|gb|AY039557.1	At5g35570.1

at5g54270	CON	MIN	HOUR	DAY	WEEK	UNIGENE	FLCDNA	TAIR
GGTTTGGTTG	69	161	70	59	8	gnl|UG|At#S11784645	gi|13926338|gb|AF372917.1	multiple non-canonical match
TCTCTCACAG	56	14	53	30	8	gnl|UG|At#S11777302	no match found	At5g54270.1

at3g20370	CON	MIN	HOUR	DAY	WEEK	UNIGENE	FLCDNA	TAIR
TGTTTGATTT	3	0	1	1	0	gnl|UG|At#S11734736	gi|21406790|gb|AY088016.1	At3g20370.1
CCTTCTCTCT	0	0	1	0	0	no match found	gi|20453332|gb|AY097389.1	non-canonical match

at2g35615	CON	MIN	HOUR	DAY	WEEK	UNIGENE	FLCDNA	TAIR
ATCAGATCAA	4	3	2	4	2	no match found	no match found	At2g35615.1

at5g35590	CON	MIN	HOUR	DAY	WEEK	UNIGENE	FLCDNA	TAIR
AGACCCCTTG	0	2	1	0	1	gnl|UG|At#S11720144	gi|20260139|gb|AY092969.1	At5g35590.1

at5g10460	CON	MIN	HOUR	DAY	WEEK	UNIGENE	FLCDNA	TAIR
GTAATGAGCT	1	1	1	1	1	gnl|UG|At#S11723625	gi|50198962|gb|BT015033.1	At5g10460.1

at5g57230	CON	MIN	HOUR	DAY	WEEK	UNIGENE	FLCDNA	TAIR
CCGGTAATGA	0	3	0	1	0	gnl|UG|At#S11717990	gi|124301041|gb|BT030035.1	non-canonical match

at5g06140	CON	MIN	HOUR	DAY	WEEK	UNIGENE	FLCDNA	TAIR
AGAGGGTTTG	2	1	4	3	0	gnl|UG|At#S11724589	gi|19424016|gb|AY080809.1	At5g06140.1

at3g55480	CON	MIN	HOUR	DAY	WEEK	UNIGENE	FLCDNA	TAIR
GTTGAGCCGT	2	1	2	1	2	gnl|UG|At#S11707949	gi|16604670|gb|AY059780.1	At3g55480.2

at3g45010	CON	MIN	HOUR	DAY	WEEK	UNIGENE	FLCDNA	TAIR
GTTCCGATGG	1	4	0	1	1	gnl|UG|At#S11730377	gi|20334785|gb|AY093993.1	At3g45010.1

at1g03760	CON	MIN	HOUR	DAY	WEEK	UNIGENE	FLCDNA	TAIR
CAAATGTGCG	0	1	1	1	0	gnl|UG|At#S11742741	gi|26452469|dbj|AK118726.1|	At1g03760.1

at2g23030	CON	MIN	HOUR	DAY	WEEK	UNIGENE	FLCDNA	TAIR
AACGGTACGT	0	1	0	0	0	gnl|UG|At#S11736730	gi|110736246|dbj|AK228137.1|	At2g23030.1

at4g01230	CON	MIN	HOUR	DAY	WEEK	UNIGENE	FLCDNA	TAIR
ATGGATATTT	1	0	0	0	0	gnl|UG|At#S11727379	no match found	non-canonical match

at1g62670	CON	MIN	HOUR	DAY	WEEK	UNIGENE	FLCDNA	TAIR
GTTACAACTG	1	0	0	0	0	no match found	no match found	At1g62670.1

at3g23760	CON	MIN	HOUR	DAY	WEEK	UNIGENE	FLCDNA	TAIR
TGATGACATT	3	1	0	0	0	gnl|UG|At#S11733683	gi|21407058|gb|AY088284.1	At3g23760.1

at5g12950	CON	MIN	HOUR	DAY	WEEK	UNIGENE	FLCDNA	TAIR
ATTGTAAAGC	0	1	0	0	0	no match found	gi|26451122|dbj|AK118034.1|	multiple non-canonical match
TGTTTAGATG	2	2	1	0	1	gnl|UG|At#S11723121	no match found	At5g12950.1

at1g73110	CON	MIN	HOUR	DAY	WEEK	UNIGENE	FLCDNA	TAIR
GACGATTGAT	3	2	1	1	0	no match found	gi|13605705|gb|AF361834.1	At1g73110.1
AGCATTTTGA	0	0	2	0	0	gnl|UG|At#S11728442	no match found	At1g45545.1

at1g78690	CON	MIN	HOUR	DAY	WEEK	UNIGENE	FLCDNA	TAIR
GTAATCAAAT	0	0	0	0	2	gnl|UG|At#S11704676	gi|15028034|gb|AY045874.1	At1g78690.1

at2g15410	CON	MIN	HOUR	DAY	WEEK	UNIGENE	FLCDNA	TAIR
GAACTCGGCA	0	0	1	0	1	no match found	no match found	At2g15410.1

at2g25070	CON	MIN	HOUR	DAY	WEEK	UNIGENE	FLCDNA	TAIR
AACAGTTAAA	0	3	1	0	0	no match found	gi|21407740|gb|AY088966.1	non-canonical match

at3g46780	CON	MIN	HOUR	DAY	WEEK	UNIGENE	FLCDNA	TAIR
AAGTCACTGA	2	8	1	1	0	no match found	gi|23505928|gb|AY143885.1	At3g46780.1
at5g18760	CON	MIN	HOUR	DAY	WEEK	UNIGENE	FLCDNA	TAIR
AACAGAGTGA	4	2	2	1	3	gnl|UG|At#S11721634	no match found	At5g18760.1
at3g24170	CON	MIN	HOUR	DAY	WEEK	UNIGENE	FLCDNA	TAIR
AAAACTCGGT	1	3	5	8	8	gnl|UG|At#S11733537	gi|62319679|dbj|AK221165.1|	At3g24170.1
at1g76130	CON	MIN	HOUR	DAY	WEEK	UNIGENE	FLCDNA	TAIR
ACGTAAGATT	2	1	2	1	2	gnl|UG|At#S24442599	gi|62320475|dbj|AK221564.1|	At1g76130.1
at2g46690	CON	MIN	HOUR	DAY	WEEK	UNIGENE	FLCDNA	TAIR
GTGGCCGTGA	0	2	2	0	1	gnl|UG|At#S35245299	gi|110736080|dbj|AK228052.1|	At2g46690.1
at5g52440	CON	MIN	HOUR	DAY	WEEK	UNIGENE	FLCDNA	TAIR
GTTTTCTCTT	1	2	2	1	0	gnl|UG|At#S11718472	gi|28973018|gb|BT005414.1	multiple canonical match
at2g33530	CON	MIN	HOUR	DAY	WEEK	UNIGENE	FLCDNA	TAIR
ACATTTCTCA	0	1	0	1	5	gnl|UG|At#S15460824	no match found	non-canonical match
at2g19390	CON	MIN	HOUR	DAY	WEEK	UNIGENE	FLCDNA	TAIR
GACGACGAAG	0	1	1	1	1	gnl|UG|At#S11707971	gi|16604626|gb|AY059758.1	At2g19390.1
at4g32770	CON	MIN	HOUR	DAY	WEEK	UNIGENE	FLCDNA	TAIR
TCATTGCTAG	3	0	2	2	0	gnl|UG|At#S11722146	gi|110741453|dbj|AK226552.1|	At4g32770.1
at1g20260	CON	MIN	HOUR	DAY	WEEK	UNIGENE	FLCDNA	TAIR
TCTTTTTTGT	4	7	2	4	5	gnl|UG|At#S18942535	gi|51968479|dbj|AK175169.1|	At1g20260.2
GACACTGCTC	0	1	0	0	1	no match found	gi|51969595|dbj|AK175727.1|	At1g20260.1
at1g12360	CON	MIN	HOUR	DAY	WEEK	UNIGENE	FLCDNA	TAIR
ATTGTAAGTG	3	2	1	2	2	gnl|UG|At#S11741800	gi|110743379|dbj|AK227584.1|	At1g12360.1
at3g18215	CON	MIN	HOUR	DAY	WEEK	UNIGENE	FLCDNA	TAIR
TTACAATGAT	1	1	1	0	2	no match found	gi|62319000|dbj|AK220821.1|	At3g18215.1
GCCTTTCTCT	0	1	0	0	0	no match found	gi|20259212|gb|AY091383.1	non-canonical match
at1g55180	CON	MIN	HOUR	DAY	WEEK	UNIGENE	FLCDNA	TAIR
CTCGGTATCC	1	0	0	0	0	gnl|UG|At#S11732588	no match found	multiple non-canonical match
at3g27470	CON	MIN	HOUR	DAY	WEEK	UNIGENE	FLCDNA	TAIR
GGAAGGGGTG	0	0	0	0	1	gnl|UG|At#S38433669	gi|27311906|gb|BT002559.1	At3g27470.1
at1g28540	CON	MIN	HOUR	DAY	WEEK	UNIGENE	FLCDNA	TAIR
AACATTCAGA	1	0	2	0	1	gnl|UG|At#S11739435	gi|21405667|gb|AY086943.1	At1g28540.1
at2g23200	CON	MIN	HOUR	DAY	WEEK	UNIGENE	FLCDNA	TAIR
AAGAAGATAG	1	1	0	1	1	gnl|UG|At#S11736688	no match found	At2g23200.1
at5g40650	CON	MIN	HOUR	DAY	WEEK	UNIGENE	FLCDNA	TAIR
CCAACAGGTG	1	1	0	1	0	gnl|UG|At#S11810571	gi|21406241|gb|AY087504.1	At5g40650.1
at2g16890	CON	MIN	HOUR	DAY	WEEK	UNIGENE	FLCDNA	TAIR
GAAGAATTTA	0	0	0	1	2	gnl|UG|At#S11738259	gi|23270363|gb|AY056244.2	At2g16890.2
at1g36510	CON	MIN	HOUR	DAY	WEEK	UNIGENE	FLCDNA	TAIR
ACTTTTGCTT	2	5	2	0	0	no match found	no match found	At1g36510.1
at5g23405	CON	MIN	HOUR	DAY	WEEK	UNIGENE	FLCDNA	TAIR

AATCATTTCC	1	2	0	0	1	gnl|UG|At#S11720837	gi|15529239|gb|AY052244.1	At5g23405.1
at3g22210	CON	MIN	HOUR	DAY	WEEK	UNIGENE	FLCDNA	TAIR
ATCGTTTTGC	5	1	2	6	1	gnl|UG|At#S11734161	gi|26451780|dbj|AK118372.1|	At3g22210.1
at4g23820	CON	MIN	HOUR	DAY	WEEK	UNIGENE	FLCDNA	TAIR
GAAGCTTGAA	0	2	1	0	1	gnl|UG|At#S20758007	gi|22136229|gb|AY128793.1	At4g23820.1
at4g12500	CON	MIN	HOUR	DAY	WEEK	UNIGENE	FLCDNA	TAIR
TGAGAGTTTT	1	0	1	2	3	gnl|UG|At#S11725647	gi|38566589|gb|BT010818.1	At4g12500.1
at3g48670	CON	MIN	HOUR	DAY	WEEK	UNIGENE	FLCDNA	TAIR
GGACTGAATA	0	1	0	0	0	no match found	gi|23197855|gb|BT000136.1	non-canonical match
at4g35760	CON	MIN	HOUR	DAY	WEEK	UNIGENE	FLCDNA	TAIR
GATAATAAAT	2	2	0	0	0	no match found	gi|21406968|gb|AY088194.1	non-canonical match
at5g35320	CON	MIN	HOUR	DAY	WEEK	UNIGENE	FLCDNA	TAIR
CACATCTACA	1	0	0	0	3	gnl|UG|At#S11720171	gi|21406502|gb|AY087764.1	At5g35320.1
at1g28610	CON	MIN	HOUR	DAY	WEEK	UNIGENE	FLCDNA	TAIR
AGTGGTAGAA	0	0	1	0	1	gnl|UG|At#S11703103	gi|13937188|gb|AF372948.1	At1g28610.2
at3g48530	CON	MIN	HOUR	DAY	WEEK	UNIGENE	FLCDNA	TAIR
AGCCATCCGG	1	13	5	2	0	gnl|UG|At#S11729728	gi|14596102|gb|AY042839.1	At3g48530.1
at1g27660	CON	MIN	HOUR	DAY	WEEK	UNIGENE	FLCDNA	TAIR
ATGGGCTGTG	1	1	0	0	0	gnl|UG|At#S11739755	no match found	At1g27660.1
at1g56720	CON	MIN	HOUR	DAY	WEEK	UNIGENE	FLCDNA	TAIR
ATTATGAGCT	2	0	0	0	0	gnl|UG|At#S34118283	gi|110736024|dbj|AK228023.1|	At1g56720.2
at2g42680	CON	MIN	HOUR	DAY	WEEK	UNIGENE	FLCDNA	TAIR
AACGTGTGCC	5	4	0	3	2	no match found	gi|21406914|gb|AY088140.1	At2g42680.1
at2g12400	CON	MIN	HOUR	DAY	WEEK	UNIGENE	FLCDNA	TAIR
TTTTGGGAAG	0	0	0	0	1	gnl|UG|At#S11739175	gi|25083237|gb|BT002043.1	multiple canonical match
at2g26800	CON	MIN	HOUR	DAY	WEEK	UNIGENE	FLCDNA	TAIR
TTGAACGGTT	1	2	0	2	0	gnl|UG|At#S11735833	gi|22136059|gb|AY128409.1	At2g26800.1
at5g55280	CON	MIN	HOUR	DAY	WEEK	UNIGENE	FLCDNA	TAIR
TAACGATAAT	2	4	3	1	0	gnl|UG|At#S11718185	gi|14334637|gb|AY034992.1	At5g55280.1
at5g38870	CON	MIN	HOUR	DAY	WEEK	UNIGENE	FLCDNA	TAIR
GTGGAGGAGG	0	2	0	1	0	no match found	no match found	At5g38870.1
at5g48710	CON	MIN	HOUR	DAY	WEEK	UNIGENE	FLCDNA	TAIR
AAAAAAAAGC	0	0	1	0	0	no match found	no match found	At5g48710.1
at5g61720	CON	MIN	HOUR	DAY	WEEK	UNIGENE	FLCDNA	TAIR
GCCTTCTTCA	0	0	0	1	0	no match found	gi|30793952|gb|BT008603.1	At5g61720.1
at3g57290	CON	MIN	HOUR	DAY	WEEK	UNIGENE	FLCDNA	TAIR
AGCAGTTGAT	3	2	0	0	2	gnl|UG|At#S11728088	gi|19310714|gb|AY079357.1	At3g57290.1
at2g29630	CON	MIN	HOUR	DAY	WEEK	UNIGENE	FLCDNA	TAIR
TTACTTTTAG	6	7	1	2	7	no match found	gi|110742111|dbj|AK226911.1|	At2g29630.1
TGTGGACCAA	0	1	0	0	0	gnl|UG|At#S15460982	gi|22136155|gb|AY128756.1	At2g29630.2
at5g43870	CON	MIN	HOUR	DAY	WEEK	UNIGENE	FLCDNA	TAIR

ATATTCTTTT 1 0 1 0 0 gnl|UG|At#S11719341 no match found At5g43870.1

at5g47650 CON MIN HOUR DAY WEEK UNIGENE FLCDNA TAIR
TTAAAGCCCC 0 0 0 0 1 gnl|UG|At#S11718956 gi|14596036|gb|AY042806.1 non-canonical match

at3g18380 CON MIN HOUR DAY WEEK UNIGENE FLCDNA TAIR
TGGATTTATT 1 0 0 3 0 no match found gi|20260285|gb|AY093042.1 At3g18380.1

at2g42900 CON MIN HOUR DAY WEEK UNIGENE FLCDNA TAIR
GCTCCACGTC 0 1 0 0 0 gnl|UG|At#S11817155 gi|46931221|gb|BT012596.1 At2g42900.1

at1g21660 CON MIN HOUR DAY WEEK UNIGENE FLCDNA TAIR
GGGGATATTG 2 0 1 0 4 gnl|UG|At#S11740895 gi|20466222|gb|AY099577.1 At1g21660.1
CGTGCGCTGT 0 0 1 0 0 no match found gi|34098884|gb|BT010382.1 non-canonical match

at5g44320 CON MIN HOUR DAY WEEK UNIGENE FLCDNA TAIR
CCTAAAAAAA 99 27 144 101 32 no match found gi|30794000|gb|BT008629.1 pseudo chromosome match

at3g48820 CON MIN HOUR DAY WEEK UNIGENE FLCDNA TAIR
GTCAGCTTTG 0 2 0 0 0 no match found gi|22136811|gb|AY133816.1 multiple non-canonical match
TCTTACCTTG 1 0 0 0 0 gnl|UG|At#S11729675 gi|19347710|gb|AY080589.1 At3g48820.1

at3g43610 CON MIN HOUR DAY WEEK UNIGENE FLCDNA TAIR
ACTCTAACAT 1 1 1 0 0 gnl|UG|At#S11730645 no match found non-canonical match

at4g16580 CON MIN HOUR DAY WEEK UNIGENE FLCDNA TAIR
TGTTGAGTTA 2 5 0 1 0 gnl|UG|At#S11724930 gi|26450941|dbj|AK117940.1| At4g16580.1

at3g18740 CON MIN HOUR DAY WEEK UNIGENE FLCDNA TAIR
AAGGAATCAA 0 0 0 0 1 no match found gi|17386133|gb|AF446880.1 multiple non-canonical match
TTGTTCTCTA 23 17 12 9 30 gnl|UG|At#S35245313 gi|21407059|gb|AY088285.1 At3g18740.1

at2g32760 CON MIN HOUR DAY WEEK UNIGENE FLCDNA TAIR
GAAATTCTCT 1 0 0 1 1 gnl|UG|At#S15460855 gi|18700112|gb|AY075661.1 At2g32760.1

at4g36780 CON MIN HOUR DAY WEEK UNIGENE FLCDNA TAIR
GTCGAGGAAG 2 5 2 0 0 gnl|UG|At#S11721473 gi|15215730|gb|AY050394.1 At4g36780.1

at4g15030 CON MIN HOUR DAY WEEK UNIGENE FLCDNA TAIR
GAGGGGATGT 0 1 0 1 0 gnl|UG|At#S11725197 no match found At4g15030.1

at2g03340 CON MIN HOUR DAY WEEK UNIGENE FLCDNA TAIR
GGATAAACAA 0 3 1 2 1 gnl|UG|At#S11740478 gi|110737968|dbj|AK229036.1| At2g03340.1

at5g53550 CON MIN HOUR DAY WEEK UNIGENE FLCDNA TAIR
CCGAGTAAAT 1 2 0 1 1 gnl|UG|At#S11718360 gi|110741695|dbj|AK226687.1| non-canonical match

at3g17800 CON MIN HOUR DAY WEEK UNIGENE FLCDNA TAIR
GTCTGTAATG 9 8 37 19 9 gnl|UG|At#S38433713 gi|17064819|gb|AY062486.1 non-canonical match

at1g02510 CON MIN HOUR DAY WEEK UNIGENE FLCDNA TAIR
AAACTTACTC 1 1 1 0 0 no match found no match found At1g02510.1

at4g14350 CON MIN HOUR DAY WEEK UNIGENE FLCDNA TAIR
TGTGTTGTTG 1 1 2 0 0 gnl|UG|At#S35230801 gi|22135802|gb|AY128279.1 At4g14350.2

at3g49470 CON MIN HOUR DAY WEEK UNIGENE FLCDNA TAIR
ACTCAGGCTG 2 1 4 0 1 gnl|UG|At#S11702642 gi|18377569|gb|AY072536.1 At3g49470.1

at3g52230 CON MIN HOUR DAY WEEK UNIGENE FLCDNA TAIR
CTGGCAAGAA 10 6 17 12 5 no match found gi|15450438|gb|AY052320.1 multiple non-canonical match

at3g54950	CON	MIN	HOUR	DAY	WEEK	UNIGENE	FLCDNA	TAIR
CTGCCCAAAA	0	1	1	0	0	gnl|UG|At#S34117112	gi|110738273|dbj|AK229196.1|	pseudo chromosome match
at5g55960	CON	MIN	HOUR	DAY	WEEK	UNIGENE	FLCDNA	TAIR
TAGATTAGTA	0	3	0	1	0	gnl|UG|At#S11718117	gi|16648986|gb|AY059863.1	At5g55960.1
at3g05210	CON	MIN	HOUR	DAY	WEEK	UNIGENE	FLCDNA	TAIR
GTGACCACAT	0	0	1	1	0	no match found	gi|21407689|gb|AY088915.1	non-canonical match
at5g42890	CON	MIN	HOUR	DAY	WEEK	UNIGENE	FLCDNA	TAIR
ATGAAGGAAC	0	3	0	0	0	gnl|UG|At#S11719440	gi|21436302|gb|AY117215.1	At5g42890.1
at2g29670	CON	MIN	HOUR	DAY	WEEK	UNIGENE	FLCDNA	TAIR
AGTTATGACT	1	0	0	0	0	gnl|UG|At#S11735121	no match found	non-canonical match
AAGACACCAT	1	2	0	0	0	no match found	gi|20465642|gb|AY096656.1	non-canonical match
TTTGTCACCA	8	13	10	0	1	gnl|UG|At#S11822854	gi|18377764|gb|AY074336.1	At2g29670.1
at5g19530	CON	MIN	HOUR	DAY	WEEK	UNIGENE	FLCDNA	TAIR
TCACAAAAAC	0	1	0	2	3	gnl|UG|At#S38433282	gi|22022543|gb|AY127003.1	At5g19530.1
at2g29640	CON	MIN	HOUR	DAY	WEEK	UNIGENE	FLCDNA	TAIR
TAACAAATTA	0	0	1	0	0	gnl|UG|At#S11735127	no match found	non-canonical match
at5g61750	CON	MIN	HOUR	DAY	WEEK	UNIGENE	FLCDNA	TAIR
ACTTCTTGCT	0	0	1	0	0	no match found	no match found	At5g61750.1
at5g37090	CON	MIN	HOUR	DAY	WEEK	UNIGENE	FLCDNA	TAIR
ATAACTAAAA	1	0	0	0	0	no match found	no match found	At5g37090.1
at4g33620	CON	MIN	HOUR	DAY	WEEK	UNIGENE	FLCDNA	TAIR
GAAGCAGAGA	0	0	0	0	1	gnl|UG|At#S34115495	gi|110741399|dbj|AK230453.1|	multiple non-canonical match
TCAAATTTTA	2	0	0	1	0	no match found	no match found	At4g33620.1
at3g22790	CON	MIN	HOUR	DAY	WEEK	UNIGENE	FLCDNA	TAIR
GTTTCTGAAA	2	1	0	0	1	gnl|UG|At#S34115467	gi|110741450|dbj|AK226550.1|	At3g22790.1
at5g49030	CON	MIN	HOUR	DAY	WEEK	UNIGENE	FLCDNA	TAIR
GCCTTCCAAT	0	0	0	1	0	no match found	gi|110736690|dbj|AK228370.1|	multiple non-canonical match
GCTGCAAAAT	0	1	0	0	0	gnl|UG|At#S11718815	gi|23296803|gb|AY142605.1	At5g49030.1
at1g20693	CON	MIN	HOUR	DAY	WEEK	UNIGENE	FLCDNA	TAIR
GAAGATTTCC	1	7	1	6	2	gnl|UG|At#S11740989	gi|21403441|gb|AY084731.1	At1g20693.1
at5g21222	CON	MIN	HOUR	DAY	WEEK	UNIGENE	FLCDNA	TAIR
TAGAATTTGT	1	2	2	0	0	gnl|UG|At#S15460581	no match found	At5g21222.1
at4g22860	CON	MIN	HOUR	DAY	WEEK	UNIGENE	FLCDNA	TAIR
TGGTATAACT	0	0	0	1	0	no match found	gi|66792639|gb|BT023431.1	At2g31720.1
TGGTTCATAG	0	1	0	0	0	gnl|UG|At#S34115420	gi|110741523|dbj|AK226597.1|	At4g22860.1
at3g51760	CON	MIN	HOUR	DAY	WEEK	UNIGENE	FLCDNA	TAIR
ACAATTTTTT	0	0	0	1	0	gnl|UG|At#S34116100	no match found	non-canonical match
at5g18800	CON	MIN	HOUR	DAY	WEEK	UNIGENE	FLCDNA	TAIR
TTTTGATTTG	13	5	1	3	2	gnl|UG|At#S11721624	gi|110735766|dbj|AK227889.1|	At5g18800.1
at3g22890	CON	MIN	HOUR	DAY	WEEK	UNIGENE	FLCDNA	TAIR
GCACCAGGAC	3	24	14	9	6	gnl|UG|At#S11733942	gi|27311864|gb|BT002538.1	At3g22890.1
at1g64710	CON	MIN	HOUR	DAY	WEEK	UNIGENE	FLCDNA	TAIR

AATATGAAAG	0	0	0	1	0	gnl|UG|At#S11730190	no match found	At1g64710.1
CAGCTGCTTC	4	3	4	1	5	no match found	gi|29824176|gb|BT006064.1	non-canonical match
at1g14600	CON	MIN	HOUR	DAY	WEEK	UNIGENE	FLCDNA	TAIR
GCGAGAAAAC	0	1	0	1	0	gnl|UG|At#S11741576	no match found	At1g14600.1
at1g62620	CON	MIN	HOUR	DAY	WEEK	UNIGENE	FLCDNA	TAIR
AAAGTAAATT	1	0	0	0	0	gnl|UG|At#S18897042	no match found	multiple canonical match
at4g11440	CON	MIN	HOUR	DAY	WEEK	UNIGENE	FLCDNA	TAIR
TCGCAAGGAG	0	0	1	0	0	gnl|UG|At#S11725819	no match found	non-canonical match
at3g22950	CON	MIN	HOUR	DAY	WEEK	UNIGENE	FLCDNA	TAIR
ATTGGCATAT	1	1	2	1	1	gnl|UG|At#S11733923	gi|20465872|gb|AY096401.1	non-canonical match
at5g55200	CON	MIN	HOUR	DAY	WEEK	UNIGENE	FLCDNA	TAIR
AGTTCTTTTT	0	0	0	1	0	no match found	gi|20148444|gb|AY081551.1	non-canonical match
ACTATGCTCT	1	0	0	1	0	gnl|UG|At#S11718193	gi|14596128|gb|AY042852.1	At5g55200.1
at3g09470	CON	MIN	HOUR	DAY	WEEK	UNIGENE	FLCDNA	TAIR
TTAAATTATG	0	0	0	0	1	gnl|UG|At#S11738043	gi|15146217|gb|AY049250.1	At3g09470.1
ACACGGAAGG	0	1	0	0	0	no match found	gi|28460680|gb|BT004735.1	At3g09470.2
at1g63290	CON	MIN	HOUR	DAY	WEEK	UNIGENE	FLCDNA	TAIR
ATGGACAAGG	1	1	1	0	0	gnl|UG|At#S11730484	no match found	multiple canonical match
at5g66610	CON	MIN	HOUR	DAY	WEEK	UNIGENE	FLCDNA	TAIR
TGGTTGGAGT	0	1	0	0	0	gnl|UG|At#S11717036	gi|110738235|dbj|AK229177.1|	At5g66610.1
at3g31960	CON	MIN	HOUR	DAY	WEEK	UNIGENE	FLCDNA	TAIR
AAATGTGTGC	1	0	0	0	0	gnl|UG|At#S11731325	no match found	At3g31960.1
at5g56130	CON	MIN	HOUR	DAY	WEEK	UNIGENE	FLCDNA	TAIR
ATACACAACT	1	2	2	0	1	gnl|UG|At#S11718100	gi|20260441|gb|AY093120.1	At5g56130.1
at3g59820	CON	MIN	HOUR	DAY	WEEK	UNIGENE	FLCDNA	TAIR
TTTTGCTGCT	0	2	0	3	3	gnl|UG|At#S28282780	no match found	At3g59820.1
at1g27020	CON	MIN	HOUR	DAY	WEEK	UNIGENE	FLCDNA	TAIR
GTGGGGTGTG	0	3	1	0	0	gnl|UG|At#S18898119	no match found	non-canonical match
TAATATCTTT	0	3	0	0	0	no match found	gi|18175957|gb|AY072136.1	At1g27020.1
GACGCTTGTC	0	0	0	0	2	no match found	gi|21436364|gb|AY117277.1	non-canonical match
at5g66320	CON	MIN	HOUR	DAY	WEEK	UNIGENE	FLCDNA	TAIR
CTAGGAGTAT	0	0	1	1	1	gnl|UG|At#S18941410	gi|22531222|gb|AY136450.1	At5g66320.2
at4g02010	CON	MIN	HOUR	DAY	WEEK	UNIGENE	FLCDNA	TAIR
AAAATATTAC	0	1	0	0	0	gnl|UG|At#S11727178	gi|32815838|gb|BT009694.1	At4g02010.1
at5g55135	CON	MIN	HOUR	DAY	WEEK	UNIGENE	FLCDNA	TAIR
AAATAGATTA	0	1	0	0	0	gnl|UG|At#S35273919	no match found	non-canonical match
at5g05990	CON	MIN	HOUR	DAY	WEEK	UNIGENE	FLCDNA	TAIR
ATCAACAAGG	0	0	0	0	1	gnl|UG|At#S11724630	gi|21404491|gb|AY085781.1	At5g05990.1
at3g03720	CON	MIN	HOUR	DAY	WEEK	UNIGENE	FLCDNA	TAIR
TACACAAGCC	0	0	0	0	1	no match found	no match found	At3g03720.1
TTTTATTCCT	2	0	0	0	2	gnl|UG|At#S11739592	no match found	non-canonical match
at4g15730	CON	MIN	HOUR	DAY	WEEK	UNIGENE	FLCDNA	TAIR
AACTTCCATA	19	10	20	8	12	gnl|UG|At#S11725076	no match found	multiple non-canonical match

at5g45600	CON	MIN	HOUR	DAY	WEEK	UNIGENE	FLCDNA	TAIR
TTGTAACTGT	1	0	0	0	1	gnl|UG|At#S11719167	gi|18175885|gb|AY072123.1	At5g45600.1
at2g01735	CON	MIN	HOUR	DAY	WEEK	UNIGENE	FLCDNA	TAIR
TGTCTTTTGT	2	0	0	1	3	gnl|UG|At#S34114536	gi|110743178|dbj|AK227479.1|	multiple non-canonical match
at1g51930	CON	MIN	HOUR	DAY	WEEK	UNIGENE	FLCDNA	TAIR
GAGATTGAGA	0	0	0	1	0	no match found	no match found	At1g51930.1
at5g23720	CON	MIN	HOUR	DAY	WEEK	UNIGENE	FLCDNA	TAIR
GAGATTCAAA	0	1	2	0	0	gnl|UG|At#S15460455	gi|34098832|gb|BT010356.1	At5g23720.1
at5g03070	CON	MIN	HOUR	DAY	WEEK	UNIGENE	FLCDNA	TAIR
GCGAACAGTC	0	2	0	0	0	gnl|UG|At#S34117193	gi|110738118|dbj|AK229115.1|	At5g03070.1
at3g16050	CON	MIN	HOUR	DAY	WEEK	UNIGENE	FLCDNA	TAIR
TGTTGGCTGA	0	0	1	3	0	gnl|UG|At#S34118214	gi|27808575|gb|BT003136.1	At3g16050.1
at5g11280	CON	MIN	HOUR	DAY	WEEK	UNIGENE	FLCDNA	TAIR
AATTGTCTAT	1	1	1	0	1	gnl|UG|At#S11723434	gi|17064977|gb|AY062565.1	At5g11280.1
at5g36950	CON	MIN	HOUR	DAY	WEEK	UNIGENE	FLCDNA	TAIR
TTGTACACTT	0	0	0	1	1	no match found	gi|17978950|gb|AY069888.1	At5g36950.1
at3g45730	CON	MIN	HOUR	DAY	WEEK	UNIGENE	FLCDNA	TAIR
CCAGCTTCCA	1	6	1	1	0	no match found	gi|26451520|dbj|AK118239.1|	At3g45730.1
at3g27270	CON	MIN	HOUR	DAY	WEEK	UNIGENE	FLCDNA	TAIR
GATTTGGTTG	0	0	1	0	0	gnl|UG|At#S18903832	gi|48310203|gb|BT014791.1	At3g27270.1
at5g16630	CON	MIN	HOUR	DAY	WEEK	UNIGENE	FLCDNA	TAIR
TGTTTCTTGA	0	2	0	1	0	gnl|UG|At#S28281793	gi|17065357|gb|AY062755.1	At5g16630.1
at4g22360	CON	MIN	HOUR	DAY	WEEK	UNIGENE	FLCDNA	TAIR
TGTTTAACTA	2	0	2	0	0	gnl|UG|At#S11723939	gi|21403665|gb|AY084955.1	At4g22360.1
TTCAAGATGA	1	0	0	0	0	no match found	gi|20258978|gb|AY091266.1	At3g43730.1
at4g16690	CON	MIN	HOUR	DAY	WEEK	UNIGENE	FLCDNA	TAIR
AAACCGGAGT	1	0	0	0	0	no match found	gi|21407090|gb|AY088316.1	multiple non-canonical match
CGAAGCAATC	2	0	5	5	1	gnl|UG|At#S11724908	no match found	At4g16690.1
at2g01340	CON	MIN	HOUR	DAY	WEEK	UNIGENE	FLCDNA	TAIR
TTTCCCGGAA	0	1	3	0	1	no match found	no match found	At2g01340.1
TCTCCACTCT	1	1	1	0	1	no match found	gi|110741089|dbj|AK226497.1|	non-canonical match
GCGGAAAGAG	1	1	0	0	0	no match found	gi|62867640|gb|BT021987.1	multiple non-canonical match
at3g52280	CON	MIN	HOUR	DAY	WEEK	UNIGENE	FLCDNA	TAIR
CTAATGACGA	0	1	0	0	0	gnl|UG|At#S11729011	no match found	non-canonical match
at1g67690	CON	MIN	HOUR	DAY	WEEK	UNIGENE	FLCDNA	TAIR
TACGGTGTTG	0	0	2	0	0	no match found	no match found	At1g67690.1
at3g47540	CON	MIN	HOUR	DAY	WEEK	UNIGENE	FLCDNA	TAIR
AATTAACATT	1	0	0	0	0	gnl|UG|At#S11729896	gi|26452467|dbj|AK118725.1|	At3g47540.1
at4g30650	CON	MIN	HOUR	DAY	WEEK	UNIGENE	FLCDNA	TAIR
TACCAAATAA	1	0	0	20	30	gnl|UG|At#S30648177	no match found	At4g30650.1
GAAGTTTTCT	0	0	2	0	2	no match found	gi|38566501|gb|BT010774.1	non-canonical match
at5g12210	CON	MIN	HOUR	DAY	WEEK	UNIGENE	FLCDNA	TAIR

ATAGATAGAG	0	3	0	0	0	gnl|UG|At#S11723193	gi|28466950|gb|BT004818.1	At5g12210.2
at3g04600	CON	MIN	HOUR	DAY	WEEK	UNIGENE	FLCDNA	TAIR
TTCGAATAGA	0	0	0	1	2	gnl|UG|At#S11739338	gi|110740618|dbj|AK226252.1|	At3g04600.2
at1g35710	CON	MIN	HOUR	DAY	WEEK	UNIGENE	FLCDNA	TAIR
TTAGGCCTTT	0	0	2	1	0	gnl|UG|At#S23657717	gi|14596040|gb|AY042808.1	At1g35710.1
at3g03740	CON	MIN	HOUR	DAY	WEEK	UNIGENE	FLCDNA	TAIR
AAGCGGAGAT	1	2	3	0	1	gnl|UG|At#S11739586	gi|13605820|gb|AF367309.1	At3g03740.1
at4g30630	CON	MIN	HOUR	DAY	WEEK	UNIGENE	FLCDNA	TAIR
GCAAGATCCA	0	2	1	0	0	gnl|UG|At#S11722510	gi|88196744|gb|BT024617.1	At4g30630.1
at4g34600	CON	MIN	HOUR	DAY	WEEK	UNIGENE	FLCDNA	TAIR
CATATCACCG	0	1	0	0	0	no match found	gi|38566571|gb|BT010809.1	non-canonical match
CAGAATGTAA	0	1	0	0	0	gnl|UG|At#S11721831	no match found	At4g34600.1
at3g59880	CON	MIN	HOUR	DAY	WEEK	UNIGENE	FLCDNA	TAIR
TCTTGTCGGG	0	1	1	1	1	gnl|UG|At#S26540484	no match found	non-canonical match
at3g13490	CON	MIN	HOUR	DAY	WEEK	UNIGENE	FLCDNA	TAIR
TGGTAAACAG	3	0	2	1	2	gnl|UG|At#S11704160	gi|14532625|gb|AY039937.1	At3g13490.1
at5g57650	CON	MIN	HOUR	DAY	WEEK	UNIGENE	FLCDNA	TAIR
TTGAAGCGTA	1	0	0	0	0	gnl|UG|At#S11717949	no match found	At5g57650.1
at1g60790	CON	MIN	HOUR	DAY	WEEK	UNIGENE	FLCDNA	TAIR
TCACATCACA	2	0	0	0	2	gnl|UG|At#S11709904	gi|17979148|gb|AY070075.1	At1g60790.1
at1g22970	CON	MIN	HOUR	DAY	WEEK	UNIGENE	FLCDNA	TAIR
GTGATAAAAG	1	0	1	0	0	gnl|UG|At#S11709850	gi|21700854|gb|AY124842.1	At1g22970.1
at2g38650	CON	MIN	HOUR	DAY	WEEK	UNIGENE	FLCDNA	TAIR
AAGCCGTGGC	1	0	0	0	0	gnl|UG|At#S11732931	gi|20259302|gb|AY091448.1	At2g38650.1
at3g62600	CON	MIN	HOUR	DAY	WEEK	UNIGENE	FLCDNA	TAIR
ATCCAAATAC	2	2	3	1	3	gnl|UG|At#S11726665	no match found	At3g62600.1
ATTAGGTTTT	0	1	0	1	0	no match found	gi|21406378|gb|AY087640.1	non-canonical match
at1g76920	CON	MIN	HOUR	DAY	WEEK	UNIGENE	FLCDNA	TAIR
ACAACAGAAA	0	2	1	2	0	gnl|UG|At#S11678785	gi|21404183|gb|AY085473.1	At1g76920.1
at4g37370	CON	MIN	HOUR	DAY	WEEK	UNIGENE	FLCDNA	TAIR
TGCAGAGCAC	0	1	2	0	0	gnl|UG|At#S11721377	gi|17473897|gb|AY065192.1	At4g37370.1
at2g25625	CON	MIN	HOUR	DAY	WEEK	UNIGENE	FLCDNA	TAIR
CAAAATTTTC	0	0	2	2	2	gnl|UG|At#S15461157	gi|14334615|gb|AY034981.1	At2g25625.2
at3g23610	CON	MIN	HOUR	DAY	WEEK	UNIGENE	FLCDNA	TAIR
GATATAAAAG	0	0	1	0	0	gnl|UG|At#S11733724	no match found	At3g23610.1
at5g23160	CON	MIN	HOUR	DAY	WEEK	UNIGENE	FLCDNA	TAIR
TGACCTGATA	0	0	0	0	3	gnl|UG|At#S35313996	no match found	pseudo chromosome match
at4g11110	CON	MIN	HOUR	DAY	WEEK	UNIGENE	FLCDNA	TAIR
CCAATTACCT	0	1	1	0	0	gnl|UG|At#S11725853	gi|110738245|dbj|AK229182.1|	At4g11110.1
at5g16240	CON	MIN	HOUR	DAY	WEEK	UNIGENE	FLCDNA	TAIR
ATGAGGAAAA	0	0	1	0	0	gnl|UG|At#S11705839	gi|15912300|gb|AY056428.1	At5g16240.1
AAAACCCAAT	0	0	1	0	0	no match found	gi|110743836|dbj|AK227771.1|	At5g16240.1

at2g24762	CON	MIN	HOUR	DAY	WEEK	UNIGENE	FLCDNA	TAIR
TGCGGTCACG	0	3	1	0	0	no match found	no match found	At2g24762.1
at5g38100	CON	MIN	HOUR	DAY	WEEK	UNIGENE	FLCDNA	TAIR
AGAAAAAAAA	10	6	12	12	8	no match found	gi|110737683|dbj|AK228891.1|	non-canonical match
at4g11540	CON	MIN	HOUR	DAY	WEEK	UNIGENE	FLCDNA	TAIR
TCTTGTTGTT	0	1	0	0	0	no match found	no match found	At4g11540.1
at1g68020	CON	MIN	HOUR	DAY	WEEK	UNIGENE	FLCDNA	TAIR
GTTTAGCTTC	1	2	2	1	0	gnl|UG|At#S11704735	gi|15028156|gb|AY046028.1	At1g68020.2
at1g71750	CON	MIN	HOUR	DAY	WEEK	UNIGENE	FLCDNA	TAIR
ACATCTTTTG	1	0	0	0	2	gnl|UG|At#S21737325	gi|21407691|gb|AY088917.1	At1g71750.1
at4g25030	CON	MIN	HOUR	DAY	WEEK	UNIGENE	FLCDNA	TAIR
ATGCTTGATG	1	8	1	2	0	gnl|UG|At#S18941799	gi|89000968|gb|BT024736.1	At4g25030.2
at2g16990	CON	MIN	HOUR	DAY	WEEK	UNIGENE	FLCDNA	TAIR
TGATTATTTG	0	1	0	0	0	gnl|UG|At#S21736306	gi|51970879|dbj|AK176369.1|	multiple non-canonical match
at5g48960	CON	MIN	HOUR	DAY	WEEK	UNIGENE	FLCDNA	TAIR
TATTTCCGCT	0	2	0	0	0	gnl|UG|At#S11718822	gi|17064895|gb|AY062524.1	multiple canonical match
at5g16330	CON	MIN	HOUR	DAY	WEEK	UNIGENE	FLCDNA	TAIR
CTTTGGAAAA	0	0	0	1	0	gnl|UG|At#S11722259	no match found	multiple non-canonical match
at3g24760	CON	MIN	HOUR	DAY	WEEK	UNIGENE	FLCDNA	TAIR
TAATTAGACG	0	1	1	0	2	gnl|UG|At#S11733325	no match found	At3g24760.1
at5g38240	CON	MIN	HOUR	DAY	WEEK	UNIGENE	FLCDNA	TAIR
TTATGTTCTT	0	0	0	0	1	no match found	no match found	At5g38240.1
at3g11660	CON	MIN	HOUR	DAY	WEEK	UNIGENE	FLCDNA	TAIR
TGAAGTGTCC	5	2	1	2	3	gnl|UG|At#S11737397	gi|28392928|gb|BT003850.1	At3g11660.1
at3g24200	CON	MIN	HOUR	DAY	WEEK	UNIGENE	FLCDNA	TAIR
TTTTAAATAA	0	0	0	1	1	gnl|UG|At#S28282918	gi|51968851|dbj|AK175355.1|	At3g24200.1
at2g37630	CON	MIN	HOUR	DAY	WEEK	UNIGENE	FLCDNA	TAIR
GAGGCTAAGA	0	0	0	0	1	gnl|UG|At#S11733193	gi|109946408|gb|BT026027.1	At2g37630.1
at2g01420	CON	MIN	HOUR	DAY	WEEK	UNIGENE	FLCDNA	TAIR
GCTGTTGCTG	1	4	1	1	1	gnl|UG|At#S11807218	gi|62320920|dbj|AK221788.1|	At2g01420.2
at2g01690	CON	MIN	HOUR	DAY	WEEK	UNIGENE	FLCDNA	TAIR
AGTTTGTAGA	2	4	4	3	4	gnl|UG|At#S18942994	no match found	At2g01690.1
at5g65660	CON	MIN	HOUR	DAY	WEEK	UNIGENE	FLCDNA	TAIR
GATGCTTCTC	1	4	1	0	1	gnl|UG|At#S18914155	gi|21405203|gb|AY086493.1	At5g65660.1
atmg01390	CON	MIN	HOUR	DAY	WEEK	UNIGENE	FLCDNA	TAIR
AAATGTTGAC	1	0	0	0	0	no match found	gi|110739854|dbj|AK230010.1|	pseudo chromosome match
GTTGGAGACC	0	1	1	0	0	gnl|UG|At#S35249512	no match found	pseudo chromosome match
at2g28410	CON	MIN	HOUR	DAY	WEEK	UNIGENE	FLCDNA	TAIR
GTGTGGTTGG	0	2	0	1	0	gnl|UG|At#S11735439	gi|26450172|dbj|AK117544.1|	At2g28410.1
at5g09680	CON	MIN	HOUR	DAY	WEEK	UNIGENE	FLCDNA	TAIR
CCTGGGTCAA	0	1	1	1	0	gnl|UG|At#S21737565	gi|34146793|gb|BT010404.1	At5g09680.1

at2g46225	CON	MIN	HOUR	DAY	WEEK	UNIGENE	FLCDNA	TAIR
ATTTTGGTCT	1	2	0	1	1	gnl|UG|At#S43850012	gi|26452760|dbj|AK118876.1|	At2g46225.1
at4g38850	CON	MIN	HOUR	DAY	WEEK	UNIGENE	FLCDNA	TAIR
TTAATACATT	0	0	0	0	1	gnl|UG|At#S30643140	gi|21404105|gb|AY085395.1	At4g38850.1
at5g56180	CON	MIN	HOUR	DAY	WEEK	UNIGENE	FLCDNA	TAIR
TGGTGAATCT	0	0	1	0	0	gnl|UG|At#S18941472	gi|28393341|gb|BT004068.1	At5g56180.2
at1g17120	CON	MIN	HOUR	DAY	WEEK	UNIGENE	FLCDNA	TAIR
CAACCTATGA	1	1	0	0	0	gnl|UG|At#S11743518	gi|21436166|gb|AY117296.1	At1g17120.1
at3g06040	CON	MIN	HOUR	DAY	WEEK	UNIGENE	FLCDNA	TAIR
GAGCTAGCTG	0	2	0	1	0	gnl|UG|At#S18942177	gi|17381069|gb|AY063991.1	At3g06040.1
at4g03150	CON	MIN	HOUR	DAY	WEEK	UNIGENE	FLCDNA	TAIR
GAGCTTTCAC	1	1	1	1	0	no match found	gi|16226696|gb|AF428466.1	At4g03150.1
at1g08020	CON	MIN	HOUR	DAY	WEEK	UNIGENE	FLCDNA	TAIR
AATTAGTGTC	1	1	0	0	0	no match found	no match found	At1g08020.1
at2g19450	CON	MIN	HOUR	DAY	WEEK	UNIGENE	FLCDNA	TAIR
AAACAACTGT	0	1	0	0	0	gnl|UG|At#S11737614	gi|15450799|gb|AY054480.1	At2g19450.1
at3g25545	CON	MIN	HOUR	DAY	WEEK	UNIGENE	FLCDNA	TAIR
TTTCTATGCT	0	2	0	0	0	gnl|UG|At#S11733138	gi|16604419|gb|AY058108.1	At3g25545.1
at2g21710	CON	MIN	HOUR	DAY	WEEK	UNIGENE	FLCDNA	TAIR
TTGGCTTGTA	0	1	0	0	0	gnl|UG|At#S11737036	no match found	At2g21710.1
at1g12760	CON	MIN	HOUR	DAY	WEEK	UNIGENE	FLCDNA	TAIR
ACCCACGACA	1	1	0	0	1	no match found	gi|23198141|gb|BT000279.1	non-canonical match
TCTCAATGAC	0	1	1	0	0	gnl|UG|At#S11741762	gi|22531063|gb|AY136370.1	multiple non-canonical match
at2g44040	CON	MIN	HOUR	DAY	WEEK	UNIGENE	FLCDNA	TAIR
TTTTGACCTC	2	1	1	0	1	gnl|UG|At#S11731624	gi|26452702|dbj|AK118845.1|	At2g44040.1
at1g32870	CON	MIN	HOUR	DAY	WEEK	UNIGENE	FLCDNA	TAIR
GAGAATAACT	0	0	0	0	1	gnl|UG|At#S11703509	gi|14334571|gb|AY034959.1	At1g32870.1
at1g02290	CON	MIN	HOUR	DAY	WEEK	UNIGENE	FLCDNA	TAIR
CTTTCTGGTT	5	3	7	11	15	gnl|UG|At#S24442797	no match found	At1g02280.1
AAAATATGAA	0	1	1	0	0	gnl|UG|At#S20791144	gi|51971824|dbj|AK176814.1|	At1g02290.1
at5g01710	CON	MIN	HOUR	DAY	WEEK	UNIGENE	FLCDNA	TAIR
GATTACACTT	1	0	0	0	0	gnl|UG|At#S11725730	gi|15810368|gb|AY056223.1	At5g01710.1
at4g26760	CON	MIN	HOUR	DAY	WEEK	UNIGENE	FLCDNA	TAIR
ACTAATGTGA	0	1	0	0	0	gnl|UG|At#S11723182	gi|21404140|gb|AY085430.1	At4g26760.1
CTAGATGAAT	0	1	0	0	0	no match found	gi|133778877|gb|BT030366.1	non-canonical match
at2g02850	CON	MIN	HOUR	DAY	WEEK	UNIGENE	FLCDNA	TAIR
GCTGTGTCAG	0	0	0	0	1	gnl|UG|At#S11740555	gi|21403117|gb|AY084407.1	At2g02850.1
at1g55265	CON	MIN	HOUR	DAY	WEEK	UNIGENE	FLCDNA	TAIR
TTCGAAAATG	1	0	0	0	0	gnl|UG|At#S11732555	gi|107738189|gb|BT025768.1	At1g55265.1
at3g18180	CON	MIN	HOUR	DAY	WEEK	UNIGENE	FLCDNA	TAIR
AATCTGAGGT	4	2	6	2	1	gnl|UG|At#S11735385	no match found	multiple canonical match

at1g55360	CON	MIN	HOUR	DAY	WEEK	UNIGENE	FLCDNA	TAIR
TAAGTAGGTG	4	6	8	4	2	gnl|UG|At#S20818586	gi|27311622|gb|BT002417.1	At1g55360.1
at3g51820	CON	MIN	HOUR	DAY	WEEK	UNIGENE	FLCDNA	TAIR
GTGGGCTGGC	0	2	2	5	0	gnl|UG|At#S11729093	gi|20148304|gb|AY081481.1	At3g51820.1
at4g24990	CON	MIN	HOUR	DAY	WEEK	UNIGENE	FLCDNA	TAIR
CACCATTTTG	1	1	0	3	0	gnl|UG|At#S34114700	gi|27765069|gb|BT003091.1	At4g24990.1
at1g72930	CON	MIN	HOUR	DAY	WEEK	UNIGENE	FLCDNA	TAIR
TTGTTTCTAG	3	5	8	4	8	gnl|UG|At#S38434068	gi|17473666|gb|AY065114.1	At1g72930.1
at4g21430	CON	MIN	HOUR	DAY	WEEK	UNIGENE	FLCDNA	TAIR
GAGTTTTGAT	0	0	1	0	0	gnl|UG|At#S11724101	gi|23296295|gb|AY142484.1	multiple non-canonical match
at3g53740	CON	MIN	HOUR	DAY	WEEK	UNIGENE	FLCDNA	TAIR
TTGTTACCAG	3	12	0	1	2	gnl|UG|At#S38433607	gi|21405387|gb|AY086677.1	At3g53740.2
at1g12580	CON	MIN	HOUR	DAY	WEEK	UNIGENE	FLCDNA	TAIR
GAAACTGACC	4	5	6	5	5	gnl|UG|At#S11705227	gi|16323175|gb|AY057692.1	At1g12580.1
at5g53570	CON	MIN	HOUR	DAY	WEEK	UNIGENE	FLCDNA	TAIR
GCTGGTCAGC	0	1	2	1	0	gnl|UG|At#S11718358	gi|62321149|dbj|AK221904.1|	At5g53570.1
at5g11790	CON	MIN	HOUR	DAY	WEEK	UNIGENE	FLCDNA	TAIR
TGTCACCACA	1	2	1	0	1	gnl|UG|At#S11723298	gi|13605683|gb|AF361823.1	At5g11790.1
at1g66090	CON	MIN	HOUR	DAY	WEEK	UNIGENE	FLCDNA	TAIR
TTTTGGGGTC	0	0	1	0	0	gnl|UG|At#S11729909	gi|21407507|gb|AY088733.1	At1g66090.1
at4g17895	CON	MIN	HOUR	DAY	WEEK	UNIGENE	FLCDNA	TAIR
TCGACTTTCT	1	2	0	0	1	gnl|UG|At#S11724697	no match found	pseudo chromosome match
at2g40490	CON	MIN	HOUR	DAY	WEEK	UNIGENE	FLCDNA	TAIR
GTATTAAAGT	1	2	3	3	1	gnl|UG|At#S11732468	gi|14334767|gb|AY035057.1	At2g40490.1
at2g32190	CON	MIN	HOUR	DAY	WEEK	UNIGENE	FLCDNA	TAIR
CAAATTATTT	1	1	0	0	1	gnl|UG|At#S38433896	gi|26451538|dbj|AK118248.1|	At2g32190.1
at1g10120	CON	MIN	HOUR	DAY	WEEK	UNIGENE	FLCDNA	TAIR
AGGATCATCA	1	0	0	0	0	no match found	gi|110738492|dbj|AK229309.1|	At1g10120.1
at4g29740	CON	MIN	HOUR	DAY	WEEK	UNIGENE	FLCDNA	TAIR
TCTTAATTAA	1	0	0	0	1	gnl|UG|At#S15460556	gi|23197941|gb|BT000179.1	At4g29740.2
at2g09850	CON	MIN	HOUR	DAY	WEEK	UNIGENE	FLCDNA	TAIR
GAAGCTGCGA	0	0	1	0	0	no match found	no match found	At2g09850.1
at3g47965	CON	MIN	HOUR	DAY	WEEK	UNIGENE	FLCDNA	TAIR
AGCTCTTACC	1	1	0	1	1	gnl|UG|At#S35255808	gi|22137157|gb|AY133594.1	At3g47965.1
at3g42780	CON	MIN	HOUR	DAY	WEEK	UNIGENE	FLCDNA	TAIR
GGTGAGAATC	1	0	0	0	0	gnl|UG|At#S11730888	no match found	At3g42780.1
at1g50020	CON	MIN	HOUR	DAY	WEEK	UNIGENE	FLCDNA	TAIR
ATGGAAGTAG	4	6	3	3	1	gnl|UG|At#S11734491	gi|13430517|gb|AF360171.1	non-canonical match
at5g55160	CON	MIN	HOUR	DAY	WEEK	UNIGENE	FLCDNA	TAIR
AAACTTATTG	13	13	10	9	4	gnl|UG|At#S11675689	gi|21403901|gb|AY085191.1	At5g55160.1
at4g29810	CON	MIN	HOUR	DAY	WEEK	UNIGENE	FLCDNA	TAIR

TTTTGTGATA	2	3	0	2	4	gnl|UG|At#S43849467	no match found	At4g29810.1
AGAGTGTTTA	0	3	0	0	1	no match found	gi|14326470|gb|AF385688.1	non-canonical match
at2g05830	CON	MIN	HOUR	DAY	WEEK	UNIGENE	FLCDNA	TAIR
AGAAAATCCC	1	2	2	0	0	no match found	gi|20466257|gb|AY099595.1	non-canonical match
GGCTTGCCCG	0	1	0	1	2	gnl|UG|At#S18900572	no match found	At2g05830.2
at3g25530	CON	MIN	HOUR	DAY	WEEK	UNIGENE	FLCDNA	TAIR
CCTGTAGCCG	1	8	3	0	0	gnl|UG|At#S11733141	gi|15146313|gb|AY049298.1	At3g25530.1
at4g15570	CON	MIN	HOUR	DAY	WEEK	UNIGENE	FLCDNA	TAIR
GCCGGAGAAG	0	0	1	1	0	gnl|UG|At#S11725103	gi|26451534|dbj|AK118246.1|	At4g15570.1
at4g30270	CON	MIN	HOUR	DAY	WEEK	UNIGENE	FLCDNA	TAIR
ATCTACAATT	6	59	9	3	3	no match found	no match found	At4g30270.1
at5g59560	CON	MIN	HOUR	DAY	WEEK	UNIGENE	FLCDNA	TAIR
ATTCAAGCTG	0	1	0	0	0	gnl|UG|At#S11717747	no match found	At5g59560.1
at1g17650	CON	MIN	HOUR	DAY	WEEK	UNIGENE	FLCDNA	TAIR
TCAATTGATA	4	1	3	0	3	gnl|UG|At#S11741280	gi|20260471|gb|AY093135.1	At1g17650.1
at3g02110	CON	MIN	HOUR	DAY	WEEK	UNIGENE	FLCDNA	TAIR
CCCGAGAAAA	2	10	5	4	1	gnl|UG|At#S11740045	gi|21406928|gb|AY088154.1	At3g02110.1
at1g68900	CON	MIN	HOUR	DAY	WEEK	UNIGENE	FLCDNA	TAIR
GTTATGATCA	0	0	0	0	1	no match found	no match found	At1g68900.1
at3g06340	CON	MIN	HOUR	DAY	WEEK	UNIGENE	FLCDNA	TAIR
ATTCTGGTGG	0	0	0	0	1	gnl|UG|At#S11738845	gi|62319270|dbj|AK220958.1|	At3g06340.1
at1g20960	CON	MIN	HOUR	DAY	WEEK	UNIGENE	FLCDNA	TAIR
GAAGAATGAT	1	2	3	2	1	gnl|UG|At#S11633473	gi|62319685|dbj|AK221168.1|	multiple non-canonical match
at1g18480	CON	MIN	HOUR	DAY	WEEK	UNIGENE	FLCDNA	TAIR
ACCAACCATA	0	3	1	1	0	gnl|UG|At#S11741204	gi|16226289|gb|AF428293.1	At1g18480.1
at1g62780	CON	MIN	HOUR	DAY	WEEK	UNIGENE	FLCDNA	TAIR
GAGAAGATAC	2	2	3	0	8	no match found	gi|23197935|gb|BT000176.1	multiple canonical match
at1g12850	CON	MIN	HOUR	DAY	WEEK	UNIGENE	FLCDNA	TAIR
CTCCCGCCAC	3	4	4	1	0	gnl|UG|At#S18921763	gi|14517375|gb|AY039522.1	At1g12850.1
at4g34030	CON	MIN	HOUR	DAY	WEEK	UNIGENE	FLCDNA	TAIR
GGAGGCGCTC	1	4	1	0	0	gnl|UG|At#S11721931	gi|14423377|gb|AF386926.1	At4g34030.1
at3g11405	CON	MIN	HOUR	DAY	WEEK	UNIGENE	FLCDNA	TAIR
ATTCAGTAAA	0	0	0	0	1	gnl|UG|At#S35253310	no match found	At3g11405.1
at3g61950	CON	MIN	HOUR	DAY	WEEK	UNIGENE	FLCDNA	TAIR
TCAGCCTTAA	1	0	0	0	0	gnl|UG|At#S15459145	gi|28950876|gb|BT005298.1	At3g61950.2
at1g53590	CON	MIN	HOUR	DAY	WEEK	UNIGENE	FLCDNA	TAIR
TATATGTTTC	0	1	1	1	0	gnl|UG|At#S11733159	gi|15451209|gb|AY054685.1	At1g53590.1
at2g31560	CON	MIN	HOUR	DAY	WEEK	UNIGENE	FLCDNA	TAIR
AGCCAGAAGT	0	2	0	0	0	no match found	gi|50897251|gb|BT015169.1	At2g31560.2
at1g78100	CON	MIN	HOUR	DAY	WEEK	UNIGENE	FLCDNA	TAIR
GACGCTTTTG	0	5	1	2	0	gnl|UG|At#S11727164	gi|15450975|gb|AY054568.1	At1g78100.1

at1g62520	CON	MIN	HOUR	DAY	WEEK	UNIGENE	FLCDNA	TAIR
TCAAGCTACA	0	1	0	0	0	gnl|UG|At#S14273799	gi|28973498|gb|BT005654.1	At1g62520.1
at3g63140	CON	MIN	HOUR	DAY	WEEK	UNIGENE	FLCDNA	TAIR
ATTTCAGCTA	46	23	42	37	13	gnl|UG|At#S11726492	gi|16649034|gb|AY059887.1	At5g53320.1
CATTTCTACG	0	0	1	1	0	no match found	gi|22136251|gb|AY128804.1	non-canonical match
at4g15880	CON	MIN	HOUR	DAY	WEEK	UNIGENE	FLCDNA	TAIR
CCATACTTCC	0	1	0	0	0	gnl|UG|At#S11725052	gi|20148438|gb|AY081548.1	At4g15880.1
at1g04810	CON	MIN	HOUR	DAY	WEEK	UNIGENE	FLCDNA	TAIR
ATTTTGTAAT	1	1	0	1	1	gnl|UG|At#S14273942	gi|28393230|gb|BT004007.1	multiple canonical match
at4g31200	CON	MIN	HOUR	DAY	WEEK	UNIGENE	FLCDNA	TAIR
AGTGCAAGAG	1	0	1	1	1	no match found	gi|38564261|gb|BT010740.1	At4g31190.1
at4g34135	CON	MIN	HOUR	DAY	WEEK	UNIGENE	FLCDNA	TAIR
GAAGAGTTTA	0	0	4	6	1	gnl|UG|At#S11703711	gi|14334981|gb|AY035164.1	At4g34135.1
at5g19820	CON	MIN	HOUR	DAY	WEEK	UNIGENE	FLCDNA	TAIR
CTTTCCTCCT	1	2	1	0	0	gnl|UG|At#S11721367	gi|15293298|gb|AY051083.1	At5g19820.1
at3g44590	CON	MIN	HOUR	DAY	WEEK	UNIGENE	FLCDNA	TAIR
GGATTCAGTC	0	0	1	0	2	no match found	gi|28466810|gb|BT004748.1	At3g44590.2
TTGAATCTGG	4	0	0	0	1	gnl|UG|At#S11730457	no match found	At3g44590.1
at2g32800	CON	MIN	HOUR	DAY	WEEK	UNIGENE	FLCDNA	TAIR
TAATTTATCT	2	1	1	0	0	gnl|UG|At#S11734334	no match found	non-canonical match
GCGGATGTAT	0	0	1	0	0	no match found	gi|13877680|gb|AF370599.1	non-canonical match
GGTCACTGAT	0	0	1	0	0	no match found	no match found	At2g32800.1
at1g27435	CON	MIN	HOUR	DAY	WEEK	UNIGENE	FLCDNA	TAIR
GGAGGAGGAA	0	0	0	1	0	no match found	gi|21407827|gb|AY089053.1	multiple canonical match
at1g66390	CON	MIN	HOUR	DAY	WEEK	UNIGENE	FLCDNA	TAIR
GGGCCACTTT	0	0	0	0	1	gnl|UG|At#S11700704	no match found	At1g66390.1
at5g11620	CON	MIN	HOUR	DAY	WEEK	UNIGENE	FLCDNA	TAIR
TTGAGGATGT	0	1	0	0	0	gnl|UG|At#S11723346	gi|28973392|gb|BT005601.1	pseudo chromosome match
at4g09160	CON	MIN	HOUR	DAY	WEEK	UNIGENE	FLCDNA	TAIR
TCCCGGTTCA	0	2	0	0	0	gnl|UG|At#S11726048	gi|110738241|dbj|AK229180.1|	At4g09160.1
at1g20890	CON	MIN	HOUR	DAY	WEEK	UNIGENE	FLCDNA	TAIR
TTTTTCAGAC	0	0	0	0	1	gnl|UG|At#S11740968	no match found	At1g20890.1
TCCACTGCCT	0	0	1	0	0	no match found	gi|46518372|gb|BT012524.1	non-canonical match
at3g56020	CON	MIN	HOUR	DAY	WEEK	UNIGENE	FLCDNA	TAIR
GAATGATCCT	10	6	14	12	20	no match found	gi|72197542|gb|DQ108751.1	multiple non-canonical match
at1g17550	CON	MIN	HOUR	DAY	WEEK	UNIGENE	FLCDNA	TAIR
GACTTGAATG	1	1	2	1	0	gnl|UG|At#S11741289	gi|26452331|dbj|AK118656.1|	At1g17550.1
at5g27710	CON	MIN	HOUR	DAY	WEEK	UNIGENE	FLCDNA	TAIR
TGCTGAAACA	1	0	2	1	0	gnl|UG|At#S18388569	gi|40823290|gb|BT011237.1	At5g27710.1
at2g31240	CON	MIN	HOUR	DAY	WEEK	UNIGENE	FLCDNA	TAIR
GAGATAGCGC	1	0	0	0	0	gnl|UG|At#S11708373	gi|17380951|gb|AY063932.1	At2g31240.1
at3g13160	CON	MIN	HOUR	DAY	WEEK	UNIGENE	FLCDNA	TAIR
ATTAAAGGAT	1	1	0	0	0	gnl|UG|At#S11815859	gi|27311982|gb|BT002597.1	At3g13160.1

at1g51350	CON	MIN	HOUR	DAY	WEEK	UNIGENE	FLCDNA	TAIR
ATTCGTCTAT	0	0	1	0	1	gnl|UG|At#S11734014	gi|20260481|gb|AY093140.1	At1g51350.1
at1g10290	CON	MIN	HOUR	DAY	WEEK	UNIGENE	FLCDNA	TAIR
AGTCAAATGT	1	4	3	2	0	gnl|UG|At#S11742005	gi|110737888|dbj|AK228995.1|	At1g10290.1
TGTTGGCAGC	1	0	3	0	0	no match found	gi|62319735|dbj|AK221193.1|	multiple non-canonical match
at1g51360	CON	MIN	HOUR	DAY	WEEK	UNIGENE	FLCDNA	TAIR
GAAGAAGCGA	0	2	0	0	0	no match found	gi|51968799|dbj|AK175329.1|	At1g51360.1
at5g51430	CON	MIN	HOUR	DAY	WEEK	UNIGENE	FLCDNA	TAIR
CCAATTCCGC	1	3	2	0	1	gnl|UG|At#S11718573	gi|110737409|dbj|AK228749.1|	At5g51430.1
at3g19010	CON	MIN	HOUR	DAY	WEEK	UNIGENE	FLCDNA	TAIR
TCCAAGACCT	1	6	3	0	0	gnl|UG|At#S18903261	gi|21406898|gb|AY088124.1	At3g19010.1
at4g04210	CON	MIN	HOUR	DAY	WEEK	UNIGENE	FLCDNA	TAIR
TCAACTTGAT	1	1	0	0	0	gnl|UG|At#S11726605	gi|21404043|gb|AY085333.1	At4g04210.1
at3g55420	CON	MIN	HOUR	DAY	WEEK	UNIGENE	FLCDNA	TAIR
GCTCACTGAA	2	0	2	0	0	gnl|UG|At#S11728436	gi|21436134|gb|AY117239.1	At3g55420.1
atcg00500	CON	MIN	HOUR	DAY	WEEK	UNIGENE	FLCDNA	TAIR
CTTTTTTTCC	0	0	1	0	0	no match found	no match found	AtCg00500
at2g33510	CON	MIN	HOUR	DAY	WEEK	UNIGENE	FLCDNA	TAIR
TGAGTGGCAT	1	1	0	1	0	no match found	gi|28393657|gb|BT004232.1	At2g33510.1
at3g10740	CON	MIN	HOUR	DAY	WEEK	UNIGENE	FLCDNA	TAIR
TCAAAAGTCT	5	4	6	3	0	gnl|UG|At#S11737666	gi|62321673|dbj|AK222175.1|	At3g10740.1
at4g12917	CON	MIN	HOUR	DAY	WEEK	UNIGENE	FLCDNA	TAIR
TCTTCTCATT	0	0	0	0	1	gnl|UG|At#S18905815	no match found	pseudo chromosome match
at1g10390	CON	MIN	HOUR	DAY	WEEK	UNIGENE	FLCDNA	TAIR
GACGAGAGCA	0	3	0	0	0	no match found	gi|27764947|gb|BT003030.1	multiple canonical match
at3g44510	CON	MIN	HOUR	DAY	WEEK	UNIGENE	FLCDNA	TAIR
AGGTCCCTAA	0	2	0	0	0	gnl|UG|At#S21736373	gi|89111893|gb|BT024836.1	At3g44510.1
at1g06110	CON	MIN	HOUR	DAY	WEEK	UNIGENE	FLCDNA	TAIR
AGAACAGATT	1	0	1	1	1	gnl|UG|At#S11742416	gi|16930490|gb|AF419599.1	At1g06110.1
CAATGTCCTA	0	1	0	0	1	no match found	gi|20466210|gb|AY099571.1	non-canonical match
at5g57240	CON	MIN	HOUR	DAY	WEEK	UNIGENE	FLCDNA	TAIR
AGAAGTGTAA	1	2	0	0	0	no match found	gi|26449626|dbj|AK117264.1|	At5g57240.1
at5g48200	CON	MIN	HOUR	DAY	WEEK	UNIGENE	FLCDNA	TAIR
TGAAGGGCAA	0	0	1	0	0	no match found	no match found	At5g48200.1
at2g37870	CON	MIN	HOUR	DAY	WEEK	UNIGENE	FLCDNA	TAIR
ATCTTATGAG	0	0	0	0	1	gnl|UG|At#S11752687	gi|15451109|gb|AY054635.1	At2g37870.1
at1g17560	CON	MIN	HOUR	DAY	WEEK	UNIGENE	FLCDNA	TAIR
GTTCCAAGAG	0	1	0	0	0	gnl|UG|At#S11741288	gi|28973234|gb|BT005522.1	At1g17560.1
at1g06680	CON	MIN	HOUR	DAY	WEEK	UNIGENE	FLCDNA	TAIR
GTCACTCCTA	76	230	99	55	8	gnl|UG|At#S11742363	gi|15912276|gb|AY056416.1	At1g06680.1
at5g66450	CON	MIN	HOUR	DAY	WEEK	UNIGENE	FLCDNA	TAIR

CCCAATCCAT	1	0	0	0	1	gnl|UG|At#S21736914	gi|34146857|gb|BT010436.1	At5g66450.1	
at4g18340	CON	MIN	HOUR	DAY	WEEK	UNIGENE	FLCDNA	TAIR	
ATGTATGCTC	0	1	0	0	0	no match found	gi|21539538|gb|AY120764.1	At4g18340.1	
at3g11290	CON	MIN	HOUR	DAY	WEEK	UNIGENE	FLCDNA	TAIR	
TGTTTTCTCT	0	0	0	1	0	gnl|UG|At#S43849756	no match found	multiple non-canonical match	
GAAGAGGTTG	0	2	0	0	0	gnl|UG|At#S11737503	no match found	At3g11290.1	
at1g01490	CON	MIN	HOUR	DAY	WEEK	UNIGENE	FLCDNA	TAIR	
AGGATGAAGA	6	5	7	5	0	gnl|UG|At#S18925701	no match found	At1g01490.1	
at3g08640	CON	MIN	HOUR	DAY	WEEK	UNIGENE	FLCDNA	TAIR	
AATATAATAA	0	1	0	0	11	gnl|UG|At#S11738307	gi|19698960|gb|AY081327.1	At3g08640.1	
at2g28315	CON	MIN	HOUR	DAY	WEEK	UNIGENE	FLCDNA	TAIR	
AAGCCAAGAA	0	0	1	0	0	gnl|UG|At#S15461050	no match found	At2g28315.1	
at4g01890	CON	MIN	HOUR	DAY	WEEK	UNIGENE	FLCDNA	TAIR	
TCTATGGCGG	0	1	0	0	0	no match found	no match found	At4g01890.1	
at1g69840	CON	MIN	HOUR	DAY	WEEK	UNIGENE	FLCDNA	TAIR	
GTGTTTGGGA	0	4	3	0	0	gnl|UG|At#S18942290	gi|20466747|gb|AY099840.1	At1g69840.1	
at1g08465	CON	MIN	HOUR	DAY	WEEK	UNIGENE	FLCDNA	TAIR	
TATGTATATA	1	0	0	0	1	gnl|UG|At#S38434394	gi|28393670|gb|BT004243.1	multiple canonical match	
at3g05545	CON	MIN	HOUR	DAY	WEEK	UNIGENE	FLCDNA	TAIR	
CTCAAGTTAT	2	2	3	1	0	gnl|UG|At#S11704585	gi|15027848|gb|AY045781.1	At3g05545.1	
at3g04350	CON	MIN	HOUR	DAY	WEEK	UNIGENE	FLCDNA	TAIR	
AGCAATATGA	2	6	3	3	1	gnl|UG|At#S11739408	gi|15450690|gb|AY052713.1	At3g04350.1	
at2g41500	CON	MIN	HOUR	DAY	WEEK	UNIGENE	FLCDNA	TAIR	
TTTGTGCAAG	0	0	2	0	0	gnl|UG|At#S15644821	gi|30794127|gb|BT008700.1	At2g41500.1	
at1g54440	CON	MIN	HOUR	DAY	WEEK	UNIGENE	FLCDNA	TAIR	
AATATGAAAC	4	0	0	0	0	gnl|UG|At#S34115214	gi|110739342|dbj|AK229747.1|	At1g54440.1	
at4g02040	CON	MIN	HOUR	DAY	WEEK	UNIGENE	FLCDNA	TAIR	
GGATTCAAAT	0	0	2	0	0	gnl|UG|At#S11727172	no match found	At4g02040.1	
at3g05625	CON	MIN	HOUR	DAY	WEEK	UNIGENE	FLCDNA	TAIR	
AATAAAGAAA	1	0	0	0	1	gnl|UG|At#S11816255	gi|27311658|gb|BT002435.1	At3g05625.1	
at5g41960	CON	MIN	HOUR	DAY	WEEK	UNIGENE	FLCDNA	TAIR	
TTCAGCTTGA	0	2	0	0	0	gnl|UG|At#S18908968	gi|32815932|gb|BT009717.1	At5g41960.1	
at4g07990	CON	MIN	HOUR	DAY	WEEK	UNIGENE	FLCDNA	TAIR	
CGCTCTAAAG	1	3	1	1	0	gnl|UG|At#S11726161	gi|21403046|gb|AY084336.1	At4g07990.1	
AATAATTCAG	2	1	0	2	1	gnl|UG|At#S20707615	gi|62321663|dbj|AK222170.1|	At4g07990.1	
at1g12300	CON	MIN	HOUR	DAY	WEEK	UNIGENE	FLCDNA	TAIR	
TAAATCTATC	0	0	0	0	1	gnl|UG|At#S34115489	gi|110741410|dbj|AK230459.1|	non-canonical match	
at2g45980	CON	MIN	HOUR	DAY	WEEK	UNIGENE	FLCDNA	TAIR	
GAATCATTCG	0	0	1	0	0	no match found	gi|17065597|gb|AY062947.1	non-canonical match	
TGTGCGTGAT	2	6	1	4	0	gnl|UG|At#S11731192	gi|13877854|gb|AF370190.1	At2g45980.1	
at1g78310	CON	MIN	HOUR	DAY	WEEK	UNIGENE	FLCDNA	TAIR	
TTAGAAACAA	1	0	0	1	0	gnl|UG|At#S11727102	gi|17529351|gb|AY065462.1	At1g78310.1	

CGGTATCTCC	0	1	0	0	0	no match found	gi|21436408|gb|AY117330.1	non-canonical match
at5g42370	CON	MIN	HOUR	DAY	WEEK	UNIGENE	FLCDNA	TAIR
GATTTTGATC	0	0	1	0	0	gnl|UG|At#S11702145	gi|13430795|gb|AF360310.1	At5g42370.1
at5g14880	CON	MIN	HOUR	DAY	WEEK	UNIGENE	FLCDNA	TAIR
GTGGCTCCTT	1	0	0	1	0	gnl|UG|At#S11722621	no match found	non-canonical match
at1g27385	CON	MIN	HOUR	DAY	WEEK	UNIGENE	FLCDNA	TAIR
CTATGGTTGT	0	1	0	2	0	gnl|UG|At#S34117694	gi|26451019|dbj|AK117980.1|	At1g27385.1
at5g03900	CON	MIN	HOUR	DAY	WEEK	UNIGENE	FLCDNA	TAIR
GAATCGCTGT	0	1	0	0	0	no match found	gi|30387512|gb|BT006578.1	non-canonical match
GCACATACTG	0	2	0	0	1	gnl|UG|At#S11808324	gi|26453261|dbj|AK119134.1|	At5g03900.2
at4g30690	CON	MIN	HOUR	DAY	WEEK	UNIGENE	FLCDNA	TAIR
TAATTGTAAC	8	11	2	1	0	gnl|UG|At#S18907668	gi|14596120|gb|AY042848.1	At4g30690.1
at1g66580	CON	MIN	HOUR	DAY	WEEK	UNIGENE	FLCDNA	TAIR
GTCCATTGGC	3	1	2	2	0	no match found	gi|15777884|gb|AY055103.1	non-canonical match
TGTTTCTTAC	14	6	22	19	38	gnl|UG|At#S18923014	gi|21406150|gb|AY087426.1	At1g66580.1
at3g55070	CON	MIN	HOUR	DAY	WEEK	UNIGENE	FLCDNA	TAIR
TTTGGCTGCT	0	1	0	1	1	gnl|UG|At#S38433595	gi|15215685|gb|AY050371.1	At3g55070.1
at5g24450	CON	MIN	HOUR	DAY	WEEK	UNIGENE	FLCDNA	TAIR
GAGAACAACT	1	1	1	0	0	no match found	no match found	At5g24450.1
at5g47760	CON	MIN	HOUR	DAY	WEEK	UNIGENE	FLCDNA	TAIR
ATTTCATTTT	5	13	5	5	1	no match found	gi|26452300|dbj|AK118640.1|	At1g49990.1
at3g04800	CON	MIN	HOUR	DAY	WEEK	UNIGENE	FLCDNA	TAIR
CTCCACCACT	0	0	1	0	0	no match found	no match found	At3g04800.1
at5g02490	CON	MIN	HOUR	DAY	WEEK	UNIGENE	FLCDNA	TAIR
TCAGATTCAC	0	0	0	3	0	gnl|UG|At#S11725538	gi|110742446|dbj|AK227090.1|	At5g02490.1
at2g35360	CON	MIN	HOUR	DAY	WEEK	UNIGENE	FLCDNA	TAIR
ATCAATGGTC	0	1	1	1	0	gnl|UG|At#S14233078	no match found	At2g35360.1
at5g51700	CON	MIN	HOUR	DAY	WEEK	UNIGENE	FLCDNA	TAIR
TTCTTCTTAT	0	0	0	0	1	gnl|UG|At#S11718546	gi|51969989|dbj|AK175924.1|	At5g51700.1
at3g05070	CON	MIN	HOUR	DAY	WEEK	UNIGENE	FLCDNA	TAIR
TAAAATCCTA	2	0	0	2	3	gnl|UG|At#S11739201	gi|21404000|gb|AY085290.1	At3g05070.1
at5g12290	CON	MIN	HOUR	DAY	WEEK	UNIGENE	FLCDNA	TAIR
TTGAGCAACC	0	0	0	0	1	no match found	gi|27311688|gb|BT002450.1	non-canonical match
ATCTATGTTG	0	0	0	1	1	gnl|UG|At#S11723175	gi|26452570|dbj|AK118777.1|	At5g12290.1
at3g24255	CON	MIN	HOUR	DAY	WEEK	UNIGENE	FLCDNA	TAIR
TTGGTTCGGT	0	0	0	0	1	no match found	gi|62321783|dbj|AK222230.1|	non-canonical match
at5g40340	CON	MIN	HOUR	DAY	WEEK	UNIGENE	FLCDNA	TAIR
TGGCTTTCTT	0	2	3	0	2	gnl|UG|At#S11719696	no match found	At5g40340.1
GGAAAATGAA	4	9	4	2	4	gnl|UG|At#S11832177	gi|16604542|gb|AY058891.1	non-canonical match
at1g04555	CON	MIN	HOUR	DAY	WEEK	UNIGENE	FLCDNA	TAIR
AGGTTTTCGT	0	0	0	1	0	gnl|UG|At#S38434427	no match found	non-canonical match
at5g04630	CON	MIN	HOUR	DAY	WEEK	UNIGENE	FLCDNA	TAIR

GTTAGTTTCA	0	0	0	1	0	no match found	no match found	At5g04630.1
at1g71800	CON	MIN	HOUR	DAY	WEEK	UNIGENE	FLCDNA	TAIR
ATGCAGCCTT	1	1	0	2	0	gnl|UG|At#S11728730	gi|110738119|dbj|AK229116.1|	At1g71800.1
at5g57040	CON	MIN	HOUR	DAY	WEEK	UNIGENE	FLCDNA	TAIR
GTTATGGGTA	2	7	1	1	3	gnl|UG|At#S11718009	gi|21407273|gb|AY088499.1	At5g57040.1
at5g45550	CON	MIN	HOUR	DAY	WEEK	UNIGENE	FLCDNA	TAIR
TTAGTGATGG	0	1	0	0	0	gnl|UG|At#S11719172	gi|62321711|dbj|AK222194.1|	non-canonical match
AATTTGGACT	3	3	2	0	4	no match found	gi|21405440|gb|AY086730.1	At5g45550.1
at3g19515	CON	MIN	HOUR	DAY	WEEK	UNIGENE	FLCDNA	TAIR
TCACGGGACG	1	2	4	0	1	gnl|UG|At#S21736929	gi|51969525|dbj|AK175692.1|	At3g19515.1
at5g67330	CON	MIN	HOUR	DAY	WEEK	UNIGENE	FLCDNA	TAIR
GATAGTAGCT	1	3	1	1	0	no match found	gi|28059554|gb|BT003406.1	non-canonical match
AAGTTCCTTC	1	0	0	2	2	gnl|UG|At#S11815863	gi|22531256|gb|AY136467.1	At5g67330.1
at4g21150	CON	MIN	HOUR	DAY	WEEK	UNIGENE	FLCDNA	TAIR
AGTTTCTTAA	1	0	1	3	3	gnl|UG|At#S11724149	no match found	At4g21150.1
AGTCACAGGT	0	5	2	0	0	no match found	gi|18958021|gb|AY079034.1	non-canonical match
at3g25590	CON	MIN	HOUR	DAY	WEEK	UNIGENE	FLCDNA	TAIR
GAGCTGATTT	0	0	2	3	0	gnl|UG|At#S11733121	gi|27311724|gb|BT002468.1	At3g25590.1
at3g42200	CON	MIN	HOUR	DAY	WEEK	UNIGENE	FLCDNA	TAIR
GATAAAATGG	0	0	1	0	0	no match found	no match found	At3g42200.1
at3g27080	CON	MIN	HOUR	DAY	WEEK	UNIGENE	FLCDNA	TAIR
GATACGGAAA	0	1	0	0	0	gnl|UG|At#S11732606	gi|21405761|gb|AY087037.1	At3g27080.1
at1g09280	CON	MIN	HOUR	DAY	WEEK	UNIGENE	FLCDNA	TAIR
GATTTAGGCA	0	4	0	0	1	gnl|UG|At#S11742110	gi|15081790|gb|AY048288.1	non-canonical match
at4g09080	CON	MIN	HOUR	DAY	WEEK	UNIGENE	FLCDNA	TAIR
CCCTCTCTAT	0	0	0	1	1	no match found	no match found	At4g09080.1
at1g36390	CON	MIN	HOUR	DAY	WEEK	UNIGENE	FLCDNA	TAIR
TTTTACGATT	0	0	1	0	0	no match found	gi|21406824|gb|AY088050.1	At1g36390.1
AATCAAAGCA	0	0	0	0	1	gnl|UG|At#S15460110	no match found	At1g36390.2
at5g24240	CON	MIN	HOUR	DAY	WEEK	UNIGENE	FLCDNA	TAIR
TTCCAAAAAA	2	0	2	2	0	gnl|UG|At#S35243957	no match found	pseudo chromosome match
at3g51140	CON	MIN	HOUR	DAY	WEEK	UNIGENE	FLCDNA	TAIR
TGACAGAGAG	4	3	2	4	4	no match found	gi|21405367|gb|AY086657.1	multiple non-canonical match
GCCCTCTTAT	0	0	2	0	0	gnl|UG|At#S11729221	no match found	At3g51140.1
at4g06599	CON	MIN	HOUR	DAY	WEEK	UNIGENE	FLCDNA	TAIR
CAAATCGTGA	1	2	0	0	1	gnl|UG|At#S15461464	no match found	At4g06599.1
at4g26850	CON	MIN	HOUR	DAY	WEEK	UNIGENE	FLCDNA	TAIR
GAGGTCCGAT	9	4	19	13	5	gnl|UG|At#S11706157	gi|30387534|gb|BT006589.1	At4g26850.1
at1g75220	CON	MIN	HOUR	DAY	WEEK	UNIGENE	FLCDNA	TAIR
TATATCAACA	2	3	1	0	3	gnl|UG|At#S11727996	gi|15724239|gb|AF412060.1	At1g75220.1
at3g02230	CON	MIN	HOUR	DAY	WEEK	UNIGENE	FLCDNA	TAIR
GAGCTGTCCA	1	3	0	4	3	gnl|UG|At#S11700584	gi|27311606|gb|BT002409.1	At3g02230.1

at5g11390	CON	MIN	HOUR	DAY	WEEK	UNIGENE	FLCDNA	TAIR
TAAGAGTCTT	1	0	0	0	0	gnl|UG|At#S11723407	gi|22530925|gb|AY136301.1	At5g11390.1
at5g36800	CON	MIN	HOUR	DAY	WEEK	UNIGENE	FLCDNA	TAIR
GTAGCCACAG	0	1	0	0	0	gnl|UG|At#S34114318	gi|62318561|dbj|AK220601.1|	At5g36710.1
at5g49945	CON	MIN	HOUR	DAY	WEEK	UNIGENE	FLCDNA	TAIR
TAGCTTACAG	0	0	0	1	0	gnl|UG|At#S11718722	no match found	At5g49945.1
at4g02500	CON	MIN	HOUR	DAY	WEEK	UNIGENE	FLCDNA	TAIR
GGTCCTAAAG	0	3	1	0	0	no match found	gi|30387558|gb|BT006601.1	non-canonical match
GATCAATAAT	1	1	2	1	4	gnl|UG|At#S11727061	gi|22655159|gb|AY140029.1	At4g02500.1
at5g55940	CON	MIN	HOUR	DAY	WEEK	UNIGENE	FLCDNA	TAIR
CAAACGAGAG	1	2	2	0	0	no match found	gi|110741033|dbj|AK226469.1|	At5g51620.2
at2g18740	CON	MIN	HOUR	DAY	WEEK	UNIGENE	FLCDNA	TAIR
AATCTAGTTT	0	1	1	1	3	gnl|UG|At#S11737794	gi|28973514|gb|BT005662.1	At2g18740.1
at3g29639	CON	MIN	HOUR	DAY	WEEK	UNIGENE	FLCDNA	TAIR
TATGCGTCAC	0	0	0	1	0	gnl|UG|At#S38433663	no match found	non-canonical match
at1g21700	CON	MIN	HOUR	DAY	WEEK	UNIGENE	FLCDNA	TAIR
TTTGGCAACA	2	0	0	1	0	gnl|UG|At#S11807201	gi|20259453|gb|AY091026.1	At1g21700.1
at3g49580	CON	MIN	HOUR	DAY	WEEK	UNIGENE	FLCDNA	TAIR
TATATAGATC	3	3	2	1	0	gnl|UG|At#S18320326	gi|26450287|dbj|AK117604.1|	At3g49580.1
at5g56860	CON	MIN	HOUR	DAY	WEEK	UNIGENE	FLCDNA	TAIR
GCTCTGTCGT	2	3	2	0	1	gnl|UG|At#S11718026	gi|57222163|gb|BT020488.1	At5g56860.1
at5g46730	CON	MIN	HOUR	DAY	WEEK	UNIGENE	FLCDNA	TAIR
AGGAAACCAA	0	0	0	1	0	gnl|UG|At#S15644906	gi|30793974|gb|BT008615.1	At5g46730.1
at5g42250	CON	MIN	HOUR	DAY	WEEK	UNIGENE	FLCDNA	TAIR
AGATGAAATT	1	0	0	0	0	no match found	gi|22136297|gb|AY128827.1	non-canonical match
TTTAACTATT	0	0	0	0	2	gnl|UG|At#S11719504	no match found	At5g42250.1
at5g06950	CON	MIN	HOUR	DAY	WEEK	UNIGENE	FLCDNA	TAIR
TTGTTTTAGT	0	1	0	1	1	gnl|UG|At#S11824130	gi|26450447|dbj|AK117686.1|	At5g06950.1
at5g52820	CON	MIN	HOUR	DAY	WEEK	UNIGENE	FLCDNA	TAIR
CAGATGAGGT	0	2	2	1	1	gnl|UG|At#S11718435	gi|28416554|gb|BT004562.1	At5g52820.1
at2g25850	CON	MIN	HOUR	DAY	WEEK	UNIGENE	FLCDNA	TAIR
ATCTCTTTAT	0	1	0	0	0	no match found	gi|14532699|gb|AY039974.1	At2g25850.2
AGGGTTTTGT	0	2	0	1	0	no match found	no match found	At2g25850.1
CATTGTTCGA	0	3	1	0	0	gnl|UG|At#S28282264	gi|110739983|dbj|AK230076.1|	At2g25850.1
at3g14980	CON	MIN	HOUR	DAY	WEEK	UNIGENE	FLCDNA	TAIR
AGTACCCAAG	0	0	0	2	0	gnl|UG|At#S11736350	no match found	non-canonical match
at2g36310	CON	MIN	HOUR	DAY	WEEK	UNIGENE	FLCDNA	TAIR
GATCAAGGCC	0	1	0	0	0	gnl|UG|At#S11733514	gi|17978870|gb|AY066040.1	At2g36310.1
at2g35605	CON	MIN	HOUR	DAY	WEEK	UNIGENE	FLCDNA	TAIR
GACTCCTTCA	0	0	0	1	0	no match found	no match found	At2g35605.1
at2g43130	CON	MIN	HOUR	DAY	WEEK	UNIGENE	FLCDNA	TAIR
GAGACCTCTG	0	2	0	0	0	gnl|UG|At#S15644853	gi|110738856|dbj|AK229494.1|	At2g43130.1

at4g21630	CON	MIN	HOUR	DAY	WEEK	UNIGENE	FLCDNA	TAIR
ATCTCATAAT	1	0	0	0	1	no match found	no match found	At4g21630.1
at3g52340	CON	MIN	HOUR	DAY	WEEK	UNIGENE	FLCDNA	TAIR
AGTGAATTGG	3	6	3	1	2	gnl|UG|At#S21737280	gi|51968931|dbj|AK175395.1|	At3g52340.1
at3g15610	CON	MIN	HOUR	DAY	WEEK	UNIGENE	FLCDNA	TAIR
GTCCGGTTCA	0	4	0	0	0	gnl|UG|At#S11736157	gi|28059686|gb|BT003420.1	At3g15610.1
at1g50710	CON	MIN	HOUR	DAY	WEEK	UNIGENE	FLCDNA	TAIR
ACTGATCACT	1	0	0	0	0	gnl|UG|At#S11734254	gi|28973208|gb|BT005509.1	At1g50710.1
at4g00570	CON	MIN	HOUR	DAY	WEEK	UNIGENE	FLCDNA	TAIR
TGGTTCCCTG	1	4	0	2	1	gnl|UG|At#S11727532	gi|23197733|gb|BT000075.1	At4g00570.1
at3g19800	CON	MIN	HOUR	DAY	WEEK	UNIGENE	FLCDNA	TAIR
ACTTCTATCG	1	1	0	1	0	gnl|UG|At#S43849880	gi|13430475|gb|AF360150.1	non-canonical match
at1g36070	CON	MIN	HOUR	DAY	WEEK	UNIGENE	FLCDNA	TAIR
GGAGCCATCA	0	1	0	0	0	no match found	gi|111074417|gb|BT026475.1	At1g36070.1
CCGTCTCTGG	0	0	1	0	0	gnl|UG|At#S11709603	gi|18086386|gb|AY065006.1	non-canonical match
at5g53190	CON	MIN	HOUR	DAY	WEEK	UNIGENE	FLCDNA	TAIR
ATGACAAGAA	0	1	0	2	0	no match found	gi|34146817|gb|BT010416.1	multiple non-canonical match
at2g15090	CON	MIN	HOUR	DAY	WEEK	UNIGENE	FLCDNA	TAIR
GGAAGACTGT	0	6	2	1	0	gnl|UG|At#S11664354	gi|110737951|dbj|AK229027.1|	multiple canonical match
at5g57580	CON	MIN	HOUR	DAY	WEEK	UNIGENE	FLCDNA	TAIR
GGAGGAGGAG	0	0	1	0	1	no match found	gi|15810500|gb|AY056289.1	multiple non-canonical match
TCTCCTTGGT	0	1	2	2	2	gnl|UG|At#S11717956	no match found	At5g57580.1
at2g45680	CON	MIN	HOUR	DAY	WEEK	UNIGENE	FLCDNA	TAIR
GCGATGGCGG	0	0	13	0	0	no match found	gi|62320545|dbj|AK221599.1|	At1g15825.1
GTCCACTCAA	0	0	1	2	0	gnl|UG|At#S11706053	gi|15810350|gb|AY056214.1	At2g45680.1
at2g47020	CON	MIN	HOUR	DAY	WEEK	UNIGENE	FLCDNA	TAIR
AGAAAAAGAT	1	1	1	0	0	no match found	gi|16930397|gb|AF419552.1	non-canonical match
at5g53160	CON	MIN	HOUR	DAY	WEEK	UNIGENE	FLCDNA	TAIR
TAATTCATCA	0	0	1	0	0	gnl|UG|At#S18911607	no match found	non-canonical match
TGTCTAATTT	1	9	2	1	1	gnl|UG|At#S20803442	gi|110736601|dbj|AK228324.1|	At5g53160.1
at3g42353	CON	MIN	HOUR	DAY	WEEK	UNIGENE	FLCDNA	TAIR
GAAAAATCAA	0	1	1	0	0	no match found	no match found	At3g42353.1
at1g50110	CON	MIN	HOUR	DAY	WEEK	UNIGENE	FLCDNA	TAIR
GGACTTGTAA	0	0	0	1	0	no match found	gi|110742292|dbj|AK227007.1|	At1g50110.1
at5g24690	CON	MIN	HOUR	DAY	WEEK	UNIGENE	FLCDNA	TAIR
AAAAAGGTTT	3	3	5	5	2	gnl|UG|At#S11720706	gi|110739957|dbj|AK230063.1|	At5g24690.1
at5g59860	CON	MIN	HOUR	DAY	WEEK	UNIGENE	FLCDNA	TAIR
TCTTTTTTTT	1	1	0	1	0	gnl|UG|At#S24442014	gi|62321622|dbj|AK222149.1|	At4g10000.2
at1g28520	CON	MIN	HOUR	DAY	WEEK	UNIGENE	FLCDNA	TAIR
ACTCGTATCT	0	1	2	1	0	gnl|UG|At#S11739440	gi|56236051|gb|BT020261.1	At1g28520.1
at5g03420	CON	MIN	HOUR	DAY	WEEK	UNIGENE	FLCDNA	TAIR
CTTAAACCGT	2	0	0	0	2	no match found	no match found	At5g03420.1

at1g08000	CON	MIN	HOUR	DAY	WEEK	UNIGENE	FLCDNA	TAIR
ATTCGCAGTG	1	1	0	0	0	gnl|UG|At#S18942616	gi|17380993|gb|AY063953.1	At1g08000.1
at2g04700	CON	MIN	HOUR	DAY	WEEK	UNIGENE	FLCDNA	TAIR
TTCATCACCT	9	7	2	5	3	gnl|UG|At#S11708524	no match found	At2g04700.1
at4g24060	CON	MIN	HOUR	DAY	WEEK	UNIGENE	FLCDNA	TAIR
GTGCTAATTA	0	1	0	1	0	no match found	gi|21406424|gb|AY087686.1	non-canonical match
at2g42560	CON	MIN	HOUR	DAY	WEEK	UNIGENE	FLCDNA	TAIR
GACAACGTTG	1	0	1	0	0	gnl|UG|At#S34116645	gi|110739181|dbj|AK229663.1|	At2g42560.1
at1g09920	CON	MIN	HOUR	DAY	WEEK	UNIGENE	FLCDNA	TAIR
AAACCAAATG	0	1	0	0	0	no match found	gi|23197977|gb|BT000197.1	multiple non-canonical match
AGACTTGCTT	1	1	0	2	3	gnl|UG|At#S11742042	gi|21406988|gb|AY088214.1	At1g09920.1
at4g17250	CON	MIN	HOUR	DAY	WEEK	UNIGENE	FLCDNA	TAIR
ATAAAACCTA	0	2	1	0	0	gnl|UG|At#S11724806	gi|16323154|gb|AY057681.1	At1g09920.1
at2g02820	CON	MIN	HOUR	DAY	WEEK	UNIGENE	FLCDNA	TAIR
AATTCTTGAT	0	1	0	1	0	gnl|UG|At#S34115442	gi|110741488|dbj|AK226575.1|	non-canonical match
at2g02120	CON	MIN	HOUR	DAY	WEEK	UNIGENE	FLCDNA	TAIR
ACTCAATCGT	0	0	0	0	1	gnl|UG|At#S11740668	gi|26450992|dbj|AK117966.1|	At2g02120.1
at1g07400	CON	MIN	HOUR	DAY	WEEK	UNIGENE	FLCDNA	TAIR
TTTATGCGTA	0	1	0	0	0	gnl|UG|At#S11742294	gi|21405566|gb|AY086856.1	At1g07400.1
at3g21610	CON	MIN	HOUR	DAY	WEEK	UNIGENE	FLCDNA	TAIR
CTGGTCGTCA	1	1	0	0	0	gnl|UG|At#S28282935	no match found	At3g21610.1
at3g53020	CON	MIN	HOUR	DAY	WEEK	UNIGENE	FLCDNA	TAIR
TACAGAAAGC	4	10	4	4	4	gnl|UG|At#S11728870	gi|30102869|gb|BT006545.1	At3g53020.1
at3g62430	CON	MIN	HOUR	DAY	WEEK	UNIGENE	FLCDNA	TAIR
AGAGAACTGA	0	1	0	0	0	no match found	no match found	At3g62430.1
at5g59800	CON	MIN	HOUR	DAY	WEEK	UNIGENE	FLCDNA	TAIR
GTCCGAGGCA	1	0	0	0	0	gnl|UG|At#S11717723	gi|50198784|gb|BT014975.1	At5g59800.1
at2g03690	CON	MIN	HOUR	DAY	WEEK	UNIGENE	FLCDNA	TAIR
TGTGTGTACT	1	0	0	0	0	gnl|UG|At#S11740428	gi|21404540|gb|AY085830.1	At2g03690.1
at1g14345	CON	MIN	HOUR	DAY	WEEK	UNIGENE	FLCDNA	TAIR
TTCGAAAAAA	0	0	1	0	0	no match found	gi|15292966|gb|AY050917.1	pseudo chromosome match
TTCTTTCATT	8	2	7	1	2	gnl|UG|At#S11741602	gi|21404580|gb|AY085870.1	At1g14345.1
at5g06300	CON	MIN	HOUR	DAY	WEEK	UNIGENE	FLCDNA	TAIR
TGGGTAACTG	0	0	1	0	1	gnl|UG|At#S11724550	gi|62318684|dbj|AK220662.1|	At5g06300.1
at1g67800	CON	MIN	HOUR	DAY	WEEK	UNIGENE	FLCDNA	TAIR
AAAAAACCAC	0	0	1	1	0	gnl|UG|At#S11729548	gi|110743197|dbj|AK227489.1|	At1g67800.1
at4g04900	CON	MIN	HOUR	DAY	WEEK	UNIGENE	FLCDNA	TAIR
TGGCTCATAT	0	1	0	0	0	gnl|UG|At#S11726412	no match found	non-canonical match
at4g03120	CON	MIN	HOUR	DAY	WEEK	UNIGENE	FLCDNA	TAIR
AAAAGGAAAA	0	0	0	3	0	no match found	gi|110743940|dbj|AK227824.1|	At4g03120.1
at3g15380	CON	MIN	HOUR	DAY	WEEK	UNIGENE	FLCDNA	TAIR
TTGGGCATTG	0	1	1	0	0	gnl|UG|At#S11736224	gi|25055019|gb|BT001974.1	At3g15380.1

at2g38810	CON	MIN	HOUR	DAY	WEEK	UNIGENE	FLCDNA	TAIR
ATTGAGGATG	0	0	1	0	1	gnl|UG|At#S15460637	gi|62319093|dbj|AK220868.1|	At2g38810.1
at2g45700	CON	MIN	HOUR	DAY	WEEK	UNIGENE	FLCDNA	TAIR
ATTATGTAGT	1	4	2	2	0	gnl|UG|At#S11731258	gi|110736828|dbj|AK228444.1|	At2g45700.1
at3g48690	CON	MIN	HOUR	DAY	WEEK	UNIGENE	FLCDNA	TAIR
CATAAATTCT	5	6	15	7	1	gnl|UG|At#S11729699	gi|110742005|dbj|AK226849.1|	non-canonical match
at2g23120	CON	MIN	HOUR	DAY	WEEK	UNIGENE	FLCDNA	TAIR
GCGACGGGTA	7	21	38	35	22	gnl|UG|At#S17006756	gi|23297117|gb|AY142639.1	At2g23120.1
at1g43790	CON	MIN	HOUR	DAY	WEEK	UNIGENE	FLCDNA	TAIR
TTCTTGGCGT	0	1	0	0	0	no match found	gi|88011141|gb|BT024569.1	At1g43790.1
at1g53510	CON	MIN	HOUR	DAY	WEEK	UNIGENE	FLCDNA	TAIR
ACAAACCCGG	0	0	3	2	0	gnl|UG|At#S11733181	gi|13507572|gb|AF360353.1	At1g53510.1
at5g09600	CON	MIN	HOUR	DAY	WEEK	UNIGENE	FLCDNA	TAIR
AAATTTGTCA	4	11	5	3	3	gnl|UG|At#S38433332	no match found	At5g09600.1
CTATCGATTT	0	5	1	0	0	gnl|UG|At#S18906676	gi|33589713|gb|BT010154.1	At4g32210.1
atcg01040	CON	MIN	HOUR	DAY	WEEK	UNIGENE	FLCDNA	TAIR
GGGTTCATAT	1	0	0	0	1	no match found	no match found	AtCg01040
at4g20910	CON	MIN	HOUR	DAY	WEEK	UNIGENE	FLCDNA	TAIR
AGCTTTTGCA	0	0	2	1	1	gnl|UG|At#S11724190	no match found	At4g20910.1
at4g25290	CON	MIN	HOUR	DAY	WEEK	UNIGENE	FLCDNA	TAIR
TTATAACTCA	2	0	0	1	0	gnl|UG|At#S11807497	gi|20258851|gb|AY091088.1	non-canonical match
at5g46410	CON	MIN	HOUR	DAY	WEEK	UNIGENE	FLCDNA	TAIR
TAGAGTAAAC	1	1	0	0	0	gnl|UG|At#S43849163	gi|26449835|dbj|AK117371.1|	At5g46410.1
CATTCTTTTG	0	3	0	0	0	no match found	gi|20465764|gb|AY096737.1	non-canonical match
at3g60410	CON	MIN	HOUR	DAY	WEEK	UNIGENE	FLCDNA	TAIR
CTTGAAAGGG	0	1	3	0	0	no match found	gi|13430777|gb|AF360301.1	At3g60410.1
TCTCTATCTG	0	1	0	0	0	gnl|UG|At#S18941946	no match found	At3g60410.2
at1g07310	CON	MIN	HOUR	DAY	WEEK	UNIGENE	FLCDNA	TAIR
TTTCTAAAAC	3	2	0	1	0	gnl|UG|At#S11742303	gi|15451131|gb|AY054646.1	At1g07310.1
at1g08040	CON	MIN	HOUR	DAY	WEEK	UNIGENE	FLCDNA	TAIR
GAACAACACA	2	1	0	0	0	gnl|UG|At#S11742230	gi|15810566|gb|AY056322.1	At1g08040.1
at1g05470	CON	MIN	HOUR	DAY	WEEK	UNIGENE	FLCDNA	TAIR
GTTTTTGCTA	0	0	0	0	1	gnl|UG|At#S18897571	no match found	multiple non-canonical match
at5g42200	CON	MIN	HOUR	DAY	WEEK	UNIGENE	FLCDNA	TAIR
AAATTAGTTT	1	0	0	1	0	gnl|UG|At#S35310341	gi|28392903|gb|BT003837.1	At5g42200.1
at1g14700	CON	MIN	HOUR	DAY	WEEK	UNIGENE	FLCDNA	TAIR
TCGGTTCACG	1	2	1	0	0	gnl|UG|At#S18896791	gi|24030192|gb|BT000877.1	At1g14700.1
at3g06670	CON	MIN	HOUR	DAY	WEEK	UNIGENE	FLCDNA	TAIR
AGATATAAGG	0	0	1	0	1	gnl|UG|At#S11738731	no match found	At3g06670.1
GTCTATCAGC	3	1	2	1	3	no match found	gi|20453089|gb|AY094413.1	non-canonical match
at3g21960	CON	MIN	HOUR	DAY	WEEK	UNIGENE	FLCDNA	TAIR
AACTGCAATT	1	0	0	0	0	no match found	no match found	At3g21960.1

at2g27970	CON	MIN	HOUR	DAY	WEEK	UNIGENE	FLCDNA	TAIR
GTTAAGAATG	0	0	0	2	0	no match found	no match found	At2g27970.1
at4g36648	CON	MIN	HOUR	DAY	WEEK	UNIGENE	FLCDNA	TAIR
GCAGGTCACC	1	1	5	5	3	no match found	gi|72198523|gb|DQ108877.1	At2g27970.1
GACTGACATC	1	0	0	1	0	gnl|UG|At#S11807837	gi|20453078|gb|AY094406.1	At2g27970.1
at1g02360	CON	MIN	HOUR	DAY	WEEK	UNIGENE	FLCDNA	TAIR
GAGCCATAAA	0	0	1	0	0	gnl|UG|At#S11742879	gi|46931225|gb|BT012598.1	At1g02360.1
at1g29060	CON	MIN	HOUR	DAY	WEEK	UNIGENE	FLCDNA	TAIR
AGATATTTTA	0	1	0	0	1	gnl|UG|At#S34117597	gi|110737335|dbj|AK228711.1|	At1g29060.1
GCATACTTGT	1	0	2	1	0	no match found	gi|21407039|gb|AY088265.1	multiple non-canonical match
at2g27710	CON	MIN	HOUR	DAY	WEEK	UNIGENE	FLCDNA	TAIR
AAAAGTCCGG	16	19	31	33	28	gnl|UG|At#S35282305	gi|16226550|gb|AF428429.1	At2g27710.3
GGTTTCAGTC	0	1	0	0	0	no match found	gi|30102815|gb|BT006518.1	non-canonical match
at2g31270	CON	MIN	HOUR	DAY	WEEK	UNIGENE	FLCDNA	TAIR
AGACAGATGT	1	0	0	1	0	gnl|UG|At#S11816045	no match found	At2g31270.1
at3g16565	CON	MIN	HOUR	DAY	WEEK	UNIGENE	FLCDNA	TAIR
AAGATGAGAA	1	1	0	0	0	no match found	gi|51870315|gb|BT015541.1	multiple non-canonical match
at3g06380	CON	MIN	HOUR	DAY	WEEK	UNIGENE	FLCDNA	TAIR
GATTATGGAT	0	1	0	0	0	no match found	gi|111074397|gb|BT026465.1	multiple non-canonical match
CAAAGAAGCG	5	1	2	2	0	gnl|UG|At#S11738830	gi|28393389|gb|BT004092.1	At3g06380.1
at4g21540	CON	MIN	HOUR	DAY	WEEK	UNIGENE	FLCDNA	TAIR
AGATGTGATG	0	3	1	0	0	gnl|UG|At#S11724084	gi|22136029|gb|AY128394.1	At4g21540.1
at3g27530	CON	MIN	HOUR	DAY	WEEK	UNIGENE	FLCDNA	TAIR
CAGTTTCCTG	1	0	1	0	1	gnl|UG|At#S11732459	gi|24111294|gb|BT001017.1	At3g27530.1
at5g57785	CON	MIN	HOUR	DAY	WEEK	UNIGENE	FLCDNA	TAIR
CAGCTTTGAC	1	2	4	0	1	no match found	gi|21405388|gb|AY086678.1	multiple canonical match
at3g52470	CON	MIN	HOUR	DAY	WEEK	UNIGENE	FLCDNA	TAIR
TTCGGTGTCA	0	4	2	2	1	gnl|UG|At#S11744361	gi|19423891|gb|AY080785.1	At3g52470.1
at3g15350	CON	MIN	HOUR	DAY	WEEK	UNIGENE	FLCDNA	TAIR
ACCTACACTA	0	2	0	0	0	no match found	gi|20856991|gb|AY102127.1	non-canonical match
ATATATATAT	0	0	0	0	1	gnl|UG|At#S18942092	gi|15146307|gb|AY049295.1	multiple non-canonical match
at2g31140	CON	MIN	HOUR	DAY	WEEK	UNIGENE	FLCDNA	TAIR
ACAGCAAATG	0	0	0	1	0	gnl|UG|At#S11734751	gi|28416604|gb|BT004587.1	At2g31140.1
at1g31300	CON	MIN	HOUR	DAY	WEEK	UNIGENE	FLCDNA	TAIR
AACTTGATAT	2	3	1	1	1	gnl|UG|At#S28282559	gi|20466068|gb|AY098946.1	At1g31300.1
at2g42390	CON	MIN	HOUR	DAY	WEEK	UNIGENE	FLCDNA	TAIR
GTCAAGAATC	0	0	0	1	0	gnl|UG|At#S15644861	gi|21403687|gb|AY084977.1	At2g42390.1
at1g31817	CON	MIN	HOUR	DAY	WEEK	UNIGENE	FLCDNA	TAIR
TCGTCGTCTC	1	1	2	0	0	gnl|UG|At#S11738347	gi|17380741|gb|AY063845.1	At1g31817.1
at5g49700	CON	MIN	HOUR	DAY	WEEK	UNIGENE	FLCDNA	TAIR
AAGAATCGTT	2	1	0	0	1	no match found	no match found	At5g49700.1
at3g51740	CON	MIN	HOUR	DAY	WEEK	UNIGENE	FLCDNA	TAIR

ACCGCCGCCG	0	1	0	0	0	gnl|UG|At#S11729108	gi|13937245|gb|AF372978.1	At3g51740.1
at1g64110	CON	MIN	HOUR	DAY	WEEK	UNIGENE	FLCDNA	TAIR
GTTAATAAGA	0	0	0	0	2	gnl|UG|At#S15450007	gi|15810166|gb|AY056097.1	multiple canonical match
at4g02890	CON	MIN	HOUR	DAY	WEEK	UNIGENE	FLCDNA	TAIR
TCTCGTTTCA	15	18	18	6	6	gnl|UG|At#S15461550	gi|24899674|gb|BT001165.1	At4g02890.2
AGGTCTTGGT	17	21	21	7	1	no match found	gi|110741186|dbj|AK230345.1|	non-canonical match
at2g27770	CON	MIN	HOUR	DAY	WEEK	UNIGENE	FLCDNA	TAIR
TTTAGGACAA	1	1	0	0	0	gnl|UG|At#S11735604	gi|26449501|dbj|AK117201.1|	At2g27770.1
at1g29160	CON	MIN	HOUR	DAY	WEEK	UNIGENE	FLCDNA	TAIR
ATTTTCCGGT	0	1	0	0	1	no match found	gi|124300951|gb|BT029990.1	At1g29160.1
at5g65460	CON	MIN	HOUR	DAY	WEEK	UNIGENE	FLCDNA	TAIR
GAATTCTCTT	0	0	0	0	1	no match found	no match found	At5g65460.1
at4g24540	CON	MIN	HOUR	DAY	WEEK	UNIGENE	FLCDNA	TAIR
CAAGTAACAT	0	2	0	0	0	gnl|UG|At#S11723553	gi|92856618|gb|BT025171.1	At4g24540.1
at3g20390	CON	MIN	HOUR	DAY	WEEK	UNIGENE	FLCDNA	TAIR
TTGGCTGATT	3	8	2	2	1	gnl|UG|At#S18901880	gi|21404746|gb|AY086036.1	At3g20390.1
at5g60980	CON	MIN	HOUR	DAY	WEEK	UNIGENE	FLCDNA	TAIR
AGGGGATTCA	0	2	0	1	0	gnl|UG|At#S11709178	gi|27764907|gb|BT003010.1	At5g60980.2
at1g33540	CON	MIN	HOUR	DAY	WEEK	UNIGENE	FLCDNA	TAIR
TATTTTATAA	2	1	0	0	0	no match found	no match found	At1g33540.1
at4g13610	CON	MIN	HOUR	DAY	WEEK	UNIGENE	FLCDNA	TAIR
AACCTCTTAA	0	0	0	1	1	no match found	no match found	At4g13610.1
at4g14790	CON	MIN	HOUR	DAY	WEEK	UNIGENE	FLCDNA	TAIR
CATTCCATAA	0	0	0	0	1	gnl|UG|At#S11725236	gi|22655092|gb|AY139995.1	At4g14790.1
at1g68050	CON	MIN	HOUR	DAY	WEEK	UNIGENE	FLCDNA	TAIR
TAATGTTAAT	0	2	9	1	5	gnl|UG|At#S11729497	gi|18086373|gb|AY064999.1	At1g68050.1
at3g53560	CON	MIN	HOUR	DAY	WEEK	UNIGENE	FLCDNA	TAIR
GTCTAGATGC	0	0	1	1	0	no match found	no match found	At3g53560.1
CTTAGAAGAC	0	0	2	3	1	gnl|UG|At#S18913866	gi|21407897|gb|AY089123.1	multiple non-canonical match
at2g07707	CON	MIN	HOUR	DAY	WEEK	UNIGENE	FLCDNA	TAIR
GCCAAGGAAG	4	2	0	1	0	gnl|UG|At#S35238116	gi|109946562|gb|BT026104.1	multiple canonical match
at4g10340	CON	MIN	HOUR	DAY	WEEK	UNIGENE	FLCDNA	TAIR
CTTCTAAGGA	71	19	56	31	13	gnl|UG|At#S11725931	no match found	At4g10340.1
TTTGCGATGC	191	383	232	141	16	no match found	gi|15809717|gb|AY054126.1	non-canonical match
at1g80910	CON	MIN	HOUR	DAY	WEEK	UNIGENE	FLCDNA	TAIR
GATTAGATTC	3	1	2	1	1	gnl|UG|At#S11703965	gi|14517359|gb|AY039514.1	At1g80910.1
at5g02120	CON	MIN	HOUR	DAY	WEEK	UNIGENE	FLCDNA	TAIR
ATTGGTCTCA	6	10	6	3	3	no match found	gi|17065539|gb|AY062846.1	At5g02120.1
at2g43750	CON	MIN	HOUR	DAY	WEEK	UNIGENE	FLCDNA	TAIR
CTCCAACAGT	1	10	2	2	2	gnl|UG|At#S11731703	no match found	multiple canonical match
at4g39090	CON	MIN	HOUR	DAY	WEEK	UNIGENE	FLCDNA	TAIR
CCCTTACATA	3	52	1	3	3	gnl|UG|At#S11721083	gi|21406359|gb|AY087621.1	non-canonical match

at2g45380	CON	MIN	HOUR	DAY	WEEK	UNIGENE	FLCDNA	TAIR
AATTCACTGG	0	3	0	0	0	gnl|UG|At#S11709597	gi|18086396|gb|AY065012.1	non-canonical match
at3g10815	CON	MIN	HOUR	DAY	WEEK	UNIGENE	FLCDNA	TAIR
TACAAAAGGG	0	1	0	0	0	gnl|UG|At#S21736054	gi|51971438|dbj|AK176621.1|	At3g10815.1
at2g35880	CON	MIN	HOUR	DAY	WEEK	UNIGENE	FLCDNA	TAIR
CCCAGTTTCT	2	3	2	0	0	gnl|UG|At#S11733608	gi|16209719|gb|AY057620.1	At2g35880.1
at1g16500	CON	MIN	HOUR	DAY	WEEK	UNIGENE	FLCDNA	TAIR
AAGAAGCCTA	1	1	2	0	0	gnl|UG|At#S11741390	no match found	multiple canonical match
at1g73930	CON	MIN	HOUR	DAY	WEEK	UNIGENE	FLCDNA	TAIR
AGAATCACAC	0	0	1	0	2	gnl|UG|At#S11728259	no match found	multiple canonical match
at5g65470	CON	MIN	HOUR	DAY	WEEK	UNIGENE	FLCDNA	TAIR
TTTGATTAGT	2	0	4	0	1	gnl|UG|At#S11717150	gi|20260535|gb|AY093167.1	At5g65470.1
at5g56350	CON	MIN	HOUR	DAY	WEEK	UNIGENE	FLCDNA	TAIR
ATTTCTTTTT	3	2	4	9	7	gnl|UG|At#S11718077	no match found	At5g56350.1
GTCCTAACCA	0	1	1	1	1	no match found	gi|17978780|gb|AY064679.1	non-canonical match
at5g24670	CON	MIN	HOUR	DAY	WEEK	UNIGENE	FLCDNA	TAIR
TTCCGCCCTC	0	1	0	0	0	gnl|UG|At#S11720708	gi|110736605|dbj|AK228326.1|	At5g24670.1
at4g15990	CON	MIN	HOUR	DAY	WEEK	UNIGENE	FLCDNA	TAIR
TTTTTTCATC	0	0	1	0	0	no match found	no match found	At4g15990.1
at1g19920	CON	MIN	HOUR	DAY	WEEK	UNIGENE	FLCDNA	TAIR
GAAAAAACTC	3	2	9	3	3	gnl|UG|At#S11741062	gi|21404811|gb|AY086101.1	At1g19920.1
at4g01990	CON	MIN	HOUR	DAY	WEEK	UNIGENE	FLCDNA	TAIR
TTCTCGTTAT	0	0	0	0	1	gnl|UG|At#S11704031	gi|14517493|gb|AY039582.1	At4g01990.1
at4g36380	CON	MIN	HOUR	DAY	WEEK	UNIGENE	FLCDNA	TAIR
TCAAATTATA	0	1	1	0	1	gnl|UG|At#S34118180	gi|110736224|dbj|AK228126.1|	multiple canonical match
at3g50740	CON	MIN	HOUR	DAY	WEEK	UNIGENE	FLCDNA	TAIR
GCACGTGGTG	0	0	1	0	0	no match found	gi|53749149|gb|BT015770.1	multiple non-canonical match
GTATCACGTG	4	9	5	1	1	gnl|UG|At#S11705012	gi|15146271|gb|AY049277.1	At3g50740.1
at1g75690	CON	MIN	HOUR	DAY	WEEK	UNIGENE	FLCDNA	TAIR
GATATTGTAA	19	5	9	6	11	no match found	gi|27311544|gb|BT002378.1	At1g75690.1
at3g43682	CON	MIN	HOUR	DAY	WEEK	UNIGENE	FLCDNA	TAIR
TTGACAAAAA	0	0	0	1	1	gnl|UG|At#S35258970	no match found	non-canonical match
at3g18440	CON	MIN	HOUR	DAY	WEEK	UNIGENE	FLCDNA	TAIR
GCCTGCACGG	0	2	1	1	0	gnl|UG|At#S11735313	gi|23198099|gb|BT000258.1	At3g18440.1
at5g17420	CON	MIN	HOUR	DAY	WEEK	UNIGENE	FLCDNA	TAIR
TAGTTGCAGT	0	1	0	0	0	no match found	no match found	At5g17420.1
at5g08330	CON	MIN	HOUR	DAY	WEEK	UNIGENE	FLCDNA	TAIR
GCCGACAACG	1	8	0	0	0	gnl|UG|At#S11724028	gi|15724267|gb|AF412074.1	At5g08330.1
at5g50390	CON	MIN	HOUR	DAY	WEEK	UNIGENE	FLCDNA	TAIR
TAACGGGTTT	0	1	0	0	0	no match found	no match found	At5g50390.1
at2g15580	CON	MIN	HOUR	DAY	WEEK	UNIGENE	FLCDNA	TAIR

TGCCCATAAG	2	3	2	2	0	gnl|UG|At#S11710121	gi|21407271|gb|AY088497.1	At2g15580.1
at5g35475	CON	MIN	HOUR	DAY	WEEK	UNIGENE	FLCDNA	TAIR
TTTTGAGAAA	0	2	0	0	0	no match found	no match found	At5g35475.1
at1g24340	CON	MIN	HOUR	DAY	WEEK	UNIGENE	FLCDNA	TAIR
AGAGATGTCT	0	4	2	1	1	gnl|UG|At#S11824234	gi|26450237|dbj|AK117578.1|	non-canonical match
at1g30120	CON	MIN	HOUR	DAY	WEEK	UNIGENE	FLCDNA	TAIR
ACTACTTAGA	1	2	5	1	0	gnl|UG|At#S11738982	gi|13605514|gb|AF361583.1	At1g30120.1
at1g30580	CON	MIN	HOUR	DAY	WEEK	UNIGENE	FLCDNA	TAIR
AAATTCGAGG	0	0	2	0	0	no match found	gi|109134110|gb|BT025965.1	non-canonical match
TCTTTGCCTT	6	3	6	7	9	gnl|UG|At#S11738814	gi|21404555|gb|AY085845.1	At1g30580.1
at5g60580	CON	MIN	HOUR	DAY	WEEK	UNIGENE	FLCDNA	TAIR
CTTTGACTCT	1	0	0	0	0	gnl|UG|At#S11717644	gi|13605862|gb|AF367330.1	At5g60580.1
GTTCCTCAGC	0	1	0	0	0	gnl|UG|At#S34117319	gi|110737877|dbj|AK228989.1|	non-canonical match
at2g46500	CON	MIN	HOUR	DAY	WEEK	UNIGENE	FLCDNA	TAIR
GTAATGACAA	1	1	0	2	0	gnl|UG|At#S11731073	gi|16930424|gb|AF419566.1	At2g46500.2
at5g59300	CON	MIN	HOUR	DAY	WEEK	UNIGENE	FLCDNA	TAIR
GCTTCGCAAG	0	1	0	0	0	gnl|UG|At#S11743888	gi|28973768|gb|BT005798.1	non-canonical match
at5g58220	CON	MIN	HOUR	DAY	WEEK	UNIGENE	FLCDNA	TAIR
TATAAAAATT	1	4	1	1	0	gnl|UG|At#S11717888	gi|17065389|gb|AY062771.1	At5g58220.1
at1g10230	CON	MIN	HOUR	DAY	WEEK	UNIGENE	FLCDNA	TAIR
GAAAAGCTAG	1	2	0	1	0	gnl|UG|At#S11742011	no match found	At1g10230.1
at5g42780	CON	MIN	HOUR	DAY	WEEK	UNIGENE	FLCDNA	TAIR
TGGCTGTCAC	0	1	0	0	0	gnl|UG|At#S11719451	gi|21403077|gb|AY084367.1	At5g42780.1
at1g14990	CON	MIN	HOUR	DAY	WEEK	UNIGENE	FLCDNA	TAIR
TAATTCCCGC	2	2	1	2	1	gnl|UG|At#S11741535	gi|26452811|dbj|AK118902.1|	At1g14990.1
at1g29420	CON	MIN	HOUR	DAY	WEEK	UNIGENE	FLCDNA	TAIR
CTCTTTTCAA	1	0	0	2	0	no match found	gi|44681405|gb|BT011637.1	non-canonical match
at3g55040	CON	MIN	HOUR	DAY	WEEK	UNIGENE	FLCDNA	TAIR
AGACCAAGTC	4	3	2	1	1	gnl|UG|At#S11728508	no match found	At3g55040.1
at3g13940	CON	MIN	HOUR	DAY	WEEK	UNIGENE	FLCDNA	TAIR
ACCGATCCAG	0	1	0	1	0	gnl|UG|At#S11702041	gi|14532855|gb|AY040052.1	At3g13940.1
at5g45930	CON	MIN	HOUR	DAY	WEEK	UNIGENE	FLCDNA	TAIR
GTGATAAACA	2	1	1	2	1	gnl|UG|At#S11719134	gi|53749175|gb|BT015783.1	At5g45930.1
at3g16000	CON	MIN	HOUR	DAY	WEEK	UNIGENE	FLCDNA	TAIR
AGGAGCCAAC	1	2	3	5	1	gnl|UG|At#S11707976	gi|30794107|gb|BT008690.1	At3g16000.1
at1g19980	CON	MIN	HOUR	DAY	WEEK	UNIGENE	FLCDNA	TAIR
CAACTGACCA	0	0	0	0	1	gnl|UG|At#S14274066	gi|28392993|gb|BT003883.1	At1g19980.1
at1g32370	CON	MIN	HOUR	DAY	WEEK	UNIGENE	FLCDNA	TAIR
GAAAGCACAC	0	1	0	0	0	no match found	gi|88011094|gb|BT024563.1	At1g32370.1
ATGACTGCAT	1	1	0	1	0	gnl|UG|At#S28282554	gi|51969041|dbj|AK175450.1|	At1g32370.2
at5g63620	CON	MIN	HOUR	DAY	WEEK	UNIGENE	FLCDNA	TAIR
CAGTGCACAC	1	0	2	0	0	gnl|UG|At#S15437378	gi|21404735|gb|AY086025.1	At5g63620.2

at1g30620	CON	MIN	HOUR	DAY	WEEK	UNIGENE	FLCDNA	TAIR
ACGAGATTTT	0	0	1	0	1	gnl|UG|At#S11738798	gi|62318958|dbj|AK220800.1|	At1g30620.1

at1g10210	CON	MIN	HOUR	DAY	WEEK	UNIGENE	FLCDNA	TAIR
GAAGCTCTGT	0	1	0	0	0	no match found	gi|23197707|gb|BT000062.1	multiple non-canonical match

at2g20130	CON	MIN	HOUR	DAY	WEEK	UNIGENE	FLCDNA	TAIR
TCTTCGTGGT	0	1	0	0	0	gnl|UG|At#S11737443	no match found	At2g20130.1

at3g46640	CON	MIN	HOUR	DAY	WEEK	UNIGENE	FLCDNA	TAIR
GCTTCTTATC	0	2	2	0	1	no match found	gi|110743671|dbj|AK227686.1|	At3g46640.1
TAGAAGCTAA	0	1	0	0	1	gnl|UG|At#S28282853	no match found	pseudo chromosome match

at2g37560	CON	MIN	HOUR	DAY	WEEK	UNIGENE	FLCDNA	TAIR
GTGTGGTAGT	0	0	1	0	0	gnl|UG|At#S11733211	gi|17979312|gb|AY070386.1	pseudo chromosome match

at4g21280	CON	MIN	HOUR	DAY	WEEK	UNIGENE	FLCDNA	TAIR
GTGATTTAGC	6	1	3	4	0	gnl|UG|At#S28281994	no match found	At4g21280.1

at3g59920	CON	MIN	HOUR	DAY	WEEK	UNIGENE	FLCDNA	TAIR
TGTCTCCGTT	8	9	9	10	1	gnl|UG|At#S11727526	gi|23197605|gb|BT000011.1	At3g59920.1

at2g37190	CON	MIN	HOUR	DAY	WEEK	UNIGENE	FLCDNA	TAIR
AGACCTAGAT	4	11	3	2	6	gnl|UG|At#S11733304	gi|13926285|gb|AF372896.1	At2g37190.1

at2g20290	CON	MIN	HOUR	DAY	WEEK	UNIGENE	FLCDNA	TAIR
ATTATGATTC	13	11	10	10	8	no match found	no match found	At2g20290.1

at3g23590	CON	MIN	HOUR	DAY	WEEK	UNIGENE	FLCDNA	TAIR
CCCTTGCCTA	0	3	0	0	0	gnl|UG|At#S11733730	gi|26449339|dbj|AK117119.1|	At3g23590.1

at2g26280	CON	MIN	HOUR	DAY	WEEK	UNIGENE	FLCDNA	TAIR
TGAGTGAAGC	0	1	1	0	2	no match found	gi|133778821|gb|BT030338.1	non-canonical match
GGAAGAGAGG	9	8	12	8	12	gnl|UG|At#S11735966	gi|110737158|dbj|AK228618.1|	At2g26280.1

at5g54520	CON	MIN	HOUR	DAY	WEEK	UNIGENE	FLCDNA	TAIR
TACGACTACA	0	1	0	0	0	gnl|UG|At#S11718263	gi|26451678|dbj|AK118320.1|	At5g54520.1

at5g53560	CON	MIN	HOUR	DAY	WEEK	UNIGENE	FLCDNA	TAIR
ATGGACAAAT	2	3	1	0	1	gnl|UG|At#S11718359	gi|21281007|gb|AY114045.1	At5g53560.1

at2g44220	CON	MIN	HOUR	DAY	WEEK	UNIGENE	FLCDNA	TAIR
TCTATATTTG	0	0	1	0	0	gnl|UG|At#S11816052	no match found	multiple canonical match

at5g17890	CON	MIN	HOUR	DAY	WEEK	UNIGENE	FLCDNA	TAIR
TTTTAAGAGT	1	4	1	2	0	gnl|UG|At#S11721848	gi|19310532|gb|AY079160.1	At5g17890.1

at1g32340	CON	MIN	HOUR	DAY	WEEK	UNIGENE	FLCDNA	TAIR
TCTGCAAAGA	0	1	1	0	0	no match found	gi|25083845|gb|BT002115.1	non-canonical match

at3g15000	CON	MIN	HOUR	DAY	WEEK	UNIGENE	FLCDNA	TAIR
TCTCCCACAG	1	1	1	2	4	gnl|UG|At#S11736345	no match found	At3g15000.1
GGAGGAGCAC	0	0	1	0	0	no match found	gi|53828642|gb|BT015868.1	non-canonical match
GTCAAAAAAA	3	1	7	5	1	no match found	gi|110740937|dbj|AK226420.1|	non-canonical match
GTTAGAACTT	3	2	4	8	4	gnl|UG|At#S20757707	gi|16226542|gb|AF428427.1	non-canonical match

| at5g17440 | CON | MIN | HOUR | DAY | WEEK | UNIGENE | FLCDNA | TAIR |
[truncated: 930,532 more chars]
