# Supplementary material for: Differential SAGE analysis in Arabidopsis uncovers increased transcriptome complexity in response to low temperature
Source: BMC Genomics. 2008 Sep 22;9:434. doi: 10.1186/1471-2164-9-434 (PMC2568001; doi:10.1186/1471-2164-9-434)
Supplement: Additional file 2 — A list of all observed SAGE tags, their abundance in each library and their assigned location in the Arabidopsis genome. [file 1471-2164-9-434-S2.pdf]

Additional File 2. A list of all observed SAGE tags, their abundance in each library and their assigned location in the Arabidopsis genome.

TAG	Control	30min	2hour	2day	1week	Assigned location(s)
GGCCTTCGCC	646	986	638	736	259	At1g29930.1:d:+837:primary
GGAGCTGTTG	290	1165	199	227	19	At2g34420.1:d:+557:primary
AGGAGAAAGA	501	455	479	348	69	At1g29910.1:d:+922:primary
AACAAATTTG	391	297	522	324	46	At3g50990.1:v:+1788:primary
CAGGTGTGGC	320	539	226	337	106	At1g67090.2:d:+197:secondary,At1g67090.1:d:+197:secondary
GCGAAAAGGA	336	202	446	416	98	At1g32060.1:d:+1414:primary,AtCg00440:d:+336:primary
ATAGAACCTT	336	394	261	227	166	At2g45180.1:X:+585:quaternary
AAAGTTCTCG	261	385	414	271	46	At5g64040.1:d:+679:primary
TCCGAATCTT	273	541	309	169	84	At4g21960.1:d:+1369:secondary
AAGGTGTGGC	236	543	302	190	29	At5g38420.1:d:+220:primary,At5g38430.1:d:+200:primary,At5g38410.1:d:+220:primary
TGTTTTTATG	333	373	253	142	54	At1g61520.1:d:+983:primary
GCACAACAAC	353	292	293	137	45	At3g54890.1:d:+755:secondary,At3g54890.2:d:+653:secondary,At3g54890.3:d:+688:secondary
CACCTGAACG	332	135	317	227	26	At1g30380.1:d:+521:secondary
TTTGCGATGC	191	383	232	141	16	At4g10340.1:d:+884:secondary
GAGGTGGTGA	194	261	147	178	48	At3g12690.1:d:+1944:primary,AtCg00830:d:+692:primary,At3g12690.2:d:+1972:primary,AtCg00830:d:+692:primary,At3g12690.3:d:+1842:primary
GTGCGTTTGT	128	162	172	178	154	At2g05520.1:d:+563:primary
TCAAATCATT	286	67	198	128	111	At4g38970.1:d:+1358:primary,At4g38970.2:d:+1362:primary
CTCTTTTCTG	112	239	202	125	105	At4g25100.3:d:+853:primary
CCTAAGATCT	289	86	218	141	47	At5g66570.1:d:+1181:secondary
TTTCTATAAA	258	268	78	64	102	At1g79040.1:d:+523:primary
CTCGGAGCCC	109	239	210	121	43	At2g34420.2:d:+371:primary,At1g29920.1:d:+389:primary
GGCAGGCAAG	180	197	116	194	33	ChrC:+104842:quaternary
GGCGAACGAC	80	261	203	123	41	No gene matches found
TGATGAGTTT	74	84	146	199	170	At3g09390.1:d:+483:primary
TTAATTTTTA	204	131	147	128	54	At4g02770.1:d:+764:primary
GTTTGAAGGA	102	316	135	74	21	At3g16640.1:d:+573:secondary
CGCCCGCCGC	92	241	90	157	35	Chr3:+14214243:quaternary,Chr2:+9286:quaternary
AGGCTTGTTC	176	50	228	115	14	At1g15820.1:d:+1012:secondary
GAAATGAAAG	44	413	70	36	11	AtCg00480:d:+643:primary
TTTGTACAAA	199	188	85	54	37	At3g61470.1:d:+901:primary
TTTACTTTCA	264	55	143	72	24	At5g14740.2:d:+1355:primary,At5g14740.1:d:+1473:primary
TTTCCTTCCT	107	319	63	36	31	At1g20620.2:d:+1791:primary,At1g20620.1:d:+1692:primary
CTTGTGATGG	161	152	137	69	19	At2g39730.1:d:+1401:primary,At2g39730.3:d:+1372:primary,At2g39730.2:d:+1372:primary
TAAAACTCTA	169	69	114	103	75	At4g37930.1:d:+1745:primary
TACATATAGA	266	41	35	33	150	ChrC:+32222:quaternary,Chr1:+8650800:quaternary
TAAAACTTTG	104	120	138	107	50	At4g05320.4:d:+1469:primary,At4g05320.3:d:+1276:primary,At4g05320.5:d:+1048:primary,At4g05320.1:d:+1276:primary
TAAGGTATAG	26	94	11	56	323	At4g12470.1:d:+618:primary
TGGCAACAGT	118	56	211	90	24	At3g47470.1:d:+1226:primary
TTGAGACAAA	124	35	146	115	78	At1g44575.2:d:+1352:primary,At1g44575.1:d:+893:primary
GTCACTCCTA	76	230	99	55	8	At1g06680.1:d:+479:primary
GATCCATACA	145	111	96	85	23	At1g20630.1:d:+86:secondary
TAAAATCTTG	130	83	146	70	17	At1g03130.1:d:+743:secondary
TAAAAAAAAA	88	85	119	85	58	At1g29360.1:p:+870:secondary,At4g20380.1:d:+14:secondary
TGAGATTCTA	131	105	98	76	23	At5g01530.1:d:+1034:secondary
TATCTTATCT	92	77	26	37	190	At1g54410.1:d:+583:primary,At1g42520.1:p:+1432:primary
GATAAGCTCA	92	141	77	81	26	At2g07080.1:p:+2393:primary
AGAGTTTGTA	54	146	87	84	38	At1g13440.1:d:+1206:primary
TCCGAATCAA	68	144	104	64	26	Chr1:+1605587:quaternary
ATATTTCTTT	48	278	22	35	23	At3g50450.1:v:+37:secondary
CCTAAAAAAA	99	27	144	101	32	Chr4:+5428421:quaternary
TAGAAGAAGA	127	91	82	56	40	AtCg00740:d:+695:primary
TGTGTTTACT	128	72	96	53	37	At2g30570.2:d:+661:primary
AACACTGCTG	48	168	84	50	27	At5g54770.1:d:+826:primary
CCTCCTGTTG	102	99	95	57	21	At3g56940.1:d:+1220:primary
AAAATGAAAA	48	213	50	47	10	At3g15590.1:d:+1776:primary
GGTTTGGTTG	69	161	70	59	8	At5g54270.1:d:+597:secondary,At1g23850.1:d:+737:secondary,At1g13410.1:v:+1371:secondary,At1g23830.1:d:+745:secondary

TGGATCTTCT	89	23	106	108	40	At5g36900.1:v:+1075:primary,At4g35090.1:d:+1654:primary
ACAAGAAAAC	67	18	77	100	103	At4g33230.1:v:+2570:primary
TTCTCTATGT	179	117	42	12	14	At2g05100.1:d:+725:primary
ATCGTTTAAT	69	31	63	58	141	At5g02960.1:d:+651:primary
TCTATCTCTC	161	73	63	32	29	At3g21055.1:d:+450:primary
TAATAGCAAA	98	127	77	43	12	At1g64720.1:d:+1320:primary
TTACATTTGT	115	82	39	31	75	At2g26500.2:d:+556:secondary,At2g26500.1:d:+588:secondary
AGTGTACGAT	63	55	41	71	106	At2g05380.1:d:+409:primary
TGAGGTGATG	67	30	68	87	83	At3g04120.1:d:+1283:primary
CCCCAGAAAC	102	48	106	68	11	At2g45960.1:d:+1137:primary
TCAAAGGCCT	86	49	76	64	59	At3g04120.1:d:+1130:secondary,At5g36226.1:p:+377:secondary,At1g13440.1:d:+1080:secondary
TTGGTCCGAC	65	50	108	71	39	At3g56240.1:d:+613:primary
TTTCATTTGA	67	102	19	38	107	At3g22120.1:d:+1155:primary
AATCTAATCC	57	51	67	87	69	At1g13930.1:d:+652:primary
TCTTTCTTTG	84	48	19	30	146	At3g51600.1:d:+626:primary
TTTTCTCTTT	93	76	58	53	44	At2g30770.1:d:+1522:primary,At2g13360.2:d:+1353:primary,At4g34470.1:v:+1121:primary,At2g13360.1:d:+1375:primary
TCCTTTTATG	90	35	54	22	120	At4g23420.2:d:+911:primary,At5g42530.1:d:+390:primary,At4g23420.1:d:+1044:primary
AATATAGAGG	104	85	30	30	69	At4g21100.1:d:+1291:secondary
TCCTTCATCG	122	50	86	31	28	At5g66570.1:d:+1212:primary
TTCTTGTCTG	78	42	50	39	100	At2g41900.1:d:+2485:primary,At2g37220.1:d:+1140:primary
GTCTCTCCAG	46	45	85	63	63	At2g37220.1:X:-158:quaternary
ACATTAAATT	94	50	74	66	14	At5g01530.1:d:+1133:primary
AACCCAGCCG	44	155	44	51	2	At3g26520.1:d:+710:secondary
ACCATCCATC	103	67	64	57	5	At3g01500.3:d:+1142:primary
TTTGACCCAA	57	61	61	77	38	At2g18900.1:d:+2681:primary,At2g25450.1:d:+1301:primary
TTACCTTTCT	103	94	49	24	12	At3g16140.1:d:+512:primary
TTTTTCCATT	72	59	60	45	35	At3g26650.1:d:+1347:primary
CAAAAAAAAA	61	45	93	44	27	At1g10740.2:d:+1792:primary,At2g38610.1:d:+1201:primary,At4g10590.2:d:+3249:primary,At1g10740.1:d:+1724:primary,At2g38610.2:d:+1263:primary
TCGTATCAAG	110	10	44	53	47	At4g31500.1:d:+358:secondary
ATTCGGAATG	87	29	76	47	25	At1g12900.1:d:+1320:primary
TCGATGTTGT	70	92	50	40	8	At5g27660.1:v:+2108:primary,At4g13990.1:d:+799:primary
TTCAGAGACT	39	14	49	73	79	At5g42270.1:d:+2364:secondary
TGTACTAAGT	9	0	0	76	168	At3g22840.1:d:+807:primary
TGTTTGTCAT	51	21	24	22	133	At2g25510.1:d:+424:secondary,At5g42530.1:d:+333:secondary
TCGAGCGCGT	6	5	6	184	46	At5g13930.1:d:+1102:primary
ATCATTCGTG	91	33	53	38	32	At3g60750.1:d:+2731:primary
GGCTAAATGG	42	86	51	49	17	At5g53300.1:d:+563:primary,At5g53300.2:d:+544:primary
AGTTTTGGTT	45	18	36	63	82	At4g18100.1:d:+579:primary
ATGTTTTTGA	77	87	36	39	4	At1g66020.1:d:+1994:primary
GCCGTTCTTA	3	222	5	7	3	Chr3:+14209944:quaternary,Chr2:+4987:quaternary
TAATGATGTA	96	74	31	32	7	At3g08940.2:d:+976:primary,At3g08940.1:d:+1061:primary
TTCATCAAAA	75	40	68	35	22	At2g30570.1:d:+748:primary
CAATTACAAA	72	34	59	48	21	At4g04640.1:d:+1379:primary
GCTTATGATG	16	191	12	11	2	At3g16770.1:d:+418:secondary
GCCTTCGTCC	23	114	54	38	3	At1g55670.1:d:+182:primary
CCAAAAATGA	48	55	53	49	26	AtCg00820:d:+251:primary
TTTCCTCTTT	77	55	45	30	22	At5g26000.1:d:+1701:primary,At5g26000.2:d:+1701:primary
GTGGTCTTAA	69	68	47	33	12	At4g03280.1:d:+810:primary,At4g03280.2:d:+909:primary
AAACAAAAAA	60	10	70	52	37	At2g39930.1:d:+2949:primary
GCAATAGAAG	31	120	35	39	2	At1g20340.1:d:+292:primary
GCACTAAGTG	53	55	53	39	24	At3g14415.1:v:+1365:primary
AAGACCAAAG	50	36	55	45	36	At1g02560.1:d:+1152:primary
CCTATGTCTC	73	38	66	29	15	At5g09660.1:d:+1265:primary
GGCCTTCGCT	53	89	41	30	5	At2g34430.1:d:+830:primary
GGCTAGATTA	49	54	60	32	20	AtCg01130:d:+3702:secondary
TCTTGCTGTG	25	119	23	28	18	At5g02380.1:d:+84:primary
TTTCTGTGTT	91	60	40	20	2	At3g16240.1:d:+892:primary
AATCAAAAGT	77	30	49	43	12	At2g30570.2:d:+595:secondary,At2g30570.1:d:+595:secondary
GATGGATTTC	33	22	55	60	41	At1g52300.1:d:+479:primary

AAAGCTTTCT	21	153	19	14	3	At1g31330.1:d:+208:primary
AGAACTTATG	43	19	38	44	65	At3g49910.1:d:+622:primary
TTTGGTTATC	37	45	36	32	59	At1g16000.1:d:+434:primary,At1g43170.1:d:+1283:primary,At1g43170.2:d:+1249:primary
ACTCAGTATG	68	56	43	17	23	At1g23310.1:d:+1710:primary
GGTCATACCA	47	80	29	32	18	AtCg01130:d:+4305:secondary
TCTTTATAAA	58	37	28	32	51	At2g41070.1:d:+1209:primary,At2g41070.2:d:+1284:primary,At1g60950.1:d:+534:primary,At2g41070.3:d:+1319:primary
TTAAAAAAAA	41	46	52	33	33	At5g49630.1:i:+3040:tertiary
TGAAAAAAAA	31	32	52	53	37	At1g18460.1:d:+1348:secondary,At3g56040.1:d:+3017:secondary
AGGCAGACTG	40	68	26	41	25	At1g07920.1:d:+1364:primary,At1g07930.1:d:+1348:primary
AGGCAGACCG	28	42	37	49	40	At5g60390.1:d:+1318:primary
AACTATCTGT	81	44	33	19	18	AtCg01130:d:+2978:secondary

TAAGCTATAG	7	19	6	9	154	At4g12480.1:d:+648:primary
GTACGGATCT	15	3	162	7	5	At2g34420.1:d:+199:secondary,At1g29930.1:d:+216:secondary,At2g34420.2:d:+199:secondary
CTTCTAAGGA	71	19	56	31	13	At4g10340.1:d:+1160:primary
TATTATCTAC	89	27	24	15	33	At1g09310.1:d:+700:primary
AGTTACTTAT	42	39	40	26	40	At1g42970.1:d:+1518:secondary
TACAGCCTCT	47	34	37	40	28	At4g05050.1:d:+845:primary
TCTCTTAAAG	29	118	16	16	6	At5g19140.1:d:+863:primary
CCTAAGAAAA	53	22	51	38	19	At1g77230.1:i:-1421:tertiary
TAACCGTTTG	19	29	79	23	32	At4g32020.1:d:+1362:secondary
CTAGGGACAA	47	73	26	29	7	At2g47400.1:d:+316:primary
GTTGCTATAA	59	27	41	30	24	At5g04140.1:d:+5215:primary,At5g04140.2:d:+5114:primary
AGAATGCTCT	23	17	34	53	52	At2g36530.1:d:+1592:secondary
AAACTTAAAT	25	39	29	50	35	At4g13940.1:d:+1707:secondary
TGCCTCACCA	39	95	22	19	1	At4g12800.1:d:+592:secondary
ATAAAAAAAA	28	30	55	37	26	At2g17845.1:v:+22:secondary
CCTCTGTTTT	47	9	51	41	27	At3g47070.1:d:+565:secondary
AGATCCATCG	45	52	46	30	1	At5g45440.1:d:+850:secondary,At5g45490.1:d:+858:secondary,At1g03130.1:d:+603:secondary
ATAATTGCTT	60	20	48	25	21	At1g03600.1:d:+717:primary
TTGCAGTTTA	22	26	31	43	51	At2g39460.1:d:+731:primary
TAAGAGTGAT	19	23	26	59	45	At2g15970.1:d:+715:secondary
AGTGTGTTTT	44	24	41	33	30	At1g79850.1:d:+609:primary
AGCTTGTTTT	33	35	33	36	35	At1g74060.1:d:+757:primary,At1g74050.1:d:+815:primary,At1g18540.1:d:+719:primary
TCAAAAAAAA	21	32	61	30	28	At1g73610.1:v:+1794:primary
TTTAATAGTA	2	1	1	14	153	At2g42540.1:d:+512:primary,At2g42540.2:d:+577:primary
TGGTCTGGTT	28	31	51	39	22	At1g30230.1:d:+807:primary
AAATTCGTCA	29	66	46	26	3	At4g23670.1:d:+551:primary
CGAGCTTCGG	23	65	42	34	4	At3g61440.1:d:+1157:primary
ATGCAAACTA	35	35	47	33	18	At1g31812.1:d:+532:primary
CGTTGTTGGA	51	35	36	36	9	At4g16980.1:d:+565:primary
TCCGAATCTA	33	62	42	13	17	At2g32270.1:i:-1349:tertiary
CTTTTTAAGG	45	25	40	30	26	At5g50920.1:d:+3120:secondary
GTTGATGCGG	27	56	34	34	15	At5g17920.1:d:+2343:primary
GAAAAAAAAA	24	35	51	44	12	At5g08110.1:v:+2736:secondary,At1g40091.1:p:+1492:secondary
GTGAAAAATG	37	21	30	39	35	At4g15000.1:d:+552:primary
AAAAAAACAT	2	0	7	87	66	At3g57910.1:v:+1502:primary,At4g14690.1:d:+796:primary
TCTCTCACAG	56	14	53	30	8	At5g54270.1:d:+1006:primary
ATTTCAGCTA	46	23	42	37	13	At5g53320.1:d:+2120:primary
TCTCTCTTAT	54	64	21	9	13	At3g15353.1:d:+352:primary
ATCGGGAGCT	48	28	17	41	25	Chr3:+14210584:quaternary
AATCTGATGA	56	29	27	36	11	At5g53440.1:v:+3841:secondary,At5g35630.1:d:+1451:secondary
ATTCCCTCAA	31	19	20	32	57	At1g55490.2:d:+2026:primary,At1g55490.1:d:+2053:primary
GTAGTGACCA	36	50	38	14	20	At4g21960.1:d:+1166:secondary
AATGTGAACT	34	24	28	32	40	At2g34480.1:d:+690:primary
CTGCAGCCTC	19	108	12	14	5	At1g12090.1:d:+391:primary
AGAATATTGT	65	12	38	34	8	At3g59790.1:v:+700:secondary
GATTCTATGT	41	12	24	30	49	At3g62250.1:d:+620:primary
TGAATATGTG	38	79	27	6	6	At3g26740.1:d:+516:primary,At3g60060.1:v:+974:primary
ATTTGCCAGA	33	60	40	22	1	At3g09260.1:d:+1742:primary

GTTAAAAAAA	24	20	43	40	28	Chr5:+13598432:quaternary,Chr5:+7751926:quaternary,Chr2:+3050644:quaternary,Chr4:+11154737:quaternary,Chr3:+2552141:quaternary,Chr2:+19040502:quaternary
TTACAAGAGG	41	13	20	19	61	At1g04270.1:d:+690:primary
TGTAAAAAAA	43	22	47	34	7	At3g08850.1:d:+4758:primary
AAGTGGTGGT	21	72	29	23	8	At1g56220.1:d:+568:primary,At1g56220.2:d:+582:primary,At1g56220.3:d:+577:primary
TTACCACCAA	46	15	34	28	30	At1g54500.1:d:+742:primary,At4g01200.1:d:+812:primary
TATAATCAAC	38	21	26	29	38	At4g38770.1:d:+1505:primary
TAATTGAAAG	46	17	31	33	25	At4g20130.1:d:+1542:primary,At2g20900.1:d:+1756:primary
TGTGTTTAAA	53	15	47	30	4	At5g40480.1:i:+1134:tertiary
GTTTATCTCT	37	56	29	18	9	At2g20260.1:d:+490:primary
GATATGAGAA	39	8	31	33	38	At1g47440.1:p:+1265:primary,At1g62750.1:d:+2593:primary
ATCAAGTTTT	30	18	35	46	20	At1g15930.2:d:+639:primary,At1g15930.1:d:+742:primary
ACTTCAGACG	2	1	3	94	48	At4g14690.1:d:+577:secondary
CAAGTTAGAA	24	8	41	54	21	At2g36880.1:d:+1660:primary
AAGATGAGGA	41	52	37	17	1	At1g60500.1:d:+1735:primary,At1g51400.1:d:+247:primary
TTTTGAGTTT	41	25	36	24	20	At5g54585.1:d:+543:primary,At3g60540.1:d:+391:primary,At3g60540.2:d:+813:primary,At5g20290.1:d:+762:primary
TTTTCTGATT	48	23	38	25	11	At4g22890.1:d:+1263:secondary,At4g22890.3:d:+1259:secondary
AAAGTAAAAA	36	36	18	23	30	Chr5:+10922344:quaternary,Chr5:+4139058:quaternary,ChrC:+46610:quaternary,Chr1:+6862914:quaternary,Chr1:+10956059:quaternary
GTTTCGCCGA	16	69	34	20	4	At5g46110.2:d:+1304:primary,At5g46110.1:d:+1297:primary
TTTGAGAGAG	31	20	27	33	32	At2g15765.1:p:+173:primary
GTTCGTTGAG	31	25	34	38	15	At1g29070.1:d:+647:secondary,At3g56750.1:d:+890:secondary
TCGGATTCGA	21	8	30	64	18	At4g27520.1:d:+1162:secondary
CAGAACATTA	47	20	42	22	10	At1g03600.1:d:-712:primary
TTGAGATATC	43	15	20	27	32	At3g63410.1:d:+1267:primary
GTGGTAAAGG	30	15	38	28	26	At3g48990.1:d:+1731:primary
TACTTACATT	56	20	27	13	21	At3g14420.1:d:+1387:primary,At3g14420.2:d:+1428:primary,At3g14420.3:d:+1520:primary
GAACAGATGA	45	28	31	18	11	At2g21170.1:d:+1104:primary
TTTCACCCAT	41	45	12	19	15	At5g24470.1:d:+1663:secondary,At4g34620.1:d:+390:secondary,At1g05385.1:d:+689:secondary
GGAGAGGAGC	17	41	32	31	11	At1g26630.1:d:+565:secondary
AATCCCTTAA	33	19	33	21	24	At5g65220.1:d:+732:primary
AGAACGCCAT	23	24	31	27	25	At5g10360.1:d:+909:primary
ACTCTTTTAA	33	21	25	26	25	At4g30960.1:d:+1919:primary
AGAGGAAACT	32	22	44	30	2	At4g09010.1:d:+1160:primary
TTAAACAAAA	12	30	51	15	22	At2g28140.1:d:+882:secondary
AGTCACATCT	30	31	30	25	13	At1g56190.1:d:+1374:primary,At3g12780.1:d:+1428:primary
TGTACTTTGT	37	23	29	16	23	At5g30510.1:d:+1421:primary
TCTTTTGAGT	28	17	22	27	34	At4g33865.1:d:+285:primary
AAGGATGCGA	3	68	9	34	14	At3g15280.1:d:+267:primary,At2g21660.2:d:+244:primary,At2g21660.1:d:+244:primary
TCGTTTGTGT	8	92	20	2	6	At2g33830.2:d:+499:primary,At2g33830.1:d:+491:primary
TTGGCCAAGA	32	20	30	21	24	At4g27090.1:d:+398:primary
TGCTACCTCC	16	60	27	21	3	At3g62290.1:d:+584:primary
TTTACGCAAT	21	18	19	28	41	At3g06700.1:d:+361:primary
GTACGGATCC	11	1	84	14	17	At2g34430.1:d:+209:secondary,At1g29920.1:d:+217:secondary,At1g29910.1:d:+203:secondary
AAAAGTCCGG	16	19	31	33	28	At2g27710.3:d:+502:primary,At2g27710.1:d:+436:primary,At2g27710.2:d:+432:primary
TGTGAAAAAA	27	18	41	32	9	At1g12660.1:v:+759:primary
TTACCAAATT	24	12	18	37	33	At4g31985.1:d:+317:primary
ACAAAAAAAA	22	27	32	23	20	At4g11560.1:d:+1925:primary,At1g71680.1:v:+2027:primary
TGAATTTGTA	17	33	37	21	15	At4g20260.1:d:+1027:primary,At4g20260.2:d:+952:primary
GCGACGGGTA	7	21	38	35	22	At2g23120.1:d:+159:primary
AACGACAAAG	26	32	25	23	16	At4g20360.1:d:+1354:primary
CTTGAACTGA	38	12	42	27	3	At2g27385.1:d:+619:primary
TTATTGAACT	27	15	13	22	45	At1g23290.1:d:+505:primary
CGTTCCCAAA	22	25	42	20	12	AtCg01130:d:+2065:secondary
TGGTTAAGAT	24	17	25	26	27	At5g28060.1:d:+667:primary
AATTGGAATG	40	29	20	21	9	At2g46820.1:d:+711:primary
TGTGATGTGT	44	23	31	14	7	At1g22690.1:d:+451:primary
CCAAGCGTGT	19	48	22	24	5	At4g40030.1:d:+434:primary,At4g40040.1:d:+442:primary

ACCAAAAAAA	26	11	40	30	10	Chr3:+1590528:quaternary,Chr1:+13369980:quaternary,Chr2:+1005686:quaternary,Chr1:+12380569:quaternary
GCAGAGTTGC	9	16	21	31	40	At3g09390.1:X:-371:quaternary
TGTTTGGACA	18	12	21	27	38	At5g26742.1:v:+3008:primary
AACCAAACCT	15	19	21	32	29	At5g06860.1:d:+1109:primary
TGCTTACCGT	31	43	34	6	2	At4g36040.1:d:+694:secondary
CCTCTGGTGA	25	52	29	10	0	At3g16420.2:d:+1065:primary,At3g16430.1:d:+1056:primary,At3g16430.2:d:+1069:primary,At3g16420.1:d:+1081:primary
GCTTGCGGAC	20	34	34	27	1	At1g37130.1:d:+2752:primary
TTTGGGTTAT	30	31	12	18	24	At3g11930.1:d:+753:primary,At3g11930.3:d:+744:primary,At3g11930.2:d:+666:primary
TGTTTTTCAT	40	10	29	8	27	At5g32593.1:p:+2942:primary,At1g31580.1:d:+641:primary
ATTCCGATTC	15	29	49	10	11	At5g24330.1:v:+427:secondary
GGATTTGAGA	17	50	23	15	9	At1g78040.1:d:+576:primary
CTTTAGTTTC	22	34	22	23	13	At1g32470.1:d:+647:primary
TTGTTGTATG	47	34	16	4	13	At1g05010.1:d:+1239:primary
TCTTATTTTG	26	13	19	16	39	At3g44010.1:d:+393:primary
GGATGACCAC	25	31	32	20	5	At2g37170.1:d:+792:primary,At2g37180.1:d:+796:primary,At3g53420.1:d:+994:primary
TGCAAGTTTG	6	36	14	32	25	At1g21310.1:d:+1592:primary
TTTCTTGTCT	13	23	13	11	52	At4g36570.1:v:+895:primary,At3g53460.1:d:+1323:primary
CTACTAAGTT	14	19	19	28	32	At3g05590.1:d:+708:primary
AAGTACCGTA	9	17	11	15	60	At2g02100.1:d:+367:primary
AACAAGCTCA	27	46	16	12	10	At3g57530.1:d:+1279:secondary,At1g34110.1:d:+751:secondary,At2g38910.1:v:+1602:secondary
CCAAGACCAC	6	13	28	43	21	At1g20440.1:d:+857:secondary
GAAAAAGCCG	27	15	38	24	6	At4g33010.1:d:+3131:primary
CCGGAGCTAG	19	23	24	23	21	At3g49010.2:d:+623:primary,At3g49010.1:d:+637:primary
TGACCCCTGA	9	49	20	24	8	At3g02470.1:d:+1354:primary
AAGAAGTTTT	17	11	27	25	30	At1g09210.1:d:+1521:primary
TAAAGTTTGA	14	3	32	45	14	At5g24120.1:d:+1873:primary
TTTATTTTTC	20	18	27	21	22	At3g55800.1:d:+1465:primary
GCACTGAGTG	19	36	35	13	5	At3g14420.1:d:+1085:secondary,At3g14420.3:d:+1218:secondary,At3g14420.2:d:+1126:secondary
AAGCTTATTT	24	29	22	19	14	At1g51200.1:d:+980:primary
GTGAGACTTG	14	30	19	27	17	At5g15200.1:d:+495:primary
AAGACACGTG	11	71	8	8	9	At5g14920.1:d:+851:primary
AGGAACACCA	14	30	22	31	10	At5g02500.1:d:+1771:secondary,At5g02490.1:d:+1746:secondary,At2g05200.1:p:+1052:secondary
AAAATATGAG	31	13	23	21	19	At4g29060.1:d:+3249:primary
TTTTTAAGTT	9	78	13	5	2	At2g05040.1:p:+2462:secondary,At1g19160.1:v:+1150:secondary
AAGTACCTTG	39	11	11	18	28	At1g18080.1:d:+1194:primary
TTCAATAGTT	3	0	10	33	60	At2g42530.1:d:+521:primary
CTAGGAGAGA	23	37	17	14	14	At1g02780.1:d:+568:primary
TTGGATCATA	39	13	21	20	11	At1g68010.1:d:+1322:secondary
GGGATTGGCT	24	19	37	13	10	At2g43970.1:d:+1932:primary,At2g43970.2:d:+1884:primary
CATTTGAAAC	34	17	26	21	4	At1g52220.1:d:+668:primary
AATGAGAATT	19	7	21	31	24	At1g26610.1:d:+405:secondary
AAATAAGTTA	14	54	14	13	6	At1g64230.1:d:+673:primary
CGAGCTCTTT	16	34	18	22	10	At4g35830.1:d:+2994:primary
GAAGAAAAAG	22	10	26	25	17	At3g61410.1:v:+354:secondary,At1g41803.1:p:+1509:secondary
GTTAATATCT	37	5	25	13	20	At2g21330.1:d:+1398:primary
AGTTTACATA	27	14	28	12	19	At2g20890.1:d:+1142:secondary
GAGCTACAAC	20	43	19	14	3	At3g47460.1:d:+3833:secondary
TGTTTCTTAC	14	6	22	19	38	At1g66580.1:d:+842:primary
GTGTGATCTC	33	16	28	14	8	At1g31812.1:d:+484:secondary
TGTGGAGTTA	26	9	31	23	10	At1g06040.2:d:+1130:secondary,At1g06040.1:d:+1048:secondary,At4g00490.1:d:+1382:secondary
TTGTTGCACT	18	18	19	17	26	At4g16720.1:d:+730:primary
TCTTATGTCA	28	17	15	10	28	At4g03520.1:d:+792:primary
GGAAGCAGAG	10	27	33	21	7	At3g53990.2:d:+549:secondary,At3g53990.1:d:+447:secondary,At1g74270.1:d:+329:secondary
TGACTCTCGT	19	13	31	19	16	At2g31570.1:d:+775:primary
GTTGAAGTCT	19	18	22	23	15	At5g64400.1:d:+616:primary
AGAGCTGGCA	14	20	27	20	16	At5g08670.1:d:+1841:primary
ATTTATGCTT	24	6	13	8	46	At4g31700.1:d:+910:primary
GGTTTCAGTT	18	20	23	24	12	At2g27720.1:d:+444:primary,At3g48350.1:d:+1203:primary

AGGATCCGTG	20	32	24	12	9	At2g09990.1:d:+319:primary,At3g04230.1:d:+205:primary,At5g18380.1:d:+270:primary
GTTTTCCCAC	20	25	10	18	24	At1g72610.1:d:+531:primary
TTTTACCTTT	25	14	12	11	35	At1g36010.1:p:+3548:primary,At4g34670.1:d:+989:primary
TGTTTATTTT	28	40	15	10	4	At1g01620.1:d:+1071:primary
TGTGTTCGTT	18	21	15	24	16	At5g44340.1:d:+1562:primary
TCTGTTTCTT	17	37	19	14	7	At5g41700.4:d:+559:primary,At5g41700.3:d:+762:primary,At5g41700.2:d:+665:primary,At5g41700.1:d:+620:primary
GGAGTTTTAC	19	13	29	25	8	At4g26190.1:d:+3271:primary
GTGCTGTTTG	23	30	16	9	15	At4g39200.1:d:+249:primary
AATCTACTTT	22	21	25	12	13	At1g64370.1:d:+716:primary
CTATACTCCC	20	49	5	9	9	ChrC:+106281:quaternary
GAGGAGTTTT	18	12	9	32	21	At5g52650.1:d:+647:primary
GTTCTGCAAA	18	12	18	18	26	At5g07970.1:d:+1172:secondary,At5g07940.1:d:+1219:secondary,At5g07980.1:v:+1330:secondary
AGACAAATGG	17	7	33	20	15	At2g23130.1:d:-708:secondary
ATTCTGAGCA	22	20	25	18	7	At1g09340.1:d:+1195:primary
ATCATCTTTG	19	33	19	10	10	At5g44020.1:d:+977:primary
TTGTTCTCTA	23	17	12	9	30	At3g18740.1:d:+434:primary
GACTTGGTGC	18	24	31	9	9	At3g08030.2:d:+1388:primary,At3g08030.1:d:+1308:primary
AGTTAATTTT	13	32	15	16	15	At2g41840.1:d:+1045:primary
AAAACAAAAA	24	0	34	25	8	At2g30810.1:d:+346:primary,At2g27750.1:v:+1057:primary,At4g00440.1:d:+3779:primary
GCTGTTTTTG	14	28	15	27	7	At1g07890.1:d:+946:primary,At1g07890.2:d:+829:primary,At1g07890.3:d:+842:primary
ACCACTCGAT	14	47	21	7	1	GI;7525047:d:X:+-34:quaternary
GTTTTGGTTC	18	5	18	29	20	At4g39800.1:d:+1904:primary
GGAGGAGACT	2	2	31	34	21	At4g17090.1:d:+1631:primary
AAAATGATTA	28	15	17	23	7	At3g02730.1:d:+767:primary
AGAGAGCTCG	21	41	16	11	1	At1g75380.3:d:+1134:primary,At1g75380.2:d:+1142:primary
TGTCTTAGCT	23	10	12	16	29	At1g09690.1:d:+593:primary
TCCGAAAAAA	17	27	28	14	3	At4g33790.1:X:--19:quaternary
CGTCGTACCG	12	40	29	5	3	At3g27690.1:d:+170:primary
GGATTAGTGA	21	13	24	20	11	At2g45470.1:d:+1422:primary
GGTGAAATTT	16	18	19	26	10	At1g19910.1:d:+684:primary
TTTTCACATT	34	22	13	13	6	At2g42220.1:d:+755:primary
GCGTCTTTTG	17	36	19	15	1	At1g52230.1:d:+70:primary
GCTGTTGAGT	20	23	14	17	14	At1g15690.1:d:+2370:secondary
GGATGCGATG	17	5	22	22	21	At4g24280.1:d:+2300:primary
TAATTTTGTC	6	30	10	16	25	At4g02380.1:d:+501:primary
TCAGGACGCA	24	11	23	19	10	At3g63490.1:d:+1291:secondary,At3g63490.2:d:+1281:secondary
TTATTATATT	28	32	11	4	12	At3g43610.1:v:+3886:primary,At1g14540.1:d:+1005:primary
TGGAGTCAAT	17	41	3	10	16	At2g38540.1:d:+368:primary
TATGTCGGAG	18	28	21	13	7	At2g27860.1:d:+1423:secondary
TATCGAAGTT	25	13	21	22	5	At5g44190.1:d:+1361:primary
AAGAAACAAA	33	0	37	14	2	At4g17615.2:d:+1109:primary,At4g17615.1:d:+903:primary
TTTTCGATTT	23	8	19	20	15	At3g53890.1:d:+449:primary
AACGCAGTTC	24	10	27	19	5	At2g10940.2:X:-1558:quaternary
ATACTGTCAA	21	15	18	22	9	At2g10940.2:d:+1564:primary,At2g10940.1:d:+1210:primary
TATGTGTTTT	18	16	18	18	15	At1g32990.1:d:+809:primary
TATTTCGAAG	14	30	10	17	13	At3g04400.1:d:+352:primary
TGGATCTTGA	20	11	18	12	23	At2g40510.1:d:+564:primary
AGGAGATTAA	14	16	18	21	15	At1g56045.1:d:+93:primary
GATAATTCGT	13	26	27	14	3	At1g76490.1:d:+2107:primary
AAGAAGCTAG	16	31	13	9	14	At1g07770.2:d:+395:primary,At1g07770.1:d:+435:primary,At3g46040.1:d:+431:primary
AAAACACAAA	12	14	14	14	28	Chr1:+55191:quaternary,Chr1:+14720370:quaternary,Chr1:+26981509:quaternary
GAAGAGATGT	18	10	21	19	14	At3g61580.1:d:+1662:secondary
AAACTTTATT	3	2	17	36	24	At3g55700.1:X:+-248:quaternary,Chr3:+13031736:quaternary
GTATATGCAA	19	14	20	12	17	At3g54050.1:d:+1351:secondary
AGTGTTGTTT	19	23	20	10	10	At3g30390.1:d:+1826:secondary
GTCTGTAATG	9	8	37	19	9	At3g17800.1:d:+1552:secondary
TCGCGTTCTA	24	9	28	19	2	At2g42750.1:d:+1216:primary
AAAAAGAAAA	20	10	15	19	18	At1g15270.1:d:+426:primary
AAGAAATTAT	14	35	23	7	3	At1g11260.1:d:+1827:primary
GCTATACAAA	24	12	14	13	18	At4g11150.1:d:+1014:primary

GGGATAACGG	18	11	23	16	13	At3g15190.1:d:+686:primary
ACCTTTTGTT	26	17	11	12	15	At2g04880.2:d:+23:secondary,At5g08280.1:d:+1474:secondary,At2g04880.1:d:+23:secondary
ACTATTGCTT	16	26	14	16	9	At1g71190.1:d:+400:secondary
TTTGTTGCTT	22	15	17	13	14	At1g58290.1:d:+1967:primary
AACAAGAGAT	21	10	14	9	27	At1g53240.1:d:+1378:primary
AAAATCCGCG	23	4	33	15	6	At1g32060.1:d:-1409:secondary
GCCTGCAGTT	13	17	25	22	4	At1g09750.1:d:+1532:primary
AAGAAATCAA	17	14	23	13	13	At5g53730.1:d:+406:secondary,At5g48820.1:v:+366:secondary,At3g23000.1:d:+1417:secondary
GGAGATTTTG	17	8	25	18	12	At5g23740.1:d:+604:primary
ATCTACAATT	6	59	9	3	3	At4g30270.1:d:+825:primary
TGTTAGTGTG	19	43	12	0	6	At2g41100.2:d:+867:primary,At2g41100.1:d:+1134:primary
AGAAAATATG	22	22	15	9	12	At2g33800.1:d:+1058:primary
GGGGCTTTCT	15	8	23	20	13	At5g50920.1:d:+3038:secondary
ATTTAGATTC	38	4	8	18	11	At3g20430.1:d:+113:secondary
CCTGTCTCAG	14	21	11	18	15	At2g42590.1:d:+1143:primary
CTTAAATTTG	14	45	10	8	2	At2g39010.1:d:+953:primary
GGTCCTTTAC	8	42	8	15	6	At1g67740.1:d:+447:primary
AAGCTTTATG	18	48	6	7	0	At1g62380.1:d:+931:primary
AAGAGTTTAG	19	10	20	19	10	At5g28750.1:d:+543:primary
TGATCCGAAT	10	6	25	22	15	At5g26717.1:v:+1050:secondary
TGAGAGTTGG	15	31	18	10	4	At1g68560.1:d:+2855:primary,At2g07490.1:p:+456:primary
CGGTGAAAAA	14	1	9	30	23	No gene matches found
AAACCAGCAA	13	30	15	16	3	At5g62350.1:d:+638:primary
ATTTGAAAAC	11	16	16	17	17	At5g44130.1:d:+855:primary
TACTTTATCA	38	11	13	10	5	At4g37980.1:d:+1263:primary
TTCTCGATTA	17	35	12	8	5	At2g46330.2:d:+647:primary,At2g46330.1:d:+490:primary
GGGTTCATTG	9	0	60	4	4	At2g05070.1:d:+565:secondary,At2g05100.1:d:+566:secondary
TGTTTGTCAA	22	6	27	10	12	At1g45130.1:d:+1721:secondary,At5g56870.1:d:+1599:secondary,At1g31580.1:d:+570:secondary
CATCTGTATG	29	7	13	12	15	At5g58330.1:d:+1556:primary,At5g58330.3:d:+1471:primary,At2g20360.1:d:+1626:primary,At5g58330.2:d:+1496:primary
AGGAGCGATG	13	27	16	17	3	At5g25460.1:d:+1076:primary
AAGGGTATCG	20	13	19	15	9	At2g42600.2:d:+3057:primary,At2g42600.1:d:+3162:primary
CCTGGACTCA	16	35	8	12	5	At1g71270.1:d:+1491:secondary,At3g48140.1:d:+300:secondary
GCACAATACT	20	20	25	6	5	At1g54780.1:d:+925:primary
CCAGCACCAC	1	18	16	30	11	At1g20450.2:d:+1010:secondary,At1g20450.1:d:+1013:secondary
CCGTGCCGGT	13	16	25	22	0	At1g10960.1:d:+332:primary
GGTATTGGAA	15	25	21	6	9	At1g05850.1:d:+1018:secondary
TCGGAGATTT	12	44	16	3	0	At5g64570.1:d:+2344:primary
TGTTTGAACT	10	14	15	20	16	At5g12140.1:d:+513:secondary
CTTGTTTATT	19	13	20	10	13	At3g23820.1:d:+1624:primary
TCTGTCAAGA	6	13	48	4	4	At1g70700.1:d:+1182:primary,At5g39520.1:d:+1201:primary
GCTTCATAGA	15	11	13	16	20	At1g65820.1:d:+684:primary
CTAAAAAAAA	16	18	16	13	11	Chr3:+16234478:quaternary,Chr2:+12528996:quaternary,Chr4:+1434523:quaternary,Chr5:+19448019:quaternary
AGGTGTATCT	3	66	3	2	0	At4g35750.1:d:+783:primary
GTGTGGACCT	16	37	9	12	0	At3g15360.1:d:+405:secondary,At4g03520.1:d:+438:secondary,At1g31020.1:d:+375:secondary,At3g06730.1:d:+357:secondary
GATAAGCTCG	11	14	15	22	12	At2g30950.1:d:+2019:primary
TGGCCCATCT	25	15	24	7	3	At1g19000.1:d:+1161:primary,At1g19000.2:d:+1334:primary
ATCAGCATCT	20	15	31	6	1	At5g14740.1:d:+1350:secondary,At5g14740.2:d:+1232:secondary
AGTTATAAGT	17	7	20	18	11	At3g62030.1:d:+1022:secondary
AAAGTTTTGA	8	12	13	19	21	At2g32060.2:d:+659:primary,At2g32060.3:d:+657:primary,At2g32060.1:d:+695:primary
TTCCAAAGGT	25	13	21	8	6	At2g22990.5:d:+1347:primary,At2g22990.1:d:+1347:primary,At2g22990.4:d:+1351:primary,At2g22990.3:d:+1349:primary,At2g22990.2:d:+1293:primary,At2g23000.1:d:+1354:primary
TAGACCGATC	14	10	23	19	6	At5g51110.1:X:-174:quaternary
GTAGTGACTC	17	13	9	13	20	At4g09320.1:d:+431:primary
AACGATCTTG	11	35	11	14	0	At5g62690.1:d:+1387:primary,At2g29550.1:d:+1351:primary,At5g62700.1:d:+1349:primary,At2g34760.1:p:+1299:primary
CCCTTTGATC	6	15	14	12	24	At5g13650.1:d:+2256:primary,At5g13650.2:d:+2285:primary
GATCTCCGAT	8	35	6	19	3	At4g37020.1:d:+730:secondary

TATTGTGATT	10	37	6	8	10	At4g29350.1:d:+577:primary
TAGAAAGAAA	27	6	27	11	0	At4g26660.1:d:+1330:secondary
GGACCACCAC	9	10	13	18	21	At3g53970.1:d:+789:primary,At2g27530.1:d:+724:primary,At2g27530.2:d:+693:primary
ATCAAAGATA	14	42	3	5	7	At2g30860.1:d:+688:primary
GCGAGTCATT	13	15	18	13	12	At1g23410.1:d:+413:primary,At2g47110.1:d:+430:primary
TATCGAACTG	6	44	15	4	2	At1g12780.1:d:+1367:primary
TAACTGTTGC	12	11	17	13	17	At5g39570.1:d:+1415:secondary,At1g74150.1:v:+1057:secondary,At3g18290.1:d:+3652:secondary
GCCCATTATT	23	10	18	9	10	At4g25100.1:d:+1093:primary,At4g25100.2:d:+957:primary
TGGATGTCGC	7	24	24	14	1	At3g01690.1:d:+1374:primary
TAATAAACAG	26	8	9	6	21	At1g70760.1:d:+736:secondary,At1g80160.1:d:+694:secondary
AAGAACCATA	16	7	17	17	13	At1g53550.1:v:+29:secondary
CATTTGGATT	11	33	9	10	7	At1g25275.1:d:+298:primary
AACACCAATC	15	10	16	17	11	At5g13630.1:d:+4142:primary
TTTCAGAAAA	11	13	20	16	9	At4g01310.1:d:+897:primary
TGTTTGGTTT	13	3	17	19	17	At3g52880.1:d:+1554:primary,At3g52690.1:v:+1733:primary
TGTTGCCGAT	10	29	15	14	1	At3g48560.1:d:+1979:primary
AGTTTATTAA	15	10	8	18	18	At3g17210.1:d:+549:primary
AACTTCCATA	19	10	20	8	12	At2g21960.1:d:+1141:secondary,At5g15620.1:v:+1713:secondary,At4g15730.1:d:+3652:secondary,At3g54160.1:v:+1655:secondary
AGAGAAGAGA	17	15	17	12	8	At1g47128.1:d:+1483:primary
ATTAATAACA	44	6	9	3	7	At1g34430.1:d:+1704:secondary
GTCTATGCTT	12	3	19	18	17	At1g06040.2:d:+1240:primary,At1g06040.1:d:+1158:primary
TTAAATTTAT	17	27	10	5	9	At5g60360.1:d:+1415:primary
TACAGAGTCG	5	56	2	5	0	At4g35770.1:d:+466:primary
ATCCATCGTT	18	13	13	12	12	At4g34710.1:d:+2765:primary,At4g34710.2:d:+2630:primary
GTCTCAAACT	14	19	14	15	6	At4g30950.1:d:+1472:primary
GTTTGTAATT	16	14	21	11	6	At3g48000.1:d:+1875:primary
AGGGCTAAGT	12	5	10	19	22	At3g11120.1:d:+80:primary
GACCAATAAT	14	14	26	9	5	At1g50720.1:v:+1023:secondary,At4g01850.1:d:+1327:secondary
AGAGTTTGTT	19	18	11	11	8	Chr2:+2179296:quaternary,Chr5:+10557010:quaternary
GTGGGGGTCG	8	26	10	19	4	At5g44580.1:d:+288:primary
GATCAAAGAG	17	31	8	9	2	At4g32260.1:d:+494:primary
TAATAAAAAA	16	11	11	16	13	At4g31250.1:i:+937:tertiary,At5g39220.1:i:+1857:tertiary
TCCTTCAAGA	9	16	11	13	18	At3g60770.1:d:+564:primary
GAGATTATCC	10	30	8	8	11	At3g11940.1:d:+414:primary,At3g11940.2:d:+365:primary,At2g37270.1:d:+401:primary
AGGTCTTGGT	17	21	21	7	1	At3g09260.1:d:+1684:secondary
ACCACTGACC	7	19	15	22	4	At2g47730.1:d:+542:primary
TTACAATAAC	14	11	6	5	30	At5g19510.1:d:+856:primary
ATGATGATGA	20	16	13	11	6	At5g15770.1:v:+813:primary,At3g25280.1:v:+2282:primary,At3g13280.1:v:+1275:primary,At5g63850.1:d:+1712:primary,At1g25988.1:v:+1258:primary,At1g02870.1:d:+656:primary
CTGAAAAAGG	5	29	9	16	7	At5g01600.1:d:+975:primary
AAAAATGGAG	18	4	14	10	20	At4g13940.1:d:-1702:secondary
GGACTCGAGG	14	12	17	14	9	At3g56800.1:d:+697:secondary
ACCCGTCTCA	11	33	11	10	1	At3g50820.1:d:+605:primary
ATTTTGAGAT	12	8	12	16	18	At3g27240.1:d:+1319:primary
TCAAAGTAGA	23	7	18	10	8	At3g18490.1:d:+1585:primary
AGTGAGCATA	20	15	17	11	3	At2g25080.1:d:+803:primary
TAGCTGTTGG	13	31	9	12	1	At1g74470.1:d:+904:primary
GTGTTAATTG	20	8	16	11	10	At5g63135.1:d:+549:primary,At5g10980.1:d:+604:primary
TGAACATATA	13	3	11	15	23	At3g23400.1:d:+1008:primary
GCCCCTGCGC	5	1	5	36	18	At3g13857.1:d:+51:primary,At5g46310.1:v:+1955:primary,At5g62990.1:v:+2175:primary,At3g13850.1:v:+1598:primary
AGGTGTTAGT	9	4	16	13	23	At2g15970.1:d:+811:primary
GTTATTTGGG	9	8	22	19	7	At1g77760.1:d:+2905:primary
TTTCAAAAAA	9	14	14	17	10	Chr4:+15358852:quaternary,Chr3:+10556050:quaternary
TATTGCTTGT	31	8	12	2	11	At5g64940.2:d:+2620:primary,At5g64940.1:d:+2675:primary
TCGAGTTATA	16	7	9	15	17	At5g51110.1:d:+805:secondary
AACAAAAAAA	17	9	16	17	5	At4g14550.1:d:+783:secondary,At1g28135.1:v:+222:secondary,At5g45120.1:v:+1826:secondary,At3g12880.1:d:+32:secondary
TCTCTCTTCT	19	8	10	11	16	At3g51260.1:d:+1122:primary,At5g52920.1:d:+1946:primary

TGTAGATTCT	12	27	14	11	0	At3g13750.1:d:+3053:primary
TTGCACCCTC	14	38	9	3	0	At1g62510.1:d:+401:primary
TCTGAAAGAG	11	8	17	11	17	At1g05490.1:d:+503:secondary
ACCTTGTGAC	9	10	14	15	15	At5g20500.1:d:+559:secondary
TGAATCTTGT	11	13	10	14	15	At4g04770.1:d:+1860:primary
TCTCGTTTCA	15	18	18	6	6	At4g02890.2:d:+795:primary,At4g02890.1:d:+1022:primary,At4g02890.3:d:+1319:primary
GATGTTCACG	16	6	15	12	14	At3g04840.1:d:+746:primary
GTCCGGTTTA	11	13	13	16	10	At2g19480.1:d:+1379:primary
TTTGAGGTGG	16	27	12	4	3	At5g43830.1:d:+970:primary
CCTCTAGTAG	13	14	11	11	13	At5g39740.1:d:+978:primary
ATTCAGTGAA	14	16	10	14	8	At5g08290.1:d:+677:primary
GGTGTCTCTT	13	15	10	14	10	At5g07090.1:d:+732:primary,At5g58420.1:d:+798:primary
CCCTTACATA	3	52	1	3	3	At4g39090.1:d:+1032:secondary
TCGTGTTTGG	15	15	15	9	8	At3g62030.1:d:+751:secondary
GAATGATCCT	10	6	14	12	20	At2g16380.1:d:+1046:secondary,At3g08520.1:v:+568:secondary,At1g05470.1:v:+893:secondary,At4g34580.1:v:+1298:secondary
GGCCTAATGA	8	6	13	24	11	At2g04030.1:d:+2502:primary,At2g04030.2:d:+2493:primary
TCTTGTCCAA	19	4	14	15	10	At1g74730.1:d:+694:secondary,At4g38260.1:d:+415:secondary,At3g42940.1:v:+613:secondary
GGAGAAGTCC	11	20	17	11	3	At1g60940.1:d:+1350:primary,At1g60940.2:d:+1214:primary
TTAAAACGGA	13	11	21	9	8	At1g42970.1:d:+1642:secondary
AATAGAGAGA	20	15	14	8	4	Chr1:+21634065:quaternary,Chr4:+1031767:quaternary
TATAGAGCCA	6	40	12	1	2	At4g37260.1:d:+1137:primary,At5g61590.1:d:+737:primary
TTCGACAAAA	11	8	19	13	10	At3g49840.1:i:-1141:tertiary
AGAATGCTCG	14	10	20	16	1	At2g47940.1:d:+2079:primary
CCCGCTTCAG	10	6	15	18	12	At2g28800.1:d:+1726:primary
AACGCACACC	16	7	18	12	7	AtCg00340:d:+2048:primary
GCGCGAAAAT	17	5	22	12	4	At4g26530.1:d:-1301:secondary
AAGTCGACGG	0	1	53	4	2	At4g25480.1:d:+726:primary
TTATGATACA	16	18	10	2	14	At3g54530.1:v:+1289:primary,AtCg00810:d:+441:primary
TTTCTGTTTT	17	11	7	7	18	At3g05560.2:d:+711:primary,At3g05560.1:d:+508:primary
TAAAGAAAAA	10	18	11	16	5	At3g03700.1:d:+1517:primary,At1g31970.1:d:+1563:primary,At3g04440.1:d:+1478:primary,At4g29190.1:d:+1139:primary
CTTGGGAAAA	8	24	13	11	4	At2g45820.1:d:+411:primary,At3g61260.1:d:+479:primary
TATCAACGTT	14	13	13	9	11	At1g22850.1:d:+1164:primary
TGTGGACTCA	5	26	9	15	5	At1g20020.1:d:+1025:primary
TGAAGAACGT	5	22	23	6	4	At1g09070.1:d:+1107:primary
AACGTTGGTG	11	22	10	8	9	At1g01100.2:d:+250:primary,At1g01100.1:d:+238:primary,At5g47700.1:d:+301:primary
TCCGGTAATT	7	10	15	11	16	At5g12890.1:d:+1637:primary
AATAAAAAAA	9	16	10	15	9	At4g25900.1:d:+1241:primary
CCGATGATGA	8	45	5	1	0	At3g47340.2:d:+2009:primary,At1g64210.1:v:+2391:primary,At3g47340.3:d:+2090:primary,At3g47340.1:d:+1916:primary
AGAGAGTTCA	7	23	6	19	4	At3g13920.1:d:+1107:primary,At1g54270.1:d:+1236:primary
CAGTCTGTTA	13	8	11	8	18	At5g67360.1:d:+2501:secondary
ATTTCTAATG	25	12	5	7	9	At5g55500.1:d:+1986:primary,At1g12800.1:d:+2534:primary
AAGAGCCGAG	10	25	8	11	4	At5g54600.1:d:+414:secondary
AGGGATGCGA	3	39	6	5	5	At4g39260.4:d:+240:primary
ATGTGGTGGA	10	24	6	6	12	At3g49110.1:d:+1077:primary,At3g49120.1:d:+1193:primary
TTTTTTTGAT	15	18	5	8	12	At2g21870.1:d:+1035:primary,At2g21870.2:d:+1035:primary
TAGTCTATCT	6	44	5	1	2	At2g15890.1:d:+727:primary
GCTAAAAGGG	17	15	12	11	3	At2g01490.1:d:+1015:primary
AATAAAAGTG	16	3	11	10	18	At1g70780.1:d:+944:primary,At3g59010.1:d:+1891:primary
TGATTGTGAA	6	27	24	0	1	At1g32920.1:d:+425:primary,At3g52790.1:d:+398:primary
CGATTAGGAG	13	9	12	15	9	At1g09130.1:d:+1154:primary
GGTTCCGTCG	4	15	22	11	5	ChrC:+82098:quaternary
GAAACCTTGA	10	21	10	11	5	At5g49720.1:d:+1977:primary
TGTGTTAAAA	16	11	19	8	3	At5g44720.2:i:+837:tertiary,At5g44720.1:i:+837:tertiary
AAAAGAAGAA	10	11	17	10	9	At4g39745.1:v:+156:secondary,At1g04410.1:d:+1347:secondary,At5g40060.1:v:+42:secondary
TGTGATGATC	9	22	17	7	2	At4g23400.1:d:+1002:primary
AATCGCGTCA	8	15	17	9	8	At3g51550.1:d:+3037:primary
TATTACTATT	8	10	6	4	29	At3g47650.1:d:+572:primary
CCTCTTGTAG	16	14	4	9	14	At3g25520.1:d:+995:primary

CGCTAACAAC	18	11	18	7	3	At1g79040.1:d:-508:secondary,At1g79040.1:d:-518:secondary
CTTGTTGATT	17	6	12	8	14	At1g65960.1:d:+1703:primary
AAGAAAAAAA	11	8	17	16	5	At1g36745.1:v:+935:primary,At5g49820.1:d:+1698:primary
ATCACCAGAA	6	24	15	8	4	At1g31810.1:X:-494:quaternary
GAAGAAGCGG	24	5	17	9	2	At1g30380.1:d:+622:primary
TATTTGGTTT	14	15	6	6	16	At1g27970.1:d:+636:primary
TCACATTAGG	13	7	11	10	16	At1g27950.1:d:+602:primary
TTGGGTCTTT	18	15	3	13	8	At1g07560.1:v:-2795:secondary,At1g07560.1:v:-2788:secondary,At1g07560.1:v:-2804:secondary
TCTCTCACTC	25	11	7	10	4	At1g07473.1:v:+1008:primary
CACTTGGATT	11	16	16	7	6	Chr5:+3474898:quaternary
GTTATGTTTA	18	8	13	3	14	At5g42980.1:d:+655:primary
TTGTGTGGTT	21	4	12	9	10	At5g25610.1:d:+1197:primary
TACAATTCGG	7	41	5	3	0	At5g20250.2:d:+2328:primary,At5g20250.1:d:+2307:primary
GTGCATTTGG	8	27	10	9	2	At4g35100.1:d:+671:secondary,At2g16850.1:d:+682:secondary
AAATCCAGCA	15	11	19	7	4	At4g27700.1:d:+947:primary
TCTTTGAATC	16	10	18	5	7	At3g62410.1:d:+546:primary
AAATTGATCT	23	4	13	9	7	At3g26570.2:d:+2028:primary,At3g26570.1:d:+1917:primary
GCACCAGGAC	3	24	14	9	6	At3g22890.1:d:+1243:primary
ATACACTGAA	21	10	12	7	6	At3g08610.1:d:+454:primary
GTTGAGATCC	9	13	11	11	12	At3g06720.2:d:+271:secondary,At3g06720.1:d:+335:secondary,At3g05720.1:v:+444:secondary,At1g56070.1:d:+2305:secondary
CAAACGTATA	9	32	13	1	1	At3g04730.1:d:+817:primary
GTTGGTCTTA	10	17	11	14	4	At2g38230.1:d:+896:primary
CTTTAGTTTG	5	10	8	13	20	At2g17360.1:d:+944:primary
CACTTGTGGT	17	12	15	9	3	At1g71695.1:d:+1186:primary
CGTTGCACTA	15	28	9	2	2	At1g16830.1:v:+24:secondary
CCCCAGAAAA	12	6	26	9	2	No gene matches found
GACCCGGTGA	8	29	7	9	2	Chr5:+1817181:quaternary
GTAAAAAAAA	9	13	13	10	10	At5g23780.1:d:+1278:primary,At1g79310.1:d:+1296:primary,At1g10990.1:d:+122:primary,At1g10990.2:d:+122:primary
CAATTTATTA	3	20	4	8	20	At2g41090.1:d:+744:primary
GACCAACCAC	18	24	6	6	1	At2g36830.1:d:+703:primary
TAAGCTAATC	15	2	10	7	21	At1g62750.1:d:+2542:secondary
GGAAATGTTT	4	39	6	4	2	At1g41760.1:p:+339:secondary,At5g37500.1:d:+225:secondary,At4g06509.1:p:+535:secondary,At1g41803.1:p:+3415:secondary,At3g23050.1:d:+684:secondary ,At4g06510.1:p:+663:secondary,At5g34867.1:p:+779:secondary
CGCTTTGATC	11	22	9	6	7	At1g33140.1:d:+568:primary
TTATCTCTCT	22	11	7	3	12	At1g04820.1:d:+1598:primary
AAATCTGGGG	7	24	11	8	4	ChrC:+125548:quaternary
GGAGTTGATG	11	6	14	14	9	At5g38470.1:d:+1336:primary
CTTTTTTGTT	14	10	7	8	15	At5g35416.1:p:+4626:primary,At1g72370.1:d:+1025:primary
ATACAAAGTA	21	5	11	4	13	At5g17290.1:i:+2134:tertiary
GTAAAGTTGT	10	13	16	10	5	At4g24770.1:d:+874:primary
AGAATGGTTG	13	23	9	8	1	At3g51730.1:d:+920:primary
GAAACCTTAA	5	9	19	9	12	At3g50230.1:v:+1876:secondary,At3g52970.1:d:+1128:secondary
GGGCACGTGG	13	12	6	20	3	At2g44430.1:d:+206:secondary
ATTCCTTCTA	33	10	4	5	2	At2g21210.1:d:+524:primary
AAATGACATT	8	6	9	12	19	At1g58983.1:d:+1035:secondary
TTGTATCCTG	15	16	8	4	10	Chr3:+1694094:quaternary
ATCACACACA	16	7	16	7	7	At5g28450.1:X:-437:quaternary,At3g15220.1:X:-188:quaternary,At2g01830.2:X:--83:quaternary
GCTCTGATGT	11	9	12	12	9	At4g33590.1:v:+2121:primary,At5g19540.1:d:+1514:primary
AGAGTTATCT	9	7	4	13	20	At4g00810.1:d:+597:primary,At4g00810.2:d:+519:primary
TGGATCTCAT	9	7	3	14	20	At3g16080.1:d:+444:primary
CACAAGCTCT	10	7	6	18	12	At3g14690.1:d:+1559:primary
AAGATTGGTC	15	10	11	8	9	At3g11510.1:d:+443:primary
AATGTTATCG	16	24	6	6	1	At1g76080.1:d:+852:primary
AATTTGTGTT	13	18	7	10	5	At1g47420.1:d:+1036:primary
GGCCTTCGCA	10	6	24	9	4	At1g29910.1:d:+824:secondary
ATTTGGGTTT	12	10	13	10	8	At1g22300.2:d:+1137:primary
ATGGTGATTA	5	25	5	9	8	At5g11740.1:d:+104:primary
GCTGAGAGAC	4	28	12	8	0	At4g22710.1:d:+1452:primary,At4g22690.1:d:+1986:primary

GCCAAACAGG	6	23	10	11	2	At4g17430.1:d:+72:secondary,At5g10450.1:d:+657:secondary
TTGTATCCAA	21	11	9	6	5	At3g16400.1:d:+1646:primary
CTCAAACCTT	6	2	17	11	16	At2g34460.1:d:+894:secondary
ATTATGATTC	13	11	10	10	8	At2g20290.1:v:+5287:primary
TTATAATTCA	21	15	4	3	9	At2g06850.1:d:+1085:primary
GACGTATTGA	13	10	9	16	4	At1g48350.1:d:+522:primary
GAAGAAAAAA	18	1	10	15	8	At1g17010.1:d:+432:primary
TAAACAATCC	18	9	14	4	7	At1g06460.1:d:+1155:secondary
TTTTGCTTGA	8	14	15	7	7	At5g47610.1:d:+558:primary,At5g22270.1:d:+343:primary
CAACAGCTCT	11	18	7	8	7	At5g47210.1:d:+1003:primary,At4g17520.1:d:+917:primary
TAATTTACAG	15	16	4	4	12	At5g47190.1:d:+858:primary
TTCAAAAAAA	11	4	20	10	6	At5g15100.1:v:+1465:secondary
AAGTTTACGG	11	3	5	20	12	At4g37910.1:d:+2308:primary
TACCAAATAA	1	0	0	20	30	At4g30650.1:d:+363:primary
TTTAAAAAAA	18	10	11	7	5	At2g45540.1:i:+9263:tertiary,At5g27030.1:i:+4204:tertiary

GTGTAATCAA	12	9	6	8	16	At2g28000.1:d:+1893:primary
GGGAGGCAAG	12	14	11	7	7	At2g21330.1:d:+1110:secondary
GAGGAGGTAA	7	12	10	9	13	At2g18020.1:d:+682:primary
AGTGTTTTCT	7	11	16	10	7	At2g02130.1:d:+386:primary
GTTGTTGTAA	7	8	13	14	9	At1g64320.1:v:+1893:primary,At1g30360.1:d:+2524:primary
GAAGCAAATA	5	3	14	10	19	At1g58643.1:v:+2213:primary,At1g58936.1:v:+2213:primary,At1g59312.1:v:+2213:primary,At1g70970.1:v:+1942:primary
GAACAGTGGC	18	7	12	9	4	At5g65480.1:d:+684:secondary
GATCTCCAGT	11	3	17	14	5	At5g65010.2:d:+1899:primary,At5g65010.1:d:+1896:primary
AGATTGAGAT	10	9	16	9	6	At5g49210.1:d:+475:primary,At5g49210.2:d:+348:primary,At3g07390.1:d:+1154:primary
GAGGTCCGAT	9	4	19	13	5	At4g26850.1:d:+1783:primary
ATAGTGGTGT	25	2	5	8	10	At4g17245.1:d:-593:secondary
ATGTAAGAAA	10	13	7	12	8	At3g58680.1:d:+666:secondary
GTGCCATTGA	9	16	11	11	3	At3g58610.1:d:+1785:primary
TACACAATTG	12	9	14	9	6	At3g43330.1:v:+2291:primary
CTTGTTTCGG	4	36	5	3	2	At3g04720.1:d:+644:primary
ATCAAAGGTG	11	17	10	9	3	At2g43030.1:d:+792:primary,At3g32310.1:p:+74:primary
TTTGATTTCT	13	9	13	6	9	At2g27600.1:d:+1604:primary,At1g06900.1:d:+2946:primary
AAAGAAATTG	10	7	9	13	11	At2g19240.1:v:+3068:primary
GATATTGTAA	19	5	9	6	11	At1g75690.1:d:+619:primary
CTGGCAAGAA	10	6	17	12	5	At1g52130.1:v:+1195:secondary,At3g52230.1:d:+634:secondary
GGAGCTTAGG	4	34	5	6	1	At1g22530.1:d:+1940:primary
GTGGTTGATT	16	9	7	12	6	At1g08200.1:d:+1410:primary
GAGGAGGCGT	3	33	2	12	0	At1g05135.1:p:+1043:primary
ATCAAAAAAA	10	11	13	8	7	Chr3:+10715256:quaternary,Chr3:+10034081:quaternary
TCGACTCTAT	10	3	9	10	17	At5g61820.1:d:+1516:primary
GATTGAAGTT	8	18	9	9	5	At5g61410.2:d:+822:primary,At1g72810.1:d:+1424:primary,At5g61410.1:d:+953:primary
AAACTTATTG	13	13	10	9	4	At5g55160.1:d:+603:primary
TTACCACAGA	10	1	12	13	13	At5g35970.1:X:+430:quaternary
CACCTGAAAA	17	2	13	14	3	At5g27970.1:i:-9251:tertiary
AACAACAAAA	10	16	11	7	5	At4g37300.1:d:+695:primary,At1g80440.1:d:+1310:primary
GAGGCTTGTA	9	11	15	11	3	At4g23850.1:d:+434:secondary
AGGATCCTTT	4	9	16	11	9	At4g21620.1:d:+525:primary
TATGTGTGCA	18	11	10	4	6	At3g61430.1:d:+1126:secondary
GAGATGCAGG	5	27	10	3	4	At3g17390.1:d:+943:primary
ATCAAAAGCG	7	6	12	9	15	At3g06700.1:X:-177:quaternary
CAGATCTTGA	18	2	19	9	1	At3g01500.1:d:+1211:primary,At3g01500.2:d:+1213:primary
CTTGTTTTCT	14	19	5	3	8	At2g35370.1:d:+684:primary,At1g73790.1:d:+384:primary
GGAAGAGAGG	9	8	12	8	12	At2g26280.1:d:+2006:primary
CAAATCAGTT	9	8	7	11	14	At1g76930.1:d:+955:primary,At1g76930.2:d:+1001:primary
GATGGTTGGG	7	4	12	23	3	At1g22410.1:d:+1822:primary
TGACTCTAAA	7	4	12	13	12	Chr3:+1849299:quaternary
TTGGTTAACC	4	21	13	7	3	AtCg00900:d:+76:primary,AtCg00900:d:+76:primary
TTACATCTAA	18	6	4	9	11	At5g42300.1:d:+391:primary
TATTGCCTCC	8	15	16	5	4	At5g18650.1:d:+1174:primary

CGTTGTTGTT	19	6	9	12	2	At4g14920.1:i:+626:tertiary
ACTCGTGTTT	11	8	9	9	11	At3g60820.1:d:+914:primary
AGATTAATTT	3	34	7	1	3	At3g57660.1:i:+732:tertiary
CAGAATAAGT	6	3	14	16	9	At3g55610.1:d:+2520:primary
AGCTTTTTGT	12	8	10	9	9	At3g13520.1:d:+420:primary
ATGTTAACCG	7	26	11	1	3	At3g12120.1:d:+697:primary
GATTTGTGGC	6	5	10	13	14	At3g08590.2:d:+1718:primary,At3g08590.1:d:+1719:primary,At1g09780.1:d:+1679:primary
AGAAAAAAAA	10	6	12	12	8	At3g05700.1:d:+949:secondary
ATGACAACTT	9	10	7	11	11	At2g26900.1:d:+1443:primary
GCTCGTGCCA	7	24	6	6	5	At2g23090.1:d:+170:primary
TGAGTGCTTG	3	11	9	17	8	At2g19110.1:d:+3610:primary,At1g02930.1:d:+808:primary
GCAAGACGAC	15	7	15	10	1	At2g05060.1:v:+1771:primary
ACTATCTTCT	13	16	9	4	6	At1g75950.1:d:+719:primary
AATTACGAGG	12	10	13	2	11	At1g65290.1:d:+594:primary
GCGAGGAAAT	1	23	11	7	6	At1g35720.1:d:+673:primary
AAATTTCTGC	14	11	16	6	1	At1g22700.2:d:+250:primary,At4g18970.1:d:+1332:primary
AATCTTCAAT	11	13	7	10	7	At1g16180.1:d:+1279:primary
GCACTTCGAA	14	12	7	11	3	ChrC:-70565:quaternary
GCTATGTTTA	13	14	6	5	9	At5g67590.1:d:+655:primary
AGGTGCTATT	12	7	17	7	4	At5g51010.1:d:-509:secondary
ATAAAACTAC	13	4	5	11	14	At5g47930.1:d:+427:secondary
GAAAGAGCCA	7	17	14	5	4	At5g47110.1:d:+817:primary
TCTTCTAATT	9	13	9	6	10	At5g27700.1:d:+457:primary
TACAGCCCTG	6	23	12	5	1	At5g11670.1:d:+1841:primary
AAGCTGAGAG	10	18	3	5	11	At4g09800.1:d:+419:primary
GATGTTGTGG	2	24	8	8	5	At3g55440.1:d:+524:secondary
AAAGCATTTG	17	12	12	3	3	At3g25690.1:d:+3139:primary
CCCTGTGACC	3	29	7	3	5	At3g17020.1:d:+523:primary
TTTGTTTTTC	9	6	17	4	11	At3g08740.1:d:+889:primary
GTTGACGCGG	7	3	16	16	5	At3g03780.2:d:+2323:primary,At3g03780.1:d:+2330:primary
CTGTTTCTGA	4	12	17	11	3	At2g28720.1:d:+475:primary,At5g22880.1:d:+447:primary,At3g46030.1:d:+472:primary
GCATAAGCAA	11	7	15	3	11	At1g79850.1:X:-603:quaternary,At1g49970.1:X:--8:quaternary,At4g21360.1:X:-3451:quaternary,At4g08108.1:X:-3446:quaternary
TTAATGTTTA	17	7	6	6	11	At1g74970.1:d:+793:primary
TGTTTTTTGC	5	28	5	9	0	At1g73330.1:d:+823:primary
GCAAATGTTC	9	3	10	14	11	At1g17220.1:d:+3516:primary
TAACAGTAAA	17	1	16	6	7	At1g12890.1:v:+1205:primary
CTTCTAAAAA	13	8	15	8	2	No gene matches found
AAAGCGGTCT	9	4	13	8	12	At5g19500.1:d:+1638:primary
ATCTCCTTCT	7	13	9	3	14	At5g14320.1:d:+647:primary
CAAGTTAAGT	9	15	9	10	3	At4g28025.1:d:+556:primary
CTCTGTTTTT	5	12	9	5	15	At4g08260.1:v:+1316:primary,At3g45755.1:v:+1292:primary,At4g24120.1:d:+2173:primary,At2g42360.1:d:+918:primary,At1g53310.1:d:+3139:primary,At2g18470.1:v:+2548:primary
ACGCAAGCCG	7	13	12	10	4	At3g47250.2:d:+1478:primary,At3g47250.1:d:+1526:primary,At3g47250.3:d:+1455:primary
CTCCAATGCT	11	16	9	7	3	At3g16370.1:d:+1172:primary
TCTGAATAAT	7	0	5	23	11	At2g37040.1:d:+2357:primary
GTGAGTTTGT	4	24	8	7	3	At2g05440.2:d:+601:primary,At2g05510.1:d:+523:primary,At2g05440.1:d:+516:primary
GAAGAATTGT	7	23	6	8	2	At1g78410.1:d:+441:primary,At3g56910.1:d:+252:primary
GGAGGATTCC	6	12	8	15	5	At1g71880.1:d:+1682:primary
ACTTTGTCTC	19	11	5	4	7	At1g11860.1:d:+1408:secondary,At1g11860.2:d:+1409:secondary
AGTTTGGACT	11	10	11	7	6	At5g60640.1:d:+1913:primary,At5g60640.2:d:+2005:primary
GAATCAGTGA	5	6	13	10	11	At5g55220.1:d:+590:secondary
ATTAATAGAA	8	22	5	3	7	At5g36005.1:p:+4551:secondary,At1g35390.1:p:+4959:secondary
ACTGGACTCG	4	12	20	4	5	At4g31500.1:d:+1480:primary
GATGGTTTCT	4	10	7	16	8	At4g24690.1:d:+2444:primary
AGATGATCAA	6	3	5	8	23	At4g15860.1:p:+1163:secondary,At4g06529.1:p:+4629:secondary,At3g12770.1:d:+938:secondary
ATATTTTCTT	10	5	2	11	17	At3g09735.1:d:+411:primary
TCCTACTGGG	9	14	9	4	9	At3g01120.1:d:+1628:secondary
AAACAAGTTA	9	3	7	18	8	At2g21160.1:d:+1051:primary
ACTCATCTTT	7	21	5	5	7	At2g18960.1:d:+3128:primary

GAGAAAGAGA	4	20	13	5	3	At1g78370.1:d:+657:primary
GTGCGGCGGA	11	20	7	5	2	At1g23390.1:d:+993:secondary
GACAACCTGA	12	9	7	13	4	At1g17880.1:d:+416:primary
AATGACAAAA	8	12	16	8	1	At1g09250.1:X:+-158:quaternary,Chr1:+10819854:quaternary
GGAGTTGCAG	7	14	13	6	5	At1g08360.1:d:+551:primary
TATATTTTTA	12	6	7	7	12	At5g60930.1:v:+4447:primary
AAATGAGTTT	11	1	6	14	12	At5g44650.1:d:+1165:primary
TATACGGCGA	7	8	22	4	3	At5g38660.1:d:+915:primary
ATTGGTGGAG	9	16	5	7	7	At5g37780.1:d:+613:primary
CTTCAAGTGG	9	12	16	4	3	At5g13180.1:d:+753:primary
AAGAAACTGG	0	34	4	2	4	At4g27440.1:d:+944:primary
TATAAAATGA	8	17	10	5	4	At4g05070.1:d:+477:primary
CAACTCCTCA	14	10	16	4	0	At3g26450.1:d:+475:primary
GTGGTGGTTA	8	24	7	5	0	At2g30620.1:d:+1297:primary
TTGTGTTTGG	4	22	1	7	10	At2g16600.1:d:+459:primary
GGAGATTACT	14	9	13	5	3	At1g42960.1:d:+611:primary
TGGTGATGAT	20	5	12	6	1	At1g33813.1:p:+927:secondary,At4g02770.1:d:+737:secondary
TCCAAAACTT	7	6	10	16	5	At1g10760.1:i:+343:tertiary
AAATTATAAG	14	11	1	6	12	At1g08520.1:d:+2488:primary
TGGCAACAAA	8	8	20	6	1	No gene matches found
ATTCCGATGA	0	26	11	5	1	At5g67300.1:d:+967:primary
TGCATTTTAT	11	15	8	6	3	At5g66570.1:d:-1176:secondary
TAATCAAAAC	10	12	7	9	5	At5g63310.1:d:+870:primary
TAAAGGAGTG	12	8	10	1	12	At5g53500.1:d:+2761:primary
GCAGGGGCAA	6	22	10	4	1	At5g49360.1:d:+2256:primary
TTTCTGGATA	5	15	6	7	10	At5g20150.1:d:+950:primary
GTTGGAGGTA	6	16	10	3	8	At5g08680.1:d:+1682:primary,At5g08690.1:d:+1635:primary
AATAAAACTG	9	19	7	8	0	At4g27450.1:d:+1053:primary
TCGAATGGAA	5	14	17	5	2	At4g21105.1:d:+277:primary
TTCAGAGAAA	3	0	7	22	11	At4g14170.1:d:+654:secondary
GAGAAAGGCT	3	20	15	5	0	At4g14030.1:d:+1386:primary
AAGCTCACCT	2	20	11	6	4	At3g25920.1:d:+688:secondary
GTTATGCCTA	6	10	11	5	11	At3g15850.1:d:+1212:primary
GGGTGTGAAT	10	11	10	7	5	At3g14067.1:v:+2831:secondary,At1g20960.1:v:+2873:secondary
AAGTGATTTT	10	8	4	11	10	At3g12390.1:d:+794:primary
CTTTTTAAAA	3	7	14	7	12	At3g11400.1:d:+1185:primary
TATCCACGGA	13	8	14	8	0	At3g05020.1:d:+652:primary
TGAAGCATTA	12	8	11	6	6	At1g78830.1:d:+1584:primary
GTTTCCAGCA	7	13	11	9	3	At1g78380.1:d:+576:primary
TGAAGTTTGT	6	18	8	9	2	At1g65980.1:d:+476:primary,At1g60740.1:d:+346:primary
TAATATAATT	6	25	4	2	6	At1g59870.1:d:+4731:primary
ATTTCGATTA	4	3	7	15	14	At1g54960.1:i:+1346:tertiary
TATAACTTGT	14	7	7	9	6	At1g16720.1:d:+1956:primary
ATCTTTGTCA	8	20	4	7	4	At1g03680.1:d:+591:primary
TTCCAACTGA	2	28	10	1	1	ChrC:+98980:quaternary
CATATAATAA	16	0	2	3	21	Chr5:+781081:quaternary
AATCATAATA	11	4	4	4	19	Chr5:+16881016:quaternary
TTAAATGTAC	13	8	6	6	9	AtCg01130:d:+2465:secondary
TCTCTTCTTT	13	10	8	7	4	At5g64820.1:v:+1220:primary
AAAGAGAGGA	6	14	12	4	6	At5g59613.1:d:+699:primary
AGTTGGATCT	3	0	0	33	6	At5g52310.1:d:+1294:primary
GCGGATGAGT	12	7	5	12	6	At5g35737.1:v:+354:secondary
TTTTGAGTCA	6	6	16	8	6	At5g19240.1:d:+646:primary
AAAGATATTC	0	0	12	13	17	At5g17460.1:d:+554:secondary
CTTGAGCTAG	8	17	12	5	0	At5g07620.1:v:+1161:primary,At2g29290.1:d:+785:primary
ACTCGAGCCT	3	32	7	0	0	At5g07440.1:d:+1327:primary
ACCTTCGAGG	4	17	11	7	3	At4g14880.2:d:+1051:secondary,At4g14880.1:d:+1106:secondary
TGCAGCATTG	8	17	7	4	6	At4g02080.1:d:+609:primary,At1g56330.1:d:+588:primary,At3g62560.1:d:+573:primary
GCCTTGCTCC	6	15	6	8	7	At4g00100.1:d:+312:primary
AAGATTAAGG	5	22	6	7	2	At3g12110.1:d:+1082:primary,At3g18780.2:d:+1203:primary
TCAGCCACTG	6	21	5	9	1	At2g47440.1:d:+1808:primary

GGCGAGGTAA	6	9	15	11	1	At2g14910.1:d:+1455:primary,At2g14910.2:d:+1633:primary
TTGATGATTC	9	13	15	3	2	At1g70410.2:d:+1078:primary,At1g70410.1:d:+1024:primary,At1g70410.3:d:+940:primary
TTGAGTTCAC	9	9	8	7	9	At1g69620.1:d:+586:primary
TAATTGCGAT	16	1	11	5	9	At1g48300.1:d:+985:secondary
AGTTTCAGTA	8	7	7	10	10	At1g30880.1:d:+549:primary
CGGGTGATGT	2	39	1	0	0	At1g28330.3:d:+721:primary,At1g28330.2:d:+550:primary,At1g28330.1:d:+443:primary
TAAGCTACAG	13	8	12	4	5	At1g16880.1:d:+902:primary
AAGTGTGTTT	9	14	10	3	6	At1g15270.1:d:+293:secondary,At5g14090.1:v:+6:secondary,At5g15510.1:d:+358:secondary
TTGGGTAAAA	10	0	9	11	12	At1g07080.1:d:+1000:primary
AACCACAACC	11	5	9	12	5	At1g02140.1:d:+698:primary
CCCTTATGTA	25	2	4	7	3	ChrC:-32217:quaternary
ATTAAAAAAA	4	12	12	5	8	Chr2:+5010899:quaternary,Chr4:+17520688:quaternary,Chr1:+3075373:quaternary,Chr2:+16703574:quaternary,Chr3:+4443800:quaternary
AAGAAAAAAG	16	5	9	5	6	AtCg01130:d:+1313:secondary,At1g63710.1:d:+864:secondary,At2g04290.1:p:+3557:secondary
GTTGATGCGA	5	4	4	21	7	At5g56030.1:d:+1568:secondary
GTTGAGCTAG	2	22	14	2	1	At5g54940.1:d:+174:primary,At5g54940.2:d:+108:primary
GTGGTATCTT	10	11	12	6	2	At5g24930.1:d:+1272:primary
AGAAGTTTAT	3	15	15	5	3	At5g08790.1:d:+1136:secondary
TGTGTTCTTG	7	4	6	10	14	At5g04800.1:d:+617:primary,At5g04800.2:d:+623:primary
TCTTAATTCA	10	2	6	9	14	At5g02960.1:X:-268:quaternary
TCAAGAGTGT	19	8	8	1	5	At4g34350.1:d:+1515:primary
AAATTTGCAC	9	7	7	13	5	At4g17245.1:X:+644:quaternary
GTTACTACAA	5	20	8	2	6	At4g16190.1:d:+1076:primary
AAGGTTACTG	5	14	11	4	7	At4g01970.1:d:+2276:secondary,At5g27850.1:d:+305:secondary
CATTTACATT	2	0	0	14	25	At3g55120.1:d:+895:primary
CGAACGCACA	7	5	10	9	10	At3g46430.1:v:+754:secondary
GATACAAATT	7	13	13	7	1	At3g15840.1:d:+806:primary
TAGACAATGA	14	9	12	5	1	At3g09250.1:d:+1063:secondary
AGAGTCACTT	8	4	11	12	6	At2g43090.1:d:+818:secondary,At4g06525.1:p:+2044:secondary
CTTGGATTCT	7	5	9	9	11	At2g38270.1:d:+969:primary
TTGTTATTTT	9	10	9	5	8	At1g76200.1:d:+499:primary
GATCCGGTTT	7	10	11	8	5	At1g74270.1:d:+512:primary
ACGGGATCAC	7	19	7	5	3	At1g49240.1:d:+1327:primary
GGGCCGAAGT	7	14	7	9	4	At1g11650.2:d:+1111:primary,At1g11650.1:d:+1112:primary
CTTTCTGGTT	5	3	7	11	15	At1g02280.1:d:+1124:primary
AAGCCGGAAT	1	18	2	17	2	ChrC:+102291:quaternary
ACTAAAAAAA	10	5	14	7	4	Chr5:+2304217:quaternary,Chr3:+9650322:quaternary,Chr3:+3299186:quaternary
TGTGTGTGAA	12	13	9	5	1	At5g59030.1:d:+684:primary
GCGTTGAGAT	5	2	10	15	8	At5g51040.1:d:+737:primary
ATTATAAAGA	10	5	7	6	12	At5g47020.1:d:+4266:secondary
TGAATGTTTT	9	8	10	8	5	At5g46520.1:v:+4458:primary
ATTGCAGAGA	3	15	13	6	3	At5g37600.1:d:+1117:secondary,At1g66200.1:d:+1132:secondary
ATTTTGTTTT	20	2	7	7	4	At5g02110.1:v:+1776:primary
TCTGAATGAT	8	3	4	6	19	At4g38130.1:d:+1824:primary
CCATTGACTA	9	14	7	6	4	At4g34110.1:d:+1953:primary
TTCAAAGTTT	1	3	15	12	9	At4g26460.1:v:+790:primary,At1g17290.1:d:+2013:primary
GGGTTGTGAT	4	3	12	10	11	At4g13510.1:d:+1668:primary
TAAAACGCTA	6	9	13	9	3	At3g23460.1:v:+62:secondary
AATAATGAAA	8	9	8	3	12	At3g10430.1:v:+1522:secondary
TTGTATGCTT	15	12	2	7	4	At3g05730.1:d:+526:primary
TACTCCGGTT	5	7	11	9	8	At2g33040.1:X:+451:quaternary
TTTCGCAACT	8	13	9	7	3	At2g05710.1:d:+3337:secondary
TTCTGTTTTT	8	5	14	7	6	At1g75460.1:d:+1760:primary,At5g55220.1:d:+1740:primary
AAATAAATTA	2	8	6	6	18	At1g23300.1:v:+2242:primary
TCCATATCGA	3	17	13	4	3	At1g21400.1:d:+1517:secondary,At5g11700.1:d:+2553:secondary
TCTATAGGCA	12	12	6	5	4	AtCg01130:d:+3937:secondary
TTGTCCGAGT	5	5	12	10	7	At5g64400.1:d:+449:secondary
GAATCAAATT	6	6	7	13	7	At5g58290.1:d:+1380:primary
TCTTTGAGAT	5	6	7	9	12	At5g51110.1:d:+859:primary
GCTCGGTTCT	10	9	8	10	2	At5g48485.1:d:+405:primary
TTTTGAGTCC	7	7	12	6	7	At5g19250.1:d:+620:primary,At5g19230.1:d:+599:primary

TTCTGTTTCC	11	5	13	6	4	At4g39330.1:d:+1292:primary
AGCCTATGTA	9	6	2	6	16	At4g30190.1:d:+3243:primary
TTACCACAAA	12	2	12	8	5	At4g19710.1:d:-3127:secondary,At4g19710.2:d:-3040:secondary
ATAACACACA	16	6	4	3	10	At4g11150.1:d:-1009:secondary
CCAGATTTGG	7	6	16	7	3	At4g10810.1:d:+441:primary
GCCATTGGAA	2	23	11	3	0	At4g02520.1:d:+394:primary
GTTTTATATA	6	4	2	2	25	At3g59540.1:d:+303:primary
ATTTTGAATG	1	1	10	10	17	At3g44450.1:d:+440:primary
TAAGCTTCTC	12	11	8	5	3	At3g27997.1:p:+618:primary
CGAAAGGTAA	14	5	7	11	2	At3g27925.1:d:+1455:primary
TCCTATCGTT	11	16	9	2	1	At3g19710.1:d:+1243:primary
GAAACTTCTG	8	11	7	9	4	At2g42100.1:v:+1167:secondary
AGTGTTAATT	3	30	2	4	0	At2g20670.1:d:+1008:primary
TTTCTTATAC	25	6	2	0	6	At2g05310.1:d:+465:primary
GACTTGAGGG	2	1	30	0	6	At1g79040.1:d:+245:secondary
AGTTCCGGTT	11	3	3	9	13	At1g79010.1:d:+932:primary
GGTGCTTGAA	0	3	4	23	9	At1g77120.1:d:+1190:primary
TCTTTGATTG	8	5	2	11	13	At1g68220.1:d:+548:primary,At3g21690.1:d:+1851:primary
TGATGACGAA	5	20	4	2	8	At1g66410.1:d:+394:secondary
TTACCAAAAA	7	4	9	11	8	At1g41810.1:v:+1042:secondary
GAGGAACTAA	1	22	9	6	1	At1g08620.1:d:+3460:primary,At1g56280.2:d:+797:primary,At1g56280.1:d:+755:primary
TAGATGTGTG	10	10	10	3	6	At1g04400.1:d:+2220:primary
AAGATCAAGG	7	19	7	4	1	At5g09810.1:d:+1085:primary
TTTGAAATAA	8	7	9	4	10	At5g08530.1:d:+1870:primary,At3g26200.1:d:+1738:primary
GGAAAGTATC	7	5	10	12	4	At5g06130.2:d:+1219:primary,At5g06130.1:d:+1433:primary
CGTGCGCCTG	3	11	18	6	0	At5g03380.1:d:+1232:primary
ATTTTGTAGG	5	0	2	13	18	At5g02030.1:d:+1835:primary
TCTTCTCGAA	5	8	9	11	5	At4g38920.1:d:+749:primary
TGCTGCTTGT	12	15	7	4	0	At4g21860.1:d:+539:primary
ACAACTACAT	17	8	7	3	3	At3g54500.2:d:+2359:primary,At3g54500.1:d:+2243:primary
TCTTTGGGGG	10	9	9	9	1	At3g16520.3:d:+1485:secondary
ACAATGACTC	12	4	6	3	13	At3g10860.1:d:+449:primary
TCTATTTCTA	15	2	8	6	7	At2g42460.1:v:+2121:primary
TCTTTGTATT	10	16	10	1	1	At2g28910.1:d:+1243:primary
GAGGCCAAGG	4	17	9	5	3	At2g26250.1:d:+1572:primary
AGCAAATAAC	6	3	18	7	4	At1g70940.1:d:+2516:primary
GATCTCTCAG	10	12	8	6	2	At1g68590.1:d:+466:primary
CAGTTAACTC	10	3	10	11	4	At1g67090.1:X:-191:quaternary
TAACTTGTGC	12	3	13	7	3	At1g51680.1:d:+1971:primary
TACCCTAGCA	11	8	5	9	5	At1g41880.1:d:+383:primary,At3g55750.1:d:+407:primary
GTTTAAGTTT	6	5	9	12	6	At1g25054.1:d:+2957:primary,At1g25141.1:d:+2885:primary
GCCTAATAAC	12	6	10	2	8	At1g15120.1:d:+494:primary
GGCAAATACC	4	14	6	8	6	At1g11910.1:d:+1508:primary
TTTGATTTAT	13	14	6	2	3	At1g11260.1:d:+1696:secondary
GCTTTTCTTC	8	9	8	10	3	At1g04440.1:d:+2095:primary
CCAAAGTGAT	11	7	10	5	4	AtCg01130:d:+1167:secondary
AGTGTGTAAT	7	1	3	4	22	At5g66400.1:d:+776:primary
GTTTCTTCAA	5	9	9	8	6	At5g55540.1:d:+42:secondary,At1g63520.1:d:+371:secondary,At1g23290.1:d:+334:secondary,At1g70600.1:d:+362:secondary
CAAAGAGGAG	10	0	13	8	6	At5g50920.1:d:+3150:primary
TAGAAGTTTG	0	2	27	5	3	At5g48250.1:d:+1658:primary
TCTTTTCTTC	9	11	11	6	0	At5g23820.1:d:+770:primary
CAGCAGGATA	11	2	12	7	5	At5g10480.1:d:+914:primary
GTTGGTACGT	2	8	3	12	12	At4g39730.1:d:+392:primary
CAATTAGAGT	13	6	8	7	3	At3g61870.2:d:+1253:primary,At3g61870.1:d:+936:primary
TGTCTCCGTT	8	9	9	10	1	At3g59920.1:d:+1610:primary
TTTTGCTTAG	10	10	5	3	9	At3g53870.1:d:+577:primary
ATTTAAGAGT	8	12	13	1	3	At3g51920.1:d:+550:primary
GCGAGAATAG	6	0	7	15	9	At3g24190.1:d:+2382:primary
CTTGGACTTG	1	6	5	21	4	At3g23810.1:d:+1114:primary
TTTTTTTTCT	7	15	6	3	6	At3g11650.1:d:+813:primary,At1g33190.1:p:+809:primary,At2g02760.1:d:+797:primary

ATTGAGTTTT	11	2	15	8	1	At3g03520.1:i:+1121:tertiary,At4g04545.1:i:+4064:tertiary
GGAGCCATCG	9	14	3	8	3	At2g34250.1:d:+1482:primary
TAATTACACT	11	4	3	3	16	At2g27680.1:d:+1225:secondary
ACGAAAAAAG	7	7	15	5	3	At1g76200.1:d:+338:secondary
ATGCAAAAAA	7	11	13	5	1	At1g69935.1:d:+870:primary
AGATGAGTTT	6	6	8	6	11	At1g59359.1:d:+866:primary,At1g58684.1:d:+866:primary
GAGCTGTACG	12	10	7	3	5	At1g57720.1:d:+1261:primary
TTCATCAGAA	6	2	10	5	14	At1g50450.1:d:+1405:primary
CTTGAATGTT	9	3	8	4	13	At1g48750.1:d:+481:primary
CTTAAAAAAA	6	4	11	10	6	At1g24470.1:i:+1456:tertiary,At3g03300.1:i:+3351:tertiary,At2g26200.1:i:+2158:tertiary
GTCTACTTCT	12	7	3	9	5	AtCg01130:d:+1574:secondary
TTCAATAAAT	5	16	8	6	1	At5g58090.1:d:+1680:primary
AATAATTTCA	8	2	7	10	9	At5g50100.1:d:+858:primary
TGATAATCTG	7	9	6	9	5	At5g38480.1:d:+959:primary
GCAACTTTGA	4	4	10	12	6	At5g27150.1:d:+2231:primary
CTTGTGATGA	7	9	6	4	10	At5g13450.1:d:+823:primary
GGAGTTTGAG	6	3	8	13	6	At5g08040.1:d:+233:primary
TAACGATCCA	6	6	5	8	11	At4g36790.1:X:+-114:quaternary
GTAAAGCAGT	9	4	10	6	7	At4g34820.1:d:+1200:primary
AGAACACTGC	5	8	9	7	7	At4g33250.1:d:+777:primary
ATTCTATTGT	5	23	2	3	3	At4g13320.1:d:+765:primary,At3g44310.1:d:+820:primary,At3g44310.2:d:+704:primary
AATGTGATGA	14	10	7	5	0	At4g11211.1:d:+409:primary,At5g17620.1:d:+1140:primary
CGGAATATCA	3	2	5	16	10	At3g63410.1:d:+1218:secondary
TAGAGCTCCT	3	10	7	10	6	At3g57890.1:d:+1829:secondary
GATCCGCCAA	5	7	16	7	1	At3g54890.1:X:-98:quaternary
GGCAGGTAAA	6	12	6	9	3	At3g52930.1:d:+992:primary
AAAAGAAAAA	7	6	10	10	3	At3g52440.1:v:+1519:primary
GGTCAGACAA	10	3	19	4	0	At3g47460.1:d:+3995:primary
AGTTTTTGTT	6	3	5	12	10	At3g28900.1:d:+464:primary
GTTTCGGTTT	2	6	3	14	11	At3g13330.1:d:+5261:primary
TTTGTTTAAA	15	4	6	7	4	At3g03490.1:i:+204:tertiary,At5g42240.1:i:+403:tertiary
AGGTGAAAGC	3	18	6	5	4	At3g02040.1:d:+1275:primary
TTGGAGAAGA	11	2	12	6	5	At2g47070.1:d:+3209:primary
CTCTTCCGTT	12	9	7	2	6	At2g44100.1:d:+1683:primary
TGTCCATATG	9	11	6	6	4	At2g20230.1:d:+1069:secondary
TTAGAGAGAT	7	12	9	4	4	At1g76820.1:d:+1038:primary
GAACTGTCAC	1	24	2	8	1	At1g65930.1:d:+1093:primary
ATTTACGTTG	12	8	7	3	6	At1g65720.1:d:+642:primary
AATTACTTTC	9	5	1	14	7	At1g56580.1:d:+593:primary
TGAGGACATC	8	8	7	4	9	At1g26880.1:d:+484:primary
AATACAGAAT	0	0	0	8	28	At1g16850.1:d:+674:primary
CTTTGATGTA	7	11	1	7	10	At1g14400.1:d:+800:primary,At1g14400.2:d:+671:primary
AAGTGTTTGT	15	4	9	6	1	Chr1:+14257177:quaternary,At2g05540.1:X:+540:quaternary,Chr3:+13394959:quaternary
ACAATGTATA	10	3	5	0	17	At5g67030.2:d:+2264:primary,At5g67030.1:d:+2260:primary
TGCTTACTTT	11	4	12	6	2	At5g65480.1:d:+1185:primary
TTGGTGTTTC	9	7	13	3	3	At5g63030.1:d:+579:primary,At3g60970.1:v:+3868:primary
CTTGTTGACT	8	10	6	4	7	At5g58110.1:d:+778:primary
TTGTCGTTTT	10	7	13	4	1	At5g57887.1:X:+524:quaternary
TTTTCACAAT	9	4	3	6	13	At5g48300.1:d:+1780:primary
TATTGTTTGT	14	15	3	1	2	At5g16520.1:d:+1934:primary,At5g12250.1:d:+1515:primary
ATGAAGATTG	10	0	8	11	6	At5g13130.1:v:+1506:secondary
ACTAATATGA	13	7	2	2	11	At4g27000.1:d:+1563:primary
TGATATAAAA	7	17	1	7	3	At4g23890.1:d:+979:primary
GAGAAAGTCG	6	18	7	2	2	At4g09000.1:d:+221:secondary
GAATTTTAGT	2	7	17	4	5	At4g08850.1:d:+3381:primary
GAAAGCTTCT	10	5	6	6	8	At4g02920.2:d:+1480:primary,At4g02920.1:d:+1398:primary
CAAATTATTG	4	2	2	5	22	At3g22235.1:d:+310:primary,At3g22240.1:d:+223:primary
AATTTCAGAA	8	8	13	2	4	At3g22133.1:p:+90:secondary
AACGAAAAAT	4	5	8	10	8	At3g19450.1:d:+1220:primary
TCAAGTAATG	4	15	3	8	5	At3g15730.1:d:+2539:primary
GATATAAGAT	12	6	8	6	3	At3g12340.2:d:+750:primary

TTGACGATAA	9	10	7	4	5	At3g01470.1:d:+1255:primary
GTTTGAGTGG	8	8	6	6	7	At3g01280.1:d:+1058:primary
TTCGACAATC	6	6	13	4	6	At2g33120.1:d:+1083:primary
TTTTTATTTC	9	12	4	6	4	At1g78900.1:d:+2226:primary,At5g30490.1:d:+1007:primary
AATTCTTATG	14	11	4	6	0	At1g70820.1:d:+1969:secondary
TTTTTTTCCA	12	6	5	3	9	At1g69860.1:v:+2495:primary,At4g00585.1:d:+542:primary
AAGCATCCGC	7	4	16	6	2	At1g64950.1:d:+1515:primary,At1g64900.1:d:+1545:primary,At1g64940.1:v:+1842:primary
TTGTTGCTCT	7	10	7	7	4	At1g61740.1:d:+1471:primary
GAAGGGTTGG	6	16	8	2	3	At1g52740.1:d:+275:primary
GTCTGGTTTA	7	6	7	12	3	At1g09640.1:d:+1639:primary
TGCCAGCGGA	5	7	12	9	2	At1g08510.1:d:+1874:primary
GTCGTTGGGT	5	9	13	5	2	At5g60600.2:d:+2387:primary,At5g60600.1:d:+2390:primary
GTGCTATTTT	5	6	5	10	8	At5g55190.1:d:+842:secondary
TAAATTTAAG	9	3	7	11	4	At5g54680.1:d:+1115:secondary
AGAAAGTTGA	1	4	6	16	7	At5g51570.1:d:+1168:primary
CCTAAGATCA	9	4	11	8	2	At5g41260.1:d:+1114:secondary
TAACATACTC	13	5	7	4	5	At5g38980.1:d:+333:primary
GAACCGTGAC	7	14	6	5	2	At5g23120.1:d:+1128:primary
AGATGGAGCT	7	8	7	10	2	At5g13120.1:d:+880:primary
GGCTAAACTT	6	10	8	6	4	At5g09650.1:d:+948:primary
TTTGGTGAAA	8	8	12	4	2	At5g06290.1:d:+975:primary
AACTCCGCTG	7	15	1	10	1	At4g28240.1:d:+401:primary
TATTGATCGT	8	8	7	7	4	At4g18110.1:v:+1258:primary
TCTGAGATGA	4	4	10	10	6	At4g13930.1:d:+1478:primary
GTCTCCACTG	5	18	5	4	2	At4g11150.1:d:+677:secondary
GCGGCTGTGT	2	3	14	7	8	At4g03400.1:d:+1963:primary
ATAGGAGAAT	4	6	12	8	4	At3g63310.1:v:+1198:secondary
CTTGATTTGT	7	6	8	7	6	At3g56010.1:d:+876:primary
CATAAATTCT	5	6	15	7	1	At3g48690.1:d:+1014:secondary
CTCAAAAAAA	2	9	13	6	4	At3g42251.1:p:-590:secondary
ATCAGGGAAT	8	17	1	5	3	At3g11630.1:d:+635:primary
AAGAGCTCTT	4	5	6	8	11	At2g46670.1:d:+552:primary,At2g46790.2:d:+1073:primary,At3g49560.1:d:+951:primary,At2g46790.1:d:+1519:primary
TTCCTATTCT	15	10	2	4	3	At2g45960.1:d:+759:secondary
TGGACACCAA	7	12	11	2	2	At2g32150.1:d:+1005:primary
AATCAAAAAA	6	5	5	10	8	At2g23400.1:v:+1487:primary
TTTGCCATAA	8	13	8	4	1	At1g76010.1:d:+1313:primary
TCATTTGCTT	10	11	5	7	1	At1g72430.1:d:+608:primary
AACATTGTGT	14	10	5	2	3	At1g64510.1:d:+679:primary
TACTACTATA	19	2	2	1	10	At1g29070.1:d:+730:primary
TCATCAAGGA	9	16	5	1	3	At1g24020.1:d:+455:primary
TAGCTGTGGG	8	9	10	5	2	At1g17370.1:d:+1596:primary
GATAACCCAA	10	4	5	8	7	At1g14980.1:d:+566:primary
AAACAATTAG	4	16	5	5	4	At1g13110.1:d:+1581:primary
CCATAAATCA	3	21	2	4	3	Chr5:+17986646:quaternary
CAAGCTCCAG	7	15	4	5	2	At5g55850.1:v:+567:secondary
CAATAACTCT	7	5	4	11	6	At5g48930.1:d:+1601:primary
AAAGAAGGAG	6	12	5	6	4	At5g42980.1:d:+397:secondary,At3g60170.1:p:+382:secondary
ACAAGAAAAA	1	4	5	7	16	At5g23470.1:v:+1006:secondary,At2g02290.1:v:+1006:secondary
GACACTCGAG	5	11	6	8	3	At5g23040.1:d:+760:primary,At5g23040.2:d:+760:primary
TCTGGGAGTG	8	11	6	4	4	At5g20900.1:d:+861:primary
TTGGAGAGAT	2	18	3	5	5	At5g14780.1:d:+1131:primary
AGTAGACTTA	11	14	5	3	0	At5g14120.1:d:+2262:primary
CCCACTCGTG	2	9	9	8	5	At5g13510.1:d:+544:primary
TTTGTGTTGA	1	22	2	4	4	At5g03545.1:d:+584:primary
TGTCAAGGAG	4	16	2	5	6	At4g34870.1:d:+329:primary
GTGTTGTCGG	4	24	1	4	0	At4g26970.1:d:+1277:secondary
TATGGTGAGA	5	15	4	4	5	At4g09670.1:d:+992:primary,At1g27450.1:d:+907:primary,At1g27450.2:d:+679:primary
TGACTTACCT	7	8	6	6	6	At3g62870.1:d:+478:primary
AGATTTTATG	5	13	4	8	3	At3g23030.1:d:+838:primary
ATTCTCATCA	4	8	9	2	10	At3g14600.1:d:+628:primary

TAGTATGCTT	11	11	6	0	5	At3g11700.1:d:+1494:primary
TCGCAAACAG	8	0	17	5	3	At2g30760.1:X:--126:quaternary
GGAGCTGTGA	1	27	4	1	0	At1g73660.1:d:+2921:secondary
GGTCAGTGCT	2	22	1	6	2	At1g69870.1:i:+1335:tertiary
TTTAGATTTC	3	5	2	11	12	At1g66410.1:d:+718:primary
TCTTTTGTTT	11	8	4	4	6	At1g64140.1:d:+2732:primary
CTGTTGTGGA	9	6	11	4	3	At1g35680.1:d:+506:primary
AATAGCTCTC	8	3	8	7	7	At1g32220.1:d:+1104:primary
AATACTCGGT	6	7	9	8	3	At1g29400.2:d:+2826:primary,At1g29400.1:d:+2785:primary
TAAAAATAAA	9	9	3	10	2	At1g22630.1:d:+596:primary
CCGTTTCTTT	5	8	4	10	6	At1g17290.1:d:+1811:secondary
AGTTGTTTTT	2	16	9	2	4	At1g15530.1:v:+2321:secondary,At1g13260.1:d:+1365:secondary,At5g24440.1:v:+291:secondary
GCTGGTTTAG	6	5	13	6	3	At1g12840.1:d:+1131:secondary,At3g45720.1:v:+354:secondary
GAATTTAGAA	5	5	13	5	4	Chr2:+18587845:quaternary
TCTAAAAAAA	7	3	6	4	12	Chr1:+21451107:quaternary
ATCACTCAAG	10	8	10	3	1	At5g66570.1:d:-1086:secondary
TGTGGTCTTA	9	12	5	4	2	At5g66190.1:d:+1102:secondary,At1g44940.1:p:+458:secondary
TGTAGTCAAA	4	20	0	7	1	At5g64260.1:d:+1035:primary
TTTACATTTT	11	4	5	5	7	At5g40580.2:d:+967:primary,At5g12910.1:v:+901:primary,At5g40580.1:d:+1037:primary
GCTTGTGGGC	6	11	4	6	5	At5g35980.1:d:+3549:primary
GAACTATTAA	12	3	6	7	4	At5g22750.1:X:-17:quaternary
ACTTGAATGA	14	3	8	2	5	At5g22340.1:d:+1259:primary
GAAGTTGGAA	8	1	9	7	7	At5g16840.1:d:+957:primary
GTTGGGATCA	4	3	5	18	2	At5g01410.1:d:+983:primary
ATAAAAGAGA	5	4	8	6	9	At4g38770.1:X:-1396:quaternary
TTTAACCAGA	5	9	11	4	3	At4g34150.1:d:+999:primary
TTCTTCGTGC	2	7	17	4	2	At4g29905.1:d:+168:primary
ATACCCAACA	5	20	4	2	1	At4g21960.1:X:-703:quaternary
TTATACCAAA	12	5	4	5	6	At4g21640.1:v:+682:secondary,At4g21650.1:d:+354:secondary
ATTTATTTAT	10	8	7	5	2	At4g16240.1:v:+104:secondary
ATAAACAATA	7	4	4	7	10	At3g61400.1:v:+1141:secondary
GGTTCTCTGC	4	0	7	5	16	At3g53460.1:X:-160:quaternary
TTGTTTTGCA	4	3	9	8	8	At3g52730.1:d:+373:primary
AGGATGATGA	8	5	5	10	4	At3g50360.1:d:+541:primary,At1g75500.1:d:+1453:primary
ATCTAGTTAT	9	4	10	6	3	At3g47070.1:d:+646:primary
ATGTTCGGAT	9	6	6	6	5	At3g06510.1:X:+351:quaternary
TGACCACAGT	9	8	5	4	6	At3g01390.1:d:+385:primary,At3g01390.2:d:+554:primary
TTAGACAAAT	6	3	6	8	9	At2g44870.1:d:+1081:primary
GGTTTGGGCT	1	5	6	17	3	At2g33680.1:v:+1708:secondary,At3g44110.2:d:+1290:secondary,At3g44110.1:d:+1457:secondary
CGTTCGAGTC	1	14	12	4	1	At2g31360.1:d:+833:primary
ATGCGAAGCT	2	10	5	13	2	At2g30490.1:d:+1232:primary
TTTGTCACCA	8	13	10	0	1	At2g29670.1:d:+1909:primary
AACCATTGGA	12	2	5	3	10	At2g27020.1:d:+977:primary
TGGAATTTGT	6	6	12	6	2	At2g19140.1:p:+944:secondary,At1g77050.1:v:+1201:secondary,At1g53580.1:d:+1018:secondary
ATGATGACGA	10	5	11	6	0	At2g04270.1:d:+1664:secondary,At2g04270.2:d:+1509:secondary,At2g04515.1:d:+514:secondary,At1g49330.1:v:+370:secondary,At2g04270.4:d:+852:secondary,At1g49330.1:v:+457:secondary,At2g04270.3:d:+852:secondary
CAAATTTATT	2	11	9	1	9	At1g30400.1:d:+799:secondary,At3g30420.1:v:+1378:secondary
CAACTCTGAT	4	10	5	9	4	At1g29370.1:d:+2813:primary
CCACATTGAG	7	6	7	3	9	At1g27400.1:d:+530:primary
GGAACTTGGC	7	3	11	9	2	At1g21065.1:d:+571:primary
AAGAAAGAAA	3	11	12	2	4	At1g18950.1:d:+2716:primary
GGGATGGTCG	2	12	12	5	0	Chr3:+17216209:quaternary,ChrC:+84475:quaternary
AATAGAAAAG	7	14	5	1	4	AtCg00770:d:+55:primary
GAACTTGCTT	6	8	13	4	0	At5g48620.1:d:+3241:primary
CTTGAGAACT	8	11	3	9	0	At5g43470.2:d:+3182:primary,At5g43470.1:d:+3188:primary
CAGTATCGTT	10	6	4	6	5	At5g42080.1:d:+2161:primary
TAGAGATACA	4	1	3	1	22	At5g24460.1:d:+1095:primary
AATCTCAGAC	6	9	7	7	2	At5g06340.1:d:+845:primary
AGGAAGGAAG	5	12	6	4	4	At5g03660.1:d:+371:primary,At3g52920.1:d:+514:primary,At3g52920.2:d:+477:primary
CTTCTGGCAG	4	11	11	3	2	At4g37200.1:d:+827:primary

GTTTCATTTT	9	6	5	8	3	At4g27720.1:d:+934:secondary
GAAAGGAAGA	5	12	3	5	6	At4g27500.1:d:+1587:primary
ATTTACATAA	8	13	8	1	1	At4g22970.1:d:+2240:secondary
TGAGTTTCAA	2	5	9	7	8	At4g01870.1:d:+1685:primary
GGAGAATCAA	4	9	5	9	4	At3g54210.1:d:+430:primary
GCACTTAAAT	7	5	3	10	6	At3g27740.1:d:+1416:primary
AATTATTCCA	5	4	6	7	9	At3g08580.1:X:-459:quaternary
TAAGCACAGA	7	8	8	4	4	At3g07460.1:d:+986:secondary,At3g07460.2:d:+975:secondary
ATTCAATTTT	6	3	7	5	10	At2g41680.1:d:+1654:primary
TTAGCGCTTG	2	22	4	2	1	At2g30870.1:d:+612:secondary
GCTCCGCTCC	5	6	8	10	2	At2g26080.1:d:+3054:primary
GCTGGACCGG	4	10	6	4	7	At2g24200.1:d:+1576:primary
GTGTTGTTTG	3	13	5	2	8	At2g21580.1:d:+195:primary,At3g30740.1:p:+59:primary
CTGTTCGTGG	10	10	3	6	2	At1g78630.1:d:+622:secondary
TCTTTGCCTT	6	3	6	7	9	At1g30580.1:d:+1426:primary
TCCTTGAGTA	11	7	4	3	6	At1g28150.1:d:+553:primary
GTAAATAATA	11	4	8	7	1	At1g15390.1:i:+606:tertiary
TGGAAGGTTT	4	1	14	11	1	At1g08640.1:d:+1173:primary
GAGACGCTGG	4	14	4	7	2	At1g02500.1:d:+863:secondary,At1g02500.2:d:+863:secondary
TTTTCACAAA	3	7	7	8	5	Chr1:+29346065:quaternary,Chr4:+2412492:quaternary,Chr1:+8278587:quaternary,Chr4:+3417458:quaternary

AAATGGGTTG	1	22	3	3	1	AtCg01130:d:+559:primary
GTTTTCTGTT	5	2	12	6	5	At5g64770.1:d:+549:primary
GGCCGTTGGT	1	2	7	14	6	At5g58070.1:d:+111:secondary
TCGATTGCAT	5	11	7	3	4	At5g54160.1:d:+1019:primary
GTCGCTCCAT	3	2	21	2	2	At5g38420.1:d:+112:secondary,At5g38410.1:d:+112:secondary,At5g38430.1:d:+92:secondary
TTATTCTAAG	12	8	4	5	1	At5g35530.1:d:+887:primary
TACTCTTACC	12	1	8	2	7	At5g32482.1:v:+1684:primary
TATGCGAAGA	4	10	4	5	7	At5g19770.1:d:+1298:primary
AAGGTCTTTT	1	7	5	7	10	At5g16130.1:d:+583:primary
AAAACTTGTC	7	5	11	3	4	At4g34700.1:d:+607:primary
TACAGAAGCT	7	9	7	2	5	At4g32940.1:d:+1741:secondary
AAGCTTGAAG	3	3	12	6	6	At4g29390.1:d:+296:primary
TTTTCTGATC	6	5	6	6	7	At4g29040.1:d:+1499:primary,At3g24340.1:v:+4178:primary
GAGCTCAAGA	6	8	10	5	1	At4g23570.1:d:+1114:primary,At4g23570.2:d:+1167:primary,At4g11260.1:d:+1187:primary
GGATTCGTTT	2	3	16	6	3	At4g18010.1:d:+2332:primary,At4g18010.2:d:+2024:primary
TAAGAAATCT	5	3	9	7	6	At4g17530.1:d:+816:primary
AAAAGAAAAT	8	2	5	10	5	At4g13010.1:d:+1245:primary,At5g46990.1:v:+1356:primary
GTTTCGAGAT	0	4	5	17	4	At4g11600.1:d:+403:secondary
TATCCGAAAT	12	1	5	6	6	At3g62040.1:d:+737:primary
CCGCCGAAGT	2	18	2	5	3	At3g53430.1:d:+78:primary
GTGTTACCGG	4	6	14	4	2	At3g45140.1:d:+2678:primary
CAGCGGGGAG	4	13	5	5	3	At3g42050.1:d:+1383:primary
ATTTTGTGTT	11	13	4	2	0	At3g20470.1:p:+678:primary
TATAGTGCTG	9	10	3	5	3	At3g16670.1:d:+727:primary
ATTTGTTTGC	7	3	8	6	6	At3g13870.1:d:+2793:primary,At3g13870.2:d:+2797:primary
AGACTGTTGA	9	14	4	3	0	At3g03460.1:X:-363:quaternary,At2g04490.1:X:-3470:quaternary
TGTACTCTTA	3	10	3	5	9	At3g02670.1:d:+738:primary,At1g65260.1:d:+1151:primary
ATGAATAATT	8	2	4	8	8	At2g43710.1:d:+1706:primary,At2g43710.2:d:+1706:primary
TGTTTCATTT	6	5	6	7	6	At2g36160.1:d:+602:secondary,At1g49660.1:d:+947:secondary,At4g15810.1:v:+3315:secondary
AAAATAGTTT	8	5	11	5	1	At2g30560.1:v:+1222:secondary
TTGTTAAACA	5	5	6	7	7	At2g21250.1:d:+1125:primary,At2g21250.2:d:+1186:primary
ATTTCGATTT	3	1	8	10	8	At1g78510.1:d:+1587:primary
ATTTCTTACC	6	11	9	1	3	At1g72150.1:d:+2051:primary
TTTCTGTATC	14	5	10	1	0	At1g67870.1:d:+1003:primary
GTCGAAGGTA	6	5	3	3	13	At1g57660.1:d:+468:primary
TAAACCCGTT	5	7	5	11	2	At1g52510.1:d:+1332:primary
TAAAGATGTT	6	11	4	5	4	At1g32790.1:d:+1364:primary
TTATTGAAAG	5	7	6	3	9	At1g27330.1:d:+420:primary
GTTGATTTAT	7	3	5	6	9	At1g12840.1:d:+1447:primary
TATTCGTCTT	8	5	2	3	12	At1g09560.1:d:+864:primary

TATCAATGGA	3	7	5	5	10	At1g08880.1:d:+702:primary
GTTGTGGGAG	6	11	3	6	4	At1g05190.1:d:+444:primary
GTTATCTTTT	10	8	5	4	3	At1g04410.1:d:+1373:primary
AGTTTAATTA	7	10	6	4	2	AtCg00650:d:+100:primary
CCGGTTTTTT	5	6	11	7	0	At5g54110.1:d:+1070:primary
ACTTTTGATG	10	5	2	5	7	At5g43750.1:d:+748:primary
AAGTTTCCGT	1	22	2	2	2	At5g40450.1:d:+8057:secondary
AGGCTTTAGC	4	9	7	7	2	At5g16470.1:d:+348:primary
GTCTGGATGA	2	8	8	7	4	At5g13440.1:d:+1015:primary
GGAACATATA	9	9	5	3	3	At4g38740.1:d:+757:primary
TGACAGGTGA	7	6	11	2	3	At4g26630.1:d:+1537:primary
CACGTCTTGC	4	1	16	6	2	At4g19680.1:d:+318:secondary,At5g01530.1:d:+884:secondary
GGGTTTTTCC	4	10	4	9	2	At4g16450.1:d:+309:primary
GTTCACCACA	1	18	3	6	1	At4g13340.1:d:+2155:primary
AGACAGAACT	7	4	7	6	5	At4g13220.1:d:+610:primary
TAAATGTGTG	11	3	2	7	6	At4g04610.1:d:+1671:primary
AAAACATATG	12	6	6	2	3	At4g03560.1:d:+2454:primary
GCTACACCAA	4	7	8	9	1	At4g00430.2:d:+601:primary
GATGTGAAGT	5	8	3	7	6	At3g61110.1:d:+170:primary,At5g40400.1:d:+2231:primary
TTTGTTTGTT	11	2	6	3	7	At3g59830.1:d:+1645:primary,At4g37800.1:d:+1125:primary
CTTCCGTGTT	7	8	5	1	8	At3g48930.1:d:+422:primary
TCTGAGGAAG	2	10	5	8	4	At3g33585.1:p:+2219:primary,At2g14310.1:p:+2315:primary,At1g15500.1:d:+1862:primary
GTCTTTGGGA	14	5	5	5	0	At3g29240.1:d:+1060:primary,At3g29240.2:d:+1073:primary
CTGCAATCAA	5	11	7	4	2	At3g22960.1:d:+1893:primary
TCCTTTTAAA	7	4	8	1	9	At3g12020.1:i:+3302:tertiary
GAACCACTCT	8	3	12	4	2	At3g11780.1:d:+620:secondary
AACTTTTGTT	2	2	6	11	8	At3g05500.1:d:+954:primary
TTATTTTCCA	7	7	3	4	8	At2g44120.1:d:+933:primary,At2g44120.2:d:+1160:primary
AATGATTTCG	4	12	6	5	2	At2g44060.2:d:+737:primary,At2g44060.1:d:+740:primary
TGAGAAATCT	7	5	7	6	4	At2g42500.2:d:+1243:primary,At2g42500.1:d:+1416:primary
ATGAATAAAA	8	6	6	4	5	At2g40460.1:d:+2039:primary
AAAAAAATCA	1	8	3	3	14	At2g32950.1:d:+1353:secondary,At2g11780.1:p:+1084:secondary
TAATAGTTCT	4	3	3	10	9	At2g32480.2:d:+1400:primary,At2g32480.1:d:+1511:primary
GCCCATTAAA	6	6	8	7	2	At1g78960.1:i:-786:tertiary
CCACAGTTTT	2	9	10	8	0	At1g78080.1:d:+1727:primary
AGGAAGAAGA	3	8	4	5	9	At1g73850.1:v:+1872:primary,At3g21860.1:v:+763:primary
CTTTGAACAG	3	14	8	2	2	At1g58290.1:d:+1702:secondary
ATTTCATTTT	5	13	5	5	1	At1g49990.1:v:+1930:primary
TGGTAGTAAC	8	4	5	3	9	At1g49970.1:d:+1401:secondary
GCATAAGACC	12	3	4	5	5	At1g45474.2:d:+780:primary,At1g45474.1:d:+756:primary
TCTCAAGAGA	5	6	5	7	6	At1g40071.1:p:+669:primary,At2g24060.1:d:+1075:primary,At1g39511.1:p:+2816:primary
AGACGCAAGG	2	2	25	0	0	At1g30380.1:d:+166:secondary
TTTACCGGGA	3	10	8	5	3	At1g29660.1:d:+1049:primary
TTAATGTTCA	5	13	6	0	5	At1g21130.1:d:+1119:primary,At1g21120.1:d:+1094:primary,At1g21130.2:d:+1207:primary
ATGAAAATCT	1	8	6	7	7	At1g16210.1:d:+876:primary,At5g64290.1:d:+2021:primary
TTTGATCTAT	9	5	3	3	9	At1g15950.1:d:+1315:primary
TGTGCCAGAA	8	2	12	6	1	At1g08190.1:d:+3243:primary
GTCCAAGCCC	3	1	7	13	4	ChrM:-361075:quaternary
TGACTTGAAA	6	1	5	9	7	At5g67360.1:d:+2617:primary
GGACAGATTC	3	8	4	10	3	At5g62300.1:d:+340:primary
GAGTAGTTAA	6	4	9	5	4	At5g58260.1:d:+769:primary
AATCAGAATG	11	1	8	6	2	At5g54380.1:d:+2764:primary
GGAGATGGGC	2	0	21	2	3	At5g54270.1:d:+394:secondary
CCGTTTCTGA	5	8	9	2	4	At5g39090.1:d:+503:primary,At5g59910.1:d:+485:primary
AGAGCGGCAT	1	15	7	5	0	At5g23010.1:d:+1244:primary
AGGAGATTAT	2	19	3	4	0	At5g22920.1:d:+1069:primary
GACTTCTTTT	3	4	7	8	6	At5g20920.2:d:+1088:primary,At5g20920.1:d:+1131:primary
TCTTAAACTC	2	10	9	6	1	At5g20190.1:d:+868:primary
TTTATTGCGG	3	6	7	6	6	At5g19690.1:d:+2616:primary
GCAGCTATCT	6	10	9	1	2	At5g12860.1:d:+1369:primary
AGCATAGAGA	6	15	6	1	0	At5g10860.1:d:+691:primary

TAGAATCTGT	9	6	7	3	3	At5g06980.2:d:+1087:primary,At5g06980.1:d:+1152:primary
TAAATAAATA	1	15	3	3	6	At5g06320.1:d:+914:secondary
ATTGGTCTCA	6	10	6	3	3	At5g02120.1:d:+285:primary
GAAAGGTTGA	2	14	4	4	4	At5g01220.1:d:+1762:primary
TTGTAAGTTT	6	9	4	7	2	At4g35230.1:d:+1769:primary
GAGATTACAC	5	7	5	3	8	At4g34670.1:d:+776:secondary
AGGTTTATGG	4	1	9	7	7	At4g28660.1:d:+588:primary
CTCATATTAA	7	2	7	3	9	At4g28410.1:i:+799:tertiary,At3g42320.1:i:+2256:tertiary
GTGCAACTAT	6	7	6	4	5	At4g22510.1:X:--161:quaternary,At4g22510.1:X:--86:quaternary
GGTTATTTGT	7	6	9	6	0	At4g19170.1:d:+1800:primary
TTAGACTCGT	7	6	5	2	8	At4g11420.1:d:+3253:primary
TGGTTACTCT	5	15	3	5	0	At4g03210.1:d:+988:primary
TGAAATTGTA	2	5	5	8	8	At4g02580.1:d:+1054:primary
TACACACAAA	2	1	13	4	8	At3g62880.1:d:+610:secondary
TAATTGGTGT	7	10	5	1	5	At3g62420.1:v:+1004:secondary
TTTGAAGTTT	4	6	7	5	6	At3g45150.1:v:+1125:primary,At5g48380.1:d:+2086:primary
TTGCAAAAAA	10	1	5	8	4	At3g44520.1:v:+797:primary
AATTTTTGTT	5	10	8	3	2	At3g30212.1:p:+531:secondary
TTTATGCTTC	4	18	4	0	2	At3g28960.1:v:+2001:primary,At5g57660.1:d:+1128:primary
TTATACCAAT	10	1	1	6	10	At3g26060.1:d:+797:primary
ACACCACGAG	1	21	4	0	2	At3g18080.1:d:+1334:primary
GATTGTAAGA	11	7	3	2	5	At3g14100.1:d:+1556:secondary
ACTAGTCCCT	2	9	2	6	9	At3g12490.2:d:+690:secondary,At3g12490.1:d:+656:secondary
CTCGAATCAG	2	10	7	7	2	At3g11450.1:d:+893:secondary
TGCCACCTCT	5	5	6	8	4	At2g47170.1:d:+644:primary
AGCAGTTCTC	2	19	5	2	0	At2g37130.1:d:+944:primary
GAGTTTTCGA	2	19	2	5	0	At2g25900.1:d:+563:primary
AATGGTTAAC	4	0	5	9	10	At2g03320.1:i:+208:tertiary
GTATCATTTG	3	13	3	4	5	At2g01250.1:d:+606:primary
ATTTATAAAT	15	3	4	5	1	At1g76100.1:d:+588:primary
GCTATTTCAA	3	11	0	9	5	At1g75750.1:d:+82:primary
GAAATGGAAA	4	9	9	3	3	At1g74920.1:d:+1698:primary
GGTTGTGAGT	3	6	5	7	7	At1g73230.1:d:+369:primary
TTGTTTCTAG	3	5	8	4	8	At1g72930.1:d:+755:primary
AAGAGGCCTT	7	11	7	2	1	At1g66200.1:X:-412:quaternary,At5g03840.1:X:-52:quaternary
ATCAGATGAT	4	2	9	5	8	At1g56010.2:i:+662:tertiary,At2g34390.1:i:+869:tertiary,At2g34390.2:i:+869:tertiary
AAGCAACTCT	6	6	6	7	3	At1g54250.1:d:+637:primary,At3g59600.1:d:+691:primary
TAATGGTTTT	3	0	0	4	21	At1g52690.1:d:+742:primary,At1g52690.2:d:+742:primary
GATCTTCCTA	7	9	4	6	2	At1g50970.1:v:+813:secondary,At3g63520.1:v:+1197:secondary,At5g19760.1:d:+995:secondary
CCACAGGGAA	14	2	10	2	0	At1g23740.1:d:+1199:primary
GGATGATCTT	4	7	7	6	4	At1g10590.2:d:+654:primary,At1g10590.3:d:+702:primary,At5g11250.1:v:+4390:primary,At1g10590.1:d:+705:primary
AGTAACGTTG	8	7	5	4	4	At1g07320.2:d:+1137:primary,At1g07320.1:d:+1143:primary
GCCAAGAACC	6	9	6	5	2	At1g01170.1:d:+250:primary
AAAAGAAATG	8	9	2	2	6	Chr4:+10812049:quaternary,Chr3:+5003702:quaternary
TAATAATTGA	7	7	2	5	6	Chr3:+6472734:quaternary
TGAGAGTTTC	3	0	6	15	3	At5g65720.1:d:+1634:primary
ACAAAGCTTT	4	10	9	0	4	At5g65670.2:d:+1298:primary,At5g65670.1:d:+1304:primary
GGGGATCCAG	5	12	1	4	5	At5g64350.1:d:+363:primary
GATGAGTTGA	3	3	4	12	5	At5g56030.1:d:+1934:primary
TCGAAACAGA	7	0	11	7	2	At5g54640.1:d:-599:primary
ATATTATTTC	3	10	3	3	8	At5g54500.1:d:+836:primary
TCCAATGGTT	12	2	7	3	3	At5g35630.1:d:+1607:primary
CCTCTGTTCA	4	14	3	5	1	At5g27700.1:d:+171:secondary,At5g51950.1:d:+738:secondary
CGCTCCAAAT	2	4	6	8	7	At5g24570.1:d:+283:primary
GCATTCGACT	5	10	8	1	3	At5g22640.1:d:+1644:secondary
TTAGAGCAAG	3	6	4	7	7	At5g13430.1:d:+1022:secondary
GCCGTTCTCA	5	4	12	4	2	At5g08650.1:d:+2020:primary
TGTTGCTTGT	3	4	5	7	8	At4g30470.1:d:+1158:primary
CCTCGTGTAC	5	14	5	3	0	At4g19860.1:d:+1783:primary
TTAAAAAAGG	6	8	5	3	5	At4g17040.1:d:+1103:primary

TCCGAAGGAA	7	7	5	7	1	At4g15802.1:d:+363:primary
CCATCTCACA	4	10	8	5	0	At4g15560.1:d:+2403:primary
TGTGTGTCCA	1	9	6	6	5	At4g12720.3:d:+958:primary,At4g12720.1:d:+1077:primary,At4g12720.2:d:+1016:primary
TATGCAAGTT	1	0	2	3	21	At4g12490.1:d:+652:primary
CACATAGTTT	5	9	4	7	2	At4g02770.1:X:-731:quaternary
CTCGACGAGA	2	8	5	8	4	At4g02230.1:d:+551:primary
GATTATACAA	12	1	4	6	4	At3g58370.1:v:+1394:primary
TGGTGGGCTC	3	4	10	6	4	At3g53990.2:d:+916:primary,At3g53990.1:d:+814:primary
AAAAATGTGG	9	5	6	5	2	At3g52220.1:d:+960:primary
TAATGATGCA	5	8	9	2	3	At3g29320.1:d:+3018:primary
AGAGAAAGTG	1	0	11	7	8	At3g28740.1:d:+1427:primary
TGTTTAGTTT	6	3	11	5	2	At3g17510.2:d:+1465:primary,At3g17510.1:d:+1924:primary
TGTAAATAAG	8	13	2	0	4	At3g16770.1:d:+1011:primary
TAGCCAACTA	5	4	8	2	8	At3g15110.1:d:+857:primary
TTGTTCGTGT	9	13	2	1	2	At3g09740.1:d:+1083:primary
GTCACTTCCT	4	9	5	4	5	At3g09200.1:d:+740:secondary,At5g67540.2:d:+1441:secondary,At5g67540.1:d:+1358:secondary
GTCTGTTTCT	9	1	9	4	4	At3g04790.1:d:+808:primary
ATGTCGACCC	4	8	8	3	4	At2g47610.1:d:+516:primary
TTATGGGCAA	9	7	3	3	5	At2g45070.1:d:+357:primary
AGTTCAAATG	1	6	16	3	1	At2g40140.1:d:+2106:primary
CTATGTTCTC	7	7	7	2	4	At2g37410.1:d:+997:primary,At2g37410.2:d:+973:primary
AGATCCAATA	9	1	7	2	8	At2g35840.2:d:+1471:primary,At2g35840.1:d:+1410:primary
ATACAGTAGA	17	6	1	1	2	At2g35260.1:d:+1377:secondary
CTTTTTCGCA	6	4	8	5	4	At2g30390.1:d:+1833:primary
TATTAAACTC	12	5	2	3	5	At2g26670.1:d:+1082:primary,At5g50970.1:d:+1706:primary
AAGGGTTCTG	4	13	7	3	0	At2g22670.2:d:+1074:primary,At2g22670.1:d:+1126:primary
AAATCTCAAA	8	2	9	5	3	At1g74960.2:d:+2199:primary
TCCTAAATCT	4	5	3	6	9	At1g74560.1:d:+1041:primary
AGTTACTCTC	7	3	8	6	3	At1g74260.1:d:+4325:primary
AGAGCTGCAC	2	10	9	3	3	At1g67700.2:d:+575:primary,At1g67700.1:d:+570:primary
TTTGATCTTT	6	4	8	3	6	At1g48170.1:d:+863:primary,At4g36800.1:d:+961:primary
ACCAATGCTT	8	2	8	6	3	At1g31800.1:d:+1920:secondary
AAGGATCTAT	4	2	5	8	8	At1g08570.1:d:+1333:primary
TTTAAACATT	8	6	2	3	8	At1g01320.1:d:+5712:primary
TGTGCGATTA	5	0	3	14	4	Chr3:-7803507:quaternary
TAAACTCTTG	4	9	10	3	0	Chr2:+3019301:quaternary
TGAAACTGGA	3	2	12	6	3	Chr2:+13317229:quaternary
TAATCATCAA	7	13	2	3	1	At5g66040.2:d:+529:primary,At5g66040.1:d:+476:primary
TGAAGAAATC	5	11	6	3	1	At5g63670.1:d:+380:primary,At1g60010.1:d:+905:primary
TAATCTTCAG	10	2	8	2	4	At5g61230.1:d:+1274:primary,At1g79080.1:d:+1958:primary
GGAAAGGTTC	3	3	6	6	8	At5g56670.1:v:+354:secondary
CCTCCACTTG	2	7	1	12	4	At5g56010.1:d:+2149:primary
GTTTCTAAGC	3	10	4	5	4	At5g54760.1:d:+481:primary
TTAAAGAAAA	9	4	4	2	7	At5g50190.1:v:+257:secondary,At4g00370.1:d:+2000:secondary
ACAGTTCGTG	5	10	5	5	1	At5g49940.1:d:+688:primary
AGATTTGTGT	8	18	0	0	0	At5g49450.1:d:+754:primary
ACTATGATCT	5	11	4	3	3	At5g47770.1:d:+1441:primary
TTCGTTTTTT	6	7	8	3	2	At5g42650.1:d:+1710:primary
AGGGTTTTTG	7	0	10	7	2	At5g36910.1:d:+640:primary
TATGTACACT	8	7	4	3	4	At5g35220.1:d:+1749:primary,At5g44290.1:d:+2462:primary
CAGACGGAGA	2	8	8	5	3	At5g25840.1:d:+650:primary
TGATCTCTAA	9	6	6	4	1	At5g23750.2:d:+762:primary,At5g23750.1:d:+773:primary
GTTTGGTTTG	4	8	4	3	7	At5g20280.1:d:+3281:primary
ACACCTTTGG	0	0	5	6	15	At5g15950.1:X:+1609:quaternary
CAACATTGTA	6	7	11	2	0	At5g14200.1:d:+1325:primary
AAATTTGTCA	4	11	5	3	3	At5g09600.1:d:+975:primary
GAACCATACA	3	6	9	5	3	At5g06310.1:v:+1807:primary
TAATGTAATG	8	11	0	3	4	At5g04590.1:d:+2014:primary
CTTCTGCTTC	7	1	10	4	4	At4g36515.1:d:+233:primary,At5g35100.1:d:+932:primary,At3g55160.1:v:+7094:primary
CCAATATTAG	4	15	4	1	2	At4g30440.1:d:+1375:primary
ATTTTACTAA	5	1	3	3	14	At4g28670.1:v:+75:secondary

ACGAAGACTT	4	11	3	4	4	At4g23850.1:d:+2114:primary
ATTGGGGAAA	4	3	3	10	6	At4g21570.1:d:+1181:primary
GAACTCTACA	2	8	6	6	4	At4g20830.2:d:+1764:primary
GAGCTTTTGT	6	11	4	5	0	At4g19160.1:d:+1947:primary,At4g19160.2:d:+1921:primary,At4g19160.3:d:+1924:primary
TGATTTTGGT	8	4	9	3	2	At4g15550.1:d:+1449:primary
TTTTTATTTT	3	13	1	4	5	At4g06590.1:p:+2061:primary,At4g12650.1:d:+1696:primary,At4g05420.1:d:+3641:primary,At5g66760.1:d:+2035:primary
TTTTTCCCCC	8	6	4	5	3	At4g02510.1:d:+4571:secondary
TTTTTTTTTT	6	8	5	6	1	At4g01440.1:d:+1348:primary,At5g47430.1:d:+3092:primary,At4g30980.1:d:+1144:primary,At5g46160.1:d:+667:primary,At5g55730.1:d:+1374:primary,At5g46160.2:d:+648:primary,At1g67310.1:v:+3712:primary,At5g66270.1:d:+1688:primary
GTTGGGTTGT	2	15	7	2	0	At3g61060.2:d:+1270:primary,At3g61060.1:d:+1258:primary
GAAAGTTATG	0	9	14	3	0	At3g56880.1:d:+855:primary
TACAGAAAGC	4	10	4	4	4	At3g53020.1:d:+282:primary
AAAATAAAAG	1	0	3	10	12	At3g50970.1:d:+575:primary
TAAAACCATA	12	4	3	0	7	At3g48420.1:d:+1133:primary
GGACAGATTT	4	8	4	8	2	At3g19820.2:d:+469:secondary,At3g45030.1:d:+316:secondary,At3g19820.1:d:+445:secondary
TAATTGAGCT	9	4	6	1	6	At3g18410.1:d:+509:primary
ACAACCAGTT	3	12	5	6	0	At3g07560.1:d:+1070:primary
GTAATGGTGG	3	19	0	4	0	At3g05220.1:d:+949:secondary,At3g05220.2:d:+762:secondary
TTTGAGTCTC	6	12	1	4	3	At3g03150.1:d:+465:primary
CAGCTCCTGG	5	15	5	1	0	At3g02180.1:d:+401:primary,At3g02180.2:d:+401:primary
GTTTCTCCTT	9	8	3	2	4	At2g44620.1:d:+427:primary
GCAACAACAG	4	13	3	5	1	At2g43680.1:d:+2215:primary,At2g43680.2:d:+2218:primary
ATACTCTTTG	7	7	6	4	2	At2g42210.1:d:+667:primary
AGACCTAGAT	4	11	3	2	6	At2g37190.1:d:+412:primary
AAAAATTATC	4	17	2	3	0	At2g36120.1:p:+804:primary
AAATAAATTT	4	3	14	2	3	At2g24820.1:d:+1729:secondary
TACTACTTCC	9	8	3	3	3	At2g20360.1:d:+1525:secondary
TTGTCGAGAT	7	7	9	2	1	At2g19310.1:d:+530:primary
CCTTAATTGT	7	9	0	1	9	At2g18030.2:X:+-87:quaternary
TCCAATAGAC	5	16	2	1	2	At2g10940.1:d:+692:secondary,At2g10940.2:d:+692:secondary
TTCATCACCT	9	7	2	5	3	At2g04700.1:d:+614:primary
GTTTCGTTAT	4	3	5	3	11	At2g02510.1:d:+427:primary
TGTTCAAGAG	7	1	10	4	4	At1g74880.1:d:+638:primary
GTCCGATTGT	8	9	3	5	1	At1g68530.1:d:+1514:primary,At1g68530.2:d:+1873:primary
TTAAGATAAT	11	3	11	1	0	At1g66100.1:d:+584:primary
AGTTTTTGGG	2	21	3	0	0	At1g62600.1:X:+-9:quaternary,Chr3:+6539388:quaternary,Chr5:+24408380:quaternary
TTGTATAGAT	5	13	7	0	1	At1g57990.1:d:+1302:primary
GACCGATTGT	10	8	5	2	1	At1g56700.1:d:+873:primary
GAAGTTGATA	3	3	9	6	5	At1g54575.1:d:+550:primary
CGTCGATCGA	3	14	3	5	1	At1g54100.2:d:+1578:primary,At1g54100.1:d:+1592:primary
AAAAACTTTA	4	1	11	6	4	At1g32550.1:d:+772:primary,At3g04630.1:d:+1146:primary,At3g04630.2:d:+1096:primary
GTTTCTTACC	8	3	4	5	6	At1g29150.1:d:+1527:primary
TGCCACTTCA	5	8	6	2	5	At1g23490.1:d:+593:primary
AAGTTGAGAG	7	6	1	7	5	At1g22780.1:d:+442:primary
GGCCCCTCGG	1	15	4	6	0	At1g21680.1:d:+2018:primary
AATTGCTTCG	1	13	9	1	2	At1g19380.1:d:+629:primary
AAATGAAAGT	10	2	5	4	5	At1g18060.1:d:+893:primary
TTTGAGGTTT	7	6	3	7	3	At1g12650.1:d:+962:primary,At4g37510.1:d:+1663:primary
TTTAATTGTT	2	15	3	1	5	At1g08730.1:i:+66:tertiary,At1g40129.1:i:+3022:tertiary
TGGCAAACTT	4	8	6	5	3	At1g07280.1:d:+1907:primary
GTTTTGTTGC	3	15	5	2	1	At1g03870.1:d:+710:primary
TAAACTACTT	11	4	6	2	2	Chr5:+22273707:quaternary
GCCAAACAAA	6	4	8	5	2	Chr3:+10909897:quaternary,Chr2:+10111782:quaternary
TTACAGGCCA	6	2	8	4	5	Chr1:-19509774:quaternary
CGGAGAAATT	2	1	1	7	14	Chr1:+12576228:quaternary
CAGTTGCTAA	4	15	3	2	1	At5g65430.1:d:+769:primary,At5g65430.2:d:+769:primary
GTTTTAGTTT	3	7	7	8	0	At5g60850.1:d:+1275:primary,At1g70980.1:d:+1757:primary
TGCACCTTCT	1	4	4	8	8	At5g60790.1:d:+2116:primary
ATTTCTTTTT	3	2	4	9	7	At5g56350.1:d:+1966:primary

AAGGAGTTGT	6	4	9	2	4	At5g54430.1:d:+981:primary
TGATGTGTAG	7	8	7	2	1	At5g53800.1:d:+1099:primary,At1g75630.1:d:+590:primary
AAGAGTTTGG	5	3	8	5	4	At5g47860.1:d:+1483:primary
ATTGATTGAA	7	4	5	4	5	At5g39950.1:d:+573:primary
GTTTCCACTT	5	7	5	3	5	At5g22990.1:v:+615:secondary,At1g20100.1:d:+1246:secondary
GCATCGTGAT	7	10	2	4	2	At5g20020.1:d:+386:primary
TCAATGTTCT	4	4	2	5	10	At5g19440.1:d:+1136:primary
GTCAATGATA	11	4	1	3	6	At5g19350.1:d:+1505:primary
ATTTGGTTAT	12	2	6	2	3	At5g16050.1:d:+1038:secondary,At4g06552.1:p:+2091:secondary
AGTACGCCTA	2	3	8	5	7	At5g14520.1:v:+2444:primary
AGGAATGTTA	5	5	1	6	8	At5g03850.1:d:+185:primary
AAAGGATTCT	5	4	7	4	5	At4g35800.1:d:+5856:primary
TTACTTCTGT	4	12	2	3	4	At4g27130.1:d:+752:primary
TCTATACTTT	11	6	3	3	2	At4g25080.1:d:+1238:primary,At4g25080.3:d:+1281:primary
AAAGACCAAA	6	2	12	4	1	At4g22890.3:d:+1379:primary,At4g22890.1:d:+1383:primary
TTAAGGACTA	8	5	6	3	3	At4g21450.1:X:+269:quaternary
AAGTGGTTCC	6	6	1	8	4	At4g18730.1:d:+544:primary
TTTTGAGACG	7	7	4	6	1	At4g16830.1:d:+1487:primary
TGCGCAGATC	3	11	7	3	1	At4g13430.1:d:+1364:secondary
AAGAGGAGCT	7	13	2	2	1	At4g08093.1:p:+958:secondary,At5g35360.1:d:+1691:secondary
GTTCGAGTAG	5	5	6	4	5	At3g62530.1:d:+580:primary
TGTTTGGCTG	5	6	3	8	3	At3g58500.1:d:+1315:primary
TGAAACCAAT	3	7	4	7	4	At3g56340.1:d:+126:primary
TTCCAACGAG	8	2	6	4	5	At3g51510.1:d:+753:primary
AAAACTCGGT	1	3	5	8	8	At3g24170.1:d:+1750:primary
CAGAAGGTGG	9	4	7	4	1	At3g21055.1:X:-444:quaternary
AAGCTCATTT	4	3	3	11	4	At3g18680.1:d:+1291:secondary
TGAATGTGAT	5	6	5	4	5	At3g17410.1:d:+1438:primary
GGACGTGCCG	7	7	10	1	0	At3g15356.1:d:+817:primary
AAAATAAAAA	3	1	11	5	5	At3g14360.1:d:+1949:secondary,At3g56040.1:d:+2812:secondary
TGGTGGGATC	2	2	19	2	0	At3g01500.3:d:+791:secondary,At3g01500.1:d:+789:secondary,At3g01500.2:d:+791:secondary,At2g06850.1:d:+800:secondary
AGATTTTCCT	3	2	2	12	6	At2g45530.1:d:+1345:primary,At4g10040.1:d:+638:primary
TTTTGATGTA	8	5	3	1	8	At2g44310.1:d:+561:primary
GTCAACCATA	10	6	2	6	1	At2g42840.2:d:+841:primary,At2g42840.1:d:+841:primary
TAATTCGTTT	6	4	9	6	0	At2g34830.1:d:+1326:primary
AGGTTTATTC	6	3	4	7	5	At2g31410.1:d:+692:primary
AATCGTCACC	5	2	2	10	6	At2g27510.1:d:+707:primary
AATAAAACTT	5	0	8	6	6	At2g24180.1:d:+1669:primary
ATGAGGGAGA	4	10	7	3	1	At1g70490.3:d:+759:primary,At1g70490.2:d:+655:primary,At1g70490.1:d:+678:primary
AGAAAGTTGG	6	9	3	3	4	At1g59900.1:d:+1282:primary
TTTCTCTAGT	12	9	1	3	0	At1g54010.1:d:+1341:primary
GATTACAGAA	5	2	5	8	5	At1g52760.1:d:+1173:primary
TGTGAATAAC	3	4	10	3	5	At1g51805.1:d:+2891:primary
ACGGTTGTGG	4	14	4	2	1	At1g22710.1:d:+1279:primary
GAAACTGACC	4	5	6	5	5	At1g12580.1:d:+1922:primary
ACATAAAAAA	3	2	10	6	3	Chr3:+21226819:quaternary,Chr2:+11163940:quaternary,Chr4:+3159573:quaternary
CTCTTAAAAA	5	3	9	3	4	Chr1:+23073550:quaternary
GTGACGCCAT	3	5	11	2	3	At5g64840.1:d:+1792:secondary
CTTTAATGGA	6	4	10	3	1	At5g63370.1:d:+2563:secondary,At3g22860.1:v:+1732:secondary,At3g56150.1:d:+1647:secondary
TAATATTTCT	9	4	5	2	4	At5g60680.1:d:+683:primary
TACCAAATAT	8	6	3	0	7	At5g59540.1:d:+1201:primary
TGGTATTCGG	5	9	3	6	1	At5g58710.1:d:+542:primary
CTGGGAAAAA	7	10	1	4	2	At5g40950.1:d:+368:primary
ACCATTCGGT	4	5	6	6	3	At5g36160.1:d:+1407:primary
TTTTGATTTG	13	5	1	3	2	At5g18800.1:d:+447:primary,At5g18800.2:d:+434:primary
AGAAATGGAG	3	9	8	3	1	At5g16390.1:d:+963:primary,At5g16390.2:d:+1188:primary
ATCAAAGTGA	8	6	7	2	1	At5g03280.1:d:+4498:primary
TCCACCCAAA	3	4	6	5	6	At4g37120.1:d:+1755:primary
TTTATTTGAA	8	6	5	1	4	At4g35820.1:v:+1563:primary,At5g51820.1:d:+2064:primary
AATCATATGA	9	5	3	3	4	At4g34230.1:d:+1273:primary,At4g34260.1:d:+2742:primary

GGCTGACACA	2	5	4	7	6	At4g27960.2:d:+659:primary,At4g27960.1:d:+521:primary
CTGCTTGTTT	4	8	4	4	4	At4g25170.1:d:+1248:primary
AATGATTTGT	4	8	5	5	2	At4g24520.1:d:+2291:primary
TAATTAAGAA	7	8	0	3	6	At4g22190.1:d:+1415:primary
ATTGGTGTCA	7	9	5	2	1	At4g16410.1:d:+535:primary
TTTTGCCCTT	8	1	4	6	5	At4g16370.1:d:+2279:primary
TGACGAGAGA	3	4	5	6	6	At4g15490.1:d:+1654:primary
CTCTTTGTGA	10	7	6	1	0	At4g15080.1:i:+2033:tertiary
AGAAATCAAA	4	8	2	7	3	At4g13840.1:d:+1447:primary
AATCAAAATG	3	7	8	2	4	At4g12040.2:d:+1250:primary,At4g12040.1:d:+862:primary
TAATCGTACA	5	1	5	7	6	At4g11175.1:d:+566:secondary
TAAATCATTG	6	7	6	4	1	At4g09830.1:d:+822:primary
GTTTGGTTTC	2	1	4	10	7	At4g03020.1:d:+1902:primary
GTACACGGCG	3	0	19	0	2	At3g61470.1:d:+451:secondary
GAGGTTGCCC	2	13	3	5	1	At3g55770.1:d:+619:primary
GCATTGAAGC	2	0	6	8	8	At3g47520.1:d:+1520:primary
GACGCTCTCG	0	3	7	7	7	At3g24503.1:d:+1476:primary
AAAAAACAAA	6	4	6	7	1	At3g23300.1:d:+2013:primary,At4g08370.1:v:+1715:primary
CTAGCTAAAT	2	5	4	2	11	At3g18820.1:d:+973:primary
AGCTCTGGGG	7	2	11	4	0	At3g13062.1:d:+1555:primary,At3g13062.3:d:+1579:primary
TTCAGACTAC	3	3	2	5	11	At3g07430.1:d:+967:primary
AAAAAAAGTT	2	4	10	4	4	At3g03890.2:d:+1181:primary,At3g03890.1:d:+1612:primary
GTTTCCAATG	4	8	5	4	3	At2g45180.1:d:+427:primary
CTTGGTGCAA	5	1	7	5	6	At2g44350.2:d:+1703:secondary,At2g44350.1:d:+1700:secondary
TTTCTAGTTT	6	10	4	2	2	At2g41410.1:d:+982:secondary
GATGAACTTC	5	8	4	5	2	At2g40880.1:d:+388:primary
CATTCGTAAC	10	5	1	4	4	At2g32690.1:p:+669:primary
CAATGTTTGT	8	3	7	1	5	At2g29450.1:d:+775:primary
GTTTGAATCT	4	3	7	4	6	At2g27960.1:d:+424:primary
TTTTGTGGAG	5	9	3	4	3	At2g24360.1:d:+1472:primary
AATACTCTTT	2	2	4	3	13	At2g23930.1:d:+440:primary
GAGAAGAAAG	0	5	17	2	0	At2g22500.1:d:+1380:primary,At3g28220.1:d:+962:primary
TTTTAGATTT	2	7	4	4	7	At2g16940.1:d:+2062:primary,At3g27360.1:d:+560:primary
TTGTTGACCA	6	7	4	3	4	At2g03440.1:d:+866:primary
TTACAGTAAT	15	2	1	0	6	At1g79600.1:d:+2387:primary
GGTGTGTTTG	5	8	3	5	3	At1g79550.1:d:+1120:secondary,At1g79550.2:d:+1081:secondary
TGCATTAATG	4	12	4	3	1	At1g75540.1:d:-1193:secondary,At1g75540.1:d:-1187:secondary
TTGGGTTCTT	0	4	17	2	1	At1g70420.1:d:+871:primary
TTTGTTTTCA	8	3	4	3	6	At1g67350.1:d:+468:primary,At1g12310.1:d:+705:primary
TTAATGACTA	10	2	2	2	8	At1g64770.1:d:+1242:primary
GACCGTGTAT	4	17	3	0	0	At1g59870.1:d:+4343:secondary
CCTCCTCCAG	5	7	6	4	2	At1g58242.1:d:+235:primary,At5g60920.1:d:+1457:primary
ACTCAAGGGG	0	3	16	4	1	At1g55450.1:d:+1162:secondary
TAAGTAGGTG	4	6	8	4	2	At1g55360.1:d:+1655:primary
TGTTGTTTCG	5	8	6	4	1	At1g48600.2:d:+1800:primary,At1g48600.1:d:+1614:primary,At3g05600.1:d:+1109:primary
TCACTCTTGC	6	6	3	4	5	At1g36160.1:v:+7259:primary
ATATTCATTG	6	0	2	5	11	At1g26630.1:d:+878:primary
AGATGATGAA	1	4	15	3	1	At1g13260.1:d:+1461:primary
ATGTTCTGTC	6	11	5	1	1	At1g10950.1:d:+1778:secondary
TTTTACAAGT	7	7	3	3	4	At1g08150.1:v:+3057:primary,At4g17245.1:d:+598:primary
ACCACTAAAA	6	4	6	6	1	No gene matches found
TTTCTGATCG	6	5	3	5	4	At5g67600.1:d:+326:primary,At4g37290.1:d:+449:primary
ACGCAAAGTA	4	9	6	1	3	At5g64120.1:d:+1080:primary
GCAACCCTGA	2	9	5	3	4	At5g60340.1:i:+785:tertiary
ATGATGGTTT	6	3	3	8	3	At5g56140.1:d:+1303:primary
GTAGTTTTAA	1	12	4	4	2	At5g54540.1:d:+966:primary
GCGTCATCTG	3	4	4	7	5	At5g52530.1:d:+3026:primary,At5g52530.2:d:+3020:primary
GAGATCAGAT	2	5	6	7	3	At5g47620.2:d:+1572:primary,At5g47620.1:d:+1589:primary,At5g47620.3:d:+1124:primary
ATGTTATTAT	4	2	3	2	12	At5g40370.1:d:+588:primary
GGAAAATGAA	4	9	4	2	4	At5g40340.1:v:+2527:secondary
AAGACGATAA	5	5	6	2	5	At5g36230.1:d:+1402:primary

AATGAGATGG	4	10	2	5	2	At5g28840.1:d:+1010:primary
ATGGTAATTA	2	12	4	1	4	At5g28050.1:d:+819:primary
CATTTATTCA	0	5	1	2	15	At5g24740.1:d:+6918:secondary
GATACGATTT	5	7	7	2	2	At5g23060.1:d:+638:primary
TTTCTTATCA	10	3	2	3	5	At5g20350.1:d:+2107:primary
GTTTACAGTT	4	4	5	8	2	At5g19290.1:d:+1154:primary
CTTGTAAATT	3	1	3	8	8	At5g15850.1:d:+1521:primary
AGTGGCGTCC	4	4	5	6	4	At5g15090.1:d:+808:primary
AATGAACCTT	6	5	4	6	2	At5g14260.1:d:+2110:primary,At5g14260.2:d:+1905:primary,At5g14260.3:d:+1990:primary
CCCAAAATAT	7	6	5	3	2	At5g13710.1:d:+1300:primary
GGTCAGCTCA	3	5	7	4	4	At5g01350.1:d:+360:primary
GGATGCAATG	4	3	10	3	3	At4g38810.2:d:+1544:primary,At4g38810.1:d:+1153:primary
TGATTGATTT	2	14	4	3	0	At4g34390.1:d:+3093:primary
TAGTAGTGCC	9	2	4	5	3	At4g27600.1:d:+1579:secondary
GTTTTGTGTT	5	3	6	4	5	At4g26480.1:v:+2436:primary,At3g51940.1:d:+1604:primary
ACTTTTTCCT	6	7	4	3	3	At4g23470.2:d:+1520:primary,At4g23470.1:d:+1172:primary
AGAGAGATAG	8	7	4	1	3	At4g21660.1:d:+1844:primary
ACTTGACAGT	0	3	8	6	6	At4g18040.1:d:+814:primary
AACCCGGCCA	6	7	5	5	0	At4g17340.1:d:+654:primary
GTAACTGAAA	4	2	8	4	5	At4g16960.1:v:+3473:secondary
GAGATCATTT	7	5	5	3	3	At4g16950.1:d:+4453:primary,At4g16950.2:d:+4474:primary
CTCTTTTAAA	2	7	2	7	5	At4g16330.1:i:+942:tertiary
ACTCGTGGAA	5	14	1	2	1	At4g13615.1:d:+31:primary
AGCTCTCAGC	2	11	1	4	5	At4g10480.1:d:+464:primary
ACTATGGCTT	1	8	12	1	1	At3g62550.1:d:+593:primary
GGCCAGGCCC	5	6	6	6	0	At3g59850.1:d:+868:primary
TATCTCCGTC	7	11	2	0	3	At3g52960.1:d:+469:primary
TGTTCAATCT	5	6	3	4	5	At3g52560.1:d:+798:primary,At3g52560.2:d:+801:primary
TTTCTCCATC	5	8	4	2	4	At3g52300.1:d:+637:primary
GAATGGAAGA	4	1	7	7	4	At3g24050.1:d:+1156:primary
AGTTGTCTTC	4	15	3	0	1	At3g19680.1:d:+1676:primary
AGCCTCTCTT	2	16	3	1	1	At3g14230.1:d:+1379:primary,At3g14230.3:d:+1364:primary,At3g14230.2:d:+1367:primary
AGAGCCAAGT	4	4	1	10	4	At3g08520.1:v:+354:secondary
CTTCGGATCT	4	9	4	5	1	At3g01540.2:d:+1750:primary,At3g01540.3:d:+1720:primary,At3g01540.1:d:+1750:primary
AGACTCTATT	5	0	3	8	7	At2g46830.2:d:+2478:primary,At2g46830.1:d:+2064:primary
AATGGCCTCA	6	3	7	4	3	At2g46820.1:d:-706:primary
TGTTTTTCAA	4	4	5	4	6	At2g44745.1:d:+745:primary
TGTGTTATTA	8	4	6	3	2	At2g40060.1:d:+958:primary
CAAAGTCAAT	3	15	0	3	2	At2g38530.1:d:+403:primary
GCTTTGTCCT	2	0	19	1	1	At2g34420.2:d:+74:secondary,At2g34420.1:d:+74:secondary
ATCTGATATG	6	1	3	4	9	At2g31380.1:d:+997:primary
TTACTTTTAG	6	7	1	2	7	At2g29630.1:d:+2137:primary
GCAAGAAAAA	2	4	5	4	8	At2g28700.1:d:+26:secondary
TGTGTCTAGT	4	4	6	7	2	At2g20860.1:d:+1308:primary
AACTTTTAAA	9	1	8	4	1	At2g05540.1:d:+704:primary
ACTCTTGACT	4	10	3	4	2	At2g05220.1:d:+183:primary,At2g04390.1:d:+174:primary
TACGTTAACA	0	18	1	2	2	At1g78820.1:d:+670:primary
GTCGTGCCAC	4	0	9	6	4	At1g72040.1:d:+1989:secondary
TGTATCCAAC	3	1	12	5	2	At1g71480.1:d:+930:primary
TAAATTTGTT	4	7	9	2	1	At1g61250.1:d:+1142:secondary,At3g62270.1:d:+79:secondary
ACGAGCTCTA	3	3	6	8	3	At1g56340.1:d:+1295:primary
TACAGAGGAG	3	14	2	1	3	At1g53885.1:d:+257:primary,At2g44670.1:d:+205:primary
GACTTGGAAC	5	0	7	6	5	At1g32080.1:d:+1441:primary
TATGATGTAT	13	1	1	1	7	At1g31580.1:d:+604:secondary
ATTTGAGTTT	3	4	7	9	0	At1g31130.1:d:+1422:secondary
AGCCCTATGC	1	12	7	2	1	At1g27730.1:d:+716:primary
TTGGAATTTT	4	7	7	5	0	At1g22890.1:d:+460:primary
CTGCGTCATC	4	4	6	4	5	At1g22610.1:d:+3188:primary
GACCTTTGGC	3	6	7	3	4	At1g14320.1:d:+667:secondary
GATTGGATTG	5	4	8	2	4	At1g14000.1:d:+1735:primary
TCTATGTTGA	7	8	0	3	5	At1g12110.1:d:+1922:primary

TCCGTTATGT	5	6	7	2	3	At1g04530.1:d:+1186:primary
AGGATGAAGA	6	5	7	5	0	At1g01490.1:d:+845:primary
AAAAAACCTA	0	6	2	7	7	ChrC:+107810:quaternary,Chr5:+15544810:quaternary
TTCTGAAAAA	3	2	9	6	2	Chr3:+13134735:quaternary
GTTGTTGTTG	4	4	5	1	8	At5g66090.1:d:+878:primary
CACGTGTGGC	1	10	7	2	2	At5g65310.1:d:+1504:primary
AGGAACGTGA	3	3	7	4	5	At5g64140.1:d:+195:primary
AGCTTATTTC	2	0	15	3	2	At5g63790.1:d:+1275:primary
GTTTACTGAA	4	14	2	2	0	At5g57020.1:d:+1659:primary
TATAACACTA	10	0	2	3	7	At5g55620.1:X:+727:quaternary
TTACAAAATT	6	9	2	5	0	At5g53170.1:d:+2635:primary
AAGGCACTGA	3	12	4	3	0	At5g47030.1:d:+434:primary
AAGTTTGATC	1	11	5	3	2	At5g46250.1:d:+1332:primary,At5g46250.2:d:+1326:primary
ATTTATGAAA	8	5	3	5	1	At5g45950.1:d:+1127:primary,At3g14270.1:d:+5962:primary
ACGATTATGT	10	2	5	1	4	At5g45430.1:d:+2567:primary
TGAGTGGATT	0	2	11	5	4	At5g42900.2:d:+963:primary,At5g42900.1:d:+966:primary
AAGAACCAAG	2	8	5	2	5	At5g42020.2:d:+1683:primary,At5g28540.1:d:+1806:primary,At5g42020.1:d:+1848:primary
TCGAGACTCC	2	16	2	2	0	At5g40450.1:d:+5966:secondary
GGAAGATGAT	4	5	8	2	3	At5g27670.1:d:+699:primary
AAAAAAAAAG	5	4	6	5	2	At5g23940.1:i:+2092:tertiary
CTGGTGCCAG	3	12	3	3	1	At5g23900.1:d:+607:primary
GGAAGAATCT	3	17	2	0	0	At5g19120.1:d:+997:primary
TAACTGTAAG	5	7	6	3	1	At5g02790.1:d:+754:primary
TATTTAATCG	1	12	5	2	2	At4g39260.1:d:+808:primary,At4g39260.2:d:+679:primary,At4g39260.3:d:+577:primary
AGAAGTTTGG	2	2	4	11	3	At4g38160.1:d:+1032:primary
AACATTGCTG	1	1	18	1	1	At4g37120.1:d:+402:secondary,At2g39730.2:d:+940:secondary,At2g39730.3:d:+940:secondary,At2g39730.1:d:+969:secondary
GAACATATGA	3	11	3	4	1	At4g34050.2:d:+196:primary
ATTTGAGTTA	8	2	3	5	4	At4g33680.1:d:+1487:primary
CAGGATTTGA	4	3	5	5	5	At4g33350.1:d:+808:primary
TAATTGTAAC	8	11	2	1	0	At4g30690.1:d:+987:primary
GAGTAGGTGG	8	8	1	4	1	At4g29020.1:d:+440:primary
TTTCTGGTAA	8	5	4	3	2	At4g14960.1:d:+1559:primary,At4g14960.2:d:+1644:primary
TAAAATAATA	1	6	4	3	8	At4g09020.1:d:+2426:primary,At2g38870.1:d:+514:primary
TATCAGTAAT	6	2	5	3	6	At4g02510.1:d:+4664:primary
AACTCGACCT	6	5	7	1	3	At4g01290.1:d:+3535:primary
GACCTCACTA	4	3	3	5	7	At3g62830.1:v:+1851:secondary
AGCGTTTGGT	1	11	4	4	2	At3g57450.1:d:+455:primary
TGCTTGGCAA	6	3	7	4	2	At3g56140.1:d:+2416:primary
GAAGAAGCTG	2	8	7	4	1	At3g26190.1:d:+1458:primary,At3g26280.1:d:+1480:primary
ATTTGTTGTG	11	5	3	1	2	At3g15480.1:d:+870:primary
TTCTTGCAGT	3	6	2	6	5	At3g13235.1:X:-270:quaternary
GTGGTATTCG	6	6	5	3	2	At3g11130.1:d:+5424:primary
CCCGAGAAAA	2	10	5	4	1	At3g02110.1:d:+1551:primary
CGGAGTGTCC	2	9	3	6	2	At3g01520.1:d:+528:primary
CTCCGTCTTT	9	4	6	1	2	At2g44920.1:d:+975:primary
GTTTGACGAT	5	3	5	5	4	At2g43360.1:d:+1319:primary
TTCAGCACCT	0	21	1	0	0	At2g40000.1:d:+204:primary
GAGATATTGG	4	2	5	3	8	At2g36990.1:d:+1774:primary
ACACACCAAA	4	5	6	4	3	At2g35635.1:d:+584:primary
TAAAAACCGT	10	3	1	1	7	At2g35490.1:d:+1248:primary
TATTGTTAAC	5	6	3	4	4	At2g26340.1:d:+834:primary
GCATTTCACA	2	8	10	1	1	At2g17820.1:d:+507:secondary
TAAGTGTAGA	11	1	3	3	4	At2g04842.1:v:+2312:secondary
TAAACTAACA	4	10	2	1	5	At2g01470.1:d:+1349:primary
ATCTGAGTTT	7	7	3	2	3	At1g69740.1:d:+1574:primary
ATAGTAATTT	6	2	6	6	2	At1g69530.1:d:+1035:primary,At1g69530.3:d:+961:primary
ACAAAAAGAA	1	1	0	9	11	At1g69220.1:d:+1977:secondary,At1g69220.2:d:+1896:secondary,At4g04990.1:v:+45:secondary
AGCTAGATAC	3	12	1	2	4	At1g65960.1:d:+1357:secondary
TAATGACCGA	6	3	5	2	6	At1g64520.1:d:+947:primary
TAAAACGAAA	9	5	4	3	1	At1g59700.1:d:+783:primary

CGGAAACAGA	4	6	3	5	4	At1g49750.1:d:+1343:primary
TTCAATGTTG	4	8	6	1	3	At1g37038.1:p:+870:secondary,At4g14430.1:d:+906:secondary
TCTTTTTTGT	4	7	2	4	5	At1g20260.2:d:+1828:primary
AAGACTCTGT	2	13	3	3	1	At1g19570.1:d:+595:primary
CCGTTTTCTC	5	7	4	5	1	At1g15810.1:d:+1744:secondary
AAGAAGAAGC	4	4	4	6	4	At1g09575.1:d:+295:secondary,At3g04870.2:d:+1890:secondary,At3g04870.1:d:+1868:secondary,At2g05210.1:d:+383:secondary,At1g36445.1:p:+85:secondary ,At1g80020.1:p:+2711:secondary
AACGCAGCCG	5	6	4	3	4	At1g04940.1:i:+1489:tertiary
ATGCCCGGGA	2	4	9	5	1	No gene matches found
GCGTACTCCT	0	10	3	4	4	ChrC:+102945:quaternary
TTGCATCAAA	2	12	3	4	0	At5g64920.1:d:+1206:primary
CTTCATTGAA	6	3	5	3	4	At5g63780.1:d:+1654:secondary
GTCTCCGGAC	4	5	3	7	2	At5g59880.1:d:+421:primary,At5g59880.2:d:+421:primary
ATTCAGTTTC	1	3	9	5	3	At5g57120.1:d:+1249:primary,At3g53640.1:v:+2397:primary
TTTAAAAACA	5	0	2	6	8	At5g53970.1:d:+1424:primary
AATGCAGTAT	5	10	4	0	2	At5g51010.1:d:+514:primary
GTTGAGTGGC	3	10	2	5	1	At5g47570.1:d:+313:primary
TCCTCGACCC	7	6	4	2	2	At5g42190.1:d:+596:secondary
GAATCTACGA	0	14	4	2	1	At5g42050.1:d:+944:primary
CTTTGAATTT	4	7	5	4	1	At5g40820.1:d:+810:secondary,At2g47450.1:d:+1386:secondary
ACATTGTTTT	3	3	4	4	7	At5g28935.1:p:+751:secondary
AAGCCCCTAT	4	2	1	4	10	At5g28920.1:X:--47:quaternary
TGGTCAAATG	4	4	2	3	8	At5g25754.1:d:+1703:primary
CATACAAAAA	2	15	2	0	2	At5g25350.1:d:+1885:primary
TGCGACTGGT	3	9	6	2	1	At5g22000.3:d:+1271:primary,At5g22000.2:d:+1218:primary,At5g22000.1:d:+1344:primary
TGCAGTAAGT	4	6	4	5	2	At5g21326.1:d:+1663:primary
TGTCTAAATT	2	7	4	5	3	At5g20790.1:d:+603:primary
TGTCTAATCG	1	6	3	6	5	At5g20570.1:d:+474:primary
ACCTGAACAA	4	1	4	4	8	At5g20180.2:d:+574:primary,At5g20180.1:d:+499:primary
TCTGTGTCAG	5	13	1	1	1	At5g17870.1:d:+46:primary
TAAATAAAAA	3	9	4	3	2	At5g15860.1:d:+1405:secondary,At1g76360.1:d:+1749:secondary,At5g15860.2:d:+1638:secondary
CCGGAGCCAT	3	6	5	3	4	At5g08300.1:d:+1005:primary
AAGAGGCACG	3	11	4	1	2	At5g02870.1:d:+420:primary
GATAGAAAAA	6	5	4	5	1	At4g34490.1:d:+1814:primary
TCTTTTGGCC	1	1	2	6	11	At4g34190.1:d:+704:secondary
TTTTGTGAAA	2	7	3	3	6	At4g26670.1:d:+810:primary
AAGCTGACAA	3	9	3	4	2	At4g26630.1:d:+664:secondary
AAGACACTTC	3	6	4	3	5	At4g24920.1:d:+339:primary
GGCGCAACCG	5	3	6	5	2	At4g19006.1:d:+1107:secondary,At5g45620.2:d:+1415:secondary,At5g45620.1:d:+1110:secondary
ATACATTGAA	4	14	1	1	1	At4g15540.1:v:+1307:primary
ACTGGTCTTG	5	7	6	1	2	At4g13770.1:d:+1471:secondary
TGGCTCCTAC	1	2	7	4	7	At4g09040.1:d:+1192:primary
TCAATGAAAA	6	0	7	3	5	At4g04900.1:v:+354:secondary
GCCGTGAACA	3	8	2	5	3	At3g59970.1:d:+1663:primary,At3g59970.3:d:+1568:primary
TAAGCTGAAC	4	6	4	4	3	At3g56460.1:d:+1176:secondary
ATCACTTCAA	6	3	7	1	4	At3g56200.1:d:+1388:primary
AGAAGCGAAG	4	6	5	6	0	At3g52220.1:d:+698:secondary
AATGTATTTG	6	4	1	1	9	At3g48730.1:d:+1524:primary,At4g34410.1:d:+1322:primary
AGCCATCCGG	1	13	5	2	0	At3g48530.1:d:+1335:primary
GATTCCAAGA	4	3	2	8	4	At3g47400.1:d:+1256:secondary
TCTGAATTTA	4	9	6	1	1	At3g47160.1:d:+1068:primary
AATTTGCGGA	1	4	4	10	2	At3g32275.1:p:+1528:primary,At3g54400.1:d:+1490:primary
GATGCTATTG	9	2	7	0	3	At3g27160.1:d:+805:primary
AAATGCAAAA	3	3	7	5	3	At3g23460.1:v:+147:secondary
TAATGAAGTT	6	3	3	5	4	At3g20050.1:d:+1973:primary
ATGGTGTAAT	5	6	2	1	7	At3g18280.1:d:+551:primary
ATAATGATTT	2	11	3	5	0	At3g16200.1:i:+782:tertiary
GTTAGAACTT	3	2	4	8	4	At3g15000.1:d:+1414:secondary
AAGGTGAAGA	2	10	4	4	1	At3g14595.1:d:+486:primary
TTTTAAGATT	6	1	7	4	3	At3g13460.1:d:+2784:primary,At3g13460.2:d:+2775:primary

AAGTGATTGT	6	7	2	5	1	At3g13200.1:d:+802:primary
ACAAGATTCC	3	6	4	4	4	At3g13110.1:d:+1297:secondary
CCCGGCCTTG	4	2	6	7	2	At3g11770.1:d:+811:primary
TGGAGAGGCT	3	6	5	1	6	At3g09630.1:d:+783:primary
TAAAAATTTT	1	3	5	8	4	At3g04550.1:d:+1446:primary
GATGGAACCA	5	11	3	1	1	At3g02090.1:d:+1438:primary,At3g02090.2:d:+1438:primary
GTTCGGTTAA	3	8	3	5	2	At2g46600.1:d:+361:primary
AAGGAGAAAG	1	9	7	3	1	At2g43500.1:v:+3287:primary
GTTGAAGGAG	4	13	2	1	1	At2g41520.2:d:+3153:primary,At5g39890.1:d:+903:primary,At2g41520.1:d:+3246:primary
AAGAAGAAGA	1	9	5	5	1	At2g39650.1:d:+844:primary,At1g07720.1:d:+1438:primary
ATTTCTGGAT	5	4	6	4	2	At2g38040.1:d:+2754:primary,At2g38040.2:d:+2762:primary
TCTTTGTTTT	6	9	4	1	1	At2g37070.1:i:+706:tertiary,At4g21215.2:i:+263:tertiary,At5g28780.1:i:+292:tertiary,At4g21215.1:i:+263:tertiary
TATTGCTGGT	7	7	2	2	3	At2g33220.1:d:+659:primary
TCATCATCGG	4	1	6	4	6	At2g27720.1:d:-439:primary,At2g27710.3:d:-461:primary,At2g27710.1:d:-395:primary,At2g27710.2:d:-391:primary
TGCCTCAAGG	4	11	3	3	0	At2g19830.1:d:+674:primary
GTTGACATCA	4	9	5	3	0	At2g18300.2:d:+1159:primary,At2g18300.1:d:+1153:primary
ATCGAAGAGC	1	7	5	6	2	At2g18110.1:d:+702:primary
TTTTGCCCCT	4	9	7	0	1	At2g17050.1:v:+4730:primary,At1g76090.1:d:+1250:primary
AGTGTTTGTG	1	5	7	5	3	At2g11910.2:d:+567:primary,At2g11910.1:d:+670:primary
GCTGCTGAAT	4	2	1	7	7	At2g03820.1:d:+1638:primary
ATTGGTTTGT	6	5	5	4	1	At2g01600.1:d:+1957:primary
CTGGCAAATT	2	8	5	4	2	At1g76810.1:d:+4261:primary
AGTCGCTAAA	1	0	4	7	9	At1g67090.1:d:+843:primary,At1g67090.2:d:+836:primary
ATCGTCGTTC	5	5	6	3	2	At1g63690.2:d:+1905:primary,At1g63690.1:d:+1905:primary
AATCTCTCTG	5	1	4	1	10	At1g58360.1:d:+1653:primary
TTTCATAGCT	5	6	0	3	7	At1g53210.1:d:+1841:primary
TCTGTTGGAT	4	3	2	4	8	At1g52270.1:d:+584:primary
CTTCCCATCT	4	4	6	3	4	At1g48620.1:d:+1737:primary
GAATCAATTT	1	3	9	6	2	At1g30630.1:d:+986:primary,At5g39270.1:v:+1568:primary
GTCCAAGATA	7	1	3	9	1	At1g27320.1:X:+294:quaternary
TTTCTCTTGT	3	1	0	1	16	At1g17745.1:d:+2257:primary,At3g28650.1:v:+2726:primary
TACCCCGGTG	2	3	3	8	5	At1g16740.1:d:+537:primary
AAATCTCTGG	7	3	7	4	0	At1g14150.1:d:+648:primary
AAGGTCAACT	4	2	6	5	4	At1g09970.1:d:+3326:primary
GATGTTGTCT	6	5	3	2	5	At1g07660.1:d:+338:primary,At5g59690.1:d:+353:primary,At5g59970.1:d:+319:primary,At1g07820.2:d:+332:primary,At1g07820.1:d:+453:primary
CATCTATTGG	10	4	3	4	0	At1g04350.1:d:+1280:primary
AGAGCTAAGT	2	9	1	4	4	No gene matches found
CATTTCACGT	2	8	6	2	2	ChrC:+126785:quaternary
CATTAGAGAG	6	0	2	2	10	Chr5:+4484260:quaternary
AAAGATAAAA	0	0	6	6	8	Chr1:+17532716:quaternary
CATTTATGAA	5	4	5	5	1	Chr1:+10875718:quaternary,Chr1:+26419615:quaternary
TTTTGGAAAA	1	2	8	7	2	Chr1:+10363922:quaternary,Chr5:+18786668:quaternary
TATTTGTTTT	4	0	3	7	6	At5g59320.1:d:+525:primary
TTTTTTGGAG	5	3	1	9	2	At5g57345.1:d:+538:primary
CGCAAAGTCA	4	6	1	8	1	At5g55290.1:v:+609:primary
ATGCACAACA	4	5	3	7	1	At5g46430.2:d:+311:primary,At5g46430.1:d:+307:primary
AACAAGGAGA	4	7	2	7	0	At5g43940.1:d:+1089:secondary,At5g24760.1:d:+1157:secondary,At5g57670.1:d:+424:secondary,At3g50690.1:d:+1001:secondary,At5g24760.2:d:+1147:secondary
AACTCTTCTC	6	3	7	0	4	At5g42280.1:v:+2864:primary
TCAGAAAAAC	4	7	1	4	4	At5g41790.1:d:+3655:secondary
TGGCAAAAAA	4	2	10	4	0	At5g39550.1:v:+877:secondary
ACTATATTTC	2	7	2	3	6	At5g23140.1:d:+931:secondary
TCACCTGTAA	1	17	0	2	0	At5g21090.1:d:+337:primary
TCTCGAACCT	1	1	0	14	4	At5g20830.1:d:+2411:primary
TTTGTTTTAA	4	8	3	1	4	At5g19780.1:d:+1546:secondary
AAAAGATAGA	10	0	2	2	6	At5g19630.1:d:+757:primary
TACGAGAATT	3	6	0	7	4	At5g17010.1:d:+1751:primary,At5g17010.3:d:+1457:primary,At5g17010.2:d:+1543:primary

GTAAGAATGT	2	1	8	4	5	At5g16970.1:d:+1040:primary,At5g16980.1:d:+885:primary
AAGAACTCGG	3	6	5	4	2	At5g14440.1:d:+809:secondary
TAGTCTGGTT	6	6	4	2	2	At5g12200.1:d:+1694:primary
ACTCACTCGA	11	5	1	3	0	At4g38840.1:d:+358:primary
GCGCAGTGCA	4	7	1	7	1	At4g34265.2:d:+74:primary,At4g34265.1:d:+74:primary
TAATATTTGA	3	7	6	3	1	At4g33905.1:d:+1019:primary,At3g04290.1:d:+1416:primary
TACCCATCGG	2	8	1	7	2	At4g32620.1:X:+750:quaternary
TTTGTATTCT	5	4	1	5	5	At4g32530.1:d:+854:primary
ATATGCACGA	7	2	3	6	2	At4g31780.1:d:+1768:primary,At4g31780.2:d:+1767:primary
AAGCTTTTAG	7	4	2	3	4	At4g31180.1:d:+1956:primary,At4g31180.2:d:+1935:primary
ACGCAACCAA	3	7	3	4	3	At4g31170.1:d:+1454:primary,At4g31170.2:d:+1552:primary
GCTTGGTCCA	4	5	5	5	1	At4g29030.1:d:+628:primary
CGTCGCTCTT	4	6	8	2	0	At4g27310.1:d:+434:primary
TTTATGTTTC	4	8	2	2	4	At4g24220.1:d:+1483:secondary
GAGATACTCA	6	7	5	2	0	At4g13520.1:d:+156:primary
TTCATTGAAG	10	3	6	1	0	At4g02120.1:d:+1013:secondary
GGATATGATC	1	7	9	2	1	At3g56310.1:d:+1232:secondary,At3g56310.2:d:+1136:secondary
GTATCACGTG	4	9	5	1	1	At3g50740.1:d:+1638:primary
AGGGACTATG	2	11	3	2	2	At3g30775.1:d:+1646:primary
CCTGCCGTTG	2	3	5	5	5	At3g29360.1:d:+1495:primary
GAGGAGGCTG	3	4	5	3	5	At3g28950.1:d:+582:primary,At1g80070.1:d:+7102:primary
AGGAAAAAAG	6	3	2	5	4	At3g26780.1:i:+1669:tertiary
TATGTAATCT	7	8	1	1	3	At3g26070.1:d:+959:secondary
TATGCTTTCT	9	4	1	2	4	At3g20550.1:d:+1136:primary,At1g48410.2:d:+3356:primary,At1g48410.1:d:+3350:primary
AGTTTTGAAG	3	3	5	6	3	At3g17040.1:i:+1070:tertiary
AATCAATCTT	3	6	4	5	2	At3g11500.1:d:+436:primary
AGGAACACAA	2	1	0	17	0	At3g09440.1:d:+1756:primary
TCTAAAACAG	4	4	4	2	6	At3g07470.1:d:+949:primary
AGAAAAGTAC	7	2	3	4	4	At3g07460.2:X:-413:quaternary
AAGGTATTTT	0	11	4	3	2	At3g02560.1:d:+571:primary,At3g02560.2:d:+572:primary
CGCTGATGAT	2	0	13	0	5	At2g47400.1:d:+239:secondary
ATTAGTCATA	4	2	0	2	12	At2g44120.2:X:-438:quaternary
GGATTTTAGC	4	13	2	1	0	At2g42870.1:d:+519:primary
ATAAATCAAC	6	3	5	5	1	At2g42690.1:d:+1307:primary
CTCAAGCCCT	3	7	4	4	2	At2g41530.1:d:+1017:primary
ATCGTTCTTA	7	2	4	5	2	At2g41250.1:d:+1129:primary
ATTGTGGCCG	2	0	9	1	8	At2g38540.1:d:+114:secondary,At2g38530.1:d:+149:secondary
ATTCCTGTTG	4	2	10	2	2	At2g37970.1:d:+719:primary
TTGAGTTTCG	2	10	5	3	0	At2g37480.1:d:+1231:primary,At2g37480.2:d:+1228:primary
AATTAGTCTC	6	7	4	1	2	At2g35680.1:d:+1746:primary
TTTTTATAAT	5	2	3	0	10	At2g34860.1:d:+698:primary
ATTTTTTTGT	2	5	3	6	4	At2g34090.1:d:+1417:primary,At2g34090.2:d:+1527:primary,At2g31610.1:d:+998:primary
AGTGAGTGGT	3	12	2	1	2	At2g33340.2:d:+1770:primary,At2g33340.1:d:+1854:primary
TTGTTTCTTA	4	5	1	2	8	At2g31610.1:d:+944:secondary,At3g08790.1:v:+235:secondary,At3g06920.1:v:+5:secondary
TTCTATGTCA	5	6	3	4	2	At2g31200.1:d:+656:secondary
CGAAAAAAAA	2	2	9	5	2	At2g30370.1:X:--68:quaternary
TTCTATCAAA	6	4	5	3	2	At2g30110.1:d:+3594:primary
CCAAAAAAAA	3	2	7	5	3	At2g24750.1:p:+1556:secondary
CTATTTTCTG	3	5	9	1	2	At2g23130.1:d:+793:primary
CACTTTGTTT	2	6	3	2	7	At2g20820.1:d:+384:primary,At2g20820.2:d:+391:primary
CTGGGTTCTG	5	1	5	4	5	At2g20270.1:d:+734:primary
GAAACGTCAA	2	1	4	10	3	At2g16500.1:d:+2425:primary,At5g51940.1:d:+503:primary
GCTGTTTTAT	2	6	9	1	2	At2g14750.1:d:+947:primary
TTTACCAAAA	2	5	3	4	6	At2g13600.1:v:+1377:secondary
TGGAATCCAG	4	3	5	3	5	At2g13560.1:d:+1956:primary
AAGAAGCAAA	5	1	9	3	2	At2g13430.1:v:+703:primary
TTAATGACGC	3	15	1	1	0	At1g78460.1:d:+686:primary
AGTGTATTTT	3	5	4	5	3	At1g73880.1:d:+1669:primary,At3g16760.2:d:+1759:primary,At3g16760.1:d:+1816:primary
AAGTACCAAG	3	11	2	2	2	At1g72020.1:d:+288:primary
TAATAGTATA	7	2	0	0	11	At1g71500.1:d:+989:secondary
AACAAGAAAC	4	1	3	4	8	At1g69630.1:v:+2106:primary,At5g15140.1:v:+1878:primary

AAAGGAAGGT	4	9	4	2	1	At1g68520.1:d:+1335:primary
TATTTGTGTG	10	3	3	0	4	At1g67860.1:d:+417:primary,At2g05810.1:d:+2172:primary
TCTCTAAACC	8	1	5	6	0	At1g67740.1:d:+193:secondary
TTTCGCAGAA	3	3	5	4	5	At1g64550.1:X:+363:quaternary
ATCTGTAATT	4	5	8	0	3	At1g53840.1:d:+1937:primary
ATACTCTGTA	11	2	0	1	6	At1g51500.1:d:+2300:primary
ATGATGAAAT	3	9	6	1	1	At1g50930.1:v:+478:secondary
TTGAACCGGA	4	3	8	4	1	At1g44835.1:d:+1027:secondary,At3g51670.1:d:+1414:secondary
AGCGTTCTCC	3	7	6	3	1	At1g43670.1:d:+1021:primary
ACGCTTAAGA	7	4	4	3	2	At1g32990.1:d:+774:secondary
AACAAACATA	5	1	4	4	6	At1g26640.1:d:+1422:secondary
GCTAATCTCC	2	4	12	1	1	At1g24330.1:v:-1444:secondary,At4g10340.1:d:-879:secondary
AGGGAGAGTT	1	14	1	3	1	At1g23130.1:d:+206:secondary
GAAAAAACTC	3	2	9	3	3	At1g19920.1:d:+1648:primary
TCAAAAGAGT	4	3	8	5	0	At1g18500.1:d:+2020:primary
CTTCTGAAAG	0	18	0	1	1	At1g16870.1:X:-16:quaternary
TGTTTCAGAA	4	6	7	2	1	At1g16560.1:d:+154:secondary,At1g16560.2:d:+185:secondary,At1g16560.3:d:+322:secondary
TTCTTTCATT	8	2	7	1	2	At1g14345.1:d:+695:primary
GTTATGATGA	1	15	3	1	0	At1g10780.1:d:+1455:primary,At5g57655.1:d:+1315:primary,At5g57655.2:d:+1353:primary
GAGAGCGAGG	5	4	4	5	2	At1g09760.1:d:+1033:secondary
TCCTTCATCT	4	11	1	3	1	At1g09430.1:d:+1396:secondary
TTAAATTAAA	2	6	4	4	4	At1g09190.1:i:+3633:tertiary
AATAAAATTG	7	1	3	7	2	At1g04500.1:d:+1470:primary,At5g39080.1:d:+1435:primary,At1g52970.1:v:+899:primary
TAATGACGTA	7	1	3	2	7	At1g04420.1:d:+1383:primary
CAGAAGCCTT	2	3	4	7	4	At1g01720.1:d:+997:primary
ACATTAAAAA	6	4	4	3	2	Chr5:+2587431:quaternary
ACCGTGCGGC	3	9	1	3	3	Chr5:+22007914:quaternary
AATGAAAGAT	4	5	2	3	5	Chr5:+13405648:quaternary
AGTAAAAAAA	6	4	4	3	2	Chr1:+6872017:quaternary,Chr3:+15978814:quaternary,Chr2:+15506757:quaternary
AAGCACATAT	1	3	1	3	11	Chr1:+15974126:quaternary,At1g61960.1:X:+1769:quaternary
GCACACTTGG	4	6	3	4	2	At5g67500.1:d:+764:primary
GTGATGATGA	0	13	2	1	3	At5g66440.1:d:+609:primary,At3g01290.1:d:+775:primary
TGTTTCTTCG	6	1	6	4	2	At5g64850.1:d:+578:primary
CCTGGTCTAG	1	7	4	5	2	At5g57330.1:d:+1312:primary
AGCTTTATGC	7	4	1	5	2	At5g56150.2:d:+684:primary,At5g56150.1:d:+691:primary
TTCTACTATT	4	2	1	3	9	At5g55450.1:d:+546:primary
TGATTTGTGT	6	6	4	3	0	At5g54145.1:d:+524:primary
TTATTTTTAT	7	3	1	2	6	At5g53880.1:d:+396:primary
AGAATCTGAC	4	3	5	6	1	At5g52280.1:d:+1805:primary,At2g35410.1:d:+977:primary
ATAGCAATTT	6	3	4	5	1	At5g49740.1:d:+2417:primary
GTTGATCAGA	1	9	7	1	1	At5g49680.1:v:+1079:secondary
GAGAATCCGA	1	10	7	1	0	At5g47390.1:d:+1188:primary
CGATGAAAAA	2	4	9	3	1	At5g47230.1:d:+1099:primary
TTCAGAATGT	4	3	7	3	2	At5g46420.1:d:+1870:primary
AGGTTGCAAG	1	9	2	4	3	At5g46020.1:d:+547:primary
AAGGAGAAGA	0	11	1	3	4	At5g28300.1:d:+1026:secondary,At1g76220.1:v:+103:secondary,At2g21190.1:d:+247:secondary,At2g14740.2:d:+946:secondary,At1g01490.1:d:+361:secondary,At2g19120.1:v:+3773:secondary,At4g03150.1:d:+257:secondary,At1g02400.1:d:+368:secondary,At2g14740.1:d:+946:secondary,At4g38790.1:d:+264:secondary
TTCTGAAGAT	9	2	1	1	6	At5g25070.1:d:+1037:secondary,At3g09080.1:v:+1343:secondary
AATCTTGTGA	4	8	4	3	0	At5g22580.1:d:+367:primary
TCTGAGACTT	3	11	2	3	0	At5g21030.1:v:+2796:secondary,At1g12230.1:d:+1364:secondary
AAGATCTTGG	4	2	9	2	2	At5g19610.1:v:+4015:secondary
GAGAATGTTT	4	6	5	2	2	At5g16710.1:d:+712:primary
TTCCTCTTTA	6	6	3	0	4	At5g14430.1:d:+2125:primary,At5g14430.2:d:+2125:primary
ACCGTGACTC	3	7	3	4	2	At5g14040.1:d:+388:primary
GGTTTCTTAG	3	5	4	5	2	At5g13950.1:d:+3048:primary
CTTGTAAAAC	8	3	4	2	2	At5g12150.1:d:+2762:primary
AGTGAACCCA	4	6	1	6	2	At5g08450.2:d:+1388:secondary,At5g08450.1:d:+1346:secondary
ACTTCGTTTT	3	8	2	4	2	At4g37630.1:d:+590:primary,At2g32170.1:d:+1669:primary,At3g61050.1:d:+1681:primary
TTATTTCCAA	2	3	3	7	4	At4g36420.1:X:+261:quaternary

ATGGAAATTA	3	6	1	7	2	At4g34630.1:d:+824:primary
AGCGTTGGGA	4	6	4	3	2	At4g34200.1:d:+1742:primary
TGTAGCATAC	9	5	2	3	0	At4g33150.1:d:+3512:primary,At4g33150.2:d:+3538:primary
AGCACTCAGA	0	14	3	2	0	At4g31550.1:d:+683:primary,At4g31550.2:d:+683:primary
TTGAAGTCTC	5	5	3	4	2	At4g25670.1:d:+1115:primary
GCCGAAGGGA	0	5	13	1	0	At4g25470.1:d:+693:primary
CAGATTCTCC	5	4	4	5	1	At4g25080.3:d:+1115:secondary,At4g25080.1:d:+1072:secondary
TACCAGACAG	8	5	6	0	0	At4g24190.2:d:+2275:primary,At4g24190.1:d:+2275:primary
TCGCTGAAGA	2	10	3	2	2	At4g23100.1:d:+1553:primary
CGAACACCGC	2	10	1	3	3	At4g19410.1:d:+898:primary
CGGATTATGG	1	3	11	1	3	At4g12290.1:d:+2411:primary
CGAGATGGGC	1	1	16	0	1	At4g10340.1:d:+537:secondary
TTTTTTCTTG	2	1	2	3	11	At4g08390.2:d:+1352:primary,At4g08390.1:d:+1411:primary
AACAAATCCA	3	3	5	8	0	At4g08290.1:d:+1096:secondary,At4g08290.2:d:+1149:secondary
ACAAAAAAAG	6	0	3	6	4	At4g03770.2:p:+3138:secondary
TAGAGGTAGA	1	7	6	1	4	At4g02260.2:d:+2961:primary,At4g02260.3:d:+3047:primary,At4g02260.1:d:+2964:primary
TCACGGTACG	4	5	3	5	2	At4g01690.1:d:+1631:primary,At4g01690.2:d:+1538:primary
AACCCTATCA	5	6	2	2	4	At4g00860.1:d:+316:primary
TCTCTCAAAA	4	3	8	4	0	At4g00430.1:X:-425:quaternary,At5g37060.1:X:-349:quaternary,At5g58460.1:X:-349:quaternary,At5g11960.1:X:-51:quaternary
AAAGAGCTCA	3	6	3	3	4	At3g60210.1:d:+641:primary
ATTCTTACTA	7	0	1	2	9	At3g53490.1:v:+1362:primary
TTTAATGTTT	3	6	2	5	3	At3g51540.1:v:+2112:primary
TACGGTTGTT	9	3	6	1	0	At3g51420.1:d:+1256:secondary
CTCTCTGTAT	9	3	2	2	3	At3g48200.1:d:+3429:primary,At5g45190.1:d:+2041:primary
TTTCAGGAAT	5	3	2	6	3	At3g47295.1:d:+187:primary
ATTTTTAAAC	3	4	7	3	2	At3g45810.1:v:+3222:primary
TTATTTACTG	6	8	2	1	2	At3g45640.1:d:+1396:primary
AATCATCGAC	4	4	6	2	3	At3g29185.1:d:+1412:primary,At3g29185.2:d:+1346:primary
TACAATCGTT	3	6	8	0	2	At3g28710.1:d:+1262:primary
TGACTGTGAT	4	0	4	7	4	At3g19450.1:d:+719:secondary,At4g16440.1:v:+964:secondary
TGATGAGCTT	3	9	3	4	0	At3g19030.1:d:+306:primary
TTCTTTAACA	8	9	1	0	1	At3g18050.1:d:+1238:primary
TCGCGCCAAT	5	7	5	2	0	At3g16460.2:d:+2163:primary,At3g16460.1:d:+2170:primary
GATTTGATTT	3	10	1	3	2	At3g12630.1:d:+669:secondary,At5g42610.1:v:+1156:secondary
GAGGGGTCGA	5	1	6	4	3	At3g10525.1:d:+606:primary
AATGTTTGTC	6	0	2	5	6	At3g06400.1:d:+3328:primary
CGAGGAAGCA	2	10	2	4	1	At3g05840.1:d:+1453:primary,At3g05840.2:d:+1403:primary
TATGCTTGTC	3	9	5	0	2	At3g05220.2:d:+1789:primary,At3g05220.1:d:+1976:primary
AAGTTAAAGG	6	7	3	2	1	At3g03100.1:X:-111:quaternary
GACAGACATT	3	6	3	5	2	At3g02080.1:d:+189:primary
TGTCGTCGTT	2	10	2	1	4	At2g43535.1:d:+462:primary
TCATAAAAGA	4	8	4	3	0	At2g42880.1:d:+2294:primary
GGTTGGTTTG	3	3	6	6	1	At2g41740.1:d:+3249:primary
CAAAATTTTG	2	5	7	4	1	At2g40220.1:v:+1457:primary
ATGGATTCTT	1	11	5	2	0	At2g39400.1:d:+990:primary
GAACTAAAAA	2	1	4	7	5	At2g33090.1:v:+1453:primary
ACAAATCCGT	3	8	4	2	2	At2g32700.1:d:+2606:primary,At2g32700.2:d:+2564:primary,At2g32700.4:d:+2582:primary,At2g32700.5:d:+2600:primary,At2g32700.3:d:+2693:primary
AACAGATTTA	4	4	3	3	5	At2g29020.1:d:+190:secondary,At3g47500.1:d:+1430:secondary
TAATTGTTGG	9	2	4	2	2	At2g28470.1:d:+2912:primary
CAAAAGCTCC	0	10	7	1	1	At2g26560.1:d:+1297:primary
ATTAAGAAGA	3	9	2	2	3	At2g25210.1:d:+98:primary
TAGTATTTTA	4	5	1	3	6	At2g20890.1:d:+1165:primary
AACCCAGAAA	2	3	5	7	2	At2g18750.1:d:+2175:primary
AACTGGACCT	7	5	2	2	3	At2g18710.1:d:+1681:secondary
TAATGTCTCT	2	6	5	3	3	At2g17840.1:d:+1558:secondary
ATGGTTATGC	2	3	8	0	6	At2g16430.2:d:+1369:primary,At2g16430.1:d:+1417:primary,At1g79340.1:d:+1300:primary
AGGAAAACTG	2	10	4	2	1	At2g15880.1:v:+2574:secondary
ATGTCTCTCC	3	11	4	1	0	At2g01450.1:d:+1612:primary

GTCACACCGA	2	11	1	5	0	At1g78020.1:d:+302:primary
TGTTCCTTCT	1	14	2	1	1	At1g75350.1:d:+127:primary
CGGTTGGTTC	1	7	3	5	3	At1g75330.1:d:+1181:primary
ATAACTGAAT	2	6	6	2	3	At1g74100.1:d:+1199:primary
GATTCACACT	5	2	6	4	2	At1g73070.1:d:+1103:secondary,At1g73060.1:d:+1122:secondary
GTTGGATTCT	1	0	16	1	1	At1g71380.1:d:+1211:primary
TGGAAGTGAT	5	5	2	4	3	At1g70310.1:d:+1125:primary
ATGATGATCT	6	5	2	2	4	At1g65860.1:d:+1447:primary
TTCTTTCAAA	8	2	4	2	3	At1g62340.1:i:+2640:tertiary
GGTATGCTTG	3	8	2	2	4	At1g50900.1:d:+352:primary
CCAAAACTTC	1	4	5	6	3	At1g43730.1:X:-239:quaternary,At5g61020.1:X:-340:quaternary
GCGACATCGG	3	4	7	3	2	At1g43700.1:d:+1291:primary
TCCAAAAAAA	4	3	4	4	4	At1g35110.1:i:+4682:tertiary
AAGCTCAGGG	3	7	3	3	3	At1g34030.1:d:+401:primary
TATTAGTCGA	8	3	1	5	2	At1g29470.1:d:+2542:primary
ATCATTTCTT	9	5	4	1	0	At1g27290.1:d:+951:primary
TTATGTTTTA	9	2	3	1	4	At1g23130.1:d:+741:primary
ATTAAGTGGC	6	0	3	4	6	At1g19350.5:d:+1450:primary,At1g19350.1:d:+1373:primary,At1g19350.4:d:+1412:primary,At1g19350.3:d:+1447:primary
CTTTTTGATT	0	8	3	5	3	At1g19180.2:d:+1242:primary,At1g19180.1:d:+1064:primary
CAACATTATA	9	0	3	2	5	At1g18730.1:d:+621:primary,At3g24240.1:v:+4201:primary
TTTCAAATGT	4	6	1	4	4	At1g17200.1:d:+719:primary
TGGGGTGAGT	0	5	4	6	4	At1g08650.1:d:+1284:primary
TTGGAGATGT	6	8	2	2	1	At1g06640.2:d:+1318:primary,At1g06640.1:d:+1189:primary
TCTCTCTTTT	5	2	6	1	5	At1g04080.1:d:+2546:primary
TAGTGTGATC	7	5	5	2	0	At1g03120.1:v:+1220:secondary
CAGAGTTTGA	4	2	8	2	3	At1g01220.1:v:+3475:primary,At3g17850.1:d:+3962:primary
ATGAAAAAAA	7	2	4	3	2	Chr5:+5205222:quaternary,ChrC:+116752:quaternary
GATCATAAGC	7	3	3	3	2	Chr4:+8074731:quaternary
CTAAGAAGTA	12	0	4	2	0	Chr4:+2658663:quaternary,Chr2:+8797262:quaternary,AtCg00280.1:X:+1542:quaternary
CCTAAGGATA	5	5	1	1	6	At5g65360.1:d:+436:primary,At5g10390.1:d:+439:primary
GCTTCTTCTC	4	7	6	1	0	At5g65207.1:d:+265:primary
TGTTTCTGTC	8	4	3	1	2	At5g63190.2:d:+2439:secondary,At5g63190.1:d:+2483:secondary
TACGATCGAA	1	9	6	1	1	At5g62720.1:d:+879:primary,At5g62720.2:d:+1365:primary
AACATTGTTG	3	7	3	2	3	At5g61170.1:d:+393:primary
TGCCAAAAGT	3	6	1	4	4	At5g58640.1:d:+914:primary
GAAAACCAAT	6	0	4	1	7	At5g57770.1:d:+270:secondary,At5g65990.1:d:+1503:secondary,At3g01410.1:d:+868:secondary
GTGGGAATTC	1	8	3	3	3	At5g48180.1:d:+973:primary
TATCGGTTCT	5	2	3	6	2	At5g45010.1:d:+465:primary
TATAGTTCTC	8	7	1	2	0	At5g39530.1:d:+1278:primary
CAGCTAACTC	7	3	6	1	1	At5g38410.1:X:-214:quaternary,At5g38420.1:X:-214:quaternary,At5g38430.1:X:-194:quaternary
AAAAAGGTTT	3	3	5	5	2	At5g24690.1:d:+1881:primary
AAAGGTTTCA	4	11	2	1	0	At5g24490.1:d:+637:primary
TTTTCGGCTT	1	15	2	0	0	At5g21020.2:d:+265:primary
AACTCAATCT	7	3	1	4	3	At5g19010.1:d:+2511:primary
ATTTTAGTGT	1	1	1	0	15	At5g15950.1:d:+1731:primary
AATACTTGTG	10	4	2	0	2	At5g15230.1:d:+612:primary
TTTGGGCTCC	4	3	5	1	5	At5g12960.1:d:+788:secondary,At4g01100.1:d:+1568:secondary
GTCTTTCGAC	4	4	4	3	3	At5g12470.1:d:+1348:primary
GAATTAGGCT	6	2	1	8	1	At5g12150.1:d:+2683:secondary
GAGGTCCACA	3	3	4	4	4	At5g11200.1:d:+1453:primary
AAACAGAAGG	4	3	5	4	2	At5g08590.1:d:+1367:primary
AACCAATGTT	6	2	6	2	2	At5g06870.1:d:+1115:primary
CTGCTCTTAT	3	3	2	4	6	At5g06110.1:d:+2103:primary
AGTTTATCAC	4	8	1	3	2	At5g04750.1:d:+340:secondary
TAAATAAACC	9	2	4	1	2	At5g01530.1:d:-1128:secondary,At5g01530.1:d:-1117:secondary
CAGAGTTCGG	2	10	4	2	0	At5g01020.1:d:+1395:primary
GAGGGTGCTG	4	8	4	1	1	At4g38800.1:d:+720:primary
ATTCGACGCA	3	12	3	0	0	At4g38470.1:d:+1574:primary
AATGAATTTC	3	5	4	3	3	At4g35490.1:d:+566:primary
TCAAAGTTTC	1	3	4	7	3	At4g35260.1:d:+1413:primary

AAGTAATGCT	8	2	0	3	5	At4g34290.1:d:+734:primary
ACTTTTATGA	5	3	2	3	5	At4g31900.1:v:+3273:secondary,At3g56490.1:d:+575:secondary
TTGTTGGCAG	5	3	3	2	5	At4g31590.1:d:+2572:secondary
TGATCTGCAA	2	4	3	7	2	At4g31300.1:d:+864:primary
AAACTTGGGA	4	7	4	3	0	At4g30310.2:X:+261:quaternary
GACTCTCACT	3	4	10	1	0	At4g26530.1:d:+932:secondary,At3g09760.1:d:+666:secondary,At2g15020.1:d:+436:secondary
GGGTCGGAGA	3	2	5	7	1	At4g24510.1:d:+1322:primary
ATCATATGTC	6	2	5	4	1	At4g23680.1:d:+684:primary
AGTTGAGTTC	2	6	6	2	2	At4g19880.1:d:+1096:primary
TAACACGGCG	4	5	4	4	1	At4g18670.1:d:+2628:primary
AACCAATATT	1	9	3	0	5	At4g15050.1:v:+1909:primary,At1g73010.1:d:+921:primary
GGGAACCGAT	1	5	1	4	7	At4g13850.2:d:+201:primary,At4g13850.1:d:+201:primary
ATGTTTCTTC	2	2	8	4	2	At4g05526.1:p:+398:secondary
TTCTGATTCT	6	2	7	3	0	At4g05180.1:d:+971:primary
TGTTAATAAT	4	4	3	2	5	At4g04040.1:d:+1917:primary
CAGGTTCTAG	1	14	1	2	0	At4g02970.1:i:-1527:tertiary
AAAGGAATCT	3	3	4	6	2	At4g02570.1:d:+2525:primary
AAAATTTACT	0	8	5	2	3	At3g62120.2:d:+1739:primary,At3g62120.1:d:+1752:primary
CTCAGTGATC	3	6	5	3	1	At3g61200.1:d:+622:primary
TGGTAAAAAA	7	4	4	2	1	At3g59010.1:d:+1790:secondary
TTGTCCTCGA	1	14	0	3	0	At3g58730.1:d:+405:primary
AGATAAGCAG	3	5	5	4	1	At3g56950.1:d:+1282:primary
CAGAACCGCT	6	3	2	5	2	At3g56680.1:d:+1308:primary
GCTGCAGCTC	1	4	3	7	3	At3g54400.1:d:+1196:secondary
GTGTTGCTAA	2	4	4	2	6	At3g54340.1:d:+811:secondary,At5g43930.1:d:+1363:secondary
CAAAGATAAA	6	3	6	1	2	At3g54150.1:d:+966:secondary,At5g01480.1:v:+1114:secondary
TTGTTACCAG	3	12	0	1	2	At3g53740.2:d:+106:primary,At3g53740.1:d:+122:primary
GATTTTGTTT	5	3	4	3	3	At3g53580.1:d:+1223:primary
AAGCAGCAAA	5	2	6	4	1	At3g52380.1:d:+1212:primary
GATGACTTTG	3	7	1	3	4	At3g50500.1:d:+1193:primary
TCTAGCACCA	2	1	3	2	10	At3g49910.1:d:-617:secondary,At1g45180.1:d:-1146:secondary
TAATACAAAA	4	12	2	0	0	At3g48360.1:d:+1365:primary
AGACACCGGA	0	11	6	1	0	At3g46620.1:d:+1032:primary
CTGTGGAACT	3	6	1	8	0	At3g46060.1:d:+840:primary
TACAATACTT	4	5	2	1	6	At3g46000.1:d:+761:primary
TTGGTGTTGA	5	2	3	4	4	At3g43980.1:d:+285:primary
AAGGTACTTT	3	11	2	2	0	At3g27330.1:i:+2625:tertiary
TGACTGATAA	3	5	4	4	2	At3g26730.1:d:+2537:primary
TAAACATCTT	9	1	1	4	3	At3g26030.1:d:+2072:primary
TAGAACCTTT	2	4	6	1	5	At3g25585.2:d:+1735:primary,At3g25585.1:d:+1603:primary
TTGGCAGAGA	4	3	5	3	3	At3g21760.1:d:+1602:primary
TATCTAATCA	8	1	3	2	4	At3g20230.1:d:+891:secondary
GTGTAACAAA	4	3	2	4	5	At3g16850.1:d:+1727:primary
AGTGGAACAT	3	5	7	1	2	At3g16250.1:d:+654:secondary
TCATCGGAAC	1	15	0	1	1	At3g15630.1:d:+206:primary
ATTGATATTC	2	9	2	2	3	At3g13120.1:d:+591:primary
AATCCCCCCA	3	3	7	4	1	At3g11780.1:d:+717:primary
TCAAAAGTCT	5	4	6	3	0	At3g10740.1:d:+2320:primary
TATCTTTAGC	2	3	5	4	4	At3g07700.1:d:+2359:primary,At3g07700.2:d:+2270:primary
TTTACTGAAA	8	1	3	1	5	At3g04920.1:d:+613:primary
TGTTCTTTCT	3	8	3	4	0	At3g02540.1:d:+1551:primary
TTGGATAAAG	2	1	6	2	7	At3g01060.3:d:+1508:primary,At3g01060.2:d:+1446:primary,At3g01060.1:d:+1533:primary
CTCATTGCTC	4	7	2	4	1	At2g47710.1:d:+552:primary
TGTCTTTTTC	4	1	4	6	3	At2g45190.1:d:+977:primary
AAATAAGTGT	4	4	4	2	4	At2g42310.1:d:+561:primary
TTATTGACAG	4	4	2	1	7	At2g41110.1:d:+696:primary
AAACTTCCAA	3	8	1	5	1	At2g39310.1:d:+1448:primary
AAGGGACTGG	1	7	4	6	0	At2g38170.2:d:+1445:primary,At2g38170.1:d:+1341:primary
TGTTTAGTAT	6	4	0	1	7	At2g33150.1:d:+1662:primary
GACAGCTCAA	0	6	8	3	1	At2g33040.1:d:+977:primary
ACGGGCAAAG	4	3	7	3	1	At2g32260.1:d:+1379:secondary

ATGACTTTTG	3	4	3	6	2	At2g30925.1:v:+761:primary,At2g22400.1:d:+2612:primary
CGCTTAAGAA	1	6	3	3	5	At2g28900.1:d:+316:primary
TTATGAACTA	5	6	0	3	4	At2g27030.2:d:+1178:primary,At2g27030.1:d:+609:primary
TGATTGTTTA	9	5	3	1	0	At2g26740.1:d:+1160:primary
GTTCAGTACG	4	9	3	1	1	At2g25910.1:d:+1180:primary
ACTCTAATTT	1	6	7	3	1	At2g22300.1:d:+3413:secondary
AAAACAAAGT	9	2	3	2	2	At2g21110.1:v:+1054:primary
GCTGAAGAAG	0	2	15	0	1	At2g12550.1:d:+272:secondary,At3g21055.1:d:+328:secondary
GACCTTCAAC	6	1	9	1	1	At2g12250.1:p:+577:primary,At5g22510.1:d:+2052:primary
TAAGGGTGTC	3	4	1	4	6	At2g05840.1:d:+1016:primary
TGGTGTAACA	6	10	2	0	0	At1g71030.1:d:+531:primary
TGTTTCGATG	3	6	6	3	0	At1g66180.1:d:+1057:primary
CAGCAGTTCC	3	7	6	2	0	At1g65845.1:d:+318:primary
TTTGTACCAG	7	8	3	0	0	At1g55330.1:d:+202:primary
TCAAATCCAA	8	2	6	2	0	At1g54470.1:v:+44:secondary
GGAGGAAGAG	4	5	7	2	0	At1g53560.1:d:+460:primary
AGATCTGTTT	4	4	2	4	4	At1g51510.1:d:+899:primary
ATTTTTCAAA	1	0	17	0	0	At1g51260.1:i:+809:tertiary
TCTGTGAAGA	1	15	1	0	1	At1g50920.1:d:+1136:secondary
ATTCTAAATA	5	4	2	4	3	At1g50640.1:d:+1251:primary
ATCTTGTCGC	3	2	9	1	3	At1g29990.1:d:+647:primary,At4g04980.1:v:+2693:primary
TTTAAAAAGA	4	8	1	1	4	At1g28290.1:d:+1272:primary
ATTCAGTAAG	7	7	2	2	0	At1g27310.1:d:+691:primary
GCATTGCAAC	4	4	6	3	1	At1g23080.1:d:+1734:primary,At1g23080.2:d:+2163:primary
GGTCAATGGC	1	4	6	3	4	At1g22750.2:d:+811:primary,At1g22750.1:d:+815:primary
AAAATGTTAA	5	2	3	3	5	At1g20320.1:d:+923:primary
AAGAAAAATA	4	4	7	3	0	At1g19660.1:d:+1175:primary
GTTTAATGCA	2	8	3	5	0	At1g19400.1:d:+1354:primary,At1g19400.2:d:+967:primary
AATGTTTTGA	0	7	5	5	1	At1g08480.1:d:+709:primary
AACCACCCTG	1	0	8	1	8	At1g07920.1:d:+1118:secondary,At1g07930.1:d:+1102:secondary,At1g07940.1:d:+1123:secondary,At5g60390.1:d:+1072:secondary
GACTGAATTC	5	4	2	4	3	At1g07110.1:d:+2386:primary
GGTAAACCAA	4	1	3	6	4	At1g04410.1:X:-292:quaternary
TACAATAAGT	6	0	4	2	6	At1g03630.1:d:+1308:primary
AAACAAAAGA	7	2	7	1	1	At1g03240.1:d:+114:primary,At4g38560.1:v:+2107:primary
GACCGACCCC	0	11	2	2	2	ChrC:+104065:quaternary
GTTCTCATCG	3	6	4	2	2	Chr5:+22031149:quaternary
GTTATTTTGA	5	2	7	3	0	Chr5:+18137671:quaternary,Chr1:+2789406:quaternary,Chr2:+12574208:quaternary,Chr2:+19370922:quaternary,Chr3:+9726097:quaternary
TAATTTTATA	5	6	0	2	4	Chr4:+18451995:quaternary,Chr4:+14869360:quaternary,Chr1:+12060618:quaternary,Chr3:+19744373:quaternary
TGCGTGTGTC	4	5	6	2	0	Chr4:+11295236:quaternary
CAAACTAGAA	3	5	4	3	2	Chr3:+8496830:quaternary,ChrC:+61660:quaternary,Chr4:+15229270:quaternary
TATCCATCAA	6	3	2	2	4	Chr3:+11452542:quaternary
CATTTGTACA	6	9	2	0	0	Chr1:+28437078:quaternary
TGCTCCATCT	3	7	1	2	4	At5g64130.1:d:+440:primary
GGCGTTGTAA	4	7	3	3	0	At5g63860.1:d:+1676:primary
TCTTCGTTGT	1	13	3	0	0	At5g63160.1:d:+1090:primary
GGAATAAAGT	4	2	2	5	4	At5g58005.1:d:+509:primary
TGTTTTTGTT	3	3	6	4	1	At5g57350.1:d:+3078:primary
TTGTTTCTGT	7	2	4	4	0	At5g54640.1:d:+604:primary
TGGATATTTT	2	4	8	3	0	At5g54250.1:d:+2258:primary
GGTTTCCTTG	2	5	3	6	1	At5g53460.1:v:+6696:primary
CTTCCTACAA	3	4	4	2	4	At5g49740.1:X:-3:quaternary,At1g30280.1:X:-3:quaternary
TTAGACTTCT	2	9	2	2	2	At5g47200.1:d:+798:primary
GTTGGTGATA	4	6	5	1	1	At5g36790.1:d:+943:primary
AGCTAATCTA	5	1	4	5	2	At5g33355.1:v:+981:primary
ATTTGGTGTT	5	0	6	4	2	At5g27390.1:i:+2547:tertiary,At3g61420.1:i:+389:tertiary,At3g02800.1:i:+766:tertiary
CACTTCCAAT	3	10	4	0	0	At5g26280.1:d:+782:primary,At2g28630.1:d:+1772:primary
CTGCAGATTT	4	4	1	5	3	At5g25510.1:d:+2093:primary
GTTATATGAT	6	4	0	0	7	At5g20520.1:d:+1412:primary

GCACCGTGAT	0	11	1	4	1	At5g20010.1:d:+401:primary
TGTGTTGTGG	4	2	7	2	2	At5g18590.2:d:+2613:secondary,At5g18590.1:d:+2558:secondary
TAAACACTAC	10	0	1	0	6	At5g17990.1:d:+1626:primary
GCTGCAAACC	3	5	5	2	2	At5g17770.1:d:+946:secondary
GGTGAGATCA	5	4	3	4	1	At5g16030.1:d:+998:secondary
ATAATAAACC	3	5	3	2	4	At5g15790.1:d:+1482:secondary,At5g15790.2:d:+1569:secondary,At4g34180.1:d:+1010:secondary
GAGAGAATAC	3	11	2	1	0	At5g15780.1:d:+500:primary
ATCATCTCGA	3	6	3	2	3	At5g15410.2:d:+2224:primary,At5g15410.1:d:+2222:primary
TAAAAGCTCT	6	4	3	2	2	At5g11500.1:d:+753:secondary
AATCCTATGG	2	1	0	4	10	At5g08180.1:d:+685:primary
ACTGAAATCT	2	4	2	3	6	At5g07470.1:d:+563:primary
TTGGCTAGTT	4	6	4	0	3	At5g06360.1:d:+908:primary
GCCCCTTACA	0	0	1	10	6	At5g05270.1:d:+811:primary,At5g05270.2:d:+752:primary
TGTTGGAACT	2	8	3	4	0	At5g03760.1:d:+1908:primary
TTTGAGAGAA	4	2	3	4	4	At4g39210.1:d:+392:secondary,At1g57850.1:v:+1145:secondary,At2g07480.1:p:+389:secondary,At2g21590.1:d:+367:secondary
AAAGCGAAGT	2	3	7	3	2	At4g32990.1:v:+625:secondary,At3g55410.1:d:+537:secondary,At2g45660.1:d:+911:secondary
TCCAAGATTA	5	6	2	0	4	At4g32410.1:d:+3633:primary
TGGATTTGGT	1	5	5	3	3	At4g32330.2:d:+1672:primary,At4g32330.1:d:+1675:primary
TTCTATAAAT	3	6	1	3	4	At4g32150.1:d:+1105:primary
GAGATACGCT	6	5	2	2	2	At4g30960.1:d:+1588:secondary
GGAATGTGCA	3	9	3	1	1	At4g27800.1:d:+1056:primary,At4g27800.2:d:+1056:primary,At4g27800.3:d:+1056:primary
GAGGATGGTG	2	6	5	2	2	At4g26840.1:d:+373:primary
TGGAAGAACA	2	4	3	6	2	At4g23630.1:d:+529:primary
TAAGTTAAAC	3	5	6	3	0	At4g23270.1:d:+2049:primary
GTCGATTTGG	0	3	10	1	3	At4g17720.1:d:+1245:primary
GCTTTAATAC	4	4	5	1	3	At4g16990.1:d:+3112:primary,At4g16990.2:d:+2562:primary,At4g16990.3:d:+2577:primary
ATAACTGAAG	3	5	3	4	2	At4g16920.1:v:+4209:primary
CAAATGATAA	5	3	5	2	2	At4g11820.1:d:+1518:primary,At4g11820.2:d:+1672:primary
GTCAAAAAAA	3	1	7	5	1	At4g11420.1:d:+917:secondary
TCGAAACCGA	1	9	3	1	3	At4g04330.1:d:+534:primary
TAGAATTCTC	2	4	4	6	1	At4g03430.1:d:+3315:primary
GAAGAGTTTG	2	6	1	5	3	At4g02450.1:d:+699:primary
AGCCATCGAA	1	2	3	10	1	At4g01560.1:d:+1220:primary
CCTCTGGGGA	4	7	6	0	0	At4g00780.1:d:+854:primary
CCTTGTTTGT	6	2	4	2	3	At3g63520.1:v:+1921:secondary,At4g22410.1:v:+431:secondary,At5g26810.1:v:+354:secondary
GAAGAACTCT	2	5	3	4	3	At3g62910.1:d:+1263:primary
CTCCAACAGT	1	10	2	2	2	At3g59760.3:d:+828:primary,At3g59760.2:d:+828:primary,At2g43750.1:d:+873:primary
AAATGGTTCC	6	2	4	1	4	At3g58700.1:d:+585:primary
TGATTATCTT	3	14	0	0	0	At3g56600.1:X:+1838:quaternary,At2g19460.1:X:+795:quaternary
AAGTTAGTAG	1	3	2	1	10	At3g52180.1:d:+1392:primary
TGACAGAGAG	4	3	2	4	4	At3g51140.1:d:+1104:secondary,At5g67140.1:d:+590:secondary
TAATCTGTAT	4	2	2	2	7	At3g47420.1:d:+1880:primary
CGCAGCCTCC	3	7	6	1	0	At3g43520.1:d:+773:primary
GTAGTCGAAG	6	2	7	2	0	At3g26290.1:d:+1435:primary
TCTTGGCTGG	0	0	5	8	4	At3g21560.1:d:+1541:primary
TGTTGAAATT	3	4	7	3	0	At3g19900.1:d:+978:primary
AAGAAGACAA	3	5	3	5	1	At3g19150.1:d:+660:secondary,At1g03790.1:d:+228:secondary,At4g22670.1:d:+299:secondary,At5g14140.1:v:+1150:secondary
ATTTGATTTC	3	7	1	3	3	At3g17820.1:d:+1169:primary
AGGTTACGAT	3	8	1	1	4	At3g16850.1:d:+1548:secondary
TACGGTAAAA	3	3	3	7	1	At3g15660.1:d:+764:primary
GGAATACATA	7	5	3	0	2	At3g14110.2:d:+1251:secondary,At3g14110.1:d:+1120:secondary
GCAGCACTGG	0	4	4	3	6	At3g07430.1:d:+744:secondary
ATGGACAAAA	4	1	5	5	2	At3g05545.1:d:+1330:secondary
TAGAACCAAC	1	6	9	1	0	At3g04210.1:d:+1850:primary
CTGTAAATTA	4	0	3	6	4	At3g02380.1:d:+1146:primary
TTCGTTTTAT	4	0	4	5	4	At2g47970.2:d:+1544:primary,At2g47970.1:d:+1337:primary
TTCCTTGTTT	5	2	1	1	8	At2g47640.2:d:+566:primary,At2g47640.3:d:+574:primary,At2g47640.1:d:+599:primary
AAGATGGAGT	3	12	1	0	1	At2g44490.1:d:+1405:primary,At3g49270.1:d:+126:primary,At4g13620.1:d:+1021:primary

AATTAAAGCT	6	2	3	3	3	At2g44350.1:d:+1793:secondary,At2g44350.2:d:+1796:secondary
TATCCGATTA	1	11	4	0	1	At2g43910.1:d:+588:primary
TTTGGCTTGA	3	3	2	2	7	At2g43180.2:d:+1933:secondary,At2g43180.4:d:+1978:secondary,At2g43180.3:d:+1901:secondary,At2g43180.1:d:+1904:secondary
TGAGCGATGA	1	7	4	1	4	At2g39770.1:d:+1156:primary
GCCGATATCG	3	3	3	5	3	At2g39670.1:X:-696:quaternary
ATTGTTTGAG	4	0	2	3	8	At2g37250.1:d:+1112:primary
ATCCTTGTCT	5	3	3	2	4	At2g37110.1:d:+965:primary
GCCTTCGCCA	11	1	0	4	1	At2g36810.1:X:-119:quaternary
TTCAACTACG	3	2	2	3	7	At2g32730.1:d:+3279:primary
ATAAGATCTT	7	7	2	1	0	At2g30170.1:d:+937:primary
AAAATGTTCT	6	4	3	3	1	At2g29340.1:d:+1121:primary
GGGGAGCAAT	0	10	2	4	1	At2g28840.1:d:+1592:primary
TATGGTTGTA	4	10	2	1	0	At2g26740.1:d:+1063:secondary
AGCTTGCCAA	5	3	5	3	1	At2g18710.1:d:+1797:primary
GAAAAGAAAA	2	1	6	2	6	At2g17972.1:d:+720:primary,At4g16080.1:v:+1584:primary,At1g24360.1:d:+898:primary
AATTTTCCGT	3	3	4	2	5	At2g17720.1:d:+1310:primary
CTCTCCAAGA	7	6	1	3	0	At2g05620.1:d:+262:primary
GCTGTCTGTT	6	4	6	1	0	At2g03010.1:v:+1316:primary,At4g04692.1:p:+679:primary,At4g04692.2:p:+543:primary
TCGTCATCAA	2	5	4	6	0	At2g02070.1:d:+1931:primary
AGTTTGTAGA	2	4	4	3	4	At2g01690.1:d:+2927:primary,At2g01690.2:d:+2930:primary
TAATCGTTTT	2	8	6	0	1	At1g80840.1:d:+1152:primary
TCTCAAAATT	4	4	3	5	1	At1g80600.1:d:+1388:primary
TCCTATCGAG	0	1	1	6	9	At1g80130.1:d:+1297:primary
TGGATTGTAT	3	5	3	4	2	At1g78880.1:d:+2191:primary,At5g39730.1:d:+735:primary
GAAACTCAGG	1	5	0	8	3	At1g73650.1:d:+861:primary,At1g73650.2:d:+861:primary,At1g73650.3:d:+861:primary
TATGTCTATC	6	2	4	2	3	At1g70160.1:d:+1800:primary
TGAATTTGAC	3	7	5	1	1	At1g69760.1:d:+764:secondary,At4g16380.1:d:+849:secondary
CTAGGTTGTA	5	9	1	2	0	At1g68840.1:d:+1252:primary
TAATGTTAAT	0	2	9	1	5	At1g68050.1:d:+2068:primary
TGAGGGATGT	4	9	2	1	1	At1g66240.1:d:+134:primary
ATCAGTATGT	11	0	3	2	1	At1g64780.1:d:+1795:primary
CAGCTGCTTC	4	3	4	1	5	At1g64710.1:d:+1212:secondary
AGAACTTTAG	1	7	5	3	1	At1g62020.1:d:+3821:primary
GTTAATGCCT	3	6	5	1	2	At1g60710.1:d:+1298:primary
TACGTAACTT	4	6	5	1	1	At1g60140.1:d:+2949:primary
CTGGAAGTCT	0	4	5	5	3	At1g56110.1:d:+1000:primary
GGATTGGCAA	3	1	3	3	7	At1g56050.1:X:+271:quaternary
TTACTCAAAA	1	4	7	5	0	At1g54690.1:d:+576:secondary
AAACTTAAGT	3	3	4	2	5	At1g51940.1:i:+984:tertiary
AAAAAAATGA	3	3	6	3	2	At1g50670.1:d:+1011:primary
ATGGAAGTAG	4	6	3	3	1	At1g50020.1:d:+628:secondary
TGGAGAGATC	2	6	2	5	2	At1g48920.1:d:+1362:primary
GTTTTACAGT	3	3	1	5	5	At1g43890.1:d:+752:primary
AACTGAAAAT	3	1	0	6	7	At1g38203.1:p:+661:secondary
TGTCTTCATC	3	8	0	4	2	At1g34000.2:d:+434:primary,At1g34000.1:d:+434:primary
ACTCATTCTC	4	4	4	2	3	At1g33680.1:d:+2578:secondary,At1g15190.1:v:+603:secondary
AAACCCAATA	4	11	1	1	0	At1g27530.1:d:+688:primary
CCAACCGTCG	4	7	4	1	1	At1g27130.1:d:+753:primary
TAAAAACTCA	6	2	4	4	1	At1g21440.1:d:+1174:primary,At2g19210.1:v:+3389:primary
GAAGATTTCC	1	7	1	6	2	At1g20693.1:d:+334:primary
GATCTTGGAT	3	0	2	10	2	At1g20220.1:d:+1317:primary
TGTGATTAAG	2	1	5	4	5	At1g18485.1:d:+576:secondary,At2g25610.1:d:+704:secondary,At1g18310.1:v:+2413:secondary
TAATTTTTTT	3	5	1	3	5	At1g18150.2:d:+2017:primary,At1g18150.1:d:+2022:primary,At5g23460.1:d:+668:primary
AATGATTCGG	1	2	7	4	3	At1g18070.1:d:+1796:primary
AGTGAGCACT	2	6	3	5	1	At1g16810.1:d:+461:primary
TAATCCTGTA	4	2	3	2	6	At1g16350.1:v:+1937:secondary
TTTAAATCGA	4	6	3	2	2	At1g14450.1:d:+346:primary,At3g61080.1:d:+1108:primary
CGCCCGCCGT	0	15	0	1	0	No gene matches found
GACGGTAGTT	3	5	2	5	1	ChrC:+103913:quaternary
CAAATCCAAA	9	0	3	1	3	Chr1:+8513252:quaternary,At4g13290.1:X:+513:quaternary,Chr5:+5837131:quaternary

ATACATAAAC	6	3	4	3	0	Chr1:+6688218:quaternary,Chr3:+7816467:quaternary,Chr2:+9598429:quaternary
ATCACTGATT	3	3	3	5	2	Chr1:+6607607:quaternary
AATTATCCAC	3	1	4	3	5	At5g67370.1:d:+1138:primary
AGACAAAAAA	2	3	5	4	2	At5g64500.1:d:+1584:primary
AAAAGCCTTC	4	2	5	3	2	At5g63060.1:v:+1329:secondary
AAAATTGATT	0	4	10	1	1	At5g62360.1:d:+787:primary
AAAACATATA	3	3	0	1	9	At5g61510.1:d:+1244:primary,At3g04240.1:d:+3298:primary
AAGCTGCAGC	1	4	4	6	1	At5g57120.1:d:+322:secondary
AAACCAAAGC	1	12	1	1	1	At5g53880.1:d:+157:secondary
CCGAAGAAAC	1	7	3	4	1	At5g52470.1:d:+960:primary
TAACCACAAC	6	3	2	2	3	At5g52240.1:d:+645:primary
TGAAGCACAA	2	4	5	4	1	At5g51720.1:d:+318:primary
AATAAGGAAG	4	0	6	1	5	At5g48970.1:X:--46:quaternary,At5g57350.1:X:-244:quaternary
TTTAATGGAA	9	1	0	1	5	At5g48490.1:d:+392:primary
TAAGTCGTCG	4	8	4	0	0	At5g46030.1:d:+511:primary
AACGAAACAA	3	2	3	5	3	At5g44330.1:v:+2031:secondary,At5g16850.1:d:+2770:secondary
ATATGAAGAA	4	5	4	1	2	At5g43450.1:d:+1167:secondary
AAGAACATAA	0	14	2	0	0	At5g42500.1:d:+342:primary
TGAGTGAAGA	5	3	5	1	2	At5g41070.1:d:+28:secondary,At1g62660.1:d:+2263:secondary,At2g20590.2:d:+806:secondary,At2g20590.1:d:+778:secondary
TGAAAAACCC	3	4	7	1	1	At5g40440.1:X:+629:quaternary
AGCTCTTGGA	6	6	2	2	0	At5g39590.1:d:+1794:primary
AATTGGAATT	0	1	3	6	6	At5g39570.1:d:+1530:primary
ATAAAACATA	3	6	2	1	4	At5g38650.1:d:+669:primary
GCTGGCTCAA	4	4	3	3	2	At5g38520.1:d:+1096:secondary
AACGAGTTGA	3	9	3	1	0	At5g25280.2:d:+803:primary,At5g25280.1:d:+806:primary
TGTGTCTTAC	6	2	2	4	2	At5g24300.1:d:+2332:primary
GAGACAGATG	0	9	1	1	5	At5g23575.1:d:+1786:primary
TCTTTTGACT	1	3	0	3	9	At5g19550.1:d:+1435:primary
CTTCTATCCC	5	7	1	3	0	At5g19190.1:d:+499:primary
TAAAATCTTT	2	10	3	1	0	At5g18630.1:d:+1457:primary,At5g18630.2:d:+1478:primary
GTGATATGAT	7	3	4	0	2	At5g18600.1:d:+570:primary
AATGAATAAT	2	9	2	0	3	At5g18310.2:d:+577:primary,At5g18310.1:d:+524:primary
AAAATGCAGC	1	3	3	7	2	At5g17230.1:d:+1418:primary
ATCAAAAGAA	5	2	3	5	1	At5g16830.1:d:+1124:primary
ATTCCGGTAT	4	4	6	2	0	At5g15350.1:d:+524:primary
AATGAACCGT	5	2	6	1	2	At5g13190.1:d:+758:primary
TGATTCTCCA	3	8	2	3	0	At5g12050.1:d:+1306:secondary,At1g09370.1:v:+1047:secondary
ACACCAAAGC	5	3	3	3	2	At5g11520.1:d:+1413:primary
GCGAAAGCTG	1	12	3	0	0	At5g11090.1:d:+750:primary
AAATAAAGAA	3	1	0	4	8	At5g08570.1:d:+1753:primary
ATGAAGTACT	0	11	2	1	2	At4g35450.3:d:+624:primary,At4g35450.1:d:+695:primary,At4g35450.2:d:+704:primary,At4g35450.4:d:+559:primary
GATTCTATTT	2	5	5	3	1	At4g34640.1:d:+1606:primary
GATGGTTTAC	1	6	2	3	4	At4g32940.1:d:+1822:primary
GCGCCATCCG	2	7	7	0	0	At4g32030.2:d:+1037:primary,At4g32030.1:d:+878:primary
AGCAGAAAAA	0	0	11	5	0	At4g31800.1:d:+506:secondary
TATGACTTTC	3	3	4	5	1	At4g27320.1:d:+1121:primary
AAAAATACAA	5	3	2	2	4	At4g25130.1:d:+897:primary
AGTATTTTTG	3	1	6	6	0	At4g24680.1:v:+5057:secondary
ATACTGCTAA	6	0	0	1	9	At4g23950.1:v:+1704:secondary
TAGCCGCTTG	6	1	2	7	0	At4g23670.1:X:-210:quaternary
CGCCTTTTGA	2	6	5	3	0	At4g21810.1:d:+857:primary
GTGGTGTTTC	0	0	0	13	3	At4g17550.1:d:+1870:primary
TCTCTTTCAG	5	5	4	1	1	At4g16880.1:d:+930:primary
AGAAGAGGAC	4	2	5	2	3	At4g14210.1:d:+1834:primary,At4g14210.2:d:+1834:primary
GATTGCAAGA	2	4	2	4	4	At4g13720.1:d:+778:primary
TAAACACTTC	8	2	1	0	5	At4g13250.1:d:+1699:primary
TGTTTATGTC	7	4	4	0	1	At4g10080.1:d:+1161:primary
AATCAAAATT	2	0	0	12	2	At4g03824.1:p:+456:secondary,At4g14690.1:d:+776:secondary

GTATACATTC	6	2	1	1	6	At4g03260.1:d:+2797:primary
AAGAAAGCCA	1	3	5	3	4	At4g01940.1:d:+776:secondary
GGCTTGGTGG	3	6	4	3	0	At3g62660.1:d:+1527:primary
TTGGGATTTG	4	5	4	2	1	At3g59060.1:d:+1521:primary
GATTGATTCA	4	5	1	4	2	At3g57380.1:d:+1102:secondary
AGTCAATAAT	4	3	0	2	7	At3g55360.1:d:+1289:primary
GAGCGACCCG	1	11	2	1	1	At3g52500.1:d:+1366:primary
AACGGCCATT	5	3	1	4	3	At3g49490.1:d:+2980:primary
TTTTGGTTCT	2	1	8	2	3	At3g47500.1:d:+1565:primary
TCTGTCTGTG	4	1	10	1	0	At3g47470.1:d:+1071:secondary
GACGCTCAGG	0	9	1	2	4	At3g46970.1:d:+2592:primary
CTTTCTTGGT	2	4	2	2	6	At3g46790.1:d:+1655:primary,At5g44320.1:d:+1895:primary
TTTGGCTGCA	3	8	2	3	0	At3g45160.1:d:+131:primary
GGCGACGTCT	3	3	6	3	1	At3g43540.2:d:+1312:primary,At3g43540.1:d:+1244:primary
GATAGTTTCT	4	4	4	3	1	At3g28040.1:d:+3122:primary,At1g57620.1:d:+788:primary
AAACAGAGTT	3	3	3	4	3	At3g27830.1:d:+879:primary,At3g05975.1:v:+1224:primary
GTATAAAAGG	6	2	2	4	2	At3g25410.1:d:+1654:primary
AGTCTAACTC	3	3	5	4	1	At3g24070.1:d:+823:primary,At1g31440.1:d:+1715:primary
GGCAAAATCG	0	0	0	11	5	At3g22840.1:X:-391:quaternary
AATCAGTATG	4	5	1	0	6	At3g22380.1:d:+5198:primary
AGGTTCCGTT	0	0	8	7	1	At3g21890.1:d:+414:primary
TTGGCTGATT	3	8	2	2	1	At3g20390.1:d:+550:primary
TCTGCTCGGT	0	6	9	1	0	At3g17790.1:d:+1067:secondary
ATCTTTTTAC	6	2	5	2	1	At3g17220.1:v:+1124:secondary
GACGCTTACG	1	11	3	1	0	At3g16770.1:d:-325:primary
ACAGAAAAGC	1	6	3	3	3	At3g15710.1:d:+595:primary,At1g52600.1:d:+559:primary
TCTTTCTGGT	4	2	7	1	2	At3g12630.1:d:+906:primary
ATTAAGAAAG	1	11	1	1	2	At3g08510.1:d:+2048:primary
GGCGTTCCAA	0	4	8	4	0	At3g02640.1:d:+630:primary,At5g11420.1:d:+501:primary
ATTATGAATT	2	2	3	4	5	At3g02190.1:d:+370:primary
TCACGATACA	4	6	3	1	2	At2g46800.2:d:+1388:primary,At2g46800.1:d:+1268:primary
TATAAAAAAA	3	3	3	2	5	At2g46020.1:v:+7154:secondary,At2g46020.2:v:+7157:secondary
TCGGTCTATT	3	4	4	4	1	At2g43100.1:d:-953:secondary
GTCATACTTA	3	0	1	4	8	At2g38210.1:d:+435:primary
AGAATCTCAA	2	3	1	6	4	At2g36290.1:d:+1257:primary,At4g30300.1:v:+1322:primary
ATTTCACCAA	5	4	3	4	0	At2g35830.1:d:+543:primary
TTCTAACCGG	5	4	6	0	1	At2g35390.2:d:+1502:primary,At2g35390.1:d:+1294:primary
TTAAACTAGG	2	0	4	4	6	At2g34660.1:d:+5437:primary
TACTTTGAAG	4	7	1	3	1	At2g33370.1:d:+366:primary
TACGGATTTT	4	1	3	3	5	At2g32920.1:d:+1481:primary
TCGAACACCA	1	11	3	1	0	At2g30520.1:d:+568:primary
GATGATTCGC	5	4	4	3	0	At2g30060.1:d:+1045:primary
CTTTTGTTTT	4	6	5	1	0	At2g29020.1:d:+638:primary
AATCTATCTT	2	2	3	3	6	At2g25520.1:d:+1432:primary,At2g28090.1:v:+1538:primary
AAAGGACGAG	5	2	2	3	4	At2g24060.1:d:+687:secondary
CACACGGTGG	0	9	4	0	3	At2g22540.1:d:+1112:primary
CAAAGTGCTC	0	5	2	6	3	At2g22170.1:d:+461:primary
CGACAATCAA	4	9	3	0	0	At2g21530.1:d:+467:primary
GTTATCCAAG	3	7	2	1	3	At2g19760.1:d:+320:primary
TATGTGAAAG	4	7	3	0	2	At2g18465.1:d:+1066:primary,At2g05084.1:v:+1095:primary,At3g32026.1:p:+1129:primary,At4g17940.1:d:+1005:primary,At5g34500.1:v:+801:primary
CTTCTACGGC	2	13	0	1	0	At2g17450.1:d:+576:primary
GTTTGGAATG	3	1	11	1	0	At2g16365.2:d:+1472:primary
ACTGTTGTTC	2	5	1	3	5	At2g16060.1:d:+658:primary
TGACTTCTGT	3	7	5	0	1	At2g14720.1:d:+2218:primary,At2g14720.2:d:+2172:primary
GAATGTTTGG	3	5	4	4	0	At2g04270.4:d:+1737:secondary,At2g04270.3:d:+1737:secondary,At2g04270.2:d:+2394:secondary,At2g04270.1:d:+2549:secondary,At5g45350.1:d:+562:secondary
GTGGAGAGAG	5	3	7	1	0	At2g03280.1:d:+896:secondary,At1g03610.1:d:+1605:secondary
AAATGAATGT	4	4	1	2	5	At1g78060.1:d:+2436:primary
TCTATAAATG	5	5	3	1	2	At1g76990.3:d:+2016:primary,At1g76990.1:d:+1809:primary,At1g76990.2:d:+1902:primary

CGTCTAAGAA	1	9	3	2	1	At1g76810.1:d:+593:secondary
CTGGGGAAGA	3	6	3	3	1	At1g75680.1:d:+1556:primary
TAGAGAGACC	3	4	3	4	2	At1g74230.1:d:+1057:primary
GAACAAAAAG	1	10	2	1	2	At1g74020.1:X:--205:quaternary
ACACAGAATG	7	2	5	1	1	At1g73310.1:v:+1138:secondary,At1g66670.1:d:+955:secondary
GGGCAACGAG	2	5	4	3	2	At1g70940.1:d:+2300:secondary
TTTCAGTATC	5	7	1	2	1	At1g67865.1:d:+357:primary
CTTTTGGGTG	9	5	0	2	0	At1g67785.1:d:+211:primary
CCATTATGCC	6	0	1	5	4	At1g66410.1:X:-568:quaternary
GAGTTTTTTT	1	6	2	2	5	At1g63940.2:d:+1560:secondary,At1g63940.3:d:+1665:secondary,At1g63940.1:d:+1578:secondary,At2g34010.1:v:+182:secondary,At1g63940.4:d:+1566:secondary
TTCTTTGATA	6	2	2	2	4	At1g61840.1:v:+2765:primary,At4g36130.1:d:+916:primary
TTTTTTTCTG	2	5	4	1	4	At1g51570.1:d:+2430:primary
GTTTACTCAA	2	4	5	3	2	At1g51160.1:d:+816:primary
GCTTGCTGTT	2	7	3	2	2	At1g50010.1:d:+1063:primary
GCAGCTTCCT	2	9	4	0	1	At1g48030.1:X:-1216:quaternary,At3g17240.3:X:-1216:quaternary
GCCACCTATG	4	1	4	7	0	At1g48030.1:d:+1495:primary,At1g48030.2:d:+1495:primary
GAACATCTGT	4	5	5	2	0	At1g44446.2:d:+1489:primary,At1g44446.3:d:+1565:primary,At1g44446.1:d:+1489:primary
GTGGATGATG	2	7	3	4	0	At1g42550.1:d:+1790:primary
AGCAACTTTC	1	6	3	1	5	At1g35670.1:d:+1691:primary
GGATTATTGA	6	0	4	4	2	At1g34190.1:d:+2022:primary
TTGAGAATTA	2	6	5	2	1	At1g28280.1:d:+922:primary
TTTTTGATTT	0	1	0	10	5	At1g27760.1:d:+1707:primary,At1g27760.2:d:+1897:primary,At1g27760.3:d:+1719:primary
GTGGCACATT	0	10	5	1	0	At1g21500.1:d:+75:primary
AAATCAATCT	4	5	2	2	3	At1g21170.1:v:+3778:primary,At2g42190.1:d:+862:primary
CTGCTTCATA	5	4	0	3	4	At1g20980.1:d:+3853:primary
AGCCTACAAA	2	3	6	1	4	At1g18790.1:v:+870:secondary
TGGAAGAAGT	2	2	1	7	4	At1g18450.1:d:+1590:primary
GTTGAGATTT	1	3	3	3	6	At1g18210.1:d:+688:primary
GTCCATTGTT	2	5	4	2	3	At1g16920.1:d:+800:primary,At2g04845.1:d:+774:primary
GCAAGTGCCA	1	6	6	2	1	At1g13930.1:d:+173:secondary
AACCTTTGGT	1	4	9	1	1	At1g13260.1:d:-1360:secondary
GTCGAAGGAA	2	2	5	2	5	At1g09590.1:d:+435:primary
CCCAAGGACA	3	2	6	0	5	At1g09200.1:d:+431:primary
AAAACTTGAC	5	3	4	4	0	At1g08450.2:d:+1266:primary,At1g08450.1:d:+1428:primary
AATCTTGTTC	4	3	2	3	4	At1g03330.1:d:+651:primary
ATCGTTTAAA	0	3	1	5	6	No gene matches found
CCCGAAGTCG	0	13	0	2	0	ChrC:+102389:quaternary
AGAATGTTTC	2	1	6	3	3	Chr5:+16094984:quaternary
GCAGGTCACC	1	1	5	5	3	Chr4:-17282157:quaternary
TGGGTGTGTA	4	2	6	1	2	Chr4:+11927318:quaternary
ATGGTTAAGT	5	0	5	4	1	Chr2:+10360483:quaternary
TATGTTGTAT	6	3	0	2	4	At5g67380.1:d:+1407:primary,At5g03560.1:d:+893:primary,At1g76950.1:d:+3899:primary
TGGGTGTTTT	7	2	4	2	0	At5g65480.1:d:+1004:secondary
GTTTGTTGTC	5	8	1	0	1	At5g65390.1:d:+599:secondary,At3g43460.1:v:+655:secondary
TTTTTCGATT	0	2	3	4	6	At5g64480.1:d:+670:primary,At1g79580.3:d:+1277:primary,At1g79580.2:d:+1350:primary,At1g79580.1:d:+1437:primary
GCACCTAAAG	2	7	2	2	2	At5g64430.1:d:+1574:primary
ATCAAAGACT	5	5	1	3	1	At5g63400.1:d:+788:primary
TCTCATAAAT	0	8	1	0	6	At5g58740.1:d:+605:primary
TGCATTCCCC	4	3	4	3	1	At5g58390.1:d:+1104:primary
TTGAGAAATT	0	10	3	2	0	At5g54500.1:d:+136:secondary
TTCCTCAGCA	5	5	0	3	2	At5g52840.1:d:+453:primary
GCCATTGTGT	0	7	4	0	4	At5g51970.2:d:+1088:primary,At5g51970.1:d:+1320:primary
GAATATAAAA	7	2	3	2	1	At5g51530.1:v:+3384:secondary,At1g61370.1:v:+66:secondary
TTACAGCCCA	5	3	2	4	1	At5g49930.1:d:+3419:primary
ATTGAAGCAA	3	1	2	2	7	At5g49910.1:d:+2336:secondary
CTAGATGAGT	3	8	2	1	1	At5g48810.1:d:+320:secondary
CTTGCTCTTG	1	11	1	1	1	At5g46860.1:d:+919:primary
GAAACGACCC	1	5	3	3	3	At5g42790.1:d:+467:primary

TAGGAAGTTA	5	1	4	4	1	At5g42310.1:d:+2450:secondary
TTGTTGTGTA	2	2	1	6	4	At5g41670.2:d:+1618:primary
AGGGTATCAA	2	8	2	0	3	At5g40855.1:v:+682:primary
TTTACTCAAA	5	1	5	1	3	At5g39350.1:v:+1741:secondary,At3g61140.1:d:+1611:secondary
TACAGTGCCA	7	4	3	1	0	At5g37360.1:d:+1081:primary
CTTGGACTAA	2	2	4	2	5	At5g36210.1:d:+2221:primary
GGTGTGAATT	3	1	7	3	1	At5g34850.1:d:+1738:secondary
TAGCTACAGA	3	6	4	1	1	At5g28530.1:v:+2218:secondary,At5g19165.1:p:+4355:secondary,At5g25040.1:v:+547:secondary
GATCGTGTCA	1	5	5	1	3	At5g25757.1:d:+1525:primary
GGCATATTTG	0	1	7	6	1	At5g24530.1:d:+1316:primary
TGCGTAAGTA	2	5	4	3	1	At5g24520.3:d:+1247:primary,At5g24520.2:d:+1192:primary,At5g24520.1:d:+1198:primary
TACAGGGTTT	4	0	5	3	3	At5g23610.1:d:+1445:primary
AGCGTGGTTT	7	3	1	3	1	At5g22640.1:d:+2600:primary
ATTGTGTTTT	1	5	4	1	4	At5g20890.1:d:+1809:primary
GCGAAAAATA	1	5	3	2	4	At5g20700.1:d:+1084:primary
AAAACATTTT	5	3	1	2	4	At5g19990.1:d:+1440:primary
GGCACCAGAC	2	4	8	1	0	At5g19370.1:d:+754:primary
GCGGGTCAAG	3	6	2	2	2	At5g18660.1:d:+1325:primary
GATTTTGATT	4	2	2	4	3	At5g16540.1:d:+253:secondary,At3g48830.1:v:+1608:secondary,At2g35733.1:v:+767:secondary,At5g16540.2:d:+114:secondary,At5g16540.3:d:+217:secondary
TTCTTTTGTT	4	5	1	2	3	At5g16210.1:d:+3810:primary,At1g01500.1:d:+1331:primary,At5g03040.1:d:+1792:primary
TCTGCTCCTC	2	12	1	0	0	At5g16030.1:d:+495:secondary
TACTTCCAAA	1	1	2	8	3	At5g14520.1:v:+2216:secondary
AACCCATATT	3	4	1	4	3	At5g13720.1:d:+908:primary
GAGATATTCC	1	2	5	6	1	At5g13655.1:v:-22:primary
GATGATTAGG	5	4	2	3	1	At5g13000.1:d:+6106:primary
TATTACAAAG	4	8	0	1	2	At5g12210.1:i:+1341:tertiary,At5g12210.2:i:+1316:tertiary
CTGACTATTG	1	3	2	3	6	At5g11770.1:d:+965:primary
GAAATTTCTC	3	2	5	4	1	At5g11060.1:d:+1744:primary
CAAAATTCGA	2	1	5	7	0	At5g10630.1:d:+2202:primary
AACAACAGAG	2	5	4	2	2	At5g09220.1:d:+1619:primary
TCTCTTGTCT	0	4	4	3	4	At5g07870.1:d:+1442:primary,At3g56290.1:d:+491:primary
CGATAGCGAA	1	6	3	3	2	At5g06700.1:d:+1654:primary
GTGGTCTCTG	0	13	2	0	0	At5g05440.1:d:+455:secondary
CCTTTTGTAA	5	4	5	1	0	At5g02840.1:d:+1214:secondary,At1g69290.1:v:+1888:secondary,At1g68980.1:v:+1795:secondary
CGAAGATTTT	1	1	6	3	4	At5g01790.1:d:+516:primary
GCACTTTGGA	2	8	3	0	2	At5g01210.1:d:+1580:primary
TAATAAAGAA	2	6	2	0	5	At4g39520.1:d:+1594:primary,At3g16190.1:d:+695:primary
ACGATGCTTA	2	12	1	0	0	At4g37830.1:d:+172:primary
GTAAAGTTCT	5	9	0	0	1	At4g37300.1:d:+274:secondary
CGAGCAAGAA	0	2	4	5	4	At4g34950.1:d:+1723:primary
TCTACGTTCA	0	9	2	2	2	At4g34720.1:d:+134:secondary
CCTTTTTACA	4	3	2	1	5	At4g34620.1:d:+634:primary
GAAATGCAAG	6	4	4	0	1	At4g30690.1:d:-982:secondary
AAAGGCCGAG	3	8	2	2	0	At4g30690.1:d:+661:secondary
AACTTGCAGC	2	6	4	2	1	At4g30220.1:d:+188:primary
CAAGAACAGT	4	2	2	4	3	At4g30200.2:d:+2501:primary,At4g30200.3:d:+2471:primary,At4g30200.1:d:+2359:primary
ACTGCTCTGA	3	4	4	3	1	At4g29950.1:d:+2853:secondary,At4g29950.2:d:+2849:secondary
TTCTTCAAAA	5	2	2	3	3	At4g29130.1:d:+1903:secondary,At4g21160.1:d:+1341:secondary,At1g73610.1:v:+366:secondary
GAGTTTAAAG	4	1	8	1	1	At4g29060.1:d:+3149:secondary
GTTAGCTCTC	7	0	4	3	1	At4g28750.1:X:+289:quaternary
TTTAGAACTG	2	0	4	6	3	At4g27520.1:X:-1016:quaternary
TGGCGGATTA	1	3	10	1	0	At4g19880.1:X:+474:quaternary
CCTCTTCTGA	6	5	0	4	0	At4g19110.1:d:+2323:primary,At4g19110.2:d:+2332:primary
GGGATACTGA	0	9	3	3	0	At4g17560.1:d:+410:secondary
CCCAAAAAAA	1	2	5	7	0	At4g16760.1:X:--8:quaternary
ACCACCTCCT	3	7	2	2	1	At4g15460.1:X:-209:quaternary,At4g15460.1:X:-251:quaternary
TTCTTGTAAA	5	4	3	1	2	At4g13780.1:d:+2588:primary
TAACTTTCGA	3	3	5	4	0	At4g13010.1:d:-1240:secondary
TAAGCACTCA	6	4	1	3	1	At4g08280.1:d:+540:primary

ATCGGAAAAT	2	7	4	0	2	At4g04340.1:d:+2437:primary,At4g04340.3:d:+2735:primary,At4g04340.2:d:+2439:primary
GATGTTCTCA	1	4	1	6	3	At4g02020.1:d:+926:secondary,At4g29840.2:d:+1578:secondary,At4g29840.1:d:+1578:secondary,At1g53330.1:v:+832:secondary
ATAAGGAGAG	4	7	2	0	2	At4g01610.1:d:+1123:primary,At4g01610.2:d:+1123:primary
ATAAAGCGGC	3	5	1	6	0	At4g00895.1:d:+313:primary
TCTTTTGGGA	6	4	4	1	0	At3g63190.1:d:+1074:primary
TAAACTGAAA	6	1	3	4	1	At3g62970.1:d:+1004:primary
TCTGTTTTAA	3	6	3	1	2	At3g60610.1:p:+1718:primary,At1g60170.1:d:+1692:primary,At5g51620.1:d:+536:primary
CACCTAACCG	2	5	3	3	2	At3g60240.1:d:+4598:primary
GTTTTGTGAA	3	3	4	5	0	At3g56800.1:d:+880:primary
CTGTGATGCT	3	2	4	3	3	At3g54300.1:d:+965:primary
AGAAGTCTCT	1	8	2	1	3	At3g54140.1:d:+1591:primary
GATGATTGTG	0	1	5	3	6	At3g52870.1:d:+1609:primary
GATGCAGCTA	1	11	3	0	0	At3g52800.1:d:+677:primary
AGTGAATTGG	3	6	3	1	2	At3g52340.1:d:+1399:primary,At3g52340.2:d:+1365:primary
CCTTGATGTT	2	11	1	1	0	At3g52060.2:d:+1018:primary,At3g52060.1:d:+1018:primary
CAAGAACTCT	2	10	2	0	1	At3g49870.1:d:+607:primary
AAATGGACTG	7	3	3	2	0	At3g49680.1:d:+1328:primary
AATAAAAATA	2	9	2	1	1	At3g48740.1:d:+960:secondary
TGTGCGTTAG	3	5	1	1	5	At3g48050.1:d:+5295:primary,At3g48050.2:d:+5215:primary
TCTTCCAGCT	2	8	4	1	0	At3g46010.1:d:+319:primary,At3g56970.1:d:+291:primary
GCTGAACCAA	1	6	5	2	1	At3g45780.1:d:+2590:primary
GCGGCACAAC	4	3	3	4	1	At3g43720.1:d:+320:primary
TATAAATGTG	5	6	3	0	1	At3g26580.1:d:+1194:primary
GTTTTGATGT	2	1	3	1	8	At3g22920.1:v:+1017:secondary,At3g25830.1:d:+507:secondary,At5g03970.1:d:+1421:secondary,At4g16730.1:v:+670:secondary,At2g43780.1:v:+722:secondary,At3g25820.1:d:+502:secondary
ATCGTTTTGC	5	1	2	6	1	At3g22210.1:d:+271:primary
GGAAAAAAAA	5	2	5	1	2	At3g19040.1:v:+4026:secondary
AATCTGAGGT	4	2	6	2	1	At3g18180.1:v:+1539:primary,At5g21930.1:d:+2774:primary
CAAATTACGA	1	3	5	4	2	At3g17810.1:d:+1485:secondary
AAACAGAAAA	3	6	4	2	0	At3g14840.2:d:+3103:primary
GGTTGATTCT	1	1	3	3	7	At3g13740.1:d:+994:primary
CCGCCTCAGT	2	5	4	3	1	At3g13470.1:d:+1679:primary
GAAGGAGCTT	3	2	4	5	1	At3g13460.1:d:+2377:secondary,At3g13460.2:d:+2368:secondary
TATTCTTTTA	2	3	2	4	4	At3g11410.1:d:+1558:primary,At1g72480.1:d:+1607:primary
TCAACCATTC	1	11	1	1	1	At3g10920.1:d:+340:primary
ACAAGTGGAT	1	11	3	0	0	At3g09860.1:d:+181:primary
ATTTGATTTG	3	4	2	3	3	At3g07940.1:d:+1499:primary,At5g65040.1:d:+567:primary
TATCGTCTCG	1	5	5	2	2	At3g06300.1:d:+1024:primary
AGCAATATGA	2	6	3	3	1	At3g04350.1:d:+2224:primary
TAGTTCCGAG	6	3	2	4	0	At3g04260.1:d:+2856:primary
GGTACCCACC	1	6	0	4	4	At3g03100.1:d:+508:primary
TGCAGCCGGT	1	8	2	2	2	At2g46900.1:d:+334:secondary
AATACATTAC	7	5	0	2	1	At2g46220.1:d:+1038:secondary
TCCCTTGTGT	4	4	1	1	5	At2g43490.1:v:+2203:secondary,At1g67430.1:d:+583:secondary
TTTCTTGTGG	5	3	2	4	1	At2g40890.1:d:+1710:primary,At5g60510.1:d:+934:primary
ATGAGTGCTG	2	0	12	1	0	At2g39730.3:d:+739:secondary,At2g39730.1:d:+768:secondary,At2g39730.2:d:+739:secondary
AACAACAATA	1	12	2	0	0	At2g38470.1:d:+1458:primary
TTCTTATTTG	5	1	1	3	5	At2g36490.1:d:+3681:secondary
AGAAACTCAA	6	5	2	1	1	At2g35800.1:X:+759:quaternary
ATCAGATCAA	4	3	2	4	2	At2g35615.1:v:+1993:primary
ATTGGTAGAG	5	5	1	1	3	At2g33210.1:d:+2008:primary,At5g11580.1:d:+1679:primary
TCTCGTTCTT	5	7	2	0	1	At2g29340.2:d:+815:primary
AGAATTTCTT	1	2	5	5	2	At2g27100.1:d:+2447:primary
AAAAAAAACA	4	3	3	2	3	At2g26720.1:v:+1369:primary,At5g41730.1:v:+2587:primary
TTTGTTTAAG	4	1	1	3	6	At2g21860.1:d:+1622:primary
ATTTTTTCCA	0	3	6	5	1	At2g21190.1:d:+1057:primary
TTTATGTATC	5	3	1	1	5	At2g21140.1:d:+1125:primary
GTGGCCACGG	0	0	10	0	5	At2g05380.1:d:+207:secondary
GGCAACTTTA	2	7	0	4	2	At2g04900.1:d:+388:primary

GGTTTGGAGA	5	5	2	3	0	At2g03440.1:d:+667:secondary
TAGGCATCGT	3	2	3	2	5	At2g03410.1:v:-1431:secondary
TATCATATAA	4	3	0	2	6	At2g02390.3:d:+858:primary,At2g02390.1:d:+813:primary
AAAAGGGTAA	5	3	3	2	2	At1g79870.1:d:+1057:primary
TGATTGCATA	6	2	3	3	1	At1g79750.1:d:+2395:primary
AGGCGTGAGG	2	4	7	1	1	At1g78670.1:d:+1310:primary
TTTCAGTTTT	2	3	4	3	3	At1g76140.1:d:+2377:primary
TTCTGTATCA	3	6	5	1	0	At1g75280.1:d:+1052:secondary,At1g09370.1:v:+1059:secondary
TACAAAGGAG	4	7	2	2	0	At1g74940.1:d:+730:primary
ATGGAAAGTT	1	12	1	1	0	At1g70890.1:d:+255:primary
TGTGTAATTG	5	1	3	4	2	At1g70870.1:v:+851:primary,At4g00400.1:d:+1678:primary
AATGTATGTA	7	7	1	0	0	At1g70830.1:d:+1145:primary,At1g70830.2:d:+1145:primary
ATCGTCCGGA	0	4	3	6	2	At1g69870.1:d:+1836:primary
AATGAAAATT	1	4	4	2	4	At1g69490.1:d:+989:primary
CAAGACACTC	2	2	5	0	6	At1g69410.1:d:+711:primary
CAAAACTTGA	1	5	5	3	1	At1g68830.1:d:+1423:primary
TACATTGGAC	1	14	0	0	0	At1g64720.1:d:+1138:secondary
AGTGGAGTGG	4	2	2	6	1	At1g64680.1:d:+803:secondary
GAGAAGATAC	2	2	3	0	8	At1g62780.1:d:+712:primary,At5g14870.1:d:+1788:primary
GTTCATTATT	6	4	3	1	1	At1g60780.1:d:+1800:primary
ATATGTTGTC	6	4	3	0	2	At1g59124.1:d:+3529:primary,At1g58807.1:d:+3478:primary
ACCTTTTCTT	6	3	3	3	0	At1g56500.1:i:+2158:tertiary
AATATAAATA	1	8	2	2	2	At1g54880.1:v:+589:secondary
ATGTAGCGTG	6	1	4	2	2	At1g50030.1:d:+7966:primary
AGTTAAAAAA	4	1	3	5	2	At1g48220.1:v:+1700:primary
ACTTAGACAA	6	2	2	0	5	At1g36730.1:d:+1967:primary
CAGGACGAAA	4	4	3	1	3	At1g35160.1:d:+816:primary
TGAAATTGGT	5	3	1	4	2	At1g32160.1:d:+1321:primary
AAGTCGAAGA	2	5	2	5	1	At1g31970.1:d:+174:secondary
AAGCTTGTTT	2	2	3	3	5	At1g26630.1:d:+850:secondary
GTTGCTCTCT	2	3	3	5	2	At1g23780.1:d:+1656:secondary,At2g30910.1:d:+306:secondary,At4g36390.1:d:+130:secondary,At2g30910.2:d:+306:secondary,At2g31300.1:d:+266:secondary
TGGTTCCCTA	1	4	3	3	4	At1g21670.1:d:+1965:primary
CAATCAATAG	0	9	0	4	2	At1g21310.1:d:+1522:secondary
GCTTTGTCTT	6	2	4	2	1	At1g21000.1:d:+1175:primary
ATGTGTGCAG	0	9	2	2	2	At1g20010.1:d:+1002:primary
TATTGGGTTG	3	6	5	1	0	At1g15980.1:d:+1234:primary
GAGGAAGCAA	0	2	4	7	2	At1g13060.1:d:+916:primary
ATGGGTGATT	2	8	1	2	2	At1g11660.1:d:+2423:primary
CTCTGTTTCG	2	3	8	1	1	At1g11580.1:d:+1819:primary
CCCGAGACTG	0	0	6	7	2	At1g10370.1:d:+709:primary
TTTCATCGAA	4	3	4	2	2	At1g08110.1:d:+792:primary,At1g08110.2:d:+1009:primary
CTAAGTTTTT	4	2	4	3	2	At1g07250.1:d:+1564:primary
ATGAAAGATG	7	4	3	1	0	At1g06570.1:d:+1293:primary
CAGATAGGTT	2	1	4	7	1	At1g06040.2:X:-710:quaternary
GTTGCGTAAG	2	4	6	2	1	At1g05580.1:X:-451:quaternary
TCATTTAAGA	4	6	2	2	1	At1g04800.1:d:+705:primary
TGAATGGAAA	5	3	5	2	0	At1g03080.1:d:+5278:primary
AAAGGTAAGT	2	6	2	3	2	At1g03040.1:d:+1163:primary
TCAGTGCTTG	1	8	4	2	0	At1g02920.1:d:+641:primary
AATGTAAGAG	3	2	4	2	4	At1g02150.1:d:+1841:primary
GTTCCAAAAA	2	1	4	5	2	Chr5:+30681:quaternary
TTATCTCAAA	2	6	3	1	2	Chr5:+1366810:quaternary
TCGGGGCTTA	2	6	4	1	1	Chr3:+20144593:quaternary
GGAAATGAAC	2	2	3	3	4	Chr2:+7838336:quaternary,Chr5:+11926294:quaternary
TTGTCGATTT	1	4	3	2	4	Chr2:+4590078:quaternary; +small RNA(AAATGTCCATGTTGTCGATTTGAG)
AAGAGAGAGG	0	0	1	6	7	Chr1:+16559300:quaternary,Chr1:+16554350:quaternary
GCAAATTTCA	2	3	6	2	1	AtMg00580:d:+1475:primary
TTATATTTCT	1	11	0	0	2	At5g66190.1:d:+1397:primary
TTTCGTCTTG	4	5	1	2	2	At5g65730.1:d:+956:primary

AAACAAATTA	3	3	3	1	4	At5g65330.1:v:+1685:primary,At3g19480.1:d:+1927:primary
TGCAAGAATT	2	5	1	2	4	At5g64200.2:d:+1180:primary
CATTTAGGTC	7	0	1	3	3	At5g62810.1:d:+1741:primary
TAAGTATCAG	4	1	3	3	3	At5g62670.1:d:+3109:secondary
AGACTTGAGT	4	1	1	4	4	At5g61770.3:d:+1139:primary,At3g57490.1:d:+915:primary,At5g61770.1:d:+1163:primary,At5g61770.2:d:+1166:primary
TCTCTTAAGT	4	5	0	1	4	At5g61660.1:d:+654:primary
TAAATCGCTA	6	5	2	0	1	At5g60460.1:d:+459:primary
GCAAGTGGTC	3	8	2	1	0	At5g58375.1:d:+297:primary
CTTGACGAAG	0	9	3	1	1	At5g58250.1:d:+340:primary
GTTATGGGTA	2	7	1	1	3	At5g57040.1:d:+487:primary
TATATCAATC	2	1	3	1	7	At5g55620.1:d:+582:secondary
GGACGGGCTG	1	8	2	3	0	At5g54810.1:d:+1239:primary
TCTCGTCCTC	3	2	2	4	3	At5g54580.1:d:+555:secondary,At2g26250.1:d:+429:secondary,At1g74850.1:d:+2336:secondary
TGTCTAATTT	1	9	2	1	1	At5g53160.1:d:+1161:primary,At5g53160.2:d:+1081:primary
GACCAAAAGC	0	1	6	5	2	At5g43260.1:d:+175:primary
AAGGCGGTGA	3	5	2	2	2	At5g43010.1:d:+1243:primary
TAATATGCAA	2	1	1	1	9	At5g42570.1:d:+822:primary,At2g17130.2:d:+1356:primary,At2g17130.1:d:+1368:primary
GATGAATTGG	2	5	4	2	1	At5g41790.1:d:+1252:secondary
AACAAAATTA	0	5	3	4	2	At5g38530.1:d:+1705:primary,At5g34480.1:p:+1881:primary
AAAGTTTCGA	3	2	5	4	0	At5g35630.1:d:-1446:secondary
ATGATTCCAG	6	2	1	2	3	At5g35620.1:d:+626:primary,At5g35620.2:d:+1045:primary
TAATGCAATT	5	3	3	2	1	At5g32386.1:p:+2026:secondary,At3g30718.1:p:+1302:secondary,At5g33381.1:p:+2765:secondary,At1g41825.1:p:+1528:secondary,At3g26744.1:v:+1959:secondary
AGCCTTCTTC	3	1	4	1	5	At5g22440.1:d:+771:primary
GTTTTTTCTA	1	3	3	4	3	At5g22090.1:d:+1699:primary
TATTTGATTA	1	6	3	3	1	At5g20230.1:d:+774:primary
TGTGTGTATC	9	0	1	0	4	At5g18580.1:d:+1687:secondary
ACTCGCTCTT	3	9	1	1	0	At5g18170.1:d:+1239:primary,At3g51950.1:d:+1095:primary
TTAAGTGTTG	2	3	0	1	8	At5g17560.1:d:+722:primary
AGTTTTTCCA	0	3	5	2	4	At5g15490.1:d:+1601:primary
TGATCTCTCG	0	5	3	3	3	At5g15220.1:d:+629:primary
GTAGATGTTG	2	8	4	0	0	At5g14130.1:d:+1256:secondary
CTTTCTAAAG	3	2	4	3	2	At5g13420.1:d:+1501:primary
GATCTTGAAT	1	3	7	2	1	At5g13150.1:v:+2799:primary,At4g17900.1:d:+1079:primary
AAATCTTGCC	3	4	2	1	4	At5g12370.1:d:+2659:primary
CTGAGACATT	1	5	2	4	2	At5g11880.1:d:+1471:primary
GCAGGTTTAG	0	3	6	3	2	At5g10745.1:d:+1333:primary
ATCATTGTGC	1	4	1	4	4	At5g09500.1:d:+333:secondary,At5g43640.1:v:+612:secondary,At5g09510.1:d:+390:secondary
ACACAGCTCG	3	5	0	4	2	At5g08540.1:d:+1280:secondary
TCGAATGTCT	1	6	5	2	0	At5g07020.1:d:+106:primary
TTATAGTGTT	4	0	4	2	4	At5g04830.1:d:+742:primary,At5g04830.2:d:+761:primary
TGCGATCGAT	4	5	0	5	0	At5g04470.1:d:+645:primary
AAAGGAGGTG	3	6	4	0	1	At5g02210.1:v:+603:secondary,At3g22380.1:d:+4587:secondary,At5g02210.1:v:+477:secondary
TTTCTTACTC	3	3	2	1	5	At4g38710.1:i:+1694:tertiary
CGTGTGTTTC	0	1	8	2	3	At4g38550.1:d:+2188:primary
ACATATGCTA	9	1	4	0	0	At4g37920.1:i:+840:tertiary
GAACATTGAA	1	3	7	1	2	At4g35920.3:d:+1511:primary,At2g20020.1:d:+2144:primary,At4g35920.1:d:+1677:primary,At4g35920.2:d:+1608:primary
GCTCTTCCTG	3	4	2	3	2	At4g35790.2:d:+2843:primary,At4g35790.1:d:+2876:primary,At4g35790.3:d:+2982:primary
GAATCAACAA	1	3	4	3	3	At4g35310.1:d:+1875:primary
TCAGTTCGTA	1	4	7	2	0	At4g35100.1:d:+1114:primary
GTGATGAATT	2	0	6	4	2	At4g35060.1:d:+876:primary
TACTTTTGAG	2	1	3	6	2	At4g31420.1:d:+1431:primary,At1g23170.1:v:+2230:primary,At4g31420.2:d:+1434:primary
CCGCGTCCTA	0	9	4	1	0	At4g29010.1:d:+1955:primary
AGGAGGAAGA	1	4	2	4	3	At4g28760.2:d:+2622:secondary,At4g28760.1:d:+2509:secondary,At5g61780.1:d:+3268:secondary,At5g07505.1:p:+915:secondary
TGAATCGGCA	2	6	1	4	1	At4g27410.1:d:+754:primary
GTGTTGTACA	7	3	2	2	0	At4g25650.1:d:+1715:primary,At4g25650.2:d:+1784:primary

CCATTGCTCT	2	7	2	1	2	At4g25570.1:d:+368:primary
TTTCAACGTC	1	4	4	2	3	At4g23430.1:d:+1117:primary,At4g23430.2:d:+1123:primary
AATATTGATA	4	4	5	0	1	At4g22990.1:i:+601:tertiary
GTTCTTTAAC	2	2	1	7	2	At4g22380.1:v:+962:primary
GTGATTTAGC	6	1	3	4	0	At4g21280.1:d:+868:primary
TGAATTGATG	3	4	3	2	2	At4g18120.1:v:+2827:primary
TAGAATGTTA	3	2	5	1	3	At4g17790.1:d:+1241:primary
CTTTTGAAAA	3	3	3	2	3	At4g17330.1:d:+3684:primary
CAGGTGTGGT	0	12	1	0	1	At4g16460.1:i:+358:tertiary
GTTTTGACAA	2	3	5	4	0	At4g16155.1:d:+1956:primary
CCCGTTTGTA	2	2	4	5	1	At4g14160.3:d:+2417:primary,At4g14160.2:d:+2535:primary,At4g14160.1:d:+2420:primary
GTCCTATTCA	2	7	2	2	1	At4g13170.1:d:+244:primary,At3g24830.1:d:+271:primary
AGATAAAACT	2	3	3	4	2	At4g12340.1:d:+754:primary
GAAGTGATGG	3	2	2	2	5	At4g11010.1:d:+671:primary
TATAGTGTTT	7	2	2	2	1	At4g10840.1:d:+2057:primary,At4g10840.2:d:+2154:primary,At4g10300.1:d:+695:primary
GTATCGAATA	3	1	4	5	1	At4g08870.1:d:+1139:primary
CAAAATCACT	3	3	4	3	1	At4g04313.1:p:+2033:secondary
CGTATGTATA	2	3	2	1	6	At4g02480.1:d:+4167:primary
GTAGATGTTT	7	0	0	3	4	At4g01850.1:X:-1234:quaternary
ATATCAACGA	2	5	5	0	2	At4g01370.1:d:+1125:primary
GGGAGTTGAG	2	5	5	2	0	At4g01050.1:d:+496:primary
AAGCTTCTTC	4	6	2	2	0	At4g00720.1:d:+1880:secondary,At5g06610.1:d:+213:secondary
AAACCTCCTT	3	4	1	4	2	At4g00270.1:X:-143:quaternary
ATTGATTGTT	5	4	1	3	1	At3g63120.1:d:+880:primary
ATGATAGCAG	3	4	0	3	4	At3g57150.1:d:+1391:primary
GAGATACGTA	5	3	2	2	2	At3g54490.1:d:+905:secondary
GAAGATCCTA	4	6	1	1	2	At3g48870.1:d:+2942:primary
TATTGTAAAA	2	3	3	2	4	At3g47960.1:d:+2035:primary
AAATTGAATT	2	7	1	2	2	At3g45430.1:v:+2441:secondary,At1g31420.1:d:+2349:secondary,At3g28670.1:d:+941:secondary
AAGGCGTCGA	3	1	3	1	6	At3g44010.1:d:+283:secondary
AGTGTTCCAA	0	4	5	3	2	At3g42553.1:p:+1900:secondary,At2g05960.1:p:+1891:secondary
ATAAAGAAGC	3	4	6	1	0	At3g27820.1:d:+1489:primary
GCTCCAGTGT	1	4	7	2	0	At3g27770.1:d:+1035:secondary
GGTTGCAAAA	1	0	2	8	3	At3g26730.1:d:-2532:secondary
AAATTAAAAT	3	5	2	2	2	At3g25770.1:d:+866:primary
CCTTTGACGA	4	2	5	1	2	At3g24160.1:d:+1158:secondary
ATAGTAATGG	6	1	3	2	2	At3g21500.1:d:+1429:secondary,At3g21500.2:d:+1432:secondary
TGATGAAGAG	5	3	3	2	1	At3g20362.1:v:+181:secondary
ATCTGTGATT	4	0	2	3	5	At3g20250.1:X:+175:quaternary
TGCTATTGCA	3	4	1	2	4	At3g18190.1:d:+1840:primary
TTTGCGTTCA	2	5	5	1	1	At3g16857.1:d:+2262:secondary
TAAATGTTTC	1	5	5	3	0	At3g16570.1:d:+606:primary
TTGTTTCTCT	0	8	2	0	4	At3g15210.1:d:+986:primary
TGTTGTTGTT	6	3	3	1	1	At3g13950.1:d:+586:primary,At5g63140.1:d:+1256:primary,At3g11600.1:d:+731:primary
CCTTTTTCTG	3	5	3	0	3	At3g13772.1:d:+2091:primary
ACGGTTGGGC	0	7	3	3	1	At3g13580.2:d:+868:primary,At3g13580.1:d:+638:primary,At3g13580.3:d:+1212:primary
TGTCCGGTAC	3	3	5	1	2	At3g11560.2:d:+2821:primary
TTTGATCCTT	6	2	2	4	0	At3g09770.1:d:+1580:primary,At3g09770.2:d:+1666:primary
AACATTAAAG	5	1	1	3	4	At3g08730.1:d:+1761:primary
TGCCCTAAAC	3	1	4	3	3	At3g08580.1:d:+1298:secondary,At3g08580.2:d:+1413:secondary
ATCTTCTGGT	3	5	2	2	2	At3g07310.1:d:+1244:primary
AAAAGTCGTC	1	1	7	3	2	At3g06350.1:d:+1900:primary
TCTCAAGGAT	5	1	4	3	1	At3g06050.1:d:+723:primary
GAAGACACAT	4	2	3	3	2	At3g03250.1:d:+1591:primary
TGGATTGTGG	2	1	5	4	2	At2g47690.1:d:+555:primary
ACTTCTTCTC	1	7	3	1	2	At2g46720.1:d:+1530:secondary,At3g10280.1:v:+442:secondary,At3g57090.1:d:+625:secondary
GTTTCTCTGT	0	0	0	0	14	At2g46110.1:d:+1303:primary
AACGTGTGCC	5	4	0	3	2	At2g42680.1:d:+299:primary
TGAGTCTCGA	3	5	6	0	0	At2g41940.1:d:+799:primary,At3g58070.1:d:+788:primary
TTTATTGGAT	10	1	2	1	0	At2g40610.1:d:+1023:secondary,At5g10630.1:d:+1441:secondary

CTCGTGGTCC	3	2	6	2	1	At2g39800.1:d:+2410:primary,At2g39800.2:d:+2046:primary
AAACTGTATT	3	0	4	3	4	At2g37770.1:d:+1167:primary,At2g23620.1:d:+849:primary
TGATTGATGT	6	2	3	2	1	At2g34315.1:v:+928:secondary,At4g39520.1:d:+1458:secondary
ACCAAGTCAA	1	8	1	4	0	At2g33450.1:d:+102:primary,At3g49480.1:v:+1648:primary
TTGTCCCAGA	1	3	4	4	2	At2g31880.1:d:+1930:primary
GATTTTTGGA	5	2	4	2	1	At2g28520.1:d:+2867:primary
CTCTTCGGAA	2	3	4	2	3	At2g26975.1:d:+507:primary
TATTTCGCCG	1	4	3	2	4	At2g25670.2:d:+1448:secondary,At2g25670.1:d:+1330:secondary
TTTGTATTGG	3	6	1	3	1	At2g24270.1:d:+1819:primary,At2g24270.2:d:+1587:primary
GCTATTTCCT	6	1	3	3	1	At2g24150.1:d:+1156:primary
ACAACTATTC	5	0	6	1	2	At2g24090.1:d:+646:primary
TTAGCCCACG	2	8	1	3	0	At2g23670.1:d:+133:primary
TTGGCCAAAA	1	3	2	3	5	At2g20450.1:d:+391:primary
TGAAACAAAA	2	1	6	3	2	At2g19660.1:v:+2033:secondary,At1g08300.1:v:+1612:secondary,At5g37620.1:v:+2042:secondary,At1g29040.1:d:+902:secondary
CTGATCCCAA	2	2	4	2	4	At2g16470.1:d:+95:secondary,At1g01450.1:v:+1204:secondary
TCAGCTTGGG	2	1	3	5	3	At2g06950.1:p:+1503:secondary
AGCCATAAGT	6	2	3	2	1	At2g05920.1:d:+2370:primary
AACAACAAAT	1	1	5	4	3	At2g04630.1:d:+640:secondary,At3g20190.1:d:+435:secondary
TGTATGGAGC	6	3	3	1	1	At2g04240.2:d:+1271:primary,At1g36105.1:p:+1804:primary,At2g04240.1:d:+812:primary
GATGGACCAG	5	2	5	0	2	At2g01870.1:d:+428:primary
CCAAATGTGT	3	4	2	4	1	At1g80410.1:d:+2997:primary
AGGCAGGATG	1	7	2	2	2	At1g80380.1:d:+974:primary,At1g80380.2:d:+1259:primary
GCTGCGGGAG	1	4	0	7	2	At1g79920.2:d:+2380:primary,At1g79920.1:d:+2380:primary
AGCTTGTACA	2	2	3	4	3	At1g79510.2:d:+1022:primary,At1g79510.1:d:+1130:primary
TTCACCTCTT	3	2	1	5	3	At1g79050.1:d:+1402:primary
CAGCTTCTTC	1	3	3	4	3	At1g78300.1:d:+885:primary
ATTTCCCTTG	2	4	5	1	2	At1g75480.1:p:+556:primary,At1g78660.2:d:+1155:primary,At1g78360.1:v:+1278:primary,At1g78660.3:d:+1256:primary,At1g19720.1:d:+2992:primary,At1g78660.1:d:+1207:primary
AATTTGGTTT	6	2	2	3	1	At1g75080.1:d:+1410:primary,At5g36880.1:d:+2442:primary
AGACAGATCT	2	4	3	3	2	At1g74360.1:d:+2464:secondary,At4g37470.1:d:+690:secondary,At1g72700.1:d:+2317:secondary,At1g72510.1:d:+788:secondary
CACGGTTGGA	4	6	4	0	0	At1g70290.1:d:+2664:primary
AATGGGGACG	2	5	6	0	1	At1g67900.2:d:+2151:primary,At1g67900.1:d:+2114:primary
ACTCATCGTG	4	5	3	2	0	At1g67250.1:d:+555:primary
GAGGAGAAAG	4	3	5	2	0	At1g67230.1:d:+1412:secondary,At2g19120.1:v:+718:secondary,At3g48710.1:d:+162:secondary
ACTCATTGCT	4	4	5	1	0	At1g66760.2:d:+1565:primary,At1g66760.1:d:+1649:primary
TTTCTTGTGA	7	2	3	1	1	At1g66150.1:d:+3014:secondary,At4g07890.1:p:+284:secondary
TGTTTGACTT	4	4	5	0	1	At1g65970.1:d:+642:secondary
AAGAAATAGA	4	4	3	3	0	At1g63970.2:d:+664:primary,At1g63970.1:d:+715:primary
AAAAAATGTT	4	2	0	4	4	At1g63055.1:d:+335:secondary
TAAATCTCAA	2	1	7	3	1	At1g62960.1:d:+1746:primary
ACTTACAGTG	4	5	4	0	1	At1g62040.1:d:+524:primary,At4g21980.1:d:+388:primary
GTCCAGTTTT	1	3	5	3	2	At1g61770.1:d:+1156:primary
AACATTTAAA	0	1	4	4	5	At1g61380.1:d:+2596:secondary
AAATTAGAAA	2	3	2	2	5	At1g61040.1:d:+2316:primary
TTGTAGCCGC	3	1	5	5	0	At1g55960.1:d:+1202:primary
TCTCTCACAA	7	1	4	2	0	At1g55790.1:v:+811:secondary,At4g32140.1:d:+1268:secondary
AAGGTCTTCT	5	3	0	0	6	At1g48830.2:d:+497:primary,At1g48830.1:d:+557:primary
AACACAATGT	8	2	1	1	2	At1g48010.1:v:+1063:secondary,At1g70760.1:d:+677:secondary
TTGAAAAAAA	1	0	4	4	5	At1g36030.1:v:+771:secondary
TTTAAGATTC	4	2	4	2	2	At1g34575.1:v:+1626:secondary
TAGTATTTAG	4	6	1	0	3	At1g34290.1:v:+1149:primary,At5g52040.2:d:+1273:primary
GGGAAGCTGA	2	3	5	4	0	At1g33600.1:d:+1225:secondary
TGTCTGCAAG	4	5	1	4	0	At1g32580.1:d:+791:primary
AGATTTGTTG	2	3	3	3	3	At1g31660.1:d:+1152:primary,At4g39880.1:d:+584:primary
GGAAAAAAGT	3	1	2	5	3	At1g31340.1:d:+565:primary
TGTTGAACTA	2	0	8	0	4	At1g29020.1:i:+2952:tertiary
ATGATGCGGC	5	7	1	1	0	At1g28600.1:X:-282:quaternary
TAATGAAGAT	0	5	5	1	3	At1g27350.1:d:+448:primary

TGGATATCAG	5	3	6	0	0	At1g27290.1:d:+828:secondary
TGGGGAATAA	3	2	2	2	5	At1g27000.1:d:+1309:secondary
AGGATGGTCC	3	2	5	3	1	At1g26850.3:d:+1949:primary,At1g26850.2:d:+2009:primary,At1g26850.1:d:+2068:primary
GGCGTCTGCG	2	5	6	1	0	At1g23950.2:d:+1174:primary,At1g23950.3:d:+1179:primary,At1g23950.1:d:+1264:primary
CTCGACGGAG	3	6	1	4	0	At1g23440.1:d:+557:primary
AGTCTCCTTT	3	6	0	2	3	At1g20050.1:d:+824:primary
GATGATGAGT	2	6	5	1	0	At1g19770.1:d:+1154:primary
GTCGAAGACC	1	0	12	1	0	At1g15820.1:d:+639:secondary
TGGTTTAAAA	1	2	5	3	3	At1g15120.1:d:+349:secondary
TTGAGATAAA	5	1	4	2	2	At1g13780.1:d:+1231:secondary
TTGAAATGAA	3	2	4	2	3	At1g12130.1:v:+1819:secondary,At1g17840.1:d:+2668:secondary
GACAAGATTG	3	3	4	3	1	At1g11970.1:v:+537:primary,At1g06430.1:d:+1949:primary
TCATAAACTT	1	9	1	1	2	At1g08980.1:d:+1317:primary
AACAATTTCA	4	3	3	3	1	At1g08490.1:d:+1613:secondary,At5g03435.1:v:+299:secondary,At2g06925.1:d:+408:secondary
GAGGTTTTGG	2	4	4	2	2	At1g07990.1:d:+748:secondary,At4g29200.1:v:+1082:secondary,At5g36030.1:v:+780:secondary,At5g11450.1:d:+927:secondary,At5g28970.1:v:+2943:secondary,At4g01000.1:d:+1221:secondary,At1g35110.1:v:+3726:secondary,At4g19310.1:v:+882:secondary
TGATTTTTTA	1	1	5	5	2	At1g07140.1:d:+1174:secondary
TTTGATTGAA	4	0	5	3	2	At1g05560.1:d:+1621:primary
TACTTCGAAG	5	3	1	2	3	At1g04480.1:d:+304:primary
GTGTTGTAGC	5	7	1	1	0	At1g02305.1:d:+1045:primary
ACGGCTCTTG	0	2	2	7	3	At1g01470.1:d:+359:primary
TAACATTCCG	0	11	2	0	0	No gene matches found
CATAAACTAA	4	3	1	3	2	ChrC:+50033:quaternary
TCACGCCGAG	4	1	6	2	0	ChrC:+140707:quaternary
AGAACACACA	3	1	8	0	1	Chr5:+21892497:quaternary
AAAACAACGA	4	3	0	3	3	Chr4:+6411057:quaternary
CAGAAAAAAA	2	4	3	2	2	Chr4:+5735248:quaternary,Chr5:+23855805:quaternary
TTTCTAAAAA	0	3	4	2	4	Chr1:-27652934:quaternary,Chr2:-2949279:quaternary
TGTGCAAGAC	2	2	5	3	1	At5g67250.1:d:+1751:primary
GCGCTCTTCT	2	5	2	2	2	At5g64740.1:d:+3193:primary
AGGAGAAGGA	4	6	3	0	0	At5g64200.1:d:+600:secondary,At5g64200.2:d:+600:secondary,At5g62390.1:d:+835:secondary
GTTCGCAAAA	0	7	2	2	2	At5g62000.2:d:+2794:primary,At5g62000.1:d:+2920:primary,At5g62000.3:d:+2794:primary
GATGGCTGGA	4	5	1	2	1	At5g61500.1:d:+1136:primary
CAGAAACACG	2	4	1	3	3	At5g59850.1:d:+228:primary
TCCTTTTTTG	3	3	3	3	1	At5g57870.2:d:+2773:primary,At5g57870.1:d:+2783:primary
GCTATGATAA	2	7	3	1	0	At5g56100.1:d:+541:primary
AGCCAATGAG	3	0	3	2	5	At5g55510.1:d:+713:primary
GAAGCCGCTG	0	2	6	3	2	At5g54710.1:d:+1762:primary
TAACACAAGA	2	3	3	2	3	At5g53310.1:d:+855:secondary
TCTTCTCTAA	3	2	1	3	4	At5g53030.2:d:+60:secondary,At5g53030.1:d:+60:secondary
GGAATATCAG	1	2	6	2	2	At5g52550.1:d:+1433:primary
GTTGCCTCCT	3	4	1	5	0	At5g51750.1:d:+2387:primary
TATGTTTGTT	8	2	2	0	1	At5g49460.1:d:+2085:secondary
TTTACAATTC	1	1	1	2	8	At5g47930.1:d:+543:primary
TAACCGCTCT	3	5	0	4	1	At5g47550.1:d:+413:primary
CTGGTGTTAT	3	5	2	3	0	At5g45980.1:v:+1301:secondary,At2g46220.1:d:+938:secondary
GAGAATTCCT	2	3	7	1	0	At5g45820.1:v:+1134:secondary
TTAATTCCAA	2	2	4	3	2	At5g44720.1:d:+1111:secondary
ACCTTGGAAT	2	0	5	3	3	At5g43460.1:d:+811:primary
GCCTCCCACT	4	1	2	5	1	At5g42990.1:d:+628:secondary
TTGAAGAAGA	3	2	3	2	3	At5g36935.1:p:+1690:secondary,At1g05180.2:d:+29:secondary,At3g02400.1:v:+1426:secondary,At4g14490.1:d:+960:secondary,At4g37440.1:d:+291:secondary,At1g05180.1:d:+70:secondary
CTATTTACAG	1	4	1	2	5	At5g35690.1:d:+2084:primary
ATATTGGCGA	3	6	1	3	0	At5g33320.1:d:+1145:primary
CTTCAGTTTC	5	1	2	3	2	At5g24810.1:d:+3373:primary
TTCGTTAAAT	2	1	4	5	1	At5g24800.1:d:+981:secondary
AACGAGCTCT	1	3	2	3	4	At5g24660.1:d:+316:primary
TACGACGACT	1	5	7	0	0	At5g24590.2:d:+1769:primary,At5g24590.1:d:+474:primary

GCTGCAGATG	0	4	1	6	2	At5g20160.1:d:+262:primary,At1g04625.1:p:+651:primary,At5g20160.2:d:+358:primary
TCTCTGCGTA	2	1	2	6	2	At5g20000.1:d:+1519:primary
AAACTGCTGC	2	3	3	4	1	At5g18940.2:d:+1254:primary,At5g18940.1:d:+1320:primary
GCGCTTCGAG	1	2	5	4	1	At5g16290.2:d:+1603:primary,At5g16290.1:d:+1588:primary
TTTTTCCAGG	2	8	1	1	1	At5g14910.1:d:+387:primary
AGTAAAAGCT	2	3	2	2	4	At5g12140.1:d:+634:primary
AAAACTTTTC	2	3	4	3	1	At5g09900.2:d:+1453:primary,At5g09900.1:d:+1484:primary
ATGGCAAGTT	0	3	1	1	8	At5g08260.1:d:+1534:primary
GCAGATCACC	1	4	2	2	4	At5g08060.1:d:+123:primary
GCTGGTTTTT	2	2	1	4	4	At5g05400.1:v:+2609:secondary,At2g41110.1:d:+622:secondary
TACTTTTAAC	3	2	2	1	5	At4g38460.1:d:+1230:primary
GGCGGTCGCA	1	6	4	1	1	At4g36850.1:d:+880:primary
AGAATTGATA	2	2	5	1	3	At4g36670.1:i:+478:tertiary
GAACAACTTC	2	4	4	2	1	At4g36250.1:d:+1459:primary
GCCCTGCGAT	3	5	0	4	1	At4g35230.1:X:-695:quaternary
GACCCAAGAG	1	6	4	2	0	At4g35000.1:d:+692:primary
TCTGGTTTAG	4	4	3	1	1	At4g34100.1:v:+3427:secondary
TAGTAACAAA	5	3	0	1	4	At4g33640.1:d:+511:primary
AAGAAAGTTT	1	1	3	6	2	At4g33500.1:d:+802:secondary,At1g06460.1:d:+1070:secondary,At3g30213.1:p:+2177:secondary
TTTTTGTTCA	4	5	1	2	1	At4g33380.1:d:+1382:primary
TAATCTGCGA	1	6	4	0	2	At4g33300.1:d:+2723:primary
TTCTTCTAAG	5	3	3	0	2	At4g33220.1:d:+1721:primary
TTTTCTATAA	4	4	0	1	4	At4g33090.1:d:+2733:primary
GAATAAAGTA	1	0	0	3	9	At4g33070.1:d:+2053:primary
GGAATCCCAA	0	6	3	3	1	At4g33050.3:d:+1836:primary,At4g33050.2:d:+1740:primary,At4g33050.1:d:+1825:primary
GTTTTTTTTT	2	0	7	1	3	At4g32551.1:d:+3205:primary,At5g66420.1:d:+2563:primary
GCTCCCCTCT	2	5	2	3	1	At4g28300.1:d:+1686:primary,At4g28300.2:d:+2066:primary
AAGACGGGAA	3	3	4	2	1	At4g28260.1:d:+1629:secondary
GGATCGTCAG	2	4	3	4	0	At4g27990.1:d:+540:primary
GGAGATGTGG	3	3	5	2	0	At4g27430.1:d:+1129:secondary
ACAAAAAACA	2	1	5	4	1	At4g27000.1:X:-221:quaternary
GCTGGGGAGC	1	6	5	1	0	At4g25260.1:d:+553:secondary
AGGTGGTGAA	3	4	2	3	1	At4g24800.1:d:+2074:primary
AGGACGCGCC	2	3	3	3	2	At4g24780.1:d:+1125:primary
CAATTTTAAT	4	2	0	3	4	At4g24590.1:d:+991:primary
GGTGATGATA	4	6	1	2	0	At4g23650.1:d:+1500:primary
CTAAGAAGGT	0	10	1	1	1	At4g21790.1:d:+744:primary
AATGATCTTG	3	4	2	2	2	At4g20890.1:d:+1372:primary
TTGCCTTTCA	6	1	1	0	5	At4g20440.1:d:+1042:primary,At4g20440.2:d:+934:primary
GTTCTGGCAA	2	7	3	1	0	At4g19420.1:d:+1293:primary,At4g19420.2:d:+1283:primary
TTTGGAGGAG	2	7	2	2	0	At4g19200.1:d:+607:primary
CAAACAAGAT	3	2	4	1	3	At4g18740.2:d:+770:primary,At4g18740.1:d:+863:primary
AAAAAAAGAA	3	2	5	3	0	At4g18600.1:d:+5971:primary
TCAATTTTGT	0	3	9	0	1	At4g18200.1:v:+3958:secondary
TGTTTTTGCA	2	3	6	1	1	At4g18030.1:d:+2233:primary
CGAAGCAATC	2	0	5	5	1	At4g16690.1:d:+988:primary
TGTTGAACTT	2	1	1	4	5	At4g14800.1:d:+828:primary
AGGATTCGAT	1	1	5	3	3	At4g14342.1:d:+349:primary
CAACGTGTGT	4	5	3	0	1	At4g14020.1:d:+462:primary
AACCTGAAGA	1	4	3	4	1	At4g13350.1:d:+2088:primary,At4g13350.2:d:+2228:primary
TAATTCGAAG	2	3	4	3	1	At4g13270.1:d:+894:primary
TAACTGTCTT	2	6	1	3	1	At4g10920.1:d:+674:primary
AAATTATTTT	2	4	7	0	0	At4g08290.2:d:+1567:primary,At4g08290.1:d:+1431:primary
TTGTTGAAAT	2	1	4	3	3	At4g02990.1:d:+1882:primary
AGATCTATTG	4	2	7	0	0	At4g02770.1:d:+631:secondary
AACAAAATCC	1	2	3	4	3	At4g01000.1:d:+1555:primary
AAGATCCCTA	5	2	4	1	1	At3g61820.1:d:+1679:primary
TAATTAAGTT	7	3	3	0	0	At3g61550.1:d:+781:primary
TACGTTCTTT	1	4	4	2	2	At3g60600.1:d:+851:primary
ATGTCAAAAT	2	5	2	3	1	At3g58160.1:i:-499:tertiary
GAGCGGTCTC	1	2	4	5	1	At3g58140.1:d:+1387:primary

GAATCAAAAC	1	4	2	2	4	At3g57930.1:d:+1005:primary
TAATTCGTGA	1	4	0	3	5	At3g57420.1:d:+2586:primary
CTCTGGGTAC	1	7	3	1	1	At3g55330.1:d:+642:primary
TGTGGAGAAG	2	3	5	2	1	At3g54660.1:d:+1574:primary
GACAAATTTG	2	2	3	6	0	At3g50300.1:d:-397:secondary
GGGTGTTGAA	2	8	2	0	1	At3g49720.1:d:+514:primary,At5g65810.1:d:+512:primary
CCTTAGAAAG	3	4	3	2	1	At3g48750.1:d:+1055:primary
GAGATCGATG	1	0	7	4	1	At3g47540.1:X:+647:quaternary
TTATCCACCT	3	4	1	2	3	At3g46740.1:d:+2529:primary
TAGCCAATTA	1	2	3	3	4	At3g44300.1:d:+1273:primary
CTGGGGTTTT	1	12	0	0	0	At3g44260.1:d:+841:primary
AGGATGGAAC	0	11	0	1	1	At3g30775.1:d:+1459:secondary
GATTAAAAAC	5	5	2	1	0	At3g28860.1:d:+3992:primary
GAACGTGTGG	0	3	4	4	2	At3g27390.1:d:+1882:primary
CAATTACTCA	3	2	2	5	1	At3g23980.1:X:--83:quaternary
TTCTTTAATG	5	1	1	1	5	At3g23920.1:d:+2091:primary
TGTTTTGATC	1	3	4	5	0	At3g23050.1:d:+1109:primary
TGTCTGTCGG	4	5	3	1	0	At3g23050.1:d:+1026:secondary
AGCCAAAGCT	5	3	3	0	2	At3g22170.1:d:+2757:primary
TGAGCTGGTA	0	2	5	4	2	At3g21690.1:d:+1686:secondary
ACCGGACACA	0	6	3	2	2	At3g20820.1:d:+323:primary
CTTGGTTAGT	4	7	2	0	0	At3g17100.2:d:+882:secondary,At3g17100.1:d:+984:secondary
TGCTATAGCA	1	6	4	0	2	At3g15580.1:d:+414:primary
AACATAAACA	3	2	3	4	1	At3g14100.1:d:+1624:secondary
AATTCGGGTC	1	6	3	3	0	At3g14067.1:v:+2593:secondary
ATTTCTGTGA	0	1	6	2	4	At3g13110.1:d:+1642:primary
GCCTTACCTG	2	4	2	4	1	At3g12760.1:d:+917:primary
TGAAGTGTCC	5	2	1	2	3	At3g11660.1:d:+807:primary
ACGAAATGGT	3	3	3	4	0	At3g09470.1:X:-82:quaternary
AATCTTGGTC	6	4	2	0	1	At3g09250.1:d:+1121:primary
TAATCATATT	0	4	4	3	2	At3g08930.2:d:+1953:primary,At3g08930.1:d:+1901:primary
AAAATAAAGA	5	1	1	3	3	At3g06230.1:v:+221:secondary
TAAATCCAGG	2	3	4	3	1	At3g03860.1:d:+1368:primary
AAAAACCCTT	4	4	2	2	1	At3g03470.1:d:+1587:primary
CCATTGAGCT	4	4	3	2	0	At3g03070.1:d:+636:primary
ATGAATGTTT	2	4	0	5	2	At3g02530.1:d:+1892:primary
TATCGGTGAA	5	2	3	2	1	At3g01370.1:d:+3190:primary
CGACATTGAA	3	6	1	2	1	At2g47380.1:d:+88:primary
AAGGAATGGA	1	7	0	2	3	At2g46505.1:d:+416:primary
TGTGCGTGAT	2	6	1	4	0	At2g45980.1:d:+1015:primary
ATTCGATCAG	1	3	2	6	1	At2g44610.1:d:+901:primary
AGTGATATTG	2	2	5	1	3	At2g44360.1:d:+611:secondary
TCAAGAAGCC	5	2	4	1	1	At2g43945.1:d:+976:primary
TTCCAAGATA	5	1	1	3	3	At2g43100.1:d:+958:primary
AAACTGATGA	1	6	3	2	1	At2g40650.1:d:+1073:primary,At4g32105.1:d:+556:primary
TTTTGATTAT	6	1	0	0	6	At2g40475.1:d:+1089:primary
ATCACATAAC	0	4	2	3	4	At2g40300.1:d:+970:primary
ATACAACATC	5	0	4	1	3	At2g39760.1:d:+952:primary,At4g34050.1:d:+1062:primary
TCTTCTCAAG	3	0	5	4	1	At2g38950.1:d:+630:secondary,At4g30890.2:d:+221:secondary
AATAGAGAAC	3	1	6	1	2	At2g38640.1:d:+766:primary
TAAACTGTGT	7	2	1	2	1	At2g37570.1:d:+2189:primary,At2g37570.2:d:+1772:primary,At5g54870.1:d:+1748:primary
CGGTTTCTGA	2	2	7	2	0	At2g37470.1:d:+442:primary
GTTACTCCAG	3	6	2	2	0	At2g36460.1:d:+812:primary
GTTGTACGTT	3	6	1	3	0	At2g36320.1:d:+626:secondary
TATCGTTTGA	3	2	4	3	1	At2g34970.1:d:+2421:primary
TTTTGGAAAG	7	2	3	0	1	At2g34720.1:d:+863:primary
GATTTCTGAG	2	1	2	4	4	At2g33590.1:d:+1446:primary
CTCAAGTGAA	2	0	5	3	3	At2g33250.1:d:+914:primary
TGCTTTGAAT	3	1	5	4	0	At2g32090.1:d:+657:primary
GGAATTTGCA	3	2	3	4	1	At2g31100.1:X:-645:quaternary,At5g20580.1:X:-97:quaternary
CATTTGGAGT	2	5	1	5	0	At2g31040.1:d:+989:primary

TATCATTTAT	2	5	3	1	2	At2g27700.1:v:+1752:secondary,At4g34090.2:d:+160:secondary,At4g34090.1:d:+160:secondary,At1g74910.1:d:+1590:secondary,At1g74910.3:d:+1588:secondary,At1g74910.2:d:+1541:secondary
TGAGACTAGT	3	1	4	3	2	At2g21330.1:d:+1374:secondary
TTGCAGTTAA	2	2	1	7	1	At2g20970.1:v:+1750:secondary,At5g02680.1:v:+358:secondary
TGAGATGCTT	0	5	4	3	1	At2g18700.1:d:+2748:primary
GTCTTACTGC	6	0	2	3	2	At2g18040.1:d:+800:primary
ATTGATTTTG	5	0	6	2	0	At2g13560.1:i:+534:tertiary,At3g05720.1:i:+532:tertiary
GCTTTCGTTT	3	2	2	5	1	At2g13440.1:X:+433:quaternary
TGATTTAAGA	5	3	0	2	3	At2g01670.1:d:+1030:primary,At3g01530.1:d:+1267:primary
TAGTGTTGAA	4	4	0	2	3	At2g01410.1:d:+1261:primary
GCAAGGCAAG	3	5	1	3	1	At2g01140.1:d:+1170:primary
AAGAATGAAT	2	4	1	2	4	At2g01060.2:d:+1330:primary,At2g01060.1:d:+1176:primary
ACAAATTGGC	0	2	4	4	3	At1g79350.1:d:+4232:primary
CACAAGAATG	2	2	5	4	0	At1g79040.1:X:+306:quaternary
TTTGGAAGTT	1	6	2	2	2	At1g74840.1:d:+1095:secondary
TCGAGAGAAT	3	1	4	3	2	At1g72550.2:d:+2019:primary,At1g72550.1:d:+2009:primary
TAAAGAATGC	5	1	3	2	2	At1g70370.1:d:+2199:primary
GTTTTTTTTG	6	2	2	1	2	At1g69160.1:d:+1286:primary
ATCAGTGATG	2	4	3	1	3	At1g68140.1:d:+1508:secondary
GTCTTCAGTT	2	6	3	2	0	At1g64510.1:X:-246:quaternary
TTTCTCATTG	6	3	1	0	3	At1g61563.1:d:+398:primary
TTGGGTGCAG	0	0	12	0	1	At1g61520.1:d:+467:secondary
TTATAGGATT	2	6	3	0	2	At1g60870.1:d:+747:primary
GGTAGAGAAT	2	2	5	3	1	At1g58100.1:d:+1429:primary
AAAAAAAAAT	5	3	4	1	0	At1g56490.1:p:+2065:primary
TCTTTGACGA	2	3	3	4	1	At1g53670.1:d:+529:primary
TTGGAGGTTC	1	9	0	2	1	At1g52400.1:d:+974:primary
AGTGTTGTTG	3	4	5	0	1	At1g51610.1:d:+1778:primary
AAGTTGTGCC	4	6	1	1	1	At1g50920.1:d:+1947:primary
TCAATGCTCT	1	4	1	2	5	At1g48650.1:d:+3689:primary,At1g16240.1:d:+841:primary
TGTTAAGGAG	2	4	1	3	3	At1g48340.1:p:-48:primary
TCAAGTTTTT	2	7	3	1	0	At1g47770.1:v:+594:secondary
AAAGCTCGAA	0	3	3	4	3	At1g44835.1:d:+1132:primary
AGAGAAAATA	2	7	2	1	1	At1g33850.1:v:+721:secondary,At4g14930.1:d:+1206:secondary,At3g58360.1:v:+1477:secondary
AACAAAAAAC	4	5	1	2	1	At1g33240.1:d:+2491:primary
GATTCTCTTG	5	1	1	1	5	At1g32470.1:X:-187:quaternary,At4g13260.1:X:-408:quaternary
AAGAAGGTGC	2	3	2	5	1	At1g32230.2:d:+2214:primary,At1g32230.1:d:+2217:primary
GAAAATCATT	4	4	1	4	0	At1g32050.1:d:+770:primary
TATTAGAAAC	6	1	3	1	2	At1g31180.1:d:+1280:primary
TAGTCTCTGG	4	1	6	0	2	At1g30690.1:d:+1771:primary
AAAATCAGTT	4	7	0	2	0	At1g28000.1:v:+1719:primary,At1g25230.1:d:+1044:primary
AGATCCTCTA	2	3	4	4	0	At1g26945.1:d:+638:primary
CGGTGATTGA	3	2	3	4	1	At1g25380.1:d:+1364:primary
TTTTATCTTT	1	3	2	1	6	At1g25350.1:d:+2583:primary,At2g33730.1:d:+2381:primary
AGATTGGTGG	1	3	1	7	1	At1g21460.1:d:+525:primary
TGGATGGTGT	2	2	7	1	1	At1g20110.1:d:+1992:secondary
TATGTATTTT	2	8	2	1	0	At1g19530.1:d:+612:primary,At4g16780.1:d:+1113:primary
TAATGTGACA	2	3	3	3	2	At1g17840.1:d:+2468:secondary
GCGATGGCGG	0	0	13	0	0	At1g15825.1:v:+1207:primary
AAGGCAGAAG	3	4	1	4	1	At1g15340.1:d:+1263:primary
CTTCTTTGTT	0	11	0	2	0	At1g13245.1:d:+193:primary
TTTACTGACG	2	3	2	3	3	At1g12270.1:d:+1858:primary
TAATAGTATC	8	0	1	0	4	At1g11860.2:d:+1537:secondary,At1g11860.1:d:+1536:secondary
GCCGTGACGC	1	1	4	7	0	At1g10960.1:d:-211:primary
AAGAAAAGTT	1	4	2	3	3	At1g08490.1:d:+1714:primary
TAATCTTTTG	2	6	0	1	4	At1g07530.1:d:+2705:primary
GCTGGTTTGG	0	10	2	1	0	At1g03090.2:d:+2042:primary,At1g03090.1:d:+1982:primary
ATCCAGATCT	6	4	1	1	1	At1g01620.1:X:-174:quaternary
GCGATAAAAA	3	1	0	3	5	No gene matches found
GAGGTGTGGC	4	1	1	6	0	No gene matches found

AACTTCAACA	2	5	1	3	1	Chr5:+23621788:quaternary,Chr1:+26703139:quaternary
GATGGAGTTC	3	4	2	2	1	Chr4:+16618310:quaternary,Chr1:+24303089:quaternary
TCGATGAAAT	3	4	2	2	1	Chr2:+3708528:quaternary,At3g61150.1:X:+554:quaternary
CGCGGGGCTC	1	6	2	3	0	At5g66140.1:d:+266:primary
AGAGAATCTT	4	3	2	3	0	At5g65750.1:d:+3286:primary
TCAGACTTCA	2	0	4	3	3	At5g65460.1:X:-49:quaternary,At5g15030.1:X:-325:quaternary
CTATAAAAAG	4	1	3	3	1	At5g65370.1:v:+1558:primary
ATGGAGTAGA	4	1	2	3	2	At5g62380.1:v:+1607:primary
TGTTTAGGGT	1	10	1	0	0	At5g62280.1:d:+748:primary
AGATTTCTGT	3	2	3	3	1	At5g61970.1:d:+2138:secondary
TTTGAGCTTT	2	2	1	4	3	At5g61780.1:d:+3502:primary
AGTGAAAGCT	1	2	1	4	4	At5g61670.2:d:+1130:secondary,At4g25340.1:d:+1019:secondary,At3g02410.1:v:+212:secondary
ATTTCATTTA	0	11	1	0	0	At5g61600.1:d:+841:primary
TACTTGTTTC	1	2	3	5	1	At5g60540.1:d:+1073:primary
TGCATCATAC	2	3	5	1	1	At5g60360.1:d:+1200:secondary
TTGTTTTTAA	3	2	2	3	2	At5g59420.1:d:+1603:primary
TAAAAACTTA	1	2	3	3	3	At5g54290.1:d:+1123:primary
TCTCGTTCAA	1	1	2	6	2	At5g53540.1:d:+1489:primary
TCAAACATTT	4	3	5	0	0	At5g53050.1:d:+1489:primary,At5g53050.2:d:+1481:primary
AGAAACAGAG	0	8	2	1	1	At5g52860.1:d:+1686:primary,At1g23710.1:d:+890:primary
TCGAATAAGA	2	2	5	0	3	At5g50960.1:d:+1300:primary
TATTGTAGCC	1	4	0	4	3	At5g47690.1:v:+5595:primary
GAGCGGAAGC	3	3	2	4	0	At5g47480.1:d:+3933:secondary
GCTTCTAGTT	1	4	1	5	1	At5g46630.2:d:+1700:primary,At5g46630.1:d:+1499:primary
CAGAAACGAT	5	2	2	1	2	At5g46000.1:v:+614:secondary,At3g17930.1:d:+554:secondary
AATTTGGACT	3	3	2	0	4	At5g45550.1:d:+642:primary
GAGAGTTTAG	1	2	4	2	3	At5g42990.1:d:+740:primary
TTTGTTGTTT	4	1	1	4	2	At5g42950.1:d:+5357:primary
AAGATCAATA	2	5	4	0	1	At5g27380.1:d:+1660:primary
GAAACTGATC	2	1	3	2	4	At5g26717.1:v:+1158:primary
CGTTCCGGAA	0	2	4	5	1	At5g26030.1:d:+1600:primary
CGCATCTTAG	2	3	0	2	5	At5g22060.1:d:+1398:primary
TTCTTTGTTC	2	8	2	0	0	At5g21170.1:d:+1071:primary
AACACAACAA	2	1	2	5	2	At5g20710.1:d:+2113:primary,At5g26751.1:d:+1482:primary
TGCTTGATAA	2	3	5	2	0	At5g20650.1:d:+551:primary
CACTTGGGAG	1	0	8	1	2	At5g20620.1:d:+1286:secondary,At5g03240.2:d:+1234:secondary,At5g03240.1:d:+1054:secondary
AACAGTCACA	0	0	3	5	4	At5g19850.1:d:+1192:secondary
AAACTTCTTT	5	5	1	1	0	At5g19150.2:i:+2642:tertiary
AACAGAGTGA	4	2	2	1	3	At5g18760.1:d:+527:primary
AAGTCTCAGC	1	4	0	6	1	At5g17190.1:d:+349:primary
GTACACAATT	1	3	4	1	3	At5g16620.1:d:-1579:secondary
GTCACTGAAA	3	0	2	4	3	At5g15910.1:d:+873:primary,At3g49770.1:v:+1484:primary
AGTTTATACT	6	0	0	2	4	At5g13850.1:d:+805:primary
AAAATGTTGC	3	2	1	1	5	At5g11950.2:d:+840:primary
GTGGATATGA	4	5	3	0	0	At5g10960.1:d:+707:primary
TTGCAGCGGT	1	2	4	4	1	At5g10920.1:d:+1709:primary
TACGGATGAG	1	6	2	1	2	At5g10350.1:d:+802:primary,At5g10350.2:d:+891:primary
TAGGACTTGA	1	1	3	3	4	At5g07350.1:d:+3318:secondary
GGGACCACTC	2	4	2	2	2	At5g05170.1:d:+3161:primary
ACTTTTGAGC	3	3	4	2	0	At5g04280.1:d:+1219:primary
GTCTATGCAC	5	3	1	3	0	At5g03650.1:d:+2351:primary
ACATTTGGAG	5	1	2	2	2	At5g03350.1:d:+812:primary
TTAAACAATT	4	1	2	4	1	At5g03240.1:d:+1221:primary,At5g03240.2:d:+1401:primary
CAATATCATC	4	5	1	1	1	At5g02760.1:d:+1422:secondary
TTTGTAAATA	6	1	1	2	2	At5g02310.1:d:+6193:primary
ACAGGTGGCA	1	5	2	4	0	At5g01750.2:d:+568:primary
TTGAGGAAAG	2	4	3	3	0	At5g01730.1:d:+3352:primary
TAGAAGAATC	6	2	3	0	1	At5g01090.1:d:+1424:primary
TGTGCGAGTC	1	9	1	1	0	At4g39940.1:d:+744:primary
GGCCGTTCTC	2	6	1	3	0	At4g37450.1:d:+627:primary
GAACTGGCTA	1	5	4	0	2	At4g35570.1:d:+378:primary

GCCTGATGGA	1	3	2	6	0	At4g34070.1:d:+1087:primary,At2g45240.1:d:+1146:primary
GTTTCTGCTA	1	1	2	1	7	At4g33950.1:d:+1156:primary
AGGATGGTTT	3	4	4	1	0	At4g32340.1:d:+699:primary
TGGCTTAAAA	4	3	0	2	3	At4g32150.1:d:+992:secondary
TTTTAGGTCT	2	4	1	3	2	At4g31580.1:d:+813:primary
TTGTGTTTTG	3	3	3	1	2	At4g31560.1:d:+488:primary
TGGAAGAATT	2	4	0	4	2	At4g30580.1:d:+1168:primary
ATGATGGGTG	0	7	1	3	1	At4g29060.1:d:+1099:secondary
AAATCTATAA	2	6	0	1	3	At4g28060.1:v:+912:secondary
GCTCACCAAA	5	1	0	5	1	At4g28060.1:v:+44:secondary
GTGTAAGTGG	0	5	4	2	1	At4g28040.2:d:+1244:secondary,At4g28040.1:d:+1166:secondary
CAGAGTTTCA	3	2	4	2	1	At4g25740.1:d:+186:secondary
TGTCGTTGGT	1	2	1	6	2	At4g25630.1:d:+1015:primary
ATGCTTGATG	1	8	1	2	0	At4g25030.2:d:+1645:primary,At4g25030.1:d:+1154:primary
TTCAAGTCCT	2	7	1	2	0	At4g23800.1:d:+1461:primary
ACTCTCACGG	3	5	2	1	1	At4g23670.1:d:+297:secondary,At4g23680.1:d:+208:secondary
ATTTGAGGGT	3	3	3	2	1	At4g22740.2:d:+1257:secondary,At4g22740.1:d:+1222:secondary
GTAATGATTA	2	0	5	0	5	At4g22580.1:d:+1540:primary
TGTATGCCGT	2	3	3	3	1	At4g21110.1:d:+466:primary
GTTTATGTTG	5	2	1	2	2	At4g21050.1:v:+1133:primary
TGGTGGGTAT	0	4	4	3	1	At4g20150.1:d:+203:primary
GATAGGGCAG	4	4	2	1	1	At4g19640.1:d:+733:primary
TTTTGAATAT	5	1	1	0	5	At4g19580.1:v:+1733:primary
GTGGGCTTGG	3	1	2	5	1	At4g17600.1:d:+537:secondary
TCCACTCTAA	6	3	2	1	0	At4g17050.1:d:+1050:primary
TCTTCTGTGT	2	9	0	1	0	At4g16520.2:d:+606:primary,At4g16520.1:d:+402:primary
TCGATATAAT	3	3	4	1	1	At4g14230.1:d:+1700:primary
ATCTATAATT	0	9	0	3	0	At4g14130.1:d:+881:primary,At5g57560.1:d:+833:primary
CTACTGGGAA	3	3	2	4	0	At4g13010.1:d:+1093:secondary
TGTTAAAAAA	2	0	4	3	3	At4g12890.1:d:+439:secondary
ACTTTGTTTT	4	1	3	1	3	At4g08520.1:d:+718:primary
ATAACTCAAA	2	2	3	2	3	At4g07440.1:v:+75:secondary,At5g35260.1:v:+76:secondary,At2g05880.1:v:+77:secondary
TCTTAAAAAA	3	2	4	2	1	At4g04840.1:d:+467:primary
TTGTGTGAGC	3	5	2	2	0	At4g03030.1:d:+1482:primary
TGTTTTCTAC	0	0	4	3	5	At4g02940.1:d:+2035:primary
AATTTGAGAT	4	3	2	0	3	At4g02840.1:d:+576:primary
GTTTACTGGT	3	5	2	1	1	At4g02400.1:d:+2886:primary,At4g26110.1:d:+943:primary
TACTGGAGTT	5	1	0	5	1	At4g00810.2:d:+475:secondary,At4g00810.1:d:+553:secondary
ATGCTGCTGA	2	6	2	1	1	At3g63400.2:d:+692:primary
GTGGAGTCGT	2	2	4	3	1	At3g62720.1:d:+1715:primary
CCACATTGAA	1	5	3	2	1	At3g62400.2:d:+84:primary,At3g62400.1:d:+111:primary
TGTGTCTAAA	0	4	4	3	1	At3g62270.1:X:-90:quaternary
GATGGAGTTT	0	2	4	3	3	At3g61230.1:d:+254:secondary
AATCGAAACC	2	5	2	2	1	At3g59780.1:d:+1994:primary
AAATTTCAAT	3	2	3	2	2	At3g57410.1:d:+3420:primary
TTCAAAACGA	0	5	5	2	0	At3g54550.1:v:+1652:primary,At1g19050.1:d:+606:primary
AGAAGAGTCT	3	2	3	2	2	At3g52880.1:d:+1501:secondary
ATGCTCTAGT	3	2	3	2	2	At3g51000.1:d:+1042:secondary
TTGAATTCGA	4	0	2	2	4	At3g50830.1:i:+434:tertiary
AGATTAATGA	4	7	1	0	0	At3g47620.1:d:+1420:primary
AAGTCACTGA	2	8	1	1	0	At3g46780.1:d:+295:primary
ACCCCATCGC	1	1	4	5	1	At3g44720.1:d:+1382:primary
TCCGGTTGCA	2	5	2	2	1	At3g44100.1:d:+319:secondary
TGAAACTTCT	3	1	3	4	1	At3g29250.1:i:+3944:tertiary
GTTTTCTTGG	1	2	3	0	6	At3g28290.1:d:+1329:primary,At3g28300.1:d:+1329:primary
TTTAAGGATC	6	0	0	3	3	At3g27430.2:d:+1037:primary,At3g27430.1:d:+1035:primary
GTGTCGTTGT	3	3	4	1	1	At3g27100.1:d:+468:primary
GTTTATGTCA	2	7	1	1	1	At3g26710.1:d:+793:primary
CCTGTAGCCG	1	8	3	0	0	At3g25530.1:d:+843:primary
TACTAGAACG	4	0	5	1	2	At3g23700.1:d:+1185:primary
TTCACTCACC	0	7	0	2	3	At3g22110.1:d:+774:primary

ATTACTTCTC	2	3	4	3	0	At3g21350.1:d:+923:primary
CGATTAAGAG	1	9	0	1	1	At3g20310.1:d:+629:secondary
ATTCTCAAAA	0	3	2	5	2	At3g20060.1:d:+700:primary
ATGACAGCAC	0	0	10	2	0	At3g16640.1:d:+501:secondary
TTTGTGAATT	2	1	3	5	1	At3g16630.2:d:+2850:primary,At3g16630.1:d:+2864:primary
GATTTGGATT	0	5	5	1	1	At3g16180.1:d:+2012:primary
AGGAGCCAAC	1	2	3	5	1	At3g16000.1:d:+2269:primary
TAGCTGTCTT	4	1	1	4	2	At3g14790.1:d:+2257:primary
AGAGCTTGAA	0	3	5	4	0	At3g12650.1:d:+751:primary
GATAATCTCT	3	0	2	3	4	At3g12600.1:d:+860:primary
AATCTGAAAT	1	1	5	4	1	At3g11900.1:d:+1464:primary
TCGCTGTGTC	1	6	1	3	1	At3g11730.1:d:+819:primary
GTTGCCTCAA	2	3	5	2	0	At3g10720.2:d:+1842:secondary,At3g10720.1:d:+1041:secondary
ACAAACTACT	2	3	3	3	1	At3g10670.1:d:+1253:primary
ACCACCTTCC	0	0	3	6	3	At3g09860.1:X:+306:quaternary
TGGCCTTTGT	3	1	2	3	3	At3g09850.1:d:+2540:primary
AATATAATAA	0	1	0	0	11	At3g08640.1:d:+1299:primary
TCAGAAATCT	2	3	5	1	1	At3g08020.1:v:+2916:primary
GCTTTGTTTT	2	4	2	2	2	At3g07510.1:d:+724:primary
TTCGAATAAG	1	7	3	1	0	At3g06850.2:d:+1544:primary,At3g06850.1:d:+1585:primary
TGAGAGACTT	4	1	4	0	3	At3g06790.1:d:+851:secondary,At3g06790.2:d:+851:secondary
TGGTTAAGTG	2	5	1	3	1	At3g05980.1:d:+1004:primary,At1g03280.1:d:+1823:primary
TAAATGTTTG	4	5	1	2	0	At3g02580.1:d:+1151:primary
AACAAAAGCC	1	0	1	0	10	At3g02480.1:d:+263:primary
TGGTGGATTA	3	1	4	2	2	At3g01910.1:d:+1484:primary
ATGGAATGCT	5	2	1	3	1	At3g01480.1:d:+1633:primary
TCTTTTTCCT	6	3	2	1	0	At3g01480.1:d:+1582:secondary
GTTTGCGCAA	3	2	3	1	3	At2g47580.1:d:+861:primary
AGGAGAAAGG	1	3	5	3	0	At2g47580.1:d:+398:secondary
TTTTTATTTA	2	6	2	1	1	At2g46280.3:d:+1283:primary,At2g46280.1:d:+1190:primary,At2g46280.2:d:+1134:primary,At4g28005.1:v:+937:primary
TTTAGGTAAG	3	1	1	3	4	At2g45990.1:d:+925:primary
GCTGTGAACA	4	3	1	3	1	At2g44160.1:d:+1577:primary
TTTCTTCTTC	3	8	1	0	0	At2g44080.1:d:+826:primary
CGAATTGGAG	3	4	3	2	0	At2g42490.1:d:+2572:primary
GAAGAATAAT	2	3	1	2	4	At2g42270.1:d:+6680:primary,At5g09890.1:d:+1892:primary
TGTGGTTTTG	5	1	2	2	2	At2g41870.1:d:+1363:primary
TACATAAAGG	5	6	0	0	1	At2g39705.1:d:+535:primary
CATATCATAA	8	3	1	0	0	At2g38310.1:d:+944:primary
TTACCAAGAG	5	5	2	0	0	At2g37340.2:d:+1192:secondary,At2g37340.1:d:+1048:secondary,At2g37340.3:d:+1255:secondary
GACAACGCTG	3	4	2	2	1	At2g36835.1:d:+450:primary
GTTCTCAAGA	2	4	0	1	5	At2g36620.1:d:+50:primary
ACAGAGTTGT	2	2	1	6	1	At2g36580.1:d:+1648:primary
ATCACTGGTA	3	0	1	3	5	At2g36145.1:d:+688:primary
GGAGGCATAA	2	5	4	1	0	At2g35680.1:d:+1477:secondary
AAGAAAAAGA	2	3	2	1	4	At2g34570.1:d:+798:primary
GATTCTCAAA	0	5	0	4	3	At2g32600.1:d:+1068:primary
ACCACATAAC	1	5	1	2	3	At2g31400.1:d:+2813:primary
GCCAATCACC	0	8	2	0	2	At2g30600.2:d:+2366:primary,At2g30600.1:d:+2389:primary
GGCTCTTTAA	0	3	4	3	2	At2g22430.1:d:+1432:primary
AGAACGATGA	0	5	3	3	1	At2g22080.1:d:+523:primary
GCCAAAGCAC	1	2	5	2	2	At2g21870.2:d:+711:secondary,At1g67120.1:d:+7051:secondary,At2g21870.1:d:+711:secondary
AGAACTTTAT	1	0	3	7	1	At2g21390.1:d:+4050:primary
ATGCCGCCTC	0	6	3	1	2	At2g20760.1:d:+854:secondary
CCAAGAAGAC	4	2	5	1	0	At2g20610.2:d:+1486:secondary,At2g20610.1:d:+1406:secondary
GTTTTTCTAC	5	1	0	3	3	At2g20580.1:d:+2942:primary
GATTTGGTAA	2	3	5	1	1	At2g18680.1:d:+818:primary
TGAAGTGGCA	2	0	5	4	1	At2g15430.1:d:+1167:primary
GCTGCTCAAG	0	0	7	1	4	At2g05380.1:d:+324:secondary,At5g08120.1:d:+1059:secondary
GTTATTTCCG	3	3	4	1	1	At2g04780.1:d:+814:primary,At2g04780.2:d:+1222:primary
ACAGTCACTT	5	5	1	0	1	At2g04410.1:d:+439:primary

GAGATGAGAA	5	1	3	2	1	At2g04036.1:p:+317:secondary,At4g11910.1:d:+1256:secondary,At4g26630.1:d:+1372:secondary,At2g10010.1:p:+317:secondary,At3g24515.1:d:+614:secondary
GATGAATATC	1	1	3	2	5	At2g01350.2:d:+1323:primary,At2g01350.1:d:+1251:primary
GATGCTGAAC	2	4	2	4	0	At1g80700.1:d:+391:primary,At1g80980.1:d:+987:primary
GCGAAGAGGA	3	0	6	3	0	At1g80530.1:X:-74:quaternary
TTACGTGGAT	1	1	4	3	3	At1g80030.1:d:+1882:primary
GCTGCGGGGA	0	3	1	7	1	At1g79930.1:d:+2388:primary
TTTAAGTTGA	5	5	1	0	1	At1g79230.1:d:+1333:primary,At4g08600.1:p:+3005:primary
AGGGGATAAA	0	5	3	1	3	At1g77130.1:d:+2053:primary
CTTGTCCTCA	2	5	4	1	0	At1g76680.2:d:+996:secondary,At1g76680.1:d:+987:secondary
ACAATGTGTG	5	0	2	3	2	At1g76570.1:d:+984:primary,At1g23800.1:d:+1828:primary
AATGATAATT	3	2	3	0	4	At1g76050.1:d:+1536:secondary,At1g76050.2:d:+1552:secondary
ATATTCAAAA	4	0	2	2	4	At1g71810.1:d:+138:secondary
GCGAAAGGGA	1	5	3	2	1	At1g71680.1:v:-538:secondary
CCAGAGGACG	1	6	1	3	1	At1g64870.1:v:+1065:primary
GGAAGACATT	4	2	3	2	1	At1g64860.1:d:+1633:primary
TAGTAGCGAG	1	8	3	0	0	At1g64370.1:d:-711:secondary
AGTTCAACGC	2	2	2	4	2	At1g64355.1:d:+740:primary
GATTATTTCA	5	2	3	1	1	At1g62880.1:d:+217:secondary
ACTTTGTATA	3	0	0	0	9	At1g62180.1:d:+1631:primary
GAAGCTACTG	1	2	3	2	4	At1g61570.1:d:+263:primary
CAGACCTGAG	1	4	2	2	3	At1g56450.1:d:+613:primary
CACCTTTAAT	6	2	1	2	1	At1g54730.3:i:+2809:tertiary,At1g54730.2:i:+2748:tertiary
AACTGCGAAA	1	3	3	5	0	At1g53750.1:d:+1208:primary
CGACTTTTTT	2	1	3	4	2	At1g53240.1:d:+1362:secondary
TTTTCAAACT	4	1	5	1	1	At1g50980.1:X:--25:quaternary,At2g45470.1:X:-1416:quaternary,At1g67140.1:X:-22:quaternary,At1g45332.1:X:-50:quaternary
TTTTAAGAAT	4	1	4	2	1	At1g50200.1:d:+3264:primary,At1g29300.1:d:+1607:primary
AGACATTTAG	1	1	3	4	3	At1g49410.1:X:+99:quaternary
GATAATTTTG	2	1	2	4	3	At1g49260.1:v:+1165:primary,At4g09570.1:d:+1671:primary
AATGGAGTCT	5	3	3	1	0	At1g49140.1:d:+403:primary
TTGTATAATT	0	10	0	1	1	At1g48240.1:d:+1199:primary
GAGGCTTTTG	8	0	1	3	0	At1g45760.1:p:+369:primary
TCCAACTAAA	1	4	0	4	3	At1g33490.1:d:+852:primary
TGGTTTTGTA	1	8	1	1	1	At1g30590.1:v:+2298:primary
TTAGTTGAAA	3	2	0	5	2	At1g28340.1:d:+2167:primary,At4g32870.1:d:+565:primary
GTAGGGTCTA	0	9	2	0	1	At1g25400.1:d:+990:secondary
CCACAACATT	0	5	2	5	0	At1g22930.1:d:+3509:primary
TTCCCACTTT	4	2	4	2	0	At1g22140.1:d:+343:primary,At1g22140.2:d:+563:primary
GATTTTTTTA	0	1	6	4	1	At1g21380.1:d:+1853:secondary
TTATCTCATC	2	1	2	2	5	At1g21080.1:d:+1536:primary
AGGCAGTTCC	1	7	1	1	2	At1g20970.1:v:+3760:secondary
AAATTGTTTT	0	3	4	2	3	At1g17130.1:d:+1233:secondary
GAACTTGACG	3	0	6	2	1	At1g16470.1:d:+680:primary
TATGAAAATT	0	4	1	2	5	At1g13910.1:d:+1293:primary,At3g26560.1:d:+3714:primary
AGGACATTTG	1	4	2	4	1	At1g12920.1:d:+1356:primary
CTCCCGCCAC	3	4	4	1	0	At1g12850.1:d:+1381:primary
TTTTGCAACA	5	0	3	1	3	At1g12440.1:d:+867:primary,At1g12440.2:d:+1306:primary
ACCCAAAAAA	0	5	2	4	1	At1g12210.1:v:+3020:primary
TCGGCACTTG	3	2	1	6	0	At1g10650.1:d:+1305:primary
GAGGCTGCAC	0	8	3	1	0	At1g10200.1:d:+503:primary
ACTTTGTAAA	4	3	1	2	2	At1g09770.1:d:+2780:primary
GTCTCAAACC	3	5	1	2	1	At1g07610.1:d:+216:primary
TGTGTGGCAC	0	10	1	0	1	At1g07140.1:d:+449:secondary
TTAACATCAT	3	4	0	0	5	At1g03475.1:d:+1346:primary
ATGAGGTCCT	3	2	5	2	0	At1g02130.1:d:+865:primary
GGAAACTGAG	2	0	5	5	0	At1g01320.1:d:+5505:secondary
GAGCTAAGTT	1	8	2	0	0	No gene matches found
AAAGTCTCGG	4	1	0	6	0	ChrC:+27722:quaternary
TACGGTTCTG	2	3	0	3	3	ChrC:+139807:quaternary

GTGCACAAAA	2	5	1	1	2	Chr5:+23867914:quaternary
AGGAACAAAA	1	0	4	2	4	Chr5:+17090546:quaternary,Chr1:+11584726:quaternary,Chr5:+16754044:quaternary,Chr5:+8213873:quaternary
AACAATAAAA	2	3	4	0	2	Chr5:+15058297:quaternary,Chr3:+20875858:quaternary,Chr3:+16446573:quaternary
AATAAGTATA	4	2	0	1	4	Chr4:+7846735:quaternary; small RNA(MIR863a)
AGATAAAGAG	3	0	2	0	6	Chr4:+76188:quaternary
AAACGAAACA	4	1	4	1	1	Chr4:+5447469:quaternary
GGTTAGTCGA	1	5	2	2	1	Chr3:+14212620:quaternary; +small RNA(GCACATGGGTTAGTCGA; CACATGGGTTAGTCGAT; CATGGGTTAGTCGATCC)
CTCGCGCATC	1	0	1	6	3	Chr2:+3300:quaternary,Chr3:+14208257:quaternary
GGAATTCAAA	3	3	1	3	1	Chr2:+17668161:quaternary
TGGATTTGAA	1	2	7	1	0	Chr2:+17450102:quaternary
AATTAAAAAA	4	1	1	1	4	Chr2:+17343809:quaternary,At1g74450.1:X:+1809:quaternary
TGTTTTATGC	0	0	5	3	3	Chr1:+3309095:quaternary
TTAAAACAAA	3	2	3	1	2	Chr1:+25170087:quaternary,Chr2:+16823482:quaternary
AGTTTAAAAA	5	0	2	2	2	Chr1:+22090022:quaternary,Chr1:+26827741:quaternary
TCGATAAAAT	2	3	5	1	0	Chr1:+11498333:quaternary
TTTCGTATAG	6	1	2	0	2	AtCg00490.1:X:-1672:quaternary,Chr2:-12673387:quaternary
TTAACAAATT	5	1	1	1	3	AtCg00160:d:+313:primary
GTTTAAATAG	6	2	1	0	2	AtCg00040:d:+173:secondary
CAGCTCAGAA	1	5	1	3	1	At5g67385.1:d:+1535:primary
GTGGAGCAGT	1	5	1	2	2	At5g65940.1:d:+1073:secondary
CCTTGTAATA	5	2	0	0	4	At5g65380.1:d:+1725:primary
TTCAATCTCA	1	4	5	1	0	At5g64860.1:d:+1912:primary
TTTTGGGGAC	4	0	4	2	1	At5g63570.1:d:+1545:primary
GAAGTCGAAA	1	3	2	3	2	At5g62440.1:d:+142:secondary
AAGAAAACTT	0	3	5	1	2	At5g60780.1:v:+2246:primary
AGGAGACACA	1	1	4	2	3	At5g59820.1:d:+405:primary
ATGGTAGCTT	4	3	3	0	1	At5g58590.1:d:+821:primary
TATTTGTACA	2	6	1	1	1	At5g57887.1:d:+482:secondary
GAATCGAAGC	1	4	4	2	0	At5g57180.2:d:+1062:primary
ATTGACTCTT	6	1	3	1	0	At5g56710.1:d:+579:primary
GGTAATTTGC	0	1	1	1	8	At5g56320.1:i:+352:tertiary
CTCTTTAAAA	3	4	3	1	0	At5g54050.1:X:--24:quaternary
CACAAAAAAA	2	3	3	2	1	At5g52820.1:X:+305:quaternary
ATTTGGTTGA	1	3	3	1	3	At5g52310.1:d:+253:secondary,At1g56300.1:d:+787:secondary
ATTTCAAATG	3	3	1	1	3	At5g51200.1:d:+5521:primary
GACGCCATTG	2	3	1	1	4	At5g50460.1:d:+61:primary
CTAATAAAGG	6	1	2	0	2	At5g49810.1:d:+3473:primary
ATTCTGTTTG	4	1	0	2	4	At5g49760.1:d:+3251:primary
CCGTGATTGT	4	0	3	4	0	At5g48930.1:d:+1539:secondary
TTTTGAGCCA	0	3	1	5	2	At5g48760.1:d:+724:primary
CAAATTTTGT	0	2	1	7	1	At5g48240.1:v:+1425:secondary
AAGCACTTTC	2	4	3	0	2	At5g47480.1:d:+4167:secondary
TTTCCACTAA	1	1	1	6	2	At5g47120.1:d:+888:primary
CTTGAAAAAA	1	2	1	3	4	At5g47040.1:d:+2057:secondary
AAGATGGTGA	3	3	4	1	0	At5g44800.1:v:+7002:primary
AGTACCCATT	0	7	0	3	1	At5g44250.1:d:+1338:primary
TTGAGACCAA	5	2	2	1	1	At5g43430.1:d:+315:secondary,At1g43775.1:p:+101:secondary
ATGTGTCGGT	2	2	2	2	3	At5g43330.1:d:+1241:primary
AGTGTTGTAT	2	0	2	3	4	At5g43150.1:d:+510:primary
TGTTCGTGTG	4	4	2	1	0	At5g39610.1:d:+1029:primary
TCCACTACTG	1	0	1	2	7	At5g35920.1:v:+423:secondary
TGTATTTTAT	6	1	0	1	3	At5g27350.1:d:+1896:primary
CATACATCAA	1	5	1	3	1	At5g27320.1:d:+1551:primary
AGAAAGTGGT	3	1	4	1	2	At5g25210.1:d:+648:secondary,At4g34230.1:d:+1230:secondary
GATGTAGATG	0	5	4	2	0	At5g24470.1:d:+1717:primary
GAAATAATGA	3	2	3	1	2	At5g24400.1:d:+1170:primary
TCATCCAACA	1	7	1	2	0	At5g23540.1:d:+977:primary
GAGATAATTG	2	2	5	1	1	At5g23250.1:d:+1209:primary
GAGAAACTTT	2	0	4	3	2	At5g20380.1:d:+1632:primary
ATTTTCAGAA	3	2	2	3	1	At5g20270.1:d:+1340:primary

TAATCAACAA	4	1	2	3	1	At5g19790.1:v:+1383:primary
CCGTTGATCA	3	6	1	1	0	At5g17560.1:d:+514:secondary
TTAATCTTAA	4	1	0	1	5	At5g16620.1:d:+1584:primary
AGCACCAAGT	5	2	1	2	1	At5g16270.1:d:+3562:primary
CTTGCTTAGC	4	3	0	0	4	At5g15750.1:d:+640:primary
CTATTGGGAC	6	1	2	2	0	At5g14970.1:d:+1090:primary
GAGTTGTGGA	0	5	1	3	2	At5g14710.1:d:+555:primary
GTGGTTTGTT	1	1	2	5	2	At5g14030.1:d:+581:primary
GAACAGTCGT	5	4	1	1	0	At5g13800.1:d:+1636:primary,At5g13800.2:d:+1520:primary
AAAGTTGAAG	1	3	4	3	0	At5g13770.1:d:+1701:secondary
TGTTTGTTGT	1	1	3	0	6	At5g12040.1:d:+1316:primary,At5g12040.2:d:+1524:primary
CAAACTATAA	4	3	3	1	0	At5g10180.1:d:+2275:primary
GTGGGTTCTA	0	3	2	3	3	At5g09590.1:d:+2251:primary
AGTGATAAAA	0	7	3	0	1	At5g08420.1:d:+1004:primary
ATATTCGGTT	1	6	3	1	0	At5g06530.1:d:+2312:primary,At5g06530.2:d:+2406:primary
GATTATGAAT	4	4	0	1	2	At5g06390.1:d:+1680:secondary,At3g21110.1:d:+1559:secondary
ATATCGAGCA	0	6	2	3	0	At5g06110.1:d:+755:secondary
GAGCAGTTTA	0	1	2	2	6	At5g05740.1:d:+1803:primary,At5g05740.2:d:+1716:primary
AGAGGTATTA	3	4	1	1	2	At5g04550.1:d:+2169:secondary
CTTCAAAAAA	4	3	2	2	0	At5g03650.1:d:+1143:secondary,At1g70950.1:d:+737:secondary
AAGTTTTGAA	2	2	5	1	1	At5g03345.1:d:+380:primary
TTTTAACATT	2	5	2	0	2	At5g02810.1:d:+2534:primary
TTTTAAAGTG	5	3	1	0	2	At5g02480.1:d:+2131:primary
TACTTTTAGT	2	3	2	4	0	At4g39350.1:d:+3627:primary
GCTTCTGTTG	0	5	3	1	2	At4g38830.1:d:+1847:primary
TTCGAAATTG	4	3	1	1	2	At4g38660.1:d:+1344:primary
ATAGAACCAA	0	4	3	2	2	At4g37870.1:d:+2358:primary
AGCAGTAAGA	1	2	4	1	3	At4g37460.1:d:+3670:primary
TTTGAAATGT	4	3	2	0	2	At4g36210.1:d:+2174:primary
ATCGTAAACC	3	2	4	1	1	At4g34980.1:d:+2454:primary
TATTAAGTTT	1	2	1	5	2	At4g34190.1:d:+755:primary
GAAGAGTTTA	0	0	4	6	1	At4g34135.1:d:+1552:primary
TGGAAAAGTT	1	2	3	5	0	At4g33240.1:d:+1715:secondary
ATGATATGTT	8	0	2	1	0	At4g31850.1:v:+3804:secondary
TGCTTTCTTT	2	0	3	4	2	At4g31530.1:d:+1093:primary
TTCATTGAAA	3	1	5	2	0	At4g30996.1:X:+580:quaternary,Chr1:+6830823:quaternary
TTTTGTGATA	2	3	0	2	4	At4g29810.1:d:+1306:primary
AGCAGCTCGA	0	8	1	1	1	At4g28610.1:d:+1141:primary
GCCACCAAAA	1	0	4	5	1	At4g28080.1:d:+5736:primary
GCTTTTGTTG	1	8	0	2	0	At4g26710.2:d:+112:primary,At4g26710.1:d:+77:primary
TCGTCTGCTT	2	4	3	2	0	At4g26690.1:d:+2576:secondary
ACATAACGGT	2	1	1	4	3	At4g26530.1:X:+532:quaternary
TTTCTGTCTT	0	1	2	2	6	At4g26370.1:d:+1261:primary,At4g26370.2:d:+1444:primary
ACCCAACCCG	1	7	3	0	0	At4g26130.1:d:+411:primary
TAACCCAAAT	0	0	4	4	3	At4g25640.1:d:+1731:primary
GGGAAAATGG	0	0	9	2	0	At4g23750.2:d:+736:secondary,At3g16770.1:d:+330:secondary,At1g43160.1:d:+383:secondary,At4g27950.1:d:+498:secondary,At4g23750.1:d:+947:secondary
GCGATACATT	1	1	6	2	1	At4g23370.1:v:+1387:secondary
GAGAAAAACA	2	0	5	3	1	At4g22410.1:v:+1167:secondary,At4g22350.1:d:+1308:secondary,At2g28490.1:d:+33:secondary,At1g34540.1:v:+2118:secondary,At4g22290.1:v:+2778:secondary
GTTTTTTTCT	1	2	3	3	2	At4g22330.1:d:+1093:primary
AGAACGTCAT	2	6	2	0	1	At4g22220.1:d:+142:primary
TATGACGATG	3	5	2	0	1	At4g21960.1:d:+1341:secondary
TATTCTCACG	2	2	5	1	1	At4g20330.1:d:+1093:primary
CGTAAGATGA	2	3	0	3	3	At4g18280.1:d:+516:primary
AAGACGGTCC	1	6	4	0	0	At4g18030.1:d:+1888:secondary
CTTTGGTTCC	6	1	2	2	0	At4g17810.1:v:+1042:primary
TCTTGTCCTT	4	2	2	2	1	At4g17100.1:p:+2625:primary
GAATAAGCTT	2	2	3	3	1	At4g16143.1:d:+1847:primary

TTTGATGATA	2	8	0	1	0	At4g15545.1:d:+760:primary
GATTCGTTTA	1	2	4	0	4	At4g15510.1:d:+923:primary,At4g15510.2:d:+1478:primary
AGATTGTGCC	0	11	0	0	0	At4g15210.2:d:+1376:primary,At4g15210.1:d:+1492:primary
TTGGATATTA	1	1	3	2	4	At4g14710.1:d:+866:primary
AATCCGAACT	1	8	2	0	0	At4g14270.1:d:+445:primary
AGAATTGTTT	5	1	1	3	1	At4g13810.1:v:+2553:secondary,At4g13900.1:p:+2509:secondary
CCGGTTTACC	2	5	2	2	0	At4g12730.1:d:+842:primary
TCGGAGGAAT	0	3	3	3	2	At4g12250.1:d:+1603:secondary
AAGAGATTTG	2	3	2	1	3	At4g11090.1:d:+1439:primary,At1g49650.1:d:+1243:primary
GATAAAAAAA	4	1	5	1	0	At4g09470.1:v:+1169:primary
TTTACTTTAA	6	0	2	3	0	At4g08340.1:i:+5656:tertiary,At1g17980.1:i:+2754:tertiary,At5g12040.1:i:+1792:tertiary,At1g17980.2:i:+2734:tertiary,At5g12040.2:i:+1792:tertiary
GATGATCTTG	1	1	4	2	3	At4g07494.1:p:+1499:primary,At3g12720.1:d:+985:primary
TATTGTTCGA	3	1	3	1	3	At4g06509.1:p:+1523:secondary,At4g06510.1:p:+1606:secondary,At2g10210.1:p:+1294:secondary,At3g33121.1:p:+1670:secondary,At1g01610.1:d:+1667:secondary
AATCAAATGT	4	3	4	0	0	At4g03415.1:v:+1888:secondary
GTTCGGGTGA	3	2	2	1	3	At4g03390.1:d:+2778:primary
AAGATGATCA	2	8	0	1	0	At4g01540.1:v:+1551:secondary,At4g18130.1:d:+734:secondary,At5g65040.1:d:+119:secondary,At1g09570.1:d:+923:secondary
CACCGTTCAG	0	6	0	5	0	At4g00730.1:d:+2750:primary
AAGAACGGAC	1	2	4	3	1	At4g00360.1:d:+1684:primary
AAAAAAACAA	0	1	1	5	4	At4g00130.1:v:+1573:primary
TAATGTACAC	3	6	0	0	2	At3g63500.1:d:+3101:secondary,At3g63500.2:d:+3600:secondary
ATCCAAATAC	2	2	3	1	3	At3g62600.1:d:+1406:primary
AATGTTTGAA	5	3	2	1	0	At3g62230.1:d:+1215:primary
AGCTGATGAA	2	3	1	1	4	At3g61890.1:d:+661:primary,At4g04260.1:v:+1203:primary
GCCTGATTTT	3	1	2	3	2	At3g61480.1:d:+1280:secondary,At5g28350.2:d:+1394:secondary,At5g28350.1:d:+1580:secondary
ATTTACCCTA	1	3	3	1	3	At3g60800.1:d:+1524:primary
TGTAAATCTG	7	1	2	0	1	At3g60340.2:d:+1235:secondary,At3g60340.1:d:+1104:secondary
GTCGACAATC	2	6	2	1	0	At3g59940.1:d:+970:primary
GTTTCTTTGA	3	1	3	1	3	At3g59280.1:d:+623:primary
TCTCGTAAAC	2	2	2	3	2	At3g58680.1:d:+738:primary
ATGAGGAGAT	0	5	2	2	2	At3g57340.2:d:+1242:primary,At3g57340.1:d:+1171:primary
TAGAGTGGAA	1	4	2	4	0	At3g56400.1:d:+1009:primary
GGTTAACTCT	0	3	6	2	0	At3g55980.1:d:+1787:secondary
CCCGTGGTCC	1	3	0	6	1	At3g55610.1:d:+2134:secondary
GACGGTCTCG	2	4	2	3	0	At3g55460.1:d:+540:primary
AGACCAAGTC	4	3	2	1	1	At3g55040.1:v:+1389:primary
TGTTCAAACC	2	5	2	2	0	At3g53110.1:d:+1593:primary
ATTGATTTAT	1	2	0	2	6	At3g52840.1:i:+4812:tertiary
TCTGGCTTTT	1	2	0	5	3	At3g52140.1:d:+4509:primary
CTCTCTAGAC	2	2	1	3	3	At3g51620.2:d:+2010:primary,At3g51620.1:d:+2554:primary
TTTGCTCGTT	0	3	5	2	1	At3g51420.1:d:+1421:secondary
TACGAACTCA	4	3	3	1	0	At3g50590.1:d:+4991:primary
AAAGTGATGG	2	5	2	2	0	At3g47460.1:d:+3910:secondary,At5g28823.1:v:+1707:secondary,At1g79250.1:d:+555:secondary
GTTCGTTGAA	2	1	2	5	1	At3g46120.1:v:+1136:secondary
AACAAGAGAA	1	3	3	2	2	At3g44880.1:d:+1825:primary
AAGAGAAAGA	1	5	1	3	1	At3g29580.1:v:+1588:primary
ACTCCAAAAT	1	1	2	4	3	At3g28690.1:d:+2094:secondary,At5g45060.1:d:+194:secondary
TCTTCTCTCT	8	2	1	0	0	At3g27060.1:d:+1212:primary
CTTTCGGTCT	0	0	11	0	0	At3g26520.1:d:+312:secondary
TAAGTTACAT	2	3	1	1	4	At3g26290.1:X:-747:quaternary
GAAGAGGCTG	2	2	4	3	0	At3g26230.1:d:+1440:primary,At3g26220.1:d:+1443:primary
TTAAATTTAA	0	3	2	2	4	At3g26040.1:v:+1885:primary
GTTCTATGGT	1	2	2	4	2	At3g23920.1:d:+1901:secondary
CAGAGTAGTT	2	4	4	1	0	At3g23080.1:d:+1475:primary
CGATCTAAAT	4	4	3	0	0	At3g22970.1:d:+1398:primary
TCACAACTCA	4	1	4	1	1	At3g21630.1:d:+1996:primary,At5g63710.1:d:+1404:primary
AACTTTGAGA	0	0	6	4	1	At3g21250.1:d:+4267:primary

AGTGTTTACT	3	5	1	2	0	At3g19820.1:d:+1624:primary,At3g19820.2:d:+1648:primary
TAATGGAATT	1	2	3	1	4	At3g17860.2:d:+1341:primary,At3g17860.1:d:+1551:primary
TCTCCAATTT	3	4	2	2	0	At3g16480.1:d:+1756:primary
TTAGATAATT	3	0	2	5	1	At3g16290.1:v:+3112:secondary
GTGGGTCGAT	2	0	4	5	0	At3g14770.1:d:+1510:primary
TCTTCTCAAA	2	4	2	1	2	At3g14540.1:d:+310:secondary,At3g14520.1:d:+409:secondary
GAGCTCGCCA	0	0	9	2	0	At3g14415.1:v:+1114:secondary,At3g14420.3:d:+967:secondary,At3g14420.1:d:+834:secondary,At3g14420.2:d:+875:secondary
AACAAAACCA	3	3	2	1	2	At3g14350.3:d:+2497:primary,At3g14350.2:d:+2713:primary,At3g14350.1:d:+2581:primary
TAAAATCACA	3	3	4	0	1	At3g14110.1:d:+1052:secondary,At3g14110.2:d:+1183:secondary
CAAAGCGGTG	1	7	0	2	1	At3g11830.1:d:+1490:primary
TAAGGAAAGC	0	10	1	0	0	At3g09922.1:d:+151:secondary
TGTTCTTTTC	0	7	3	1	0	At3g09840.1:d:+1879:primary
CTACTGAAGG	1	0	1	2	7	At3g09450.1:v:+2523:secondary
AGTCCTAATT	4	0	3	3	1	At3g08680.2:X:+1027:quaternary
CACTAGGAAG	4	3	0	4	0	At3g07760.1:d:+617:primary,At3g07760.2:d:+618:primary
AGGTTTCTCC	1	3	0	5	2	At3g07110.1:d:+245:secondary
AATCTGTCCT	2	3	1	4	1	At3g07100.1:d:+4003:primary
GAATACGCAG	0	4	3	4	0	At3g06650.1:d:+1663:primary
CCGAGAAGAA	2	0	4	3	2	At3g05090.2:d:+2984:primary
TATACATACA	6	2	1	0	2	At3g04910.1:d:+2573:primary
AGAATGGTGG	0	6	1	4	0	At3g04340.1:d:+2943:primary
GATGATTTTG	2	1	4	2	2	At3g03600.1:d:+850:primary
AAAACCATCC	3	3	3	2	0	At3g02780.1:d:+862:primary
AGCCGGAAGA	0	5	3	2	1	At3g02520.1:d:+845:primary
GAATTCAATT	0	5	3	3	0	At3g02330.1:v:+1937:secondary,At1g58310.1:v:+2315:secondary
GAGCTGTCCA	1	3	0	4	3	At3g02230.1:d:+1020:primary
AAGATTCTTT	3	5	1	0	2	At2g48070.1:d:+643:primary
TGATTTGATA	5	2	1	0	3	At2g46390.1:d:+224:primary
TGCTGAAGAA	2	2	4	3	0	At2g46260.1:d:+425:secondary,At5g45650.1:d:+138:secondary
GATCCATCCT	1	2	3	3	2	At2g45670.2:d:+1869:primary,At2g45670.1:d:+1855:primary
CGAATGGTTA	0	3	1	5	2	At2g44860.1:d:+810:secondary
CTTCAAGTTC	1	3	3	2	2	At2g43950.1:d:+987:primary,At2g43950.2:d:+1234:primary,At2g43950.3:d:+922:primary
ATGCACTTGA	1	4	2	4	0	At2g43160.2:d:+2960:primary,At2g43160.1:d:+3000:primary,At2g43160.3:d:+2955:primary
TTGTACAGAG	6	1	1	0	3	At2g43070.1:d:+1959:primary
AAGAAGAAAA	3	1	4	0	3	At2g43000.1:v:+385:secondary,At1g60440.1:d:+20:secondary,At5g07790.1:d:+743:secondary,At3g27330.1:d:+2153:secondary,At5g29646.1:p:+3077:secondary
GTGGTGATTG	1	1	4	1	4	At2g42490.1:d:+1208:secondary
ATTGTAGCCG	2	5	2	2	0	At2g41560.1:d:+3011:primary
CAGAAGCCAT	2	3	1	3	2	At2g38650.1:X:+417:quaternary
ACTTGAATGT	2	5	1	1	2	At2g38440.1:d:+1738:secondary,At2g33630.1:d:+1544:secondary
TGATTGTTAT	0	1	1	3	6	At2g36530.1:d:+1669:primary
AGATTTTTGA	5	1	3	2	0	At2g35520.2:d:+481:primary,At2g35520.1:d:+478:primary
TCCTCCTCTG	1	7	1	2	0	At2g35050.1:d:+3718:primary
TGGGAAATGG	3	2	3	0	3	At2g33430.1:d:+983:primary
TAGTGTTTTT	1	1	3	4	2	At2g32710.1:d:+1071:secondary,At1g79270.1:d:+2252:secondary,At2g32710.2:d:+1025:secondary
TAACCAAAAC	0	3	2	5	1	At2g32580.1:d:+867:primary
TCATCTGTGT	0	3	4	2	2	At2g32380.1:d:+698:primary
GGATTGGCAG	2	5	3	1	0	At2g32180.1:d:+287:primary,At2g32650.1:d:+333:primary,At2g32650.2:d:+432:primary
TGAACCCATT	3	0	0	6	2	At2g29530.1:d:+456:primary
TTCCAAACTT	0	4	2	2	3	At2g29210.1:d:+2829:primary,At5g08370.1:d:+1818:primary
CGAGGAGGAA	1	2	4	2	2	At2g28740.1:d:+271:primary
CAAGATGAAG	2	4	4	1	0	At2g26690.1:d:+696:primary
CCCTCCATTG	1	3	4	1	2	At2g26500.1:d:+682:primary,At2g26500.2:d:+650:primary
CTAAGATCAC	4	2	3	2	0	At2g26430.1:d:+1474:primary
TCTTCTTCCG	2	3	3	3	0	At2g26210.1:d:+863:primary
TTTCTCTTCT	0	10	0	1	0	At2g23370.1:i:+732:tertiary
GTGGAAAACT	2	1	4	0	4	At2g23310.1:d:+1017:primary,At2g23310.2:d:+1014:primary
TATTACAGCT	3	3	1	0	4	At2g23130.1:d:+713:secondary
GAAATTGTAC	2	3	4	2	0	At2g22720.1:d:+2074:primary,At2g22720.2:d:+2169:primary,At2g22720.3:d:+2054:primary

AAATTTGGGA	0	1	3	4	3	At2g21130.1:X:-291:quaternary
TGACTAATAA	4	3	0	2	2	At2g20570.1:d:+1536:primary
TTGATCAATC	2	3	2	2	2	At2g18410.1:d:+779:primary
TATTGGTGTG	2	3	4	0	2	At2g18160.1:d:+1146:secondary
TCAATGCTTC	5	2	1	3	0	At2g17695.1:d:+769:primary
AAAGAGAAAA	3	1	3	1	3	At2g17220.2:d:+1733:primary,At2g17220.1:d:+1727:primary
AACTGATGTC	3	4	1	3	0	At2g17200.1:d:+1814:primary
AGGGATCTTC	2	3	2	4	0	At2g15695.1:d:+1493:primary
TTTTTCCGTT	3	0	3	3	2	At2g14880.1:d:+772:secondary
GCAGAGGTAC	0	0	11	0	0	At2g13360.2:d:+1072:secondary,At2g13360.1:d:+1094:secondary
ATGCATCAAA	0	4	4	3	0	At2g12280.1:d:+113:primary,At1g50480.1:d:+1819:primary
CTTTTTGCTC	2	3	2	2	2	At2g11000.1:d:+2243:primary
GTTCGACAAA	3	5	1	2	0	At2g07783.1:p:+1353:secondary
AAACATCAAA	4	2	2	1	2	At2g03930.2:v:+1162:primary
CCAGTTTTGA	0	5	3	3	0	At2g03890.2:d:+2127:primary,At2g03890.1:d:+2487:primary
AACCTTTTTT	2	2	4	1	2	At2g03470.1:d:+1646:primary,At2g03470.2:d:+1620:primary
TTTCTCTCAA	7	1	1	2	0	At2g03230.1:v:+935:secondary,At5g08270.1:v:+1668:secondary
GGCGAGAAGA	0	5	2	4	0	At2g02160.1:d:+1612:primary
GCGGTTTACT	2	3	2	3	1	At2g02040.1:d:+1824:primary
ATAATTCAGA	1	3	2	2	3	At2g01110.1:d:+1352:secondary
TATCTTGTAG	6	1	2	1	1	At2g01110.1:d:+1200:secondary
TATATTCTTG	5	5	1	0	0	At1g80180.1:X:+759:quaternary
AACTAAAGTC	3	6	1	1	0	At1g80180.1:d:+534:secondary
TTACGAAGAG	2	5	2	2	0	At1g79970.2:d:+1043:primary,At1g79970.1:d:+979:primary
AAAGCCAATC	3	1	2	3	2	At1g79720.1:d:+1599:primary
GGAATGTAAG	4	1	2	2	2	At1g79430.2:d:+1183:primary,At1g79430.1:d:+1273:primary
TAATGCTCAA	1	2	6	1	1	At1g78700.1:d:+1578:primary
CGTTGATGAC	1	7	2	1	0	At1g78680.1:d:+797:primary
TGTACTTATA	1	5	4	0	1	At1g76600.1:d:+880:secondary
ACTCGTAAAG	4	3	4	0	0	At1g76030.1:d:+1265:primary
GTTTCTTCTT	1	1	5	1	3	At1g75240.1:d:+122:secondary,At4g12800.1:d:+482:secondary,At2g20770.1:d:+1552:secondary
TTCTTCTGCC	4	4	3	0	0	At1g74670.1:d:+263:primary
TCTTTGTTGT	1	2	1	2	5	At1g74520.1:d:+638:primary
TTCTCAGGTC	1	8	2	0	0	At1g73655.1:d:+569:secondary
ACTTTATGAA	3	2	4	2	0	At1g72160.1:d:+1737:primary
TACATTTTGG	2	3	1	1	4	At1g72030.1:d:+808:secondary
AAGGTTGATG	0	9	2	0	0	At1g68670.1:d:+1013:primary
GGAATGGTTC	1	4	2	1	3	At1g68370.1:d:+1554:primary
ATGGGATATG	0	9	0	2	0	At1g67280.1:d:+925:primary
AGGTTTTTGC	0	1	4	5	1	At1g67080.1:d:+1008:primary
CCAGAGAAAG	2	1	4	3	1	At1g66900.1:d:+1337:primary
TAACCTGAAG	1	4	2	2	2	At1g65660.1:d:+1701:primary
ACATATCAAG	3	1	0	2	5	At1g65270.3:d:+1313:primary,At1g65270.1:d:+1164:primary
AAGTTGTTTT	3	1	4	1	2	At1g65010.1:d:+4129:primary
CTCCATCAAT	3	0	3	1	4	At1g64490.1:d:+547:primary
TGTTCAACAA	4	2	2	0	3	At1g63910.1:d:+794:primary,At3g20300.1:d:+1644:primary
ACATTGACTT	2	2	3	2	2	At1g63460.1:d:+677:primary
GATATGAAGA	1	0	0	4	6	At1g61800.1:d:+1383:primary
TCACTCCCAC	1	4	4	2	0	At1g59124.1:d:-3514:secondary,At1g58807.1:d:-3463:secondary,At1g66850.1:d:-133:secondary,At1g59124.1:d:-3524:secondary,At1g58807.1:d:-3473:secondary
AAGGTTCTTT	1	7	1	1	1	At1g58030.1:d:+2171:primary
TATTTGTAAC	1	0	2	2	6	At1g54610.1:d:+1989:primary
ATTCGAACAA	0	1	3	4	3	At1g54570.1:d:+2183:primary
AGCCTCTATC	2	3	1	2	3	At1g53910.1:d:+1367:primary
AATCTTGCAA	2	4	2	3	0	At1g53520.1:d:+937:primary
GAGAAGAGAA	3	1	2	3	2	At1g53190.1:d:+1694:primary,At2g15530.2:d:+2670:primary,At2g15530.1:d:+2657:primary
GCTCGTTATC	0	7	2	2	0	At1g52870.1:d:+792:primary,At1g52870.2:d:+792:primary
GTCATTGATG	1	3	3	2	2	At1g51470.1:d:+1412:primary,At1g47600.1:d:+1440:primary
TAGTGAAAGT	1	2	4	2	2	At1g50570.1:d:+1402:secondary
AGTCTCTTTA	2	2	4	2	1	At1g50250.1:d:+2243:primary
TGAAACTAAA	0	1	4	3	3	At1g49450.1:d:+1823:primary

TGATCGGAGA	0	6	3	2	0	At1g45688.1:d:+830:primary,At1g45688.2:d:+950:primary
AAGGCTGTGA	1	4	2	3	1	At1g45000.1:d:+1199:secondary
CATTGGAGGT	0	7	2	0	2	At1g44800.1:d:+1304:primary
GAATTGATTA	2	7	2	0	0	At1g42698.1:p:+249:secondary
ATAAACAAAA	1	2	3	2	3	At1g36310.1:X:+291:quaternary,Chr2:+15073880:quaternary
GTTGTAGTTT	2	0	3	3	3	At1g36310.1:d:+1374:primary
TTTTAAAAAA	3	2	2	1	3	At1g35760.1:p:+2646:secondary
CCAGAGCTCC	1	8	1	1	0	At1g33590.1:d:+675:primary
CCCCCTCCAC	2	7	1	0	1	At1g32200.2:d:+1176:primary,At1g32200.1:d:+1176:primary
AGGAAACTCT	3	3	3	2	0	At1g31930.2:d:+2854:primary
GAGATGAATT	2	1	2	2	4	At1g30000.1:d:+2310:primary
ATAAAGTTCA	0	1	5	1	4	At1g29790.1:d:+1521:primary
AACCTTGCTT	0	6	3	1	1	At1g27100.1:d:+1802:secondary
TCGAAGACGA	4	2	4	0	1	At1g27080.1:v:+2019:primary
AAATGTAAGT	5	3	3	0	0	At1g24100.1:d:+1489:primary,At2g03650.1:p:+1055:primary
TATTGCATCA	5	1	1	2	2	At1g23080.2:d:+2084:secondary,At1g47740.2:d:+1251:secondary,At1g47740.1:d:+1303:secondary
GCTCAAGAAG	0	10	1	0	0	At1g22530.1:d:+120:secondary,At5g31758.1:p:+176:secondary,At3g49990.1:d:+940:secondary
GCCAAGTGGA	0	8	2	1	0	At1g21980.1:d:+651:secondary,At4g33670.1:d:+969:secondary,At2g34880.1:v:+2650:secondary
TGATTGTAGT	2	2	3	2	2	At1g21520.1:v:+888:primary
AAATGGCCTT	3	5	1	0	2	At1g21400.1:d:+1611:secondary
AAAGCCTACA	2	7	1	1	0	At1g20696.1:d:+483:primary
AAAAGTTTTT	1	1	2	5	2	At1g20450.2:d:+1498:primary,At1g20450.1:d:+1501:primary
ATGCAAACGC	2	6	1	2	0	At1g19740.1:d:+372:primary
AAGAATAAAG	2	0	2	3	4	At1g19580.1:d:+1312:primary
CTTTGTTCAA	2	1	2	1	5	At1g19450.1:d:+1814:primary
GATTAATTAA	2	5	4	0	0	At1g19020.1:d:+480:primary
TAGTTTCTTT	1	2	2	4	2	At1g17940.1:i:+1247:tertiary
CACGTTAAGT	0	5	5	1	0	At1g17710.1:v:+1270:secondary
TCAATTGATA	4	1	3	0	3	At1g17650.1:d:+1142:primary
TAAATATTTG	5	5	0	1	0	At1g15280.2:d:+2217:secondary,At1g15280.1:d:+2236:secondary
ACCCGCTAAG	1	5	2	1	2	At1g14870.1:d:+516:primary
CAGAGAGTGT	1	3	3	2	2	At1g14810.1:d:+907:secondary
TCGCTTGCAC	0	5	1	4	1	At1g14650.1:d:+2440:primary
GAAAGGATTC	5	0	2	2	2	At1g14345.1:d:-690:secondary
TAAAATGTCT	5	3	2	1	0	At1g14280.1:d:+1396:primary
TGCTTCCAAG	2	6	1	2	0	At1g14210.1:d:+680:primary,At5g01030.1:d:+2421:primary,At5g01030.2:d:+2463:primary
TGTTTGGGGA	2	4	2	2	1	At1g12860.1:v:+2984:secondary,At4g15180.1:d:+6164:secondary
ATGGGGTATG	0	6	3	1	1	At1g11840.3:d:+638:primary,At1g11840.2:d:+628:primary,At1g11840.1:d:+663:primary
ATTTCCTGAC	2	1	2	2	4	At1g11475.1:d:+491:secondary
AATGGATTAA	2	2	0	2	5	At1g11390.1:d:+1470:secondary
AGTGTACCAA	3	3	2	3	0	At1g10360.1:d:+861:secondary
GAAATCTCTG	5	1	1	1	3	At1g09130.1:i:+2287:tertiary
AATAAAAAAG	1	3	3	4	0	At1g08830.1:d:+764:primary
TAAAGAAAGA	1	5	3	1	1	At1g08070.1:d:+2385:primary,At5g04050.1:d:+2874:primary
TCCTGAGAAT	4	4	2	1	0	At1g07570.2:d:+1567:primary
TACCCACTTT	2	5	3	1	0	At1g07230.1:d:+1858:primary
ATTGGTAATT	4	2	3	1	1	At1g06400.1:d:+858:primary
AATAAAAGTC	2	1	2	5	1	At1g06180.1:d:+890:primary
AAACGTTGAT	2	2	4	2	1	At1g04940.1:i:-1484:tertiary
TTTAGTCCAA	3	1	4	3	0	At1g03120.1:v:+1360:primary
GGTTCCAGAT	1	0	1	7	2	At1g02820.1:d:+347:primary
TTCCTTCAAA	2	1	3	2	2	No gene matches found
GTTAGCTCTA	5	0	3	2	0	No gene matches found
CTCTAAAAAA	2	1	4	2	1	Chr5:+25415867:quaternary,Chr2:+11724880:quaternary
AAAAAATGGA	1	4	1	2	2	Chr5:+21612610:quaternary
ATTGTTGTGC	2	1	2	3	2	Chr4:-15503975:quaternary
TCCTCTTGTT	1	9	0	0	0	Chr3:+15717685:quaternary
CATAGGTAAA	5	0	1	0	4	Chr3:+11819875:quaternary
TTGTTATGAA	1	4	1	3	1	Chr1:+18142615:quaternary,Chr2:+9447245:quaternary
AAAGAATTTT	1	5	1	1	2	Chr1:+13655813:quaternary,At4g23910.1:X:+289:quaternary
TATCCCTGTG	1	6	2	0	1	At5g67490.1:d:+597:primary

CAACGTGTAA	0	6	3	0	1	At5g67480.1:d:+968:primary,At5g67480.2:d:+1078:primary
TAGATTTTTG	4	1	2	3	0	At5g65840.1:d:+999:primary
TATGTATTAT	3	3	1	0	3	At5g65120.1:d:+1168:primary,At5g35735.1:d:+1369:primary
GTTTGTTTTA	1	4	0	4	1	At5g65020.1:d:+1091:primary
TATGGCTTGA	2	5	2	1	0	At5g64300.1:d:+1301:primary
AGCAAATTGT	2	1	2	1	4	At5g60960.1:d:+1695:secondary
GTAGTGGTCG	4	2	1	1	2	At5g59780.3:d:+728:primary,At5g59780.1:d:+884:primary,At5g59780.2:d:+789:primary
AAACCACATA	5	2	1	2	0	At5g59370.1:d:+908:secondary
TGTTGTTGTA	3	1	4	2	0	At5g58870.1:d:+2644:primary
CATTATTTTG	4	3	1	0	2	At5g58060.1:d:+835:primary
GAGTGGTTGC	4	3	1	1	1	At5g57850.1:d:+1329:primary
GTAATCCAAG	2	3	3	1	1	At5g56600.1:d:+331:primary
TACAATGAAA	3	5	2	0	0	At5g56210.1:v:+2162:primary
CGGGAGAAGG	0	2	0	5	3	At5g55920.1:d:+2087:primary
GACGGATTTT	0	3	0	3	4	At5g55920.1:d:+1724:secondary
TAACGATAAT	2	4	3	1	0	At5g55280.1:d:+1194:primary
AACTTATCGA	1	4	2	2	1	At5g54960.1:d:+643:secondary,At3g05690.1:d:+930:secondary,At5g64770.1:d:+292:secondary
AACGAACAAT	3	2	2	1	2	At5g54870.1:d:+1557:secondary,At2g09850.1:p:+1178:secondary
GATTATGCGA	4	4	0	1	1	At5g54510.1:d:+1789:primary
AACTTGTGTT	3	1	3	1	2	At5g53650.1:d:+480:primary
GAAAGAAGGT	1	3	2	3	1	At5g52960.1:d:+552:primary
ACTCTTTCTT	2	0	7	1	0	At5g52860.1:d:+348:secondary,At5g44530.1:v:+622:secondary
GCTGGGTTTG	2	2	2	3	1	At5g52510.1:d:+1912:primary
GAACGGGGCT	4	4	1	1	0	At5g51020.1:d:+657:primary
AAACTGTTTG	3	5	1	1	0	At5g48930.1:d:+1323:secondary
ACAACTTGCC	1	1	1	3	4	At5g48540.1:d:+679:primary
GATGTCTTTA	1	1	2	4	2	At5g47880.1:d:+1698:primary
AAATTGTATA	2	4	1	0	3	At5g47780.1:d:+1987:primary
TTGATCAATA	0	1	2	1	6	At5g46970.1:v:+1322:primary,At2g35960.1:d:+814:primary
AAGTATCATC	3	1	0	2	4	At5g46580.1:d:+2136:primary
CAACTTCCAC	3	5	2	0	0	At5g46290.1:d:+1200:primary
ATGATACTTA	2	2	1	2	3	At5g45130.1:d:+966:primary
AAAGAATTTC	0	0	1	5	4	At5g44110.1:d:+1029:primary
TAAAAACGGT	1	2	3	3	1	At5g44100.1:d:+2080:primary
GAACCTTGAA	2	3	1	2	2	At5g42960.1:d:+719:primary
GGGGCTGGAG	1	1	8	0	0	At5g42680.1:d:+579:secondary,At2g30570.2:d:+313:secondary,At2g30570.1:d:+313:secondary
TCCATTTCCT	3	3	3	1	0	At5g42100.2:d:+1263:primary,At5g42100.1:d:+1243:primary
AACTCCGATC	2	1	3	3	1	At5g41685.1:d:+257:primary
AATCTTTTTA	3	1	2	2	2	At5g41520.1:d:+786:primary
GTGATTGCTA	0	1	2	3	4	At5g41010.1:X:+108:quaternary
GTCTGATCCT	0	7	2	0	1	At5g40890.1:d:+2236:secondary,At1g43444.1:p:+1986:secondary,At1g36720.1:p:+1599:secondary
ATAAAAGAGC	0	7	1	1	1	At5g40450.1:d:+6736:secondary
GAAAGACAAA	3	2	2	2	1	At5g40200.1:d:+1930:primary
GCCAAAATCC	0	8	0	2	0	At5g39160.1:d:+346:primary,At5g39190.1:d:+346:primary,At5g39130.1:d:+340:primary
CTCTTCGTTG	3	3	1	2	1	At5g37720.1:d:+1108:primary
GCTCTTAAGT	1	2	6	1	0	At5g37380.1:d:+1812:primary,At5g37380.2:d:+1728:primary
CCTTACAAAA	0	0	7	2	1	At5g37260.1:v:+1146:primary
AAACTGTCAA	3	2	2	1	2	At5g36660.1:p:+1961:secondary,At5g36737.1:p:+1961:secondary
GGATCTATTC	3	4	2	1	0	At5g35790.1:d:+1646:primary
CTCGGGATGA	1	8	0	0	1	At5g30440.1:p:+993:primary
TCTTTTGTTA	5	4	1	0	0	At5g28770.1:d:+1146:primary,At5g28770.2:d:+1113:primary
ATGAGAAAGA	3	0	5	2	0	At5g28580.2:p:+1725:secondary,At2g43710.1:d:+1034:secondary,At2g43710.2:d:+1034:secondary
GAGAGAGTGA	3	4	3	0	0	At5g28500.1:d:+806:primary
GTCATTTTGT	3	2	0	2	3	At5g27760.1:X:+364:quaternary,Chr4:+14785385:quaternary
AAGGAACCAA	0	0	4	3	3	At5g27630.1:d:+2360:secondary
CTTGGCTTGC	1	4	2	1	2	At5g26760.2:d:+2461:primary,At5g26760.1:d:+2671:primary
CGATTTTATA	1	3	3	2	1	At5g26667.2:X:+225:quaternary
TCATAACCGA	4	0	3	0	3	At5g24300.1:d:-2327:secondary
TAATTTTCTG	0	0	1	3	6	At5g24160.1:X:-486:quaternary
ATAAAAATAA	1	4	0	3	2	At5g23890.1:d:+2981:primary
AGCATTTTTC	0	1	2	6	1	At5g23630.1:X:+1194:quaternary

AGCATAATTT	0	5	0	2	3	At5g22210.2:X:+535:quaternary
TCTCAAGTTT	2	2	2	2	2	At5g20730.1:d:+4182:secondary,At5g20730.3:d:+4137:secondary,At1g60130.1:v:+354:secondary,At5g20730.2:d:+4108:secondary
GGCACCGCAA	0	0	0	6	4	At5g17050.1:d:+1114:primary
CGATCCTTAT	1	3	1	2	3	At5g16990.1:d:+327:secondary,At1g73030.1:d:+771:secondary,At5g19580.1:d:+387:secondary
TAAACTATAA	7	1	1	1	0	At5g16400.1:d:+834:primary
GCCGTTCCAA	3	4	1	2	0	At5g16250.1:d:+673:primary
GGAGTGTCAA	4	1	3	2	0	At5g16040.1:X:-329:quaternary
TAAGAAGGAA	4	4	2	0	0	At5g15680.1:v:+6958:secondary
GAATTTACTT	0	5	3	1	1	At5g13980.1:d:+2451:primary,At5g13980.2:d:+2451:primary
TATATGTATA	4	5	0	0	1	At5g13655.1:v:+1572:primary,At5g40760.1:d:+1784:primary,At5g56870.1:d:+2419:primary,At1g55280.1:d:+1365:primary,At5g02680.1:v:+1210:primary
TTTGTTTGTC	3	1	0	2	4	At5g13640.1:d:+2716:primary
TGCAAATACT	3	3	2	1	1	At5g13240.1:d:+980:primary
TACTGGAAAA	2	1	3	2	2	At5g13190.1:d:+669:secondary
GCGATTAAGG	5	0	0	1	4	At5g10830.1:d:-1071:secondary
CATCAGATTG	0	7	0	3	0	At5g10780.1:d:+546:primary
TTGTTGTTGT	2	3	3	2	0	At5g07260.1:d:+1777:primary,At1g52150.2:d:+3298:primary,At5g40590.1:d:+818:primary,At1g52150.1:d:+3295:primary,At3g01670.1:d:+2639:primary
AGAGGGTTTG	2	1	4	3	0	At5g06140.1:d:+1267:primary
GTGTCTTTCT	3	2	4	1	0	At5g05700.1:d:+2079:secondary
GAGATATCTT	4	1	1	2	2	At5g05370.1:d:+225:primary
AACTGGGAAA	2	4	3	1	0	At5g04490.1:d:+876:primary
GTGTCTGCTC	1	3	4	1	1	At5g04410.1:d:+1912:secondary
ATCGCATATA	3	2	1	1	3	At5g02310.1:d:-6188:secondary
AAACTTGTCT	2	0	3	3	2	At4g39980.1:d:+1893:primary
TACATAGGTT	4	5	0	1	0	At4g37610.1:d:+1357:primary
ACTTGCTTTG	1	4	1	2	2	At4g36970.1:d:+1460:primary
AGTTTGTTTG	3	1	5	0	1	At4g36900.1:d:+957:primary
GTCTGAGGCG	2	2	3	2	1	At4g36860.1:d:+1830:primary,At4g36860.2:d:+1804:primary
ACCGGTGGAT	0	4	5	1	0	At4g36800.1:d:+701:secondary
AGACAGGAAG	0	3	4	3	0	At4g36690.3:d:+1607:primary,At4g36690.1:d:+1607:primary,At4g36690.2:d:+1607:primary
ATGAATTCAA	3	1	1	3	2	At4g36680.1:d:+1573:primary
GCGGATTTTC	2	3	1	4	0	At4g36540.2:d:+843:primary,At4g36540.1:d:+849:primary
ATGAAGTCCC	0	1	5	3	1	At4g36530.2:d:+1208:primary,At4g36530.1:d:+1088:primary
CTAAATTACT	1	1	4	1	3	At4g36220.1:X:-852:quaternary
TTAAAAAGGT	3	2	1	1	3	At4g36080.1:d:+7117:secondary,At1g51200.1:d:+761:secondary
TGGGGAGAAA	0	1	2	4	3	At4g35300.1:d:+2528:primary,At4g35300.2:d:+2498:primary
AAATCCGCAG	2	1	3	3	1	At4g35090.1:d:+1457:secondary
TGCAGAATCT	1	4	4	0	1	At4g34530.1:d:+1052:primary
CAACACCGGT	1	2	4	1	2	At4g33000.2:d:+823:primary,At4g33000.1:d:+788:primary
GTATACAAAG	0	10	0	0	0	At4g32480.1:d:+1092:primary
TACCCGGTTG	1	6	2	0	1	At4g32285.1:d:+1023:primary
TGAATTCTTG	1	5	1	3	0	At4g32040.1:d:+1254:primary
GCAAGAGATG	1	3	4	2	0	At4g30210.1:d:+2147:primary,At4g30210.2:d:+2147:primary
GTGTCAGAAT	2	8	0	0	0	At4g29580.1:v:+1690:primary
ATTCTCTCTT	4	2	2	1	1	At4g29480.1:d:+521:primary
ATCTTGCTCT	1	3	1	3	2	At4g28440.1:d:+687:primary
TGGTGGTTGT	4	3	2	1	0	At4g27710.1:d:+1777:primary
TTTCTAAGTA	2	0	1	5	2	At4g27560.1:d:+1537:primary
GCAAGCTTAG	1	4	2	3	0	At4g27450.1:X:-270:quaternary
GCCGGAAACC	0	9	1	0	0	At4g27280.1:d:+157:primary
GGAAGCAAAG	3	2	3	0	2	At4g25620.1:d:+1776:primary
TCTTACTTGG	3	4	0	1	2	At4g24830.1:d:+1557:primary
TACAAAAAAA	0	3	1	2	4	At4g24560.1:d:+2148:secondary
AAGTTTGTAG	3	1	2	3	1	At4g23860.1:d:+1710:primary,At4g23860.2:d:+1718:primary
GAAGCAGCCA	0	6	1	3	0	At4g23170.1:d:+632:primary
GCTGCTCTTC	1	5	1	2	1	At4g22670.1:d:+1369:primary
TCCCAGAAAC	0	6	1	2	1	At4g22490.1:d:+146:primary
TAAGTTATGT	0	0	6	2	2	At4g22030.1:i:+1480:tertiary
AAAATGTGGA	0	10	0	0	0	At4g20850.1:d:+3041:secondary

AAACCCTTTT	1	2	2	4	1	At4g19960.1:d:+2492:primary,At3g10120.1:d:+740:primary
AACAAAAAAT	0	1	2	5	2	At4g19620.1:v:+21:secondary
TTAGTTTCAC	2	3	1	3	1	At4g18590.1:d:+500:primary
AAGACAGTCG	2	5	0	2	1	At4g16760.1:d:+1483:primary
TTTTGATTAA	2	0	2	2	4	At4g16620.1:i:+581:tertiary
TATTGATGGT	2	3	0	2	3	At4g16490.1:d:+1662:primary
CGTAACGTTT	0	9	1	0	0	At4g16260.1:d:+478:secondary
GAGCTGTTGA	6	1	0	2	1	At4g14810.1:v:+436:secondary
ATGCTGGATG	0	4	3	2	1	At4g13640.1:d:+975:secondary
TGTTGAAGAA	2	6	1	1	0	At4g13360.1:d:+1337:secondary,At3g25810.1:v:+589:secondary,At2g11610.1:p:+797:secondary,At3g18060.1:d:+376:secondary,At1g31970.1:d:+582:secondary
AAGCTAGATG	4	4	2	0	0	At4g12880.1:d:+435:primary
TGGGTCATTG	1	3	2	3	1	At4g12110.1:d:+1240:primary
TTTGGTCTTT	1	0	3	0	6	At4g10450.1:d:+800:primary
GACTTACAGT	0	6	1	1	2	At4g10300.1:d:+333:secondary
GCTATATTGA	1	4	2	1	2	At4g08180.2:d:+2948:primary,At4g08180.1:d:+2951:primary,At4g08180.3:d:+2948:primary
ATTCATCAAA	1	5	3	1	0	At4g07803.1:X:+1260:quaternary,Chr3:+3591206:quaternary
CCGCGGTGAA	3	5	1	1	0	At4g06566.1:p:+4393:secondary
CCAAGTTTGG	0	7	2	1	0	At4g06477.1:p:+3960:secondary
TAGAAATTTT	0	2	6	2	0	At4g03110.2:d:+1797:primary,At4g03110.1:d:+1709:primary
AAACATAGTC	1	7	1	0	1	At4g02770.1:X:-758:quaternary
GAGTCTCTTC	0	5	2	2	1	At3g60320.1:d:+2490:primary
AGATCATAAT	3	0	0	3	4	At3g59140.1:v:+4866:secondary
TGTTGGGGGG	1	3	3	1	2	At3g59090.1:d:+1513:primary,At3g59090.2:d:+1430:primary
GATGAATTCC	2	5	1	1	1	At3g59020.1:d:+2956:secondary
ATATTCCCAG	3	1	3	3	0	At3g58170.1:d:+583:primary
ATCAAATCAA	1	3	2	2	2	At3g57650.1:d:+1613:primary,At2g02950.1:d:+1501:primary
TTGTATCTAC	0	0	2	1	7	At3g57020.1:d:+1170:primary
TAAATGAGTC	3	1	3	0	3	At3g56500.1:v:+1133:primary,At1g55260.1:d:+700:primary
TCTCGATTAT	4	2	2	2	0	At3g56190.1:d:+1335:secondary
TCCGACTCTT	3	4	1	1	1	At3g56190.1:d:+1111:secondary,At2g24750.1:p:+2007:secondary
AAATGGCTAA	0	1	9	0	0	At3g55980.1:d:+2119:primary
AAAAAGATTG	2	0	1	3	4	At3g55280.1:d:+374:primary
TTTACTTTTT	0	2	2	1	5	At3g55250.1:d:+875:secondary
TTTCAAAAAC	4	0	2	2	2	At3g54680.1:d:+1051:primary
TCTCGTTGAT	1	2	2	2	3	At3g54470.1:d:+1590:primary
CATAGTTGAC	2	1	1	5	1	At3g54350.2:d:+2706:primary,At3g54350.1:d:+2566:primary
AAACAGAAAG	3	3	1	1	2	At3g54170.1:d:+1169:primary,At4g10950.1:v:+1754:primary
TTTGTTTATT	2	2	1	2	3	At3g53630.1:d:+963:primary
CTCTGGACAA	3	4	1	1	1	At3g53470.2:d:+385:primary
TTATCTTTCA	4	5	0	0	1	At3g53280.1:d:+1649:primary
TTCAATCAGC	2	1	0	6	1	At3g53260.1:d:+1603:primary
TAAATGAATC	3	4	3	0	0	At3g52850.1:d:+2115:primary
GTAGTAGCTA	3	5	1	0	1	At3g52070.1:d:+377:primary
GATTTCATCA	2	4	3	1	0	At3g51840.1:d:+1256:primary
ACGAAATTAA	2	2	2	3	1	At3g50370.1:X:-785:quaternary
GAAGTCTGCA	1	3	5	0	1	At3g49890.1:d:+705:primary
TGTGGTTGGT	3	2	1	2	2	At3g49260.2:d:+1698:secondary,At3g49260.1:d:+1612:secondary
TGATTGTTGT	1	5	3	0	1	At3g48780.1:d:+1807:primary
TAACAACAAT	2	2	2	1	3	At3g48380.2:d:+1986:primary,At3g48380.1:d:+2001:primary
GAGCTCCTCT	2	6	1	0	1	At3g44890.1:d:+103:primary,At1g01640.1:d:+706:primary,At1g01640.2:d:+664:primary
ATCATCCATC	4	2	1	2	1	At3g44690.1:v:+706:secondary
ATTTGTAAAG	1	2	1	4	2	At3g44190.1:d:+1460:primary
TTTCTTCTTT	4	3	0	2	1	At3g43240.1:d:+2567:primary,At2g03750.1:d:+1200:primary
GTATCAAAAG	3	0	1	1	5	At3g29800.1:v:+8:secondary,At4g13550.1:v:+2840:secondary
TAGTAAACGT	1	2	5	2	0	At3g28730.1:d:+2070:secondary
TATGTTTGAA	4	2	2	1	1	At3g27370.1:v:+605:primary,At5g14540.1:d:+1830:primary
GAGAAGAAAA	3	1	0	2	4	At3g27010.1:d:+1180:primary
ACATAAAATC	3	6	1	0	0	At3g26740.1:d:-511:secondary,At1g10480.1:d:-1527:secondary
TCTTCATTTG	2	1	3	1	3	At3g24590.1:d:+928:primary,At5g62550.1:d:+1720:primary

GACTACTAGA	1	2	1	2	4	At3g23990.1:d:+1811:primary
GCAACGGTTA	0	5	2	3	0	At3g21650.1:d:+2107:primary
GATAATGATA	4	0	2	0	4	At3g21250.1:d:+4230:secondary
GCATCACACA	1	6	3	0	0	At3g20910.1:d:+411:secondary
TTTGATGTTG	4	2	2	2	0	At3g20100.1:d:+1591:primary
TCGTAACAAT	1	0	2	0	7	At3g19740.1:d:+1386:primary
AATTTGCTCA	4	1	1	2	2	At3g19720.1:d:+2418:primary,At3g19720.2:d:+2310:primary,At3g59990.2:d:+1649:primary,At3g59990.1:d:+1583:primary
TACACTCGAT	3	2	2	1	2	At3g19490.1:d:+1994:secondary
TCCAAGACCT	1	6	3	0	0	At3g19010.1:d:+691:primary,At3g19010.2:d:+691:primary
TGAAAGTGTA	3	2	1	3	1	At3g17650.1:d:+2286:primary
GATCGTTATG	2	1	4	0	3	At3g15210.1:d:+899:secondary
GACTTAGATT	2	1	4	3	0	At3g13750.1:d:-3048:secondary
TCTGCAAAGG	1	8	1	0	0	At3g13410.1:d:+286:primary
TCTTTACGTC	2	1	2	3	2	At3g13235.1:d:+1473:primary
ATCTCTTAAG	0	7	2	1	0	At3g10970.1:d:+1343:primary,At3g10970.2:d:+1343:primary
AAAACAAAAG	2	2	2	1	3	At3g10410.1:d:+1597:primary,At3g06710.1:v:+1452:primary
AGGAACGTTA	1	4	2	3	0	At3g10090.1:d:+210:primary
CTAGATTTAC	1	0	2	4	3	At3g09650.1:X:+2555:quaternary
GCAATTCCAA	1	4	2	3	0	At3g09300.1:d:+1544:primary
TACAAGACAG	1	4	2	2	1	At3g08690.1:d:+482:primary
TAGGGCTTTC	3	2	1	3	1	At3g07810.1:d:+2377:primary,At3g07810.2:d:+2310:primary
GTCTATCAGC	3	1	2	1	3	At3g06670.1:d:+3152:secondary
CAAAGAAGCG	5	1	2	2	0	At3g06380.1:d:+1556:primary
ACAGCTCTAT	0	1	7	1	1	At3g05800.1:d:+709:primary
TTGTGTAGCC	6	3	1	0	0	At3g04740.1:d:+5448:primary
TGTAAGGTTG	4	1	3	2	0	At3g04520.1:d:+1208:primary
AACTTGAAGG	1	8	1	0	0	At3g02170.1:d:+3009:primary
CCCCTATTGA	2	3	2	0	3	At3g01860.2:d:+507:primary,At3g01860.1:d:+782:primary
TATAGAATGA	5	0	2	1	2	At3g01810.1:d:+3090:primary

CGAGTGCACT	1	4	1	2	2	At2g47390.1:d:+3035:secondary
TGTGTACACT	3	3	2	1	1	At2g46540.1:d:+417:primary
GTCCTCAAAG	1	2	3	2	2	At2g45790.1:d:+1016:primary
TGTTGGCAAA	1	4	1	2	2	At2g45160.1:d:+1994:primary
AAAATGGAAA	1	1	5	3	0	At2g44900.1:v:+3516:primary,At2g30560.1:v:+1298:primary
GTGCGACTTT	1	1	4	2	2	At2g44525.1:d:+673:primary
GTGGCAATGC	2	2	2	3	1	At2g43820.1:d:+1127:primary
TATAATGGTT	3	4	1	1	1	At2g43530.1:d:+313:primary,At3g12710.1:d:+1256:primary
GAAAAGAAGA	1	4	2	1	2	At2g43350.1:d:+809:primary,At3g63070.1:d:+4420:primary,At1g19840.1:v:+1176:primary
CAGTGTCTCG	2	8	0	0	0	At2g43060.1:d:+501:primary
TGATTTTTGT	3	3	3	1	0	At2g43010.1:d:+1733:primary,At2g43010.2:d:+1727:primary
CAGGCCGCGA	2	4	4	0	0	At2g43010.1:d:+1404:secondary,At2g43010.2:d:+1404:secondary
TAAAGATCTC	2	3	1	2	2	At2g41960.1:d:+4005:primary
AACAATGAGT	4	2	1	1	2	At2g41120.1:d:+873:secondary,At4g01540.1:v:+1449:secondary
GTATTAAAGT	1	2	3	3	1	At2g40490.1:d:+1214:primary
AGCTGAGGGA	1	2	1	4	2	At2g39390.1:d:+139:primary
GTAGAGAACC	1	3	3	2	1	At2g38800.1:d:+1784:primary
AGGAAAAAAA	2	1	4	2	1	At2g38770.1:d:+4756:primary
TAACATTTTC	3	1	4	2	0	At2g37450.1:d:+1332:primary
CTGCTTGACG	0	6	1	3	0	At2g36910.1:d:+3701:primary
GTGGAGGTGC	0	1	4	2	3	At2g36880.1:d:+951:secondary
TCATCATCGA	0	1	8	0	1	At2g35910.1:d:+544:primary
ACTCTGCTTT	2	3	1	1	3	At2g35790.1:d:+751:primary
TCTTTATCTT	1	1	2	0	6	At2g35110.1:d:+4177:primary
AACTTCTGAG	4	3	1	2	0	At2g33600.1:d:+1192:primary
TTCCAGAAAA	1	1	4	4	0	At2g33300.1:v:+529:secondary
TCTTGGTTTC	1	5	1	2	1	At2g31440.1:v:+1161:secondary,At3g54270.1:d:+1439:secondary
CTTTTAGGAC	1	3	4	0	2	At2g29190.1:d:+3580:primary
AATTTGTAAC	4	3	2	0	1	At2g28990.1:v:+3193:secondary,At2g26270.1:v:+1832:secondary,At5g56460.1:d:+292:secondary
GGTTTGTGTT	4	2	3	1	0	At2g28950.1:d:+1041:primary

CCCGAGTTAT	1	5	1	1	2	At2g28550.2:d:+1402:primary,At2g28550.1:d:+1402:primary
GCTTGATGCA	3	3	2	2	0	At2g27190.1:d:+1413:primary
TATGGGATCA	3	2	2	0	3	At2g26910.1:d:+4399:secondary,At4g28530.1:d:+1136:secondary
TAAAGTATTA	4	1	1	0	4	At2g26330.1:d:+3084:primary
CAAATGGGAT	1	6	3	0	0	At2g25290.1:v:+654:secondary,At4g18200.1:v:+3842:secondary
TTGTGTTGTT	3	3	2	1	1	At2g25060.1:d:+682:primary
TGTTAAGTAT	6	1	1	1	1	At2g24860.1:d:+642:secondary
TGTTTAGCTT	0	6	0	0	4	At2g24390.1:d:+684:primary
GAAAGAACCA	0	3	1	2	4	At2g24120.2:d:+3327:primary,At2g24120.1:d:+3201:primary
TCAAAGGCAA	4	3	2	1	0	At2g22430.1:d:+1410:secondary
GTGCTTGAAG	4	0	1	3	2	At2g21970.1:d:+740:secondary
AAAATGTATT	5	2	2	1	0	At2g21340.1:d:+1866:primary,At2g21340.2:d:+1857:primary
CTTGCCTCGG	0	3	4	3	0	At2g20960.1:d:+1956:primary
GTTGTTGATC	2	2	5	0	1	At2g20630.1:d:+1249:primary
AAAAGTACGT	1	7	1	1	0	At2g20470.1:v:+2280:primary,At1g03900.1:d:+462:primary
CTGGAAGAAA	2	3	5	0	0	At2g19790.1:d:+414:primary
TAATAGTGTC	4	2	3	1	0	At2g17390.1:d:+1174:primary
GGTCGTGGAA	2	1	3	1	3	At2g17340.1:d:+986:primary
AGTTAGCTGA	0	9	1	0	0	At2g17230.1:d:+869:primary
TACCAACAAA	4	3	2	0	1	At2g16800.1:d:+1261:primary
GCTCGAAGCT	1	4	2	2	1	At2g16740.1:d:+570:secondary
GTGCTACATA	3	1	1	3	2	At2g16070.2:d:+1133:primary,At2g16070.1:d:+1336:primary
GGATGCTTAA	1	1	1	4	3	At2g15620.1:d:+1637:primary
AAAAAGTGGA	4	2	3	1	0	At2g14080.1:v:+4259:primary
GCATTTCTCA	0	1	8	1	0	At2g11290.1:p:+1507:primary
GAAGCTGCTC	2	6	1	1	0	At2g06925.1:d:+495:primary
GTTTTTGTCT	0	4	1	4	1	At2g06290.1:p:+2154:secondary
TCTGATGAGG	2	5	2	1	0	At2g04520.1:d:+454:primary
GGATGTTTCT	3	1	3	3	0	At2g01290.1:d:+1118:primary,At1g71100.1:d:+822:primary
TAGTGCTTTA	2	1	3	1	3	At2g01190.1:d:+2476:primary
ATACAAGAAG	3	5	0	2	0	At1g80940.2:d:+856:primary,At1g80940.1:d:+817:primary
AAAATCTAAG	2	1	4	1	2	At1g80180.1:X:-754:quaternary
AAAACGATGC	3	5	1	1	0	At1g79850.1:d:+181:secondary
TACAGCCCTT	3	6	0	0	1	At1g79750.1:d:+2119:secondary
ACAAAATATA	0	5	2	2	1	At1g78850.1:d:+1154:primary
TGATTTTATT	5	2	2	0	1	At1g76670.1:d:+1609:primary,At1g79060.1:d:+1762:primary
TGCGTATGCG	4	0	4	2	0	At1g76490.1:d:+1963:secondary
TGCTTCACTA	1	5	2	2	0	At1g75380.1:d:+232:secondary,At1g75380.3:d:+219:secondary,At1g75380.2:d:+227:secondary
ATCAAGGCGG	2	2	2	1	3	At1g75300.1:d:+703:primary,At1g75660.1:d:+3174:primary
TTATCTTTTG	3	1	1	1	4	At1g75210.1:d:+2203:primary
GGTTTATGTT	3	0	2	2	3	At1g74740.1:d:+2134:primary,At3g28005.1:p:+669:primary,At3g29805.1:p:+539:primary
TGTGTGTTCT	8	2	0	0	0	At1g73920.2:d:+2523:primary,At1g73920.1:d:+2518:primary
CGTAATTTCC	1	2	3	2	2	At1g73660.1:d:+3821:primary
TTTTACGTGC	3	2	2	0	3	At1g72710.1:d:+1766:primary
GGTCTTCTTG	0	2	6	0	2	At1g71260.1:d:+875:primary
AAGACAATAT	3	2	1	1	3	At1g71040.1:d:+1804:primary
ATTCATTCGT	2	1	3	2	2	At1g70600.1:d:+693:primary
GTTGATAGAG	2	1	3	0	4	At1g69295.1:d:+1466:primary
TTTTGCTTTA	4	4	2	0	0	At1g68720.1:d:+4190:primary
AATCTACCAT	5	0	3	1	1	At1g68010.1:d:+1471:primary
AAAACAAAAC	1	0	5	2	2	At1g67160.1:v:+598:secondary,At4g12810.1:v:+631:secondary
AGAAGCTTTT	3	0	3	1	3	At1g66670.1:d:+1174:primary
CAGACGTAGT	1	3	4	2	0	At1g65490.1:d:+234:primary
GTCGCATAAG	1	4	2	1	2	At1g64810.1:d:+1513:primary
CCAAGGATGG	3	2	4	1	0	At1g63770.1:d:+2724:primary,At1g63770.2:d:+2720:primary
GTGTCTAGAA	0	0	2	5	3	At1g62570.1:d:+1607:primary
CTGTTCGAAA	1	6	2	0	1	At1g62390.1:d:+2308:primary
ACTGCATCTC	0	3	6	1	0	At1g61100.1:d:+2505:primary
GATGTTATTA	4	5	0	0	1	At1g58180.2:d:+855:primary
GCTATGGCGG	0	10	0	0	0	At1g56200.1:d:+41:primary
ATAAGATTCC	0	6	2	2	0	At1g55920.1:d:+1140:primary

TGATGTCAAA	3	1	4	2	0	At1g55520.1:d:+632:secondary,At1g55520.2:d:+632:secondary
TCCATTCTTG	2	1	1	3	3	At1g55000.1:d:+944:primary
AACAAAATTC	3	3	1	2	1	At1g53730.1:d:+2358:primary
TGTAAGTGTA	1	5	1	2	1	At1g53320.1:d:+1276:secondary
AAAAATAAAA	2	1	2	2	3	At1g53200.2:d:+1010:secondary,At1g53200.1:d:+1027:secondary
CAAAAGGAAG	0	3	4	3	0	At1g52370.1:d:+650:primary,At2g01910.1:d:+1736:primary
TCCTATGCCC	0	6	1	1	2	At1g52200.1:d:+431:primary
ACGGACCAAG	0	2	4	3	1	At1g51720.1:d:+2214:primary
TGTATGTCTC	2	0	1	1	6	At1g48210.1:d:+1390:primary
CATACATATA	5	1	1	0	3	At1g43980.1:i:+2602:tertiary
AAACTAACAA	1	4	2	0	3	At1g42697.1:X:-1638:quaternary,At3g22120.1:X:-1096:quaternary,At3g33145.1:X:--223:quaternary
TGAACAAATT	1	0	3	4	2	At1g42150.1:p:+3444:secondary
TAAACCCACA	2	2	2	2	2	At1g34360.1:d:+1128:primary
TAAGAGGTTT	2	1	3	3	1	At1g34270.1:d:+1606:primary,At5g05230.1:d:+1429:primary
CATCCGTCTG	0	5	2	2	1	At1g33290.1:d:+1258:primary,At1g33290.2:d:+1030:primary
ACTCTGAGAA	1	1	3	4	1	At1g33110.1:d:+1493:primary
TTAAGTAGGT	0	6	1	1	2	At1g33000.1:v:+947:primary,At4g17950.1:d:+1726:primary
GACCAAGACT	2	1	2	2	3	At1g32900.1:d:+1863:primary,At4g17780.1:v:+1211:primary
TGAGTCCATT	1	6	1	1	1	At1g32400.2:d:+1215:primary,At1g32400.1:d:+1057:primary
AGTATGATCA	4	2	2	1	1	At1g32070.2:d:+963:primary
GTTTAATCTT	1	1	0	4	4	At1g31660.1:X:+245:quaternary
GACTGGATTG	1	2	2	3	2	At1g30090.1:d:+1594:primary
CTGCTGATAT	0	4	3	1	2	At1g29900.1:d:+3427:secondary
AACATAAGAG	2	4	1	2	1	At1g29395.1:d:+559:primary
TAGAAATTAT	2	6	1	1	0	At1g26920.1:d:+592:secondary
ACTGCACCAA	1	0	2	6	1	At1g26880.1:X:-161:quaternary
TTATATCTCA	4	4	0	0	2	At1g25440.1:d:+1440:primary
AGATTACACT	3	0	2	3	2	At1g22450.1:d:+756:primary
CTTCGACCGT	4	2	0	2	2	At1g22190.1:d:+1428:primary
TTCATATTTT	1	2	3	4	0	At1g21240.1:d:+2339:primary
TCTTTGTCTT	1	4	5	0	0	At1g21100.1:d:+1343:primary
TTTCTGATCA	3	2	4	1	0	At1g20510.1:d:+1844:primary
ACAAAATTTT	0	5	4	1	0	At1g20370.1:d:+1845:primary
GATTGATGTA	3	2	3	1	1	At1g18720.1:d:+763:secondary
AATGTAGTTC	1	8	1	0	0	At1g18570.1:d:+1541:primary
TTCTTCGTAT	6	1	1	0	2	At1g16340.1:d:+243:secondary
AATGTGCTCC	2	1	4	2	1	At1g16010.1:d:+1669:primary
TGATTAACTT	3	1	0	4	2	At1g15860.1:d:+828:primary
GCTCTTCTAC	2	6	2	0	0	At1g15740.1:d:+2075:secondary
TGACATTGGA	3	3	1	3	0	At1g14920.1:d:+1964:secondary
AGAAAGTAAT	1	1	3	1	4	At1g14820.3:d:+859:primary,At1g14820.2:d:+1225:primary,At1g14820.1:d:+1123:primary
ATTGTAAGTG	3	2	1	2	2	At1g12360.1:d:+2398:primary
GCTCGACCGG	3	3	3	1	0	At1g12090.1:X:-385:quaternary,At1g62510.1:X:-395:quaternary
AAAGAAGAAT	3	5	2	0	0	At1g11930.1:d:+1030:primary,At1g11930.2:d:+1024:primary
TAAAATTCTG	0	3	7	0	0	At1g11050.1:d:+2192:primary
ACAGGTATCT	4	3	1	0	2	At1g10670.2:d:+1299:primary,At1g10670.1:d:+1306:primary
AGTCAAATGT	1	4	3	2	0	At1g10290.1:d:+3133:primary
GAGCTGTATG	2	2	1	5	0	At1g09640.1:d:+1259:secondary
GAATTCTAGT	5	2	3	0	0	At1g09415.1:d:+572:primary
AATCTTCGAG	0	6	4	0	0	At1g07135.1:d:+394:primary
TACACTCTCT	3	3	2	2	0	At1g06150.1:d:+724:secondary
TAGCCCAGAA	2	2	3	3	0	At1g05500.1:v:+2064:secondary
CGAAGAAGCT	0	4	4	0	2	At1g05070.1:d:+620:primary
CCTCTTCTTA	2	3	3	1	1	At1g04850.1:d:+1337:primary
GCTACGAAAA	6	0	2	2	0	At1g04820.1:X:-784:quaternary
AATATACAAT	3	4	2	0	1	At1g04300.1:d:+3571:primary
GGAGCCATTC	2	2	3	1	2	At1g04280.1:d:+957:primary
TTTGGATTTT	1	2	7	0	0	At1g02840.2:d:+1162:primary,At2g07110.1:v:+1182:primary,At3g46430.1:v:+987:primary
GCAATCTTTT	2	3	1	3	1	At1g02280.1:X:-226:quaternary
GGATGTTGAC	2	2	4	0	2	At1g02140.1:X:-245:quaternary
ACTACATAGA	6	1	0	0	3	At1g01910.2:d:+1424:primary,At1g01910.1:d:+1276:primary

GGCCTTCGCG	3	3	3	0	0	No gene matches found
GGCCCTCGCC	0	3	0	6	0	No gene matches found
CTCCTTTCTG	2	2	1	3	1	No gene matches found
GGCCTTCACC	1	4	1	3	0	No gene matches found
CAGGTGCGGC	1	4	0	2	2	No gene matches found
ATTTCTTAAA	2	3	2	1	1	No gene matches found
GGGTAGAGCC	1	4	2	1	1	ChrC:-69226:quaternary,At3g19840.1:X:-283:quaternary
AGCTTTAGCA	0	6	2	0	1	ChrC:+53581:quaternary
GCAAGACTCC	0	6	0	3	0	Chr5:-8469157:quaternary
AGACCAAAAA	4	2	1	1	1	Chr5:+4198663:quaternary,Chr1:+6029308:quaternary
TTTAAATGCA	1	2	2	3	1	Chr5:+15815478:quaternary,Chr5:+5415908:quaternary,Chr5:+15811730:quaternary
AATAACAATA	3	3	0	1	2	Chr5:+14449119:quaternary,At5g42250.1:X:+-172:quaternary,Chr2:+2865519:quaternary
ACATATTCTA	3	0	3	1	2	Chr4:-2411975:quaternary
TGCCCTGCGC	1	2	0	5	1	Chr4:-15965205:quaternary
CACCCGAACG	2	1	5	1	0	Chr4:-13709562:quaternary
AACTGATTTT	3	2	1	0	3	Chr4:+4881902:quaternary,At3g66654.2:X:+294:quaternary
GCTACTAAAA	2	3	2	2	0	Chr4:+13647457:quaternary
TTTGTTTGAA	3	2	3	0	1	Chr3:+17366537:quaternary
TTTCTTAAAA	4	0	1	3	1	Chr3:+15369618:quaternary,Chr1:+28999693:quaternary,Chr1:+18349248:quaternary
GTTTCCACTA	3	2	1	2	1	Chr2:-14812068:quaternary
TGAAAGTTGA	2	2	2	2	1	Chr2:+6989089:quaternary
TCAAGGCCTA	2	2	1	1	3	Chr2:+6976664:quaternary
AAAGAGGACC	2	3	2	1	1	Chr2:+3497557:quaternary
AGGCTTAAAA	5	0	2	1	1	Chr2:+2702909:quaternary,Chr4:+4703231:quaternary
TAAAAAAACC	3	3	2	1	0	Chr2:+11952377:quaternary,Chr4:+6426776:quaternary,Chr2:+6585548:quaternary
TTACAAAAAA	3	2	1	0	3	Chr1:+3637607:quaternary,Chr3:+12894662:quaternary,Chr5:+9279494:quaternary,Chr1:+22856833:quaternary,Chr4:+17291174:quaternary,Chr1:+11442591:quaternary
TTCTATAAAA	2	0	3	3	1	Chr1:+26342869:quaternary,Chr3:+12694125:quaternary
AGCAAATTTG	0	1	3	4	1	Chr1:+11407541:quaternary,Chr3:+16275424:quaternary,Chr5:+13111371:quaternary,Chr5:+8726125:quaternary
ACGACTGGGA	1	3	3	2	0	AtMg00040:d:+794:primary
GAGAACGTAG	2	3	0	3	1	At5g67510.1:d:+468:primary
TTACTCGAGC	2	4	1	1	1	At5g65110.1:d:+2029:primary
CCGCTCCACT	2	4	1	2	0	At5g64960.1:d:+1274:secondary
GTGATTGGTT	3	0	2	2	2	At5g64220.1:d:+3497:primary
AAAATCGACC	3	1	2	3	0	At5g63530.1:d:+745:secondary,At3g17630.1:v:+3075:secondary
GACCAGGAGC	1	3	0	3	2	At5g62740.1:d:+851:primary,At3g17060.1:d:+942:primary
TTCACTCACA	0	1	5	1	2	At5g61380.1:d:+2553:primary
TTAAATACAT	1	2	0	1	5	At5g61210.1:d:+1278:primary
GAGAAAAGTG	0	0	1	7	1	At5g61030.1:d:+922:primary
TGAGTCAGAT	2	2	3	1	1	At5g60430.1:d:+242:secondary
AGGGTAATAG	2	4	2	0	1	At5g59840.1:d:+853:primary
CATCTCCGGT	3	0	1	4	1	At5g58470.2:d:+1541:secondary
TTAGGGACTT	0	1	5	3	0	At5g58430.1:d:+2205:primary
CTGCTGTATC	1	4	2	2	0	At5g57990.1:d:+2727:primary
GTTTCAAAGT	2	1	5	0	1	At5g57815.1:d:+365:primary
CGGAAGCGCC	0	7	1	1	0	At5g56980.1:d:+970:secondary
ATGTTTTACC	5	1	3	0	0	At5g56350.1:i:+794:tertiary
TATTTTTGAG	3	2	1	2	1	At5g54280.1:d:+3425:secondary
TCACTTTAAA	4	1	4	0	0	At5g54080.1:d:+1565:primary
GAAGATCCCT	1	3	2	1	2	At5g52530.1:i:+514:tertiary,At5g52530.2:i:+492:tertiary
GATCCTCTCT	1	2	3	1	2	At5g52520.1:d:+1760:primary
AGAATTTTAT	1	2	1	2	3	At5g51300.1:d:+2650:primary
GTTCAATAGG	1	3	2	1	2	At5g51070.1:d:+2533:secondary
TAATTATATT	2	2	0	0	5	At5g49890.1:d:+2802:primary
CCAAGACAAA	2	1	1	4	1	At5g48760.1:X:-205:quaternary
GAGAGGAAGC	3	4	1	1	0	At5g47890.1:d:+78:primary
AATTGTAAGC	1	1	2	2	3	At5g47750.1:d:+2126:primary
TAGCATCGAA	0	3	6	0	0	At5g47240.1:d:+1327:primary

GCGGGTGGAG	0	3	1	4	1	At5g46800.1:d:+927:primary
TCTCCAATTG	1	4	2	2	0	At5g45390.1:d:+754:secondary,At4g08593.1:v:+1025:secondary
AAAGGATCTG	2	6	1	0	0	At5g43700.1:d:+752:primary
AACTCCTTAG	2	2	1	2	2	At5g42820.1:d:+1083:primary
GTTTAGCAGT	1	3	3	1	1	At5g42620.1:d:+2762:primary
ATGGAAGAGG	1	2	0	4	2	At5g42540.1:d:+3024:primary
ACTTAGTCGT	2	2	3	1	1	At5g41940.1:d:+1662:secondary
AATTTCTCGA	1	2	5	1	0	At5g41800.1:d:+1433:primary
ATGGTGTGAA	0	3	2	1	3	At5g41020.1:d:+546:secondary
TTTATCTAAT	1	2	1	0	5	At5g40770.1:d:+1114:secondary,At5g08200.1:d:+748:secondary
CGTTGAACTG	1	2	3	3	0	At5g40170.1:d:+2278:primary
TCACTTTCGA	1	0	1	7	0	At5g39850.1:d:+827:primary
ACCACAAACA	1	2	1	3	2	At5g36700.1:X:-386:quaternary,At5g36790.1:X:-386:quaternary
GCTTCTGCTG	2	3	3	0	1	At5g36170.2:d:+1400:primary,At5g36170.3:d:+1422:primary,At5g36170.1:d:+1403:primary
CTCGCAACAG	1	3	4	1	0	At5g35160.1:v:+2157:secondary
GAGATTTGTC	1	0	5	2	1	At5g34850.1:d:+1775:primary,At4g36550.1:d:+1541:primary
CTCAAAAGAG	1	3	2	0	3	At5g33355.1:v:+944:secondary
TTTCTCATTA	3	4	2	0	0	At5g32450.1:d:+1052:secondary
TAAAAAGCCA	4	0	3	1	1	At5g29000.1:d:+778:secondary,At5g29000.2:d:+873:secondary,At3g13040.1:d:+1115:secondary,At3g13040.2:d:+969:secondary
GCCCAAAAAA	2	1	2	3	1	At5g28624.1:X:-3853:quaternary
GGAGCTGCTG	0	9	0	0	0	At5g28237.2:d:+675:secondary,At5g28237.1:d:+675:secondary
GCTTCCTGAT	3	2	2	1	1	At5g26710.1:d:+1957:secondary,At1g25570.1:d:+196:secondary
GACTGGTATT	1	8	0	0	0	At5g26360.1:d:+1516:primary,At3g45110.1:v:+496:primary
GGATGGGGAA	2	1	3	0	3	At5g25980.1:d:+1790:primary,At5g25980.2:d:+1794:primary
GACAAGAAAA	1	1	2	2	3	At5g25920.1:v:+1806:secondary
GCAAAACCCT	1	4	3	0	1	At5g24890.1:d:+717:primary
CTTACAAACT	1	0	5	3	0	At5g24450.1:i:+3913:tertiary
AGAGAGTATA	3	2	2	1	1	At5g23450.1:d:+2669:primary,At3g63520.1:v:+2353:primary,At5g23450.2:d:+2863:primary
AAGAACAGTG	4	3	2	0	0	At5g22080.1:d:+654:primary
ATGATAAAAA	5	1	2	1	0	At5g22035.1:v:+796:secondary
TCTCTAAAAT	0	0	2	3	4	At5g20935.1:d:+359:primary,At1g65780.1:v:+3986:primary
GGTAAATCAG	0	2	4	3	0	At5g20070.1:d:+1284:primary
ATTGTCGATC	2	6	1	0	0	At5g20050.1:d:+759:primary
AGAAAAAAAG	2	2	2	1	2	At5g19390.2:d:+2982:primary,At5g19390.1:d:+2975:primary
TTCATCCTGG	1	4	4	0	0	At5g19330.2:d:+1932:primary,At5g19330.1:d:+2036:primary
TATGGAATGA	3	1	2	3	0	At5g17510.1:d:+1596:primary
TTCAAATGCA	1	2	2	3	1	At5g16760.1:d:+1375:primary
GTTTTCCAGA	1	4	2	1	1	At5g16650.1:d:+868:primary
GCGGTGGCGG	1	3	2	3	0	At5g15870.1:v:+2545:secondary,At4g38030.1:v:+867:secondary
TGTTTAAATA	3	5	1	0	0	At5g15260.1:d:+1330:primary,At3g01170.1:d:+1163:primary
AACTAGAGAA	3	3	0	1	2	At5g14800.1:d:+965:primary
AGCATAGGAG	1	7	0	0	1	At5g14170.1:d:+1458:primary
AATAATTGGG	2	4	0	1	2	At5g13620.1:v:+194:secondary
ACTACTTTGG	4	3	2	0	0	At5g13090.1:d:+1032:primary
CAGTGTGCGA	0	5	2	1	1	At5g13010.1:d:+3566:secondary
AGATCAGTTG	2	3	2	0	2	At5g11910.1:d:+893:primary
GGACAATCTG	1	1	4	2	1	At5g11710.1:d:+1914:primary
CAGAAGAAGA	1	5	1	2	0	At5g11100.1:v:+64:secondary,At5g11450.1:d:+281:secondary,At4g12540.1:v:+400:secondary
TGTGTTATGA	0	2	4	1	2	At5g10860.1:X:+431:quaternary
ATTATGAAAA	0	3	2	3	1	At5g10695.1:d:+512:primary
CTAAGAGGGT	2	3	3	0	1	At5g10400.1:d:+408:primary
GCACTTTTCG	0	1	3	3	2	At5g09870.1:v:+3009:primary
TTAGATTTCT	1	5	0	0	3	At5g09370.2:i:+391:tertiary,At5g09370.1:i:+391:tertiary
TAATTTTACA	2	5	0	2	0	At5g09320.1:i:+1935:tertiary
TTTTTATTGT	0	0	1	3	5	At5g08640.1:d:+1221:primary
ATTAGTTTCC	2	0	5	2	0	At5g08520.1:d:+1657:primary
GCCGACAACG	1	8	0	0	0	At5g08330.1:d:+88:primary
ATGACCCAAA	2	2	2	2	1	At5g05210.1:d:+1032:primary
TTTGATCTGA	3	3	1	1	1	At5g05010.1:d:+1368:primary

TCTTGTGTGT	4	1	0	1	3	At5g04950.1:d:+1099:primary
GCTTCTTAGA	4	1	2	2	0	At5g04810.1:d:+2756:primary
TCATTGAACC	0	5	1	2	1	At5g04600.1:d:+464:primary
TCGAGCTTCA	0	6	2	1	0	At5g04040.1:d:+1855:primary
GCAATTCTAC	1	3	2	3	0	At5g03630.1:d:+1132:primary
TGAACGTTGT	0	2	1	4	2	At5g02280.1:d:+575:secondary,At5g44040.1:d:+390:secondary
TAATTAACGA	4	4	0	1	0	At4g40060.1:d:+1359:primary
CATTGTTGTT	7	1	1	0	0	At4g39900.1:d:+149:secondary,At4g08350.1:v:+2267:secondary
TTTTTCTTTT	3	4	0	0	2	At4g39750.1:v:+1049:primary,At5g14880.1:v:+3130:primary
GCGAGAGACT	1	4	2	2	0	At4g38680.1:d:+752:primary
CGTTTTACAT	3	0	4	2	0	At4g37760.1:d:+1964:primary,At5g23530.1:d:+977:primary
GTCGAGGAAG	2	5	2	0	0	At4g36780.1:d:+1210:primary
GTAAGAGTTC	4	1	3	1	0	At4g35350.1:d:+1220:primary,At4g35350.2:d:+1316:primary
TAACCATACT	3	2	0	2	2	At4g35080.1:d:+1398:primary,At4g35080.2:d:+1294:primary
GACGAAATTT	0	5	2	2	0	At4g34920.1:d:+730:primary,At4g34930.1:d:+1099:primary
AGCTCAAGTT	2	0	3	3	1	At4g34730.1:d:+1320:secondary
TTTGTTATAA	5	1	0	0	3	At4g34290.1:i:+707:tertiary,At2g35510.1:i:+729:tertiary,At1g61890.1:i:+1878:tertiary
GCCCGTGGGA	2	3	4	0	0	At4g34138.1:d:+1300:primary
TCATTGTAAA	3	4	2	0	0	At4g33750.1:d:+499:primary,At1g50630.1:d:+1611:primary
AACTAGTAGT	1	0	0	0	8	At4g33130.1:X:+542:quaternary,At3g07050.1:X:+439:quaternary
AACTGGATTC	2	1	2	3	1	At4g33110.1:d:+1310:primary,At4g33120.1:d:+1297:primary
ACCACATAAA	2	6	0	0	1	At4g33030.1:d:+1578:primary
ACAAAGAAAA	1	1	2	2	3	At4g32100.1:i:+425:tertiary
TGTTTGACTC	0	4	5	0	0	At4g32060.1:d:+1399:primary
ATTTTTTCAC	1	3	1	3	1	At4g31900.1:i:+3548:tertiary
ATTTGTTCAA	2	1	1	5	0	At4g31870.1:v:+1148:primary
GTCGTCGTAA	1	3	5	0	0	At4g31340.1:d:+1459:primary
TACACAATAA	2	2	1	2	2	At4g31040.1:d:+1777:primary
CCTTTGTGTT	1	1	2	1	4	At4g30190.1:d:+3188:secondary
GTGCCTACTA	2	2	2	1	2	At4g29720.1:d:+1648:primary
TCGAGACAGA	0	6	3	0	0	At4g27690.1:d:+801:primary
ATAACTCCAC	1	0	4	3	1	At4g27520.1:d:+1295:secondary
TTTCTGTTTC	3	3	1	2	0	At4g24820.1:d:+1300:primary,At5g45510.1:d:+3979:primary,At4g24820.2:d:+1300:primary
CGAGGTCAAA	5	4	0	0	0	At4g23470.2:d:-1468:secondary,At4g23470.1:d:-1120:secondary
AACAACGCTT	2	2	3	1	1	At4g22130.1:X:-606:quaternary
GTGCTAGACA	1	8	0	0	0	At4g21960.1:d:+806:secondary
TAAAAAAGAA	2	3	1	2	1	At4g21450.2:d:+1425:secondary,At4g21450.1:d:+1164:secondary
ACCATATTAT	3	0	1	2	3	At4g21210.1:d:+1377:primary
TAAAGAGCTC	1	4	0	1	3	At4g20010.1:d:+1272:primary
CAAAATATTT	1	4	2	2	0	At4g19350.1:d:+797:primary
AGGGGTATGT	2	0	3	0	4	At4g18700.1:d:+1779:primary
AAAAGATCCA	1	4	3	1	0	At4g17615.2:d:-369:primary
CCGCTTTTAT	0	6	3	0	0	At4g17490.1:d:+833:primary
CAACGAAGTA	2	3	2	1	1	At4g17110.1:v:+1408:primary
AAGCTGGAGA	1	2	3	2	1	At4g16630.1:d:+412:secondary,At4g09840.1:d:+427:secondary
GAAGAAGAAC	3	4	2	0	0	At4g16060.1:d:+825:primary
TGAAGGAGAG	3	3	2	0	1	At4g16060.1:d:+416:secondary
TATTGTTGTT	4	1	1	0	3	At4g15900.1:d:+1691:primary
TTTGAAAATG	3	3	1	1	1	At4g15520.1:d:+898:primary,At5g67280.1:d:+2212:primary
CCAAAAATGG	0	2	4	3	0	At4g15110.1:d:+1740:primary
TAGAGTAGTA	5	2	1	1	0	At4g14500.1:d:+1646:primary
GACCAGATTT	0	1	6	1	1	At4g14040.1:d:+1734:secondary
TGATGTAACC	3	4	2	0	0	At4g12600.1:d:+373:primary
GAATGCGTGA	1	5	1	2	0	At4g11850.1:d:+2625:primary,At4g11840.1:d:+2386:primary,At4g11830.1:d:+3356:primary,At4g11830.2:d:+3452:primary
TTGATCGTTG	2	3	3	1	0	At4g11330.1:d:+1313:secondary
GCAGCTCTTA	0	0	5	2	2	At4g09750.1:d:+1032:primary
AAGCTATACC	5	0	3	1	0	At4g08685.1:d:+588:secondary
GATGGATGTT	1	2	1	2	3	At4g07410.1:d:+2476:primary
TGCTTCATCG	4	1	2	1	1	At4g06500.1:p:-4095:secondary

TACCAATGGG	0	4	2	0	3	At4g05150.1:d:+1194:primary
TAGTGAATAT	1	5	1	0	2	At4g04830.1:d:+528:primary
AAAGAAAAAA	2	1	2	3	1	At4g04270.1:p:+2430:primary
ATTCAGTTCT	2	6	0	1	0	At4g02620.1:d:+407:primary
GATATGTTTC	2	2	2	1	2	At4g02600.1:d:+1874:primary,At5g44720.2:d:+569:primary
CGGCTTCTGC	0	6	2	1	0	At4g02510.1:d:+2948:secondary
GATCAATAAT	1	1	2	1	4	At4g02500.1:d:+1747:primary
AATTATGCAG	2	2	1	4	0	At4g01900.1:d:+772:primary
TAGATTCAGT	4	4	1	0	0	At4g01595.1:X:--172:quaternary
AAAAAATGCA	1	2	4	2	0	At4g00330.1:d:+1264:primary
CAGTCAAGTT	3	2	3	1	0	At3g63080.1:d:+1005:primary
AATATGCACT	3	2	3	0	1	At3g62600.1:X:-354:quaternary,At5g33406.1:X:--41:quaternary
AAACATCAGC	1	0	3	2	3	At3g62270.1:d:+2751:primary
TAAAGTCTCG	1	1	3	3	1	At3g60370.1:d:+922:primary
TATCAAAACA	2	4	0	1	2	At3g59380.1:d:+1094:primary
GTTTTGGCTA	3	3	3	0	0	At3g58040.1:d:+1514:primary
GGTCCAACAT	0	4	2	1	2	At3g56240.1:X:-433:quaternary
GCAGTACATT	1	4	2	1	1	At3g55830.1:d:+1022:primary
ATTTAACAAA	3	0	2	3	1	At3g55630.2:d:+1624:primary,At3g55630.3:d:+1660:primary,At3g55630.1:d:+1589:primary
ACAACATCTG	4	1	1	2	1	At3g55260.1:d:+1805:primary
GATCGATGGT	2	3	3	0	1	At3g55240.1:d:+588:primary
ACACGTGTTT	0	4	1	3	1	At3g55130.1:d:+139:secondary
ATGGAAGAAT	2	4	1	1	1	At3g54620.1:d:+1381:primary
TCATATAAAT	4	0	1	3	1	At3g54280.1:i:+6805:tertiary
TTGCGGCACA	1	3	0	4	1	At3g53610.2:d:+717:primary,At3g53610.1:d:+754:primary
ACAGAGTAGT	1	3	1	1	3	At3g52990.1:d:+1683:primary
TTTTGCGAAT	1	1	4	1	2	At3g52950.1:d:+1923:primary
TACAGATATA	4	4	1	0	0	At3g52840.1:d:+2322:primary
TTCGGTGTCA	0	4	2	2	1	At3g52470.1:d:+622:primary
GTGGGCTGGC	0	2	2	5	0	At3g51820.1:d:+872:primary
TAGATCTTGA	1	1	4	1	2	At3g51660.1:d:+560:primary,At2g19060.1:d:+1706:primary
AATAGTCGAA	2	2	4	1	0	At3g51610.1:d:+883:primary
TCTTTAGCAT	4	2	3	0	0	At3g51520.1:d:+1151:secondary
AGATCCAACC	1	5	1	1	1	At3g51370.1:d:+1209:primary,At3g51370.2:d:+1101:primary
CAATGTAACA	0	0	0	3	6	At3g50370.1:d:+5908:secondary,At5g06760.1:d:+786:secondary
TATATAGATC	3	3	2	1	0	At3g49580.1:d:+401:primary
GACCAAATCT	1	2	1	1	4	At3g49570.1:d:+310:primary
AAGGTCAGAA	0	1	6	1	1	At3g49010.2:d:+253:secondary,At3g49010.1:d:+267:secondary,At4g30100.1:d:+1384:secondary
ATTACAAAGG	3	1	1	2	2	At3g48810.1:v:+1132:secondary
AGAATATACC	0	5	0	1	3	At3g48170.1:d:+1621:primary
AAAGTTTGTA	0	4	3	1	1	At3g47590.1:d:+969:primary
TTCTTAGAAA	2	5	1	1	0	At3g47140.1:v:+1055:primary,At2g45810.1:d:+2118:primary
GTGCAAGCGG	0	3	3	1	2	At3g46440.1:d:+1221:primary
CACACAAAAA	1	1	4	1	2	At3g46420.1:i:-442:tertiary
CACAAAATCA	2	4	0	1	2	At3g46360.1:v:+1210:primary,At3g08720.1:d:+1755:primary,At3g08720.2:d:+1945:primary
CCAGCTTCCA	1	6	1	1	0	At3g45730.1:d:+351:primary
TTTTAATTTT	2	5	2	0	0	At3g45260.1:d:+1787:primary
TTTTGTGATG	2	0	4	2	1	At3g45030.1:d:+600:primary
AATAATTGGA	3	1	3	0	2	At3g44990.1:d:+1027:primary
TTTTTTTGTT	3	1	2	0	3	At3g44710.1:d:+1642:primary
ATTCACCAGT	0	5	0	4	0	At3g44690.1:v:+1150:secondary
CTCTTGATAT	2	3	2	1	1	At3g43670.1:d:+2230:primary
AGAAATTGGT	1	4	1	1	2	At3g33545.1:p:+304:primary
GATAAATTCA	0	5	2	1	1	At3g28940.1:d:+434:primary
GAAGAATGAT	1	2	3	2	1	At3g28870.1:v:+1410:secondary,At1g20960.1:v:+6858:secondary,At2g42270.1:d:+6511:secondary,At1g61500.1:v:+1742:secondary,At1g49540.1:d:+1335:secondary
TATGAGTATC	5	3	0	0	1	At3g28050.1:d:+1267:primary
GTATCTTTGA	0	8	1	0	0	At3g27090.1:d:+768:primary
TAGTCAACAG	4	2	0	1	2	At3g26530.1:X:-91:quaternary,At1g35650.1:X:-91:quaternary,At1g08740.1:X:-91:quaternary,At5g44890.1:X:-91:quaternary,At2g29240.1:X:-91:quaternary

AACATCATCA	2	1	1	1	4	At3g26250.1:v:+859:secondary,At3g26240.1:d:+1900:secondary
AGAAAGCTTC	2	2	2	2	1	At3g25480.1:d:+861:primary
AGAAAAAAAT	2	4	1	1	1	At3g24630.1:v:+2730:secondary
GAATCTTCAG	3	4	1	1	0	At3g24320.1:d:+2774:secondary
CCAAGACCGA	0	0	3	2	4	At3g23820.1:X:+2077:quaternary
GTTTACAAGT	1	3	1	4	0	At3g23450.1:p:+1452:primary
TTGGAGAAAT	0	3	5	0	1	At3g22980.1:v:+3471:primary
TAATTTCTCT	0	0	0	3	6	At3g22840.1:d:+755:secondary
GAGGACTCTA	1	4	2	1	1	At3g22440.1:d:+1160:primary
TCGCTACGGT	1	1	3	3	1	At3g21865.1:d:+876:primary
AAAAAAACTA	0	1	2	3	3	At3g21110.1:d:+1614:secondary
GCTCCGGAGA	0	4	3	1	1	At3g20680.1:d:+623:primary
TGGATTAGTA	4	1	0	3	1	At3g20630.1:v:+2878:primary
GAAGACAGAT	1	0	4	2	2	At3g19100.1:d:+2144:primary
AAAGAATCTA	3	1	4	0	1	At3g18210.1:d:+1599:primary
TAGTGAGTTC	1	3	1	4	0	At3g18060.1:d:+1882:primary
TTTTGGATAA	1	0	4	1	3	At3g17170.1:d:+1032:primary
CCGATACATC	4	2	2	1	0	At3g16520.3:d:+1616:primary
TACTTTGACT	1	2	2	2	2	At3g16170.1:d:+1897:primary
TCTCCCACAG	1	1	1	2	4	At3g15000.1:d:+1544:primary
GTCAAGAAAC	1	7	0	1	0	At3g14990.2:d:+302:primary,At3g14990.1:d:+373:primary
TCGTCTCTCC	2	4	1	2	0	At3g14220.1:d:+1168:primary
AAAAAGCAGA	2	5	0	1	1	At3g13840.1:v:+2009:primary,At5g05780.1:d:+1010:primary,At1g11820.1:v:+2058:primary
TTTCTTTTTT	1	3	1	3	1	At3g13270.1:v:+2115:primary,At1g30440.1:d:+2118:primary
TACTGGCTGA	2	2	3	0	2	At3g13070.1:v:+2305:secondary
TTATGGAACA	3	0	0	4	2	At3g12930.1:d:+1066:primary
AACTCTGCAA	0	4	0	4	1	At3g11710.1:d:+1671:primary
CCTTATGTAT	0	3	1	0	5	At3g11400.1:d:-1180:secondary
ATCGAAGTTA	1	0	2	5	1	At3g10690.1:d:+3072:primary
GTTTGAAGTA	3	1	2	1	2	At3g10520.1:d:+779:primary
TCTGACTTCA	0	4	1	2	2	At3g10420.1:d:+2280:primary,At3g10420.2:d:+2187:primary
AGTTACTGAC	0	2	4	0	3	At3g10405.1:d:+782:primary
ATCACTTCAC	4	0	2	2	1	At3g09210.1:d:+1084:secondary
TTGTAAGATA	2	2	1	2	2	At3g08650.2:d:+1964:primary,At3g08650.1:d:+2111:primary
AGACTTGAGG	1	2	2	2	2	At3g06760.1:d:+697:primary
AACTAAGCAA	0	1	3	3	2	At3g06410.1:d:+1353:primary
AAGACTTCAA	2	3	1	3	0	At3g05530.1:d:+1316:secondary,At3g15330.1:v:+624:secondary
ATTAGTACCA	4	1	1	0	3	At3g05350.1:d:+1923:primary
GACTAGTGGG	1	4	2	2	0	At3g05165.2:d:+1404:primary,At3g05165.1:d:+1288:primary
AAGCTCTTTA	2	1	5	1	0	At3g03790.1:d:+3944:primary,At3g03790.2:d:+3953:primary
ATTGAATGTA	4	0	2	1	2	At3g02900.1:d:+518:primary
TAATAATGGC	1	4	2	1	1	At3g02750.1:d:+2695:primary
GGAGATTGTA	0	6	1	2	0	At3g02260.1:d:+15096:primary
GAAAATGGAT	2	6	1	0	0	At3g01340.1:d:+686:primary
GCCAGGATGC	1	1	2	3	2	At2g47910.2:d:+800:primary,At2g47910.1:d:+690:primary
CTATTGGGTT	1	4	3	1	0	At2g46220.1:d:-1033:secondary
TTTGCAAAGT	2	4	0	1	2	At2g45790.1:i:+1183:tertiary
ATTATGTAGT	1	4	2	2	0	At2g45700.1:d:+2330:primary
TTTGTATTTT	3	0	2	2	2	At2g45640.1:d:+610:primary,At5g55530.3:d:+1561:primary,At5g55530.2:d:+1589:primary,At5g55530.1:d:+1667:primary
CTCTGTTCTT	0	3	2	3	1	At2g45620.1:d:+2440:primary
CCGTACCCGT	0	3	3	2	1	At2g45560.1:d:+1661:primary
GTTGGTTTTG	1	3	2	2	1	At2g45350.1:v:+2256:secondary,At4g23540.1:d:+1001:secondary,At1g07135.1:d:+21:secondary,At2g31830.1:v:+292:secondary,At5g62700.1:d:+905:secondary,At5g12250.1:d:+882:secondary,At4g34190.1:d:+395:secondary,At4g20890.1:d:+928:secondary
ATAAGGTTTC	4	2	1	2	0	At2g44870.1:d:+1002:secondary
ACTATAAAAA	4	2	0	3	0	At2g44640.1:d:+1451:secondary
GTTTGTAACT	2	3	2	2	0	At2g43520.1:d:+389:primary,At5g38520.1:d:+1307:primary
GTTTGTGTAA	2	5	0	1	1	At2g43510.1:d:+402:primary,At1g47540.1:d:+334:primary
ATGAGATTTT	2	1	1	5	0	At2g43010.1:d:+1693:secondary,At2g35800.1:d:+1845:secondary,At2g43010.2:d:+1687:secondary
GCTACTTCAA	0	5	2	2	0	At2g42780.1:d:+1083:primary

GCGTTGGCGA	0	6	2	1	0	At2g42580.1:d:+2085:primary
AATCACCATA	1	5	2	1	0	At2g42190.1:d:-857:secondary
CCGGAAAATA	0	0	5	3	1	At2g41905.1:X:+-238:quaternary
GGTCGTTCAG	1	4	3	0	1	At2g41790.1:d:+2879:primary
GTTTCGTTAA	0	0	3	3	3	At2g41720.1:X:--27:quaternary,At1g17830.1:X:-36:quaternary
ACCGGACGGT	2	2	2	3	0	At2g40940.1:d:+2155:primary
TTACGCTATA	2	1	3	0	3	At2g40100.1:d:+979:primary
GTTGATGCTG	0	2	0	4	3	At2g39990.1:d:+618:primary
GAAGCAGAGG	0	5	4	0	0	At2g39950.1:d:+2033:primary
TTGGCGAGAA	1	5	2	1	0	At2g39870.1:d:+1064:primary
TAGTGGCCTT	2	2	3	1	1	At2g39570.1:d:+1609:primary
AACGTTATGC	2	7	0	0	0	At2g39570.1:d:+1328:secondary
TATCCTCTTG	1	5	1	1	1	At2g39080.1:d:+1182:secondary
GTGCTAGAGG	1	2	4	0	2	At2g38290.1:d:+1492:primary,At2g38290.2:d:+1571:primary
GATATTGTTA	2	2	3	0	2	At2g37940.1:d:+1425:primary
ATGTTTGGCA	1	2	3	3	0	At2g37760.1:d:+1193:primary,At2g37760.2:d:+1366:primary
ATACCGGCGT	2	3	3	1	0	At2g36060.1:d:+471:primary,At2g36060.2:d:+474:primary
TGTATGAAAC	1	1	1	4	2	At2g36020.1:d:+536:secondary
AGGTACCTCT	1	3	2	1	2	At2g35780.1:d:+1295:primary
GGCTGCCTGA	3	0	4	1	1	At2g34420.2:X:-193:quaternary,At1g29920.1:X:-211:quaternary,At1g29910.1:X:-197:quaternary
GTGGCGTCCT	0	1	6	1	1	At2g34300.1:d:+2413:primary
TCACGTCTTT	1	3	3	0	2	At2g33410.1:d:+1487:primary
CTTGTCTATA	2	2	4	1	0	At2g32870.1:d:+1506:primary
TTTTTGGATG	3	1	0	1	4	At2g32730.1:d:+805:secondary,At3g63460.1:d:+3644:secondary,At3g63460.2:d:+3638:secondary
TACTGATCTC	3	5	1	0	0	At2g32710.2:d:+1327:primary,At2g32710.1:d:+1373:primary
ACATTTTCTC	6	0	3	0	0	At2g32540.1:v:+2667:primary
AAAGCTAAGT	2	3	3	1	0	At2g32450.1:d:+2518:primary,At1g05150.1:d:+2625:primary
GCTGTTGATT	1	6	2	0	0	At2g31670.1:d:+429:primary
AGAAAAAACT	1	3	1	2	2	At2g31620.1:v:+1325:secondary
TGAACGCTGT	2	3	2	1	1	At2g31390.1:d:+857:secondary
TGAGGCGACA	1	2	2	3	1	At2g30980.1:d:+1272:primary
ACTGAGACGT	2	3	3	1	0	At2g30570.1:X:-72:quaternary
TATTTGAGTT	4	0	3	2	0	At2g30200.1:d:+1261:primary,At2g30200.2:d:+1203:primary
GCCGAGTCAC	2	3	1	3	0	At2g27820.1:d:+1175:primary
ACTGATGTGT	2	2	3	0	2	At2g26920.1:d:+2168:primary
TCATATCTTC	1	2	2	3	1	At2g26460.1:d:+679:secondary
AGGCGGCATA	0	1	1	5	2	At2g25620.1:d:+1579:primary
TTTCCACTGT	2	2	0	3	2	At2g25430.1:d:+2350:primary
TTCTTTATTC	2	0	0	2	5	At2g24440.1:i:+1275:tertiary,At4g15215.1:i:+591:tertiary
GTGAAAATCA	1	1	2	3	2	At2g24330.1:d:+1636:primary
GAGACCGCAA	1	2	1	5	0	At2g23760.2:d:+1452:secondary,At2g23760.1:d:+1362:secondary
AGTTATAATG	3	3	3	0	0	At2g23450.1:d:+2348:primary,At2g23450.2:d:+2427:primary
AGAAAGCTAC	4	1	2	1	1	At2g21950.1:d:+1425:primary
ATCAAGCACA	0	6	1	1	1	At2g21600.1:d:+639:primary
AATAGTGATC	1	0	1	2	5	At2g21320.1:d:+754:primary
TGGTGTTTGG	1	5	1	1	1	At2g21130.1:d:+493:primary
TGAAAGTTGT	1	2	1	4	1	At2g20990.1:d:+1056:primary
TGAATGCTGA	2	2	4	1	0	At2g20830.1:d:+724:secondary,At3g26230.1:d:+586:secondary
CCTCGAGATT	1	1	3	2	2	At2g20120.1:X:--63:quaternary,At3g16640.1:X:-399:quaternary
TTTTCTGAAA	3	0	2	3	1	At2g19610.1:d:+1739:primary,At2g19610.2:d:+1662:primary
TGATGTATGT	4	1	1	2	1	At2g19350.1:d:+649:primary,At1g12250.1:d:+942:primary
ATATAATTGG	1	3	2	1	2	At2g19220.1:X:--143:quaternary,At3g04290.1:X:-478:quaternary
TTAACGACTC	1	2	2	1	3	At2g18590.1:v:+1540:secondary
AATTGCTACA	1	1	4	2	1	At2g18160.1:d:+326:secondary
TTTATGTTTT	3	3	0	3	0	At2g17030.1:d:+1350:secondary,At4g28390.1:d:+1398:secondary,At2g26010.1:v:+694:secondary,At4g21300.1:v:+176:secondary
TGCCCATAAG	2	3	2	2	0	At2g15580.1:d:+550:primary
GGAAGACTGT	0	6	2	1	0	At2g15120.1:p:+1490:primary,At2g15090.1:d:+1484:primary
TTGTCAAAAG	2	1	1	2	3	At2g14880.1:X:-360:quaternary,At1g36933.1:X:-744:quaternary
AGCCTTGGTA	2	2	0	3	2	At2g11890.1:d:+1022:primary

GACGCTAGCG	0	4	0	2	3	At2g10410.1:p:+250:primary
AACGTAATGC	1	5	0	2	1	At2g07688.1:p:+1034:primary,AtCg00020:d:+998:primary
AGATTGGAAG	0	6	1	1	1	At2g04550.2:d:+1041:primary
CTTGTATCGG	1	3	0	2	3	At2g01720.1:d:+1465:primary
TCTGAAGTTT	0	7	1	1	0	At2g01570.1:d:+1680:primary
CGAGACAAGA	4	1	2	2	0	At2g01510.1:v:+2043:primary
AAATTGACGT	2	3	0	3	1	At2g01180.1:d:+1363:secondary,At2g01180.2:d:+1578:secondary
AGTTGAGAAC	0	3	2	2	2	At1g79810.2:d:+1408:secondary,At1g79810.1:d:+1404:secondary
GGCCTGTGAG	1	2	3	2	1	At1g78915.1:d:+1325:primary
GGCGGTTCCA	0	3	5	1	0	At1g78600.1:d:+900:primary
CTTTAGATCT	4	2	0	0	3	At1g78170.1:d:+904:primary
GACGAAGATG	0	7	1	1	0	At1g77800.1:d:+3073:secondary,At1g32690.1:d:+33:secondary,At1g09770.1:d:+591:secondary
CCAAGCTTAG	3	4	1	1	0	At1g77490.1:d:+1079:primary
TATCCAAACT	1	4	0	2	2	At1g76200.1:X:+-141:quaternary,Chr1:+23621400:quaternary
ATGCACACGC	2	4	2	1	0	At1g75460.1:d:+398:secondary
TATATCAACA	2	3	1	0	3	At1g75220.1:d:+1860:primary
TATCCGAGTA	2	4	1	0	2	At1g74270.1:d:+353:secondary
TTAGAACTTT	2	3	1	0	3	At1g73840.1:d:+1236:primary
TAAAAATTTG	1	1	2	4	1	At1g73670.1:d:+2195:primary
CAACATAACA	3	1	1	3	1	At1g72570.1:i:+1517:tertiary
AATCTTGACA	1	4	4	0	0	At1g72450.1:d:+603:primary
GACCTGATTG	0	1	1	4	3	At1g72440.1:d:+2911:primary
AAACTTTTTG	0	0	1	5	3	At1g72320.2:d:+2722:primary,At1g72320.3:d:+2713:primary
ATGTACACAA	4	4	0	1	0	At1g70900.1:d:+766:secondary
ACAAATCCAG	1	6	0	1	1	At1g70620.2:d:+2921:primary,At1g70620.1:d:+2698:primary
ACGATATGTA	2	1	0	1	5	At1g70610.1:d:+2291:primary
AATAGGTGAT	5	2	0	0	2	At1g70200.1:d:+1759:primary
TTTTCAAAAA	0	3	1	3	2	At1g69780.1:d:+1207:primary
GAGGATGATT	0	5	3	0	1	At1g69240.1:d:+1178:secondary,At3g10990.1:v:+347:secondary,At4g38870.1:v:+1070:secondary,At5g67220.1:d:+1097:secondary
GGAAAAAATG	4	1	3	1	0	At1g68730.1:v:+995:secondary
TTAAATAAAA	0	8	1	0	0	At1g68730.1:v:+97:secondary
AATTGAATAA	2	7	0	0	0	At1g68600.1:i:+1959:tertiary,At5g03780.1:i:+1531:tertiary
AGAATGTTGT	0	6	1	2	0	At1g68440.1:d:+663:primary
TAACAAACGA	2	1	2	1	3	At1g67890.1:d:+2752:primary
AAGAAAGAAT	3	3	0	1	2	At1g67730.1:d:+1162:primary
GAACGTATAC	2	4	2	1	0	At1g65800.1:d:+2727:primary
GCTGGATCAA	2	2	3	1	1	At1g65410.1:d:+1308:primary
TTGGTTATAA	2	4	3	0	0	At1g64680.1:d:+1021:primary
AATTTTTTCC	1	1	2	4	1	At1g64650.1:d:+1227:primary
TTGCGTTTTT	2	1	2	4	0	At1g64550.1:d:+2226:secondary
GGTGAATTTG	1	2	0	4	2	At1g59218.1:d:+3532:primary
AGATAAGTTT	1	3	1	4	0	At1g58380.1:d:+949:primary
GCCCTGAGAG	1	5	1	2	0	At1g57680.1:d:+1316:primary,At1g57680.2:d:+1269:primary
TTATTCGATC	4	1	1	2	1	At1g55460.1:d:+1469:primary
GGTGTTAAAA	1	0	3	4	1	At1g52730.1:d:+1499:primary,At1g52730.2:d:+1353:primary
TACTCGTCAA	5	0	0	4	0	At1g52590.1:d:+703:primary
GATAAAAAGG	0	3	2	4	0	At1g52380.1:d:+1267:secondary
CACTGCATTC	1	5	2	1	0	At1g51980.1:d:+1215:primary
GCAAATGTTT	2	1	5	0	1	At1g51950.1:d:+1054:primary
TGTTTTTTCT	1	2	2	2	2	At1g51520.1:d:+2036:primary,At5g66920.1:d:+1980:primary,At1g51520.2:d:+1958:primary
GTCATTGACG	4	0	4	1	0	At1g51400.1:X:-103:quaternary
ATCCGAAGGA	2	2	2	1	2	At1g50600.1:d:+1884:primary
ATGGGAATGT	4	1	3	1	0	At1g48900.1:d:+1595:primary,At1g15310.1:d:+1522:primary
GGGAAGTGTC	2	2	4	1	0	At1g48300.1:d:+708:secondary
TATACGGGTA	4	2	0	1	2	At1g48090.1:d:+12674:primary
ATTTTGAAAA	3	1	1	3	1	At1g47750.1:d:+914:secondary
TCCACAAAAA	1	2	1	3	2	At1g45050.1:d:+709:primary
CGACCAGCAA	0	1	7	0	1	At1g44770.1:d:+839:primary
TTTCTACCGA	1	3	1	1	3	At1g44100.1:d:+1865:secondary
GGCTGAGCTC	0	0	6	2	1	At1g42970.1:d:+619:secondary

ACTTTTGCTT	2	5	2	0	0	At1g36510.1:v:+1809:primary
TAATATGGCT	5	1	1	0	2	At1g33811.1:d:+1210:primary
ACCCAAGCAG	2	3	3	0	1	At1g33040.1:d:+586:primary
GTTGGTAAAA	1	2	2	3	1	At1g32800.1:v:+1972:primary,At3g50370.1:d:+6767:primary
AGAAATCTCG	0	5	1	1	2	At1g32130.1:d:+838:primary
AATGACTATA	4	2	2	0	1	At1g31812.1:d:+307:secondary
GTGTTTCTCT	0	0	2	0	7	At1g30680.1:d:+2403:primary
ACTACTTAGA	1	2	5	1	0	At1g30120.1:d:+1245:primary
TAAAAAGAGG	6	0	2	0	1	At1g28250.1:d:+669:primary
TAAATTTCGG	3	4	1	1	0	At1g27200.1:d:+2121:primary
AAAGCGATAG	8	0	1	0	0	At1g27080.1:v:-1990:secondary
GTGTGGATGA	2	5	1	1	0	At1g26670.1:d:+663:primary
TGGATTCTTG	0	4	3	2	0	At1g26580.1:d:+2639:primary
GTTTGCATTC	1	1	1	5	1	At1g25260.1:d:+882:secondary
ATGGGGACTG	2	2	4	0	1	At1g23870.1:d:+2914:primary
AACCAAAGCT	1	4	1	1	2	At1g22770.1:d:+3637:primary
AAGACGAGGT	5	0	2	2	0	At1g22270.1:d:+417:primary
TTAATCATCC	4	4	0	1	0	At1g22160.1:d:+636:primary
TGGTCTGAAT	1	1	5	0	2	At1g21830.1:X:+637:quaternary
TTTTTTTTTA	2	0	4	1	2	At1g21510.1:v:+1557:primary
TTCGTTTTTA	4	2	1	2	0	At1g21050.1:d:+849:primary
GCCTCAGTAA	1	2	3	3	0	At1g20340.1:d:+94:secondary
AGATGTTGTT	4	2	1	2	0	At1g20330.1:d:+1080:primary
TAGTCTGTTG	2	2	4	0	1	At1g20110.1:d:+2060:primary
TATGTTAGTT	1	2	3	2	1	At1g20100.1:i:+589:tertiary
CTCCTAAACG	1	3	2	3	0	At1g19715.1:d:+1988:primary
ATCAAGAGTA	4	4	1	0	0	At1g18470.1:d:+1535:primary,At1g18470.2:d:+1679:primary
AGAGAGATCT	1	0	2	3	3	At1g17940.1:d:+1401:primary
CGCGGCAATA	3	1	1	4	0	At1g17340.1:d:+2923:primary
ATGTTTGGAA	1	3	3	1	1	At1g17080.1:d:+376:primary
ATGGTTATGT	6	3	0	0	0	At1g16410.1:d:+1502:primary
CTCATAGTGG	2	2	1	2	2	At1g16320.1:d:+912:primary
ATGTCAATTT	4	3	1	0	1	At1g15880.1:d:+974:primary
TTTGCAGTTG	1	4	0	4	0	At1g15750.2:d:+3373:secondary,At3g17550.1:v:+354:secondary,At1g15750.1:d:+3402:secondary
ATAAATATGA	0	9	0	0	0	At1g15670.1:d:+1201:secondary
ATCTGATAAG	4	1	2	0	2	At1g15290.1:d:+4677:primary
AACAACTTAA	4	1	2	2	0	At1g14610.1:d:+3571:primary
GTTTGGTTGG	2	2	2	2	1	At1g13820.1:d:+1189:primary
TTGAAGGTGG	4	2	1	2	0	At1g12910.1:d:+1048:primary,At3g26640.1:d:+965:primary
GATTAGACAA	3	1	0	4	1	At1g12810.1:d:+572:primary
TAATCTTGTA	1	1	4	2	1	At1g12120.1:d:+1844:primary
GTCTCACCAT	1	4	3	0	1	At1g11720.1:i:+2781:tertiary
ATATGTTATT	1	3	1	0	4	At1g11310.1:d:+2109:primary
CTATTTTTTC	4	1	2	2	0	At1g11120.1:i:+2613:tertiary,At1g24706.1:i:+10991:tertiary,At5g12150.1:i:+2264:tertiary
AGTTGTCGAT	1	0	2	1	5	At1g10870.1:X:--47:quaternary
TTTTACTGTC	4	1	1	0	3	At1g10630.1:d:+743:primary
TCGTCGGAGA	1	2	3	2	1	At1g10470.1:d:+571:primary
TATTTTATTT	3	3	1	2	0	At1g09250.1:d:+874:primary
TTTTAAGAAA	4	2	0	1	2	At1g08980.1:i:+415:tertiary
TGCCGAGGGA	0	3	1	2	3	At1g08780.1:d:+289:primary
TGAGTGGGCT	0	9	0	0	0	At1g07440.1:d:+563:primary
TGTCTTAGTT	0	0	3	3	3	At1g07420.2:d:+1206:primary,At1g07420.1:d:+1009:primary
GCTCTAGTTT	2	4	2	1	0	At1g07210.1:d:+1059:secondary
GATCCTTTTT	3	2	4	0	0	At1g07140.1:d:+1075:secondary,At2g47070.1:d:+334:secondary
GTCGCCGAAT	2	1	3	1	2	At1g06530.1:d:+895:primary
GTCATACTCG	4	2	1	1	1	At1g06130.2:d:+1166:primary,At1g06130.1:d:+1187:primary
AGAATGATCC	0	3	5	1	0	At1g04850.1:d:+542:secondary
GTTTCAATGG	0	5	2	2	0	At1g04780.1:d:+2416:secondary,At4g17410.1:d:+1337:secondary,At5g64170.1:d:+500:secondary
AAGAAATTGT	1	5	2	1	0	At1g04040.1:d:+545:primary
AAACAAGAAA	1	0	1	3	4	At1g02960.2:d:+1095:secondary,At1g02960.1:d:+1095:secondary,At5g10300.1:d:+474:secondary
CTTCTGACAG	1	4	0	4	0	At1g02170.1:d:+1006:secondary

GGTTGCACCA	4	1	1	3	0	At1g01770.1:d:+2052:primary
AACGCAGAAA	0	4	4	0	1	At1g01550.1:d:+916:primary
GAATGATCTA	2	4	1	1	1	At1g01430.1:d:+1436:primary
TTTTTTGTTG	1	2	2	2	2	At1g01230.1:d:+723:primary
CTTACTATAA	5	1	0	1	1	No gene matches found
CGCCTTCGCC	0	1	3	4	0	No gene matches found
GGCAGGCAAA	2	3	1	2	0	No gene matches found
AAACCGTAAG	0	4	0	3	1	ChrM:+364705:quaternary,ChrC:+105214:quaternary
TATTTGGATA	3	3	0	2	0	ChrC:+76392:quaternary
GATATTGGAT	4	3	1	0	0	Chr5:+9789529:quaternary,Chr3:+1292419:quaternary
TTTTTTTCAA	4	1	1	1	1	Chr5:+9496989:quaternary,Chr3:+14410417:quaternary
AATAAGTAAA	2	3	1	1	1	Chr5:+8665127:quaternary
AGCAAAAAAA	2	0	2	3	1	Chr5:+389722:quaternary
GCAAAAAAAA	0	3	3	2	0	Chr5:+26056994:quaternary,Chr2:+1210524:quaternary,Chr5:+8439951:quaternary
CCTAATCTGC	2	3	1	2	0	Chr4:-1201225:quaternary
GGCCACAAAA	2	0	4	1	1	Chr4:+7303726:quaternary,Chr2:+7912675:quaternary
TTGTAAAAGT	0	5	1	1	1	Chr4:+2605617:quaternary
GCCAAAAAAA	3	2	1	1	1	Chr4:+15227685:quaternary,Chr5:+20188482:quaternary
ACACAAAACA	3	1	1	3	0	Chr3:+904179:quaternary,Chr1:+7908085:quaternary
ATAATAAAAA	1	1	1	1	4	Chr3:+14236785:quaternary,Chr4:+9698621:quaternary
AAATTAAAAA	0	2	1	3	2	Chr3:+11405199:quaternary,Chr5:+15779030:quaternary,At1g74450.1:X:+-95:quaternary
TTGGATAAAA	3	0	2	3	0	Chr3:+11350352:quaternary,Chr5:+20429721:quaternary,Chr1:+7700150:quaternary,At2g38950.1:X:+-110:quaternary
AATCTAAAAA	3	3	1	1	0	Chr2:+925782:quaternary,Chr1:+22045574:quaternary
TTTGTAAAAA	1	1	3	1	2	Chr2:+833447:quaternary,Chr2:+833334:quaternary
GATTTGTCAT	2	2	1	1	2	Chr2:+16089854:quaternary
CAAGGAATAG	4	1	1	0	2	Chr2:+12238243:quaternary
AGAAAAAGAC	0	5	0	2	1	Chr1:+8646315:quaternary,At3g51720.1:X:+-52:quaternary
CACCAAAAAA	2	0	2	3	1	Chr1:+7907981:quaternary,At3g26400.1:X:+-188:quaternary
AACTAAAAAC	0	1	2	4	1	Chr1:+4113352:quaternary
TGTATTCGAT	0	0	2	5	1	Chr1:+26041140:quaternary
TTTCTGTTGA	1	2	1	2	2	Chr1:+25384357:quaternary
ACAAATACAA	2	3	1	2	0	Chr1:+16971344:quaternary,Chr4:+9419459:quaternary
GAATCTCTCG	2	1	1	3	1	AtCg01130:d:+1472:secondary
TACATCTGTA	2	0	1	3	2	AtCg00630.1:X:+-69:quaternary
TGTAAACGTA	5	1	1	0	1	AtCg00510.1:X:+246:quaternary
CCCGTGTGTA	2	1	4	1	0	At5g66860.1:d:+729:primary
GTTGCTTCAG	0	5	2	1	0	At5g66510.1:d:+621:primary
TAATTTAAGT	1	2	2	2	1	At5g66490.1:d:+500:primary
AAATTGCGTT	2	3	1	1	1	At5g66250.1:d:+1256:primary,At5g66250.2:d:+1595:primary
AAGATAAACC	0	3	0	3	2	At5g66240.2:d:+1270:primary,At5g66240.1:d:+1175:primary
AGTTCAGGAG	0	6	1	1	0	At5g66210.2:d:+1529:primary,At5g66210.1:d:+1557:primary
AAATTCGTCT	0	1	2	2	3	At5g65930.2:d:+3924:primary,At5g65930.1:d:+3921:primary
TCGCAGTTGA	2	1	2	1	2	At5g65930.1:d:+2366:secondary,At2g35155.1:d:+1048:secondary,At5g65930.2:d:+2369:secondary
AAGTGATAGT	1	3	2	0	2	At5g64330.1:d:+2472:secondary
TTTAGTTCCA	0	3	1	2	2	At5g63790.1:d:+802:secondary
GTAATCTCGA	3	5	0	0	0	At5g63470.1:d:+742:primary
TGTGTATATA	4	0	1	1	2	At5g63180.1:d:+1338:primary
TAACCTCGCG	2	1	1	3	1	At5g61420.1:d:+1740:primary,At5g61420.2:d:+1360:primary
ATTGTAAAAA	4	0	1	2	1	At5g61270.1:d:+1251:primary
AAATTAGTTA	3	2	1	1	1	At5g61170.1:X:+286:quaternary
GAATTGATGC	2	1	0	4	1	At5g60750.1:d:+1211:primary
GATCGAACAA	2	0	4	1	1	At5g59613.1:X:-15:quaternary
TTTAAGATAT	3	0	3	0	2	At5g59310.1:d:+573:primary
AAAAAAGAAA	2	2	2	0	2	At5g59060.1:v:+741:secondary
AAAGCTCTGA	2	3	2	0	1	At5g58950.1:d:+1808:primary
AAGGAGTTGA	0	2	3	0	3	At5g58230.1:d:+1506:primary
CAGCTTTGAC	1	2	4	0	1	At5g57785.1:d:+512:primary,At3g01650.1:d:+1596:primary
TCTAACTTGA	1	4	3	0	0	At5g57760.1:d:+488:primary
GCTCTGTCGT	2	3	2	0	1	At5g56860.1:d:+1284:primary
AAGAAGAGCT	4	2	1	0	1	At5g56360.1:d:+2252:primary

AAAATCTTAC	4	2	0	1	1	At5g56290.1:d:+2352:primary
GGTGCATTGG	2	2	3	1	0	At5g56240.1:d:+3251:primary
TCCTGTGAAC	3	0	2	3	0	At5g56170.1:d:+250:primary
ATTTAACATA	3	1	1	0	3	At5g54600.1:d:+792:primary,At5g54600.2:d:+735:primary
TCGTTTTCCT	2	3	1	1	1	At5g54390.1:d:+1554:primary
AACACGAGAC	0	6	2	0	0	At5g54310.1:d:+1396:primary
TCTGAGCATC	1	2	0	0	5	At5g53400.1:d:+996:primary
AGTTGCAGAG	2	0	3	2	1	At5g51890.1:d:+1075:primary
ACAAGAAGAA	4	2	1	0	1	At5g51350.1:v:+2371:secondary,At4g34300.1:v:+1066:secondary,At4g31910.1:d:+117:secondary
TATAAAGCAT	1	3	0	0	4	At5g51230.1:d:-2354:secondary,At5g51230.2:d:-2351:secondary
CCAGGAGTTC	2	2	0	3	1	At5g51120.1:d:+684:primary
ATGATGTGGT	0	2	3	3	0	At5g50210.1:d:+2403:primary
GCTCTTTGTG	2	1	3	1	1	At5g49970.2:d:+1623:primary,At5g49970.1:d:+1639:primary
CTTGTCAAGC	1	3	0	3	1	At5g48790.1:d:+1124:primary
TCAAAATTAA	4	0	1	1	2	At5g48790.1:d:+1061:secondary
TTCTCTCTTC	2	4	0	2	0	At5g48480.1:d:+348:primary
TGAAGGAGCT	1	7	0	0	0	At5g48150.2:d:+1467:primary,At5g48150.1:d:+1392:primary
GGTCGACAAA	1	1	3	2	1	At5g48020.1:d:+1271:primary
TGATAGAACG	1	0	2	2	3	At5g47640.1:d:+857:primary
GGGACACATA	1	4	0	2	1	At5g47310.1:d:+453:primary
TATTTTTCTA	0	2	2	1	3	At5g47080.1:d:+1256:primary,At5g59060.1:v:+982:primary,At3g54760.1:v:+3172:primary,At5g47080.2:d:+1446:primary,At2g19400.1:d:+1958:primary
GCTTTGACCA	1	4	1	1	1	At5g45410.1:d:+868:primary
AACAGTATGA	0	7	1	0	0	At5g44260.1:d:+1252:primary
GCCATTTCAA	4	1	0	1	2	At5g43130.1:d:+2255:primary
AGTCTTTTCA	0	0	2	4	2	At5g42270.1:d:+2247:secondary
CTTGGTTTGG	2	1	3	0	2	At5g42150.1:d:+1221:primary
ATCATATCAG	0	5	2	0	1	At5g41520.1:d:+82:secondary
AAACGTTAGG	3	3	2	0	0	At5g41220.1:X:+1159:quaternary
AATGCTTGTG	3	1	2	1	1	At5g40500.1:d:+576:primary
AATCTGAAAG	0	8	0	0	0	At5g40450.1:d:+3743:secondary,At5g40450.1:d:+1757:secondary
ACGAGGAGGA	4	2	1	0	1	At5g39510.1:d:+710:primary
TAAACGAAGA	2	3	1	1	1	At5g38840.1:d:+2027:primary
GAGATAGTTA	0	3	2	3	0	At5g38830.1:X:+357:quaternary
GAAGAAGAAA	2	2	2	2	0	At5g37150.1:v:+2851:secondary,At4g21500.1:v:+354:secondary,At3g43315.1:p:+591:secondary,At5g52090.1:v:+2362:secondary,At1g19835.1:d:+259:secondary,At4g37280.1:d:+873:secondary,At2g06980.1:p:+1385:secondary,At2g26660.1:v:+1175:secondary,At2g31220.1:d:+114:secondary,At3g50370.1:d:+1672:secondary,At1g72960.1:d:+1519:secondary
TTCTTTGAAA	3	1	0	3	1	At5g35650.1:v:+915:secondary
AGAAAGCAGG	2	3	2	1	0	At5g35200.1:d:+1877:primary
CAAAACAAAA	2	0	3	2	1	At5g33382.1:p:+4401:secondary,At1g61180.1:d:+1307:secondary,At1g61190.1:v:+1506:secondary
AAGGGTCTCA	1	2	3	1	1	At5g28770.1:d:+622:secondary,At5g28770.2:d:+589:secondary
AATCCAAGGT	0	4	4	0	0	At5g28630.1:d:+79:primary
AGGCTTTTAG	0	0	2	1	5	At5g28580.2:p:+270:secondary,At5g50920.1:d:+2775:secondary
CTCTCATCTA	2	2	2	1	1	At5g27290.1:d:+1096:secondary,At1g33270.1:d:+1805:secondary,At1g33270.2:d:+1980:secondary
TATATGTCAA	0	3	2	2	1	At5g26340.1:d:+1880:primary
CTCATCGCCT	3	3	2	0	0	At5g25100.1:d:+1931:primary,At5g10840.1:d:+1883:primary
TGTGCAAACG	1	1	5	1	0	At5g24340.1:d:-1685:secondary
TTCTAAGCAA	2	1	4	1	0	At5g24314.1:d:+388:primary
CTTGGTTGTT	1	1	1	4	1	At5g24120.1:d:+783:secondary
ACTGAAACGG	1	2	2	1	2	At5g23535.1:d:+783:primary
ATCAGTGGTC	2	1	1	0	4	At5g23395.1:d:+931:primary
TAAACATTAT	2	2	0	1	3	At5g22950.1:d:+894:primary
TAACACCAGT	4	0	3	0	1	At5g22875.1:d:+442:primary
TTTTCCGGTT	2	1	1	2	2	At5g22300.1:d:+970:primary
GCTTGTCCTT	3	1	1	1	2	At5g21430.1:d:+639:primary
GTTGACATCC	2	3	1	0	2	At5g20060.1:d:+844:primary,At5g20060.2:d:+1293:primary
CCAAATTTGG	3	1	2	2	0	At5g19930.1:d:+994:secondary
CTGCAGTGAC	2	0	1	2	3	At5g19550.1:d:+1291:secondary
GCGACTCAAC	1	0	4	0	3	At5g18380.1:d:+69:secondary
TCAATCAAAC	2	2	0	3	1	At5g18140.1:d:+1009:primary

TTTTAAGAGT	1	4	1	2	0	At5g17890.1:d:+4977:primary
ATCAATTTAT	1	2	0	2	3	At5g17530.2:d:+2138:primary,At5g17530.1:d:+2134:primary,At3g20020.1:d:+1782:primary
TGAAAAGACA	2	3	2	1	0	At5g17310.2:d:+1582:secondary,At5g17310.1:d:+1300:secondary
TGTTTTTGAG	1	3	2	2	0	At5g16510.1:d:+1329:primary,At5g16510.2:d:+1390:primary
CTCGGTTACT	0	8	0	0	0	At5g16340.1:d:+1201:primary
AATATTGGGA	3	1	1	2	1	At5g15610.1:d:+1603:primary,At5g15610.2:d:+1516:primary
AAGAGCTGTT	0	0	1	2	5	At5g15520.1:d:+463:primary
TATCTTTGTT	2	0	0	2	4	At5g14640.1:d:+1571:primary,At4g18190.1:v:+1557:primary
GAGGAGGATG	1	5	0	0	2	At5g13780.1:d:+540:secondary
GCTTTAGATG	2	3	2	1	0	At5g13570.1:d:+1570:primary
TACGGACAGA	1	1	1	2	3	At5g13540.2:d:+2667:primary
TTTTATTAGA	2	4	0	1	1	At5g12050.1:d:+1386:primary
TTGTTTCGTC	1	3	3	1	0	At5g11880.1:d:+1109:secondary,At1g12230.1:d:+1436:secondary
ATGATCGGAG	0	8	0	0	0	At5g11070.1:d:+218:primary
AGATGCAAAG	6	0	1	1	0	At5g09990.1:d:-385:secondary,At5g09990.1:d:-395:secondary
CTTAATACTT	1	6	1	0	0	At5g08670.1:d:+1722:secondary
AAGTTCGTGA	1	3	1	2	1	At5g08130.1:d:+1506:secondary
ATGCTGTCAG	0	6	2	0	0	At5g08080.1:d:+821:primary
ATGCCTCATA	1	0	1	0	6	At5g07810.1:d:+3563:primary
GCAACAAGCT	3	0	3	2	0	At5g07180.1:d:+857:secondary
CAATTTTTAT	2	2	1	1	2	At5g06600.2:d:+3736:primary,At5g06600.1:d:+3737:primary
AATTGAATTG	1	2	2	2	1	At5g06280.3:d:+553:primary
GCAGGCAATT	1	1	3	1	2	At5g06260.1:d:+1521:primary
ATGATTGGCT	1	0	1	3	3	At5g05740.2:d:+1570:secondary,At5g05740.1:d:+1657:secondary,At3g31425.1:p:+729:secondary
AAAAGATTAG	1	6	1	0	0	At5g05690.1:d:+1001:secondary,At5g35390.1:v:+1557:secondary
TCATTAAGTT	0	2	3	2	1	At5g05590.1:d:+1022:primary,At1g07780.1:d:+1085:primary,At1g07780.3:d:+1886:primary,At1g07780.2:d:+1040:primary,At5g05590.2:d:+1251:primary
TATAAAAGAA	2	3	0	0	3	At5g04170.1:d:+1428:primary
CTCACCGTCC	2	3	2	1	0	At5g04170.1:d:+1184:secondary
TACAGACCAA	2	2	2	0	2	At5g03910.1:d:+1995:secondary
TACTAAAGTT	1	3	1	1	2	At5g03455.1:d:+523:secondary
GTTTTAAAAA	1	0	4	2	1	At5g02880.1:d:+4608:primary
GGGGATGAAG	2	2	0	3	1	At5g01820.1:d:+1359:primary
AAGAAGAAAC	2	4	0	1	1	At4g39150.1:d:+1034:primary,At3g25890.1:d:+61:primary
TAAATCTCGG	3	3	1	1	0	At4g38860.1:d:+478:primary
TCCGCCACTT	0	0	5	1	2	At4g38770.1:d:+1355:secondary
TGAGTGTGTA	4	1	3	0	0	At4g38690.1:d:+1125:primary
GTCGTATGCA	4	0	1	2	1	At4g38430.1:d:+1763:primary
GGTCTCATAA	2	2	3	0	1	At4g37520.1:d:+1190:primary
ATGAATATAA	0	7	0	0	1	At4g36670.1:d:+1594:primary
CGGTGGAGTT	0	4	2	2	0	At4g36020.1:d:+759:primary
CTTAGTTTTA	1	4	1	1	1	At4g35630.1:d:+1435:primary
TTGCTACGTT	0	2	2	2	2	At4g35260.1:d:+1383:secondary
GATGTTCGAC	0	3	2	2	1	At4g34700.1:d:+593:secondary
GCCGATAACT	0	3	4	1	0	At4g34215.2:d:+1039:secondary
AAACTTGACT	2	3	3	0	0	At4g33750.1:X:+605:quaternary
TGACTACTAC	3	3	2	0	0	At4g33660.1:d:+435:primary
AGATCGGTTG	2	2	1	3	0	At4g33630.1:d:+2173:secondary
GTCTCTCTGC	1	0	4	2	1	At4g32600.1:d:+1941:primary
TAAATAAACA	0	1	1	0	6	At4g31860.1:d:+1502:secondary,At2g27160.1:v:+203:secondary,At4g31860.2:d:+1587:secondary
GGTTTGTTCC	0	2	2	2	2	At4g31490.1:d:+3287:primary
AAATGTCTCA	2	5	0	0	1	At4g31480.1:d:+2695:primary
TAATTATATA	0	0	0	1	7	At4g31390.1:d:+2256:primary
AGGAGGACTA	0	5	3	0	0	At4g31290.1:d:+780:primary
ACCAAATATG	2	3	2	1	0	At4g30993.2:d:+738:secondary,At4g30993.1:d:+738:secondary
AAGAAAACAT	2	0	2	2	2	At4g30990.1:v:+6998:primary
GAGCTTGTAC	0	7	0	1	0	At4g30960.1:d:+651:secondary,At3g61010.1:d:+1130:secondary
GCAGAAGGGT	1	3	1	3	0	At4g29350.1:d:-572:secondary
ATAGAGAATG	5	1	1	1	0	At4g29220.1:d:+1958:secondary,At4g08333.1:p:+1324:secondary
GGGTCTTGAA	2	1	3	2	0	At4g27830.1:d:+1139:primary
ATGATTGTTG	0	2	3	2	1	At4g27720.1:d:+1386:primary

GGGCAAGGAA	2	1	3	1	1	At4g27700.1:d:+783:secondary
TTGAGTTCAG	1	1	3	2	1	At4g27430.1:d:+3378:primary
AAAAAAAAGA	0	2	4	2	0	At4g27290.1:i:+1493:tertiary
TATCACTCTG	3	2	1	1	1	At4g27040.1:d:+1425:secondary
TACCATTTAA	1	0	2	2	3	At4g27030.1:v:+1455:secondary,At1g02520.1:v:+4462:secondary,At4g03730.1:p:+1520:secondary
GGAACTTTTG	1	0	2	4	1	At4g26650.1:d:+2254:secondary,At4g34440.1:d:+1516:secondary
TCTATTTGTT	4	3	1	0	0	At4g26570.2:d:+979:primary,At4g26570.1:d:+967:primary
TTTTTTCTTT	0	3	0	1	4	At4g26000.1:d:+1730:primary
GTATTCGAAA	3	3	1	1	0	At4g25880.1:d:+3148:primary,At4g25880.2:d:+3121:primary
AATTTTGATG	0	2	3	2	1	At4g25280.1:d:+810:primary
GATTAAAGGC	1	5	0	2	0	At4g24750.1:d:+471:primary
ATACAACTCT	1	1	1	3	2	At4g24580.1:i:+755:tertiary
AACACTTTAG	4	0	1	0	3	At4g23600.2:d:+1168:primary,At4g23600.1:d:+1394:primary
AGAGAGAAGG	1	4	1	0	2	At4g21990.1:d:+980:primary
AGTTTCTTAA	1	0	1	3	3	At4g21150.1:d:+2409:primary
AAAAATGTAT	2	2	0	0	4	At4g20070.1:d:+1727:primary
AAGCACAATC	2	4	0	2	0	At4g19530.1:d:+3691:primary
TGTAAACCAC	2	1	2	2	1	At4g19350.1:d:+736:secondary
GTTTTGCTTT	0	2	2	3	1	At4g18710.1:d:+1763:primary
GTTTAGAGAA	2	0	3	1	2	At4g17510.1:d:+939:primary
GGTCAACCTT	1	5	1	1	0	At4g17460.1:d:+997:primary
AGAAAAGCTA	0	4	1	1	2	At4g17030.1:d:+579:secondary
TGTTGAGTTA	2	5	0	1	0	At4g16580.1:d:+1723:primary
ACCAACTTCT	0	3	4	1	0	At4g16515.1:d:+492:primary
GGGAGAAAGA	0	3	3	2	0	At4g16130.1:d:+2421:secondary
ATCAATGCCT	2	6	0	0	0	At4g14716.1:d:+640:primary
TGTTTTTAAT	2	2	2	1	1	At4g14550.1:d:+844:secondary
TTGTCGTGTT	0	6	1	1	0	At4g14145.1:d:+674:primary
GCGCGATCAA	2	2	2	1	1	At4g13840.1:X:-840:quaternary
TTGTGGTTGT	1	0	2	4	1	At4g10360.1:d:+972:primary
TCGAAGACTG	2	5	1	0	0	At4g10120.1:d:+2946:primary
GATTGTGGTA	2	2	2	2	0	At4g09150.1:d:+3385:primary
TAATAATAAT	1	0	2	0	5	At4g09130.1:v:+965:secondary
AACCAGGCCT	1	2	1	4	0	At4g08980.3:d:+1066:primary,At4g08980.2:d:+1543:primary,At4g08980.1:d:+1087:primary
GCAGGTCAAA	2	0	0	5	1	At4g08108.1:p:+746:secondary,At2g18440.1:d:+293:secondary
TCTTAGCTCC	1	3	2	1	1	At4g04720.1:d:+1925:primary
ACATACAGTG	1	3	1	1	2	At4g04620.1:d:+514:primary,At4g04620.2:d:+419:primary
ATTTTGGTTT	2	2	1	0	3	At4g02390.1:d:+1244:secondary
TTCAAGCGTA	2	4	1	0	1	At4g01590.1:d:+995:primary
TGCTTGTGTC	2	1	0	2	3	At4g01100.1:d:+1651:primary
TGGTTCCCTG	1	4	0	2	1	At4g00570.1:d:+1878:primary
CAGTGTGGTA	1	4	3	0	0	At4g00460.1:i:+1395:tertiary
ATCATTGTAT	3	0	1	2	2	At3g63410.1:d:+1154:secondary
GCTGATCACT	1	0	4	3	0	At3g62820.1:d:+699:primary
TGCGTCTAAT	1	3	2	1	1	At3g62130.1:d:+1672:primary
TTACTGAAAG	2	0	1	3	2	At3g61810.1:v:+1796:primary,At2g47490.1:d:+1481:primary
TCATCAATGA	1	1	4	1	1	At3g61210.1:d:+899:primary
GAGGAAGGAG	0	3	2	2	1	At3g61150.1:d:+2431:primary
TAAAACTCAT	4	1	2	0	1	At3g60250.1:d:+1299:primary
TAAATCTGAA	1	5	0	2	0	At3g59900.1:d:+706:primary
TTTTGCTGCT	0	2	0	3	3	At3g59820.1:d:+2354:primary
GAGTCATAGA	1	1	0	1	5	At3g58690.1:d:+1478:primary
GCTTGAAGAG	0	2	3	2	1	At3g58670.1:d:+864:primary
TGAAAGAAAA	2	0	1	2	3	At3g58340.1:v:+1180:secondary
TGGAGTATGA	0	2	3	2	1	At3g57610.1:d:+1359:primary
AGATTATGTC	1	1	6	0	0	At3g57560.1:d:+1106:primary
ATCAGTTTGC	3	0	3	0	2	At3g57230.1:d:+829:secondary
AAGTGCAACA	1	7	0	0	0	At3g56150.1:d:+2442:primary
TGTGCCACGG	0	7	0	1	0	At3g56130.1:d:+308:primary
GCATTTCGAT	1	1	2	2	2	At3g56090.1:d:+792:secondary

GGAAATCCTT	1	2	0	2	3	At3g56070.1:d:+543:primary
GTTGAGCCGT	2	1	2	1	2	At3g55480.2:d:+3400:primary,At3g55480.1:d:+3472:primary
AAACAGTAAA	2	3	0	2	1	At3g55000.1:d:+1042:primary
CTTCTTTATG	0	4	0	1	3	At3g53530.1:d:+894:secondary,At5g65450.1:v:+2232:secondary
GTTAGTGACT	2	4	1	0	1	At3g53520.2:d:+1228:primary,At3g53520.1:d:+1226:primary
TTTCTGTGAC	1	3	0	1	3	At3g53000.1:d:+1374:primary
TTATTCTTTT	2	1	2	1	2	At3g51660.1:d:+489:secondary
GATTACGGTT	0	0	1	6	1	At3g51240.1:d:+877:primary,At4g06569.1:p:+2179:primary
GACTGGGAAG	0	6	1	1	0	At3g50950.1:d:+2586:primary,At3g50950.2:d:+2610:primary
ATTGTTTCAG	3	2	1	1	1	At3g50270.1:d:+1359:primary
ATAAGTGGTT	1	0	4	3	0	At3g49670.1:d:+3416:primary
ACTCAGGCTG	2	1	4	0	1	At3g49470.1:d:+645:primary
CTTTGTGAAC	1	2	0	2	3	At3g48960.1:v:+1304:primary,At1g49470.1:d:+1111:primary
ATTGATACAT	4	2	0	1	1	At3g48940.1:i:+456:tertiary,At3g46740.1:i:+1957:tertiary
AAAGCCTTTT	1	1	2	2	2	At3g48800.1:v:+1318:secondary
TAAGGTTTTG	2	1	2	2	1	At3g47530.1:v:+2608:primary,At5g57460.1:d:+2196:primary
TTCCAAAATC	1	1	2	3	1	At3g47520.1:d:-1515:secondary
AGAATCATTA	1	4	1	2	0	At3g45980.1:d:+721:secondary
GCCCAATAAG	2	1	0	2	3	At3g44430.1:d:+632:primary
CTTAATGAGG	0	4	2	0	2	At3g44010.1:X:-157:quaternary
AGTTTTTCTA	1	2	2	2	1	At3g43800.1:d:+735:primary
TAATGTATGT	2	1	3	1	1	At3g42220.1:p:+1333:secondary,At5g47240.1:d:+1256:secondary
TCCTAATCTT	3	3	2	0	0	At3g42150.2:d:+332:primary,At3g42150.1:d:+547:primary
ATGTGGTCGA	3	1	2	2	0	At3g32980.1:d:+1067:secondary
TCGTTTTGCA	1	3	3	1	0	At3g28920.1:d:+1253:primary
TTTGCGAAAC	0	1	2	2	3	At3g25230.1:d:+1683:primary
TTGACTATAA	2	0	1	2	3	At3g24010.1:d:+837:primary
TTACTTTGTG	4	2	0	2	0	At3g23940.1:d:+2003:primary
TTTTCTTTTT	2	3	1	0	2	At3g23280.2:d:+1637:primary,At3g23280.1:d:+1640:primary,At5g56800.1:v:+1390:primary
AAGAACATTT	1	1	3	1	2	At3g22510.1:d:+465:primary
TTTTGAATGT	3	1	1	1	2	At3g22480.1:d:+677:primary,At3g22480.2:d:+592:primary
TGTTTTTGGA	3	0	2	3	0	At3g22300.1:d:+935:primary
ACTGGAATCG	0	2	2	3	1	At3g22150.1:X:+2732:quaternary
TAGCTCTTAA	2	1	4	0	1	At3g21790.1:v:+1825:secondary
TGGTTTTGCG	1	2	1	3	1	At3g21410.1:v:+1012:secondary
CATTCGCAGT	1	1	2	2	2	At3g21215.1:d:+1292:primary
TAATATTTTT	1	5	1	1	0	At3g20770.1:d:+2211:primary
GCATTATTTT	2	0	3	2	1	At3g20230.1:d:+624:secondary
AATGAGTTTC	1	3	2	2	0	At3g19760.1:d:+1024:primary
TATTGCTTAT	1	2	2	1	2	At3g19553.1:d:+1759:primary
TCACGGGACG	1	2	4	0	1	At3g19515.1:d:+1669:primary
AAATCATTTG	0	6	0	1	1	At3g18820.1:d:+445:secondary
TCCAATCTGT	2	2	4	0	0	At3g18290.1:d:+3832:primary
ACGGCTAGAT	0	5	3	0	0	At3g18140.1:d:+1044:primary
AAAAAATGAT	3	1	2	2	0	At3g17340.1:d:+2656:primary,At5g59390.1:v:+1483:primary
TTACACAGAA	1	3	2	2	0	At3g17205.1:v:+3347:primary
AATAGAACCA	2	4	1	1	0	At3g17040.1:d:+1837:primary
TCTAATGCTT	1	1	0	4	2	At3g16660.1:X:+459:quaternary,Chr4:+10981584:quaternary
GTCAGAAGTC	0	6	2	0	0	At3g15530.1:d:+868:primary,At3g15530.2:d:+870:primary
AAAGCTCAGA	1	3	2	1	1	At3g15115.1:d:+1098:secondary,At1g11310.1:d:+1911:secondary
TCTGACCCTT	2	4	1	1	0	At3g14930.2:d:+1473:primary,At3g14930.3:d:+1374:primary,At3g14930.1:d:+1391:primary
AGGAAGTAAG	3	0	2	1	2	At3g14280.1:d:+1104:primary
GGTTGGCAAA	2	0	2	1	3	At3g13930.1:d:+2056:primary
TGATCTTGTG	0	3	2	1	2	At3g13860.1:d:+1833:primary
TGGTAAACAG	3	0	2	1	2	At3g13490.1:d:+2041:primary
CTTATGGGTT	2	0	2	2	2	At3g13300.1:d:+4229:primary,At3g13300.2:d:+4124:primary
AACCAAGCAG	4	0	0	1	3	At3g12670.1:X:-22:quaternary
CACTCTATGA	0	4	2	2	0	At3g12480.1:d:+1232:primary
GACTCTCCTC	0	3	1	4	0	At3g11080.1:d:+1654:secondary,At4g38510.2:d:+1453:secondary,At4g38510.1:d:+1423:secondary
AGTCAGCTGA	1	3	1	2	1	At3g10760.1:d:+1304:primary
TAATTCATCT	2	0	0	2	4	At3g10610.1:d:+717:primary

CTCACTGTTT	0	4	1	1	2	At3g10300.2:d:+1307:primary,At3g10300.3:d:+1094:primary
GACTATATTT	1	2	2	1	2	At3g09820.1:d:+775:secondary,At3g09820.2:d:+968:secondary
GAAGAGTGAT	2	0	2	1	3	At3g09090.1:d:+2171:secondary,At1g07200.1:d:+1061:secondary,At2g44860.1:d:+598:secondary
ATGGTGTTCT	2	4	1	0	1	At3g09040.1:v:+2527:secondary,At2g43100.1:d:+815:secondary
CCTCCCTAAG	3	1	2	1	1	At3g08580.1:X:-396:quaternary
CCACCATATG	0	4	2	1	1	At3g08530.1:d:+5077:primary
TCTTCGGTAA	3	2	2	0	1	At3g07870.1:d:+1518:primary
TCTCGTGTCT	2	4	0	0	2	At3g07680.1:d:+783:secondary
CAAAAGTACG	1	1	0	4	2	At3g07090.1:d:+859:primary
CAGGTTCGAG	0	6	1	1	0	At3g06730.1:d:+475:secondary
GTCGAGGCGT	1	3	1	2	1	At3g06430.1:d:+1401:primary
GATTTGTGTC	1	0	3	4	0	At3g06070.1:d:+559:secondary
TAGTCGAAGA	1	5	2	0	0	At3g05900.1:d:+1628:primary
CTCAAGTTAT	2	2	3	1	0	At3g05545.1:d:+1404:primary
TGAGCTTTTT	2	2	1	1	2	At3g05090.1:d:+2841:primary
TTATTTCTGA	0	5	2	0	1	At3g04470.1:d:+1423:primary
CTTGCGAGGG	0	4	0	2	2	At3g03920.1:d:+291:primary
CAACAAAGCT	0	3	4	0	1	At3g03870.2:d:+1013:secondary,At5g18130.2:d:+1595:secondary,At5g18130.1:d:+906:secondary
CATTACTTAT	1	1	0	3	3	At3g03440.1:d:+1502:primary
ACAAGTGCTG	4	2	0	2	0	At3g03070.1:X:-281:quaternary
TGGTACCTGT	0	8	0	0	0	At3g02550.1:d:+1058:primary
AACCTTATCC	2	4	1	1	0	At3g02360.2:d:+1324:primary,At3g02360.1:d:+1169:primary
AAACCGTGGC	2	2	1	1	2	At3g02350.1:d:+1827:primary
GCCTTGCAGT	1	2	2	2	1	At3g02250.1:d:+2464:primary
GGGGGAATAA	2	0	2	3	1	At3g02050.1:d:+2520:primary
GCGCTTCAGG	1	4	0	2	1	At3g01780.1:d:+3571:primary
TGATGGAATT	1	1	1	5	0	At3g01160.1:d:+908:secondary,At1g64640.1:d:+580:secondary
TATTTTTCTT	2	1	0	4	1	At2g48140.1:d:+784:primary,At4g20410.1:d:+1122:primary
GTGTGGCCAC	0	4	2	1	1	At2g47470.2:d:+589:primary,At2g47470.1:d:+589:primary
ATGCTCACCA	1	4	0	0	3	At2g46900.1:d:+1954:secondary
CTTTGTAGCT	0	1	0	2	5	At2g46680.1:d:+1228:primary
ACAATCCATA	1	2	2	0	3	At2g45710.1:d:+520:primary
TTCTTTTTTA	0	3	0	2	3	At2g45040.1:d:+1197:primary,At3g17520.1:d:+1016:primary
TTGAAATATT	0	0	4	1	3	At2g44940.1:d:+1123:primary
CTTCAATCTA	4	3	0	1	0	At2g44130.1:d:+1335:primary
CGTACCTATT	1	1	2	3	1	At2g44100.1:d:+1583:secondary
GGAGAGATAA	1	0	0	4	3	At2g44090.1:d:+2425:primary
GGAAGGATAA	2	2	2	2	0	At2g43970.2:X:-428:quaternary
TTGGCGTTAC	0	7	1	0	0	At2g43150.1:d:+52:primary
AGTGGTTGAA	0	0	2	1	5	At2g42530.1:X:-238:quaternary,At1g79550.2:X:-73:quaternary
AAAAAATTAT	0	0	1	3	4	At2g42450.1:d:+1912:primary,At1g67360.1:d:+1063:primary,At1g67360.2:d:+1039:primary
CGACAGGAGT	1	2	4	1	0	At2g41630.1:d:+1016:primary
GAATCAGCTA	2	1	2	1	2	At2g40935.1:d:+656:primary,At2g40935.2:d:+717:primary
TTGCACTCTT	0	2	4	1	1	At2g40430.1:d:+1310:primary
GTTTTATAAA	2	3	2	0	1	At2g40290.1:d:+1377:secondary,At2g40290.2:d:+1465:secondary
TCTCTGATGT	2	4	1	1	0	At2g39780.1:d:+846:primary
GCTCAATCAC	3	2	0	1	2	At2g39670.1:d:+1432:primary,At2g39670.2:d:+1441:primary
ATAATGACGT	2	0	4	0	2	At2g37710.1:d:+2266:primary
AGACAAAGAA	1	0	2	1	4	At2g37680.1:v:+1363:primary
AGATGGGTTC	2	1	1	3	1	At2g36870.1:d:+865:primary
GCCGCTATTC	0	3	2	3	0	At2g36800.1:d:+1212:primary
GGATTCTTAG	3	1	3	0	1	At2g36630.1:d:+1435:primary
AACAAATTGA	3	0	2	3	0	At2g34920.1:i:+3831:tertiary
AGTGAGATCA	3	1	3	1	0	At2g34620.1:d:+715:primary
AGGAAGACTG	1	1	5	1	0	At2g34420.2:d:+155:secondary,At2g34420.1:d:+155:secondary
GGATGATATA	2	2	1	0	3	At2g31570.1:d:+750:secondary
ACAAAAGAAA	0	5	1	2	0	At2g30440.1:d:+1390:secondary,At2g16910.1:d:+1931:secondary
CTCACTAAAT	3	0	1	1	3	At2g30140.1:d:+1485:primary
TGTTGGGAAG	1	4	2	0	1	At2g29980.2:d:+1152:primary,At2g29980.1:d:+1089:primary
TAGCTGTTGA	0	4	2	0	2	At2g29400.1:d:+1205:primary

GACTGGATCT	2	2	3	0	1	At2g29390.3:d:+1054:primary,At2g29390.2:d:+789:primary,At2g29390.1:d:+833:primary
ATCTAAAAAA	1	2	3	2	0	At2g29060.1:i:+2217:tertiary
TGTGAAGCCC	0	0	2	4	2	At2g28310.2:d:+1394:primary,At2g28310.1:d:+1464:primary
AAATCACTGC	0	1	1	2	4	At2g26500.1:d:-158:primary,At2g26500.2:d:-126:primary
ACTGGAGCTT	0	6	1	1	0	At2g26240.1:d:+373:primary
ATGTTTCATA	2	2	0	0	4	At2g26060.1:d:+1290:primary
CTCTTATCTT	2	3	0	1	2	At2g25970.1:d:+2012:primary
AAAAAGGCGA	0	7	1	0	0	At2g25490.1:d:+1343:primary
GGGATTGAAA	3	3	1	1	0	At2g25250.1:d:+629:primary
GTAGTAGTAT	1	4	0	0	3	At2g24790.1:d:+970:primary
TTCTTAAAAG	2	1	1	0	4	At2g24590.1:d:+911:primary
GCCAAAGCAG	2	1	2	3	0	At2g24020.1:d:+114:primary
TTCCTTTTGA	0	8	0	0	0	At2g23810.1:d:+13:primary
GCCAAGAACT	0	5	1	1	1	At2g23610.1:d:+607:primary
CGACCTGATC	2	3	1	1	1	At2g23420.1:d:+1609:primary
TTCGTTTTCT	1	2	3	2	0	At2g23390.1:d:+1645:primary
TTGATTGTTA	3	4	1	0	0	At2g23320.2:d:+922:secondary,At2g23320.1:d:+924:secondary
ATGGTTTTGA	1	5	0	1	1	At2g20920.1:d:+592:primary
GTTCGAAAGG	1	3	2	2	0	At2g20530.1:d:+289:primary
AGAAAGTTTA	0	2	1	3	2	At2g20040.1:d:+1217:secondary
AAAAGAGAAG	4	0	1	1	2	At2g19910.1:v:+729:secondary,At2g14880.1:d:+592:secondary
ATCCTGTTTC	2	0	2	1	3	At2g19520.1:d:+1759:primary
ACTCTGCAAA	1	1	1	2	3	At2g19480.1:d:-1374:secondary
AAATTTTAAT	0	1	4	2	1	At2g18440.1:d:+702:primary
AAGATCCAAG	2	4	2	0	0	At2g18160.1:d:+689:secondary
AAGATCCACG	0	7	0	1	0	At2g17880.1:d:+613:primary
AAAGGCGGCG	1	1	3	2	1	At2g17560.1:d:+99:primary
CAAGTTACTG	0	2	3	2	1	At2g17110.1:d:+2647:primary
ACTTGGATTT	2	2	3	1	0	At2g15860.1:d:+1693:primary
AGCAATACTA	2	2	3	1	0	At2g15580.1:i:+653:tertiary
TGGCATAAGT	4	2	0	2	0	At2g15050.1:d:+476:primary
GGATGATTCA	3	1	2	1	1	At2g14680.1:d:+1995:primary
CATACACACG	3	0	1	2	2	At2g14610.1:d:+542:primary
CAGCTAGGAA	2	3	2	1	0	At2g14045.2:d:+121:primary,At2g14045.1:d:+118:primary
CTCCCCAGGG	2	0	2	3	1	At2g07777.1:v:+1337:primary
AGAAGAATTA	2	2	3	1	0	At2g07660.1:p:+2881:secondary,At5g62680.1:d:+1388:secondary,At4g03840.1:p:+2172:secondary,At5g29075.1:p:+5365:secondary,At2g01024.1:p:+110:secondary
TTCTAGTTAA	2	0	1	1	4	At2g07340.1:d:+533:primary,At2g07340.2:d:+530:primary
GGGATTCCGT	3	0	1	3	1	At2g04760.1:p:+1870:secondary
TGCAAAAAAA	2	3	0	1	2	At2g04080.1:d:+1538:secondary
TAGATGTAGT	2	1	0	1	4	At2g03390.1:d:+1254:primary
ATTTTTATTT	0	5	2	1	0	At2g02650.1:v:+1707:primary
ACATATTCAT	4	4	0	0	0	At2g01850.1:d:+968:primary
GCTCACATCG	1	3	2	1	1	At2g01820.1:d:+2569:primary
GCTGTTGCTG	1	4	1	1	1	At2g01420.2:d:+1777:primary,At2g01420.1:d:+1765:primary
GATTAGATTC	3	1	2	1	1	At1g80910.1:d:+1710:primary
GATAATGCAG	2	0	2	2	2	At1g80860.2:d:+633:primary
TCTCGGTGAA	0	2	2	2	2	At1g80770.1:d:+1251:primary
GAATGGTGGA	1	2	4	1	0	At1g80360.1:d:+1216:primary
GTGATGACCA	0	1	0	7	0	At1g80230.1:d:+597:secondary
TTCCGTGATT	0	0	2	2	4	At1g80130.1:d:+1042:secondary
GCAACTCAAT	0	2	2	2	2	At1g80040.2:d:+1175:primary,At1g80040.1:d:+936:primary
ATTTGGTCCA	3	0	4	1	0	At1g79530.1:d:+1597:primary,At5g60300.2:d:+2464:primary,At5g60300.1:d:+2434:primary
TAATTCTGTA	3	4	0	1	0	At1g79380.1:d:+1697:primary
ACGCTCACAG	1	3	0	1	3	At1g78900.1:d:-2170:secondary
TACTTGCCGA	1	0	2	3	2	At1g78895.1:d:+757:primary
GTTTAGACTT	3	3	0	1	1	At1g78890.1:d:+614:primary
GTAACATCAA	0	1	2	1	4	At1g78670.1:i:-613:tertiary
ACTGTGTTGG	0	1	1	5	1	At1g78570.1:d:+1883:secondary
GACGCTTTTG	0	5	1	2	0	At1g78100.1:d:+116:primary

TAGATAATCA	2	3	1	0	2	At1g77930.1:d:+1098:primary
GACGCCAGAG	0	1	5	2	0	At1g77590.1:d:+1980:secondary,At1g22710.1:d:+894:secondary
TGTCTAACAA	0	5	3	0	0	At1g76970.1:X:-42:quaternary,At4g39260.1:X:-635:quaternary
GCTTTTTTGT	2	2	3	1	0	At1g76660.1:d:+1853:primary
GAATGGAAAC	3	2	2	1	0	At1g76410.1:d:+779:primary
AAAGAAAATA	0	1	3	1	3	At1g76240.1:d:+1076:primary
ACGTAAGATT	2	1	2	1	2	At1g76130.1:d:+1425:primary
GCTGAGGAAG	1	0	2	1	4	At1g75990.1:d:+1496:primary
GTATTGAACG	2	1	0	4	1	At1g75500.1:d:+1154:secondary
ATTTTGCAGC	2	1	3	2	0	At1g74850.1:d:+2869:primary
TATGATGAAT	1	1	2	1	3	At1g74790.1:d:+2274:primary
AGCTTTGAGA	1	1	3	2	1	At1g74720.1:d:+3422:primary
GAAGAGAGAA	1	3	1	2	1	At1g74150.1:v:+1426:secondary,At4g00740.1:d:+1926:secondary,At5g04530.1:d:+205:secondary
GTGGTTTGGT	0	2	2	2	2	At1g74070.1:d:+1165:primary
CAGAAGGCAA	2	5	0	1	0	At1g73830.1:d:+752:primary
GATCCGATTT	1	7	0	0	0	At1g73500.1:d:+425:primary
TTCTCTATTG	2	2	0	0	4	At1g73490.1:d:+985:primary
GCGTTTCTAC	0	4	3	1	0	At1g73480.1:d:+1336:primary
GCTCAATCGA	1	1	3	3	0	At1g73430.1:d:+2804:primary
GGAGTCAAGA	1	3	1	1	2	At1g72970.1:d:+1833:primary
TTAATTACTC	1	0	3	0	4	At1g72770.1:d:+2197:secondary
GAGTCTCACC	2	1	1	1	3	At1g71170.1:d:+969:secondary
ATGTTGATGA	0	0	6	1	1	At1g70640.1:d:+656:primary
TGTGTGTGTG	3	3	2	0	0	At1g69910.1:d:+2099:primary
GAAGAAGAAG	1	3	3	1	0	At1g69200.1:d:+1648:primary,At3g02860.1:d:+870:primary,At3g02860.2:d:+907:primary,At4g38440.1:d:+4318:primary
AACTATGGAT	0	1	3	0	4	At1g68680.1:d:+453:secondary
CGAGGCCTTT	1	1	5	0	1	At1g68520.1:X:-184:quaternary
AAAGAGTTCG	2	3	0	1	2	At1g68300.1:d:+582:primary
GTCCATTGGC	3	1	2	2	0	At1g66580.1:d:+654:secondary
TTTACCAGTC	0	5	2	0	1	At1g65840.1:d:+2060:secondary,At5g05490.1:d:+1865:secondary,At5g05490.2:d:+1039:secondary
TGCTCCTTTC	1	2	1	0	4	At1g65500.1:d:+379:primary
AGAGGTGAGT	1	3	2	0	2	At1g65430.1:d:+1959:primary
AAGAGTCTTG	2	0	2	4	0	At1g65230.1:d:+592:primary
TAGTCTTTGT	1	1	1	0	5	At1g64350.1:d:+1225:primary
ATCTATGGGT	1	2	0	3	2	At1g63940.2:d:+1789:primary,At1g63940.3:d:+1894:primary,At1g63940.1:d:+1807:primary,At1g63940.4:d:+1795:primary
TCCGGCTTGA	4	1	1	2	0	At1g63690.1:d:+1707:secondary,At1g63690.2:d:+1707:secondary
AAGATGACTG	3	2	3	0	0	At1g63130.1:d:+2139:primary
GGGTCGCTTT	0	0	0	5	3	At1g60190.1:d:+1912:primary
AATTAGGGTT	3	4	1	0	0	At1g60160.1:v:+2892:secondary
GCCGCAATTC	1	3	3	0	1	At1g59830.1:d:+988:primary,At1g59830.2:d:+1050:primary
GAGGTTCTGA	1	5	2	0	0	At1g59770.1:p:+765:primary,At1g03970.1:d:+873:primary
GGTGAGTGTT	0	3	3	1	1	At1g58410.1:d:+651:secondary,At1g58602.1:d:+1141:secondary
AGAATACAAA	2	1	1	1	3	At1g56600.1:d:+1356:primary
CAGTCTAAAC	3	1	2	2	0	At1g56220.3:d:-572:secondary,At1g56220.2:d:-577:secondary,At1g56220.1:d:-563:secondary
TGTATCGACA	3	2	1	1	1	At1g56050.1:X:+305:quaternary
AAATCTCGGT	2	4	0	1	1	At1g55530.1:d:+1377:primary
TTCTGTTTGT	1	4	2	1	0	At1g55520.2:d:+1096:primary,At1g55520.1:d:+1093:primary,At2g22000.1:d:+172:primary
TATTTATTTA	0	6	1	1	0	At1g55390.1:d:+873:secondary,At1g55450.1:d:+1083:secondary,At2g01990.1:v:+306:secondary
TCAAAGTTGT	1	1	6	0	0	At1g53240.1:d:+1325:secondary
CATAGAAAAT	0	2	4	0	2	At1g52890.1:d:+1118:secondary
CAAGTATATA	4	2	1	0	1	At1g52780.1:d:+3441:primary
GCGCGGTGAG	0	6	1	1	0	At1g52410.1:d:+2193:primary,At1g52410.2:d:+2205:primary
GCAGAAAGCT	1	0	5	0	2	At1g52210.1:p:+861:secondary
AGGAAAAAGA	1	3	1	2	1	At1g52140.1:d:+358:primary
TGATGTTTCT	2	0	3	1	2	At1g52000.1:d:+2389:primary
TCATCTATAA	2	3	1	1	1	At1g51730.1:d:+1077:primary
GGGAGAAGGC	2	1	3	2	0	At1g51690.1:d:+1960:primary,At1g51690.2:d:+1957:primary
CTTTTGCTGA	2	1	0	3	2	At1g51630.1:d:+1447:primary
ATAGTAAAAA	3	2	0	1	2	At1g51580.1:d:+1932:primary,At5g35540.1:v:+1331:primary

GTTTGGAGGA	1	6	1	0	0	At1g51520.1:d:+624:secondary,At1g02610.1:v:+754:secondary,At1g51520.2:d:+624:secondary
GTTTTCCTGT	2	1	3	2	0	At1g50740.1:d:+698:primary
ATTGCCATTG	3	0	5	0	0	At1g50370.1:d:+1157:primary
AATTTCAGAC	0	2	2	4	0	At1g49660.1:X:+1097:quaternary
TGATTCTTGT	0	3	2	1	2	At1g49430.1:d:+2233:primary
AAAAATTAAT	3	0	2	2	1	At1g49270.1:v:+2758:primary,At1g02500.1:d:+1505:primary,At1g25310.1:v:+1128:primary,At1g02500.2:d:+1505:primary
GTCTCTTAGG	0	4	2	2	0	At1g48490.1:d:+3040:primary
TAGACTCAAG	0	2	3	1	2	At1g48450.1:d:+1339:primary
CACCCAAAAG	0	4	1	3	0	At1g47720.1:X:+519:quaternary
TGATTGTGAT	3	1	1	0	3	At1g45976.1:d:+996:primary
GAATAGAGTA	1	3	3	0	1	At1g45201.2:d:+1096:primary
GAGATTCAGT	1	1	2	3	1	At1g44000.1:d:+746:primary
TGGTGGTTAT	1	1	2	3	1	At1g43710.1:d:+1381:secondary
TAATTTTGGA	2	5	1	0	0	At1g34370.1:d:+1508:primary,At1g34370.2:d:+1553:primary
TTGCTGCGTG	2	3	2	0	1	At1g33780.1:d:+1011:primary
ACTAATTGTG	1	3	2	1	1	At1g33050.1:d:+2330:primary
AACTTGATAT	2	3	1	1	1	At1g31300.1:d:+985:primary
GCTGCTGGTG	2	3	1	2	0	At1g31190.1:d:+990:primary,At2g40190.1:d:+1164:primary
ACGTGAAGAA	2	1	2	1	2	At1g30890.1:d:+1014:primary
GAACACATAC	3	0	4	0	1	At1g30370.1:v:+2193:primary
GAATGTTGTG	1	2	4	0	1	At1g30270.1:d:+1991:primary
TCCTGAGAGA	1	2	2	2	1	At1g29150.1:d:+1360:secondary
TTGTTATGTG	4	4	0	0	0	At1g28580.1:d:+1331:secondary,At1g28580.2:d:+1598:secondary
CATCTTCCAT	0	6	1	1	0	At1g25682.1:d:+805:primary
AGAGATGTCT	0	4	2	1	1	At1g24340.1:v:+2439:secondary
TGTGTGTGTA	1	4	3	0	0	At1g24260.1:d:+884:primary,At1g24260.2:d:+863:primary
GACTCAAAAG	1	7	0	0	0	At1g24160.1:d:+1574:primary
TTTCTGTAAC	3	3	0	0	2	At1g23820.2:d:+1226:secondary,At1g23820.1:d:+1222:secondary
ATCTCCGGCA	3	1	2	1	1	At1g23310.1:X:-53:quaternary
GGTATTTCTC	1	6	0	0	1	At1g22930.1:d:+2834:secondary
AAATTTTATT	2	5	1	0	0	At1g22570.1:v:+2141:secondary
GATTTAAAAG	1	2	3	2	0	At1g21830.1:d:+861:primary
GATCTGGTTC	1	1	2	2	2	At1g21770.1:d:+161:primary
TAATTTGTAT	4	2	0	0	2	At1g21590.1:d:+2759:primary
CCTCTCTTTG	0	1	7	0	0	At1g21110.1:d:+1340:primary
TTGAGAAATG	3	0	1	2	2	At1g20816.1:d:+610:primary
ACCATCCCGA	1	1	3	2	1	At1g20440.1:d:+638:secondary
TTAAGCTCAA	4	0	1	1	2	At1g19140.1:i:+896:tertiary,At1g19140.2:i:+847:tertiary
TACAAACAAA	3	1	2	1	1	At1g17850.1:v:+1749:secondary
TGCCAATAAG	4	2	1	0	1	At1g17145.1:d:+1319:primary
GCCACCGGTT	0	2	0	6	0	At1g17100.1:d:+34:secondary
ATGCCTTTTC	3	2	0	2	1	At1g17060.1:d:+451:secondary
TTAATGAAAT	0	2	3	1	2	At1g16840.3:d:+802:primary,At1g16840.2:d:+1224:primary
AGGAACTTCT	1	2	2	0	3	At1g16700.1:d:+756:primary
TGCTTCGAGG	1	1	3	2	1	At1g16140.1:v:+1330:secondary,At1g33270.1:d:+1492:secondary,At1g33270.2:d:+1667:secondary
GTGGAAAGAC	1	2	3	2	0	At1g16080.1:d:+846:primary
AGGAAAAAAT	3	3	1	1	0	At1g15400.2:d:+526:primary
AGGTTCTCTA	3	0	1	3	1	At1g15200.1:d:+1305:primary
TAATTCCCGC	2	2	1	2	1	At1g14990.1:d:+622:primary
GTCACACTCA	4	0	0	3	1	At1g11915.1:d:+1038:primary
TAGAGGACGT	1	2	1	2	2	At1g11750.1:d:+760:primary
CAAACGCTTT	1	0	4	3	0	At1g11700.1:X:+880:quaternary
GTCTTTCTTA	0	2	2	0	4	At1g11210.1:d:+1001:primary
GCTCGTAAAT	0	2	3	2	1	At1g10840.2:d:+1020:primary,At1g10840.1:d:+1038:primary
TACAGAAGAT	1	4	2	0	1	At1g10720.1:d:+1487:primary
CTCTTTCTTT	1	4	1	0	2	At1g10500.1:d:+500:primary
TAACTCTTTG	1	2	3	1	1	At1g09230.1:i:+3055:tertiary
CTTCATATAG	3	3	1	0	1	At1g09060.1:d:+2951:primary,At1g09060.2:d:+2991:primary
TGGTCCTGAA	0	3	3	1	1	At1g08550.1:d:+1124:primary
AGGCAGGACT	2	2	1	3	0	At1g08540.1:d:+1614:primary

GTTCGGTTTG	1	5	1	0	1	At1g08260.1:v:+1611:secondary,At5g04290.1:v:+4969:secondary,At2g27120.1:v:+1578:secondary
GGCTTGGTTG	2	1	2	2	1	At1g07940.1:X:-727:quaternary
AGGATTCATC	1	1	4	0	2	At1g07560.1:v:-2996:secondary
TTTCAGTAAA	1	1	1	0	5	At1g07390.1:v:+2558:secondary
TTGGTTATCT	2	1	3	1	1	At1g07140.1:d:+1025:secondary
TAACAAACCA	2	3	2	1	0	At1g07080.1:d:+883:secondary
TGTAACGAGT	1	1	0	2	4	At1g07040.1:d:+1437:primary
GGAGGTCTCA	2	2	2	2	0	At1g06950.1:d:+2967:primary
AAACCAAGTT	4	1	1	2	0	At1g06670.1:d:+4974:secondary
AGTTTTCTAG	0	5	2	1	0	At1g06230.1:d:+2551:primary,At1g06230.2:d:+2498:primary
AGCTTCATCC	1	1	2	4	0	At1g06200.1:d:+873:secondary
GCCACAGGCG	2	4	1	1	0	At1g06060.1:d:+881:secondary
TAGAGCTGAA	1	1	3	1	2	At1g05720.1:d:+509:primary
GCTTCTTGAT	1	2	1	2	2	At1g05680.1:d:+392:secondary,At4g32530.1:d:+712:secondary
TATGAACCCC	0	2	0	3	3	At1g04870.1:d:+1341:primary,At1g04870.2:d:+1351:primary
GGTTTAAGTG	2	2	1	0	3	At1g04750.1:d:+647:primary
TAAGTTTCCA	0	1	4	1	2	At1g04620.1:d:+1503:primary
TTTATCAATA	0	3	1	0	4	At1g04120.1:d:+5126:primary
GATGCCGGAC	2	0	4	2	0	At1g02680.1:i:+1313:tertiary
AAGCCTTGTT	1	1	2	2	2	At1g01930.1:d:+1982:primary
AAGCCTTAAT	0	1	2	3	2	At1g01730.1:d:+812:primary
GCGCAACAAC	1	2	4	0	0	No gene matches found
CGCCCGCCGA	1	3	1	1	1	No gene matches found
CCCTTTTCTG	1	2	2	1	1	No gene matches found
AATTTGAGCA	5	1	0	1	0	No gene matches found
TCCGAATCTG	1	3	1	1	1	No gene matches found
GTAGAACCTT	1	1	1	2	2	No gene matches found
CCTATTGGCG	2	1	4	0	0	ChrC:-71879:quaternary
TTCGGTTTTG	2	1	3	1	0	ChrC:+9324:quaternary
ACATTGAGAT	1	4	2	0	0	Chr5:+25735935:quaternary
TCACTTAAAA	2	0	1	0	4	Chr5:+19552440:quaternary
CCACCTTGGT	0	6	0	0	1	Chr5:+18908278:quaternary
GCAGTTTGTT	1	5	0	0	1	Chr4:-17281681:quaternary,Chr4:-1434983:quaternary
TTTAGCAAAA	0	1	2	1	3	Chr4:+6669506:quaternary,Chr5:+15045921:quaternary
AATTATTAGA	2	2	0	1	2	Chr4:+14676445:quaternary
AAACAAAGTG	1	4	1	1	0	Chr4:+12361373:quaternary
ATGATATTAA	1	2	2	0	2	Chr4:+11045528:quaternary,Chr3:+22592964:quaternary,Chr5:+3251133:quaternary,Chr4:+2268284:quaternary
TACACACACA	3	1	0	0	3	Chr3:+374394:quaternary,Chr1:+4432796:quaternary
CACAACAACA	5	0	2	0	0	Chr3:+3588429:quaternary,Chr2:+9490693:quaternary
CCCGCGGCTT	0	1	3	1	2	Chr3:+14208389:quaternary
TTGGCGAAGA	1	3	1	2	0	Chr2:+9297427:quaternary
TTGACCAAAA	4	0	2	1	0	Chr2:+7612494:quaternary
GTGGTAACGG	0	1	1	4	1	Chr2:+4056:quaternary,Chr3:+14209013:quaternary
AATTCTGCTT	1	1	5	0	0	Chr2:+16652895:quaternary
ACATAACAAA	0	1	5	0	1	Chr2:+1649989:quaternary
TCGACAACCG	1	2	3	1	0	Chr1:-24303076:quaternary,Chr1:-24303084:quaternary,Chr4:-16618305:quaternary,Chr4:-16618297:quaternary
GTTCAAAAAA	0	2	2	2	1	Chr1:+5193122:quaternary,Chr5:+20420896:quaternary
ATAACCTGGA	0	0	4	3	0	Chr1:+4948127:quaternary
CCCCTAACTA	0	1	3	3	0	Chr1:+24888161:quaternary
TTAATGAAAA	0	2	1	2	2	Chr1:+2190993:quaternary
GCAGCATCAG	2	1	3	1	0	Chr1:+20987064:quaternary
TACTCGCCGG	0	3	2	1	1	Chr1:+17298643:quaternary
AAACTTTTTC	1	0	3	2	1	Chr1:+1131290:quaternary
GACATATTTT	1	1	1	3	1	AtCg00380:d:+341:primary
GGGTAACCCC	1	0	3	1	2	AtCg00210.1:X:+261:quaternary
TTTCGTGGTG	3	0	2	0	2	At5g67630.1:d:+1530:primary
GGGAAGTTCT	0	2	1	4	0	At5g67580.2:d:+1036:primary,At5g67580.1:d:+1115:primary
AATTGCCTTA	1	2	3	0	1	At5g67130.1:d:+1604:secondary
AAGAAAACAA	0	1	3	1	2	At5g66890.1:v:+1688:secondary,At3g28640.1:v:+1861:secondary

CACACATATA	1	5	1	0	0	At5g66880.1:d:+1329:primary
ACAGCTAAAT	1	2	3	1	0	At5g66730.1:d:+2270:primary
AGATAAAGAA	0	1	0	1	5	At5g66610.1:i:+1348:tertiary
TGTATCAGTG	1	2	1	1	2	At5g65890.1:X:+-80:quaternary
TAGCTTTCAA	4	1	1	0	1	At5g65740.1:d:+1141:primary
GATGCTTCTC	1	4	1	0	1	At5g65660.1:d:+171:primary
AACTTGGTTC	3	0	2	2	0	At5g65600.1:v:+1858:secondary
TTTGATTAGT	2	0	4	0	1	At5g65470.1:d:+1811:primary
CGAAAGAATT	2	1	1	3	0	At5g65050.1:d:+855:primary
GCCGCGAAGT	2	2	2	1	0	At5g64730.1:d:+190:secondary
GAAAGAAAAA	1	0	3	2	1	At5g64450.1:v:+1315:primary
TGAAGATGCA	0	2	2	3	0	At5g64420.1:d:+3954:primary
GACACTTACC	2	0	2	0	3	At5g64420.1:d:+3022:secondary,At5g46710.1:d:+348:secondary
TGTAATCCAG	1	0	3	1	2	At5g64250.2:d:+1416:secondary,At5g64250.1:d:+1248:secondary
GCCGTTAGAG	0	2	2	2	1	At5g63840.1:d:+2796:primary
TACTGTAATG	1	1	1	1	3	At5g63530.1:d:+1092:secondary
TCGTGGTGGC	4	3	0	0	0	At5g63110.1:d:+1443:primary
TCATTGATTT	1	2	1	3	0	At5g62980.1:d:+534:primary
AAAGCTCTTG	0	2	3	1	1	At5g62640.1:d:+1720:primary
AGTGTTCTTG	1	2	1	0	3	At5g62575.1:d:+354:primary
GATGTTTAAA	1	1	2	2	1	At5g62540.1:d:+807:primary
AGAGGCATTT	1	2	2	1	1	At5g62090.2:d:+2570:primary,At5g62090.1:d:+2971:primary
TCTACACCGC	1	2	1	1	2	At5g61790.1:d:+658:primary
TAATAAACAT	1	1	0	1	4	At5g61140.1:d:+21:secondary
AGCATTCATC	1	0	0	1	5	At5g61020.1:d:+1727:primary,At5g61020.2:d:+1741:primary
CATCCTCGAT	0	1	3	2	1	At5g61020.1:d:+1297:secondary,At5g61020.2:d:+1311:secondary
CAAATAAAGT	2	2	2	1	0	At5g61010.1:d:+2730:secondary
CCATTGGATT	1	1	2	1	2	At5g60450.1:d:+3062:primary
GCCGCCGTTC	1	0	3	2	1	At5g59870.1:d:+258:primary
AAGAGAAGGT	1	4	1	1	0	At5g59730.1:d:+1608:primary
TATAAAAATT	1	4	1	1	0	At5g58220.1:d:+1106:primary
CTTGTTGTTG	3	1	1	1	1	At5g58120.1:d:+3237:secondary
AAGTATGGTC	1	2	2	0	2	At5g57800.1:d:+2241:primary
TCTCCTTGGT	0	1	2	2	2	At5g57580.1:d:+2398:primary
TAGGTTTTTT	0	5	1	1	0	At5g57180.1:d:+1845:primary
TTCAAATAAA	1	1	0	4	1	At5g56430.1:X:-329:quaternary
CCAAAATACA	1	2	1	2	1	At5g56360.1:d:+434:secondary
GGTCCAGCAG	1	3	2	1	0	At5g56150.1:d:+200:secondary,At5g56150.2:d:+193:secondary
TTCTTCAGTA	2	4	0	1	0	At5g55930.1:d:+2669:primary
ATGAGGAATG	1	4	0	1	1	At5g55670.1:d:+2006:primary,At5g56760.1:d:+947:primary
TGTAGCTAGT	2	3	1	1	0	At5g55390.1:d:+4120:primary
TTTGTGTTCT	3	2	0	0	2	At5g54740.1:d:+803:primary,At4g11120.1:d:+1442:primary
TACAAAATAA	1	0	1	2	3	At5g54130.1:v:+1276:primary
CTTTGAACTT	0	2	2	0	3	At5g53860.2:d:+1538:primary,At5g53860.1:d:+1478:primary
AGAATGAGAG	3	2	0	1	1	At5g53620.2:d:+609:secondary,At4g22415.1:p:+1300:secondary,At5g53620.1:d:+559:secondary
ATGGACAAAT	2	3	1	0	1	At5g53560.1:d:+279:primary
GTCGTTTTCT	2	1	4	0	0	At5g53480.1:d:+3046:secondary
AGCTTTTTTG	0	2	4	0	1	At5g53290.1:d:+1403:primary,At1g49120.1:v:+1572:primary
GTACAATGAT	2	3	1	0	1	At5g53000.1:d:+1190:primary
TTTCACAGAG	1	1	1	2	2	At5g52200.1:d:+646:primary
CATTCATTCC	1	4	0	1	1	At5g51960.1:d:+331:secondary
CCAATTCCGC	1	3	2	0	1	At5g51430.1:d:+2474:primary
GAGTCTGTGC	0	2	2	2	1	At5g50850.1:d:+1054:secondary
AAACGCTTTA	1	2	1	3	0	At5g50810.1:d:+299:primary
ACTGCAAATC	2	1	0	3	1	At5g50430.1:d:+1019:primary
GGTTTTATAT	1	4	1	1	0	At5g50380.1:d:+2394:primary
GAAAATGAAT	1	0	3	1	2	At5g50375.1:d:+552:secondary
GTGAAAGTTT	1	1	1	4	0	At5g49580.1:v:+2472:secondary
GCTAAGGCAG	0	5	1	1	0	At5g49540.1:d:+354:primary
TTATGCCTTT	0	0	0	2	5	At5g49330.1:X:+955:quaternary
GAAACTAAAT	0	2	1	2	2	At5g49280.1:d:+710:primary

GGTAAGGTTG	0	4	0	3	0	At5g49160.1:d:+4441:primary
GCAATAGCAC	2	1	2	2	0	At5g48900.1:d:+1403:primary
GCAGATTTGG	0	1	1	4	1	At5g48880.2:d:+997:primary,At5g48880.1:d:+782:primary,At1g61340.1:d:+552:primary
AAGGGAAAAA	1	1	1	1	3	At5g47455.6:d:+611:primary,At5g47455.5:d:+621:primary,At5g47455.3:d:+519:primary,At5g47455.4:d:+517:primary
TAAATCTTTT	1	3	0	2	1	At5g47020.1:d:+4309:primary
GGAGAAGTTG	1	2	3	1	0	At5g46210.1:d:+445:secondary
GTGATAAACA	2	1	1	2	1	At5g45930.1:d:+1169:primary
CCTTATTCCT	3	1	2	0	1	At5g45680.1:d:+584:primary
AAAAAGATTA	0	0	0	2	5	At5g45180.1:X:--20:quaternary,At3g17520.1:X:-1010:quaternary
GAGGTGATGA	1	4	1	0	1	At5g44860.1:d:+868:primary
TAAGGATTTT	0	0	0	2	5	At5g44565.1:d:+509:primary
CATATAAAAA	1	0	2	1	3	At5g44480.1:v:+127:secondary,At2g32620.1:v:+2728:secondary
AGACCGGAGA	0	4	2	1	0	At5g44200.1:d:+746:primary
ACGGGTTTTA	2	2	1	2	0	At5g44150.1:d:+946:primary,At1g72820.1:d:+1607:primary
TGTGATAAAA	1	0	0	4	2	At5g44110.1:X:-365:quaternary
CTCTAACAGT	5	1	1	0	0	At5g44100.1:X:--235:quaternary
GCTTCCCTCC	1	3	3	0	0	At5g44070.1:d:+1460:primary
CTGCCGTTTG	1	0	0	4	2	At5g42760.1:d:+981:primary
GAATTGGGTA	1	2	2	1	1	At5g42420.1:d:-1388:secondary
TAAAAGTTTG	2	1	0	3	1	At5g42240.1:d:+1585:primary
GACGAGGTTG	0	2	2	3	0	At5g42070.1:d:+458:primary
GTTTTGGACT	1	2	3	0	1	At5g41790.1:d:+4030:primary
CTCTTTGCCC	1	2	2	0	2	At5g40770.1:d:+883:secondary
AAGACCCTGT	0	7	0	0	0	At5g40450.1:d:+7454:secondary
AACCAACAAA	2	3	0	2	0	At5g40450.1:d:+3029:secondary
ATGTACTTAA	1	5	1	0	0	At5g40450.1:d:+2276:secondary
GAAGTACAAA	1	6	0	0	0	At5g40450.1:d:+112:secondary
TGGCTTTCTT	0	2	3	0	2	At5g40340.1:v:+3073:primary
TGTGATTATT	0	3	1	0	3	At5g40150.1:d:+1434:primary
TTCGAAGCAA	1	1	3	1	1	At5g39820.1:X:-621:quaternary
AAATGGATGT	1	2	2	2	0	At5g39050.1:d:+1373:primary
GAATATGGCA	1	5	1	0	0	At5g38710.1:v:+1813:secondary,At2g28910.1:d:+211:secondary
ATTCTCTGTT	1	3	2	0	1	At5g38460.1:d:+1534:primary
GTGGCCTGAG	1	0	5	1	0	At5g38410.1:X:-106:quaternary
GCGATATATA	2	1	1	2	1	At5g37510.2:d:+2501:primary,At5g37510.1:d:+2587:primary
TTGTTCATCT	1	0	0	2	4	At5g37250.1:v:+1387:primary,At5g37270.1:v:+1350:primary
CTGGCTTCAA	1	4	0	1	1	At5g36290.1:d:+925:primary,At5g36290.2:d:+965:primary
TACAAAAAGA	3	2	2	0	0	At5g35610.1:v:+744:primary
GTCGTTGTTT	0	5	2	0	0	At5g32450.1:d:+1105:primary
TTGAATACAA	2	1	0	2	2	At5g31884.1:p:+447:primary,At3g07050.1:d:+1625:primary
AAGGAGATCG	2	3	2	0	0	At5g31662.1:p:+5118:primary,At3g43681.1:p:+5336:primary,At4g04157.1:p:+3798:primary
ATAAAATGTC	3	0	1	2	1	At5g30510.1:X:-200:quaternary
TAAATTTTCA	1	3	2	1	0	At5g29720.1:p:+3490:secondary,At4g33620.1:v:+1600:secondary,At5g06020.1:v:+610:secondary
TAGATGGAGA	1	2	3	1	0	At5g28300.1:d:+1978:primary
TTTTGTAAGT	1	1	0	2	3	At5g27760.1:d:+394:primary
GTGTAATTTT	3	0	2	1	1	At5g27600.1:d:+2224:primary
AAGTTAAAAA	1	2	0	2	2	At5g27430.1:d:+704:primary
TGGATTTGTT	4	0	1	2	0	At5g27310.1:v:+150:secondary,At4g35300.2:d:+2431:secondary,At4g06594.1:p:+4022:secondary,At2g27790.1:v:+1470:secondary,At4g35300.1:d:+2461:secondary
TTCACTTAAA	1	4	1	1	0	At5g27260.1:i:-375:tertiary
TCACACTTGT	1	1	3	2	0	At5g26340.1:X:-926:quaternary
ACATTTCTCA	0	1	0	1	5	At5g26010.1:i:+451:tertiary
TGTTTTTTTT	0	3	1	2	1	At5g25810.1:d:+986:primary,At1g13900.1:d:+2166:primary,At3g52710.1:d:+1286:primary,At5g08141.1:v:+1055:primary
AAGAGGAGAA	2	2	0	3	0	At5g25780.1:d:+2116:primary
GGATATAGAG	1	3	1	0	2	At5g25265.1:d:+957:primary
TGTTGATCGA	3	0	3	0	1	At5g25070.1:d:+2392:primary

TGAATTGAGT	1	3	1	1	1	At5g24430.1:d:+2005:primary
AGTCGGATGG	0	1	1	4	1	At5g23310.1:d:+807:primary
TATAGATGAT	5	1	0	1	0	At5g22800.1:d:+3038:primary
GTCCTCCTTG	3	3	0	1	0	At5g22360.1:d:+798:primary
GATTCAAACC	1	2	2	1	1	At5g22210.1:d:+174:primary
TGGTTTATCA	4	0	2	1	0	At5g22050.2:d:+1116:primary,At5g22050.1:d:+1072:primary
TTGGCTTTAA	1	1	1	4	0	At5g21040.1:d:+1899:primary
AACCTGACAG	1	3	0	2	1	At5g20920.2:X:-244:quaternary
CAAACCCACT	0	2	2	2	1	At5g20610.1:d:+2978:secondary
AATCTTCCAA	3	3	0	1	0	At5g19350.1:d:+1482:secondary,At4g37210.1:d:+1683:secondary,At1g03060.1:d:+8788:secondary,At4g02660.1:d:+9150:secondary,At4g37210.2:d:+1675:secondary
AAAACCGACA	0	2	2	3	0	At5g19180.1:d:+1463:primary
CTTTCACAGT	2	3	2	0	0	At5g18670.1:d:+1660:primary
AGCTTTTTTC	5	1	1	0	0	At5g18020.1:d:+429:primary,At5g18010.1:d:+332:primary
GCACTTGCTG	1	3	2	1	0	At5g17640.1:d:+1612:primary
TAAGGCTTCT	0	3	1	2	1	At5g17610.1:d:+520:primary
CTTTGTATTT	0	0	3	3	1	At5g17460.1:d:+343:secondary
AGGAGAGAGA	1	3	1	1	1	At5g17430.1:X:-41:quaternary
TAGAATTTTA	0	0	1	3	3	At5g17310.1:d:+1485:primary,At5g17310.2:d:+1767:primary
ACACGGAAAC	1	5	1	0	0	At5g16730.1:d:+1088:secondary
ATTCTCCGTG	3	3	1	0	0	At5g16120.1:d:+1067:primary
GCTTAGTAGA	0	7	0	0	0	At5g16110.1:d:+902:primary
TATCACTCAC	0	2	0	3	2	At5g15980.1:d:+2319:secondary
AGTCTTTTGG	2	1	1	2	1	At5g14790.1:d:+1530:primary
TGGTTTATGT	1	2	1	2	1	At5g14410.1:d:+502:primary
TGCCCATATT	1	2	2	2	0	At5g14180.1:d:+1346:primary
GCTATGGGGA	1	4	0	1	1	At5g14050.1:d:+1686:primary
GGGCCTGTTG	1	0	2	2	2	At5g13650.1:d:+1515:secondary,At5g13650.2:d:+1544:secondary
TATCGTTGTT	0	4	2	1	0	At5g13010.1:d:+3945:primary
CTTACTTTCA	1	1	0	5	0	At5g12920.1:X:-22:quaternary
TGATTGTTCC	1	1	5	0	0	At5g12440.1:d:+2472:primary
GAAAGAAAAT	0	2	0	1	4	At5g12400.1:v:+3170:secondary,At5g35340.1:p:+481:secondary,At3g23070.1:d:+2838:secondary
TAATCTAAAG	3	2	0	1	1	At5g11700.1:d:+4452:primary
GAATTTATTG	2	0	1	1	3	At5g11560.1:X:-523:quaternary
GGTCCTTTCA	2	2	2	0	1	At5g11560.1:d:+2942:primary
AACTCAAACA	1	2	1	1	2	At5g11480.1:d:+699:secondary,At5g25220.1:d:+1111:secondary
AAATTTCTTT	0	3	1	2	1	At5g11450.1:d:+959:primary
ATACAACAAT	1	3	1	1	1	At5g10770.1:d:+1690:primary,At1g40087.1:v:+1867:primary,At3g30200.1:v:+2025:primary
ATAGTTTATT	0	0	1	2	4	At5g10740.1:d:+1496:primary
TCTTGTTCTT	5	1	0	1	0	At5g10560.1:d:+2348:primary
ATGTATCTTT	1	1	2	1	2	At5g10290.1:d:+2150:primary
GTTCCGGAGG	2	0	2	2	1	At5g09670.2:d:+2006:primary,At5g09670.1:d:+2737:primary
TGTTTCTATG	3	3	1	0	0	At5g09310.1:d:+570:primary
TTGGCTTCAG	0	4	1	2	0	At5g08560.1:d:+1938:primary
GTAAGTTCTA	1	1	1	2	2	At5g08470.1:d:+3552:primary
TGGTTCGTGT	0	2	3	1	1	At5g08410.1:d:+417:primary
TGTATAGAGA	0	1	1	0	5	At5g08380.1:d:+1314:primary,At4g07516.1:p:+3520:primary,At4g08995.1:p:+763:primary,At1g17390.1:v:+1012:primary
TTTATTCCTT	2	1	0	0	4	At5g07150.1:v:+2426:primary
TATAAAGTTT	2	2	1	1	1	At5g06440.2:d:+1872:primary,At5g06440.1:d:+1684:primary
TTTGGGGTCA	2	2	1	2	0	At5g05480.1:d:+1961:secondary
ACTATATACA	5	1	0	0	1	At5g05200.1:d:+1802:secondary
TTGTATAAAA	1	3	0	0	3	At5g04720.1:d:+2510:primary
TTAATGATCC	5	0	1	0	1	At5g04440.1:d:+987:primary
CACAAATTTG	2	0	1	2	2	At5g04290.1:v:+849:secondary
CGTAGCTTTT	3	2	2	0	0	At5g04110.1:d:+1943:primary
TTGTTACCTA	2	2	1	1	1	At5g03520.1:d:+978:primary
GCTAAATTGT	1	2	1	1	2	At5g03460.1:d:+192:primary
TGTCTGATAC	0	1	1	2	3	At5g03030.1:d:+539:primary
TCTCTCTGTC	5	0	1	0	1	At5g01890.1:d:+2199:secondary,At1g20430.1:d:+445:secondary

GTTTGTTTGT	4	0	1	1	1	At5g01590.1:d:+1800:secondary
GCAAGACGCT	0	0	7	0	0	At5g01530.1:d:+642:secondary
GCCTATTGAA	3	2	2	0	0	At5g01460.1:d:+1976:primary
ATGCTATAAA	3	1	0	1	2	At5g01015.1:d:+463:primary,At3g60980.1:d:+1242:primary
TAAACTGTAG	4	2	1	0	0	At4g39900.1:d:+1000:primary
AATCTAAGAC	2	1	2	0	2	At4g39800.1:d:+1623:secondary
AATTTGTGCC	2	1	1	2	1	At4g39540.2:d:+1011:primary,At4g39540.1:d:+1166:primary
GAGCCAAGGA	0	6	0	1	0	At4g39100.1:d:+384:primary
CGGTTCTGAA	0	4	1	2	0	At4g39050.1:d:+3170:primary
GTCATTAGAA	2	1	2	2	0	At4g38810.1:X:-421:quaternary
CTTGAATGTG	0	2	3	2	0	At4g38750.1:d:+3248:primary
GAGGAGGAGA	0	3	2	2	0	At4g38630.1:d:+784:primary
ACGATTAATT	0	3	1	1	2	At4g38270.1:d:+2217:primary
TAATCAGTTA	1	5	1	0	0	At4g38090.1:d:+733:primary,At4g38090.2:d:+831:primary
TACAAGAAAA	1	1	1	2	2	At4g38030.1:v:+2518:secondary,At4g27030.1:v:+1349:secondary,At5g21030.1:v:+142:secondary
TAATGCTTTC	1	1	1	2	2	At4g36980.1:X:-174:quaternary,At4g25510.1:X:-55:quaternary,At5g06580.1:X:-14:quaternary
ACGATAAAGT	0	4	2	1	0	At4g36410.1:d:+515:primary
TAAAGAGTCA	1	2	1	2	1	At4g36195.1:d:+1554:primary
TACGGCTGCG	0	1	4	1	1	At4g36010.1:d:+1078:primary
GACAACACAA	2	2	1	2	0	At4g35250.1:d:+953:primary
AACACAGACG	0	5	1	1	0	At4g35230.1:d:+1512:secondary
TTGGCTCGTA	2	2	2	0	1	At4g34620.1:X:-126:quaternary
CTTTTCAGGG	0	1	5	0	1	At4g34620.1:d:+308:secondary
CCAATCTTGC	0	0	1	5	1	At4g34550.1:v:+1206:secondary
TATCTTGGTA	3	1	0	1	2	At4g34260.1:d:+2680:secondary
AAAGAATTGG	2	2	1	2	0	At4g34220.1:d:+2370:primary
ACTCCGAGAC	1	4	0	2	0	At4g34120.1:d:+354:secondary
GGGAATGGGA	3	1	2	1	0	At4g34000.2:d:+1386:primary
ACGAAACCAA	0	3	4	0	0	At4g33980.1:d:+603:primary
TAGTAAAATT	1	2	2	1	1	At4g33945.1:d:+1631:primary
TGTTTGTTCC	1	2	1	3	0	At4g33580.1:d:+1172:secondary
ATTCCTTACA	2	2	0	1	2	At4g33380.1:d:-1377:secondary,At5g44950.1:v:-914:secondary
TACGACTATC	4	0	0	2	1	At4g32980.1:d:+2201:primary
AAACTGAAAA	0	2	0	3	2	At4g32820.1:v:+5943:primary,At4g26020.1:d:+900:primary,At5g50370.1:d:+816:primary
TACCAAAAAA	0	0	3	1	3	At4g32810.1:i:+1034:tertiary,At1g68170.1:i:+921:tertiary,At4g22800.1:i:+655:tertiary
TCATTGCTAG	3	0	2	2	0	At4g32770.1:d:+1558:primary
TCAAACAATA	2	2	2	0	1	At4g32590.2:d:+532:primary,At4g32590.3:d:+524:primary,At4g32590.1:d:+512:primary
AACGTTTTTA	0	2	1	2	2	At4g31800.1:d:+1026:primary
GAGAGAAGAG	1	0	2	2	2	At4g31490.1:d:+3062:secondary
ATTTTTAGTG	1	2	3	1	0	At4g31470.1:v:+1108:secondary,At1g79280.1:v:+6686:secondary
TTTTGATGAT	3	1	2	0	1	At4g30780.1:d:+2068:primary
ACTTTATATG	3	1	0	0	3	At4g30600.1:d:+2184:secondary
AAAAGGAAAT	2	2	0	2	1	At4g30490.1:d:+1861:secondary
TGTTTTTGCC	1	1	1	2	2	At4g30150.1:d:+6214:primary
TCTAGTAATA	4	0	1	1	1	At4g29070.1:d:+1044:primary
AAGATTATGT	2	2	1	2	0	At4g28860.1:d:+1483:primary
TATCGTTGTA	2	3	2	0	0	At4g28400.1:d:+976:primary
GAGTCTCCGC	1	5	1	0	0	At4g27900.1:d:+474:primary,At4g27900.2:d:+427:primary
TGAATTTCAG	1	3	3	0	0	At4g27820.1:d:+1379:primary
GAAGTTCCTA	0	1	4	1	1	At4g25730.1:d:+2720:primary
TACTTTTTCA	2	4	1	0	0	At4g25370.1:d:+499:primary
TTCCAGTTTT	1	1	0	3	2	At4g25340.1:d:+678:secondary
ACCAATCTTG	0	1	4	0	2	At4g25100.2:d:+632:secondary,At4g25100.3:d:+632:secondary,At4g25100.1:d:+768:secondary
TACCAAATGT	0	3	2	1	1	At4g25080.2:d:+1136:primary
TCTAAAAGTT	2	0	3	1	1	At4g24160.2:d:+1564:primary,At4g24160.1:d:+1483:primary
TATTGTGGAA	3	2	1	1	0	At4g23890.1:d:+678:secondary,At5g11920.1:d:+1812:secondary
GTTCTCGCTC	1	2	3	1	0	At4g23670.1:X:-231:quaternary
AATATTTTTC	1	1	2	3	0	At4g23290.1:d:+1941:primary,At4g23290.2:d:+2112:primary
AAAAAAAAAC	1	1	3	2	0	At4g21970.1:i:+561:tertiary
ACAATTTGCT	1	4	2	0	0	At4g21560.1:d:+1149:secondary,At4g21560.3:d:+1430:secondary,At4g21560.2:d:+1132:secondary
AGTCACAGGT	0	5	2	0	0	At4g21150.1:d:+1581:secondary

ACTCCGTATG	0	3	3	1	0	At4g20860.1:d:+1482:primary
TTACTTGATT	2	0	2	1	2	At4g20090.1:v:+2490:secondary
GTTTAGGTTT	1	2	2	2	0	At4g19660.1:v:+2275:primary
GTTTCACCGG	1	2	2	1	1	At4g19450.1:d:+1608:primary
GAGAAGCGTC	0	3	3	0	1	At4g18880.1:d:+1031:primary
TTTGTATACT	0	1	1	1	4	At4g18810.1:d:+1926:primary
TTTTTAAAAA	1	3	1	1	1	At4g18780.1:d:+3194:primary
TGGGAGGCAG	0	3	0	2	2	At4g17940.1:d:+781:secondary
TGCCAAGGCT	1	2	1	2	1	At4g17830.1:d:+1306:primary
CAGAGTAATC	1	1	1	3	1	At4g17260.1:d:+1231:primary
GAGGCCTCTG	4	0	1	2	0	At4g17170.1:d:+571:primary
TTCTTCTTTA	2	3	1	0	1	At4g16380.1:d:+1073:primary
CTCTCACACT	2	3	0	1	1	At4g16210.1:d:+720:primary
AAAAAAGCTC	0	0	5	2	0	At4g16045.1:v:+1972:primary
AAATCTAAAT	2	2	0	1	2	At4g15020.1:d:+2709:primary
TTAGCAGTTT	2	1	2	1	1	At4g14455.1:X:+331:quaternary
ATAGTGCGGA	1	2	2	2	0	At4g14440.1:d:+645:primary
GTCGTGGCTA	0	2	4	1	0	At4g14070.1:d:+2261:primary
TGTTTTTTTG	4	0	1	2	0	At4g13050.1:d:+1424:primary
TGAGAGTTTT	1	0	1	2	3	At4g12500.1:d:+692:primary
GTCAGCGATA	0	5	1	1	0	At4g12420.1:d:+1637:primary
TAAATCCTCA	2	3	2	0	0	At4g11860.1:d:+2189:primary
AATCCAACAA	1	2	1	0	3	At4g10750.1:d:+1225:primary
ATAAAGCACC	4	0	1	0	2	At4g10340.1:d:-1155:secondary
AGCCGTCCCT	1	2	2	0	2	At4g10320.1:d:+3573:primary
GTCAAAAATA	2	0	3	2	0	At4g10220.1:v:+13:secondary
TAAATAAAGT	3	1	3	0	0	At4g09890.1:d:+444:primary
GACTTGCTGA	0	2	3	0	2	At4g09510.1:d:+1875:primary,At4g09510.2:d:+1896:primary
TTATAATACA	4	1	1	0	1	At4g08920.1:d:+2358:primary
CTAAATTGAT	1	3	2	1	0	At4g08810.1:d:+1733:primary
AGGATCAGAT	3	0	3	0	1	At4g08340.1:v:+738:secondary,At4g33430.1:d:+2435:secondary
TGGCTCGTGC	1	3	2	0	1	At4g08230.1:d:+390:primary
GCTTCACTTT	0	2	2	1	2	At4g08170.1:d:+1084:primary,At4g08170.2:d:+1515:primary
GCGAAAGGAG	1	0	2	3	1	At4g08050.1:p:+4221:secondary
GATAGTGAGT	1	0	2	1	3	At4g07686.1:p:+294:primary
AATCTTTCCT	1	3	1	0	2	At4g05390.1:d:+1322:primary
AAAGGTTCAC	1	3	1	1	1	At4g04950.1:d:+1247:primary
AAGCCAATGC	2	0	1	3	1	At4g04850.1:d:+1939:secondary
AATTTTCGAT	1	0	1	3	2	At4g02930.1:d:+1694:secondary
TAAAATATGG	3	1	0	0	3	At4g02790.1:d:+1345:primary
TAGAAGAGAG	1	0	2	2	2	At4g02480.1:d:+3962:secondary
GACTGGCGAG	3	1	1	0	2	At4g01210.1:d:+3153:primary
TATGAACTCT	4	0	2	1	0	At4g00710.1:d:+2459:primary
GCAATCACTG	1	0	4	2	0	At3g62690.1:d:+1021:primary
AGATCGATCT	0	0	1	5	1	At3g62670.1:v:+601:secondary
GATATTTTGA	2	0	2	1	2	At3g62350.1:v:+478:secondary
TTTATTACAG	4	0	0	0	3	At3g61430.1:d:+1229:primary
ATTCCATTGG	1	4	1	1	0	At3g60750.1:d:+1904:secondary
GTTGATGATT	2	3	2	0	0	At3g60510.1:d:+1273:primary
CATCAACGTC	0	6	1	0	0	At3g60300.1:d:+894:primary
TATGAGGATG	3	2	1	0	1	At3g59960.1:v:+1344:secondary,At5g59250.1:d:+1790:secondary
TTTTAGAAAC	1	3	2	1	0	At3g59920.1:d:+1589:secondary
CACAACCTAT	2	0	1	2	2	At3g59870.1:d:+1173:primary
GCTGGGAGAC	0	3	2	2	0	At3g59280.1:d:+207:secondary,At5g03300.1:d:+835:secondary
TAGATGGAAA	1	1	1	3	1	At3g58970.1:d:+1640:primary
ATAAAAAATG	0	1	2	1	3	At3g58830.1:d:+1131:primary
GTTTTGTGGC	1	4	1	1	0	At3g57870.1:d:+139:primary
TTGAGATTCT	1	5	0	1	0	At3g57520.3:d:+2466:primary,At3g57520.2:d:+2494:primary,At3g57520.1:d:+2528:primary
ATATAATACA	3	2	0	0	2	At3g57400.1:d:+1421:primary
AGCAGTTGAT	3	2	0	0	2	At3g57290.1:d:+1307:primary
AAGATAAAAT	2	1	2	2	0	At3g57060.1:v:+170:secondary

ATGGCTAAGT	0	5	0	2	0	At3g56790.1:v:+1028:primary,At5g21274.1:d:+507:primary
ACCATATTTT	3	2	0	1	1	At3g56630.1:d:+1567:primary
AATCTTACAA	1	3	1	2	0	At3g56060.1:d:+1858:primary
ATTGTATGAT	3	0	2	1	1	At3g55170.2:d:+488:primary,At3g55170.1:d:+508:primary
CTTCCTACAG	1	2	4	0	0	At3g54890.3:d:+902:primary,At3g54890.1:d:+969:primary,At3g54890.2:d:+867:primary
CGTTGAAGTG	1	6	0	0	0	At3g54810.2:d:+1159:primary,At3g54810.1:d:+1276:primary
AGAAAATCTG	1	4	0	2	0	At3g54720.1:d:+2151:primary
TGAAACAGAT	0	3	0	2	2	At3g54640.1:d:+1017:primary
TGTTCACGAT	3	2	0	1	1	At3g54440.1:d:+3329:primary
AGGAATTATC	0	1	1	5	0	At3g54280.1:d:+2009:secondary,At2g15690.1:d:+2063:secondary
TCACATTTTG	2	4	0	1	0	At3g53850.1:d:+731:primary
GAGAGTGTAG	3	2	2	0	0	At3g53370.1:d:+474:primary,At5g52560.1:d:+1953:primary
AAAATTGGTC	1	3	1	1	1	At3g52580.1:d:+469:primary
TTTATGCAAG	1	1	3	1	1	At3g52430.1:d:+1889:primary
TTTGCAAGGT	1	2	3	1	0	At3g51980.1:d:+1401:primary
AGAATCAGTG	3	3	0	1	0	At3g51850.1:d:+2035:primary
GGCCAAAAAA	1	0	3	3	0	At3g51830.1:d:+1310:primary
TTCAGTATTC	0	1	0	1	5	At3g51260.1:d:+1041:secondary
TAAAGAATCA	3	1	0	3	0	At3g50790.1:d:+1347:primary
AGGGTGGTGG	0	2	3	1	1	At3g50670.2:d:+2081:primary,At3g50670.1:d:+1183:primary
TATTTGCTTC	1	4	1	0	1	At3g50210.2:d:+1276:primary,At3g50210.1:d:+1306:primary
GCGGAAATGC	0	3	3	1	0	At3g50060.1:d:+854:primary
GTCAAGAGTC	0	2	1	1	3	At3g49990.1:d:+1427:primary
GTGGCTCAGA	2	1	3	1	0	At3g49800.1:d:+1633:primary
GTTTGTTTTG	3	0	1	3	0	At3g49800.1:d:+1572:secondary,At3g43920.1:v:+1680:secondary,At2g26320.1:v:+180:secondary
ATGAAACTTC	1	2	1	2	1	At3g48890.1:d:+679:primary
TATACAAATT	1	1	1	1	3	At3g48570.1:d:+457:primary
AAGCAAAAAT	0	1	5	1	0	At3g48190.1:i:+12787:tertiary
TGGTGGTGGT	2	1	2	1	1	At3g48070.1:d:+1315:primary
GCGTATGAAC	0	5	1	0	1	At3g47370.1:d:+91:primary,At3g47370.2:d:+62:primary
TATGAGACCA	0	6	1	0	0	At3g46600.1:d:+1550:primary,At3g46600.2:d:+1471:primary
TGAACCAGGC	0	3	3	0	1	At3g46530.1:d:+2634:secondary
GTTCCGATGG	1	4	0	1	1	At3g45010.1:d:+1476:primary
TGCGTGAGAC	0	0	3	2	2	At3g44450.1:X:+567:quaternary
TCACTCCTAT	1	2	2	0	2	At3g44300.1:d:+1085:secondary,At4g30030.1:v:+556:secondary
CTTGTACAAA	0	3	1	2	1	At3g43835.1:p:+2249:secondary
TCCTGTTAGC	2	3	2	0	0	At3g43720.1:X:-102:quaternary
AGAGAGTTCT	4	1	1	1	0	At3g43300.1:d:+5161:primary
TCATTAATTA	3	3	1	0	0	At3g42980.1:v:+1446:primary,At3g57090.1:d:+785:primary
CAGCAACAAA	2	2	1	1	1	At3g42790.1:d:+878:primary
GCTTCACCTA	0	1	1	5	0	At3g42178.1:p:+4584:secondary,At2g38240.1:d:+1526:secondary
TGTCTATGAG	2	5	0	0	0	At3g30842.1:v:+257:secondary
GTCAGTGGTA	1	0	1	1	4	At3g29200.1:d:+1316:primary
GGCCATCCAT	3	0	1	3	0	At3g28740.1:d:+1210:secondary
CTTTACCAGA	1	4	0	1	1	At3g28670.1:d:+1630:primary
GAGAGGTAAT	2	0	2	2	1	At3g27570.1:d:+1289:primary
AAGAGTCCCT	1	1	2	3	0	At3g27260.1:d:+3107:primary
GAGAGGAGGA	0	4	1	2	0	At3g27210.1:d:+776:primary
GCTCAATGTA	1	4	2	0	0	At3g26810.1:d:+2078:primary
GAAGCTCCGC	3	3	1	0	0	At3g26520.1:X:-205:quaternary
AAGCCTCCTC	2	1	3	1	0	At3g26290.1:X:+752:quaternary
TATGTAAAAA	0	0	0	2	5	At3g26080.1:d:+907:primary
TTTAGATCTT	0	2	0	3	2	At3g24506.1:d:+554:primary
AAGAGTTTGT	3	4	0	0	0	At3g23900.1:d:+3284:primary
GCTGTGATAA	0	7	0	0	0	At3g23600.1:d:+331:primary
TTTTGGAAAC	0	1	3	2	1	At3g23410.1:d:+2402:primary
ATCTAAACAT	5	0	0	0	2	At3g23325.1:d:+441:primary
TATTTTGTTT	2	1	1	1	2	At3g22660.1:d:+1023:primary,At3g28340.1:d:+1480:primary
TAATTAGGAG	3	2	0	0	2	At3g22430.1:d:-641:primary
AGTTTTGTTG	1	1	5	0	0	At3g20600.1:d:+765:primary

TATGTATGTA	1	1	1	1	3	At3g19990.1:d:+1383:primary,At3g14050.1:d:+2567:primary
GAAAGGGAAG	2	1	0	1	3	At3g19980.1:d:+1012:primary
CCTCCTAATA	1	3	1	1	1	At3g19860.1:d:+731:primary
AGCTCCAGCT	0	2	3	2	0	At3g19670.1:d:+3011:primary
GGGGTTGAAT	2	2	3	0	0	At3g19400.1:d:+987:secondary
GAAAGAGAAC	0	1	1	3	2	At3g18870.1:d:+662:primary,At4g10110.1:d:+641:primary
GCTTCTGGCT	1	3	2	1	0	At3g18035.1:d:+671:primary
TGTGGGCACT	1	1	0	4	1	At3g17840.1:d:+2167:primary,At1g01310.1:d:+590:primary
GTCTCAAAAT	2	0	2	1	2	At3g17800.1:d:+38:secondary,At1g22300.3:d:+1499:secondary,At1g22300.1:d:+1151:secondary
TAAAATAAAA	2	1	3	1	0	At3g17150.1:i:+1158:tertiary,At1g27090.1:i:+830:tertiary,At4g10770.1:i:+1426:tertiary
TATATCCTTT	0	2	2	1	2	At3g17100.2:d:+1034:primary,At3g17100.1:d:+1136:primary
ACCTCATATC	1	3	1	2	0	At3g17090.1:d:+1263:primary
GGCTTGGTTT	2	1	2	2	0	At3g16920.1:d:+1112:secondary,At5g34860.1:v:+902:secondary
AAACGTGAAA	3	0	1	3	0	At3g16400.1:d:-1641:secondary,At1g73000.1:v:-1263:secondary,At1g73000.1:v:-1251:secondary
TAAGTTAAAT	4	1	0	2	0	At3g16300.1:v:+1449:secondary,At5g52900.1:d:+1168:secondary,At3g16290.1:v:+315:secondary
GACGCGACCG	0	4	3	0	0	At3g15810.1:d:+339:primary
TCTTTCTTAA	4	0	1	1	1	At3g15680.1:d:+888:primary
GATGCAACAA	1	0	1	3	2	At3g14810.1:v:+2891:primary
TCTTAGAATT	2	0	0	3	2	At3g14720.1:d:+1756:primary
TGCTCTTGTC	2	1	2	2	0	At3g14650.1:d:+1993:secondary
TATTTTCTTG	1	4	0	0	2	At3g14310.1:d:+2059:primary
AGTTTTGTTT	2	2	3	0	0	At3g13480.1:v:+1397:primary,At1g77510.1:d:+1630:primary
TTAATCTTTC	1	3	1	1	1	At3g12920.1:d:+1165:primary
AGTATTTTGT	2	5	0	0	0	At3g12460.1:v:+1241:primary,At1g79245.1:p:+4525:primary
GACATTTACA	0	5	0	1	1	At3g12260.1:d:+183:primary
TAGGAATCAA	1	3	1	1	1	At3g11910.1:d:+3466:primary
TTTCTAAGGT	1	1	2	2	1	At3g11480.1:d:+724:secondary,At5g26742.1:v:+2900:secondary
AGAATTTTGG	1	2	2	2	0	At3g11450.1:d:+2018:primary
TTTTAATCAA	1	4	0	0	2	At3g11200.2:d:+1119:secondary,At3g11200.1:d:+971:secondary
GATGGAGGAT	2	1	3	1	0	At3g11110.1:v:+931:secondary
TCAGCTTTCG	3	1	3	0	0	At3g10620.1:d:+625:secondary
ATTAAGAACC	1	0	1	2	3	At3g10572.1:d:+1201:primary
TATTTGTCAA	1	2	1	0	3	At3g10260.2:d:+984:primary,At3g10260.1:d:+1083:primary,At3g10260.3:d:+1339:primary
AGAAGTGAGA	4	1	0	2	0	At3g10160.1:d:+1527:primary
TGGACAGTTT	0	4	2	0	1	At3g07950.1:d:+1104:secondary
TGTTTGATGA	3	2	1	1	0	At3g07880.1:d:+1174:primary
ATCGTCTATG	3	2	1	0	1	At3g07780.1:d:+1892:primary,At1g74820.1:v:+1457:primary
TTCAAGAACT	1	3	0	2	1	At3g07680.1:d:+914:primary
ATTTAAAAAA	0	1	4	0	2	At3g07610.1:v:+129:secondary,At3g60270.1:v:+37:secondary
GAACGATAAC	1	3	2	0	1	At3g07360.2:d:+1437:primary,At3g07360.1:d:+1440:primary
TGTCGTGCCA	1	5	0	0	1	At3g07350.1:d:+1044:primary
TCTGTTTGTT	2	2	3	0	0	At3g07270.2:d:+1421:primary,At3g07270.1:d:+1788:primary,At5g21070.1:d:+899:primary
TAGATGATTT	5	0	0	1	1	At3g07250.1:i:+559:tertiary
CCCGTCTTCT	1	2	2	2	0	At3g06820.1:d:+1538:primary
TAGACCTATA	3	0	1	1	2	At3g06680.1:X:+253:quaternary
TATTTATGGT	3	1	0	1	2	At3g06483.1:X:+452:quaternary
AGAACTGGTG	0	2	3	2	0	At3g06410.1:d:+877:secondary
TTTCCGGTCT	1	2	2	1	1	At3g06060.1:d:+1183:primary
ATTTACTTTG	6	0	1	0	0	At3g05280.1:d:+1079:primary
TAAAATCCTA	2	0	0	2	3	At3g05070.1:d:+615:primary
TCATAAGCAA	1	2	0	2	2	At3g05040.1:v:+2175:secondary
AACTTTAAGA	1	1	2	1	2	At3g04940.1:d:+1188:primary
GTACGACAAC	0	0	6	0	1	At3g04120.1:d:+1075:secondary,At1g13440.1:d:+1025:secondary
AAGCGGAGAT	1	2	3	0	1	At3g03740.1:d:+1468:primary
TTAATTTCAT	3	0	0	1	3	At3g02420.1:d:+1194:primary
GAATCAATGA	2	1	1	0	3	At3g01980.2:d:+1187:primary,At3g01980.1:d:+1082:primary
CAATGCAGAT	2	0	3	2	0	At2g48160.1:d:+4518:primary
GCCAGAGGAC	0	2	2	2	1	At2g48010.1:d:+1268:primary,At3g49100.1:d:+321:primary
TTGAAAGAGA	0	1	1	5	0	At2g47900.1:d:+1724:primary
AATGATGATG	0	2	3	0	2	At2g47890.2:d:+1331:primary,At2g47890.1:d:+1248:primary
TGATTTGCTT	2	0	2	2	1	At2g47180.1:d:+1240:primary

GCTATGAATC	0	6	0	1	0	At2g46490.1:d:+283:primary
GAAAGTGTTG	1	4	1	1	0	At2g45260.1:d:+1337:primary,At4g34080.1:d:+1039:primary
GTTTCCGCAA	1	1	0	5	0	At2g44950.1:d:+3248:primary,At4g20040.1:d:+1401:primary
ATGCATCGAA	2	3	1	0	1	At2g44750.1:d:+925:primary,At2g44750.2:d:+931:primary
TGTATACTGA	2	1	1	1	2	At2g44210.1:d:+1668:primary
AAACTAGAAG	0	3	4	0	0	At2g44065.2:d:+915:primary,At2g44065.1:d:+906:primary
TCTTTTTCTT	3	1	0	1	2	At2g43810.1:d:+474:primary
TGTTAGATTA	4	2	0	1	0	At2g43550.1:d:+493:primary
AACTTTTTGT	0	3	1	1	2	At2g42620.1:d:+1963:primary
CAGCTTTTTA	2	4	0	0	1	At2g41620.1:d:+2751:primary
AATGAAATTT	1	1	2	3	0	At2g41120.1:d:+947:secondary,At5g26630.1:v:+1131:secondary
TAACTAAATC	0	0	2	4	1	At2g41040.1:d:+1200:primary
TGAATTTTTT	1	4	1	1	0	At2g41020.1:d:+1688:primary,At2g41020.2:d:+1964:primary
TTGAAGCTCC	0	6	0	0	1	At2g40840.1:d:+2794:primary
TATCAGAAGT	0	5	2	0	0	At2g40810.2:d:+1131:secondary,At2g40810.1:d:+1151:secondary
ATTTCAAAAA	0	3	3	1	0	At2g40180.1:i:+1264:tertiary,At2g38940.1:i:+448:tertiary
TGTATGTTTT	1	0	1	3	2	At2g40170.1:d:+483:primary,At4g01940.1:d:+975:primary
TTGTGTCGTC	1	2	2	1	1	At2g39940.1:d:+2145:secondary
TGAATTCCCC	1	2	4	0	0	At2g39850.1:d:+2323:primary
CGAGAAGCGT	0	4	0	2	1	At2g39750.1:d:+2184:primary
CTTATTTGCT	3	0	3	1	0	At2g39705.1:d:+411:secondary
GTTGTATTAT	1	1	2	2	1	At2g39700.1:d:+1299:primary
TCTCTCACCA	2	1	2	2	0	At2g39190.2:d:+2513:primary,At2g39190.1:d:+2505:primary
CCCCGGTCTC	2	3	1	1	0	At2g38120.1:d:+1716:secondary
TTTTTTTAAA	0	2	1	1	3	At2g37730.1:v:+2223:secondary
ACGGATTTAG	1	1	5	0	0	At2g37710.1:d:+1971:secondary,At4g11090.1:d:+873:secondary
GAACGTTGCT	0	5	1	1	0	At2g37640.1:d:+767:primary
GCTCCGCCGC	0	0	0	3	4	At2g36620.1:X:-44:quaternary
TGGTATTGAA	1	5	1	0	0	At2g36180.1:v:+646:secondary,At5g17470.1:v:+649:secondary
AAGAACTATA	2	1	1	2	1	At2g36130.1:d:+637:primary
CCCAGTTTCT	2	3	2	0	0	At2g35880.1:d:+1203:primary
TTAATCCCAT	4	1	2	0	0	At2g35820.1:d:+753:primary
AGTGTGCTTC	1	2	1	1	2	At2g35810.1:d:+631:primary
TCGATTTGTT	2	3	0	2	0	At2g35470.1:d:+624:primary
TAGCTCTCTA	3	2	0	2	0	At2g35260.1:d:-1372:secondary,At4g27720.1:d:-1027:secondary
GGCAGTTGAT	2	0	2	2	1	At2g34810.1:d:+1798:primary
TATGTGCTCT	1	5	1	0	0	At2g34750.1:d:+1492:primary
TAAAACGTCT	1	0	1	0	5	At2g34480.1:d:+575:secondary
AGACTCATTA	3	0	2	0	2	At2g34470.1:d:+1071:primary
ACTTCAACTG	1	1	4	1	0	At2g34160.1:d:+321:primary
ACTGTGTTTC	1	4	0	2	0	At2g34040.1:d:+1773:primary
TCACTCTACC	0	4	3	0	0	At2g33830.2:X:-54:quaternary
TTGTGTTCTG	1	2	3	1	0	At2g33700.1:d:+1993:primary
ACCATAATAT	2	0	1	4	0	At2g33570.1:d:+1629:secondary
TGTGTGAGGG	4	0	1	2	0	At2g33255.1:X:+327:quaternary
GACGATGCAG	1	1	3	0	2	At2g32950.1:d:+1964:primary
ACTCATTGCG	1	2	2	1	1	At2g31750.1:d:+1121:secondary
ATCCGAAGTC	1	1	5	0	0	At2g31450.1:d:+1183:primary
GTTGCTCAGA	1	1	1	3	1	At2g30100.1:d:+1251:secondary
TTTTCACAGA	1	5	1	0	0	At2g30050.1:d:+429:secondary
AATTCGCATA	0	1	3	1	2	At2g29100.1:v:+2940:secondary
ACTCTCGGAG	3	1	2	1	0	At2g29080.1:d:+2207:secondary
GTGTATGCAA	1	2	1	2	1	At2g28800.1:X:-320:quaternary
AAACTGTTAA	1	5	0	1	0	At2g28480.1:v:+967:secondary,At1g26790.1:v:+1273:secondary,At5g40450.1:d:+7181:secondary
GCAACAAGAA	0	5	0	1	1	At2g27730.1:d:+102:primary
TCAATGTAAA	2	1	3	1	0	At2g27580.1:d:+796:primary
AACCAGTACT	3	0	4	0	0	At2g26910.1:d:+4493:primary
TTTGTTTCTC	1	1	3	2	0	At2g26680.1:d:+1203:primary
CAAATAGGAC	0	5	1	1	0	At2g26530.1:d:+1171:primary
TATTTGTGTA	0	1	2	3	1	At2g26140.1:d:+2394:primary
GCAATCCTCC	1	1	2	3	0	At2g25920.1:d:+997:primary

AAATTATAGT	2	2	1	0	2	At2g25170.1:d:+4347:primary
GCGACGGTAC	3	1	1	0	2	At2g23980.1:d:+2060:primary
AAGCACCGTC	3	1	2	1	0	At2g23600.1:d:+510:primary
TGAAGAAGAA	1	0	2	1	3	At2g22880.1:v:+927:primary
TCGCAGCTGA	2	0	1	1	3	At2g22870.1:d:+893:secondary
TTTTCAAAAC	1	1	4	1	0	At2g22740.2:i:+2852:tertiary,At3g21480.1:i:+3749:tertiary
AAGCTCGGGT	0	3	1	2	1	At2g22010.1:d:+4131:primary
GTAAAGAGTG	1	1	0	4	1	At2g21620.1:d:+489:primary,At2g21620.2:d:+482:primary
TCAGGTCCAC	1	2	2	1	1	At2g21520.1:X:+3:quaternary
TAATAATTTT	1	3	0	3	0	At2g21385.1:d:+1159:primary
CTAACAGAAA	1	0	3	2	1	At2g21380.1:d:+3496:secondary
TTGTGTACTA	1	2	1	0	3	At2g21270.1:d:+1105:primary
TTTGGGAATT	2	3	2	0	0	At2g21050.1:d:+1836:primary
AATAAAAATC	1	0	1	2	3	At2g20420.1:d:+1621:primary
TCTTCAATAA	0	6	1	0	0	At2g19800.1:d:+936:primary
ATAGTTTTGC	0	1	1	3	2	At2g18690.1:d:+1394:primary
TTTGTTCACT	0	3	0	1	3	At2g17780.1:i:+145:tertiary
TATTCATAAA	1	3	1	0	2	At2g15290.1:d:+945:primary
CCTTATTTCT	2	1	2	1	1	At2g14890.1:d:+859:primary
ATGAAATTAT	3	0	1	2	1	At2g14300.1:p:+3614:secondary
GGAATAAGAA	2	0	1	4	0	At2g14170.1:d:+1979:primary
CAGCACCACC	1	5	1	0	0	At2g13200.1:p:+2141:primary
GCGAATGGAT	2	2	2	0	1	At2g12390.1:p:+4439:primary,At4g33520.1:d:+855:primary
GCCAAGGAAG	4	2	0	1	0	At2g07707.1:v:+808:primary,At2g07718.1:v:+1445:primary,AtMg00480:d:+458:primary
CAAGAATTTG	1	4	2	0	0	At2g06530.1:d:+542:primary
AGAGCATCTG	1	4	1	0	1	At2g04280.1:d:+1697:secondary,At5g03050.1:d:+161:secondary
GGATAAACAA	0	3	1	2	1	At2g03340.1:d:+1872:primary
TTGTCTTCAG	3	2	0	1	1	At2g02790.1:v:+2443:primary
GAAAATGTGT	2	2	1	2	0	At2g02470.1:d:+940:primary,At3g03000.1:d:+758:primary
AAATAAGTTT	0	3	1	2	1	At2g01650.1:d:+1515:primary
GATCACATTC	0	3	0	3	1	At1g80550.1:d:+1749:primary,At4g12750.1:d:+3497:primary
GGCGAACGGA	1	0	3	1	2	At1g79850.1:X:-89:quaternary
TACACGTCCA	1	2	1	0	3	At1g79730.1:X:+312:quaternary
CGGCAAAACA	0	3	3	1	0	At1g78995.1:d:+701:primary
ATAAATTGGA	2	1	2	2	0	At1g78870.2:d:+595:primary,At1g78870.1:d:+698:primary
CTGCAAATTA	0	3	2	1	1	At1g78670.1:d:+1012:secondary
GGTCTGAATC	1	3	2	0	1	At1g78420.1:d:+1734:primary
AATTTGCTTC	2	2	0	2	1	At1g77765.1:v:+1013:secondary,At3g12630.1:d:+834:secondary
GAAGCCTCTG	1	5	0	1	0	At1g77540.1:d:+330:primary
AGTAACAAAA	3	2	0	2	0	At1g77260.1:d:+2392:primary
ATACCGGGGA	2	2	2	1	0	At1g77260.1:d:+2168:secondary
GTTGTAGTAG	0	7	0	0	0	At1g77210.1:d:+1205:primary
GGGAAGAAGA	2	2	1	1	1	At1g77180.1:d:+1461:primary
AGAGGTCCAA	0	4	2	0	1	At1g76890.2:d:+1042:primary,At1g76890.1:d:+762:primary
TGCTTTCAAC	0	4	1	1	1	At1g76590.1:d:+736:primary
AACACCGTGG	0	6	1	0	0	At1g76010.1:d:+820:secondary
GGAGAATTCT	0	4	1	1	1	At1g75800.1:d:+1281:primary
TTTGGTGAAC	4	0	2	1	0	At1g75690.1:X:-100:quaternary
AACAATGATG	0	3	2	2	0	At1g75500.1:X:-410:quaternary
CTTCTCTAGT	2	2	1	0	2	At1g75270.1:d:+767:primary
CAAAAAACAT	2	0	2	0	3	At1g74490.1:X:-73:quaternary
TAATTTTTTA	1	4	1	1	0	At1g74440.1:d:+1151:primary
GGGCTTCATT	2	0	2	3	0	At1g74045.1:v:+1432:primary
TTGTGGGTGG	0	1	3	1	2	At1g73870.1:d:+983:primary
AAAGTCCTGA	2	2	2	1	0	At1g73600.1:d:+1501:primary,At1g73600.2:d:+1498:primary
GACGATTGAT	3	2	1	1	0	At1g73110.1:d:+1325:primary
GTACTTGAAT	2	0	2	2	1	At1g72770.1:d:+2341:secondary
TCAAAGCTAC	1	0	2	0	4	At1g72610.1:d:+247:secondary
GCACCGGCGT	2	1	2	2	0	At1g72340.1:d:+996:secondary
ATATAGCATA	1	4	1	1	0	At1g71970.1:d:+945:primary
TGGGCTTTGA	1	2	1	2	1	At1g71340.1:d:+764:secondary

GCTATTTTGC	0	1	1	2	3	At1g71220.1:d:+5075:primary
GACCTCTTGA	2	1	2	2	0	At1g71010.1:d:+4938:primary
TTTGATTTGG	1	2	4	0	0	At1g70770.1:d:+2228:secondary
GAACTTCGAG	1	2	2	1	1	At1g70520.1:d:+1952:primary
TCTAGAACTG	1	0	1	2	3	At1g70510.1:i:-3753:tertiary
AAAAGTCTCC	1	4	1	1	0	At1g70280.1:d:+1850:primary,At1g70280.2:d:+1765:primary
GAGGAGTACA	3	2	0	2	0	At1g70230.1:d:+1516:primary
GTGTTTGGGA	0	4	3	0	0	At1g69840.1:d:+199:primary,At1g69840.2:d:+277:primary,At1g69840.4:d:+166:primary,At1g69840.3:d:+541:primary
GGACCCGTCT	1	0	1	1	4	At1g69490.1:d:+760:secondary
CTTTCAAATC	0	0	0	2	5	At1g69070.1:d:+2654:primary,At2g02570.1:d:+753:primary,At2g02570.2:d:+753:primary
CGCTGTCCCA	1	0	3	2	1	At1g69070.1:d:+1855:secondary
AATCACAACC	1	5	0	1	0	At1g68840.1:d:-1247:secondary
AATCTTCTTT	0	3	1	0	3	At1g68820.1:d:+1754:primary
TCTACATATG	2	3	1	0	1	At1g67840.1:d:+2029:primary,At1g67840.2:d:+2033:primary
GCTGATGATG	1	0	3	2	1	At1g67550.1:d:+2092:secondary,At1g21730.1:d:+1783:secondary
GTGACCCCAG	0	7	0	0	0	At1g67230.1:d:+347:secondary
GCTTCTTGGG	3	0	1	3	0	At1g67130.1:v:+214:secondary
TATCAACTAT	2	1	1	1	2	At1g66750.1:d:+1260:primary
TAATTAATTA	2	2	2	0	1	At1g66330.2:d:+1449:primary,At1g66330.1:d:+1459:primary
GCAGTGGTAA	1	3	2	1	0	At1g66245.1:v:+1380:secondary,At1g64370.1:d:+538:secondary
TTTCAATGTT	3	0	0	1	3	At1g66200.1:d:+1429:primary
GCTCAGTTCT	2	1	2	1	1	At1g64790.1:d:+6779:secondary,At3g02540.1:d:+1517:secondary
AAGCTGAAAC	0	2	4	1	0	At1g63980.1:d:+1096:primary
TAAAATAGAT	5	0	0	1	1	At1g63660.2:d:+1850:primary,At1g63660.1:d:+1799:primary
CCAATTGATT	1	2	1	2	1	At1g63360.1:v:+2984:secondary
GGGTTTCCAG	2	2	1	1	1	At1g63180.1:d:+1224:primary
AAGCCGAAGA	0	5	1	1	0	At1g62855.1:d:+302:primary
TAGAAAAAAA	1	2	3	1	0	At1g62420.1:i:+2832:tertiary
ACATAAAGGT	2	0	2	2	1	At1g61250.1:d:-1137:secondary
AATTTTTACT	0	3	0	2	2	At1g61250.1:d:+1201:primary
TGGATCTTGG	2	1	2	2	0	At1g60850.1:d:+1098:primary,At1g76580.1:d:+3301:primary,At1g60850.3:d:+1141:primary,At1g60850.2:d:+1085:primary
AGAGCCACTT	1	4	1	1	0	At1g60260.1:p:+2576:primary
CTGAATCAGC	1	3	2	0	1	At1g59610.1:d:+2518:primary
AAGAGCAACA	1	2	2	1	1	At1g58230.1:v:+1183:secondary
TAAAGTTGTT	1	2	3	1	0	At1g58110.1:d:+1573:primary
TGATCTTCCT	2	2	1	1	1	At1g56000.1:d:+1311:secondary
AGCTACCGTT	1	2	2	0	2	At1g55900.1:d:+1303:primary
TCCGAACCAG	0	1	1	3	2	At1g55500.1:d:-2326:secondary
GTACTGTTAT	1	2	3	1	0	At1g55310.1:d:+1104:primary
TTTTGCTTTT	1	4	2	0	0	At1g55160.1:d:+694:primary
GATTTATCTA	1	1	3	1	1	At1g54830.1:d:+930:secondary,At1g54830.3:d:+1338:secondary,At1g54830.2:d:+852:secondary,At2g27900.1:d:+387:secondary
AATTTATGGA	0	5	2	0	0	At1g54740.1:d:+808:primary
CTTGATAGTT	3	1	1	0	2	At1g54220.1:d:+1986:primary
TACATAAAGA	3	3	0	0	1	At1g54210.1:d:+456:primary
AGACCGAGAC	1	2	2	1	1	At1g54030.1:d:+1195:primary
TATCAAAAAA	2	2	2	1	0	At1g53030.1:d:+323:secondary
TTTCTGTTTG	0	1	3	1	2	At1g52380.1:d:+1671:primary
GTTTAGCAGA	3	4	0	0	0	At1g52190.1:d:+1560:primary
ATAGGAAACC	2	1	2	2	0	At1g51680.1:X:-305:quaternary
TTTATCTGTT	2	0	1	1	3	At1g50440.2:d:+1207:primary,At1g50440.1:d:+1147:primary
CTTTCTGTTC	0	2	0	4	1	At1g49760.1:d:+1905:primary
TATGTTGTAG	2	0	1	1	3	At1g48840.1:d:+2352:secondary
CTCAAAATGG	1	4	0	1	1	At1g48320.1:d:+519:primary
GCTGATGAAA	1	1	0	5	0	At1g48040.1:d:+511:secondary,At4g34030.1:d:+838:secondary,At3g07920.1:v:+354:secondary,At5g20920.2:d:+76:secondary,At5g20920.1:d:+116:secondary
TACACATACA	0	2	0	2	3	At1g47890.1:v:+3667:primary,At1g54320.1:d:+1275:primary
TAAAAAAACA	2	0	2	2	1	At1g47550.1:v:+3012:primary

GTTTCCATAG	5	0	1	1	0	At1g47400.1:d:+389:primary
CCAAGTAAAG	0	5	1	1	0	At1g46912.1:v:+184:secondary
ATAACAAAAG	1	3	3	0	0	At1g45207.2:i:+955:tertiary
AATATTCGGT	0	3	2	1	1	At1g44920.1:d:+792:primary
TTGTGTATTG	1	1	2	2	1	At1g43690.1:d:+2209:primary,At5g65860.1:d:+1170:primary
TGGTGGCGTG	1	1	2	1	2	At1g43620.1:d:+2222:primary,At1g43620.2:d:+2032:primary
TCAGCTTATG	1	4	0	0	2	At1g43560.1:d:+323:primary
ATTGAAAATT	1	1	1	2	2	At1g43150.1:p:+455:secondary,At5g27640.1:d:+2357:secondary
ATGTCGAAGC	1	4	2	0	0	At1g42990.1:d:+635:primary
TATCTTGTTG	0	3	4	0	0	At1g41890.1:p:+3407:secondary,At2g06050.2:d:+1420:secondary,At4g16295.1:v:+845:secondary,At2g06050.1:d:+1329:secondary,At3g55740.2:d:+1407:secondary,At3g55740.1:d:+1449:secondary
CTTATTTATC	2	0	0	0	5	At1g35620.1:d:+1447:primary
TGTTCTTATT	2	3	0	0	2	At1g35612.1:X:+1143:quaternary,Chr2:+17153665:quaternary,Chr5:+8810143:quaternary
AAACCAGTCA	4	1	1	1	0	At1g35580.1:d:+1769:primary,At1g35580.2:d:+1768:primary
GGAGCTTGTA	2	2	2	0	1	At1g34430.1:d:+1507:secondary
AGTTTCAATA	4	1	0	0	2	At1g33950.1:i:+3362:tertiary
TGAACTTGTA	3	1	2	0	1	At1g33520.1:d:+1733:primary
AGCCTCCTCT	1	5	1	0	0	At1g32810.1:v:+2315:primary
GAAAGAGACT	0	2	3	0	2	At1g32190.1:d:+1581:primary
AAAGGAAGCT	0	2	1	2	2	At1g30530.1:d:+1564:primary
TTCCAGTTGC	1	1	2	0	3	At1g29700.1:d:+1347:primary
ACATCGACTG	1	2	1	3	0	At1g29250.1:d:+376:primary
TCAACATCAT	0	6	0	0	1	At1g28670.1:d:+939:primary
GGAGAGATTG	0	6	1	0	0	At1g28660.1:d:+563:primary,At1g28660.2:d:+537:primary
TTTGTGGTGA	2	1	1	1	2	At1g28490.1:d:+908:primary
TAAAGATTAG	2	2	0	2	1	At1g27430.1:d:+4628:primary,At1g24300.1:d:+4289:primary,At4g26430.1:d:+1189:primary
AAAAAGGGTT	4	1	0	0	2	At1g27300.1:d:+810:secondary
TTGCTACGCC	0	5	0	2	0	At1g25550.1:d:+807:primary
TCCTATTCTG	0	1	0	2	4	At1g25540.1:d:+2871:primary
AAACAGAGAA	2	0	3	1	1	At1g24490.1:d:+3026:secondary,At5g07850.1:v:+1958:secondary
AGAAAATACT	1	3	0	1	2	At1g24330.1:v:+984:secondary
TACGTGAAAG	0	4	0	2	1	At1g24180.1:d:+1227:primary
AATCAGAGAG	1	4	0	1	1	At1g23890.1:d:+1291:primary
TGGAGAGGAC	1	6	0	0	0	At1g23400.1:d:+1211:primary
ATCGGAGAAA	2	1	2	2	0	At1g23040.1:d:+512:primary
TCTTGTGTGC	0	4	3	0	0	At1g22790.1:d:+953:primary
TACGTAGGCC	0	4	1	1	1	At1g22760.1:d:+1618:secondary
TAATGGGTTG	2	1	0	2	2	At1g22740.1:d:+950:primary
CAGCGCCAAT	1	5	0	1	0	At1g22430.1:d:+992:primary
AGCCTCGTAC	0	3	3	0	1	At1g22280.1:d:+655:primary,At1g22280.2:d:+652:primary
GGGGATATTG	2	0	1	0	4	At1g21660.1:d:+1952:primary
GAGAAACATC	1	1	0	1	4	At1g21600.1:d:+1001:primary
AAGCTCGAAA	1	2	2	2	0	At1g20650.1:d:+1697:primary
TTTTGTATAA	1	3	1	0	2	At1g20225.1:d:+871:primary
CTCTCTGCCT	3	2	0	0	2	At1g19860.1:d:+1584:primary
GGAAAATATG	5	1	1	0	0	At1g19310.1:d:+918:primary
AAAGCTGTGA	1	3	1	0	2	At1g18270.1:d:+4125:primary
TTTCGAATTC	4	1	0	2	0	At1g18250.1:d:+828:primary
AAGGCTGGAG	0	5	0	2	0	At1g18170.1:d:+657:primary
CGTTGATAGG	3	2	1	1	0	At1g17580.1:d:+4919:primary
TGTTGGCTTG	2	0	3	2	0	At1g15410.1:d:+1790:primary
TTGGTGTTTG	2	1	1	1	2	At1g14980.1:d:+427:secondary
GTGTGTCATC	2	2	1	1	1	At1g14400.1:X:-13:quaternary
AGCTCGGCGT	1	2	3	1	0	At1g13930.1:d:-168:primary
AGATCACTGA	4	0	0	2	1	At1g13560.1:d:+1275:primary,At1g13560.2:d:+1230:primary
ATCAACGCTT	4	2	1	0	0	At1g13090.1:d:+1217:primary
TGTTTTCAAA	1	0	2	3	1	At1g12820.1:d:+2129:secondary
CACAGGGAAA	1	4	0	2	0	At1g12050.1:d:+1347:primary

GAACCGTCCT	2	1	2	2	0	At1g11720.1:d:+3027:primary
TATGCATTCA	1	2	1	2	1	At1g11430.1:d:+411:primary
TTGCAGAAGG	1	4	1	0	1	At1g11360.1:d:+900:primary
TCAACTAACA	1	1	3	0	2	At1g11300.1:i:+2093:tertiary
CTTTTAGAGC	1	2	0	1	3	At1g11240.1:d:+719:primary
AACTATCATC	0	2	2	0	3	At1g10410.1:d:+1801:primary
GCTGACCTAG	0	2	0	3	2	At1g10180.1:d:+2346:primary
AGACTTGCTT	1	1	0	2	3	At1g09920.1:d:+920:primary
CTTCGCTGCT	2	0	2	3	0	At1g09620.1:d:+3321:primary
GTTGAAGGTA	0	1	2	4	0	At1g09486.1:p:+1231:primary,At1g57860.1:d:+459:primary,At1g31355.1:p:+783:primary
GTTGGTTTTT	1	0	3	3	0	At1g09330.1:d:+824:primary
AGATTCAGCT	1	2	3	0	1	At1g09270.1:d:+1765:primary
ATGATCGAGG	1	2	2	0	2	At1g09240.1:d:+991:secondary
TATGTCCATA	2	0	1	2	2	At1g08845.1:d:+587:primary
AGGAAGAAAA	3	1	0	1	2	At1g08400.1:d:+2327:primary,At1g13960.1:d:+1765:primary,At1g13960.2:d:+1826:primary
GGGATTATGA	1	2	2	1	1	At1g07790.1:d:+319:primary
CCTAGAGGTC	1	6	0	0	0	At1g06700.1:d:+997:primary
ACAATATATG	2	1	1	0	3	At1g06650.2:d:+1221:primary,At1g06650.1:d:+1150:primary
ACTCGCAAAT	0	1	2	3	1	At1g06220.2:d:+3438:primary,At1g06220.1:d:+3305:primary
TGGGCTCAAG	1	1	0	3	2	At1g06000.1:d:+560:primary
CAGGATGTGT	2	0	1	4	0	At1g05680.1:d:+1194:primary
ATCTAGTTAA	0	0	3	4	0	At1g05570.1:i:+826:tertiary,At3g12020.1:i:+2416:tertiary
TTATACATAT	3	0	0	0	4	At1g05385.1:d:+700:primary
GCATCCTCTT	0	1	3	1	2	At1g05270.1:d:+968:primary
GCGAAATTAC	3	3	0	0	1	At1g05200.1:d:+3094:primary
CAAGAGAGTT	1	2	3	1	0	At1g05020.1:d:+1055:primary
TACTAATAAA	1	5	1	0	0	At1g04780.1:d:+2737:primary
ACGTCAATAT	0	0	2	0	5	At1g04690.1:d:+1356:primary
AAAGGATCGG	1	1	0	5	0	At1g04250.1:d:+752:primary
AACAAAGAGA	1	2	1	0	3	At1g03290.1:d:+1630:primary
CCTTCTCAGA	2	3	2	0	0	At1g03140.1:d:+1323:primary
TAAAGAGAAA	2	2	0	2	1	At1g02890.1:d:+3866:primary
GTGTATACCT	3	0	2	1	1	At1g02160.1:d:+392:primary
CTTGAAGCCT	3	1	2	0	1	At1g01970.1:d:+1500:primary
GGATATGAAT	0	0	2	3	2	At1g01520.1:v:+1255:primary
AGATGGGAGC	0	6	0	0	1	At1g01320.1:d:+1651:secondary
TTGACGGTAT	2	2	1	1	1	At1g01090.1:d:+973:primary
TCTTGTCTCT	1	0	2	3	1	At1g01060.1:d:+2535:primary,At1g01060.2:d:+2645:primary
TCTCTCCAGT	2	0	0	0	4	No gene matches found
CCCCAAAAAA	2	2	2	0	0	No gene matches found
TGCTTAAAAA	2	0	2	2	0	No gene matches found
CATAGAATAC	1	2	0	3	0	No gene matches found
ATAGAACCCT	0	2	1	3	0	No gene matches found
GGCCTTTGCC	3	2	0	1	0	No gene matches found
AGTGTACGAG	0	2	1	1	2	No gene matches found
GGCCTCCGCC	1	3	0	1	1	No gene matches found
ATAGAGCCTT	2	2	0	2	0	No gene matches found
GGCCTTAGCC	0	0	2	3	1	No gene matches found
CAAGTCGAAC	0	2	1	3	0	ChrM:-363218:quaternary
TCCTCGAAAA	1	1	1	1	2	ChrC:+84038:quaternary
AGCTGTTCTT	0	5	1	0	0	ChrC:+5123:quaternary
TCCCTCTCGT	0	5	0	0	1	ChrC:+102696:quaternary; +small RNA(CCATGTCCCTCTCGTGT; CATGTCCCTCTCGTGTG)
GAAAAAACAA	0	0	5	1	0	Chr5:+1924615:quaternary,Chr4:+13770670:quaternary,Chr1:+12810977:quaternary
AACTGCAACT	1	0	2	2	1	Chr5:+18921603:quaternary
GATAAACTGA	2	0	4	0	0	Chr5:+12935068:quaternary,At2g37620.1:X:+436:quaternary
AACAGATTTG	0	3	1	2	0	Chr4:+8241538:quaternary,Chr4:+9819897:quaternary
GAGGAGGCAC	0	6	0	0	0	Chr4:+7846275:quaternary
AGACTATGGA	0	3	1	0	2	Chr4:+7719046:quaternary
GCTATAGATA	1	3	0	0	2	Chr4:+458938:quaternary
TGAGACAAAA	0	1	1	3	1	Chr4:+4311319:quaternary
TGCTGAAAAA	2	1	1	2	0	Chr4:+4206238:quaternary

AAAACCAAAG	0	0	5	0	1	Chr4:+2625709:quaternary
TGGTCTCTTA	4	0	1	0	1	Chr4:+17767253:quaternary,Chr3:+17564024:quaternary
GTTCCAAAAG	1	0	3	2	0	Chr4:+15779635:quaternary
ATCATATAGT	2	0	2	0	2	Chr4:+15226361:quaternary
TAAGTAAGTA	1	2	0	1	2	Chr3:+812128:quaternary
CTTTGAAAAA	1	0	1	2	2	Chr3:+404043:quaternary
CACTTCCATT	1	2	2	0	1	Chr3:+3129598:quaternary
TTCCAAAAAA	2	0	2	2	0	Chr3:+15755384:quaternary
GCCTCTGTGC	2	0	3	1	0	Chr3:+14208939:quaternary,Chr2:+3982:quaternary
GTTATCAAAA	0	0	2	3	1	Chr2:+8155912:quaternary
TTTACTAAAA	2	2	1	1	0	Chr2:+1250932:quaternary,Chr5:+25109487:quaternary
TGTAGCCTTT	1	2	3	0	0	Chr1:+8981160:quaternary,At2g39660.1:X:+604:quaternary
ACATAAAATT	1	2	0	2	1	Chr1:+7016235:quaternary,Chr1:+26524006:quaternary,Chr3:+8574322:quaternary,Chr2:+4866414:quaternary,Chr2:+19000958:quaternary
AAGCAAATAA	1	3	2	0	0	Chr1:+4027720:quaternary,Chr1:+22959512:quaternary
ACCATAAAAA	2	2	2	0	0	Chr1:+3441576:quaternary
TTATTCTTAA	1	0	4	0	1	Chr1:+28909964:quaternary,Chr5:+13490599:quaternary
ATTTGGGTTA	1	0	4	1	0	Chr1:+26683113:quaternary
TGGCACAGTT	0	0	4	2	0	Chr1:+25806221:quaternary
AAACAAGATT	2	1	0	1	2	Chr1:+174664:quaternary,Chr3:+21151937:quaternary,Chr5:+14580478:quaternary
AATCTAATCA	2	2	1	0	1	Chr1:+16715354:quaternary
TAGTCGAAGC	1	1	3	0	1	Chr1:+16403864:quaternary
CACTACCATC	2	0	1	2	1	Chr1:+10309062:quaternary,Chr1:+10300669:quaternary
ATTACCTTAT	1	4	1	0	0	AtCg01090:d:+82:primary
AACGGAAGAG	1	2	0	3	0	AtCg00860:d:+1793:secondary,AtCg00860:d:+1793:secondary,At1g43850.1:d:+3128:secondary
TTCAAGCAAG	1	5	0	0	0	AtCg00750:d:+95:primary
AGGGATTCCT	2	0	3	1	0	AtCg00530.1:X:+780:quaternary
GATAGTAGCT	1	3	1	1	0	At5g67330.1:d:+1419:secondary
GCTCAAGTTA	1	1	3	0	1	At5g67240.1:v:+2609:primary
TTCCAATTTC	2	0	2	1	1	At5g67070.1:d:+735:primary
AACAACAAGT	1	2	2	0	1	At5g66180.1:v:+1453:primary
TTCAAGTCCA	1	1	2	1	1	At5g66120.2:d:+1309:primary,At5g66120.1:d:+887:primary
GTTTCGATTA	0	4	0	1	1	At5g66052.1:d:+222:primary
CAGAAAACCG	0	2	1	3	0	At5g66030.2:d:+2490:primary,At5g66030.1:d:+2559:primary
TAACTATGTA	2	3	1	0	0	At5g65760.1:d:+1665:primary
CTTACGAAGC	0	4	1	0	1	At5g65620.1:d:+1744:primary
GTGGGTCTGA	1	1	1	1	2	At5g64930.1:d:+1694:primary
TGTACAGTAT	2	1	1	2	0	At5g64770.1:d:+465:secondary
GAGAAAGGAA	0	2	1	2	1	At5g64750.1:d:+1556:primary,At2g37230.1:d:+2200:primary
AATTTAATAT	1	2	0	2	1	At5g64620.1:d:+642:primary
TAAAACATAG	0	1	1	0	4	At5g64580.1:d:+2653:primary
CAGTTTCAGG	0	0	1	4	1	At5g64400.1:d:-611:secondary
GACAGGGATT	0	1	1	2	2	At5g64270.1:d:+3559:secondary
TAATGATTAT	1	3	1	0	1	At5g64240.1:d:+1519:secondary,At5g64240.2:d:+1364:secondary
GATTTTGTAA	2	1	0	1	2	At5g64170.1:d:+2158:primary,At4g30070.1:d:+395:primary
GTTTTCTTAG	0	4	1	0	1	At5g64040.1:d:+297:secondary
AAGATAAAGT	0	3	1	2	0	At5g63810.1:d:+2356:primary
TCAAGGCTCT	1	2	3	0	0	At5g63200.1:d:+1932:primary
ATAATGACGA	1	1	3	0	1	At5g63130.1:d:+710:primary
GTGGAGGTCG	1	2	1	1	1	At5g62440.1:d:+608:primary
AAGAAGAAAG	0	1	0	5	0	At5g62210.1:d:+119:primary,At1g15165.1:v:+1549:primary,At4g30550.1:d:+494:primary
TGAAATGAAA	1	0	2	2	1	At5g61620.1:v:+1297:secondary,At1g29700.1:d:+1189:secondary
AAGTAATTTC	3	2	0	1	0	At5g61580.1:d:+1736:primary
ATTTGAGATG	1	2	1	1	1	At5g61520.1:d:+1671:secondary
AGAAGATTGT	0	3	2	1	0	At5g61330.1:d:+1330:primary
CTTGGCTCAA	0	0	1	4	1	At5g60960.1:X:+1754:quaternary
GCGAGGGCAT	0	4	2	0	0	At5g60570.1:v:+1402:primary
ATTGAGAGGT	0	0	4	0	2	At5g60390.1:d:+670:secondary,At1g07940.1:d:+721:secondary,At1g07920.1:d:+716:secondary,At1g07930.1:d:+700:secondary,At4g18050.1:v:+3471:secondary
GTTGTTGAGA	1	0	2	0	3	At5g60390.1:d:+1261:secondary

GATAAGCTTT	0	3	3	0	0	At5g59960.1:d:+1425:secondary
GTGTCCCGAC	1	1	3	0	1	At5g59890.1:d:+388:primary,At5g59890.2:d:+341:primary
TGTTTCTTGT	0	1	1	2	2	At5g59740.1:d:+1393:primary
TGGTGAGAAA	3	0	1	1	1	At5g59610.1:v:+1227:secondary
ACATCTTAAT	2	1	1	0	2	At5g59440.2:d:+1059:primary,At5g59440.1:d:+1070:primary
TTATATTATC	2	4	0	0	0	At5g59160.1:d:+1265:primary,At5g59160.2:d:+1570:primary
TATAGTTAGC	2	1	1	0	2	At5g58787.1:d:+1217:primary
TGTAATATAA	2	1	0	2	1	At5g58610.1:v:+3658:secondary,At4g03080.1:d:+3065:secondary
GTAGAATTTG	1	1	1	2	1	At5g56900.1:d:+2178:primary,At5g56900.2:d:+2082:primary
CTCAAAAATA	1	3	2	0	0	At5g56840.1:d:+425:primary,At1g49010.1:d:+596:primary
GATGTCCTCG	0	3	1	1	1	At5g56680.1:d:+1561:primary
TTCTCAATGT	0	1	0	0	5	At5g56420.1:X:-779:quaternary
ATACACAACT	1	2	2	0	1	At5g56130.1:d:+996:primary
TCAAAATTTT	2	1	2	0	1	At5g56050.1:v:+354:secondary
AATCTAGTTG	1	3	2	0	0	At5g55660.1:d:+2352:secondary,At1g55500.1:d:+257:secondary
TGGCACCCTC	1	1	0	3	1	At5g55070.1:d:+672:secondary
CCAAACAGGC	0	5	0	0	1	At5g54960.1:d:+1500:primary
TTTAGAGTGT	0	5	0	0	1	At5g54800.1:d:+1435:primary
GACGCGTGAT	1	1	2	2	0	At5g54750.1:d:+583:primary
TTGGGGAAAA	2	3	0	0	1	At5g54630.1:d:+1481:primary
GTTAACTCGG	1	0	0	4	1	At5g54580.1:d:+809:primary
GCTGTTCTTC	0	1	2	2	1	At5g53860.2:d:+1234:secondary,At5g53860.1:d:+1174:secondary
GGTTCAAGAC	1	4	0	0	1	At5g53850.1:d:+1563:primary,At5g53850.2:d:+1580:primary,At5g53850.3:d:+1611:primary
TTCCTTTTGT	1	0	2	1	2	At5g53440.1:v:+4012:primary
GACTCTATGT	1	4	0	0	1	At5g53350.1:d:+1654:primary
GCCATATTGT	2	4	0	0	0	At5g52920.1:d:+1605:secondary
CAGATGAGGT	0	2	2	1	1	At5g52820.1:d:+1398:primary
GTTTTCTCTT	1	2	2	1	0	At5g52440.1:d:+207:primary,At1g65670.1:v:+2205:primary
GTAAATCACT	1	5	0	0	0	At5g51550.1:d:+502:primary
GATATTTGAG	1	1	1	1	2	At5g51540.1:v:+2949:secondary
ATTTATCATT	1	1	1	0	3	At5g51400.1:d:+955:primary,At5g56075.1:v:+1477:primary
GAAGGGTATC	0	2	2	2	0	At5g51140.1:d:+1301:secondary
CCGCGGTTGT	1	0	5	0	0	At5g50730.1:v:+475:secondary
AAAGGTGCTG	3	1	1	1	0	At5g50580.2:d:+1127:primary,At5g50680.1:d:+1013:primary,At5g50580.1:d:+1121:primary
CAAGTTAGAT	0	0	0	3	3	At5g50360.1:d:+972:primary
AGATAAGCCT	2	2	2	0	0	At5g49950.1:d:+1761:secondary
AGCTTGCAGA	1	1	1	0	3	At5g49780.1:v:+2740:secondary,At1g20100.1:d:+1020:secondary
AAATTTCTAA	1	0	0	1	4	At5g49080.1:d:+2001:primary
TTCCAGCTTA	1	4	1	0	0	At5g48160.1:d:+1483:primary
AGAATCTGCG	0	3	3	0	0	At5g47830.2:d:+606:primary,At5g47830.1:d:+566:primary
TACGATCAAG	1	2	2	0	1	At5g46330.1:d:+2442:secondary,At2g18940.1:d:+1993:secondary
AACTAAATGT	0	2	1	2	1	At5g46210.1:d:+1893:primary
TAAGAGTTTT	1	2	1	0	2	At5g46170.1:d:+1563:primary
AAAGCTTCTT	3	3	0	0	0	At5g45890.1:d:+1059:primary,At2g33470.2:d:+671:primary,At2g33470.1:d:+787:primary
TGAGTATTAG	3	1	2	0	0	At5g45800.1:d:+2164:primary
GACAAACCGA	1	3	1	1	0	At5g45490.1:d:+1077:primary
TTTCCTTCAA	2	1	1	1	1	At5g45160.1:X:+599:quaternary
CTTCCAGTGA	2	2	1	0	1	At5g44800.1:v:+1111:secondary
GCCTAAGCTT	1	2	1	2	0	At5g44785.1:d:+1437:primary,At5g44785.2:d:+1443:primary
TACTAGACAC	2	0	1	1	2	At5g44580.1:X:+364:quaternary
GAAAAGGCAA	2	2	2	0	0	At5g43560.1:d:+1745:secondary,At5g43560.2:d:+1375:secondary
TGTACTAAAT	0	0	3	0	3	At5g42520.1:d:+1323:primary
CATTAAAGAA	1	2	1	1	1	At5g42390.1:d:+3624:secondary
GGACCTCGAG	1	1	3	0	1	At5g42130.1:d:+1134:primary
GTTGGGATGT	0	4	2	0	0	At5g42090.1:d:+845:primary
TCAGTGTTGA	0	1	3	2	0	At5g41350.1:d:+912:primary
AACAAATTGG	3	1	0	2	0	At5g41310.1:X:--207:quaternary
AGGAATCTCC	0	1	2	2	1	At5g40480.1:v:+5995:secondary
AAATTGAAAC	0	6	0	0	0	At5g40450.1:d:+1583:secondary,At3g60670.1:v:+275:secondary
GCAAAGGAAA	0	3	1	1	1	At5g40200.1:d:+504:secondary,At4g26930.1:v:+1887:secondary

AAAATATTAT	1	2	0	1	2	At5g39830.2:d:+1368:primary,At5g39830.1:d:+1410:primary
TTTATGCAGT	2	1	1	0	2	At5g39800.1:d:+565:primary,At5g40080.1:d:+541:primary
TAAGAAAAAA	0	2	1	0	3	At5g39290.1:i:+341:tertiary,At5g07800.1:i:+1491:tertiary,At5g39270.1:i:+340:tertiary,At3g61160.2:i:+1752:tertiary,At3g61160.1:i:+1883:tertiary
GTACAAAACT	0	1	3	1	1	At5g38220.1:d:+1331:primary,At5g38220.2:d:+1321:primary
TCTTGCTTAA	2	2	1	0	1	At5g37920.1:v:+1512:secondary,At2g20630.1:d:+1192:secondary
AAACCATCAA	0	2	0	1	3	At5g36940.1:d:+1976:primary
TTTTGTTCTA	2	1	0	0	3	At5g35460.1:d:+1286:primary
GAACGTGCTT	3	1	1	0	1	At5g35450.1:d:+2925:primary
AGATGATGAT	2	1	1	2	0	At5g34834.1:p:+1091:secondary,At1g74800.1:d:+2133:secondary
TCAATCATTA	0	1	3	2	0	At5g33624.1:X:-2305:quaternary
GTGGTTGATA	1	1	0	0	4	At5g32481.1:X:-1842:quaternary
ATGCTGAAGC	0	4	1	1	0	At5g32440.1:d:+662:primary
ACCTGAAAGG	0	4	1	1	0	At5g28900.1:d:+1584:primary
CTTATTGGGT	1	2	0	2	1	At5g28370.1:d:+1667:secondary,At5g28460.1:d:+1682:secondary,At3g61520.1:d:+1654:secondary
TAATGGTACT	3	0	1	1	1	At5g27640.1:d:+2404:secondary
AAGAGTATAA	2	1	1	1	1	At5g27560.1:d:+1158:secondary
ATGGGAAATA	3	0	0	0	3	At5g27450.2:d:+1665:primary,At5g27450.1:d:+1425:primary
GGCTTCTTCA	0	6	0	0	0	At5g27420.1:d:+225:secondary
TTTGATAGAT	1	1	3	1	0	At5g27240.1:v:+4106:primary
GAAAGCAGAA	0	3	2	0	1	At5g25630.1:d:+1390:primary
CGAATGTAAT	2	1	1	2	0	At5g25250.1:d:+1513:primary
AATGATTTGG	2	3	1	0	0	At5g23860.1:d:+1325:primary
AAGGTGTGGA	1	4	1	0	0	At5g23790.1:d:+53:primary
ACTGATGATA	1	4	1	0	0	At5g23760.1:d:+136:primary
TTTTTCCAAG	2	1	3	0	0	At5g23670.1:d:+1917:primary
GGGGTGGCGG	1	0	3	2	0	At5g23440.1:X:-148:quaternary
CTTTTTTCGG	0	2	1	3	0	At5g22740.1:d:+983:primary
TGAAGTGGGA	0	2	3	1	0	At5g22630.1:d:+1686:primary
GAGAGTGGTA	1	5	0	0	0	At5g22310.1:d:+1593:primary
ATTTATTCAT	1	1	0	0	4	At5g22035.1:i:+2349:tertiary
AATCCAAAGG	1	4	1	0	0	At5g21050.1:v:+835:secondary,At3g51550.1:d:+2793:secondary,At4g05613.1:p:+1419:secondary
ATGGGTGTGT	1	2	2	1	0	At5g20165.1:d:+291:primary
TACTATATGA	3	1	1	0	1	At5g19855.1:d:+893:primary
GGCTTCTCCT	0	4	1	1	0	At5g19560.1:v:-1744:secondary
TCACAAAAAC	0	1	0	2	3	At5g19530.1:d:+1153:primary
GCTGGCTATT	0	2	0	3	1	At5g19450.1:X:-294:quaternary
TTGTCTGGAT	2	0	1	0	3	At5g19450.1:d:+1861:primary,At5g19450.2:d:+2006:primary
GAAATGTTTT	0	3	1	2	0	At5g19090.1:d:+1817:primary
GGATTCCCAT	1	0	3	0	2	At5g18550.1:d:+394:secondary,At2g30860.1:d:+505:secondary
TTGGTGATTG	1	2	1	2	0	At5g18490.1:d:+1353:primary
GGTGTTTCTC	0	1	2	2	1	At5g18400.1:d:+1048:primary,At5g18400.2:d:+964:primary
TTCTTGGTCC	3	1	1	1	0	At5g18120.1:d:+1480:primary
TAATGTGGAG	2	3	1	0	0	At5g18110.1:d:+785:secondary
TTTGAGTCCG	1	1	2	0	2	At5g17780.1:d:+1397:primary
GAAGTCGAGA	1	2	0	3	0	At5g17380.1:d:+1422:primary
GCGACGACCG	0	3	2	1	0	At5g17280.1:d:+240:primary
TCAATGCTAA	0	0	0	1	5	At5g17220.1:X:+564:quaternary
AGATTTGAAA	0	1	2	2	1	At5g16880.3:d:+1470:primary,At5g16880.1:d:+1547:primary,At5g16880.2:d:+1547:primary
TTCTGAACAT	0	3	1	1	1	At5g16810.1:d:+1361:primary
CTGCTGAGAC	1	2	1	1	1	At5g16780.1:d:+2899:primary
AGATCGATGT	0	6	0	0	0	At5g16730.1:d:+1568:secondary,At3g02930.1:v:+1885:secondary
TAATTAAGTA	3	0	0	0	3	At5g16300.2:d:+3318:secondary,At5g16300.1:d:+3420:secondary
TAGAGATTGG	1	2	1	1	1	At5g16140.1:v:+1102:primary
ACAAAGGGAA	1	4	1	0	0	At5g16070.1:d:+1550:primary
TAAACAAAAG	2	2	1	0	1	At5g15900.1:d:+33:secondary
GCCTTGCCAG	1	1	0	2	2	At5g15740.1:d:+2366:primary
GATTGAAGCT	1	2	0	0	3	At5g15650.1:d:+1078:primary
GGATTGCACC	1	1	1	2	1	At5g15550.2:d:+759:secondary,At5g15550.1:d:+759:secondary
ATTGGCTTTT	0	0	4	1	1	At5g15460.1:d:+656:primary,At5g15460.2:d:+713:primary
ATTCATCCTT	2	3	0	0	1	At5g15320.1:d:+351:primary

TGGTGGCATC	1	1	4	0	0	At5g14740.2:d:+842:secondary,At5g14740.1:d:+960:secondary
TCTGCTGAAT	1	3	1	0	1	At5g14680.1:d:+623:secondary
GTCCCAAGGT	3	0	0	3	0	At5g14590.1:d:+1466:primary
GAAAGCTTTG	0	1	2	3	0	At5g14540.1:X:-1195:quaternary
TCAGATTTCC	1	0	4	1	0	At5g14540.1:d:+1751:secondary
TCGCGAAAGA	1	2	0	1	2	At5g14520.1:X:-171:quaternary
ATGTTTCGCG	0	0	4	2	0	At5g13750.2:d:+1567:primary
AGGCGGAGCT	1	2	2	0	1	At5g13340.1:v:+607:secondary,At5g55190.1:d:+711:secondary
CTACATTTGT	1	0	5	0	0	At5g13300.1:i:+2730:tertiary
CTTTCTTCTC	2	1	1	2	0	At5g13260.1:d:+1827:primary
TGTTTAGATG	2	2	1	0	1	At5g12950.1:d:+2814:primary
GGGGCTGTTG	0	4	1	1	0	At5g12310.1:d:+468:secondary,At5g24630.1:v:+1440:secondary,At2g38185.4:d:+1696:secondary
TATAATAATT	1	4	0	0	1	At5g12230.1:d:+1281:primary
GCGGTTTTGT	0	6	0	0	0	At5g11870.1:d:+759:primary,At5g64410.1:d:+2133:primary
GGTTTAATCT	0	1	2	1	2	At5g11650.1:d:+1376:primary
GAGTGTCTTA	1	0	2	1	2	At5g10330.1:d:+1364:primary,At1g71920.1:d:+1225:primary
TTCTGCTAGT	2	0	1	3	0	At5g09250.1:d:+512:primary
CCTTGAGCTG	1	2	2	0	1	At5g08185.1:d:+558:primary
TCGACAAATT	1	2	2	1	0	At5g08160.2:d:+1073:primary,At5g08160.1:d:+1181:primary,At2g35070.1:d:+200:primary
AATCTTTGTG	2	1	1	1	1	At5g07900.1:d:+1290:primary,At3g20080.3:d:+2072:primary
AATAATTCAG	2	1	0	2	1	At5g07120.1:X:--9:quaternary
AGAAATATGC	2	1	2	1	0	At5g06980.1:d:+913:secondary,At5g06980.2:d:+848:secondary
ATCTACTCCT	2	1	2	1	0	At5g06660.1:d:+834:secondary
AACACAAAAG	3	2	1	0	0	At5g06640.1:v:+553:primary,At5g37620.1:v:+2160:primary,At1g70330.1:d:+1505:primary
GCTGAGCCGG	1	1	3	0	1	At5g06460.1:d:+2893:primary
CTCAATGTAC	3	0	0	0	3	At5g06240.1:d:+684:primary
AAATATCTCA	0	6	0	0	0	At5g05930.2:d:+1242:primary,At5g05930.1:d:+1341:primary
AGTTGCTTGA	2	2	2	0	0	At5g05690.1:X:+312:quaternary
ATATATGAGT	1	1	0	0	4	At5g05660.1:d:+2971:primary,At1g72110.1:d:+1585:primary,At3g58420.1:v:+1235:primary
AGGACATACT	2	0	2	1	1	At5g05580.1:d:+1492:secondary
ACTTCACATA	1	2	0	2	1	At5g05110.1:d:+943:primary
ACGCAATTTT	1	1	2	2	0	At5g05100.1:d:+1068:primary
TTGACAAGAA	0	1	1	3	1	At5g05000.1:d:+867:primary,At5g05000.3:d:+1023:primary,At5g05000.2:d:+806:primary
GGACCATATC	1	3	0	1	1	At5g03680.1:v:+143:secondary,At5g61060.1:d:+953:secondary,At3g04090.1:d:+670:secondary
GCCTAATAAG	1	3	1	0	1	At5g03540.1:d:+2185:primary
CGATACCTGA	3	3	0	0	0	At5g03230.1:d:+415:primary
TAACTTAAGA	0	1	1	2	2	At5g03160.1:d:+1638:secondary
TGTCTTAGTA	2	0	3	1	0	At5g02740.1:d:+963:secondary,At5g02740.2:d:+1329:secondary
TGGTGAAGTG	3	1	0	1	1	At5g02740.1:d:+1067:primary,At5g02740.2:d:+1433:primary
TATTCCTTCC	3	1	1	1	0	At5g02530.1:d:+996:primary
AGTAAGCACT	0	2	2	2	0	At5g02150.1:d:+1035:primary
TAAATACTGA	3	0	2	0	1	At5g01800.1:d:+902:secondary
ACACAAGAAT	2	3	1	0	0	At5g01450.1:i:+2625:tertiary
AACACTCATC	0	1	4	0	1	At5g01010.1:d:+1473:primary
TCATTGTTCT	1	2	1	2	0	At4g39980.1:d:+1809:secondary
CTCAGGAGAT	1	1	1	0	3	At4g39960.1:d:+915:primary
TACAGTTTAT	3	0	1	2	0	At4g39910.1:d:+1521:primary
TTATCTGAAT	1	1	2	1	1	At4g39690.1:d:+2215:secondary
GATCTATGAA	0	0	2	0	4	At4g39680.1:d:+2413:primary
TTTTGAGGGA	0	1	3	2	0	At4g39170.1:d:+250:secondary
ATTACAAACC	2	1	0	2	1	At4g39060.1:v:+1854:primary
TTGTCAATTG	0	6	0	0	0	At4g38480.1:d:+1102:secondary
CTTCAAAATC	2	1	1	1	1	At4g38020.1:d:+1084:primary
GAACGGCTAG	2	0	0	3	1	At4g37990.1:d:+1072:primary
TTCTGGTTGT	3	0	0	1	2	At4g37680.1:d:+1282:primary,At4g38320.1:d:+1247:primary
GAAAATAAAA	0	0	3	1	2	At4g37320.1:d:+1558:primary
GTTGAGACTG	1	0	2	1	2	At4g37180.1:X:-564:quaternary
TTAGTCCAGC	1	1	1	1	2	At4g37000.1:d:+743:primary
TACTAAAAAA	1	0	1	2	2	At4g36810.1:d:+1362:secondary

TCTTTATTCA	2	1	0	0	3	At4g36760.2:d:+2138:primary,At4g36760.1:d:+2123:primary
CTTGAAGGCT	1	2	1	1	1	At4g36180.1:d:+3483:primary
ATAGTAGTAA	2	1	0	1	2	At4g36140.1:v:+5388:primary
TGGTTAGAGT	3	0	1	2	0	At4g35840.1:d:+909:primary
TTTGTGTGTT	2	2	1	0	1	At4g35580.1:d:+1640:primary
AGTTGGCTTT	1	4	1	0	0	At4g35470.1:d:+2304:secondary
TATCAATGTG	2	1	1	1	1	At4g35470.1:d:+2064:secondary,At1g56090.1:d:+1149:secondary
AAAACTCCTT	1	0	3	2	0	At4g35100.1:d:+470:secondary
CAGTTGATCG	0	1	3	1	1	At4g35080.1:d:-1393:secondary,At4g35080.2:d:-1289:secondary
CTTCACGGGG	1	0	0	2	3	At4g34740.1:d:+1661:primary
AACATAATTT	0	0	3	2	1	At4g34650.1:v:+1880:primary
GGAGGCGCTC	1	4	1	0	0	At4g34030.1:d:+1597:primary
AACAACAACT	0	1	1	1	3	At4g33700.1:d:+1675:primary
CTCCCTCTTC	3	0	1	1	1	At4g33666.1:d:+147:primary
ATAATCCCAA	4	1	0	1	0	At4g33580.1:d:-1167:secondary
ATCAAGAAGG	0	5	0	1	0	At4g33510.1:d:+1338:primary
AAATAGAGAT	0	2	0	2	2	At4g33410.1:d:+1343:primary
TCGGAAGACC	1	2	1	1	1	At4g32850.2:d:+2476:primary,At4g32850.3:d:+2476:primary,At4g32850.1:d:+2476:primary,At4g32850.5:d:+2476:primary,At4g32850.6:d:+2476:primary
AGAGATTTCG	0	0	1	4	1	At4g32720.1:d:+1394:primary
ATTAAAGTCT	0	3	1	1	1	At4g32570.1:d:+1242:secondary
ATAACACAGA	0	1	2	3	0	At4g32540.1:v:+748:secondary
TATGTGGTGC	4	1	1	0	0	At4g32350.1:d:+2473:primary
CTATCGATTT	0	5	1	0	0	At4g32210.1:p:+3043:primary
AGGGTTCAGT	2	1	1	1	1	At4g32175.1:v:-1108:secondary
TGTGTTTGTG	1	2	2	1	0	At4g31090.1:d:+826:primary,At5g57310.1:v:+1052:primary
AAGATTACCA	1	3	1	0	1	At4g30935.1:d:+1608:primary
CGTGTATATT	0	1	2	0	3	At4g30825.1:v:+3144:secondary
TCTGAACTCG	1	0	2	3	0	At4g30810.1:d:+1440:primary
TGAAATAGTG	0	1	2	2	1	At4g30600.1:d:+2237:primary
CTGTTGTTTT	1	4	0	0	1	At4g30600.1:d:+2083:secondary
AAAAGATGCA	1	0	1	2	2	At4g30480.2:d:+1040:primary,At4g30480.1:d:+1068:primary
AACAAATTTA	2	1	1	2	0	At4g29690.1:d:+385:secondary
TAATGACAGT	0	0	0	1	5	At4g29660.1:d:+625:primary
TGCCACAAGC	1	0	0	3	2	At4g29510.1:d:+1054:primary
TATGCATTGA	0	4	0	0	2	At4g29160.1:d:+812:primary,At4g29160.2:d:+1155:primary
ATTTGAAGTA	3	1	1	0	1	At4g29120.1:d:+1112:primary
GTATGAACTC	2	3	1	0	0	At4g28830.1:d:+735:primary
TAATTGTTGA	1	1	1	1	2	At4g28730.1:d:+732:primary
GTGGTATGCA	3	0	2	1	0	At4g28706.1:d:+1365:primary,At4g28706.3:d:+1371:primary,At4g28706.2:d:+1374:primary
ACAATTTGTT	0	0	2	2	2	At4g28540.1:d:+1901:primary
TGGGTCATTT	1	3	0	2	0	At4g28510.1:d:+983:primary
TTTGGTGATG	0	2	0	1	3	At4g28480.1:d:+680:primary,At3g29810.1:d:+1451:primary
TTGAAGTTCA	1	1	1	3	0	At4g28200.1:d:+2162:secondary
GATGGTCTGA	0	4	1	1	0	At4g27870.1:d:+2252:primary
AAAATAGAGT	0	4	2	0	0	At4g27750.1:d:+1018:primary
TGTGTCTCTT	1	0	4	0	1	At4g27652.1:d:+418:primary
ACTATGTTTG	2	1	2	1	0	At4g27470.1:X:+991:quaternary
GGTTGTTTAT	0	1	2	0	3	At4g26940.2:d:+1831:primary,At4g26940.1:d:+1929:primary
ACCCAGAACT	0	4	1	0	1	At4g26870.1:d:+1423:primary
CGAACGAGCC	1	1	1	2	1	At4g26555.1:d:+604:primary
TGAAATTCAC	1	0	2	2	1	At4g26520.1:d:+1218:primary
GAGTGAGGAA	1	0	2	1	2	At4g26140.1:i:+2566:tertiary,At4g26140.2:i:+2566:tertiary,At3g58570.1:i:+2322:tertiary
TTTGTCCACG	3	1	0	1	1	At4g25700.1:d:+751:primary
ATTGGGGTTC	0	1	0	3	2	At4g25510.1:v:+800:secondary,At3g08850.1:d:+1168:secondary
CGCGTTGGTG	0	1	0	4	1	At4g25340.1:d:+1376:primary
CCACACGTGC	0	2	2	2	0	At4g25230.2:d:+1800:primary,At4g25230.1:d:+2038:primary
TCTTCTGAGT	2	1	1	2	0	At4g24550.2:i:+2246:tertiary,At4g24550.1:i:+2161:tertiary
ATTGTGTTTG	2	1	1	2	0	At4g24500.1:d:+1304:primary,At4g24500.2:d:+1148:primary,At4g02720.1:d:+1517:primary

AATCTCTCTA	1	3	1	0	1	At4g24330.1:d:+1627:primary
TGAATGTCCT	0	5	1	0	0	At4g24240.1:d:+1428:primary
ATGAAGCTCT	2	3	0	0	1	At4g23890.1:X:-973:quaternary
CCTTGTTTCC	1	1	1	0	3	At4g23760.1:d:+689:primary
TAAGTTTAAA	3	0	0	1	2	At4g23150.1:i:+2157:tertiary,At4g23230.1:i:+2134:tertiary
ATTGCAGCTT	1	2	0	2	1	At4g22820.1:d:+666:primary,At4g22820.2:d:+991:primary
TAAAAAAAAC	2	1	2	1	0	At4g22753.1:v:+1322:secondary
TTCGTTTTGA	2	0	3	1	0	At4g22520.1:v:+848:secondary
GTATACATAA	2	1	0	1	2	At4g21960.1:d:+1403:primary
ACACCAACTT	1	1	3	0	1	At4g21650.1:d:+468:secondary
GTCAATAATC	1	1	3	0	1	At4g21580.1:d:+1122:primary
CCACAGTCTC	0	0	2	2	2	At4g21215.1:d:+871:primary,At4g21215.2:d:+877:primary
TTGTATTCTA	2	1	0	2	1	At4g21090.2:d:+965:primary,At4g21090.1:d:+889:primary
GCGAGAATCG	0	1	3	2	0	At4g20850.1:d:+3802:primary
CACAACTGCT	1	2	1	2	0	At4g20480.1:d:+719:primary
AGAGAGGTTG	3	2	0	1	0	At4g20270.1:d:+3090:primary
ATGCGAAATG	2	2	2	0	0	At4g19600.1:d:+1884:primary
ACTGGTGTGA	0	4	2	0	0	At4g19120.2:d:+1561:secondary,At1g26850.2:d:+1781:secondary,At4g18030.1:d:+1660:secondary,At1g13860.2:d:+1963:secondary,At5g30269.1:p:+14409:secondary,At5g30269.1:p:+2030:secondary,At2g03480.1:d:+1855:secondary,At1g13860.3:d:+2043:secondary,At1g13860.1:d:+2043:secondary,At1g13860.4:d:+2043:secondary,At3g51070.1:v:+2749:secondary,At4g19120.1:d:+1712:secondary,At2g34300.1:d:+2148:secondary,At2g03480.2:d:+1822:secondary,At1g26850.1:d:+1840:secondary
GGAGACAGTG	0	3	2	1	0	At4g18930.1:d:+606:secondary
AATCTATAGC	0	1	1	2	2	At4g18905.1:d:+1835:primary
TATCAGATCT	1	4	1	0	0	At4g18710.1:d:+637:secondary
TCTAGTCTCT	4	0	2	0	0	At4g17600.1:d:+956:primary
TGGTTGTGGG	0	0	6	0	0	At4g17230.1:d:+1970:primary
GAGATGAAAG	0	3	1	0	2	At4g17090.1:d:+1345:secondary
GATGGAGAAG	2	1	3	0	0	At4g16660.1:d:+2252:primary
TTTTGGGGAT	1	1	1	0	3	At4g16563.1:d:+1684:primary
TTTCCATTAA	1	2	1	1	1	At4g16146.1:d:+479:primary
GATGAAAACT	0	1	1	3	1	At4g15475.1:d:+2013:secondary
AGCGGCATAG	0	5	0	0	1	At4g15470.1:d:+131:primary
TCTCAAATGT	3	2	0	1	0	At4g14990.1:d:+3193:primary
TTGGTTTGGA	2	1	1	1	1	At4g14570.1:d:+41:secondary,At3g55560.1:d:+198:secondary
AAAGGATCCG	0	3	3	0	0	At4g14560.1:d:+595:primary
TGACAACTCA	2	4	0	0	0	At4g14420.1:d:+323:primary
GAGTGGGTTG	1	1	0	3	1	At4g13670.1:d:+1140:primary
AGAGCAATAA	4	0	0	1	1	At4g13590.1:d:+1315:secondary
CTCTCTCTCT	0	4	0	1	1	At4g13550.1:i:+3771:tertiary
TATCATATGA	2	3	0	0	1	At4g13530.2:d:+906:primary,At4g13530.1:d:+974:primary
GAAGAGAATC	1	3	2	0	0	At4g12560.1:d:+603:primary,At1g54200.1:d:+996:primary
TAAACTTTTG	2	0	1	1	2	At4g11970.2:d:+1490:secondary
TAGCTCTCAT	1	2	2	0	1	At4g11960.1:d:+1126:secondary
AACACAAAAA	1	2	1	1	1	At4g11610.1:d:+2847:secondary,At3g13780.1:d:+40:secondary
TAAGAACTTT	2	4	0	0	0	At4g11450.1:d:+503:secondary,At4g33180.1:d:+1075:secondary
TTTGTGTTAC	2	1	1	1	1	At4g11380.1:d:+2911:secondary
TAATTTTAAC	1	3	1	0	1	At4g11280.1:d:+1707:primary
AAGGAAGAGA	1	3	2	0	0	At4g10970.2:d:+697:primary,At1g72800.1:d:+1084:primary,At4g10970.1:d:+588:primary,At4g10970.3:d:+697:primary
TACTGAATGT	2	2	1	0	1	At4g10925.1:d:+1052:primary,At4g10925.2:d:+1068:primary
GAAAATTGTA	0	0	0	2	4	At4g10730.1:d:+2463:primary
ATGAGATCGG	0	1	2	2	1	At4g10610.1:d:+239:secondary
TCAGGTGATG	1	1	1	2	1	At4g10570.1:d:+2938:primary
GTTTCCAAAA	1	2	0	3	0	At4g09460.1:d:+932:primary
CCCGATATTT	0	4	0	2	0	At4g08350.1:v:+3126:secondary
CGCTCTAAAG	1	3	1	1	0	At4g07990.1:d:+1081:primary
AGGAACACGA	1	1	2	2	0	At4g06594.1:p:+2312:secondary,At3g12580.1:d:+1768:secondary,At1g38710.1:p:+2373:secondary,At3g42115.1:p:+2699:secondary
TCATCGATGT	3	3	0	0	0	At4g06477.1:X:-1635:quaternary
TCAAGGCTTG	2	1	3	0	0	At4g05590.1:d:+511:primary

ATGTTAATGG	0	3	1	1	1	At4g05190.1:d:+2005:secondary,At5g07830.1:d:+1832:secondary
AAGAAACCTG	1	1	2	1	1	At4g04780.1:d:+1132:primary
GATCGTGACA	2	1	3	0	0	At4g04470.1:d:+620:secondary
GACCCGAAAA	2	0	0	3	1	At4g04350.1:d:+1852:secondary
CTCCAAGAGG	1	3	2	0	0	At4g03960.1:d:+709:secondary
TTTAATAGTT	1	3	0	1	1	At4g03200.1:d:+2612:primary
GGTTTTTCTT	0	0	1	2	3	At4g02590.1:d:+1167:primary,At3g03260.1:d:+2336:primary
CTCACAATCA	2	1	1	1	1	At4g02490.1:X:-1730:quaternary
GCTTTTCGAT	1	0	2	3	0	At4g02420.1:v:+2514:secondary
GAGTGTAATT	0	2	3	0	1	At4g02290.1:d:+443:secondary
GGGTCCTCTA	0	4	0	0	2	At4g02050.1:d:+1316:primary
TTAGTGATGA	4	2	0	0	0	At4g01410.1:d:+905:primary
TATTTGGCTA	0	2	1	2	1	At4g01026.1:d:+1050:primary
ATGATTAATT	2	0	2	2	0	At4g00880.1:d:+765:primary
ATCCTTTTCC	2	1	1	2	0	At4g00660.2:d:+2219:secondary,At4g00660.1:d:+2262:secondary
TAATGGTTGC	3	1	0	1	1	At4g00040.1:d:+1255:secondary
TTTCTCAAAA	0	3	1	1	1	At3g63500.2:d:+998:secondary,At3g63500.1:d:+499:secondary
GATTTTTTTT	1	2	1	2	0	At3g63460.1:d:+3441:secondary,At2g38770.1:d:+4687:secondary,At3g63460.2:d:+3435:secondary
AGAGTGAGTG	0	2	3	1	0	At3g63210.1:d:+1253:primary
ACTATTTCCA	1	0	1	3	1	At3g62360.1:d:+3962:primary
TAAGTTGAGT	3	1	2	0	0	At3g61750.1:v:+1780:secondary
GACGTCTTTT	1	4	0	0	1	At3g61600.2:d:+1718:primary
AAAGTTGCAC	0	1	3	0	2	At3g61240.1:d:+2235:secondary,At3g43220.1:d:+2343:secondary
TTAATACTTG	2	2	1	1	0	At3g60860.1:d:+5435:primary
ACACAGATGC	2	3	0	1	0	At3g60860.1:d:+4909:secondary
GATTAGTAAG	1	0	4	1	0	At3g60520.1:d:+672:primary
GTTCACGGGA	0	3	1	2	0	At3g59840.1:d:+70:primary
ACTTAAAAAA	2	1	1	2	0	At3g58790.1:i:+1879:tertiary
CTCCTGTTGT	1	1	2	1	1	At3g58030.2:d:+1576:primary,At3g58030.1:d:+1764:primary,At3g58030.3:d:+1582:primary
ATGACTTGAA	2	0	4	0	0	At3g57890.1:d:+2100:primary
ATGACTCTTT	3	1	0	0	2	At3g57880.1:d:+2645:primary
TAACAAGAAA	1	4	0	1	0	At3g57680.1:v:+2229:secondary
CCATTCCGCA	0	3	2	0	1	At3g57530.1:d:+1436:primary
CGTTCATTTT	2	1	1	2	0	At3g57410.1:d:+3308:secondary
ATTCTAGCCG	0	4	2	0	0	At3g57330.1:d:+2983:primary
GTTAATAACC	1	0	0	4	1	At3g57040.1:d:+1326:primary
TGTATACTTT	1	0	0	1	4	At3g57030.1:d:+1333:primary
TGGGTCTTTG	2	0	1	3	0	At3g56860.2:d:+1858:primary
TGATAATCTA	2	0	4	0	0	At3g56710.1:d:+761:primary
AATCCGATTC	1	1	1	2	1	At3g56570.1:v:+976:primary
TGTTGTTCCA	1	3	1	1	0	At3g56360.1:d:+256:primary
CCAAGGCTGC	0	1	2	3	0	At3g56240.1:d:+128:secondary,At1g47930.1:p:+846:secondary
GGAGTGGAGA	0	3	3	0	0	At3g56170.1:d:+976:primary
TGTATGAAAT	2	2	0	1	1	At3g55470.1:d:+734:secondary
TAAGTACTAA	3	1	0	0	2	At3g55430.1:d:+1552:primary
AGCAAGTAAT	2	0	0	3	1	At3g55250.1:d:+931:primary
TGATAGAAGA	1	2	3	0	0	At3g54930.1:v:+2002:primary
AGATGTAATG	4	1	1	0	0	At3g54890.1:X:-312:quaternary,At4g09316.1:X:-1563:quaternary,At1g69770.1:X:-296:quaternary
TCTTCAGACT	2	2	0	1	1	At3g54840.1:d:+738:primary
TATTGTCCAT	1	0	1	1	3	At3g54690.1:d:+1230:primary
TCCACTATGG	1	4	0	1	0	At3g54600.1:d:+727:primary
GCAGAGTTGG	0	3	2	0	1	At3g54560.1:d:+257:primary,At2g21230.2:d:+1305:primary,At2g21230.1:d:+1305:primary
ATTGTACTCT	3	1	1	0	1	At3g54360.1:d:+1525:primary
GACTTCAAGT	0	4	1	1	0	At3g53800.1:d:+1330:primary
CTTAGAAGAC	0	0	2	3	1	At3g53560.1:d:+1111:secondary,At1g54270.1:d:+786:secondary
AACACGGCAA	3	0	2	1	0	At3g53420.1:X:-34:quaternary
TGACCCCAAA	2	2	0	1	1	At3g52950.1:d:-1918:secondary
AGCATATCTA	0	2	2	1	1	At3g52400.1:d:+1173:primary
TTTCATTTTT	2	1	1	2	0	At3g52200.1:d:+2144:primary
CAACTTCATC	1	0	2	2	1	At3g52090.1:d:+199:primary
GATTTATGGT	3	1	0	1	1	At3g51710.1:v:+2245:primary,At2g37620.1:d:+1443:primary

ATCAGAGACT	1	3	1	0	1	At3g51430.1:d:+1072:primary
TTTCCTCTAA	2	3	0	1	0	At3g51390.1:d:+1231:primary
TATCCAGAGC	3	2	1	0	0	At3g51150.1:d:+2020:secondary
ATGCTGTGGG	2	3	1	0	0	At3g50690.1:d:+1166:primary
TTTGTTTGAT	1	3	1	0	1	At3g50670.2:d:+1404:secondary
GACATATCTT	1	3	0	2	0	At3g50570.1:d:+709:primary
AACTTTCTCA	1	2	2	0	1	At3g50440.1:d:+785:primary
ATGATGAGGA	0	3	2	0	1	At3g50240.1:d:+3202:primary
ACAACCAGGA	1	1	3	0	1	At3g50000.1:d:+905:primary
TAAAAAGCTA	1	1	4	0	0	At3g49310.1:X:-191:quaternary
TATTAAAAAT	1	0	0	1	4	At3g48830.1:i:+4002:tertiary
AGAGATATGT	1	1	3	1	0	At3g48100.1:d:+918:secondary
TGTATCTATC	2	0	2	1	1	At3g48060.1:d:+4876:primary
CCAGGTATGG	0	5	0	0	1	At3g47860.1:d:+956:primary
TTATGCTCTT	0	3	0	0	3	At3g47800.1:d:+1210:secondary
GCAACTTATG	4	0	1	1	0	At3g47640.1:d:+909:primary
GATCGGGTGA	0	3	0	2	1	At3g47560.2:d:+771:secondary,At3g47560.1:d:+657:secondary
TGTAGATCCA	2	0	3	0	1	At3g47080.1:d:+1929:primary
CAAAAACAAT	2	1	2	0	1	At3g47000.1:d:+1942:primary
GAAAAGCTTC	2	0	3	1	0	At3g46630.1:d:+471:primary
ATTTTTTGTG	0	2	4	0	0	At3g46530.1:d:+2875:primary
TTCAGATTGT	1	0	1	1	3	At3g45770.1:d:+1354:primary,At3g45770.2:d:+1330:primary
TTTTTGTTTA	2	1	1	1	1	At3g45420.1:v:+2804:primary
GGTTTGATTT	1	2	1	2	0	At3g44670.1:d:+3561:primary
TTTTATCTAT	2	1	1	0	2	At3g44480.1:d:+4004:primary,At3g44630.1:d:+3993:primary,At3g44630.2:d:+4052:primary
AAGAATTCAA	1	1	1	1	2	At3g44330.1:d:+1853:primary
AAGGTAAATT	1	3	1	1	0	At3g44200.1:d:+3174:primary
CAGAACAAAT	0	0	1	4	1	At3g44110.2:d:+1564:primary,At3g44110.1:d:+1731:primary
AAACAAAGAG	1	0	3	1	1	At3g44100.1:d:+591:primary
ATTGAGTCTG	3	1	1	0	1	At3g43955.1:p:+4501:primary
TTTTCTCCAA	1	2	2	0	1	At3g43860.1:d:+1803:secondary,At4g27260.1:d:+2110:secondary
ACCACTTGAT	2	1	1	1	1	At3g43810.1:d:+771:primary
TTGTACCCTA	1	1	2	0	2	At3g33530.1:d:+4178:primary
ACTCTTAGCT	2	0	2	0	2	At3g30390.1:d:+1891:primary
ATGATATTAT	3	2	1	0	0	At3g29220.1:v:+726:secondary,At5g16030.1:d:+1198:secondary
GAGACTAGCT	0	3	3	0	0	At3g29000.1:d:+580:primary
TTCTGAGTCT	1	0	2	3	0	At3g28715.1:v:+1401:secondary
AAAAAAGAGA	0	3	0	1	2	At3g28530.1:v:+496:primary
TGCAAGTTAT	1	1	1	2	1	At3g28480.1:d:+1141:primary
GATTATATTC	3	2	1	0	0	At3g28200.1:d:+800:primary
GTGTTCAAGA	1	2	2	0	1	At3g28180.1:d:+2148:primary
TTGCGCTTTG	1	1	2	2	0	At3g28100.1:d:+1179:secondary
TGAAGCCATT	1	1	0	3	1	At3g27040.1:v:+138:secondary
GTTACACAAG	2	2	1	1	0	At3g26890.2:d:+2502:primary,At3g26890.1:d:+2517:primary
CTTAGATTGA	2	1	1	2	0	At3g26744.1:X:-202:quaternary
AAACAAAGTT	2	2	1	0	1	At3g26660.1:v:+986:primary
GTCCTGCTCT	0	5	0	0	1	At3g26380.1:d:+1982:primary
AAACTGTTTT	1	1	1	2	1	At3g26240.1:d:+3289:primary
TAATTTTGAA	1	1	2	2	0	At3g26210.1:d:+1692:secondary
TCATTTTCTC	1	1	0	1	3	At3g26090.1:d:+1605:primary
GGTGGAGTTC	2	1	2	1	0	At3g25840.1:d:+3076:primary,At3g25826.1:p:+2242:primary
CTTGGTTTAA	2	2	1	1	0	At3g25660.1:d:+1816:secondary
GGGCGTTTCA	1	1	0	4	0	At3g24927.1:p:+85:primary
AATCTGAATA	1	2	2	0	1	At3g24160.1:d:+1528:primary
GAGACTTCTC	1	2	1	0	2	At3g23750.1:d:+2699:primary
CCAAGATTCT	1	3	1	0	1	At3g23690.1:d:+1285:primary
TTTGTGGCTA	1	1	2	0	2	At3g23570.1:d:+928:primary
GCTTGAATAT	0	0	1	3	2	At3g23560.1:d:+1721:primary
ATTGGCATAT	1	1	2	1	1	At3g22950.1:d:+636:secondary
AGAAGAAGAC	1	1	1	2	1	At3g22530.1:d:+891:primary
TTTGAAGGAG	2	0	2	1	1	At3g22310.1:d:+1591:primary

GATTCTAGAG	2	1	1	1	1	At3g22290.1:d:-1332:secondary
GAGTAGTAGG	2	2	0	1	1	At3g22270.1:d:+2878:primary
ATGAGACAGA	1	1	1	0	3	At3g22231.1:d:+298:primary
CCCACCACCA	0	3	1	2	0	At3g22120.1:d:+512:secondary
AAACATCAGT	2	0	1	2	1	At3g21670.1:d:+1857:primary
TGTTTATTCG	2	2	0	1	1	At3g21550.1:d:+671:primary
GAAACTAGGA	2	2	1	0	1	At3g21530.1:d:+1442:primary
GGTCCCGCAT	0	0	1	5	0	At3g21150.1:d:+845:primary
TTGAACGGTA	3	0	3	0	0	At3g20800.1:d:+1232:primary
CGCTATGGAC	1	1	1	1	2	At3g20500.1:d:+1197:primary
AGCTCAAGGA	2	0	1	2	1	At3g20330.1:d:+1499:primary
CCTGTGATGG	0	3	2	1	0	At3g19430.1:d:+1507:secondary
CACGATAACT	2	1	0	3	0	At3g19240.1:d:+1976:primary
CCTTATATAT	3	1	0	0	2	At3g18890.1:d:+2222:primary
GAGAAGGAGT	0	4	0	1	1	At3g18830.1:d:+891:primary
CATTTTGTGG	0	2	1	1	2	At3g18030.1:d:+971:primary
CCGATATATG	0	4	0	0	2	At3g17880.1:d:+1009:primary
ACCAGAATAA	1	2	2	0	1	At3g16910.1:d:+1622:primary
GTTACAGGCG	0	4	0	2	0	At3g16840.1:d:+105:secondary
GATCTGGATC	0	4	2	0	0	At3g16720.1:d:+762:primary
ATTTGCATTA	2	1	2	1	0	At3g16220.1:d:+904:primary
GCGTCTCTTG	0	0	6	0	0	At3g16140.1:d:+65:secondary
TGTTTTAAGG	0	3	1	2	0	At3g16100.1:d:+755:primary
CAGCCTCCCA	1	2	2	1	0	At3g15880.2:d:+3511:primary
TTCAAGAAAA	1	1	2	0	2	At3g15820.1:i:+2088:tertiary
GTCTTGTGCT	1	3	1	1	0	At3g15770.1:d:+677:primary
TTGTTAAAAA	1	1	3	1	0	At3g15602.1:p:+2177:primary,At3g43526.1:p:+2257:primary,At2g07780.1:p:+2278:primary,At5g13340.1:v:+1400:primary
TTCAACAACA	4	0	0	2	0	At3g15354.1:d:+2728:secondary
CAGATGATTC	1	2	2	1	0	At3g15060.1:d:+778:primary
ACTGAAAATC	0	1	1	2	2	At3g14860.2:d:+1736:primary,At3g14860.1:d:+1746:primary
AACACGTGAT	5	0	1	0	0	At3g14620.1:d:+1780:primary
ATTCAAAAAA	2	0	2	2	0	At3g14330.1:i:+818:tertiary
TTCACCCAAC	0	5	0	1	0	At3g14240.1:d:+1985:primary
AGTGTATTAA	0	1	2	0	3	At3g14180.1:d:+1649:primary
ACGAGGTAGC	1	1	1	2	1	At3g13550.1:d:+583:primary
TAAGCTCGGT	0	1	2	0	3	At3g13360.1:d:+1412:primary
ATTGTAGCTA	0	6	0	0	0	At3g12630.1:d:+494:secondary
CCGTGATAGC	0	5	1	0	0	At3g12500.1:d:+741:primary
GATTCTTTTT	0	0	3	2	1	At3g11850.2:d:+2325:primary,At3g11850.1:d:+1941:primary
GAGTATTCAG	2	2	1	1	0	At3g11670.1:d:+2227:primary
TGCACAAACA	1	0	2	2	1	At3g11397.1:d:+883:primary
GGCCCGTCAA	0	4	1	1	0	At3g10850.1:d:+652:primary
CGTTCTTCCT	1	3	1	1	0	At3g10800.1:d:+2061:primary
TAACGTAATC	1	1	2	1	1	At3g09925.1:v:+233:secondary
GGTTCAAAAT	1	0	1	3	1	At3g09880.1:d:+2012:primary
TAAATCCAAA	2	3	1	0	0	At3g09450.1:v:+2912:secondary
ACACCAGTAG	2	3	1	0	0	At3g08940.2:d:-971:secondary,At3g08940.1:d:-1018:secondary,At3g08940.2:d:-933:secondary,At3g08940.1:d:-1056:secondary
GCGAGTTCAA	2	0	1	2	1	At3g08570.1:X:-124:quaternary,At1g32550.1:X:-28:quaternary
GAGCACAAAA	0	1	3	2	0	At3g08550.1:i:-677:tertiary,At4g21610.1:i:-1055:tertiary
CTTGAATTTG	1	2	2	1	0	At3g08010.1:d:+1250:primary
TTTGCTCGAG	0	1	4	0	1	At3g07880.1:d:+817:secondary
TGCCACAAAC	1	0	3	2	0	At3g07760.2:d:-613:secondary,At3g07760.1:d:-612:secondary
TTAGCAAGGG	2	2	1	0	1	At3g07750.1:d:+989:secondary,At3g07750.2:d:+990:secondary
GTTGGATCTG	3	1	0	1	1	At3g07670.1:d:+1674:secondary
AGAACTTTAC	1	3	2	0	0	At3g07220.1:d:+1045:primary
GTGAAACATA	0	1	2	0	3	At3g07170.1:d:+1002:primary
GCGGCCTTCT	2	0	3	1	0	At3g07100.1:d:+3755:secondary
AACTCTTTAG	1	2	1	2	0	At3g06590.1:d:+715:primary
TGACTTCATT	1	1	2	2	0	At3g06190.2:d:+1471:primary,At3g06190.1:d:+1804:primary

TACAAGGAGC	0	3	2	0	1	At3g05970.1:d:+2115:primary
GAAACGGCTT	3	0	1	2	0	At3g05700.1:d:+1054:primary
AGGATAACTT	2	0	2	1	1	At3g05675.1:d:+533:secondary,At1g77260.1:d:+1382:secondary,At3g05675.2:d:+740:secondary
GCTGCACCAG	1	1	2	1	1	At3g04830.2:d:+824:primary,At3g04830.1:d:+851:primary
GTTGACTGAT	1	1	1	2	1	At3g04420.1:d:+694:primary
ATGAGTTCTC	0	3	2	0	1	At3g04070.1:d:+1169:primary
ATGGAAGAAA	0	3	0	2	1	At3g04030.1:d:+482:secondary,At3g04030.2:d:+459:secondary
GAGTATACTG	0	4	0	1	1	At3g03940.1:d:+2485:primary
GAAACATTAA	1	2	1	1	1	At3g02880.1:d:+1572:primary
AAGTCATCTG	1	2	0	1	2	At3g02870.1:d:+979:secondary
ATTGTGGAGA	0	3	1	2	0	At3g02760.1:d:+1575:primary
TTTGGTTGTA	0	1	1	1	3	At3g02220.1:d:+760:primary
GCAGGGCTTG	0	1	0	4	1	At3g01820.1:d:+800:primary
AGTTGTTGTA	1	2	0	0	3	At3g01770.1:d:+2164:primary
GTTGATAACT	3	0	2	0	1	At3g01440.1:d:+755:primary
ATTCGCTATG	0	1	3	0	2	At2g48110.1:v:+4375:primary
TTCGATGAGC	0	4	1	0	1	At2g47590.1:d:+1235:primary
GCGGCTGGTT	0	5	1	0	0	At2g47450.1:d:+701:secondary
GGAATGGAAG	0	3	0	2	1	At2g47420.1:d:+939:primary
TCTGTAACCG	0	2	2	1	1	At2g47410.1:d:+5350:primary
ATGATAGTGG	0	2	0	2	2	At2g47330.1:d:+2378:primary
CCCTACAACG	1	1	1	0	3	At2g46450.1:X:+347:quaternary
AAGCTACTAA	1	2	1	1	1	At2g46340.1:d:+3334:primary
TATGTTGGAT	2	3	0	1	0	At2g46250.1:d:+1665:primary
GTTTTGATGA	0	4	2	0	0	At2g46225.1:i:+1953:tertiary
AAACGTGAGA	0	1	1	2	2	At2g46170.1:d:+1055:primary
TTGTCTTCCG	0	3	2	1	0	At2g46080.1:d:+976:primary
GATAGCAGCA	1	2	2	0	1	At2g45910.1:d:+2970:primary
GTCTTATCAG	0	4	1	1	0	At2g45740.2:d:+334:primary,At2g45740.1:d:+347:primary
TATCAATTTA	0	0	0	6	0	At2g45660.1:d:+1271:primary
CTCTGATTTT	0	0	0	3	3	At2g45570.1:d:+1648:primary
AATGTACTCT	0	4	1	1	0	At2g45520.1:d:+650:secondary
ATGTAACAAT	1	1	0	1	3	At2g45340.1:d:+2229:primary
CAAAGGGGCT	0	0	4	2	0	At2g44610.1:X:-225:quaternary
CACTCTCAGA	3	3	0	0	0	At2g43790.1:d:+1230:primary
TAGTCCTTAA	0	3	1	1	1	At2g43400.1:d:+1952:primary
GAAAACTATC	2	1	2	0	1	At2g42770.1:d:+950:primary
TCTGAATATT	2	1	2	0	1	At2g42280.2:d:+1587:primary,At2g42280.1:d:+1506:primary
TAAAAATATG	2	0	2	1	1	At2g41460.1:d:+1711:primary
TGATAGATCT	2	2	0	0	2	At2g41230.1:d:+461:secondary
ACCTCGGCTG	0	1	2	3	0	At2g40660.1:d:+1198:primary
GATCCCTGTA	1	2	3	0	0	At2g40316.1:d:+1189:primary
TATATGATAA	3	1	0	1	1	At2g40270.2:d:+1719:primary,At2g40270.1:d:+1761:primary
TCCTAAAAAA	1	0	2	3	0	At2g39855.2:d:+736:primary,At2g39855.1:d:+967:primary
CCGGTGAAGG	0	4	1	0	1	At2g39810.1:d:+2666:primary
AGAGAACCTT	3	0	1	0	2	At2g39030.1:d:+499:secondary
AAACTAAAAA	0	2	2	0	2	At2g38960.2:v:+2062:secondary,At1g68980.1:v:+172:secondary,At2g38960.1:v:+1953:secondary,At2g37310.1:v:+205:secondary
GTGTGCTTCA	2	3	0	1	0	At2g38620.1:d:+462:secondary,At1g04690.1:d:+900:secondary,At3g54180.1:d:+496:secondary
TCCTGATCGA	1	1	1	1	2	At2g38450.1:d:+401:primary
GGAGCCGTTG	1	3	0	2	0	At2g37840.2:d:-1848:secondary,At2g37840.1:d:-1563:secondary
AGACGAGTCC	1	3	2	0	0	At2g37790.1:d:+949:primary
AGACACACGG	2	0	3	1	0	At2g37760.3:d:+934:primary
AAAACGTCAA	3	0	1	1	1	At2g37020.1:d:+964:secondary
CTGCTTTCGG	0	2	0	1	3	At2g36885.2:d:+769:primary,At2g36885.1:d:+772:primary
TATGATAAAT	0	4	1	1	0	At2g36690.1:v:+1447:secondary
TTATGCTCTA	1	2	1	1	1	At2g36680.1:d:+886:primary
GACATAAGTT	0	2	1	3	0	At2g36080.1:d:+861:primary
TTTCAGGGGA	1	1	2	1	1	At2g36070.1:d:+1752:primary
TGGAAAAAAA	1	2	2	0	1	At2g35860.1:d:+1493:secondary
GCAAGCATTC	1	4	1	0	0	At2g35795.1:d:+186:primary

AGATTAAAAT	3	1	1	1	0	At2g35620.1:d:+1929:primary
CGGATGAGAT	1	2	2	0	1	At2g35450.1:d:+1060:primary
TCATAAAAGC	2	2	0	2	0	At2g35100.1:d:+1689:primary
ATTACTTAGA	0	6	0	0	0	At2g34590.1:d:+1216:primary
TTGCTGCCAT	1	0	0	1	4	At2g32850.1:d:+2243:primary,At2g32850.2:d:+2070:primary
AACCAAAAAA	2	0	3	1	0	At2g32780.1:v:+2011:secondary,At3g42190.1:v:+59:secondary
GAGATTTTCT	0	4	1	1	0	At2g32500.1:d:+774:secondary,At3g02260.1:d:+9308:secondary
ACAGTGAAGA	2	2	1	1	0	At2g32240.1:d:+3112:primary
TACCAATGAG	2	1	1	2	0	At2g32230.1:d:+1790:primary
GAAGAGTAAC	2	0	3	1	0	At2g32100.1:d:+844:primary
AGCCTTGAAC	1	2	1	1	1	At2g32090.2:d:+435:secondary,At2g32090.1:d:+513:secondary
TAACCAAAAT	1	1	1	2	1	At2g32040.1:d:+1830:secondary
ATCTTACTCA	0	6	0	0	0	At2g31810.2:d:+1227:primary,At2g31810.1:d:+1224:primary,At2g31810.3:d:+1224:primary
GGATTTTGTT	0	3	0	3	0	At2g31610.1:d:+853:secondary,At3g44440.1:v:+840:secondary
GAGTTTTGGT	1	0	3	1	1	At2g31180.1:d:+682:primary,At3g45850.1:d:+3281:primary
GCTTCACGAT	0	1	2	3	0	At2g30960.1:d:+1343:secondary
TATAGTGGCA	1	3	0	1	1	At2g30700.1:d:+1894:primary
TTTAGTTCTG	2	1	2	1	0	At2g30620.1:X:+914:quaternary
TTTTGGCTAC	1	1	2	2	0	At2g30440.1:d:+1783:primary
TGTTGTTTTC	2	2	0	0	2	At2g30150.1:v:+21:secondary,At3g58750.1:d:+1858:secondary
TTAGGTGTTG	2	1	1	2	0	At2g29900.1:d:+1202:primary
TTCTGTTTCT	2	2	0	1	1	At2g29350.1:d:+1034:primary,At2g29350.2:d:+1038:primary,At1g17180.1:d:+786:primary,At5g51950.1:d:+1901:primary,At3g16880.1:v:+1878:primary
GTTAATCACA	2	4	0	0	0	At2g29290.1:d:+570:secondary
GTTTTGTTAA	1	0	1	1	3	At2g28990.1:v:+2131:secondary
GTGTGTTTTT	3	2	0	0	1	At2g28370.1:d:+877:primary
AGAAGGTAGC	3	2	0	0	1	At2g28305.1:d:+642:primary
TTCTCATCAT	3	2	0	0	1	At2g26930.1:d:+1603:primary,At3g60180.1:d:+793:primary
TAATTTTTTG	1	1	4	0	0	At2g26930.1:d:+1185:secondary,At5g24550.1:v:+2140:secondary,At1g53990.1:v:+1527:secondary,At5g62260.1:v:+14:secondary
GGATTTTCCT	1	0	2	2	1	At2g26590.1:d:+1119:primary
ATTGCTGCAA	1	1	1	2	1	At2g26500.1:d:-417:secondary,At2g26500.2:d:-385:secondary,At1g05750.1:d:-984:secondary
TTACATTTTT	2	1	1	1	1	At2g26230.1:d:+1322:primary
CAAAATTTTC	0	0	2	2	2	At2g25625.2:d:+725:primary,At2g25625.1:d:+734:primary
AATCTTTTGA	1	2	1	2	0	At2g25520.1:d:+1369:secondary
GAGCATAGAA	0	0	5	0	1	At2g25450.1:d:+948:secondary
TTCAAGACCT	0	1	2	1	2	At2g25450.1:d:+1244:secondary
GAATGAGATG	1	1	3	0	1	At2g25290.1:v:+2550:secondary,At4g32070.1:v:+2660:secondary
CATATGAGAG	1	2	1	1	1	At2g25180.1:d:+1785:primary
ACAAGAAGTA	2	3	1	0	0	At2g25110.1:d:+826:primary
TGGATAAATT	0	1	3	1	1	At2g24420.2:d:+1649:primary,At2g24420.1:d:+1740:primary
ATTTGAGATT	1	4	0	0	1	At2g23290.1:d:+1083:primary
GTGCGTTTGC	0	2	1	1	2	At2g22480.1:d:+1548:primary
AGGACTTTCT	1	3	0	2	0	At2g22430.1:d:+1060:secondary
TGGACTCTTC	1	1	2	1	1	At2g22230.1:d:+754:secondary
CAAGAACAAA	0	3	1	2	0	At2g22125.1:d:+945:primary
AAGAAGAGCA	2	0	2	1	1	At2g21960.1:d:+1216:primary
AGTGAAAGGT	0	4	0	1	1	At2g21630.1:d:+2124:primary,At5g10950.1:d:+619:primary
CGTGTCGATT	0	1	3	1	1	At2g21370.2:X:+323:quaternary
TTATCCAATA	3	1	0	0	2	At2g21180.1:d:+549:primary
AGGAGAATAT	1	0	0	3	2	At2g21160.1:d:-1046:secondary
TCAACGTGTG	1	0	4	1	0	At2g20950.4:d:+1849:primary,At2g20950.2:d:+1768:primary,At2g20950.3:d:+1774:primary,At2g20950.1:d:+1819:primary
AGTGACAATT	1	0	3	0	2	At2g20830.1:d:+1841:primary
AAAGAGAATT	1	0	1	1	3	At2g20610.1:d:+1645:primary,At2g20610.2:d:+1725:primary
GTGGAAGAGT	2	1	0	2	1	At2g20330.1:d:+1811:primary
TGTTTACAAG	2	0	3	1	0	At2g20120.1:d:+1050:primary
CCTATTGAAT	1	1	3	0	1	At2g19230.1:v:+267:secondary,At5g24260.1:d:+2459:secondary,At5g24250.1:v:+226:secondary
GGAGTTATAT	0	3	0	1	2	At2g19180.1:d:+664:primary
GCGAAGAATG	1	2	1	1	1	At2g18940.1:d:+2341:secondary
AATCTAGTTT	0	1	1	1	3	At2g18740.1:d:+264:primary

GTTGAGGAAG	4	1	0	1	0	At2g18540.1:v:+1353:secondary,At2g25800.1:d:+933:secondary
TGAAGGGTAA	2	3	0	0	1	At2g18280.1:d:+1621:primary
GATGTAATCA	2	1	1	2	0	At2g18090.1:d:+3037:primary
AGAAGAGCCT	2	2	0	1	1	At2g17410.1:d:+2652:primary
TTGGATTTTG	2	0	1	0	3	At2g16780.1:d:+1429:primary
AGTTATCTGA	4	2	0	0	0	At2g16380.1:d:+1633:primary
GAAGGTCATT	1	2	2	1	0	At2g16170.1:v:+842:primary
ATCTCGCAGA	0	2	2	2	0	At2g15240.1:d:+1162:primary
TCGTTGCATT	2	2	0	2	0	At2g15050.2:d:+264:primary
TTAGTTACGT	0	0	0	3	3	At2g14880.1:d:+808:secondary
GACGTTTATA	2	0	2	2	0	At2g14260.2:d:+1339:primary,At2g14260.1:d:+1287:primary
CACTTTTGAA	1	1	3	1	0	At2g13790.1:d:+2094:primary
GAAGCTGTTG	2	3	1	0	0	At2g11520.1:d:+1698:secondary,At5g65110.1:d:+417:secondary
ATTGTGTTTC	1	3	0	2	0	At2g09970.1:d:+465:primary
AATAAGAAGA	1	2	0	2	1	At2g07460.1:p:+1893:primary
TAATCTCTCT	0	0	2	1	3	At2g07130.1:p:+1202:primary,At1g51710.1:d:+1516:primary
AAAAGAAATT	1	1	4	0	0	At2g06912.1:p:+624:primary
TCGGAATCTT	1	2	3	0	0	At2g06910.1:p:+24:secondary
GAAGCTTCTT	0	4	2	0	0	At2g06255.1:d:+495:primary,At5g19220.1:d:+1275:primary
GGGATGTATA	1	1	3	0	1	At2g04880.1:d:+1967:secondary,At2g04880.2:d:+1895:secondary
CCAAGTCGAT	2	2	0	1	1	At2g04690.1:d:+733:primary
CTAGTGGAAG	0	4	1	0	1	At2g04380.1:X:--71:quaternary
TGATATTGGA	3	2	0	0	1	At2g03980.1:d:+1260:primary
TTTTTGTCTA	0	4	0	1	1	At2g03510.1:d:+1280:primary
ATGAATGAGG	2	0	0	1	3	At2g03120.1:d:+1160:primary
ACAATAATTA	0	2	1	1	2	At2g02450.1:d:+1169:primary,At2g02450.2:d:+1274:primary
AGAAACCCAT	3	1	2	0	0	At2g02400.1:d:+1172:primary
AGAGTCGAGC	0	4	2	0	0	At2g02220.1:v:+3220:secondary,At3g29210.1:v:+1416:secondary
GAAATGTGAA	0	2	2	0	2	At2g02050.1:d:+187:primary
TGTCTTTTGT	2	0	0	1	3	At2g01735.1:d:+1222:secondary,At1g14930.1:d:+559:secondary
AAGGTGCTGG	0	5	1	0	0	At2g01540.1:d:+658:primary,At2g28400.1:d:+458:primary
TTAAGTTTGG	1	2	0	1	2	At2g01190.1:d:+2363:secondary
AAGACTGTTT	1	2	0	2	1	At1g80930.1:d:+2914:primary
GACACTGAGT	1	5	0	0	0	At1g80780.1:d:+351:primary,At1g80780.2:d:+520:primary
TTTGTTGTGT	2	1	0	1	2	At1g80480.1:d:+1511:primary,At3g48650.1:p:+1435:primary
TCGAAAGACG	1	3	1	0	1	At1g80350.1:d:+1593:primary
ATCTTATCCT	2	4	0	0	0	At1g80300.1:d:+1713:primary
GAAGATTAAC	2	0	3	1	0	At1g80210.1:v:+1559:secondary
TGACCTTGGA	0	4	1	1	0	At1g80020.1:p:+3140:primary
GATCGGGTGT	0	4	1	0	1	At1g79940.1:d:+1924:primary
GGTAACACCT	1	4	1	0	0	At1g79830.1:d:+220:secondary
ATGAAAACGA	2	2	2	0	0	At1g79730.1:d:+1400:secondary,At1g65440.1:d:+185:secondary
AACACTTCTC	1	0	1	0	4	At1g79140.1:d:+592:secondary
TAAAACCGAA	0	0	3	2	1	At1g78230.1:d:+2209:primary
GTTCTTGAAA	1	2	1	1	1	At1g77940.1:d:+598:primary
AAGATAGGGA	0	3	1	0	2	At1g77060.1:d:+1051:primary
TGTGTTTTCT	2	2	1	0	1	At1g76250.1:i:+1767:tertiary
GATCCACCAA	0	3	1	2	0	At1g76070.1:d:+1019:primary
CTCTCAAGCT	1	4	0	1	0	At1g75440.1:d:+465:primary
CGGGTTATTT	0	5	0	1	0	At1g75400.1:d:+1757:primary
AGGCTTTAAT	0	3	1	2	0	At1g75370.1:d:+1851:primary
GGACCACTTC	1	2	1	2	0	At1g75100.1:d:+1957:primary
TTTGTGAATC	2	0	0	3	1	At1g74870.1:v:+1355:secondary
AGATTGTGGA	1	2	2	1	0	At1g74380.1:d:+1680:primary
GTAAATGAAT	1	4	0	1	0	At1g74090.1:d:+1140:primary
ATTGCTATTT	1	2	1	2	0	At1g73760.1:d:+1248:primary
GCAATAGTCT	3	1	0	2	0	At1g73730.1:d:+1902:primary
ATCCATCATC	0	1	1	2	2	At1g73000.1:v:+373:secondary,At5g45290.1:d:+2022:secondary,At1g14440.2:d:+259:secondary,At1g14440.1:d:+354:secondary
GAACAAGTGA	0	3	2	1	0	At1g72690.1:d:+182:primary
ATTATTAATC	2	1	1	1	1	At1g71920.1:i:+1148:tertiary,At5g10330.1:i:+1368:tertiary

GAAGATCTTA	2	2	2	0	0	At1g71340.1:d:+1954:primary
TGTTGCTTTC	3	1	0	1	1	At1g70160.1:d:+1488:secondary
TTTGGCTTTG	2	0	2	2	0	At1g69980.1:d:+801:primary
GACCAGAATT	1	4	0	1	0	At1g69960.1:d:+996:primary
ACACTGGCTC	1	3	0	1	1	At1g69830.1:d:+2370:primary
TCCATCGGAC	0	3	0	3	0	At1g68570.1:d:+1500:primary
ACAACAACAA	3	2	1	0	0	At1g68030.1:v:+1727:primary
GTTTAGCTTC	1	2	2	1	0	At1g68020.2:d:+2749:primary,At1g68020.1:d:+2826:primary
ACATTGTGTA	3	2	0	0	1	At1g67720.1:d:+3086:primary
GCTGAGTGGT	1	2	1	2	0	At1g67500.1:v:+6009:primary
GTTCTGCAAG	1	1	1	1	2	At1g67430.1:X:+189:quaternary
TTGCTGCGGT	2	2	1	1	0	At1g67325.1:d:+1079:primary
ATATTGAAAT	3	2	0	1	0	At1g67230.1:d:+2076:secondary,At5g18250.1:d:+113:secondary
CAGTTCGGGC	0	3	0	0	3	At1g66940.1:d:+893:primary
CATTAAATTT	0	2	1	1	2	At1g66550.1:v:+1231:secondary
TATAAAAGGC	3	2	0	1	0	At1g65590.1:d:+1841:primary
ACTGAAGTAA	2	0	3	0	1	At1g65020.1:d:+1098:secondary
TTTATGTCTT	4	0	2	0	0	At1g64390.1:d:+2495:primary
GGCTCCAACC	1	2	2	1	0	At1g64355.1:d:+267:secondary
CTCTTTCCTG	0	4	1	1	0	At1g64200.1:v:+916:secondary
CTCCTCCCAC	2	1	1	2	0	At1g63900.1:d:+1297:primary
TATGCAGACA	1	2	2	1	0	At1g63830.1:d:+601:primary,At1g63830.2:d:+555:primary
AATGAGGATT	1	3	1	1	0	At1g63700.1:v:+3205:primary
GCTAATAGCT	0	0	2	3	1	At1g62040.1:d:+203:secondary
ACCGGTTCAA	2	1	0	2	1	At1g61900.1:d:+1745:primary,At1g61900.2:d:+1738:primary
AAGGATCTGA	3	1	2	0	0	At1g61190.1:v:+2616:primary,At1g61310.1:d:+2287:primary
GCTACATACA	2	0	3	0	1	At1g60950.1:d:+202:secondary
AGGCTCTCTG	3	2	0	1	0	At1g60780.1:d:+1773:secondary
TTTCATCAAA	0	3	2	0	1	At1g60640.1:d:+423:secondary,At2g39690.1:d:+609:secondary
GTGAGCGATT	1	2	1	1	1	At1g60600.1:X:+238:quaternary
CCGGCCTTCA	1	2	0	2	1	At1g60550.1:d:+968:primary
GAGGCCATCA	3	2	1	0	0	At1g60010.1:d:+626:secondary
AGTGAGTTTG	2	1	2	0	1	At1g58360.1:d:+1541:secondary
AACCAAAGTT	1	0	1	2	2	At1g58290.1:X:-1440:quaternary,At1g63080.1:X:-530:quaternary,At1g07670.1:X:-722:quaternary,At1g63070.1:X:-560:quaternary
CTGATTGAGC	1	5	0	0	0	At1g58200.2:d:+1776:primary,At1g58200.1:d:+1759:primary
AGAGGAAATA	1	1	2	1	1	At1g58080.1:d:+979:primary
ATGCAACAGA	0	2	3	0	1	At1g56660.1:d:+994:secondary
AATCTAAAAT	0	5	0	0	1	At1g56420.1:v:+1214:secondary,At5g40450.1:d:+893:secondary
AAAGCTGTAT	1	2	1	0	2	At1g56180.1:d:+1483:primary
CGCTTCTTCT	2	0	4	0	0	At1g56045.1:d:-88:primary
TAAAATACCA	2	3	1	0	0	At1g56010.1:d:+1220:secondary,At1g56010.2:d:+1205:secondary
TAGTCGAGGT	2	0	0	3	1	At1g55890.1:d:+1394:primary
GTTCAGTCCA	1	2	1	1	1	At1g55840.1:d:+1164:primary
GAAAGCGATT	0	2	3	0	1	At1g55340.1:d:+955:primary
GTTTACGAAA	0	6	0	0	0	At1g55280.1:d:+142:secondary
TTTTATCAAA	3	1	2	0	0	At1g55220.1:v:+1064:primary
ACACTCTTTT	4	2	0	0	0	At1g54820.1:d:+1382:primary,At1g29030.1:d:+1784:primary
GACTGTTTCT	2	1	3	0	0	At1g54710.1:d:+3283:primary
AAAGTCTACG	0	2	1	2	1	At1g54610.1:X:-412:quaternary
TTATAAAAAA	0	3	0	2	1	At1g54560.1:i:+7033:tertiary
GTTTTTGAGC	2	2	1	1	0	At1g54290.1:d:+579:primary
GGCAAATTAA	4	1	1	0	0	At1g54060.1:d:+1622:primary
GACAAGCTCG	1	1	3	1	0	At1g53645.1:d:+1582:primary
ACCGTACTAG	0	1	2	2	1	At1g53500.1:d:+2011:primary
TGAGCTCTAA	1	0	2	1	2	At1g52590.1:d:+552:secondary
TGACACAAAC	1	3	2	0	0	At1g52380.1:d:+663:secondary
GAGAAAGTAA	0	2	1	2	1	At1g52310.1:d:+1918:primary
AGCCTGATAT	3	1	0	1	1	At1g52290.1:d:+1675:primary
GGTCCGGGTC	0	1	1	1	3	At1g51700.1:d:+525:secondary
AGTTATGGTT	0	4	1	1	0	At1g51500.1:d:+1770:secondary

AATCTCAAAA	0	1	2	2	1	At1g51470.1:i:+1712:tertiary
AAAGGCAAAT	2	1	2	1	0	At1g51110.1:d:+1068:secondary
TTTTTCATTT	2	4	0	0	0	At1g50360.1:v:+3927:primary,At2g01480.1:d:+2576:primary,At2g03550.1:d:+907:primary
TATTTGTTAT	3	1	0	1	1	At1g50030.1:i:+8222:tertiary,At1g32380.1:i:+493:tertiary
TACGCTACTG	2	1	0	2	1	At1g49600.1:d:+1300:primary
ATTTTTTATA	1	0	1	1	3	At1g48850.1:d:+1561:secondary
ATGTTCAAGC	3	0	1	1	1	At1g48600.1:d:+1246:secondary,At1g48600.2:d:+1432:secondary
AAGAAAAACA	4	0	1	1	0	At1g48490.1:d:+2910:secondary
TGAAATGAGA	1	0	0	3	2	At1g48460.1:d:+1264:primary
GTACCTCAAC	2	1	0	1	2	At1g47490.2:d:+1464:primary,At1g47490.1:d:+1351:primary,At1g47500.1:d:+1389:primary
TTTTAGGAAA	0	0	2	1	3	At1g47370.1:v:+1052:secondary
AGAAAGTTAA	0	5	0	0	1	At1g47260.1:d:+705:secondary
GTGAAAAGGA	0	1	2	3	0	At1g45904.1:p:+194:secondary
GTCTCCTCGT	0	1	3	1	1	At1g44910.1:i:+5621:tertiary
AAATATTTGG	3	0	2	1	0	At1g44350.1:d:+1711:secondary
GACTTAGTTG	0	2	0	4	0	At1g44170.2:d:+1696:primary
ATTTTTTGTT	2	2	0	2	0	At1g43850.1:d:+3653:primary
GATGAGAAAG	1	4	1	0	0	At1g42540.1:v:+3018:secondary
GGGATTATCA	0	0	0	2	4	At1g41850.1:p:+3741:primary
CAGCTTCTTG	0	6	0	0	0	At1g38710.1:p:+488:secondary,At4g01897.1:d:+372:secondary
AACTCGTCGT	1	1	3	1	0	At1g36630.1:p:+5251:secondary
GAAAGTCACA	0	3	1	0	2	At1g36370.1:d:+1736:primary
TTTCAGTAGT	2	3	0	0	1	At1g36320.1:d:+1444:primary
CTTCAAAGTT	2	2	1	1	0	At1g36180.1:d:+4891:secondary,At5g22950.1:d:+873:secondary
AGTCCTGTAT	3	1	0	0	2	At1g35510.1:d:+2112:primary
ACGTTGAGGA	0	2	2	1	1	At1g35183.1:v:+382:primary,At1g13640.1:d:+2188:primary
TAAGACTACC	1	3	0	2	0	At1g34640.1:d:+514:secondary
ATTCGTGATT	0	4	1	0	1	At1g34220.1:d:+2032:secondary
GTGGATTATA	1	0	0	2	3	At1g33330.1:d:+1041:primary
AAATGAATAC	1	4	1	0	0	At1g32700.2:d:+1226:primary,At1g32700.1:d:+979:primary
GAGCTGCTAT	1	2	2	0	1	At1g32500.1:i:+1841:tertiary
CTTTAATGTA	0	2	3	1	0	At1g32210.1:d:+462:primary
TGATAAAAAA	0	2	3	0	1	At1g32140.1:i:+1329:tertiary
ATCTACGAAA	1	1	2	2	0	At1g31920.1:d:+1853:primary
TATTACTTCA	2	1	1	0	2	At1g31910.1:d:+1482:primary
GTTTTGCCAC	1	1	1	1	2	At1g31480.1:d:+3045:primary
GTTTGGAAAG	0	4	2	0	0	At1g30910.1:d:+882:primary
ACTAGCTCTC	3	1	1	0	1	At1g30520.1:d:+1724:primary
CACAATGCTA	2	0	1	0	3	At1g30400.1:d:+4376:secondary,At2g38740.1:d:+1004:secondary
TGACTTGATT	1	0	2	2	1	At1g30130.1:d:+1258:primary
AATTGTAAAG	1	0	3	1	1	At1g27850.1:d:+3639:primary
ATTTGAAAAA	0	0	1	4	1	At1g27770.2:d:+479:secondary,At1g27770.1:d:+479:secondary,At3g26300.1:v:+2038:secondary
GCAAAATCCC	0	2	1	3	0	At1g27390.1:d:+687:primary
CAAGTCGGAC	0	5	0	1	0	At1g27320.1:d:+2017:secondary
GTTCAGTGCC	2	3	1	0	0	At1g27300.1:d:+874:primary
TGAATAGATC	2	1	1	1	1	At1g27210.1:X:+232:quaternary
GACAGCAAGA	2	3	0	1	0	At1g27090.1:d:+1099:primary
GGAGAAGCAG	0	1	5	0	0	At1g27050.1:d:+1190:primary
AAGTCCAGGA	0	1	2	2	1	At1g26830.1:d:+2189:primary
AGTTTTGTAC	0	3	0	2	1	At1g26690.1:d:+783:primary
TAAAGAGTTG	1	0	3	2	0	At1g26640.1:d:+1394:secondary
AACCAAAAGG	2	2	1	1	0	At1g26160.1:d:+891:primary
TTGTAAGTAG	0	3	2	0	1	At1g25400.1:d:+1194:primary
GGCCGATCCT	0	0	2	3	1	At1g25350.1:d:+545:secondary
TTTTGTAGAA	1	0	0	1	4	At1g25260.1:d:+928:primary
TAGTGTGCAA	0	6	0	0	0	At1g25097.1:X:-360:quaternary,At1g25170.1:X:-360:quaternary,At1g24996.1:X:-360:quaternary,At1g24822.1:X:-360:quaternary
AAGAAAGAGA	0	3	1	2	0	At1g24706.1:d:+5030:primary,At2g47950.1:d:+278:primary
ACTACTCTGT	0	3	1	0	2	At1g24510.1:d:+603:primary,At1g24510.2:d:+854:primary
TTCTTATGCT	2	2	0	0	2	At1g23820.2:d:+1308:primary,At1g23820.1:d:+1304:primary
ACAGATGGAT	0	0	6	0	0	At1g23310.1:d:+1413:secondary

TGAGACGAAT	1	1	2	2	0	At1g23180.1:X:+704:quaternary
CATAGGCGAA	1	4	0	1	0	At1g23130.1:d:-736:secondary
GTGAAAAAAA	2	2	0	0	2	At1g22590.1:i:-1770:tertiary
GTGTGGACAC	1	2	0	0	3	At1g21750.1:d:+1275:primary,At1g21750.2:d:+1275:primary
AGCCAACATC	0	1	2	1	2	At1g21640.1:d:+2963:primary
ACCCGTTCGG	1	2	0	2	1	At1g21630.1:d:+3877:primary
ATGCGTTGTG	1	0	4	1	0	At1g21520.1:v:+799:secondary
TTGATGGAGA	0	0	3	3	0	At1g21410.1:d:+1254:primary
CAAAGCAAAG	3	1	1	0	1	At1g20925.1:v:+61:secondary
AAGATAGTCC	1	0	2	2	1	At1g20920.1:d:+880:secondary
TCTCGTAACT	1	2	3	0	0	At1g20190.1:d:+639:primary
CCTTCTACTT	1	3	0	0	2	At1g19360.1:d:+1646:primary
TCAAAATGAT	0	1	2	2	1	At1g19080.1:d:+791:primary
TCTGTTGTGT	0	0	0	3	3	At1g18800.1:d:+1183:primary
ATTAAAGGAA	1	3	0	1	1	At1g18660.3:d:+1684:primary,At1g18660.1:d:+1776:primary,At1g18660.4:d:+1867:primary,At1g18660.2:d:+1675:primary
TGAGTAGTGT	4	1	0	0	1	At1g18650.1:d:+685:primary
ACAAGCGTTC	1	3	2	0	0	At1g18620.1:d:+1903:secondary,At5g42765.1:d:+928:secondary
TGGTTTCATT	1	1	2	2	0	At1g18530.1:v:+1034:primary
ATCATCCAGA	2	2	2	0	0	At1g18490.1:d:+443:secondary
TAATTCTTGT	0	3	1	1	1	At1g18330.1:d:+1421:primary
GATTTCATAC	1	0	1	2	2	At1g17890.3:d:+1238:primary,At1g17890.1:d:+1264:primary,At1g17890.2:d:+1325:primary
CCATTCCTAC	2	2	2	0	0	At1g17730.1:d:+639:secondary,At1g73030.1:d:+645:secondary
TTAAATTCCT	0	2	1	0	3	At1g17710.1:i:+407:tertiary,At5g35930.1:i:+5084:tertiary
TAGCGAGACC	0	2	2	1	1	At1g17520.1:d:+790:primary
GAAGAAGTGA	1	3	0	2	0	At1g17490.1:d:+184:primary
GAAGAGTATC	1	0	1	1	3	At1g17330.1:v:+972:primary
CCAAATTCTG	0	0	1	3	2	At1g17170.1:d:+333:primary
TCTATTCTTT	0	4	0	1	1	At1g16860.1:d:+2119:primary
GAAGTGAAAG	0	2	2	1	1	At1g16710.1:d:+2470:secondary,At3g03950.2:d:+263:secondary,At2g04890.1:d:+1420:secondary,At3g03950.1:d:+264:secondary,At5g66570.1:d:+396:secondary
GTGAAATAAA	2	3	0	1	0	At1g16170.1:d:+640:primary
TCAAAACTAA	2	1	2	1	0	At1g15780.1:d:+4067:primary
TATTACGAGT	1	3	2	0	0	At1g15260.1:d:+534:primary
GTTTTCCAAA	1	1	1	2	1	At1g15170.1:d:+1647:primary
GAAATATCAA	2	0	1	1	2	At1g15080.1:d:+1096:primary
TTTTCTTGGG	2	2	0	0	2	At1g14830.1:d:+2172:primary
TCTCTGGTTG	1	1	2	1	1	At1g14620.1:d:+916:primary
ATCTCTGCTA	2	1	1	1	1	At1g14570.1:d:+1501:primary,At1g14570.2:d:+1488:primary
CTCTTTGTGC	1	2	2	0	1	At1g14270.2:d:+1307:secondary,At1g14270.1:d:+1114:secondary
AAGCGAGGTT	0	2	2	2	0	At1g13670.1:X:+761:quaternary
TACTTGCTCA	0	4	1	1	0	At1g13450.1:d:+1425:primary
ACCACTGTCC	0	1	5	0	0	At1g13440.1:d:+612:secondary
TATTTTACCA	1	3	1	1	0	At1g13380.1:d:+533:primary
TTTTCTGTGA	0	2	1	0	3	At1g13320.1:d:+1995:primary
GATCTTATTG	1	3	0	1	1	At1g13280.1:d:+897:secondary
GCACAGGCGA	1	2	1	0	2	At1g13020.1:d:+1693:primary
CTTCTGGAGA	2	1	2	1	0	At1g12470.1:d:+3098:secondary
TCAAGTTATC	0	4	0	2	0	At1g12200.1:d:+1454:primary,At5g44980.1:d:+1365:primary,At5g49110.1:v:+5290:primary
AAATAGATGA	0	3	0	1	2	At1g10910.1:v:+2387:secondary
TTTGTAACGC	1	1	2	2	0	At1g10170.1:d:+3820:primary
TCGTCAGTGG	0	2	2	2	0	At1g09340.1:d:-1190:secondary
ATACAGTGGA	2	3	0	1	0	At1g08900.1:d:+1292:primary
GATATGGAGG	0	5	1	0	0	At1g08630.2:d:+1045:primary,At1g08630.3:d:+1037:primary,At1g08630.1:d:+1182:primary
TTGAAATTTG	2	1	1	0	2	At1g08600.1:d:+4521:primary
GAAGGGATCG	0	5	0	1	0	At1g08315.1:d:+897:primary
TGGTGGCTAC	2	0	2	2	0	At1g08200.1:d:+1308:secondary,At5g08710.1:d:+1142:secondary
TGTTTGTGAA	1	1	1	1	2	At1g08190.1:d:+3112:secondary
CAGAAGCACG	1	0	5	0	0	At1g07770.2:d:+178:secondary,At1g07770.1:d:+218:secondary
CTATAGGTTA	1	1	1	1	2	At1g07710.1:X:-1524:quaternary

GACACAGCCC	1	1	0	2	2	At1g07670.1:d:+2484:primary
ATTATTTATG	2	2	0	1	1	At1g07470.1:d:+1403:secondary
TTTCTAAAAC	3	2	0	1	0	At1g07310.1:d:+1407:primary
TTCAAGATTC	1	2	2	0	1	At1g07010.1:d:+1255:primary
GATATCAGAA	3	1	1	1	0	At1g06210.2:d:+1466:primary,At1g06210.1:d:+1286:primary
GTTACCGGGG	1	2	0	2	1	At1g06190.1:d:+305:secondary
ATAGTCTTTG	0	2	1	2	1	At1g05890.1:d:+2143:primary
GTTCTGGCTA	0	2	3	0	1	At1g05830.1:d:+3512:primary
AAGATCTGGT	1	3	1	0	1	At1g05120.1:v:+2892:secondary
AAATAATTTC	3	2	0	0	1	At1g04950.2:d:+1971:secondary,At1g04950.1:d:+2067:secondary
GAAATTTCCA	1	1	2	1	1	At1g04860.1:d:+335:secondary
AAATTTCATT	0	0	2	2	2	At1g04860.1:d:+3313:primary
ATCTCAAACT	1	1	2	2	0	At1g04820.1:d:+1241:secondary
AGGGAAAACT	0	1	2	3	0	At1g04710.1:d:+1491:primary
AACAGTTGGT	1	1	0	3	1	At1g04640.1:d:+690:primary
AAGAGATATC	0	2	2	1	1	At1g04510.1:d:+1746:primary
GATGGTGCAC	1	1	2	1	1	At1g04170.1:d:+635:primary
GCGTGAATCC	0	1	2	2	1	At1g03850.2:d:+336:primary
CTTCTCCCCT	0	4	0	1	1	At1g03220.1:d:+327:primary
AAGTATGCGC	0	4	0	2	0	At1g02840.3:d:+597:primary,At1g02840.1:d:+601:primary
TTTGTAATCA	3	3	0	0	0	At1g02730.1:d:+3801:primary
GGATTCATAT	3	3	0	0	0	At1g02640.1:d:+2325:primary
TAACAATTTT	1	3	2	0	0	At1g02340.1:d:+1217:primary
GTCGAGATAT	1	2	2	1	0	At1g02100.1:d:+1295:primary,At1g02100.2:d:+1222:primary,At1g02100.3:d:+1216:primary
TTTTCTAATT	2	3	1	0	0	At1g02080.1:d:+7264:primary
GGAAAGTTAG	0	4	2	0	0	At1g01800.1:d:+553:primary
ATTTTTTTTG	0	3	1	1	1	At1g01780.1:v:+1072:primary,At4g22430.1:v:+1501:primary
TCACTTATTA	0	2	0	1	3	At1g01540.1:d:+1835:primary,At1g01540.2:d:+1798:primary
TATAATGAAT	2	4	0	0	0	At1g01140.1:d:+1506:primary,At1g01140.2:d:+1512:primary,At1g01140.3:d:+1518:primary
CGCAGGCAAG	3	0	1	1	0	No gene matches found
GTGTAAAAAA	0	3	0	2	0	No gene matches found
CAGGTGTGGG	0	4	0	0	1	No gene matches found
GTTGTATTAA	1	1	2	0	1	No gene matches found
GCGCGAAAAC	1	3	1	0	0	No gene matches found
GCACACAACA	1	1	2	1	0	No gene matches found
TAGAACTCTA	4	1	0	0	0	No gene matches found
AGGCTAAAAA	1	0	1	2	1	No gene matches found
TACCTGAACG	1	0	1	3	0	No gene matches found
CCTCCTGTGA	0	4	1	0	0	No gene matches found
CCCGAATCTT	0	3	1	1	0	No gene matches found
GCGAAAGGAC	1	0	3	0	1	No gene matches found
AGAGTTCTCG	2	2	0	1	0	No gene matches found
CAAATCAGCT	0	0	1	1	3	No gene matches found
CGGGTGTGGC	0	0	1	4	0	No gene matches found
TAGGTGTGGC	1	1	1	2	0	No gene matches found
CCTAAGATCG	2	1	1	1	0	No gene matches found
GTCCTTCGCC	0	5	0	0	0	No gene matches found
GGCGTTCGCC	1	3	1	0	0	No gene matches found
GAACAAACTC	0	5	0	0	0	No gene matches found
TCGAAAAGGA	1	0	3	1	0	No gene matches found
CAGAGTGAAC	0	4	0	0	1	No gene matches found
CGCCTAAGGA	0	3	1	1	0	ChrM:-362154:quaternary
CATACAAATT	1	4	0	0	0	ChrC:+46305:quaternary,Chr2:+9826540:quaternary
ACCTGATCAA	0	1	1	1	2	ChrC:+107716:quaternary
AAGTCTTGAA	0	3	1	0	1	Chr5:-6573699:quaternary
ATCCATACAT	3	0	2	0	0	Chr5:+8125066:quaternary,Chr3:+17472602:quaternary
TTCCAAGAAA	2	1	1	0	1	Chr5:+26353162:quaternary
TATTAAAAAA	1	1	0	1	2	Chr5:+25679705:quaternary,Chr1:+24729720:quaternary,Chr5:+3184423:quaternary,Chr1:+9927495:quaternary,Chr1:+24597083:quaternary,Chr5:+9120345:quaternary,At5g67385.1:X:+850:quaternary,Chr1:+16692867:quaternary,Chr4:+17384764:quaternary
GATCACGTTC	0	0	0	2	3	Chr5:+20856886:quaternary

TGAAACAATG	0	1	1	2	1	Chr5:+15271025:quaternary
GCTTCAGATA	2	0	2	0	1	Chr5:+15211608:quaternary,ChrC:+32045:quaternary
GGCTGAACGA	1	1	1	2	0	Chr4:-12521759:quaternary
AGATCATAAA	3	0	0	2	0	Chr4:+4957418:quaternary,Chr3:+7505306:quaternary
TTCGTACAAA	1	3	0	1	0	Chr4:+20804:quaternary,Chr2:+12181428:quaternary
TACAAATTTG	2	1	1	1	0	Chr4:+14373924:quaternary,Chr5:+22066073:quaternary
CACACGTTCA	1	1	1	2	0	Chr4:+12989139:quaternary
AAGATGATTA	0	3	1	0	1	Chr4:+12258695:quaternary
TAAATTTAAA	0	1	2	1	1	Chr4:+10019018:quaternary,Chr1:+26913040:quaternary,Chr3:+16087123:quaternary
TTCTTCTAAA	1	0	2	2	0	Chr3:-1774000:quaternary
GGTTAAAAAA	1	2	0	1	1	Chr3:+4961409:quaternary,Chr4:+1214989:quaternary
ACAGCAAAAG	2	0	1	1	1	Chr3:+4886857:quaternary
CGTGATGCGT	2	0	2	1	0	Chr3:+3173237:quaternary
TCAAAAGACT	2	1	0	1	1	Chr3:+21016134:quaternary
ACTCAAAAAA	1	0	2	2	0	Chr3:+17893280:quaternary
CCTGTCGGGA	1	1	0	2	1	Chr3:+14211891:quaternary,Chr2:+6934:quaternary
TGTAAGTATG	2	1	1	0	1	Chr3:+14208720:quaternary,Chr2:+3763:quaternary
AAATTGTAGT	1	3	0	0	1	Chr3:+10585751:quaternary
ATCCAAAAAA	0	1	1	3	0	Chr3:+10545898:quaternary,Chr5:+16168467:quaternary
ACCTTAGAGA	0	0	4	1	0	Chr3:+10299299:quaternary,Chr3:+10299332:quaternary
ATTCAAACTT	0	1	2	1	1	Chr2:+8663948:quaternary
GAGCAACTTG	3	0	0	2	0	Chr2:+4113172:quaternary,Chr2:+4114692:quaternary
GGAGCAGATT	0	3	1	1	0	Chr2:+3333882:quaternary
TCTTTGTTGG	0	3	0	2	0	Chr2:+2921255:quaternary
CTTGGGACAT	0	0	4	0	1	Chr2:+2889320:quaternary
CTGACATTTT	0	5	0	0	0	Chr2:+15964734:quaternary
TTCCGTGCGA	0	2	2	1	0	Chr1:-24915734:quaternary,Chr1:-24915727:quaternary
AATGAAGTAA	1	1	2	1	0	Chr1:+27156143:quaternary
CAGTGACCGA	0	2	2	1	0	Chr1:+25578254:quaternary
TTTACACGTG	2	0	3	0	0	Chr1:+24888307:quaternary,At5g57660.1:X:+-69:quaternary
GAATCCAAGT	1	3	0	0	1	Chr1:+22466162:quaternary
TTGACCCAAA	0	2	0	2	1	Chr1:+16972412:quaternary
GAACATAGAA	1	1	1	0	2	Chr1:+13172882:quaternary,Chr1:+19501178:quaternary
TCACAAAGTA	0	4	1	0	0	Chr1:+10304117:quaternary
CCTGTTGAAA	1	0	2	2	0	AtCg00740:d:+532:secondary
CACTTCTGGG	0	1	1	3	0	AtCg00280:d:+398:secondary
CCTCATATCA	2	1	2	0	0	At5g67390.1:d:+878:primary
AAGTTCCTTC	1	0	0	2	2	At5g67330.1:d:+1873:primary
GAGTCGAGAG	1	2	1	1	0	At5g66820.1:d:+900:primary
TTCCTAAAAA	0	1	1	1	2	At5g66800.1:d:-610:secondary
GGAAGAGATG	2	0	2	1	0	At5g66690.1:d:+702:secondary,At2g17972.1:d:+315:secondary,At5g26310.1:d:+681:secondary
TGTACATTTG	1	0	2	2	0	At5g66160.2:d:+1339:secondary,At5g66160.1:d:+1261:secondary
ACTTGGAAGT	1	0	3	1	0	At5g65770.1:v:+1654:secondary
GTCGGCCTGT	1	0	4	0	0	At5g65710.1:v:-3283:secondary,At1g65490.1:d:-229:secondary
TACATATGTT	2	0	0	1	2	At5g65480.1:d:+1151:secondary
TATTACACAC	1	1	2	1	0	At5g64813.1:d:+1720:primary
GGCCTGACCC	1	0	3	0	1	At5g64540.1:v:+1117:secondary
TTTCTTTAGC	0	0	0	1	4	At5g64480.1:d:+618:secondary
ACCAAGATGA	0	4	0	1	0	At5g64110.1:d:+1003:primary
GTGAAAGCGA	0	2	1	1	1	At5g64030.1:d:+2691:primary
TCGAGTATGG	2	2	1	0	0	At5g63800.1:d:+2108:secondary
AGTTTTCTGA	1	2	0	1	1	At5g63770.1:d:+2665:secondary,At3g31440.1:p:+4825:secondary
TGGAATGTTG	0	2	1	1	1	At5g63610.1:d:+1789:primary
TGGTTTCTTT	0	1	2	1	1	At5g63530.1:d:+1202:primary
CTCAAGGAGA	0	2	1	2	0	At5g63460.1:d:+518:primary,At5g63460.2:d:+506:primary
TTGTTTGTTC	4	0	0	0	1	At5g63050.1:d:+1416:primary
TCTGTCTAAA	1	2	1	0	1	At5g62990.1:v:-551:secondary
TTGTCTTTTG	0	4	0	1	0	At5g62910.1:d:+1109:primary
CGGTTTTATG	0	1	3	1	0	At5g62470.2:d:+1685:primary,At5g62470.1:d:+1452:primary
AAGCTTTATA	1	1	1	1	1	At5g62430.1:d:+986:primary
GTGCTATCGT	0	2	2	1	0	At5g62390.1:d:+946:secondary

AGTAATGCTC	0	1	2	0	2	At5g62350.1:d:+508:secondary
TTCAAGTCAG	0	2	1	0	2	At5g61240.1:v:+1422:secondary
TAATAATGGA	1	1	0	1	2	At5g61150.1:d:+2188:primary,At5g61150.2:d:+2182:primary
GAGTTACCTC	0	5	0	0	0	At5g61140.1:d:+6328:primary
GTGTGTGTGT	1	4	0	0	0	At5g61130.1:d:+274:primary
CTTAATACAA	3	0	1	0	1	At5g61010.1:d:+2814:primary
ACTTTCGGAA	0	5	0	0	0	At5g60710.1:d:+2236:primary
ACTTATGTTC	1	0	0	2	2	At5g60620.1:d:+1245:primary
AATCAATGGG	3	0	0	1	1	At5g60540.1:i:+1209:tertiary
AAGCTTAGCT	0	4	0	0	1	At5g60270.1:v:+2473:secondary
TTATGTGTTG	1	0	3	0	1	At5g60160.1:d:+1610:primary,At3g28450.1:d:+2068:primary
TTTATCGAGT	1	0	1	1	2	At5g59920.1:v:+2453:secondary
CCACGAAAAA	0	1	3	0	1	At5g59880.1:d:-416:secondary,At5g59880.2:d:-416:secondary
CAAAGGAGAA	3	1	1	0	0	At5g59650.1:v:+1325:secondary,At1g55270.1:d:+1633:secondary,At3g17900.1:d:+2638:secondary
GCAAATCCTG	2	1	0	2	0	At5g59480.2:d:+693:primary,At5g59480.1:d:+683:primary
GCTACTGGAC	1	1	2	1	0	At5g59430.1:d:+1812:primary,At5g59430.2:d:+2006:primary
TTAAGACACA	3	0	1	0	1	At5g59310.1:X:-158:quaternary
TCTCAATGAA	0	0	0	0	5	At5g59180.1:X:+289:quaternary
GAGGAAATCA	1	2	1	0	1	At5g58490.1:d:+965:primary
CGGATGTCAA	0	1	3	1	0	At5g58020.1:d:+795:secondary
TTCCTGATGT	1	1	1	2	0	At5g57930.2:d:+1227:primary,At5g57930.1:d:+1231:primary
GATCGGGTTT	0	4	1	0	0	At5g57710.1:d:+2468:primary
AGTTTTTGTA	0	5	0	0	0	At5g57480.1:v:+1530:secondary
ACGAGAGACC	1	2	0	0	2	At5g57440.1:d:+925:secondary
GCACTTGTGT	0	0	3	2	0	At5g57360.1:d:+1891:primary
TGGCACTGTT	0	3	1	0	1	At5g57280.1:d:+675:primary
GGAATAGTGT	1	1	2	1	0	At5g57050.1:d:+1967:primary
TATAGAATCT	1	1	0	0	3	At5g57030.1:d:+1811:primary
GGAGAAGTAG	0	2	2	1	0	At5g56890.1:d:+3083:secondary
GTGCAATGGC	0	2	2	1	0	At5g56630.1:d:+1585:primary
GAAGTGCAGT	0	2	1	2	0	At5g55910.1:d:+1396:primary
TATATTTAGA	1	0	0	1	3	At5g55740.1:v:+2880:secondary,At5g40820.1:d:+3653:secondary
TGTAGTATTT	1	0	1	1	2	At5g55580.1:d:+1992:primary
TTGTATCTCC	2	0	1	0	2	At5g55530.2:d:+1469:secondary,At5g55530.3:d:+1441:secondary,At5g55530.1:d:+1547:secondary
AGGAAATTTG	1	2	2	0	0	At5g55510.1:X:-238:quaternary
TCAAGCTGTT	1	1	2	1	0	At5g55300.1:d:+2989:primary
TACTCGTTTG	1	3	0	0	1	At5g55130.1:d:+1609:primary
AACATAGTTA	0	1	4	0	0	At5g54730.1:d:+2471:primary
ATGACCATTT	0	2	1	1	1	At5g54220.1:v:+396:secondary,At4g22470.1:d:+1135:secondary,At4g09300.1:d:+852:secondary
CCGAGTAAAT	1	2	0	1	1	At5g53550.1:v:+2340:secondary
GCTCGAGAGA	0	3	1	1	0	At5g52870.1:d:+878:primary
TTTGGCTTCT	0	2	1	1	1	At5g52420.1:d:+513:secondary
GCTCATTCAT	1	3	0	0	1	At5g51880.1:d:+871:primary
GAACGAGATG	2	3	0	0	0	At5g51640.1:d:+1543:primary
CAAACGAGAG	1	2	2	0	0	At5g51620.2:d:+130:primary
AGCTAGGTAA	1	3	1	0	0	At5g51560.1:d:+2135:primary
AAAACAATGT	1	1	0	2	1	At5g51220.1:d:+1124:primary,At5g39140.1:v:+1048:primary,At5g39200.1:v:+1195:primary,At5g39170.1:v:+1054:primary
AAGAAGAGTT	0	1	3	0	1	At5g51070.1:d:+3192:primary
GGCACCAAAG	0	1	3	1	0	At5g50920.1:X:-1393:quaternary
TTGATTGATG	3	0	2	0	0	At5g50900.1:d:+1814:primary
AAACAGAGAG	2	0	0	2	1	At5g50840.1:d:+1262:primary,At5g50840.2:d:+1301:primary
CCAGTTTAGT	0	0	4	1	0	At5g50450.1:X:-864:quaternary
GTGAAGCATC	0	2	0	3	0	At5g50320.1:d:+1720:secondary
AACTATGGCG	0	2	1	0	2	At5g50310.1:d:+2169:primary
TCTTGGCCGA	0	1	2	1	1	At5g50150.1:d:+1513:primary
AATGGTCTCA	1	0	2	0	2	At5g49840.1:v:+2222:primary
CTACTCCACA	0	3	1	0	1	At5g49760.1:d:-3246:secondary
AAATAAAAAA	2	1	0	1	1	At5g49750.1:v:+2134:secondary
TCTTCTTGAT	1	2	1	1	0	At5g49720.1:i:+1472:tertiary
GACAATAGTT	2	1	1	1	0	At5g49555.1:d:+1803:primary

AACTAGAGAT	1	1	0	0	3	At5g49510.1:d:+702:primary
AGAATGATGT	0	3	1	1	0	At5g49430.1:v:+1690:secondary,At2g33830.1:d:+397:secondary,At2g33830.2:d:+405:secondary,At1g22060.1:d:+2990:secondary,At2g47410.1:d:+1883:secondary
CTGAGTATGT	2	1	0	0	2	At5g49230.1:d:+792:primary
AACATTGTGA	1	2	0	0	2	At5g48630.1:d:+686:primary
TGAAGTTCAG	2	2	1	0	0	At5g48580.1:d:+156:primary
TGGTCCTTCT	1	1	1	0	2	At5g48385.1:d:+113:secondary,At3g25190.1:d:+722:secondary,At2g13360.1:d:+1326:secondary,At2g13360.2:d:+1304:secondary
TACTCATTAA	2	0	0	1	2	At5g48230.2:d:+1765:primary,At5g48230.1:d:+1503:primary
TATTGCGATT	3	2	0	0	0	At5g47100.1:d:+1093:primary
GCGGAACAAC	2	0	1	1	1	At5g47040.1:d:+2680:primary
TTGGAAAATT	1	1	1	0	2	At5g46990.1:v:+258:secondary
GTCTGTTATC	0	5	0	0	0	At5g46710.1:d:+614:primary
TATATCTGCA	1	3	1	0	0	At5g46240.1:d:+2236:primary
AAACAGTATG	3	0	2	0	0	At5g45590.1:d:+587:primary
CTACGATAAA	1	0	2	1	1	At5g45010.1:X:-215:quaternary
TAACAACGAT	1	2	0	1	1	At5g44790.1:d:+3277:primary
TGATATTTAG	2	3	0	0	0	At5g44680.1:d:+1250:primary
GTTTTTAGCA	2	0	2	0	1	At5g44575.1:d:+367:primary
TATACACATA	1	1	1	0	2	At5g44560.1:d:+914:primary,At4g29590.1:d:+1017:primary
CATATTTCTG	1	1	3	0	0	At5g44420.1:d:+376:primary
TAGATTAATG	2	1	0	0	2	At5g44210.1:d:+10:secondary,At5g41870.1:v:+1726:secondary
TGGGTTCCCC	2	1	2	0	0	At5g43920.1:v:+2231:primary
TAAACATAAG	1	0	0	1	3	At5g43860.1:d:+997:primary
GAGCTTTTTT	0	2	1	2	0	At5g43720.1:d:+1306:primary
GAATTCAGAG	0	0	4	1	0	At5g43720.1:d:+1216:secondary
CTTAAAACAG	0	1	0	3	1	At5g43450.1:d:+1302:primary
GGTTAGAGAG	2	1	2	0	0	At5g43270.3:d:+1450:primary,At5g43270.2:d:+1498:primary,At5g43270.1:d:+1573:primary
GCATTGGGTT	2	2	1	0	0	At5g43190.1:d:+1120:primary
TATCGTCTCA	0	1	2	1	1	At5g42810.1:d:+1618:primary
TTGTGAGGAT	4	0	0	0	1	At5g42770.1:d:+819:primary
CCTTTCGATA	1	0	1	1	2	At5g42765.1:X:-279:quaternary,At3g04480.1:X:-531:quaternary
GCTCAAACCG	0	1	1	3	0	At5g41970.1:d:+1062:primary
AGAGAAATGT	2	0	2	0	1	At5g41560.1:d:+439:primary
TAAACCACGA	0	0	1	3	1	At5g41190.1:v:+1859:secondary
TTTTTCGGTA	1	2	1	0	1	At5g41020.1:X:+1937:quaternary
ATGCTCTTTG	1	2	1	0	1	At5g40930.1:d:+284:primary
GAGTTCACAA	2	2	0	1	0	At5g40890.1:d:+2899:primary
AGACTGTTTC	0	5	0	0	0	At5g40450.1:d:+5054:secondary,At3g47570.1:d:+108:secondary
TAATCTTAAA	3	1	1	0	0	At5g39860.1:d:+594:primary
AATTTCACAA	1	2	2	0	0	At5g39680.1:d:+2527:secondary
GGGGTTAGGT	1	3	0	1	0	At5g39590.1:d:+1521:secondary
CCTGCCTTTG	0	3	0	1	1	At5g39320.1:d:+1502:primary
ATGAGCTTCT	0	2	0	3	0	At5g39040.1:d:+1853:primary
ATTGAAAAAA	0	1	1	0	3	At5g38340.1:v:+972:secondary,At3g25510.1:v:+4551:secondary
AAGCTGCTCT	0	4	0	1	0	At5g38140.1:d:+611:primary
CTACAACGCG	0	0	1	1	3	At5g37020.1:d:+2588:primary
GAGAGAGGAA	2	2	0	1	0	At5g35840.1:d:+3482:primary
TACTGTTCTC	1	2	0	1	1	At5g35740.1:d:+530:primary
GATATCTCCA	3	1	0	1	0	At5g35330.1:d:+830:secondary,At5g35330.2:d:+824:secondary
TACTTTGATT	1	0	1	0	3	At5g34834.1:p:+1165:primary
CTTCGCTTCC	1	1	3	0	0	At5g30510.1:d:+1198:secondary
AATTTGGCAG	0	1	0	2	2	At5g30390.1:p:+951:secondary,At2g27090.1:d:+2498:secondary
TACGTTGACG	1	1	3	0	0	At5g30269.1:p:+10921:secondary
TAAATCTGCT	3	0	1	1	0	At5g28640.1:d:+1115:primary
AACGTTTCAT	0	2	2	1	0	At5g28020.2:d:+1212:primary,At5g28020.1:d:+1517:primary
AATTTTAATA	0	2	0	3	0	At5g28020.1:d:+1488:secondary,At5g28020.2:d:+1183:secondary
ATTGAGTATT	0	5	0	0	0	At5g27882.1:p:+663:secondary,At2g05990.1:d:+1133:secondary,At4g07720.1:p:+1891:secondary,At2g05990.2:d:+1166:secondary
TGAAATTTTG	1	0	3	1	0	At5g27830.1:d:+1038:primary

TTACAAGATA	3	0	0	1	1	At5g27820.1:d:+562:primary
ATAAGTTATG	0	3	0	0	2	At5g27730.1:d:+1576:primary
CATCTGTTGT	1	0	1	1	2	At5g27120.1:d:+1092:primary
GTTATAAACA	4	0	0	0	1	At5g26800.1:v:+956:primary
GACCAGAGGC	1	0	2	1	1	At5g26780.1:d:+1715:primary,At5g26780.3:d:+1851:primary,At5g26780.2:d:+1763:primary
GAAAGTGTGT	1	1	1	1	1	At5g26210.1:d:+813:primary
GTGGAAAATG	0	0	3	0	2	At5g26160.1:v:+3275:secondary
TACAGTCCTG	0	1	1	2	1	At5g25880.1:d:+1738:primary
AGTTTTTTTT	0	0	1	2	2	At5g25220.1:d:+1708:primary
AGCTCGAAAA	1	3	1	0	0	At5g25210.1:d:+726:primary
AACCAAACCA	0	2	1	1	1	At5g24990.1:d:+58:secondary,At5g25010.1:v:+354:secondary
GGTGGTGTTC	0	2	1	2	0	At5g24650.1:d:+219:primary
GTTCTTCTCG	1	0	3	0	1	At5g24260.1:d:+2502:primary
TACTGTTGTC	2	0	1	1	1	At5g23940.1:d:+1808:primary
CTCGATTGCT	1	1	2	1	0	At5g23820.1:d:-765:secondary
CTCGCTTATA	1	1	0	1	2	At5g23330.1:d:+986:primary
GGAAATTCAG	1	2	1	1	0	At5g22920.1:d:-1064:secondary
TCGAAAAGTT	1	2	1	0	1	At5g22770.1:d:+3296:primary,At5g22770.3:d:+3226:primary,At5g22780.1:d:+3295:primary
CAGAGAGTCG	1	1	3	0	0	At5g22690.1:d:+3209:primary
AGGTCTTAGG	0	1	2	2	0	At5g22460.1:d:+1002:primary,At5g22460.2:d:+991:primary
TTGTTTGTGT	2	0	2	1	0	At5g22140.1:d:+1401:primary,At5g22140.2:d:+1286:primary
GGATTGAAGC	0	2	1	0	2	At5g22120.1:d:+1094:primary
GGGATGATGG	0	2	2	1	0	At5g22040.1:d:+656:primary
TAGAATTTGT	1	2	2	0	0	At5g21222.1:d:+2761:primary
GCAATGGCAA	1	0	2	1	1	At5g20960.1:d:+4280:primary
AAATCAGCAA	0	0	4	1	0	At5g20730.2:d:+4262:primary
TTAAAACATT	1	1	0	2	1	At5g20620.1:d:+1445:primary
AAAATGGAGT	1	1	2	0	1	At5g20600.1:d:+1689:primary
TAGAGGTGTG	0	1	1	2	1	At5g20590.1:d:+1686:secondary
TAATAAAATG	0	2	1	0	2	At5g20080.1:d:+1177:primary
AGACAACCGA	2	1	0	1	1	At5g19990.1:d:-1435:secondary
GTAAGCTTGT	1	2	2	0	0	At5g19900.1:d:+1770:primary
TTGTACAAAT	2	1	0	0	2	At5g19740.1:d:-1475:secondary
CAAAATCACG	1	3	0	0	1	At5g19590.1:d:+241:primary
GCCGAGAGTT	0	5	0	0	0	At5g19450.1:d:+906:secondary,At5g19450.2:d:+1051:secondary
GATGAAGATG	0	1	3	1	0	At5g19190.1:d:+159:secondary,At4g26630.1:d:+480:secondary,At3g07120.1:d:+925:secondary,At3g16730.1:v:+1680:secondary
CTTTCTCTTC	1	0	1	1	2	At5g18440.1:v:+697:secondary
TGGTTACATT	0	1	0	1	3	At5g18250.1:d:+304:secondary
TGATCCTGTT	0	2	1	1	1	At5g17930.1:v:+2472:secondary
TATTTTTTTA	1	1	2	0	1	At5g17520.1:d:+1476:primary,At4g04960.1:d:+2214:primary
AGATTCATCT	1	0	2	2	0	At5g17300.1:d:+1612:primary
AAATCAGGGA	1	3	0	0	1	At5g16780.1:d:+336:secondary
CTTCTGCTTT	1	2	0	1	1	At5g16220.1:d:+1607:primary
GGTCAAATAA	0	0	0	4	1	At5g15860.2:X:-297:quaternary,At2g44780.1:X:--221:quaternary
ACTCGCAGAC	0	0	5	0	0	At5g15840.1:X:-253:quaternary
TGTCGAAAAT	0	2	1	2	0	At5g15802.1:d:+143:primary
ATCCTGGAAT	1	1	2	1	0	At5g15550.1:d:+1408:primary,At5g15550.2:d:+1492:primary
GAACTACTGG	0	1	2	1	1	At5g15530.1:d:+886:secondary
GTTATGGCCT	1	2	1	1	0	At5g15230.1:d:+181:secondary
GGAAGTTTAC	1	0	1	2	1	At5g15150.1:X:--113:quaternary
TAAAACTACT	3	1	0	1	0	At5g14950.1:d:+3697:primary
TTGAGTATGA	0	4	0	1	0	At5g14120.1:d:+1998:secondary
ACAGCGATGT	0	3	2	0	0	At5g13750.1:d:+1370:primary
GTCACAAAAA	1	0	1	2	1	At5g13480.1:v:+1273:secondary
CAATAATAAT	0	5	0	0	0	At5g13460.1:d:+1465:primary,At4g11650.1:d:+883:primary
TAGATTCAAA	0	5	0	0	0	At5g13140.1:d:+263:primary
TGAGCTCAGG	0	5	0	0	0	At5g13050.1:d:+913:primary
AATGGAAGGA	0	5	0	0	0	At5g12120.1:d:+1815:secondary
TGAAGGATTT	0	3	1	0	1	At5g11890.1:d:+859:primary
TGTCACCACA	1	2	1	0	1	At5g11790.1:d:+1208:primary

TTTGATGAGA	0	1	0	3	1	At5g11760.1:d:+779:primary
GATTATGACC	1	2	2	0	0	At5g11480.1:d:+973:primary
CCATTCTTTT	1	2	1	1	0	At5g11330.1:d:+1229:primary
GAGGATAAGT	0	1	3	1	0	At5g11240.1:d:+1470:secondary,At4g00650.1:d:+1023:secondary,At5g14370.1:d:+168:secondary
AAGACAAAGA	4	0	1	0	0	At5g11060.1:d:+1525:secondary,At3g25510.1:v:+1690:secondary
CATAGCTTCC	1	0	2	0	2	At5g10940.1:d:+2481:primary
GTCTGAAATG	2	0	1	1	1	At5g10610.1:d:+1481:primary
GAAACAGATG	0	2	2	1	0	At5g10490.1:d:+1524:primary
GTAATGAGCT	1	1	1	1	1	At5g10460.1:d:+837:primary
TCGGAGAATC	0	3	1	1	0	At5g10150.1:d:+1022:secondary
AAGTCCTTTG	0	2	1	1	1	At5g10010.1:d:+1487:primary
ACACTTTAAT	2	1	0	1	1	At5g09960.1:d:+597:primary
TTCACATTTC	0	0	1	2	2	At5g09225.1:d:+455:primary
CAAAGGTCAG	0	0	1	1	3	At5g08580.1:X:+213:quaternary
ACCACGCCAG	2	0	0	2	1	At5g08550.1:v:+3028:secondary
GAATCCGCAG	0	1	2	2	0	At5g08430.1:d:+1895:primary
ATCGATCTTG	0	1	4	0	0	At5g08290.1:d:+341:secondary
CAGGATTCGC	0	2	2	1	0	At5g08190.1:d:+508:primary
GAAACGCTCA	0	3	1	0	1	At5g07460.1:d:+730:primary
CTTACAGTTT	1	0	0	3	1	At5g07350.1:d:+3351:primary
AATCTGAATG	2	2	0	0	1	At5g07340.1:d:+1820:primary
CTTGTATATA	1	2	1	0	1	At5g06600.2:d:+1663:secondary,At5g06600.1:d:+1664:secondary,At3g17110.1:p:+741:secondary
GTAAGGATTT	0	1	3	1	0	At5g06390.1:d:+1791:secondary
TATCTGGTAC	0	1	1	1	2	At5g06220.1:d:+2539:primary
GTTGATGCAA	0	2	1	1	1	At5g05950.1:d:+598:primary
AAAGGAAGAA	1	0	2	2	0	At5g05880.1:v:+2091:secondary
TGTATAAAAC	1	1	1	0	2	At5g05730.1:d:+1928:secondary
GCGGAGTCTT	1	2	1	0	1	At5g05520.1:d:+1693:primary
ATATGTTTGT	1	3	0	0	1	At5g05190.1:d:+2392:secondary
CTAAACCAAA	0	4	1	0	0	At5g05080.1:d:+758:secondary
GGCGTGCGCC	1	2	1	1	0	At5g04990.1:d:+1540:primary
GACACTGGAA	1	2	1	1	0	At5g04930.1:d:+3356:secondary
TATGAATCAG	0	0	2	2	1	At5g03880.1:d:+643:secondary
ACTATTGTCA	1	2	1	0	1	At5g03730.1:d:+2892:primary
GAGATGGACC	2	2	1	0	0	At5g03470.1:d:+1679:primary
TGCGAATCTT	1	4	0	0	0	At5g03360.1:v:+1736:secondary
TATATTTTCT	0	2	1	1	1	At5g03140.1:d:+2381:primary,At1g74530.1:d:+1074:primary,At1g74530.2:d:+1078:primary
CTCAAGCAAC	1	1	1	1	1	At5g02970.1:d:+1981:primary
TTGGGTTGGG	0	0	2	0	3	At5g02890.1:d:+975:secondary,At2g41840.1:d:+533:secondary
GCACGGGCAT	0	1	2	2	0	At5g01920.1:d:+926:secondary
GAATCCCGGA	0	0	1	1	3	At5g01160.1:d:+1167:secondary
CAGCTTGGCG	1	0	3	0	1	At5g01020.1:d:+1140:secondary
AGAGAAAAAA	2	0	1	1	1	At4g40010.1:v:+220:secondary,At2g39410.1:d:+56:secondary,At2g39410.2:d:+56:secondary
TGTGATCACG	1	4	0	0	0	At4g39970.1:d:+877:primary
GGAGTCACTT	0	3	0	1	1	At4g39850.1:d:+4226:primary
TCCTATCTCC	1	2	1	0	1	At4g39640.1:d:+1665:primary,At4g39640.2:d:+1630:primary
TAAACTATAG	4	0	0	0	1	At4g39160.1:v:+1986:secondary
TTCGTCAAAG	2	0	3	0	0	At4g38970.2:d:+1259:secondary,At4g38970.1:d:+1255:secondary
GGGCGGCAGA	0	0	5	0	0	At4g38970.1:d:+1128:secondary,At4g38970.2:d:+1132:secondary
TATGCGAGTG	3	1	1	0	0	At4g38860.1:X:+737:quaternary
TCCACCTAAA	2	0	0	1	2	At4g38770.1:d:+662:secondary
TCCGCCTAAG	2	0	2	0	1	At4g38770.1:d:+1013:secondary
AGCAATGGAT	1	0	1	1	2	At4g38620.1:d:+1084:primary
TGATGTCATA	0	2	0	2	1	At4g38580.1:d:+605:primary
ATGATTCTCA	1	3	0	1	0	At4g38540.1:d:+1232:primary
AGGAAGGCAA	1	2	1	0	1	At4g38490.1:d:+559:primary,At3g04080.1:d:+1766:primary
GCCGACACAA	2	0	0	2	1	At4g38460.1:d:-1201:secondary
GTCAAAAGCA	2	3	0	0	0	At4g38420.1:d:+1446:primary
TAAACACAAA	0	1	0	3	1	At4g38260.1:d:+970:secondary
ACAAATTTTG	1	0	0	2	2	At4g38110.1:d:+1526:primary
TAGCAGTGAA	0	3	1	1	0	At4g37980.1:d:+662:secondary,At4g07760.1:p:+3896:secondary

AAAATAACCA	1	0	1	1	2	At4g37910.1:X:-820:quaternary
AAGTTTTTTA	1	1	0	2	1	At4g37880.1:d:+1458:primary
GTTCTCTTCA	2	1	2	0	0	At4g37800.1:X:-603:quaternary
TTTCTTAAGG	0	3	1	0	1	At4g37590.1:d:+1738:primary
TGAAGGAAGA	1	1	2	0	1	At4g37550.1:d:+1261:primary
TCTTTCATCT	0	5	0	0	0	At4g37540.1:d:+296:primary
GCTTGAGTTC	1	2	0	1	1	At4g37130.1:d:+1720:primary
TGAAGCATTT	1	2	1	1	0	At4g37120.1:d:+941:secondary,At4g02940.1:d:+1753:secondary,At1g43502.1:p:+1931:secondary
CCTGACAAAG	0	1	2	2	0	At4g36870.1:d:+2274:primary
AGCAGCCAAG	2	1	0	2	0	At4g36400.2:d:+1740:primary,At4g36400.1:d:+1713:primary
CTCATCGCAA	0	2	2	1	0	At4g36390.1:d:+1591:primary
CAGAGTCACA	0	1	3	0	1	At4g36190.1:d:+1514:primary
TTTTGTTTTT	0	1	1	2	1	At4g35987.1:v:+1155:primary,At2g10237.1:p:+1121:primary,At2g20280.1:d:+1164:primary
TAATGTGTTG	1	0	2	1	1	At4g35920.1:d:+1570:secondary,At4g35920.2:d:+1501:secondary,At2g31890.1:d:+2091:secondary,At4g35920.3:d:+1404:secondary
GAAGAAGCCC	3	1	0	1	0	At4g35785.1:d:+721:primary,At4g35785.2:d:+746:primary
TGAACCAATG	1	4	0	0	0	At4g35470.1:X:+559:quaternary
AAGGATGGTG	2	3	0	0	0	At4g35220.1:d:+597:primary
ATCCGATGAT	0	3	0	0	2	At4g34760.1:d:+401:primary
ATTTTGTAGC	2	0	1	0	2	At4g34610.1:v:+1936:secondary,At2g16400.1:d:+1953:secondary
TTTACAAAAA	2	3	0	0	0	At4g34570.1:d:+1625:secondary
ATCAAGAATC	0	3	0	2	0	At4g34400.1:v:+1188:secondary,At2g15530.1:d:+2416:secondary,At4g37780.1:d:+514:secondary,At2g15530.2:d:+2429:secondary
TTGTTGATGC	2	0	1	1	1	At4g34310.2:d:+2786:secondary,At4g34310.1:d:+2786:secondary
GGTGTGAAAA	2	1	0	1	1	At4g34230.1:d:+639:secondary
TTGAATTACA	2	2	1	0	0	At4g34040.1:d:+2598:primary
AGTATCCAAG	0	3	1	1	0	At4g34020.1:d:+1444:primary
AGAGAAAAAC	3	0	2	0	0	At4g33900.1:v:+1753:primary
ATCACATTGC	2	1	2	0	0	At4g33640.1:d:+473:secondary
AAACACTTAC	2	1	0	1	1	At4g33530.1:d:+2720:primary
TGAACCTGGA	1	1	1	1	1	At4g33500.1:d:+1994:primary
GCAGCAATAC	0	1	2	1	1	At4g33300.1:i:+497:tertiary
TTATATGTAG	3	0	1	0	1	At4g33060.1:d:+1715:primary
TGAAAAAAAT	1	2	1	1	0	At4g32960.1:d:+896:primary
TTGAACAAAT	2	2	0	1	0	At4g32650.1:d:+1988:primary,At4g32650.2:d:+1848:primary
CAGTGATGCC	0	4	1	0	0	At4g32460.1:d:+504:primary,At4g32460.2:d:+475:primary
CAAGCCAAGT	2	1	1	1	0	At4g32330.2:d:+921:secondary,At2g20670.1:d:+774:secondary,At4g32330.1:d:+924:secondary
CCCGAATAAT	2	0	0	2	1	At4g32320.1:d:+1096:primary
TACATAATGA	2	0	3	0	0	At4g32250.2:d:+2125:secondary,At4g32250.1:d:+1985:secondary
ACCTCGCCGC	1	0	4	0	0	At4g32020.1:d:+583:secondary
AGATTAAATA	0	0	2	2	1	At4g32020.1:d:+1474:primary
AAGAAGCAAG	0	4	1	0	0	At4g31880.1:d:+1211:primary,At4g00450.1:d:+6493:primary
TATATTCGTT	2	3	0	0	0	At4g31720.2:d:+711:primary,At4g31720.1:d:+728:primary
TCAGAAGACT	2	2	1	0	0	At4g31650.1:v:+171:secondary,At1g76150.1:d:+1027:secondary,At2g25920.1:d:+451:secondary
ATCAGGCCGT	2	3	0	0	0	At4g31510.1:d:+56:primary
AATAAGTTAC	0	3	2	0	0	At4g30280.1:d:+921:primary
TATTTTCTAT	1	3	1	0	0	At4g29780.1:d:+1961:primary
GCTATGTATA	0	0	2	0	3	At4g29110.1:v:+1142:primary
TACTCAAAAA	1	1	0	3	0	At4g28810.1:i:+1938:tertiary
TCCTCCATAA	1	0	0	2	2	At4g28480.1:X:+587:quaternary
ACCGCATATT	0	0	1	1	3	At4g28440.1:d:-682:secondary
TTTATGTGTC	3	2	0	0	0	At4g28270.1:d:+782:primary
AAGCTCAGAA	1	1	0	1	2	At4g28200.1:d:+1335:secondary,At3g47450.2:d:+322:secondary,At3g47450.1:d:+322:secondary
ATCAACTTGC	2	0	3	0	0	At4g28070.1:d:-1637:secondary
TAATTGTAAA	0	2	3	0	0	At4g28030.1:d:+836:primary
GCAGCGGGAT	0	0	0	4	1	At4g27680.1:d:+1324:secondary
GTCCTTGTGT	2	0	2	1	0	At4g27585.1:d:+1557:primary,At3g25080.1:v:+1382:primary
CTGGGTTCAG	1	1	3	0	0	At4g27540.1:d:+762:primary
GCTGAATATA	0	0	2	1	2	At4g27340.1:d:+1877:primary
GAGAATTATA	1	3	1	0	0	At4g27320.1:X:-143:quaternary
GTTCTTGTTT	0	1	3	1	0	At4g27010.1:v:+2507:secondary

AACGCTACAC	1	3	1	0	0	At4g26970.1:d:+2963:secondary
CACTCGATTG	0	3	1	1	0	At4g26910.2:d:+1219:secondary,At4g26910.3:d:+1076:secondary,At4g26910.1:d:+1318:secondary
TAAGGGTTGA	1	0	0	2	2	At4g26860.1:X:--118:quaternary
TATAATGTTA	2	0	0	0	3	At4g26860.1:d:+827:primary
TAAAACCTTG	0	3	1	0	1	At4g26770.1:d:+1663:primary
TTGTATCTTT	1	2	2	0	0	At4g26650.1:d:-2043:secondary
TCACTCGGAG	1	0	3	0	1	At4g26450.1:v:+2418:secondary
GCCGTTCAGC	1	2	1	1	0	At4g26400.2:d:+1204:primary,At4g26400.1:d:+1221:primary
AGCTTTGGAG	3	0	0	1	1	At4g26370.2:d:+464:secondary,At4g26370.1:d:+519:secondary
TGGTGCATCA	1	1	1	1	1	At4g26160.1:d:+767:primary
GTGGATCAAT	1	1	1	2	0	At4g26090.1:d:+3463:primary
ACTGTTCGTG	1	4	0	0	0	At4g25910.1:d:+792:primary
TTTCTGTGGT	1	3	0	0	1	At4g25900.1:d:+1034:secondary
ATTAGTTCGT	1	2	1	0	1	At4g25550.1:d:+734:secondary
TGTTTCATTC	2	0	2	0	1	At4g25550.1:d:+1003:primary
AGACCTTCCA	0	1	3	1	0	At4g25500.2:d:+212:primary,At4g25500.1:d:+365:primary
AGATAGAATA	0	0	2	2	1	At4g25490.1:X:-1108:quaternary
CCAAACTATG	0	0	2	3	0	At4g25100.1:X:-353:quaternary
CACCATTTTG	1	1	0	3	0	At4g24990.1:d:+633:primary
GGTTCTTCTG	0	2	3	0	0	At4g24860.1:d:+1691:secondary,At1g62130.1:v:+1855:secondary
TGGACCACTC	0	2	1	2	0	At4g24740.2:d:+1109:primary,At4g24740.1:d:+1286:primary
TTACTACTTC	3	0	1	0	1	At4g24700.1:d:+521:secondary
ACGTTCACGA	0	2	2	0	1	At4g24550.1:d:+1246:primary,At4g24550.2:d:+1327:primary
TTTGACTAAA	2	0	2	1	0	At4g24520.1:X:-198:quaternary
AAACGTTTAA	3	1	1	0	0	At4g24275.1:d:+526:primary
GGATAGACGG	2	0	0	1	2	At4g24090.1:d:+818:primary
TGGTGAAAAA	2	1	0	2	0	At4g24060.1:d:-660:secondary
GCTTAAACAA	1	0	3	1	0	At4g23980.1:d:+2305:primary
TAAGGTTCAA	0	3	1	1	0	At4g23870.1:d:+773:primary
GAGGACCATT	0	0	5	0	0	At4g23750.2:d:+1319:primary,At4g23750.1:d:+1530:primary
AATTTGATGT	0	0	0	0	5	At4g22880.1:d:+1195:primary
GTTTCAGTTT	1	3	1	0	0	At4g22540.1:d:+2507:primary,At4g22540.2:d:+1927:primary
CTTTATGAAA	2	1	1	0	1	At4g22340.2:d:+1503:primary,At4g22340.1:d:+1581:primary
GTAATCAATC	0	1	3	1	0	At4g22310.1:d:+294:primary
CGGTAGTCAA	0	5	0	0	0	At4g22190.1:d:+254:secondary
ATTACTATAG	3	1	0	0	1	At4g22000.1:d:+538:primary
GAGAAAAAAA	0	2	2	1	0	At4g21550.1:d:+469:secondary
ACCAGAAAAG	2	1	2	0	0	At4g21190.1:d:+1119:secondary
TGGGGGATTG	1	2	0	2	0	At4g20980.1:d:+1585:primary
ATATTTACCT	3	0	0	0	2	At4g20960.1:d:+1444:primary
TACAAAAATA	0	2	1	2	0	At4g20730.1:v:+44:secondary
GAGAATATGG	0	3	1	0	1	At4g20300.2:d:+1408:primary
CTGCAGTGAA	0	0	2	2	1	At4g20020.2:d:+1272:primary
TGTGTATAAT	2	0	2	0	1	At4g19840.1:d:+882:primary
TTTGGTGGTC	1	1	2	1	0	At4g19120.2:d:+2225:primary,At4g19120.1:d:+2376:primary
GGTTAATCCG	0	2	1	0	2	At4g18593.1:d:+428:primary
GAAGTTCTCG	1	0	3	1	0	At4g18240.1:d:+143:secondary
AAGTATATTT	2	1	0	0	2	At4g18220.1:v:+1562:primary
GACAAGTGCT	2	3	0	0	0	At4g17730.1:d:+1064:primary
GAAGCTGAAA	0	2	2	1	0	At4g17486.1:d:+904:primary
TCTCTCACGT	2	2	1	0	0	At4g17360.1:d:+1016:primary
TGAATTCATA	0	1	0	2	2	At4g17280.1:d:+1480:secondary
TACACTCCTT	1	1	2	0	1	At4g17270.1:d:+1475:secondary
CTACTGTTTG	2	1	2	0	0	At4g17220.1:i:+864:tertiary
TAAGAGAAGT	3	0	2	0	0	At4g17080.1:v:+2095:secondary
ATTTGCCTTT	0	4	1	0	0	At4g16935.1:p:+123:primary
AGATTGACCT	1	4	0	0	0	At4g16920.1:v:+1333:secondary,At4g16860.1:d:+1111:secondary,At4g16960.1:v:+1351:secondary,At4g16940.1:v:+1330:secondary,At4g16890.1:v:+1339:secondary
TTTGTAATTA	1	2	0	0	2	At4g16860.1:d:+3593:primary
GTTTCAGTAT	0	0	5	0	0	At4g16740.1:d:+501:secondary

GTTGGAGTCT	0	0	1	1	3	At4g16390.1:d:+2098:primary
GAGTGCTCTT	0	0	4	0	1	At4g16190.1:d:+968:secondary
TACTGGAAAC	0	1	1	1	2	At4g16170.1:d:+1130:secondary
CATAGTCTTG	2	1	1	1	0	At4g16140.1:d:-624:primary
GACGGTTGCG	1	1	1	2	0	At4g15940.1:d:+417:primary
CATCTTCAAA	3	0	1	1	0	At4g15930.1:d:+659:primary,At3g26486.1:p:+300:primary
ACACAATGGT	2	0	0	2	1	At4g15790.1:d:+710:primary
AGGTTGGTTA	1	3	1	0	0	At4g15765.1:v:+1401:primary
TTGGAGGAGC	1	2	0	2	0	At4g15610.1:d:+550:primary
ACTCACCATA	0	0	2	2	1	At4g15475.1:d:-1970:secondary
GCAAGAGTAG	1	2	1	0	1	At4g15420.1:d:+1770:primary
AGCAAACTCA	1	0	3	0	1	At4g15230.1:d:+4068:primary
TCTGCTATTT	1	1	0	1	2	At4g15080.1:d:+2828:primary
AATGTAATGA	1	0	2	0	2	At4g15075.1:v:+1091:primary
TTGACACACT	0	0	0	2	3	At4g14690.1:d:-771:secondary,At1g29475.1:p:-850:secondary
GCGATGGCCG	0	0	0	4	1	At4g14690.1:d:-425:secondary
TAAAACAGTA	2	3	0	0	0	At4g14620.1:d:+1502:primary
ATCAATGTTA	1	1	0	1	2	At4g14365.1:d:+1355:primary
TTAATTCAAA	1	3	0	1	0	At4g13340.1:d:+434:secondary,At2g25590.1:v:+1431:secondary
GGTCAACTTC	1	2	0	1	1	At4g12590.1:d:+496:primary
TTGCTCGCTC	0	0	0	0	5	At4g12545.1:d:+223:secondary,At4g12550.1:d:+232:secondary,At3g58770.1:v:+2216:secondary,At4g12480.1:d:+405:secondary
GTTTCGACTA	2	0	2	1	0	At4g12390.1:d:+817:primary
GGTATTTTGA	0	3	0	2	0	At4g12060.1:d:+416:primary
TACGTACTTT	2	0	1	1	1	At4g11880.1:i:+1716:tertiary
AAACTGTGTC	0	1	4	0	0	At4g11600.1:d:+938:primary
GAACGGATCG	1	3	0	1	0	At4g11320.1:d:+1224:primary
GAACCAGTCA	1	1	1	0	2	At4g11160.1:d:+2394:primary
TAAAGAATGT	0	0	0	3	2	At4g10610.1:d:+1156:primary
TACGGCTTTA	1	1	0	3	0	At4g09980.1:d:+3147:primary
TGACCTTTTA	2	0	2	1	0	At4g09840.1:d:+748:secondary
TTGTATGACT	1	3	0	0	1	At4g08950.1:d:+1164:primary
TTTAGTTTTT	1	2	1	0	1	At4g08099.1:p:+1994:secondary
AACTTTGCCG	1	1	1	2	0	At4g07390.1:d:+614:primary
AAGAATACAA	0	3	2	0	0	At4g06676.1:v:+1101:secondary
AAGAACTTTG	0	2	2	1	0	At4g04940.1:d:+2443:secondary,At3g59990.2:d:+1200:secondary,At3g59990.1:d:+1134:secondary
TTTCTATAAT	1	0	1	3	0	At4g04310.1:p:+2845:secondary
GTCTCTTCTT	0	0	2	2	1	At4g03695.1:X:--208:quaternary
AAATTGGAAG	1	1	1	1	1	At4g03560.1:X:+369:quaternary,At5g49530.1:X:+396:quaternary
AACAGCCGAC	0	1	1	1	2	At4g03490.1:v:+101:secondary
CGATCCCTTT	0	3	1	1	0	At4g03190.1:d:+1595:secondary
ATTTTAGATG	1	0	1	1	2	At4g03180.1:d:+853:secondary
GAAGATTGCC	1	1	1	1	1	At4g03000.2:d:+2846:primary,At4g03000.1:d:+2551:primary
TCATATGCAG	1	1	2	0	1	At4g02910.1:X:-62:quaternary
TAGCTGGGTA	0	1	0	2	2	At4g02725.1:d:+372:primary
TGAAACAGGT	0	3	1	1	0	At4g02610.1:d:+711:secondary
CTAAAGCTAA	0	2	0	0	3	At4g02580.1:d:+857:secondary
TGGATGAGAT	0	0	2	2	1	At4g02130.1:d:+1585:primary
ATTTTTGCCA	0	3	0	0	2	At4g02030.1:d:+2203:primary
CTCAATCGCT	0	1	2	1	1	At4g01995.1:d:+747:primary
GCACAGATGT	0	3	2	0	0	At4g01810.1:d:+2502:secondary
AATCACGTCG	0	2	1	1	1	At4g01800.1:v:+2479:primary
TCTCACATTT	1	1	0	1	2	At4g01710.1:d:+499:primary
AAGGTTTAGG	2	1	1	1	0	At4g01460.1:d:+1356:primary,At4g39280.1:d:+1253:primary
ACCGATATGT	1	2	1	1	0	At4g01450.3:d:+1157:primary,At4g01450.1:d:+1201:primary,At4g01450.2:d:+1255:primary
AAATGGTTGG	1	2	1	0	1	At4g01310.1:X:-246:quaternary
GCGAAAGTAC	0	5	0	0	0	At4g01250.1:d:+486:primary
TTGTAGCACA	1	2	2	0	0	At4g01110.1:v:+1569:primary
GCCATTATAC	1	0	1	2	1	At4g01070.1:d:+1298:primary
AGTCAAGAAG	0	2	2	1	0	At4g01060.1:d:+108:secondary,At4g24230.2:d:+981:secondary,At4g24230.1:d:+956:secondary,At4g01060.2:d:+159:secondary

TTGCTTAACA	2	0	1	1	1	At4g00990.1:X:+173:quaternary
TTCCTCTCTA	1	2	0	1	1	At4g00720.1:d:+2079:primary
TAGTTTTTAA	0	1	0	2	2	At4g00620.1:d:+1440:primary
GTTTTTGTAC	1	3	0	1	0	At4g00355.3:d:+1195:primary,At4g00355.1:d:+1101:primary,At4g00355.2:d:+1088:primary
CTTGTGTGTG	2	0	0	2	1	At4g00270.1:d:+1208:primary
GCTGACCATC	0	1	2	2	0	At4g00180.1:d:+899:primary
CCAAATCCCA	0	2	2	0	1	At3g63490.1:d:+790:secondary,At3g63490.2:d:+790:secondary
GGATCCAAAA	0	1	2	0	2	At3g63420.1:d:+425:primary
AAGAGATGCT	1	2	1	1	0	At3g63260.2:d:+1308:primary,At3g63260.1:d:+1284:primary
TCGGGAGTGT	1	2	1	0	1	At3g63170.1:d:+1036:secondary
GCTGATGATA	0	2	1	0	2	At3g62920.1:d:+292:primary
GATGGGAGCT	1	3	1	0	0	At3g62770.2:d:+1323:primary,At3g62770.1:d:+1301:primary
TAAAACCCCA	0	4	0	0	1	At3g61870.1:d:+121:secondary,At3g61870.2:d:+158:secondary
GAGGTTTTTT	1	2	2	0	0	At3g61790.1:d:+1277:primary
TTGTAGTATC	2	0	0	0	3	At3g61770.1:d:+1055:primary
GCTAAATGAT	1	1	3	0	0	At3g61580.1:d:+1552:secondary
AATAAAATCA	1	2	0	2	0	At3g61130.1:d:+2340:primary
TATCCGATTT	2	0	1	2	0	At3g60840.1:v:+2345:primary,At2g01680.1:d:+1762:primary
ACCGTTAGAC	0	4	1	0	0	At3g60530.1:d:+366:primary
TCGGGGATGG	0	2	2	1	0	At3g60200.1:d:+775:primary
TGACCCCAGA	0	1	2	2	0	At3g59810.1:d:+565:primary
CGGCTCGTTT	0	1	1	2	1	At3g59800.1:d:+122:primary
TGTGTACAAA	0	1	2	2	0	At3g59420.1:d:+2058:secondary
ATTCCTTCTG	2	1	2	0	0	At3g58990.1:d:+766:secondary
TCCTCTCGAA	0	2	2	0	1	At3g58990.1:d:+1014:primary
AACTGAGACT	2	0	2	1	0	At3g58790.1:d:+1970:secondary,At1g14660.1:d:+2103:secondary
TATTTTGTAT	3	1	0	0	1	At3g58530.1:d:+1372:primary,At1g30730.1:v:+2181:primary,At4g35760.1:d:+1477:primary,At1g14740.1:d:+2465:primary
AAATAAGCGA	1	2	0	1	1	At3g57785.1:d:+560:secondary
ATCTCTTATG	1	2	1	1	0	At3g57550.1:d:+1669:primary
TATTGTTTTG	0	1	0	2	2	At3g57540.1:d:+1239:primary,At1g76520.2:d:+1433:primary,At1g76520.1:d:+1369:primary
GTATTTGGTT	2	0	2	1	0	At3g57070.1:d:+1567:primary
AGTCCGATTG	1	0	0	1	3	At3g56850.1:d:+1703:primary
TTGTTCTTCA	2	2	0	0	1	At3g56760.1:d:+1631:primary,At3g26020.1:d:+1991:primary
GATGAAAGAA	1	2	1	0	1	At3g56750.1:d:+1425:primary
AATGTAAAGA	0	3	0	1	1	At3g56690.1:d:+3363:primary
TTTTCTTGCT	0	1	2	1	1	At3g56190.1:d:+1429:primary
GCACATAGAG	2	1	1	1	0	At3g56120.1:d:+1588:primary
AAAGTAGTAT	1	0	0	1	3	At3g56090.1:d:+1096:primary
CTTTTCAATG	0	1	2	1	1	At3g55990.1:d:-968:secondary
CCTGAATGGT	2	0	2	1	0	At3g55960.1:d:+1009:primary
TGAGACTACA	1	1	0	0	3	At3g55880.2:d:+944:primary
TTTTTGCTGA	1	3	0	0	1	At3g55740.1:d:+1591:primary,At1g41890.1:p:+3550:primary,At3g55740.2:d:+1549:primary
TTCTTGAGAT	2	0	2	1	0	At3g55560.1:d:+723:primary
AGCTTTGTTT	1	1	0	0	3	At3g55530.1:d:+142:secondary
ATTTGTTTGG	2	0	1	2	0	At3g55520.1:d:+857:secondary,At1g01260.1:d:+946:secondary
TTATTCAAGT	1	1	0	1	2	At3g55270.1:d:+2981:primary
GCTTACAGAT	0	4	1	0	0	At3g55200.1:d:+3478:primary
GGAATATTGT	2	1	0	1	1	At3g55110.1:d:+2418:primary
TATGGTTTTA	0	0	2	2	1	At3g55005.1:d:+974:primary
TAGTGAATAA	1	1	2	0	1	At3g54920.1:d:+1135:primary
CTGTTTTCAA	0	1	1	1	2	At3g54670.1:i:+5881:tertiary
TTTTTGACCC	2	1	0	2	0	At3g54110.1:d:+1057:primary
GCGATTCCGA	0	2	1	1	1	At3g53990.2:X:-115:quaternary
GATCTCTATG	1	0	0	1	3	At3g53620.1:d:+891:primary
TGTCCCTGTA	1	1	2	0	1	At3g53540.1:d:+3177:primary
CATCAAAGGC	0	0	0	2	3	At3g53460.1:X:-95:quaternary
AACAAACTAG	1	2	2	0	0	At3g53320.1:i:+2657:tertiary
ATAATTGATT	0	2	2	1	0	At3g53130.1:d:+1685:secondary
GCGCTGCAGT	1	2	1	1	0	At3g52890.1:d:+3109:primary,At3g52890.2:d:+2669:primary

GTGGCGGAGC	0	0	3	2	0	At3g52800.1:d:-335:primary,At5g58020.1:d:-84:primary
GTACACCAAG	1	4	0	0	0	At3g52720.1:d:+480:primary
TGATAAAAAT	0	3	1	1	0	At3g52370.1:d:+1419:primary
GACAAGTTCG	0	1	3	1	0	At3g52360.1:d:+323:primary
AAGACTAGTC	1	0	3	1	0	At3g52230.1:d:+469:secondary
TCTGTTGCAT	2	0	1	0	2	At3g52155.1:d:+791:primary
TTCAGTCTCG	0	0	3	2	0	At3g51890.1:d:+924:primary
AACAATTTGG	0	1	2	1	1	At3g51860.1:d:+1382:secondary
GTCATTTGCA	0	0	1	1	3	At3g51800.1:d:+991:primary
GCAGATTTTT	1	0	1	1	2	At3g51680.1:X:--2:quaternary
ATAAAGACAG	2	0	1	2	0	At3g51630.1:d:+2271:primary
AAAACCAAAA	0	2	0	1	2	At3g51540.1:v:+202:secondary
AGCGGCTTTT	0	1	2	2	0	At3g51270.1:v:+1758:secondary
GATGCTAAGC	2	1	1	0	1	At3g51160.1:d:+1176:primary
AGGAGGTTGA	0	2	3	0	0	At3g50930.1:d:+2003:primary
TAATCCACAT	0	2	1	1	1	At3g50830.1:d:+750:secondary
ACTATTTTGT	1	1	2	0	1	At3g50750.1:d:+1094:secondary
GGGAAGACGA	3	0	0	2	0	At3g49810.1:d:+2075:primary
GATGCTGACT	1	3	0	0	1	At3g49790.1:d:+1447:primary
TATCTCAACA	0	3	1	0	1	At3g49780.1:X:+383:quaternary
TAGTTAGGCT	0	1	2	1	1	At3g49530.1:d:+1720:primary
TACACACGAG	2	2	1	0	0	At3g49220.1:d:+1618:primary
TTTCTTCAAA	1	2	0	2	0	At3g49080.1:d:+1593:primary,At4g34900.1:v:+4552:primary
GATCACAAGA	1	3	0	1	0	At3g48880.1:d:+1095:primary,At3g48880.2:d:+869:primary
TAAGTTTCAC	2	2	1	0	0	At3g48860.2:d:+1927:primary
AATGTTTGTT	1	1	0	1	2	At3g48760.1:i:+816:tertiary,At1g20080.1:i:+816:tertiary
CACTATCGAA	0	5	0	0	0	At3g48680.1:d:+462:primary
AGTATGTCAA	0	1	1	1	2	At3g48610.1:d:+1565:primary
TCTTACGATG	1	1	1	0	2	At3g48500.1:d:+1803:primary
AGAATTTATC	0	2	2	1	0	At3g48340.1:d:+368:secondary
TTTACTCATT	1	0	2	1	1	At3g48310.1:d:+1618:primary
TAGTTTTCTT	0	2	1	2	0	At3g47900.1:d:+1358:primary
GCACAACACT	3	1	1	0	0	At3g47470.1:d:+986:secondary
CAAATAGGTA	1	0	1	1	2	At3g46920.1:v:+3929:secondary
GCTTCTTATC	0	2	2	0	1	At3g46640.1:d:+1086:primary
GAGGCCAGAG	0	3	2	0	0	At3g46510.1:d:+1487:secondary
TGTTTAAAAA	0	3	2	0	0	At3g46350.1:v:+50:secondary,At5g49920.1:d:+1451:secondary
ATGAACCATT	1	3	1	0	0	At3g46100.1:d:+1507:primary
AAGGCTATTA	2	1	0	1	1	At3g45030.1:X:-292:quaternary
TTGAATCTGG	4	0	0	0	1	At3g44590.1:d:+582:primary
GTTGATTGCA	2	0	1	2	0	At3g44020.1:d:+410:primary
CCTTAAAAAA	2	0	1	1	1	At3g43230.1:d:-179:primary
TTAAAATTAT	0	1	0	3	1	At3g43220.1:d:+3037:primary,At5g11260.1:d:+770:primary
ATCTATTTAT	1	0	1	0	3	At3g42182.1:p:+2428:secondary
GGGTGAAACC	0	0	3	1	1	At3g33025.1:p:-1081:secondary,At3g15210.1:d:-981:secondary
GGTTCCCTTT	0	2	2	1	0	At3g33000.1:p:+288:primary,AtCg00150:d:+288:primary
TTTGTTAAAA	2	2	1	0	0	At3g32904.1:v:+197:secondary
TAAATCTTGC	0	1	4	0	0	At3g32112.1:p:+1309:secondary
TTCTAAAAAA	2	1	0	1	1	At3g31403.1:p:+483:secondary
AAAGTTCTCA	1	1	1	2	0	At3g30837.1:p:+2547:primary
TAGATGACAC	0	5	0	0	0	At3g30745.1:p:+3684:secondary,At3g42803.1:p:+1235:secondary,At2g22440.1:v:+997:secondary
ATCTCAGTGG	2	0	1	1	1	At3g30300.1:X:+658:quaternary
CTTCCTCCAC	1	4	0	0	0	At3g29390.1:d:+1623:primary
CACGCTTTGT	0	1	2	2	0	At3g29200.1:X:-387:quaternary
TCTGACCAAG	4	1	0	0	0	At3g28690.1:d:+2127:primary
TTACATTTAT	1	1	1	2	0	At3g28160.1:p:+2091:primary
AGTTTTGCTG	2	0	2	1	0	At3g28100.1:i:-3110:tertiary
GGAGGCTGAT	2	0	2	0	1	At3g27750.1:d:+561:primary
TCTTAATTCC	2	2	0	0	1	At3g27670.1:d:+5725:primary
ATCTATTCAT	3	0	0	1	1	At3g27490.1:v:+400:secondary
TCATCTCCAT	3	0	0	1	1	At3g27050.1:d:+640:primary

TATTTTGTAA	1	1	1	2	0	At3g26630.1:d:+1586:primary,At1g34770.1:d:+1694:primary,At1g34770.2:d:+777:primary
TCATTCGCTC	0	0	5	0	0	At3g26570.2:d:+1519:secondary,At3g26570.1:d:+1408:secondary
GAAATTTTAA	1	2	1	1	0	At3g26520.1:d:+1187:primary,At1g42110.1:p:+1758:primary,At2g15940.1:p:+1666:primary
AGCTAAAGGA	1	0	3	1	0	At3g25970.1:v:+1036:secondary,At4g29210.1:d:+1791:secondary,At4g29210.2:d:+1914:secondary
TAGTGTTCTT	0	1	0	0	4	At3g25940.1:d:+578:primary
ACAACTCTCA	0	4	0	1	0	At3g25760.1:d:+108:primary
GAGCTGATTT	0	0	2	3	0	At3g25590.1:d:+1594:primary
CAATATCTAG	3	2	0	0	0	At3g25580.1:d:+816:primary
CTCAATGGAC	0	3	2	0	0	At3g25150.1:d:+1283:secondary
TTGCTGCTTT	0	1	0	1	3	At3g25070.1:d:+757:primary
GGTCGAGCAA	1	4	0	0	0	At3g24570.1:d:+743:secondary
ATAAAGTTTA	2	3	0	0	0	At3g24550.1:d:+1930:primary
AAGGCCTTAT	1	2	1	1	0	At3g23490.1:d:+769:secondary
CTTTAATTAT	1	1	1	1	1	At3g22740.1:d:+875:primary
TAAAGATACC	3	0	0	0	2	At3g22370.1:d:+1416:primary
AGGGATCGTG	0	4	0	1	0	At3g22330.1:d:+1532:primary
GATATAGTTT	1	1	2	0	1	At3g22290.1:d:+1337:primary
GCTGTGGTGT	2	2	0	1	0	At3g22270.1:d:+2663:secondary,At4g17080.1:v:+1288:secondary
GATTTACTTA	0	2	0	1	2	At3g22200.1:d:+1213:primary
GTTTCTCTAT	0	0	1	3	1	At3g22150.1:d:+1694:secondary
ATCTATTTGG	2	1	0	1	1	At3g22120.1:d:+1102:secondary
TTTATGTCAT	3	0	2	0	0	At3g21270.1:d:+966:primary
ATAATACAAA	2	1	2	0	0	At3g20790.1:d:+1192:secondary
ACCCAACGGG	1	2	1	1	0	At3g20390.1:X:+-152:quaternary
TGTTTGATTT	3	0	1	1	0	At3g20370.1:d:+1288:primary
ACGCTTCTGT	0	3	2	0	0	At3g20340.1:d:+455:primary
GTCAATGGGC	0	3	1	0	1	At3g20310.1:d:+1580:primary
TATAATTCGT	1	2	0	1	1	At3g20270.2:d:+1668:secondary
AGAAGTAGAA	0	1	1	2	1	At3g20240.1:d:+1362:primary
TGGGTAGAAG	1	2	0	1	1	At3g19780.1:d:+3179:primary
AGTTTCTGGT	1	0	1	2	1	At3g19460.1:d:+1025:primary
GCCTGACTAC	2	2	1	0	0	At3g19380.1:d:+1432:secondary
GTTTCACATA	0	3	0	1	1	At3g19130.1:d:+1561:primary
TAAGAAGCAG	2	2	0	0	1	At3g19120.1:d:+1437:primary
TCTTCAGCAA	1	4	0	0	0	At3g18900.1:i:+1074:tertiary
CCTTTGAACT	0	0	2	3	0	At3g18620.1:d:+1805:primary
GACAGGTCCG	0	0	1	3	1	At3g18420.1:d:+661:primary
CATCTGCATT	0	3	1	0	1	At3g18390.1:d:+2149:primary
AACCTTGGCT	1	0	0	2	2	At3g18240.1:d:+1362:primary,At3g18240.2:d:+1451:primary
TTACAATGAT	1	1	1	0	2	At3g18215.1:d:+928:primary
TTCATATAAT	1	1	1	1	1	At3g17510.1:d:+1691:secondary,At3g17510.2:d:+1232:secondary
CGGTCAATGT	1	2	1	0	1	At3g17310.1:d:+2692:primary,At3g17310.2:d:+2582:primary
AAGAAGCTCA	0	3	1	1	0	At3g16940.1:d:+2682:primary
CCTCAACCTA	0	1	1	1	2	At3g16830.1:d:+3329:primary
CCACTCACCA	2	3	0	0	0	At3g16450.2:d:+941:primary,At3g16450.1:d:+993:primary
TCAATCGAGC	1	2	0	1	1	At3g16080.1:i:-481:tertiary
TTCAATTAGT	0	3	1	1	0	At3g15950.1:d:+2396:primary
GAGGAGCAAA	0	2	2	0	1	At3g15570.1:d:+1557:primary
GTGCCCAGAA	0	2	3	0	0	At3g15360.1:d:-400:primary
AAGCCAAGGA	0	2	0	3	0	At3g15090.1:d:+1150:primary
CCAAGAGAGC	1	2	1	0	1	At3g14920.1:d:+1406:primary
AGGAGCAGCA	1	0	2	2	0	At3g14860.2:d:+1515:secondary,At3g14860.1:d:+1525:secondary
AGAGGCCATT	0	2	1	0	2	At3g14840.2:d:+2446:secondary,At1g53420.1:v:+2748:secondary
AGCTCAAAAA	3	0	0	2	0	At3g14720.1:d:-287:secondary
GTTTGGACCC	1	2	1	0	1	At3g14660.1:d:+480:secondary
TGGGCTTCTT	0	3	1	1	0	At3g14590.1:v:+1611:secondary,At1g27930.1:d:+674:secondary
ATGAAAATGG	1	3	1	0	0	At3g14290.1:d:+538:primary
ACTTACATCT	2	0	1	1	1	At3g14205.1:X:+347:quaternary
CCATATCATA	1	2	0	2	0	At3g14010.1:d:+2029:primary
TGTTTGTATA	2	1	1	0	1	At3g13890.1:v:+1865:primary,At5g05610.2:d:+1012:primary,At5g05610.1:d:+1107:primary
TAAACACATT	0	0	0	0	5	At3g13790.1:d:+1804:primary

TTGATGGTTT	2	1	1	1	0	At3g13480.1:v:+559:secondary
TAATGGATAC	1	3	0	1	0	At3g13450.1:d:+1293:primary
TTTTGTGTTG	2	1	1	1	0	At3g13440.1:d:+1904:primary
TTCTTTAAAA	2	0	2	0	1	At3g13070.1:i:+3072:tertiary
GAATCGCTTC	0	4	1	0	0	At3g12830.1:d:+424:secondary
TTTGTTAGTG	2	1	0	0	2	At3g12700.1:d:+1534:primary
AATCCGTAAG	0	1	3	1	0	At3g12630.1:d:-829:secondary
TTAATAACAA	2	3	0	0	0	At3g12610.1:d:+1144:secondary
TATCATTGGA	0	5	0	0	0	At3g12570.1:d:+1615:primary,At3g12570.2:d:+1894:primary,At3g12570.3:d:+1722:primary
CTTTAACTTT	0	1	3	0	1	At3g12300.1:d:+788:secondary
GTTCTTTAGG	0	2	1	0	2	At3g12100.1:d:+1301:primary
ACAGCCCATC	2	3	0	0	0	At3g12020.1:v:+3172:secondary
TACATACATC	2	1	0	0	2	At3g11950.1:d:+2956:primary
TATTGCCATT	1	3	1	0	0	At3g11820.1:d:+1112:primary,At3g11820.2:d:+1012:primary
AATTTACACG	2	0	0	2	1	At3g11590.1:d:+2192:primary
TTCTCGAAAA	1	2	1	1	0	At3g11380.1:v:+2141:primary
TGAAGAGGGT	2	0	1	2	0	At3g11270.1:d:+1098:primary
AGCTTAACTT	0	2	1	2	0	At3g10915.2:d:+937:primary,At3g10915.3:d:+989:primary,At3g10915.1:d:+944:primary
TATAAACCGG	0	3	1	1	0	At3g10770.2:d:+812:primary,At3g10770.1:d:+812:primary
ATACACATAT	1	1	0	0	3	At3g10520.1:d:+625:secondary
GCCTTTAAAA	0	1	3	0	1	At3g10450.1:d:+1499:primary
TTTCTCGGAT	2	2	0	1	0	At3g10370.1:d:+2171:primary
GTTGTTCTTG	2	2	0	0	1	At3g10050.1:d:+1872:primary
AGGAAAGAAG	0	3	2	0	0	At3g09980.1:d:+425:primary
CTACATCGAA	0	0	1	1	3	At3g09730.1:v:+2018:primary
TCGGATTCAA	1	0	1	1	2	At3g09720.1:d:+1944:primary
AGATAATACT	1	1	1	0	2	At3g09650.1:d:+2454:primary
AAAACTAGTC	1	3	0	1	0	At3g09410.2:X:--34:quaternary
CTTATCTTCT	1	2	0	1	1	At3g09140.1:v:+2013:primary,At1g69340.1:d:+1966:primary
ATCAATGACG	1	2	1	0	1	At3g08920.1:d:+393:primary
AATGGTTGTA	2	0	2	1	0	At3g08680.2:d:+2527:primary,At3g08680.1:d:+2452:primary
CTCAGAGAGG	1	0	1	0	3	At3g08505.2:d:+1177:primary,At3g08505.1:d:+1147:primary
GGGTGAGATT	0	1	3	0	1	At3g07790.1:d:+1217:primary
AGAGAGTAAG	0	4	0	1	0	At3g07670.1:d:+1787:primary,At5g64160.1:d:+376:primary
AAGGAGATTT	0	3	1	0	1	At3g07660.1:d:+2886:primary
AGTAGGGTCG	1	2	2	0	0	At3g07565.1:v:+1333:primary
AAGCTAAAGG	1	2	0	1	1	At3g07130.1:d:+374:secondary,At5g16730.1:d:+1861:secondary
GAGTTCTCTT	1	2	0	0	2	At3g06780.1:d:+150:secondary
GTTATACTCC	1	1	1	1	1	At3g06720.1:d:+1921:primary,At3g06720.2:d:+1847:primary
TTTGCAGAAA	0	0	0	1	4	At3g06530.1:v:+6005:secondary
TATATAGTCC	1	2	1	0	1	At3g06170.1:d:+1254:primary
GTCCTGCACC	1	2	1	1	0	At3g06080.2:d:+1707:primary,At3g06080.1:d:+1841:primary
CAGGAAATGA	0	1	2	0	2	At3g05937.1:v:+1160:primary
GAAAATGTAC	0	3	1	1	0	At3g05910.1:d:+1294:primary
GATTATAATG	1	2	1	0	1	At3g05400.1:d:+1256:secondary
GAAAAGTTTC	1	1	3	0	0	At3g05370.1:d:+2524:primary,At5g23990.1:v:+2792:primary
TGCCAAGAGG	0	4	0	1	0	At3g05200.1:d:+1495:primary
GAGGTAGTTT	0	3	0	1	1	At3g05060.1:d:+995:secondary,At3g29770.1:d:+561:secondary
TTTGAAATGA	2	1	0	1	1	At3g05000.1:d:+720:primary
GCCCAAACAA	0	1	1	3	0	At3g04890.1:d:+750:secondary
CCTAGCTCAA	0	5	0	0	0	At3g04810.1:d:+1210:primary,At3g04810.2:d:+1126:primary
TGCTTCAGCA	0	2	1	2	0	At3g04780.1:d:+369:primary
TATCAATAAC	1	1	0	0	3	At3g04000.1:d:+917:primary
AATCAAAACA	2	0	1	1	1	At3g03650.1:d:+545:secondary
CATTGTTTTG	0	2	1	1	1	At3g03270.2:d:+551:primary
CTCTTTTGTG	1	2	2	0	0	At3g02920.1:i:+1763:tertiary
AAGACAGGTG	1	3	1	0	0	At3g02830.1:d:+1089:primary
TGTGACTTGA	1	0	1	1	2	At3g02540.1:X:-60:quaternary
GATGCCATCT	3	0	1	0	1	At3g02450.1:d:+1991:primary
TCGAGGCATC	1	0	2	2	0	At3g02150.2:d:+1190:primary,At2g16280.1:d:+1537:primary
GGTGTTGTTT	0	2	2	1	0	At3g01910.1:d:+1165:secondary

TGTCTCCTGC	1	1	0	2	1	At3g01370.1:d:+3012:secondary
AAGCTTCTAT	2	0	0	0	3	At3g01345.1:d:+1509:primary
TAAGCTCCTT	3	0	2	0	0	At3g01180.1:d:+2669:primary
TCCTGGAGCT	0	3	1	1	0	At2g48020.2:d:+1447:primary,At2g48020.1:d:+1463:primary
GTAATGTCTA	1	1	0	0	3	At2g47990.1:d:+1754:primary
TAATATTAAT	0	3	1	0	1	At2g47930.1:d:+577:primary
TCCACCGCCA	0	0	3	1	1	At2g47730.1:d:+191:secondary
TCTCAGTTTT	1	1	1	0	2	At2g47600.1:d:+1852:primary
TAAACCCAAT	3	1	0	0	1	At2g47450.1:d:+1547:primary
ATGCTGGCTG	1	1	1	0	2	At2g47450.1:d:+1422:secondary
TGCATATAAA	1	1	2	0	1	At2g47440.1:X:+1403:quaternary
TAAAAGCTTT	1	3	0	0	1	At2g47320.1:d:+748:primary
CAATAAGATT	1	0	0	1	3	At2g47115.1:d:+992:secondary
TAAATTTTCC	2	0	1	2	0	At2g46735.1:d:+859:secondary
GTGGCCGTGA	0	2	2	0	1	At2g46690.1:d:+494:primary
GACGAACATC	3	1	0	1	0	At2g46650.1:d:+257:primary
AACATTCTAC	0	0	0	2	3	At2g46510.1:X:-2506:quaternary,At5g06760.1:X:-576:quaternary
TTAATTTTGT	0	4	0	1	0	At2g46440.1:d:+1840:primary
GACTTCGTTC	1	1	1	1	1	At2g46260.1:d:+1733:primary
ATTTTGGTCT	1	2	0	1	1	At2g46225.1:d:+1208:primary
GAAACCCATT	1	0	2	2	0	At2g46100.1:d:+613:secondary
TTTGAATGAT	1	1	1	1	1	At2g46070.1:d:+1185:primary
AAAAGATCAC	0	3	1	0	1	At2g46060.2:d:+2733:primary,At2g46060.1:d:+2690:primary
TAATGAAAAA	1	1	3	0	0	At2g45920.1:d:+1436:primary
GGCCTTCATA	1	0	1	0	3	At2g45850.2:d:+1615:secondary,At2g45850.1:d:+1747:secondary,At4g04525.1:v:+354:secondary
TCTTGAAACA	1	0	0	3	1	At2g45790.1:d:+833:secondary
TTTTACGAAC	2	0	1	0	2	At2g45600.1:d:+1220:primary
ATGTTGTTGT	0	5	0	0	0	At2g45130.1:d:+817:primary
ATGATTGCTC	0	1	2	2	0	At2g44530.1:d:+1219:primary
TGATGTTGAT	1	0	2	1	1	At2g44300.1:d:+711:primary
GGGATCTCAA	0	2	1	1	1	At2g44150.1:d:+1568:primary
ACTGTTGGAC	1	0	2	2	0	At2g44120.2:d:+874:secondary,At2g44120.1:d:+647:secondary,At3g06420.1:d:+294:secondary
TTTTGACCTC	2	1	1	0	1	At2g44040.1:d:+1253:primary
CTCACTTGTT	0	2	1	2	0	At2g43900.1:d:+4026:primary
CTTCGTTTTG	0	1	0	3	1	At2g43650.1:d:+2179:primary
TGGTTCTGGA	0	0	1	4	0	At2g43620.1:d:+673:primary
GTGTTATAAA	1	0	1	1	2	At2g43240.1:d:+2541:primary
GACGTGCTTG	1	3	0	1	0	At2g42980.1:v:+837:secondary,At4g00300.1:d:+2302:secondary
GTGTCTTCCC	1	0	0	3	1	At2g42910.1:d:+964:primary
ATGATGGTCA	0	0	0	4	1	At2g42890.1:d:+2534:secondary,At2g42890.2:d:+2412:secondary,At1g56380.1:v:+1269:secondary,At1g54410.1:d:+368:secondary
ATAATTAAAG	0	4	0	0	1	At2g42810.1:d:+1760:primary
GCGATGTCTT	0	0	2	0	3	At2g42540.2:d:+88:secondary,At1g43030.1:p:+3972:secondary,At2g42530.1:d:+76:secondary
GCCTTTCTAT	0	1	1	2	1	At2g42300.1:d:+1474:primary
GTATTTGATT	0	0	0	3	2	At2g41945.1:d:+722:primary
ATATTTTGTG	2	1	1	0	1	At2g41710.1:d:+1540:primary,At2g41710.2:d:+1555:primary
GAGAGCCTAT	3	0	0	2	0	At2g41705.1:d:+1570:primary
CCTCTCTCTT	1	3	1	0	0	At2g41440.1:i:+5718:tertiary
TTTGATTGAT	0	2	2	1	0	At2g41410.1:d:+808:secondary
TCTAAGATCT	3	0	1	1	0	At2g40750.1:d:+313:secondary
AATTGATTTA	0	0	1	4	0	At2g40610.1:d:+1089:primary
GTGTTCTTAT	1	1	2	0	1	At2g40475.1:d:+921:secondary
GGACTGGAGA	1	1	2	1	0	At2g40410.1:d:+1034:primary,At2g40410.2:d:+1037:primary
GCTATTCACT	1	1	1	0	2	At2g40360.1:d:+2269:primary
ATGGAAAAAA	2	1	2	0	0	At2g40090.1:d:+606:secondary,At4g17850.1:v:+555:secondary
TGGAAGATCC	0	4	0	1	0	At2g39630.1:d:+1090:primary,At2g39630.2:d:+1092:primary
ATAGAACATT	2	2	0	1	0	At2g39620.1:v:+3109:primary
AAGTAGGTCC	0	3	1	1	0	At2g39470.1:d:+460:secondary
TGGAATTGAA	0	2	1	2	0	At2g39140.1:d:+1242:primary
AAGCAACTTG	0	0	2	2	1	At2g38950.1:d:+2422:primary
AAGCTTATCA	0	0	2	1	2	At2g38670.1:d:+1296:primary

GCTCCTTCGC	1	1	1	2	0	At2g38240.1:d:+2357:primary
TCAAAAGTTT	2	2	1	0	0	At2g38080.1:d:+1895:primary
TGGATTTTGA	1	1	1	1	1	At2g37690.1:d:+2342:primary
CGATGCACGT	1	3	0	0	1	At2g37680.1:i:+2071:tertiary
CTACGTTGCA	1	2	2	0	0	At2g37540.1:d:+960:primary
TTCTTGGGAC	0	0	2	2	1	At2g37520.1:d:+2949:primary
TATTGTGAAC	0	3	1	0	1	At2g37500.1:i:+2736:tertiary
GGGATGCGAT	2	1	0	1	1	At2g37500.1:d:+1422:secondary
AAGAGAAACT	1	1	0	2	1	At2g37280.1:v:+1860:secondary,At1g76740.1:d:+1745:secondary,At3g53480.1:d:+1674:secondary,At2g03310.1:d:+492:secondary
TGGAATACAT	1	2	0	0	2	At2g37250.1:d:+949:secondary
TTTTTGTTGC	3	0	0	0	2	At2g37080.1:d:+2131:primary
GGAATGCTTG	1	1	1	2	0	At2g37050.1:d:+2980:primary
GATTTAGTGA	1	3	0	1	0	At2g36720.1:d:+3417:primary
TAAGATGTGT	1	3	1	0	0	At2g36410.2:d:+925:primary,At2g36410.1:d:+934:primary
TGGGGTCCAG	0	2	1	1	1	At2g36220.1:d:+896:primary
GAAGCAAGTA	3	1	1	0	0	At2g36000.1:d:+1140:primary
GGATTCCTAT	1	2	0	1	1	At2g35940.1:d:+2184:primary,At2g35940.2:d:+2056:primary
TTCTATTTGT	4	1	0	0	0	At2g35860.1:d:+1560:primary
CTTTTAAGGA	2	0	2	1	0	At2g34780.1:v:+787:secondary
GACTTTGAAG	4	0	0	0	1	At2g34640.1:d:+1692:primary
GCCCATCTGC	0	2	3	0	0	At2g34420.2:X:-365:quaternary
TCAATGCAAA	2	1	1	1	0	At2g33740.2:d:+455:primary
ATTCTGCTTG	1	1	2	1	0	At2g33340.2:d:+469:secondary,At2g33340.1:d:+469:secondary
CCACTATCAA	0	2	1	2	0	At2g33170.1:d:+3454:primary
AGTGACCTCA	3	0	1	1	0	At2g32810.1:d:+2653:secondary
GATCAAACTC	3	0	2	0	0	At2g32770.2:d:+1895:secondary
GTTTTTTAAA	1	2	0	0	2	At2g32400.1:d:+135:secondary,At1g36600.1:p:+1521:secondary
TTGCAGAGCT	0	5	0	0	0	At2g31350.2:d:+952:primary,At2g31350.1:d:+955:primary
ACTTGAAACT	0	0	2	2	1	At2g31280.1:d:+2972:primary
CATTGAACAA	0	3	0	1	1	At2g31070.1:d:+1666:primary
ACCCAAGTTA	1	1	0	2	1	At2g31050.1:v:-1415:secondary
TTTCAGCCAA	0	2	0	0	3	At2g30970.1:d:+1473:primary
GACGCAGAGA	1	2	1	1	0	At2g30720.1:d:+1446:primary
CTTTGGTCAA	1	2	2	0	0	At2g30550.2:d:+1724:primary
TCTCATTGAT	1	3	0	1	0	At2g30010.1:d:+1122:primary
GAAAAGTCTG	0	1	3	0	1	At2g29960.1:X:-335:quaternary
TTTGTTTTCT	0	3	1	1	0	At2g29620.1:v:+3011:primary,At5g49830.1:d:+2640:primary,At1g75850.1:d:+2619:primary
GTTCATAGAA	2	1	2	0	0	At2g29360.1:d:+621:primary
CTTGAGAAAT	1	2	1	1	0	At2g29320.1:d:+838:primary
TTAGGAGCAA	1	0	2	0	2	At2g29170.1:v:+471:secondary
ACCACCAAAC	1	0	2	1	1	At2g28605.1:d:+722:primary
GAGAAAGAGG	1	2	1	1	0	At2g28390.1:d:+618:secondary,At3g11760.1:v:+1376:secondary,At3g51890.1:d:+352:secondary
TGGTGTGATT	3	0	1	1	0	At2g28190.1:d:+670:primary
ATTTGTAAGA	0	2	0	1	2	At2g28100.1:d:+1654:primary
TTGTTGTAAG	5	0	0	0	0	At2g27810.2:d:+2440:primary,At2g27810.1:d:+2587:primary
TCTAGTAAAC	1	1	1	0	2	At2g27650.1:i:+2609:tertiary
GACCTAGTCA	0	3	0	1	1	At2g27450.1:d:+805:primary,At2g27450.2:d:+794:primary
TAAGAAGACA	0	3	2	0	0	At2g27402.1:d:+542:primary
CCTCGACTCT	1	2	0	2	0	At2g27360.1:d:+1157:primary
AATTTCAAAA	0	2	2	1	0	At2g27320.1:i:+830:tertiary
TTGGATTCAT	1	1	1	2	0	At2g27060.1:d:+3064:primary
GAAGATCTGC	1	0	2	1	1	At2g26990.1:d:+1205:primary
TTGAACGGTT	1	2	0	2	0	At2g26800.1:d:+1298:primary,At2g26800.2:d:+1446:primary
CTTGCACGAA	0	3	1	1	0	At2g26770.1:d:+1571:primary,At2g26770.2:d:+1524:primary
AACAAAAGAG	1	2	1	0	1	At2g26570.1:d:+2221:primary
AACAAAAAGC	2	2	1	0	0	At2g26540.1:d:+1317:primary
CATACGAGTT	0	2	2	1	0	At2g26300.1:d:+1271:primary
TCGCTAGGAC	2	3	0	0	0	At2g26110.1:d:+623:secondary
TGCTGTGGTC	0	3	2	0	0	At2g26070.1:d:+824:primary
TAAACGTATT	0	1	1	0	3	At2g25930.1:d:+2598:primary

CCTTGTCACA	0	1	1	1	2	At2g25670.1:d:+1614:primary,At2g25670.2:d:+1732:primary
TCCATTGTTG	0	1	2	2	0	At2g25650.1:d:+1507:primary
ATGTTTGGTC	1	0	1	2	1	At2g25480.1:d:+1567:primary
GTGGTGATGA	2	2	1	0	0	At2g23950.1:d:+320:secondary
AAGAGAGATG	0	1	0	2	2	At2g23910.1:d:+991:primary
TTTACTTGTT	2	1	2	0	0	At2g23320.2:d:+1189:primary,At2g23320.1:d:+1191:primary
TTCAAAAGAT	1	2	2	0	0	At2g22980.1:d:+1033:primary
AAGAAGCTGT	1	1	1	1	1	At2g22910.1:d:+1887:primary
AATTTGTTTC	0	4	1	0	0	At2g22880.1:v:+737:secondary
GGAGGTCCAC	0	0	3	1	1	At2g22840.1:d:+1457:primary
TGGCTTAATA	2	2	1	0	0	At2g22660.1:d:+2632:secondary
ACTTGCTCGT	0	3	1	1	0	At2g22360.1:d:+926:primary
GGCCACTGAT	0	1	0	2	2	At2g21660.1:d:+108:secondary,At2g21660.2:d:+108:secondary
GCCAGCTGTT	1	2	0	2	0	At2g21340.1:d:+815:secondary,At3g22290.1:d:+982:secondary,At2g21340.2:d:+806:secondary
ATCACGGTGG	1	4	0	0	0	At2g21185.1:d:+235:primary
GAGACTAGCG	2	2	1	0	0	At2g21170.1:X:-90:quaternary
GAAGGATATC	1	1	2	1	0	At2g20890.1:d:+488:secondary
TGTGCATTTA	2	2	0	1	0	At2g20740.3:d:+485:primary,At2g20740.1:d:+901:primary,At2g20740.2:d:+771:primary
TAATGATCCA	4	0	1	0	0	At2g20180.1:d:+1852:primary,At2g20180.2:d:+1780:primary,At3g10890.1:v:+1929:primary
TGAATGATGC	0	2	2	1	0	At2g20140.1:d:+1366:primary
GGAGGTTTTT	0	4	0	0	1	At2g19810.1:d:+1262:primary
AAGCACAAGG	0	2	1	1	1	At2g19270.1:d:+1014:primary
TTAGATAAAA	0	1	0	1	3	At2g18230.1:d:+865:primary
CATCTTTGAT	3	0	1	1	0	At2g18160.1:d:+1066:secondary
AAAAAAGTAT	3	1	0	0	1	At2g17790.1:d:+2758:primary
TAAATTCCAG	0	2	3	0	0	At2g17730.1:d:+1134:primary
TGTTGTTGAT	1	1	1	0	2	At2g17050.1:v:+3374:secondary
GGTTGTCAAA	1	0	1	3	0	At2g16920.1:d:+3604:primary
ACAACAAAAA	0	0	4	1	0	At2g15610.1:v:+1321:secondary
AAGATCCTGC	0	4	0	1	0	At2g15560.1:d:+1601:primary
AGTTTACTCT	0	1	2	0	2	At2g15320.1:d:+1240:primary
ACCCCTCTTA	2	0	1	1	1	At2g15240.1:d:+1048:secondary
GACACAAGTG	1	0	2	1	1	At2g14740.1:d:-2083:secondary,At2g14740.2:d:-1990:secondary
AAATGGTTTA	0	1	0	1	3	At2g14280.1:p:+265:secondary,At3g10120.1:d:+499:secondary,At4g26300.1:d:+2139:secondary
CATAAACCCT	1	0	1	2	1	At2g14255.1:d:+898:primary
TGTCACAGTT	2	1	0	0	2	At2g13840.1:d:+1346:primary
TGTTTAATTA	0	1	0	1	3	At2g13610.1:d:+2064:primary
GAGAAGTTGG	1	2	0	1	1	At2g13540.1:d:+2624:primary
TATTCACAAA	2	1	1	1	0	At2g13300.1:p:+2226:primary,At5g51180.1:d:+1633:primary,At5g51180.2:d:+1532:primary
TTTCCCGCTT	1	3	1	0	0	At2g07050.1:d:+2711:primary
GGAATTGATT	0	1	1	3	0	At2g06922.1:p:+986:primary,At1g11400.1:d:+985:primary,At1g11400.2:d:+1021:primary
GAGGAAGAAG	0	2	2	0	1	At2g06210.1:d:+3262:primary,At2g06210.2:d:+3342:primary
TTTCTTGTAA	1	1	3	0	0	At2g05990.1:d:+1555:primary,At2g05990.2:d:+1588:primary
AGAAAATCCC	1	2	2	0	0	At2g05830.2:d:+809:secondary,At2g05830.1:d:+789:secondary
TATTGCATTA	1	1	1	0	2	At2g05520.1:d:+526:secondary
ATAGTTTTTC	1	3	0	1	0	At2g05410.1:v:+385:secondary
ACCATCAAAA	1	0	1	2	1	At2g04842.1:v:+2419:secondary
GTTTGTGTTT	3	1	0	1	0	At2g04795.1:d:+478:primary
GGAAAGAACT	1	3	1	0	0	At2g04750.1:v:+905:secondary,At2g28950.1:d:+867:secondary
ATAAACCACC	1	1	1	1	1	At2g04360.1:d:+1111:primary
TTCTTGTTTT	1	1	0	3	0	At2g03760.1:d:+1216:primary
AACAAAGCAG	2	2	0	1	0	At2g03640.1:d:+1524:primary
AATTTATTTG	2	1	1	1	0	At2g03350.1:d:+697:primary
TTTGATTAAA	1	1	2	1	0	At2g03310.1:d:+803:primary
TAACTTCTTA	1	3	0	0	1	At2g03220.1:d:+1777:primary
ATATATGGAA	4	1	0	0	0	At2g03140.1:d:+5673:secondary
GGGTAGGTGA	0	1	0	4	0	At2g02970.1:d:+2121:primary
CCGTTTTGGT	0	3	1	1	0	At2g02590.1:d:+830:primary,At4g15920.1:d:+536:primary

TTTTCTCTAT	3	1	0	0	1	At2g02560.1:i:+3709:tertiary,At5g35950.1:i:+1322:tertiary,At5g63910.1:i:+1498:tertiary,At5g07940.1:i:+5531:tertiary,At5g11750.1:i:+791:tertiary
AAAAATGTTT	0	2	0	2	1	At2g02500.1:d:+989:primary
AAAAACAAAA	1	1	1	1	1	At2g02480.1:d:+3886:primary
TGTAACCACT	1	0	1	0	3	At2g02090.1:d:+2562:primary
CTTGGAACCG	1	1	0	1	2	At2g01970.1:d:+1774:primary
AATTTTTATT	2	1	1	1	0	At2g01810.1:v:+2805:primary
ATTGTTAAAA	0	3	0	2	0	At2g01710.1:d:+1073:primary
TTTCCCGGAA	0	1	3	0	1	At2g01340.1:d:+923:primary
ATTTTGTGGA	2	1	1	0	1	At2g01175.1:d:+1099:secondary,At2g11170.1:p:+3101:secondary
CCAGAAGAAG	3	1	0	1	0	At2g01090.1:d:+84:primary
TGGGGACTGG	2	1	0	1	1	At1g80790.1:d:+1839:primary
AGTTCAGGGC	0	4	1	0	0	At1g80750.1:d:+252:primary
GGTACTCATT	1	0	0	4	0	At1g80720.1:d:+921:primary
CAGAATCCAT	0	1	3	0	1	At1g80710.1:d:+1542:secondary,At3g54620.1:d:+1198:secondary,At1g18485.1:d:+2331:secondary
TCATCTGCGG	0	2	1	1	1	At1g80620.1:d:+998:primary
TATTGTCTGT	3	1	1	0	0	At1g79830.1:d:+3062:primary
TTGCTACCAA	1	2	2	0	0	At1g79750.1:X:-330:quaternary,At5g61250.1:X:-91:quaternary
GTTACATAAG	4	0	0	0	1	At1g79640.1:v:+2785:primary
TAATGCCAGC	1	3	0	0	1	At1g79560.1:d:+2884:secondary
CTTAGTTTCC	1	1	3	0	0	At1g79190.1:d:+2916:secondary,At2g14170.1:d:+1925:secondary
CAGAGTTTGC	1	1	1	2	0	At1g79090.1:d:+2438:primary,At1g79090.2:d:+2516:primary
TTATAATGAA	3	0	2	0	0	At1g79000.1:d:+2967:secondary
GATTAATGGT	0	4	1	0	0	At1g78920.1:d:+2957:primary
TTATCAAACT	2	0	2	1	0	At1g78810.1:d:+1604:primary,At1g78810.2:d:+1661:primary
TATTCTCAGT	0	2	1	1	1	At1g78620.2:d:+1086:secondary,At1g78620.1:d:+1059:secondary
GACGTTAACT	0	1	4	0	0	At1g78590.1:d:+890:primary
TAACCCAATA	0	1	1	2	1	At1g78580.1:d:+2945:secondary
TGAATGTTGT	1	1	0	2	1	At1g78290.1:d:+2169:primary,At3g09180.1:d:+1543:primary,At1g78290.2:d:+2068:primary
TCACACGATT	2	3	0	0	0	At1g78270.1:d:+1643:primary
AAAACTAAAA	0	1	1	1	2	At1g77080.6:v:+1185:primary,At1g77080.5:d:+581:primary
TGAGACTAAA	1	0	3	0	1	At1g77010.1:v:+307:secondary
ACAACAGAAA	0	2	1	2	0	At1g76920.1:d:+1358:primary
CTTTGCAGAG	0	5	0	0	0	At1g76850.1:d:+3185:primary
GATACATTAT	0	1	2	0	2	At1g76690.1:d:+1313:primary
TTTTGTTAAT	1	1	0	0	3	At1g76300.1:d:+655:primary
GATGAACAAA	2	1	1	1	0	At1g76260.1:d:+472:secondary
CCGGATGAAG	2	0	2	0	1	At1g76150.1:d:+1171:primary
TGTTTTGGGA	0	0	1	1	3	At1g75900.1:d:+1124:secondary
TCTTGCAGGC	1	2	2	0	0	At1g75760.1:d:+1096:primary
GTGGTGTGTG	1	0	1	1	2	At1g75580.1:d:+645:primary
GGTTTGGTAT	3	0	2	0	0	At1g75410.1:d:+1707:secondary
GAGATGGTGA	1	2	1	1	0	At1g75390.1:d:+1156:primary
TATGATTGTA	3	0	0	2	0	At1g74530.2:i:+1051:tertiary,At1g74530.1:i:+1051:tertiary,At4g00070.1:i:+390:tertiary
GACACAATGA	1	2	1	1	0	At1g74250.1:v:+2337:secondary
TTTCAGCAAC	0	1	2	0	2	At1g73990.1:d:+1989:primary
ATCAAAAAGC	1	0	1	2	1	At1g73240.1:d:+1915:primary
AAGGCTCTTA	0	4	0	0	1	At1g72920.1:d:+577:primary,At1g72940.1:d:+617:primary
AGTGTGTTTG	0	3	1	1	0	At1g72750.1:d:+1034:primary
ATGAAGAGAG	2	1	0	1	1	At1g72420.1:X:--55:quaternary,At1g76980.1:X:-840:quaternary
GCCAGGAGAA	1	0	2	0	2	At1g72370.1:d:+847:secondary
TCTACCTCGT	1	0	2	2	0	At1g72340.1:d:-829:secondary
CAATGCTTTG	1	1	3	0	0	At1g72170.1:d:+450:primary
TCCTTCCTCC	1	1	2	1	0	At1g72060.1:d:+185:primary
CCTCCTCGGT	1	2	1	0	1	At1g72050.2:d:+1201:primary,At1g72050.1:d:+1434:primary
TGTAATGCGA	2	0	1	0	2	At1g71780.1:d:+841:primary
ACAAGTTCTA	1	0	2	2	0	At1g71490.1:d:+2151:secondary
AGTTTTTGGC	1	1	1	2	0	At1g71140.1:v:+1926:secondary,At1g32010.1:v:+1909:secondary
AACCGTGCAC	2	1	2	0	0	At1g71090.1:d:+1853:primary
AAGAAATTGC	1	0	3	0	1	At1g70900.1:d:+1126:primary

TTGTATGTTT	0	5	0	0	0	At1g70850.2:d:+1124:primary,At1g70850.1:d:+1120:primary
AATACATCAA	3	2	0	0	0	At1g70770.1:d:+1876:secondary
TAATGTGTTT	1	3	1	0	0	At1g70670.1:d:+973:primary,At1g59850.1:v:+2124:primary
GCTTGTGTCA	0	4	0	1	0	At1g70660.1:d:+428:primary
CTTACCTCGC	2	2	1	0	0	At1g70300.1:d:+2693:primary
GACCGTCCAC	0	2	0	1	2	At1g70070.1:d:+3532:primary
TAACTAAAAA	0	1	1	1	2	At1g69860.1:v:-2119:secondary
TTTTTTTTAT	1	0	0	1	3	At1g69640.1:d:+1210:primary,At5g13360.1:v:+2440:primary
AACTGAGAGA	1	1	1	1	1	At1g69570.1:d:+1264:primary
AGAACGGGTT	0	4	1	0	0	At1g69270.1:d:+1609:secondary
GGCATTGTAG	0	0	0	3	2	At1g68790.1:d:+3575:primary
TTCCTTTCGG	1	0	1	3	0	At1g68780.1:d:+1759:primary
TGTCCTATCG	3	1	0	1	0	At1g68760.1:d:+677:primary
TAACCTATGT	2	2	0	0	1	At1g68580.2:d:+2448:primary
GGAAAAGCTT	0	2	3	0	0	At1g67970.1:d:+1013:secondary,At1g22680.1:v:+530:secondary,At5g01230.1:d:+91:secondary,At5g01230.2:d:+140:secondary
ACATTACCAA	0	2	1	1	1	At1g67910.1:d:+833:secondary
TACATACAAG	3	0	2	0	0	At1g67530.1:d:+2550:primary
ACCGACCAGC	1	0	2	2	0	At1g67330.1:d:+1113:primary
AATGTGTTTT	0	3	0	1	1	At1g67300.2:d:+1665:primary,At5g38720.1:d:+1085:primary,At1g67300.1:d:+1679:primary
TATTGGTTGA	3	0	1	0	1	At1g66840.1:v:+862:secondary
TTCAGTGGGT	2	1	0	0	2	At1g66680.1:d:+1111:primary,At3g42725.1:d:+595:primary
CGGAAATGCT	3	1	1	0	0	At1g66530.1:d:+1791:primary
TATGACATTG	1	2	0	2	0	At1g66345.1:v:+331:secondary
ACCCCAACGA	0	0	2	2	1	At1g66330.1:X:-262:quaternary,At2g32460.1:X:-275:quaternary
TATGTTGTTG	0	3	0	0	2	At1g65390.1:d:+1254:primary,At1g65390.2:d:+1322:primary,At1g65380.1:d:+2325:primary,At2g02840.1:v:+887:primary
AGAATGGATA	1	0	3	1	0	At1g65290.1:d:+526:secondary
GAAATCTATA	2	2	0	1	0	At1g65140.1:v:+1153:secondary,At3g29170.1:d:+244:secondary
GAAGGGCCTT	0	1	3	1	0	At1g64970.1:d:+982:secondary,At3g21190.1:d:+1414:secondary
TTGATCTTTT	0	1	2	2	0	At1g64830.1:v:+1998:primary
TCTTGCTCCG	0	2	1	0	2	At1g64660.1:d:+1233:primary
AAAATGATCA	0	0	1	0	4	At1g63750.1:d:+1319:secondary
TTGGTGAATG	1	1	0	2	1	At1g63260.1:d:+1306:primary,At1g63260.2:d:+1098:primary
CTCAAATATT	0	2	0	2	1	At1g63010.1:d:+1740:primary,At1g63010.2:d:+1758:primary
ATGCCCTGAT	0	2	3	0	0	At1g62540.1:d:+1599:primary
TCCACAACCT	0	1	1	2	1	At1g62460.1:p:+296:secondary,At1g04350.1:d:+791:secondary,At4g09455.1:p:+296:secondary,At3g55610.1:d:+2474:secondary
TAATAGGCCA	0	3	1	1	0	At1g62300.1:d:+1566:primary
TAGGCAACGC	1	0	1	2	1	At1g62045.1:d:+470:primary
CTTACCACGA	0	1	1	2	1	At1g61870.1:d:+1586:primary
ATACTAGACT	0	2	2	0	1	At1g61790.1:v:+1015:primary
AGTGAGAGGA	0	1	1	1	2	At1g61780.1:X:-130:quaternary
TCCTTTTTTT	2	1	1	1	0	At1g61330.1:v:+1763:secondary
AGAACGAGCT	1	1	2	1	0	At1g61140.1:d:+3864:primary
TCACAACTGC	1	4	0	0	0	At1g60800.1:d:+1905:primary
AAGATCTCCA	1	3	1	0	0	At1g60220.1:d:+1364:primary
GAATTTGCTT	0	3	1	0	1	At1g60200.1:d:+1215:secondary
GACTGTTACT	1	0	0	2	2	At1g60000.1:d:+644:primary
GATTGGTTAC	1	3	1	0	0	At1g59910.1:d:+2943:primary
TTTGCTTCTT	3	1	0	1	0	At1g59580.2:d:+1311:secondary,At3g09240.1:v:+1881:secondary,At1g59580.1:d:+1374:secondary,At1g30790.1:d:+1228:secondary,At4g19140.1:d:+1003:secondary
AAGGTCTTAA	2	1	0	0	2	At1g58983.1:X:-167:quaternary,At1g59359.1:X:-167:quaternary,At1g58684.1:X:-167:quaternary
ATGAGAGAAA	1	0	2	1	1	At1g58410.1:d:+1489:secondary,At4g29670.1:d:+16:secondary
GAGACAGAAC	2	1	1	0	1	At1g58120.1:d:+1839:primary,At5g58620.1:d:+2036:primary
TCTTTTTAAA	1	0	2	2	0	At1g57770.1:d:+1872:primary
AATTTAGGTA	1	0	1	0	3	At1g57540.2:d:+582:primary,At1g57540.1:d:+473:primary
TATGAGTACA	2	1	1	1	0	At1g56710.1:d:+236:secondary
AAGTTGCTAA	3	0	0	1	1	At1g56340.1:d:+367:secondary,At1g80740.1:d:+1577:secondary
TAGCAATCCT	1	0	2	2	0	At1g55910.1:d:+1094:primary

TTTTAACCAA	0	4	0	0	1	At1g55860.1:d:+11574:primary
TAAATGATTA	1	0	2	1	1	At1g55600.1:i:+778:tertiary
GAGCTGAGAA	2	0	2	0	1	At1g55530.1:d:+1165:secondary
ACTCTGTTGA	2	0	1	0	2	At1g55500.1:d:+2331:primary
TCAATAATCT	5	0	0	0	0	At1g54370.1:d:+1748:primary
TTTGGTGTCC	1	1	2	1	0	At1g54350.1:d:+2238:primary
AATGGTGGTG	1	2	0	2	0	At1g54170.1:d:+1991:primary
TATATATACT	2	2	0	0	1	At1g54130.1:d:+2473:primary
TCATTCAATA	2	0	1	2	0	At1g53850.1:d:+957:primary
CTTGAGATTT	0	3	1	0	1	At1g53780.1:d:+988:primary
ACAAACCCGG	0	0	3	2	0	At1g53510.1:d:+1501:primary
GCTCCTGAGT	0	2	2	0	1	At1g53430.1:d:+2622:primary
CTTATGGCCC	0	3	1	0	1	At1g53290.1:d:+794:secondary,At3g14960.1:d:+790:secondary
GGAAGAGAAG	2	0	1	1	1	At1g53180.1:d:+823:primary,At3g57180.1:d:+1644:primary,At5g20130.1:d:+547:primary
AGCTCTCAAA	2	1	0	1	1	At1g52930.1:d:+929:primary
TTCTTGGATT	1	2	0	1	1	At1g52760.1:d:-1136:secondary
AGACCTTAAA	1	1	1	0	2	At1g52420.1:d:+2344:secondary
TAAGTTTTTC	0	3	1	1	0	At1g51805.1:i:+2542:tertiary,At4g33760.1:i:+2904:tertiary,At3g49210.1:i:+2762:tertiary
ATAAGTATGA	2	2	0	0	1	At1g51370.1:d:+392:secondary,At1g51370.2:d:+305:secondary
CCGTTTTCTT	0	2	2	1	0	At1g51350.1:i:+1515:tertiary
TCCTTCGTCC	2	0	0	2	1	At1g51070.1:d:+786:primary
GGTTAATATC	1	0	0	3	1	At1g50730.1:v:+3429:secondary
TACCTTGTGA	0	1	2	0	2	At1g50430.1:d:+925:primary
TTCTCCGGCA	1	1	1	2	0	At1g50320.1:d:+133:primary
CAACGAAAAT	1	1	1	2	0	At1g50200.1:X:-123:quaternary
TAAGAACGTT	1	0	1	0	3	At1g50170.1:d:+751:primary
GATTTGTAAT	1	1	1	2	0	At1g49820.1:d:+1436:primary
AGTGGAGCTT	0	2	2	1	0	At1g49670.1:d:+2022:primary
GTTATGGGAT	0	0	3	0	2	At1g49630.1:d:+2823:primary,At1g49630.3:d:+2823:primary,At1g49630.2:d:+2823:primary,At3g19170.1:d:+2847:primary
TTGCTAATCA	0	0	2	2	1	At1g49380.1:d:+1826:primary
TAAATTAGAA	0	0	0	0	5	At1g49005.1:v:+1027:primary,At4g39210.1:d:+1807:primary
TGTTATGCGT	1	0	1	2	1	At1g48950.1:d:+1921:primary
TAAAACTGAA	1	2	1	1	0	At1g48790.1:d:+1905:primary
GTACAGCGCC	4	0	1	0	0	At1g48430.1:d:+1838:primary
CTTTGAGAGG	1	0	1	2	1	At1g47830.1:X:+283:quaternary
TGTTGAGAAT	2	0	1	2	0	At1g47395.1:d:+179:secondary
CCTTCTTCCT	0	1	1	2	1	At1g46768.1:d:+794:primary
AAGGTGAGGC	0	4	1	0	0	At1g46480.1:v:+1114:primary
GTGGTCTTGT	0	1	2	1	1	At1g45145.1:d:+400:primary
GCTTATTTTG	1	0	2	1	1	At1g44960.1:d:+895:primary
TTAAGAGTTC	1	0	0	2	2	At1g44830.1:v:+1163:secondary
GGGAAATGGA	1	3	1	0	0	At1g44750.2:d:+1078:primary,At1g44750.1:d:+855:primary
AACTTTGGAA	1	1	2	1	0	At1g41893.1:p:+797:secondary,At3g42433.1:p:+112:secondary,At1g42100.1:p:+819:secondary
ATTTTGTAAA	0	1	0	2	2	At1g41803.1:p:+2206:secondary,At1g62490.1:v:+1643:secondary,At4g10760.1:d:+2183:secondary
AGCGAGGTTC	0	4	0	1	0	At1g35340.1:d:+220:primary
GCTGCGACGG	0	3	2	0	0	At1g35140.1:d:+780:primary
TTCTACAAAG	3	2	0	0	0	At1g34630.1:d:+1741:primary
AAAGGGTTCC	1	1	1	2	0	At1g34550.1:v:+2797:secondary
TGAAAGTATT	0	2	1	1	1	At1g34260.1:d:+4691:primary
CTATGGAGTT	0	3	1	1	0	At1g34210.1:d:+2270:secondary
GTTAGGAATT	1	3	0	1	0	At1g33470.1:d:+1739:primary,At1g33470.2:d:+1707:primary
CCTCTCTAGT	1	0	3	0	1	At1g32530.1:X:+721:quaternary
GTACATTTTG	0	2	2	1	0	At1g32360.1:d:+1621:primary
TTTTGGTACC	3	1	0	1	0	At1g32070.1:d:+773:primary
CTGATTTCCC	0	1	4	0	0	At1g32060.1:d:+1182:secondary
ACTCGAGAGA	0	1	2	1	1	At1g32050.1:X:+455:quaternary
GACTCGACGG	0	1	3	0	1	At1g31860.1:d:+677:primary
GACGCAATCA	0	2	1	1	1	At1g31850.3:d:+1879:primary,At1g31850.2:d:+1825:primary,At1g31850.1:d:+1934:primary
ATTTGAATAA	2	0	1	0	2	At1g31670.1:i:+3797:tertiary,At3g48030.1:i:+1115:tertiary
AAGCAGTGAA	0	0	2	2	1	At1g31500.1:d:+1266:primary,At1g31500.2:d:+1269:primary,At1g31500.3:d:+1343:primary

GAGGCTCTTC	1	2	0	2	0	At1g31380.1:v:+852:primary
GGCAGACCGG	1	3	1	0	0	At1g30490.1:d:+2581:primary
GGACAAAAGA	1	1	0	2	1	At1g30470.1:d:+2606:primary
GAGAAGGAAA	0	3	0	2	0	At1g30460.1:v:+2365:secondary
ATTGGATTCT	1	2	1	0	1	At1g30320.1:d:+1672:primary
CATATGTTTG	3	1	0	0	1	At1g30260.1:d:+481:primary
TCAAAGTTAA	0	0	2	1	2	At1g29930.1:X:+1094:quaternary
TAACGCTTGG	1	2	0	1	1	At1g29900.1:d:+3859:primary
CTCACCACGG	1	1	1	2	0	At1g29890.1:d:+1654:primary
GGTAAGCAGG	1	1	2	1	0	At1g29850.1:d:+173:primary
CAATTCTTGA	2	0	2	1	0	At1g29800.1:d:+1595:primary
CTTACTAATA	4	0	0	1	0	At1g29760.1:d:+1740:primary
AACCGAGCCA	0	1	3	1	0	At1g29520.1:d:+177:secondary,At2g46680.1:d:+1049:secondary
GTTTCCGTAG	0	0	4	0	1	At1g29070.1:d:+452:secondary,At1g19450.1:d:+1408:secondary
AGGCGTGTGG	0	2	0	1	2	At1g28440.1:d:+2947:primary
GAGAAAGGTA	0	3	2	0	0	At1g28400.1:d:+799:primary
GTCCGTGAAG	0	1	1	1	2	At1g28200.1:d:+839:secondary
TTATTGCACT	0	2	0	2	1	At1g28060.1:d:+2449:secondary
TCTATCCAAG	0	2	3	0	0	At1g28050.1:d:+1672:primary
CCACTAGACG	1	0	3	0	1	At1g27850.1:d:-3634:secondary
TAATGGATGT	1	1	2	0	1	At1g27595.1:d:+3525:primary
GGAAATAAAA	0	3	1	1	0	At1g27030.1:d:+1202:primary
GATTGTTTCC	1	3	0	1	0	At1g26940.1:d:+919:primary
TTTTGTGCGA	0	3	1	1	0	At1g26550.1:d:+225:primary
GTCTTTGCTT	0	5	0	0	0	At1g26270.1:d:+1442:primary
GGTTGTATAT	1	1	0	1	2	At1g26180.1:d:+1228:primary
GCTCCGGAAT	0	3	2	0	0	At1g26150.1:d:+2201:primary
CTCTAAGTTT	2	1	0	2	0	At1g25490.1:d:+1653:primary
TAAAAAACCG	1	0	1	2	1	At1g25170.1:i:+1490:tertiary,At1g24822.1:i:+1490:tertiary,At1g25097.1:i:+1490:tertiary
GACAGAAGAT	1	2	1	0	1	At1g24909.1:d:+510:secondary,At5g57890.1:d:+720:secondary,At1g25220.1:d:+695:secondary,At1g25155.1:d:+510:secondary,At1g25083.1:v:+860:secondary,At1g24807.1:v:+899:secondary
GAAATAAAGA	1	1	0	2	1	At1g24575.1:d:+462:secondary,At4g14370.1:v:+4567:secondary
TTTTTTCGGA	0	4	1	0	0	At1g24450.1:d:+654:primary
ATTTAGTGTT	4	0	0	0	1	At1g24050.1:i:+844:tertiary
TTGCAAAGAT	1	2	0	2	0	At1g23860.2:X:-75:quaternary
CTGTTTTCCA	2	2	0	1	0	At1g23860.1:d:+835:secondary,At1g49030.1:d:+407:secondary,At2g26210.1:d:+138:secondary,At1g23860.2:d:+833:secondary
TAGTTATAGC	0	1	1	3	0	At1g23760.1:d:+149:secondary
AATCTTTCTC	1	2	0	0	2	At1g23360.2:d:+1262:primary,At1g23360.1:d:+969:primary,At1g23360.3:d:+1087:primary
CAATAAACTT	0	4	1	0	0	At1g23110.1:v:+1118:secondary
AGGCCCGAGT	0	2	2	1	0	At1g23090.1:d:+1893:secondary
CCAATAATAA	0	4	1	0	0	At1g23030.1:d:+1932:primary
GTTTTTAATT	0	2	2	0	1	At1g22590.1:d:+529:primary,At1g22590.2:d:+534:primary
GTTCTTCTAA	1	3	1	0	0	At1g22360.1:d:+1455:primary
AAAGTCTGAA	0	3	2	0	0	At1g22300.3:d:+1577:primary,At1g22300.1:d:+1229:primary
GTTGTGCCGC	0	1	4	0	0	At1g22250.1:X:+508:quaternary
TTTCGTTGAA	2	0	1	1	1	At1g22210.1:d:+1184:primary,At3g32394.1:v:+995:primary
TAATGTCTTT	2	2	0	1	0	At1g22070.1:d:+1486:primary
TGAGGTTTCT	1	3	1	0	0	At1g21910.1:d:+1082:primary
ATGTAAATAT	2	3	0	0	0	At1g21730.1:d:+3166:primary
TTTGTATGTG	5	0	0	0	0	At1g21080.1:i:+3284:tertiary,At2g44110.1:i:+1318:tertiary,At2g44110.2:i:+1331:tertiary
TAAAGATCCT	1	3	0	1	0	At1g20640.2:d:+2964:primary,At1g20640.1:d:+3022:primary
CATAACGAAG	1	2	0	2	0	At1g20200.1:d:+1456:primary
CAGTGGTGGA	1	4	0	0	0	At1g19600.1:d:+1015:primary
GGCTCCTGGT	1	2	0	1	1	At1g19485.1:d:+1553:secondary,At4g34700.1:d:+293:secondary
AATCTTTGGT	0	2	2	1	0	At1g19220.1:d:+3570:primary
TAAAATATAT	4	1	0	0	0	At1g19150.1:d:+980:primary
TAATTTCTGT	1	1	1	0	2	At1g19110.1:d:+2505:primary
TTTTCTCAAA	3	0	1	1	0	At1g18980.1:d:+707:primary
GAGCAAATCT	0	1	3	1	0	At1g18950.1:X:+1247:quaternary

ATTCGGTCCT	1	1	2	1	0	At1g18730.1:d:-117:primary
ACAACATTCT	0	3	2	0	0	At1g18620.1:d:+3206:primary
AACAGTAATT	1	3	1	0	0	At1g18600.1:d:+1354:primary
ACCAACCATA	0	3	1	1	0	At1g18480.1:d:+1262:primary
TGACTAAAAA	0	1	3	0	1	At1g18180.1:i:+338:tertiary
TTTTGTTTAC	2	1	0	0	2	At1g18160.1:d:+3414:primary
GGAACTATCG	0	1	0	2	2	At1g18060.1:d:+834:secondary,At4g18420.1:v:+39:secondary
GAAGAATCGG	0	0	4	0	1	At1g18060.1:d:+513:secondary
TAAAACTCGT	2	1	2	0	0	At1g17980.2:d:+2578:primary,At1g17980.1:d:+2562:primary
AAAATTGATC	2	2	1	0	0	At1g17840.1:d:+2409:secondary
TTTTTGCAAT	3	1	0	1	0	At1g17580.1:d:+536:secondary,At3g58160.1:v:+739:secondary
GACTTGAATG	1	1	2	1	0	At1g17550.1:d:+2250:primary
AAAAGAAACA	0	0	3	0	2	At1g17540.1:v:+2920:primary
GGAAAAAGTA	0	4	0	1	0	At1g17210.1:d:+2966:primary
ATTTTTTGTC	2	2	0	1	0	At1g16970.1:i:+1638:tertiary
GATGGTGCCC	0	2	0	3	0	At1g16610.1:d:+572:primary,At1g16610.2:d:+533:primary
AGCAAACTCC	0	0	2	2	1	At1g15610.1:v:+481:secondary
CAGGTGAAGA	1	3	0	1	0	At1g15290.1:d:+4319:secondary
AAATTGAATA	1	1	1	2	0	At1g15130.1:d:+2709:primary
AGTATTTCTT	0	2	0	0	3	At1g14880.1:d:+613:primary
GCTTGCATTT	1	4	0	0	0	At1g14670.1:d:+1735:primary
AGATTCTTGA	0	3	1	1	0	At1g14590.1:d:+1210:primary,At5g37290.1:d:+675:primary
GCTTTAGCTT	0	1	1	1	2	At1g14320.1:d:+936:primary
TTTGAGCTAA	1	3	0	0	1	At1g13280.1:d:+1119:primary
GCCAGACAAC	0	1	0	2	2	At1g13270.2:d:+1151:primary,At1g13270.1:d:+1048:primary
GAAAGTGTAT	0	0	5	0	0	At1g13080.2:d:+1511:primary,At2g37280.1:v:+5032:primary,At1g13080.1:d:+1563:primary
AACCACTCAA	1	1	1	2	0	At1g12770.1:v:+1593:secondary
GCTCCAATGG	0	2	2	1	0	At1g12500.1:d:+806:primary
GGACAGTCAC	2	2	0	1	0	At1g12000.1:d:+1405:secondary
GTGATTTCTT	2	0	3	0	0	At1g11730.1:v:+895:secondary,At3g56940.1:d:+828:secondary
AGTACTTGGC	0	2	1	2	0	At1g11700.1:d:+576:primary
GAATTTCTAT	0	4	1	0	0	At1g11530.1:d:+120:primary
GCGAAAGCCG	0	3	1	1	0	At1g11480.1:d:+1725:primary
CGATGAAAAG	3	1	1	0	0	At1g11020.1:d:+1350:primary
ATGAAATGCT	2	0	0	1	2	At1g10900.1:X:+420:quaternary,At1g10900.1:X:+429:quaternary
GCTGTTTTGG	0	4	0	0	1	At1g10760.1:d:+3691:primary
GATCCTCAGG	0	5	0	0	0	At1g10760.1:d:+238:secondary
AAGATCACAA	2	0	0	2	1	At1g10710.1:v:+42:secondary
TCCTTAGATT	2	3	0	0	0	At1g10600.1:v:+1160:secondary
TACAAGGGTT	0	0	2	0	3	At1g10590.2:d:+332:secondary,At1g10590.1:d:+383:secondary,At1g10590.3:d:+380:secondary
TGTTAGTTCT	1	1	1	1	1	At1g10585.1:d:+606:primary
TGGATGAAGT	0	4	1	0	0	At1g10070.1:d:+1127:primary,At4g23895.1:d:+512:primary
GATTATATGG	0	1	0	2	2	At1g10030.1:d:+669:primary
TCGTTGCGGG	0	3	0	1	1	At1g09970.1:d:+2693:secondary,At1g09970.2:d:+2616:secondary
CCCTTGATGG	0	1	1	1	2	At1g09940.1:d:+2013:primary
TTATTTCATA	1	0	0	1	3	At1g09890.1:v:+2404:primary
TTACAGAAAA	0	4	0	0	1	At1g09850.1:d:+1383:primary
TAGTGTCCTC	0	1	1	2	1	At1g09815.1:d:+562:primary
GGCTCCCGGG	0	5	0	0	0	At1g09740.1:d:+468:primary
TAGGTGGAAC	2	2	0	1	0	At1g09645.1:d:+542:primary
ATGTATCATT	1	1	1	2	0	At1g09570.1:d:+3759:primary
CTGATGGCTG	0	4	1	0	0	At1g09570.1:d:+3247:secondary
GATTTAGGCA	0	4	0	0	1	At1g09280.2:d:+1105:secondary,At1g09280.1:d:+1123:secondary
TGAAACATTA	3	0	1	0	1	At1g08910.1:d:+2702:primary
ATTCGTTCCG	0	1	1	2	1	At1g08880.1:X:-384:quaternary
CAACTTTGTT	0	1	3	1	0	At1g08800.1:d:+3974:primary
CTAAACAAAA	2	1	1	0	1	At1g08790.1:v:+1407:primary
TTGGGTGTTG	1	1	2	1	0	At1g08700.1:d:+1408:secondary
CTGCCGAGCG	0	0	3	2	0	At1g08570.1:d:-1211:secondary
TCCTCCTCCT	0	2	0	0	3	At1g08510.1:d:+1696:secondary
GGATGAATAT	1	3	0	1	0	At1g08080.1:X:-342:quaternary

TTGGTCAGTG	0	1	0	2	2	At1g07830.1:d:+748:primary
TCTTGGTTCT	3	2	0	0	0	At1g07750.1:d:+968:primary
TGTGTAACAG	1	1	0	1	2	At1g07640.2:d:+1477:primary,At1g07640.1:d:+1357:primary
GGTTCAAACT	1	1	0	1	2	At1g07170.1:X:+494:quaternary
TCACGTTGCT	0	1	3	1	0	At1g06630.1:d:+1410:primary
CAGCGATGAT	0	1	0	3	1	At1g06550.1:d:+1276:secondary
TGGACCTTCC	0	1	2	2	0	At1g06470.2:d:+1754:primary,At1g06470.1:d:+1682:primary
TCTGAAGAGA	2	1	1	0	1	At1g06390.1:d:+1309:secondary,At1g35910.1:d:+499:secondary,At5g04780.1:v:+2297:secondary,At1g06390.2:d:+1281:secondary,At1g75170.1:d:+336:secondary
AGAGATTTAT	1	1	1	1	1	At1g06360.1:d:+936:primary
TTTTAATGGT	1	2	1	1	0	At1g06010.1:d:+387:primary
GTTTTTAGTC	2	0	1	1	1	At1g05850.1:d:+1391:primary
CTGGATTTAG	0	3	1	0	1	At1g05805.1:d:+1037:primary
AACAGGTTTC	4	0	1	0	0	At1g05790.1:d:+2076:primary
CTCTTGATCA	1	1	1	1	1	At1g05590.1:d:+1991:primary
GGACCTTCTC	0	4	1	0	0	At1g05575.1:d:+312:primary
TAAATGGCGT	2	1	1	1	0	At1g05570.1:d:+5812:primary
ACAAGACTAC	0	3	0	1	1	At1g05520.1:d:+2477:primary
AAAGCTATGG	0	3	1	0	1	At1g05010.1:d:+1082:secondary
GCTGAAAACA	0	4	1	0	0	At1g04990.1:d:+1513:primary,At1g04990.2:d:+1512:primary
GGTTTTGTCT	0	2	2	0	1	At1g04985.1:d:+785:primary
AACAAAAAGA	1	2	0	1	1	At1g04950.1:d:+625:secondary,At2g17120.1:d:+1405:secondary,At2g05080.1:v:+3670:secondary,At1g04950.2:d:+529:secondary
ATTGGATCTC	0	1	1	2	1	At1g04270.1:X:-89:quaternary
GTTCTCTTTT	1	4	0	0	0	At1g04140.1:d:+2387:primary,At1g04140.2:d:+2387:primary
ACGTGTTGAA	3	1	1	0	0	At1g04130.1:d:+1005:primary
TGGAGATCAA	0	0	3	2	0	At1g03380.1:d:+3453:primary
TGTGGAAAAA	2	1	0	2	0	At1g03370.1:d:+993:secondary,At5g38380.1:d:+404:secondary,At5g38380.2:d:+404:secondary
TGGTAAGTGA	2	0	3	0	0	At1g03130.1:d:+829:primary
AAATTGATAT	3	0	1	0	1	At1g02910.1:d:+1505:primary
AATGATTTCA	1	2	0	1	1	At1g02890.1:d:+3728:secondary,At5g51110.1:d:+640:secondary
GAGTCTGCAA	0	1	1	3	0	At1g02850.1:d:+1107:primary,At1g02850.2:d:+1188:primary,At1g02850.3:d:+1116:primary,At1g02850.4:d:+1107:primary
AAAGTTTTCT	1	0	1	3	0	At1g02720.2:d:+224:secondary,At1g02720.1:d:+224:secondary,At2g42630.1:v:+780:secondary,At4g04740.1:d:+2433:secondary
GACATTATCA	0	3	1	1	0	At1g02690.2:d:+818:secondary,At4g02150.1:d:+765:secondary,At1g02690.1:d:+818:secondary,At4g38250.1:d:+1029:secondary
TTTTGAGTGA	2	1	1	1	0	At1g02690.1:d:+1859:primary,At1g02690.2:d:+1862:primary
GAGTGGCAAG	0	0	3	1	1	At1g02475.1:d:+235:primary
TTTTTTAAGA	2	2	0	0	1	At1g02410.1:d:+1027:primary
GTCACCTGAT	0	2	3	0	0	At1g01750.1:d:+432:primary,At2g43770.1:d:+964:primary
CAGAGGAACC	0	1	2	1	1	At1g01090.1:X:-160:quaternary
ATCGTAAGAC	2	0	1	1	1	At1g01080.1:d:+967:primary
TTAATGTCAA	1	0	3	0	0	No gene matches found
GACCTTCGCC	0	2	2	0	0	No gene matches found
TCGAGCGCGC	0	0	0	4	0	No gene matches found
TTGCTATAAC	1	0	1	0	2	No gene matches found
AGGCTTGCTC	1	1	1	1	0	No gene matches found
TGTATAGGTT	2	1	1	0	0	No gene matches found
CTGACTAAAA	2	0	1	1	0	No gene matches found
GAGGCGGTGA	2	2	0	0	0	No gene matches found
GGCCTTCGTC	0	2	2	0	0	No gene matches found
CACCTGAGCG	0	0	1	1	2	No gene matches found
TGCCTTCGCC	0	2	1	0	1	No gene matches found
GGAGCTGTCG	0	3	0	1	0	No gene matches found
CAGGTGTGGA	1	3	0	0	0	No gene matches found
TCACTCCTAC	3	0	1	0	0	No gene matches found
AAGGTGCGGC	1	0	0	3	0	No gene matches found
TTCGGAGCCC	2	1	1	0	0	No gene matches found
GGAGCCTTCT	2	0	1	0	1	No gene matches found

CACCTGGACG	2	0	0	2	0	No gene matches found
AGTTACGTTA	1	2	0	1	0	No gene matches found
GTACGGATGA	0	0	4	0	0	No gene matches found
CGCCCGCCTC	0	4	0	0	0	No gene matches found
GGGAAAAGGA	0	0	1	2	1	No gene matches found
AGGGGAAAGA	0	2	0	1	1	No gene matches found
AGGAGAAGAG	0	0	3	1	0	No gene matches found
GCGCAAAAAA	2	0	1	1	0	No gene matches found
GGGCTTCGCC	2	0	0	0	2	No gene matches found
AACGACTATT	1	2	0	0	1	No gene matches found
CTCTTTTCCG	1	1	0	1	1	No gene matches found
TGATGAGTTA	1	0	1	1	1	No gene matches found
CGGTGTAAAA	0	0	0	3	1	No gene matches found
CGGTTATGCA	0	1	1	2	0	ChrC:+99593:quaternary
AGCGTGAAAG	0	4	0	0	0	ChrC:+97947:quaternary
TAGCATTCAG	1	3	0	0	0	ChrC:+85028:quaternary
TGGCTCTTTA	0	3	0	1	0	ChrC:+127301:quaternary
CCCCTTATGC	0	0	1	3	0	ChrC:+102166:quaternary; +small RNA(CATCATGCCCCTTATGC)
TACTTGCCGG	0	0	2	0	2	Chr5:-6663684:quaternary
AGCTTGGGCA	0	2	1	1	0	Chr5:-21452022:quaternary
TCCGAGTCTT	1	2	1	0	0	Chr5:-14798228:quaternary
TATAATGATT	1	1	0	0	2	Chr5:+9181158:quaternary,At3g07880.1:X:+359:quaternary
TAAAGAAAAG	1	0	1	1	1	Chr5:+8444589:quaternary,Chr5:+7724524:quaternary
TATCTTTACA	2	1	0	1	0	Chr5:+6225574:quaternary
TGGACAACCT	0	2	1	1	0	Chr5:+5449147:quaternary
CATTAAAAAA	1	0	3	0	0	Chr5:+3865625:quaternary,Chr3:+21717632:quaternary,Chr5:+22134198:quaternary,Chr1:+9033056:quaternary
AAGCTGGACT	0	2	1	1	0	Chr5:+26943418:quaternary
GGGACAAACG	0	1	0	1	2	Chr5:+26584805:quaternary
AATGATGATA	0	1	1	1	1	Chr5:+24715532:quaternary
TTCGTTGAGG	0	3	0	0	1	Chr5:+24399520:quaternary
TTGGCAAAGT	1	2	0	1	0	Chr5:+22390057:quaternary
TTGAATCGTT	1	0	1	1	1	Chr5:+21670728:quaternary
GAACCAAAAA	1	0	1	0	2	Chr5:+20565672:quaternary
AACTTTTTTG	1	0	1	1	1	Chr5:+17309226:quaternary,Chr1:+17065183:quaternary,Chr5:+12320180:quaternary,Chr2:+12826938:quaternary
GGCACGACAT	1	1	0	2	0	Chr5:+16729170:quaternary
ATTGAATAGA	1	2	1	0	0	Chr5:+15230278:quaternary,Chr3:+3583286:quaternary
GTTAATAAAA	1	0	0	1	2	Chr5:+13804246:quaternary
TAAATCAAAT	1	1	1	1	0	Chr5:+10385833:quaternary,At5g02840.1:X:+-213:quaternary
CGAAAAGGAG	2	0	0	2	0	Chr5:+10291932:quaternary
CATATCCCTT	2	1	1	0	0	Chr4:+815028:quaternary
TACAAATTAA	1	0	1	2	0	Chr4:+7863685:quaternary,Chr1:+22808153:quaternary
GTGGAGTACA	0	2	0	1	1	Chr4:+18574582:quaternary
ATACTTCAGT	0	3	0	1	0	Chr4:+17779627:quaternary,ChrC:+48064:quaternary
TAAGCCACAA	0	0	1	3	0	Chr4:+17092102:quaternary
AATGTGAAAT	1	1	1	0	1	Chr4:+15625189:quaternary
CTCCATTATA	3	1	0	0	0	Chr4:+14327603:quaternary
TTGAACAAAA	1	1	1	1	0	Chr4:+14256087:quaternary,Chr2:+18007382:quaternary
AACTTCTTCT	1	1	1	0	1	Chr4:+13271602:quaternary,Chr1:+22847008:quaternary
TTGTAAAAAA	1	1	0	2	0	Chr4:+1213291:quaternary,Chr1:+4772307:quaternary
CAAAAAGGTA	1	1	0	2	0	Chr4:+10967439:quaternary
AGTTTTCTAA	0	2	0	0	2	Chr4:+10721241:quaternary,Chr1:+18234979:quaternary,Chr3:+8426526:quaternary,At1g08280.1:X:+-169:quaternary
ATCCCGAAAA	1	1	1	1	0	Chr3:-17461465:quaternary
AGTAGAAAGA	2	1	1	0	0	Chr3:+9716835:quaternary
GCGAAAAAAA	0	0	1	3	0	Chr3:+7920044:quaternary,Chr2:+12006644:quaternary
TACTTGGTAA	2	1	0	0	1	Chr3:+3620259:quaternary
AATTTTAAAA	0	0	1	1	2	Chr3:+2774533:quaternary,Chr5:+25321549:quaternary,Chr4:+5193444:quaternary,Chr1:+22525471:quaternary,Chr5:+16574124:quaternary,Chr1:+9914318:quaternary,At1g27150.1:X:+274:quaternary

GAACCAATTG	2	0	1	1	0	Chr3:+23299735:quaternary
AAAAACTCTA	3	0	1	0	0	Chr3:+22671160:quaternary,Chr4:+16724567:quaternary,Chr5:+3507132:quaternary
GATGTTGTTA	2	1	0	0	1	Chr3:+21132866:quaternary
AAATAGTCAA	0	1	2	0	1	Chr3:+18938529:quaternary,Chr1:+13243050:quaternary,Chr1:+16872979:quaternary,Chr2:+4671388:quaternary
AACGTTCCCA	1	0	3	0	0	Chr3:+1522093:quaternary
AACAAAAATA	0	1	1	2	0	Chr3:+10192726:quaternary,Chr4:+1781180:quaternary,Chr5:+19196645:quaternary,Chr2:+7130716:quaternary,Chr1:+14306367:quaternary
GGATGATAAA	1	0	2	1	0	Chr2:-12024695:quaternary
TTTTTTCAAA	1	2	0	1	0	Chr2:+794795:quaternary
TTGGGAAACA	0	1	0	0	3	Chr2:+7688642:quaternary,At1g55930.1:X:+317:quaternary
TATTTAAAAA	0	2	1	1	0	Chr2:+5773091:quaternary,Chr3:+19163488:quaternary,Chr3:+19168576:quaternary
TTCGTTAAAA	1	2	1	0	0	Chr2:+559548:quaternary
TTTCGCAAAA	0	1	1	2	0	Chr2:+5111016:quaternary
TTGGAGAAGC	0	1	2	1	0	Chr2:+19527839:quaternary; small RNA(MIR164a)
AAATAGAAAG	0	0	0	0	4	Chr2:+19372752:quaternary
GTGCAAGTAA	0	1	0	1	2	Chr2:+19075857:quaternary
TCCACTGTTA	2	0	2	0	0	Chr2:+18290343:quaternary,ChrC:+43036:quaternary
CTTCTCTAAC	0	2	2	0	0	Chr2:+12223679:quaternary
TTCGAATCTT	0	0	1	1	2	Chr2:+11486360:quaternary
CTGAAAAAAA	0	1	1	1	1	Chr2:+10367712:quaternary,Chr3:+18074555:quaternary,Chr5:+1482922:quaternary
AATTGAGAAA	1	1	1	1	0	Chr2:+10324476:quaternary,At4g36195.1:X:+287:quaternary
GCTCATCACA	1	2	0	0	1	Chr1:-2586301:quaternary
TCGCATTCAT	1	0	2	1	0	Chr1:+9586017:quaternary
TCAATACATT	3	0	0	1	0	Chr1:+6634196:quaternary
GTTGAAAAAA	0	0	1	3	0	Chr1:+447712:quaternary
TGTGTAAAAA	2	0	1	1	0	Chr1:+4213358:quaternary
TAAAATCCAA	1	0	2	1	0	Chr1:+4193390:quaternary
CTTCATTTGA	0	3	0	0	1	Chr1:+3758633:quaternary
AAGACGATCT	2	1	1	0	0	Chr1:+3545124:quaternary
GGCTCGATTT	1	0	1	1	1	Chr1:+29606886:quaternary
TAAACTCTAT	0	0	2	0	2	Chr1:+29429553:quaternary
TAAAGGGTTT	3	0	1	0	0	Chr1:+28639264:quaternary,Chr2:+4789789:quaternary
TGTTATACAA	4	0	0	0	0	Chr1:+27431891:quaternary
AAAATAGTAC	1	0	1	0	2	Chr1:+24573089:quaternary
ATAAGTTTAT	0	0	1	1	2	Chr1:+22136336:quaternary,Chr4:+16652810:quaternary
ATAAAAAAAG	1	0	2	1	0	Chr1:+2165757:quaternary,Chr5:+8404864:quaternary
TGCGTGAAAA	0	0	1	1	2	Chr1:+19707344:quaternary
TCTCGTCATT	2	2	0	0	0	Chr1:+12410458:quaternary
GACAAAAAAA	1	0	1	1	1	Chr1:+12288737:quaternary,Chr5:+1523922:quaternary,Chr5:+19932195:quaternary,Chr5:+1619918:quaternary,At3g22180.1:X:+1563:quaternary
GAAATCATAA	3	1	0	0	0	Chr1:+11020674:quaternary
CGGAGGAACA	0	3	0	1	0	AtCg00720:d:-84:primary
GGATTGGCCG	0	1	3	0	0	AtCg00670:d:+289:secondary
AAGGGACTCA	0	2	0	1	1	AtCg00590.1:X:--2:quaternary
CTCGATAGAA	1	0	3	0	0	AtCg00530.1:X:+-70:quaternary
CATAAGAGTT	1	0	3	0	0	AtCg00510.1:X:-241:quaternary
TGTACAAGCT	0	1	3	0	0	AtCg00490:d:+1281:primary
TCCGAATAGA	0	0	2	0	2	AtCg00300.1:X:+-155:quaternary
TGAATTCTGC	0	0	0	3	1	AtCg00210.1:X:--2:quaternary,Chr3:-1139746:quaternary
ATGAGATCAC	0	1	0	0	3	At5g67560.1:d:+1020:primary
GAAGACGAAC	1	0	2	1	0	At5g67350.1:d:+947:primary
TGATGAAGTT	0	3	0	1	0	At5g67260.1:d:+948:primary
ACCCGTCTTA	0	0	3	1	0	At5g66570.1:d:+579:secondary
TTTTTAAGTA	0	1	0	0	3	At5g66550.1:d:+737:primary
GTCAATCCCC	1	2	0	1	0	At5g66490.1:d:+138:secondary
ATGATGCTAT	2	1	1	0	0	At5g66290.1:d:+706:primary
GGTAACGCAG	0	2	1	1	0	At5g66200.1:d:+2076:primary
CTCAACACAC	0	4	0	0	0	At5g66170.1:d:+352:primary,At4g09390.1:v:+1492:primary
ACTCTATCAG	2	1	0	0	1	At5g66100.1:d:+1480:primary
GTGCGTCTTT	0	2	0	1	1	At5g66000.1:d:+927:secondary

TAATAGAAAA	0	2	0	1	1	At5g65970.1:X:-456:quaternary
ACTTGGGTTT	1	0	2	1	0	At5g65950.1:d:+2685:primary
TACATTACTA	0	2	1	0	1	At5g65490.1:d:+2078:secondary
CCTCGCTCAA	0	1	1	0	2	At5g65390.1:d:+120:secondary
TTTCTTGATT	0	1	1	1	1	At5g65140.1:d:+482:secondary,At1g04890.1:v:+90:secondary,At1g78090.1:d:+425:secondary
TTCTGGCTTG	1	0	0	1	2	At5g65010.2:X:+531:quaternary
GAATCTCCAG	0	0	2	2	0	At5g65010.2:d:-1894:secondary,At5g65010.1:d:-1891:secondary
CCGGTGGTGT	1	2	0	1	0	At5g65000.2:d:+966:primary,At5g65000.1:d:+883:primary
GATTCTTCCA	0	3	1	0	0	At5g64940.2:d:+1483:secondary,At5g64940.1:d:+1483:secondary,At5g10970.1:v:+354:secondary,At3g23145.1:p:+1052:secondary
GCGAAGAAGA	0	3	1	0	0	At5g64816.1:d:+152:primary,At5g64816.2:d:+204:primary
TAATACACAA	2	0	1	0	1	At5g64780.1:d:+789:primary
GGTCTCTGTT	0	0	2	2	0	At5g64550.1:d:+2400:primary
CTACTGGTAT	0	2	1	0	1	At5g64340.1:d:+2374:primary
ATATTTCATT	1	0	1	2	0	At5g64070.1:d:+4400:primary
ATGTGATATT	0	2	1	0	1	At5g64070.1:d:+4303:secondary
GAGGAACGGT	1	0	0	1	2	At5g64050.1:d:+1727:primary
ACAAAGGAAT	1	3	0	0	0	At5g63910.1:d:+1448:primary
CACTCAAAAT	0	1	3	0	0	At5g63580.1:d:+1053:primary
CAAAATCAAT	1	0	2	1	0	At5g63490.1:d:+1474:primary
CTTATGCGAC	2	1	0	0	1	At5g63440.1:d:+1010:primary,At5g63440.2:d:+882:primary
TCTTCTGAAT	0	2	2	0	0	At5g63150.1:d:+503:primary
AAGAATTGGT	1	0	1	2	0	At5g62790.1:d:+1581:primary
TGAGTCTGTA	1	1	2	0	0	At5g62660.1:v:+689:secondary
TTGAAAAAAT	0	1	1	0	2	At5g62650.1:d:+1746:secondary
AATTAGAAAT	1	0	1	1	1	At5g62390.1:d:+1774:primary
CAGTATCAGT	1	1	0	0	2	At5g62220.1:v:+1626:primary
GCTAACACTG	0	2	0	2	0	At5g62070.1:d:+1089:primary,At5g07240.1:d:+1046:primary
AAGATTGGAA	0	1	2	0	1	At5g62030.1:d:+1917:primary
TCTATTGAAT	2	1	1	0	0	At5g61880.1:d:+736:primary,At5g61880.2:d:+746:primary
ATACGCGTCA	2	0	0	1	1	At5g61830.1:d:+1070:primary
ATCCCACCAC	0	3	1	0	0	At5g61640.1:d:+407:primary
ATCCAAGATT	0	2	1	0	1	At5g61520.1:d:+1796:primary
GACCAAGATT	0	3	0	1	0	At5g61420.1:d:+1245:secondary,At5g61420.2:d:+865:secondary
TTTTCCAATC	2	1	0	0	1	At5g61060.1:X:-570:quaternary,At4g35420.1:X:-232:quaternary,At2g17330.1:X:-1590:quaternary,At5g52000.1:X:-98:quaternary
GAGACATCAG	0	3	0	1	0	At5g60860.1:d:+581:primary
TTAAGAAAAT	0	0	2	1	1	At5g60540.1:i:+1090:tertiary,At2g45540.1:i:+9289:tertiary
GAGATCCTGC	0	1	1	1	1	At5g60410.2:d:+2637:primary,At5g60410.1:d:+2637:primary
AACCGCCCAC	1	2	0	0	1	At5g60120.1:d:+1802:primary
TATCCATCCA	2	1	0	1	0	At5g59920.1:v:+1535:secondary,At4g36630.1:d:+1204:secondary,At4g36630.2:v:+1461:secondary,At3g56860.2:d:+1541:secondary,At3g56860.3:d:+1541:secondary,At3g56860.1:d:+1541:secondary
AGGACCTTGC	0	0	2	0	2	At5g59880.1:d:+312:secondary,At5g59880.2:d:+312:secondary
GGTTAGGTCA	0	4	0	0	0	At5g59550.1:d:+1084:primary
CATTGGTTTG	2	1	0	0	1	At5g59540.1:X:-337:quaternary
TACTTCTTTA	1	2	0	1	0	At5g59140.1:d:+455:primary
TTGTTCTCTC	2	0	1	0	1	At5g58930.1:d:+2062:primary
TTTTGGCTTA	2	0	0	1	1	At5g58920.1:d:+785:primary
GTCTTTGAAA	0	2	1	0	1	At5g58510.1:d:+2544:primary
TTAAGTGTGA	4	0	0	0	0	At5g58140.3:d:+3097:secondary,At5g58140.1:d:+3041:secondary,At5g52530.1:d:+1729:secondary,At5g58140.2:d:+3097:secondary,At5g52530.2:d:+1723:secondary
AGTCCTCCAC	1	2	0	1	0	At5g58140.3:d:+2793:secondary,At5g58140.2:d:+2793:secondary,At5g58140.1:d:+2737:secondary
ATAAGCTCAC	3	1	0	0	0	At5g57887.1:d:-520:secondary
TAAATTATGG	3	0	1	0	0	At5g57780.1:d:+710:primary
GTGAAGCAGG	1	2	1	0	0	At5g57700.1:d:+1116:primary,At5g57700.2:d:+1099:primary
AATCAAGAAT	1	0	2	1	0	At5g57400.1:v:+293:secondary,At3g16250.1:d:+988:secondary
AACCGTCGCG	0	3	1	0	0	At5g57370.1:d:+687:primary
GACTACTCGT	1	1	0	2	0	At5g57340.1:d:+890:primary,At5g57340.2:d:+1284:primary
CCGGTAATGA	0	3	0	1	0	At5g57230.1:v:+694:secondary

GCTACATTTC	1	2	1	0	0	At5g57130.1:d:+3026:primary
GATAAAGTTT	0	3	1	0	0	At5g57080.1:v:+625:secondary
AGGAAACCTA	0	1	2	0	1	At5g56990.1:v:+1195:secondary,At2g17270.1:d:+9:secondary
ATTCATTAGC	0	0	2	2	0	At5g56940.1:d:+486:primary
GAAACCGGTT	1	1	2	0	0	At5g56890.1:d:+3564:primary
CTGGGAAGCT	0	2	1	1	0	At5g56360.1:d:+446:secondary
GTCCTAACCA	0	1	1	1	1	At5g56350.1:d:+1399:secondary
AGCTGTAGGA	1	3	0	0	0	At5g56300.1:d:+707:secondary
AACTTGGGAA	1	1	2	0	0	At5g56190.1:d:+1601:primary,At5g56190.2:d:+1683:primary
TACACACCCA	1	1	2	0	0	At5g56150.2:d:-679:secondary,At5g56150.1:d:-686:secondary
ATTTAATGTT	1	2	0	0	1	At5g56140.1:d:-1298:secondary
ATTGGTCAGT	1	0	0	2	1	At5g56030.1:d:+443:secondary
AAGCAGCTCC	2	0	0	2	0	At5g56010.1:X:+2107:quaternary
ATTGGACAGT	1	2	0	1	0	At5g56010.1:d:+457:secondary,At1g56620.1:v:+1083:secondary
GATGAGCTGA	1	0	1	1	1	At5g56000.1:d:+1928:primary
TAGATTAGTA	0	3	0	1	0	At5g55960.1:d:+2171:primary
TTAAATCTGG	0	1	2	0	1	At5g55850.1:v:+508:secondary
CAATTTCGTT	0	0	1	1	2	At5g55660.1:d:+2652:primary
CAACAACTGA	0	3	0	0	1	At5g55660.1:d:+1409:secondary,At4g18210.1:d:+384:secondary
CTACCTTATG	1	0	2	1	0	At5g55200.1:d:-1098:secondary
TATTGTTTTT	0	3	0	0	1	At5g55020.1:v:+2363:primary,At3g33520.1:d:+1418:primary
ATGGTTCTGG	0	2	0	1	1	At5g54910.1:d:+2066:primary
CCGTGAAACC	1	2	1	0	0	At5g54855.1:d:+494:primary
TTTGATCTAA	0	1	1	1	1	At5g54840.1:d:+630:primary,At5g54840.2:d:+630:primary
GATCACCTCA	1	0	3	0	0	At5g54270.1:d:-389:primary
CGATGTCGGT	0	0	3	0	1	At5g54030.1:v:+1245:secondary
GCTGGTCAGC	0	1	2	1	0	At5g53570.1:d:+1932:primary
ATTATAATAG	0	1	0	0	3	At5g53450.2:d:+2190:primary,At5g53450.1:d:+2357:primary
AAGAATAAGG	1	2	0	0	1	At5g53420.1:d:+1004:primary
AGTGGGCACA	1	1	0	2	0	At5g52900.1:d:+1059:secondary
TTACCAAAAT	1	0	0	1	2	At5g52590.1:d:+1330:primary
AAATTTCTAG	0	0	0	4	0	At5g52450.1:d:+1704:primary
TAGTTGGAAA	0	1	0	1	2	At5g52430.1:d:+1662:secondary
ATACGAGTGT	1	1	1	0	1	At5g52100.1:d:+1062:primary
CTTTGTAAAA	1	1	1	1	0	At5g51830.1:d:+1363:primary
ATATAAAAAA	1	2	0	1	0	At5g51800.1:X:+1679:quaternary,Chr4:+14887100:quaternary
CCGGAGATAC	1	1	1	0	1	At5g51460.2:d:+1496:primary,At5g51460.3:d:+1499:primary,At5g51460.1:d:+1618:primary
CGTGTTCTGA	0	2	1	0	1	At5g51300.2:d:+2424:primary
GGAATATTTT	0	4	0	0	0	At5g51190.1:d:+641:primary
ATTTTGATTG	1	3	0	0	0	At5g51150.1:d:+1732:primary
GCTTCTTCGG	0	0	1	2	1	At5g50920.1:X:-406:quaternary,At4g38770.1:X:-743:quaternary
ATGAACAACA	3	1	0	0	0	At5g50380.1:d:+1674:secondary,At4g29100.1:d:+532:secondary
ACATTTTGGC	0	0	3	1	0	At5g50375.1:i:+964:tertiary
GCGTCTTCTT	1	3	0	0	0	At5g50270.1:v:+1870:primary,At1g54520.1:d:+121:primary
GTCAATGTGC	0	3	0	1	0	At5g49990.1:d:+1393:primary
AAGAATCGTT	2	1	0	0	1	At5g49700.1:v:+1287:primary
GCTGGCTCTG	0	1	3	0	0	At5g49650.1:d:+1601:primary,At5g49650.2:d:+1596:primary
CACATCAAAT	0	4	0	0	0	At5g49560.1:v:+84:secondary,At5g49570.1:d:+2245:secondary
AGATGAAAGT	0	2	0	1	1	At5g49410.1:d:+879:primary
GCAGGGCAAG	0	3	0	1	0	At5g49020.1:d:+1362:secondary,At5g49020.2:d:+1357:secondary,At1g08680.2:d:+2090:secondary,At1g08680.1:d:+2093:secondary
TATTTACGTG	1	1	1	1	0	At5g48330.1:d:+1469:primary
AAGCAAAAAA	1	0	1	1	1	At5g48270.1:v:+1712:primary,At5g03580.1:v:+1024:primary
GACCGATTTA	4	0	0	0	0	At5g48230.2:d:+1730:secondary,At5g48230.1:d:+1468:secondary
TGGACGCGGT	0	1	1	2	0	At5g48030.1:d:+1225:primary
AGGAAATAAA	1	2	1	0	0	At5g47820.2:d:+3469:primary
GACAAAAATG	0	3	1	0	0	At5g47690.1:v:+4179:secondary
TGGAAAGAAA	3	0	0	0	1	At5g47490.1:d:+2482:secondary,At5g47480.1:d:+2443:secondary
GTCTCTTACC	1	2	0	1	0	At5g47435.1:d:+774:primary
AACTAGTCCT	2	2	0	0	0	At5g47370.1:d:+1221:primary
AGTTTTAAAT	1	0	1	1	1	At5g47040.1:i:+3424:tertiary

AACAACAACA	0	1	1	0	2	At5g46915.1:v:+1529:primary
TATTTTCAAT	0	0	1	2	1	At5g46470.1:d:+1338:secondary,At5g46260.1:v:+1676:secondary,At2g18550.1:v:+12:secondary
GCCTTCAAGG	0	1	3	0	0	At5g46420.1:d:+316:secondary,At1g61100.1:d:+1133:secondary
ACTTTGCGCC	0	3	0	0	1	At5g46330.1:d:+3328:primary
ATCAAACTGG	2	1	1	0	0	At5g46250.1:d:+999:secondary,At5g46250.2:d:+999:secondary,At3g20050.1:d:+1735:secondary
TTTAATCTCA	1	1	1	1	0	At5g46070.1:d:+3221:primary
GAAACCTCGG	0	4	0	0	0	At5g45750.1:d:+662:primary
TCCTTGACAA	1	1	0	0	2	At5g45620.1:d:+1437:primary,At5g45620.2:d:+1742:primary
TGAAAAGCAA	0	2	0	2	0	At5g45550.1:X:+272:quaternary,Chr3:+3081083:quaternary,Chr1:+25815816:quaternary
GTGGCTCACT	2	1	1	0	0	At5g45310.1:d:+1049:primary
GTCTTAGGAT	3	0	1	0	0	At5g45300.1:d:+2104:primary
CTCATTGCCA	0	0	1	2	1	At5g45280.2:d:+1111:primary,At5g45280.1:d:+1104:primary
CACACGGTTA	1	1	1	1	0	At5g45250.1:v:+3984:secondary
ATACAGGTGG	0	2	0	1	1	At5g45040.1:d:+304:secondary
GAAAACGTGC	3	1	0	0	0	At5g44710.1:d:+392:primary
AACTTGAATG	1	0	2	0	1	At5g44500.1:d:+1000:primary
GATTGATGAG	0	1	0	2	1	At5g43760.1:d:+1667:primary
AAAACCGGTT	1	1	1	1	0	At5g43660.1:v:+1761:primary,At4g22300.1:v:+2107:primary
GAAGAACAAA	0	0	1	1	2	At5g43630.1:d:+2879:primary
ATCTTATACT	2	0	1	1	0	At5g43460.1:d:+718:secondary
AGAATATGAT	2	1	1	0	0	At5g43280.1:d:+1037:primary
AGGAACTTTT	1	0	0	1	2	At5g43250.1:v:+842:secondary,At1g51980.1:d:+914:secondary
TTCTTGTCAA	2	1	1	0	0	At5g42880.1:v:+2571:primary
ATGATGATAA	2	1	1	0	0	At5g42870.1:d:+2808:primary
AACTCTGCTT	1	2	0	0	1	At5g42730.1:p:+955:primary
GAAGAGACCA	0	0	2	0	2	At5g42530.1:X:-47:quaternary
TCTGAGCTTG	0	1	1	2	0	At5g42480.1:d:+2539:primary
GCTGAGCGAC	0	1	1	2	0	At5g42470.1:d:+1118:primary
GAAATAACTT	1	2	0	0	1	At5g42420.1:d:+1393:primary
AAAAGACAAC	0	3	1	0	0	At5g42320.1:v:+1837:secondary
CAAGTGTCGC	0	0	3	1	0	At5g42270.1:d:+1959:secondary
GGTTTAGGAT	0	3	1	0	0	At5g42050.1:d:+403:secondary
GGGAAGAGTG	0	2	1	1	0	At5g42030.1:d:+379:primary
GAAGCTGGTC	0	3	0	1	0	At5g41990.1:d:+1894:secondary
TTATATGACT	1	0	0	0	3	At5g41810.2:d:+1135:primary,At5g41810.1:d:+1157:primary
CAACTTAGGT	0	2	2	0	0	At5g41750.2:d:+3536:primary
GACACAGTCC	0	0	0	1	3	At5g41670.1:d:+1532:primary
TTTTGTTAGA	1	1	2	0	0	At5g41600.1:d:+925:primary
GAATACAACT	1	0	1	2	0	At5g41360.1:d:+2440:primary
AATTATCTCT	0	1	2	0	1	At5g41340.1:d:+781:primary
AGATCCTTTT	1	2	1	0	0	At5g41210.1:d:+667:primary
GTCTCCCTTC	0	1	1	1	1	At5g40850.1:d:+1159:secondary
TTTAAAGATC	0	2	1	0	1	At5g40670.1:d:+1066:primary
TTGTTCAAAG	1	1	2	0	0	At5g40640.1:d:+934:secondary,At2g44920.2:d:+779:secondary,At2g44920.1:d:+891:secondary
CTCCAAAGAG	1	3	0	0	0	At5g40450.1:d:+3444:secondary
AGATTCTGAG	0	2	1	1	0	At5g40340.1:v:+62:secondary,At1g59520.3:d:+243:secondary,At1g59520.1:d:+243:secondary,At1g59520.2:d:+243:secondary
TGCTTTTTTC	0	2	1	1	0	At5g40200.1:i:+1496:tertiary
AGAATGGGAG	1	2	1	0	0	At5g39990.1:d:+1413:secondary,At1g80930.1:d:+969:secondary
ACATTGTATG	2	1	1	0	0	At5g39660.1:d:+1854:primary
TATAGTTCTG	1	2	1	0	0	At5g39520.1:d:+889:secondary
GGCTTCCTGG	0	2	0	1	1	At5g39410.1:d:+945:secondary
AAGATCCTAT	2	1	1	0	0	At5g39030.1:v:+2799:secondary
CCCATCAGAT	1	1	2	0	0	At5g39020.1:v:+2609:secondary,At3g23510.1:d:+834:secondary,At5g39030.1:v:+2621:secondary
GACGGCTGTG	2	0	2	0	0	At5g38980.1:d:-149:primary
GGCTTCGCCA	1	1	2	0	0	At5g38970.1:X:-410:quaternary
ATGTCAAAAA	1	1	1	0	1	At5g38770.1:v:+669:secondary,At3g50180.1:v:+616:secondary
CGACAACAGA	0	0	1	1	2	At5g38690.1:d:+680:secondary
ATTATCAACC	2	1	1	0	0	At5g38365.1:p:+4409:secondary,At5g51730.1:v:+1495:secondary
AAACAAAGAA	1	1	1	0	1	At5g37830.1:d:+1551:secondary
TGAGAAAATG	0	0	1	1	2	At5g37475.1:d:+868:primary

TTTCTGAAGC	1	0	2	0	1	At5g37380.1:d:+619:secondary,At1g78290.2:d:+1195:secondary,At5g37380.2:d:+535:secondary,At5g46260.1:v:+3691:secondary,At1g78290.1:d:+1296:secondary,At5g46500.1:v:+1111:secondary
ATAGGTTTGC	1	1	0	1	1	At5g37350.2:d:+1837:primary,At5g37350.1:d:+1748:primary
GCACGTGCTT	1	1	0	2	0	At5g36250.1:d:+1364:primary
AGACGTATTA	1	0	2	0	1	At5g36160.1:d:+1268:secondary
AGAAAACTTA	1	0	1	1	1	At5g36070.1:v:+145:secondary,At5g36060.1:v:+1215:secondary
TGTTTTAACA	2	0	1	0	1	At5g35890.1:v:+1620:primary
AATTGAACCA	0	1	1	1	1	At5g35730.1:d:+1707:primary
GATATTTATC	1	1	1	0	1	At5g35695.1:v:+419:secondary
GACCCATACA	0	0	4	0	0	At5g35630.1:d:+1326:secondary
AGACCCCTTG	0	2	1	0	1	At5g35590.1:d:+443:primary
CTTGACTGGG	1	0	3	0	0	At5g35530.1:d:+633:secondary,At2g31610.1:d:+639:secondary
GTTGAAGGAC	2	1	1	0	0	At5g35370.1:v:+2841:secondary
TGGTTAGCTT	3	0	1	0	0	At5g35330.2:d:+1070:secondary,At5g35330.1:d:+1076:secondary
CACATCTACA	1	0	0	0	3	At5g35320.1:d:+999:primary
TTCATCTTAG	1	2	0	1	0	At5g35170.1:d:+1430:secondary
ACCTAAGCTT	2	0	1	1	0	At5g33370.1:d:+1189:primary
ATGAATATAT	0	2	0	0	2	At5g32433.1:X:-1415:quaternary,At1g68070.1:X:-336:quaternary
CTCGGAGCCA	1	1	2	0	0	At5g30460.1:p:-1209:secondary,At5g32358.1:p:-4221:secondary
AGCGAAGTAC	1	2	1	0	0	At5g29053.1:p:+3278:secondary
ATCTTCAGTT	1	0	2	1	0	At5g28850.2:d:+2180:secondary,At5g28850.1:d:+2271:secondary
AATAAAGAAC	1	1	0	2	0	At5g28675.1:p:+2564:secondary
AAAAGAAAGA	0	4	0	0	0	At5g28545.1:p:+637:secondary,At1g48740.1:d:+1103:secondary,At1g16800.1:v:+5779:secondary
AAGCCCTGAA	1	1	0	1	1	At5g28495.1:p:+1034:secondary,At2g46230.1:d:+731:secondary
ACTTACTTCA	1	2	0	1	0	At5g28350.2:d:+3287:primary,At3g61480.1:d:+3203:primary,At5g28350.1:d:+3473:primary
ACGTTTCCAC	2	0	0	1	1	At5g28290.1:d:+2001:primary
CTTGCGACGT	1	0	2	1	0	At5g28220.1:d:+1200:primary
TGAAAAGATG	0	1	0	1	2	At5g28056.1:p:+1015:secondary
CCTGTTGATG	0	3	1	0	0	At5g27990.1:d:+597:primary
AGGATGCTAA	0	3	0	1	0	At5g27840.1:d:+588:primary
TGCTGAAACA	1	0	2	1	0	At5g27710.1:d:+1138:primary
TCAAATCAAA	0	0	3	1	0	At5g27606.1:v:+1442:secondary,At2g10510.1:p:+3321:secondary
TGACAAATAA	1	2	0	1	0	At5g27450.2:d:+1594:secondary,At5g27450.1:d:+1354:secondary
GATCTATGTG	0	2	1	0	1	At5g27430.1:i:+253:tertiary
GTCAAGTAGT	0	2	1	1	0	At5g27360.1:d:+1332:primary
TAACACTTTT	1	0	0	0	3	At5g27230.1:i:+1790:tertiary
ACCTCAAGCT	0	1	2	1	0	At5g26920.1:d:+1258:primary
GTAGAAACGT	0	4	0	0	0	At5g26880.1:d:+677:primary
TGTATTTGAA	4	0	0	0	0	At5g26770.1:d:+1350:primary,At5g53480.1:d:+3769:primary
GAGACTCGCT	0	3	0	1	0	At5g26570.1:d:+3282:primary
TTGGTTGTAA	1	1	0	2	0	At5g26270.1:v:+1230:secondary
AAGACAGCAA	1	2	1	0	0	At5g26220.1:d:+715:primary
AAGTCAAGCG	1	3	0	0	0	At5g26040.2:d:+1125:secondary,At5g26040.1:d:+1041:secondary
TGGTGGCTCA	1	3	0	0	0	At5g25560.1:d:+976:primary
CAAGAAGTGC	0	2	2	0	0	At5g25440.1:d:+937:primary
AACCAGAGAC	2	2	0	0	0	At5g25415.1:d:+49:secondary,At1g14000.1:d:+1470:secondary,At5g49470.1:d:+2353:secondary,At5g49470.2:d:+2473:secondary
TCCGGCCAGC	0	1	2	1	0	At5g25110.1:d:+1468:primary
GTCTGTTCTC	0	2	2	0	0	At5g25060.1:d:+3106:primary
TTCCGAAATT	0	0	1	2	1	At5g24810.1:d:+2601:secondary
TTTAATGTCT	1	2	1	0	0	At5g24620.1:d:+1481:primary
GTTTTCTCTC	0	3	0	1	0	At5g24320.1:v:+2804:primary,At5g24320.2:v:+2816:primary
GACCCTCCCT	0	0	2	0	2	At5g24300.1:d:+2074:secondary
GTTTATTATT	2	1	0	0	1	At5g24270.1:v:+1361:primary
GATCTTGATG	1	0	1	1	1	At5g23690.1:d:+1397:secondary
AATCATTTCC	1	2	0	0	1	At5g23405.1:d:+333:primary,At5g23405.2:d:+337:primary
TGCATCAGAG	0	3	0	1	0	At5g23210.1:d:+780:primary
GGAAAGCTGC	0	2	1	0	1	At5g23080.1:d:+2014:secondary
TCTGTATCAA	0	4	0	0	0	At5g23050.1:d:+2293:primary

AAAATGAGCA	0	3	0	0	1	At5g22920.1:d:+172:secondary,At1g79830.1:d:+1127:secondary
TATTCCTATC	3	0	0	0	1	At5g22830.1:d:+1374:secondary
GTTGTTATGG	1	1	0	0	2	At5g22410.1:d:+90:secondary
TCGACGAGGA	0	1	2	1	0	At5g22390.1:d:+851:primary
TTTTGGAAGA	0	0	0	3	1	At5g22020.1:d:+1114:primary
GCTTCTCTTT	2	1	1	0	0	At5g21990.1:d:+1712:primary
TCCAATTATG	1	0	1	1	1	At5g21482.1:d:+1839:primary
TCTGAAAAAA	1	0	0	3	0	At5g21280.1:X:-85:quaternary
GCCCTAGGCT	0	3	1	0	0	At5g21160.1:d:+2088:primary
TTATTGGTAT	2	2	0	0	0	At5g21105.1:d:+1801:primary
TGGTTGTTTC	0	1	1	1	1	At5g21010.1:d:+1484:primary
TGGTCACCTT	1	0	2	0	1	At5g20920.1:d:+1097:secondary,At5g20920.2:d:+1054:secondary
TATATGAGGC	1	2	0	1	0	At5g20840.1:d:+3274:primary
TGGTTTATAT	0	0	0	1	3	At5g20610.1:d:+3687:primary
TCCAGATTTG	0	2	1	0	1	At5g20510.1:d:+968:secondary
AACCTTTTGC	1	1	0	0	2	At5g20420.1:v:+4446:secondary
ATGTCTTTGC	0	2	1	1	0	At5g20410.1:d:+1624:primary
TCCGAGCCCG	0	2	0	0	2	At5g20400.1:d:+656:primary
TTCATTCTGC	2	1	1	0	0	At5g20350.1:d:+1847:secondary
CCACTCCTGT	0	1	3	0	0	At5g19980.1:d:+989:primary
TGAACTTAAA	0	0	1	2	1	At5g19875.1:d:+582:primary
AAAGTCTTGT	0	0	3	0	1	At5g19840.1:d:+1882:primary
CTTTCCTCCT	1	2	1	0	0	At5g19820.1:d:+3404:primary
AAGTAGAGTT	0	1	2	1	0	At5g19150.1:d:+1178:primary,At5g19150.2:d:+1165:primary
GTAATTAGCA	1	1	1	1	0	At5g19130.1:d:+2255:secondary,At5g19130.2:d:+2232:secondary
AATTAATTCA	0	0	1	1	2	At5g19050.1:d:+1181:primary
AACGGAAAGG	1	2	1	0	0	At5g19030.2:d:+410:secondary
TTTTTAGGTC	1	3	0	0	0	At5g18850.1:d:+412:primary
ATCACATATG	1	2	0	0	1	At5g18640.1:v:+1685:primary
TTTCATATGT	1	3	0	0	0	At5g18520.1:d:+1219:primary
CAAGGAGAAA	0	3	0	0	1	At5g18440.1:v:+1951:secondary
ACCCGATTAA	0	1	1	1	1	At5g18260.1:d:+1416:primary
TACTGTAGTT	1	0	2	1	0	At5g18200.1:d:+1268:primary
TCAAGGAAAA	1	2	0	1	0	At5g18190.1:d:+2012:primary
TCTTAAAAAG	1	0	0	1	2	At5g18110.1:d:+1049:primary
ATAGCACAAG	1	2	0	1	0	At5g17860.1:d:+1288:primary
GTTCTGTTTT	1	1	1	0	1	At5g17840.1:d:+592:primary
ACCTACCCTA	1	1	2	0	0	At5g17770.1:d:+1235:primary
GAAGAATACG	2	1	0	0	1	At5g17670.1:d:+425:primary
TTTTTGTTTC	1	0	1	1	1	At5g17550.1:d:+875:primary
GAGATGATGA	1	2	1	0	0	At5g17160.1:d:+1587:primary
GAAGATGTTA	0	0	2	2	0	At5g16610.1:d:+2027:primary,At5g16610.2:d:+2260:primary
AAAACCATTC	1	1	0	1	1	At5g16440.1:d:+852:primary
AGTTACGTTT	2	0	1	1	0	At5g16370.1:d:+1785:secondary
GCGACCAGTC	2	0	0	1	1	At5g16200.1:d:+511:primary
AGAAACATCC	2	1	1	0	0	At5g16040.1:d:+1578:primary
GATTGTTCCG	2	1	0	0	1	At5g15980.1:d:+2443:primary
AATCTTTCTT	0	1	0	3	0	At5g15880.1:d:+1205:primary
AATATGATTA	0	0	2	0	2	At5g15860.1:d:+1468:primary,At5g15860.2:d:+1701:primary
AGTCTCAAAA	1	1	1	1	0	At5g15730.1:d:+1522:primary
AGAATTCGGA	0	0	2	1	1	At5g15490.1:X:-1517:quaternary
TTCGTTGCTG	0	3	1	0	0	At5g14530.1:d:+945:primary
CATATCAAAA	0	0	1	3	0	At5g14490.1:v:+739:secondary
TTGTTTTTTG	1	0	0	2	1	At5g14240.1:d:+1004:secondary,At5g63060.1:v:+649:secondary,At1g25480.1:v:+132:secondary
TTCAATAAAA	1	1	2	0	0	At5g13800.1:X:-591:quaternary
GAAAGCTTCA	1	1	1	0	1	At5g13660.1:d:+1883:primary
GAGAGCAGAA	1	0	1	2	0	At5g13160.1:d:+1163:secondary
TTAGGGAGGA	0	2	1	0	1	At5g13100.1:d:+1268:primary
GTTATAATAA	1	1	1	0	1	At5g13070.1:d:+641:primary
TGTTTAATTT	1	1	0	0	2	At5g12410.1:i:+147:tertiary,At1g34770.1:i:+115:tertiary
AGGCCCTTTT	3	0	0	1	0	At5g12310.1:d:+1083:primary

AAGTTTTGTA	1	0	2	1	0	At5g12140.1:X:-192:quaternary
AATCAGAAGT	2	0	0	2	0	At5g11960.1:i:+2354:tertiary
TTTCTCCAAA	1	1	1	1	0	At5g11900.1:d:+909:primary
GAAGTGTAAA	2	0	0	1	1	At5g11810.1:d:+1137:primary
TAATGTATCA	2	2	0	0	0	At5g11800.1:d:+2114:primary
GCATCTCCAC	0	1	1	2	0	At5g11770.1:X:-347:quaternary
CGTACGTGGA	1	1	1	0	1	At5g11680.1:d:+719:primary
TTTCCTCCCT	0	3	0	1	0	At5g11640.1:d:-208:secondary
GCCGCAGCAA	1	2	0	0	1	At5g11570.1:v:+1608:primary,At2g34357.1:d:+3209:primary
AATTGTCTAT	1	1	1	0	1	At5g11280.1:d:+813:primary
AAAGTCCTCG	1	1	0	1	1	At5g10810.1:X:-70:quaternary
TAGATTTGCT	1	0	1	0	2	At5g10760.1:d:+1598:secondary
CCGAATGAAA	2	0	0	2	0	At5g10710.2:d:+998:primary,At5g10710.1:d:+1098:primary
CCAGGTGGGG	0	3	0	1	0	At5g10540.1:d:+1768:secondary
AAAACTATTT	1	2	1	0	0	At5g10450.1:d:+1314:primary
TAGTATAATG	2	0	0	0	2	At5g10070.1:d:+1020:primary,At5g10070.2:d:+1026:primary
AAGGTATCAT	0	0	0	1	3	At5g09930.1:v:+2525:secondary
TTAAATGAAT	2	0	1	0	1	At5g09920.1:d:+650:primary
GTGCAGCAAG	0	2	1	1	0	At5g09900.2:d:+316:secondary,At5g09900.1:d:+347:secondary
CTCTCTCTAT	1	2	0	0	1	At5g09830.1:d:+252:primary
TATGTTGCCA	0	3	1	0	0	At5g09810.1:d:+506:secondary
GTGGTGGGAA	0	2	1	1	0	At5g09670.2:d:+1266:secondary,At5g09670.1:d:+2309:secondary,At5g09670.2:d:+1578:secondary,At1g31130.1:d:+1001:secondary,At5g64550.1:d:+1756:secondary,At5g09670.1:d:+1997:secondary,At5g64550.1:d:+1447:secondary
GCTGCACCAC	1	1	0	1	1	At5g09620.1:d:+1650:primary
TCTATGGAGA	0	0	1	0	3	At5g09320.1:d:+2371:primary
TGTTGTATCT	1	0	1	2	0	At5g09230.2:i:+705:tertiary,At5g09230.4:i:+650:tertiary,At5g09230.3:i:+710:tertiary,At5g09230.5:i:+725:tertiary,At5g09230.6:i:+725:tertiary,At5g09230.1:i:+705:tertiary
TACGTTATTC	1	0	1	0	2	At5g08750.1:d:+1569:primary
GTTGGGTTAC	1	0	1	1	1	At5g08620.1:d:+1562:primary
ACTTGCCTGA	1	1	1	1	0	At5g08580.1:d:+998:secondary
GCTAAGATCT	0	0	4	0	0	At5g08570.1:d:+1190:secondary
GAAATAAAAT	1	1	1	1	0	At5g08530.1:d:+1636:secondary,At1g52120.1:v:+697:secondary
GGTTTTCTGT	0	1	2	0	1	At5g07890.1:d:+1384:primary
ATTGACTCCA	0	2	1	1	0	At5g07740.1:p:+7753:primary
TTCTTGATGG	0	2	0	1	1	At5g07370.2:d:+865:primary,At5g07370.4:d:+1604:primary,At5g07370.3:d:+1139:primary,At5g07370.1:d:+1014:primary
TCGAACTTTT	1	0	1	2	0	At5g07350.1:d:+3230:secondary
AAGGTTTTGT	0	2	1	1	0	At5g07220.1:d:+500:secondary,At5g56080.1:v:+1555:secondary
GGCGTGGCCA	1	2	0	1	0	At5g07110.1:d:+614:secondary
CAGCAGCAGA	2	2	0	0	0	At5g07030.1:d:+1278:primary
AGCTTCCAAT	0	3	0	0	1	At5g06780.1:d:+1286:primary
AGATCAAGAA	1	2	0	1	0	At5g06420.1:d:+1195:primary,At5g06420.2:d:+1771:primary
GCCTTGGCCA	1	1	0	1	1	At5g06160.1:d:+1295:primary
AAGGAACACA	0	0	2	1	1	At5g06140.1:X:-248:quaternary,At2g40120.1:X:-146:quaternary
GTACATCAAG	1	2	1	0	0	At5g06060.1:d:+637:primary
AAATGGGATT	1	1	0	1	1	At5g05870.1:d:+1446:primary
GCTCTTCCAC	0	2	1	1	0	At5g05830.1:d:+729:primary
TTGATGAACC	1	1	1	1	0	At5g05800.1:d:+1624:primary
GTCAATAATA	0	1	1	0	2	At5g05720.1:v:-1746:secondary
CCTTCATAGA	2	1	0	1	0	At5g05670.2:d:+919:primary,At5g05670.1:d:+874:primary
TTGAATCTCA	1	2	1	0	0	At5g05600.1:d:+1237:secondary
TTATGTCCTT	1	1	1	1	0	At5g05450.1:d:+1934:primary
GTGATTGTTT	1	2	1	0	0	At5g05110.1:d:+898:secondary
AGATTACTGA	0	1	1	1	1	At5g04910.1:v:+847:secondary
GTGAGAAATG	2	1	0	1	0	At5g04710.1:d:+1744:primary
AATGCAGGAG	3	0	1	0	0	At5g04480.1:i:+3301:tertiary,At4g21150.1:i:+1094:tertiary
GAAATCACTC	0	3	0	1	0	At5g04430.2:d:+1004:secondary,At5g04430.1:d:+941:secondary
CCTAAGATCC	0	2	0	1	1	At5g04360.1:d:+1014:secondary
TACTTGATCA	3	0	0	0	1	At5g04220.2:d:+1944:primary,At5g04220.1:d:+1971:primary

ATCTTTCTTC	0	3	1	0	0	At5g04140.1:d:+723:secondary,At5g04140.2:d:+622:secondary
GGTGTCATTA	1	1	0	1	1	At5g03970.1:d:+1442:primary
ATTATCTAGG	2	1	1	0	0	At5g03940.1:d:+1870:primary
TAATAAACAA	0	1	0	1	2	At5g03910.1:d:+2177:primary
AAGAGAGTTG	0	2	0	1	1	At5g03910.1:d:+1569:secondary,At3g51100.1:d:+652:secondary,At2g14415.1:p:+520:secondary
TACTTATATA	2	1	0	0	1	At5g03880.1:d:+1173:primary
CTTAAACCGT	2	0	0	0	2	At5g03420.1:v:+2449:primary
AAGCCGGAGA	1	2	0	1	0	At5g03415.1:d:+1392:primary
GAATTTAATG	0	1	1	1	1	At5g02860.1:v:-2251:secondary
GAGAAGAAGG	0	1	1	2	0	At5g02850.1:d:+962:primary,At3g59450.1:v:+977:primary,At3g59440.1:d:+672:primary
ATTCTTGACC	1	0	1	1	1	At5g02710.1:X:+333:quaternary
AGCTTAGAGA	0	1	3	0	0	At5g02610.1:d:+104:primary
AATCAATCGT	0	1	3	0	0	At5g02550.1:d:+434:primary
AGATGCATTG	1	1	1	1	0	At5g02540.1:d:+1184:primary
TTTTTAATGG	2	1	0	0	1	At5g02050.1:d:+1010:primary
ATCGTGTTCT	1	1	1	1	0	At5g02040.2:d:+915:primary,At5g02040.1:d:+925:primary
GTTCAGGTTA	1	2	1	0	0	At5g02020.1:d:+462:primary,At5g02020.2:d:+462:primary
GATCAACAAG	2	1	0	1	0	At5g01880.1:d:+1314:primary
AAGGAGAGTA	0	2	2	0	0	At5g01810.1:d:+1678:primary
GGGCGTTGTA	0	4	0	0	0	At5g01740.1:d:+483:primary
ATGCAGCTTG	1	0	1	2	0	At5g01410.1:d:+830:secondary
TAATTCGAGT	1	0	1	1	1	At5g01160.1:d:+1377:primary
TTCGCGTTAT	2	1	0	0	1	At5g01090.1:d:+1151:secondary
GCGGAGATGG	0	2	1	0	1	At4g39950.1:d:+1113:primary
TTGCAGATTC	1	0	1	1	1	At4g39840.1:d:-112:primary
CTAGTGATTT	1	0	3	0	0	At4g39470.1:d:+1277:primary,At5g51670.1:v:+1988:primary
TCTGAAACGT	1	1	1	0	1	At4g38890.1:v:+2498:secondary
AGTATACGTA	1	0	0	0	3	At4g38770.1:X:-1235:quaternary
GAGAAGCCTA	1	1	0	1	1	At4g38710.1:d:+1135:primary
TACAAGAAAC	1	2	1	0	0	At4g38600.1:d:+5611:primary,At4g38600.2:d:+5508:primary
TTGAATCCTT	1	0	2	1	0	At4g38495.1:d:+466:primary
TATTTATCTA	0	1	0	0	3	At4g38480.1:d:+1474:primary
CAAGAAGCGA	0	3	0	1	0	At4g38470.1:i:-1202:tertiary
ATTTCTTATT	0	2	0	0	2	At4g38360.1:d:+1888:primary,At4g38360.2:d:+1883:primary
TTGGTACACT	0	0	1	0	3	At4g38350.1:v:+3700:secondary
CTATCACATC	1	0	0	1	2	At4g38225.2:d:+1103:primary,At4g38225.1:d:+1039:primary
ATGTTGGTGT	1	0	0	1	2	At4g38040.1:d:+680:secondary
TACACCTCTT	1	2	1	0	0	At4g38040.1:d:+1419:primary
AACACACTCT	2	0	0	0	2	At4g37920.1:v:+2390:secondary,At1g50190.1:p:+530:secondary
TACCACTTCA	0	3	1	0	0	At4g37870.1:d:+1618:secondary
TGTATGTATA	3	0	0	0	1	At4g37820.1:d:+2025:primary
GAACCATCTC	2	0	1	1	0	At4g37800.1:d:+499:secondary
TGAAACAGAA	2	1	1	0	0	At4g37750.1:d:+2096:primary
AGGTGGCGGG	0	0	2	2	0	At4g37720.1:v:+505:secondary
ATTAGTCCCA	0	0	2	2	0	At4g37530.1:d:+1177:primary
ATAATTTAGC	3	0	0	1	0	At4g37460.1:d:+955:secondary
GTCGTGGAAA	1	1	2	0	0	At4g37270.1:d:+2499:secondary
TTTTGTAATG	2	0	0	2	0	At4g37210.1:d:+1714:primary,At4g37210.2:d:+1706:primary
TGAATGAAGG	1	1	2	0	0	At4g37180.1:X:+569:quaternary
TGCTATGATA	1	1	2	0	0	At4g36440.1:d:+1484:primary
ACAAACAAAA	0	0	3	0	1	At4g36370.1:d:+383:primary
TGCCGAGAGC	0	3	0	1	0	At4g36360.1:d:+2201:primary,At4g36360.2:d:+2201:primary
AGGTGCGAGG	1	0	2	1	0	At4g36280.1:d:+1836:secondary,At4g36270.1:v:+2043:secondary,At4g36290.1:d:+1783:secondary
AATGATGTGT	1	2	1	0	0	At4g36220.1:d:+1499:secondary
TTTGATAAAA	0	0	1	1	2	At4g36160.1:d:+1331:primary
TACACTCCGA	0	0	3	1	0	At4g35830.1:d:+2715:secondary
TATAATTTTC	1	2	1	0	0	At4g35780.1:d:+1874:primary
TACAGGGCTT	0	3	1	0	0	At4g35770.1:i:+964:tertiary
GATAATAAAT	2	2	0	0	0	At4g35760.1:d:+1064:secondary
CCGATATTTT	0	1	2	1	0	At4g35480.1:X:-760:quaternary
ATTTATCCAA	0	1	1	0	2	At4g35460.1:d:+1426:primary

GGACCTTCTT	1	1	1	0	1	At4g35320.1:d:+461:secondary
TAAATGAAAG	1	1	1	1	0	At4g35310.1:X:+598:quaternary
CAGACTAAAA	1	0	1	2	0	At4g35090.1:X:-399:quaternary
TAGCATCAGG	0	0	2	2	0	At4g34910.1:d:+2111:secondary,At1g17810.1:d:+612:secondary,At1g17810.2:d:+330:secondary
TATGCAGTAA	0	0	3	0	1	At4g34730.1:d:+1476:primary
TCGATGTGAA	0	0	4	0	0	At4g34670.1:d:+452:secondary,At5g11370.1:v:+1431:secondary
TTCTGAATCT	3	0	1	0	0	At4g34660.1:d:+1424:primary
CAGACTTGTG	2	1	1	0	0	At4g34450.1:d:+2537:primary
CTTTCTTGTT	0	1	0	2	1	At4g34412.1:v:+991:primary
CAAATCAGCA	0	3	0	1	0	At4g34350.1:X:-378:quaternary
TTATAATCAC	1	0	1	0	2	At4g34240.1:d:+1768:primary
ATGCAGGAAG	1	1	1	0	1	At4g34240.1:d:+1326:secondary
AAGCAGATGT	0	2	0	1	1	At4g34180.1:d:+913:secondary
TGTATTTTTC	0	0	0	2	2	At4g33890.1:d:+1478:primary,At4g33890.2:d:+1418:primary
TTTTCTTAGT	1	0	1	1	1	At4g33780.1:d:+882:primary
CAAATCAGGA	0	0	1	1	2	At4g33700.1:i:+1313:tertiary
TAATCTAAAT	0	2	0	1	1	At4g33480.1:d:+1217:secondary
TAGAAAATGA	0	0	4	0	0	At4g33040.1:d:+698:primary
TTGAGAAAAA	2	1	0	1	0	At4g32820.1:v:+2944:secondary
TGAAAATTCC	0	2	1	1	0	At4g32760.1:v:+2865:primary
AAGATGTTCT	1	2	0	1	0	At4g32530.1:X:-361:quaternary
TTTTGTTGTA	3	0	0	0	1	At4g32300.1:d:+3017:primary
AAATGTTTGT	0	2	1	1	0	At4g31600.1:d:+1473:primary,At2g23010.2:d:+1370:primary,At1g75470.1:v:+1569:primary,At2g23010.1:d:+1370:primary
TGAAGTGTGT	2	0	1	1	0	At4g31450.1:d:+1529:secondary
CATCTCTGGA	1	1	2	0	0	At4g31410.1:d:+1377:primary
CTTATTTCAT	1	3	0	0	0	At4g31240.2:d:+1408:primary,At4g31240.1:d:+1676:primary
AGTGCAAGAG	1	0	1	1	1	At4g31190.1:p:+355:primary
GCTGAAGAGT	2	1	0	1	0	At4g30993.2:d:+1558:primary,At4g30993.1:d:+1651:primary
ATTATTATTT	0	3	0	0	1	At4g30970.1:v:+1055:primary
CTTCTATAAA	0	2	1	1	0	At4g30970.1:i:+1619:tertiary
TCTTCTTATC	0	0	0	0	4	At4g30890.2:d:+2334:primary,At4g30890.1:d:+2109:primary
CTTATGCTAA	0	4	0	0	0	At4g30760.2:d:+212:primary,At4g30760.1:d:+212:primary
CACTATTTAT	0	2	0	2	0	At4g30750.1:d:+757:primary
GAAGTTTTCT	0	0	2	0	2	At4g30650.1:d:+99:secondary
GGATGGTATT	0	2	0	2	0	At4g30600.1:X:-297:quaternary
ATGAACGCGG	1	2	0	1	0	At4g30330.1:d:+349:primary
GACAGCAAAC	0	4	0	0	0	At4g30140.1:d:+211:primary
AAACCGAGAA	0	1	0	1	2	At4g29820.1:d:+1174:primary
AGAGTGTTTA	0	3	0	0	1	At4g29810.1:d:+1269:secondary
TTCTCTTGCT	0	3	1	0	0	At4g29670.2:d:+715:primary
TAGTTTATTT	0	1	2	0	1	At4g29500.1:d:+672:primary
GTGTCTCAAG	0	0	4	0	0	At4g29020.1:d:+154:secondary
GCAAGCCTTC	2	0	1	1	0	At4g28740.1:d:+1028:primary
TTTTGGTTTG	1	1	0	2	0	At4g28715.1:v:+2628:primary,At5g26900.1:v:+1956:primary
GCCCAAGTGG	0	3	1	0	0	At4g28703.1:d:+177:primary
GCATAATATA	2	0	1	0	1	At4g28250.1:d:+1222:primary
CCTTTAGCAG	2	1	1	0	0	At4g28100.1:d:+936:primary
AGACTTGTGA	1	0	3	0	0	At4g28080.1:d:+5329:secondary
AGCAGACCAC	0	4	0	0	0	At4g28080.1:d:+3211:secondary
TCAGAATTTT	0	0	2	1	1	At4g28060.1:v:+1001:primary
ATATCTGTTG	0	0	2	1	1	At4g27657.1:d:+84:primary
TTCCGGAGCT	2	2	0	0	0	At4g27640.1:d:+3047:primary
GAAACCAACG	1	2	1	0	0	At4g27240.1:d:+478:secondary
CAATTTACAT	1	1	2	0	0	At4g27180.1:i:+709:tertiary
TAACTTCTCT	0	2	1	0	1	At4g26900.1:d:+2053:secondary
TTTTAAGACC	1	0	3	0	0	At4g26860.1:X:-498:quaternary
GATGATAAAA	0	0	2	2	0	At4g26650.1:d:+2048:secondary
GGAGTGCGGT	0	2	1	1	0	At4g26610.1:d:+1413:primary
TCCAAAAGTT	0	0	0	4	0	At4g26600.1:d:+2263:primary
GGTATGTCGG	1	0	2	0	1	At4g26410.1:d:+775:primary

ATAATATCTG	0	1	0	0	3	At4g26230.1:d:+640:primary
CGTTTCAGAT	0	1	2	1	0	At4g26210.2:d:+497:primary,At4g26210.1:d:+511:primary
TGAAGATTCT	1	0	1	1	1	At4g26190.1:d:+463:secondary
ATGTTTCGTG	0	1	2	1	0	At4g26120.1:v:+2217:secondary
GGAGCTGGTG	0	4	0	0	0	At4g26100.3:d:+3378:secondary,At4g17900.1:d:+149:secondary
AAATTTGGTC	1	2	1	0	0	At4g26100.1:d:+1732:secondary,At4g26100.3:d:+1732:secondary
TCGAGATCCA	1	2	0	1	0	At4g26080.1:d:+1390:primary
AATGCTAAAG	0	2	2	0	0	At4g25900.1:d:-1236:secondary,At3g62570.1:d:-1344:secondary
GGACAGTGAC	2	0	0	1	1	At4g25720.1:d:+951:primary
AGGAAAAGTA	0	0	2	2	0	At4g25640.1:X:-245:quaternary
ATTTCTCAGA	0	2	1	1	0	At4g25515.1:d:+1474:primary,At4g25520.1:d:+2318:primary
CTTTTACCGC	0	0	4	0	0	At4g25490.1:d:+725:primary
TGAAGAAGCC	1	1	1	0	1	At4g25210.1:d:+477:primary
TGCTTAGGTC	0	3	0	0	1	At4g24972.1:d:+714:primary
TATATCTCAA	0	0	2	0	2	At4g24940.1:d:+1178:primary
GCAGTGTCAG	1	1	0	1	1	At4g24840.1:d:+1976:primary
AACTAAAACA	1	2	1	0	0	At4g24805.1:d:+766:primary
CTGAGGATAC	0	2	2	0	0	At4g24680.1:v:+4836:secondary
ATTTGTTTTG	0	3	0	1	0	At4g24390.2:d:+2126:primary
ATCAACAACT	0	0	2	0	2	At4g24160.1:d:+1308:secondary,At4g24160.2:d:+1389:secondary
GAAGCTTGAA	0	2	1	0	1	At4g23820.1:d:+1249:primary
ATTTTCCCAA	0	4	0	0	0	At4g23330.1:v:+1458:secondary
TTCCAATGTC	0	3	0	1	0	At4g22480.1:p:-494:primary
TGTTTAACTA	2	0	2	0	0	At4g22360.1:d:+1611:primary
TTTACTTATC	1	2	1	0	0	At4g22290.1:i:+3535:tertiary,At4g22280.1:i:+458:tertiary,At4g22280.2:i:+454:tertiary,At1g31500.1:i:+2157:tertiary,At1g31500.2:i:+2175:tertiary,At1g31500.3:i:+2140:tertiary
AACAATAGAT	1	1	0	0	2	At4g22260.1:d:+1266:primary
AGAATTATCC	0	0	1	2	1	At4g22240.1:d:+1266:primary
GTTATTATGC	0	1	1	1	1	At4g21910.2:d:+1705:primary,At4g21910.3:d:+1747:primary,At4g21910.1:d:+1729:primary
TGTGTGAAGT	1	2	0	1	0	At4g21710.1:d:+3368:primary
GACTGGTTTT	1	1	1	0	1	At4g21570.1:d:+1072:secondary
AGATGTGATG	0	3	1	0	0	At4g21540.1:v:+4090:primary
CTAACTTCTA	0	0	4	0	0	At4g21460.1:d:+1509:secondary
AGTGAATGAA	3	0	0	1	0	At4g20940.1:d:+3132:primary
AGCTTTTGCA	0	0	2	1	1	At4g20910.1:d:+3041:primary
GTCAACCTAA	1	2	1	0	0	At4g20870.1:d:+560:primary
GGGCGAATAG	0	0	1	3	0	At4g20830.1:d:+1508:secondary,At4g20830.2:d:+1501:secondary
TCTTTCCTTT	2	0	1	0	1	At4g20400.1:d:+3203:primary
GCTCTCGCTT	0	0	4	0	0	At4g20360.1:d:+388:secondary
TACAATAGCA	1	1	0	0	2	At4g19710.2:d:+3045:primary,At4g19710.1:d:+3132:primary
TACAGAGTCA	1	2	0	0	1	At4g19670.1:d:+1595:primary
GTTGAAATTT	1	1	2	0	0	At4g19610.1:v:+2772:secondary
TATGCTCAAT	2	0	0	1	1	At4g19510.1:i:+4462:tertiary
TCTATTCCTT	1	0	1	0	2	At4g19500.1:v:+3975:secondary
ACTAAATGTC	1	0	2	1	0	At4g19220.1:v:+1134:secondary,At5g19860.1:d:+769:secondary
TCACGTGTTT	0	2	0	1	1	At4g19100.1:v:+1467:primary
TTCAGTGTCA	1	1	2	0	0	At4g18570.1:d:+2092:primary
AAAGAATGAG	0	2	2	0	0	At4g18170.1:d:+1217:primary
GTTTAGTTGC	0	2	0	1	1	At4g18070.1:d:+948:primary,At4g18070.2:d:+707:primary
TCAACAATAT	3	1	0	0	0	At4g17970.1:d:+917:primary
TCGACTTTCT	1	2	0	0	1	At4g17895.1:X:+258:quaternary
TTTATTAGTT	1	2	0	1	0	At4g17670.1:d:+772:primary
TCAGTGGTGC	0	3	0	0	1	At4g17610.1:v:+5854:secondary
ACATCTCCGA	1	2	1	0	0	At4g17560.1:d:-405:primary
TTCCGTGTGG	0	3	1	0	0	At4g17230.1:d:+1763:secondary
AAGAACATAG	0	2	1	1	0	At4g17070.1:d:+1069:primary
GCTTCACCAC	2	0	2	0	0	At4g16980.1:d:+374:secondary,At3g16960.1:v:+432:secondary
TGAACGTTTC	1	2	0	1	0	At4g16920.1:v:+3893:secondary,At4g16860.1:d:+3440:secondary,At4g16950.1:d:+3688:secondary,At4g16950.2:d:+3688:secondary
AAAATAATCT	0	3	1	0	0	At4g16860.1:i:+3401:tertiary

CTCAAAGAGT	2	1	1	0	0	At4g16530.1:d:+2506:primary
TAATGGTCGG	2	2	0	0	0	At4g16444.1:d:+572:primary
ATGGAACAGC	0	1	0	2	1	At4g16380.1:d:-844:secondary
ATCCCTCCAA	1	3	0	0	0	At4g16330.1:d:+891:primary
AGCTCCCTCA	0	1	1	0	2	At4g16070.1:d:+2210:primary
AAGGCTTTAT	0	0	0	0	4	At4g15520.1:X:+554:quaternary
GGTTCAATTG	1	1	0	1	1	At4g15415.1:d:+2264:primary,At4g15415.2:d:+2287:primary
GGGTTCCCTC	0	2	0	2	0	At4g15410.1:d:+1228:primary
TGAATTTCAA	1	1	0	1	1	At4g15093.1:d:+1033:primary
GCCTTGAGCC	1	1	0	2	0	At4g14920.1:v:+3654:primary
CTGGATCTTT	0	1	2	0	1	At4g14910.1:d:+887:secondary
TCGGTATATA	1	1	0	0	2	At4g14905.2:d:+1486:primary,At4g14905.1:d:+1257:primary
GCGACTCTTC	0	2	0	0	2	At4g14890.1:d:+59:primary
GCTCCTGGAC	0	1	2	0	1	At4g14680.1:d:+1178:primary
TGAGTCGCAG	1	0	0	3	0	At4g14605.1:d:+1626:primary
TAACCCAAAC	1	2	1	0	0	At4g14520.1:d:+720:primary,At4g14520.2:d:+750:primary
CCAATGTGGC	0	1	1	2	0	At4g14410.2:d:+936:primary,At4g14410.1:d:+908:primary
GTTCACTCAT	1	3	0	0	0	At4g14385.2:d:+688:primary,At4g14385.1:d:+657:primary
CTTGGGATAT	1	2	0	0	1	At4g14360.1:d:+1928:primary
TGTGTTGTTG	1	1	2	0	0	At4g14350.2:d:+2117:primary,At4g14350.1:d:+2021:primary
TGCTATCTAT	0	1	0	2	1	At4g14300.1:d:+1522:primary
ACAATGTCAT	1	1	1	0	1	At4g14240.1:d:+1164:primary
GAGACGTATT	1	0	2	1	0	At4g14040.1:d:+1816:primary
AAAGCCACTA	0	0	4	0	0	At4g13970.1:v:+2689:primary
GTTCGATTCG	1	0	1	1	1	At4g13940.1:d:+1908:primary
AGAAAATCTC	1	0	0	2	1	At4g13730.2:d:+1498:primary,At4g13730.1:d:+1455:primary,At1g52240.1:d:+1289:primary
CCACATCAAC	0	1	2	1	0	At4g13040.1:d:+780:primary
AGCGAGAATT	0	3	0	1	0	At4g13020.2:d:+1258:primary,At4g13020.1:d:+1542:primary
TGCTTTCTAT	0	0	1	0	3	At4g12830.1:d:+1481:primary
CATTGTTCGA	0	3	1	0	0	At4g12790.1:X:-53:quaternary
ACACTATTAG	1	0	2	0	1	At4g12610.1:X:+223:quaternary
TTGTGACAAG	3	0	0	1	0	At4g12350.1:d:+18:secondary,At2g36890.1:d:+60:secondary
GACCAAAAAA	0	0	2	2	0	At4g12290.1:d:+32:secondary
ATCTCCCGGT	0	2	0	2	0	At4g12290.1:d:+2218:secondary
ACAACTTGGG	1	1	2	0	0	At4g11980.1:d:+1085:primary
AGAGAGTAAA	1	1	2	0	0	At4g11820.2:X:-346:quaternary
ATGAAGAAGA	1	1	0	0	2	At4g11810.1:v:+1807:secondary,At1g74660.1:d:+81:secondary,At1g54460.1:d:+550:secondary,At3g56410.1:d:+208:secondary,At5g03310.1:v:+354:secondary, At5g61710.1:v:+354:secondary,At3g56410.2:d:+76:secondary,At3g10380.1:d:+1023:secondary,At5g57480.1:v:+1861:secondary
AAGCTCCAAG	0	2	1	1	0	At4g11770.1:v:+886:secondary,At3g54760.1:v:+1699:secondary,At4g23580.1:v:+832:secondary
TATATAATGT	0	3	0	0	1	At4g11670.1:i:+3175:tertiary
GAGGAGTGTA	0	1	2	1	0	At4g11570.1:d:+760:primary,At4g11570.2:d:+1031:primary
GTCGATGCTC	0	2	0	2	0	At4g11360.1:d:+140:primary
GCAGGCAAGA	1	0	1	1	1	At4g10780.1:v:+1428:secondary
CCACCATCTA	0	1	3	0	0	At4g10710.1:d:+3376:primary
ATTGTACAAA	0	0	1	1	2	At4g10540.1:X:-303:quaternary
AACGGATAAT	0	3	0	1	0	At4g10450.1:X:-229:quaternary
GTCTGGTTCG	0	1	2	1	0	At4g10130.1:d:+781:primary
TCTTGGAAGA	0	3	0	1	0	At4g10030.1:d:+1194:primary
TAAACAAAAA	0	3	0	1	0	At4g09850.1:v:+532:primary
TTTGATTTCC	0	2	0	0	2	At4g09730.1:d:+1388:primary
ATTTAAGGAC	1	2	0	0	1	At4g09620.1:X:+714:quaternary
ATTATGTAAA	0	0	2	1	1	At4g09580.1:d:+1253:primary
TCAAACATCT	0	1	1	2	0	At4g09550.1:d:+141:primary
GAGCTTAGTG	2	0	1	1	0	At4g09350.1:d:+643:primary
CTTCTTCTAT	3	0	0	0	1	At4g09255.1:p:+1501:secondary,At4g09205.1:p:+1503:secondary,At4g09313.1:p:+2018:secondary
GGCTTCCAAG	0	1	2	1	0	At4g08930.1:d:+1132:primary
ACAGCAATGG	0	0	1	3	0	At4g08900.1:d:+1075:primary
TTTACTTAAA	2	2	0	0	0	At4g08620.1:i:+5422:tertiary
TGCAATGGTC	0	3	1	0	0	At4g07933.1:X:-461:quaternary,At5g06600.2:X:-313:quaternary
GATGCACATA	1	0	1	1	1	At4g07874.1:p:+756:secondary,At3g19480.1:d:+1843:secondary

ATCTCGTTTG	1	2	0	1	0	At4g07825.1:d:+1321:primary
CAAATCGTGA	1	2	0	0	1	At4g06599.1:d:+881:primary
AGGAAAAAGT	3	0	0	0	1	At4g05020.1:d:+1873:secondary,At5g27200.1:v:+971:secondary
AATCTTTGTA	1	3	0	0	0	At4g04925.1:d:+580:primary
TTATTTTGCT	3	0	0	1	0	At4g04910.1:d:+2434:secondary
AAACTACCAA	0	2	1	0	1	At4g04760.1:v:+1867:primary
AGCTCTGTAA	1	0	0	1	2	At4g04570.1:d:+1908:primary
ACTTCAACTA	0	0	2	2	0	At4g03420.1:d:+1283:primary
GAGCTTTCAC	1	1	1	1	0	At4g03150.1:d:+536:primary
CCTCGGGGGA	0	3	0	0	1	At4g02950.1:v:-17:primary
TCGTTGTATT	1	0	0	2	1	At4g02950.1:v:+160:secondary
CAAACCTAAG	1	0	1	1	1	At4g02920.2:d:-1428:secondary,At4g02920.1:d:-1346:secondary
TCTTGGTCTG	0	0	2	1	1	At4g02860.1:d:+928:secondary
GCTTATATAT	2	0	0	0	2	At4g02640.1:d:+1370:primary,At4g02640.2:d:+1389:primary
AAGCCCGGTT	0	3	0	1	0	At4g02540.1:d:+2344:primary
GGTCCTAAAG	0	3	1	0	0	At4g02500.1:d:+1005:secondary
TCTCTGGTGC	0	2	2	0	0	At4g02440.1:d:+1048:primary
TCCATTGCAG	2	0	1	0	1	At4g02420.1:v:+2547:primary,At3g02610.1:d:+1308:primary
ATTGAGAGAA	0	1	1	2	0	At4g02370.1:d:+592:primary
AAGGTGGTTT	1	2	0	1	0	At4g02340.1:d:+860:primary
CTTTCTAGCT	1	1	1	1	0	At4g01935.1:v:+1128:secondary
GTTATAGTTT	0	3	0	0	1	At4g01915.2:d:+1054:primary,At4g01915.1:d:+971:primary,At4g01915.3:d:+613:primary
GTGTATACGA	0	1	2	1	0	At4g01880.1:d:+1469:primary
AGATGCAAAA	2	1	1	0	0	At4g01730.1:v:+2014:primary
TAATCAAATT	0	3	0	1	0	At4g01600.1:d:+710:primary
ACCACAAAAA	0	2	1	1	0	At4g01410.1:d:-900:secondary
TGAGCTGTTG	2	2	0	0	0	At4g01390.1:X:-37:quaternary
TGTACCTTTA	0	2	1	0	1	At4g00420.1:d:+938:primary,At4g00420.2:d:+941:primary
CCCTCCCCCT	1	1	0	2	0	At4g00165.1:d:+158:primary
GAGATGTCGC	0	3	1	0	0	At4g00150.1:d:+1840:primary
TTGATATATT	2	1	0	0	1	At4g00120.1:d:+658:secondary,At5g51540.1:v:+934:secondary,At2g10910.1:p:+4155:secondary
AGGCGTATTT	1	0	0	2	1	At4g00090.1:d:+1433:primary
AATTATGATA	1	0	1	0	2	At4g00050.1:d:+1381:primary
TAAGCCTCAT	0	1	2	0	1	At3g66658.1:d:+2038:primary,At3g66658.2:d:+2034:primary
CCAGTTGGAC	0	1	1	2	0	At3g63270.1:d:+1427:primary
GAAAAGTTTT	0	1	3	0	0	At3g63180.1:X:-538:quaternary,At2g46960.2:X:-46:quaternary
AGCAATGAAT	1	1	0	0	2	At3g62980.1:d:+2116:primary
AACCGGTTGA	0	1	2	1	0	At3g62570.1:d:+1539:primary,At1g64050.1:d:+2224:primary
TTACCAAGAT	2	1	0	1	0	At3g62450.1:v:+726:secondary
AGTTTGAATG	1	2	0	0	1	At3g61880.1:d:+1549:primary,At2g46660.1:d:+1481:primary
TTGCTCTAAA	0	0	2	1	1	At3g61750.1:X:-762:quaternary
TAAAAAAACT	0	3	0	0	1	At3g61710.1:d:+1721:primary,At3g61710.2:d:+1825:primary
TTGTGTTGAT	1	0	0	0	3	At3g61650.1:v:+2128:primary
TCCTTTGAAA	0	1	2	1	0	At3g61510.1:X:-359:quaternary
TGTGCTTCCG	0	0	2	2	0	At3g61430.1:d:+427:secondary,At2g45960.1:d:+383:secondary
AGATCTTAAA	0	0	1	1	2	At3g61390.1:d:-122:primary,At3g61390.2:d:-122:primary
TAAAGACTCG	0	1	0	1	2	At3g61240.2:d:+2008:primary
TGTGTTTGTC	3	0	0	1	0	At3g60910.1:d:+828:primary
ACACCTTTTG	1	2	1	0	0	At3g60790.1:v:+861:secondary
TGTTTTTATA	1	0	2	0	1	At3g60740.1:i:+1593:tertiary
GTTCTGAATT	0	0	2	2	0	At3g60600.1:d:+651:secondary
TCAAAGATTT	1	3	0	0	0	At3g60590.1:d:+987:primary,At3g60590.2:d:+1089:primary
GAATTAGGTC	1	1	1	1	0	At3g60480.1:d:+424:primary
CTTGAAAGGG	0	1	3	0	0	At3g60410.1:d:+1098:primary
CTGCAAAGAA	1	1	1	1	0	At3g60350.1:d:+3112:secondary
TTCGTTGAGC	3	0	0	0	1	At3g60240.1:d:+1110:secondary
GAATCTGCGT	1	2	0	0	1	At3g60190.1:d:+1612:primary
TCTTGTCGGG	0	1	1	1	1	At3g59880.1:v:+588:secondary
AACTTACAAA	1	1	0	0	2	At3g59330.1:d:+277:secondary
ACGAGAGTTT	1	1	2	0	0	At3g59300.1:d:+1564:secondary
CTTCAGAAAG	0	0	0	2	2	At3g58890.1:i:-793:tertiary

AGTTTCTCTT	0	2	1	0	1	At3g58630.1:d:+1235:primary
ACGAAAAGGA	0	0	2	2	0	At3g58410.1:v:+703:secondary
CGGCAACTCC	0	3	0	1	0	At3g58050.1:d:+3293:primary
AGCGCTGAAT	1	2	0	1	0	At3g57990.1:d:+404:primary
AATGTGTATA	0	0	0	1	3	At3g57800.1:d:+1597:primary,At3g57800.2:d:+1565:primary
TGAACGGGTT	0	2	0	0	2	At3g57790.1:v:+1624:secondary
CATAACGCCG	0	2	0	1	1	At3g57570.1:d:+3259:primary
TTCCTTTTTT	0	0	2	2	0	At3g57500.1:v:+846:primary
TTGCCTTCCG	0	2	0	1	1	At3g57080.1:d:+668:primary
GCGTCGTCGG	0	0	4	0	0	At3g56880.1:d:+133:secondary
GAGTTCCTGG	1	2	0	1	0	At3g56840.1:d:+1355:primary
CCATACCAGT	0	2	0	1	1	At3g56590.1:d:+1461:secondary
TCTCTCTTTC	0	1	0	1	2	At3g56580.2:d:+1257:secondary,At3g56580.1:d:+1383:secondary
TGACGTTGCG	1	1	0	1	1	At3g56510.1:d:+884:secondary
ATCCTCTCGA	3	0	1	0	0	At3g56190.1:X:-370:quaternary
TAATGGTCAA	0	2	1	0	1	At3g55530.1:d:+1029:secondary
ATGAATCTAA	1	0	0	1	2	At3g55520.1:d:+785:secondary
AATTATTTTC	0	0	1	1	2	At3g55510.1:d:+2130:primary
GCTCACTGAA	2	0	2	0	0	At3g55420.1:d:+846:primary
GATGATGTAT	1	0	1	0	2	At3g55320.1:v:+4914:primary
GAGTTCCTAA	0	2	1	1	0	At3g55140.1:d:+1030:primary,At3g55140.2:d:+1188:primary
CTTGAGGACG	1	2	1	0	0	At3g55130.1:d:+2130:primary
TTGTTTGCCT	1	1	1	1	0	At3g55010.1:d:+1492:primary,At2g27930.1:v:+1025:primary,At3g50830.1:d:+1006:primary,At3g55010.2:d:+1599:primary
AAAGAGGGAG	0	1	1	2	0	At3g54980.1:d:+2746:primary
AAATCTGATG	1	2	0	0	1	At3g54960.1:d:+1711:primary
GAAAAACTAG	0	2	1	1	0	At3g54860.1:d:+1774:primary
GATTCTCTTA	0	3	0	1	0	At3g54826.1:d:+561:primary
GTGGCTCAGT	1	1	2	0	0	At3g54820.1:d:+481:primary
TGCAATTCTT	1	0	1	2	0	At3g54540.1:d:+2485:primary
AGTACAATCC	1	2	0	0	1	At3g54400.1:X:-519:quaternary
CTACTCCGGC	0	0	1	1	2	At3g54400.1:d:+1075:secondary
CTAGAATTAA	1	0	1	1	1	At3g54360.1:d:+613:secondary
GCTGGCGTCA	1	2	1	0	0	At3g54200.1:d:+610:primary
CACATATAGA	1	0	0	1	2	At3g54150.1:i:+953:tertiary
AAGATCAAAG	3	0	1	0	0	At3g53750.1:d:+1135:primary
GTGATGCAGA	0	3	1	0	0	At3g53700.1:d:+2145:secondary
TATAGAAATG	2	2	0	0	0	At3g53570.2:d:+1689:primary
TATAGAGCAA	1	2	0	0	1	At3g53400.1:d:+1861:primary
ATTTATAATT	0	3	0	0	1	At3g53340.1:v:+1067:secondary
TTTGATTAGG	0	0	1	2	1	At3g53270.3:d:+1243:primary,At3g53270.1:d:+1202:primary,At3g53270.4:d:+1307:primary,At3g53270.2:d:+1393:primary
GAAGCGATTC	2	0	0	1	1	At3g52630.1:v:+700:secondary
TGTTCTTAAT	2	1	0	0	1	At3g52420.1:v:+615:secondary
AGCTTCTTCT	2	0	1	0	1	At3g52100.1:v:+2482:secondary,At4g15810.1:v:+2436:secondary
CTTTATTGTA	1	2	0	0	1	At3g52050.2:d:+1663:primary,At3g52050.1:d:+1508:primary
GCAAATCCAT	1	1	1	0	1	At3g52040.1:d:+127:primary
TTTGGGGAAC	0	0	0	1	3	At3g51870.1:d:+1304:primary
AATGAGGGAG	0	2	0	0	2	At3g51860.1:d:+1525:primary
AAAGCAGAAA	1	0	3	0	0	At3g51710.1:v:+210:secondary
GACAAAGGTA	1	0	1	2	0	At3g51700.1:d:+919:secondary
TTACCTTCAC	0	3	0	0	1	At3g51400.1:d:+929:secondary
AAGAGTAATC	0	2	0	2	0	At3g51310.1:d:+2423:primary
GGTTGCTTTC	1	0	2	1	0	At3g51250.1:d:+1311:primary
GAGGCTAGCT	2	1	1	0	0	At3g51110.1:d:+1873:primary
GAGATTCCGT	0	1	1	1	1	At3g50900.1:X:+611:quaternary
GTGTGTCTAG	0	2	1	0	1	At3g50530.1:d:+2067:primary
CTGCTAATGT	0	1	1	1	1	At3g49920.1:v:+508:secondary,At1g54170.1:d:+447:secondary
ACGGATTCAA	1	1	1	1	0	At3g49670.1:d:+2901:secondary
AAGTGAATCG	0	2	1	1	0	At3g49260.1:d:+832:secondary,At3g49260.2:d:+918:secondary
AACCAAAATC	0	3	0	0	1	At3g49140.1:d:+3755:primary

TTGAGATATA	2	1	1	0	0	At3g48760.1:d:+1440:primary
GGTTTGAAAT	1	2	0	1	0	At3g48440.1:v:+1753:secondary,At5g15030.1:d:+779:secondary
AATCATCTGA	1	1	1	1	0	At3g48195.1:d:+1401:secondary
ATTTCAGGTT	1	0	0	3	0	At3g48140.1:d:-295:secondary
AGCTCTTACC	1	1	0	1	1	At3g47965.1:d:+217:primary
GAATCACGAG	1	0	1	1	1	At3g47833.1:d:+533:primary
CTTCAGAGAC	1	3	0	0	0	At3g47810.2:d:+462:secondary,At3g47810.1:d:+417:secondary,At3g47810.3:d:+469:secondary
AAAGTTTAGA	0	2	1	0	1	At3g47630.1:d:+1449:primary
ATCTATAAAA	2	0	0	1	1	At3g47600.1:i:+771:tertiary
AATAATAGAT	3	1	0	0	0	At3g47460.1:d:+3755:secondary
GTAAAGTCTG	1	3	0	0	0	At3g47040.1:i:+1745:tertiary
TTGCTCTTCT	0	2	0	0	2	At3g46830.1:d:+855:primary
ATCGATCAGC	0	0	1	3	0	At3g46560.1:d:+115:primary
AAATTTCTTG	2	1	1	0	0	At3g46420.1:v:+1722:secondary,At4g09680.1:d:+1664:secondary
ACGTAGGACG	1	0	0	3	0	At3g46180.1:d:+1193:primary
AGGAAACATA	1	0	1	1	1	At3g46070.1:v:+666:secondary
TTCTTAGGCT	2	2	0	0	0	At3g45620.1:d:+1314:primary
GCCACGCGAT	2	0	1	1	0	At3g45050.1:d:+414:primary
TACATAACAA	0	1	1	0	2	At3g45040.1:d:+1807:primary,At5g29050.1:v:+1069:primary,At4g06490.1:v:+1023:primary
GGTTTTTGTA	0	0	1	2	1	At3g44400.1:v:+3705:primary,At1g79490.1:d:+2690:primary
AATGAATGTT	1	1	2	0	0	At3g44140.1:v:+117:primary,At4g27880.1:d:+1485:primary
TCTCAAAAAA	1	1	1	0	1	At3g43730.1:p:+2411:secondary
TGTAGACTTT	1	3	0	0	0	At3g43670.1:d:+2157:secondary
AAACATATAA	0	2	0	0	2	At3g43652.1:p:+1716:secondary,At2g13230.1:p:+3442:secondary,At1g54350.1:d:+2220:secondary
AGTCCAATGG	0	1	3	0	0	At3g43590.1:d:+1352:secondary
TTTGTGATTT	1	1	0	1	1	At3g43440.1:d:+921:primary
TGAACCATTT	0	2	0	1	1	At3g42052.1:p:+5245:secondary
TCATCATTGG	1	0	1	0	2	At3g31900.1:v:+589:secondary,At5g23740.1:d:+477:secondary,At3g32280.1:v:+874:secondary,At4g12920.1:v:+1426:secondary
GATCCAAGAC	0	4	0	0	0	At3g30380.1:v:+1596:secondary
AAAAACCAAA	1	0	2	0	1	At3g30180.1:d:+305:secondary
TTACAAATCC	2	0	0	2	0	At3g29575.1:d:+740:primary
TTCTGTGAAC	2	1	1	0	0	At3g29310.1:d:+1804:primary
GGATGTGTTT	1	2	1	0	0	At3g29290.1:d:+1806:primary
GCGGAGCGGA	0	3	0	1	0	At3g29140.1:v:+627:primary
TAAGACACAA	2	1	0	1	0	At3g29120.1:X:+870:quaternary
ACTAAAAAAG	0	1	1	2	0	At3g29075.1:d:+946:primary
GCTACAAAGT	1	2	0	1	0	At3g28690.1:d:+377:secondary
GAGAAAAGGA	1	3	0	0	0	At3g28590.1:v:+120:secondary,At3g05150.1:d:+76:secondary
GAACTTGACT	1	0	1	1	1	At3g28570.1:v:+2048:primary,At2g46810.1:d:+1230:primary
TCTCCAGCTG	0	1	2	1	0	At3g28540.1:d:+1282:primary
CACTCTCTTC	1	2	0	1	0	At3g27883.1:p:+5213:primary,At5g49630.1:d:+1539:primary,At3g28945.1:p:+5385:primary
AGAAGTTTTA	0	1	1	2	0	At3g27880.1:d:+816:secondary
TTTGTTGAAA	0	1	2	1	0	At3g27830.1:d:+830:secondary
TTACCTTTGA	0	0	2	0	2	At3g27310.1:d:+814:primary
GCTACATTTG	1	1	0	2	0	At3g27300.1:d:+1658:primary
GCTTCAACTT	2	1	1	0	0	At3g27180.1:d:+1422:primary
TTTGTTGGGA	1	2	1	0	0	At3g27020.1:d:+1979:primary
ATGAATAGAG	0	1	0	1	2	At3g26840.1:d:+2152:secondary
CACATTCTTC	1	0	1	1	1	At3g26670.1:d:+1585:primary,At3g26670.2:d:+1744:primary,At3g26670.3:d:+1642:primary
AAAGTGAAAT	2	2	0	0	0	At3g26420.1:d:+3021:primary
CAAAGAAAAA	1	1	1	1	0	At3g26390.1:v:+241:secondary
AATTAAACAT	1	3	0	0	0	At3g26200.1:d:-1733:secondary
CAAACGTGCT	0	3	0	1	0	At3g26100.2:d:+1738:primary,At3g26100.1:d:+1858:primary
AATACAACTC	1	1	1	1	0	At3g25860.1:d:+1244:primary
GGCCACATAC	0	0	1	2	1	At3g25660.1:d:+1893:primary
GTTGCGATGC	0	2	2	0	0	At3g25585.1:d:+583:secondary,At3g25585.2:d:+715:secondary,At1g80280.1:d:+509:secondary
TGATGTTCTG	2	1	1	0	0	At3g25570.1:d:+1448:primary
TTGTTGTAAA	1	2	0	1	0	At3g25500.1:d:+3361:primary
TTTCCTTCCC	0	1	1	0	2	At3g25485.1:X:-3523:quaternary
GATCGCCGGG	1	1	0	1	1	At3g25040.1:d:+725:primary

TAATTAGACG	0	1	1	0	2	At3g24760.1:d:+1266:primary
GTGGTGGTGA	1	2	0	1	0	At3g24490.1:d:+929:primary
TTGGGTATTG	2	1	0	1	0	At3g24080.1:d:+1329:primary
AACTTGAAGA	0	0	1	0	3	At3g23980.1:d:+2675:primary
ACTTCAAAAA	1	1	0	1	1	At3g23970.1:X:--154:quaternary,At2g29620.1:X:-632:quaternary
TGATGACATT	3	1	0	0	0	At3g23760.1:d:+524:primary
GCTCGCAGGC	1	2	0	1	0	At3g23660.1:d:+2198:primary
CCAAGGGTTT	1	1	1	1	0	At3g23550.1:d:+1349:primary
ATAGGTTAAT	0	0	3	1	0	At3g23540.1:X:+519:quaternary,Chr2:+17577708:quaternary
GCTCTTGGAT	0	2	1	1	0	At3g23510.1:d:+2731:secondary,At3g16260.1:d:+931:secondary,At3g23530.1:d:+2752:secondary
ACTTAGCCAA	0	3	0	1	0	At3g22845.1:d:+455:primary
ACGTCAGACG	0	0	0	0	4	At3g22840.1:d:+608:secondary
GTTTCTGAAA	2	1	0	0	1	At3g22790.1:v:+5524:primary
TTCTTAAAAA	1	1	1	1	0	At3g22650.1:v:+1677:secondary
AGCCATCGTC	0	2	0	1	1	At3g22425.1:d:+1035:primary,At3g22425.2:d:+926:primary
AGTTCGATAG	0	2	0	2	0	At3g22420.1:d:+1517:primary,At3g22420.2:d:+1694:primary
GAAGTCGCGA	0	3	1	0	0	At3g22370.1:d:+788:secondary,At3g22360.1:v:+954:secondary
ACTCGAGGAA	0	4	0	0	0	At3g22270.1:d:-2301:secondary,At3g22270.1:d:-2305:secondary
GGAGAAACAG	1	1	1	1	0	At3g22190.1:v:+2035:primary
CTTGCATTTT	0	2	2	0	0	At3g22160.1:d:+1077:primary
TAACTAAAGG	0	2	0	1	1	At3g22120.1:X:-1149:quaternary
TAAAGTTCGT	1	3	0	0	0	At3g21630.1:X:-247:quaternary
GAAGACATCT	1	1	0	1	1	At3g21200.1:d:+668:primary
GAATGATATT	0	1	2	1	0	At3g21060.1:d:+2097:secondary
TTCATATATT	1	0	1	0	2	At3g20800.1:i:+799:tertiary
AGTTATGAGC	0	0	0	3	1	At3g20720.1:d:+3587:primary
TATATGAATA	1	0	0	2	1	At3g20570.1:d:+801:primary,At3g51090.1:d:+1227:primary
AGAAAGAGAG	1	0	0	1	2	At3g20500.1:d:+645:secondary,At5g55820.1:v:+937:secondary
GCAATGGGTT	0	1	1	2	0	At3g20470.1:p:+604:secondary
GATGCTGCTG	1	3	0	0	0	At3g20410.1:d:+1499:primary
ATAACTTTTC	0	1	1	2	0	At3g20290.1:d:+1978:primary
TACGTAAGCC	1	0	0	0	3	At3g20270.1:d:+2266:primary,At3g20270.2:d:+3244:primary
TACAACTACT	0	3	0	0	1	At3g20000.1:d:+816:primary
AAATTCGGAC	0	3	1	0	0	At3g19930.1:d:+1622:primary
AAAATCCAAA	1	1	0	2	0	At3g19515.1:d:+355:secondary
ACCGGCAAGG	0	3	1	0	0	At3g19420.1:d:+1384:primary
GGGCTGAAGC	2	0	1	1	0	At3g19340.1:d:+1536:primary
GAGCTTTATG	1	1	1	0	1	At3g19300.1:d:+360:secondary,At5g26600.2:d:+1550:secondary
TTTCCACTTC	1	1	1	0	1	At3g19260.1:d:+1175:primary
ACCACTTCCC	0	1	2	0	1	At3g19170.1:X:-20:quaternary
ATGAATAGTT	1	2	0	1	0	At3g19090.1:v:+2140:primary,At2g48000.1:d:+1637:primary
GTGTCTCAAA	0	0	2	0	2	At3g19030.1:d:+129:secondary
ATCTCTTTCT	2	2	0	0	0	At3g18800.1:d:+620:primary
TGTGAATTTC	0	0	2	0	2	At3g18450.1:d:+682:primary
GCCTGCACGG	0	2	1	1	0	At3g18440.1:d:+1575:primary
TCTGATGAGC	2	0	1	0	1	At3g18430.1:d:+485:primary
TGGATTTATT	1	0	0	3	0	At3g18380.1:d:+1423:primary,At3g18380.2:d:+1406:primary
AGAAACCCTA	0	2	2	0	0	At3g18280.1:d:+258:secondary
GCCGAGGGAC	0	0	1	3	0	At3g18130.1:d:+66:primary
AGCTATGAAC	0	2	0	0	2	At3g18040.1:d:+1747:primary,At3g18040.2:d:+2084:primary
ACGTTCAAGC	0	0	0	2	2	At3g18000.1:d:+1444:primary
TTTGACTTTA	1	1	1	0	1	At3g17860.2:d:+34:secondary,At1g67920.1:d:+398:secondary
TCCTTGCCAG	1	2	0	1	0	At3g17670.1:i:+714:tertiary
ATCTTCCACA	2	1	1	0	0	At3g17470.1:d:+1710:primary
GAATCTGGTA	1	1	1	1	0	At3g17450.1:d:+2862:primary
TTCTTTTACT	3	0	0	1	0	At3g17440.1:d:+1175:primary
TAGAAGCCCT	1	1	0	1	1	At3g17240.1:d:+1605:primary,At3g17240.3:d:+1605:primary
TAGTAGAGAT	0	0	1	2	1	At3g17205.1:v:+3130:secondary,At2g26060.1:d:+507:secondary
ACCCAAAAAC	0	1	1	1	1	At3g17160.1:d:+856:primary
GTGGCGGTTT	0	4	0	0	0	At3g17050.1:p:+1370:primary
GTTTCAATTA	1	3	0	0	0	At3g16950.1:d:+2119:primary

AGTTCTCCTT	2	2	0	0	0	At3g16850.1:d:+1409:secondary
GAGAAATACA	1	0	1	1	1	At3g16760.1:d:+1521:secondary,At3g16760.2:d:+1464:secondary
GTTCTGGATA	0	4	0	0	0	At3g16740.1:d:+1161:primary
TATAGTCCGT	2	0	0	1	1	At3g16560.1:d:+1839:secondary
AACGTTTGTG	0	0	2	1	1	At3g16400.1:d:+1497:secondary
CCTTTACCTC	1	3	0	0	0	At3g16310.1:d:+996:primary
AATATAGATC	2	1	1	0	0	At3g16130.1:d:+1881:primary
TGTTGGCTGA	0	0	1	3	0	At3g16050.1:d:+1115:primary
AAAAAGATGA	0	2	2	0	0	At3g16010.1:d:+2032:primary
AAACTGTAAG	0	1	1	1	1	At3g15790.1:d:+797:primary
GTCCGGTTCA	0	4	0	0	0	At3g15610.1:d:+1106:primary
TTCTTGAAAA	1	2	1	0	0	At3g15590.1:d:+625:secondary,At2g04160.1:v:+75:secondary
AGATTGACAA	0	3	0	1	0	At3g15500.1:d:+722:primary
CCTGATTTGG	0	2	1	1	0	At3g15480.1:X:+492:quaternary
TGGAACGATG	1	0	1	1	1	At3g15460.1:d:+1007:primary
AGCTCAATAG	0	2	1	1	0	At3g15353.1:X:-173:quaternary
GACGTTGGTG	0	0	4	0	0	At3g15353.1:d:+196:secondary
TCAAACCAAG	0	2	0	0	2	At3g15260.1:d:+885:primary,At3g15260.2:d:+885:primary
AAAATCTGTA	1	1	0	0	2	At3g15095.1:X:+2234:quaternary
AAAAAAATTG	1	1	1	0	1	At3g15095.1:d:+1342:primary
CAGAATGCTT	0	2	1	1	0	At3g15070.1:d:+1521:secondary
CACATTGAAG	0	1	1	1	1	At3g15030.2:d:+1589:primary
AATCAAGAGA	0	2	0	1	1	At3g14720.1:d:+1519:secondary
CTCTGAAATC	0	0	2	2	0	At3g14620.1:d:+1607:secondary,At2g39220.1:d:+755:secondary
GATGGTGTGG	0	2	2	0	0	At3g14560.1:d:+630:secondary
CTTCTGGAAA	0	2	1	1	0	At3g13750.1:d:+2478:secondary,At4g00440.1:d:+983:secondary,At1g74690.1:d:+931:secondary
AATCATCTTC	1	2	1	0	0	At3g13700.1:d:+1937:secondary,At3g24030.1:d:+840:secondary,At3g13710.1:d:+1977:secondary
GGGGTGGTGA	2	2	0	0	0	At3g13530.1:d:+2459:secondary
TATGTTGCTG	2	1	0	0	1	At3g13445.1:d:+1087:primary
TTCCATTTTG	0	1	0	3	0	At3g13226.1:d:+1295:primary
TCCAAGAGTT	0	3	0	1	0	At3g13060.2:d:+1839:primary,At3g13060.1:d:+1925:primary
TCCAAAACCA	1	0	1	2	0	At3g13040.1:d:+1468:primary,At3g13040.2:d:+1322:primary
TGTATAAGGG	0	1	0	2	1	At3g12980.1:d:+5259:primary
GGTTAGGCCA	0	2	1	1	0	At3g12810.1:d:+6030:primary
TATCTTGTGA	0	3	1	0	0	At3g12740.1:d:+1206:secondary
GATATGAATG	1	0	0	1	2	At3g12670.1:d:+1979:primary
ATGATATAAC	1	1	0	0	2	At3g12620.1:d:+1360:primary,At5g06750.1:d:+1332:primary
TTGGGGATCT	3	0	0	1	0	At3g12400.1:d:+1494:primary
ACTTATTACG	1	2	1	0	0	At3g12360.1:d:+1707:primary
TTAAAAAAAT	0	0	2	1	1	At3g12340.1:v:+2503:primary
TTAGATTCAA	2	2	0	0	0	At3g12320.1:d:+674:primary
AGCATCGCAG	0	1	1	1	1	At3g12270.1:d:+1682:primary
ACAGAAGATG	0	2	1	0	1	At3g12250.2:d:+1568:primary,At3g12250.1:d:+1494:primary,At3g12250.3:d:+1705:primary
TTCCCCTCCA	1	2	0	1	0	At3g12200.1:d:+1575:primary
CATAACAGAG	1	3	0	0	0	At3g12080.1:d:+1460:primary
GTGAGCTTGT	2	1	1	0	0	At3g11810.1:d:+961:primary
GATGGTGCCG	0	1	3	0	0	At3g11420.1:d:+1611:secondary
TGATACATCA	3	0	0	0	1	At3g11170.1:d:+1258:primary
AACTGTTGCT	0	0	2	1	1	At3g11020.1:d:+1366:primary
TCTCTAAGGC	1	0	1	0	2	At3g10730.1:d:+1233:secondary
TAAATCTAGA	0	3	1	0	0	At3g10720.2:d:+2019:primary,At3g10720.1:d:+1218:primary
CCTCGGGCCG	0	1	1	1	1	At3g10540.1:d:+1806:primary
TAATTTTCAG	1	1	2	0	0	At3g10250.1:d:+1268:primary,At3g10250.2:d:+1195:primary
ACAAGTCGAC	2	1	0	1	0	At3g10060.1:d:+488:primary
TAAATTTGCT	0	3	1	0	0	At3g09922.1:d:+504:primary
CTTATAAATC	0	3	0	1	0	At3g09920.1:d:+2569:primary
GAGATTCCGA	0	0	1	0	3	At3g09830.2:d:+1617:primary,At3g09830.1:d:+1684:primary
TGAGTTAAGT	2	0	0	1	1	At3g09760.1:d:+1759:primary
ATGAGAGACA	0	0	2	2	0	At3g09590.1:v:+320:secondary
TTGATGTTCT	2	2	0	0	0	At3g09570.1:d:+1288:primary

TTAGATTTTT	0	4	0	0	0	At3g09410.3:i:+1413:tertiary,At3g05840.1:i:+276:tertiary,At3g09410.1:i:+1429:tertiary,At3g05840.2:i:+255:tertiary
GAATCGTTGT	1	1	1	1	0	At3g09085.1:d:+519:primary
AAAGACCTTG	0	3	0	1	0	At3g09050.1:d:+531:primary
ACTGGAAACC	0	3	1	0	0	At3g09000.1:d:+1751:primary
AATTTGATAA	1	2	0	1	0	At3g08890.1:d:+700:primary
TCAAGAGTTT	2	0	1	0	1	At3g08780.1:d:+1269:primary
TGTCAGAAGG	3	0	0	0	1	At3g08730.1:X:-363:quaternary
TATATGTGGA	2	2	0	0	0	At3g08620.1:X:+405:quaternary,Chr4:+6553798:quaternary
GCCCAACACT	0	3	0	1	0	At3g07640.1:d:+786:primary
TAGTTAACCT	1	0	2	1	0	At3g07510.1:X:-20:quaternary,At1g76240.1:X:-1070:quaternary
GCTAACCAGA	2	0	1	1	0	At3g07180.1:d:+1814:secondary,At3g07180.2:d:+1379:secondary
AAAAAAATAA	1	1	1	1	0	At3g07010.1:i:+1873:tertiary
ACCTCTTACT	0	3	0	0	1	At3g07010.1:d:+1381:primary
GAGTCTTGCA	0	0	2	0	2	At3g06980.1:d:+2569:primary
GTTGAGCTTC	0	1	2	1	0	At3g06960.2:d:+1768:primary,At3g06960.1:d:+1454:primary
CAAAGCTACT	1	2	0	1	0	At3g06940.1:p:+2921:primary
TAATCCTGTC	1	1	2	0	0	At3g06750.1:d:+233:primary
GAGAATTAGT	1	2	0	1	0	At3g06480.1:d:+3543:primary
TAAAATCGTT	1	0	3	0	0	At3g06250.1:d:+2937:primary
TAATTAACAA	2	0	2	0	0	At3g06240.1:d:+1663:primary
CTTCGAATTA	0	0	2	2	0	At3g06145.1:v:+847:secondary
TATAGATAAC	3	0	0	1	0	At3g06140.1:d:+1338:primary
GAGTTTATGT	0	0	1	1	2	At3g05990.1:d:+1918:primary
AAGAGAGTGG	1	2	1	0	0	At3g05937.1:v:+810:secondary
GTTTCAAGTT	0	1	2	1	0	At3g05880.1:i:+563:tertiary
ATGATTATAT	0	1	0	1	2	At3g05760.1:d:+687:secondary
ATGATACAAG	1	1	1	0	1	At3g05670.1:d:+3041:primary
GCAGAACACT	0	1	2	0	1	At3g05520.1:d:+925:primary
GCACCTGAAG	0	2	1	0	1	At3g04910.1:d:+1029:secondary
TGACCTTAAG	0	0	3	0	1	At3g04840.1:d:+595:secondary
ATGATCTCCC	0	4	0	0	0	At3g04640.1:d:+270:primary
GCTGAAGCTG	1	1	0	2	0	At3g04610.1:d:+1690:primary
GGTTACATTA	0	0	1	2	1	At3g04140.1:d:+2200:primary
GATTTGCTCC	0	4	0	0	0	At3g03990.1:d:+493:primary
AGAGGTAGAG	1	0	2	0	1	At3g03770.1:d:+2720:primary
ACCCAGAAAT	1	0	0	2	1	At3g03680.1:d:-3232:secondary
TAATCTTCTG	1	2	1	0	0	At3g03680.1:d:+3237:primary
AGAAACAATC	0	0	2	0	2	At3g03640.1:d:+1506:primary
TCATCGACTC	0	1	1	0	2	At3g03570.1:d:+2153:primary
GACAAGGTTG	0	1	2	1	0	At3g03330.1:d:+903:primary
AGATTTGAAT	1	1	1	1	0	At3g03305.1:d:+2352:primary
GCATACTTGT	1	0	2	1	0	At3g03305.1:d:+1840:secondary,At1g29060.1:d:+585:secondary
ATAATAATTG	1	3	0	0	0	At3g03300.1:v:+4343:secondary
ATGAAATCCC	0	0	0	2	2	At3g03160.1:d:+367:primary
GTAAGAACGT	1	1	1	1	0	At3g03080.1:d:+1033:primary,At5g17000.1:d:+1067:primary,At5g16990.1:d:+1169:primary
AATCTCTCTC	1	2	0	0	1	At3g02990.1:d:+962:secondary
TTAAATCAAC	4	0	0	0	0	At3g02790.1:d:+533:primary
TTATGTAAAC	2	0	2	0	0	At3g02720.1:d:+1249:primary
GAAGCTAAAC	1	0	1	2	0	At3g02700.1:d:+859:primary
ATTTGTCCAA	0	0	1	1	2	At3g02350.1:d:-1742:secondary,At3g02350.1:d:-1737:secondary
GATCTGTTTC	2	1	0	1	0	At3g01790.2:d:+835:primary
CTTCCAAGTA	1	2	0	0	1	At3g01790.1:d:+471:primary
TTTCATAAAT	2	0	0	0	2	At3g01760.1:v:+2183:primary
TGAGCTTTAA	1	1	0	2	0	At3g01640.1:d:+1187:primary
GGATTCCCAA	0	1	3	0	0	At3g01560.1:d:+1701:primary
ATTGGATATC	1	0	1	0	2	At3g01410.1:X:-196:quaternary
TTTAGGTTGT	3	0	1	0	0	At3g01150.1:d:+1351:primary
CCACCAAGAT	0	1	1	1	1	At3g01090.2:d:+2175:primary,At3g01090.1:d:+1956:primary
TAAGAGAGTA	1	1	0	0	2	At3g01050.1:X:+348:quaternary
TTTTTTGTTT	0	2	1	0	1	At2g48130.1:d:+642:primary,At4g37780.1:d:+959:primary

TGATGTGAAT	0	2	0	1	1	At2g47850.1:d:+1783:primary
GTGATTTATA	0	0	2	0	2	At2g47760.1:d:+1461:primary
CTCGAGCTCG	2	2	0	0	0	At2g47650.1:d:+1183:primary
GAAGGAATAT	0	4	0	0	0	At2g47550.1:d:+1445:secondary,At3g05610.1:d:+1619:secondary,At3g14310.1:d:+1524:secondary
TTCATAAAAG	0	2	2	0	0	At2g47310.1:d:+1640:primary
GTAATAAAAG	0	0	3	0	1	At2g47230.1:d:+2209:primary,At1g76790.1:d:+1203:primary
TCACAATCTC	1	1	2	0	0	At2g46930.1:d:+1336:primary
CTTTTGGGAC	0	1	1	1	1	At2g46910.1:d:+841:primary
TTTTTGAGAG	2	2	0	0	0	At2g46710.1:d:+1568:primary
ATGGTGAAGC	0	2	2	0	0	At2g46510.1:d:+2140:primary
GTAATGACAA	1	1	0	2	0	At2g46500.2:d:+2113:primary,At2g46500.1:d:+2286:primary
CAGGAAGGTC	0	4	0	0	0	At2g46470.1:d:+1027:primary
TTAACTTTGT	0	3	0	1	0	At2g46430.1:d:+2243:primary
TGTTTATGAG	0	2	0	2	0	At2g46220.1:d:+270:secondary
ACTGTTTACA	1	2	0	1	0	At2g46030.1:d:+630:secondary
GAGCTGTTGG	2	1	0	0	1	At2g45720.1:d:+1768:primary
GGTAAAAAAA	0	0	2	1	1	At2g45590.1:d:+1607:secondary
ATTCTACTAA	0	4	0	0	0	At2g45520.1:d:+175:secondary
AGAAGTGCAG	1	1	1	1	0	At2g45330.1:X:+291:quaternary
ACCCTTGCCG	0	2	0	2	0	At2g45300.1:d:+1438:primary
TGGTGGAAAG	3	1	0	0	0	At2g45250.1:d:+639:primary
TAACGGTGTC	1	1	2	0	0	At2g44980.1:v:+3063:primary,At2g44980.2:v:+3120:primary
AAGAATAATT	3	0	1	0	0	At2g44950.1:d:+3112:secondary
CAGTTTGATT	1	1	1	1	0	At2g44920.2:d:+972:primary
TGTTTTCAGT	0	2	0	1	1	At2g44820.2:X:+607:quaternary,Chr1:+16672126:quaternary,Chr1:+357552:quaternary
ATAACCTTTA	0	3	0	1	0	At2g44520.1:d:+1381:primary
AACATCGTAC	2	0	0	0	2	At2g44370.1:X:-593:quaternary,At5g55130.1:X:-608:quaternary,At2g17740.1:X:-326:quaternary
AGTTTATCAA	1	0	1	0	2	At2g44350.2:d:+1865:primary,At2g44350.1:d:+1862:primary
TCGACATCTG	1	1	2	0	0	At2g44060.2:d:+80:secondary,At2g44060.1:d:+83:secondary
ATCACGTATG	1	0	1	2	0	At2g44050.1:d:+749:primary
TCCAAGAGAA	2	0	0	1	1	At2g43980.1:d:+1483:primary
ACTGCTTTGA	0	2	1	1	0	At2g43640.1:d:+375:primary
GCTACACTTC	2	0	0	0	2	At2g43480.1:d:+581:primary,At1g23100.1:d:+364:primary
GATTTAACTC	0	0	2	1	1	At2g43280.1:X:+368:quaternary
GAGGATGTGA	0	4	0	0	0	At2g43080.1:d:+972:primary
AAGTTCGGAG	0	4	0	0	0	At2g42870.1:d:+406:secondary
TGAATTAAAA	3	0	0	1	0	At2g42760.1:d:+933:primary,At5g12460.1:v:+2037:primary
TTTGAGTTAT	1	1	1	0	1	At2g42700.1:d:+2444:primary,At5g47060.1:d:+866:primary
GCCTCGATTC	1	2	1	0	0	At2g42500.1:d:+1017:secondary,At2g42500.2:d:+844:secondary
ATCACGTTCT	1	2	1	0	0	At2g42150.1:v:-2416:secondary
ATCGGTTGAA	1	0	0	2	1	At2g41980.1:v:+1496:secondary
ATGAACATTG	0	3	0	1	0	At2g41730.1:d:+375:primary
AGATCCAAGA	0	0	2	2	0	At2g41620.1:d:+2551:secondary,At1g69520.1:v:+100:secondary
TTAACTGTAA	1	1	0	1	1	At2g41600.1:d:+998:primary,At2g41600.2:d:+766:primary
ACAAAGAGAC	0	0	0	2	2	At2g41190.1:d:+1886:secondary,At1g72690.1:d:+167:secondary,At5g42390.1:d:+2327:secondary
GTTAAGCTTC	1	0	0	3	0	At2g41140.1:d:+1946:primary
AAAGATAGGT	0	3	1	0	0	At2g41100.1:d:+981:secondary,At2g41100.2:d:+714:secondary
AATTAGTTTC	1	2	0	1	0	At2g40765.1:i:+680:tertiary
CCCCAAGTTG	3	0	1	0	0	At2g40490.1:d:-1209:secondary
AGTCGGAGGA	0	2	1	1	0	At2g40330.1:d:+586:primary
GAATGAGTTT	0	2	1	0	1	At2g40150.1:d:+1305:primary
GCAGCTGCAG	0	3	0	1	0	At2g40140.1:d:+1504:secondary,At1g17930.1:d:+1837:secondary
TAAATAAAAC	0	1	2	0	1	At2g40095.1:d:+770:primary
ATCAAGGAAG	0	2	2	0	0	At2g39970.1:d:+1115:primary
TATCCAAGAG	0	3	0	0	1	At2g39920.1:d:+1129:primary
TTTTTGCAGA	0	3	1	0	0	At2g39890.1:d:+1656:primary
GAAAAAGCAA	0	1	3	0	0	At2g39870.1:X:+742:quaternary,Chr1:+7803874:quaternary
AGACGGTCAA	0	1	0	2	1	At2g39805.1:d:+1001:primary
GTTCAGAGAT	1	2	0	0	1	At2g39740.1:d:+1612:primary
AGACCCATCG	0	1	1	2	0	At2g39030.1:d:+740:secondary
ATGAAGCTAG	1	3	0	0	0	At2g39000.3:d:+745:primary,At2g39000.1:d:+852:primary,At2g39000.2:d:+856:primary

TACTCGTTAA	1	2	0	0	1	At2g38940.1:d:+1390:primary
AAGAAAGCGT	1	1	1	1	0	At2g38880.1:d:+223:primary,At2g38880.2:d:+209:primary
GAAAATGCTA	1	0	2	1	0	At2g38330.1:d:+1577:secondary
AACCAGAAAC	1	0	1	2	0	At2g38280.2:d:+1278:secondary,At4g14150.1:d:+1715:secondary,At2g47700.1:d:+1204:secondary,At2g38280.1:d:+1278:secondary
GACGATGACG	0	0	3	0	1	At2g38230.1:d:+602:secondary
TCTAATTTAC	0	0	1	2	1	At2g37990.1:d:+1023:secondary
CTCTTTAACT	0	0	0	3	1	At2g37975.1:d:+329:primary
GGAACCGATT	0	3	1	0	0	At2g37920.1:d:+371:primary
ATCAAAGTCA	1	2	0	0	1	At2g37910.1:v:+684:secondary
TTCAGAGATT	2	1	0	1	0	At2g37880.1:d:+857:secondary,At1g47830.1:d:+623:secondary
AAACTTAAAA	0	0	1	1	2	At2g37330.1:d:+968:secondary
GAAGGATACC	0	1	0	2	1	At2g37240.1:d:+608:primary
TGAAGTTTCT	0	0	1	1	2	At2g37025.1:d:+1385:primary
TTTATTTGGC	0	2	0	1	1	At2g36960.2:d:+2605:secondary,At2g36960.1:d:+2612:secondary
GATCCTGCTC	2	1	1	0	0	At2g36895.1:d:+405:primary
GAAACAGGTG	1	3	0	0	0	At2g36850.1:d:+4821:primary
ATGGGACCAC	1	0	1	0	2	At2g36810.1:X:--23:quaternary
GACTTGCAAG	0	4	0	0	0	At2g36810.1:i:+7996:tertiary
AGATGGGGAG	0	0	0	3	1	At2g36750.1:d:+1284:primary
ATCAGAGCTT	1	1	0	2	0	At2g36740.1:d:+1271:primary
GTGTATGCAC	1	2	1	0	0	At2g36390.1:d:+2516:primary
TTAGCGAATC	0	1	0	1	2	At2g36060.2:X:+386:quaternary
GGAGACAATA	0	4	0	0	0	At2g35800.1:d:+2558:primary
ACGGAAGCAG	0	2	1	1	0	At2g35630.1:d:+3901:secondary,At1g68720.1:d:+2738:secondary
GCAAGAAAAC	0	0	0	1	3	At2g35060.1:X:--21:quaternary
TCAAGAGCAA	1	3	0	0	0	At2g35040.1:d:+1503:primary
AAGGCACAGT	0	1	1	2	0	At2g34660.1:d:+2991:secondary
TATTTTATGT	1	0	1	0	2	At2g34490.1:d:+1768:primary
TTTCTGTGGA	1	0	1	2	0	At2g34450.1:d:+625:primary
ACACAGAACA	1	0	1	0	2	At2g34060.1:d:-1097:secondary
TAGAATATGT	1	0	3	0	0	At2g33770.1:d:+3969:primary
TATTACTTGA	1	2	1	0	0	At2g32940.1:v:+2749:secondary,At1g79245.1:p:+3836:secondary,At1g60860.1:v:+658:secondary,At3g59030.1:d:+1008:secondary
AGGTATTTTA	1	3	0	0	0	At2g32900.1:d:+2389:primary
CTTGTGTTTG	1	0	1	2	0	At2g32880.1:d:+1140:primary
TAATTTATCT	2	1	1	0	0	At2g32800.1:d:+2616:secondary
CTGGGCTTGT	0	1	2	1	0	At2g32640.1:d:+1705:primary,At2g32640.2:d:+1769:primary
GGGAAAGATA	2	0	2	0	0	At2g32560.1:d:+1171:primary
GTCTACTCTT	1	0	2	1	0	At2g32530.1:d:+2261:primary
GGACTTTCTG	2	2	0	0	0	At2g32520.1:d:+734:secondary,At4g11830.1:d:+2459:secondary,At4g11830.2:d:+2555:secondary
TATTTTTTTC	1	0	1	1	1	At2g32260.1:d:+1357:secondary
TGTATCTCTC	1	2	0	0	1	At2g32080.1:d:+1245:secondary,At2g32080.2:d:+1242:secondary
TTTGCTTCTC	1	0	1	1	1	At2g31890.1:d:+1625:secondary
ATAAATTTTA	1	0	1	1	1	At2g31830.1:v:+143:secondary
TTTACAATAA	2	2	0	0	0	At2g31800.1:d:+1553:secondary
CAGAGCTGAG	2	2	0	0	0	At2g31660.1:d:+3265:primary
GAGTAATAGT	2	1	0	0	1	At2g31620.1:v:+1416:secondary
CAATTCTTTA	1	0	1	0	2	At2g31260.1:d:+3161:primary
AAACGGAGTT	0	1	1	2	0	At2g30800.1:d:+4195:secondary,At2g30790.1:v:+17:secondary
CAAATCGCCT	2	0	1	0	1	At2g29310.1:X:+319:quaternary
TGAATGGGCA	1	2	1	0	0	At2g29300.1:d:+545:primary,At2g29310.1:d:+525:primary
TAATTTTAGG	0	0	2	1	1	At2g29060.1:X:+1963:quaternary
AAATCATTGT	0	2	1	1	0	At2g29000.1:v:+1710:secondary
AACTGCTTGA	1	1	2	0	0	At2g28890.1:d:+2038:primary
TAAAACGTTT	0	4	0	0	0	At2g28630.1:d:+1525:secondary,At3g21660.1:v:+1966:secondary
TGAGAAAAAA	0	1	2	1	0	At2g28560.1:X:-380:quaternary,At1g26370.1:X:--8:quaternary,At1g76550.1:X:-152:quaternary
TATGGAAGCG	1	2	0	1	0	At2g28540.1:d:+1760:primary
AGGCGGCACA	0	3	0	1	0	At2g28200.1:d:+856:primary
AAAGGTTGGA	1	2	1	0	0	At2g27900.1:d:+3537:primary
ACCATCATTA	0	2	1	1	0	At2g27860.1:d:+1091:secondary

AAGAACGGTA	0	3	0	1	0	At2g27830.1:d:+520:primary,At1g24540.1:d:+1528:primary
TATTGGATTC	1	1	2	0	0	At2g27500.1:d:+1375:primary
TGTCCTAAAA	1	1	0	1	1	At2g27430.1:X:--177:quaternary
CATCTTTCAC	0	1	1	1	1	At2g27395.1:v:+23:secondary
AACTATTCAA	3	0	0	0	1	At2g27170.1:v:+1708:secondary
CTACAAGTTT	1	1	1	0	1	At2g27050.1:d:+2204:primary
CGAAACTGCA	0	1	2	1	0	At2g27020.1:X:-175:quaternary
GTGATGGAAA	0	0	0	2	2	At2g26960.1:v:+472:secondary,At5g62990.1:v:+1832:secondary,At5g37320.1:v:+874:secondary,At4g34960.1:d:+616:secondary,At3g05190.1:d:+1829:secondary
ATTGTTTGAT	1	0	1	0	2	At2g26550.1:v:+1745:primary
AACGCTCTTA	0	0	2	1	1	At2g26500.1:d:+422:secondary,At2g26500.2:d:+390:secondary
TGAGTGAAGC	0	1	1	0	2	At2g26280.1:d:+1673:secondary
TACAGATCAG	1	1	1	1	0	At2g26270.1:v:+2148:primary
ACCCAAGGTG	2	2	0	0	0	At2g26110.1:d:+1138:primary
ATGATGAATA	2	0	1	0	1	At2g26040.1:v:+913:primary,At5g38380.1:d:+1286:primary,At5g38380.2:d:+1196:primary
AAGTTCTAAA	2	0	1	1	0	At2g25760.2:d:+2303:primary,At2g25760.1:d:+2294:primary
GTTTTGGTGA	0	1	2	1	0	At2g25660.1:d:+6520:primary
AAGCGATCAT	1	1	1	1	0	At2g25570.1:d:+981:primary
GGGTTCCACT	1	1	1	1	0	At2g25450.1:d:+370:secondary
GGAGTTCTTC	0	0	0	3	1	At2g25100.1:d:+408:secondary
GGATTTGTTT	1	0	2	0	1	At2g25070.1:d:+1503:primary,At4g10640.1:d:+1407:primary
AACAGTTAAA	0	3	1	0	0	At2g25070.1:d:+1038:secondary
TGCGGTCACG	0	3	1	0	0	At2g24762.1:d:+453:primary
GCTCTTGAAA	0	2	1	1	0	At2g24640.1:d:+2150:primary
CGCTACTACA	2	2	0	0	0	At2g24500.1:d:+1030:primary
CCCGAAGGAG	0	0	1	2	1	At2g24100.1:d:+1247:primary
CCAGAGTTTG	1	1	0	1	1	At2g24050.1:d:+2411:primary
TTTCCAAGAT	0	4	0	0	0	At2g23790.1:d:+1046:primary
ATGCTACGCT	1	3	0	0	0	At2g23780.1:d:+675:primary
AAGAAGATAG	1	1	0	1	1	At2g23200.1:d:+2399:primary
AGTTCTTAGC	2	0	1	0	1	At2g23150.1:d:+1599:primary
GAAACGCTGG	1	3	0	0	0	At2g23140.1:v:+2924:secondary
GACAAAGTTC	0	4	0	0	0	At2g23070.1:d:+1149:primary
TCGAAAAAAA	0	1	0	1	2	At2g23030.1:X:--101:quaternary
AAGTGAAAGG	1	0	2	1	0	At2g22900.1:d:+1522:primary
TGGGTGTTTA	1	3	0	0	0	At2g22830.1:d:+1851:primary
AATCGGATTC	0	2	1	1	0	At2g22795.1:d:+1290:primary
AGCTAGAAAG	0	3	0	0	1	At2g22660.1:d:+2759:primary
AAGCTTGTTC	2	0	0	2	0	At2g22570.1:d:+867:primary
ACATTTGAGG	1	2	0	0	1	At2g22490.1:d:+1412:primary
TGATCTGATA	0	2	0	0	2	At2g22450.1:d:+1642:primary
TGAAACAATT	1	1	1	1	0	At2g21970.1:d:+815:primary
TCTCTTGGAC	0	1	1	1	1	At2g21790.1:d:+2675:primary
CACCGGTGGT	0	0	3	0	1	At2g21530.1:d:+239:secondary
GACACGAAGA	0	3	0	1	0	At2g21410.1:d:+2170:primary
TGAACACATT	1	1	2	0	0	At2g21370.2:d:+1477:primary,At2g21370.1:d:+1483:primary
GCCACACTGT	3	0	1	0	0	At2g21300.1:d:+2817:primary
GCATTTTTAA	0	1	1	2	0	At2g21150.1:d:+1155:primary
AGTCTTAAAA	2	0	2	0	0	At2g21120.1:d:+1404:primary
CAAGTGTCAC	0	1	0	0	3	At2g20360.1:d:+1312:secondary
TTTCCTTATT	1	2	0	0	1	At2g20270.1:d:+622:secondary
AGTTGATCTG	1	1	2	0	0	At2g20000.1:d:+2823:primary
CAGATACTAA	1	0	2	1	0	At2g19520.1:i:+1718:tertiary
GACGACGAAG	0	1	1	1	1	At2g19390.1:d:+4001:primary
TCTCCGACTG	1	1	2	0	0	At2g19385.1:d:+975:primary
ATTTGCTTTA	0	0	1	0	3	At2g19220.1:v:+213:secondary
ATGGGGTTGG	1	1	1	0	1	At2g18960.1:d:-3123:secondary
TCAGAACATC	2	0	0	0	2	At2g18950.1:d:+1603:primary
TTTTGTTCAT	0	1	1	0	2	At2g18915.2:d:+2173:primary,At2g18915.1:d:+1934:primary
TTATTGTTAA	2	0	2	0	0	At2g18820.1:p:+255:secondary,At3g32029.1:p:+397:secondary

TCTATGCCTC	0	1	0	2	1	At2g18510.1:d:+1161:primary
GAAGCCAAGT	0	2	1	1	0	At2g18400.1:d:+149:primary
GTTCGTATTG	1	2	1	0	0	At2g17720.1:d:-1305:secondary
GAAGTGTCAG	2	0	1	1	0	At2g17540.2:d:+966:primary,At2g17540.1:d:+954:primary
CGTACCTTTC	2	0	1	0	1	At2g17500.2:d:+1566:primary,At2g17500.3:d:+1498:primary,At2g17500.1:d:+1549:primary
AAGGAGAGAG	2	0	0	1	1	At2g17350.1:d:+314:primary
AATTTATTGT	0	1	0	0	3	At2g17280.1:d:+1038:primary
AACTAAAAAA	1	0	0	1	2	At2g17250.1:d:+1854:secondary,At1g12630.1:v:+1296:secondary
GCTCCAGAAT	0	0	4	0	0	At2g17040.1:d:+790:primary
TTATCTCCAA	0	1	1	2	0	At2g17030.1:d:+1466:primary
GTTCCTGTCT	2	1	1	0	0	At2g16950.1:d:+2970:primary,At4g19070.1:d:+733:primary
AGACTTCTTA	1	1	1	1	0	At2g16850.1:d:+913:secondary
TGATTGGAAC	0	3	0	0	1	At2g16710.1:d:+393:secondary
GGGAGAACTT	1	0	2	1	0	At2g16405.1:d:+1540:primary
AGGAGACAAT	0	0	1	2	1	At2g16400.1:d:+2048:secondary
GTTCTAGAGC	1	1	0	2	0	At2g16370.1:X:+279:quaternary
TAGTATGTCT	0	3	1	0	0	At2g15960.1:d:+349:primary
ATTGCAGATT	0	2	1	1	0	At2g15910.1:X:-44:quaternary
AAGAATATTA	0	2	0	1	1	At2g15910.1:d:+1364:secondary
TAGGAGAAGA	0	2	0	2	0	At2g15890.1:d:+443:secondary
GTCTCTACTC	0	1	0	2	1	At2g15830.1:d:+191:primary
AAGAAGGTAA	1	1	1	1	0	At2g15570.1:d:+529:primary,At3g22430.1:d:+1202:primary
CGTGAATCAG	1	0	0	3	0	At2g15000.1:d:+386:primary
AAAGCAAAAG	1	2	1	0	0	At2g14850.1:d:+1323:primary
TGGTGTGCAA	0	0	2	1	1	At2g14660.1:X:+615:quaternary
CACTTTTCAT	0	1	2	0	1	At2g14530.1:d:+1469:primary
GCCAGAGGCA	0	1	3	0	0	At2g14260.1:d:+1039:secondary,At2g14260.2:d:+1091:secondary
ACTGCAAAAA	1	1	1	1	0	At2g14190.1:p:+442:secondary
TGTCAATTTT	2	0	1	0	1	At2g13820.1:d:+611:primary
TCAAATCTTG	3	0	1	0	0	At2g13680.1:i:-6761:tertiary
GCAAAAGCTG	0	1	2	0	1	At2g13650.1:d:+1246:primary
AGCGAAGAAG	1	1	0	1	1	At2g13560.1:d:+1761:secondary,At5g51540.1:v:+2670:secondary
GGGGCTGAAA	0	0	4	0	0	At2g13360.2:d:+967:secondary,At2g13360.1:d:+989:secondary
TTTTGCAAAA	2	0	1	1	0	At2g13080.1:p:+5716:primary
TCTGCGATGA	2	0	1	0	1	At2g12650.1:p:+1160:secondary,At2g32980.1:d:+711:secondary
TTAACAAAAA	1	1	2	0	0	At2g12470.1:p:+1587:secondary
CGGCTCTTGC	1	0	2	1	0	At2g10940.2:d:+152:secondary,At2g10940.1:d:+152:secondary
CTTCCTTCCT	1	3	0	0	0	At2g07775.1:v:+1061:primary
TGTATTTGAT	0	0	2	1	1	At2g07690.1:d:+2344:primary
CTATTGATGA	1	2	1	0	0	At2g07360.1:d:+3863:primary
TTGAAACATA	0	0	1	3	0	At2g06990.1:d:+3239:primary,At4g01660.1:d:+1786:primary
ATCAACTTCC	0	2	2	0	0	At2g06885.1:p:+471:secondary
ATGTTTATGC	0	2	0	0	2	At2g06850.1:d:+1051:secondary
TACAATTGTG	1	2	1	0	0	At2g06025.1:d:+1140:secondary
GAGATCACTA	0	3	0	0	1	At2g05940.1:d:+64:secondary
GGCTTGCCCG	0	1	0	1	2	At2g05830.2:d:+1496:primary,At2g05830.1:d:+1476:primary
TTTATCTATT	1	1	0	0	2	At2g05550.1:p:+408:secondary
GTGGTGGATT	1	0	0	2	1	At2g05380.1:d:+153:secondary
ATTGCCGACA	2	1	1	0	0	At2g04790.1:d:+416:primary,At2g04790.2:d:+428:primary
TCACAAAAAA	1	0	1	0	2	At2g04720.1:X:-1301:quaternary
TAGAGGAAAT	0	1	0	1	2	At2g04530.1:d:+1008:primary
GTCCACGCAG	1	1	0	0	2	At2g04350.2:d:+1929:primary,At2g04350.1:d:+1978:primary
AAGGCAACAC	0	3	1	0	0	At2g04160.1:v:+2181:secondary
GCAGAGTTTT	0	1	2	1	0	At2g04110.1:p:+461:secondary
TTATGTGAGA	0	2	0	0	2	At2g04040.1:d:+686:primary
GGAGAGAACC	0	2	1	0	1	At2g04030.2:d:+1656:secondary,At2g04030.1:d:+1665:secondary
CTAGCATTGA	2	1	0	1	0	At2g03730.1:d:+1143:primary
GTTGAGAATC	2	1	0	0	1	At2g03640.1:d:+1378:secondary
TAGTAGCTTC	2	1	0	0	1	At2g03620.1:d:+1464:primary

TATATATATT	0	1	1	1	1	At2g03160.1:v:+1401:primary,At3g24120.1:d:+1057:primary,At3g24120.2:d:+1198:primary
TTGCCATACC	0	1	1	2	0	At2g03150.1:d:+2533:secondary
GAGATTGGTT	0	1	2	0	1	At2g02990.1:d:+954:primary
GGTACTCACG	1	2	1	0	0	At2g02800.1:d:+954:primary,At2g02800.2:d:+954:primary
CTAATATACG	0	2	0	2	0	At2g02710.3:d:+1395:primary,At2g02710.2:d:+1596:primary,At2g02710.1:d:+1405:primary
AGCTGAAACT	0	3	0	0	1	At2g01860.1:d:+956:secondary
ATTTTGGAAA	0	1	1	1	1	At2g01640.1:d:+609:primary
TCTCCACTCT	1	1	1	0	1	At2g01340.1:d:+817:secondary
TCTGTTCTAG	1	2	1	0	0	At1g80890.1:d:+420:secondary
GCATAATAAA	0	0	0	3	1	At1g80830.1:d:+1804:primary
GCTTATTCAT	3	0	0	0	1	At1g80680.1:d:+3564:primary
AACTAAAGTA	2	0	1	0	1	At1g80670.1:X:-137:quaternary
GATTTGAAGC	2	0	1	1	0	At1g80640.1:d:+1450:primary,At3g49055.1:v:+2184:primary
GGGTTTCAGT	0	1	1	2	0	At1g80530.1:d:+1428:primary
CACCTTTAGG	0	3	1	0	0	At1g80510.1:d:+1815:primary
TTTGCAGTGG	1	2	0	1	0	At1g80490.1:d:+3406:primary,At1g80490.2:d:+3438:primary
AGAGACATCT	2	0	1	1	0	At1g80460.1:d:+1744:primary
TGTTTATACG	0	0	3	0	1	At1g80230.1:d:+843:primary
GACGGCGCTA	1	0	2	1	0	At1g80180.1:X:-109:quaternary
GAGCCAATAG	1	1	1	1	0	At1g80050.1:X:-162:quaternary
TTGCTTCTTT	1	2	0	0	1	At1g79975.1:d:+982:primary,At1g79975.2:d:+927:primary
CTTGATCTCA	2	0	2	0	0	At1g79840.1:d:+1146:secondary
GTGATGATGG	0	2	2	0	0	At1g79670.2:v:+2492:primary
AAGAACGAAT	1	0	0	1	2	At1g79610.1:d:+1678:primary
CTGAAGATTG	1	0	0	1	2	At1g79280.1:v:+4543:secondary
ACAAAATGCA	0	3	0	1	0	At1g79010.1:d:+562:secondary
GTATTTTGAT	1	1	2	0	0	At1g78830.1:X:-1578:quaternary
AAGTCCTCAC	1	1	1	1	0	At1g78560.1:d:+1291:secondary
TCTACTTATT	0	4	0	0	0	At1g78240.1:d:+2209:primary
AATTGGCCAA	1	1	0	1	1	At1g78230.1:d:+2065:secondary
AAAAGAATAG	1	1	0	2	0	At1g78200.1:d:+1167:primary,At1g78200.2:d:+1088:primary
TTTGGTCAAC	0	1	1	0	2	At1g78070.2:d:+1578:secondary,At4g21370.1:v:+697:secondary
CTGATGAACT	1	0	1	2	0	At1g77080.4:d:+266:primary,At1g77080.3:d:+224:primary,At5g65060.1:d:+254:primary
AGAGTTTCAA	1	2	1	0	0	At1g76960.1:d:+207:secondary
TTCACGATGG	1	3	0	0	0	At1g76900.1:d:+1352:primary,At1g76900.2:d:+1451:primary
AGAATTTAAA	1	2	0	0	1	At1g76880.1:d:+1889:primary,At1g55720.1:v:+2028:primary
TGGTTAAGAA	1	1	0	1	1	At1g76825.1:v:+201:secondary
ACATTGGGAG	0	4	0	0	0	At1g76650.1:d:+283:secondary
TCCTTTTCTG	1	2	1	0	0	At1g76460.1:d:+1998:primary
TCTTCCAATA	0	1	1	2	0	At1g76350.1:d:+2702:primary
TCATTGTAGA	2	0	0	1	1	At1g76060.1:d:+693:primary
TAAAAGTATT	1	3	0	0	0	At1g75960.1:d:+1787:primary
AACTAAAAGT	1	1	1	0	1	At1g75550.1:v:+1072:primary
ATGGTATAAA	0	0	1	1	2	At1g75410.1:d:+2075:primary
AGAGAGGTAA	1	2	1	0	0	At1g75380.1:d:+1147:primary
GAACAAATTT	1	1	1	1	0	At1g75310.1:v:+4670:secondary
TTTCACAGCT	0	2	1	1	0	At1g74950.1:d:+604:primary
AATGTTCATA	0	0	2	0	2	At1g74710.1:d:+1969:primary,At1g74710.2:d:+2063:primary
TTTATGCAGA	0	2	2	0	0	At1g74640.1:d:+841:primary
GAGACAAATT	0	1	1	2	0	At1g74450.1:d:+1531:primary
ATTTGTCGAC	2	1	0	0	1	At1g74260.1:X:-891:quaternary
TGCCAAGAAT	0	1	1	2	0	At1g73980.1:d:+2174:primary
TCAAAATGGA	0	0	1	2	1	At1g73530.1:d:+685:primary
TTGAAAGGTT	1	2	1	0	0	At1g73440.1:d:+785:primary
CCTATTTATT	1	3	0	0	0	At1g73260.1:d:+908:primary
TTTATAGAGA	0	1	1	0	2	At1g73175.1:p:+1382:primary,At5g35756.1:p:+5689:primary,At5g53940.1:d:+476:primary
GAAGCCAATT	0	0	1	2	1	At1g72990.1:X:-276:quaternary,At5g35912.1:X:-1357:quaternary,At4g07540.1:X:-1593:quaternary
CGAGTAGAGA	0	1	0	1	2	At1g72930.1:d:+398:secondary
ATTTGGCCAA	1	2	0	1	0	At1g72880.2:d:+1304:primary,At1g72880.1:d:+1402:primary
AATCCGGATC	1	1	1	1	0	At1g72790.1:d:+1744:primary
ATTTTGCTTT	1	0	0	1	2	At1g72730.1:d:+1569:primary

TACTAAGTAA	2	0	0	0	2	At1g72645.1:d:+772:primary
TCAAACCGTG	0	1	1	1	1	At1g72440.1:d:+2509:secondary,At1g12470.1:d:+2992:secondary
TCATATGAAG	0	2	0	0	2	At1g72330.1:d:+1923:primary
AAGATGAACT	0	3	0	1	0	At1g72330.1:d:+1778:secondary,At1g35647.1:p:+4584:secondary,At4g06620.1:p:+1667:secondary
ATTTTTCCTA	1	1	0	2	0	At1g72230.1:d:+867:primary
TCACCGTATA	1	2	0	0	1	At1g71980.1:d:+1491:primary
ACGGTTCTCG	0	1	3	0	0	At1g71880.1:d:+1292:secondary
ATGCAGCCTT	1	1	0	2	0	At1g71800.1:d:+1521:primary
AAAGCAAAAA	1	2	0	0	1	At1g71750.1:d:+374:secondary,At4g12340.1:d:+703:secondary,At4g10730.1:d:+2323:secondary,At1g71060.1:v:+813:secondary
GGACTCAAGA	0	2	2	0	0	At1g71730.1:d:+562:primary
TTGTACAATG	0	0	2	1	1	At1g71720.1:d:+1493:primary
TCGTTATACT	1	1	1	0	1	At1g71710.1:d:+2151:primary
GAATATGCAA	2	1	1	0	0	At1g71697.1:d:+1293:secondary,At3g02420.1:d:+611:secondary
CTAGGCCACT	2	0	0	2	0	At1g71500.1:d:+1174:primary
TACGAGATCA	1	1	1	1	0	At1g71270.1:d:+2233:primary
ATTCTTCTGT	1	2	0	0	1	At1g70810.1:d:+492:primary,At1g70800.1:d:+450:primary
TCGAATAACT	3	0	1	0	0	At1g70790.1:d:+838:primary,At1g70790.2:d:+794:primary
TACTCTCGAA	1	1	1	1	0	At1g70750.1:X:+582:quaternary
TTTCCGGAGT	0	0	2	1	1	At1g70640.1:d:-430:primary
TTGTTGTCGT	2	1	1	0	0	At1g70290.1:X:+577:quaternary
AGACATCCGA	2	0	1	1	0	At1g70250.1:X:+1354:quaternary
CGTGTAAGGT	2	0	1	0	1	At1g70210.1:d:+1898:primary
GACGAAGCAG	0	3	1	0	0	At1g70180.2:d:+1213:primary,At1g70180.1:d:+1201:primary
GTTTCTTCTC	0	3	1	0	0	At1g69990.1:v:+2062:secondary
TCTTGTTACG	0	2	1	1	0	At1g69935.1:d:+669:secondary
TAATATAAAA	0	3	1	0	0	At1g69880.1:d:+676:primary
TCATACACAG	0	3	0	0	1	At1g69740.1:d:+985:secondary,At5g56580.1:d:+607:secondary
ATCTTTGATG	2	1	0	1	0	At1g69700.1:d:+628:primary
ATTGGTTATC	0	1	1	1	1	At1g69360.1:d:+3223:primary
TGATAGACAA	2	0	0	1	1	At1g69160.1:d:-1281:secondary
TAACCTATCA	0	0	1	0	3	At1g69010.1:d:+1208:primary
GGGTTTTTGG	0	2	0	2	0	At1g68890.1:d:+540:secondary
TGGAGAAAGA	2	1	0	1	0	At1g68730.1:v:+695:secondary
TTAAAATCCT	1	2	0	1	0	At1g68560.1:X:-1839:quaternary
AGTTGGGATT	1	0	3	0	0	At1g68540.1:d:+888:primary
TCTCTGTTTT	2	0	0	2	0	At1g68310.1:d:+604:primary
TAACTGTGAA	1	2	0	1	0	At1g68190.1:d:+1238:primary
TCTTCGGCGA	1	2	0	1	0	At1g68130.1:d:+1450:primary
AAAAGGATCA	0	2	0	1	1	At1g68060.1:d:+1875:primary
GTTTATGTCT	2	0	1	1	0	At1g67940.1:d:+1040:secondary
AGACACGTGT	0	2	2	0	0	At1g67900.1:X:+654:quaternary
AGTTTAGGAG	0	1	2	1	0	At1g67340.1:d:+1005:secondary
AAGCTACGGC	0	1	0	2	1	At1g67230.1:d:+3456:primary
TTTAAATCTA	1	2	1	0	0	At1g67050.1:d:+1116:primary
TTACCAGTTA	1	1	1	0	1	At1g66970.1:d:+2159:secondary
GTTAAATAAA	0	1	2	0	1	At1g66850.1:d:+444:primary,At2g27240.1:v:+1975:primary,At2g33810.1:d:+896:primary
ATCCTTATAC	0	0	3	1	0	At1g66740.1:d:+837:primary
GGCCACTAAA	0	0	4	0	0	At1g66730.1:X:--135:quaternary
ATCCAAATAT	1	3	0	0	0	At1g66710.1:p:+1270:primary
TGTTTAGATA	1	0	2	1	0	At1g66540.1:d:+1582:primary
AGGCTCTACT	0	3	1	0	0	At1g66430.1:d:+1234:primary
TACTTTTGTG	3	0	1	0	0	At1g66260.1:d:+1081:primary
TTAAGAAACC	1	3	0	0	0	At1g66160.1:d:+1302:primary,At1g66160.2:d:+1254:primary
TGGAACTAAT	0	2	1	1	0	At1g65845.1:d:+181:secondary
TTTCTCGTCT	2	1	1	0	0	At1g65840.1:d:+46:secondary
ATTTTGATTT	3	1	0	0	0	At1g65790.1:v:+3024:primary,At2g38360.1:d:+937:primary
TGTTGGGTTT	0	2	0	1	1	At1g65700.1:d:+312:primary
CTTCAGCAGT	0	0	0	1	3	At1g64970.1:d:+1348:secondary,At4g14880.2:d:+529:secondary,At4g14880.1:d:+584:secondary
TTTCTTTTGT	2	1	1	0	0	At1g64670.1:d:+1621:primary
GAACTTGAAA	0	1	1	2	0	At1g64430.1:d:+1758:primary

TTTCTTGGTT	0	2	1	1	0	At1g64040.1:d:+1282:primary
TTGTGCACCT	1	3	0	0	0	At1g63800.1:d:+865:primary
AACACCTCTA	1	1	0	1	1	At1g63780.1:d:+959:primary
GTTTTGTTTA	1	1	1	0	1	At1g63690.1:i:+1853:tertiary,At1g63690.2:i:+1853:tertiary,At5g53680.1:i:+508:tertiary,At3g07570.1:i:+1840:tertiary
TTTTGGTACT	1	0	2	0	1	At1g63170.1:d:+1566:primary
CTGGAGACTT	0	3	0	1	0	At1g63090.1:d:+944:primary
TCATTGTTGA	0	4	0	0	0	At1g62830.1:d:+2326:primary
GGAGGCAAAG	1	3	0	0	0	At1g62770.1:d:+553:primary
TCTATATAGA	1	1	1	0	1	At1g62740.1:d:+1964:primary
TAATATAGAT	0	3	0	1	0	At1g62420.1:v:+1935:secondary
TTCCGATTGA	0	0	1	3	0	At1g62360.1:d:+1252:primary
GTCTCCGTTG	0	2	1	1	0	At1g61890.1:d:+1084:primary
TGTAATAGTT	0	0	1	1	2	At1g61800.1:X:+346:quaternary
TGACCTCCAA	2	1	0	1	0	At1g61350.1:X:+2050:quaternary
AAAAAACATT	0	0	2	1	1	At1g61280.1:v:+1001:primary
ATCCTCTTTC	2	0	2	0	0	At1g61000.1:v:+3256:secondary
TCACATCACA	2	0	0	0	2	At1g60790.1:d:+1751:primary
GGGAAAGACA	1	1	1	0	1	At1g60740.1:d:+317:secondary
GTGAAGCTAT	1	0	1	2	0	At1g60660.1:X:+611:quaternary
GAGAAAACGA	1	2	1	0	0	At1g60640.1:d:+858:primary
AGAATCGCCA	0	2	1	1	0	At1g60430.1:d:+846:primary
TGGGCAGCTT	2	0	0	2	0	At1g60230.1:d:+1368:primary
CGAAAGAAAA	0	1	1	0	2	At1g60060.1:X:+-216:quaternary
GTAGTGAAGA	0	2	1	1	0	At1g59960.1:d:+873:primary
GCTTAAAAGT	0	0	3	1	0	At1g59820.1:d:+1623:secondary
GAGCAGCTCG	1	2	1	0	0	At1g59750.1:d:+400:secondary,At1g59750.2:d:+400:secondary
TGAAATAAAA	1	1	1	1	0	At1g59520.1:X:+440:quaternary,At1g27880.1:X:+-113:quaternary,Chr4:+18412310:quaternary,At4g35600.1:X:+-244:quaternary,Chr1:+2908016:quaternary,At3g50890.1:X:+-13:quaternary
AGCAGTTCGG	0	2	0	1	1	At1g58280.2:d:+931:primary,At1g58280.1:d:+816:primary
TTCTTTGATT	1	1	0	2	0	At1g57760.1:v:+1544:primary
TGCGGAGGAA	1	1	0	2	0	At1g56660.1:d:+848:secondary
AGGATGTATC	0	2	1	0	1	At1g56660.1:d:+550:secondary
GAGCAGGCAA	0	3	0	1	0	At1g56610.1:d:+1543:primary
ACCTACACGA	1	3	0	0	0	At1g56150.1:d:+358:primary
CATAGACACA	0	2	2	0	0	At1g55920.1:d:+274:secondary,At1g24350.1:d:+695:secondary
ATTTGATTGA	1	1	0	1	1	At1g55760.1:d:+1711:primary
TTAGTTTCTT	2	1	0	0	1	At1g55690.3:d:+2485:primary,At1g55690.2:d:+2283:primary
TAAATAATGT	0	2	0	2	0	At1g55420.1:v:+11:secondary,At4g34900.1:v:+2029:secondary,At1g07300.1:v:+820:secondary
CTTTGGTGGA	0	0	4	0	0	At1g55350.3:d:+467:secondary,At1g55350.2:d:+467:secondary,At1g55350.1:d:+467:secondary,At1g55350.4:d:+673:secondary,At4g19170.1:d:+1365:secondary
ATTTGATGAT	2	0	1	1	0	At1g55300.1:d:+868:primary
TTCTTCTACT	1	2	0	1	0	At1g55190.1:d:+691:secondary
TAAGATAACA	0	1	0	0	3	At1g55150.1:d:+1777:primary
GGAACTACAA	0	1	1	2	0	At1g54790.2:d:+1458:primary
TCTATGTTAC	0	3	0	0	1	At1g54680.1:v:+1319:primary
TGGTTACAAG	0	2	0	1	1	At1g54500.1:d:+432:secondary
ATGGAGAGAC	1	2	1	0	0	At1g54460.1:d:+1114:primary
AATATGAAAC	4	0	0	0	0	At1g54440.1:d:+2188:primary
TAGTATGTTG	0	1	1	2	0	At1g54150.1:d:+1092:secondary
TGGTGGCAGT	1	0	2	1	0	At1g54090.1:d:+2138:primary
CACAACAAGA	0	3	1	0	0	At1g53990.1:v:+1086:secondary,At5g53420.1:d:+650:secondary
AGGGACTAAT	1	1	1	0	1	At1g53480.1:d:+696:primary
GCTCCAGAGT	0	2	1	0	1	At1g53440.1:v:+2859:primary
TGGTTTTCTT	0	1	3	0	0	At1g53320.1:d:+1386:primary
AGCTCACATC	1	2	0	1	0	At1g53230.1:d:+1733:primary
GCGACGTATC	1	3	0	0	0	At1g53170.1:d:+577:primary
ACCGACATAT	1	0	2	1	0	At1g53050.1:d:+2487:primary
GTACGTCACA	0	1	2	1	0	At1g52790.1:v:+1399:secondary

GAAATGGATA	0	2	1	0	1	At1g52610.1:p:+941:secondary
TAAAGATTAA	1	1	0	0	2	At1g51760.1:d:+1508:primary
TATCCCCAAT	1	0	0	2	1	At1g51660.1:d:+1565:primary
GCAAAAATAA	1	0	1	1	1	At1g50810.1:p:+851:secondary,At2g15650.1:p:+794:secondary
ATTCCTGCTT	0	2	1	1	0	At1g50380.1:d:+2287:primary
GCTTTAACAG	0	4	0	0	0	At1g49975.1:d:+60:primary
ACTGCAATTT	0	2	0	2	0	At1g49730.2:i:+1034:tertiary,At1g49730.3:i:+852:tertiary,At1g49730.1:i:+1029:tertiary
TAGCTTTTAA	1	1	1	0	1	At1g49320.1:d:+891:primary
ATCTTGTTAT	2	0	0	1	1	At1g49270.1:v:+921:secondary
TCCAGAATTA	1	1	1	0	1	At1g48440.1:X:+278:quaternary
AACACGTCAG	0	3	1	0	0	At1g48420.1:d:+929:primary
CAGTTGGATA	1	0	0	3	0	At1g48380.1:d:+1257:primary
TACCTGGCTC	2	1	1	0	0	At1g48260.1:d:+1488:primary
AAATGATCAC	1	1	0	0	2	At1g48170.1:d:+593:secondary
AAGCTTCTGG	1	3	0	0	0	At1g48050.1:d:+992:secondary,At4g23800.1:d:+698:secondary,At1g58200.2:d:+376:secondary,At1g58200.1:d:+359:secondary
ACTGGTGTTA	0	3	0	0	1	At1g47970.1:d:+75:primary
GAAACTTTGA	1	1	1	1	0	At1g47640.1:d:+860:primary
GAGAAACTCA	1	0	0	2	1	At1g47590.1:p:+350:secondary,At4g37660.1:d:+510:secondary
AAGAGTCCAC	0	3	1	0	0	At1g47330.1:d:+1661:secondary
TCTGTCAAAA	2	0	2	0	0	At1g47250.1:i:+1081:tertiary
AAACATATGA	1	0	1	0	2	At1g45332.1:i:+1334:tertiary
TGTATAAGCT	0	1	0	1	2	At1g45160.1:d:+3417:primary
ATGCAACTAA	1	0	2	0	1	At1g45130.1:i:-2306:tertiary
ATTACAACTG	0	2	1	0	1	At1g44860.1:v:+1371:primary,At4g03977.1:v:+1327:primary,At1g21930.1:d:+504:primary
GCGAGTTGGT	1	0	2	0	1	At1g43900.1:d:+831:secondary
AGGTTTGCTT	1	3	0	0	0	At1g43800.1:d:+1179:secondary
ACATCATATT	0	4	0	0	0	At1g43667.1:X:-489:quaternary,At4g03520.1:X:-431:quaternary,At5g46140.1:X:-362:quaternary
GGTGTATATA	0	0	0	0	4	At1g43160.1:d:+879:primary
TGGACAGGAC	0	1	2	0	1	At1g43130.1:d:+787:primary
TAAGAAAATA	0	1	0	2	1	At1g42630.1:v:+1033:secondary
GTGGTCGTTT	2	2	0	0	0	At1g42003.1:X:--12:quaternary
TTCTTTTAAT	2	1	1	0	0	At1g41830.1:d:+1891:primary
TGACCTTGAT	0	0	4	0	0	At1g38221.1:p:+2493:primary
GGGTTATTCG	0	1	2	1	0	At1g38131.1:d:+977:secondary,At2g16070.1:d:+938:secondary,At2g16070.2:d:+735:secondary,At1g38065.1:d:+449:secondary
CCTAAAAAAG	0	2	1	1	0	At1g37020.1:v:+1194:secondary
TGCGTTTGTA	2	1	1	0	0	At1g36360.1:p:-2444:secondary
GCAGACTGCA	0	1	2	0	1	At1g36360.1:p:+37:secondary,At2g18950.1:d:+1454:secondary
AAACACCAAT	1	0	1	0	2	At1g36160.1:v:+4786:secondary,At4g11510.1:v:+769:secondary
CTCCAAATGT	2	2	0	0	0	At1g36050.1:d:+1489:primary
GATGTCTGGC	1	3	0	0	0	At1g35515.1:i:+866:tertiary
AAGGAAAGGA	1	0	2	1	0	At1g34340.1:X:+452:quaternary
TAATAAAGCA	1	1	0	1	1	At1g33790.1:d:+1564:primary
TGGGGGTTGG	2	1	1	0	0	At1g33240.1:X:-507:quaternary
CATTTGAGGT	2	1	0	1	0	At1g33180.1:X:+-15:quaternary
AATTTGTACA	2	0	1	1	0	At1g32930.1:X:-377:quaternary
AGTAGTCTTG	1	1	2	0	0	At1g32750.1:d:+4912:primary
GAGCAGTTGT	1	0	0	1	2	At1g32490.1:d:+3524:primary
TAACTCTTGT	1	1	0	0	2	At1g32440.1:d:+1671:primary
AGTACCGTTA	0	3	0	0	1	At1g32090.1:d:+2443:primary
TCGTCGTCTC	1	1	2	0	0	At1g31817.1:d:+1044:primary
CTCTTCGATT	1	3	0	0	0	At1g31730.1:d:+2256:primary
AACAAAGCCG	1	1	1	1	0	At1g31650.1:d:+1870:primary
TCGGAGTTTT	0	1	1	1	1	At1g31490.1:d:+1655:primary
TCGCTCACTA	0	1	1	1	1	At1g31330.1:d:+76:secondary
CTAGCATATG	3	0	0	0	1	At1g31230.1:d:+2878:secondary
TCTGCTAAAA	1	0	1	0	2	At1g31200.1:v:+642:secondary
TCAAGTTTTC	0	0	2	1	1	At1g31166.1:p:-32:primary
TCAACCCAAA	2	1	1	0	0	At1g31160.1:d:+626:primary
TAGAAAAAGA	0	1	0	1	2	At1g30755.1:X:--8:quaternary,At1g32090.1:X:-253:quaternary

AGCATCAAAA	1	0	1	2	0	At1g30450.3:d:+3123:primary
ATGGTCAAAA	1	1	0	1	1	At1g30450.1:d:+3362:primary,At1g30450.2:d:+3358:primary,At5g27270.1:d:+2653:primary
ATAAAGAGAT	2	1	1	0	0	At1g30400.1:d:+4798:primary
TTAAAATAAA	1	2	0	0	1	At1g30300.1:d:+1311:primary
TGTGTGATCT	1	0	3	0	0	At1g30200.1:d:+1534:primary,At1g30200.2:d:+1545:primary
TGAACTGCGG	1	0	1	1	1	At1g29750.1:d:+1652:secondary,At1g29750.2:d:+1314:secondary
TTTTTTTTTC	1	2	1	0	0	At1g29140.1:d:+659:primary,At2g26190.1:d:+1873:primary
TTGTTGATGT	4	0	0	0	0	
						At1g29120.2:d:+1270:secondary,At1g62390.1:d:+1376:secondary,At3g02790.1:d:+484:secondary,At1g29120.1:d:+1230:secondary
ATTTTCTTGT	1	1	0	0	2	At1g29120.1:d:+1756:primary,At1g29120.2:d:+1712:primary
AACATTCAGA	1	0	2	0	1	At1g28540.1:d:+410:primary
ACTCGTATCT	0	1	2	1	0	At1g28520.1:d:+1684:primary
TCCTCCAAGT	0	4	0	0	0	At1g28380.1:d:+1348:primary
GGGACGTTAC	0	2	2	0	0	At1g28370.1:d:+173:primary
GTCACTCTCA	0	3	0	1	0	At1g28240.1:d:+2193:primary
ATAATATCCT	0	1	1	0	2	At1g28120.1:d:+1320:primary
ATCTCTTGAC	1	2	1	0	0	At1g28110.1:d:+1463:primary
TGGAACATTC	1	0	2	1	0	At1g28090.2:d:+1859:primary
AAATATTGGA	1	2	1	0	0	At1g28010.1:d:+3863:primary
ACGATCGTTG	0	0	2	1	1	At1g27950.1:d:+76:secondary
GGGCATTATA	1	0	2	0	1	At1g27930.1:d:+1381:primary
TTTTATTCCT	2	0	0	0	2	At1g27595.1:i:+3380:tertiary
ATAGAACCTG	0	1	2	0	1	At1g27520.1:i:-317:tertiary
ATGCAAAGAT	2	1	0	1	0	At1g27210.1:X:+288:quaternary
TTTTTAAATA	0	4	0	0	0	At1g27150.1:d:+1569:primary
GTGGGGTGTG	0	3	1	0	0	At1g27020.1:d:+1157:secondary
GAAACGTATT	0	1	3	0	0	At1g26650.1:d:+1293:primary
GAAATGAAGA	0	3	0	1	0	At1g26300.1:d:+694:primary,At5g17260.1:v:+1639:primary
TTTCTCGGAG	2	1	1	0	0	At1g26220.1:d:+21:primary
GGCTGCCAAT	0	3	1	0	0	At1g26110.1:d:+795:primary
CGACCCACGA	0	1	2	1	0	At1g25390.1:d:+1598:primary
TTGTAATGTT	0	1	2	0	1	At1g24560.1:d:+2403:primary
CTGCGTCTAT	1	1	0	0	2	At1g24267.1:d:+1369:primary
AGATTTTAAG	0	0	3	1	0	At1g23980.1:d:+1124:primary
GATAGTACAG	1	2	1	0	0	At1g23960.1:d:+965:primary
ATGTATCTAC	2	1	1	0	0	At1g23900.2:d:+3153:primary,At1g23900.1:d:+3083:primary
GCTTGTGTTA	0	3	0	0	1	At1g23260.1:d:+382:primary
AGCCGATGTT	0	1	2	1	0	At1g22990.1:d:+534:primary
TATCAGCTTA	2	1	0	1	0	At1g22910.2:d:+1113:primary,At1g22910.1:d:+1312:primary,At1g22910.3:d:+1316:primary
ATTCTGCTAC	0	0	0	1	3	At1g22760.1:i:+832:tertiary
GTCTATCGCC	0	2	0	2	0	At1g22650.1:d:+1587:primary
AGAGTGATGC	2	1	0	1	0	At1g22640.1:d:+775:secondary
CCTGCTGGCT	1	0	1	0	2	At1g22300.3:X:-248:quaternary
GACAACGAGC	1	1	0	1	1	At1g22200.1:d:+1272:primary
TATATAAAGT	0	4	0	0	0	At1g22060.1:d:+6023:primary
GTATGATGAT	0	3	1	0	0	At1g21910.1:d:+677:secondary
AACTTTAAGG	0	3	0	1	0	At1g21760.1:d:+687:secondary
CAAAAGGCTA	1	0	0	3	0	At1g21690.1:d:+917:secondary,At1g21690.2:d:+881:secondary
ATCGTTTATG	0	0	2	1	1	At1g21525.1:p:+330:primary
AAAGCAGAAT	0	3	0	0	1	At1g21380.1:d:+1977:secondary
ATCTTGAAAG	0	4	0	0	0	At1g21130.2:d:+948:secondary
GGAGAGAGTA	1	3	0	0	0	At1g21000.1:d:+650:secondary
GTGGTAGTAA	2	0	1	0	1	At1g20970.1:v:+481:secondary,At5g38520.1:d:+1254:secondary
TTTATTACAA	2	2	0	0	0	At1g20860.1:v:+496:secondary,At1g55660.1:v:+239:secondary,At4g00315.1:v:+18:secondary
CCTTTGGAAG	0	3	0	1	0	At1g20840.1:d:+2246:primary
AAGCTATGCA	1	3	0	0	0	At1g20830.1:d:+870:primary
GATACTCGTT	0	2	1	0	1	At1g20760.1:d:+3080:primary
TAGAGTTGTT	1	0	2	1	0	At1g20750.1:i:+2217:tertiary
TCGTTTAGAA	1	1	0	2	0	At1g20575.1:d:+909:primary
TGGTCCCAAA	0	3	1	0	0	
						At1g20510.2:d:+1829:secondary,At5g28624.1:p:+3233:secondary,At5g38383.1:p:+4452:secondary,At1g20510.1:d:+1766:secondary

GTGGTGACGA	0	2	1	1	0	At1g20450.1:X:-178:quaternary
GATCGACCAA	1	0	1	0	2	At1g20310.1:d:+709:primary,At1g74030.1:d:+1654:primary
TCGCTTTAGG	0	0	1	3	0	At1g20220.1:d:+1278:secondary
AATATGGAAT	2	0	0	0	2	At1g20160.1:d:+2374:primary
TTGATCAAAA	1	1	0	0	2	At1g19660.1:X:-223:quaternary
GCTCTGCTTC	2	1	0	1	0	At1g19525.1:d:+364:primary
TTTTCAGATC	2	0	0	1	1	At1g19440.1:d:+1850:primary
TCCGTTGGGT	0	0	2	2	0	At1g19170.1:d:+1479:primary
ATTCTGCTTC	1	0	1	2	0	At1g19140.1:d:+1061:primary,At1g19140.2:d:+1015:primary
GAGCAAGATC	1	1	2	0	0	At1g19000.2:d:+626:secondary,At1g19000.1:d:+631:secondary
ACGCTCTCAC	1	1	1	1	0	At1g18890.1:d:+1607:secondary
GTGGTCTTAT	0	2	1	0	1	At1g18720.1:X:-195:quaternary
TGAAGAACGC	0	2	1	1	0	At1g18670.1:v:+1921:secondary
AATGTGATCT	2	1	0	0	1	At1g18590.1:d:+1095:primary
AAGCCTTGGC	1	2	0	0	1	At1g18580.1:d:+1795:primary
AACGATATGC	0	0	1	3	0	At1g18460.1:d:+1884:primary
GGTTCTTAAA	1	0	1	1	1	At1g18310.1:v:+1211:secondary
GACAAGGACT	2	1	1	0	0	At1g18260.1:d:+1728:primary
CGGTTGTCGA	0	0	2	1	1	At1g18070.1:d:+1470:secondary
TGCAAGGGTT	0	2	0	0	2	At1g17710.1:v:+804:secondary,At1g73010.1:d:+569:secondary
GAAGAAAGTA	0	3	0	1	0	At1g17500.1:d:+3803:primary
AGTCTTATAG	0	2	1	0	1	At1g17440.1:d:+2292:primary,At1g17440.2:d:+2391:primary
GTTTGTGCTT	0	3	0	1	0	At1g17280.1:d:+958:primary
TGAAAACTCA	1	2	1	0	0	At1g17250.1:X:-82:quaternary
GAACCAGTTG	0	0	0	2	2	At1g17210.1:d:+2266:secondary,At5g58600.1:d:+1222:secondary,At5g58600.2:d:+1307:secondary
CCTGACCGAA	1	0	2	0	1	At1g17160.1:i:+1526:tertiary
TGTTAAATTT	2	1	1	0	0	At1g17145.1:i:+1379:tertiary
AACAAATTTC	1	1	1	1	0	At1g17140.1:d:+1482:primary,At1g17140.2:d:+1426:primary
CTTATTATTG	0	0	0	4	0	At1g17050.1:X:+666:quaternary,Chr4:+3805843:quaternary
CGTTGAGGAA	0	1	2	1	0	At1g16870.1:d:+1529:primary
ATTTTTTTAT	0	1	1	2	0	At1g16780.1:i:+2032:tertiary
CGAAACCATT	1	1	1	0	1	At1g16560.2:d:+1395:primary,At1g16560.1:d:+1364:primary,At1g16560.3:d:+1532:primary
AAGAAGCCTA	1	1	2	0	0	At1g16500.1:d:+792:primary,At1g40093.1:p:+2341:primary,At3g18680.2:d:+1150:primary,At1g41730.1:p:+2341:primary,At5g32267.1:p:+2280:primary
TGGAACCGGT	0	2	1	1	0	At1g16460.2:d:+1037:primary
TGACTAAACA	1	0	2	0	1	At1g16330.1:v:+2007:primary
GATGGCACCA	1	2	1	0	0	At1g16270.1:d:+3108:primary
CGAGTATGAC	0	0	0	3	1	At1g16190.1:v:-1690:secondary
TTCGCCTTGG	0	4	0	0	0	At1g15920.1:d:+771:primary
GCCGATGGGC	0	0	4	0	0	At1g15820.1:d:+422:secondary
GCAACCATTG	1	0	2	1	0	At1g15810.1:d:+1816:secondary
ATTATTACGC	1	2	0	0	1	At1g15710.1:d:+835:primary
TACTATTTGG	0	4	0	0	0	At1g15670.1:d:+1499:primary
AAGATTCTGA	1	0	2	1	0	At1g15430.2:d:+1332:primary,At1g15430.1:d:+914:primary
AGTGTGATCA	2	1	1	0	0	At1g15390.1:d:+716:primary
AGATGATTAC	1	1	2	0	0	At1g15290.1:d:+2195:secondary
AATAGGAGCT	0	0	1	2	1	At1g15280.2:d:+2270:primary,At1g15280.1:d:+2289:primary
GAAAGTCCTG	1	2	1	0	0	At1g15280.2:d:+1734:secondary,At1g15280.1:d:+1753:secondary
TTGGGTTGGC	0	2	1	1	0	At1g14920.1:d:+1731:secondary
AGCAACATAA	2	1	1	0	0	At1g14910.1:d:+2147:primary
GTGAAGAACA	1	2	0	1	0	At1g14900.1:d:+318:primary
TCGGTTCACG	1	2	1	0	0	At1g14700.1:d:+991:primary
CAGAACGCGC	1	1	1	1	0	At1g14690.1:i:+1763:tertiary
GAACTAACAA	2	1	0	0	1	At1g14685.2:d:+1089:primary,At1g14685.1:d:+1047:primary,At1g14685.3:d:+1310:primary
AAAAGTAATT	0	2	0	1	1	At1g14580.1:d:+1933:primary
ACAGAAAACA	1	0	0	2	1	At1g14500.1:i:-647:tertiary
CATTTGCAAA	1	0	1	1	1	At1g14410.1:d:+787:primary
GGATGGAGGT	0	2	2	0	0	At1g14400.1:d:+365:secondary,At5g62540.1:d:+414:secondary,At1g14400.2:d:+236:secondary
TCTAGTCTAT	3	0	0	1	0	At1g14380.2:d:+2105:secondary,At1g14380.1:d:+2291:secondary
ACTCCTGGTG	0	3	1	0	0	At1g14370.1:d:+1420:secondary
TAACCGATAC	1	0	3	0	0	At1g13770.1:d:+1423:primary

GGATCCGGAT	0	0	3	1	0	At1g13640.1:d:+1993:secondary,At5g66400.1:d:+552:secondary
TGATTCAGTC	0	0	1	3	0	At1g13350.1:X:+537:quaternary
TGATAAAGAA	1	2	0	0	1	At1g13250.1:d:+1254:primary
GTAGAGTCCA	1	2	0	0	1	At1g13000.1:d:+1657:primary
AAGAATCTTG	2	1	0	0	1	At1g12930.1:d:+3272:primary
AGAGACTCTA	1	1	0	2	0	At1g12660.1:i:+1284:tertiary
GGAAATCAGC	1	1	2	0	0	At1g12270.1:d:-1853:secondary
AATAATTCCT	0	1	1	0	2	At1g12140.1:d:+1406:primary
GCATTGCAAT	0	0	0	3	1	At1g12130.1:v:+1341:secondary
ACTCAAGAGC	0	1	2	0	1	At1g11910.1:d:+1244:secondary
AAAGGCACAT	0	2	0	2	0	At1g11850.1:d:+39:primary,At1g11850.2:d:+39:primary
CAAAAAAAGA	0	2	1	1	0	At1g11510.1:v:+160:secondary,At2g40475.1:d:+93:secondary
CTTCGTTCCA	0	2	1	0	1	At1g11260.1:d:-1691:secondary
TATAAAAAAT	0	2	1	1	0	At1g11180.1:d:+1105:primary,At5g62230.1:v:+3628:primary,At4g21460.1:d:+1571:primary
ACATCTGATA	0	4	0	0	0	At1g11160.1:d:+2215:secondary,At1g15125.1:v:+1387:secondary
GAAAAATGTT	0	0	2	1	1	At1g11090.1:d:+1088:primary
GTACTATGAA	0	0	2	1	1	At1g10940.1:d:+1695:primary
AAGATAGTGG	1	2	0	1	0	At1g10940.1:d:+1288:secondary
CTTTAGGCTT	2	1	0	0	1	At1g10830.1:d:+1162:primary
GTTTAAGTCG	0	2	0	2	0	At1g10820.2:d:+1497:primary,At1g10820.1:d:+1471:primary
GTCAAAGTCA	0	1	1	1	1	At1g10660.2:d:+1351:primary,At1g10660.1:d:+1322:primary,At1g10660.3:d:+1346:primary,At1g10660.4:d:+1377:primary
CAAATATTTT	0	0	1	3	0	At1g10490.1:i:+4532:tertiary
ATTCACTACG	1	2	0	1	0	At1g10450.1:d:+3652:primary
TGTTGGCAGC	1	0	3	0	0	At1g10290.1:d:+3101:secondary,At3g13330.1:d:+2352:secondary
GAAAAGCTAG	1	2	0	1	0	At1g10230.1:d:+366:primary
CGCTGTGATG	3	0	0	1	0	At1g09980.1:d:+2682:primary
AGTTTGCTAA	0	0	1	2	1	At1g09660.1:d:+1149:primary,At1g09660.2:d:+1195:primary
TTATTTAAAA	0	0	2	1	1	At1g09500.2:d:+1582:primary,At1g09500.1:d:+1116:primary
AATAAAGCAG	0	1	2	1	0	At1g09415.1:d:+345:secondary
GAGAGGAATG	0	1	3	0	0	At1g08720.1:d:+2660:primary
AGTGAGACCA	1	3	0	0	0	At1g08660.1:d:+1337:primary,At1g08660.2:d:+1423:primary
TCGAAGGAAC	0	0	2	2	0	At1g08630.3:i:+114:tertiary,At1g08630.1:i:+358:tertiary,At1g08630.2:i:+1382:tertiary
ACTACTGTCG	0	0	1	1	2	At1g08490.1:X:-344:quaternary
AAAATGTAAA	0	1	0	1	2	At1g08250.1:d:+1334:primary
AACTGGAGAA	1	3	0	0	0	At1g08060.1:d:+5525:secondary,At1g08060.2:d:+5257:secondary
TATCTTTTTC	1	1	0	1	1	At1g07990.1:d:+2765:primary
GTTGTGGAGA	2	0	2	0	0	At1g07920.1:d:+1307:secondary,At1g07930.1:d:+1291:secondary,At1g07940.1:d:+1312:secondary
TTACATTGTG	0	0	2	2	0	At1g07910.1:d:+3113:secondary,At5g04410.1:d:+2169:secondary
GACATCAAAC	0	0	3	0	1	At1g07890.2:d:+563:secondary,At1g07890.1:d:+680:secondary,At1g07890.3:d:+576:secondary
TGGGAGTTGT	1	0	2	1	0	At1g07700.3:d:+1046:primary,At1g07700.1:d:+571:primary,At1g07700.2:d:+914:primary
TACTATGTTG	0	4	0	0	0	At1g07630.1:d:+2168:primary
ACTCTCGGGG	0	1	1	2	0	At1g07510.1:d:+2250:primary
TATCTTTAGG	1	0	1	2	0	At1g07150.1:d:+1290:secondary
TGGCTCAACC	0	2	1	0	1	At1g07130.1:d:+369:secondary
AAGCCGTGGT	1	0	0	3	0	At1g06780.1:d:+1934:secondary
TCGTATATGT	0	3	0	0	1	At1g06660.1:d:+1783:secondary
GGAGCTGTTA	1	2	0	1	0	At1g06540.1:d:+199:secondary
AACAGTCCAC	0	2	2	0	0	At1g06310.1:d:+1524:primary
ATGATGATGT	2	0	1	0	1	At1g06250.1:d:+1244:primary,At3g03460.1:v:+1893:primary
GAATTGCTCT	0	1	1	1	1	At1g06240.1:d:+1433:primary
GTTTTAAACC	0	0	1	3	0	At1g06200.1:d:+926:primary
AGAACAGATT	1	0	1	1	1	At1g06110.1:d:+1520:primary
TATTTGTCTC	1	1	0	0	2	At1g05430.1:d:+946:primary,At3g43000.1:p:+2172:primary
CTCAGAAAGA	1	0	0	2	1	At1g05410.1:d:+1370:primary
ACATTGAAGA	2	2	0	0	0	At1g05320.1:d:+2542:primary
CCCAGAAGAA	2	0	1	1	0	At1g05210.1:d:+817:primary
AAACAGAGCT	1	1	0	1	1	At1g05160.1:d:+2007:primary
CTTGTATTAT	0	0	1	1	2	At1g05140.1:d:+1385:primary
TGGATAGAGA	0	3	0	1	0	At1g05060.1:d:+810:primary
GGAAATGATG	2	2	0	0	0	At1g04980.1:d:+1292:primary

TAGTGATGGG	0	2	0	0	2	At1g04940.1:d:+653:secondary
ATACTCAATA	1	1	1	0	1	At1g04900.1:d:+1709:secondary
AACCACAAAA	1	0	2	1	0	At1g04860.1:d:+2235:secondary
ATTTTGTAAT	1	1	0	1	1	At1g04810.1:d:+3130:primary,At3g46090.1:d:+576:primary
GCAAACTACA	0	0	3	1	0	At1g04690.1:d:-1346:secondary,At1g04690.1:d:-1351:secondary
GACTGTAAGG	0	3	1	0	0	At1g04590.2:d:+1214:primary,At1g04590.1:d:+1204:primary
GTTGGTTCGA	0	2	0	1	1	At1g04540.1:v:+1557:secondary
GGTTCTTTCG	0	4	0	0	0	At1g04430.1:d:+1673:primary
CCTCTCTTAA	0	3	1	0	0	At1g04410.1:d:+1135:secondary,At5g43330.1:d:+1109:secondary
AAAGCTAGGT	1	3	0	0	0	At1g04300.1:d:+1206:secondary
ATCATCGTCC	0	3	1	0	0	At1g04270.1:d:+362:secondary
GGGCGATTTC	0	2	0	2	0	At1g04260.1:d:+137:primary
AATCTCATCA	0	2	0	2	0	At1g04210.1:d:+4163:primary
AGATTCTCCC	1	1	1	0	1	At1g04190.1:d:+1095:primary
TTCATTTGTT	0	0	4	0	0	At1g04160.1:v:+2099:secondary
CTTGTTTCTG	1	1	1	0	1	At1g04070.1:d:+411:primary
AAATTTTTGC	0	1	0	3	0	At1g03960.2:d:+2258:primary,At1g03960.1:d:+2106:primary
AGCTTGAAGA	1	0	1	0	2	At1g03950.1:d:+799:primary
GAAACTGCGT	0	0	4	0	0	At1g03730.1:X:-42:quaternary
ATGTTTGCTT	1	1	2	0	0	At1g03610.1:d:+1367:secondary
GTCGTGGTAG	0	1	1	2	0	At1g03530.1:d:+2377:primary
GAAGGAGTTG	0	2	1	1	0	At1g02960.2:d:+1382:primary,At1g02960.1:d:+1355:primary
TTTACAAATT	0	2	0	2	0	At1g02930.1:i:+559:tertiary,At3g58780.1:i:+2628:tertiary
TTTGATGATG	0	1	2	0	1	At1g02750.1:d:+550:primary
TGAATCAGTG	0	1	2	1	0	At1g02400.1:X:+425:quaternary
AACACAGACA	3	0	1	0	0	At1g02400.1:i:+1609:tertiary
TGAAATGTAT	1	2	1	0	0	At1g02400.1:d:+1249:secondary
CGATGAGAAG	1	1	0	2	0	At1g02340.1:d:+1022:secondary
TTTGCAGACT	1	1	1	0	1	At1g02330.1:d:+945:primary
CTTGTCTCAC	0	0	1	1	2	At1g02270.1:d:+1439:secondary
AGACCGATGC	1	1	2	0	0	At1g02150.1:d:+1692:secondary
GCAATGTCTG	2	0	1	0	1	At1g02130.1:d:+556:secondary
TAAATTTGAG	1	1	1	0	1	At1g02110.1:d:+2093:primary
CCCTTCACTT	1	1	2	0	0	At1g01920.1:d:+1705:primary
TAGTGTACCA	1	0	1	1	1	At1g01610.1:d:+1741:primary
ATGAAGAAAA	0	0	2	1	1	At1g01600.1:d:+928:primary
TATGGTTTAT	1	2	1	0	0	At1g01560.1:d:+979:secondary
GTGCTCGACA	0	1	0	3	0	At1g01300.1:d:+594:primary
GAACCTTCTA	0	1	1	2	0	At1g01120.1:d:+1528:primary
CAGGAACCTG	1	2	0	1	0	At1g01050.1:d:+445:primary
CAAAGAGATT	0	2	1	1	0	At1g01040.1:d:+5955:primary
AGTTACCTAT	1	0	0	2	0	No gene matches found
GGATAACGGA	1	0	0	0	2	No gene matches found
AGTGTACGAA	0	0	1	0	2	No gene matches found
TTTGCGATGT	0	2	0	1	0	No gene matches found
TCCGAATCAG	1	2	0	0	0	No gene matches found
ATAGAACCTA	1	0	1	0	1	No gene matches found
GACGGGAAAA	1	0	1	1	0	No gene matches found
AGTGGTTAAT	0	0	0	1	2	No gene matches found
AGCAAATCCA	0	0	3	0	0	No gene matches found
GAATCAGTGG	0	1	0	2	0	No gene matches found
CAAATTAAGG	0	0	3	0	0	No gene matches found
TGAGTAACCA	0	2	1	0	0	No gene matches found
GGGCCTTCGC	0	3	0	0	0	No gene matches found
ATCCGAAGTT	2	0	1	0	0	No gene matches found
AAAGTGTGGC	0	1	0	2	0	No gene matches found
GCGCGTTTGT	0	0	1	0	2	No gene matches found
GCTACCTCTC	2	0	1	0	0	No gene matches found
CGCCTGAACG	1	0	1	1	0	No gene matches found
TAGACTCTAT	0	0	2	0	1	No gene matches found
GGCCACATAA	0	1	1	0	1	No gene matches found

GAGGTGGGAA	1	2	0	0	0	No gene matches found
TCGTATCAGG	1	0	1	1	0	No gene matches found
GTGCAAACTA	0	1	1	1	0	No gene matches found
GCGAAAAGGG	0	1	1	1	0	No gene matches found
GAGCAACTCT	0	0	2	0	1	No gene matches found
GCCGTCCTTA	0	3	0	0	0	No gene matches found
ACAGAACCTT	1	1	1	0	0	No gene matches found
AGGGTGTGGC	0	2	1	0	0	No gene matches found
GCACAACGAC	0	1	2	0	0	No gene matches found
TGTTTGGACG	2	0	1	0	0	No gene matches found
TGAGGTGAGG	0	0	2	0	1	No gene matches found
TTGAAGTCTA	0	0	3	0	0	No gene matches found
GGCAAATCCA	0	1	1	0	1	No gene matches found
TCTCCCAAAA	0	0	0	2	1	No gene matches found
GGCCTTCGAC	2	0	1	0	0	No gene matches found
TTGAGTTCCA	1	0	1	0	1	No gene matches found
GCAAAAATGA	0	1	1	0	1	No gene matches found
GGCCTTCCCC	1	0	1	1	0	No gene matches found
AACAGATGAG	2	0	1	0	0	No gene matches found
GGCCTGCGCC	1	1	1	0	0	No gene matches found
ATGCAGACTA	0	1	1	0	1	No gene matches found
TCTATCTCTA	1	0	1	0	1	No gene matches found
CGAGCTCTAA	1	0	0	2	0	No gene matches found
CTACCAAGAG	0	1	0	2	0	No gene matches found
TTTGTACAAT	1	1	1	0	0	No gene matches found
CTAATTGCTT	1	0	1	1	0	No gene matches found
CAGGCGTGGC	0	2	0	1	0	No gene matches found
GCACTAAGCG	1	1	0	1	0	No gene matches found
TGCAGTTTGC	0	2	0	1	0	No gene matches found
CGTGTTTACT	2	0	0	0	1	No gene matches found
TTGGTTACCG	1	0	1	1	0	No gene matches found
GTAGTGACCC	0	0	0	2	1	No gene matches found
CCTCCTGTCG	0	2	0	1	0	No gene matches found
TGAGGTTCTA	0	2	0	1	0	No gene matches found
ATAGGACCTT	0	2	1	0	0	No gene matches found
GGCTTTCGCC	0	1	1	1	0	No gene matches found
GGACAAATGG	0	1	1	1	0	No gene matches found
TTCCAACGAA	1	0	2	0	0	No gene matches found
GCACAACAGC	0	1	0	2	0	No gene matches found
TTCCGATAAA	1	0	0	1	1	No gene matches found
CAGTTGTGGC	0	0	0	3	0	No gene matches found
CGTCCTGTTG	0	0	2	1	0	No gene matches found
GTGGCCTTAA	1	1	1	0	0	No gene matches found
CTACAAAAAA	0	0	3	0	0	No gene matches found
GATCCATACG	0	1	2	0	0	No gene matches found
CCTCCTGTGG	0	1	0	2	0	No gene matches found
TAATAGCAAG	2	1	0	0	0	No gene matches found
GCATCTGGTT	0	3	0	0	0	No gene matches found
ACCATTCGTG	1	0	0	1	1	No gene matches found
AACGCAAAAA	1	1	0	1	0	No gene matches found
GGCTAGATGG	0	1	2	0	0	No gene matches found
ATAGAACCTC	2	1	0	0	0	No gene matches found
GCTCAGTATG	0	0	2	1	0	No gene matches found
TCTCTATAAA	0	0	1	0	2	No gene matches found
CAGGAGTGGC	1	1	1	0	0	No gene matches found
TCAAGGGCCT	1	1	0	0	1	No gene matches found
GCGGTCTTAA	0	0	2	1	0	No gene matches found
GGCCTACGCC	2	1	0	0	0	No gene matches found
AGCTTAAGAA	1	0	0	2	0	No gene matches found
CTAGAAACAT	1	1	1	0	0	GI;7525047:d:X:--39:quaternary
CCGTAAACGA	0	0	1	2	0	ChrM:-362477:quaternary

AGCAGGTTGA	0	1	2	0	0	ChrM:-11263:quaternary
AGTAACGATA	2	0	0	1	0	ChrM:-10681:quaternary
CTTTCCGTCG	2	0	0	1	0	ChrM:+218077:quaternary
CAAATCTACA	1	0	0	2	0	ChrC:-69889:quaternary
CCAGAACGAA	0	0	1	1	1	ChrC:-123216:quaternary
AATACAGCAT	0	0	3	0	0	ChrC:+78364:quaternary
TATGGGGGCT	0	1	0	1	1	ChrC:+106519:quaternary
CTCCACTTGG	0	0	1	1	1	ChrC:+103651:quaternary,Chr2:+12712584:quaternary
TTATCTATCT	0	2	0	0	1	ChrC:+103285:quaternary,Chr1:+3937311:quaternary
CCGCGAATCC	0	0	1	1	1	ChrC:+101959:quaternary
CATCCTCATA	2	0	0	0	1	Chr5:-23076857:quaternary
CATTGTCGAA	0	0	1	2	0	Chr5:-17826525:quaternary,Chr5:-17826529:quaternary
CCTAAAAGAA	0	1	0	1	1	Chr5:-13352732:quaternary
CGAGCAGAAA	1	0	0	2	0	Chr5:-12745732:quaternary
CATCTTATCT	1	0	1	1	0	Chr5:+980574:quaternary,Chr3:+16529789:quaternary,Chr4:+6226569:quaternary
AATTTTCTAA	0	0	1	1	1	Chr5:+9032680:quaternary
TAAGCAATCA	0	0	1	1	1	Chr5:+8958437:quaternary
TTGAAACAAT	0	1	0	0	2	Chr5:+8052158:quaternary,Chr5:+8942002:quaternary
GTATACAAAA	1	1	1	0	0	Chr5:+6187585:quaternary
TTGGTGGATC	0	3	0	0	0	Chr5:+4629683:quaternary
GCAACGAGAC	0	2	1	0	0	Chr5:+3080273:quaternary
ACAAAGAATT	0	2	0	1	0	Chr5:+26549971:quaternary,Chr4:+1022055:quaternary
ATTAAAAACA	0	0	2	1	0	Chr5:+25906514:quaternary,Chr2:+10366901:quaternary,Chr1:+11722496:quaternary
TATTTACAAA	1	2	0	0	0	Chr5:+24965797:quaternary,Chr3:+11201136:quaternary
CAATTCAAAT	0	1	1	1	0	Chr5:+24085087:quaternary
TAAATTTACA	0	1	1	0	1	Chr5:+23868571:quaternary,Chr3:+6673157:quaternary
AAAATATAAT	1	1	0	0	1	Chr5:+22225184:quaternary,Chr2:+9941360:quaternary,Chr1:+21075764:quaternary,Chr2:+416259:quaternary,Chr2:+10753420:quaternary
AATTAATTGT	2	0	0	0	1	Chr5:+22169512:quaternary,Chr1:+3075746:quaternary
TTGTTTTCCA	1	0	1	0	1	Chr5:+20941000:quaternary
TTAATTTTAG	0	0	1	1	1	Chr5:+19992623:quaternary
ATAAATTAAA	1	0	0	2	0	Chr5:+19538244:quaternary,Chr3:+3415586:quaternary
TTGTCCTTTT	1	1	0	1	0	Chr5:+18989837:quaternary,ChrC:+56719:quaternary
ACCGACAAAA	0	1	1	1	0	Chr5:+18260879:quaternary
GGTTGGCTCG	0	2	0	1	0	Chr5:+17492358:quaternary
TTTCCATAAA	1	1	1	0	0	Chr5:+16932400:quaternary,Chr1:+29947249:quaternary
TTTCAAAATT	1	1	1	0	0	Chr5:+16700029:quaternary,Chr3:+10356786:quaternary,Chr5:+2676068:quaternary,Chr2:+9493492:quaternary,Chr4:+14852462:quaternary
AAGGTGGGGA	1	0	1	1	0	Chr5:+16588257:quaternary
ATACATAAAT	1	2	0	0	0	Chr5:+16355635:quaternary,At2g46220.1:X:+601:quaternary
CAAACTTGAG	0	3	0	0	0	Chr5:+15397171:quaternary
TTTTTCCATA	0	0	1	0	2	Chr5:+14277516:quaternary,Chr1:+1915984:quaternary
ATATGTTATG	2	0	0	0	1	Chr5:+13532112:quaternary
AGAGTTTACA	1	0	0	1	1	Chr5:+10597191:quaternary
AACCGGACGG	1	2	0	0	0	Chr4:-7785854:quaternary
GACTACGGGA	1	0	1	1	0	Chr4:-2327564:quaternary
TGATTAGTTC	0	0	1	1	1	Chr4:-10278794:quaternary
TGTTCAAAAA	0	1	1	1	0	Chr4:+9732419:quaternary
ACCATACAAA	1	0	2	0	0	Chr4:+862871:quaternary,Chr5:+20150332:quaternary
GAATATTAAA	0	1	0	2	0	Chr4:+7969890:quaternary,Chr5:+9883427:quaternary,Chr2:+16573087:quaternary,Chr5:+19583553:quaternary
TCAATAAACA	2	0	0	1	0	Chr4:+6552909:quaternary,Chr5:+26662209:quaternary
ATAATGAAAA	1	2	0	0	0	Chr4:+60413:quaternary,At4g25820.1:X:+747:quaternary,Chr3:+4077531:quaternary,Chr2:+1928080:quaternary,Chr5:+26526989:quaternary,Chr2:+3575231:quaternary
GAACATATGT	1	0	0	0	2	Chr4:+5932806:quaternary
ATAATATGCA	1	1	1	0	0	Chr4:+5716203:quaternary
ATTAATTTAG	1	0	0	2	0	Chr4:+5424689:quaternary,At5g45380.1:X:+459:quaternary
TAAAACTAAT	0	2	1	0	0	Chr4:+5087105:quaternary
TTATTACTTG	0	2	1	0	0	Chr4:+4431327:quaternary
AGTTTTTAAA	0	0	1	2	0	Chr4:+4096460:quaternary

ATTGGTAACT	0	0	0	1	2	Chr4:+2505498:quaternary
TTTTAAGTCA	2	1	0	0	0	Chr4:+2037670:quaternary,Chr1:+18702573:quaternary,Chr3:+8808542:quaternary
TTATGGTCCA	1	0	0	1	1	Chr4:+2037096:quaternary
AAACCTTCTA	1	1	0	1	0	Chr4:+16810163:quaternary; -small RNA(ATAGAAGGTTTCATGTGATTCTTT)
TTATCTCTCA	1	1	0	1	0	Chr4:+15512425:quaternary,Chr1:+18234591:quaternary
CTAAATTGTT	0	0	0	1	2	Chr4:+14943638:quaternary
CAGACCATTA	0	1	0	0	2	Chr4:+14344972:quaternary
AATGTATTGT	0	0	0	0	3	Chr4:+14325564:quaternary
TATAATATTA	0	1	0	0	2	Chr4:+11899271:quaternary,Chr2:+9701160:quaternary
CGAGATTCTA	0	0	1	2	0	Chr4:+11298606:quaternary
GAAAAAACAC	1	0	1	1	0	Chr4:+10167113:quaternary
CGTCGCTTCT	1	2	0	0	0	Chr3:-14214238:quaternary,Chr2:-9281:quaternary
CTGAGACAAA	0	0	1	1	1	Chr3:-12933609:quaternary
GGCCAAAACA	0	0	1	0	2	Chr3:+848958:quaternary
TATAATGTGT	1	0	2	0	0	Chr3:+7577231:quaternary
TTGCTGTAAT	2	0	1	0	0	Chr3:+6993871:quaternary
TAATAGTAAA	0	1	1	1	0	Chr3:+697548:quaternary
AGCAAAAGTT	1	1	1	0	0	Chr3:+6443445:quaternary
AATGTATGAT	0	3	0	0	0	Chr3:+3690726:quaternary,Chr4:+13684011:quaternary
ATTAAAGAAA	2	0	0	1	0	Chr3:+22725981:quaternary,Chr4:+5031137:quaternary,Chr1:+18205280:quaternary
TGAAGTGAAG	0	3	0	0	0	Chr3:+22704519:quaternary,Chr4:+10579451:quaternary,At1g68440.1:X:+1262:quaternary
GAAATTGTGT	1	0	1	1	0	Chr3:+21818473:quaternary
GCATTAAATT	0	0	2	1	0	Chr3:+21229357:quaternary
CTAGCAACTT	2	1	0	0	0	Chr3:+20776076:quaternary,At1g03420.1:X:+935:quaternary
AATTTAATAA	0	2	0	0	1	Chr3:+20506717:quaternary
AGATACCAAT	3	0	0	0	0	Chr3:+17849702:quaternary
ATAAACTCAA	0	0	1	1	1	Chr3:+14696261:quaternary
AGGAGAGATG	0	2	0	1	0	Chr3:+14658647:quaternary
AGATTAAATT	0	1	2	0	0	Chr3:+14572999:quaternary
CAAACGAACG	0	0	0	2	1	Chr3:+14210668:quaternary,Chr2:+5711:quaternary
TTCCAAACAA	1	1	1	0	0	Chr3:+13108318:quaternary
GGTTTAACGA	0	0	2	1	0	Chr3:+11196371:quaternary
TATTTTCTCT	1	2	0	0	0	Chr3:+11088607:quaternary,Chr1:+18101202:quaternary,Chr1:+19377699:quaternary
GCCAAACGAA	1	0	0	1	1	Chr2:-16503121:quaternary
CTGCAGCGTT	0	1	2	0	0	Chr2:-11820062:quaternary
TACACAACAA	0	1	1	1	0	Chr2:+886199:quaternary,Chr2:+896122:quaternary
AGAATATGAG	1	0	0	1	1	Chr2:+8444580:quaternary,Chr5:+9818275:quaternary
TAATTTGCGT	0	0	1	2	0	Chr2:+8043397:quaternary,Chr2:+8043379:quaternary
CAAATTTCTT	1	1	0	1	0	Chr2:+7628558:quaternary
TTGGCAAAAA	0	1	2	0	0	Chr2:+7063027:quaternary
CAGCTAAAAA	1	0	1	1	0	Chr2:+6932947:quaternary
CCACTCATTT	1	0	1	1	0	Chr2:+655384:quaternary
TGACCTGATA	0	0	0	0	3	Chr2:+3621175:quaternary
TGAACTAAAA	1	1	1	0	0	Chr2:+3534283:quaternary,Chr4:+5957295:quaternary
GTGGATAAAA	0	0	1	2	0	Chr2:+18833811:quaternary,Chr5:+8361523:quaternary
GAAAAAAAGA	0	0	2	1	0	Chr2:+16959768:quaternary,At4g21865.1:X:+-99:quaternary,Chr5:+1567561:quaternary,Chr1:+28921031:quaternary,Chr1:+9201655:quaternary,Chr2:+16258604:quaternary
CTTTTAAAAA	1	0	2	0	0	Chr2:+16024149:quaternary,Chr2:+4111002:quaternary,Chr1:+26805165:quaternary
GTCTCATAAT	1	0	1	0	1	Chr2:+1593672:quaternary,Chr1:+7289138:quaternary
ACTACATCAT	1	0	1	0	1	Chr2:+15770943:quaternary
CGAAACAAAA	1	0	0	0	2	Chr2:+15693484:quaternary,Chr4:+17001891:quaternary
TCCTTCAGAA	1	1	1	0	0	Chr2:+13847370:quaternary
TAATTCTTCT	0	0	2	1	0	Chr2:+13322317:quaternary
TTGAGACGAA	1	0	1	1	0	Chr2:+12931998:quaternary
AAGACCAAAA	1	0	1	0	1	Chr2:+11940031:quaternary,Chr1:+8873776:quaternary,Chr4:+17638864:quaternary
AAGGATTATA	1	0	0	1	1	Chr2:+10473339:quaternary
GGCAAAAAAA	1	0	0	1	1	Chr2:+10455374:quaternary
TTTTCATTAC	0	1	0	0	2	Chr2:+10076053:quaternary
GCTCTTAAAG	1	1	1	0	0	Chr1:-7166:quaternary
GATTTAACTA	1	2	0	0	0	Chr1:-10145550:quaternary
TGTACTAAAA	1	1	0	0	1	Chr1:+9500259:quaternary

TAATTTACAT	1	1	0	0	1	Chr1:+9412363:quaternary,Chr1:+13290213:quaternary
TTTTCACATA	1	2	0	0	0	Chr1:+8544714:quaternary,Chr4:+11238012:quaternary,Chr4:+11231035:quaternary,Chr2:+12286289:quaternary,Chr1:+8542688:quaternary,Chr4:+1560215:quaternary
TATCACGAGA	2	0	1	0	0	Chr1:+766028:quaternary
GACTTGCGAC	0	3	0	0	0	Chr1:+7289583:quaternary
ATTACAAAAA	2	0	0	0	1	Chr1:+5280287:quaternary,ChrM:+114527:quaternary,Chr2:+3370925:quaternary,Chr2:+3505093:quaternary
TTGAGATTTT	0	1	1	0	1	Chr1:+4292150:quaternary
AAAGTAAAAC	1	0	0	2	0	Chr1:+4116844:quaternary,Chr1:+20047532:quaternary
GTGCGTTGTA	0	0	0	2	1	Chr1:+29125951:quaternary
TGAGCTATCT	0	2	0	0	1	Chr1:+24758945:quaternary
AGAGTTTACC	2	0	0	0	1	Chr1:+2445136:quaternary
AAAAAACCTT	1	1	1	0	0	Chr1:+24274036:quaternary
AACTTGAGCT	0	3	0	0	0	Chr1:+23642745:quaternary,Chr4:+15308788:quaternary
GGTTTTTATG	0	1	1	0	1	Chr1:+23243963:quaternary
ATATGTTCAT	1	2	0	0	0	Chr1:+22934809:quaternary
AATCCAAAAA	0	0	1	2	0	Chr1:+22756397:quaternary
ATACACTCCA	3	0	0	0	0	Chr1:+22465766:quaternary
AGTCGAAAAA	0	1	1	0	1	Chr1:+22321927:quaternary
AATCAGTGAA	2	0	0	0	1	Chr1:+22154167:quaternary
ATATTCATTA	1	1	0	0	1	Chr1:+20980417:quaternary
TTATTTTTAA	2	0	0	1	0	Chr1:+19487183:quaternary,Chr3:+4623462:quaternary,Chr1:+7638980:quaternary
AAAAACATTG	0	1	0	0	2	Chr1:+19478135:quaternary,Chr2:+13404734:quaternary
CAACTGCCAA	0	2	1	0	0	Chr1:+19419955:quaternary
ATCACTTACT	2	1	0	0	0	Chr1:+16870670:quaternary
CGTCTTTTAC	1	1	0	1	0	Chr1:+13920819:quaternary,At3g14010.1:X:+315:quaternary,Chr5:+8526862:quaternary
TCCTTATAAA	1	1	1	0	0	Chr1:+13817330:quaternary,Chr3:+11479604:quaternary
ATCACATAAA	0	1	0	0	2	Chr1:+12601769:quaternary
TAAAACTCTG	1	0	1	1	0	Chr1:+12232553:quaternary
CTATGGGGTG	0	3	0	0	0	AtCg00860:d:+4321:secondary,AtCg00860:d:+4321:secondary
TCAACATATT	0	2	0	1	0	AtCg00720:d:+89:primary
GTGCTAGAAC	0	3	0	0	0	AtCg00680:d:+1409:primary
CATAAATGGC	0	0	1	2	0	AtCg00160.1:X:+-92:quaternary
ATGAAAGAAG	0	2	0	0	1	At5g67640.1:v:+694:primary
TCGTTTTTGC	1	0	0	1	1	At5g67360.1:X:-2495:quaternary
GAATGAATTT	1	0	1	1	0	At5g67320.1:i:+2709:tertiary
AAGCAGCTGA	0	1	2	0	0	At5g67250.1:d:+669:secondary
TCTTTGGACT	1	0	0	2	0	At5g67070.1:d:+707:secondary
GTTTCTACTT	1	2	0	0	0	At5g67010.1:v:+441:secondary,At5g05200.1:d:+1867:secondary
TTGTACTTTG	2	0	1	0	0	At5g66730.1:d:+2230:secondary
TAGATAAGTT	1	2	0	0	0	At5g66675.1:d:+1384:primary
CGGGGGTAAC	0	1	2	0	0	At5g66650.1:d:+1201:primary
TTCCAGAAAC	2	0	0	1	0	At5g66600.1:v:+1183:secondary,At1g21280.1:v:+981:secondary
GTTGGTGTTC	1	2	0	0	0	At5g66590.1:d:+252:primary
TGGACACAAA	1	2	0	0	0	At5g66410.1:d:+495:primary
TCATAAATTG	1	2	0	0	0	At5g66340.1:v:+16:secondary
CTAGGAGTAT	0	0	1	1	1	At5g66320.2:d:+1543:primary,At5g66320.1:d:+1373:primary
TAAAAAAAAG	0	1	0	2	0	At5g66270.1:i:+749:tertiary
CTTGGAACAG	0	1	2	0	0	At5g66190.1:d:+790:secondary,At5g04290.1:v:+2391:secondary
TGTACATTTA	0	0	3	0	0	At5g66160.1:d:+1233:secondary,At5g66160.2:d:+1311:secondary
AGGTATAGGC	0	3	0	0	0	At5g66070.1:X:+163:quaternary
TACCTGAGAG	2	0	1	0	0	At5g66050.1:d:+1239:primary,At5g66050.2:d:+1547:primary
CAACAAGAGC	0	3	0	0	0	At5g66030.2:d:+601:secondary,At5g66030.1:d:+601:secondary,At1g20960.1:v:+1486:secondary
AGATCCTGAC	1	1	0	1	0	At5g65990.1:d:+1335:secondary
CTCCGCCGGT	0	3	0	0	0	At5g65925.1:d:+188:primary
CCTTTTAACG	0	0	0	2	1	At5g65900.1:d:+2071:primary
ATTGAAACTA	0	1	0	2	0	At5g65640.1:d:+1144:primary
GCTTCGGTAG	0	3	0	0	0	At5g65630.1:d:+1663:primary
TGATTTCAAG	1	2	0	0	0	At5g65490.1:d:+2049:secondary
AATCTTGAAT	1	1	1	0	0	At5g65410.1:d:+925:primary

GTTAAGCATA	2	1	0	0	0	At5g65260.1:d:+710:primary
TTACATCTTT	0	1	0	0	2	At5g65205.1:d:+930:primary
GAATCAAAAA	1	2	0	0	0	At5g64980.1:d:+1117:secondary
TACTTTGTTT	1	2	0	0	0	At5g64880.1:d:+471:primary
GAACCGAACC	0	0	2	0	1	At5g64840.1:d:+2377:primary
TGATTGGAGG	0	2	0	0	1	At5g64730.1:d:+739:primary
TTCTCTGGCT	0	1	0	1	1	At5g64680.1:d:+534:primary,At5g64680.2:d:+717:primary
CCGTTTTAAA	0	1	1	1	0	At5g64660.1:d:+1311:primary
TTGTTTCTCC	1	1	0	1	0	At5g64650.1:d:+582:primary
AGACCCGATA	0	0	1	1	1	At5g64570.1:d:+1863:secondary
TTGAGATTGT	1	0	1	0	1	At5g64200.1:d:+1152:secondary,At4g20310.1:d:+1646:secondary
TCGCTGGTGC	1	1	1	0	0	At5g63980.1:d:+1008:primary
ACCGTCCGCA	0	0	0	2	1	At5g63940.1:d:+2021:primary
AAGGTCCAGA	1	2	0	0	0	At5g63870.1:d:+1017:primary,At5g63870.2:d:+1032:primary,At5g63870.3:d:+1120:primary
GTGATGCTGT	0	2	0	0	1	At5g63680.1:d:+1591:primary
TTAAAGAGGC	0	2	0	1	0	At5g63650.1:d:+1267:primary
CAGTGCACAC	1	0	2	0	0	At5g63620.2:d:+1085:primary,At5g63620.1:d:+1125:primary
TGTTTATCTA	1	0	1	0	1	At5g63510.1:d:+846:primary
GCTAAGGGAG	0	0	1	1	1	At5g63310.1:d:+701:secondary
GTTAATTTGT	1	0	1	1	0	At5g63190.2:d:+2541:primary,At5g63190.1:d:+2585:primary
GAAGTAACGG	0	1	2	0	0	At5g62940.1:d:+866:secondary
AACACGTTTC	1	1	1	0	0	At5g62930.1:d:+802:primary
TTCTTGCCGT	1	2	0	0	0	At5g62920.1:d:+134:primary
ATATTCTTCT	0	0	0	2	1	At5g62890.2:d:+1266:primary
GTGAGTTTGC	1	1	1	0	0	At5g62580.1:d:+2140:primary
TTCTCAAAAA	0	2	0	1	0	At5g62530.1:i:+1444:tertiary,At1g19890.1:i:+322:tertiary
GCTTGGGCTA	0	3	0	0	0	At5g62460.1:d:+907:primary
AAACACTTCA	0	2	1	0	0	At5g62370.1:v:+3763:primary,At5g34856.1:p:+5536:primary
ATGACTACTA	0	1	0	2	0	At5g62310.1:X:+268:quaternary
GTGAAATACC	0	2	0	0	1	At5g62190.1:d:+1227:primary
GCTTCAACAA	0	0	2	1	0	At5g62140.1:d:+12:primary
ACACAGGACC	0	3	0	0	0	At5g62050.1:d:+1274:primary
GATGTAAATT	0	1	0	2	0	At5g61960.1:d:+2600:primary
AATGATCTCG	1	1	0	1	0	At5g61900.1:d:+1816:primary,At5g61900.3:d:+1814:primary
TGGAATACGA	0	0	0	1	2	At5g61820.1:d:+959:secondary
ATCCTCGTAT	1	0	2	0	0	At5g61670.2:d:+947:secondary,At5g61670.1:d:+990:secondary
ATGTTAAATG	0	0	2	0	1	At5g61480.1:v:+3448:secondary,At3g27600.1:v:+865:secondary
AACTAATACT	0	1	1	0	1	At5g61450.1:d:+1557:primary
TGTTTGTACC	1	2	0	0	0	At5g61060.1:d:+1758:primary
GAAGAAACTG	0	0	0	2	1	At5g61040.1:d:+2314:primary
AGGGGATTCA	0	2	0	1	0	At5g60980.2:d:+1085:primary,At5g60980.1:d:+1002:primary
TTGCTTTGTA	0	3	0	0	0	At5g60940.1:d:+1492:primary,At3g44820.1:v:+2462:primary,At5g60940.2:d:+1541:primary
TTATTGTCCA	0	1	1	1	0	At5g60550.1:X:+299:quaternary
ATTTCTGCAA	0	1	0	2	0	At5g60450.1:d:+2712:secondary,At1g50490.1:d:+715:secondary
CTATGAATGA	0	1	1	1	0	At5g60250.1:d:+1753:secondary
AAGAGAGGTT	0	2	1	0	0	At5g60170.1:d:+2846:primary
CTCACATTTC	1	1	0	1	0	At5g60020.1:d:+453:secondary,At4g32600.1:d:+1408:secondary
GAGTTAATCG	0	1	1	0	1	At5g59750.1:d:+1246:primary
AGACCAAACA	0	0	2	0	1	At5g59290.1:X:-286:quaternary,At3g18524.1:X:-832:quaternary
TCATCTAAGC	1	0	2	0	0	At5g59080.1:d:+323:primary
GCAAGTACTT	0	2	0	1	0	At5g58800.1:d:+705:primary
ATCGGCTTTT	0	0	1	2	0	At5g58760.1:d:+1828:primary
CGTAGACTCT	0	1	1	1	0	At5g58730.1:d:+1137:primary
TATTGAATAT	1	1	0	0	1	At5g58570.1:d:+446:primary
GGGGAAACCG	0	1	0	2	0	At5g58470.1:d:+1310:secondary,At5g58470.2:d:+1310:secondary
ACTCCAATCT	0	3	0	0	0	At5g58380.1:d:+1034:primary
AATGCATCTC	3	0	0	0	0	At5g58260.1:d:+685:secondary
TTATAAAGAA	1	1	0	0	1	At5g58240.2:d:+722:secondary
GCAACAAAAA	0	1	2	0	0	At5g58190.1:d:+1160:primary,At5g58190.2:d:+1119:primary
ACAACAATAC	0	0	2	1	0	At5g58100.1:d:+2881:primary
CAAACTACAG	1	0	0	1	1	At5g58070.1:d:+898:secondary

CCTAATATTA	0	3	0	0	0	At5g57990.1:d:+1778:secondary
AGAAGCTCAA	0	0	1	2	0	At5g57960.1:d:+1428:primary,At4g24040.1:d:+1798:primary
CTTATGTAGA	0	1	1	1	0	At5g57887.1:d:-477:secondary,At2g26480.1:d:-797:secondary
AAGAGTTGCT	0	2	0	1	0	At5g57860.1:d:+397:primary,At5g57860.3:d:+439:primary,At5g57860.2:d:+405:primary
AACATTCGTC	0	3	0	0	0	At5g57770.1:X:-16:quaternary
GAGTTTTGCC	0	0	3	0	0	At5g57630.1:d:+1262:primary
GATACATTGT	1	1	0	1	0	At5g57565.1:X:+247:quaternary
CAAAATTCCG	0	0	2	0	1	At5g57560.1:X:-926:quaternary
ACACATACCA	0	0	2	1	0	At5g57460.1:d:-2191:secondary
TTATTATTGA	0	1	0	0	2	At5g57300.1:i:+1042:tertiary
AGAAGTGTAA	1	2	0	0	0	At5g57240.1:d:+1319:primary
TTTTGTAACT	0	0	1	1	1	At5g57160.1:d:+3768:primary
TGAACCAGGT	1	1	1	0	0	At5g57130.1:d:+2746:secondary
CTTTTCCATT	1	0	2	0	0	At5g57110.2:d:+1852:secondary,At1g14630.1:v:+952:secondary,At5g57110.1:d:+1836:secondary
TTTCTATAGA	2	1	0	0	0	At5g57050.1:i:+1470:tertiary
TACAAACAAG	2	0	1	0	0	At5g57035.1:d:+925:secondary
ATAGACTTGC	0	0	3	0	0	At5g57000.1:d:+760:primary
ATACAGATTA	1	0	2	0	0	At5g56980.1:d:+1577:primary
AAGAACTCTA	2	1	0	0	0	At5g56660.1:d:+1320:primary
ATCTCGTTGT	1	1	1	0	0	At5g56590.1:d:+1672:primary
TTGGTTATGT	0	2	1	0	0	At5g56540.1:d:+379:primary
AGCAGCAACA	2	0	1	0	0	At5g56500.1:d:+2167:primary
CTTCATCTGT	0	0	1	1	1	At5g56500.1:d:+1769:secondary,At2g22530.1:v:+1802:secondary
GCCGTTTAGT	0	1	1	1	0	At5g56340.1:d:+1163:primary
TCAAGAAACT	0	0	2	0	1	At5g56320.1:d:+702:primary
GTGAGAAGAA	0	1	0	2	0	At5g56260.1:d:+587:secondary,At3g13810.1:d:+966:secondary,At3g50700.1:d:+936:secondary
GCCGAGCCAG	0	3	0	0	0	At5g56190.2:d:+1303:secondary,At5g56190.1:d:+1221:secondary
TAAGAGATTA	0	0	0	2	1	At5g56160.1:d:+1094:primary,At5g62880.1:d:+817:primary
GTTGACGCTA	1	0	0	2	0	At5g56000.1:d:+1562:secondary,At5g56010.1:d:+1582:secondary
ATGAAGTTGA	0	2	1	0	0	At5g55860.1:v:+1381:primary
CCCAACTTGA	1	0	0	2	0	At5g55660.1:d:+2601:secondary
CACGAGCACT	0	1	2	0	0	At5g55400.1:v:+2306:secondary
CTATTAAATT	1	0	1	0	1	At5g55380.1:d:+1171:primary
TGAATCTGCG	1	0	1	1	0	At5g55000.2:d:+908:primary
TCTTTGTTCT	0	2	0	1	0	At5g54980.1:d:+582:primary
AAGGATATCT	0	1	0	1	1	At5g54970.1:d:+65:primary
CACTTTCGGT	0	3	0	0	0	At5g54900.1:d:+961:primary
TCGCCTTGAC	3	0	0	0	0	At5g54660.1:d:+635:primary
AGGGGAGCAG	0	2	0	1	0	At5g54600.1:d:-771:secondary,At5g54600.2:d:-714:secondary
GATGAGAAAA	0	0	3	0	0	At5g54570.1:v:+1599:secondary
AGATAGTTAA	1	0	0	1	1	At5g54540.1:X:+614:quaternary
GGTCTGATTC	0	3	0	0	0	At5g53980.1:d:+507:primary
AAACTCTCTG	2	0	0	1	0	At5g53900.2:d:+1342:primary,At5g53900.1:d:+1662:primary
TAAAGCAAAG	1	1	0	0	1	At5g53890.1:d:+3413:primary
CTGATGAAGG	0	3	0	0	0	At5g53850.3:d:+1142:secondary,At5g53850.2:d:+1094:secondary,At5g53850.1:d:+1094:secondary
GACCTACTGA	2	0	0	1	0	At5g53770.1:d:+1658:primary
TTGAAGATAA	0	2	0	0	1	At5g53580.1:d:+1045:primary
TTCACGGCTG	0	0	2	0	1	At5g53390.1:d:+115:secondary,At3g21055.1:d:+280:secondary
TTTCACTGGC	0	1	2	0	0	At5g53300.1:d:+212:secondary,At5g53300.2:d:+193:secondary
AATGACTGAG	1	0	1	1	0	At5g53050.2:X:-140:quaternary
TTCACTGGAT	1	0	1	0	1	At5g52980.1:d:+451:primary
ATTGGAATAT	2	0	0	1	0	At5g52900.1:X:-346:quaternary
TTTACTTTTA	2	0	0	1	0	At5g52900.1:d:+1224:primary
ACGCCTCTGT	0	1	0	1	1	At5g52820.1:i:+307:tertiary
TGAAGATGGT	0	0	2	0	1	At5g52540.1:d:+1462:primary
TAATAAACGA	0	0	2	1	0	At5g52370.1:d:+754:primary
GTGAGGCGGT	1	0	1	0	1	At5g52300.1:d:+1785:primary
ATTAATACAA	0	0	1	0	2	At5g52250.1:d:+1233:primary
TGCTGTTGTT	1	0	1	1	0	At5g52190.1:d:+750:primary
CCGATACAAT	0	1	1	0	1	At5g52060.1:d:+793:primary
TTAGGAGTTT	0	2	0	1	0	At5g51980.1:d:+1573:primary

TTCCCGCTGG	0	2	0	1	0	At5g51880.1:d:+830:secondary
TACGAAGAAT	0	2	1	0	0	At5g51795.1:v:+1509:secondary
TGTTGAGAGT	0	2	0	1	0	At5g51795.1:v:+1183:secondary,At3g23085.1:p:+1029:secondary,At5g46195.1:p:+95:secondary,At5g39070.1:p:+45:secondary,At1g55460.1:d:+1114:secondary
CGAAAAGGAC	1	0	2	0	0	At5g51590.1:X:-218:quaternary
GCAAACATAT	0	1	2	0	0	At5g51290.1:d:+2137:primary
TCAAAATATT	0	0	1	2	0	At5g51280.1:d:+2099:primary
TCGATGGAAA	0	3	0	0	0	At5g51170.1:d:+284:primary
CACAGGTCCC	1	2	0	0	0	At5g50920.1:d:+1657:secondary
AGATCTTATC	0	1	0	0	2	At5g50870.1:X:+253:quaternary
ACCGATCCGA	0	0	0	3	0	At5g50800.1:d:+996:primary
TAAGATCTGC	0	1	0	0	2	At5g50665.1:v:+742:primary,At5g50565.1:v:+742:primary
CTTTTATTAG	1	0	0	0	2	At5g50550.1:d:+1302:primary
AGTTGTTTGT	0	1	1	0	1	At5g50510.1:v:+7:secondary,At1g67830.1:d:+857:secondary,At5g50620.1:v:+7:secondary,At5g50500.1:v:+525:secondary
TTATTTTAAA	2	0	0	0	1	At5g50450.1:d:+1246:primary,At4g29230.1:v:+2239:primary
GCATTACTCA	1	0	2	0	0	At5g50340.1:d:+474:secondary
GAAACTGGGG	0	0	2	1	0	At5g50020.1:d:+1373:primary
CCATACCCTG	1	1	1	0	0	At5g50000.1:d:+1001:primary
ATTGTTGATG	0	0	2	0	1	At5g49980.1:d:+2099:primary
GGAAGATGTG	2	0	1	0	0	At5g49460.1:d:+1937:secondary
CCTTAGAATC	1	1	1	0	0	At5g49400.1:d:+957:primary
TGAGAGTTCA	0	2	1	0	0	At5g49360.1:d:+256:secondary
TTCTTCGTCT	1	1	0	1	0	At5g49140.1:v:+362:secondary,At1g13190.1:d:+1922:secondary
GGAAGCGGTA	0	1	2	0	0	At5g48990.1:d:+780:primary
GTTTGTTCTG	1	1	0	0	1	At5g48880.1:i:+1328:tertiary,At5g48880.2:i:+693:tertiary,At4g00750.1:i:+2335:tertiary
GTTCTTGAAG	0	1	0	1	1	At5g48870.1:d:+192:primary
GTGTGGTTGA	0	2	1	0	0	At5g48730.1:v:+1783:secondary
TAAACTTTGG	0	1	0	2	0	At5g48655.1:X:-51:quaternary
TAACACTGTT	3	0	0	0	0	At5g48640.1:d:+903:primary
AAGCAAAAAG	0	2	0	0	1	At5g48590.1:d:+873:primary,At1g43810.1:v:+727:primary
TGTTCTAAAG	0	2	0	0	1	At5g48545.1:d:+339:primary
ATTGTGAGAG	2	1	0	0	0	At5g48360.1:d:+2352:primary
ACGAAAGGGA	0	2	0	1	0	At5g48220.1:d:+918:primary
CAGCTACAAA	0	1	0	2	0	At5g47970.1:d:+1911:primary
CTTCAGTCGT	1	2	0	0	0	At5g47910.1:d:+2666:secondary
TACACAAAAC	1	1	0	0	1	At5g47860.1:d:+1402:secondary
ACGGGCTGTG	0	2	0	1	0	At5g47840.1:d:+50:primary
GAGTATGAGA	1	1	0	0	1	At5g47770.1:d:+1163:secondary,At1g29800.1:d:+857:secondary
ATCTTCTGGA	2	1	0	0	0	At5g47680.1:d:+1035:secondary
AATCTCTTTG	0	0	1	1	1	At5g47580.1:d:+2535:primary,At2g17590.1:d:+1934:primary
AACGTTGAAA	0	0	2	1	0	At5g47520.1:d:+749:secondary
AGTGGTAATG	2	0	0	1	0	At5g47480.1:d:+4323:primary
AACAAGAAAA	0	0	2	1	0	At5g47140.1:i:+553:tertiary,At4g05090.1:i:+1391:tertiary
TCTGGTCACC	0	2	0	0	1	At5g47120.1:d:-883:secondary
ATGTTAAAAA	1	0	1	0	1	At5g46910.1:v:+1684:secondary,At3g53360.1:v:+2467:secondary
TTGCTCCACC	0	2	0	1	0	At5g46750.1:d:+874:primary
CTAAGTATCA	1	1	1	0	0	At5g46690.1:d:+910:primary
AAGATGTAGG	1	0	0	2	0	At5g46560.1:d:+1396:primary
AGAGTGCCAT	1	1	0	1	0	At5g46490.1:d:+1305:primary
CGAAAGGTGC	2	0	0	1	0	At5g46470.1:i:+5927:tertiary
CATTCTTTTG	0	3	0	0	0	At5g46410.1:d:+1344:secondary
CGTAAAAAAA	2	0	0	1	0	At5g46080.1:X:--226:quaternary
AGCAGTTTCT	0	1	2	0	0	At5g46050.1:d:+1597:primary
ATGATCATCA	1	1	1	0	0	At5g46010.1:v:+391:secondary,At5g10830.1:d:+1076:secondary,At5g57735.1:p:+61:secondary,At3g45080.1:v:+640:secondary,At4g39180.1:d:+699:secondary,At2g36890.1:d:+767:secondary,At1g15730.1:d:+1000:secondary,At5g40780.1:d:+195:secondary,At1g64405.1:d:+470:secondary,At2g40260.1:v:+1435:secondary,At5g47225.1:v:+147:secondary
TTATGTATAA	1	0	0	1	1	At5g45990.1:v:+2559:primary
GTGCTGGCCC	0	0	0	3	0	At5g45900.1:d:+1614:primary

TTGTGAGGAG	1	0	0	1	1	At5g45650.1:d:+2420:primary
TTCCGTAAGG	0	1	0	1	1	At5g45500.1:d:+1239:primary
TCCTTCTTCT	1	1	1	0	0	At5g45480.1:d:+2950:primary
ATCTGAATAA	1	0	0	1	1	At5g45460.1:d:+150:secondary
CCTCTATATC	0	1	2	0	0	At5g45340.1:d:+1719:primary,At5g45340.2:d:+1812:primary
ACCGAGAATT	1	1	0	0	1	At5g45260.1:d:+3598:secondary
TCAGTTCTGA	0	3	0	0	0	At5g45160.1:d:+2546:secondary
GTGGTGTATC	0	1	2	0	0	At5g45060.1:d:+3615:primary
ATCTGAATCT	1	1	1	0	0	At5g44820.1:d:+1031:primary
CCTCAATCCA	0	1	1	1	0	At5g44800.1:v:+611:secondary
AATTGTCTTT	2	0	0	1	0	At5g44750.1:d:+3711:primary
CTTTGTTGGT	1	1	0	0	1	At5g44730.1:d:+934:primary,At5g44730.2:d:+743:primary
AAATTTCAAA	0	0	0	1	2	At5g44630.1:i:+1933:tertiary,At2g31170.1:i:+1294:tertiary
ACTAAACCTG	0	2	1	0	0	At5g44450.1:d:+1178:primary
AAAACTTCCT	0	1	1	1	0	At5g44410.1:d:+104:secondary
TCAAAAAAAT	1	0	1	1	0	At5g44390.1:d:+1825:primary
AGAGAGCATT	1	1	0	1	0	At5g44316.1:v:+1642:secondary
TTTTCAGTTG	0	1	0	2	0	At5g44313.1:v:+189:secondary,At5g03950.1:v:+222:secondary,At5g40110.1:v:+222:secondary,At3g45120.1:v:+149:secondary,At5g27590.1:v:+300:secondary, At2g41760.1:d:+822:secondary
GGTCCTATTT	0	3	0	0	0	At5g44240.1:d:+3004:primary
TCACCGGGAG	0	2	1	0	0	At5g44210.1:d:+651:primary
CACTCATATA	1	1	0	0	1	At5g44190.1:d:-1356:secondary
GCTCCGATTG	2	1	0	0	0	At5g44180.1:d:+4792:secondary
AAATATTTGT	1	1	0	0	1	At5g44090.1:d:+2280:primary
ACTTCCGTTG	0	2	0	1	0	At5g43960.2:d:+1343:primary,At5g43960.1:d:+1391:primary
TTAAAAATAG	1	1	0	1	0	At5g43930.1:d:+2507:primary
TTTTGGTGGG	0	2	1	0	0	At5g43920.1:v:+1697:secondary
AACTGATAAA	0	1	1	0	1	At5g43910.1:i:+232:tertiary,At5g43910.3:i:+232:tertiary,At5g43910.2:i:+232:tertiary
GATTTGCTGG	1	2	0	0	0	At5g43850.1:d:+232:primary
TGTCCCTCTG	0	0	1	1	1	At5g43780.1:d:+1370:secondary
GATCAAAAAT	0	1	0	2	0	At5g43670.1:d:+2285:primary
AAAAGCTTCT	0	2	1	0	0	At5g43560.2:d:+498:secondary,At5g56870.1:d:+2263:secondary,At3g30582.1:p:+278:secondary,At5g43560.1:d:+868:secondary
GGATTACCAA	0	2	1	0	0	At5g43500.2:d:+1362:primary,At5g43500.1:d:+1588:primary
TTGTTTGAAG	0	2	1	0	0	At5g43430.1:d:+807:primary
TAACAGAAAA	0	2	0	0	1	At5g43410.1:v:+227:secondary,At1g08840.1:v:+4398:secondary,At2g34730.1:d:+1676:secondary
ATTTCCTTCG	0	2	0	1	0	At5g43380.1:d:+1388:primary
AGTACCCGGT	0	2	1	0	0	At5g43060.1:d:+1308:primary
ATGATTTCTC	0	0	2	1	0	At5g42940.1:d:+2263:primary
TAACATTTTT	0	1	0	1	1	At5g42920.2:d:+2529:primary,At5g42920.1:d:+2606:primary
ATGAAGGAAC	0	3	0	0	0	At5g42890.1:d:+165:primary
TCACTTGTTT	1	1	1	0	0	At5g42830.1:d:+1336:primary
ATCGTCCTTC	0	0	3	0	0	At5g42765.1:X:-260:quaternary
GTTCCAAAAC	0	2	1	0	0	At5g42690.1:i:+2155:tertiary
AGAGCTCGAT	0	1	0	2	0	At5g42680.1:d:+645:primary
CCTCAAAAGC	1	0	1	1	0	At5g42650.1:X:-1388:quaternary
GCCTCTCTAC	1	1	0	0	1	At5g42460.1:d:+1234:primary,At2g17830.1:d:+1313:primary
TATTGAGAAG	1	0	0	0	2	At5g42360.1:d:+1972:primary,At3g45240.1:d:+1355:primary
AAAATGTCTT	2	1	0	0	0	At5g41940.1:d:+1799:primary
AATGCAAACA	0	2	0	1	0	At5g41790.1:i:+4073:tertiary
GGATTTTGCT	0	0	1	2	0	At5g41720.1:d:+314:secondary
TATGTAAGTA	0	0	0	2	1	At5g41370.1:d:+2651:primary
AGCACTTGAA	0	0	1	2	0	At5g41270.1:d:+168:secondary
ATAGTTAGCT	0	0	1	1	1	At5g41070.1:d:+1323:primary
CATCTAACGG	0	1	1	1	0	At5g40830.1:d:+1133:primary
GACAAGGAGA	0	2	1	0	0	At5g40820.1:X:-721:quaternary,At3g56040.1:X:-186:quaternary
CGTGAGAAAA	0	0	1	0	2	At5g40770.1:d:+1179:secondary
CTCCGGCTCC	0	1	1	1	0	At5g40730.1:d:+176:primary
CCAACAGGTG	1	1	0	1	0	At5g40650.1:d:+723:primary

AGAGAATGTG	0	0	1	0	2	At5g40540.1:d:+1344:primary
TATGGTTATC	1	2	0	0	0	At5g40490.1:d:+1438:secondary
TGACTGTTGC	0	3	0	0	0	At5g40450.1:d:+3254:secondary
CTCAACAAAA	0	1	0	1	1	At5g40250.1:v:+1658:secondary
AGTGATTTCT	0	0	1	2	0	At5g40210.1:d:+1075:primary
GAACGTCTTC	1	2	0	0	0	At5g40190.1:d:+155:primary
GACAGCTAGA	0	2	0	1	0	At5g39862.1:p:+1908:secondary,At2g01550.1:p:+3860:secondary,At3g43357.1:p:+4036:secondary
GATAGGCTTG	1	0	1	1	0	At5g39860.1:d:-589:secondary
TTTTTCAAAA	0	1	2	0	0	At5g39700.1:v:+400:secondary,At5g33340.1:v:+1683:secondary,At3g44440.1:v:+675:secondary
CCAAATCATT	0	1	0	2	0	At5g39620.1:i:+1423:tertiary
TTTGAATTTT	0	1	0	2	0	At5g39450.1:d:+2002:primary
ACAAAGAGTA	1	1	0	0	1	At5g39170.1:X:-205:quaternary,At5g58390.1:X:-220:quaternary,At5g39200.1:X:-199:quaternary,At5g39140.1:X:-199:quaternary
CTTTCCCAAT	0	0	2	1	0	At5g39155.1:p:+3894:secondary,At1g76580.1:d:+3120:secondary,At1g20980.1:d:+3496:secondary,At5g39185.1:p:+3894:secondary
CCCTTAGGAA	0	1	1	1	0	At5g38980.1:d:-328:secondary
CTTTGTGTTT	1	0	0	0	2	At5g38970.1:d:+1326:primary,At5g38970.3:d:+1325:primary,At5g38970.2:d:+1383:primary
GTGGAGGAGG	0	2	0	1	0	At5g38870.1:p:+157:primary
ATATCTGTGC	1	2	0	0	0	At5g38860.1:d:+822:primary
GAGGTTGATA	0	0	0	2	1	At5g38640.1:d:+2268:primary
GACCGTAATA	1	1	0	1	0	At5g38620.1:X:--151:quaternary
TTTCTTATTA	0	1	0	0	2	At5g38600.1:d:+1975:secondary
AGGAACCTTG	0	0	1	0	2	At5g38530.1:d:-1567:secondary
CAAAAAACAA	2	0	0	0	1	At5g38450.1:i:+832:tertiary,At3g47730.1:i:+1589:tertiary
GTGGCTTGAG	3	0	0	0	0	At5g38430.1:X:-86:quaternary,At5g38420.1:X:-106:quaternary
TGTACGGCCG	2	1	0	0	0	At5g37790.1:d:+1773:primary
TGGTTATGAT	1	0	0	0	2	At5g37381.1:p:+1580:secondary
TTGTTTAACT	0	3	0	0	0	At5g37370.2:d:+1375:primary,At5g37370.1:d:+1399:primary,At2g34290.1:v:+1459:primary
CTGTACCAAA	0	2	0	0	1	At5g37350.2:d:+1437:secondary,At5g37350.1:d:+1348:secondary
AATTCAACTC	0	2	0	0	1	At5g36130.1:v:+105:secondary
TTGATAACTA	0	0	1	1	1	At5g35930.1:i:+2263:tertiary
ATCTCTCTTG	1	1	0	0	1	At5g35930.1:d:+3568:primary
AATAAGAGCA	0	1	0	0	2	At5g35910.1:d:+2281:primary
CGGTTCCCGT	0	2	0	0	1	At5g35732.1:d:+178:primary
GAAGAAGATG	0	1	1	0	1	At5g35715.1:v:+1569:primary,At2g25770.1:d:+543:primary,At5g56530.1:d:+1127:primary
ACGCGTTTGA	0	2	0	1	0	At5g35700.1:d:+2266:primary
CTTTCAGTTT	0	0	2	0	1	At5g35330.1:X:-257:quaternary
TATTGGTGAT	3	0	0	0	0	At5g35210.2:d:+4475:secondary
TAGGTACACT	1	1	0	0	1	At5g35210.1:d:+4976:primary,At5g35210.2:d:+5397:primary
AATGGCCGAG	0	0	2	1	0	At5g35170.1:d:+2117:primary
TTTTTCCAAA	1	1	0	1	0	At5g34930.1:d:+994:secondary
TTGGGAGATT	0	1	0	2	0	At5g34870.1:v:+1073:secondary
AGAATCACAC	0	0	1	0	2	At5g33436.1:p:+1159:primary,At1g73930.1:d:+2464:primary
GGAACAAGTT	0	2	0	0	1	At5g32800.1:p:+249:primary
AAAATATTGA	0	0	1	1	1	At5g31637.1:p:+329:secondary
GACAATGAAA	1	0	1	1	0	At5g30490.1:d:+954:secondary,At5g03560.1:d:+577:secondary,At4g15890.1:d:+1528:secondary
TTGTGTTTTA	1	2	0	0	0	At5g30450.1:p:+835:secondary
ATAATAGAAA	1	0	0	1	1	At5g30269.1:p:+13534:secondary,At5g30269.1:p:+1165:secondary
GCTAAATCAA	0	3	0	0	0	At5g29022.1:p:+335:secondary,At5g17580.1:d:+865:secondary
ATCAAAAGCT	0	1	1	1	0	At5g28920.1:X:-68:quaternary
GAAAGGTGGA	0	0	3	0	0	At5g28840.1:d:+811:secondary,At1g71110.1:d:+138:secondary,At5g15340.1:v:+1645:secondary
TGGAGATCTC	0	1	1	1	0	At5g28740.1:d:+2606:primary
AGAAGAAAGA	2	0	1	0	0	At5g28635.1:p:+1774:secondary,At5g06110.1:d:+1412:secondary,At3g49730.1:d:+1163:secondary,At5g55875.1:p:+866:secondary,At2g07010.1:p:+866:secondary
CCCCAGAAGC	1	0	1	0	1	At5g28545.1:p:+1992:secondary
AAAGAAATGA	0	0	1	0	2	At5g28535.1:p:+1056:secondary
GAAAACGGAT	1	0	2	0	0	At5g28510.1:v:+1629:secondary,At3g09260.1:d:+1300:secondary
CAACAAGCTC	0	0	2	1	0	At5g28145.1:p:+1027:secondary
GATTTGTTTT	1	1	1	0	0	At5g27860.1:d:+695:primary

AATTTAACTG	1	1	1	0	0	At5g27830.1:d:+1001:secondary
AATTGCTGAA	0	1	1	0	1	At5g27740.1:d:+990:primary
TTGGGTATAC	0	0	2	1	0	At5g27540.1:i:+4541:tertiary,At4g21880.1:i:+2268:tertiary
GAAGAGACAC	1	1	1	0	0	At5g27470.1:d:+906:primary
CCAAAAGACC	1	2	0	0	0	At5g27460.1:v:+1774:secondary
TCTCCCTCAT	1	2	0	0	0	At5g27400.1:d:+824:secondary
AGGTTAAGTA	1	2	0	0	0	At5g27280.1:d:+571:secondary
GCCGGAGGAT	0	2	1	0	0	At5g27070.1:v:+575:secondary,At5g26870.1:d:+93:secondary,At5g27050.1:v:+443:secondary
ATCGTCAAAA	1	0	1	1	0	At5g26980.1:X:-201:quaternary,At5g07360.1:X:-300:quaternary,At3g55480.1:X:-15:quaternary
GTGGAGTATT	2	0	0	0	1	At5g26980.1:d:+1595:primary
ATCACATCTT	1	1	1	0	0	At5g26860.1:d:+2643:primary
AAATTCAAGT	1	1	1	0	0	At5g26830.1:d:+2198:secondary,At4g34630.1:d:+640:secondary
CTCCTAATAG	2	1	0	0	0	At5g26740.1:d:+1572:primary,At5g26740.2:d:+1608:primary
TTTCATTTTG	1	2	0	0	0	At5g26670.1:i:+2347:tertiary,At5g26670.2:i:+2349:tertiary
AATTCAACTG	0	2	1	0	0	At5g26667.2:X:-220:quaternary
GTAATATCCA	0	2	1	0	0	At5g26360.1:d:+153:secondary
GTTTCTCTAA	1	0	1	0	1	At5g26283.1:p:+669:secondary,At2g07788.1:p:+523:secondary,At2g13250.1:p:+1831:secondary,At5g28624.1:p:+802:secondary,At2g13260.1:p:+554:secondary ,At5g38383.1:p:+2016:secondary
TTGCCACCCA	0	2	0	1	0	At5g26240.1:d:+2578:secondary
TCTTAATTTT	0	2	0	1	0	At5g26230.1:v:+1419:primary
TAACAAAAAG	1	2	0	0	0	At5g25800.1:d:+1571:primary
ATCAAAGAAT	0	2	1	0	0	At5g25770.2:d:+1357:primary,At5g25770.1:d:+1575:primary
TCACAGAGAG	0	1	1	0	1	At5g25590.1:d:+2163:primary
AAGAAACTAT	1	1	0	1	0	At5g25570.1:d:+498:primary,At5g25570.2:d:+396:primary
GCGGGCGATG	0	2	1	0	0	At5g25440.1:d:+712:secondary
ATAGAATTAA	0	0	0	2	1	At5g25360.1:X:-151:quaternary,At1g75550.1:X:-716:quaternary
TCTTCTATTA	0	2	0	1	0	At5g25360.1:d:+453:primary
CCGTTTATAT	0	3	0	0	0	At5g25270.1:v:+1791:secondary,At5g06620.1:v:+898:secondary
TTCAAGGTTG	0	1	0	2	0	At5g25260.1:d:+71:secondary,At5g05480.1:d:+1420:secondary
TGTCCCAATC	0	0	1	1	1	At5g25150.1:X:+245:quaternary
AATAAAACGA	0	2	0	1	0	At5g24890.1:X:-154:quaternary
GTAATGGTCC	0	0	0	2	1	At5g24850.1:d:+1584:primary
TACAACAAGA	1	1	0	1	0	At5g24830.1:d:+2443:primary
CATAGCTGTC	0	1	2	0	0	At5g24810.1:d:+3226:secondary
TATAGGCCAA	0	1	1	0	1	At5g24800.1:d:+1063:secondary
TGAGAATCCA	0	1	0	1	1	At5g24760.2:X:-292:quaternary
TTTTGGCTAT	1	2	0	0	0	At5g24740.1:d:+10022:primary
AAAAATCATT	1	1	1	0	0	At5g24710.1:v:+4647:primary
TTGATCTCCG	0	3	0	0	0	At5g24610.1:d:+574:primary
GCTGTTTCTT	0	1	1	1	0	At5g24600.1:d:+598:primary
GAGAACAACT	1	1	1	0	0	At5g24450.1:d:+1474:primary
GCGTCTCTCT	0	0	0	1	2	At5g24420.1:d:+573:secondary
TCTTATGTAA	0	0	0	1	2	At5g24390.1:X:-1574:quaternary
TGTTCCAGAG	0	1	1	1	0	At5g24340.1:d:+1690:primary
CTCTTCTCTG	1	0	2	0	0	At5g24310.2:d:+276:secondary,At4g16150.1:v:+481:secondary,At4g17565.1:v:+1443:secondary,At5g24310.1:d:+424:secondary,At1g18290.1:v:+612:secondary
TATACTTGTT	1	1	0	0	1	At5g24030.1:d:+2316:primary
CACACCAAAA	1	0	2	0	0	At5g23955.1:p:+3303:secondary
TAGAAACAAG	0	1	0	0	2	At5g23890.1:d:-2976:secondary
TAATCTCGTA	0	2	0	0	1	At5g23880.1:d:+2332:primary
TGATAAAACA	1	0	1	0	1	At5g23870.3:d:+1358:secondary,At5g23870.2:d:+1358:secondary,At5g23870.1:d:+1358:secondary
TCTTTTCTTT	0	3	0	0	0	At5g23840.2:d:+836:primary,At5g23840.1:d:+746:primary
TAACAATGAA	1	1	0	1	0	At5g23820.1:d:+418:secondary
GAGATTCAAA	0	1	2	0	0	At5g23720.1:d:+2718:primary,At5g23720.2:d:+2724:primary
TATTTGCGAG	0	3	0	0	0	At5g23690.1:d:+1650:primary
TAACTGTAAC	0	3	0	0	0	At5g23660.1:d:+918:primary
CGAAGAAGAA	1	2	0	0	0	At5g23590.1:d:+656:secondary,At5g27140.1:v:+1472:secondary
TTTTCTCGTG	1	0	0	0	2	At5g23210.2:d:+1348:primary

GAGGCCTTTT	1	0	2	0	0	At5g23150.1:d:+4689:primary
ATCTCTCAAA	2	0	0	1	0	At5g23140.1:d:+891:secondary
GCACCGAAAA	0	2	1	0	0	At5g23080.1:X:--65:quaternary
TCCTTCATTT	0	1	1	0	1	At5g23080.1:d:+3038:primary
GATGATGCTT	1	0	0	1	1	At5g22700.1:d:+1544:primary
GTGGTGGTTG	0	1	0	2	0	At5g22620.1:d:+1256:primary
TAACTTTAAA	1	0	2	0	0	At5g22590.1:v:+297:secondary
TGCTTAAACA	0	2	0	1	0	At5g22500.1:d:+1561:secondary
ATTTAATTTT	0	1	0	1	1	At5g22480.1:d:+1581:primary
GGGCATCTAG	0	2	0	0	1	At5g22350.1:d:+1188:primary
GAGGTTGAGA	0	1	0	2	0	At5g22320.1:d:+1343:primary
ATAGACAAAC	0	1	2	0	0	At5g22290.1:d:+963:primary
TATAACAAAA	0	0	0	1	2	At5g21160.1:i:+4008:tertiary,At3g57540.1:i:+934:tertiary
GCATCTTCAC	0	1	2	0	0	At5g21105.1:d:+1398:secondary,At4g13885.1:v:+1091:secondary
CAACAAGTCT	1	1	0	1	0	At5g20980.1:d:+2537:primary
GATGAAATAG	1	1	1	0	0	At5g20660.1:d:+2556:primary,At3g61450.1:d:+683:primary
GATTAAAAAA	0	1	0	2	0	At5g20460.1:i:+78:tertiary
GATGAGCCAT	2	0	1	0	0	At5g20350.1:d:+1452:secondary,At2g37450.1:d:+1137:secondary
GTTTCTTAAA	1	0	1	0	1	At5g20330.1:d:+1513:primary
AACAATAGGA	0	1	0	1	1	At5g20300.1:d:+2257:primary
AAGAAGATCC	0	0	2	1	0	At5g20290.1:d:+682:secondary
TGTTCGTTTT	0	0	1	1	1	At5g20270.1:d:+1122:secondary,At3g03580.1:d:+1461:secondary
GAACTTCATA	1	0	1	1	0	At5g20140.1:d:+1091:primary
CATCAAACGA	0	1	1	1	0	At5g20090.1:d:+373:secondary
AACTAAATAA	1	1	1	0	0	At5g19960.1:d:+1292:primary
TATGGCATAA	1	0	1	1	0	At5g19860.1:X:+316:quaternary
TTTGTACATA	0	1	0	0	2	At5g19860.1:d:+648:secondary
AGAGATAATC	0	0	3	0	0	At5g19760.1:d:+732:secondary
TTTCTTACCT	1	0	1	0	1	At5g19750.1:d:+928:primary
AAGTACAAAT	0	3	0	0	0	At5g19700.1:X:-1703:quaternary
ATTGCTCTCC	0	2	0	0	1	At5g19570.1:d:+196:primary
GCTGGAAACA	1	2	0	0	0	At5g19490.1:v:+926:primary,At5g14480.1:d:+515:primary
GAATTGAAGG	0	0	2	0	1	At5g19460.1:d:+1371:primary
ATATTTCCTT	0	3	0	0	0	At5g19220.1:i:+2156:tertiary
GAAGCACCCA	0	1	0	1	1	At5g19000.1:v:+1098:secondary,At2g40660.1:d:+807:secondary
AAAGGAGAAT	1	0	1	0	1	At5g18920.1:d:+246:primary
GTTGGAACTA	0	1	0	0	2	At5g18900.1:d:+963:primary
TCGTTCGTTT	0	1	1	1	0	At5g18770.1:d:+1648:primary
GTTTCCTAGT	2	0	1	0	0	At5g18525.1:d:+607:secondary
ATGGTCACTT	0	2	0	1	0	At5g18525.1:d:+1743:primary
ACCTAAAGCC	2	1	0	0	0	At5g18500.1:d:+1638:primary
GATCTCGTTT	0	2	1	0	0	At5g18280.1:d:+1585:primary
TTTTCCGATT	2	1	0	0	0	At5g18230.1:i:+5466:tertiary
TTTGTATATA	1	0	0	0	2	At5g18230.1:d:+2733:primary
GAAGACGGTG	0	0	1	2	0	At5g18150.1:v:+413:secondary
ATGAAGCTTT	1	1	0	1	0	At5g18065.1:v:+1381:primary,At1g12460.1:d:+3103:primary
GATGCTCCTA	0	0	1	2	0	At5g17900.1:d:+1412:primary
AGGCAATAAT	1	2	0	0	0	At5g17710.1:d:+821:primary,At5g17710.2:d:+842:primary
TAGAGTCGAG	0	2	0	1	0	At5g17690.1:d:+1572:secondary
TTGGAGAAAA	1	1	0	1	0	At5g17460.1:X:-1082:quaternary
TAAACAACAC	0	0	0	1	2	At5g17030.1:d:+1401:primary
ACTGCAGCAG	0	1	1	1	0	At5g17020.1:d:+3157:primary
GAAGAGTCCC	0	0	1	2	0	At5g17000.1:X:-330:quaternary,At5g16970.1:X:-330:quaternary,At5g16980.1:X:-330:quaternary,At5g54810.1:X:-257:quaternary
TTACAATTCG	0	0	1	1	1	At5g16950.1:X:+534:quaternary
TAGCGTAAGT	0	3	0	0	0	At5g16780.1:X:+458:quaternary
GACCATTTAA	0	0	2	0	1	At5g16715.1:d:+2967:primary
GGCAAGATAA	0	1	2	0	0	At5g16680.1:d:+3556:primary
TGTTTCTTGA	0	2	0	1	0	At5g16630.1:d:+2647:primary
TTCGAAGCTG	0	0	2	1	0	At5g16570.1:X:-91:quaternary,At5g37600.1:X:-91:quaternary
AAGACAGGAG	1	2	0	0	0	At5g16540.3:d:+1071:primary,At5g16540.1:d:+1009:primary,At5g16540.2:d:+870:primary

CCGTCGTTGG	0	2	0	1	0	At5g16505.1:p:+1881:primary
AAATAGAAAA	1	1	0	0	1	At5g16450.1:i:+110:tertiary,At1g17370.1:i:+2385:tertiary,At5g16450.2:i:+126:tertiary,At1g56500.1:i:+1310:tertiary,At5g53950.1:i:+775:tertiary
TCAGTTTCTC	0	2	1	0	0	At5g16260.1:d:+1228:secondary
AAAAGATTTC	0	2	1	0	0	At5g16160.1:d:+441:primary
TTTTGCTGGC	0	0	3	0	0	At5g16010.1:d:+591:primary
GACTTGAGCC	0	0	0	1	2	At5g15950.1:d:+1268:secondary
AAGCAGTAAA	0	2	1	0	0	At5g15810.1:d:+1925:primary
TAGATTCATA	1	2	0	0	0	At5g15800.1:i:+1704:tertiary
AACAAATTCG	0	2	1	0	0	At5g15490.1:d:+1237:secondary
AATAGGGTTG	0	1	1	1	0	At5g15450.1:d:+2607:primary
TACAACTCTT	1	1	0	0	1	At5g15390.1:d:+1340:primary
GATTCACTCA	2	1	0	0	0	At5g15310.1:d:+713:primary
GTTCTTCGCC	1	1	1	0	0	At5g15080.1:d:+1710:primary
TGAATTTTAT	0	0	1	1	1	At5g15030.1:d:+1017:primary
AGAGCTTGCA	0	2	0	0	1	At5g14770.1:v:+284:secondary,At4g21210.1:d:+637:secondary
TAATACTTTT	1	1	1	0	0	At5g14730.1:d:+898:secondary
AATAATGCAA	1	0	1	1	0	At5g14400.1:v:+990:secondary
GAAAACAACT	0	2	0	1	0	At5g14390.1:d:+840:primary
ATATTGCTAT	0	3	0	0	0	At5g14370.1:d:+1116:primary
AAGTTCCTGT	2	1	0	0	0	At5g14240.1:X:-314:quaternary,At5g37450.1:X:--149:quaternary
AATGAAAACT	0	1	2	0	0	At5g14230.1:d:+1927:primary
TTGGACATAG	0	3	0	0	0	At5g14060.1:d:+1589:primary
AAAATCCACT	0	1	2	0	0	At5g13850.1:d:-800:secondary
TGTTTGCAGC	1	2	0	0	0	At5g13740.1:i:+775:tertiary,At1g06560.1:i:+3901:tertiary
ATACTGCATA	0	2	0	1	0	At5g13740.1:d:+1159:primary
TTACGAACAT	0	2	0	0	1	At5g13710.1:X:-186:quaternary
AGTTTACTCA	1	2	0	0	0	At5g13690.1:d:+2475:primary
CCTATTTGTC	0	1	0	1	1	At5g13430.1:d:+1155:primary
AGTTACACTC	2	1	0	0	0	At5g13370.1:d:+1946:primary
TGTAACAATT	0	0	1	2	0	At5g13330.1:d:+837:primary
TCCAGACGTT	0	0	2	1	0	At5g13300.1:d:+2851:primary
AGAAAGTCGC	1	1	0	0	1	At5g13100.1:d:+1172:secondary
AAGTTAGTAT	1	2	0	0	0	At5g12400.1:v:+5425:primary
GACGACCAAG	0	1	2	0	0	At5g12240.1:v:+354:primary
ATAGATAGAG	0	3	0	0	0	At5g12210.2:d:+855:primary,At5g12210.1:d:+883:primary
ATGAAGATAC	2	0	0	0	1	At5g12140.1:d:+616:secondary
AGAAAGATGT	1	1	1	0	0	At5g12110.1:d:+697:primary
GAAGATTTCG	0	3	0	0	0	At5g11970.1:d:+153:primary
CGAGGTCTCG	0	3	0	0	0	At5g11550.1:d:+529:primary
CGGTGCTGAT	0	1	0	2	0	At5g11480.1:X:-15:quaternary
GGCCAAGTCT	1	0	0	1	1	At5g11150.1:d:+905:primary
GTGGTGTTCG	1	1	0	0	1	At5g11110.1:d:+2461:primary
TTCAGATCTC	0	1	1	0	1	At5g11000.1:d:+812:primary
GACTATGACT	0	2	1	0	0	At5g10810.1:d:+158:primary
GCTGATGCAT	0	0	1	1	1	At5g10790.1:d:+1744:primary
AGCCCTTCCA	0	2	1	0	0	At5g10750.1:d:+125:secondary
TCAATGAAGT	0	2	0	1	0	At5g10750.1:d:+1048:secondary,At3g20770.1:d:+1581:secondary
TGTGAGTACA	1	2	0	0	0	At5g10690.1:d:+1661:primary
TCAACATATG	1	0	1	0	1	At5g10650.1:d:+1567:secondary,At5g25120.1:v:+1516:secondary
AGGAGGGAGA	1	2	0	0	0	At5g10560.1:X:-193:quaternary
TGTATTTATT	2	0	0	0	1	At5g10470.1:v:+4239:secondary
ATTTCGTGTG	0	3	0	0	0	At5g10430.1:d:+606:primary
CCTTTCATCT	0	3	0	0	0	At5g10380.1:d:+571:primary
GTGGAATCGG	1	0	2	0	0	At5g10370.1:d:+5306:primary
GGAGGATGTG	0	0	3	0	0	At5g10360.1:d:+245:secondary
GTTGAGGCAA	1	0	1	1	0	At5g10160.1:d:+473:secondary
GGCGATATCC	0	2	0	1	0	At5g10110.1:d:+994:secondary
GATTTCCTTT	0	1	2	0	0	At5g10020.1:d:+3450:primary
AATATGAAGC	1	1	0	0	1	At5g09880.1:d:+1634:primary

ATGGATCCTC	0	0	1	1	1	At5g09870.1:v:+2055:secondary,At5g64740.1:d:+2230:secondary
GATCTTCTTT	0	1	1	1	0	At5g09820.1:d:+761:primary
GGAAAGATCT	0	3	0	0	0	At5g09770.1:d:+438:primary
CCTGGGTCAA	0	1	1	1	0	At5g09680.1:d:+665:primary
TCGTATGGTT	0	1	1	1	0	At5g09530.1:d:+1326:secondary
AGTTTATATA	0	2	0	0	1	At5g09390.1:d:+1202:primary
TGAAGAACCT	0	2	0	1	0	At5g09350.1:d:+3990:primary
TTCTCAGATG	0	2	1	0	0	At5g09300.1:d:+1375:primary,At5g09300.2:d:+1278:primary
ACCCGAGAAG	0	2	1	0	0	At5g08770.1:d:+775:primary
AGTATTTCGA	1	0	0	1	1	At5g08610.1:X:+399:quaternary
GGACTTGACA	1	1	0	0	1	At5g08610.1:d:+2516:primary
CTACAAAGCA	0	1	2	0	0	At5g08570.1:d:-1748:secondary
CTTTGCTTCT	0	1	1	1	0	At5g08450.2:d:+2996:primary,At5g08450.1:d:+2954:primary
CTCACTTGGC	0	2	1	0	0	At5g08450.1:d:+1020:secondary,At5g08450.2:d:+1062:secondary
ACATTAGCGA	0	1	0	1	1	At5g08400.1:d:+813:secondary
AACAAAAAAG	1	1	0	1	0	At5g08390.1:i:+554:tertiary
ACCAAAGAAA	2	0	1	0	0	At5g08380.1:d:+966:secondary,At2g28220.1:v:+2879:secondary
CCTGCAAAAT	1	2	0	0	0	At5g08370.1:d:+1251:secondary
AGGCATCCGA	0	1	1	1	0	At5g08335.1:d:+673:secondary
GTTCTGTAAA	1	0	0	2	0	At5g08139.1:d:+1265:secondary
CTTTGTAGTG	2	1	0	0	0	At5g07360.1:d:+2273:primary
AACTCACATC	0	0	2	0	1	At5g07320.1:v:+1983:primary
ATAAATATGT	1	1	0	0	1	At5g07220.1:d:+1185:primary
TCATTCATTT	0	2	0	1	0	At5g07130.1:d:+1767:primary
TATGTAGCTA	0	0	1	1	1	At5g07000.1:d:+1509:primary
TTGTTTTAGT	0	1	0	1	1	At5g06950.1:d:+1450:primary,At5g06950.2:d:+1433:primary
TTTTATTGAA	1	1	0	0	1	At5g06710.2:d:+1410:primary,At5g06710.1:d:+1288:primary
CTTCTTCTTA	0	2	0	0	1	At5g06620.1:v:+1460:primary,At3g02060.1:d:+2692:primary
TATGTTTCTA	1	2	0	0	0	At5g06580.1:d:+1760:primary
GATATACAGA	0	0	3	0	0	At5g06420.2:d:+1273:secondary,At1g01350.1:v:+832:secondary,At5g06420.1:d:+697:secondary
ACACAGTGAA	0	1	1	1	0	At5g06120.1:X:+227:quaternary
AGAGTAGCTC	1	1	1	0	0	At5g05980.1:d:+1682:secondary
ATGAAGAGGT	0	2	1	0	0	At5g05750.1:d:+1216:primary
TAGTCACTCA	1	2	0	0	0	At5g05680.1:d:+2526:primary
AAGTTAAGCC	0	3	0	0	0	At5g05540.1:d:+1159:primary
GGATATGGCC	0	2	1	0	0	At5g05450.1:d:+1415:secondary
TTTGATGTCG	0	0	2	1	0	At5g05410.1:d:+963:primary
GTTCTTAGGT	1	0	0	2	0	At5g05270.2:d:-747:secondary,At5g05270.1:d:-806:secondary
TAAATAAATG	3	0	0	0	0	At5g05250.1:d:+908:primary
GGGGCTGATG	0	0	0	2	1	At5g05220.1:d:+442:primary
GTTTTGCTGA	1	0	2	0	0	At5g05080.1:d:+1211:primary
TGAAAGAAGA	0	1	0	2	0	At5g04740.1:d:+733:primary
TAACTTTAAC	1	1	1	0	0	At5g04520.1:v:+1352:primary
GTTCCTTTTT	1	0	0	0	2	At5g04510.1:i:+1031:tertiary,At5g04510.2:i:+1001:tertiary,At1g16650.1:i:+1208:tertiary
GTTTTTGTAT	1	1	0	0	1	At5g04480.1:d:+3658:primary
AAAAAATATA	0	0	0	2	1	At5g04480.1:d:+1633:secondary,At1g35330.1:v:+1412:secondary
CTCTTGTATG	1	1	1	0	0	At5g04320.1:d:+1862:primary
TTCAGAAAAA	0	0	0	1	2	At5g04310.1:i:+2734:tertiary
TCCTCTTCGA	0	2	0	1	0	At5g04230.1:d:+1742:primary
GGCTCCACCA	3	0	0	0	0	At5g04190.1:d:+1098:primary
ATCAGTGTAC	1	1	1	0	0	At5g04090.1:d:+1020:primary,At5g04090.2:d:+1234:primary
GCACATACTG	0	2	0	0	1	At5g03900.2:d:+1759:primary,At5g03900.1:d:+1991:primary
ATGACTACTC	0	2	1	0	0	At5g03740.1:d:+979:primary
GAGGCGGACC	0	0	1	2	0	At5g03490.1:d:+1231:primary
GAGCACAAAC	0	0	1	2	0	At5g03455.1:d:+583:primary
ATCTTGGAAC	0	1	1	1	0	At5g03455.1:d:+385:secondary
ATATGCTTAT	1	0	0	0	2	At5g03430.1:d:+1892:primary
ATCAAGTCAG	0	2	0	1	0	At5g03320.1:d:+1259:primary
CTTAATTAAG	0	0	1	1	1	At5g03200.1:d:+1211:primary
CTTCAAACTC	1	0	1	0	1	At5g03190.1:d:+1710:primary
CTAAATGTTT	1	0	1	1	0	At5g02980.1:v:+27:secondary

GGCAAGGCCT	0	3	0	0	0	At5g02800.1:d:+981:primary
TTATTAATAG	1	0	0	0	2	At5g02710.1:d:+850:secondary
ATCGGAGATG	0	0	1	0	2	At5g02710.1:d:+303:secondary
TGTTGTCTAA	0	2	0	0	1	At5g02630.1:d:+1018:primary
TCAGATTCAC	0	0	0	3	0	At5g02490.1:d:+2228:primary
TGCAAATGCT	1	2	0	0	0	At5g02350.1:v:+1610:secondary,At5g02330.1:v:+1610:secondary,At2g13900.1:d:+1324:secondary
TTATCTCACA	1	1	1	0	0	At5g02290.1:d:+1546:primary,At5g02290.2:d:+1544:primary
GAGACTGCAC	0	1	1	0	1	At5g02270.1:d:+495:primary
GTTTCTTCGA	0	0	1	0	2	At5g02260.1:d:+1214:primary
GTAATATGGT	2	0	0	0	1	At5g02250.1:d:+2694:primary
GGCCGAATCA	1	0	0	1	1	At5g02130.1:d:+1169:primary
GGGTGATCTC	0	3	0	0	0	At5g02100.1:d:+1349:primary
AAGGAGTCGT	0	2	1	0	0	At5g02010.1:d:+1940:primary
TTTTGCGAAG	0	2	0	0	1	At5g01960.1:d:+1454:secondary
ACTTAGAGTG	2	0	0	0	1	At5g01890.1:d:+3371:primary
TTCATCTCAT	1	0	2	0	0	At5g01680.1:v:+678:secondary,At3g56490.1:d:+431:secondary
CTCGCCTCTG	1	0	2	0	0	At5g01470.1:d:+787:primary
TATAGAACTT	0	1	0	0	2	At5g01450.1:d:+1840:primary
CCGATCCCTA	2	1	0	0	0	At5g01390.1:d:+964:primary
TGGATTAGTT	0	1	0	2	0	At5g01270.1:d:+2810:primary
CTCACGTACC	0	2	1	0	0	At5g01240.2:d:+1103:primary,At5g01240.1:d:+1409:primary
TCATTACATT	0	3	0	0	0	At5g01210.1:d:+191:secondary,At3g19970.1:d:+836:secondary
GAACTCCTCT	0	2	1	0	0	At5g01075.1:d:+190:primary
GAGATTTCGA	0	1	1	1	0	At4g40045.1:d:+84:secondary
TATGCCGCTT	0	1	1	0	1	At4g40010.1:v:+1452:primary
ATCATTATAA	1	2	0	0	0	At4g39870.1:d:+1524:primary
TACCTCGTTC	0	2	1	0	0	At4g39840.1:d:+1460:primary
TATTTTGAGT	1	1	1	0	0	At4g39820.1:d:+1565:primary
TATTCTCTCA	1	2	0	0	0	At4g39780.1:d:+1185:primary
ATGAGAGCTA	1	1	0	1	0	At4g39770.1:d:+1125:primary
AACAGCTGTG	0	1	1	1	0	At4g39690.1:d:-2210:secondary
ATGATTTGGA	0	0	1	2	0	At4g39580.1:v:+1485:primary
AGAAGAAAAC	0	1	1	0	1	At4g39340.1:v:+935:secondary,At3g52140.1:d:+3735:secondary
GAGGGCTCAA	0	3	0	0	0	At4g39340.1:v:+1096:primary
GCGCCGTTAC	0	0	3	0	0	At4g39090.1:d:+590:secondary
TAATTGAAAA	3	0	0	0	0	At4g39050.1:i:+4432:tertiary
GTGACTCCCG	0	1	1	1	0	At4g38970.1:d:+892:secondary,At4g38970.2:d:+892:secondary
GTGCCAAGGA	0	0	3	0	0	At4g38970.1:d:+567:secondary,At4g38970.2:d:+567:secondary,At2g21330.1:d:+549:secondary
GCAACAAAAT	1	0	0	2	0	At4g38900.1:d:+1636:primary,At4g38900.2:d:+1693:primary
GGCTTTATGT	1	1	0	1	0	At4g38790.1:d:+557:primary
CCCTCCGCCG	0	0	1	1	1	At4g38770.1:d:+1298:secondary
TGACCGGGCT	0	2	0	1	0	At4g38680.1:X:-746:quaternary
TGTAGAAAAA	1	1	0	1	0	At4g38670.1:d:+1105:secondary
AAATGTTTAT	1	2	0	0	0	At4g38550.1:d:+2033:secondary
TTCAGAGACA	0	0	0	2	1	At4g38540.1:d:+1149:secondary
CCACTCAGCT	0	1	2	0	0	At4g38520.2:d:+1316:primary,At4g38520.1:d:+1051:primary
TTCCAGAACT	0	2	0	0	1	At4g38500.1:d:+1947:secondary
CTCATTTATG	0	1	0	0	2	At4g38370.1:d:+927:secondary
GCCCAAAGTA	1	0	1	1	0	At4g38350.1:v:+3683:secondary
TTAACTCAAC	0	1	2	0	0	At4g38350.1:v:+3571:secondary
TTTTTCCTAA	1	1	0	1	0	At4g38340.1:v:+2953:secondary
AAGTTGATTC	2	0	1	0	0	At4g38180.1:d:+2815:primary
GCGCAGTTTC	0	0	2	1	0	At4g37930.1:X:-73:quaternary
TTCCTTACTC	0	1	0	1	1	At4g37920.1:i:+969:tertiary
CTTATCTCCT	2	0	0	0	1	At4g37910.1:d:+2186:secondary
TTAGTTACTT	0	2	0	0	1	At4g37790.1:d:+939:primary
CGCCATCCTC	0	2	1	0	0	At4g37760.1:d:+1234:secondary
TAATACTTTG	1	0	1	0	1	At4g37660.1:d:+736:primary
GGAGCCGGTA	0	1	2	0	0	At4g37470.1:d:+252:secondary
GAAACCAAAA	1	0	0	0	2	At4g37380.1:v:+2614:secondary,At4g37370.1:d:+30:secondary
TGCAGAGCAC	0	1	2	0	0	At4g37370.1:d:+1473:primary

TTTTTAATTA	2	0	1	0	0	At4g37330.1:d:+1546:primary
CATATATGAT	0	0	1	1	1	At4g37310.1:d:+1659:secondary,At1g05710.4:d:+12:secondary
AGAGTTGAAA	1	0	2	0	0	At4g37180.2:d:+1272:primary,At4g37180.1:d:+1251:primary
GATTTATATT	0	0	1	2	0	At4g37170.1:v:+391:secondary,At2g28530.1:d:+760:secondary
AGTTCAATTT	1	0	0	2	0	At4g37080.2:d:+2146:primary,At4g37080.1:d:+1895:primary
AAGATGATGA	0	1	2	0	0	At4g37010.1:d:+548:primary
TGAAATACTT	1	0	1	0	1	At4g36990.1:d:+1487:primary
TCCATCAAGA	1	2	0	0	0	At4g36980.1:d:+464:secondary
AGCTTGGACG	1	2	0	0	0	At4g36960.1:d:+1312:primary
TTCTTGGTCA	0	1	1	0	1	At4g36920.1:d:+1520:primary
GCCAGATGTT	0	2	1	0	0	At4g36890.1:d:+1518:secondary
TGTCTGTAAT	1	1	0	0	1	At4g36810.1:d:+1409:primary
GAGACTTCTA	0	2	0	0	1	At4g36650.1:v:+1852:primary
ACAAAGTACA	0	0	3	0	0	At4g36480.1:d:+965:secondary
TCAAATTATA	0	1	1	0	1	At4g36380.1:d:+1879:primary,At4g03630.1:v:+1348:primary
AAGTTAAGCT	0	1	0	0	2	At4g36240.1:d:+830:secondary
GATAACGAAA	2	1	0	0	0	At4g36030.1:X:-217:quaternary
ATAGTGTCAA	1	2	0	0	0	At4g35980.1:d:+487:primary
TTCTGGACCA	1	0	0	1	1	At4g35940.1:d:+1410:primary
GCCTGCTATT	1	2	0	0	0	At4g35860.1:d:+624:primary
TGGATCTTTC	1	0	2	0	0	At4g35770.1:d:+210:secondary
AGGAGAAAAA	0	2	0	1	0	At4g35560.1:d:+2522:primary
TCTCTCCACA	1	0	0	2	0	At4g35470.1:d:+2095:secondary
CGGCAACAGT	1	0	2	0	0	At4g35440.1:X:-558:quaternary
GAGCGTGGTT	0	3	0	0	0	At4g35430.1:d:+262:primary,At4g35440.1:d:+2387:primary
GGCCGTGGAA	0	0	1	2	0	At4g35360.1:d:+1050:primary
AAGAAGACGA	0	1	1	0	1	At4g35295.1:v:+786:secondary,At3g10120.1:d:+318:secondary,At3g50630.1:d:+737:secondary,At1g23340.1:d:+156:secondary,At3g62150.1:v:+439:secondary
CTTATATGTA	2	0	0	0	1	At4g35270.1:d:+3156:primary
GTGGAGAAAG	2	0	1	0	0	At4g35240.1:v:+3058:primary
CCGTTCAACC	0	0	1	1	1	At4g34890.1:d:+3819:primary
AGTCTCAGCG	0	0	2	0	1	At4g34870.1:d:+155:secondary
ATAAGGGAGC	0	3	0	0	0	At4g34770.1:d:+438:primary
TGGAGTCCGG	0	1	1	0	1	At4g34680.1:d:+698:primary
GTGATGGTGA	0	3	0	0	0	At4g34460.3:d:+1109:primary,At4g34460.1:d:+1109:primary,At4g34460.2:d:+1294:primary
TGGCTGATAG	0	1	1	1	0	At4g34460.2:X:-237:quaternary
GTGATGAAAC	2	0	0	0	1	At4g34240.2:d:+1233:primary
GGATGTGACG	0	1	0	1	1	At4g34160.1:d:+1568:primary
TTTGGATCAT	1	0	1	0	1	At4g34090.2:d:+2031:secondary,At5g65790.1:d:+568:secondary
ATGCTCCTGT	0	0	1	0	2	At4g33865.1:X:-35:quaternary
AAAGGAGCAA	0	1	0	0	2	At4g33820.1:d:+1214:secondary,At1g50620.1:d:+817:secondary
GTGGAATTGC	0	1	2	0	0	At4g33760.1:d:+1834:primary
CAACAACTTA	0	0	1	1	1	At4g33630.1:d:+2539:secondary,At4g33630.2:d:+2543:secondary,At5g17980.1:v:+17:secondary,At5g50290.1:v:+1733:secondary
TCAAATTTTA	2	0	0	1	0	At4g33620.1:v:+2789:primary
GTGAGTCACA	1	1	1	0	0	At4g33565.1:d:+994:primary
CGTCTATGAC	0	2	0	0	1	At4g33560.1:d:+399:primary
TTACTCGTGG	0	0	0	0	3	At4g33350.1:X:+249:quaternary
TGGTATCAAA	1	1	0	0	1	At4g33280.1:v:+1417:secondary
AATTGTAGTG	1	1	0	1	0	At4g33270.1:d:+1659:primary
AGGCAATACA	1	0	1	1	0	At4g33240.1:d:+5014:primary
TGATTCAGTA	1	0	0	0	2	At4g33240.1:d:+1868:secondary
TCAAAGTTCC	1	1	0	1	0	At4g33210.1:v:+3111:secondary
GGATAACATC	2	1	0	0	0	At4g32940.1:i:+2327:tertiary
TAAATTTCGA	1	1	1	0	0	At4g32900.1:d:+827:primary,At4g32900.2:d:+819:primary
AACCCTCCTA	1	2	0	0	0	At4g32840.1:d:+1490:primary
TTCCAAATAA	0	2	1	0	0	At4g32830.1:d:+1088:primary
TTTCATAAAA	0	0	0	1	2	At4g32810.1:i:+1008:tertiary,At5g41970.1:i:+1093:tertiary
CAAAATGCTC	0	1	1	0	1	At4g32690.1:d:+401:primary

TTTCAGATAT	1	0	1	1	0	At4g32680.1:d:+1026:primary
TTCGGAATTT	1	1	0	1	0	At4g32640.1:d:+3369:primary
CAGGCTAAAT	0	3	0	0	0	At4g32480.1:d:+789:secondary
AGAAAAAGCG	0	2	0	1	0	At4g32420.1:d:+892:secondary
GAATCACCTT	0	0	2	1	0	At4g32410.1:X:-692:quaternary
CGTACCAATT	0	1	1	0	1	At4g32290.1:d:+732:primary
GTCGTTGTCG	0	2	0	1	0	At4g32130.1:d:+587:primary
CGTATCCCTT	0	0	1	1	1	At4g32120.1:d:+1383:primary
AAATAAATAA	0	1	0	0	2	At4g32000.1:i:+1215:tertiary
TGTATATGAC	2	0	0	0	1	At4g31990.2:d:+1470:primary,At4g31990.1:d:+1476:primary
GGTACACAAT	1	0	1	1	0	At4g31700.1:d:+880:secondary
ATCTTTTAAT	0	0	1	0	2	At4g31450.1:d:+1875:primary
CTCAGTCAGA	1	1	1	0	0	At4g31410.2:d:+642:primary
ATCATTTAAT	0	1	1	0	1	At4g31310.1:d:+665:primary
CTAATAAACA	0	2	0	0	1	At4g31210.1:d:+3867:primary
AAGAAGAATC	1	0	2	0	0	At4g31110.1:d:+1519:secondary,At1g76070.1:d:+846:secondary,At1g78230.1:d:+984:secondary,At2g20290.1:v:+4890:secondary,At1g49770.1:d:+194:secondary
TCCTCACTTG	0	2	1	0	0	At4g30900.1:d:+1271:primary
AAAAGGAAGA	1	1	1	0	0	At4g30880.1:X:+350:quaternary
ATTTCGATGA	0	0	2	0	1	At4g30800.1:d:+738:primary
AACTGTTTCC	0	3	0	0	0	At4g30720.1:d:+1910:secondary,At1g66800.1:d:+829:secondary
TAAGCTCTGA	1	1	0	1	0	At4g30710.1:d:+2820:primary,At4g30710.2:d:+2820:primary
GCAAGATCCA	0	2	1	0	0	At4g30630.1:d:+1097:primary
CAGAATTTGT	0	1	1	1	0	At4g30620.1:d:+260:primary
ATCGGTCCAG	0	3	0	0	0	At4g30210.2:d:+1832:secondary,At5g53460.1:v:+2355:secondary,At4g30210.1:d:+1832:secondary
GAAATGTTCC	0	1	1	1	0	At4g30200.2:X:-1186:quaternary
CCTTGTTTCT	1	2	0	0	0	At4g30190.1:X:-296:quaternary,At1g79570.1:X:-20:quaternary
TCATTTAGTC	0	2	0	1	0	At4g30160.1:d:+2601:secondary
GAAGCTGCAT	0	2	1	0	0	At4g30130.1:d:+2399:primary
TGATTCATCA	1	0	1	1	0	At4g30110.1:v:+3290:primary
AAAGAAAAAT	0	3	0	0	0	At4g30060.1:i:+1635:tertiary
TGTAAGATGT	0	0	1	1	1	At4g29950.1:d:+3069:primary,At4g29950.2:d:+3065:primary
TGAGGTTGCA	1	0	1	1	0	At4g29670.1:d:+1063:primary
CACAGAAAGT	0	2	0	0	1	At4g29520.1:d:+902:primary
AATGCACTGT	2	0	0	1	0	At4g29360.1:d:+1796:primary,At4g29360.2:d:+1751:primary
GTTGTCCAAG	0	0	3	0	0	At4g29350.1:d:+326:secondary
TACTACATTC	2	0	1	0	0	At4g29260.1:d:+948:primary
AGCCTCAAAG	0	1	1	1	0	At4g29210.2:d:+2173:secondary,At4g29210.1:d:+2050:secondary
GTGTTTTCGA	0	0	3	0	0	At4g29190.1:d:+501:secondary
CCGGTGGCAG	0	1	0	2	0	At4g29140.1:d:+1565:primary
TTTTACCAAC	2	0	0	1	0	At4g29130.1:d:+2103:secondary
AAGAGCTCTG	1	1	0	1	0	At4g29080.1:d:+861:primary
GGCGCTAAGC	0	1	1	0	1	At4g29050.1:d:+2003:primary
GGAATGACTG	0	2	0	1	0	At4g28910.1:d:+1488:primary,At4g28910.2:d:+1527:primary
GCTTGCTACT	0	2	0	1	0	At4g28900.1:p:+1380:secondary
AAAGTGTATG	1	0	2	0	0	At4g28880.1:d:+1625:primary
GCGATTGGGA	1	0	1	1	0	At4g28770.1:d:+502:secondary
TTTGATCAGA	1	0	1	0	1	At4g28485.1:v:+687:secondary
AACATTTTTG	1	0	0	2	0	At4g28450.1:d:+1539:primary
AACCTTCTCC	0	3	0	0	0	At4g28410.1:d:+161:secondary,At1g45130.1:d:+455:secondary,At5g56870.1:d:+342:secondary,At4g26140.1:d:+357:secondary,At4g26140.2:d:+357:secondary,At1g14840.1:d:+256:secondary,At3g52840.1:d:+344:secondary
TGAACATTTC	1	0	2	0	0	At4g28370.1:d:+1311:secondary
AATCATTTTG	1	1	1	0	0	At4g28350.1:v:+2412:secondary,At3g45110.1:v:+354:secondary
GGCGAGAGAC	0	1	2	0	0	At4g28310.1:d:+200:secondary
AAGCTGACAT	1	0	1	1	0	At4g28220.1:d:+1952:secondary,At5g66620.1:v:+469:secondary
GAAGAATGAA	0	1	1	1	0	At4g28200.1:d:+408:secondary
GCTGTTGCAC	0	0	2	1	0	At4g28090.1:v:+2401:primary,At2g24040.1:d:+131:primary
GCTAAAAAAA	1	0	2	0	0	At4g28080.1:i:+127:tertiary
AGCTGTATCT	0	0	1	1	1	At4g28070.1:d:+1735:secondary

TAGTTTGCTT	1	1	1	0	0	At4g27760.1:d:+1326:primary
TGTCAGGAGA	2	0	0	1	0	At4g27650.1:d:+1262:primary
CGTGATTGTC	2	0	0	0	1	At4g27490.1:d:+769:primary
AATCCTTGGC	2	0	0	1	0	At4g27450.1:X:+358:quaternary
AATAGTTCTA	0	0	0	2	1	At4g27370.1:i:+3702:tertiary
ATTCGTCGTT	0	0	0	0	3	At4g27360.1:d:+483:primary
AGAGCTGTTG	1	1	0	1	0	At4g27210.1:p:-1807:secondary
TTAACGCCAG	0	1	1	1	0	At4g27180.1:d:+2371:primary
ATCTTGAACC	1	0	1	0	1	At4g27090.1:X:-93:quaternary
AGCTTGTGAA	0	2	0	0	1	At4g27060.1:d:+2686:primary
GCAAAGAATC	0	1	1	0	1	At4g27010.1:v:+6407:secondary,At3g18890.1:d:+1027:secondary
AGTGCACCTT	0	1	2	0	0	At4g26940.2:d:+1706:secondary,At4g26940.1:d:+1804:secondary
CTATAATTGA	1	0	0	2	0	At4g26780.1:d:+1387:primary
ACTCTAGAGA	1	1	0	0	1	At4g26740.1:X:--172:quaternary,At1g66070.1:X:-289:quaternary
CACTCTTCTT	0	2	0	0	1	At4g26530.1:i:+113:tertiary
ACTTTTGATT	1	0	1	1	0	At4g26450.1:i:+4829:tertiary
TCATTGAACT	0	0	2	1	0	At4g26300.1:d:+2112:secondary
TAATACTCTT	1	0	0	1	1	At4g26240.1:d:+852:primary
TACACTATTA	1	0	0	0	2	At4g25990.2:d:+1430:primary
TGAAGAAAGG	0	0	1	0	2	At4g25970.1:d:+1839:secondary,At5g57190.1:v:+2008:secondary
AAAAGTAATA	1	1	0	1	0	At4g25810.1:d:+939:primary
CTGGCGACAC	1	1	1	0	0	At4g25660.1:d:+692:primary
GTTCTCAACA	0	2	1	0	0	At4g25640.1:d:+1244:secondary
CAGCAGTGAC	0	1	2	0	0	At4g25610.1:d:+1485:secondary
ACAACTAAAA	0	0	0	2	1	At4g25433.1:v:+986:secondary
TCGTTTTATG	0	0	0	1	2	At4g25433.1:v:+706:secondary
GAACGAAATG	0	1	1	1	0	At4g25360.1:d:+1835:primary
TTATAACTCA	2	0	0	1	0	At4g25290.1:v:+2201:secondary
TCAAAAACAT	1	0	0	0	2	At4g25270.1:X:-760:quaternary
GTAAACTTTC	0	3	0	0	0	At4g25090.1:i:+1165:tertiary
ACAACAAGAA	1	1	1	0	0	At4g24910.1:v:+184:secondary,At4g11080.1:d:+695:secondary
TGACGCAGCA	0	0	1	0	2	At4g24690.1:d:-2380:secondary
TGATTATGAT	2	0	0	0	1	At4g24670.1:d:+1388:primary,At4g24670.2:d:+1570:primary
ATGACGTGGC	0	0	2	1	0	At4g24660.1:d:+684:secondary
GCTCTTTATA	0	2	1	0	0	At4g24570.1:d:+925:primary
CCTATTCTGA	0	0	3	0	0	At4g24510.1:d:+82:secondary,At2g17790.1:d:+1018:secondary
TATTATAGAA	0	2	0	0	1	At4g24470.1:d:+1153:secondary,At4g24470.2:d:+1089:secondary
TATGAATTCC	0	0	1	1	1	At4g24280.1:d:-2295:secondary
AGATAATCCA	1	1	0	0	1	At4g24140.1:d:+1742:primary
ATGTAACGAC	1	1	0	1	0	At4g24020.1:d:+2949:primary
CATAATGTTT	0	0	3	0	0	At4g23900.1:i:+762:tertiary
GCGCTGTCTC	0	1	2	0	0	At4g23850.1:X:+315:quaternary
AATTTGGTTG	2	0	1	0	0	At4g23850.1:i:+561:tertiary
GAACGAGATC	0	1	2	0	0	At4g23850.1:d:+1952:secondary
TAAAATTATG	0	0	0	1	2	At4g23790.1:d:+1423:primary
GCCAGCACTA	0	2	0	1	0	At4g23650.1:d:+950:secondary,At5g53310.1:d:+501:secondary
AATCTAATCT	1	1	0	1	0	At4g23540.1:d:+2028:primary
TTGCTTTCCT	0	1	2	0	0	At4g23530.1:d:+1478:primary
CATAAGATCT	1	0	2	0	0	At4g23350.1:X:-131:quaternary,At4g23370.1:X:-131:quaternary,At4g23360.1:X:-131:quaternary
AAGACGTAAC	0	1	2	0	0	At4g23180.1:X:+462:quaternary,Chr5:+8784652:quaternary
GCTTGTACAG	0	3	0	0	0	At4g23060.1:d:+1473:primary
TTTGGTGGCA	0	2	1	0	0	At4g23040.1:d:+1132:secondary,At4g11740.1:d:+1197:secondary
TCGTCGAGGC	2	1	0	0	0	At4g22990.1:d:+2171:primary
TGCATTCAAC	1	0	1	0	1	At4g22830.1:d:+772:primary
AATTGTTCTG	1	0	1	1	0	At4g22780.1:d:+58:secondary
CGGTGTGGCG	0	2	1	0	0	At4g22720.2:d:+1106:primary,At4g22720.1:d:+1160:primary
GCAGAGATTG	0	0	2	0	1	At4g22690.1:d:+1593:secondary,At4g22710.1:d:+1059:secondary,At5g05870.1:d:+982:secondary
TGTGATCGAG	0	1	0	2	0	At4g22570.1:d:+627:primary
AATCAAAGAT	2	0	0	1	0	At4g22550.1:d:+756:primary
TGAAAACGTT	0	1	2	0	0	At4g22530.1:d:+946:primary
GTGAATCTCC	1	0	0	1	1	At4g22200.1:d:+2666:primary

TTGATTGAAA	0	2	0	0	1	At4g22170.1:v:-1501:secondary,At2g22122.1:d:-502:secondary
TTCGTTGGTC	1	2	0	0	0	At4g21870.1:d:+129:primary
GACTTCTTGT	0	1	0	2	0	At4g21700.1:v:+3660:primary
CTGGCGGCTG	0	2	1	0	0	At4g21660.1:d:+1136:secondary
ATGTTATAAT	0	1	2	0	0	At4g21650.1:d:+2280:primary
GTCTGAGCAG	1	1	1	0	0	At4g21610.1:d:+411:primary
TACAGTACAT	1	1	0	0	1	At4g21540.1:i:+4180:tertiary
CTACTTCCAG	0	0	1	1	1	At4g21430.1:v:+1038:secondary
AATGTGAAGC	0	2	0	1	0	At4g21410.1:d:+2172:primary
TTATACACTT	0	0	0	0	3	At4g21060.1:i:+406:tertiary
AACTTGACAA	2	0	0	0	1	At4g20940.1:d:-3016:secondary,At5g38850.1:d:-1901:secondary
ATTCAGACTT	0	2	1	0	0	At4g20880.1:d:+1428:primary
GAGTTTGGAC	1	0	1	1	0	At4g20840.1:v:+1976:primary
ATGCGTACTG	0	1	2	0	0	At4g20780.1:d:+633:primary
CATTCTACTA	0	1	1	0	1	At4g20760.1:d:+742:primary
CCACCACATA	0	0	0	2	1	At4g20440.2:d:+883:secondary,At4g20440.1:d:+887:secondary
GGTAGTTTAT	0	3	0	0	0	At4g20260.1:i:+722:tertiary,At4g20260.2:i:+676:tertiary
CTTGCGATGC	0	3	0	0	0	At4g19720.1:X:-543:quaternary
TCTTCTTGAC	0	1	0	1	1	At4g19700.1:d:+979:primary
AAGCAAGTTA	0	2	0	1	0	At4g19350.1:X:-163:quaternary,At2g02730.2:X:--16:quaternary
AGGCTTCGTC	1	1	0	1	0	At4g19020.1:d:+3932:primary
TGTCTGTGGA	0	0	0	2	1	At4g19006.1:X:-319:quaternary
AATCTATCCT	1	0	0	1	1	At4g19006.1:d:+1375:primary
AAGCAAGGGA	0	3	0	0	0	At4g19003.1:d:+326:primary,At4g19003.2:d:+490:primary
TATATGTAGA	0	2	0	0	1	At4g18975.1:d:+935:secondary
CACAAGTAGG	0	0	3	0	0	At4g18480.1:d:+942:primary
TAGCAAGTTT	0	1	1	1	0	At4g18380.1:d:+1492:primary
CAACGGAAGT	0	2	0	1	0	At4g18060.1:d:+704:secondary
GAACTAATTT	0	2	0	1	0	At4g18030.1:X:-207:quaternary,At5g17820.1:X:--70:quaternary
AGATAAATAA	1	1	0	0	1	At4g17880.1:d:+2085:primary
TGACGATCTC	1	1	0	0	1	At4g17770.1:d:+2240:primary
GACTGGTGAA	1	1	0	0	1	At4g17640.1:d:+1276:primary
TGTTAATGGC	2	0	1	0	0	At4g17340.1:X:+560:quaternary
CAGGATTTGG	0	1	1	0	1	At4g17300.1:d:+1617:primary
ATCCACGATT	0	1	0	2	0	At4g17240.1:X:+308:quaternary
GGTCCTGGAG	2	0	0	1	0	At4g16980.1:d:-489:secondary
CGAGGACAAA	0	2	1	0	0	At4g16860.1:d:+500:secondary,At5g23570.1:d:+840:secondary,At4g16940.1:v:+719:secondary,At4g16920.1:v:+722:secondary,At4g16900.1:v:+731:secondary, At4g16960.1:v:+740:secondary
ATAACGTCCA	1	2	0	0	0	At4g16830.1:d:+1263:secondary
AACTCCAACA	1	0	2	0	0	At4g16810.1:v:+1842:secondary
AGAAAGCAAT	0	2	1	0	0	At4g16800.1:d:+476:primary
GAGAAAAAGA	1	0	2	0	0	At4g16740.1:d:+1619:secondary
TAGCCCTGCG	0	0	0	1	2	At4g16720.1:X:-201:quaternary
GAAGAAAAAC	2	0	1	0	0	At4g16720.1:d:+643:secondary,At1g04600.1:v:+4973:secondary
GCTTGTAAGA	0	1	1	0	1	At4g16695.2:d:+342:primary,At4g16695.1:d:+350:primary
TGGAGGTTTC	0	0	3	0	0	At4g16490.1:d:+1528:secondary
AAGGCGGTTG	0	2	1	0	0	At4g16442.1:d:+273:primary
ATTCCAACGT	2	0	0	1	0	At4g16430.1:d:+1507:primary
AGATATTCAA	0	1	0	0	2	At4g16420.2:d:+1614:primary,At4g16420.3:d:+1623:primary,At4g16420.1:d:+1626:primary,At3g31377.1:p:+1040:primary
TCTTGTTCGG	0	0	3	0	0	At4g16410.1:d:+144:secondary
AGGAGAAAGT	1	0	1	1	0	At4g16370.1:X:-127:quaternary
ATTTATTCTC	1	0	2	0	0	At4g16260.1:i:+177:tertiary
ACATTGTAAA	2	0	0	0	1	At4g16240.1:v:+78:secondary
AGATGCTGTA	1	1	0	1	0	At4g16170.1:d:+1156:primary
AGAGATCTTC	1	0	1	1	0	At4g16140.1:d:+629:primary
AGCATCCTTT	0	1	2	0	0	At4g16015.1:v:+382:secondary,At2g13920.1:p:+1131:secondary,At2g13900.1:d:+1533:secondary
AGACAAACCA	1	0	0	2	0	At4g15930.1:d:+431:secondary
GATTTAATTG	0	2	1	0	0	At4g15780.1:d:+727:primary
CATCTAAGAG	0	2	1	0	0	At4g15700.1:X:+634:quaternary,Chr5:+13777017:quaternary

TATGTTGTCT	1	2	0	0	0	At4g15680.1:d:+567:primary
TTTCTATTTC	2	0	0	0	1	At4g15630.1:d:+734:primary
GTTGCAACTG	0	0	2	1	0	At4g15560.1:d:+1932:secondary,At5g16250.1:d:+386:secondary
AGGAAGACAA	0	1	2	0	0	At4g15530.2:X:+238:quaternary
GAGCTGAGTT	1	0	1	1	0	At4g15090.1:d:+2903:primary
GAGAAACAGA	0	3	0	0	0	At4g14965.1:d:+612:primary
CTTACTACTG	1	0	0	1	1	At4g14900.1:d:+1687:primary
TTGGAGGAAC	1	0	2	0	0	At4g14740.2:d:+1272:primary,At4g14740.1:d:+1272:primary
CACTTTGCAG	0	1	2	0	0	At4g14713.1:d:+657:primary,At4g14713.2:d:+657:primary
TGAATCTCAA	0	2	0	1	0	At4g14590.1:v:+1696:primary
GTGACTACGA	1	0	1	1	0	At4g14540.1:d:+492:primary
TTTTGGGAGA	0	0	2	1	0	At4g14430.1:d:+846:secondary
CTACAGCGCT	0	1	1	0	1	At4g14400.3:d:+1965:primary,At4g14400.2:d:+1895:primary,At4g14400.1:d:+1978:primary
TGTACTTCAA	0	2	1	0	0	At4g14340.1:d:+1904:primary
TGGTGTTTTG	2	0	1	0	0	At4g14310.2:d:+3080:primary
AATCTTTATG	0	1	0	2	0	At4g14010.1:d:+389:primary,At5g46570.1:d:+1883:primary
TTGGATTAAA	0	0	0	2	1	At4g13680.1:v:+1522:primary
AACTGCGGCT	0	0	1	1	1	At4g13670.1:d:-1135:secondary
GCCTTTTGTT	0	3	0	0	0	At4g13660.1:d:+1137:primary
TAATCCGAAA	0	0	0	2	1	At4g13560.1:d:+528:primary
TTTTGAGTTC	2	0	0	0	1	At4g13270.1:d:+705:secondary
CCAATTCTCT	1	1	1	0	0	At4g12980.1:d:+808:primary
GGGCTATGTT	1	2	0	0	0	At4g12780.1:d:+1539:primary
CTTTTTACGC	0	1	1	1	0	At4g12710.1:X:+1079:quaternary
CCTCTGTTTA	0	1	2	0	0	At4g12570.1:d:+2971:primary
GCTAAGGAGG	0	1	1	1	0	At4g12460.1:v:+2584:primary
TGGCTCGAGT	0	0	2	1	0	At4g12390.1:d:+613:secondary
AAGACAAAGG	0	3	0	0	0	At4g12160.1:p:+431:primary
GCAGACTTCT	0	3	0	0	0	At4g12080.1:d:+1255:secondary
ATGTGAAAGA	1	0	1	1	0	At4g12000.1:d:+1195:primary
ATCAAACCTT	1	2	0	0	0	At4g11880.1:i:+2478:tertiary
AATTTCGGCT	0	0	3	0	0	At4g11590.1:v:-1277:secondary
TTCATATGTT	0	2	0	0	1	At4g11400.1:v:+377:secondary
TTCTAGGTTC	1	0	0	0	2	At4g11270.1:d:+4431:primary
ATTCGTTTCC	0	0	1	2	0	At4g11175.1:d:-561:primary
ATTAGGTTTA	2	0	1	0	0	At4g11175.1:d:+689:primary
TGTAAGTGTC	2	0	1	0	0	At4g10920.1:d:+623:secondary,At2g27110.1:d:+2887:secondary,At2g27110.2:d:+3073:secondary
TAAGTTTTGA	0	0	2	1	0	At4g10895.1:d:+277:secondary,At1g03475.1:d:+1251:secondary,At4g15660.1:v:+758:secondary
ATTTTTACAA	1	0	1	1	0	At4g10890.1:i:+754:tertiary
AAACAAATTT	0	1	0	0	2	At4g10730.1:i:+4954:tertiary
TAAATTTCGT	0	1	1	0	1	At4g10380.1:d:+194:secondary
AATGTTTTTG	1	2	0	0	0	At4g10290.1:d:+302:primary,At3g09800.2:d:+1001:primary
TACTTGAGCT	0	3	0	0	0	At4g10270.1:d:+318:primary,At4g10265.1:d:+285:primary
TTGTAATCTA	0	0	1	0	2	At4g10180.1:d:+1753:primary
GAAAATTAGT	1	0	1	0	1	At4g10090.1:d:+984:primary
AACAACGAAA	0	0	0	2	1	At4g10060.1:X:+459:quaternary,Chr2:+4970056:quaternary
GTGTTGAGAT	1	0	0	0	2	At4g10050.1:d:+1108:primary
TCTTTTTTTT	1	1	0	1	0	At4g10000.2:d:+1252:primary
TCCATTGAAG	0	2	1	0	0	At4g09840.1:d:+906:secondary,At1g70410.2:d:+692:secondary,At1g70410.1:d:+638:secondary,At1g70410.3:d:+554:secondary,At3g27290.1:d:+1249:secondary
ATATAGCTAT	1	0	1	0	1	At4g09760.3:d:+1430:primary,At4g09760.1:d:+1257:primary,At4g09760.2:d:+1209:primary
ATCAAAGAGA	0	0	1	1	1	At4g09570.1:d:+1360:secondary
GCGGTGAAAT	1	0	2	0	0	At4g09520.1:d:+1529:primary,At3g30841.1:d:+1547:primary
TAAAATTTGA	0	2	0	1	0	At4g09020.1:X:+503:quaternary,Chr4:+1891261:quaternary,Chr2:+1345543:quaternary
ATGGAACCAG	1	1	1	0	0	At4g08920.1:d:-2353:secondary
ATGATGACTT	0	1	1	0	1	At4g08610.1:p:+230:secondary
CTAAAGAAAT	0	1	0	1	1	At4g08455.1:X:+653:quaternary
TTCTTCACCC	1	1	0	0	1	At4g08410.1:v:+2862:primary,At2g16930.1:d:+531:primary,At1g59590.1:d:+135:primary,At2g16930.2:d:+492:primary

AAGCTACACA	0	2	1	0	0	At4g08310.1:d:+707:primary
CCTAATTTTG	0	2	1	0	0	At4g08300.1:i:+2439:tertiary
AAGAGAAAAA	0	0	2	0	1	At4g07960.1:d:+2395:secondary,At2g31865.2:d:+1362:secondary,At2g31865.1:d:+1332:secondary
ACTTCATTAC	0	3	0	0	0	At4g07696.1:X:-1031:quaternary
AATAAGTTGC	0	0	0	2	1	At4g06746.1:d:+424:primary
AAGAAACATT	1	1	0	0	1	At4g06556.1:p:+5177:primary
GATGTTGGTC	0	2	0	0	1	At4g06546.1:p:+6807:secondary,At5g11500.1:d:+417:secondary
TTAAGCAAAA	1	0	1	1	0	At4g06519.1:p:+1344:secondary
TTTTCAACTT	1	1	0	0	1	At4g05560.1:v:+912:primary,At3g30700.1:v:+1077:primary,At4g06607.1:p:+1100:primary,At1g40390.1:v:+2043:primary,At2g09900.1:v:+1092:primary,At3g30690.1:v:+1077:primary
GGATGAAATT	0	1	0	2	0	At4g05410.1:d:+735:secondary
TGGCTCTGTG	0	1	1	1	0	At4g04925.1:X:-859:quaternary
TTGATTCTAC	0	2	0	1	0	At4g04890.1:d:+2270:primary
GGTGCTTCTT	1	1	0	1	0	At4g04860.1:d:+753:secondary
CTTGCATTTA	0	0	1	0	2	At4g04840.1:i:+730:tertiary
ACTATTTAAA	0	1	0	0	2	At4g04800.1:d:+684:primary
CTTTGTATCA	0	3	0	0	0	At4g04710.1:v:+2384:primary
TTTTGGTGAA	0	0	3	0	0	At4g04630.1:d:+1054:primary
ATGTTTTTCC	1	0	1	1	0	At4g04040.1:X:+275:quaternary
GTTTGGTTTT	1	0	0	1	1	At4g03740.1:v:+434:secondary,At5g54010.1:d:+45:secondary,At3g07810.1:d:+2000:secondary,At5g44450.1:d:+1105:secondary,At1g50580.1:v:+386:secondary,At3g29630.1:d:+121:secondary,At3g07810.2:d:+1933:secondary
GCTCTGATGA	0	0	2	1	0	At4g03430.1:d:+2984:secondary,At4g09680.1:d:+1853:secondary
TAGCCAGTAG	0	1	1	0	1	At4g03280.2:d:-473:primary,At4g03280.1:d:-374:primary
GAAGCTTTCT	0	0	1	1	1	At4g03140.1:v:+1331:secondary,At3g56580.1:d:+513:secondary,At1g08570.1:d:+1216:secondary,At3g56580.2:d:+387:secondary,At1g54200.1:d:+583:secondary
AAAAGGAAAA	0	0	0	3	0	At4g03120.1:d:+874:primary
GCTTTCTTGG	0	1	1	1	0	At4g03100.1:d:+376:secondary
AGGTGCTAAG	0	1	0	2	0	At4g03020.1:d:+1240:secondary
ACAAAAGTTT	0	0	1	2	0	At4g03010.1:v:+1852:primary
AATGTTGTTG	0	0	1	1	1	At4g02980.1:d:+732:secondary,At3g26660.1:v:+108:secondary
TAAATGTTGG	0	1	1	0	1	At4g02930.1:d:+246:secondary
ATCGCTGCTC	1	0	1	1	0	At4g02770.1:X:-427:quaternary
AACCTTGAAC	0	1	0	2	0	At4g02680.1:d:+3262:primary
AAGCTACAAA	1	2	0	0	0	At4g02500.1:d:-1742:secondary
AAACCCAAAC	1	1	0	0	1	At4g02440.1:d:-995:secondary,At3g10860.1:d:-444:secondary
AACTCAAACG	0	0	1	1	1	At4g02420.1:v:+2419:secondary
GCCTTTTTCA	0	1	2	0	0	At4g02410.1:d:+365:secondary
TTGATATATA	0	1	2	0	0	At4g02410.1:d:+2154:primary
TGATTAATTC	0	1	1	1	0	At4g02330.1:d:+1682:primary
AACTATGAAG	0	2	1	0	0	At4g02150.1:d:+1825:primary
TGAGCTACGC	1	1	0	1	0	At4g02060.1:d:+2111:secondary
CGTTTTTATG	0	2	1	0	0	At4g01975.1:p:+2025:secondary
TTTGTCTCTC	1	1	1	0	0	At4g01960.1:d:+1043:primary,At1g29420.1:v:+842:primary
ATTGTCAGCT	0	2	0	1	0	At4g01925.1:v:+1841:primary,At1g76730.1:d:+875:primary
AAACCACATT	2	1	0	0	0	At4g01920.1:X:-956:quaternary,At2g01510.1:X:-831:quaternary,At2g42500.2:X:-346:quaternary,At4g01925.1:X:-367:quaternary,At4g01910.1:X:-826:quaternary
TTTCTGAACC	0	0	2	1	0	At4g01860.2:d:+4104:secondary,At4g01860.1:d:+4228:secondary
CGATAAAGTG	1	1	0	1	0	At4g01850.1:X:+1388:quaternary
GCTCGCAGGG	0	0	2	0	1	At4g01850.1:d:+991:secondary
TGGTTACAGT	3	0	0	0	0	At4g01800.1:v:+955:secondary
TCAGATCATT	1	0	0	0	2	At4g01160.1:v:+2266:primary
AAAACTCCCC	0	2	1	0	0	At4g01090.1:d:+2576:primary
TTATGTTTTC	2	0	1	0	0	At4g01040.1:d:+1327:primary
GTTTCAATCA	0	2	0	0	1	At4g00955.1:d:+972:secondary
TGTTATAAAA	0	1	0	0	2	At4g00830.1:d:+1737:primary
ATGGTAACCG	0	1	2	0	0	At4g00830.1:d:+1597:secondary
TGACTCTGCG	0	1	1	1	0	At4g00740.1:d:+2253:secondary,At1g53310.1:d:+2810:secondary
TGGTGATGAA	0	1	0	2	0	At4g00730.1:d:+2527:secondary

AAACGGAATC	0	1	2	0	0	At4g00720.1:X:-436:quaternary
ATAAGAAAGC	1	2	0	0	0	At4g00390.1:d:+803:primary
GATGTTTTGA	1	1	1	0	0	At4g00238.1:d:+1082:primary
CACTTTAGAA	1	1	1	0	0	At4g00060.1:d:+3247:primary
CTCTACTCAG	1	0	0	1	1	At4g00050.1:d:+1175:secondary
GTCAGTGGGA	0	0	2	0	1	At3g63520.1:v:+1434:secondary,At4g16150.1:v:+2771:secondary
TGATTTACTA	2	1	0	0	0	At3g63490.2:d:+1377:secondary,At3g63490.1:d:+1387:secondary
CGAGTGGTCG	0	0	2	0	1	At3g63410.1:d:+453:secondary
AAGTTTTAGC	0	0	2	0	1	At3g63190.1:X:--50:quaternary
GAACGCGAGT	1	0	1	1	0	At3g63110.1:d:+1072:primary
AGAAAAGAAG	2	1	0	0	0	At3g62930.1:v:+982:secondary,At5g58320.2:d:+1060:secondary,At5g58320.1:d:+1183:secondary,At4g35710.1:v:+783:secondary,At4g35690.1:v:+780:secondary
AAAGTGTAAG	1	0	1	1	0	At3g62820.1:d:+662:secondary,At2g26590.1:d:+1099:secondary
GGGTCTGGAA	0	3	0	0	0	At3g62740.1:v:+1460:secondary,At3g62750.1:v:+1439:secondary
AATGGGAGAT	0	2	0	1	0	At3g62720.1:d:-1710:secondary
GTGGGTCTCG	0	0	2	1	0	At3g62290.1:d:+174:secondary
AGTATTGTCG	0	1	2	0	0	At3g62220.1:d:+1120:primary
GTTACTTTCT	0	2	1	0	0	At3g62110.1:d:+1544:primary
GATGGGTTGC	0	1	0	2	0	At3g61990.1:d:+819:primary
GCGCTCGCGT	0	1	0	2	0	At3g61980.1:d:+404:primary
ATTTTCACTG	1	1	0	1	0	At3g61460.1:d:+553:primary
TGATGGCTTA	0	1	2	0	0	At3g61320.1:d:+1381:primary
AACATCTGCA	0	3	0	0	0	At3g61260.1:X:-24:quaternary,At5g64560.1:X:-539:quaternary
TTTGAGAAAA	1	2	0	0	0	At3g61250.1:d:+1354:primary
GGTCAACTAA	0	1	1	0	1	At3g61220.1:d:+537:primary
GTGTGTTTGT	1	1	0	0	1	At3g61190.1:d:+812:primary
AGCGTAGATG	1	0	2	0	0	At3g60970.1:v:+354:secondary
TGGAATATGA	0	3	0	0	0	At3g60810.2:d:+497:secondary,At3g60810.1:d:+512:secondary
GTCTCCGGTT	1	1	1	0	0	At3g60630.1:d:+1643:primary
TCAGAAAGAA	0	3	0	0	0	At3g60590.3:v:+1919:primary
GCACTAGTAA	1	0	1	0	1	At3g60440.1:d:+895:secondary
CTCTTCTTTC	0	2	1	0	0	At3g60390.1:d:+1258:primary
GTTGTGTAGC	1	0	1	1	0	At3g60360.1:d:+897:primary
GACTATGCCT	1	2	0	0	0	At3g60340.1:d:+295:secondary,At1g20850.1:d:+643:secondary,At3g60340.2:d:+426:secondary
TCCGTCCAAT	1	0	2	0	0	At3g60230.1:v:+244:secondary,At5g48920.1:v:+1097:secondary
ACATCGAAAA	1	0	0	0	2	At3g59660.1:X:-300:quaternary,At5g23390.1:X:--130:quaternary
TTTATAAGTC	0	1	0	0	2	At3g59410.1:i:+6477:tertiary
TTATCCTAAT	0	0	1	2	0	At3g59360.2:i:+2671:tertiary,At3g59360.1:i:+2650:tertiary
ACACCAACTC	0	2	0	0	1	At3g59360.2:d:+1674:primary,At3g59360.1:d:+1645:primary
AGCATTGTGG	1	2	0	0	0	At3g59350.1:d:+1343:primary,At3g59350.2:d:+1226:primary
TTGTTGTGTT	2	0	1	0	0	At3g59220.1:d:+730:primary
AACCCTAATT	1	0	0	2	0	At3g59190.1:d:+25:secondary
TCGCTAGACC	0	2	0	1	0	At3g59110.1:d:+2068:primary
AGATGGAAGA	1	0	2	0	0	At3g59050.1:d:+1751:primary
TTGTAATGAT	0	0	0	1	2	At3g58790.1:d:+2314:primary
AAAAGACATC	0	1	0	1	1	At3g58570.1:i:+1376:tertiary
TCCCTGGGGT	2	0	1	0	0	At3g58560.1:d:+2199:primary
TTGTAAACTT	0	3	0	0	0	At3g58510.2:d:+1777:primary,At3g03855.1:p:+3769:primary,At3g58510.1:d:+1617:primary
GCTTTGCAAA	0	0	1	1	1	At3g58290.1:v:+1608:primary
CGTCTAGTAA	1	1	1	0	0	At3g58220.1:v:+1999:primary,At3g27120.1:d:+746:primary
AGATGCTCTC	0	3	0	0	0	At3g58110.1:X:+2384:quaternary
ATGCTCTCAT	0	3	0	0	0	At3g58100.1:d:+715:primary
GGTCGTCGAT	0	1	2	0	0	At3g57970.1:v:-109:primary
ATAAGCTATG	0	0	1	1	1	At3g57940.1:d:+3372:primary
GTTTTGGTCA	0	0	2	0	1	At3g57810.1:d:+1011:primary,At3g57810.2:d:+1081:primary
TTTCAAAGCT	0	2	1	0	0	At3g57785.1:d:+622:primary
TACAAATCTA	1	1	0	1	0	At3g57680.1:v:+1973:secondary,At1g33870.1:v:+1252:secondary
CCATTGTGTT	0	3	0	0	0	At3g57410.1:i:+7579:tertiary
GGTGGGTTTT	0	1	0	2	0	At3g57410.1:d:+3227:secondary,At3g07130.1:d:+1381:secondary
CCTGTTTGAG	0	2	1	0	0	At3g57340.2:X:+1481:quaternary

TGAAGAATGG	0	1	0	1	1	At3g57270.1:d:+937:primary
GGCCTATAGG	0	0	0	2	1	At3g57260.1:d:+1216:primary
AGCTTGATGA	1	2	0	0	0	At3g57220.1:d:-900:secondary,At1g15690.1:d:-2365:secondary
TCCAACAATC	1	1	0	0	1	At3g57220.1:d:+1436:primary
TTGCAACTGC	1	0	0	2	0	At3g57000.1:d:+675:primary
TATGATCCGG	0	1	1	0	1	At3g56740.1:d:+1256:secondary
TTTTGAGACC	2	0	1	0	0	At3g56720.1:d:+1507:primary,At3g57380.1:d:+1258:primary
CTGAGCATAA	1	1	0	1	0	At3g56660.1:v:+2366:primary
ACCTACGAGA	0	1	0	1	1	At3g55640.1:d:+1158:secondary
ATGAAGAAAG	1	0	1	1	0	At3g55605.1:d:+760:primary
TAAGCCAGAA	0	3	0	0	0	At3g55520.1:d:+446:secondary
GAACATCTCA	1	0	1	0	1	At3g55510.1:d:+2064:secondary,At5g53480.1:d:+3102:secondary
TCTTCTTCTT	0	1	0	0	2	At3g55450.1:d:+1336:primary
TCAAAGAGCA	0	1	1	1	0	At3g55410.1:d:+3133:primary
TTATAACCAA	1	1	0	1	0	At3g55360.1:X:-176:quaternary
TTTGGCTGCT	0	1	0	1	1	At3g55070.1:d:+1584:primary
GTTTTAGTGG	0	2	0	0	1	At3g55070.1:d:+1567:secondary
ATTTTGCCAA	0	2	1	0	0	At3g54970.1:d:+1093:primary,At3g54970.2:d:+1073:primary
ATATCCTTTA	0	0	0	1	2	At3g54910.1:d:+1218:secondary
AGACAGAAAG	0	0	2	0	1	At3g54790.1:d:+1463:secondary
TCTAGATTTC	0	3	0	0	0	At3g54740.1:d:+1564:primary
GTTCTTTGTT	0	1	1	0	1	At3g54700.1:d:+181:secondary
CCAAGCCGCA	0	1	0	2	0	At3g54670.1:d:+3812:primary
TTTATCATTG	1	2	0	0	0	At3g54650.1:d:+1942:primary
TTGTAATGCT	0	1	0	1	1	At3g54380.1:d:+1273:primary,At3g54380.2:d:+1287:primary
GCACAGCAAC	1	1	1	0	0	At3g54280.1:X:+3:quaternary
TTTCTTTGTT	1	0	1	1	0	At3g54070.1:v:+2183:primary,At4g36450.1:v:+1514:primary
ACTCAAGCCA	0	0	1	1	1	At3g54060.1:v:+2107:primary
TAATACTAGT	0	0	0	1	2	At3g54050.1:d:+1503:primary
AGGCATACTA	1	0	1	0	1	At3g54030.1:d:-1842:secondary
AGAGAGATAT	2	0	0	0	1	At3g54010.2:d:+2131:primary,At3g54010.1:d:+2061:primary
GAAAAACTTA	1	0	1	0	1	At3g53950.1:d:+1801:primary
ATTGGGCATC	2	0	0	0	1	At3g53720.1:X:+504:quaternary
TGAAGTCAAA	0	1	1	1	0	At3g53710.1:d:+621:secondary,At5g12410.1:d:+485:secondary
GCAACAACAC	0	2	0	0	1	At3g53540.1:d:+2693:secondary,At3g10070.1:d:+297:secondary
GTACATTTGG	0	0	2	1	0	At3g53420.1:d:+902:secondary
TTACATTTTC	2	0	0	1	0	At3g53250.1:v:+866:secondary,At1g80850.1:v:+1489:secondary
ATGAAATAAA	1	0	1	1	0	At3g53230.1:d:+2632:primary
ACAAGCCAAA	1	0	1	1	0	At3g52910.1:v:+188:secondary
CTACAACATT	0	1	1	0	1	At3g52910.1:i:+2972:tertiary
AATGCTCCAA	0	0	1	0	2	At3g52880.1:X:-202:quaternary
AGGCTTTTCC	2	0	0	1	0	At3g52860.1:d:+421:primary
CTTCCGGAAA	0	2	0	1	0	At3g52840.1:d:-2303:secondary
TGTACACAGT	1	0	1	0	1	At3g52640.1:d:+2061:secondary,At3g52640.2:d:+2148:secondary
TGGAAAATTA	0	2	0	0	1	At3g52290.1:X:-798:quaternary
GCATCAGTGA	1	1	0	1	0	At3g52190.1:d:+1190:primary
AATAAAATAA	0	1	1	0	1	At3g52160.1:d:+1414:primary
CTACATCCAA	1	0	0	1	1	At3g52150.1:X:+234:quaternary
TAGAGCAGCA	0	0	3	0	0	At3g52130.1:v:+536:secondary
GGTGATTATA	0	1	2	0	0	At3g52120.1:d:+1100:primary
GGAGGTTCCA	0	1	1	1	0	At3g52050.2:X:+316:quaternary
CTTCACACGT	1	0	0	1	1	At3g51895.1:d:+2004:primary
CCCCCCTGAG	0	1	2	0	0	At3g51730.1:d:+797:secondary
ACTGGGACGA	0	1	0	1	1	At3g51580.1:d:+1424:primary
AACAAATTAG	1	0	1	1	0	At3g51530.1:d:+1596:secondary
GTGATGAATG	0	2	0	1	0	At3g51510.1:d:+106:secondary
ATGTTTCTCT	0	1	2	0	0	At3g51440.1:d:+1241:primary,At4g22270.1:d:+1426:primary
CACTTATCAC	0	1	1	0	1	At3g51050.1:d:+1980:primary
GGTAAGGTTA	0	0	2	0	1	At3g50760.1:d:+926:primary
ATTTTTCAAT	0	0	2	1	0	At3g50750.1:d:+1263:primary
CCGTTTGGAG	0	2	1	0	0	At3g50660.1:d:+1425:primary

TAATTCAACT	2	0	1	0	0	At3g50630.1:d:+876:primary
CTCTTAATAC	2	0	1	0	0	At3g50550.1:d:+417:primary,At3g50550.2:d:+287:primary
GAATGGTAGA	3	0	0	0	0	At3g50420.1:v:+2098:secondary,At5g39862.1:p:+2481:secondary,At2g01550.1:p:+4433:secondary,At1g26330.1:v:+769:secondary,At3g58090.1:p:+573:secondary
TGGAAAGCTG	1	2	0	0	0	At3g50380.1:d:+9070:primary
CGTTTGATAT	2	1	0	0	0	At3g50360.1:d:-434:secondary
GATACAATGC	0	0	0	1	2	At3g50240.1:d:+382:secondary,At3g07780.1:d:+1714:secondary
TATTCAATGG	1	2	0	0	0	At3g49940.1:d:+188:primary
TTATGTCAAG	0	0	0	2	1	At3g49850.1:d:+1163:primary
TGGGGATTAT	2	0	0	1	0	At3g49710.1:v:+2501:secondary
CTATGTAGAA	1	0	1	1	0	At3g49240.1:X:+2165:quaternary
GAAGGATTAA	2	0	0	1	0	At3g49160.1:d:+2424:primary
TAGTATCAGG	1	0	0	1	1	At3g48710.1:d:+1570:primary
CAGAATGAAA	1	0	1	1	0	At3g48510.1:d:+839:secondary,At1g67230.1:d:+2243:secondary
AATATGCGAG	0	0	2	0	1	At3g48420.1:i:+1214:tertiary
AGATTCTATT	2	0	1	0	0	At3g48360.1:X:--36:quaternary
TATGATCACT	0	3	0	0	0	At3g48185.1:d:+504:primary
TACACTATAA	2	0	0	0	1	At3g48150.1:d:+1875:primary
CAATTGGTGG	1	1	1	0	0	At3g48110.2:d:+3046:primary,At3g48110.1:d:+3046:primary
TTGTATGATC	2	1	0	0	0	At3g48090.1:d:+2031:primary,At3g48080.1:d:+2042:primary
TGTGTATCTC	0	0	0	1	2	At3g48050.1:d:+5183:secondary,At3g48050.2:d:+5103:secondary
TTCAAACTCT	0	1	0	0	2	At3g47990.1:d:+1516:primary
GTGGAAAAGC	0	0	1	0	2	At3g47930.1:d:+1854:primary
AATGTTGCAA	2	1	0	0	0	At3g47910.1:d:+3473:secondary
TAAAGCGATT	1	2	0	0	0	At3g47833.1:d:-528:secondary
TGATTACGTG	2	0	1	0	0	At3g47610.1:d:+1452:primary
TAGAAGATGG	1	1	0	1	0	At3g47610.1:d:+1247:secondary
ATACACGGTG	0	3	0	0	0	At3g47550.6:d:+1124:secondary,At3g47550.3:d:+970:secondary,At3g47550.5:d:+1157:secondary,At3g47550.1:d:+1040:secondary,At3g47550.2:d:+963:secondary,At3g47550.4:d:+1234:secondary
CTGGAATCAC	0	1	0	2	0	At3g47520.1:d:+948:secondary
CCTCGGCCTC	1	1	1	0	0	At3g47470.1:d:+283:secondary
TTGAAGCCAC	3	0	0	0	0	At3g47430.1:d:+211:primary
TCCACAAATA	0	2	0	0	1	At3g46960.1:v:+4467:secondary
TAAAGTACAC	0	2	0	0	1	At3g46850.1:v:+1903:secondary
TGAAGGCTCA	0	1	0	2	0	At3g46610.1:d:+2119:primary
CGTACCACGC	1	1	1	0	0	At3g46490.1:v:+1454:secondary
TTCAATAACA	0	3	0	0	0	At3g46430.1:v:+596:secondary
TCGGTTGGCT	0	0	3	0	0	At3g46370.1:v:+2342:secondary,At5g19120.1:d:+438:secondary
ATTCTACATT	1	0	0	0	2	At3g45970.1:d:+979:primary
ACAAGAAGCC	0	2	0	0	1	At3g45970.1:d:+652:secondary
TAAATTCACA	1	2	0	0	0	At3g45960.1:d:+6:secondary,At3g45970.1:d:+6:secondary,At3g61350.1:d:+1251:secondary
GATGCCAATG	0	0	0	0	3	At3g45850.1:d:+2674:secondary
CTCCTAGATC	1	2	0	0	0	At3g45780.1:d:+2314:secondary
AAGAATCAAA	0	1	0	2	0	At3g45630.1:d:+2163:secondary,At2g11320.1:p:+1620:secondary,At5g09450.1:d:+402:secondary
CTCTGTTATC	2	1	0	0	0	At3g45600.1:d:+737:secondary
TTATTATATG	2	0	0	0	1	At3g45530.1:v:+2703:primary,At1g76960.1:d:+334:primary
TTCTTTGATC	1	0	2	0	0	At3g45310.1:i:+697:tertiary
TTATCACGAT	2	0	0	0	1	At3g45300.1:d:+1586:secondary
CCTCGACAAT	0	3	0	0	0	At3g45300.1:d:+1055:secondary
AAGGAATTGG	0	2	1	0	0	At3g45210.1:d:+407:primary
ACAACAAAAG	0	2	0	0	1	At3g45200.1:v:+1075:primary
ATCCGAAAAG	1	1	1	0	0	At3g44990.1:d:+931:secondary
AACTTGTTTT	0	1	1	0	1	At3g44970.1:v:+354:secondary
CAAGAAGACT	0	1	1	0	1	At3g44750.1:d:+731:primary
ACAGGCCGTT	0	0	1	0	2	At3g44600.1:d:+1731:secondary
GGATTCAGTC	0	0	1	0	2	At3g44590.2:d:+358:primary
ACCTTGATGC	0	1	1	0	1	At3g44340.1:d:+2997:primary
TTTTGTATTT	1	0	1	1	0	At3g44060.1:v:+2036:primary,At3g50780.1:d:+1926:primary
TCTGTTGGTT	1	1	1	0	0	At3g43870.1:v:+1390:primary

CGTATTGTTG	0	1	1	1	0	At3g43726.1:p:+244:primary
ACTCTAACAT	1	1	1	0	0	At3g43610.1:v:+3685:secondary
AAAGAAGGTA	0	0	1	1	1	At3g43600.1:d:+4119:primary
TTGGTGGTGA	1	0	1	1	0	At3g43450.1:v:+1207:secondary,At1g64570.1:v:+2320:secondary,At4g16250.1:v:+3405:secondary
TGCTGTTCAC	1	0	2	0	0	At3g43360.1:p:+1986:primary
TGGAATTTAT	1	1	1	0	0	At3g43357.1:p:+692:secondary
TTTGATCGAA	2	1	0	0	0	At3g43270.1:d:+1931:primary
AATGTAAAGT	0	1	1	1	0	At3g43230.1:d:+1510:secondary
TTTCTACAAA	2	0	0	1	0	At3g42924.1:p:+793:primary
TCTACATTGA	1	1	0	1	0	At3g42922.1:p:+243:secondary,At4g05582.1:p:+242:secondary
GATTGGTGAA	2	1	0	0	0	At3g42720.1:p:+1709:secondary,At2g42850.1:d:+169:secondary,At4g08016.1:p:+153:secondary,At5g28165.1:p:+6369:secondary,At2g06720.1:p:+1547:secondary,At1g64550.1:d:+284:secondary
TTCACAATCA	1	1	0	1	0	At3g42670.1:X:-47:quaternary
GATCCCAGTT	0	3	0	0	0	At3g42660.1:v:+2327:secondary
GTCAGAGCTA	0	0	0	1	2	At3g42432.1:p:+1520:primary,At3g46210.1:d:+1029:primary
GATGCTGATG	1	1	1	0	0	At3g42236.1:p:+2212:primary,At5g35148.1:p:+1242:primary
TTTGTCTAAA	1	1	1	0	0	At3g42230.1:X:+2851:quaternary
GAACCGAGGG	0	1	1	1	0	At3g42170.1:p:+2828:primary
CTGTTTCTAG	0	3	0	0	0	At3g33083.1:p:+912:secondary,At4g18780.1:d:+2459:secondary
TCACAAAACA	1	1	1	0	0	At3g33011.1:p:+1712:secondary,At3g32340.1:p:+1693:secondary,At3g42182.1:p:+2530:secondary,At4g08094.1:p:+43:secondary,At4g07840.1:p:+1739:secondary,At3g32305.1:p:+1702:secondary,At3g42471.1:p:+1675:secondary,At3g21260.2:d:+257:secondary,At4g03810.1:p:+1645:secondary,At3g21260.1:d:+226:secondary
TCAAACAAAA	0	1	1	0	1	At3g32375.1:p:+1696:primary
AAGAAGATTA	0	1	0	1	1	At3g32230.1:p:+5016:primary,At3g48430.1:d:+4094:primary
GGTTCGAATC	0	0	1	2	0	At3g32200.1:v:+929:secondary
GAAGAGAAAG	0	0	3	0	0	At3g32092.1:p:+3697:primary,At4g06602.1:p:+3145:primary
TTTTACATTT	0	1	1	0	1	At3g31373.1:p:+950:secondary,At5g38910.1:v:+1169:secondary,At3g31377.1:p:+286:secondary,At3g32095.1:p:+372:secondary
GATCTTGAAG	0	2	0	1	0	At3g30840.1:v:+27:secondary
GCAAGGAAAA	1	0	2	0	0	At3g30838.1:p:+281:secondary,At5g34846.1:p:+824:secondary
AGATGTGTCT	1	2	0	0	0	At3g30815.1:p:+1348:primary
ATTCCCACAT	1	1	1	0	0	At3g30708.1:p:+403:secondary
CATTCTTTAT	1	1	0	0	1	At3g30400.1:p:+2126:secondary,At1g29520.1:d:+694:secondary
GAGATGGATG	1	1	1	0	0	At3g30180.1:d:+1306:primary,At1g47750.1:d:+1246:primary
ATTGTCTCAA	0	1	0	1	1	At3g30122.1:p:+2247:primary
ATCCAACAAT	1	1	1	0	0	At3g29792.1:X:+4750:quaternary,Chr5:+11652260:quaternary
GTTTTGGTTT	2	1	0	0	0	At3g29365.1:p:+595:secondary
ATCGTAAAAA	3	0	0	0	0	At3g29255.1:p:+1323:secondary,At3g10200.1:d:+1827:secondary
AGCATTGTAC	0	2	0	0	1	At3g29180.1:d:+1253:primary
GATCTGGCTA	0	2	0	0	1	At3g29075.1:d:+421:secondary
CAATCTCTGG	0	0	1	0	2	At3g29034.1:d:+145:primary
AAACCAGTTC	0	2	0	1	0	At3g28940.1:X:+395:quaternary
AAAAGACATT	1	0	0	1	1	At3g28925.1:v:+69:secondary
GTTCTAATTT	0	2	0	1	0	At3g28910.1:d:+1258:primary
GAAACTTCTC	0	0	3	0	0	At3g28865.1:p:+4492:secondary,At1g76740.1:d:+2099:secondary
AGCTCAAGAA	1	0	0	1	1	At3g28865.1:p:+2587:secondary
GATATAAAAT	1	1	1	0	0	At3g28780.1:d:+2000:primary
TTATTCTCCA	0	3	0	0	0	At3g28550.1:d:+1929:secondary,At3g54580.1:d:+2103:secondary,At3g54590.1:v:+2378:secondary,At3g28250.1:v:+461:secondary
AAGATAATGA	2	0	1	0	0	At3g28270.1:d:+1588:primary,At3g28270.2:d:+1046:primary
GTCCACGAGT	1	2	0	0	0	At3g28130.1:d:+686:secondary,At3g28130.2:d:+631:secondary
CTGGAAAGAA	0	2	1	0	0	At3g27560.1:d:+1301:primary
CAGTTTCCTG	1	0	1	0	1	At3g27530.1:d:+2544:primary
AAAGATCTGA	1	0	0	1	1	At3g27290.1:d:+1602:secondary
GTAAATAAAT	0	0	1	2	0	At3g27280.1:d:+1192:primary
ACAACTTTTA	0	1	1	0	1	At3g27190.1:X:+377:quaternary
TTGTCGTGCA	0	3	0	0	0	At3g26980.1:d:+587:secondary
TCTGGAACCG	0	1	2	0	0	At3g26950.1:d:+1409:primary
TCAGGTCGTA	1	0	2	0	0	At3g26932.1:X:-784:quaternary

TCGACTATGA	0	2	1	0	0	At3g26900.1:X:-178:quaternary
GTTGGTCTTG	1	1	0	1	0	At3g26820.1:v:+2221:secondary
CGAAGTAATC	0	0	3	0	0	At3g26740.1:d:+285:secondary
TCAATAATAT	0	1	1	0	1	At3g26445.1:v:+60:secondary
TGTAAAGCTT	0	1	2	0	0	At3g26370.1:d:+1803:primary
GGCAATGGCA	0	2	0	1	0	At3g26290.1:d:+963:secondary,At3g26300.1:v:+1286:secondary
AGAATGCGAG	0	2	1	0	0	At3g26000.1:d:+1246:primary
AATTATATCA	2	1	0	0	0	At3g25740.1:d:+1055:primary
TATCAAGTCT	0	2	0	1	0	At3g25690.1:d:+947:secondary
GACAAGGACG	0	2	0	1	0	At3g25600.1:d:+180:primary
GAGTTATCAG	1	1	1	0	0	At3g25560.1:d:+1967:secondary,At3g25560.2:d:+2018:secondary
AATCTGAACC	1	0	2	0	0	At3g25540.1:d:+1135:primary
ATCAAGAACA	1	2	0	0	0	At3g25400.1:d:+499:primary
CATCACAACT	1	1	0	0	1	At3g25190.1:d:+739:primary
ATTTCTCTAA	1	2	0	0	0	At3g24440.1:d:+1881:primary
TAAAGACTTA	1	0	2	0	0	At3g24350.1:d:+1212:secondary
TAGAGATCAC	1	1	0	1	0	At3g24270.1:v:+968:primary
GATTGATCTT	2	1	0	0	0	At3g24150.1:d:+1461:primary
AACTTCTGGG	1	2	0	0	0	At3g24040.1:d:+1117:primary
CTAGGAACCT	0	2	0	1	0	At3g23990.1:d:+1124:secondary
GATGCAATTG	1	0	2	0	0	At3g23910.1:d:+1291:primary
TGAATCAAAA	0	1	1	1	0	At3g23780.1:v:+3668:primary,At5g55030.1:d:+2123:primary
GCTAGATTAG	0	0	1	1	1	At3g23660.1:d:+1642:secondary
GCTATTGAGT	0	0	1	2	0	At3g23590.1:d:+847:secondary
CCCTTGCCTA	0	3	0	0	0	At3g23590.1:d:+3752:primary
AGTCAATACA	1	0	1	0	1	At3g23580.1:d:+1014:primary
CACGAGTTCT	0	0	2	1	0	At3g23295.1:v:+764:secondary
GGAATATCTC	2	1	0	0	0	At3g23150.1:d:+2174:secondary
GACTGGCCGG	0	3	0	0	0	At3g23070.1:d:+1238:secondary,At4g18010.2:d:+887:secondary,At4g18010.1:d:+1096:secondary
TTGGGCAATA	1	0	2	0	0	At3g23050.1:d:-1021:secondary
GTACTCAATA	1	0	0	0	2	At3g23000.1:d:+1508:primary
GCTCTTCTTT	0	0	2	1	0	At3g22680.1:d:+483:primary
TGACCTTTAC	0	1	0	0	2	At3g22670.1:v:+2127:secondary
TCTGTAGAAG	0	1	0	0	2	At3g22630.1:d:+521:primary
GCAATCTTCT	0	1	0	1	1	At3g22600.1:d:+576:primary
AGAAAAGAAA	1	0	1	1	0	At3g22104.1:d:+1074:primary
TTTTGATTTT	0	0	0	0	3	At3g22090.1:d:+411:primary,At5g56520.1:d:+436:primary
ATTGAAAACA	0	1	0	2	0	At3g21750.1:d:+1738:primary
ATGGCTAAAC	1	1	0	1	0	At3g21720.1:d:+350:secondary,At1g46192.1:p:+3035:secondary,At4g22830.1:d:+625:secondary
CTAAAACGTT	0	0	2	1	0	At3g21510.1:d:+658:secondary,At1g34355.1:d:+3370:secondary
GAACTTCTCA	0	0	2	1	0	At3g21340.1:d:+795:secondary,At1g53210.1:d:+1748:secondary
GGTATCAAGA	0	1	2	0	0	At3g21290.1:d:+3969:primary
AGTGTTGGAA	1	1	1	0	0	At3g21160.1:d:+1385:primary
AACCATAAAA	1	1	0	1	0	At3g21150.1:d:+19:secondary
GGAGATCAAA	0	0	2	1	0	At3g21100.1:d:+2242:primary
GTCATCGATG	0	2	0	1	0	At3g21055.1:X:-127:quaternary
CCCCTGATGA	0	2	0	1	0	At3g20920.1:d:+877:primary
CTCGGAGCAG	0	0	0	3	0	At3g20810.1:d:-1463:secondary,At3g20810.2:d:-1528:secondary
GGTCAATGCT	1	2	0	0	0	At3g20770.1:X:+2330:quaternary
CGTAGCAAAG	0	1	0	0	2	At3g20630.1:v:+2109:secondary
ATCTAGCGCT	1	1	1	0	0	At3g20350.1:d:+2302:primary
GCTGAAGATG	1	0	2	0	0	At3g20320.2:d:+1062:primary,At3g20320.1:d:+976:primary
TCGATTTGTA	1	1	1	0	0	At3g20300.1:d:+1622:secondary
AACAACTTTG	0	0	1	2	0	At3g19970.1:d:+1528:primary
ATATATACAA	0	1	0	0	2	At3g19960.1:d:+911:secondary,At1g50360.1:v:+906:secondary
TAAAGTTTTA	1	1	0	1	0	At3g19870.1:d:+3409:primary
GATTATACAC	0	1	0	1	1	At3g19850.1:X:+625:quaternary
ACTTCTATCG	1	1	0	1	0	At3g19800.1:d:+391:secondary
ATGGGTTTTA	1	0	0	1	1	At3g19790.1:v:+1024:secondary
TGGGTTTGGA	0	3	0	0	0	At3g19700.1:d:+2716:secondary,At4g19985.1:d:+753:secondary
GTCCAAGTAG	1	1	1	0	0	At3g19640.1:d:+1391:primary

AAACAGACAT	0	2	0	0	1	At3g19630.1:d:+1190:secondary
TACAGTTGTA	0	1	1	0	1	At3g19553.1:d:+1635:secondary
TTGAACGCAA	0	1	1	1	0	At3g19520.1:d:+880:primary
TTCACATTAA	0	2	0	1	0	At3g19400.1:d:+1222:primary
GACTTGCCTG	0	2	0	1	0	At3g19330.2:d:+1303:primary,At3g19330.3:d:+1297:primary,At3g19330.1:d:+1394:primary
GTGAAGGATG	1	0	0	1	1	At3g19290.1:d:+1460:primary
TAAGCTTGGG	0	1	0	2	0	At3g19220.1:d:+634:primary
TGTAATTGTG	3	0	0	0	0	At3g19080.1:i:+1094:tertiary
TAACTGTTAT	1	0	1	1	0	At3g19050.1:v:+8578:secondary
TTTATGTTTA	1	0	1	0	1	At3g19000.1:d:+1321:primary
AAGAAAAGCT	1	1	0	1	0	At3g18900.1:v:+1330:secondary,At2g40955.1:v:+862:secondary,At1g09900.1:v:+2109:secondary,At4g15096.1:v:+862:secondary,At3g55160.1:v:+6118:seconda ry
TTGTCTTAAT	0	0	1	0	2	At3g18640.1:v:+2401:primary
AAAGCAAAGA	0	0	0	1	2	At3g18600.1:d:+1289:primary
GTGGAAATGG	0	1	2	0	0	At3g18520.2:d:+1773:primary,At3g18520.1:d:+1761:primary
ATGGTGGACC	0	0	0	1	2	At3g18510.1:d:+411:primary
ATGGTTGTCT	0	0	1	0	2	At3g18350.1:d:+696:secondary
TAACGTCCTG	1	0	0	1	1	At3g18230.1:d:+2095:primary
CTTGAGACAC	0	0	1	1	1	At3g18165.1:d:+724:primary
TAATATTTAT	2	0	0	0	1	At3g18110.1:d:+4398:primary,At5g46875.1:v:+1205:primary
GCCTCTCCTC	0	3	0	0	0	At3g18080.1:d:+54:secondary
AGAAATGCCC	0	1	1	0	1	At3g17970.1:d:+1333:primary
CGGGTGTGTG	0	1	1	0	1	At3g17940.1:d:+979:primary
TGTTTGCGTT	1	1	1	0	0	At3g17900.1:d:+2911:primary
ATTTGATCTT	1	0	0	0	2	At3g17780.1:d:+542:primary
CTTAACTTGA	0	1	0	2	0	At3g17590.1:d:+859:primary
CCAAGTTAAG	1	0	2	0	0	At3g17510.1:d:+1415:secondary,At3g17510.2:d:+956:secondary
TTGTTTGAGT	0	2	1	0	0	At3g17300.1:d:+352:primary
CTCACGATCC	1	0	0	2	0	At3g17130.1:v:+613:primary
GTTGCTTACA	1	2	0	0	0	At3g17020.1:X:-143:quaternary
TATCCACCTT	0	1	1	1	0	At3g16860.1:d:+2035:primary,At5g18410.1:d:+3880:primary
TTAGGGGTTA	2	0	0	0	1	At3g16857.2:d:+2409:primary,At4g14250.1:v:+2986:primary
AAATTTTAAA	1	0	1	0	1	At3g16857.2:d:+2368:secondary
TAATGGTTCA	1	0	1	0	1	At3g16800.1:d:+1333:primary,At3g16800.2:d:+1316:primary
TGCAAAGAAG	0	1	2	0	0	At3g16785.1:d:+3470:primary
AAAGTGAAGG	0	1	0	2	0	At3g16780.1:d:+433:primary
TTGCATATTA	0	3	0	0	0	At3g16710.1:v:+1773:primary
CCAAGAAGTA	0	1	0	2	0	At3g16660.1:X:+550:quaternary
AATAAGGTCT	1	2	0	0	0	At3g16660.1:d:+718:primary
TTTATAGTAG	1	1	0	0	1	At3g16640.1:d:+882:primary
GGAAGAAGAC	1	1	1	0	0	At3g16470.1:d:+1129:primary
GAAGAGCCGA	0	0	3	0	0	At3g16420.1:d:+937:secondary,At3g16430.1:d:+919:secondary,At3g16420.2:d:+921:secondary,At3g16430.2:d:+932:secondary
TGCTTCCAAA	1	0	0	2	0	At3g16260.1:d:+2684:primary
AAGACATTTC	2	0	1	0	0	At3g16250.1:d:+1058:secondary
AGCAGGAAGA	0	0	2	1	0	At3g16000.1:d:+761:secondary
TAGTTTTTTA	1	0	1	0	1	At3g15860.1:v:+1059:secondary,At2g35760.1:d:+917:secondary
AAAATTTTGA	0	1	0	0	2	At3g15800.1:v:+1975:primary,At2g32740.1:v:+2239:primary
CTTTCTGCCT	0	2	0	1	0	At3g15750.1:d:+707:primary,At1g34570.1:d:+784:primary
TCATAATTCT	0	0	2	0	1	At3g15440.1:v:+988:secondary
CCTATGATCA	1	2	0	0	0	At3g15290.1:d:+624:secondary,At5g36870.1:v:+4830:secondary
CGTTTCTTCT	0	1	0	1	1	At3g15210.1:d:+755:secondary
TATTATTGTT	2	0	0	0	1	At3g15080.1:d:+1036:primary,At3g25290.1:d:+1308:primary
TCTGAATCTT	0	1	1	1	0	At3g14980.1:v:+2402:secondary
TATGTAAATG	0	0	0	1	2	At3g14900.1:d:+1937:primary
GAATGTCACT	2	0	0	1	0	At3g14800.2:p:+2936:primary
AGAGAATGAA	1	1	1	0	0	At3g14770.1:X:-257:quaternary,At1g70410.1:X:-243:quaternary
AATTAAGGAA	0	0	1	1	1	At3g14440.1:d:+2291:primary
CTAGTCACGT	0	3	0	0	0	At3g14370.1:d:+1313:primary
TTGTCGGAAT	0	1	2	0	0	At3g14340.1:d:+105:secondary,At5g11540.1:v:+889:secondary

GAGATTGAAC	1	1	0	1	0	At3g14205.1:d:+3102:secondary
GATCCAGTAT	1	0	0	2	0	At3g14160.1:d:+1169:primary
AGAGCAACCT	0	1	1	0	1	At3g14010.1:X:-310:quaternary
GGACCTGGCC	0	2	0	1	0	At3g13990.1:v:+2886:secondary
TCTGTAACAC	1	0	0	1	1	At3g13930.1:d:+1714:secondary
GCGAATTATT	0	3	0	0	0	At3g13910.1:d:+55:secondary
TAACCTCAAT	0	2	0	0	1	At3g13670.1:d:+2005:primary
TGTTATTTGT	0	0	0	2	1	At3g13570.1:d:+1114:primary
GGAAATCTGA	1	0	1	1	0	At3g13530.1:d:+1175:secondary,At5g27650.1:d:+1675:secondary
AGAGGAGATT	0	1	1	0	1	At3g13460.2:X:-255:quaternary
TTTGCGGTCC	1	2	0	0	0	At3g13320.1:d:+1539:primary
CTTGGTTTTG	0	1	0	2	0	At3g13230.1:d:+428:primary
CAGGCTGGGA	0	3	0	0	0	At3g13222.1:d:+1589:primary
GTGATGTGAA	1	0	1	1	0	At3g13050.1:i:-1236:tertiary
TTCCAGGATT	0	0	1	1	1	At3g12940.1:d:+2169:primary
TCTGAGATCT	0	0	0	1	2	At3g12930.1:d:-978:secondary
ATAGGAAGCA	0	2	1	0	0	At3g12750.1:d:+1317:primary
CTTCTTCAAT	1	0	0	1	1	At3g12640.1:d:+2169:primary
AGCTTACTCA	0	1	1	0	1	At3g12630.1:X:+1165:quaternary
TTCTATCTTT	0	1	0	0	2	At3g12510.1:d:+546:primary
CTACTGAAGT	1	0	0	1	1	At3g12490.2:d:+587:secondary,At3g12490.1:d:+553:secondary
GCGAGGAGCA	0	2	0	1	0	At3g12380.1:d:+2167:primary
GAGGGTTTCA	0	1	0	1	1	At3g12180.1:d:+720:primary
TAAATGTTGC	0	2	0	0	1	At3g12140.1:d:+1557:secondary,At3g12140.2:d:+1575:secondary
GGGAAGCAGA	1	1	1	0	0	At3g12130.1:d:+897:secondary
GCAAAGTTCG	0	2	0	1	0	At3g12050.1:d:+191:primary,At3g12050.2:d:+191:primary
TAAACAGCTC	0	2	0	0	1	At3g11930.3:d:-739:secondary,At3g11930.2:d:-661:secondary,At3g11930.1:d:-748:secondary
AATCACCGTG	1	1	1	0	0	At3g11930.3:d:-272:primary,At3g11930.2:d:-272:primary
CTTGTTTATA	0	2	0	1	0	At3g11910.1:d:+1644:secondary
CAATAGGTTG	2	0	0	0	1	At3g11890.2:d:+1567:primary,At3g11890.1:d:+1519:primary
TGTTGGATTT	1	0	0	1	1	At3g11540.1:d:+3423:primary
AAGTAGAAAA	0	2	0	1	0	At3g11480.1:i:+625:tertiary
TGAACAAAAA	1	1	0	0	1	At3g11480.1:d:+392:secondary,At1g65810.1:v:+299:secondary
AACGTAACTA	0	1	1	1	0	At3g11420.1:d:+2073:primary
AAAGAAGTGC	0	1	2	0	0	At3g11402.1:v:+360:secondary
CTTCAGTGAG	0	0	2	0	1	At3g10980.2:d:+2366:secondary,At3g10980.1:d:+2347:secondary
AAGTCACTCC	0	2	1	0	0	At3g10860.1:d:+305:secondary,At5g11040.1:d:+3251:secondary
TTATTGGACC	0	1	1	1	0	At3g10840.1:d:+1467:primary
CTTGATCTGA	1	1	1	0	0	At3g10730.1:d:+1524:primary
GGTATCTCCG	0	1	1	1	0	At3g10490.2:d:+1221:primary,At3g10490.1:d:+1257:primary
AAGTTTCATA	1	1	1	0	0	At3g10260.2:d:+754:secondary,At3g10260.3:d:+1109:secondary,At3g10260.1:d:+853:secondary
GAGGCTAGGC	0	0	2	0	1	At3g10070.1:d:+1741:secondary
GAAAACTTCA	0	0	3	0	0	At3g09970.1:d:+842:secondary
TCTTTGAAGA	0	1	1	0	1	At3g09720.1:d:+1650:secondary,At3g26930.1:v:+1083:secondary
GGTAAGACAC	0	2	1	0	0	At3g09680.1:d:+84:secondary,At5g02960.1:d:+93:secondary
TCTTGTATGT	3	0	0	0	0	At3g09580.1:d:+1509:primary
AACCACAACA	0	0	1	2	0	At3g09540.1:d:+725:primary
TCATTCAGAT	0	1	0	1	1	At3g09470.1:d:+1582:secondary
ACTTTGATAA	2	0	1	0	0	At3g09200.1:d:+1270:primary
TTACCAGAGA	2	0	0	0	1	At3g09180.1:d:-1538:secondary
TCTGGGTTGG	1	1	0	1	0	At3g09100.2:d:+2384:primary
GTGAGGGTTT	1	1	1	0	0	At3g08680.1:d:-2447:secondary,At3g08680.2:d:-2522:secondary
TAAGCTTTCC	0	0	1	1	1	At3g08610.1:X:--241:quaternary
ATTCGCTTTA	0	2	0	0	1	At3g08610.1:d:+233:secondary
GTGGGAGTGG	1	1	1	0	0	At3g08040.1:d:+1498:primary
CATCTCGGAA	0	3	0	0	0	At3g07980.1:d:+3696:primary
CAACCGAATT	0	2	1	0	0	At3g07780.1:d:+1247:secondary,At5g48160.1:d:+1254:secondary
CCTAAGATTT	1	1	1	0	0	At3g07470.1:X:-162:quaternary,At3g07460.2:X:-172:quaternary
TCTCGTTGCT	1	1	1	0	0	At3g07280.1:p:+818:secondary
TCACAGCTCT	0	2	1	0	0	At3g07190.1:d:+554:primary

TTTACGATTA	1	0	0	0	2	At3g07180.1:d:+2038:primary,At3g07180.2:d:+1603:primary
CGCCTCCTCC	0	0	0	3	0	At3g07110.1:d:+840:primary
CTTGATTGCT	1	1	0	0	1	At3g07020.1:d:+2093:primary,At3g07020.2:d:+2419:primary
ATCAAAAGCA	0	1	1	1	0	At3g06880.1:X:-836:quaternary
GAGCAGCCGG	0	1	2	0	0	At3g06810.1:d:+2478:primary
GGGAGTGAAG	1	2	0	0	0	At3g06540.1:d:+1729:primary
TACGTATGTA	0	2	0	0	1	At3g06440.1:d:+2006:primary
TGCTACAGCA	3	0	0	0	0	At3g06420.1:d:+450:primary
GCACAATGTT	0	1	0	1	1	At3g06370.1:d:+1065:secondary
TAATATTGTC	0	1	0	0	2	At3g06310.1:d:+581:primary
TCAGTTTTTG	2	0	1	0	0	At3g06290.1:d:+5206:primary
CCTTTAGCGA	1	2	0	0	0	At3g06130.1:d:+1632:primary
ATGAAAGGTG	2	0	1	0	0	At3g06070.1:X:-272:quaternary
GAGCTAGCTG	0	2	0	1	0	At3g06040.1:d:+324:primary,At3g06040.2:d:+736:primary
TACTCTTCCT	2	1	0	0	0	At3g06035.1:d:+646:primary
AGAACGGTGA	0	1	1	1	0	At3g05900.1:d:+323:secondary
GAAGAAGAGT	2	1	0	0	0	At3g05780.1:v:+1437:secondary,At1g69020.1:d:+1536:secondary,At4g33540.1:d:+965:secondary
TAGACCTAGA	0	1	1	1	0	At3g05760.1:d:+254:secondary
AGTTTACAAA	0	0	2	1	0	At3g05610.1:X:-1148:quaternary
CCCAAGACCG	2	1	0	0	0	At3g05230.1:d:+588:primary
GTCTTTTCAA	1	0	1	0	1	At3g05180.1:d:+809:primary
TTAATTTTTT	1	1	0	1	0	At3g05150.1:d:+1487:secondary
AGAAGTGGGC	0	1	1	1	0	At3g05090.2:d:+2802:secondary
TCTAGTTGGA	0	1	1	0	1	At3g04920.1:d:-608:secondary
ACCAACAGAC	1	1	1	0	0	At3g04920.1:d:+150:secondary
TTCGAATAGA	0	0	0	1	2	At3g04600.2:d:+1283:primary,At3g04600.1:d:+1283:primary
AACTAGAAAA	0	3	0	0	0	At3g04560.1:d:+1377:secondary
AGTATGATCC	1	1	0	0	1	At3g04450.1:v:+1678:primary
CACTGTACTT	0	2	1	0	0	At3g03960.1:d:+1696:primary
GTTGGCTGAT	0	1	0	2	0	At3g03880.1:d:+662:secondary
AATGTAGTAT	0	3	0	0	0	At3g03870.2:d:+1143:secondary,At1g71970.1:d:+887:secondary
TAAATGCCAA	2	1	0	0	0	At3g03820.1:v:+733:secondary
TTTATAGTAC	0	0	0	0	3	At3g03776.1:X:--2:quaternary
GCTATAATAG	1	1	1	0	0	At3g03210.1:d:+1490:primary
CAAGTGCAGT	2	0	1	0	0	At3g03150.1:d:+267:secondary
TTGTAACATT	2	1	0	0	0	At3g03070.1:d:+597:secondary
GCGACTTCAG	1	1	0	1	0	At3g02570.1:d:+1015:primary,At5g36810.1:v:+354:primary
GCCTAGAAAG	0	2	1	0	0	At3g02555.1:d:+579:primary
CCGGTCTTGT	0	0	1	0	2	At3g02480.1:d:-258:primary
TTGAAAAGTC	0	2	0	0	1	At3g02260.1:d:+9176:secondary
AAAGCTCTTA	2	0	0	0	1	At3g02200.2:d:+511:primary,At4g30310.2:d:+1633:primary,At4g30310.3:d:+1438:primary,At4g30310.1:d:+1598:primary,At3g02200.1:d:+511:primary
TGTTTTGAAA	0	0	1	0	2	At3g02150.1:X:+304:quaternary
TACATTTTAT	1	1	0	1	0	At3g02140.1:d:+1173:primary
TATCTAATGT	2	1	0	0	0	At3g02120.1:d:+437:primary
ACTTCGTCAA	0	0	1	0	2	At3g02080.1:d:+128:secondary
TGGAGGAGAT	1	1	1	0	0	At3g02040.1:d:+1037:secondary
TGTAATATAT	2	1	0	0	0	At3g02030.1:d:+1764:secondary
TATATTGTCA	0	0	2	0	1	At3g01950.1:X:+396:quaternary
ATGGACAAGG	1	1	1	0	0	At3g01850.2:d:+522:primary,At1g63290.1:d:+527:primary
GCAGTCAACG	1	1	1	0	0	At3g01690.1:d:-1200:secondary
TTCGATTGAA	0	1	1	1	0	At3g01680.1:d:+2438:primary
TCATTCGATA	1	2	0	0	0	At3g01460.1:d:+5914:secondary
AAATTTTGGG	0	0	1	1	1	At3g01460.1:d:+2345:secondary,At5g47060.1:d:+801:secondary
ACAATATTGT	1	0	2	0	0	At3g01370.1:i:+2748:tertiary
ATTAAAGGTA	3	0	0	0	0	At3g01360.1:i:+956:tertiary
TAGTGTGTGT	1	1	0	0	1	At3g01320.1:d:+4405:primary
TAATCATATC	0	3	0	0	0	At3g01310.1:X:-425:quaternary
TTTCTTCGTC	1	0	1	0	1	At3g01300.1:d:+1866:primary
GAATTCAAAC	0	1	2	0	0	At3g01160.1:d:+972:primary
TAGCAAACCT	1	2	0	0	0	At3g01140.1:v:+1567:primary

TGATGATGAG	0	0	3	0	0	At3g01040.1:i:+1105:tertiary
ATCACAACAG	1	0	1	0	1	At3g01040.1:d:+2308:primary
AAATATCTAT	2	0	0	1	0	At2g47980.1:d:+3596:primary
TTTCAGAAGA	0	3	0	0	0	At2g47860.1:d:+1876:primary,At2g47860.2:d:+2039:primary,At3g05160.1:d:+897:primary
TTTTAGATTC	0	0	0	1	2	At2g47800.1:d:+4639:primary
ATATACTTGA	0	0	0	0	3	At2g47770.1:d:+974:primary
GGAGAGAGAT	1	1	0	0	1	At2g47700.1:d:+1232:primary
GTGAATTCAC	0	0	1	1	1	At2g47650.1:X:-73:quaternary
TAGCTGTGAC	0	2	1	0	0	At2g47510.1:d:+1243:primary
GCTTCTTATT	0	2	0	1	0	At2g47500.1:d:+3220:primary
ACTCGCCGCC	1	0	1	1	0	At2g47400.1:d:-311:secondary
ATCCGACCAA	0	3	0	0	0	At2g47270.1:d:+196:primary
TTGATTTTTA	0	2	0	1	0	At2g47220.1:i:+1122:tertiary,At1g19650.1:i:+1215:tertiary
CTAATAACTT	0	2	1	0	0	At2g46910.1:i:+1289:tertiary
TTCTGGTGGA	0	1	0	1	1	At2g46900.1:d:+2133:primary
GGGATGTTAC	0	1	1	1	0	At2g46800.1:d:+791:secondary,At2g46800.2:d:+911:secondary
TCAAAGTCCA	0	3	0	0	0	At2g46640.1:v:+945:secondary
TCAGTGATTA	1	1	0	0	1	At2g46630.1:p:+1148:primary
GAAGAACAAG	0	0	3	0	0	At2g46535.1:d:+573:primary,At3g61840.1:d:+690:primary
GTGATGGGAA	0	1	2	0	0	At2g46340.1:d:+1055:secondary
TTTGGTTTTT	0	1	0	1	1	At2g46000.1:d:+818:primary
TTGAAGCTAG	1	1	0	1	0	At2g45860.1:d:+325:primary
GTCCACTCAA	0	0	1	2	0	At2g45680.1:d:+1543:primary
ATTATAGTTT	1	2	0	0	0	At2g45590.1:d:+2320:primary
TTACAAAACG	1	0	1	1	0	At2g45400.1:X:-219:quaternary
AATTCACTGG	0	3	0	0	0	At2g45380.1:d:+1334:secondary
CGAAGATTGA	1	2	0	0	0	At2g45330.1:d:+855:primary
CACCTTTGGT	1	2	0	0	0	At2g45140.1:d:+1062:secondary
ATCTCTACAA	3	0	0	0	0	At2g44980.2:v:+1731:secondary,At2g44980.1:v:+1674:secondary
GAAAGCCCCA	0	0	2	1	0	At2g44830.1:X:+1093:quaternary
AAATGTTCTT	0	1	0	2	0	At2g44820.2:d:+851:secondary,At3g18730.1:d:+354:secondary,At2g44820.1:d:+738:secondary
GAGTTTAACA	0	2	1	0	0	At2g44770.1:d:+1000:primary,At2g33390.1:d:+466:primary
AATATAATAG	1	0	0	0	2	At2g44760.1:d:+1682:primary
ACGATGCGTC	1	2	0	0	0	At2g44730.1:d:+95:primary
AAACCGCAGA	2	1	0	0	0	At2g44680.1:d:+975:primary,At2g44680.2:d:+972:primary,At2g41050.1:d:+1328:primary
ATCGAGCTCC	1	0	1	1	0	At2g44640.1:d:+1373:secondary
GTGAAGAAGA	2	1	0	0	0	At2g44510.1:d:+929:primary,At5g60400.3:d:+342:primary,At5g60400.1:d:+142:primary,At1g35540.1:v:+2097:primary,At5g60400.2:d:+158:primary
TTTTTACGAT	0	3	0	0	0	At2g44500.2:d:+2168:primary,At2g44500.1:d:+2040:primary
TTATAAGATC	0	1	1	1	0	At2g44430.1:d:-94:primary
AACAAATGTT	1	0	1	0	1	At2g44360.1:d:+730:secondary
TTCTGACTTC	0	2	1	0	0	At2g44350.1:d:-315:primary,At2g44350.2:d:-318:primary
ATAAGCACTT	0	2	0	1	0	At2g44200.1:d:+1021:secondary
TTCCTTATCT	1	2	0	0	0	At2g43920.1:d:+895:primary
GGACCAAGGG	0	0	1	0	2	At2g43910.1:d:+215:secondary
GACAAACTCA	0	1	1	0	1	At2g43850.1:d:+1588:primary,At2g43850.2:d:+1588:primary
TTCGAGGGCA	0	1	1	1	0	At2g43760.1:d:+356:primary
AAAACATAAC	3	0	0	0	0	At2g43560.1:d:+921:primary
TACGACAAAA	0	0	2	0	1	At2g43540.1:X:+-118:quaternary
ACTAAAACTT	0	1	1	0	1	At2g43510.1:d:-397:primary
TTTGACAACT	0	0	2	0	1	At2g43490.1:v:+2768:primary
CTGAAGATTT	1	0	1	1	0	At2g43400.1:X:+368:quaternary
GAACAATACT	0	1	1	0	1	At2g43200.1:v:+2152:secondary
ATCAGTATTT	2	1	0	0	0	At2g42890.1:d:+2979:primary,At2g42890.2:d:+2857:primary
GAACTCCTCC	0	1	1	1	0	At2g42840.2:d:+194:secondary,At2g17050.1:v:+2599:secondary,At2g42840.1:d:+194:secondary
AGACCACAAC	0	2	1	0	0	At2g42790.1:d:+1436:primary
TGCGTTATAT	0	1	0	1	1	At2g42400.1:d:+1411:primary
AAGTGTGATG	0	1	2	0	0	At2g42170.1:d:+954:primary
TCTATGTGTA	1	0	1	0	1	At2g42040.1:d:+1154:primary
CTAAGAGTCC	0	2	0	1	0	At2g41960.1:d:+1576:secondary

CACAGATAAA	0	0	0	3	0	At2g41945.1:d:+533:secondary
GCTGCAGAGG	2	0	0	1	0	At2g41905.1:d:+141:secondary,At2g31210.1:d:+1258:secondary
AGGGAGAAGA	0	2	0	0	1	At2g41720.2:d:+606:secondary,At1g74250.1:v:+1198:secondary,At2g41720.1:d:+936:secondary
CAACAGAACC	1	0	0	0	2	At2g41710.2:d:+889:secondary,At2g41710.1:d:+874:secondary
GTATTAGATC	1	2	0	0	0	At2g41430.4:d:+182:primary
CGGCAAAAGT	0	0	0	3	0	At2g41410.1:X:-762:quaternary
AAGCTTATTA	0	1	1	0	1	At2g41250.1:X:-213:quaternary
CTTGTACTTT	0	1	0	0	2	At2g41220.1:d:+5093:primary
GACACACAAA	0	0	0	1	2	At2g41060.1:i:+2072:tertiary
AATAATCTTG	0	1	1	0	1	At2g41010.1:d:+858:primary
GGAAAGAAAT	1	1	1	0	0	At2g40980.1:d:+1829:primary
TGTGTTATCA	2	1	0	0	0	At2g40860.1:d:+2061:primary
ATAAATGCAA	1	0	1	1	0	At2g40770.1:d:+5184:primary
TTTTTCTTGA	0	1	1	1	0	At2g40670.1:v:+926:secondary
TCATAAGATG	0	1	1	1	0	At2g40660.1:d:+452:secondary
CTTTCCGCCA	0	2	0	1	0	At2g40640.1:d:+801:primary,At2g40640.2:d:+801:primary
AACGACCAAA	1	0	2	0	0	At2g40610.1:d:+731:secondary
TTTGCTTTAT	0	0	1	1	1	At2g40550.1:i:+1060:tertiary
CCGACTTCTA	0	0	1	2	0	At2g40540.1:d:-2582:secondary
TCGTTAGAGT	0	0	1	0	2	At2g40510.1:d:+319:secondary
GGACTTTTCG	2	0	1	0	0	At2g40460.1:d:+1843:secondary
TAGTTAGGTA	0	1	0	2	0	At2g40340.1:d:+1292:primary
GAAACAATAA	1	0	1	1	0	At2g40280.1:d:+1746:primary
TAAATGTAAA	0	3	0	0	0	At2g40110.1:d:+656:primary,At2g40110.2:d:+727:primary
TATTGAATCT	1	0	1	1	0	At2g39970.1:X:-304:quaternary
GAACAGTGAA	1	0	1	0	1	At2g39795.1:d:+784:primary
GTACATTAAC	1	0	0	0	2	At2g39670.1:X:+701:quaternary
CCTCTGCAAG	0	1	0	2	0	At2g39450.1:d:+1134:primary
CAGTTTAGTG	1	0	0	1	1	At2g39360.1:d:+2477:primary
ACGGCTAAGG	0	0	3	0	0	At2g39170.1:d:+605:secondary
TCATTGATTG	2	1	0	0	0	At2g39130.1:d:+1633:primary
TTGTTGACTG	1	0	0	1	1	At2g39020.1:d:+603:primary
TATCGCCTAC	0	1	1	0	1	At2g38960.1:v:-50:primary,At2g38960.2:v:-50:primary
TTCTAAGATT	0	0	0	1	2	At2g38860.1:d:+1193:secondary,At2g38860.2:d:+1216:secondary
AAGAGTTACG	0	2	0	1	0	At2g38700.1:d:+1193:primary
TGTATTGCTA	2	0	1	0	0	At2g38680.1:v:+1516:primary
GTATCGGGAA	0	3	0	0	0	At2g38550.1:d:+837:primary
CCAGGAAGGG	0	0	0	1	2	At2g38465.1:d:+177:primary
ATGAGTGCGC	0	1	1	1	0	At2g38440.1:d:+4468:primary
CTGGAGCCGT	0	0	2	0	1	At2g38170.1:d:+1114:secondary,At2g38170.2:d:+1114:secondary,At2g38170.3:d:+1114:secondary
CATTGATGCA	0	0	2	1	0	At2g38020.1:d:+2463:primary
TCTTCTCCTC	1	1	1	0	0	At2g37330.1:d:+858:secondary
AACGGTTGAT	0	2	1	0	0	At2g37080.1:d:+1377:secondary
AGGTGATCAT	0	2	0	0	1	At2g37035.1:d:+1646:primary
ACAAACATAT	0	1	2	0	0	At2g36960.1:X:+-103:quaternary
CAGTTACTGG	0	0	2	1	0	At2g36930.1:X:+217:quaternary
TCACTTATGC	0	1	1	1	0	At2g36670.2:X:-8:quaternary
AATGATGAAA	1	0	2	0	0	At2g36670.2:d:+1789:primary,At2g36670.1:d:+1804:primary
AGAGTTGTAG	0	0	0	3	0	At2g36500.1:d:+997:secondary
GAGGGTAGGG	2	1	0	0	0	At2g36420.1:v:+1487:secondary
TCTAAATAGG	1	1	1	0	0	At2g36400.1:d:+1586:primary
AACTGTTATT	0	0	1	1	1	At2g36380.1:X:-395:quaternary
TATCTCTTTG	1	1	0	0	1	At2g36360.1:d:+1789:primary
TCTTAGAATC	0	2	0	1	0	At2g36305.1:v:+1114:primary
CTTGCTGGTA	0	3	0	0	0	At2g36300.1:d:+633:primary
AACTTAGCAC	0	3	0	0	0	At2g36050.1:d:+626:primary
AATCCAACTA	1	0	1	1	0	At2g35940.1:d:+404:secondary,At2g35940.2:d:+276:secondary
TTTACAGAAT	1	0	1	0	1	At2g35870.1:d:+248:primary,At2g04620.1:v:+3080:primary
AATAAATTGA	1	0	1	0	1	At2g35820.1:d:+638:secondary,At2g31460.1:v:+402:secondary

TGACCATTTT	1	0	0	1	1	At2g35700.1:v:+977:secondary
GGCTTGTTCG	0	2	1	0	0	At2g35650.1:d:+1524:primary
ATCAATGGTC	0	1	1	1	0	At2g35360.1:d:+912:primary
GCTTTTCATA	1	1	0	0	1	At2g35320.1:d:+1091:primary
CGTTGACTCT	1	1	0	1	0	At2g35240.1:d:+736:primary
TTCCGTTGCG	1	2	0	0	0	At2g35060.1:d:+2003:primary
AAACCAGAGT	1	1	0	0	1	At2g35035.1:d:+730:primary
TGAAATCTCC	0	2	0	1	0	At2g35020.1:d:+1586:primary
GAACAGCTCT	2	0	1	0	0	At2g35010.1:d:+630:primary
TTGCTTCAAA	1	2	0	0	0	At2g34900.2:d:+1288:primary
ATTATTACAA	0	1	1	0	1	At2g34900.1:d:+493:secondary,At2g34900.2:d:+456:secondary,At4g21240.1:v:+718:secondary
AACCTCTAAT	0	0	0	0	3	At2g34830.1:X:-637:quaternary,At2g41600.1:X:-504:quaternary
ATTCCGGGTA	0	1	1	0	1	At2g34780.1:v:+4119:secondary
GATGGGCTAT	0	3	0	0	0	At2g34690.1:d:+505:primary
AAACCTTAAA	1	1	0	0	1	At2g34680.1:d:+4986:primary
ATGTGTTGGT	0	3	0	0	0	At2g34560.2:d:+985:primary,At2g34560.1:d:+993:primary
TGTAGTTATA	1	0	1	0	1	At2g34170.1:d:+1781:primary
AAACAATAAT	1	1	0	1	0	At2g33850.1:d:+843:primary
AAAAATCCAA	1	0	2	0	0	At2g33840.1:i:+1874:tertiary
TTTTTGTTGT	0	1	0	1	1	At2g33680.1:v:+2371:secondary
CCCTGGAAGT	1	2	0	0	0	At2g33620.3:d:+1181:secondary,At2g33620.2:d:+1587:secondary,At2g33620.1:d:+1298:secondary
TATAGACACT	2	0	0	0	1	At2g33585.1:v:+1006:secondary
TGAGTGGCAT	1	1	0	1	0	At2g33510.1:d:+717:primary
AATTGGGTTT	0	2	1	0	0	At2g33480.1:d:+519:primary
CTAAAAAGAG	1	1	0	0	1	At2g33430.1:X:+472:quaternary
TGAGAGGTAT	0	1	0	0	2	At2g33430.1:d:+435:secondary
GAGAATGTTC	0	1	1	1	0	At2g33310.2:d:+842:primary,At2g33310.1:d:+957:primary
TGACTATCAT	0	1	0	0	2	At2g32980.1:d:+1163:primary
TAAGTAAACA	0	0	0	0	3	At2g32970.1:i:+3518:tertiary
GAAATTCTCT	1	0	0	1	1	At2g32760.1:d:+1586:primary
TAGTAAAACT	0	0	3	0	0	At2g32520.1:d:+1069:primary
TAAAACATTT	0	0	1	1	1	At2g32500.1:d:+891:primary
AAAAAAGGAA	0	2	0	1	0	At2g32490.1:i:+868:tertiary
GTGGATGCGA	0	3	0	0	0	At2g32410.1:d:+320:secondary,At5g52640.1:d:+1603:secondary
AAGATGTGTA	1	0	0	2	0	At2g32295.1:d:+1417:primary
GGGAAACTGA	0	3	0	0	0	At2g32240.1:d:+1154:secondary,At3g22520.1:d:+367:secondary
CTTCTGAAGA	1	2	0	0	0	At2g32210.1:d:+301:primary
CAAATTATTT	1	1	0	0	1	At2g32190.1:d:+324:primary
GAGGTCTGAA	0	0	2	1	0	At2g32070.1:d:+784:primary
CATAGGATGG	0	0	0	2	1	At2g31820.1:v:+2205:secondary
GAAGGAGAGA	2	0	0	1	0	At2g31790.1:d:+1307:primary
GATGCTTGCC	0	2	0	1	0	At2g31740.1:d:+1762:primary
TGTCTGGTTT	2	1	0	0	0	At2g31510.1:d:+1983:primary
GGACAACAAC	0	0	2	1	0	At2g31350.2:i:-1757:tertiary,At2g31350.1:i:-1757:tertiary
CCTTAGGAAT	0	1	2	0	0	At2g31260.1:X:-132:quaternary
TTGATCTTGT	1	1	0	0	1	At2g31230.1:d:+797:primary
ATTTGATTCA	1	0	2	0	0	At2g30910.1:d:+1535:primary,At2g30910.2:d:+1535:primary
TGTTTTTATT	0	2	0	0	1	At2g30695.2:i:+1519:tertiary,At2g30695.1:i:+1531:tertiary
TTTTTGAGAA	1	1	1	0	0	At2g30695.1:d:+405:primary,At2g30695.2:d:+557:primary
TATGTTGTTC	1	1	1	0	0	At2g30590.1:d:+1599:primary
AAATCCAAAA	1	1	1	0	0	At2g30505.1:d:+458:secondary,At1g34190.1:d:+1637:secondary
TTGATATAGT	2	0	0	0	1	At2g30500.1:d:+1684:primary
GAACTGGCTG	0	2	0	1	0	At2g30470.1:d:+2437:primary
TGGAATAAGG	0	0	1	1	1	At2g30390.1:d:+1714:secondary
AACTACTCTT	1	2	0	0	0	At2g30330.1:d:+666:secondary
AATATTGAGA	0	0	0	0	3	At2g30270.1:d:+748:primary
ATCAATCAGA	0	2	1	0	0	At2g30250.1:d:+876:primary
TGGATAAGAT	0	3	0	0	0	At2g30230.1:v:+1078:primary
TGTATCAAAT	0	1	0	2	0	At2g29910.2:d:+1681:primary,At2g29910.1:d:+1603:primary
TTCTTCATTG	0	1	0	2	0	At2g29700.1:d:+467:primary
AGCTTTTTAT	0	0	0	1	2	At2g29680.1:d:+796:secondary,At2g29680.2:d:+828:secondary

AAGACACCAT	1	2	0	0	0	At2g29670.1:d:+1785:secondary
GCTTGTAGTC	1	1	0	1	0	At2g29650.1:d:+1307:primary,At2g29650.2:d:+1344:primary,At2g29650.3:d:+1166:primary
TGACTGTGGA	0	1	0	1	1	At2g29510.1:d:+2386:primary
TAAACCTATA	1	1	0	1	0	At2g29170.1:v:+917:secondary,At1g46120.1:p:+438:secondary,At1g46120.1:p:+2491:secondary
GAAAACGTTG	0	1	1	1	0	At2g29170.1:v:+609:secondary
AATATTCAAA	0	0	3	0	0	At2g28990.1:v:+3044:secondary
GCTCGTTTAC	1	1	1	0	0	At2g28930.1:d:+1536:secondary
GAATCAGCAA	0	2	0	1	0	At2g28830.1:d:+1855:primary
TAACCAAGCC	1	1	0	1	0	At2g28820.1:v:+581:primary
GTAAAGGATT	0	1	1	1	0	At2g28740.1:X:+531:quaternary
TTGACAGAAG	1	1	1	0	0	At2g28605.1:X:-428:quaternary,Chr5:-5799615:quaternary
TGTCAGGGAA	0	0	1	1	1	At2g28600.1:d:+211:secondary
GTGTGGTTGG	0	2	0	1	0	At2g28410.1:d:+374:primary
TTAAGCATTG	1	0	1	0	1	At2g28320.1:d:+2827:primary
AGAGTGTTGG	0	1	0	1	1	At2g28080.1:d:+1157:primary
ATGATATCTT	0	0	0	0	3	At2g28056.1:d:+981:primary
TGAGTTTGGT	1	0	1	1	0	At2g27970.1:d:+423:secondary,At1g65370.1:d:+488:secondary
AAAAGGTTAT	0	1	0	0	2	At2g27760.1:d:+1506:primary
CAAACACACT	1	1	0	1	0	At2g27690.1:d:+1651:primary
AGTTGATGAT	0	2	0	0	1	At2g27680.1:X:-227:quaternary
ATTTCAATAT	1	0	0	1	1	At2g27590.1:d:+1300:primary
TTTTCGTGCA	1	0	0	1	1	At2g27420.1:d:+1229:primary
ACGTGGATCT	0	2	1	0	0	At2g27080.2:d:+847:primary,At2g27080.1:d:+862:primary
TAGATGATAA	0	0	1	1	1	At2g26975.1:X:+715:quaternary,Chr3:+156334:quaternary
GGGTAAGTTT	1	0	0	0	2	At2g26910.1:d:+2283:secondary
TATAATGTCT	1	2	0	0	0	At2g26890.1:i:+7932:tertiary
TTATGTTCTA	0	0	1	1	1	At2g26860.1:d:+1308:primary,At5g45020.1:d:+1159:primary,At2g26860.2:d:+1383:primary
TTAACAAGAG	0	2	1	0	0	At2g26780.1:d:+258:secondary
TTTCTATTAC	1	0	0	0	2	At2g26650.1:d:+758:secondary
GGTGGAAAGG	0	1	0	1	1	At2g26460.1:d:+1528:primary
CTTGGAAGGT	0	2	1	0	0	At2g26440.1:d:+483:primary
TGTCGTGGTT	0	2	0	1	0	At2g26360.1:X:-628:quaternary
ACTAAAAACC	1	0	1	0	1	At2g26260.1:d:+1828:primary
ATCTGATTTT	1	0	0	0	2	At2g26100.1:d:+1329:primary
CAGGTGTGAC	0	2	0	1	0	At2g25910.1:i:+826:tertiary
AGGGTTTTGT	0	2	0	1	0	At2g25850.1:d:+2373:primary
GAATTGAACA	0	1	0	2	0	At2g25830.1:d:+947:primary
CGCTGAAACA	0	1	2	0	0	At2g25740.1:d:+1673:primary
TCTACTTGTA	1	1	0	0	1	At2g25730.1:d:+7680:primary
TTTGTGATTA	1	0	2	0	0	At2g25680.1:v:+1843:secondary
ATTAGATTTA	0	0	2	0	1	At2g25605.1:d:+715:primary
TTTCTTACAT	2	0	1	0	0	At2g25510.1:d:+371:secondary,At5g42530.1:d:+280:secondary
GTCGTTGTTG	1	1	0	0	1	At2g25310.1:d:+583:secondary
GGATGATGAG	0	0	2	1	0	At2g24830.1:d:+691:secondary,At5g46880.1:v:+511:secondary
TATACATAAA	2	0	0	0	1	At2g24810.1:i:+57:tertiary,At1g19320.1:i:+103:tertiary
GTTTTTGCTC	0	0	1	1	1	At2g24750.1:p:+5733:secondary,At2g24720.1:v:+2235:secondary,At4g03100.1:d:+885:secondary,At2g24710.1:v:+2235:secondary
CTTAATTCTG	1	1	0	1	0	At2g24490.1:d:+1051:primary
GCTCGGACTT	0	0	2	0	1	At2g24430.2:X:-265:quaternary
CAGATCTCAG	1	2	0	0	0	At2g24280.1:d:+2702:primary
TAATGAGAAT	2	0	1	0	0	At2g24190.1:v:+1283:secondary,At5g13640.1:d:+1480:secondary
TTGTAAAGAT	3	0	0	0	0	At2g24020.1:i:+516:tertiary
TCTGCGTGTA	1	0	1	0	1	At2g23940.1:d:+715:primary,At5g46850.1:v:+1638:primary
CCAGAAGAAT	0	1	1	0	1	At2g23840.1:d:+808:primary
CGCGGTTCTT	1	0	0	2	0	At2g23670.1:X:-127:quaternary
ATTCTGTCTT	0	2	0	0	1	At2g23580.1:d:+884:primary,At3g48740.1:d:+1190:primary
AATGTAATGC	1	2	0	0	0	At2g23350.1:d:+1801:primary
AATGTTCACT	0	1	0	0	2	At2g23310.2:X:+402:quaternary
TCTCTGTGTT	1	1	1	0	0	At2g23310.2:d:+321:secondary,At2g23310.1:d:+321:secondary
TAAGGGCTAT	0	2	1	0	0	At2g23120.1:d:-154:primary
GCTGATTTCT	1	0	1	1	0	At2g23090.1:X:-164:quaternary

AATAAATTTG	1	2	0	0	0	At2g22830.1:X:-477:quaternary,At1g35450.1:X:-213:quaternary
AGCTTAAAAA	0	0	1	1	1	At2g22780.1:i:+657:tertiary
CATTAAACTA	0	0	1	2	0	At2g22610.1:X:+614:quaternary
TTCTTGATTT	0	1	0	1	1	At2g22320.1:v:+667:primary,At2g17530.1:d:+1439:primary
GGAGTTGTTG	1	2	0	0	0	At2g22270.1:d:-421:primary
TTATGGCTGC	0	1	1	0	1	At2g22010.1:d:+3999:secondary,At3g59810.1:d:+422:secondary
TTTCTCTAAA	1	1	0	0	1	At2g21920.1:i:+435:tertiary,At1g16900.1:i:+2542:tertiary
ATTTGCTTTG	1	1	1	0	0	At2g21860.1:d:+502:secondary,At1g09820.1:d:+1860:secondary,At1g26620.1:v:+3151:secondary
AGAAGGAGTT	1	2	0	0	0	At2g21800.1:d:+1288:primary
TCTTTGAAAA	0	0	2	0	1	At2g21540.1:i:+2865:tertiary,At4g39160.1:i:+527:tertiary
TGATAATGGC	1	1	0	1	0	At2g21540.1:d:+1776:primary
GCATTCTTGA	0	0	1	2	0	At2g21520.1:d:+1444:primary
GCCGCCAAGA	1	0	1	0	1	At2g21385.1:d:+596:secondary
TGTTCGTGCA	1	1	1	0	0	At2g21240.2:d:+720:primary,At2g21240.1:d:+810:primary
CGCTTCTGAA	0	2	0	1	0	At2g21080.1:d:+1004:primary
CGTTTTTGTT	0	0	1	2	0	At2g20940.1:d:+508:primary
ATGAAGATGA	0	1	1	1	0	At2g20725.1:d:+1108:primary,At4g23380.1:v:+1498:primary
ACTCACCCTA	1	0	1	1	0	At2g20670.1:X:-495:quaternary
ACGTGTACAG	1	1	1	0	0	At2g20610.2:X:-274:quaternary
TTGCAGACCA	1	0	1	0	1	At2g20560.1:d:+1236:primary
TCTCAAAGGA	0	2	0	1	0	At2g20190.1:d:+4095:primary
CCAATGATCC	0	2	1	0	0	At2g19940.2:d:+992:primary,At2g19940.1:d:+992:primary
GGGTTTTGTT	0	3	0	0	0	At2g19880.1:d:+1244:primary
ACCTGATCTA	0	0	0	0	3	At2g19870.1:d:+2035:primary
ATTGGACGGA	0	0	3	0	0	At2g19810.1:d:+393:secondary,At4g29190.1:d:+360:secondary,At5g53810.1:d:+1100:secondary,At2g25900.1:d:+422:secondary
AAAACATAAA	1	0	1	1	0	At2g19780.1:d:+1244:primary
AAAACAACAA	1	0	1	0	1	At2g19755.1:p:+175:primary
TCATTTGTCT	1	0	1	1	0	At2g19720.1:d:+513:secondary
GGAATTCGTA	2	0	0	1	0	At2g19680.1:d:+134:primary
TTGATACATA	3	0	0	0	0	At2g19600.1:d:+1425:secondary
ATGTAAAAAA	0	1	2	0	0	At2g19220.1:v:+93:secondary
ATCTTCACTC	2	0	1	0	0	At2g18990.1:d:+741:primary
TTTTGTTACC	1	1	0	1	0	At2g18840.1:d:+1056:primary
ATGCTGATGA	1	0	1	1	0	At2g18790.1:d:+3697:primary
AGGATTGTGT	2	1	0	0	0	At2g18770.1:d:+898:primary
GATCGGTTTT	0	1	0	2	0	At2g18670.1:d:+1037:primary
TTTCAAGACA	1	0	0	0	2	At2g18250.1:i:+1144:tertiary
CTGGTGGAAT	0	1	0	1	1	At2g18240.2:d:+944:primary,At2g18240.1:d:+947:primary
GAGGAACCAG	1	0	0	2	0	At2g18170.1:d:+1193:primary
ATTTTGTCTT	1	0	1	1	0	At2g18160.1:d:+1200:primary
AGAAACCTGT	1	1	1	0	0	At2g18100.1:d:+694:secondary,At2g41700.1:d:+5597:secondary
GTAGAAGAAG	1	2	0	0	0	At2g18030.1:d:+718:primary,At2g18030.2:d:+863:primary
ATCGGTGGAG	1	1	1	0	0	At2g17980.1:d:+1868:primary
GCATCCTTGG	0	0	0	3	0	At2g17930.1:d:+11373:primary,At4g36080.1:d:+11505:primary
AATCACTTTC	0	0	1	1	1	At2g17900.1:d:+1510:primary
TATTTGCTCA	0	2	0	1	0	At2g17820.1:X:-376:quaternary
GTCTGTAACT	1	1	0	0	1	At2g17790.1:d:+2664:secondary
CTTGATTCTT	1	0	0	1	1	At2g17630.1:d:+1317:primary
GCTGAAAGAG	0	0	3	0	0	At2g17525.1:d:+1944:primary
TGATTGTTTG	0	0	1	1	1	At2g17380.1:d:+717:primary
TTTGGAACAG	0	1	1	0	1	At2g17265.1:d:+782:primary
GCGTCGCTTT	0	3	0	0	0	At2g17240.1:d:+50:primary
GCTGGACAAA	0	2	1	0	0	At2g17140.1:d:+2661:primary,At5g24920.1:v:+567:primary
GTATGCACTT	1	0	2	0	0	At2g17033.2:d:+1262:secondary,At2g17033.1:d:+1314:secondary,AtCg00490:d:+923:secondary
TCATTAATCA	1	1	0	0	1	At2g17030.1:d:-604:secondary
TGTTTCGGTT	2	0	0	1	0	At2g16900.1:d:+994:primary,At5g07160.1:v:+946:primary
GAAGAATTTA	0	0	0	1	2	At2g16890.2:d:+1420:primary
TTGGAAACAA	2	0	0	1	0	At2g16860.1:d:+1036:primary
GCAAAGACTG	0	0	1	1	1	At2g15790.1:d:+1413:primary
GCCAATGGGG	0	1	2	0	0	At2g15480.1:d:+1075:primary

AATGTGCTTG	0	3	0	0	0	At2g14900.1:d:+305:primary
CAGGCAAACA	0	3	0	0	0	At2g14170.1:d:+1501:secondary
AGACAATGTA	0	0	2	1	0	At2g14120.2:d:+2718:primary,At2g14120.1:d:+2718:primary
TATTTGATTT	0	0	1	1	1	At2g14030.1:p:+2307:secondary
TTTCAATAAT	0	0	1	2	0	At2g13890.1:p:+2504:secondary,At5g49290.1:v:+2222:secondary
AAGAAGATCT	1	2	0	0	0	At2g13370.1:d:+5115:primary
CTCGTTTGGG	1	0	1	0	1	At2g13360.2:d:+927:secondary,At2g13360.1:d:+949:secondary
ATTCCTTCAA	2	0	0	1	0	At2g13350.1:d:+1373:secondary
AGACTCAAAA	1	1	1	0	0	At2g13175.1:X:-538:quaternary,At5g10070.1:X:--15:quaternary,At2g10070.2:X:--50:quaternary
CTTATTGTTT	0	2	0	1	0	At2g13100.1:d:+1378:primary
TAGACAAGGA	1	2	0	0	0	At2g12720.1:p:+924:secondary,At1g63960.1:v:+791:secondary,At1g10522.1:d:+746:secondary
GACAAATGTA	1	1	0	1	0	At2g11320.1:p:+6671:primary,At1g22860.1:d:+3138:primary
GCAATAAACT	0	1	1	1	0	At2g11230.1:p:+4066:primary
GTCGAAGCCA	0	1	1	1	0	At2g11190.1:p:+1970:primary
ATGACAAGAA	0	1	0	2	0	At2g10465.1:v:+364:secondary,At5g16600.1:d:+446:secondary,At5g35260.1:v:+478:secondary,At2g05880.1:v:+478:secondary,At5g53190.1:v:+1048:secondary,At4g07440.1:v:+478:secondary
CTTTTATTAA	0	1	2	0	0	At2g07790.1:p:+259:secondary,At4g02600.1:d:+753:secondary,At5g59340.1:v:+34:secondary,At1g33380.1:p:+158:secondary
CTCAGTAGTT	2	1	0	0	0	At2g07777.1:v:+381:secondary
CCTCTTTATA	0	2	1	0	0	At2g07640.1:v:+648:primary,At3g12800.1:d:+707:primary
AATTTGATGA	2	1	0	0	0	At2g07210.1:p:+1415:secondary,At4g03680.1:v:+769:secondary
TAAATACTAA	3	0	0	0	0	At2g07140.1:v:+1790:primary,At1g56460.1:d:+1701:primary
TTATCACGAA	1	0	1	1	0	At2g07100.1:p:+2314:secondary
CTTTACTCTT	1	2	0	0	0	At2g07050.1:d:+2517:secondary
GGCCAACAAA	0	3	0	0	0	At2g06210.2:d:+1000:secondary,At2g06210.1:d:+920:secondary
GGAGCCTAAG	0	2	0	1	0	At2g05715.1:v:+776:secondary
GTGTTAATTT	2	0	0	1	0	At2g05630.1:d:+613:primary
GATCCTCTTG	0	0	0	1	2	At2g05610.1:p:+442:secondary
GCGGTGGGTG	1	1	1	0	0	At2g04940.1:d:+1299:primary
TGTGTTGAAA	1	0	2	0	0	At2g04900.1:i:+1006:tertiary
TGATTTGGAA	0	0	1	1	1	At2g04840.1:v:+1635:secondary
CGCTGGTGTC	1	1	1	0	0	At2g04750.1:v:+2513:secondary
CACCATTTGC	0	0	1	2	0	At2g04740.1:X:+483:quaternary
CCAAGTCGTA	1	0	2	0	0	At2g04690.1:i:+1529:tertiary
TGTTAGACTA	0	0	0	1	2	At2g04660.1:d:+2807:primary
CGTTTTTCAT	0	0	2	1	0	At2g04050.1:v:+835:secondary
GCAGGAGAAA	0	2	0	1	0	At2g04030.1:d:+950:secondary,At2g04030.2:d:+950:secondary
ATCATTTTCT	3	0	0	0	0	At2g04010.1:p:+890:secondary,At3g33160.1:p:+892:secondary,At3g32060.1:p:+694:secondary,At1g68790.1:d:+3420:secondary
ACAAGGCTCC	0	1	2	0	0	At2g03890.2:d:+1835:secondary,At2g03890.1:d:+2195:secondary
ATGATCCTTT	1	0	2	0	0	At2g03870.1:d:+253:primary,At2g03870.2:d:+338:primary,At1g16340.1:d:+876:primary
AAGTACGAAG	0	2	0	1	0	At2g03750.1:d:+783:secondary
CACACACATA	1	1	0	0	1	At2g03290.1:d:+581:primary
GGCATCAACA	1	0	2	0	0	At2g03240.1:d:+2678:primary
AAGGAGGAGA	0	0	3	0	0	At2g03230.1:v:+424:secondary,At5g52650.1:d:+419:secondary
GTGGCGGATT	1	0	2	0	0	At2g03200.1:d:+1258:secondary,At1g05135.1:p:+917:secondary
CTGTGAGAAT	0	0	1	1	1	At2g03120.1:d:-232:primary
GAAGAGGAAC	1	0	2	0	0	At2g02910.1:d:+1552:primary
GACTACCTGA	1	1	1	0	0	At2g02780.1:d:+2335:primary
TTCTTCAGCA	1	1	0	1	0	At2g02370.1:d:+1251:secondary,At3g17770.1:d:+737:secondary
CTCAGAGACT	1	0	0	2	0	At2g02110.1:p:+2862:secondary,At5g18633.1:p:+3657:secondary,At5g25205.1:p:+3885:secondary
AGAATGGGTA	0	0	1	1	1	At2g02100.1:d:-293:secondary
CGTGAGTGCA	0	0	1	1	1	At2g02100.1:d:+179:secondary
GCTGCTTCTT	0	0	1	1	1	At2g02070.1:d:+40:secondary
GAGACATATC	0	0	2	1	0	At2g01890.1:d:+1200:primary
ATCACTTCTT	0	0	1	2	0	At2g01690.1:X:-509:quaternary
AAAGAAGAAA	2	0	0	0	1	At2g01320.4:d:+1224:secondary,At5g17270.1:v:+3050:secondary,At5g16570.1:d:+1114:secondary,At2g01320.2:d:+1224:secondary,At2g01320.3:d:+1224:secondary,At1g17830.1:d:+1047:secondary,At2g21370.1:d:+283:secondary,At2g01320.1:d:+1224:secondary,At1g42350.1:p:+3083:secondary
GGCCTCGACG	0	0	0	3	0	At2g01170.1:X:+515:quaternary

GCAGTGATGC	1	1	0	0	1	At2g01150.1:d:+437:secondary,At3g01930.2:d:+755:secondary,At3g01930.1:d:+517:secondary
CTGAAAAGGA	0	1	1	0	1	At2g01140.1:d:+1003:secondary
CACTCTCCCT	1	0	1	0	1	At2g01120.1:X:-178:quaternary
TGTTTCTTAA	2	1	0	0	0	At1g81020.1:p:+678:primary
AGATGTTGTG	1	1	1	0	0	At1g80970.1:d:+1225:primary,At1g80710.1:d:+1654:primary
GAGAAACAGG	1	2	0	0	0	At1g80930.1:d:+713:secondary
TCTGAGATTG	0	0	2	0	1	At1g80810.1:v:+2772:secondary,At1g43860.1:d:+1144:secondary,At2g17900.1:d:+450:secondary,At2g21460.1:p:+5099:secondary
TTTTTTGAAC	0	2	0	0	1	At1g80610.1:d:+794:primary
ACATATTTCA	1	1	0	1	0	At1g80480.1:d:+1208:secondary
CTGGTCTGCG	1	1	1	0	0	At1g80310.1:d:+1552:primary
CTTTTCTTGT	0	0	3	0	0	At1g80260.1:d:+2926:primary
ACATTATTGT	0	1	0	0	2	At1g79780.1:i:+411:tertiary
AGGTTGAACT	1	0	0	2	0	At1g79730.1:d:+1531:primary
GTTCCTTCCT	0	1	1	1	0	At1g79730.1:d:+1363:secondary
TGACCGAAAT	0	1	1	1	0	At1g79650.1:d:+1192:secondary,At1g79650.3:d:+1115:secondary,At1g79650.2:d:+1184:secondary
TTAGCTGTAG	1	0	2	0	0	At1g79630.2:d:+1433:primary,At1g79630.1:d:+1963:primary
TTTCAACACA	1	0	1	1	0	At1g79620.1:d:+2515:primary
ATTTTTAAGT	0	0	1	0	2	At1g79440.1:d:+1792:primary
ATGGGTAAAG	1	2	0	0	0	At1g79390.1:d:+250:secondary
TCCATTCGCT	0	0	0	0	3	At1g79150.1:v:+1497:secondary
GTTAAGCTCA	0	0	0	1	2	At1g79140.1:d:+766:primary
AACCGAAAAT	1	0	0	1	1	At1g79010.1:X:-269:quaternary
GAGAAGCCGG	0	0	0	3	0	At1g78930.1:d:+1685:primary
CGTTGGATAT	0	0	3	0	0	At1g78830.1:d:+318:secondary,At1g65010.1:d:+458:secondary
TCGTTGAATC	0	0	1	1	1	At1g78800.1:d:+1155:primary
TTTACAATAT	3	0	0	0	0	At1g78570.1:d:+915:secondary
AAAATCATAT	1	1	0	0	1	At1g78560.1:d:+1467:primary
GTGCGGGAGC	0	0	2	1	0	At1g78460.1:X:-100:quaternary
ATCTCACACG	0	1	2	0	0	At1g78390.1:d:+497:secondary,At1g13790.1:v:+1276:secondary
GAAGATGTAT	2	1	0	0	0	At1g78290.2:d:+1338:secondary,At1g78290.1:d:+1439:secondary
TAAGGACTTT	0	2	0	0	1	At1g78270.1:X:-926:quaternary
TAGGAGATAA	1	0	0	0	2	At1g78210.1:d:+887:primary
TTTGCCAAAA	2	0	0	1	0	At1g78190.1:v:+483:secondary
GTTTCAAAAA	2	0	0	0	1	At1g78180.1:d:+400:secondary
TAGAATAAAA	2	1	0	0	0	At1g78110.1:X:+1397:quaternary
ACTGGCCCAA	1	1	0	1	0	At1g78080.1:d:+1600:secondary
TTAGGACAAG	0	2	0	0	1	At1g78000.2:d:+1930:primary,At1g78000.1:d:+1945:primary
GCAAGGACAA	0	2	1	0	0	At1g77770.1:d:+756:secondary,At1g77770.2:d:+768:secondary
GTTTAATTTC	2	0	0	0	1	At1g77620.1:i:+2898:tertiary,At3g03380.1:i:+2889:tertiary,At1g54000.1:i:+1475:tertiary,At1g54010.1:i:+1579:tertiary
TTTGGATTCT	0	2	1	0	0	At1g77610.1:d:+740:secondary
TCTACGTTGT	0	2	0	0	1	At1g77440.1:d:+646:primary
CAGTCAGCCA	1	1	1	0	0	At1g77420.1:d:+1155:primary
AATTGTGATA	1	0	0	1	1	At1g77350.1:d:+541:primary
AGAAGCTAAA	0	0	1	1	1	At1g77310.1:i:+2591:tertiary
AGCGGTTGTT	0	2	0	0	1	At1g77000.1:d:+1050:primary
TGGCTGCCTC	1	0	2	0	0	At1g76955.1:d:+471:primary
TTAATGAGTG	1	2	0	0	0	At1g76930.2:d:+974:secondary,At1g76930.1:d:+928:secondary
TGGTTGGACC	1	2	0	0	0	At1g76720.1:v:+3211:secondary,At1g76810.1:d:+3209:secondary,At1g76825.1:v:+1420:secondary
ATAAGCTTGA	0	1	1	0	1	At1g76400.1:d:+1525:primary,At2g06140.1:v:+2364:primary
CTTGCTTAAC	0	1	1	0	1	At1g76110.1:d:+1246:secondary
AATATGTAAG	1	2	0	0	0	At1g76100.1:X:-582:quaternary
GGCGTTCGGG	0	1	1	0	1	At1g75980.1:d:+916:primary
TGTTCACTGT	0	1	2	0	0	At1g75860.1:v:+354:secondary
TGTATTGCTG	0	1	1	0	1	At1g75860.1:v:+1005:secondary
TATCAAGTTG	0	1	1	1	0	At1g75780.1:d:+1487:primary
CTCGAAGGGT	0	0	2	1	0	At1g75640.1:v:+3687:secondary
TGACATAGTC	1	0	0	0	2	At1g75510.1:d:+912:primary
GCTTCTGCTT	0	0	1	1	1	At1g75390.1:d:+943:secondary,At4g21670.1:d:+1942:secondary

AATAAAATGT	1	1	0	1	0	At1g75240.1:X:+1459:quaternary,ChrC:+128691:quaternary,Chr5:+21634434:quaternary,Chr5:+8848133:quaternary,Chr1:+24408709:quaternary,Chr5:+9933981:quaternary
GATCTTCGGA	0	2	1	0	0	At1g75140.1:d:+1878:primary
GAGGCGGTCT	0	1	1	1	0	At1g74970.1:d:+507:secondary
AAATGCCTCT	0	2	0	0	1	At1g74770.1:v:+3753:primary,At3g02290.2:d:+1831:primary,At3g02290.1:d:+1344:primary
AAGAATGCAA	1	0	2	0	0	At1g74690.1:d:+2003:secondary,At2g40770.1:d:+2789:secondary
TTGTCTTGTC	0	0	2	0	1	At1g74510.1:d:+1781:primary,At1g74510.2:d:+2106:primary
ATTGGGGTCA	1	1	0	1	0	At1g74510.1:d:+1654:secondary,At1g74510.2:d:+1979:secondary
TGAAACTTAG	0	2	1	0	0	At1g74325.1:v:+1028:secondary
AGTTTCATCT	1	0	0	1	1	At1g74230.1:i:+511:tertiary
GAAGGATACA	0	3	0	0	0	At1g74210.1:d:+933:primary
ATCTCAAAAA	0	0	2	0	1	At1g74180.1:v:+477:secondary
CCGGCGAAAC	0	3	0	0	0	At1g74050.1:d:+130:secondary
GTGCGATGGT	0	2	1	0	0	At1g74020.1:d:+281:secondary
GAATTATGTT	2	0	0	0	1	At1g73970.1:d:+2800:primary
TAGACACCGT	0	2	1	0	0	At1g73950.1:d:+1430:primary
ATGAAAGATT	0	1	0	2	0	At1g73930.1:d:+788:secondary,At5g57930.2:d:+750:secondary,At5g57930.1:d:+754:secondary
TTAGAAACTG	2	0	1	0	0	At1g73820.1:d:+784:primary
ATTTACATCT	0	0	0	1	2	At1g73810.1:X:+752:quaternary
CCGGCAAGAT	0	2	1	0	0	At1g73760.1:d:+970:secondary
TAATGTGTAC	2	0	0	0	1	At1g73540.1:d:+897:primary
GTCTGAAGCC	0	0	0	3	0	At1g73390.1:d:+1265:primary,At1g73390.3:d:+1467:primary,At1g73390.2:d:+1439:primary
ACAGTGACAC	0	0	3	0	0	At1g73330.1:X:-817:quaternary,At5g62390.1:X:-1022:quaternary
TTTGGTAAAA	2	0	1	0	0	At1g73200.1:v:+2809:secondary,At2g21440.1:d:+1290:secondary
GACTCATTAA	1	1	1	0	0	At1g73165.1:X:-181:quaternary
GATCTCTTGT	0	2	1	0	0	At1g72830.1:d:+1638:primary,At1g72830.2:d:+1641:primary
TAAAGAGTAG	0	1	0	1	1	At1g72700.1:d:+3800:primary
TCTCTCTTGA	0	1	1	1	0	At1g72680.1:d:+930:primary
ACCAAAGCCT	1	1	1	0	0	At1g72390.1:d:+3547:secondary
CCGGTTCGTG	0	1	2	0	0	At1g72175.1:d:+651:primary
AACTGAACTG	0	0	1	1	1	At1g72030.1:d:+876:secondary
CGGTCTTTGT	1	1	1	0	0	At1g71980.1:d:+670:secondary
TGAAAATGAA	0	1	1	0	1	At1g71960.1:d:+2210:primary
GCTGATGAAT	0	3	0	0	0	At1g71950.1:d:+140:secondary,At5g28160.1:v:+885:secondary
AAATATTTTG	1	0	1	1	0	At1g71940.1:d:+1263:primary
GCCTAACATT	0	0	2	1	0	At1g71940.1:d:+1051:secondary
TTGCTATCTG	1	1	0	1	0	At1g71840.1:d:+1470:primary
GTATCTTTCC	2	1	0	0	0	At1g71820.1:d:+2651:primary
ACATCTTTTG	1	0	0	0	2	At1g71750.1:d:+611:primary
CTCTGTTTTG	1	0	1	1	0	At1g71260.1:d:+829:secondary,At4g13220.1:d:+128:secondary,At1g74310.1:d:+3023:secondary
AATCTGCAGA	0	1	2	0	0	At1g70760.1:i:+211:tertiary,At3g26780.1:i:+1589:tertiary
AGGAGAAAGC	1	1	1	0	0	At1g70750.1:d:+725:secondary
ACGGGGACAG	0	1	2	0	0	At1g70530.1:v:+2469:primary
GTTGTAAACT	1	2	0	0	0	At1g70480.1:d:-1402:secondary,At5g64510.1:d:-1051:secondary
TGCCGATTTC	0	0	3	0	0	At1g70420.1:X:-793:quaternary
CTTCTTGGAT	0	1	1	0	1	At1g70320.1:v:+9399:secondary
GATCAACTAT	3	0	0	0	0	At1g70260.1:X:+424:quaternary
AGGAACTAGA	0	2	1	0	0	At1g70200.1:d:+1173:secondary
GGTGAGACTT	1	1	1	0	0	At1g70090.1:d:+1160:secondary
TCTCCTTCAT	2	1	0	0	0	At1g69790.1:d:+1220:primary
AGGGCAGAAT	0	2	0	1	0	At1g69730.1:d:+2257:primary
TTTAGGAAAA	1	1	0	0	1	At1g69570.1:d:+925:secondary,At2g03170.1:v:+49:secondary
ATTTTAGCCT	1	1	1	0	0	At1g69550.1:d:+2347:primary
AACAACATAT	1	2	0	0	0	At1g69510.2:d:+647:primary,At1g69510.1:d:+544:primary,At1g69510.3:d:+513:primary
AAGAGATGTA	0	3	0	0	0	At1g69460.1:d:+517:primary
CTTGTGCATA	1	0	1	0	1	At1g69450.1:v:+2334:secondary
TGGGCTTAAA	0	0	2	1	0	At1g69410.1:d:-695:secondary,At1g69410.1:d:-706:secondary
GTTCCTTGGT	1	0	0	2	0	At1g69330.1:d:+664:secondary,AtCg00020:d:+647:secondary
TGTGTTATCT	0	1	2	0	0	At1g69330.1:d:+1328:primary
GTCCGGCTGA	0	2	0	0	1	At1g69280.1:d:+650:primary

CTTGCAGACC	1	2	0	0	0	At1g69250.2:d:+1306:primary,At1g69250.1:d:+1208:primary
ACTAAGTGTT	2	1	0	0	0	At1g69040.1:d:+1553:primary,At1g69040.2:d:+1559:primary
ATATTTCGTC	1	2	0	0	0	At1g68920.1:d:+2069:primary,At1g68920.2:d:+2066:primary
TTTCCTTCTT	0	2	0	0	1	At1g68800.1:v:+354:secondary,At1g23240.1:d:+48:secondary,At1g23240.2:d:+515:secondary
TATACCAATA	3	0	0	0	0	At1g68725.1:v:+1158:secondary
GTGTAATAAG	1	2	0	0	0	At1g68600.1:d:+1713:primary
GAACCAATAA	0	1	1	0	1	At1g68120.1:d:+840:primary,At2g01930.2:d:+1298:primary,At2g01930.1:d:+930:primary
ACCCGAACCG	0	0	3	0	0	At1g68010.1:d:+1120:secondary
GTCACAGATA	0	2	1	0	0	At1g68000.1:d:+430:primary
GATCATTTGG	0	1	1	1	0	At1g67960.1:d:+2070:primary
GAGGGAAGTA	0	2	0	1	0	At1g67830.1:d:+920:primary
TCGAGTTCTT	0	1	1	1	0	At1g67660.1:d:+1107:primary
GTCAAGAAGA	1	2	0	0	0	At1g67650.1:d:+1418:primary,At5g48720.1:v:+1595:primary,At1g67680.1:d:+1514:primary
TGTCTCATAT	0	2	1	0	0	At1g67640.1:v:+1761:primary
GAGTCACTGG	0	3	0	0	0	At1g67560.1:d:+2741:primary
TTGAAGTAGG	0	2	1	0	0	At1g67480.1:d:+1375:primary
GAATTCAACG	0	3	0	0	0	At1g67400.1:d:+880:primary
CAATGGGTGA	0	2	0	1	0	At1g67360.1:d:+602:secondary,At1g67360.2:d:+578:secondary
TCATCTGTTA	0	3	0	0	0	At1g66890.1:d:+459:primary
GTCAAATTTT	1	0	1	1	0	At1g66840.1:v:+2127:secondary
TTAGTAAAAA	3	0	0	0	0	At1g66730.1:i:-3451:tertiary,At4g18530.1:i:-2808:tertiary
TAAATAGAGT	2	0	1	0	0	At1g66550.1:i:+607:tertiary
TTTGTATAAA	1	1	0	0	1	At1g66200.1:X:-469:quaternary
GCTGTCTCTT	0	1	1	0	1	At1g66150.1:d:+3193:primary
GTTAATGATT	2	1	0	0	0	At1g66070.1:i:+306:tertiary
TTTTTGGTTC	0	1	0	0	2	At1g66070.1:d:+1094:primary
GAAGTTGGTG	0	0	0	3	0	At1g66020.1:d:+1359:secondary,At4g20210.1:v:+1692:secondary
ATTTATTACA	0	2	0	0	1	At1g65820.1:d:-679:secondary
CATATCATTT	0	1	0	1	1	At1g65790.1:v:+2914:secondary
GTCCTCCTAA	0	0	2	0	1	At1g65440.1:d:+671:secondary
CATATCTGAC	1	0	0	2	0	At1g65370.1:d:+1122:primary
AAGCAAAATT	0	1	0	0	2	At1g65320.1:d:+1837:primary
CATACTAAGT	2	0	0	1	0	At1g65280.1:d:+1933:primary
GAGATGGAAC	0	0	2	1	0	At1g65220.1:d:+1105:primary
AGGACTCAGA	0	0	1	2	0	At1g65030.1:d:+1053:primary
GGAATGTACT	0	1	1	1	0	At1g64990.1:d:+1309:primary,At4g27630.1:d:+837:primary,At4g27630.2:d:+1306:primary
GATTAACTCA	0	1	1	1	0	At1g64970.1:d:+1328:secondary
TTATCTGGTA	0	0	2	0	1	At1g64970.1:d:+1295:secondary
TCCGAATCGA	0	1	1	1	0	At1g64920.1:X:-600:quaternary
AGGAGCACAC	0	1	0	2	0	At1g64880.1:d:+1345:secondary
GGACAAGTCT	1	2	0	0	0	At1g64850.1:d:+38:primary
TAACCAAAAG	1	2	0	0	0	At1g64640.1:d:+849:secondary
GCAGCCTCAC	0	1	1	1	0	At1g64610.1:d:+2084:primary,At1g64610.2:d:+1882:primary
GAGCAGCAAG	0	0	2	1	0	At1g64500.1:d:+752:primary
GGCTACAACA	0	1	2	0	0	At1g64450.1:X:-1174:quaternary
GAAAGAGATC	0	1	1	1	0	At1g64400.1:d:+1984:primary
ATGACTGTAA	0	2	1	0	0	At1g64280.1:d:+1995:secondary
TGATGAGTGA	1	0	1	1	0	At1g64185.1:d:+186:secondary
ACTCTACAGT	0	0	0	2	1	At1g64100.1:v:+354:secondary
GCCCGACCCA	0	1	2	0	0	At1g63840.1:d:+472:primary
GAACAAACAC	1	2	0	0	0	At1g63810.1:d:+3030:primary
ACTTCTGAGA	0	1	2	0	0	At1g63640.2:d:+3191:primary,At1g63640.1:d:+3191:primary
ATTTGTAATC	1	0	0	0	2	At1g63500.1:d:+1679:primary
TTATCGGCGG	0	2	0	0	1	At1g63470.1:d:+1166:primary
GAAAAAGCTC	0	0	2	1	0	At1g63055.1:d:+294:secondary
TTCTGCTTAG	0	2	0	1	0	At1g62970.1:d:+2600:primary
GATATTGATG	1	2	0	0	0	At1g62810.1:d:+1722:primary
TTATGTATTA	1	1	0	0	1	At1g62790.1:d:+618:secondary,At2g42350.1:v:+10:secondary,At1g62790.2:d:+596:secondary
GCCTTCTTTA	0	0	2	1	0	At1g62500.1:d:+1221:secondary
GTGGAATCAC	0	2	1	0	0	At1g62430.1:d:+1313:secondary
AACTAAACCC	1	1	1	0	0	At1g62420.1:X:-739:quaternary

GCATTTGTAG	0	2	0	0	1	At1g62330.1:d:+2075:primary
GATTTTCTTT	1	1	0	1	0	At1g62305.2:d:+1358:primary
AATAAAAAAC	0	1	1	0	1	At1g62210.1:d:+197:secondary,At2g36710.1:d:+1294:secondary
GATACATTGG	1	0	1	0	1	At1g61990.1:d:+1664:primary
TCCAAAGGAT	0	2	0	1	0	At1g61970.1:d:+1321:secondary,At1g62150.1:d:+1418:secondary
TGATCTCATA	0	1	1	0	1	At1g61380.1:d:+2646:primary
TTCTAGACTG	3	0	0	0	0	At1g61300.1:d:+2129:primary
GATGTTTCAA	1	0	1	1	0	At1g61180.1:d:+2615:primary
ACTTGCTCAT	1	1	1	0	0	At1g60860.1:v:+2758:primary
CCATTTGGAA	0	2	0	1	0	At1g60810.1:d:+764:primary
TTAAAAGTTG	1	2	0	0	0	At1g60670.1:d:+1401:secondary
GCCAAGTATT	0	0	2	1	0	At1g60600.1:d:+1427:primary
GGCGCATATG	0	3	0	0	0	At1g60260.1:p:+1694:secondary
GAAATGAGAG	0	1	1	0	1	At1g59970.1:d:+1283:primary
TGAAGAAGAT	0	2	1	0	0	At1g59850.1:v:+1898:secondary,At1g11100.1:d:+1785:secondary,At5g15780.1:d:+386:secondary
GACGAGAGCA	0	3	0	0	0	At1g59660.1:d:+2734:primary,At1g10390.1:d:+3266:primary
GGTTGCACTT	0	3	0	0	0	At1g59610.1:d:+900:secondary
GAGCTTTGTT	1	1	1	0	0	At1g59580.2:d:+1392:primary,At1g59580.1:d:+1455:primary
GAGTAGCACA	1	0	1	0	1	At1g59520.1:d:+1367:primary,At1g59520.3:d:+1367:primary
GGTGTGTGTT	0	1	2	0	0	At1g59124.1:d:+793:secondary,At1g59218.1:d:+654:secondary,At1g58848.1:v:+980:secondary,At1g58807.1:d:+654:secondary
GCGAAGAAGG	1	1	0	1	0	At1g58807.1:d:+3239:secondary,At1g59124.1:d:+3290:secondary
CGTCATCCTT	0	0	2	1	0	At1g58440.1:d:+1098:primary
GCCCAATGCA	0	0	1	2	0	At1g58270.1:d:+1033:primary
GTTGAATTTC	2	0	0	1	0	At1g58060.1:i:+4374:tertiary
TCATCAGAGA	0	3	0	0	0	At1g58040.1:p:+563:primary
GCATTACACT	0	2	0	0	1	At1g57870.1:d:+1344:primary
ATCTTGATTG	0	0	1	2	0	At1g57790.1:d:+1072:primary
TGCAACAGTC	1	0	1	0	1	At1g57660.1:d:+334:secondary,At1g31355.1:p:+585:secondary,At1g09486.1:p:+1033:secondary,At1g57860.1:d:+325:secondary
TGTTAATCTT	0	1	0	1	1	At1g57610.2:d:+1202:primary,At1g57610.1:d:+1295:primary
GTTGCTGGAG	0	1	1	0	1	At1g57560.1:d:+127:secondary,At2g23380.1:v:+2874:secondary,At4g01680.1:d:+260:secondary
ATGAAACAGA	1	1	0	1	0	At1g56660.1:d:+778:secondary,At4g18020.1:d:+1763:secondary,At4g18020.2:d:+1806:secondary
AGAAGGAACA	1	0	0	1	1	At1g56660.1:d:+406:secondary
TCTCTTGGTA	2	0	0	0	1	At1g56660.1:d:+1748:primary
CCGGTCAAAA	2	1	0	0	0	At1g56590.1:d:+1302:primary
CTGGCAAAAA	1	0	0	1	1	At1g56540.1:v:+1713:secondary
GAGAGCCCAA	2	0	0	1	0	At1g56310.1:v:+2259:primary
TGGTAACTTT	0	2	1	0	0	At1g56300.1:X:+363:quaternary
TAATATTCGG	1	1	0	0	1	At1g56230.1:d:+2312:primary
GACGACGACA	0	2	0	1	0	At1g56145.1:d:+3075:primary
AGAGGTATCT	0	0	2	0	1	At1g56070.1:d:+2158:secondary
AATGAAACCC	1	0	0	0	2	At1g55980.1:X:--38:quaternary
CGACGGGCGT	0	0	3	0	0	At1g55920.1:d:+855:secondary
TTGATTATTG	1	1	1	0	0	At1g55860.1:d:+2734:secondary
GCTCACCGGC	0	1	1	0	1	At1g55350.4:d:+7094:primary,At1g55350.2:d:+6867:primary,At1g55350.1:d:+6888:primary,At1g55350.3:d:+6954:primary
AACAAGTTTG	1	1	0	1	0	At1g55090.1:i:+335:tertiary
AAAAAAATAG	1	0	2	0	0	At1g54730.3:i:+4583:tertiary,At1g54730.2:i:+4522:tertiary
TGGGTCTCCT	0	2	1	0	0	At1g54510.1:d:+1927:primary
TACAAAGGTT	1	0	0	2	0	At1g54390.2:d:+1236:primary,At1g54390.3:d:+1263:primary,At1g54390.1:d:+1346:primary,At1g54390.4:d:+1150:primary
AGCTTTAATG	2	0	1	0	0	At1g54380.1:d:+1627:primary
GAAAAGATGT	0	1	0	2	0	At1g54370.1:d:+1571:secondary,At4g32940.1:d:+472:secondary
TTGTTAGAGA	3	0	0	0	0	At1g54115.1:v:+2321:secondary
GGTCCAATAT	0	0	2	0	1	At1g54030.1:X:+529:quaternary
CTACAGTTTC	0	1	1	0	1	At1g53770.1:d:+1823:primary
AGTGGAACAA	1	1	0	1	0	At1g53760.1:v:+756:primary
AAATTTTGGA	0	0	1	0	2	At1g53710.1:v:+1561:secondary
TATATGTTTC	0	1	1	1	0	At1g53590.1:d:+2395:primary
TCACTGGTAC	0	3	0	0	0	At1g53580.2:d:+327:primary

AAAATGAATA	0	0	0	0	3	At1g53570.1:d:+2188:secondary,At1g65330.1:v:+1020:secondary,At1g53570.2:d:+2185:secondary
AAGCTTCTCA	1	1	0	1	0	At1g53500.1:d:+761:secondary,At4g35270.1:d:+2864:secondary
TTTGATTAGC	2	1	0	0	0	At1g53390.1:d:+3631:primary
AGTATCTTAG	1	1	1	0	0	At1g53280.1:d:+1373:secondary
AAAAAAAAGT	0	1	1	1	0	At1g53240.1:X:-349:quaternary
ACCTCCAAGA	1	0	1	0	1	At1g53160.2:d:+367:primary,At1g53160.1:d:+365:primary
ATACTTCTTT	0	3	0	0	0	At1g53060.1:v:+1564:primary
TTTCCGTATT	2	1	0	0	0	At1g53050.1:i:+1033:tertiary
AACGAGAGAA	1	0	2	0	0	At1g52950.1:v:+1984:secondary
TAAGTTTAAT	0	0	1	1	1	At1g52890.1:d:+1247:primary
GGCAATCATT	1	1	1	0	0	At1g52825.1:d:+197:primary
TGTGCTCAGA	0	1	0	1	1	At1g52540.1:d:+1194:primary
AAGCAACATA	2	0	1	0	0	At1g52420.1:d:+2296:secondary
TACTTATTGA	1	0	0	1	1	At1g52200.1:d:+313:secondary
AGAAGATCAA	1	1	0	1	0	At1g51960.1:v:+963:secondary,At3g44190.1:d:+592:secondary,At3g57190.1:d:+1263:secondary
ATACTAAAAA	1	0	1	1	0	At1g51940.1:X:--51:quaternary,At4g14310.1:X:-1559:quaternary
GTAACAGTTA	1	0	1	1	0	At1g51780.1:d:+1464:primary,At4g16850.1:d:+1093:primary
TGAGGATGCT	0	0	1	1	1	At1g51745.1:d:+1392:secondary
TTGGCTCTTG	1	1	1	0	0	At1g51600.2:d:+1146:primary,At1g51600.1:d:+1184:primary
CAAAATCTCA	0	1	1	1	0	At1g51560.1:d:+1318:primary
ATCATCCACA	0	1	2	0	0	At1g51440.1:X:+-83:quaternary
GAGTTGTCTC	0	1	1	0	1	At1g51390.1:d:+1039:primary
TTTTAACTGT	2	1	0	0	0	At1g51340.1:d:+1577:primary,At1g51340.2:d:+1600:primary
AATCTTTTCG	0	0	3	0	0	At1g51300.1:v:+1169:secondary
TGACTTTTTG	0	0	1	2	0	At1g51300.1:v:+1052:secondary
AAGAAGATGC	0	2	1	0	0	At1g51175.1:p:+1337:secondary
ATATAATTTT	0	3	0	0	0	At1g51110.1:d:+1425:primary,At3g01420.1:d:+2222:primary
AAGGCAAGAA	0	1	0	1	1	At1g51085.1:p:+109:primary
GCATTTGGTG	0	3	0	0	0	At1g50940.1:d:+947:primary
TAGTTGAAAT	0	1	1	0	1	At1g50910.1:v:+2325:primary
AACCCACTTG	0	3	0	0	0	At1g50750.1:d:+392:secondary
TCCTTAAACA	1	2	0	0	0	At1g50660.1:d:+2608:primary
ATTTGTTCCT	1	1	1	0	0	At1g50500.1:d:+2575:primary
ACATAGGAAA	1	1	0	1	0	At1g50240.1:d:+881:secondary
TGTAAAACGG	0	0	0	1	2	At1g50200.1:X:-148:quaternary
AGAAAAAGAT	1	1	1	0	0	At1g50200.1:d:+1432:secondary
GCAGCTTGGG	0	2	0	1	0	At1g50140.1:d:+1435:primary
CAATAATTTG	1	1	0	0	1	At1g50120.1:d:+2126:primary
CTTATCGCCA	1	0	1	0	1	At1g49950.2:d:+1324:primary
TTTGTAATGG	1	0	0	0	2	At1g49850.1:d:+965:primary
ACTTATCACA	2	0	0	1	0	At1g49630.3:i:+7123:tertiary,At1g49630.1:i:+7123:tertiary
TCTTTAAAAA	0	1	0	2	0	At1g49430.1:i:+1471:tertiary,At4g17580.1:i:+708:tertiary
TGGCTCAAAA	2	1	0	0	0	At1g49420.1:v:+574:secondary
TGTTATTCAT	1	2	0	0	0	At1g49405.1:v:+1137:secondary,At5g59290.1:d:+1284:secondary
TCTTTAGCTA	1	2	0	0	0	At1g49200.1:d:+836:primary
TAAATCTATA	0	1	0	2	0	At1g49170.1:d:+538:secondary
ACACGGCGGC	1	2	0	0	0	At1g48990.1:d:+470:secondary,At3g44720.1:d:+904:secondary,At4g14020.1:d:+264:secondary
TTGAGTTCGC	0	1	0	2	0	At1g48920.1:d:+1106:secondary
AACAAAATGC	1	2	0	0	0	At1g48860.2:d:+1239:primary,At1g48860.1:d:+1345:primary
GAAGATGCAA	0	0	1	2	0	At1g48650.1:d:+1696:secondary
TCTTCAGCTA	0	2	1	0	0	At1g48540.1:d:+1078:secondary,At1g48540.2:d:+1078:secondary
CAAGATGTGC	2	0	0	1	0	At1g48520.1:d:+1241:secondary,At1g48520.2:d:+1253:secondary,At1g48520.3:d:+1253:secondary
ATTGGATAAA	1	1	0	0	1	At1g48090.2:d:+1082:secondary,At1g48090.1:d:+1082:secondary
TGTTTATCGA	2	0	1	0	0	At1g47960.1:i:+1234:tertiary
AGATGAAATG	0	0	1	1	1	At1g47900.1:d:+3218:primary
TGATGCATCT	0	0	0	3	0	At1g47710.1:d:+1423:primary
TGTTTAAAAG	1	0	0	2	0	At1g47570.1:X:+-193:quaternary
TATGTCGCCC	0	2	0	1	0	At1g47570.1:d:+1631:primary
CCTGAAATGG	0	2	0	1	0	At1g47420.1:d:+366:secondary
CCTGTTTCAC	1	1	0	0	1	At1g47210.1:d:+1154:primary
GCGATTCTTT	1	2	0	0	0	At1g47128.1:d:+86:secondary

GCATCAAGCA	0	2	0	0	1	At1g47056.1:v:+1788:secondary
GAAATGCAAA	1	1	1	0	0	At1g45249.2:d:+1278:primary
TCACAGATCT	0	1	0	1	1	At1g44790.1:d:+591:primary
CCGCCGTGGC	2	0	0	1	0	At1g44350.1:d:+1440:secondary
ATTTTGCTGT	2	1	0	0	0	At1g44110.1:d:+1502:primary
GCACAACAAG	2	0	1	0	0	At1g43990.1:p:+3033:secondary,At3g32405.1:p:+5557:secondary,At5g32593.1:p:+2635:secondary
TTGATGTATG	1	1	1	0	0	At1g43910.1:d:+1553:secondary
GACGACTCTA	0	0	2	0	1	At1g43860.1:d:+1159:primary
TCAAAATAAA	1	0	1	0	1	At1g43835.1:p:+78:secondary,At1g56660.1:d:+115:secondary
ATCAACCTTC	0	0	0	2	1	At1g43710.1:d:+1714:primary
AAAAAAACGG	0	1	2	0	0	At1g43690.1:d:-2204:secondary
TTTCTGAAAG	0	0	3	0	0	At1g43690.1:d:+1335:secondary
GAGTTTCCTC	1	1	1	0	0	At1g43245.1:d:+1511:primary
GGCTTAACAT	0	0	3	0	0	At1g43190.1:d:+1613:primary
GTCGCTTCCA	0	0	3	0	0	At1g43170.1:d:+1189:secondary,At1g43170.2:d:+1155:secondary
GACCGAATAT	0	0	1	0	2	At1g43160.1:d:+716:secondary
AACAAACTTG	1	0	0	2	0	At1g43120.1:p:+156:secondary
AAAAAAAGGG	0	2	1	0	0	At1g43080.1:v:+1324:secondary,At1g43090.1:v:+1324:secondary,At1g43100.1:v:+1324:secondary
AGTGTGCAAC	2	0	0	0	1	At1g42970.1:X:+385:quaternary
AAAGCTCTCT	1	2	0	0	0	At1g42480.1:i:-1854:tertiary
TAACAAAAAA	1	0	0	2	0	At1g41930.1:p:+3034:secondary
GGGTGTATCT	0	1	2	0	0	At1g41893.1:p:+42:secondary,At1g42100.1:p:+79:secondary,At3g29782.1:p:+1364:secondary,At2g13240.1:p:+1166:secondary
GCAAGGGCTG	0	2	1	0	0	At1g37130.1:d:-2747:secondary
ATAACAGAAT	0	0	1	2	0	At1g37015.1:v:-1045:secondary,At5g22610.1:v:-1921:secondary
AGTATTACTT	1	0	0	0	2	At1g36580.1:v:+945:secondary
AATCTGGTGA	0	2	1	0	0	At1g36380.1:d:+613:primary
CTCCACAAAG	0	3	0	0	0	At1g36050.1:i:-1186:tertiary
TTAGGCCTTT	0	0	2	1	0	At1g35710.1:d:+3608:primary
TAGCATTTCC	1	2	0	0	0	At1g35375.1:X:-233:quaternary
CTCTTGATAA	0	1	1	0	1	At1g35240.1:v:+1114:secondary
ATTTTGCTAC	3	0	0	0	0	At1g34430.1:d:+1634:secondary
AAAGCTCTCG	1	0	1	1	0	At1g34320.1:d:+1927:primary
GAGAAAGGTG	0	3	0	0	0	At1g34315.1:v:+1041:secondary,At2g15410.1:p:+994:secondary
CATCTATGTT	0	2	0	0	1	At1g34300.1:d:+2401:primary
AGAATTGAAA	0	0	0	1	2	At1g34210.1:i:+576:tertiary
TGTAGTGTAA	0	0	1	0	2	At1g34150.1:d:+1464:primary
TTTGAGAAGC	0	2	1	0	0	At1g33990.1:d:+1065:primary
AACTTCTCAA	0	1	0	2	0	At1g33960.1:d:+710:secondary
TATTTTATAA	2	1	0	0	0	At1g33540.1:v:+1811:primary
GGATTAAGAC	0	1	2	0	0	At1g33420.1:d:+2466:primary
TCTTAAGCAA	0	1	0	0	2	At1g33400.1:d:+2384:primary
AGAGCAGCCA	0	2	0	1	0	At1g33330.1:d:+759:secondary
GGCTGAGATG	0	1	1	0	1	At1g33270.2:d:+1416:secondary,At1g33270.1:d:+1241:secondary
AGTTTCTGGG	2	0	1	0	0	At1g33240.1:i:+717:tertiary
CGTTTCAAGT	1	0	1	0	1	At1g32990.1:X:-367:quaternary,ChrM:-354607:quaternary
TTACTGGCGA	0	0	0	2	1	At1g32860.1:d:+1383:primary
TTTTTTTTAA	0	3	0	0	0	At1g32690.1:d:+701:primary
ATGACTGCAT	1	1	0	1	0	At1g32370.2:d:+663:primary
GTTAGACTAG	1	0	2	0	0	At1g32360.1:i:+1609:tertiary
ACAATGTCTT	0	2	1	0	0	At1g32260.1:d:+738:primary
CAAGAAAAAA	1	0	1	1	0	At1g32100.1:d:+1100:primary
ATGAATAGAC	1	1	1	0	0	At1g31812.1:X:-88:quaternary
TTGATGTCGT	0	1	1	0	1	At1g31420.1:d:+924:secondary,At5g18780.1:v:+1129:secondary
TTCCTTCATC	1	0	0	1	1	At1g31420.1:d:+2423:primary
AAAATATGGA	0	1	1	1	0	At1g31010.1:d:+1242:primary
TATTTTTTTT	0	3	0	0	0	At1g30825.1:d:+1172:primary,At4g19230.1:d:+1859:primary,At4g19230.2:d:+1861:primary
GAGATCATTG	2	0	1	0	0	At1g30820.1:d:+1856:primary
TAGAAACAAA	0	0	2	1	0	At1g30790.1:d:+1061:secondary
GCGGAACTGA	0	2	0	1	0	At1g30640.1:v:+1851:secondary
GTGATATATA	0	1	0	0	2	At1g30540.1:d:+1284:primary

ATATGATGAT	1	0	0	0	2	At1g30510.2:d:+1382:primary,At1g30510.3:d:+1291:primary,At1g30510.1:d:+1438:primary
ATATAATTTC	0	2	0	0	1	At1g30410.1:X:+229:quaternary
ATCTTTAACA	2	1	0	0	0	At1g30400.1:i:+4918:tertiary
GTTTGGTTGA	2	1	0	0	0	At1g30400.1:d:+3555:secondary
GATGAAGCAA	1	1	0	0	1	At1g30280.1:d:+1038:secondary,At5g40880.1:d:+607:secondary,At1g18620.1:d:+2777:secondary,At1g29750.2:d:+1718:secondary,At1g29750.1:d:+2056:secondary,At5g50370.1:d:+376:secondary
CACTTCCGGT	1	0	1	1	0	At1g30260.1:d:+336:secondary
TTCATTGTCA	2	0	0	0	1	At1g30210.1:d:+1605:primary,At1g30210.2:d:+1486:primary
CCACACAAGC	1	1	0	1	0	At1g29810.1:d:+790:primary
GAGAACAATA	0	0	0	1	2	At1g29750.2:d:+3008:primary,At1g29750.1:d:+3346:primary
CTCTTTTCAA	1	0	0	2	0	At1g29420.1:v:+704:secondary
GGAGCCATTG	0	0	0	1	2	At1g29310.1:d:+1414:primary
GATATGCGGT	1	2	0	0	0	At1g29260.1:d:+815:primary
TAGCAAGAGT	0	1	0	1	1	At1g29220.1:d:+1221:primary
GAAAGACCCT	1	0	0	1	1	At1g28600.1:d:+945:primary
TTCTTTTCTT	0	0	0	1	2	At1g28580.2:d:+557:secondary
TGTTGTGTGT	0	0	1	1	1	At1g28570.2:d:+1079:primary,At1g28570.1:d:+1253:primary
TCTCGAGTAT	0	1	0	1	1	At1g28350.1:d:+2211:primary
ATGAGATCAA	2	0	0	0	1	At1g28300.1:d:+387:secondary,At5g35380.1:v:+928:secondary,At5g14220.1:d:+812:secondary
GACAGTCAAT	0	2	1	0	0	At1g27980.1:d:+1678:secondary
TCTTTATTAC	1	1	1	0	0	At1g27910.1:d:+2660:primary
TGCGTTGTTC	1	0	2	0	0	At1g27840.1:d:+1509:primary
CCTGTTGCTG	0	2	0	1	0	At1g27770.1:d:+3166:primary,At1g27770.2:d:+2944:primary
TCCAAAAACT	0	0	1	1	1	At1g27695.1:d:+345:primary
TATGACCAAA	1	0	1	1	0	At1g27630.1:d:+1312:secondary
ATGCTTCCAG	0	2	1	0	0	At1g27510.1:v:+826:secondary
CTATGGTTGT	0	1	0	2	0	At1g27385.1:d:+462:primary
GCAGATAAAC	0	3	0	0	0	At1g27190.1:d:+1738:primary
TAATATCTTT	0	3	0	0	0	At1g27020.1:d:+1189:primary
TGTAGTGTCC	2	0	1	0	0	At1g27000.1:d:+1261:secondary
ACAGACACCA	2	0	0	1	0	At1g26640.1:d:+1716:primary
TGAACCGTGT	0	0	3	0	0	At1g26630.1:d:+386:secondary
AATTTTGACA	1	1	0	0	1	At1g26620.1:v:+1377:secondary
TTTCTCTTTG	0	1	1	1	0	At1g26450.1:d:+1063:primary
TGTAGGTAGT	0	0	2	1	0	At1g26210.1:d:+954:primary
CCGGGGTTTG	0	1	1	1	0	At1g26090.1:d:+1330:secondary
AGCTCGGTTT	0	2	0	1	0	At1g25520.1:d:+188:primary
ATTCCGGTTG	0	1	0	2	0	At1g25510.1:d:+1328:primary
CCATCATCTA	1	2	0	0	0	At1g25420.3:d:+885:primary,At1g25420.2:d:+1032:primary
GAGGCTGCTC	0	0	3	0	0	At1g25270.1:v:+539:secondary,At5g28610.1:d:+21:secondary
GTCCAATCAT	0	1	0	0	2	At1g25220.1:d:+241:secondary
GACTGAAGAG	0	2	1	0	0	At1g24880.1:i:+2810:tertiary,At1g25054.1:i:+2811:tertiary,At1g25141.1:i:+2811:tertiary,At1g24793.1:i:+2811:tertiary,At1g25210.1:i:+2811:tertiary
TTTTGTGTCG	1	0	1	1	0	At1g24460.1:v:+5844:primary
GGTTAGACTT	0	0	2	0	1	At1g24170.1:d:+1232:primary
GAGGTTTATA	1	0	0	2	0	At1g24145.1:d:+498:primary
GACGCAATTT	0	1	1	1	0	At1g23390.1:d:+1526:primary
GATAGCTTCA	0	1	2	0	0	At1g23310.1:d:+1677:secondary
GTTACAACTT	0	1	1	0	1	At1g23230.1:v:+5290:secondary,At1g58310.1:v:+2185:secondary
TAAAATTTTG	1	0	1	0	1	At1g23180.1:d:+2935:primary
AGTCTGCAAA	2	0	0	1	0	At1g23110.1:v:+354:secondary
ATTACATCGT	0	0	2	1	0	At1g22920.2:d:+1317:primary,At1g22920.1:d:+1219:primary
TTACCAATGG	1	0	0	0	2	At1g22882.1:d:+2225:primary
TTTTGCAAGA	0	1	0	1	1	At1g22870.1:v:+3367:primary
GATCTATGCA	1	0	1	0	1	At1g22870.1:i:+949:tertiary
TTTGTGAATG	1	1	0	0	1	At1g22620.1:i:+5120:tertiary,At4g06634.1:i:+356:tertiary
TGGATTACTT	0	0	1	0	2	At1g22570.1:v:+1945:secondary
AAGGTGTGGG	0	2	1	0	0	At1g22130.1:v:+1225:secondary
TCAGACTTCT	1	1	1	0	0	At1g21840.1:d:+1010:primary

TGGCACCATC	1	2	0	0	0	At1g21830.1:d:+612:secondary
TTTGGCAACA	2	0	0	1	0	At1g21700.1:d:+2454:primary
GATACTTGAC	0	1	0	1	1	At1g21650.1:i:+3507:tertiary
GTTTATAAAG	0	2	1	0	0	At1g21610.2:d:+2340:primary,At1g21610.1:d:+2343:primary
TTCAGTCAAA	0	0	0	1	2	At1g21370.2:d:+1582:secondary,At1g21370.1:d:+1663:secondary
TGTTGTCGAA	0	2	0	1	0	At1g21370.1:d:+1539:secondary,At1g21370.2:d:+1458:secondary
GAAGCGATCG	0	3	0	0	0	At1g21360.1:d:+265:primary
TATGATATAA	2	0	0	0	1	At1g21350.2:d:+1060:primary,At1g21350.3:d:+960:primary,At1g21350.1:d:+1056:primary
TATTTCTTTT	0	1	0	1	1	At1g21250.1:d:+2472:secondary
GAACCTTAAT	1	0	1	0	1	At1g21200.1:X:+1716:quaternary
TGAATTCTCT	0	2	1	0	0	At1g21200.1:d:+1082:primary
GTGAAAATGT	0	0	2	1	0	At1g20925.1:v:+564:secondary
AGTTTTGACT	1	1	0	1	0	At1g20900.1:d:+314:secondary
TTCTACAGCT	0	2	1	0	0	At1g20880.1:d:+1287:primary
ATTTTGCTAA	1	0	1	1	0	At1g20870.1:d:+1542:primary
AAGAAATTAA	0	0	2	1	0	At1g20850.1:d:+1253:primary
CTCACAGACT	0	0	2	0	1	At1g20693.1:X:+377:quaternary
CCGAAAAATA	1	2	0	0	0	At1g20630.1:d:+1290:primary
CAAGAGTTAG	0	0	1	2	0	At1g20450.1:X:+719:quaternary
TTTCTTCTGA	1	0	1	1	0	At1g20430.1:d:+619:primary
TACTCTTTTT	0	1	1	0	1	At1g20300.1:d:+1814:secondary
TTCATATTGT	2	0	0	0	1	At1g20225.1:d:+791:secondary,At2g18915.2:d:+2134:secondary,At2g18915.1:d:+1895:secondary
CTCATTTCTT	0	3	0	0	0	At1g20090.1:d:+334:primary
ATTTGGTTAA	1	0	0	2	0	At1g20080.1:v:+2427:primary
TTAACTGTGT	0	0	0	0	3	At1g19960.1:v:+636:secondary
TTAGGGAAAA	0	3	0	0	0	At1g19940.1:d:+1340:secondary
CTGATTTGTA	2	0	1	0	0	At1g19850.1:d:+3211:primary
TGTCCTCAAA	1	0	2	0	0	At1g19835.1:d:+2912:primary
TTCAATGAAA	0	1	2	0	0	At1g19740.1:X:+1329:quaternary,Chr4:+5430932:quaternary
GCGTGGGTAA	2	0	1	0	0	At1g19720.1:d:+2845:secondary
GGCTAGGCTT	0	1	1	1	0	At1g19300.1:d:+1113:primary
TTGAGAGGAT	0	1	1	0	1	At1g18900.2:d:+2921:primary,At1g18900.1:d:+2760:primary
GACAAGACTT	0	2	1	0	0	At1g18880.1:d:+1773:primary
AACCATTGAT	0	1	0	1	1	At1g18750.1:v:+1953:primary
TATTACAATC	0	1	0	0	2	At1g18720.1:d:+861:primary
CTCATACATT	0	2	1	0	0	At1g18680.1:d:+615:primary
CGTCCCGGAT	0	1	1	1	0	At1g18490.1:d:+859:secondary
GAGCTTCCTA	2	0	1	0	0	At1g18450.1:d:+1387:secondary
GTCCCTTTGA	1	1	1	0	0	At1g18400.1:d:+840:primary
GGAACTGTTG	0	3	0	0	0	At1g18070.1:d:+1151:secondary
GTAATTGAGT	1	0	1	1	0	At1g18010.1:X:+1594:quaternary
GGTTGTTAGC	0	0	1	2	0	At1g17880.1:d:+334:secondary
TCAAACTTAA	0	2	0	1	0	At1g17860.1:d:+217:primary
TGATGATGAA	2	0	0	0	1	At1g17780.1:d:+1394:primary
AATAAAACTA	0	2	0	0	1	At1g17720.2:d:+111:secondary,At1g17720.1:d:+111:secondary
ATGTAACCAG	0	2	1	0	0	At1g17720.1:d:+1900:primary,At1g17720.2:d:+1897:primary
TTTGCTTTCT	0	0	1	1	1	At1g17690.1:d:+2434:primary
GAAACGGAAA	0	1	1	0	1	At1g17640.1:v:+1375:primary
AAGTTCTTCG	1	1	1	0	0	At1g17400.1:v:+354:secondary
TCATAAAATA	0	0	1	1	1	At1g17350.1:d:+838:primary
AAGAACCTTT	0	3	0	0	0	At1g17250.1:X:-1832:quaternary,At1g06200.1:X:-248:quaternary
TGAGGTTTGT	1	0	1	0	1	At1g17190.1:d:+823:primary
ATTATTACGA	0	1	1	0	1	At1g17110.1:d:+2840:primary
CATAAAAAAA	1	0	0	1	1	At1g17010.1:i:+836:tertiary
CATTTCAGAG	1	0	2	0	0	At1g17000.1:v:-2636:secondary,At2g01100.1:d:-1136:secondary
TTGGTGGATT	0	3	0	0	0	At1g16930.1:v:+1796:primary
GGGGTTATAA	0	1	0	1	1	At1g16920.1:X:-471:quaternary
ACTATCTGAG	1	0	1	0	1	At1g16730.1:v:+985:secondary
AAACAGCCAT	2	1	0	0	0	At1g16710.1:d:+5277:primary
GGAGCTTGAA	0	0	3	0	0	At1g16680.1:d:+1641:primary
TGAAGATGAT	0	0	0	1	2	At1g16550.1:p:+2065:primary

TATAAATTCT	0	1	1	1	0	At1g16520.1:i:+1810:tertiary
AATATCAATC	1	1	1	0	0	At1g16400.1:d:+1849:primary
CACACATCCA	2	0	0	1	0	At1g16260.1:v:+2392:secondary
TGTATGTATT	1	0	1	1	0	At1g15830.1:v:+2100:primary,At2g47210.1:d:+1536:primary
TTGGTAGAAA	0	0	1	1	1	At1g15480.1:d:+1942:primary
GATTTATGTG	1	1	0	0	1	At1g15060.1:d:+2210:primary
TTCACAAGTT	1	1	0	1	0	At1g15000.1:d:+1433:primary
AAAAAGATCA	2	0	0	1	0	At1g14840.1:d:+1676:primary
ATGAGTTCAG	0	0	1	2	0	At1g14800.1:v:+1465:primary
TGTTGATGAA	0	0	1	1	1	At1g14770.1:d:+1599:primary,At1g14770.2:d:+1637:primary
AAGCTGAGAA	0	1	1	1	0	At1g14740.1:d:+2190:secondary
ACTTATTCGT	0	2	0	0	1	At1g14720.1:d:+985:primary
AAGATGATCT	1	2	0	0	0	At1g14600.1:X:-54:quaternary,At3g43890.1:X:-1538:quaternary,At4g18670.1:X:-823:quaternary,At5g26000.1:X:-295:quaternary
GGAAATGCGT	0	0	2	1	0	At1g14510.1:d:+939:primary
TGCTAGGCCA	0	2	0	1	0	At1g14460.1:d:+3510:primary
GAAATCTCCT	1	1	0	0	1	At1g14460.1:d:+291:secondary,At3g25840.1:d:+1434:secondary
AATGAAAAGA	1	0	0	1	1	At1g14390.1:v:+3014:primary
TCGGAGATGA	0	1	1	1	0	At1g14310.1:d:+646:primary
ATGTTCACCA	0	3	0	0	0	At1g14290.1:d:+791:primary
GGGCTAGGTT	2	1	0	0	0	At1g14200.1:d:+724:primary
CAGGACTAGA	0	0	0	2	1	At1g14130.1:d:+1042:primary
CTGGCGAATC	0	1	2	0	0	At1g14120.1:d:+996:primary
TCAAACACTT	2	1	0	0	0	At1g14020.1:d:+1752:primary
AAGATTGCAA	0	1	1	1	0	At1g13810.1:v:+1171:primary
TATCTAAACC	0	2	0	1	0	At1g13690.1:d:+833:primary
TTTAACACTC	1	0	0	0	2	At1g13580.1:d:+948:secondary
GCCGCACCGC	0	3	0	0	0	At1g13300.1:d:+962:primary
GATACTTTCT	1	0	0	1	1	At1g13280.1:d:+1071:secondary,At1g22080.1:v:+1732:secondary
TGATTGATGA	0	1	0	1	1	At1g13070.1:p:+757:secondary,At3g53290.1:v:+892:secondary,At3g26160.1:d:+776:secondary,At1g13080.1:d:+789:secondary,At1g13080.2:d:+737:secondary
TATTCGTTAT	0	1	0	2	0	At1g12990.1:d:+1963:primary
GGGTTACCAG	0	1	2	0	0	At1g12780.1:d:+1243:secondary
ACCCACGACA	1	1	0	0	1	At1g12760.1:d:+1300:secondary
ATAACCCAAG	1	0	0	1	1	At1g12660.1:i:+1926:tertiary
GTGTTAAGCT	0	0	1	0	2	At1g12640.1:d:+1374:primary
TAGTGATGAT	1	0	1	0	1	At1g12530.1:v:+848:primary
TTCAGATTAA	0	1	1	0	1	At1g12390.1:d:+765:primary
TTGGGAATAT	1	0	1	0	1	At1g12350.1:d:+1240:primary
GAGGAAACCG	1	0	2	0	0	At1g12330.1:d:+1330:primary
ATTGTACCGA	3	0	0	0	0	At1g12230.1:d:+1507:primary
GAAGATGGAT	0	1	2	0	0	At1g12220.1:v:+867:secondary,At2g30050.1:d:+730:secondary
AAGTAAAAAA	1	0	2	0	0	At1g12160.1:i:+1350:tertiary
TCCTAAACCA	1	0	2	0	0	At1g12010.1:d:+571:secondary,At1g62380.1:d:+582:secondary
GTCTAAATTT	1	1	1	0	0	At1g12000.1:d:+2004:primary
AATCAATAAT	1	0	0	1	1	At1g11990.1:v:+2123:secondary,At5g57230.1:v:+1006:secondary
GGATATGTGA	0	1	2	0	0	At1g11860.2:d:+1260:secondary,At1g11860.1:d:+1259:secondary
TTAATGAGAG	0	3	0	0	0	At1g11680.1:d:+1244:primary
GTTTTTTGTT	0	0	1	1	1	At1g11610.1:v:+1967:secondary,At5g44670.1:d:+1777:secondary,At2g01460.1:v:+606:secondary
TTATTTATTG	3	0	0	0	0	At1g11580.1:d:-1814:secondary
AAACAAGCCC	0	0	1	0	2	At1g11490.1:v:+1448:secondary
TTGATCTGAT	0	0	1	1	1	At1g11475.1:d:+279:secondary
TTGCCTGAGA	1	0	1	0	1	At1g11390.1:d:+2342:primary
TTTTATTGTA	1	1	0	0	1	At1g11340.1:v:+197:secondary
AGCCTCGCCG	0	1	0	1	1	At1g11330.1:d:+2546:primary
TTACACAAAC	0	1	1	1	0	At1g11280.2:d:+2690:primary,At1g11280.3:d:+2654:primary,At1g11280.1:d:+2549:primary
AAACAGAGCA	1	1	1	0	0	At1g11240.1:d:+599:secondary
TGCGTCAAAG	0	0	3	0	0	At1g11200.1:d:+729:primary
AGCTTTACTT	1	0	2	0	0	At1g11180.1:d:-1100:secondary
ATCATAATGA	0	2	0	1	0	At1g11080.1:d:+1905:primary,At3g60380.1:d:+2218:primary

TGAGATTTTA	1	0	1	1	0	At1g11050.1:d:+1899:secondary
GGAATCATAG	0	0	2	1	0	At1g10900.1:d:+2321:primary
ATGAAACAGC	1	1	0	1	0	At1g10870.1:d:+2581:primary
CTCCTCCTGA	0	2	0	1	0	At1g10720.1:d:+944:secondary
CGCAACTAGC	0	0	1	0	2	At1g10630.1:d:+574:secondary
ACCGTGCGGT	0	1	1	0	1	At1g10520.1:v:+2412:primary
GAGCAAAACT	0	1	2	0	0	At1g10430.1:d:+1034:primary
GATACAAACG	0	0	3	0	0	At1g10310.1:d:+407:primary
GATCTTATAT	1	1	0	0	1	At1g10150.1:d:+1741:primary
TTGATACAAG	0	3	0	0	0	At1g10140.1:d:+370:primary
TGATGAGTCT	0	1	0	1	1	At1g10095.1:v:+653:secondary
CATCCTCATC	0	1	0	1	1	At1g09840.3:X:-237:quaternary
AATACAAACA	0	0	0	1	2	At1g09840.1:d:+1731:secondary,At1g09840.3:d:+1865:secondary,At1g09840.2:d:+1655:secondary,At1g28000.1:v:+1667:secondary
ACACCACCAT	2	1	0	0	0	At1g09820.1:d:+2107:primary
AGGAGACTCC	0	1	1	0	1	At1g09795.1:d:+1250:primary
GTCGTACTGG	0	1	0	0	2	At1g09590.1:d:+190:secondary,At1g57860.1:d:+214:secondary,At1g57660.1:d:+223:secondary
GATTGTTTAT	0	0	0	1	2	At1g09580.1:d:+835:primary
TCAATGGCGT	0	2	1	0	0	At1g09530.2:d:+1448:primary,At1g09530.1:d:+1408:primary
TTTTTCGATC	0	1	2	0	0	At1g09520.1:d:+923:primary
AACAAACCAA	1	0	0	2	0	At1g09510.1:d:+1301:secondary
CTCTGGAGGC	0	0	1	2	0	At1g09350.1:d:+772:primary
TCCTCGGATG	0	0	3	0	0	At1g09340.1:d:+1082:secondary
ACATAGTGCG	0	0	1	2	0	At1g09320.1:d:+1855:primary
ATAGTTAGAG	0	1	0	2	0	At1g09310.1:d:-695:secondary
TCTGGGGTTG	1	2	0	0	0	At1g09140.1:d:+563:primary
GGTGGTTTCG	0	0	3	0	0	At1g09070.1:d:+1022:secondary
CTAGAGTGGA	0	2	1	0	0	At1g08920.1:d:+1445:primary,At1g08920.2:d:+1466:primary
AAATAATAAA	0	2	0	1	0	At1g08840.1:v:+4593:primary
AATAAAGATG	1	1	0	0	1	At1g08710.1:d:+1097:primary
TATTTCACCT	1	1	1	0	0	At1g08700.1:d:+1552:primary
TGATTAATGC	0	2	0	0	1	At1g08560.1:d:+881:primary
TTGAGTTTGT	0	2	0	0	1	At1g08490.1:X:-445:quaternary,At1g21830.1:X:-110:quaternary
GTTACTGAAA	0	3	0	0	0	At1g08450.2:X:--2:quaternary
TTTCCTTCCA	2	0	1	0	0	At1g08300.1:v:-1731:secondary
GAACAACACA	2	1	0	0	0	At1g08040.1:d:+1278:primary
ATTCGGGCAG	1	0	0	1	1	At1g08010.1:d:+1061:primary
GTTTTAAATT	0	0	0	2	1	At1g07890.2:i:+574:tertiary,At1g07890.3:i:+669:tertiary,At1g07890.1:i:+969:tertiary,At2g41960.1:i:+552:tertiary
CTCTTGCAGT	0	1	0	0	2	At1g07840.1:d:+1215:primary,At1g07840.2:d:+1294:primary
CTGATAATTT	0	3	0	0	0	At1g07620.1:v:+3801:primary
CATTTTTATT	1	0	1	1	0	At1g07490.1:d:+515:primary
TCTTTCTACT	2	0	1	0	0	At1g07350.2:d:+1454:primary,At1g07350.1:d:+1370:primary
GAAAGTTTTT	2	0	0	1	0	At1g07200.1:d:+1797:primary
TGAAAGACGA	0	1	1	0	1	At1g07180.1:d:+918:primary
ATAAAACCTA	0	2	1	0	0	At1g07160.1:X:--37:quaternary
GACAAGAATC	1	1	0	0	1	At1g07140.1:d:+1125:secondary,At4g32400.1:d:+651:secondary
CCCTCTCAAG	0	2	1	0	0	At1g07090.1:d:+396:primary
AACTTCAGGT	0	1	0	2	0	At1g07010.1:d:+1207:secondary
CCTGGTTCTA	1	1	1	0	0	At1g06890.1:d:+983:primary
GAATGCACTG	1	0	2	0	0	At1g06870.1:d:+1471:primary
TAACATTGTT	0	0	2	0	1	At1g06770.2:d:+2165:primary,At1g06770.1:d:+1750:primary
TAAAGAGTCC	0	2	0	1	0	At1g06770.1:d:+749:secondary,At1g06770.2:d:+1164:secondary
TAGCAGCTTG	0	1	2	0	0	At1g06500.1:d:+340:primary
CTTGAATCTC	2	1	0	0	0	At1g06410.1:d:+2488:primary
TCCAAAAGAT	0	1	1	0	1	At1g06390.2:d:+1431:primary,At1g06390.1:d:+1605:primary
AGCCTGAAAA	1	0	1	1	0	At1g06190.1:d:+923:primary
TGGGTCACAA	0	1	1	1	0	At1g06180.1:d:-885:secondary
GAGCTCACAA	0	1	1	1	0	At1g06160.1:d:+108:secondary,At3g29635.1:v:+611:secondary
TTTGGGGAAG	0	1	2	0	0	At1g06080.1:d:+762:primary
TTGGATTATC	0	1	0	2	0	At1g05940.1:d:+1703:primary

TAGCCAGCAA	0	0	2	1	0	At1g05850.1:d:+521:secondary
GGAATAGATT	0	2	1	0	0	At1g05820.1:v:-1309:secondary
AACTGTAATT	0	2	0	1	0	At1g05820.1:v:+30:secondary
TCTAATTTTG	1	1	1	0	0	At1g05785.2:d:+639:primary,At1g05785.1:d:+652:primary
GTTGGCAATG	0	0	1	1	1	At1g05710.1:d:+760:primary,At1g05710.3:d:+1021:primary,At1g05710.4:d:+649:primary,At1g05710.2:d:+1087:primary
TATACATTTG	1	1	0	0	1	At1g05630.1:X:+595:quaternary
TTCTTTGTCT	0	2	0	1	0	At1g05577.1:v:+1658:primary,At2g39900.1:d:+843:primary
GACATATAAA	0	0	2	0	1	At1g05350.1:v:+1803:secondary,At1g66830.1:d:+1655:secondary
GGGTTTACCG	0	0	2	0	1	At1g05205.1:d:+74:primary
TGGTTTCTGC	0	1	0	2	0	At1g05180.2:d:+1392:primary,At1g05180.1:d:+1531:primary
TAATCTTCAA	0	1	2	0	0	At1g05010.1:X:-193:quaternary
TCTTTGAAAT	0	2	0	1	0	At1g04970.1:d:+1274:primary,At1g04970.2:d:+845:primary
GATTGGCTAT	0	1	1	0	1	At1g04940.1:i:+1524:tertiary
TCCGGTTTGC	0	0	2	1	0	At1g04790.1:d:+1952:primary
CAGAAGCTTA	1	1	0	0	1	At1g04550.2:d:+990:primary,At1g04550.1:d:+1144:primary
AAAATTGTTA	2	0	1	0	0	At1g04520.1:d:+1312:primary
AGTACGCCAA	0	0	0	1	2	At1g04410.1:X:-80:quaternary
CAACAAAAAA	0	0	0	1	2	At1g04160.1:v:+1255:secondary
AAGCAATCGA	1	0	2	0	0	At1g04120.1:d:+2881:secondary
GAAGCAAGTG	0	1	0	2	0	At1g04080.1:d:+1366:secondary
CAAATGTGCG	0	1	1	1	0	At1g03760.1:d:+1301:primary
ACAGACAGCT	1	1	1	0	0	At1g03740.1:d:+2713:primary
GTGTGGACCC	1	0	1	1	0	At1g03680.1:d:+446:secondary
TTAACAATTT	1	1	1	0	0	At1g03610.1:X:+575:quaternary,Chr3:+11468034:quaternary,Chr3:+16032268:quaternary
TTTTACTCCG	0	3	0	0	0	At1g03440.1:d:+1019:primary
ATATAATACG	2	0	0	0	1	At1g03400.1:d:+1177:primary
GAACTCACCG	1	0	1	1	0	At1g03130.1:d:+350:secondary
TTTTGCTTCA	0	1	2	0	0	At1g03060.1:d:+11274:primary
GTTGTTTCAT	3	0	0	0	0	At1g03050.1:v:+2546:primary
TGGACGCTCG	0	1	2	0	0	At1g02930.1:X:-367:quaternary,At1g02920.1:X:-370:quaternary
GACTTCTGAA	1	2	0	0	0	At1g02910.1:X:-189:quaternary
AAGTTGTTTG	0	1	0	1	1	At1g02900.1:d:+557:primary
TACATTTGTT	1	0	1	0	1	At1g02670.1:i:+2660:tertiary
ATATGAACTC	0	3	0	0	0	At1g02660.1:d:+2061:primary
AAACTTACTC	1	1	1	0	0	At1g02510.1:v:+1510:primary
GAATTAGGTT	1	1	0	0	1	At1g02460.1:i:+1700:tertiary
TCGCCTAAAA	0	2	0	1	0	At1g02405.1:v:+354:secondary
TTGGATTTGT	1	1	0	0	1	At1g02350.1:v:+968:secondary,At5g35076.1:p:+395:secondary
CTTATAAGAG	1	0	1	1	0	At1g02340.1:X:+542:quaternary
AGGACTTTGT	0	0	2	0	1	At1g02140.1:d:+546:secondary
ATTCATTCGA	0	1	1	1	0	At1g02000.1:d:+1620:primary
ATGAAAAAAG	1	0	0	2	0	At1g01980.1:v:+2426:primary
GGAAGAGATT	0	1	0	0	2	At1g01960.1:d:+5371:primary
AGCTGAGTGA	1	0	1	0	1	At1g01940.1:i:-275:tertiary
AATGACTTTG	1	0	1	0	1	At1g01940.1:d:-421:secondary,At4g37040.1:d:-795:secondary
TACACTCAGA	0	1	0	2	0	At1g01940.1:d:+609:primary
CCCTCACGTT	0	1	0	1	1	At1g01650.1:d:+1443:secondary
TAATTTTTCA	3	0	0	0	0	At1g01630.1:d:+1088:primary
CAAAAAACGG	0	2	1	0	0	At1g01510.1:d:+1324:primary
GAAGCAAGGA	0	2	1	0	0	At1g01240.3:d:+1308:primary,At1g01240.2:d:+735:primary,At1g01240.1:d:+655:primary
TTGCCTGCAA	0	2	1	0	0	At1g01070.2:d:+535:secondary,At1g01070.1:d:+413:secondary,At3g58050.1:d:+1550:secondary
CGATCCGAAT	0	0	0	2	0	No gene matches found
GAAAGGTTCA	0	0	0	1	1	No gene matches found
AAGGTGTGAC	0	1	1	0	0	No gene matches found
CTCAGCCCTT	0	2	0	0	0	No gene matches found
TAACCGTTGG	0	0	2	0	0	No gene matches found
AGACTTATTG	0	0	1	1	0	No gene matches found
CTGGATCATA	0	0	0	0	2	No gene matches found
CTGTGCCGGT	1	0	1	0	0	No gene matches found
ACGCAGACTG	1	0	0	1	0	No gene matches found

AAACCCTTCG	0	2	0	0	0	No gene matches found
TAAACTCATA	0	0	1	1	0	No gene matches found
GTACGGATAT	0	0	1	1	0	No gene matches found
GTGCATTGGT	2	0	0	0	0	No gene matches found
AACGAATTTG	1	0	1	0	0	No gene matches found
TGTTTTTACG	0	2	0	0	0	No gene matches found
TAAGGTGTAG	0	1	0	0	1	No gene matches found
TTACGGAAAA	0	1	0	0	1	No gene matches found
CAGGTGGGAC	0	0	1	0	1	No gene matches found
CGAATTTATT	0	2	0	0	0	No gene matches found
GACTCACACT	0	1	1	0	0	No gene matches found
TGGGCTGGCC	2	0	0	0	0	No gene matches found
CTCTAGAATG	0	0	1	0	1	No gene matches found
AGGCGAAAGA	1	0	0	1	0	No gene matches found
TATCACTCCG	0	0	0	2	0	No gene matches found
TTTGCGATGA	0	2	0	0	0	No gene matches found
GTTCGGAATG	0	0	1	1	0	No gene matches found
GTTGACCAGC	0	2	0	0	0	No gene matches found
CCCCTACATA	0	2	0	0	0	No gene matches found
TCAAAGGCGT	1	0	1	0	0	No gene matches found
ATGCCTCAAA	0	1	0	1	0	No gene matches found
CTACAACTAA	2	0	0	0	0	No gene matches found
ATGGACAATA	0	0	2	0	0	No gene matches found
CACTTGTGGC	0	2	0	0	0	No gene matches found
TGCAGCCTCA	0	2	0	0	0	No gene matches found
ATTCTGTGTT	0	2	0	0	0	No gene matches found
CGTACTAAGT	0	0	0	1	1	No gene matches found
GGCCTTCGTT	0	2	0	0	0	No gene matches found
GACTTTCCTC	0	2	0	0	0	No gene matches found
GATAAGCCCA	1	0	0	1	0	No gene matches found
GGAGGGGAGC	0	1	0	1	0	No gene matches found
CTTGACCCAA	0	1	0	1	0	No gene matches found
CCTCTGATGT	1	0	0	1	0	No gene matches found
TTTACAGTTA	1	0	0	0	1	No gene matches found
GGCGAACGAG	0	0	2	0	0	No gene matches found
CAAATCAGGT	0	0	2	0	0	No gene matches found
TTTCACCCAG	2	0	0	0	0	No gene matches found
TGAGGTGACG	0	1	1	0	0	No gene matches found
TGATGAGTAT	1	0	1	0	0	No gene matches found
CGGACTAGCT	0	0	2	0	0	No gene matches found
ACGCAGCCGC	0	2	0	0	0	No gene matches found
CCCGAAAAAA	1	1	0	0	0	No gene matches found
TGTGGTCCAG	0	0	2	0	0	No gene matches found
GGCCTTCGAA	2	0	0	0	0	No gene matches found
CATTTCACGG	1	1	0	0	0	No gene matches found
AGGATATTGT	0	0	0	2	0	No gene matches found
CCTGGACTTG	0	0	1	1	0	No gene matches found
GAAATCCGCG	0	0	1	1	0	No gene matches found
CTGGTGTGGC	0	2	0	0	0	No gene matches found
CCCAACCCAC	0	0	0	1	1	No gene matches found
GCGGGATCAC	0	0	2	0	0	No gene matches found
GTTACTGAAG	0	2	0	0	0	No gene matches found
CCAAAATGAT	0	2	0	0	0	No gene matches found
CTTGAGGACT	1	1	0	0	0	No gene matches found
GCCGATCTTA	0	2	0	0	0	No gene matches found
ATACTGTCGA	0	1	0	0	1	No gene matches found
AGACAGGTCT	0	0	0	2	0	No gene matches found
GGTTACTACA	0	1	1	0	0	No gene matches found
TGGCAGCAGT	0	0	2	0	0	No gene matches found
TTTCTAAGGA	0	0	1	1	0	No gene matches found
AGTCGCATCT	1	1	0	0	0	No gene matches found

CTTCTGATGA	0	1	0	1	0	No gene matches found
GGCGAAGGAC	0	0	2	0	0	No gene matches found
TGGAGACAAA	0	0	2	0	0	No gene matches found
GGAATGCTCT	0	0	0	1	1	No gene matches found
CCTGCCGTAG	0	0	2	0	0	No gene matches found
CCTGCCTCAG	0	0	1	0	1	No gene matches found
TCCTATCGCT	1	0	1	0	0	No gene matches found
GTTCATCGAA	0	0	1	0	1	No gene matches found
GTGAAGTCTA	2	0	0	0	0	No gene matches found
ACACGGACTT	0	2	0	0	0	No gene matches found
AGGACCAAAG	1	0	1	0	0	No gene matches found
GTAATAGAAG	0	0	1	1	0	No gene matches found
ACCTGAACGA	0	2	0	0	0	No gene matches found
TGGCACGAGT	0	0	1	1	0	No gene matches found
TGTACTAAGG	0	0	0	0	2	No gene matches found
CGATGCGATG	0	0	0	2	0	No gene matches found
TTGGTCCGGC	0	0	2	0	0	No gene matches found
AGATCTAATG	1	1	0	0	0	No gene matches found
GGGAGAAGCA	0	1	0	1	0	No gene matches found
TGGGACTTGA	0	2	0	0	0	No gene matches found
ACTGGGCTCG	0	1	1	0	0	No gene matches found
GCCCCTGCGA	0	0	0	1	1	No gene matches found
TCTCTCCAGG	1	0	0	1	0	No gene matches found
GGAAATGTTC	0	1	0	1	0	No gene matches found
TCCGAGTCAA	0	2	0	0	0	No gene matches found
CGTTGTTCCC	0	0	0	2	0	No gene matches found
CCTTTTAAGG	1	0	0	1	0	No gene matches found
TGTACTTTTA	1	0	0	0	1	No gene matches found
GCCTAATGAA	0	0	0	0	2	No gene matches found
TCCGAATCCT	0	0	0	1	1	No gene matches found
GTTGCCATAA	0	1	0	0	1	No gene matches found
AAGTGCCTTG	0	0	0	2	0	No gene matches found
GGGCAGACTG	1	0	1	0	0	No gene matches found
CCCCAGAAAG	0	0	1	1	0	No gene matches found
CTTCTGAAGT	0	2	0	0	0	No gene matches found
CGCCCGCCTG	0	2	0	0	0	No gene matches found
CTCGGAGACC	1	1	0	0	0	No gene matches found
CCGAGCGCGT	0	0	1	1	0	No gene matches found
CGGCTGCTTC	0	2	0	0	0	No gene matches found
TAGAAGAGGA	0	1	1	0	0	No gene matches found
ATGTTTGCGT	0	0	2	0	0	No gene matches found
CAGGTGGGGA	1	0	1	0	0	No gene matches found
GAGCAAAAAA	1	0	0	1	0	No gene matches found
TGGACCTTCT	2	0	0	0	0	No gene matches found
CAGAGCATTA	1	0	0	1	0	No gene matches found
CGTGAAAAAA	1	0	0	1	0	No gene matches found
GAGACACGTG	1	1	0	0	0	No gene matches found
CGCCCGCAAA	1	0	1	0	0	No gene matches found
CCGACTCTAT	0	0	0	1	1	No gene matches found
GGATTAGCGA	0	0	1	0	1	No gene matches found
AGTCACGTCT	0	2	0	0	0	No gene matches found
AAAGGTCTCG	0	2	0	0	0	No gene matches found
GAAATGAACC	1	0	0	1	0	No gene matches found
GCAGTTTTAA	0	1	0	1	0	No gene matches found
TGACTCTCAA	0	1	0	0	1	No gene matches found
TGGAAAGGTT	0	1	1	0	0	No gene matches found
CTAGGAGCCC	1	0	0	1	0	No gene matches found
GAACTATTAG	1	0	1	0	0	No gene matches found
TGAGATGATG	0	1	0	1	0	No gene matches found
CGTTTTGTAA	0	1	1	0	0	No gene matches found
TGCCCGCCGC	0	1	0	1	0	No gene matches found

TGAACGCCAT	1	1	0	0	0	No gene matches found
CAAACAATCC	0	1	1	0	0	No gene matches found
CCGAAAAAAA	0	0	1	1	0	No gene matches found
TGGTCAAAAA	0	0	1	0	1	No gene matches found
GATAAGCTCC	0	1	1	0	0	No gene matches found
AGGATCCGCG	0	1	0	1	0	No gene matches found
TTACTTGAAA	0	1	0	0	1	No gene matches found
AGTTTTGGTG	0	0	0	0	2	No gene matches found
CTTCTAGGGA	1	0	0	1	0	No gene matches found
GCTGTTCTTA	0	2	0	0	0	No gene matches found
CCTAAGAGCT	0	0	2	0	0	No gene matches found
CAGGGTGTGG	0	1	0	1	0	No gene matches found
AGGCAGACTA	0	0	0	0	2	No gene matches found
TAAAACTTGG	0	0	2	0	0	No gene matches found
AGCGAACGAC	0	1	0	1	0	No gene matches found
GACCTGAACG	0	0	1	1	0	No gene matches found
GTGCGGATCT	0	0	2	0	0	No gene matches found
CGACTTTTTG	0	2	0	0	0	No gene matches found
GCACAATGCT	0	1	1	0	0	No gene matches found
AAGGTGGGAC	1	0	1	0	0	No gene matches found
TAGGAGTTTT	0	0	0	1	1	No gene matches found
GAAAGCTTCG	1	1	0	0	0	No gene matches found
CGAACGAGAG	0	1	1	0	0	No gene matches found
TTGTTAACAG	0	0	1	0	1	No gene matches found
TTTCGATGTG	0	0	1	1	0	No gene matches found
ATAACTGAGT	1	0	0	0	1	No gene matches found
CTTGCGATGG	0	1	0	1	0	No gene matches found
GGGCTTTCTT	1	0	0	0	1	No gene matches found
GAGAGGGTGC	0	0	1	0	1	No gene matches found
AACACTGCCG	0	1	0	0	1	No gene matches found
AAGACCGTGG	0	1	1	0	0	No gene matches found
CTTTGTATGC	0	0	0	0	2	No gene matches found
TTTCTGGTGA	0	1	1	0	0	No gene matches found
TCCTGTTTAA	0	1	0	0	1	No gene matches found
TCCGAACCTT	0	0	1	1	0	No gene matches found
GGCAGGCAGA	0	1	1	0	0	No gene matches found
TCTACTTTCA	2	0	0	0	0	No gene matches found
CGTCGTTGGA	0	1	0	1	0	No gene matches found
TTATGCGTTA	1	0	1	0	0	No gene matches found
AGACACTCAC	1	0	0	1	0	No gene matches found
GATTCTTAGG	1	0	0	0	1	No gene matches found
GGTTTACATA	1	0	1	0	0	No gene matches found
TATAATCGAC	1	0	0	1	0	No gene matches found
GTTATCTCTA	0	1	1	0	0	No gene matches found
GCTTCTTAAA	0	1	0	1	0	No gene matches found
CACTTGGAAT	0	1	1	0	0	No gene matches found
TCGAATAAAA	1	0	1	0	0	No gene matches found
AAAAAGCCGT	1	0	1	0	0	No gene matches found
CGAGGTGATG	0	0	1	0	1	No gene matches found
GCACAAGAAC	1	1	0	0	0	No gene matches found
CAGACACGTG	0	1	0	1	0	No gene matches found
CCCCTTCGCC	1	0	0	1	0	No gene matches found
GAGGTGGCGA	0	2	0	0	0	No gene matches found
AGCCGTAATG	1	0	0	1	0	No gene matches found
TGCACTCCTA	1	1	0	0	0	No gene matches found
GGAGCTGTAG	0	2	0	0	0	No gene matches found
TAAGCTATGG	1	0	0	0	1	No gene matches found
TGGTCTTAAG	1	0	0	1	0	No gene matches found
GACCACAACC	0	0	1	0	1	No gene matches found
GTGCGAAGCT	0	0	0	2	0	No gene matches found
TATGTTGAAA	0	2	0	0	0	No gene matches found

GTGCGTCTGT	0	1	0	1	0	No gene matches found
TGACGAGTTT	0	0	0	1	1	No gene matches found
GCAACAGTTA	1	0	1	0	0	No gene matches found
GGCAGGCGAG	0	2	0	0	0	No gene matches found
CCAAAGGCCT	0	1	0	1	0	No gene matches found
GTCGCTCCTA	0	0	0	2	0	No gene matches found
TCCGAATCAT	0	1	1	0	0	No gene matches found
TCCGGTTTAT	1	0	0	1	0	No gene matches found
GCCACTCCTA	1	1	0	0	0	No gene matches found
AAGGTTCTCG	0	1	0	1	0	No gene matches found
AATTGTGACA	0	1	1	0	0	No gene matches found
GTACGGATAC	0	0	2	0	0	No gene matches found
CCTCGCGTAC	0	0	1	1	0	No gene matches found
ACACTAAGTG	0	0	1	1	0	No gene matches found
ATACGTCTAC	0	2	0	0	0	No gene matches found
CGCCCGCAGC	0	1	0	1	0	No gene matches found
GCGAACGACG	1	0	0	0	1	No gene matches found
GTCAAGGCCT	1	1	0	0	0	No gene matches found
AGGCTGTTCA	1	0	0	1	0	No gene matches found
TTCGGCTTTG	1	1	0	0	0	No gene matches found
GTGGGTGTGC	0	1	1	0	0	No gene matches found
CGATGTAGCT	0	0	1	0	1	No gene matches found
GATCCTATGT	0	0	0	2	0	No gene matches found
AGCACTGCTG	1	0	0	1	0	No gene matches found
TCTTGCTGAG	0	2	0	0	0	No gene matches found
AGGAGACCGT	0	2	0	0	0	No gene matches found
TAGGCTATAG	0	0	0	1	1	No gene matches found
CAGACTAGAC	0	0	1	0	1	No gene matches found
CGGAGAAAGG	1	0	0	1	0	No gene matches found
CACACTGCTG	1	0	1	0	0	No gene matches found
CACCTGAAGG	0	0	0	2	0	No gene matches found
GGAAGACGAA	1	0	1	0	0	No gene matches found
GCCGCTCTTA	0	2	0	0	0	No gene matches found
GTTTACGAAG	0	2	0	0	0	No gene matches found
GCTTCAGACG	0	0	0	2	0	No gene matches found
GGAGGAAACT	0	0	1	0	1	No gene matches found
TCAAATCGTT	0	1	0	1	0	No gene matches found
TCTTGCCGTG	0	0	1	0	1	No gene matches found
TCTACTGACG	0	0	0	1	1	No gene matches found
GAGGGAATAC	0	1	1	0	0	No gene matches found
CAACGTATAC	0	2	0	0	0	No gene matches found
ACAGGTTTAG	0	0	0	1	1	No gene matches found
GCCGTTCCTA	0	2	0	0	0	No gene matches found
GCCATAAAAA	0	0	0	2	0	No gene matches found
GAGTTCGTGG	0	2	0	0	0	No gene matches found
TGGCACAGTA	2	0	0	0	0	No gene matches found
ACTGATATGA	1	0	0	1	0	No gene matches found
AAGGCGAAGT	0	0	1	0	1	No gene matches found
GGGCAACAGT	2	0	0	0	0	No gene matches found
TCACTAAGTG	1	0	0	1	0	No gene matches found
ACAAAGGCCT	0	0	1	0	1	No gene matches found
AGTGTACTTT	0	0	0	1	1	No gene matches found
GCAGTGACCA	0	1	0	1	0	No gene matches found
TGTGGCCACT	0	1	0	1	0	No gene matches found
CCACTAAGTT	0	0	0	1	1	No gene matches found
GTTCGTTGGG	1	0	0	0	1	No gene matches found
TGGTCTTGGT	0	1	1	0	0	No gene matches found
TGAATCTCGT	0	0	1	0	1	No gene matches found
CTAGGGACAT	0	2	0	0	0	No gene matches found
CACCTAAAAA	1	0	1	0	0	No gene matches found

GCTTGCTGCT	0	2	0	0	0	No gene matches found
GGCGAGCGAC	1	1	0	0	0	No gene matches found
GCATTTCCAC	0	2	0	0	0	ChrM:-351127:quaternary
GTTGGCCCTC	0	2	0	0	0	ChrM:-29701:quaternary
CTGAGTCACA	1	0	0	0	1	ChrM:-288198:quaternary
GCGCAAAAGC	0	1	1	0	0	ChrM:-288059:quaternary
GGTTTTTTCA	0	1	1	0	0	ChrM:+337396:quaternary
CTTGGAACGG	0	1	0	1	0	ChrM:+205738:quaternary
GTTGGAGACC	0	1	1	0	0	ChrM:+181852:quaternary,Chr2:+3438317:quaternary,ChrM:+47687:quaternary
AGTGGATCAG	0	2	0	0	0	ChrM:+122616:quaternary
CAGGATGGGG	1	0	1	0	0	ChrC:-56714:quaternary
TCCGGTTCCC	1	0	1	0	0	ChrC:+77151:quaternary,Chr5:+25520671:quaternary
CCTACTATAA	1	0	0	0	1	ChrC:+65715:quaternary
CGAGATAACG	1	0	1	0	0	ChrC:+138461:quaternary
TTGTCAAAGA	1	0	0	0	1	ChrC:+130844:quaternary
GGTTGAGAAC	0	2	0	0	0	ChrC:+129519:quaternary
CGTATTCTTA	0	1	1	0	0	ChrC:+123912:quaternary
CCACTGAAAG	0	0	0	0	2	ChrC:+103819:quaternary
GCTGTCGTCA	1	1	0	0	0	ChrC:+102018:quaternary; +small RNA(GCATGGCTGTCGTCAGC; GTGCATGGCTGTCGTCA)
ACGTTAAATT	0	1	1	0	0	Chr5:-9368343:quaternary
AAGTTTCCGC	0	2	0	0	0	Chr5:-8771712:quaternary
GGTGATGTGT	0	1	0	1	0	Chr5:-23018177:quaternary
CTTAGCTAGT	0	0	0	0	2	Chr5:-18781342:quaternary
ATGTGTGGAT	0	0	2	0	0	Chr5:-172003:quaternary
CATTGTTGAA	1	1	0	0	0	Chr5:-16223044:quaternary,Chr5:-17831185:quaternary,At4g10800.1:X:-1370:quaternary
GGTTTGGTGA	1	0	1	0	0	Chr5:-15330449:quaternary
AGTGTATAAT	0	1	0	0	1	Chr5:-14973738:quaternary,Chr5:-3732635:quaternary
TATCTAAAAA	1	1	0	0	0	Chr5:-14458907:quaternary,Chr4:-2349670:quaternary,Chr5:-14518207:quaternary
GCTGGTAGAA	0	2	0	0	0	Chr5:-14248338:quaternary
TGTTGTATGT	0	1	0	1	0	Chr5:-11468054:quaternary
AACTATAATT	0	2	0	0	0	Chr5:+9635147:quaternary
AAGTATTCTC	1	1	0	0	0	Chr5:+9305314:quaternary
TCTGTTTCTC	2	0	0	0	0	Chr5:+9268913:quaternary
AAAACATACG	0	2	0	0	0	Chr5:+9134905:quaternary,Chr1:+7402269:quaternary
ATAACGCCAT	0	1	1	0	0	Chr5:+714684:quaternary
CAATAAATTT	0	1	1	0	0	Chr5:+7060038:quaternary
ACAATTGTCT	0	0	2	0	0	Chr5:+4634825:quaternary
GCCTGCTGGC	0	1	1	0	0	Chr5:+4122103:quaternary
AGTTGTCTTT	0	1	0	0	1	Chr5:+4086406:quaternary,Chr5:+23804477:quaternary
TAGTAATAAA	1	0	0	0	1	Chr5:+3480983:quaternary
TGTGGACCAT	0	2	0	0	0	Chr5:+26615342:quaternary
AAACTTTGTT	0	0	1	1	0	Chr5:+26274078:quaternary
GCTGTCACTA	0	2	0	0	0	Chr5:+24684735:quaternary
TATAAAACAA	0	0	0	0	2	Chr5:+24433340:quaternary
ATCAAAATAG	1	0	0	1	0	Chr5:+24336768:quaternary,Chr2:+8141235:quaternary
TTTTACTTTA	1	0	0	0	1	Chr5:+24323011:quaternary
ATAGGAGTTA	1	0	0	0	1	Chr5:+24263300:quaternary
AAAATAAGAA	1	0	0	0	1	Chr5:+23492819:quaternary
ATCAATACTT	1	0	0	1	0	Chr5:+23375479:quaternary
TTTACACATA	0	2	0	0	0	Chr5:+23255448:quaternary,Chr5:+14297152:quaternary
AACCGAACCT	0	0	0	2	0	Chr5:+21824829:quaternary
AAAATGATCT	1	1	0	0	0	Chr5:+21423266:quaternary
GCTCACTTTG	0	1	1	0	0	Chr5:+21163093:quaternary
AATCCTAAAA	0	0	0	2	0	Chr5:+20973303:quaternary
CAAATTTATA	0	2	0	0	0	Chr5:+20961525:quaternary,Chr5:+6443658:quaternary,Chr1:+4338190:quaternary,Chr4:+16055131:quaternary,Chr3:+2101939:quaternary
TTAGAAAAAA	1	0	1	0	0	Chr5:+2075550:quaternary,Chr2:+11240716:quaternary
CACAAGAAAA	0	1	1	0	0	Chr5:+20558586:quaternary
CTCCGGTTTA	0	1	1	0	0	Chr5:+19847882:quaternary
TGTGATAAAC	0	2	0	0	0	Chr5:+19524454:quaternary
GCTATATAAG	1	1	0	0	0	Chr5:+19412359:quaternary

TAAACTCTCT	0	2	0	0	0	Chr5:+19037861:quaternary,At1g76880.1:X:+-179:quaternary
TAAATCAAAA	1	1	0	0	0	Chr5:+18979519:quaternary,Chr2:+15144532:quaternary,Chr3:+6721132:quaternary
ATATAAGATT	2	0	0	0	0	Chr5:+1866941:quaternary
TCTATACAAA	1	0	0	1	0	Chr5:+17789738:quaternary
ATTATAACGA	0	1	0	1	0	Chr5:+17533581:quaternary; small RNA(MIR166f)
TTCAATCAAA	0	2	0	0	0	Chr5:+17291269:quaternary
GTTTGGTTCG	0	0	1	1	0	Chr5:+16764368:quaternary
ACCATTGTTC	0	1	1	0	0	Chr5:+16134215:quaternary
TCCAAATCTT	0	1	1	0	0	Chr5:+14918355:quaternary
CTGCCCAAAA	0	1	1	0	0	Chr5:+14324686:quaternary
TAAACTTGTA	0	2	0	0	0	Chr5:+14264721:quaternary
AAAAAGCTAG	0	0	0	2	0	Chr5:+13845651:quaternary
GATTTTTTAT	0	0	1	0	1	Chr5:+132004:quaternary,At2g16700.1:X:+513:quaternary
TAATTACTTT	0	1	0	0	1	Chr5:+13067401:quaternary,Chr5:+13878914:quaternary,Chr1:+21177207:quaternary
CCTTGAGAAA	0	1	0	1	0	Chr5:+12696668:quaternary
TCTTCTTAAA	1	0	0	0	1	Chr5:+1142479:quaternary
TTTTAGACAT	0	2	0	0	0	Chr5:+10736157:quaternary
AAAAATATCA	0	1	1	0	0	Chr5:+10295570:quaternary,Chr5:+14079690:quaternary,Chr3:+2884308:quaternary,Chr5:+14072665:quaternary
GCCTAATAAA	1	0	0	0	1	Chr4:-9830100:quaternary
GTCCGTTGAG	0	0	0	0	2	Chr4:-6356191:quaternary
GACTGACATC	1	0	0	1	0	Chr4:-17280746:quaternary
GGAACAGTGT	0	0	1	1	0	Chr4:-12923230:quaternary
AAGCTTAAAT	0	0	2	0	0	Chr4:-11893059:quaternary
ATTTGTTTAA	1	1	0	0	0	Chr4:+9999065:quaternary
TAATCAACAC	0	1	0	1	0	Chr4:+9483048:quaternary
GAGCCACTTG	1	1	0	0	0	Chr4:+8985920:quaternary
GCAAATACTT	0	0	1	0	1	Chr4:+8985768:quaternary
CCTTTTGATC	0	0	0	1	1	Chr4:+8460311:quaternary
TGTGAAAAGA	0	0	2	0	0	Chr4:+8387016:quaternary
TTTAGGAATT	1	0	0	1	0	Chr4:+8346721:quaternary,Chr4:+12335292:quaternary
TTGTGTAAAA	0	0	0	1	1	Chr4:+8176047:quaternary
AACATATTAA	1	1	0	0	0	Chr4:+6735406:quaternary
GTTAAGCTCG	0	1	0	0	1	Chr4:+6616317:quaternary
ATATTTCTCT	0	2	0	0	0	Chr4:+6507567:quaternary
AGTTTTAGTT	0	0	0	2	0	Chr4:+6199646:quaternary
TTCCCTCTTT	0	2	0	0	0	Chr4:+5630254:quaternary,Chr3:+4042990:quaternary
ATAATTCGTG	2	0	0	0	0	Chr4:+559492:quaternary
TTACAAAAAT	0	1	0	0	1	Chr4:+5432529:quaternary,Chr1:+18994362:quaternary,Chr1:+768788:quaternary,Chr1:+14011668:quaternary,Chr2:+18145037:quaternary,Chr1:+27599436:quaternary
TGAAAAAAAG	0	1	0	0	1	Chr4:+5143441:quaternary,Chr5:+21595674:quaternary
AAAAAACAAT	0	1	0	0	1	Chr4:+434139:quaternary
TAATCAAATC	1	0	0	1	0	Chr4:+3334490:quaternary
ACATTCATTA	1	0	0	1	0	Chr4:+2178905:quaternary
AAAATCATCT	0	2	0	0	0	Chr4:+18455354:quaternary
TCACTAAAAA	0	0	0	1	1	Chr4:+180298:quaternary
CAAAAAAAAG	0	0	1	1	0	Chr4:+17652146:quaternary
CCTAACAAAA	0	0	0	2	0	Chr4:+17526417:quaternary
GTTTGAAGGG	0	2	0	0	0	Chr4:+17382206:quaternary
GACGAGGATG	0	1	1	0	0	Chr4:+17350850:quaternary
TGTTGGTTGG	0	1	0	0	1	Chr4:+16880720:quaternary
CAAAAAAACA	1	1	0	0	0	Chr4:+16524559:quaternary,Chr3:+21563389:quaternary
AGTCACAAAA	0	1	0	1	0	Chr4:+16279393:quaternary
GGCTACTTCT	0	1	1	0	0	Chr4:+14956700:quaternary
ATAGTCAATA	0	0	1	0	1	Chr4:+14674040:quaternary
TTAAGACTTA	0	0	0	0	2	Chr4:+14267509:quaternary,At2g03670.1:X:+-119:quaternary
TTATTGTCAA	0	0	0	1	1	Chr4:+14082154:quaternary,Chr1:+26047960:quaternary
ACCTAAAAAA	1	0	0	1	0	Chr4:+13639221:quaternary,Chr3:+11956993:quaternary,Chr4:+6553065:quaternary
GGAAATCATT	0	0	2	0	0	Chr4:+11541509:quaternary
TAAGACATAT	1	1	0	0	0	Chr4:+11521937:quaternary

TTTAATCAAA	0	1	0	0	1	Chr4:+11373134:quaternary
TAGTCTGTTT	1	1	0	0	0	Chr4:+11210904:quaternary
AACGTAACAA	1	1	0	0	0	Chr4:+10428330:quaternary
GAGAACATTA	1	0	0	0	1	Chr3:-8594554:quaternary
CTTTTTAGGA	0	2	0	0	0	Chr3:-8065343:quaternary,At5g15320.1:X:-438:quaternary
TGAGGTATTC	0	0	0	1	1	Chr3:-7259800:quaternary
CAATTCCACA	0	0	1	0	1	Chr3:-6727795:quaternary
GTTTCGTCGC	0	0	1	1	0	Chr3:-5055768:quaternary
GTCGTGACCG	0	2	0	0	0	Chr3:-18399567:quaternary
GTTTAACCAC	0	0	0	1	1	Chr3:-1648917:quaternary
TCTCTTAGAG	0	2	0	0	0	Chr3:-15697566:quaternary
GAACAGTAGA	0	0	0	2	0	Chr3:-1522322:quaternary
AAGCAACTAA	0	1	1	0	0	Chr3:-14544873:quaternary
TGTCTCATTA	0	1	0	0	1	Chr3:-10867790:quaternary,Chr5:-15186008:quaternary,Chr2:-950710:quaternary,Chr2:-112532:quaternary
ATTTCTAAAA	1	0	1	0	0	Chr3:+9988760:quaternary,Chr5:+4776562:quaternary,At2g19160.1:X:+-129:quaternary
TCTAATGGTT	0	1	1	0	0	Chr3:+9512182:quaternary
TGAATCTCTT	0	0	1	1	0	Chr3:+9397022:quaternary,At5g23400.1:X:+-100:quaternary
AAACCAACAA	0	0	0	2	0	Chr3:+7803544:quaternary
TGGTTAAAAC	2	0	0	0	0	Chr3:+7321366:quaternary
GGATTGTAGT	0	1	0	1	0	Chr3:+7274814:quaternary
AAATAAGAGA	0	0	0	0	2	Chr3:+7093783:quaternary
CAAAGTAAGG	0	1	1	0	0	Chr3:+6564771:quaternary,ChrM:+339940:quaternary
ATTAATAAAA	0	2	0	0	0	Chr3:+6555628:quaternary,Chr5:+21210926:quaternary,At4g09800.1:X:+-234:quaternary
AAAAGTATAC	0	1	1	0	0	Chr3:+5754801:quaternary
TGATTTGTAA	0	1	0	0	1	Chr3:+5272909:quaternary,Chr3:+18582535:quaternary
TCGAATCATT	1	0	1	0	0	Chr3:+5080782:quaternary
GATGTGAGAT	1	0	1	0	0	Chr3:+4978446:quaternary
AGATCAAAGT	0	1	1	0	0	Chr3:+4582826:quaternary
TAACGTTGAC	1	0	0	1	0	Chr3:+4462178:quaternary
ATACCAAAAA	1	1	0	0	0	Chr3:+4234803:quaternary
AGAAAAGAGT	0	0	1	0	1	Chr3:+2364429:quaternary,At5g11010.3:X:+398:quaternary
GTCAAGTTTT	0	0	1	0	1	Chr3:+2351387:quaternary
TTCCAAATTA	0	0	1	1	0	Chr3:+22724132:quaternary
CTTCTCAAAA	2	0	0	0	0	Chr3:+22408464:quaternary
CAAAGCGTGT	0	2	0	0	0	Chr3:+22139943:quaternary
TATCTGTAGT	1	0	0	0	1	Chr3:+22058323:quaternary
TCGGAAAAAA	1	0	0	1	0	Chr3:+21567774:quaternary
GCGATGTATC	1	1	0	0	0	Chr3:+21132443:quaternary
TTGATTCATT	0	2	0	0	0	Chr3:+20657416:quaternary
GCAACAATAA	0	2	0	0	0	Chr3:+19819546:quaternary
GCTTTCACTA	2	0	0	0	0	Chr3:+19712624:quaternary
GAAGTAAAAA	0	2	0	0	0	Chr3:+19698280:quaternary,Chr1:+15644549:quaternary
ACTCTTTAAA	2	0	0	0	0	Chr3:+19609984:quaternary,Chr1:+4809242:quaternary
GGCCAAGCCC	0	0	1	1	0	Chr3:+19578079:quaternary
GGAATGAAAG	1	1	0	0	0	Chr3:+18714890:quaternary
AGACTTTATT	0	0	0	1	1	Chr3:+1747873:quaternary
TAGAAGCTAA	0	1	0	0	1	Chr3:+17195972:quaternary
CCATAACTTA	0	2	0	0	0	Chr3:+16800316:quaternary,Chr5:+26757299:quaternary
TATCACGTAT	1	0	0	0	1	Chr3:+16800193:quaternary
TTTTTTCCCA	0	1	0	0	1	Chr3:+16410413:quaternary
TCTTTCTAAA	0	1	0	0	1	Chr3:+15946207:quaternary,Chr2:+17152316:quaternary
GAGGAGGTGT	0	2	0	0	0	Chr3:+14790978:quaternary
AGAGCAAACC	0	1	1	0	0	Chr3:+14695961:quaternary,Chr1:+16350468:quaternary
TGATTTCTAT	2	0	0	0	0	Chr3:+14410495:quaternary
CAATGCAAAC	0	1	0	0	1	Chr3:+14231301:quaternary,Chr5:+26250060:quaternary,Chr3:+14217922:quaternary,Chr3:+14218426:quaternary
ATACTCTGTT	2	0	0	0	0	Chr3:+13960400:quaternary,Chr4:+7587837:quaternary
TTCTCTATGA	0	1	1	0	0	Chr3:+13394162:quaternary
CCGTCTTCTC	0	2	0	0	0	Chr3:+12834709:quaternary

TGAGCTTTGA	1	1	0	0	0	Chr3:+12161989:quaternary
TTTAGTCAAA	1	0	0	0	1	Chr3:+1215122:quaternary,Chr3:+8270534:quaternary
GTTCGAAGGA	0	2	0	0	0	Chr3:+11906012:quaternary
GTGATTTTGC	1	0	1	0	0	Chr3:+11481766:quaternary
AGTCAATGTC	0	1	0	1	0	Chr3:+10639447:quaternary
AAACTTCACT	0	1	1	0	0	Chr3:+10302114:quaternary
TAAGGAGTGT	1	0	0	1	0	Chr3:+10301506:quaternary
GAATGGAAAA	0	0	0	1	1	Chr3:+10114707:quaternary
TCGGTACGCT	1	0	1	0	0	Chr2:-6054:quaternary,Chr3:-14211011:quaternary
CCGATACGCG	1	1	0	0	0	Chr2:-5622:quaternary,Chr3:-14210579:quaternary
GGAAATGGAG	0	1	0	1	0	Chr2:-17777343:quaternary
CGTGATGTGT	0	1	1	0	0	Chr2:-15957998:quaternary
GTCGGATAAA	0	0	0	2	0	Chr2:-15285125:quaternary
CTAAGATCTT	0	1	1	0	0	Chr2:-15023450:quaternary
AAAGGTCTCT	0	0	2	0	0	Chr2:-14449940:quaternary
CGTAATAATG	0	1	1	0	0	Chr2:-11819842:quaternary,At5g64390.1:X:-369:quaternary
ACAATTTACA	1	1	0	0	0	Chr2:+9694342:quaternary
AACCACAATC	1	1	0	0	0	Chr2:+9527087:quaternary
GTCAACAATG	0	2	0	0	0	Chr2:+9417161:quaternary
TCATTTGTTT	0	1	0	0	1	Chr2:+9100068:quaternary
ATAGAAGATT	1	0	1	0	0	Chr2:+9047651:quaternary
GCTAAAAGGA	0	0	0	2	0	Chr2:+8556384:quaternary
GAACTTAAAT	1	1	0	0	0	Chr2:+8023307:quaternary
CACAACAAAA	0	0	0	2	0	Chr2:+7863130:quaternary
TTACTCTAAA	0	2	0	0	0	Chr2:+7719195:quaternary,Chr1:+19148929:quaternary,Chr5:+10736718:quaternary
AATGAAAGAA	1	0	0	1	0	Chr2:+7688143:quaternary
TGCTTCGTAT	1	0	0	0	1	Chr2:+7647562:quaternary
TGCTTCCACT	0	1	1	0	0	Chr2:+7530569:quaternary
GAAAATCTCA	2	0	0	0	0	Chr2:+7190237:quaternary,Chr2:+6060018:quaternary,Chr5:+17345089:quaternary
GTTTCCAATT	1	1	0	0	0	Chr2:+7071929:quaternary
ATTTGAGTAA	0	2	0	0	0	Chr2:+6922596:quaternary,Chr5:+8498322:quaternary
TAAAGCTTAT	0	0	0	0	2	Chr2:+6461758:quaternary
TAGCTGATAA	2	0	0	0	0	Chr2:+6096837:quaternary
CAAAATATGC	0	1	1	0	0	Chr2:+4602400:quaternary
AATATTCAAG	0	0	0	1	1	Chr2:+4465123:quaternary
AAGTGTGATC	0	0	0	2	0	Chr2:+4090698:quaternary
TGCTTGTCAT	0	0	0	0	2	Chr2:+3933500:quaternary
GATTTGTGAC	1	0	0	0	1	Chr2:+3535818:quaternary
AGGGGGAAGC	1	0	0	1	0	Chr2:+3497151:quaternary,ChrM:+106591:quaternary
CAGTACTTTG	0	2	0	0	0	Chr2:+3457158:quaternary,ChrM:+66532:quaternary
TGCAGTTCCT	0	0	2	0	0	Chr2:+3371273:quaternary,Chr4:+12995525:quaternary,ChrM:+114875:quaternary,Chr2:+3505441:quaternary
GTTCTTTGAA	0	1	1	0	0	Chr2:+2074479:quaternary,Chr1:+11929572:quaternary,Chr4:+1043513:quaternary
CTTTTTTCAT	0	2	0	0	0	Chr2:+2035166:quaternary
TGTCTAAAAA	1	0	1	0	0	Chr2:+19195424:quaternary
CCTTTGTTTT	0	0	1	1	0	Chr2:+17785133:quaternary,Chr2:+835309:quaternary
GCTATCTCTC	1	1	0	0	0	Chr2:+17013899:quaternary
ATTTGAAATA	0	1	1	0	0	Chr2:+16567067:quaternary
GAAATTGCTT	0	1	0	0	1	Chr2:+16152297:quaternary,Chr1:+16360769:quaternary,Chr3:+9085229:quaternary,Chr3:+9096488:quaternary
ATTCAATAAC	0	0	1	1	0	Chr2:+14073084:quaternary
TTTAAGAAAC	0	2	0	0	0	Chr2:+13918011:quaternary
AAAACATCTT	0	2	0	0	0	Chr2:+13341391:quaternary
TTGTGTTATC	1	0	0	0	1	Chr2:+13269781:quaternary,At1g04610.1:X:+536:quaternary,At3g07430.1:X:+1062:quaternary
AGAGAAACTA	1	1	0	0	0	Chr2:+12950373:quaternary
ATTTCAAGTG	0	0	1	0	1	Chr2:+12929583:quaternary
TTGGAAAAAA	0	0	2	0	0	Chr2:+12530067:quaternary,Chr3:+11616921:quaternary,Chr1:+10143617:quaternary
TTAAAGTTTA	0	0	0	1	1	Chr2:+12208774:quaternary
TGTTTTCAGA	1	0	0	1	0	Chr2:+12098667:quaternary,Chr1:+23653696:quaternary
ATTCGCTAAA	0	2	0	0	0	Chr2:+12033844:quaternary
CTTTTTAAGA	1	1	0	0	0	Chr2:+10802776:quaternary,At3g07790.1:X:+1930:quaternary

TGTTTTGGGT	2	0	0	0	0	Chr2:+10586851:quaternary,Chr2:+1257317:quaternary
ACTACAAGTA	0	0	0	0	2	Chr2:+10066983:quaternary
TCGAGACCGA	0	0	1	0	1	Chr1:-9347891:quaternary
TATAGGCATA	0	0	1	0	1	Chr1:-6426957:quaternary
TCTGTATTGA	0	2	0	0	0	Chr1:-5223257:quaternary
AGAATATTGC	1	0	0	1	0	Chr1:-3393112:quaternary
TTTATTGGAA	1	0	0	1	0	Chr1:-28008505:quaternary
TTGAAGTCAA	0	1	0	1	0	Chr1:-27155349:quaternary
TGACTGATTT	1	0	0	1	0	Chr1:-26522085:quaternary
TGGTTTTATG	0	1	1	0	0	Chr1:-24533955:quaternary,Chr4:-16397220:quaternary
TTCGTTTTAA	0	1	0	1	0	Chr1:-22785897:quaternary
CTAATACTGA	1	0	0	0	1	Chr1:-21056062:quaternary
ACAATGGCCT	0	0	1	1	0	Chr1:-20313187:quaternary
CCACCAAAAA	0	0	2	0	0	Chr1:-16275524:quaternary
GCAAATCATT	1	0	1	0	0	Chr1:-12872566:quaternary
TTTTGGATCG	0	1	1	0	0	Chr1:-10428932:quaternary
TTTACTGACA	1	1	0	0	0	Chr1:-10185853:quaternary
CAGAATCAAA	1	0	1	0	0	Chr1:+9349361:quaternary
AGTTTTTGGT	1	1	0	0	0	Chr1:+8591443:quaternary,Chr1:+15374445:quaternary,Chr3:+18580260:quaternary,Chr1:+15276348:quaternary,Chr1:+15292682:quaternary,Chr1:+15390786:quaternary,Chr1:+15272043:quaternary,Chr1:+15378918:quaternary,Chr1:+15370140:quaternary,Chr1:+15280821:quaternary
AATCCCTAAA	1	0	1	0	0	Chr1:+81:quaternary
CATTAATTAA	0	1	0	1	0	Chr1:+7523153:quaternary
TTTCATCCCT	0	1	1	0	0	Chr1:+6263944:quaternary
TGCGAAAAAA	1	0	1	0	0	Chr1:+6118255:quaternary
TTCAGTAGAA	0	1	0	0	1	Chr1:+5586081:quaternary
AAATATCGGT	0	2	0	0	0	Chr1:+5552556:quaternary
ACTACATAAA	0	0	0	1	1	Chr1:+521923:quaternary
TGAAAATTAA	0	1	0	1	0	Chr1:+4514700:quaternary
GGGAAAAAAA	0	1	0	0	1	Chr1:+432166:quaternary
TTGGTTAAAA	0	1	0	0	1	Chr1:+4211271:quaternary
AGAGTACAAC	2	0	0	0	0	Chr1:+4092558:quaternary
AAAGTGATCG	0	1	1	0	0	Chr1:+3884602:quaternary
GGTCGTACCA	0	1	0	1	0	Chr1:+3484310:quaternary
GGATGATTTT	0	0	0	1	1	Chr1:+3478527:quaternary
ATCGTCGACT	0	2	0	0	0	Chr1:+3431100:quaternary
ACATTTTGCA	0	0	1	1	0	Chr1:+29682240:quaternary
CATCATAATA	0	1	1	0	0	Chr1:+29418544:quaternary
GGATCCCTCT	0	0	1	1	0	Chr1:+29229273:quaternary
TTGAGTGGTT	0	0	1	1	0	Chr1:+29189592:quaternary
AGTTTTTGGA	0	2	0	0	0	Chr1:+28353985:quaternary
ATCACTAATC	0	0	1	0	1	Chr1:+28005568:quaternary
GGGAAGTCTT	1	0	1	0	0	Chr1:+27991298:quaternary
ATCAGAGAAT	0	0	0	1	1	Chr1:+27057060:quaternary,At1g69420.1:X:+1111:quaternary
TGTAATGTGT	2	0	0	0	0	Chr1:+26697038:quaternary,Chr3:+3386732:quaternary,Chr4:+160303:quaternary,Chr4:+10055206:quaternary,Chr2:+8551957:quaternary
ATGCGATCGT	1	0	1	0	0	Chr1:+26241957:quaternary,Chr1:+24846800:quaternary
GTCAAAATGA	1	0	1	0	0	Chr1:+26239555:quaternary,Chr5:+24948633:quaternary
GTTATAAAAA	1	0	1	0	0	Chr1:+26164740:quaternary,At1g50170.1:X:+391:quaternary,Chr5:+20152307:quaternary
TAGATATAGA	2	0	0	0	0	Chr1:+25675293:quaternary
ATGCTCCAAA	0	1	0	1	0	Chr1:+25540430:quaternary
TGATAGAAAG	1	0	0	0	1	Chr1:+25035444:quaternary
TAGTGATGTA	1	1	0	0	0	Chr1:+25029975:quaternary
TTTTCGCCAA	0	0	2	0	0	Chr1:+24924993:quaternary
CAGACGTACT	0	1	0	1	0	Chr1:+24353733:quaternary
ATGGCAACAT	1	0	0	1	0	Chr1:+23988403:quaternary
AATTCATCCG	1	0	1	0	0	Chr1:+23924319:quaternary
AAGAATTACA	0	1	1	0	0	Chr1:+23847391:quaternary
AAGCTTACAC	0	0	1	1	0	Chr1:+23391524:quaternary
AAAATCAGGT	1	1	0	0	0	Chr1:+23292439:quaternary
GATGACAAAA	0	0	2	0	0	Chr1:+23142351:quaternary

CTTTGAAAGA	1	1	0	0	0	Chr1:+22071386:quaternary
ACCATTTCAA	0	1	1	0	0	Chr1:+2203785:quaternary
TCCCATTTGT	0	2	0	0	0	Chr1:+19969009:quaternary
AGAGATACAT	0	1	0	0	1	Chr1:+19443027:quaternary
TGTTTAATTG	1	0	1	0	0	Chr1:+19361851:quaternary,Chr3:+16345369:quaternary,Chr3:+19121284:quaternary
TAGGAACACA	1	0	1	0	0	Chr1:+19169326:quaternary
TTAAATGCAA	0	0	1	1	0	Chr1:+18353375:quaternary,At3g09180.1:X:+482:quaternary
AAAGGATAAA	0	0	1	0	1	Chr1:+17965075:quaternary
TAAATTATGT	0	1	1	0	0	Chr1:+17694574:quaternary,Chr5:+24084851:quaternary,Chr1:+25626662:quaternary,Chr2:+8181465:quaternary
TATGTAGTAT	1	0	1	0	0	Chr1:+17687904:quaternary
TCCCTTAAAG	0	1	1	0	0	Chr1:+17676494:quaternary
TTCGGTTAAT	0	0	1	1	0	Chr1:+16843189:quaternary,At2g25870.1:X:+349:quaternary
TATTTTAAGA	2	0	0	0	0	Chr1:+16421461:quaternary,Chr1:+21703755:quaternary,Chr3:+9472877:quaternary,Chr4:+5630394:quaternary,Chr4:+1568928:quaternary,Chr1:+28708327:quaternary,Chr5:+15367758:quaternary
GCTGCCAATA	1	1	0	0	0	Chr1:+16347008:quaternary,Chr5:+8208026:quaternary
TCAGTTGGTA	0	0	2	0	0	Chr1:+16282664:quaternary,ChrM:+271237:quaternary
ATATGTTTTA	0	0	2	0	0	Chr1:+1548987:quaternary
GATCCTTAAA	0	0	2	0	0	Chr1:+14920579:quaternary
TCAATGAACA	1	1	0	0	0	Chr1:+14865317:quaternary
ATTACTATTC	1	0	0	1	0	Chr1:+1433259:quaternary
GAATTATCAA	0	0	0	2	0	Chr1:+13767127:quaternary
ATGAAAATCA	1	0	1	0	0	Chr1:+12730791:quaternary,Chr5:+7413365:quaternary
AATATGAATA	0	2	0	0	0	Chr1:+12643425:quaternary
ATATTTCTTC	0	1	0	1	0	Chr1:+12440620:quaternary
TTTAGAAAAA	0	0	2	0	0	Chr1:+12182571:quaternary,Chr4:+5438119:quaternary,Chr3:+7228951:quaternary
TTCTAAGAAA	0	0	1	0	1	Chr1:+12143857:quaternary
AAACAATTCT	1	0	1	0	0	Chr1:+12126807:quaternary
TAATTTGTTA	0	0	1	1	0	Chr1:+12097406:quaternary,Chr1:+22984185:quaternary,Chr3:+676861:quaternary
AAACCGATCA	0	0	1	0	1	Chr1:+11029530:quaternary
TCACGATCTA	1	0	0	1	0	Chr1:+10959719:quaternary
GTTTTGTTCG	1	1	0	0	0	Chr1:+10756992:quaternary
GTGACAGTTT	1	0	0	1	0	Chr1:+10452918:quaternary
GTGATTGACT	1	0	0	1	0	Chr1:+10300408:quaternary,Chr1:+10308802:quaternary
ATAATAAGAA	0	0	1	0	1	Chr1:+10139534:quaternary,Chr5:+23256414:quaternary,Chr5:+24988723:quaternary
ATCAAGTTAC	0	0	1	1	0	AtMg00570:d:+478:primary,At2g07716.1:p:+549:primary
TATACGCGAT	1	0	0	1	0	AtCg01130:d:+5338:primary
GCGAGATTTA	1	0	0	0	1	AtCg01130:d:+1949:secondary
TACTTGTTTT	1	1	0	0	0	AtCg01070:d:+17:primary
AAACAACTCG	1	1	0	0	0	AtCg01060:d:+215:primary
GGGTTCATAT	1	0	0	0	1	AtCg01040:d:+771:primary
TACTCGGGTG	1	0	0	1	0	AtCg00905:d:+102:primary,AtCg00905:d:+360:primary
AATCTAGGAA	2	0	0	0	0	AtCg00890:d:+715:secondary,AtCg00890:d:+715:secondary
GCCCAACGAC	0	0	0	1	1	AtCg00730:d:+96:primary
GTGTCTAATA	0	0	1	0	1	AtCg00470.1:X:+520:quaternary
GCTGAGTGAT	0	0	1	1	0	AtCg00350:d:+303:secondary
TTTGTTGCCA	0	2	0	0	0	AtCg00330:d:+270:primary
GGCTCGCCGC	1	0	0	0	1	AtCg00280:d:+777:secondary
CCGGATTAAT	0	0	1	0	1	AtCg00280:d:+170:secondary
AAGTTACAAA	0	1	0	0	1	AtCg00270:d:-172:primary
GATTGGCCAG	0	0	0	0	2	AtCg00270:d:+188:secondary
ATCCCTATAG	0	1	0	1	0	AtCg00120:d:+472:primary
ATATTTCAAA	0	1	0	0	1	AtCg00050:d:+206:primary
ACGCAACCTA	1	0	0	1	0	At5g67610.1:X:-454:quaternary
ATATCTTGCA	0	0	2	0	0	At5g67560.1:d:+706:secondary
GTGGTGAGAG	0	0	1	0	1	At5g67470.1:d:+2497:primary
CTCGCTCGTG	0	2	0	0	0	At5g67450.1:d:+281:primary
TTCTTGAAGC	0	2	0	0	0	At5g67440.1:d:+1657:primary,At4g39955.1:d:+1179:primary
TTATGAGTTT	0	1	1	0	0	At5g67400.1:d:+1075:primary
CTTCAGAAAA	0	0	1	1	0	At5g67380.1:i:+2086:tertiary

GATCACAACT	0	1	0	1	0	At5g67360.1:d:+1098:secondary
ACCTCCGAGA	0	2	0	0	0	At5g67320.1:d:+1568:primary
AGCCCAGGAA	0	0	2	0	0	At5g67300.1:d:+511:secondary
GTGGTGAAGC	1	0	1	0	0	At5g67290.1:d:+823:primary
TTCGATTTCA	0	1	0	1	0	At5g67280.1:i:+2070:tertiary
GGTTAAAGAT	0	0	1	1	0	At5g67230.1:d:+1144:secondary,At4g03913.1:p:+204:secondary,At4g14720.1:d:+1230:secondary
TATGTGTTGT	1	0	1	0	0	At5g67150.1:d:+1420:primary
CAATGGCGAG	1	0	1	0	0	At5g67030.1:d:+1453:secondary,At5g67030.2:d:+1453:secondary
AGCATTGGAT	0	0	1	1	0	At5g66950.1:d:+2622:primary
TTCCTCCCAT	0	1	0	0	1	At5g66930.1:d:+684:primary,At5g66930.2:d:+573:primary
CTATGTCTCG	0	0	1	0	1	At5g66910.1:X:+939:quaternary
CCAAAGAATT	2	0	0	0	0	At5g66900.1:d:+2578:primary
ACATCAGAAG	0	1	1	0	0	At5g66810.1:d:+2065:primary
GTTTCCTAAA	2	0	0	0	0	At5g66800.1:X:+642:quaternary
GATTCAACTT	0	1	1	0	0	At5g66720.2:d:+1023:primary,At5g66720.1:d:+1032:primary
CTTCTTGATT	0	1	1	0	0	At5g66660.1:X:-1073:quaternary,At5g11360.1:X:-413:quaternary,At3g20780.1:X:-327:quaternary,At5g58050.1:X:-227:quaternary
AGCACGTCCA	0	2	0	0	0	At5g66610.1:X:+449:quaternary
TGTAATATTA	0	0	0	1	1	At5g66580.1:X:+771:quaternary
AAGATGATAT	0	2	0	0	0	At5g66540.1:d:+440:secondary
TTGAAGAAAG	0	0	2	0	0	At5g66540.1:d:+1704:primary
TCAGGTGAAC	1	0	0	1	0	At5g66470.1:d:+1068:secondary
CCCAATCCAT	1	0	0	0	1	At5g66450.1:d:+646:primary
TGAGATCGAG	0	2	0	0	0	At5g66420.1:d:+1565:secondary
ATGACCTGGC	0	0	0	1	1	At5g66400.1:X:-71:quaternary
GTGTCCGGCG	1	0	0	1	0	At5g66330.1:d:+1341:primary
TTACATAAAA	1	1	0	0	0	At5g66290.1:X:-333:quaternary
TTTGTTGATA	1	0	1	0	0	At5g66250.1:d:+1181:secondary,At5g66250.2:d:+1520:secondary
TAAATGAGGC	0	1	0	1	0	At5g66240.1:d:+1070:secondary,At5g66240.2:d:+1165:secondary
GAAGACAATT	0	1	0	1	0	At5g66130.1:d:+1235:secondary,At3g56590.1:d:+494:secondary
CAGGTTTCAC	0	0	2	0	0	At5g66040.1:d:+324:secondary,At5g66040.2:d:+377:secondary
GCGACGAAGA	1	1	0	0	0	At5g66030.2:d:+1629:secondary,At5g66030.1:d:+1629:secondary
GTCAAGCTAA	0	0	0	1	1	At5g65950.1:X:+1729:quaternary
ATTTAAAGTG	0	2	0	0	0	At5g65925.1:d:-76:primary
CCTTGCTTCT	0	1	1	0	0	At5g65910.1:d:+814:primary
GAATGTCTTT	0	1	0	1	0	At5g65840.1:d:+529:secondary
CTTCTGATGC	1	0	1	0	0	At5g65780.1:d:+2181:primary,At5g03440.2:d:+699:primary,At5g03440.1:d:+639:primary
AACTACCATC	1	1	0	0	0	At5g65770.1:v:+3214:secondary
CGTTTGTTGT	0	2	0	0	0	At5g65750.1:d:+508:secondary
GGAAGTAGGT	1	0	0	1	0	At5g65710.1:v:-184:primary
ATTTTTGGAG	0	1	1	0	0	At5g65687.1:d:+1061:primary
TTGCACTTTA	1	1	0	0	0	At5g65610.1:i:-1155:tertiary,At5g65610.1:i:-1163:tertiary
TTATAAACCA	1	1	0	0	0	At5g65610.1:i:+1266:tertiary
TAGCACAAAA	0	1	0	0	1	At5g65450.1:v:+2438:secondary
GAGAATCGTG	0	0	2	0	0	At5g65430.1:d:+285:secondary,At5g65430.2:d:+285:secondary
ATTTGCCTGT	0	1	1	0	0	At5g65420.1:i:+890:tertiary
TTCCCAAGGA	0	1	1	0	0	At5g65300.1:d:+337:primary
TGTTCTTATG	0	2	0	0	0	At5g65230.1:d:+1060:secondary
TAGAGAAGGG	1	1	0	0	0	At5g65165.1:d:+365:secondary,At1g69760.1:d:+613:secondary
TGTAAATAAA	2	0	0	0	0	At5g65010.2:i:+472:tertiary,At5g65010.1:i:+472:tertiary
AAGAAATGTA	0	0	0	1	1	At5g64980.1:d:+1240:primary
ATTTATCACC	1	0	0	0	1	At5g64940.2:i:+4376:tertiary,At5g64940.1:i:+4376:tertiary,At3g06580.1:i:+1404:tertiary
ACCACTCTGT	1	0	1	0	0	At5g64940.1:d:-2670:secondary,At5g64940.2:d:-2615:secondary,At5g64940.1:d:-2661:secondary,At5g64940.2:d:-2606:secondary
TTGGCTTCTA	0	2	0	0	0	At5g64930.1:d:+1093:secondary,At5g40450.1:d:+5797:secondary,At5g11210.1:v:+2670:secondary
GCACAATACA	0	1	0	1	0	At5g64910.1:v:+2187:primary
GAATTATCTT	0	2	0	0	0	At5g64830.1:d:+1320:primary
GTTGGAGATC	0	0	1	0	1	At5g64740.1:d:+2876:secondary
CAATTGCTGG	0	0	1	1	0	At5g64740.1:d:+2463:secondary
AAGAGAGATT	0	1	1	0	0	At5g64740.1:d:+1858:secondary,At5g09870.1:v:+1683:secondary,At5g27020.1:v:+459:secondary

GGCAAGGGGA	1	0	0	0	1	At5g64650.1:X:-210:quaternary
AGGTTTTAGG	0	1	0	1	0	At5g64640.1:X:+708:quaternary
AGATGGTTTT	0	0	0	1	1	At5g64630.2:d:+1717:primary,At5g64630.1:d:+1783:primary,At5g64630.3:d:+1696:primary
ACCCTATACA	0	0	1	0	1	At5g64610.1:X:-2:quaternary
GAAACTAGTG	1	1	0	0	0	At5g64580.1:d:+2451:secondary
TGATCAGAGG	0	1	0	1	0	At5g64460.2:d:+490:primary,At5g64460.1:d:+514:primary
ATTAAGATGT	1	0	0	1	0	At5g64410.1:i:+1983:tertiary
TGAGAGCTCG	0	1	1	0	0	At5g64390.2:d:-3263:secondary,At5g64390.1:d:-2724:secondary
GATCTCAATC	0	1	1	0	0	At5g64370.1:d:+1124:primary
CGAAGCTGAC	0	1	1	0	0	At5g64360.1:d:+200:secondary,At5g09540.1:v:+473:secondary,At5g64360.3:d:+200:secondary,At5g64360.2:d:+135:secondary
TGTATTGCCG	0	1	0	1	0	At5g64340.1:d:+240:secondary,At1g56130.1:v:+405:secondary
TGGGTTGAAG	0	1	0	1	0	At5g64330.1:d:+2684:secondary
CTCGGCGCTA	0	0	1	1	0	At5g64280.1:d:+1107:primary
ATTTGAACAT	0	2	0	0	0	At5g64250.2:d:+491:secondary,At5g64250.1:d:+323:secondary,At2g20280.1:d:+338:secondary
CTCGGTCTGA	0	2	0	0	0	At5g64180.1:d:+328:primary
TTGTGTGACA	0	1	1	0	0	At5g64170.1:d:+556:secondary
ACAAATTATA	1	0	1	0	0	At5g64150.1:i:+1273:tertiary
TGAGAAAAAT	1	1	0	0	0	At5g64090.1:d:+1468:primary
CAAGCTTCAC	0	0	0	1	1	At5g64080.1:d:+423:primary,At1g77830.1:v:+1371:primary,At5g64080.2:d:+423:primary
TAAAGAAACT	1	0	0	1	0	At5g63800.1:d:+2479:primary
TTCGAACTGT	1	1	0	0	0	At5g63800.1:d:+136:secondary
CCGCAGCCAT	1	0	1	0	0	At5g63770.1:d:+2257:secondary
TGTGTTTATT	0	0	1	1	0	At5g63660.1:v:+778:primary
ACATCGAGGG	0	1	0	0	1	At5g63640.1:d:+944:primary
TTAATCCGAG	0	1	1	0	0	At5g63370.1:d:+2889:primary
TCTGATTTAT	0	0	1	1	0	At5g63280.1:i:+349:tertiary,At1g20240.1:i:+735:tertiary
AAAGATGCAA	0	1	1	0	0	At5g63220.1:d:+762:primary
CAAGGAGGTG	0	1	1	0	0	At5g63200.1:d:+242:secondary
AGATGTTTAT	1	0	1	0	0	At5g63190.2:d:+2420:secondary,At5g63190.1:d:+2464:secondary
GAGGAAGAGG	0	1	1	0	0	At5g63080.1:d:+1357:primary,At2g45000.1:d:+1906:primary
ATGTGGTCCG	0	1	0	1	0	At5g63020.1:d:+1418:secondary
AAAAGCAGAG	0	1	1	0	0	At5g62900.1:d:+467:primary
TACGAATCGT	1	0	1	0	0	At5g62760.1:d:+2076:secondary,At5g62760.2:d:+1969:secondary
GTGCTAGTTA	1	0	1	0	0	At5g62740.1:d:+772:secondary
TTACCACCGT	0	0	1	0	1	At5g62710.1:d:+1544:secondary
AAGGATCTGC	1	0	1	0	0	At5g62630.1:d:+1676:primary
GATTAGGTGA	0	0	1	1	0	At5g62575.2:d:+608:primary
TCACAGAAGA	0	2	0	0	0	At5g62570.1:d:+1421:primary
TTGCCGGAGC	0	2	0	0	0	At5g62560.1:d:+1092:secondary
GCTAAAGAGA	0	0	2	0	0	At5g62360.1:d:+651:secondary
ATTCTAGCCC	0	1	0	1	0	At5g62350.1:d:-620:secondary,At5g62350.1:d:-633:secondary
TGTTGGTTCC	0	0	1	0	1	At5g62300.1:X:-8:quaternary
AAAGTGAGAC	0	0	0	2	0	At5g62290.1:d:+749:primary
CCAAAGATCT	1	0	0	1	0	At5g62220.1:v:+735:secondary
GCTCTAAAGC	0	0	0	1	1	At5g62200.1:d:+404:primary
GCAGACATTT	1	1	0	0	0	At5g62170.1:d:+2190:primary
TTGCTCTCTG	2	0	0	0	0	At5g61930.1:d:+330:secondary,At1g27150.1:d:+830:secondary
ATGCGAGTAG	0	0	2	0	0	At5g61890.1:d:+705:secondary,At1g55850.1:d:+1125:secondary
GGCCAAGAGT	1	1	0	0	0	At5g61810.1:d:+1458:secondary
AAAGTAAGCT	0	1	0	0	1	At5g61760.1:d:+1165:primary
GAGCAATGGC	1	0	0	1	0	At5g61580.1:d:+1412:secondary,At4g21200.1:v:+998:secondary,At4g38060.2:d:+807:secondary
GGACTTGCTC	0	1	0	0	1	At5g61530.2:d:+1148:primary,At5g61530.1:d:+1208:primary
GTGAAGTCGC	0	1	0	1	0	At5g61460.1:d:+3220:secondary
GACGGTCGTT	0	0	1	1	0	At5g61410.1:d:+546:secondary,At5g61410.2:d:+415:secondary
TCTTCCACCA	0	0	0	1	1	At5g61380.1:X:+1483:quaternary,Chr2:+7254600:quaternary
ATTGTGCAGT	0	0	0	1	1	At5g61220.1:d:+498:primary
ACTATTTGTT	0	0	0	0	2	At5g60990.1:i:+2388:tertiary
TGGGTGATTT	1	1	0	0	0	At5g60490.1:d:+855:primary
TTGTCTCCCT	0	1	0	1	0	At5g60390.1:d:-665:secondary
GATTCTCCAT	0	2	0	0	0	At5g60260.1:v:+1196:primary

AAATCAAAAA	1	0	1	0	0	At5g60230.2:d:+992:secondary,At5g60230.1:d:+638:secondary
TTCCAAAGAT	1	0	1	0	0	At5g60210.1:d:+1890:primary
TTTTTTAATG	0	0	1	1	0	At5g60200.1:d:+934:primary
GGAGTGAATA	0	2	0	0	0	At5g60190.1:d:+516:primary
AATTGGGTTC	0	0	2	0	0	At5g59910.1:X:+820:quaternary
TCTCCTTCCT	0	2	0	0	0	At5g59900.1:X:-1669:quaternary,At1g54160.1:X:-64:quaternary,At1g78640.1:X:-480:quaternary
ATGACTGCAA	0	0	1	0	1	At5g59890.1:d:+153:secondary,At3g45990.1:v:+364:secondary,At5g59880.1:d:+186:secondary,At5g59890.2:d:+106:secondary,At5g59880.2:d:+186:secondary
TTTTCTCTTC	1	0	1	0	0	At5g59730.1:d:+921:secondary,At2g28220.1:v:+446:secondary,At4g35100.1:d:+1101:secondary
ACATATCACA	0	1	0	1	0	At5g59710.1:d:+1890:secondary
AGTAAAGTTT	0	0	1	1	0	At5g59700.1:d:+2592:primary
TTTTTCTTTG	0	1	0	0	1	At5g59660.1:v:+3293:primary,At4g12520.1:v:+793:primary
GTTGTGATTT	0	1	1	0	0	At5g59660.1:v:+3046:secondary,At5g58550.1:d:+500:secondary
GCCAGTGTTC	0	0	2	0	0	At5g59613.1:d:+21:secondary,At3g46430.1:v:+371:secondary
GTTCTCTTAA	0	0	1	0	1	At5g59500.1:d:+1421:primary
CCCTTTGTTT	1	1	0	0	0	At5g59480.1:i:+930:tertiary,At5g59480.2:i:+930:tertiary
AAGGAGGCTA	0	1	0	0	1	At5g59450.1:d:+1815:primary
AAAATCCTCA	0	1	1	0	0	At5g59370.1:d:+652:secondary,At3g55270.1:d:+2843:secondary
CCTAACTATA	0	1	0	0	1	At5g59180.1:d:+455:secondary
TCACGAACTG	0	1	1	0	0	At5g59130.1:v:+2694:secondary
GTTTCAGGAA	0	1	0	0	1	At5g59070.1:d:+1282:primary
TTGTTCGGAA	0	1	0	1	0	At5g59030.1:d:+487:secondary
AAATTCTCGA	0	1	0	1	0	At5g59010.1:d:+1209:primary
AGTGACCCCT	0	0	0	1	1	At5g59000.1:X:+-38:quaternary
GTCGCCCTGT	0	0	0	2	0	At5g59000.1:d:+625:primary
GGTACCTGTT	0	1	1	0	0	At5g58980.1:v:+2339:primary
GAGAGTGTCG	1	1	0	0	0	At5g58960.1:d:+1622:primary,At5g58960.2:d:+1613:primary
GAAATGGAAT	0	0	1	1	0	At5g58710.1:d:+371:secondary,At3g15920.1:d:+1519:secondary
AGACAAATGA	0	0	2	0	0	At5g58510.1:d:+2421:secondary
ACAGGGACAG	1	0	0	1	0	At5g58450.1:d:+3267:primary
TATGGCACAG	1	0	1	0	0	At5g58440.1:d:+1937:primary
TATTGTGTAT	1	0	1	0	0	At5g58430.1:d:+2084:secondary
TCCTGTTGAA	0	0	2	0	0	At5g58420.1:d:-738:secondary
TAGTAGACCC	0	0	0	1	1	At5g58410.1:X:+431:quaternary
TTTAGACAAG	0	1	0	0	1	At5g58410.1:d:+5559:primary
GTGAAATGAC	0	1	1	0	0	At5g58350.1:X:+537:quaternary
TGTTTAATAA	1	0	0	0	1	At5g58350.1:d:+2130:primary
AGATCAGGGA	1	1	0	0	0	At5g58320.2:d:+1244:primary
TAAATAAGAA	0	0	1	1	0	At5g58320.1:d:+2043:primary
GTAATAATTA	0	1	0	1	0	At5g58290.1:X:+357:quaternary,Chr3:+11044129:quaternary
ATGCCTGGTT	1	0	1	0	0	At5g58200.1:d:+390:secondary
AAAAGAGCCG	0	1	0	1	0	At5g58150.1:d:+2736:primary
AAGCGATCAA	1	0	1	0	0	At5g58130.1:d:+2498:primary
GCTTCAGAAG	0	1	0	0	1	At5g58130.1:d:+1833:secondary
TTCAAAGGCT	0	0	0	0	2	At5g58005.1:d:-504:secondary
GGCATTGGAT	0	2	0	0	0	At5g57940.1:d:+2053:primary,At5g57940.3:d:+1701:primary,At5g57940.2:d:+1901:primary
TTTTATAATA	1	1	0	0	0	At5g57900.1:d:+944:primary,At5g20930.1:d:+2571:primary
AGATAAGATC	1	0	0	1	0	At5g57890.1:d:+1066:primary
CGGCGTGGTT	0	0	0	2	0	At5g57830.1:d:+1312:primary
TTGGAATTGA	1	1	0	0	0	At5g57750.1:v:+1156:primary
ACTAAGGAAA	1	0	1	0	0	At5g57660.1:d:-1123:secondary
GAGGTAAGTT	0	0	2	0	0	At5g57630.1:i:+1958:tertiary
GGAGGAGGAG	0	0	1	0	1	At5g57580.1:d:+1920:secondary,At1g06170.2:d:+164:secondary,At1g74260.1:d:+1579:secondary,At1g06170.1:d:+133:secondary,At5g08600.1:v:+354:secondary
GTCCAGTTAC	0	1	0	1	0	At5g57410.1:d:+1108:primary
GAGTCTCTTG	1	0	1	0	0	At5g57345.1:X:-295:quaternary
ATATTCACTA	1	1	0	0	0	At5g57320.1:v:+541:secondary,At3g29770.1:d:+1316:secondary
GCCGAATCTT	0	1	0	1	0	At5g57300.1:i:+2009:tertiary

GTACTCTACT	1	0	1	0	0	At5g57270.1:d:+1675:primary,At5g57270.3:d:+1472:primary,At5g57270.2:d:+1690:primary
ATTGTCCAGA	0	1	1	0	0	At5g57260.1:v:+5212:primary
CTTGAACATA	1	1	0	0	0	At5g57210.1:d:+2183:primary
TGATTGCAAA	0	1	1	0	0	At5g56980.1:X:+1739:quaternary,At5g56980.1:X:+1734:quaternary
TGGAGATACT	0	0	0	1	1	At5g56950.1:d:+1425:primary
CCTTATGATG	0	1	0	1	0	At5g56910.1:d:+630:primary
CGAAGCATCG	0	1	0	0	1	At5g56900.1:d:+1975:secondary,At5g56900.2:d:+1879:secondary
TGGTATACGG	1	1	0	0	0	At5g56860.1:d:+887:secondary,At5g40060.1:v:+3641:secondary
GATTTGTGAT	0	1	0	1	0	At5g56800.1:v:+790:secondary,At1g50980.1:v:+937:secondary,At5g56430.1:v:+814:secondary,At3g14020.1:v:+1514:secondary,At3g43425.1:p:+592:secondary
GGATTAAAGC	0	2	0	0	0	At5g56750.1:d:+1106:primary
ACTATCATAG	0	0	1	0	1	At5g56730.1:v:+3384:primary
TCATATACCA	0	2	0	0	0	At5g56360.1:d:+1761:secondary
AGAAGTCCGG	1	0	0	1	0	At5g56300.1:d:+917:secondary
TAAAAGATCC	1	1	0	0	0	At5g56280.1:d:+981:primary
AGTAGATTAC	1	0	1	0	0	At5g56270.1:d:+2647:primary
ACGCACATAC	1	0	1	0	0	At5g56210.1:v:+1042:secondary
GGAATGGGAT	1	0	0	0	1	At5g56110.1:v:+964:secondary,At4g02740.1:d:+1101:secondary
AATACTTTTT	0	2	0	0	0	At5g56050.1:v:+1640:primary
ATTGGTCAAT	0	2	0	0	0	At5g56000.1:d:+437:secondary
AGTTTGAGGA	0	2	0	0	0	At5g55960.1:d:+1270:secondary,At1g68790.1:d:+1169:secondary
TTTAGATCAT	0	0	0	2	0	At5g55840.1:v:+4374:primary
TTACTTTCAA	0	0	1	0	1	At5g55840.1:v:+1351:secondary
AGAATCAAAA	0	0	0	2	0	At5g55780.1:v:+1666:secondary,At5g48390.1:v:+1179:secondary
TGACTGGTAG	0	2	0	0	0	At5g55700.1:d:+1310:primary
AATCTCAGTT	1	1	0	0	0	At5g55640.1:d:+574:primary
GATGATTCTC	0	0	0	0	2	At5g55630.2:X:+411:quaternary
CTGGATATTT	0	0	0	1	1	At5g55620.1:X:+835:quaternary
GGTAGTTCAA	0	1	0	1	0	At5g55610.1:d:+857:primary,At5g55610.2:d:+990:primary
AAAACTTTGT	0	0	1	0	1	At5g55550.1:d:+1948:secondary
ATCCCAGTTC	0	0	1	1	0	At5g55480.1:d:+2047:primary
CTACCAAAAA	0	1	1	0	0	At5g55390.1:d:+740:secondary
GAATTTTTTA	0	0	0	1	1	At5g55270.1:v:+9:secondary
TGTTGCCAAA	1	0	1	0	0	At5g55260.1:d:+1238:primary
AACCTAACAG	0	1	1	0	0	At5g55210.1:d:+432:primary
ACTATGCTCT	1	0	0	1	0	At5g55200.1:d:+1103:primary
GAGGTTACTA	0	2	0	0	0	At5g55120.1:d:+1524:primary
TTAGCTGATA	1	1	0	0	0	At5g55100.2:d:+2598:primary,At5g55100.1:d:+2598:primary
GAGTTGCATT	0	0	1	1	0	At5g55070.1:d:+1833:primary
TCTCAGAAGT	1	0	0	0	1	At5g54890.1:d:+1182:primary
GGAGCAGGAT	0	1	0	0	1	At5g54860.1:d:+1419:primary
TTCCTGGGAT	0	0	1	0	1	At5g54770.1:d:+800:secondary
ATGGTCCTTT	1	0	1	0	0	At5g54770.1:d:+740:secondary,At1g79000.1:d:+4501:secondary
AAGACCTTTT	1	1	0	0	0	At5g54670.1:d:+2674:primary
TTCTATTCTA	1	0	1	0	0	At5g54530.1:d:+677:primary
TTTAGACTTA	0	1	1	0	0	At5g54490.1:d:+358:primary
CGTTGTCTTC	0	0	1	0	1	At5g54470.1:d:+272:primary
GACAGTAACT	0	2	0	0	0	At5g54380.1:d:+2218:secondary
CTCAGAGCAT	1	0	1	0	0	At5g54270.1:d:+553:secondary
TGTTCCCTAA	1	1	0	0	0	At5g54180.1:d:+967:secondary
TCTCTTAACA	1	1	0	0	0	At5g54180.1:d:+1464:primary
ATCGCTCAAG	1	0	0	0	1	At5g54100.1:d:+1171:primary
GCTACAAATT	0	2	0	0	0	At5g53820.1:d:-487:secondary
ACTCTCTGGA	1	1	0	0	0	At5g53650.1:d:+228:secondary
GATGATCATC	0	0	1	1	0	At5g53560.1:d:+177:secondary
TAATATTTCA	0	2	0	0	0	At5g53530.1:d:+173:primary
GCTTCGCTTC	0	2	0	0	0	At5g53490.1:d:+25:primary,At5g53490.2:d:+25:primary
TTTCTGACGT	0	1	0	1	0	At5g53480.1:X:+1309:quaternary
TTAATAGGAG	0	1	1	0	0	At5g53480.1:d:+3585:secondary
GGACCGGTTT	0	0	1	1	0	At5g53470.1:d:+747:primary

AATGATCATA	1	1	0	0	0	At5g53450.2:d:+1844:secondary,At5g53450.1:d:+2011:secondary
GTTCTTCCTA	0	1	1	0	0	At5g53330.1:d:+675:primary
GAGGGCGATT	0	0	1	0	1	At5g53200.1:d:-190:primary
GAAGTTGAAG	0	1	1	0	0	At5g53130.1:d:+2143:primary
CCACAGCTAA	0	2	0	0	0	At5g53060.1:d:+1167:primary
GCGATGCTTT	1	1	0	0	0	At5g52970.1:d:+15:primary,At5g52965.1:v:+1133:primary
TTACATTTAG	0	1	0	0	1	At5g52910.1:d:+3522:primary
TCACAGAAAA	0	2	0	0	0	At5g52790.1:d:+639:secondary,At5g21326.1:d:+1099:secondary
CCTCCAATGA	0	2	0	0	0	At5g52750.1:d:+95:primary
ACCAAAGGAC	0	1	0	1	0	At5g52520.1:d:-1755:secondary
GAATCTACCC	0	2	0	0	0	At5g52380.1:d:+595:primary
GAGACTTTCA	0	1	0	1	0	At5g52370.1:X:-463:quaternary,At1g19060.1:X:-41:quaternary
CAAATCAGTA	0	0	1	0	1	At5g52340.1:v:+2059:secondary
AGAAGGGAGC	0	0	0	0	2	At5g52300.1:d:+237:secondary
CAAGTTAAAA	1	1	0	0	0	At5g52290.1:X:-923:quaternary
ATGAGTAGGA	0	1	0	1	0	At5g52210.1:d:+854:primary,At5g52210.2:d:+932:primary
AAGCAAATAT	1	0	1	0	0	At5g52140.1:v:+1570:primary
TTTTGTGCTT	1	0	0	0	1	At5g52070.1:d:+1091:primary,At3g25170.1:d:+468:primary
TGAAGATAGT	2	0	0	0	0	At5g52050.1:d:+839:secondary,At5g20350.1:d:+2051:secondary
GTGAATTGAG	0	0	1	0	1	At5g52010.1:d:+1367:secondary
GACAGAGACC	1	1	0	0	0	At5g51840.1:d:+770:secondary
TCAATGGCCT	0	1	1	0	0	At5g51810.1:d:+838:secondary,At2g22930.1:d:+293:secondary,At5g48940.1:d:+282:secondary
TTGAAGTTAA	0	0	1	1	0	At5g51770.1:v:+1090:secondary
TTGTGAATCC	0	1	1	0	0	At5g51730.1:i:+125:tertiary
ATGAGGGTTT	1	0	1	0	0	At5g51620.2:d:-73:primary,At5g51620.1:d:-73:primary
TTGGAGCTCT	0	1	0	1	0	At5g51600.1:d:+766:secondary,At5g64290.1:d:+1546:secondary
CAAACGCTAC	1	0	0	1	0	At5g51500.1:v:+503:secondary
GAAGAGATCA	0	1	0	1	0	At5g51460.3:d:+1319:secondary,At2g34160.1:d:+84:secondary,At5g51460.1:d:+1438:secondary,At1g69070.1:d:+589:secondary,At5g51460.2:d:+1316:secondary
TTCTCTTTTG	0	0	1	0	1	At5g51420.1:v:+2128:primary
TTGGCTATGG	0	1	0	1	0	At5g51410.1:d:+786:secondary,At5g51410.2:d:+786:secondary
TATTTGCCAT	1	0	0	1	0	At5g51400.1:d:+413:secondary
CGGTAAGGTG	0	0	2	0	0	At5g51280.1:X:+1046:quaternary
TCTCAACGAT	1	0	0	1	0	At5g51260.1:d:+953:primary
CTCAACCCTC	0	0	1	0	1	At5g51110.1:X:-9:quaternary
GGAAACTGTA	0	0	0	2	0	At5g51100.1:d:+805:primary
TATGGTTTTC	0	0	0	0	2	At5g51060.1:i:+1049:tertiary,At5g54910.1:i:+2390:tertiary
TGATTATAGT	1	1	0	0	0	At5g50950.2:d:+1687:secondary
ACATCAAACG	0	0	0	2	0	At5g50920.1:d:+2424:secondary
TCGACACAAA	0	0	1	0	1	At5g50915.2:d:+937:primary,At5g50915.1:d:+937:primary
TCCAGCGTAT	1	1	0	0	0	At5g50420.1:d:+1782:primary
GGATCAGCTA	0	1	1	0	0	At5g50410.1:X:-6:quaternary
TCAAGTGTGG	0	1	1	0	0	At5g50390.1:v:+856:secondary,At5g48430.1:v:+1663:secondary
AGCAGGATCT	0	2	0	0	0	At5g50330.1:d:+1330:primary
TGTAGTAAAA	1	0	1	0	0	At5g50280.1:v:+2686:secondary,At5g50290.1:v:+205:secondary
GGAAAGTAGT	0	2	0	0	0	At5g50250.1:d:+703:primary
CCCTTCCTAA	0	1	0	1	0	At5g50130.2:d:+1057:primary
TAACATTGAT	0	0	0	2	0	At5g50110.1:d:+849:primary
AAGATGGAGG	0	2	0	0	0	At5g49970.2:d:+762:secondary,At5g02810.1:d:+1418:secondary,At1g27750.1:d:+2057:secondary,At5g49970.1:d:+762:secondary,At1g76010.1:d:+664:secondary
TTTGACTTGA	0	1	0	1	0	At5g49945.1:d:+1607:secondary
CGCTCAGAAG	1	1	0	0	0	At5g49940.1:i:-465:tertiary
AGTTCATCTG	1	1	0	0	0	At5g49930.1:d:+2293:secondary
TACAATAAAC	0	0	1	0	1	At5g49910.1:d:+2395:secondary
CCTAGAAAAA	1	1	0	0	0	At5g49830.1:i:+2494:tertiary
AATCTCCATA	1	0	1	0	0	At5g49730.1:d:+1383:primary
GGGAAATTGA	0	1	0	0	1	At5g49720.1:X:-141:quaternary
TACAAGCTCA	1	0	1	0	0	At5g49680.1:v:+2359:secondary
TGAAATCTCA	0	0	1	1	0	At5g49665.1:d:+1605:primary

TATTTAAATA	0	0	0	0	2	At5g49660.1:d:+3157:primary
AGTCTGTAAG	0	1	0	1	0	At5g49570.1:X:-244:quaternary
CAAATTAAAT	1	0	0	0	1	At5g49560.1:v:+1274:secondary,At1g35390.1:p:+5117:secondary,At5g36005.1:p:+4709:secondary
CTGTTGCCCA	0	2	0	0	0	At5g49530.1:d:+822:secondary
GATTGCTTCA	1	1	0	0	0	At5g49520.1:d:+1235:secondary,At5g41510.1:v:+26:secondary
AGAAAGAAAG	0	0	0	2	0	At5g49500.1:v:+1453:secondary,At1g48900.1:d:+1233:secondary,At2g13520.1:p:+2605:secondary,At5g26600.2:d:+1518:secondary,At1g15980.1:d:+1074:secondary,At2g13490.1:p:+2407:secondary,At4g22760.1:v:+954:secondary
AATAGAAAAA	1	0	0	0	1	At5g49430.1:X:--66:quaternary
ATTAGGGTTT	1	0	0	1	0	At5g49300.1:v:+800:primary
TTACAAGGAA	0	0	1	0	1	At5g49290.1:v:+2625:secondary,At1g54480.1:v:+1611:secondary
TGTAACCAAA	0	1	0	1	0	At5g49215.1:d:+1725:primary,At1g52990.1:v:+1584:primary
GTGGTGGAGG	0	0	1	1	0	At5g49100.1:d:+1243:primary
ACCAGGGGCA	0	0	0	2	0	At5g49030.1:X:-99:quaternary
TATTTGTACT	1	1	0	0	0	At5g49020.2:d:+1683:secondary,At5g49020.1:d:+1688:secondary
CTTATCTATT	0	1	1	0	0	At5g49015.1:X:-210:quaternary,Chr1:-28713222:quaternary
TAAGCTCCTG	0	1	0	1	0	At5g49015.1:d:+489:primary
TATTTCCGCT	0	2	0	0	0	At5g48960.1:d:+1963:primary,At3g45450.1:v:+1680:primary
CTGTATGATT	1	0	0	1	0	At5g48930.1:d:+1578:secondary
ATATCACATA	1	0	0	0	1	At5g48860.1:v:+1235:primary
ATAACATTGG	0	2	0	0	0	At5g48830.1:d:+1107:primary
AAAAAGTTTC	0	1	1	0	0	At5g48780.1:v:+2744:secondary
AACTCAACTC	0	1	0	0	1	At5g48730.1:v:+1197:secondary
TGGACAGTCT	0	1	1	0	0	At5g48620.1:d:+2936:secondary
TTAAACAACA	2	0	0	0	0	At5g48600.1:d:+2234:secondary,At4g04760.1:v:+1066:secondary
AGTCTTTGAA	0	0	1	1	0	At5g48570.1:d:+1926:primary
CTGACTACAC	1	0	1	0	0	At5g48490.1:d:+229:secondary
TGACGTAAAA	0	0	0	0	2	At5g48470.1:d:+1399:primary
TCGAAAGCTA	1	1	0	0	0	At5g48440.1:X:-285:quaternary
TCATTATATT	0	0	0	1	1	At5g48430.1:v:+1906:primary
TGCAGGAGCA	0	2	0	0	0	At5g48385.1:d:-1872:secondary
GGAACTTCTA	0	1	1	0	0	At5g48385.1:d:+1877:primary
AAGGTGTTGA	2	0	0	0	0	At5g48340.1:d:+1790:primary
AGTCAGTCAT	0	2	0	0	0	At5g48230.1:d:+1329:secondary,At5g48230.2:d:+1591:secondary
GGAGCTGGGA	0	0	2	0	0	At5g48180.1:d:+813:secondary
TCATAAGGTT	0	1	0	1	0	At5g48060.1:d:+3205:primary
CACAAAGTCG	0	0	0	1	1	At5g47970.1:d:-1906:secondary
GAGTTATCTG	0	1	1	0	0	At5g47910.1:d:+3141:primary
TCATTCGTTT	1	1	0	0	0	At5g47900.1:v:+1856:secondary
TGTGTGTCAC	0	2	0	0	0	At5g47720.2:d:+1370:primary
CCTCTCTCAG	0	2	0	0	0	At5g47710.1:d:+433:primary
ATGCTTACAG	2	0	0	0	0	At5g47630.1:d:+630:secondary
CTCTGTGTCA	0	1	1	0	0	At5g47630.1:d:+497:secondary
TTCCTCGCCG	0	0	1	1	0	At5g47560.1:X:-338:quaternary
TCTTTAAGCT	0	0	0	2	0	At5g47540.1:v:+1198:secondary
AGACTAAAAA	0	1	0	1	0	At5g47510.1:d:+1046:primary
GAGCTTGGAT	0	0	0	1	1	At5g47490.1:d:+4168:secondary
AGTTACCCCA	2	0	0	0	0	At5g47480.1:d:+4147:secondary,At5g56560.1:v:+128:secondary
AATCTAGAAG	0	1	0	1	0	At5g47455.5:d:-96:primary,At5g47455.4:d:-96:primary,At5g47455.1:d:-96:primary,At5g47455.2:d:-98:primary,At5g47455.6:d:-96:primary,At5g47455.3:d:-96:primary
TCGAAAAGTC	1	0	0	1	0	At5g47445.1:p:+1324:primary
GTGGATGTTG	0	1	1	0	0	At5g47435.1:d:-769:secondary,At5g47435.2:d:-769:secondary
AGTCGTTTCT	0	1	0	0	1	At5g47360.1:v:+1764:secondary
TTGGTGTACG	0	1	1	0	0	At5g47220.1:d:+244:primary
AATTGGAAAC	0	1	0	0	1	At5g47180.2:d:+873:primary,At5g47180.1:d:+694:primary
ATATGAAAGA	0	1	0	1	0	At5g47140.1:d:+1828:primary
GTCATCATTC	1	0	0	0	1	At5g47030.1:d:+340:secondary,At2g26910.1:d:+1645:secondary
AAAATTCAAA	0	0	0	1	1	At5g47000.1:d:+1327:primary
TAAGAAACCA	0	1	0	0	1	At5g46920.1:d:+2246:primary
ATCAATGACC	1	0	0	0	1	At5g46800.1:X:-257:quaternary

CCTTCCGATT	0	1	1	0	0	At5g46790.1:d:+116:primary
GAGGTGGTTC	0	0	1	0	1	At5g46730.1:d:+827:secondary
GTTACCATAA	0	1	0	0	1	At5g46700.1:d:+357:primary
GAGCTGTGAG	0	1	0	1	0	At5g46620.1:d:+360:primary
TTTGTGAGGC	1	1	0	0	0	At5g46520.1:v:+4005:secondary
TCTATATATG	0	0	0	0	2	At5g46510.1:d:+4641:primary
AAGGATAAAA	0	0	2	0	0	At5g46470.1:d:+4201:secondary
AAATGTTGAT	0	0	2	0	0	At5g46450.1:v:+4213:primary
TAGAGTAAAC	1	1	0	0	0	At5g46410.1:d:+2468:primary
AAAGCCGATG	0	2	0	0	0	At5g46400.1:d:+3120:primary,At3g48590.1:d:+270:primary
CTATGAAACT	0	0	0	1	1	At5g46390.2:d:+1483:primary,At5g46390.1:d:+1574:primary
TCAACTTTGT	1	0	1	0	0	At5g46390.1:d:-1569:secondary,At5g46390.2:d:-1478:secondary,At5g38890.1:d:-371:secondary
AGGACACACA	0	0	1	0	1	At5g46280.1:d:+2216:primary
ATACTTTGCT	1	0	0	1	0	At5g46230.1:d:+480:primary
GAGAATCATC	0	0	1	0	1	At5g46220.1:v:+1028:secondary,At1g55690.1:d:+1467:secondary,At1g55690.2:d:+1343:secondary,At5g65850.1:v:+959:secondary,At1g55690.3:d:+1467:secondary
TTTAGATATG	1	0	0	0	1	At5g46190.1:d:+2210:primary
GTGATACTTG	0	2	0	0	0	At5g46180.1:d:+868:secondary
CGGGTAACTA	1	0	0	0	1	At5g46160.1:X:-459:quaternary
GTGTTGCACA	0	0	2	0	0	At5g46150.2:d:+1475:primary
TTGCTCCATT	0	1	0	1	0	At5g46150.1:d:+1133:primary
CAACCAAAAA	0	0	0	2	0	At5g46090.1:v:+172:secondary
AACAAGAACG	0	0	1	0	1	At5g45840.1:v:+2363:secondary
GGAAACACAT	0	0	2	0	0	At5g45820.1:v:+265:secondary,At5g27000.1:d:+2412:secondary
AGGGACATTA	0	2	0	0	0	At5g45775.1:d:+97:primary,At5g45775.2:d:+96:primary
CGTGAGGAAT	0	0	0	0	2	At5g45740.1:v:+411:primary
TATGCAAATC	0	0	1	0	1	At5g45730.1:v:+2177:secondary
GCTGTACAGT	0	2	0	0	0	At5g45660.1:d:+666:primary
GAAGAAGTAA	0	0	1	1	0	At5g45605.1:p:+4664:secondary,At5g11390.1:d:+946:secondary,At4g15640.1:d:+685:secondary,At1g77050.1:v:+1572:secondary,At1g32590.1:p:+3824:secondary
TTGTAACTGT	1	0	0	0	1	At5g45600.1:d:+1012:primary
AAGAAATCAG	0	0	0	1	1	At5g45500.1:d:+420:secondary
AGTTACAACA	0	0	0	1	1	At5g45480.1:X:-2944:quaternary,At1g69935.1:X:-160:quaternary
AACTTTTACA	0	1	0	0	1	At5g45390.1:d:+1228:primary
GTGTGTACTA	0	1	1	0	0	At5g45360.1:d:+870:primary
CCTTTTGGTA	0	1	1	0	0	At5g45340.1:d:+1289:secondary,At5g45340.2:d:+1289:secondary
TAGAGAAAAC	0	0	1	0	1	At5g45330.1:v:+2282:primary
GTCAACATTC	1	0	0	0	1	At5g45270.1:d:+573:primary
TTCAGTCCAA	1	1	0	0	0	At5g45200.1:X:-272:quaternary
TCTGATATTC	1	0	0	1	0	At5g45200.1:i:+1132:tertiary
AAAAGCAAGA	1	0	1	0	0	At5g45190.1:d:+240:secondary,At1g29940.1:d:+2431:secondary
GTGAGATTTT	0	2	0	0	0	At5g45190.1:d:+1662:secondary
CGAAGCAGTT	1	0	1	0	0	At5g45170.1:d:+1049:primary
AAGCATATCT	0	0	0	0	2	At5g45113.1:v:+954:primary
ACTCAGTACG	0	1	0	0	1	At5g44930.2:i:+1676:tertiary,At5g44930.1:i:+1679:tertiary
TGAGATAGAA	1	1	0	0	0	At5g44870.1:v:+3963:primary
ATTTGACAAG	1	0	1	0	0	At5g44800.1:v:+2983:secondary
ACACGAGCTA	0	1	0	1	0	At5g44750.1:d:+3675:secondary
AGGATTTCGT	1	0	1	0	0	At5g44720.1:d:+1178:primary
TTCTACTCAA	0	0	1	0	1	At5g44690.1:v:+179:secondary
TCTCTACAGT	1	0	1	0	0	At5g44630.1:v:-1185:secondary
TGAATTTCCT	0	1	0	0	1	At5g44610.1:d:+679:primary
TTGTGTGGTG	1	0	0	1	0	At5g44510.1:v:+3772:secondary,At5g58340.1:v:+1935:secondary
AGTCCTCAAT	0	0	1	0	1	At5g44380.1:i:+865:tertiary
ATTCGTCTTT	0	1	1	0	0	At5g44380.1:d:+1763:primary

GTTGGTTTCG	0	0	2	0	0	At5g44340.1:d:+909:secondary,At5g16630.1:d:+2271:secondary,At1g75780.1:d:+843:secondary,At1g59870.1:d:+4104:secondary
GATATAAAAA	2	0	0	0	0	At5g44330.1:v:-72:primary,At5g34450.1:v:-71:primary
AACCAACATT	1	1	0	0	0	At5g44316.1:v:+2033:primary
TGAATCTTTT	0	2	0	0	0	At5g44140.1:v:+1482:primary
AAAAAATAAT	0	0	2	0	0	At5g44030.1:i:+4027:tertiary
GCTTACAAGT	0	2	0	0	0	At5g44020.1:d:+721:secondary
ATGTTAGCGT	0	0	2	0	0	At5g43940.1:d:+541:secondary
GGAGAGGAAT	0	1	1	0	0	At5g43880.1:d:+2566:primary
ATATTCTTTT	1	0	1	0	0	At5g43870.1:d:+1574:primary
TCTCCAACTC	0	0	1	1	0	At5g43745.1:d:+2675:primary
AAAGAGGGGG	0	0	0	2	0	At5g43745.1:d:+2277:secondary
GCTGTTATAT	0	1	0	0	1	At5g43740.2:d:+2362:secondary,At5g43740.1:d:+2362:secondary
CTCAGAAATA	1	0	1	0	0	At5g43710.1:d:+1719:primary
AAGCTGCCCA	0	0	1	1	0	At5g43630.1:d:+2179:secondary
TTTAATATTA	0	2	0	0	0	At5g43610.1:v:+1865:secondary,At5g06170.1:v:+1883:secondary
TGCTTTCTCA	0	1	0	1	0	At5g43580.1:v:+671:secondary
ATTGGGGTTG	0	1	1	0	0	At5g43560.2:d:+615:secondary,At5g43560.1:d:+985:secondary
CCACATTCTG	0	1	0	0	1	At5g43560.1:d:+3453:primary,At5g43560.2:d:+3083:primary
TTGTGTTATA	0	0	0	1	1	At5g43490.1:v:+994:primary,At5g48610.1:v:+2024:primary
ATGAGAGAAG	0	0	0	1	1	At5g43470.1:d:+1673:secondary,At1g53350.1:v:+1833:secondary,At5g43470.2:d:+1673:secondary,At1g59124.1:d:+1643:secondary,At1g58602.1:d:+2006:secondary,At1g58848.1:v:+1530:secondary,At1g58400.1:v:+1824:secondary,At1g10920.1:d:+1628:secondary,At1g58390.1:v:+1845:secondary,At5g48620.1:d:+1620:secondary,At1g58807.1:d:+1504:secondary,At1g59218.1:d:+1504:secondary
GTAGAAAAGT	1	0	0	1	0	At5g43405.1:v:+142:secondary
AAAATTTGAT	1	1	0	0	0	At5g43370.1:v:+2194:primary,At2g38750.1:d:+1146:primary
ACGAGAAATA	0	1	0	0	1	At5g43350.1:d:+1641:primary
GGCACATTAG	0	0	2	0	0	At5g43330.1:d:-1236:secondary
TCCGGGGATT	0	1	0	1	0	At5g43320.1:d:+822:primary
TATTCAATAT	0	0	0	0	2	At5g43310.1:d:+3768:primary
ATCTGAGCCT	0	1	1	0	0	At5g43310.1:d:+1973:secondary
GAATTGGCTT	0	2	0	0	0	At5g43280.1:d:+588:secondary
ACTGATTTGA	1	0	1	0	0	At5g43260.1:X:-169:quaternary,At3g30582.1:X:-411:quaternary
ACAACTCAAC	0	1	0	1	0	At5g43130.1:d:+1075:secondary
AAGAATTTAA	1	1	0	0	0	At5g43065.1:p:+3915:secondary,At4g16155.1:d:+1839:secondary
ACGACAAGGT	0	1	0	0	1	At5g42990.1:d:+523:secondary,At1g45050.1:d:+529:secondary
CCACTAATCA	0	2	0	0	0	At5g42950.1:d:+2891:secondary
TTACAAGGTA	0	2	0	0	0	At5g42860.1:d:+1094:primary
GCTCGTCTTA	0	2	0	0	0	At5g42765.1:d:+42:secondary,At4g14746.1:v:+354:secondary
GCTCCATTGT	0	0	1	0	1	At5g42720.1:d:+1123:primary
TGATTGATAA	0	0	0	2	0	At5g42645.1:p:+4176:secondary
AGAACAACTT	1	0	0	1	0	At5g42630.1:d:+276:secondary
ATGTGTAAAT	1	0	1	0	0	At5g42540.1:i:-4585:tertiary
ACCAACCACA	0	0	2	0	0	At5g42370.1:X:-283:quaternary
TATTGAAAAG	0	0	1	1	0	At5g42350.1:d:+1970:primary
AAGCAGACGT	2	0	0	0	0	At5g42310.1:d:+2184:secondary
GTGGCGCCGC	0	1	1	0	0	At5g42250.1:d:+190:secondary
TTTAACTATT	0	0	0	0	2	At5g42250.1:d:+1307:primary
TACAATTCAG	0	1	0	0	1	At5g42235.1:X:-470:quaternary
GCTCAAATGA	1	0	0	0	1	At5g42220.1:d:+2604:primary
GCAATGTAAA	1	1	0	0	0	At5g42200.1:X:+976:quaternary
AAATTAGTTT	1	0	0	1	0	At5g42200.1:d:+696:primary
GGAATTTCAG	0	0	0	1	1	At5g42150.1:d:+717:secondary,At1g54920.2:d:+1488:secondary,At1g54920.1:d:+671:secondary
TTGGTTTCTC	1	0	0	1	0	At5g42120.1:v:+1054:secondary,At2g19200.1:v:+585:secondary,At5g16530.1:v:+242:secondary
TGTCTAAGCA	0	0	2	0	0	At5g42060.1:d:+449:primary
GAAGCTAATG	2	0	0	0	0	At5g42060.1:d:+417:secondary
TTCAGCTTGA	0	2	0	0	0	At5g41960.1:d:+558:primary
GGAGTCTTTA	0	0	0	2	0	At5g41950.1:d:+1713:secondary
GAGAGTAGCT	0	1	1	0	0	At5g41770.1:d:+1922:primary
CCAAGACCTT	0	0	1	1	0	At5g41760.1:d:+1027:primary
AGTAGCCGCA	0	2	0	0	0	At5g41690.1:X:--14:quaternary

GAAAGGTTAC	0	0	1	0	1	At5g41650.1:i:+508:tertiary
ATATTGTTTG	0	0	1	1	0	At5g41410.1:d:+2206:primary
CATTCTAGAA	0	1	0	1	0	At5g41350.1:d:+690:secondary
CTTTCTTCAC	2	0	0	0	0	At5g41270.1:d:+596:primary
CTCTGAAACT	0	2	0	0	0	At5g41260.1:d:-277:primary
ACCTAAATGC	0	1	1	0	0	At5g41260.1:d:+627:secondary
CAAGACAGAG	0	2	0	0	0	At5g41260.1:d:+1347:primary
ATGTGAATTG	0	1	0	1	0	At5g41220.1:X:-1154:quaternary
GTTTCTCTTC	1	0	0	1	0	At5g41120.1:d:+2051:primary
ATGATAAGAC	0	2	0	0	0	At5g41080.2:d:+1121:primary,At5g41080.1:d:+1029:primary
AAGCAGAAAC	0	0	1	1	0	At5g41050.1:d:+246:primary
ATTTTGTAAC	0	2	0	0	0	At5g40810.1:d:+1325:primary
TCGTATGGCG	0	2	0	0	0	At5g40720.1:d:+1431:primary
TGGAAGAATA	0	1	1	0	0	At5g40690.1:d:+643:primary,At4g16280.1:d:+4715:primary,At4g16280.3:d:+2070:primary
GATCGAGAAT	0	1	1	0	0	At5g40670.1:d:-548:secondary
AGAAGAATCA	0	0	2	0	0	At5g40670.1:d:-1061:secondary,At5g40670.1:d:-1055:secondary
ACATTGATGT	0	0	1	1	0	At5g40660.1:d:+1070:primary
TAAAGATATA	1	1	0	0	0	At5g40650.1:i:+1488:tertiary
GAGGTTCTTC	1	0	1	0	0	At5g40530.2:d:+188:primary,At5g40530.1:d:+187:primary
TGACATCTTT	0	0	1	1	0	At5g40490.1:d:+1585:primary
CTTGTCTACT	0	2	0	0	0	At5g40450.1:X:-587:quaternary
TCTTACAATA	0	0	0	1	1	At5g40400.1:d:+1144:secondary
TGCTTTTTAA	1	0	0	0	1	At5g40380.1:d:+714:secondary,At3g20800.1:d:+1064:secondary
ATTAAAAGTT	0	2	0	0	0	At5g40310.1:i:+1785:tertiary
AAAATTTGTC	0	0	1	1	0	At5g40210.1:i:+1025:tertiary
AGCCTTAGAT	0	0	1	1	0	At5g40150.1:X:-1428:quaternary
TTCTTTTTTC	1	0	0	1	0	At5g39980.1:v:+2661:primary
TAGTTTATGA	0	0	0	1	1	At5g39900.1:d:+2013:primary
AATTTGTGCT	1	0	0	1	0	At5g39830.1:d:+292:secondary,At5g39830.2:d:+292:secondary
GGTTTTGGTT	0	0	0	1	1	At5g39740.1:i:+224:tertiary,At1g53580.2:i:+260:tertiary,At1g53580.1:i:+260:tertiary
GCTGATCCGG	1	0	1	0	0	At5g39660.1:d:+225:secondary,At5g39660.2:d:+198:secondary
ACCTGCCCTT	0	2	0	0	0	At5g39550.1:v:+1989:primary,At1g66050.1:v:+1998:primary
TATATGTATT	1	1	0	0	0	At5g39360.1:d:+996:primary,At1g14230.1:d:+1802:primary
ATATTCAATT	0	1	1	0	0	At5g39330.1:v:+1140:secondary
TCTCCACCGT	1	1	0	0	0	At5g38990.1:d:+2070:primary
AGTTTGATCA	0	2	0	0	0	At5g38800.1:d:+145:primary,At1g80870.1:d:+962:primary
AACTCAGGAA	1	0	1	0	0	At5g38640.1:d:+1444:secondary,At5g18550.1:d:+1479:secondary
TTTAAAGGGA	0	1	0	0	1	At5g38560.1:d:-2682:secondary
ATATAATGTA	2	0	0	0	0	At5g38560.1:d:+2715:primary
AGCTTTCTGC	1	1	0	0	0	At5g38510.1:v:+1203:primary
GAGTTAGCTT	0	1	1	0	0	At5g38380.2:d:+823:secondary,At5g38380.1:d:+913:secondary
GAGAGATTAC	1	1	0	0	0	At5g38285.1:p:+430:secondary
TTTGCTTTCA	0	1	1	0	0	At5g38230.1:p:+303:secondary,At5g35210.2:d:+4415:secondary
GTGTTCCTCC	1	1	0	0	0	At5g38210.1:d:+2015:primary
CAGAGGATGC	1	0	1	0	0	At5g38200.1:d:+1321:primary
TTTTAAGTTT	0	1	0	0	1	At5g38080.1:v:+1116:primary
TTAAATCTTC	0	1	1	0	0	At5g38050.1:v:+1245:secondary
TTGCTTTTGT	0	1	1	0	0	At5g38035.1:p:+595:secondary
CCCTTCATCG	0	0	1	0	1	At5g37910.1:v:+728:secondary
TTGATTCCCA	1	1	0	0	0	At5g37890.1:d:+859:primary
ACTGCTCTTC	0	1	0	1	0	At5g37850.1:d:+797:primary
ATCATAACTT	0	0	2	0	0	At5g37620.1:v:+1498:secondary,At2g19660.1:v:+1474:secondary,At2g28500.1:v:+1040:secondary
TAGGTTAACG	0	0	0	1	1	At5g37570.1:v:+2347:primary
GTCAGCTTTG	0	2	0	0	0	At5g37570.1:v:+1166:secondary,At3g48820.2:d:+1300:secondary,At3g48820.1:d:+1331:secondary
CATAATCAAC	0	0	0	0	2	At5g37520.1:v:-278:primary
TGTGTGGTTA	0	2	0	0	0	At5g37360.1:i:+1232:tertiary
TGGCTCTCGT	0	0	1	0	1	At5g37180.1:v:-1825:secondary
AAGTGGTGTT	0	2	0	0	0	At5g37145.1:p:+803:secondary
TTCCTGTCCT	0	1	1	0	0	At5g37050.1:v:+214:secondary
TGGTTTAAGA	0	2	0	0	0	At5g37000.1:v:+2438:primary

TTGTACACTT	0	0	0	1	1	At5g36950.1:d:+1797:primary
GAATGACTCT	0	1	0	0	1	At5g36925.1:d:+260:primary
AGGTAAAAGG	0	2	0	0	0	At5g36880.1:d:+1974:secondary
TTCCCTCAAA	0	1	0	1	0	At5g36870.1:i:+11997:tertiary
TGGGGAAAAA	0	0	1	1	0	At5g36820.1:v:-1469:secondary,At5g36730.1:v:-1469:secondary
GGCTCCACTT	0	1	1	0	0	At5g36230.1:d:+1107:secondary
AAGAACTGTA	0	2	0	0	0	At5g36000.1:v:-1675:secondary
TGTTGTTATA	2	0	0	0	0	At5g35995.1:d:+1519:primary
CTCTTTGCTT	0	0	0	2	0	At5g35695.1:v:+1077:secondary,At1g18080.1:d:+843:secondary
TACAAACATA	0	1	1	0	0	At5g35690.1:d:+2028:secondary
ACAAGAAAGC	2	0	0	0	0	At5g35660.1:X:-1513:quaternary,At2g44590.1:X:-319:quaternary
GAGATTTGTT	0	1	0	0	1	At5g35570.1:d:+2286:primary
TTTTGAGAAA	0	2	0	0	0	At5g35475.1:v:+1235:primary
AGGAAGGTAA	0	2	0	0	0	At5g35430.1:d:+1803:secondary,At1g72790.1:d:+718:secondary
GCGCAACCAA	0	2	0	0	0	At5g35331.1:p:+1019:secondary,At5g64030.1:d:+327:secondary
TCAACATTAT	0	1	0	0	1	At5g35240.1:p:+171:secondary,At1g74750.1:v:+3063:secondary
GCTCATTATA	2	0	0	0	0	At5g35210.2:X:-383:quaternary,At5g35220.1:X:-254:quaternary
GAATTTGAAC	0	0	1	1	0	At5g35210.1:d:+3934:secondary,At4g14190.1:d:+70:secondary,At5g35210.2:d:+3934:secondary
TGACATATGT	0	1	0	1	0	At5g35180.1:d:+2484:primary
TTGGTGACCG	0	0	1	0	1	At5g35160.1:v:+2560:primary
ATTATAAACA	2	0	0	0	0	At5g35100.1:X:-262:quaternary,At5g26360.1:X:--45:quaternary
TAACACATAT	1	1	0	0	0	At5g35080.1:d:+1171:primary
ATTTATGCAT	0	0	0	1	1	At5g34623.1:p:+940:secondary,At5g23630.1:d:+2273:secondary
GGAGACCATC	0	1	1	0	0	At5g33438.1:p:+1589:primary,At2g35900.1:d:+546:primary
TGATTTGAGA	0	1	0	1	0	At5g32825.1:X:-2214:quaternary
GTTGAGTGAA	0	1	1	0	0	At5g32512.1:X:-1920:quaternary,At3g20770.1:X:-1955:quaternary
TATCTTACCT	1	1	0	0	0	At5g32512.1:X:-1342:quaternary
ACCGTGTCTC	0	1	1	0	0	At5g30510.1:d:+968:secondary
TTAATTTTGA	1	0	1	0	0	At5g30500.1:i:+1330:tertiary,At1g68750.1:i:+3884:tertiary,At5g62050.1:i:+1907:tertiary
ACACCAGTTG	2	0	0	0	0	At5g29031.1:X:+707:quaternary
TTTATTGGGA	0	2	0	0	0	At5g29000.2:d:+1324:secondary,At5g29000.1:d:+1229:secondary
TGTTAGGGTT	0	0	1	1	0	At5g28935.1:X:-4484:quaternary
AAGTTTTCAA	1	1	0	0	0	At5g28927.1:p:+2096:primary
AGAGTAACAA	0	1	0	0	1	At5g28850.2:X:-541:quaternary
AAAGGAAAAG	1	0	0	1	0	At5g28760.1:i:+1479:tertiary
TAGATCTTGC	0	0	2	0	0	At5g28672.1:p:-1367:secondary
GATCCGCTGC	0	2	0	0	0	At5g28635.1:p:+1536:secondary,At1g31710.1:d:+1685:secondary
TTTGAAAAAA	0	0	0	1	1	At5g28610.1:X:+-231:quaternary,Chr1:+9924769:quaternary
GAAGTGGTGG	0	1	0	0	1	At5g28610.1:d:+235:primary,At4g05360.1:v:+2458:primary
TTTTCTAAAA	1	0	0	1	0	At5g28605.1:p:+1885:secondary,At2g06870.1:p:+2898:secondary
GTGGGGGTTG	0	0	2	0	0	At5g28600.1:v:+1203:secondary
GATTCTTCAA	0	1	1	0	0	At5g28500.1:d:+101:secondary
AGCTTGCGCC	1	1	0	0	0	At5g28380.1:v:+442:secondary
TCAAAGGCTT	0	0	1	1	0	At5g28290.1:d:-873:secondary
ATTGGTTTCT	0	1	1	0	0	At5g28150.1:d:+1220:primary
TATAATGAAA	0	0	0	1	1	At5g27947.1:p:+1629:secondary
ACGATTTTAA	0	1	0	0	1	At5g27930.2:d:+1313:secondary,At5g27930.1:d:+1228:secondary
GATAACAACT	0	1	0	0	1	At5g27882.1:p:+97:secondary
AGCATATTTC	0	1	1	0	0	At5g27750.1:d:+1507:primary
TTTACTAGTT	1	0	0	0	1	At5g27630.1:i:+916:tertiary
ACGAGAGTAA	1	1	0	0	0	At5g27620.1:d:+1356:primary
AAACATTTGT	1	0	0	1	0	At5g27610.1:v:+3260:primary
GTATAATAAT	0	1	0	0	1	At5g27460.1:v:+1970:primary
AAGCTTAATT	0	1	1	0	0	At5g27400.1:d:+642:secondary
TAGTTGATTC	0	0	1	0	1	At5g27330.1:d:+2100:primary
CTTAATTTGT	0	2	0	0	0	At5g27270.1:d:+1525:secondary
TTTCCGGATT	0	0	0	2	0	At5g27210.1:i:+761:tertiary
TTTTCCTTCA	0	2	0	0	0	At5g26980.1:d:+1255:secondary,At1g35420.1:d:+1091:secondary
GCCAAGAACA	1	0	0	1	0	At5g26940.4:d:+1113:primary,At5g26940.3:d:+1035:primary,At5g26940.1:d:+1054:primary,At5g26940.2:d:+1593:primary

AGAATTGAGA	1	1	0	0	0	At5g26910.1:d:+2733:primary
GGACAATCAC	0	2	0	0	0	At5g26900.1:v:+1007:secondary,At5g56240.1:d:+2569:secondary
ATAACAAAGT	0	1	1	0	0	At5g26742.1:v:+2223:secondary
GATTTATGAT	0	1	0	1	0	At5g26667.1:d:+792:primary,At5g54830.1:d:+2291:primary
AACCTAGCCC	1	0	1	0	0	At5g26340.1:X:-616:quaternary
TTTTGCTATC	1	1	0	0	0	At5g26260.1:d:+1217:secondary
AAAGGAACAT	0	0	0	1	1	At5g26180.1:d:+1795:primary
CATCTGCAAG	0	2	0	0	0	At5g26000.1:X:-253:quaternary
CCTTCGTTTC	0	0	0	2	0	At5g25890.1:X:+272:quaternary
GCAAGAACTT	0	1	0	1	0	At5g25800.1:i:+2243:tertiary
AAACTCGTCG	0	0	1	0	1	At5g25770.1:X:-40:quaternary
CTTATAACCC	0	2	0	0	0	At5g25620.1:d:+1953:primary
ATATATTGTT	0	0	0	1	1	At5g25540.1:d:+720:primary
CCTCCTGGAT	1	1	0	0	0	At5g25520.2:d:+2800:secondary,At5g25520.1:d:+3007:secondary
AGGAGTTTTC	0	0	1	1	0	At5g25470.1:d:+255:secondary,At5g25470.2:d:+222:secondary
CTTCTCGTTG	0	0	2	0	0	At5g25460.1:d:+272:secondary
CATTTTTGTT	1	0	1	0	0	At5g25390.2:i:+408:tertiary,At5g25390.1:i:+382:tertiary
AATCGTGTTA	2	0	0	0	0	At5g25305.1:X:--250:quaternary
GCAGATCTGA	2	0	0	0	0	At5g25280.2:d:-798:secondary,At5g25280.1:d:-801:secondary
AGAGTCAAAA	0	0	1	1	0	At5g25270.1:v:+2239:primary
GCTTCTCTTC	0	2	0	0	0	At5g25190.1:d:+355:primary
TTTTCTTTGG	1	0	1	0	0	At5g25170.1:d:+1260:primary
AAGAGCAGCA	0	1	1	0	0	At5g25160.1:d:+725:secondary,At4g32790.1:v:+1788:secondary
TTAATATATG	1	1	0	0	0	At5g25130.1:d:+1234:primary,At5g25140.1:d:+1290:primary
GAGGTGGTGG	0	1	0	1	0	At5g25120.1:v:+969:secondary,At4g33010.1:d:+2524:secondary,At5g25180.1:v:+969:secondary,At4g04296.1:p:+683:secondary,At2g36120.1:p:+539:secondary,At5g58220.1:d:+737:secondary
AGACTTTTGC	0	2	0	0	0	At5g25050.1:d:+1675:primary
GTTTCTCTTT	0	0	0	2	0	At5g25040.1:v:+1057:secondary,At2g22790.1:v:+544:secondary
TCATACTAAT	1	0	0	1	0	At5g24980.1:d:+9:primary
TGTAGTTGTA	0	0	1	1	0	At5g24940.1:v:+1172:secondary
ATGATGGGAT	0	1	0	0	1	At5g24870.1:d:+1863:primary,At5g24870.2:d:+1753:primary
ATGAATCAAA	0	1	0	0	1	At5g24760.2:d:+1450:primary,At5g24760.1:d:+1460:primary
GAAATTGCTG	0	1	1	0	0	At5g24750.1:d:+1513:primary
AAAAGGGTTT	1	0	0	0	1	At5g24740.1:i:+5121:tertiary
TTAATTCTTA	0	1	0	0	1	At5g24740.1:i:+17761:tertiary,At4g12910.1:i:+1618:tertiary,At3g21810.1:i:+3522:tertiary
TGTTATGTCA	0	1	0	1	0	At5g24740.1:d:+3591:secondary
GCTTGGACAG	0	0	0	1	1	At5g24350.1:d:+1379:secondary,At2g30110.1:d:+3067:secondary
CTTGGATTCA	0	0	1	0	1	At5g24340.1:i:+2133:tertiary,At2g22260.1:i:+1158:tertiary
CAAGGAAAGA	0	1	0	1	0	At5g24270.1:v:+1031:secondary
TATAAGAATG	0	0	1	0	1	At5g24210.1:d:+1273:primary,At1g52660.1:v:+1731:primary
TCACATCAAA	0	0	0	1	1	At5g24200.1:v:+1463:secondary
AAAATGTTGG	0	1	1	0	0	At5g24160.1:d:+1489:primary
AACCCTCGTC	1	0	0	1	0	At5g24150.1:d:+1354:primary
CGTCATCCAG	0	0	2	0	0	At5g24150.1:d:+1054:secondary
CTCAATCCGC	0	0	2	0	0	At5g24105.1:d:+253:primary
GTTTTTTAGT	0	1	0	1	0	At5g23940.1:d:+1774:secondary
CCAATTGCCA	0	1	1	0	0	At5g23900.1:d:+498:secondary
ATTAAGAATT	1	0	1	0	0	At5g23590.1:d:+1197:primary
CTGTGGCGAC	1	0	0	1	0	At5g23575.1:d:+741:secondary
AAAGAAGAGA	0	0	0	1	1	At5g23570.1:d:+1641:secondary,At5g16780.1:d:+1616:secondary,At1g58561.1:p:+2747:secondary,At1g47360.1:p:+2560:secondary,At2g07717.1:p:+4864:secondary,At1g09575.1:d:+565:secondary
AATAATTTGT	1	1	0	0	0	At5g23520.1:v:+1901:primary
TAAATCGAGT	1	1	0	0	0	At5g23440.1:d:+401:primary
CGAGTTTCAG	0	0	1	0	1	At5g23400.1:d:+1902:primary
ATCTTGGTCA	1	1	0	0	0	At5g23390.1:d:+1915:primary
TGACAGAAGT	1	1	0	0	0	At5g23340.1:d:+1243:secondary
GTACTTCATT	0	2	0	0	0	At5g23240.1:d:+1516:primary

GCCGCCAGGG	0	0	0	1	1	At5g23140.1:d:-347:primary
GGAGTTTTTT	0	0	0	1	1	At5g23080.1:i:+267:tertiary
CTGTAAAAGA	0	1	1	0	0	At5g23020.1:d:+1292:primary
ATTTGGCGTG	0	0	2	0	0	At5g23010.1:d:-1154:secondary
CCAGTCTAGG	0	0	0	2	0	At5g22860.1:d:+1540:primary
AGAAGCTTCC	0	1	1	0	0	At5g22770.3:d:+1700:secondary,At5g22770.1:d:+1770:secondary,At5g22770.2:d:+1770:secondary,At5g22780.1:d:+1771:secondary,At3g50780.1:d:+1340:secondary
AAAATGTTAT	1	0	0	1	0	At5g22760.1:d:+3683:secondary
GAGTCACGGA	0	2	0	0	0	At5g22500.1:d:+1319:secondary
CCGTGGAAGT	0	0	0	1	1	At5g22450.1:v:+3188:primary
GAGGAGAAGC	0	1	0	0	1	At5g22440.1:d:+566:secondary,At1g68890.1:d:+2152:secondary,At1g01720.1:d:+676:secondary
GTCGTGAGAT	0	0	1	1	0	At5g22330.1:d:+1699:primary
TATTCCGGCG	0	0	0	2	0	At5g22300.1:d:-965:secondary
CTAGTCTTTC	0	1	1	0	0	At5g22280.1:d:+256:primary
ATGCAATTAG	1	0	0	1	0	At5g22270.1:X:-220:quaternary
TGGCCAGATG	0	2	0	0	0	At5g22220.1:d:+1469:primary
ACTTCAGATT	1	0	1	0	0	At5g22100.1:i:+282:tertiary
ATGGAACGTT	1	1	0	0	0	At5g22000.2:d:+1063:secondary,At5g22000.1:d:+1189:secondary,At5g22000.3:d:+1116:secondary
CCCATTTTTT	0	0	1	1	0	At5g21970.1:i:-269:tertiary,At4g04840.1:i:-725:tertiary
ACTCGTAGGC	0	0	2	0	0	At5g21482.1:d:-1834:secondary
GCTATACTTT	0	0	2	0	0	At5g21482.1:d:+1646:secondary,At3g43126.1:p:+3168:secondary
CATCAAAAAC	1	0	1	0	0	At5g21160.1:X:--36:quaternary,At5g55510.1:X:-319:quaternary
GCGTCTCGAA	0	0	1	0	1	At5g21090.1:d:+150:secondary,At5g08690.1:d:+30:secondary
TGTTTCAGTT	0	0	0	2	0	At5g21080.1:v:+3335:primary
TTAGACAACG	1	1	0	0	0	At5g21010.1:d:+1265:secondary
TGTTTTAAGT	0	0	2	0	0	At5g20740.1:d:+730:secondary
AAAAAGAAGA	1	0	0	1	0	At5g20610.1:d:+1115:secondary
ATCAAGCTGC	0	0	0	1	1	At5g20510.1:d:+1064:primary
CAACAAGTGG	1	1	0	0	0	At5g20490.1:d:+4540:primary
TGTTTGCCAT	0	2	0	0	0	At5g20490.1:d:+437:secondary,At4g00232.1:v:+985:secondary
TTGAGAGAGT	0	1	1	0	0	At5g20450.1:X:-266:quaternary
TTCCAGATCA	2	0	0	0	0	At5g20450.1:i:+272:tertiary
TCTCCCTTAG	0	2	0	0	0	At5g20410.1:d:+1519:secondary
GAATTTGGGG	0	2	0	0	0	At5g20300.1:i:+407:tertiary
TTGTAAGACA	1	0	0	0	1	At5g20290.1:X:-112:quaternary
CCTCTGCTAG	0	2	0	0	0	At5g20250.2:d:+1792:secondary,At5g20250.1:d:+1771:secondary
ACCGTTCCTC	0	0	2	0	0	At5g20230.1:d:+385:secondary
AAACAGCTCT	0	0	1	1	0	At5g20220.1:i:+584:tertiary
CCGGAAGCCG	1	0	1	0	0	At5g20220.1:d:+1138:primary
CGAGGCACAA	0	0	0	1	1	At5g20180.1:d:-494:primary,At5g20180.2:d:-569:primary
AAGCATCCAG	0	0	1	0	1	At5g20165.1:X:-36:quaternary
TTCTTGAGTA	1	1	0	0	0	At5g20040.1:d:+1682:primary
GTCACTCCCA	0	2	0	0	0	At5g19920.1:X:-107:quaternary
TAATGTTTGG	1	0	0	1	0	At5g19790.1:v:+1144:secondary
GCCTGTTGTT	0	0	0	0	2	At5g19770.1:d:+1043:secondary
GTACCAGGCT	0	0	0	0	2	At5g19750.1:d:+406:secondary
GATATTTATT	1	1	0	0	0	At5g19680.1:d:+1215:primary
GATTGAAGGA	0	1	0	1	0	At5g19620.1:d:+1493:secondary,At1g11190.1:d:+413:secondary
TACAAACAGT	1	0	1	0	0	At5g19540.1:d:+1453:secondary
ATGATTTTGA	0	1	1	0	0	At5g19430.1:d:+592:primary
GCTGAAAGAC	0	0	1	1	0	At5g19420.1:d:+2869:secondary
TGGTGAATAC	1	1	0	0	0	At5g19420.1:d:+1170:secondary
GAAATAGTTG	2	0	0	0	0	At5g19410.1:v:+1266:secondary
TTGGTGAAGA	0	1	0	0	1	At5g19400.1:d:+3348:primary
TTCCTGTTTG	0	2	0	0	0	At5g19340.1:d:+750:primary
GTAGACGTGC	0	2	0	0	0	At5g19220.1:i:+1326:tertiary
AAGAGATTTT	0	1	0	0	1	At5g19097.1:p:+3149:secondary
CCGAAATCTG	0	0	2	0	0	At5g19090.1:d:+212:secondary,At5g19090.2:v:+562:secondary

AACGAAGCGG	0	1	0	0	1	At5g19070.1:d:+1042:primary
TAAAATGATT	2	0	0	0	0	At5g18970.1:d:+725:secondary
TTTAGAGTAT	0	1	0	0	1	At5g18960.1:v:+3143:primary
TTCTTAGGGG	0	2	0	0	0	At5g18890.1:v:+1689:secondary,At5g18860.1:d:+2736:secondary
ATCGAAACTG	0	0	2	0	0	At5g18880.1:v:+123:secondary
CATCAGAAGA	0	0	0	0	2	At5g18760.1:X:--134:quaternary,At1g79480.1:X:-1143:quaternary
AAACTGTACA	0	0	1	0	1	At5g18710.1:v:-2366:secondary,At4g13510.1:d:-1663:secondary
TGACAAAGTG	2	0	0	0	0	At5g18700.1:v:+4629:secondary
GCCGTGTCAC	0	1	0	1	0	At5g18680.1:d:+1055:primary
ACCTCGTTGG	0	1	1	0	0	At5g18600.1:X:-421:quaternary
ACCGTTCTTA	0	2	0	0	0	At5g18390.1:d:-480:secondary,At5g16860.1:v:-1556:secondary
AATCATAATC	1	1	0	0	0	At5g18290.1:d:+1061:primary
AACACAGAGG	1	0	0	1	0	At5g18240.4:d:+1103:secondary,At5g18240.3:d:+1147:secondary,At5g18240.5:d:+1097:secondary,At5g18240.1:d:+1178:secondary,At5g18240.2:d:+1160:secondary
AGAATAAGTA	0	2	0	0	0	At5g18160.1:v:-668:secondary,At5g47240.1:d:-1251:secondary
ATGATCCGAC	1	1	0	0	0	At5g18030.1:d:+365:primary
ACAATTATAA	0	1	0	0	1	At5g17930.1:i:+2584:tertiary
GCAGGAGGTG	0	1	0	1	0	At5g17900.1:X:+348:quaternary
TCTTTATTGG	0	1	0	1	0	At5g17880.1:d:+3173:primary
GGTTTGTATA	0	0	0	1	1	At5g17850.1:v:+2067:secondary
TAGCATAACA	2	0	0	0	0	At5g17790.1:d:+2330:primary
GGACATTGCT	0	0	0	0	2	At5g17760.1:d:+1283:primary,At5g17760.2:d:+1476:primary
AAGATAAACG	0	1	0	0	1	At5g17660.1:X:-147:quaternary,At5g15540.1:X:-56:quaternary
TTTGGGAAGC	0	2	0	0	0	At5g17650.1:d:+650:primary
CTTCGGAGCC	0	2	0	0	0	At5g17530.2:i:+3217:tertiary,At5g17530.1:i:+3217:tertiary
GACCACCACA	0	0	0	0	2	At5g17260.1:i:+746:tertiary
TATCTTAAAA	0	1	0	1	0	At5g17260.1:i:+1684:tertiary
GAAACAATCG	0	1	0	0	1	At5g17070.1:d:+744:primary
CTAGAAAATG	0	0	2	0	0	At5g16780.1:d:+1163:secondary
AATTATAAAT	1	0	0	0	1	At5g16750.1:d:+2740:primary
TAAAACGTAT	0	0	2	0	0	At5g16730.1:d:+2608:primary
TTGTCAGAAG	1	1	0	0	0	At5g16720.1:d:+465:secondary
AAAAAGAGAT	0	0	1	0	1	At5g16680.1:X:-108:quaternary
GACACATCAA	1	1	0	0	0	At5g16650.1:d:+587:secondary
ATCAGGCTTT	2	0	0	0	0	At5g16590.1:d:+1928:primary
ATGATACTAA	1	0	0	0	1	At5g16510.2:d:-1351:secondary,At5g16510.1:d:-1290:secondary
TATCGCACAT	0	2	0	0	0	At5g16490.1:v:+673:primary
TGTGATGTGA	0	2	0	0	0	At5g16480.1:d:+1092:primary
CAAATGAAAA	2	0	0	0	0	At5g16460.1:v:+264:secondary
TCTTTTGTTG	0	1	1	0	0	At5g16460.1:v:+1897:primary
GAAGTGTAGC	0	0	1	0	1	At5g16420.1:v:+2414:primary
TTCCTTATAA	1	1	0	0	0	At5g16310.1:d:+1320:secondary
AGAAGGAAAC	0	0	1	1	0	At5g16310.1:d:+1037:secondary,At5g66520.1:v:+1908:secondary
AGAATTGTTC	0	1	0	1	0	At5g16280.1:d:+3849:primary
CTACAGTTAG	0	1	1	0	0	At5g16260.1:d:+1643:primary
ACTAATATCT	1	0	0	0	1	At5g16230.1:i:+1396:tertiary
CAGGCTCTTG	0	2	0	0	0	At5g16190.1:d:+1468:primary
TCGTAAGCAA	1	0	1	0	0	At5g16060.1:d:+204:primary
TTTGAGAATG	0	2	0	0	0	At5g16000.1:d:+2294:primary
GGTTTTAAAA	0	0	1	0	1	At5g15870.1:v:+1364:secondary
CAGTCTAGTT	0	0	1	1	0	At5g15850.1:X:-374:quaternary
TTATTTTTAC	0	0	1	0	1	At5g15820.1:X:+1464:quaternary,Chr2:+9919991:quaternary
TCAGAAATAT	0	1	1	0	0	At5g15710.1:d:+1710:primary
TGTAAACCCG	1	0	0	1	0	At5g15700.1:d:+3608:primary
ACTTGTTTTA	0	0	1	1	0	At5g15570.1:v:+1574:primary
TCAGAAAAAA	1	0	1	0	0	At5g15460.2:X:-187:quaternary
TACTGGTTAA	0	1	0	1	0	At5g15270.1:d:+2099:secondary
GAGATTTAAC	0	2	0	0	0	At5g15260.1:X:-903:quaternary
CAATTCCATT	0	2	0	0	0	At5g15160.1:d:+546:primary
ATAGTAAAAT	0	0	1	0	1	At5g15120.1:i:+1133:tertiary

GTCGGAGTTA	0	2	0	0	0	At5g15120.1:d:-385:primary
CACCAGAAAA	0	0	1	0	1	At5g14970.1:d:-1085:secondary
AGAACAATGG	0	1	0	0	1	At5g14940.1:v:+1743:secondary
AAGTGGTGGA	0	2	0	0	0	At5g14930.2:d:+1625:primary
GTGGCTCCTT	1	0	0	1	0	At5g14880.1:v:+2403:secondary
TTTTTTTTGA	2	0	0	0	0	At5g14880.1:i:+1763:tertiary
ACTCAAGAAC	2	0	0	0	0	At5g14820.1:v:+928:secondary,At3g62470.1:d:+693:secondary
ACAGACTTTC	0	0	0	2	0	At5g14760.1:d:+2119:primary
GATATGATTT	1	0	0	0	1	At5g14720.1:d:+1047:secondary
TCGTGGTTTA	1	0	0	0	1	At5g14660.2:d:+1176:primary,At5g14660.1:d:+1178:primary
AGGCGATGAT	0	1	1	0	0	At5g14610.1:d:+2117:primary
TGGGTCTTCT	0	0	0	1	1	At5g14570.1:d:+1302:primary,At5g14570.2:d:+1302:primary
CACGAGCCTC	1	0	1	0	0	At5g14520.1:v:+564:secondary
CTGTTAAACA	0	2	0	0	0	At5g14470.1:X:-473:quaternary,At4g21660.1:X:--1:quaternary
TGGCTGACCG	0	1	1	0	0	At5g14450.1:d:+1144:primary
GAACACCTTC	0	2	0	0	0	At5g14430.1:d:+1684:secondary,At5g14430.2:d:+1684:secondary
GCCTTTGGTT	0	2	0	0	0	At5g14420.1:d:+1441:primary,At5g14420.4:d:+1423:primary,At5g14420.3:d:+1429:primary,At5g14420.2:d:+1537:primary
ATATCTAATT	0	1	0	0	1	At5g14380.1:X:-845:quaternary
GTGGTGGTAG	0	1	1	0	0	At5g14370.1:X:-162:quaternary
TTGTAATATA	0	0	1	0	1	At5g14360.1:v:+1008:secondary
GATCAGTGCC	0	2	0	0	0	At5g14270.1:d:+1562:secondary
ATCTCAATCA	0	0	0	0	2	At5g14250.1:d:-1208:secondary,At4g11170.1:v:-613:secondary
TAAACAGCGA	0	0	1	1	0	At5g14250.1:d:+1372:primary
GAAGCAATTG	0	2	0	0	0	At5g14220.1:d:+1390:primary
GCCAGATCGG	1	0	0	1	0	At5g14210.1:d:+1782:secondary
TCGTGTTGCT	0	1	1	0	0	At5g14080.1:d:+1721:primary
TCACAAAAAG	0	0	0	0	2	At5g13970.1:d:+1155:primary
GACCGGATGG	0	1	1	0	0	At5g13960.1:d:+1933:primary
GCTCCTTCTC	0	1	0	0	1	At5g13930.1:d:+349:secondary
GTTAATTGTC	0	0	0	1	1	At5g13890.1:i:+1181:tertiary,At5g13890.3:i:+1181:tertiary
TTGTATCATT	1	0	0	0	1	At5g13850.1:d:+739:secondary
GAAAAAGGGG	0	0	0	1	1	At5g13820.1:d:+2469:primary
GATATCGCTT	1	1	0	0	0	At5g13810.1:d:+818:primary
TCATATCTTT	0	2	0	0	0	At5g13760.1:X:+-178:quaternary,Chr1:+2656840:quaternary
GACACAATCC	0	2	0	0	0	At5g13760.1:d:+1970:primary
AGCAAGCTTT	0	0	2	0	0	At5g13655.1:v:+280:secondary,At5g54770.1:d:+550:secondary
TTAATTTCTC	1	0	1	0	0	At5g13650.1:i:+3744:tertiary,At5g13650.2:i:+3770:tertiary
ATGCTCAACT	1	0	0	0	1	At5g13640.1:d:+2580:secondary
TACTTGAGCC	0	0	1	0	1	At5g13630.1:d:+3662:secondary
CGATTCATTA	0	0	1	0	1	At5g13540.2:d:-2662:secondary
TCCTTGCCGC	0	1	0	1	0	At5g13500.3:d:+1187:primary,At5g13500.1:d:+1312:primary,At5g13500.2:d:+1234:primary
TATTTGTTAA	2	0	0	0	0	At5g13500.1:d:+818:secondary,At5g13500.2:d:+740:secondary,At5g13500.3:d:+693:secondary
AATCCTCAGA	0	2	0	0	0	At5g13480.1:v:+2274:secondary,At2g33835.1:d:+675:secondary
CTTTACTTGT	1	0	0	0	1	At5g13480.1:i:+2649:tertiary,At5g42240.1:i:+118:tertiary,At1g12680.1:i:+1787:tertiary
AGGAACAGAA	0	2	0	0	0	At5g13460.1:d:+393:secondary
TGTGGTAGCC	0	1	0	0	1	At5g13320.1:d:+1701:primary
TATCAAAACC	1	1	0	0	0	At5g13290.1:d:+1828:primary,At5g13290.2:d:+1433:primary
TTCTTTATAC	2	0	0	0	0	At5g13280.1:d:+1580:primary
AAAGTTCTAG	0	0	2	0	0	At5g13260.1:i:-2129:tertiary
AAAAACTTAA	0	0	0	1	1	At5g13260.1:i:+3047:tertiary
TTCGTTTTTG	0	1	0	1	0	At5g13220.2:d:+1212:primary,At5g13220.3:d:+795:primary,At5g13220.1:d:+742:primary
GGTTTTGTGA	0	0	0	2	0	At5g13200.1:d:+810:primary
ATCGGAAGAA	2	0	0	0	0	At5g13180.1:d:-748:secondary
AAAATGAAGA	0	1	0	1	0	At5g13090.1:d:+109:secondary
ATCTGATGAT	1	1	0	0	0	At5g13080.1:d:+695:primary
ACTGATATTC	0	0	0	1	1	At5g13060.1:d:+2162:primary
CAGACGCCTG	0	0	1	0	1	At5g13030.1:d:-1880:secondary
GATCGTTGAA	0	2	0	0	0	At5g13030.1:d:+1071:secondary,At5g57625.1:d:+441:secondary
ATCTCTCAGC	2	0	0	0	0	At5g13020.1:d:-1122:secondary

AACTAGCTCT	0	1	0	0	1	At5g13020.1:d:+1127:primary
AGAGTATGGC	1	0	1	0	0	At5g12910.1:v:+414:secondary,At5g66920.1:d:+1861:secondary
TCACAACACC	0	1	0	0	1	At5g12860.1:X:-93:quaternary
AATGGTGCAA	0	2	0	0	0	At5g12850.1:d:+2264:primary
CAAGATGTTG	0	2	0	0	0	At5g12480.1:d:+1641:primary
AAAAAAACAG	0	0	0	0	2	At5g12430.1:X:--102:quaternary,At1g68880.1:X:--2:quaternary
CTTAAATCAA	1	0	1	0	0	At5g12430.1:d:+2494:secondary
ACTTTTCAGA	1	0	0	1	0	At5g12320.1:d:+502:primary
ATCTATGTTG	0	0	0	1	1	At5g12290.1:d:+2055:primary
TTGCTCTCAG	1	0	1	0	0	At5g12250.1:X:-576:quaternary
CATATGAGGA	0	2	0	0	0	At5g12220.1:d:+1748:primary
GGAGGAACAA	0	0	2	0	0	At5g12170.1:v:+1074:primary
TGCCGCTTGG	0	0	2	0	0	At5g12130.1:d:+1463:primary
AATGCATCCT	0	0	1	0	1	At5g12120.1:d:+1374:secondary
TCCGAATATT	0	0	1	1	0	At5g12090.1:v:+1898:primary
ATCAGTCATT	0	0	1	0	1	At5g12040.2:d:+1400:secondary,At5g12040.1:d:+1192:secondary
CTGCTCTCTG	0	0	2	0	0	At5g11670.1:d:-1836:secondary
CGGTGTTTAG	0	2	0	0	0	At5g11560.1:d:+2317:secondary
TATGGACTAA	1	0	1	0	0	At5g11410.1:v:+1162:primary
CGAGAATATG	0	1	0	1	0	At5g11350.1:d:+1463:secondary
TGCAGACAAA	1	0	0	1	0	At5g11340.1:d:+418:primary
AGTGAAACAA	0	1	1	0	0	At5g11330.1:d:+887:secondary,At4g04316.1:p:+1969:secondary
AAAGCTATAG	0	0	0	0	2	At5g11180.1:v:+2574:secondary,At5g11210.1:v:+2523:secondary
AACAAATATT	0	0	1	1	0	At5g11100.1:v:+2377:primary
CATAGACTAT	1	0	0	1	0	At5g11040.1:d:+3785:primary
GCCAAGAATC	1	0	1	0	0	At5g11030.1:d:+1653:primary
TTTTTTTATA	0	0	0	0	2	At5g11010.2:d:+1359:primary,At5g11010.3:d:+1232:primary,At5g11010.1:d:+1445:primary
GAAACCAGAA	0	0	1	1	0	At5g10970.1:v:+1517:primary
GTCGGCCATC	0	0	2	0	0	At5g10830.1:d:+684:secondary,At3g02690.1:d:+56:secondary
AAACATTTCG	0	1	0	1	0	At5g10820.1:d:+1587:primary
TTAATTAAAA	0	0	0	1	1	At5g10800.1:i:+424:tertiary
CTTCAACATT	0	0	0	2	0	At5g10760.1:d:+1664:primary
CGTCAGTGGC	0	2	0	0	0	At5g10700.1:v:+640:secondary
AACACCAAAG	0	1	1	0	0	At5g10560.1:X:--99:quaternary,At5g42860.1:X:-125:quaternary,At1g31130.1:X:-806:quaternary,At4g22680.1:X:-519:quaternary
GTCGTTGAGC	0	0	0	2	0	At5g10530.1:v:+2299:primary
TTTTCGATTC	0	0	1	0	1	At5g10460.1:i:+226:tertiary
CTCGCTTACA	0	0	2	0	0	At5g10450.1:d:+522:secondary
TCTGTGGAGC	0	1	0	0	1	At5g10360.1:X:-307:quaternary
TGGATCTCCT	0	0	0	1	1	At5g10340.1:v:-716:secondary
GTGATGCTCT	0	1	1	0	0	At5g10300.1:d:+773:primary
ATTAGAAAAA	1	0	0	1	0	At5g10210.1:v:+111:secondary
TTAAGAAAGA	0	0	0	2	0	At5g10070.1:d:+783:secondary,At2g05240.1:p:+839:secondary,At2g10520.1:p:+836:secondary
ACATTCCTAA	1	1	0	0	0	At5g09850.1:d:+1657:primary
TGAATCGAGT	0	0	1	0	1	At5g09850.1:d:+1177:secondary
TTATAAAGCA	0	1	1	0	0	At5g09830.1:X:--128:quaternary
GAAAGATTAC	0	0	0	2	0	At5g09760.1:d:+1478:secondary
AAGCTGTTTA	1	0	1	0	0	At5g09760.1:d:+1425:secondary
TGATCTGTGC	1	0	1	0	0	At5g09740.1:d:+1240:primary
GTGGTGGAAG	0	0	2	0	0	At5g09670.2:d:+1113:secondary,At5g09670.1:d:+1844:secondary,At5g64550.1:d:+1219:secondary,At3g11840.1:d:+1221:secondary
TAATAAACTC	0	1	0	1	0	At5g09450.1:X:-1354:quaternary,At5g09450.1:X:-1341:quaternary,At5g09450.1:X:-1335:quaternary,At5g09450.1:X:-1366:quaternary
AGACACATAG	0	1	0	0	1	At5g09450.1:d:+1431:primary
TGAGAGGACA	0	2	0	0	0	At5g09410.1:d:+2564:primary
GTTCATATAT	0	0	0	0	2	At5g09400.1:i:+3634:tertiary,At2g28900.1:i:+609:tertiary,At5g25370.1:i:+2845:tertiary
CAGTTTTGAC	0	1	1	0	0	At5g09380.1:v:+1117:secondary
TACATCAAAA	0	0	1	0	1	At5g09280.1:v:+1286:secondary
TTAAGCGTTG	0	0	0	2	0	At5g09270.1:d:+659:primary
TGTTGAATCT	2	0	0	0	0	At5g09240.2:d:+526:primary,At5g09240.3:d:+515:primary,At5g09240.1:d:+441:primary

GATTTGAATA	0	1	0	1	0	At5g08780.1:d:+243:secondary
TATTTATGTA	1	0	0	0	1	At5g08720.1:d:+2267:primary
GTTTTTTGCA	0	0	0	2	0	At5g08550.1:v:+3484:primary
TCCAATCTAT	0	1	0	0	1	At5g08500.1:d:+234:secondary
TTTGGTTTCA	0	0	0	0	2	At5g08500.1:d:+1500:secondary,At1g06820.1:d:+412:secondary,At2g46380.1:v:+164:secondary
GATGATACTG	0	0	0	0	2	At5g08500.1:d:+1211:secondary,At5g23575.1:d:+1426:secondary,At1g34380.1:d:+1174:secondary
CAAAGATCAG	1	0	0	1	0	At5g08415.1:d:+888:primary
AACTCAACTT	1	1	0	0	0	At5g08390.1:v:+3078:primary
ACAAAACACA	1	0	0	1	0	At5g08310.1:v:+4081:primary
TTCTTCACAG	1	1	0	0	0	At5g08230.1:d:+4498:primary
TACTTTGAAT	1	0	0	0	1	At5g08139.1:d:+1462:primary
AACGATTTTG	0	1	1	0	0	At5g08130.1:d:+2034:primary
GAATATAAAG	0	2	0	0	0	At5g08100.2:d:+1006:primary,At5g08100.1:d:+819:primary
GATGTTTCCA	0	0	2	0	0	At5g08030.1:d:+1195:primary
TCATTAGAGA	0	0	1	0	1	At5g08020.1:v:+2251:secondary
GAAGAAATAC	2	0	0	0	0	At5g08010.1:d:+1678:primary
GTGTGTGTGC	0	2	0	0	0	At5g08000.1:d:+138:primary
TTCACAGCTG	0	0	0	2	0	At5g07990.1:d:+941:primary
TAGCATTACT	0	1	1	0	0	At5g07970.1:d:+3630:primary
TCTTAAGAGT	1	1	0	0	0	At5g07960.1:d:+437:primary
CTAAAGAGAG	0	1	0	0	1	At5g07920.1:d:+2484:secondary
TTGTATTGAA	2	0	0	0	0	At5g07840.1:d:+1259:primary
AATCTTTATA	0	2	0	0	0	At5g07820.1:d:+1753:primary
TAAACGCAGA	0	2	0	0	0	At5g07810.1:d:+1484:secondary,At4g01000.1:d:+475:secondary
GCTATGAATT	1	0	0	1	0	At5g07800.1:X:-34:quaternary
AAGCTCAAAA	1	0	0	1	0	At5g07790.1:d:+863:secondary,At2g06490.1:p:+2192:secondary,At3g43571.1:p:+1142:secondary,At3g30218.1:p:+3334:secondary
GATCATCTTC	1	0	1	0	0	At5g07730.1:d:+720:primary
CCCAAGATTA	0	2	0	0	0	At5g07700.1:v:+1409:secondary
TGGACTCCCT	0	1	0	1	0	At5g07590.1:d:+1330:primary
AAGAGCTTCA	0	1	0	1	0	At5g07580.1:d:+606:primary
GATTCTACGT	0	0	0	2	0	At5g07390.1:d:+1813:secondary
TTTTTGGTTT	2	0	0	0	0	At5g07370.4:d:+757:secondary,At4g03820.2:d:+1423:secondary,At4g05160.1:d:+800:secondary
TGGGAATCGA	0	1	0	1	0	At5g07350.1:d:+2148:secondary,At5g11960.1:d:+57:secondary,At3g01670.1:d:+2119:secondary
TGGGTGGATT	0	2	0	0	0	At5g07250.1:d:+816:secondary
TTCTTGTAAG	0	2	0	0	0	At5g06980.2:i:+483:tertiary,At4g01550.1:i:+831:tertiary,At5g06980.1:i:+545:tertiary
TCGAAGGTAT	2	0	0	0	0	At5g06940.1:v:+826:secondary
TTTCTTGGAG	1	1	0	0	0	At5g06920.1:v:+840:secondary
CTTGATGAGG	0	1	0	0	1	At5g06910.1:d:+665:primary
TGAGGAGTTT	0	0	1	0	1	At5g06790.1:X:-718:quaternary
CAACTTAACA	1	0	0	0	1	At5g06760.1:d:+832:primary,At4g08876.1:v:+1002:primary
AAAGAAGCGG	0	0	1	0	1	At5g06760.1:d:+325:secondary
TACTGGAGAA	1	1	0	0	0	At5g06580.1:d:+1614:secondary,At1g55320.1:v:+1994:secondary
CAAATCAGTC	0	1	0	0	1	At5g06520.1:X:-935:quaternary,At5g06520.1:X:-548:quaternary
AGAGCTTTCT	0	2	0	0	0	At5g06360.1:d:-243:secondary
AAGAGTCATC	0	0	0	0	2	At5g06360.1:d:+270:secondary
TCTCTACTAT	0	1	1	0	0	At5g06350.1:d:+2307:primary
AACTCGTCTA	0	0	0	1	1	At5g06340.1:d:+728:secondary
TGGGTAACTG	0	0	1	0	1	At5g06300.1:d:+815:primary
TGTCATTGTC	1	0	0	1	0	At5g06270.1:d:+927:primary
TGTAAGAGAT	1	1	0	0	0	At5g06210.1:d:+537:primary
TCACTTAACA	0	2	0	0	0	At5g06110.1:d:+1186:secondary,At3g11450.1:d:+1234:secondary
TTAAACATCA	0	1	0	1	0	At5g06060.1:i:+1000:tertiary
TGTATTCATA	0	0	0	2	0	At5g05987.1:i:+755:tertiary
GTTAAGATGG	0	0	0	2	0	At5g05987.1:d:+789:primary
GGAGAGAAGA	0	0	0	2	0	At5g05840.1:v:+1089:secondary,At4g31270.1:d:+690:secondary
TTTGTCCTTA	0	1	0	0	1	At5g05780.1:X:+487:quaternary
ACTAGAGAAG	0	1	1	0	0	At5g05660.1:X:-388:quaternary
GGAATGTTTT	0	1	0	1	0	At5g05630.1:d:+1205:secondary,At2g27590.1:d:+529:secondary,At1g31820.1:d:+955:secondary
TAATGAGCTT	0	1	0	0	1	At5g05570.1:i:+1830:tertiary,At5g55310.1:i:+2836:tertiary
ATGGCGCTCC	1	0	1	0	0	At5g05570.1:d:+3058:secondary

GCAGGATATC	0	0	0	0	2	At5g05570.1:d:+1686:secondary
TAATTCAATT	1	0	0	1	0	At5g05560.1:i:+10558:tertiary,At5g07770.1:i:+1347:tertiary
AGCCTGTGTT	1	1	0	0	0	At5g05510.1:v:+895:secondary
AAGATTATTG	1	0	1	0	0	At5g05450.1:d:+434:secondary,At5g43450.1:d:+853:secondary
GTATCATTCT	0	1	1	0	0	At5g05440.1:d:+1045:primary
GTTAGAACTC	0	1	0	1	0	At5g05380.1:v:+500:secondary
ATCTTCTGCT	1	0	0	1	0	At5g05370.1:X:-64:quaternary
TTTCTGAAAT	0	0	0	0	2	At5g05210.1:d:+171:secondary,At5g17270.1:v:+3244:secondary
TTTTTTTATG	1	0	0	1	0	At5g05180.2:d:+1392:primary,At5g05180.1:d:+1464:primary
TCTGTCCGAC	0	1	1	0	0	At5g05160.1:d:+1452:primary
ATCCAAAGGA	0	2	0	0	0	At5g05140.1:d:+1491:primary
CAGCTTATGA	0	2	0	0	0	At5g05090.1:d:+498:primary
GCTATCTCCT	2	0	0	0	0	At5g05060.1:d:+360:primary
CTTTTATCCT	1	0	1	0	0	At5g04940.1:d:+2692:primary,At5g04940.2:d:+2608:primary
CAATCTATTG	1	1	0	0	0	At5g04900.1:d:+1154:primary
GGTTCAAGTC	1	0	0	1	0	At5g04870.1:d:+1682:primary
GTTTACTAAC	1	1	0	0	0	At5g04820.1:d:+273:secondary
GACTGGACTG	0	1	1	0	0	At5g04760.1:d:+738:primary
GCTTGTTTGT	0	0	2	0	0	At5g04750.1:d:-335:secondary,At3g22350.1:v:-1389:secondary
TTTCTGTCAA	0	2	0	0	0	At5g04700.1:X:-595:quaternary
CTTTACCAGC	1	0	0	0	1	At5g04590.1:X:+357:quaternary
TGCATTTTGC	0	1	1	0	0	At5g04540.1:i:+1409:tertiary
TATAGACTAG	0	1	1	0	0	At5g04540.1:d:+2836:primary
TTTAATTGGC	0	2	0	0	0	At5g04530.1:d:+1649:secondary
ACACACCGTG	2	0	0	0	0	At5g04440.1:d:-982:secondary
GGCTTCGGGA	1	1	0	0	0	At5g04440.1:d:+729:secondary
AGGCTAAAAC	0	2	0	0	0	At5g04420.1:d:+1132:secondary
GCTTGTCTTT	1	0	0	0	1	At5g04360.1:d:+2998:primary
ATGCTCTTTA	0	1	0	0	1	At5g04330.1:d:+529:secondary,At4g32175.1:v:+1113:secondary
TTATTCTAAA	1	0	0	0	1	At5g04290.1:v:+6:secondary
CCTAAAATCT	1	0	0	1	0	At5g04290.1:i:-3955:tertiary
TTGATGTCAA	0	2	0	0	0	At5g04260.1:d:+421:primary
TAACGATTGG	0	0	2	0	0	At5g04240.1:X:+2209:quaternary
TAATATCTGG	0	1	0	1	0	At5g04240.1:d:+4068:secondary
TGGTTAAAAA	0	0	0	1	1	At5g04235.1:p:+2204:secondary
GTACACAAAA	0	0	2	0	0	At5g04180.1:d:+454:secondary
ACATTCTCAT	2	0	0	0	0	At5g04130.1:d:+2164:primary
AGCTTCAAGA	0	1	0	1	0	At5g04060.1:d:+1886:primary
TATAATGTCA	0	0	2	0	0	At5g03905.1:d:+692:primary
GGTAGGACTG	0	0	2	0	0	At5g03850.1:d:+113:secondary,At5g64140.1:d:+123:secondary
ATGCGTGCGT	0	1	0	1	0	At5g03730.2:d:+1714:secondary,At5g03730.1:d:+1714:secondary
AATACTGGCT	0	0	0	1	1	At5g03710.1:i:+188:tertiary
ATGATGAAAA	1	1	0	0	0	At5g03640.1:v:+2589:secondary
TTGTGAGAAG	0	0	2	0	0	At5g03620.1:v:+1615:primary
ATTCAACAAG	2	0	0	0	0	At5g03500.1:v:+867:secondary
AAAAGCTTTC	1	1	0	0	0	At5g03430.1:d:+1244:secondary,At4g13820.1:d:+2590:secondary
TTCGGTTCTG	0	1	1	0	0	At5g03420.1:v:+144:secondary
TGTATATGTT	0	1	1	0	0	At5g03370.1:d:+652:primary
GAAGAGAGAG	0	2	0	0	0	At5g03290.1:d:+666:primary,At5g21970.1:d:+1384:primary
TTTGGATCAA	0	0	2	0	0	At5g03170.1:d:+572:primary
CACCGTTCTC	2	0	0	0	0	At5g03120.1:d:+549:primary
GCGAACAGTC	0	2	0	0	0	At5g03070.1:d:+1543:primary
TTATATTTTC	1	0	1	0	0	At5g02940.1:d:+2494:primary
GATGCTGTTC	0	0	2	0	0	At5g02880.1:d:+829:secondary,At5g51830.1:d:+1118:secondary
ACTGCTCCAG	0	0	2	0	0	At5g02870.1:d:+147:secondary,At1g79670.2:v:+2421:secondary,At1g79670.1:d:+2195:secondary
TGAATTATTA	0	0	0	1	1	At5g02820.1:d:+1479:primary
GATTGAGGAA	0	0	0	2	0	At5g02790.1:d:+666:secondary
TAAGACATCA	0	0	2	0	0	At5g02770.1:d:+1396:primary
TTGTTGATTG	0	0	0	0	2	At5g02590.1:d:+752:primary
GGATTATCAT	0	0	1	0	1	At5g02550.1:X:-428:quaternary
GTTCTTATTA	2	0	0	0	0	At5g02500.1:d:+484:secondary,At5g02490.1:d:+459:secondary

CGTGTAATGT	0	0	1	1	0	At5g02460.1:d:+1460:primary
GCTTGTAGTA	0	1	0	0	1	At5g02440.1:v:-696:secondary
ATGTTTTCTG	0	0	1	1	0	At5g02410.1:d:+1720:primary
AAAATTCTGT	0	1	0	1	0	At5g01970.1:d:+1106:primary
CTCAAACGAG	0	0	1	0	1	At5g01920.1:d:+1607:secondary
TTGTGTAGTC	2	0	0	0	0	At5g01800.1:d:+1030:primary
TAAATGCTTT	0	1	0	0	1	At5g01770.1:v:+4567:primary
GTACCGTTGT	0	1	1	0	0	At5g01720.1:d:+2285:primary
CAAAATATAT	1	0	0	0	1	At5g01690.1:v:+32:secondary
AGAGACAGAG	0	0	1	0	1	At5g01660.1:v:+2262:primary
CTTGTTTTTG	1	0	1	0	0	At5g01660.1:v:+207:secondary
CGAGGAGGTG	0	1	0	1	0	At5g01620.1:d:+1097:primary,At5g01620.2:d:+1041:primary
CCTCTCTCGC	0	0	1	1	0	At5g01600.1:d:+705:secondary
GATTTTGCTC	0	1	1	0	0	At5g01560.1:X:-301:quaternary
TTCGGTACAG	1	0	0	1	0	At5g01510.1:d:+1762:primary
CCAAACAGCA	0	1	0	1	0	At5g01500.1:d:+1182:primary
GAATCGGATC	0	2	0	0	0	At5g01360.1:d:+1329:primary
ACGTTAAACG	1	1	0	0	0	At5g01350.1:i:+867:tertiary
CCATTAGAAC	0	0	0	1	1	At5g01340.1:d:+929:primary
CGCTTTGATG	0	1	1	0	0	At5g01180.1:d:+1808:primary
GACAACAATT	1	1	0	0	0	At5g01080.1:v:+325:secondary
TAGATGATTA	0	2	0	0	0	At4g40080.1:d:+1248:primary
GATTCTAAAG	0	1	0	1	0	At4g39990.1:d:+233:primary
TTAGCAAACT	0	2	0	0	0	At4g39955.1:d:+976:secondary
TTTTTAGCTT	1	1	0	0	0	At4g39955.1:d:+1071:secondary,At3g33197.1:p:+752:secondary
GAGGTAATTT	0	0	0	2	0	At4g39920.1:d:+1343:secondary
GTTACATCCT	0	1	0	1	0	At4g39910.1:d:+1263:secondary
ACGGTGCGGT	1	0	1	0	0	At4g39840.1:d:+117:secondary
TTGTTCTCCG	0	0	1	1	0	At4g39800.1:X:-160:quaternary
GCTAGAGCCA	1	1	0	0	0	At4g39800.1:d:+534:secondary
TACTTGATAA	0	1	0	1	0	At4g39740.1:d:+753:primary
GTGATCGCCG	0	0	1	1	0	At4g39730.1:d:-387:primary
ATGAAGTTAC	0	0	0	0	2	At4g39720.1:d:+961:primary
ATTTTGATTA	0	2	0	0	0	At4g39660.1:X:+409:quaternary
GTTTGATTTA	1	1	0	0	0	At4g39550.1:d:+807:secondary
ATTCATTTGA	1	1	0	0	0	At4g39530.1:v:+3186:primary,At1g31120.1:d:+3087:primary
CTTAAACAAT	1	0	0	0	1	At4g39520.1:d:+1131:secondary
GGCTTAGAGT	0	1	1	0	0	At4g39510.1:d:+1671:primary
CATCCTGAAA	0	2	0	0	0	At4g39480.1:i:+1698:tertiary
AAAGTCCTCT	1	1	0	0	0	At4g39450.1:d:-1075:secondary
TGAGTGAGTG	1	0	1	0	0	At4g39450.1:d:+4660:primary
CACATTGTAA	0	1	0	1	0	At4g39300.2:i:+1262:tertiary
ATTTGTGTCA	2	0	0	0	0	At4g39150.1:i:+2060:tertiary
GCACGTGTCC	0	0	2	0	0	At4g38970.2:d:+1078:secondary,At2g21330.1:d:+1056:secondary,At4g38970.1:d:+1074:secondary
AATGTAAGCT	0	0	0	1	1	At4g38960.1:d:+987:primary
TCTAACGGTC	0	2	0	0	0	At4g38930.2:d:+492:primary,At4g38930.1:d:+512:primary
ATCTCTCACC	0	0	0	2	0	At4g38880.1:v:+1263:secondary
ACCATCGACG	0	0	2	0	0	At4g38740.1:d:+144:secondary
ATCAAGAGAT	0	1	0	1	0	At4g38700.1:d:+673:primary,At5g11240.1:d:+1897:primary
TTCTTGACGA	0	1	1	0	0	At4g38640.1:d:+1715:primary
TTGTGTACGA	0	0	1	1	0	At4g38570.1:d:+988:secondary
GAGGATGGAG	1	1	0	0	0	At4g38540.1:d:+933:secondary
AATGCAGACT	0	1	0	1	0	At4g38440.1:d:+310:secondary,At2g15410.1:p:+4777:secondary
TTCACCAAAA	0	1	0	1	0	At4g38240.1:X:-90:quaternary
CAGCCTCCTT	1	1	0	0	0	At4g38240.1:d:+1464:secondary,At1g65550.1:v:+232:secondary,At5g04380.1:v:+194:secondary,At4g38240.2:d:+1567:secondary
ATGATGACGG	1	0	1	0	0	At4g38140.1:v:+469:secondary
GATATCTAGC	0	1	0	1	0	At4g38100.1:d:+92:primary
TAAAGTCTTG	1	0	0	0	1	At4g38060.2:d:+1310:primary
TCGACATCAG	0	0	0	1	1	At4g38050.1:X:--166:quaternary,At1g07220.1:X:-215:quaternary
AAGTACAAGA	0	0	2	0	0	At4g37930.1:d:+1663:secondary

GATTCGAAAA	1	1	0	0	0	At4g37890.2:d:+2178:primary,At4g37890.1:d:+2251:primary
TAATAAGAGA	0	0	1	0	1	At4g37800.1:i:+1064:tertiary
ATGTCTTATG	2	0	0	0	0	At4g37800.1:d:+1087:secondary
GGGAAACTCC	0	2	0	0	0	At4g37740.1:d:+1530:primary
GATCCCAATA	0	0	2	0	0	At4g37530.1:d:+747:secondary,At4g03280.2:d:+764:secondary,At4g03280.1:d:+665:secondary
GACCCAACAA	0	2	0	0	0	At4g37520.1:d:+887:secondary
ACAAAGCCAG	0	1	1	0	0	At4g37510.1:X:-228:quaternary
ATTCATTCCC	0	0	0	1	1	At4g37490.1:d:+1509:primary
TGTACTAAAG	1	1	0	0	0	At4g37480.1:v:+2405:primary
TGTAGGCCTA	1	1	0	0	0	At4g37430.1:d:+1597:primary
TAAATTTCTT	0	1	0	0	1	At4g37280.1:d:+1145:primary
TTTACATCTA	1	0	0	0	1	At4g37180.2:d:-1267:secondary,At4g37180.1:d:-1246:secondary
ATGCATCATC	0	2	0	0	0	At4g37100.1:v:+3117:secondary
TAGGTAGCAG	0	1	0	1	0	At4g37090.1:d:-754:secondary
TATCAGATAT	0	1	0	1	0	At4g37090.1:d:+759:primary
TATTATATAA	1	1	0	0	0	At4g36990.1:d:+1393:secondary
GGATAATAAC	0	1	0	1	0	At4g36980.1:d:+1842:primary
TATTAGAATA	0	1	0	0	1	At4g36910.1:d:+907:primary
TTGTGATTCT	0	1	1	0	0	At4g36790.1:d:+1681:primary
TTTCTGGACC	0	1	0	0	1	At4g36760.1:d:+2108:secondary,At4g36760.2:d:+2123:secondary
TTTCTTTATA	0	1	1	0	0	At4g36750.1:d:+1064:primary
AAGCCGGTAG	0	1	1	0	0	At4g36730.2:d:+882:primary,At4g36730.1:d:+888:primary
TGTAATACTA	1	1	0	0	0	At4g36640.1:d:+1241:primary
AAACGCAATT	0	0	2	0	0	At4g36630.1:X:-374:quaternary,At1g05350.1:X:-193:quaternary
GAAGAAGACG	0	1	1	0	0	At4g36630.1:d:+3081:primary
CTTTACAGTT	0	1	0	1	0	At4g36560.1:v:+1128:secondary,At5g32630.1:p:+4092:secondary
GAGAGCAATC	0	1	0	0	1	At4g36520.1:v:+3118:secondary
GTGGGATAGT	0	2	0	0	0	At4g36520.1:v:+1921:secondary,At1g41803.1:p:+3370:secondary,At2g10210.1:p:+254:secondary,At3g33121.1:p:+642:secondary
CGTGATCTCG	0	1	1	0	0	At4g36520.1:i:+4220:tertiary
TACATCAAAT	2	0	0	0	0	At4g36490.1:d:+1922:primary,At3g49920.1:v:+1334:primary
GTGATGTCAT	0	1	0	1	0	At4g36380.1:X:--69:quaternary
TGCATATATG	0	0	0	0	2	At4g36290.1:d:+2056:primary
ACTGCTGCAA	0	0	2	0	0	At4g36210.2:d:+1875:primary
TAGACCAAAG	2	0	0	0	0	At4g36160.1:i:+2081:tertiary
AGTTTCTTAG	0	1	0	1	0	At4g36150.1:v:+3963:secondary,At5g42900.1:d:+464:secondary,At3g15240.1:d:+747:secondary,At5g42900.2:d:+464:secondary
AAATGTTGAA	1	1	0	0	0	At4g36140.1:v:+1456:secondary
ATTGGTCAAG	0	0	2	0	0	At4g36130.1:d:+544:secondary
TGTTACTACT	2	0	0	0	0	At4g36040.1:d:+1055:primary
GTTTCCATAA	2	0	0	0	0	At4g35970.1:X:+485:quaternary
AAGAAGTGAA	0	2	0	0	0	At4g35900.1:d:+1063:primary
CCAATAGTAT	0	0	1	0	1	At4g35880.1:d:+1603:primary
GCTTCTTCGT	2	0	0	0	0	At4g35850.1:d:+1485:primary
TGAAACGGTT	1	1	0	0	0	At4g35800.1:d:+5831:secondary
ATTTAAGAGA	1	1	0	0	0	At4g35800.1:d:+5562:secondary
TTTTAGGTCA	0	0	1	1	0	At4g35800.1:d:+5550:secondary
GTGAAGGAGA	0	0	2	0	0	At4g35785.2:X:-702:quaternary
TAAGGTACGT	0	0	1	1	0	At4g35770.1:d:-461:secondary
TGAGGAGGAA	0	0	2	0	0	At4g35750.1:d:+631:secondary,At4g28190.1:d:+832:secondary
AAGGAAGTAG	0	0	1	1	0	At4g35600.1:d:+1286:primary
TTTGTTTTTG	0	1	0	0	1	At4g35590.1:v:+1580:secondary
TTTTCAACTC	1	0	1	0	0	At4g35540.1:d:+1666:primary
AGAATTCACA	1	0	0	0	1	At4g35470.1:d:+294:secondary,At2g17440.1:d:+228:secondary
GTCTTTCTTT	1	0	0	0	1	At4g35470.1:d:+2422:secondary
AAATCAAAGA	0	2	0	0	0	At4g35420.1:d:+1079:primary
GTTATTAAAA	0	0	0	1	1	At4g35320.1:X:-733:quaternary
AAAGATGAAG	0	0	0	2	0	At4g35290.2:d:+3006:primary,At4g35290.1:d:+2903:primary
GTAAAGCTTT	0	0	0	1	1	At4g35240.1:i:+1373:tertiary,At3g55860.1:i:+561:tertiary,At5g05170.1:i:+136:tertiary
GTTTTTCATT	0	1	1	0	0	At4g35140.1:d:+1687:primary
GAGGCCACAA	0	1	0	1	0	At4g35050.1:d:+1179:primary

GCTAATCAGA	0	0	0	1	1	At4g35040.1:d:+991:primary
ATGAGAGGAA	0	0	0	2	0	At4g34990.1:d:+680:primary
TGATGATCTG	0	1	1	0	0	At4g34960.1:d:+1032:primary
GTTTTGGACC	1	0	1	0	0	At4g34910.1:d:+2232:secondary,At5g21950.1:v:+535:secondary
GGTTTAATGA	0	2	0	0	0	At4g34790.1:v:+354:secondary
GAAAGAGGAA	2	0	0	0	0	At4g34730.1:d:+958:secondary
TTGTGGAACG	0	0	1	0	1	At4g34710.1:X:-2539:quaternary
ATGAGATCCA	1	0	0	1	0	At4g34710.1:d:+2753:secondary,At4g34710.2:d:+2618:secondary
CCGTATCTTG	1	1	0	0	0	At4g34710.1:d:+2545:secondary,At4g34710.2:d:+2410:secondary
GCAGAAACCC	0	0	2	0	0	At4g34660.1:X:-272:quaternary
TCGAATCTGA	0	1	1	0	0	At4g34580.1:v:+1840:secondary
AAGAGGTATG	1	1	0	0	0	At4g34560.1:d:+781:primary
GTTTTGTTTG	1	1	0	0	0	At4g34555.1:d:+254:primary
TGTTTACTGA	1	0	0	1	0	At4g34490.1:d:+1442:secondary
TAATGCGTGT	1	1	0	0	0	At4g34430.2:X:+716:quaternary
GCCTTTCCAC	0	2	0	0	0	At4g34430.1:d:+2982:primary,At4g34430.2:d:+2980:primary,At4g34430.4:d:+2985:primary,At4g34430.3:d:+2974:primary
GATTAAGTTA	0	2	0	0	0	At4g34420.1:i:+1290:tertiary
CAAATCAAGC	0	0	0	2	0	At4g34412.1:i:+1225:tertiary
AAAGATCAGA	0	0	0	1	1	At4g34290.1:d:+550:secondary
CGGGTGGTAG	0	2	0	0	0	At4g34250.1:d:+1207:primary
ACATTGTTTC	1	0	0	1	0	At4g34240.2:d:+1220:secondary
GCGCCACGAC	0	1	0	1	0	At4g34190.1:X:-117:quaternary
TTCCGCAACT	1	0	1	0	0	At4g34190.1:d:+222:secondary
TAAGCCTTAA	0	0	0	0	2	At4g34100.1:v:+3891:primary
TGATTGTCGA	0	0	0	2	0	At4g34090.2:i:+191:tertiary,At4g34090.1:i:+191:tertiary
AATTTCACAC	2	0	0	0	0	At4g34040.1:d:+2321:secondary
CGACTTTTAA	0	0	2	0	0	At4g33980.1:X:+390:quaternary
AGTACCCTTT	0	2	0	0	0	At4g33940.1:d:+644:primary
TTCCCATTTG	0	0	1	1	0	At4g33910.1:d:+806:primary
ATTGTCGACT	0	0	1	0	1	At4g33865.1:X:-80:quaternary
GAGGACGAGA	0	0	2	0	0	At4g33810.1:v:+2233:secondary
ATCGGTCCAC	1	0	1	0	0	At4g33650.1:d:+2874:primary
AACCAAGTAT	2	0	0	0	0	At4g33590.1:X:-301:quaternary
CACATTCTGA	0	0	1	0	1	At4g33560.1:d:-394:primary
CACCTACTTG	0	1	0	0	1	At4g33480.1:d:+1323:primary
TCCTGGATCC	1	1	0	0	0	At4g33470.1:d:+1028:primary
GTGATGCAGC	0	0	0	2	0	At4g33460.1:d:+848:primary
AATCTTCTAG	0	2	0	0	0	At4g33420.1:d:+1074:primary
TGAATCGTAT	1	0	1	0	0	At4g33260.1:d:+1559:primary
GATTCTGAAA	0	0	0	1	1	At4g33240.1:d:+493:secondary
TGAAGTGGAT	0	1	0	1	0	At4g33200.1:i:+835:tertiary
TACCCTCGTT	1	0	1	0	0	At4g33200.1:d:+4147:primary
GTGCTTTTGC	0	1	1	0	0	At4g33150.1:d:+3008:secondary,At4g33150.2:d:+3034:secondary
TTTACCTTCT	0	0	1	0	1	At4g33140.1:i:+1625:tertiary
GAAGTGACTT	0	2	0	0	0	At4g33110.1:d:+1013:secondary
TTTTTCGAGA	1	0	1	0	0	At4g33100.1:d:+464:primary
GACCCACAAT	0	0	2	0	0	At4g33010.1:d:+2926:secondary
TTTCGGTTCT	0	1	0	0	1	At4g32980.1:i:+990:tertiary
GATTTTATAC	0	2	0	0	0	At4g32980.1:d:+779:secondary,At5g19220.1:d:+734:secondary
ACACGACAGC	0	1	0	0	1	At4g32950.1:v:+1365:primary
GCAAATACAT	0	0	1	0	1	At4g32930.1:d:+773:secondary
GTTTGAGATA	1	0	1	0	0	At4g32920.1:d:+4692:primary
GACGAGAACG	0	0	0	1	1	At4g32920.1:d:+4153:secondary
CCGCTTTGAG	0	0	1	1	0	At4g32850.2:d:-2471:secondary,At4g32850.1:d:-2471:secondary,At4g32850.6:d:-2471:secondary,At4g32850.5:d:-2471:secondary,At4g32850.3:d:-2471:secondary,At4g32850.4:d:-2471:secondary
CCAAACCAAC	2	0	0	0	0	At4g32760.1:v:+2481:secondary
GTCGGTTTTG	0	1	0	1	0	At4g32750.1:d:+1295:primary
GTTATACTTG	0	0	1	0	1	At4g32610.1:i:+2193:tertiary
CGAGTCTCCA	0	1	0	1	0	At4g32590.1:d:-507:primary,At4g32590.3:d:-519:primary,At4g32590.2:d:-527:primary
ATTTGAAAAT	0	2	0	0	0	At4g32570.1:i:+615:tertiary

GAAATATACG	0	1	1	0	0	At4g32560.1:d:+1068:primary
TCAGAAGGAG	1	1	0	0	0	At4g32420.1:d:+1420:primary
TGTTATGAAG	0	1	0	1	0	At4g32400.1:d:+1190:primary
GCTTTTTGCT	1	1	0	0	0	At4g32390.1:v:+525:secondary,At4g34100.1:v:+1457:secondary
CAAATCCAGC	0	1	1	0	0	At4g32350.1:d:+1661:secondary
ATTCTGTCCA	1	0	1	0	0	At4g32330.1:d:-1670:secondary,At4g32330.2:d:-1667:secondary
TCATAATCTT	1	0	1	0	0	At4g32240.1:d:+506:primary
ATGTCAACAA	0	0	2	0	0	At4g32150.1:X:-299:quaternary
CTCCCACAAT	0	2	0	0	0	At4g32050.1:d:-634:secondary,At4g38470.1:d:-1569:secondary
GAAGCAAACC	0	0	1	0	1	At4g31990.1:X:+635:quaternary
CATACGAGAA	1	0	1	0	0	At4g31920.1:d:+1776:primary
TGTTCTTCAT	0	0	1	0	1	At4g31877.1:d:+523:secondary
GACATAAAGC	0	1	0	0	1	At4g31860.2:d:+1644:primary,At4g31860.1:d:+1559:primary
GGTTTGGTTT	0	1	1	0	0	At4g31820.1:d:+562:secondary
GTGGATTGCT	0	1	1	0	0	At4g31810.1:d:+1185:primary
AAACCTCGGT	0	1	0	1	0	At4g31805.1:v:-1224:secondary
GAGATTCTGT	0	0	0	1	1	At4g31790.1:d:+728:primary,At4g31790.2:d:+886:primary
TCGCTAACAA	0	2	0	0	0	At4g31500.1:X:+888:quaternary
ACAGGTTTTA	0	0	1	1	0	At4g31450.1:X:-234:quaternary,At1g10370.1:X:-297:quaternary
GAATTTTTTT	1	0	1	0	0	At4g31450.1:i:+145:tertiary,At3g45140.1:i:+532:tertiary
TAATAGTTGC	0	2	0	0	0	At4g31440.1:v:+1825:primary
AAGAAGAGTG	0	1	0	0	1	At4g31430.2:d:+1625:primary,At4g31430.1:d:+1617:primary
GACAGTCGAA	0	2	0	0	0	At4g31360.1:d:+147:secondary
ACTTTTGTGA	1	0	1	0	0	At4g31310.1:d:+622:secondary
TAAAACTCAA	0	0	1	0	1	At4g31240.2:d:+1341:secondary,At4g31240.1:d:+1609:secondary,At5g63770.1:d:+892:secondary
AAAGGAGGGG	0	2	0	0	0	At4g31210.1:i:-5166:tertiary
ATATCTTTTT	0	2	0	0	0	At4g31210.1:i:+4925:tertiary
CTCAAAAGTT	0	1	0	1	0	At4g31210.1:d:+2664:secondary,At1g18330.1:d:+508:secondary,At3g10113.1:v:+667:secondary
AGCAGTGGGG	0	0	1	1	0	At4g31160.1:v:+5844:secondary
ATGCCTATAA	1	1	0	0	0	At4g31140.1:d:+1683:primary
AGATGTCTGG	1	1	0	0	0	At4g30996.1:d:+132:primary
GATTAAAGTT	0	1	0	0	1	At4g30990.1:v:+5593:secondary
ATTCAACACG	0	0	1	1	0	At4g30970.1:v:+902:secondary
CTTGTGGGAG	0	0	0	2	0	At4g30930.1:d:+1073:primary
AAATTCGATA	1	1	0	0	0	At4g30920.1:d:+1189:primary
AGTTGCTATC	0	0	0	0	2	At4g30800.1:d:+717:secondary
CTGATCGATG	1	0	1	0	0	At4g30690.1:d:-582:secondary
TTCTTTGGTT	1	0	1	0	0	At4g30510.1:d:+1203:primary
GTTCGGATCA	0	2	0	0	0	At4g30490.1:X:-161:quaternary
ATGTCGATCG	1	0	1	0	0	At4g30410.1:d:+703:primary,At4g30410.2:d:+928:primary
ATGATTGACT	0	0	0	0	2	At4g30360.1:d:+2372:primary
AATGGAGCTT	0	1	1	0	0	At4g30270.1:X:-164:quaternary
TATGTCTCTG	1	0	1	0	0	At4g30260.1:d:+909:primary
TGCCAAATGC	0	0	0	0	2	At4g30190.1:X:-322:quaternary
CACATTCTCT	1	0	1	0	0	At4g30190.1:d:+3214:secondary,At5g48150.1:d:+733:secondary,At5g48150.2:d:+808:secondary
GAAACTTAAA	1	1	0	0	0	At4g30170.1:d:+1168:primary
GCTTGTTTAA	0	1	1	0	0	At4g30160.1:d:+3323:primary
AGAAGCTTAA	2	0	0	0	0	At4g30160.1:d:+3300:secondary
TCAGGTTCTT	1	1	0	0	0	At4g30100.1:d:+4217:primary
AACTGTTGAA	0	1	1	0	0	At4g30100.1:d:+4066:secondary
TTTGGAATCA	0	0	0	1	1	At4g30080.1:d:+2034:primary
CAATTTCAAA	0	0	2	0	0	At4g30020.1:d:+2432:primary,At2g17160.1:v:+1086:primary,At5g04910.1:v:+1252:primary
CTAAGGAGCA	0	0	2	0	0	At4g30010.1:d:+327:primary
GGCTCCAATG	0	1	1	0	0	At4g30000.1:d:+2132:primary
AGAAAGCAAA	0	2	0	0	0	At4g29950.2:d:+96:secondary,At4g29950.1:d:+96:secondary
TGGAGAAACT	0	2	0	0	0	At4g29900.1:d:+2761:primary
TCGCCCAGGT	0	1	1	0	0	At4g29900.1:d:+2064:secondary
TTCGTACTCG	0	2	0	0	0	At4g29870.1:d:+363:primary
ATGTCATCAT	1	1	0	0	0	At4g29860.1:d:+1318:secondary
AACAATTCGG	2	0	0	0	0	At4g29830.1:d:+1001:primary
AGTAAAACAA	0	0	1	0	1	At4g29820.1:d:+1100:secondary,At1g31570.1:p:+5298:secondary,At2g12210.1:p:+5209:secondary

ATGGTATAAG	0	1	1	0	0	At4g29790.1:d:+3931:primary
ATTTGTAATT	0	1	0	0	1	At4g29750.1:v:+2901:primary
TCTTAATTAA	1	0	0	0	1	At4g29740.2:d:+1673:primary,At4g29740.1:d:+1763:primary
GGAGATCGAA	0	0	2	0	0	At4g29500.1:d:+498:secondary,At2g33860.1:d:+1313:secondary
TACATCCCAA	0	1	1	0	0	At4g29440.1:d:+3368:secondary
AAGATGTGAT	0	0	1	0	1	At4g29400.1:v:-1331:secondary
TTCAATTACA	2	0	0	0	0	At4g29400.1:v:+1336:primary
GGTAAGGTAC	0	0	0	0	2	At4g29390.1:d:+85:secondary
AGCTCCAAGT	0	2	0	0	0	At4g29380.1:d:+4072:primary
TGCGAGGTCG	0	0	2	0	0	At4g29350.1:d:+143:secondary
TAGAAAAGGA	2	0	0	0	0	At4g29200.1:v:+712:secondary
GATCGAGTTG	1	0	1	0	0	At4g29190.1:d:+1064:secondary
AAACAGATCC	0	0	2	0	0	At4g29160.2:d:+874:secondary,At2g19830.1:d:+541:secondary,At4g29160.1:d:+531:secondary,At1g04810.1:d:+1074:secondary
ATATTGGTTA	0	1	0	0	1	At4g29070.1:d:+578:secondary,At2g16640.1:d:+2998:secondary,At3g16620.1:d:+2627:secondary
GGAATAGAAG	1	1	0	0	0	At4g28820.1:d:+469:primary
GACTCATAGC	1	0	1	0	0	At4g28760.1:d:+2942:primary,At4g28760.2:d:+3055:primary
GAAACACCGG	0	1	1	0	0	At4g28640.1:d:+715:primary
CATTTTCATT	0	1	1	0	0	At4g28630.1:d:+1938:primary
AATTTCTAGG	0	0	1	0	1	At4g28620.1:v:+2799:primary
GAATAACTTA	0	1	1	0	0	At4g28570.1:d:+2438:primary
TATTGGGATT	0	0	0	0	2	At4g28510.1:i:+440:tertiary
TTCTTTTATG	2	0	0	0	0	At4g28480.1:X:+-128:quaternary
AAATCCATTA	1	0	0	0	1	At4g28470.1:v:+3024:secondary
ATGACAAAAA	0	1	1	0	0	At4g28450.1:d:+824:secondary
TTCCCAGAGA	1	0	0	1	0	At4g28360.1:d:+1058:secondary
CCTCCTCCTC	0	0	2	0	0	At4g28300.2:d:+1288:secondary,At4g28300.1:d:+908:secondary,At2g16630.1:d:+519:secondary
TCTTCATAAA	0	1	1	0	0	At4g28280.1:v:-1182:secondary
GGTTAAAAAT	0	1	0	1	0	At4g28260.1:d:+1973:primary
TGTCGTGTGT	0	0	2	0	0	At4g28260.1:d:+1690:secondary
AAGAAAGATT	0	0	2	0	0	At4g28230.1:d:+1507:primary
TATCACAATT	0	1	0	0	1	At4g28210.1:d:+909:primary
CCTCTACGTC	1	0	0	1	0	At4g28200.1:X:+283:quaternary
TCCGTTACAC	1	0	0	1	0	At4g28080.1:d:+5130:secondary
AGAAGAGGTT	0	1	1	0	0	At4g28050.1:d:+1275:primary
ATTGGGGAGG	0	1	1	0	0	At4g28030.1:X:-165:quaternary
TACAAATGAT	0	0	1	0	1	At4g28010.1:d:+2846:primary
GATCTGTGGA	0	0	1	0	1	At4g27940.1:d:+1544:primary
TACCTTTCTC	1	1	0	0	0	At4g27860.1:i:-1071:tertiary
AGGTAAAAAG	1	0	0	0	1	At4g27840.1:d:+1164:primary
GGACCAGTTT	0	1	1	0	0	At4g27780.1:d:+871:primary
CCTGGGGGAT	1	0	1	0	0	At4g27760.1:d:-1321:secondary
AAGAAGTTCA	0	1	0	1	0	At4g27550.1:v:+1137:secondary,At5g47990.1:d:+415:secondary
AAACAAGTCT	0	0	0	2	0	At4g27520.1:X:-883:quaternary,At3g14090.1:X:-835:quaternary
AAGAGAACAA	0	0	0	2	0	At4g27380.1:d:+421:primary,At4g30670.1:d:+182:primary
TGGATTATTG	0	0	0	2	0	At4g27350.1:d:+439:primary
CAAAAAACCT	0	1	0	0	1	At4g27240.1:d:+981:primary
TTTGATTGCA	0	2	0	0	0	At4g27180.1:X:-107:quaternary,At5g40450.1:X:-3827:quaternary
GTTGCTTTAG	1	0	0	1	0	At4g27120.1:X:+541:quaternary,At4g27120.1:X:+439:quaternary
AAATACCATA	0	2	0	0	0	At4g27120.1:d:+1153:secondary
TTTGTTTATA	0	2	0	0	0	At4g27110.1:v:+2736:primary
AGCTTTTCTC	0	0	1	1	0	At4g27090.1:X:-26:quaternary
GGGTAGGAAG	1	0	1	0	0	At4g27090.1:d:+331:secondary
GAAAGCTCGT	0	0	2	0	0	At4g27090.1:d:+129:secondary
AAAAAGATAG	0	1	0	0	1	At4g27080.1:d:+1498:primary
GAGTGAGCCA	0	0	0	2	0	At4g27030.1:v:+1265:secondary,At5g52340.1:v:+2420:secondary
TCTATTTAAA	2	0	0	0	0	At4g27010.1:v:+8170:primary
CCTCTTTTCT	0	0	1	1	0	At4g26980.1:d:+1180:primary
CCTTTCCCAC	1	0	1	0	0	At4g26850.1:d:+1242:secondary
TGTTAAACTA	0	1	1	0	0	At4g26740.1:i:+1295:tertiary
CTTCCAAACC	1	1	0	0	0	At4g26700.1:d:+1426:primary

TTGGGCGGAT	0	0	1	1	0	At4g26640.2:X:-443:quaternary
GGGAGAGTAC	0	0	2	0	0	At4g26640.1:d:+1468:primary,At4g26640.2:d:+1756:primary
AAAAATATTT	0	1	1	0	0	At4g26590.1:i:+1810:tertiary
TCCCGGAGAG	0	1	1	0	0	At4g26480.1:v:+2239:secondary
GAACAAGGGA	0	2	0	0	0	At4g26420.1:d:+593:secondary,At5g40090.1:d:+826:secondary,At1g49180.1:d:+933:secondary
GATGGCTTGA	0	1	0	0	1	At4g26360.1:X:-2977:quaternary
TTTTATGATA	0	1	0	0	1	At4g26330.1:v:+2927:secondary,At3g35707.1:p:+607:secondary,At3g33225.1:p:+1716:secondary
CACAGTTGTA	0	1	1	0	0	At4g26310.1:d:+645:primary
TGAGAAGCCT	0	0	0	2	0	At4g26190.1:d:+679:secondary
AAAAGCGGGC	0	0	1	0	1	At4g26190.1:d:+1454:secondary
ATTATTGTAA	0	0	0	1	1	At4g26150.1:X:+823:quaternary
AAATTTTGTT	0	0	1	1	0	At4g26150.1:d:+1162:primary
TGTTTTAAAA	1	0	1	0	0	At4g26120.1:v:+2491:primary
AGACAGCACG	0	0	1	0	1	At4g26110.1:d:-938:secondary
TAAGGTTGGG	0	0	1	0	1	At4g25990.2:d:+980:secondary,At4g25990.1:v:+1330:secondary
TTGAGACTTC	0	0	0	1	1	At4g25990.2:d:+1406:secondary,At4g25990.1:v:+1711:secondary
TAAAATTGTC	0	1	0	0	1	At4g25980.1:v:-329:primary
CAAATAAGGT	0	0	2	0	0	At4g25970.1:d:+2269:primary
ATAGAGGGAA	0	1	0	1	0	At4g25960.1:d:+3243:primary
TCCTCTTTAC	0	1	0	0	1	At4g25920.1:d:+445:secondary,At3g06320.1:d:+212:secondary
GTGAACACAA	1	1	0	0	0	At4g25900.1:d:+1167:secondary
TGCATATAGA	0	1	0	0	1	At4g25880.2:i:+4215:tertiary,At4g25880.1:i:+4215:tertiary
AATTTGTAAT	0	1	1	0	0	At4g25840.1:d:+1082:primary
AGTTACTGTA	0	1	0	1	0	At4g25835.1:d:+1163:primary
GTCCTCCTCT	1	1	0	0	0	At4g25830.1:d:+536:primary
TCCCTAAGAT	1	0	0	1	0	At4g25710.1:d:+1127:primary
GTAATATCTG	1	0	1	0	0	At4g25690.2:d:+747:primary,At4g25690.1:d:+1274:primary
CAGTCACCAA	1	0	1	0	0	At4g25680.1:d:+889:primary
GCGGTGTTGA	0	0	2	0	0	At4g25650.1:d:+1509:secondary,At4g25650.2:d:+1578:secondary
TTTCAGTGTA	0	1	1	0	0	At4g25440.1:d:+1790:primary
GGAAAACCCA	0	0	1	1	0	At4g25340.1:d:+1202:secondary
CTTTTGCAAA	0	1	1	0	0	At4g25300.1:d:+1211:primary,At4g25300.2:d:+1290:primary
AAATCTAAAA	1	0	0	1	0	At4g25190.1:v:+1991:primary
ATCCAACCAC	0	1	0	0	1	At4g25130.1:d:+569:secondary
CTAAGACGGT	0	2	0	0	0	At4g25080.3:d:+447:secondary,At4g25080.1:d:+447:secondary,At4g25080.2:d:+447:secondary
GCTAAGACTT	0	0	1	1	0	At4g24972.1:X:+696:quaternary,Chr3:+4975483:quaternary
AATGTTAAAA	1	0	0	0	1	At4g24930.1:d:+1073:primary
GAGAAGGCCT	1	1	0	0	0	At4g24900.1:X:+272:quaternary
AGGATTCTTT	1	0	1	0	0	At4g24900.1:i:+1325:tertiary
CATCTGGAAG	0	0	2	0	0	At4g24810.1:d:+1469:primary
GTAGATTCCC	1	0	1	0	0	At4g24770.1:X:-111:quaternary
GAAGGGTGTG	0	1	1	0	0	At4g24760.1:d:+1049:primary
GGGTTCACCA	1	0	1	0	0	At4g24730.2:d:+1254:primary,At4g24730.1:d:+898:primary
CCAGCGACGT	0	1	1	0	0	At4g24680.1:v:+3066:secondary
TCTATGATTT	0	1	0	1	0	At4g24620.1:d:+2124:secondary
CAAGTTCTCG	1	0	1	0	0	At4g24610.1:d:+638:secondary
AAATTGTATC	1	0	0	1	0	At4g24560.1:d:+3626:primary
CAAGTAACAT	0	2	0	0	0	At4g24540.1:d:+359:primary
TGATTCCAAG	1	1	0	0	0	At4g24440.2:d:+426:primary,At4g24440.1:d:+463:primary
CTTCTTACCC	1	0	0	1	0	At4g24410.1:v:+1039:secondary,At2g44630.1:d:+407:secondary
CTTAGATTCG	0	2	0	0	0	At4g24370.1:d:+285:secondary
TAGAGAGCCA	1	0	0	1	0	At4g24290.1:d:+1919:primary,At4g24290.2:d:+1554:primary
GCTGCTCTTA	0	0	1	1	0	At4g24280.1:d:+2084:secondary,At2g07744.1:p:+1846:secondary,At4g26600.1:d:+66:secondary
GGGATTTGGA	0	1	0	0	1	At4g24270.2:d:+1704:primary,At4g24270.1:d:+1704:primary
AACGGAAAAG	1	0	0	1	0	At4g24100.1:d:+1369:secondary,At3g61610.1:d:+1151:secondary
CACCCCCAAT	0	1	1	0	0	At4g24060.1:d:-1350:secondary
GTGCTAATTA	0	1	0	1	0	At4g24060.1:d:+1182:secondary
TAGCTAAGAA	1	1	0	0	0	At4g24050.1:d:+1213:secondary,At5g18570.1:d:+1719:secondary
TTGTTTTGTT	0	1	0	1	0	At4g24015.1:d:+336:primary
GATGATGAAG	1	0	0	1	0	At4g23940.1:d:+2539:primary
TATGTGAAGT	1	0	0	1	0	At4g23930.2:d:+552:primary,At4g23930.1:d:+617:primary

CTTGGATTAA	0	0	0	1	1	At4g23910.1:i:+147:tertiary
ATTTCAATAC	0	0	1	0	1	At4g23810.1:d:+1303:primary
GGAACATCAA	0	1	0	0	1	At4g23740.1:d:+1603:primary
TTGTGTGTGG	1	0	0	1	0	At4g23730.1:d:+1294:primary
GGAAACCACG	0	0	1	1	0	At4g23680.1:d:+492:secondary
ACCTTCTGGT	2	0	0	0	0	At4g23660.2:d:+1585:primary,At4g23660.1:d:+1818:primary
AACTATTACT	0	1	0	1	0	At4g23640.1:d:+2492:primary
GACGTCTAGC	0	1	1	0	0	At4g23493.1:d:+386:primary
AGAAGTCGTC	0	1	0	1	0	At4g23330.1:i:+1248:tertiary
TCTGAAAATA	0	0	0	1	1	At4g23300.1:d:+2048:primary
ACTTGGTCTC	0	1	0	1	0	At4g23180.1:d:+1713:primary
AGAAATTTGG	0	1	1	0	0	At4g23100.1:d:+515:secondary,At1g43850.1:d:+1229:secondary,At4g30230.1:v:+133:secondary
TGTTTGATCA	0	1	0	1	0	At4g23010.1:d:+256:secondary,At4g31070.1:v:+970:secondary,At5g08070.1:v:+772:secondary
CGAATGATGC	0	1	0	1	0	At4g23000.1:v:+1494:secondary
GTCTCATAGT	0	1	0	0	1	At4g22920.1:d:+1243:primary
GAGCTTCCAG	0	0	1	1	0	At4g22890.2:d:+780:primary
AGGAGAACAA	1	1	0	0	0	At4g22760.1:v:+1074:secondary,At5g54930.1:d:+147:secondary
AAAATGCTCG	0	2	0	0	0	At4g22756.1:d:+1142:primary
GTGTAAGATA	0	2	0	0	0	At4g22750.1:d:+397:primary
TTAATCGCCG	0	0	2	0	0	At4g22690.1:d:-1981:secondary
AATCTAATCG	0	0	0	0	2	At4g22580.1:X:-921:quaternary
GCTTCAAAGA	0	0	0	0	2	At4g22520.1:v:+354:secondary,At4g22610.1:d:+113:secondary
TGAGTTCCCT	0	1	1	0	0	At4g22350.1:X:+377:quaternary
CAGATATATG	0	2	0	0	0	At4g22350.1:d:+1746:primary
TTTATGAAAA	0	0	2	0	0	At4g22214.1:i:+181:tertiary
GGGTTCCCAC	0	0	2	0	0	At4g22150.1:d:+823:primary
GTCAAAAGAA	0	0	1	1	0	At4g22120.1:d:+2438:primary
AAAATAAAAT	0	0	1	0	1	At4g22080.1:i:+1678:tertiary,At4g22090.1:i:+1785:tertiary
TAAGATCAAC	0	1	1	0	0	At4g22070.1:v:+2118:secondary
TGTATTTTTA	1	1	0	0	0	At4g22030.1:v:+2495:primary,At1g19670.1:d:+1129:primary
GACAGCGATC	0	1	0	1	0	At4g22010.1:d:+1536:primary
ATTTTTTTTT	1	0	0	1	0	At4g21920.1:v:+1202:primary
TGAGCAATTT	1	1	0	0	0	At4g21910.3:i:+1357:tertiary,At4g21910.1:i:+1418:tertiary
TTGCAACAAA	1	0	1	0	0	At4g21880.1:i:-3536:tertiary,At4g21880.1:i:-3524:tertiary
TTATGTTCCA	1	1	0	0	0	At4g21850.1:d:+629:primary,At4g21850.2:d:+733:primary
AAATGGAAAA	0	0	1	0	1	At4g21800.1:d:+1022:secondary,At4g21800.2:d:+1041:secondary
GGCTCGGATT	0	0	1	0	1	At4g21760.1:d:+405:secondary,At1g56110.1:d:+822:secondary
GGGCATCCGT	0	1	1	0	0	At4g21710.1:X:+292:quaternary
ATCTCATAAT	1	0	0	0	1	At4g21630.1:v:+2915:primary
CTGTTGTTAC	0	0	0	1	1	At4g21605.1:p:+1160:primary,At1g62800.1:d:+1237:primary,At1g62800.2:d:+1243:primary
AAGTGTAATG	1	0	1	0	0	At4g21560.2:d:+1236:secondary,At4g24650.1:d:+76:secondary,At4g21560.3:d:+1534:secondary,At4g21560.1:d:+1253:secondary
TATTTTAGTC	1	0	1	0	0	At4g21510.1:d:+1022:primary
ATCTTCACGT	1	1	0	0	0	At4g21480.1:v:+1647:secondary,At1g11260.1:d:+1434:secondary
GCTGCTTCAC	1	1	0	0	0	At4g21470.1:d:+1102:primary
GTTTTCAATT	0	1	0	1	0	At4g21380.1:d:+854:secondary
TACAAAAGAA	0	1	0	0	1	At4g21350.1:v:+1634:secondary,At2g03330.1:v:+1864:secondary
CTCTCTGTTC	0	2	0	0	0	At4g21160.1:d:+814:secondary,At4g21160.2:d:+852:secondary,At4g21160.3:d:+1014:secondary,At4g21160.4:d:+1014:secondary
CAAAAGAATC	2	0	0	0	0	At4g21160.1:d:+1705:primary
AGCTAATCAT	1	0	0	0	1	At4g21150.1:d:-2404:secondary,At4g21150.1:d:-2391:secondary
ATCTGAGAGA	0	0	0	2	0	At4g21105.1:d:-95:primary
TATGAATGAA	0	1	0	0	1	At4g20960.1:d:+1423:secondary
CTCAGCTAAT	0	1	0	1	0	At4g20820.1:v:+1546:primary
ACAAGAAACC	0	0	1	1	0	At4g20490.1:p:+2961:primary
ATAGCACGAT	2	0	0	0	0	At4g20440.1:X:-1036:quaternary
GAAAAATGAA	0	0	1	1	0	At4g20420.1:d:+627:primary
GAACTTGACC	0	0	0	2	0	At4g20380.2:d:+1012:primary,At4g20380.3:d:+1022:primary,At4g20380.1:d:+1051:primary
TCCGTTGATG	0	1	0	1	0	At4g20380.1:d:+755:secondary,At4g20380.2:d:+716:secondary,At4g20380.3:d:+726:secondary
AGGCTTCTTC	1	0	1	0	0	At4g20360.1:d:-318:secondary
GTTCGATCTC	0	2	0	0	0	At4g20170.1:d:+1477:primary

CTTTCTTATT	0	1	1	0	0	At4g20030.1:d:+541:secondary
TTTGACTTTT	0	0	0	2	0	At4g19985.1:d:+1010:primary
AATTCTTTGT	0	1	0	0	1	At4g19870.2:d:+1461:primary
AGATTTATTT	0	1	0	1	0	At4g19850.1:v:+1014:secondary
CGGCTGGTGT	0	1	1	0	0	At4g19840.1:d:+740:secondary
AACTTGATCT	0	1	0	0	1	At4g19645.2:d:+870:primary,At4g19645.1:d:+906:primary
ATAGGAACAG	1	1	0	0	0	At4g19540.1:d:-403:primary
AGTTGCTTCG	0	2	0	0	0	At4g19540.1:d:+777:primary
ATTGAAAAAG	0	0	1	1	0	At4g19515.1:d:+1213:secondary
AAAGGAAAGC	0	2	0	0	0	At4g19500.1:v:+1506:secondary
TCGAGCTAAT	1	0	0	0	1	At4g19490.1:d:+3253:primary,At4g19490.2:d:+3253:primary
ATTCATTATT	0	1	0	1	0	At4g19420.2:i:+2892:tertiary,At4g19420.1:i:+2892:tertiary
AGAACTGGTT	0	1	1	0	0	At4g19400.1:d:+349:secondary,At1g73740.1:d:+1017:secondary,At5g15620.1:v:+1769:secondary
TTAAAAGACA	0	1	1	0	0	At4g19290.1:v:+1122:primary
GTCTAAAAAC	0	2	0	0	0	At4g19185.1:d:+1266:primary
GGTTTGATCC	0	0	0	2	0	At4g19180.1:d:+206:secondary
TTTCTGCAAA	1	0	0	0	1	At4g19130.1:d:+1319:secondary,At5g45400.1:v:+2809:secondary
GGTTCCGTTT	0	2	0	0	0	At4g19100.1:v:+818:secondary
GTAATAAGTT	1	0	1	0	0	At4g19050.1:v:+4631:primary
ACCTTCACGA	1	0	1	0	0	At4g18975.1:d:+682:secondary
ACGGCTCATC	0	0	2	0	0	At4g18950.1:d:+1673:primary
CTTCTTGGTA	2	0	0	0	0	At4g18830.1:d:+1374:primary
ATTATTCGTA	1	1	0	0	0	At4g18820.1:X:+304:quaternary
TGATTCAGCA	0	2	0	0	0	At4g18820.1:d:+3080:primary
AAATTGGAGT	0	2	0	0	0	At4g18760.1:v:+1654:secondary
ATCGGAATGT	0	1	0	1	0	At4g18700.1:d:+954:secondary,At5g45810.1:v:+1039:secondary
GAAAATTTCT	0	0	1	0	1	At4g18600.1:d:+3889:secondary
GAAGGCGATG	0	0	1	1	0	At4g18520.1:d:+1557:secondary
TTTTTGTGAA	1	1	0	0	0	At4g18372.1:d:+531:primary,At1g60770.1:d:+1722:primary
TTATTGCTAA	1	1	0	0	0	At4g18370.1:d:+486:secondary,At1g30820.1:d:+621:secondary
ATCTTTGGCT	2	0	0	0	0	At4g18370.1:d:+182:secondary
GAATAAAATT	1	0	0	1	0	At4g18370.1:d:+1197:secondary
TTGTTGTAAC	1	0	1	0	0	At4g18360.1:X:+-209:quaternary
TGAATTTGCA	0	0	2	0	0	At4g18335.1:v:+1226:secondary
AAGCTGCAAA	0	0	1	1	0	At4g18290.1:d:+1862:primary
AAAATGGAAT	1	1	0	0	0	At4g18260.1:d:+438:secondary
TCCGGTTGTT	0	2	0	0	0	At4g18240.1:d:+2771:primary
CAAATTTACC	0	1	1	0	0	At4g18230.1:d:+426:primary
ATAGAATGGA	0	1	0	1	0	At4g18200.1:v:+2470:secondary
CGCCGTTTCC	0	1	1	0	0	At4g18130.1:d:+3005:secondary
TTTTTGATGG	1	0	0	1	0	At4g18010.1:d:+1943:secondary,At4g18010.2:d:+1635:secondary,At1g43785.1:p:+597:secondary
GCGGCCACCG	0	1	1	0	0	At4g17900.1:d:-188:secondary,At5g49130.1:v:-466:secondary
CCTTCGGAGT	0	2	0	0	0	At4g17870.1:d:+118:primary
GTATATACTC	0	1	0	1	0	At4g17840.1:d:+1439:primary
ACAAATGAAA	0	0	2	0	0	At4g17820.1:p:+1222:secondary,At4g08405.1:p:+392:secondary,At4g12426.1:p:+1199:secondary,At3g42883.1:p:+1095:secondary,At2g03080.1:p:+859:secondary,At4g20180.1:p:+1774:secondary
TTTTGTTTTC	0	0	0	2	0	At4g17650.1:d:+1088:primary
CTCGTTTCAT	1	1	0	0	0	At4g17640.1:d:+756:secondary,At4g37840.1:v:+594:secondary
ATTAACGGTA	0	0	1	0	1	At4g17600.1:d:-951:secondary
CAAAGATTGC	0	0	0	1	1	At4g17540.1:d:+955:primary
TTCCAGAATG	0	1	1	0	0	At4g17470.1:d:+730:primary
AACTTGATTC	0	2	0	0	0	At4g17430.1:d:+1322:secondary,At1g11510.1:v:+1120:secondary
TGTTTCTAAA	0	0	1	1	0	At4g17380.1:i:+658:tertiary
GGAGTTGCTG	0	2	0	0	0	At4g17370.1:d:+1175:primary
GAGTAGCGGC	0	0	2	0	0	At4g17340.1:d:+460:secondary
CCCCCAACAA	1	1	0	0	0	At4g17210.1:v:+149:secondary,At2g33350.1:v:+1782:secondary
TATAAAGCAA	0	0	1	0	1	At4g17200.1:v:+596:secondary
ACCCAAGTTG	0	1	0	1	0	At4g17180.1:d:+1139:secondary
GGACCCCAAA	0	0	2	0	0	At4g17160.1:v:+76:secondary
ATTGATTTAA	0	0	1	0	1	At4g17160.1:i:+898:tertiary

ATTTGCAGAA	1	0	0	0	1	At4g17030.1:d:+717:primary
CTTCGATTCT	1	0	0	1	0	At4g17020.1:d:+1696:secondary
AGTGGATTCG	0	1	1	0	0	At4g16990.1:d:+2648:secondary,At4g16990.3:d:+2113:secondary,At4g16990.2:d:+2098:secondary
CCGCCTATGT	0	0	2	0	0	At4g16980.1:d:+224:secondary
AAACCCTTAG	1	1	0	0	0	At4g16970.1:d:+2573:primary
ATTATATATC	0	0	0	0	2	At4g16950.2:d:+4397:secondary
AATTATCCAA	0	0	1	0	1	At4g16920.1:i:+2881:tertiary
CGTAATCTCC	0	2	0	0	0	At4g16900.1:v:+1887:secondary,At4g16860.1:d:+1761:secondary,At4g16950.2:d:+1775:secondary,At4g16920.1:v:+1980:secondary,At4g16950.1:d:+1775:secondary
AGAGAAGAGG	0	2	0	0	0	At4g16880.1:X:-651:quaternary
GTGGTACAAA	1	1	0	0	0	At4g16870.1:p:+3728:secondary
GAACCTGAAG	0	0	1	0	1	At4g16700.1:d:+759:secondary,At5g11730.1:d:+1400:secondary
ATGTCTTGTG	0	1	1	0	0	At4g16670.1:d:+1404:primary
AAAATTGAAA	0	1	0	1	0	At4g16600.1:v:+272:secondary
CTTAGTGTGG	1	0	1	0	0	At4g16566.1:d:+285:secondary
CTCTCGGCTC	1	1	0	0	0	At4g16480.1:d:+1686:primary
CGGCAAAAGC	0	1	0	1	0	At4g16440.1:v:+1243:secondary
GCAGAAACAA	0	1	0	1	0	At4g16400.1:v:+1204:primary
GAGTATGGGT	1	1	0	0	0	At4g16360.1:d:+1333:primary
GCGGTTTGTA	1	0	0	0	1	At4g16340.1:d:+5388:secondary
AATCTAGTGA	0	2	0	0	0	At4g16295.1:v:+1160:secondary
ACTGAAAAAA	0	0	1	1	0	At4g16180.1:X:--2:quaternary
GAAAGAGGAC	0	0	0	1	1	At4g16180.1:v:+1456:secondary,At1g31150.1:d:+1578:secondary
GGATTTGAAA	0	0	0	1	1	At4g16143.1:X:-178:quaternary
GCTCTTTCTT	0	2	0	0	0	At4g16141.1:v:+1014:primary
CGGACTAGGA	0	1	1	0	0	At4g16130.1:d:+2837:primary
TGTGTCTTGA	0	0	1	1	0	At4g15990.1:v:+997:secondary
TCTACCGATG	0	0	1	0	1	At4g15870.1:v:+891:secondary,At5g09510.1:d:+225:secondary
ATATAAACAA	1	0	0	0	1	At4g15830.1:d:+1002:secondary
TCTTGAAAGG	0	1	0	1	0	At4g15770.1:d:+414:primary
TTAGGGAAAT	0	1	1	0	0	At4g15760.1:d:+884:secondary
ACCATACATT	0	0	1	0	1	At4g15760.1:d:+1373:primary
AGTCTCCATC	0	1	1	0	0	At4g15700.1:d:+399:primary,At4g15670.1:d:+247:primary,At4g15690.1:d:+367:primary
CATTTAAGAA	0	0	0	1	1	At4g15670.1:X:+506:quaternary
CTTTTGGTTT	0	0	1	0	1	At4g15660.1:v:+855:secondary
AACACATTCT	0	0	1	0	1	At4g15640.1:d:+1296:primary
TAATTGTCTA	1	0	0	0	1	At4g15620.1:d:+727:primary
TATGGACCAT	1	0	0	1	0	At4g15610.1:i:+584:tertiary,At5g19140.1:i:+495:tertiary
GCCGGAGAAG	0	0	1	1	0	At4g15570.1:d:+2519:primary
TTTGTATCCC	0	0	0	1	1	At4g15475.1:d:-2037:secondary
TGCCGAAGCC	2	0	0	0	0	At4g15475.1:d:+2042:primary
GAGGAACACT	0	0	1	1	0	At4g15430.1:d:+2375:primary
GAAGAGACTT	1	0	0	1	0	At4g15360.1:d:+1412:primary
ACTGCTTTTG	0	2	0	0	0	At4g15140.1:d:+663:primary
TTTTAGAAAA	0	2	0	0	0	At4g15090.1:d:+1418:secondary
GAGGGGATGT	0	1	0	1	0	At4g15030.1:d:+592:primary
CTCCAACATC	1	0	0	0	1	At4g14950.2:d:+1634:primary,At4g14950.1:d:+1598:primary,At4g14950.3:d:+1631:primary
GCAAGGCAGC	1	0	1	0	0	At4g14880.2:d:+859:secondary,At4g14880.1:d:+914:secondary
ATCAAAACTA	1	0	1	0	0	At4g14880.2:d:+1296:primary,At4g14880.1:d:+1351:primary
GCTTCCGCTT	0	0	1	0	1	At4g14880.1:X:-73:quaternary
GAGGATACTC	0	1	0	0	1	At4g14746.1:v:+1085:secondary
TGTCTGCCAT	0	0	0	1	1	At4g14690.1:X:+671:quaternary
GACTAGCAAA	0	2	0	0	0	At4g14620.1:X:-642:quaternary
CTAATGTGCC	0	1	0	1	0	At4g14550.1:d:+1472:primary
GGATAGAAAT	0	1	0	0	1	At4g14380.1:d:+855:primary
TCCTCGAATT	0	1	0	1	0	At4g14340.1:d:+1759:secondary
TATCAACCTC	0	1	0	0	1	At4g14220.1:d:+1114:primary
ATTTCCGGGT	0	2	0	0	0	At4g14040.1:X:-112:quaternary
TCGATGTTGG	0	1	1	0	0	At4g13950.1:X:-64:quaternary,At5g20740.1:X:-1050:quaternary
AAGGTAAGAT	1	0	1	0	0	At4g13950.1:d:+408:primary

AGAATCTTTT	0	0	2	0	0	At4g13920.1:d:+2696:primary
TTTATTTGTT	1	0	1	0	0	At4g13800.1:d:+1497:primary,At5g05400.1:v:+3238:primary
ATGTCTTTTG	0	0	0	2	0	At4g13770.1:d:+1688:primary
TTCCCTAAAA	2	0	0	0	0	At4g13670.1:i:-1470:tertiary
GACCAAACCT	0	0	1	1	0	At4g13650.1:d:+3292:secondary
AGCAAGTGTA	0	0	2	0	0	At4g13630.1:d:+1808:primary
AACCTCTTAA	0	0	0	1	1	At4g13610.1:v:+4980:primary
GGTGGTTCTA	0	2	0	0	0	At4g13540.1:X:+3:quaternary
GGGACACTTT	0	0	2	0	0	At4g13510.1:d:+1337:secondary
CCAAATTAGG	1	0	0	1	0	At4g13500.1:d:+214:secondary
GTTGTGGAAG	0	0	0	1	1	At4g13430.1:d:+960:secondary
AGAACAAGTT	0	0	2	0	0	At4g13420.1:d:+127:secondary
AGAGATCACC	0	1	0	1	0	At4g13345.1:d:+1685:secondary,At4g13345.2:d:+1685:secondary
GATCAAAAAA	0	0	1	0	1	At4g13263.1:v:+1223:primary
GTCCACATTT	1	0	1	0	0	At4g13260.1:d:+1812:primary
ATTCAGTATC	1	1	0	0	0	At4g13160.1:d:+960:primary
TTTGCTCTTG	0	2	0	0	0	At4g13140.1:d:+300:primary
GATGACACTC	0	1	0	0	1	At4g12900.1:d:+525:primary
AACTCAATTT	2	0	0	0	0	At4g12810.1:v:+1664:primary
TGGCATTCAT	0	0	0	2	0	At4g12770.1:d:+2952:primary
CTAAGGAAAG	0	2	0	0	0	At4g12770.1:d:+1573:secondary,At4g12780.1:d:+234:secondary
TATGACCCTG	1	1	0	0	0	At4g12650.1:d:+313:secondary
AATCAAGCAA	1	1	0	0	0	At4g12420.1:d:+1155:secondary,At2g43500.1:v:+2568:secondary
TTTGAGGAGT	0	1	0	1	0	At4g12400.1:d:+1992:primary,At3g23250.1:d:+965:primary
CTTTGAATTA	0	0	1	1	0	At4g12010.1:i:+1819:tertiary
AATCAAGAAA	0	0	1	0	1	At4g12010.1:d:+3272:secondary,At2g42400.1:d:+1287:secondary
AGAAAGTAAA	0	0	0	1	1	At4g11990.1:X:-219:quaternary
TTCACAAAAA	2	0	0	0	0	At4g11990.1:d:+1362:primary
TTTGCAAATG	0	0	0	0	2	At4g11800.1:X:-32:quaternary
GAATGGAATC	0	1	0	1	0	At4g11800.1:d:+2603:primary
ATTCTTTTTG	1	0	1	0	0	At4g11745.1:v:+829:secondary
CGTTTGGAGA	0	1	0	0	1	At4g11740.1:d:+1705:primary
GATTCAACGC	1	0	0	0	1	At4g11630.1:d:-611:primary,At5g11750.1:d:-656:primary
AGAGTGACTT	0	1	1	0	0	At4g11420.1:d:+2328:secondary
AAAAAGGGAA	0	0	2	0	0	At4g11420.1:d:+2057:secondary,At5g22010.1:d:+156:secondary,At2g38770.1:d:+1104:secondary
GGTGCTATTT	1	1	0	0	0	At4g11220.1:d:+541:primary
CAGAAAGAGA	1	0	0	1	0	At4g11170.1:v:+3532:secondary
CCAATTACCT	0	1	1	0	0	At4g11110.1:d:+3086:primary
TCCTTCTTTG	0	0	0	1	1	At4g11020.1:i:+1046:tertiary,At2g46930.1:i:+458:tertiary
CAAACATCTG	0	2	0	0	0	At4g10890.1:d:+1405:secondary
GTTGGGACCT	0	0	1	1	0	At4g10790.1:d:+119:primary
TTGCTTTCGT	0	1	1	0	0	At4g10760.1:d:+2149:secondary
ATGCCACAAA	0	0	2	0	0	At4g10750.1:d:+1191:secondary
GCTGAAAGCG	0	0	2	0	0	At4g10610.1:d:+988:secondary
TTTTTATTCT	1	0	1	0	0	At4g10560.1:v:+2566:primary,At1g35960.1:p:+5233:primary,At2g13980.1:v:+800:primary
TCAATTCTAG	0	1	0	0	1	At4g10470.1:d:+1389:primary
CTTTTACAAA	1	1	0	0	0	At4g10430.1:d:+783:primary,At4g10430.2:d:+798:primary,At4g10430.3:d:+777:primary
CGAACTCAAC	0	0	0	1	1	At4g10400.1:d:+1459:primary
TGATTGAGCT	0	2	0	0	0	At4g10170.1:d:+719:primary,At4g10170.2:d:+600:primary
TGGAATACAA	1	0	1	0	0	At4g10120.1:d:+2641:secondary
GACAAAGAAG	0	2	0	0	0	At4g10100.1:d:+133:primary,At4g10100.2:d:+127:primary
GCCGGTTTTG	1	0	1	0	0	At4g10080.1:d:+1051:secondary
AACTGCTTTT	0	0	0	0	2	At4g10040.1:d:+506:secondary
ATCTCTTTTG	0	1	1	0	0	At4g09965.1:v:+1129:primary,At1g15910.1:d:+2165:primary
AGTGTAGACA	2	0	0	0	0	At4g09680.1:d:+3427:primary
TTCAAGGATT	0	0	0	2	0	At4g09630.1:d:+2191:primary
GTCTTTTCTG	0	0	2	0	0	At4g09620.1:i:+1230:tertiary
GTGTTAATGG	1	1	0	0	0	At4g09620.1:d:+197:primary
TTTCTCTGTA	0	0	0	0	2	At4g09200.1:i:+663:tertiary,At4g09310.1:i:+582:tertiary,At4g09250.1:i:+1295:tertiary
TCCCGGTTCA	0	2	0	0	0	At4g09160.1:d:+1697:primary
GTGAATGCAC	0	0	0	0	2	At4g09150.1:i:+4043:tertiary

CCCTCTCTAT	0	0	0	1	1	At4g09080.1:v:+1830:primary
GTGTAAATTT	0	0	2	0	0	At4g09020.1:d:+1612:secondary,At4g17900.1:d:+1047:secondary
GGATTGAAAT	0	0	2	0	0	At4g09020.1:d:+126:secondary
CCATCTTCTT	0	2	0	0	0	At4g08960.1:d:+1356:primary
ATCAACCAAT	0	1	1	0	0	At4g08953.1:p:+197:primary
ATCGGTCTCC	0	1	1	0	0	At4g08850.2:d:+2827:primary
GTTTGAGGTG	0	2	0	0	0	At4g08620.1:i:+4996:tertiary
TCTTCATCCA	0	1	0	0	1	At4g08540.1:X:+640:quaternary
TAATGTATAA	0	1	0	0	1	At4g08500.1:d:+2384:primary
CCTCACTTCT	0	0	2	0	0	At4g08330.1:d:+453:secondary
GAAGTTGCCA	0	0	0	1	1	At4g08262.1:p:+5271:primary
ATTATTACTT	0	0	1	1	0	At4g08110.1:p:+1812:secondary,At5g35540.1:v:+807:secondary
AAAGAAGAAG	0	0	2	0	0	At4g08053.1:p:+2463:secondary,At4g39090.1:d:+832:secondary,At4g18770.1:d:+114:secondary
TCCAAGGTGT	2	0	0	0	0	At4g07950.1:d:+335:primary,At1g01210.1:d:+283:primary
CGGCGGCATC	0	1	0	0	1	At4g07825.1:d:+603:secondary
TTGATACTGA	0	1	1	0	0	At4g07810.1:p:+5989:primary,At1g73630.1:d:+410:primary
TTGCATCATT	0	0	1	0	1	At4g07810.1:p:+3303:secondary
ATTCAGTTAC	0	0	1	1	0	At4g07733.1:p:+4740:secondary,At5g23810.1:d:+145:secondary,At3g33530.1:d:+2878:secondary,At2g13020.1:p:+5672:secondary,At3g42436.1:v:+1257:secondary,At5g48660.1:v:+354:secondary,At5g33202.1:p:+1027:secondary
TTTGTTTAAT	0	2	0	0	0	At4g07675.1:v:+1159:primary,At3g50860.1:d:+923:primary
TTATAGATGT	0	0	1	1	0	At4g07666.1:v:+156:secondary
AATTACAAGA	0	0	2	0	0	At4g07492.1:p:+38:secondary
TTTATTGAAT	1	0	0	0	1	At4g07475.1:p:+1506:primary
AGTTTTTTTG	0	1	0	1	0	At4g06748.1:X:+1822:quaternary
GTGGCTCCCT	0	0	0	1	1	At4g06744.1:d:+1419:primary
TTTATGCTTA	1	0	0	0	1	At4g06658.1:p:+1115:secondary
TTAGTATATC	0	0	0	1	1	At4g06636.1:p:+1476:secondary,At2g06410.1:p:+1436:secondary,At1g56170.1:d:+737:secondary,At3g42945.1:p:+1166:secondary,At3g09165.1:p:+1839:secondary,At1g34620.1:p:+1938:secondary,At4g04547.1:p:+665:secondary
ATAGAACCGA	0	1	0	0	1	At4g06634.1:X:--80:quaternary
GCAAGCAAAA	0	1	0	1	0	At4g06634.1:d:+1111:primary
ACAGAGAAGA	0	2	0	0	0	At4g06628.1:p:+616:secondary,At1g35647.1:p:+236:secondary
TATTATTTTT	1	1	0	0	0	At4g06614.1:p:+1157:primary
AGATAAAAAA	0	0	0	1	1	At4g06598.1:p:+365:secondary
AATGGATTCT	1	0	0	1	0	At4g06589.1:X:-1338:quaternary
ATTACAGAGA	0	0	2	0	0	At4g06576.1:p:+1849:primary
AACATTGACT	1	1	0	0	0	At4g06573.1:p:+1186:secondary
TCAACTATTG	0	0	1	1	0	At4g06553.1:X:-2374:quaternary
GATGTTGATA	1	1	0	0	0	At4g06540.1:p:+2659:primary
GTTGGCGATT	0	0	1	1	0	At4g06517.1:p:+1105:secondary,At5g32107.1:p:+1935:secondary,At5g32511.1:p:+1105:secondary,At1g40310.1:p:+1081:secondary,At5g32228.1:p:+1105:secondary,At5g31719.1:p:+1935:secondary,At5g32306.1:p:+1105:secondary,At4g06720.1:p:+1055:secondary,At3g53720.1:d:+1908:secondary,At2g09187.1:p:+5437:secondary,At5g32197.1:p:+1105:secondary
AAAGGATGCC	1	0	1	0	0	At4g06500.1:p:+4100:secondary,At1g04120.1:d:+5016:secondary
AATGATGACT	0	0	2	0	0	At4g06497.1:p:+2493:secondary
AGCGTGGTTC	0	1	0	0	1	At4g05583.2:p:+841:secondary
AGGCTTGTTA	1	0	1	0	0	At4g05581.1:v:+830:secondary
GCTGCTGCGG	0	0	2	0	0	At4g05530.1:d:+740:secondary
TCTCCTCTTT	0	1	0	0	1	At4g05470.1:v:+2180:primary
GATGTTGGAT	1	1	0	0	0	At4g05450.1:X:+-122:quaternary
AAATGATCAA	0	0	0	1	1	At4g05450.1:d:+1001:primary
GAGAATGTGA	0	1	0	1	0	At4g05440.1:d:+881:primary,At4g05495.1:p:+281:primary
TTAAAATATA	0	1	0	0	1	At4g05430.1:v:+949:secondary
TTAAAAAAGA	1	0	1	0	0	At4g05410.1:d:+1229:secondary
ATTCGAGTTA	0	2	0	0	0	At4g05400.1:d:+74:primary
TCTAATGATT	0	2	0	0	0	At4g05390.1:X:-597:quaternary,At2g33030.1:X:-265:quaternary,At4g12910.1:X:-441:quaternary
TTTGAGCTTA	0	0	1	1	0	At4g05340.1:v:+646:secondary
AAACATTTTA	0	0	1	0	1	At4g05210.1:i:+402:tertiary,At5g27560.1:i:+828:tertiary,At5g39940.1:i:+2324:tertiary
ATGAAAGGGT	0	1	0	1	0	At4g05160.1:d:+1264:primary
TCAAAAATGA	0	0	0	1	1	At4g05076.1:X:-72:quaternary

ATGGAGAAAT	0	2	0	0	0	At4g05060.1:d:+851:primary
TATCGTTTCA	0	0	1	0	1	At4g05050.1:d:+800:secondary
ACGTGGGGGG	1	1	0	0	0	At4g05040.2:X:-518:quaternary
ATATTGAAGG	1	0	0	0	1	At4g05030.1:v:+898:primary
GTCTTTTTTT	0	1	0	1	0	At4g05020.1:d:+2164:primary
TGAATATAAC	0	2	0	0	0	At4g05010.1:v:+972:primary
TGGATCACGA	0	2	0	0	0	At4g05010.1:v:+586:secondary
AGATTCAAAC	0	0	1	0	1	At4g04980.1:v:+2350:secondary
GTGGTTAGCA	1	0	0	1	0	At4g04970.1:d:+5482:primary
GGGTTTGTCT	1	0	0	0	1	At4g04750.1:v:+1792:secondary,At4g16210.1:d:+475:secondary
GTTCTTAAAC	0	0	2	0	0	At4g04650.1:X:-410:quaternary,At4g32070.1:X:-1364:quaternary,At2g25290.1:X:-1253:quaternary,At1g67090.1:X:-503:quaternary
CTCAGGAAAA	0	1	1	0	0	At4g04570.1:d:+892:secondary
TAACTTGGGG	2	0	0	0	0	At4g04490.1:v:+2397:secondary
AATCTTTGTC	0	1	1	0	0	At4g04480.1:v:+1712:primary
TCCGGATCTT	0	2	0	0	0	At4g04450.1:i:-1107:tertiary
AGCAAACATA	1	1	0	0	0	At4g04440.1:p:+1686:primary
TGGGAGCTTC	0	0	1	0	1	At4g04360.1:v:+666:secondary
AAACCAAATA	0	1	1	0	0	At4g04296.1:X:+-92:quaternary
TCAACTTGAT	1	1	0	0	0	At4g04210.1:d:+529:primary
TTGCTGCTTG	0	1	1	0	0	At4g04130.1:i:+2558:tertiary
GCTCTCAACA	1	0	1	0	0	At4g04120.1:p:+681:secondary,At3g17790.1:d:+1470:secondary
TCCAGAAAAA	0	0	1	1	0	At4g04000.1:p:+1292:secondary
TAGAACAAGT	1	0	0	1	0	At4g03960.1:d:+921:primary
TGAAAAAGAA	1	0	0	0	1	At4g03890.1:v:+380:secondary,At1g17665.1:v:+688:secondary
GGTGAACGAC	0	1	1	0	0	At4g03830.1:v:-1625:secondary
ACATAGTATA	1	1	0	0	0	At4g03813.1:p:-944:secondary,At2g14940.1:p:-2372:secondary,At1g21050.1:d:-844:secondary
TTTTCGATTA	0	0	2	0	0	At4g03700.1:p:+146:secondary
CAAAGGAAGA	0	2	0	0	0	At4g03695.1:X:+-223:quaternary
GTAAGGAAGA	0	2	0	0	0	At4g03590.1:v:+978:secondary,At1g08310.1:d:+1137:secondary
AAATATCACT	2	0	0	0	0	At4g03520.1:d:+672:secondary
CTAATTTTTA	1	1	0	0	0	At4g03380.1:i:+3572:tertiary
GTCCCGGTGA	0	0	2	0	0	At4g03280.2:d:+596:secondary,At4g03280.1:d:+497:secondary
TCTCAGCAAA	0	1	0	1	0	At4g03230.1:v:+3174:primary,At1g19330.1:d:+839:primary
TACAATAATG	2	0	0	0	0	At4g03060.1:d:+1540:primary
AGAGGAGAAT	0	0	1	0	1	At4g02990.1:d:+1777:secondary
AAAATATGTA	1	1	0	0	0	At4g02980.1:d:+807:primary
CCTGTCCCAC	1	0	1	0	0	At4g02930.1:d:+1319:secondary
AAGAGCTTAT	0	1	1	0	0	At4g02880.1:d:+1689:primary
AAAGGCTCTA	0	0	1	0	1	At4g02850.1:d:+944:primary
AACACTCATT	0	0	0	0	2	At4g02840.1:d:+208:secondary
TTCTTCAAGA	0	0	0	1	1	At4g02830.1:d:+188:primary
CAGACTAATA	0	0	0	2	0	At4g02820.1:d:-943:secondary
ACGACATCAG	0	1	0	0	1	At4g02790.1:d:+1271:secondary
AGAGAAGGTC	1	0	1	0	0	At4g02770.1:d:+433:secondary
ACCCTTTCGG	0	2	0	0	0	At4g02720.1:d:+1282:secondary
CGAGGAGAGG	0	2	0	0	0	At4g02710.1:v:+3454:secondary
GGCATCCACA	0	0	1	1	0	At4g02600.1:d:-1869:secondary
GTTGAATTAG	0	0	1	1	0	At4g02560.1:d:+3057:primary
CATATACTCA	0	1	0	1	0	At4g02550.1:X:-239:quaternary,At5g43320.1:X:-20:quaternary
GTAAACTGGG	1	0	0	1	0	At4g02480.1:d:+3437:secondary
TATTAGATTA	0	0	0	0	2	At4g02465.1:v:+980:secondary
TTGAAGATTA	1	0	0	1	0	At4g02280.1:d:+2847:primary
GTCAAAAGAG	0	0	2	0	0	At4g02260.2:d:+706:secondary,At4g02260.3:d:+706:secondary,At4g02260.1:d:+706:secondary
GAAGACAATA	0	1	0	1	0	At4g02200.2:d:+562:secondary,At5g34770.1:p:+1754:secondary,At4g02200.1:d:+562:secondary
AGCCTCAAAA	0	2	0	0	0	At4g02190.1:v:+2671:primary
GTCATTTTTA	0	0	2	0	0	At4g02075.1:d:+812:primary
GGATTCAAAT	0	0	2	0	0	At4g02040.1:v:+1096:primary
AGGCGGATAA	0	1	0	1	0	At4g02020.1:d:+2327:primary
CACACTCGTT	0	0	0	1	1	At4g01985.1:d:+1814:primary
ACTTTCTTGA	0	1	0	1	0	At4g01980.1:X:-146:quaternary

AATGGCCTTG	0	2	0	0	0	At4g01950.1:X:+1260:quaternary,At4g01950.1:X:+1248:quaternary
CGACTAAAAG	0	0	2	0	0	At4g01860.2:X:+-100:quaternary
TAAAACAACT	1	1	0	0	0	At4g01790.1:v:+49:secondary,At3g48670.2:d:+2229:secondary,At4g01780.1:v:+2041:secondary,At3g48675.1:v:+101:secondary,At3g48670.1:d:+2270:secondary,At2g46560.1:d:+5684:secondary
GAAACAAACA	0	0	1	0	1	At4g01770.1:v:+1716:secondary
TAGCTTTGAA	2	0	0	0	0	At4g01720.1:i:+2345:tertiary
AAGTTGTTGA	0	1	0	1	0	At4g01700.1:d:+995:primary
TTGCAAGTGG	1	0	1	0	0	At4g01690.2:d:+1059:secondary,At5g20560.1:v:+1237:secondary,At4g01690.1:d:+1059:secondary
AGTTTCAAAC	2	0	0	0	0	At4g01600.1:i:+703:tertiary
AAGAGCAATG	1	1	0	0	0	At4g01570.1:d:+2194:primary
CAGGAGCCTG	0	2	0	0	0	At4g01480.1:d:+422:primary
TGGTCAAATC	1	1	0	0	0	At4g01460.1:d:+1150:secondary
GAGAAAGAAT	0	1	1	0	0	At4g01420.1:i:+136:tertiary
ACTTGGAGAC	1	1	0	0	0	At4g01400.1:v:+3615:primary
CCTCTAGGGG	0	1	0	0	1	At4g01390.1:v:+1134:primary
TAATTATGGT	1	0	0	0	1	At4g01360.1:d:+1299:primary
TCACATATAA	0	1	0	0	1	At4g01330.1:X:-210:quaternary,Chr4:-5498234:quaternary
CAACAGTGAT	1	1	0	0	0	At4g01330.1:d:+187:secondary,At4g29090.1:v:+2374:secondary
AATAACAAAA	1	1	0	0	0	At4g01220.2:i:+905:tertiary,At4g01220.1:i:+905:tertiary,At3g22410.1:i:+281:tertiary
TGAAGAGGTG	1	0	0	0	1	At4g01220.1:d:+1420:primary
TCTCTGAGGT	1	0	0	0	1	At4g01120.1:d:+1010:primary
CTTTGGCAAC	0	0	2	0	0	At4g01100.1:d:+813:secondary
AAAAATTGAA	0	0	1	1	0	At4g01060.1:d:+228:primary,At4g01060.2:d:+279:primary
CCCGCTTCAA	1	1	0	0	0	At4g01030.1:X:-670:quaternary
TTTGGGCGAG	0	0	0	2	0	At4g01030.1:v:+969:secondary,At1g49080.1:p:+20:secondary,At3g46487.1:p:+16:secondary,At4g34000.1:d:+1147:secondary,At4g34000.2:d:+1147:secondary
AGTCAAGCTT	0	0	2	0	0	At4g01010.1:X:--2:quaternary
AACGTGGACA	0	1	1	0	0	At4g01000.1:X:-326:quaternary
ATTACCGAGT	1	0	1	0	0	At4g00990.1:d:+2740:secondary
TATGTTTTAC	0	0	0	0	2	At4g00955.1:d:+1119:primary
ATACATCGTA	1	0	0	1	0	At4g00950.1:X:-289:quaternary
ACGACATCAA	0	0	1	1	0	At4g00950.1:d:+407:secondary
GCTCAAATAA	1	1	0	0	0	At4g00760.1:d:+1043:primary
GATGCAGAGG	0	2	0	0	0	At4g00755.1:d:+1197:primary,At4g00755.2:d:+1069:primary
GTCGTCGCTT	0	1	0	0	1	At4g00752.1:d:+1295:secondary
TATTAGTAGG	1	0	0	1	0	At4g00740.1:d:+2287:primary
TCGCATCATA	0	0	1	1	0	At4g00710.1:d:+2017:secondary
TATGCTTAAG	0	0	1	1	0	At4g00670.1:i:+380:tertiary; -small RNA(TTTAGACTTAAGCATACATGGCAAAT)
GAATCTTTCC	0	0	1	1	0	At4g00585.1:d:+514:secondary
ATAGTATTCC	0	0	0	1	1	At4g00560.3:d:+1187:primary,At4g00560.2:d:+972:primary,At4g00560.1:d:+1096:primary
TTAGCTAGAA	1	1	0	0	0	At4g00530.1:v:+641:secondary
CAATCAAAAA	0	1	0	1	0	At4g00440.1:d:+1880:secondary,At5g55893.1:v:+1434:secondary
ACTTCGTGAA	0	0	1	1	0	At4g00430.1:d:+1303:primary
ATTATGCAAT	0	0	1	0	1	At4g00413.1:p:+85:primary
GATTATGTCT	0	2	0	0	0	At4g00340.1:d:+2679:primary
TGAAATATTT	1	0	0	1	0	At4g00310.1:d:+1121:primary
GTGACTCCGA	1	0	0	1	0	At4g00270.1:d:+77:secondary,At2g25650.1:d:+183:secondary
GACGACATCT	1	0	1	0	0	At4g00230.1:d:+1822:primary
ACGGTTATCG	0	1	0	1	0	At4g00030.1:d:+249:primary
TAATTTTTCT	1	0	0	1	0	At3g63530.1:d:+1151:primary
CGTGACTGTG	0	0	2	0	0	At3g63520.1:v:+1835:secondary
CCTCATCTGT	1	0	0	0	1	At3g63420.2:X:+238:quaternary
GGATGTTCTG	0	0	2	0	0	At3g63410.1:d:+924:secondary
TATTACTAGA	0	1	0	1	0	At3g63410.1:d:+1249:secondary
CTGAGAGCTT	0	0	2	0	0	At3g63390.1:d:+572:primary
GGTTTGGTGC	0	0	1	0	1	At3g63340.1:d:+2046:secondary
TGGAGTGTTG	1	0	0	1	0	At3g63330.1:d:+818:secondary,At2g01450.1:d:+693:secondary
GCAAACCAGC	0	1	1	0	0	At3g63180.1:d:+2828:primary
AGCATTCCAG	0	1	1	0	0	At3g63150.1:d:+1823:primary

CTTACTTCTG	0	0	2	0	0	At3g63140.1:d:+936:secondary
CATTTCTACG	0	0	1	1	0	At3g63140.1:d:+1128:secondary
CATTAAAAAG	0	1	0	1	0	At3g63010.1:X:+1522:quaternary
GCCAATACTT	1	0	0	1	0	At3g63000.1:d:+1455:primary
CTTTTGGGCT	0	1	0	1	0	At3g62940.1:d:+972:primary,At3g62940.2:d:+983:primary
AATAGTAGTA	1	0	0	0	1	At3g62840.1:d:+663:secondary
ATCGTCCAGG	0	0	1	0	1	At3g62810.1:d:+260:primary
AGAAGAACAC	1	0	0	0	1	At3g62760.1:v:+418:secondary,At5g22330.1:d:+1500:secondary,At2g12730.1:p:+1581:secondary
TCTTGTAATT	0	1	0	1	0	At3g62740.1:v:+314:secondary
ATCTAAGGTC	0	1	0	1	0	At3g62700.1:d:+4141:primary
GACTCTTTGG	0	1	1	0	0	At3g62630.1:d:+1171:primary
ATTAGGTTTT	0	1	0	1	0	At3g62600.1:d:+1292:secondary
GATTATTTTT	0	0	1	1	0	At3g62590.1:d:+2175:primary
ACCATCGATA	1	0	1	0	0	At3g62580.1:d:+521:secondary
ATAAAACCTT	0	2	0	0	0	At3g62520.1:p:+304:secondary,At4g27597.1:p:+304:secondary
CTAATTGTTG	0	1	0	1	0	At3g62460.1:d:+676:primary
GGTGTGAATG	1	0	1	0	0	At3g62380.1:v:+1722:secondary
TCGAAGGAAA	1	1	0	0	0	At3g62360.1:d:+3312:secondary,At1g34050.1:v:+1861:secondary,At1g24590.1:v:+1129:secondary
GAAATAAGCA	0	2	0	0	0	At3g62300.1:d:+2306:primary
AGTTTGGAAA	0	1	1	0	0	At3g62130.1:d:+923:secondary,At2g43750.1:d:+744:secondary
GTTGCAACCG	1	1	0	0	0	At3g62030.1:d:+1086:primary
TACTTTTGTC	0	0	0	0	2	At3g62000.1:d:+1054:primary
AAATCTGGAT	0	2	0	0	0	At3g61860.1:d:+184:primary
TCAATTTTTC	1	0	0	1	0	At3g61830.1:d:+2201:primary
GAGCGCGTGT	0	1	1	0	0	At3g61820.1:d:+815:secondary
TCAAAACCTT	0	0	2	0	0	At3g61810.1:v:+601:secondary,At1g44980.1:v:+245:secondary,At1g68940.1:v:+3468:secondary,At3g27980.1:v:+440:secondary
GGAGAACGAT	0	0	1	1	0	At3g61780.1:v:+3591:secondary
CTCGTCGTTT	1	0	0	1	0	At3g61720.1:v:+2580:secondary
AACAGAGATG	0	1	0	0	1	At3g61700.1:v:+546:secondary,At4g02820.1:d:+1160:secondary
TCGGGTATGG	0	2	0	0	0	At3g61700.1:v:+1181:primary
GGCGGTAATG	0	1	1	0	0	At3g61690.1:d:-4185:secondary
ATCATCGGTT	1	0	0	1	0	At3g61690.1:d:+4190:primary
AAAGAGGTTT	0	2	0	0	0	At3g61670.1:d:+2118:primary
GGATGTGGAT	0	1	1	0	0	At3g61630.1:d:+997:primary
CAGTGGTGTT	0	0	0	2	0	At3g61620.1:d:+298:primary
GGTGGACCTT	0	0	0	2	0	At3g61600.2:d:+279:secondary,At3g61600.1:d:+279:secondary
ACTTTTCGAT	0	1	1	0	0	At3g61600.1:d:+2168:primary
GCTCTTTTTT	0	0	2	0	0	At3g61580.1:d:+1795:primary
ATCTTCTGGG	1	1	0	0	0	At3g61530.1:d:+822:primary,At3g61530.2:d:+1164:primary
TTTGGCCTAA	0	0	2	0	0	At3g61480.1:X:+274:quaternary,At5g28350.1:X:+274:quaternary
GTGCTTGGTA	1	0	0	0	1	At3g61370.1:d:+594:secondary
AGGAAGAGCC	0	2	0	0	0	At3g61300.1:d:+2379:secondary
GAAACAAACG	0	0	2	0	0	At3g61270.1:d:+452:secondary
ATAAATAGAT	0	0	1	0	1	At3g61240.1:d:-2230:secondary
CAAGGAAATG	0	2	0	0	0	At3g61160.2:d:+1502:primary,At3g61160.1:d:+1612:primary
AGAATGCTTT	0	1	1	0	0	At3g61150.1:d:+677:secondary
CCAGATCGGC	0	1	1	0	0	At3g61140.1:d:+1356:secondary
GACATTATTG	0	1	1	0	0	At3g61070.1:d:+799:primary
ATAAGTGAAT	0	2	0	0	0	At3g61010.1:X:-14:quaternary
TTTTTTGATA	0	0	0	0	2	At3g60870.1:v:+1413:secondary
ACAGGATTCG	1	1	0	0	0	At3g60830.1:d:+1092:primary
GAGAATGTCT	0	1	1	0	0	At3g60680.1:d:+1640:primary
TGGGGATGTT	0	0	1	1	0	At3g60620.1:X:+431:quaternary
GAGTTTGATT	1	1	0	0	0	At3g60620.1:d:+1324:secondary
TGTCTTATTA	0	0	1	1	0	At3g60520.1:X:-364:quaternary
ACATTTAGGA	0	0	0	2	0	At3g60500.1:d:+663:secondary,At3g60500.2:d:+685:secondary
CGATGAGTTT	0	0	0	1	1	At3g60470.1:v:-1369:secondary
ATCCTGTCAG	0	2	0	0	0	At3g60450.1:d:+799:primary
TGTTTTGATG	1	0	0	0	1	At3g60440.1:d:+1154:primary
AGACTCAGAA	0	2	0	0	0	At3g60320.1:d:+1783:secondary

CCGGAGAAGA	0	1	0	0	1	At3g60220.1:d:+1251:primary
TAACCAATGG	1	0	0	0	1	At3g60150.1:v:-1103:secondary
GTGGGACGAA	0	0	0	2	0	At3g60130.1:d:+1590:primary
TTCAATTGTT	1	0	0	1	0	At3g60080.1:d:+1387:primary
AGCAGATGAG	0	1	0	1	0	At3g60080.1:d:+1118:secondary
CTGCAGGATC	0	2	0	0	0	At3g60070.1:d:+449:secondary,At5g05200.1:d:+1610:secondary
GTTGAGATGC	0	2	0	0	0	At3g60030.1:d:+2350:primary
GACGTTGGGG	1	0	1	0	0	At3g59970.2:d:+1137:primary
AATTCGTAAT	0	0	2	0	0	At3g59950.2:d:+2032:secondary,At3g59950.1:d:+2266:secondary
ATGTTCAACA	0	1	1	0	0	At3g59950.1:d:+1340:secondary,At3g59950.2:d:+1336:secondary
AAGGATCATT	1	0	0	0	1	At3g59920.1:d:-1584:secondary
AAAGCGGACG	0	0	1	1	0	At3g59910.1:d:+2002:primary
CCAAGTTAAA	1	0	0	1	0	At3g59890.2:d:+1214:primary,At3g59890.1:d:+1182:primary
GTTTTTCAGT	0	0	0	1	1	At3g59790.1:v:-1717:secondary
AGATGAAAAA	1	0	0	0	1	At3g59670.1:v:+2290:secondary,At1g13410.1:v:+1300:secondary
TCACCAAACA	1	1	0	0	0	At3g59650.1:d:+349:primary
GGAAGCTATT	0	1	0	0	1	At3g59640.1:d:+375:primary
AAATTCTCTT	0	0	0	1	1	At3g59530.2:X:+509:quaternary
CTACCAACAG	1	0	0	0	1	At3g59520.1:d:+1374:primary
AAACTCAAAC	1	1	0	0	0	At3g59430.2:X:-170:quaternary,At1g22460.1:X:--8:quaternary,At5g18970.1:X:-126:quaternary,At3g60210.1:X:-174:quaternary
ATAGAAGTTG	0	2	0	0	0	At3g59410.1:i:+242:tertiary
GTTTAAAATT	1	0	0	1	0	At3g59340.1:i:+775:tertiary
GTAAAAAGAG	1	0	1	0	0	At3g59290.1:d:+3360:primary
ATTTGAGATC	0	0	1	0	1	At3g59280.1:i:+321:tertiary,At4g34412.1:i:+1143:tertiary
TGAATTTTTA	0	0	1	0	1	At3g59230.1:v:+311:secondary
TTTTCATTTG	2	0	0	0	0	At3g59230.1:v:+2181:primary
AGATTGGTAG	0	1	0	1	0	At3g59150.1:d:+1662:primary
ATGCTAAACT	1	0	1	0	0	At3g59090.1:d:-1508:secondary,At3g59090.2:d:-1425:secondary
TGTCAATACT	1	0	0	0	1	At3g59060.2:X:+318:quaternary
TAATATAACT	0	1	0	1	0	At3g59050.1:X:--69:quaternary
GATGCGTCTG	0	1	1	0	0	At3g59040.2:d:+1798:primary,At3g59040.1:d:+1421:primary
AGCAGTCCTT	0	2	0	0	0	At3g58790.1:d:+1317:secondary
GTCTAATTCC	0	1	0	0	1	At3g58780.1:i:+1901:tertiary,At4g27830.1:i:+2441:tertiary
ATGTCATATA	1	1	0	0	0	At3g58760.1:d:-1557:secondary
TACTTTTGAT	0	1	1	0	0	At3g58750.1:d:+1977:primary
AACTGAAGTA	1	0	0	0	1	At3g58650.1:d:+2342:primary
CAAAAAGGCC	1	0	1	0	0	At3g58620.1:X:+332:quaternary; +small RNA (TTACATGCAAAAAGGCCAATAAG)
TTGGTTTAAA	0	2	0	0	0	At3g58620.1:d:+1547:secondary
GTTGACAACT	0	0	2	0	0	At3g58610.1:d:+1631:secondary,At2g14630.1:v:+354:secondary
GCCTCAATTC	0	2	0	0	0	At3g58500.1:d:+1020:secondary
AACAGCAAAA	1	0	0	1	0	At3g58470.1:X:--51:quaternary
TGTGTTGGAT	1	0	1	0	0	At3g58470.1:d:+897:primary
AAAGATTCCT	0	0	0	2	0	At3g58290.1:X:--2:quaternary
GTGGAGAGGA	1	0	1	0	0	At3g58180.1:d:+691:secondary,At3g62530.1:d:+425:secondary
TTGTTAATTG	0	1	1	0	0	At3g58160.1:v:+4265:secondary
GCGAAAAGAT	1	0	1	0	0	At3g58120.1:d:+1273:primary
GTTTGAGAAA	0	1	1	0	0	At3g58020.1:d:+716:primary
ATATCTTATC	1	1	0	0	0	At3g58010.1:d:+831:primary,At2g42130.4:d:+769:primary,At2g42130.2:d:+836:primary,At2g42130.3:d:+832:primary,At2g42130.5:d:+1135:primary
TTATCAACGA	0	0	0	0	2	At3g57980.1:v:+98:secondary
AGAAGCTTTC	0	0	1	1	0	At3g57940.1:d:+1389:secondary,At4g00490.1:d:+831:secondary,At4g27100.1:v:+1938:secondary
TAGCCACTCT	1	0	1	0	0	At3g57830.1:d:+2392:primary
ATTCAAGTGA	1	0	0	1	0	At3g57760.1:d:+1208:primary
TGTCTGAGGT	0	1	0	1	0	At3g57750.1:d:+817:primary
CGTTGTACTT	0	1	0	1	0	At3g57720.1:d:+1790:primary
CAACCTTTTC	0	1	0	1	0	At3g57700.1:v:+1379:secondary
GAGACGAGAG	0	1	1	0	0	At3g57630.1:d:+2350:primary,At3g57630.2:d:+2376:primary,At3g53040.1:d:+1347:primary
TTCATTTTTT	0	0	0	1	1	At3g57600.1:d:+1069:primary
GGACGCTTTC	0	1	1	0	0	At3g57520.1:d:+837:secondary,At3g57520.3:d:+837:secondary,At3g57520.2:d:+837:secondary
AATAGTAAAC	0	1	0	1	0	At3g57470.1:d:+2444:primary

GGTAGATATT	1	0	0	0	1	At3g57460.1:i:+2148:tertiary
TAGACACTGT	0	2	0	0	0	At3g57410.1:X:-288:quaternary
TCACAATGAG	0	0	0	0	2	At3g57350.1:v:+3340:primary
CTGCTATTCT	0	2	0	0	0	At3g57300.1:d:+1240:secondary
AGAAATTGTT	2	0	0	0	0	At3g57200.1:i:+1429:tertiary
CATCAATTAT	0	2	0	0	0	At3g57130.1:v:+903:secondary,At5g04620.1:d:+1042:secondary,At5g04620.2:d:+672:secondary,At2g41370.1:d:+805:secondary
CAAGCATACC	0	1	0	1	0	At3g57050.3:d:+1469:primary,At3g57050.1:d:+1406:primary,At3g57050.2:d:+1338:primary
GATATGAGTT	1	0	0	0	1	At3g57030.1:d:+1251:secondary,At1g12640.1:d:+126:secondary,At3g44240.1:v:+465:secondary
CGAGTCCGGC	0	0	2	0	0	At3g57020.1:d:+737:secondary
CATATCGAAC	1	0	0	1	0	At3g56940.1:d:-1000:secondary
CCAATTGGCT	2	0	0	0	0	At3g56860.3:d:+729:secondary,At1g22300.1:d:+678:secondary,At3g56860.2:d:+729:secondary,At1g22300.2:d:+678:secondary,At3g56860.1:d:+729:secondary,At1g22300.3:d:+678:secondary
ATACAATGCT	0	1	0	1	0	At3g56800.1:X:-644:quaternary
AAGGCTTTCT	0	1	0	1	0	At3g56780.1:v:-584:secondary
TCAAATCTTC	0	1	0	1	0	At3g56720.1:d:+851:secondary
GTCGCTATAA	0	1	0	1	0	At3g56630.1:d:-1562:secondary
TGTGAGATAC	1	0	1	0	0	At3g56580.2:d:-1352:secondary,At3g56580.1:d:-1478:secondary
TTAGCTTGTT	0	0	0	0	2	At3g56580.2:d:+1357:primary,At3g56580.1:d:+1483:primary
AATTGACCTT	0	1	0	1	0	At3g56500.1:v:+116:secondary
CAGCTCTGTG	0	0	1	1	0	At3g56480.1:d:+1602:primary
GCTCTCTTCT	0	1	0	0	1	At3g56420.1:v:+900:primary
TTTTTTCACC	1	0	1	0	0	At3g56370.1:d:+3136:primary
AATAAAGCTT	0	0	0	0	2	At3g56260.1:d:+771:secondary
CGTCCTAATA	0	1	0	0	1	At3g56190.1:i:+1052:tertiary
ATGAGCTACT	1	0	1	0	0	At3g56170.1:d:+732:secondary
GATCCAAGTG	0	0	1	1	0	At3g56160.1:d:+933:secondary
ATTGATTCAG	0	1	1	0	0	At3g56160.1:d:+1240:primary
GCGAGACCTG	0	2	0	0	0	At3g56110.1:d:+320:primary
TGACTTTACC	0	1	0	1	0	At3g56050.1:d:+567:primary
TTTGCTACTA	0	1	1	0	0	At3g56010.1:d:+620:secondary
ATTGGACCGA	0	0	2	0	0	At3g55980.1:d:+853:secondary,At1g21130.1:d:+970:secondary,At1g21100.1:d:+946:secondary,At1g21110.1:d:+970:secondary,At1g21130.2:d:+1058:secondary,At1g21120.1:d:+945:secondary
GGTTTGGTTA	0	2	0	0	0	At3g55940.1:v:+2204:primary
GAAGTAGTGC	0	0	1	0	1	At3g55920.1:d:+620:primary
ATGATGCAGT	1	0	1	0	0	At3g55850.1:d:+1796:primary,At3g55850.2:d:+2044:primary
CATTCACTTA	0	1	0	1	0	At3g55810.1:v:+2089:primary
TACCACAAGA	0	1	1	0	0	At3g55760.2:d:+1844:primary,At3g55760.1:d:+1730:primary
ATCTGTGTGT	0	1	1	0	0	At3g55600.1:d:+679:primary
CGGTTGGTGG	0	0	2	0	0	At3g55580.1:d:+1491:primary
ATCAAAGATG	0	1	1	0	0	At3g55540.1:v:+363:secondary
TGAAAGAGCT	0	2	0	0	0	At3g55470.1:d:+396:secondary
ACCCTTGTAC	1	1	0	0	0	At3g55450.1:d:-821:primary
GTTTCTGTTC	0	2	0	0	0	At3g55400.1:d:+1562:primary
GATACGAGCT	0	0	0	1	1	At3g55380.1:d:+409:primary
ACTCTTCTCA	0	0	1	1	0	At3g55290.2:d:+781:primary,At3g55290.1:d:+793:primary
AAAACGATTT	1	1	0	0	0	At3g55240.1:d:+400:secondary
AAACAGTTGC	0	0	1	0	1	At3g55220.1:v:+4175:secondary
ATATGGAGAA	0	1	0	0	1	At3g55210.1:v:+1096:primary
AGAGCGGAAC	1	1	0	0	0	At3g55050.1:d:+1116:primary,At3g55050.2:d:+1233:primary
GTTAGTGACA	0	2	0	0	0	At3g55030.1:d:+505:secondary,At1g19450.1:d:+1557:secondary
TCACTGATTG	0	2	0	0	0	At3g54830.1:v:+1800:secondary
ATCTTCATCC	0	0	1	1	0	At3g54820.1:d:+364:secondary,At2g37170.1:d:+328:secondary,At2g37180.1:d:+332:secondary
ACATTTCACT	0	1	0	1	0	At3g54710.1:d:+1486:primary
TTTAACAAAA	0	0	1	1	0	At3g54670.1:i:+1902:tertiary
CCTTTCCGTT	1	0	1	0	0	At3g54550.1:v:+354:secondary
AGTAAAGAGG	1	0	1	0	0	At3g54530.1:X:--54:quaternary
CCCTTTTGTA	0	0	1	1	0	At3g54500.2:d:+2106:secondary
TATTCCAAGG	0	1	0	1	0	At3g54480.1:d:+869:primary

CTTGGTGACT	0	1	1	0	0	At3g54360.1:d:+870:secondary
TGGGAGATGA	0	0	0	2	0	At3g54300.1:X:-315:quaternary
GAAGAACTTA	0	0	0	2	0	At3g54300.1:d:+917:secondary
ACATTTGGTG	0	2	0	0	0	At3g54290.1:d:+1196:primary
AACAATGGTG	1	0	1	0	0	At3g54260.1:d:+1188:primary
TCTGACAATA	0	2	0	0	0	At3g54230.1:v:+3654:secondary
TCGTGGAACA	1	1	0	0	0	At3g54220.1:d:+2074:primary
TGTGGTCCAA	0	0	2	0	0	At3g54190.1:d:+1665:primary
GGAAAACAGT	1	1	0	0	0	At3g54100.1:d:+2253:primary
TGTGTAAGCT	1	1	0	0	0	At3g54080.1:d:+1309:secondary
CGGCTATAGG	0	1	0	1	0	At3g54040.1:d:+197:secondary,At2g30150.1:v:+1312:secondary
GGAAGATCTC	1	0	1	0	0	At3g54030.1:d:+1847:primary
TAAATTTGGC	0	1	1	0	0	At3g54010.2:d:-2126:secondary,At3g54010.1:d:-2056:secondary
TACCTTATAA	0	0	1	0	1	At3g53980.1:d:+491:primary,At3g53980.2:d:+456:primary
ACATTCGAGG	0	0	1	1	0	At3g53940.1:d:+1259:primary
ATCTTCTAGA	0	1	0	1	0	At3g53920.1:d:+1640:primary
TATATGCTGG	1	1	0	0	0	At3g53900.1:d:+733:primary,At3g53900.2:d:+817:primary
CTTCAGTTCA	0	1	0	0	1	At3g53890.1:d:+174:secondary,At5g61500.1:d:+895:secondary
AGACGGAAAA	0	0	2	0	0	At3g53880.1:i:-207:tertiary
TAGCCATCTT	1	0	1	0	0	At3g53870.1:d:-520:secondary
ACGACTCAGA	0	0	0	1	1	At3g53870.1:d:+57:secondary,At5g47690.1:v:+5167:secondary
TTATAGTAGC	0	1	0	0	1	At3g53860.1:d:+766:primary,At2g31600.1:d:+818:primary,At2g31600.2:d:+874:primary
GTTAAAGTGG	0	2	0	0	0	At3g53720.1:d:+2663:secondary
TAGAGGATAT	0	0	0	2	0	At3g53720.1:d:+2544:secondary
TTAATGAAGA	0	1	0	0	1	At3g53630.1:d:+897:secondary
TGGATCACTC	1	1	0	0	0	At3g53570.3:d:+1372:primary,At3g53570.1:d:+1600:primary
GTCTAGATGC	0	0	1	1	0	At3g53560.1:d:+1267:primary
ATCTTCAAAG	0	1	1	0	0	At3g53540.1:d:+966:secondary
TCCATCCATC	1	0	0	1	0	At3g53510.1:d:+1257:secondary
GATGCTCACA	1	0	0	1	0	At3g53490.1:v:+778:secondary,At2g44710.1:d:+1240:secondary,At4g21326.1:v:+1211:secondary
TGTATGTGTA	0	2	0	0	0	At3g53410.1:d:+830:secondary,At5g03200.1:d:+1046:secondary
TAACTTTTGT	1	0	0	0	1	At3g53360.1:v:+2074:secondary,At2g34680.1:d:+2180:secondary,At3g01513.1:v:+1105:secondary
GAAGCTGAGC	0	0	0	2	0	At3g53350.3:d:+1210:primary,At3g53350.2:d:+1236:primary,At3g53350.1:d:+1233:primary
AAAGAATCAA	0	1	0	1	0	At3g53330.1:v:+1738:primary
AAGGTTTGTT	1	0	1	0	0	At3g53130.1:i:+3916:tertiary,At5g14340.1:i:+222:tertiary,At2g21770.1:i:+2144:tertiary,At5g09870.1:i:+2043:tertiary,At4g21470.1:i:+716:tertiary,At3g53140.1:i:+1044:tertiary,At4g37440.1:i:+863:tertiary
AAGAAGATGG	1	1	0	0	0	At3g53060.1:v:+510:primary,At3g30187.1:p:+707:primary
AACAAAGTGC	0	2	0	0	0	At3g53030.1:d:+1392:primary
AATCCTTTCA	0	1	0	1	0	At3g52940.1:d:+1317:primary,At3g52940.2:d:+1308:primary
GGATCTGCCA	0	0	1	0	1	At3g52920.1:d:-509:primary,At3g52920.2:d:-472:primary
GATCTTACCT	1	1	0	0	0	At3g52900.1:d:+528:primary
CATTTGTTGA	0	2	0	0	0	At3g52890.1:d:+1531:secondary,At3g52890.2:d:+1091:secondary
GAGGGAGGTA	1	1	0	0	0	At3g52880.1:d:+1276:secondary
TTGGCCTTCA	0	2	0	0	0	At3g52820.1:d:+1311:primary
ATGAACATCG	0	0	1	1	0	At3g52740.1:d:+100:primary
CGCCGTAATT	0	1	1	0	0	At3g52730.1:d:+161:secondary
TGTCACGTGC	0	2	0	0	0	At3g52525.1:d:+443:primary
CTTGTTCATC	1	0	0	1	0	At3g52260.1:d:+1224:primary,At3g52260.2:d:+1210:primary
AATCTTAAAA	0	0	0	1	1	At3g52250.1:i:+522:tertiary,At2g37690.1:i:+3416:tertiary
ATCAATACCG	0	2	0	0	0	At3g52140.1:d:+4145:secondary
TAAATGATGG	2	0	0	0	0	At3g51980.1:X:+475:quaternary
ATGGTGGAAA	0	0	2	0	0	At3g51895.1:d:+1833:secondary,At2g41080.1:d:+962:secondary
ATCGGTGTTC	0	1	1	0	0	At3g51730.1:d:+409:secondary
TTTAGCTATT	0	0	2	0	0	At3g51670.1:d:+1563:primary
TTAGTGACCG	0	0	1	1	0	At3g51600.1:d:+174:secondary
CCACCTTGTT	1	0	0	1	0	At3g51530.1:d:+1490:secondary,At4g36140.1:v:+3377:secondary
TGTTCTTGCT	0	2	0	0	0	At3g51480.1:d:+2972:primary
ACACGACCGT	1	1	0	0	0	At3g51420.1:d:-640:secondary
ATCTAAACAC	0	0	0	0	2	At3g51360.1:i:+1201:tertiary

CTCCATTCTA	0	0	2	0	0	At3g51300.1:d:+892:primary
AAATGGTTTG	1	1	0	0	0	At3g51260.1:d:+940:secondary
AGAAAACAAA	1	1	0	0	0	At3g51230.1:v:+1181:primary
GCCCTCTTAT	0	0	2	0	0	At3g51140.1:d:+1366:primary
ATGCGTATCC	1	1	0	0	0	At3g51130.1:d:+1311:primary
CCGCTTATAT	1	0	0	1	0	At3g51120.1:X:-1852:quaternary
GATCAGGTTC	1	0	1	0	0	At3g51120.1:d:+3896:secondary
AGGTTTATTT	0	1	1	0	0	At3g50930.1:d:-191:primary
GCCGGCGGAG	0	0	0	2	0	At3g50890.1:d:+397:primary
GATGTACATT	0	0	0	1	1	At3g50810.1:i:+937:tertiary
GCTTAATCAT	0	2	0	0	0	At3g50800.1:d:+334:primary
GGGAGTGGTT	0	0	2	0	0	At3g50750.1:X:-804:quaternary
AGATTGAAGA	0	0	0	1	1	At3g50690.1:d:+845:secondary,At3g49430.1:d:+137:secondary,At5g65770.1:v:+2632:secondary,At5g25070.1:d:+1882:secondary,At1g76220.1:v:+1033:secondary
AAAGGAGTTG	0	2	0	0	0	At3g50660.1:d:+705:secondary
AACTCGATAT	2	0	0	0	0	At3g50560.1:d:+963:primary
GAGGTGTGAT	0	0	0	2	0	At3g50520.1:d:+526:primary
GCCAGAGAGA	0	0	2	0	0	At3g50370.1:d:+6421:secondary
GGAACAACTT	1	0	1	0	0	At3g50370.1:d:+5794:secondary,At1g71970.1:d:+284:secondary
ATTATTATTC	1	1	0	0	0	At3g50340.1:d:+1340:primary
TGAGGAAGGA	0	1	1	0	0	At3g49990.1:d:+350:secondary
CCGATCCGTA	0	0	2	0	0	At3g49910.1:d:+197:secondary
TGTTGGCGGG	0	1	1	0	0	At3g49880.1:d:+1472:primary
GCTCTCCTTC	1	1	0	0	0	At3g49780.1:d:+102:primary
TGATGAAGAA	1	1	0	0	0	At3g49640.1:d:+981:secondary
GATTTCGAGT	0	0	1	1	0	At3g49620.1:d:+1195:primary
AAGTGGCTCC	0	0	1	0	1	At3g49490.1:X:-864:quaternary
TCTTTTTTTG	1	1	0	0	0	At3g49460.1:v:+905:secondary
TACTCTCGTG	0	2	0	0	0	At3g49400.1:X:+507:quaternary,At3g49400.1:X:+513:quaternary
ACCTCGTTGT	0	2	0	0	0	At3g49350.1:d:+1962:secondary
TATGGTTATT	0	2	0	0	0	At3g49310.1:d:+1579:primary
AGGGCGTTAA	0	1	0	0	1	At3g49240.1:d:+1596:primary
AACCAGATGA	0	0	2	0	0	At3g49210.1:d:+1485:primary
TTTAAAGTTC	0	1	0	0	1	At3g49055.1:v:+109:secondary,At5g06810.1:v:+1907:secondary
TTCTCTACTC	1	1	0	0	0	At3g49050.1:d:+1977:primary
GAACTCGTCA	0	0	1	0	1	At3g49010.2:d:+541:secondary,At3g49010.1:d:+555:secondary
TCCATTTATT	0	1	1	0	0	At3g48980.1:i:+2310:tertiary,At4g33090.1:i:+1651:tertiary
TCAACAAAAA	1	1	0	0	0	At3g48940.1:v:+243:secondary,At1g58130.1:p:+169:secondary
TGTAATTTGT	0	1	1	0	0	At3g48940.1:v:+1050:secondary,At5g56720.1:v:+354:secondary
GCTGATGTTG	0	0	1	1	0	At3g48930.1:X:-75:quaternary
AGCAAGCTCA	1	1	0	0	0	At3g48770.1:X:-342:quaternary,At1g26230.1:X:-20:quaternary
CGATGTGTGG	0	0	2	0	0	At3g48730.1:d:+1249:secondary
AGCCTAACAG	0	0	1	1	0	At3g48710.1:d:-1565:secondary
GAAAAAGGTG	0	2	0	0	0	At3g48470.1:d:+2316:secondary,At4g28050.1:d:+801:secondary,At4g17120.1:v:+4014:secondary
GATTCTGGTT	0	0	1	1	0	At3g48280.1:d:+1557:primary
CCATCATAAT	0	0	0	0	2	At3g48190.1:d:-11483:secondary
GAAACGTGTC	0	2	0	0	0	At3g48110.2:X:+474:quaternary
TTTCAGATGA	1	0	1	0	0	At3g48070.1:d:-1310:secondary,At3g03790.2:d:-2433:secondary,At3g03790.1:d:-2424:secondary
TCTGGTTCTA	0	2	0	0	0	At3g48030.1:X:-19:quaternary
ATCGAGATGT	0	0	1	1	0	At3g48020.1:v:+158:secondary,At1g70230.1:d:+1291:secondary
AGAAAGTGTA	0	1	0	1	0	At3g48000.1:d:+1217:secondary
CTTTGGAATA	0	2	0	0	0	At3g47920.1:v:+1321:secondary,At1g70505.1:v:+1491:secondary
GCTGAGTGAA	0	1	0	0	1	At3g47870.1:v:+1787:primary
TATATTACTG	0	0	1	0	1	At3g47780.1:X:+598:quaternary
ATTTGCTATG	1	0	0	0	1	At3g47780.1:d:+2120:secondary,At1g52730.2:d:+1268:secondary,At1g52730.1:d:+1414:secondary
AGTTAATAGA	1	0	0	1	0	At3g47700.1:d:+2597:primary
CCACACAACC	0	0	1	1	0	At3g47650.1:d:-282:primary
GACTCAAAAA	0	0	2	0	0	At3g47650.1:d:+492:secondary,At3g43357.1:p:+2282:secondary,At4g15093.1:d:+177:secondary
TTCTCAATGC	0	1	1	0	0	At3g47650.1:d:+287:secondary

AGTGAGAATT	1	0	0	1	0	At3g47620.1:X:--40:quaternary
ATTTAAAAGG	1	0	0	0	1	At3g47590.1:X:-215:quaternary
GTTTAGTTAG	0	0	0	1	1	At3g47460.1:i:+3634:tertiary
ACCTGGTCCA	0	2	0	0	0	At3g47390.1:i:+1929:tertiary
CAAAACAGAA	2	0	0	0	0	At3g47370.1:i:+818:tertiary,At3g47370.2:i:+799:tertiary
TGTTAATATA	1	0	0	0	1	At3g47300.1:d:+757:primary
GGGAGAAGAA	0	0	2	0	0	At3g47290.1:d:+1380:primary
TAAAGCCAAA	0	0	1	1	0	At3g47090.1:v:+3718:primary,At3g07600.1:d:+706:primary
TTCTTATTCG	1	1	0	0	0	At3g47080.1:X:-471:quaternary,At2g10400.1:X:-80:quaternary,At4g03981.1:X:-205:quaternary
TACTGTGTTT	1	0	0	1	0	At3g47010.1:i:+727:tertiary
TTTAGTGATT	0	1	0	0	1	At3g46950.1:d:+1568:secondary
GCGATTCATC	1	0	0	1	0	At3g46940.1:d:+147:secondary,At1g33990.1:d:+483:secondary
AAAGAGCTGA	1	1	0	0	0	At3g46930.1:d:+1587:primary
CCTATGTCCC	0	1	0	0	1	At3g46840.1:v:+498:secondary
TCAGAGATAC	0	0	0	1	1	At3g46820.1:d:+1468:secondary
AATTATATAT	0	1	0	0	1	At3g46800.1:v:+2494:primary
CAATGCATTA	0	2	0	0	0	At3g46700.1:v:+1466:secondary
TATCATCTAT	0	1	0	0	1	At3g46690.1:d:+467:secondary,At5g12350.1:d:+1205:secondary
GGTTAGAGGA	0	0	1	1	0	At3g46590.1:d:+165:secondary
TCCTCCTCCA	0	1	0	0	1	At3g46550.1:d:+290:primary,At2g33630.1:d:+1956:primary
AAGTCTTACA	0	1	1	0	0	At3g46540.1:d:+775:primary
GAAATGTTTG	0	0	1	0	1	At3g46310.1:v:+677:secondary
TTAATGTTGT	1	0	0	0	1	At3g46270.1:X:-94:quaternary
GTCGTCAGGT	1	0	0	1	0	At3g46130.1:v:+1109:primary
ATAAGTTTCT	1	0	0	0	1	At3g46070.1:v:+1148:primary
TTCATCAAAT	1	0	1	0	0	At3g45640.1:d:+882:secondary
TTTTAAAGAA	1	0	1	0	0	At3g45450.1:v:+1559:secondary
TAATTGAGAG	0	0	0	2	0	At3g45440.1:X:-1357:quaternary
ACCATCGCCA	0	1	0	1	0	At3g45420.1:v:+2572:secondary
GATGATGGAT	1	0	0	1	0	At3g45280.1:d:+499:secondary,At5g17930.1:v:+747:secondary,At5g28487.1:p:+3367:secondary,At1g03670.1:v:+2245:secondary,At2g16330.1:v:+354:secondary,At5g37830.1:d:+3101:secondary,At3g42620.1:v:+143:secondary,At1g45080.1:v:+354:secondary,At2g05480.1:v:+354:secondary,At2g16160.1:v:+354:secondary,At2g12140.1:v:+354:secondary
GTTCTAATGA	0	2	0	0	0	At3g45190.1:v:+2107:primary
GGTCACCTCA	0	0	0	1	1	At3g45140.1:d:+2247:secondary
GTAATTGGTA	0	0	1	0	1	At3g45100.1:d:+1638:primary
AATTGTTCTT	0	0	1	1	0	At3g45090.1:i:+834:tertiary,At3g45090.2:i:+786:tertiary
ATGTACAATG	0	0	1	0	1	At3g45050.4:d:-765:secondary,At3g45050.3:d:-936:secondary,At3g45050.2:d:-789:secondary
AGCACCGAGC	1	1	0	0	0	At3g45050.2:d:+794:secondary,At3g45050.4:d:+770:secondary,At3g45050.3:d:+941:secondary
TAATAAATAC	1	0	0	0	1	At3g44970.1:v:+2021:primary
TCGCAAAAGT	0	1	0	0	1	At3g44730.1:v:+3775:secondary
AGAAGTTGGC	0	1	0	1	0	At3g44713.1:v:+2367:primary
TTTTCGTCTT	1	0	0	1	0	At3g44680.1:d:+1573:primary
CTTGTTAAGG	0	1	0	0	1	At3g44630.1:d:+3380:secondary,At3g02360.2:d:+511:secondary,At3g02360.1:d:+356:secondary,At3g44400.1:v:+3126:secondary,At3g44480.1:d:+3374:secondary,At3g44670.1:d:+3179:secondary,At3g44630.3:d:+3442:secondary,At3g44630.2:d:+3439:secondary
AGGTCCCTAA	0	2	0	0	0	At3g44510.1:d:+470:primary
GTGGAGAAGA	2	0	0	0	0	At3g44425.1:p:+655:secondary
TGCTCTGTAT	1	1	0	0	0	At3g44370.1:v:+1783:primary
TATGTGTTTA	1	0	1	0	0	At3g44340.1:i:+5929:tertiary
AACAACTTCT	0	0	0	2	0	At3g44325.1:p:+2963:secondary,At4g17430.1:d:+31:secondary
AAGTTTCGTT	0	1	0	1	0	At3g44280.1:d:+138:primary
AGTTTCGGTA	0	1	1	0	0	At3g44100.1:X:-70:quaternary
ATAATCGAGA	0	2	0	0	0	At3g44050.1:d:+3439:primary
AAGCTAGCCG	0	1	0	1	0	At3g44045.1:p:+117:secondary,At1g12935.1:p:+96:secondary,At4g13455.1:p:+117:secondary,At3g47165.1:p:+116:secondary,At3g42883.1:p:+2258:secondary,At2g04130.1:p:+1479:secondary,At4g08405.1:p:+1550:secondary,At4g12426.1:p:+2359:secondary,At5g56605.1:p:+225:secondary,At3g50490.1:p:+117:secondary,At4g17820.1:p:+2403:secondary,At4g17005.1:p:+42:secondary,At5g28466.1:p:+363:secondary,At4g20180.1:p:+2944:secondary,At5g36685.1:p:+132:secondary,At5g36775.1:p:+136:secondary,At2g44790.1:d:+432:secondary
ACCAGAAAAA	1	0	0	0	1	At3g44040.1:v:+648:secondary

GAGTTGATGA	1	0	1	0	0	At3g44030.1:p:+727:secondary,At3g30520.1:v:+715:secondary,At1g43720.1:v:+439:secondary,At1g35820.1:v:+631:secondary
AAGTTGGTGC	0	0	0	1	1	At3g44010.1:X:-22:quaternary
TAATAGTTGA	0	2	0	0	0	At3g43860.1:X:+885:quaternary
AGAAAGGTGA	0	1	1	0	0	At3g43625.1:p:+326:secondary,At2g41570.1:v:+988:secondary,At3g57586.1:p:+638:secondary
ATCCAAGAAA	0	0	1	1	0	At3g43573.1:p:+4590:primary
TTTCCTACCT	0	2	0	0	0	At3g43330.1:i:+1997:tertiary,At5g56700.1:i:+1058:tertiary,At5g06390.1:i:+1943:tertiary
GAACACATCC	0	0	0	1	1	At3g43240.1:X:-27:quaternary
GGATGACCAA	0	0	1	0	1	At3g43220.1:i:+4347:tertiary
TTGGTTTGAT	0	1	0	1	0	At3g43126.1:p:+1485:secondary,At3g32226.1:p:+1480:secondary,At5g29580.1:p:+1065:secondary,At3g30680.1:p:+1479:secondary,At5g59640.1:p:+1232:secondary,At4g03900.1:p:+1479:secondary,At3g30335.1:p:+1373:secondary,At5g45085.1:p:+1479:secondary,At4g06569.1:p:+193:secondary,At3g32230.1:p:+2761:secondary,At5g29056.1:p:+1474:secondary,At1g35590.1:p:+1479:secondary,At4g08000.1:p:+1176:secondary,At5g33234.1:p:+1373:secondary,At3g30790.1:p:+1871:secondary,At2g11670.1:p:+1463:secondary,At1g42510.1:p:+1516:secondary,At3g42305.1:p:+321:secondary,At1g36200.1:p:+1479:secondary,At1g40123.1:p:+1359:secondary,At5g28926.1:p:+1479:secondary
TTGAAGAAAA	0	1	1	0	0	At3g43050.1:p:+1657:secondary,At5g03750.1:v:+182:secondary,At1g10760.1:d:+2273:secondary
AAATTACCTT	1	1	0	0	0	At3g42860.1:d:+1394:primary
TTCTTGTCTA	1	1	0	0	0	At3g42837.1:p:+1571:secondary,At1g13195.1:d:+492:secondary
AAAGGACATT	0	2	0	0	0	At3g42791.1:p:+30:secondary,At3g54930.1:v:+1650:secondary
GAACCACTTC	0	0	1	1	0	At3g42790.1:X:-167:quaternary
GTGGAAGGAA	0	0	2	0	0	At3g42722.1:v:+1031:primary
GTGAAACAGA	0	0	0	1	1	At3g42660.1:v:+3376:secondary
AACTCAAAAA	0	0	1	1	0	At3g42653.1:p:+1263:secondary,At3g30790.1:p:+1663:secondary
CCTGTATTTA	0	1	1	0	0	At3g42630.1:d:+1437:primary
TTCTGACAAA	0	0	1	0	1	At3g42553.1:p:+942:secondary,At2g05960.1:p:+933:secondary
ATATGCAGCA	1	0	1	0	0	At3g42438.1:p:-1707:secondary,At2g26660.1:v:-1385:secondary
TGGAAAGTTG	0	2	0	0	0	At3g42383.1:p:+1007:secondary,At5g34865.1:p:+1172:secondary
GAAAAATCAA	0	1	1	0	0	At3g42353.1:p:+438:primary
GAAGAGCTAT	1	0	0	1	0	At3g42150.2:d:-327:primary,At3g42150.1:d:-542:primary
AAGCGTTTTC	0	0	0	2	0	At3g42086.1:p:+1940:secondary
CCTATCTCTT	0	2	0	0	0	At3g33235.1:p:+2245:primary
GTAAAACTTG	0	1	1	0	0	At3g33035.1:p:+1327:secondary,At5g51890.1:d:+875:secondary,At2g06060.1:p:+339:secondary
AAACAGAAGA	1	0	1	0	0	At3g32964.1:p:+2635:secondary,At3g02710.1:d:+1017:secondary,At1g74860.1:d:+1224:secondary,At2g25730.1:d:+2990:secondary,At5g07380.1:v:+1405:secondary
TTTCTGCAAT	0	2	0	0	0	At3g32940.1:d:+2006:primary
ACACATCATT	0	0	0	1	1	At3g32940.1:d:+1134:secondary
ATCTCTATCC	0	1	0	1	0	At3g32425.1:X:-2784:quaternary,At1g51730.1:X:-127:quaternary
GCTAAAGCCA	0	0	1	0	1	At3g32375.1:p:+715:secondary,At1g54680.1:v:+1152:secondary,At3g31906.1:p:+705:secondary,At1g16080.1:d:+81:secondary
AGTTTGAAGC	0	1	0	1	0	At3g32164.1:p:+4688:primary,At5g32060.1:p:+4834:primary
AAGGAGACAC	0	2	0	0	0	At3g32043.1:p:+4768:secondary,At1g20150.1:v:+763:secondary,At3g04260.1:d:+1823:secondary
AAGCTGTTGG	1	1	0	0	0	At3g32029.1:p:+618:secondary,At3g25520.1:d:+684:secondary,At3g51570.1:v:+3277:secondary,At5g39740.1:d:+664:secondary
AAAACATTGG	1	1	0	0	0	At3g32022.1:p:+3140:secondary,At3g04070.1:d:+447:secondary
AAATTCGAAT	0	0	1	1	0	At3g31945.1:p:+3685:secondary
GAGGTTTGAT	1	1	0	0	0	At3g31904.1:p:+5977:secondary,At2g14300.1:p:+4654:secondary,At3g30560.1:v:+4378:secondary,At3g32320.1:p:+3234:secondary,At3g32373.1:p:+4615:secondary,At4g03690.1:v:+1666:secondary
GTTACAAAAA	0	0	0	2	0	At3g31395.1:p:+543:secondary,At1g61690.1:d:+212:secondary,At5g59580.1:d:+663:secondary
GCTCAACACC	0	1	0	1	0	At3g31370.1:v:+587:secondary,At1g22540.1:d:+2009:secondary
TACAAAAAAC	0	0	1	1	0	At3g31365.1:X:-2514:quaternary
GGAGAACCTT	0	0	1	0	1	At3g30836.1:p:+1181:secondary
CAGTCCAGGA	1	0	0	1	0	At3g30821.1:p:+661:secondary
GAAAACTCTA	1	0	0	0	1	At3g30737.1:p:+4161:primary
CTGGTAAAAA	0	0	0	0	2	At3g30737.1:p:+3981:secondary,At4g21450.2:d:+1385:secondary,At4g21450.1:d:+1124:secondary
GAAGGTGGCT	0	2	0	0	0	At3g30727.1:p:+1664:primary
GGTCGGGCTT	0	2	0	0	0	At3g30720.1:d:+353:primary
GTCTAGTTGG	0	1	1	0	0	At3g30710.1:X:-196:quaternary
AGAAGAAAGC	0	1	0	1	0	At3g30465.1:p:+4860:primary
TAGTATTTTC	0	0	0	0	2	At3g30460.1:X:+472:quaternary,Chr1:+25623981:quaternary

TCTGAATCAA	0	2	0	0	0	At3g30418.1:p:+5377:secondary
TAGAAGAAAC	0	0	2	0	0	At3g30411.1:p:+3318:secondary
CCTAGTTTTT	0	2	0	0	0	At3g30310.1:v:+1090:secondary
AAAAACTTCT	0	0	0	1	1	At3g30145.1:p:+4301:secondary,At3g06280.1:v:+266:secondary
TCTTTGAGAA	1	0	0	0	1	At3g29800.1:v:+2160:primary
TCTTACTTTT	0	1	0	1	0	At3g29720.1:v:+164:secondary
AGATGCCAAT	0	0	1	1	0	At3g29642.1:p:-1321:secondary
GGCTCACAAC	0	0	2	0	0	At3g29590.1:v:+881:secondary,At1g70770.1:d:+1073:secondary
ATTGATCGTT	0	0	0	1	1	At3g29590.1:v:+1901:primary
GTTATACAAA	0	1	0	0	1	At3g29400.1:X:-1030:quaternary
TGGAGATTAT	0	2	0	0	0	At3g29320.1:d:+1446:secondary,At4g30700.1:d:+2383:secondary
AGGAAAAAGG	0	0	2	0	0	At3g29290.1:d:+815:secondary
GATGCTTGTT	0	1	1	0	0	At3g29240.2:d:+178:secondary,At4g12070.1:d:+1722:secondary,At3g29240.1:d:+165:secondary
TTCAATGGTT	0	0	0	2	0	At3g29160.3:d:+1653:primary,At3g29160.2:d:+1703:primary
ATGAAACTTG	0	0	0	2	0	At3g29130.1:X:-244:quaternary,At2g43945.1:X:--129:quaternary
TGTATTACTT	1	0	1	0	0	At3g29130.1:v:+1055:secondary
GAGTGGAAGA	0	2	0	0	0	At3g28930.1:d:+456:primary
AGACGTATCT	1	0	0	1	0	At3g28865.1:p:+5035:secondary
TCCTGATTGT	1	0	1	0	0	At3g28850.1:d:+1236:primary
TACTACTATT	1	0	0	0	1	At3g28720.1:d:+2152:primary
ATTGTTACAC	0	0	1	1	0	At3g28480.1:X:-662:quaternary
CTACTCATTG	1	0	1	0	0	At3g28460.1:d:+1391:primary
AATCTTTATT	0	2	0	0	0	At3g28250.1:v:+504:primary,At2g43660.2:d:+280:primary,At2g43670.1:d:+270:primary,At2g43660.1:d:+289:primary
AAGTAAAGAC	1	1	0	0	0	At3g28100.1:d:+1219:primary
GGCAATACGT	1	1	0	0	0	At3g28080.1:d:+835:primary,At3g28080.2:d:+827:primary
TCTACTCCGA	0	2	0	0	0	At3g27990.1:i:+2963:tertiary
GAATGGTATT	0	0	2	0	0	At3g27990.1:d:+1406:primary
AACTAATTTG	1	0	0	1	0	At3g27990.1:d:+1295:secondary,At3g55060.1:v:+222:secondary
ATTCAACAAA	1	0	0	1	0	At3g27965.1:p:+17:secondary
GAAGCAAAGA	1	1	0	0	0	At3g27930.1:d:+735:primary
TTATTCGTGG	0	1	1	0	0	At3g27925.1:d:+521:secondary
GAAGCTTGGT	1	0	0	0	1	At3g27886.1:p:+866:primary
GGTTGTGAGA	0	0	2	0	0	At3g27883.1:p:+5098:secondary,At3g28945.1:p:+5270:secondary
TTTAAGATTT	1	0	1	0	0	At3g27820.1:d:+76:secondary
GCACGTCTCT	0	2	0	0	0	At3g27820.1:d:+721:secondary
TTTTGATGTT	2	0	0	0	0	At3g27800.1:v:+34:secondary
CTGTTTATTT	1	0	0	1	0	At3g27730.1:v:+3736:secondary
AAACACAGAA	1	0	0	1	0	At3g27700.1:d:+2686:primary,At3g27700.2:d:+2686:primary
GGAGAGGTCA	0	2	0	0	0	At3g27650.1:d:+881:primary
TTCTGAAGAA	0	0	2	0	0	At3g27610.1:i:+725:tertiary,At3g27310.1:i:+575:tertiary
GGTATGTATA	0	1	0	0	1	At3g27460.1:d:+1273:primary
GAACCAAACC	0	0	1	1	0	At3g27430.2:d:+806:secondary,At4g29250.1:d:+154:secondary,At5g40580.1:d:+837:secondary,At3g27430.1:d:+806:secondary,At3g18950.1:d:+428:secondary, At1g32330.1:d:+1301:secondary,At5g40580.2:d:+767:secondary,At4g17460.1:d:+305:secondary
TGCTATGAAC	0	1	0	0	1	At3g27380.1:d:+417:primary
TACTTAAAAA	1	0	0	0	1	At3g27340.1:i:+700:tertiary
GTGGTGGATG	1	0	1	0	0	At3g27320.1:d:+626:secondary
CTTCTAGCGG	0	1	0	1	0	At3g27230.1:d:+1129:primary
TCCGATTCTT	0	1	0	1	0	At3g27220.1:d:+338:secondary
TTCGTTGTTG	0	0	2	0	0	At3g27200.1:d:+512:secondary,At1g13440.1:d:+477:secondary
TCAGTGGCCA	0	1	0	1	0	At3g27170.1:d:+2288:primary
GAGAGCCCCA	0	0	2	0	0	At3g27160.1:d:-800:primary
ATAATATTTT	1	1	0	0	0	At3g27050.1:i:+1116:tertiary
TGATTGGTGG	0	1	0	0	1	At3g27000.1:d:+1476:primary
GTTTAGCACC	1	1	0	0	0	At3g26935.1:v:+1694:secondary
CAGGAGTAGG	1	1	0	0	0	At3g26910.1:d:+2061:primary
TATGAACCTA	0	0	0	1	1	At3g26850.1:d:+1352:secondary
TGGAAGGCTG	0	0	1	0	1	At3g26780.1:d:+2396:secondary,At1g80280.1:d:+2091:secondary
ATGACATTAG	0	2	0	0	0	At3g26720.1:d:+2482:primary
ATGCAGAGAC	0	1	0	1	0	At3g26618.1:d:+1159:primary

GTTTTTGATG	0	0	2	0	0	At3g26600.1:d:+2257:primary,At3g14980.1:v:+4121:primary
CCGACCAGAA	0	0	1	1	0	At3g26520.1:d:+116:secondary
TCCTATCTTT	2	0	0	0	0	At3g26450.1:i:+1221:tertiary; +small RNA(AAGAACATGTCCTATCTTTATTTA)
ACTAGTTCGT	0	1	1	0	0	At3g26420.1:d:+1690:secondary,At3g26430.1:d:+1687:secondary
TTCATAGTTT	0	0	1	1	0	At3g26360.1:v:+942:secondary
GTGGCCGAAG	0	0	1	1	0	At3g26320.1:v:+1758:primary
GAATATATAA	0	0	0	1	1	At3g26300.1:v:+1871:secondary
AGAGATCTCG	0	0	1	1	0	At3g26300.1:v:+1077:secondary
GGAAACATCG	0	0	2	0	0	At3g26290.1:d:+107:secondary,At4g03415.1:v:+981:secondary
TATATCTCCT	0	1	0	1	0	At3g26240.1:d:+3225:secondary
ATCTCTCCAA	0	1	0	1	0	At3g26220.1:d:+169:secondary,At5g56930.1:d:+1211:secondary
ATAGGTAACC	0	1	1	0	0	At3g26210.1:X:+793:quaternary
TTCCGGTTCT	1	0	1	0	0	At3g26180.2:d:+1366:primary,At3g26180.1:d:+1768:primary
TTCCATTGTG	0	1	1	0	0	At3g26080.1:i:+1009:tertiary
ACCTTTCTAC	0	2	0	0	0	At3g26070.1:d:+1212:primary
GTATTATTGA	0	0	0	1	1	At3g26060.1:d:-792:secondary
ACCTTCTTAT	1	0	0	1	0	At3g26060.1:d:+749:secondary
TGCCGAGAGG	0	2	0	0	0	At3g25815.1:X:-2465:quaternary
CTCTAAGCTC	1	0	1	0	0	At3g25800.1:d:+1635:primary
ATTGTGCCAG	0	1	1	0	0	At3g25680.1:d:+752:primary
AAGAGCAAAC	0	1	0	1	0	At3g25650.1:v:+1235:primary
TTTCTATGCT	0	2	0	0	0	At3g25545.1:d:+951:primary
AGTTGATTTG	0	0	1	1	0	At3g25430.1:X:+722:quaternary
TGATTACAAA	0	0	1	0	1	At3g25290.1:X:+542:quaternary
AAAGCTGTTG	0	1	0	1	0	At3g25220.1:d:+98:primary
GAACAGAGAA	0	0	2	0	0	At3g25185.1:p:+1816:primary
AGCTTCGTTC	0	0	1	1	0	At3g25010.1:v:+646:secondary,At5g04530.1:d:+1447:secondary
CAGGTCTTTT	0	0	1	1	0	At3g24870.1:v:+6161:primary
GATACAGATG	0	0	0	2	0	At3g24800.1:d:+1315:primary
AGGTTCCTAG	0	1	0	0	1	At3g24770.1:d:+512:secondary
TAATTTTAAA	1	1	0	0	0	At3g24700.1:v:+168:secondary
CACACACAAA	0	0	0	2	0	At3g24610.1:v:+2119:primary
ATATATGATC	1	0	0	0	1	At3g24520.1:d:+1399:primary
TTTTTATGTC	2	0	0	0	0	At3g24350.1:X:+-218:quaternary,At1g53190.1:X:+404:quaternary
GACAACTGAA	1	1	0	0	0	At3g24315.1:d:+1122:primary
CCTCGGCTAA	0	1	0	1	0	At3g24300.1:d:+1701:primary
TTTTAAATAA	0	0	0	1	1	At3g24200.1:d:+1835:primary
GGACGATATG	0	0	2	0	0	At3g24170.1:X:-38:quaternary
AGTTGGGTTG	0	1	0	0	1	At3g24110.1:v:+993:primary
ATTTGGTCAT	1	1	0	0	0	At3g24000.1:v:+2686:primary
CCACAACCTG	1	0	0	1	0	At3g23920.1:d:-2051:secondary
CCACAATCTA	0	0	2	0	0	At3g23920.1:d:-1896:secondary
CTACTACTTT	0	0	0	0	2	At3g23920.1:d:+2056:secondary
AAGAAAACCA	0	1	1	0	0	At3g23890.1:d:+3632:primary
TCCTCTAAGC	0	2	0	0	0	At3g23830.1:d:+196:secondary,At3g23830.2:d:+163:secondary
CTAATGTGAG	0	0	1	0	1	At3g23820.1:d:+1433:secondary
AGACGATTTA	0	0	0	1	1	At3g23810.1:X:-1125:quaternary
CAGCTTAAAC	0	2	0	0	0	At3g23730.1:d:+335:primary
TTCATCTAAA	1	0	0	1	0	At3g23690.1:X:-361:quaternary
TTGGGGTCTG	2	0	0	0	0	At3g23670.1:d:+4124:primary
AATATGAAGT	1	0	0	1	0	At3g23640.1:d:+3041:primary
GACTTGGTTA	0	1	0	0	1	At3g23620.1:d:+754:secondary
GTGATCAGCT	0	2	0	0	0	At3g23540.1:i:+3807:tertiary
CACAATCTTG	0	0	1	0	1	At3g23530.1:d:+3121:primary
ATTATGCATT	0	1	0	0	1	At3g23480.1:d:+1569:primary
ATAATTCATA	2	0	0	0	0	At3g23410.1:d:+2348:secondary
TTTAGTTATT	0	0	0	1	1	At3g23320.1:v:+174:secondary
ATCAATTACC	1	1	0	0	0	At3g23310.1:d:+1844:primary
TGACTTTAAA	0	0	1	0	1	At3g23295.1:v:-783:secondary
CCCTTTCCTA	1	0	0	1	0	At3g23295.1:v:+788:secondary
GGGAAAATAT	0	1	1	0	0	At3g23220.1:v:+383:secondary,At1g19640.1:d:+700:secondary,At1g19680.1:d:+1715:secondary

TTGATTCATC	0	0	2	0	0	At3g23210.1:d:+1140:primary
ATGATGAAAC	1	0	0	0	1	At3g23090.1:d:+1029:primary
GATTTCTTCA	1	0	0	1	0	At3g23080.1:d:+86:secondary,At2g03440.1:d:+150:secondary
GATTTGGTGG	1	1	0	0	0	At3g23080.1:d:+1133:secondary
TGTTTTGTTT	0	0	1	1	0	At3g22990.1:d:+1571:primary
TTGTACCAAA	1	0	0	0	1	At3g22980.1:X:-3115:quaternary
GTTTCGCCGG	0	2	0	0	0	At3g22960.1:d:-133:primary
TTATGTAAAA	1	1	0	0	0	At3g22900.1:d:+667:primary
TTGTATTAGA	0	2	0	0	0	At3g22750.1:d:+498:secondary
ATGTTACTGT	1	0	0	0	1	At3g22740.1:i:+1952:tertiary
ATTTAGTAGC	1	0	0	0	1	At3g22370.1:d:-1411:secondary
TTCTTGTTCC	1	1	0	0	0	At3g22320.1:d:+532:primary
TATATTGACT	1	0	0	0	1	At3g22260.2:d:+1052:primary
GTATCCAAGT	1	1	0	0	0	At3g22237.1:p:+417:primary
GAAGTTCTTG	0	0	1	1	0	At3g22220.1:d:+2252:primary
TTAAGAGAAG	0	2	0	0	0	At3g22170.1:d:+600:secondary,At1g50360.1:v:+3022:secondary,At4g19990.1:v:+691:secondary,At4g06474.1:p:+169:secondary
GCAAAGCCAC	0	0	0	1	1	At3g22140.1:p:-614:primary
GAGATTCATA	1	0	1	0	0	At3g22133.1:X:+-171:quaternary,Chr3:+22033993:quaternary
GTGTAGGCGG	0	1	1	0	0	At3g22120.1:X:-506:quaternary
TTGAATACTT	0	1	1	0	0	At3g22070.1:d:+707:primary
CCAAGCTAGT	1	0	1	0	0	At3g22070.1:d:+561:secondary
AAAGAATTTA	1	0	1	0	0	At3g22040.1:i:+362:tertiary
TGGTGGGATG	2	0	0	0	0	At3g21760.1:X:--248:quaternary
CTGGTCGTCA	1	1	0	0	0	At3g21610.1:d:+466:primary
TTTGGCAAAA	0	1	0	1	0	At3g21600.2:i:+1464:tertiary,At3g21600.1:i:+1507:tertiary
ATCGATGTTT	0	0	1	0	1	At3g21560.1:d:+1276:secondary
TCAAGCAAAT	0	0	0	1	1	At3g21540.1:d:+2687:secondary
AAGTCAAATT	1	1	0	0	0	At3g21540.1:d:+1529:secondary,At5g17890.1:d:+3032:secondary
GCAAACACAA	0	0	1	0	1	At3g21520.1:d:+102:secondary
ACTACAATTA	0	2	0	0	0	At3g21510.1:d:+737:primary
ACTCTTTTGG	1	1	0	0	0	At3g21500.2:d:+494:secondary,At3g21500.1:d:+494:secondary
CACGGAACTG	0	0	1	0	1	At3g21465.1:d:-1244:secondary
CTACATTAGC	0	1	0	0	1	At3g21465.1:d:+1249:primary
TTTGACGGTT	0	0	0	2	0	At3g21390.1:d:+1086:primary
ACTATATAAA	1	0	1	0	0	At3g21340.1:i:+2283:tertiary
GTTCCAGTTT	0	0	1	1	0	At3g21320.1:v:+1527:secondary
GGTTCTGTTG	1	0	1	0	0	At3g21310.1:d:+976:secondary
GACACAACTC	2	0	0	0	0	At3g21310.1:d:+1526:primary
GATGGATCTC	0	0	2	0	0	At3g21260.1:d:+287:primary,At3g21260.2:d:+318:primary
GTGTTGAGAC	0	0	1	0	1	At3g21140.1:d:+1227:primary
TGTTCTGAAG	0	1	0	1	0	At3g21090.1:d:+2207:primary
CTGATGCATT	0	0	1	0	1	At3g20970.1:d:+837:primary
TAACAGCAAA	0	0	1	0	1	At3g20870.1:i:+2466:tertiary
TGCAATAATT	1	0	0	0	1	At3g20870.1:d:+1074:primary
TCGAACGGTG	0	0	1	1	0	At3g20770.1:X:-1789:quaternary
TTCCTTGAAA	0	0	0	1	1	At3g20760.1:v:+24:secondary
ACTTGAATAC	1	0	0	0	1	At3g20750.1:v:+1262:secondary
TTGCTACTCC	0	0	2	0	0	At3g20740.1:d:+1390:primary
GTGTGATCAA	1	1	0	0	0	At3g20700.1:v:+1660:primary
GAAATCAGTT	0	2	0	0	0	At3g20660.1:v:+1613:secondary
AGAAGGCTTG	0	0	1	1	0	At3g20650.1:d:+1008:secondary,At3g20650.2:d:+1005:secondary
AACATATCAA	0	0	0	1	1	At3g20610.1:v:+94:secondary,At1g64360.1:d:+452:secondary
TCTGAACCCT	0	2	0	0	0	At3g20540.1:d:+3095:primary
GACCGAATCA	0	1	1	0	0	At3g20470.1:X:-672:quaternary
GTTGCAAAAA	1	0	1	0	0	At3g20440.1:i:+1335:tertiary,At2g27980.1:i:+3383:tertiary
CTTAGATTTG	0	1	0	1	0	At3g20400.1:v:+986:primary
GATTATCCAT	0	2	0	0	0	At3g20362.1:v:+521:primary
TTTGTATATG	1	0	1	0	0	At3g20340.1:d:+373:secondary,At1g09440.1:v:+1933:secondary
AGCAGCACCG	0	1	1	0	0	At3g20310.1:d:+547:secondary,At1g50640.1:d:+560:secondary
TCTTGTGACA	0	0	1	1	0	At3g20220.1:v:+687:secondary

GGGAGGAGTT	0	2	0	0	0	At3g20080.2:d:+2289:secondary,At3g20935.1:p:+598:secondary,At3g20083.1:p:+609:secondary,At3g20080.3:d:+1098:secondary,At3g20935.1:p:+618:secondary,At3g20087.1:p:+555:secondary,At4g15380.1:d:+621:secondary,At3g20110.1:d:+752:secondary,At3g20100.1:d:+614:secondary,At3g20080.1:d:+637:secondary,At3g20950.1:v:+960:secondary,At4g15350.1:d:+577:secondary,At3g20080.2:d:+612:secondary,At3g20940.1:d:+613:secondary
CGTTTAAAGG	1	0	0	1	0	At3g20060.1:X:-41:quaternary
GAGGGCGAAG	0	0	0	2	0	At3g20050.1:d:+1147:secondary
CGTTACCCGA	0	1	0	0	1	At3g20040.1:v:+2102:primary
CAAACATTCT	0	1	1	0	0	At3g19910.1:d:+1107:primary
GTCATTGTCT	0	0	1	0	1	At3g19870.1:d:+2076:secondary,At3g52930.1:d:+938:secondary
CAAAATGTAT	0	0	1	1	0	At3g19850.1:d:+1862:secondary
TTTGTGATAA	0	1	1	0	0	At3g19660.1:d:+428:primary
TAAACATCTA	1	0	1	0	0	At3g19590.1:d:+650:primary
AAGGTTTTAG	0	0	1	1	0	At3g19510.1:d:+2323:primary
CTAGTGGGCA	1	1	0	0	0	At3g19390.1:d:+1377:primary
GAAATCTAAT	0	1	0	0	1	At3g19370.1:d:+1475:primary
CTTTGAGTGT	0	1	0	1	0	At3g19280.1:d:+1636:primary
TCTCTGAGAT	0	0	1	0	1	At3g19280.1:d:+1397:secondary
AATCGGCTCT	1	0	1	0	0	At3g19250.1:i:+134:tertiary
AATATAGAAA	0	0	1	1	0	At3g19150.1:d:+892:primary
CTTAACAATT	2	0	0	0	0	At3g19100.1:i:+3209:tertiary
CTGATGAACG	0	0	1	1	0	At3g19100.1:d:-1663:primary
AAAACCAATA	0	1	0	0	1	At3g19040.1:i:+6352:tertiary
TATCTTCCTT	0	2	0	0	0	At3g18990.1:d:+1051:primary,At3g28020.1:v:+1258:primary
TTCTTTGAAT	0	0	0	1	1	At3g18980.1:d:+1425:secondary
CATTGGATTC	0	1	0	0	1	At3g18970.1:d:+1822:primary
GAAGAGTATG	0	1	0	0	1	At3g18940.1:d:+885:primary
TTCTTTCAGT	1	1	0	0	0	At3g18910.1:d:+1234:primary
TGGAAAACAA	0	1	1	0	0	At3g18860.1:d:+1879:primary,At3g18860.2:d:+1879:primary
AAAACCAAAC	0	0	0	2	0	At3g18840.1:i:+2275:tertiary
CTGCTTGGTG	0	2	0	0	0	At3g18780.2:d:-1198:secondary,At3g18780.1:d:-1198:secondary
TTACAAAAGC	0	0	1	1	0	At3g18610.1:i:+2967:tertiary
TTTTTATGCA	0	2	0	0	0	At3g18560.1:d:+787:primary
GAGCAGAGGA	0	1	0	0	1	At3g18480.1:d:+2191:primary
TGAAAGACTA	0	1	0	1	0	At3g18370.1:d:+2393:secondary
GTAATGGAAT	1	0	0	1	0	At3g18295.1:X:+320:quaternary,Chr5:+1707172:quaternary
GCACAAGCAG	0	1	0	1	0	At3g18165.1:d:-719:primary
AAATAAGTTG	0	1	0	0	1	At3g18070.1:v:-1369:secondary
ATGCGTTTGT	1	0	1	0	0	At3g18020.1:v:+2113:secondary
CTTGAAAAGA	0	0	1	0	1	At3g18010.1:i:+1332:tertiary
GAAAAAAACA	0	1	1	0	0	At3g17980.1:d:+647:secondary
ACTTGAGACT	0	0	0	0	2	At3g17920.1:v:+3696:primary
TTTTTTGGTG	0	0	0	2	0	At3g17860.1:d:+1417:secondary,At3g17860.2:d:+1207:secondary
TCTCTGCTCT	1	1	0	0	0	At3g17820.1:d:+93:secondary
CCCACTGCCT	1	0	0	1	0	At3g17770.1:X:-13:quaternary
ATCCCTTCTC	1	0	0	0	1	At3g17770.1:d:+1788:primary
GTCGAAGAAA	0	0	2	0	0	At3g17740.1:d:+3331:primary,At4g00540.1:d:+1446:primary
TTGTTGGTTC	0	2	0	0	0	At3g17700.1:d:+1297:primary
ATCGTTAAGA	0	0	1	1	0	At3g17670.1:i:-709:tertiary
TGTTCTAAGC	0	0	2	0	0	At3g17609.2:d:+167:primary,At3g17609.1:d:+123:primary
GAAGAAAACA	0	1	0	1	0	At3g17580.1:v:+362:secondary,At1g06670.1:d:+4566:secondary,At1g48330.1:d:+64:secondary,At5g19610.1:v:+3867:secondary
GACCCTCCTT	0	2	0	0	0	At3g17420.1:d:+1660:primary
GATGAAATTA	0	1	1	0	0	At3g17185.1:d:+84:primary
CATAAATAAT	1	0	0	1	0	At3g17180.1:v:+1855:primary
ATGTATTCAT	0	0	1	0	1	At3g17150.1:i:+1132:tertiary
GATCCACGTT	0	1	1	0	0	At3g17070.1:d:+778:primary
AATACGGATT	1	1	0	0	0	At3g16990.1:X:+430:quaternary
GATGGTAATT	0	0	0	2	0	At3g16940.1:X:+487:quaternary
CACACCAAGA	0	0	1	0	1	At3g16920.1:X:+601:quaternary

TGTGTTTTGC	1	0	0	1	0	At3g16900.1:v:+978:primary
GCTCTCTCTT	1	1	0	0	0	At3g16870.1:d:+622:primary,At3g06740.1:d:+657:primary
CTGAATCAAT	0	0	1	0	1	At3g16840.1:d:+2110:primary
GATGATATTT	1	1	0	0	0	At3g16800.1:d:+1103:secondary,At3g16800.2:d:+1086:secondary,At3g27810.1:d:+815:secondary,At1g21870.1:v:+1181:secondary
TAATTCAAAA	0	0	1	1	0	At3g16780.1:X:+390:quaternary,Chr5:+19838818:quaternary
AAATGTTTGA	0	1	1	0	0	At3g16760.2:X:+435:quaternary
CGTGTGTGTA	2	0	0	0	0	At3g16760.2:d:+1685:secondary,At3g16760.1:d:+1742:secondary
ACCCACTTAC	0	0	0	2	0	At3g16760.1:d:-1811:secondary,At3g16760.2:d:-1754:secondary
GTACAAAAAA	0	2	0	0	0	At3g16730.1:v:+2929:primary
AATAAGGTCA	0	2	0	0	0	At3g16670.1:d:+650:secondary
CAAACCCTGG	2	0	0	0	0	At3g16660.1:X:-454:quaternary
GTGAGGTTAA	0	2	0	0	0	At3g16620.1:d:+353:secondary
ATTCTGAGAG	1	0	1	0	0	At3g16520.3:X:-325:quaternary
TCTTCAAGAC	1	0	1	0	0	At3g16500.1:d:+1163:secondary
TAGTTGTAGA	1	0	1	0	0	At3g16270.1:d:+2549:primary
AACCCAGCAC	0	0	2	0	0	At3g16240.1:d:+699:secondary
ACTTCAAATT	0	0	2	0	0	At3g16220.1:i:+1027:tertiary
AATCCATCCT	1	0	1	0	0	At3g16160.1:v:+1791:primary
CCAAGGCCCG	0	1	1	0	0	At3g16060.1:d:+1866:secondary
CTGCAGTTTT	0	2	0	0	0	At3g16000.1:X:-71:quaternary
CTTCAAACTT	1	1	0	0	0	At3g16000.1:d:+2069:secondary
TCGAGCAGAA	0	0	0	1	1	At3g15940.1:d:+660:secondary
CTATTCTTTG	2	0	0	0	0	At3g15940.1:d:+2548:primary
GTGATTGTTG	0	0	1	0	1	At3g15860.1:v:+1228:primary
ACAAGCTGAG	1	0	1	0	0	At3g15850.1:d:-939:secondary
AGAAGAATGC	0	2	0	0	0	At3g15820.1:d:+828:primary
TTAGAAATTT	0	0	0	2	0	At3g15810.1:X:+642:quaternary
TAAGTAGTCA	2	0	0	0	0	At3g15760.1:d:+813:primary
CAGCGTACAA	1	0	1	0	0	At3g15680.1:d:-883:secondary
TAACACTTCT	0	0	2	0	0	At3g15620.1:d:+1847:primary
TGTAATAACA	0	1	1	0	0	At3g15570.1:X:-627:quaternary
TTATGGTTTT	0	0	1	1	0	At3g15540.1:d:+812:primary,At1g18610.1:v:+2560:primary
CATTATATAA	0	2	0	0	0	At3g15510.1:d:+1362:primary,At3g62680.1:d:+1043:primary
TTTGTATGAA	1	1	0	0	0	At3g15430.1:d:+1881:secondary,At3g15430.2:d:+1988:secondary
ACTTCACTCA	0	2	0	0	0	At3g15410.1:d:+1651:primary
GTTGCTGTAG	1	0	1	0	0	At3g15395.1:d:+280:primary,At3g15395.2:d:+120:primary
TTGGGCATTG	0	1	1	0	0	At3g15380.1:d:+2008:primary
CGTCGGTGGA	0	0	1	1	0	At3g15360.1:d:-795:secondary
GATCCTCTTT	0	0	1	1	0	At3g15355.1:d:+1921:primary
GTGAATCGGC	0	1	0	1	0	At3g15352.1:d:+212:primary
ACCTACACTA	0	2	0	0	0	At3g15350.1:d:+1364:secondary,At3g15350.2:d:+1432:secondary
TTGTTATCTA	0	0	0	1	1	At3g15290.1:d:+1144:primary
GTGCCTGGTG	1	0	0	1	0	At3g15180.1:d:+1358:primary
ACTTTGCTTT	0	1	0	0	1	At3g15160.1:d:+1870:primary
ATCCATAAAT	0	1	0	1	0	At3g15140.1:d:+1118:secondary
TTTTCGAGGG	0	2	0	0	0	At3g15095.1:d:+845:secondary
TCTTTCACCA	0	2	0	0	0	At3g15040.1:d:+472:secondary
CCACAGTAAC	1	0	0	0	1	At3g15000.1:d:-1409:secondary
CATTCTGGGC	0	1	1	0	0	At3g15000.1:d:+1284:secondary
AGTACCCAAG	0	0	0	2	0	At3g14980.1:v:+3537:secondary
CTCCACGCAA	0	0	1	1	0	At3g14910.1:d:+1165:primary
TACGTATAGA	2	0	0	0	0	At3g14840.2:i:+1053:tertiary
TGAGGTAGAC	0	1	0	0	1	At3g14690.1:d:+610:secondary
TTGGATTACG	0	0	1	1	0	At3g14660.1:d:+2025:primary
ACCCGTCCGT	0	1	1	0	0	At3g14517.1:X:--123:quaternary
TTTCTGTAGG	1	0	0	0	1	At3g14430.1:d:+402:primary
GGCTACTTCC	0	0	2	0	0	At3g14420.1:d:+394:secondary,At3g14420.3:d:+527:secondary,At3g14420.2:d:+435:secondary
GTTAGCTCGA	0	0	1	1	0	At3g14415.1:v:-1360:secondary
GGCTACTTCA	0	0	2	0	0	At3g14415.1:v:+674:secondary
AAGCCTCTAC	1	0	0	0	1	At3g14400.1:d:+2323:primary

TAAATTCTTT	0	0	0	1	1	At3g14350.1:d:+2320:secondary,At3g14350.3:d:+2236:secondary,At3g14350.2:d:+2452:secondary
GAAGAAGTTG	0	0	0	1	1	At3g14300.1:v:+1425:secondary,At2g11345.1:v:+608:secondary,At3g53290.1:v:+1503:secondary,At1g35970.1:p:+5563:secondary
ATGATCAAAT	0	1	1	0	0	At3g14280.1:d:+503:secondary,At5g28415.1:p:+448:secondary
CTGGAAGGAG	1	0	0	0	1	At3g14230.3:d:+1244:secondary,At3g14230.2:d:+1247:secondary,At3g14230.1:d:+1259:secondary
ACTTTCACAC	0	0	1	1	0	At3g14225.1:v:+1198:secondary
GTGACTAATG	0	2	0	0	0	At3g14210.1:i:+422:tertiary,At1g65040.2:i:+1238:tertiary,At1g65040.1:i:+2806:tertiary
TGCAGCTTTT	0	2	0	0	0	At3g14205.1:d:+2605:secondary
GATTCAATTC	0	0	1	1	0	At3g14150.1:d:+1260:primary
TACTATCAGT	2	0	0	0	0	At3g14100.1:d:+1381:secondary,At1g54080.2:d:+1367:secondary,At1g54080.1:d:+1355:secondary
ACCTTTACCA	0	0	1	1	0	At3g14075.1:d:+1676:secondary
GCTAAGCTCT	1	0	1	0	0	At3g14070.1:v:+2674:secondary,At4g34138.1:d:+212:secondary,At3g14067.1:v:+354:secondary
GTTATCAAAT	1	0	0	0	1	At3g14067.1:v:+2963:primary
TAGCAGAGGA	0	2	0	0	0	At3g14010.1:d:+1019:secondary
ACCGATCCAG	0	1	0	1	0	At3g13940.1:d:+1233:primary
AAAATTTAAT	1	1	0	0	0	At3g13880.1:v:+2597:secondary
GACATCGATG	0	1	0	0	1	At3g13880.1:v:+1631:secondary,At2g31410.1:d:+153:secondary
ATATTGGTCG	0	2	0	0	0	At3g13870.2:d:+553:secondary,At3g13870.1:d:+549:secondary
TAATTCTTCC	1	1	0	0	0	At3g13845.1:d:+316:primary
GAAGTAACTA	0	1	0	0	1	At3g13800.1:d:+810:primary
GTCAGAAGAC	0	1	1	0	0	At3g13740.1:d:-974:secondary
TAGAATCTTT	0	0	0	1	1	At3g13682.1:X:-250:quaternary,At2g05845.1:X:-1439:quaternary
CTTCGCCGTG	0	0	2	0	0	At3g13580.1:d:+449:secondary,At3g13580.3:d:+1023:secondary,At3g13580.2:d:+679:secondary,At2g01250.1:d:+455:secondary
CTTCAAGAAG	1	0	0	1	0	At3g13540.1:d:+870:primary,At4g28490.1:d:+2927:primary
TTGTAGCTTT	1	1	0	0	0	At3g13490.1:d:+1861:secondary,At4g21360.1:p:+3697:secondary
CCGTGAGCGA	0	0	2	0	0	At3g13460.2:X:-188:quaternary
CCAACCAAGA	0	1	1	0	0	At3g13450.1:d:+1166:secondary,At3g43610.1:v:+2683:secondary
AATCAGGAGC	1	0	0	1	0	At3g13350.1:d:+961:primary
CATAAAACAT	1	1	0	0	0	At3g13290.1:d:+4155:secondary
GTGAAGATTC	1	0	1	0	0	At3g13280.1:v:+1212:secondary
ACTTTGACGA	1	0	0	1	0	At3g13228.1:d:+998:primary
ATTAATGATG	1	1	0	0	0	At3g13190.1:d:+549:primary,At5g23470.1:v:+1656:primary,At3g13190.2:d:+515:primary
ATTAAAGGAT	1	1	0	0	0	At3g13160.1:d:+973:primary
GTTATATTGG	0	1	0	0	1	At3g13150.1:d:+1796:primary
TGCAACTTAC	1	0	0	0	1	At3g13062.1:d:+1453:secondary,At3g13062.3:d:+1477:secondary
CCTTTCTTTG	0	0	0	0	2	At3g13050.1:i:+1185:tertiary
TTATTCAGGA	1	0	0	0	1	At3g13000.2:d:+2128:primary,At3g13000.1:d:+2006:primary
TCTGAGAACT	0	2	0	0	0	At3g12980.1:d:+2776:secondary
GCAATCGAAC	0	0	1	0	1	At3g12950.1:d:+1988:primary
TTTCTAGATT	0	1	0	0	1	At3g12930.1:d:+983:secondary,At4g16280.1:d:+2643:secondary
ACTCCTGCTA	1	0	1	0	0	At3g12775.1:v:+1728:primary
CTCATTACCT	1	0	0	0	1	At3g12740.1:d:+1446:secondary
GTTTACAACT	1	0	0	0	1	At3g12730.1:d:+46:secondary,At4g30290.1:d:+823:secondary,At5g37110.1:v:+58:secondary,At2g13560.1:d:+657:secondary
GTTCGTCGAG	1	0	1	0	0	At3g12710.1:d:+962:secondary
CAAGTTTGAC	0	1	0	0	1	At3g12680.1:d:+1623:primary
AAGGTACTCT	0	0	1	0	1	At3g12610.1:d:+791:secondary
CATAAGACTT	0	1	1	0	0	At3g12600.1:d:-855:secondary
AACCTAGGCT	0	1	0	1	0	At3g12590.1:d:+4013:primary
ACGCAGTCCA	1	1	0	0	0	At3g12520.1:d:+1898:primary
GAGAATCAAC	0	1	0	0	1	At3g12380.1:d:+1052:secondary,At3g51120.1:d:+128:secondary,At1g76310.1:v:+948:secondary
ATCTTGGGTC	2	0	0	0	0	At3g12300.1:d:+866:primary
ATAAGTTCAT	0	0	1	1	0	At3g12300.1:d:+636:secondary,At3g32897.1:p:+1437:secondary,At1g23960.1:d:+750:secondary
GTAGCAAATA	0	0	1	0	1	At3g12280.1:d:+3142:primary
GTTGTCTTTA	0	1	0	1	0	At3g12180.1:d:+472:secondary
TTTAATTTCG	1	0	0	0	1	At3g12145.1:d:+902:primary
GTCAATCATA	0	0	2	0	0	At3g12140.2:X:-286:quaternary,At5g67380.1:X:-68:quaternary
ACATAACGGA	1	0	1	0	0	At3g12130.1:X:-898:quaternary
TGTGTGAATC	0	1	0	0	1	At3g12130.1:d:+1228:primary

AAAATTGGAT	1	0	0	0	1	At3g12000.1:d:+1330:primary
GAAATCCACC	0	0	0	2	0	At3g11964.1:v:+5275:secondary
GATGAGAAGA	0	1	0	1	0	At3g11964.1:v:+2619:secondary,At2g04270.2:d:+2972:secondary,At2g04270.1:d:+3127:secondary,At2g04270.4:d:+2315:secondary
GTTAACGAAT	0	2	0	0	0	At3g11960.1:d:+4248:primary,At3g11960.2:d:+4054:primary
GAGTGTATGT	0	1	0	1	0	At3g11950.1:i:+1868:tertiary
AATTAAATAG	2	0	0	0	0	At3g11800.1:d:+925:primary,At3g47170.1:v:+2077:primary
TTCTGGAAGA	0	2	0	0	0	At3g11800.1:d:+512:secondary
GGTTGCGCAG	1	1	0	0	0	At3g11620.2:d:+988:primary,At3g11620.3:d:+1165:primary,At3g11620.1:d:+990:primary
AATTTGAACT	1	0	1	0	0	At3g11590.1:d:+2154:secondary
TATTGATATT	0	0	0	1	1	At3g11510.1:i:+281:tertiary
AAGGTGAAGG	0	0	2	0	0	At3g11510.1:d:+251:secondary,At5g18390.1:d:+1150:secondary,At3g52820.1:d:+947:secondary
TCGAAAGCGA	1	0	0	1	0	At3g11490.1:v:+1593:secondary
GATTTGATCT	0	0	0	1	1	At3g11480.1:d:+998:secondary,At5g28490.1:v:+354:secondary,At3g46710.1:v:+2586:secondary
AGCGATGCGC	0	0	1	0	1	At3g11450.1:X:-1748:quaternary
TAAACATTAC	1	0	0	1	0	At3g11420.1:d:+2022:secondary
AAACAGAGGA	1	0	1	0	0	At3g11380.1:i:+824:tertiary
CGGTAAAAAA	1	0	0	0	1	At3g11370.1:d:+111:secondary
GAAGAGGTTG	0	2	0	0	0	At3g11290.1:d:+1385:primary
ATACCCAAAA	1	0	1	0	0	At3g11240.1:d:+1940:primary
CTCTCATTCA	0	0	1	0	1	At3g11220.1:d:+749:primary
TACGCCCAAT	0	0	2	0	0	At3g11130.1:d:+5204:secondary
AACCCTTTTG	0	0	0	1	1	At3g11080.1:d:+2823:primary,At3g11010.1:d:+2643:primary
ACAATTCTCA	1	0	0	1	0	At3g10950.1:v:+115:secondary
ATCGGTGATT	0	0	1	1	0	At3g10910.1:d:+960:primary
TTTTTTCATT	0	1	0	1	0	At3g10880.1:v:+1363:secondary
AAACCGGAGC	0	1	1	0	0	At3g10870.1:d:+745:primary
TTAAGGATGA	0	0	0	0	2	At3g10790.1:v:+874:secondary
GAAGAGCTCA	0	2	0	0	0	At3g10640.2:d:+1011:primary,At3g10640.1:d:+758:primary
ACAGGATCTC	0	1	0	1	0	At3g10630.1:d:+1490:secondary
TCGGAAGTCA	1	1	0	0	0	At3g10550.1:d:+1820:primary
GAGTGTATAG	0	1	0	0	1	At3g10510.1:v:+423:secondary
AATAACAGCA	1	0	1	0	0	At3g10450.1:d:+1049:secondary,At2g22990.3:d:+1101:secondary,At2g22990.5:d:+1101:secondary,At2g22990.2:d:+1047:secondary,At2g22990.1:d:+1101:secondary,At2g22990.4:d:+1101:secondary
AAGCGGATTG	2	0	0	0	0	At3g10440.1:v:+2180:secondary
GTTAAAGCTT	0	2	0	0	0	At3g10430.1:v:+147:secondary
ATTTTCATAA	1	1	0	0	0	At3g10380.1:d:+3408:primary
TTCACCAGAA	1	1	0	0	0	At3g10350.1:d:+762:primary
ATTTGTGTTT	0	0	2	0	0	At3g10340.1:d:+2433:primary
CCACTGACCT	0	0	2	0	0	At3g10330.1:X:+350:quaternary
CTGGAGATTT	1	0	1	0	0	At3g10330.1:d:+763:primary
CTAGGAGAGT	0	2	0	0	0	At3g10250.2:d:+543:secondary,At3g10250.1:d:+616:secondary
ACAAAGGGGA	0	0	2	0	0	At3g10230.1:d:+1640:primary
ATGTAGTTTC	0	0	0	0	2	At3g10160.1:d:+686:secondary
TATACTGCAG	0	0	1	1	0	At3g10140.1:d:+1477:primary
GTTTCTACGT	1	1	0	0	0	At3g10140.1:d:+1059:secondary
GGATTGAATT	0	0	0	2	0	At3g10070.1:d:+1796:primary,At3g14360.1:d:+2359:primary
CTATAATCCA	2	0	0	0	0	At3g09970.1:d:+1173:primary
CACTGGTCTG	0	2	0	0	0	At3g09925.1:v:+1247:primary
AAATTATTAT	0	0	0	2	0	At3g09890.1:d:+907:primary
TTTAAGAAAA	0	0	0	1	1	At3g09880.1:d:+395:secondary
GATGTAATTA	1	0	0	0	1	At3g09820.1:d:+1411:secondary,At3g09820.2:d:+1604:secondary
AGGATAAGAA	0	2	0	0	0	At3g09670.1:d:+2126:primary
CTCTCCTAGC	0	2	0	0	0	At3g09670.1:d:+1149:secondary
GACTTGGGTT	1	0	1	0	0	At3g09660.1:v:+2847:secondary
TGGGAATTGT	0	1	1	0	0	At3g09600.1:d:+1050:primary
TAGGAGTTAA	0	1	0	1	0	At3g09560.2:d:+3320:primary,At3g09560.1:d:+3366:primary
GTTGCATCTT	0	2	0	0	0	At3g09470.2:d:+318:secondary,At3g09470.1:d:+319:secondary
TTGTATGCGT	0	0	0	1	1	At3g09440.1:X:-1446:quaternary,At1g69670.1:X:-11:quaternary
CTAATATTCG	1	0	0	1	0	At3g09350.1:d:+509:primary

AAAGCATATA	0	1	0	0	1	At3g09165.1:p:+2283:secondary,At1g34620.1:p:+2382:secondary
TTGTTCTTGA	2	0	0	0	0	At3g09035.1:d:+1489:primary
TAAAATCTAA	0	1	0	1	0	At3g09035.1:d:+1424:secondary,At2g20320.1:v:+1720:secondary
TTCCTCAAGA	0	0	0	2	0	At3g08980.1:d:+649:primary
ATTCCCTACA	0	2	0	0	0	At3g08955.1:d:+582:primary
AAGACCGAAG	1	1	0	0	0	At3g08950.1:d:+917:secondary
GGTACCCGGG	0	0	2	0	0	At3g08940.1:d:+84:secondary,At3g08940.2:d:+84:secondary
TAATGATGTG	1	0	1	0	0	At3g08940.1:d:+1023:secondary,At3g08940.2:d:+938:secondary
AATTGAGTTT	0	0	0	1	1	At3g08910.1:i:+814:tertiary,At1g43650.1:i:+936:tertiary
TCCAAGATGT	0	1	1	0	0	At3g08860.1:d:+1506:primary
TTGAAACCGG	1	1	0	0	0	At3g08840.3:d:+2714:primary,At3g08840.2:d:+2687:primary
CTTCCACCCT	0	2	0	0	0	At3g08770.1:X:+171:quaternary
AAGTACTCGT	0	0	0	1	1	At3g08760.1:d:+1575:primary
TTTGACAATG	0	1	1	0	0	At3g08670.1:d:+1863:primary
GAGAGACCAC	0	0	2	0	0	At3g08600.1:X:-1443:quaternary
TTATTTATAG	0	0	0	0	2	At3g08580.2:d:+1559:primary,At3g08580.1:d:+1444:primary
AAAACTCCCA	0	1	0	0	1	At3g08550.1:d:+1142:primary
TTTTAGGTAA	0	0	0	1	1	At3g07920.1:v:+247:secondary
CTGGAACATT	1	1	0	0	0	At3g07870.1:d:+1299:secondary
AAAGGGAGAA	0	0	1	1	0	At3g07770.1:d:+2145:primary
CTTTGTGATA	0	1	0	0	1	At3g07750.2:d:+1093:primary,At3g07750.1:d:+1092:primary
TAAATATAAA	0	1	1	0	0	At3g07740.2:d:+1818:primary,At3g07740.1:d:+1732:primary
GCTGCAGTCG	0	1	0	1	0	At3g07720.1:d:+315:primary
CCTACACCAA	0	0	0	1	1	At3g07670.1:d:-1669:secondary
CGAAGAGTTT	0	2	0	0	0	At3g07630.1:d:+393:primary,At3g07630.2:d:+388:primary
GTTCTCTGTT	0	2	0	0	0	At3g07525.1:d:+678:primary,At3g07525.2:d:+646:primary
GTGAATTTCA	1	0	0	1	0	At3g07490.1:v:+744:secondary,At3g15200.1:v:+2065:secondary
CGAGGCTAAA	0	1	1	0	0	At3g07470.1:d:+335:secondary
TTTTCGCCAT	1	1	0	0	0	At3g07400.1:d:+3241:primary
TTGTTATCCG	0	1	0	0	1	At3g07340.1:d:+1745:primary
TCAGAAACCT	0	0	2	0	0	At3g07320.1:d:+1347:secondary
TTTTTCCTTC	0	2	0	0	0	At3g07290.1:v:+406:secondary
ACCACTTATC	0	1	1	0	0	At3g07280.1:X:-812:quaternary
TTCTTTTCTG	0	0	0	1	1	At3g07280.1:p:+917:secondary
GATCAGAAAG	0	1	1	0	0	At3g07280.1:p:+2122:primary
CGCCGTGTTT	1	0	1	0	0	At3g07210.1:d:+1147:secondary
GTGCCGCAGG	0	0	2	0	0	At3g07195.1:v:+1004:secondary
CGTATTGGCT	0	1	0	0	1	At3g07160.1:d:+5520:primary
CACCATCTTC	0	1	0	1	0	At3g07140.2:d:+1743:primary,At3g07140.1:d:+1743:primary
CTCGACGATA	0	2	0	0	0	At3g07020.1:d:+1797:secondary,At3g07020.2:d:+1844:secondary,At1g05770.1:d:+178:secondary
CTGCAAAAAA	0	1	0	1	0	At3g06990.1:i:+505:tertiary
ATGTCCCAAA	0	0	0	1	1	At3g06980.1:d:+2379:secondary
GTCTTTGTCT	1	1	0	0	0	At3g06950.1:d:+924:primary
TAGATAGTAT	0	0	0	0	2	At3g06910.1:d:+1578:primary
CTTTAAACTA	2	0	0	0	0	At3g06880.1:i:+5280:tertiary
TTCTGGGCTG	1	0	1	0	0	At3g06860.1:d:+2074:primary
TGTAAACAAA	0	0	1	0	1	At3g06770.3:d:+1676:primary,At3g06770.2:d:+1782:primary,At3g06770.1:d:+1632:primary
AGATATAAGG	0	0	1	0	1	At3g06670.1:d:+3312:primary
AATGAAAGCC	0	2	0	0	0	At3g06580.1:d:+1356:primary
CGTTCATCTA	0	1	0	0	1	At3g06320.1:X:+373:quaternary
CGAGAGGGTG	0	0	1	1	0	At3g06250.1:d:+2571:secondary
TTCCTCTTGA	0	1	0	1	0	At3g06210.1:d:+2532:primary
GATTATGTTA	1	1	0	0	0	At3g06200.1:d:+1126:primary
TATAACTCCG	1	0	1	0	0	At3g06145.1:v:+946:primary
ACAGCTCCGG	0	0	2	0	0	At3g06100.1:d:+756:primary
AAGATATGTT	1	0	0	0	1	At3g06020.1:v:+742:primary
ATTTTAAGAA	0	2	0	0	0	At3g05710.2:d:+1531:primary,At3g05710.1:d:+1528:primary
TACATTAGCT	0	2	0	0	0	At3g05680.1:d:+6328:primary
TCTTAACTCA	0	0	1	0	1	At3g05675.1:d:+1561:primary,At3g05675.2:d:+1768:primary
CCACGACCTG	0	1	1	0	0	At3g05640.1:d:+1417:primary,At3g05640.2:d:+1364:primary
TGTGATCAAG	0	1	1	0	0	At3g05630.1:d:+3132:primary

AATAAAGAAA	1	0	0	0	1	At3g05625.1:d:+1017:primary
AAAGTGACTG	0	0	1	0	1	At3g05590.1:d:+323:secondary
ACTGGCAAGG	0	0	1	0	1	At3g05590.1:d:+245:secondary,At5g27850.1:d:+227:secondary
TCTTTACTTT	0	0	1	1	0	At3g05570.1:v:+776:secondary
GTACTGTACG	0	0	0	2	0	At3g05550.1:i:+717:tertiary
AACTCTTATC	1	0	0	0	1	At3g05510.2:d:+1534:primary,At3g05510.1:d:+1661:primary
GACAATGTTA	1	1	0	0	0	At3g05420.2:d:+1696:primary,At3g05420.1:d:+1693:primary
GTGCTTGGTG	0	2	0	0	0	At3g05400.1:d:+1439:primary
TCCGCAACAT	1	1	0	0	0	At3g05280.1:d:+791:secondary
AACTCAAAAC	0	2	0	0	0	At3g05270.1:d:+1146:secondary,At3g25910.1:d:+781:secondary
AAGAGCAAAA	0	0	2	0	0	At3g05240.1:v:+1941:secondary
GTGACCACAT	0	0	1	1	0	At3g05210.1:d:+1300:secondary
GAAACTCTTT	0	0	0	1	1	At3g05180.1:d:+19:secondary,At5g37910.1:v:+1249:secondary
AGAAGAAACG	0	2	0	0	0	At3g05170.1:d:+1146:primary
TATCTCAATT	1	0	1	0	0	At3g05155.1:i:+336:tertiary
GTACATTTTA	0	0	1	0	1	At3g05150.1:d:+1352:secondary,At4g20430.1:v:+3015:secondary
TTTAGTCTGT	0	0	1	0	1	At3g05140.1:d:+414:secondary
GTCTACTCAC	0	0	1	0	1	At3g05070.1:d:-610:secondary
TCAACCTTCT	0	1	0	1	0	At3g05050.1:d:+1758:primary
GTGCTAAAAA	1	0	0	1	0	At3g05030.1:X:-79:quaternary
GAAAGACTAA	0	1	1	0	0	At3g05020.1:d:+174:secondary
TAAAAGTCTA	1	0	1	0	0	At3g04980.1:v:+4094:primary
TGAACTAAAG	0	0	1	0	1	At3g04970.1:d:+1058:secondary,At3g04970.2:d:+1143:secondary
TTATTACAGA	1	0	1	0	0	At3g04930.1:d:-1873:secondary,At5g41750.1:d:-3680:secondary
CGCAGCAGAT	0	0	0	1	1	At3g04920.1:X:+269:quaternary
TCGAGAGCTG	0	0	1	1	0	At3g04890.1:d:+637:secondary,At2g01650.1:d:+518:secondary
AGATTAGGTG	0	2	0	0	0	At3g04770.2:d:+1003:primary,At3g04770.1:d:+1080:primary
TCTCTCCTTT	1	0	0	1	0	At3g04690.1:v:+389:secondary,At5g13730.1:d:+1458:secondary,At3g01260.1:d:+370:secondary
TGGAGCGATG	2	0	0	0	0	At3g04670.1:d:+1492:secondary
TCCTTTGGAA	0	0	1	1	0	At3g04650.1:i:+584:tertiary
TTATGGCTTC	0	1	0	0	1	At3g04580.2:d:+2674:primary,At3g04580.1:d:+2765:primary
GATGATGAAC	1	0	0	0	1	At3g04530.1:d:+804:primary
CAAGTTAGAG	0	0	1	1	0	At3g04460.1:d:+1389:primary
GCAATGCATC	0	2	0	0	0	At3g04440.1:d:+333:secondary,At1g21630.1:d:+1876:secondary,At3g03700.1:d:+372:secondary
AGGCTTGTGA	2	0	0	0	0	At3g04360.1:d:+480:secondary,At5g49665.1:d:+1120:secondary
GTTTGGTTGT	0	1	1	0	0	At3g04260.1:d:+338:secondary,At2g39700.1:d:+1244:secondary
AGGACCCGGC	0	0	2	0	0	At3g04240.1:d:+3115:secondary
GGCCGAGGCT	0	0	1	0	1	At3g04120.1:d:+397:secondary,At5g48060.1:d:+2736:secondary
TAATAATTTA	0	0	0	0	2	At3g04110.1:d:+2591:primary
GGCTTATTTT	0	1	0	0	1	At3g04050.1:v:+2319:primary
GAAGTGATTG	1	0	0	0	1	At3g04010.1:d:+1021:primary
TTGCTTCCTC	0	0	2	0	0	At3g03940.1:X:-390:quaternary
CAAAACTCTA	1	0	0	0	1	At3g03940.1:i:+1433:tertiary
GGAAGCTAAA	0	0	2	0	0	At3g03910.1:d:+1320:primary
GAGACTGATT	0	1	1	0	0	At3g03880.1:d:+756:primary
TGAATAGATA	0	2	0	0	0	At3g03850.1:d:+360:primary
TGAAACACTT	1	1	0	0	0	At3g03790.1:d:+2533:secondary,At3g03790.2:d:+2542:secondary
AGTTACTTAG	0	0	0	0	2	At3g03730.1:v:+1363:secondary
TGTAAAAAAC	0	1	0	0	1	At3g03600.1:X:-844:quaternary
TTCTTTTTCT	0	1	1	0	0	At3g03550.1:d:+1425:primary
GGCCAAATGG	0	1	0	1	0	At3g03530.1:d:+1103:secondary
AGATCGTTGA	0	1	1	0	0	At3g03520.1:d:+1551:primary
TTAATATTTT	0	1	0	0	1	At3g03380.1:d:+3587:primary
GTGTCTATGT	0	2	0	0	0	At3g03380.1:d:+3107:secondary
TATGTTACTC	1	1	0	0	0	At3g03340.1:d:+1413:primary
TCCGGAAAAA	0	0	2	0	0	At3g03260.1:d:+1486:secondary
AGAGAGGGAA	0	0	0	0	2	At3g03250.1:X:-57:quaternary
GATAATAAGA	1	0	1	0	0	At3g03240.1:X:-242:quaternary,At5g44610.1:X:-608:quaternary
GCCACACCCT	0	1	1	0	0	At3g03120.1:d:+862:secondary
GCCGATTCTT	0	2	0	0	0	At3g03110.1:d:+3250:primary
TGTAGTCACT	0	1	0	0	1	At3g03090.1:d:+1729:primary

TTCACACTTT	0	1	0	0	1	At3g03060.1:v:+2309:primary
ATTCATAGGT	1	0	1	0	0	At3g03050.1:d:+3937:primary
TATACAAAAA	0	1	1	0	0	At3g03040.1:v:+49:secondary
GATTTGTACA	0	0	1	0	1	At3g03010.1:d:+776:primary
TCTATAGCTT	0	0	0	0	2	At3g02780.1:X:-225:quaternary,At3g51760.1:X:-61:quaternary
ATGTGATGTT	0	1	1	0	0	At3g02750.1:X:-729:quaternary
ACTACAAAAA	0	0	1	1	0	At3g02740.1:X:--191:quaternary,Chr5:-13988828:quaternary
GTCGTCTTCT	1	0	0	0	1	At3g02740.1:d:+1440:primary
GGACCAGATA	2	0	0	0	0	At3g02710.1:d:+1728:primary
GCGAAGAGGG	0	0	0	1	1	At3g02660.1:d:+1294:primary
AGACTGCTTA	1	1	0	0	0	At3g02630.1:d:+927:primary
GTCTTTTTCG	0	1	1	0	0	At3g02600.1:d:+840:primary
TGTCTCTCTC	1	0	1	0	0	At3g02580.1:d:+1090:secondary
CACGATCGTT	1	0	1	0	0	At3g02470.1:d:+764:secondary
ACTGGTCTGG	1	0	0	0	1	At3g02470.1:d:+1170:secondary
ATACCAAAAC	0	0	0	1	1	At3g02260.1:X:-1040:quaternary,At1g08125.1:X:-78:quaternary,At3g20760.1:X:-236:quaternary
ACCAATAATC	1	0	1	0	0	At3g02220.1:X:-137:quaternary
ATAAGCTTTC	0	2	0	0	0	At3g02170.1:d:+2613:secondary
GGCCAAACTA	0	1	0	1	0	At3g02030.1:X:+-234:quaternary
CCGTTGATAA	0	2	0	0	0	At3g01970.1:d:+618:primary
GGGTCGACAA	1	0	0	0	1	At3g01970.1:d:+327:secondary
ATGGCAGAGA	0	0	1	1	0	At3g01960.1:v:+612:secondary,At1g33900.1:v:+1146:secondary
TCTCTGGATA	1	1	0	0	0	At3g01860.2:d:+331:secondary,At3g01860.1:d:+606:secondary
ATTTCTTTTA	0	1	0	0	1	At3g01850.2:i:+309:tertiary,At3g01850.1:i:+309:tertiary
GCGGCAACAC	0	0	2	0	0	At3g01810.1:d:-3085:secondary
TGCAAGTCCA	0	1	0	0	1	At3g01800.1:d:+891:secondary
GGTCAGGGCC	0	2	0	0	0	At3g01770.1:d:+1345:secondary
ATCATTCGAG	0	0	1	1	0	At3g01720.1:d:+694:secondary
TGTGTGGTTT	1	0	1	0	0	At3g01680.1:d:+2411:secondary,At1g22050.1:d:+760:secondary
ACACTATTAA	0	0	1	0	1	At3g01550.1:d:+1386:primary
GTGTTTGCTT	0	0	2	0	0	At3g01500.2:d:+591:secondary,At3g01500.3:d:+591:secondary,At2g28120.1:d:+308:secondary,At5g14740.2:d:+642:secondary,At3g01500.1:d:+589:secondary, At5g14740.1:d:+760:secondary
TCGCAACAGT	1	0	1	0	0	At3g01490.1:X:-254:quaternary
CACTACGTTC	0	0	2	0	0	At3g01490.1:d:+1530:primary
CCCTGCCTTG	0	2	0	0	0	At3g01430.1:d:+204:primary
ACGATGTTCT	0	0	2	0	0	At3g01410.1:d:+1131:secondary,At5g06400.1:v:+196:secondary
GTGGATAAAT	0	1	0	0	1	At3g01400.1:d:+891:primary
TGAAATTATT	1	0	1	0	0	At3g01380.1:v:+3275:secondary
ATGGTCGAAG	0	1	0	1	0	At3g01370.1:d:+2231:secondary
AGAAGCATCA	0	0	1	0	1	At3g01360.1:d:+1570:secondary,At3g47570.1:d:+59:secondary,At1g58420.1:d:+119:secondary
AGAGCACATA	0	0	1	1	0	At3g01330.1:d:+1131:primary
AAAGAATTGT	0	2	0	0	0	At3g01310.1:d:+737:secondary,At5g15070.1:d:+722:secondary
TGGTAGTGAA	0	1	0	1	0	At3g01310.1:d:+2290:secondary,At5g17320.1:v:+1618:secondary
ATGAATCATA	1	0	1	0	0	At3g01200.1:d:+1263:primary
CCTTTGGTCT	0	2	0	0	0	At3g01180.1:X:-1323:quaternary
TCTTTGGAAT	0	1	0	1	0	At3g01180.1:d:+2589:secondary
AAAGAACACA	2	0	0	0	0	At3g01100.1:d:+2344:primary
GAAGTCTGGG	0	2	0	0	0	At2g48160.1:d:+2264:secondary
GTTAGCTACT	0	1	0	0	1	At2g48110.1:v:+2952:secondary
AAATGAAATC	0	1	0	1	0	At2g48100.1:d:+1259:primary,At2g48100.2:d:+1241:primary
GCCAACTTTC	0	1	0	1	0	At2g47940.1:X:-538:quaternary
CTGTTCTGAT	1	1	0	0	0	At2g47920.1:d:+917:primary
AACTCGTCTT	0	0	0	1	1	At2g47880.1:X:+836:quaternary
TTCTGCTTTC	0	0	0	1	1	At2g47690.1:d:+487:secondary
TCTCTTATTG	0	0	2	0	0	At2g47630.1:i:+1961:tertiary
ACGACGAAAG	0	1	1	0	0	At2g47460.1:d:+1180:secondary
CTGAGAAGGA	0	2	0	0	0	At2g47390.1:d:+1697:secondary
ACTAGTCGTC	0	1	1	0	0	At2g47350.1:d:+1438:primary,At2g47350.2:d:+1438:primary
CAAAACGTGG	0	0	1	0	1	At2g47330.1:X:-66:quaternary
TCTCATACGA	0	0	2	0	0	At2g47320.1:X:+335:quaternary

TGTCTTCTTA	0	0	1	1	0	At2g47250.1:i:+1969:tertiary
GATGTGAGAA	0	0	0	2	0	At2g47240.1:i:+4766:tertiary
CAGCATTCTG	0	0	0	2	0	At2g47200.1:d:+530:secondary
GAATAATCGC	1	0	1	0	0	At2g47130.1:X:+872:quaternary
GTCAATAGCA	2	0	0	0	0	At2g47090.1:X:-189:quaternary
GGCTACACCT	0	2	0	0	0	At2g47060.2:d:+976:primary,At2g47060.1:d:+976:primary
GAGTTTCTGG	0	0	1	1	0	At2g47010.1:d:+802:secondary,At3g21480.1:d:+515:secondary,At5g13570.1:d:+908:secondary,At2g47010.2:d:+802:secondary
TGCAATGAGA	0	2	0	0	0	At2g46990.1:d:+400:primary
GTTGTGTCAC	0	1	0	0	1	At2g46920.1:d:+3233:primary,At2g46920.2:d:+2787:primary
TATCGAGGCA	0	1	0	0	1	At2g46720.1:d:+2129:primary
ATTATGAAGA	2	0	0	0	0	At2g46680.1:d:+742:secondary,At2g03810.2:d:+213:secondary,At4g00430.1:d:+1285:secondary
GCTACTGGAA	1	0	0	1	0	At2g46580.1:d:+629:primary
CCTCTTCCTC	0	2	0	0	0	At2g46550.1:d:+384:secondary
TGCAAGTTAG	0	1	0	0	1	At2g46520.1:d:+3143:secondary
ACTACGGTAG	0	2	0	0	0	At2g46370.2:d:+1065:primary,At2g46370.1:d:+1148:primary
TCGATTTCTA	0	1	0	1	0	At2g46270.2:d:+1379:secondary,At2g46270.1:d:+1448:secondary,At3g49240.1:d:+111:secondary
AAGTAGCATC	0	1	0	0	1	At2g46260.1:d:+1243:secondary
GGCTATACTA	0	2	0	0	0	At2g46250.1:d:+273:secondary,At5g13640.1:d:+30:secondary
AAACTAATCT	0	0	0	2	0	At2g46225.1:i:+1876:tertiary
TAATTATCTT	0	2	0	0	0	At2g46225.1:d:+1161:secondary
GTTCATATAA	1	0	1	0	0	At2g46130.2:X:+452:quaternary
TGTTGTGTGC	0	2	0	0	0	At2g46130.1:d:-201:primary,At2g46130.2:d:-125:primary
GAGCCTTCAA	0	1	1	0	0	At2g46110.1:d:+1149:secondary
GGCCATCATA	0	0	2	0	0	At2g46090.1:d:+724:primary
AGGTGAGATC	0	1	1	0	0	At2g46020.2:v:+6307:secondary,At2g46020.1:v:+6304:secondary
AAGAAACAGA	1	0	0	1	0	At2g45900.1:v:+1588:secondary,At1g09160.2:d:+1087:secondary,At3g27870.1:d:+1193:secondary,At3g18540.1:v:+529:secondary,At1g09160.1:d:+1150:secondary
AAAGTGAAAA	0	1	1	0	0	At2g45900.1:v:+1303:secondary,At4g32190.1:d:+1150:secondary
AGAGCTGGGA	0	0	2	0	0	At2g45880.1:X:-166:quaternary
GAATCTAGGA	0	0	1	0	1	At2g45850.1:d:+1443:secondary,At5g33415.1:p:+1605:secondary,At2g45850.2:d:+1311:secondary
TTTGACCCAG	1	0	0	0	1	At2g45830.1:d:+858:secondary,At2g45830.2:d:+961:secondary
CAGCAGAAGA	1	1	0	0	0	At2g45810.1:d:-2070:secondary
ATATGAACCT	1	0	0	1	0	At2g45810.1:d:+2075:secondary
CTTCCACAAG	1	1	0	0	0	At2g45770.1:d:+928:primary
CAGATGAGTT	0	0	2	0	0	At2g45730.1:d:+1322:primary
GCAGTTGTAT	1	0	0	0	1	At2g45630.2:v:+1441:secondary
GGCTAAGCAA	0	1	0	1	0	At2g45590.1:d:+1864:secondary
ATTCCAGATG	0	2	0	0	0	At2g45440.1:d:+1203:primary
AGAGGTGATG	0	0	1	0	1	At2g45330.1:d:+282:secondary,At5g23600.1:v:+438:secondary
AGTTTTTTTC	0	0	0	2	0	At2g45280.1:d:+1274:primary
GGGCCGTCAT	0	1	0	0	1	At2g45210.1:d:+786:secondary
GTAAGATCCG	0	1	1	0	0	At2g45150.1:d:+1308:primary,At2g45150.3:d:+1299:primary,At2g45150.2:d:+1473:primary
TCTTTTTCTC	0	0	0	2	0	At2g45135.1:v:+1340:secondary
GGGGCACTTG	0	2	0	0	0	At2g45135.1:v:+1181:secondary
AAGGTGTATT	1	1	0	0	0	At2g45060.1:d:+1168:primary
ACACAAGGAA	0	0	1	1	0	At2g45030.1:d:+2155:primary
AGAAGACTTT	0	0	1	1	0	At2g45010.1:X:+-21:quaternary
ACTTCATTGA	2	0	0	0	0	At2g45010.1:d:+1038:secondary,At2g45010.2:d:+1624:secondary
TCAGATAAAA	1	1	0	0	0	At2g45000.1:i:-2692:tertiary
ATTGGATTTA	0	1	0	1	0	At2g44950.1:i:+1852:tertiary,At5g03345.1:i:+386:tertiary
AGACTATTGT	0	0	1	1	0	At2g44900.1:v:+3294:secondary
GACTGTAATG	0	0	2	0	0	At2g44870.1:X:-628:quaternary
GCTTCGTTTT	0	0	0	0	2	At2g44850.1:v:+1777:primary
GAAGGATAAG	0	1	0	1	0	At2g44820.1:d:+161:secondary,At2g44820.2:d:+274:secondary
TGGAACCACT	0	1	0	1	0	At2g44790.1:d:+909:primary
GTTGGAGTTG	0	0	2	0	0	At2g44540.1:d:+1204:primary
TTTGCCCCTT	0	1	0	0	1	At2g44430.1:d:+2012:primary
AAAATGAGAT	0	1	0	1	0	At2g44230.1:d:+1487:primary

TGAATGGGAA	0	2	0	0	0	At2g44210.1:d:+524:secondary
GAGTGAAGAG	1	0	0	1	0	At2g44210.1:d:+1624:secondary
GCATACTCGC	0	1	1	0	0	At2g44190.1:d:+1632:primary
ACCATCTTGG	1	0	1	0	0	At2g44090.1:d:+2161:secondary
CCACTACATT	1	0	0	1	0	At2g43800.1:d:+2572:primary
TCAAGAGAGA	0	0	1	0	1	At2g43770.1:d:+137:secondary,At3g13682.1:v:+2663:secondary
TAATAAGTGA	0	0	0	1	1	At2g43590.1:d:+838:primary
TTTGCTCATT	2	0	0	0	0	At2g43590.1:d:+364:secondary,At2g43580.1:d:+382:secondary
CTTTCGCCGG	0	1	0	1	0	At2g43540.1:d:+124:primary
GTGAGAACCA	0	0	1	1	0	At2g43490.1:v:+2560:secondary
ACTTACACAT	2	0	0	0	0	At2g43330.1:d:+1842:primary
TTTCGTGTTC	1	0	1	0	0	At2g43250.1:d:+2348:secondary
AATTTGCCTA	0	0	0	1	1	At2g43230.1:d:-1637:secondary
AAGCCAAAGG	0	1	1	0	0	At2g43210.1:d:+980:primary,At2g43210.2:d:+958:primary
GCGTCTTTAG	1	0	1	0	0	At2g43180.1:d:+1021:secondary,At2g43180.4:d:+1021:secondary,At2g43180.2:d:+1021:secondary,At2g43180.3:d:+1021:secondary
GAGACCTCTG	0	2	0	0	0	At2g43130.1:d:+474:primary
TATGGTCCAA	0	1	0	0	1	At2g43110.1:d:+827:primary
ATTCAACAAT	1	1	0	0	0	At2g43070.1:d:+1706:secondary
ATCTCTACGA	0	2	0	0	0	At2g43020.1:d:+1689:primary
AAGAACCTAT	0	0	1	0	1	At2g42980.1:v:+1985:primary,At3g29090.1:d:+1107:primary
ACAATGAAAA	0	1	0	0	1	At2g42955.1:v:+724:secondary
TGTATATAAC	0	2	0	0	0	At2g42890.1:d:+2927:secondary,At2g42890.2:d:+2805:secondary,At2g42900.1:v:+199:secondary
CGATTACTGG	0	0	1	1	0	At2g42840.1:d:+633:secondary,At2g42840.2:d:+633:secondary
TTGTTCTGAG	0	1	0	1	0	At2g42790.1:i:+875:tertiary
ACATTTACAT	0	0	0	0	2	At2g42750.1:d:-1211:secondary
GACAACGTTG	1	0	1	0	0	At2g42560.1:d:+1989:primary
TGGTCAACTT	0	1	0	1	0	At2g42520.1:d:+1613:primary
CATCATCATC	0	1	1	0	0	At2g42380.1:d:+1232:primary,At2g42380.2:d:+1265:primary
GATATAGCAG	2	0	0	0	0	At2g42320.1:d:+1810:primary
TATTTCAAAG	1	0	0	0	1	At2g42230.1:d:+1708:primary,At2g42230.2:d:+1708:primary
TCTGCCTCAA	1	1	0	0	0	At2g42220.1:d:-750:primary
TCAGATTTTA	0	1	0	1	0	At2g42070.1:d:+1175:secondary
AGAGGACATT	1	0	1	0	0	At2g42010.1:d:+3293:primary
AACGTCAACG	1	0	1	0	0	At2g41940.1:d:+90:secondary
TGAAGAATGA	0	0	1	1	0	At2g41910.1:v:+1213:secondary
GTCATCAAAA	0	0	2	0	0	At2g41900.1:d:+2169:secondary,At1g04520.1:d:+15:secondary
AGAGCCCTTG	0	0	2	0	0	At2g41870.1:d:+1192:secondary
TCCAGAAGTG	0	1	1	0	0	At2g41835.1:d:+1123:primary
GTTTCCATAT	2	0	0	0	0	At2g41800.1:i:+205:tertiary
TAATGTTCCA	1	1	0	0	0	At2g41770.1:d:+2690:primary
TTTGTGCAAG	0	0	2	0	0	At2g41500.1:d:+1886:primary
ATCAACACAC	0	0	0	1	1	At2g41470.1:X:-395:quaternary
CACATCACAA	1	1	0	0	0	At2g41370.1:i:+766:tertiary,At2g34890.1:i:+1791:tertiary
CTGTTAAACC	0	0	1	1	0	At2g41310.1:d:+1501:primary
TTCAGACTTC	0	0	1	1	0	At2g41250.1:d:+977:secondary
GCCGGCGAGC	1	1	0	0	0	At2g41170.1:d:+989:primary
GCTTCGTTAT	1	0	1	0	0	At2g41160.1:d:+775:primary
AAGTGTAATA	1	0	0	0	1	At2g41000.1:d:+1015:primary
GCTAAGGATT	0	0	0	2	0	At2g40960.1:d:+1189:primary
TTTAGTTCAG	0	1	1	0	0	At2g40950.1:d:+1845:primary
AACCTTCTCG	0	0	1	1	0	At2g40900.1:d:+885:secondary
ATCTCCGAGG	0	1	1	0	0	At2g40880.1:d:+174:secondary
GATGCTTTCT	0	2	0	0	0	At2g40830.1:d:+394:primary,At2g40830.3:d:+375:primary,At2g40830.2:d:+471:primary,At4g00480.1:d:+1503:primary
AATCTTTTCC	0	1	0	0	1	At2g40800.1:d:+1398:primary
GAATGCTTCA	0	1	0	0	1	At2g40750.1:d:+408:secondary,At4g28180.1:v:+1101:secondary
AATGTGAATT	1	0	0	1	0	At2g40690.1:d:+1483:primary
TGGACGAAGT	0	1	1	0	0	At2g40650.1:d:+587:secondary
AAGACCATCT	0	1	1	0	0	At2g40620.1:d:+1107:primary
TGTCCAAAAA	0	0	2	0	0	At2g40570.1:d:-1092:secondary

TAGCGCTCAA	0	0	1	1	0	At2g40570.1:d:+1817:primary
TTTAGCTTCA	1	0	1	0	0	At2g40540.1:d:+2658:primary
AACTCCCATC	0	1	1	0	0	At2g40530.1:d:-657:secondary,At2g40530.1:d:-665:secondary
GGTGTGATTC	0	0	1	1	0	At2g40400.2:d:+2284:primary
ATTTCATTTG	0	1	1	0	0	At2g40400.1:d:+2046:secondary,At2g40400.2:d:+2046:secondary
GTGCGTTTAG	0	1	0	1	0	At2g40380.1:d:+570:primary
GGAGTGATGA	1	0	0	0	1	At2g40360.1:d:+420:secondary
GGGTAAATGG	0	1	0	1	0	At2g40350.1:d:+254:primary
GCTAAGCTAC	0	0	1	1	0	At2g40290.2:d:+1172:secondary,At2g40290.1:d:+1084:secondary
AGCAGCAGAT	0	1	0	1	0	At2g40140.1:d:+1877:secondary
TATTCTGATC	0	0	1	0	1	At2g40080.1:d:+279:primary
TTTATTACTC	0	0	0	0	2	At2g40070.1:X:-994:quaternary
GTAACTTGTC	0	1	1	0	0	At2g40070.1:d:+1895:primary
GGATTTGATC	1	0	0	0	1	At2g40010.1:d:+934:primary
GAGGAGAAGA	0	1	0	1	0	At2g39960.1:d:+153:primary
TCCAAAGCTT	1	0	0	0	1	At2g39950.1:X:-526:quaternary
AAGTTTGATG	0	1	0	0	1	At2g39920.1:d:+994:secondary,At2g20540.1:v:+816:secondary
CATATGGTCG	1	0	0	1	0	At2g39840.1:d:+1375:primary
AGAATATCTT	0	0	1	1	0	At2g39710.1:d:+1369:primary
AGAGGTCCTT	1	0	0	1	0	At2g39690.1:d:+1519:primary
AACTGGGGTC	0	0	2	0	0	At2g39580.1:i:+3064:tertiary
ACTTTATTAC	0	1	1	0	0	At2g39500.1:d:+193:primary
TTTGCAGAGA	0	0	1	0	1	At2g39470.1:i:+536:tertiary
TCTCCGGCTA	0	2	0	0	0	At2g39460.1:d:+130:secondary
TCATTGGTGA	0	1	1	0	0	At2g39350.1:d:+2049:secondary,At4g29360.1:d:+1057:secondary,At4g29360.2:d:+921:secondary
TAATGAAACC	0	1	1	0	0	At2g39340.1:d:+4046:primary
ATGAAATATT	1	1	0	0	0	At2g39340.1:d:+3303:secondary
TCGACTAAAT	1	0	1	0	0	At2g39330.1:d:+1467:secondary
AGTTTCTGTG	1	0	0	0	1	At2g39260.1:d:+3760:secondary
AGAAATTGAG	1	0	0	1	0	At2g39230.1:v:+145:secondary
TTGATTTTGT	0	1	0	0	1	At2g39210.1:d:+1080:primary,At1g72850.1:v:+1941:primary
TGAAGAGTAG	1	0	0	0	1	At2g39090.1:d:+1742:primary
TACGACACAG	0	0	0	1	1	At2g39010.1:d:-467:secondary
ACAGTTAGAC	0	0	1	0	1	At2g38860.2:X:-169:quaternary
TAGTTTGGTG	1	1	0	0	0	At2g38860.1:d:+1374:primary,At2g38860.2:d:+1397:primary
CTCGTTGTTT	1	1	0	0	0	At2g38840.1:d:+2092:primary
CATACGTAGA	0	2	0	0	0	At2g38820.1:d:+1337:primary,At2g38820.2:d:+1470:primary
ATTGAGGATG	0	0	1	0	1	At2g38810.1:d:+668:primary,At2g38810.2:d:+571:primary,At2g38810.3:d:+700:primary
GATGATCAAA	0	0	1	1	0	At2g38790.1:d:+750:primary,At1g73310.1:v:+1526:primary
TAAGTGTTGT	0	1	0	1	0	At2g38780.1:d:+1421:primary
ATTTGGTTTC	0	1	0	0	1	At2g38695.1:v:+1344:primary
GTTTATCCTA	0	1	1	0	0	At2g38660.1:d:+1250:primary
CGCTAGGAAG	0	1	1	0	0	At2g38530.1:d:-398:secondary
GCGTAACCAA	0	0	1	1	0	At2g38480.1:d:+1215:primary
TCGAGATTCT	1	0	1	0	0	At2g38430.1:X:-61:quaternary
GCAAAGGGAA	1	1	0	0	0	At2g38400.1:d:+1046:secondary
TTGGGGATAT	1	0	0	0	1	At2g38390.1:d:+1105:primary
GAATCAATCT	0	1	0	0	1	At2g38280.2:d:+2128:secondary,At2g38280.1:d:+2128:secondary
GGCTAAGGGA	0	0	1	0	1	At2g38270.1:d:+768:secondary
AACAAAACAA	0	0	0	0	2	At2g38255.1:i:+1207:tertiary
TTAAAGTTAA	1	1	0	0	0	At2g38255.1:d:+868:primary
CTAAGACTAT	2	0	0	0	0	At2g38130.2:d:+770:secondary,At2g38130.1:d:+817:secondary
AGAGATTAAT	0	2	0	0	0	At2g38120.1:i:+2596:tertiary
TTCCAGAAGC	0	0	2	0	0	At2g38050.1:d:+1248:primary
CTTAGGGACT	0	1	0	0	1	At2g38040.2:d:+1918:secondary,At2g38040.1:d:+1918:secondary
GAAGAGGAGG	0	1	0	1	0	At2g38025.1:d:+591:primary
ACTACTGTTG	1	0	0	0	1	At2g37940.1:d:-1420:secondary
CAATGCTAAG	0	1	1	0	0	At2g37900.1:v:+2118:secondary
ACTGCCAAAC	0	1	0	1	0	At2g37860.2:d:+1135:primary
ATGGATCGGA	0	1	1	0	0	At2g37840.1:d:+1959:primary,At2g37840.2:d:+2244:primary
TCTTCTTCCT	0	1	0	1	0	At2g37790.1:i:+637:tertiary

CTTGGGATTT	0	0	1	0	1	At2g37760.1:d:+1179:secondary,At2g37760.2:d:+1352:secondary
TTGTGGAATG	0	1	1	0	0	At2g37670.1:v:+2092:secondary,At1g77830.1:v:+1151:secondary,At2g02760.1:d:+282:secondary
GTGTTCACCG	0	0	2	0	0	At2g37580.1:d:+459:primary
GTACTCTTTC	0	0	1	1	0	At2g37520.1:X:-14:quaternary
TTTTGTATAG	2	0	0	0	0	At2g37520.1:d:+2897:secondary
AAGGTTTAGA	0	0	0	1	1	At2g37510.1:i:+456:tertiary
ATAAAGTAAT	1	1	0	0	0	At2g37440.1:d:+1099:secondary,At2g37440.2:d:+1013:secondary
GATCTGAAAT	0	0	0	2	0	At2g37320.1:v:+2321:primary
ACGTTGAATA	0	0	1	1	0	At2g37310.1:v:+2485:primary
ACCACACCCC	0	1	1	0	0	At2g37150.2:X:-47:quaternary
TTCACTTTAC	0	2	0	0	0	At2g37120.1:d:+554:primary
ATTATATCAT	0	0	0	0	2	At2g36810.1:i:+7549:tertiary,At1g05860.1:i:+857:tertiary
TTTTGTGTTT	1	0	0	1	0	At2g36570.1:d:+2222:primary
TTTCCCTTAC	0	1	0	0	1	At2g36530.1:X:-252:quaternary
CATCCAGCCT	0	1	0	1	0	At2g36490.1:d:+3932:primary
TCAACATTCG	0	2	0	0	0	At2g36490.1:d:+2232:secondary
ATGTGGGATT	0	1	0	0	1	At2g36470.1:d:+1340:primary
GACGAGGATT	0	1	1	0	0	At2g36450.1:v:+756:secondary,At1g06200.1:d:+633:secondary
GGCGGACCAT	0	2	0	0	0	At2g36400.1:d:+1308:secondary
GATTAAAACT	0	2	0	0	0	At2g36390.1:i:+1771:tertiary
GTGCTGCAGT	0	1	0	1	0	At2g36350.1:d:+2657:primary
TTACCTCGCT	0	2	0	0	0	At2g36350.1:d:+1085:secondary
CGTCGGAGAT	1	0	0	1	0	At2g36240.1:X:+1617:quaternary
CCCTTCTCAC	1	0	0	0	1	At2g36160.1:X:+277:quaternary
AAAATAGGCC	0	0	2	0	0	At2g36160.1:d:+477:secondary
TAGTACGGAG	1	1	0	0	0	At2g36080.1:X:-186:quaternary
AAGCTTGTGC	0	2	0	0	0	At2g36000.2:d:+960:primary
ATTCTTGCAT	0	1	1	0	0	At2g35930.1:d:+1353:primary
GAGTAATTAA	0	0	1	1	0	At2g35640.1:v:+1504:primary
ACTGAAACGT	1	1	0	0	0	At2g35540.1:v:+1209:secondary
GACTTGGAGT	0	1	1	0	0	At2g35510.1:d:+1445:secondary
CGACAGGTAA	0	2	0	0	0	At2g35340.1:v:+3558:secondary,At1g32490.1:d:+3156:secondary
TTTATCAAAA	1	0	1	0	0	At2g35310.1:d:+688:secondary
GATGAATAAA	0	1	0	0	1	At2g35270.1:v:+1477:secondary
TTCTGGTAAT	1	1	0	0	0	At2g35190.1:d:+1570:primary
AAGTTCTCGC	1	1	0	0	0	At2g35155.1:d:+220:secondary
ATATATACCA	0	0	0	0	2	At2g35030.1:X:+2238:quaternary
ACCAACAAAA	0	0	1	1	0	At2g35000.1:d:+1601:primary
AACTGCTTCG	1	0	0	1	0	At2g34960.1:d:+1869:primary
CATCCTCAAT	1	1	0	0	0	At2g34860.1:d:-659:secondary
GTTCAAGCTA	0	1	0	1	0	At2g34840.1:d:+987:primary
AATTGGTATA	1	0	0	0	1	At2g34810.1:d:+1369:secondary
CCCAACCTAC	0	1	1	0	0	At2g34770.1:d:+799:primary
CAGAGTCAAC	0	0	1	1	0	At2g34730.1:d:+2197:primary
AGTTCATTGG	1	0	0	1	0	At2g34720.1:X:-413:quaternary,At2g42570.1:X:-28:quaternary
GTCCAAAAAA	0	0	2	0	0	At2g34710.1:X:--92:quaternary
TTCGTGAATT	0	0	1	1	0	At2g34710.1:d:+2812:primary
AGAGGAAGTC	2	0	0	0	0	At2g34670.1:d:+1593:secondary
CGAATCTAGC	0	2	0	0	0	At2g34630.1:d:+1216:primary
GAAGAGTTTC	0	0	2	0	0	At2g34630.1:d:+1086:secondary,At4g28570.1:d:+2051:secondary,At1g09860.1:d:+26:secondary
GAGATATTAA	0	1	0	1	0	At2g34610.1:d:+1072:primary
TGTGTTTCTT	1	1	0	0	0	At2g34600.1:d:+208:primary
CGAACAATCT	0	2	0	0	0	At2g34520.1:d:+159:primary
AAACACGTTA	0	1	0	1	0	At2g34490.1:X:-1762:quaternary
AGTGTGATTA	0	0	1	1	0	At2g34490.1:d:+1607:secondary
GGACAGATAC	0	0	0	2	0	At2g34460.1:d:+535:secondary
GTGATTCGGC	0	1	0	1	0	At2g34430.1:d:-163:primary
ACTCTTAAAA	0	0	1	0	1	At2g34357.1:i:-5138:tertiary
GTTGAAGGCT	0	1	0	1	0	At2g34357.1:d:+2881:secondary
CGTCTTTAGA	0	2	0	0	0	At2g34070.1:i:+724:tertiary
TTACCTTCAA	0	0	2	0	0	At2g34070.1:i:+1644:tertiary

TATATTACAT	1	0	0	0	1	At2g34070.1:d:+1256:primary
TATGAAGTGA	1	1	0	0	0	At2g33980.1:d:+1843:secondary
TTGCTAGACA	1	0	0	1	0	At2g33860.1:d:+1965:primary
GTTTGATGAG	0	1	0	1	0	At2g33840.1:d:+967:primary
AGCTCAAACT	1	1	0	0	0	At2g33835.1:d:+1932:primary
GCCTTGGCCG	0	0	2	0	0	At2g33830.2:d:+96:secondary,At2g33830.1:d:+94:secondary
AGGTAGAAGG	0	1	1	0	0	At2g33610.1:d:+1239:primary
TATCATTTTT	0	2	0	0	0	At2g33570.1:d:+1700:primary
TTTTGAAATT	0	1	0	1	0	At2g33560.1:d:+1421:primary,At3g44690.1:v:+4369:primary
ATCGGTCAGA	0	0	0	2	0	At2g33540.1:d:+4100:primary
TGATGCAGCC	1	0	0	0	1	At2g33440.1:d:+792:primary
TTGAAGACAG	1	0	0	1	0	At2g33435.1:d:+318:secondary
TAACTGTCTG	0	0	1	1	0	At2g33400.1:X:-1134:quaternary
GTCGGTGAGT	0	1	0	1	0	At2g33290.1:d:+2199:primary
TCATTAAAAA	0	0	1	1	0	At2g33240.1:v:+6123:primary
CACCAAAACC	0	0	2	0	0	At2g33150.1:d:+1528:secondary
TGAAACTTTG	0	0	1	1	0	At2g33020.1:v:+3053:secondary,At3g33005.1:p:+1038:secondary
GAAAAGAAGT	0	0	2	0	0	At2g32920.1:d:+910:secondary,At1g71270.1:d:+950:secondary,At1g71300.1:d:+791:secondary,At3g16200.1:d:+403:secondary
GTATCTCAAG	0	1	1	0	0	At2g32760.1:d:+830:secondary,At4g37120.1:d:+191:secondary
ACTGTTGGAT	1	1	0	0	0	At2g32720.1:d:+104:primary
ACAACCCCGG	0	1	0	0	1	At2g32710.2:d:+654:secondary,At2g32710.1:d:+700:secondary
GCATTTACAA	0	0	2	0	0	At2g32690.1:p:+196:secondary
AATCTTCTGG	1	0	1	0	0	At2g32440.1:d:+985:primary
ACATTGTTCT	1	0	0	1	0	At2g32415.1:d:+1849:secondary
TATATTGATT	0	2	0	0	0	At2g32410.1:d:+1838:primary
TCGACAGCGA	0	1	0	1	0	At2g32390.1:d:+2414:primary
TTGACAAAAA	0	0	0	1	1	At2g32350.1:v:+1241:secondary
AATTGTTTTT	0	0	0	1	1	At2g32220.1:d:+486:primary
GTTGGTGTGC	1	0	0	0	1	At2g32120.1:d:+1784:primary,At2g32120.2:d:+1797:primary
AGTTACGAAT	0	0	0	2	0	At2g32080.1:d:+1451:primary,At2g32080.2:d:+1448:primary
TCAGGAGTAG	0	0	1	0	1	At2g32010.1:d:+2538:primary
GGAGGTCCTT	0	1	0	0	1	At2g32000.1:d:+2520:primary
TATATAGTTT	0	2	0	0	0	At2g31960.1:v:+6267:secondary
GCTTTCTTCT	0	0	2	0	0	At2g31840.1:d:+998:primary
TGGAAGTTGT	0	1	0	0	1	At2g31820.1:v:+1264:secondary,At1g64860.1:d:+858:secondary,At4g08220.1:p:+323:secondary
AGAAGAGAGC	0	1	0	1	0	At2g31800.1:X:-71:quaternary
TATGTCAACC	0	1	1	0	0	At2g31580.1:d:+1837:primary
AGCCAGAAGT	0	2	0	0	0	At2g31560.2:d:+466:primary
CAGTTTGGGC	0	2	0	0	0	At2g31510.1:X:-286:quaternary
TTGCTGTGTT	0	0	0	0	2	At2g31340.1:d:+2061:primary
CTGAAAAGCT	1	1	0	0	0	At2g31280.1:d:+2390:secondary,At4g25730.1:d:+1534:secondary
AGACAGATGT	1	0	0	1	0	At2g31270.1:d:+1818:primary
TATTGTTAGG	0	0	1	1	0	At2g31260.1:X:-1012:quaternary
TCTTTCAGAG	1	0	0	1	0	At2g31200.1:d:+122:secondary
AATCCACAAT	1	1	0	0	0	At2g31190.1:d:+1479:primary
TTGCTAAAAA	0	0	0	1	1	At2g31170.1:i:+1508:tertiary
TAGACCCTTC	0	1	0	1	0	At2g31170.1:d:+65:secondary
AGTCAGATTA	2	0	0	0	0	At2g31150.1:d:+1987:primary
AATACGCCCC	0	0	1	1	0	At2g31130.1:d:+364:secondary
GCAGATCTTT	1	0	1	0	0	At2g31070.1:d:+306:secondary
GCAGATGGAG	0	1	0	1	0	At2g31040.1:d:+866:secondary
AGGATTCTGA	0	0	0	1	1	At2g31020.1:d:+1348:secondary,At1g73930.1:d:+2347:secondary,At5g55300.1:d:+446:secondary
TTCTCACAGG	0	2	0	0	0	At2g31010.1:d:+2784:secondary
ATTCCATAAC	1	0	0	1	0	At2g30960.1:d:+958:secondary
GTTTTAATGA	0	0	0	0	2	At2g30900.1:v:+390:secondary,At5g29720.1:p:+653:secondary,At5g36275.1:p:+595:secondary
AAGAACAGAG	1	0	0	1	0	At2g30880.2:d:+1916:secondary,At2g30880.1:d:+1900:secondary
TCTCTTCGGT	0	0	1	1	0	At2g30800.1:d:+4147:secondary
ATCACTCCTA	0	1	0	1	0	At2g30790.1:v:+762:secondary
TAGCTGAAGT	0	0	1	0	1	At2g30760.1:X:--89:quaternary,At3g57260.1:X:-531:quaternary
AAATTTGGGG	0	1	0	0	1	At2g30710.1:d:+1843:primary,At3g18295.1:d:+715:primary

GTGCTTGCTG	0	0	1	0	1	At2g30710.1:d:+1612:secondary
TCTCTCAGCA	2	0	0	0	0	At2g30640.1:X:-1775:quaternary
ATACCCGTCA	1	0	1	0	0	At2g30550.2:d:-1383:primary
GTTTATTCTA	0	0	0	0	2	At2g30460.1:v:+1223:secondary
TTGCTAGGAC	0	1	1	0	0	At2g30370.1:v:+1151:secondary
ATGCTACAGC	0	1	1	0	0	At2g30260.1:d:+592:primary
CGTTTGGCAT	0	1	0	0	1	At2g30160.1:d:+1194:primary
TTTTGAGTCG	2	0	0	0	0	At2g30060.1:d:+832:secondary
TAATTTTATG	1	0	0	1	0	At2g29995.1:X:+339:quaternary
ATGACCGATT	0	0	0	0	2	At2g29990.1:v:+2104:secondary
GTCACGATGA	1	1	0	0	0	At2g29980.2:d:+882:secondary,At1g64090.1:d:+143:secondary,At2g29980.1:d:+897:secondary
ATGAATTGAT	1	0	1	0	0	At2g29950.1:v:+982:secondary
TGTTTTCTAA	0	0	1	0	1	At2g29670.1:i:+1721:tertiary,At3g17950.1:i:+129:tertiary
TTGGTTTGTC	0	0	2	0	0	At2g29660.1:d:+1372:primary
AACTCCTTCA	1	1	0	0	0	At2g29570.1:d:+742:primary
GTTCACAGAT	1	1	0	0	0	At2g29210.1:d:+2219:secondary
ATGACTACAA	2	0	0	0	0	At2g29000.1:v:+565:secondary,At1g20020.1:d:+780:secondary
AGAGGTAAGA	2	0	0	0	0	At2g28950.1:X:-458:quaternary
TAAAGAAGAA	0	2	0	0	0	At2g28930.1:d:+1650:secondary,At3g27100.1:d:+295:secondary,At2g21040.1:v:+505:secondary,At2g21100.1:d:+392:secondary
GGACTATCAA	0	0	2	0	0	At2g28840.1:d:+1459:secondary
ATCGGTTACT	0	2	0	0	0	At2g28800.2:d:+1061:secondary,At2g28800.1:d:+1061:secondary,At5g66750.1:d:+2459:secondary
AGCTCGGATC	1	1	0	0	0	At2g28670.1:d:+1081:primary
CAATGGATCC	0	0	2	0	0	At2g28510.1:d:+1271:primary
TTACGTGTTA	0	0	1	0	1	At2g28500.1:v:+1213:secondary
ACTCCGTTTC	0	2	0	0	0	At2g28430.1:d:+162:primary
TACAAACAAC	0	1	1	0	0	At2g28380.1:d:+1198:primary
TCCTATGAAA	1	1	0	0	0	At2g28310.2:d:-1389:secondary,At2g28310.1:d:-1459:secondary
GCAAGGAGTG	0	0	0	1	1	At2g28310.2:d:+1193:secondary,At2g28310.1:d:+1263:secondary
GTTGTGGTTG	0	1	1	0	0	At2g28290.2:d:+9898:primary
GAGGAAGTAG	0	1	1	0	0	At2g28250.1:d:+826:secondary,At5g59440.1:d:+850:secondary,At5g59440.2:d:+839:secondary
ATTATGATTT	0	1	0	0	1	At2g28180.1:i:+1526:tertiary,At3g52180.1:i:+1136:tertiary
AACTAAATTA	1	0	0	1	0	At2g28130.1:i:+2251:tertiary
TGGGCTGTGT	0	1	1	0	0	At2g28130.1:d:+1408:primary
CCAAGAGGTA	0	2	0	0	0	At2g28110.1:d:+842:primary
GATATGTTTA	2	0	0	0	0	At2g28040.1:d:+1315:primary
GTTAAGAATG	0	0	0	2	0	At2g27970.1:d:+509:primary
TTCTTTTTTT	1	0	0	1	0	At2g27940.1:d:+740:primary,At5g11160.1:d:+852:primary
TTTAGGACAA	1	1	0	0	0	At2g27770.1:d:+1165:primary
TTTGGCGGTG	0	0	2	0	0	At2g27580.1:X:-177:quaternary,At1g53260.1:X:-441:quaternary,At1g53260.1:X:-405:quaternary
GAAGAGAAGC	0	0	1	0	1	At2g27530.2:d:+585:secondary,At3g14230.1:d:+866:secondary,At2g27530.1:d:+616:secondary,At2g11060.1:p:+1779:secondary,At1g31570.1:p:+5352:secondary,At1g53720.1:d:+1606:secondary,At5g66520.1:v:+1336:secondary,At3g14230.3:d:+851:secondary,At4g16330.1:d:+299:secondary,At3g14230.2:d:+854:secondary,At5g45990.1:v:+816:secondary,At2g12210.1:p:+5263:secondary
TAACTATCTT	1	0	1	0	0	At2g27385.1:d:+587:secondary
CTGCTTCTTA	0	2	0	0	0	At2g27350.4:X:-341:quaternary
TCAACAAGAA	0	0	1	0	1	At2g27170.1:v:+3274:primary
ATTCTCAAAG	0	1	1	0	0	At2g27140.1:d:+756:primary
GAGATTTAAG	0	2	0	0	0	At2g27110.2:d:+3230:primary,At2g27110.1:d:+3044:primary
GAGACTTAAG	1	0	0	0	1	At2g27110.1:X:+348:quaternary
GTATGGGTGG	0	0	1	1	0	At2g27100.1:d:+975:secondary,At5g63020.1:d:+542:secondary
TTCTTCTGTT	1	1	0	0	0	At2g27040.1:d:+2894:primary
TAATGACAAA	0	2	0	0	0	At2g27030.3:d:+361:primary
CAAAGAAGGC	0	2	0	0	0	At2g27020.1:d:+676:secondary
AAACCCACGG	0	0	1	0	1	At2g26975.1:d:-502:secondary
AAACCATTTT	1	1	0	0	0	At2g26930.1:d:+1343:secondary
CTCTTTTTGT	0	1	1	0	0	At2g26710.1:d:+1832:primary
TATGAAAAAT	0	1	0	0	1	At2g26660.1:v:+1390:primary
AGAGGAGAGT	0	0	2	0	0	At2g26650.1:d:+2254:secondary
GCGTTGCAAG	0	1	0	0	1	At2g26600.2:d:+864:primary,At2g26600.1:d:+1084:primary
ATGAGCAAAC	1	0	1	0	0	At2g26590.1:i:+1471:tertiary

CAGCAGCCTG	0	0	0	2	0	At2g26580.2:d:+168:secondary,At2g26580.1:d:+127:secondary
GCAGCTGCTC	0	1	1	0	0	At2g26580.1:d:+194:primary,At2g26580.2:d:+235:primary
CTCTTATAAA	0	2	0	0	0	At2g26560.1:d:+1231:secondary
TTGGCCTACT	1	1	0	0	0	At2g26470.1:d:+1621:primary
CCTACCTTTT	0	2	0	0	0	At2g26350.1:d:+1349:primary
TGTAATTTCA	1	1	0	0	0	At2g26330.1:i:+1376:tertiary
AGCCATCAGA	0	1	0	1	0	At2g26220.1:p:-386:secondary
TAACTTGGCT	1	0	1	0	0	At2g26170.2:d:+2073:primary,At5g48320.1:d:+2953:primary,At2g26170.1:d:+1966:primary
ACCCATTCGA	0	0	1	0	1	At2g26135.1:v:-673:secondary,At5g08300.1:d:-1000:secondary
GACTTTGTTA	0	1	0	0	1	At2g26070.1:i:+927:tertiary
TCGAGTTAAT	1	1	0	0	0	At2g25840.2:d:+468:primary,At2g25840.1:d:+468:primary,At2g25840.3:d:+432:primary
GAGCCAAACA	0	1	1	0	0	At2g25800.1:d:+2907:primary
CTTGTGATTC	0	1	0	1	0	At2g25800.1:d:+1081:secondary,At5g59030.1:d:+611:secondary
GACACAACTA	0	1	1	0	0	At2g25710.2:d:+1217:primary,At2g25710.1:d:+1209:primary
TTTGATATGT	0	0	1	1	0	At2g25670.1:i:+1552:tertiary,At2g25670.2:i:+1562:tertiary,At2g34210.1:i:+3555:tertiary
GGAAGATTAG	0	2	0	0	0	At2g25580.1:v:+1039:secondary,At4g32190.1:d:+2104:secondary
ATGCACGCTT	0	0	0	1	1	At2g25510.1:d:+162:secondary
TCTGATTTTC	0	2	0	0	0	At2g25430.1:d:+543:secondary,At3g13260.1:v:+576:secondary
GAATACAACA	2	0	0	0	0	At2g25380.1:i:+568:tertiary
GTAGAGAAAT	1	1	0	0	0	At2g25355.1:d:+771:primary
TCTACATCAG	1	0	0	1	0	At2g25350.1:v:+2506:secondary
AAATGAAAAA	0	1	0	0	1	At2g25340.1:v:+71:secondary
TTTCATCTAT	1	0	0	0	1	At2g25310.1:d:+1021:primary
ATATTTTGGA	0	0	2	0	0	At2g25290.1:v:+2642:primary
AACTAAAGAT	0	1	0	0	1	At2g25280.1:d:+985:secondary,At2g39620.1:v:+171:secondary,At2g41140.1:d:+1274:secondary
AAGACGACAC	1	1	0	0	0	At2g25170.1:d:+3964:secondary
AGTTTTACTT	0	0	0	0	2	At2g25170.1:d:+1099:secondary
CGAGAAAAGA	1	0	1	0	0	At2g25100.1:d:+857:primary
TATATTTCTG	1	0	0	0	1	At2g25000.1:d:+896:primary
CCATTAACCA	0	0	2	0	0	At2g24850.1:d:+1371:primary
GTTAAACTGT	1	0	1	0	0	At2g24830.1:d:+1609:secondary
TAAATATTTA	1	1	0	0	0	At2g24650.1:i:+4097:tertiary,At1g11000.1:i:+934:tertiary,At2g03140.1:i:+1480:tertiary,At2g05840.1:i:+1818:tertiary
TTTTATATCA	0	2	0	0	0	At2g24600.2:d:+2041:primary
GTTTTAAGAT	0	0	1	0	1	At2g24580.1:d:+1137:primary
GGGCGTGGCT	0	0	2	0	0	At2g24420.1:d:+727:secondary,At2g24420.2:d:+727:secondary,At5g35630.1:d:+1229:secondary
AGGGGGATTT	0	0	1	1	0	At2g24390.1:d:+576:secondary
ACCAAAAGAT	1	0	0	0	1	At2g24300.1:d:+1210:secondary,At4g31000.1:d:+1319:secondary,At2g24300.2:d:+1081:secondary
TTTATTGGCA	1	0	0	1	0	At2g24290.1:X:+592:quaternary
TTCTCTATAT	1	0	0	0	1	At2g24290.1:d:+764:primary
AATGCCACGA	1	0	1	0	0	At2g24285.1:v:+861:secondary
CATTGTTCTA	0	0	2	0	0	At2g24270.2:d:+855:secondary,At2g24270.1:d:+1087:secondary
AGGATCGCGT	0	1	1	0	0	At2g24270.1:d:+1430:secondary,At2g24270.2:d:+1198:secondary
ATCTCTAGCA	1	1	0	0	0	At2g24250.1:d:+1297:primary
GCTGGGATTA	0	0	2	0	0	At2g24060.1:d:+72:secondary,At1g60190.1:d:+1312:secondary
AATTATGAGA	0	0	0	2	0	At2g23770.1:v:+2391:primary
TGGCCGAGAT	1	0	1	0	0	At2g23690.1:d:+341:primary
AATCGCTAGA	0	2	0	0	0	At2g23680.1:d:+384:primary
TCGATGTTGA	0	2	0	0	0	At2g23640.1:d:-112:primary
ATGCTTTTGT	0	0	1	0	1	At2g23570.1:i:+312:tertiary
GAGAATTATT	0	1	0	1	0	At2g23430.1:d:+349:primary
TCTTCTAAAA	0	0	1	1	0	At2g23330.1:p:+1786:secondary
CAAAAAAAAC	0	2	0	0	0	At2g23220.1:i:+695:tertiary
GACTATGCCA	0	2	0	0	0	At2g23170.1:d:+1696:primary
GCTCGGTGCT	0	0	1	0	1	At2g23170.1:d:+1570:secondary,At2g36800.1:d:+896:secondary
GATCATCCAT	0	0	1	0	1	At2g23080.2:d:+1121:primary,At2g23080.1:d:+1050:primary
CCTGCATCGA	1	1	0	0	0	At2g22800.1:d:+736:primary
GAAATGAATA	0	2	0	0	0	At2g22690.1:d:+546:secondary
ATGGCGTCCT	0	1	1	0	0	At2g22660.1:d:-2627:secondary
ATCACTGCTG	0	0	0	2	0	At2g22650.1:d:+34:secondary
TTGATCTCCA	0	0	1	1	0	At2g22475.2:d:+1441:primary,At2g22475.1:d:+1058:primary

GGCTAACAAA	0	1	0	1	0	At2g22470.1:d:+414:primary
ATCAGAACAC	0	0	0	1	1	At2g22400.1:d:+1508:secondary
TTTTCTCCAT	0	0	0	1	1	At2g22390.1:p:+729:primary
TGAGAGGTTA	0	1	1	0	0	At2g22300.1:d:+2864:secondary
TCTTCCGAGC	0	0	1	1	0	At2g22170.1:d:+357:secondary
ACAAAAAAAC	0	1	0	1	0	At2g22140.1:X:-144:quaternary
TAAGATATAT	0	0	0	0	2	At2g22122.1:d:+507:primary
TCCAGCAGCT	0	2	0	0	0	At2g22120.1:d:+728:primary
TTCTCTGATA	1	1	0	0	0	At2g22100.1:d:+1191:primary
AAGAAGATTT	0	1	0	0	1	At2g22070.1:d:+2050:secondary,At5g29580.1:p:+2892:secondary,At3g08020.1:v:+2697:secondary,At1g18990.1:d:+1352:secondary
GGCCAAGAAC	0	0	2	0	0	At2g21950.1:X:-1470:quaternary
ATAAGAATCT	0	0	1	1	0	At2g21920.1:X:-388:quaternary
GCGGCGATTC	0	2	0	0	0	At2g21860.1:d:+16:secondary
TGTGGTGTGT	1	0	0	1	0	At2g21850.1:d:+1559:secondary
TAATATTAAA	0	2	0	0	0	At2g21820.1:d:+309:secondary
TTTTTGCAAA	0	0	2	0	0	At2g21740.1:v:+941:secondary,At3g15120.1:v:+5960:secondary
AAGCTGCAGT	1	0	0	0	1	At2g21650.1:d:+366:secondary,At1g40150.1:p:+1361:secondary
TATTTGGGTG	1	0	0	0	1	At2g21640.1:d:+423:primary
GAGAAACTTG	0	1	1	0	0	At2g21620.1:d:+453:secondary,At2g21620.2:d:+446:secondary
GACTTTGTTC	0	0	1	0	1	At2g21590.1:d:+808:secondary,At3g24460.1:d:+893:secondary,At4g13345.2:d:+1163:secondary,At4g13345.1:d:+1163:secondary,At2g11510.1:p:+894:secondary,At5g04800.1:d:+360:secondary,At5g04800.2:d:+366:secondary
AACAAAGGCG	0	0	0	2	0	At2g21590.1:d:+1588:primary
TCAGAGAACA	1	0	1	0	0	At2g21560.1:d:+686:primary
AGGAGTTTGG	0	0	0	0	2	At2g21550.1:v:+1051:secondary
ACTTCTTGGT	0	2	0	0	0	At2g21530.1:d:-234:primary
TAAATGTGAG	0	2	0	0	0	At2g21520.1:X:+-152:quaternary
ATGGTGAGCA	0	0	1	1	0	At2g21470.1:d:+2004:primary,At2g21470.2:d:+2229:primary
TTCTTAAAGA	0	2	0	0	0	At2g21350.1:d:+425:primary
ATGATACCCT	0	1	1	0	0	At2g21280.1:d:+692:primary
GAGAGGAAAT	0	2	0	0	0	At2g21250.1:d:+942:secondary,At2g21250.2:d:+1003:secondary,At1g17350.1:d:+535:secondary
CGGTGGAACT	0	0	1	0	1	At2g21070.1:d:+839:secondary,At2g39670.2:d:+1077:secondary,At2g39670.1:d:+1068:secondary
GCGAGAGAAT	0	1	1	0	0	At2g21060.1:d:+504:primary
TTTACTTGAA	2	0	0	0	0	At2g20990.1:i:+509:tertiary
TTTTTCACTA	0	2	0	0	0	At2g20980.1:i:+1387:tertiary
ACTGCCGGTC	1	1	0	0	0	At2g20900.3:d:+1422:primary,At2g20900.2:d:+1438:primary
TACTTCAGGG	0	1	1	0	0	At2g20840.1:d:+845:primary
GTTGAAGATC	0	2	0	0	0	At2g20810.1:d:+1655:primary,At5g54880.1:d:+1169:primary
TAAGAAAGTG	0	0	1	0	1	At2g20760.1:d:+1618:primary
GGAGGATGAT	0	0	0	2	0	At2g20725.1:d:+286:secondary,At1g66070.1:d:+318:secondary
GGGAAGTATG	0	1	0	1	0	At2g20690.1:d:+791:primary
TCCAAAATCC	0	1	1	0	0	At2g20660.1:v:+811:primary
GAAGAAGTTT	0	0	1	1	0	At2g20640.1:p:+398:secondary,At4g35900.1:d:+341:secondary,At1g61980.1:d:+136:secondary,At5g08630.1:d:+719:secondary
GTTAAACATT	0	0	1	0	1	At2g20620.1:v:+996:secondary
CTCTTCGACA	0	1	1	0	0	At2g20610.1:d:+253:secondary,At1g12310.1:d:+168:secondary,At2g20610.2:d:+253:secondary
GGACGATTAA	0	0	1	1	0	At2g20570.1:d:-1531:secondary
CTTTTCTCAT	0	1	0	1	0	At2g20495.1:d:+907:primary
GACATCATTA	0	1	1	0	0	At2g20420.1:d:+1040:secondary,At3g07290.1:v:+2679:secondary
TGGACTAATA	1	0	1	0	0	At2g20410.1:d:+1286:primary
TAAGTTAGAT	0	2	0	0	0	At2g20400.1:d:+1700:primary
ATTGAGAGTC	1	1	0	0	0	At2g20390.1:d:+784:primary
GAAGAGTGAG	0	1	0	1	0	At2g20370.1:d:+2199:primary
GAGTCTCTTT	0	1	1	0	0	At2g20320.1:v:+3114:secondary
GTTAGGTTCT	2	0	0	0	0	At2g20300.1:d:+2676:secondary,At5g09280.1:v:+183:secondary
CCCCAATGGT	0	2	0	0	0	At2g20280.1:d:+549:secondary
TAGTATCTGT	0	0	1	0	1	At2g20060.1:d:+1086:primary
TGGGCTCTTG	0	1	1	0	0	At2g20000.1:X:-215:quaternary
AGAATGAGTT	0	0	1	1	0	At2g19970.1:d:+491:primary
GTTGGTTTTA	1	0	0	1	0	At2g19950.1:d:+2504:primary,At5g50720.1:d:+290:primary

ATCAAGATGA	0	0	0	1	1	At2g19910.1:v:+1507:secondary,At1g68520.1:d:+844:secondary
TACCGATTTA	1	0	0	0	1	At2g19870.1:d:+1755:secondary
TTCACCCCAA	0	1	0	1	0	At2g19710.1:d:+2946:primary
GATATGTTTT	0	0	0	1	1	At2g19700.1:d:+618:primary
CAAAGCTGGT	0	2	0	0	0	At2g19580.1:d:+718:primary
TGGCCATTGC	0	1	1	0	0	At2g19570.1:d:+453:primary
GTTCACTGGT	0	1	1	0	0	At2g19480.1:d:+1024:secondary,At5g02900.1:v:+539:secondary
TCTCTTTGGA	0	0	0	1	1	At2g19340.2:d:+778:primary
GAACCCCTAG	0	0	2	0	0	At2g19160.1:d:+1778:secondary
TTTCTACTCT	0	1	0	0	1	At2g18940.1:d:+2655:primary,At5g06800.1:d:+1377:primary
TATAAACTCA	0	0	1	0	1	At2g18910.1:d:+613:primary
GAAGCAACCA	1	1	0	0	0	At2g18730.1:d:+1529:primary
AGCAACAGAA	1	1	0	0	0	At2g18700.1:d:+2522:secondary,At4g06638.1:p:+1624:secondary,At5g63900.1:v:+949:secondary,At1g36690.1:p:+1375:secondary,At4g06549.1:p:+2522:secondary
TGACCCGGAT	0	0	1	1	0	At2g18650.1:v:+1034:secondary
TAGTCTGTCT	0	1	0	0	1	At2g18600.1:d:+673:primary
TGTCTTAAAA	2	0	0	0	0	At2g18540.1:i:+1336:tertiary
CTTGACTAAT	0	1	0	1	0	At2g18390.1:d:+720:primary
GATTGTTGAA	0	0	0	2	0	At2g18380.1:i:+440:tertiary,At5g10480.1:i:+1080:tertiary
CTCCAAGATC	0	1	1	0	0	At2g18350.1:d:+365:primary
TGATGTGACC	1	1	0	0	0	At2g18330.1:d:+2237:primary
AAATATTTGA	1	1	0	0	0	At2g18330.1:d:+1473:secondary,At4g07334.1:p:+2136:secondary,At1g15730.1:d:+1267:secondary
TACAGTTTAC	1	1	0	0	0	At2g18196.1:X:-562:quaternary,At1g29700.1:X:-105:quaternary
TTTGATTTGT	0	0	1	1	0	At2g18180.1:v:+2265:primary
GTAGTACTGG	1	0	1	0	0	At2g18050.1:d:-705:secondary,At2g18050.2:d:-642:secondary
GCCTAAGGTT	0	0	1	0	1	At2g18020.1:d:+635:secondary
CTACCAAATT	0	1	0	0	1	At2g17850.1:v:+616:secondary,At3g45850.1:d:+2192:secondary
CAATGTCTTC	0	0	1	1	0	At2g17820.1:d:+3692:primary
ACTGGCTATC	0	1	1	0	0	At2g17760.1:d:+1459:primary
AGATCTTTGT	2	0	0	0	0	At2g17640.1:d:+1206:primary
ATGAGAACAG	1	0	0	0	1	At2g17630.1:d:+924:secondary
TAGTAGTAAC	0	0	0	1	1	At2g17580.1:d:+2433:primary
TCTATTAAAA	1	0	0	0	1	At2g17580.1:d:+1355:secondary
GAAACTGGAG	0	1	0	1	0	At2g17580.1:d:+1222:secondary
AAAGCCACAG	0	2	0	0	0	At2g17570.1:d:+704:primary
GTAACTGTAT	2	0	0	0	0	At2g17550.1:d:+2834:primary
AATTTTTATC	0	1	0	1	0	At2g17510.1:i:+3899:tertiary
GAGGTAGTAG	0	1	1	0	0	At2g17510.1:d:+2835:primary
CATTTCGCCT	0	0	2	0	0	At2g17480.1:d:+1499:primary
ATTCTTCACA	0	1	0	1	0	At2g17440.1:d:+1904:primary
ATAAAATCAC	1	0	1	0	0	At2g17420.1:d:+1098:primary
GTCTCAAAAG	0	0	1	1	0	At2g17370.1:d:+1253:secondary
GGTCGCGGGA	0	1	1	0	0	At2g17320.1:d:+969:primary
GATTGGTCTA	0	0	2	0	0	At2g17090.1:v:+780:secondary
CTTGACCTTG	0	1	1	0	0	At2g17036.1:v:+269:secondary
TTTGTGATGC	0	2	0	0	0	At2g16980.2:d:+1111:primary,At2g16980.1:d:+1111:primary
GGAAAACAGA	1	0	0	1	0	At2g16910.1:i:-2089:tertiary
AAATGAAACT	0	0	0	1	1	At2g16895.1:X:+-15:quaternary
ACGAACCAGT	0	0	1	0	1	At2g16890.2:d:-1415:secondary
TTCGTATAAA	0	2	0	0	0	At2g16850.1:d:+1136:primary
CCTCTTACTC	1	1	0	0	0	At2g16790.1:d:+531:primary
ATACAGGAGG	0	2	0	0	0	At2g16740.1:d:-216:primary
GCAAAACCAG	0	0	1	1	0	At2g16720.1:d:+611:secondary
TGATTCAAAA	1	1	0	0	0	At2g16680.1:p:+3280:secondary
ATACAATATC	1	0	0	0	1	At2g16660.1:d:+1736:primary
TTAGAGTGAG	0	1	0	0	1	At2g16640.1:d:+3767:primary
ACTGCTATTC	1	1	0	0	0	At2g16630.1:d:+1068:primary
ATCCGATGAG	0	1	1	0	0	At2g16580.1:v:+669:primary
GCTCTGTCTT	1	0	1	0	0	At2g16380.1:d:+1239:secondary,At5g35736.1:p:+567:secondary

ATATATCATT	2	0	0	0	0	At2g16300.1:v:+511:secondary
AGAACAATTC	0	1	1	0	0	At2g16060.1:X:-198:quaternary
ATCGGATCCG	0	0	1	0	1	At2g15970.1:d:+124:secondary
TGTTATTACT	1	0	0	1	0	At2g15910.1:d:+1481:primary
AACTAAAGCA	0	1	0	0	1	At2g15890.1:X:-406:quaternary
TGTTAGAGAA	0	1	0	0	1	At2g15820.1:d:+1531:secondary
ATCAAATTTG	0	1	1	0	0	At2g15820.1:d:+1089:secondary
CAAGCAAATC	0	0	1	1	0	At2g15640.1:v:+84:secondary
TGGGTAGCGC	0	1	1	0	0	At2g15560.1:d:+1039:secondary
GAACTCGGCA	0	0	1	0	1	At2g15410.1:p:+5709:primary
ATAGTTTTGT	0	0	0	0	2	At2g15400.1:d:+877:secondary,At5g56210.1:v:+1880:secondary
TACACAAAGA	0	2	0	0	0	At2g15350.1:v:+1142:secondary,At2g15370.1:v:+1415:secondary,At1g14070.1:v:+1328:secondary
AAGTGAGGCT	0	0	1	0	1	At2g15290.1:X:-138:quaternary
GCACAATTGA	0	2	0	0	0	At2g14990.1:p:+2890:secondary,At4g14580.1:v:+1622:secondary
AATTTTCAGG	0	2	0	0	0	At2g14910.2:X:-680:quaternary
GAAAATTTGT	0	1	0	1	0	At2g14860.1:v:+1390:primary
TATATCACGA	0	0	0	1	1	At2g14860.1:v:+1273:secondary
GGACTTCTGT	0	1	0	1	0	At2g14740.2:d:+1995:secondary,At2g14740.1:d:+2088:secondary
TAATAACACT	0	1	0	0	1	At2g14520.1:d:+1409:primary,At3g06730.1:d:+845:primary
TTTCCACAAC	1	0	0	1	0	At2g14220.1:p:+2863:primary
CTCTTCTCGA	1	1	0	0	0	At2g14110.1:v:+949:primary
AAGACCCGAA	1	0	1	0	0	At2g13975.1:d:+512:primary
GTTGCTTTAA	1	1	0	0	0	At2g13840.1:X:-171:quaternary
CTGGCACTGA	0	1	0	1	0	At2g13800.1:d:+1741:primary
ATTGAATAAA	1	0	1	0	0	At2g13710.1:p:+736:secondary
AGGTGGTTCC	0	1	0	0	1	At2g13680.1:v:+454:secondary
CGTTTGGCTA	0	0	1	1	0	At2g13660.1:X:+540:quaternary
GTTTGGCTCT	0	1	1	0	0	At2g13610.1:X:-268:quaternary
AGTAACTTTT	0	0	0	2	0	At2g13550.1:d:+1106:secondary
AAATGATTCA	0	1	0	1	0	At2g13490.1:p:+1451:secondary,At2g17910.1:p:+1598:secondary
CCAAACTTTT	1	0	1	0	0	At2g13410.1:p:+3456:primary
AGTCGAGCGC	0	1	1	0	0	At2g13370.1:d:+3476:secondary
TCATATATGT	1	1	0	0	0	At2g13143.1:p:+596:primary
TGGAAGATGA	0	0	1	0	1	At2g13000.1:X:-3594:quaternary,At3g30335.1:X:-2277:quaternary,At4g17420.1:X:-323:quaternary,At1g42510.1:X:-2444:quaternary
TTTGAGATGC	1	0	1	0	0	At2g12870.1:p:+2037:primary
ATGAATCAAC	0	0	0	1	1	At2g12860.1:p:+330:secondary
AGTTTGTTTC	1	0	0	0	1	At2g12800.1:p:+4704:secondary
CACAAGAATA	2	0	0	0	0	At2g12760.1:X:-3385:quaternary,At2g04720.1:X:-159:quaternary,At4g05585.1:X:-4986:quaternary
TCTTGGAACA	0	0	0	1	1	At2g12480.1:d:+476:secondary,At5g42240.1:d:+417:secondary
AAAGATCTTG	1	0	1	0	0	At2g12470.1:p:+4240:secondary,At2g06840.1:p:+3935:secondary
ATACATCAAA	0	1	0	0	1	At2g12200.1:d:+109:primary
ATTCTTATTA	0	1	0	0	1	At2g11810.1:d:+1461:primary,At3g46820.1:d:+1549:primary
ATATTTTTGA	0	2	0	0	0	At2g11640.1:p:+459:secondary,At2g42880.1:d:+588:secondary
GCTTAAAAAA	1	0	0	1	0	At2g11550.1:p:+2970:secondary,At5g32521.1:p:+3032:secondary,At2g12390.1:p:+513:secondary,At2g11590.1:p:+2974:secondary,At4g07760.1:p:+691:secondary,At3g33045.1:p:+896:secondary
TTTCACAAAA	0	1	0	0	1	At2g11390.1:p:+464:primary
AGTAAGTCTT	1	1	0	0	0	At2g11240.1:X:-896:quaternary
GTTTACGAGA	0	2	0	0	0	At2g11235.1:p:+1295:secondary
CAGCTACAGA	1	0	0	1	0	At2g11115.1:p:+185:primary
CAAGTGTCCT	2	0	0	0	0	At2g10940.2:d:+1205:secondary
TACATTACAA	1	0	0	0	1	At2g10800.1:p:+2332:secondary
GAGATTTTGG	2	0	0	0	0	At2g10760.1:p:+1380:secondary,At1g79220.1:d:+764:secondary
AATAAATTAA	1	0	0	1	0	At2g10510.1:p:+3259:secondary
GAAATCCAAG	0	1	1	0	0	At2g10510.1:p:+2465:secondary
GTAAATGTAG	0	0	0	0	2	At2g10400.1:p:+2694:primary
TATGTGAAAT	1	0	0	0	1	At2g10232.1:p:+1124:primary
AACTTTTCAA	0	0	2	0	0	At2g10020.1:v:+227:secondary
CCATCTTCAT	1	1	0	0	0	At2g07775.1:v:+933:secondary
AAAGGAAGAC	0	1	0	1	0	At2g07769.1:p:+109:primary

ATATATTTGC	1	0	1	0	0	At2g07751.1:v:+937:primary
GACGGAGCAA	0	2	0	0	0	At2g07723.1:p:+205:secondary,AtMg00180:d:+205:secondary
GGTGCAGGAT	0	1	1	0	0	At2g07715.1:v:+1758:primary
CACCGGCCCC	0	1	1	0	0	At2g07709.1:p:+5120:secondary
TAGAGATCCG	0	1	0	1	0	At2g07706.1:i:+737:tertiary,At1g20150.1:i:+778:tertiary
GAACTACTAT	0	2	0	0	0	At2g07695.1:v:+583:secondary,AtMg00160:d:+233:secondary
AAGTGAAGGA	0	1	1	0	0	At2g07693.1:p:+159:secondary,At5g35840.1:d:+2901:secondary
TTCTGCTATA	0	0	1	0	1	At2g07620.1:p:+3323:secondary,At1g30150.1:p:+432:secondary,At1g32100.1:d:+1061:secondary
AGGAAATACA	0	0	0	0	2	At2g07320.1:v:+1441:secondary
TAGAGCACAC	1	0	0	1	0	At2g06910.1:p:+742:secondary,At4g34215.2:d:+972:secondary
TACCAGATCG	0	1	1	0	0	At2g06850.1:d:+542:secondary
GCTTATTTTT	0	0	1	1	0	At2g06790.1:p:+1841:secondary
GTTCGAAAAA	0	1	0	0	1	At2g06780.1:p:+1648:secondary
TATTTGATGA	0	0	1	0	1	At2g06600.1:p:+1067:secondary
AATGTTCTCT	0	2	0	0	0	At2g06560.1:p:+3542:secondary
ATGATGATAC	1	0	0	1	0	At2g06555.1:v:+1236:primary
CTTGATTTGC	1	0	0	0	1	At2g06510.1:d:+2008:primary,At2g06510.2:d:+1904:primary
GTTTACAATC	0	1	1	0	0	At2g06430.1:p:+980:secondary,At2g15140.1:p:+980:secondary,At4g07967.1:p:+426:secondary,At4g07967.1:p:+4044:secondary,At3g32389.1:p:+741:secondary,At2g11040.1:p:+3040:secondary,At3g32900.1:v:+270:secondary,At2g04980.1:p:+976:secondary,At3g19820.1:d:+862:secondary,At2g15190.1:p:+980:secondary,At2g02210.1:p:+981:secondary,At2g11040.1:p:+2800:secondary,At3g19820.2:d:+886:secondary,At3g32900.1:v:+29:secondary,At4g07967.1:p:+658:secondary
AAAGAGGCAG	0	1	1	0	0	At2g06045.1:X:--70:quaternary
CAATGTTTAA	0	0	1	1	0	At2g06020.1:i:-429:tertiary
TGAAAGCACA	0	1	0	0	1	At2g06010.1:d:+653:primary
GAATGTGTCT	0	1	0	0	1	At2g06005.1:d:+1183:primary,At3g46980.2:d:+1533:primary,At3g46980.1:d:+1457:primary,At2g06005.2:d:+1113:primary
CACTCGCCAA	0	0	1	1	0	At2g05990.2:d:+325:secondary,At2g05990.1:d:+292:secondary
TATGAGTGCC	1	1	0	0	0	At2g05940.1:d:+1105:primary
GAGGAGTGGT	0	0	0	2	0	At2g05810.2:X:-190:quaternary
GGTCAAACGC	0	2	0	0	0	At2g05790.1:d:+346:primary
ATTTGTTGGT	0	1	0	1	0	At2g05635.1:v:+1082:primary
TGTCTATGAT	1	0	0	1	0	At2g05370.1:i:+839:tertiary
AAACTTTAGA	0	1	0	1	0	At2g05320.1:d:+1293:primary
GTTTCTCCAG	0	1	1	0	0	At2g05270.1:d:+358:primary,At2g32910.1:d:+2087:primary
ATATTAACCA	1	0	0	1	0	At2g05210.1:d:+1234:primary
TTATGGTTTA	1	1	0	0	0	At2g05185.1:d:+627:primary
GCCGTCAGAC	0	2	0	0	0	At2g05170.1:d:+2139:secondary
ACAGGTTCAT	1	0	0	1	0	At2g05070.1:d:+1002:primary
CTACAACTAT	0	1	1	0	0	At2g04890.1:d:+1536:secondary
GAACTGTCAG	0	1	0	1	0	At2g04865.1:v:+2425:primary
AAACAAAAAT	1	0	1	0	0	At2g04760.1:X:+3:quaternary,Chr5:+18533165:quaternary,Chr1:+25844265:quaternary,Chr3:+16038243:quaternary,Chr5:+15192857:quaternary,Chr2:+16303711:quaternary
TTGTAGATAT	0	0	0	1	1	At2g04740.1:d:+1844:primary
TGAGAGAGCT	1	1	0	0	0	At2g04700.1:d:+592:secondary,At3g14020.1:v:+1275:secondary,At3g48190.1:d:+7705:secondary
AATTGAGTTC	0	0	0	1	1	At2g04650.1:d:+1409:primary
GAGATTGGTG	1	0	1	0	0	At2g04550.3:d:+995:primary,At2g04550.1:d:+993:primary
TCTGGGAAAA	0	1	0	1	0	At2g04550.3:d:+525:secondary,At2g04550.1:d:+525:secondary,At2g04550.2:d:+525:secondary,At1g58210.1:v:+1134:secondary,At2g02480.1:d:+1793:secondary
ATTGTGGTTG	0	0	2	0	0	At2g04400.1:d:+1097:secondary
TATTGTTGCT	1	1	0	0	0	At2g04305.1:d:+1660:primary
AATGCAAATA	1	1	0	0	0	At2g03930.2:v:+1122:secondary
AACTGTGAGA	1	1	0	0	0	At2g03800.1:d:+959:secondary
GAATTTCAAA	1	1	0	0	0	At2g03750.1:X:-1194:quaternary
AGACTTGAAA	0	0	2	0	0	At2g03670.1:d:+1883:primary
GCTTCTTCTG	0	0	1	0	1	At2g03530.1:d:+1135:primary,At1g26770.1:d:+141:primary
GTGTTGGATC	0	0	0	2	0	At2g03510.1:d:+1060:secondary
TCTACTAAAA	0	1	1	0	0	At2g03500.1:i:-1446:tertiary
GTGGCACTCC	0	2	0	0	0	At2g03500.1:d:+1184:primary
ACCACCTCCG	0	0	2	0	0	At2g03440.1:X:-661:quaternary

AGAATGCTCA	0	1	0	0	1	At2g03380.1:v:-1262:secondary
TGATAATGTA	0	2	0	0	0	At2g03270.1:d:+2122:primary
AGTTTTTCAA	0	0	0	2	0	At2g03180.1:v:+337:secondary
AAGAAATTTG	0	0	1	1	0	At2g03170.1:v:+588:secondary
ATGCAGAATG	0	0	1	0	1	At2g03070.1:d:+1770:primary
CTTGCTTTCT	0	0	2	0	0	At2g03040.1:v:+1205:primary,At2g03030.1:v:+1200:primary
AAACAGAGCC	1	0	0	0	1	At2g03010.1:v:+1053:secondary,At5g56890.1:d:+1649:secondary,At4g16530.1:d:+2284:secondary
GAGCCATTAC	1	0	0	1	0	At2g02960.1:d:+1016:primary,At2g02960.5:d:+1327:primary,At2g02960.4:d:+1025:primary,At2g02960.2:d:+1012:primary,At2g02960.3:d:+1226:primary
GAAATCGAGA	0	0	1	1	0	At2g02950.1:d:+862:secondary
AACGAGCTCA	1	0	0	1	0	At2g02930.1:X:-211:quaternary,At4g02520.1:X:-188:quaternary
TATTCATCTT	0	0	0	2	0	At2g02890.1:v:+741:secondary
TTCACTCATC	0	1	0	0	1	At2g02700.1:v:+179:secondary
GAACCGTACC	0	0	0	0	2	At2g02390.1:d:+612:secondary,At2g02390.3:d:+657:secondary
TCTTCTAATG	0	1	1	0	0	At2g02230.1:d:+1061:secondary,At3g07970.1:d:+100:secondary
TCTCACCTTG	0	0	1	0	1	At2g02100.1:X:+428:quaternary
GTCTGATACA	0	2	0	0	0	At2g02080.1:d:+1068:primary
AAACGAACCA	0	0	1	1	0	At2g02020.1:d:+1043:primary
CGTGTCAGCT	1	1	0	0	0	At2g01980.1:d:+3362:primary
TCGTCAGCGA	0	1	1	0	0	At2g01940.1:d:+1279:primary
GCCACATAGT	0	1	0	1	0	At2g01910.1:d:-1731:secondary
GCTCAGATCC	0	1	1	0	0	At2g01750.1:d:+212:secondary,At1g14840.1:d:+269:secondary
GAATCAGTCT	0	0	0	0	2	At2g01640.1:d:-539:primary
AGATTGCAAT	0	1	1	0	0	At2g01620.1:d:+856:primary
GCCTAATAGA	1	0	0	0	1	At2g01590.1:d:-332:primary
TTCTCAAAGC	0	2	0	0	0	At2g01530.1:d:+538:primary
TTTGTGATGA	1	0	1	0	0	At2g01400.1:d:+636:secondary,At1g49130.1:d:+177:secondary,At5g45570.1:v:+2793:secondary,At4g08430.1:v:+2454:secondary,At5g33250.1:p:+959:secondary,At5g28235.1:v:+1962:secondary,At1g42590.1:p:+3046:secondary,At3g32966.1:p:+2902:secondary
GCGGAAAGAG	1	1	0	0	0	At2g01340.1:d:+670:secondary,At1g80150.1:d:+1251:secondary
ACAGTTTTCA	1	0	1	0	0	At2g01320.4:d:+1806:primary,At2g01320.1:d:+1806:primary,At2g01320.2:d:+1806:primary,At2g01320.3:d:+1806:primary
GGAAACGTTT	0	0	2	0	0	At2g01220.2:d:+1359:primary,At2g01220.1:d:+1356:primary
ATGCCTCCAC	0	0	0	0	2	At2g01190.1:d:+1831:secondary,At1g07360.1:d:+1208:secondary
AAGAGTGGAT	1	0	1	0	0	At2g01180.2:X:-1572:quaternary
ATTACTACCC	0	0	1	1	0	At2g01100.2:d:+1049:primary,At2g01100.3:d:+1415:primary
CTCTTGTCTG	1	0	1	0	0	At2g01080.1:i:+933:tertiary,At1g29820.1:i:+1931:tertiary
AATTTCGATT	1	1	0	0	0	At2g01080.1:d:+736:primary
GGATACCATA	0	2	0	0	0	At1g80950.1:d:+1111:primary
CCTCTGTGAA	0	1	0	1	0	At1g80900.1:d:+1467:primary
AAGGAGTTAC	0	0	1	0	1	At1g80810.1:v:+2831:primary
AGAGTTCGTA	0	0	1	0	1	At1g80770.1:i:-1823:tertiary
CTTAACATAC	1	1	0	0	0	At1g80760.1:d:+899:primary
GGAGCTTCCA	0	0	2	0	0	At1g80740.1:d:+730:secondary,At5g63790.1:d:+279:secondary,At4g08033.1:p:+759:secondary,At1g44970.1:d:+505:secondary
CAGGCAAAGG	1	1	0	0	0	At1g80740.1:d:+2356:secondary
ACATTTTGAA	0	1	1	0	0	At1g80670.1:d:+1266:primary
TGAAGACAAA	1	0	1	0	0	At1g80650.1:d:+606:primary
ACTATATATA	0	1	0	0	1	At1g80580.1:v:+1295:secondary
GTTCTGCACC	0	0	2	0	0	At1g80560.1:d:+1008:primary
TGTTTCCAGG	0	1	0	0	1	At1g80460.1:d:+1186:secondary
ATGGGTTGGA	0	0	1	1	0	At1g80360.1:d:+756:secondary
GTTGGAGCTG	0	1	1	0	0	At1g80290.1:d:+1006:secondary,At1g48640.1:v:+519:secondary
GCTGCACAGT	0	0	0	1	1	At1g80270.1:d:+1833:secondary,At1g77860.1:v:+740:secondary,At1g80270.2:d:+1806:secondary
GTCTTGAATC	1	0	0	0	1	At1g80245.1:d:+624:primary,At1g80245.2:d:+677:primary
TAACAATTAT	0	0	1	1	0	At1g80190.1:v:+1091:primary
GCGGGTCTTC	0	0	2	0	0	At1g80180.1:d:+115:secondary,At2g34180.1:v:+411:secondary
TTTTCACAGG	0	2	0	0	0	At1g80030.3:d:+868:secondary,At1g80030.1:d:+868:secondary,At1g80030.2:d:+933:secondary
CAGACAGAGA	0	1	0	0	1	At1g79990.1:d:+2610:primary
CAGGACGCCA	0	0	1	0	1	At1g79930.1:d:+1771:secondary,At1g79920.1:d:+1764:secondary,At1g79920.2:d:+1764:secondary
ATGAGAGCAT	1	1	0	0	0	At1g79910.1:d:+1137:primary

TTTCGTTCAT	0	0	1	0	1	At1g79740.1:v:+2076:primary
ATGTCGGAGT	1	0	0	0	1	At1g79740.1:v:+1758:secondary
AGATACTAAC	0	1	0	1	0	At1g79690.1:d:+2180:secondary
CTCTTAAGAA	0	2	0	0	0	At1g79660.1:d:+170:primary
GATGCTGCAT	0	0	1	1	0	At1g79570.1:d:+3226:secondary
CTAGAAAAGA	1	0	1	0	0	At1g79560.1:d:+1984:secondary,At3g42120.1:v:+657:secondary,At4g30030.1:v:+237:secondary
GTTGCTGATA	0	0	2	0	0	At1g79530.1:X:-231:quaternary
ATTTACATTG	1	1	0	0	0	At1g79470.1:d:+1720:primary
TATTTTTTAT	1	0	0	1	0	At1g79410.1:d:+1726:primary
AGAAATGGCG	0	0	1	1	0	At1g79280.1:v:+3646:secondary
AAAGAATCTG	0	2	0	0	0	At1g79280.1:v:+2547:secondary
ATTTCATTTC	0	1	0	1	0	At1g79250.1:d:+1876:primary
ACTTGATTTT	0	0	0	1	1	At1g79245.1:p:+2150:secondary
GCTGAAAGAA	1	1	0	0	0	At1g79200.1:d:+325:primary
TTGTTTTTAT	0	1	1	0	0	At1g79150.1:v:+182:secondary,At3g30715.1:p:+1165:secondary
CTGCCGTTAA	1	0	1	0	0	At1g79040.1:d:-240:primary
CGAGCTTTAA	0	1	1	0	0	At1g79000.1:d:+5245:primary
TTGATTCTCA	0	2	0	0	0	At1g78860.1:v:+1799:secondary
CATCAATAAC	2	0	0	0	0	At1g78770.1:d:+2237:primary
TGAACCTTAA	1	0	1	0	0	At1g78730.1:d:+1357:primary
TACATTTTCA	1	0	0	0	1	At1g78700.1:d:+1499:secondary
GTAATCAAAT	0	0	0	0	2	At1g78690.1:d:+1013:primary
TAATGAATAC	0	1	0	1	0	At1g78630.1:d:+981:primary
GACTTGTGGT	1	1	0	0	0	At1g78620.2:i:+1145:tertiary,At1g78620.1:i:+1145:tertiary
GAGTCATTAA	0	1	1	0	0	At1g78610.1:d:+2323:primary
GTCGAGGAGC	0	0	2	0	0	At1g78570.1:d:+1742:secondary
TTTATCCTCA	0	0	1	0	1	At1g78550.1:d:+560:secondary,At2g35670.1:d:+2220:secondary
CACCGCTTTG	0	0	0	2	0	At1g78370.1:X:-250:quaternary
TTAGAAACAA	1	0	0	1	0	At1g78310.1:d:+1252:primary
ACCTCTGGAA	0	1	0	1	0	At1g78240.1:X:-368:quaternary
TACGGTGCTA	1	1	0	0	0	At1g78180.1:d:+1000:secondary
CACCCACACG	1	0	1	0	0	At1g78170.1:d:+702:secondary
GACTCCGATT	1	0	1	0	0	At1g78070.2:d:+213:secondary,At1g78070.1:d:+214:secondary
TTATTTCAAA	0	1	0	0	1	At1g78050.1:d:+1523:secondary
AAACGAGGAA	0	0	1	1	0	At1g78050.1:d:+1289:secondary
TCTGATCTGC	0	0	1	1	0	At1g78040.1:d:-560:secondary,At1g78040.1:d:-571:secondary
GATCGATGTC	0	0	0	1	1	At1g78030.1:i:+633:tertiary
GCTGTGAGTG	1	1	0	0	0	At1g78010.1:d:+1242:primary
GACGCACTCT	0	0	1	1	0	At1g77840.1:d:+1246:primary
ACTAGCCAAT	0	1	1	0	0	At1g77770.2:d:+1027:secondary,At1g77770.1:d:+917:secondary
CGGACCACCG	2	0	0	0	0	At1g77760.1:d:+2745:secondary
GCGGCGGAAT	0	1	1	0	0	At1g77740.1:d:+608:secondary,At1g51550.1:d:+20:secondary
AATTCCCTGG	0	1	0	0	1	At1g77670.1:d:+912:primary,At5g01860.1:v:+1117:primary
AAGGTCGTAA	0	1	1	0	0	At1g77660.1:v:+1330:primary,At1g21920.1:d:+1242:primary
CGGAAAAAAA	0	1	1	0	0	At1g77620.1:X:--8:quaternary
ACAAGAAATC	1	0	0	1	0	At1g77580.2:d:+2422:primary,At1g77580.1:d:+3047:primary
GCTGGACCAG	0	1	1	0	0	At1g77480.2:d:+975:primary,At1g77480.1:d:+975:primary
GCGGATAGAC	0	0	1	1	0	At1g77450.1:X:--54:quaternary
GCTTTGTACG	0	0	1	0	1	At1g77450.1:d:+250:primary
AGAGTCCAAT	0	1	1	0	0	At1g77410.1:d:+2564:primary
TAAAACAAAA	0	1	0	0	1	At1g77390.1:v:+2147:primary
TGCTGCAACA	0	1	1	0	0	At1g77380.1:d:+1803:secondary
TATCGAAACC	0	0	0	2	0	At1g77350.1:X:+396:quaternary
GTTGCGGAAC	0	1	0	1	0	At1g77300.1:d:+5199:primary
TTAAGACAAT	1	0	0	1	0	At1g77270.1:d:+2429:primary
CTTCTAAACG	0	1	0	0	1	At1g77250.1:d:+1689:primary
TTATCAAAAA	0	0	2	0	0	At1g77160.1:v:+127:secondary,At2g06060.1:p:+684:secondary,At3g60920.1:v:+289:secondary
GTTTTCTATT	1	0	0	0	1	At1g77110.1:i:+1950:tertiary
ATGTTTTTGG	1	0	1	0	0	At1g77110.1:i:+1843:tertiary
CTTCTCAGGA	0	0	2	0	0	At1g76990.3:d:+1387:secondary,At1g76990.2:d:+1273:secondary,At1g76990.1:d:+1180:secondary
GAGATGGGTG	0	2	0	0	0	At1g76880.1:d:+712:secondary

TTATTTCGTT	0	2	0	0	0	At1g76825.1:v:+2472:primary,At1g76720.1:v:+4338:primary
AAGAGAAGAA	1	0	1	0	0	At1g76780.1:v:+2695:secondary,At4g39270.2:d:+1574:secondary,At1g35150.1:v:+499:secondary,At5g24300.1:d:+440:secondary,At4g39270.1:d:+1574:secondary
GTTCCAATAC	2	0	0	0	0	At1g76760.1:d:+401:secondary
TGATTGAAGC	0	1	1	0	0	At1g76680.1:d:+948:secondary,At1g76680.2:d:+957:secondary
TACATTGAAA	0	0	2	0	0	At1g76630.1:v:+3943:secondary
GGAGAGTGTT	0	2	0	0	0	At1g76560.1:d:+64:primary
AAGCTAATTC	1	0	0	0	1	At1g76510.2:X:-10:quaternary
GTCGACTGTT	0	0	1	1	0	At1g76510.1:d:+1500:primary,At1g76510.2:d:+1479:primary
GTTATACAGC	0	2	0	0	0	At1g76460.1:d:+1256:secondary
TTACAGATAG	0	1	1	0	0	At1g76450.1:d:+231:secondary
AAGGGAAAAT	0	0	0	0	2	At1g76405.1:d:+514:primary,At1g76405.2:d:+543:primary
GATTTGACTC	0	0	2	0	0	At1g76390.1:d:+70:secondary,At2g21110.1:v:+210:secondary
GCTACAGGTC	0	2	0	0	0	At1g76360.1:d:+1148:secondary
ATCAGGAAGT	1	0	1	0	0	At1g76200.1:d:-494:secondary
ATCTTGTAAT	1	0	0	0	1	At1g76190.1:v:+787:secondary
GACGGCTATA	0	2	0	0	0	At1g76160.1:d:+1523:primary
TCAGACAGTT	0	0	0	2	0	At1g75950.1:d:+472:secondary
TCGTTGATCA	0	1	0	1	0	At1g75790.1:X:-16:quaternary,At5g25757.1:X:-19:quaternary
ATCGTTGACC	0	1	1	0	0	At1g75710.1:d:+671:primary,At5g47070.1:d:+1348:primary
GAGCTGATGG	1	0	1	0	0	At1g75640.1:X:-168:quaternary
AAACAACAAG	0	0	1	0	1	At1g75540.1:d:+886:secondary
TACAAAATCC	2	0	0	0	0	At1g75540.1:d:+1199:primary
ACGGAGGGTC	1	0	1	0	0	At1g75490.1:v:-174:primary
ATACGGTAAC	1	0	1	0	0	At1g75460.1:d:-393:primary
CTTTCAAAGC	0	1	1	0	0	At1g75410.1:d:+1373:secondary
ACTCATTCAT	1	1	0	0	0	At1g75360.1:v:+1528:primary
GGCTAATCTG	0	1	0	1	0	At1g75350.1:d:-122:primary
ACGAATTGGA	0	1	0	1	0	At1g75310.1:v:+3400:secondary
GTGGTAAATT	0	1	1	0	0	At1g75280.1:d:-1031:secondary
GATCCACATC	0	0	2	0	0	At1g75260.1:v:+2381:primary
GGAAGAAAAA	0	1	1	0	0	At1g75190.1:d:+711:primary
TACTGGAATA	1	1	0	0	0	At1g75180.3:d:+881:primary,At1g75180.2:d:+1437:primary
GAAGAACGGT	0	1	1	0	0	At1g75170.1:d:+509:secondary
TGCAAAACAT	0	0	0	1	1	At1g74810.1:v:+2464:secondary
GTAGCTTATG	0	0	0	1	1	At1g74780.1:d:+1766:primary
GTTGAGGAAA	0	1	1	0	0	At1g74690.1:d:+1493:secondary
AAACTGCAGA	1	0	0	1	0	At1g74690.1:d:+1314:secondary,At1g17495.1:p:+2442:secondary,At1g48470.1:d:+963:secondary
TCTGTTTTCT	0	0	1	1	0	At1g74680.1:d:+1770:primary
GAAGCTACAC	0	2	0	0	0	At1g74650.1:d:+1152:primary
GTTCGTACAA	0	0	0	1	1	At1g74550.1:d:+1613:primary
AGTATATAGG	0	0	0	0	2	At1g74470.1:d:+502:secondary,At1g65440.1:d:+4232:secondary
AATAAGAATA	1	0	1	0	0	At1g74410.1:d:+1012:primary
ATTTAGTTTG	0	0	1	0	1	At1g74325.1:v:+1325:secondary
AGGTTCCGAG	0	1	1	0	0	At1g74290.1:v:+1366:primary
TGCTTTTTAG	1	0	0	1	0	At1g74260.1:d:+4254:secondary
AAGGTTAGTG	1	0	1	0	0	At1g74240.1:d:+1129:primary
GTTTCTTTCT	1	0	1	0	0	At1g74200.1:d:+2102:primary
CTATAAAAAA	0	1	1	0	0	At1g74140.1:i:+1599:tertiary
ACGAAGTGAT	0	1	0	0	1	At1g74060.1:X:+360:quaternary
TGACAGAGAC	1	1	0	0	0	At1g74055.1:d:+497:primary
GTCCTCTCTC	0	2	0	0	0	At1g73940.1:d:+271:primary
TGAACAAGAA	1	1	0	0	0	At1g73920.2:d:+1465:secondary,At1g73920.1:d:+1460:secondary
GATTCCACAG	0	2	0	0	0	At1g73830.1:d:-747:secondary
ATTGTACATA	0	1	0	0	1	At1g73800.1:d:+774:primary
TTCATTAGTG	0	2	0	0	0	At1g73680.1:d:+2035:primary
TTATTACCTA	1	0	0	1	0	At1g73600.1:X:+574:quaternary
GAGAAGGTCT	0	2	0	0	0	At1g73570.1:v:+1882:secondary
ATCCGTCAGT	0	0	1	1	0	At1g73550.1:v:+658:primary

GCCTTGGACT	0	1	0	0	1	At1g73450.1:d:+2891:primary
GTTTTAGTAT	0	0	2	0	0	At1g73100.1:X:+2252:quaternary,Chr2:+9734531:quaternary
GACTCAGATT	0	2	0	0	0	At1g73080.1:d:+2887:primary
TATTCCAGTG	0	0	2	0	0	At1g73070.1:d:+3429:primary,At1g73060.1:d:+3448:primary
CGGTTCAAAT	0	1	0	1	0	At1g72990.1:d:+2282:secondary
CGAGTCCTTA	0	0	1	1	0	At1g72930.1:d:-606:secondary
AAGGAACTAA	0	1	1	0	0	At1g72910.1:d:+588:primary
TGTTTTTGAC	0	1	1	0	0	At1g72890.1:v:+1579:secondary,At1g72900.1:d:+65:secondary
TAACCTCGTG	1	0	0	1	0	At1g72840.1:d:+3795:primary,At3g49020.1:v:+1670:primary
GGTCAGACGA	0	1	0	1	0	At1g72750.1:d:+241:secondary
AACTATGTTC	0	2	0	0	0	At1g72740.1:d:+1064:primary
GGTCGATGCA	0	1	0	1	0	At1g72650.2:d:+2095:secondary,At1g72650.1:d:+2077:secondary
GAAGTTTGAC	1	0	1	0	0	At1g72650.1:d:+2389:primary,At1g72650.2:d:+2407:primary
AGCTGTAATG	1	0	0	0	1	At1g72470.1:d:+2144:primary
TCTGTGTCCA	0	0	2	0	0	At1g72440.1:X:-400:quaternary
GGAACTAAAA	0	0	2	0	0	At1g72390.1:X:+1927:quaternary,Chr5:+19548321:quaternary
CAAAGGATAA	0	1	0	1	0	At1g72320.1:d:+2248:primary
TGTGATCTTT	1	0	1	0	0	At1g72210.1:d:+1291:primary
ATTAGAGTTC	0	0	1	0	1	At1g72180.1:d:+3224:primary
CTTAAAAACA	0	0	0	2	0	At1g72150.1:d:+927:secondary
GTGGTACATT	0	0	2	0	0	At1g72150.1:d:+1337:secondary,At1g22530.1:d:+1679:secondary
GTATCAGTCA	0	1	1	0	0	At1g72140.1:d:+1504:primary
GTGTTGTTTT	0	0	0	0	2	At1g72110.1:d:+928:secondary,At2g04110.1:p:+608:secondary
CTTGAATGAC	0	1	1	0	0	At1g72010.1:d:+1543:primary
TGCCCCAACC	1	1	0	0	0	At1g71870.1:X:-269:quaternary,At1g03940.1:X:--18:quaternary
AATCTCATTC	0	1	0	0	1	At1g71850.1:v:+2000:secondary
TTACTTGACT	0	2	0	0	0	At1g71830.1:d:+1969:primary
TATCAGCTTC	1	1	0	0	0	At1g71810.1:d:+2133:primary
TGACCATCTT	0	1	1	0	0	At1g71790.1:d:+912:primary
TATTATTGGA	1	0	1	0	0	At1g71695.1:i:+738:tertiary,At4g24710.1:i:+1736:tertiary
GAATTGAGGA	0	0	2	0	0	At1g71440.1:d:+1290:secondary
TTGTCTCTTC	0	1	0	0	1	At1g71430.1:d:+631:primary
GAAGGGAAAG	0	1	0	1	0	At1g71430.1:d:+128:secondary,At5g35331.1:p:+1752:secondary,At4g10280.1:d:+386:secondary
ATCGTAAGTA	1	0	0	1	0	At1g71410.1:X:+1360:quaternary
GGAAAGTATA	0	1	1	0	0	At1g71310.2:d:+735:primary,At1g71310.3:d:+1151:primary
TTTTAACTAG	0	0	1	0	1	At1g71240.1:d:+2576:primary
GAAGAAAGGA	1	0	0	1	0	At1g71190.1:d:+822:primary
GTCGATAGAG	1	1	0	0	0	At1g71070.1:d:+1337:primary
TATCAAAAGC	0	0	0	0	2	At1g70885.1:p:+199:primary
GGATGCAATT	0	0	2	0	0	At1g70780.1:d:+653:secondary
ATGATTGGTG	0	1	0	0	1	At1g70760.1:d:-372:primary
CGTAAATAGC	1	0	1	0	0	At1g70750.1:X:-577:quaternary
GGACTTTTGG	0	0	2	0	0	At1g70730.1:d:+1423:primary
CACACACACA	1	0	0	0	1	At1g70670.1:d:+595:secondary,At2g20070.1:v:+233:secondary,At5g16020.1:v:+2699:secondary,At1g68480.1:v:+215:secondary
GACGTATCGG	0	0	0	1	1	At1g70600.1:d:+110:secondary,At1g23290.1:d:+82:secondary
TCCATAACTT	0	0	0	2	0	At1g70590.1:d:+1186:primary
AAGGCTACGA	0	2	0	0	0	At1g70570.1:d:+1381:primary
TAGATGAGTT	0	1	1	0	0	At1g70550.2:d:+1549:primary,At1g70550.1:d:+1721:primary
ATGAGCTTTA	0	1	1	0	0	At1g70500.1:v:+1747:primary
ATTCTGGGAT	0	1	0	0	1	At1g70480.1:d:+1270:secondary
TGTAAGGTGA	1	1	0	0	0	At1g70470.1:d:+471:primary
TTTTATCTAA	1	0	0	1	0	At1g70380.1:v:+96:secondary
GCCTGCAAGT	0	1	0	1	0	At1g70350.1:d:+330:secondary
CTTATTGGGA	0	2	0	0	0	At1g70330.1:d:+1202:secondary
GACGATGAAG	0	1	0	1	0	At1g70320.1:v:+6729:secondary,At1g73870.1:d:+580:secondary
CAAGGAATTG	0	1	0	1	0	At1g70300.1:d:+2369:secondary
CAACCACCCG	0	1	0	0	1	At1g70270.1:d:+186:primary
CTTGGTCTAG	0	1	0	0	1	At1g70120.1:v:+1262:primary
AATAATTTTC	0	2	0	0	0	At1g69900.1:d:+1261:primary

TCATTCTAGT	0	1	1	0	0	At1g69890.1:d:+892:primary
GTTGTGGGTG	0	0	1	1	0	At1g69880.1:d:+443:secondary,At3g19515.1:d:+1074:secondary
AACACAAAGA	0	2	0	0	0	At1g69850.1:d:+1165:primary
AGAGACATTA	0	1	0	0	1	At1g69800.1:d:+1236:primary
AACACTGTTG	0	1	1	0	0	At1g69770.1:d:+2586:primary
CAAACCTTTT	0	0	1	0	1	At1g69770.1:d:+2247:secondary,At4g06509.1:p:+1706:secondary
ATCTTATTTC	0	2	0	0	0	At1g69680.1:d:+981:primary
GATGAAGATA	0	1	1	0	0	At1g69630.1:v:+354:secondary
CCGATCTTGT	0	1	1	0	0	At1g69530.2:X:-777:quaternary,Chr2:-15288521:quaternary
AACGTGCGTT	0	1	1	0	0	At1g69510.3:d:+248:secondary,At1g69510.2:d:+382:secondary,At1g69510.1:d:+279:secondary
TTTTACTGCT	0	1	1	0	0	At1g69440.1:d:+2962:primary
GGAGCTGATG	0	2	0	0	0	At1g69440.1:d:+2194:secondary,At4g15955.1:d:+563:secondary
TCTGCAATGG	1	1	0	0	0	At1g69410.1:d:+520:secondary
TATATATATA	0	1	0	0	1	At1g69390.1:d:+869:primary
GAGGAAATAA	0	0	0	1	1	At1g69310.1:i:+3408:tertiary
AGCCGTTGGC	0	0	1	0	1	At1g69295.1:X:-416:quaternary
TAGATCATCC	0	1	0	0	1	At1g69260.1:d:+1132:primary
TTTATTTTCC	0	1	1	0	0	At1g69220.2:d:+2547:primary,At1g69220.1:d:+2628:primary
GATGAGCTTG	0	0	2	0	0	At1g69210.1:d:+529:secondary,At2g15620.1:d:+1289:secondary
ATATACAAAG	0	0	0	0	2	At1g69090.1:v:+996:secondary
AAGGACAAAT	0	0	1	0	1	At1g69060.1:d:+1989:primary
ATCGAAGAGG	1	0	0	1	0	At1g68790.1:d:+3322:secondary
GAAGAGACTA	0	2	0	0	0	At1g68750.1:X:-86:quaternary,At1g52830.1:X:--82:quaternary
GCTGATCAAC	1	1	0	0	0	At1g68740.1:d:+1758:secondary,At2g43970.1:d:+191:secondary,At2g43970.2:d:+191:secondary
TTTTCAGTAA	0	1	1	0	0	At1g68600.1:d:+1065:secondary
GCACAATCCC	0	0	1	1	0	At1g68590.1:d:+381:secondary
AATGTTATTT	0	0	0	1	1	At1g68570.1:X:+628:quaternary,Chr5:+14124883:quaternary,At1g73460.1:X:+382:quaternary
AACCGTCACC	0	0	1	0	1	At1g68570.1:d:+1161:secondary,At1g23935.1:v:+804:secondary
CGCTTAAGAC	0	1	0	1	0	At1g68550.1:d:+309:primary,At1g68550.2:d:+323:primary
GCCATCGCCG	0	0	2	0	0	At1g68530.2:d:+1077:secondary,At1g68530.1:d:+1077:secondary,At5g33340.1:v:+663:secondary
TGGTGAAGTT	0	1	1	0	0	At1g68400.1:d:+1916:primary
AGTTTCTAGA	0	1	1	0	0	At1g68260.1:d:+281:secondary
TCAATACTGA	1	1	0	0	0	At1g68150.1:d:+838:secondary
TCCACTATGC	0	1	1	0	0	At1g68070.1:d:+1081:secondary
TAAATCGAGA	2	0	0	0	0	At1g67930.1:d:+2691:secondary
AATTTACGCG	0	1	1	0	0	At1g67910.1:d:+912:secondary
GCAAGGGGTG	0	1	0	1	0	At1g67850.2:d:+1568:primary,At1g67850.1:d:+1564:primary,At1g24570.1:d:+966:primary
GAACTTTGTG	1	0	1	0	0	At1g67840.2:d:+1723:secondary,At1g67840.1:d:+1719:secondary,At5g22800.1:d:+2286:secondary
ATTATTCCAT	0	1	0	0	1	At1g67810.1:d:+731:primary
AAAAAACCAC	0	0	1	1	0	At1g67800.1:d:+1791:primary,At1g67800.2:d:+1653:primary,At1g67800.3:d:+1551:primary
TTGTCCCTGG	0	1	1	0	0	At1g67750.1:d:+1377:primary
TACGGTGTTG	0	0	2	0	0	At1g67690.1:v:+2593:primary
TGCGCCAGCC	0	1	0	1	0	At1g67550.1:d:+2571:primary
TTGGGGAAGT	0	1	1	0	0	At1g67470.1:d:+973:primary
ATGTAAAAGA	0	1	1	0	0	At1g67440.1:d:+1506:primary
TTCTTCCCAT	1	1	0	0	0	At1g67410.1:X:-433:quaternary,At3g05390.1:X:-9:quaternary
TTTTTAAAAG	1	1	0	0	0	At1g67320.1:d:+1447:primary
TTAATTTTAA	1	0	0	1	0	At1g67310.1:v:+242:secondary
TCCATCAATG	0	0	0	1	1	At1g67210.1:d:+1644:primary
TTGTTGAAAA	1	0	0	1	0	At1g67190.1:d:+1415:primary
TTACTTTCCA	1	0	0	0	1	At1g67180.1:i:-856:tertiary
AAATACAGAC	2	0	0	0	0	At1g67120.1:X:-62:quaternary,At1g19450.1:X:-320:quaternary
TTAAATTAGC	1	0	1	0	0	At1g67080.1:d:+912:secondary,At5g35360.1:d:+482:secondary
TTAAGTTACA	0	2	0	0	0	At1g67050.1:d:+692:secondary
CCAGTATTAT	0	0	0	1	1	At1g67050.1:d:+1044:secondary
AATCTTCTAT	1	1	0	0	0	At1g66990.1:p:+617:primary,At1g66980.1:d:+3284:primary
GTACTGCGGA	0	1	1	0	0	At1g66900.1:d:+847:secondary
AAGCTTTCTC	0	2	0	0	0	At1g66860.1:d:-1315:secondary
TTAGTTCCTT	1	1	0	0	0	At1g66620.1:v:+1116:secondary
TGAAGAAAAA	1	0	1	0	0	At1g66360.1:X:+-14:quaternary,At3g27110.2:X:+-96:quaternary,Chr5:+24426195:quaternary,Chr3:+15306565:quaternary

TATTGGACCA	0	0	1	1	0	At1g66345.1:v:+2098:secondary
TTTGAAGCTG	1	0	0	1	0	At1g66200.1:X:-91:quaternary,At4g14905.2:X:-1388:quaternary
CTTTATGGTA	0	0	1	0	1	At1g66130.1:d:+1235:primary
AAAACTTAAA	0	2	0	0	0	At1g66060.1:v:+1713:primary
CAGCGTATGA	0	0	1	0	1	At1g65950.1:d:+1672:primary
CAAAAACTAT	0	0	2	0	0	At1g65930.1:d:+971:secondary,At3g11964.1:v:+23:secondary
TAGAGAAATG	1	0	0	1	0	At1g65920.1:d:+2816:primary
ATTCGAGGAA	0	0	1	0	1	At1g65860.1:X:-210:quaternary
AACATTGCAC	1	1	0	0	0	At1g65660.1:d:+431:secondary
GAAGTGACAG	0	2	0	0	0	At1g65580.1:d:+2744:primary
ACGATTTCTT	1	0	0	0	1	At1g65540.1:v:+2992:primary
AAGCTTGGTC	1	0	0	0	1	At1g65540.1:v:+2724:secondary
TTTGTACTTC	1	1	0	0	0	At1g65520.1:d:+730:secondary
CATCGTGGAA	0	1	1	0	0	At1g65510.1:d:+173:primary
TGGAGGTTCT	0	0	1	0	1	At1g65490.1:d:+177:secondary
TAGCTACTAC	0	0	1	0	1	At1g65480.1:i:+933:tertiary
AGCTTTCTTA	0	0	1	1	0	At1g65390.1:d:+115:secondary,At1g65390.2:d:+191:secondary
AATATGTTAC	1	1	0	0	0	At1g65310.1:d:+872:primary
GCTGAAGAAC	0	0	2	0	0	At1g65270.1:d:+921:secondary,At1g65270.3:d:+921:secondary,At2g43910.1:d:+78:secondary,At1g65270.2:d:+921:secondary
TTCGTATTAA	1	1	0	0	0	At1g65190.1:d:+1430:primary
ATCGGAGCCG	0	0	1	1	0	At1g65060.2:d:+412:secondary,At1g65060.1:d:+412:secondary
AAAGAGTACT	0	0	0	1	1	At1g65060.2:d:+1306:primary,At1g65060.1:d:+1306:primary
AAAACGTTTC	0	0	1	0	1	At1g65020.1:X:+343:quaternary
AACTTGAAAA	0	1	1	0	0	At1g65010.1:d:+872:secondary
CCTCAGCTGG	0	0	0	1	1	At1g64990.1:d:+553:secondary
GTATTTATTG	1	0	0	0	1	At1g64960.1:v:+3981:secondary
TAAATATGAG	0	0	0	1	1	At1g64960.1:v:+3916:secondary
GTCCAACTTT	0	0	0	1	1	At1g64890.1:d:+1635:secondary
TAACATCGGA	1	0	0	0	1	At1g64880.1:d:+1916:primary
TAGAGTGAGT	1	1	0	0	0	At1g64840.1:d:+1069:secondary
GCAAAGAAAA	1	0	0	1	0	At1g64790.1:d:+3179:secondary
TTCTTTCAAG	1	1	0	0	0	At1g64760.1:d:+1366:primary
GATTAGTTAC	0	0	0	1	1	At1g64750.1:d:+536:primary
TACTCCAAAA	0	1	0	0	1	At1g64740.1:d:+1301:primary
CTCTTTTCTC	0	2	0	0	0	At1g64700.1:d:+193:primary
AACGTGAACC	0	0	1	1	0	At1g64500.1:X:+1292:quaternary
ACCTCAAAAA	2	0	0	0	0	At1g64440.1:i:+309:tertiary
GGATTATATT	1	0	0	0	1	At1g64440.1:d:+1351:primary
AATCCGAAAC	1	1	0	0	0	At1g64340.1:v:+657:secondary
CTTGCAACTT	0	0	1	0	1	At1g64250.1:p:+1059:secondary
GATACAATGA	0	0	0	2	0	At1g64140.1:d:+2288:secondary
AGTTTTATTA	0	1	0	0	1	At1g64130.1:v:+974:primary
GTTAATAAGA	0	0	0	0	2	At1g64110.1:d:+2780:primary,At1g64110.2:d:+2798:primary,At5g42710.1:v:+2938:primary
AATGTTATTA	1	0	0	0	1	At1g64090.1:d:+1067:primary
CGTCTTAGCT	0	0	0	1	1	At1g63900.1:X:+-114:quaternary
GGAAAGGCTG	0	2	0	0	0	At1g63880.1:d:+2643:primary
CAAGAGTTCT	0	0	1	1	0	At1g63800.1:d:+165:secondary
ATATCACAAA	1	1	0	0	0	At1g63750.1:d:+2738:secondary
CCTTGGTGGA	1	0	1	0	0	At1g63700.1:v:+354:secondary
AGCTAAGAAC	1	0	0	0	1	At1g63670.1:d:+2027:primary
TCATATAGAT	1	0	0	0	1	At1g63640.2:i:+1535:tertiary,At1g63640.1:i:+1535:tertiary,At1g48470.1:i:+215:tertiary
GAATCCATCA	0	2	0	0	0	At1g63500.1:d:-1674:secondary
AACATTTCCC	0	0	1	0	1	At1g63500.1:d:+1567:secondary
CTGTATTGCT	1	0	1	0	0	At1g63160.1:d:+1157:primary
TACCAGTACG	0	2	0	0	0	At1g63120.1:d:+892:primary
TTAAAGTTGT	0	0	0	2	0	At1g63070.1:v:+2250:secondary
AAATGAAAAG	1	0	1	0	0	At1g62940.1:i:+1962:tertiary,At1g33990.1:i:+869:tertiary
TTTGTTAATT	1	1	0	0	0	At1g62890.1:v:+196:secondary
TAGTGGAAAA	0	0	0	2	0	At1g62860.1:v:+2071:secondary
TTATTCGAAA	0	1	1	0	0	At1g62840.1:v:+1012:secondary

CTATTCGACA	0	1	1	0	0	At1g62820.1:d:+139:primary
TTAGTATATT	0	0	0	0	2	At1g62790.2:d:+632:primary,At1g62790.1:d:+654:primary
CGTTTTAAGA	0	1	0	0	1	At1g62740.1:d:+1913:secondary
GAGAGCAATG	0	1	1	0	0	At1g62730.1:d:+1091:primary
CGGTGGTTAT	0	1	0	0	1	At1g62710.1:d:+1460:primary
AAAAAATAAA	1	0	1	0	0	At1g62420.1:v:+2108:secondary
ACTTAATGGA	1	0	0	1	0	At1g62380.1:d:+218:secondary
ATTTGGTGAT	1	0	0	0	1	At1g62370.1:v:+1046:primary
TATTGGTTAG	1	0	0	0	1	At1g62330.1:d:+1946:secondary,At1g01400.1:v:+1185:secondary
GAGAGGTTTT	0	0	1	0	1	At1g62290.1:d:+1665:primary
GTATACTCTG	1	0	0	0	1	At1g62200.1:d:+1845:primary
TCGTTTGTGA	0	1	0	0	1	At1g62180.1:d:+1479:secondary
GTGACAAGGA	0	1	0	0	1	At1g62110.1:d:+1557:primary
AGAAGTGAAA	0	0	1	1	0	At1g61960.1:d:+1580:primary
ACCTTGGAAA	0	1	1	0	0	At1g61840.1:v:-2300:secondary
TCATTAGACC	0	0	0	0	2	At1g61800.1:d:+1084:secondary
TATTTTTTTG	1	0	1	0	0	At1g61780.1:d:+493:primary
CAACGATACA	0	0	1	0	1	At1g61770.1:d:+979:secondary
TGCAAAATTT	0	0	2	0	0	At1g61710.1:i:+1118:tertiary
TTACTCGAGT	1	0	0	1	0	At1g61690.1:d:+3513:primary
GTCTATGTCT	0	2	0	0	0	At1g61660.2:d:+1308:primary,At1g61660.1:d:+1209:primary
ATCATATTTC	0	2	0	0	0	At1g61660.1:i:+1188:tertiary,At1g61660.2:i:+1188:tertiary
ATGTAACCAC	0	0	0	1	1	At1g61620.1:d:+1043:primary
AAACTGCTGA	0	0	2	0	0	At1g61560.1:d:+1598:secondary,At1g66200.1:d:+983:secondary
TCTACAAAAG	2	0	0	0	0	At1g61370.1:v:+2814:secondary
AATGTTCCAA	0	0	1	1	0	At1g61360.1:d:+2508:primary
ATCTTCTTTA	1	0	1	0	0	At1g61240.1:d:+1666:primary,At1g61240.2:d:+1758:primary
ATCATTGGTA	0	0	2	0	0	At1g61215.1:d:+2076:primary
AAAAGTCACT	0	2	0	0	0	At1g61210.1:d:+4012:primary
ACATTAAGAA	1	1	0	0	0	At1g61120.1:d:+2370:secondary
GCTGATCTCT	1	1	0	0	0	At1g61100.1:X:-70:quaternary,At3g20680.1:X:-29:quaternary
CCAAATCAAA	0	0	1	1	0	At1g61100.1:d:+1104:secondary
CCCACAAAGC	0	2	0	0	0	At1g61040.1:d:+1335:secondary
TTTAGTAATA	0	0	0	0	2	At1g61010.1:d:+2232:primary
TATAGTTCTT	0	2	0	0	0	At1g60960.1:d:+1411:primary
GCTGTGACAC	1	0	0	1	0	At1g60950.1:d:-197:primary
TTTAGTTCTC	0	0	0	1	1	At1g60900.1:d:+1956:primary
ATTTGACTCG	1	1	0	0	0	At1g60890.1:d:+2880:primary
GCTGGTGAGT	0	1	0	0	1	At1g60780.1:d:+1532:secondary,At1g10730.1:v:+1608:secondary
TCATTTAAGT	1	0	0	0	1	At1g60730.2:d:+1147:primary,At1g60730.1:d:+1143:primary
CTCGTATGGA	0	0	0	1	1	At1g60640.1:d:+441:secondary
TGGAGATATC	0	2	0	0	0	At1g60630.1:X:-380:quaternary
GGCCAAGGTA	0	2	0	0	0	At1g60620.1:d:+1047:secondary
TAGGCACTTG	1	0	0	1	0	At1g60610.2:d:+2014:primary,At1g60610.1:d:+1586:primary
GTACCGAGCG	1	0	0	1	0	At1g60560.2:d:+1611:primary,At1g60560.1:d:+1690:primary
TAAACAACCA	1	0	0	0	1	At1g60300.1:v:+1518:secondary,At4g01290.1:d:+3301:secondary
TGATTGATGC	0	1	1	0	0	At1g60090.1:v:+2168:primary
GAGATTATTC	0	1	0	1	0	At1g60020.1:p:+2510:secondary
TCAGAGAGTT	0	2	0	0	0	At1g59910.1:d:+2740:secondary
GCAACAACCA	0	0	1	1	0	At1g59890.1:d:+3613:primary
TAGTTACATA	0	1	0	0	1	At1g59840.1:d:+1047:primary
TGGAGTTTGG	0	0	1	0	1	At1g59770.1:X:-530:quaternary
GAGATTGCTC	0	1	1	0	0	At1g59760.1:d:+2851:primary
ACTGTTGTCT	0	0	0	1	1	At1g59710.1:d:+1106:primary
ACTGAATATG	0	0	0	0	2	At1g59580.2:d:+684:secondary,At1g43170.1:d:+972:secondary,At1g59580.1:d:+747:secondary,At1g43170.2:d:+938:secondary,At1g10210.1:d:+685:secondary,At2g43790.1:d:+776:secondary,At2g46070.1:d:+648:secondary
AGTTTTTGAC	0	2	0	0	0	At1g59560.1:d:+1235:primary
AAATGAAGTT	0	1	0	1	0	At1g59453.1:v:+5604:secondary,At1g59077.1:v:+2412:secondary,At1g58766.1:v:+2412:secondary
TTGGTTTGGG	0	0	2	0	0	At1g59359.1:d:+389:secondary,At1g58684.1:d:+389:secondary,At1g58380.1:d:+472:secondary,At1g58983.1:d:+401:secondary

TATAAATTGT	1	0	1	0	0	At1g58983.1:d:+1098:primary
AGTAACGTTA	0	0	1	1	0	At1g58889.1:p:+211:secondary,At4g02960.1:p:+70:secondary,At1g59265.1:p:+70:secondary
ACCGTTTATG	0	1	0	1	0	At1g58350.1:d:+2699:primary
GTTTAAGCGT	0	1	0	1	0	At1g58250.1:d:+7700:secondary
CAGTCAATGG	0	2	0	0	0	At1g58220.1:d:+2101:primary,At5g45570.1:v:+3335:primary
GGGTGAAATT	0	1	1	0	0	At1g58060.1:X:-316:quaternary
TATAAGACAC	0	0	0	0	2	At1g58060.1:d:+4437:primary
AGAAGAAAAA	0	0	1	0	1	At1g58020.1:p:+3208:secondary,At1g76780.1:v:+1030:secondary,At4g20520.1:v:+471:secondary,At4g20725.1:p:+169:secondary,At5g61620.1:v:+658:secondary,At2g06820.1:v:+961:secondary,At1g54510.1:d:+178:secondary
ACTTTAAAAG	1	1	0	0	0	At1g57680.2:X:+1564:quaternary,At1g57680.2:X:+1570:quaternary
GAGGAAAGCT	0	1	0	0	1	At1g57630.1:v:+791:secondary
CAATCTTTGA	0	2	0	0	0	At1g57565.1:v:+1360:primary
ATTATGAGCT	2	0	0	0	0	At1g56720.2:d:+1896:primary,At1g56720.1:d:+1577:primary
CCTTGGACTT	0	0	1	0	1	At1g56650.1:d:+548:primary
CTTTTAATTT	0	0	0	2	0	At1g56610.1:d:+37:secondary,At4g18570.1:d:+123:secondary
TGACGTTGAT	0	1	1	0	0	At1g56510.1:d:+1438:secondary
ACCACTTTGA	1	0	1	0	0	At1g56410.1:v:+1599:secondary,At5g02490.1:d:+1329:secondary,At5g02500.1:d:+1354:secondary
GTTTTGTGGA	0	1	1	0	0	At1g56360.1:v:+2016:primary
TTCAATTGTC	1	0	1	0	0	At1g56300.1:d:+684:secondary
CCGTAAGTAG	1	0	1	0	0	At1g56220.2:d:-486:secondary,At1g56220.3:d:-481:secondary,At1g56220.1:d:-472:secondary
TAAAATGACT	0	1	1	0	0	At1g56000.1:d:+1240:secondary
ATGAATTTCA	0	0	2	0	0	At1g55940.1:d:+1465:secondary
TATAGAAAGA	0	0	0	0	2	At1g55930.1:d:+2179:primary
TATTCAGATT	1	1	0	0	0	At1g55880.2:d:+1674:primary,At1g55880.1:d:+1566:primary
TTCTCTGACC	0	2	0	0	0	At1g55870.1:d:+1957:primary
TGCTTTTACT	1	1	0	0	0	At1g55810.1:d:+1243:primary,At1g55810.2:d:+1749:primary,At1g55810.3:d:+1345:primary
TTTCTGAGCT	0	2	0	0	0	At1g55730.1:i:+1579:tertiary
ACATTTCTTT	0	1	0	0	1	At1g55610.1:v:+4094:secondary
ACATTTTAGA	0	0	0	1	1	At1g55550.1:v:+3221:primary
GCTTCAGCTG	0	1	0	1	0	At1g55540.1:d:+2445:primary
TGATGCACTT	1	0	1	0	0	At1g55535.1:i:+737:tertiary,At1g55535.2:i:+730:tertiary
AATTCGACGG	0	2	0	0	0	At1g55460.1:d:+411:secondary
ACTAAACACT	0	0	0	0	2	At1g55320.1:v:+2681:primary
TGTTTGCGAT	0	0	1	1	0	At1g55260.1:d:+679:secondary
CTTTCACATT	0	0	0	2	0	At1g55180.1:v:+21:secondary,At5g54670.1:d:+1604:secondary,At2g07190.1:v:+1773:secondary
CTGGTTACTC	0	0	1	1	0	At1g54990.1:d:+1419:primary
GGAATTCAGG	0	2	0	0	0	At1g54920.2:d:+518:secondary
TCGAGTAACA	0	2	0	0	0	At1g54850.1:d:+187:primary
GATAGGGTTT	1	0	0	1	0	At1g54560.1:v:+182:secondary,At5g27510.1:v:+992:secondary
AAATCCTCTA	0	1	1	0	0	At1g54490.1:d:+2402:primary
TTTACAGAAA	0	0	0	1	1	At1g54490.1:d:+1197:secondary
GTAATTGTAT	0	2	0	0	0	At1g54360.2:d:+1626:primary,At1g54360.1:d:+1557:primary
ACACCCGTTT	0	0	1	1	0	At1g54350.1:d:-2215:secondary
GATGGATAAG	0	0	0	1	1	At1g54310.2:v:+1942:secondary,At1g09880.1:v:+967:secondary
TGTTTGTCTT	0	2	0	0	0	At1g54215.1:v:+1010:primary,At1g28390.1:d:+1576:primary
CTAGCAAACC	0	2	0	0	0	At1g54140.1:d:+275:primary
ACCGAGCACG	1	0	0	0	1	At1g54120.1:d:+408:primary
TTGTTGTTAT	1	0	1	0	0	At1g54060.1:d:+1584:secondary
GTTGTGTGAG	0	0	1	1	0	At1g54040.2:d:+1235:primary,At1g54040.1:d:+1177:primary
TTTTAAAGCT	0	1	0	0	1	At1g53870.1:d:+719:primary,At1g53870.2:d:+637:primary,At1g53890.1:d:+719:primary
AGATCTAACG	0	0	1	0	1	At1g53800.1:d:+1566:primary
TATTAATGTG	1	1	0	0	0	At1g53720.1:i:+1982:tertiary
TTCTTGTCTT	0	1	1	0	0	At1g53660.1:v:+997:secondary
TTGAAACCTG	0	0	2	0	0	At1g53450.2:d:+1353:primary,At1g53450.1:d:+1353:primary
AGAAGAGGAA	0	2	0	0	0	At1g53440.1:v:+1830:secondary,At1g27300.1:d:+120:secondary
AACTCTTCAC	0	0	1	1	0	At1g53380.1:i:+1424:tertiary
ATCGCCGTTT	0	1	0	1	0	At1g53380.1:d:+1617:primary
TTTGTGCCTC	2	0	0	0	0	At1g53240.1:d:+1252:secondary
GACTCTTCGG	0	2	0	0	0	At1g53200.2:d:+1804:primary,At1g53200.1:d:+1821:primary
TAAGTTCAAG	1	1	0	0	0	At1g53180.1:d:+152:secondary

GCTGGTGTTT	0	1	0	1	0	At1g53140.1:d:+2539:primary
TGCCTATGTA	0	1	0	0	1	At1g53060.1:v:+1339:secondary
TGATAGAGAG	1	1	0	0	0	At1g53040.1:d:+1913:primary,At1g53040.2:d:+1824:primary
TCTAAATCTG	0	2	0	0	0	At1g52905.1:d:+149:primary
GCTGCTGCTT	0	0	1	0	1	At1g52870.2:d:+113:secondary,At1g52870.1:d:+113:secondary
GTGACGATGA	0	1	0	1	0	At1g52710.1:d:+359:primary
AGGCGTTTGA	0	1	0	1	0	At1g52510.1:d:+563:secondary
CAACATTAAA	1	0	1	0	0	At1g52430.1:v:+3284:secondary
TAACCAAAAA	0	0	0	1	1	At1g52390.1:v:+1052:primary
GATGAAAAAA	1	1	0	0	0	At1g52340.1:X:-177:quaternary
GTGTGCATCT	0	1	1	0	0	At1g52280.1:d:+572:primary
GATGACTTAA	0	0	0	2	0	At1g52160.1:d:+2667:primary
ACCGTTTTAC	1	1	0	0	0	At1g52150.1:X:-208:quaternary
TCACGGCTAT	1	0	0	1	0	At1g52100.1:d:+1389:primary
TGTCTTATCA	1	1	0	0	0	At1g52040.1:d:+1578:primary
TAAAAAACTT	0	1	0	1	0	At1g51990.1:v:+1875:primary,At1g51990.2:v:+1875:primary
TCTTCTCCGT	2	0	0	0	0	At1g51965.1:v:+2390:secondary
AAGCAAGTGG	1	0	1	0	0	At1g51940.1:d:+2026:primary
TTTCAGTTTA	0	1	1	0	0	At1g51810.1:v:+3036:secondary
TTAACAGAGA	0	1	1	0	0	At1g51790.1:d:+2257:primary,At3g20660.1:v:+2124:primary
CAACGCGAAG	0	0	2	0	0	At1g51710.1:d:+1426:secondary
TAAACGCGGT	1	1	0	0	0	At1g51700.1:d:-1114:secondary
GATCAAAAGT	0	1	1	0	0	At1g51630.1:d:+327:secondary
ATCATCCAAC	1	1	0	0	0	At1g51570.1:X:-2424:quaternary
GAGGAATCGG	0	2	0	0	0	At1g51550.1:d:+768:secondary
ACAGTGGATG	0	0	1	0	1	At1g51550.1:d:+1627:primary
GTGAGGTTCC	0	0	2	0	0	At1g51460.1:d:+1524:secondary
CAGTTACAAA	2	0	0	0	0	At1g51370.1:d:-1638:secondary
TGAAGTGTAT	0	2	0	0	0	At1g51360.1:X:+-131:quaternary,ChrC:+140155:quaternary
GAAGAAGCGA	0	2	0	0	0	At1g51360.1:d:+412:primary
ATTCGTCTAT	0	0	1	0	1	At1g51350.1:d:+2225:primary
AACAAAGCTA	0	1	1	0	0	At1g51140.1:d:+1393:primary
CCTCCTGCTG	0	0	0	1	1	At1g51070.1:d:+752:secondary
ATATGAATAT	1	0	0	0	1	At1g50840.1:d:+3492:primary
GCGCTTGAAG	0	1	1	0	0	At1g50730.1:v:+2961:secondary
TTAATTTTCA	0	1	1	0	0	At1g50670.1:d:+790:secondary
AGAACAACAA	0	0	1	0	1	At1g50630.1:d:+150:secondary
TGGTACCCTT	0	1	0	1	0	At1g50510.1:d:+1283:primary
AATCATCATC	0	1	0	1	0	At1g50460.1:d:+1844:primary
AGCTGCATTC	0	0	0	1	1	At1g50410.1:d:+3191:primary
GAAACACCAT	0	0	2	0	0	At1g50300.1:d:+353:secondary,At4g39980.1:d:+1344:secondary
ATCGGGACAG	0	0	2	0	0	At1g50300.1:d:+1166:primary
TTTATGGAGC	0	1	1	0	0	At1g50290.1:d:+497:primary
GAAGGCTCAA	0	1	0	0	1	At1g50260.1:d:+2049:primary
TGTCAAAAAA	1	0	0	1	0	At1g50200.1:X:--35:quaternary
GTTGAAACTT	0	1	1	0	0	At1g50080.1:v:+223:secondary,At1g50090.1:v:+1691:secondary
AGAATCTCTG	1	1	0	0	0	At1g50030.1:d:+7779:secondary
AGGAATTCAG	1	1	0	0	0	At1g50000.1:i:+695:tertiary
AGTCAAAGAA	1	1	0	0	0	At1g49920.1:X:-1058:quaternary
GAACTTCTAT	1	0	1	0	0	At1g49880.1:d:+621:primary
CCCTCCTCCA	0	0	2	0	0	At1g49750.1:d:+232:secondary,At1g49750.1:d:+259:secondary
CCATTGGAAA	0	0	2	0	0	At1g49730.3:d:+1446:primary,At1g49730.2:d:+1261:primary,At1g49730.1:d:+1256:primary
CAGCAGCAGC	0	1	0	1	0	At1g49720.1:d:+790:primary
AGATTGGTGA	0	1	1	0	0	At1g49710.1:d:+1817:primary
TCAGTGGCTA	0	0	1	0	1	At1g49670.1:X:-172:quaternary
TGAATTATTT	0	0	1	1	0	At1g49490.1:v:+2954:secondary,At1g73610.1:v:+1070:secondary
GATTTACCAA	0	2	0	0	0	At1g49450.1:d:+470:secondary
TTAAGATACT	1	0	0	0	1	At1g49400.1:d:+641:primary
TATGTACGGA	1	1	0	0	0	At1g49340.1:d:+6008:secondary,At1g49340.2:d:+6008:secondary
CGAGAAGATT	0	1	1	0	0	At1g49230.1:d:+576:primary
GGTTCCAGGG	0	2	0	0	0	At1g48790.1:d:+699:secondary

ATTTCTTTCG	0	2	0	0	0	At1g48790.1:d:+1044:secondary
AACAAACAAA	0	0	0	1	1	At1g48730.1:X:-315:quaternary,At1g07350.2:X:--39:quaternary,At5g56580.1:X:--12:quaternary,At2g39240.1:X:--178:quaternary,At4g36440.1:X:-257:quaternary
GGCTAATTGA	1	0	0	0	1	At1g48600.1:X:+364:quaternary
GAAGCTCAAG	0	2	0	0	0	At1g48540.2:d:+3212:primary,At1g48540.1:d:+3248:primary
GAAACAGAGG	1	0	0	1	0	At1g48480.1:d:+1454:primary
AGGTCTTGGC	0	0	0	1	1	At1g48260.1:d:+227:secondary,At3g17510.1:d:+539:secondary
GCTACTTCCT	0	1	0	1	0	At1g48175.1:d:+572:primary,At5g07430.1:d:+1152:primary
TTTTGTTATC	0	1	1	0	0	At1g48175.1:d:+554:secondary,At5g19640.1:v:+2406:secondary
CAGTTTCCTA	1	1	0	0	0	At1g48140.1:d:+291:primary
ATGTGGCTTT	1	0	0	0	1	At1g48120.1:d:+602:secondary
TAGGATTTCA	1	0	0	1	0	At1g48090.1:i:+3748:tertiary,At1g48090.2:i:+3748:tertiary
TGAAGCAGCT	1	0	0	1	0	At1g48050.1:d:+303:secondary,At4g24490.1:v:+2101:secondary
GCCTAGTCAA	1	1	0	0	0	At1g47860.1:p:+2607:secondary
TAGAGATGGA	1	1	0	0	0	At1g47760.1:v:+763:secondary
GGCACCAAAA	0	0	1	1	0	At1g47740.2:d:-519:secondary,At1g47740.1:d:-571:secondary
GTTACATACA	0	0	0	0	2	At1g47710.1:X:+1009:quaternary
ATAAGTTCCT	1	1	0	0	0	At1g47620.1:v:+1270:secondary
TCTTTACCAT	0	1	0	1	0	At1g47520.1:p:+1182:secondary,At3g02730.1:d:+646:secondary
TGAGTTTATT	0	0	1	1	0	At1g47410.1:v:+1006:primary
AAATGAAACC	0	0	1	1	0	At1g47370.1:v:+1846:primary,At4g25260.1:d:+871:primary
TTGAGTATCT	0	0	1	0	1	At1g47340.1:d:+1277:secondary
AACAAGTTTT	0	1	1	0	0	At1g47310.1:d:+1529:primary,At2g26760.1:d:+1524:primary
TCATTCACAA	0	1	0	1	0	At1g47310.1:d:+1291:secondary
TTAGCATTAC	0	0	0	1	1	At1g47280.1:X:-188:quaternary
TTTATTGCAA	0	0	0	1	1	At1g47280.1:X:+396:quaternary
GGTATCGTCG	0	2	0	0	0	At1g47270.1:d:+983:secondary
TCAAGAAGAT	0	2	0	0	0	At1g47240.1:d:+1726:primary
GGGTAAAAGC	0	0	2	0	0	At1g47128.1:d:+1018:secondary
AGCATTTTGA	0	0	2	0	0	At1g45545.1:v:+3038:primary
ACTGGAAGCT	0	0	1	0	1	At1g45474.2:d:+653:secondary,At1g45474.1:d:+629:secondary
ACACAAGGCA	0	1	1	0	0	At1g45332.1:d:+2293:primary
ATTGTTGGAC	0	1	0	1	0	At1g45332.1:d:+1891:secondary,At2g45030.1:d:+1753:secondary
AAGAATTTGC	0	0	1	1	0	At1g45170.1:d:+872:primary
GAACCTGGAA	1	0	1	0	0	At1g45170.1:d:+618:secondary
TAGACCATCT	1	0	1	0	0	At1g45150.1:d:+2036:primary
AAACCATCTC	0	1	0	0	1	At1g44910.1:i:+1010:tertiary
AAAAAAATGT	0	1	1	0	0	At1g44900.1:d:+2873:primary
ATTTCACAAA	0	0	2	0	0	At1g44820.1:d:+1577:primary
TACAATCAAA	1	0	0	1	0	At1g44110.1:X:-122:quaternary,At4g10810.1:X:-435:quaternary
TGTTAACCAA	0	0	0	0	2	At1g43850.1:d:+3576:secondary
TATAGTGGTC	0	1	0	1	0	At1g43580.1:d:+939:primary
ACGATTATCC	0	2	0	0	0	At1g43240.1:p:+1007:secondary,At5g67240.1:v:+685:secondary
ATCAATCATC	1	1	0	0	0	At1g43205.1:v:+1189:primary,At4g07523.1:v:+1189:primary,At1g43230.1:v:+1148:primary
TCCAAGAACA	1	1	0	0	0	At1g43190.1:d:+1252:secondary
CAGATGATAC	0	0	1	0	1	At1g43150.1:p:+3658:secondary
TAGCATAAAA	0	2	0	0	0	At1g42970.1:d:-1637:secondary
GGGATCTTCT	1	0	0	1	0	At1g42440.1:d:+514:secondary
AACTTGTACA	0	0	0	0	2	At1g42440.1:d:+2439:primary
GCCAATGGAT	0	2	0	0	0	At1g42150.1:p:+4021:primary,At3g33035.1:p:+3408:primary
GTAACAGCGG	0	0	2	0	0	At1g41880.1:d:+303:secondary
TAAACAAAGG	0	2	0	0	0	At1g41840.1:X:-1963:quaternary,At5g66190.1:X:-166:quaternary
CCAAGAATAG	1	1	0	0	0	At1g41740.1:p:+2001:secondary,At4g08872.1:p:+461:secondary,At3g32895.1:p:+4138:secondary,At4g07668.1:p:+2119:secondary,At2g06890.1:p:+3710:secondary
TCCATATGGA	0	0	1	0	1	At1g37405.1:p:+5094:secondary
ATGTTCGTAA	0	0	0	2	0	At1g37130.1:d:+2117:secondary
TCACGAGGAG	1	1	0	0	0	At1g37130.1:d:+1203:secondary
AAGGTGGTGA	2	0	0	0	0	At1g37110.1:p:+1172:secondary

GCTAAGCTTA	0	0	1	1	0	At1g37045.1:v:+381:secondary,At5g61480.1:v:+1325:secondary,At2g06840.1:p:+31:secondary,At1g41930.1:p:+8907:secondary,At5g29762.1:p:+31:secondary,At1g42160.1:p:+52:secondary,At3g42430.1:v:+381:secondary,At2g13740.1:p:+31:secondary,At5g05470.1:d:+1040:secondary
ACTCTTACTT	0	0	2	0	0	At1g36960.1:v:+724:secondary
CTTCACAAAA	1	0	0	0	1	At1g36936.1:p:+855:primary
AGATTTCTCA	0	2	0	0	0	At1g36890.1:X:-1234:quaternary,At1g20530.1:X:--38:quaternary
TGAATGTCTC	1	0	0	0	1	At1g36675.1:v:+1173:primary
TATGGTTGAT	1	0	1	0	0	At1g36500.1:p:+1184:primary
TTCTTTCTTG	0	0	1	0	1	At1g36390.2:i:+1919:tertiary
ATCATCTTCG	0	2	0	0	0	At1g36110.1:X:-1471:quaternary
TTCCTGTCAA	0	0	1	1	0	At1g36070.1:X:-2:quaternary
TTTTATCTAC	1	0	1	0	0	At1g36030.1:v:+894:primary,At2g30150.1:v:+2088:primary
TTCAATTCGA	0	1	0	1	0	At1g35840.1:p:+2176:secondary
TCGACGGCGG	0	2	0	0	0	At1g35780.1:d:+184:secondary
AGAAAATCAA	0	0	1	1	0	At1g35720.1:d:+565:secondary
GCACGCAAGC	0	0	1	1	0	At1g35710.1:d:-3603:secondary
ACACAATAGA	0	1	0	0	1	At1g35710.1:d:+2645:secondary
AAGATGAGAA	1	1	0	0	0	At1g35647.1:p:+1559:secondary,At5g16270.1:d:+2647:secondary,At3g16540.1:v:+601:secondary,At3g24080.1:d:+838:secondary,At2g13490.1:p:+3348:secondary,At4g06628.1:p:+1947:secondary
TAGACAAGAA	0	2	0	0	0	At1g35620.1:d:+985:secondary,At4g27880.1:d:+697:secondary,At1g02280.1:d:+816:secondary
TCTACTGTAA	0	2	0	0	0	At1g35580.2:X:-180:quaternary
ATCACTCACT	0	0	0	1	1	At1g35560.1:X:-1091:quaternary
TCCTTTAAAA	1	0	0	0	1	At1g35510.1:i:-2045:tertiary
TTTGCTTTGT	0	1	0	1	0	At1g35480.1:p:+747:secondary
GTTCTATTCC	0	1	1	0	0	At1g35350.1:v:+1851:primary
AAGCAGCTTC	0	0	1	1	0	At1g35220.1:v:+1966:secondary
AATGCTCTTT	0	2	0	0	0	At1g35190.1:d:+1097:primary
TAACATTTGA	0	0	1	1	0	At1g35180.1:d:+797:primary
ATCTACGATC	0	2	0	0	0	At1g35170.1:d:+317:primary,At1g45010.1:d:+326:primary
TTTCTGTTAC	1	0	1	0	0	At1g34780.1:d:+1255:primary,At3g23170.1:d:+646:primary
AGAAGATTAC	0	0	1	0	1	At1g34750.1:d:+1084:primary
AATCAAAAGG	1	0	1	0	0	At1g34550.1:X:--60:quaternary,At4g36090.1:X:--116:quaternary
AGAAAAACAA	1	0	0	1	0	At1g34550.1:v:+1195:secondary
CAGAAAATGT	1	0	0	1	0	At1g34420.1:v:+3553:secondary
AATGTTGAAA	0	1	1	0	0	At1g34130.1:X:+347:quaternary
AAGACGATCC	0	0	1	1	0	At1g34020.1:d:+993:primary
CTCTTGGACT	0	0	2	0	0	At1g33960.1:d:+1227:primary
TAATTTGTTT	0	1	0	0	1	At1g33890.1:v:+324:secondary
GGCTTAGGTC	0	1	0	1	0	At1g33890.1:v:+1415:secondary,At3g54400.1:d:+671:secondary
ATTATTTGTG	1	0	0	0	1	At1g33800.1:d:+1159:primary
ATTCTAGCAA	0	2	0	0	0	At1g33700.1:d:+1793:secondary
TCATTTGCGA	0	0	1	1	0	At1g33560.1:d:+2576:primary
GCATTGAGTG	1	1	0	0	0	At1g33480.1:v:+1855:primary
GTGGTACAAG	2	0	0	0	0	At1g33440.1:d:+181:secondary,At5g49740.1:d:+1863:secondary,At3g05190.1:d:+645:secondary
AAGAGTTTTT	0	1	1	0	0	At1g33360.1:d:+2236:primary
AGGTTGTAAC	0	1	1	0	0	At1g33265.1:d:+618:secondary
TGATTTGCAT	0	1	0	0	1	At1g33260.1:d:+1545:primary,At1g33260.2:d:+1542:primary
TTGCTTCTCG	0	0	2	0	0	At1g33170.1:X:+364:quaternary
CGGTGGTTTC	0	0	1	0	1	At1g33110.1:i:+927:tertiary
TTTTTCGTTT	0	1	0	0	1	At1g33060.2:d:+2012:primary,At1g33060.1:d:+2000:primary
GAGTAAAACT	0	0	0	1	1	At1g32900.1:d:+455:secondary,At5g44110.1:d:+361:secondary
CAGATTCTTC	1	0	0	0	1	At1g32850.1:i:+1614:tertiary
CCCAGGCGGA	0	1	1	0	0	At1g32740.1:d:+407:primary
TACTGTTTAG	0	2	0	0	0	At1g32700.1:d:+276:secondary,At1g32700.2:d:+523:secondary
CATCTCCGTT	0	0	1	1	0	At1g32610.1:d:+1188:primary
TAAACTGTAA	0	1	1	0	0	At1g32550.1:d:+625:secondary
AGTGATGATG	0	2	0	0	0	At1g32450.1:d:+1934:primary
GAACAATGGG	0	1	0	0	1	At1g32390.1:v:+647:secondary,At3g03940.1:d:+1990:secondary

AAGTTTCCAG	1	0	1	0	0	At1g32375.1:v:+840:secondary,At1g62380.1:d:+94:secondary
TCTATCGATC	0	0	0	2	0	At1g32375.1:v:+1348:secondary
TCTGCAAAGA	0	1	1	0	0	At1g32340.1:d:+2096:secondary
TTTACTACAT	1	1	0	0	0	At1g32250.1:v:+1144:primary
TTTGAGCATT	1	0	1	0	0	At1g32060.1:d:-1177:secondary
TAAGTTGGCG	1	0	0	1	0	At1g31885.1:v:+1148:primary
GCTTTGGAAA	0	0	1	1	0	At1g31830.2:d:+1746:primary,At1g31830.1:d:+1928:primary
AACAAACATC	1	1	0	0	0	At1g31810.1:d:+3606:primary
TTTGCTTCCT	0	0	0	1	1	At1g31800.1:d:+1599:secondary,At5g50375.1:d:+596:secondary
ATCTTATTAT	0	2	0	0	0	At1g31730.1:d:+618:secondary,At4g39190.1:v:+1567:secondary
TTCCTAGCCA	0	2	0	0	0	At1g31710.1:d:+1913:secondary,At1g12480.1:d:+877:secondary
TTTACTTTTC	0	1	1	0	0	At1g31680.1:v:+271:secondary
TTACCCCAAT	2	0	0	0	0	At1g31670.1:i:-2779:tertiary
TCTTTCAAAA	0	0	2	0	0	At1g31670.1:i:+2784:tertiary,At4g32551.1:i:+3418:tertiary
ATAGTGTATC	0	0	0	0	2	At1g31660.1:X:+225:quaternary
GAAAGCCTCA	0	1	1	0	0	At1g31600.1:d:+1541:primary,At1g31600.3:d:+1496:primary,At1g31600.2:d:+956:primary
TCTCAGCTCA	1	0	1	0	0	At1g31550.1:d:+1116:primary
CATCAGCTAG	0	1	0	0	1	At1g31460.1:d:+1632:primary
CACCTGAACA	1	0	1	0	0	At1g31430.1:X:-805:quaternary
TGTTTCTGGG	1	0	0	1	0	At1g31420.1:d:+1061:secondary,At2g35620.1:d:+763:secondary,At3g10500.1:d:+1694:secondary
CTATGATAGC	0	1	1	0	0	At1g31150.1:d:+2149:primary
AGAGAGAGAA	1	1	0	0	0	At1g31070.2:d:+1720:primary
GAACAACTCT	0	0	1	1	0	At1g31020.1:d:+592:primary
GAGAGAGAGA	1	1	0	0	0	At1g30975.1:p:+168:primary
GCAAAACTGA	0	2	0	0	0	At1g30820.1:X:-41:quaternary
TATTAAAGTT	1	0	0	0	1	At1g30730.1:v:+1949:secondary
AAAAGAAAAG	0	1	1	0	0	At1g30720.1:d:+1660:primary
GCGTAGAACC	0	1	1	0	0	At1g30690.1:d:-1264:secondary
ACGAGATTTT	0	0	1	0	1	At1g30620.1:d:+1751:primary
AAATTCGAGG	0	0	2	0	0	At1g30580.1:d:+1126:secondary
AAGCTTGGCT	0	1	0	1	0	At1g30570.1:v:+2743:secondary
TCTCAATGAC	0	1	1	0	0	At1g30560.1:v:+1495:secondary,At2g02720.1:d:+953:secondary,At1g14420.1:d:+803:secondary
GGCTGAACAC	1	0	0	1	0	At1g30380.1:d:-161:primary
GGAAGGCACC	0	1	1	0	0	At1g30360.1:d:+374:secondary
TTTGGCCTCG	0	1	0	1	0	At1g30330.1:d:+3245:primary
TGTTGCCTTT	1	0	0	0	1	At1g30280.1:d:+1613:primary
GTGTCAATAG	0	0	2	0	0	At1g30230.1:d:-688:secondary
TTTGTGTAAA	0	1	1	0	0	At1g30150.1:p:+2306:secondary,At4g08120.1:p:+948:secondary,At5g29646.1:p:+6547:secondary,At1g43723.1:p:+623:secondary,At4g06614.1:p:+578:secondar y
TGATGCAGGC	0	0	1	1	0	At1g30120.1:d:+1047:secondary
AGCAAGTTTT	0	0	2	0	0	At1g30010.1:d:+1928:secondary
GCGCAAGCGA	0	0	1	0	1	At1g29980.2:d:+1115:primary,At1g29980.1:d:+1120:primary
AATACGGTAC	1	0	0	0	1	At1g29965.1:X:+593:quaternary
GCCCACCTGC	1	0	0	0	1	At1g29920.1:X:-383:quaternary
GTGTCTATAT	1	1	0	0	0	At1g29880.1:d:+1683:primary
CTTCTTAACA	0	1	0	1	0	At1g29840.1:v:+1183:secondary
ATCACAAATC	0	0	2	0	0	At1g29670.1:d:+923:secondary,At5g13150.1:v:+403:secondary
GCAAAATCAT	1	0	0	1	0	At1g29650.1:p:+3343:secondary
ATTTCGTAAT	0	1	0	1	0	At1g29550.1:d:+898:primary
AGATTATTCT	0	1	0	1	0	At1g29530.1:d:+1037:primary
GTCTGCTCCA	0	2	0	0	0	At1g29470.1:d:+270:secondary,At3g43230.1:d:+1209:secondary,At5g27410.1:d:+50:secondary,At1g71410.1:d:+681:secondary
AAGCAGAAGC	0	1	0	0	1	At1g29400.1:d:+141:secondary,At1g29400.2:d:+182:secondary
AACTTTCTAA	1	0	0	1	0	At1g29355.1:v:+330:secondary,At3g29270.2:d:+1804:secondary,At3g29270.1:d:+936:secondary
TTCTTCTCTT	0	0	1	0	1	At1g29350.1:d:+2920:primary
GAAGAAGGTG	0	2	0	0	0	At1g29340.1:d:+1476:primary
GGAAGCGTTG	0	2	0	0	0	At1g29280.1:v:+1172:primary
GAGTGATACG	1	0	0	0	1	At1g29170.1:v:+3389:secondary
CTTTTTCACT	0	2	0	0	0	At1g29170.1:i:+909:tertiary,At5g62920.1:i:+1055:tertiary
ATTTTCCGGT	0	1	0	0	1	At1g29160.1:v:+829:primary

TACAGATGAA	0	0	1	1	0	At1g29120.2:d:-1707:secondary,At1g29120.1:d:-1751:secondary
AGATATTTTA	0	1	0	0	1	At1g29060.1:d:+755:primary
AACCGTCTTG	0	1	0	0	1	At1g29050.1:d:+865:secondary
GAACCAACTT	0	2	0	0	0	At1g29050.1:d:+1236:primary
GAGACGACGG	0	1	0	1	0	At1g29040.2:d:+238:primary
GATAAGTTCC	0	1	0	0	1	At1g28680.1:d:+1354:primary
AGTGGTAGAA	0	0	1	0	1	At1g28610.2:d:+1449:primary,At1g28610.1:d:+1503:primary
TGTTAATCTC	1	1	0	0	0	At1g28420.1:X:+1017:quaternary
AGGATGAGGA	1	1	0	0	0	At1g28323.1:p:+1517:secondary,At3g03760.1:d:+797:secondary,At2g10610.1:p:+97:secondary,At3g53630.1:d:+412:secondary,At1g45070.1:p:+452:secondary
TTTGTAAAGC	0	0	1	1	0	At1g28320.1:d:+2221:primary
AGGTTCCTGG	0	0	1	1	0	At1g28060.1:d:+631:secondary
TCATCTTCAT	0	1	1	0	0	At1g27850.1:d:+2371:secondary
GTGGAACTGA	0	2	0	0	0	At1g27770.1:d:+503:secondary,At1g27770.2:d:+503:secondary
CTATAAGGAG	0	2	0	0	0	At1g27670.1:d:+541:primary
TTCATTAAAA	1	0	0	1	0	At1g27660.1:i:+1832:tertiary
ATGGGCTGTG	1	1	0	0	0	At1g27660.1:d:+1747:primary
GAAGTGGAAA	0	1	0	1	0	At1g27650.1:d:+882:primary
AGGAAGATTG	1	0	1	0	0	At1g27610.1:v:+255:secondary,At2g38180.1:d:+351:secondary,At1g52830.1:d:+404:secondary
TTGTTGGGAT	0	0	1	0	1	At1g27540.1:d:+1490:primary
GATATATAGT	0	0	0	0	2	At1g27520.1:d:+1991:primary
GGGTGGGACG	1	1	0	0	0	At1g27480.1:d:+816:primary
GATGGAGGTT	0	0	0	1	1	At1g27470.1:d:+2447:primary
TACCAAGTTT	1	0	0	1	0	At1g27440.1:X:+667:quaternary
AAAGGAATTG	0	2	0	0	0	At1g27410.1:d:+997:primary
TTTCTTCGTA	1	1	0	0	0	At1g27380.2:X:--67:quaternary
AAAGCAAGAA	1	0	0	1	0	At1g27370.2:d:+79:secondary
TCTACGACTA	0	2	0	0	0	At1g27370.1:d:+2695:primary,At1g27370.2:d:+1773:primary
GTCTCTGGGA	0	1	0	1	0	At1g27340.1:d:+1209:primary
ATTTAGGATA	2	0	0	0	0	At1g27320.1:X:+234:quaternary
GGAATGGATC	1	0	0	1	0	At1g27300.1:d:+588:secondary
AGCTTTGTGT	0	1	1	0	0	At1g27200.1:d:+1086:secondary
CTCACACAAA	1	1	0	0	0	At1g27100.1:X:-581:quaternary
TGCCGACGCC	1	1	0	0	0	At1g27100.1:X:-423:quaternary
CCTCTTGTGA	1	0	1	0	0	At1g27060.1:v:+1562:secondary
ATGGTAATTG	0	1	0	1	0	At1g27030.1:d:+1141:secondary
GACGCTTGTC	0	0	0	0	2	At1g27020.1:d:+599:secondary
TTTGCAATAA	1	1	0	0	0	At1g27000.1:X:+312:quaternary
GGTACCAAGT	0	1	0	1	0	At1g26930.1:d:+1295:primary
GGTTTTCGTT	0	2	0	0	0	At1g26920.1:d:+875:primary,At1g43845.1:p:+402:primary,At1g15560.1:p:+1631:primary
GATAAGTTTT	2	0	0	0	0	At1g26910.1:d:+841:primary
TTATTAACAA	2	0	0	0	0	At1g26890.1:v:+1541:primary
TTTAGACCAA	0	1	0	0	1	At1g26870.1:v:+1181:secondary,At5g04420.1:d:+1872:secondary
GGTCGTGTCC	1	0	0	1	0	At1g26800.1:d:+577:secondary
TCTGTAGCTA	1	0	0	0	1	At1g26750.1:d:+380:secondary
CTTCTCTTGA	0	1	0	1	0	At1g26740.1:d:+613:primary
GAGGAGATAT	0	1	1	0	0	At1g26580.1:d:+2446:secondary,At5g08540.1:d:+656:secondary,At5g38850.1:d:+81:secondary
ATAAAGAAAA	1	0	1	0	0	At1g26530.1:i:+466:tertiary
AACAATACTC	0	0	1	0	1	At1g26520.1:v:+1344:primary
CCCAGTGTTT	1	1	0	0	0	At1g26460.1:d:+2070:primary
AAATCGGTTT	1	0	1	0	0	At1g26420.1:d:+1728:primary
ATCTCAACTC	1	1	0	0	0	At1g26340.1:d:-652:secondary,At5g24010.1:d:-1281:secondary
AGAAGTCACA	1	1	0	0	0	At1g26230.1:X:+255:quaternary
GAAATGACCA	1	1	0	0	0	At1g26210.1:d:+484:secondary,At5g17920.1:d:+414:secondary
GCAGAAAGAA	0	2	0	0	0	At1g26150.1:i:+1624:tertiary
TCCTTTACGT	0	1	0	1	0	At1g25450.1:X:+678:quaternary
GAGGAGCTAG	0	1	1	0	0	At1g25440.1:d:+787:secondary
GATCTCTGAA	1	1	0	0	0	At1g25320.1:d:+1726:primary
CTAATCCCTT	1	0	0	1	0	At1g25280.2:d:+1884:primary,At1g25280.1:d:+1947:primary
TTATCTTTCT	0	2	0	0	0	At1g25210.1:d:+2926:primary

GGAAAAGCTC	0	0	2	0	0	At1g25155.1:d:+365:secondary,At1g72370.1:d:+262:secondary,At1g25220.1:d:+550:secondary,At1g25083.1:v:+715:secondary,At1g24807.1:v:+754:secondary,At5g57890.1:d:+575:secondary,At3g42240.1:v:+568:secondary,At1g24909.1:d:+365:secondary
AAAGACCTCA	1	0	1	0	0	At1g24610.1:d:+1407:secondary
CCCTCTTTGC	0	0	0	0	2	At1g24580.1:d:+370:primary
AACTCTTGGA	0	0	1	0	1	At1g24575.1:X:+493:quaternary
GCAACTTCTC	1	0	1	0	0	At1g24560.1:d:+2203:secondary,At3g50820.1:d:+119:secondary
TCTTAAGGCG	0	0	1	1	0	At1g24490.1:d:+3060:primary
TACGTAGTAG	1	0	0	0	1	At1g24310.1:d:+1377:primary
AAGAAGGTGG	0	0	1	1	0	At1g24300.1:d:+4093:secondary,At4g14670.1:v:+2222:secondary,At1g27430.1:d:+4432:secondary
TGCAGGCAAG	1	0	0	0	1	At1g24250.1:i:+835:tertiary,At1g24220.1:i:+3463:tertiary,At5g10360.1:i:+1251:tertiary
TTCCCCTTGT	0	1	0	0	1	At1g24240.1:d:+634:primary,At5g11750.1:d:+661:primary
AGAAAATGCT	0	0	0	0	2	At1g24190.1:d:+2423:secondary,At1g08125.1:d:+616:secondary,At3g02100.1:v:+141:secondary
TCTTTGCTGT	0	1	0	1	0	At1g24120.1:d:+1045:primary,At1g59980.1:d:+1086:primary
CGCTTCCGTC	0	0	0	2	0	At1g24090.1:i:+2278:tertiary
TTGTTGTCTT	1	1	0	0	0	At1g24050.1:d:+658:primary
GCCGTCTGCG	0	1	1	0	0	At1g23970.2:d:+1172:primary,At1g23970.1:d:+1177:primary
GCCATTGAAG	0	2	0	0	0	At1g23900.1:d:+1047:secondary,At1g23900.2:d:+1117:secondary,At2g24720.1:v:+1317:secondary,At1g23730.1:d:+561:secondary
GCTTGGCCAA	0	1	1	0	0	At1g23850.1:d:+1250:secondary
ATTGTAAAAC	0	0	1	0	1	At1g23830.1:d:+1022:primary
GTTCTACAGT	0	1	0	1	0	At1g23800.1:i:+1731:tertiary
GTATAAAAAA	0	1	0	1	0	At1g23600.1:v:+1508:primary
ATTCTTACAA	1	0	1	0	0	At1g23540.1:v:+2792:primary
ACCAGTAGGC	0	0	2	0	0	At1g23490.1:X:+352:quaternary
CATTACAAAC	1	1	0	0	0	At1g23340.1:X:-603:quaternary
ACTCAGATGA	1	1	0	0	0	At1g23280.1:v:+1087:primary
TATGACCAAT	1	0	0	0	1	At1g23110.1:v:+210:secondary
TCAAGCTCCA	0	2	0	0	0	At1g23060.1:d:+811:primary,At1g23060.2:d:+761:primary
ATCTTTATAA	0	0	0	0	2	At1g23020.1:d:+1657:primary
TTTGAATTAT	1	0	1	0	0	At1g22985.1:d:+678:primary
GTGATAAAAG	1	0	1	0	0	At1g22970.1:d:+712:primary
TGCCGAGTAA	0	1	0	1	0	At1g22950.1:v:+1508:primary
GCCCCTCCAA	0	2	0	0	0	At1g22890.1:d:+202:secondary
TATTTATCAT	2	0	0	0	0	At1g22885.1:d:+431:primary
GGCGAAAGCA	1	0	1	0	0	At1g22810.1:d:+834:primary
TATTTCGTAT	0	1	0	0	1	At1g22800.1:d:+1323:secondary
GTTCCTCTTT	0	1	0	1	0	At1g22770.1:i:+2659:tertiary
CGCTGGTGTG	0	1	0	1	0	At1g22770.1:d:+2082:secondary
GAGCTTCTAT	0	2	0	0	0	At1g22750.2:d:+540:secondary,At1g22750.1:d:+540:secondary,At3g12800.1:d:+138:secondary
AAAGTTAATC	0	0	2	0	0	At1g22570.1:v:+2418:primary
GTGTCTTAAG	0	2	0	0	0	At1g22380.1:X:-591:quaternary
ATGTTGGATG	2	0	0	0	0	At1g22335.1:v:-448:secondary
TTAAGAAAAA	0	0	2	0	0	At1g22335.1:v:+1062:primary
TACAAAACAT	1	0	0	1	0	At1g22310.2:d:+1789:primary,At1g22310.1:d:+1674:primary
AAATCCTAAA	0	1	0	0	1	At1g22180.3:d:+1638:primary,At1g22180.1:d:+1001:primary,At1g22180.2:d:+1007:primary
AGAGATAAAA	1	0	1	0	0	At1g22060.1:d:+1531:secondary
AAGCTTATGA	1	0	0	0	1	At1g22040.1:d:+1653:secondary,At1g15990.1:v:+162:secondary
AGAAAAAGAA	1	0	0	0	1	At1g22015.1:d:+288:secondary
GTCAGTTTGC	0	1	0	0	1	At1g21900.1:d:+323:primary
CTGACCAGGT	0	1	1	0	0	At1g21880.2:d:+1157:primary
GCTCTACTAA	0	1	1	0	0	At1g21780.1:d:+674:primary
ACTTTACCGT	0	1	0	0	1	At1g21760.1:d:+1603:primary
TTTACATTGT	0	1	0	1	0	At1g21720.1:d:+668:primary
GAAACGATGT	1	0	1	0	0	At1g21660.1:i:+2973:tertiary
ATTACACAAG	1	0	1	0	0	At1g21650.1:i:+6934:tertiary
TAGTCTCTGT	0	1	0	0	1	At1g21580.1:d:+4230:secondary
ACTCAAGACT	0	0	0	1	1	At1g21560.1:d:+1336:secondary
TATACATCAA	2	0	0	0	0	At1g21520.1:v:+152:secondary,At1g12220.1:v:+2144:secondary
TATTATTACC	1	0	1	0	0	At1g21450.1:d:+2098:secondary
GGTGAGCTCA	0	0	2	0	0	At1g21312.1:v:+549:secondary,At4g35100.1:d:+134:secondary

GGATAACAAC	0	1	0	0	1	At1g21160.1:d:+570:secondary
TTTTGATGAA	0	2	0	0	0	At1g21150.1:d:+1427:primary
ATTGGGCAAA	0	1	1	0	0	At1g21140.1:X:+976:quaternary
TGCGCGGCCA	0	1	1	0	0	At1g21130.1:X:-51:quaternary
TACTCTTCAA	0	2	0	0	0	At1g21080.1:X:-87:quaternary
ATTCTACTAT	2	0	0	0	0	At1g21060.1:d:+1502:primary
TGAAACGATT	1	0	1	0	0	At1g21050.1:d:-761:secondary
GTTTTTAAAA	0	0	1	0	1	At1g21020.1:v:+2737:secondary
CAACAAACAT	0	1	0	0	1	At1g20910.1:d:+1505:primary
TGTTCTAAAA	0	1	1	0	0	At1g20620.2:i:+428:tertiary,At1g20620.1:i:+428:tertiary
AAAATAAATA	0	0	1	1	0	At1g20600.1:d:+250:secondary,At1g77765.1:v:+850:secondary,At5g45980.1:v:+1177:secondary
TAAGCTTTGA	1	0	0	1	0	At1g20580.1:d:+508:primary
TCTGGGATCT	0	2	0	0	0	At1g20540.1:d:+735:secondary
TAGTTTGTTT	0	1	0	1	0	At1g20490.1:d:+1524:primary
TTTTCTGACT	1	0	1	0	0	At1g20480.1:i:+1954:tertiary
CATAAGAGGA	0	0	1	1	0	At1g20440.1:X:+780:quaternary
GATCGACTAG	0	1	0	1	0	At1g20380.1:d:+2187:primary
GTGGATGGGA	0	1	1	0	0	At1g20270.1:d:+1048:primary
GACACTGCTC	0	1	0	0	1	At1g20260.1:d:+1511:primary
AGAGGAGGTT	0	0	2	0	0	At1g20150.1:v:+1074:secondary,At5g60290.1:v:+576:secondary
ATGTGGCTGT	0	0	1	0	1	At1g20100.1:d:+772:secondary,At5g34846.1:p:+20:secondary
CAGAACACTA	0	1	0	0	1	At1g19970.1:d:+645:primary
TGTAGTGAAA	0	0	0	1	1	At1g19960.1:v:+586:secondary
AAAATGAAAT	1	1	0	0	0	At1g19950.1:d:+513:primary,At1g09410.1:v:+2957:primary
TATATGGTTT	0	1	0	0	1	At1g19940.1:d:+1733:primary
TTGGAGGAGG	1	0	1	0	0	At1g19930.1:v:+79:secondary
GATCCAAGGG	0	0	0	1	1	At1g19920.1:X:-236:quaternary
CGGTTTTTGC	1	0	0	1	0	At1g19880.1:d:+1442:primary
CAGCCAACAC	0	1	1	0	0	At1g19870.1:d:+2252:primary
TCTCTTGTCG	0	1	0	1	0	At1g19800.3:d:+1233:primary,At1g19800.2:d:+1205:primary,At1g19800.1:d:+1140:primary
GGAAAACTCG	0	0	1	0	1	At1g19770.1:d:+536:secondary,At2g21830.1:d:+112:secondary
CTTTTTTTAT	0	1	0	0	1	At1g19730.1:d:+535:primary
AGTTATTTTG	0	1	0	1	0	At1g19480.1:d:+1360:primary
GTTGTTTTTT	0	0	0	1	1	At1g19440.1:d:+1733:secondary
AAGAGGCGGA	1	1	0	0	0	At1g19430.1:d:+935:secondary
TTTTTTTTTG	1	1	0	0	0	At1g19220.1:d:+3328:secondary
GCTCATCTGG	0	0	2	0	0	At1g19150.1:d:+814:secondary
TCTTGGTCCT	1	1	0	0	0	At1g19130.1:i:+779:tertiary
GGAGAACAGG	0	1	0	1	0	At1g19110.1:d:+532:secondary
AATCTGTTTT	0	1	0	0	1	At1g18990.1:d:+1632:primary
GAGTTAATTT	0	2	0	0	0	At1g18950.1:d:+994:secondary
GGGACTGCTG	0	1	1	0	0	At1g18830.1:v:+1646:secondary,At1g60170.1:d:+838:secondary
AGTCTTTTTC	0	0	2	0	0	At1g18770.1:v:+694:primary,At1g49780.1:d:+1400:primary
GAATGGCTGA	0	0	1	1	0	At1g18660.1:d:+779:secondary,At1g18660.2:d:+678:secondary,At1g18660.3:d:+687:secondary,At1g18660.4:d:+779:secondary
ATTGGAGATG	0	1	1	0	0	At1g18640.2:d:+873:primary,At1g18640.1:d:+873:primary
CAACTTCGAT	0	0	1	1	0	At1g18510.1:v:-36:primary
TTCTCTTATC	0	0	0	0	2	At1g18510.1:v:+963:secondary
AGCAACAAAA	1	1	0	0	0	At1g18480.1:X:+-185:quaternary
GAAGACAAAG	0	2	0	0	0	At1g18380.1:v:+969:secondary,At5g45340.2:d:+845:secondary,At1g46552.1:p:+4216:secondary,At5g45340.1:d:+845:secondary
GTATTAAGAT	0	0	1	0	1	At1g18080.1:d:+894:secondary
TATGTCGGAA	0	0	2	0	0	At1g18050.1:X:-173:quaternary
TTCGCCGGCG	0	2	0	0	0	At1g18000.1:d:+789:primary,At1g18010.1:d:+689:primary
AGGAGAGGGA	0	0	2	0	0	At1g17990.1:X:-265:quaternary
CGAAAAGCCT	1	1	0	0	0	At1g17990.1:d:+1095:primary,At1g18020.1:d:+858:primary
AGTTTCATAA	0	0	0	0	2	At1g17970.1:i:+2126:tertiary
TCCATTGCGT	0	0	2	0	0	At1g17970.1:d:+1159:primary
TGACTTCTCT	0	2	0	0	0	At1g17940.1:X:-56:quaternary,At1g55035.1:X:-645:quaternary,At2g01570.1:X:-93:quaternary,At5g07810.1:X:-161:quaternary
GTGCGTTCTT	0	0	0	2	0	At1g17910.1:v:+3029:primary

TTGTCTGGTC	0	1	0	1	0	At1g17880.1:X:-9:quaternary
GATCAGCCAG	0	1	1	0	0	At1g17760.1:d:+2103:primary
TACCAGGGTG	0	2	0	0	0	At1g17730.1:d:-832:secondary
TTCAGGGATT	0	1	1	0	0	At1g17730.1:d:-794:secondary
CTTTTGTTTA	0	1	0	1	0	At1g17730.1:d:+837:primary,At2g27920.1:d:+1602:primary,At2g27920.2:d:+1304:primary
TGCGTCAACG	1	0	0	0	1	At1g17610.1:d:+1320:secondary
AAGTTACTCG	1	1	0	0	0	At1g17510.1:d:+396:primary
AATTCTTGAT	0	1	0	1	0	At1g17340.1:i:+721:tertiary
AATTGGCGGT	0	0	0	2	0	At1g17340.1:d:-2918:secondary
GTTCTTCTTC	0	0	1	1	0	At1g17270.1:d:+1804:primary,At3g17000.1:d:+979:primary
CATCGCTCCA	0	0	1	0	1	At1g17200.1:d:-497:secondary
CAACCTATGA	1	1	0	0	0	At1g17120.1:d:+1726:primary
AGGATATTTG	0	0	0	0	2	At1g17090.1:d:+400:secondary,At4g23700.1:d:+1838:secondary
GAAGACCGTT	0	0	2	0	0	At1g17070.1:d:+1911:primary
GCATACTTCT	0	1	0	1	0	At1g16900.1:X:-72:quaternary,At5g53300.1:X:-133:quaternary,At3g44215.1:X:-3667:quaternary
AACGAAAATG	0	0	0	1	1	At1g16900.1:d:+334:secondary
TGGCAGGAGA	0	0	0	1	1	At1g16900.1:d:+2704:primary
TTCATAGCTT	0	0	1	0	1	At1g16830.1:i:+1540:tertiary,At5g45190.1:i:+481:tertiary
CAATATGGTT	2	0	0	0	0	At1g16780.1:d:+2473:primary
TGTCTCGGTT	0	1	0	0	1	At1g16700.1:d:-751:secondary
GTTTCAAACA	0	0	2	0	0	At1g16670.1:d:+1545:primary
GATTTGAAAG	0	1	0	1	0	At1g16670.1:d:+1261:secondary
GAATGAAGCT	0	1	0	0	1	At1g16650.1:d:+1413:primary
TCGAAATTGC	0	0	1	1	0	At1g16610.1:d:+474:secondary,At1g16610.2:d:+435:secondary,At1g43005.1:p:+53:secondary
AGTCTGTGTA	0	0	1	0	1	At1g16570.1:d:+1592:primary
CTTCTAGAGA	0	0	1	1	0	At1g16560.1:d:+914:secondary,At1g16560.3:d:+1082:secondary,At3g19300.1:d:+1495:secondary,At4g04500.1:d:+1609:secondary,At1g16560.2:d:+945:secondary
TAATAATACA	1	0	1	0	0	At1g16530.1:i:+761:tertiary
TAGAACAAAG	0	0	0	1	1	At1g16430.1:d:+324:primary
TATGCCGCCC	0	0	2	0	0	At1g16410.1:d:+1317:secondary,At1g16400.1:d:+1331:secondary
TGTGAGGAAC	1	0	0	1	0	At1g16390.1:d:+1365:primary
GACTCTAGCC	0	0	2	0	0	At1g16330.1:v:+1703:secondary
GCGTTAGTTG	0	2	0	0	0	At1g16300.1:d:+1363:primary
TTGTGTGATT	1	0	0	0	1	At1g16190.1:v:+1695:secondary
AAAGAGAACA	2	0	0	0	0	At1g16110.1:d:+1954:secondary,At1g16120.1:v:+2298:secondary
GCTTTGGAGA	0	1	0	0	1	At1g16020.1:d:+1670:primary,At1g16020.2:d:+1631:primary
AAGACTTGAC	0	0	0	1	1	At1g15960.1:d:+1780:primary
AATGAAGGAT	0	0	0	2	0	At1g15940.1:d:+3055:primary
AACTATCTGA	0	0	1	0	1	At1g15910.1:i:+467:tertiary
CATTTGTGGC	0	1	1	0	0	At1g15810.1:d:+1522:secondary
TCCTCTGCGG	0	2	0	0	0	At1g15810.1:d:+1051:secondary
TTGGTCTATG	1	1	0	0	0	At1g15530.1:v:+2400:secondary
AGTGATTGAT	0	1	0	1	0	At1g15420.1:d:+910:primary
TTGAGACTTT	0	1	0	1	0	At1g15415.1:d:+434:secondary
CGGTTGTTGC	0	2	0	0	0	At1g15400.1:d:+397:primary
GAAGTGGATA	0	2	0	0	0	At1g15340.1:d:+834:secondary
TGCTTATCCA	0	1	0	0	1	At1g15250.1:d:+192:primary
ATGTCACAAA	1	1	0	0	0	At1g15240.1:d:+1550:secondary,At2g33580.1:d:+488:secondary
TTTTCAGCAG	0	1	0	1	0	At1g15180.1:d:+1162:primary
CTCAAAGAGA	1	0	0	0	1	At1g15130.1:d:+1823:secondary,At2g42600.2:d:+2613:secondary,At5g12440.1:d:+1146:secondary,At2g42600.1:d:+2718:secondary
TTTGGTAAGT	1	1	0	0	0	At1g15050.1:i:+636:tertiary,At4g27680.1:i:+1638:tertiary
GACACTGCTG	1	0	0	1	0	At1g14970.1:i:+2052:tertiary
GCCATCGAAG	0	1	0	1	0	At1g14810.1:X:-310:quaternary
CACACGCTTG	0	0	1	0	1	At1g14810.1:d:+1355:primary
AACTCCAAGA	0	2	0	0	0	At1g14770.2:X:-81:quaternary,At5g10240.1:X:-122:quaternary,At2g01240.1:X:-74:quaternary
CATATATATA	0	0	0	0	2	At1g14710.2:d:+2486:primary,At1g33100.1:v:+2219:primary,At2g24000.1:v:+2263:primary,At1g52880.1:d:+1357:primary,At1g14710.1:d:+2306:primary
GTGGTGGTGG	1	0	1	0	0	At1g14710.1:d:+629:secondary,At1g67050.1:d:+815:secondary,At5g55550.2:d:+661:secondary,At5g46730.1:d:+137:secondary,At3g62090.1:d:+232:secondary,

At3g61870.2:d:+955:secondary,At3g43575.1:p:+395:secondary,At1g14710.2:d:+809:secondary,At1g13740.1:d:+1275:secondary,At5g55550.3:d:+490:secondary,At4g08395.1:v:+597:secondary,At3g32385.1:p:+3651:secondary,At5g03160.1:d:+1525:secondary,At3g56770.1:v:+979:secondary,At5g55550.1:d:+490:secondary,At1g02710.1:v:+481:secondary

TGTGCTTAAA	0	0	0	2	0	At1g14690.1:i:+3892:tertiary
TTCACTTTTT	0	0	0	2	0	At1g14650.1:d:+853:secondary,At1g65580.1:d:+1493:secondary
GCGAGAAAAC	0	1	0	1	0	At1g14600.1:d:+546:primary
ATTGTTAGAC	0	1	1	0	0	At1g14560.1:d:+1680:primary
TTCAGAGAAT	0	1	0	1	0	At1g14530.1:d:+1013:secondary,At1g14530.2:d:+1013:secondary,At3g11325.1:v:+1396:secondary
CTTTGAGTTT	0	0	2	0	0	At1g14410.1:i:+657:tertiary
GAATCACTTA	0	2	0	0	0	At1g14390.1:v:+2979:secondary
TATATGGGGA	0	2	0	0	0	At1g14380.2:d:+595:secondary,At1g14380.1:d:+595:secondary
GCAGCTTCTT	0	1	0	1	0	At1g14345.1:d:+34:secondary,At4g19500.1:v:+354:secondary
AAGTGTGATA	0	1	0	1	0	At1g14340.1:d:+936:secondary
ATGATGCTGA	0	1	0	0	1	At1g14340.1:d:+1006:primary
GATACCATCT	0	1	0	0	1	At1g14330.1:d:+1781:primary
AGAGGAACTT	0	1	0	1	0	At1g14320.1:d:-657:secondary,At1g14320.1:d:-662:secondary
CTCAAGAGGC	0	0	2	0	0	At1g14320.1:d:+499:secondary
GGAATACTCG	0	0	0	1	1	At1g14280.1:d:+807:secondary
ACAGTAATGT	0	0	0	1	1	At1g14270.2:d:+1445:primary,At1g14270.1:d:+1252:primary
TTTCTTAAAT	0	0	0	1	1	At1g14170.1:d:+1504:primary
GATGTGAAGC	0	1	0	0	1	At1g14070.1:v:+1765:secondary,At2g15350.1:v:+1570:secondary,At2g15370.1:v:+1849:secondary
CATAGACAGA	0	2	0	0	0	At1g14040.1:v:+2577:primary
GTTGAGTTTC	0	1	1	0	0	At1g14010.1:d:+578:secondary,At5g52430.1:d:+896:secondary
GTTCGCAAAT	0	0	1	0	1	At1g14000.1:d:-1352:secondary
AAAACAAGAA	1	1	0	0	0	At1g13990.1:d:+509:primary
AAACCTTCAA	0	0	1	1	0	At1g13970.1:d:+835:secondary
AAAAAGATTC	0	1	0	1	0	At1g13970.1:d:+2018:primary
AGGAACCTGA	0	1	0	1	0	At1g13950.1:i:-668:tertiary
TCGAAAAGCC	0	0	0	2	0	At1g13820.1:d:+1010:secondary
ATTATCCCCA	0	0	0	2	0	At1g13790.1:v:+2620:secondary
AAACACTGAT	1	1	0	0	0	At1g13780.1:d:+1474:primary
GTAGTTTCTT	1	0	1	0	0	At1g13740.1:d:+1239:secondary,At1g47565.1:p:+2316:secondary
AGAGATTTCT	0	0	0	1	1	At1g13470.1:d:+1140:primary
AGTCCCTCAA	0	1	1	0	0	At1g13440.1:X:-34:quaternary
AGCTTAAGGT	0	1	0	0	1	At1g13440.1:d:+259:secondary,At3g26600.1:d:+1239:secondary
GTGGCTTCTC	0	0	1	1	0	At1g13390.2:d:+520:primary,At1g13390.1:d:+443:primary
GCCACCGTCA	0	1	1	0	0	At1g13350.1:d:+178:secondary
GGGAAGAAGC	0	0	1	1	0	At1g13340.1:d:+79:secondary,At3g42850.1:v:+1464:secondary,At2g38290.2:d:+1463:secondary,At5g19220.1:d:+173:secondary,At1g49600.1:d:+1188:secondary,At3g14870.1:d:+490:secondary,At2g38290.1:d:+1384:secondary
AGATTTTGTA	0	0	1	1	0	At1g13320.1:d:+1898:secondary
ACGACACATC	0	0	2	0	0	At1g13245.1:X:-187:quaternary
AAGAGAGTGA	0	1	1	0	0	At1g13195.1:d:+854:secondary
GGATAATTGA	0	0	1	0	1	At1g13170.1:X:-201:quaternary
AAGCCTAAAA	0	0	2	0	0	At1g13160.1:X:+-243:quaternary
AACCTGAGTA	0	2	0	0	0	At1g13120.1:d:+791:secondary,At1g77810.1:d:+815:secondary,At1g77810.2:d:+797:secondary
TCAATTAGGG	0	0	1	1	0	At1g13100.1:X:-711:quaternary,At1g13100.1:X:-797:quaternary
AGTAAAGGAC	0	1	0	1	0	At1g13100.1:X:+716:quaternary
AGTTTCTATT	0	1	1	0	0	At1g12990.1:d:+1828:secondary
GTGGTGCAGC	0	2	0	0	0	At1g12970.1:d:+1433:primary
AAAACGCAAA	0	1	1	0	0	At1g12930.1:i:+2000:tertiary
AGGTATGTTT	0	0	1	0	1	At1g12930.1:i:+1319:tertiary,At1g51590.1:i:+331:tertiary
TTTTTGGACA	0	1	0	0	1	At1g12870.1:i:+1554:tertiary
TCACAAAATG	1	0	0	0	1	At1g12840.1:d:-1442:secondary
TGTCTTATCT	0	0	1	0	1	At1g12840.1:d:+1019:secondary
ATGCGAACAC	0	1	1	0	0	At1g12790.1:d:+640:primary
GTCTGCCTTT	0	0	1	0	1	At1g12700.1:i:+2501:tertiary
TAAATAGAAT	1	0	0	1	0	At1g12680.1:d:+1845:primary
TGTTTTATAT	1	1	0	0	0	At1g12640.1:d:+424:secondary,At1g63050.1:d:+395:secondary
TTCTTAAGTT	0	2	0	0	0	At1g12630.1:v:+1220:secondary,At5g06980.2:d:+397:secondary,At5g06980.1:d:+459:secondary
CTTGTGGTGG	0	2	0	0	0	At1g12540.1:d:-679:secondary

GTGCTCAAAA	1	1	0	0	0	At1g12530.1:v:+520:secondary
AAGGGAATGC	1	1	0	0	0	At1g12430.1:d:+2804:primary
AACAATTCGA	0	0	1	0	1	At1g12400.1:d:+227:secondary,At1g27060.1:v:+101:secondary
CAGCCGTTGA	0	0	1	1	0	At1g12380.1:d:+2465:primary
CTTGTCTTAT	1	1	0	0	0	At1g12320.1:d:+812:primary
TAATATATTA	1	1	0	0	0	At1g12240.1:d:+2208:primary
TGACAACATT	0	1	0	0	1	At1g12020.1:d:+830:primary
TAACAATCCA	0	0	1	0	1	At1g11925.1:v:+908:primary
TCTCTTGCAT	1	1	0	0	0	At1g11910.1:d:+406:secondary,At5g64220.1:d:+3188:secondary
GGAGGCATTG	1	0	0	0	1	At1g11870.3:d:+1539:primary,At1g11870.2:d:+1559:primary,At1g11870.1:d:+1553:primary
TGACTTCAAA	0	1	0	0	1	At1g11820.1:v:+1685:secondary
ATGTGTTTTT	1	0	0	1	0	At1g11760.1:i:+1227:tertiary
AAATAAATAT	2	0	0	0	0	At1g11460.1:i:+279:tertiary
TTAGGTCATA	0	1	1	0	0	At1g11410.1:d:+2362:primary
GAGAAAGTGT	0	2	0	0	0	At1g11390.1:d:+1428:secondary
GAAGTTTGAT	0	1	1	0	0	At1g11380.1:d:+1016:primary
ACGAGCCACT	0	0	0	0	2	At1g11210.1:X:-367:quaternary
ATGCCTAAAA	0	0	0	2	0	At1g11170.1:i:+2437:tertiary,At1g11170.2:i:+2126:tertiary
TGTTGGGCCT	0	0	1	0	1	At1g10950.1:d:+2197:secondary
GAGCTTGATT	1	0	0	1	0	At1g10930.1:d:+3707:primary
AACTACGTAC	1	0	0	0	1	At1g10910.1:v:-2382:secondary
ACCCTGACTC	0	2	0	0	0	At1g10850.1:p:+2129:secondary
GCGTTCTCTT	0	0	2	0	0	At1g10730.1:v:+1798:secondary,At3g62610.1:d:+111:secondary
GAAACGTTTC	0	1	0	0	1	At1g10700.1:d:+1119:primary
TTTATGTATA	1	1	0	0	0	At1g10690.1:d:+606:primary
TTGTTTGGAA	0	1	1	0	0	At1g10670.1:d:+265:secondary,At1g10670.2:d:+258:secondary
TATATCATCT	1	0	0	0	1	At1g10640.1:d:+1222:primary
AGTTCCAGGA	0	2	0	0	0	At1g10560.1:d:+2057:primary
TTATGTCCGT	1	0	1	0	0	At1g10330.1:v:+1830:secondary
ATCCATCAAT	0	0	1	1	0	At1g10155.1:v:+983:secondary
ATGTTAATCT	0	1	0	1	0	At1g10130.1:i:+569:tertiary
CAACCTGTAG	0	2	0	0	0	At1g10090.1:i:+4289:tertiary
AGATTGTTGC	2	0	0	0	0	At1g10020.1:d:+1950:primary
AGTCACTGAG	0	2	0	0	0	At1g10020.1:d:+1628:secondary
CTTCACTCTT	0	0	1	0	1	At1g09970.2:d:+2387:secondary,At1g09970.1:d:+2464:secondary
CTTTTGACAA	0	2	0	0	0	At1g09870.1:d:+1425:primary
ATATTGGTTG	0	1	0	1	0	At1g09830.1:d:+1617:primary
GTCGAAAATT	0	0	0	2	0	At1g09815.1:i:+329:tertiary
AATTGCTTTA	0	1	1	0	0	At1g09810.1:d:+1341:primary
TTATGCGTAG	1	0	1	0	0	At1g09760.1:d:+1190:secondary
GCCGGGATAC	0	0	2	0	0	At1g09750.1:d:+1265:secondary
TGCAGCAATC	0	0	0	0	2	At1g09690.1:d:+328:secondary
ATGACGAAAC	1	0	1	0	0	At1g09650.1:d:+1229:secondary,At1g79070.1:d:+425:secondary
GTGTCATTTG	0	0	1	0	1	At1g09640.1:d:+1079:secondary
GCTGCTGGAG	0	1	1	0	0	At1g09540.1:d:+276:secondary,At4g02880.1:d:+1215:secondary
TAAGACTTAA	1	0	0	1	0	At1g09500.1:d:-1111:secondary,At1g09500.2:d:-1577:secondary
GTTTAGATGA	0	1	0	1	0	At1g09430.1:d:+786:secondary,At5g04730.1:v:+553:secondary
CTCTTAACAT	1	0	0	0	1	At1g09240.1:d:+1225:primary
AGACTGTTGG	0	2	0	0	0	At1g09230.1:i:+1698:tertiary
AGCGGTGGCG	0	0	0	1	1	At1g09210.1:X:-113:quaternary
GAAGGAAAAA	0	2	0	0	0	At1g09160.1:d:+1468:primary,At2g15100.1:p:+4894:primary,At1g09160.2:d:+1405:primary
CCATAGAAAA	0	1	1	0	0	At1g09070.1:X:+1276:quaternary
TGCTTGCGCT	0	1	0	1	0	At1g09020.1:d:+1921:primary
AAAGCGGATG	0	1	1	0	0	At1g08970.3:d:+872:primary,At1g08970.1:d:+430:primary,At1g08970.2:d:+430:primary,At1g08970.4:d:+872:primary
TGCCAAACCA	0	2	0	0	0	At1g08845.1:d:+261:secondary
GAAATGAAGG	1	0	1	0	0	At1g08800.1:d:+3544:secondary
TAAATTTGGG	0	0	2	0	0	At1g08650.1:d:+1002:secondary
GTCCAGATAG	0	0	1	1	0	At1g08570.1:d:+746:secondary,At5g63890.2:d:+966:secondary,At5g63890.1:d:+977:secondary
GAAATAAGCG	1	0	1	0	0	At1g08530.1:d:+516:primary
ACTCTGGAGT	0	2	0	0	0	At1g08510.1:d:+1381:secondary

GTCAGGGCTT	0	1	1	0	0	At1g08500.1:i:+587:tertiary
GCAAAAAGGA	1	0	1	0	0	At1g08470.1:X:-536:quaternary
CTGTAGCTGT	0	1	0	1	0	At1g08470.1:d:+1136:primary
TATGTATATA	1	0	0	0	1	At1g08465.1:p:+3558:primary,At5g63700.1:v:+2247:primary
GCCCTTGAAG	0	2	0	0	0	At1g08300.1:v:+891:secondary,At5g35930.1:d:+1419:secondary
GTGGTAAGGT	1	0	0	0	1	At1g08220.1:i:+485:tertiary
ATTTGAGGTG	0	0	0	2	0	At1g08200.1:X:-142:quaternary,At5g27882.1:X:-1115:quaternary
TCTGGGATTC	1	0	1	0	0	At1g08200.1:X:-109:quaternary,At2g27860.1:X:-109:quaternary
CGTATCTCCA	0	2	0	0	0	At1g08160.1:d:+403:primary
GGAGTTGAGT	0	0	2	0	0	At1g08090.1:d:+940:secondary,At1g08100.1:d:+913:secondary
TATATAAATA	0	0	0	0	2	At1g08060.1:d:+6492:primary,At1g08060.2:d:+6224:primary
AATTAGTGTC	1	1	0	0	0	At1g08020.1:d:+456:primary
ATTCGCAGTG	1	1	0	0	0	At1g08000.1:d:+1103:primary,At1g08000.2:d:+1114:primary
CTGCCCAAAC	0	0	2	0	0	At1g07990.1:d:+1159:secondary
ACAGATGCAG	0	1	1	0	0	At1g07980.1:d:+624:primary
GTTTTCAGCA	0	0	1	0	1	At1g07960.1:d:+643:primary,At1g07960.3:d:+622:primary,At1g07960.2:d:+1035:primary
AGATTTTGAG	0	0	1	1	0	At1g07910.1:d:+3342:primary
AAGCTTTCTG	0	0	1	0	1	At1g07890.1:d:+864:secondary,At5g45060.1:d:+1198:secondary,At1g07890.3:d:+760:secondary,At1g07890.2:d:+747:secondary
TGCTGACCGA	0	1	1	0	0	At1g07830.1:d:+454:secondary
GCGGTCACCG	0	1	1	0	0	At1g07820.1:X:-447:quaternary
GGAAGGCTTC	0	2	0	0	0	At1g07810.1:d:+2879:primary
GTCCATAATG	0	1	1	0	0	At1g07770.1:X:-41:quaternary
AGGCAACGAG	0	1	1	0	0	At1g07600.1:v:+935:primary
TAATCCCAAG	0	1	0	0	1	At1g07520.1:d:+2117:primary
AAACAAGGTG	0	1	0	1	0	At1g07476.1:v:+936:secondary,At1g28470.1:d:+581:secondary,At1g61660.1:d:+1118:secondary,At5g29646.1:p:+7324:secondary,At1g61660.2:d:+1118:secondary
ATACCAGTTT	1	0	1	0	0	At1g07390.1:v:+3400:primary
TAATTTTATC	0	2	0	0	0	At1g07240.1:d:+1605:primary
AATTTTTGGT	0	0	0	1	1	At1g07220.1:v:+1895:secondary,At1g36230.1:v:+1246:secondary
GCTGGGACTG	1	1	0	0	0	At1g07130.1:d:-151:secondary
TATTGCTTTT	0	0	0	1	1	At1g07000.1:d:+1969:primary
TTCTCAAGGA	0	2	0	0	0	At1g06980.1:d:+229:primary
AGAGTGATTC	0	0	0	2	0	At1g06900.1:X:--21:quaternary
CATTGGATTT	0	1	1	0	0	At1g06840.1:i:+1922:tertiary
AAGCATCATT	1	0	0	0	1	At1g06840.1:d:+2743:primary
CCATTCAATA	0	1	0	0	1	At1g06820.1:d:+1603:primary
GTATTCTTAA	0	2	0	0	0	At1g06720.1:d:+2956:primary
TTAATGATTT	0	0	1	1	0	At1g06670.1:d:+1200:secondary,At5g57140.1:d:+996:secondary
ATATAATTAG	1	1	0	0	0	At1g06550.1:d:+1322:primary
ATTTGAAGGA	1	1	0	0	0	At1g06530.1:d:+654:secondary
ATTTGATATT	1	1	0	0	0	At1g06450.1:d:+1384:primary,At1g31510.1:v:+1494:primary
AGTGGGTCTT	0	0	0	0	2	At1g06380.1:d:+937:primary
CAGATGATCA	0	0	0	1	1	At1g06360.1:X:-205:quaternary,At1g21990.1:X:-281:quaternary
GCGGTTACGC	0	1	1	0	0	At1g06230.2:d:+1900:secondary,At1g06230.1:d:+1953:secondary
GTTGGGTGTT	0	0	0	2	0	At1g06230.1:d:+1744:secondary,At1g06230.2:d:+1691:secondary,At3g21750.1:d:+1682:secondary
AAGGACCTCA	0	2	0	0	0	At1g06220.2:d:+2255:secondary,At4g36760.1:d:+1616:secondary,At1g06220.1:d:+2122:secondary
GTTGAGTACT	0	0	2	0	0	At1g06135.1:d:+316:primary
CAATGTCCTA	0	1	0	0	1	At1g06110.1:d:+1469:secondary
AAGGGTGTTC	1	1	0	0	0	At1g05910.1:d:+3688:primary
TATGTGTACT	0	0	0	0	2	At1g05870.1:d:+764:primary
TAGTCTCGAG	1	1	0	0	0	At1g05820.1:v:+1806:primary
TTGGGGACAA	0	0	1	0	1	At1g05805.1:d:-1005:secondary
AAAAATGAAA	2	0	0	0	0	At1g05700.1:v:+51:secondary
TTATTGTTTT	0	0	0	1	1	At1g05670.1:d:+25:secondary,At4g22870.1:v:+213:secondary
GAGGATGTGT	0	0	1	1	0	At1g05670.1:d:+1149:secondary
TATTCAACTT	0	1	1	0	0	At1g05630.1:d:+3757:primary
ATCCTGCGAC	0	1	0	1	0	At1g05620.1:d:+860:primary
GAAAAGATGG	0	0	1	1	0	At1g05620.1:d:+386:secondary,At4g04540.1:v:+2051:secondary,At4g04570.1:d:+1772:secondary
TTATTTATTT	0	1	1	0	0	At1g05420.1:v:+1090:secondary

TAAAGACTAT	0	0	0	1	1	At1g05380.1:d:+1782:primary
GCCGCAAGAC	0	1	1	0	0	At1g05340.1:X:-8:quaternary
TGTTGCTGTT	0	1	0	1	0	At1g05340.1:d:+299:primary
GGTTTCAGCT	0	1	0	1	0	At1g05030.1:d:+1392:primary
GAGATATTCG	0	0	1	1	0	At1g05000.1:d:+653:secondary,At3g23800.1:d:+1060:secondary,At1g15150.1:d:+801:secondary,At1g15170.1:d:+800:secondary,At1g15160.1:v:+1092:secondary
TAAAAAGAAG	0	1	0	0	1	At1g04960.1:d:+1231:primary
TATGCTCTCT	0	0	0	0	2	At1g04950.1:d:+2129:primary,At1g04950.2:d:+2033:primary
TCTTGCCATT	1	0	0	1	0	At1g04830.1:X:-277:quaternary
TCTCATCAAG	0	1	0	1	0	At1g04770.1:d:+678:primary
AAAGGTTGGT	1	0	1	0	0	At1g04680.1:X:-431:quaternary
TTCCTTGCCG	0	2	0	0	0	At1g04630.1:d:+240:secondary,At1g51350.1:d:+1528:secondary
ATATTGCTAA	0	1	1	0	0	At1g04610.1:d:+1365:primary
AAGCACAAAG	0	0	1	0	1	At1g04570.1:X:-987:quaternary
CACCTTGTGC	0	0	1	0	1	At1g04570.1:d:+1652:primary
AAATTTCTGT	1	0	0	0	1	At1g04390.1:d:+2867:primary
AGGAGAGTAA	0	1	1	0	0	At1g04360.1:X:-510:quaternary,At4g35785.2:X:-127:quaternary
GGTATTGTTG	0	0	0	2	0	At1g04270.1:X:+504:quaternary
CTTAATGGTC	1	1	0	0	0	At1g04240.1:d:+854:primary
TATAATACTA	0	0	0	1	1	At1g04220.1:d:+1657:primary
TGGGAATCCA	0	0	1	1	0	At1g04210.1:d:-1087:secondary,At1g62570.1:d:-1277:secondary
TACACTGATT	0	0	1	1	0	At1g04010.1:i:+1745:tertiary
AACACCAAAA	0	1	0	1	0	At1g04000.1:d:+250:primary
AAAGAAAGAA	0	0	0	1	1	At1g03990.1:v:+2094:secondary
CTTGCGGACC	1	0	0	1	0	At1g03930.1:d:+992:primary
CTGGTCCACC	1	0	0	1	0	At1g03910.1:v:+2227:secondary
AATCTTAGCC	0	0	1	0	1	At1g03890.1:d:-880:secondary,At3g61770.1:d:-1050:secondary
AAACCCTAAA	0	1	0	1	0	At1g03860.2:d:+577:primary,At1g03860.3:d:+604:primary,At1g03860.1:d:+517:primary
GGTTTGGGGC	0	1	0	1	0	At1g03830.1:v:+3241:secondary
AATGCTTGAA	0	0	1	1	0	At1g03780.1:d:+2421:primary,At1g03780.2:d:+2256:primary
TAACTTGCTG	0	2	0	0	0	At1g03760.1:X:-130:quaternary
TGTTTTTGTG	0	2	0	0	0	At1g03700.1:d:+631:primary
ATAGTTGGGA	0	1	0	0	1	At1g03680.1:X:-222:quaternary
GCTAGAGCTT	1	1	0	0	0	At1g03590.1:d:+1030:primary,At3g29350.1:d:+309:primary,At3g29350.2:d:+309:primary
GATTTGGATG	0	0	0	2	0	At1g03530.1:d:+654:secondary
GATTTTGGAT	0	1	0	1	0	At1g03495.1:p:+1467:primary,At1g03940.1:d:+1333:primary
CGTCTGTTAC	0	2	0	0	0	At1g03475.1:X:+388:quaternary,At1g03475.1:X:+396:quaternary
TATAAATGTT	0	2	0	0	0	At1g03457.1:d:+1601:primary,At1g03457.2:d:+1609:primary
GTATGGTTTC	1	0	1	0	0	At1g03380.1:d:+3086:secondary,At3g53180.1:d:+1091:secondary
TGAAGACGAG	0	1	0	1	0	At1g03370.1:d:+5689:primary
CGATTGATGA	0	1	0	0	1	At1g03350.1:d:+437:primary
AGGTGTCAGT	0	0	0	2	0	At1g03260.1:d:+957:primary
GATTCCTTGA	0	2	0	0	0	At1g03250.1:d:+513:primary
GCTTATTAGT	1	0	0	0	1	At1g03190.1:d:+2406:primary,At1g03190.2:d:+2469:primary
TACTAGTACA	0	0	0	1	1	At1g03110.1:d:+1535:primary
CCACCTGCTG	0	1	1	0	0	At1g03080.1:d:+2292:secondary
CTTTGAATTC	1	0	0	0	1	At1g03055.1:d:+790:primary,At1g03055.2:d:+943:primary
TAATTTAATA	0	0	0	0	2	At1g02965.1:v:+1279:primary,At2g18250.1:v:+914:primary
TGATGTGAAG	0	0	2	0	0	At1g02940.1:v:+1085:secondary,At4g01870.1:d:+1317:secondary
TGTGCTTGCT	0	1	0	1	0	At1g02860.1:d:+1090:primary
ACATTTAATT	1	0	0	0	1	At1g02770.1:i:+549:tertiary
GCCGAGAGAC	0	2	0	0	0	At1g02700.1:d:+458:primary
ATATCAAAAA	0	0	1	1	0	At1g02670.1:v:+1186:secondary
GCTATATTCG	1	0	0	1	0	At1g02560.1:d:+623:secondary
AAATATGTTT	1	0	0	0	1	At1g02560.1:d:+1082:secondary
GTTATAATTA	0	2	0	0	0	At1g02510.1:v:+1485:secondary,At1g63990.1:d:+1117:secondary
TCATCAAGCC	0	0	2	0	0	At1g02500.1:d:+770:secondary,At1g02500.2:d:+770:secondary
TTGTTCTTAT	2	0	0	0	0	At1g02500.1:d:+1480:secondary,At1g02500.2:d:+1480:secondary
TTAGACAAAA	0	0	0	2	0	At1g02350.1:v:+300:secondary
CAAGAGGACT	0	2	0	0	0	At1g02300.1:X:+275:quaternary

AAAATATGAA	0	1	1	0	0	At1g02290.1:v:+1735:primary
ATGTAATGTA	1	0	0	0	1	At1g02150.1:X:+-71:quaternary
GTCTTGTCGC	1	0	1	0	0	At1g02120.1:d:-1608:secondary
AAGGGGGATG	0	2	0	0	0	At1g02090.1:d:+745:primary
TTCACGTCAT	0	0	2	0	0	At1g01990.1:d:+867:primary
TTCAAAGGAG	1	1	0	0	0	At1g01960.1:d:+4989:secondary
GGGGTCTTCT	2	0	0	0	0	At1g01910.2:d:+1212:secondary
GAGGATGAGT	1	1	0	0	0	At1g01910.1:d:+624:secondary,At1g01910.2:d:+624:secondary
TTCCATATAT	1	0	0	0	1	At1g01820.1:d:+871:primary
GTCTTAATCT	0	2	0	0	0	At1g01790.1:d:+1879:primary
AGTGTTTTTG	1	1	0	0	0	At1g01780.1:v:+983:secondary,At3g02130.1:d:+891:secondary
TAGCTCAACA	1	1	0	0	0	At1g01725.1:d:+247:primary
AGACCCGTGG	1	0	1	0	0	At1g01710.1:d:+1282:secondary
TTGCAGTGAA	2	0	0	0	0	At1g01660.1:v:+391:secondary
TTTGGTGCTC	0	0	2	0	0	At1g01620.1:X:-364:quaternary,At4g00430.1:X:-327:quaternary
GATGAAACAA	0	2	0	0	0	At1g01550.1:d:+375:secondary,At3g43444.1:p:+2854:secondary
GAAGCCGCGA	1	0	1	0	0	At1g01540.2:d:+1528:secondary,At1g01540.1:d:+1565:secondary
GCCGTTATAC	1	0	0	1	0	At1g01420.1:v:+1499:primary
AAAATATCCA	1	1	0	0	0	At1g01400.1:v:+1298:primary
CTCAAGATAA	0	1	1	0	0	At1g01390.1:d:+1543:primary
TTCGTGTAGA	0	0	1	1	0	At1g01290.1:d:+600:primary
TATATTTTCA	1	0	0	1	0	At1g01250.1:d:+681:primary
GGCGGATATA	0	1	0	1	0	At1g01180.1:v:+1091:secondary
AAGTAATAGT	0	1	0	0	1	At1g01140.2:i:+2082:tertiary,At1g01140.1:i:+2082:tertiary,At1g01140.3:i:+2082:tertiary
TACAGGACAG	0	0	0	1	0	No gene matches found
GTGGCTTTAT	0	0	0	1	0	No gene matches found
CGTTTCAGTT	1	0	0	0	0	No gene matches found
ACCTTGGACA	0	1	0	0	0	No gene matches found
TCCATCCCCC	0	0	0	0	1	No gene matches found
AGTGGTTGCG	0	0	1	0	0	No gene matches found
AGAGTCTGTA	0	0	0	1	0	No gene matches found
CCTGTCCCAG	0	1	0	0	0	No gene matches found
TATCAGAGTC	0	0	1	0	0	No gene matches found
ATCCGATTGT	0	1	0	0	0	No gene matches found
GACTTGCTAG	0	1	0	0	0	No gene matches found
TATGGCATAG	1	0	0	0	0	No gene matches found
TGAGGTGTCC	0	1	0	0	0	No gene matches found
AGAGCGGTCT	0	1	0	0	0	No gene matches found
GCCCGTTCAG	0	1	0	0	0	No gene matches found
AGAGGGGAGA	1	0	0	0	0	No gene matches found
ATCCGAAGAA	0	0	0	1	0	No gene matches found
GGGTGAAAAA	0	0	1	0	0	No gene matches found
ATCTCGATCT	0	1	0	0	0	No gene matches found
ATCATAGACG	1	0	0	0	0	No gene matches found
ACAGCCTCGC	1	0	0	0	0	No gene matches found
AGGCTCATTT	0	0	0	1	0	No gene matches found
TTCAGAGGCT	0	0	0	1	0	No gene matches found
AGAGGTGTGT	0	0	1	0	0	No gene matches found
GCCACAGGGG	0	0	0	1	0	No gene matches found
TCTAAGAGAT	0	0	1	0	0	No gene matches found
CGGAGTTGAG	0	0	1	0	0	No gene matches found
CCAGAGCTGA	0	0	1	0	0	No gene matches found
CTATGGCTGC	1	0	0	0	0	No gene matches found
CGAGCTCGAT	0	0	0	1	0	No gene matches found
AGGTTGTGCC	0	1	0	0	0	No gene matches found
TAATTCGTCT	0	0	0	1	0	No gene matches found
TACGTCGGAG	0	1	0	0	0	No gene matches found
GACCCGCCAA	0	1	0	0	0	No gene matches found
AAGTGTCTGT	0	0	0	1	0	No gene matches found
ATAACGTCCG	0	0	0	1	0	No gene matches found
GTCTCTCCTA	0	1	0	0	0	No gene matches found

CTACGTTAGG	0	0	1	0	0	No gene matches found
CAGTTTGCCG	0	0	1	0	0	No gene matches found
CCCGCTCAGA	0	0	1	0	0	No gene matches found
GGTGATGCGG	1	0	0	0	0	No gene matches found
TTTGCAGTGA	0	1	0	0	0	No gene matches found
GGGTGGTCGA	0	0	0	1	0	No gene matches found
GACCTCTTAA	0	1	0	0	0	No gene matches found
CCGATGATGG	0	1	0	0	0	No gene matches found
CGCTTGATGC	0	1	0	0	0	No gene matches found
CCGTATTCAC	1	0	0	0	0	No gene matches found
GTCGACCAAA	1	0	0	0	0	No gene matches found
GCTCGACAAA	0	1	0	0	0	No gene matches found
ACAAGGACTT	0	0	0	1	0	No gene matches found
TGGTATCAAG	0	0	1	0	0	No gene matches found
TAGAGGTAGG	1	0	0	0	0	No gene matches found
GTGACTCAGC	0	0	0	0	1	No gene matches found
TGATCTCCCG	0	0	1	0	0	No gene matches found
ATGCAATCTA	0	1	0	0	0	No gene matches found
CATTAGACTA	1	0	0	0	0	No gene matches found
TTGCTACGAA	1	0	0	0	0	No gene matches found
GACGCAGCCG	0	0	0	1	0	No gene matches found
GATGTTATCG	1	0	0	0	0	No gene matches found
AAACTTATGG	0	0	1	0	0	No gene matches found
CGGTTTTCAT	0	0	0	1	0	No gene matches found
TGGTGGCTAG	0	0	1	0	0	No gene matches found
TTTACTGAGA	1	0	0	0	0	No gene matches found
TTCTTGTCCG	0	1	0	0	0	No gene matches found
ATAAGACTAC	0	0	0	1	0	No gene matches found
TGGAGTTTCG	0	0	0	0	1	No gene matches found
GAATTCGTCA	0	1	0	0	0	No gene matches found
GGTGGGTAAA	1	0	0	0	0	No gene matches found
AATAGTCCAG	0	1	0	0	0	No gene matches found
TGTTCATTCC	1	0	0	0	0	No gene matches found
CCCGGGGAAA	1	0	0	0	0	No gene matches found
ATTCGGAGGG	0	0	1	0	0	No gene matches found
GCCTTTGTAT	0	1	0	0	0	No gene matches found
AAACCGTGGT	0	0	0	1	0	No gene matches found
AAGGTGTAGC	1	0	0	0	0	No gene matches found
TGATCTGTGA	0	1	0	0	0	No gene matches found
ACTAACTCTA	1	0	0	0	0	No gene matches found
GAGGTCATCG	1	0	0	0	0	No gene matches found
CCGTGAAAAA	0	0	0	1	0	No gene matches found
GTTGAACTTT	0	0	1	0	0	No gene matches found
TGTCCGAGTA	0	0	1	0	0	No gene matches found
GAGGTGGGAG	0	0	0	1	0	No gene matches found
GGCGTCATTA	0	0	0	1	0	No gene matches found
CGACCAGCAG	0	1	0	0	0	No gene matches found
GGCGATCATT	0	0	0	0	1	No gene matches found
AGGTATCTGT	1	0	0	0	0	No gene matches found
TGAGAGACAG	0	0	1	0	0	No gene matches found
GTACATAAAC	0	0	0	1	0	No gene matches found
CCCAACCATA	0	0	1	0	0	No gene matches found
CAGCGGCAGA	0	0	1	0	0	No gene matches found
GATGAAAAAG	0	1	0	0	0	No gene matches found
GGGAGATCAA	0	0	1	0	0	No gene matches found
TTCGTCAAAA	0	0	1	0	0	No gene matches found
AGTCTCCTTG	0	1	0	0	0	No gene matches found
CTTCTGTTTA	0	0	1	0	0	No gene matches found
TTCGTTGCTT	0	0	0	1	0	No gene matches found; small RNA(GGTCATGTTCGTTGCTTGTCGCT)
ACAGTCCATA	1	0	0	0	0	No gene matches found

AAGTGGAGGT	0	0	0	1	0	No gene matches found
AAATGAGGGA	0	0	0	1	0	No gene matches found
CAAATCAGAG	0	0	0	0	1	No gene matches found
TTATGAGCTA	0	1	0	0	0	No gene matches found
CAGACCTGAT	0	1	0	0	0	No gene matches found
GTTTGCGAGA	0	0	0	1	0	No gene matches found
GCCCTTCGAC	0	1	0	0	0	No gene matches found
GGCAGGCAGG	0	0	1	0	0	No gene matches found
CACGTTAGAT	0	1	0	0	0	No gene matches found
CAACTTATTG	1	0	0	0	0	No gene matches found
TTATAGCGAG	0	1	0	0	0	No gene matches found
ACGGCTGTGG	0	1	0	0	0	No gene matches found
GTGCTGCTTT	1	0	0	0	0	No gene matches found
GGCGAAAAGG	0	0	1	0	0	No gene matches found
ATGGTAGTAA	0	0	0	1	0	No gene matches found
AGACCAGAGT	0	1	0	0	0	No gene matches found
GACCCGGTGT	0	1	0	0	0	No gene matches found
CCCGAGTATG	0	1	0	0	0	No gene matches found
TCGGAGATTG	0	0	0	1	0	No gene matches found
TGCGTGTGTA	0	0	1	0	0	No gene matches found
CAACGAAAAA	1	0	0	0	0	No gene matches found
AGAGCGGGAT	0	1	0	0	0	No gene matches found
TATCACTTCT	0	0	0	1	0	No gene matches found
CTCCCACGTT	0	1	0	0	0	No gene matches found
GATTTCGAGG	0	1	0	0	0	No gene matches found
TCTATTCAAA	0	0	0	0	1	No gene matches found
ACAGCAGATG	0	1	0	0	0	No gene matches found
TGTTGCGATG	1	0	0	0	0	No gene matches found
GCGCATTTGG	0	0	1	0	0	No gene matches found
AGCAGGCAAG	1	0	0	0	0	No gene matches found
GCTATTCCTG	0	1	0	0	0	No gene matches found
ACATCGAGCA	0	0	0	0	1	No gene matches found
ATTCGAGCAG	1	0	0	0	0	No gene matches found
GCTTTGTCCC	0	0	0	1	0	No gene matches found
TAAGACGAAG	0	1	0	0	0	No gene matches found
GCAAGCGGTC	0	0	0	1	0	No gene matches found
CGAAAGAACT	1	0	0	0	0	No gene matches found
CGAGTTGATG	0	0	0	1	0	No gene matches found
CCCCAGAAAT	0	1	0	0	0	No gene matches found
CTGATGGATA	1	0	0	0	0	No gene matches found
TACTCGTCAG	0	1	0	0	0	No gene matches found
GGGCAGTTTG	0	0	0	0	1	No gene matches found
GCTTCTGAAG	0	1	0	0	0	No gene matches found
AAGTCGAAAT	0	0	0	1	0	No gene matches found
CGGTGAAAAG	0	0	0	0	1	No gene matches found
GGGCTGTCTG	1	0	0	0	0	No gene matches found
TATCGGAGTT	0	0	0	1	0	No gene matches found
AGATCCGAAT	0	0	1	0	0	No gene matches found
AGACTCGAGG	0	0	0	1	0	No gene matches found
AATGCTCGGT	0	0	1	0	0	No gene matches found
TCCTATAAAA	0	0	0	1	0	No gene matches found
AGGGATCGCG	0	1	0	0	0	No gene matches found
CCAGTCGATC	0	1	0	0	0	No gene matches found
ATGCCTCTTC	0	0	1	0	0	No gene matches found
AGTAACGGTG	0	0	0	1	0	No gene matches found
AGCTCTTTTC	0	0	1	0	0	No gene matches found
GGGTTGCGAT	0	0	1	0	0	No gene matches found
CATTACTTCG	0	0	0	0	1	No gene matches found
AGACGCAGTT	0	0	0	1	0	No gene matches found
ACGCAGCCTC	0	1	0	0	0	No gene matches found
CGGTTTCCTT	0	1	0	0	0	No gene matches found

TCGAAACTGA	0	1	0	0	0	No gene matches found
GTTTATCGCT	1	0	0	0	0	No gene matches found
CTACAGGTAC	0	1	0	0	0	No gene matches found
AAGTAGGTTT	0	1	0	0	0	No gene matches found
TAGCTGAGAA	0	0	0	0	1	No gene matches found
AAGGGTGGGA	0	1	0	0	0	No gene matches found
TATTGATCGC	0	1	0	0	0	No gene matches found
TCACGACGCA	1	0	0	0	0	No gene matches found
GATCTCATAA	0	1	0	0	0	No gene matches found
TGTCAGGAGG	0	1	0	0	0	No gene matches found
CGAACACTTC	0	1	0	0	0	No gene matches found
ATCTTATCGA	1	0	0	0	0	No gene matches found
TTTCGAGTCC	0	0	0	1	0	No gene matches found
CTCTCTCCAG	0	0	0	1	0	No gene matches found
GATCATTGCA	0	1	0	0	0	No gene matches found
CTACTGGGAG	0	1	0	0	0	No gene matches found
CATTCGCCGC	0	1	0	0	0	No gene matches found
TACATCTAGA	0	0	1	0	0	No gene matches found
TGAGCTTATG	1	0	0	0	0	No gene matches found
GCGCGGAAGT	0	0	0	1	0	No gene matches found
TCTTATTTCG	1	0	0	0	0	No gene matches found
ACCAGCCATA	0	1	0	0	0	No gene matches found
TGATTTTCCG	0	1	0	0	0	No gene matches found
GGCCTGCTTG	1	0	0	0	0	No gene matches found
CCGAAAATAC	0	1	0	0	0	No gene matches found
ACCAAGTTTT	1	0	0	0	0	No gene matches found
TAACACTCCG	0	1	0	0	0	No gene matches found
TACGGAGTCG	0	1	0	0	0	No gene matches found
CCAAGCGTGC	0	0	0	1	0	No gene matches found
GTCTGTAGAC	0	0	0	1	0	No gene matches found
GCGGGGTTCC	0	0	1	0	0	No gene matches found
AGAATGTATA	0	0	0	0	1	No gene matches found
TTTCTGTGCT	0	0	1	0	0	No gene matches found
AAAGGCCTCT	0	1	0	0	0	No gene matches found
ACTCGCTGAT	0	1	0	0	0	No gene matches found
AATCCCGCAA	0	0	0	0	1	No gene matches found
GTAGTCGATG	0	0	0	1	0	No gene matches found
GCTGGGGATA	0	0	1	0	0	No gene matches found
TCGGGTTCGA	0	0	0	1	0	No gene matches found
ACGGTGGATA	0	1	0	0	0	No gene matches found
TGACTCCAAG	0	0	0	1	0	No gene matches found
GAGCGTTTGT	0	0	0	0	1	No gene matches found
GTCCAATATA	0	0	1	0	0	No gene matches found
CTAGGATCAA	0	0	1	0	0	No gene matches found
GCTCGAGGCT	0	0	0	0	1	No gene matches found
CAAGGGGTGA	0	0	0	0	1	No gene matches found
TGAAGTCGCA	0	0	0	0	1	No gene matches found
CATAGTGGTA	0	0	0	1	0	No gene matches found
GTGAAGCATT	0	0	1	0	0	No gene matches found
TGTCAGGAGT	0	1	0	0	0	No gene matches found
TCTGCGCGGC	0	0	0	1	0	No gene matches found
CTGAACTTAT	0	0	1	0	0	No gene matches found
TTATCGAAAG	0	0	0	0	1	No gene matches found
CTGCACCTCT	0	1	0	0	0	No gene matches found
ACTCAGCTCG	0	0	1	0	0	No gene matches found
GGCACCTCAC	0	0	1	0	0	No gene matches found
GTAATAAAGG	0	1	0	0	0	No gene matches found
GATATGTCTC	0	0	0	1	0	No gene matches found
GCTATACTGA	0	0	0	1	0	No gene matches found
TAAGTCGCCG	0	0	0	1	0	No gene matches found
CGTCGCGATA	1	0	0	0	0	No gene matches found

CAGGTGCTGA	0	1	0	0	0	No gene matches found
GGAGCTTTGA	1	0	0	0	0	No gene matches found
GCTCCGGACT	0	0	1	0	0	No gene matches found
TTACAGATCC	1	0	0	0	0	No gene matches found
GGGCTTTAGC	1	0	0	0	0	No gene matches found
TGCGTGTGAA	0	0	0	1	0	No gene matches found
AATAAACTGC	0	0	1	0	0	No gene matches found
AAAAGGACGG	0	1	0	0	0	No gene matches found
TTCGACCCAA	0	0	0	0	1	No gene matches found
TAGCCGCTTC	0	0	0	1	0	No gene matches found
TTGAGGTGTA	0	0	0	1	0	No gene matches found
CTCTAACCGG	1	0	0	0	0	No gene matches found
GTTGCACCAC	1	0	0	0	0	No gene matches found
CTTTAGTATG	0	0	0	1	0	No gene matches found
GGTTGCGTTG	0	0	0	1	0	No gene matches found
GTCTTCGTCC	0	1	0	0	0	No gene matches found
TCCATTCGGT	0	0	1	0	0	No gene matches found
AGAGTTTACG	0	1	0	0	0	No gene matches found
AGGGAAGGGT	0	0	1	0	0	No gene matches found
ACAAAGGGTG	0	0	0	1	0	No gene matches found
GTAGTGATGG	1	0	0	0	0	No gene matches found
CGTTGCGCTA	1	0	0	0	0	No gene matches found
CCCCTGAACG	0	0	0	1	0	No gene matches found
AAGTTAGTAC	0	0	1	0	0	No gene matches found
TCCCTTCGAA	0	0	1	0	0	No gene matches found
AAGCAACTCC	0	0	0	1	0	No gene matches found
GATAAAAGTG	0	0	1	0	0	No gene matches found
GATTTGCTCA	0	0	0	1	0	No gene matches found
TTACGTAGCA	0	0	0	1	0	No gene matches found
TGGTAGATCA	1	0	0	0	0	No gene matches found
AGCCGCAATG	0	0	1	0	0	No gene matches found
GATTCATTCG	0	1	0	0	0	No gene matches found
AATGCAGTTT	0	1	0	0	0	No gene matches found
AGCAGTCATT	0	0	0	0	1	No gene matches found
TGCTCTCCAA	0	0	0	0	1	No gene matches found
ATTCGGGGTA	0	0	1	0	0	No gene matches found
GAAGGGCTAA	0	1	0	0	0	No gene matches found
AGAGAGCGAG	1	0	0	0	0	No gene matches found
GACAACGTGA	0	0	0	1	0	No gene matches found
ATTGCCAGAT	0	1	0	0	0	No gene matches found
GTGTCGGAAT	0	0	0	0	1	No gene matches found
GGATAAGTGA	0	0	1	0	0	No gene matches found
TTCACCGCTT	0	0	1	0	0	No gene matches found
TCTAGTCGAG	1	0	0	0	0	No gene matches found
GTTGATGCGT	0	1	0	0	0	No gene matches found
CATTTCACGA	1	0	0	0	0	No gene matches found
AGAATCCGCG	1	0	0	0	0	No gene matches found
ACCAGCGTGT	0	1	0	0	0	No gene matches found
CGTTGTGAGT	1	0	0	0	0	No gene matches found
GTGCGTTCGT	1	0	0	0	0	No gene matches found
GCCTGGCCCA	0	1	0	0	0	No gene matches found
TGAAGGGTAG	0	0	1	0	0	No gene matches found
TGTCTCCAAT	0	0	1	0	0	No gene matches found
TATACGGAGA	0	1	0	0	0	No gene matches found
ATTTCACTGG	0	0	0	1	0	No gene matches found
CGAAAAAGCC	1	0	0	0	0	No gene matches found
TGGTATTCGC	1	0	0	0	0	No gene matches found
GGTTGTAATT	0	0	0	1	0	No gene matches found
GTTAGCTCAA	1	0	0	0	0	No gene matches found
TGTAATAGTG	0	1	0	0	0	No gene matches found
AGGAACCATC	0	0	1	0	0	No gene matches found

GGCGTTCGCT	0	1	0	0	0	No gene matches found
TGTGGAGTCT	0	0	0	1	0	No gene matches found
CAGAGTTGCT	0	1	0	0	0	No gene matches found
AGGTTTCTGA	0	1	0	0	0	No gene matches found
CACTGGGTGA	0	0	0	1	0	No gene matches found
ACTGGTGAAC	1	0	0	0	0	No gene matches found
ACCCATTAAA	0	0	0	1	0	No gene matches found
CGAGATTTCG	0	0	0	1	0	No gene matches found
CGAGATTACT	0	0	1	0	0	No gene matches found
GGGATACGGT	0	0	1	0	0	No gene matches found
ATGACTACGA	0	0	0	0	1	No gene matches found
CAAAGGTCGG	1	0	0	0	0	No gene matches found
GGCCTAAGAA	0	0	1	0	0	No gene matches found
GCGGATGATG	0	1	0	0	0	No gene matches found
CCCCGGTTAA	0	1	0	0	0	No gene matches found
AGGTTGCCTA	0	1	0	0	0	No gene matches found
AAAGCGGACA	0	0	1	0	0	No gene matches found
TGGTAGACAA	0	0	0	1	0	No gene matches found
ACCAGTCGTC	0	1	0	0	0	No gene matches found
TGTTTGAGGA	0	0	1	0	0	No gene matches found
TCACCGCTGA	0	1	0	0	0	No gene matches found
CTGTGAGAGT	0	0	0	1	0	No gene matches found
AGGACGCCAT	0	0	0	1	0	No gene matches found
ACTCGTTGCT	0	0	0	1	0	No gene matches found
GTGGTCCGAC	1	0	0	0	0	No gene matches found
GGAGTAATGT	0	0	0	0	1	No gene matches found
ATACTCGTTT	0	1	0	0	0	No gene matches found
CCCCCGAAAC	0	0	1	0	0	No gene matches found
AGGGCACGTG	1	0	0	0	0	No gene matches found
CCAGGCCGAT	1	0	0	0	0	No gene matches found
ATAGAGCCTC	0	1	0	0	0	No gene matches found
ACCACTTGAG	0	1	0	0	0	No gene matches found
TAGTGACCAC	0	0	1	0	0	No gene matches found
CAGATCGTGA	0	0	0	1	0	No gene matches found
GTGCGTTGAA	0	0	0	0	1	No gene matches found
AGTAGCCGCG	0	1	0	0	0	No gene matches found
ACACGCTTTA	1	0	0	0	0	No gene matches found
ATGGTCGAAT	0	1	0	0	0	No gene matches found
GGTTGACCAC	0	1	0	0	0	No gene matches found
TGTAGCCGCC	1	0	0	0	0	No gene matches found
GGAAGTTTGG	0	0	0	0	1	No gene matches found
CGCAAGTCAG	0	1	0	0	0	No gene matches found
ATCGGGGAAT	0	0	0	0	1	No gene matches found
TAAACGCTAG	1	0	0	0	0	No gene matches found
CCTCGCGAGA	0	0	0	1	0	No gene matches found
GGGAGTTGCA	0	0	0	0	1	No gene matches found
GACGCCTTTT	0	1	0	0	0	No gene matches found
CTGGGAGCCC	0	1	0	0	0	No gene matches found
GCTGTGACGC	0	0	1	0	0	No gene matches found
TATTTGTGGC	0	0	0	0	1	No gene matches found
ACACCTCTCT	0	0	0	1	0	No gene matches found
GCTGAGAGAG	0	0	0	1	0	No gene matches found
CTCGCCGCAC	0	0	0	1	0	No gene matches found
TCACTCTTAA	0	0	1	0	0	No gene matches found
TGAATGGTGG	1	0	0	0	0	No gene matches found
TTAACCCAGA	1	0	0	0	0	No gene matches found
TCACTCGTGC	0	0	0	0	1	No gene matches found
ACAGTTATGG	0	0	1	0	0	No gene matches found
AGGTTATTCG	0	1	0	0	0	No gene matches found
TCAGGAAGCA	0	0	1	0	0	No gene matches found

GTTCCCGGGG	0	0	0	1	0	No gene matches found
CTACAAGAGG	0	0	0	1	0	No gene matches found
TCGGATGCGA	0	0	1	0	0	No gene matches found
CTTCCTGCAG	0	0	0	0	1	No gene matches found
GGCGGGCAAG	0	0	0	1	0	No gene matches found
CAATAAGACT	0	1	0	0	0	No gene matches found
ATTCACGGCA	0	0	1	0	0	No gene matches found
GAAACGATAA	0	0	1	0	0	No gene matches found
GGCGACACAA	0	1	0	0	0	No gene matches found
GTCGAAGGGA	0	1	0	0	0	No gene matches found
CGTTTTAGAT	0	1	0	0	0	No gene matches found
AATTACACGA	0	0	1	0	0	No gene matches found
TGGAGCCGGT	0	1	0	0	0	No gene matches found
TTTTTTTGTA	0	0	0	1	0	No gene matches found
GCCGTTTTTG	0	1	0	0	0	No gene matches found
GTCTGTCCAG	1	0	0	0	0	No gene matches found
CCCATCTAGA	0	0	0	0	1	No gene matches found
AGAGGGCTCG	0	0	1	0	0	No gene matches found
CGACAATCGA	0	0	1	0	0	No gene matches found
TCCGCATCTT	0	1	0	0	0	No gene matches found
TCGCTCAATA	0	0	1	0	0	No gene matches found
CAACTGCCAT	0	0	1	0	0	No gene matches found
GTGGAATGGG	0	0	1	0	0	No gene matches found
CTCCTGCTGT	1	0	0	0	0	No gene matches found
CTCCTGCGGC	0	1	0	0	0	No gene matches found
CAGAGTTAGA	0	0	1	0	0	No gene matches found
TGCAGTCATA	0	0	1	0	0	No gene matches found
CCTCCTGGAG	0	1	0	0	0	No gene matches found
AGTTAGTGTG	0	1	0	0	0	No gene matches found
TCCAAAGAGT	0	0	1	0	0	No gene matches found
GATCACATCC	0	0	0	1	0	No gene matches found
AAAAGTCCGA	0	0	0	0	1	No gene matches found
TGACCCCGAA	0	0	0	0	1	No gene matches found
CCGATAAAAA	0	0	1	0	0	No gene matches found
AGGCTGGATG	0	1	0	0	0	No gene matches found
TCTCCGGACA	0	1	0	0	0	No gene matches found
GGTGAGATCC	1	0	0	0	0	No gene matches found
GTGCTACAAA	0	0	0	1	0	No gene matches found
GGCGAAGGGC	0	0	1	0	0	No gene matches found
GCTCAACGTA	0	1	0	0	0	No gene matches found
GCCGATGTAT	0	1	0	0	0	No gene matches found
CTTCTGAGCA	0	0	1	0	0	No gene matches found
GTGTCCCAAG	0	0	1	0	0	No gene matches found
CAGGTGAGGC	0	0	0	0	1	No gene matches found
TAGATATCCT	0	1	0	0	0	No gene matches found
CGTTCAAGAA	0	0	1	0	0	No gene matches found
TTCTCGCTCT	1	0	0	0	0	No gene matches found
TGGAAGGAGA	0	0	0	1	0	No gene matches found
GTGACTTTCA	1	0	0	0	0	No gene matches found
CAGCTGAACG	1	0	0	0	0	No gene matches found
CCGGTGAACA	0	0	0	1	0	No gene matches found
GGCAAAGGGA	1	0	0	0	0	No gene matches found
GACGACAATT	0	1	0	0	0	No gene matches found
TTGGTACGCC	0	0	1	0	0	No gene matches found
CTGGATTCTC	0	1	0	0	0	No gene matches found
AAACCAAGGC	0	1	0	0	0	No gene matches found
GGCCCAACAT	0	0	0	1	0	No gene matches found
TAACCTTTTG	0	1	0	0	0	No gene matches found
TAAGGGAGTG	0	0	0	1	0	No gene matches found
TAGGGGAGGA	0	0	0	1	0	No gene matches found
TAGTGGCTAC	0	0	0	1	0	No gene matches found

TCTGTGTACT	0	0	0	0	1	No gene matches found
ACAGCCAGTT	0	1	0	0	0	No gene matches found
ATCAAAGACC	0	1	0	0	0	No gene matches found
TCCATCGTCT	0	0	1	0	0	No gene matches found
GGTACAAGTG	0	0	0	0	1	No gene matches found
CGCGTTAAGT	0	1	0	0	0	No gene matches found
CCAAGACCGC	0	0	0	1	0	No gene matches found
GTGAGATCAA	1	0	0	0	0	No gene matches found
AGCCCTATCA	0	0	0	0	1	No gene matches found
GAATCGTTTA	0	0	0	0	1	No gene matches found
ATTCCTCTCT	0	0	1	0	0	No gene matches found
TTTTCTCTGC	0	1	0	0	0	No gene matches found
TAACAAGTCT	0	1	0	0	0	No gene matches found
CGCAAAGTAC	0	1	0	0	0	No gene matches found
GTGTCTTAAT	0	1	0	0	0	No gene matches found
CTCAAAAGCG	0	1	0	0	0	No gene matches found
GTCGTTCAGG	0	0	0	1	0	No gene matches found
AGACACTGCT	0	0	0	0	1	No gene matches found
CTGCTCCATA	0	0	0	1	0	No gene matches found
CGAAAAAGGA	0	0	0	0	1	No gene matches found
ACGGGATCCA	1	0	0	0	0	No gene matches found
GTCCTGCGAT	0	0	0	1	0	No gene matches found
CCGGAGCTAT	0	0	1	0	0	No gene matches found
CAATCTGTTA	0	1	0	0	0	No gene matches found
GGCAAATAGC	0	0	0	0	1	No gene matches found
TTACCGCCAA	0	0	0	0	1	No gene matches found
AGGAGTACAA	0	0	0	0	1	No gene matches found
GTTTGGACAC	0	0	1	0	0	No gene matches found
TTAGATTTAT	0	0	1	0	0	No gene matches found
TATGACAATG	1	0	0	0	0	No gene matches found
ACAGAGAAGC	1	0	0	0	0	No gene matches found
CGTGCGCCGG	0	0	1	0	0	No gene matches found
GGAAATGCCA	0	0	1	0	0	No gene matches found
CTGATGTCCC	0	1	0	0	0	No gene matches found
CTGCTACACC	1	0	0	0	0	No gene matches found
AGATTCGTGT	0	1	0	0	0	No gene matches found
ATCCACAGGA	1	0	0	0	0	No gene matches found
TTGTTTCGGC	0	1	0	0	0	No gene matches found
ACCGTGTTGG	0	1	0	0	0	No gene matches found
GCAGGGGTCG	0	1	0	0	0	No gene matches found
GTACGGATCA	0	0	1	0	0	No gene matches found
GATTTTCCCA	0	0	0	1	0	No gene matches found
ACAGGCCGCT	0	1	0	0	0	No gene matches found
TCTCATAAGC	1	0	0	0	0	No gene matches found
TTGGAGTTCC	0	1	0	0	0	No gene matches found
CGGTTATGTG	0	0	0	1	0	No gene matches found
ATCCCCTTCT	0	1	0	0	0	No gene matches found
TGGATACTTG	0	0	0	0	1	No gene matches found
GCTTTGCCAG	0	0	1	0	0	No gene matches found
ATGCTTGACG	0	1	0	0	0	No gene matches found
CCAGAGGATG	0	1	0	0	0	No gene matches found
CGACCTCCGC	0	1	0	0	0	No gene matches found
CCAGGTGATT	0	0	0	1	0	No gene matches found
TGCCCATATG	0	0	1	0	0	No gene matches found
TACGATCGTT	0	0	1	0	0	No gene matches found
ACTGTATAAG	0	1	0	0	0	No gene matches found
TATGCTTATT	0	0	0	0	1	No gene matches found
CCGGAGCCAA	0	1	0	0	0	No gene matches found
AGAATGGCTC	0	0	1	0	0	No gene matches found
AACCCGGCCG	0	0	0	1	0	No gene matches found
ATTCTGTTCG	1	0	0	0	0	No gene matches found

GGCCTTGCGC	0	1	0	0	0	No gene matches found
TAAAGAGCAC	1	0	0	0	0	No gene matches found
GAGAGATGGT	0	0	0	0	1	No gene matches found
GGATGCGGTG	0	1	0	0	0	No gene matches found
TATGCTGAGA	0	1	0	0	0	No gene matches found
AGGGGTTCTG	0	1	0	0	0	No gene matches found
ATCACCCACG	0	0	0	1	0	No gene matches found
CAGGTGGGCA	0	1	0	0	0	No gene matches found
GGAGACGACA	0	0	1	0	0	No gene matches found
TCTTCAGGAC	1	0	0	0	0	No gene matches found
AAAGGTCTCC	0	0	1	0	0	No gene matches found
CAATAGAGTA	0	1	0	0	0	No gene matches found
AGGGTTAAGT	0	0	1	0	0	No gene matches found
GATTTCCGAG	0	0	1	0	0	No gene matches found
GTAGGGTGTG	0	1	0	0	0	No gene matches found
CGGCTGAACG	0	0	0	0	1	No gene matches found
ATCCGAAAGG	0	0	0	1	0	No gene matches found
GAGACAGCTG	0	1	0	0	0	No gene matches found
TTCTTGCACT	1	0	0	0	0	No gene matches found
TTTCGATGGA	1	0	0	0	0	No gene matches found
GGAATTGGCT	0	0	1	0	0	No gene matches found
TCGAAATGAA	0	1	0	0	0	No gene matches found
TGCACGTCCA	0	0	0	0	1	No gene matches found
AGAGAACGAT	0	0	0	1	0	No gene matches found
GCGCAGTGCT	0	0	0	1	0	No gene matches found
ACGATGCGGC	0	1	0	0	0	No gene matches found
GCAGTAAGGG	0	0	1	0	0	No gene matches found
TCGTCAGTAG	0	1	0	0	0	No gene matches found
GGCCGTCTCA	0	1	0	0	0	No gene matches found
GGCGAACGGC	0	1	0	0	0	No gene matches found
ACTCGTGAAG	0	1	0	0	0	No gene matches found
TGGTAACTAA	0	0	1	0	0	No gene matches found
CATCTGACAG	0	0	0	0	1	No gene matches found
AGGGGAAAAC	1	0	0	0	0	No gene matches found
TACAGCCTCC	1	0	0	0	0	No gene matches found
TGACATTAGA	0	1	0	0	0	No gene matches found
CCTGTTGATT	0	0	1	0	0	No gene matches found
CTGCGGCCTC	0	1	0	0	0	No gene matches found
GAAAGTATCG	1	0	0	0	0	No gene matches found
TTACATTGGA	0	0	1	0	0	No gene matches found
TGAATTGGAG	0	0	0	1	0	No gene matches found
GAGCATATCC	0	0	1	0	0	No gene matches found
TTGAAGGAGG	1	0	0	0	0	No gene matches found
TAACCTCCTT	0	0	0	0	1	No gene matches found
GTCTCGGCCA	0	0	1	0	0	No gene matches found
AGACCTCATA	0	1	0	0	0	No gene matches found
TGTTGGTGGA	1	0	0	0	0	No gene matches found
CTCCACACAG	0	0	1	0	0	No gene matches found
TAAAGTCTCA	0	0	0	1	0	No gene matches found
TTCAGAGACC	0	0	0	0	1	No gene matches found
GTGCAACCAT	0	1	0	0	0	No gene matches found
TTCTAGTCTG	0	1	0	0	0	No gene matches found
GAGGGTAAAG	0	0	0	0	1	No gene matches found
TATATCGGCT	0	0	0	1	0	No gene matches found
TCAGAACCTT	0	1	0	0	0	No gene matches found
GTGCAATGGA	0	1	0	0	0	No gene matches found
CAATAAGTGT	0	0	1	0	0	No gene matches found
CTGGCAGAGT	0	0	1	0	0	No gene matches found
AGAGTGTAGA	0	0	0	0	1	No gene matches found
TTAACTGAGG	1	0	0	0	0	No gene matches found
TGTTAGCGTG	0	1	0	0	0	No gene matches found

GCAAGCGGAG	0	0	1	0	0	No gene matches found
TTGTGACTGG	0	1	0	0	0	No gene matches found
TGCGATTGTA	0	0	1	0	0	No gene matches found
TTTCGCAATT	1	0	0	0	0	No gene matches found
AAGCAGTATG	0	1	0	0	0	No gene matches found
CTCACTTCCA	0	0	0	0	1	No gene matches found
CCATATTAGC	0	1	0	0	0	No gene matches found
GCCTAAAAAA	0	0	1	0	0	No gene matches found
CGCAATATGA	0	0	1	0	0	No gene matches found
GGTGAGGTTG	0	1	0	0	0	No gene matches found
ATATTCTTTG	0	1	0	0	0	No gene matches found
GCTGACGCGG	0	0	1	0	0	No gene matches found
GAGCTTGCAC	0	1	0	0	0	No gene matches found
CAAACAGTGT	0	1	0	0	0	No gene matches found
CTATGGTCGT	1	0	0	0	0	No gene matches found
TATTATGTAC	0	1	0	0	0	No gene matches found
GGTTCGCCGA	1	0	0	0	0	No gene matches found
GTTCTCACAG	0	1	0	0	0	No gene matches found
GAGGTCGCGG	0	1	0	0	0	No gene matches found
GCCCAGGATT	1	0	0	0	0	No gene matches found
TAATCATCCA	1	0	0	0	0	No gene matches found
AGAACAGGTA	1	0	0	0	0	No gene matches found
CAATTAAGGT	0	1	0	0	0	No gene matches found
GGGAACGGAT	0	1	0	0	0	No gene matches found
CTACAAGGAA	0	1	0	0	0	No gene matches found
ACAGGTGTCT	1	0	0	0	0	No gene matches found
CGTCTAAGAT	0	1	0	0	0	No gene matches found
GCTCTTAGTG	0	1	0	0	0	No gene matches found
TAGTTCAACT	0	0	1	0	0	No gene matches found
TAATGTTGGC	0	1	0	0	0	No gene matches found
TGCCAATGCC	0	0	0	0	1	No gene matches found
TGTGCTCTTG	0	0	1	0	0	No gene matches found
GTCGTCGGGT	0	1	0	0	0	No gene matches found
CCTCGTTGAT	0	0	0	1	0	No gene matches found
AAGGGAATCG	0	0	0	1	0	No gene matches found
CCGTTTCCGA	0	0	1	0	0	No gene matches found
TTTCCAGTCT	0	1	0	0	0	No gene matches found
CTATAATTGG	0	1	0	0	0	No gene matches found
TCGACCTCTC	0	1	0	0	0	No gene matches found
AGGATGATCA	0	0	0	0	1	No gene matches found
GCTGTGGGAC	0	0	1	0	0	No gene matches found
TTCGCCGAGG	0	0	1	0	0	No gene matches found
ACGGTGGCAT	0	1	0	0	0	No gene matches found
GCGTCGCTTA	0	0	0	1	0	No gene matches found
GGGAACCTTG	0	0	0	0	1	No gene matches found
CTTGTAGAAC	0	0	1	0	0	No gene matches found
TAGCTCGGGC	0	0	1	0	0	No gene matches found
CCTGTTTTAT	0	1	0	0	0	No gene matches found
GGAGAGTTGA	0	1	0	0	0	No gene matches found
GACTTGGGGC	1	0	0	0	0	No gene matches found
GGGAGCTGTT	0	1	0	0	0	No gene matches found
ATACGAAGTA	0	0	1	0	0	No gene matches found
GGAGCGGGGG	1	0	0	0	0	No gene matches found
GGGCAGTTAC	1	0	0	0	0	No gene matches found
AGCTTCGAGG	0	0	0	1	0	No gene matches found
CGTATGTCTC	1	0	0	0	0	No gene matches found
CCTATGTCTA	0	0	1	0	0	No gene matches found
TGGCACGTGG	0	0	1	0	0	No gene matches found
TAATCCCATA	0	1	0	0	0	No gene matches found
CCCAGGCTCG	0	0	0	1	0	No gene matches found
TCCAATTGGG	0	1	0	0	0	No gene matches found

CCCGATCTTG	0	0	0	0	1	No gene matches found
GAGATATGTC	0	0	1	0	0	No gene matches found
CGTTGAGGAG	1	0	0	0	0	No gene matches found
TATGTTACCC	0	0	0	1	0	No gene matches found
TAGTCTTATC	0	0	0	0	1	No gene matches found
CAGCGGGGAC	0	1	0	0	0	No gene matches found
CAGATCAGTT	0	0	0	0	1	No gene matches found
GACAAGAGAT	0	0	0	1	0	No gene matches found
AACATCTCGA	0	0	1	0	0	No gene matches found
AAGGTCCCTA	0	0	0	1	0	No gene matches found
TCGCATAAGT	0	1	0	0	0	No gene matches found
GACGTATGAG	0	1	0	0	0	No gene matches found
CTTCTGGCAT	0	1	0	0	0	No gene matches found
CCTCCGTGTT	0	0	0	1	0	No gene matches found
TACGAGGTTG	0	0	0	1	0	No gene matches found
CGCGGCAAAA	0	1	0	0	0	No gene matches found
CTCTCTCATA	0	0	0	1	0	No gene matches found
GCTCTGGAAG	0	0	1	0	0	No gene matches found
AAAAGTCTGC	0	1	0	0	0	No gene matches found
CTCTCCAGAC	0	1	0	0	0	No gene matches found
AATGAATGTC	1	0	0	0	0	No gene matches found
ATATCAATGG	0	0	0	1	0	No gene matches found
GAAGTCCACA	0	1	0	0	0	No gene matches found
AGGGATCTCC	0	1	0	0	0	No gene matches found
ACGTGTTTGA	0	0	1	0	0	No gene matches found
CACACCGGAG	0	0	0	1	0	No gene matches found
TTGATGGGGA	0	0	1	0	0	No gene matches found
AGTGGAGCGA	0	0	1	0	0	No gene matches found
AGTGGATTGT	0	0	1	0	0	No gene matches found
GGAAAACAAG	0	0	1	0	0	No gene matches found
GTCTAGTACA	1	0	0	0	0	No gene matches found
CGCCCGCCAC	0	0	0	1	0	No gene matches found
TGGAATGTCT	0	0	0	0	1	No gene matches found
CCGGGTTTTT	0	0	0	1	0	No gene matches found
TAGATGGAAT	0	0	0	0	1	No gene matches found
TATTATGTCA	0	1	0	0	0	No gene matches found
AAAGGCGTTG	0	0	0	1	0	No gene matches found
CTTGTGACGA	0	1	0	0	0	No gene matches found
TCTATGCTTA	0	0	1	0	0	No gene matches found
CAACCAATTC	0	0	0	0	1	No gene matches found
CCGCTCCTGG	0	1	0	0	0	No gene matches found
AATAAGAGTG	1	0	0	0	0	No gene matches found
AAGGGGTGGC	0	0	0	1	0	No gene matches found
TGTACGCTTG	0	0	1	0	0	No gene matches found
CTCCCTAATG	0	0	0	1	0	No gene matches found
TCCCGTCATT	0	1	0	0	0	No gene matches found
TCTTCATTAA	1	0	0	0	0	No gene matches found
AATTGCTCCG	0	1	0	0	0	No gene matches found
AAGGTCAACG	0	0	1	0	0	No gene matches found
AGGCTTAGCT	1	0	0	0	0	No gene matches found
CCTCTGGGAG	1	0	0	0	0	No gene matches found
TGTTACCTCC	0	0	0	1	0	No gene matches found
TAGGATAAAG	0	0	1	0	0	No gene matches found
AACCCTTCAT	0	1	0	0	0	No gene matches found
AACTCCGATG	1	0	0	0	0	No gene matches found
TTACTTCTGC	0	0	0	0	1	No gene matches found
CAAAGTGTCC	1	0	0	0	0	No gene matches found
TCCAGATTAG	0	0	0	0	1	No gene matches found
GAGCCGACTA	0	0	1	0	0	No gene matches found
CTAGCAGATG	0	0	0	1	0	No gene matches found

CCCCCACTTG	0	1	0	0	0	No gene matches found
ACTCCCTCAA	0	0	0	1	0	No gene matches found
CGCCTCCAGA	0	0	1	0	0	No gene matches found
CTTGGCGCAA	0	1	0	0	0	No gene matches found
TGCAATAATG	0	1	0	0	0	No gene matches found
TTTATGGACG	0	1	0	0	0	No gene matches found
CTCAAACTGG	0	0	1	0	0	No gene matches found
CAAGGCCCTG	0	0	1	0	0	No gene matches found
CAAATTGTCG	0	0	1	0	0	No gene matches found
TCAGATTTGG	0	0	0	0	1	No gene matches found
CTCGGTGCCC	0	1	0	0	0	No gene matches found
GATCTCCTGA	0	1	0	0	0	No gene matches found
TAAGATTCGT	0	0	1	0	0	No gene matches found
ACTGCGTTTT	0	0	0	0	1	No gene matches found
GGTCGCAATC	0	1	0	0	0	No gene matches found
CCTGCAGCTG	0	0	1	0	0	No gene matches found
TATATGGTAG	0	1	0	0	0	No gene matches found
CGCCTATGTA	0	0	0	1	0	No gene matches found
AAGAGCTCGG	0	1	0	0	0	No gene matches found
ATCACGTGGT	0	0	0	0	1	No gene matches found
ATCGTCGTTT	0	0	1	0	0	No gene matches found
CGAAAGTAAC	0	1	0	0	0	No gene matches found
TCACGCGGCT	0	0	1	0	0	No gene matches found
CTAGCTAAAG	0	0	1	0	0	No gene matches found
AATTTGGGAC	0	0	0	1	0	No gene matches found
TTGTTTGCCA	0	0	0	0	1	No gene matches found
TCCACGACCC	0	0	1	0	0	No gene matches found
TGTGTTACGA	1	0	0	0	0	No gene matches found
CAGTATCTCC	0	1	0	0	0	No gene matches found
AAGGGATTTG	1	0	0	0	0	No gene matches found
TGGGGGCTTA	0	0	0	0	1	No gene matches found
ATCGTTTAAG	0	0	1	0	0	No gene matches found
GGGGGGGTCG	0	1	0	0	0	No gene matches found
GTGTCTACGA	1	0	0	0	0	No gene matches found
GTAAGTTGTG	0	0	0	1	0	No gene matches found
GTTGTCATCG	0	1	0	0	0	No gene matches found
AGACAGTAGA	1	0	0	0	0	No gene matches found
TTACACAATT	0	0	0	1	0	No gene matches found
AATAGAGCCA	0	0	1	0	0	No gene matches found
TGGGGAGATG	1	0	0	0	0	No gene matches found
ATGTGCGTAG	0	0	1	0	0	No gene matches found
CCCTTCGCGA	1	0	0	0	0	No gene matches found
AGACGCAGGG	0	0	1	0	0	No gene matches found
AAAGCGATGC	0	0	1	0	0	No gene matches found
TTCTGTATGC	0	1	0	0	0	No gene matches found
GTCCGGAGCA	0	1	0	0	0	No gene matches found
TATGCTGATG	0	0	1	0	0	No gene matches found
CGTTTGGACA	0	0	0	1	0	No gene matches found
CCAGCGAAAA	1	0	0	0	0	No gene matches found
GATTCTATGA	0	0	1	0	0	No gene matches found
GCAGGGCTGA	0	0	1	0	0	No gene matches found
ACCATAACTT	0	1	0	0	0	No gene matches found
CAGCTTTGCA	1	0	0	0	0	No gene matches found
GCCTTCGTCT	0	1	0	0	0	No gene matches found
ACTTCGGACG	0	0	0	0	1	No gene matches found
ACCACCCTAT	1	0	0	0	0	No gene matches found
GTCTCCAATG	0	0	1	0	0	No gene matches found
CGTATAGATG	0	0	0	1	0	No gene matches found
ACCCGTGTTT	0	1	0	0	0	No gene matches found
GGCTATGGAC	0	1	0	0	0	No gene matches found
ACTTCGTTGA	0	0	0	1	0	No gene matches found

CCTTCGCCGA	0	0	0	1	0	No gene matches found
AGCAGCTCGG	1	0	0	0	0	No gene matches found
AAAGCGGTCG	1	0	0	0	0	No gene matches found
CTTGACTGAA	0	0	0	1	0	No gene matches found
TGTCCATAGG	0	1	0	0	0	No gene matches found
CTCCAGGAAA	0	0	0	1	0	No gene matches found
CTAACCTTAA	0	0	0	1	0	No gene matches found
GTGCGGCACA	0	0	0	1	0	No gene matches found
GTGGGGATTC	0	0	0	1	0	No gene matches found
GACCCGTGAT	0	0	1	0	0	No gene matches found
GGCCAGTAAA	0	0	1	0	0	No gene matches found
TGACCGTTTG	0	0	1	0	0	No gene matches found
AAGGTCCTGA	1	0	0	0	0	No gene matches found
GCAGGGGTAC	0	0	1	0	0	No gene matches found
GGCGGGCCAT	0	1	0	0	0	No gene matches found
GATTCGGTTT	0	1	0	0	0	No gene matches found
GCCCTTGACT	0	0	0	1	0	No gene matches found
GCTACTGCGG	0	0	1	0	0	No gene matches found
TGGACTGAAG	0	0	0	0	1	No gene matches found
CGTCTAAAAA	1	0	0	0	0	No gene matches found
CGCACGCCGC	0	0	1	0	0	No gene matches found
ACTCCAACTT	0	0	1	0	0	No gene matches found
TAGGCCGATC	1	0	0	0	0	No gene matches found
ACACAGCCTC	0	1	0	0	0	No gene matches found
GCACCAAGTG	0	1	0	0	0	No gene matches found
CTGAGAGGGT	0	0	0	1	0	No gene matches found
CTTGTAATTA	0	0	0	1	0	No gene matches found
GATGTGGAGC	0	1	0	0	0	No gene matches found
AGGGGGATCT	0	0	0	0	1	No gene matches found
AAGTGGTCAG	0	1	0	0	0	No gene matches found
CTCGGAGCCT	0	0	0	1	0	No gene matches found
GTAACCTGGA	0	0	0	0	1	No gene matches found
TTGCGCAAAC	0	0	1	0	0	No gene matches found
AGCATAATCA	0	0	0	0	1	No gene matches found
GAGGAATAAG	0	1	0	0	0	No gene matches found
AGTAGTTATG	0	0	0	0	1	No gene matches found
GTGGTGTATT	0	1	0	0	0	No gene matches found
GATAATCCGT	1	0	0	0	0	No gene matches found
CCTTTGATAT	0	0	0	0	1	No gene matches found
CAAGAGTGAT	0	0	0	1	0	No gene matches found
GGTGTAATCA	0	0	0	0	1	No gene matches found
TAACCGCCCT	0	1	0	0	0	No gene matches found
CTGTACCAGA	0	0	0	0	1	No gene matches found
CCTCTTTTAA	0	0	1	0	0	No gene matches found
GATCTCCGTT	0	0	1	0	0	No gene matches found
GGGAAGGCTG	0	1	0	0	0	No gene matches found
AGCAGAGGAG	1	0	0	0	0	No gene matches found
ATGATTCGCG	1	0	0	0	0	No gene matches found
GCTTAGAGTC	0	0	0	0	1	No gene matches found
CCCTAATGTA	0	0	1	0	0	No gene matches found
CCTGTCAAGA	0	0	0	1	0	No gene matches found
CGATGCGATA	0	0	1	0	0	No gene matches found
CGAGCTCTTA	0	0	0	1	0	No gene matches found
CTCGACGATG	0	0	1	0	0	No gene matches found
TCCGATGCTA	1	0	0	0	0	No gene matches found
ACCCTTCAGA	0	0	1	0	0	No gene matches found
TTTGCACGGA	0	0	0	1	0	No gene matches found
AATGTTCGCC	1	0	0	0	0	No gene matches found
TGGATCTTCC	1	0	0	0	0	No gene matches found
CGAAGTGTCC	0	0	0	1	0	No gene matches found
GGAATTCCAC	0	0	0	1	0	No gene matches found

TATGCGAGAA	0	1	0	0	0	No gene matches found
CTCGGAGCGA	0	0	1	0	0	No gene matches found
TAAGCCAGCA	1	0	0	0	0	No gene matches found
CGAAAGAATA	0	0	0	0	1	No gene matches found
GGGAGGCAAA	0	1	0	0	0	No gene matches found
TCGAACACCG	0	0	0	1	0	No gene matches found
GTGGGCTGTC	0	1	0	0	0	No gene matches found
ACAGGTGGAG	0	1	0	0	0	No gene matches found
AGACTCTATG	0	0	1	0	0	No gene matches found
CTGTCAGTGG	0	0	1	0	0	No gene matches found
ATTGCCAGAG	1	0	0	0	0	No gene matches found
AGTGGATTGG	0	0	0	0	1	No gene matches found
TCCGCAGCCG	1	0	0	0	0	No gene matches found
CCCCAGAGAA	1	0	0	0	0	No gene matches found
CAGCGCTCAA	0	1	0	0	0	No gene matches found
TTTCGGACAA	1	0	0	0	0	No gene matches found
TCCTTGTATG	0	0	1	0	0	No gene matches found
GGTATGCTGC	0	1	0	0	0	No gene matches found
ACGCCACGAG	0	1	0	0	0	No gene matches found
GACAGCCCTT	0	0	0	0	1	No gene matches found
CCTAGGATCT	0	0	1	0	0	No gene matches found
ACCACTGGCC	1	0	0	0	0	No gene matches found
CCGCGGCTGT	0	0	0	1	0	No gene matches found
GTGACGCGAC	0	0	0	1	0	No gene matches found
TAAATTCCGG	0	0	1	0	0	No gene matches found
CGTTGTTGCA	0	0	1	0	0	No gene matches found
TCAACCGTCA	0	0	0	1	0	No gene matches found
ATCGCCCAAG	0	0	0	1	0	No gene matches found
TCTCCTGTTG	1	0	0	0	0	No gene matches found
AATGACTCGG	0	0	0	1	0	No gene matches found
AGGTCGACCT	1	0	0	0	0	No gene matches found
TCCGATCTTT	1	0	0	0	0	No gene matches found
AAATTTGCGC	0	1	0	0	0	No gene matches found
TACGATAAAT	0	0	0	1	0	No gene matches found
GCGAGGTTTA	0	0	0	0	1	No gene matches found
AGGCGTGCGC	0	0	1	0	0	No gene matches found
ATGTCAGCTA	1	0	0	0	0	No gene matches found
CGAGCGAAGG	1	0	0	0	0	No gene matches found
GATCTATGAG	0	0	0	0	1	No gene matches found
TGGGTCGATA	0	0	0	1	0	No gene matches found
ACGTGATTAT	0	1	0	0	0	No gene matches found
CCAAAGTGAC	0	0	0	1	0	No gene matches found
AAATAGCTCA	0	1	0	0	0	No gene matches found
CTTCTGAGGA	0	0	0	1	0	No gene matches found
AGTGTACGTT	0	0	0	0	1	No gene matches found
CGTGTGATGG	1	0	0	0	0	No gene matches found
CTAGTGCTCC	0	0	1	0	0	No gene matches found
GACTTGACGG	0	0	1	0	0	No gene matches found
GCTAGATCCG	0	0	0	1	0	No gene matches found
GTGTCAGACA	0	1	0	0	0	No gene matches found
ATGGACCTCG	0	0	0	0	1	No gene matches found
GCACCACAAC	1	0	0	0	0	No gene matches found
GTGACAAATG	0	0	0	0	1	No gene matches found
ACTCTAAAAA	0	0	1	0	0	No gene matches found
GGGATGGTGA	1	0	0	0	0	No gene matches found
ATAAAACGGA	0	1	0	0	0	No gene matches found
TATCGAAGTA	1	0	0	0	0	No gene matches found
ACCTGTGGCA	0	1	0	0	0	No gene matches found
TTACTGTGAT	0	1	0	0	0	No gene matches found
CAGTGGTGGC	1	0	0	0	0	No gene matches found
CAAACGTGTA	0	1	0	0	0	No gene matches found

ATGGTAGGCC	0	0	1	0	0	No gene matches found
CTTTCAGTTG	0	1	0	0	0	No gene matches found
GTTTAACCAG	0	0	0	1	0	No gene matches found
TTCAGAGAGG	0	0	0	1	0	No gene matches found
TGTACTAGGT	0	0	0	0	1	No gene matches found
TATCCGAAGT	0	0	1	0	0	No gene matches found
CGAATTTTCA	0	0	0	1	0	No gene matches found
AGTAGCTCTC	0	0	0	1	0	No gene matches found
GACAAGCTCA	0	1	0	0	0	No gene matches found
GCGAAAACGG	0	0	0	0	1	No gene matches found
GACGTAAGGA	0	1	0	0	0	No gene matches found
TGGTGTTTCC	1	0	0	0	0	No gene matches found
GATAGGCTCG	1	0	0	0	0	No gene matches found
GCGTTGCACT	1	0	0	0	0	No gene matches found
CAATTGGAGT	0	0	1	0	0	No gene matches found
CGATAATCTG	0	0	0	1	0	No gene matches found
CTTGCTTCGG	0	0	0	1	0	No gene matches found
AGTGAAGTAT	0	1	0	0	0	No gene matches found
GTAGGTGTTG	0	1	0	0	0	No gene matches found
GGGTGAGGGC	0	0	1	0	0	No gene matches found
GTCAGGGACT	1	0	0	0	0	No gene matches found
CGTACTTTGT	0	0	1	0	0	No gene matches found
TAAGCCGTGA	1	0	0	0	0	No gene matches found
TCGCGTCTAG	0	0	1	0	0	No gene matches found
GAAGTCAGTT	0	0	0	1	0	No gene matches found
CAACTCTTGC	0	1	0	0	0	No gene matches found
CAGTAGCAGA	0	0	0	0	1	No gene matches found
ACTGTGAGTG	0	1	0	0	0	No gene matches found
GGAGTGGACA	0	0	1	0	0	No gene matches found
GGCTTATCAC	0	1	0	0	0	No gene matches found
GGGGAAAGGA	0	0	0	0	1	No gene matches found
TATCAACGCT	0	0	0	0	1	No gene matches found
CAGTGTGGCC	0	1	0	0	0	No gene matches found
CCTGTTTATT	0	1	0	0	0	No gene matches found
TGAAAACCGT	0	0	0	1	0	No gene matches found
ATTAGGAATG	0	0	1	0	0	No gene matches found
GAAGGACGAG	0	0	0	1	0	No gene matches found
TTTGTGGAGC	0	1	0	0	0	No gene matches found
GTTGTTGAAA	0	0	1	0	0	No gene matches found
TCCCTTTATG	0	0	0	0	1	No gene matches found
CCTCCAGTAG	0	1	0	0	0	No gene matches found
TGCGTACCGT	0	0	1	0	0	No gene matches found
GGACAGATCT	0	0	1	0	0	No gene matches found
TAGTCTGCTA	0	0	0	1	0	No gene matches found
GATGCGATGG	0	0	0	1	0	No gene matches found
GCTAAATGGA	0	0	0	1	0	No gene matches found
ACACTGTATA	1	0	0	0	0	No gene matches found
CGCTCCCAAA	0	1	0	0	0	No gene matches found
TCACCAGGAC	0	1	0	0	0	No gene matches found
TTGCCACAAA	0	0	1	0	0	No gene matches found
TGGGGTCAGA	0	0	1	0	0	No gene matches found
ATTGGGAATG	0	0	1	0	0	No gene matches found
TATGATGTGG	1	0	0	0	0	No gene matches found
GAGGTGGGGA	0	0	0	1	0	No gene matches found
CGTTTAATTA	0	1	0	0	0	No gene matches found
GTAACAGGTG	0	0	0	0	1	No gene matches found
GAGCTGTGCG	0	1	0	0	0	No gene matches found
GGTATGCTCG	0	1	0	0	0	No gene matches found
GGGAGTGAAT	0	0	1	0	0	No gene matches found
CCCGGCCTAA	1	0	0	0	0	No gene matches found

GTAGAACTTT	1	0	0	0	0	No gene matches found
CCAAGCTCTA	0	1	0	0	0	No gene matches found
CTTGTGATGT	0	1	0	0	0	No gene matches found
GGCCTCATAA	0	1	0	0	0	No gene matches found
GCGACAACAG	0	1	0	0	0	No gene matches found
GATCCCCAGT	1	0	0	0	0	No gene matches found
CTTTCAAAGG	0	1	0	0	0	No gene matches found
CCAGCTTATT	0	1	0	0	0	No gene matches found
TCAATGTTGT	0	0	1	0	0	No gene matches found
TTACGGATCC	0	0	1	0	0	No gene matches found
CACCATATGG	0	0	1	0	0	No gene matches found
TCTATAGGCT	0	0	1	0	0	No gene matches found
TCGTAGACGT	1	0	0	0	0	No gene matches found
CACTGCGGTT	0	1	0	0	0	No gene matches found
TGTTGCCAAT	0	1	0	0	0	No gene matches found
GTGGTATACA	0	0	0	1	0	No gene matches found
GGCTGAGTGA	0	0	0	1	0	No gene matches found
GGAAGGTACG	0	1	0	0	0	No gene matches found
GTAGGAAACC	0	1	0	0	0	No gene matches found
TAAAATCCTG	0	0	1	0	0	No gene matches found
GCAAATCCAG	1	0	0	0	0	No gene matches found
GGGGAACTGA	0	0	1	0	0	No gene matches found
CTCCCTGATC	1	0	0	0	0	No gene matches found
ACGTTTTTGA	0	1	0	0	0	No gene matches found
TGTAGGTAAG	1	0	0	0	0	No gene matches found
CACTTCTTCA	0	0	1	0	0	No gene matches found
GAGGATGGGG	0	1	0	0	0	No gene matches found
GGCAAAACCG	0	0	0	0	1	No gene matches found
AGTAAAGCTC	0	0	1	0	0	No gene matches found
ACAAAGAGGC	0	0	0	0	1	No gene matches found
AACTTGGTGC	0	0	1	0	0	No gene matches found
GGAGCCTGTT	0	0	0	1	0	No gene matches found
TAGCTTATTA	0	1	0	0	0	No gene matches found
TGGCAACGGT	0	0	0	1	0	No gene matches found
TGTACTTGGA	0	0	1	0	0	No gene matches found
TTGGACTTGA	0	0	0	1	0	No gene matches found
GGTCTGGATC	0	1	0	0	0	No gene matches found
GGTAGCAGGG	0	1	0	0	0	No gene matches found
GTTGTTAAAA	0	0	0	0	1	No gene matches found
GCGTTCGTCC	0	0	1	0	0	No gene matches found
ATTACGAGGC	0	1	0	0	0	No gene matches found
GCGGAGTTGC	0	0	0	0	1	No gene matches found
ATGTTTGTCA	0	0	0	0	1	No gene matches found
ACCGATCCAT	0	1	0	0	0	No gene matches found
ACGGCTGGTT	0	1	0	0	0	No gene matches found
AGACCGAGCT	0	0	1	0	0	No gene matches found
TTCCGGTGCT	0	1	0	0	0	No gene matches found
GCAGGTGGTG	0	0	0	1	0	No gene matches found
CACCCGAAAA	0	0	0	1	0	No gene matches found
AGGTTGAGAG	0	1	0	0	0	No gene matches found
TACCAAGTGT	1	0	0	0	0	No gene matches found
TGGAATAGTA	0	0	0	0	1	No gene matches found
TCCGAATCTC	0	1	0	0	0	No gene matches found
TATTTCAGTC	0	0	0	0	1	No gene matches found
TATAACTTGG	0	0	1	0	0	No gene matches found
CGCTTACCGT	0	0	0	1	0	No gene matches found
CTCTTCGGTA	0	1	0	0	0	No gene matches found
GAATTCGGAC	0	0	1	0	0	No gene matches found
GATGTCGAAC	0	0	1	0	0	No gene matches found
TGTGATGGTT	0	1	0	0	0	No gene matches found
TAAACTACTG	0	0	0	1	0	No gene matches found

GCGCTAGACA	0	0	0	1	0	No gene matches found
CAACTCAGTT	0	0	0	1	0	No gene matches found
GGTATGGAAG	1	0	0	0	0	No gene matches found
GGGCGTTTGT	0	0	0	1	0	No gene matches found
TCCACCTATG	1	0	0	0	0	No gene matches found
CAAGAGGAGT	0	1	0	0	0	No gene matches found
GCCTCTCCAG	0	1	0	0	0	No gene matches found
TAGGCTCCTT	0	0	0	1	0	No gene matches found
GTTACCGCAC	0	0	0	0	1	No gene matches found
CTCTCAGCTC	0	1	0	0	0	No gene matches found
ACATCCGTGA	0	1	0	0	0	No gene matches found
TCTCAGACGG	0	0	0	1	0	No gene matches found
CAGTGACCTA	0	1	0	0	0	No gene matches found
GCCCATCTCA	0	0	1	0	0	No gene matches found
TGAGATCGTT	0	0	0	0	1	No gene matches found
AGGTGGGGGG	0	0	1	0	0	No gene matches found
CCCTGCTTGT	0	0	0	1	0	No gene matches found
TGGCTGTGTT	0	1	0	0	0	No gene matches found
GCTCAAGGGG	0	0	0	1	0	No gene matches found
CGAGCTTCTG	0	1	0	0	0	No gene matches found
GAATTCGTAC	0	0	0	1	0	No gene matches found
AATCGTTATG	0	0	0	0	1	No gene matches found
CGATTTGTGT	0	0	0	0	1	No gene matches found
TCTAAGGGAG	0	0	0	1	0	No gene matches found
GGTTACCGAA	0	0	0	0	1	No gene matches found
AGTCCGGCCA	1	0	0	0	0	No gene matches found
ATCGGACCGT	0	0	0	1	0	No gene matches found
CTCATATTAG	1	0	0	0	0	No gene matches found
AGGGAAAGTG	0	0	1	0	0	No gene matches found
GGTCGTGCCA	0	1	0	0	0	No gene matches found
CCCGTGCGTA	0	0	0	0	1	No gene matches found
GGTCCATACA	0	1	0	0	0	No gene matches found
TGTGATAAAG	0	0	0	1	0	No gene matches found
TATGAGTACG	0	0	0	0	1	No gene matches found
ATTTGACAAA	0	0	0	0	1	No gene matches found
TAGCTACAAC	1	0	0	0	0	No gene matches found
GGGGAGTTGA	0	0	0	1	0	No gene matches found
AGGGGTGTTC	0	0	0	0	1	No gene matches found
TTGGAAAACC	0	1	0	0	0	No gene matches found
TTCAGTCAGC	0	0	0	1	0	No gene matches found
CGTTCCAAAG	0	1	0	0	0	No gene matches found
CTGTCGAGAT	0	0	0	1	0	No gene matches found
AGAATGAGGA	0	0	0	0	1	No gene matches found
GGAATCTCTC	1	0	0	0	0	No gene matches found
ATTAAAGCCT	0	1	0	0	0	No gene matches found
TTAAAGCGGA	0	1	0	0	0	No gene matches found
GATAGGTCCG	0	1	0	0	0	No gene matches found
GCAATCTAGG	0	1	0	0	0	No gene matches found
GTATCAAAAA	0	1	0	0	0	No gene matches found
AGGATCCATC	0	0	1	0	0	No gene matches found
GCCATCGTGT	1	0	0	0	0	No gene matches found
AAGCAAAAGC	0	0	1	0	0	No gene matches found
ATTCACCCAT	1	0	0	0	0	No gene matches found
CGCTCATCGA	0	0	1	0	0	No gene matches found
CGTTTCTTAC	0	0	0	1	0	No gene matches found
AGACTGCTGA	1	0	0	0	0	No gene matches found
AATAAGTGTT	1	0	0	0	0	No gene matches found
GGTGCTGTTG	0	1	0	0	0	No gene matches found
GGCGAATCTT	0	0	0	0	1	No gene matches found
GATCGGGCTA	0	0	0	0	1	No gene matches found
AGGTGTGGTT	0	1	0	0	0	No gene matches found

CCAATAGAAG	0	0	1	0	0	No gene matches found
ATTTGCTGGC	0	0	0	1	0	No gene matches found
CAGGATAAGT	0	0	0	0	1	No gene matches found
GCTTGGATAG	0	1	0	0	0	No gene matches found
GATATCTGCC	0	1	0	0	0	No gene matches found
CAGGGCGAAA	0	1	0	0	0	No gene matches found
AAACAGGGTT	0	1	0	0	0	No gene matches found
ATATAACGGA	0	0	1	0	0	No gene matches found
GGGGCTAAAA	1	0	0	0	0	No gene matches found
GGTTCATAAA	0	0	0	0	1	No gene matches found
GCTCGGAGCC	0	1	0	0	0	No gene matches found
GAATGGATGT	0	0	0	1	0	No gene matches found
CTTCAGTGGA	0	1	0	0	0	No gene matches found
AGCTTATACT	0	0	0	0	1	No gene matches found
GGGAATAACC	0	0	0	0	1	No gene matches found
CTTCTGGCAC	0	1	0	0	0	No gene matches found
CAGTCGTCTA	0	0	0	1	0	No gene matches found
CCCGAGACGT	0	0	0	0	1	No gene matches found
TTCTATAAGT	1	0	0	0	0	No gene matches found
GATTGGATCG	0	1	0	0	0	No gene matches found
GCAATCCTAC	1	0	0	0	0	No gene matches found
GCCCCCTACA	0	0	0	1	0	No gene matches found
GCTCTGCCTC	0	1	0	0	0	No gene matches found
ACGGCGATTA	0	1	0	0	0	No gene matches found
GGTCATAGCA	0	1	0	0	0	No gene matches found
GAGAGATTGC	0	0	1	0	0	No gene matches found
CTTGCGCATC	0	0	0	1	0	No gene matches found
ATCTGGGCAC	0	0	0	1	0	No gene matches found
TAACCTGTGC	1	0	0	0	0	No gene matches found
GACACTTTAG	0	0	1	0	0	No gene matches found
TCCGAAGATT	0	0	0	1	0	No gene matches found
TTTCTAGTCT	0	1	0	0	0	No gene matches found
ATATTCGGTA	0	1	0	0	0	No gene matches found
GCCGGCAACG	0	1	0	0	0	No gene matches found
GTCTCTCCGG	0	0	1	0	0	No gene matches found
AACTCCCGGG	0	0	0	1	0	No gene matches found
GAGTTTACGG	0	0	1	0	0	No gene matches found
TAGGACTACG	1	0	0	0	0	No gene matches found
CGTGGAGTTA	0	1	0	0	0	No gene matches found
GGGTCTTGGT	0	0	0	1	0	No gene matches found
GTGGCGTAAG	0	0	0	1	0	No gene matches found
GCCACTGACC	0	0	0	1	0	No gene matches found
CTAACTTTTC	1	0	0	0	0	No gene matches found
GTCGAAACGA	0	1	0	0	0	No gene matches found
GGAGGTCTCG	0	0	0	1	0	No gene matches found
AAAGCTCGGT	0	0	0	0	1	No gene matches found
ACCGAATCAA	0	0	0	1	0	No gene matches found
TGGTCAGATG	0	0	0	1	0	No gene matches found
CAGATGTGGC	0	0	0	1	0	No gene matches found
CCTAGATTGA	0	1	0	0	0	No gene matches found
ATGGTCTTAA	0	0	0	1	0	No gene matches found
GGCGAACGCC	0	0	0	0	1	No gene matches found
GATAAATTAA	0	0	1	0	0	No gene matches found
GGCCTTCAAC	0	0	0	1	0	No gene matches found
TTTGACCCGA	0	0	1	0	0	No gene matches found
CTAAGGAGTA	0	0	0	1	0	No gene matches found
CTTTTTAGGG	0	1	0	0	0	No gene matches found
ACAGCCCTGT	0	1	0	0	0	No gene matches found
AGACGCAGGA	0	0	1	0	0	No gene matches found
CCGTCTCTCC	0	1	0	0	0	No gene matches found
ACAACGTCCA	1	0	0	0	0	No gene matches found

TTTGCCTTAA	1	0	0	0	0	No gene matches found
CCATTGCGTT	0	0	0	1	0	No gene matches found
GAGATCGTTT	0	0	1	0	0	No gene matches found
TGGTAAAGGT	1	0	0	0	0	No gene matches found
GCAGAGTTGA	0	0	1	0	0	No gene matches found
ACCACTCGAA	0	0	1	0	0	No gene matches found
GCCAGAAACC	0	1	0	0	0	No gene matches found
GGGCCGAAGC	0	0	0	1	0	No gene matches found
TGGATTATGG	0	1	0	0	0	No gene matches found
CGTGCAAATC	0	0	0	1	0	No gene matches found
GCTGGTATTA	0	0	0	0	1	No gene matches found
GAGCTTTCGA	0	1	0	0	0	No gene matches found
TTTCGCAACG	0	1	0	0	0	No gene matches found
TTTCCTGTTT	0	0	1	0	0	No gene matches found
TCCGCTGGCT	1	0	0	0	0	No gene matches found
CTGATTTGGG	0	1	0	0	0	No gene matches found
TGAGACTTGA	0	0	0	0	1	No gene matches found
AAGTAGGTCA	0	1	0	0	0	No gene matches found
GCGAAACCTG	1	0	0	0	0	No gene matches found
GCTATACGAG	1	0	0	0	0	No gene matches found
GGCACCGCGA	0	0	1	0	0	No gene matches found
CCCGAGAAAC	1	0	0	0	0	No gene matches found
TTGCGCTATA	0	0	0	1	0	No gene matches found
GATACTGACG	1	0	0	0	0	No gene matches found
AAGCTTTACG	0	0	0	1	0	No gene matches found
TTTGCACAAA	0	0	0	0	1	No gene matches found
GAAACTGCGG	0	0	1	0	0	No gene matches found
AGAGTAGTTA	0	0	0	0	1	No gene matches found
GGTTACCTTT	0	1	0	0	0	No gene matches found
CGAGATCTAG	0	1	0	0	0	No gene matches found
CAGCCTGTTA	1	0	0	0	0	No gene matches found
TAAATAAGTA	0	1	0	0	0	No gene matches found
CACGGTCTTG	1	0	0	0	0	No gene matches found
CGGTGATTGT	0	1	0	0	0	No gene matches found
GGCAGCACCG	0	1	0	0	0	No gene matches found
GTGACATAGG	1	0	0	0	0	No gene matches found
AAAGGGAGGA	0	1	0	0	0	No gene matches found
ATTCGGAAAG	0	0	0	1	0	No gene matches found
GAACATCGAA	0	0	0	1	0	No gene matches found
CAACGCCGGT	0	0	0	1	0	No gene matches found
GGCTGTGCCC	0	0	1	0	0	No gene matches found
GTCCAAGCCA	0	0	1	0	0	No gene matches found
GCTACTAATT	0	1	0	0	0	No gene matches found
TTCTGAGTTT	1	0	0	0	0	No gene matches found
GAGATTCCTA	0	1	0	0	0	No gene matches found
AGCTTTTTCG	0	0	1	0	0	No gene matches found
CGATCAATAG	0	1	0	0	0	No gene matches found
TGCGCGTGAT	0	0	0	0	1	No gene matches found
ACCGAACTCT	0	0	0	1	0	No gene matches found
CCGAGACGGT	0	1	0	0	0	No gene matches found
AATATTACAT	0	0	0	0	1	No gene matches found
GACGACCGAG	0	0	0	0	1	No gene matches found
GATTGGGTGT	0	0	1	0	0	No gene matches found
CGAAATTGTA	0	0	0	1	0	No gene matches found
GGTTCAGATA	0	0	0	1	0	No gene matches found
GGGCTGAGTT	0	0	0	1	0	No gene matches found
TGGAAGGTTC	0	0	0	1	0	No gene matches found
CCTGGCAACG	0	1	0	0	0	No gene matches found
CCTTTGGGTG	0	1	0	0	0	No gene matches found
GTAATAGTAC	0	0	0	0	1	No gene matches found

GCGGAATCAT	0	0	0	0	1	No gene matches found
CTACTAAAAG	0	0	1	0	0	No gene matches found
CACTTTGTAT	1	0	0	0	0	No gene matches found
TTCCCTATGT	0	1	0	0	0	No gene matches found
GAACAGTTGC	0	1	0	0	0	No gene matches found
GGATTGTTTC	0	1	0	0	0	No gene matches found
AGCTCGAATT	0	0	0	1	0	No gene matches found
CATAACCTAT	0	0	0	0	1	No gene matches found
CGAGATCACT	1	0	0	0	0	No gene matches found
CCATTGGCTA	0	1	0	0	0	No gene matches found
CGTTCACTAC	0	1	0	0	0	No gene matches found
AGAGCTTGGA	0	1	0	0	0	No gene matches found
AGCAAGTTTG	0	0	1	0	0	No gene matches found
CGTGTTTAAA	0	0	1	0	0	No gene matches found
TGTTCGCAAA	0	0	0	0	1	No gene matches found
CCCCGCCGCC	0	0	0	1	0	No gene matches found
TCGCTACGGC	0	0	1	0	0	No gene matches found
CAGTTTCAGT	0	0	0	0	1	No gene matches found
GTAAAGTCGA	0	1	0	0	0	No gene matches found
AGAGTTTTAC	0	0	0	1	0	No gene matches found
ATCGAGTTGA	0	1	0	0	0	No gene matches found
TCGGTATCTC	0	0	1	0	0	No gene matches found
TCTTACTTCA	0	0	0	0	1	No gene matches found
CAAATGCTTT	0	0	0	1	0	No gene matches found
GGGGGCTTTC	0	0	0	0	1	No gene matches found
ACGCACACTG	0	0	0	1	0	No gene matches found
GACTTAAAAG	0	1	0	0	0	No gene matches found
TTCCCACAGT	0	0	0	1	0	No gene matches found
GACAATCTGG	1	0	0	0	0	No gene matches found
TATGTTGGCC	0	1	0	0	0	No gene matches found
GTGTTAGCAG	0	0	1	0	0	No gene matches found
TGGTAACGGG	1	0	0	0	0	No gene matches found
GGGCGTTAGC	0	1	0	0	0	No gene matches found
CTCGGGGACC	0	1	0	0	0	No gene matches found
TGGACAGATC	0	0	0	1	0	No gene matches found
GGATTGTTTG	0	0	1	0	0	No gene matches found
CCACAGTGAG	0	0	1	0	0	No gene matches found
GTTAATATCC	0	0	0	1	0	No gene matches found
AAGACACGCG	0	1	0	0	0	No gene matches found
TAGCGAGTAT	0	1	0	0	0	No gene matches found
AATATAGGGG	0	0	0	1	0	No gene matches found
GTCACAGGCG	1	0	0	0	0	No gene matches found
TTTAACCTTA	1	0	0	0	0	No gene matches found
GACAAATTCG	0	0	0	1	0	No gene matches found
CACCGAATTC	0	1	0	0	0	No gene matches found
AACCAGGCCA	0	0	1	0	0	No gene matches found
ATGGGCCTAC	0	1	0	0	0	No gene matches found
TAAGGTAGAA	0	0	0	0	1	No gene matches found
AAGAATCACG	0	0	1	0	0	No gene matches found
TTATCTCTGA	1	0	0	0	0	No gene matches found
AGTATGGCTT	0	0	1	0	0	No gene matches found
ACCTCGGTTT	0	1	0	0	0	No gene matches found
ACGGGAGAGA	0	0	1	0	0	No gene matches found
CTCGGAGCAC	0	0	0	0	1	No gene matches found
TCCTATCGTG	0	1	0	0	0	No gene matches found
CACTCTCCGA	0	1	0	0	0	No gene matches found
AAAGGATAGA	0	0	1	0	0	No gene matches found
TTTGCGACGC	0	1	0	0	0	No gene matches found
CATAATCTGG	0	1	0	0	0	No gene matches found
GCGGTTGTAA	0	1	0	0	0	No gene matches found
TGAAGCTGCC	0	0	1	0	0	No gene matches found

TTCCGAATTC	0	0	1	0	0	No gene matches found
AGACCGTGGG	1	0	0	0	0	No gene matches found
CTGTCGTGGA	0	0	0	0	1	No gene matches found
CGGTCCTATA	0	1	0	0	0	No gene matches found
TCCAGAATGT	0	1	0	0	0	No gene matches found
GGAGCAGAAA	1	0	0	0	0	No gene matches found
GTCTTCCGTT	0	0	0	0	1	No gene matches found
CGGATCTTGA	0	0	0	1	0	No gene matches found
CGGCACATAG	0	1	0	0	0	No gene matches found
ATTTAACTGT	0	1	0	0	0	No gene matches found
AGAGTGACAT	0	1	0	0	0	No gene matches found
TTTGGGGTCC	0	0	1	0	0	No gene matches found
GGAGGGACAA	1	0	0	0	0	No gene matches found
TTCCAGGAAA	0	0	1	0	0	No gene matches found
CTTTATGTAC	0	1	0	0	0	No gene matches found
CCTCAAGAGA	0	1	0	0	0	No gene matches found
TTGCAGTTCA	0	0	1	0	0	No gene matches found
GGCGGCCGCA	0	1	0	0	0	No gene matches found
GACGAACCGA	1	0	0	0	0	No gene matches found
AGTGTGATAA	1	0	0	0	0	No gene matches found
ATGGAAGCAA	1	0	0	0	0	No gene matches found
GGGGTATCAA	0	0	1	0	0	No gene matches found
TCTGAGAGAC	0	1	0	0	0	No gene matches found
TAATCATACT	1	0	0	0	0	No gene matches found
AGTGGGCTTT	0	0	1	0	0	No gene matches found
TGCACCCTCA	1	0	0	0	0	No gene matches found
CGGTCAAAAA	0	0	1	0	0	No gene matches found
CCCGACCAGC	0	0	1	0	0	No gene matches found
GTGTCACATT	0	1	0	0	0	No gene matches found
TTCCCGCTAA	0	1	0	0	0	No gene matches found
CTGTGTGAGT	1	0	0	0	0	No gene matches found
CGTATGGAAT	0	0	0	1	0	No gene matches found
CATAAGCTCA	0	0	1	0	0	No gene matches found
TTCAGAGACG	0	0	0	1	0	No gene matches found
ATTCCGAGCA	0	0	0	1	0	No gene matches found
GGCCAAACGA	1	0	0	0	0	No gene matches found
TGTGGAAAAC	1	0	0	0	0	No gene matches found
GGTTAGCTTC	0	1	0	0	0	No gene matches found
AAGGTGGGGC	0	1	0	0	0	No gene matches found
AGAACGCCAG	0	0	0	0	1	No gene matches found
ATGTTCGGAA	0	0	0	1	0	No gene matches found
AGGTCCATCG	0	0	1	0	0	No gene matches found
AGGGACTACG	0	1	0	0	0	No gene matches found
GCGCATTCAA	1	0	0	0	0	No gene matches found
TAGAGACAAG	0	0	0	0	1	No gene matches found
CGTCATCGGT	0	0	0	0	1	No gene matches found
GGCGAAAGAT	0	0	1	0	0	No gene matches found
TCATTAAATC	0	0	0	1	0	No gene matches found
ACTGATTCGG	0	0	0	1	0	No gene matches found
GGCTGTTCTT	1	0	0	0	0	No gene matches found
GCAAAGGTCC	0	0	1	0	0	No gene matches found
CTTCATAGAG	0	0	1	0	0	No gene matches found
TAAAGAGCCG	0	0	0	0	1	No gene matches found
ATCTTGCCTT	0	1	0	0	0	No gene matches found
AGACTTGGGG	0	1	0	0	0	No gene matches found
AGGCAGACGA	0	0	0	1	0	No gene matches found
TGAACTACTT	0	0	0	1	0	No gene matches found
GCACCAGGAA	0	1	0	0	0	No gene matches found
GCCTATGATG	0	1	0	0	0	No gene matches found
AAGGAGTGGC	0	1	0	0	0	No gene matches found
TTCGGTACAC	1	0	0	0	0	No gene matches found

AGCGAGCGTT	1	0	0	0	0	No gene matches found
CTTTAGTGGA	0	1	0	0	0	No gene matches found
GGACAAACCA	0	1	0	0	0	No gene matches found
GGGTGGAATG	0	0	1	0	0	No gene matches found
TGCGCCAACC	1	0	0	0	0	No gene matches found
GCACAGATCT	0	0	0	1	0	No gene matches found
AGAACGTGTA	0	1	0	0	0	No gene matches found
AGGAGCGCAC	1	0	0	0	0	No gene matches found
TTCGGGTCGA	0	0	1	0	0	No gene matches found
AAGTCTGGGG	0	0	1	0	0	No gene matches found
TGCTACTCCG	0	1	0	0	0	No gene matches found
ATGTGTTTCT	0	0	0	1	0	No gene matches found
TCAATGCCTC	0	0	0	1	0	No gene matches found
GCTATTGGAT	1	0	0	0	0	No gene matches found
GGTCTAAGTG	0	0	0	0	1	No gene matches found
TATGGTGGGA	0	0	0	1	0	No gene matches found
AACGGTGTTG	0	0	1	0	0	No gene matches found
GGAGGGGATG	1	0	0	0	0	No gene matches found
CCTAAGTCGA	1	0	0	0	0	No gene matches found
CGTTGTGGAG	0	0	1	0	0	No gene matches found
GCAGGGTTGC	1	0	0	0	0	No gene matches found
AGACGATTAA	1	0	0	0	0	No gene matches found
TCCACAGTCA	0	0	0	0	1	No gene matches found
TTGCGTTTCT	0	1	0	0	0	No gene matches found
TGATTGAGTG	1	0	0	0	0	No gene matches found
TCTAACACCA	0	0	1	0	0	No gene matches found
TTTTAGTGGG	0	0	1	0	0	No gene matches found
ATGTTGGATC	0	0	0	0	1	No gene matches found
ATGTAATGCT	1	0	0	0	0	No gene matches found
TTTCGTGTCT	0	1	0	0	0	No gene matches found
AGATGCGATG	1	0	0	0	0	No gene matches found
CGGTAGTCAC	0	1	0	0	0	No gene matches found
AGGAGGGCGA	0	0	0	0	1	No gene matches found
TTACAAGCCC	0	0	1	0	0	No gene matches found
GAGGTGCCCA	0	1	0	0	0	No gene matches found
ACCATTCGGG	0	0	1	0	0	No gene matches found
TCGTATCTGT	0	1	0	0	0	No gene matches found
CTGGCGATTG	1	0	0	0	0	No gene matches found
GGGAGAGATC	0	1	0	0	0	No gene matches found
GTTATGCCTT	0	0	1	0	0	No gene matches found
CTCGGCCATT	0	0	1	0	0	No gene matches found
TAAAACTGTA	1	0	0	0	0	No gene matches found
ACCTTTATGA	0	0	0	1	0	No gene matches found
CCCGGATCTG	0	0	1	0	0	No gene matches found
CACCGCCTTG	0	0	0	1	0	No gene matches found
CCTATTTCAA	0	0	0	0	1	No gene matches found
GTTCCGAGAT	0	1	0	0	0	No gene matches found
TTGGCTCTAA	0	0	1	0	0	No gene matches found
CTTTACTTTC	0	0	1	0	0	No gene matches found
CTGTACTAAG	0	0	0	0	1	No gene matches found
GCCCTTCGCT	0	0	0	1	0	No gene matches found
GACACGAGAC	0	1	0	0	0	No gene matches found
CCTTCGTGGT	0	1	0	0	0	No gene matches found
AGGTTGTAAA	0	0	1	0	0	No gene matches found
TACTTCTGTA	0	1	0	0	0	No gene matches found
GGAGCGGTTG	0	1	0	0	0	No gene matches found
TCTTCGATTG	0	0	0	0	1	No gene matches found
AATTGGGGCA	0	0	1	0	0	No gene matches found
TCTGTCGAAA	0	0	1	0	0	No gene matches found
TGGTCTTGAG	0	0	1	0	0	No gene matches found
TCATCTCACA	0	0	1	0	0	No gene matches found

CCTTCCAGCT	0	1	0	0	0	No gene matches found
GCTCGATATC	1	0	0	0	0	No gene matches found
TGTGTAGACC	1	0	0	0	0	No gene matches found
CCGCTCTTAT	1	0	0	0	0	No gene matches found
CAGTGCCACT	0	1	0	0	0	No gene matches found
CTAGGGACAG	0	1	0	0	0	No gene matches found
TTTTTGAGGC	0	0	1	0	0	No gene matches found
ACTGCGCAGG	0	0	0	0	1	No gene matches found
TAAAGACCGA	0	0	1	0	0	No gene matches found
TCGAAGTGCC	0	0	0	0	1	No gene matches found
TTTCCGTGTT	0	0	0	1	0	No gene matches found
GACGCCTCCT	0	0	1	0	0	No gene matches found
CAGGTGTGTG	0	1	0	0	0	No gene matches found
AGGTTGTTTT	0	0	0	0	1	No gene matches found
TGCCTCGCCA	0	0	0	1	0	No gene matches found
ATTCGTCAAA	0	0	1	0	0	No gene matches found
TGGAAGCTTC	0	0	0	1	0	No gene matches found
ATTGCGATGC	0	1	0	0	0	No gene matches found
TTTCGTTCCA	0	1	0	0	0	No gene matches found
TCTGGACCAG	0	0	0	1	0	No gene matches found
AGGGGTATCT	0	0	1	0	0	No gene matches found
GGCTCTCCAG	0	0	0	1	0	No gene matches found
CTGTGGCGAA	0	0	1	0	0	No gene matches found
CTTACCTCCA	0	1	0	0	0	No gene matches found
GCCGTGAACG	0	1	0	0	0	No gene matches found
CCCTTATATA	1	0	0	0	0	No gene matches found
TTGCTTGCAC	0	0	0	1	0	No gene matches found
TCGAACCATA	0	0	0	1	0	No gene matches found
TTCGACTTCG	0	0	1	0	0	No gene matches found
GCTGTTGAGG	0	0	0	0	1	No gene matches found
TTTTCGTATT	0	0	1	0	0	No gene matches found
GCCCGATGGA	0	0	0	1	0	No gene matches found
CCGCAAATCG	0	0	1	0	0	No gene matches found
AGGGTCAACT	0	0	0	1	0	No gene matches found
GTTTTGAGGG	0	0	1	0	0	No gene matches found
GCAGCTTCCC	0	0	1	0	0	No gene matches found
AGCGTGTGTC	0	1	0	0	0	No gene matches found
GGCGAGGTAT	0	1	0	0	0	No gene matches found
ACAGTTCGGA	0	0	1	0	0	No gene matches found
TTTCCCGTCT	0	0	0	0	1	No gene matches found
GACCTTCGCT	0	0	1	0	0	No gene matches found
TTTACTTCAG	1	0	0	0	0	No gene matches found
GATCCGCCAG	0	1	0	0	0	No gene matches found
GTTGAGGTCT	0	0	1	0	0	No gene matches found
TCCTGCTGTG	0	0	0	0	1	No gene matches found
AGCTTATGTA	0	1	0	0	0	No gene matches found
GGAGTTCCGA	0	0	0	1	0	No gene matches found
AGGGCTAAGA	0	0	0	0	1	No gene matches found
AACCCAGGAG	0	1	0	0	0	No gene matches found
GTTTACTGAG	0	1	0	0	0	No gene matches found
CGTGCCAGAA	0	0	0	1	0	No gene matches found
TTAAGACCGG	0	0	0	0	1	No gene matches found
CGCTGAGTGG	0	0	1	0	0	No gene matches found
GAGAGTTTCG	0	0	1	0	0	No gene matches found
GTTAGGACTA	0	0	1	0	0	No gene matches found
CAAGCTCTTT	0	1	0	0	0	No gene matches found
GCAAATGTTG	0	0	1	0	0	No gene matches found
CGTCGTGCCG	0	1	0	0	0	No gene matches found
TTAGAACGGA	0	1	0	0	0	No gene matches found
AATGACAGAT	0	0	0	0	1	No gene matches found

GAAGACCATC	0	0	1	0	0	No gene matches found
AAGGGTTTGG	0	0	0	0	1	No gene matches found
GAGGCAACAC	0	0	1	0	0	No gene matches found
CGACAAGATT	1	0	0	0	0	No gene matches found
ACAAGACGCA	0	0	1	0	0	No gene matches found
ATAGCAAATC	1	0	0	0	0	No gene matches found
GTGGAAAAAA	0	0	0	0	1	No gene matches found
AAGGATGCAA	0	0	0	1	0	No gene matches found
TCTTCGATGT	0	0	1	0	0	No gene matches found
CAGACACTCT	1	0	0	0	0	No gene matches found
CGAGATTCCT	0	0	0	0	1	No gene matches found
GTAGGAATGT	0	0	0	1	0	No gene matches found
GCCCTCGTCC	1	0	0	0	0	No gene matches found
GTGGTCTTAG	0	0	0	1	0	No gene matches found
ATTTGCATTC	1	0	0	0	0	No gene matches found
GCCATACTTA	0	0	0	1	0	No gene matches found
AGCTCGCAGC	0	0	1	0	0	No gene matches found
GTGCGTCCGT	0	0	1	0	0	No gene matches found
TCGCTGTGAA	0	0	0	1	0	No gene matches found
GTGTCACGTG	0	0	1	0	0	No gene matches found
TCTGGCTCCC	0	0	0	1	0	No gene matches found
GGAGATGTGA	1	0	0	0	0	No gene matches found
TTGCAAAGAG	0	0	0	0	1	No gene matches found
CAATTACGAA	0	1	0	0	0	No gene matches found
TCTCAACGAC	0	0	0	0	1	No gene matches found
GGGATGGTTT	0	0	0	0	1	No gene matches found
TGTCATTACT	0	0	0	1	0	No gene matches found
CGTTATAGGC	0	0	0	1	0	No gene matches found
TCTTTCGGAT	0	0	0	0	1	No gene matches found
CCCGGCATTT	0	0	1	0	0	No gene matches found
GTTGAAGGGG	0	1	0	0	0	No gene matches found
ATGGCCTGAG	0	1	0	0	0	No gene matches found
AGTCGAGGCT	0	0	0	1	0	No gene matches found
TTTGGGGTGG	0	1	0	0	0	No gene matches found
CTCGCAGCCT	0	0	0	1	0	No gene matches found
GCTCCTGCTG	0	1	0	0	0	No gene matches found
GTCTGTGCTT	0	1	0	0	0	No gene matches found
GCACGGACAT	0	1	0	0	0	No gene matches found
CAGGTAGTGG	0	0	1	0	0	No gene matches found
CTACTACGGC	0	1	0	0	0	No gene matches found
CACTTGGATC	0	0	0	0	1	No gene matches found
CTAGAAGTTC	0	1	0	0	0	No gene matches found
AAACCCGACC	0	0	0	0	1	No gene matches found
GGACCATACA	0	0	1	0	0	No gene matches found
GTCTCTCGCA	0	0	1	0	0	No gene matches found
TGAGTCAACG	0	0	1	0	0	No gene matches found
TGGCAGACCG	1	0	0	0	0	No gene matches found
AAAACCCGAT	0	0	1	0	0	No gene matches found
TCAAGTAACG	0	0	1	0	0	No gene matches found
TACACGGCGG	0	0	1	0	0	No gene matches found
GACATTTCTG	0	0	0	0	1	No gene matches found
CTGGATGGAT	0	0	0	1	0	No gene matches found
ACGCAAAGTC	0	1	0	0	0	No gene matches found
CAAGCTAATA	0	0	0	1	0	No gene matches found
TTAATGCGCC	0	0	1	0	0	No gene matches found
CCTCTGGGTG	0	1	0	0	0	No gene matches found
ACTGAAGCAT	0	1	0	0	0	No gene matches found
CTACCTAAAA	1	0	0	0	0	No gene matches found
GGTCTAGATG	0	0	1	0	0	No gene matches found
TACTTCGCCG	0	0	0	1	0	No gene matches found
GCTGTTCACG	0	0	0	1	0	No gene matches found

CGACATCAAT	0	0	0	1	0	No gene matches found
GGGAATCTGG	0	0	0	1	0	No gene matches found
TGAAGCTGTA	0	1	0	0	0	No gene matches found
GAGCGGACCG	0	1	0	0	0	No gene matches found
TCTTTAACGA	0	0	0	1	0	No gene matches found
AGAGAAAGGG	0	0	1	0	0	No gene matches found
ACCTCTGCTG	1	0	0	0	0	No gene matches found
GTCTATGCCC	1	0	0	0	0	No gene matches found
TACCAACGAG	0	0	0	1	0	No gene matches found
ACCAGAATGG	1	0	0	0	0	No gene matches found
CGAGCTGTGA	0	0	1	0	0	No gene matches found
TGTGATAACT	0	1	0	0	0	No gene matches found
ACGTCTTTGG	1	0	0	0	0	No gene matches found
GTCCGCACAA	0	1	0	0	0	No gene matches found
GTCCAGGTGC	0	0	1	0	0	No gene matches found
TACCCCTCGG	1	0	0	0	0	No gene matches found
CAACATATTG	0	0	1	0	0	No gene matches found
AGGGGGCTCT	0	0	0	1	0	No gene matches found
CGCCCGCTGC	0	0	0	1	0	No gene matches found
ATAATGCTGA	0	0	1	0	0	No gene matches found
TGGCGACAGT	0	0	0	1	0	No gene matches found
TTTCTAAGCG	1	0	0	0	0	No gene matches found
TTTGAGGGAT	0	0	0	0	1	No gene matches found
TGCTAATCTG	1	0	0	0	0	No gene matches found
CCTATGGAAG	1	0	0	0	0	No gene matches found
GACAACCCGA	1	0	0	0	0	No gene matches found
ACCCAGGCCG	0	0	1	0	0	No gene matches found
GGAAGGAAGG	0	0	0	1	0	No gene matches found
GGGGTCTTGA	0	0	1	0	0	No gene matches found
TGTCTTCTGT	0	1	0	0	0	No gene matches found
GACACCACAA	0	0	1	0	0	No gene matches found
TGCGTCGTAA	0	0	0	0	1	No gene matches found
GCAAGCCCTC	0	0	0	1	0	No gene matches found
AAACCCAAGC	0	1	0	0	0	No gene matches found
CAGATGCGAT	0	0	0	0	1	No gene matches found
GCTGACCAAG	0	0	1	0	0	No gene matches found
ATCAACTTGA	0	0	1	0	0	No gene matches found
CACTGTGGTA	0	0	0	1	0	No gene matches found
ACACTTACCT	1	0	0	0	0	No gene matches found
GACCAATGAT	0	0	0	1	0	No gene matches found
CCGCCGAAGC	0	1	0	0	0	No gene matches found
TCTGTGTTGA	0	0	0	0	1	No gene matches found
GGTGGCGTCC	0	0	1	0	0	No gene matches found
TATCTATCTG	0	0	0	0	1	No gene matches found
AGCATACTTG	0	0	0	1	0	No gene matches found
GTAGTGCACT	0	1	0	0	0	No gene matches found
ATTGTCGATG	0	1	0	0	0	No gene matches found
AGAGTGTAAT	0	0	0	0	1	No gene matches found
CTGGAACTAA	0	1	0	0	0	No gene matches found
TAAAAGTCTG	1	0	0	0	0	No gene matches found
TCAGATGGAG	0	0	0	0	1	No gene matches found
ACGAGTCATT	0	0	0	0	1	No gene matches found
GTTCCCATCG	0	1	0	0	0	No gene matches found
TTGAGCTAGA	1	0	0	0	0	No gene matches found
AATGGCGATC	0	1	0	0	0	No gene matches found
CAGAAGGCGG	0	0	0	1	0	No gene matches found
CTTAATAAAA	0	0	1	0	0	No gene matches found
ACTTGGCTGG	0	0	0	1	0	No gene matches found
GTGGTTAAGA	0	1	0	0	0	No gene matches found
CATCGACTAT	0	0	0	0	1	No gene matches found
TCAAAGGTCT	0	0	0	0	1	No gene matches found

CTGTGTCGGT	0	0	0	1	0	No gene matches found
CTTCTGGCTA	0	0	0	1	0	No gene matches found
CCAGCATTAA	0	0	0	1	0	No gene matches found
ACTACTATGA	0	1	0	0	0	No gene matches found
GGTGAGGTCA	0	1	0	0	0	No gene matches found
CCAGTTAGAA	0	0	0	0	1	No gene matches found
CAAGGTATAG	0	0	0	0	1	No gene matches found
CTGAGCTAGA	0	0	0	1	0	No gene matches found
ACTAATACGA	1	0	0	0	0	No gene matches found
CGCAAATTAA	0	0	1	0	0	No gene matches found
GTACACGGGA	0	0	1	0	0	No gene matches found
GTTTGAGCGA	0	0	0	0	1	No gene matches found
ACGATTATGA	1	0	0	0	0	No gene matches found
GGAATTGGGT	0	0	0	0	1	No gene matches found
CCCGTGCGGA	0	0	1	0	0	No gene matches found
GGTCGCAAAA	1	0	0	0	0	No gene matches found
GTTGCTACTC	1	0	0	0	0	No gene matches found
AAAAGTTCTT	0	1	0	0	0	No gene matches found
CCTCTCACAG	0	0	1	0	0	No gene matches found
ACCTTTGACG	0	0	1	0	0	No gene matches found
CTCGAATGAC	0	0	0	1	0	No gene matches found
TTTGCCCAAA	0	1	0	0	0	No gene matches found
AAATGGAAGT	0	0	1	0	0	No gene matches found
ATAATGAGTG	0	0	0	0	1	No gene matches found
AAATCTTACC	0	1	0	0	0	No gene matches found
AGACAATGGG	0	0	0	0	1	No gene matches found
TGGCCGAAGA	0	0	1	0	0	No gene matches found
CTGCGAACAC	0	0	0	0	1	No gene matches found
TATTGCCTTC	0	1	0	0	0	No gene matches found
TCCTATTCAT	1	0	0	0	0	No gene matches found
CGAGAGCTCA	0	0	0	1	0	No gene matches found
GGGAGATTAT	0	1	0	0	0	No gene matches found
TAAACTCTCG	0	0	0	1	0	No gene matches found
GCCCTGAGAA	1	0	0	0	0	No gene matches found
TATTATGACA	0	0	0	0	1	No gene matches found
CGGAATATCG	0	0	0	1	0	No gene matches found
CACTTCACGA	0	1	0	0	0	No gene matches found
TACATTAGGG	0	0	0	0	1	No gene matches found
GGCTCAAAAA	0	0	0	0	1	No gene matches found
ACTTTAATAC	0	0	0	0	1	No gene matches found
CTTTGAATAT	0	0	0	1	0	No gene matches found
AACTTACCAC	0	1	0	0	0	No gene matches found
TATAGTGGCG	0	0	0	0	1	No gene matches found
GTCAGCTCTA	1	0	0	0	0	No gene matches found
ACGCTTTCGG	0	1	0	0	0	No gene matches found
GAGTGTCACT	1	0	0	0	0	No gene matches found
GCCGTTAGAA	0	0	1	0	0	No gene matches found
GGGTAAAATG	0	0	0	0	1	No gene matches found
CAGTTTCAGC	0	0	1	0	0	No gene matches found
AGAAAGCGCC	0	0	1	0	0	No gene matches found
TATTACGATA	1	0	0	0	0	No gene matches found
ATCACACACT	1	0	0	0	0	No gene matches found
TAGAATACAA	0	0	0	1	0	No gene matches found
CGTGAGAGTC	0	0	1	0	0	No gene matches found
GACTAAGGGA	0	0	0	0	1	No gene matches found
CCCGCGGCCG	1	0	0	0	0	No gene matches found
GCGGCGACCG	0	1	0	0	0	No gene matches found
CGAACATATA	0	0	0	1	0	No gene matches found
GGACCCATCG	1	0	0	0	0	No gene matches found
ACTCATAGGG	0	0	0	0	1	No gene matches found
GGGATAGCGG	1	0	0	0	0	No gene matches found

GTAATGTTAT	1	0	0	0	0	No gene matches found
TGATTCTCCT	0	1	0	0	0	No gene matches found
GTTCTATAAA	0	0	1	0	0	No gene matches found
GAGGTTATCC	0	1	0	0	0	No gene matches found
CCCACTGACC	0	0	0	1	0	No gene matches found
TGCAGAGAGG	0	0	1	0	0	No gene matches found
CCAACCGTTG	0	1	0	0	0	No gene matches found
GCCTCAGAGA	0	0	1	0	0	No gene matches found
GTCGAAGAAC	0	0	1	0	0	No gene matches found
GAACAAAGTG	0	1	0	0	0	No gene matches found
AGTTGCCGAT	0	1	0	0	0	No gene matches found
AAGGGTATCA	0	0	1	0	0	No gene matches found
GGCAGGGAGG	0	0	1	0	0	No gene matches found
CTGTCGTTTT	0	1	0	0	0	No gene matches found
GCCCTCGAGA	0	1	0	0	0	No gene matches found
GGAACGCCAT	0	0	0	1	0	No gene matches found
CCAGCACCGC	1	0	0	0	0	No gene matches found
TCCTACTGGA	0	0	1	0	0	No gene matches found
TTTCGAGTGA	0	1	0	0	0	No gene matches found
CCCTTGGTCC	0	0	1	0	0	No gene matches found
TCGAACACAC	0	0	1	0	0	No gene matches found
GAATAAGTTG	0	1	0	0	0	No gene matches found
CTCGGAGTTT	0	1	0	0	0	No gene matches found
GGTGTTGAGT	0	0	0	0	1	No gene matches found
GGTACAGCTG	0	0	0	0	1	No gene matches found
AATATGGGAG	0	0	1	0	0	No gene matches found
TTGACCTCGA	0	0	1	0	0	No gene matches found
GGAGAGTAAC	0	0	1	0	0	No gene matches found
CCTGAACTGA	0	0	1	0	0	No gene matches found
AATAGCTATC	0	0	1	0	0	No gene matches found
GTCATTCCTA	0	1	0	0	0	No gene matches found
TGTACAATCG	0	1	0	0	0	No gene matches found
GTTGACCCAA	0	0	1	0	0	No gene matches found
GAAGTGTAAG	1	0	0	0	0	No gene matches found
ACGGTTATGA	0	0	1	0	0	No gene matches found
GGTTTGGCTG	1	0	0	0	0	No gene matches found
TGGTGGGACG	0	0	0	1	0	No gene matches found
GAACCAGTAG	0	0	0	0	1	No gene matches found
GTTTACCCAC	0	0	0	0	1	No gene matches found
GGATTGGGTG	0	1	0	0	0	No gene matches found
CGGGTGATTT	0	1	0	0	0	No gene matches found
GTTGGTAGAG	0	0	0	1	0	No gene matches found
TTGGAACGAC	0	1	0	0	0	No gene matches found
GAAACCTTGC	0	0	1	0	0	No gene matches found
GATTGTAGTC	0	1	0	0	0	No gene matches found
GGAGTGCACC	0	0	0	1	0	No gene matches found
TCGCCTTAAA	0	1	0	0	0	No gene matches found
TTTGTTGCTC	0	0	0	0	1	No gene matches found
GACTGTACTG	0	0	1	0	0	No gene matches found
GTTTTCCCCC	0	1	0	0	0	No gene matches found
GACTTGGAGC	1	0	0	0	0	No gene matches found
GGTGGATTTC	0	1	0	0	0	No gene matches found
AGTTCAATGT	0	0	0	1	0	No gene matches found
TGAAAAAGTC	0	0	0	1	0	No gene matches found
GGCTGGGAAG	0	1	0	0	0	No gene matches found
GAACCGTAAC	0	1	0	0	0	No gene matches found
CAAAGAGATA	0	0	0	1	0	No gene matches found
GGACTCGGGG	0	0	0	1	0	No gene matches found
AAACAACCAT	0	0	1	0	0	No gene matches found
CCCTCCGCCA	0	0	1	0	0	No gene matches found
TTGTGATGAC	0	0	0	0	1	No gene matches found

AGGCAGATGA	1	0	0	0	0	No gene matches found
CAGCAGCAAT	0	0	0	0	1	No gene matches found
TCTACAGAAT	0	0	0	0	1	No gene matches found
ATCACTCGTG	1	0	0	0	0	No gene matches found
AGACTCGGTA	1	0	0	0	0	No gene matches found
ATTCTAAAGG	1	0	0	0	0	No gene matches found
AACCTTATAC	0	0	1	0	0	No gene matches found
GACCTTCACC	0	1	0	0	0	No gene matches found
GCAGAAAACT	0	1	0	0	0	No gene matches found
GTGAGCCGTA	0	0	0	1	0	No gene matches found
TAAGGGATAG	0	0	0	0	1	No gene matches found
TGGATCCTGA	0	1	0	0	0	No gene matches found
TCGTGTATGG	0	1	0	0	0	No gene matches found
GAGTACCTTG	0	1	0	0	0	No gene matches found
GCTCGTGTTT	0	0	0	1	0	No gene matches found
CGAGCCAAGG	0	0	0	1	0	No gene matches found
TTTAGTTGCT	0	0	0	1	0	No gene matches found
ACTTGGACAT	0	0	1	0	0	No gene matches found
CTATTTCCTA	1	0	0	0	0	No gene matches found
ATGCGTGCGG	0	1	0	0	0	No gene matches found
GCGTCGCTTC	0	1	0	0	0	No gene matches found
CAACTGTCAG	0	0	1	0	0	No gene matches found
ACGATTGGTC	0	0	0	1	0	No gene matches found
GTGATTCCAG	0	0	0	1	0	No gene matches found
GAGATTGTCC	0	1	0	0	0	No gene matches found
GCCCTGACAG	0	1	0	0	0	No gene matches found
GTAACCCGAT	0	0	1	0	0	No gene matches found
GGTCTGGCAA	0	1	0	0	0	No gene matches found
TTCCACCCCT	0	0	1	0	0	No gene matches found
CACCCAAAGT	0	1	0	0	0	No gene matches found
AGAACCGATG	0	0	1	0	0	No gene matches found
GGCGAACGTA	0	1	0	0	0	No gene matches found
CATAAGCTCG	1	0	0	0	0	No gene matches found
GTGGTGATTA	0	0	0	0	1	No gene matches found
CGGCAGACTG	1	0	0	0	0	No gene matches found
AGTCTGCGTA	0	0	1	0	0	No gene matches found
ATTGTATTGG	1	0	0	0	0	No gene matches found
GCAGGTTCAT	0	0	1	0	0	No gene matches found
CGATTAAGAT	0	1	0	0	0	No gene matches found
GCAGCCTTCG	0	1	0	0	0	No gene matches found
GTTCACTTTT	0	1	0	0	0	No gene matches found
TCGAGTGGAA	0	0	1	0	0	No gene matches found
ACTACTGCTT	0	0	0	1	0	No gene matches found
ACGCGAGCCG	0	0	0	0	1	No gene matches found
GCCTCTAAAA	0	1	0	0	0	No gene matches found
TTCAGGATTG	0	1	0	0	0	No gene matches found
TAACAGGTGA	1	0	0	0	0	No gene matches found
TCTCTTAGTG	0	0	1	0	0	No gene matches found
CGTTGCTGGA	0	0	0	0	1	No gene matches found
TGGGCTAAGT	0	0	0	1	0	No gene matches found
TCACATCATT	0	0	1	0	0	No gene matches found
AGGAATTGAC	0	0	0	0	1	No gene matches found
GAAGGAGCGG	1	0	0	0	0	No gene matches found
TTTGCGGTGC	0	1	0	0	0	No gene matches found
GATTTCTATG	0	0	1	0	0	No gene matches found
AAAGCTGGCA	0	1	0	0	0	No gene matches found
GACTCGGTGA	0	1	0	0	0	No gene matches found
TGTACGACAA	0	0	1	0	0	No gene matches found
CCTATGCCTC	0	0	1	0	0	No gene matches found
CTCTCAGACG	0	0	1	0	0	No gene matches found

GCACTAAGTA	1	0	0	0	0	No gene matches found
TCCGCTTCAG	0	0	1	0	0	No gene matches found
TGACGGTATG	1	0	0	0	0	No gene matches found
AAGTTGACGG	0	0	1	0	0	No gene matches found
GGCATTAAGC	0	0	0	0	1	No gene matches found
ACAGAGGAGC	1	0	0	0	0	No gene matches found
CCTATTAAGG	1	0	0	0	0	No gene matches found
GTGGTCCTAA	0	1	0	0	0	No gene matches found
TATTGTTCGT	0	1	0	0	0	No gene matches found
TATGAACCTG	0	1	0	0	0	No gene matches found
TGCCGTCTTA	0	1	0	0	0	No gene matches found
AAACCTGGGG	0	1	0	0	0	No gene matches found
CAGAGTTGAA	0	0	1	0	0	No gene matches found
TCATTCGACT	0	0	0	1	0	No gene matches found
ATAGGGACAA	0	1	0	0	0	No gene matches found
GCTTACAGTG	0	1	0	0	0	No gene matches found
ATCCCTAAGA	0	1	0	0	0	No gene matches found
GGTCAGGCCC	0	0	1	0	0	No gene matches found
TGTCTAAGTA	0	0	1	0	0	No gene matches found
AGGGATCGTT	0	0	0	0	1	No gene matches found
TAAGCGATAG	0	1	0	0	0	No gene matches found
GCGAGAAGGA	0	0	0	1	0	No gene matches found
TCCCAACGAG	0	0	0	1	0	No gene matches found
GATCGTTGAG	0	0	1	0	0	No gene matches found
TGCGTTTACT	0	0	0	1	0	No gene matches found
GCTCGGTGTA	0	1	0	0	0	No gene matches found
CGAGACATCT	0	0	1	0	0	No gene matches found
GATCTCAATT	0	0	1	0	0	No gene matches found
TCTTGGGCTG	0	1	0	0	0	No gene matches found
TCCTGCAGTT	0	0	1	0	0	No gene matches found
TCACAGCCTT	0	1	0	0	0	No gene matches found
AAATTCGTGA	0	0	0	1	0	No gene matches found
GGCCTTCTCC	1	0	0	0	0	No gene matches found
AATGAAAACG	0	1	0	0	0	No gene matches found
CGCTTAGAAT	0	1	0	0	0	No gene matches found
ATGCTGGAGG	0	0	1	0	0	No gene matches found
CTCACCACGA	0	0	0	1	0	No gene matches found
GTTCTGGGAG	0	1	0	0	0	No gene matches found
CAATAGTTCT	1	0	0	0	0	No gene matches found
GGATGGCCAC	0	1	0	0	0	No gene matches found
AAATCGGGTC	0	0	0	1	0	No gene matches found
GCAACCTTGA	0	1	0	0	0	No gene matches found
AGGAGTGTTA	0	0	0	0	1	No gene matches found
AGTCATATCT	0	1	0	0	0	No gene matches found
AAAAGGATAA	0	0	0	1	0	No gene matches found
CAACTTAAGC	0	1	0	0	0	No gene matches found
TGCAAGGTTG	0	1	0	0	0	No gene matches found
CACTATGGAT	1	0	0	0	0	No gene matches found
GGGTGAGGAG	0	1	0	0	0	No gene matches found
TGGAACGGTT	0	0	0	0	1	No gene matches found
TTAATAAACC	0	0	1	0	0	No gene matches found
GCTGTTTATA	0	0	0	0	1	No gene matches found
CGCTGTTGGA	0	0	0	1	0	No gene matches found
ATAATACGTT	0	1	0	0	0	No gene matches found
GATGGAGTCC	0	0	0	1	0	No gene matches found
CTTGTATATT	0	1	0	0	0	No gene matches found
CTGGCGAAAA	0	0	0	1	0	No gene matches found
CCAATGTAAA	0	0	0	1	0	No gene matches found
TTAGACGTGA	0	1	0	0	0	No gene matches found
GGTGAAAAAA	0	0	0	0	1	No gene matches found
CCCACTCCTG	1	0	0	0	0	No gene matches found

AGAATATCCT	0	0	1	0	0	No gene matches found
CCTAAGATAC	0	0	1	0	0	No gene matches found
GGCGAGGAAG	0	0	0	1	0	No gene matches found
ATCGGGAACT	0	0	0	1	0	No gene matches found
TTGTCACGAC	0	1	0	0	0	No gene matches found
ACACTGTCAA	0	0	1	0	0	No gene matches found
GTCATTGGAA	0	1	0	0	0	No gene matches found
CGGCCGAAGG	0	0	0	1	0	No gene matches found
CCGGTGTGGA	0	1	0	0	0	No gene matches found
CTTACAGAAA	1	0	0	0	0	No gene matches found
GTAAGTTATG	0	0	1	0	0	No gene matches found
TGCGTTGATT	0	1	0	0	0	No gene matches found
TCGCTTGTGA	0	1	0	0	0	No gene matches found
ACTCCCTTAA	0	0	0	1	0	No gene matches found
ATATCACGAG	0	0	1	0	0	No gene matches found
ATCCACCGTT	0	0	0	1	0	No gene matches found
ACAAAAGCCC	0	0	1	0	0	No gene matches found
CGCAAAAAAA	0	0	1	0	0	No gene matches found
TTCGAAGTTT	0	0	0	1	0	No gene matches found
ACCCGATCAA	0	0	0	0	1	No gene matches found
ATGGATCAGT	0	1	0	0	0	No gene matches found
TGGATCTTAC	0	0	0	0	1	No gene matches found
TTTCGTTCCT	0	1	0	0	0	No gene matches found
GGATTGTTGT	0	0	0	0	1	No gene matches found
TGGGGGATGT	0	1	0	0	0	No gene matches found
CTTGCGCTTG	0	1	0	0	0	No gene matches found
CACAGTAGAA	0	1	0	0	0	No gene matches found
CCAGCTTACA	0	1	0	0	0	No gene matches found
ATGGTGATTG	0	1	0	0	0	No gene matches found
CACCTGAAGA	0	0	1	0	0	No gene matches found
TTCAGGGACT	0	0	0	0	1	No gene matches found
ACCCGGTAAG	0	1	0	0	0	No gene matches found
AGATTAATAT	0	1	0	0	0	No gene matches found
AGAAATTGGC	0	1	0	0	0	No gene matches found
TAAGAGTGAA	0	0	0	1	0	No gene matches found
TTCGAGTAGA	0	0	0	1	0	No gene matches found
GTGTCTCTAC	1	0	0	0	0	No gene matches found
AGAGGGGCAT	0	0	1	0	0	No gene matches found
CTGATCAAGC	0	1	0	0	0	No gene matches found
TAGCCCCGAA	0	0	0	0	1	No gene matches found
GAGAGTTAGC	0	1	0	0	0	No gene matches found
CCTAAGAATA	0	1	0	0	0	No gene matches found
ATTGGGTCAA	0	0	1	0	0	No gene matches found
GGCTGAATTC	0	0	1	0	0	No gene matches found
GTGTCCGAGT	0	0	0	0	1	No gene matches found
TGAACCTAGA	0	1	0	0	0	No gene matches found
TCTGAGCCTT	0	0	1	0	0	No gene matches found
ATCAACGCTG	0	0	0	1	0	No gene matches found
ATCTGCCAGA	1	0	0	0	0	No gene matches found
GATCCGGAAA	0	0	0	1	0	No gene matches found
GCTGGCCCGG	0	1	0	0	0	No gene matches found
CCGATCCGTG	0	0	0	0	1	No gene matches found
ACTATCGCGT	0	0	0	0	1	No gene matches found
ACCATCGGTC	0	0	1	0	0	No gene matches found
ATGATATAGC	0	0	0	0	1	No gene matches found
CGTTTGTCAA	0	0	1	0	0	No gene matches found
GGTTCATTTT	0	1	0	0	0	No gene matches found
TAGATTCAGA	1	0	0	0	0	No gene matches found
TCGGCTAGTT	1	0	0	0	0	No gene matches found
CTGAAAAGGG	0	1	0	0	0	No gene matches found
GCCTTCGTCA	1	0	0	0	0	No gene matches found

AAACAGGTCA	1	0	0	0	0	No gene matches found
GCCGAATCAA	0	0	0	1	0	No gene matches found
CAGAAGGTTT	1	0	0	0	0	No gene matches found
TGCCGTCGTT	0	1	0	0	0	No gene matches found
TCACGGAACG	0	1	0	0	0	No gene matches found
AAGCCCCTAC	1	0	0	0	0	No gene matches found
CTACTAGGTT	0	0	0	1	0	No gene matches found
GTTGTCGTAA	0	0	1	0	0	No gene matches found
CGAGCTCCTT	0	0	0	0	1	No gene matches found
ATTTCTAACG	0	0	1	0	0	No gene matches found
TGTAAGGAAT	0	0	0	0	1	No gene matches found
AATGTGAGCT	1	0	0	0	0	No gene matches found
CGCGCGCCGC	0	0	1	0	0	No gene matches found
CTTGAAGTGA	1	0	0	0	0	No gene matches found
TCAGAGGCCT	0	0	0	0	1	No gene matches found
CTAGGGGCAA	0	1	0	0	0	No gene matches found
CTTTTAAGGG	0	0	1	0	0	No gene matches found
TGGATCTGAG	0	1	0	0	0	No gene matches found
TGTGGAGAAT	0	0	1	0	0	No gene matches found
CGCAGTGCAG	0	1	0	0	0	No gene matches found
GACAGGGTTG	0	1	0	0	0	No gene matches found
CCTCATAAAT	0	0	0	0	1	No gene matches found
AAGTTAGCTC	0	0	0	1	0	No gene matches found
AGCGCAGTTC	0	0	0	1	0	No gene matches found
ATCATTTGTG	0	0	1	0	0	No gene matches found
CAGGGGGGAC	0	0	0	0	1	No gene matches found
CCTAACAAAC	0	0	1	0	0	No gene matches found
CGCTAACACT	1	0	0	0	0	No gene matches found
GCCCTGCGAC	0	0	0	1	0	No gene matches found
TAGTCTATCC	0	1	0	0	0	No gene matches found
ATACACAACG	0	0	0	0	1	No gene matches found
GCAGCTATCA	0	1	0	0	0	No gene matches found
GTTGCTATAC	0	0	1	0	0	No gene matches found
GGAGGCAGAA	0	0	1	0	0	No gene matches found
CGTCGGACCG	0	0	1	0	0	No gene matches found
CCAGTAGGCG	0	0	1	0	0	No gene matches found
TCAGACCTCT	0	1	0	0	0	No gene matches found
GACCTTGCTT	0	0	0	1	0	No gene matches found
CCCACTGCTG	0	0	1	0	0	No gene matches found
TCCTTTTAAT	1	0	0	0	0	No gene matches found
GTGATACTCG	0	1	0	0	0	No gene matches found
TGCGGACTCA	0	0	0	0	1	No gene matches found
AGAGCGATGC	0	0	1	0	0	No gene matches found
TAAAGTTGTG	1	0	0	0	0	No gene matches found
GGCAGGCACG	0	0	0	1	0	No gene matches found
GTTCCCAGCA	0	0	0	1	0	No gene matches found
TGGTATTGGA	0	0	0	1	0	No gene matches found
GTTTGACTAA	0	1	0	0	0	No gene matches found
TTATTGTCTA	0	0	0	0	1	No gene matches found
GGCCAAACAA	0	0	1	0	0	No gene matches found
CCTCCTGTTC	0	1	0	0	0	No gene matches found
TGAATTTCGA	0	1	0	0	0	No gene matches found
TAGAAAGCAT	0	1	0	0	0	No gene matches found
CCTATGTCTT	0	1	0	0	0	No gene matches found
AACACCGAAT	0	0	0	0	1	No gene matches found
GGAGTCTGTT	1	0	0	0	0	No gene matches found
CGTAAGATCT	0	0	0	0	1	No gene matches found
ATACTCCGGC	0	1	0	0	0	No gene matches found
CCAGGTGGGT	0	1	0	0	0	No gene matches found
ACTGGTCCGG	0	0	1	0	0	No gene matches found
TATCTGTTGC	1	0	0	0	0	No gene matches found

CGATAAGGTG	0	0	0	1	0	No gene matches found
TGAGGAGGCT	0	1	0	0	0	No gene matches found
TGGATTGGCT	0	1	0	0	0	No gene matches found
CCTGGTCTAT	0	1	0	0	0	No gene matches found
TAGCATAATG	0	0	0	0	1	No gene matches found
TCTCTTCCGT	0	0	0	0	1	No gene matches found
GTGCTGTGTG	0	1	0	0	0	No gene matches found
AAATCCGCGG	0	0	1	0	0	No gene matches found
ACGCTACGGT	0	0	0	1	0	No gene matches found
TTGCATCTCG	0	0	0	0	1	No gene matches found
GTTATTTGGA	0	0	1	0	0	No gene matches found
GTGACGCTTA	0	0	0	0	1	No gene matches found
CTCATACATC	0	1	0	0	0	No gene matches found
GGACTTTTAC	0	0	0	1	0	No gene matches found
GTGGATCGGA	1	0	0	0	0	No gene matches found
GGCCTCGCCA	0	1	0	0	0	No gene matches found
TATTGAAAGG	0	0	0	0	1	No gene matches found
AAGTGAGTTT	0	0	0	0	1	No gene matches found
AGCCTGTTTT	0	0	0	1	0	No gene matches found
TGTTGCTAAA	1	0	0	0	0	No gene matches found
CTCCATTGGC	0	0	0	1	0	No gene matches found
TACCTATTAA	1	0	0	0	0	No gene matches found
GGTTGTGGGC	1	0	0	0	0	No gene matches found
TGTCCGGACT	0	1	0	0	0	No gene matches found
ACGCTTTCTA	0	0	1	0	0	No gene matches found
GACCCCACTA	0	0	0	0	1	No gene matches found
AGTGTCGATT	0	0	0	0	1	No gene matches found
GTTGATAGAA	0	0	0	0	1	No gene matches found
CGAGGAAGAA	0	0	1	0	0	No gene matches found
GCTCCGCTCA	0	0	1	0	0	No gene matches found
TTCGCCGTGG	0	0	1	0	0	No gene matches found
CAGGATTTGT	0	1	0	0	0	No gene matches found
AAGCACTTGG	0	0	1	0	0	No gene matches found
CTCTCGATTA	0	1	0	0	0	No gene matches found
GACCTGTGGC	0	1	0	0	0	No gene matches found
TAATTGGGCT	0	0	0	1	0	No gene matches found
CTTACTTGCT	0	1	0	0	0	No gene matches found
CGTGTTTTCA	0	0	0	0	1	No gene matches found
CAGAAGCCTG	1	0	0	0	0	No gene matches found
CCAGAGATAG	0	0	1	0	0	No gene matches found
GTCGACGCGG	0	0	0	1	0	No gene matches found
GCACAGTTGC	0	1	0	0	0	No gene matches found
CTCATTAAAA	0	0	0	1	0	No gene matches found
GATGGGTTGT	0	0	0	1	0	No gene matches found
ATAATTGGGT	0	0	1	0	0	No gene matches found
TACACTCCAG	0	0	0	1	0	No gene matches found
GTAACTCACC	0	0	0	1	0	No gene matches found
GGGAGACTAA	0	0	0	1	0	No gene matches found
TGCCTCAGGG	0	0	0	1	0	No gene matches found
CTTCTTGGGC	0	0	1	0	0	No gene matches found
GCTCTGGCAA	0	1	0	0	0	No gene matches found
GTCCAAATAC	0	0	0	1	0	No gene matches found
TATCAACGTA	0	1	0	0	0	No gene matches found
TACTTCAAGT	0	1	0	0	0	No gene matches found
ATAATCCGCG	0	0	1	0	0	No gene matches found
AACGGTTGTT	1	0	0	0	0	No gene matches found
CCTCCGAATT	0	0	0	0	1	No gene matches found
ATGATCGGGA	0	0	1	0	0	No gene matches found
GCGGAGATGC	0	0	0	0	1	No gene matches found
GAATAGTTGG	0	0	1	0	0	No gene matches found

CGCGACTGGT	0	1	0	0	0	No gene matches found
GTGTGGAAGT	0	0	1	0	0	No gene matches found
AGGGCGGAGG	0	0	0	0	1	No gene matches found
TCTGTACAAA	1	0	0	0	0	No gene matches found
TGGGGCCCAA	0	0	1	0	0	No gene matches found
TTGCATCCTC	0	1	0	0	0	No gene matches found
GGGAGCGAAC	0	0	0	1	0	No gene matches found
TGAACCAGAC	0	0	0	1	0	No gene matches found
AGGCACTCTG	0	0	0	0	1	No gene matches found
GTTCTTTCGT	0	0	0	1	0	No gene matches found
AGGCCTTGGT	0	0	1	0	0	No gene matches found
GGGGTCATTA	1	0	0	0	0	No gene matches found
AATAAAAGCG	1	0	0	0	0	No gene matches found
TGAGCAATGG	0	0	0	0	1	No gene matches found
GCAGAATTGC	0	1	0	0	0	No gene matches found
GGCCTTGGCC	0	0	0	1	0	No gene matches found
TCGAGCGCGG	0	0	0	1	0	No gene matches found
AGAAACGTTG	0	0	0	1	0	No gene matches found
ACAGTAACAT	0	1	0	0	0	No gene matches found
AATCCCGGTG	0	0	1	0	0	No gene matches found
GTAGACTTAC	0	1	0	0	0	No gene matches found
GGGGAAGTCC	1	0	0	0	0	No gene matches found
GGTGATGGTA	0	1	0	0	0	No gene matches found
CGAGAATAGT	0	0	0	1	0	No gene matches found
CCTCGGGTAC	0	1	0	0	0	No gene matches found
GGACTACTGG	0	0	1	0	0	No gene matches found
GGTTAATACA	0	1	0	0	0	No gene matches found
ATCATCGATG	0	0	0	0	1	No gene matches found
CACTTGAACG	0	0	1	0	0	No gene matches found
TATTGCTTGC	1	0	0	0	0	No gene matches found
TTGCGATGGT	0	1	0	0	0	No gene matches found
GAGCGAGATC	0	0	1	0	0	No gene matches found
ATGCAGTTCC	0	0	0	1	0	No gene matches found
ATCGTACAGA	0	0	1	0	0	No gene matches found
ATGCCTAAGA	1	0	0	0	0	No gene matches found
TCTGGGACTT	0	1	0	0	0	No gene matches found
TCGAATGTAC	0	0	1	0	0	No gene matches found
ATACGCTGAA	0	0	0	1	0	No gene matches found
TGTGAGACTT	1	0	0	0	0	No gene matches found
TTAAGTGTCG	0	0	0	1	0	No gene matches found
TCCCAAAGGG	1	0	0	0	0	No gene matches found
TCCGATCAAA	0	0	1	0	0	No gene matches found
CCAGAATGAA	0	0	1	0	0	No gene matches found
GGGTCAGACA	0	0	1	0	0	No gene matches found
TGTCTAACTC	0	1	0	0	0	No gene matches found
TGTAGCCCCA	0	0	0	0	1	No gene matches found
AAACGATAAT	0	0	0	0	1	No gene matches found
GAATTCCGTG	0	0	0	1	0	No gene matches found
TGCCACGTAT	0	0	0	1	0	No gene matches found
TCAACGGCCT	0	0	0	1	0	No gene matches found
GAACTAAGTG	0	1	0	0	0	No gene matches found
TCAATCATTC	0	0	1	0	0	No gene matches found
TGTCTTTAAA	1	0	0	0	0	No gene matches found
CCAGGACGAA	0	1	0	0	0	No gene matches found
TTGGTAGATG	0	0	1	0	0	No gene matches found
GATCATCGGA	0	0	0	1	0	No gene matches found
TGTGTACAGT	0	0	0	0	1	No gene matches found
TAGACTCAGA	0	0	0	0	1	No gene matches found
TAAGAGCAAA	0	0	0	0	1	No gene matches found
TCACTTGAGA	0	0	0	1	0	No gene matches found
GGAGCTGCTC	0	0	1	0	0	No gene matches found

TCTCGTTCTC	1	0	0	0	0	No gene matches found
AAGCTAGTAG	0	0	0	1	0	No gene matches found
AGCGCCCTGC	0	1	0	0	0	No gene matches found
ATTCATACAA	0	0	0	1	0	No gene matches found
GTAAAAACCT	0	0	0	1	0	No gene matches found
TTGGAATGAA	0	1	0	0	0	No gene matches found
CTCGGAGTCC	0	1	0	0	0	No gene matches found
GCGCCTCGAA	0	0	1	0	0	No gene matches found
AAGTTCCCGT	0	1	0	0	0	No gene matches found
AGGTTGCGAG	0	1	0	0	0	No gene matches found
CGCAGTAAGA	0	0	1	0	0	No gene matches found
ATGACCATCT	0	0	0	0	1	No gene matches found
CCTTTGGGAG	0	0	1	0	0	No gene matches found
TGCGTGTGTG	0	0	1	0	0	No gene matches found
CGCCCGTCGC	0	1	0	0	0	No gene matches found
CCGATGTTGT	0	0	1	0	0	No gene matches found
CGCCATCCGG	0	0	1	0	0	No gene matches found
ACACAAAGCG	0	1	0	0	0	No gene matches found
GAAACGCAAG	0	1	0	0	0	No gene matches found
TCTATCGCCG	0	0	0	1	0	No gene matches found
TCCCAAGCCT	0	0	0	1	0	No gene matches found
CGAACACAAA	0	0	1	0	0	No gene matches found
TTGGGCCAAG	0	0	0	0	1	No gene matches found
TGGGTACTCT	0	1	0	0	0	No gene matches found
TTACACGCCA	0	0	0	0	1	No gene matches found
CGCTTTGACC	0	0	0	1	0	No gene matches found
CGGAAGTGAT	0	0	1	0	0	No gene matches found
TACAATGATT	0	0	0	0	1	No gene matches found
ATCCAAACGG	0	0	0	0	1	No gene matches found
TCGGTCCGAC	0	0	1	0	0	No gene matches found
CCGGCAAGAA	0	0	1	0	0	No gene matches found
AGCGATCGAC	1	0	0	0	0	No gene matches found
GCATTCCACA	0	0	0	0	1	No gene matches found
GGAATAATCT	0	1	0	0	0	No gene matches found
TTGGGGATCC	0	1	0	0	0	No gene matches found
TGTAGAGTTA	0	0	1	0	0	No gene matches found
CAGACACCGG	1	0	0	0	0	No gene matches found
GACCATCTGT	0	1	0	0	0	No gene matches found
ACAAGCGCTC	0	0	0	1	0	No gene matches found
CTTTAGCTTC	0	0	1	0	0	No gene matches found
CCAGACAGGC	0	1	0	0	0	No gene matches found
AAAGGTATAG	0	0	0	0	1	No gene matches found
CTTTTCGGAA	0	1	0	0	0	No gene matches found
TCGTCCGAGT	0	1	0	0	0	No gene matches found
CTTGAACTTT	0	0	0	0	1	No gene matches found
GACGATGGCG	0	0	0	0	1	No gene matches found
TGCGATAGTA	0	0	0	0	1	No gene matches found
ACTGCAAGTT	1	0	0	0	0	No gene matches found
AAAGCCACTT	0	0	0	0	1	No gene matches found
GGCTAGACTA	1	0	0	0	0	No gene matches found
TCTTGAGTCG	0	0	1	0	0	No gene matches found
TCTATGGCTT	0	0	1	0	0	No gene matches found
CAATAACTAT	0	0	1	0	0	No gene matches found
CAGCTTTAAG	0	0	1	0	0	No gene matches found
TGGCACCAGT	0	1	0	0	0	No gene matches found
AGGATGATTA	0	1	0	0	0	No gene matches found
GCAAGTGCCG	0	0	1	0	0	No gene matches found
ACAATGCTTC	0	1	0	0	0	No gene matches found
TCAGGTATAG	0	0	0	1	0	No gene matches found
GTGTTCAAGG	0	1	0	0	0	No gene matches found
TGCACTTGCT	0	0	0	0	1	No gene matches found

CCCCAGGAAC	0	1	0	0	0	No gene matches found
TGGATGTACA	0	0	0	0	1	No gene matches found
GACCCAGCCG	0	0	0	1	0	No gene matches found
TTAATCGGAT	0	1	0	0	0	No gene matches found
ATTAACGCTA	1	0	0	0	0	No gene matches found
GAGGGTTGCT	0	0	1	0	0	No gene matches found
CTCACCGTCT	0	1	0	0	0	No gene matches found
ACCCTTAAAA	0	0	0	0	1	No gene matches found
TTGTCTATGT	0	0	1	0	0	No gene matches found
TTAAGCGTAG	0	1	0	0	0	No gene matches found
ACGAAGTAAG	0	0	0	0	1	No gene matches found
TGATGAGGAC	1	0	0	0	0	No gene matches found
ATGGGTGCGT	0	0	0	1	0	No gene matches found
CCAGCATTGA	0	1	0	0	0	No gene matches found
AGCAGCTTTT	0	1	0	0	0	No gene matches found
TGCATACGGT	0	1	0	0	0	No gene matches found
GACTTCTGAG	0	1	0	0	0	No gene matches found
CACTTTGACT	0	0	1	0	0	No gene matches found
TAAGTGATTA	0	0	0	0	1	No gene matches found
CCCAACCCCG	0	0	0	1	0	No gene matches found
GGATATGACA	0	0	0	1	0	No gene matches found
ATATGTTGAC	0	1	0	0	0	No gene matches found
GTCCGAGAGT	0	1	0	0	0	No gene matches found
CTGATGTTTT	0	0	1	0	0	No gene matches found
CGGCAGACCG	1	0	0	0	0	No gene matches found
CCCATCCTCG	1	0	0	0	0	No gene matches found
GACTCTATGA	0	1	0	0	0	No gene matches found
GAACGATCCT	0	0	0	0	1	No gene matches found
TTCCCAGAGG	0	1	0	0	0	No gene matches found
GACTTCGTCC	0	1	0	0	0	No gene matches found
AACTGCTGTC	0	0	0	0	1	No gene matches found
CACTTTGGAC	1	0	0	0	0	No gene matches found
GATACTAAGT	0	0	0	0	1	No gene matches found
GAAGCTTCGC	0	0	1	0	0	No gene matches found
CTAGGACCTT	0	0	0	1	0	No gene matches found
AGAGATGCTC	0	0	1	0	0	No gene matches found
TTAGAGCGAG	0	0	0	1	0	No gene matches found
AGCGAATCTA	0	0	1	0	0	No gene matches found
GTCCAGGCCC	0	1	0	0	0	No gene matches found
CTATGTAGAG	0	0	0	0	1	No gene matches found
TGTGTCTACT	0	0	0	1	0	No gene matches found
GGCACAACAA	1	0	0	0	0	No gene matches found
GGCCGACACA	0	0	0	1	0	No gene matches found
GTTAGTGCCA	0	1	0	0	0	No gene matches found
TCTGAGACAT	1	0	0	0	0	No gene matches found
GAGAAGCTCG	1	0	0	0	0	No gene matches found
TACTAGGTAG	0	1	0	0	0	No gene matches found
TACGATTCGG	0	1	0	0	0	No gene matches found
AGGGTTGATG	1	0	0	0	0	No gene matches found
CTGCAGTTTA	0	0	0	0	1	No gene matches found
TATAAGCTCG	0	0	0	1	0	No gene matches found
GCTTCCATCT	0	1	0	0	0	No gene matches found
CAAGTCGAAG	0	1	0	0	0	No gene matches found
GAGCTACACG	1	0	0	0	0	No gene matches found
AATAGGGAGA	1	0	0	0	0	No gene matches found
CCGCTTTTGT	0	0	1	0	0	No gene matches found
ATCAGGGGGG	0	0	1	0	0	No gene matches found
AAACAGTGTG	0	1	0	0	0	No gene matches found
AGATTTCAGT	0	0	0	1	0	No gene matches found
TCCTTCGTCG	0	0	1	0	0	No gene matches found
GGTGAAGTCT	0	0	0	1	0	No gene matches found

GGAGAGACTC	1	0	0	0	0	No gene matches found
ACAGACCAAG	0	0	1	0	0	No gene matches found
CCCTCGGCTG	0	0	0	0	1	No gene matches found
CGCACCTTCT	0	0	1	0	0	No gene matches found
AGACGCTGAT	0	1	0	0	0	No gene matches found
CCTCTACTAG	0	0	0	1	0	No gene matches found
TTATCTTTAA	0	1	0	0	0	No gene matches found
CGCCCGCGCG	0	0	1	0	0	No gene matches found
GAAAGGGCCA	1	0	0	0	0	No gene matches found
GGCCCCCCGG	0	0	0	1	0	No gene matches found
GTGTTCGGAT	0	0	0	1	0	No gene matches found
CTGATTTTTC	0	0	1	0	0	No gene matches found
GGATGATCCT	0	1	0	0	0	No gene matches found
GAGGTGGTTT	1	0	0	0	0	No gene matches found
AAGGGGATCG	0	0	0	1	0	No gene matches found
TTGCGATGCA	0	1	0	0	0	No gene matches found
TAGACCGATG	0	0	1	0	0	No gene matches found
GCTGGTACGC	0	0	1	0	0	No gene matches found
TTGGGGAAAG	0	0	1	0	0	No gene matches found
GTCGAAGTCT	0	0	0	1	0	No gene matches found
GATTCGTTAA	0	0	0	0	1	No gene matches found
GTGAGACCTG	0	1	0	0	0	No gene matches found
CCGCAACCAC	0	0	0	0	1	No gene matches found
CGGGGAGGAT	0	0	0	0	1	No gene matches found
CACGAGAACG	1	0	0	0	0	No gene matches found
CAAGCTACTT	0	1	0	0	0	No gene matches found
GGCCATCCGG	0	1	0	0	0	No gene matches found
GCCTTCGCTA	0	1	0	0	0	No gene matches found
CTGTTCGGAT	0	0	1	0	0	No gene matches found
GAAAAGGCCT	0	0	0	0	1	No gene matches found
AACGGCTGCG	0	0	1	0	0	No gene matches found
CACAATCAAC	0	1	0	0	0	No gene matches found
GACGAAGATC	0	1	0	0	0	No gene matches found
ATGAGTTGAG	0	0	0	0	1	No gene matches found
ACCTGCCCCT	0	1	0	0	0	No gene matches found
GCTCTTCGTG	0	0	0	1	0	No gene matches found
TCCGAGGCTT	0	1	0	0	0	No gene matches found
ACGGAAACCG	0	0	1	0	0	No gene matches found
GGCTACCTCA	0	0	1	0	0	No gene matches found
GTGGAAGGGA	0	0	1	0	0	No gene matches found
CAGGCGTCGA	0	0	1	0	0	No gene matches found
GCAACGCTCC	1	0	0	0	0	No gene matches found
AGGATGGAGC	0	1	0	0	0	No gene matches found
CCCTGAACGA	1	0	0	0	0	No gene matches found
TGACCCCTGC	0	1	0	0	0	No gene matches found
GGTCGAGGTG	0	1	0	0	0	No gene matches found
GATGTTCAGA	0	0	0	0	1	No gene matches found
AACCGATTGT	1	0	0	0	0	No gene matches found
GAAAGGTCGT	0	0	1	0	0	No gene matches found
CCAGATGAAT	0	0	0	0	1	No gene matches found
CTCGGGAGCG	1	0	0	0	0	No gene matches found
GATTACGAGG	0	0	0	0	1	No gene matches found
TTATAGAATT	0	1	0	0	0	No gene matches found
CATCCGTAAC	0	0	0	1	0	No gene matches found
GAACGGGGCC	0	1	0	0	0	No gene matches found
GCGAGTCAAT	0	0	0	0	1	No gene matches found
GGGGCATCCG	1	0	0	0	0	No gene matches found
GGCGGTGGCA	0	0	1	0	0	No gene matches found
TTGCGATGAC	0	1	0	0	0	No gene matches found
CGCCCGCAAT	0	1	0	0	0	No gene matches found

AGGGGGGAAG	0	0	1	0	0	No gene matches found
ATAGACCCTT	0	1	0	0	0	No gene matches found
CGGTGTCACT	0	1	0	0	0	No gene matches found
CTCAGAGCCC	0	0	0	1	0	No gene matches found
TCTGGGAGGA	1	0	0	0	0	No gene matches found
TAAGTCCACG	0	0	0	1	0	No gene matches found
GAGCTAATAG	0	0	0	1	0	No gene matches found
AGTGTATGAT	0	0	0	0	1	No gene matches found
GGAAGTCTCT	0	0	0	1	0	No gene matches found
CATCTGAACG	1	0	0	0	0	No gene matches found
TGAGTGGATG	0	1	0	0	0	No gene matches found
TCTCCACCGC	0	0	1	0	0	No gene matches found
TCAAGTCATT	0	0	1	0	0	No gene matches found
CATTCGGAAC	0	0	1	0	0	No gene matches found
CGGAAATGAG	0	0	1	0	0	No gene matches found
CAGACTAGTC	0	0	0	1	0	No gene matches found
ACCCCTCTCT	1	0	0	0	0	No gene matches found
TGCAATCGTT	0	0	1	0	0	No gene matches found
TTTCGGAAAA	0	0	1	0	0	No gene matches found
CTCACTCGTG	1	0	0	0	0	No gene matches found
CACAAGTGGA	0	1	0	0	0	No gene matches found
CCCTTTTACA	0	0	1	0	0	No gene matches found
CTTATGAGGT	0	1	0	0	0	No gene matches found
TAATAGCTAA	0	1	0	0	0	No gene matches found
ACCGACAAAG	0	1	0	0	0	No gene matches found
AGTTACCGAC	0	0	1	0	0	No gene matches found
CGCGAACGAC	1	0	0	0	0	No gene matches found
TCTATTCCTA	0	0	0	0	1	No gene matches found
CACTGCGTTC	0	1	0	0	0	No gene matches found
CGTCGTAGAG	0	0	1	0	0	No gene matches found
GCAGGACAAC	0	0	1	0	0	No gene matches found
GTCATTCCAA	1	0	0	0	0	No gene matches found
TGTGTCTTTC	1	0	0	0	0	No gene matches found
AGCAGCACAT	0	0	0	1	0	No gene matches found
CCTCCGATTC	0	0	1	0	0	No gene matches found
GGCCTTCGGA	0	0	1	0	0	No gene matches found
AATGGACTGG	0	0	0	1	0	No gene matches found
TGGGCGACGT	1	0	0	0	0	No gene matches found
GTTGGTACGC	0	0	0	1	0	No gene matches found
CGCTTAATAT	0	0	0	0	1	No gene matches found
TTCGATACAT	0	1	0	0	0	No gene matches found
AAGGCGTGGC	0	0	1	0	0	No gene matches found
TAGTGGATCC	0	0	1	0	0	No gene matches found
ATTGCAGATC	0	1	0	0	0	No gene matches found
TGACTCAGCG	0	0	0	0	1	No gene matches found
CGGATGAGTA	0	0	0	0	1	No gene matches found
GTCTCTGAAG	0	0	1	0	0	No gene matches found
TAGGAGAAAT	0	0	1	0	0	No gene matches found
GCTTGCGGTC	1	0	0	0	0	No gene matches found
GTGGTGAAGG	0	1	0	0	0	No gene matches found
TTGGATAAGC	0	0	1	0	0	No gene matches found
TATCCGTCTG	0	0	1	0	0	No gene matches found
GTGCCTCTGG	1	0	0	0	0	No gene matches found
TGTATTAACG	0	0	0	1	0	No gene matches found
TAAGAGCGAT	0	0	0	1	0	No gene matches found
CCCGTATGTA	1	0	0	0	0	No gene matches found
AAGGGTATAG	0	0	0	1	0	No gene matches found
GGATCGCCGA	0	0	1	0	0	No gene matches found
TCAGGACGCG	0	0	1	0	0	No gene matches found
TAGCAGCCAA	0	1	0	0	0	No gene matches found
CTCAGGGAAT	0	0	1	0	0	No gene matches found

CCAGATATAG	0	0	1	0	0	No gene matches found
GCTGATGCGG	0	0	0	1	0	No gene matches found
GATCCTTATT	0	0	1	0	0	No gene matches found
CGCCAGGAGG	0	1	0	0	0	No gene matches found
AAGACACGTA	0	1	0	0	0	No gene matches found
AGACATCAGT	0	0	0	0	1	No gene matches found
CGGAGTTCGG	0	1	0	0	0	No gene matches found
ATAGAGAGAC	0	0	0	1	0	No gene matches found
TAGGCAAATG	0	0	1	0	0	No gene matches found
CAGTTAGTAG	0	0	0	0	1	No gene matches found
ACAGTTCCCG	0	0	0	1	0	No gene matches found
CCATACTCAT	0	0	0	0	1	No gene matches found
TTCAGTCTAA	0	0	0	0	1	No gene matches found
ATTCCAGTTT	0	1	0	0	0	No gene matches found
AAAGCGGTCA	0	0	0	1	0	No gene matches found
GGACGTGCGA	0	0	0	1	0	No gene matches found
GGGGACTATG	0	0	1	0	0	No gene matches found
ACAGATTCAG	0	1	0	0	0	No gene matches found
ACCCTCGAGG	1	0	0	0	0	No gene matches found
GTCCAGCTTA	0	0	0	1	0	No gene matches found
AATGATACGG	0	1	0	0	0	No gene matches found
AGATGAGCAC	0	0	0	1	0	No gene matches found
GTGTAAGTAG	1	0	0	0	0	No gene matches found
GGCAAGCAAG	0	0	1	0	0	No gene matches found
GATAAGCACA	0	0	1	0	0	No gene matches found
CGTGTGGTGA	0	0	1	0	0	No gene matches found
ATCCAGACGT	0	1	0	0	0	No gene matches found
CCCTCTGATC	0	1	0	0	0	No gene matches found
GGCTGCCTTA	1	0	0	0	0	No gene matches found
AGGTGATTTT	0	0	1	0	0	No gene matches found
GAGACTAGTC	0	0	1	0	0	No gene matches found
GGCTAAATGC	0	1	0	0	0	No gene matches found
TAGACTTCTC	0	1	0	0	0	No gene matches found
TAATTCGTTC	0	0	0	1	0	No gene matches found
TGCTTGTAGC	0	1	0	0	0	No gene matches found
CGCCCGGAAA	1	0	0	0	0	No gene matches found
TGAGAGTTGA	0	0	1	0	0	No gene matches found
AAATGGCCCT	0	1	0	0	0	No gene matches found
TAGTGTGCCA	1	0	0	0	0	No gene matches found
ACCGCTCACG	0	0	1	0	0	No gene matches found
ACCTTGTCGC	0	0	0	1	0	No gene matches found
ATTCCTGCTC	0	0	0	0	1	No gene matches found
TCCGAATCAC	0	1	0	0	0	No gene matches found
TCTTACTGAT	0	0	0	0	1	No gene matches found
ATGGTTAAGC	0	0	0	1	0	No gene matches found
AATGTGCGGA	0	0	0	0	1	No gene matches found
TCTGACACGT	0	0	0	1	0	No gene matches found
GCCTAGATTA	0	0	1	0	0	No gene matches found
GTGGGCGATT	0	0	0	0	1	No gene matches found
GTTGAGGTCC	0	0	0	0	1	No gene matches found
AGGTTGGACT	0	0	1	0	0	No gene matches found
TAGCAGGATA	0	0	0	0	1	No gene matches found
TCTAGAGAAC	0	0	1	0	0	No gene matches found
TTGTCTCAAA	0	0	1	0	0	No gene matches found
ATTGCGAGTT	0	0	1	0	0	No gene matches found
AACGAGGCGA	0	0	1	0	0	No gene matches found
CTAAGCGAAA	0	0	1	0	0	No gene matches found
AAGGCTGATG	0	1	0	0	0	No gene matches found
GGAGAGGTGG	0	0	1	0	0	No gene matches found
TAGAATTCCC	0	0	0	1	0	No gene matches found
GACCGTGGAG	0	1	0	0	0	No gene matches found

TACCAAATAG	0	1	0	0	0	No gene matches found
GGACCACTCT	0	0	0	0	1	No gene matches found
TCTTTTCTGA	0	1	0	0	0	No gene matches found
AACCGAGAGG	0	1	0	0	0	No gene matches found
TATCCGACTA	0	1	0	0	0	No gene matches found
GCGGCTAGAT	0	0	1	0	0	No gene matches found
GCAGCAAAAT	0	0	0	1	0	No gene matches found
AGTTGGGTCG	0	0	1	0	0	No gene matches found
CCACATTCAA	0	0	1	0	0	No gene matches found
GAGACAACGC	0	0	1	0	0	No gene matches found
CGAAGAACGT	0	1	0	0	0	No gene matches found
GTCAAAGTCG	0	1	0	0	0	No gene matches found
GGGATAACGA	0	0	1	0	0	No gene matches found
ACTCTTTAAG	0	0	0	0	1	No gene matches found
GTGGTATTCC	0	0	1	0	0	No gene matches found
GGCAGCTCGA	0	1	0	0	0	No gene matches found
TCTACGAAAA	1	0	0	0	0	No gene matches found
GTAGCTGGTT	0	1	0	0	0	No gene matches found
GGACGTGCGG	0	0	1	0	0	No gene matches found
CACAGATGAG	1	0	0	0	0	No gene matches found
AAACCGCCAA	1	0	0	0	0	No gene matches found
GAACCGTCAA	0	0	0	1	0	No gene matches found
CGAGCGTTTA	0	0	1	0	0	No gene matches found
TCAGGACCCA	0	0	1	0	0	No gene matches found
TCGCATTAGG	0	0	0	1	0	No gene matches found
GGAGGCATCG	0	1	0	0	0	No gene matches found
AAATTTCCGC	0	1	0	0	0	No gene matches found
GGCGGACCGT	0	1	0	0	0	No gene matches found
AGAACTCGGG	1	0	0	0	0	No gene matches found
ACCCTTGCTT	0	0	0	0	1	No gene matches found
AAAACGTGGC	0	0	0	1	0	No gene matches found
TTTACGCATT	0	0	0	0	1	No gene matches found
TGATCTGAAT	0	0	1	0	0	No gene matches found
GGTTGATAAC	0	0	1	0	0	No gene matches found
TAAACCTCGT	0	0	1	0	0	No gene matches found
TAGCTACTGA	0	1	0	0	0	No gene matches found
AGTAGACTTG	0	1	0	0	0	No gene matches found
CGGTTTGTAT	0	0	0	0	1	No gene matches found
TTGCGTTTGT	0	1	0	0	0	No gene matches found
TCTGACCCAA	0	1	0	0	0	No gene matches found
AAGAGAACGG	0	0	1	0	0	No gene matches found
GTCGTTGATG	0	1	0	0	0	No gene matches found
CGTCGTACGA	0	0	1	0	0	No gene matches found
GGGGTTCCCA	0	1	0	0	0	No gene matches found
CACGTGTGGG	0	0	1	0	0	No gene matches found
GTTGTTATGC	1	0	0	0	0	No gene matches found
GCAGGGCAAA	0	1	0	0	0	No gene matches found
TCCAAAACCT	1	0	0	0	0	No gene matches found
TCAAAGGCAT	0	0	0	1	0	No gene matches found
GGTCATACCG	0	0	1	0	0	No gene matches found
CGAGCTTCGT	0	1	0	0	0	No gene matches found
TAAGCTGTAG	0	0	0	0	1	No gene matches found
GACTTCAGGA	0	0	0	0	1	No gene matches found
TTTCGATCAC	0	0	1	0	0	No gene matches found
CTTTGGACAG	0	1	0	0	0	No gene matches found
GGTGCAGTAC	0	1	0	0	0	No gene matches found
GGAAGAGTCT	0	1	0	0	0	No gene matches found
CCCCAAAATT	0	0	1	0	0	No gene matches found
TCGATGTTGC	0	0	0	1	0	No gene matches found
CTGAGAGAGG	1	0	0	0	0	No gene matches found
GTTGTTATGA	0	0	0	0	1	No gene matches found

AGGCCTTCGC	0	1	0	0	0	No gene matches found
GGAGCTCAGG	0	1	0	0	0	No gene matches found
AGGCATCCGC	1	0	0	0	0	No gene matches found
ATTGAGACAT	0	0	0	0	1	No gene matches found
ATCAAGGTCA	0	1	0	0	0	No gene matches found
GAGTGTGGTA	0	1	0	0	0	No gene matches found
CAGTCCGCGA	0	1	0	0	0	No gene matches found
ACGTTTGGAA	0	0	1	0	0	No gene matches found
TTCTGGAGAT	0	1	0	0	0	No gene matches found
GTCTGTAGTG	0	0	0	0	1	No gene matches found
GCGATTGTAA	1	0	0	0	0	No gene matches found
ACCACTCGAG	1	0	0	0	0	No gene matches found
AATGGTCGAG	1	0	0	0	0	No gene matches found
CACAGTGCTC	1	0	0	0	0	No gene matches found
GGGGTCTTAA	1	0	0	0	0	No gene matches found
ACCGCGAACT	0	0	1	0	0	No gene matches found
TTCCTATAAA	1	0	0	0	0	No gene matches found
CGTTATTATG	0	1	0	0	0	No gene matches found
TCGGAATTAC	0	0	1	0	0	No gene matches found
GTTGGACGTT	0	0	0	0	1	No gene matches found
TTCAAGAGGG	0	0	1	0	0	No gene matches found
TATTGTCCCT	1	0	0	0	0	No gene matches found
CGTTCCAAAC	0	1	0	0	0	No gene matches found
AGCTAGGTAC	0	1	0	0	0	No gene matches found
GGGAGTGTAC	1	0	0	0	0	No gene matches found
ATGATATGTC	1	0	0	0	0	No gene matches found
ACGTCGACTG	0	0	0	1	0	No gene matches found
CAACAGCTTT	1	0	0	0	0	No gene matches found
TCGATGAGAT	1	0	0	0	0	No gene matches found
CCGAGGAGGA	0	0	1	0	0	No gene matches found
GGAGATACTA	0	0	0	1	0	No gene matches found
CGAGCTTCAG	0	0	1	0	0	No gene matches found
GCAGACGACC	0	1	0	0	0	No gene matches found
GCTCCGCCCC	0	0	0	0	1	No gene matches found
GTCACTGTGG	0	0	1	0	0	No gene matches found
ATGGTGGGTG	0	0	1	0	0	No gene matches found
AAAGTTCCGG	1	0	0	0	0	No gene matches found
CGGGGTGGAA	0	1	0	0	0	No gene matches found
GTGGCCGTGT	0	1	0	0	0	No gene matches found
CTTGAATCGA	1	0	0	0	0	No gene matches found
CCTCTATTAA	1	0	0	0	0	No gene matches found
GGAGTGGCAG	0	0	0	1	0	No gene matches found
TTCTTTCGAA	1	0	0	0	0	No gene matches found
ATTTGCCACA	0	1	0	0	0	No gene matches found
TATTGCTGAT	1	0	0	0	0	No gene matches found
TAAACAACAG	0	0	0	0	1	No gene matches found
AATCGAGTTG	0	0	1	0	0	No gene matches found
TTTTCGATGC	1	0	0	0	0	No gene matches found
AAAAAAGCCG	0	1	0	0	0	No gene matches found
GTCTCCTTAT	0	1	0	0	0	No gene matches found
TTTGCAAGGC	0	0	0	1	0	No gene matches found
CTGGTTCGCC	0	1	0	0	0	No gene matches found
TTGTTAACCG	1	0	0	0	0	No gene matches found
TTGGCTCATA	0	1	0	0	0	No gene matches found
CCACAGTTGA	0	0	1	0	0	No gene matches found
CCTTGCGGAC	0	0	0	1	0	No gene matches found
ATGCAGTAAG	0	0	0	0	1	No gene matches found
TGACCCGTGA	0	1	0	0	0	No gene matches found
CAGGTGTAGG	0	1	0	0	0	No gene matches found
TCTGTGTCCG	0	1	0	0	0	No gene matches found

TGGCGGGATC	0	0	1	0	0	No gene matches found
GGGATACTTT	0	0	0	0	1	No gene matches found
TGCCCGATTT	0	0	0	1	0	No gene matches found
TCTCCTAAAG	0	1	0	0	0	No gene matches found
TATCACCCTG	1	0	0	0	0	No gene matches found
CGCTAACAAA	1	0	0	0	0	No gene matches found
TTTATGCAGG	0	0	0	0	1	No gene matches found
GCCCGCCGCC	0	1	0	0	0	No gene matches found
GGTCTCCGAT	0	1	0	0	0	No gene matches found
CCTCTTGTAC	0	0	1	0	0	No gene matches found
CGGAAATGGA	0	0	1	0	0	No gene matches found
TGCAGTTATG	0	0	0	0	1	No gene matches found
TACCGAACAC	0	1	0	0	0	No gene matches found
GGGACGGAAG	0	0	1	0	0	No gene matches found
ACTTAACCCT	0	0	1	0	0	No gene matches found
CCCGAAGCCG	0	1	0	0	0	No gene matches found
TTACGTTTGG	0	0	0	0	1	No gene matches found
GGCCGTTGGA	1	0	0	0	0	No gene matches found
AAGACGAGGA	0	1	0	0	0	No gene matches found
CTTTCGAAAA	0	0	0	1	0	No gene matches found
CAAGACATAG	1	0	0	0	0	No gene matches found
AGGTCTCTAG	0	0	1	0	0	No gene matches found
GCGAGGCGGT	0	0	0	1	0	No gene matches found
CCGACCGTCG	0	0	1	0	0	No gene matches found
TGCGAACGAC	0	0	1	0	0	No gene matches found
AGGTTTATGA	0	0	1	0	0	No gene matches found
TATGTGCGCA	0	0	0	1	0	No gene matches found
CATCGCACTG	0	0	1	0	0	No gene matches found
GGCGTTGGTT	0	0	0	0	1	No gene matches found
TAAGTACACT	0	0	0	0	1	No gene matches found
GGAGTTGCAC	0	1	0	0	0	No gene matches found
GCGACTGTAC	0	1	0	0	0	No gene matches found
CTGTTTGACG	0	1	0	0	0	No gene matches found
TGTAGATGAG	0	0	1	0	0	No gene matches found
TTACGATAAC	0	0	0	0	1	No gene matches found
ATTGTGTCAG	0	1	0	0	0	No gene matches found
TCCCACAACG	0	0	0	1	0	No gene matches found
CGTTCTTAGG	0	1	0	0	0	No gene matches found
TCACGAGTTG	0	0	1	0	0	No gene matches found
GCAGAGTGAG	0	0	0	1	0	No gene matches found
TCTCGAACGA	0	0	0	1	0	No gene matches found
GCCTTGAGAG	0	1	0	0	0	No gene matches found
CACCTAACAG	0	0	0	1	0	No gene matches found
CGCTAACAAG	0	0	0	1	0	No gene matches found
AATAGAAAGG	0	1	0	0	0	No gene matches found
GGGATCGGCT	0	0	1	0	0	No gene matches found
AAACAAAGTC	1	0	0	0	0	No gene matches found
TCCGGAAATT	0	0	1	0	0	No gene matches found
TTTGTACAGA	1	0	0	0	0	No gene matches found
GGAGATTACC	0	0	1	0	0	No gene matches found
ACATATGCGA	0	0	1	0	0	No gene matches found
CGAGTTGAGA	0	0	0	1	0	No gene matches found
TCGCAAACGC	0	0	0	1	0	No gene matches found
AGCGTTGTAT	0	0	0	0	1	No gene matches found
CACTTGGAGT	0	0	1	0	0	No gene matches found
GAGCGTGGCT	0	0	1	0	0	No gene matches found
CGAGTAAAAA	0	0	0	1	0	No gene matches found
CTTAACCCGT	0	1	0	0	0	No gene matches found
TGGATCGACG	0	0	1	0	0	No gene matches found
TAGCCAGAGT	1	0	0	0	0	No gene matches found
AACTATGTGT	0	1	0	0	0	No gene matches found

CTCTTGGGAA	0	1	0	0	0	No gene matches found
GGACGTCACA	0	1	0	0	0	No gene matches found
TGTGCCCCCA	0	0	0	1	0	No gene matches found
GGTAAAGGTA	1	0	0	0	0	No gene matches found
GTACCGATCC	0	0	1	0	0	No gene matches found
TTTTGAGTTG	0	0	0	0	1	No gene matches found
TATAGCCTAA	1	0	0	0	0	No gene matches found
TCTTGGCCTC	1	0	0	0	0	No gene matches found
CTCTTACATA	0	0	1	0	0	No gene matches found
AGTATGCTAC	0	1	0	0	0	No gene matches found
ATCCTGGATT	0	0	0	1	0	No gene matches found
CGTCTTTGTT	0	1	0	0	0	No gene matches found
ACTTGCTTTA	0	0	0	0	1	No gene matches found
TTACCACCGA	0	0	1	0	0	No gene matches found
TGGCAAGAGT	0	0	1	0	0	No gene matches found
AACGTTTCTA	0	0	0	1	0	No gene matches found
GGTCGATGAG	0	0	0	0	1	No gene matches found
GGCCGTCGCC	0	0	1	0	0	No gene matches found
AACGACATAG	0	0	0	0	1	No gene matches found
CCCCAGAAGA	0	0	1	0	0	No gene matches found
CAGGACTTTG	1	0	0	0	0	No gene matches found
GGGAAAGTCT	0	1	0	0	0	No gene matches found
ATAAGCGGTT	0	0	0	0	1	No gene matches found
TTGGTCCTAT	0	1	0	0	0	No gene matches found
AGAGCGATTA	0	0	0	0	1	No gene matches found
TGGACCTTGA	0	0	0	1	0	No gene matches found
TTCAGATAGC	0	0	1	0	0	No gene matches found
GGAATTGCTT	0	0	0	0	1	No gene matches found
GGATGATCAA	0	0	0	1	0	No gene matches found
CTGGCAGAAT	0	1	0	0	0	No gene matches found
CTCCCTAAAG	0	0	0	0	1	No gene matches found
AACGTAACGC	0	0	0	1	0	No gene matches found
TAGGTGTTGT	0	0	0	1	0	No gene matches found
CGAACGCACT	0	1	0	0	0	No gene matches found
CGGATTTCTC	0	0	1	0	0	No gene matches found
AAGTGATGGT	1	0	0	0	0	No gene matches found
AGAAGCCTTC	0	1	0	0	0	No gene matches found
GAATTTGTAC	0	0	0	1	0	No gene matches found
GTACGGATCG	0	0	1	0	0	No gene matches found
CGAAAAGAGA	0	0	0	1	0	No gene matches found
GGTAAGCTCA	0	0	0	1	0	No gene matches found
CCGTTTCTGG	1	0	0	0	0	No gene matches found
CAAAGAGGCG	0	0	0	0	1	No gene matches found
TCGGGACGCA	0	0	1	0	0	No gene matches found
GATTGACCTT	1	0	0	0	0	No gene matches found
TAAGTCGCAC	0	0	1	0	0	No gene matches found
GGGAAAGCCC	0	0	1	0	0	No gene matches found
GGCCTGAACC	0	0	1	0	0	No gene matches found
GGAACGCACA	0	1	0	0	0	No gene matches found
GGAGCTGGGG	1	0	0	0	0	No gene matches found
AGATCCATAG	0	0	1	0	0	No gene matches found
AGCAAACTCG	0	0	0	0	1	No gene matches found
GCATTATGAT	0	1	0	0	0	No gene matches found
TGGAAGCCCT	0	1	0	0	0	No gene matches found
GAACAGGATC	0	0	1	0	0	No gene matches found
GTTCCAGAAG	1	0	0	0	0	No gene matches found
GCAACCGTGA	0	1	0	0	0	No gene matches found
TTTACGTTTC	0	0	0	0	1	No gene matches found
AGTTCAACTA	0	0	1	0	0	No gene matches found
GCGTCTTTCG	0	0	0	1	0	No gene matches found
TCTGTCACCC	1	0	0	0	0	No gene matches found

TGTATGTCCC	0	1	0	0	0	No gene matches found
GATCGGTCAG	0	0	0	1	0	No gene matches found
AGCGAGAATA	0	1	0	0	0	No gene matches found
CTTCTAAATA	0	1	0	0	0	No gene matches found
TTACAAGGGG	0	0	0	1	0	No gene matches found
ATTTCTAAGA	0	0	1	0	0	No gene matches found
CAACGAGAAT	0	0	1	0	0	No gene matches found
TCGTTCAAGA	0	1	0	0	0	No gene matches found
GTCCAAGATG	0	0	0	1	0	No gene matches found
CAAGAGACTC	0	0	1	0	0	No gene matches found
CATTTCGCCG	0	0	1	0	0	No gene matches found
TGAGTAAGAG	0	0	0	0	1	No gene matches found
CACTGATCTC	0	1	0	0	0	No gene matches found
GACTGTACTT	1	0	0	0	0	No gene matches found
TGAGGCGATG	0	0	0	1	0	No gene matches found
CTAAACAATA	0	0	0	0	1	No gene matches found
AAGGACGCGA	0	1	0	0	0	No gene matches found
CAGGTGTGTA	0	1	0	0	0	No gene matches found
CCCGTAGTAC	0	0	1	0	0	No gene matches found
CTGTCCCAGA	1	0	0	0	0	No gene matches found
CGCTTAGTAC	0	1	0	0	0	No gene matches found
ATTTCGAGTA	0	0	0	0	1	No gene matches found
TCTTCACTAC	0	1	0	0	0	No gene matches found
TGAAACTACA	0	0	1	0	0	No gene matches found
CTCGACAAAA	0	1	0	0	0	No gene matches found
CAGTGTTGTT	1	0	0	0	0	No gene matches found
TAATTGACTC	0	0	0	0	1	No gene matches found
GGGTTCCAAA	1	0	0	0	0	No gene matches found
CTTAACTCAT	0	0	0	0	1	No gene matches found
CATCCGTCAA	0	0	0	0	1	No gene matches found
TAGTCTATAG	0	0	0	0	1	No gene matches found
GGAAAGTTCT	0	0	0	1	0	No gene matches found
GCTTTCATCC	0	1	0	0	0	No gene matches found
CCTCCGTTTT	0	0	0	1	0	No gene matches found
TACCTTCAAC	1	0	0	0	0	No gene matches found
AAAACGTGAA	0	0	1	0	0	No gene matches found
AACACCACTG	0	1	0	0	0	No gene matches found
GGAGATGTAG	0	0	0	1	0	No gene matches found
CACACCGGTC	0	1	0	0	0	No gene matches found
TAGCAAAACG	0	0	1	0	0	No gene matches found
CGCAAATTGT	0	0	1	0	0	No gene matches found
AGGCAGACAG	0	0	0	0	1	No gene matches found
GAACCCTGCT	0	0	0	0	1	No gene matches found
GGTGCGTTTG	0	1	0	0	0	No gene matches found
AAAACGTGCT	0	0	1	0	0	No gene matches found
GCACTTCGGA	0	1	0	0	0	No gene matches found
GTATCCATAC	0	0	0	1	0	No gene matches found
CCTATAGGCA	0	0	0	0	1	No gene matches found
GTAGCTGGTA	0	1	0	0	0	No gene matches found
GCCTTAATAC	0	0	0	1	0	No gene matches found
GCCGTTCTCG	1	0	0	0	0	No gene matches found
CACGAAAGCT	0	1	0	0	0	No gene matches found
GAGGTAGTGA	0	0	1	0	0	No gene matches found
CTCTTGTCAG	0	0	1	0	0	No gene matches found
CGATACTTGG	0	0	0	1	0	No gene matches found
AGGAATGCTA	0	0	0	1	0	No gene matches found
ACAGAGTGGA	0	0	1	0	0	No gene matches found
AAAACCACCC	0	1	0	0	0	No gene matches found
ATACACAGTA	0	0	0	0	1	No gene matches found
TTGTTACTTC	1	0	0	0	0	No gene matches found
GATAGAATGA	0	1	0	0	0	No gene matches found

ATACGCTCAA	0	0	0	1	0	No gene matches found
GCAAAACGGA	0	0	0	1	0	No gene matches found
GACCCCATCG	0	0	0	0	1	No gene matches found
CACAAGGAGA	1	0	0	0	0	No gene matches found
CGTAAATCTG	0	0	0	1	0	No gene matches found
GTCTCAAAAA	0	1	0	0	0	No gene matches found
GCTGGTTTGA	0	1	0	0	0	No gene matches found
CCTCTAAGGC	0	0	0	0	1	No gene matches found
TACAATCCGG	0	1	0	0	0	No gene matches found
TCGCAGTTTA	0	0	0	1	0	No gene matches found
GGCCTTCGAT	1	0	0	0	0	No gene matches found
CCAATACATC	0	0	1	0	0	No gene matches found
TGAGTGGCAA	1	0	0	0	0	No gene matches found
GGTCCAACTT	0	0	0	1	0	No gene matches found
TCCGAGCCCA	0	0	0	1	0	No gene matches found
AAGGAGTGAT	0	0	1	0	0	No gene matches found
GACCGTGTAG	0	1	0	0	0	No gene matches found
CTACTGTCAA	1	0	0	0	0	No gene matches found
GAGAAGTGGC	0	0	0	0	1	No gene matches found
TGTATCACTG	1	0	0	0	0	No gene matches found
GATGCGACAA	0	1	0	0	0	No gene matches found
GTATCAAAGG	1	0	0	0	0	No gene matches found
TGCATTAGGG	0	0	1	0	0	No gene matches found
GCCCTTCGCC	0	1	0	0	0	No gene matches found
AGGCTCGTTC	0	1	0	0	0	No gene matches found
GAGCGTTCCA	0	0	1	0	0	No gene matches found
TGGGGTCTTT	0	0	0	0	1	No gene matches found
AATTCGGGCA	1	0	0	0	0	No gene matches found
TAATTCGAGG	0	0	0	1	0	No gene matches found
TTCGAAGCCG	0	1	0	0	0	No gene matches found
TACGGCCCTT	0	1	0	0	0	No gene matches found
TCTAGTCGTG	0	1	0	0	0	No gene matches found
TCCTCAGCAC	0	1	0	0	0	No gene matches found
TGTCGTGGCA	0	1	0	0	0	No gene matches found
AAACGAAGCA	0	0	0	1	0	No gene matches found
CCGGTCTACC	0	1	0	0	0	No gene matches found
CTTGTTTCAG	0	1	0	0	0	No gene matches found
CATCCTGTTG	1	0	0	0	0	No gene matches found
CGGGAAAATC	0	0	0	0	1	No gene matches found
GTTCTTCTCA	0	0	0	1	0	No gene matches found
AACCCAGGCC	0	1	0	0	0	No gene matches found
CTTGGGCTAA	1	0	0	0	0	No gene matches found
CCCTACAGCG	0	1	0	0	0	No gene matches found
CTCAGCTACC	0	0	1	0	0	No gene matches found
ATTTGCCAGG	0	1	0	0	0	No gene matches found
CCCAGACTGG	0	1	0	0	0	No gene matches found
ATAGCACCTT	0	1	0	0	0	No gene matches found
CAACGGAGCC	0	0	1	0	0	No gene matches found
CGACAAGGTC	1	0	0	0	0	No gene matches found
AGCGAGCATC	0	0	1	0	0	No gene matches found
CCACAACTCA	0	1	0	0	0	No gene matches found
GTACTGGCAA	0	1	0	0	0	No gene matches found
CGTCTCCGGA	1	0	0	0	0	No gene matches found
ATGGAAAGCT	0	1	0	0	0	No gene matches found
GCCTTCTCCA	0	1	0	0	0	No gene matches found
ACCTTACATA	0	1	0	0	0	No gene matches found
ACTTGATGCA	1	0	0	0	0	No gene matches found
GAGGACTCCA	0	0	1	0	0	No gene matches found
AGCCTTCGCC	0	1	0	0	0	No gene matches found
AGGTATTTGA	0	1	0	0	0	No gene matches found

TCGGATCAAG	0	0	1	0	0	No gene matches found
ATACAGTGAG	0	1	0	0	0	No gene matches found
CAGCACTGGG	0	0	0	0	1	No gene matches found
TATGTGGATA	1	0	0	0	0	No gene matches found
ATTTCGATGT	0	0	1	0	0	No gene matches found
TTTCGGTTTA	0	0	0	0	1	No gene matches found
TGCATCAAAA	0	0	0	1	0	No gene matches found
TCCTTGATGG	1	0	0	0	0	No gene matches found
GATCTTCGGG	0	1	0	0	0	No gene matches found
TATACTCACG	0	1	0	0	0	No gene matches found
TAATAACTCT	1	0	0	0	0	No gene matches found
AAGATGCTGG	0	1	0	0	0	No gene matches found
ACCAAACAGG	0	1	0	0	0	No gene matches found
TCCTACGCCC	0	0	1	0	0	No gene matches found
CCAGTATCGT	0	1	0	0	0	No gene matches found
AGACCTAGAC	0	1	0	0	0	No gene matches found
ATTACTCGAT	0	1	0	0	0	No gene matches found
TCGAGGAAAA	0	0	1	0	0	No gene matches found
CTCCCGCCGC	0	1	0	0	0	No gene matches found
AGTCTGGTGA	1	0	0	0	0	No gene matches found
ATCGCGAAAA	0	1	0	0	0	No gene matches found
CGTGTTGTGG	0	0	0	1	0	No gene matches found
GGGGCGGGTA	0	0	1	0	0	No gene matches found
TGTTCCTGAC	0	0	0	0	1	No gene matches found
TAGGTATAGT	0	0	0	0	1	No gene matches found
GATGCTGCTC	0	1	0	0	0	No gene matches found
TCTGGGTAGG	0	0	1	0	0	No gene matches found
AAGCAACTGA	0	0	0	0	1	No gene matches found
AGTATTGTGG	0	0	1	0	0	No gene matches found
GTAGAGTTGT	1	0	0	0	0	No gene matches found
TCGTATCCTG	1	0	0	0	0	No gene matches found
AATCTTATCT	0	1	0	0	0	No gene matches found
AGGAGATGAG	1	0	0	0	0	No gene matches found
GCCCCCAGAT	0	0	0	1	0	No gene matches found
TGGAACTAAG	0	0	0	0	1	No gene matches found
AGTTTGTGTG	0	1	0	0	0	No gene matches found
ATTTGAGCCC	0	0	1	0	0	No gene matches found
CCGATTGCCG	1	0	0	0	0	No gene matches found
TGCGAGGCAA	0	0	0	1	0	No gene matches found
CGGATGTAAT	0	0	0	1	0	No gene matches found
AATGCTGCCT	0	0	1	0	0	No gene matches found
AGTGTGCTTT	0	1	0	0	0	No gene matches found
GGTTCAACGC	0	0	0	0	1	No gene matches found
AGAACGCTCT	1	0	0	0	0	No gene matches found
GATGTTAGCA	1	0	0	0	0	No gene matches found
ATTAGCATCT	0	0	1	0	0	No gene matches found
ACGGCTCTAT	0	0	1	0	0	No gene matches found
ATGCCCGAAA	0	1	0	0	0	No gene matches found
ACTCTTTTAG	1	0	0	0	0	No gene matches found
ATTCGAAATG	1	0	0	0	0	No gene matches found
ATCAAACGTT	0	0	1	0	0	No gene matches found
TAGGGTATAG	0	0	0	1	0	No gene matches found
AAAGTTCTGG	0	1	0	0	0	No gene matches found
CAGATCTGGA	0	0	1	0	0	No gene matches found
TAGGAACTTT	0	0	0	1	0	No gene matches found
ACGCAGACTT	0	1	0	0	0	No gene matches found
GGTATGCCTA	1	0	0	0	0	No gene matches found
AACTAGGAAG	0	1	0	0	0	No gene matches found
CGGTGAGAAA	0	0	0	0	1	No gene matches found
ACGAAAGATG	0	0	0	1	0	No gene matches found
TTGGGTTAGC	0	1	0	0	0	No gene matches found

GGCGCACGAC	0	0	0	0	1	No gene matches found
CCGGTGTTAT	0	0	1	0	0	No gene matches found
CACCTGAACT	0	0	1	0	0	No gene matches found
ATTAAAGGCT	0	1	0	0	0	No gene matches found
CGCAGCCACG	0	1	0	0	0	No gene matches found
CGAGCTGTAA	0	1	0	0	0	No gene matches found
GTGCCCAGCT	1	0	0	0	0	No gene matches found
GTTCAGTGCT	0	1	0	0	0	No gene matches found
GAGTACTAGG	0	0	0	1	0	No gene matches found
GAACGAGTGA	0	0	1	0	0	No gene matches found
GAGTGATTGT	0	0	0	0	1	No gene matches found
AGCCTGTGTA	0	0	0	0	1	No gene matches found
GGGAAATGCT	0	0	0	1	0	No gene matches found
GCCTGGTTTA	1	0	0	0	0	No gene matches found
CCAAATCCGT	0	0	0	1	0	No gene matches found
TGCCTTCATC	0	0	0	1	0	No gene matches found
CGCTTAGACT	0	1	0	0	0	No gene matches found
GAGTAGTTGA	1	0	0	0	0	No gene matches found
AAAGTTCCCG	0	0	0	1	0	No gene matches found
TATGAACCGA	0	1	0	0	0	No gene matches found
AGAACCTCAA	0	0	1	0	0	No gene matches found
ATGCCCGGAT	1	0	0	0	0	No gene matches found
AGCAATTATG	1	0	0	0	0	No gene matches found
TACAGCGCCA	0	0	1	0	0	No gene matches found
GAATCAAAGC	0	0	1	0	0	No gene matches found
TTGTTACCTC	0	0	0	0	1	No gene matches found
GCACTCCTAC	1	0	0	0	0	No gene matches found
GTACGAATCT	0	0	1	0	0	No gene matches found
CGAGTGAAAA	0	0	0	0	1	No gene matches found
GCAACAGCAG	0	1	0	0	0	No gene matches found
TAATGTAATC	0	0	1	0	0	No gene matches found
AGGTAGTAGA	1	0	0	0	0	No gene matches found
GTGTGGACGC	0	0	1	0	0	No gene matches found
TCTTATGCCA	1	0	0	0	0	No gene matches found
GCATTAAAGC	0	0	0	1	0	No gene matches found
TGAGTACAAG	0	0	1	0	0	No gene matches found
CCCGTGGTCT	0	1	0	0	0	No gene matches found
TTTTCGAGTT	0	0	1	0	0	No gene matches found
CCCGTGGGAA	0	0	1	0	0	No gene matches found
GATATGAACG	0	0	0	1	0	No gene matches found
CCCTCCATAG	0	0	1	0	0	No gene matches found
TAACGAAAAT	0	0	1	0	0	No gene matches found
TCCCTCATCG	0	0	1	0	0	No gene matches found
TTTAGAATGC	1	0	0	0	0	No gene matches found
TCCAGACCTG	0	0	1	0	0	No gene matches found
ACATCCATCG	0	1	0	0	0	No gene matches found
CGTCAGATTG	0	1	0	0	0	No gene matches found
GGGTTCCACC	0	0	1	0	0	No gene matches found
ATTTGACGCA	0	1	0	0	0	No gene matches found
TCCCGCTATT	0	0	0	1	0	No gene matches found
AAGGAGAACC	0	0	1	0	0	No gene matches found
TTGGCCGAGA	0	0	1	0	0	No gene matches found
TTGTGCTACT	0	0	0	1	0	No gene matches found
CTGGTGTTTC	0	0	1	0	0	No gene matches found
AGTTGCCAGA	0	0	1	0	0	No gene matches found
AGAGTAGAAC	1	0	0	0	0	No gene matches found
GGTTGATCCT	0	0	0	1	0	No gene matches found
TACCCCCGGT	0	0	0	0	1	No gene matches found
CGCGGATGAG	0	0	0	1	0	No gene matches found
CTTTGTATAA	1	0	0	0	0	No gene matches found
CCCGACTCTT	0	0	1	0	0	No gene matches found

ACCTAGTCAG	0	0	0	0	1	No gene matches found
TCCTGCTGGG	0	1	0	0	0	No gene matches found
CTGTCAAGCT	0	1	0	0	0	No gene matches found
TTAATCCCAA	0	1	0	0	0	No gene matches found
TGTGCGATGC	1	0	0	0	0	No gene matches found
CTATGCATCG	1	0	0	0	0	No gene matches found
GTATACAGAG	0	1	0	0	0	No gene matches found
CTGTTGACTG	0	1	0	0	0	No gene matches found
CACTTGTAGT	0	0	1	0	0	No gene matches found
GCTATTGCAC	0	0	0	1	0	No gene matches found
AGCCTCTGTT	0	1	0	0	0	No gene matches found
GCATCATAAT	0	0	1	0	0	No gene matches found
CTAAATCAAC	0	0	0	1	0	No gene matches found
TGCTACTATT	0	0	1	0	0	No gene matches found
GAGGCCGAAG	0	0	0	1	0	No gene matches found
GGGTTCCTAG	0	1	0	0	0	No gene matches found
CTACCTCATC	1	0	0	0	0	No gene matches found
CACCAGGACT	0	0	1	0	0	No gene matches found
CAGTCTAGTG	1	0	0	0	0	No gene matches found
CTTTGCGCCA	0	1	0	0	0	No gene matches found
CGGAGAAAGA	1	0	0	0	0	No gene matches found
TTGATGCGGC	1	0	0	0	0	No gene matches found
GGAGTGACTC	0	0	0	0	1	No gene matches found
TACTTGACAT	1	0	0	0	0	No gene matches found
ATGGCCGAAG	0	0	0	1	0	No gene matches found
ACTGGATAAA	0	0	1	0	0	No gene matches found
TGAACTGGAT	0	0	0	1	0	No gene matches found
ACTGGGGGTG	0	0	1	0	0	No gene matches found
GTCTCGCCGA	0	1	0	0	0	No gene matches found
CCCGTTCTCA	0	1	0	0	0	No gene matches found
GAACCGAGAG	0	0	1	0	0	No gene matches found
GGTATTTCCT	0	0	1	0	0	No gene matches found
CCTGGACTCG	0	1	0	0	0	No gene matches found
GCAATATATG	0	0	1	0	0	No gene matches found
CGCTCCGAAT	0	1	0	0	0	No gene matches found
TTGGTCCGAT	0	0	1	0	0	No gene matches found
TACACTCCGT	0	0	1	0	0	No gene matches found
AATACTGATA	0	0	0	1	0	No gene matches found
GAAAACTATT	0	0	0	0	1	No gene matches found
TAGTTGGCCA	0	0	1	0	0	No gene matches found
GTTTACTCAC	1	0	0	0	0	No gene matches found
GTCACGTGTT	0	0	0	0	1	No gene matches found
CCCAGTCCAG	0	0	0	1	0	No gene matches found
AATGACGAAA	0	0	1	0	0	No gene matches found
AACTGGAATG	0	0	0	1	0	No gene matches found
GATGGTCAAC	0	0	0	1	0	No gene matches found
GCGCCAGGTC	0	1	0	0	0	No gene matches found
CCTATTGCTT	0	0	1	0	0	No gene matches found
CCCGCTGAGC	0	0	0	1	0	No gene matches found
CCGGTCCACA	0	0	0	0	1	No gene matches found
AAGAGACGAG	0	0	0	1	0	No gene matches found
GGAATTGCTC	0	1	0	0	0	No gene matches found
AGCTGTTGAG	0	0	0	0	1	No gene matches found
TAGTTGCACT	0	0	1	0	0	No gene matches found
CCGGGTGGCA	0	0	0	1	0	No gene matches found
AATGCAGTAG	0	1	0	0	0	No gene matches found
GGACACATAC	0	0	1	0	0	No gene matches found
ATCCAGACGC	0	0	0	0	1	No gene matches found
ATATTCTTTC	0	1	0	0	0	No gene matches found
GCTCAGTTAT	0	0	0	1	0	No gene matches found
AGGTTACTCC	0	1	0	0	0	No gene matches found

GGAAGTTAGA	0	0	1	0	0	No gene matches found
GAGATTGGCT	0	0	0	0	1	No gene matches found
GGTAAAACGA	0	0	0	1	0	No gene matches found
AGTTGGGTCT	0	0	0	1	0	No gene matches found
CAACGAAGCT	0	1	0	0	0	No gene matches found
CGCTGATGAC	0	0	1	0	0	No gene matches found
TGAGGTGATT	0	1	0	0	0	No gene matches found
GGAGTGTGCA	0	0	0	1	0	No gene matches found
TCCGAAGAAA	0	0	1	0	0	No gene matches found
TTGGATCGGA	0	0	1	0	0	No gene matches found
GGGACACATT	0	0	1	0	0	No gene matches found
GCTAAAAGGC	0	0	1	0	0	No gene matches found
CCTTGGCTGT	0	1	0	0	0	No gene matches found
GCGACATAGA	0	0	0	0	1	No gene matches found
ATCATATCGG	0	0	0	0	1	No gene matches found
TAACCCAAAA	0	0	0	1	0	No gene matches found
ATCTCCCTCT	1	0	0	0	0	No gene matches found
GTCCACCGAG	0	0	0	0	1	No gene matches found
AGTGGTTAAC	0	0	0	0	1	No gene matches found
TAGCTGGAGG	0	0	0	1	0	No gene matches found
TTCCTTGGGT	1	0	0	0	0	No gene matches found
AGTGAAATAA	0	0	1	0	0	No gene matches found
AACGTAGTGC	0	0	0	0	1	No gene matches found
GGCTACCTCC	0	1	0	0	0	No gene matches found
GTCAGCATCT	0	0	1	0	0	No gene matches found
CTATACTCCG	0	1	0	0	0	No gene matches found
AGTTTTTACA	0	1	0	0	0	No gene matches found
CTATCAAAAG	0	0	0	1	0	No gene matches found
ACAGTGACAT	0	1	0	0	0	No gene matches found
TCGCGTTGAT	0	0	0	1	0	No gene matches found
CTCTTTAAGA	0	0	1	0	0	No gene matches found
GAAAGTTCTC	0	1	0	0	0	No gene matches found
GAATGATCCC	0	1	0	0	0	No gene matches found
CTCATAGTGT	1	0	0	0	0	No gene matches found
TGCGGCTGCG	0	0	1	0	0	No gene matches found
GAATATCTCG	0	1	0	0	0	No gene matches found
CTCGACAAGA	0	0	1	0	0	No gene matches found
GCCGCAATAG	0	1	0	0	0	No gene matches found
CATTTGATCG	0	0	0	1	0	No gene matches found
CGGAGGAGAC	0	0	0	1	0	No gene matches found
TGACGAAGAG	0	1	0	0	0	No gene matches found
GTCGATGCGT	0	0	1	0	0	No gene matches found
AGCGTACGAT	0	0	0	1	0	No gene matches found
AAAGAATCGA	1	0	0	0	0	No gene matches found
CTCGAATAGT	1	0	0	0	0	No gene matches found
GTTACTCTTA	1	0	0	0	0	No gene matches found
GGCCAAAAGC	0	0	1	0	0	No gene matches found
TGTCTGTCAT	0	1	0	0	0	No gene matches found
GGGGAGGTAA	0	0	0	1	0	No gene matches found
TTAGCGCAAG	1	0	0	0	0	No gene matches found
GCCCTAGTTT	0	1	0	0	0	No gene matches found
CAGGTGGGCC	0	0	0	1	0	No gene matches found
GGTTGAATCT	0	0	0	0	1	No gene matches found
GCTTTTTCGT	0	0	0	0	1	No gene matches found
GGCGATGTAA	0	0	1	0	0	No gene matches found
TGGGGTGATG	0	0	0	1	0	No gene matches found
GGTCTTCGCC	0	0	0	1	0	No gene matches found
TTGGTACGTT	1	0	0	0	0	No gene matches found
CCTATTCTTG	0	1	0	0	0	No gene matches found
GCACCAGGAT	0	0	1	0	0	No gene matches found
TCTGTGTGGG	1	0	0	0	0	No gene matches found

CGAAATTAAA	0	1	0	0	0	No gene matches found
GGATAAAGTG	0	0	0	1	0	No gene matches found
TGACTCGTTT	0	0	1	0	0	No gene matches found
AGGCAGACCT	0	0	1	0	0	No gene matches found
TCTCTCGCCT	0	1	0	0	0	No gene matches found
CCCGAATCTA	0	1	0	0	0	No gene matches found
TGCGTTATTC	1	0	0	0	0	No gene matches found
GTGCGTTGGT	0	1	0	0	0	No gene matches found
GCCTTCGCTT	1	0	0	0	0	No gene matches found
ACTGCGAAGC	0	1	0	0	0	No gene matches found
CTAAAACGCT	0	1	0	0	0	No gene matches found
ATCACTGATA	0	0	1	0	0	No gene matches found
GGAAGTATGT	1	0	0	0	0	No gene matches found
TCCGGTATAG	0	0	0	0	1	No gene matches found
TGACGGGTGA	0	0	1	0	0	No gene matches found
TGGCCCCTCT	0	0	1	0	0	No gene matches found
GGCATTTTTA	0	0	1	0	0	No gene matches found
AAAGGCCTTC	0	0	0	0	1	No gene matches found
TAGGAAGGTA	0	0	0	0	1	No gene matches found
GCACGGAGTG	0	0	1	0	0	No gene matches found
GTGCGGCGGC	0	1	0	0	0	No gene matches found
CTGGTTCATA	0	0	1	0	0	No gene matches found
GGAGTTGAGA	0	0	0	1	0	No gene matches found
TCTGGGGTTT	0	0	0	1	0	No gene matches found
CAGGGGCCGA	1	0	0	0	0	No gene matches found
CATCGATCCG	0	0	1	0	0	No gene matches found
TTTTGCCTTC	0	1	0	0	0	No gene matches found
GTCTGAAGGA	1	0	0	0	0	No gene matches found
GCGACGGATA	0	0	1	0	0	No gene matches found
CCTCCTGGTG	0	0	1	0	0	No gene matches found
ATGTCTGATA	0	1	0	0	0	No gene matches found
CACCGTTCCT	0	1	0	0	0	No gene matches found
GGCTTCCTCA	0	1	0	0	0	No gene matches found
ATCTGGCATA	1	0	0	0	0	No gene matches found
GGGCCACTTC	0	0	0	1	0	No gene matches found
GGAACTTTAC	1	0	0	0	0	No gene matches found
GATGGTCCAG	0	1	0	0	0	No gene matches found
AGTGTGCGAT	0	1	0	0	0	No gene matches found
ATGTCCCTCC	0	0	0	0	1	No gene matches found
AACCCAGCCA	0	0	1	0	0	No gene matches found
GCCCTTGCGC	0	0	0	0	1	No gene matches found
CCCTTCGAGG	0	0	0	1	0	No gene matches found
AAGTATCGGT	0	0	0	0	1	No gene matches found
GAGGTATTAA	0	1	0	0	0	No gene matches found
GCATCTTTGT	1	0	0	0	0	No gene matches found
CACGTTGGTT	0	0	1	0	0	No gene matches found
ATTTGACACG	0	0	1	0	0	No gene matches found
TGAGCTGGCA	0	0	1	0	0	No gene matches found
GGAGTCACAG	0	1	0	0	0	No gene matches found
CAAGTTGGAA	0	0	0	1	0	No gene matches found
GCAGGGGAAA	0	1	0	0	0	No gene matches found
GCTATGAGTT	0	1	0	0	0	No gene matches found
GAGGTATCGG	0	1	0	0	0	No gene matches found
CTTGGTGCGG	0	0	0	1	0	No gene matches found
CTTTTCCGCC	0	0	1	0	0	No gene matches found
TACGCGTGAT	0	1	0	0	0	No gene matches found
TGTCGGTTCT	0	1	0	0	0	No gene matches found
TTACCTCTCT	0	0	1	0	0	No gene matches found
AGTGTCCTTG	0	1	0	0	0	No gene matches found
TAAATCACGC	0	0	1	0	0	No gene matches found

TAAAATCGGT	0	1	0	0	0	No gene matches found
GTCAAATACC	0	0	0	1	0	No gene matches found
TGGGCTTGCC	0	1	0	0	0	No gene matches found
TCCTTCTATG	0	0	0	0	1	No gene matches found
ACCCAAGCGG	0	1	0	0	0	No gene matches found
TGTCTAGATA	0	0	0	0	1	No gene matches found
CAACACCGGG	0	0	1	0	0	No gene matches found
TGATTGGCAA	0	0	0	0	1	No gene matches found
GGGGGCAGCC	0	0	1	0	0	No gene matches found
TCGCTGCCAT	0	1	0	0	0	No gene matches found
AACCCTGTCA	0	1	0	0	0	No gene matches found
GAGGGGTTGG	0	1	0	0	0	No gene matches found
GTGGAATTAA	0	0	0	0	1	No gene matches found
GTGGTGGGTA	0	0	0	1	0	No gene matches found
AGAACTTACG	0	1	0	0	0	No gene matches found
AGGCTAAGGA	0	1	0	0	0	No gene matches found
TGTGAAAGAA	1	0	0	0	0	No gene matches found
CTCACACAAT	0	1	0	0	0	No gene matches found
ATACGGCGTG	0	1	0	0	0	No gene matches found
GGTGCTGGAA	0	0	0	0	1	No gene matches found
GCCTTGAGCG	0	1	0	0	0	No gene matches found
TAGCCTATCT	0	1	0	0	0	No gene matches found
ATTTTAGTGC	0	0	0	0	1	No gene matches found
CGCTTAGACC	0	1	0	0	0	No gene matches found
CGCCCGGCGC	0	0	0	0	1	No gene matches found
GCTGGGATCA	0	0	0	1	0	No gene matches found
AGTCCGAGCA	0	0	1	0	0	No gene matches found
CAGTCTATCT	0	0	1	0	0	No gene matches found
TTGAGACTAA	0	0	0	0	1	No gene matches found
CGTCATCAAA	0	1	0	0	0	No gene matches found
GTGGGCTCGA	1	0	0	0	0	No gene matches found
TGTCTTTAGA	0	0	1	0	0	No gene matches found
TTATGTGACC	0	0	1	0	0	No gene matches found
GCCTTTAGCA	0	0	0	0	1	No gene matches found
CCTCCCACAG	0	0	0	1	0	No gene matches found
CACTCTGTGA	0	0	0	1	0	No gene matches found
GGTTATGTGA	0	0	1	0	0	No gene matches found
GGAGATCACT	0	1	0	0	0	No gene matches found
TCGGGATTTG	0	1	0	0	0	No gene matches found
TCCGAAAGGT	0	0	0	0	1	No gene matches found
TGCGATAGCA	0	0	1	0	0	No gene matches found
GTAGTGAAAT	0	0	0	0	1	No gene matches found
AGCGAAGATT	0	0	0	0	1	No gene matches found
CAGGAGTGTG	0	0	0	0	1	No gene matches found
TAACCTGTAT	1	0	0	0	0	No gene matches found
AACTCGCCAG	0	0	1	0	0	No gene matches found
CAGTATCGCT	0	1	0	0	0	No gene matches found
ACCGTCCACC	1	0	0	0	0	No gene matches found
CCATACTCCC	0	1	0	0	0	No gene matches found
GGATAGTCTG	1	0	0	0	0	No gene matches found
GAACCTCGGC	0	1	0	0	0	No gene matches found
TAACCGTTAT	0	0	1	0	0	No gene matches found
ATGTATTGTA	1	0	0	0	0	No gene matches found
GCGGGTGATG	0	1	0	0	0	No gene matches found
CAATCTTCGA	0	0	1	0	0	No gene matches found
GTCCGATTGA	1	0	0	0	0	No gene matches found
CAGGTGTAGC	0	1	0	0	0	No gene matches found
TTTGGAATTG	0	1	0	0	0	No gene matches found
TGATTTCTAA	0	0	1	0	0	No gene matches found
GCTTAAGATG	0	1	0	0	0	No gene matches found
TCTGCGGATC	0	0	0	1	0	No gene matches found

TCTCCCATTG	1	0	0	0	0	No gene matches found
CCTCTTATAA	0	0	0	0	1	No gene matches found
AGTCAAATTG	0	1	0	0	0	No gene matches found
ATGAAGGTGT	0	1	0	0	0	No gene matches found
CCTTGCTGTG	0	1	0	0	0	No gene matches found
TAATAGCAGA	1	0	0	0	0	No gene matches found
TCGTAAGACC	1	0	0	0	0	No gene matches found
ATCGCTTAAT	0	0	0	1	0	No gene matches found
GAACAGATGG	0	1	0	0	0	No gene matches found
CAACTGTGCG	0	1	0	0	0	No gene matches found
CCTGTGTCAG	0	1	0	0	0	No gene matches found
TGATCGGCAT	0	1	0	0	0	No gene matches found
TTGGTGTTAT	0	1	0	0	0	No gene matches found
CGCTCCAATT	0	1	0	0	0	No gene matches found
GCCTGCAGGG	0	0	1	0	0	No gene matches found
GAGTGGTATT	0	1	0	0	0	No gene matches found
GCGTGTGAAC	0	0	0	1	0	No gene matches found
AAGCGATTTT	0	0	1	0	0	No gene matches found
GGATGCGATC	0	0	1	0	0	No gene matches found
TCTCACTTAT	0	0	1	0	0	No gene matches found
GTGTTTCTGT	0	1	0	0	0	No gene matches found
GGCCTGTTGT	0	0	0	0	1	No gene matches found
CCCAGTTTCA	0	1	0	0	0	No gene matches found
TTAGGAGCAG	0	0	0	1	0	No gene matches found
GCTGGCGTCT	0	1	0	0	0	No gene matches found
TCTGACTTCG	0	0	0	1	0	No gene matches found
AGAGTACGAT	0	0	0	0	1	No gene matches found
TCGAGTTCAC	0	0	1	0	0	No gene matches found
TGACTGTGCG	0	1	0	0	0	No gene matches found
GAATTTCTCG	0	1	0	0	0	No gene matches found
ACGAATACAT	0	1	0	0	0	No gene matches found
CAGTTGCTAT	0	1	0	0	0	No gene matches found
GAATGGCTCT	0	1	0	0	0	No gene matches found
ACTTCGACGC	0	0	0	0	1	No gene matches found
CCAGGGGTAA	0	1	0	0	0	No gene matches found
ACCGGCAGGA	0	1	0	0	0	No gene matches found
GGAACTAAGT	0	0	0	0	1	No gene matches found
TAGGACGAAA	1	0	0	0	0	No gene matches found
CAGAGTGGTA	0	0	0	1	0	No gene matches found
TTCTACCATT	0	0	0	0	1	No gene matches found
CCTGGACCCA	0	1	0	0	0	No gene matches found
TTCCCGATTA	0	1	0	0	0	No gene matches found
TACACAATTC	0	0	1	0	0	No gene matches found
ACAGTGATGG	1	0	0	0	0	No gene matches found
CGTGTTTTCT	0	1	0	0	0	No gene matches found
CGTTTCATAT	1	0	0	0	0	No gene matches found
AAATCCTCTC	0	1	0	0	0	No gene matches found
CTCCTGAACG	0	0	0	1	0	No gene matches found
TTAGACTCGA	0	0	0	0	1	No gene matches found
GGAGGCAGAC	0	1	0	0	0	No gene matches found
CAGGCAACAC	0	1	0	0	0	No gene matches found
CAGCTAGGAG	0	1	0	0	0	No gene matches found
GGTGCTCGTC	1	0	0	0	0	No gene matches found
TAGTGTCGAA	0	0	0	0	1	No gene matches found
TAATTGCTGG	0	0	0	0	1	No gene matches found
GAATCAGGGA	0	0	1	0	0	No gene matches found
TAAGAGGAGT	0	0	1	0	0	No gene matches found
CTTCTAACGA	0	0	0	0	1	No gene matches found
CCACAAGATT	0	1	0	0	0	No gene matches found
CAGTGAAGTT	1	0	0	0	0	No gene matches found
GTTGCCTAAA	0	0	1	0	0	No gene matches found

TCCCTGTAAA	0	0	1	0	0	No gene matches found
GCCAAAGTCA	1	0	0	0	0	No gene matches found
CTCCAAGTGG	0	1	0	0	0	No gene matches found
CTTGATCTTT	1	0	0	0	0	No gene matches found
GACAGATTGT	1	0	0	0	0	No gene matches found
CGGATTTGGT	1	0	0	0	0	No gene matches found
GCACGGGTGT	0	0	1	0	0	No gene matches found
CAACATCGTA	1	0	0	0	0	No gene matches found
GCGACGGGGA	1	0	0	0	0	No gene matches found
CCAGCTGATT	0	1	0	0	0	No gene matches found
GATTAGTGAG	0	0	0	1	0	No gene matches found
ACGGTGTTCT	0	1	0	0	0	No gene matches found
GGTAATCGAG	0	0	1	0	0	No gene matches found
CTTATGACAT	0	1	0	0	0	No gene matches found
TGGCAAACTG	1	0	0	0	0	No gene matches found
TACACTGCTC	0	0	0	0	1	No gene matches found
GCGTCCGCGA	1	0	0	0	0	No gene matches found
GTCGGGAGCT	0	0	0	1	0	No gene matches found
TCCGAAGGAG	0	0	0	0	1	No gene matches found
TGCTAGTGTG	0	1	0	0	0	No gene matches found
AATGGTTGGG	1	0	0	0	0	No gene matches found
GACGGTAGTA	0	0	0	1	0	No gene matches found
GCCTTAAAAG	0	1	0	0	0	No gene matches found
AGAGGACGAG	0	0	1	0	0	No gene matches found
CACACAGGGT	0	1	0	0	0	No gene matches found
GGTGTTTATC	0	0	0	0	1	No gene matches found
TGACTGTAAA	0	1	0	0	0	No gene matches found
TGCTAAGGAG	0	0	1	0	0	No gene matches found
GTGTATCAAA	0	0	1	0	0	No gene matches found
TTGATCCCGC	0	1	0	0	0	No gene matches found
GCAGAGTGCT	0	0	1	0	0	No gene matches found
GCACACAAAG	0	0	0	1	0	No gene matches found
TTTCGCCGAC	1	0	0	0	0	No gene matches found
TCTCGATAAT	0	0	1	0	0	No gene matches found
GACTCGGTGC	0	0	0	1	0	No gene matches found
TTATTCGAAT	0	0	1	0	0	No gene matches found
GTCAGAGACT	0	0	1	0	0	No gene matches found
GCGAGTCAAC	0	0	0	0	1	No gene matches found
TTGTCTACAG	0	0	1	0	0	No gene matches found
TGAGATATCT	0	0	0	1	0	No gene matches found
ACTTGTTGTG	0	1	0	0	0	No gene matches found
AGTGAATAGG	0	0	0	1	0	No gene matches found
CTGCAGCATT	0	0	0	1	0	No gene matches found
TGCGTCTAAA	0	0	1	0	0	No gene matches found
TACAACTAGG	1	0	0	0	0	No gene matches found
CGAGCTGTTG	0	1	0	0	0	No gene matches found
ATCGTCCGAA	0	1	0	0	0	No gene matches found
AGCATCGTAC	0	1	0	0	0	No gene matches found
CAGCGGCTCC	0	1	0	0	0	No gene matches found
CTTTTTGAGG	0	0	0	1	0	No gene matches found
CCCCTTAAAG	0	1	0	0	0	No gene matches found
GGAGTTTTTC	0	0	1	0	0	No gene matches found
CGAAGACTTT	0	0	1	0	0	No gene matches found
GAATTTAGAC	0	1	0	0	0	No gene matches found
GGGTCAAACG	0	0	0	1	0	No gene matches found
GATTGGTAAG	0	0	1	0	0	No gene matches found
TCCGAACCTA	0	1	0	0	0	No gene matches found
GCCGAAGGAA	0	0	0	1	0	No gene matches found
CACGAGAATG	0	1	0	0	0	No gene matches found
TAACGGTTTG	0	1	0	0	0	No gene matches found
ATGACTTTCG	0	1	0	0	0	No gene matches found

ACACCTCGTG	0	0	0	1	0	No gene matches found
AAGGCGCCCG	1	0	0	0	0	No gene matches found
GCAATGTGAA	0	0	1	0	0	No gene matches found
AGTGCGTTTT	0	0	0	1	0	No gene matches found
TAAATTAGGA	0	0	0	1	0	No gene matches found
TGATCCTGTA	1	0	0	0	0	No gene matches found
CAGCCTCCCG	0	0	0	1	0	No gene matches found
CTGTTTTCTG	0	0	0	0	1	No gene matches found
GCCATCGCAG	0	0	1	0	0	No gene matches found
CAAAGCGGCG	0	0	0	1	0	No gene matches found
AGTTGCCTTC	0	1	0	0	0	No gene matches found
GGCTCGGTTT	0	0	0	1	0	No gene matches found
GGCCTGAGCC	0	1	0	0	0	No gene matches found
GCTGCCTGAT	1	0	0	0	0	No gene matches found
GTCACGCAGA	0	0	0	1	0	No gene matches found
TATTCCGGTT	0	0	0	1	0	No gene matches found
CAAACTATTG	0	0	0	0	1	No gene matches found
TTATCCCTCT	0	1	0	0	0	No gene matches found
TGTGTAACTT	0	0	0	1	0	No gene matches found
CCTCTAGAGA	0	0	0	0	1	No gene matches found
CCCTCGGATG	0	0	1	0	0	No gene matches found
GAGGCGCAAA	0	0	0	1	0	No gene matches found
CCTTAGATCT	0	0	0	0	1	No gene matches found
TATCCACCAA	0	0	1	0	0	No gene matches found
AGAGGTCAAA	0	0	0	1	0	No gene matches found
GAAAAGCCCT	0	0	0	1	0	No gene matches found
CTCATTGAAG	0	0	0	1	0	No gene matches found
CGGGAAAGAA	0	0	0	0	1	No gene matches found
CAAGTGTGGC	0	1	0	0	0	No gene matches found
TATCTCCGGC	0	1	0	0	0	No gene matches found
TACTTCAACG	0	0	0	0	1	No gene matches found
CTGTTGACCA	0	1	0	0	0	No gene matches found
GAGAAAGCCG	0	0	1	0	0	No gene matches found
AACGACAGGG	0	0	1	0	0	No gene matches found
TTTCTAGTTG	0	0	1	0	0	No gene matches found
CAGAACATTG	1	0	0	0	0	No gene matches found
CCTGGTCTAA	0	0	1	0	0	No gene matches found
TTGGATACGG	0	0	0	0	1	No gene matches found
CGACCGCCGC	0	1	0	0	0	No gene matches found
CTTTCTGATC	0	1	0	0	0	No gene matches found
GTTTCGCAGA	0	0	0	1	0	No gene matches found
AACGCAGTCG	0	1	0	0	0	No gene matches found
GGGTTTATTC	0	1	0	0	0	No gene matches found
GCGCCGTCTC	0	1	0	0	0	No gene matches found
GCGTTTTATC	1	0	0	0	0	No gene matches found
GACCTACGGA	0	1	0	0	0	No gene matches found
TCACTTATGG	0	0	0	1	0	No gene matches found
TCCTTGCAGT	0	0	0	1	0	No gene matches found
CCAGATTCTC	0	1	0	0	0	No gene matches found
AAGTAATGTA	0	1	0	0	0	No gene matches found
CGTTGTCGGA	1	0	0	0	0	No gene matches found
AGGAAACCTG	0	0	1	0	0	No gene matches found
CCTGGGTGTG	0	0	0	1	0	No gene matches found
ATGACGACTT	0	0	0	0	1	No gene matches found
ACCGTGCTGG	0	1	0	0	0	No gene matches found
TGGCAGCTGA	1	0	0	0	0	No gene matches found
AAAGCGGTTT	0	0	1	0	0	No gene matches found
CGCCCGCCAA	0	1	0	0	0	No gene matches found
CAGTTCGGGG	0	0	1	0	0	No gene matches found
CTCAGTGATG	0	0	1	0	0	No gene matches found

GCTCCCACTC	0	0	1	0	0	No gene matches found
CCGCAGAGAG	0	0	1	0	0	No gene matches found
AATGGATCAG	0	1	0	0	0	No gene matches found
GTGTAACAGA	0	0	0	1	0	No gene matches found
CTAAGAGCCC	0	0	0	1	0	No gene matches found
TGTACTTTCT	1	0	0	0	0	No gene matches found
TGCGAGTGGT	0	0	1	0	0	No gene matches found
AATTGGAGCA	1	0	0	0	0	No gene matches found
AGGATGTACT	0	1	0	0	0	No gene matches found
ACAGACATTG	0	0	0	0	1	No gene matches found
CCAAGCAGCA	0	1	0	0	0	No gene matches found
CGAGGTCCAA	0	0	0	1	0	No gene matches found
CCAAAGTAGA	0	0	0	0	1	No gene matches found
TTGAATACAT	1	0	0	0	0	No gene matches found
GAAACTTGTC	0	0	0	0	1	No gene matches found
CGTTGCAATA	0	0	1	0	0	No gene matches found
ACACACCAAG	0	0	1	0	0	No gene matches found
CAACGTGTAT	0	1	0	0	0	No gene matches found
GGGATGGGCT	0	0	1	0	0	No gene matches found
GGGACCAATC	0	0	0	0	1	No gene matches found
TCCCTACTGG	0	0	1	0	0	No gene matches found
CGCTCGGCGA	0	0	0	1	0	No gene matches found
AGAGGTCCGA	0	1	0	0	0	No gene matches found
AAGGATGGCT	0	0	0	1	0	No gene matches found
CCACATTGAT	0	1	0	0	0	No gene matches found
ACCAGGAGAC	1	0	0	0	0	No gene matches found
TATACACTTA	0	0	0	0	1	No gene matches found
GCAGCTGACT	0	1	0	0	0	No gene matches found
CGGAACATTA	0	0	1	0	0	No gene matches found
GGAAGCAAAT	1	0	0	0	0	No gene matches found
CAATAAGTTA	0	1	0	0	0	No gene matches found
CGGAAGACAG	0	1	0	0	0	No gene matches found
GTATACGCAA	1	0	0	0	0	No gene matches found
GATCCATACT	0	1	0	0	0	No gene matches found
TTGTTCTCGC	1	0	0	0	0	No gene matches found
GTCAAGTGGT	0	0	0	0	1	No gene matches found
ACGACAAGCA	1	0	0	0	0	No gene matches found
CCTCTCCAGG	0	0	1	0	0	No gene matches found
ATAGTGGGGT	1	0	0	0	0	No gene matches found
GGCGACGACT	0	1	0	0	0	No gene matches found
AGGAAGACCA	1	0	0	0	0	No gene matches found
CCTAAGTAGA	0	0	1	0	0	No gene matches found
ATCCCCTGAA	0	0	0	1	0	No gene matches found
TGCTCCCAAG	0	1	0	0	0	No gene matches found
GCACGGAGCG	0	0	1	0	0	No gene matches found
AAAGGGGACT	1	0	0	0	0	No gene matches found
CGATACTCCC	0	1	0	0	0	No gene matches found
TCCAGACAAA	1	0	0	0	0	No gene matches found
AGAACGCCGT	0	1	0	0	0	No gene matches found
AATCCCGAAG	1	0	0	0	0	No gene matches found
CATCAATCTT	1	0	0	0	0	No gene matches found
GGGAATACAT	1	0	0	0	0	No gene matches found
GTTGAAAAGC	0	0	1	0	0	No gene matches found
AAGGGGAATG	1	0	0	0	0	No gene matches found
TTATGAGCCC	0	0	0	0	1	No gene matches found
GTAATTCAGA	0	0	0	0	1	No gene matches found
GCAGCCTTTG	0	1	0	0	0	No gene matches found
GGGGCCTGAG	0	1	0	0	0	No gene matches found
CCTATAGAGA	0	0	1	0	0	No gene matches found
AGATGAATTA	0	0	1	0	0	No gene matches found
TATGTCGGAC	0	0	0	1	0	No gene matches found

AACGGGTTGA	0	0	0	1	0	No gene matches found
GGTTTTGACA	0	1	0	0	0	No gene matches found
CGTACCTAAA	0	1	0	0	0	No gene matches found
GGTACACCAA	0	0	1	0	0	No gene matches found
GCAGGTGCAA	0	1	0	0	0	No gene matches found
AGCGTTTAAT	1	0	0	0	0	No gene matches found
TGTCCTATCA	0	0	0	0	1	No gene matches found
TGGATTGGGA	0	0	0	1	0	No gene matches found
GGCTTACATT	0	0	0	1	0	No gene matches found
CGGATCTTCT	0	0	1	0	0	No gene matches found
GAGGATAATT	0	0	1	0	0	No gene matches found
AGAGCCACTA	0	0	1	0	0	No gene matches found
ATCCCTTATG	0	1	0	0	0	No gene matches found
TCAATGTGTG	0	0	0	0	1	No gene matches found
CGATGCATCA	0	0	1	0	0	No gene matches found
GCACTGAGGG	0	1	0	0	0	No gene matches found
ATCTAGACAT	0	0	0	0	1	No gene matches found
GGAGCCTGTA	0	0	1	0	0	No gene matches found
CGGATGGTTA	0	0	0	0	1	No gene matches found
CGGAGGGTGC	0	0	1	0	0	No gene matches found
GTGTCTGTTC	0	0	1	0	0	No gene matches found
AATTCGAGCA	0	0	1	0	0	No gene matches found
GACAAGGCCC	0	0	0	0	1	No gene matches found
ACTATGGCTC	0	1	0	0	0	No gene matches found
GCTAAACTCC	1	0	0	0	0	No gene matches found
TTTTACCAGT	1	0	0	0	0	No gene matches found
CTTAGCCTTA	0	0	0	0	1	No gene matches found
TTACGAGAGG	0	0	0	0	1	No gene matches found
TAATGAAAGG	0	1	0	0	0	No gene matches found
CGAATGGTTG	0	0	0	1	0	No gene matches found
CACAGATCAG	0	1	0	0	0	No gene matches found
TTGTACGCTT	1	0	0	0	0	No gene matches found
TTAGCGCTAG	0	1	0	0	0	No gene matches found
ATAGTACAGG	0	0	1	0	0	No gene matches found
CTTGAAATAG	0	0	0	1	0	No gene matches found
TATTAGACTC	0	0	0	1	0	No gene matches found
TACAGAGAGC	0	1	0	0	0	No gene matches found
TGTGCAACTG	0	0	1	0	0	No gene matches found
ATTGGACAGA	0	1	0	0	0	No gene matches found
CAGACGGTCG	0	1	0	0	0	No gene matches found
GGAATCCCCA	0	1	0	0	0	No gene matches found
GAAGGCTCAC	0	0	0	1	0	No gene matches found
GGGCCAGAGC	0	0	0	1	0	No gene matches found
ATCGGTAGGA	1	0	0	0	0	No gene matches found
AGGGAGATAG	0	1	0	0	0	No gene matches found
ATTCCGTAGA	0	0	1	0	0	No gene matches found
GAGCGTTAGC	0	1	0	0	0	No gene matches found
GTTGAGTAGC	0	1	0	0	0	No gene matches found
TGCTAGAATG	0	1	0	0	0	No gene matches found
GAGGTCAGAT	0	0	0	1	0	No gene matches found
ATCCAAGTTA	0	0	0	0	1	No gene matches found
TGGTGCGAAA	0	0	1	0	0	No gene matches found
GGGATATTTG	1	0	0	0	0	No gene matches found
GGGGCAAGGA	0	1	0	0	0	No gene matches found
GAGAGCGTAG	0	0	0	1	0	No gene matches found
TCTATGTTGC	0	1	0	0	0	No gene matches found
AGGCGTTAGG	0	1	0	0	0	No gene matches found
ACCACTGATC	0	1	0	0	0	No gene matches found
CAGTCGTCCA	0	0	0	1	0	No gene matches found
TGAGGGGTAA	0	1	0	0	0	No gene matches found
TTGCGCGTTT	0	0	1	0	0	No gene matches found

TACTTTGTCT	1	0	0	0	0	No gene matches found
CGGGAACCAC	0	0	0	1	0	No gene matches found
AATGATCCTT	1	0	0	0	0	No gene matches found
TACATATAGG	1	0	0	0	0	No gene matches found
GATAATTCTC	0	1	0	0	0	No gene matches found
ATTTTGGACC	0	1	0	0	0	No gene matches found
ATTTATGCTG	0	0	0	0	1	No gene matches found
GGTAAAATAA	0	0	1	0	0	No gene matches found
GATCTGAGAA	0	0	0	0	1	No gene matches found
TGTGGTGACA	0	0	1	0	0	No gene matches found
TCTTGCTGGG	0	0	0	1	0	No gene matches found
CCGCAGAAAG	0	0	0	1	0	No gene matches found
ATGGGGTGTG	0	0	1	0	0	No gene matches found
GATCCGTACA	0	1	0	0	0	No gene matches found
AGGATCCGGA	0	0	0	1	0	No gene matches found
TCCCCCAAGT	0	0	1	0	0	No gene matches found
CTTAAGTGGG	0	0	0	0	1	No gene matches found
GGGTAGAGAC	1	0	0	0	0	No gene matches found
GGGATGCAGG	0	1	0	0	0	No gene matches found
TGAATTTGGC	0	0	0	1	0	No gene matches found
TCTAAGAGTT	1	0	0	0	0	No gene matches found
TTCGGAATAG	0	0	1	0	0	No gene matches found
CTGCAGCCCC	0	1	0	0	0	No gene matches found
ATAGCTCAGG	0	0	1	0	0	No gene matches found
GGGAGTTAGC	0	1	0	0	0	No gene matches found
CAACGAAGAG	0	0	1	0	0	No gene matches found
ATCCTGTTAA	0	1	0	0	0	No gene matches found
CTGCGGAATG	0	1	0	0	0	No gene matches found
TAGTGGAGGG	0	1	0	0	0	No gene matches found
AATCGCGTCG	0	0	0	1	0	No gene matches found
TAATTTAAGG	0	0	0	1	0	No gene matches found
GTCGGAGCCC	0	1	0	0	0	No gene matches found
GTCTCAAACG	0	0	0	0	1	No gene matches found
CTCGTGGGGC	0	0	1	0	0	No gene matches found
GTGCGACTTG	0	0	1	0	0	No gene matches found
AAGGCCATTG	0	0	0	0	1	No gene matches found
AAGACTGGTC	0	0	0	0	1	No gene matches found
TTCCTGGGAC	0	0	1	0	0	No gene matches found
AGCTGGACCT	1	0	0	0	0	No gene matches found
AGGCAACAGT	1	0	0	0	0	No gene matches found
CCTTTCGTAA	0	0	0	1	0	No gene matches found
ATCGGGAGCC	0	1	0	0	0	No gene matches found
GGTAGCGTAG	0	1	0	0	0	No gene matches found
CTACTGAGTT	0	0	1	0	0	No gene matches found
CGCTTCCGCC	0	0	0	1	0	No gene matches found
GGAAGACGAC	0	0	1	0	0	No gene matches found
GGAAAAACTT	0	0	0	0	1	No gene matches found
CCCCAGAGAC	0	0	0	1	0	No gene matches found
GGCGTTCTCC	0	1	0	0	0	No gene matches found
TTGTGTCGTT	0	0	0	1	0	No gene matches found
GCGACTTTGA	1	0	0	0	0	No gene matches found
CAACGTGTAG	0	1	0	0	0	No gene matches found
AGGTGATTTC	0	0	0	0	1	No gene matches found
ACACCGATTA	0	0	0	0	1	No gene matches found
CCTCTAAGGA	0	0	1	0	0	No gene matches found
CATCTTGGAA	0	1	0	0	0	No gene matches found
AGGCAGGCCG	0	1	0	0	0	No gene matches found
AACGCGACCG	1	0	0	0	0	No gene matches found
GGTTCCGTCC	0	0	0	1	0	No gene matches found
GAGCGGAGAT	0	0	0	0	1	No gene matches found
CCTTCTGTTC	0	1	0	0	0	No gene matches found

GTCTTACATC	0	1	0	0	0	No gene matches found
CTGGCAAATC	1	0	0	0	0	No gene matches found
CTGGCAAGAG	0	0	0	1	0	No gene matches found
TGGATCGCGC	0	1	0	0	0	No gene matches found
AGAATGGATT	0	0	0	1	0	No gene matches found
ATTTACTGAC	0	0	1	0	0	No gene matches found
GGAGACTGAG	0	0	0	0	1	No gene matches found
ATGATGGAAT	0	1	0	0	0	No gene matches found
CGCCCATATT	0	0	1	0	0	No gene matches found
GCAAGAGCCA	0	0	0	0	1	No gene matches found
TGTGGAGGTA	0	0	1	0	0	No gene matches found
AATGCTCGTG	0	0	0	1	0	No gene matches found
TTCTTCGCGC	0	0	0	1	0	No gene matches found
CACGAATCAA	0	1	0	0	0	No gene matches found
TATCCTATCT	0	0	0	0	1	No gene matches found
GTTTAACGTG	1	0	0	0	0	No gene matches found
GTTTCAACGG	0	1	0	0	0	No gene matches found
CCTTGATAAA	0	1	0	0	0	No gene matches found
TGGGAACATA	0	0	0	0	1	No gene matches found
ATTACACCCA	0	1	0	0	0	No gene matches found
AGCTCTGCGG	0	1	0	0	0	No gene matches found
TTGTACAAAG	0	0	0	0	1	No gene matches found
GGCGAACGAT	0	1	0	0	0	No gene matches found
CAGGAGGATG	0	0	0	1	0	No gene matches found
GCCCGTCTGC	0	1	0	0	0	No gene matches found
ACGAGCAGAA	0	0	1	0	0	No gene matches found
GTGTGATGAC	0	0	1	0	0	No gene matches found
GTACAGATCT	0	0	1	0	0	No gene matches found
TCTGTGAAGG	0	1	0	0	0	No gene matches found
ATTGTTCAAG	0	0	0	1	0	No gene matches found
CCAAATCCTG	0	0	1	0	0	No gene matches found
GTCTGTCTGA	0	1	0	0	0	No gene matches found
GGTCCAAAAA	0	0	0	1	0	No gene matches found
GGAAATGCCC	1	0	0	0	0	No gene matches found
TGAACGGCTA	0	0	0	1	0	No gene matches found
TTATTGGTCC	0	1	0	0	0	No gene matches found
TGATTGGTGT	0	0	0	0	1	No gene matches found
CAGGTGTGCC	1	0	0	0	0	No gene matches found
CTGCGCCTAT	0	1	0	0	0	No gene matches found
GATTACTCAA	0	0	1	0	0	No gene matches found
CCCCCGCCGC	0	0	0	1	0	No gene matches found
AGTGGGGCCG	0	0	1	0	0	No gene matches found
GTCGAAGGTG	0	0	0	0	1	No gene matches found
CATCGAAGTT	0	0	0	1	0	No gene matches found
TCCAGACGTA	0	0	1	0	0	No gene matches found
AGCTGTGGGA	0	0	1	0	0	No gene matches found
CAACTGTTTG	0	0	0	1	0	No gene matches found
CCTTGTATAT	1	0	0	0	0	No gene matches found
GTGGTAGCTT	0	0	0	1	0	No gene matches found
CGCGGAGCCC	0	1	0	0	0	No gene matches found
GGAGGAGGCC	0	0	1	0	0	No gene matches found
GAACATATAG	1	0	0	0	0	No gene matches found
CCTTATATAA	1	0	0	0	0	No gene matches found
AATAACGTTG	0	0	1	0	0	No gene matches found
TGGGATTCGA	0	0	1	0	0	No gene matches found
TAGCCAAAAC	0	0	1	0	0	No gene matches found
ATGCTCTAGC	0	1	0	0	0	No gene matches found
CATATCCCTA	0	0	0	0	1	No gene matches found
CGTTAAGATA	1	0	0	0	0	No gene matches found
CGGCTTAAAA	0	0	0	1	0	No gene matches found

GATGCTCACG	0	0	0	0	1	No gene matches found
TAATGTAGTG	0	0	1	0	0	No gene matches found
GTCGTCGTGA	0	0	1	0	0	No gene matches found
GGATCGAATC	0	0	0	1	0	No gene matches found
GGCGCTTCGC	0	0	1	0	0	No gene matches found
TACTTCTTAC	0	0	0	1	0	No gene matches found
GTTCAGTAAG	0	1	0	0	0	No gene matches found
GGTGGCGTCG	0	0	0	0	1	No gene matches found
GCTATGTCGG	0	1	0	0	0	No gene matches found
AGGATCCTTC	0	0	1	0	0	No gene matches found
GATGAGCTCA	0	0	0	1	0	No gene matches found
GCATTAGGCT	0	0	1	0	0	No gene matches found
AGTTTCGGTT	0	0	0	0	1	No gene matches found
TCAAAGCTTG	0	0	1	0	0	No gene matches found
CCAAAATGTA	0	1	0	0	0	No gene matches found
CCCTGATCGA	0	1	0	0	0	No gene matches found
CGAAAAAAAT	1	0	0	0	0	No gene matches found
CAGGTATGGC	0	0	1	0	0	No gene matches found
TAGGCTTTGA	1	0	0	0	0	No gene matches found
GTTTCTGATC	0	0	0	1	0	No gene matches found
GTCACCCCTA	0	0	0	1	0	No gene matches found
AGGAAGTAGA	1	0	0	0	0	No gene matches found
GTAGTGACTT	0	1	0	0	0	No gene matches found
CCTCATACCA	0	0	1	0	0	No gene matches found
TAGTACCGAT	1	0	0	0	0	No gene matches found
AGTTGACCTG	0	0	0	1	0	No gene matches found
TATAGATAGC	0	1	0	0	0	No gene matches found
GTCTTAGGTA	1	0	0	0	0	No gene matches found
TGTCTCACAG	0	1	0	0	0	No gene matches found
CACCGTGATC	0	0	1	0	0	No gene matches found
GGAGGGGCTC	0	0	1	0	0	No gene matches found
AGGTCGACGG	0	0	1	0	0	No gene matches found
TGGCTCATCC	0	1	0	0	0	No gene matches found
TTAGCTGTAT	0	1	0	0	0	No gene matches found
AGAAGGCTCG	0	1	0	0	0	No gene matches found
TACCTGTAAG	0	1	0	0	0	No gene matches found
TGAGGATACT	0	0	0	0	1	No gene matches found
GGCGAAGATA	1	0	0	0	0	No gene matches found
TAGCCAGTAA	0	0	1	0	0	No gene matches found
TGAACCTTGT	0	1	0	0	0	No gene matches found
CAACCGTTTG	0	0	0	0	1	No gene matches found
TGAAGAACCC	0	1	0	0	0	No gene matches found
AGCTACCGCT	0	0	0	1	0	No gene matches found
CCTCTTCGGA	0	0	1	0	0	No gene matches found
GGCGGTCGCT	0	1	0	0	0	No gene matches found
GATAATTCGG	0	1	0	0	0	No gene matches found
GAATCACTGG	0	0	1	0	0	No gene matches found
CGGTAGTAAC	0	1	0	0	0	No gene matches found
GTACGGATTC	0	0	1	0	0	No gene matches found
TACAGTTCGG	0	1	0	0	0	No gene matches found
GACGGATTTC	0	0	0	0	1	No gene matches found
CCGAAGATCT	1	0	0	0	0	No gene matches found
CCTCTGGCGG	0	0	0	1	0	No gene matches found
CTATTTACTG	0	0	1	0	0	No gene matches found
GGAATGTGGC	1	0	0	0	0	No gene matches found
TACTGGAGTG	0	0	1	0	0	No gene matches found
ATTTGTAGGA	0	0	0	0	1	No gene matches found
GATGCCCGGC	0	0	1	0	0	No gene matches found
TGCTTCGGGG	0	0	0	0	1	No gene matches found
TTCGCGATGC	0	1	0	0	0	No gene matches found
GGGGATGCGA	0	1	0	0	0	No gene matches found

TGCAATACTT	0	1	0	0	0	No gene matches found
GGGAAACATT	0	0	0	0	1	No gene matches found
GACGTCCGCC	1	0	0	0	0	No gene matches found
ACGAGATTAT	0	1	0	0	0	No gene matches found
GGTTTGACGA	1	0	0	0	0	No gene matches found
TATGTTGTGG	0	0	0	0	1	No gene matches found
TGAACCGAAC	0	0	1	0	0	No gene matches found
CTCCTATCTT	0	0	1	0	0	No gene matches found
GTCTCCCCAG	0	0	0	1	0	No gene matches found
TCTATCTCGC	0	0	1	0	0	No gene matches found
AGGCTTGTGC	0	1	0	0	0	No gene matches found
ACACTTTTCG	1	0	0	0	0	No gene matches found
GAGTCCTGGC	0	0	0	0	1	No gene matches found
ACCACCCCCT	0	1	0	0	0	No gene matches found
GTCACTCAAA	0	0	1	0	0	No gene matches found
ATGTGGTGAA	0	1	0	0	0	No gene matches found
GGAGAACAAA	1	0	0	0	0	No gene matches found
AGGCCTGTTC	1	0	0	0	0	No gene matches found
GTTGCTCAGT	0	0	1	0	0	No gene matches found
ATTTCGTGGA	1	0	0	0	0	No gene matches found
CCTAGGGGGA	0	0	0	0	1	No gene matches found
AATCGGGTCA	0	0	1	0	0	No gene matches found
CGAAGAGGAG	1	0	0	0	0	No gene matches found
GAAAGGATCT	0	1	0	0	0	No gene matches found
GAAGCTTATT	1	0	0	0	0	No gene matches found
AGTTCCGGAT	1	0	0	0	0	No gene matches found
AGGGTTAAAT	0	0	0	0	1	No gene matches found
AGAAGTTGGA	0	0	1	0	0	No gene matches found
TGTGCAAGTT	0	0	0	0	1	No gene matches found
TCTCTTAGGC	1	0	0	0	0	No gene matches found
AAGCAAACCT	0	0	0	0	1	No gene matches found
ACAAACTGGC	0	1	0	0	0	No gene matches found
GTGGGGGTGG	0	0	1	0	0	No gene matches found
CAAGAGTCTG	0	0	0	1	0	No gene matches found
ATTGGAGCCG	0	1	0	0	0	No gene matches found
TATCCGAAAA	0	0	0	1	0	No gene matches found
ATGTCCGAAT	0	0	1	0	0	No gene matches found
ATGAACGCAG	0	0	0	1	0	No gene matches found
TTGTCCGCGA	0	1	0	0	0	No gene matches found
AACGCATAAC	0	0	1	0	0	No gene matches found
GACAGTATGA	0	1	0	0	0	No gene matches found
AGACTTAAAT	0	1	0	0	0	No gene matches found
ATTGACCCAA	0	0	0	0	1	No gene matches found
GTTTGGCTAT	0	1	0	0	0	No gene matches found
ACACAATACT	1	0	0	0	0	No gene matches found
TGCTTAATAA	0	0	0	1	0	No gene matches found
CGCCCGCCTT	0	1	0	0	0	No gene matches found
GTAACTGAAT	0	0	0	0	1	No gene matches found
AAGGGGGACG	0	0	1	0	0	No gene matches found
TTGAGGTATC	0	0	0	0	1	No gene matches found
AGTACGCCTC	0	0	0	0	1	No gene matches found
AGTCGTGGGA	0	0	1	0	0	No gene matches found
GGCATTCGAC	0	1	0	0	0	No gene matches found
TGCGCGAGGA	0	0	1	0	0	No gene matches found
TTCGGTAAGT	0	0	1	0	0	No gene matches found
GATCCGGTCT	0	0	0	0	1	No gene matches found
CCGTCTATAT	0	1	0	0	0	No gene matches found
CTGGGCAAAT	0	0	0	0	1	No gene matches found
GCCCAACCAC	1	0	0	0	0	No gene matches found
CTCTCCGATC	0	0	1	0	0	No gene matches found
TCACCCAACA	0	0	0	0	1	No gene matches found

CAGGGTAGGC	0	0	0	1	0	No gene matches found
ACCCGTCTCG	0	1	0	0	0	No gene matches found
CTCGTAGCCC	0	0	1	0	0	No gene matches found
ATGTTCTTAG	0	0	1	0	0	No gene matches found
TACAGATATG	0	1	0	0	0	No gene matches found
GCAGGAATTG	0	0	1	0	0	No gene matches found
GAAAATACGA	0	0	1	0	0	No gene matches found
GCAGTCGAAG	0	1	0	0	0	No gene matches found
GGAAAAGCCG	0	0	0	0	1	No gene matches found
TGCACTAAGT	0	0	0	0	1	No gene matches found
GACAGCTCAG	0	0	1	0	0	No gene matches found
GGTCGAACAT	0	1	0	0	0	No gene matches found
GGCGATGACG	0	1	0	0	0	No gene matches found
GCCACTCGAT	1	0	0	0	0	No gene matches found
TCCTCTAGAG	0	0	0	1	0	No gene matches found
GAACATCAGT	1	0	0	0	0	No gene matches found
CTTACCAAAT	0	1	0	0	0	No gene matches found
AATGGGTAAC	0	1	0	0	0	No gene matches found
ATTGTCCGGA	0	0	0	1	0	No gene matches found
AGGCTTGGTC	1	0	0	0	0	No gene matches found
AAGCGTGATA	0	0	1	0	0	No gene matches found
CGCCAACTGA	0	0	1	0	0	No gene matches found
TCCGATCTGT	0	0	1	0	0	No gene matches found
TCTCCGACTA	1	0	0	0	0	No gene matches found
ACGGATAAAA	0	0	0	1	0	No gene matches found
TGCTTGGACT	0	1	0	0	0	No gene matches found
GCCGTCTGAC	0	0	1	0	0	No gene matches found
TAAGGGGTCA	0	0	0	1	0	No gene matches found
GATGTTCGAG	0	1	0	0	0	No gene matches found
GCCAACAGCT	0	1	0	0	0	No gene matches found
ACCATCGCCG	0	0	0	1	0	No gene matches found
TTACCTTTGG	0	0	0	1	0	No gene matches found
GCTGTGATGC	0	0	0	1	0	No gene matches found
AAGGATGGGG	0	1	0	0	0	No gene matches found
GGAGTCTCAG	0	0	0	1	0	No gene matches found
CGCGGTTGGA	0	0	0	1	0	No gene matches found
CACAAGCTCA	0	0	1	0	0	No gene matches found
TAGAGAGATG	0	1	0	0	0	No gene matches found
GCAGCATCCG	0	0	0	1	0	No gene matches found
AATTAGGCTC	0	0	0	0	1	No gene matches found
GGCCTTCGGT	0	1	0	0	0	No gene matches found
TCGCTGTTGG	1	0	0	0	0	No gene matches found
CGAACTAAAA	0	0	1	0	0	No gene matches found
CTGGCTTGAG	0	0	0	1	0	No gene matches found
TATGTGGGGC	1	0	0	0	0	No gene matches found
TCAGGGACCA	0	0	1	0	0	No gene matches found
ACTTCTGTGT	0	1	0	0	0	ChrM:-361476:quaternary
ATCCCCGCTC	0	0	1	0	0	ChrM:-360232:quaternary
AGCTAGCGTT	0	1	0	0	0	ChrM:-351604:quaternary
TACGTAGGGC	0	0	0	1	0	ChrM:-29257:quaternary
CAAGTCGATC	0	0	0	0	1	ChrM:-289631:quaternary
CTCCCACGGC	0	1	0	0	0	ChrM:-289258:quaternary,Chr1:-10928316:quaternary
GCCTGAATGC	0	0	0	1	0	ChrM:-285151:quaternary
AAACACGAGC	0	0	0	1	0	ChrM:-200611:quaternary
CTCTCAGAAG	1	0	0	0	0	ChrM:-188045:quaternary
GAACTCTAAG	0	1	0	0	0	ChrM:-187030:quaternary
AAACGGGCAT	0	0	1	0	0	ChrM:-186501:quaternary
CTATTCCACA	0	1	0	0	0	ChrM:-185867:quaternary
AAATGTTGAC	1	0	0	0	0	ChrM:-180700:quaternary,Chr5:-7179884:quaternary,Chr2:-3437165:quaternary,ChrM:-46535:quaternary,Chr2:-13134622:quaternary
GACTAAACGG	0	1	0	0	0	ChrM:-140382:quaternary,Chr2:-3396763:quaternary

CGAGTGCTCC	0	1	0	0	0	ChrM:+46610:quaternary,ChrM:+180775:quaternary,Chr2:+3437240:quaternary
ATTCGTAATT	0	1	0	0	0	ChrM:+46540:quaternary,Chr4:+17527106:quaternary,Chr2:+3437170:quaternary,Chr2:+2224352:quaternary,ChrM:+180705:quaternary
ATCGCGCACT	1	0	0	0	0	ChrM:+362810:quaternary
CTTGAGCGCA	1	0	0	0	0	ChrM:+287667:quaternary
GAATTTTTCC	0	0	1	0	0	ChrM:+274901:quaternary
GTCTTCCTAC	0	1	0	0	0	ChrM:+263870:quaternary
CTGAAAAGAC	0	0	0	1	0	ChrM:+258587:quaternary
AAAACAGCTT	0	1	0	0	0	ChrM:+248623:quaternary
AGCACACCCA	0	1	0	0	0	ChrM:+246765:quaternary
CACACGAGGA	0	1	0	0	0	ChrM:+217978:quaternary
TTACGAGGCT	0	0	0	1	0	ChrM:+204525:quaternary,ChrM:+29084:quaternary
AGCGATATCG	0	0	0	0	1	ChrM:+156802:quaternary,Chr2:+3413334:quaternary
TCTCATTAGC	0	1	0	0	0	ChrM:+150334:quaternary,Chr2:+3406766:quaternary
AATCGAAGCG	0	0	1	0	0	ChrM:+141402:quaternary
CAAACATAAC	0	0	0	0	1	ChrM:+123502:quaternary
AATTGTCGGA	0	0	1	0	0	ChrC:-32420:quaternary
CTAAGAATAG	0	0	1	0	0	ChrC:+95563:quaternary
GAACAATATG	1	0	0	0	0	ChrC:+93488:quaternary,Chr1:+23372933:quaternary
GCCAAGGGTA	0	0	1	0	0	ChrC:+67491:quaternary
AAGCAAATTT	1	0	0	0	0	ChrC:+64685:quaternary
ATCGAACATT	0	1	0	0	0	ChrC:+51937:quaternary
ATTAACTAAT	0	1	0	0	0	ChrC:+45493:quaternary
CATAACTTCC	0	0	0	1	0	ChrC:+452:quaternary
ACAGAAGGGA	0	1	0	0	0	ChrC:+44339:quaternary,Chr2:+3436302:quaternary,ChrM:+179837:quaternary,ChrM:+45672:quaternary
TAGGTTCCAG	0	0	0	1	0	ChrC:+41711:quaternary
CCCAGACTGA	0	1	0	0	0	ChrC:+37641:quaternary; -small RNA(TCAGTCTGGGCATGGAT)
ATCATAAGCA	0	0	0	0	1	ChrC:+3469:quaternary
TCAAGATAAA	0	1	0	0	0	ChrC:+31075:quaternary
CTGGGCTCTA	0	0	1	0	0	ChrC:+26956:quaternary
TTCGGCAGTA	0	0	1	0	0	ChrC:+19013:quaternary
TACAGAATGA	0	0	0	0	1	ChrC:+141498:quaternary
AAAACGTGAC	0	0	1	0	0	ChrC:+139674:quaternary,Chr5:+9967061:quaternary
GATTCAGCAG	0	0	1	0	0	ChrC:+133812:quaternary; -small RNA(TGCTGCTGAATCCATGG; CTGCTGCTGAATCCATG; CTGCTGAATCCATGGGC)
GGTTACTTCA	0	0	0	1	0	ChrC:+132373:quaternary
AACGAAGAAA	0	0	0	1	0	ChrC:+130711:quaternary
AGCTGTCCCG	0	1	0	0	0	ChrC:+129027:quaternary,ChrM:+105339:quaternary
GTATTCCGGA	0	0	1	0	0	ChrC:+122505:quaternary
AAACCTTGAC	0	0	1	0	0	ChrC:+119686:quaternary
AACATCACAG	0	1	0	0	0	ChrC:+118669:quaternary
TTCGAGTATC	0	1	0	0	0	ChrC:+118098:quaternary
CTCGGACACA	0	1	0	0	0	ChrC:+117506:quaternary
CGACATATAA	0	0	0	0	1	ChrC:+112291:quaternary
GAGAGTTCGA	0	0	0	1	0	ChrC:+101019:quaternary; +small RNA(TCTCATGGAGAGTTCGA)
GGATTCATTG	0	0	0	1	0	Chr5:-9858352:quaternary
GCTGAACCAT	0	0	1	0	0	Chr5:-9166667:quaternary
CATTGTGATT	0	1	0	0	0	Chr5:-8990693:quaternary
CCTTGGCTGA	0	1	0	0	0	Chr5:-896815:quaternary
GCCCACAAAA	0	0	0	1	0	Chr5:-889050:quaternary
TCTTTATAAT	0	0	0	1	0	Chr5:-8810138:quaternary
TTTGCGTTAA	0	1	0	0	0	Chr5:-8698648:quaternary,Chr1:-16358111:quaternary
TGTGCTTGTC	0	0	1	0	0	Chr5:-8689425:quaternary,Chr5:-8689442:quaternary,Chr5:-8689433:quaternary
ACGCACAAAT	0	0	0	1	0	Chr5:-8617491:quaternary
AATATAAGAT	0	0	1	0	0	Chr5:-8441894:quaternary
TCCTATTGCA	0	0	0	0	1	Chr5:-8427302:quaternary
TACATATGGA	0	0	0	0	1	Chr5:-830870:quaternary,Chr2:-4368037:quaternary
AGTAACTAAA	0	0	1	0	0	Chr5:-8293710:quaternary
TTGCTGCCGT	0	0	1	0	0	Chr5:-8176361:quaternary
GTGAATACTG	0	0	0	1	0	Chr5:-8157176:quaternary
CAAACTTCGA	1	0	0	0	0	Chr5:-7917707:quaternary
GGTTCATTGA	1	0	0	0	0	Chr5:-742877:quaternary,Chr5:-10058868:quaternary

CTTGGAATGA	0	0	1	0	0	Chr5:-7383338:quaternary,Chr4:-15139679:quaternary
TATTCAGGTT	0	1	0	0	0	Chr5:-7319240:quaternary
TGGTAGTCAA	0	0	0	0	1	Chr5:-7111881:quaternary
CCACAATTGG	0	1	0	0	0	Chr5:-6574156:quaternary
ATTAGTCTGC	0	1	0	0	0	Chr5:-6400236:quaternary,At1g55920.1:X:--98:quaternary
TCTGTGGTGC	0	0	1	0	0	Chr5:-5968102:quaternary
AGAAATGATA	0	0	0	0	1	Chr5:-579585:quaternary
GGTCTTTATG	0	0	0	0	1	Chr5:-5616614:quaternary
ACGCCTCAAA	0	1	0	0	0	Chr5:-4546985:quaternary
TGGCTGTGAT	1	0	0	0	0	Chr5:-312846:quaternary
TTGAAACTTG	0	0	1	0	0	Chr5:-3119791:quaternary,Chr4:-10513710:quaternary
TACAATAGTT	0	0	1	0	0	Chr5:-2948113:quaternary
AGAGTATGAT	0	0	0	0	1	Chr5:-278851:quaternary,Chr5:-10386391:quaternary
GTAGTGGCCA	1	0	0	0	0	Chr5:-26780779:quaternary
TTTCGAGATG	0	0	0	1	0	Chr5:-26588338:quaternary
ATTGACTAAA	1	0	0	0	0	Chr5:-26336915:quaternary
TGTCTGTAAA	1	0	0	0	0	Chr5:-26298112:quaternary
CGTTTTACAA	0	1	0	0	0	Chr5:-26106235:quaternary
AGGATGATCG	0	0	0	0	1	Chr5:-25868608:quaternary
CAGTGGATAA	0	0	1	0	0	Chr5:-25680455:quaternary
AATGTGTGCA	0	0	0	1	0	Chr5:-25136541:quaternary
ACTCGTGGAT	0	1	0	0	0	Chr5:-25008465:quaternary,Chr5:-25008472:quaternary
GATGACTCTA	0	1	0	0	0	Chr5:-24987723:quaternary
CACCATTTGA	0	1	0	0	0	Chr5:-24920756:quaternary
GACGGAGAAT	0	0	1	0	0	Chr5:-24402845:quaternary
AGGGCTTTCT	0	0	0	1	0	Chr5:-24353590:quaternary
GGCAATAAAA	1	0	0	0	0	Chr5:-24255453:quaternary
TACACATACC	0	0	1	0	0	Chr5:-23867513:quaternary
CAACCTATCA	0	1	0	0	0	Chr5:-23866818:quaternary
GTTTGAGTTT	0	0	0	1	0	Chr5:-23386709:quaternary
TAGTAGTAAG	0	0	0	0	1	Chr5:-23316822:quaternary
AAGACACAAA	1	0	0	0	0	Chr5:-23112260:quaternary
GAAAAATGTA	0	0	0	0	1	Chr5:-21637198:quaternary,Chr4:-18127370:quaternary
GTTAATTAAA	0	0	0	1	0	Chr5:-21570214:quaternary,Chr3:-19738512:quaternary
ATCGCAGAGA	0	1	0	0	0	Chr5:-21233017:quaternary
TGTATTACAA	0	0	0	0	1	Chr5:-20310567:quaternary
CTTAATTTGA	0	1	0	0	0	Chr5:-20004436:quaternary
CGCATTTTCT	0	1	0	0	0	Chr5:-19760661:quaternary
CCTTTATTTC	1	0	0	0	0	Chr5:-19759875:quaternary
AGTCACAATC	0	1	0	0	0	Chr5:-19684269:quaternary
TGGTAAAAAT	0	0	0	1	0	Chr5:-19121059:quaternary
AAGGAGAAGG	0	1	0	0	0	Chr5:-18765111:quaternary
CATTTGGATA	1	0	0	0	0	Chr5:-18599009:quaternary
TCACTTGATT	0	0	0	1	0	Chr5:-18350572:quaternary
TTACCAAGAA	0	0	1	0	0	Chr5:-18232906:quaternary
TGTTGCGGAG	0	1	0	0	0	Chr5:-17989631:quaternary
GTCGAAGGAT	0	0	1	0	0	Chr5:-17986641:quaternary
GTATCACGTT	0	0	0	0	1	Chr5:-17979097:quaternary
TGGACGTGAT	0	0	0	1	0	Chr5:-17965937:quaternary
GTTTCAACTG	0	1	0	0	0	Chr5:-17960901:quaternary
TCTATCTTCT	1	0	0	0	0	Chr5:-17784722:quaternary
TTCTGAATTA	0	1	0	0	0	Chr5:-17749635:quaternary
AGAAGTTAAG	0	1	0	0	0	Chr5:-17552811:quaternary,At5g46840.1:X:-720:quaternary
TTACAATTAA	1	0	0	0	0	Chr5:-17543099:quaternary
TGGAGCGTGA	0	1	0	0	0	Chr5:-17447673:quaternary
GCACCAGAAC	0	1	0	0	0	Chr5:-17021031:quaternary
TATCCGAATA	0	0	1	0	0	Chr5:-16970819:quaternary,Chr1:-30163677:quaternary
TAATTGAACC	0	1	0	0	0	Chr5:-1655951:quaternary
TGCCTTCAAC	0	1	0	0	0	Chr5:-16411247:quaternary
ATCTTATCCA	1	0	0	0	0	Chr5:-15325522:quaternary
GTAAAGTTAA	0	0	0	1	0	Chr5:-14333265:quaternary,Chr5:-12093136:quaternary

GCCCGTTTAC	0	0	0	1	0	Chr5:-14307942:quaternary
TAATGGCTGC	0	1	0	0	0	Chr5:-142422:quaternary
TGCTATTGAA	0	0	1	0	0	Chr5:-13709914:quaternary,Chr5:-13709906:quaternary
GATTAGTTCA	0	1	0	0	0	Chr5:-13576455:quaternary
GAGACAACGG	0	1	0	0	0	Chr5:-13249998:quaternary
TGGCCACCCA	0	1	0	0	0	Chr5:-12396357:quaternary
TTGGTTGGGA	0	1	0	0	0	Chr5:-11224977:quaternary
TTGTGATACA	1	0	0	0	0	Chr5:-1119047:quaternary
GTTATGATAA	0	1	0	0	0	Chr5:-10389045:quaternary
GGCTAAATGA	0	0	0	1	0	Chr5:-10295900:quaternary
GAGTAATAAC	0	0	0	0	1	Chr5:-10040007:quaternary,Chr5:-10040020:quaternary
TGTTTTGAGC	0	0	1	0	0	Chr5:+998277:quaternary
ATTATGGCTT	0	0	1	0	0	Chr5:+9966364:quaternary
TGAGAACACA	0	1	0	0	0	Chr5:+992400:quaternary
TTTAACCCAA	0	0	1	0	0	Chr5:+9919388:quaternary
TTTACGTTCA	0	1	0	0	0	Chr5:+9899653:quaternary,Chr1:+10315109:quaternary,Chr4:+5359946:quaternary
TCAACACGTG	0	1	0	0	0	Chr5:+9819999:quaternary
TAAAATGATA	1	0	0	0	0	Chr5:+9800255:quaternary,Chr3:+8400403:quaternary
TAGTTACAAG	0	1	0	0	0	Chr5:+9748485:quaternary
TTACTAAAAA	0	0	0	0	1	Chr5:+9707330:quaternary
TACTTATATT	1	0	0	0	0	Chr5:+9496882:quaternary,Chr3:+6158969:quaternary,Chr5:+25282912:quaternary
AGGTCCGATC	0	0	0	1	0	Chr5:+9337316:quaternary
CAACTTAAAA	0	1	0	0	0	Chr5:+9298968:quaternary
TAGAAGAAAG	0	0	1	0	0	Chr5:+925986:quaternary
ATCAAGGTTG	0	0	1	0	0	Chr5:+9217850:quaternary,Chr5:+10618378:quaternary
CTTTCTTGCC	0	1	0	0	0	Chr5:+9133368:quaternary
ATCGACCTTC	0	1	0	0	0	Chr5:+9089296:quaternary
TAATTAACTC	0	0	0	1	0	Chr5:+9035122:quaternary
TGATGTTGAA	0	1	0	0	0	Chr5:+8995234:quaternary,Chr2:+6882942:quaternary
TTTGCGACTT	0	1	0	0	0	Chr5:+8995192:quaternary
CTTTTGCAGT	0	0	0	1	0	Chr5:+8990698:quaternary
TTTTTTCAAG	0	0	0	0	1	Chr5:+897208:quaternary; small RNA(MIR822a)
TGAGAGCAAA	0	0	0	1	0	Chr5:+8957835:quaternary
AAAATTAGAG	0	0	0	1	0	Chr5:+8874442:quaternary,Chr3:+17527615:quaternary
CTCGTCATTC	0	0	0	0	1	Chr5:+8790612:quaternary
TCTTTAACTA	0	1	0	0	0	Chr5:+8762368:quaternary,At3g17970.1:X:+368:quaternary
AATCATTCAT	0	1	0	0	0	Chr5:+8730696:quaternary
TAAGCTAAAA	1	0	0	0	0	Chr5:+8692781:quaternary
GTCTACAATT	0	1	0	0	0	Chr5:+8686500:quaternary
ATCACAAGGA	0	0	0	1	0	Chr5:+8617496:quaternary
ATCTTGGTGC	0	0	0	1	0	Chr5:+8498007:quaternary
CAACTGGTTA	0	0	0	0	1	Chr5:+8474064:quaternary,Chr5:+8474030:quaternary
ATGATTATAC	0	0	0	0	1	Chr5:+8422714:quaternary
GAAATTCAGA	0	0	0	1	0	Chr5:+8345113:quaternary
AAATTTATGC	0	0	0	1	0	Chr5:+8224147:quaternary,Chr3:+22087316:quaternary
AAACCAAAAC	0	0	0	1	0	Chr5:+806592:quaternary
TGGGAGAATT	0	1	0	0	0	Chr5:+8051756:quaternary
TGATGAATGC	0	0	0	1	0	Chr5:+801626:quaternary
CAAGTCCAAA	0	1	0	0	0	Chr5:+7981343:quaternary
GTTATTGAGA	0	1	0	0	0	Chr5:+7979899:quaternary
TGAATGCTTA	1	0	0	0	0	Chr5:+7887507:quaternary
TTGAGCTAAA	1	0	0	0	0	Chr5:+7861401:quaternary,ChrM:+323680:quaternary
TACCTAGCGA	1	0	0	0	0	Chr5:+7860646:quaternary
ATGAACAGCA	1	0	0	0	0	Chr5:+7793672:quaternary
ACTACAAACA	0	1	0	0	0	Chr5:+7740344:quaternary
GGCTTGTTTT	0	0	1	0	0	Chr5:+7643812:quaternary
ACAATAATGG	0	0	0	0	1	Chr5:+7498814:quaternary
TGAGTTGTGA	0	0	0	1	0	Chr5:+7358303:quaternary,Chr5:+19517549:quaternary,At5g20150.1:X:+-200:quaternary
GGTTTAAAAA	0	0	1	0	0	Chr5:+7321341:quaternary
ATTCCCATTC	0	0	1	0	0	Chr5:+7319245:quaternary
TGTCGTTTCA	0	0	1	0	0	Chr5:+6795072:quaternary

ATGTTGTAAT	0	0	0	0	1	Chr5:+6767134:quaternary
TGTTTAACCA	0	0	0	0	1	Chr5:+6669951:quaternary
CATTGAAAAC	1	0	0	0	0	Chr5:+6610362:quaternary
TGTTTGAGTT	0	0	1	0	0	Chr5:+6232624:quaternary,Chr3:+8072413:quaternary
AGAGTTTGTG	0	0	1	0	0	Chr5:+619097:quaternary
TTCGAAAAAA	0	0	1	0	0	Chr5:+6167432:quaternary
ATACAAAGGC	0	1	0	0	0	Chr5:+6158735:quaternary
TCCTTCACCA	0	1	0	0	0	Chr5:+6158692:quaternary
TTTAGGTATC	0	0	1	0	0	Chr5:+6155557:quaternary
AGACTGAGAT	0	1	0	0	0	Chr5:+608561:quaternary
GTTGAAGTTT	1	0	0	0	0	Chr5:+5948388:quaternary
GTTGGTTGGT	0	0	0	0	1	Chr5:+589077:quaternary,At5g45670.1:X:+507:quaternary
ATTAAGAGTT	0	0	1	0	0	Chr5:+581568:quaternary,Chr4:+14548558:quaternary
TTCACATTTA	1	0	0	0	0	Chr5:+5799655:quaternary,Chr2:+129694:quaternary
TCGAGGACCA	0	1	0	0	0	Chr5:+5746003:quaternary
GAGCTTTCTG	0	0	0	0	1	Chr5:+5645347:quaternary
TGTGTTTAGA	1	0	0	0	0	Chr5:+5617756:quaternary
TAGCTTTCGA	0	0	0	1	0	Chr5:+5511946:quaternary
AATCTATTCA	0	0	0	0	1	Chr5:+5479959:quaternary
TCGTGTATTC	0	1	0	0	0	Chr5:+5380374:quaternary
AAGACGAGAA	0	0	0	1	0	Chr5:+5299486:quaternary
CTTGTGTGGC	0	0	1	0	0	Chr5:+5263349:quaternary
TAACTATGAA	0	1	0	0	0	Chr5:+525851:quaternary
ACCATACATC	0	0	1	0	0	Chr5:+5232759:quaternary
AGACTGAAAT	1	0	0	0	0	Chr5:+5165928:quaternary
GAATTTATTC	0	1	0	0	0	Chr5:+5043736:quaternary
TACACAACAG	0	0	0	1	0	Chr5:+5042681:quaternary,At1g55340.1:X:+291:quaternary
GGGAAATGGC	0	0	1	0	0	Chr5:+4946797:quaternary
ATGATTGAGA	0	1	0	0	0	Chr5:+4917110:quaternary
TAAATCAAAC	0	0	0	0	1	Chr5:+4734852:quaternary,Chr1:+8706494:quaternary
TACTTGAATT	0	0	1	0	0	Chr5:+4709825:quaternary
ATAGAAAAAG	1	0	0	0	0	Chr5:+4619214:quaternary
TGAGCAATCT	0	1	0	0	0	Chr5:+4484810:quaternary
AAACACAAAC	0	0	0	1	0	Chr5:+4459756:quaternary,Chr3:+9300147:quaternary
TTTTTCGTTG	0	1	0	0	0	Chr5:+443122:quaternary
TGTTGAAAAA	0	1	0	0	0	Chr5:+4359953:quaternary,At3g01070.1:X:+690:quaternary
CAAATTAAAA	0	0	0	0	1	Chr5:+4092490:quaternary,Chr5:+9248252:quaternary,At5g38210.1:X:+917:quaternary,Chr4:+18392799:quaternary
ATGCTCCTTT	0	0	0	0	1	Chr5:+4091150:quaternary
TTACCCTTCT	0	1	0	0	0	Chr5:+4021455:quaternary
TAAACAATAA	0	0	0	1	0	Chr5:+3600991:quaternary,Chr5:+6838777:quaternary,Chr4:+14688220:quaternary
AATTTAAAAA	0	0	1	0	0	Chr5:+3528305:quaternary,Chr5:+13608922:quaternary
CATTATACAA	0	0	0	0	1	Chr5:+3506864:quaternary,Chr3:+5254913:quaternary
CTATTATTTT	1	0	0	0	0	Chr5:+3476262:quaternary
TTTGATACTC	0	1	0	0	0	Chr5:+3327185:quaternary
AGTTTCAAAA	1	0	0	0	0	Chr5:+3229717:quaternary,Chr4:+8594222:quaternary
TTATGGGCAT	0	1	0	0	0	Chr5:+32273:quaternary
CTTATTCCAT	0	0	1	0	0	Chr5:+3191817:quaternary
CGTATCCACA	0	0	1	0	0	Chr5:+3062812:quaternary
AACGAGATCT	1	0	0	0	0	Chr5:+2940803:quaternary
TAACCGTAAC	0	0	0	0	1	Chr5:+2937877:quaternary
TTTGCGCTTG	0	1	0	0	0	Chr5:+279464:quaternary
ATTTAAAATC	0	0	0	0	1	Chr5:+2752637:quaternary
TGTGCAACAA	0	0	0	0	1	Chr5:+2752035:quaternary,Chr1:+20025169:quaternary
TAAACTGATA	1	0	0	0	0	Chr5:+26951049:quaternary
TATCATCCTC	0	0	0	0	1	Chr5:+26901248:quaternary
CAAATGCATA	0	0	1	0	0	Chr5:+2687844:quaternary
CAAAAACCAC	0	0	0	1	0	Chr5:+26770140:quaternary,Chr2:+383101:quaternary
CATTATGTAA	1	0	0	0	0	Chr5:+26765214:quaternary,Chr3:+12320317:quaternary,At5g67020.1:X:+1585:quaternary
AAAAAAGCCA	0	1	0	0	0	Chr5:+26631624:quaternary
TTACCTTGAT	1	0	0	0	0	Chr5:+26623874:quaternary

TAATTTAATG	0	1	0	0	0	Chr5:+2656732:quaternary
AACTTGCCTG	0	0	1	0	0	Chr5:+26554091:quaternary
TTCTGCATAT	0	0	0	0	1	Chr5:+26553933:quaternary,Chr3:+7362338:quaternary
CAATTGATCT	0	0	1	0	0	Chr5:+26499449:quaternary
TTGCTTAAAA	0	0	0	1	0	Chr5:+26383548:quaternary
GAATTAATTA	0	1	0	0	0	Chr5:+2612078:quaternary
TCTCTGTTAA	0	1	0	0	0	Chr5:+26057112:quaternary
TAACAGTGTC	0	1	0	0	0	Chr5:+26055118:quaternary
GTTTGCAAAA	0	0	1	0	0	Chr5:+26046301:quaternary
ATCGCTCTAT	0	0	0	1	0	Chr5:+25955797:quaternary
TATAAAATTG	0	0	0	0	1	Chr5:+25953322:quaternary,Chr3:+11983233:quaternary,Chr5:+25073180:quaternary
CGTAAAAATA	0	0	1	0	0	Chr5:+25765318:quaternary
AATGGCAATA	0	0	1	0	0	Chr5:+25720455:quaternary
TATCTTATCC	0	0	0	0	1	Chr5:+25718722:quaternary
TAATCCAAAC	0	1	0	0	0	Chr5:+25693386:quaternary
ATGGAACCAA	0	0	1	0	0	Chr5:+25616022:quaternary
ACAAAAACAC	1	0	0	0	0	Chr5:+25253357:quaternary,Chr2:+106514:quaternary
TAAACAGCAA	1	0	0	0	0	Chr5:+25102640:quaternary
CATCTATTTA	1	0	0	0	0	Chr5:+25012033:quaternary
AAAAACAACA	0	1	0	0	0	Chr5:+24993239:quaternary,Chr2:+17427114:quaternary
TGTTGTCAAA	0	0	0	1	0	Chr5:+249520:quaternary
TTTACCTGGA	0	1	0	0	0	Chr5:+24789254:quaternary
TGTTCTTTCA	0	0	0	1	0	Chr5:+24659969:quaternary,Chr1:+29789676:quaternary
TGTCTAAGGA	1	0	0	0	0	Chr5:+24603730:quaternary,At4g15450.1:X:+281:quaternary
CATTACATAA	1	0	0	0	0	Chr5:+24551658:quaternary,Chr4:+5447140:quaternary
CCGGTGATGA	0	1	0	0	0	Chr5:+24549724:quaternary
ATTTGTTGTA	0	0	0	0	1	Chr5:+24508023:quaternary
TTTGATGGTT	0	0	0	1	0	Chr5:+2444708:quaternary,Chr1:+24442510:quaternary
CACTCTGATT	0	0	1	0	0	Chr5:+24402850:quaternary
TGACATACTC	0	1	0	0	0	Chr5:+24368408:quaternary
TGGGGTCCGG	0	0	1	0	0	Chr5:+2432693:quaternary
GTATATATGA	0	0	0	1	0	Chr5:+24212929:quaternary
ATACATTTGT	0	0	0	0	1	Chr5:+24182838:quaternary
TTTAGTAAGA	0	0	0	0	1	Chr5:+23957505:quaternary,Chr2:+1664325:quaternary
CGATCTCTAA	1	0	0	0	0	Chr5:+23914151:quaternary
TCACGTGTGA	0	1	0	0	0	Chr5:+23849339:quaternary
AAGCATTTTA	0	1	0	0	0	Chr5:+23841552:quaternary
CCTAATTCTA	0	1	0	0	0	Chr5:+23724882:quaternary,Chr1:+25321127:quaternary
AAACAATAAA	0	0	0	1	0	Chr5:+23635429:quaternary,Chr1:+26248815:quaternary,Chr1:+13070404:quaternary,Chr5:+10272210:quaternary,Chr2:+293459:quaternary,Chr1:+18418857:quaternary,Chr1:+7093390:quaternary
TTTTTGCACA	0	1	0	0	0	Chr5:+23386120:quaternary
CATTTGCTTC	1	0	0	0	0	Chr5:+23314938:quaternary
TGGCTTAAGA	0	0	1	0	0	Chr5:+23256176:quaternary
AGTATGAAAA	1	0	0	0	0	Chr5:+23253044:quaternary,Chr3:+10443572:quaternary
TGGCAAAAGC	1	0	0	0	0	Chr5:+23111377:quaternary,Chr3:+10202738:quaternary
AAGTCTCGTG	1	0	0	0	0	Chr5:+2290695:quaternary
ACATTTATAA	0	0	0	0	1	Chr5:+22693935:quaternary,Chr1:+4700269:quaternary,Chr3:+17908431:quaternary
AAAACCTCCA	0	0	1	0	0	Chr5:+22652053:quaternary
ATCACAAAAT	1	0	0	0	0	Chr5:+22341401:quaternary,Chr5:+24476475:quaternary,Chr5:+10524300:quaternary
ACACATTTGA	0	0	1	0	0	Chr5:+22299118:quaternary
TTTTTCCGAA	0	0	0	1	0	Chr5:+22243477:quaternary
ATAATCAAAT	0	1	0	0	0	Chr5:+22187847:quaternary,At2g39030.1:X:+1188:quaternary
GTTACTAGGA	0	0	1	0	0	Chr5:+22133488:quaternary
TAAATTCGTT	0	0	1	0	0	Chr5:+22037275:quaternary
GAACATAAAA	0	0	1	0	0	Chr5:+21950314:quaternary,Chr5:+20986253:quaternary
TCTATTGTAG	0	1	0	0	0	Chr5:+21891842:quaternary
AGAAGGTTAA	0	1	0	0	0	Chr5:+21443402:quaternary,Chr3:+10602889:quaternary
TTAAAAAGTG	0	0	1	0	0	Chr5:+21423834:quaternary
CCTCTAGTTT	0	0	0	0	1	Chr5:+21362246:quaternary
ATTGTCGCTG	0	0	0	0	1	Chr5:+21287815:quaternary

TCTCAGTATG	0	1	0	0	0	Chr5:+21094831:quaternary
TCAAGTTGTA	1	0	0	0	0	Chr5:+21063094:quaternary
ACATTACTGA	0	1	0	0	0	Chr5:+21058485:quaternary
AATTGAAAAA	0	0	0	0	1	Chr5:+20986151:quaternary
TGAACAATCA	0	0	0	1	0	Chr5:+20775920:quaternary
TACATTTAAA	0	0	0	0	1	Chr5:+20519533:quaternary
CACAAAAAGC	1	0	0	0	0	Chr5:+20487266:quaternary
GCTGAAAAAA	0	1	0	0	0	Chr5:+20429325:quaternary
ATCCATTATA	0	0	1	0	0	Chr5:+2042436:quaternary
CAACTAAAAA	0	1	0	0	0	Chr5:+20394421:quaternary
TTTAATTAAA	1	0	0	0	0	Chr5:+20311645:quaternary,Chr1:+24533532:quaternary
TTTTTCATAC	0	0	1	0	0	Chr5:+20187244:quaternary
AAGGATATTT	0	0	1	0	0	Chr5:+20150232:quaternary
GTTTGCTACA	1	0	0	0	0	Chr5:+19988870:quaternary
TTAGCTTATT	0	0	1	0	0	Chr5:+19807782:quaternary
ATGAAGTAAA	0	0	1	0	0	Chr5:+19769681:quaternary
CTTTAAATAG	0	0	0	1	0	Chr5:+19761338:quaternary
TCTCGCTGCT	0	0	1	0	0	Chr5:+19729843:quaternary,Chr3:+4839732:quaternary
TGAACTTATT	0	0	0	0	1	Chr5:+19684115:quaternary
AATCTAATAT	1	0	0	0	0	Chr5:+19650014:quaternary
ATTATCCAGG	0	0	1	0	0	Chr5:+19641883:quaternary
AAAAGGTTAG	0	0	0	1	0	Chr5:+19640920:quaternary
ACATACCACA	1	0	0	0	0	Chr5:+19591023:quaternary
GTTGGTGCTC	0	1	0	0	0	Chr5:+19538514:quaternary
TGAGGCCAGA	0	1	0	0	0	Chr5:+1951145:quaternary
CCAAACTTAA	0	1	0	0	0	Chr5:+19448857:quaternary
GATTCTACTG	0	0	0	1	0	Chr5:+19412052:quaternary
TAAACTAAAA	0	1	0	0	0	Chr5:+19236478:quaternary,At3g16390.1:X:+-226:quaternary,Chr3:+4406568:quaternary
TCTTGTGATG	0	0	0	1	0	Chr5:+1915481:quaternary,Chr1:+19377871:quaternary,Chr1:+19377944:quaternary,Chr1:+11473342:quaternary
ATAGAAAAAA	0	1	0	0	0	Chr5:+19058977:quaternary
CAAAGAATTT	0	0	0	1	0	Chr5:+19026802:quaternary
ATTAAGTGAA	1	0	0	0	0	Chr5:+1900349:quaternary,Chr5:+22107346:quaternary
ATCTCAAACG	0	0	0	0	1	Chr5:+18961748:quaternary
AATATGGCTT	0	0	1	0	0	Chr5:+18808707:quaternary
TCATTGCATT	0	0	0	0	1	Chr5:+18775961:quaternary
TCCAAGCTCA	0	0	0	0	1	Chr5:+1867295:quaternary,At2g47640.1:X:+295:quaternary
TTTTTCTTAG	1	0	0	0	0	Chr5:+18551540:quaternary
TCACTCTCAA	0	1	0	0	0	Chr5:+18551258:quaternary
ACTCTTGTTG	0	1	0	0	0	Chr5:+18433820:quaternary
GGCTTCGGTA	0	1	0	0	0	Chr5:+18329675:quaternary
GCAAGAACTC	0	0	1	0	0	Chr5:+18329034:quaternary
AGAATGAGAA	0	1	0	0	0	Chr5:+18311820:quaternary
TGTGGTAGTA	0	0	0	0	1	Chr5:+18112095:quaternary,Chr5:+5785109:quaternary
GAAACGTAAT	0	1	0	0	0	Chr5:+18002733:quaternary
ATTCCATCCG	0	1	0	0	0	Chr5:+17998011:quaternary
TGTTGTAGAC	0	0	0	0	1	Chr5:+17978483:quaternary
GGTTATATAG	0	1	0	0	0	Chr5:+17827280:quaternary
TACGACGATT	0	1	0	0	0	Chr5:+17808851:quaternary
TGAAACTCTT	0	0	1	0	0	Chr5:+17793682:quaternary,Chr4:+11361608:quaternary,Chr4:+8064346:quaternary,Chr5:+16374429:quaternary,Chr3:+22130294:quaternary,Chr2:+708168:quaternary,Chr1:+9816923:quaternary,Chr1:+22454323:quaternary,Chr1:+16328626:quaternary
AAAACTATAG	0	0	1	0	0	Chr5:+17787185:quaternary
TCTTTTAAAA	1	0	0	0	0	Chr5:+17759177:quaternary
ATCCATTGAA	0	1	0	0	0	Chr5:+17412637:quaternary,Chr3:+22752106:quaternary
TCCAATGGTG	0	0	1	0	0	Chr5:+17376406:quaternary; +small RNA(AGCATGTCCAATGGTGG)
GCGTTGTGCT	0	0	1	0	0	Chr5:+17361795:quaternary
TCGAAAACTG	0	0	1	0	0	Chr5:+17337325:quaternary
AACTTCAAAA	0	1	0	0	0	Chr5:+17292068:quaternary,Chr2:+10820724:quaternary,Chr1:+12738731:quaternary
CAAACAATTT	0	0	0	0	1	Chr5:+17177570:quaternary,Chr5:+24785191:quaternary,Chr1:+22620688:quaternary
CGTATTGCTT	0	0	0	1	0	Chr5:+17165207:quaternary

TAACACTTAA	1	0	0	0	0	Chr5:+16961905:quaternary
TTTGCAAAGA	0	0	0	1	0	Chr5:+16897502:quaternary
GAATAAAGAA	1	0	0	0	0	Chr5:+16860922:quaternary,Chr1:+7249456:quaternary
TCCTTTGGTT	1	0	0	0	0	Chr5:+16765609:quaternary,Chr1:+8163700:quaternary,Chr5:+14073172:quaternary
TATAGTCTCA	1	0	0	0	0	Chr5:+16648624:quaternary
TGTGTTCGAA	0	0	0	1	0	Chr5:+16640972:quaternary
CTTTGGTTAG	0	0	0	0	1	Chr5:+16575371:quaternary
TATAATATTC	0	1	0	0	0	Chr5:+16216877:quaternary,Chr5:+13261178:quaternary,Chr4:+12830404:quaternary,Chr3:+12801285:quaternary
CTTGATGTAA	0	1	0	0	0	Chr5:+16192820:quaternary
AGAAAAATCC	0	1	0	0	0	Chr5:+16152202:quaternary,Chr1:+8167372:quaternary,At5g31770.1:X:+-210:quaternary
TATATAGTAA	0	0	0	0	1	Chr5:+15932406:quaternary,Chr3:+5686951:quaternary,At2g16595.1:X:+269:quaternary
TTACATAAAG	0	1	0	0	0	Chr5:+15915938:quaternary
ATCACCTGAA	0	1	0	0	0	Chr5:+15885129:quaternary
AAGCATATAT	0	0	0	1	0	Chr5:+15879575:quaternary
CAAGGGTGGT	0	0	0	1	0	Chr5:+15852624:quaternary
TTATGTCCTC	0	1	0	0	0	Chr5:+15827079:quaternary
TATGAAAAAA	0	0	0	0	1	Chr5:+15818182:quaternary,Chr5:+15822377:quaternary
CTCATATAAT	0	0	0	0	1	Chr5:+15798665:quaternary
TATTAATGCA	1	0	0	0	0	Chr5:+15780564:quaternary,Chr3:+10489410:quaternary
GAGAAAAAAG	0	0	1	0	0	Chr5:+15721621:quaternary,Chr3:+17391599:quaternary
AGGATTATAG	0	1	0	0	0	Chr5:+1566787:quaternary
CATAATACAT	0	1	0	0	0	Chr5:+15649645:quaternary,Chr1:+1455600:quaternary
TGTTGCGAAA	0	0	1	0	0	Chr5:+15597437:quaternary
TCTCCCTTCT	0	1	0	0	0	Chr5:+15408984:quaternary
TCTATGTCGA	0	1	0	0	0	Chr5:+15383290:quaternary
TAATATCAAA	0	1	0	0	0	Chr5:+15336370:quaternary,At2g36950.1:X:+-109:quaternary
TTCCTTCCTT	0	0	1	0	0	Chr5:+15300561:quaternary
CAAATTTGTT	0	0	1	0	0	Chr5:+15211703:quaternary,Chr5:+1268265:quaternary,Chr2:+2131863:quaternary
ACTCTAAAGA	0	0	0	0	1	Chr5:+15199341:quaternary
AAACTATTGT	0	1	0	0	0	Chr5:+1519328:quaternary
AATAACCCAC	0	1	0	0	0	Chr5:+15167150:quaternary
CTTATGATGT	1	0	0	0	0	Chr5:+15051981:quaternary
TAAAAAACAT	0	0	0	0	1	Chr5:+14918383:quaternary,At3g30722.1:X:+1883:quaternary
AGAGTTTATA	0	0	1	0	0	Chr5:+14881798:quaternary
TTTTACTTAA	0	0	0	0	1	Chr5:+14819858:quaternary,Chr5:+24560175:quaternary
TTGATTTCAT	0	0	0	1	0	Chr5:+14803697:quaternary
TGACAAAAAA	1	0	0	0	0	Chr5:+14734219:quaternary,Chr3:+8235183:quaternary,Chr5:+26367442:quaternary
CCAAGATCTT	0	0	1	0	0	Chr5:+14671500:quaternary
AAACCAGCAG	0	0	0	1	0	Chr5:+14670307:quaternary
GCTTTGTTCC	0	0	0	1	0	Chr5:+14670066:quaternary
TGTGATCTCT	0	0	0	1	0	Chr5:+14559031:quaternary
GATTGTCAAA	0	0	0	0	1	Chr5:+14460408:quaternary,Chr1:+9004868:quaternary,Chr1:+12914885:quaternary,Chr5:+14519708:quaternary,Chr3:+15331053:quaternary
ATCATTCTTT	1	0	0	0	0	Chr5:+14331545:quaternary
TTGCAAATTA	0	1	0	0	0	Chr5:+14307965:quaternary
CGTTTTGATC	0	0	0	0	1	Chr5:+14243837:quaternary
CCTAGGAAAA	0	0	0	1	0	Chr5:+14218285:quaternary
AGATGAGCAA	0	0	1	0	0	Chr5:+1401405:quaternary
CGTTTTAGCT	0	0	0	0	1	Chr5:+13999348:quaternary
TTTGTGATGG	0	0	0	1	0	Chr5:+13827204:quaternary
AACGAGAGAT	0	0	0	1	0	Chr5:+13726038:quaternary
ATGTCTTAAA	1	0	0	0	0	Chr5:+13698506:quaternary
AGAGTTGATG	0	0	0	1	0	Chr5:+13669583:quaternary
GTCACGCCCT	1	0	0	0	0	Chr5:+13388959:quaternary
AATCCTTTAA	0	1	0	0	0	Chr5:+13336755:quaternary,Chr5:+12230069:quaternary,Chr5:+16961879:quaternary
TTGGTGTCGA	0	0	1	0	0	Chr5:+13137089:quaternary,Chr5:+13074902:quaternary
GAATGCCAAA	0	0	1	0	0	Chr5:+13116826:quaternary,Chr1:+13559913:quaternary
TCCTTCTAAA	0	0	0	0	1	Chr5:+13100046:quaternary,Chr1:+16191133:quaternary,Chr1:+16229287:quaternary
TTGTTTGAAT	0	0	0	1	0	Chr5:+12991523:quaternary,Chr5:+12990808:quaternary,Chr5:+12991098:quaternary
TTTTGTTGAC	0	1	0	0	0	Chr5:+12930138:quaternary

AAACATTCAT	1	0	0	0	0	Chr5:+12929667:quaternary
CTTAACAAAA	0	1	0	0	0	Chr5:+12885741:quaternary,Chr5:+12843022:quaternary
AAGACAGTAA	0	0	0	0	1	Chr5:+1282367:quaternary
ATTTTGTACT	0	0	1	0	0	Chr5:+1278843:quaternary,Chr1:+17395300:quaternary
GAGTTTAGAT	0	0	1	0	0	Chr5:+12748493:quaternary
CCAAGACCAA	0	0	1	0	0	Chr5:+12706584:quaternary,Chr1:+15652927:quaternary
TGTGAAACTG	1	0	0	0	0	Chr5:+12696074:quaternary
TTTGTGTTCC	0	1	0	0	0	Chr5:+12673023:quaternary
TCGATGTAGT	0	1	0	0	0	Chr5:+12652025:quaternary
ATGTGGTAGA	0	0	0	1	0	Chr5:+12622857:quaternary
AAATACCTAA	0	1	0	0	0	Chr5:+12461744:quaternary,At5g35732.1:X:+600:quaternary
GTTGGTAACT	0	1	0	0	0	Chr5:+12438548:quaternary
CAATATTCCG	0	1	0	0	0	Chr5:+12437093:quaternary
TCACAACGTT	1	0	0	0	0	Chr5:+123821:quaternary,At5g05970.1:X:+308:quaternary
AGGTCCACAA	1	0	0	0	0	Chr5:+12354880:quaternary
TAAATAGAAA	0	0	1	0	0	Chr5:+12332555:quaternary,Chr3:+19640897:quaternary,Chr3:+15844756:quaternary,Chr3:+22429714:quaternary,Chr1:+21372188:quaternary
AGTGGTAAAT	0	0	0	1	0	Chr5:+12321590:quaternary
AGGCTTGAAA	1	0	0	0	0	Chr5:+12204084:quaternary,Chr2:+4158957:quaternary,Chr5:+11125850:quaternary,Chr5:+12355338:quaternary
GTGATCAGAA	0	0	0	1	0	Chr5:+12016968:quaternary
ACTCAGTATA	0	1	0	0	0	Chr5:+11886609:quaternary
AACTACGAAG	0	1	0	0	0	Chr5:+11781993:quaternary
CAGGCACAAA	0	1	0	0	0	Chr5:+11672114:quaternary,At1g15170.1:X:+-58:quaternary
TATCTCAAAT	0	0	0	0	1	Chr5:+11568168:quaternary,Chr2:+5947580:quaternary
CATAAGAAAA	1	0	0	0	0	Chr5:+1142389:quaternary,Chr2:+4770758:quaternary
ACTGTAAAAA	0	0	0	1	0	Chr5:+11349947:quaternary
AACACTCTAT	0	0	0	0	1	Chr5:+11340936:quaternary,Chr5:+13281401:quaternary,Chr5:+12489344:quaternary
TAACATTTGG	1	0	0	0	0	Chr5:+11295349:quaternary
TTTTATGAGA	0	1	0	0	0	Chr5:+11234346:quaternary,Chr3:+10174272:quaternary
GATGATTCTG	0	0	0	0	1	Chr5:+11192670:quaternary
TGAAAAAAGT	0	0	1	0	0	Chr5:+1115777:quaternary
CCTCTTGTAA	0	0	1	0	0	Chr5:+10893596:quaternary
GAAATAAAAA	0	1	0	0	0	Chr5:+10882469:quaternary
GTTACCAAAA	1	0	0	0	0	Chr5:+10842065:quaternary
TTTTTCCGGG	0	1	0	0	0	Chr5:+10828516:quaternary
GTTCTTTTAA	0	1	0	0	0	Chr5:+10822072:quaternary,Chr3:+15033429:quaternary
GCTAGTGCTT	0	0	0	1	0	Chr5:+10763108:quaternary
TAAATTAAGA	0	0	0	0	1	Chr5:+10674427:quaternary,Chr2:+1140752:quaternary
TCAAATACAT	0	1	0	0	0	Chr5:+10646981:quaternary
TCACCAAATT	0	0	0	1	0	Chr5:+10547371:quaternary
AATTAAATAC	0	0	0	0	1	Chr5:+10539407:quaternary
ATTGCAAAAA	0	1	0	0	0	Chr5:+10524033:quaternary
GATATTAATA	1	0	0	0	0	Chr5:+1046338:quaternary,At1g14345.1:X:+848:quaternary,Chr4:+16431519:quaternary
AGGAGGGAAG	0	0	0	0	1	Chr5:+10455767:quaternary
AAATTGATAG	0	0	0	0	1	Chr5:+10395809:quaternary
GTAAACTGAT	0	0	0	1	0	Chr5:+10378672:quaternary,Chr3:+4080138:quaternary
AGGTCCATCA	0	0	0	1	0	Chr5:+10337986:quaternary,Chr5:+10335458:quaternary
CAACTCTGGT	0	1	0	0	0	Chr5:+10298569:quaternary
GTCTGGTCTA	0	1	0	0	0	Chr5:+10215105:quaternary
TTGGATTCTC	0	1	0	0	0	Chr5:+1002453:quaternary,Chr3:+10861435:quaternary
AATCCTGGTT	0	0	1	0	0	Chr5:+10002259:quaternary
TATGTATAGG	1	0	0	0	0	Chr4:-9544846:quaternary
ACTATACTGG	1	0	0	0	0	Chr4:-9518207:quaternary,Chr4:-9538185:quaternary,Chr4:-9538173:quaternary,Chr4:-9499583:quaternary,Chr4:-9518219:quaternary
GTGTTTTACG	0	1	0	0	0	Chr4:-8933481:quaternary
TGTGTGTTGA	0	0	0	1	0	Chr4:-8881253:quaternary,Chr4:-8881243:quaternary,At5g60750.1:X:-987:quaternary
GAATAATTAA	0	0	0	0	1	Chr4:-8624507:quaternary
TTTACTACAA	0	1	0	0	0	Chr4:-8447315:quaternary
GTGGTGACAA	0	0	1	0	0	Chr4:-8404134:quaternary
TATCGACGTT	0	0	0	1	0	Chr4:-8252791:quaternary

GTTGAAGATG	0	1	0	0	0	Chr4:-7887544:quaternary
GTAACTCTTG	1	0	0	0	0	Chr4:-7563297:quaternary
TCTTCTCATT	0	0	0	0	1	Chr4:-7563188:quaternary,Chr1:-19522873:quaternary,Chr1:-19522869:quaternary
GTCACTCCTC	0	0	1	0	0	Chr4:-749496:quaternary,Chr4:-749488:quaternary
GAACTAAGAA	0	0	0	1	0	Chr4:-7427527:quaternary
GGGGTGACCA	0	0	1	0	0	Chr4:-7314045:quaternary
CGGAATGTAC	1	0	0	0	0	Chr4:-7153987:quaternary
CAGAACAAGT	0	0	0	1	0	Chr4:-6936725:quaternary
CAAGCTAGAC	0	0	1	0	0	Chr4:-6823068:quaternary
GTCCATTGTC	0	1	0	0	0	Chr4:-6793117:quaternary
AGGAGATCTA	0	0	1	0	0	Chr4:-6584136:quaternary
GACGAATCTT	0	0	0	0	1	Chr4:-5394984:quaternary
ACAGTGGATT	0	1	0	0	0	Chr4:-5108895:quaternary
TAGAAGATGT	0	0	1	0	0	Chr4:-5087066:quaternary
TTTACATTCA	0	0	1	0	0	Chr4:-4878396:quaternary
AAAATTCGAT	0	0	1	0	0	Chr4:-4871752:quaternary,Chr3:-5420217:quaternary,Chr4:-14851408:quaternary
CTTGACCTTA	0	0	0	1	0	Chr4:-4871601:quaternary
ACATTAGACT	1	0	0	0	0	Chr4:-4867123:quaternary
CGTTGTAGAG	0	0	0	1	0	Chr4:-4668127:quaternary
GAGGGATTTT	0	0	1	0	0	Chr4:-450781:quaternary
TCAAACTCTA	0	0	1	0	0	Chr4:-3880928:quaternary
CCTTACTCAA	0	0	0	0	1	Chr4:-3837011:quaternary
AACAAATGAG	1	0	0	0	0	Chr4:-3665689:quaternary,Chr1:-24918626:quaternary
TCTTCAGATA	0	1	0	0	0	Chr4:-3514001:quaternary
AGCCCATTCT	1	0	0	0	0	Chr4:-329814:quaternary
ATAGCAATTG	1	0	0	0	0	Chr4:-3030292:quaternary
GGTACAAGTA	0	0	1	0	0	Chr4:-2841087:quaternary
AGAACATTCC	0	0	1	0	0	Chr4:-2571179:quaternary
ACAAACACAA	1	0	0	0	0	Chr4:-2508004:quaternary
TAAGCATCAA	0	0	0	0	1	Chr4:-2353272:quaternary
GTGGAAGTTG	0	0	0	1	0	Chr4:-2264135:quaternary
GGCGTCGGGT	0	0	1	0	0	Chr4:-18517603:quaternary
GATGCAAATT	0	0	1	0	0	Chr4:-18455072:quaternary
GCCGTGTCTA	0	1	0	0	0	Chr4:-18454164:quaternary,At3g24490.1:X:--172:quaternary
TTCTCGTCTG	0	0	0	1	0	Chr4:-18392762:quaternary
GGGGAGGCAA	0	0	1	0	0	Chr4:-17694959:quaternary
GCAGAGTGAC	0	0	0	0	1	Chr4:-17688202:quaternary
TGGTCCAGAT	0	0	1	0	0	Chr4:-1761254:quaternary
CTTGCCATCT	1	0	0	0	0	Chr4:-17358085:quaternary
AACTGAAGTT	0	1	0	0	0	Chr4:-17319222:quaternary
TTGTATGCGC	0	1	0	0	0	Chr4:-16891526:quaternary
AGGGGTTACG	0	0	1	0	0	Chr4:-16101830:quaternary
TGACATTCAT	0	0	0	0	1	Chr4:-15381050:quaternary,Chr4:-11586422:quaternary,Chr4:-11586435:quaternary
GCGACAACGT	0	1	0	0	0	Chr4:-1516269:quaternary
ATGGGAGCTT	1	0	0	0	0	Chr4:-15000706:quaternary
GACAGGCAAG	0	0	0	0	1	Chr4:-14515123:quaternary
ATCAAATCCA	0	0	0	1	0	Chr4:-14454466:quaternary
TCTCTCGTAT	0	1	0	0	0	Chr4:-14408789:quaternary
GGAATTTACA	0	0	0	1	0	Chr4:-14304433:quaternary
TCCAAATCTA	0	1	0	0	0	Chr4:-14031475:quaternary
GGTGGTCCAA	0	0	1	0	0	Chr4:-13889288:quaternary
TCTCTTAAGC	0	1	0	0	0	Chr4:-1348023:quaternary
TTAATCATAT	1	0	0	0	0	Chr4:-1328438:quaternary
ATTGGGTTGT	0	0	0	0	1	Chr4:-12871920:quaternary,Chr3:-6089129:quaternary
ACAACATATG	1	0	0	0	0	Chr4:-12289444:quaternary
ATTCAGGAAT	1	0	0	0	0	Chr4:-12153560:quaternary
TAATTTGAGA	0	0	1	0	0	Chr4:-11898449:quaternary,Chr2:-12397937:quaternary,Chr5:-25282907:quaternary,Chr3:-22974702:quaternary
CAAAAAGCAT	1	0	0	0	0	Chr4:-11847742:quaternary
TCCATAATAT	0	0	0	1	0	Chr4:-11687255:quaternary
GTCATCGAAT	0	0	0	1	0	Chr4:-11599811:quaternary

CTATAGCATT	0	0	0	0	1	Chr4:-10769206:quaternary
ATGACAACGA	0	0	1	0	0	Chr4:-10515186:quaternary
TGACAACAGT	0	0	0	0	1	Chr4:-10497514:quaternary
TCTATATCTC	0	0	0	0	1	Chr4:+9943330:quaternary
GAGAGCTAAG	0	1	0	0	0	Chr4:+9897925:quaternary
TTGAGAAAAG	0	0	0	0	1	Chr4:+9860176:quaternary,Chr1:+21523205:quaternary
AATAGTTGGA	0	0	1	0	0	Chr4:+9839990:quaternary
TATACTCCCA	0	1	0	0	0	Chr4:+9758897:quaternary
TTAGCTATAG	0	1	0	0	0	Chr4:+9755557:quaternary
AAACAGTTTT	0	1	0	0	0	Chr4:+9665524:quaternary
TAATAATTAA	1	0	0	0	0	Chr4:+9613128:quaternary,Chr3:+13234885:quaternary,Chr5:+5883444:quaternary,Chr3:+5079454:quaternary,Chr3:+12869943:quaternary
CTCTAGAGTT	0	0	1	0	0	Chr4:+9546042:quaternary
AAGATTCTAT	0	1	0	0	0	Chr4:+9475802:quaternary
AAAAAATATT	1	0	0	0	0	Chr4:+9439186:quaternary,Chr5:+21547808:quaternary,Chr1:+16827446:quaternary,Chr2:+4630384:quaternary,Chr5:+8766205:quaternary,Chr5:+18871686:quaternary,Chr4:+628531:quaternary,Chr5:+18900310:quaternary,Chr1:+16906712:quaternary,Chr1:+8115252:quaternary,Chr4:+1350079:quaternary,Chr1:+4785937:quaternary,Chr1:+23131597:quaternary,Chr1:+9816691:quaternary,Chr3:+16462264:quaternary,At5g51230.1:X:+429:quaternary,Chr3:+15948767:quaternary,Chr5:+17382746:quaternary,Chr2:+12808563:quaternary,Chr1:+13231750:quaternary,Chr1:+19089149:quaternary,Chr3:+7652230:quaternary,Chr2:+13274484:quaternary,Chr2:+6107005:quaternary,Chr4:+6178840:quaternary,Chr1:+23217826:quaternary,Chr3:+10864034:quaternary,Chr5:+9590907:quaternary,Chr3:+16891187:quaternary,Chr1:+11700170:quaternary,Chr2:+500478:quaternary,Chr3:+3716756:quaternary,Chr2:+14341354:quaternary
TTTCATTTTA	0	0	1	0	0	Chr4:+9424092:quaternary
TTGGCTAGGT	1	0	0	0	0	Chr4:+9297980:quaternary,Chr2:+3284224:quaternary
AAAAAAGGGT	0	0	1	0	0	Chr4:+9278412:quaternary
TACTTAACTA	0	0	1	0	0	Chr4:+9217949:quaternary
TAAAAAGTAC	1	0	0	0	0	Chr4:+9068467:quaternary
TAAAAGGTAC	1	0	0	0	0	Chr4:+9068391:quaternary
TAAAAAACTA	0	1	0	0	0	Chr4:+9066107:quaternary,Chr5:+1115441:quaternary,Chr3:+15978965:quaternary
ATACGCAACT	0	1	0	0	0	Chr4:+9043158:quaternary
TTATGAAAAA	0	0	0	0	1	Chr4:+9033669:quaternary
AATGAAAAAA	0	0	1	0	0	Chr4:+8970174:quaternary,Chr5:+11349744:quaternary,Chr4:+47697:quaternary,At5g43560.2:X:+-83:quaternary
CCAAATGAAA	0	0	0	0	1	Chr4:+8943878:quaternary
GACTAAATGG	0	0	0	1	0	Chr4:+8941855:quaternary,Chr1:+26521431:quaternary
AATGAATAAC	0	1	0	0	0	Chr4:+8924783:quaternary
TAAACCAATT	0	0	0	1	0	Chr4:+8803076:quaternary
TTGATCTAGA	1	0	0	0	0	Chr4:+8782564:quaternary
CAAATTCAAT	0	0	0	1	0	Chr4:+8753825:quaternary,Chr5:+14718205:quaternary
ATTACTGTGA	1	0	0	0	0	Chr4:+8486105:quaternary
AGTGATAAAG	0	1	0	0	0	Chr4:+8435322:quaternary,Chr3:+11734593:quaternary
GGTTAGTCTC	0	0	0	1	0	Chr4:+8419549:quaternary
TCATAAAAAA	0	0	0	0	1	Chr4:+8409391:quaternary,Chr5:+26036405:quaternary,At1g78650.1:X:+288:quaternary
GATACGTAAA	0	0	1	0	0	Chr4:+8386846:quaternary
CCAGAGGTAG	1	0	0	0	0	Chr4:+8347314:quaternary
AGTAGTTAAA	0	0	0	1	0	Chr4:+8308170:quaternary
CCCTACACAA	0	0	1	0	0	Chr4:+8143660:quaternary
ATTCACAGAA	1	0	0	0	0	Chr4:+8088130:quaternary
ATGATGTGGC	0	0	0	1	0	Chr4:+7961216:quaternary
AATCCTATTA	0	0	1	0	0	Chr4:+7960094:quaternary,Chr4:+11139265:quaternary
TAGCAGTAAA	1	0	0	0	0	Chr4:+7929558:quaternary
TTCAAAGAAG	0	0	0	0	1	Chr4:+7845561:quaternary
AACATTAATG	0	0	1	0	0	Chr4:+7845406:quaternary
AGTTTAATTT	0	0	1	0	0	Chr4:+7812264:quaternary,Chr1:+11472693:quaternary
CATAGCATAA	1	0	0	0	0	Chr4:+7720869:quaternary,Chr5:+21550549:quaternary
ATATGAACTT	0	0	0	1	0	Chr4:+7718303:quaternary
ACTGACTCTT	1	0	0	0	0	Chr4:+7707245:quaternary
ACCATACTCA	0	1	0	0	0	Chr4:+7530021:quaternary
GTTGTGTCAT	0	1	0	0	0	Chr4:+7407568:quaternary
TAATCTGCAT	0	0	0	0	1	Chr4:+7385231:quaternary,Chr1:+28323980:quaternary
ATCACGAATT	0	1	0	0	0	Chr4:+7356749:quaternary
AGTGTGTTTA	0	0	1	0	0	Chr4:+734874:quaternary

AAAATATTTG	0	0	0	0	1	Chr4:+7324515:quaternary
ACAACCAGCT	0	0	1	0	0	Chr4:+7262698:quaternary
CCGTTTTTTC	0	1	0	0	0	Chr4:+7262132:quaternary
AAAAACTTTG	0	0	1	0	0	Chr4:+7228697:quaternary,Chr5:+2441085:quaternary,Chr4:+5142741:quaternary
GTCTACTATT	0	0	0	0	1	Chr4:+721680:quaternary
AGCAAGTAAA	0	1	0	0	0	Chr4:+6990448:quaternary,Chr3:+20360033:quaternary
TAATGTAATT	0	0	0	0	1	Chr4:+6974476:quaternary
CAGTATTTTA	0	1	0	0	0	Chr4:+6942289:quaternary,Chr1:+22882459:quaternary
AATTAAAATG	0	1	0	0	0	Chr4:+6933950:quaternary,Chr5:+24405748:quaternary
TAATCTGTTA	1	0	0	0	0	Chr4:+6831870:quaternary,At3g16980.1:X:+208:quaternary,Chr1:+28750855:quaternary,Chr3:+15622482:quaternary
TGTACAAAAA	0	0	1	0	0	Chr4:+6821230:quaternary
CTTCAAACTA	0	0	1	0	0	Chr4:+6770536:quaternary
TTCTCAATAG	0	1	0	0	0	Chr4:+6743248:quaternary
CCTTCAAAAA	0	0	1	0	0	Chr4:+6672903:quaternary,Chr5:+5479831:quaternary
CAAGTTTCAA	0	1	0	0	0	Chr4:+6616499:quaternary
TATATTATTT	0	1	0	0	0	Chr4:+6554497:quaternary
TTGGGATATC	0	0	0	1	0	Chr4:+6489347:quaternary
TAAAAAACGT	1	0	0	0	0	Chr4:+6488282:quaternary
ATTCAGATTT	0	0	1	0	0	Chr4:+6312555:quaternary
CCTTATTTTG	0	0	0	1	0	Chr4:+6267680:quaternary,Chr1:+18083072:quaternary
GACCTTTGTC	0	1	0	0	0	Chr4:+6243845:quaternary
ATATATGCAA	0	1	0	0	0	Chr4:+6227477:quaternary
ATATTTTCAA	0	0	0	0	1	Chr4:+5929042:quaternary,Chr4:+13279356:quaternary
AGTTAGCAAC	0	0	1	0	0	Chr4:+5839184:quaternary,Chr4:+2182794:quaternary
TTTCTTCTAA	1	0	0	0	0	Chr4:+5780452:quaternary
TGTATGTTTC	0	1	0	0	0	Chr4:+5692137:quaternary,Chr3:+7280478:quaternary
GATGTTTCTT	1	0	0	0	0	Chr4:+5472557:quaternary,At4g12690.1:X:+-70:quaternary
GTATGGAACA	1	0	0	0	0	Chr4:+5447820:quaternary
TTATTATCAA	0	1	0	0	0	Chr4:+5442560:quaternary,Chr5:+17662466:quaternary,Chr5:+15213695:quaternary,Chr3:+22672036:quaternary
AGATGCAATT	0	1	0	0	0	Chr4:+5353435:quaternary,Chr2:+11407237:quaternary
TGGGATCAAA	0	0	0	1	0	Chr4:+5313921:quaternary
ATCGTTAAAG	0	1	0	0	0	Chr4:+5221367:quaternary
TTTACTTGTA	0	1	0	0	0	Chr4:+5087912:quaternary
AGTTCAAAAA	0	1	0	0	0	Chr4:+5059031:quaternary,Chr5:+12298733:quaternary,Chr2:+2400485:quaternary,Chr4:+1104140:quaternary
GTGAAGCTAA	0	0	0	0	1	Chr4:+5048383:quaternary
TTTGGAATGC	0	0	0	0	1	Chr4:+5034355:quaternary,Chr2:+5254719:quaternary
CATTATTCAA	0	0	1	0	0	Chr4:+4885629:quaternary,Chr4:+2398522:quaternary
GAAGGGCTGT	1	0	0	0	0	Chr4:+4868134:quaternary
TGACAAAATT	0	0	1	0	0	Chr4:+4609483:quaternary,Chr1:+14840372:quaternary,Chr3:+12719008:quaternary,Chr2:+4204509:quaternary,Chr2:+4216905:quaternary
AAAGCTCCTG	1	0	0	0	0	Chr4:+4445132:quaternary,Chr4:+5917987:quaternary
AATCACAAAA	0	0	0	0	1	Chr4:+437937:quaternary,Chr5:+3345705:quaternary
TAAAAGATGT	0	0	1	0	0	Chr4:+4314214:quaternary
CAAATATAAA	0	1	0	0	0	Chr4:+4216638:quaternary
AGATCAATAA	0	0	1	0	0	Chr4:+4210685:quaternary,Chr2:+7972187:quaternary
GCCTATCTCC	0	0	1	0	0	Chr4:+4206331:quaternary
ATTTCACGTT	0	1	0	0	0	Chr4:+392036:quaternary,Chr4:+2270014:quaternary
ATTCACGAGT	1	0	0	0	0	Chr4:+3881263:quaternary
TAGAATTCTA	0	0	0	0	1	Chr4:+3818092:quaternary,Chr5:+9846971:quaternary
ATCGATATAA	0	0	0	1	0	Chr4:+3522759:quaternary
TTTCTTCTTG	0	1	0	0	0	Chr4:+3233857:quaternary,Chr4:+3257755:quaternary,Chr4:+3231334:quaternary,Chr4:+3247832:quaternary,Chr4:+3195190:quaternary,Chr4:+3215628:quaternary,Chr4:+3220499:quaternary,Chr4:+3245261:quaternary,Chr4:+3226493:quaternary,Chr4:+3240364:quaternary,Chr4:+3236190:quaternary,Chr4:+3250130:quaternary,Chr4:+3242935:quaternary,Chr4:+3228791:quaternary
CTAGTATATT	0	0	1	0	0	Chr4:+311445:quaternary,Chr5:+2006614:quaternary
ATAAAGGATA	0	1	0	0	0	Chr4:+2769442:quaternary
AACTGGGTTC	0	0	0	1	0	Chr4:+2768903:quaternary

TAACCAAAGA	0	1	0	0	0	Chr4:+2716784:quaternary
CTTGAATCTT	0	1	0	0	0	Chr4:+2648511:quaternary
TTAACTTTTA	0	1	0	0	0	Chr4:+2584223:quaternary,Chr2:+15145026:quaternary
AGAATATATT	0	1	0	0	0	Chr4:+2552222:quaternary,Chr1:+17351140:quaternary,Chr4:+7209519:quaternary,Chr5:+14770889:quaternary,Chr5:+21155073:quaternary
AGCCAAAAAA	0	0	0	1	0	Chr4:+2470728:quaternary
TGCAACAAAA	1	0	0	0	0	Chr4:+2412139:quaternary,Chr2:+11835266:quaternary
TACGGGAAGA	0	1	0	0	0	Chr4:+2181369:quaternary
TATCTCCGAT	1	0	0	0	0	Chr4:+1996878:quaternary
GGCCTTGACC	0	0	1	0	0	Chr4:+189965:quaternary
GATCTCAGTG	0	0	0	1	0	Chr4:+18578029:quaternary
ATAACGAAAT	0	0	1	0	0	Chr4:+18565024:quaternary,Chr3:+8168209:quaternary,Chr2:+19620555:quaternary
CGTTTAATAA	0	0	0	1	0	Chr4:+18454143:quaternary
TACAGACAAA	1	0	0	0	0	Chr4:+18324351:quaternary
TATGCATCAA	1	0	0	0	0	Chr4:+1810197:quaternary
AAGCTTTCCT	0	0	0	1	0	Chr4:+17893407:quaternary,Chr5:+10704283:quaternary
AGCTAAAAAA	0	0	1	0	0	Chr4:+17762235:quaternary
ATTAATTAAG	0	0	0	0	1	Chr4:+17760732:quaternary,Chr4:+17318040:quaternary,Chr5:+11231671:quaternary,Chr1:+23081293:quaternary,Chr4:+14956849:quaternary,Chr1:+22833220:quaternary
AGACGATGAT	1	0	0	0	0	Chr4:+17670127:quaternary,Chr1:+10397580:quaternary,Chr2:+930693:quaternary
TAATTGGTTT	0	0	0	0	1	Chr4:+17605498:quaternary,Chr4:+7221915:quaternary
ATGAGATTTG	0	0	1	0	0	Chr4:+17497418:quaternary
AAAAAGGTCG	0	0	0	0	1	Chr4:+17439090:quaternary,Chr2:+12309168:quaternary
ATCTCGTTCT	0	1	0	0	0	Chr4:+17149822:quaternary
AACACCAATG	0	0	1	0	0	Chr4:+17003657:quaternary
AGTTATCAAA	0	0	1	0	0	Chr4:+16920509:quaternary
TAATGTGTAG	1	0	0	0	0	Chr4:+16745971:quaternary
GTTTAGTGAT	0	0	1	0	0	Chr4:+16652720:quaternary
CTCTCGGTGC	0	1	0	0	0	Chr4:+1661068:quaternary
AACTCTTTTG	0	0	1	0	0	Chr4:+16527167:quaternary
TTGGTGATAG	0	0	0	1	0	Chr4:+16471242:quaternary
GTTAAACAAA	0	0	0	0	1	Chr4:+16362784:quaternary
GGCTAGAAAA	1	0	0	0	0	Chr4:+16168381:quaternary
AGGACTCATA	0	0	1	0	0	Chr4:+16101727:quaternary
CCTACGATCT	0	0	1	0	0	Chr4:+15918957:quaternary
ATCATTCGAA	0	0	1	0	0	Chr4:+15823874:quaternary,Chr1:+10952007:quaternary
GTTTTTTTTA	0	1	0	0	0	Chr4:+15815523:quaternary
ATTGATTGGT	0	0	0	0	1	Chr4:+156792:quaternary
TGCATTGTGA	0	1	0	0	0	Chr4:+15660016:quaternary
AGTGTTTCTA	1	0	0	0	0	Chr4:+15400013:quaternary
TTAACATAAT	0	1	0	0	0	Chr4:+1533055:quaternary,Chr1:+24943115:quaternary,Chr1:+20958170:quaternary
AGATGATAAC	0	0	0	0	1	Chr4:+15330012:quaternary
TGACTTTTCC	0	1	0	0	0	Chr4:+15192890:quaternary,Chr1:+8299302:quaternary
TCTTAAAAAT	0	0	0	0	1	Chr4:+15061176:quaternary
GTTGTACAAA	0	1	0	0	0	Chr4:+14824922:quaternary
CTGTTAATTT	0	0	0	1	0	Chr4:+14795035:quaternary
TAGAGTGATT	0	0	1	0	0	Chr4:+14786129:quaternary
ATGATATCCA	0	1	0	0	0	Chr4:+14674064:quaternary
GAAAGGTAAA	0	0	1	0	0	Chr4:+14636564:quaternary
TTTATCCTGA	0	0	0	1	0	Chr4:+14582419:quaternary
ATGCTGTTGA	0	1	0	0	0	Chr4:+14578149:quaternary
TCACAACCAA	0	1	0	0	0	Chr4:+14551883:quaternary,Chr4:+4677725:quaternary
AGAGACCAAA	1	0	0	0	0	Chr4:+14374473:quaternary,Chr1:+13435862:quaternary
TATCATTACA	0	0	0	0	1	Chr4:+14356344:quaternary
GGATTCATTT	0	0	0	0	1	Chr4:+14328783:quaternary
GTTTTCTAAC	0	0	0	0	1	Chr4:+14258461:quaternary
TCTCCTAATT	0	0	0	0	1	Chr4:+14099213:quaternary,Chr4:+13014392:quaternary
ACTACTACTT	1	0	0	0	0	Chr4:+14002832:quaternary
AAAAGAAAGC	0	0	0	0	1	Chr4:+1339950:quaternary

ACAAGCTTTA	0	0	0	1	0	Chr4:+13393928:quaternary
CTTTTTAATC	0	0	0	1	0	Chr4:+1338398:quaternary
TTTCCCGGAG	0	0	1	0	0	Chr4:+13333245:quaternary
AATTGATCAT	1	0	0	0	0	Chr4:+13279002:quaternary,Chr1:+12740875:quaternary
GACCAAACCA	0	0	1	0	0	Chr4:+13249792:quaternary
CAAAATTTTA	0	0	0	1	0	Chr4:+13232324:quaternary,Chr2:+15229103:quaternary,Chr5:+15246728:quaternary
TGACCCTTGA	0	0	1	0	0	Chr4:+13196217:quaternary
ATTAGTTTCG	0	0	1	0	0	Chr4:+13020668:quaternary,Chr2:+1346867:quaternary
AAAATCAATA	0	0	0	0	1	Chr4:+12990454:quaternary,Chr2:+17495557:quaternary,Chr1:+19813157:quaternary,Chr4:+7526147:quaternary,At5g24360.1:X:+-196:quaternary,Chr5:+9166672:quaternary
GATTGAGGTG	0	1	0	0	0	Chr4:+12987194:quaternary
TCACTGTTGC	0	1	0	0	0	Chr4:+12858465:quaternary
ATTAGGCAGC	1	0	0	0	0	Chr4:+12857497:quaternary
TAGATTAACG	0	0	0	0	1	Chr4:+12845300:quaternary
GAAAAACTAA	0	1	0	0	0	Chr4:+12777237:quaternary,At2g40116.1:X:+566:quaternary,Chr2:+13070283:quaternary,Chr4:+1397594:quaternary
ATATATAATT	0	1	0	0	0	Chr4:+12764531:quaternary,Chr1:+23102448:quaternary,Chr5:+3725610:quaternary
GTCTAATGAA	0	1	0	0	0	Chr4:+1271299:quaternary
CGTACAAAAT	0	0	1	0	0	Chr4:+12621212:quaternary
AAACATAAAA	0	0	1	0	0	Chr4:+12549812:quaternary,Chr4:+2185223:quaternary,Chr2:+1500865:quaternary,Chr5:+16085937:quaternary,Chr1:+14176471:quaternary,Chr1:+14176390:quaternary
CCTAAGAGAA	0	0	1	0	0	Chr4:+1253514:quaternary
TCACTGAAAT	0	0	1	0	0	Chr4:+12530761:quaternary
GTTGATCGAA	0	1	0	0	0	Chr4:+1246044:quaternary
CCTTGGTTTG	1	0	0	0	0	Chr4:+12378717:quaternary
TGACAAAGTT	0	1	0	0	0	Chr4:+12297722:quaternary
TACAGATTGG	0	1	0	0	0	Chr4:+12232815:quaternary
GACACAAAAT	0	0	0	1	0	Chr4:+1213230:quaternary
AAAGATTTGC	0	0	0	1	0	Chr4:+12052836:quaternary,At3g23930.1:X:+443:quaternary
AATGAGAATG	0	0	1	0	0	Chr4:+12026382:quaternary,Chr3:+1852147:quaternary,Chr1:+5136023:quaternary
TGAGAGTTGT	0	1	0	0	0	Chr4:+11971539:quaternary
TACACTAAAC	0	1	0	0	0	Chr4:+11906505:quaternary,Chr1:+15927497:quaternary,Chr5:+22276769:quaternary
TTTCGAAAAA	0	0	0	0	1	Chr4:+11867097:quaternary,At4g22890.3:X:+-209:quaternary
AGTGGACAAT	0	0	0	1	0	Chr4:+11722393:quaternary
GGATATGTTG	0	0	0	0	1	Chr4:+11668381:quaternary
GTGATGATAA	1	0	0	0	0	Chr4:+11650403:quaternary,Chr5:+16983289:quaternary
AATCAGTCTC	0	0	1	0	0	Chr4:+11524956:quaternary
TTTGAGTTCT	0	0	1	0	0	Chr4:+11374791:quaternary,Chr1:+4188603:quaternary
TGTGTTGGTG	0	1	0	0	0	Chr4:+11109702:quaternary,At4g11211.1:X:+546:quaternary
GCTTCCTTGA	0	0	0	1	0	Chr4:+10808489:quaternary
GACTTATCTT	0	0	0	1	0	Chr4:+10702591:quaternary,Chr5:+5661260:quaternary
ATCCACATAT	0	0	0	1	0	Chr4:+10600005:quaternary
TCTCTTGAGT	0	0	0	0	1	Chr4:+10427896:quaternary,Chr4:+10430163:quaternary,At1g68585.1:X:+-194:quaternary,Chr4:+558114:quaternary
TGTACATATG	1	0	0	0	0	Chr4:+10424224:quaternary
ACTTTAAAAA	0	0	0	1	0	Chr4:+10375109:quaternary,Chr5:+15090484:quaternary,Chr1:+8476503:quaternary
TAAATAAATT	0	1	0	0	0	Chr4:+10363939:quaternary,Chr1:+27872589:quaternary
ATTGGCTCAT	0	0	1	0	0	Chr4:+10335721:quaternary,At1g51350.1:X:+-97:quaternary
TTGAGATATG	0	0	0	0	1	Chr4:+10310782:quaternary
GATAGTTTGA	0	0	1	0	0	Chr4:+10223850:quaternary
AAAAAGTATT	0	0	0	0	1	Chr4:+10140083:quaternary
ATAAAACAAT	1	0	0	0	0	Chr4:+10113109:quaternary,Chr1:+367951:quaternary,Chr5:+12203214:quaternary
TGTTTCATAC	0	0	1	0	0	Chr3:-9877715:quaternary
AGAAGATTCT	0	0	1	0	0	Chr3:-9349959:quaternary
GTAGAAAAAA	0	0	0	0	1	Chr3:-9285788:quaternary
GACACTAAAA	1	0	0	0	0	Chr3:-899525:quaternary
ATTCATCGAA	0	0	1	0	0	Chr3:-8295230:quaternary
CGGAGAAGAA	0	0	0	0	1	Chr3:-8233196:quaternary

TCTTGTAACT	0	0	1	0	0	Chr3:-7986805:quaternary,Chr3:-7986811:quaternary
TGTAAGAATG	0	1	0	0	0	Chr3:-7598059:quaternary
TAGATGTTTA	0	0	1	0	0	Chr3:-5995157:quaternary
TTAATTCCTT	0	1	0	0	0	Chr3:-5695060:quaternary
ACTCTTGTTA	0	1	0	0	0	Chr3:-5354410:quaternary
CAGAAGATTA	0	0	1	0	0	Chr3:-5174148:quaternary
TGTTGGGATC	0	0	1	0	0	Chr3:-5150651:quaternary
CCTTATTACA	0	0	0	0	1	Chr3:-4779085:quaternary
CGTGTAATGG	0	0	1	0	0	Chr3:-4376853:quaternary
ACTAGAAAAA	0	0	0	0	1	Chr3:-4077575:quaternary,Chr3:-11993909:quaternary,Chr3:-11995772:quaternary,Chr3:-11988323:quaternary,Chr3:-11992048:quaternary,Chr3:-11986118:quaternary,Chr1:-28670526:quaternary,Chr3:-11990185:quaternary
TCAAGCGTGT	0	0	0	1	0	Chr3:-3524435:quaternary
GCATAATCAA	0	0	1	0	0	Chr3:-2906609:quaternary
ATTACGGAAC	0	1	0	0	0	Chr3:-2847843:quaternary
ACACGGATCA	0	0	0	1	0	Chr3:-2570912:quaternary
TGGCTATCGA	0	0	1	0	0	Chr3:-22330951:quaternary,Chr3:-1055725:quaternary
TTTCAATAAA	0	1	0	0	0	Chr3:-22240789:quaternary
TTTGGGTGAT	0	0	1	0	0	Chr3:-22102613:quaternary
TAGCCAAACG	0	0	1	0	0	Chr3:-22088516:quaternary
GACACACCGG	0	0	1	0	0	Chr3:-21991842:quaternary
TTGGTCGTCG	0	0	1	0	0	Chr3:-2167027:quaternary
TTCCTATTTC	0	1	0	0	0	Chr3:-20770977:quaternary
AGTTGCATTT	0	1	0	0	0	Chr3:-20584062:quaternary
GCGAGATTTT	0	0	1	0	0	Chr3:-20536823:quaternary
TTTCTGTGTC	1	0	0	0	0	Chr3:-19883547:quaternary
GTTGGTCTCA	0	0	1	0	0	Chr3:-19740017:quaternary
TTGTAAATAT	0	0	0	0	1	Chr3:-19712193:quaternary,Chr3:-17869581:quaternary,Chr3:-16999702:quaternary
TAAACGGCCT	0	0	0	1	0	Chr3:-19447673:quaternary
TAAATTGATC	0	1	0	0	0	Chr3:-19079679:quaternary,Chr1:-21045963:quaternary
AAACTTCGTT	0	1	0	0	0	Chr3:-18904193:quaternary
GATGTACTCT	0	0	0	1	0	Chr3:-18903957:quaternary
GAACGATGTG	1	0	0	0	0	Chr3:-18718049:quaternary
TGAGTGGTAA	1	0	0	0	0	Chr3:-18581085:quaternary,Chr5:-19120398:quaternary
GTTGTGAGTG	1	0	0	0	0	Chr3:-18520033:quaternary
ACCGTAAACG	0	1	0	0	0	Chr3:-18400080:quaternary
ATTCTGATGT	0	0	1	0	0	Chr3:-18399246:quaternary
TCAATCATTT	0	0	0	1	0	Chr3:-18148766:quaternary
AAAAAGGCTT	0	0	0	1	0	Chr3:-1808440:quaternary
GGTTGGAGAT	0	0	0	0	1	Chr3:-18055581:quaternary,Chr3:-18055594:quaternary
CAAGCACGTC	0	0	0	0	1	Chr3:-17901609:quaternary
TGTGTGCGAA	0	0	0	1	0	Chr3:-17563060:quaternary
ATCTACTTCT	1	0	0	0	0	Chr3:-17466073:quaternary
TCAGAGTAGA	0	0	1	0	0	Chr3:-17252274:quaternary,Chr1:-3307433:quaternary
GCTCCATCAG	0	1	0	0	0	Chr3:-16766348:quaternary
GACTGGGAAA	0	0	0	1	0	Chr3:-1657489:quaternary
GTTCGACCTA	0	0	0	1	0	Chr3:-16566234:quaternary
TTTAGGTTTA	1	0	0	0	0	Chr3:-16448768:quaternary,Chr3:-16448772:quaternary,Chr1:-3243328:quaternary
GCTGGCTTAG	0	0	1	0	0	Chr3:-16122836:quaternary
ACCGCACAAA	0	0	0	1	0	Chr3:-15620070:quaternary
AGTGTGAGAT	0	0	1	0	0	Chr3:-15424122:quaternary,Chr3:-15430375:quaternary,Chr3:-15428921:quaternary,Chr3:-15422676:quaternary
CAAAAAGGGA	1	0	0	0	0	Chr3:-14771577:quaternary,Chr5:-12509927:quaternary
CTTGGGAAAT	0	1	0	0	0	Chr3:-14486505:quaternary
TTAGGGACAA	0	0	1	0	0	Chr3:-14382917:quaternary
CGCGTCACTA	0	0	1	0	0	Chr3:-14213398:quaternary,Chr2:-8441:quaternary
AACGTTTAGC	0	0	1	0	0	Chr3:-1397279:quaternary
CGTCTTTTGC	1	0	0	0	0	Chr3:-13645423:quaternary
AAAATGAGTC	0	0	0	1	0	Chr3:-13546395:quaternary
GGGTTGGTTG	1	0	0	0	0	Chr3:-13055676:quaternary
TTAGAACCTT	1	0	0	0	0	Chr3:-12686371:quaternary
GATATGACGA	0	0	1	0	0	Chr3:-12647103:quaternary

TTATAAAGGA	0	1	0	0	0	Chr3:-12144441:quaternary,Chr3:-1773639:quaternary
CAACGATAAA	0	0	1	0	0	Chr3:-11922390:quaternary
TATAACTGTG	1	0	0	0	0	Chr3:-11801319:quaternary
TGCTTCAGCC	0	1	0	0	0	Chr3:-11760557:quaternary,Chr5:-757650:quaternary
CTTGAGCTGA	0	1	0	0	0	Chr3:-11742755:quaternary
ACTAGACCCT	0	0	0	0	1	Chr3:-11391694:quaternary
AACGTATCAA	0	0	1	0	0	Chr3:-10870487:quaternary
TACACTAAAA	0	1	0	0	0	Chr3:-10751311:quaternary,Chr3:-10751322:quaternary
TGTGCTATTT	0	1	0	0	0	Chr3:-10470739:quaternary
ATTTGTGCTT	0	1	0	0	0	Chr3:-10403272:quaternary
AGTCTTAGAT	0	1	0	0	0	Chr3:-10018232:quaternary
TTTAAAAGAT	0	1	0	0	0	Chr3:+9951228:quaternary,Chr5:+21229612:quaternary
GATGGAAAAA	0	0	0	0	1	Chr3:+9879780:quaternary,At4g08180.1:X:+711:quaternary
GAGTTCTCAC	0	0	1	0	0	Chr3:+9784660:quaternary; -small RNA(GTGAGAACTCCATGGGG)
GATGTTGTTC	0	0	1	0	0	Chr3:+9784249:quaternary,Chr2:+18825906:quaternary
AAACACAAAG	0	0	0	0	1	Chr3:+9745121:quaternary
TTATACTGTT	0	0	0	1	0	Chr3:+9723151:quaternary,Chr5:+16661598:quaternary
ATACTTGGGT	1	0	0	0	0	Chr3:+9704364:quaternary
ATAACCCAAA	1	0	0	0	0	Chr3:+9587339:quaternary
GGGTTTTCAC	0	0	1	0	0	Chr3:+9581796:quaternary
ACTTGGATTA	0	1	0	0	0	Chr3:+9468073:quaternary
CTCGGTCAGT	0	1	0	0	0	Chr3:+9465805:quaternary
TCTCATTTGA	0	0	0	0	1	Chr3:+9444606:quaternary,Chr3:+9452869:quaternary
AGAGTTTGTC	0	1	0	0	0	Chr3:+9363079:quaternary
AAGTCGTGAA	0	0	1	0	0	Chr3:+9330163:quaternary
CAGTCAAATG	0	0	1	0	0	Chr3:+9324324:quaternary
GAAAAAACAT	0	0	0	1	0	Chr3:+9321591:quaternary
TGCTTAAGAA	1	0	0	0	0	Chr3:+9121930:quaternary
GTGTATAGGT	1	0	0	0	0	Chr3:+9116559:quaternary,Chr3:+9105820:quaternary
ACCGTAATCT	0	1	0	0	0	Chr3:+9116213:quaternary
ACAGTAACTG	0	1	0	0	0	Chr3:+9105457:quaternary
AACAATTCCT	0	1	0	0	0	Chr3:+9097308:quaternary,Chr1:+25719839:quaternary
CTCTTTTATG	1	0	0	0	0	Chr3:+8951526:quaternary
TGCCTTGTTT	1	0	0	0	0	Chr3:+8934101:quaternary
AATTATGAAA	0	0	1	0	0	Chr3:+8892490:quaternary
ATCTGAGTCT	1	0	0	0	0	Chr3:+8818556:quaternary
TTCCGAAAAA	0	1	0	0	0	Chr3:+8785163:quaternary,Chr3:+8785122:quaternary
TCTTTAACCA	1	0	0	0	0	Chr3:+8780065:quaternary
TGTACACGGG	0	1	0	0	0	Chr3:+8760409:quaternary
AGAGATTGGC	0	0	0	0	1	Chr3:+8690363:quaternary
TCAATTTGTT	1	0	0	0	0	Chr3:+8591522:quaternary
CATCTGATGA	1	0	0	0	0	Chr3:+8585280:quaternary
ATAAAAAAGA	0	0	0	1	0	Chr3:+853203:quaternary,Chr1:+25384275:quaternary
TTGGATCAAT	0	1	0	0	0	Chr3:+8518636:quaternary,Chr2:+11762409:quaternary
ATTAATTTTT	0	1	0	0	0	Chr3:+8408859:quaternary
AGACTTAAAA	0	0	1	0	0	Chr3:+840876:quaternary
TAGAAACTGA	0	0	1	0	0	Chr3:+8337097:quaternary
ACGACGAAAA	0	0	0	1	0	Chr3:+8332467:quaternary,Chr4:+17313083:quaternary
AGAAATCAAC	0	0	0	0	1	Chr3:+8305424:quaternary
ATCTCACTAA	0	1	0	0	0	Chr3:+8249347:quaternary
AGATTCGTCA	0	1	0	0	0	Chr3:+8211505:quaternary
TCCAATTGTT	0	0	1	0	0	Chr3:+8198672:quaternary
ATAAAGAGGC	1	0	0	0	0	Chr3:+8157354:quaternary
TCGTTGATTA	1	0	0	0	0	Chr3:+8078139:quaternary
CCTTCGGTTT	0	1	0	0	0	Chr3:+8042913:quaternary
CAAAAGGTTT	0	0	0	1	0	Chr3:+7932067:quaternary
TCGACGTTGT	0	0	1	0	0	Chr3:+7862944:quaternary,Chr3:+7853852:quaternary
GTCTAATAAA	0	0	0	1	0	Chr3:+7848887:quaternary,AtCg00490.1:X:+-153:quaternary,Chr4:+8962515:quaternary,Chr3:+7858921:quaternary
TTTAGACTTC	0	0	0	1	0	Chr3:+7741753:quaternary

GAGTATCATT	0	0	0	0	1	Chr3:+7703626:quaternary,At5g15810.1:X:+602:quaternary
AGAGACTAAA	1	0	0	0	0	Chr3:+7683477:quaternary
GTTTCTTAGA	0	1	0	0	0	Chr3:+7666500:quaternary
GAAAAAAATT	0	1	0	0	0	Chr3:+7647900:quaternary,Chr1:+27671780:quaternary,Chr4:+1883860:quaternary,Chr3:+19093758:quaternary,Chr2:+18275391:quaternary
TACAAAATGA	0	1	0	0	0	Chr3:+7637932:quaternary,Chr4:+5298419:quaternary
CTTGATTGGT	0	0	1	0	0	Chr3:+7633167:quaternary
CACGCTCTCA	0	0	0	1	0	Chr3:+7558417:quaternary
AGTGTTACTA	0	1	0	0	0	Chr3:+7521071:quaternary
CCTATATTTC	0	1	0	0	0	Chr3:+7510089:quaternary
TGGCCACATT	0	1	0	0	0	Chr3:+7491972:quaternary
TCCAATCAAA	0	1	0	0	0	Chr3:+7477076:quaternary
TTTGATAGTC	0	0	1	0	0	Chr3:+7386077:quaternary
ACAATCAGAA	0	0	0	0	1	Chr3:+7338234:quaternary
TTTGAGCCAA	0	0	1	0	0	Chr3:+7323061:quaternary
TACAGTAATT	0	0	1	0	0	Chr3:+7287288:quaternary
AACAATGGTA	1	0	0	0	0	Chr3:+7180522:quaternary
ATGGATGTGG	0	0	0	1	0	Chr3:+7145186:quaternary
ATTGAAATCG	0	0	0	1	0	Chr3:+7112379:quaternary,Chr4:+14191131:quaternary
AAAAGCAGGG	0	0	1	0	0	Chr3:+6774851:quaternary
AGTGTTTCGA	0	0	0	0	1	Chr3:+6719296:quaternary
TAGCTGCTGG	0	1	0	0	0	Chr3:+6621080:quaternary
AATCTTGATT	1	0	0	0	0	Chr3:+6619995:quaternary,At2g32040.1:X:+558:quaternary
GTTAACCAAT	0	1	0	0	0	Chr3:+6618503:quaternary,Chr1:+22885278:quaternary
GAGTGATGGA	0	1	0	0	0	Chr3:+6495677:quaternary
GAAGGGGCTT	0	0	0	0	1	Chr3:+6469757:quaternary,Chr3:+1428082:quaternary
CAAACTAAAA	0	0	1	0	0	Chr3:+6463950:quaternary
TAATTAGTTA	0	0	1	0	0	Chr3:+6437648:quaternary,Chr2:+4801404:quaternary,Chr2:+7536592:quaternary,Chr3:+15496677:quaternary
CTCGAGCGGC	0	0	1	0	0	Chr3:+63:quaternary
GGTTATGGGT	0	1	0	0	0	Chr3:+6179181:quaternary
TGGGCTTTAA	0	1	0	0	0	Chr3:+6039160:quaternary
TAACCTTCAA	0	1	0	0	0	Chr3:+6016596:quaternary,Chr2:+3266025:quaternary
TAACTCTCTT	0	0	0	0	1	Chr3:+5898841:quaternary,At3g22340.1:X:+1093:quaternary,Chr2:+1995723:quaternary
GTAGATCTAG	1	0	0	0	0	Chr3:+5720079:quaternary
CATTTAGCAT	0	0	0	1	0	Chr3:+5719549:quaternary,At5g08535.1:X:+379:quaternary
TCTCTAGAAT	0	0	1	0	0	Chr3:+5646715:quaternary,Chr2:+6816385:quaternary
TTGTTGCGCT	0	1	0	0	0	Chr3:+5611547:quaternary
TCGTACTTGT	0	1	0	0	0	Chr3:+5603591:quaternary
CGGCCCACGG	0	1	0	0	0	Chr3:+558435:quaternary
TACAAACGTA	0	0	0	1	0	Chr3:+5569770:quaternary
TATGGTTAAA	0	0	1	0	0	Chr3:+5320051:quaternary,At2g02510.1:X:+447:quaternary,Chr4:+14841914:quaternary,Chr2:+15399747:quaternary
CCAAAAATAA	0	0	1	0	0	Chr3:+5273004:quaternary,Chr5:+19237997:quaternary
ATAACGCACA	0	0	0	1	0	Chr3:+5156186:quaternary
CAAAAAACTT	0	1	0	0	0	Chr3:+5133332:quaternary,Chr3:+22139513:quaternary
GATGATGGGT	0	0	1	0	0	Chr3:+5124571:quaternary
GGTCAACCCT	0	1	0	0	0	Chr3:+5061102:quaternary
TCTCTCACGA	1	0	0	0	0	Chr3:+4873707:quaternary
TGTGTTGTTT	0	0	1	0	0	Chr3:+4844077:quaternary
CTTGATGTAT	0	1	0	0	0	Chr3:+4750886:quaternary
CCCTCCTTTT	0	0	1	0	0	Chr3:+4700364:quaternary
TGTGGATATG	0	1	0	0	0	Chr3:+4623932:quaternary,Chr2:+9445106:quaternary
ATCAAACATA	0	0	1	0	0	Chr3:+4581832:quaternary,Chr5:+18308658:quaternary,Chr1:+22807629:quaternary
TCCATATCAT	1	0	0	0	0	Chr3:+4532120:quaternary,Chr4:+14636950:quaternary
TAAACTCAAA	0	0	0	1	0	Chr3:+4524758:quaternary,Chr4:+11639219:quaternary
GTGGTTAGAT	0	0	1	0	0	Chr3:+4374530:quaternary
GAACAAATCA	1	0	0	0	0	Chr3:+4361501:quaternary
ATACGATGGT	0	1	0	0	0	Chr3:+4290432:quaternary
TGCTTCAAGG	0	1	0	0	0	Chr3:+421961:quaternary
ATTCGAAACA	0	0	1	0	0	Chr3:+4145920:quaternary

CATTAATTAG	0	1	0	0	0	Chr3:+4145000:quaternary,Chr3:+3507601:quaternary
CTCTCTCCAA	0	0	0	1	0	Chr3:+4138647:quaternary
GTGCAATCGC	0	0	0	0	1	Chr3:+4137613:quaternary
CAAAGAATCG	0	1	0	0	0	Chr3:+4082516:quaternary
GATTATATTA	0	1	0	0	0	Chr3:+3693057:quaternary
ATATCTGTAA	1	0	0	0	0	Chr3:+3690446:quaternary
GACCACATTA	1	0	0	0	0	Chr3:+3678256:quaternary,Chr2:+18634238:quaternary
TTAATAGTAA	0	0	0	0	1	Chr3:+3524224:quaternary,Chr2:+7909391:quaternary,Chr4:+14128879:quaternary
AATCTCTTTC	1	0	0	0	0	Chr3:+3437440:quaternary
TATGTCCAAA	0	1	0	0	0	Chr3:+3356433:quaternary,Chr1:+24707732:quaternary
GCCTCCAAAT	1	0	0	0	0	Chr3:+3285003:quaternary
TAACAGTATC	1	0	0	0	0	Chr3:+2704138:quaternary
TATTCGGTTT	0	0	0	1	0	Chr3:+2665654:quaternary
ACAACAACGT	0	0	0	1	0	Chr3:+2614479:quaternary
CAACTTGTTG	0	0	0	0	1	Chr3:+2614103:quaternary,Chr1:+27310633:quaternary,Chr1:+7351979:quaternary
CTTCAAATTG	0	0	0	0	1	Chr3:+2579471:quaternary
CATAGAATAA	0	0	0	0	1	Chr3:+251051:quaternary
ATCACTCAGA	0	0	0	1	0	Chr3:+23448892:quaternary
GGTTAACCCT	0	0	0	0	1	Chr3:+23352856:quaternary
GATAATGTAA	0	0	0	0	1	Chr3:+23308569:quaternary,Chr5:+7225093:quaternary
TACTATACGA	1	0	0	0	0	Chr3:+23291925:quaternary
TGTTATTGTG	0	0	0	0	1	Chr3:+23242517:quaternary
AAACTTGAGT	0	0	0	0	1	Chr3:+23189620:quaternary
CCTACAAAAA	0	0	1	0	0	Chr3:+23176885:quaternary
CATCTAAAAG	0	0	1	0	0	Chr3:+23128146:quaternary
TAAACACTCG	0	0	1	0	0	Chr3:+23060184:quaternary
ATAATTATCC	0	0	1	0	0	Chr3:+22987969:quaternary
ATTTGTGGAT	0	1	0	0	0	Chr3:+22964698:quaternary
GTTTCATTAA	0	1	0	0	0	Chr3:+2295719:quaternary,Chr1:+12939268:quaternary
TTTAGTGACT	1	0	0	0	0	Chr3:+22878263:quaternary
TATTGTAAAT	0	1	0	0	0	Chr3:+22685955:quaternary,Chr3:+20831958:quaternary,Chr2:+5624341:quaternary
AAACGTTGTA	0	1	0	0	0	Chr3:+22664300:quaternary
AACAAAACGA	0	0	0	1	0	Chr3:+22338272:quaternary
GGTGATGAGA	0	1	0	0	0	Chr3:+22301749:quaternary
ACTCTAAAAG	0	0	0	0	1	Chr3:+22202880:quaternary,Chr1:+11287801:quaternary
TACCGCCTAA	0	1	0	0	0	Chr3:+22184802:quaternary; +small RNA(CATGTACCGCCTAAGCT)
TGATTGGTAT	0	0	0	0	1	Chr3:+2217169:quaternary,Chr2:+2167557:quaternary
AGTAATAAAA	0	0	0	1	0	Chr3:+22019401:quaternary
TAAAACGTTG	0	1	0	0	0	Chr3:+21842774:quaternary,Chr1:+17591195:quaternary
AAGAAGAGAA	0	0	0	0	1	Chr3:+21618723:quaternary,Chr5:+2838679:quaternary
CTTGCATAGC	0	0	0	1	0	Chr3:+21566690:quaternary
TCTCCTACTA	0	0	1	0	0	Chr3:+21430230:quaternary
GTGATAAACG	0	0	0	1	0	Chr3:+21375145:quaternary
GGTTTTGTTA	0	1	0	0	0	Chr3:+21358259:quaternary
CAACTTCTTT	0	1	0	0	0	Chr3:+2132119:quaternary,At3g50550.1:X:+320:quaternary
TTCTGAATTC	0	0	1	0	0	Chr3:+20746520:quaternary
ATGGTATAAT	1	0	0	0	0	Chr3:+20741339:quaternary
ACTCTAGTTT	0	0	0	1	0	Chr3:+20695970:quaternary,Chr5:+25031755:quaternary
ACTTTGTCTT	0	0	0	1	0	Chr3:+20587551:quaternary
AGAATGGAAA	0	0	1	0	0	Chr3:+2058664:quaternary,At1g01790.1:X:+-132:quaternary
CTAACAGAGA	0	1	0	0	0	Chr3:+20585757:quaternary
TACTTACTCC	0	1	0	0	0	Chr3:+20493891:quaternary
TTACTATAAA	1	0	0	0	0	Chr3:+204889:quaternary
AAAACCATTA	0	0	0	0	1	Chr3:+20468184:quaternary
TGAGTTTGTC	0	0	0	1	0	Chr3:+20443443:quaternary
ATTCCTCTGT	0	0	0	0	1	Chr3:+20302587:quaternary
CCAAAAATCC	1	0	0	0	0	Chr3:+20283643:quaternary
GCCAGAATAA	0	0	1	0	0	Chr3:+20168895:quaternary
TTTGCGCAAA	0	0	1	0	0	Chr3:+20150600:quaternary
AAAAAAGTGT	0	1	0	0	0	Chr3:+20021510:quaternary,Chr5:+15798512:quaternary
CAATGATAAT	0	1	0	0	0	Chr3:+20020200:quaternary,Chr5:+12721455:quaternary

TATTTGGATT	0	1	0	0	0	Chr3:+20006282:quaternary,Chr5:+19308816:quaternary,Chr4:+17346055:quaternary
ATACAAACTA	0	0	0	1	0	Chr3:+20002866:quaternary
AATATATGGA	0	1	0	0	0	Chr3:+1999492:quaternary,Chr4:+12764992:quaternary,Chr5:+1308715:quaternary
TGTCTCACTT	1	0	0	0	0	Chr3:+19811513:quaternary
AATCGTTTTT	0	0	0	0	1	Chr3:+197039:quaternary,Chr1:+24692017:quaternary,Chr1:+10028238:quaternary
TGTTAATAGT	0	0	0	0	1	Chr3:+19547373:quaternary
TGGGAAAAAA	0	0	0	0	1	Chr3:+19473716:quaternary
GGAACACATA	1	0	0	0	0	Chr3:+19473530:quaternary
ATTTAAATGA	0	0	1	0	0	Chr3:+19300944:quaternary,Chr5:+17239114:quaternary
AATCGAGAGA	0	1	0	0	0	Chr3:+19278560:quaternary,Chr3:+14356453:quaternary
GCAAATGAAA	0	0	0	1	0	Chr3:+19050334:quaternary
GAAAGAAAGA	0	1	0	0	0	Chr3:+18935823:quaternary,Chr1:+19223466:quaternary,Chr3:+18935871:quaternary,Chr1:+906462:quaternary
TTCGTAAAAA	0	0	0	1	0	Chr3:+18831544:quaternary
AAAAGTGTTG	0	0	1	0	0	Chr3:+18792753:quaternary
CTAATAAAAA	0	0	0	0	1	Chr3:+18681330:quaternary
TAAGACTATA	0	0	1	0	0	Chr3:+18580288:quaternary
AGTTACAAAA	0	1	0	0	0	Chr3:+18527662:quaternary
GTCATCGTGG	0	1	0	0	0	Chr3:+18481006:quaternary
GCCCATTATA	0	0	1	0	0	Chr3:+18469973:quaternary
TCAAATGAAT	0	0	0	0	1	Chr3:+18441866:quaternary,Chr4:+11351289:quaternary
GTTATCGTTG	0	0	1	0	0	Chr3:+18398813:quaternary
TTCCAAGTAA	0	1	0	0	0	Chr3:+18350927:quaternary
AAAACGACAC	0	0	0	1	0	Chr3:+18347886:quaternary,Chr5:+5222928:quaternary
GACATTTTTG	0	0	0	1	0	Chr3:+18258829:quaternary
TTCGTTTCGT	0	1	0	0	0	Chr3:+18055435:quaternary
AAAATAGTAT	0	1	0	0	0	Chr3:+18016715:quaternary
GTTTATGCTT	0	0	0	1	0	Chr3:+17913397:quaternary,Chr1:+19918767:quaternary
AAAAGATTTG	0	1	0	0	0	Chr3:+17878017:quaternary
AATTTTACTC	0	0	0	0	1	Chr3:+17652056:quaternary
TGAAACCAAA	0	0	1	0	0	Chr3:+17467651:quaternary,Chr3:+20599562:quaternary
ATTCAGGATT	0	0	0	1	0	Chr3:+17456919:quaternary
CTAATATTTT	0	1	0	0	0	Chr3:+17409489:quaternary
AAAATGAAAG	0	1	0	0	0	Chr3:+17357434:quaternary,Chr2:+2960868:quaternary,At1g51070.1:X:+-59:quaternary,Chr4:+17304582:quaternary
CAATTATTGA	0	1	0	0	0	Chr3:+17182919:quaternary
TAAACCCTGT	0	0	1	0	0	Chr3:+17056258:quaternary
GTTGTTGGAA	0	0	1	0	0	Chr3:+16975297:quaternary
ATGGATAAGA	0	1	0	0	0	Chr3:+16826170:quaternary
TTTCTACCAA	0	0	0	1	0	Chr3:+16816221:quaternary
GGTTTACTCC	0	0	0	1	0	Chr3:+16686839:quaternary
CTTTTTTCCA	0	0	0	1	0	Chr3:+16533399:quaternary
TGGTCGACTC	0	0	0	1	0	Chr3:+16491685:quaternary
TAAATCAATC	1	0	0	0	0	Chr3:+16460838:quaternary
TAAAAAACTG	0	0	1	0	0	Chr3:+16460429:quaternary
AATTGTTGTC	1	0	0	0	0	Chr3:+16441109:quaternary,Chr1:+20470028:quaternary
ATAATATCTT	0	0	0	0	1	Chr3:+16378070:quaternary
TAAGACTTTG	0	0	0	1	0	Chr3:+16268681:quaternary
TGGACGCAAT	0	0	0	0	1	Chr3:+1608673:quaternary
AGGGTTTTTT	0	0	0	1	0	Chr3:+16054635:quaternary
AATTAAACTA	0	1	0	0	0	Chr3:+15952411:quaternary,Chr1:+10777666:quaternary
TCTTTAAGTT	0	1	0	0	0	Chr3:+1590359:quaternary
AATGGAGTGT	0	0	1	0	0	Chr3:+1590312:quaternary
TCGTTTGTGG	0	1	0	0	0	Chr3:+15853911:quaternary
TTCTGCATCT	0	0	0	0	1	Chr3:+15652554:quaternary
TCACTTAGTG	0	0	1	0	0	Chr3:+15622782:quaternary
AGTTTGTTTT	0	0	0	1	0	Chr3:+1548281:quaternary,Chr3:+11961907:quaternary,Chr2:+6408641:quaternary,Chr2:+16616191:quaternary
GAAAGCAAGT	0	0	1	0	0	Chr3:+15481420:quaternary,Chr3:+10967113:quaternary,Chr3:+15466133:quaternary
CTGCTTTTAT	0	1	0	0	0	Chr3:+15355022:quaternary
ACTATTGATA	1	0	0	0	0	Chr3:+1533981:quaternary

AAAGAAACAT	0	0	0	1	0	Chr3:+15303255:quaternary
GGTTTGGATG	0	0	0	1	0	Chr3:+15217993:quaternary
GAACATAGTG	0	0	0	0	1	Chr3:+1518121:quaternary
TATACTCACT	1	0	0	0	0	Chr3:+15104350:quaternary,Chr4:+3559898:quaternary
AAACTGGTAA	0	0	1	0	0	Chr3:+14898742:quaternary,Chr4:+1775301:quaternary
TCAAACTTGG	0	0	1	0	0	Chr3:+14894447:quaternary
AAACTTCTCG	1	0	0	0	0	Chr3:+14788650:quaternary
CACTAATATC	1	0	0	0	0	Chr3:+14630520:quaternary,Chr5:+21739816:quaternary
GAATCACAAA	0	1	0	0	0	Chr3:+14604458:quaternary
TTCACTAAAA	1	0	0	0	0	Chr3:+14604159:quaternary
AATAAAAGCT	0	0	0	0	1	Chr3:+14603829:quaternary
ACAAGTCTAT	1	0	0	0	0	Chr3:+14585000:quaternary
ACTAAAAAGA	0	0	1	0	0	Chr3:+14573441:quaternary,Chr4:+2657072:quaternary
GTTGGCAAAA	0	0	0	0	1	Chr3:+14544878:quaternary
ATGGCTGAAA	0	1	0	0	0	Chr3:+14504907:quaternary
TGAGTGTGGA	0	0	0	1	0	Chr3:+14497720:quaternary
TTATTATTTG	0	0	0	1	0	Chr3:+14494016:quaternary,Chr3:+4438632:quaternary
AGTTAGTTTC	0	0	1	0	0	Chr3:+14487021:quaternary
GATGTATGTA	0	0	1	0	0	Chr3:+1444656:quaternary
TAGAAAGCAG	0	1	0	0	0	Chr3:+14431292:quaternary
TCAAGTTTAG	1	0	0	0	0	Chr3:+14217209:quaternary,At5g35180.1:X:+270:quaternary,Chr3:+14184122:quaternary
AGGTTGTGAT	0	0	1	0	0	Chr3:+14150134:quaternary
GTGAACAACT	0	0	0	0	1	Chr3:+14077833:quaternary
TCATAATTCA	0	0	1	0	0	Chr3:+14076963:quaternary
TAGTCGTTAA	0	1	0	0	0	Chr3:+13990655:quaternary
CCGTTTGTGA	0	0	0	0	1	Chr3:+13760346:quaternary
GTCTCAAATT	0	0	0	1	0	Chr3:+13718666:quaternary
TAGTAACGGA	0	1	0	0	0	Chr3:+13601309:quaternary,Chr3:+14188450:quaternary,Chr3:+13601819:quaternary,Chr3:+13864460:quaternary,Chr3:+13857002:quaternary,Chr3:+13850180:quaternary,Chr3:+13846147:quaternary,Chr3:+13686074:quaternary,Chr3:+13859047:quaternary,Chr3:+13826470:quaternary,Chr3:+14198167:quaternary,Chr3:+13597267:quaternary,Chr3:+13689132:quaternary,Chr3:+13858544:quaternary,Chr3:+14187460:quaternary,Chr3:+13880567:quaternary,Chr3:+13878546:quaternary,Chr3:+13856495:quaternary,Chr3:+13821422:quaternary,Chr3:+13879560:quaternary,Chr3:+13598799:quaternary,Chr3:+14196182:quaternary,Chr3:+13824958:quaternary,Chr3:+13861101:quaternary,Chr3:+13880064:quaternary,Chr3:+13600799:quaternary,Chr3:+14184971:quaternary,Chr3:+13872682:quaternary,Chr3:+14191411:quaternary,Chr3:+13820543:quaternary,Chr3:+14192402:quaternary,Chr3:+13688628:quaternary,Chr3:+13687082:quaternary,Chr3:+13873732:quaternary,Chr3:+13851151:quaternary,Chr3:+13863446:quaternary,Chr3:+13881075:quaternary,Chr3:+13874749:quaternary,Chr3:+14186467:quaternary,Chr3:+13865476:quaternary,Chr3:+13685568:quaternary,Chr3:+13858014:quaternary
TGATACCTTT	0	1	0	0	0	Chr3:+13600012:quaternary,Chr3:+14189640:quaternary,Chr3:+13880779:quaternary,Chr3:+14186677:quaternary,Chr3:+13822643:quaternary,Chr3:+13686282:quaternary,Chr3:+14196879:quaternary,Chr4:+3055393:quaternary,Chr3:+13859816:quaternary,Chr3:+13856199:quaternary,Chr3:+14185665:quaternary,Chr3:+13536617:quaternary,Chr3:+13687271:quaternary,Chr3:+13601523:quaternary,Chr3:+13870389:quaternary,Chr3:+13877494:quaternary,Chr3:+13849376:quaternary,Chr4:+3056856:quaternary,Chr3:+13599504:quaternary,Chr3:+13598502:quaternary,Chr3:+13873945:quaternary,Chr3:+13827190:quaternary,Chr3:+14186171:quaternary,Chr3:+14195886:quaternary,Chr4:+3055891:quaternary,Chr4:+3054895:quaternary,Chr3:+13846866:quaternary,Chr5:+11186506:quaternary,Chr3:+14187164:quaternary,Chr3:+13859259:quaternary,Chr3:+13879263:quaternary,Chr3:+13858753:quaternary,Chr5:+11186004:quaternary,Chr3:+13847372:quaternary,Chr3:+13851872:quaternary,Chr3:+13881285:quaternary,Chr3:+14194097:quaternary,Chr5:+11185000:quaternary,Chr3:+13596479:quaternary,Chr3:+13688839:quaternary,Chr3:+13687773:quaternary,Chr3:+13825164:quaternary,Chr3:+13688333:quaternary,Chr3:+13677718:quaternary,Chr3:+13821249:quaternary,Chr3:+13820752:quaternary,Chr3:+14193596:quaternary,Chr4:+3060342:quaternary,Chr3:+14190141:quaternary,Chr3:+14198861:quaternary,Chr3:+13880273:quaternary,Chr3:+13876989:quaternary,Chr3:+13868713:quaternary,Chr3:+14191619:quaternary,Chr4:+3059844:quaternary,Chr4:+3059346:quaternary,Chr3:+13878758:quaternary,Chr3:+13601013:quaternary,Chr3:+13857717:quaternary,Chr3:+13874962:quaternary,Chr3:+13597991:quaternary,Chr3:+14194584:quaternary,Chr3:+14189143:quaternary,Chr4:+3057354:quaternary,Chr3:+14197871:quaternary,Chr3:+14195380:quaternary,Chr5:+12042911:quaternary,Chr3:+14193099:quaternary,Chr4:+3054397:quaternary,Chr3:+13848870:quaternary,Chr3:+14196392:quaternary,Chr3:+13869218:quaternary,Chr5:+11188015:quaternary,Chr4:+3057852:quaternary,Chr3:+14192612:quaternary,Chr3:+14190628:quaternary,Chr3:+13876484:quaternary,Chr3:+14192106:quaternary,Chr3:+13854689:quaternary,Chr3:+13855197:quaternary,Chr4:+3058848:quaternary,Chr3:+13862802:quaternary,Chr3:+13861314:quaternary,Chr3:+13865689:quaternary,Chr5:+11185502:quaternary,Chr3:+13600502:quaternary,Chr3:+13689341:quaternary,Chr3:+13871876:quaternary,Chr3:+13846360:quaternary,Chr3:+13857212:quaternary,Chr3:+13878252:quaternary,Chr3:+14191115:quaternary,Chr5:+11187511:quaternary,Chr3:+14188154:quaternary,Chr3:+13686787:quaternary,Chr5:+11187009:quaternary,Chr3:+14188660:quaternary,Chr3:+13685779:quaternary,Chr3:+13856707:quaternary,Chr3:+13860321:quaternary,Chr3:+13855702:quaternary,Chr5:+11188517:quaternary,Chr4:+3056389:quaternary,Chr4:+3058350:quaternary
AATGAAACAA	1	0	0	0	0	Chr3:+13492836:quaternary
TGAGTGTGAA	0	0	0	1	0	Chr3:+13232921:quaternary
ATTACAAAGA	0	0	0	0	1	Chr3:+13227714:quaternary,Chr1:+13768327:quaternary
GAGACATACA	0	0	1	0	0	Chr3:+13156434:quaternary
GAAGAAAAGT	0	0	1	0	0	Chr3:+13064400:quaternary
GGAAAAGCAA	0	0	0	0	1	Chr3:+13050727:quaternary

TGGCTAGGTT	1	0	0	0	0	Chr3:+12962005:quaternary
TGAGCTCAAG	0	1	0	0	0	Chr3:+12904536:quaternary,Chr3:+22050697:quaternary
TGCTGCAAAA	0	1	0	0	0	Chr3:+12901321:quaternary
ATCGATTTAT	0	0	0	0	1	Chr3:+12875344:quaternary
AGACGAAGAA	0	1	0	0	0	Chr3:+12853245:quaternary
ATTTGAATGA	0	1	0	0	0	Chr3:+1242003:quaternary,Chr3:+11227193:quaternary
GTTGATTTAA	1	0	0	0	0	Chr3:+1241965:quaternary
ATTCTACAAA	0	0	0	0	1	Chr3:+12340963:quaternary,Chr2:+2265935:quaternary
CAATCAAAAC	0	0	0	0	1	Chr3:+12290115:quaternary
TGAAAAAGGA	0	1	0	0	0	Chr3:+12243579:quaternary
AGAGATTTAA	0	1	0	0	0	Chr3:+12096209:quaternary
AACAAAAGAA	0	1	0	0	0	Chr3:+11976461:quaternary
AGTCCTAAAG	0	1	0	0	0	Chr3:+11899461:quaternary
AGGATGAAGG	0	0	0	0	1	Chr3:+11843534:quaternary
GTGAGAATCT	0	0	1	0	0	Chr3:+11752567:quaternary
TGGATTTTAT	0	1	0	0	0	Chr3:+11737893:quaternary
ACTTTTCCTT	0	1	0	0	0	Chr3:+11716843:quaternary
ATCCATCCTT	1	0	0	0	0	Chr3:+11626879:quaternary
TTTGGCAACT	0	1	0	0	0	Chr3:+11489267:quaternary
GGTCAATGGA	0	0	0	0	1	Chr3:+11486742:quaternary
AGTAACAAGA	0	0	1	0	0	Chr3:+11391515:quaternary
CAAGTGTTTA	0	0	0	0	1	Chr3:+1138582:quaternary,Chr4:+1876851:quaternary,Chr1:+3852425:quaternary,Chr2:+12850370:quaternary,Chr3:+10402986:quaternary
GTGTGTCGAG	1	0	0	0	0	Chr3:+11377809:quaternary
AATCCAAAAT	0	0	1	0	0	Chr3:+11369697:quaternary,Chr1:+21735968:quaternary
TGTCTGTGCA	0	1	0	0	0	Chr3:+11351628:quaternary
GAAGTCAATG	0	0	1	0	0	Chr3:+11272930:quaternary
AAAACATCTA	0	1	0	0	0	Chr3:+11220131:quaternary
AAGTAAGTTA	0	1	0	0	0	Chr3:+11203252:quaternary
AAGTTTAAGT	0	0	0	1	0	Chr3:+11183993:quaternary
TTTTGGCCCG	0	1	0	0	0	Chr3:+11164544:quaternary
AGACACAAGA	0	0	0	0	1	Chr3:+11096257:quaternary,At3g62410.1:X:+692:quaternary
GTAAAATAGT	0	0	1	0	0	Chr3:+11073969:quaternary
TGTGTATAAA	1	0	0	0	0	Chr3:+11064646:quaternary
GTCCTCTTCT	0	1	0	0	0	Chr3:+11055602:quaternary
CTTGGAATTT	0	1	0	0	0	Chr3:+10998120:quaternary
TGGTACAAAT	1	0	0	0	0	Chr3:+10938490:quaternary
AGCCAAAAAG	0	0	0	0	1	Chr3:+10925726:quaternary
GCTTGTTCAC	1	0	0	0	0	Chr3:+1091326:quaternary
AAACTAGTGA	0	0	1	0	0	Chr3:+10827013:quaternary,Chr4:+12291233:quaternary,At5g22300.1:X:+337:quaternary
TATCCAAAAA	0	0	0	1	0	Chr3:+10744536:quaternary,Chr5:+1283168:quaternary
ATTATCTTTA	0	1	0	0	0	Chr3:+10684965:quaternary
AGTTATATAG	1	0	0	0	0	Chr3:+10605307:quaternary
AACTATATGT	0	0	1	0	0	Chr3:+10558910:quaternary,Chr1:+13488477:quaternary
GTTGAGATAG	0	0	1	0	0	Chr3:+10553381:quaternary,Chr3:+10571614:quaternary
AGATACATAG	0	0	0	0	1	Chr3:+10138172:quaternary
AACACTGGCT	0	1	0	0	0	Chr3:+10077346:quaternary
GTAAGCTGTA	0	1	0	0	0	Chr3:+10076933:quaternary
AAGTGAAAAA	0	0	1	0	0	Chr3:+10068127:quaternary,Chr3:+9666528:quaternary,Chr5:+2611255:quaternary,Chr1:+4966770:quaternary
GAACCTAGAA	0	0	1	0	0	Chr3:+10022827:quaternary
CGCTACCTCC	0	1	0	0	0	Chr2:-9559039:quaternary
TCAACTTTTA	0	0	0	1	0	Chr2:-9431366:quaternary,Chr4:-1770409:quaternary
GCGTATGTTT	0	1	0	0	0	Chr2:-9399013:quaternary
ATTCATACCA	1	0	0	0	0	Chr2:-9386780:quaternary,Chr2:-9386771:quaternary,At4g26890.1:X:--147:quaternary
CTCAGGGGTT	0	1	0	0	0	Chr2:-9264005:quaternary
TGTTTTATGA	0	1	0	0	0	Chr2:-8717155:quaternary
TTATAACAAT	0	0	1	0	0	Chr2:-8682999:quaternary
GGATAATTTT	0	0	1	0	0	Chr2:-8674832:quaternary
TACCAGTTTT	1	0	0	0	0	Chr2:-814500:quaternary,Chr5:-1867638:quaternary
TGGAGGAGAG	0	1	0	0	0	Chr2:-807671:quaternary

TCGCCTTTCT	1	0	0	0	0	Chr2:-804116:quaternary
GCGTTGAATT	0	0	1	0	0	Chr2:-7959355:quaternary
GGAGCTGTGG	1	0	0	0	0	Chr2:-7736784:quaternary
CTGCACAACA	0	0	0	1	0	Chr2:-7703206:quaternary
TCATACTAGT	0	1	0	0	0	Chr2:-6803090:quaternary
TAAAATTCTC	0	1	0	0	0	Chr2:-645139:quaternary
GCTGAATGAT	0	0	0	0	1	Chr2:-565027:quaternary
GCTGTCTAAA	0	1	0	0	0	Chr2:-5058650:quaternary
CACTTCTGGC	0	1	0	0	0	Chr2:-4949176:quaternary
TGGGAGGTTT	1	0	0	0	0	Chr2:-477198:quaternary
GTTCTAGCTC	1	0	0	0	0	Chr2:-4716252:quaternary
GGAACTTTAG	0	1	0	0	0	Chr2:-4592114:quaternary,Chr1:-16889049:quaternary,Chr1:-16889044:quaternary
TACATTTTTA	0	0	0	0	1	Chr2:-3803560:quaternary
GGCATTACAA	0	1	0	0	0	Chr2:-2931461:quaternary
AAAACACAGA	0	0	0	0	1	Chr2:-2888985:quaternary
TTGATTATTT	0	0	1	0	0	Chr2:-2631334:quaternary,Chr2:-2659651:quaternary
TCGTGGCTAA	0	0	1	0	0	Chr2:-2528831:quaternary
TGGTCTGGTA	0	0	0	1	0	Chr2:-2486453:quaternary
AGACAAACGG	0	1	0	0	0	Chr2:-2152459:quaternary,Chr2:-2157738:quaternary
TGTGTGCAAG	0	1	0	0	0	Chr2:-19219424:quaternary
CTATTGAGGA	1	0	0	0	0	Chr2:-18655930:quaternary
GAATAAGCAA	0	0	0	0	1	Chr2:-1863672:quaternary
AAAACCCAAT	0	0	1	0	0	Chr2:-18577700:quaternary
GTTGATTGAA	0	0	0	1	0	Chr2:-18482188:quaternary,At2g38010.2:X:--2:quaternary
ATGAGGTTGA	0	0	0	1	0	Chr2:-18404163:quaternary
GATGCGAAGT	0	0	0	0	1	Chr2:-18198853:quaternary
GTATCATTCG	0	1	0	0	0	Chr2:-1791764:quaternary,Chr2:-17177959:quaternary
GCCATTCCGG	1	0	0	0	0	Chr2:-17785098:quaternary
TAAAGGGGAG	0	0	1	0	0	Chr2:-16557853:quaternary
ACGATACATT	0	0	1	0	0	Chr2:-16466410:quaternary
TAGACTACTT	1	0	0	0	0	Chr2:-16298207:quaternary
GGCCGAGCAT	0	1	0	0	0	Chr2:-15785847:quaternary
ATTTCCTATT	0	0	0	1	0	Chr2:-15733081:quaternary
GAGTGTATAT	0	0	0	0	1	Chr2:-15302439:quaternary
TTAGCTTAAA	0	0	0	0	1	Chr2:-15288053:quaternary
GTGGATTCTG	0	0	0	1	0	Chr2:-15225181:quaternary
TGCGAATCAA	0	0	1	0	0	Chr2:-15136378:quaternary
ATTCTATGTT	0	0	1	0	0	Chr2:-14952887:quaternary
CACGTTAAAA	0	0	1	0	0	Chr2:-1491348:quaternary
AACCAATGTA	0	1	0	0	0	Chr2:-1470451:quaternary
TGGCTTGAAA	0	0	0	1	0	Chr2:-14177173:quaternary
TCGAGAAGAC	0	0	0	1	0	Chr2:-1414129:quaternary
ATTGCTAAAA	1	0	0	0	0	Chr2:-13992897:quaternary,Chr1:-22807329:quaternary
AGTTTGAGAT	1	0	0	0	0	Chr2:-1385444:quaternary
TGCTTGCCGT	0	1	0	0	0	Chr2:-1375131:quaternary,Chr2:-1375206:quaternary
GGCCGAAGCC	1	0	0	0	0	Chr2:-13710337:quaternary
TAGTGAAGAT	0	0	0	0	1	Chr2:-13469785:quaternary
GTTTTGGAAC	1	0	0	0	0	Chr2:-13340761:quaternary,Chr2:-13340765:quaternary
TAACTCATTT	1	0	0	0	0	Chr2:-13249555:quaternary,At2g34610.1:X:-1283:quaternary
TGAGAAGAAT	0	0	1	0	0	Chr2:-13235848:quaternary
TAAATCTCAT	1	0	0	0	0	Chr2:-1315165:quaternary
ACCCCACTAT	0	0	0	0	1	Chr2:-13078493:quaternary
TATACTCTTG	0	0	0	0	1	Chr2:-12971938:quaternary,Chr5:-18084421:quaternary
TGATTGCGAA	0	1	0	0	0	Chr2:-12779866:quaternary
TGAAAAGCCC	0	1	0	0	0	Chr2:-12673341:quaternary
TGTTCGTTTA	0	1	0	0	0	Chr2:-1255219:quaternary
GACCCAAAAA	0	0	0	0	1	Chr2:-12393305:quaternary,Chr5:-13934113:quaternary,Chr2:-15146688:quaternary
TTGGTGGATA	0	0	1	0	0	Chr2:-12357940:quaternary
GTTTGAAAAC	0	0	0	1	0	Chr2:-12201365:quaternary
TAGGAGTTTC	0	0	0	0	1	Chr2:-11729277:quaternary
TACCGTTTGT	0	0	1	0	0	Chr2:-11461722:quaternary

TTGCGATGCT	0	1	0	0	0	Chr2:-11317793:quaternary
GCTAATTACT	0	0	0	0	1	Chr2:-11170431:quaternary
ATTCGTCTCA	0	0	1	0	0	Chr2:-11114833:quaternary,Chr2:-11114839:quaternary
TAAGAGAGAT	0	0	1	0	0	Chr2:-10684032:quaternary,Chr1:-29948839:quaternary
TGCAGAGCTA	0	1	0	0	0	Chr2:-10667621:quaternary
GCAGATTGGA	0	1	0	0	0	Chr2:-10511713:quaternary
TATCGGAAAT	0	0	1	0	0	Chr2:-10324793:quaternary
TGTATGCTGA	0	0	0	1	0	Chr2:-10287764:quaternary
TGATCAAAAA	0	0	0	1	0	Chr2:-10091248:quaternary
CTCCACGAAC	1	0	0	0	0	Chr2:+9993057:quaternary,At1g62380.1:X:+-185:quaternary
CAAAAGATTA	0	0	0	0	1	Chr2:+9990904:quaternary
GGATTTATTA	0	0	0	0	1	Chr2:+9988486:quaternary
CTCATCAAAA	1	0	0	0	0	Chr2:+9867617:quaternary
GTAGGCTTCT	0	0	0	1	0	Chr2:+9862696:quaternary
CTCAAACCTG	0	0	0	1	0	Chr2:+9862442:quaternary
AAGCTACCAT	0	1	0	0	0	Chr2:+9858520:quaternary
TACCCAAATT	0	0	0	1	0	Chr2:+9826220:quaternary
AAGTAGTTGT	0	1	0	0	0	Chr2:+9781525:quaternary
AACTCCTAAG	0	0	0	1	0	Chr2:+9647243:quaternary
GATTCCATTT	0	1	0	0	0	Chr2:+9558442:quaternary,Chr2:+8285027:quaternary,Chr5:+15304767:quaternary
ATAAAAGATC	1	0	0	0	0	Chr2:+9547686:quaternary,Chr2:+5689817:quaternary
TTTTTTGGAC	0	0	0	0	1	Chr2:+9464550:quaternary
ATCAAGGTAC	1	0	0	0	0	Chr2:+9345736:quaternary,Chr1:+15605267:quaternary
TTCAACTAAA	0	0	0	1	0	Chr2:+9299007:quaternary
TGTAATCAAC	0	0	1	0	0	Chr2:+9233621:quaternary
TAAAAATTTA	0	1	0	0	0	Chr2:+9046071:quaternary,Chr4:+11551143:quaternary,Chr3:+11203932:quaternary
AAAGTATATT	0	0	1	0	0	Chr2:+9039640:quaternary,Chr2:+9573521:quaternary,Chr1:+16905131:quaternary,Chr5:+18620590:quaternary
TCTTTAAATT	0	0	0	0	1	Chr2:+8970797:quaternary,At5g10920.1:X:+584:quaternary
CGACGAAAAC	0	0	1	0	0	Chr2:+8939157:quaternary
GAACTGAGCC	1	0	0	0	0	Chr2:+8938100:quaternary
TGGGTGTGGT	1	0	0	0	0	Chr2:+8860147:quaternary,Chr5:+7890600:quaternary
TTCTCTTATA	0	1	0	0	0	Chr2:+8830176:quaternary
TGAATGTTCT	0	0	0	0	1	Chr2:+8665702:quaternary
TATTTGGAGA	0	1	0	0	0	Chr2:+8543282:quaternary,Chr5:+19573143:quaternary
CATCTCAAGC	0	1	0	0	0	Chr2:+8542173:quaternary
GGCCTGGCTC	0	0	1	0	0	Chr2:+853038:quaternary,Chr4:+815452:quaternary
AACATCAAGT	1	0	0	0	0	Chr2:+8075890:quaternary
GCACAACATA	1	0	0	0	0	Chr2:+8064624:quaternary
TTACGTATAA	0	0	0	0	1	Chr2:+8018883:quaternary
TAAACAATAC	0	0	1	0	0	Chr2:+7991667:quaternary
GATCCTGAAA	0	0	1	0	0	Chr2:+7784831:quaternary
GTTGTACGCT	0	1	0	0	0	Chr2:+7713194:quaternary
CAAATTACTA	1	0	0	0	0	Chr2:+7647166:quaternary
TGCTTGTCTG	0	0	0	0	1	Chr2:+7586449:quaternary
TACCAACCTA	1	0	0	0	0	Chr2:+7357034:quaternary
TTGTGACCAA	0	1	0	0	0	Chr2:+731514:quaternary
CAAATTTTGA	0	0	0	0	1	Chr2:+7211850:quaternary,Chr5:+729696:quaternary
TGTCTCCTTT	0	0	0	0	1	Chr2:+7178906:quaternary
ATATTACACA	1	0	0	0	0	Chr2:+7156126:quaternary
AGAAACTCTC	0	0	0	0	1	Chr2:+7103419:quaternary
TAAACTATGT	1	0	0	0	0	Chr2:+7092626:quaternary,At2g11520.1:X:+360:quaternary
TAAATAGCCA	0	0	0	0	1	Chr2:+7006423:quaternary
TAAACCTCTG	1	0	0	0	0	Chr2:+6968983:quaternary
TTGTACAAAA	0	1	0	0	0	Chr2:+6898583:quaternary
CCCTCAATAA	0	0	0	0	1	Chr2:+6748618:quaternary
AAACAATGAG	1	0	0	0	0	Chr2:+6745073:quaternary
TAAATAAGAC	1	0	0	0	0	Chr2:+6696991:quaternary
CATCCGTCGA	0	0	1	0	0	Chr2:+6671938:quaternary
GGAATGCAAA	0	0	1	0	0	Chr2:+6636046:quaternary
TTAATAAATT	0	0	1	0	0	Chr2:+6531020:quaternary

AGAGTTTGGA	0	0	0	0	1	Chr2:+6508365:quaternary
GACAAAATCG	0	0	0	0	1	Chr2:+6506386:quaternary
GATTGGAATG	0	1	0	0	0	Chr2:+6500747:quaternary
GGCCTCTGAG	0	0	0	0	1	Chr2:+6450729:quaternary
ATGCAGAAAA	0	1	0	0	0	Chr2:+6436088:quaternary
CAAGTGGAGA	1	0	0	0	0	Chr2:+6435551:quaternary,At3g11600.1:X:+872:quaternary
TTTGGAATTA	0	1	0	0	0	Chr2:+6406436:quaternary
ACGCTATATA	0	1	0	0	0	Chr2:+6404573:quaternary
TGATTTCTGT	0	1	0	0	0	Chr2:+6361689:quaternary
TATGATAAGA	0	1	0	0	0	Chr2:+6331736:quaternary
ATATTCGTGG	1	0	0	0	0	Chr2:+6323294:quaternary
GAGCAAAGCG	0	0	0	1	0	Chr2:+625158:quaternary
ATCTACGATT	0	1	0	0	0	Chr2:+6247766:quaternary
AAGAATATAT	0	0	0	0	1	Chr2:+6216294:quaternary
ATCAACTTTT	0	0	0	1	0	Chr2:+6199894:quaternary,Chr2:+6202341:quaternary,Chr2:+6226052:quaternary,Chr3:+16753294:quaternary
ATTCCCTCTA	1	0	0	0	0	Chr2:+6063612:quaternary
AAATAAAAGC	1	0	0	0	0	Chr2:+5954009:quaternary,Chr3:+10083922:quaternary
TGAAGTTGGA	1	0	0	0	0	Chr2:+5799117:quaternary
TGATCTCTCA	1	0	0	0	0	Chr2:+5746224:quaternary
GGATTGAGAA	0	1	0	0	0	Chr2:+5726270:quaternary
ATACATTAAA	0	0	0	1	0	Chr2:+5725621:quaternary,Chr4:+6438902:quaternary
CATCTCTGAT	0	1	0	0	0	Chr2:+5688588:quaternary,Chr5:+8453352:quaternary
TCGGATTCTT	0	0	1	0	0	Chr2:+5653943:quaternary
CTCGGGAGCT	0	0	0	1	0	Chr2:+5627:quaternary
TAAGAGGAGA	0	0	0	0	1	Chr2:+5550458:quaternary
ATCCAAAGAG	0	0	1	0	0	Chr2:+5520654:quaternary
CAATTTTGGG	0	0	0	1	0	Chr2:+5454343:quaternary,Chr5:+23136367:quaternary,At4g03500.1:X:+-12:quaternary
CCATAGTTTT	0	0	1	0	0	Chr2:+5453535:quaternary
ATCTTGGAAG	0	1	0	0	0	Chr2:+531455:quaternary
TCAAAATAAC	0	0	0	1	0	Chr2:+5300320:quaternary,Chr5:+20087608:quaternary
TAGGTTTAAA	0	1	0	0	0	Chr2:+5196836:quaternary
TTCAAGTAAA	0	0	0	1	0	Chr2:+512486:quaternary
TAATTATAAA	0	0	0	0	1	Chr2:+5119458:quaternary,Chr1:+18218421:quaternary
AAGAGAAGGA	0	1	0	0	0	Chr2:+481850:quaternary
AAAGGAGAGA	0	0	0	0	1	Chr2:+4803356:quaternary
CCCTCTCTCT	0	0	1	0	0	Chr2:+4799896:quaternary
TGTTATTTAA	0	1	0	0	0	Chr2:+4792214:quaternary,Chr3:+16099288:quaternary,Chr3:+13533117:quaternary,Chr3:+16074436:quaternary,Chr3:+16053830:quaternary
GCAAGAAGAA	0	0	1	0	0	Chr2:+4769834:quaternary
ACGTCAGATT	0	0	0	1	0	Chr2:+449666:quaternary
TGTACATAAA	1	0	0	0	0	Chr2:+436986:quaternary
TCTGCGGCTA	0	1	0	0	0	Chr2:+4230815:quaternary,Chr4:+11115050:quaternary
AGAGTTTGGT	0	0	1	0	0	Chr2:+422570:quaternary
GTGTTAATTA	1	0	0	0	0	Chr2:+4219026:quaternary
AGATAAGACA	0	0	1	0	0	Chr2:+4133613:quaternary
AGAACTCAAA	0	0	0	1	0	Chr2:+4037933:quaternary
AAGGAGCTAG	0	0	1	0	0	Chr2:+399890:quaternary
GACCCATCGC	0	1	0	0	0	Chr2:+379017:quaternary
GAGACACCAT	0	1	0	0	0	Chr2:+3789693:quaternary
AGAACAGAAA	0	0	1	0	0	Chr2:+3736228:quaternary
ACCTTTTGTC	0	0	0	1	0	Chr2:+3722679:quaternary
TGGGTTGAGA	0	1	0	0	0	Chr2:+3496389:quaternary,ChrM:+105829:quaternary
AGACTTTTTG	0	0	1	0	0	Chr2:+3424663:quaternary,ChrM:+168199:quaternary,At4g26000.1:X:+670:quaternary
ATACGGACTC	1	0	0	0	0	Chr2:+3424526:quaternary,ChrM:+168062:quaternary
AAAAGGCCTG	0	1	0	0	0	Chr2:+3374521:quaternary,Chr2:+3508689:quaternary,ChrM:+118123:quaternary
ATTGATTAGC	0	0	0	0	1	Chr2:+3365261:quaternary
TCAATCCTAG	0	1	0	0	0	Chr2:+3326473:quaternary
GCTTGGTAAT	0	1	0	0	0	Chr2:+3280717:quaternary
CGGTTACTTC	0	0	0	1	0	Chr2:+3264192:quaternary
CTGGTAAGAC	0	0	0	1	0	Chr2:+3262656:quaternary

AAGAGTCGAG	0	1	0	0	0	Chr2:+3256481:quaternary
GATGGGTTCT	0	0	1	0	0	Chr2:+3137286:quaternary
TAAAACCAAA	0	0	1	0	0	Chr2:+3000436:quaternary,Chr2:+15771193:quaternary
TCTATTTTTC	1	0	0	0	0	Chr2:+294664:quaternary,Chr2:+11674522:quaternary,Chr4:+14779779:quaternary
GGAGATCACA	0	0	0	0	1	Chr2:+2866798:quaternary
AGGGAAGAAG	0	1	0	0	0	Chr2:+2865537:quaternary
ACATTGGTGC	0	0	1	0	0	Chr2:+2858295:quaternary
ACAACAACAG	0	0	0	1	0	Chr2:+2787269:quaternary
TCTTTCCTTG	0	0	1	0	0	Chr2:+2702587:quaternary,Chr5:+12203754:quaternary,Chr4:+4702921:quaternary
GAGCATTTGT	0	1	0	0	0	Chr2:+2666016:quaternary,Chr4:+18247609:quaternary,Chr2:+2637616:quaternary
AATAATTGAA	0	0	1	0	0	Chr2:+2358228:quaternary,Chr5:+24658701:quaternary,Chr1:+7015961:quaternary
AGGAAGATTT	0	0	1	0	0	Chr2:+2297040:quaternary
ACCAAAACAT	0	1	0	0	0	Chr2:+2209376:quaternary,At3g29780.1:X:+3:quaternary
AACAAATCTA	0	0	1	0	0	Chr2:+2180285:quaternary
CTAATTGCTA	1	0	0	0	0	Chr2:+215508:quaternary
TTATTTCTTT	0	1	0	0	0	Chr2:+2041538:quaternary
AAAAATATGA	0	0	1	0	0	Chr2:+2018260:quaternary
CTCTCTCTTG	0	1	0	0	0	Chr2:+1991549:quaternary
TGTGTTACTT	0	0	0	0	1	Chr2:+19481750:quaternary,Chr5:+17577089:quaternary
CTAAATGATA	0	0	1	0	0	Chr2:+19475898:quaternary,Chr3:+20019881:quaternary
ATGAGCAAAA	0	0	1	0	0	Chr2:+19410708:quaternary
TATAAATCAA	0	0	0	0	1	Chr2:+19363723:quaternary,ChrC:+31120:quaternary,Chr2:+14219898:quaternary
AGAATTGTCT	0	0	1	0	0	Chr2:+193062:quaternary
TGTTGATTCA	0	1	0	0	0	Chr2:+19235237:quaternary
GTATTGAATG	0	1	0	0	0	Chr2:+19183702:quaternary
GTTGAGTTTT	0	1	0	0	0	Chr2:+19166520:quaternary,Chr2:+15965931:quaternary
TGGTGGTTAA	0	0	1	0	0	Chr2:+19058411:quaternary
ATTTCGAGAT	0	0	0	0	1	Chr2:+19029025:quaternary
TAATCTTTAT	1	0	0	0	0	Chr2:+19027693:quaternary,Chr3:+7558011:quaternary,Chr1:+26523559:quaternary,Chr3:+6978096:quaternary,Chr4:+12306226:quaternary
TAGACGATAA	0	1	0	0	0	Chr2:+19017227:quaternary
CCATACAAAA	0	0	0	0	1	Chr2:+19006954:quaternary
TTAAAACTTA	0	0	1	0	0	Chr2:+18971917:quaternary,Chr1:+10168341:quaternary
TATTCAATAC	0	0	1	0	0	Chr2:+1896561:quaternary,Chr3:+19485368:quaternary
CACTATTTAC	0	1	0	0	0	Chr2:+18923550:quaternary
CATCAGTAAT	0	0	0	0	1	Chr2:+18820632:quaternary
AATACTGCTG	1	0	0	0	0	Chr2:+18606017:quaternary
TTATCACCAC	0	0	1	0	0	Chr2:+18542251:quaternary
ATTTCAAATT	0	1	0	0	0	Chr2:+18489435:quaternary,Chr5:+12412581:quaternary,Chr4:+18254661:quaternary,Chr3:+9873640:quaternary
TTCGTGTTAC	0	0	0	0	1	Chr2:+18366114:quaternary
CACCAAATAA	0	0	0	0	1	Chr2:+18359187:quaternary,Chr1:+24653440:quaternary
TCAGTATGTC	0	1	0	0	0	Chr2:+18059567:quaternary
TGATAAGAAT	0	0	1	0	0	Chr2:+18024485:quaternary,Chr1:+26355986:quaternary,Chr2:+18033117:quaternary
TATATCTATC	0	0	0	1	0	Chr2:+18012969:quaternary
GCAACTAAAG	0	1	0	0	0	Chr2:+17945168:quaternary
TAAAACTCGA	1	0	0	0	0	Chr2:+17836606:quaternary,Chr2:+18589512:quaternary
AATTAAAGAA	0	1	0	0	0	Chr2:+17752614:quaternary
GAAGTTAAAT	0	1	0	0	0	Chr2:+17695030:quaternary
AGGATTTACT	0	0	1	0	0	Chr2:+1768323:quaternary
GAGAATGAGA	1	0	0	0	0	Chr2:+17649243:quaternary
CTAAAAAGAA	0	0	0	0	1	Chr2:+1761:quaternary
ATCCAAAGGT	1	0	0	0	0	Chr2:+17450936:quaternary
TTACTTCGGT	1	0	0	0	0	Chr2:+17445372:quaternary
TAAATCTAAT	0	0	1	0	0	Chr2:+17318510:quaternary
ATATTTTCTG	0	0	0	1	0	Chr2:+17257920:quaternary
GATTCTAGAT	0	1	0	0	0	Chr2:+17226339:quaternary
AGTGTATAAA	0	0	0	0	1	Chr2:+17155287:quaternary
AGTGTTTTTA	0	0	0	0	1	Chr2:+17142207:quaternary,Chr3:+10153241:quaternary
CCTACTTGAT	0	1	0	0	0	Chr2:+17115460:quaternary,Chr2:+5688995:quaternary,Chr2:+11850816:quaternary,Chr1:+12307973:quaternary,Chr2:+5690298:quaternary

AAGAATTCTC	0	0	1	0	0	Chr2:+17108380:quaternary,ChrM:+225607:quaternary,Chr4:+7642858:quaternary
AGTTTTTTAA	0	0	1	0	0	Chr2:+16903394:quaternary,Chr1:+19708419:quaternary,Chr1:+10490279:quaternary,Chr4:+5782675:quaternary,Chr2:+11974188:quaternary
AGACCTTCTG	0	1	0	0	0	Chr2:+16870600:quaternary
ATTGTTTATT	0	0	0	1	0	Chr2:+1684658:quaternary,Chr1:+24585775:quaternary,Chr5:+20960916:quaternary
AAACATTAAT	0	0	1	0	0	Chr2:+16546099:quaternary,Chr3:+518214:quaternary
AAGAGTCGAT	0	0	1	0	0	Chr2:+1648889:quaternary
TTTGCATAAT	0	1	0	0	0	Chr2:+16442980:quaternary
ACGCATATAC	0	0	0	1	0	Chr2:+16347320:quaternary; small RNA(MIR160a)
TAAAAAATAA	0	1	0	0	0	Chr2:+16296765:quaternary,Chr5:+16883656:quaternary,Chr2:+12301210:quaternary
GATGTACAAA	1	0	0	0	0	Chr2:+16262083:quaternary
ATAAGAAAGA	0	1	0	0	0	Chr2:+16261727:quaternary
TATTTTTATA	0	0	0	0	1	Chr2:+16222791:quaternary,Chr4:+10515191:quaternary,Chr1:+21139435:quaternary,Chr4:+13305988:quaternary
CATCTTATTT	0	0	1	0	0	Chr2:+16049985:quaternary
ACAAATAGTA	0	0	0	0	1	Chr2:+16027588:quaternary
TCTATGATAT	0	0	0	0	1	Chr2:+16022583:quaternary
CAGATTATTG	1	0	0	0	0	Chr2:+15979299:quaternary
TATCTTCACT	0	0	1	0	0	Chr2:+15967250:quaternary,Chr5:+17447678:quaternary
TCCATCTCTC	0	1	0	0	0	Chr2:+1587872:quaternary
TACATTATCA	0	1	0	0	0	Chr2:+15834155:quaternary,At4g23860.2:X:+-164:quaternary,At2g05750.1:X:+2616:quaternary
AACGAATCGA	0	0	0	1	0	Chr2:+15832727:quaternary
ACACAAGAGA	1	0	0	0	0	Chr2:+15741482:quaternary
AGTTTTTAAC	1	0	0	0	0	Chr2:+15714787:quaternary
GAAAATCCAA	0	0	1	0	0	Chr2:+15613868:quaternary
AATCATTAAC	0	0	0	0	1	Chr2:+15560313:quaternary
AACATTTTTT	0	0	0	1	0	Chr2:+15526466:quaternary,Chr1:+26898746:quaternary
CACTTGAATT	0	1	0	0	0	Chr2:+15524893:quaternary
GTTATTTCAA	0	0	1	0	0	Chr2:+15506880:quaternary
TAAAAGTGTT	0	0	0	1	0	Chr2:+15502003:quaternary,Chr2:+6610882:quaternary
CTTTCCATTT	0	1	0	0	0	Chr2:+15501720:quaternary
TGAGTTGATG	0	0	0	0	1	Chr2:+15394313:quaternary
CAAGTTTGTG	0	0	0	1	0	Chr2:+15288526:quaternary
CCTAAAAAAT	0	0	0	0	1	Chr2:+15107778:quaternary
TTTCATTGAT	0	1	0	0	0	Chr2:+14999703:quaternary
CTTAGATTCA	0	0	0	1	0	Chr2:+14998984:quaternary,Chr5:+18695021:quaternary
GCTTATGAGT	0	1	0	0	0	Chr2:+14869573:quaternary
ACCATCTATC	0	0	1	0	0	Chr2:+14833842:quaternary
ACCAAAACAA	0	0	0	0	1	Chr2:+14713623:quaternary
TAAATGTGCA	1	0	0	0	0	Chr2:+14706248:quaternary
CAATAAAAAA	0	1	0	0	0	Chr2:+14705195:quaternary
TAAATCTGAT	0	0	1	0	0	Chr2:+14684686:quaternary
CAACTGGTCG	0	1	0	0	0	Chr2:+14596462:quaternary
AAATGAGACA	0	1	0	0	0	Chr2:+14363418:quaternary
ATTTCCTTAA	0	0	0	0	1	Chr2:+14354247:quaternary
ATGAGGAGAA	0	0	0	1	0	Chr2:+14346706:quaternary
GTGATTAAAA	1	0	0	0	0	Chr2:+14316179:quaternary
ATCTCTAATT	0	0	1	0	0	Chr2:+14187727:quaternary,Chr5:+1046602:quaternary
TATATAAGAA	0	1	0	0	0	Chr2:+13993095:quaternary,At4g17340.1:X:+641:quaternary
TATTCAACCA	0	0	0	0	1	Chr2:+13918064:quaternary
GATCGTGTCC	0	1	0	0	0	Chr2:+13917791:quaternary
CCTCTGGAGA	0	1	0	0	0	Chr2:+13830397:quaternary
TACTTTTGTT	0	1	0	0	0	Chr2:+13811429:quaternary,Chr3:+17089389:quaternary
TTTAACCAAA	0	0	1	0	0	Chr2:+13718945:quaternary,Chr5:+9090929:quaternary
TTATAATGTA	1	0	0	0	0	Chr2:+13698834:quaternary
AGCCCGTGGT	0	1	0	0	0	Chr2:+13697150:quaternary
TGGTGATTGG	1	0	0	0	0	Chr2:+13654650:quaternary
AAACTATATT	0	0	1	0	0	Chr2:+13586495:quaternary,Chr1:+10221659:quaternary
CGATTAGGAT	0	1	0	0	0	Chr2:+13583755:quaternary
AGAGCAAAAA	0	0	0	1	0	Chr2:+13486217:quaternary
TTTCATAGCA	0	1	0	0	0	Chr2:+13479309:quaternary

TTATACCGAA	0	0	0	0	1	Chr2:+13253537:quaternary
TGAACAAATA	0	0	0	0	1	Chr2:+13212574:quaternary
ACCAACCACC	0	0	0	1	0	Chr2:+13115994:quaternary
TGGTATTTAA	0	1	0	0	0	Chr2:+12986420:quaternary
TGATATGGTA	1	0	0	0	0	Chr2:+12965385:quaternary
GAAAACTTTG	1	0	0	0	0	Chr2:+1295949:quaternary,Chr2:+13145416:quaternary
AAAACTCGGG	0	0	0	0	1	Chr2:+12874089:quaternary
GATGTTGACA	0	0	0	0	1	Chr2:+12787327:quaternary
TTAGTGCAAC	0	1	0	0	0	Chr2:+12730107:quaternary
TTTTGTCCAC	0	0	1	0	0	Chr2:+12501222:quaternary
TCCTAAATTT	0	0	1	0	0	Chr2:+12355063:quaternary
TTTTTTCGCA	0	0	1	0	0	Chr2:+122811:quaternary
ATGATAATAA	0	0	1	0	0	Chr2:+12270824:quaternary,Chr1:+26755625:quaternary
CCCATTAATA	0	1	0	0	0	Chr2:+1222323:quaternary
TTGTGTTAAA	0	0	0	0	1	Chr2:+12188936:quaternary
ATGGTTAAAA	1	0	0	0	0	Chr2:+1215149:quaternary,Chr2:+1221831:quaternary
ATTCACATCT	1	0	0	0	0	Chr2:+12144062:quaternary
GTGAATGTGA	0	1	0	0	0	Chr2:+12085837:quaternary
CATATAAACA	0	1	0	0	0	Chr2:+12085454:quaternary,Chr1:+29498724:quaternary,Chr5:+7397144:quaternary,Chr1:+14334247:quaternary,At3g43357.1:X:+-111:quaternary,Chr1:+16530520:quaternary
CAAAAGAATA	0	0	0	1	0	Chr2:+12083821:quaternary,Chr3:+20535701:quaternary
GGACACAAGT	0	0	1	0	0	Chr2:+11961290:quaternary
GAAAACCAAC	0	0	0	1	0	Chr2:+11900745:quaternary
AAAAATATCG	0	0	1	0	0	Chr2:+11896566:quaternary,Chr1:+9914535:quaternary
GAATAAAAAA	0	0	1	0	0	Chr2:+11779066:quaternary,Chr4:+6934284:quaternary
GCTAGCAAAT	1	0	0	0	0	Chr2:+11736515:quaternary
TAAGAAAAAG	0	1	0	0	0	Chr2:+11551868:quaternary,Chr2:+15733086:quaternary
CAGGTTAAGT	0	1	0	0	0	Chr2:+1126012:quaternary
CATAATGTGA	0	0	1	0	0	Chr2:+11101072:quaternary,Chr4:+16058571:quaternary,Chr4:+5714131:quaternary
TTTCCTTCGT	0	1	0	0	0	Chr2:+11021360:quaternary
TAGTGAGTGG	0	0	1	0	0	Chr2:+10921380:quaternary
ACCAACTCAA	0	1	0	0	0	Chr2:+10832398:quaternary
ATCTAATCTT	0	1	0	0	0	Chr2:+10660201:quaternary
TGTAAACGTT	0	0	1	0	0	Chr2:+10587698:quaternary
TAGGTCATTT	0	1	0	0	0	Chr2:+10468492:quaternary
GGACATTATC	0	1	0	0	0	Chr2:+10421809:quaternary
ATGGTTTAAT	0	0	0	1	0	Chr2:+10360947:quaternary,Chr1:+8040045:quaternary
CACAAAAACA	0	0	1	0	0	Chr2:+10347600:quaternary
CTGAAATTTA	0	1	0	0	0	Chr2:+10345117:quaternary,Chr3:+19993283:quaternary
GTGCATTTGT	0	0	0	1	0	Chr2:+10330774:quaternary
TATCTTAGCG	0	1	0	0	0	Chr2:+10288157:quaternary
ATATCTCTTT	0	1	0	0	0	Chr2:+10154545:quaternary,Chr2:+13257012:quaternary
ATTTGCCAGC	0	1	0	0	0	Chr2:+10090792:quaternary
GAAGTTCATT	0	0	1	0	0	Chr2:+10075043:quaternary
AGTATTGCTT	0	0	0	0	1	Chr1:-9995722:quaternary
TGAGTTTCAT	0	0	1	0	0	Chr1:-9960257:quaternary
TCCTATTTTG	0	0	1	0	0	Chr1:-9792984:quaternary
TAAAAAGCCG	1	0	0	0	0	Chr1:-9500640:quaternary
TATTCATATC	0	1	0	0	0	Chr1:-9499849:quaternary
TGATCTGACG	0	1	0	0	0	Chr1:-9461565:quaternary,Chr5:-7469360:quaternary
TTCCGCTCTG	0	0	0	1	0	Chr1:-9075861:quaternary
TAGCAAATCC	0	0	1	0	0	Chr1:-9017961:quaternary
AGATGGGTAG	0	0	1	0	0	Chr1:-8575846:quaternary
TCGTTTGTGC	0	1	0	0	0	Chr1:-8173256:quaternary
TTCTAGATTA	1	0	0	0	0	Chr1:-7884113:quaternary
GTTTGAATGA	0	1	0	0	0	Chr1:-7698365:quaternary
GCACAACATT	0	0	1	0	0	Chr1:-7570277:quaternary
CATATGGAAT	0	0	0	1	0	Chr1:-6334867:quaternary
AGTGTTGTCT	0	1	0	0	0	Chr1:-5031493:quaternary
GGGACTTTCA	1	0	0	0	0	Chr1:-4028207:quaternary

TAAGGTGAAC	1	0	0	0	0	Chr1:-4021288:quaternary
GCAGCTGGGT	0	1	0	0	0	Chr1:-3824755:quaternary
TGGTTATATT	0	0	0	0	1	Chr1:-3530442:quaternary,Chr5:-16641162:quaternary
TCTTACTGCA	1	0	0	0	0	Chr1:-3426052:quaternary
GTTGTTATAA	0	0	1	0	0	Chr1:-3420727:quaternary
GTCAAGTCCC	0	1	0	0	0	Chr1:-30119894:quaternary
GGAATATGGG	0	0	0	0	1	Chr1:-29229268:quaternary
TGTAGCTAGA	1	0	0	0	0	Chr1:-29055498:quaternary
TTTCGTTGAG	0	0	0	1	0	Chr1:-29016953:quaternary
TGAGTTGAAA	0	0	0	0	1	Chr1:-28994539:quaternary
TGCCCTAGCA	0	0	1	0	0	Chr1:-28830674:quaternary
CCTTAAGTAT	0	1	0	0	0	Chr1:-28382847:quaternary
AATCCGAAAT	0	0	0	1	0	Chr1:-28328246:quaternary
CAAAGTTATT	1	0	0	0	0	Chr1:-2818590:quaternary,Chr1:-6263669:quaternary,Chr5:-17744623:quaternary,Chr5:-17744629:quaternary,Chr4:-7313306:quaternary
ATACAACCCT	1	0	0	0	0	Chr1:-27537109:quaternary
ATCAGGGGAT	1	0	0	0	0	Chr1:-26577847:quaternary
CTTAAGATCT	0	0	0	1	0	Chr1:-26244650:quaternary
TAGTCTATTA	0	0	1	0	0	Chr1:-25744568:quaternary
ACTCCATACA	1	0	0	0	0	Chr1:-2538249:quaternary
AACAGAGATT	0	0	1	0	0	Chr1:-25363053:quaternary,Chr5:-17611652:quaternary
TTTGCCTTTA	1	0	0	0	0	Chr1:-25283056:quaternary
GACTTCGAAG	0	0	0	1	0	Chr1:-25176830:quaternary
GGTTTTTTTA	0	0	0	1	0	Chr1:-25059656:quaternary,Chr5:-16999224:quaternary,Chr1:-25059664:quaternary
CTTTAGTTGA	0	0	1	0	0	Chr1:-24580160:quaternary
TGGCCTAAAA	0	0	1	0	0	Chr1:-24538157:quaternary
AGATGTTAAA	0	0	1	0	0	Chr1:-24218625:quaternary
TCCAAAAGGA	0	0	1	0	0	Chr1:-23798208:quaternary
CTATCAAAAC	1	0	0	0	0	Chr1:-23621318:quaternary
AAGACAGCTC	0	1	0	0	0	Chr1:-23478248:quaternary,At4g06598.1:X:-2521:quaternary,Chr1:-23478261:quaternary
CATTTGGATG	0	1	0	0	0	Chr1:-23397458:quaternary,Chr3:-7470492:quaternary
TAAACCAGCT	0	0	0	1	0	Chr1:-23391301:quaternary
TGGATATGTG	1	0	0	0	0	Chr1:-23251631:quaternary
ATTTGCCGAT	0	0	0	1	0	Chr1:-22959507:quaternary
ATCTATACAA	0	0	0	1	0	Chr1:-22260967:quaternary
GGATATAAGC	0	1	0	0	0	Chr1:-21917520:quaternary
ATGCAAGTTT	0	0	1	0	0	Chr1:-2187515:quaternary
CGCCATCATC	0	0	1	0	0	Chr1:-21704259:quaternary
TTGCTCCTGC	0	0	0	1	0	Chr1:-21534802:quaternary
CATTTATGAC	0	0	0	0	1	Chr1:-21045418:quaternary
ACAAGTAACA	0	1	0	0	0	Chr1:-20599111:quaternary
TCGTTCATCT	0	0	0	0	1	Chr1:-20508308:quaternary
GAAACGTGTA	0	1	0	0	0	Chr1:-20010404:quaternary
GCTAGTCTTG	0	1	0	0	0	Chr1:-19561105:quaternary
CCGAATTTTT	0	0	1	0	0	Chr1:-19371409:quaternary
TAACCGTGAA	1	0	0	0	0	Chr1:-19358047:quaternary
GGCAGCAAGA	0	0	0	1	0	Chr1:-19296883:quaternary,Chr3:-7520340:quaternary
AAGTTGCATC	0	0	0	0	1	Chr1:-18998842:quaternary
GATTGTAACT	0	1	0	0	0	Chr1:-18950678:quaternary,Chr2:-19264144:quaternary
TTTGGTGATC	0	1	0	0	0	Chr1:-18817551:quaternary,Chr2:-13668163:quaternary
ACCACCTTCT	0	1	0	0	0	Chr1:-18335795:quaternary
AACAAAGTTG	0	0	1	0	0	Chr1:-17676548:quaternary,Chr1:-23962809:quaternary
TTTGCACATT	1	0	0	0	0	Chr1:-17646900:quaternary
AGTGGATTTG	0	0	0	1	0	Chr1:-17394138:quaternary
TCCTCTGTGA	0	1	0	0	0	Chr1:-17025719:quaternary
TATTTTTGGA	1	0	0	0	0	Chr1:-16871892:quaternary
TCATTTTGGT	0	0	0	0	1	Chr1:-1678901:quaternary,Chr5:-21922316:quaternary
AAGTGGTAGT	0	0	1	0	0	Chr1:-16723075:quaternary
GCACATTAGG	0	0	0	1	0	Chr1:-16638673:quaternary
TGGTAAGAAA	0	1	0	0	0	Chr1:-1612985:quaternary,Chr1:-25632121:quaternary
AACTGTATGA	1	0	0	0	0	Chr1:-16118008:quaternary

ATCTCAGGAT	0	0	0	1	0	Chr1:-15884002:quaternary
TGTGGATGAC	0	0	1	0	0	Chr1:-14217064:quaternary
GGGAATGTTT	0	1	0	0	0	Chr1:-13303044:quaternary
CACAAGCTAT	0	0	1	0	0	Chr1:-13238253:quaternary
AAAACTATCT	1	0	0	0	0	Chr1:-12476540:quaternary,Chr1:-10518671:quaternary
GGGGAAAATA	0	0	0	0	1	Chr1:-116631:quaternary
GGCTTCTTGA	1	0	0	0	0	Chr1:-11322063:quaternary,Chr1:-16495193:quaternary,Chr1:-11322059:quaternary
TAGTTCACAA	0	0	0	1	0	Chr1:-11285511:quaternary
CGTGAATCCA	0	0	0	1	0	Chr1:-11137707:quaternary
GGTTGTTGGA	0	0	1	0	0	Chr1:-11083866:quaternary
TCGAAAGGAG	0	0	1	0	0	Chr1:-11032227:quaternary,Chr1:-12517305:quaternary
TAATTTGGGG	1	0	0	0	0	Chr1:-11006414:quaternary,Chr5:-22187842:quaternary
CCCGCTTCGT	0	0	0	0	1	Chr1:-10614555:quaternary
TGTGCTAAGT	0	0	0	0	1	Chr1:-10600553:quaternary
TAAGCTAACG	0	1	0	0	0	Chr1:-10554080:quaternary
AAACCTTTGG	0	0	0	0	1	Chr1:+9925682:quaternary
AACTTTGAAA	0	1	0	0	0	Chr1:+9915396:quaternary,Chr3:+13394932:quaternary
TGTGTTCAAA	1	0	0	0	0	Chr1:+9906181:quaternary
TGTACGTAAA	1	0	0	0	0	Chr1:+9867852:quaternary
TTGGGAAATA	0	0	0	0	1	Chr1:+9460105:quaternary,Chr1:+11703615:quaternary
TATCGTTGAT	0	0	1	0	0	Chr1:+9347029:quaternary
GAAATGTGGT	0	0	1	0	0	Chr1:+903688:quaternary
TTATGAGGCA	0	1	0	0	0	Chr1:+896831:quaternary
GGCCTGTGGG	0	1	0	0	0	Chr1:+8961449:quaternary
TGCAATACTA	0	0	0	1	0	Chr1:+8926463:quaternary
GGACACGTGT	0	1	0	0	0	Chr1:+8924324:quaternary
CCGGAATTGG	0	0	1	0	0	Chr1:+8888675:quaternary
CCATTTTTTA	0	0	1	0	0	Chr1:+8765132:quaternary
CAAGAAAAGA	0	0	0	1	0	Chr1:+8725533:quaternary,Chr2:+8706154:quaternary
TCCCTCTCTT	0	1	0	0	0	Chr1:+8591965:quaternary
GCCAAATTCT	0	0	0	1	0	Chr1:+8545558:quaternary
TGGATGAAGC	0	0	1	0	0	Chr1:+8542572:quaternary
CCGAGACAAA	0	0	1	0	0	Chr1:+8540189:quaternary
CAACAAAAAT	0	0	1	0	0	Chr1:+8537656:quaternary
CGGCATCACT	0	0	0	0	1	Chr1:+8387478:quaternary
GTTTGTGATA	0	0	0	0	1	Chr1:+8311188:quaternary
AGGAGACATC	0	1	0	0	0	Chr1:+8298286:quaternary
TTTTTCCCTC	0	0	0	1	0	Chr1:+8278449:quaternary
GAGAGTGAAC	0	1	0	0	0	Chr1:+8173514:quaternary
CAAACGAAAA	0	0	1	0	0	Chr1:+8145557:quaternary,Chr3:+10106040:quaternary
GGGAAGGAGT	0	0	0	0	1	Chr1:+8113189:quaternary
ACCAAAGCCC	0	1	0	0	0	Chr1:+8060684:quaternary
AACTTAAATT	0	0	1	0	0	Chr1:+7946325:quaternary
TTATCTGAAA	1	0	0	0	0	Chr1:+7845828:quaternary
ACTTTATAAA	0	0	0	0	1	Chr1:+7831578:quaternary
CAAAGTCGAT	0	1	0	0	0	Chr1:+7701609:quaternary
AAACTGATTT	0	1	0	0	0	Chr1:+767832:quaternary
GCCTCCTTGT	1	0	0	0	0	Chr1:+7458180:quaternary
TTCATCGAAA	0	0	0	1	0	Chr1:+7456506:quaternary
GATCGGTTAA	0	0	0	1	0	Chr1:+7369607:quaternary
ACCTAAAGCA	0	0	0	0	1	Chr1:+7365717:quaternary
TATATGAGAC	1	0	0	0	0	Chr1:+7310618:quaternary,Chr1:+25065039:quaternary
GAGTTTTATT	0	1	0	0	0	Chr1:+7237897:quaternary
ATCGAACAAG	0	0	0	1	0	Chr1:+7230231:quaternary
TATCATCTTA	0	1	0	0	0	Chr1:+6874855:quaternary
TTTTAGTTTG	0	0	0	0	1	Chr1:+6763464:quaternary,Chr3:+6280640:quaternary
CTAAAAAAAC	0	0	1	0	0	Chr1:+6728683:quaternary,Chr1:+9653003:quaternary
GCCTTGGAAT	0	0	1	0	0	Chr1:+6695721:quaternary
ATAGTCAAAA	0	1	0	0	0	Chr1:+6615683:quaternary
CTTAAATGTT	0	0	0	0	1	Chr1:+6520162:quaternary,Chr1:+8862857:quaternary,Chr3:+7259901:quaternary
ATTCTCCAAT	0	0	1	0	0	Chr1:+6475663:quaternary

TGGCTAAGAT	0	0	0	0	1	Chr1:+6472485:quaternary
TTTCAAAGCC	0	0	0	1	0	Chr1:+6462617:quaternary
AATGTTCAAA	0	1	0	0	0	Chr1:+6458945:quaternary
GCTCGTGCCG	0	1	0	0	0	Chr1:+6366576:quaternary,Chr4:+1159071:quaternary
GTTTTATAAT	0	0	0	1	0	Chr1:+6141953:quaternary,Chr1:+23172377:quaternary,Chr4:+4091039:quaternary,Chr1:+18910880:quaternary
AGTCTAGCTC	1	0	0	0	0	Chr1:+6139033:quaternary
TCTCAGAATT	0	0	0	1	0	Chr1:+5967810:quaternary
TGTATAAACG	1	0	0	0	0	Chr1:+590300:quaternary
GAATGAGAAT	0	1	0	0	0	Chr1:+5828530:quaternary
TTGTGCACCA	1	0	0	0	0	Chr1:+5656107:quaternary
ATAACTTTAT	0	0	0	0	1	Chr1:+5646078:quaternary
TTACTCTTAA	1	0	0	0	0	Chr1:+5614108:quaternary
CATCGTTTTC	0	0	0	0	1	Chr1:+5507080:quaternary,Chr5:+6907114:quaternary
AGTTTTCGTA	0	1	0	0	0	Chr1:+5350241:quaternary
GTCCCAAAAA	0	0	0	1	0	Chr1:+5324815:quaternary
GTGTTGCTTG	1	0	0	0	0	Chr1:+5156941:quaternary
GTTCTCTATC	0	1	0	0	0	Chr1:+5147218:quaternary
TTAGCTCATT	0	0	0	1	0	Chr1:+5135113:quaternary,At5g16470.1:X:+691:quaternary
ACACATCCAC	1	0	0	0	0	Chr1:+5051796:quaternary
TAAAACTCCA	0	0	0	1	0	Chr1:+5022914:quaternary
TGAATTATAT	0	1	0	0	0	Chr1:+4964954:quaternary,Chr1:+4941416:quaternary
GTATCCAAAG	0	0	1	0	0	Chr1:+4860028:quaternary
AAGAATATCA	0	1	0	0	0	Chr1:+4627085:quaternary
GCTTGATTCA	0	0	1	0	0	Chr1:+4412093:quaternary
CTCAGAAAAA	0	0	0	0	1	Chr1:+4315952:quaternary
AAATTGAAAA	0	1	0	0	0	Chr1:+430928:quaternary,Chr3:+23221055:quaternary,Chr1:+18214761:quaternary
AATGAATCAC	0	0	0	0	1	Chr1:+4225869:quaternary
TCTCCTTAGC	1	0	0	0	0	Chr1:+4171623:quaternary
TTCTTATTAG	1	0	0	0	0	Chr1:+4112937:quaternary,Chr3:+12020344:quaternary,Chr5:+9395868:quaternary,Chr1:+13908056:quaternary,Chr2:+6670038:quaternary,Chr5:+15224688:quaternary,Chr5:+11396767:quaternary,Chr1:+16600509:quaternary,Chr3:+11377366:quaternary,Chr2:+5748361:quaternary,Chr3:+10866818:quaternary
TCCTTGGAAT	0	1	0	0	0	Chr1:+405561:quaternary
AGATTGTGAA	0	1	0	0	0	Chr1:+4038738:quaternary,Chr3:+4868761:quaternary,Chr3:+4881157:quaternary
TTCATCAGAG	0	0	0	1	0	Chr1:+3829180:quaternary
TTGAGAGCTA	1	0	0	0	0	Chr1:+3809149:quaternary
GAGGAGCTGA	0	1	0	0	0	Chr1:+3745421:quaternary
CATTTTTAAA	0	0	0	0	1	Chr1:+369066:quaternary
CTTATTATCT	1	0	0	0	0	Chr1:+3531629:quaternary
TATGATACCG	0	1	0	0	0	Chr1:+3497619:quaternary
AGAAAACGAT	0	0	0	0	1	Chr1:+3482759:quaternary
TTTTTCCAAT	0	1	0	0	0	Chr1:+3420613:quaternary
AGAAAAAACA	0	0	1	0	0	Chr1:+3324614:quaternary
ACTCTGTCAT	0	0	1	0	0	Chr1:+3195561:quaternary
CTTGGGAAAC	0	1	0	0	0	Chr1:+30430672:quaternary
ATTATATGAA	0	0	1	0	0	Chr1:+30382162:quaternary,Chr3:+17877916:quaternary
TTATCGAAAA	0	0	0	0	1	Chr1:+30369636:quaternary
CAGCATCTTC	0	0	0	0	1	Chr1:+30353325:quaternary,Chr5:+22660210:quaternary
AGAAATGAAC	0	0	0	1	0	Chr1:+30257629:quaternary
TTTTAAACTA	0	1	0	0	0	Chr1:+30042027:quaternary
GTACATAATA	0	0	1	0	0	Chr1:+29971480:quaternary
ATTTATCTGC	1	0	0	0	0	Chr1:+29949878:quaternary,Chr5:+14198743:quaternary
GTTTGTTTAA	1	0	0	0	0	Chr1:+2988528:quaternary
ATTATGACCA	1	0	0	0	0	Chr1:+29441960:quaternary
ATTTAGGAGT	0	0	1	0	0	Chr1:+29283945:quaternary
TCTCTCACTG	0	0	0	1	0	Chr1:+29280798:quaternary
ATCATTGCAA	0	0	0	0	1	Chr1:+29269851:quaternary
AACAGTGCTG	0	0	0	1	0	Chr1:+29237444:quaternary
TAACTTAAAT	0	0	0	0	1	Chr1:+29202866:quaternary,Chr2:+17627804:quaternary
GGTGTTGTTG	0	1	0	0	0	Chr1:+29202405:quaternary
AGCAGGTGAT	0	0	0	0	1	Chr1:+29070372:quaternary

ATCTTTGGGA	1	0	0	0	0	Chr1:+29041445:quaternary
ACTTATGAGA	0	1	0	0	0	Chr1:+28905475:quaternary,Chr3:+22799323:quaternary
TGCATAAACA	1	0	0	0	0	Chr1:+28873720:quaternary
CAACTCCGAA	0	0	1	0	0	Chr1:+28867431:quaternary
AGTTAGTTTT	0	0	0	0	1	Chr1:+28848246:quaternary
AACCTTTGGC	0	0	0	1	0	Chr1:+28842154:quaternary
GTATAAACAG	0	1	0	0	0	Chr1:+28449248:quaternary
TTGTAAGGAT	0	0	0	0	1	Chr1:+28359365:quaternary
GCATATGGTT	0	0	1	0	0	Chr1:+28330412:quaternary
GCATTATTGG	0	1	0	0	0	Chr1:+28250064:quaternary
GTTTAGATGT	0	0	0	1	0	Chr1:+28216817:quaternary
TGCAAATCCA	1	0	0	0	0	Chr1:+28179793:quaternary
TACATCGTCT	0	0	0	0	1	Chr1:+28087413:quaternary
CACTTCCAAA	0	0	0	1	0	Chr1:+28061988:quaternary
ATGTAATTTT	0	0	0	1	0	Chr1:+28058955:quaternary
ACCAAAAATC	1	0	0	0	0	Chr1:+27872539:quaternary
TTGGCTGTAA	0	1	0	0	0	Chr1:+27747120:quaternary
ATTTGTCATT	0	1	0	0	0	Chr1:+27668694:quaternary,Chr2:+1686722:quaternary
GGAATAAATA	1	0	0	0	0	Chr1:+27412324:quaternary
AATGAAAGTG	1	0	0	0	0	Chr1:+27228298:quaternary
TAGTATAATT	0	1	0	0	0	Chr1:+27180594:quaternary,Chr1:+4291377:quaternary
ATGATCAAAA	0	1	0	0	0	Chr1:+27155718:quaternary
ACTTCTTCTA	0	1	0	0	0	Chr1:+27143453:quaternary
AGAAAATTTC	0	0	1	0	0	Chr1:+2712664:quaternary
TAGATTTGTC	1	0	0	0	0	Chr1:+27096104:quaternary
AAAGAATGAA	0	0	1	0	0	Chr1:+27088993:quaternary
TAACAGTTTT	1	0	0	0	0	Chr1:+27074386:quaternary
CTTAGCAAGT	0	0	1	0	0	Chr1:+27074070:quaternary
TCAAAATGTC	0	0	0	0	1	Chr1:+26743434:quaternary
GAAAAAAAAG	1	0	0	0	0	Chr1:+26723779:quaternary,Chr1:+23906382:quaternary
GATTTTCCTG	0	0	1	0	0	Chr1:+26620274:quaternary
AAGTGATATG	1	0	0	0	0	Chr1:+26545729:quaternary
GGCCTTCAAA	0	0	1	0	0	Chr1:+26523018:quaternary
CACACACTCT	0	1	0	0	0	Chr1:+26491917:quaternary,Chr5:+20938138:quaternary
ACGGTGCCGG	1	0	0	0	0	Chr1:+26460057:quaternary
TTGGTGGGTA	1	0	0	0	0	Chr1:+26452991:quaternary,Chr4:+6135676:quaternary
GAAGAATGGA	0	0	1	0	0	Chr1:+26452511:quaternary
AGATTTATTA	0	0	0	0	1	Chr1:+26378863:quaternary
TTAGTAATAA	0	1	0	0	0	Chr1:+26356237:quaternary
TTATTTTTCA	0	0	1	0	0	Chr1:+26349203:quaternary
AGAGTCACGA	1	0	0	0	0	Chr1:+26328704:quaternary
ACCATTAAAA	0	0	0	0	1	Chr1:+26317868:quaternary
CTACTTCAAA	1	0	0	0	0	Chr1:+26276931:quaternary
TGATTAAAAA	0	1	0	0	0	Chr1:+26180198:quaternary,Chr1:+25744316:quaternary
GACACATCTG	1	0	0	0	0	Chr1:+26179520:quaternary
AGACGACAAA	1	0	0	0	0	Chr1:+26171572:quaternary
CTTAAGAACT	0	0	1	0	0	Chr1:+26121596:quaternary
TAAAACACTT	0	0	1	0	0	Chr1:+26064646:quaternary
AGACAAACTA	0	0	1	0	0	Chr1:+26014298:quaternary,Chr4:+2110145:quaternary
GGTTGTGTGT	1	0	0	0	0	Chr1:+26003416:quaternary
CTCATCATCA	0	0	1	0	0	Chr1:+25977792:quaternary
AAAACATTAA	0	1	0	0	0	Chr1:+25874099:quaternary
TAACTTTGAG	0	1	0	0	0	Chr1:+25778953:quaternary
GGTGTGTTGA	0	1	0	0	0	Chr1:+25723473:quaternary
TTTTTCCGCC	0	1	0	0	0	Chr1:+25701381:quaternary,Chr1:+25701674:quaternary,Chr5:+2072229:quaternary,Chr5:+19840715:quaternary,Chr3:+2138643:quaternary,Chr3:+2138940:quaternary,Chr1:+25701780:quaternary,Chr1:+25701487:quaternary
GTGACTAGGA	1	0	0	0	0	Chr1:+25623838:quaternary
ATTAGAGAAA	0	0	1	0	0	Chr1:+25613242:quaternary,Chr1:+4523525:quaternary
AAAATAAGAT	1	0	0	0	0	Chr1:+25585184:quaternary

GACACTTAAA	1	0	0	0	0	Chr1:+25553713:quaternary,Chr5:+6838548:quaternary
TTCTAAGAAG	0	0	1	0	0	Chr1:+25408108:quaternary
GTCTTGATAG	0	0	0	0	1	Chr1:+25362852:quaternary,Chr5:+9175147:quaternary
TTTTCTGTCG	0	0	1	0	0	Chr1:+25242297:quaternary
AACATTTCTG	0	0	1	0	0	Chr1:+25072592:quaternary
GCTCCCAACA	0	0	0	1	0	Chr1:+25036487:quaternary
ATAATTTTGC	0	0	0	0	1	Chr1:+24972724:quaternary
TAAAATTGAA	0	0	0	1	0	Chr1:+24941735:quaternary
CCTATCAATC	0	0	0	0	1	Chr1:+24925189:quaternary
ATAGGAAAAC	0	0	0	1	0	Chr1:+24851392:quaternary
CTACATTTTA	1	0	0	0	0	Chr1:+24803404:quaternary
GTTTGGCCTA	0	0	0	1	0	Chr1:+24785441:quaternary
TTTTATTCCA	0	0	0	0	1	Chr1:+24771460:quaternary
CTTGGAAAAT	0	0	1	0	0	Chr1:+24766445:quaternary,At1g76320.1:X:+345:quaternary,Chr4:+16745818:quaternary
TAAGAACTAA	0	0	0	1	0	Chr1:+24688477:quaternary,Chr5:+4591122:quaternary
GACTCAAGCT	0	0	1	0	0	Chr1:+24658567:quaternary
TTCTTTGTTA	0	1	0	0	0	Chr1:+24540056:quaternary
ATAGAATCTT	0	0	1	0	0	Chr1:+24445461:quaternary
ATAACCATTC	0	1	0	0	0	Chr1:+24400943:quaternary
CCCGCGTCCA	0	1	0	0	0	Chr1:+24351879:quaternary
TAAAGAAGTT	0	0	1	0	0	Chr1:+24349550:quaternary
GTCAACTTTT	0	0	1	0	0	Chr1:+2432272:quaternary
GGACATCATA	0	0	0	1	0	Chr1:+24256633:quaternary
GTAGATAAAA	0	0	0	1	0	Chr1:+24221732:quaternary
CCTTGTTAAA	0	0	0	0	1	Chr1:+24016592:quaternary
TTTGTTGGGT	0	1	0	0	0	Chr1:+24012917:quaternary,Chr1:+1887925:quaternary
TGCCTAAGAT	0	1	0	0	0	Chr1:+23978866:quaternary
CACCATCGAG	0	1	0	0	0	Chr1:+2392788:quaternary
AATTGTTGTA	0	1	0	0	0	Chr1:+23911104:quaternary
ATTCTATAAA	1	0	0	0	0	Chr1:+23900578:quaternary
TATTTTTCTC	0	0	0	0	1	Chr1:+2385952:quaternary
AATCTGAGAG	0	1	0	0	0	Chr1:+23834096:quaternary
TATTATCCAC	0	0	0	1	0	Chr1:+23725859:quaternary,Chr5:+3168904:quaternary
GTAAATCCAA	0	1	0	0	0	Chr1:+23686070:quaternary
TCTTTCTTCG	0	0	0	1	0	Chr1:+23602405:quaternary,Chr2:+4014867:quaternary
GAAAAGTTTA	0	1	0	0	0	Chr1:+23419067:quaternary
AAGGGAAGAG	0	0	1	0	0	Chr1:+23391435:quaternary
CATTAAAACG	0	0	1	0	0	Chr1:+23368228:quaternary
AATGTGACTA	0	0	0	0	1	Chr1:+23348166:quaternary,Chr5:+19172157:quaternary
AAACTTTGGT	0	1	0	0	0	Chr1:+23300329:quaternary,Chr4:+2097058:quaternary,Chr1:+16661755:quaternary,Chr1:+23300129:quaternary
TGAAATTCTA	1	0	0	0	0	Chr1:+23147625:quaternary
TAGTAAATTA	0	1	0	0	0	Chr1:+23139670:quaternary
TAGGATTTGA	0	1	0	0	0	Chr1:+23114242:quaternary
TGCTGCTAAC	0	0	0	0	1	Chr1:+23039414:quaternary
AGAGAGAAAC	0	0	1	0	0	Chr1:+22989146:quaternary
CATAATTTAT	0	1	0	0	0	Chr1:+22959148:quaternary
GAACTATGTG	0	0	0	0	1	Chr1:+22894901:quaternary
AGTTGGCGAA	0	1	0	0	0	Chr1:+22805976:quaternary
ATTTTGGCTA	0	0	1	0	0	Chr1:+22773268:quaternary
TCACATAAGG	0	0	0	1	0	Chr1:+22731864:quaternary
TAAAACTGTG	0	0	1	0	0	Chr1:+22717667:quaternary
TACTTACATA	0	1	0	0	0	Chr1:+22640810:quaternary
ACTCATATTA	0	1	0	0	0	Chr1:+22634977:quaternary,Chr4:+9444923:quaternary
GAAACATTGG	0	1	0	0	0	Chr1:+22616058:quaternary
TCTCAAAGTA	0	1	0	0	0	Chr1:+22545281:quaternary
ATCTTGAAAA	0	0	0	1	0	Chr1:+22426801:quaternary,Chr3:+18762504:quaternary
CAAGCTTTAT	0	0	1	0	0	Chr1:+22400035:quaternary
GTTTGGGTGG	0	0	0	1	0	Chr1:+22260672:quaternary
AACAATGAGG	0	1	0	0	0	Chr1:+22090434:quaternary
GATCCATCAT	0	1	0	0	0	Chr1:+22080556:quaternary

TTTCCTTTAC	0	0	0	1	0	Chr1:+21979655:quaternary
GAGATTTTAG	0	1	0	0	0	Chr1:+21831171:quaternary,Chr1:+21794913:quaternary
TAAAAAGCTG	0	1	0	0	0	Chr1:+21828311:quaternary,Chr1:+21792053:quaternary
AAATGGTCAC	1	0	0	0	0	Chr1:+21746796:quaternary
AATCCTAATG	0	0	0	0	1	Chr1:+21685251:quaternary,Chr5:+9930989:quaternary
ATATAATACC	0	0	0	0	1	Chr1:+21674950:quaternary
TTAAAAACTG	0	0	0	0	1	Chr1:+21632632:quaternary
GCCGAAGAGA	1	0	0	0	0	Chr1:+21589065:quaternary
TCCATTTCCG	0	0	1	0	0	Chr1:+21540875:quaternary
TCTCTCATAA	0	0	1	0	0	Chr1:+21522872:quaternary
TTTATACAAT	0	0	0	0	1	Chr1:+21425484:quaternary,Chr1:+7289214:quaternary,Chr3:+11370078:quaternary,Chr5:+26073314:quaternary
AACATTTGAA	0	1	0	0	0	Chr1:+21393064:quaternary
GTTAAATTTG	0	1	0	0	0	Chr1:+213461:quaternary,At5g11090.1:X:+-133:quaternary
TTACCGTTTT	0	1	0	0	0	Chr1:+21277146:quaternary,Chr1:+21280184:quaternary,Chr1:+21299704:quaternary,Chr5:+2632734:quaternary,Chr1:+21298173:quaternary,Chr1:+21272619:quaternary,Chr1:+21301053:quaternary,Chr1:+21283222:quaternary,Chr1:+21274128:quaternary,Chr1:+21281693:quaternary,Chr1:+21275637:quaternary,Chr1:+21295293:quaternary,Chr1:+21293944:quaternary,Chr1:+21286286:quaternary,Chr1:+21287818:quaternary,Chr1:+21278655:quaternary,Chr1:+21305466:quaternary,Chr1:+21306815:quaternary,Chr1:+21302585:quaternary,Chr1:+21290882:quaternary,Chr1:+21296824:quaternary,Chr1:+21292413:quaternary,Chr1:+21309887:quaternary,Chr1:+21289351:quaternary,Chr1:+21284754:quaternary,Chr1:+21303934:quaternary
ACATTGCAGA	0	0	1	0	0	Chr1:+21232929:quaternary
GATAAAAAGA	0	1	0	0	0	Chr1:+21111268:quaternary,Chr3:+10422981:quaternary
ACAAGTGCCA	0	1	0	0	0	Chr1:+21091321:quaternary
TAAACTAGGG	0	0	0	1	0	Chr1:+2107675:quaternary
TATATAGGTT	0	1	0	0	0	Chr1:+21055480:quaternary
TTAATCAATA	0	0	1	0	0	Chr1:+21053721:quaternary,Chr3:+18313428:quaternary
TGGACTCTTT	1	0	0	0	0	Chr1:+21049319:quaternary
TACTTGTTGC	0	0	0	0	1	Chr1:+21042646:quaternary
AAGAGGAGGA	0	0	1	0	0	Chr1:+20942220:quaternary
AACAGCTCGA	0	1	0	0	0	Chr1:+20935480:quaternary
ATTTTGAAGA	1	0	0	0	0	Chr1:+20914704:quaternary,At1g64230.1:X:+537:quaternary,Chr5:+24085891:quaternary,Chr1:+28659742:quaternary
TCATTCTCCA	0	0	0	1	0	Chr1:+20914500:quaternary
GTTATCACTT	0	0	0	1	0	Chr1:+20599538:quaternary,Chr5:+3454894:quaternary
AAGGCTGAAG	0	0	1	0	0	Chr1:+20508204:quaternary
TCTCTTTCAC	0	0	1	0	0	Chr1:+20500906:quaternary,Chr3:+4618711:quaternary
TTATAAGCAA	0	1	0	0	0	Chr1:+20492196:quaternary
GTCTGATTTA	1	0	0	0	0	Chr1:+20473302:quaternary
ATGTATGCAG	0	0	0	1	0	Chr1:+20416056:quaternary,Chr5:+1268972:quaternary,ChrM:+282617:quaternary
ACAACATTAA	1	0	0	0	0	Chr1:+20237821:quaternary
ATAATGATTA	1	0	0	0	0	Chr1:+20168419:quaternary
TGATCAGATT	0	1	0	0	0	Chr1:+20130019:quaternary,Chr1:+20117454:quaternary,Chr2:+14092715:quaternary
ACCGTGATTA	0	1	0	0	0	Chr1:+20129663:quaternary,Chr1:+20117098:quaternary
CAACACTAAC	0	0	0	1	0	Chr1:+20047881:quaternary
ATAATTTTTG	1	0	0	0	0	Chr1:+20023914:quaternary,At5g50160.1:X:+466:quaternary
AGACAAACAA	0	0	0	1	0	Chr1:+1999224:quaternary,Chr5:+17516435:quaternary
AATGTGGACT	0	1	0	0	0	Chr1:+19781718:quaternary
GTGATTCACA	0	1	0	0	0	Chr1:+19606502:quaternary
GGCTGAAATT	0	0	0	0	1	Chr1:+19372536:quaternary,Chr3:+6014672:quaternary
AGTTAATGAA	1	0	0	0	0	Chr1:+19168385:quaternary
TAAAATTTAA	1	0	0	0	0	Chr1:+19015318:quaternary
CAATGAACTC	1	0	0	0	0	Chr1:+18980088:quaternary
TAAAGCAAGC	0	0	1	0	0	Chr1:+18962961:quaternary
TTTGACCCAT	1	0	0	0	0	Chr1:+1881777:quaternary,At2g41340.1:X:+366:quaternary
TTATTTTTAG	0	0	1	0	0	Chr1:+18773539:quaternary
GTTACGAAAG	0	1	0	0	0	Chr1:+18772279:quaternary,Chr4:+11871539:quaternary
TCAACCATAA	1	0	0	0	0	Chr1:+18763085:quaternary
AGTTGTAGAG	0	1	0	0	0	Chr1:+18750891:quaternary
TTTTAACTTT	0	0	0	1	0	Chr1:+18609343:quaternary,Chr5:+26211214:quaternary
ATATCTGGGG	1	0	0	0	0	Chr1:+18605977:quaternary
TTTGGTAAGA	0	1	0	0	0	Chr1:+18605846:quaternary,Chr2:+16334676:quaternary

ATACATAATC	0	0	1	0	0	Chr1:+18565117:quaternary
CACACCAATG	0	0	0	1	0	Chr1:+18454476:quaternary
CACACTTAAA	0	0	1	0	0	Chr1:+18441014:quaternary
TATATCGTTT	1	0	0	0	0	Chr1:+18348747:quaternary,At5g08320.1:X:+349:quaternary
GTATCATAAA	0	0	1	0	0	Chr1:+18324218:quaternary
AAGTAGTTCC	0	0	1	0	0	Chr1:+18245667:quaternary
CAAAGTTTAA	0	0	0	1	0	Chr1:+18168311:quaternary
CTTGAATCAA	0	0	1	0	0	Chr1:+18036322:quaternary
CAGAACCATA	0	0	1	0	0	Chr1:+17984317:quaternary
GAACTTGAAC	0	0	1	0	0	Chr1:+17905810:quaternary
TTAATATCTT	0	0	1	0	0	Chr1:+17699786:quaternary
GTTATGAATA	0	1	0	0	0	Chr1:+17671534:quaternary
AAGGGATTTT	0	1	0	0	0	Chr1:+17636107:quaternary
GATTTTTTAA	0	1	0	0	0	Chr1:+17580983:quaternary,Chr3:+10297471:quaternary
AGGAATCTTC	0	0	1	0	0	Chr1:+17573712:quaternary
TGATAAAAGG	0	0	0	1	0	Chr1:+17486397:quaternary
CAAATAGATA	0	0	0	0	1	Chr1:+1747959:quaternary,Chr3:+6023529:quaternary
TGAATCTGCA	0	0	1	0	0	Chr1:+17433612:quaternary
TAATTTGACC	0	0	0	1	0	Chr1:+17414325:quaternary
ATTCGTTAAA	1	0	0	0	0	Chr1:+17413781:quaternary
CCTATTAAAA	0	1	0	0	0	Chr1:+1739566:quaternary
AATGAATTTT	0	0	0	0	1	Chr1:+17395254:quaternary
AGATAGTACT	0	0	0	0	1	Chr1:+17160344:quaternary
CTCATTAACA	0	0	1	0	0	Chr1:+17137295:quaternary
GCTAAATACT	0	0	0	1	0	Chr1:+17073304:quaternary
CTCATTCGTA	1	0	0	0	0	Chr1:+16969600:quaternary,Chr3:+17080683:quaternary,Chr1:+16594451:quaternary,Chr5:+13638104:quaternary,Chr5:+3115561:quaternary,Chr3:+12663353:quaternary,Chr2:+6686792:quaternary,Chr5:+2188093:quaternary,Chr2:+5878160:quaternary,Chr5:+15403965:quaternary,Chr2:+6861006:quaternary,Chr5:+26501506:quaternary
TACCTCACTA	1	0	0	0	0	Chr1:+16888728:quaternary,Chr1:+43206:quaternary
ATGCGTGTTT	0	1	0	0	0	Chr1:+16888265:quaternary
CTGAAGATCG	0	1	0	0	0	Chr1:+16821424:quaternary
TGTCGACAAA	0	1	0	0	0	Chr1:+16801322:quaternary
TGTTCAACCA	0	1	0	0	0	Chr1:+16770534:quaternary
TGGTATCTTT	0	1	0	0	0	Chr1:+16706372:quaternary
AGCTTATCTC	0	0	1	0	0	Chr1:+16706337:quaternary
ATGAGATTAT	0	1	0	0	0	Chr1:+16693377:quaternary
CTGACGTTTT	0	1	0	0	0	Chr1:+1660837:quaternary
AAGTAAATTA	0	0	0	1	0	Chr1:+16406706:quaternary
ACAACAAGAC	1	0	0	0	0	Chr1:+16396181:quaternary
AAATGACGTT	0	1	0	0	0	Chr1:+16365705:quaternary
ATGCCACAGC	0	0	1	0	0	Chr1:+1616855:quaternary
CAGAACCGTC	1	0	0	0	0	Chr1:+16149496:quaternary,Chr1:+16148766:quaternary
GGTGGTCCAT	0	0	1	0	0	Chr1:+16148079:quaternary
TCTACGCATT	0	0	1	0	0	Chr1:+16118013:quaternary
ATGATGTTAC	0	0	0	1	0	Chr1:+1605436:quaternary
TGGTTGTGAT	1	0	0	0	0	Chr1:+16025148:quaternary,Chr4:+2827810:quaternary,Chr4:+16565348:quaternary
AGAAAAATTA	0	0	1	0	0	Chr1:+15909390:quaternary,Chr5:+1670041:quaternary,Chr4:+5181484:quaternary
TCGTTAAACA	0	1	0	0	0	Chr1:+15893285:quaternary
ATCATTCGGT	0	0	0	1	0	Chr1:+1588894:quaternary
CAATTGCAAA	0	0	0	0	1	Chr1:+15863568:quaternary
TCGAGGAAGT	0	1	0	0	0	Chr1:+1578022:quaternary
GTTTGAAAGA	0	0	0	1	0	Chr1:+15555806:quaternary
TGAAAATAAA	1	0	0	0	0	Chr1:+1553205:quaternary,At1g59590.1:X:+1195:quaternary,Chr3:+16749553:quaternary,Chr1:+8987775:quaternary,Chr4:+15477846:quaternary
GTCATACATA	0	0	0	1	0	Chr1:+1549103:quaternary
TTCAGTTGCA	0	0	0	0	1	Chr1:+146402:quaternary
ATAATGATCC	0	1	0	0	0	Chr1:+14333985:quaternary,ChrC:+47981:quaternary,At5g63550.1:X:+618:quaternary
AGTTTATAAA	0	0	0	0	1	Chr1:+14302474:quaternary,Chr5:+25804045:quaternary,Chr5:+17983931:quaternary
TACTCAACCT	0	0	0	0	1	Chr1:+14044167:quaternary
ATAAGTTTTA	0	0	0	1	0	Chr1:+13884186:quaternary

GATGATGTGT	0	0	1	0	0	Chr1:+1383069:quaternary
TTTAGACAAA	0	0	1	0	0	Chr1:+13391209:quaternary
TGAAATGTTT	0	0	0	1	0	Chr1:+13389565:quaternary
ATGAAGATTT	1	0	0	0	0	Chr1:+13352336:quaternary
AATCCTTAAC	0	1	0	0	0	Chr1:+13325101:quaternary
ATCATTTTTG	0	0	0	1	0	Chr1:+13237503:quaternary,Chr2:+1725007:quaternary
AAATCATATT	0	0	0	0	1	Chr1:+13107490:quaternary,Chr4:+17743538:quaternary
CCAAAAATAT	0	1	0	0	0	Chr1:+13087253:quaternary,Chr3:+17061490:quaternary
ACCTAATTGA	0	1	0	0	0	Chr1:+12926307:quaternary
CCTCTTGCAC	0	1	0	0	0	Chr1:+12919743:quaternary,Chr3:+12669763:quaternary
GTAAAAGAGA	0	0	0	0	1	Chr1:+12832940:quaternary,Chr4:+10547346:quaternary
TGTAATCATT	0	1	0	0	0	Chr1:+1274475:quaternary
TTCCTCGTTC	0	0	0	1	0	Chr1:+12728710:quaternary
TGAAAGTGGA	0	0	1	0	0	Chr1:+12630539:quaternary,At3g54820.1:X:+441:quaternary
TTAAGGTAAC	1	0	0	0	0	Chr1:+12601027:quaternary
TTTATACACC	0	0	0	0	1	Chr1:+12549037:quaternary
GACCGTGGTC	0	1	0	0	0	Chr1:+12479929:quaternary
TTAAGATTTA	0	1	0	0	0	Chr1:+12478787:quaternary
AGTACGTTGA	0	0	1	0	0	Chr1:+12382755:quaternary
ACAATCTTCT	0	1	0	0	0	Chr1:+12361769:quaternary
TTCTAAATTA	0	0	0	1	0	Chr1:+12290805:quaternary,Chr3:+8111387:quaternary
ATATACATAT	0	0	0	0	1	Chr1:+12060991:quaternary
AGGGAGTTTT	0	0	0	0	1	Chr1:+12056455:quaternary
CTTCGTCTTG	0	0	0	1	0	Chr1:+12031518:quaternary
ACGTGTCGCA	1	0	0	0	0	Chr1:+11979827:quaternary
TCCAATTAGT	0	1	0	0	0	Chr1:+11867873:quaternary
TACTGTTGCT	1	0	0	0	0	Chr1:+11824939:quaternary
CAGAAAAGAA	0	0	1	0	0	Chr1:+11803819:quaternary
GAATATATAG	0	0	0	1	0	Chr1:+11794125:quaternary
TGCCTTCGCT	0	1	0	0	0	Chr1:+11670691:quaternary
GTGAAAAAAT	0	0	0	1	0	Chr1:+11636283:quaternary
AACAAATACA	1	0	0	0	0	Chr1:+11619316:quaternary
TCAAGAGCAT	0	0	0	1	0	Chr1:+11592453:quaternary
CTTTACACTT	0	1	0	0	0	Chr1:+11579579:quaternary
TTTTCGAAAA	0	1	0	0	0	Chr1:+11571846:quaternary
TGAGATATTC	0	1	0	0	0	Chr1:+11473542:quaternary
AACCTCCTTT	1	0	0	0	0	Chr1:+11435650:quaternary
GACTTCAAAT	0	0	1	0	0	Chr1:+11380676:quaternary,Chr4:+2658573:quaternary,Chr4:+8780428:quaternary
AGAATAATTA	0	1	0	0	0	Chr1:+1135529:quaternary
CCTTGAATTT	0	0	0	1	0	Chr1:+11309679:quaternary
TGGAAGAGTA	0	0	1	0	0	Chr1:+11289456:quaternary
ATAGTCGAAG	0	1	0	0	0	Chr1:+11193235:quaternary
AGAAATGAGA	0	0	1	0	0	Chr1:+11140036:quaternary
TGAATATGAA	0	0	0	0	1	Chr1:+11131222:quaternary,At3g16785.1:X:+619:quaternary
AATTTACAGA	0	0	0	1	0	Chr1:+11089308:quaternary
TCTCCCTTCC	0	0	1	0	0	Chr1:+11072850:quaternary
GGTCAACTAT	1	0	0	0	0	Chr1:+11070471:quaternary
TGTTTTCTGC	0	1	0	0	0	Chr1:+10964598:quaternary,Chr1:+1101156:quaternary
AGAGTTTGAT	0	1	0	0	0	Chr1:+10942699:quaternary
TCCTTTAACA	1	0	0	0	0	Chr1:+10897341:quaternary
TGGTGTTGAC	0	0	0	0	1	Chr1:+10840431:quaternary
CAAAGTGTAA	0	0	0	0	1	Chr1:+10820049:quaternary
AGAGGAGAAA	0	0	0	0	1	Chr1:+10819593:quaternary
TGGAAGAGAC	0	0	0	1	0	Chr1:+10802273:quaternary
AGCATCCAAC	0	0	1	0	0	Chr1:+10704408:quaternary
GAGATGTATT	0	0	0	1	0	Chr1:+10694352:quaternary
GAGAACCAAC	0	0	0	1	0	Chr1:+10590878:quaternary
ATTGATCAAT	0	1	0	0	0	Chr1:+10581168:quaternary,At2g23000.1:X:+-245:quaternary
GCATTTTATT	0	1	0	0	0	Chr1:+10569904:quaternary
TTCGAATCAA	0	1	0	0	0	Chr1:+10557751:quaternary,Chr5:+26764841:quaternary,Chr2:+13276287:quaternary

AAAGAACTAT	0	0	0	0	1	Chr1:+10476509:quaternary
CCCACATAAT	0	1	0	0	0	Chr1:+10370829:quaternary
AGTTTGGTAT	0	0	0	0	1	Chr1:+10322245:quaternary,Chr3:+16315683:quaternary
CTTGCTCTGA	0	0	1	0	0	Chr1:+10250462:quaternary
TGCATTTTTT	0	1	0	0	0	Chr1:+1022753:quaternary
TTGAAACAAA	0	0	0	1	0	Chr1:+10195529:quaternary,Chr2:+2149227:quaternary,Chr1:+6016303:quaternary,Chr4:+16783558:quaternary
GCCACATAAA	0	1	0	0	0	Chr1:+10188172:quaternary,Chr4:+10649526:quaternary
GAGTTGTATT	0	0	1	0	0	AtMg01320:d:+1029:secondary,At2g07733.1:p:+107:secondary
GACTCCCAGG	0	1	0	0	0	AtMg00670:d:+436:primary
CTATCCTTAC	0	1	0	0	0	AtMg00620:d:+34:primary,AtMg00620:d:+34:primary
AAACGATCGA	1	0	0	0	0	AtMg00590:d:+800:primary
CTTACTATCA	0	0	0	0	1	AtMg00550:d:+268:secondary,At2g07714.1:v:+696:secondary,At1g79730.1:d:+200:secondary
CTATGGATGT	0	1	0	0	0	AtMg00510:d:+365:secondary,At2g07709.1:p:+2392:secondary
GGCTTCATTT	0	1	0	0	0	AtMg00410:d:+818:primary,At2g07699.1:p:+818:primary
GGTAGCTTGG	0	0	0	1	0	AtMg00310:d:+123:secondary,At2g07737.1:p:+263:secondary
TTCCCAGAAA	0	0	1	0	0	AtMg00180:d:+1087:primary,At2g07723.1:p:+2046:primary
GCAATTAGGA	1	0	0	0	0	AtMg00160:d:+60:secondary,At2g07695.1:v:+410:secondary
CAATACTTTT	0	0	0	1	0	AtMg00090:d:+718:primary
ATGTTCATAT	0	0	0	0	1	AtCg01130:d:+223:secondary,AtCg01130:d:+223:secondary,At2g07739.1:v:+570:secondary,AtMg00370:d:+220:secondary
GTGTTCTTCG	1	0	0	0	0	AtCg01110:d:+71:secondary
GGATCTTCGT	0	0	0	1	0	AtCg01110:d:+693:primary
GTACGGGGTT	0	0	0	0	1	AtCg01080:d:+360:primary
GAATAAGGTT	0	1	0	0	0	AtCg00900.1:X:+683:quaternary
GGTGGGGCAA	0	1	0	0	0	AtCg00890:d:+190:secondary,AtCg00890:d:+190:secondary
CGAAATTATA	0	1	0	0	0	AtCg00890:d:+1030:primary,AtCg00890:d:+1030:primary
GAAGAATTGA	0	0	0	1	0	AtCg00870:d:+20:secondary,AtCg00870:d:+20:secondary
CGGGTTCCCC	1	0	0	0	0	AtCg00730:d:-91:primary
TAACAGGATT	1	0	0	0	0	AtCg00680:d:+473:secondary
ACTCGTTTAG	1	0	0	0	0	AtCg00680:d:+199:secondary
CCTACTTTTT	0	1	0	0	0	AtCg00680:d:+1078:secondary,At5g45670.1:d:+625:secondary,At3g50910.1:d:+282:secondary
GACTAGCTGT	0	1	0	0	0	AtCg00570:d:+56:primary
GTCTCTGCGG	0	1	0	0	0	AtCg00500:d:+835:secondary
CTTTTTTTCC	0	0	1	0	0	AtCg00500:d:+1442:primary
TACTACTTAC	0	0	0	1	0	AtCg00360.1:X:-1379:quaternary
CCCCCTTATC	0	0	0	1	0	AtCg00350:d:+1114:secondary
CTATTTGGGA	0	0	0	1	0	AtCg00340:d:+269:secondary
GAAAATTACA	0	0	1	0	0	AtCg00330:d:+134:secondary
ATAAAATCCC	0	0	1	0	0	AtCg00300.1:X:+-90:quaternary
TATGGTTAGG	1	0	0	0	0	AtCg00280:d:+713:secondary
GTGCTACTGT	1	0	0	0	0	AtCg00270:d:+647:secondary
GGAAGAGGTT	0	0	0	1	0	AtCg00190:d:+1536:secondary
AGATTTATGC	0	0	0	1	0	AtCg00180:d:+1895:primary
GGCATTACAG	1	0	0	0	0	AtCg00170:d:+3335:primary
TAAAACATCA	0	0	0	0	1	AtCg00170:d:+2849:secondary,At3g33004.1:p:+1226:secondary
AGAACTCGGA	0	1	0	0	0	AtCg00170:d:+1691:secondary,At3g33004.1:p:+70:secondary
CCCAAGTTCT	0	0	1	0	0	AtCg00150:d:+116:secondary,At3g33000.1:p:+116:secondary
AGTATTGTCC	0	1	0	0	0	AtCg00120.1:X:--2:quaternary
AATAGTCATA	0	0	0	1	0	AtCg00050.1:X:--45:quaternary
GCAGATAGGT	0	1	0	0	0	At5g67540.1:d:+1550:primary,At5g67540.2:d:+1633:primary
TAAATGATTG	0	0	1	0	0	At5g67490.1:d:+123:secondary
TTGAAGCAAG	0	0	1	0	0	At5g67480.1:d:+418:secondary,At5g67480.2:d:+528:secondary
TGTAGTATAT	0	0	1	0	0	At5g67470.1:X:-1152:quaternary
ACTCGTTTTT	0	0	0	1	0	At5g67470.1:i:+1868:tertiary,At4g35420.1:i:+1032:tertiary
GATAATAATG	0	1	0	0	0	At5g67460.1:X:--235:quaternary,At5g55300.1:X:--30:quaternary
TCCTTTATCT	0	1	0	0	0	At5g67420.1:d:+776:primary
TTGGAGAAGT	0	0	1	0	0	At5g67410.1:v:+839:secondary
TCAAAGAACA	0	0	0	0	1	At5g67410.1:v:+1474:primary
GACCCAACCA	0	0	1	0	0	At5g67400.1:d:+785:secondary

AAGCCTGAAG	0	0	0	0	1	At5g67385.1:X:+955:quaternary
ACGACAATGG	0	1	0	0	0	At5g67380.1:X:--27:quaternary
GCGCACGCTT	0	0	0	1	0	At5g67380.1:d:+1173:secondary
ATGGTAAGTT	0	0	0	1	0	At5g67340.1:i:+444:tertiary,At3g50140.1:i:+1312:tertiary,At3g50190.1:i:+1424:tertiary,At1g73720.1:i:+4374:tertiary,At1g07290.1:i:+892:tertiary
ATACACATCT	0	0	0	1	0	At5g67340.1:d:+2306:primary
AGTTTTTAAT	0	0	0	1	0	At5g67310.1:i:+943:tertiary
GACCTTTGAT	0	1	0	0	0	At5g67310.1:d:+2724:primary
CCCAAATCTG	0	0	0	1	0	At5g67310.1:d:+1476:secondary
TCCTGGTATG	0	1	0	0	0	At5g67310.1:d:+1352:secondary
ACGGAGATGC	0	0	1	0	0	At5g67300.1:d:+907:secondary
GAGTCCTGAA	0	1	0	0	0	At5g67300.1:d:+114:secondary,At2g23290.1:d:+144:secondary
TGATTTTACT	1	0	0	0	0	At5g67290.1:i:+887:tertiary,At2g31510.1:i:+3362:tertiary
GTCGGATCCG	0	0	0	1	0	At5g67280.1:X:-906:quaternary
CAAAACACCC	0	0	1	0	0	At5g67280.1:d:+912:secondary
ATGATTCTCC	0	1	0	0	0	At5g67240.1:v:+1924:secondary
AAACTTGTAC	1	0	0	0	0	At5g67240.1:v:+1119:secondary,At1g26720.1:v:+117:secondary
ACCGAGACTT	0	0	1	0	0	At5g67230.1:d:+1785:primary
GTGGAGCCAC	1	0	0	0	0	At5g67230.1:d:+1223:secondary
TGGGGTTGGA	0	0	1	0	0	At5g67200.1:d:+2124:primary
GAAGGCGGCG	0	1	0	0	0	At5g67190.1:v:+354:primary
GACGATGATG	0	0	0	0	1	At5g67170.2:d:+375:secondary,At5g37475.1:d:+850:secondary,At5g67170.1:d:+383:secondary,At5g26860.1:d:+780:secondary,At4g18300.1:v:+1878:secondary
AGAATCACGC	1	0	0	0	0	At5g67140.1:i:+1044:tertiary
ACCGCCATTA	0	0	1	0	0	At5g67140.1:d:+430:secondary
CAAGCACACA	0	0	1	0	0	At5g67100.1:v:+4511:secondary,At5g20910.1:d:+880:secondary
TTTCATCTAA	0	1	0	0	0	At5g67100.1:v:+2745:secondary,At1g36110.1:p:+1477:secondary
AATACAAAAC	0	1	0	0	0	At5g67090.1:v:+2749:primary
AATACAATTT	0	1	0	0	0	At5g67090.1:v:+2701:secondary
ATCTCAAATC	0	1	0	0	0	At5g67080.1:v:+1553:secondary,At5g56270.1:d:+2189:secondary
GCAGCTTCGT	0	1	0	0	0	At5g67070.1:d:+29:secondary
GAGAGTCTTT	0	0	0	1	0	At5g67060.1:d:-1034:secondary
AAAGCTCTAG	0	0	1	0	0	At5g67050.1:d:+759:primary
TGTTTAGTCC	1	0	0	0	0	At5g67030.1:d:+217:secondary,At5g67030.2:d:+217:secondary
TTTCACTCAG	1	0	0	0	0	At5g67010.1:v:+7:secondary
TACGTTAAGT	0	0	0	1	0	At5g66990.1:v:+253:secondary
CGGAGATTAA	0	0	1	0	0	At5g66980.1:i:+127:tertiary
AGCAGTTAGC	0	1	0	0	0	At5g66950.1:d:-1809:secondary
TAGTGTTTGT	0	1	0	0	0	At5g66910.1:i:+834:tertiary
TCATCGTCAA	0	0	0	0	1	At5g66890.1:v:+1880:secondary
ACATCCTCAG	0	0	1	0	0	At5g66850.1:d:+2125:primary
TGGTCGAGGA	1	0	0	0	0	At5g66840.1:X:-262:quaternary
GTTTTTAAGG	0	1	0	0	0	At5g66830.1:v:+957:secondary,At4g08970.1:p:+1918:secondary
AGAATGAGAC	1	0	0	0	0	At5g66830.1:v:+1092:secondary
TCTATTGCTT	1	0	0	0	0	At5g66820.1:d:+161:secondary
TTTCATCGAT	1	0	0	0	0	At5g66800.1:d:+743:primary
CTCCAGCTCT	0	1	0	0	0	At5g66770.1:d:+1375:primary
TGAACAGGCC	0	0	1	0	0	At5g66760.1:d:-1965:secondary
TGCTCCTCTG	0	1	0	0	0	At5g66760.1:d:-1861:secondary
AACGTAGTAG	1	0	0	0	0	At5g66730.1:d:+2040:secondary
GAAGGCAATA	0	0	0	1	0	At5g66710.1:v:+804:secondary,At1g53270.1:v:+152:secondary
TGTTTTTTAG	0	0	1	0	0	At5g66700.1:i:-679:tertiary
ATGAGCCCGT	0	0	0	1	0	At5g66680.1:d:+1122:primary
GCTGGTCTGA	0	0	1	0	0	At5g66650.1:d:+666:secondary,At5g11520.1:d:+1322:secondary
GAGAGAGTAC	1	0	0	0	0	At5g66640.1:i:+1728:tertiary
AATCAGATCA	0	1	0	0	0	At5g66640.1:d:+1284:primary
CTTGGCTTAG	0	1	0	0	0	At5g66640.1:d:+1045:secondary,At5g66630.1:d:+1838:secondary,At5g66610.1:d:+1244:secondary
TCTGAACAGA	0	1	0	0	0	At5g66630.1:d:+1654:secondary
AAGATTGAGA	0	0	1	0	0	At5g66620.1:v:+1644:secondary,At5g66630.1:d:+1546:secondary
TGGTTGGAGT	0	1	0	0	0	At5g66610.1:d:+1321:primary

GCCACAAAGT	0	0	1	0	0	At5g66570.1:d:-85:primary
GCAGCCTCTC	0	0	1	0	0	At5g66570.1:d:+90:secondary
TCATCAACTT	0	0	1	0	0	At5g66560.1:d:-215:secondary
AAGAAAGTGA	0	0	0	1	0	At5g66560.1:d:+1972:primary,At1g32390.1:v:+1332:primary,At2g12430.1:p:+549:primary
GGTAGAGAGT	0	0	0	1	0	At5g66540.1:X:-20:quaternary
AGAGAGCACG	0	1	0	0	0	At5g66540.1:d:+965:secondary
GAGAGAGATT	0	0	0	0	1	At5g66520.1:v:+1473:secondary
TTTGCGTATT	0	0	1	0	0	At5g66500.1:d:+15:secondary
GGGATTTGTA	0	0	1	0	0	At5g66490.1:X:-132:quaternary,At5g22740.1:X:-517:quaternary
TGAGTTATAA	0	0	1	0	0	At5g66460.1:d:+1736:secondary
ACACCAACTG	1	0	0	0	0	At5g66420.1:X:-490:quaternary
AGCAAATGTC	1	0	0	0	0	At5g66420.1:d:+496:secondary
CAAACAAAGC	0	0	1	0	0	At5g66390.1:X:-499:quaternary,At1g62680.1:X:-902:quaternary
ATATTACATC	0	1	0	0	0	At5g66360.1:d:+1125:primary,At5g66360.2:d:+1209:primary
GTGTCAGCAA	0	0	0	0	1	At5g66310.1:d:+2840:secondary
TCTAACCTGA	1	0	0	0	0	At5g66310.1:d:+2482:secondary
TCTGATAAAG	0	1	0	0	0	At5g66310.1:d:+1504:secondary,At3g51150.1:d:+1414:secondary
TCTAAAACGG	0	0	1	0	0	At5g66260.1:v:+112:secondary
GCTAGCTGCT	0	0	1	0	0	At5g66250.2:i:+1492:tertiary,At5g66250.1:i:+1490:tertiary
TTTGTGACAG	0	0	0	1	0	At5g66240.2:d:+1063:secondary,At5g66240.1:d:+968:secondary
CACACACTCA	0	1	0	0	0	At5g66240.1:i:+1636:tertiary,At5g66240.2:i:+1642:tertiary
AAAAAATCAC	0	0	0	0	1	At5g66210.1:X:-160:quaternary
ATTCAGAGAA	0	0	1	0	0	At5g66210.1:d:+1386:secondary,At5g66210.2:d:+1358:secondary
GCTGCTGCTA	0	0	0	0	1	At5g66190.1:d:+154:secondary,At1g79470.1:d:+301:secondary
ACGATGTCGG	0	0	1	0	0	At5g66150.1:v:+520:secondary
TGGAGAGATA	1	0	0	0	0	At5g66150.1:i:+5133:tertiary
CCAAGGCAAT	1	0	0	0	0	At5g66140.1:X:-43:quaternary
CCATAGAAAC	0	0	1	0	0	At5g66120.2:d:+1121:secondary,At1g68690.1:p:+2863:secondary,At5g66120.1:d:+699:secondary
TGAAAACAAG	0	0	1	0	0	At5g66120.2:d:+1035:secondary,At5g23955.1:p:+3423:secondary,At5g66120.1:d:+613:secondary
AAAGTCTTCG	0	0	1	0	0	At5g66120.1:d:+191:secondary,At5g66120.2:d:+613:secondary,At5g35735.1:d:+763:secondary,At2g47990.1:d:+950:secondary
TGATCAATTC	1	0	0	0	0	At5g66110.1:v:+714:secondary,At5g04690.1:d:+156:secondary
CATCTCACAC	0	0	0	1	0	At5g66090.1:d:-847:secondary
TGGAGAGTTT	0	0	0	1	0	At5g66055.2:d:+840:primary,At5g66055.1:d:+840:primary
TTTACTGTTT	0	0	0	0	1	At5g66052.1:d:+130:secondary,At3g04080.1:d:+604:secondary,At5g18280.1:d:+575:secondary
GTTTCCGGCA	0	1	0	0	0	At5g66050.2:d:+724:secondary,At5g66050.1:d:+416:secondary
CGGTACCATC	0	0	1	0	0	At5g66050.1:X:-151:quaternary
AATCGAGGAG	0	0	1	0	0	At5g66040.2:d:+175:secondary,At5g66040.1:d:+185:secondary
TTTGGGAGCG	0	1	0	0	0	At5g66010.1:d:+127:primary
TTTTGTTGTC	0	1	0	0	0	At5g66005.2:d:+1168:primary
GAGTTAGATA	0	0	1	0	0	At5g66000.1:d:+726:secondary,At1g03050.1:v:+1137:secondary
GCTTACGGAG	0	0	1	0	0	At5g65990.1:d:+923:secondary
TATGCCAACA	0	1	0	0	0	At5g65970.1:d:+1812:primary
TTTGGAGGTG	0	0	1	0	0	At5g65960.1:d:+1209:primary
GCAGTGAAGC	0	1	0	0	0	At5g65950.1:d:+1817:secondary
GCAAACGGAC	0	0	1	0	0	At5g65940.1:d:+1356:primary
AGAGCAATCC	0	0	1	0	0	At5g65930.2:d:+3074:secondary,At2g40430.1:d:+463:secondary,At5g65930.1:d:+3071:secondary
GCTACCACAG	0	1	0	0	0	At5g65930.1:d:+1018:secondary,At5g65930.2:d:+1018:secondary
CATTGGTCTG	0	1	0	0	0	At5g65920.1:X:+2110:quaternary
TTTGGATGTG	0	0	0	1	0	At5g65920.1:d:+1307:primary
TGCCATACTA	0	1	0	0	0	At5g65900.1:d:-2066:secondary
TTTGTACTAT	1	0	0	0	0	At5g65880.1:d:+254:primary
TTTTAATTAG	0	0	1	0	0	At5g65850.1:v:+1524:secondary,At5g14360.1:v:+1045:secondary,At1g22330.1:d:+29:secondary
AGCGGTGAGA	0	0	0	1	0	At5g65840.1:X:-279:quaternary
GAGTGACTTA	0	0	1	0	0	At5g65830.1:X:-909:quaternary
CTTCGTCGTT	1	0	0	0	0	At5g65830.1:X:-194:quaternary
CCTGTTTACT	0	1	0	0	0	At5g65830.1:d:+915:secondary
CACTGTACGA	0	0	0	0	1	At5g65820.1:X:-1952:quaternary
TTAAGGAGCA	0	0	1	0	0	At5g65750.1:d:+3152:secondary
AGGAAAGTAG	1	0	0	0	0	At5g65710.1:v:+3288:secondary
TTTTCGCCTT	0	1	0	0	0	At5g65687.1:i:+1035:tertiary

TGTTAGAGAG	0	1	0	0	0	At5g65660.1:X:-65:quaternary
TTTTGAATTT	0	0	1	0	0	At5g65620.1:i:+3062:tertiary,At1g24070.1:i:+1455:tertiary
TGCTGAAGAG	0	0	1	0	0	At5g65620.1:d:-1739:secondary
TTGGGATCAA	0	0	0	1	0	At5g65600.1:v:+874:secondary
ATGTCCTCGG	0	0	0	1	0	At5g65590.1:d:+589:primary
GCGTAAGCAG	0	0	0	0	1	At5g65570.1:v:+2849:primary
GCAGCAATTT	1	0	0	0	0	At5g65540.1:d:-1637:secondary
TGTAAAAGAG	0	0	0	1	0	At5g65540.1:d:+1972:secondary
GCTACAACAG	1	0	0	0	0	At5g65520.1:d:+99:primary
TGTTAATCTA	0	1	0	0	0	At5g65510.1:i:+1045:tertiary
GTGATAATAG	0	0	0	1	0	At5g65500.1:i:+494:tertiary
ACGGAGAAGA	0	1	0	0	0	At5g65495.1:d:+99:primary
AACAATTTTG	0	1	0	0	0	At5g65490.1:d:+1370:secondary
GCAAACAAGT	0	0	0	1	0	At5g65460.1:X:--34:quaternary,At3g25460.1:X:-1206:quaternary
GAATTCTCTT	0	0	0	0	1	At5g65460.1:v:+4377:primary
TTATTTGACA	0	1	0	0	0	At5g65460.1:i:+1552:tertiary
GAGAAACAAT	0	0	1	0	0	At5g65440.1:d:+2682:secondary
GCGAAACAGG	0	0	1	0	0	At5g65430.2:d:+688:secondary,At5g65430.1:d:+688:secondary
AACACTCTGT	0	1	0	0	0	At5g65420.1:d:+230:secondary
AATCGCGCAA	0	0	0	1	0	At5g65360.1:X:-410:quaternary
GGTTTTTCGA	0	0	1	0	0	At5g65350.1:d:+454:primary
TCGACTTTGA	1	0	0	0	0	At5g65310.1:X:-977:quaternary,At5g16210.1:X:-136:quaternary
GAACACCACA	0	0	1	0	0	At5g65310.1:X:-621:quaternary
AAAAGATCAG	0	1	0	0	0	At5g65280.1:X:+732:quaternary
TGTTGTAACT	0	0	0	1	0	At5g65270.1:d:+728:primary
AATGAAGTTT	0	0	0	0	1	At5g65240.1:v:+2292:primary
AACATTGCAA	0	0	1	0	0	At5g65240.1:v:+1582:secondary,At5g10290.1:d:+1417:secondary
TTATTTGTGT	0	1	0	0	0	At5g65230.1:d:+1085:primary
AAACATTGCA	0	1	0	0	0	At5g65210.2:d:+1460:secondary,At5g65210.1:d:+1222:secondary
TATTGATAAG	0	0	0	0	1	At5g65210.1:d:+1520:primary,At5g65210.2:d:+1758:primary
GTTTTCACGA	0	0	0	0	1	At5g65210.1:d:+148:secondary
AAAGATTTTC	0	0	0	1	0	At5g65205.1:d:+812:secondary,At2g17630.1:d:+1295:secondary
ATTCGGCTCT	0	1	0	0	0	At5g65200.1:d:+1212:secondary
ACTAGCAAGA	0	0	1	0	0	At5g65140.1:d:+542:secondary
ATGAAGATAA	0	0	1	0	0	At5g65100.1:v:+672:secondary
TTTTGATCAA	0	1	0	0	0	At5g65100.1:v:+1331:secondary
TTACTTGCTT	0	0	1	0	0	At5g65080.1:i:+1312:tertiary
TTTTAAAATC	0	1	0	0	0	At5g65080.1:d:+854:primary
TGATTCAAGA	0	1	0	0	0	At5g65050.1:i:+623:tertiary
TCAAAGATCA	0	0	0	0	1	At5g65050.1:d:+330:secondary,At5g65060.1:d:+220:secondary
ATTGATTCAA	0	0	1	0	0	At5g65010.2:i:-141:tertiary,At3g05630.1:i:-3970:tertiary,At5g65010.1:i:-141:tertiary
TTGGATGGAA	0	0	1	0	0	At5g65010.2:d:+383:secondary,At5g65010.1:d:+383:secondary
ATCTTTCAGA	1	0	0	0	0	At5g65010.1:d:-1447:secondary,At5g65010.2:d:-1435:secondary,At5g65010.1:d:-1435:secondary,At5g65010.2:d:-1447:secondary
AACAAAGCTC	0	0	1	0	0	At5g64990.1:X:-53:quaternary
CTCGTTTTCT	0	1	0	0	0	At5g64990.1:i:+672:tertiary
TGGTCAGTTG	0	0	0	1	0	At5g64960.1:d:+781:secondary
AAAATGTCAT	1	0	0	0	0	At5g64960.1:d:+356:secondary,At5g10270.1:d:+339:secondary
GTTGGCATTG	0	1	0	0	0	At5g64950.1:d:+1076:primary
CAAATAGCTT	0	1	0	0	0	At5g64940.1:i:-1576:tertiary,At5g64940.2:i:-1576:tertiary
ACCATTGAAA	0	0	1	0	0	At5g64905.1:d:+274:primary
GAATTTTTAA	0	0	1	0	0	At5g64900.1:i:+320:tertiary
CTTATGTCTC	0	0	1	0	0	At5g64870.1:d:+326:secondary
GTTGGACGAT	0	1	0	0	0	At5g64860.1:d:+1197:secondary
ACAGAGCCTT	0	0	1	0	0	At5g64840.1:d:+1061:secondary
TGTGTTTTGT	1	0	0	0	0	At5g64810.1:i:+737:tertiary,At3g17160.1:i:+409:tertiary,At5g49330.1:i:+987:tertiary,At2g31650.1:i:+5162:tertiary
AGAGGATGAA	0	0	1	0	0	At5g64760.1:d:+1686:primary
TGGTCTTTTT	0	0	1	0	0	At5g64760.1:d:+1612:secondary
CTTGGAGACG	0	0	1	0	0	At5g64740.1:X:+1019:quaternary
ATGGAGAGGT	0	0	0	1	0	At5g64730.1:d:+316:secondary

AATTGATTAC	0	1	0	0	0	At5g64670.1:d:+573:primary
ATATGTGTCG	0	0	1	0	0	At5g64660.1:X:-1305:quaternary,At3g02440.1:X:-260:quaternary
ACAGCGATTA	0	1	0	0	0	At5g64610.1:d:+1171:primary
ATGTGCGCAA	0	0	0	1	0	At5g64600.1:d:-445:secondary
GCAAGAGCAG	0	1	0	0	0	At5g64600.1:d:+1287:primary
TTCTATGAGA	0	0	0	0	1	At5g64510.1:d:+1470:primary
AAAGTTTGGA	0	1	0	0	0	At5g64490.1:d:+888:primary
AAGTTAATGT	0	0	0	0	1	At5g64470.2:d:+1421:primary,At5g64470.1:d:+1398:primary
AATGAAAAGC	0	0	1	0	0	At5g64450.1:v:+1266:secondary
TTATTTCGAT	0	1	0	0	0	At5g64440.1:d:+1479:primary
GTACTGCGGC	0	0	1	0	0	At5g64430.1:X:-1494:quaternary
AATTAAGCCA	0	0	0	1	0	At5g64430.1:d:+28:secondary
GGATGATCAG	0	0	1	0	0	At5g64430.1:d:+166:secondary,At1g51310.1:d:+1101:secondary,At5g16560.1:d:+934:secondary
AAAATTCAGA	0	1	0	0	0	At5g64420.1:d:+3143:secondary,At3g12340.1:v:+176:secondary
AGTATGAACT	0	0	0	0	1	At5g64410.1:i:+1915:tertiary
AACTGGTTTT	0	0	0	1	0	At5g64410.1:d:+1863:secondary
GAACTGAGAT	0	1	0	0	0	At5g64390.1:X:+374:quaternary
CCTTCATCCT	0	0	1	0	0	At5g64390.1:d:+2620:secondary,At5g64390.2:d:+3159:secondary
GCTCTGAACA	0	1	0	0	0	At5g64380.1:i:+1327:tertiary
CTTGCAAAAG	0	0	0	1	0	At5g64380.1:d:+392:primary
CGGTCTTGGT	1	0	0	0	0	At5g64340.1:d:+1098:secondary
AGAGGAAACG	0	0	1	0	0	At5g64330.1:d:+1919:secondary
AAATAACGTA	0	0	0	1	0	At5g64270.1:X:+4065:quaternary
CCGTCTAGAT	0	0	1	0	0	At5g64260.1:d:-1030:primary
ACTTATAGCT	0	0	1	0	0	At5g64240.2:d:+1102:secondary,At5g64240.1:d:+1189:secondary
ATGGACACAA	1	0	0	0	0	At5g64240.1:d:+1692:secondary,At1g07380.1:v:+2292:secondary,At5g64240.2:d:+1537:secondary
ACAATGACCC	0	0	0	0	1	At5g64190.1:v:+1473:secondary
AATAGTTGAA	0	0	1	0	0	At5g64170.1:i:+548:tertiary
TAATCTACGA	0	0	0	1	0	At5g64150.1:X:-37:quaternary
AGCGAGCATA	0	0	1	0	0	At5g64130.1:d:+307:secondary
GCCAAGCCAC	0	0	1	0	0	At5g64120.1:d:+124:secondary
GGAGCTGTTT	0	1	0	0	0	At5g64090.1:d:+1104:secondary
GAGATTACCA	1	0	0	0	0	At5g64050.1:X:-306:quaternary
GCCGCCATTA	1	0	0	0	0	At5g64040.1:X:-102:quaternary
GGTTTAATCA	0	0	0	1	0	At5g64020.1:i:+268:tertiary
TAAATGGAAC	0	0	0	0	1	At5g64020.1:d:+988:primary
ATAATTTCCC	0	0	1	0	0	At5g64010.1:d:+762:primary
GAGCTTATAT	0	0	1	0	0	At5g64000.1:i:+125:tertiary,At1g17020.1:i:+372:tertiary
ACTTATCCAG	0	0	1	0	0	At5g63980.1:d:+855:secondary
TTTATACTTC	1	0	0	0	0	At5g63970.1:d:+1393:primary
TCTTTTGAAA	1	0	0	0	0	At5g63960.1:v:+1440:secondary
GCGAATAATT	0	1	0	0	0	At5g63930.1:d:+3376:primary
TGGGAGATTT	0	1	0	0	0	At5g63930.1:d:+2843:secondary,At3g27860.1:v:+538:secondary
TAATTTATAT	0	1	0	0	0	At5g63920.1:i:+6180:tertiary
AAGATTAGGG	0	0	0	0	1	At5g63920.1:i:+3655:tertiary
ACTGTACAAT	1	0	0	0	0	At5g63890.1:d:+1363:primary,At5g63890.2:d:+1352:primary
ATTCGATTTT	0	0	0	1	0	At5g63870.2:i:+173:tertiary,At5g63870.3:i:+173:tertiary,At5g63870.1:i:+147:tertiary,At4g36080.1:i:+5301:tertiary
CCTGATTGTG	0	0	0	1	0	At5g63860.1:X:-162:quaternary
TGGATGGAGA	0	0	1	0	0	At5g63860.1:d:+1170:secondary
CTTATGCCGT	0	0	1	0	0	At5g63850.1:d:+1456:secondary
CTATGTGGTT	0	0	1	0	0	At5g63850.1:d:+1132:secondary
GAAGAAGGAA	0	0	0	0	1	At5g63840.1:d:+63:secondary
TATGTAATAA	0	0	0	1	0	At5g63830.1:d:+72:secondary
AGTCTTGAAA	0	0	0	1	0	At5g63820.1:v:+1002:primary
GAGAGGAGAT	0	0	1	0	0	At5g63800.1:d:+1856:secondary,At1g19940.1:d:+334:secondary,At1g13990.1:d:+448:secondary,At2g44540.1:d:+183:secondary
GAAGCAGAGA	0	0	0	0	1	At5g63800.1:d:+1740:secondary,At1g01260.1:d:+2019:secondary,At4g33620.1:v:+2524:secondary
GACGTGTCAT	0	0	0	1	0	At5g63780.1:d:+2127:primary
ATGTTATATT	0	0	0	0	1	At5g63770.1:X:+645:quaternary,Chr2:+18293257:quaternary,At3g26100.1:X:+-142:quaternary
CAGAGTATGA	0	1	0	0	0	At5g63750.1:d:+1353:primary

TTTCTGAATT	0	1	0	0	0	At5g63720.1:i:+330:tertiary
CGGTTGAAGA	0	1	0	0	0	At5g63700.1:v:+1072:secondary,At5g22570.1:d:+144:secondary
AGCGTATCGT	0	0	0	0	1	At5g63670.1:d:+177:secondary
GCTTCCATCG	0	1	0	0	0	At5g63660.1:v:+535:secondary
GTACTTAAAG	1	0	0	0	0	At5g63650.1:d:+1001:secondary
TCGAGATGAG	1	0	0	0	0	At5g63630.1:v:+1714:secondary
GTTACAAAAT	0	1	0	0	0	At5g63630.1:v:+1320:secondary,At5g42690.1:v:+1053:secondary,At2g21730.1:v:+1498:secondary,At3g28520.1:v:+1835:secondary,At2g21890.1:v:+1495:secondary
CGGTGGTGAA	0	1	0	0	0	At5g63580.1:d:+104:secondary
AGGATCATTA	0	1	0	0	0	At5g63550.1:d:-955:primary
AAAAGACTGA	0	1	0	0	0	At5g63550.1:d:+999:primary
CAGTGTTTGT	0	0	1	0	0	At5g63530.1:X:+940:quaternary
CTGCGATCAT	0	0	1	0	0	At5g63530.1:d:+734:secondary
GCATTTGCTG	0	0	1	0	0	At5g63530.1:d:+1178:secondary
GATGTGAACT	0	0	0	1	0	At5g63510.1:i:+1000:tertiary
GAGTAATTCA	0	1	0	0	0	At5g63480.1:d:-690:secondary
AAGCCAAAAA	1	0	0	0	0	At5g63480.1:d:+730:primary
GCCGCTACTT	1	0	0	0	0	At5g63470.1:X:-736:quaternary
TCCTTGTAGA	0	1	0	0	0	At5g63450.1:d:+1610:primary
GCTGGTGGGC	0	0	1	0	0	At5g63450.1:d:+1528:secondary
GATCTTCGAT	0	1	0	0	0	At5g63440.2:i:+1282:tertiary,At5g63440.1:i:+1282:tertiary
ACTAGTTTTT	0	0	1	0	0	At5g63420.1:X:+273:quaternary
GAAAGAACAG	0	1	0	0	0	At5g63420.1:d:+2038:primary
CGTCCAGGGG	0	1	0	0	0	At5g63420.1:d:+1885:secondary
AGATCCACCG	0	0	0	1	0	At5g63400.1:X:-99:quaternary
ATCCTTGAAG	0	1	0	0	0	At5g63380.1:i:+1715:tertiary
AAAGGTTATG	0	0	0	1	0	At5g63380.1:d:+1280:primary
ATGAGTTTCT	0	1	0	0	0	At5g63370.1:d:+2843:secondary
TGTAAATATG	0	0	0	0	1	At5g63370.1:d:+2459:secondary
AAGATCAAGA	0	0	0	1	0	At5g63370.1:d:+1256:secondary,At5g20370.1:v:+865:secondary,At2g06150.1:p:+949:secondary
GCCCTTCGTA	0	0	1	0	0	At5g63350.1:d:+1045:primary
TAGAAATAAT	1	0	0	0	0	At5g63330.1:d:+1332:primary
ATAATGGGGA	0	1	0	0	0	At5g63320.1:v:+1192:primary
ACTTGCATAT	1	0	0	0	0	At5g63310.1:i:+836:tertiary
TAGCTAGAGC	0	0	0	0	1	At5g63300.1:v:+1368:primary
ACAAGTGATC	0	1	0	0	0	At5g63300.1:v:+1199:secondary
TGAGGTACCT	0	0	0	0	1	At5g63290.1:d:+1397:primary
TTGATGTCTT	0	0	1	0	0	At5g63280.1:d:+318:secondary
ATCCTCAATC	0	0	0	1	0	At5g63280.1:d:+1314:primary
TTGAGAAGCC	0	0	1	0	0	At5g63260.1:d:+1468:secondary
GACGCATCAA	0	0	1	0	0	At5g63190.1:d:+1797:secondary,At5g63190.2:d:+1753:secondary
AGGTTGTGAA	0	1	0	0	0	At5g63190.1:d:+1225:secondary,At5g63190.2:d:+1181:secondary,At4g24800.1:d:+1183:secondary,At1g51420.1:v:+1210:secondary
AGAGGACGGT	0	0	1	0	0	At5g63180.1:X:-164:quaternary
ACTGCAAGCA	0	0	0	1	0	At5g63180.1:d:+620:secondary,At1g73960.1:d:+3660:secondary
GCTTTAGCAG	0	1	0	0	0	At5g63160.1:X:-89:quaternary
GCTTCACTCA	0	1	0	0	0	At5g63160.1:X:-61:quaternary
TCAAGCAAAA	0	0	0	0	1	At5g63160.1:d:+804:secondary
CTTATGGTAA	1	0	0	0	0	At5g63140.1:d:+1055:secondary
AAAACTACAC	0	0	0	0	1	At5g63120.2:X:-189:quaternary,At4g23640.1:X:--26:quaternary
ATATTTCTAA	0	0	0	0	1	At5g63120.2:d:+2127:primary,At5g63120.1:d:+2314:primary
ACAATGCTAA	0	0	0	1	0	At5g63120.2:d:+1744:secondary,At5g63120.1:d:+1931:secondary
TAGTGTCTTT	1	0	0	0	0	At5g63120.1:i:+1583:tertiary,At5g63120.2:i:+1583:tertiary
CATTAAACAA	1	0	0	0	0	At5g63090.4:X:-669:quaternary
GACTGTGTGT	1	0	0	0	0	At5g63090.4:d:+974:secondary,At5g63090.1:d:+970:secondary,At5g63090.3:d:+1100:secondary,At5g63090.2:d:+1029:secondary
GACTGTTCTT	0	0	0	0	1	At5g63090.4:d:+1018:primary,At5g63090.2:d:+1073:primary,At5g63090.3:d:+1144:primary,At5g63090.1:d:+1014:primary
TCAAGATAAG	0	1	0	0	0	At5g63080.1:X:-550:quaternary

TGGTTATCAT	0	0	1	0	0	At5g63040.1:d:+1358:primary,At5g63040.2:d:+1557:primary
AAGACCAAGA	0	0	0	1	0	At5g63020.1:X:-536:quaternary,At2g42390.1:X:-280:quaternary
TTGTTGTACT	1	0	0	0	0	At5g63010.1:d:+1146:primary
AGCTTCAGTT	0	1	0	0	0	At5g63000.1:X:-18:quaternary
ATTTCACAAC	0	0	0	0	1	At5g63000.1:X:+286:quaternary
AAGAAAGCAA	0	0	1	0	0	At5g63000.1:d:+572:primary
GAGCTATTCC	1	0	0	0	0	At5g62980.1:d:+126:secondary
CTATGGAGCC	1	0	0	0	0	At5g62960.1:d:+1122:primary
AGACCACCAC	0	0	1	0	0	At5g62940.1:X:-772:quaternary
CCAAAGACTT	0	0	1	0	0	At5g62940.1:d:+1017:primary
GAGAGTTTTC	0	0	0	1	0	At5g62900.1:d:+377:secondary
TTCTGATTTG	0	0	0	1	0	At5g62890.1:i:+2785:tertiary,At1g04680.1:i:+2289:tertiary,At5g62890.2:i:+2785:tertiary,At2g05630.1:i:+610:tertiary
GTGTCAGTTT	0	0	0	1	0	At5g62860.1:v:-349:secondary
TCTTTGTTTG	1	0	0	0	0	At5g62850.1:v:+211:secondary
GCAGAAGCAG	0	0	1	0	0	At5g62810.1:d:+1473:secondary
TTTCAGTTGA	0	0	1	0	0	At5g62760.1:X:--81:quaternary
TCGCTTGGTG	0	1	0	0	0	At5g62720.2:d:-1360:secondary,At5g62720.1:d:-874:secondary
ATCTGAAAAA	0	0	0	1	0	At5g62720.2:d:+950:secondary
CACAGAGAAC	0	0	1	0	0	At5g62670.1:d:+2805:secondary
GCGATGATGA	0	1	0	0	0	At5g62670.1:d:+2321:secondary,At1g60990.1:v:+354:secondary,At1g61000.1:v:+3657:secondary
ATGAGAAAAG	0	0	1	0	0	At5g62660.1:v:+23:secondary,At1g78490.1:d:+803:secondary,At3g54950.1:d:+2042:secondary
AAAAAACGGA	0	0	0	1	0	At5g62650.1:X:-74:quaternary
CTCTCAATAG	1	0	0	0	0	At5g62650.1:d:+1824:primary
AGATGACACA	0	1	0	0	0	At5g62620.1:d:+2426:primary
TGAGAGCAAG	0	0	0	1	0	At5g62610.1:d:+646:primary
TCTTATGACA	0	0	0	0	1	At5g62600.1:d:+2444:primary
CTTACTTGTT	0	1	0	0	0	At5g62600.1:d:+1682:secondary
TCTAATGTTT	0	0	1	0	0	At5g62560.1:d:+1970:primary
ATAGTGATGA	0	0	0	1	0	At5g62520.2:d:+1182:secondary,At5g62520.1:d:+1128:secondary
AACGGTGCTG	0	1	0	0	0	At5g62490.1:d:+350:primary
GCAAAACAAG	0	0	1	0	0	At5g62470.2:d:+708:secondary,At5g62470.1:d:+475:secondary
GTCTAATATC	0	1	0	0	0	At5g62470.1:d:+950:secondary,At5g62470.2:d:+1183:secondary
GTTGAGATGT	0	0	1	0	0	At5g62460.1:i:+312:tertiary
GTGCTGCTAG	0	0	1	0	0	At5g62440.1:d:+382:secondary
TTACTCTGTG	0	0	0	0	1	At5g62410.1:i:-2431:tertiary
GCAAAACTAG	0	1	0	0	0	At5g62410.1:d:+3200:primary
ACTTTGTTCC	0	1	0	0	0	At5g62400.1:v:+1442:primary
TACTGACGGA	0	0	1	0	0	At5g62360.1:d:+523:secondary
AGAGACTGCG	0	0	1	0	0	At5g62360.1:d:+374:secondary
ATCTTGTATT	0	0	1	0	0	At5g62270.1:d:+1349:primary
TTCACGAGTT	0	0	1	0	0	At5g62230.1:v:+3471:secondary,At5g50115.1:v:+907:secondary
GATTGAGATT	0	0	0	1	0	At5g62220.1:X:-432:quaternary,At4g11680.1:X:-61:quaternary,At3g61230.1:X:--61:quaternary
GGTGAAAAAT	0	0	1	0	0	At5g62190.1:d:+976:secondary
GCTCGGTTGA	0	0	1	0	0	At5g62190.1:d:+1103:secondary
GTAATACACA	0	1	0	0	0	At5g62180.1:X:-257:quaternary
GTTACTGCAC	0	1	0	0	0	At5g62130.1:d:+949:secondary
AAAGATATGA	1	0	0	0	0	At5g62090.1:d:+2129:secondary,At4g10590.2:d:+2332:secondary,At3g60240.1:d:+4144:secondary,At4g10570.1:d:+2274:secondary,At4g10590.1:d:+2332:secondary,At5g62090.2:d:+1728:secondary
TCTCAGAGAA	0	0	1	0	0	At5g62000.2:d:+2392:secondary,At5g62000.3:d:+2392:secondary,At5g62000.1:d:+2518:secondary,At3g33575.1:p:+2574:secondary
GGAGCAATTT	1	0	0	0	0	At5g61990.1:d:+3323:primary
AGACATAACT	0	1	0	0	0	At5g61980.1:v:+1209:secondary
TGAATTGTAA	0	0	0	1	0	At5g61970.1:i:-3238:tertiary
GGAGATCCTA	0	0	0	0	1	At5g61960.1:X:-12:quaternary
ACCATACTAA	0	0	0	0	1	At5g61910.2:X:-312:quaternary
AGAGGAGTGT	0	0	1	0	0	At5g61840.1:d:+1406:primary
CATTTGTGTT	0	0	1	0	0	At5g61830.1:i:+763:tertiary
AAGCTACAAT	0	1	0	0	0	At5g61810.1:d:+676:secondary
GAGCGGTTTG	0	1	0	0	0	At5g61800.1:d:+1275:primary

TTTGGGCCTG	0	0	1	0	0	At5g61790.1:d:+525:secondary
ATTTCTTCTT	0	0	0	0	1	At5g61780.1:i:+4671:tertiary
TCTTCCCCAT	0	0	0	0	1	At5g61770.2:d:-1105:secondary,At5g61770.3:d:-1078:secondary,At5g61770.1:d:-1102:secondary
CTTCCCTACA	0	1	0	0	0	At5g61770.2:d:+267:secondary,At5g61770.1:d:+267:secondary,At5g61770.3:d:+240:secondary
AACGTTTGTC	0	0	0	0	1	At5g61760.1:d:+1082:secondary
ACTTCTTGCT	0	0	1	0	0	At5g61750.1:v:+1332:primary
CTACTTAGGG	0	0	0	1	0	At5g61740.1:v:+2907:secondary
TGTGTTACAA	0	0	1	0	0	At5g61730.1:i:+2941:tertiary
GCCTTCTTCA	0	0	0	1	0	At5g61720.1:d:+302:primary
AGGAAGAGGT	1	0	0	0	0	At5g61700.1:v:+2776:secondary,At2g26790.1:v:+1771:secondary,At1g69340.1:d:+982:secondary,At1g12970.1:d:+1294:secondary,At4g06474.1:p:+256:secondary
TGATTGACTT	0	0	0	1	0	At5g61690.1:i:+2371:tertiary
AATTTGGAAA	0	0	0	1	0	At5g61605.1:d:+74:secondary
CTTGAGTTAG	0	1	0	0	0	At5g61570.1:d:+1001:primary
AATCGTTGTG	1	0	0	0	0	At5g61560.1:d:+2740:primary
TGGCGAGAAG	0	0	1	0	0	At5g61530.1:d:+1110:secondary,At5g61530.2:d:+1050:secondary
GCAAGAGCAC	0	0	1	0	0	At5g61510.1:X:-300:quaternary
GTGAGCTTTG	0	0	1	0	0	At5g61510.1:d:+975:secondary
AACCTGATGA	1	0	0	0	0	At5g61500.1:d:+685:secondary
AGACCATACT	0	0	1	0	0	At5g61490.1:v:+1529:primary
GCCTGCAAAA	0	0	0	0	1	At5g61460.1:d:+2806:secondary
CAAAAGAAGT	0	1	0	0	0	At5g61450.1:d:+1416:secondary
TGCTGCCTTT	0	1	0	0	0	At5g61440.1:d:+631:primary
TAGAACTGAG	1	0	0	0	0	At5g61430.1:X:-353:quaternary
TCCATTAATT	0	0	1	0	0	At5g61430.1:d:+1210:primary
GTTTCCAAGG	0	0	1	0	0	At5g61420.1:d:+897:secondary,At5g61420.2:d:+517:secondary
GTTGTATCTA	0	0	0	1	0	At5g61410.1:i:+2094:tertiary,At5g61410.2:i:+1963:tertiary
TCGGTCAACC	0	0	1	0	0	At5g61410.1:d:+852:secondary,At5g61410.2:d:+721:secondary
AAAGAAACAC	0	0	0	1	0	At5g61390.1:d:+1632:primary
AGATTCTTTT	0	0	0	0	1	At5g61370.1:d:+1769:primary
TAGGATTTAA	0	0	1	0	0	At5g61350.1:v:+2932:secondary
GAAGATGATG	0	1	0	0	0	At5g61330.1:d:+279:secondary,At5g61940.1:v:+369:secondary,At4g11740.1:d:+783:secondary,At2g17150.1:d:+249:secondary
AGGGATCCAA	0	1	0	0	0	At5g61330.1:d:+1032:secondary
TCGCCATTTT	1	0	0	0	0	At5g61320.1:X:-6:quaternary
AGCCTAAGAG	1	0	0	0	0	At5g61270.1:d:+757:secondary
AGAAAAGAGA	1	0	0	0	0	At5g61270.1:d:+1178:secondary,At2g46050.1:v:+1921:secondary,At2g05170.1:d:+2605:secondary,At1g28420.1:d:+1192:secondary
CCGATATTTA	0	0	0	1	0	At5g61260.1:v:+2155:secondary
ATTCGAAGAA	0	1	0	0	0	At5g61260.1:v:+2133:secondary
GTGTGAAATC	1	0	0	0	0	At5g61250.2:d:+1663:primary,At5g61250.1:d:+1619:primary
AATGTATCCG	0	1	0	0	0	At5g61230.1:d:-1269:secondary
CATCGAACAA	0	0	0	0	1	At5g61200.1:v:+1418:secondary
GGAGTGTATA	0	0	0	0	1	At5g61190.1:i:+4091:tertiary
TTTAAGTTGT	0	0	0	0	1	At5g61190.1:d:+2959:primary
GCTAGGAAGG	0	0	0	0	1	At5g61170.1:d:+243:secondary
ATCAGTGAAT	0	1	0	0	0	At5g61160.1:X:+1584:quaternary
GGGGTAGAGT	1	0	0	0	0	At5g61150.1:d:+532:secondary,At5g61150.2:d:+532:secondary
ATTTTTTTAA	0	0	0	1	0	At5g61150.1:d:+2171:secondary,At5g61150.2:d:+2165:secondary
AGGTAAGTTT	0	1	0	0	0	At5g61140.1:i:+2827:tertiary
CGTGTGATCA	0	1	0	0	0	At5g61140.1:d:-6323:secondary
TCAATGTCAA	0	0	0	0	1	At5g61140.1:d:+1888:secondary,At1g62130.1:v:+2754:secondary
GTTTTTGTTG	0	0	1	0	0	At5g61090.1:v:+288:secondary
GAGCTCCATA	0	1	0	0	0	At5g61010.1:d:+2310:secondary
TCTTCCGGGT	0	1	0	0	0	At5g61000.1:d:+1825:primary
AGAATGTTCC	1	0	0	0	0	At5g60980.2:d:+953:secondary,At5g60980.1:d:+870:secondary
ATACGGATCA	1	0	0	0	0	At5g60970.1:v:+1393:primary
ATCAAGCAAA	1	0	0	0	0	At5g60960.1:X:-1689:quaternary
TTCACGGCAC	1	0	0	0	0	At5g60950.1:d:+476:secondary
AACGCTGCTT	1	0	0	0	0	At5g60930.1:X:-3:quaternary

TGGATGTTCT	0	0	0	1	0	At5g60930.1:v:+3608:secondary
AACTATCTAT	0	0	0	0	1	At5g60930.1:v:+3015:secondary
ACGCAATTGT	0	0	1	0	0	At5g60920.1:X:-171:quaternary
CAGCTATCTA	0	0	1	0	0	At5g60910.1:i:+2768:tertiary
CTAAGCTCAA	0	0	1	0	0	At5g60910.1:d:+387:secondary
AATAATTAAT	0	0	1	0	0	At5g60910.1:d:+1058:primary
AGAAATGTTA	0	0	0	0	1	At5g60900.1:v:+2475:secondary
TTGCAAAGAA	0	0	1	0	0	At5g60890.1:d:+356:secondary,At2g26695.1:v:+77:secondary
TTTTCATATG	1	0	0	0	0	At5g60870.1:i:+881:tertiary,At5g60870.2:i:+861:tertiary
CCACATAAAT	0	0	1	0	0	At5g60850.1:d:-1270:secondary
CATACTGGAG	1	0	0	0	0	At5g60850.1:d:+989:secondary
AACGGAGTTG	0	0	0	1	0	At5g60850.1:d:+157:secondary
GCTTGGGATT	1	0	0	0	0	At5g60820.1:d:+1255:primary
TAAATGTGAA	1	0	0	0	0	At5g60810.1:v:+448:secondary
CAAATATATC	0	1	0	0	0	At5g60800.1:d:+1070:primary
TGGCATTTTT	0	0	0	1	0	At5g60770.1:v:+2255:primary
AAAGGAACTA	0	0	0	1	0	At5g60760.1:d:-1135:secondary
GGTGATCTCA	0	0	1	0	0	At5g60730.1:d:+1259:primary
TTGGACCGAC	0	0	1	0	0	At5g60720.1:X:-437:quaternary
AAATTCTGAA	0	0	0	1	0	At5g60700.1:d:+2218:primary
TTGAAGAGAT	0	0	0	1	0	At5g60690.1:d:+3772:primary
TGAGTTATGA	0	0	0	0	1	At5g60690.1:d:+3134:secondary
CGGCGGTAAC	1	0	0	0	0	At5g60680.1:d:-465:primary
AAGGAGTAGG	0	0	1	0	0	At5g60680.1:d:+512:secondary
GTTCATTTAG	1	0	0	0	0	At5g60660.1:d:+693:secondary,At4g00060.1:d:+50:secondary,At2g39010.1:d:+727:secondary
GAATTTCCCA	0	0	0	1	0	At5g60660.1:d:+1066:primary
TTCTGATGAA	0	1	0	0	0	At5g60610.1:d:+759:secondary,At3g49350.1:d:+925:secondary
GGATGCATTG	0	0	0	0	1	At5g60600.1:d:+2227:secondary,At5g60600.2:d:+2224:secondary
GATGGGATTA	0	1	0	0	0	At5g60600.1:d:+1106:secondary,At5g60600.2:d:+1106:secondary
CTTTGACTCT	1	0	0	0	0	At5g60580.1:d:+1525:primary,At5g60580.2:d:+1546:primary,At5g60580.3:d:+1868:primary
TCTAAGACTG	0	1	0	0	0	At5g60550.1:d:-281:secondary
AATCCATTAA	0	1	0	0	0	At5g60540.1:X:-280:quaternary,At2g35470.1:X:-21:quaternary,At1g06660.1:X:--39:quaternary
AAATTCGAAA	0	0	1	0	0	At5g60470.1:v:+1919:primary
CAGAGTTTGT	0	1	0	0	0	At5g60450.1:d:+1479:secondary
GGGAGCGGAT	0	0	1	0	0	At5g60360.1:d:+1128:secondary
CATTAATCCA	0	1	0	0	0	At5g60340.1:X:-204:quaternary
GCTGTGACTT	0	0	0	0	1	At5g60340.1:v:+619:secondary
TTTGTTACTC	0	0	0	0	1	At5g60320.1:v:+2340:primary
GGAAAAGGAG	0	0	1	0	0	At5g60300.1:d:+1080:secondary,At5g60300.2:d:+1110:secondary
GAAGAAGTGG	0	0	1	0	0	At5g60290.1:v:+738:secondary,At1g59700.1:d:+421:secondary,At4g24560.1:d:+1880:secondary,At1g59670.1:d:+416:secondary
TTTTGTAAAA	0	0	0	0	1	At5g60290.1:i:+596:tertiary
AATCTTTCAG	0	0	1	0	0	At5g60280.1:v:+1023:secondary
TTTCTGCACA	0	1	0	0	0	At5g60270.1:v:+2769:primary,At4g23000.1:v:+2761:primary
GTGGTTTTGA	1	0	0	0	0	At5g60260.1:v:+710:secondary,At1g01960.1:d:+1742:secondary
AGTTTTGCTA	0	1	0	0	0	At5g60250.1:d:+1546:secondary,At4g19670.1:d:+1312:secondary,At2g26135.1:v:+1360:secondary
CTCTCTGCTG	1	0	0	0	0	At5g60210.1:d:+1618:secondary
GGCCTGTATC	0	0	0	1	0	At5g60170.1:d:+920:secondary
ATCTCAGAAA	0	0	0	1	0	At5g60170.1:d:+2519:secondary
CGCTTCAGTT	0	1	0	0	0	At5g60150.1:v:+566:secondary
ACAGTCCTAT	0	0	0	0	1	At5g60100.1:v:+1585:primary
GTTAGAGGCT	1	0	0	0	0	At5g60050.1:d:+1589:primary
TCTTCTTGCC	1	0	0	0	0	At5g60040.1:i:+6367:tertiary
TCTATAAGAA	0	0	0	0	1	At5g60040.1:i:+5227:tertiary
TGTGACAAAA	0	0	1	0	0	At5g60010.1:v:+969:secondary
GAGATCGATT	0	0	1	0	0	At5g60010.1:v:+728:secondary
TGTAACTAAA	0	0	0	1	0	At5g60010.1:v:+134:secondary
TTTTCTGTTC	0	0	0	0	1	At5g60010.1:i:+2445:tertiary
TTGCTAAACT	0	0	1	0	0	At5g59990.1:v:+1160:secondary,At5g35965.1:p:+3326:secondary,At3g44980.1:v:+161:secondary
GCGGTCACAG	0	0	1	0	0	At5g59970.1:X:-313:quaternary
CCTTGTGGTA	0	0	0	1	0	At5g59950.1:d:+1168:primary,At5g59950.3:d:+1064:primary,At5g59950.2:d:+1279:primary

TTTTTCTGAT	0	0	0	0	1	At5g59940.1:v:+2273:secondary
TTTTGTGAAG	0	0	0	1	0	At5g59930.1:v:+2547:secondary
TTTTCTGATG	0	1	0	0	0	At5g59930.1:v:+2291:secondary
AGATTCTTCT	0	0	0	0	1	At5g59920.1:v:-1095:secondary,At4g34280.1:d:-2396:secondary
CTTCGCCAAC	0	1	0	0	0	At5g59910.1:d:-480:secondary
GGTTGGTTAA	0	0	1	0	0	At5g59900.1:v:+952:secondary
CAGAAAGCTG	0	1	0	0	0	At5g59900.1:v:+2658:primary
ACTTGCCTCT	0	0	0	1	0	At5g59850.1:X:-90:quaternary
TGAAAACGCG	0	0	1	0	0	At5g59820.1:d:-219:primary
AGAATGCAAA	0	0	1	0	0	At5g59810.1:d:+406:secondary
GTCCGAGGCA	1	0	0	0	0	At5g59800.1:d:+992:primary
TGTTCAAAGC	0	0	0	1	0	At5g59720.1:d:+246:primary
CACCTGAGAG	0	1	0	0	0	At5g59710.1:i:-319:tertiary
CAAGTTCTAT	0	0	0	0	1	At5g59710.1:d:+1859:secondary
ACTTGAAAAA	0	1	0	0	0	At5g59680.1:v:+239:secondary
ATAGTGATGT	0	0	1	0	0	At5g59650.1:v:+3490:primary
TTGCCAGAGG	1	0	0	0	0	At5g59650.1:v:+253:secondary
CGCTGTTGTT	0	0	0	1	0	At5g59616.1:v:+167:secondary
TCCGTTTATG	0	0	1	0	0	At5g59616.1:v:+142:secondary
AGTTTCCAAC	0	0	1	0	0	At5g59613.1:d:+452:secondary
AGAGAAGTAC	0	1	0	0	0	At5g59590.1:X:-423:quaternary
AACAATGTAA	1	0	0	0	0	At5g59590.1:d:+369:secondary
CCAAAGATGA	0	0	0	0	1	At5g59580.1:X:-179:quaternary
ATTCAAGCTG	0	1	0	0	0	At5g59560.1:d:+1005:primary,At5g59560.2:d:+1147:primary
GATAGGAGAG	1	0	0	0	0	At5g59540.1:X:-282:quaternary
GAGCTCTTGT	0	0	0	1	0	At5g59540.1:d:+824:secondary,At1g59900.1:d:+1249:secondary
GATTGCGTGA	0	0	0	1	0	At5g59540.1:d:+643:secondary
TATCAGAATA	0	1	0	0	0	At5g59530.1:d:+1295:primary
TTCTTCGTTT	0	0	0	0	1	At5g59520.1:d:+1161:primary
AATAGCATAA	0	0	0	0	1	At5g59500.1:d:-1416:secondary
GATGTTGTGA	0	1	0	0	0	At5g59500.1:d:+438:secondary
TGTTGTTGGT	0	1	0	0	0	At5g59450.1:d:+673:secondary
GCGTTCGGGA	0	1	0	0	0	At5g59430.1:d:+705:secondary,At5g59430.2:d:+899:secondary
GAGTAGGTGT	1	0	0	0	0	At5g59420.1:d:-1376:secondary
ACAGTCGGCT	1	0	0	0	0	At5g59400.2:d:-776:secondary,At5g59400.1:d:-784:secondary
TTTGACAGAG	0	0	0	1	0	At5g59400.2:d:+300:secondary,At5g59400.1:d:+308:secondary
TCTGTGAGTG	0	1	0	0	0	At5g59400.1:d:+789:primary,At5g59400.2:d:+781:primary
ACGACCAAAA	0	1	0	0	0	At5g59370.1:X:+-168:quaternary
TCTCTAACAA	1	0	0	0	0	At5g59370.1:d:-713:secondary
TTTCCTCCGT	0	0	0	1	0	At5g59360.1:v:+759:secondary
AGAGATGCTG	1	0	0	0	0	At5g59350.1:d:+993:primary
TGATGTGAGA	0	0	0	0	1	At5g59350.1:d:+323:secondary
AACATTTTGA	0	1	0	0	0	At5g59340.1:v:+1126:primary,At4g25315.1:d:+556:primary
TTAAAGATAA	0	0	0	0	1	At5g59330.1:v:+895:primary
GCTTCGCAAG	0	1	0	0	0	At5g59300.1:v:+452:secondary
AGCAAATTAA	0	0	1	0	0	At5g59290.1:X:-433:quaternary
GGTAGCTGTT	0	1	0	0	0	At5g59250.1:d:+1304:secondary
TTTATGGATT	0	0	0	1	0	At5g59240.1:d:+805:primary
AGTTTCTTGT	0	1	0	0	0	At5g59200.1:v:+2005:secondary,At3g33555.1:p:+601:secondary,At5g57050.1:d:+238:secondary
GAACTCGGTG	0	0	1	0	0	At5g59200.1:v:+1809:secondary
ATTAATTGTT	0	0	1	0	0	At5g59200.1:v:+1074:secondary
TTGCAGTTGA	0	0	1	0	0	At5g59160.2:d:+1448:secondary,At5g45030.1:d:+871:secondary
GATTCTTCAC	0	0	1	0	0	At5g59160.1:X:+316:quaternary
GACCCTGCCG	0	0	1	0	0	At5g59160.1:d:+228:secondary,At5g59160.2:d:+258:secondary
GTCTTGCATC	1	0	0	0	0	At5g59150.1:d:-904:secondary
TGATGCGACA	0	0	0	1	0	At5g59130.1:X:-61:quaternary
ATCAAATCTG	0	0	0	1	0	At5g59130.1:v:+1965:secondary,At1g60460.1:v:+1542:secondary
AAACAAAAAG	0	0	1	0	0	At5g59130.1:i:+714:tertiary
GCGAAAGGAA	0	0	0	1	0	At5g59100.1:d:+46:secondary
TGGACCCGAT	0	0	0	1	0	At5g59100.1:d:+1790:secondary
CCTATTGTAT	0	1	0	0	0	At5g59050.2:d:+3014:primary,At5g59050.1:d:+1302:primary

ACAAAGATTG	0	0	1	0	0	At5g59050.1:d:+495:secondary,At5g59050.2:d:+525:secondary
AACCTCAAGA	0	0	0	0	1	At5g59030.1:X:-678:quaternary
TAATAAATGA	1	0	0	0	0	At5g58990.1:d:+769:primary
GAAAAGCTTG	0	0	0	1	0	At5g58990.1:d:+331:secondary,At2g21250.1:d:+507:secondary,At5g51100.1:d:+788:secondary,At2g21250.2:d:+486:secondary,At1g49920.1:v:+2160:secondary,At2g32820.1:v:+1248:secondary,At5g51090.1:v:+21:secondary,At2g21260.1:d:+508:secondary,At5g52370.1:d:+362:secondary
TCACTTAAAT	0	1	0	0	0	At5g58940.1:v:+1839:primary
AAACATTCCG	0	0	0	1	0	At5g58930.1:X:-1773:quaternary
ATGGACTATA	0	1	0	0	0	At5g58930.1:d:+1960:secondary
GCGTTGAAGG	0	0	1	0	0	At5g58930.1:d:+1779:secondary
TTAAGGATCA	0	0	0	0	1	At5g58910.1:v:+891:secondary
GAGGAAACTA	1	0	0	0	0	At5g58900.1:d:+722:primary
AGCATCAAAT	1	0	0	0	0	At5g58880.1:v:+417:secondary
ATGACGAAAA	0	1	0	0	0	At5g58880.1:v:+3746:primary
GAGAATTTGA	0	1	0	0	0	At5g58880.1:v:+2382:secondary,At5g07950.1:d:+766:secondary
ATTCACATTA	0	1	0	0	0	At5g58860.1:d:+1594:primary
GGAATGAAGG	0	0	0	1	0	At5g58830.1:d:+241:secondary
AAAAATAAAT	0	0	0	1	0	At5g58820.1:v:+2783:primary
CGCCCCCAAA	0	0	0	1	0	At5g58760.1:X:-352:quaternary
AACTTAGAAA	0	0	0	1	0	At5g58720.1:d:+1236:secondary
AGAAATATAT	0	0	0	0	1	At5g58700.1:d:+1953:primary
GGAGGTATCT	0	0	1	0	0	At5g58670.1:d:+1200:secondary
TTCAGTGCCA	0	0	0	1	0	At5g58590.1:X:-306:quaternary
TTATAATTAC	0	1	0	0	0	At5g58575.1:X:+412:quaternary,Chr2:+3943142:quaternary
GAGATCGCAG	0	1	0	0	0	At5g58560.1:d:+604:secondary
GCCACAACAC	0	0	1	0	0	At5g58550.1:d:+2476:primary
AACTTTTTTT	0	1	0	0	0	At5g58520.1:X:+577:quaternary
TGAATTACTG	0	1	0	0	0	At5g58510.1:X:-28:quaternary
AGCTATGACA	0	0	0	0	1	At5g58510.1:i:+4460:tertiary
GCAAAAGAAT	0	1	0	0	0	At5g58510.1:d:+1138:secondary,At1g50810.1:p:+1187:secondary,At2g15650.1:p:+1130:secondary
TTGCTATAAA	0	0	1	0	0	At5g58470.2:X:-656:quaternary,At5g28510.1:X:-302:quaternary
ACCTTTTGGT	0	0	0	1	0	At5g58470.1:d:+1990:secondary,At5g58470.2:d:+2142:secondary
CCAGTGCTCC	0	0	0	0	1	At5g58450.1:d:-2604:secondary
GCGCAACAGA	0	1	0	0	0	At5g58450.1:d:+3106:secondary
GCGGAGAATG	1	0	0	0	0	At5g58430.1:d:+114:secondary
GAGTTGGAAG	0	0	0	0	1	At5g58420.1:X:+-42:quaternary
AGCTCGTGCA	0	1	0	0	0	At5g58380.1:X:-105:quaternary
ATCCTGAACC	0	1	0	0	0	At5g58360.1:v:+766:secondary
CCCTCACGAG	0	0	1	0	0	At5g58330.2:d:+780:secondary,At5g58330.1:d:+840:secondary,At5g58330.3:d:+755:secondary
CCGGTCGAAG	1	0	0	0	0	At5g58330.1:d:+1237:secondary,At5g58330.3:d:+1152:secondary,At5g58330.2:d:+1177:secondary
GATGATGTTG	0	1	0	0	0	At5g58300.1:d:+2067:primary
TCCTTAATAA	1	0	0	0	0	At5g58290.1:d:-1375:secondary
GAGATGGTAG	0	1	0	0	0	At5g58290.1:d:+327:secondary,At1g35350.1:v:+1242:secondary
GGAATATACT	1	0	0	0	0	At5g58260.1:X:-179:quaternary
AATTTAGCGT	1	0	0	0	0	At5g58250.1:d:-335:primary
CCTCATTTTA	0	1	0	0	0	At5g58240.2:d:+761:secondary
GCGACGGAGG	0	0	1	0	0	At5g58240.1:d:+21:secondary,At5g21900.1:v:+435:secondary
AGAGGATGGT	0	0	0	0	1	At5g58230.1:d:+1467:secondary
TATCAAAATT	0	0	1	0	0	At5g58220.1:d:+1067:secondary
TCGAATAAAG	0	0	0	0	1	At5g58210.1:d:+1850:secondary,At3g46340.1:v:+2337:secondary
TAAATGGAAG	1	0	0	0	0	At5g58200.1:d:+1059:primary
AATCCTACGG	0	0	0	1	0	At5g58180.1:v:-598:secondary
AAAATAGTCA	0	0	1	0	0	At5g58170.1:X:-228:quaternary
TTTGGTCAAT	1	0	0	0	0	At5g58160.1:v:+4519:secondary,At5g66675.1:d:+779:secondary
TAGAGCCCCT	0	1	0	0	0	At5g58140.1:d:+1598:secondary,At5g58140.4:d:+1598:secondary,At5g58140.3:d:+1654:secondary,At5g58140.2:d:+1654:secondary
GCAGAGGATT	0	0	1	0	0	At5g58130.1:d:+1192:secondary,At1g22730.1:d:+321:secondary
GTGGGTATTG	1	0	0	0	0	At5g58120.1:d:+565:secondary
CTTTTTACAA	0	0	0	0	1	At5g58100.1:i:-3024:tertiary
CTATACAAAT	0	0	0	0	1	At5g58100.1:d:+668:secondary
AGATTTCTGC	1	0	0	0	0	At5g58100.1:d:+2758:secondary,At4g17220.1:d:+441:secondary

AATTTGTTTT	0	0	1	0	0	At5g58070.1:d:+1018:primary
GTTTGGTCAG	0	1	0	0	0	At5g58040.1:i:+4405:tertiary
ATCTTGCAGA	0	0	1	0	0	At5g58040.1:d:+3449:primary,At4g37040.1:d:+800:primary
GAAGAGCAAT	0	0	0	1	0	At5g58030.1:i:+804:tertiary
GGAACATTCA	0	0	0	0	1	At5g58030.1:d:+530:primary
CGCTTGTTTT	0	0	0	1	0	At5g58000.1:d:-2855:secondary
AGCTCGTTTT	0	0	0	1	0	At5g57960.1:d:+23:secondary
GATGTGGATG	0	1	0	0	0	At5g57950.1:d:+456:primary
GTTGGCAATC	0	0	1	0	0	At5g57930.2:d:+1173:secondary,At5g57930.1:d:+1177:secondary
GCAAGATCCT	0	1	0	0	0	At5g57910.1:d:+733:primary
TCGATCACGA	0	0	1	0	0	At5g57880.1:v:+1985:primary
AAATCTTTAA	0	0	0	1	0	At5g57870.2:i:+1940:tertiary,At5g57870.1:i:+1938:tertiary
GGGTTTTTAG	0	0	1	0	0	At5g57850.1:d:+1248:secondary
GAATCAGTTT	0	0	0	0	1	At5g57830.1:X:-643:quaternary
CATCACTCTT	0	0	1	0	0	At5g57780.1:d:+588:secondary
CTCACTCTCT	1	0	0	0	0	At5g57770.1:d:+1168:primary
ATTTACCCAA	0	0	0	1	0	At5g57730.1:X:-104:quaternary
GCCGTGAGAT	0	0	1	0	0	At5g57710.1:d:+2249:secondary
CAGAAGAATG	0	0	1	0	0	At5g57685.1:X:+1065:quaternary
CTATGCATAA	1	0	0	0	0	At5g57660.1:d:-239:primary
AAAGGTTCTT	0	0	0	0	1	At5g57660.1:d:+614:secondary
TTGAAGCGTA	1	0	0	0	0	At5g57650.1:v:+472:primary
TATGATAACC	1	0	0	0	0	At5g57640.1:v:+744:secondary
ACGTTAAAGT	0	0	1	0	0	At5g57580.1:X:-850:quaternary
AATGGCCTGG	0	0	1	0	0	At5g57580.1:d:+1701:secondary
GATTCACTAG	0	0	1	0	0	At5g57565.1:d:+998:primary
TAGTTCCTCT	0	0	0	0	1	At5g57560.1:X:-520:quaternary
GCCGTTTTTA	0	1	0	0	0	At5g57550.1:i:+356:tertiary
ATCTATAACT	0	1	0	0	0	At5g57550.1:d:+827:primary
TGTTTCCTCG	0	0	0	0	1	At5g57550.1:d:+715:secondary
CTACGCTCAA	0	1	0	0	0	At5g57490.1:d:+605:primary
TTTTACACTA	0	0	0	0	1	At5g57440.1:d:+1121:primary
TCAAGAACCT	0	0	1	0	0	At5g57380.1:X:-737:quaternary,At3g24440.1:X:-926:quaternary
TCCAGAAGCT	0	0	1	0	0	At5g57380.1:v:+1153:secondary,At5g48310.1:v:+3142:secondary
ATGGTTCCTG	0	0	0	1	0	At5g57350.1:X:-6:quaternary
AATGGTGAAA	0	1	0	0	0	At5g57350.1:X:-49:quaternary,At5g59960.1:X:-483:quaternary
AAAAAGTGTT	0	0	0	1	0	At5g57350.1:i:+460:tertiary
GTTTTAGACT	0	0	1	0	0	At5g57350.1:d:+3053:secondary,At1g25540.1:d:+2816:secondary
GTTTTGATCA	0	0	1	0	0	At5g57350.1:d:+2076:secondary,At1g04450.1:v:+1025:secondary,At4g11250.1:v:+1105:secondary,At4g02235.1:v:+989:secondary,At5g65330.1:v:+1111:secondary
TATGGGCTTT	0	0	1	0	0	At5g57345.1:i:+594:tertiary
GGAACAAAGA	0	0	0	1	0	At5g57340.1:d:+386:secondary,At5g57340.2:d:+780:secondary
TAGAAATTCC	0	0	1	0	0	At5g57300.1:d:+759:primary
CCACAAGTTT	0	1	0	0	0	At5g57300.1:d:+210:secondary
AACCTCGTTT	0	1	0	0	0	At5g57280.1:d:+467:secondary
TACACTGCTG	0	0	0	0	1	At5g57260.1:X:-451:quaternary
GTCACAGGGA	0	1	0	0	0	At5g57220.1:d:+624:secondary
ATTGTTTTGA	0	1	0	0	0	At5g57220.1:d:+1632:secondary
AAAGTTGAAA	0	0	0	0	1	At5g57160.1:i:+3859:tertiary
CAGTTTTTGC	0	0	1	0	0	At5g57150.2:d:+1575:primary
AGATTGTGTA	0	1	0	0	0	At5g57110.2:i:+1092:tertiary,At5g57110.1:i:+1092:tertiary
AACACGCCAC	0	1	0	0	0	At5g57110.1:d:+2970:primary,At5g57110.2:d:+2986:primary
GATCATACCA	0	1	0	0	0	At5g57100.1:d:+809:secondary
TCGGTCTGCT	0	0	1	0	0	At5g57040.1:d:+375:secondary
CCTACAGTTA	0	0	0	1	0	At5g57035.1:d:+178:secondary
GCAAGGACTT	0	1	0	0	0	At5g57015.1:d:+1050:secondary
AAACCTCTTT	0	0	0	0	1	At5g56990.1:i:+761:tertiary,At5g02910.1:i:+1339:tertiary
GCACGAACAA	0	0	1	0	0	At5g56980.1:X:-1571:quaternary
TTCAATCTCT	0	1	0	0	0	At5g56960.1:v:+310:secondary,At2g38100.1:v:+1812:secondary
GCTGAGAGTA	0	0	1	0	0	At5g56930.1:X:-562:quaternary

TAGATGAAAC	0	0	1	0	0	At5g56930.1:d:+2067:primary
TTGAGGCAAA	0	0	1	0	0	At5g56920.1:d:+466:secondary
TCTCAGCTAA	1	0	0	0	0	At5g56910.1:d:+427:secondary
GAACACCAGT	1	0	0	0	0	At5g56870.1:d:-734:secondary
TCGAAATGGA	0	0	0	0	1	At5g56870.1:d:+1820:secondary
AGGAGCTTGT	0	0	0	1	0	At5g56850.1:d:+407:primary
AATCGTTGGC	0	1	0	0	0	At5g56760.1:X:-419:quaternary
TGACACTAGG	0	0	0	0	1	At5g56760.1:d:+725:secondary
TTACGACTAC	1	0	0	0	0	At5g56750.1:X:+-180:quaternary,Chr4:+15023119:quaternary
ACATATGGTT	0	0	1	0	0	At5g56750.1:d:-275:primary
TGAGAACCGA	1	0	0	0	0	At5g56740.1:d:+1222:primary
GAACAAGAAC	0	1	0	0	0	At5g56730.1:v:+432:secondary,At1g47056.1:v:+1695:secondary,At2g30910.2:d:+1344:secondary,At2g30910.1:d:+1344:secondary,At3g23010.1:v:+99:secondary,At5g37450.1:v:+2079:secondary
CCGCAGAGAT	0	1	0	0	0	At5g56730.1:v:+3078:secondary
ACCTCCTTTC	0	0	0	0	1	At5g56660.1:d:-700:secondary,At1g51780.1:d:-771:secondary,At5g56650.1:d:-687:secondary
AATTATAATT	1	0	0	0	0	At5g56650.1:d:+1515:primary
ACGAACTCTA	0	0	1	0	0	At5g56650.1:d:+1307:secondary
AGACAGAGTT	0	1	0	0	0	At5g56590.1:d:-1667:secondary
GACCGTGGAT	0	0	1	0	0	At5g56580.1:d:+489:secondary
TCACACCTCC	1	0	0	0	0	At5g56550.1:d:+305:primary
CTCTTTTGGT	0	1	0	0	0	At5g56510.1:v:+2047:secondary
TTACAAATTA	0	1	0	0	0	At5g56480.1:v:+928:primary
AAGCAAAGAA	0	0	0	0	1	At5g56460.1:d:+998:secondary,At2g40150.1:d:+121:secondary
CTAATTCACA	0	0	1	0	0	At5g56460.1:d:+1623:primary
TGCTTCTTAT	0	1	0	0	0	At5g56420.2:d:+1509:primary,At5g56420.1:d:+1527:primary
CTGTTCACTC	0	0	0	0	1	At5g56380.1:d:+1861:primary
AGAGTTGAGG	0	0	0	1	0	At5g56360.1:i:+1207:tertiary
ACGGCACAGA	0	0	0	1	0	At5g56350.1:X:-442:quaternary
CTCAGTGGTG	0	0	1	0	0	At5g56350.1:d:+1192:secondary
AGCTCTTCAA	0	1	0	0	0	At5g56300.1:d:+1128:secondary
TTCTTCTGAA	0	0	1	0	0	At5g56290.1:i:+2430:tertiary,At5g63110.1:i:+712:tertiary
GGTCCAGTCT	1	0	0	0	0	At5g56290.1:d:+897:secondary,At5g51550.1:d:+267:secondary
AACGAGATGG	0	0	1	0	0	At5g56280.1:d:+909:secondary
GAATCAGAGA	0	1	0	0	0	At5g56280.1:d:+813:secondary,At2g20540.1:v:+364:secondary
TTTCTCTATA	1	0	0	0	0	At5g56250.2:d:+2772:primary,At3g32190.1:v:+1842:primary,At3g22560.1:d:+581:primary,At5g56250.1:d:+2933:primary
GAAAGAAGAA	0	0	0	0	1	At5g56250.2:d:+1437:secondary,At2g20440.1:d:+470:secondary,At5g56250.1:d:+1598:secondary,At5g25930.1:d:+2413:secondary,At1g11950.1:d:+312:secondary
ATCTCACTTT	0	0	1	0	0	At5g56220.1:d:+2861:secondary
TGGGAACAGC	0	0	0	1	0	At5g56210.1:X:-653:quaternary
TGGTGAATCT	0	0	1	0	0	At5g56180.2:d:+1581:primary,At5g56180.1:d:+1441:primary
ATAGTTGCTT	1	0	0	0	0	At5g56150.2:d:-188:primary,At5g56150.1:d:-195:primary
ATATGTACAA	0	1	0	0	0	At5g56140.1:d:+812:secondary
GGTCAAAGAG	0	1	0	0	0	At5g56075.1:v:-461:primary
TCGAGTAAGA	0	0	1	0	0	At5g56030.1:d:+1889:secondary
AGAAACCTCC	0	0	1	0	0	At5g56010.1:d:-870:secondary,At5g56030.1:d:-856:secondary
GATACCGAGC	0	1	0	0	0	At5g56010.1:d:-1365:secondary,At5g56030.1:d:-1351:secondary
AGTGGGATTT	1	0	0	0	0	At5g56010.1:d:+875:secondary,At5g56030.1:d:+861:secondary,At5g56000.1:d:+855:secondary
TAGAGGACTT	0	0	0	1	0	At5g56000.1:X:-1206:quaternary
ACTCTTCAAT	0	1	0	0	0	At5g55990.1:d:+794:primary
TAAAGCTCTA	0	1	0	0	0	At5g55930.1:d:-903:secondary
GCTATTCTTT	0	1	0	0	0	At5g55920.1:X:-17:quaternary
GGTTTGTGAT	1	0	0	0	0	At5g55900.1:i:+521:tertiary
GATCTCGAGA	0	1	0	0	0	At5g55900.1:d:+629:primary
ATGGTTCAGA	0	0	1	0	0	At5g55900.1:d:+452:secondary
AAATTGATTT	0	1	0	0	0	At5g55870.1:v:+958:primary
AGCAATTGTG	0	0	0	1	0	At5g55840.1:v:+3321:secondary
TCACAACGTC	0	0	0	0	1	At5g55820.1:v:+3467:secondary,At5g55820.1:v:+3278:secondary

GCTGCTAAAA	0	1	0	0	0	At5g55790.1:v:+1342:primary
GTTTCGATAA	0	0	0	1	0	At5g55790.1:v:+102:secondary
TAATGGGAAT	0	0	1	0	0	At5g55780.1:v:+767:secondary
CCAAAATATC	0	1	0	0	0	At5g55770.1:v:-2456:secondary
TGTTTGTTGC	0	0	0	1	0	At5g55740.1:v:+2749:secondary
CTGTTACATT	0	0	0	1	0	At5g55670.1:X:-487:quaternary
CTCAGCATCC	0	0	0	1	0	At5g55660.1:d:-2271:secondary
TTGAAAAGGG	0	0	0	1	0	At5g55660.1:d:+1067:secondary
GAGCATAACA	0	0	0	1	0	At5g55640.1:X:+414:quaternary
CCTTGTCCTT	0	1	0	0	0	At5g55630.2:d:+713:primary,At5g55630.1:d:+921:primary
TAAAACCACT	1	0	0	0	0	At5g55600.1:d:+3177:primary
ATTCATTAGA	0	0	0	0	1	At5g55580.1:d:-1966:secondary
TATTTGGTCA	0	0	1	0	0	At5g55580.1:d:+1380:secondary
GAGAAGATTC	0	0	0	0	1	At5g55570.1:d:+411:primary
ATGACGATGA	1	0	0	0	0	At5g55570.1:d:+139:secondary,At1g21380.1:d:+1101:secondary,At2g25650.1:d:+1105:secondary
TATCCCTAAT	0	0	0	1	0	At5g55550.1:d:+2027:primary
GATCGTTATA	0	0	0	1	0	At5g55540.1:X:-86:quaternary
GTATGAAGAC	1	0	0	0	0	At5g55540.1:X:+1518:quaternary
AAGTGATCCA	0	0	1	0	0	At5g55540.1:d:+4121:primary
TTTCTCACAG	1	0	0	0	0	At5g55540.1:d:+2737:secondary
CAACAGTTTA	0	0	1	0	0	At5g55530.3:d:+1179:secondary,At5g55530.1:d:+1285:secondary,At5g55530.2:d:+1207:secondary
TGGTAATAAA	0	1	0	0	0	At5g55530.2:X:+1764:quaternary
ATGAGCAGGG	0	1	0	0	0	At5g55530.1:d:+592:secondary,At5g55530.3:d:+486:secondary,At5g55530.2:d:+514:secondary,At3g62700.1:d:+1887:secondary
GATTGGGATA	0	0	0	0	1	At5g55510.1:X:-166:quaternary
GATTATGGTC	0	1	0	0	0	At5g55507.1:d:+155:primary
AAGAGCCTTG	0	0	1	0	0	At5g55500.1:d:+1814:secondary
GACAGCTTTG	0	0	1	0	0	At5g55500.1:d:+1093:secondary,At5g66520.1:v:+1298:secondary
TAGATGACAA	0	1	0	0	0	At5g55490.1:X:-133:quaternary
ATTCTTTAGG	0	0	0	1	0	At5g55490.1:v:+796:secondary
CCACAGTTGG	0	1	0	0	0	At5g55490.1:v:+254:secondary
TTTGTAGCTT	1	0	0	0	0	At5g55490.1:v:+1407:secondary
TAACATCAAC	1	0	0	0	0	At5g55460.1:d:+110:primary
AAGAACTGAG	1	0	0	0	0	At5g55450.1:d:+391:secondary
TGCTAGTGCA	0	1	0	0	0	At5g55400.1:X:-138:quaternary
GATTAGTTTC	1	0	0	0	0	At5g55390.1:d:+1487:secondary
GAGGAAGAAC	0	0	0	1	0	At5g55320.1:v:+1655:secondary,At5g55330.1:v:+354:secondary
AAGTGGTTTT	0	1	0	0	0	At5g55320.1:v:+1075:secondary
TTTCTGGAAT	0	1	0	0	0	At5g55310.1:i:+3172:tertiary
ACAATACTGT	0	1	0	0	0	At5g55310.1:d:+1751:secondary
TTCCTATTGC	0	0	0	0	1	At5g55310.1:d:+1004:secondary
CTTTTTCTAG	0	1	0	0	0	At5g55290.1:X:-107:quaternary
AATGTCGCCA	0	0	0	0	1	At5g55260.1:d:-365:primary
GGGTTTTTAT	0	0	0	1	0	At5g55250.1:X:+427:quaternary
ATCTTTCAGT	0	0	0	0	1	At5g55230.1:d:+2177:primary
AGTTCTTTTT	0	0	0	1	0	At5g55200.1:d:+1029:secondary
TCTTGAGAGT	1	0	0	0	0	At5g55140.1:d:+439:primary
TATTTACTCT	0	0	1	0	0	At5g55135.1:v:+410:secondary,At4g21323.1:v:+2821:secondary
AAATAGATTA	0	1	0	0	0	At5g55135.1:i:+113:tertiary
ATCAGTGATT	0	0	0	1	0	At5g55130.1:d:+650:secondary
AAAAGCACCG	0	0	0	1	0	At5g55100.2:d:+2329:secondary,At5g55100.1:d:+2329:secondary
AATCAACAAA	0	1	0	0	0	At5g55090.1:v:+2010:secondary
GACCGATAAG	0	1	0	0	0	At5g55060.1:v:+1973:primary
TTCTTCATTA	1	0	0	0	0	At5g55050.1:i:+892:tertiary
TGGTGCTCTT	0	0	0	0	1	At5g55050.1:d:+816:secondary,At3g06980.1:d:+2211:secondary
CAATGTGCCA	0	0	0	1	0	At5g55000.2:d:+215:secondary,At5g55000.1:d:+215:secondary
GAGACGAGCT	0	0	0	0	1	At5g55000.1:d:+85:secondary,At5g54990.1:v:+1493:secondary,At5g55000.2:d:+85:secondary
AGGAATAGCA	0	1	0	0	0	At5g54910.1:X:-308:quaternary
TGAGTGATGT	1	0	0	0	0	At5g54890.1:d:+1104:secondary
AAACAAACTT	0	0	0	0	1	At5g54850.1:d:+103:primary
ATGGTTTTAA	0	1	0	0	0	At5g54830.1:d:-2077:secondary

AAGAATGGAG	0	1	0	0	0	At5g54780.1:d:+1252:primary,At3g46380.1:v:+971:primary
ACGGATATGA	0	0	1	0	0	At5g54770.1:d:+265:secondary
CGGGATAAAC	0	0	1	0	0	At5g54710.1:d:+1323:secondary
TAAAACAATA	0	0	0	1	0	At5g54690.1:i:+520:tertiary
TCGCATCAAG	1	0	0	0	0	At5g54690.1:d:+562:secondary
ACGAGTAGTA	0	1	0	0	0	At5g54660.1:d:+49:secondary
TCAGGTTTTA	1	0	0	0	0	At5g54650.1:d:+3276:primary,At5g54650.2:d:+3050:primary
AACAAAGAAA	0	0	0	1	0	At5g54610.1:d:+720:secondary
TCAAGTTTGG	0	1	0	0	0	At5g54600.1:d:+286:secondary,At5g54600.2:d:+286:secondary,At4g28670.1:v:+544:secondary
AAGCTAAAAG	0	0	1	0	0	At5g54540.1:d:+810:secondary
TACGACTACA	0	1	0	0	0	At5g54520.1:d:+1268:primary
GTGATAAGTT	0	1	0	0	0	At5g54440.1:d:-3561:secondary
TTTAATGCAG	1	0	0	0	0	At5g54430.1:X:-137:quaternary
GGACAAAGAA	0	0	0	1	0	At5g54430.1:i:+1262:tertiary
TCAAACCATT	1	0	0	0	0	At5g54420.1:v:+829:secondary,At5g54420.1:v:+767:secondary,At4g06607.1:p:+463:secondary
TGAATAAAAA	0	0	0	0	1	At5g54390.1:d:+61:secondary
GAGGTCCTGG	0	0	1	0	0	At5g54340.1:v:+432:secondary
ATTCGTTTTC	0	0	0	1	0	At5g54340.1:v:+1039:secondary
TTTTGCATTC	0	0	0	0	1	At5g54320.1:v:+954:secondary,At3g43970.1:v:+724:secondary
GGCTGGCTTT	0	0	1	0	0	At5g54310.1:d:+945:secondary
TATCAAATCT	0	1	0	0	0	At5g54290.1:i:+668:tertiary
TTTTGCATAA	0	1	0	0	0	At5g54280.1:i:+1756:tertiary
AGGATGACTT	0	0	1	0	0	At5g54280.1:d:+3834:secondary
TATATATACA	0	0	0	0	1	At5g54230.1:i:+245:tertiary,At1g56510.1:i:+1023:tertiary
TGTTCGATGA	0	0	0	1	0	At5g54206.1:p:+105:secondary,At3g16610.1:v:+520:secondary
TTCCTTTGTA	0	0	0	0	1	At5g54200.1:X:-27:quaternary,At1g57980.1:X:-209:quaternary
TCTAACATAC	0	1	0	0	0	At5g54200.1:d:+2359:primary
AAGACACCGG	0	1	0	0	0	At5g54190.1:d:+937:primary
AGAGATCTTT	0	0	0	1	0	At5g54180.1:d:-635:secondary,At4g19650.1:v:-2426:secondary
GCTCAAATCA	0	1	0	0	0	At5g54170.1:d:+1081:secondary
GCCTATTATT	0	0	0	0	1	At5g54160.1:X:+-202:quaternary
ATTAGGAAGA	0	1	0	0	0	At5g54160.1:i:+1000:tertiary
AATATGGCAT	0	0	1	0	0	At5g54160.1:d:-860:secondary
GACTACTTGT	0	0	1	0	0	At5g54160.1:d:-1014:secondary
GGCTTCTGTG	0	1	0	0	0	At5g54130.1:v:+693:secondary,At5g47790.1:d:+1117:secondary
GAAATCGGAA	0	0	0	1	0	At5g54110.1:d:+282:secondary
CTAGCAGGAT	0	0	0	1	0	At5g54100.1:X:-43:quaternary,At4g27585.1:X:-43:quaternary
AGCTTCCAAA	0	0	1	0	0	At5g54090.1:i:+1875:tertiary
AAAGCCATCG	0	1	0	0	0	At5g54090.1:d:+2773:primary
AATAGAATGC	0	0	0	1	0	At5g54080.2:d:+2097:primary
AAGGTTGCAA	0	0	0	1	0	At5g54040.1:v:+1507:secondary
GCTAATTATT	0	0	1	0	0	At5g53950.1:v:+1961:primary
TGTCCTTGGT	0	0	0	0	1	At5g53950.1:v:+1780:secondary,At2g36850.1:d:+4767:secondary
AAGATAGTCT	0	1	0	0	0	At5g53930.1:d:+1747:primary
ATATGAAACT	0	0	0	0	1	At5g53920.1:d:+1262:primary
AGCCTAGAAG	0	1	0	0	0	At5g53890.1:d:+3243:secondary
TTGAATCCGA	1	0	0	0	0	At5g53890.1:d:+2189:secondary
ATATTAAATT	0	1	0	0	0	At5g53830.1:d:+773:primary
GTACTTATTT	0	0	0	1	0	At5g53810.1:i:+711:tertiary
TGGCTATTAA	1	0	0	0	0	At5g53770.1:i:+502:tertiary
TATGGTTTTG	0	1	0	0	0	At5g53750.1:v:+1094:secondary
TTTTTATTCA	0	0	0	0	1	At5g53740.1:i:+1584:tertiary
TCCCGATCTT	0	0	0	0	1	At5g53730.1:X:-551:quaternary
TCTCACCCAA	1	0	0	0	0	At5g53720.1:v:+354:secondary
GAGTAGGATG	0	0	1	0	0	At5g53660.1:i:-133:tertiary
TATGTTTTAA	0	1	0	0	0	At5g53640.1:v:+171:secondary,At1g65640.1:v:+313:secondary
GAGATCCAGA	0	1	0	0	0	At5g53620.2:d:+533:secondary,At5g60930.1:v:+3150:secondary,At5g53620.1:d:+483:secondary,At3g01480.1:d:+1043:secondary
CTGCGTTTGC	0	0	0	0	1	At5g53600.1:v:+568:secondary
TTCAAGAAGT	0	0	0	1	0	At5g53590.1:d:+615:primary
TCCCTTGCAG	0	0	0	1	0	At5g53560.1:d:-274:secondary

GAGGTATTGA	1	0	0	0	0	At5g53550.1:v:-459:primary
ATCATAGACT	1	0	0	0	0	At5g53550.1:v:+906:secondary,At4g30940.1:d:+1748:secondary,At4g03290.1:d:+741:secondary
TGCTGTTGCT	0	0	0	1	0	At5g53550.1:v:+692:secondary
CCATAACCCG	0	0	0	1	0	At5g53480.1:d:+2713:secondary
AGGCAGACCA	0	0	0	0	1	At5g53460.1:v:-851:primary
GATGTACCAA	0	0	1	0	0	At5g53460.1:v:+6382:secondary,At4g07806.1:p:+1861:secondary
AGGATGGCAA	1	0	0	0	0	At5g53460.1:v:+6286:secondary,At3g32445.1:p:+1097:secondary
GCTTTGGATC	0	0	1	0	0	At5g53460.1:v:+4479:secondary
CCTCAGCGCC	0	0	1	0	0	At5g53450.1:d:+1480:secondary,At5g53450.2:d:+1313:secondary
AACTAATGGT	0	0	1	0	0	At5g53440.1:X:-704:quaternary
GTACTGGAGA	0	0	1	0	0	At5g53440.1:v:+958:secondary,At1g03080.1:d:+2212:secondary
AATTAGAGAG	0	1	0	0	0	At5g53430.1:d:+3604:primary
GGTCTGCAAA	0	0	0	0	1	At5g53430.1:d:+2427:secondary
CAGAACAAAA	0	0	1	0	0	At5g53430.1:d:+1875:secondary
TTTCTTCCTT	0	0	0	1	0	At5g53420.1:d:-999:secondary
ACTATGTTGG	0	0	0	1	0	At5g53390.1:i:+548:tertiary,At5g12290.1:i:+2544:tertiary
TTATTACCTC	0	0	1	0	0	At5g53370.1:d:+1718:primary
ATGTCGGATA	0	0	1	0	0	At5g53370.1:d:+1570:secondary
GTAAGATCTG	0	0	0	0	1	At5g53360.1:d:+1325:primary,At3g47830.1:v:+1129:primary
GCGCCTCTCT	1	0	0	0	0	At5g53350.1:d:-1281:secondary
TAGATGAAGG	1	0	0	0	0	At5g53340.1:d:+1246:primary
TTTGAGAACG	0	0	1	0	0	At5g53330.1:d:+632:secondary
AAAGAATCCA	0	0	1	0	0	At5g53320.1:d:+2057:secondary,At3g29310.1:d:+1730:secondary
ATGTAGGTGG	1	0	0	0	0	At5g53300.2:X:-94:quaternary
GTGGCATACC	0	0	0	0	1	At5g53290.1:d:+1019:secondary
TGTTTTATCA	0	0	0	1	0	At5g53280.1:i:+909:tertiary,At4g33270.1:i:+1398:tertiary
CTTTGTTCTG	1	0	0	0	0	At5g53250.1:d:+419:primary,At3g21540.1:d:+2858:primary
ACTTGGAGAA	0	1	0	0	0	At5g53220.1:v:+1676:secondary
CTCTTTTCTT	0	1	0	0	0	At5g53190.1:i:+81:tertiary
GCACCACCAT	0	1	0	0	0	At5g53180.1:d:+1328:primary
GTATCGAGTT	1	0	0	0	0	At5g53170.1:d:+2209:secondary
GGCACAGAGA	0	0	0	1	0	At5g53170.1:d:+1882:secondary
GAATGCAACA	1	0	0	0	0	At5g53150.1:d:+148:secondary,At5g23990.1:v:+902:secondary
TCCCCAACAG	1	0	0	0	0	At5g53140.1:d:-1668:secondary
TGGAAACAGA	0	0	1	0	0	At5g53140.1:d:+1673:secondary,At2g16640.1:d:+1342:secondary
GATTGTTGAG	0	1	0	0	0	At5g53135.1:p:+2148:secondary,At2g28000.1:d:+577:secondary
TTCTGATTAA	0	1	0	0	0	At5g53100.1:v:+745:secondary,At4g27760.1:d:+498:secondary,At5g53090.1:v:+742:secondary
CATTTTTAAT	0	0	1	0	0	At5g53100.1:i:+1038:tertiary
ATTTTTATCG	0	1	0	0	0	At5g53070.1:i:+423:tertiary,At5g18840.1:i:+1869:tertiary
TAAAAGCACT	0	0	1	0	0	At5g53050.2:X:-112:quaternary
CTTTGATTGC	0	1	0	0	0	At5g53045.1:d:+114:primary
GAAGATAAAA	0	0	1	0	0	At5g53040.1:v:+1591:primary
GTTGCACAAA	0	0	0	1	0	At5g53030.2:d:+1391:secondary
GTCAGACGGG	0	1	0	0	0	At5g53010.1:d:+2855:primary
ATCCAAAAAC	0	0	0	1	0	At5g53010.1:d:+2384:secondary
CCAATCAGGT	0	0	0	1	0	At5g52960.1:d:-470:secondary
CTCAACTATC	0	1	0	0	0	At5g52960.1:d:+475:secondary
TTTCTACTCA	0	0	0	0	1	At5g52940.1:v:+893:secondary
GAAGAGGAGC	0	1	0	0	0	At5g52900.1:d:+840:secondary
TGAATGTGTC	0	0	0	1	0	At5g52900.1:d:+1010:secondary
AGTGCATCAG	0	1	0	0	0	At5g52890.1:d:+1142:primary
CGTTGCTTGG	0	0	1	0	0	At5g52880.1:X:-18:quaternary
CTTTGGAATC	0	0	0	1	0	At5g52850.1:v:+3299:secondary
AGTTCGTGTC	0	1	0	0	0	At5g52850.1:v:+2911:secondary
GAAGAGGAAG	0	1	0	0	0	At5g52830.1:v:+1224:secondary,At4g01985.1:d:+128:secondary,At5g02920.1:v:+354:secondary,At4g01570.1:d:+26:secondary,At5g32598.1:p:+1865:secondary,At3g31945.1:p:+3382:secondary,At5g29022.1:p:+1273:secondary,At5g30207.1:p:+55:secondary,At2g13230.1:p:+1233:secondary,At5g31355.1:p:+713:secondary
AAATGAAGGA	0	0	0	0	1	At5g52810.1:d:+897:primary
AGCTATCATA	1	0	0	0	0	At5g52730.1:v:+1003:secondary
TTAAGATAAA	1	0	0	0	0	At5g52710.1:d:+491:secondary
TGAATATGCG	1	0	0	0	0	At5g52660.2:i:+510:tertiary,At5g52660.1:i:+494:tertiary

GTGGAGTGAA	0	0	0	1	0	At5g52660.1:d:+1049:primary,At5g52660.2:d:+1068:primary
AACATATTTA	0	0	1	0	0	At5g52650.1:i:+1054:tertiary
ATAGGACAAT	0	0	0	1	0	At5g52640.1:d:+475:secondary
TTGCTGTATG	1	0	0	0	0	At5g52640.1:d:+2035:primary
CTTCCTCTTT	0	0	1	0	0	At5g52600.1:X:-72:quaternary
CGTGATATTC	0	0	1	0	0	At5g52590.1:d:+667:secondary
TTCGTTCACG	1	0	0	0	0	At5g52570.1:d:+769:secondary
ATCACTCCAA	0	0	1	0	0	At5g52545.1:v:+589:primary
ATAACAGATA	0	1	0	0	0	At5g52530.2:d:+2894:secondary,At5g52530.1:d:+2900:secondary
ATGCAATCCA	1	0	0	0	0	At5g52530.1:d:+1894:secondary,At5g52530.2:d:+1888:secondary
TACTTCCCAC	0	0	1	0	0	At5g52520.1:d:+376:secondary
TTGAATAGTT	0	0	0	0	1	At5g52510.1:d:+724:secondary
GTGGCTGCGC	1	0	0	0	0	At5g52510.1:d:+1075:secondary
GTCACGCTCA	0	0	0	1	0	At5g52480.1:v:+1545:primary
GATATAGAGG	0	1	0	0	0	At5g52460.1:v:+1240:primary,At2g45630.1:d:+784:primary
TAGAGAAGTT	0	0	0	1	0	At5g52450.1:X:-323:quaternary
GAGAACAAAG	0	1	0	0	0	At5g52410.1:d:+2113:primary,At5g52410.2:d:+2135:primary
TCCAAGCTGC	0	0	1	0	0	At5g52370.1:d:+500:secondary
TTTTCTCTAG	0	0	1	0	0	At5g52340.1:v:+714:secondary
AGACGAGTTT	1	0	0	0	0	At5g52320.1:X:-458:quaternary
ACTTCTCACC	0	0	0	1	0	At5g52310.1:d:-1289:secondary
AGGCACTGGC	0	0	0	0	1	At5g52300.1:d:+579:secondary
AGACAAAACC	0	0	1	0	0	At5g52300.1:d:+1275:secondary
CTCGCTCAGC	0	1	0	0	0	At5g52280.1:d:-1800:secondary
AACCAATTAT	0	0	0	1	0	At5g52270.1:v:+1172:secondary,At4g05635.1:p:+3825:secondary
CAAAAGACAA	0	1	0	0	0	At5g52230.1:X:-305:quaternary
GCCCGAAAGA	0	0	1	0	0	At5g52230.1:X:-1704:quaternary
CAACGGATGT	0	0	1	0	0	At5g52190.1:d:+516:secondary
TATGGGAAGT	0	0	0	0	1	At5g52160.1:d:+435:secondary
CTTCCTTCTC	0	0	1	0	0	At5g52120.1:X:-3:quaternary,At3g17600.1:X:-66:quaternary,At3g43355.1:X:-267:quaternary
GTATGGCTAT	0	1	0	0	0	At5g52120.1:i:+1695:tertiary
CGGGTGACTT	0	0	1	0	0	At5g52120.1:d:+889:primary
CATTCAGGTA	0	1	0	0	0	At5g52110.1:X:--66:quaternary
TTTAACAGTG	1	0	0	0	0	At5g52100.1:d:-850:secondary
TCTGGAATCT	0	1	0	0	0	At5g52090.1:X:-982:quaternary
TCAGATCGAA	0	0	0	1	0	At5g52070.1:d:+465:secondary
AGGAGGTCTT	1	0	0	0	0	At5g52050.1:d:+1606:primary
AAAATAGTGC	0	0	1	0	0	At5g52040.1:d:+1434:primary
AATCTACCAA	0	1	0	0	0	At5g51900.1:v:+744:secondary,At4g39810.1:v:+107:secondary
AAGAAGACAT	0	0	0	0	1	At5g51880.1:d:+715:secondary
TGTGTTTGAG	1	0	0	0	0	At5g51860.1:X:--146:quaternary
AGAGTGAAAA	0	0	0	1	0	At5g51850.1:d:+420:secondary
CTGCTAAGTT	0	0	1	0	0	At5g51840.1:d:+443:secondary
CCTAACATCA	0	0	0	1	0	At5g51820.1:X:--13:quaternary
GTCCTTTCGA	0	0	1	0	0	At5g51820.1:d:+2013:secondary
GTGAAATGCA	1	0	0	0	0	At5g51810.1:d:+1268:primary
TCGTGGGCGA	0	1	0	0	0	At5g51770.1:v:+2052:secondary
TTAAAGCTAT	0	0	0	0	1	At5g51750.1:d:+2134:secondary
CATAAGTGAT	1	0	0	0	0	At5g51730.1:v:+1574:secondary,At3g49250.1:d:+1146:secondary
CATAGTAGTG	0	0	0	0	1	At5g51720.1:i:+352:tertiary
ATTATAGAAT	1	0	0	0	0	At5g51710.1:X:--20:quaternary
TTCTTCTTAT	0	0	0	0	1	At5g51700.1:d:+809:primary
TTGACTTTAT	0	0	0	0	1	At5g51690.1:d:+3181:primary
GGTATGGTTG	0	1	0	0	0	At5g51690.1:d:+2601:secondary,At2g39830.1:d:+464:secondary
GTACAAAGTA	0	0	1	0	0	At5g51660.1:d:-2258:secondary
TAGAAACAGG	1	0	0	0	0	At5g51640.1:i:+648:tertiary
CAATATTTGT	0	1	0	0	0	At5g51630.1:d:+2936:secondary,At5g51630.2:d:+2955:secondary
AAAAGAATAC	0	0	0	0	1	At5g51600.1:X:--18:quaternary
TGAGGGACAT	0	1	0	0	0	At5g51570.1:d:+891:secondary
AGTGCATAGC	0	0	0	0	1	At5g51560.1:d:-2088:secondary
GGCTGACCTG	0	0	0	1	0	At5g51540.1:v:+3180:secondary

GTGAAGGCAT	1	0	0	0	0	At5g51540.1:v:+2419:secondary
AATTATGATG	0	1	0	0	0	At5g51540.1:i:+262:tertiary
GAGGAACGCA	0	0	0	1	0	At5g51510.1:d:+446:primary
GGAGCCTTTA	0	0	1	0	0	At5g51470.1:v:+143:secondary,At5g17690.1:d:+562:secondary,At5g47630.1:d:+612:secondary
TGCCTGATGA	0	0	0	1	0	At5g51450.1:d:+1773:primary
AGCTAAAGTT	0	0	0	0	1	At5g51430.1:i:+1811:tertiary
GAAGGTCTGG	0	0	0	1	0	At5g51410.2:d:+1083:secondary,At3g16415.1:p:+591:secondary,At5g51410.1:d:+1083:secondary,At3g20290.1:d:+455:secondary
TGTTGTCTTT	0	0	0	0	1	At5g51410.1:d:+1293:primary
AGAGCTTATA	0	1	0	0	0	At5g51390.1:d:+279:primary
TGCCCTGAAA	1	0	0	0	0	At5g51380.1:d:+1565:primary
TGAGTCAAAG	0	0	1	0	0	At5g51350.1:X:-685:quaternary
TCGACCGCTT	0	1	0	0	0	At5g51340.1:d:+1064:primary
TGAATGTGGT	0	0	0	1	0	At5g51290.1:d:+2015:secondary
CCAGCAGAGA	0	0	1	0	0	At5g51280.1:d:+1631:secondary
CTCTTTTAGT	0	1	0	0	0	At5g51220.1:i:+564:tertiary
TGCCTAAATA	0	0	0	0	1	At5g51210.1:i:-488:tertiary
CAATGTATGA	1	0	0	0	0	At5g51210.1:d:+609:primary
ATCTTAAGCA	0	1	0	0	0	At5g51200.1:d:+2011:secondary
AGGATAGTGG	0	0	0	0	1	At5g51140.1:i:+2077:tertiary
TAAGCACTAA	0	0	1	0	0	At5g51130.1:v:+1591:secondary
GCTGCTCGTC	0	1	0	0	0	At5g51080.1:d:+1233:primary
ATCGAATTCG	0	1	0	0	0	At5g51070.1:d:-163:primary
AGGGTCCACG	0	1	0	0	0	At5g51050.1:v:+1417:secondary
CCTGATGTGA	0	0	1	0	0	At5g51010.1:d:+356:secondary
TCTTTATATA	0	1	0	0	0	At5g50950.2:i:+2733:tertiary,At5g50950.1:i:+2790:tertiary,At5g50950.1:i:+2733:tertiary,At5g50950.2:i:+2790:tertiary,At2g47510.1:i:+2469:tertiary
CAGCAATTTA	0	1	0	0	0	At5g50950.2:d:-1637:secondary,At5g50950.2:d:-1641:secondary
TCATTAAGAA	0	0	0	0	1	At5g50950.1:i:+2049:tertiary,At5g50950.2:i:+2049:tertiary
AATACGATTT	1	0	0	0	0	At5g50910.1:v:+249:primary
AAATGTAACA	0	1	0	0	0	At5g50890.1:v:+1663:secondary
ATCGATGGTT	0	0	1	0	0	At5g50850.1:X:--76:quaternary
AACGGAAGAA	0	0	0	0	1	At5g50820.1:d:+23:secondary
TTAAGATAGG	0	1	0	0	0	At5g50790.1:d:+1102:primary
ATCCAGAACA	0	1	0	0	0	At5g50780.1:v:+939:secondary
ACTCTGATTG	0	1	0	0	0	At5g50670.1:d:+1016:primary,At5g50570.1:d:+1027:primary,At5g50570.2:d:+958:primary
AGTTTGACTG	0	0	1	0	0	At5g50640.1:X:-87:quaternary,At5g50530.1:X:-87:quaternary
AATTGTATAT	0	1	0	0	0	At5g50640.1:v:+2145:secondary,At5g48780.1:v:+2661:secondary
AACAGGATTC	0	1	0	0	0	At5g50640.1:i:+460:tertiary,At5g50530.1:i:+471:tertiary
AGACTGCACG	0	0	0	1	0	At5g50630.1:X:-362:quaternary,At5g50520.1:X:-362:quaternary
TACGAAATTC	0	1	0	0	0	At5g50630.1:X:+789:quaternary,At5g50520.1:X:+789:quaternary
GAAAGATCAG	0	0	1	0	0	At5g50530.1:d:+570:secondary,At5g50640.1:v:+909:secondary
GGCAGCCGCT	0	0	1	0	0	At5g50530.1:d:+1571:primary
AAGTAGATTT	0	1	0	0	0	At5g50510.1:X:-494:quaternary,At5g50620.1:X:-494:quaternary
TAAACAATGT	1	0	0	0	0	At5g50480.1:d:+704:primary
GTGAGCGGTA	0	0	1	0	0	At5g50450.1:d:+1056:secondary
TATAATCTAC	0	0	1	0	0	At5g50430.1:X:--159:quaternary
GTTTTTGGTC	0	0	0	0	1	At5g50430.1:i:+956:tertiary
TTCTGTTCTG	0	0	0	0	1	At5g50430.1:i:+1017:tertiary
AATGTTATCT	0	1	0	0	0	At5g50410.1:X:--89:quaternary
TGTATTTCCT	0	0	1	0	0	At5g50410.1:i:+1070:tertiary
TAGCTGTATC	0	0	0	0	1	At5g50410.1:d:+888:primary
ATTTCTTCGA	0	1	0	0	0	At5g50400.1:v:+2186:primary
TAACGGGTTT	0	1	0	0	0	At5g50390.1:v:+2565:primary
TAATTCATCA	0	0	1	0	0	At5g50380.1:X:-1470:quaternary
AGAGAGGAGG	0	1	0	0	0	At5g50380.1:d:+811:secondary
TTTCTAAGGC	1	0	0	0	0	At5g50375.1:d:+1153:primary
GGACTGAGAG	0	0	1	0	0	At5g50375.1:d:+1078:secondary
CGGTGAGAGA	1	0	0	0	0	At5g50350.1:X:-208:quaternary
CCTTATCAAG	0	0	0	0	1	At5g50350.1:d:+1533:primary

ATCTAATCTG	0	1	0	0	0	At5g50340.1:X:-108:quaternary
ATTGAGTTTG	0	0	1	0	0	At5g50330.1:i:+666:tertiary,At3g45740.1:i:+1951:tertiary
GTCGAGATGC	0	0	1	0	0	At5g50320.1:d:+1550:secondary,At1g30340.1:p:+390:secondary
TAGGAATTTG	0	0	0	1	0	At5g50310.1:d:-2164:secondary
CAGATTTGTG	0	1	0	0	0	At5g50310.1:d:+887:secondary
GAAATGGAAG	0	0	1	0	0	At5g50310.1:d:+1027:secondary
AATGCTCTCG	0	0	0	0	1	At5g50300.1:d:+1750:secondary
ACTTACAATA	0	1	0	0	0	At5g50280.1:v:+2124:secondary
TATGCAAATG	1	0	0	0	0	At5g50260.1:d:+646:secondary
GGGAGAGGAA	0	1	0	0	0	At5g50260.1:d:+1215:primary
GCAGGTTCCC	0	1	0	0	0	At5g50250.1:X:-111:quaternary
AGCTCCTTCT	0	0	1	0	0	At5g50230.1:X:-101:quaternary
TATGGTCGCT	0	0	1	0	0	At5g50230.1:d:+1412:secondary
AAGTTGCCTA	0	0	0	0	1	At5g50200.1:d:+563:primary,At5g50200.3:d:+577:primary,At5g50200.2:d:+586:primary
TTCCCTAAAT	0	0	0	0	1	At5g50180.1:d:+1404:primary
CGAGAGAATA	0	0	0	1	0	At5g50150.1:i:+1082:tertiary
CGAAAGCGTT	0	0	1	0	0	At5g50130.1:d:+701:secondary,At3g05710.2:d:+597:secondary,At5g50130.2:d:+719:secondary,At3g05710.1:d:+594:secondary
TATTTTGAAC	0	0	0	1	0	At5g50130.1:d:+1246:primary
GAACTATAAA	0	0	0	0	1	At5g50100.1:d:+365:secondary,At3g61190.1:d:+232:secondary
TGAGTGGCTA	1	0	0	0	0	At5g50090.2:d:+651:primary,At5g50090.1:d:+669:primary
CGGTCTTCTC	0	0	0	0	1	At5g50000.1:X:+-62:quaternary
GTACCTTGAG	0	1	0	0	0	At5g49960.1:v:+2881:secondary
TCTCTTGAGC	0	1	0	0	0	At5g49960.1:i:+2883:tertiary
TAGCTTACAG	0	0	0	1	0	At5g49945.1:d:+1817:primary
ACTCCAGCTG	0	0	1	0	0	At5g49940.1:d:-683:secondary
TCCAAGTTCT	0	0	1	0	0	At5g49940.1:d:+438:secondary
AGATCGATGG	0	0	0	1	0	At5g49940.1:d:+386:secondary
ACCAAGATCA	0	0	0	0	1	At5g49910.1:d:+1494:secondary
TTTGTCTAGT	1	0	0	0	0	At5g49890.1:i:+2009:tertiary
TCTTCAAATG	0	0	0	1	0	At5g49880.1:i:-1944:tertiary
GTGAATACTC	1	0	0	0	0	At5g49880.1:d:+1788:secondary,At3g25580.1:d:+266:secondary,At2g18990.1:d:+260:secondary
TCGAAGTAGG	0	0	1	0	0	At5g49870.1:X:-449:quaternary,At5g49860.1:X:-398:quaternary,At5g49850.1:X:-449:quaternary
CTTCTAGGAA	1	0	0	0	0	At5g49840.1:v:+1054:secondary
AAACTCTCTC	0	0	1	0	0	At5g49830.1:d:+1739:secondary,At3g31356.1:p:+807:secondary
GCTTACTCAC	0	0	1	0	0	At5g49820.1:i:+856:tertiary
TGAAGGAGGG	1	0	0	0	0	At5g49820.1:d:+1591:secondary
ACGAATCAAT	0	1	0	0	0	At5g49780.1:v:+2922:primary
ACCCATTTGT	0	0	1	0	0	At5g49780.1:i:+840:tertiary,At5g49750.1:i:+780:tertiary
AAATTAGAAT	0	1	0	0	0	At5g49750.1:v:+2156:primary
TTGCGGTTCT	0	0	1	0	0	At5g49740.1:d:+1241:secondary,At5g49730.1:d:+1233:secondary
TAATCACCAA	0	1	0	0	0	At5g49730.1:X:-181:quaternary
GTGATATGAC	0	1	0	0	0	At5g49730.1:X:-161:quaternary,At5g49740.1:X:-161:quaternary
GAATGCTATC	1	0	0	0	0	At5g49730.1:d:+805:secondary
TTAAGGAGCT	0	1	0	0	0	At5g49720.1:d:+719:secondary
TGCATCACAG	0	0	0	1	0	At5g49720.1:d:+1655:secondary
TATAAAGAAT	0	0	0	0	1	At5g49710.3:d:+941:primary,At5g49710.1:d:+938:primary
ATCTCTGTGA	0	1	0	0	0	At5g49710.1:d:+912:secondary,At5g49710.3:d:+915:secondary
CATTACAACA	1	0	0	0	0	At5g49690.1:X:--230:quaternary
CTTTATTGAA	0	0	1	0	0	At5g49690.1:d:+10:secondary
TAAGATTAAT	0	1	0	0	0	At5g49670.1:i:+2047:tertiary
GTTGATGTAA	0	0	0	1	0	At5g49630.1:X:-147:quaternary
GTGATGATCA	0	0	1	0	0	At5g49600.1:v:+793:secondary,At3g18280.1:d:+57:secondary
GAGATTGCCA	0	0	1	0	0	At5g49580.1:X:-110:quaternary
ACCAAATTTA	1	0	0	0	0	At5g49580.1:v:+1092:secondary
GAAAAGAGGT	0	1	0	0	0	At5g49570.1:X:--29:quaternary
TATGAAGATC	0	1	0	0	0	At5g49570.1:d:+448:secondary
TCTATTCAAT	0	0	0	1	0	At5g49560.1:v:+1232:secondary,At3g33230.1:v:+72:secondary,At4g09360.1:v:+1064:secondary
TAATCAACTT	0	1	0	0	0	At5g49555.1:d:+1317:secondary
ACTAGATTTT	0	0	0	1	0	At5g49530.1:d:+2246:primary
CTATCACTAG	0	1	0	0	0	At5g49520.1:d:+1675:primary

CCGCCAATCT	0	0	0	0	1	At5g49500.1:X:-246:quaternary,At1g48900.1:X:-246:quaternary
AAAATATGTG	0	0	1	0	0	At5g49500.1:v:+1988:secondary
ACTCATAGAA	0	0	0	1	0	At5g49460.1:d:-2190:secondary,At5g49460.1:d:-2184:secondary,At5g49460.1:d:-2194:secondary
GGTGACGATA	0	0	0	0	1	At5g49460.1:d:-1932:secondary
TCTATATCTT	0	1	0	0	0	At5g49460.1:d:+2199:primary
AGATCTCCAG	0	0	1	0	0	At5g49450.1:d:+450:secondary
GCAAACGCAG	1	0	0	0	0	At5g49450.1:d:+320:secondary
CATATGAGGC	0	0	1	0	0	At5g49430.1:X:-64:quaternary,At1g23400.1:X:-651:quaternary
GCAGTTGACA	0	1	0	0	0	At5g49430.1:v:+3648:secondary,At2g15030.1:p:+136:secondary
AACTTATGGG	0	0	1	0	0	At5g49430.1:v:+3529:secondary
GAAAAAGCTG	1	0	0	0	0	At5g49430.1:v:+1876:secondary
CACATACATA	0	0	0	1	0	At5g49430.1:i:+755:tertiary
TCTAATTCAA	0	1	0	0	0	At5g49400.1:d:+91:secondary,At1g11580.1:d:+44:secondary
AGAGCATCCG	0	0	0	1	0	At5g49360.1:d:+1884:secondary
GATTTGGCTC	0	0	0	0	1	At5g49220.1:d:+1447:primary
GTTTTTGTTA	0	0	0	0	1	At5g49200.1:v:+2023:primary,At4g27100.1:v:+2142:primary
AACAACTCGG	0	1	0	0	0	At5g49190.1:X:-66:quaternary
AGACAGGCCA	0	1	0	0	0	At5g49160.1:d:-3375:secondary
TAAGATAAGG	0	1	0	0	0	At5g49160.1:d:+2706:secondary
CCTTTGTTTG	0	1	0	0	0	At5g49110.1:v:-3585:secondary,At5g49110.1:v:-3578:secondary,At4g20140.1:d:-2726:secondary
AGGCTTAATT	0	0	0	0	1	At5g49030.1:i:+5590:tertiary
TAGCACATAT	0	0	0	0	1	At5g49030.1:i:+1959:tertiary
GCTGCAAAAT	0	1	0	0	0	At5g49030.1:d:+3127:primary
TGGGTGATAC	0	0	0	1	0	At5g49020.2:d:-1791:secondary,At5g49020.1:d:-1796:secondary
ATTTCTTGGT	0	0	1	0	0	At5g48930.1:d:+1036:secondary
ATGCCCCGGT	0	0	0	1	0	At5g48920.1:v:-635:secondary
CCATTGATAT	1	0	0	0	0	At5g48920.1:v:+919:secondary
GTGGATATAA	0	1	0	0	0	At5g48920.1:v:+1382:secondary
CTCGTGTATG	0	0	1	0	0	At5g48910.1:d:+1945:primary
TGTAAATGTT	0	0	1	0	0	At5g48900.1:i:+1785:tertiary
AGATCACAAG	0	1	0	0	0	At5g48840.1:d:+94:secondary,At5g41140.1:d:+2474:secondary
TTTGTGTGTA	0	1	0	0	0	At5g48840.1:d:+1112:primary,At5g67610.1:d:+1679:primary
TCTTTTGGAG	0	1	0	0	0	At5g48820.1:X:-369:quaternary
ATTAGATTCA	1	0	0	0	0	At5g48790.1:i:+1145:tertiary
TACTTACAAG	0	1	0	0	0	At5g48780.1:v:+2724:secondary
GCAAATTAAA	0	0	0	1	0	At5g48770.1:v:+2545:secondary
CTTGGCCGTC	0	0	1	0	0	At5g48760.1:d:+97:secondary
GACCTATTCA	0	0	1	0	0	At5g48760.1:d:+257:secondary
AGATGTTATA	0	0	0	0	1	At5g48740.1:X:-53:quaternary
AAAAAAGCTG	0	1	0	0	0	At5g48740.1:d:+2603:secondary
AAAAAAAAGC	0	0	1	0	0	At5g48710.1:v:+1176:primary
ATGATACACA	0	0	0	1	0	At5g48680.1:d:+773:primary
ATACCGCCGA	0	1	0	0	0	At5g48657.1:X:-197:quaternary
AGATTTTCTT	0	0	0	0	1	At5g48657.1:d:+203:secondary
GAGCCGAAAG	0	1	0	0	0	At5g48657.1:d:+1263:primary,At5g48657.2:d:+676:primary
TGTCCCTTTA	0	1	0	0	0	At5g48655.2:d:+646:primary,At5g48655.1:d:+646:primary,At5g48655.3:d:+996:primary
CTCACAGTCG	0	1	0	0	0	At5g48655.1:X:-79:quaternary
TCGTGTATGC	0	1	0	0	0	At5g48655.1:X:+301:quaternary
CACCACACTC	0	1	0	0	0	At5g48630.1:X:-81:quaternary
CCAAACTTCT	0	1	0	0	0	At5g48630.1:d:+345:secondary
ATTGCGAAAA	0	0	1	0	0	At5g48620.1:d:+2689:secondary
GGATTTGAAT	0	0	1	0	0	At5g48610.1:v:+372:secondary
TTGCTGTAAA	0	1	0	0	0	At5g48570.1:X:-77:quaternary
AAGAAGGGTG	0	1	0	0	0	At5g48570.1:d:+1237:secondary
AAAAAAAACG	0	0	0	0	1	At5g48500.1:X:+-247:quaternary,Chr4:+5229056:quaternary
AGCCAGGATG	0	0	1	0	0	At5g48485.1:d:+274:secondary
TTTAGAAGTT	0	0	1	0	0	At5g48480.1:i:+573:tertiary
ATGCTGTGTT	0	0	1	0	0	At5g48470.1:i:+1680:tertiary
GTACTAGGAA	0	1	0	0	0	At5g48440.2:d:+1328:primary
ATGACTGGAG	0	1	0	0	0	At5g48410.1:d:+600:secondary,At1g18870.1:d:+460:secondary

TAAAACTATA	0	0	1	0	0	At5g48390.1:v:+3180:secondary
GTGAAGAGCG	0	0	0	1	0	At5g48385.1:d:-94:primary
AGTGAAGCAA	0	1	0	0	0	At5g48385.1:d:-290:secondary
GCAGCACAAC	0	1	0	0	0	At5g48385.1:d:+1813:secondary
GTTTCACGTC	0	1	0	0	0	At5g48385.1:d:+1261:secondary
GCAAACGGTT	0	0	1	0	0	At5g48380.1:d:+1483:secondary
TGATTATTTG	0	1	0	0	0	At5g48375.1:i:+566:tertiary,At1g02140.1:i:+472:tertiary
CGCTCGAGGA	0	0	1	0	0	At5g48370.1:X:-121:quaternary
GATGCAGAAG	1	0	0	0	0	At5g48370.1:d:+1333:primary
AAGTTATTGA	0	1	0	0	0	At5g48340.1:d:+358:secondary
ATATATTGTC	0	0	0	0	1	At5g48310.1:v:+2386:secondary
TTACTAGCAT	0	1	0	0	0	At5g48300.1:d:-562:primary
ATCCTTAAAT	0	0	1	0	0	At5g48250.1:d:+1569:secondary
AGGATAAATC	1	0	0	0	0	At5g48230.1:X:-215:quaternary
TTGCCTGGAT	1	0	0	0	0	At5g48200.1:v:-397:secondary
TAGACAATGT	0	0	0	0	1	At5g48200.1:v:+358:secondary
TGAAGGGCAA	0	0	1	0	0	At5g48200.1:v:+1440:primary
CGGCTCTTCC	1	0	0	0	0	At5g48180.1:X:-698:quaternary
TGCCAAAGCT	0	0	0	1	0	At5g48150.2:d:+594:secondary,At5g56440.1:v:+472:secondary,At5g48150.1:d:+519:secondary
AGCGCAAACT	0	0	0	1	0	At5g48120.1:d:+452:secondary
GGTTTTAATT	0	0	1	0	0	At5g48110.1:i:+1921:tertiary,At5g51200.1:i:+7294:tertiary
TTCGGTGTCC	0	0	0	0	1	At5g48110.1:i:+1691:tertiary
AACCAAACCC	0	0	0	0	1	At5g48100.1:X:-9:quaternary
TGAGGAGGGT	0	0	0	0	1	At5g48060.1:d:-2714:secondary
ACCAAAATGA	0	0	1	0	0	At5g48060.1:d:-2621:secondary
AAGCCCAAAA	1	0	0	0	0	At5g48060.1:d:+689:secondary
CTGGATGTAG	0	1	0	0	0	At5g48060.1:d:+2452:secondary
GATGTCGAGA	0	1	0	0	0	At5g48050.1:X:-1018:quaternary
ATTCGAGTAG	0	1	0	0	0	At5g48020.1:X:-242:quaternary,At5g49830.1:X:-495:quaternary
GTGTTTGGAG	0	0	1	0	0	At5g48000.3:d:+1454:primary,At5g48000.2:d:+1295:primary,At5g48000.1:d:+1435:primary
AAGAGAAATT	0	0	1	0	0	At5g47990.1:d:+803:secondary
TTTCCTGATT	0	0	1	0	0	At5g47990.1:d:+1624:primary
AGCACAACTT	0	0	0	1	0	At5g47970.1:i:+3004:tertiary
TGAGTTTACT	0	0	0	0	1	At5g47930.1:i:+392:tertiary
TAACTCAATG	0	1	0	0	0	At5g47900.1:v:-1918:secondary
TAATAAAAAC	1	0	0	0	0	At5g47880.1:X:-173:quaternary
TGGAGAGTGA	0	0	0	1	0	At5g47880.1:d:+1540:secondary
TGAATAAATA	0	0	0	1	0	At5g47840.1:i:+964:tertiary
AGATTGACTC	0	0	0	0	1	At5g47840.1:i:+2288:tertiary,At2g18876.2:i:+774:tertiary,At2g18876.1:i:+750:tertiary
AAGGGAAGAA	0	0	0	0	1	At5g47815.1:p:-3834:secondary
AACCGCCTTG	0	0	1	0	0	At5g47810.1:X:-537:quaternary
GGATAATATG	1	0	0	0	0	At5g47800.1:X:+630:quaternary
TTACAAATGT	0	1	0	0	0	At5g47800.1:i:-1887:tertiary
ATGCACAAGC	0	1	0	0	0	At5g47800.1:d:+1626:primary,At1g30475.1:d:+730:primary
GTACGTTTGT	0	1	0	0	0	At5g47790.1:d:+480:secondary,At1g47606.1:p:+493:secondary
CAAATGTAAA	1	0	0	0	0	At5g47730.1:d:+1624:primary
ACAATGGAGG	0	0	1	0	0	At5g47680.1:d:+2205:secondary,At3g22430.1:d:+920:secondary
CGGTTTGCAA	0	0	1	0	0	At5g47680.1:d:+1244:secondary
TGATATTACA	0	0	0	0	1	At5g47670.1:d:+914:secondary
AACAAGTACT	1	0	0	0	0	At5g47660.1:v:+1422:secondary
TTAAAGCCCC	0	0	0	0	1	At5g47650.1:d:+666:secondary
TGTCGAACCA	0	0	0	1	0	At5g47570.1:X:+321:quaternary
TGTCACTCAC	0	0	1	0	0	At5g47560.1:X:+-121:quaternary,At5g47560.1:X:+-114:quaternary
GATGACGTCA	1	0	0	0	0	At5g47560.1:d:+479:secondary
AATGTTGTAA	0	0	0	0	1	At5g47540.1:v:+1629:primary
ACAAGTGTAA	0	1	0	0	0	At5g47530.1:i:-1147:tertiary
GCAACGTGGA	0	0	1	0	0	At5g47520.1:X:-403:quaternary
ATAATGAATG	0	0	0	0	1	At5g47520.1:d:+876:primary,At1g55100.1:p:+714:primary
GAGACATCGG	0	0	0	0	1	At5g47520.1:d:+463:secondary
TATAAAGATT	0	1	0	0	0	At5g47500.1:d:+717:secondary,At3g43700.1:d:+863:secondary

AAGAGTATTA	0	0	0	0	1	At5g47455.3:d:+419:secondary,At5g47455.6:d:+511:secondary,At5g47455.5:d:+521:secondary,At3g57420.1:d:+958:secondary,At5g47455.2:d:+466:secondary,At5g47455.4:d:+417:secondary
TGAGGACACT	1	0	0	0	0	At5g47455.2:i:+669:tertiary,At5g47455.1:i:+667:tertiary,At5g47455.5:i:+667:tertiary,At5g47455.6:i:+667:tertiary,At5g47455.3:i:+667:tertiary,At5g47455.4:i:+667:tertiary
ATCCGTGTAT	0	0	1	0	0	At5g47455.2:i:+1155:tertiary,At5g47455.5:i:+1153:tertiary,At5g47455.3:i:+1153:tertiary,At5g47455.1:i:+1153:tertiary,At5g47455.4:i:+1153:tertiary,At5g47455.6:i:+1153:tertiary
ACTAGTTTGA	0	0	0	0	1	At5g47455.2:d:+643:primary
AAAAAGACAA	0	1	0	0	0	At5g47445.1:p:+938:secondary
CGATAGAGAA	0	1	0	0	0	At5g47440.1:v:+1566:primary
CGATTGCTGA	0	1	0	0	0	At5g47435.2:d:+1124:secondary
GCATCCCTTA	1	0	0	0	0	At5g47435.1:d:+609:secondary,At5g47435.2:d:+609:secondary
TTTGGCGACG	0	0	0	1	0	At5g47420.1:d:-929:secondary
AGAGAGAATC	1	0	0	0	0	At5g47420.1:d:+934:primary
CTCAGAAGTA	0	0	0	1	0	At5g47390.1:d:+624:secondary,At5g52660.1:d:+443:secondary,At3g10580.1:v:+760:secondary,At4g09450.1:d:+398:secondary,At5g52660.2:d:+459:secondary
CATACTTAGC	0	0	0	0	1	At5g47380.1:v:+1531:secondary
TTGAGATAAT	0	0	0	1	0	At5g47340.1:v:-1531:secondary
AGAAACTTCC	0	0	1	0	0	At5g47340.1:v:+1536:primary
CAATATATGT	0	0	0	0	1	At5g47330.1:d:+1046:primary
TGAGCTAATG	0	1	0	0	0	At5g47320.1:d:-844:secondary,At5g47320.1:d:-838:secondary
TGCGTTGTAA	0	1	0	0	0	At5g47320.1:d:+645:secondary
ACAAACAAAG	0	0	0	1	0	At5g47320.1:d:+287:secondary
ATTGTTTGAA	0	0	0	0	1	At5g47310.1:d:+68:secondary
GCTCAACTAC	0	1	0	0	0	At5g47250.1:X:-27:quaternary
CACCTACGTG	0	1	0	0	0	At5g47230.1:d:-1094:secondary
ACATTCACGG	0	0	1	0	0	At5g47220.1:X:+985:quaternary
GTACTGGCAT	0	1	0	0	0	At5g47210.1:d:+686:secondary,At2g07687.1:d:+729:secondary
GCTATGACTG	0	0	1	0	0	At5g47200.1:d:+614:secondary
GTGGGTTTGA	0	1	0	0	0	At5g47190.1:i:+821:tertiary
GGGCTTCAGT	0	0	1	0	0	At5g47130.1:v:+484:secondary
TGAATTGAAA	0	1	0	0	0	At5g47130.1:v:+1061:primary
GCGTTATTCT	0	0	1	0	0	At5g47110.1:d:+95:secondary
TCGTAAAGCC	0	0	1	0	0	At5g47100.1:X:-18:quaternary
GCTAGGCCTG	0	1	0	0	0	At5g47090.1:d:+496:secondary
AAATAGTAAA	0	0	0	0	1	At5g47090.1:d:+1198:primary
GAAATCCCCA	0	0	0	1	0	At5g47060.1:d:+158:secondary
CAGTGAGCTT	0	1	0	0	0	At5g47050.1:d:+1339:secondary
ACTACAGAGA	0	0	0	0	1	At5g47020.1:d:+970:secondary
CTGTTCACGG	0	0	1	0	0	At5g47010.1:d:+3838:secondary
CAGCAGTAGC	0	1	0	0	0	At5g46910.1:v:+2090:secondary
TCAGGGGCAA	0	0	1	0	0	At5g46880.1:v:+2877:primary
ACTCCTTGAT	0	1	0	0	0	At5g46860.1:X:-149:quaternary
TTGTTAAATG	0	0	0	1	0	At5g46760.1:v:+2254:primary
TTGAAGCAGA	0	1	0	0	0	At5g46760.1:v:+1600:secondary
CTCTTTCATA	1	0	0	0	0	At5g46740.1:d:+2331:secondary
AGGAAACCAA	0	0	0	1	0	At5g46730.1:d:+891:primary
GCTTCTTGTT	0	1	0	0	0	At5g46700.1:d:+281:secondary
AATTCGTCAG	0	0	0	1	0	At5g46660.1:v:-718:secondary,At5g46660.1:v:-729:secondary
GTGGATGCAT	0	0	1	0	0	At5g46630.2:d:+226:secondary,At5g46630.1:d:+226:secondary
CTAGTTCTGT	0	0	0	0	1	At5g46620.1:i:+727:tertiary
ATATCTTTAA	0	1	0	0	0	At5g46610.1:v:+2045:secondary
ATTCCTTTTG	1	0	0	0	0	At5g46590.1:v:-1666:secondary
AGCTTCTCTA	0	0	0	1	0	At5g46580.1:d:+1769:secondary
AGAGAGCTCA	0	0	0	0	1	At5g46580.1:d:+1757:secondary
TCCTGACCAA	1	0	0	0	0	At5g46560.1:d:+1289:secondary
TACAGAAGAA	0	0	0	0	1	At5g46560.1:d:+1144:secondary,At4g14980.1:d:+107:secondary
AAGAAAAAGG	0	0	0	0	1	At5g46550.1:d:+598:primary

ATGAGATGAT	0	0	0	1	0	At5g46540.1:v:+2083:secondary
AGAATCCATT	0	0	1	0	0	At5g46510.1:i:+2625:tertiary,At3g24760.1:i:+1327:tertiary,At5g46520.1:i:+2736:tertiary
TAGGTGCAAT	0	1	0	0	0	At5g46470.1:d:+857:secondary
TTCTTTGAGT	1	0	0	0	0	At5g46470.1:d:+4165:secondary,At1g71330.1:v:+389:secondary
AAGTTGCGTT	0	1	0	0	0	At5g46450.1:v:+1140:secondary
AACGACGCCG	0	0	0	1	0	At5g46440.1:d:+314:primary
GGAGATACTT	0	0	0	1	0	At5g46420.1:d:+1556:secondary,At2g13540.1:d:+2059:secondary
TGTCTCTCTT	0	1	0	0	0	At5g46410.1:d:+1961:secondary,At3g24400.1:p:+2060:secondary,At1g34630.1:d:+751:secondary
ATTGCTTCAA	0	0	0	1	0	At5g46400.1:i:+4361:tertiary
TTTACCTTCA	0	0	1	0	0	At5g46380.1:i:+841:tertiary
ACTAAGACCG	0	1	0	0	0	At5g46350.1:d:+662:secondary
CTGACAACTG	0	0	1	0	0	At5g46340.1:d:+1704:primary
TAAAAAGAAA	0	0	1	0	0	At5g46320.1:v:+197:secondary,At1g10620.1:v:+308:secondary
GTCATTAACT	0	1	0	0	0	At5g46270.1:v:+4188:primary
TTTCCAACAG	0	1	0	0	0	At5g46270.1:v:+4043:secondary
ACTCTTGTTT	0	0	0	1	0	At5g46250.2:i:+1360:tertiary,At5g46250.1:i:+1360:tertiary
CCTTTGTGGA	0	1	0	0	0	At5g46250.1:d:+816:secondary,At5g46250.2:d:+816:secondary
GAACAGCTTC	0	0	1	0	0	At5g46250.1:d:+1182:secondary,At5g39660.1:d:+1199:secondary,At5g46250.2:d:+1176:secondary,At5g39660.2:d:+1172:secondary
CTCTATAAAT	0	1	0	0	0	At5g46240.1:X:-576:quaternary
ACATACAAGA	0	0	0	0	1	At5g46240.1:d:+1152:secondary
TTTGCTACAA	0	0	0	0	1	At5g46220.1:v:-2155:secondary
TTAATTGTTA	0	1	0	0	0	At5g46220.1:i:+2754:tertiary
AAAAACCTTA	0	1	0	0	0	At5g46220.1:i:+2113:tertiary
ATCTCTAAGC	0	0	1	0	0	At5g46210.1:d:+1679:secondary
TTGACTTTTT	0	0	1	0	0	At5g46200.1:i:+1737:tertiary
GATTTTGACA	0	1	0	0	0	At5g46190.1:d:+758:secondary,At4g10540.1:v:+1382:secondary
GAAGTGTGTT	0	0	0	0	1	At5g46190.1:d:+2129:secondary
ATATCTCAAT	0	0	1	0	0	At5g46180.1:d:+1651:primary
GGGAGAGGTA	0	1	0	0	0	At5g46150.2:d:+1297:secondary
GTTAAGTAGT	0	0	0	1	0	At5g46110.2:d:-1299:secondary,At5g46110.1:d:-1292:secondary
TCACTAGCAA	0	0	1	0	0	At5g46110.2:d:+932:secondary,At5g46110.1:d:+925:secondary
ACTGATATGG	1	0	0	0	0	At5g46110.1:d:+1191:secondary,At5g46110.2:d:+1198:secondary,At1g35405.1:p:+3442:secondary
TTTGTTTCAG	1	0	0	0	0	At5g46090.1:v:+1141:secondary
TGAGGAACTC	0	0	0	0	1	At5g46070.1:d:-2811:secondary
GCCCGGGGAA	0	1	0	0	0	At5g46070.1:d:+1367:secondary
AAAAGCCTCG	0	0	0	0	1	At5g46050.1:d:+1543:secondary
ATTTTCGATA	0	0	1	0	0	At5g46040.1:v:+2349:secondary
TCTCTGTCAA	1	0	0	0	0	At5g46030.1:d:+457:secondary
AGCTCGCTGC	0	0	1	0	0	At5g46020.1:X:-24:quaternary
CAAGTGGGTT	0	1	0	0	0	At5g45960.1:d:+880:primary
GTCGATGGGA	0	0	1	0	0	At5g45860.1:v:+898:secondary
AGGATCAGAA	0	1	0	0	0	At5g45840.1:X:-43:quaternary
AAAGCATTTT	0	0	1	0	0	At5g45840.1:i:+1100:tertiary,At3g56120.1:i:+1438:tertiary
ATCTCGTCTC	0	0	0	1	0	At5g45830.1:d:+906:primary
AGTGGGGAAC	0	0	1	0	0	At5g45830.1:d:+749:secondary
TGGAACACCT	0	0	0	1	0	At5g45820.1:v:+866:secondary
TATCGGCACT	0	0	0	0	1	At5g45800.1:X:-107:quaternary
ACAAAATTTC	0	0	0	0	1	At5g45720.1:v:+3528:primary
AATGAGGTTT	0	1	0	0	0	At5g45710.2:d:+1437:primary,At5g45710.1:d:+1361:primary
AGCCTCCTTT	0	0	1	0	0	At5g45680.1:d:-579:secondary
TGGTAGAAGA	1	0	0	0	0	At5g45680.1:d:+182:secondary
TCTCTGCTCG	0	0	0	1	0	At5g45610.1:X:-98:quaternary,At3g25990.1:X:-159:quaternary
GATTTAGCCC	0	0	1	0	0	At5g45610.1:d:+1843:primary
TTCTGTTACT	1	0	0	0	0	At5g45576.1:p:+783:secondary,At5g28232.1:p:+1363:secondary
AATGCTGATC	0	0	1	0	0	At5g45510.1:d:+3803:secondary
CTGAAAAAGA	0	0	0	0	1	At5g45510.1:d:+1318:secondary
GCTTGATAAC	0	1	0	0	0	At5g45500.1:d:+929:secondary
AGACTTCTCT	1	0	0	0	0	At5g45480.1:X:-1477:quaternary
CTGGCGAAAG	0	1	0	0	0	At5g45480.1:d:+2541:secondary
TTCCTCAGAC	0	0	0	0	1	At5g45430.1:d:+2316:secondary

TTCATTAGAA	0	0	0	1	0	At5g45400.1:v:+7:secondary
GCTATGGCTA	0	0	1	0	0	At5g45350.1:d:+490:secondary,At5g17650.1:d:+570:secondary
CGTTGTTTGA	0	0	0	1	0	At5g45320.1:v:+607:secondary
GCGACATAAG	0	0	0	0	1	At5g45300.1:d:+2068:secondary
AAACTGTGTT	0	1	0	0	0	At5g45300.1:d:+1486:secondary
ATGATTGTTT	0	1	0	0	0	At5g45250.1:v:+4216:primary,At3g55390.1:d:+755:primary
AAACAGTTGG	1	0	0	0	0	At5g45250.1:v:+3277:secondary
ATATGAAAAA	0	0	0	1	0	At5g45250.1:v:+2455:secondary
AACATAAAAG	0	0	0	0	1	At5g45240.1:v:+1513:secondary
GAAGCTATGA	0	1	0	0	0	At5g45200.1:v:+3583:secondary,At4g36250.1:d:+95:secondary
GGTCCTCAAA	0	0	0	1	0	At5g45160.1:d:+139:secondary
ATTTACTAGG	0	1	0	0	0	At5g45140.1:i:+3956:tertiary
CAAGCTACTA	0	0	0	1	0	At5g45140.1:d:+3506:primary
AAACTGCACC	1	0	0	0	0	At5g45110.1:d:+2170:primary
GCACAAGTTT	0	0	0	0	1	At5g45105.1:v:+716:secondary
GAATGAAGTA	0	0	0	1	0	At5g45105.1:v:+12:secondary
ACCATTTTTT	0	0	1	0	0	At5g45100.1:d:+955:primary,At5g45100.2:d:+1156:primary
TTGAATGAAG	1	0	0	0	0	At5g45090.1:v:+990:secondary,At2g37150.1:d:+307:secondary,At2g37150.2:d:+820:secondary
TTTTCATAGC	1	0	0	0	0	At5g45060.1:d:+3089:secondary
TAAACAATTT	0	1	0	0	0	At5g45050.1:d:+1601:secondary,At5g45050.2:d:+1601:secondary
AGACTTCATA	0	0	0	0	1	At5g45030.1:d:+1976:primary
CCCAGAGACT	0	1	0	0	0	At5g44910.1:v:-1228:secondary
AGAGACTTGA	1	0	0	0	0	At5g44910.1:v:+1340:primary
TTTTTATCGG	0	0	1	0	0	At5g44870.1:v:+3646:secondary
AGAACCAAGA	0	0	1	0	0	At5g44870.1:v:+3514:secondary,At3g51120.1:d:+2393:secondary
AAGAACTGCG	0	0	1	0	0	At5g44870.1:v:+3087:secondary
AAACATCTAC	0	0	0	0	1	At5g44870.1:v:+2934:secondary
TCTAAAGTTC	0	0	0	1	0	At5g44870.1:v:+2166:secondary
GAATAAGTGT	1	0	0	0	0	At5g44840.1:v:+1039:secondary,At5g44830.1:v:+892:secondary,At5g27530.1:v:+1126:secondary
TGTACAATTT	0	0	0	1	0	At5g44800.1:v:+95:secondary
GCGCAGAATG	0	1	0	0	0	At5g44800.1:v:+4738:secondary
TATCATTTGC	1	0	0	0	0	At5g44770.1:v:+2458:primary
AAACTGCTAA	0	1	0	0	0	At5g44760.1:v:+1741:secondary
GGCCTTGACT	0	0	1	0	0	At5g44750.1:d:-3054:secondary
AAACAATATA	1	0	0	0	0	At5g44690.1:v:+2881:primary,At3g43950.1:v:+1471:primary
TTGTAATTGT	1	0	0	0	0	At5g44680.1:d:+1129:secondary,At1g53520.1:d:+919:secondary
GGACTTGGAG	0	0	0	1	0	At5g44630.1:v:+1617:secondary
TTGGATTCCT	0	0	1	0	0	At5g44620.1:d:+482:secondary
AACTGTTCTT	0	0	1	0	0	At5g44580.1:d:+123:secondary
ACGATAGAGA	1	0	0	0	0	At5g44560.1:d:+514:secondary,At1g77220.1:d:+1334:secondary
AAGCCTATCA	0	1	0	0	0	At5g44520.1:X:--30:quaternary
GACTAATTAT	1	0	0	0	0	At5g44520.1:d:+850:secondary
CACACCAAAC	0	0	1	0	0	At5g44510.1:X:-26:quaternary
TGTCAAATCA	0	0	0	1	0	At5g44490.1:v:+1614:secondary,At1g37000.1:v:+848:secondary
AGTACACGAG	0	0	0	1	0	At5g44450.1:X:-305:quaternary
TCCAAATTTG	1	0	0	0	0	At5g44430.1:v:+955:secondary,At4g04340.3:d:+1895:secondary,At2g44990.1:v:+142:secondary,At4g22120.1:d:+1449:secondary,At4g04340.1:d:+1597:secondary,At4g04340.2:d:+1599:secondary
CATATATGTT	1	0	0	0	0	At5g44430.1:v:+679:secondary,At2g31030.1:v:+219:secondary
ATATCTTCTA	0	1	0	0	0	At5g44430.1:v:+143:secondary
GAAAAAAAAC	0	0	1	0	0	At5g44430.1:i:+104:tertiary
TAACGGGCCT	0	1	0	0	0	At5g44410.1:X:+1637:quaternary
GAAGATTAGA	0	0	0	1	0	At5g44410.1:d:+738:primary
GCTAAAAGCA	0	0	1	0	0	At5g44410.1:d:+370:secondary
CATCAAACAA	0	1	0	0	0	At5g44400.1:d:+1630:primary
GCCAGCTTCA	0	0	1	0	0	At5g44260.1:d:+1074:secondary
GGTTTGGATA	0	0	0	1	0	At5g44200.1:d:+248:secondary
GTCACTTCAG	0	1	0	0	0	At5g44190.1:d:+824:secondary,At1g22240.1:v:+1161:secondary
GGATTAGCAT	0	0	1	0	0	At5g44180.1:d:+4991:primary
AGAAAATATA	0	0	0	0	1	At5g44160.1:i:+561:tertiary
ATCATCAAGA	0	1	0	0	0	At5g44160.1:d:+1358:primary

GAAATCGATG	0	0	1	0	0	At5g44090.1:d:+491:secondary
TAGATTCTGT	0	0	1	0	0	At5g44070.1:d:+885:secondary
AATACAAAAA	0	1	0	0	0	At5g44060.1:d:+208:secondary
AATTAAATCT	0	0	0	1	0	At5g44050.1:d:+1592:primary
GAACCAAACA	0	1	0	0	0	At5g44030.1:d:+72:secondary,At5g47370.1:d:+388:secondary,At3g22170.1:d:+1816:secondary
AGATCAGAGA	0	1	0	0	0	At5g44020.1:d:+543:secondary
TTGTGGAAAA	0	0	0	0	1	At5g44005.1:d:+194:primary
GGAATTCAAG	0	1	0	0	0	At5g44000.1:d:+455:primary
AAGAATTGTG	1	0	0	0	0	At5g43990.2:d:+1820:secondary,At5g43990.1:d:+1756:secondary
TCCAATTGTA	0	0	1	0	0	At5g43980.1:d:+1158:primary
AACATCGGGC	0	0	1	0	0	At5g43960.1:d:+945:secondary,At5g43960.2:d:+897:secondary
TGTCCTATGT	0	1	0	0	0	At5g43950.1:d:+1843:secondary,At5g01120.1:v:+702:secondary
AAGTTATGGG	0	0	1	0	0	At5g43940.1:d:+1469:secondary
GTACACTTTA	1	0	0	0	0	At5g43930.1:i:+629:tertiary
TGTTGCTCGT	0	0	0	1	0	At5g43910.1:d:+328:secondary,At5g43910.2:d:+328:secondary,At5g43910.3:d:+328:secondary
TACTGTTCTT	0	1	0	0	0	At5g43900.1:X:-396:quaternary
ATCCGATATT	0	0	1	0	0	At5g43900.1:X:-244:quaternary
TTTGCAAAAA	0	0	0	1	0	At5g43900.1:i:+4112:tertiary
ACAGAGGAAT	0	1	0	0	0	At5g43900.1:d:+4524:secondary
TTCCTTTAGT	0	1	0	0	0	At5g43900.1:d:+4135:secondary,At1g08680.1:d:+1161:secondary,At1g08680.2:d:+1161:secondary
TCTTTAAAAT	1	0	0	0	0	At5g43900.1:d:+1570:secondary
AAACGATTAA	0	0	1	0	0	At5g43870.1:i:+2929:tertiary
TACATAAACG	0	1	0	0	0	At5g43830.1:X:-320:quaternary
TAACGAAAGA	1	0	0	0	0	At5g43830.1:X:-259:quaternary
GAAATCTCTA	0	1	0	0	0	At5g43830.1:X:-180:quaternary
TTTGAGTTAC	0	1	0	0	0	At5g43830.1:d:+909:secondary
AAATTAGAAG	1	0	0	0	0	At5g43810.1:i:-550:tertiary
AGTTTATTCA	1	0	0	0	0	At5g43770.1:v:+173:secondary
GATTAGTCTT	0	1	0	0	0	At5g43760.1:d:-1662:secondary
GAGCCATCGA	0	0	1	0	0	At5g43760.1:d:+1461:secondary
GATGTAAAAC	0	0	1	0	0	At5g43745.1:X:+526:quaternary
ATGGAGTCAA	1	0	0	0	0	At5g43740.2:d:+161:secondary,At5g43740.1:d:+161:secondary,At5g35046.1:p:+3351:secondary,At4g08131.1:p:+413:secondary,At3g30819.1:p:+3105:secondary
ATCTGGTTTT	0	0	1	0	0	At5g43680.1:i:+663:tertiary,At2g18110.1:i:+256:tertiary
GTTTTCTCAA	0	0	1	0	0	At5g43680.1:d:+1103:primary
TAACAGAACC	1	0	0	0	0	At5g43600.1:d:-1791:secondary
TAATTAATAA	0	0	0	0	1	At5g43600.1:d:+1796:primary
GCTAATGGAG	1	0	0	0	0	At5g43600.1:d:+1553:secondary,At2g29010.1:p:+2654:secondary,At5g16900.1:v:+2298:secondary,At1g51880.1:v:+2298:secondary,At1g49100.1:v:+2322:secondary
CAATAGCTTC	0	0	0	1	0	At5g43560.2:d:-1370:secondary,At5g43560.1:d:-1740:secondary
GCGGTGCGGA	0	0	0	0	1	At5g43560.1:d:+3373:secondary,At5g43560.2:d:+3003:secondary
TGTTAATTTT	1	0	0	0	0	At5g43530.1:i:+3267:tertiary
GCAAAACCAA	0	0	1	0	0	At5g43500.1:d:+238:secondary,At3g02260.1:d:+10561:secondary,At1g28010.1:d:+911:secondary,At2g16430.1:d:+311:secondary,At2g16430.2:d:+358:secondary
GAGTATCTGA	0	0	1	0	0	At5g43490.1:X:-203:quaternary
CATCCACATC	0	1	0	0	0	At5g43470.1:X:-258:quaternary
ATTGGTTTGA	0	0	0	0	1	At5g43470.1:i:+2431:tertiary,At5g43470.2:i:+2431:tertiary,At5g48620.1:i:+1773:tertiary
GAAGAGGATC	0	0	1	0	0	At5g43470.1:d:+2534:secondary,At5g55390.1:d:+3133:secondary,At5g43470.2:d:+2534:secondary
AACAAGATCC	0	0	0	1	0	At5g43450.1:d:+427:secondary,At3g45710.1:d:+1730:secondary
CAGGCAAGCT	0	1	0	0	0	At5g43440.1:i:+969:tertiary
CAGCTGATCA	0	1	0	0	0	At5g43440.1:d:+893:primary
AATGAATCTT	1	0	0	0	0	At5g43400.1:d:+2195:primary,At5g43390.1:v:+2324:primary
GTTTTTGCTT	0	0	0	1	0	At5g43350.1:d:+988:secondary
GAATTTAGTG	0	1	0	0	0	At5g43320.1:X:-481:quaternary
GGTTTCTTCC	0	1	0	0	0	At5g43270.1:d:+1220:secondary,At5g43270.2:d:+1145:secondary,At5g43270.3:d:+1097:secondary
TTTTCTCATT	0	1	0	0	0	At5g43250.1:v:+223:secondary,At2g16390.1:v:+3334:secondary,At5g59050.2:d:+626:secondary
AACAAGAATA	1	0	0	0	0	At5g43220.1:v:+1473:primary
ATCAAGTTTC	0	0	0	1	0	At5g43185.1:X:+-129:quaternary

CTTCTTGCTC	0	1	0	0	0	At5g43170.1:d:+181:primary,At5g04340.1:d:+241:primary
ACGTGAGTTC	0	1	0	0	0	At5g43150.1:d:+180:secondary
TATGAATTTT	0	1	0	0	0	At5g43140.1:d:+937:primary
TTCCAAGAGT	0	0	1	0	0	At5g43130.1:X:-300:quaternary
GGGATAGATT	0	0	0	0	1	At5g43120.1:v:+1317:secondary
TATTGTTTGG	0	1	0	0	0	At5g43110.1:v:+59:secondary
ATGGATCTTG	0	1	0	0	0	At5g43105.1:p:+2846:secondary
AGTTAGACAT	0	1	0	0	0	At5g43100.1:d:+1622:primary
TATTAATTTT	0	0	1	0	0	At5g43065.1:p:+2789:secondary
GTGCCCACCT	0	0	1	0	0	At5g42980.1:d:+249:secondary
TCTATGTCTT	1	0	0	0	0	At5g42970.1:d:+1424:primary
AATTTGCAAG	0	1	0	0	0	At5g42965.1:v:+944:secondary
GGGAAACTGT	0	0	0	0	1	At5g42950.1:X:+-245:quaternary
AATTCCATAG	0	0	1	0	0	At5g42940.1:d:+2160:secondary
ATGCTATAAT	1	0	0	0	0	At5g42860.1:X:+-85:quaternary
GAGCCGTTGC	0	0	1	0	0	At5g42820.2:X:-250:quaternary
ACTCGTATCA	0	0	1	0	0	At5g42810.1:d:-672:secondary
GAGATGATTT	0	1	0	0	0	At5g42810.1:d:+259:secondary
TCGTCAAAAG	0	0	0	0	1	At5g42800.1:d:+957:secondary
CTAGCACAAC	0	0	0	0	1	At5g42800.1:d:+1126:primary
TGGCTGTCAC	0	1	0	0	0	At5g42780.1:d:+368:primary
CTAGTTATCG	0	0	0	1	0	At5g42765.1:d:+909:secondary
GAAGAGTTAC	1	0	0	0	0	At5g42750.1:d:+1044:primary
TCAAACTTTT	0	0	0	1	0	At5g42740.1:d:+1393:primary
GTTAGATCAG	0	0	1	0	0	At5g42720.1:d:-1067:secondary
CATCAAAAGT	0	1	0	0	0	At5g42710.1:v:+1961:secondary,At1g15110.1:d:+1645:secondary
GTTCTTGGAA	0	1	0	0	0	At5g42670.1:v:+703:secondary
TTTTAAACTG	0	1	0	0	0	At5g42660.1:d:+1655:primary
TACGCAAGGC	0	0	1	0	0	At5g42660.1:d:+1540:secondary
CCGTCAACGG	0	0	1	0	0	At5g42650.1:d:+463:secondary
TGTTGTGGTC	0	0	0	1	0	At5g42650.1:d:+1394:secondary
AACTCTGCAG	0	0	1	0	0	At5g42620.1:d:+2041:secondary,At5g23040.1:d:+572:secondary,At5g23040.2:d:+572:secondary,At4g18490.1:v:+892:secondary
TTTCATTTCA	0	0	0	0	1	At5g42610.1:v:+1579:primary
TCAAAGAGTT	0	0	0	0	1	At5g42600.1:d:+446:secondary,At4g34580.1:v:+886:secondary
TTCTTGGACG	0	1	0	0	0	At5g42600.1:d:+373:secondary
AGAATGACAT	1	0	0	0	0	At5g42560.1:d:+495:primary
AAGATGGGGA	0	0	0	1	0	At5g42540.1:d:+2527:secondary
CAGATTCCTG	0	0	0	1	0	At5g42540.1:d:+1041:secondary
TACAGGAGTT	0	0	1	0	0	At5g42520.1:d:+1004:secondary
TCATTAGATA	1	0	0	0	0	At5g42490.1:X:-265:quaternary
AATTGGATAA	0	0	0	0	1	At5g42480.1:i:+668:tertiary
GAAGGTTGTA	0	0	0	1	0	At5g42460.1:d:+553:secondary,At1g62600.1:d:+1067:secondary
TGAGCCGAAC	0	0	0	1	0	At5g42400.1:v:+4391:primary
TGGAATGCAG	0	0	1	0	0	At5g42400.1:v:+1737:secondary
AACACCTCCC	0	0	0	1	0	At5g42390.1:d:-4240:secondary
GCCTAGTGGG	0	0	0	0	1	At5g42390.1:d:+4245:primary
ATGGACTTTT	0	0	1	0	0	At5g42390.1:d:+3182:secondary
ATTCTGCAAA	0	0	0	0	1	At5g42390.1:d:+2984:secondary
GATTTTGATC	0	0	1	0	0	At5g42370.1:d:+1739:primary
GGACATTAAG	0	0	0	1	0	At5g42340.1:v:+1882:primary,At3g08710.1:d:+538:primary
GATGTTTTGT	1	0	0	0	0	At5g42330.1:d:+732:primary
ATCTCTGTAG	0	1	0	0	0	At5g42320.1:v:+1911:secondary
TGAATAGTGG	0	0	1	0	0	At5g42320.1:i:+1755:tertiary
GGTCTTGAGC	0	0	0	0	1	At5g42300.1:d:+297:secondary
TCAATGTATA	0	1	0	0	0	At5g42280.1:v:+2747:secondary
AAGCCCCACT	0	1	0	0	0	At5g42280.1:v:+2223:secondary
TATCTATCTA	0	0	0	0	1	At5g42280.1:v:+1235:secondary,At4g04520.1:p:+244:secondary,At1g55430.1:v:+1145:secondary,At1g47816.1:p:+313:secondary
GAGAAGCAGT	1	0	0	0	0	At5g42260.1:v:+1093:secondary,At5g44640.1:v:+1093:secondary
TTTCGACAGG	0	1	0	0	0	At5g42250.1:i:+1196:tertiary

AGATGAAATT	1	0	0	0	0	At5g42250.1:d:+1169:secondary
CGACCAGAAG	1	0	0	0	0	At5g42240.1:d:-769:secondary,At5g42230.1:d:-816:secondary,At5g42230.1:d:-811:secondary,At5g42240.1:d:-764:secondary
TTAACCAAAT	0	0	1	0	0	At5g42220.1:i:+1047:tertiary
AAATGGCTTC	0	0	1	0	0	At5g42210.1:v:+679:secondary
AACATTTTCT	0	1	0	0	0	At5g42210.1:v:+1104:secondary,At4g19700.1:d:+97:secondary
TCTCTTACAG	0	0	0	1	0	At5g42190.1:X:-234:quaternary
ACAGCAACCT	0	1	0	0	0	At5g42190.1:X:-199:quaternary
TCGAAGCTGC	0	0	0	1	0	At5g42190.1:d:+240:secondary
TTCGATCCAG	0	0	0	1	0	At5g42190.1:d:+1277:primary
TTTTTTTGGT	0	0	0	0	1	At5g42170.1:v:+1655:primary
TGGTAGTGCC	1	0	0	0	0	At5g42160.1:v:+110:secondary
GGTCGATGCC	0	0	1	0	0	At5g42150.1:d:+853:secondary
TGAACAGCTT	0	0	0	1	0	At5g42140.1:X:-978:quaternary
TCTAGAATTG	1	0	0	0	0	At5g42140.1:X:-136:quaternary
ATTGTTTAAA	0	0	0	0	1	At5g42140.1:d:+3369:primary
GGGCAAGGGT	0	0	1	0	0	At5g42140.1:d:+1710:secondary
TCGACATTCT	0	0	0	0	1	At5g42080.2:X:-85:quaternary,At2g44590.1:X:-85:quaternary
TTCAAACCGT	0	0	1	0	0	At5g42080.2:X:--10:quaternary
GTACCGAGCT	0	1	0	0	0	At5g42080.1:X:-132:quaternary
GAGAGACGCA	0	0	1	0	0	At5g42080.1:d:+1967:secondary
GTCTGTGCTG	0	0	1	0	0	At5g42080.1:d:+1811:secondary
GTATCGTTGT	0	0	0	1	0	At5g42050.1:d:-821:secondary
TAGATTTTGA	0	0	0	1	0	At5g42010.1:d:+2152:primary
CACAAGGTCC	1	0	0	0	0	At5g42000.1:d:+515:secondary
GAGGCAATTG	0	0	1	0	0	At5g41970.1:d:+474:secondary
TGTTTATCTC	1	0	0	0	0	At5g41950.1:i:+2234:tertiary,At5g13740.1:i:+2544:tertiary
TCGCCTTCGC	0	0	1	0	0	At5g41940.1:d:-1475:secondary
GGGGAGAATC	0	0	1	0	0	At5g41940.1:d:+1480:secondary
AGGCTGTTAA	1	0	0	0	0	At5g41910.1:X:+233:quaternary,At5g41910.1:X:+226:quaternary
TGATTGATGG	0	1	0	0	0	At5g41850.1:v:+886:secondary
ATCAACATCA	0	1	0	0	0	At5g41840.1:X:-191:quaternary
GATATTGTCT	0	1	0	0	0	At5g41840.1:v:+2003:secondary
AACCCCAATT	0	0	1	0	0	At5g41790.1:d:-3354:secondary
TGAAAGAATT	0	0	1	0	0	At5g41790.1:d:+827:secondary
AGACTTATCG	0	0	0	1	0	At5g41790.1:d:+3584:secondary
GAAAACAGAG	0	0	1	0	0	At5g41755.1:p:+902:secondary,At3g48200.1:d:+2432:secondary,At3g51910.1:v:+1054:secondary
AACAGTGGCC	0	1	0	0	0	At5g41750.1:d:+1631:secondary,At5g41750.2:d:+1631:secondary
AACTGATGAT	0	0	0	0	1	At5g41740.1:d:+3484:primary
AAGTGGATGT	1	0	0	0	0	At5g41740.1:d:+2877:secondary,At5g17240.1:v:+1461:secondary,At5g41750.2:d:+2996:secondary,At5g41750.1:d:+2996:secondary
TATAACTTTG	0	1	0	0	0	At5g41740.1:d:+2302:secondary
ACAAAGCTTG	0	0	0	1	0	At5g41710.1:p:+1555:secondary,At2g33240.1:v:+600:secondary,At1g04600.1:v:+549:secondary
GGCTAAACAA	0	0	1	0	0	At5g41700.4:d:+512:secondary,At5g41700.1:d:+573:secondary,At5g41700.3:d:+715:secondary,At5g41700.2:d:+618:secondary
TTTCATTGGC	0	0	1	0	0	At5g41700.4:d:+161:secondary,At4g27960.1:d:+170:secondary,At5g41700.3:d:+171:secondary,At4g27960.2:d:+308:secondary,At5g41700.1:d:+222:secondary,At5g41700.2:d:+267:secondary,At1g64230.1:d:+225:secondary
TGTGCAATCT	0	0	1	0	0	At5g41700.3:d:-647:secondary,At5g41700.1:d:-505:secondary,At5g41700.2:d:-550:secondary,At5g41700.4:d:-444:secondary
TACAAAACCG	0	0	1	0	0	At5g41700.1:d:+510:secondary,At5g41700.3:d:+652:secondary,At1g64230.1:d:+513:secondary,At5g41700.4:d:+449:secondary,At5g41700.2:d:+555:secondary
CAGAGAAACT	0	0	1	0	0	At5g41690.1:v:+2083:primary
CGTTAGGTTA	0	0	0	0	1	At5g41690.1:i:+182:tertiary
GAAACATTGA	0	0	0	1	0	At5g41670.2:X:-1612:quaternary
CTTTACTGAC	0	0	0	0	1	At5g41670.1:X:-1526:quaternary
TTTAACTTGC	0	0	0	0	1	At5g41650.1:d:+540:primary
TCTGAGGATT	0	0	0	1	0	At5g41600.1:X:+438:quaternary
ATCTTCTTTG	0	0	1	0	0	At5g41590.1:v:+1137:primary

TTTATGTCAA	1	0	0	0	0	At5g41580.1:i:+2015:tertiary,At1g78870.2:i:+622:tertiary,At4g17910.1:i:+4405:tertiary,At1g78870.1:i:+622:tertiary
AAAACTTGTG	1	0	0	0	0	At5g41550.1:v:+726:secondary
GAATAGACCA	0	0	1	0	0	At5g41510.1:v:+772:secondary
CTTATTGCCT	0	0	0	1	0	At5g41470.1:v:+845:primary
GTAAATTTTG	0	0	0	0	1	At5g41460.1:d:+1631:primary
ATAAATTAAT	0	1	0	0	0	At5g41450.1:v:+988:primary
CTCGAAGAGC	0	1	0	0	0	At5g41410.1:d:+1230:secondary
GCTGGAACCA	0	0	0	1	0	At5g41400.1:d:+225:primary
ACTTTTTTTA	0	1	0	0	0	At5g41360.1:i:+413:tertiary,At2g45380.1:i:+323:tertiary,At1g50090.1:i:+75:tertiary
CTGGTTCTAG	0	0	0	0	1	At5g41360.1:d:+2030:secondary,At1g34315.1:v:+771:secondary,At5g41370.1:d:+2014:secondary
GATCTGTTGA	0	1	0	0	0	At5g41315.1:v:+2255:secondary,At1g50390.1:v:+547:secondary,At1g06030.1:d:+749:secondary
AGTTGTCTAA	0	1	0	0	0	At5g41310.1:X:-153:quaternary
AGATCATTGA	1	0	0	0	0	At5g41260.1:d:+1143:secondary
GCGGGAGCTC	0	1	0	0	0	At5g41240.1:d:+1205:primary
AGTAATTTCA	0	0	0	0	1	At5g41240.1:d:+1179:secondary
CTCAAGTGTA	1	0	0	0	0	At5g41220.1:X:-411:quaternary
AAATTGGATG	0	1	0	0	0	At5g41220.1:X:--22:quaternary
GATCTTCCTC	0	0	0	0	1	At5g41220.1:d:+1410:secondary,At4g27440.1:d:+197:secondary
CTTGCTACAC	0	0	0	0	1	At5g41190.1:v:+1741:secondary
ATATTGAAGT	0	0	0	0	1	At5g41190.1:v:+1462:secondary
CCTTGGATAA	0	1	0	0	0	At5g41180.1:d:+2258:primary
ATCTCTATAC	0	0	0	0	1	At5g41170.1:d:+456:secondary
TGTGTTATAA	1	0	0	0	0	At5g41150.1:i:+1313:tertiary
GTTACACTCT	1	0	0	0	0	At5g41150.1:d:+3146:secondary
AAGATAATAC	0	0	1	0	0	At5g41150.1:d:+2333:secondary,At3g15520.1:d:+424:secondary,At5g49410.1:d:+540:secondary
CAGCTTGAAG	0	1	0	0	0	At5g41150.1:d:+1385:secondary
TGATAATCTC	1	0	0	0	0	At5g41140.1:d:+2649:secondary
AAAACATAGC	0	0	1	0	0	At5g41130.1:X:--36:quaternary,At5g18980.1:X:-106:quaternary
TCGGGGAAGT	0	1	0	0	0	At5g41100.2:d:+1901:primary
GTTTGTTTAT	1	0	0	0	0	At5g41100.1:d:+2002:primary
TACACTGCCT	0	1	0	0	0	At5g41080.2:d:-1044:secondary,At5g41080.1:d:-952:secondary
AGCTTCAGAA	0	0	0	1	0	At5g41020.1:X:-540:quaternary
TAAGGTATGG	1	0	0	0	0	At5g41020.1:X:-1932:quaternary
TTTTGAGTGT	0	0	0	1	0	At5g41020.1:X:-1521:quaternary
TCGATGGCGA	0	0	1	0	0	At5g41000.1:d:-1959:secondary
TAGTGTAACA	0	0	0	1	0	At5g40980.1:d:+350:primary
AAAACCAACG	0	1	0	0	0	At5g40970.1:d:+155:primary
GAACTTAGTG	1	0	0	0	0	At5g40950.1:d:-363:primary
TTTGTTTTTT	0	0	0	1	0	At5g40910.1:v:+259:secondary,At3g42230.1:p:+168:secondary,At2g30810.1:d:+220:secondary,At1g21650.1:v:+5317:secondary
GAATATCTAC	0	1	0	0	0	At5g40910.1:v:+2031:secondary
ATTTTGACAA	0	0	0	1	0	At5g40870.1:d:+1803:primary
TCTTTGTAGT	0	1	0	0	0	At5g40870.1:d:+1634:secondary
ATTGATGGTT	0	0	0	0	1	At5g40820.1:d:+7822:secondary
GAAAAGGCGA	0	1	0	0	0	At5g40820.1:d:+6010:secondary
ACGGTTTTGA	1	0	0	0	0	At5g40810.1:i:-2207:tertiary
ATGATGAGAA	0	0	1	0	0	At5g40780.2:d:+173:secondary,At2g02960.2:d:+262:secondary,At5g48380.1:d:+566:secondary,At2g02960.1:d:+266:secondary,At1g68410.1:d:+265:secondary,At4g38590.1:d:+98:secondary,At2g02960.4:d:+275:secondary,At4g24190.2:d:+1529:secondary,At2g02960.5:d:+471:secondary,At1g14110.1:v:+688:secondary,At1g14080.1:d:+396:secondary,At4g24190.1:d:+1529:secondary,At2g02960.3:d:+476:secondary
TAAACTATAT	0	1	0	0	0	At5g40760.1:i:+1446:tertiary
TACATTGCCT	1	0	0	0	0	At5g40760.1:d:-1398:secondary
TGGTCTAATT	0	1	0	0	0	At5g40740.1:d:+631:secondary,At3g12740.1:d:+743:secondary
TTCAGGAGGA	0	1	0	0	0	At5g40710.1:d:+682:primary
AGGATTGGTC	1	0	0	0	0	At5g40610.1:d:+878:primary
TCGGATGTGT	0	1	0	0	0	At5g40590.1:d:+331:secondary,At3g26190.1:d:+912:secondary,At3g26200.1:d:+951:secondary
GCAAAAACGC	0	0	0	0	1	At5g40550.1:d:+1092:primary
ATCCGAATCA	0	0	1	0	0	At5g40520.1:X:-1247:quaternary
AACTAATTGA	0	0	1	0	0	At5g40520.1:i:+2392:tertiary,At5g57140.1:i:+474:tertiary

GTGAGCAGAA	0	1	0	0	0	At5g40520.1:d:+1754:primary
AAAACTTTGG	0	0	1	0	0	At5g40460.1:d:+615:primary
GCCATCACCT	0	1	0	0	0	At5g40450.1:X:-3731:quaternary
TATTGGAGAG	0	1	0	0	0	At5g40450.1:d:+4019:secondary
TTTTACCTCT	0	0	0	0	1	At5g40410.1:i:-1905:tertiary
CTAAACACAA	1	0	0	0	0	At5g40390.1:d:+2336:primary
ATTTTGATCT	0	0	1	0	0	At5g40390.1:d:+1857:secondary,At5g64370.1:d:+255:secondary
TTGGTTTTGT	0	0	1	0	0	At5g40380.1:i:+745:tertiary,At2g24020.1:i:+746:tertiary,At3g18820.1:i:+749:tertiary
GTGGCCAGGT	0	1	0	0	0	At5g40340.1:v:+770:secondary,At3g05430.1:d:+564:secondary
ATGGAGCCAA	0	0	0	1	0	At5g40340.1:v:+2344:secondary
ATGAAGTTAA	0	1	0	0	0	At5g40340.1:v:+1615:secondary
CGGTGTAGAG	0	0	1	0	0	At5g40330.1:d:+511:primary
ATTCTTTCTA	0	1	0	0	0	At5g40300.1:d:+455:primary
ACCCGTTGTT	0	0	0	1	0	At5g40280.1:d:+1449:primary
CTGGTTCCTG	0	0	0	1	0	At5g40250.1:v:+1841:primary
AAGGATCCGA	0	0	0	1	0	At5g40240.1:X:-507:quaternary
TAAATATTGA	1	0	0	0	0	At5g40180.1:v:+174:secondary
CATATATTTC	0	1	0	0	0	At5g40155.1:i:+249:tertiary
AGTCCGTGTA	1	0	0	0	0	At5g40155.1:d:-195:secondary
GTGATGATGC	0	0	0	0	1	At5g40110.1:v:+643:secondary,At5g03950.1:v:+643:secondary
TTAACGAGAG	0	0	1	0	0	At5g40100.1:d:+1714:secondary
AGTGAACCTT	0	1	0	0	0	At5g40090.1:X:-262:quaternary
TTTATGCGGT	0	0	0	0	1	At5g40090.1:X:+1530:quaternary
AGATGGTGCT	1	0	0	0	0	At5g40060.1:v:-2904:secondary
CATTGACATA	1	0	0	0	0	At5g40060.1:v:+4002:primary,At5g61480.1:v:+3823:primary
ATCTCAGCTA	1	0	0	0	0	At5g40000.1:v:+1122:primary
GTTTGATACA	0	1	0	0	0	At5g39980.1:X:-1423:quaternary,At5g29053.1:X:-1991:quaternary
ATCTTCCCAA	0	0	0	1	0	At5g39970.1:d:-1332:secondary
GGAGTAAGAA	0	0	0	0	1	At5g39970.1:d:+1466:secondary
GAAAGACACG	0	1	0	0	0	At5g39960.1:v:+1913:secondary
GGGTGCACGG	0	1	0	0	0	At5g39940.1:d:+993:primary
AAACTCAATA	0	1	0	0	0	At5g39910.1:X:-101:quaternary,At5g12250.1:X:-765:quaternary
GTTCAAGAAC	1	0	0	0	0	At5g39850.1:d:-822:primary
CTGCAATCCA	0	0	1	0	0	At5g39800.1:i:+507:tertiary,At5g40080.1:i:+465:tertiary
AAACTTGTGA	0	0	0	0	1	At5g39785.2:d:+1756:primary,At5g39785.1:d:+1753:primary
AAGAAGACTC	0	0	1	0	0	At5g39785.1:d:+1574:secondary,At5g39785.2:d:+1574:secondary
TTTACTTTCC	1	0	0	0	0	At5g39770.1:i:+6046:tertiary
AATTCGCCGA	0	0	1	0	0	At5g39760.1:d:+777:primary
TCTGTGACAA	0	0	0	0	1	At5g39680.1:d:+2337:secondary
AATAATCTGC	0	1	0	0	0	At5g39670.1:d:+879:primary
AATAACTTAT	1	0	0	0	0	At5g39660.1:d:+1831:secondary
ATATTTTTTT	0	1	0	0	0	At5g39640.1:v:+78:secondary
TAACGAAATT	0	1	0	0	0	At5g39580.1:X:-605:quaternary
AATAGAAAGC	0	0	0	1	0	At5g39570.1:i:+1324:tertiary
TTCAAGGTGA	1	0	0	0	0	At5g39570.1:d:+364:secondary,At3g23260.1:v:+844:secondary
ATCCTCATCA	0	0	0	1	0	At5g39550.1:X:-506:quaternary,At3g05110.1:X:-16:quaternary,At1g57820.2:X:-500:quaternary,At1g66040.1:X:-506:quaternary,At1g57800.1:X:-500:quaternary
AAGCATAGGG	1	0	0	0	0	At5g39520.1:d:+701:secondary
TCAACTGAAT	0	1	0	0	0	At5g39500.1:d:+4063:primary
GAGGTAAAGG	0	0	1	0	0	At5g39460.1:v:+2549:primary
TGAACTGTGA	0	0	0	1	0	At5g39400.1:d:+999:secondary
GCAGGAAAAG	0	0	0	1	0	At5g39400.1:d:+596:secondary,At4g02680.1:d:+1614:secondary
GAATCACTCC	0	1	0	0	0	At5g39390.1:v:+512:secondary,At5g20480.1:d:+177:secondary,At3g63440.1:v:+687:secondary,At1g09995.1:d:+645:secondary
TTGTCGAAGA	0	0	1	0	0	At5g39320.1:d:-1380:secondary,At2g24210.1:v:-2621:secondary
AAATGTGAAG	0	0	1	0	0	At5g39260.1:X:--189:quaternary
AAAAATTTGT	0	0	1	0	0	At5g39240.1:v:+855:primary
TTGAGAATCC	0	1	0	0	0	At5g39220.1:d:+1029:secondary,At3g21465.1:d:+230:secondary,At4g04316.1:p:+2016:secondary
TGACAGATTC	0	0	0	1	0	At5g39210.1:d:+133:primary
ATGAAAGAAA	0	1	0	0	0	At5g39155.1:p:+695:secondary,At5g39185.1:p:+695:secondary
GCTGTTCTCT	1	0	0	0	0	At5g39060.1:p:+1878:primary

GGTCTTTGGA	0	1	0	0	0	At5g39020.1:v:+2071:secondary
TTGTTGATCC	0	1	0	0	0	At5g39020.1:v:+1728:secondary,At1g03700.1:d:+552:secondary
GGAGATGTTT	0	1	0	0	0	At5g38950.1:v:+354:secondary
GTGCGTTGAG	1	0	0	0	0	At5g38950.1:v:+175:secondary
CTAAAAGAGA	1	0	0	0	0	At5g38940.1:X:-856:quaternary,Chr3:-1592548:quaternary
GATCTACAGA	0	1	0	0	0	At5g38940.1:d:+640:primary
GACATTAGAC	0	0	0	1	0	At5g38900.1:X:--24:quaternary
GTCCTATTCC	1	0	0	0	0	At5g38890.1:d:+206:secondary
CCGCTCTTGA	0	1	0	0	0	At5g38880.1:d:+2794:primary
ACCGGGCTTT	0	0	1	0	0	At5g38840.1:d:+1381:secondary
TGATATGAAT	0	1	0	0	0	At5g38810.1:v:+1406:primary
GAGGCTAAAA	0	0	0	0	1	At5g38800.1:X:-29:quaternary
TGTATAATGT	0	0	0	0	1	At5g38730.1:v:+964:secondary
AAGAACGCCT	0	1	0	0	0	At5g38720.1:d:+692:secondary
CTCTGGCAAT	0	0	0	1	0	At5g38705.1:X:-2468:quaternary,At4g05585.1:X:-4401:quaternary,At4g06752.1:X:-1584:quaternary,At2g12740.1:X:-2673:quaternary,At4g10580.1:X:-2464:quaternary,At5g27885.1:X:-2771:quaternary,At4g08138.1:X:-2631:quaternary,At3g31970.1:X:-2677:quaternary,At2g13860.1:X:-2433:quaternary,At3g29076.1:X:-2897:quaternary,At2g01022.1:X:-1342:quaternary,At3g30846.1:X:-1838:quaternary,At2g12750.1:X:-2572:quaternary,At4g08105.1:X:-2548:quaternary,At2g12760.1:X:-2867:quaternary,At2g14650.1:X:-2479:quaternary,At2g04670.1:X:-2482:quaternary,At2g13830.1:X:-2667:quaternary,At1g42605.1:X:-2427:quaternary
AGGTTCCTCT	0	1	0	0	0	At5g38700.1:v:+558:secondary
GAACTGTACA	0	1	0	0	0	At5g38690.1:i:+3499:tertiary
ACGTTCCAAG	0	0	0	1	0	At5g38670.1:v:+666:secondary
TAGTCACCAC	0	0	0	0	1	At5g38650.1:d:+520:secondary
CCTCCTGGAC	1	0	0	0	0	At5g38640.1:d:+522:secondary
ATACAACAGC	0	0	1	0	0	At5g38610.1:v:+1239:primary
AGTAACTAAC	0	1	0	0	0	At5g38610.1:v:+1094:secondary,At1g72270.1:v:+8912:secondary
TGTAGAACAT	0	0	0	1	0	At5g38590.2:d:+1537:primary,At5g38590.1:d:+1601:primary
ATTCTTTAAA	0	0	0	1	0	At5g38550.1:i:+1912:tertiary,At5g38540.1:i:+1014:tertiary
GATCTGATAA	1	0	0	0	0	At5g38540.1:v:+592:secondary
TTATAAGTCA	0	1	0	0	0	At5g38540.1:v:+1922:secondary
TTCCAAACTA	0	0	0	1	0	At5g38530.1:d:+1621:secondary
GAAGCAATTT	0	0	0	0	1	At5g38530.1:d:+1289:secondary
GACGGCAATG	0	0	0	1	0	At5g38520.1:d:+49:secondary,At2g01029.1:p:+8869:secondary
GAAGTGATCC	0	0	1	0	0	At5g38490.1:v:+1698:primary
AATGGTGAGC	0	0	0	1	0	At5g38450.1:X:-579:quaternary
TATTTTTTGT	0	0	1	0	0	At5g38450.1:i:+465:tertiary,At2g37270.1:i:+549:tertiary
TGCAATAAAA	0	0	0	1	0	At5g38410.1:i:+257:tertiary
TAATGGCAAA	0	1	0	0	0	At5g38396.1:i:+1047:tertiary,At4g13960.1:i:+947:tertiary,At2g28170.1:i:+1234:tertiary
ATAAAACATT	0	1	0	0	0	At5g38386.1:v:+1392:secondary,At5g38390.1:v:+1533:secondary
ATTGTACTCA	1	0	0	0	0	At5g38386.1:i:-848:tertiary,At5g38386.1:i:-852:tertiary
GAAATGTATT	0	1	0	0	0	At5g38383.1:p:+842:secondary,At2g13250.1:p:+658:secondary
GGAGAAAGGT	0	0	0	1	0	At5g38360.1:v:+1465:primary
TGAGCAAGAA	1	0	0	0	0	At5g38320.1:v:+78:secondary
TGAATTTAAA	0	0	0	1	0	At5g38310.1:v:+1293:primary
AAACGTGATT	0	0	1	0	0	At5g38300.1:d:+277:primary
GCTTATTGTT	1	0	0	0	0	At5g38290.1:d:+261:secondary,At5g38290.2:d:+279:secondary
GGGTTACGAT	0	1	0	0	0	At5g38270.1:v:+1357:secondary
GAATCATACT	0	1	0	0	0	At5g38250.1:X:-876:quaternary
TTATGTTCTT	0	0	0	0	1	At5g38240.1:v:+2391:primary
ATCTTGTTGT	1	0	0	0	0	At5g38240.1:v:+1113:secondary,At4g35820.1:v:+519:secondary,At5g49690.1:d:+1423:secondary
GAGATGTTCA	0	0	1	0	0	At5g38210.1:d:-1362:secondary
TTGCAACTCC	0	0	1	0	0	At5g38210.1:d:+710:secondary,At5g03740.1:d:+774:secondary
AGGATAAGAC	0	1	0	0	0	At5g38190.1:v:+1453:secondary
AAACTTGTTT	0	0	0	1	0	At5g38160.1:d:+272:primary
GAGTAGACTC	1	0	0	0	0	At5g38150.1:v:+2207:secondary
ACAGTAATGG	0	1	0	0	0	At5g38150.1:v:+1200:secondary
GGCTAGGCTG	0	1	0	0	0	At5g38130.1:d:+1056:secondary
GCTCTGCAAC	0	0	1	0	0	At5g38060.1:v:+29:secondary
TAAATAAATC	1	0	0	0	0	At5g38040.1:v:+1894:primary
TGTTTTGCGT	1	0	0	0	0	At5g38040.1:i:+524:tertiary
ACAAGAATGA	1	0	0	0	0	At5g38035.1:p:+1946:secondary,At1g05090.1:v:+1711:secondary,At4g20720.1:d:+1521:secondary

TCGAATCTTT	0	0	1	0	0	At5g38030.1:d:+1625:secondary,At5g35917.1:v:+1045:secondary
ATTGTAATTT	0	1	0	0	0	At5g38020.1:d:+1401:primary
TTTGTGACAA	0	0	1	0	0	At5g37970.1:v:+997:secondary,At3g49550.1:v:+517:secondary
CATATTTATA	1	0	0	0	0	At5g37970.1:i:+154:tertiary,At5g56950.1:i:+2645:tertiary
CTTTCACTAT	1	0	0	0	0	At5g37940.1:d:+252:secondary,At5g38000.1:d:+258:secondary
TTTGGTTGCA	1	0	0	0	0	At5g37900.1:v:+546:secondary
AGTTAACTCT	0	1	0	0	0	At5g37890.1:X:-188:quaternary
TGCTTTGCCT	0	0	1	0	0	At5g37870.1:v:+590:secondary,At2g24150.1:d:+820:secondary
TGTTCTTGGT	1	0	0	0	0	At5g37860.1:i:+281:tertiary,At2g30920.1:i:+2093:tertiary
GAACGGGACA	0	0	1	0	0	At5g37830.1:d:+3520:primary
TAATTAAGTG	1	0	0	0	0	At5g37800.1:v:+1695:primary
TCTTGGAGCT	0	0	1	0	0	At5g37780.1:X:-75:quaternary
GCGTAGCTCA	0	1	0	0	0	At5g37780.1:X:-244:quaternary
ATCAACGAGG	0	0	1	0	0	At5g37780.1:d:+245:secondary,At3g03630.1:d:+488:secondary,At1g66410.1:d:+225:secondary
AGGAGTAAGT	1	0	0	0	0	At5g37740.1:d:+762:primary
AACACCTCTT	1	0	0	0	0	At5g37720.1:d:+1033:secondary,At5g54600.2:d:+719:secondary,At5g54600.1:d:+776:secondary
AAAAAAAGAG	0	0	0	0	1	At5g37710.1:v:+2007:secondary
CGATTCTATG	0	1	0	0	0	At5g37710.1:v:+1327:secondary
TGTTATAAAT	1	0	0	0	0	At5g37690.1:d:+1350:primary
ATGGTGAAGT	0	0	0	0	1	At5g37660.1:d:-278:primary
TTCTTGGCTA	1	0	0	0	0	At5g37660.1:d:+724:primary
TATCAATCTG	0	0	0	1	0	At5g37620.1:v:+1755:secondary
CCGGGTCAGT	0	0	0	1	0	At5g37600.1:d:+661:secondary
TGCGATGCGT	0	0	1	0	0	At5g37600.1:d:+352:secondary
AGAAGCAAAG	1	0	0	0	0	At5g37600.1:d:+178:secondary,At1g66200.1:d:+193:secondary
CATTCACCAC	0	0	0	0	1	At5g37600.1:d:+1370:primary
CTTGGTGCAG	0	1	0	0	0	At5g37540.1:d:+1240:primary
ACTTACAAAA	0	1	0	0	0	At5g37530.1:d:+1443:primary
GCGTTGTGGA	0	0	1	0	0	At5g37510.2:d:-79:primary,At5g37510.1:d:-79:primary
AAACTTAGTT	0	1	0	0	0	At5g37442.1:p:+465:secondary
ATGATGATGG	0	0	0	1	0	At5g37430.1:v:+2011:primary,At1g03620.1:v:+1322:primary
TCAAGAGAGT	1	0	0	0	0	At5g37415.1:v:+1094:primary
AGATGGGAAA	0	1	0	0	0	At5g37410.1:v:+2067:primary
CTCAATACTT	1	0	0	0	0	At5g37400.1:v:+1900:primary
GAGTATTGTT	1	0	0	0	0	At5g37381.1:p:+1416:secondary
GAACCAAAGA	0	1	0	0	0	At5g37380.2:d:+1411:secondary,At5g37380.1:d:+1495:secondary,At5g42490.1:v:+3341:secondary
CTTATGAGAA	0	1	0	0	0	At5g37380.2:d:+1189:secondary,At5g37380.1:d:+1273:secondary
AGAGTAATAG	0	0	1	0	0	At5g37360.1:X:-145:quaternary
GCAAATCAGT	0	0	1	0	0	At5g37360.1:d:+852:secondary
GGAGCAAAAC	1	0	0	0	0	At5g37340.2:d:+1493:primary,At5g37340.1:d:+1479:primary
GAAACCGAAA	0	0	1	0	0	At5g37280.1:v:+354:secondary
CAAATGCAAG	0	0	0	1	0	At5g37170.1:v:+444:secondary
TTGATGTTAA	0	0	1	0	0	At5g37170.1:v:+1218:primary
CCCACAAAGA	0	0	0	1	0	At5g37125.1:p:+528:secondary
TTTATTTTCA	1	0	0	0	0	At5g37100.1:v:+1366:secondary
ATAACTAAAA	1	0	0	0	0	At5g37090.1:v:+926:primary
AAATGTGTTG	0	0	1	0	0	At5g37090.1:v:+474:secondary,At5g36890.1:v:+1868:secondary,At2g10234.1:p:+793:secondary
TCTGTGAGGA	1	0	0	0	0	At5g37060.1:X:-646:quaternary
TACCGACTTA	0	0	0	1	0	At5g37055.1:d:+394:secondary
AGTTGGTTCA	0	1	0	0	0	At5g37050.1:v:+607:secondary,At1g24640.1:p:+1562:secondary
TGTTTGTGCT	0	0	0	0	1	At5g37050.1:v:+1165:secondary
GAATTAAAAA	0	0	1	0	0	At5g37040.1:v:+132:secondary
ATGAGAAAAA	0	0	0	1	0	At5g37030.1:v:+820:secondary,At4g07806.1:p:+3322:secondary
GGTCATCACC	0	0	1	0	0	At5g37020.1:d:-2434:secondary,At4g23980.1:d:-2057:secondary
CAGATGTTGA	0	0	1	0	0	At5g37020.1:d:+377:secondary
ATTTTCTTGG	0	1	0	0	0	At5g37000.1:v:+949:secondary
GTCTTTTTGG	0	0	1	0	0	At5g36980.1:v:+1169:secondary,At3g61430.1:d:+327:secondary
GCTGAAATTT	0	0	0	1	0	At5g36935.1:p:+9209:secondary
GCCAAAGCAA	0	1	0	0	0	At5g36930.1:X:-465:quaternary
TATCACTTCG	0	1	0	0	0	At5g36925.1:X:-212:quaternary
GAAATGCATA	0	0	1	0	0	At5g36880.1:d:+362:secondary

TTCTCTATTT	0	1	0	0	0	At5g36880.1:d:+24:secondary
TGTGAAACAA	0	0	0	1	0	At5g36735.1:v:+1203:secondary
TTTTCGTTTC	0	1	0	0	0	At5g36730.1:v:+2026:primary,At5g36820.1:v:+2026:primary
GTAGCCACAG	0	1	0	0	0	At5g36710.1:d:+311:primary
CTCCATTAGA	0	0	0	1	0	At5g36700.1:X:-47:quaternary,At5g36790.1:X:-47:quaternary
TTTCTAGAAA	0	1	0	0	0	At5g36690.1:v:+2417:primary,At5g36780.1:v:+2417:primary
ATGAAGAGTT	0	0	1	0	0	At5g36670.1:v:+1066:secondary,At5g36740.1:v:+1066:secondary
AAAATGAGAA	0	0	1	0	0	At5g36670.1:i:+3038:tertiary,At5g36740.1:i:+3038:tertiary
TTAGAGTAAG	0	1	0	0	0	At5g36300.1:p:+1220:secondary,At3g42560.1:v:+252:secondary
GTGAAGATGG	0	0	1	0	0	At5g36296.1:p:+2667:primary
CATATTTTTT	0	0	0	1	0	At5g36270.1:v:+1445:primary
GTTAGGTTTG	0	1	0	0	0	At5g36223.1:p:+589:secondary,At3g30400.1:p:+840:secondary,At1g35390.1:p:+583:secondary,At2g21040.1:v:+169:secondary,At4g06595.1:p:+845:secondary,At5g36005.1:p:+583:secondary
TTAATGCAAA	0	1	0	0	0	At5g36220.1:d:+1564:primary
CATTTGAAGA	0	0	1	0	0	At5g36210.1:X:--73:quaternary
AAAGATTCTG	0	0	1	0	0	At5g36160.1:d:+825:secondary,At5g55280.1:d:+926:secondary,At1g52190.1:d:+986:secondary
GCAGAGTTTG	0	1	0	0	0	At5g36160.1:d:+711:secondary
AAAGAACAAA	0	0	0	1	0	At5g36125.1:p:+3918:primary,At4g04600.1:p:+3902:primary,At4g04280.1:p:+3700:primary,At4g36610.1:d:+845:primary
TTTGGTGTAT	0	1	0	0	0	At5g36090.1:i:+50:tertiary
GTTTTGAGGA	0	0	1	0	0	At5g36050.1:v:+417:secondary,At4g19300.1:v:+417:secondary,At1g35090.1:v:+417:secondary,At5g28980.1:v:+417:secondary
GATGTCAATC	0	1	0	0	0	At5g36020.1:v:+1542:secondary,At4g19320.1:v:+1542:secondary,At4g23530.1:d:+172:secondary,At5g28970.1:v:+1527:secondary,At3g43920.1:v:+4548:secondary,At1g52087.1:v:+1716:secondary,At1g35110.1:v:+2409:secondary
ACCTTCAACA	1	0	0	0	0	At5g36015.1:X:-44:quaternary
ACAACTACTT	0	0	1	0	0	At5g36015.1:p:+50:secondary
TTTTACCCCA	0	0	0	1	0	At5g35980.1:i:+6796:tertiary
TCTCCCAGAA	1	0	0	0	0	At5g35980.1:d:+3176:secondary
TGGTCTTTTG	0	0	0	1	0	At5g35980.1:d:+1489:secondary
CAGACCCTGG	0	0	0	0	1	At5g35970.1:d:+3084:secondary
GCTGGAGCAA	0	0	1	0	0	At5g35970.1:d:+1654:secondary
TGTGTTCTGA	0	0	0	1	0	At5g35965.1:p:+1404:secondary
ATTTTTCTCC	0	1	0	0	0	At5g35960.1:v:+1153:secondary
CAAGGTCCAG	0	1	0	0	0	At5g35930.1:X:-34:quaternary
ACTCAAAAGT	1	0	0	0	0	At5g35930.1:d:+3253:secondary,At5g28950.1:v:+589:secondary
TTTTCTGAAT	0	0	1	0	0	At5g35930.1:d:+1019:secondary
CGGTTGCAAC	0	1	0	0	0	At5g35917.1:v:+1780:secondary
ATTATCAAAA	0	0	0	0	1	At5g35890.1:v:+297:secondary
AGAATATAAT	0	0	0	0	1	At5g35890.1:v:+1522:secondary
CTTTTCCGCA	0	0	0	1	0	At5g35840.1:d:+1466:secondary
TGATGATAAA	0	0	1	0	0	At5g35830.1:v:+590:secondary
GTGTTAAGAA	0	0	0	1	0	At5g35796.1:p:+419:secondary,At1g65190.1:d:+490:secondary
TTACAAAAGG	0	0	1	0	0	At5g35790.1:d:+310:secondary
ACGGGAAACC	1	0	0	0	0	At5g35777.1:p:+1216:secondary
CTTCGTTGCA	0	1	0	0	0	At5g35770.1:i:+3151:tertiary,At5g53010.1:i:+3423:tertiary
GTGACCAGCT	0	1	0	0	0	At5g35756.1:X:-898:quaternary
GAAACTTTTC	0	0	0	0	1	At5g35756.1:p:+456:secondary
ATAGGAAGCG	1	0	0	0	0	At5g35750.1:X:+623:quaternary
GATTAAAGGA	0	0	0	0	1	At5g35720.1:p:+831:primary,At5g52110.1:d:+873:primary
CGATGAGAGA	0	1	0	0	0	At5g35690.1:X:-134:quaternary,At1g05410.1:X:-455:quaternary
TAGATGAATG	0	0	0	0	1	At5g35640.1:i:+397:tertiary
CTCTTGGTAC	1	0	0	0	0	At5g35630.1:d:-1078:secondary
TCAGATGAGA	0	1	0	0	0	At5g35630.1:d:+170:secondary
TCTATCGCAT	0	0	0	0	1	At5g35620.2:X:+321:quaternary
GTCTCGAACT	0	0	0	1	0	At5g35602.1:p:-377:secondary,At3g30216.1:p:-374:secondary
TTGAGTGTAG	0	0	0	1	0	At5g35590.1:X:-30:quaternary
AAGCCGAGAA	0	1	0	0	0	At5g35580.1:v:+976:secondary
GATGTAATTT	0	0	0	0	1	At5g35580.1:i:-998:tertiary
GCATAGCTGA	1	0	0	0	0	At5g35570.1:X:-466:quaternary

TATACGTAAA	0	1	0	0	0	At5g35560.1:d:+2538:primary
AAAAGTAATG	0	1	0	0	0	At5g35555.1:X:-1963:quaternary,At2g13370.1:X:--49:quaternary,At2g22340.1:X:-561:quaternary
TTGAGAAATA	0	0	0	1	0	At5g35555.1:p:+1809:secondary
TGGATCGTAT	0	0	0	1	0	At5g35550.1:X:-637:quaternary
ATAAACGAAT	0	0	1	0	0	At5g35535.1:p:+4581:secondary
ACCCAGTATG	0	0	1	0	0	At5g35535.1:p:+1261:secondary
CAAATCAGAT	0	0	0	0	1	At5g35510.1:v:+546:secondary,At3g62060.1:d:+1021:secondary
CCTATTTTGG	1	0	0	0	0	At5g35490.1:d:+597:primary
TTGGCAATAC	1	0	0	0	0	At5g35475.1:v:+976:secondary,At5g22800.1:d:+2308:secondary
AATCTGGTCA	0	0	1	0	0	At5g35475.1:v:+208:secondary,At2g29080.1:d:+2046:secondary
TTGAAATGTA	0	0	1	0	0	At5g35450.1:i:+1156:tertiary,At5g43470.1:i:+2586:tertiary,At5g43470.2:i:+2586:tertiary
TGAGCTTTGT	0	0	0	0	1	At5g35430.1:d:+2457:primary
GGGTATATTG	0	1	0	0	0	At5g35425.1:p:+584:secondary
TTCGCTCATC	0	1	0	0	0	At5g35416.1:X:-455:quaternary
GAGGATGTTT	0	0	0	0	1	At5g35416.1:p:+3731:secondary,At3g43357.1:p:+2323:secondary,At5g51820.1:d:+896:secondary
GTAGACGTAA	1	0	0	0	0	At5g35410.1:d:+1297:primary
CAGTGGTGGT	0	0	1	0	0	At5g35360.1:d:+354:secondary
TCTTTTATTT	0	0	0	0	1	At5g35348.1:X:+-141:quaternary,At3g17350.1:X:+545:quaternary,Chr3:+21119359:quaternary
ATTGATGAAG	0	1	0	0	0	At5g35341.1:p:+579:secondary,At4g09760.1:d:+780:secondary,At2g19480.1:d:+722:secondary,At2g04760.1:p:+963:secondary,At4g09760.3:d:+764:secondary, At1g71697.1:d:+960:secondary,At4g09760.2:d:+732:secondary
ATTTGGGATG	0	0	1	0	0	At5g35331.1:X:-3290:quaternary
AACCAGGCCC	1	0	0	0	0	At5g35331.1:X:-203:quaternary
AAAATCCAGT	0	0	0	1	0	At5g35330.1:X:-369:quaternary,At2g13610.1:X:-1739:quaternary
TCGCTTCTCC	0	1	0	0	0	At5g35320.1:X:+-2:quaternary
TTTTGTTCAA	0	1	0	0	0	At5g35300.1:v:+342:secondary
TTTTTCGCTA	0	1	0	0	0	At5g35220.1:i:+1031:tertiary
AACAATACAA	0	0	1	0	0	At5g35205.1:p:+486:secondary
TTTGCTCAAA	0	0	0	1	0	At5g35205.1:p:+4310:secondary
CAGAGGCAGT	0	0	0	0	1	At5g35205.1:p:+2500:secondary
TTATAGTCGG	0	0	0	1	0	At5g35180.1:d:-2479:secondary
TTTAACACTA	0	0	0	0	1	At5g35170.1:i:+3148:tertiary
GTGCCGTTCT	1	0	0	0	0	At5g35170.1:i:+1940:tertiary
GATGGCTTCT	0	0	1	0	0	At5g35170.1:d:+591:secondary
GAGGCAAAGG	0	0	0	1	0	At5g35140.1:p:+1494:primary,At1g65810.1:v:+3890:primary
TAGCAAAAAA	0	0	1	0	0	At5g35130.1:X:-919:quaternary
AACGTTTAAA	0	0	1	0	0	At5g35076.1:p:+1999:secondary,At4g07806.1:p:+2223:secondary
GTTTGCTTAT	0	0	0	0	1	At5g35073.1:p:+1243:primary
GTGATTTCTC	1	0	0	0	0	At5g35045.1:X:-2512:quaternary
GTTGTTGAAC	0	0	1	0	0	At5g34965.1:p:+405:secondary
TGCTTAAGGT	0	1	0	0	0	At5g34940.2:d:+1631:primary,At5g34940.3:d:+1797:primary,At5g34940.1:d:+1782:primary
TTAATATGAA	0	0	0	0	1	At5g34940.1:d:+327:secondary
CTCCTTGACT	0	1	0	0	0	At5g34930.1:d:+1963:primary
TCTCTTGGGA	0	1	0	0	0	At5g34880.1:p:+1739:primary
AAGAAGGAAT	0	0	1	0	0	At5g34863.1:p:+3182:secondary,At1g08810.2:d:+9:secondary,At3g33070.1:p:+3293:secondary,At1g08810.1:d:+287:secondary,At1g43785.1:p:+3260:secondary
ACAAGCTAGT	0	0	0	1	0	At5g34858.1:p:+493:secondary,At3g46950.1:d:+1384:secondary
AAACAATGTT	1	0	0	0	0	At5g34857.1:p:+628:secondary
GTGATGGATC	0	0	0	1	0	At5g34851.1:p:+4936:secondary,At3g19890.1:v:+519:secondary,At2g15800.1:d:+1097:secondary
CAATACACTT	1	0	0	0	0	At5g34848.1:X:+-130:quaternary
AATCTGATCC	1	0	0	0	0	At5g34848.1:p:+357:secondary
AACACCATCA	1	0	0	0	0	At5g34835.1:p:+665:primary
AGGGAAGTGA	0	0	1	0	0	At5g34833.1:p:+298:secondary,At3g26560.1:d:+3390:secondary
GAGGAGGCGC	0	1	0	0	0	At5g33715.1:v:+807:primary
GCACAACAGT	0	0	0	1	0	At5g33436.1:X:--138:quaternary
CGGTGAAGAA	0	0	0	1	0	At5g33434.1:p:+941:secondary
TTTGGTCAAA	1	0	0	0	0	At5g33428.1:p:-2793:secondary
TGGATGTGGA	1	0	0	0	0	At5g33424.1:p:-1030:secondary
CTTGAAAACT	0	0	0	1	0	At5g33422.1:p:-1962:secondary,At2g36330.1:v:-1395:secondary,At5g33422.1:p:-1981:secondary,At5g33422.1:p:-1972:secondary

CTATTAAGTA	0	1	0	0	0	At5g33420.1:p:+2145:primary
TTAGCAAACA	0	0	0	1	0	At5g33415.1:X:-2779:quaternary
GTTTTCTTTT	0	0	1	0	0	At5g33405.1:p:+1001:primary
AATAGTATAA	0	1	0	0	0	At5g33400.1:X:--32:quaternary
CAATCTAGGA	0	1	0	0	0	At5g33385.1:X:+2724:quaternary
AGAACAATGA	0	1	0	0	0	At5g33384.1:p:+1495:primary,At1g38280.1:p:+1568:primary
TTGGATTTTA	0	0	1	0	0	At5g33381.1:p:+4231:secondary,At5g29805.1:p:+236:secondary,At5g29720.1:p:+4380:secondary,At1g41825.1:p:+2606:secondary
GTTCGATTCA	0	0	0	0	1	At5g33330.1:v:+887:primary
CTCGGAGCCG	0	0	1	0	0	At5g33306.1:p:+334:secondary
TTCGTTGAGA	0	1	0	0	0	At5g33280.1:X:-450:quaternary
AAGTTAAAAG	0	1	0	0	0	At5g33280.1:d:+1651:secondary
GGGCTTTCCT	0	1	0	0	0	At5g33259.1:p:+38:secondary
TTGCTCTGTT	0	1	0	0	0	At5g33258.1:X:+705:quaternary
ATAGACTTTT	1	0	0	0	0	At5g33237.1:p:+126:secondary
GACAAGCATA	0	1	0	0	0	At5g33226.1:p:+1259:secondary,At1g46192.1:p:+1287:secondary
ATTACAGAAA	1	0	0	0	0	At5g33050.1:p:+2001:secondary
TCTTGTCCAG	0	1	0	0	0	At5g32825.1:X:-12:quaternary
GGGAAATTAT	0	0	1	0	0	At5g32825.1:p:+345:secondary
AAAGTTATGA	0	1	0	0	0	At5g32825.1:p:+2602:secondary
TATATATGAG	1	0	0	0	0	At5g32825.1:p:+1461:secondary
CTATTAAAAA	0	0	1	0	0	At5g32800.1:X:+643:quaternary,Chr5:+7027034:quaternary
TTTAATCAAT	0	1	0	0	0	At5g32630.1:p:+872:secondary
TATGGTTGAA	0	0	1	0	0	At5g32630.1:p:+1541:secondary,At3g42100.1:v:+2894:secondary
GATGCTAAAA	1	0	0	0	0	At5g32630.1:p:+1389:secondary,At2g45550.1:v:+1131:secondary,At3g42100.1:v:+2742:secondary
CTCCTTGTAT	0	1	0	0	0	At5g32616.1:p:+663:secondary
TAGTAGCAAA	0	1	0	0	0	At5g32593.1:p:+2074:secondary
TAAAGACACA	0	0	1	0	0	At5g32580.1:p:+485:secondary
ACTTCAACAA	0	0	0	0	1	At5g32576.1:p:+140:secondary,At5g58940.1:v:+265:secondary
GATGGTTCGA	0	0	1	0	0	At5g32540.1:p:+2488:secondary,At4g05300.1:v:+1676:secondary,At2g06860.1:v:+3194:secondary,At2g01029.1:p:+7331:secondary,At2g01040.1:p:+1419:secondary,At1g54926.1:v:+1337:secondary,At2g15040.1:p:+2752:secondary,At4g00140.1:v:+1370:secondary,At2g21460.1:p:+2194:secondary,At4g08095.1:p:+1275:secondary,At5g32481.1:p:+1916:secondary,At2g01029.1:p:+4397:secondary,At3g33189.1:p:+662:secondary
GCAAGTTACA	0	0	1	0	0	At5g32521.1:p:+4586:secondary
TGAGCTTCGC	0	1	0	0	0	At5g32495.1:X:-1375:quaternary
AGGTGGTGAT	0	0	1	0	0	At5g32487.1:p:+141:secondary,At2g06760.1:p:+2881:secondary
TGTTGTTTGA	0	0	0	0	1	At5g32485.1:p:+483:primary
ATTAATAGAG	0	1	0	0	0	At5g32481.1:p:+4186:primary,At4g06543.1:p:+1003:primary
CCTTAAGTAG	0	1	0	0	0	At5g32450.1:d:-1100:secondary
AAAGACGCCA	0	0	1	0	0	At5g32440.1:d:+557:secondary
TTGGATCCAT	0	1	0	0	0	At5g32440.1:d:+26:secondary
ATATTCTTTA	0	1	0	0	0	At5g32436.1:X:-1345:quaternary
CAATTCGTTT	1	0	0	0	0	At5g32433.1:X:-325:quaternary
ACACTCAGAG	0	0	0	1	0	At5g32386.1:p:-3209:secondary
CTCACTCCTA	0	0	1	0	0	At5g32386.1:p:-2021:secondary
TCAATCACCT	0	1	0	0	0	At5g32169.1:v:+1519:primary,At5g53895.1:v:+995:primary
TCAAGCATTT	1	0	0	0	0	At5g32060.1:p:+2697:secondary
TACGTCAAAA	0	1	0	0	0	At5g32002.1:p:+3180:secondary
GAGACAAAGC	1	0	0	0	0	At5g31702.1:p:+1108:secondary,At5g37510.1:d:+1900:secondary,At5g37510.2:d:+1900:secondary
TTAGTAAAAT	0	0	1	0	0	At5g31651.1:p:+631:secondary
AAAAGAAGGA	0	0	1	0	0	At5g31412.1:v:+783:secondary,At5g39533.1:p:+872:secondary,At5g28145.1:p:+889:secondary
TGAATGATGT	0	0	1	0	0	At5g31122.1:p:+730:secondary
AGTTGAATAT	0	1	0	0	0	At5g31032.1:p:+135:secondary
TTGTACAAGA	0	0	0	0	1	At5g30721.1:p:+421:secondary
TATATCTTTT	0	1	0	0	0	At5g30584.1:p:+1853:secondary,At1g21270.1:d:+2292:secondary
TCTTCTTACA	0	0	0	0	1	At5g30520.1:v:+354:secondary,At3g42470.1:p:+4605:secondary,At1g21065.1:d:+350:secondary
TGGAAGAAGC	0	0	1	0	0	At5g30510.1:d:+491:secondary,At4g03300.1:v:+2629:secondary
TTGTGTTGTG	0	1	0	0	0	At5g30450.1:p:+609:secondary,At4g06652.1:p:+635:secondary,At4g06652.1:p:+550:secondary
AACACTGAAA	0	0	1	0	0	At5g30420.1:p:+2391:secondary,At2g36920.1:d:+296:secondary
AGTTGTGCCT	0	0	0	1	0	At5g30380.1:p:-970:secondary
TGTGGACACT	0	0	0	0	1	At5g30269.1:p:+2104:secondary

GATAATGAGA	1	0	0	0	0	At5g29762.1:p:+1322:secondary
TTTTGCTTGG	0	0	0	0	1	At5g29646.1:X:-3357:quaternary,At2g15070.1:X:-3351:quaternary
TCGTCTGTGT	0	1	0	0	0	At5g29646.1:X:-3071:quaternary
AGTTCAGGCG	0	1	0	0	0	At5g29624.1:v:-394:primary
GAGTCCACAA	0	0	0	0	1	At5g29613.1:v:+182:secondary
GGAAGATGAA	0	0	1	0	0	At5g29562.1:p:+4518:primary
GAAGAGAATT	0	0	1	0	0	At5g29562.1:p:+2758:secondary
ATATTGATCT	0	1	0	0	0	At5g29090.1:i:+297:tertiary,At1g73480.1:i:+2210:tertiary
TTTTCGCAAT	0	0	0	0	1	At5g29075.1:X:-5359:quaternary
ATGATGCTCT	0	1	0	0	0	At5g29053.1:p:+392:secondary,At2g36810.1:v:+439:secondary,At2g45500.1:d:+227:secondary
GTTCCAGCTA	0	1	0	0	0	At5g29043.1:p:+2963:secondary
CTTTTTAATT	0	0	1	0	0	At5g29030.1:p:+1473:secondary
GTTTATGAAA	0	1	0	0	0	At5g29020.1:v:+1941:secondary
AATTACATCA	1	0	0	0	0	At5g29015.1:p:+1209:secondary
TCTTCCTCTA	0	0	1	0	0	At5g28990.1:v:+537:secondary
GATCACACCA	0	0	0	0	1	At5g28980.1:v:+645:secondary,At3g42500.1:v:+354:secondary,At1g35090.1:v:+645:secondary,At2g10370.1:v:+645:secondary,At4g19300.1:v:+645:secondary,At4g03977.1:v:+609:secondary,At5g36050.1:v:+645:secondary,At3g50970.1:d:+293:secondary
CTACACTAAA	0	0	1	0	0	At5g28935.1:X:-3348:quaternary
TGTTTTTCTG	0	0	0	1	0	At5g28935.1:p:+3354:secondary
GATGAATTTG	0	1	0	0	0	At5g28935.1:p:+2450:secondary,At3g21250.1:d:+273:secondary,At5g33389.1:p:+91:secondary,At5g01230.2:d:+809:secondary,At2g30320.1:v:+1808:secondary,At5g29032.1:p:+319:secondary,At5g01230.1:d:+499:secondary
GAGGATTCTT	0	0	1	0	0	At5g28935.1:p:+2296:secondary,At3g18910.1:d:+561:secondary,At3g25460.1:v:+893:secondary
TTGAGGATAC	0	1	0	0	0	At5g28935.1:p:+2053:secondary
TAAGTTTTAT	0	0	0	0	1	At5g28935.1:p:+1436:secondary,At1g79245.1:p:+4389:secondary
TTCCCCAAAA	0	0	1	0	0	At5g28930.1:v:-1100:secondary
TGTGCTTACT	0	0	0	0	1	At5g28930.1:i:+2822:tertiary,At5g08560.1:i:+2016:tertiary,At4g05420.1:i:+3032:tertiary,At2g17480.1:i:+828:tertiary
AAAAAACTGT	0	0	1	0	0	At5g28927.1:p:+454:secondary
AAGTCGGGCT	0	0	1	0	0	At5g28920.1:d:+893:secondary
GTGACCAGAA	1	0	0	0	0	At5g28897.1:X:+3:quaternary
TATTTTGATG	0	1	0	0	0	At5g28850.2:i:+2659:tertiary,At5g28850.1:i:+2659:tertiary
TTGATAATGG	0	0	0	1	0	At5g28850.2:d:+791:secondary,At5g28850.1:d:+791:secondary,At5g28900.1:d:+842:secondary
AATCTTTTAG	0	1	0	0	0	At5g28850.1:d:+2360:primary,At5g28850.2:d:+2269:primary
CAAAAGAGAA	1	0	0	0	0	At5g28850.1:d:+2005:secondary,At4g21020.1:d:+591:secondary,At5g28850.2:d:+1914:secondary
AGAGCCATAA	0	0	1	0	0	At5g28750.1:X:--91:quaternary
ATCCAAGGAA	0	0	1	0	0	At5g28696.1:p:+292:secondary,At5g32345.1:p:+4378:secondary,At4g06554.1:p:+291:secondary,At4g07664.1:p:+238:secondary,At3g32894.1:p:+121:secondary,At4g08132.1:p:+291:secondary,At1g37867.1:p:+292:secondary,At2g13020.1:p:+145:secondary,At4g07491.1:p:+259:secondary,At3g42913.1:p:+167:secondary,At5g35046.1:p:+479:secondary,At5g33424.1:p:+143:secondary,At4g04276.1:p:+259:secondary,At5g31087.1:p:+292:secondary,At2g11050.1:p:+237:secondary,At2g11660.1:p:+273:secondary,At3g31358.1:p:+292:secondary,At5g35142.1:p:+293:secondary,At3g42178.1:p:+289:secondary,At4g07935.1:p:+286:secondary,At4g03885.1:p:+613:secondary,At5g33383.1:p:+273:secondary,At3g43144.1:p:+795:secondary,At2g12590.1:p:+189:secondary,At5g33385.1:p:+213:secondary,At4g07780.1:p:+259:secondary
TATGTGTTAA	1	0	0	0	0	At5g28672.1:p:+567:secondary
CTTCCTCGCT	0	1	0	0	0	At5g28650.1:X:-45:quaternary
GTACACTACC	0	0	1	0	0	At5g28640.1:X:-594:quaternary
GTGATCTTGA	1	0	0	0	0	At5g28635.1:p:+4193:secondary,At2g24660.1:p:+2674:secondary,At1g46552.1:p:+3442:secondary,At3g29156.1:p:+3558:secondary,At1g56675.1:p:+3278:secondary,At4g09146.1:p:+3278:secondary,At2g13940.1:p:+3323:secondary
TGACGTATGT	0	1	0	0	0	At5g28635.1:p:+2525:secondary
AGTCAAAAGG	0	0	1	0	0	At5g28624.1:X:-2103:quaternary
CATTGAAAAA	0	1	0	0	0	At5g28622.1:p:+806:primary
GTTTTTAAGC	0	0	1	0	0	At5g28622.1:p:+586:secondary
GTGGCGTGGA	0	0	0	1	0	At5g28610.1:d:-230:secondary
GCGGTGTTGT	0	0	1	0	0	At5g28600.1:v:+2014:secondary
GTTGAGGGAG	0	0	0	1	0	At5g28550.1:X:-65:quaternary
TGTTTATTCT	1	0	0	0	0	At5g28550.1:v:+1021:secondary
TCGAAATTAT	0	1	0	0	0	At5g28540.1:d:+235:secondary
AACCAAAATT	0	0	0	0	1	At5g28535.1:p:+380:secondary,At1g42390.1:p:+2125:secondary
ATTGCAAAAG	0	0	1	0	0	At5g28530.1:v:+2495:secondary

ACTGCTAATC	0	0	1	0	0	At5g28530.1:v:+2408:secondary
AAGCTAAACT	0	0	0	1	0	At5g28527.1:p:+318:secondary,At3g27570.1:d:+1082:secondary,At2g18330.1:d:+867:secondary
ACAAATTGGA	0	0	0	0	1	At5g28523.1:p:+3138:secondary
GTCCTAATCA	0	0	0	0	1	At5g28520.1:v:+862:secondary
ACAAATTTAA	0	0	1	0	0	At5g28450.1:v:+1268:primary
TTTGGTGAAT	0	0	0	0	1	At5g28380.1:v:+260:secondary
TAAAAAAGTC	0	0	0	0	1	At5g28350.1:i:-2789:tertiary,At5g28350.2:i:-2723:tertiary
TTTTTAGGTT	0	1	0	0	0	At5g28350.1:i:+663:tertiary,At3g61480.1:i:+491:tertiary,At5g28350.2:i:+597:tertiary
CTCTCCTGCA	0	0	0	0	1	At5g28340.1:v:+810:secondary,At3g46180.1:d:+904:secondary,At5g28380.1:v:+675:secondary
TTGGAGGCAG	0	1	0	0	0	At5g28340.1:v:+1461:secondary,At1g15520.1:v:+751:secondary,At5g28380.1:v:+1326:secondary
GAAAAGAAAC	0	0	1	0	0	At5g28320.1:v:+2703:secondary,At3g61780.1:v:+3099:secondary,At1g49190.1:v:+1581:secondary,At5g28400.1:v:+2838:secondary
TTTCATCAGA	0	1	0	0	0	At5g28300.1:d:+790:secondary
AACTTTCAAA	0	0	1	0	0	At5g28295.1:v:+970:secondary
TGCTTCTTTT	1	0	0	0	0	At5g28280.1:p:+403:secondary
CATTTTTAAC	0	1	0	0	0	At5g28263.1:p:+14173:secondary
CATAACTAGT	1	0	0	0	0	At5g28141.1:p:+767:secondary,At4g28760.1:d:+1856:secondary,At1g35745.1:p:+1090:secondary,At3g31422.1:p:+1419:secondary,At4g28760.2:d:+1969:secondary,At5g28673.1:p:+1573:secondary,At2g35710.2:d:+61:secondary
CCAAATGGTG	1	0	0	0	0	At5g28070.1:v:+420:secondary,At5g33395.1:p:+1478:secondary
AATGGAACGA	0	0	1	0	0	At5g28067.1:p:+1000:primary
AGGTTTGGTT	0	0	0	0	1	At5g28056.1:p:+4246:secondary
AGAAAAAGGA	0	0	1	0	0	At5g28053.1:p:+638:secondary,At2g13940.1:p:+914:secondary
AGCACCACCT	0	0	0	0	1	At5g28040.1:X:-1152:quaternary
CTCTCGGTGT	0	1	0	0	0	At5g28040.1:d:+1158:secondary,At2g06240.1:p:+1727:secondary
CTTACAAAAA	0	0	1	0	0	At5g27970.1:d:+4455:primary
CAATTGTTGG	0	0	1	0	0	At5g27970.1:d:+3598:secondary
TGTTTTATTC	0	1	0	0	0	At5g27960.1:v:+1761:primary
TATCAATAGA	0	1	0	0	0	At5g27950.1:d:-3094:secondary
AATTTTATAT	1	0	0	0	0	At5g27945.1:v:+1734:primary,At5g27570.1:v:+1682:primary
GGAATTTCTT	0	0	0	0	1	At5g27925.1:p:+3756:primary
CTCCATTAAT	0	0	0	0	1	At5g27925.1:p:+1236:secondary
TTTCGTTTTC	0	0	0	1	0	At5g27920.1:i:+2052:tertiary
TTTCTGATTC	0	0	1	0	0	At5g27920.1:d:+1206:secondary
ACAGGATTAG	0	1	0	0	0	At5g27860.1:X:-227:quaternary
AGTATTAAAA	1	0	0	0	0	At5g27850.1:d:+855:primary
ATACTGTCAG	0	1	0	0	0	At5g27730.1:i:-2456:tertiary
ATGGATAAAT	0	1	0	0	0	At5g27700.1:i:+152:tertiary
GGCCAATTAA	0	1	0	0	0	At5g27690.1:d:+892:primary
GAGCTTAGAA	0	0	0	1	0	At5g27680.1:d:+2834:primary
GAAGCCGAAG	0	0	0	1	0	At5g27680.1:d:+2252:secondary
TATTACAATT	0	0	1	0	0	At5g27650.1:d:+3424:primary
GTGAGAGCGA	0	0	0	1	0	At5g27630.1:d:+91:secondary
TGTGGTAAAA	1	0	0	0	0	At5g27600.1:i:+4063:tertiary
GAAACTTGAT	0	1	0	0	0	At5g27600.1:d:+851:secondary
TCGGTTCCCC	0	1	0	0	0	At5g27600.1:d:+1409:secondary
TAATTTGCAT	0	0	1	0	0	At5g27590.1:v:+1775:primary
TGTCAAATTC	1	0	0	0	0	At5g27560.1:d:+165:secondary
AAAATATCGA	0	0	0	0	1	At5g27560.1:d:+1313:primary
AAATCAATGT	0	1	0	0	0	At5g27560.1:d:+1045:secondary,At5g58270.1:d:+1377:secondary
TTTCAGGTTC	0	1	0	0	0	At5g27550.1:v:+1680:secondary,At1g29760.1:d:+1155:secondary
AATGGAAGAC	0	0	1	0	0	At5g27530.1:v:+350:secondary
GAAAATGGAG	0	0	0	0	1	At5g27530.1:v:+1497:secondary
TTGCAATGAT	0	0	0	0	1	At5g27505.1:p:+832:secondary,At3g44205.1:p:+1576:secondary,At5g28580.2:p:+1549:secondary
AATTTTGTTG	1	0	0	0	0	At5g27500.1:v:+24:secondary
GTGTTTAAAA	0	1	0	0	0	At5g27480.1:p:+1514:secondary
TGGATCTTGT	0	0	1	0	0	At5g27470.1:d:+519:secondary,At2g03380.1:v:+1267:secondary
AAACATAACA	0	0	0	1	0	At5g27460.1:v:+1899:secondary
CCTCTGAGAA	0	0	0	0	1	At5g27450.2:d:+994:secondary,At5g27450.1:d:+754:secondary
GAACTGGAAG	0	0	1	0	0	At5g27420.1:d:+1386:secondary,At4g30730.1:v:+383:secondary
AGAGTGGTGC	0	1	0	0	0	At5g27410.1:d:-79:secondary

TGTTTGCGGA	1	0	0	0	0	At5g27400.1:d:+1270:primary
TTCCTGATTT	0	0	0	1	0	At5g27390.1:v:+2065:primary,At1g40081.1:p:+164:primary
ATTAATACAC	1	0	0	0	0	At5g27370.1:v:+1159:primary
CTACTTGGGG	0	0	0	0	1	At5g27350.1:d:+1321:secondary
TTCTGAGAGA	0	0	1	0	0	At5g27330.1:X:-2094:quaternary
TTGAAGATTG	1	0	0	0	0	At5g27330.1:d:+328:secondary,At4g34650.1:v:+1156:secondary
GTGTGATTGC	1	0	0	0	0	At5g27300.1:v:+553:secondary
TCGAAATTTT	0	1	0	0	0	At5g27300.1:i:+1649:tertiary
AGCTATATGA	1	0	0	0	0	At5g27290.1:d:+1204:secondary
ACAATATAGG	1	0	0	0	0	At5g27270.1:d:-1418:secondary
ACTTCTAGGA	0	0	0	0	1	At5g27200.1:v:+1043:primary
AAACATTACA	1	0	0	0	0	At5g27140.1:v:+1922:primary,At5g41310.1:v:+3705:primary
ATTCAAACTA	0	1	0	0	0	At5g27120.1:X:+-195:quaternary
GGATGCAGCA	0	0	1	0	0	At5g27110.1:v:+2240:secondary,At5g42870.1:d:+1788:secondary,At1g67870.1:d:+617:secondary,At1g67870.1:d:+689:secondary,At1g67870.1:d:+761:secondary,At1g67870.1:d:+809:secondary
TTTTGGCTTG	1	0	0	0	0	At5g27080.1:v:+1929:secondary,At4g35230.1:d:+698:secondary
TGATAGTTAA	0	0	0	1	0	At5g27060.1:v:+3678:primary
TCTCCATTGG	0	1	0	0	0	At5g27035.1:p:+136:primary
GTTTACTCCT	0	1	0	0	0	At5g27010.1:v:+120:secondary
AACACACCAG	1	0	0	0	0	At5g27000.1:d:+3011:primary
ATAATGAGTA	0	0	0	0	1	At5g26990.1:v:+1160:primary
TAGTGCTCGC	0	0	1	0	0	At5g26980.1:X:-525:quaternary
CTCGCGCGGC	0	0	1	0	0	At5g26960.1:d:+431:secondary
GTGGGAGAGA	0	0	1	0	0	At5g26860.1:d:-103:primary
CTAGAGAAAG	0	0	0	0	1	At5g26860.1:d:+2538:secondary
ATGAGACTAC	0	1	0	0	0	At5g26850.1:d:+2795:primary
ATTGTTAGAA	0	1	0	0	0	At5g26830.1:X:+299:quaternary
TCTTACGGCG	0	1	0	0	0	At5g26820.1:d:+1764:primary
GAAGAGAATG	0	1	0	0	0	At5g26775.1:p:+3714:secondary,At2g27470.1:d:+686:secondary
AACAAACTTA	0	1	0	0	0	At5g26770.2:X:-312:quaternary,At1g55730.1:X:-133:quaternary
CCTCCAGAAG	0	0	0	0	1	At5g26751.1:d:+1210:secondary
TATCCTCCTC	0	0	1	0	0	At5g26742.1:X:-208:quaternary
AACCTCCAAA	1	0	0	0	0	At5g26742.1:X:-10:quaternary
GGTTATCTAC	1	0	0	0	0	At5g26740.2:d:+536:secondary,At5g26740.1:d:+500:secondary
GATCAACTAA	0	0	0	1	0	At5g26730.1:i:+861:tertiary
TATAAGATGA	1	0	0	0	0	At5g26717.1:v:+546:secondary,At5g26692.1:v:+562:secondary
CGTGACCCGG	0	0	1	0	0	At5g26710.1:d:+1178:secondary
GAGAGGCCGA	0	1	0	0	0	At5g26700.1:X:-165:quaternary
AAAACACAAG	0	0	1	0	0	At5g26690.1:X:+466:quaternary,At1g53250.1:X:+446:quaternary,Chr1:+2769446:quaternary,Chr4:+11871018:quaternary
GATCAGTTCA	0	1	0	0	0	At5g26680.1:d:+681:secondary
AATGCAGATT	0	0	0	1	0	At5g26670.2:d:+1037:primary,At5g26670.1:d:+1118:primary
CCTGTGGTTG	1	0	0	0	0	At5g26630.1:v:+885:secondary
AGTTAATAAT	0	0	1	0	0	At5g26600.2:i:+1662:tertiary
AAACTATTTG	0	0	1	0	0	At5g26600.2:d:+1799:primary
ATCAAAAGAG	0	0	0	1	0	At5g26570.1:i:-221:tertiary
CGTTTTCACC	0	1	0	0	0	At5g26360.1:X:-18:quaternary
TTTTCAACTA	0	1	0	0	0	At5g26340.1:i:+367:tertiary
GTATGCACAT	0	0	0	1	0	At5g26330.1:i:+617:tertiary
TTTTCCAGTC	0	0	0	1	0	At5g26310.1:d:+85:secondary,At5g66690.1:d:+106:secondary
CATCAAAACC	0	0	1	0	0	At5g26310.1:d:+358:secondary
TTTCACTTTA	0	0	0	1	0	At5g26290.1:v:+655:secondary
GCGGTATGAC	0	0	0	0	1	At5g26240.1:d:-2838:secondary
AACGAGTTTG	0	0	1	0	0	At5g26236.1:p:+3087:secondary
TTCCTCCGGG	0	1	0	0	0	At5g26180.2:d:+2179:primary
ACAAATAATC	0	0	1	0	0	At5g26170.1:v:+1269:secondary,At1g24470.1:v:+298:secondary
AAACTTTCTA	0	0	0	1	0	At5g26120.1:i:+2434:tertiary
CGGGAATGTG	0	0	1	0	0	At5g26110.1:d:+789:primary
CAATTTATGA	0	0	0	0	1	At5g26050.1:v:+897:secondary

GAACATCTTT	0	0	0	0	1	At5g26040.1:d:+1708:primary,At5g26040.2:d:+1792:primary
CGGATTTGAT	0	0	0	0	1	At5g26010.1:v:+1552:primary
AGCAAGAAGC	0	0	1	0	0	At5g26000.2:X:-216:quaternary,At5g48375.1:X:-34:quaternary
AACCTGATGT	0	1	0	0	0	At5g26000.2:X:-2:quaternary
AGGGAATATG	0	0	1	0	0	At5g26000.2:d:+985:secondary,At5g26000.1:d:+985:secondary
CTCCTGGTCC	0	0	1	0	0	At5g26000.1:d:+1184:secondary,At5g13070.1:d:+194:secondary,At5g26000.2:d:+1184:secondary
TGAGGCTCTT	0	0	0	1	0	At5g25980.1:d:-1638:secondary,At5g25980.2:d:-1642:secondary
AAGCTTCTTG	0	1	0	0	0	At5g25980.1:d:+40:secondary,At5g25980.2:d:+40:secondary
GTCTTGTGGT	1	0	0	0	0	At5g25955.1:p:+443:primary
AAATTGTCCA	1	0	0	0	0	At5g25955.1:p:+233:secondary
AAAGAGGTCT	1	0	0	0	0	At5g25930.1:d:+2958:primary
GCAGGCATTG	1	0	0	0	0	At5g25900.1:d:+1420:primary
AATGATTTGA	0	0	0	1	0	At5g25890.1:i:+216:tertiary
GCTGCTTGAA	0	0	1	0	0	At5g25880.1:d:-1165:secondary,At5g25880.1:d:-1173:secondary,At5g11670.1:d:-1276:secondary,At5g11670.1:d:-1268:secondary
TCTTTTCAAA	1	0	0	0	0	At5g25860.1:v:+1339:secondary
AAATGAATTT	0	1	0	0	0	At5g25830.1:v:+1508:primary
CCTCTTTTGA	0	0	0	0	1	At5g25820.1:d:+140:secondary
TCTGCTTATC	0	1	0	0	0	At5g25790.1:v:+987:secondary
TGATTTGATT	0	0	1	0	0	At5g25770.1:d:+274:secondary,At4g29610.1:v:+240:secondary
GTCGGTCTCT	0	1	0	0	0	At5g25757.1:d:+682:secondary,At5g25754.1:d:+727:secondary
TGTTCTGTCT	0	1	0	0	0	At5g25757.1:d:+245:secondary,At4g21550.1:d:+51:secondary,At5g25754.1:d:+290:secondary
ATCTACGTTG	0	0	1	0	0	At5g25757.1:d:+1435:secondary,At5g25754.1:d:+1480:secondary
CCCCAAGTTT	0	0	0	1	0	At5g25757.1:d:+1122:secondary,At5g25754.1:d:+1167:secondary
CTAACTGGAA	0	1	0	0	0	At5g25752.1:d:+413:primary
GTAGTGGCGA	0	0	1	0	0	At5g25610.1:d:+83:secondary
TCTTGCCACG	0	1	0	0	0	At5g25510.1:d:+1732:secondary
TCGAGAGTTT	0	0	1	0	0	At5g25490.1:d:+752:primary
TTGATGGCGA	0	1	0	0	0	At5g25480.1:d:+776:primary
AGGATAATTC	0	1	0	0	0	At5g25475.2:d:+239:primary,At5g25475.1:d:+263:primary
GCATAGAAAG	1	0	0	0	0	At5g25460.1:d:-1071:secondary
CCTGAAAAAA	0	0	0	1	0	At5g25425.1:v:+1105:primary
GTTTTGATTA	0	1	0	0	0	At5g25420.1:i:+497:tertiary
TGATGGGTTT	0	0	1	0	0	At5g25415.1:d:+554:primary
TTTGTGTTTA	0	0	0	0	1	At5g25380.1:v:+29:secondary
GGGTAAGTGA	1	0	0	0	0	At5g25360.1:i:+368:tertiary
GAAACTTCTT	0	0	1	0	0	At5g25340.1:d:+707:secondary,At4g04280.1:p:+343:secondary,At1g64280.1:d:+2477:secondary
TAAGAAATAG	0	0	1	0	0	At5g25305.1:p:+20:secondary,At1g29440.1:v:+965:secondary
GTGGCTGATT	0	0	1	0	0	At5g25265.1:X:-3:quaternary
AGACTTGTTC	0	1	0	0	0	At5g25110.1:d:+402:secondary
CATTCAGTAG	0	0	0	0	1	At5g24970.1:d:+1925:primary
AGAGACGTGG	0	0	1	0	0	At5g24915.1:p:+854:secondary,At1g76780.1:v:+3838:secondary
GCAGACAATA	0	1	0	0	0	At5g24890.1:X:-89:quaternary
TTTTATTTGT	1	0	0	0	0	At5g24860.1:d:+468:secondary,At1g42150.1:p:+2764:secondary,At1g41930.1:p:+881:secondary,At1g36620.1:p:+1313:secondary
CAGGATCTAT	0	0	1	0	0	At5g24850.1:i:+1405:tertiary
TTTCAGACCA	1	0	0	0	0	At5g24810.1:X:-181:quaternary,At1g79860.1:X:--140:quaternary
CTGCTTTCAT	0	0	0	0	1	At5g24800.1:d:-976:secondary
AGCAGACAAA	0	0	0	1	0	At5g24800.1:d:+1220:primary
CGTTCCAAAT	0	1	0	0	0	At5g24775.1:p:+3081:primary
TCGGAATTTT	0	0	1	0	0	At5g24775.1:p:+171:secondary
AAAATCCTCT	0	1	0	0	0	At5g24770.1:d:+384:secondary
TCACAGATTC	1	0	0	0	0	At5g24760.1:d:+1298:secondary,At5g24760.2:d:+1288:secondary,At1g24430.1:d:+324:secondary
TTTACTGTCA	0	0	1	0	0	At5g24740.1:X:-25:quaternary
GGATGGATGC	0	1	0	0	0	At5g24740.1:d:+2324:secondary
GACTCTGAAA	1	0	0	0	0	At5g24680.1:d:+1648:primary
TTCCGCCCTC	0	1	0	0	0	At5g24670.1:d:+953:primary
AAGAGTGAAG	0	0	0	0	1	At5g24640.1:d:+32:secondary,At2g41730.1:d:+29:secondary
AAACATAATC	1	0	0	0	0	At5g24620.1:d:+1413:secondary
GACTTCCCAG	0	0	0	1	0	At5g24620.1:d:+102:secondary
TCTGCGACAA	0	0	1	0	0	At5g24610.1:d:+291:secondary

GTTAAGCTTA	0	1	0	0	0	At5g24590.1:X:-431:quaternary
TCAAAAAGAA	0	0	0	0	1	At5g24580.2:i:+1268:tertiary,At5g24580.1:i:+1150:tertiary
CACTGCCAAG	0	1	0	0	0	At5g24580.1:d:+520:primary,At5g24580.2:d:+635:primary
AGCCCGGCCA	0	0	0	1	0	At5g24530.1:d:+1014:secondary
AAGTTTCCAA	0	1	0	0	0	At5g24520.3:X:-27:quaternary
AATGTAGGAG	0	0	0	0	1	At5g24510.1:v:+531:secondary
AATCTCTTCG	0	0	0	1	0	At5g24500.1:X:-26:quaternary
ATGAACCAGA	0	1	0	0	0	At5g24500.1:d:+899:primary
ATTTAGCAAA	0	1	0	0	0	At5g24450.1:i:+268:tertiary
AGAACTTATT	0	0	0	1	0	At5g24450.1:d:-1455:secondary
ATGTAAAAAG	0	1	0	0	0	At5g24430.1:d:+1983:secondary
AAATGACAGA	0	1	0	0	0	At5g24420.1:X:-685:quaternary
AAGCACAACA	1	0	0	0	0	At5g24420.1:d:+756:secondary
AATGGTGTTT	1	0	0	0	0	At5g24420.1:d:+1068:secondary,At3g33154.1:p:+2495:secondary
TAAGGCATAG	0	0	0	0	1	At5g24390.1:X:-603:quaternary
TGAAGATCGT	0	1	0	0	0	At5g24390.1:X:+-193:quaternary
TAAGTTTGGT	0	0	0	0	1	At5g24390.1:d:+2185:secondary
AAGGCCTTAC	0	1	0	0	0	At5g24350.1:d:+1528:secondary
GATGTGAAGG	0	1	0	0	0	At5g24340.1:d:+370:secondary,At2g45880.1:d:+687:secondary
TTTTGAAAGA	0	0	1	0	0	At5g24313.1:d:+356:primary
TTATGGAATT	0	0	1	0	0	At5g24290.2:d:+1600:primary,At5g24290.1:d:+1734:primary
TATGAGAACT	0	1	0	0	0	At5g24280.1:v:+741:secondary
GAATCTCAGA	0	0	0	0	1	At5g24240.1:X:-119:quaternary
AGGAAAAGTT	0	0	1	0	0	At5g24240.1:X:-1019:quaternary
GCCAAACCAA	0	1	0	0	0	At5g24160.1:d:-1484:secondary
CCGCCTAGCA	1	0	0	0	0	At5g24150.1:X:-154:quaternary
CGAATGCTCT	1	0	0	0	0	At5g24080.1:X:-261:quaternary
TTTCAATTAT	1	0	0	0	0	At5g24030.1:i:+2733:tertiary,At1g71860.2:i:+1376:tertiary,At1g71860.1:i:+1376:tertiary
TTATAGAGCA	0	1	0	0	0	At5g24030.1:i:+1457:tertiary
TGCTTCAGCT	0	1	0	0	0	At5g24010.1:d:+2400:primary
GAAGGACTTG	0	0	1	0	0	At5g23960.1:v:+1817:secondary,At3g21790.1:v:+2094:secondary,At4g20010.1:d:+1069:secondary,At1g31010.1:d:+893:secondary,At3g21780.1:v:+26:secondary
TTAATGGTTT	0	0	0	1	0	At5g23940.1:X:--93:quaternary
GTCCAAATAT	0	1	0	0	0	At5g23940.1:X:-1196:quaternary
ATTTTATTAG	0	0	0	0	1	At5g23920.1:d:+170:primary
ACGTGCGTTT	0	0	1	0	0	At5g23900.1:X:-164:quaternary
GCCAGATCGT	0	0	0	0	1	At5g23890.1:d:+2445:secondary
TCCAAATGTG	1	0	0	0	0	At5g23870.2:d:+1538:secondary
TCCAGTTATA	0	0	0	0	1	At5g23870.1:X:--241:quaternary,At1g49140.1:X:-161:quaternary
TAATAGCTTT	0	1	0	0	0	At5g23850.1:d:+1770:primary
GGGAGGCAGG	1	0	0	0	0	At5g23800.1:v:+354:secondary
GTAAAAAAGA	1	0	0	0	0	At5g23770.1:v:+1664:secondary
AACAACAAAG	0	1	0	0	0	At5g23710.1:v:+564:secondary
GCGATTTCAA	0	0	1	0	0	At5g23690.1:d:+128:secondary,At2g40630.1:d:+1261:secondary
AAGTTGATGA	0	0	0	0	1	At5g23680.1:d:+1155:secondary
GAAGGGGAGA	0	0	0	1	0	At5g23670.1:d:+986:secondary,At3g44093.1:p:+1027:secondary
GTTTGAAAAA	0	1	0	0	0	At5g23650.1:v:+748:secondary
CTACTGTTTT	0	1	0	0	0	At5g23630.1:i:+5217:tertiary
GGAGCGGTTA	0	0	0	1	0	At5g23630.1:d:+3441:primary
TTGGAGTTGC	1	0	0	0	0	At5g23630.1:d:+2474:secondary
TCTCTAAGGG	0	1	0	0	0	At5g23610.1:X:-187:quaternary
TCATATGATA	1	0	0	0	0	At5g23580.1:d:+1624:secondary,At5g02820.1:d:+1082:secondary
CCACAGAAGC	0	0	0	0	1	At5g23520.1:v:+1312:secondary
CTTCATACAA	0	1	0	0	0	At5g23490.1:d:+2249:primary,At5g23510.1:d:+511:primary
CGTCTGGCAT	0	1	0	0	0	At5g23490.1:d:+1138:secondary
GATATAAGCC	1	0	0	0	0	At5g23460.1:d:-663:secondary
GAGAAGTGAT	0	0	0	0	1	At5g23450.2:d:+2650:secondary,At5g23450.1:d:+2456:secondary
TCGTTGTTCA	0	0	0	1	0	At5g23430.2:X:+469:quaternary
AATAGAGGTT	0	1	0	0	0	At5g23430.2:d:+3315:primary,At5g23430.1:d:+3318:primary
AGTGACTTCC	0	1	0	0	0	At5g23420.1:d:+441:primary

TAATGTTGTT	0	0	0	0	1	At5g23405.2:i:+685:tertiary,At5g23405.1:i:+678:tertiary
GTGTGATTTG	1	0	0	0	0	At5g23405.2:i:+1001:tertiary,At5g23405.1:i:+994:tertiary
AAATTCTCCA	0	1	0	0	0	At5g23390.1:X:-274:quaternary
TTAACGATGA	0	1	0	0	0	At5g23370.1:X:-910:quaternary,At3g55970.1:X:-317:quaternary
AGCAAGAGAT	0	1	0	0	0	At5g23370.1:d:+916:primary
GTGTGTATAG	0	1	0	0	0	At5g23320.1:v:+715:secondary
AAGCAATTGG	0	1	0	0	0	At5g23300.1:d:+1519:primary,At5g27043.1:p:+201:primary
AGTTGGGTTA	0	0	1	0	0	At5g23210.2:X:+-227:quaternary
TCAAAATCAA	0	1	0	0	0	At5g23210.2:d:+19:secondary,At5g23210.1:d:+19:secondary
CCAAACGAAT	0	0	0	1	0	At5g23170.1:d:+337:secondary
TTAGAGACCT	0	1	0	0	0	At5g23150.1:d:+2685:secondary
TTTAGTGTTT	0	1	0	0	0	At5g23140.1:d:+961:secondary
CAGCCAGCCC	0	1	0	0	0	At5g23110.1:v:+9688:secondary
CATATGTCTC	0	0	0	1	0	At5g23110.1:v:+6473:secondary
TTCTCAAGGC	1	0	0	0	0	At5g23090.3:d:+314:primary,At5g23090.1:d:+394:primary
GTGGGTCAGG	0	0	1	0	0	At5g23090.2:d:+788:primary
AATTTGTATT	0	0	0	0	1	At5g23040.2:i:+2310:tertiary
AATGATTGTG	0	0	1	0	0	At5g23010.1:i:+1628:tertiary,At2g06005.1:i:+907:tertiary,At3g20620.1:i:+2067:tertiary,At2g06005.2:i:+876:tertiary
TTTATGTTAG	1	0	0	0	0	At5g23000.1:d:-881:secondary
GGGATTTGAA	0	1	0	0	0	At5g23000.1:d:+833:secondary
TAGGATTCTG	0	1	0	0	0	At5g22940.1:d:+1667:primary
TCAGCTTGAA	0	0	0	0	1	At5g22930.1:v:+791:secondary
TCTTGTCTGT	0	0	0	1	0	At5g22875.1:i:+442:tertiary
GAGGCAGGTG	0	0	0	1	0	At5g22840.1:d:+1645:primary
GAAACCAATT	0	0	0	1	0	At5g22820.1:X:--122:quaternary
AAGTCAGCTT	0	0	0	0	1	At5g22810.1:v:-1615:secondary
ATACGGACCG	0	0	1	0	0	At5g22790.1:d:+1585:secondary
AGACGCATTG	0	0	1	0	0	At5g22780.1:X:-62:quaternary,At5g22770.2:X:-62:quaternary
GAAGCTAAAA	0	0	1	0	0	At5g22770.3:i:+4312:tertiary,At5g22770.1:i:+4312:tertiary,At5g22770.2:i:+4312:tertiary
CTTCAAGAAT	0	1	0	0	0	At5g22770.2:d:+331:secondary,At5g22770.1:d:+331:secondary,At5g22780.1:d:+332:secondary,At5g22770.3:d:+261:secondary
CTTGAATTTT	0	0	1	0	0	At5g22770.1:i:+6687:tertiary,At5g22770.2:i:+6687:tertiary,At5g22770.3:i:+6687:tertiary
GAATTCGAAC	0	1	0	0	0	At5g22760.1:d:+4378:secondary
GATGGAACGT	1	0	0	0	0	At5g22760.1:d:+1264:secondary
GATTATTCCA	1	0	0	0	0	At5g22750.1:i:+2693:tertiary
TTATGCCTAA	1	0	0	0	0	At5g22690.1:i:-3441:tertiary
AGACACGAAC	1	0	0	0	0	At5g22670.1:v:+1281:secondary
GTTGAATGCA	1	0	0	0	0	At5g22640.1:i:+2962:tertiary
AAATATATTG	0	1	0	0	0	At5g22640.1:i:+1751:tertiary
CTAGAAATGA	0	0	0	0	1	At5g22640.1:d:-2294:secondary
CAATTAGAGA	1	0	0	0	0	At5g22600.1:i:+1241:tertiary
AGTATTATCA	0	1	0	0	0	At5g22570.1:X:--12:quaternary
GTTCTTGATG	0	1	0	0	0	At5g22560.1:v:+810:secondary,At1g16170.1:d:+255:secondary,At4g02070.1:v:+2439:secondary,At4g22200.1:d:+1069:secondary
TGTTAATAAA	0	1	0	0	0	At5g22555.1:i:+326:tertiary
TGTTAGGTCC	0	0	1	0	0	At5g22510.1:X:+582:quaternary
CATATGATAT	0	0	0	1	0	At5g22500.1:X:+324:quaternary,Chr2:+12977441:quaternary,Chr1:+27668031:quaternary
GGTGATGAGG	0	1	0	0	0	At5g22500.1:d:-1556:secondary
TAAAACTCTT	1	0	0	0	0	At5g22490.1:i:+1329:tertiary,At5g46340.1:i:+3249:tertiary,At5g43175.1:i:+800:tertiary
GTCTCCTTGT	0	0	0	0	1	At5g22460.2:X:-23:quaternary
TCGTCTACAT	1	0	0	0	0	At5g22450.1:X:-66:quaternary
GCTTGGGCTT	0	1	0	0	0	At5g22450.1:v:+169:secondary
AAAAGCCCTC	1	0	0	0	0	At5g22420.1:v:+1209:secondary
TTCTTCCTGT	0	0	1	0	0	At5g22400.1:d:+1774:primary
CCAGGACGCA	0	1	0	0	0	At5g22355.1:d:+1865:secondary,At5g59940.1:v:+2112:secondary
CCGGTTAGAT	0	0	1	0	0	At5g22350.1:X:-189:quaternary
AGTTCACGCT	1	0	0	0	0	At5g22340.1:d:-551:secondary
TACCTGTCGA	0	0	0	1	0	At5g22340.1:d:+556:secondary
GAGAATTTTA	0	0	0	1	0	At5g22330.1:d:+472:secondary
AGAGAATCAG	0	1	0	0	0	At5g22300.1:d:-182:primary

AGTCTCTGGA	1	0	0	0	0	At5g22280.1:i:+1028:tertiary
TTCCTTGCCT	0	0	0	0	1	At5g22270.1:X:-46:quaternary
TTTTATAAAC	0	1	0	0	0	At5g22270.1:i:+221:tertiary
TCCTTCACGG	0	0	1	0	0	At5g22260.1:d:+567:secondary
TTTTATTCAA	0	1	0	0	0	At5g22250.1:d:+6:primary
AGTCATCAAA	0	1	0	0	0	At5g22220.2:i:-1604:tertiary,At5g22220.1:i:-1604:tertiary
TGAAACTGCA	0	0	0	0	1	At5g22220.2:d:+1871:secondary,At5g22240.1:v:+29:secondary
CTTCCAGAAT	0	0	0	1	0	At5g22220.1:d:+994:secondary,At5g22220.2:d:+994:secondary
AGTTTTTATG	1	0	0	0	0	At5g22160.1:v:+1813:primary
ATCTGCAAAT	0	0	1	0	0	At5g22130.1:d:+1618:primary
ATTACAACTA	0	1	0	0	0	At5g22080.1:i:+313:tertiary
CTGCAAAAGG	0	0	0	0	1	At5g22080.1:i:+1125:tertiary,At1g27690.1:i:+1042:tertiary
CAGAGTCGTC	0	1	0	0	0	At5g22060.1:X:-202:quaternary
AAGGGTAAGC	0	0	0	0	1	At5g22060.1:d:+1123:secondary,At3g44110.1:d:+1153:secondary
TCATCTCAAT	0	0	0	0	1	At5g22050.2:d:-1111:secondary
TATACCGGTA	1	0	0	0	0	At5g22050.1:d:+992:secondary,At5g22050.2:d:+1016:secondary
TCCTCTCGGT	0	0	0	1	0	At5g22030.2:X:-295:quaternary
AGAACTTATA	0	0	0	0	1	At5g22030.1:i:+2695:tertiary,At5g22030.2:i:+2695:tertiary
TTGCGGGGAA	0	0	1	0	0	At5g22030.1:d:+1988:primary,At5g22030.2:d:+2056:primary
TCTGTACAAC	0	0	1	0	0	At5g22010.1:X:+212:quaternary
GGGTAAGAAG	0	1	0	0	0	At5g22000.2:d:+563:secondary,At5g04290.1:v:+3359:secondary,At5g22000.1:d:+689:secondary,At5g22000.3:d:+616:secondary
GCATCAAAAA	0	0	1	0	0	At5g21970.1:i:+199:tertiary
TTAATAAAAA	1	0	0	0	0	At5g21950.1:v:+1573:primary
TCTTTCTTAT	1	0	0	0	0	At5g21950.1:v:+1221:secondary
ACCTCAAAGA	0	0	1	0	0	At5g21950.1:v:+1117:secondary
CCAAAAACAT	0	0	0	1	0	At5g21535.1:X:-347:quaternary
AAACACATAT	0	0	0	0	1	At5g21482.1:i:+1752:tertiary
ATCAATGAAG	0	0	1	0	0	At5g21274.1:d:+225:secondary
TGGAAATCCA	0	1	0	0	0	At5g21222.1:d:+789:secondary
TGTTCATAGA	0	0	1	0	0	At5g21170.1:d:+890:secondary
AGTGCTGTTT	0	1	0	0	0	At5g21160.1:i:+1389:tertiary
AAAAAACTTA	0	1	0	0	0	At5g21105.1:i:+3089:tertiary
AACCGGAACC	1	0	0	0	0	At5g21105.1:d:+1699:secondary
ATTTCTGGGT	0	1	0	0	0	At5g21100.1:d:+1543:primary
TGAGCTTTCG	0	0	0	1	0	At5g21090.1:d:-145:primary
TCCTCCAGAG	1	0	0	0	0	At5g21090.1:d:+286:secondary,At5g14880.1:v:+1330:secondary
AGTCCAGGTC	1	0	0	0	0	At5g21060.1:X:-37:quaternary
ACCACCAGAC	0	0	0	1	0	At5g21060.1:X:-196:quaternary
TCTGTGGATG	0	0	0	0	1	At5g21060.1:d:+933:primary
AATCTACTGT	0	1	0	0	0	At5g21060.1:d:+607:secondary
AGTCTTGGTT	0	1	0	0	0	At5g21040.1:X:+2100:quaternary
AACCTGAGTG	0	1	0	0	0	At5g21030.1:v:+3110:primary
GGACCAAAGC	1	0	0	0	0	At5g20990.1:d:+2001:primary
AGAGAAAACA	0	0	0	1	0	At5g20970.1:d:+688:secondary
GCAATTGTGG	0	0	1	0	0	At5g20960.2:d:+4357:primary
AGTGAATAAA	0	0	0	1	0	At5g20960.1:i:+939:tertiary,At5g20960.2:i:+939:tertiary
CAGCTTATTA	0	0	1	0	0	At5g20960.1:d:-4275:secondary,At5g20960.2:d:-4275:secondary
CTGATAACTT	0	1	0	0	0	At5g20950.1:d:+1455:primary,At5g20950.2:d:+1474:primary
TTATGCAATA	1	0	0	0	0	At5g20920.1:d:+642:secondary,At5g20920.2:d:+599:secondary,At5g28937.1:p:+147:secondary
AATTACCAAC	0	1	0	0	0	At5g20910.1:d:+954:secondary
GAAACAATCT	0	1	0	0	0	At5g20910.1:d:+1373:secondary
TGTTCCTTCC	0	1	0	0	0	At5g20890.1:i:+1417:tertiary
ACCAAGGTTG	0	1	0	0	0	At5g20890.1:d:+944:secondary
ACTAAAAAGC	0	0	1	0	0	At5g20885.1:X:--250:quaternary,At1g27670.1:X:--123:quaternary
AAGTTAGCAA	0	0	0	1	0	At5g20880.1:p:+1062:primary
GCGAGCTTAT	0	0	0	0	1	At5g20850.1:X:-55:quaternary
TTTGTATATT	1	0	0	0	0	At5g20820.1:d:+539:primary
CATTGGATGA	0	0	1	0	0	At5g20800.1:p:+243:secondary
CCTACAGGCT	0	0	0	1	0	At5g20790.1:X:-942:quaternary

CCGCCGTTAC	0	0	0	1	0	At5g20790.1:X:-597:quaternary
GGCAAAATCA	0	0	0	0	1	At5g20750.1:v:-187:primary
ATGAATGTCA	0	0	1	0	0	At5g20730.2:i:+2532:tertiary,At5g20730.1:i:+2603:tertiary,At5g20730.3:i:+2603:tertiary
CTTACTAAAA	0	0	1	0	0	At5g20700.1:d:+118:secondary
TTAATGGCGT	0	1	0	0	0	At5g20680.1:d:+1638:secondary
TTCTGTGTTC	0	0	0	0	1	At5g20670.1:d:+751:primary
GAAGCAGAGC	0	1	0	0	0	At5g20660.1:d:+2322:secondary
GATTTCCTCG	1	0	0	0	0	At5g20660.1:d:+1541:secondary
TCTTGTTCAA	0	0	1	0	0	At5g20640.1:v:+704:secondary
CTACTGCTTC	0	0	0	1	0	At5g20610.1:d:-3596:secondary
ATGGGCAGCC	0	0	1	0	0	At5g20610.1:d:-2973:secondary
TGTCTGCTCT	1	0	0	0	0	At5g20610.1:d:+3601:secondary
TTCGAGAGAT	0	0	1	0	0	At5g20610.1:d:+1725:secondary
AGTACTTCAT	0	0	0	1	0	At5g20580.2:i:+2804:tertiary,At5g20580.1:i:+2842:tertiary
TCTGTTGAAG	1	0	0	0	0	At5g20580.1:d:+669:secondary,At4g14800.1:d:+561:secondary,At5g20580.2:d:+631:secondary,At2g29880.1:v:+1236:secondary
AAGCATATAC	0	0	0	0	1	At5g20580.1:d:+1259:primary,At5g20580.2:d:+1221:primary
TAATTTTGGC	1	0	0	0	0	At5g20450.1:i:+1318:tertiary
AGTCTGAATG	0	1	0	0	0	At5g20420.1:v:+679:secondary
GGACATCAAA	0	0	1	0	0	At5g20420.1:v:+4565:primary
TTTATATTAA	0	0	1	0	0	At5g20410.1:d:+1505:secondary,At5g22900.1:v:+3043:secondary
TTCGATGTGA	0	0	1	0	0	At5g20390.1:d:+805:secondary
CTATTTTAGT	0	1	0	0	0	At5g20380.1:i:+1960:tertiary
GAAGTCATTT	0	0	1	0	0	At5g20380.1:d:+980:secondary
GAGAAGCAAA	0	1	0	0	0	At5g20360.1:v:+354:secondary,At5g27680.1:d:+2569:secondary
TTCTCTGCTG	0	0	1	0	0	At5g20350.1:i:+719:tertiary
CAGTCTCTCA	0	0	1	0	0	At5g20340.1:v:+804:secondary
TTTGATGCAA	1	0	0	0	0	At5g20340.1:v:+1101:primary
TGAATAGAGT	0	1	0	0	0	At5g20310.1:v:+1533:secondary
TAGAACTCCA	0	0	0	1	0	At5g20290.1:X:-32:quaternary
GTGGTGATAT	0	0	0	0	1	At5g20280.1:d:+1394:secondary
CGGCAACGGC	0	0	1	0	0	At5g20270.1:d:+247:secondary
ATACTCTGCA	0	1	0	0	0	At5g20250.2:d:-1787:primary,At5g20250.1:d:-1766:primary
CCTCCCAGCA	0	1	0	0	0	At5g20230.1:d:-315:primary
ATGTGGCAGT	0	0	1	0	0	At5g20230.1:d:+320:secondary
GTCTCAACTA	0	0	0	1	0	At5g20220.1:i:-1418:tertiary
GATGTAGAAG	1	0	0	0	0	At5g20220.1:i:+809:tertiary
CAAGGCCTTG	0	0	0	1	0	At5g20220.1:i:+1730:tertiary
CTGAAGTTCT	0	0	0	1	0	At5g20200.1:i:+1089:tertiary
ATCTAGGCAC	0	0	0	1	0	At5g20200.1:d:-2472:secondary
CCCTTCACAA	0	0	1	0	0	At5g20200.1:d:+2083:secondary
TCTCGAGTCC	0	1	0	0	0	At5g20190.1:d:+50:secondary
TTTAGTTCTT	1	0	0	0	0	At5g20170.1:d:+2061:primary
TGTTTAGTTA	0	0	1	0	0	At5g20165.1:i:+328:tertiary
CACATATCTG	0	0	1	0	0	At5g20165.1:d:+167:secondary
TCTTGTTTCG	0	1	0	0	0	At5g20120.1:d:+748:primary
ATATTTGATG	0	0	1	0	0	At5g20110.1:i:+1261:tertiary
ACTAATTCCA	0	0	1	0	0	At5g20100.1:X:-686:quaternary,Chr5:-18601301:quaternary
TTTGAACCCC	1	0	0	0	0	At5g20090.1:X:-282:quaternary
TCTTTTTTTA	0	1	0	0	0	At5g20090.1:i:+774:tertiary
TTGCAAATCT	0	0	1	0	0	At5g20060.2:d:+887:secondary,At5g20060.1:d:+438:secondary
GAAAAGCTGA	0	0	0	1	0	At5g20060.1:d:+696:secondary,At3g13740.1:d:+814:secondary,At5g20060.2:d:+1145:secondary
CGTGGAAAGC	0	0	1	0	0	At5g20040.2:d:+1450:primary
GAAAGACTTT	0	0	0	0	1	At5g19990.1:d:+1376:secondary,At1g03760.1:d:+1020:secondary
ATATGGTAGA	0	0	1	0	0	At5g19960.1:d:+576:secondary
TATCTTATTA	0	0	0	0	1	At5g19950.1:d:+1495:secondary
TTCTGCGTAT	0	0	1	0	0	At5g19920.1:i:+784:tertiary
GAAACCCCAC	0	1	0	0	0	At5g19910.1:X:-373:quaternary,At5g28285.1:X:-3470:quaternary
TCCTAGGAGG	0	1	0	0	0	At5g19910.1:d:+691:primary
GCTTCGCCAG	0	0	0	1	0	At5g19910.1:d:+43:secondary
GAGACACCAA	0	0	0	0	1	At5g19900.1:X:-621:quaternary

ATTTGAGTCG	0	1	0	0	0	At5g19900.1:d:+863:secondary
CACAACTCTG	1	0	0	0	0	At5g19850.1:X:-152:quaternary
GGAGCCCATC	0	0	1	0	0	At5g19850.1:d:+947:secondary
AAGCAGCAGC	0	1	0	0	0	At5g19790.1:v:+654:secondary,At3g04960.1:d:+992:secondary
CTTCACTTGT	1	0	0	0	0	At5g19740.1:d:+2068:primary
ATATTTATTA	0	0	0	1	0	At5g19730.1:i:+713:tertiary,At1g72160.1:i:+1539:tertiary,At2g15610.1:i:+1163:tertiary
AACATCCAAA	0	0	1	0	0	At5g19690.1:X:+397:quaternary
AGGTGTATGA	0	1	0	0	0	At5g19660.1:X:-12:quaternary
ATCACACAAC	0	1	0	0	0	At5g19660.1:i:+1224:tertiary
GTCACCAACT	0	0	0	1	0	At5g19660.1:d:+2669:secondary,At1g58030.1:d:+389:secondary
AAATGGTTTT	1	0	0	0	0	At5g19600.1:d:+2194:primary
TAAACCTTTT	0	0	1	0	0	At5g19580.1:d:+2059:primary
AGTGATACTG	0	0	0	1	0	At5g19580.1:d:+1244:secondary
GTTCCTCCCT	1	0	0	0	0	At5g19540.1:d:-45:primary
ATTCTGTGCT	0	0	1	0	0	At5g19540.1:d:-1448:secondary
GCGGTGAGCT	0	1	0	0	0	At5g19540.1:d:+50:secondary
CCCGACCTGG	0	0	1	0	0	At5g19540.1:d:+1130:secondary
AAGCAAGTCT	0	0	0	1	0	At5g19530.1:d:+817:secondary
AAAGCAAGAC	1	0	0	0	0	At5g19520.1:d:-1818:secondary
GATCTCTTTG	1	0	0	0	0	At5g19510.1:d:+384:secondary
AGTGTACCCA	0	1	0	0	0	At5g19500.1:d:+932:secondary
TATGGCGGTG	0	1	0	0	0	At5g19480.1:v:+223:secondary
TACGATATGA	0	0	1	0	0	At5g19450.1:d:+1812:secondary,At5g19450.2:d:+1957:secondary
GTCGGGAAAG	0	0	1	0	0	At5g19440.1:X:+420:quaternary
AGGCAAGTGG	0	0	1	0	0	At5g19420.1:d:+2371:secondary
GCTTCCTGCT	0	0	1	0	0	At5g19410.1:v:+354:secondary
ATCTTAAAAA	0	0	0	1	0	At5g19400.1:d:+554:secondary,At1g60900.1:d:+221:secondary
CCTTTTTACG	0	0	1	0	0	At5g19310.1:i:+2638:tertiary
GAAGAGAAGG	1	0	0	0	0	At5g19310.1:d:+2873:primary
GGCCTCATCT	0	0	1	0	0	At5g19310.1:d:+1434:secondary
AAGATACGGA	0	0	0	1	0	At5g19300.1:d:+139:secondary
CTTTTAGTGT	0	1	0	0	0	At5g19290.1:X:--81:quaternary
GATTCTCCGA	0	0	1	0	0	At5g19290.1:d:+351:secondary
GCGATATTTT	0	0	0	0	1	At5g19280.1:d:+180:secondary
AAGAAACATC	0	0	1	0	0	At5g19240.1:d:-641:primary
TAATCCATCC	0	1	0	0	0	At5g19220.1:X:-67:quaternary
GGAAGAGTAT	0	0	0	0	1	At5g19210.1:d:+1597:primary,At5g19210.2:d:+1569:primary
ACATCTACTA	0	1	0	0	0	At5g19200.1:d:+1085:primary
ATGAATGGAA	0	1	0	0	0	At5g19165.1:p:+964:secondary,At1g50920.1:d:+1419:secondary
AGACTTCTTC	0	1	0	0	0	At5g19160.1:v:+2149:primary
ATTGCTTGTA	0	1	0	0	0	At5g19130.1:d:+2421:secondary,At5g19130.2:d:+2398:secondary
AGAGAACTAA	0	0	1	0	0	At5g19097.1:p:+4148:secondary
CCTAATAACA	0	0	1	0	0	At5g19090.2:v:+1155:secondary,At5g19090.1:d:+805:secondary
TCATACTCTT	1	0	0	0	0	At5g19080.1:d:+1318:primary
GAATCAAAGT	0	0	0	1	0	At5g19030.1:d:+659:primary
ATTATCTAAA	0	0	1	0	0	At5g19020.1:v:+2497:secondary
ACAGGATCTG	1	0	0	0	0	At5g19000.1:v:+1958:secondary
ATCTGCATCT	0	0	0	1	0	At5g18970.1:d:+835:secondary
GAAAATCAGA	1	0	0	0	0	At5g18950.1:v:+1555:secondary,At3g04960.1:d:+1484:secondary,At5g48657.1:d:+1029:secondary,At5g48657.2:d:+515:secondary,At5g60940.1:d:+1040:secondary,At5g60940.2:d:+1089:secondary
TACTCTGGAT	0	0	0	0	1	At5g18890.1:v:+2382:primary
TTTGGAAAGA	1	0	0	0	0	At5g18880.1:v:+1066:primary
GGCGGTGGTG	0	0	1	0	0	At5g18860.1:d:+996:secondary
ATTAGTTCTA	0	0	0	1	0	At5g18860.1:d:+3237:primary
TGAATGCAAA	0	0	1	0	0	At5g18810.1:i:+1326:tertiary
TCCTCTTCAC	1	0	0	0	0	At5g18790.1:d:+202:primary
GCAAAAAAAT	0	0	1	0	0	At5g18740.1:v:+2590:primary,At4g20110.1:d:+2278:primary
GAGGTCGTCC	0	1	0	0	0	At5g18700.1:v:+1381:secondary
CTTCACTTTC	0	1	0	0	0	At5g18670.1:d:+670:secondary
GATCAGAATG	0	0	1	0	0	At5g18650.1:i:-707:tertiary

GGAGAATATT	0	0	1	0	0	At5g18650.1:d:+613:secondary
AACTGAGCAG	0	0	0	0	1	At5g18633.1:p:+1241:secondary
GTCCATAAAC	0	1	0	0	0	At5g18620.1:d:+2998:primary,At5g18620.2:d:+2998:primary
GAGCAGAAGA	0	1	0	0	0	At5g18620.1:d:+2658:secondary,At3g06400.1:d:+2597:secondary,At5g18620.2:d:+2658:secondary
GTAGTTTCAA	0	0	0	0	1	At5g18610.1:i:-1254:tertiary
AGATGAGACT	0	0	0	0	1	At5g18590.2:d:+2760:secondary,At5g18590.1:d:+2705:secondary
GTGGCTCTGG	0	1	0	0	0	At5g18590.1:d:+1676:secondary,At5g18590.2:d:+1731:secondary
ACATTATAAA	0	0	0	0	1	At5g18580.1:i:-1309:tertiary
ATTCAGCCAA	0	1	0	0	0	At5g18570.1:d:+1977:primary
TATTGAACAA	0	0	0	1	0	At5g18530.1:d:+2388:primary
AAGAGGTTGG	0	0	1	0	0	At5g18525.1:i:+1380:tertiary
GAAAGAGAAT	0	0	1	0	0	At5g18480.1:d:+1388:secondary
CAAATCAGAC	0	0	0	1	0	At5g18475.1:X:-426:quaternary,At4g14230.1:X:-124:quaternary
GAACCTGCTG	0	0	1	0	0	At5g18470.1:d:+1255:secondary
ATGTCAAGAT	0	1	0	0	0	At5g18460.1:d:+1337:primary
GCTTACGATG	0	1	0	0	0	At5g18450.1:v:+564:secondary
TGTCCGGTCT	0	0	0	0	1	At5g18450.1:v:+1599:secondary
AGAGTAAAAA	0	1	0	0	0	At5g18440.1:v:+1834:secondary
AGCAGTATAA	1	0	0	0	0	At5g18440.1:v:+1735:secondary
AACCTTAGCT	1	0	0	0	0	At5g18430.1:v:+1413:primary
CAGAATAGGC	0	0	0	0	1	At5g18420.3:d:+1194:primary,At5g18420.2:d:+1208:primary,At5g18420.1:d:+1205:primary
AGCCTGCACT	0	0	1	0	0	At5g18420.3:d:+1065:secondary,At5g18420.2:d:+1079:secondary,At5g18420.1:d:+1079:secondary
CCGTTTTGTC	0	0	1	0	0	At5g18410.1:X:-99:quaternary
CGCTTTGTGA	0	1	0	0	0	At5g18390.1:d:+572:secondary
TAGATCAAGC	0	0	0	0	1	At5g18390.1:d:+485:secondary,At4g12840.1:v:+1675:secondary
GAGCTATTAA	1	0	0	0	0	At5g18320.1:X:-1126:quaternary
TGTTTCAATT	1	0	0	0	0	At5g18320.1:i:+380:tertiary,At4g16770.1:i:+533:tertiary
AATCGAAAAG	1	0	0	0	0	At5g18310.1:d:+478:secondary,At5g18310.2:d:+531:secondary
TTTCAACAAA	0	1	0	0	0	At5g18300.1:v:+1233:primary
TTACTTTTTT	0	1	0	0	0	At5g18300.1:v:+1056:secondary,At1g31840.1:v:+354:secondary
GACTTTGAGA	0	0	0	1	0	At5g18280.1:d:+1483:secondary
TTAGTTAATA	1	0	0	0	0	At5g18270.2:d:+1255:primary,At5g18270.1:d:+1255:primary
GGGAGTTTGT	0	0	1	0	0	At5g18230.1:d:+1227:secondary
AGTGCTTGGA	0	1	0	0	0	At5g18190.1:X:-179:quaternary
CTTGGTCCGA	0	0	1	0	0	At5g18190.1:d:+646:secondary
AAACAGAGAT	1	0	0	0	0	At5g18190.1:d:+1396:secondary
AAGTTTGACG	1	0	0	0	0	At5g18190.1:d:+1168:secondary
CTTGTGAGGG	1	0	0	0	0	At5g18180.1:v:+511:secondary,At3g09162.1:d:+457:secondary
TCTAGCTAAA	0	1	0	0	0	At5g18150.1:v:+971:primary
TAGAGGAGGG	0	0	0	1	0	At5g18150.1:v:+930:secondary
GCATCTTCAA	0	1	0	0	0	At5g18140.1:d:-527:primary
AAGTTACGAA	0	0	0	1	0	At5g18130.2:d:+237:secondary,At5g18130.1:d:+237:secondary
TACTCTGAGC	0	0	1	0	0	At5g18130.2:d:+1873:primary,At5g18130.1:d:+1184:primary
CACCTATCAC	1	0	0	0	0	At5g18120.1:X:+-12:quaternary,Chr1:+10702092:quaternary
TCAATACTTT	0	0	1	0	0	At5g18080.1:v:+769:secondary
AAGGCTGGTT	1	0	0	0	0	At5g18070.1:X:+-64:quaternary
ATCATTGGAG	0	0	1	0	0	At5g18050.1:X:-364:quaternary
ATCCAACATT	0	1	0	0	0	At5g18050.1:d:+370:primary,At5g14850.1:d:+1550:primary,At5g14850.2:d:+1550:primary
TGGTCTAGAC	0	0	1	0	0	At5g17990.1:d:+1092:secondary
CCGCAGAGAA	0	0	1	0	0	At5g17970.1:v:+310:secondary,At1g19720.1:d:+2245:secondary,At5g04320.1:d:+1728:secondary
CATTGTTTGC	1	0	0	0	0	At5g17970.1:v:+1830:secondary,At1g75330.1:d:+1018:secondary
TTATTCATTT	0	1	0	0	0	At5g17930.1:X:-117:quaternary
TAGGAGGCAA	0	0	0	1	0	At5g17930.1:v:+286:secondary
AAAAGCGAAA	0	1	0	0	0	At5g17930.1:v:+220:secondary
TAGTGTAATG	0	1	0	0	0	At5g17920.1:i:+3641:tertiary
ACCTCTCGCC	0	0	1	0	0	At5g17920.1:d:+1740:secondary
AAGCATTGCC	0	0	0	0	1	At5g17910.1:X:+1636:quaternary
AGAGCTGCAG	0	0	0	1	0	At5g17900.1:i:+1814:tertiary
GCAATACCAG	0	1	0	0	0	At5g17900.1:d:-696:primary
GTCAGTTTAG	0	0	1	0	0	At5g17890.1:d:+2347:secondary
GCGATAGAAG	1	0	0	0	0	At5g17880.1:X:-68:quaternary

AAGCTTACAA	0	1	0	0	0	At5g17880.1:d:+899:secondary
TGCTGACATC	1	0	0	0	0	At5g17820.1:d:+406:secondary
GTTAATCATA	1	0	0	0	0	At5g17820.1:d:+1254:primary
TTTGTTTAAC	0	1	0	0	0	At5g17800.1:d:+1066:primary
GCTAATTACC	0	0	0	0	1	At5g17760.1:d:+941:secondary,At5g17760.2:d:+941:secondary
TCTCCTCAGT	0	0	0	1	0	At5g17750.1:X:-440:quaternary
GATCCTAAAC	0	1	0	0	0	At5g17750.1:i:+1045:tertiary
TGAATATATA	1	0	0	0	0	At5g17740.1:v:+2346:primary
GAGATATGTA	0	0	1	0	0	At5g17725.1:X:-976:quaternary
TAATTAAAAA	0	0	0	1	0	At5g17720.1:v:+39:secondary
GGAGATGAAC	0	1	0	0	0	At5g17700.1:d:+1093:secondary
GAAGTTTTGA	0	0	0	1	0	At5g17680.1:v:+1599:secondary
GTGGTGACCA	0	1	0	0	0	At5g17650.1:X:-100:quaternary
GTTTTGGTCC	0	0	0	0	1	At5g17620.1:d:+852:secondary
AATAGGAGTC	0	0	0	0	1	At5g17610.1:X:+301:quaternary
TCTTCTCTGT	0	1	0	0	0	At5g17540.1:d:+1399:primary
GAGAGAAAGT	1	0	0	0	0	At5g17530.1:d:-1877:secondary,At3g43357.1:p:-2892:secondary,At5g17530.2:d:-1877:secondary
TAATCTCTAT	0	0	0	0	1	At5g17520.1:X:-137:quaternary
GCTCGAACTG	0	0	1	0	0	At5g17520.1:d:+934:secondary
GGGAAAGTCG	0	1	0	0	0	At5g17490.1:d:+773:secondary
TGTGTATGTA	0	1	0	0	0	At5g17490.1:d:+1944:primary,At2g17650.1:d:+1878:primary
TGAGCCAACC	0	0	0	0	1	At5g17450.2:d:+482:primary,At5g17450.1:d:+512:primary
AAAGGGGTGA	0	1	0	0	0	At5g17450.2:d:+185:secondary,At3g53930.1:d:+1778:secondary,At5g17450.1:d:+215:secondary
ACCGCTACTA	0	0	0	0	1	At5g17440.1:d:+1292:primary
GGTTTATATA	0	0	0	0	1	At5g17430.1:i:+1128:tertiary
TAGTTGCAGT	0	1	0	0	0	At5g17420.1:d:+3352:primary
GGTGGAATCG	0	0	1	0	0	At5g17380.1:d:+1079:secondary
GAGCTTGCCA	1	0	0	0	0	At5g17370.1:X:-44:quaternary
CCCCGTGACC	1	0	0	0	0	At5g17370.1:X:-35:quaternary
TTTTGCTATT	0	1	0	0	0	At5g17350.1:d:+674:secondary
AACGGTGATC	0	0	1	0	0	At5g17290.1:d:+527:primary
CAGAAAAGTG	0	0	0	0	1	At5g17270.1:v:+3012:secondary
GCCTAAAGAT	0	0	1	0	0	At5g17240.1:v:+1298:secondary
AACACCAAGC	0	0	0	0	1	At5g17230.1:i:+1613:tertiary
AGAAGACAGT	0	1	0	0	0	At5g17210.2:d:+778:primary,At5g17210.1:d:+699:primary
CAACAACATC	0	1	0	0	0	At5g17170.1:d:+72:primary,At1g04890.1:v:+2135:primary
TTCAGAGTGT	0	0	1	0	0	At5g17125.1:p:-2457:secondary,At1g34967.1:p:-2457:secondary
GTGCGAGTAA	1	0	0	0	0	At5g17050.1:X:-98:quaternary
GAACCACTAT	0	0	0	0	1	At5g17050.1:X:-506:quaternary
CTATTAAAGG	0	0	0	1	0	At5g17040.1:i:+515:tertiary,At1g56650.1:i:+369:tertiary
ACACCGCCTC	0	0	1	0	0	At5g17040.1:d:+821:secondary,At5g17050.1:d:+965:secondary
TTCTGTTTTG	0	0	0	0	1	At5g17040.1:d:+1374:primary
TATTTTGCTA	0	0	0	0	1	At5g17010.2:X:-346:quaternary
TACTTTGAGA	0	0	1	0	0	At5g16990.1:d:+859:secondary
GGTTTGTATT	1	0	0	0	0	At5g16980.1:X:-657:quaternary,At2g06210.2:X:-107:quaternary,At5g16990.1:X:-683:quaternary
TTCACAAGCA	0	1	0	0	0	At5g16970.1:X:-71:quaternary
CACGGGCGTA	0	0	1	0	0	At5g16970.1:d:+787:secondary,At5g16980.1:d:+632:secondary
TGGGATGATC	0	1	0	0	0	At5g16960.1:d:+791:primary
AGGACTCTTT	0	0	0	0	1	At5g16920.1:d:-157:secondary
TAAAAGAAAA	1	0	0	0	0	At5g16920.1:d:+943:primary
AATGCTTTGC	1	0	0	0	0	At5g16910.1:d:+3674:primary
TCCTTTATTT	0	1	0	0	0	At5g16890.1:i:+961:tertiary,At2g23510.1:i:+1284:tertiary
AACTCCACCT	0	0	1	0	0	At5g16870.1:d:+638:primary
GATTTAAAGA	0	0	0	0	1	At5g16850.1:d:+2744:secondary
CCTACTTATT	0	1	0	0	0	At5g16830.1:d:+866:secondary
TGCAGCAGAA	0	0	1	0	0	At5g16820.1:d:+481:primary,At5g16820.2:d:+481:primary
GCTGCATTGC	1	0	0	0	0	At5g16800.1:d:+986:primary
TCTGAGAAAT	0	1	0	0	0	At5g16760.1:d:+1172:secondary
GTATTGATGA	0	0	0	0	1	At5g16750.1:X:-346:quaternary
ATTCAACCGC	0	1	0	0	0	At5g16750.1:d:+2037:secondary,At5g24900.1:d:+1060:secondary

ATAAGATTTA	0	1	0	0	0	At5g16740.1:v:+1859:primary
GAGGACTAAC	0	1	0	0	0	At5g16720.1:d:+1772:primary
GATTTCTTGT	0	0	1	0	0	At5g16715.1:i:+470:tertiary,At1g09230.1:i:+1656:tertiary
GGCCGCTATT	0	0	1	0	0	At5g16715.1:d:-2962:secondary
TCTACATATT	0	1	0	0	0	At5g16660.1:d:+5:primary
TCGATGCTGT	1	0	0	0	0	At5g16630.1:d:+1420:secondary
GCTTTGTCTG	0	1	0	0	0	At5g16630.1:d:+1215:secondary
CGAAAATGGG	0	1	0	0	0	At5g16610.1:d:+565:secondary,At5g16610.2:d:+798:secondary
AACCTACCAA	0	1	0	0	0	At5g16610.1:d:+284:secondary,At5g16610.2:d:+284:secondary
GATCCTTACA	0	0	1	0	0	At5g16570.1:d:+1058:secondary,At1g20620.2:d:+63:secondary,At1g20620.1:d:+63:secondary,At5g37600.1:d:+1093:secondary,At4g35090.1:d:+76:secondary
TATGCAGTAT	0	1	0	0	0	At5g16560.1:d:+1633:primary
CAAATGATGG	1	0	0	0	0	At5g16520.1:X:-516:quaternary
CTTATAATTT	0	0	0	0	1	At5g16510.2:d:-1385:secondary,At5g16510.1:d:-1324:secondary
TTTTAGCAGT	0	0	0	1	0	At5g16505.1:p:+515:secondary,At3g56380.1:v:+415:secondary
TCTGATCAAA	0	1	0	0	0	At5g16470.1:X:-686:quaternary
TCAAGCTGCT	1	0	0	0	0	At5g16470.1:X:+646:quaternary
ATGGATCTGC	1	0	0	0	0	At5g16470.1:d:-295:primary
ATTTCTGGTG	0	1	0	0	0	At5g16420.1:v:+2323:secondary,At5g16430.1:v:+7:secondary
TTCGATGAAA	1	0	0	0	0	At5g16420.1:v:+1305:secondary,At1g22830.1:v:+184:secondary
GATGGTTACT	0	0	1	0	0	At5g16420.1:v:+1155:secondary
TGTTTAATCC	0	0	0	1	0	At5g16410.1:d:+1408:primary
GTGCCGATTC	1	0	0	0	0	At5g16390.2:X:-32:quaternary
GGGCTTTTAA	0	1	0	0	0	At5g16390.2:X:-266:quaternary
AATCTTTTTG	0	0	1	0	0	At5g16380.1:d:+983:primary
AAGATGACTC	0	0	0	1	0	At5g16360.1:d:+731:primary
CTGTACTCTT	1	0	0	0	0	At5g16300.2:d:+2756:secondary,At5g16300.1:d:+2756:secondary
AACCAAAAAC	0	0	0	0	1	At5g16300.1:d:+3305:secondary,At5g16300.2:d:+3203:secondary
AAAGTGAAAG	0	1	0	0	0	At5g16280.1:d:+3137:secondary,At1g10920.1:d:+282:secondary,At1g13980.1:d:+4303:secondary
GGTTTCATTT	0	0	1	0	0	At5g16250.1:d:+140:secondary
ATGAGGAAAA	0	0	1	0	0	At5g16240.1:d:+1039:primary
TTTCTGATGA	0	0	0	1	0	At5g16210.1:X:-624:quaternary
GAGCGAGCTC	0	0	1	0	0	At5g16180.1:v:+1140:secondary
CTTTTTTTGT	0	0	0	0	1	At5g16160.1:i:+732:tertiary
CACTGGATAT	0	1	0	0	0	At5g16150.1:d:+1555:secondary,At5g16150.3:d:+1670:secondary,At5g16150.2:d:+1539:secondary
TTTCCAGGTG	0	0	0	0	1	At5g16140.1:X:-21:quaternary
AAGCTATGCT	0	0	0	0	1	At5g16130.1:d:+500:secondary
TCGTCTGGTT	0	0	1	0	0	At5g16120.1:d:-1062:secondary
AAACGACGCG	0	0	0	1	0	At5g16070.1:d:+802:secondary
GGCTGTAGTT	0	0	1	0	0	At5g16050.1:d:+988:secondary
TTTTGGTTGT	1	0	0	0	0	At5g16050.1:d:+1082:secondary
AATTTCAAAT	1	0	0	0	0	At5g16020.1:v:+582:secondary,At5g50130.2:d:+1042:secondary,At5g50130.1:d:+1031:secondary
GTGTTCTTGA	0	1	0	0	0	At5g16000.1:d:+291:secondary
TACGCAGATT	0	0	1	0	0	At5g15960.1:i:+465:tertiary
AATATGAGAA	0	0	0	1	0	At5g15940.1:d:+1100:primary
TGTGGCTTTG	0	0	0	1	0	At5g15930.1:X:+323:quaternary
GTACTAAAAA	0	1	0	0	0	At5g15930.1:i:+1357:tertiary
GATCCTGATA	0	0	1	0	0	At5g15930.1:d:+1167:primary
AACGGTCCTT	1	0	0	0	0	At5g15920.1:d:+3181:primary
AAAGAAATCG	0	0	1	0	0	At5g15920.1:d:+2722:secondary
GGTATGGATT	1	0	0	0	0	At5g15880.1:i:+858:tertiary
CTCACTCCCT	0	0	1	0	0	At5g15880.1:d:+218:secondary,At5g67000.1:v:+282:secondary
CAACAACAAG	0	0	0	1	0	At5g15830.1:d:+88:secondary,At3g47850.1:d:+666:secondary,At5g59120.1:d:+554:secondary
ACAAATATTC	0	1	0	0	0	At5g15830.1:d:+521:secondary
GTTCAGTGTT	1	0	0	0	0	At5g15790.2:d:+1588:primary,At5g15790.1:d:+1501:primary
AAACTTTTTA	0	1	0	0	0	At5g15790.2:d:+1420:secondary,At5g52340.1:v:+175:secondary,At5g15790.1:d:+1333:secondary
ATGAACGTTT	0	0	1	0	0	At5g15760.1:X:+464:quaternary
GGCCTTGTCT	0	0	1	0	0	At5g15710.1:d:+760:secondary
AGCAGATTAT	0	1	0	0	0	At5g15700.1:i:+5182:tertiary
TGGAGAAACA	0	0	1	0	0	At5g15700.1:d:+352:secondary,At2g41560.1:d:+2660:secondary

TTTGCGATCG	0	0	1	0	0	At5g15680.1:X:-3058:quaternary
TATTTCATTT	0	0	0	0	1	At5g15660.1:v:+119:secondary
TCTCTCCTAC	0	1	0	0	0	At5g15660.1:i:-1176:tertiary
ACCATCCCAA	0	0	1	0	0	At5g15650.1:d:+659:secondary,At1g78940.1:d:+567:secondary,At3g02230.1:d:+681:secondary
TCAGAGGAGC	0	0	1	0	0	At5g15640.1:i:-2248:tertiary
AAATCTTATA	0	0	0	1	0	At5g15640.1:d:+1466:primary
TCTTGGGGAA	0	1	0	0	0	At5g15620.1:v:+1531:secondary
TATCAGCCGC	0	1	0	0	0	At5g15610.1:d:+1221:secondary
AACTGGAAGG	1	0	0	0	0	At5g15580.1:d:+3194:primary
TCAAAATTTA	0	1	0	0	0	At5g15560.1:v:+1467:primary
AAATGTGGAG	0	0	1	0	0	At5g15550.2:i:+3083:tertiary,At5g15550.1:i:+3083:tertiary
TTTGTTGAAC	0	0	1	0	0	At5g15540.1:X:-122:quaternary,At4g17090.1:X:-374:quaternary
AGCATTAAAA	0	0	0	0	1	At5g15540.1:X:--11:quaternary
TTGAAGATCA	0	0	1	0	0	At5g15540.1:d:+533:secondary,At1g08620.1:d:+1145:secondary,At4g20400.1:d:+1129:secondary
GAGGAGGAGG	0	0	0	1	0	At5g15540.1:d:+5204:primary
AGCGGTGATT	0	1	0	0	0	At5g15510.1:d:-143:primary
GTGGAAAACG	0	0	1	0	0	At5g15500.1:d:+1076:secondary,At5g15500.2:d:+762:secondary
TTTGACAGAT	0	0	1	0	0	At5g15450.1:i:+1039:tertiary
AAGATGAGTC	0	1	0	0	0	At5g15430.1:v:+1279:secondary,At1g42780.1:p:+1459:secondary,At4g07563.1:p:+1478:secondary,At2g11840.1:p:+1559:secondary,At4g37820.1:d:+917:secondary
ATTTGCCTCC	0	0	0	1	0	At5g15410.1:d:+1577:secondary,At5g15410.2:d:+1579:secondary
CGGTAATGGC	0	0	0	0	1	At5g15410.1:d:+1415:secondary,At5g15410.2:d:+1417:secondary
TAGTCTTTGG	0	0	0	1	0	At5g15400.1:i:+1138:tertiary
AAGCAAGCGT	0	1	0	0	0	At5g15390.1:d:-1335:secondary
AACATCAACA	0	0	0	0	1	At5g15380.1:v:+834:secondary,At3g30875.1:p:+2449:secondary
GCGAGATGGT	0	1	0	0	0	At5g15330.1:d:+556:primary
AAGCTCAAGG	0	0	1	0	0	At5g15320.1:d:+206:secondary
TAATCAGTTC	0	0	1	0	0	At5g15270.1:d:-1874:secondary
TCCTCTTGTA	0	1	0	0	0	At5g15270.1:d:+1917:secondary
GACTATGTGT	1	0	0	0	0	At5g15250.1:v:+81:secondary
TCTTCATTTA	0	1	0	0	0	At5g15250.1:v:+2439:secondary
CTTCTCGCAC	0	1	0	0	0	At5g15210.1:d:+799:primary
TGTATATAAA	0	0	0	0	1	At5g15190.2:d:+568:primary,At5g15190.1:d:+692:primary
GGACCGAGGC	0	0	0	1	0	At5g15170.1:d:+1962:primary
GCTGCAAGAA	0	1	0	0	0	At5g15120.1:d:+878:primary
TTTTATACGT	0	0	0	0	1	At5g15100.1:v:+1482:secondary
ATTGCCTCTG	0	0	0	0	1	At5g15090.1:X:-81:quaternary
GCAAGCTCTT	0	0	0	0	1	At5g15080.1:d:+1580:secondary,At5g59700.1:d:+731:secondary
GGACAATCCT	0	1	0	0	0	At5g15050.1:d:+1128:primary
GAAGGTGTAC	0	0	0	0	1	At5g15020.1:d:+3943:primary
TGCTGTTAAT	0	0	1	0	0	At5g15000.1:i:+243:tertiary,At5g11960.1:i:+1670:tertiary
AAAAGATCAA	0	0	0	1	0	At5g14980.1:v:+1615:primary
GTGGTACCAA	0	0	1	0	0	At5g14980.1:v:+1318:secondary,At5g14990.1:v:+154:secondary
ACGTCATTAA	0	0	1	0	0	At5g14950.1:i:+669:tertiary
AACTGTCAAA	0	0	1	0	0	At5g14950.1:d:+3650:secondary
GAAATACGAG	0	0	0	1	0	At5g14950.1:d:+3481:secondary
TTGAGCTTCT	1	0	0	0	0	At5g14950.1:d:+2606:secondary
ATGGGGTAAC	0	0	1	0	0	At5g14950.1:d:+1694:secondary
TTGGCGTAAC	0	1	0	0	0	At5g14920.1:d:-846:secondary
GAGGTTGGCA	1	0	0	0	0	At5g14910.1:X:+342:quaternary
TCTGAAGGAG	1	0	0	0	0	At5g14910.1:d:-373:primary
CTTTTTGTTT	0	0	1	0	0	At5g14880.1:v:+2839:secondary
TGAAACCGGA	0	1	0	0	0	At5g14880.1:v:+2086:secondary
TTTACAAAGC	1	0	0	0	0	At5g14880.1:v:+1099:secondary
TGTGAGCTTG	0	0	0	1	0	At5g14840.1:X:-859:quaternary
CTTTCATTAT	0	0	0	1	0	At5g14840.1:p:+1362:secondary
GCACAAGGAT	1	0	0	0	0	At5g14820.1:v:+1717:secondary,At3g62540.1:d:+1370:secondary,At5g47430.1:d:+2138:secondary,At5g50780.1:v:+1387:secondary
ACTATCTTAC	0	1	0	0	0	At5g14790.1:d:-1525:secondary

ATCTACCAAA	1	0	0	0	0	At5g14790.1:d:+1352:secondary
CCTCTCACGG	0	0	0	1	0	At5g14780.1:d:+843:secondary
ACAGGCTTCA	0	0	1	0	0	At5g14780.1:d:+739:secondary
CCTAACCAGG	0	0	0	0	1	At5g14780.1:d:+1050:secondary
CCTGTTTACG	0	0	0	0	1	At5g14760.1:d:+1360:secondary
CACTGTGCCC	0	0	1	0	0	At5g14740.2:d:-837:secondary,At5g14740.1:d:-955:secondary
TACTTTGGGC	0	0	0	1	0	At5g14740.2:d:-637:secondary,At5g14740.1:d:-755:secondary
CTACTACAAC	0	1	0	0	0	At5g14720.1:d:+2384:primary
ATTTAGAGAG	0	0	1	0	0	At5g14720.1:d:+1659:secondary
GAAGTAATTC	0	0	0	0	1	At5g14710.1:i:-191:tertiary
AGAGTTAGCT	1	0	0	0	0	At5g14700.1:d:+1219:primary
ATCTTCTCCT	0	0	1	0	0	At5g14690.1:X:-574:quaternary,At3g48690.1:X:-961:quaternary
GTGTTTAATC	0	0	0	1	0	At5g14660.2:d:+484:secondary,At5g14660.1:d:+484:secondary
TGTTGGTAAT	0	0	1	0	0	At5g14660.1:X:+294:quaternary
AAGGTACATT	0	1	0	0	0	At5g14620.1:d:+2103:primary
TGAATTGTTG	0	1	0	0	0	At5g14550.2:i:+1772:tertiary,At5g14550.1:i:+2093:tertiary
GCTTTAGCCC	0	1	0	0	0	At5g14550.2:d:+1018:primary
GGGTTCCCAA	0	1	0	0	0	At5g14540.1:d:+1530:secondary
GCATCGTAGA	0	0	0	0	1	At5g14520.1:X:-190:quaternary
TACTGTTTCA	0	1	0	0	0	At5g14510.1:d:+1026:primary
TCCGATGGAT	0	0	1	0	0	At5g14480.1:d:+214:secondary,At3g27540.1:d:+185:secondary
TGATGATAGT	0	1	0	0	0	At5g14470.1:d:+1191:secondary
ACCCACCTTT	0	0	0	1	0	At5g14460.1:v:-1403:secondary
GCTACTGGTC	0	1	0	0	0	At5g14460.1:v:+1428:secondary
TCTCTTGCCC	0	0	1	0	0	At5g14430.1:d:+824:secondary,At5g14430.2:d:+824:secondary
TAATTAGAAA	0	0	0	0	1	At5g14400.1:v:+2136:primary,At3g30250.1:v:+1357:primary
GGGGTGTGAA	0	0	1	0	0	At5g14380.1:X:--17:quaternary
TGTTGTGAAT	0	0	1	0	0	At5g14380.1:d:+830:secondary,At2g16060.1:d:+338:secondary
TTCTATGTAT	0	1	0	0	0	At5g14360.1:v:+281:secondary
TGAATGAGTC	0	0	1	0	0	At5g14330.1:X:--91:quaternary
TATTCCTTCT	0	1	0	0	0	At5g14330.1:d:+458:primary
GCAGCCCTTG	0	0	0	1	0	At5g14320.1:X:-78:quaternary
AGTTCTCTTA	0	1	0	0	0	At5g14310.1:d:+1602:primary
TCTCAGCTTG	0	1	0	0	0	At5g14290.1:v:+921:secondary
TACTATATAT	0	1	0	0	0	At5g14280.1:v:+2115:secondary
CCCAGCACCA	0	0	1	0	0	At5g14260.1:d:+1658:secondary,At5g14260.3:d:+1658:secondary,At5g14260.2:d:+1658:secondary
TGATACCTGG	0	0	1	0	0	At5g14240.1:X:-342:quaternary
TGCAACTCGA	0	1	0	0	0	At5g14240.1:d:+1159:primary
TAAGTTTAGA	0	1	0	0	0	At5g14240.1:d:+1070:secondary
TGAGCAGTTG	0	1	0	0	0	At5g14210.1:X:-187:quaternary,At4g16760.1:X:-274:quaternary
GGTGTTACTG	0	0	1	0	0	At5g14180.1:d:+1244:secondary
TAGTTATATG	0	1	0	0	0	At5g14140.1:i:+904:tertiary
ATACACCAAA	0	1	0	0	0	At5g14090.1:i:+914:tertiary
ATGAGTAAAA	0	0	0	1	0	At5g14080.1:X:-445:quaternary
GCGGAGAACT	0	0	1	0	0	At5g14080.1:X:-298:quaternary
GTAATTGATG	0	1	0	0	0	At5g14080.1:X:+450:quaternary
AATGTGTTTC	0	0	0	0	1	At5g14070.1:d:+149:secondary
GAATCTCCGA	0	0	1	0	0	At5g14040.1:d:+109:secondary
CTTACTTTGA	0	0	1	0	0	At5g14020.1:i:+1122:tertiary
GATGCGCCAA	0	0	1	0	0	At5g14020.1:d:+841:secondary
TTGATGATGA	0	0	0	1	0	At5g14020.1:d:+1322:primary
GTTCTAGGGG	0	1	0	0	0	At5g14000.1:d:+931:primary
GAGAAGAACG	1	0	0	0	0	At5g13990.1:d:+129:secondary
TAATTGATGT	0	0	1	0	0	At5g13950.1:i:+2391:tertiary
AAACTTTCCA	0	1	0	0	0	At5g13950.1:d:+435:secondary
TTTCTAAAGT	0	0	0	0	1	At5g13950.1:d:+2986:secondary,At5g45800.1:d:+129:secondary
ATGTACCAGC	0	0	0	0	1	At5g13930.1:d:+562:secondary
TCGTCTTCTG	0	0	1	0	0	At5g13930.1:d:+467:secondary
TGCGACAAGT	0	0	0	1	0	At5g13930.1:d:+265:secondary
TTATCGTTAA	0	0	1	0	0	At5g13870.1:X:-140:quaternary
ATGAGATAGA	0	0	1	0	0	At5g13870.1:d:+711:secondary,At2g01850.1:d:+471:secondary

TGCCTCTGCA	0	0	0	1	0	At5g13870.1:d:+209:secondary
ACTTGTACCA	1	0	0	0	0	At5g13840.1:X:-89:quaternary
TCAAAGGTTG	0	1	0	0	0	At5g13840.1:d:+1540:primary
AGCTTTTTCA	0	0	0	1	0	At5g13830.1:i:+456:tertiary,At1g53580.2:i:+1944:tertiary
TGCCTCTCCC	0	0	0	1	0	At5g13790.1:X:-15:quaternary
TCATAAGCAT	0	0	0	0	1	At5g13770.1:d:+2330:primary
GTCGGTGTAT	0	0	1	0	0	At5g13770.1:d:+1641:secondary
TCACGAGCTT	0	1	0	0	0	At5g13760.1:d:-1965:secondary
TAAAACACAA	0	1	0	0	0	At5g13720.1:X:+-203:quaternary
GGACCGGCGA	0	0	0	1	0	At5g13710.1:X:-39:quaternary
AGTTTTGAAA	0	0	0	0	1	At5g13710.1:i:+1617:tertiary
AAAATCTCTA	0	1	0	0	0	At5g13680.1:d:+3960:primary
AACTTAATAA	0	0	0	1	0	At5g13680.1:d:+2884:secondary
GTCCTCTTTA	0	0	1	0	0	At5g13655.1:v:+740:secondary
AGCTATCTTA	0	0	1	0	0	At5g13650.2:i:+2701:tertiary,At5g13650.1:i:+2675:tertiary
GGAAGCCTCT	0	0	0	1	0	At5g13650.2:d:+1188:secondary,At5g13650.1:d:+1159:secondary
TGACTCTTGC	1	0	0	0	0	At5g13640.1:i:+3622:tertiary
GCGCAGCAAG	0	0	1	0	0	At5g13640.1:d:-2711:secondary
GAAGCTGTGG	0	0	0	0	1	At5g13630.1:d:+2612:secondary
AGGCTGCTTT	0	0	1	0	0	At5g13630.1:d:+2042:secondary
CTCTCCACAA	0	0	0	0	1	At5g13630.1:d:+1563:secondary
GACTTTTTTT	1	0	0	0	0	At5g13620.1:v:+1388:primary
AAAATAGCTA	0	1	0	0	0	At5g13620.1:v:+112:secondary
TCAAATCCTT	0	0	0	1	0	At5g13610.1:i:+1149:tertiary
CGGACAGTAA	0	1	0	0	0	At5g13600.1:v:+2269:primary
TGGAAGAAAA	0	0	0	1	0	At5g13590.1:v:+2206:secondary,At5g37875.1:p:+1841:secondary
TCCACTTTCA	0	0	0	1	0	At5g13590.1:v:+2182:secondary
TAGTGTTTAA	0	0	1	0	0	At5g13560.1:X:+1519:quaternary
CAAAAACGTT	0	0	0	1	0	At5g13550.1:d:+1051:primary
TTGGAGCGAC	0	0	0	1	0	At5g13540.2:i:+513:tertiary,At5g13540.1:i:+513:tertiary
TTGTGGGAGT	1	0	0	0	0	At5g13540.1:d:+1648:primary
GTCTCGAGGA	0	0	0	0	1	At5g13520.1:d:+2266:secondary
TCCATTGGTT	1	0	0	0	0	At5g13500.2:i:+2098:tertiary,At5g13500.1:i:+2367:tertiary,At5g13500.3:i:+2291:tertiary
ATGGGTGGTG	0	0	0	1	0	At5g13490.1:d:+374:primary
CTGAGTGTGT	0	0	1	0	0	At5g13475.1:p:+5318:secondary
GTCTCCGCCA	0	1	0	0	0	At5g13470.1:d:-216:primary
CTTCCGTTGT	0	0	0	1	0	At5g13470.1:d:+221:primary
TCAATCAGGA	0	0	0	0	1	At5g13450.1:d:+720:secondary
ACACTCGGAC	0	0	0	0	1	At5g13440.1:X:-669:quaternary
TCTGCAAGTA	0	0	1	0	0	At5g13430.1:d:+529:secondary,At5g13440.1:d:+580:secondary
CCAGCGCTTA	0	1	0	0	0	At5g13410.1:d:+344:primary
TGTTTCCTCA	0	0	1	0	0	At5g13400.1:d:+647:secondary,At1g26920.1:d:+348:secondary
GTTGTGACTT	0	1	0	0	0	At5g13400.1:d:+2985:primary
GTGCTTCCCA	0	0	1	0	0	At5g13400.1:d:+1381:secondary
GGCAACTATC	0	1	0	0	0	At5g13390.1:d:+590:secondary
TTTTATTTTG	0	1	0	0	0	At5g13380.1:d:+593:secondary
GGAAGCAATA	0	0	0	0	1	At5g13360.1:v:+1199:secondary
TGTTGAATTT	0	0	0	0	1	At5g13350.1:v:+2451:primary
AACTCACCTC	0	0	0	1	0	At5g13330.1:d:+444:secondary,At4g21900.1:d:+2569:secondary
ACAAACTCAG	0	1	0	0	0	At5g13300.1:X:-375:quaternary
GACTTGGCCA	0	0	1	0	0	At5g13270.1:X:-1226:quaternary
AATGTACTTT	0	0	0	1	0	At5g13260.1:i:+1498:tertiary,At5g25420.1:i:+1273:tertiary
TCGGCAAAGA	0	0	0	1	0	At5g13230.1:v:+3111:primary
GTCTTGTAGT	1	0	0	0	0	At5g13230.1:v:+1465:secondary
TTGTTTAATT	0	0	0	0	1	At5g13220.3:i:+974:tertiary,At5g13220.2:i:+974:tertiary,At5g54920.1:i:+1406:tertiary,At5g13220.1:i:+974:tertiary
GAAAGGAAAG	0	0	1	0	0	At5g13210.1:d:+1524:secondary,At3g24780.1:v:+1967:secondary
CTGGGAGTGC	1	0	0	0	0	At5g13190.1:X:-78:quaternary
AGGTGGTTTG	0	0	1	0	0	At5g13180.1:d:-439:secondary
TGGCGATGTT	0	0	1	0	0	At5g13160.1:X:+725:quaternary
AGAATGAAAA	0	0	0	1	0	At5g13160.1:d:+832:secondary

ATTTTTCACC	0	0	1	0	0	At5g13150.1:v:+967:secondary
AGATATCAGA	0	0	0	0	1	At5g13100.1:d:-1167:secondary
AAAGGCACTG	0	0	1	0	0	At5g13100.1:d:+1093:secondary
TATTTTTCGA	0	1	0	0	0	At5g13090.1:X:-1026:quaternary
AAATCATCGA	0	1	0	0	0	At5g13090.1:d:+482:secondary,At1g12020.1:d:+358:secondary
AAAACAGCAA	1	0	0	0	0	At5g13050.1:X:--59:quaternary
GAAGTTTTTA	0	0	1	0	0	At5g13030.1:i:+1661:tertiary
GATGAGTATC	0	0	1	0	0	At5g13030.1:d:+1393:secondary,At1g17070.1:d:+67:secondary
AATATCAGCC	0	0	0	1	0	At5g13000.1:X:-73:quaternary
GAGGAAGCGT	0	0	1	0	0	At5g13000.1:d:+4325:secondary
CTTGAAAAAT	0	0	1	0	0	At5g12980.1:i:+1852:tertiary
CGATGCACTT	0	0	0	0	1	At5g12980.1:d:+811:primary
TCTATATTTC	1	0	0	0	0	At5g12950.1:d:-2809:secondary
ATTATCCACA	0	1	0	0	0	At5g12930.1:d:+542:secondary
AGTTCCCAAT	0	1	0	0	0	At5g12930.1:d:+1025:primary
CTGCCAAAAT	0	0	1	0	0	At5g12920.1:X:--33:quaternary
AATACTTTTC	0	0	1	0	0	At5g12920.1:i:+2348:tertiary
TAGTTGTGTG	0	1	0	0	0	At5g12900.1:i:+726:tertiary
TTTCCTCTCT	0	0	1	0	0	At5g12870.1:i:+1211:tertiary
GCAAGGAGCT	0	0	1	0	0	At5g12860.1:d:+292:secondary
GGACACACTC	0	0	1	0	0	At5g12860.1:d:+1249:secondary
TTCACAACAC	1	0	0	0	0	At5g12850.1:d:-2259:secondary
AATGATGCTG	0	1	0	0	0	At5g12850.1:d:+344:secondary,At2g25910.1:d:+802:secondary,At3g01100.1:d:+864:secondary
CAATGAGAAG	0	1	0	0	0	
At5g12840.1:d:+796:primary,At5g12840.3:d:+944:primary,At5g12840.2:d:+923:primary,At5g12840.4:d:+870:primary						
GCAGTTTCTA	0	0	1	0	0	At5g12470.1:d:+1116:secondary
GAAGAATGTA	1	0	0	0	0	At5g12460.1:v:+858:secondary
AGCAACCGAT	1	0	0	0	0	At5g12440.1:X:-246:quaternary
TGAAGAAACA	0	0	1	0	0	At5g12430.1:d:+3726:primary
TTTCCCTTAT	0	1	0	0	0	At5g12390.1:d:+621:secondary
ATGAAATCCT	0	1	0	0	0	At5g12380.1:v:+877:secondary
AGATTCTAGA	0	1	0	0	0	At5g12370.1:i:+3189:tertiary
CAAACTTTCG	1	0	0	0	0	At5g12370.1:i:+2531:tertiary
GTTGACAAAG	0	1	0	0	0	At5g12350.1:d:+3157:primary
CTTCCCTTGA	0	0	1	0	0	At5g12330.1:X:-20:quaternary,At2g31100.1:X:--49:quaternary
TTGAGCAACC	0	0	0	0	1	At5g12290.1:d:+1133:secondary
ATGGAAAGAA	0	0	1	0	0	At5g12270.1:v:+1396:secondary
GTGTCAAACG	0	1	0	0	0	At5g12260.1:d:+884:primary
AATAGTAACG	1	0	0	0	0	At5g12230.1:X:-178:quaternary
AAATTTCCAC	0	0	1	0	0	At5g12230.1:i:+2119:tertiary,At1g06150.1:i:+4309:tertiary
AATTCTTTTG	0	1	0	0	0	At5g12230.1:d:+427:secondary,At5g19480.1:v:+442:secondary,At5g17040.1:d:+620:secondary
TGATGAGTGT	0	0	1	0	0	At5g12200.1:d:+848:secondary
GCCATCCATC	0	0	0	1	0	At5g12180.1:d:+1003:secondary,At4g07517.1:p:+1493:secondary
TCTTTGTCGT	0	0	0	0	1	At5g12150.1:X:+-114:quaternary
TGGACTCCAT	0	0	0	0	1	At5g12130.1:i:+1681:tertiary
AGCGGGCAAC	1	0	0	0	0	At5g12120.1:d:+619:secondary
AACAAGCTGA	1	0	0	0	0	At5g12080.2:d:+2260:primary,At5g12080.1:d:+2221:primary
ACGGTGTGTT	0	0	1	0	0	At5g12020.1:d:+448:primary
CGTTTAATGA	0	1	0	0	0	At5g12010.1:d:+1249:primary
AAACATTGTC	0	1	0	0	0	At5g12000.1:v:+2796:primary
AACTGATCAA	0	1	0	0	0	At5g11980.1:d:+1427:secondary,At1g76700.1:d:+2228:secondary
TTACTCTAAT	1	0	0	0	0	At5g11960.1:d:+1305:primary
TTGCATCGCA	0	0	1	0	0	At5g11950.1:d:+685:primary
GAATCTAAAA	0	0	1	0	0	At5g11910.1:X:-178:quaternary
CCTGATCTTA	0	0	0	0	1	At5g11880.1:X:-32:quaternary
AAACACCACA	0	1	0	0	0	At5g11850.1:d:+2317:primary
GGAGCAGTTA	0	1	0	0	0	At5g11850.1:d:+2059:secondary
TAAATATGCA	0	0	0	0	1	At5g11830.1:v:+698:secondary
GGGTGACCCG	1	0	0	0	0	At5g11810.1:d:+801:secondary
ACTTATTTGC	0	0	1	0	0	At5g11790.1:i:+785:tertiary
TCAGCTTGGA	0	0	0	1	0	At5g11720.1:d:+256:secondary

ACTGAATAAG	0	1	0	0	0	At5g11700.1:X:-170:quaternary
TGAGTTTTAC	0	0	0	1	0	At5g11700.1:i:+7919:tertiary
ATCGTTAATG	0	0	0	0	1	At5g11700.1:i:+4422:tertiary
TAAACTGATC	0	1	0	0	0	At5g11700.1:i:+1534:tertiary
GAAAGTTTAA	0	0	0	1	0	At5g11690.1:d:+761:primary
ATAATTGCCT	0	0	0	1	0	At5g11690.1:d:+716:secondary
GACCAAGGGT	0	0	1	0	0	At5g11670.1:d:+1480:secondary,At5g25880.1:d:+1377:secondary
TTGAGGATGT	0	1	0	0	0	At5g11620.1:X:+356:quaternary
ACTGGGTATG	0	1	0	0	0	At5g11610.1:i:+1722:tertiary,At5g11610.2:i:+1722:tertiary
TCGGATTCTA	0	0	1	0	0	At5g11600.1:d:+136:secondary
GGATCTCTAA	0	1	0	0	0	At5g11590.1:v:+1052:primary
CCGCAAACAA	0	0	1	0	0	At5g11570.1:v:+105:secondary,At2g43140.1:v:+969:secondary
TCGAATGAGA	0	0	1	0	0	At5g11540.1:v:+2548:primary
TTCTTGGTCT	0	0	0	1	0	At5g11530.1:d:+3485:primary,At4g16030.1:v:+691:primary
GCTGATCGTA	0	0	1	0	0	At5g11520.1:d:+1133:secondary
CAGAGTCATT	0	1	0	0	0	At5g11490.1:d:+2429:primary
ATCATAAAAA	0	0	0	1	0	At5g11470.1:v:+540:secondary
CCATCCAGTC	0	0	0	0	1	At5g11470.1:v:+2766:primary
GTTTTGGTGG	0	0	1	0	0	At5g11460.1:d:+1387:primary
GATGATCATT	1	0	0	0	0	At5g11460.1:d:+1311:secondary,At2g17090.1:v:+123:secondary
GATCCTTGTA	1	0	0	0	0	At5g11450.1:d:-922:secondary
TATGCGTCAC	0	0	0	1	0	At5g11430.1:i:+2469:tertiary
ATACTGCTGC	0	0	1	0	0	At5g11430.1:d:+2225:primary
TGTTAGCCAA	0	1	0	0	0	At5g11420.1:i:+1805:tertiary
TGCCCAAGAC	0	0	1	0	0	At5g11420.1:d:+402:secondary
TAAGAGTCTT	1	0	0	0	0	At5g11390.1:d:+2233:primary
AGCAAAATCC	0	1	0	0	0	At5g11380.1:d:+988:secondary,At5g02010.1:d:+1514:secondary
GATAAATGCT	0	1	0	0	0	At5g11360.1:d:+722:secondary
TGTACCTTGT	0	0	1	0	0	At5g11340.1:i:+913:tertiary
ACGGAATTGG	0	0	0	0	1	At5g11330.1:d:+1185:secondary,At2g23390.1:d:+801:secondary
CAATTAAAAA	1	0	0	0	0	At5g11320.2:d:+1116:primary
ACCTCGGAAA	0	1	0	0	0	At5g11300.1:X:-247:quaternary
ACATTTCCAA	0	0	1	0	0	At5g11280.1:d:+308:secondary
TAATGTGGTT	0	0	1	0	0	At5g11250.1:v:+4125:secondary
AACCTTAGTA	1	0	0	0	0	At5g11250.1:v:+2964:secondary,At4g28780.1:d:+1124:secondary
ATACATTGCA	0	0	0	0	1	At5g11250.1:v:+2203:secondary
ACTGATGAAT	0	0	0	1	0	At5g11180.1:X:-310:quaternary
ATAAATTGAT	0	0	1	0	0	At5g11180.1:v:+3384:primary
TTGGTTTGTA	0	0	0	1	0	At5g11180.1:v:+2721:secondary
CCTTCTTAAA	0	1	0	0	0	At5g11170.1:d:+1371:secondary,At5g11170.2:d:+1116:secondary
ACAAATTTGG	0	1	0	0	0	At5g11100.1:v:+1912:secondary,At2g26650.1:d:+1002:secondary
ATTTGTAATA	0	1	0	0	0	At5g11060.1:d:+1638:secondary
AGCAAAAGCA	0	0	1	0	0	At5g11030.1:d:+1334:secondary
ACAACAAATC	0	0	1	0	0	At5g10970.1:v:-1512:secondary,At4g23030.1:v:-2086:secondary
CGTGATGCCG	0	1	0	0	0	At5g10950.1:X:-98:quaternary
AAAGCTTCTA	0	1	0	0	0	At5g10940.1:X:--75:quaternary
TTACTTTCAG	0	0	1	0	0	At5g10940.1:d:+917:secondary
ACCAGACGAT	1	0	0	0	0	At5g10930.1:X:-1501:quaternary
GCACGGTGAT	0	0	1	0	0	At5g10930.1:d:+1507:primary
CATCCAACTT	0	1	0	0	0	At5g10910.1:d:+516:primary
ATTGGAATGG	0	0	0	0	1	At5g10860.1:d:+645:secondary
GCGAAGATCA	0	0	1	0	0	At5g10840.1:X:-322:quaternary,At5g25100.1:X:-322:quaternary
CCTAGACACT	0	0	1	0	0	At5g10840.1:X:-295:quaternary
TTGGTCTTAA	0	0	1	0	0	At5g10840.1:d:+537:secondary,At5g25100.1:d:+585:secondary
CTCGGATTTC	0	0	0	1	0	At5g10840.1:d:+1154:secondary
GGGATCCACC	0	0	1	0	0	At5g10820.1:X:-250:quaternary
TGAGAAGCAT	1	0	0	0	0	At5g10800.1:X:+445:quaternary
CTTGTGTCTG	0	1	0	0	0	At5g10800.1:d:+1519:secondary,At5g25060.1:d:+1731:secondary
CACACTTATT	0	1	0	0	0	At5g10770.1:X:-966:quaternary
TCGTATATAT	0	1	0	0	0	At5g10760.1:X:-1382:quaternary
TTACGATTTC	0	0	0	1	0	At5g10760.1:d:+1190:secondary

ATATATTTAA	0	1	0	0	0	At5g10750.1:d:+1173:primary
TCATCGCCTT	0	0	1	0	0	At5g10745.1:d:+158:secondary
GAATACAACG	0	0	1	0	0	At5g10730.1:d:+989:primary
ATAGGGTTTC	0	0	1	0	0	At5g10710.1:i:+280:tertiary,At5g10710.2:i:+185:tertiary
CAAATAACTT	0	0	0	0	1	At5g10650.1:X:-567:quaternary
TGAGCTGTGT	0	1	0	0	0	At5g10650.1:d:+1584:primary
GAAAACCAAA	0	0	0	1	0	At5g10630.1:d:+1376:secondary
AATCTCTACT	1	0	0	0	0	At5g10620.1:v:+942:secondary
CCCAGAAATG	0	1	0	0	0	At5g10600.1:d:+1708:primary
AAGGACAACG	0	0	0	1	0	At5g10580.2:d:+240:secondary,At5g10580.1:d:+256:secondary
GTTACTTCGG	0	1	0	0	0	At5g10570.1:d:+883:primary
TTTCTTCATT	1	0	0	0	0	At5g10560.1:d:+1310:secondary
CCTCCGGTGA	0	0	0	1	0	At5g10550.1:v:+2031:secondary
TGTAATTTTA	1	0	0	0	0	At5g10540.1:i:+2768:tertiary,At1g06020.1:i:+540:tertiary
CTTTCTTCAA	0	0	0	1	0	At5g10540.1:d:+2230:secondary,At3g45330.1:v:+556:secondary,At3g45410.1:d:+191:secondary,At5g60320.1:v:+556:secondary
TATCCTCTTC	0	1	0	0	0	At5g10530.1:v:+2230:secondary
ACGGAGAAAG	0	0	0	0	1	At5g10510.1:i:-442:tertiary
AAGGTGCAAA	0	1	0	0	0	At5g10480.1:X:-51:quaternary,At1g12820.1:X:-799:quaternary
TGCTGCATTT	0	0	0	1	0	At5g10470.1:X:-343:quaternary
TACACATACT	0	1	0	0	0	At5g10450.1:X:-90:quaternary,At5g35065.1:X:-1530:quaternary
ATCACTGAAG	0	1	0	0	0	At5g10420.1:i:+1050:tertiary
ACTCTTGTCT	0	1	0	0	0	At5g10380.1:X:+-89:quaternary
TTATTCAGGT	1	0	0	0	0	At5g10370.1:d:+4577:secondary,At4g01020.1:d:+4568:secondary
ATCTTGAATA	0	0	1	0	0	At5g10360.1:X:-25:quaternary
GTATCTCTTC	0	1	0	0	0	At5g10350.2:i:+572:tertiary,At5g10350.1:i:+572:tertiary
CAAGGATGGC	0	0	1	0	0	At5g10310.1:X:-79:quaternary
GTTTGATCTC	0	1	0	0	0	At5g10310.1:d:+376:primary
AAACAAAAAC	0	0	0	1	0	At5g10290.1:X:-420:quaternary,At4g00940.1:X:--148:quaternary,At2g06640.1:X:-854:quaternary,At2g06710.1:X:-851:quaternary,At3g24140.1:X:--53:quaternary,At1g17090.1:X:-562:quaternary
TTGTAGAATG	0	0	1	0	0	At5g10280.1:d:+1357:primary
GATTATAACA	0	0	0	1	0	At5g10230.1:X:-855:quaternary
GAACGGTGCA	0	0	0	1	0	At5g10190.1:d:+1086:primary
GCTGGTTTAA	0	0	1	0	0	At5g10180.1:d:+442:secondary
TGATGAATTT	0	1	0	0	0	At5g10180.1:d:+2160:secondary
TTGTTTTTTT	1	0	0	0	0	At5g10150.1:d:+1390:primary
GACATTGGAC	0	0	1	0	0	At5g10140.1:X:-113:quaternary
TGGGAGCAGA	0	1	0	0	0	At5g10140.1:d:+618:primary
AAGTTTCAAA	0	0	0	0	1	At5g10110.1:d:+296:secondary,At5g22920.1:d:+367:secondary
TATCATATGC	0	1	0	0	0	At5g10050.1:d:+835:primary
AGCTCAGGTT	0	1	0	0	0	At5g10030.1:i:+1688:tertiary
AGACATTGCA	0	1	0	0	0	At5g10030.1:d:+1216:secondary
TCGTAGTCGT	0	0	1	0	0	At5g09990.1:d:-309:secondary
ATATATTGAT	1	0	0	0	0	At5g09990.1:d:+400:primary
GGGCTAGCTA	0	0	1	0	0	At5g09970.1:d:+1755:primary
GACAGTGATC	0	0	1	0	0	At5g09960.1:X:+274:quaternary
CACTTCCATA	0	1	0	0	0	At5g09950.1:X:-1922:quaternary
TTACATTCGT	0	0	0	0	1	At5g09950.1:v:+2515:secondary
CAATTCAAAA	0	0	1	0	0	At5g09880.1:X:--47:quaternary,At5g16280.1:X:-248:quaternary,At5g39490.1:X:-905:quaternary
CAATTACAAG	1	0	0	0	0	At5g09870.1:v:-209:primary
AACTCCCTGA	0	0	1	0	0	At5g09870.1:v:+1941:secondary
AGACGGGCAT	0	0	1	0	0	At5g09860.1:d:-1970:secondary
AATACGCTTT	0	0	1	0	0	At5g09860.1:d:+1975:primary
CTGGAGTTTC	0	1	0	0	0	At5g09840.1:d:+2189:secondary
AACCTTATGC	0	0	1	0	0	At5g09720.1:X:-357:quaternary
GTTACATTTT	0	1	0	0	0	At5g09690.3:d:+1154:primary,At5g09690.1:d:+1232:primary,At5g09690.2:d:+1176:primary
CAAATTCTGA	0	0	1	0	0	At5g09650.1:d:+584:secondary
TATACTCGAT	0	0	0	1	0	At5g09640.1:i:+740:tertiary
CAGGCAATTA	0	1	0	0	0	At5g09630.1:d:+1352:primary
GAACAGCAGA	0	0	1	0	0	At5g09620.1:d:+1344:secondary
TCTGGTGGTT	0	0	0	1	0	At5g09590.1:d:+2100:secondary,At1g79640.1:v:+624:secondary,At4g32210.1:p:+2431:secondary

TCTACTCCAG	0	0	0	1	0	At5g09580.1:v:+1790:primary
TTTTGATCAT	0	0	1	0	0	At5g09560.1:v:+19:secondary,At4g01160.1:v:+1992:secondary
ATCGTGACAG	1	0	0	0	0	At5g09470.1:v:+1011:secondary
TTGCAATATC	0	0	1	0	0	At5g09460.1:X:-446:quaternary
CCAAATCCAA	0	0	1	0	0	At5g09460.1:X:-1201:quaternary
CTCTCTCAAA	0	0	0	1	0	At5g09460.1:v:+1557:primary
CAGCAGTTTC	0	1	0	0	0	At5g09410.1:i:+3627:tertiary
GTTATTGGGA	1	0	0	0	0	At5g09410.1:d:+1291:secondary
GTTCCCCGAA	0	1	0	0	0	At5g09400.1:X:+723:quaternary
GATGCCATAT	0	1	0	0	0	At5g09400.1:d:-241:primary
GTCTGAGTAT	0	0	1	0	0	At5g09400.1:d:+2733:primary
CTTCTTATAT	1	0	0	0	0	At5g09400.1:d:+1722:secondary
GTAAAAAAGT	0	0	0	0	1	At5g09390.1:X:+-153:quaternary
GCTTGACAGT	0	0	1	0	0	At5g09390.1:d:+456:secondary
CCAAGGACGG	0	0	1	0	0	At5g09330.1:d:+946:secondary
AGACATAGAC	0	0	0	0	1	At5g09330.1:d:+1450:primary
TAATCATAAC	0	0	1	0	0	At5g09320.1:X:-165:quaternary
ATTGTAAAGC	0	1	0	0	0	At5g09280.1:v:+637:secondary,At5g12950.1:d:+2373:secondary,At1g30350.1:v:+823:secondary
AGTGAGAAAG	0	0	1	0	0	At5g09270.2:i:+1503:tertiary
GAGTCTCTCT	0	0	0	0	1	At5g09260.1:v:+1144:primary
TTCTGCAAGT	0	0	0	0	1	At5g09240.2:d:+491:secondary,At5g09240.1:d:+406:secondary,At5g09240.3:d:+480:secondary
AGCCGCTCTG	0	0	0	1	0	At5g09230.5:d:-993:secondary,At5g09230.4:d:-1191:secondary,At5g09230.3:d:-1094:secondary,At5g09230.2:d:-1017:secondary,At5g09230.6:d:-1004:secondary,At5g09230.1:d:-1056:secondary
AAAAATTAAG	0	0	1	0	0	At5g09210.1:i:+2280:tertiary
CTCTCCGGAA	0	0	1	0	0	At5g08790.1:d:+1241:primary
TAATGTTTGA	0	0	0	1	0	At5g08780.1:i:+331:tertiary,At5g61670.2:i:+1837:tertiary
CTCTTTCACT	0	1	0	0	0	At5g08780.1:i:+122:tertiary
AAAGAACAAG	0	0	1	0	0	At5g08780.1:d:+1555:secondary,At1g30900.1:v:+1995:secondary
ATTAAGTTAC	1	0	0	0	0	At5g08740.1:X:--25:quaternary
GTAGTGATGT	0	0	0	1	0	At5g08710.1:d:+1417:primary
TGTAGTTACA	0	1	0	0	0	At5g08710.1:d:+1160:secondary
TTGCTGAGAT	0	0	0	0	1	At5g08690.1:d:+1516:secondary
AGTGGGTGTG	0	0	0	0	1	At5g08670.1:X:-25:quaternary
TTTGGTTGTG	0	1	0	0	0	At5g08660.1:d:+2015:primary
AAAATCCAGC	0	1	0	0	0	At5g08630.1:d:+2326:primary
CCCTTGGAGC	0	0	1	0	0	At5g08630.1:d:+1534:secondary
CTCATTACTC	0	0	0	0	1	At5g08600.1:v:-94:primary
TCAAAGAGGC	0	0	1	0	0	At5g08590.1:d:+1150:secondary
GTGGTGATCG	1	0	0	0	0	At5g08580.1:X:-203:quaternary,At4g14430.1:X:-71:quaternary
AACGCATCGT	0	1	0	0	0	At5g08565.1:d:+212:primary
CTGCTCTTCT	0	0	0	1	0	At5g08550.1:X:-539:quaternary
CAGAATAAGG	0	0	1	0	0	At5g08550.1:v:+1629:secondary
AATCCAAGGG	0	1	0	0	0	At5g08550.1:i:+1667:tertiary
GCTGGAAGGA	0	1	0	0	0	At5g08535.2:d:+406:primary,At5g08535.1:d:+404:primary
ACCCATTTCT	1	0	0	0	0	At5g08530.1:d:+351:secondary
GCCTGTGCAA	0	0	1	0	0	At5g08530.1:d:+1501:secondary
AATCTATCGT	0	1	0	0	0	At5g08510.1:d:+194:secondary
TCAGGTGCAT	0	0	0	0	1	At5g08510.1:d:+1779:primary
ATCGATCTCT	0	0	1	0	0	At5g08510.1:d:+1078:secondary,At1g68930.1:v:+1893:secondary
CTTGGTTTTC	0	1	0	0	0	At5g08500.1:i:+1252:tertiary
TTTTTGTATG	0	1	0	0	0	At5g08490.1:v:+1138:secondary,At1g18750.1:v:+1705:secondary
CTCAGCTGGA	0	1	0	0	0	At5g08450.1:X:-151:quaternary,At1g65440.1:X:-55:quaternary
ATTCCCTCAG	0	0	0	0	1	At5g08440.1:d:-826:secondary
AAACCCCAGA	0	0	1	0	0	At5g08430.1:d:+1362:secondary
AGGAAAATTA	1	0	0	0	0	At5g08420.1:i:+1020:tertiary
TAGGTGTTTT	1	0	0	0	0	At5g08380.1:X:-253:quaternary,At3g14225.1:X:--66:quaternary
ATGCCAAGAC	0	0	0	1	0	At5g08380.1:d:+550:secondary,At1g67325.1:d:+645:secondary
CCGGGATCAC	1	0	0	0	0	At5g08370.1:d:+506:secondary
TTTGCTTCTA	1	0	0	0	0	At5g08340.1:d:+1024:secondary,At5g08340.2:d:+1024:secondary
GCTTCTCCGA	0	1	0	0	0	At5g08320.1:d:+105:primary
AAATCGCTAG	0	0	1	0	0	At5g08280.1:X:-48:quaternary

CACTGAAAGT	0	0	1	0	0	At5g08260.1:d:+1062:secondary
TCTATTTGAC	0	0	0	1	0	At5g08210.1:d:+366:primary
TGTGTAATCT	0	0	1	0	0	At5g08185.1:d:+134:secondary
TCTTCGTGCT	0	0	0	1	0	At5g08180.1:X:+406:quaternary
GTACTGTACA	0	1	0	0	0	At5g08180.1:i:+590:tertiary
CACTCTCTGG	0	0	0	0	1	At5g08160.1:d:+697:secondary,At5g08160.2:d:+589:secondary
ATTATGATGA	0	1	0	0	0	At5g08150.1:v:+709:secondary,At1g76190.1:v:+619:secondary
TTCAAGACCA	1	0	0	0	0	At5g08141.1:v:+391:secondary
CGCTTTGGTT	0	0	1	0	0	At5g08130.1:X:-530:quaternary
CTCAAATGAA	1	0	0	0	0	At5g08120.1:X:-330:quaternary
TATGTATCTA	1	0	0	0	0	At5g08120.1:d:+1286:primary
AAGAAATTGG	1	0	0	0	0	At5g08120.1:d:+1184:secondary,At3g56550.1:v:+1551:secondary
CATAAATTCC	0	1	0	0	0	At5g08110.1:X:-239:quaternary
TGATTTAGCA	0	0	0	1	0	At5g08080.1:i:-905:tertiary
AAGGGTTTGA	0	0	0	1	0	At5g08050.1:X:+623:quaternary
AACCTCGGAG	0	0	0	1	0	At5g08040.1:X:-102:quaternary
GGTTTGTAAA	0	0	1	0	0	At5g08020.1:v:+2367:primary
TCATTTCAAT	1	0	0	0	0	At5g07990.1:i:+557:tertiary
TGTGTAGTCA	1	0	0	0	0	At5g07990.1:d:+572:secondary
AGGATTATTT	1	0	0	0	0	At5g07960.1:d:-432:secondary
TTGGCCAGAG	1	0	0	0	0	At5g07960.1:d:-316:primary
GATCCATATA	1	0	0	0	0	At5g07940.1:d:+4824:primary
TAGCTCCAGT	0	1	0	0	0	At5g07940.1:d:+4442:secondary,At5g07980.1:v:+4490:secondary
ATATAACTGG	0	1	0	0	0	At5g07920.1:i:+3128:tertiary
GATCAGTTTC	0	1	0	0	0	At5g07910.1:d:+1096:primary
AACTCTTGAT	1	0	0	0	0	At5g07880.1:v:+1370:secondary
CATCTGTTAT	1	0	0	0	0	At5g07870.1:X:+-208:quaternary
GGAAGCTCTC	0	0	1	0	0	At5g07860.1:d:+1145:primary
GAGACTTGGC	0	0	0	0	1	At5g07850.1:v:+540:secondary,At3g26070.1:d:+646:secondary
AACAAGTGAA	1	0	0	0	0	At5g07810.1:X:--70:quaternary
AACTCCTACA	0	1	0	0	0	At5g07800.1:d:+1386:primary
AAGCAAACTA	0	0	1	0	0	At5g07790.1:d:-2241:secondary
AATCTGTCTT	1	0	0	0	0	At5g07790.1:d:+557:secondary
TACAATAAGA	0	1	0	0	0	At5g07760.1:i:+1839:tertiary
AAGATAGTAC	0	0	1	0	0	At5g07720.1:d:-1158:secondary
TAGTTGAGAA	0	0	1	0	0	At5g07710.1:d:+1661:primary
AAGAACCTTC	0	1	0	0	0	At5g07685.1:p:-1930:secondary
AATACTTAAC	1	0	0	0	0	At5g07670.1:d:+1706:primary
AATGAAAAGG	1	0	0	0	0	At5g07630.1:v:+1765:primary
GCGAATTCCT	0	0	0	0	1	At5g07630.1:v:+1338:secondary
TTCAGTGGCC	0	0	1	0	0	At5g07590.1:d:+506:secondary
TAAGCTATAA	0	0	0	0	1	At5g07570.1:v:-87:primary
AGTTTTTACT	0	1	0	0	0	At5g07570.1:v:+4993:secondary,At4g08940.1:d:+1046:secondary
TATATGTGTA	0	1	0	0	0	At5g07475.1:d:+680:primary
CCTAGACCAG	1	0	0	0	0	At5g07470.1:X:-417:quaternary
CAGAAGTTGT	0	1	0	0	0	At5g07460.1:d:+433:secondary,At4g11270.1:d:+2471:secondary
AATGAGCAAT	1	0	0	0	0	At5g07440.1:d:-1053:secondary
AAAGTTCTTC	1	0	0	0	0	At5g07370.4:d:+131:secondary,At5g07370.3:d:+48:secondary,At1g02730.1:d:+3769:secondary
CAGCAAGAAC	1	0	0	0	0	At5g07350.1:X:-236:quaternary
TAGATGAAGA	0	1	0	0	0	At5g07330.1:d:-321:primary
TCTTCGTTGA	0	0	0	0	1	At5g07320.1:v:+1903:secondary
AATGCCTACA	0	1	0	0	0	At5g07300.1:d:+1332:primary
GAACAGAGAG	0	1	0	0	0	At5g07270.1:d:+1517:primary
TTTTCCTTGA	0	0	1	0	0	At5g07210.1:v:+427:secondary
CTCTTCAAGT	0	0	0	0	1	At5g07190.1:d:-685:secondary
GGATAGGTGA	1	0	0	0	0	At5g07180.1:d:+2946:primary
AAGTCCAATA	0	0	1	0	0	At5g07170.1:v:+2080:primary
GTTTTGAGCT	1	0	0	0	0	At5g07120.1:d:+1923:primary
TTAAGAGCTC	0	0	1	0	0	At5g07110.1:d:+1066:primary
CGCCTATACA	0	0	0	1	0	At5g07100.1:d:+1072:primary,At5g07100.2:d:+1261:primary
CCTTGGCTTC	0	1	0	0	0	At5g07080.1:d:-35:primary

TGCTTGCAGT	0	0	1	0	0	At5g07070.1:X:-1403:quaternary
AGTACAGTAA	0	0	0	0	1	At5g07070.1:d:+1641:primary
AAAATTAGTC	1	0	0	0	0	At5g07060.1:v:+1447:secondary
ATCCATCGTC	0	0	1	0	0	At5g07010.1:X:-1505:quaternary
TGTGAAGCTA	0	1	0	0	0	At5g07010.1:d:+1389:primary
AGATGATGCG	0	0	0	1	0	At5g07000.1:X:-131:quaternary
GGGTCAGCAG	0	0	0	1	0	At5g06970.1:X:-547:quaternary
TGGCTACGCT	0	0	1	0	0	At5g06970.1:d:+2874:primary
GAGAAGCTAG	0	0	1	0	0	At5g06970.1:d:+2646:secondary
GCGATGGGGA	0	0	1	0	0	At5g06960.1:d:+1112:secondary,At5g06960.2:d:+1094:secondary,At2g34300.1:d:+149:secondary
TTGCGAGAGA	0	0	1	0	0	At5g06930.1:d:+865:secondary
TGTTGACCAG	0	0	0	1	0	At5g06930.1:d:+1217:primary
GCCATCTCCA	0	0	1	0	0	At5g06920.1:v:+414:secondary
TTCCAGTTCG	0	0	0	0	1	At5g06870.1:d:+774:secondary,At5g06860.1:d:+774:secondary
GGACCCTCAA	0	0	1	0	0	At5g06860.1:d:+170:secondary
GAATTCAACA	0	0	0	1	0	At5g06839.1:i:+3240:tertiary
AACAAAGATG	0	0	1	0	0	At5g06839.1:d:+460:secondary,At4g39210.1:d:+1613:secondary,At1g80460.1:d:+1219:secondary
TCTTAAGTAC	0	1	0	0	0	At5g06810.1:v:+3578:primary
ATGCGTTAAT	0	0	0	0	1	At5g06805.1:v:+391:secondary
CATAGTTTGA	0	0	0	1	0	At5g06790.1:v:+1074:primary
TGGATCAGGA	0	0	0	0	1	At5g06780.1:X:-35:quaternary
CCAGATTTTG	0	0	0	1	0	At5g06760.1:X:-169:quaternary
AAAGCACCTG	0	0	1	0	0	At5g06710.2:d:+1205:secondary,At5g06710.1:d:+1083:secondary
GCTTTTCTTT	0	1	0	0	0	At5g06660.1:d:+516:secondary
ATCGAGGCAA	0	0	1	0	0	At5g06610.1:d:+1163:primary
TTGCTTGTAG	0	1	0	0	0	At5g06600.2:i:+1802:tertiary,At5g08390.1:i:+5781:tertiary,At5g06600.1:i:+1800:tertiary
GTGAAGATAA	0	0	1	0	0	At5g06600.1:d:+1322:secondary,At5g06600.2:d:+1321:secondary,At3g16940.1:d:+531:secondary
AAGCTTCAAA	0	0	1	0	0	At5g06590.1:d:+439:secondary,At3g49290.1:d:+364:secondary
ATATCGTTTG	0	1	0	0	0	At5g06580.1:X:-76:quaternary
GTTTCTACTC	0	0	1	0	0	At5g06570.1:d:+936:primary
TTCTTCTCCG	0	0	0	1	0	At5g06560.1:X:-676:quaternary
GGATTGCAAA	0	0	0	1	0	At5g06560.1:d:+2020:secondary
ACAAATTCAA	0	0	0	1	0	At5g06550.1:d:+1363:primary
TCTCTCAAGC	0	1	0	0	0	At5g06540.1:X:-1426:quaternary
ACAACGAGTG	0	0	0	0	1	At5g06530.2:X:-353:quaternary
CTCGCCGGAG	0	0	1	0	0	At5g06530.2:d:+2132:secondary,At5g06530.1:d:+2132:secondary
TCCATTAATG	0	0	1	0	0	At5g06520.1:v:+927:secondary
TTTCAACTAG	1	0	0	0	0	At5g06510.1:d:+1145:primary,At5g06510.2:d:+1416:primary,At5g06510.3:d:+1291:primary
CCATCCAAAG	0	1	0	0	0	At5g06480.1:X:-52:quaternary
CATCAAATTC	0	0	0	1	0	At5g06480.1:d:+470:primary
ACAATCTCAA	0	1	0	0	0	At5g06450.1:d:+385:primary
GTTAGAAATA	0	0	1	0	0	At5g06440.2:d:+1522:secondary,At5g06440.1:d:+1334:secondary
ATCTGTTCCT	1	0	0	0	0	At5g06410.1:X:-222:quaternary,At3g48200.1:X:-145:quaternary
TTTTAGTTTA	0	1	0	0	0	At5g06410.1:d:+905:primary
TATTCTCAAA	0	1	0	0	0	At5g06400.1:v:-3842:secondary
TATCCACATA	0	0	1	0	0	At5g06400.1:v:-3151:secondary
TCAGAAAATG	0	0	1	0	0	At5g06400.1:v:+3086:secondary
GAATTAGAAG	1	0	0	0	0	At5g06390.1:d:+1594:secondary,At2g40940.1:d:+690:secondary
CCTTAACTGT	0	1	0	0	0	At5g06370.1:d:+405:primary
GAGATTGAAA	0	0	0	1	0	At5g06370.1:d:+27:secondary
TATCCGGAAT	0	1	0	0	0	At5g06340.1:d:-323:secondary
GCAGATGCCT	1	0	0	0	0	At5g06340.1:d:+328:secondary
AAGCATTGAT	0	1	0	0	0	At5g06320.1:d:+979:secondary
GAGGATTTCT	0	0	0	0	1	At5g06320.1:d:+1075:primary,At2g15160.1:p:+1750:primary,At5g05640.1:v:+1617:primary
ACGACTCTAA	0	0	1	0	0	At5g06280.3:d:+114:secondary,At5g06280.1:d:+105:secondary
AAAACGCCAC	1	0	0	0	0	At5g06270.1:d:+570:secondary
GAAAGTGCAG	0	0	0	1	0	At5g06265.1:i:+505:tertiary
ACTTGGCTAT	0	1	0	0	0	At5g06265.1:d:-630:secondary
CTTTCAGATT	0	0	0	1	0	At5g06265.1:d:+76:secondary
ACATTTGGTT	0	1	0	0	0	At5g06265.1:d:+635:primary
GGCCTTCGTG	0	1	0	0	0	At5g06260.1:X:+-85:quaternary

AGGGTCTGCA	0	0	1	0	0	At5g06240.1:d:+474:secondary
ACCAACTGCT	0	0	0	1	0	At5g06230.1:d:+1468:primary
CATCAAGCTC	0	0	0	0	1	At5g06220.1:d:+1532:secondary
AGACTACAAA	0	0	0	1	0	At5g06180.1:d:-1462:secondary
AAGTATATGG	0	1	0	0	0	At5g06160.1:d:+631:secondary
AGTCTGAAAA	0	1	0	0	0	At5g06160.1:d:+1028:secondary,At2g33435.1:d:+459:secondary
CTTGCTGCTT	0	0	0	0	1	At5g06150.1:d:+1125:primary
GCAAGTATCC	0	1	0	0	0	At5g06130.2:X:-8:quaternary
TTTACTATAG	0	0	0	0	1	At5g06130.2:X:-234:quaternary
ACCCCCGCTT	0	0	0	0	1	At5g06130.2:d:+1032:secondary,At5g06130.1:d:+1246:secondary
GTTTTGTTGT	0	1	0	0	0	At5g06080.1:v:+947:secondary,At2g02660.1:v:+1749:secondary,At3g18210.1:d:+964:secondary
CTTGAATGAT	0	0	1	0	0	At5g06060.1:d:+214:secondary
GAAAAACAAA	0	0	1	0	0	At5g06030.1:v:+1188:primary
GAATGCAAAA	0	0	0	1	0	At5g06020.1:v:+728:secondary
TTGGGGTCTC	0	0	0	0	1	At5g06000.1:d:+959:primary
ATCAACAAGG	0	0	0	0	1	At5g05990.1:d:+727:primary
CAAGCATAAG	0	0	0	1	0	At5g05987.1:d:+574:secondary
GCAATAAAAT	1	0	0	0	0	At5g05980.1:d:+1651:secondary
AAGAGTACCA	0	0	1	0	0	At5g05970.1:d:-2525:secondary
ACATATGTAT	1	0	0	0	0	At5g05930.1:X:-192:quaternary
TTTTCCAATA	0	1	0	0	0	At5g05930.1:i:+2558:tertiary,At5g05930.2:i:+2524:tertiary
AGATTTCTTG	0	1	0	0	0	At5g05930.1:d:+866:secondary,At5g05930.2:d:+767:secondary
AGGTGAAAAG	1	0	0	0	0	At5g05930.1:d:+1258:secondary,At5g05930.2:d:+1159:secondary
TTGTTTCTTT	0	0	0	0	1	At5g05890.1:d:+1404:primary
GTTAGATATG	0	0	0	0	1	At5g05870.1:d:+833:secondary
TTGGAAGAGG	0	0	1	0	0	At5g05850.1:d:+1426:primary
GCTTAAAGGC	0	0	1	0	0	At5g05810.1:X:--56:quaternary
CCCAAAAGTA	1	0	0	0	0	At5g05790.1:d:+875:primary
ATGTTTTGTT	0	0	1	0	0	At5g05760.1:i:+1441:tertiary
ATGAAGATCT	1	0	0	0	0	At5g05760.1:d:+1070:secondary
GCTTCATTGC	0	0	1	0	0	At5g05740.1:X:-235:quaternary
TTTTTGGACT	0	0	1	0	0	At5g05730.1:i:+470:tertiary
TCACCAGTGA	0	0	1	0	0	At5g05720.1:v:+2140:primary
CTTGATATTG	0	0	1	0	0	At5g05690.1:d:+514:secondary
TCTCTTATCT	0	0	1	0	0	At5g05670.2:X:-206:quaternary
ACGTGACGTT	0	0	0	0	1	At5g05660.1:X:+444:quaternary
GACCTAGACC	0	0	1	0	0	At5g05660.1:d:+1598:secondary
GGAAGTGTGT	0	1	0	0	0	At5g05610.2:d:+800:secondary,At2g46740.1:d:+992:secondary,At5g05610.1:d:+800:secondary
TCTCATACCC	1	0	0	0	0	At5g05610.1:X:-401:quaternary
GGCAAACGGC	0	1	0	0	0	At5g05600.1:i:-1997:tertiary
TTGCGATGAT	0	1	0	0	0	At5g05600.1:i:+2214:tertiary
GGATTTGCCT	0	0	1	0	0	At5g05600.1:d:-1232:secondary
AACAAGAAGA	0	0	0	1	0	At5g05600.1:d:+118:secondary,At1g60610.1:d:+1263:secondary,At1g02040.1:v:+436:secondary,At1g60610.2:d:+1691:secondary,At2g36720.1:d:+570:secondary,At1g09940.1:d:+1676:secondary,At5g23600.1:v:+1089:secondary
GATTCTTAAC	0	1	0	0	0	At5g05580.1:d:-503:primary,At3g43870.1:v:-163:primary,At3g11170.1:d:-498:primary
TGTCTCTAAA	1	0	0	0	0	At5g05570.1:i:+2496:tertiary
TTTATTTATC	0	0	0	0	1	At5g05570.1:d:+3658:primary,At5g55330.1:v:+1806:primary
AGAACAAATC	0	1	0	0	0	At5g05570.1:d:+2500:secondary
CTGTTGGTTC	0	1	0	0	0	At5g05570.1:d:+1993:secondary
CTAACTATGG	0	1	0	0	0	At5g05560.1:d:+4055:secondary,At2g07717.1:p:+5263:secondary
CTTACAACAA	0	0	1	0	0	At5g05550.2:X:--121:quaternary
GTTGTTACTC	1	0	0	0	0	At5g05550.2:i:+841:tertiary,At5g05550.1:i:+841:tertiary
ATGCAGAAGC	0	1	0	0	0	At5g05540.1:d:+982:secondary,At5g24240.1:v:+1975:secondary,At4g25440.1:d:+1205:secondary,At5g49200.1:v:+1405:secondary,At5g51980.1:d:+1206:secondary
TTTTTCATAA	0	1	0	0	0	At5g05530.1:d:+523:primary
ACACTGTGAG	0	0	0	0	1	At5g05520.1:d:-1688:secondary
TCCGTGGATT	0	0	0	0	1	At5g05520.1:d:-1417:secondary,At5g05520.1:d:-1427:secondary
GAGAGGCACA	1	0	0	0	0	At5g05510.1:v:+810:secondary,At5g50920.1:d:+2190:secondary
TTTGGCGAAC	0	0	1	0	0	At5g05510.1:v:+1497:primary

CTTCTTATAC	1	0	0	0	0	At5g05470.1:d:-550:secondary,At5g05470.1:d:-544:secondary
GTTAGTAGTA	0	0	0	0	1	At5g05470.1:d:+1256:primary
GCAGCTGAAC	0	1	0	0	0	At5g05450.1:d:-1287:secondary
TGAAAACAGA	1	0	0	0	0	At5g05410.1:d:+673:secondary
TACACCACCA	0	0	1	0	0	At5g05330.1:X:-224:quaternary
GAAGCTCTCG	0	1	0	0	0	At5g05320.1:d:+227:secondary,At4g13890.1:d:+150:secondary,At4g38540.1:d:+183:secondary
AAGATGAAGG	1	0	0	0	0	At5g05310.1:d:+1678:primary,At5g05310.3:d:+1510:primary,At5g05310.2:d:+1621:primary
ACTAAAAGTC	0	1	0	0	0	At5g05300.1:v:+679:secondary,At2g02240.1:v:+1696:secondary
CTAAGACCAC	0	0	0	1	0	At5g05250.1:X:--123:quaternary
GAGGGATACC	0	0	1	0	0	At5g05240.1:d:+889:secondary
TTTTGGGGGA	1	0	0	0	0	At5g05200.1:X:-26:quaternary
CCTAAGCTTC	0	0	0	0	1	At5g05170.1:d:+2532:secondary
GCACACAACC	0	0	0	1	0	At5g05110.1:X:-139:quaternary
TCCGAGTAGA	0	0	1	0	0	At5g05100.1:d:+844:secondary
TGTAAGAAAA	0	0	0	0	1	At5g05090.1:X:+-238:quaternary
TGTTGATAAT	0	0	0	1	0	At5g05020.1:d:+628:secondary
TCTTTAGATG	1	0	0	0	0	At5g05010.1:d:+411:secondary,At4g10970.1:d:+129:secondary,At4g10970.2:d:+238:secondary,At4g10970.3:d:+238:secondary
AAGGAAAAAT	0	0	1	0	0	At5g04980.1:d:+1164:primary
GTCGACTTGG	1	0	0	0	0	At5g04970.1:X:-195:quaternary
AACTTCACCG	0	1	0	0	0	At5g04970.1:d:+1826:primary
TCACAGGGAA	0	0	1	0	0	At5g04930.1:d:-1247:secondary
GATGTTCTGG	0	1	0	0	0	At5g04930.1:d:+3924:primary
CGACAAACAA	0	0	1	0	0	At5g04910.1:v:-1247:secondary
TTCCAGGCTT	0	1	0	0	0	At5g04910.1:v:+1176:secondary
TCCGGAGCAA	0	1	0	0	0	At5g04900.1:d:+837:secondary
GTTCAGAAAC	0	1	0	0	0	At5g04885.1:d:+1920:primary
ACTAGGAAAT	1	0	0	0	0	At5g04880.1:p:+1123:secondary
TGTATATAAT	0	1	0	0	0	At5g04870.1:i:+2621:tertiary,At4g28130.1:i:+1949:tertiary,At1g48700.1:i:+327:tertiary
TTCTTTAAAC	0	0	1	0	0	At5g04870.1:d:+1362:secondary
AATGTAAGTT	1	0	0	0	0	At5g04810.1:i:+3130:tertiary,At3g10940.1:i:+896:tertiary
TCGCCGGCCT	0	0	1	0	0	At5g04810.1:d:-2348:secondary
AAGGCTGCAT	0	1	0	0	0	At5g04780.1:v:+2133:secondary
AGGGGGTTTG	0	1	0	0	0	At5g04770.1:d:+1869:primary
TATATCCAGA	1	0	0	0	0	At5g04750.1:d:+227:secondary
GAAACTGCCA	0	0	0	1	0	At5g04670.1:d:+2158:primary
ACCAAAACTA	0	0	1	0	0	At5g04660.1:X:+1686:quaternary
GTTAGTTTCA	0	0	0	1	0	At5g04630.1:v:+2276:primary
CAAAACATCG	0	0	0	0	1	At5g04590.1:X:-352:quaternary
TACGGTCTTG	1	0	0	0	0	At5g04590.1:d:-1718:secondary,At4g03330.1:d:-399:secondary
GGATTCGAGA	0	0	0	1	0	At5g04590.1:d:+1924:secondary
GATAAGGTTA	0	1	0	0	0	At5g04590.1:d:+1816:secondary
GTTTTGTGGG	0	1	0	0	0	At5g04590.1:d:+1329:secondary
CGAGCGATGA	0	1	0	0	0	At5g04560.1:d:-3724:secondary
AGAGGAAGTT	0	0	1	0	0	At5g04560.1:d:+6366:primary
AATTACCAGG	1	0	0	0	0	At5g04560.1:d:+4672:secondary,At1g10840.1:d:+591:secondary,At1g10840.2:d:+573:secondary
CCACCTGAGG	1	0	0	0	0	At5g04560.1:d:+3084:secondary
ACATTGAAAA	1	0	0	0	0	At5g04540.1:X:--112:quaternary
GTATCGTCGT	1	0	0	0	0	At5g04530.1:X:-1441:quaternary
GAGCTCTTCT	0	1	0	0	0	At5g04530.1:d:+76:secondary
TTCGACACTC	0	0	1	0	0	At5g04530.1:d:+409:secondary
AGATACTGAA	0	0	1	0	0	At5g04510.2:d:+2100:primary,At5g04510.1:d:+2014:primary
AGTGCTCCTT	0	0	1	0	0	At5g04510.1:X:+313:quaternary
TGTACTTGCT	0	0	0	1	0	At5g04460.1:d:+2488:primary
TAAGCAAAGG	0	0	0	0	1	At5g04460.1:d:+2417:secondary,At2g05020.1:p:+2705:secondary
TTCTTCATCT	1	0	0	0	0	At5g04420.1:d:+662:secondary,At5g08720.1:d:+1546:secondary
GTTACTATTT	0	0	1	0	0	At5g04420.1:d:+2129:secondary
TTTCCCCCAC	1	0	0	0	0	At5g04410.1:d:+2276:primary
GATCGCAACT	0	0	0	1	0	At5g04400.1:v:-959:secondary
TGTCTCAACA	0	0	0	0	1	At5g04370.1:d:+1382:secondary,At1g07430.1:d:+1241:secondary
CTATTTCGAG	0	0	0	0	1	At5g04360.1:i:+2942:tertiary

ACAATGAAAC	0	0	1	0	0	At5g04360.1:d:+2198:secondary
CACCTGAAAG	0	0	1	0	0	At5g04320.1:d:-850:secondary
TTGTTCTTAA	0	0	0	1	0	At5g04310.1:d:+167:secondary
CAAGAGTTTA	0	0	0	1	0	At5g04290.1:v:+974:secondary
GGCTCGTGTT	0	1	0	0	0	At5g04290.1:v:+1085:secondary
GCTCTAGAGG	0	0	1	0	0	At5g04280.1:d:+364:secondary
ATCAAATGGC	0	0	0	0	1	At5g04270.1:d:+781:primary
ACTCACCCAT	0	1	0	0	0	At5g04240.1:X:+2241:quaternary
TTTAACAACG	0	1	0	0	0	At5g04240.1:d:-4198:secondary
AGATTGCTCG	1	0	0	0	0	At5g04235.1:X:-1871:quaternary
TTGCTTAGGT	0	0	0	1	0	At5g04150.1:v:+909:secondary
TGAATGATAA	0	0	0	1	0	At5g04150.1:v:+1343:primary
TAGATCAAAT	0	0	0	0	1	At5g04140.2:d:+5055:secondary,At5g04140.1:d:+5156:secondary
TGGAAAAAAC	0	0	1	0	0	At5g04140.1:d:+4885:secondary,At5g04140.2:d:+4784:secondary
ACTGGTGGCT	0	0	1	0	0	At5g04140.1:d:+4695:secondary,At5g04140.2:d:+4594:secondary
TTCTTGATTC	1	0	0	0	0	At5g04110.1:d:+1838:secondary,At4g20940.1:d:+3042:secondary,At3g58290.1:v:+916:secondary,At1g64480.1:v:+112:secondary
GGTGAAATAT	0	0	0	1	0	At5g04090.1:X:-30:quaternary
AGAGATCAAA	0	0	1	0	0	At5g04080.1:d:+123:primary
CTTTGCTTGA	0	1	0	0	0	At5g04060.1:i:+405:tertiary
GTTTTGCGGC	0	1	0	0	0	At5g04050.1:d:+80:secondary
ATAGGAAAAT	0	0	0	1	0	At5g04050.1:d:+2488:secondary
GGACGACCAC	0	1	0	0	0	At5g04050.1:d:+1733:secondary
TCTTGAAGAA	0	1	0	0	0	At5g04050.1:d:+1305:secondary
ACTCTGTTTC	0	0	0	0	1	At5g04040.1:i:+526:tertiary,At1g10870.1:i:+6621:tertiary,At4g01935.1:i:+961:tertiary
AAGAACAATA	0	0	1	0	0	At5g04020.1:d:+4553:secondary
GGAACCCGAT	1	0	0	0	0	At5g04020.1:d:+3540:secondary
GGGTTGGACT	1	0	0	0	0	At5g03990.1:d:+1026:primary
AGAGGTTCAT	0	1	0	0	0	At5g03970.1:X:-1363:quaternary
ACTGGACAAG	1	0	0	0	0	At5g03940.1:d:+960:secondary
GCGGAAATCG	0	0	1	0	0	At5g03900.2:X:+-17:quaternary
GAATCGCTGT	0	1	0	0	0	At5g03900.2:d:+213:secondary,At5g03900.1:d:+213:secondary
TCTCTTCAAA	1	0	0	0	0	At5g03890.1:v:+1216:primary
TATTAGATTT	0	1	0	0	0	At5g03890.1:v:+1128:secondary
ACCTTAACCA	0	0	0	0	1	At5g03850.1:X:-38:quaternary
AACTTTAAAA	0	0	1	0	0	At5g03790.1:i:+216:tertiary
GTCAAGATTA	0	1	0	0	0	At5g03780.1:d:+1289:primary
GAGAATCACA	0	0	1	0	0	At5g03770.1:d:+1473:primary
GTGAAGGCGA	0	1	0	0	0	At5g03770.1:d:+1142:secondary
ATATGCACAT	0	0	0	0	1	At5g03730.2:d:+2693:primary
GAGCAAGACT	0	0	0	0	1	At5g03730.1:d:+1933:secondary,At5g03730.2:d:+1933:secondary
CTGCTTAGAA	0	0	0	0	1	At5g03680.1:v:+2233:secondary
GAGGAAGATT	1	0	0	0	0	At5g03640.1:v:+1602:secondary
TTAGGCAATG	0	0	0	1	0	At5g03610.1:d:-542:primary
ACAACTCAAA	0	1	0	0	0	At5g03610.1:d:+547:primary
GGCTTGCTTC	0	0	0	1	0	At5g03555.1:X:+2011:quaternary
ACCAAAGACA	0	1	0	0	0	At5g03545.1:d:+11:secondary
CAAACCAATA	0	0	1	0	0	At5g03540.1:d:+1560:secondary
TCAATCGCAA	0	0	1	0	0	At5g03520.1:d:+746:secondary
AGATTGCACA	0	0	1	0	0	At5g03500.1:X:-12:quaternary
TAATGTTAAA	0	0	1	0	0	At5g03500.1:v:+1956:primary
CGGAGAGATT	0	0	0	0	1	At5g03480.1:v:+770:secondary
AAAGGGGCAA	0	1	0	0	0	At5g03470.1:d:+365:secondary
TTTAAGAAGA	0	1	0	0	0	At5g03470.1:d:+350:secondary
AAGTGATTGC	0	0	0	0	1	At5g03455.1:i:+1182:tertiary
TATGTAAAGC	0	1	0	0	0	At5g03450.1:d:+2009:primary
AAGATATATA	0	0	1	0	0	At5g03440.2:d:+630:secondary,At4g10090.1:d:+557:secondary,At5g03440.1:d:+570:secondary
TTAATGAAAC	0	0	0	0	1	At5g03400.1:v:+1335:primary
AGGGCCTTTT	1	0	0	0	0	At5g03390.1:d:+1360:primary
AAACCTTATT	0	1	0	0	0	At5g03360.1:v:+2292:secondary
TCGGGATTAA	1	0	0	0	0	At5g03350.1:d:+512:secondary

TCGATCTGCG	0	1	0	0	0	At5g03345.1:d:-375:secondary
AAATCCGAAT	0	0	1	0	0	At5g03345.1:d:-330:secondary
GCTGTGTCTA	0	0	1	0	0	At5g03340.1:d:+1440:secondary
ACAAAATCAC	0	0	0	0	1	At5g03330.1:X:+-148:quaternary
CTAGTGTTCG	0	1	0	0	0	At5g03320.1:d:-441:primary
ACTTGTCTTG	0	0	0	1	0	At5g03310.1:v:+856:primary
GCAGCAAATC	1	0	0	0	0	At5g03290.1:d:+68:secondary
GATGCTCAAA	0	1	0	0	0	At5g03280.1:d:+2009:secondary
TTGCAAGAGG	0	0	0	1	0	At5g03270.1:i:+613:tertiary
TGTGTGCCTA	1	0	0	0	0	At5g03180.1:X:-341:quaternary
ACACATAAAA	0	1	0	0	0	At5g03180.1:d:+1821:primary
GATATGTATT	1	0	0	0	0	At5g03150.1:d:+1956:primary
AAAATGAGAG	0	1	0	0	0	At5g03130.1:v:+447:secondary,At1g07120.1:d:+158:secondary,At1g69560.1:d:+806:secondary
TCCGGTCTTT	0	0	1	0	0	At5g03120.1:d:+512:secondary
TAGGAGGAAA	0	1	0	0	0	At5g03120.1:d:+298:secondary
GGTCAAGTCC	0	0	0	1	0	At5g03110.1:d:+274:primary
TGGTGAAAAG	0	0	1	0	0	At5g03100.1:X:+563:quaternary
TATTCTAGGG	0	1	0	0	0	At5g03080.1:d:+536:primary
TTCAACTGAT	1	0	0	0	0	At5g03060.1:X:-184:quaternary
AACCTTTCTC	0	1	0	0	0	At5g03050.1:d:+723:primary
TAGCTCGGGA	0	0	1	0	0	At5g03040.1:X:-443:quaternary
TGGAATATCA	0	0	0	0	1	At5g02930.1:v:+1928:primary
AAGTAACTTC	0	0	0	1	0	At5g02890.1:X:-486:quaternary
AGTTGATAAG	0	0	0	0	1	At5g02870.1:X:+360:quaternary
AACACTTAAG	0	0	1	0	0	At5g02840.2:d:+954:secondary,At5g02840.1:d:+954:secondary
ATCGAACGTC	0	1	0	0	0	At5g02840.2:d:+1207:primary
CAAGAGAGAA	0	0	0	1	0	At5g02830.1:d:+2178:secondary
TTTTTGGGCT	1	0	0	0	0	At5g02810.1:X:-158:quaternary
GTTTATCTCC	0	1	0	0	0	At5g02810.1:i:+1936:tertiary
TTTGGAGAAG	0	0	1	0	0	At5g02810.1:d:+848:secondary,At5g64220.1:d:+1550:secondary,At2g41190.1:d:+1424:secondary,At2g46790.1:d:+576:secondary,At2g46790.2:d:+130:secondary
ATGTCATCTC	0	1	0	0	0	At5g02810.1:d:+736:secondary
TCGCTGAGAG	0	0	0	1	0	At5g02810.1:d:+1985:secondary
TCGGTAGCAA	0	0	0	1	0	At5g02810.1:d:+1817:secondary
CAAAAAGAGA	0	0	1	0	0	At5g02790.1:d:+392:secondary
GTTTATTCCG	0	0	1	0	0	At5g02780.1:X:+169:quaternary
AACCAAGCCT	0	0	0	0	1	At5g02780.1:X:+-153:quaternary,Chr1:+14547451:quaternary
GACTTCTAAT	1	0	0	0	0	At5g02770.1:X:-69:quaternary
CAATATTCGC	0	0	0	0	1	At5g02760.1:d:+1858:secondary
GGCATCCAAT	0	0	0	1	0	At5g02740.2:d:-1428:secondary,At5g02740.1:d:-1062:secondary
TTTTAAGAAG	0	0	1	0	0	At5g02720.1:v:+772:secondary
GTAGATAACT	0	1	0	0	0	At5g02690.1:v:+453:secondary
TGGAACAAAG	1	0	0	0	0	At5g02670.1:X:--19:quaternary
CTTTAATTCT	0	0	1	0	0	At5g02630.1:X:-1496:quaternary
GTGATTGTTC	1	0	0	0	0	At5g02630.1:d:+787:secondary,At5g56630.1:d:+1329:secondary
AGTTAATGTT	0	0	1	0	0	At5g02600.2:i:+829:tertiary,At5g02600.1:i:+829:tertiary
TTGTAAAATT	1	0	0	0	0	At5g02600.2:d:+1266:primary
CCGTCTCTGA	0	0	1	0	0	At5g02570.1:v:+703:secondary
TTCTATTAGC	0	1	0	0	0	At5g02560.1:d:+333:primary
GGAACGCCTC	0	0	0	0	1	At5g02550.1:d:+293:secondary
AGGAGATTGA	0	1	0	0	0	At5g02540.1:i:-547:tertiary
TTGTAAGCGT	1	0	0	0	0	At5g02500.1:X:-1446:quaternary,At5g02490.1:X:-1446:quaternary
AAATTGTGGC	0	0	0	1	0	At5g02500.1:d:+376:secondary
AATTCCCCTG	0	0	0	1	0	At5g02480.1:d:-1950:secondary,At1g18420.1:v:-1018:secondary
GAGGAACAAT	0	0	1	0	0	At5g02480.1:d:+1819:secondary
ATGATGCTTA	0	0	0	0	1	At5g02470.3:d:+777:primary,At5g02470.1:d:+761:primary,At5g02470.2:d:+864:primary
ATCGGCCACA	0	0	1	0	0	At5g02450.1:X:+378:quaternary
GTCTGACGCA	0	0	0	1	0	At5g02440.1:v:+950:secondary
TACCTTACTT	0	1	0	0	0	At5g02410.1:d:+597:secondary
GAGCAATAAT	0	0	1	0	0	At5g02370.1:d:+1665:primary

ACCTAGATAT	0	0	0	0	1	At5g02360.1:X:-670:quaternary,At5g57685.1:X:-759:quaternary
TATATGTCGA	0	0	1	0	0	At5g02350.1:v:+2130:secondary,At2g39800.2:d:+1566:secondary,At2g39800.1:d:+1930:secondary
TTCGTCAGAG	1	0	0	0	0	At5g02310.1:d:+4823:secondary
AAAGAGGCGA	1	0	0	0	0	At5g02230.2:d:+797:primary,At2g07747.1:p:+147:primary,At5g02230.1:d:+832:primary
GCATAAGAGC	1	0	0	0	0	At5g02200.1:d:-928:secondary
TTTAAAGACT	0	1	0	0	0	At5g02200.1:d:+999:secondary
CAAGCTCGTG	1	0	0	0	0	At5g02120.1:d:-280:secondary
TTTTGGATCT	0	1	0	0	0	At5g02060.1:d:+786:primary
ATCAATTCTA	1	0	0	0	0	At5g02030.1:i:+856:tertiary
TGATGGATGG	0	0	0	0	1	At5g01990.1:X:--35:quaternary
CTCATTGTAT	0	1	0	0	0	At5g01980.1:d:+1292:primary
TAAAGCTTTG	0	1	0	0	0	At5g01950.1:i:+1088:tertiary
GAAAAAATAT	0	1	0	0	0	At5g01950.1:d:+2747:primary
GACTTGATTA	0	1	0	0	0	At5g01920.1:X:+1795:quaternary
GGATTGGTAG	0	0	0	0	1	At5g01920.1:d:+2717:secondary
AGCCATCTTC	0	1	0	0	0	At5g01910.1:v:+600:secondary
TCCAAATCCA	0	1	0	0	0	At5g01840.1:d:+73:primary
GAAACTGACG	0	0	1	0	0	At5g01830.1:d:+1630:secondary
ATGACAACCA	0	0	1	0	0	At5g01800.1:d:+261:secondary
ACTCTGGGAC	0	0	1	0	0	At5g01770.1:v:+2584:secondary
AGAATTTTCA	1	0	0	0	0	At5g01730.1:i:+880:tertiary
GTTTGGCGAT	0	1	0	0	0	At5g01730.1:d:+299:secondary
GATTACACTT	1	0	0	0	0	At5g01710.1:d:+1230:primary
GGACCGTGAC	0	1	0	0	0	At5g01700.1:X:-55:quaternary
TTTAAACAAA	0	0	0	0	1	At5g01670.2:d:+361:secondary
AAGACTAAAC	1	0	0	0	0	At5g01670.2:d:+1065:primary,At5g01670.1:d:+984:primary
TCAACTTCCT	0	0	1	0	0	At5g01660.1:X:-129:quaternary
AATAGGGAAC	0	0	0	0	1	At5g01650.1:i:+1040:tertiary
CTGAGAAGTT	0	0	0	0	1	At5g01600.1:d:+888:secondary,At2g40300.1:d:+506:secondary
ATGTTTCTGA	1	0	0	0	0	At5g01590.1:i:+840:tertiary
TGGTGTGTAC	1	0	0	0	0	At5g01590.1:d:-1771:secondary
GGCTTATGAA	0	0	0	0	1	At5g01590.1:d:+1898:primary
ATTTAACTAG	0	0	1	0	0	At5g01540.1:d:+2258:primary
TAACGCCGGT	1	0	0	0	0	At5g01530.1:d:-637:secondary
GGTACTCGGG	0	0	1	0	0	At5g01530.1:d:+193:secondary
TGTATAAACT	0	0	0	1	0	At5g01520.2:d:+861:primary,At5g01520.1:d:+861:primary
GGAGAAGAGA	0	1	0	0	0	At5g01500.1:d:+72:secondary,At1g01650.1:d:+66:secondary
ATGAGCATTT	0	1	0	0	0	At5g01480.1:v:+1811:secondary
GTGGTAATGT	0	0	0	1	0	At5g01480.1:v:+1390:secondary
ATCTGTGCAA	0	0	0	1	0	At5g01460.1:d:+1768:secondary
GTCAGACATA	0	1	0	0	0	At5g01450.1:X:-374:quaternary
TTGTCGCGAA	0	0	1	0	0	At5g01450.1:X:-199:quaternary
ATGATAAGTG	0	1	0	0	0	At5g01440.1:v:+1565:secondary
AAGCGTCAAA	1	0	0	0	0	At5g01430.1:d:+370:primary,At3g49420.1:d:+327:primary
GATGTCGTCA	0	0	1	0	0	At5g01410.1:d:+227:secondary,At2g38210.1:d:+307:secondary,At2g38230.1:d:+140:secondary
GAATGAAGAT	0	0	0	1	0	At5g01400.1:X:-828:quaternary
TATTGTAGCA	1	0	0	0	0	At5g01400.1:d:+4535:primary
TTCTTCATTC	0	0	0	1	0	At5g01360.1:d:+136:secondary
TACGGCGGAA	0	0	0	1	0	At5g01340.1:X:-199:quaternary
CATCTTGTAT	1	0	0	0	0	At5g01335.1:p:+2462:secondary,At3g60290.1:v:+1365:secondary
TCAGAAGCTG	1	0	0	0	0	At5g01310.1:v:+3016:primary
TAGATTATTT	1	0	0	0	0	At5g01300.1:d:+678:primary
TGGCACCTCT	0	1	0	0	0	At5g01270.1:d:+2338:secondary
TTTACTGAGG	0	1	0	0	0	At5g01260.2:d:+841:primary
ATAAAAGTTT	0	0	0	1	0	At5g01260.1:d:+1150:primary
AGGCTTGCTT	0	0	1	0	0	At5g01250.1:d:+859:secondary
TGGAAACTGG	0	0	1	0	0	At5g01250.1:d:+1345:primary
TGGTGACTTA	0	0	1	0	0	At5g01230.2:d:+1165:primary,At5g01230.1:d:+855:primary
AGTCCAAAAA	0	0	1	0	0	At5g01230.1:i:+559:tertiary,At5g01230.2:i:+608:tertiary
TTTGAACAAA	0	1	0	0	0	At5g01230.1:i:+1734:tertiary,At5g01230.2:i:+1783:tertiary
TTTTGGGACC	0	0	0	1	0	At5g01220.1:d:+1573:secondary

AATCCAACCA	1	0	0	0	0	At5g01210.1:X:+-60:quaternary
TGGTAGATAA	1	0	0	0	0	At5g01190.1:X:+375:quaternary
TTAGACCAAC	1	0	0	0	0	At5g01160.1:d:-1372:secondary
TTTTCCAGAC	0	0	0	0	1	At5g01130.1:v:+1906:primary
AGTCCAAAGT	0	0	0	1	0	At5g01100.1:d:+1963:primary
TATACTACTT	1	0	0	0	0	At5g01060.1:v:-1451:secondary
GATGCACATT	0	1	0	0	0	At5g01050.1:d:+1603:primary
GGGCTTGGCT	0	0	1	0	0	At5g01040.1:d:+1103:secondary,At1g54360.1:d:+1102:secondary,At2g38780.1:d:+530:secondary,At1g54360.2:d:+1171:secondary
CTGCGAGGCA	0	0	1	0	0	At5g01020.1:d:+631:secondary,At3g04710.1:d:+437:secondary
GGTTTCTTAA	1	0	0	0	0	At4g40100.1:v:+951:secondary
ACCCTTCAAC	0	0	1	0	0	At4g40080.1:d:+200:secondary
GCGGGTGAAG	0	1	0	0	0	At4g40050.1:d:+115:secondary
AATGTACATT	0	0	0	0	1	At4g40045.1:d:+703:primary
CTGTGCTTGC	0	0	1	0	0	At4g40040.1:d:+364:secondary
AATGGCACAA	0	0	1	0	0	At4g40030.1:X:-189:quaternary
CTGTCTTAGC	1	0	0	0	0	At4g40030.1:d:+356:secondary
AGCCTGTGCA	0	0	1	0	0	At4g40020.1:X:-419:quaternary
CACCTGAATC	0	0	0	1	0	At4g40000.1:X:-2:quaternary
ACCAAGCAAA	0	0	0	0	1	At4g40000.1:d:+2094:primary
ACATCCCTGC	0	0	1	0	0	At4g39970.1:X:-21:quaternary
TAATGTGTAA	1	0	0	0	0	At4g39955.1:d:+656:secondary,At4g08480.1:d:+2726:secondary,At1g15825.1:v:+781:secondary,At2g03050.1:d:+985:secondary
AACCGGAAAT	0	1	0	0	0	At4g39950.1:d:-664:secondary
TATATGGGTG	0	0	0	0	1	At4g39920.1:d:+1395:secondary
AAACCTCGTC	0	0	0	0	1	At4g39910.1:d:+606:secondary,At5g47030.1:d:+241:secondary
GATGGGAAGA	0	0	1	0	0	At4g39880.1:d:+257:secondary,At2g11010.1:v:+350:secondary
GGAGAGCCTG	0	1	0	0	0	At4g39860.2:d:+602:primary,At4g39860.1:d:+645:primary
TCAATAAATA	0	0	0	0	1	At4g39860.1:X:+-165:quaternary,Chr2:+6170527:quaternary,Chr3:+6531924:quaternary
ATCTATCTGT	0	1	0	0	0	At4g39850.1:i:+7502:tertiary
TTTGTGAGAA	0	0	1	0	0	At4g39850.1:i:+4117:tertiary
TTTAGGGACA	0	1	0	0	0	At4g39850.1:d:+3874:secondary
ATCACTGGTG	0	0	0	0	1	At4g39850.1:d:+1329:secondary
TAAAATTAAT	0	1	0	0	0	At4g39795.1:d:+531:primary
TGGAATCTTC	0	0	0	0	1	At4g39780.1:d:+820:secondary
TTGACATCAA	0	1	0	0	0	At4g39745.1:v:+762:secondary,At3g59120.1:v:+1096:secondary
TTAATGTGTG	0	0	0	1	0	At4g39745.1:v:+1200:secondary
CAATCTTCAA	0	1	0	0	0	At4g39720.1:d:+28:secondary
AAAAGGTAAA	0	0	0	0	1	At4g39680.1:X:-647:quaternary
GAAGCATCGC	0	0	0	1	0	At4g39670.1:d:+619:primary
AAGTCCTCCT	0	0	1	0	0	At4g39660.1:d:-1356:secondary
GGAACGTTTT	0	0	0	0	1	At4g39660.1:d:+1361:primary
ACTTGATGAT	0	0	1	0	0	At4g39650.1:v:+2400:secondary
CTAGGGTAAT	0	0	0	0	1	At4g39630.1:d:+923:primary
CTGAAGAAGA	0	1	0	0	0	At4g39620.1:v:+2121:secondary
AGAATTAACT	0	0	1	0	0	At4g39600.1:v:+1856:primary
GGTTTCATAT	0	0	0	0	1	At4g39580.1:v:+1133:secondary
GAATGATTTG	1	0	0	0	0	At4g39540.1:d:+269:secondary,At5g02680.1:v:+707:secondary,At5g16050.1:d:+907:secondary,At4g39540.2:d:+114:secondary
CAACTGCATC	0	0	1	0	0	At4g39520.1:d:+569:secondary
CTTAATCATC	0	0	0	0	1	At4g39510.1:d:+581:secondary
GTCTTAAAGT	0	0	0	1	0	At4g39480.1:d:+3003:primary,At2g23180.1:d:+1557:primary
TGTCGGGGTT	0	0	1	0	0	At4g39470.1:d:+1165:secondary
ACATATGTTT	0	0	0	0	1	At4g39470.1:d:+1123:secondary
GTGAATGGAT	0	0	0	0	1	At4g39450.1:d:+2524:secondary,At3g59890.1:d:+300:secondary,At3g59890.2:d:+332:secondary,At2g44040.1:d:+262:secondary
TGAAAAATGT	0	0	0	0	1	At4g39450.1:d:+2477:secondary
GGGATATCTT	0	0	0	1	0	At4g39420.1:v:+1576:secondary
TTCTCGTTAG	0	1	0	0	0	At4g39410.1:X:+323:quaternary
TAATGATTAA	0	1	0	0	0	At4g39410.1:i:+1167:tertiary,At1g11880.1:i:+1451:tertiary

TTTAAGGAGA	0	0	0	1	0	At4g39400.1:d:+3628:secondary
GCGAGGCTGA	0	0	1	0	0	At4g39400.1:d:+3256:secondary
AAATCCAGTA	0	0	1	0	0	At4g39400.1:d:+3196:secondary
GAAGGAGACC	0	0	1	0	0	At4g39400.1:d:+2522:secondary,At4g25420.1:d:+480:secondary
AAGACAATTC	0	1	0	0	0	At4g39390.2:d:+1022:primary,At4g39390.1:d:+1100:primary
TGTGTGCTCT	0	0	0	1	0	At4g39380.1:d:+1904:primary
GTCTACAAAC	0	0	1	0	0	At4g39350.1:d:-371:secondary
GTTATACTAA	1	0	0	0	0	At4g39350.1:d:+3588:secondary
TTTGTTAAGA	0	1	0	0	0	At4g39350.1:d:+2422:secondary
TTGCTGTTAA	0	0	1	0	0	At4g39330.1:d:+641:secondary,At3g12950.1:d:+859:secondary,At5g26800.1:v:+616:secondary,At2g12650.1:p:+780:secondary
AATGATCCGG	0	0	1	0	0	At4g39330.1:d:+1131:secondary
GAAGAGTTAG	0	0	0	0	1	At4g39270.1:d:+48:secondary,At4g39270.2:d:+48:secondary,At4g28220.1:d:+1264:secondary,At1g24460.1:v:+3612:secondary
AGGCTGCAGT	0	0	1	0	0	At4g39250.1:v:+740:secondary
GTTTGGGATC	0	1	0	0	0	At4g39240.1:d:+941:primary
TCGAATTCTC	1	0	0	0	0	At4g39220.1:i:+1028:tertiary
ATTTGCAATA	0	0	0	1	0	At4g39220.1:d:+924:primary
ATCAAGTACA	0	1	0	0	0	At4g39220.1:d:+637:secondary
AGACGCCAGA	0	1	0	0	0	At4g39220.1:d:+616:secondary
GCGGTATCCG	0	0	0	0	1	At4g39210.1:d:+1111:secondary
GCGCCGAAGA	0	0	0	1	0	At4g39200.1:d:+132:secondary
ATCCCAAGTG	0	1	0	0	0	At4g39190.1:v:+634:secondary
TGAAAGAGTT	0	1	0	0	0	At4g39180.1:d:+984:secondary,At1g68810.1:d:+822:secondary,At2g16380.1:d:+634:secondary
GCTTTTGTGA	0	1	0	0	0	At4g39170.1:d:+1667:primary
AAACGTTTAT	0	0	1	0	0	At4g39150.1:X:-81:quaternary
TAAAGTTACA	0	1	0	0	0	At4g39140.1:d:+1670:primary,At4g39140.4:d:+1606:primary,At4g39140.3:d:+1697:primary,At4g39140.2:d:+1693:primary
AGCCCTCCGG	1	0	0	0	0	At4g39080.1:d:+4060:primary
AATCCAAACC	0	1	0	0	0	At4g39070.1:d:+874:primary
TGGGTACTTG	0	0	0	0	1	At4g39050.1:d:+3073:secondary
TGTCGCTCAT	0	1	0	0	0	At4g39050.1:d:+1484:secondary
ACAGTATCCC	0	1	0	0	0	At4g39030.1:d:+1369:primary
TCTCCGGCTG	0	0	0	0	1	At4g38990.1:v:+657:secondary
GGTTCACTGG	0	0	0	1	0	At4g38980.1:X:+1213:quaternary
AGCACATACG	0	1	0	0	0	At4g38980.1:d:+628:primary
CCCTCCTTAG	0	0	0	1	0	At4g38970.1:X:-250:quaternary
TCCGTTTTTT	0	1	0	0	0	At4g38960.1:d:+857:secondary
TCTTGTATAT	0	0	0	0	1	At4g38950.1:d:+2567:primary
AGAATGATTG	1	0	0	0	0	At4g38950.1:d:+1243:secondary
CCACAAGGAA	0	1	0	0	0	At4g38920.1:d:-744:secondary
AGCTTTCCTC	0	0	0	1	0	At4g38910.1:i:+921:tertiary
ATCTAGATTG	0	1	0	0	0	At4g38870.1:v:+1747:primary
TTAATACATT	0	0	0	0	1	At4g38850.1:d:+488:primary
ATCAGTTGAA	0	0	1	0	0	At4g38840.1:X:-352:quaternary
GGTGATGAGC	0	0	0	1	0	At4g38810.1:d:+372:secondary,At4g38810.2:d:+763:secondary
GAGGACAAAG	0	0	0	0	1	At4g38790.1:d:+450:secondary
GTTCCTGAGA	1	0	0	0	0	At4g38780.1:d:+6814:secondary,At1g80070.1:d:+6958:secondary
AAGGTGTTTG	0	0	1	0	0	At4g38780.1:d:+3709:secondary
GGGGGTTTTG	1	0	0	0	0	At4g38770.1:X:-250:quaternary
ATACATCACA	0	0	0	0	1	At4g38750.1:i:+634:tertiary
TTATTCAAAA	0	0	0	1	0	At4g38750.1:i:+2843:tertiary
TTATTCTACA	0	1	0	0	0	At4g38750.1:d:+1955:secondary
AAGTTAGGGA	0	0	0	1	0	At4g38740.1:X:-312:quaternary
TGCCAGGGAG	0	0	1	0	0	At4g38740.1:d:+318:secondary
AAGCTGAGCT	0	1	0	0	0	At4g38710.1:d:+149:secondary
CTCAAACAGA	0	1	0	0	0	At4g38700.1:X:-62:quaternary
TCAATCTTAT	0	1	0	0	0	At4g38700.1:X:-539:quaternary
AGCAGATAGA	0	0	0	1	0	At4g38680.1:X:-1347:quaternary
GACTGGAAGG	1	0	0	0	0	At4g38680.1:X:+1382:quaternary
TGTAAATCTT	1	0	0	0	0	At4g38670.1:d:+1173:primary

GTTGCTGGTC	0	0	0	0	1	At4g38650.1:d:+470:secondary,At5g49720.1:d:+1768:secondary
TGAGGCTTCA	0	0	0	0	1	At4g38650.1:d:+1754:primary
GTTTCTTCGT	0	1	0	0	0	At4g38640.1:d:+589:secondary,At3g58610.1:d:+314:secondary
TTCCTTCTGG	0	0	1	0	0	At4g38630.1:d:+602:secondary
AAAGTATAAG	0	1	0	0	0	At4g38620.1:d:+812:secondary
GACTTTATCA	0	0	0	1	0	At4g38600.2:X:-41:quaternary
TTGGCAGCAG	0	0	0	1	0	At4g38600.2:X:-25:quaternary
GCTGCTTGCC	0	0	0	1	0	At4g38590.1:d:-93:primary
GTGCGTTGAT	1	0	0	0	0	At4g38540.1:X:-6:quaternary
ACTTGTCCCA	0	0	1	0	0	At4g38520.2:X:-210:quaternary
AACTCTATCG	0	1	0	0	0	At4g38510.1:d:+509:secondary,At4g38510.2:d:+539:secondary
AAAATTTGAA	0	1	0	0	0	At4g38500.1:i:+811:tertiary
TAGGAATAAC	1	0	0	0	0	At4g38480.1:d:+796:secondary
AGATAACCTT	1	0	0	0	0	At4g38470.1:d:+533:secondary
GCTCCGAATT	0	1	0	0	0	At4g38460.1:d:-732:secondary
GGTGAATGCT	1	0	0	0	0	At4g38460.1:d:+737:secondary
TATAATCACG	0	0	0	1	0	At4g38440.1:i:-2038:tertiary
CGACGATCAC	0	1	0	0	0	At4g38400.1:d:+848:primary
TAAATGAAGA	1	0	0	0	0	At4g38390.1:d:+1822:primary
GCTGCTCTTG	0	0	0	0	1	At4g38390.1:d:+1497:secondary,At2g48020.1:d:+94:secondary,At5g62370.1:v:+3117:secondary,At3g07300.1:d:+1027:secondary,At4g03330.1:d:+889:secondary
TGTGATCCTT	0	0	1	0	0	At4g38320.1:d:+913:secondary,At4g37680.1:d:+948:secondary
TGTTGCTGCC	0	0	1	0	0	At4g38290.1:d:-13:primary
TTCTGGTAGT	0	1	0	0	0	At4g38290.1:d:+248:primary,At2g40710.1:d:+203:primary
TGGTGGATGC	0	0	1	0	0	At4g38280.1:d:+611:primary
GAAGGAACAC	0	0	1	0	0	At4g38260.1:d:+842:secondary
TACAACATAT	0	0	1	0	0	At4g38250.1:d:+1601:secondary
CGGTCCAAAA	0	0	0	1	0	At4g38240.2:X:-227:quaternary
CGAGCTAAAA	0	0	1	0	0	At4g38240.2:i:+2123:tertiary,At4g38240.1:i:+2094:tertiary
ACCACAATGA	1	0	0	0	0	At4g38220.1:d:+1249:primary,At4g38220.2:d:+1258:primary
TATGGGTGCA	0	0	0	1	0	At4g38200.1:d:+5073:primary
AGCAATATGT	0	0	0	1	0	At4g38170.1:d:+1909:primary
GATTCTACAC	1	0	0	0	0	At4g38160.2:i:+1306:tertiary
CATTCTAATA	0	1	0	0	0	At4g38150.1:d:+1138:secondary
CAACGAATAA	1	0	0	0	0	At4g38130.1:X:-235:quaternary,At3g10700.1:X:-393:quaternary
TGAATGTATC	0	0	0	1	0	At4g38130.1:X:--206:quaternary
GAAAAAGAGT	0	0	1	0	0	At4g38050.1:d:+2192:primary
CAGATGGACC	0	0	1	0	0	At4g38050.1:d:+2035:secondary
TACGGAATCT	0	0	1	0	0	At4g38030.1:v:+771:secondary,At2g36990.1:d:+1550:secondary
AGTATATTTT	0	1	0	0	0	At4g38010.1:v:+1339:secondary
TTTCTCCAAG	0	0	0	1	0	At4g38000.1:d:+608:secondary
CCCAGGGACA	0	1	0	0	0	At4g37980.1:d:-282:primary
AAGTATCACG	0	0	1	0	0	At4g37980.1:d:+595:secondary
AAATATTAAA	0	0	0	1	0	At4g37970.1:i:+611:tertiary
TGATGAGAAA	0	0	0	1	0	At4g37950.1:i:+1129:tertiary
TACTCGGTGG	1	0	0	0	0	At4g37950.1:d:+903:secondary
GCAGAAACCT	0	0	1	0	0	At4g37930.1:d:+484:secondary
GAATGTATAG	0	1	0	0	0	At4g37930.1:d:+1725:secondary,At5g63160.1:d:+727:secondary
AAGTCGAGGA	0	0	1	0	0	At4g37930.1:d:+1607:secondary
TAGGGTCCAG	0	0	0	0	1	At4g37920.1:v:+2551:primary
ATGATACAGA	1	0	0	0	0	At4g37900.1:v:+1102:secondary
GAGAGAAACT	0	0	1	0	0	At4g37890.1:d:+707:secondary,At4g37890.2:d:+634:secondary
GCCGGCGGAA	0	1	0	0	0	At4g37830.1:X:-166:quaternary
TATTGTATGC	0	1	0	0	0	At4g37820.1:d:+1934:secondary,At1g01150.1:v:+611:secondary
AGCTGGAAAT	1	0	0	0	0	At4g37810.1:d:+346:primary
TCTGTGATGC	0	0	0	1	0	At4g37750.1:d:+518:secondary
ACTGAACAAA	0	1	0	0	0	At4g37730.1:d:+1023:primary
ACCTTCCTGG	0	0	1	0	0	At4g37700.1:d:+153:secondary,At3g15730.1:d:+2376:secondary
ATTAATGGAA	0	0	0	0	1	At4g37690.1:X:+1750:quaternary
CAGCACTTGC	0	0	0	1	0	At4g37685.1:v:-739:secondary

AAGAAGCTAA	0	0	1	0	0	At4g37670.2:d:+1795:primary,At4g37670.1:d:+1878:primary
GCCTTATCTT	1	0	0	0	0	At4g37670.1:d:+276:secondary,At4g37670.2:d:+276:secondary
AAACTTGGAT	0	0	1	0	0	At4g37590.1:d:+517:secondary,At5g66990.1:v:+1329:secondary,At1g04940.1:d:+467:secondary
ACTCGGGTGG	0	1	0	0	0	At4g37580.1:d:-1074:secondary
TGGCCAAAAA	0	0	1	0	0	At4g37580.1:d:+554:secondary
CTGGTGGCTG	0	0	1	0	0	At4g37580.1:d:+480:secondary,At2g23060.1:d:+260:secondary
GCAGCAGACT	0	1	0	0	0	At4g37560.1:d:+138:secondary
GAACTCTGCA	0	0	0	1	0	At4g37510.1:X:+-36:quaternary,At4g37510.1:X:+-48:quaternary
GATCGATGAG	0	0	1	0	0	At4g37510.1:d:+501:secondary
ATCGGTATGA	0	0	0	0	1	At4g37510.1:d:+1550:secondary
TAAGAAAGTT	0	1	0	0	0	At4g37490.1:i:+1905:tertiary
GACTGTGCAA	0	1	0	0	0	At4g37490.1:d:+1200:secondary,At3g17370.1:v:+418:secondary
TCGCCACCGA	0	1	0	0	0	At4g37470.1:X:-125:quaternary
ACATATTACT	0	0	1	0	0	At4g37460.1:d:+3271:secondary
GATTAGGCGA	0	1	0	0	0	At4g37460.1:d:+1780:secondary
AGGTGGAGAT	0	0	1	0	0	At4g37430.1:d:-260:primary
TATAGCGGCG	0	0	1	0	0	At4g37430.1:d:+787:secondary
GAGATTTTGT	0	0	0	1	0	At4g37380.1:v:+1678:secondary
TGCAAAGCAC	0	0	1	0	0	At4g37340.1:d:+1475:primary
AACACGTGGA	0	0	0	0	1	At4g37320.1:X:-661:quaternary
ATGGTAGCCG	0	0	1	0	0	At4g37320.1:d:+571:secondary
ATTGCTCATT	0	0	0	1	0	At4g37310.1:d:+886:secondary
TTCGGTTATC	0	0	0	0	1	At4g37300.1:i:+274:tertiary
GACCTTTAAT	1	0	0	0	0	At4g37270.1:d:+3935:primary
AGAGGTGGTG	0	1	0	0	0	At4g37240.1:d:+389:primary
GTATCAGCTA	0	0	1	0	0	At4g37210.2:d:+1298:secondary,At4g37210.1:d:+1306:secondary
AGGAAGATAC	0	0	0	1	0	At4g37210.1:d:-1678:secondary,At4g37210.2:d:-1670:secondary
GGAATAAGTT	0	0	1	0	0	At4g37150.1:d:+858:primary
AATAACAAGG	1	0	0	0	0	At4g37120.1:d:-1726:secondary
TGGTAGATCG	1	0	0	0	0	At4g37120.1:d:+499:secondary
TCATTATTTG	0	0	0	0	1	At4g37120.1:d:+1731:secondary
TCTTTAAATA	0	0	0	1	0	At4g37120.1:d:+1703:secondary
GATCTCTCTC	0	0	1	0	0	At4g37110.1:d:+231:secondary
TAAGAACTTG	0	0	0	1	0	At4g37110.1:d:+1451:primary
TACGTACTCT	0	0	1	0	0	At4g37110.1:d:+1374:secondary
ATGTTGGAAA	0	0	0	0	1	At4g37060.1:v:+1943:primary
TGATTAGGGT	0	0	0	1	0	At4g37020.1:X:-418:quaternary,At4g36490.1:X:-366:quaternary
TTCTTCCCAC	1	0	0	0	0	At4g37020.1:i:+724:tertiary
AACTTGAGGG	0	0	1	0	0	At4g37010.1:i:-946:tertiary
GTTGATCTCA	0	1	0	0	0	At4g37000.1:d:+214:secondary,At4g16040.1:v:+201:secondary,At3g01670.1:d:+997:secondary,At5g66980.1:v:+354:secondary
GAACGGAAGC	0	0	1	0	0	At4g36960.1:d:+250:secondary
TGGTTGTGAC	0	1	0	0	0	At4g36950.1:p:+3465:primary
AGACCTTCTT	0	0	1	0	0	At4g36920.1:d:+1439:secondary,At2g04620.1:v:+3047:secondary
GAATTTTCTG	0	0	0	1	0	At4g36910.1:X:-21:quaternary,At2g29160.1:X:-806:quaternary,At5g48120.1:X:-740:quaternary
ATTACGTAGA	1	0	0	0	0	At4g36900.1:d:+647:secondary
CGGCTTAAAC	0	1	0	0	0	At4g36880.1:X:-670:quaternary
AATCAAATAC	1	0	0	0	0	At4g36870.1:i:+3106:tertiary
AGCGTGATGG	0	1	0	0	0	At4g36860.2:d:+1036:secondary,At3g26780.1:d:+815:secondary,At4g36860.1:d:+1062:secondary,At5g65580.1:v:+504:secondary
TCTATTAACT	0	0	1	0	0	At4g36860.1:X:-43:quaternary
CTTCAAGCAG	0	0	0	0	1	At4g36850.1:d:+791:secondary
CGTGAGATGG	0	0	1	0	0	At4g36850.1:d:+108:secondary
AACTTTGAAT	0	1	0	0	0	At4g36770.1:d:+1526:primary
GCTGCAATAG	0	1	0	0	0	At4g36750.1:d:+862:secondary
TGAAGTTTGC	1	0	0	0	0	At4g36720.1:v:+643:secondary
GAAGAGCACT	0	1	0	0	0	At4g36710.1:d:+1726:primary
AAAGAGAGAC	0	0	0	1	0	At4g36690.2:d:+246:secondary,At4g36690.1:d:+246:secondary,At4g36690.3:d:+246:secondary
AAAACGACGT	0	0	1	0	0	At4g36670.1:d:+1077:secondary,At5g08440.1:d:+445:secondary
TCAACTTGAA	0	0	1	0	0	At4g36650.1:v:+1190:secondary,At5g50050.1:d:+302:secondary
TTCAGAAGAA	0	0	1	0	0	At4g36640.1:d:+139:secondary

TAAATGTTTT	0	0	0	0	1	At4g36630.1:i:+4059:tertiary,At4g36630.2:i:+3966:tertiary,At4g23060.1:i:+1667:tertiary
ACTTTTAAAA	0	0	1	0	0	At4g36620.1:v:+1193:secondary
TATAATCAAA	0	0	0	0	1	At4g36580.1:v:-2233:secondary
GACTTGTCTG	0	0	0	0	1	At4g36540.1:d:+120:secondary,At4g36540.2:d:+120:secondary
ACTGAAACTC	0	0	0	1	0	At4g36530.2:X:-69:quaternary
AGGACAATGC	0	0	0	1	0	At4g36530.2:X:-186:quaternary
AAAGAGGTAG	0	0	0	1	0	At4g36530.1:d:+523:secondary,At4g36530.2:d:+643:secondary
GGCAGGCAAT	0	1	0	0	0	At4g36520.1:v:+1709:secondary
ATCATCGTTT	0	0	0	1	0	At4g36515.1:i:+548:tertiary
ATTTTCCTTT	0	1	0	0	0	At4g36510.1:v:+567:secondary,At5g35940.1:v:+1770:secondary
CATTAGCCAC	1	0	0	0	0	At4g36480.1:d:+1206:secondary
ATCGCGAGAG	0	0	0	1	0	At4g36410.1:X:+-191:quaternary
TATTAGTAGA	0	0	0	1	0	At4g36400.2:d:+205:secondary,At4g36400.1:d:+206:secondary
TTTGTATTGA	0	1	0	0	0	At4g36290.1:i:+482:tertiary
TGCATATTTG	0	0	0	1	0	At4g36280.1:d:+2107:primary
GAGGGTATCG	1	0	0	0	0	At4g36250.1:d:-1454:secondary
AATGAAGAGA	0	0	1	0	0	At4g36250.1:d:+1121:secondary,At5g49030.1:d:+1990:secondary
ATCATTAAAA	1	0	0	0	0	At4g36240.1:d:+890:primary
CTTTTTCACC	1	0	0	0	0	At4g36230.1:X:+696:quaternary,Chr5:+20431345:quaternary
TCGAGCTCAC	0	0	1	0	0	At4g36220.1:X:-535:quaternary
TCCGCTCGGT	0	0	0	1	0	At4g36220.1:X:-380:quaternary
GTGATATCAA	0	1	0	0	0	At4g36180.1:d:+3037:secondary,At4g29380.1:d:+443:secondary
TTGATGCACT	0	1	0	0	0	At4g36160.1:X:--241:quaternary
ATACAACAAA	0	0	0	0	1	At4g36160.1:i:+816:tertiary,At3g54960.1:i:+2231:tertiary
GATCCTAATG	0	0	1	0	0	At4g36150.1:v:+4021:secondary
GCATATCTGA	0	1	0	0	0	At4g36140.1:v:+4812:secondary,At4g29750.1:v:+2732:secondary
GTTGATTTCG	0	0	1	0	0	At4g36140.1:v:+3900:secondary
GAGAAAAGAA	0	0	1	0	0	At4g36120.1:v:+3633:secondary
CGTTTTGAGT	0	1	0	0	0	At4g36120.1:v:+3379:secondary
AGGAGACTCT	0	0	0	1	0	At4g36120.1:v:+2887:secondary
AATGGGAATG	0	0	0	1	0	At4g36120.1:v:+2268:secondary
GAGTTGATGT	0	0	0	1	0	At4g36090.1:d:+600:secondary
GAGGAGGAAG	1	0	0	0	0	At4g36090.1:d:+1623:primary
ATGGTGGTAA	0	1	0	0	0	At4g36080.1:d:+9743:secondary
TTTATTCATA	0	1	0	0	0	At4g36060.2:d:+215:secondary
CCGCTTACTG	0	0	1	0	0	At4g36040.1:d:+425:secondary
GAAGTCAAGA	0	1	0	0	0	At4g35950.1:d:+955:primary
GTAATGATAC	0	0	0	0	1	At4g35920.3:X:-461:quaternary
AACTGCGACG	0	0	1	0	0	At4g35920.1:X:-39:quaternary
CTTATCAAGG	1	0	0	0	0	At4g35910.1:d:+1599:secondary
AATATATTAT	0	0	0	0	1	At4g35890.1:d:+1906:primary
ATACACAAAA	0	0	0	1	0	At4g35870.1:d:-2357:secondary
AGATTCTGTG	0	0	0	1	0	At4g35870.1:d:+2362:primary
AAGAATTGCA	0	0	0	1	0	At4g35860.1:i:+1271:tertiary
AATCGATCTT	0	1	0	0	0	At4g35850.1:i:+207:tertiary
GATCAAAACT	0	1	0	0	0	At4g35830.1:d:+1518:secondary
TGGGGTATAC	0	0	1	0	0	At4g35800.1:X:-99:quaternary
AAGTTGTCAA	0	1	0	0	0	At4g35780.1:d:+1326:secondary
CCTACACACG	0	1	0	0	0	At4g35770.1:X:+261:quaternary,Chr5:+12467449:quaternary
GATCATCATC	0	0	0	0	1	At4g35730.1:d:+823:secondary,At3g11570.1:d:+218:secondary,At5g06230.1:d:+54:secondary
AAGGAAATTG	0	0	0	1	0	At4g35730.1:d:+451:secondary,At1g13880.1:d:+937:secondary,At5g06400.1:v:+2991:secondary
AGGAGGATGG	0	0	1	0	0	At4g35680.1:d:+1670:primary
GTGAGTCTTG	0	1	0	0	0	At4g35640.1:i:+2314:tertiary
AAAGATTTCC	0	1	0	0	0	At4g35630.1:d:+1413:secondary
TGTGGTCGGT	0	0	1	0	0	At4g35600.1:d:-1281:secondary
TTGCTGAACT	0	0	0	0	1	At4g35590.1:v:+1066:secondary
CTAACATTGC	1	0	0	0	0	At4g35580.1:X:-192:quaternary
AATGAAGAAA	0	0	1	0	0	At4g35580.1:i:+1912:tertiary
AGACACAAAG	0	0	0	0	1	At4g35580.1:d:+1569:secondary
TAGAAGTTGG	0	0	0	1	0	At4g35560.1:i:+3028:tertiary
CAAGTCTAAC	0	0	0	0	1	At4g35540.1:d:-1661:secondary

ATGTATCTAG	0	1	0	0	0	At4g35530.1:d:+947:primary
AGCCTAAACT	1	0	0	0	0	At4g35520.1:d:+1565:secondary
ATTAAAAGTA	0	0	1	0	0	At4g35480.1:d:+766:primary
TCTCGTGACG	0	0	0	1	0	At4g35470.1:d:-2417:secondary
TTTGTCTATC	1	0	0	0	0	At4g35470.1:d:+2181:secondary
GAGATTGTCT	0	0	1	0	0	At4g35470.1:d:+1869:secondary
GTTGAAATAT	1	0	0	0	0	At4g35460.1:X:+584:quaternary
GCAGCTTTGG	0	0	1	0	0	At4g35460.1:d:+1096:secondary,At4g06537.1:p:+745:secondary
AGCGTTTGCC	0	0	0	0	1	At4g35450.1:d:+119:secondary
GTCGGTTTGA	0	0	0	1	0	At4g35390.1:d:+720:primary
AGGTTTTCGT	0	0	0	1	0	At4g35380.1:d:+925:secondary
AAAGTCTTGA	0	0	0	1	0	At4g35380.1:d:+4366:secondary,At1g61390.1:v:+292:secondary
GTCATCTTTT	0	0	0	1	0	At4g35380.1:d:+3950:secondary
TAAATCCGTT	0	1	0	0	0	At4g35380.1:d:+120:secondary
GCTTGATTGT	0	1	0	0	0	At4g35370.1:v:+857:secondary,At4g18905.1:d:+733:secondary
GATCCATACC	0	1	0	0	0	At4g35370.1:v:+654:secondary
GATCCCAAAA	1	0	0	0	0	At4g35370.1:v:+374:secondary
AGTTTTATAT	1	0	0	0	0	At4g35335.1:i:+783:tertiary
GCTCTTTTAA	0	0	1	0	0	At4g35335.1:d:+637:secondary,At2g03800.1:d:+546:secondary
CAAATTATAT	0	0	0	0	1	At4g35320.1:d:+739:primary
AATATTACAC	1	0	0	0	0	At4g35295.1:v:+853:secondary
ATGGAGAAAG	0	0	0	0	1	At4g35290.2:d:+984:secondary,At5g20320.1:d:+2783:secondary,At4g35290.1:d:+881:secondary
GTATGAAGTC	0	1	0	0	0	At4g35270.1:X:-444:quaternary
TTGCAGTTTG	1	0	0	0	0	At4g35270.1:i:+2282:tertiary
ATAAAGGCAA	1	0	0	0	0	At4g35270.1:d:+1696:secondary,At2g17150.1:d:+1800:secondary
CGGTATTCGA	0	1	0	0	0	At4g35260.1:d:+943:secondary
TAAGCAACAC	0	0	0	1	0	At4g35250.1:X:-79:quaternary
ATTTCAGCTG	0	0	1	0	0	At4g35250.1:X:--38:quaternary
GACACACAGG	0	0	0	0	1	At4g35250.1:d:+861:secondary
TATTGCATAC	1	0	0	0	0	At4g35230.1:X:-429:quaternary
GATTATCTAC	0	0	0	1	0	At4g35210.1:v:-122:secondary
AAAGAAAAGC	1	0	0	0	0	At4g35190.1:d:+412:secondary
TGGATCTCTA	0	0	0	0	1	At4g35180.1:d:+1263:secondary
AAGATGAAGA	1	0	0	0	0	At4g35170.1:v:+616:primary
GCTATTACCA	0	1	0	0	0	At4g35140.1:X:-212:quaternary
TCATCATCAG	0	0	1	0	0	At4g35140.1:X:--194:quaternary
GAACAGCATC	1	0	0	0	0	At4g35130.1:v:+1850:secondary
ATGGTTTAGT	0	1	0	0	0	At4g35110.1:d:+1121:primary,At4g35110.2:d:+1046:primary
GTGGTTGTTG	0	0	0	1	0	At4g35090.1:X:-737:quaternary
GTCCACGCTC	1	0	0	0	0	At4g35090.1:d:+535:secondary
CTGAGAAGTA	0	0	0	0	1	At4g35090.1:d:+1280:secondary
GTATCAACAG	0	0	0	1	0	At4g35080.2:d:+798:secondary,At4g35080.1:d:+815:secondary
GTTGGGATCC	0	0	0	0	1	At4g35050.1:X:-33:quaternary
ACAGAGAATG	1	0	0	0	0	At4g35030.1:i:+488:tertiary
AACGAAATTT	0	0	1	0	0	At4g35030.1:d:-543:secondary
GGATTCAGGT	0	0	1	0	0	At4g34910.1:d:+1068:secondary,At3g03530.1:d:+318:secondary
GTTCTCAATG	0	0	1	0	0	At4g34900.1:v:+718:secondary
AGAGCTCTTC	0	0	1	0	0	At4g34860.1:d:+2531:primary
TTGGTATGGT	1	0	0	0	0	At4g34860.1:d:+2048:secondary
TTGCGGTTTA	0	1	0	0	0	At4g34800.1:v:+427:secondary
ACTTTGAAAT	0	0	0	0	1	At4g34800.1:v:+210:secondary
TTGGATTGGC	0	0	1	0	0	At4g34750.1:d:+383:primary
TCAGTCATAT	0	0	0	1	0	At4g34730.1:i:+3787:tertiary
TTGAACGTTT	0	0	1	0	0	At4g34720.1:d:+1008:primary
GTTGGGAGAA	0	0	0	1	0	At4g34710.1:d:+162:secondary,At4g34710.2:d:+124:secondary
GTGAAGCAGA	0	0	0	1	0	At4g34700.1:d:+241:secondary
AGTTTAAGAA	0	0	0	1	0	At4g34690.1:v:+847:secondary
ATAGAACTTT	1	0	0	0	0	At4g34660.1:i:+1647:tertiary
GGTTACTTCT	0	0	0	0	1	At4g34660.1:d:+1054:secondary
TGGTACAAAA	0	1	0	0	0	At4g34650.1:v:+680:secondary,At4g30890.1:d:+506:secondary,At4g30890.2:d:+731:secondary,At5g15100.1:v:+1190:secondary

CTTTCCACTG	0	0	1	0	0	At4g34620.1:X:-44:quaternary
TGGCTGATAC	0	0	0	0	1	At4g34610.1:v:+1720:secondary
CAGAATGTAA	0	1	0	0	0	At4g34600.1:d:+478:primary
CATATCACCG	0	1	0	0	0	At4g34600.1:d:+313:secondary
TCCAAAGAGA	1	0	0	0	0	At4g34590.1:d:+1141:secondary
GCCTTTGTGA	0	0	0	1	0	At4g34580.1:v:+1608:secondary
TAGCACTATC	1	0	0	0	0	At4g34540.1:d:+2668:secondary
AATACAGAAA	0	0	0	1	0	At4g34520.1:d:-1170:secondary
AACAACTCTA	1	0	0	0	0	At4g34510.1:d:+313:secondary
GATGAAACTT	0	0	1	0	0	At4g34500.1:i:+1634:tertiary
GAGATCATTA	0	1	0	0	0	At4g34500.1:d:+1183:secondary
ACTATCTTCG	0	0	0	0	1	At4g34490.1:X:--64:quaternary
TAAACGCAAA	0	0	0	0	1	At4g34480.1:d:+1115:primary
GATGATATAT	1	0	0	0	0	At4g34460.2:i:+1308:tertiary,At4g34460.1:i:+1362:tertiary,At4g34460.3:i:+1362:tertiary
[truncated: 877,715 more chars]
