# Supplementary material for: Differential SAGE analysis in Arabidopsis uncovers increased transcriptome complexity in response to low temperature
Source: BMC Genomics. 2008 Sep 22;9:434. doi: 10.1186/1471-2164-9-434 (PMC2568001; doi:10.1186/1471-2164-9-434)
Supplement: Additional file 3 — SAGE data represented by AGI for those genes whose expression is differentially regulated by low temperature (p < 0.01). [file 1471-2164-9-434-S3.pdf]

Additional file 3: SAGE data represented by AGI for those genes whose expression is differentially regulated by low temperature (p<0.01). The tag counts were normalised to 50,000 per library to facilitate comparative expression analysis.

```
LOCUS: AT2G34420
DESCRIPTION:  chlorophyll A-B binding protein / LHCII type I (LHB1B2)
DATA:                        Control 30min  2hours  2days  1week  p-value     B&H         Pos    Fold change relative to control (log2)
SENSE COUNTS:                339     1023   247     317    35     4.50e-249   4.14e-246          0.000  1.593  -0.457  -0.097  -3.276
  GENES:
  AT2G34420.2
    SENSE COUNTS:            3       0      26      2      1      7.72e-12    5.90e-11           0.000  0.000  3.115   -0.585  -1.585
    TAGS:
      d+2     AGGAAGACTG     1       0      5       1      0      8.03e-02    1.29e-01    155    0.000  0.000  2.322   0.000   0.000
      d+2     GCTTTGTCCT     2       0      21      1      1      1.79e-10    2.21e-09    74     0.000  0.000  3.392   -1.000  -1.000
  AT2G34420.1
    SENSE COUNTS:            339     1023   247     317    35     4.50e-249   4.81e-246          0.000  1.593  -0.457  -0.097  -3.276
    TAGS:
      d+1     GGAGCTGTTG     336     1023   221     315    34     9.00e-259   1.51e-255   557    0.000  1.606  -0.604  -0.093  -3.305
      d+2     AGGAAGACTG     1       0      5       1      0      8.03e-02    1.29e-01    155    0.000  0.000  2.322   0.000   0.000
      d+2     GCTTTGTCCT     2       0      21      1      1      1.79e-10    2.21e-09    74     0.000  0.000  3.392   -1.000  -1.000

LOCUS: AT4G12470
DESCRIPTION:  protease inhibitor/seed storage/lipid transfer protein (LTP) family protein, similar to pEARLI 1 (Accession No. L43080)
DATA:                        Control 30min  2hours  2days  1week  p-value     B&H         Pos    Fold change relative to control (log2)
SENSE COUNTS:                30      82     12      77     580    2.61e-244   1.20e-241          0.000  1.451  -1.322  1.360   4.273
  GENES:
  AT4G12470.1
    SENSE COUNTS:            30      82     12      77     580    2.61e-244   1.40e-241          0.000  1.451  -1.322  1.360   4.273
    TAGS:
      d+1     TAAGGTATAG     30      82     12      77     580    2.61e-244   2.19e-241   618    0.000  1.451  -1.322  1.360   4.273

LOCUS: AT2G42540
DESCRIPTION:  cold-responsive protein / cold-regulated protein (cor15a), identical to cold-regulated protein cor15a (Arabidopsis thaliana) GI
DATA:                        Control 30min  2hours  2days  1week  p-value     B&H         Pos    Fold change relative to control (log2)
SENSE COUNTS:                2       0      1       19     274    1.22e-185   3.74e-183          0.000  0.000  -1.000  3.248   7.098
  GENES:
  AT2G42540.2
    SENSE COUNTS:            2       0      1       19     274    1.22e-185   3.26e-183          0.000  0.000  -1.000  3.248   7.098
    TAGS:
      d+1     TTTAATAGTA     2       0      1       19     274    1.22e-185   6.83e-183   577    0.000  0.000  -1.000  3.248   7.098
  AT2G42540.1
    SENSE COUNTS:            2       0      1       19     274    1.22e-185   4.35e-183          0.000  0.000  -1.000  3.248   7.098
    TAGS:
      d+1     TTTAATAGTA     2       0      1       19     274    1.22e-185   6.83e-183   512    0.000  0.000  -1.000  3.248   7.098

LOCUS: AT3G22840
DESCRIPTION:  chlorophyll A-B binding family protein / early light-induced protein (ELIP), identical to early light-induced protein; ELIP (Arabidopsis
thaliana) GI
DATA:                        Control 30min  2hours  2days  1week  p-value     B&H         Pos    Fold change relative to control (log2)
SENSE COUNTS:                10      0      0       109    318    1.66e-164   3.82e-162          0.000  0.000  0.000   3.446   4.991
  GENES:
  AT3G22840.1
    SENSE COUNTS:            10      0      0       109    318    1.66e-164   3.55e-162          0.000  0.000  0.000   3.446   4.991
    TAGS:
      d+1     TGTACTAAGT     10      0      0       105    301    3.04e-154   1.02e-151   807    0.000  0.000  0.000   3.392   4.912
      d+2     TAATTTCTCT     0       0      0       4      10     2.17e-05    1.08e-04    755    0.000  0.000  0.000   2.000   3.322
      d+2     ACGTCAGACG     0       0      0       0      7      3.14e-05    1.48e-04    608    0.000  0.000  0.000   0.000   2.807

LOCUS: AT4G12480
DESCRIPTION:  protease inhibitor/seed storage/lipid transfer protein (LTP) family protein, identical to pEARLI 1 (Accession No. L43080)
```

```
DATA:                         Control 30min  2hours  2days  1week  p-value     B&H         Pos    Fold change relative to control (log2)
SENSE COUNTS:                 8       16     6       12     276    1.41e-156   2.59e-154          0.000  1.000  -0.415  0.585  5.109
  GENES:
  AT4G12480.1
    SENSE COUNTS:             8       16     6       12     276    1.41e-156   2.51e-154          0.000  1.000  -0.415  0.585  5.109
    TAGS:
      d+1      TAAGCTATAG     8       16     6       12     276    1.41e-156   5.92e-154   648    0.000  1.000  -0.415  0.585  5.109

LOCUS: AT5G13930
DESCRIPTION:  chalcone synthase / naringenin-chalcone synthase, identical to SP|P13114
DATA:                         Control 30min  2hours  2days  1week  p-value     B&H         Pos    Fold change relative to control (log2)
SENSE COUNTS:                 6       4      7       256    84     6.82e-130   1.05e-127          0.000  -0.585 0.222   5.415  3.807
  GENES:
  AT5G13930.1
    SENSE COUNTS:             6       4      7       256    84     6.82e-130   1.04e-127          0.000  -0.585 0.222   5.415  3.807
    TAGS:
      d+1      TCGAGCGCGT     6       4      6       255    82     1.56e-131   4.37e-129   1102   0.000  -0.585 0.000   5.409  3.773
      d+2      ATGTACCAGC     0       0      0       0      1      1.65e-01    2.35e-01    562    0.000  0.000  0.000   0.000  0.000
      d+2      TCGTCTTCTG     0       0      1       0      0      4.55e-01    5.09e-01    467    0.000  0.000  0.000   0.000  0.000
      d+2      GCTCCTTCTC     0       0      0       0      1      4.65e-01    5.17e-01    349    0.000  0.000  0.000   0.000  0.000
      d+2      TGCGACAAGT     0       0      0       1      0      3.09e-01    4.06e-01    265    0.000  0.000  0.000   0.000  0.000

LOCUS: ATCG00480
DESCRIPTION:  chloroplast-encoded gene for beta subunit of ATP synthase
DATA:                         Control 30min  2hours  2days  1week  p-value     B&H         Pos    Fold change relative to control (log2)
SENSE COUNTS:                 51      362    78      49     19     4.97e-126   6.53e-124          0.000  2.827  0.613   -0.058 -1.424
  GENES:
  ATCG00480.1
    SENSE COUNTS:             51      362    78      49     19     4.97e-126   6.65e-124          0.000  2.827  0.613   -0.058 -1.424
    TAGS:
      d+1      GAAATGAAAG     51      362    78      49     19     4.97e-126   1.19e-123   643    0.000  2.827  0.613   -0.058 -1.424

LOCUS: AT3G50450
DESCRIPTION:  hypersensitive response protein 1 (HR1), identical to HR1 (Arabidopsis thaliana) GI
DATA:                         Control 30min  2hours  2days  1week  p-value     B&H         Pos    Fold change relative to control (log2)
SENSE COUNTS:                 55      244    24      48     41     3.48e-75    4.00e-73           0.000  2.149  -1.196  -0.196 -0.424
  GENES:
  AT3G50450.1
    SENSE COUNTS:             55      244    24      48     41     3.48e-75    4.14e-73           0.000  2.149  -1.196  -0.196 -0.424
    TAGS:
      v+2      ATATTTCTTT     55      244    24      48     41     3.48e-75    6.50e-73    37     0.000  2.149  -1.196  -0.196 -0.424

LOCUS: AT3G16770
DESCRIPTION:  encodes a member of the ERF (ethylene response factor) subfamily B-2 of ERF/AP2 transcription factor family (RAP2.3). The protein
contains one AP2 domain. There are 5 members in this subfamily including RAP2.2 AND RAP2.12.
DATA:                         Control 30min  2hours  2days  1week  p-value     B&H         Pos    Fold change relative to control (log2)
SENSE COUNTS:                 27      178    15      15     10     3.02e-74    3.09e-72           0.000  2.721  -0.848  -0.848 -1.433
  GENES:
  AT3G16770.1
    SENSE COUNTS:             27      178    15      15     10     3.02e-74    3.23e-72           0.000  2.721  -0.848  -0.848 -1.433
    TAGS:
      d+1      TGTAAATAAG     9       11     2       0      7      1.36e-02    2.41e-02    1011   0.000  0.290  -2.170  0.000  -0.363
      d+2      GCTTATGATG     18      167    13      15     3      6.54e-76    1.37e-73    418    0.000  3.214  -0.469  -0.263 -2.585

LOCUS: AT1G30380
DESCRIPTION:  photosystem I reaction center subunit psaK, chloroplast, putative / photosystem I subunit X, putative / PSI-K, putative (PSAK),
identical to SP|Q9SUI5; strong similarity to SP|P36886 Photosystem I reaction center subunit psaK, chloroplast precursor (Photo
DATA:                         Control 30min  2hours  2days  1week  p-value     B&H         Pos    Fold change relative to control (log2)
SENSE COUNTS:                 413     123    398     327    49     8.89e-69    8.18e-67           0.000  -1.747 -0.053  -0.337 -3.075
  GENES:
```

AT1G30380.1														
SENSE COUNTS:		413	123	398	327	49	8.89e-69	8.65e-67		0.000	-1.747	-0.053	-0.337	-3.075
TAGS:														
d+1	GAAGAAGCGG	27	4	18	12	3	3.35e-05	1.56e-04	622	0.000	-2.755	-0.585	-1.170	-3.170
d+2	CACCTGAACG	384	118	353	315	46	6.80e-61	8.16e-59	521	0.000	-1.702	-0.121	-0.286	-3.061
d+2	AGACGCAAGG	2	1	27	0	0	6.85e-15	1.26e-13	166	0.000	-1.000	3.755	0.000	0.000

LOCUS: AT4G14690
DESCRIPTION: chlorophyll A-B binding family protein / early light-induced protein, putative, strong similarity to early light-induced protein; ELIP (Arabidopsis thaliana) GI

DATA:		Control	30min	2hours	2days	1week	p-value	B&H	Pos	Fold change relative to control (log2)				
SENSE COUNTS:		2	0	4	131	87	5.05e-67	4.22e-65		0.000	0.000	1.000	6.033	5.443
GENES:														
AT4G14690.1														
SENSE COUNTS:		2	0	4	131	87	5.05e-67	4.50e-65		0.000	0.000	1.000	6.033	5.443
TAGS:														
X+4	TGTCTGCCAT	0	0	0	1	1	3.25e-01	4.21e-01	671	0.000	0.000	0.000	0.000	0.000
d+2	ACTTCAGACG	2	0	3	130	86	5.29e-67	8.89e-65	577	0.000	0.000	0.585	6.022	5.426
d+2	GCTCAGGGCG	0	0	1	0	0	4.55e-01	5.13e-01	220	0.000	0.000	0.000	0.000	0.000

LOCUS: AT3G51600
DESCRIPTION: nonspecific lipid transfer protein 5 (LTP5), identical to SP|Q9XFS7 Nonspecific lipid-transfer protein 5 (LTP 5) {Arabidopsis thaliana}

DATA:		Control	30min	2hours	2days	1week	p-value	B&H	Pos	Fold change relative to control (log2)				
SENSE COUNTS:		97	42	22	42	262	8.67e-66	6.65e-64		0.000	-1.208	-2.140	-1.208	1.434
GENES:														
AT3G51600.1														
SENSE COUNTS:		97	42	22	42	262	8.67e-66	7.14e-64		0.000	-1.208	-2.140	-1.208	1.434
TAGS:														
d+1	TCTTTCTTTG	97	42	21	41	262	1.04e-66	1.59e-64	626	0.000	-1.208	-2.208	-1.242	1.434
d+2	TTAGTGACCG	0	0	1	1	0	5.21e-01	5.71e-01	174	0.000	0.000	0.000	0.000	0.000

LOCUS: AT5G14740
DESCRIPTION: carbonic anhydrase 2 / carbonate dehydratase 2 (CA2) (CA18), nearly identical to SP|P42737 Carbonic anhydrase 2 (EC 4.2.1.1) (Carbonate dehydratase 2) {Arabidopsis thaliana}

DATA:		Control	30min	2hours	2days	1week	p-value	B&H	Pos	Fold change relative to control (log2)				
SENSE COUNTS:		330	61	197	107	44	1.29e-64	9.13e-63		0.000	-2.436	-0.744	-1.625	-2.907
GENES:														
AT5G14740.2														
SENSE COUNTS:		330	61	197	107	44	1.29e-64	9.20e-63		0.000	-2.436	-0.744	-1.625	-2.907
TAGS:														
d+1	TTTACTTTCA	306	48	159	99	43	1.50e-63	2.10e-61	1355	0.000	-2.672	-0.945	-1.628	-2.831
d+2	ATCAGCATCT	23	13	34	8	1	7.63e-06	4.23e-05	1232	0.000	-0.823	0.564	-1.524	-4.524
d+2	TGGTGGCATC	1	0	4	0	0	9.82e-02	1.56e-01	842	0.000	0.000	2.000	0.000	0.000
AT5G14740.1														
SENSE COUNTS:		330	61	197	107	44	1.29e-64	9.86e-63		0.000	-2.436	-0.744	-1.625	-2.907
TAGS:														
d+1	TTTACTTTCA	306	48	159	99	43	1.50e-63	2.10e-61	1473	0.000	-2.672	-0.945	-1.628	-2.831
d+2	ATCAGCATCT	23	13	34	8	1	7.63e-06	4.23e-05	1350	0.000	-0.823	0.564	-1.524	-4.524
d+2	TGGTGGCATC	1	0	4	0	0	9.82e-02	1.56e-01	960	0.000	0.000	2.000	0.000	0.000

LOCUS: AT5G66570
DESCRIPTION: oxygen-evolving enhancer protein 1-1, chloroplast / 33 kDa subunit of oxygen evolving system of photosystem II (PSBO1) (PSBO), identical to SP

DATA:		Control	30min	2hours	2days	1week	p-value	B&H	Pos	Fold change relative to control (log2)				
SENSE COUNTS:		476	118	341	239	134	4.80e-63	3.15e-61		0.000	-2.012	-0.481	-0.994	-1.829
GENES:														
AT5G66570.1														
SENSE COUNTS:		476	118	341	239	134	4.80e-63	3.21e-61		0.000	-2.012	-0.481	-0.994	-1.829
TAGS:														
d+1	TCCTTCATCG	141	43	95	43	50	2.47e-17	5.61e-16	1212	0.000	-1.713	-0.570	-1.713	-1.496

```
      d+2      CCTAAGATCT     335     75      242     195     84      2.54e-47        2.25e-45        1181    0.000   -2.159  -0.469  -0.781  -1.996
      d+2      ACCCGTCTTA     0       0       3       1       0       9.46e-02        1.50e-01        579     0.000   0.000   1.585   0.000   0.000
      d+2      GCAGCCTCTC     0       0       1       0       0       4.55e-01        5.16e-01        90      0.000   0.000   0.000   0.000   0.000

LOCUS: AT3G50990
DESCRIPTION:  peroxidase, putative, similar to peroxidase ATP6a (Arabidopsis thaliana) gi|1429215|emb|CAA67310
DATA:                         Control 30min   2hours  2days   1week   p-value         B&H             Pos     Fold change relative to control (log2)
SENSE COUNTS:                 453     260     581     449     82      3.95e-62        2.42e-60                0.000   -0.801  0.359   -0.013  -2.466
  GENES:
  AT3G50990.1
    SENSE COUNTS:             453     260     581     449     82      3.95e-62        2.49e-60                0.000   -0.801  0.359   -0.013  -2.466
    TAGS:
      v+1      AACAAATTTG     453     260     581     449     82      3.95e-62        5.10e-60        1788    0.000   -0.801  0.359   -0.013  -2.466

LOCUS: AT1G20620
DESCRIPTION:  catalase 3 (SEN2), almost identical to catalase 3 SP
DATA:                         Control 30min   2hours  2days   1week   p-value         B&H             Pos     Fold change relative to control (log2)
SENSE COUNTS:                 124     280     71      49      56      1.75e-58        1.01e-56                0.000   1.175   -0.804  -1.339  -1.147
  GENES:
  AT1G20620.1
    SENSE COUNTS:             124     280     71      49      56      1.75e-58        9.86e-57                0.000   1.175   -0.804  -1.339  -1.147
    TAGS:
      d+1      TTTCCTTCCT     124     280     70      49      55      2.09e-58        2.34e-56        1692    0.000   1.175   -0.825  -1.339  -1.173
      d+2      GAGGGTTTCG     0       0       0       0       0       6.15e-01        6.40e-01        666     0.000   0.000   0.000   0.000   0.000
      i+3      TGTTCTAAAA     0       0       1       0       0       7.06e-01        7.15e-01        428     0.000   0.000   0.000   0.000   0.000
      d+2      AGGGATATTC     0       0       0       0       1       1.65e-01        2.40e-01        408     0.000   0.000   0.000   0.000   0.000
  AT1G20620.2
    SENSE COUNTS:             124     280     71      49      56      1.75e-58        1.04e-56                0.000   1.175   -0.804  -1.339  -1.147
    TAGS:
      d+1      TTTCCTTCCT     124     280     70      49      55      2.09e-58        2.34e-56        1791    0.000   1.175   -0.825  -1.339  -1.173
      d+2      GAGGGTTTCG     0       0       0       0       0       6.15e-01        6.40e-01        666     0.000   0.000   0.000   0.000   0.000
      i+3      TGTTCTAAAA     0       0       1       0       0       7.06e-01        7.15e-01        428     0.000   0.000   0.000   0.000   0.000
      d+2      AGGGATATTC     0       0       0       0       1       1.65e-01        2.40e-01        408     0.000   0.000   0.000   0.000   0.000

LOCUS: AT1G15820
DESCRIPTION:  chlorophyll A-B binding protein, chloroplast (LHCB6), nearly identical to Lhcb6 protein (Arabidopsis thaliana) GI
DATA:                         Control 30min   2hours  2days   1week   p-value         B&H             Pos     Fold change relative to control (log2)
SENSE COUNTS:                 205     43      272     160     25      1.90e-55        1.03e-53                0.000   -2.253  0.408   -0.358  -3.036
  GENES:
  AT1G15820.1
    SENSE COUNTS:             205     43      272     160     25      1.90e-55        1.02e-53                0.000   -2.253  0.408   -0.358  -3.036
    TAGS:
      d+2      AGGCTTGTTC     204     43      254     159     25      1.25e-50        1.31e-48        1012    0.000   -2.246  0.316   -0.360  -3.029
      d+2      GTCGAAGACC     1       0       13      1       0       5.56e-07        4.10e-06        639     0.000   0.000   3.700   0.000   0.000
      d+2      GGTTGGGTGG     0       0       1       0       0       4.55e-01        5.25e-01        565     0.000   0.000   0.000   0.000   0.000
      d+2      GCCGATGGGC     0       0       4       0       0       5.58e-03        1.17e-02        422     0.000   0.000   2.000   0.000   0.000

LOCUS: AT2G05100
DESCRIPTION:  chlorophyll A-B binding protein / LHCII type II (LHCB2.1) (LHCB2.3), identical to Lhcb2 protein (Arabidopsis thaliana) GI
DATA:                         Control 30min   2hours  2days   1week   p-value         B&H             Pos     Fold change relative to control (log2)
SENSE COUNTS:                 207     102     46      16      25      1.86e-48        9.51e-47                0.000   -1.021  -2.170  -3.693  -3.050
  GENES:
  AT2G05100.1
    SENSE COUNTS:             207     102     46      16      25      1.86e-48        9.48e-47                0.000   -1.021  -2.170  -3.693  -3.050
    TAGS:
      d+1      TTCTCTATGT     207     102     46      16      25      1.86e-48        1.84e-46        725     0.000   -1.021  -2.170  -3.693  -3.050

LOCUS: AT5G02960
DESCRIPTION:  40S ribosomal protein S23 (RPS23B), ribosomal protein S23, Fragaria x ananassa, PIR
DATA:                         Control 30min   2hours  2days   1week   p-value         B&H             Pos     Fold change relative to control (log2)
```

SENSE COUNTS: 80 27 70 80 253 2.47e-48 1.20e-46 0.000 -1.567 -0.193 0.000 1.661
GENES:
AT5G02960.1
SENSE COUNTS: 80 27 70 80 253 2.47e-48 1.20e-46 0.000 -1.567 -0.193 0.000 1.661
TAGS:
d+1 ATCGTTTAAT 80 27 70 80 253 2.47e-48 2.31e-46 651 0.000 -1.567 -0.193 0.000 1.661

LOCUS: AT1G31330
DESCRIPTION: photosystem I reaction center subunit III family protein, contains Pfam profile
DATA: Control 30min 2hours 2days 1week p-value B&H Pos Fold change relative to control (log2)
SENSE COUNTS: 24 134 22 20 6 4.83e-46 2.22e-44 0.000 2.481 -0.126 -0.263 -2.000
GENES:
AT1G31330.1
SENSE COUNTS: 24 134 22 20 6 4.83e-46 2.25e-44 0.000 2.481 -0.126 -0.263 -2.000
TAGS:
d+1 AAAGCTTTCT 24 134 21 19 5 4.16e-47 3.49e-45 208 0.000 2.481 -0.193 -0.337 -2.263
d+2 TCGCTCACTA 0 0 1 1 1 8.43e-01 8.45e-01 76 0.000 0.000 0.000 0.000 0.000

LOCUS: AT3G09390
DESCRIPTION: metallothionein protein, putative (MT2A), identical to Swiss-Prot
DATA: Control 30min 2hours 2days 1week p-value B&H Pos Fold change relative to control (log2)
SENSE COUNTS: 85 73 162 276 305 1.35e-45 5.91e-44 0.000 -0.220 0.930 1.699 1.843
GENES:
AT3G09390.1
SENSE COUNTS: 85 73 162 276 305 1.35e-45 6.02e-44 0.000 -0.220 0.930 1.699 1.843
TAGS:
d+1 TGATGAGTTT 85 73 162 276 305 1.35e-45 1.08e-43 483 0.000 -0.220 0.930 1.699 1.843

LOCUS: AT2G42530
DESCRIPTION: cold-responsive protein / cold-regulated protein (cor15b), nearly identical to cold-regulated gene cor15b (Arabidopsis thaliana) GI
DATA: Control 30min 2hours 2days 1week p-value B&H Pos Fold change relative to control (log2)
SENSE COUNTS: 3 0 11 45 107 5.28e-45 2.21e-43 0.000 0.000 1.874 3.907 5.157
GENES:
AT2G42530.1
SENSE COUNTS: 3 0 11 45 107 5.28e-45 2.26e-43 0.000 0.000 1.874 3.907 5.157
TAGS:
d+1 TTCAATAGTT 3 0 11 45 107 5.28e-45 4.03e-43 521 0.000 0.000 1.874 3.907 5.157

LOCUS: AT4G38970
DESCRIPTION: fructose-bisphosphate aldolase, putative, strong similarity to plastidic fructose-bisphosphate aldolase (EC 4.1.2.13) from Nicotiana paniculata (NPALDP1) (GI
DATA: Control 30min 2hours 2days 1week p-value B&H Pos Fold change relative to control (log2)
SENSE COUNTS: 333 58 229 178 199 1.18e-42 4.72e-41 0.000 -2.521 -0.540 -0.904 -0.743
GENES:
AT4G38970.1
SENSE COUNTS: 333 58 229 178 199 1.18e-42 4.68e-41 0.000 -2.521 -0.540 -0.904 -0.743
TAGS:
d+1 TCAAATCATT 331 58 220 177 199 2.47e-42 1.80e-40 1358 0.000 -2.513 -0.589 -0.903 -0.734
d+2 TTCGTCAAAG 2 0 3 0 0 1.23e-01 1.88e-01 1255 0.000 0.000 0.585 0.000 0.000
d+2 GGGCGGCAGA 0 0 5 0 0 1.10e-03 3.09e-03 1128 0.000 0.000 2.322 0.000 0.000
d+2 GTGACTCCCG 0 0 1 1 0 7.90e-01 7.95e-01 892 0.000 0.000 0.000 0.000 0.000
AT4G38970.2
SENSE COUNTS: 333 58 229 178 199 1.18e-42 4.86e-41 0.000 -2.521 -0.540 -0.904 -0.743
TAGS:
d+1 TCAAATCATT 331 58 220 177 199 2.47e-42 1.80e-40 1362 0.000 -2.513 -0.589 -0.903 -0.734
d+2 TTCGTCAAAG 2 0 3 0 0 1.23e-01 1.88e-01 1259 0.000 0.000 0.585 0.000 0.000
d+2 GGGCGGCAGA 0 0 5 0 0 1.10e-03 3.09e-03 1132 0.000 0.000 2.322 0.000 0.000
d+2 GTGACTCCCG 0 0 1 1 0 7.90e-01 7.95e-01 892 0.000 0.000 0.000 0.000 0.000

LOCUS: AT1G29910

```
DESCRIPTION:  chlorophyll A-B binding protein 2, chloroplast / LHCII type I CAB-2 / CAB-140 (CAB2A), identical to SP|P04778 Chlorophyll A-B binding
protein 2, chloroplast precursor (LHCII type I CAB-2) (CAB-140) (LHCP) {Arabidopsis thaliana}
DATA:                         Control 30min   2hours  2days   1week   p-value         B&H             Pos     Fold change relative to control (log2)
SENSE COUNTS:                 591     404     559     494     131     2.66e-41        1.02e-39                0.000   -0.549  -0.080  -0.259  -2.174
  GENES:
  AT1G29910.1
    SENSE COUNTS:             591     404     559     494     131     2.66e-41        1.02e-39                0.000   -0.549  -0.080  -0.259  -2.174
    TAGS:
      d+1      AGGAGAAAGA     580     399     533     482     124     4.51e-40        3.16e-38        922     0.000   -0.540  -0.122  -0.267  -2.226
      d+2      GGCCTTCGCA     11      5       26      12      7       4.32e-04        1.46e-03        824     0.000   -1.138  1.241   0.126   -0.652

LOCUS: AT3G47470
DESCRIPTION:  chlorophyll A-B binding protein 4, chloroplast / LHCI type III CAB-4 (CAB4), identical to SP|P27521 Chlorophyll A-B binding protein 4,
chloroplast precursor (LHCI type III CAB-4) (LHCP) {Arabidopsis thaliana}
DATA:                         Control 30min   2hours  2days   1week   p-value         B&H             Pos     Fold change relative to control (log2)
SENSE COUNTS:                 144     49      248     125     43      2.87e-40        1.06e-38                0.000   -1.555  0.784   -0.204  -1.744
  GENES:
  AT3G47470.1
    SENSE COUNTS:             144     49      248     125     43      2.87e-40        1.06e-38                0.000   -1.555  0.784   -0.204  -1.744
    TAGS:
      d+1      TGGCAACAGT     136     49      235     124     43      2.73e-37        1.70e-35        1226    0.000   -1.473  0.789   -0.133  -1.661
      d+2      TCTGTCTGTG     4       0       11      1       0       9.79e-04        2.82e-03        1071    0.000   0.000   1.459   -2.000  0.000
      d+2      GCACAACACT     3       0       1       0       0       2.51e-01        3.42e-01        986     0.000   0.000   -1.585  0.000   0.000
      d+2      CCTCGGCCTC     1       0       1       0       0       8.46e-01        8.47e-01        283     0.000   0.000   0.000   0.000   0.000

LOCUS: AT3G15590
DESCRIPTION:  DNA-binding protein, putative, similar to DNA-binding protein (Triticum aestivum) GI
DATA:                         Control 30min   2hours  2days   1week   p-value         B&H             Pos     Fold change relative to control (log2)
SENSE COUNTS:                 55      187     55      65      17      1.45e-38        5.13e-37                0.000   1.766   0.000   0.241   -1.694
  GENES:
  AT3G15590.1
    SENSE COUNTS:             55      187     55      65      17      1.45e-38        5.17e-37                0.000   1.766   0.000   0.241   -1.694
    TAGS:
      d+1      AAAATGAAAA     55      187     55      65      17      1.45e-38        9.74e-37        1776    0.000   1.766   0.000   0.241   -1.694

LOCUS: AT3G16640
DESCRIPTION:  translationally controlled tumor family protein, similar to translationally controlled tumor protein GB
DATA:                         Control 30min   2hours  2days   1week   p-value         B&H             Pos     Fold change relative to control (log2)
SENSE COUNTS:                 119     277     161     105     38      1.78e-36        6.07e-35                0.000   1.219   0.436   -0.181  -1.647
  GENES:
  AT3G16640.1
    SENSE COUNTS:             119     277     161     105     38      1.78e-36        6.14e-35                0.000   1.219   0.436   -0.181  -1.647
    TAGS:
      d+1      TTTATAGTAG     1       0       0       0       1       6.74e-01        6.88e-01        882     0.000   0.000   0.000   0.000   0.000
      d+2      GTTTGAAGGA     118     277     150     102     37      5.83e-38        3.77e-36        573     0.000   1.231   0.346   -0.210  -1.673
      d+2      ATGACAGCAC     0       0       11      2       0       8.97e-06        4.85e-05        501     0.000   0.000   3.459   1.000   0.000
      d+2      TTGGTGTACC     0       0       0       1       0       3.09e-01        4.07e-01        92      0.000   0.000   0.000   0.000   0.000

LOCUS: AT3G54890
DESCRIPTION:  chlorophyll A-B binding protein / LHCI type I (CAB), identical to chlorophyll A/B-binding protein (Arabidopsis thaliana) GI
DATA:                         Control 30min   2hours  2days   1week   p-value         B&H             Pos     Fold change relative to control (log2)
SENSE COUNTS:                 411     257     330     190     80      2.18e-35        7.16e-34                0.000   -0.677  -0.317  -1.113  -2.361
  GENES:
  AT3G54890.3
    SENSE COUNTS:             411     257     330     190     80      2.18e-35        6.86e-34                0.000   -0.677  -0.317  -1.113  -2.361
    TAGS:
      d+1      CTTCCTACAG     1       1       4       0       0       1.70e-01        2.40e-01        902     0.000   0.000   2.000   0.000   0.000
      d+2      GCACAACAAC     409     256     326     190     80      7.60e-35        4.56e-33        688     0.000   -0.676  -0.327  -1.106  -2.354
      i+3      ATGCAACATT     1       0       0       0       0       4.28e-01        5.19e-01        344     0.000   0.000   0.000   0.000   0.000
  AT3G54890.1
```

```
    SENSE COUNTS:             411    257    330    190    80     2.18e-35       7.07e-34              0.000  -0.677  -0.317  -1.113  -2.361
    TAGS:
      d+1     CTTCCTACAG      1      1      4      0      0      1.70e-01       2.40e-01       969    0.000  0.000   2.000   0.000   0.000
      d+2     GCACAACAAC      409    256    326    190    80     7.60e-35       4.56e-33       755    0.000  -0.676  -0.327  -1.106  -2.354
      i+3     ATGCAACATT      1      0      0      0      0      4.28e-01       5.19e-01       344    0.000  0.000   0.000   0.000   0.000
  AT3G54890.2
    SENSE COUNTS:             411    257    330    190    80     2.18e-35       7.29e-34              0.000  -0.677  -0.317  -1.113  -2.361
    TAGS:
      d+1     CTTCCTACAG      1      1      4      0      0      1.70e-01       2.40e-01       867    0.000  0.000   2.000   0.000   0.000
      d+2     GCACAACAAC      409    256    326    190    80     7.60e-35       4.56e-33       653    0.000  -0.676  -0.327  -1.106  -2.354
      i+3     ATGCAACATT      1      0      0      0      0      4.28e-01       5.19e-01       344    0.000  0.000   0.000   0.000   0.000

LOCUS: AT2G02100
DESCRIPTION:  plant defensin-fusion protein, putative (PDF2.2), plant defensin protein family member, personal communication, Bart Thomma
(Bart.Thomma@agr.kuleuven.ac.be); similar to SWISS-PROT
DATA:                         Control 30min  2hours 2days  1week  p-value        B&H            Pos    Fold change relative to control (log2)
SENSE COUNTS:                 10     14     14     21     109    7.24e-34       2.30e-32              0.000  0.485   0.485   1.070   3.446
  GENES:
  AT2G02100.1
    SENSE COUNTS:             10     14     14     21     109    7.24e-34       2.21e-32              0.000  0.485   0.485   1.070   3.446
    TAGS:
      X+4     TCTCACCTTG      0      0      1      0      1      3.96e-01       5.02e-01       428    0.000  0.000   0.000   0.000   0.000
      d+1     AAGTACCGTA      10     14     12     20     107    2.52e-33       1.32e-31       367    0.000  0.485   0.263   1.000   3.420
      d+2     CGTGAGTGCA      0      0      1      1      1      5.61e-01       6.10e-01       179    0.000  0.000   0.000   0.000   0.000

LOCUS: AT4G25480
DESCRIPTION:  encodes a member of the DREB subfamily A-1 of ERF/AP2 transcription factor family (CBF3). The protein contains one AP2 domain. There are
six members in this subfamily, including CBF1, CBF2, and CBF3. This gene is involved in response to low temperature an
DATA:                         Control 30min  2hours 2days  1week  p-value        B&H            Pos    Fold change relative to control (log2)
SENSE COUNTS:                 0      0      59     5      3      1.47e-33       4.51e-32              0.000  0.000   5.883   2.322   1.585
  GENES:
  AT4G25480.1
    SENSE COUNTS:             0      0      59     5      3      1.47e-33       4.37e-32              0.000  0.000   5.883   2.322   1.585
    TAGS:
      d+1     AAGTCGACGG      0      0      59     5      3      1.47e-33       7.97e-32       726    0.000  0.000   5.883   2.322   1.585

LOCUS: AT5G64040
DESCRIPTION:  photosystem I reaction center subunit PSI-N, chloroplast, putative / PSI-N, putative (PSAN), SP
DATA:                         Control 30min  2hours 2days  1week  p-value        B&H            Pos    Fold change relative to control (log2)
SENSE COUNTS:                 302    341    462    376    83     6.81e-33       2.02e-31              0.000  0.175   0.613   0.316   -1.863
  GENES:
  AT5G64040.1
    SENSE COUNTS:             302    341    462    376    83     6.81e-33       1.97e-31              0.000  0.175   0.613   0.316   -1.863
    TAGS:
      d+1     AAAGTTCTCG      302    338    461    376    82     3.85e-33       1.96e-31       679    0.000  0.162   0.610   0.316   -1.881
      d+2     GTTTTCTTAG      0      3      1      0      1      2.14e-01       2.97e-01       297    0.000  1.585   0.000   0.000   0.000

LOCUS: AT4G21960
DESCRIPTION:  peroxidase 42 (PER42) (P42) (PRXR1), identical to SP|Q9SB81 Peroxidase 42 precursor (EC 1.11.1.7) (Atperox P42) (PRXR1) (ATP1a/ATP1b)
{Arabidopsis thaliana}
DATA:                         Control 30min  2hours 2days  1week  p-value        B&H            Pos    Fold change relative to control (log2)
SENSE COUNTS:                 363    529    388    254    189    3.08e-32       8.86e-31              0.000  0.543   0.096   -0.515  -0.942
  GENES:
  AT4G21960.1
    SENSE COUNTS:             363    529    388    254    189    3.08e-32       8.67e-31              0.000  0.543   0.096   -0.515  -0.942
    TAGS:
      d+1     GTATACATAA      2      0      0      1      3      4.33e-01       5.23e-01       1403   0.000  0.000   0.000   -1.000  0.585
      d+2     TCCGAATCTT      316    475    344    234    150    3.29e-31       1.58e-29       1369   0.000  0.588   0.122   -0.433  -1.075
      d+2     TATGACGATG      3      4      2      0      1      4.52e-01       5.45e-01       1341   0.000  0.415   -0.585  0.000   -1.585
      d+2     GTAGTGACCA      41     43     42     19     35     7.61e-02       1.23e-01       1166   0.000  0.069   0.035   -1.110  -0.228
```

```
      d+2      GTGCTAGACA     1       7       0       0       0       1.46e-03        3.93e-03        806     0.000   2.807   0.000   0.000   0.000

LOCUS: AT4G33230
DESCRIPTION:  pectinesterase family protein, contains Pfam profile
DATA:                         Control 30min   2hours  2days   1week   p-value         B&H             Pos     Fold change relative to control (log2)
SENSE COUNTS:                 77      15      85      138     185     1.63e-31        4.54e-30                0.000   -2.360  0.143   0.842   1.265
  GENES:
  AT4G33230.1
    SENSE COUNTS:             77      15      85      138     185     1.63e-31        4.36e-30                0.000   -2.360  0.143   0.842   1.265
    TAGS:
      v+1      ACAAGAAAAC     77      15      85      138     185     1.63e-31        8.05e-30        2570    0.000   -2.360  0.143   0.842   1.265

LOCUS: AT4G35750
DESCRIPTION:  Rho-GTPase-activating protein-related, contains weak similarity to Rho-GTPase-activating protein 1 (GTPase-activating protein rhoOGAP)
(Rho-related small GTPase protein activator) (CDC42 GTPase-activating protein) (p50-rhoGAP) (Swiss-Prot
DATA:                         Control 30min   2hours  2days   1week   p-value         B&H             Pos     Fold change relative to control (log2)
SENSE COUNTS:                 3       57      3       2       0       5.13e-30        1.39e-28                0.000   4.248   0.000   -0.585  0.000
  GENES:
  AT4G35750.1
    SENSE COUNTS:             3       57      3       2       0       5.13e-30        1.34e-28                0.000   4.248   0.000   -0.585  0.000
    TAGS:
      d+1      AGGTGTATCT     3       57      3       2       0       5.13e-30        2.33e-28        783     0.000   4.248   0.000   -0.585  0.000

LOCUS: AT1G06680
DESCRIPTION:  photosystem II oxygen-evolving complex 23 (OEC23), JBC 14
DATA:                         Control 30min   2hours  2days   1week   p-value         B&H             Pos     Fold change relative to control (log2)
SENSE COUNTS:                 88      202     110     76      14      7.73e-30        2.03e-28                0.000   1.199   0.322   -0.212  -2.652
  GENES:
  AT1G06680.1
    SENSE COUNTS:             88      202     110     76      14      7.73e-30        1.97e-28                0.000   1.199   0.322   -0.212  -2.652
    TAGS:
      d+1      GTCACTCCTA     88      202     110     76      14      7.73e-30        3.42e-28        479     0.000   1.199   0.322   -0.212  -2.652

LOCUS: AT1G29930
DESCRIPTION:  chlorophyll A-B binding protein 2, chloroplast / LHCII type I CAB-2 / CAB-140 (CAB2B), identical to SP|P04778 Chlorophyll A-B binding
protein 2, chloroplast precursor (LHCII type I CAB-2) (CAB-140) (LHCP) {Arabidopsis thaliana}
DATA:                         Control 30min   2hours  2days   1week   p-value         B&H             Pos     Fold change relative to control (log2)
SENSE COUNTS:                 748     866     713     1022    468     1.04e-29        2.66e-28                0.000   0.211   -0.069  0.450   -0.677
  GENES:
  AT1G29930.1
    SENSE COUNTS:             748     866     713     1022    468     1.04e-29        2.59e-28                0.000   0.211   -0.069  0.450   -0.677
    TAGS:
      X+4      TCAAAGTTAA     0       0       2       1       3       2.08e-01        2.89e-01        1094    0.000   0.000   1.000   0.000   1.585
      d+1      GGCCTTCGCC     748     866     711     1021    465     4.71e-30        2.20e-28        837     0.000   0.211   -0.073  0.449   -0.686

LOCUS: AT4G10340
DESCRIPTION:  chlorophyll A-B binding protein CP26, chloroplast / light-harvesting complex II protein 5 / LHCIIc (LHCB5), identical to SP|Q9XF89
Chlorophyll A/B-binding protein CP26, chloroplast precursor (Light-harvesting complex II protein 5) (LHCB5) (LHCIIc) {Arabid
DATA:                         Control 30min   2hours  2days   1week   p-value         B&H             Pos     Fold change relative to control (log2)
SENSE COUNTS:                 304     352     337     238     52      1.22e-29        3.03e-28                0.000   0.212   0.149   -0.353  -2.547
  GENES:
  AT4G10340.1
    SENSE COUNTS:             304     352     337     238     52      1.22e-29        2.97e-28                0.000   0.212   0.149   -0.353  -2.547
    TAGS:
      d+1      CTTCTAAGGA     82      16      62      43      23      6.87e-12        9.95e-11        1160    0.000   -2.358  -0.403  -0.931  -1.834
      d+2      TTTGCGATGC     221     336     258     195     28      6.78e-34        3.93e-32        884     0.000   0.604   0.223   -0.181  -2.981
      d+2      CGAGATGGGC     1       0       17      0       1       4.96e-09        4.93e-08        537     0.000   0.000   4.087   0.000   0.000

LOCUS: AT1G16850
DESCRIPTION:  expressed protein
```

```
DATA:                         Control 30min   2hours  2days   1week   p-value         B&H             Pos     Fold change relative to control (log2)
SENSE COUNTS:                 0       0       0       11      50      8.63e-29        2.09e-27                0.000   0.000   0.000   3.459   5.644
  GENES:
  AT1G16850.1
    SENSE COUNTS:             0       0       0       11      50      8.63e-29        2.05e-27                0.000   0.000   0.000   3.459   5.644
    TAGS:
      d+1     AATACAGAAT      0       0       0       11      50      8.63e-29        3.72e-27        674     0.000   0.000   0.000   3.459   5.644

LOCUS: AT1G12090
DESCRIPTION:  protease inhibitor/seed storage/lipid transfer protein (LTP) family protein, similar to 14 kDa polypeptide (Catharanthus roseus) GI
DATA:                         Control 30min   2hours  2days   1week   p-value         B&H             Pos     Fold change relative to control (log2)
SENSE COUNTS:                 22      94      13      19      8       1.52e-28        3.59e-27                0.000   2.095   -0.759  -0.212  -1.459
  GENES:
  AT1G12090.1
    SENSE COUNTS:             22      94      13      19      8       1.52e-28        3.54e-27                0.000   2.095   -0.759  -0.212  -1.459
    TAGS:
      d+1     CTGCAGCCTC      22      94      13      19      8       1.52e-28        6.38e-27        391     0.000   2.095   -0.759  -0.212  -1.459

LOCUS: AT5G42270
DESCRIPTION:  FtsH protease, putative, similar to FtsH protease GI
DATA:                         Control 30min   2hours  2days   1week   p-value         B&H             Pos     Fold change relative to control (log2)
SENSE COUNTS:                 45      12      59      107     144     8.30e-28        1.91e-26                0.000   -1.907  0.391   1.250   1.678
  GENES:
  AT5G42270.1
    SENSE COUNTS:             45      12      59      107     144     8.30e-28        1.89e-26                0.000   -1.907  0.391   1.250   1.678
    TAGS:
      d+2     TTCAGAGACT      45      12      54      101     141     1.11e-26        3.97e-25        2364    0.000   -1.907  0.263   1.166   1.648
      d+2     AGTCTTTTCA      0       0       2       5       3       4.65e-02        7.73e-02        2247    0.000   0.000   1.000   2.322   1.585
      d+2     CAAGTGTCGC      0       0       3       1       0       9.46e-02        1.50e-01        1959    0.000   0.000   1.585   0.000   0.000

LOCUS: AT3G21055
DESCRIPTION:  photosystem II 5 kD protein, putative, identical to Swiss-Prot
DATA:                         Control 30min   2hours  2days   1week   p-value         B&H             Pos     Fold change relative to control (log2)
SENSE COUNTS:                 186     64      71      44      52      1.22e-27        2.74e-26                0.000   -1.539  -1.389  -2.080  -1.839
  GENES:
  AT3G21055.1
    SENSE COUNTS:             186     64      71      44      52      1.22e-27        2.72e-26                0.000   -1.539  -1.389  -2.080  -1.839
    TAGS:
      d+1     TCTATCTCTC      186     64      70      44      52      8.54e-28        3.26e-26        450     0.000   -1.539  -1.410  -2.080  -1.839
      d+2     ACCGCTACCT      0       0       1       0       0       4.55e-01        5.29e-01        133     0.000   0.000   0.000   0.000   0.000

LOCUS: AT5G40450
DESCRIPTION:  expressed protein
DATA:                         Control 30min   2hours  2days   1week   p-value         B&H             Pos     Fold change relative to control (log2)
SENSE COUNTS:                 8       67      6       7       4       1.33e-27        2.91e-26                0.000   3.066   -0.415  -0.193  -1.000
  GENES:
  AT5G40450.1
    SENSE COUNTS:             8       74      6       7       4       9.43e-32        2.59e-30                0.000   3.209   -0.415  -0.193  -1.000
    TAGS:
      d+2     AAGTTTCCGT      1       19      2       2       3       4.94e-07        3.70e-06        8057    0.000   4.248   1.000   1.000   1.585
      d+2     AAGACCCTGT      0       6       0       0       0       9.09e-04        2.65e-03        7454    0.000   2.585   0.000   0.000   0.000
      d+2     ATAAAAGAGC      0       6       1       1       1       4.45e-02        7.40e-02        6736    0.000   2.585   0.000   0.000   0.000
      d+2     TCGAGACTCC      2       14      2       2       0       1.02e-04        4.21e-04        5966    0.000   2.807   0.000   0.000   0.000
      d+2     TATTGGAGAG      0       0       0       0       0       6.15e-01        6.39e-01        4019    0.000   0.000   0.000   0.000   0.000
      d+2     AATCTGAAAG      0       7       0       0       0       2.70e-04        9.80e-04        3743    0.000   2.807   0.000   0.000   0.000
      d+2     CTCCAAAGAG      1       2       0       0       0       2.43e-01        3.34e-01        3444    0.000   1.000   0.000   0.000   0.000
      d+2     TGACTGTTGC      0       2       0       0       0       9.14e-02        1.46e-01        3254    0.000   1.000   0.000   0.000   0.000
      d+2     AACCAACAAA      2       2       0       2       0       4.35e-01        5.25e-01        3029    0.000   0.000   0.000   0.000   0.000
      d+2     ATGTACTTAA      1       4       1       0       0       1.14e-01        1.78e-01        2276    0.000   2.000   0.000   0.000   0.000
      d+2     GAAGTACAAA      1       5       0       0       0       1.37e-02        2.43e-02        112     0.000   2.322   0.000   0.000   0.000
```

```
LOCUS: AT2G33830
DESCRIPTION:  dormancy/auxin associated family protein, contains Pfam profile
DATA:                         Control 30min   2hours  2days   1week   p-value         B&H             Pos     Fold change relative to control (log2)
SENSE COUNTS:                 9       80      24      2       10      2.52e-27        5.39e-26                0.000   3.152   1.415   -2.170  0.152
  GENES:
  AT2G33830.2
    SENSE COUNTS:             9       80      24      2       10      2.52e-27        5.39e-26                0.000   3.152   1.415   -2.170  0.152
    TAGS:
      d+1      TCGTTTGTGT     9       80      22      2       10      5.69e-28        2.22e-26        499     0.000   3.152   1.290   -2.170  0.152
      d+2      GCCTTGGCCG     0       0       2       0       0       1.21e-01        1.88e-01        96      0.000   0.000   1.000   0.000   0.000
  AT2G33830.1
    SENSE COUNTS:             9       80      24      2       10      2.52e-27        5.50e-26                0.000   3.152   1.415   -2.170  0.152
    TAGS:
      d+1      TCGTTTGTGT     9       80      22      2       10      5.69e-28        2.22e-26        491     0.000   3.152   1.290   -2.170  0.152
      d+2      GCCTTGGCCG     0       0       2       0       0       1.21e-01        1.88e-01        94      0.000   0.000   1.000   0.000   0.000

LOCUS: AT3G22120
DESCRIPTION:  protease inhibitor/seed storage/lipid transfer protein (LTP) family protein, similar to SP|Q00451|PRF1_LYCES 36.4 kDa proline-rich
protein Lycopersicon esculentum, proline-rich cell wall protein (Medicago sativa) GI
DATA:                         Control 30min   2hours  2days   1week   p-value         B&H             Pos     Fold change relative to control (log2)
SENSE COUNTS:                 79      91      23      55      193     5.39e-27        1.13e-25                0.000   0.204   -1.780  -0.522  1.289
  GENES:
  AT3G22120.1
    SENSE COUNTS:             79      91      24      55      193     1.30e-26        2.62e-25                0.000   0.204   -1.719  -0.522  1.289
    TAGS:
      X+4      CGCATTGCAC     0       0       1       0       0       4.55e-01        5.33e-01        1367    0.000   0.000   0.000   0.000   0.000
      d+1      TTTCATTTGA     77      89      21      52      192     2.97e-28        1.19e-26        1155    0.000   0.209   -1.874  -0.566  1.318
      d+2      ATCTATTTGG     2       0       0       1       1       6.87e-01        7.01e-01        1102    0.000   0.000   0.000   -1.000  -1.000
      d+2      CCCACCACCA     0       2       1       2       0       3.87e-01        4.94e-01        512     0.000   1.000   0.000   1.000   0.000

LOCUS: AT5G19140
DESCRIPTION:  auxin/aluminum-responsive protein, putative, strong similarity to auxin down-regulated protein ARG10 (Vigna radiata) GI
DATA:                         Control 30min   2hours  2days   1week   p-value         B&H             Pos     Fold change relative to control (log2)
SENSE COUNTS:                 33      103     17      22      10      5.96e-27        1.22e-25                0.000   1.642   -0.957  -0.585  -1.722
  GENES:
  AT5G19140.1
    SENSE COUNTS:             33      103     17      22      10      5.96e-27        1.25e-25                0.000   1.642   -0.957  -0.585  -1.722
    TAGS:
      d+1      TCTCTTAAAG     33      103     17      22      10      5.96e-27        2.18e-25        863     0.000   1.642   -0.957  -0.585  -1.722

LOCUS: AT1G61520
DESCRIPTION:  chlorophyll A-B binding protein / LHCI type III (LHCA3.1), nearly identical to PSI type III chlorophyll a/b-binding protein GI
DATA:                         Control 30min   2hours  2days   1week   p-value         B&H             Pos     Fold change relative to control (log2)
SENSE COUNTS:                 387     327     294     197     98      6.12e-27        1.22e-25                0.000   -0.243  -0.397  -0.974  -1.981
  GENES:
  AT1G61520.1
    SENSE COUNTS:             387     327     294     197     98      6.12e-27        1.26e-25                0.000   -0.243  -0.397  -0.974  -1.981
    TAGS:
      d+1      TGTTTTTATG     386     327     281     197     97      3.42e-27        1.28e-25        983     0.000   -0.239  -0.458  -0.970  -1.993
      i+3      ATGTTCAAAA     1       0       0       0       0       4.28e-01        5.19e-01        487     0.000   0.000   0.000   0.000   0.000
      d+2      TTGGGTGCAG     0       0       13      0       1       6.58e-08        5.73e-07        467     0.000   0.000   3.700   0.000   0.000

LOCUS: AT1G67090
DESCRIPTION:  ribulose bisphosphate carboxylase small chain 1A / RuBisCO small subunit 1A (RBCS-1A) (ATS1A), identical to SP|P10795 Ribulose
bisphosphate carboxylase small chain 1A, chloroplast precursor (EC 4.1.1.39) (RuBisCO small subunit 1A) {Arabidopsis thaliana}
DATA:                         Control 30min   2hours  2days   1week   p-value         B&H             Pos     Fold change relative to control (log2)
SENSE COUNTS:                 372     473     255     476     206     1.34e-26        2.62e-25                0.000   0.347   -0.545  0.356   -0.853
  GENES:
  AT1G67090.2
```

SENSE COUNTS:		372	473	255	476	206	1.34e-26	2.66e-25		0.000	0.347	-0.545	0.356	-0.853
TAGS:														
d+1	AGTCGCTAAA	1	0	4	9	16	4.51e-05	2.02e-04	836	0.000	0.000	2.000	3.170	4.000
d+2	CAGGTGTGGC	371	473	251	467	190	1.88e-28	7.70e-27	197	0.000	0.350	-0.564	0.332	-0.965
AT1G67090.1														
SENSE COUNTS:		372	473	255	476	206	1.34e-26	2.61e-25		0.000	0.347	-0.545	0.356	-0.853
TAGS:														
d+1	AGTCGCTAAA	1	0	4	9	16	4.51e-05	2.02e-04	843	0.000	0.000	2.000	3.170	4.000
d+2	CAGGTGTGGC	371	473	251	467	190	1.88e-28	7.70e-27	197	0.000	0.350	-0.564	0.332	-0.965

LOCUS: AT4G30650
DESCRIPTION: hydrophobic protein, putative / low temperature and salt responsive protein, putative, similar to SP|Q9ZNQ7 Hydrophobic protein RCI2A (Low temperature and salt responsive protein LTI6A) {Arabidopsis thaliana}; contains Pfam profile PF01679

DATA:		Control	30min	2hours	2days	1week	p-value	B&H	Pos	Fold change relative to control (log2)				
SENSE COUNTS:		1	0	2	27	56	1.48e-26	2.84e-25		0.000	0.000	1.000	4.755	5.807
GENES:														
AT4G30650.1														
SENSE COUNTS:		1	0	2	27	56	1.48e-26	2.83e-25		0.000	0.000	1.000	4.755	5.807
TAGS:														
d+1	TACCAAATAA	1	0	0	27	53	1.72e-26	6.02e-25	363	0.000	0.000	0.000	4.755	5.728
d+2	GAAGTTTTCT	0	0	2	0	3	8.60e-02	1.38e-01	99	0.000	0.000	1.000	0.000	1.585

LOCUS: AT4G18100
DESCRIPTION: 60S ribosomal protein L32 (RPL32A), ribosomal protein L32, human, PIR1

DATA:		Control	30min	2hours	2days	1week	p-value	B&H	Pos	Fold change relative to control (log2)				
SENSE COUNTS:		52	15	40	87	147	1.78e-26	3.34e-25		0.000	-1.794	-0.379	0.743	1.499
GENES:														
AT4G18100.1														
SENSE COUNTS:		52	15	40	87	147	1.78e-26	3.34e-25		0.000	-1.794	-0.379	0.743	1.499
TAGS:														
d+1	AGTTTTGGTT	52	15	40	87	147	1.78e-26	6.10e-25	579	0.000	-1.794	-0.379	0.743	1.499

LOCUS: AT5G15950
DESCRIPTION: adenosylmethionine decarboxylase family protein, contains Pfam profile

DATA:		Control	30min	2hours	2days	1week	p-value	B&H	Pos	Fold change relative to control (log2)				
SENSE COUNTS:		1	0	6	9	55	2.42e-26	4.45e-25		0.000	0.000	2.585	3.170	5.781
GENES:														
AT5G15950.1														
SENSE COUNTS:		1	0	6	9	55	2.42e-26	4.46e-25		0.000	0.000	2.585	3.170	5.781
TAGS:														
d+1	ATTTTAGTGT	1	0	1	0	26	9.45e-16	1.87e-14	1731	0.000	0.000	0.000	0.000	4.700
X+4	ACACCTTTGG	0	0	5	8	26	1.81e-10	2.22e-09	1609	0.000	0.000	2.322	3.000	4.700
d+2	GACTTGAGCC	0	0	0	1	3	6.27e-02	1.02e-01	1268	0.000	0.000	0.000	0.000	1.585

LOCUS: AT1G79040
DESCRIPTION: photosystem II 10 kDa polypeptide, identical to photosystem II 10 kDa polypeptide, chloroplast (precursor) SP

DATA:		Control	30min	2hours	2days	1week	p-value	B&H	Pos	Fold change relative to control (log2)				
SENSE COUNTS:		303	236	124	93	193	2.23e-25	4.02e-24		0.000	-0.361	-1.289	-1.704	-0.651
GENES:														
AT1G79040.1														
SENSE COUNTS:		303	236	124	93	193	2.23e-25	4.04e-24		0.000	-0.361	-1.289	-1.704	-0.651
TAGS:														
d+1	TTTCTATAAA	299	235	86	88	183	1.35e-33	7.56e-32	523	0.000	-0.347	-1.798	-1.765	-0.708
X+4	CACAAGAATG	2	1	5	5	0	2.23e-01	3.08e-01	306	0.000	-1.000	1.322	1.322	0.000
d+2	GACTTGAGGG	2	0	33	0	10	1.28e-15	2.50e-14	245	0.000	0.000	4.044	0.000	2.322

LOCUS: AT2G30570
DESCRIPTION: photosystem II reaction center W (PsbW) protein-related, similar to photosystem II reaction center W protein SP

DATA:		Control	30min	2hours	2days	1week	p-value	B&H	Pos	Fold change relative to control (log2)				
SENSE COUNTS:		323	124	235	180	126	2.65e-25	4.69e-24		0.000	-1.381	-0.459	-0.844	-1.358

```
  GENES:
  AT2G30570.2
    SENSE COUNTS:              237    89     160    132    87     6.66e-19      9.02e-18                    0.000  -1.413  -0.567  -0.844  -1.446
    TAGS:
      d+1      TGTGTTTACT      148    63     106    73     66     6.84e-10      7.37e-09      661           0.000  -1.232  -0.482  -1.020  -1.165
      d+2      AATCAAAAGT      89     26     54     59     21     4.50e-10      5.18e-09      595           0.000  -1.775  -0.721  -0.593  -2.083
  AT2G30570.1
    SENSE COUNTS:              175    61     129    107    60     1.49e-15      1.52e-14                    0.000  -1.520  -0.440  -0.710  -1.544
    TAGS:
      d+1      TTCATCAAAA      86     35     75     48     39     1.67e-06      1.08e-05      748           0.000  -1.297  -0.197  -0.841  -1.141
      d+2      AATCAAAAGT      89     26     54     59     21     4.50e-10      5.18e-09      595           0.000  -1.775  -0.721  -0.593  -2.083

LOCUS: AT2G05380
DESCRIPTION:  glycine-rich protein (GRP3S), identical to cDNA glycine-rich protein 3 short isoform (GRP3S)  GI
DATA:                          Control 30min  2hours 2days  1week  p-value       B&H           Pos           Fold change relative to control (log2)
SENSE COUNTS:                  74     48     56     100    199    1.12e-24      1.94e-23                    0.000  -0.624  -0.402  0.434   1.427
  GENES:
  AT2G05380.1
    SENSE COUNTS:              74     48     56     100    199    1.12e-24      2.00e-23                    0.000  -0.624  -0.402  0.434   1.427
    TAGS:
      d+1      AGTGTACGAT      73     48     45     98     190    4.87e-24      1.54e-22      409           0.000  -0.605  -0.698  0.425   1.380
      d+2      GTGGCCACGG      0      0      11     0      8      9.75e-06      5.22e-05      207           0.000  0.000   3.459   0.000   3.000
      d+2      GTGGTGGATT      1      0      0      2      1      3.00e-01      4.03e-01      153           0.000  0.000   0.000   1.000   0.000

LOCUS: AT5G52310
DESCRIPTION:  low-temperature-responsive protein 78 (LTI78) / desiccation-responsive protein 29A (RD29A)
DATA:                          Control 30min  2hours 2days  1week  p-value       B&H           Pos           Fold change relative to control (log2)
SENSE COUNTS:                  3      0      0      45     10     4.41e-24      7.51e-23                    0.000  0.000   0.000   3.907   1.737
  GENES:
  AT5G52310.1
    SENSE COUNTS:              3      0      0      45     10     4.41e-24      7.74e-23                    0.000  0.000   0.000   3.907   1.737
    TAGS:
      d+1      AGTTGGATCT      3      0      0      45     10     4.41e-24      1.42e-22      1294          0.000  0.000   0.000   3.907   1.737

LOCUS: AT1G44575
DESCRIPTION:  photosystem II 22kDa protein, chloroplast / CP22 (PSBS), identical to photosystem II 22 kDa protein, chloroplast (precursor) SP
DATA:                          Control 30min  2hours 2days  1week  p-value       B&H           Pos           Fold change relative to control (log2)
SENSE COUNTS:                  144    30     162    159    141    5.85e-22      9.79e-21                    0.000  -2.263  0.170   0.143   -0.030
  GENES:
  AT1G44575.2
    SENSE COUNTS:              144    30     162    159    141    5.85e-22      1.01e-20                    0.000  -2.263  0.170   0.143   -0.030
    TAGS:
      d+1      TTGAGACAAA      143    30     162    159    140    6.96e-22      2.05e-20      1352          0.000  -2.253  0.180   0.153   -0.031
      d+2      TACTTAAATT      0      0      0      0      1      1.65e-01      2.43e-01      1312          0.000  0.000   0.000   0.000   0.000
      d+2      CTGCTTACTT      1      0      0      0      0      4.28e-01      5.34e-01      54            0.000  0.000   0.000   0.000   0.000
  AT1G44575.1
    SENSE COUNTS:              144    30     162    159    141    5.85e-22      9.94e-21                    0.000  -2.263  0.170   0.143   -0.030
    TAGS:
      d+1      TTGAGACAAA      143    30     162    159    140    6.96e-22      2.05e-20      893           0.000  -2.253  0.180   0.153   -0.031
      d+2      TACTTAAATT      0      0      0      0      1      1.65e-01      2.43e-01      853           0.000  0.000   0.000   0.000   0.000
      d+2      CTGCTTACTT      1      0      0      0      0      4.28e-01      5.34e-01      61            0.000  0.000   0.000   0.000   0.000

LOCUS: AT3G26520
DESCRIPTION:  tonoplast intrinsic protein, putative, similar to tonoplast intrinsic protein GI
DATA:                          Control 30min  2hours 2days  1week  p-value       B&H           Pos           Fold change relative to control (log2)
SENSE COUNTS:                  51     136    62     71     3      9.73e-22      1.60e-20                    0.000  1.415   0.282   0.477   -4.087
  GENES:
  AT3G26520.1
    SENSE COUNTS:              51     136    62     71     3      9.73e-22      1.63e-20                    0.000  1.415   0.282   0.477   -4.087
    TAGS:
```

```
     d+2     AACCCAGCCG     51      136     49      70      3       6.21e-24       1.93e-22       710     0.000   1.415   -0.058  0.457   -4.087
     d+2     CTTTCGGTCT     0       0       12      0       0       3.96e-08       3.54e-07       312     0.000   0.000   3.585   0.000   0.000
     d+2     CCGACCAGAA     0       0       1       1       0       5.21e-01       5.69e-01       116     0.000   0.000   0.000   0.000   0.000

LOCUS: AT3G61470
DESCRIPTION:  chlorophyll A-B binding protein (LHCA2), identical to Lhca2 protein (Arabidopsis thaliana) GI
DATA:                          Control 30min   2hours  2days   1week   p-value        B&H            Pos     Fold change relative to control (log2)
SENSE COUNTS:                  233     165     115     74      69      1.03e-21       1.66e-20               0.000   -0.498  -1.019  -1.655  -1.756
 GENES:
 AT3G61470.1
   SENSE COUNTS:               233     165     115     74      69      1.03e-21       1.70e-20               0.000   -0.498  -1.019  -1.655  -1.756
   TAGS:
     d+1     TTTGTACAAA     230     165     94      74      66      3.75e-24       1.24e-22       901     0.000   -0.479  -1.291  -1.636  -1.801
     d+2     GTACACGGCG     3       0       21      0       3       5.68e-10       6.24e-09       451     0.000   0.000   2.807   0.000   0.000

LOCUS: AT4G31700
DESCRIPTION:  40S ribosomal protein S6 (RPS6A), ribosomal protein S6, Arabidopsis thaliana, PID
DATA:                          Control 30min   2hours  2days   1week   p-value        B&H            Pos     Fold change relative to control (log2)
SENSE COUNTS:                  28      5       15      12      82      1.25e-21       1.98e-20               0.000   -2.485  -0.900  -1.222  1.550
 GENES:
 AT4G31700.1
   SENSE COUNTS:               28      5       15      12      82      1.25e-21       2.03e-20               0.000   -2.485  -0.900  -1.222  1.550
   TAGS:
     d+1     ATTTATGCTT     27      5       14      11      82      1.33e-22       3.99e-21       910     0.000   -2.433  -0.948  -1.295  1.603
     d+2     GGTACACAAT     1       0       1       1       0       7.18e-01       7.24e-01       880     0.000   0.000   0.000   0.000   0.000

LOCUS: AT4G35770
DESCRIPTION:  senescence-associated protein (SEN1), identical to senescence-associated protein GI
DATA:                          Control 30min   2hours  2days   1week   p-value        B&H            Pos     Fold change relative to control (log2)
SENSE COUNTS:                  6       51      5       6       0       1.74e-21       2.71e-20               0.000   3.087   -0.263  0.000   0.000
 GENES:
 AT4G35770.1
   SENSE COUNTS:               6       51      5       6       0       1.74e-21       2.78e-20               0.000   3.087   -0.263  0.000   0.000
   TAGS:
     i+3     TACAGGGCTT     0       2       1       0       0       2.47e-01       3.37e-01       964     0.000   1.000   0.000   0.000   0.000
     d+1     TACAGAGTCG     5       49      2       6       0       6.98e-22       2.02e-20       466     0.000   3.293   -1.322  0.263   0.000
     X+4     CCTACACACG     0       0       0       0       0       6.15e-01       6.37e-01       261     0.000   0.000   0.000   0.000   0.000
     d+2     TGGATCTTTC     1       0       2       0       0       3.07e-01       4.12e-01       210     0.000   0.000   1.000   0.000   0.000

LOCUS: AT3G55120
DESCRIPTION:  chalcone-flavanone isomerase / chalcone isomerase (CHI), identical to SP|P41088
DATA:                          Control 30min   2hours  2days   1week   p-value        B&H            Pos     Fold change relative to control (log2)
SENSE COUNTS:                  2       0       0       19      44      4.56e-21       6.99e-20               0.000   0.000   0.000   3.248   4.459
 GENES:
 AT3G55120.1
   SENSE COUNTS:               2       0       0       19      44      4.56e-21       7.18e-20               0.000   0.000   0.000   3.248   4.459
   TAGS:
     d+1     CATTTACATT     2       0       0       19      44      4.56e-21       1.30e-19       895     0.000   0.000   0.000   3.248   4.459

LOCUS: AT1G04270
DESCRIPTION:  40S ribosomal protein S15 (RPS15A), Strong similarity to Oryza 40S ribosomal protein S15. ESTs gb|R29788,gb|ATTS0365 come from this gene
DATA:                          Control 30min   2hours  2days   1week   p-value        B&H            Pos     Fold change relative to control (log2)
SENSE COUNTS:                  47      13      23      28      109     9.39e-21       1.42e-19               0.000   -1.854  -1.031  -0.747  1.214
 GENES:
 AT1G04270.1
   SENSE COUNTS:               47      13      23      28      109     9.39e-21       1.44e-19               0.000   -1.854  -1.031  -0.747  1.214
   TAGS:
     d+1     TTACAAGAGG     47      11      22      26      109     7.61e-23       2.32e-21       690     0.000   -2.095  -1.095  -0.854  1.214
     X+4     GGTATTGTTG     0       0       0       2       0       4.80e-02       7.97e-02       504     0.000   0.000   0.000   1.000   0.000
     d+2     ATCATCGTCC     0       2       1       0       0       2.47e-01       3.39e-01       362     0.000   1.000   0.000   0.000   0.000
```

```
LOCUS: AT2G15970
DESCRIPTION:  cold-acclimation protein, putative (FL3-5A3), similar to cold acclimation WCOR413-like protein gamma form (Hordeum vulgare)
gi|18449100|gb|AAL69988; similar to stress-regulated protein SAP1 (Xerophyta viscosa) gi|21360378|gb|AAM47505; identical to cDNA co
DATA:                         Control 30min  2hours  2days  1week  p-value     B&H         Pos    Fold change relative to control (log2)
SENSE COUNTS:                 32      23     46      99     122    1.38e-20    2.05e-19           0.000  -0.476  0.524  1.629  1.931
  GENES:
  AT2G15970.1
    SENSE COUNTS:             32      23     46      99     122    1.38e-20    2.08e-19           0.000  -0.476  0.524  1.629  1.931
    TAGS:
      d+1      AGGTGTTAGT     10      3      17      18     41     2.39e-07    1.91e-06    811    0.000  -1.737  0.766  0.848  2.036
      d+2      TAAGAGTGAT     22      20     28      81     80     4.39e-15    8.19e-14    715    0.000  -0.138  0.348  1.880  1.862
      d+2      ATCGGATCCG     0       0      1       0      1      3.96e-01    5.02e-01    124    0.000  0.000   0.000  0.000  0.000

LOCUS: AT4G12490
DESCRIPTION:  protease inhibitor/seed storage/lipid transfer protein (LTP) family protein, similar to pEARLI 1 (Accession No. L43080)
DATA:                         Control 30min  2hours  2days  1week  p-value     B&H         Pos    Fold change relative to control (log2)
SENSE COUNTS:                 1       0      2       4      37     2.19e-20    3.20e-19           0.000  0.000   1.000  2.000  5.209
  GENES:
  AT4G12490.1
    SENSE COUNTS:             1       0      2       4      37     2.19e-20    3.25e-19           0.000  0.000   1.000  2.000  5.209
    TAGS:
      d+1      TATGCAAGTT     1       0      2       4      37     2.19e-20    6.03e-19    652    0.000  0.000   1.000  2.000  5.209

LOCUS: AT1G52690
DESCRIPTION:  late embryogenesis abundant protein, putative / LEA protein, putative, similar to SP|P13934 Late embryogenesis abundant protein 76 (LEA
76) {Brassica napus}; contains Pfam profile PF02987
DATA:                         Control 30min  2hours  2days  1week  p-value     B&H         Pos    Fold change relative to control (log2)
SENSE COUNTS:                 3       0      0       5      38     7.96e-20    1.14e-18           0.000  0.000   0.000  0.737  3.663
  GENES:
  AT1G52690.1
    SENSE COUNTS:             3       0      0       5      38     7.96e-20    1.17e-18           0.000  0.000   0.000  0.737  3.663
    TAGS:
      d+1      TAATGGTTTT     3       0      0       5      37     1.11e-19    3.01e-18    742    0.000  0.000   0.000  0.737  3.624
      d+2      TTTGTTTGCT     0       0      0       0      1      1.65e-01    2.37e-01    646    0.000  0.000   0.000  0.000  0.000
      X+4      AAAACGAGAA     0       0      0       0      0      6.15e-01    6.42e-01    -172   0.000  0.000   0.000  0.000  0.000
  AT1G52690.2
    SENSE COUNTS:             3       0      0       5      38     6.20e-21    9.61e-20           0.000  0.000   0.000  0.737  3.663
    TAGS:
      d+1      TAATGGTTTT     3       0      0       5      37     1.11e-19    3.01e-18    742    0.000  0.000   0.000  0.737  3.624
      d+2      TTTGTTTGCT     0       0      0       0      1      1.65e-01    2.37e-01    646    0.000  0.000   0.000  0.000  0.000

LOCUS: AT4G27520
DESCRIPTION:  plastocyanin-like domain-containing protein, similar to PIR|JC7196 phytocyanin-related protein Pn14 {Ipomoea nil}; contains Pfam profile
PF02298
DATA:                         Control 30min  2hours  2days  1week  p-value     B&H         Pos    Fold change relative to control (log2)
SENSE COUNTS:                 25      7      37      92     33     1.43e-19    2.02e-18           0.000  -1.837  0.566  1.880  0.401
  GENES:
  AT4G27520.1
    SENSE COUNTS:             25      7      37      92     33     1.43e-19    2.07e-18           0.000  -1.837  0.566  1.880  0.401
    TAGS:
      d+2      ATAACTCCAC     1       0      4       4      1      1.69e-01    2.39e-01    1295   0.000  0.000   2.000  2.000  0.000
      d+2      TCGGATTCGA     24      7      33      88     32     7.68e-19    1.98e-17    1162   0.000  -1.778  0.459  1.874  0.415

LOCUS: AT1G09310
DESCRIPTION:  expressed protein, contains Pfam profile PF04398
DATA:                         Control 30min  2hours  2days  1week  p-value     B&H         Pos    Fold change relative to control (log2)
SENSE COUNTS:                 103     23     27      20     60     2.08e-19    2.90e-18           0.000  -2.163  -1.932 -2.365 -0.780
  GENES:
  AT1G09310.1
```

```
    SENSE COUNTS:            103    23     27     20     60     2.08e-19      2.97e-18             0.000  -2.163 -1.932 -2.365 -0.780
    TAGS:
      X+4     GGAAACCTCT     0      0      0      0      1      1.65e-01      2.40e-01      835    0.000  0.000  0.000  0.000  0.000
      d+1     TATTATCTAC     103    23     26     20     59     1.20e-19      3.20e-18      700    0.000  -2.163 -1.986 -2.365 -0.804
      d+2     GAGAGTTAGT     0      0      1      0      0      4.55e-01      5.22e-01      665    0.000  0.000  0.000  0.000  0.000

LOCUS: AT1G28330
DESCRIPTION:  dormancy-associated protein, putative (DRM1), identical to dormancy-associated protein (Arabidopsis thaliana) GI
DATA:                        Control 30min  2hours 2days  1week  p-value       B&H           Pos    Fold change relative to control (log2)
SENSE COUNTS:                2      34     1      0      0      6.51e-19      8.94e-18             0.000  4.087  -1.000 0.000  0.000
  GENES:
  AT1G28330.3
    SENSE COUNTS:            2      34     1      0      0      6.51e-19      9.05e-18             0.000  4.087  -1.000 0.000  0.000
    TAGS:
      d+1     CGGGTGATGT     2      34     1      0      0      6.51e-19      1.71e-17      721    0.000  4.087  -1.000 0.000  0.000
  AT1G28330.2
    SENSE COUNTS:            2      34     1      0      0      6.51e-19      9.17e-18             0.000  4.087  -1.000 0.000  0.000
    TAGS:
      d+1     CGGGTGATGT     2      34     1      0      0      6.51e-19      1.71e-17      550    0.000  4.087  -1.000 0.000  0.000
  AT1G28330.1
    SENSE COUNTS:            2      34     1      0      0      6.51e-19      8.93e-18             0.000  4.087  -1.000 0.000  0.000
    TAGS:
      d+1     CGGGTGATGT     2      34     1      0      0      6.51e-19      1.71e-17      443    0.000  4.087  -1.000 0.000  0.000

LOCUS: AT4G39090
DESCRIPTION:  cysteine proteinase RD19a (RD19A) / thiol protease, identical to cysteine proteinase RD19a, thiol protease SP
DATA:                        Control 30min  2hours 2days  1week  p-value       B&H           Pos    Fold change relative to control (log2)
SENSE COUNTS:                3      45     4      4      5      7.00e-19      9.47e-18             0.000  3.907  0.415  0.415  0.737
  GENES:
  AT4G39090.1
    SENSE COUNTS:            3      45     4      4      5      7.00e-19      9.36e-18             0.000  3.907  0.415  0.415  0.737
    TAGS:
      d+2     CCCTTACATA     3      45     1      4      5      6.89e-21      1.93e-19      1032   0.000  3.907  -1.585 0.415  0.737
      d+2     GCGCCGTTAC     0      0      3      0      0      2.70e-02      4.62e-02      590    0.000  0.000  1.585  0.000  0.000

LOCUS: AT3G04120
DESCRIPTION:  glyceraldehyde-3-phosphate dehydrogenase, cytosolic (GAPC) / NAD-dependent glyceraldehyde-3-phosphate dehydrogenase, identical to
SP|P25858 Glyceraldehyde 3-phosphate dehydrogenase, cytosolic (EC 1.2.1.12) {Arabidopsis thaliana}
DATA:                        Control 30min  2hours 2days  1week  p-value       B&H           Pos    Fold change relative to control (log2)
SENSE COUNTS:                77     26     75     120    150    9.12e-19      1.22e-17             0.000  -1.566 -0.038 0.640  0.962
  GENES:
  AT3G04120.1
    SENSE COUNTS:            77     26     75     120    150    9.12e-19      1.20e-17             0.000  -1.566 -0.038 0.640  0.962
    TAGS:
      d+1     TGAGGTGATG     77     26     75     120    149    1.87e-18      4.49e-17      1283   0.000  -1.566 -0.038 0.640  0.952
      X+4     TTGCCTTTTG     0      0      0      0      1      1.65e-01      2.36e-01      683    0.000  0.000  0.000  0.000  0.000

LOCUS: AT5G24460
DESCRIPTION:  expressed protein
DATA:                        Control 30min  2hours 2days  1week  p-value       B&H           Pos    Fold change relative to control (log2)
SENSE COUNTS:                4      0      3      1      39     1.13e-18      1.49e-17             0.000  0.000  -0.415 -2.000 3.285
  GENES:
  AT5G24460.1
    SENSE COUNTS:            4      0      3      1      39     1.13e-18      1.47e-17             0.000  0.000  -0.415 -2.000 3.285
    TAGS:
      d+1     TAGAGATACA     4      0      3      1      39     1.13e-18      2.79e-17      1095   0.000  0.000  -0.415 -2.000 3.285

LOCUS: AT1G03130
DESCRIPTION:  photosystem I reaction center subunit II, chloroplast, putative / photosystem I 20 kDa subunit, putative / PSI-D, putative (PSAD2),
similar to SP|P12353 Photosystem I reaction center subunit II, chloroplast precursor (Photosystem I 20 kDa subunit) (PSI-D)
```

```
DATA:                         Control 30min   2hours  2days   1week   p-value         B&H             Pos     Fold change relative to control (log2)
SENSE COUNTS:                 153     72      166     98      30      1.22e-18        1.58e-17                0.000   -1.087  0.118   -0.643  -2.350
  GENES:
  AT1G03130.1
    SENSE COUNTS:             153     72      166     98      30      1.22e-18        1.57e-17                0.000   -1.087  0.118   -0.643  -2.350
    TAGS:
      d+1     TGGTAAGTGA      2       0       3       0       0       1.23e-01        1.88e-01        829     0.000   0.000   0.585   0.000   0.000
      d+2     TAAAATCTTG      150     72      162     97      30      1.41e-17        3.24e-16        743     0.000   -1.059  0.111   -0.629  -2.322
      d+2     GAACTCACCG      1       0       1       1       0       7.18e-01        7.25e-01        350     0.000   0.000   0.000   0.000   0.000

LOCUS: AT5G02380
DESCRIPTION:  metallothionein protein 2B (MT-2B), identical to SWISS-PROT
DATA:                         Control 30min   2hours  2days   1week   p-value         B&H             Pos     Fold change relative to control (log2)
SENSE COUNTS:                 28      104     25      38      32      1.70e-18        2.17e-17                0.000   1.893   -0.163  0.441   0.193
  GENES:
  AT5G02380.1
    SENSE COUNTS:             28      104     25      38      32      1.70e-18        2.17e-17                0.000   1.893   -0.163  0.441   0.193
    TAGS:
      d+1     TCTTGCTGTG      28      104     25      38      32      1.70e-18        4.14e-17        84      0.000   1.893   -0.163  0.441   0.193

LOCUS: AT1G20340
DESCRIPTION:  plastocyanin, similar to plastocyanin GI
DATA:                         Control 30min   2hours  2days   1week   p-value         B&H             Pos     Fold change relative to control (log2)
SENSE COUNTS:                 36      106     42      58      3       2.94e-18        3.71e-17                0.000   1.558   0.222   0.688   -3.585
  GENES:
  AT1G20340.1
    SENSE COUNTS:             36      106     42      58      3       2.94e-18        3.70e-17                0.000   1.558   0.222   0.688   -3.585
    TAGS:
      d+1     GCAATAGAAG      35      105     39      54      3       1.11e-18        2.78e-17        292     0.000   1.585   0.156   0.626   -3.544
      d+2     GCCTCAGTAA      1       1       3       4       0       4.61e-01        5.14e-01        94      0.000   0.000   1.585   2.000   0.000

LOCUS: AT3G59540
DESCRIPTION:  60S ribosomal protein L38 (RPL38B), 60S RIBOSOMAL PROTEIN L38 - Lycopersicon esculentum, EMBL
DATA:                         Control 30min   2hours  2days   1week   p-value         B&H             Pos     Fold change relative to control (log2)
SENSE COUNTS:                 6       3       2       2       44      3.01e-18        3.74e-17                0.000   -1.000  -1.585  -1.585  2.874
  GENES:
  AT3G59540.1
    SENSE COUNTS:             6       3       2       2       44      3.01e-18        3.74e-17                0.000   -1.000  -1.585  -1.585  2.874
    TAGS:
      d+1     GTTTTATATA      6       3       2       2       44      3.01e-18        7.12e-17        303     0.000   -1.000  -1.585  -1.585  2.874

LOCUS: AT2G36530
DESCRIPTION:  enolase, identical to SWISS-PROT
DATA:                         Control 30min   2hours  2days   1week   p-value         B&H             Pos     Fold change relative to control (log2)
SENSE COUNTS:                 26      14      38      77      103     3.05e-18        3.74e-17                0.000   -0.893  0.547   1.566   1.986
  GENES:
  AT2G36530.1
    SENSE COUNTS:             26      14      38      77      103     3.05e-18        3.75e-17                0.000   -0.893  0.547   1.566   1.986
    TAGS:
      d+1     TGATTGTTAT      0       0       1       4       10      7.67e-04        2.32e-03        1669    0.000   0.000   0.000   2.000   3.322
      d+2     AGAATGCTCT      26      14      37      73      93      1.49e-15        2.88e-14        1592    0.000   -0.893  0.509   1.489   1.839

LOCUS: AT5G01530
DESCRIPTION:  chlorophyll A-B binding protein CP29 (LHCB4), identical to CP29 (Arabidopsis thaliana) GI
DATA:                         Control 30min   2hours  2days   1week   p-value         B&H             Pos     Fold change relative to control (log2)
SENSE COUNTS:                 259     135     199     196     66      3.29e-18        3.98e-17                0.000   -0.940  -0.380  -0.402  -1.972
  GENES:
  AT5G01530.1
    SENSE COUNTS:             259     135     199     196     66      3.29e-18        4.00e-17                0.000   -0.940  -0.380  -0.402  -1.972
    TAGS:
```

d+1 ACATTAAATT 108 43 82 91 25 1.76e-10 2.19e-09 1133 0.000 -1.329 -0.397 -0.247 -2.111
d+2 TGAGATTCTA 151 92 109 105 41 1.69e-08 1.57e-07 1034 0.000 -0.715 -0.470 -0.524 -1.881
d+2 GCAAGACGCT 0 0 7 0 0 3.86e-05 1.77e-04 642 0.000 0.000 2.807 0.000 0.000
d+2 GGTACTCGGG 0 0 1 0 0 4.55e-01 5.11e-01 193 0.000 0.000 0.000 0.000 0.000

LOCUS: AT4G30270
DESCRIPTION: MERI-5 protein (MERI-5) (MERI5B) / endo-xyloglucan transferase / xyloglucan endo-1,4-beta-D-glucanase (SEN4), identical to endo-xyloglucan transferase gi
DATA: Control 30min 2hours 2days 1week p-value B&H Pos Fold change relative to control (log2)
SENSE COUNTS: 6 51 10 4 5 3.43e-18 4.10e-17 0.000 3.087 0.737 -0.585 -0.263
GENES:
AT4G30270.1
SENSE COUNTS: 6 51 10 4 5 3.43e-18 4.12e-17 0.000 3.087 0.737 -0.585 -0.263
TAGS:
d+1 ATCTACAATT 6 51 10 4 5 3.43e-18 8.00e-17 825 0.000 3.087 0.737 -0.585 -0.263

LOCUS: AT4G31500
DESCRIPTION: cytochrome P450 83B1 (CYP83B1), Identical to Cytochrome P450 (SP
DATA: Control 30min 2hours 2days 1week p-value B&H Pos Fold change relative to control (log2)
SENSE COUNTS: 131 19 71 78 92 1.36e-17 1.60e-16 0.000 -2.785 -0.884 -0.748 -0.510
GENES:
AT4G31500.1
SENSE COUNTS: 131 19 71 78 92 1.36e-17 1.62e-16 0.000 -2.785 -0.884 -0.748 -0.510
TAGS:
d+1 ACTGGACTCG 4 10 22 5 8 2.95e-03 7.11e-03 1480 0.000 1.322 2.459 0.322 1.000
X+4 TCGCTAACAA 0 1 0 0 0 2.54e-01 3.44e-01 888 0.000 0.000 0.000 0.000 0.000
d+2 TCGTATCAAG 127 8 49 73 84 1.47e-24 4.94e-23 358 0.000 -3.989 -1.374 -0.799 -0.596

LOCUS: AT5G14920
DESCRIPTION: gibberellin-regulated family protein, similar to SP|P46689 Gibberellin-regulated protein 1 precursor {Arabidopsis thaliana}; contains Pfam profile PF02704
DATA: Control 30min 2hours 2days 1week p-value B&H Pos Fold change relative to control (log2)
SENSE COUNTS: 12 62 8 11 16 3.53e-17 4.11e-16 0.000 2.369 -0.585 -0.126 0.415
GENES:
AT5G14920.1
SENSE COUNTS: 12 62 8 11 16 3.53e-17 4.15e-16 0.000 2.369 -0.585 -0.126 0.415
TAGS:
d+1 AAGACACGTG 12 62 8 11 16 3.53e-17 7.91e-16 851 0.000 2.369 -0.585 -0.126 0.415

LOCUS: AT3G16240
DESCRIPTION: delta tonoplast integral protein (delta-TIP), identical to delta tonoplast integral protein (delta-TIP) (GI
DATA: Control 30min 2hours 2days 1week p-value B&H Pos Fold change relative to control (log2)
SENSE COUNTS: 105 52 47 27 3 8.42e-17 9.68e-16 0.000 -1.014 -1.160 -1.959 -5.129
GENES:
AT3G16240.1
SENSE COUNTS: 105 52 47 27 3 8.42e-17 9.79e-16 0.000 -1.014 -1.160 -1.959 -5.129
TAGS:
d+1 TTTCTGTGTT 105 52 44 27 3 3.58e-17 7.91e-16 892 0.000 -1.014 -1.255 -1.959 -5.129
d+2 AACCCAGCAC 0 0 2 0 0 1.21e-01 1.87e-01 699 0.000 0.000 1.000 0.000 0.000
d+2 TGAACCCAGC 0 0 1 0 0 4.55e-01 5.24e-01 355 0.000 0.000 0.000 0.000 0.000

LOCUS: AT1G55490
DESCRIPTION: RuBisCO subunit binding-protein beta subunit, chloroplast / 60 kDa chaperonin beta subunit / CPN-60 beta, identical to SWISS-PROT
DATA: Control 30min 2hours 2days 1week p-value B&H Pos Fold change relative to control (log2)
SENSE COUNTS: 35 16 22 44 102 1.08e-16 1.23e-15 0.000 -1.129 -0.670 0.330 1.543
GENES:
AT1G55490.1
SENSE COUNTS: 35 16 22 44 102 1.08e-16 1.24e-15 0.000 -1.129 -0.670 0.330 1.543
TAGS:
d+1 ATTCCCTCAA 35 16 22 44 102 1.08e-16 2.36e-15 2053 0.000 -1.129 -0.670 0.330 1.543

AT1G55490.2
SENSE COUNTS: 35 16 22 44 102 1.08e-16 1.23e-15 0.000 -1.129 -0.670 0.330 1.543
TAGS:
d+1 ATTCCCTCAA 35 16 22 44 102 1.08e-16 2.36e-15 2026 0.000 -1.129 -0.670 0.330 1.543

LOCUS: AT1G34430
DESCRIPTION: dihydrolipoamide S-acetyltransferase, putative, similar to dihydrolipoamide S-acetyltransferase (LTA2) (Arabidopsis thaliana) GI
DATA: Control 30min 2hours 2days 1week p-value B&H Pos Fold change relative to control (log2)
SENSE COUNTS: 56 6 12 4 13 1.33e-16 1.49e-15 0.000 -3.222 -2.222 -3.807 -2.107
GENES:
AT1G34430.1
SENSE COUNTS: 56 6 12 4 13 1.33e-16 1.50e-15 0.000 -3.222 -2.222 -3.807 -2.107
TAGS:
d+2 ATTAATAACA 51 5 10 4 12 1.51e-16 3.25e-15 1704 0.000 -3.350 -2.350 -3.672 -2.087
d+2 CGATGAATCT 0 0 0 0 0 6.15e-01 6.30e-01 1669 0.000 0.000 0.000 0.000 0.000
d+2 ATTTTGCTAC 3 0 0 0 0 2.13e-02 3.69e-02 1634 0.000 0.000 0.000 0.000 0.000
d+2 GGAGCTTGTA 2 1 2 0 1 8.10e-01 8.13e-01 1507 0.000 -1.000 0.000 0.000 -1.000
d+2 GTTGAAGAAG 0 0 0 0 0 6.15e-01 6.54e-01 350 0.000 0.000 0.000 0.000 0.000

LOCUS: AT1G55670
DESCRIPTION: photosystem I reaction center subunit V, chloroplast, putative / PSI-G, putative (PSAG), identical to SP|Q9S7N7; similar to SP|Q00327 Photosystem I reaction center subunit V, chloroplast precursor (PSI-G) (Photosystem I 9 kDa protein) {Hordeum vulgare}; c
DATA: Control 30min 2hours 2days 1week p-value B&H Pos Fold change relative to control (log2)
SENSE COUNTS: 26 100 60 52 5 2.28e-16 2.53e-15 0.000 1.943 1.206 1.000 -2.379
GENES:
AT1G55670.1
SENSE COUNTS: 26 100 60 52 5 2.28e-16 2.54e-15 0.000 1.943 1.206 1.000 -2.379
TAGS:
d+1 GCCTTCGTCC 26 100 60 52 5 2.28e-16 4.85e-15 182 0.000 1.943 1.206 1.000 -2.379

LOCUS: AT3G55700
DESCRIPTION: UDP-glucoronosyl/UDP-glucosyl transferase family protein, glucuronosyl transferase homolog, Lycopersicon esculentum, PIR
DATA: Control 30min 2hours 2days 1week p-value B&H Pos Fold change relative to control (log2)
SENSE COUNTS: 3 1 18 49 43 2.32e-16 2.54e-15 0.000 -1.585 2.585 4.030 3.841
GENES:
AT3G55700.1
SENSE COUNTS: 3 1 18 49 43 2.32e-16 2.56e-15 0.000 -1.585 2.585 4.030 3.841
TAGS:
X+4 AAACTTTATT 3 1 18 49 43 2.32e-16 4.81e-15 -248 0.000 -1.585 2.585 4.030 3.841

LOCUS: AT4G12800
DESCRIPTION: photosystem I reaction center subunit XI, chloroplast (PSI-L) / PSI subunit V, identical to Photosystem I reaction center subunit XI, chloroplast precursor (PSI-L) (PSI subunit V) (Swiss-Prot
DATA: Control 30min 2hours 2days 1week p-value B&H Pos Fold change relative to control (log2)
SENSE COUNTS: 45 83 24 26 1 3.24e-16 3.51e-15 0.000 0.883 -0.907 -0.791 -5.492
GENES:
AT4G12800.1
SENSE COUNTS: 45 83 24 26 1 3.24e-16 3.54e-15 0.000 0.883 -0.907 -0.791 -5.492
TAGS:
d+2 TGCCTCACCA 45 83 24 26 1 3.24e-16 6.64e-15 592 0.000 0.883 -0.907 -0.791 -5.492

LOCUS: AT3G01500
DESCRIPTION: carbonic anhydrase 1, chloroplast / carbonate dehydratase 1 (CA1), nearly identical to SP|P27140 Carbonic anhydrase, chloroplast precursor (EC 4.2.1.1) (Carbonate dehydratase) {Arabidopsis thaliana}
DATA: Control 30min 2hours 2days 1week p-value B&H Pos Fold change relative to control (log2)
SENSE COUNTS: 139 59 92 91 9 4.61e-16 4.93e-15 0.000 -1.236 -0.595 -0.611 -3.949
GENES:
AT3G01500.1
SENSE COUNTS: 20 1 21 12 1 1.71e-05 5.08e-05 0.000 -4.322 0.070 -0.737 -4.322
TAGS:

d+1 CAGATCTTGA 20 1 21 12 1 1.71e-05 8.63e-05 1211 0.000 -4.322 0.070 -0.737 -4.322
AT3G01500.2
SENSE COUNTS: 20 1 21 12 1 1.71e-05 5.07e-05 0.000 -4.322 0.070 -0.737 -4.322
TAGS:
d+1 CAGATCTTGA 20 1 21 12 1 1.71e-05 8.63e-05 1213 0.000 -4.322 0.070 -0.737 -4.322
AT3G01500.3
SENSE COUNTS: 119 58 71 79 8 1.21e-12 9.81e-12 0.000 -1.037 -0.745 -0.591 -3.895
TAGS:
d+1 ACCATCCATC 119 58 71 79 8 1.21e-12 1.83e-11 1142 0.000 -1.037 -0.745 -0.591 -3.895

LOCUS: AT2G45960
DESCRIPTION: plasma membrane intrinsic protein 1B (PIP1B) / aquaporin PIP1.2 (PIP1.2) / transmembrane protein A (TMPA), identical to plasma membrane intrinsic protein 1B SP
DATA: Control 30min 2hours 2days 1week p-value B&H Pos Fold change relative to control (log2)
SENSE COUNTS: 135 50 121 99 24 7.09e-16 7.50e-15 0.000 -1.433 -0.158 -0.447 -2.492
GENES:
AT2G45960.1
SENSE COUNTS: 135 50 121 99 24 7.09e-16 7.66e-15 0.000 -1.433 -0.158 -0.447 -2.492
TAGS:
d+1 CCCCAGAAAC 118 42 118 94 19 2.31e-16 4.85e-15 1137 0.000 -1.490 0.000 -0.328 -2.635
d+2 TTCCTATTCT 17 8 2 5 5 7.03e-03 1.38e-02 759 0.000 -1.087 -3.087 -1.766 -1.766
X+4 CCAAATGTTA 0 0 1 0 0 4.55e-01 5.12e-01 412 0.000 0.000 0.000 0.000 0.000

LOCUS: AT3G49910
DESCRIPTION: 60S ribosomal protein L26 (RPL26A), 60S RIBOSOMAL PROTEIN L26, Brassica rapa, EMBL
DATA: Control 30min 2hours 2days 1week p-value B&H Pos Fold change relative to control (log2)
SENSE COUNTS: 49 16 44 61 116 9.81e-16 1.03e-14 0.000 -1.615 -0.155 0.316 1.243
GENES:
AT3G49910.1
SENSE COUNTS: 49 16 44 61 116 9.81e-16 1.05e-14 0.000 -1.615 -0.155 0.316 1.243
TAGS:
d+1 AGAACTTATG 49 16 42 61 116 5.26e-16 1.05e-14 622 0.000 -1.615 -0.222 0.316 1.243
d+2 CCGATCCGTA 0 0 2 0 0 1.21e-01 1.85e-01 197 0.000 0.000 1.000 0.000 0.000

LOCUS: AT2G46110
DESCRIPTION: ketopantoate hydroxymethyltransferase family protein, similar to SP|Q9Y7B6 3-methyl-2-oxobutanoate hydroxymethyltransferase (EC 2.1.2.11) (Ketopantoate hydroxymethyltransferase) {Emericella nidulans}; contains Pfam profile PF02548
DATA: Control 30min 2hours 2days 1week p-value B&H Pos Fold change relative to control (log2)
SENSE COUNTS: 0 0 1 0 25 9.86e-16 1.02e-14 0.000 0.000 0.000 0.000 4.644
GENES:
AT2G46110.1
SENSE COUNTS: 0 0 1 0 25 9.86e-16 1.04e-14 0.000 0.000 0.000 0.000 4.644
TAGS:
d+1 GTTTCTCTGT 0 0 0 0 25 7.86e-19 2.00e-17 1303 0.000 0.000 0.000 0.000 4.644
d+2 GAGCCTTCAA 0 0 1 0 0 7.06e-01 7.16e-01 1149 0.000 0.000 0.000 0.000 0.000

LOCUS: AT2G39730
DESCRIPTION: ribulose bisphosphate carboxylase/oxygenase activase / RuBisCO activase, identical to SWISS-PROT
DATA: Control 30min 2hours 2days 1week p-value B&H Pos Fold change relative to control (log2)
SENSE COUNTS: 188 133 165 96 34 1.20e-15 1.23e-14 0.000 -0.499 -0.188 -0.970 -2.467
GENES:
AT2G39730.1
SENSE COUNTS: 188 133 165 96 34 1.20e-15 1.26e-14 0.000 -0.499 -0.188 -0.970 -2.467
TAGS:
d+1 CTTGTGATGG 186 133 152 95 34 1.07e-14 1.93e-13 1401 0.000 -0.484 -0.291 -0.969 -2.452
d+2 ATGAGTGCTG 2 0 13 1 0 2.60e-06 1.62e-05 768 0.000 0.000 2.700 -1.000 0.000
AT2G39730.2
SENSE COUNTS: 188 133 165 96 34 1.20e-15 1.25e-14 0.000 -0.499 -0.188 -0.970 -2.467
TAGS:
d+1 CTTGTGATGG 186 133 152 95 34 1.07e-14 1.93e-13 1372 0.000 -0.484 -0.291 -0.969 -2.452

```
      d+2      ATGAGTGCTG     2      0      13     1      0      2.60e-06      1.62e-05      739    0.000  0.000  2.700  -1.000 0.000
  AT2G39730.3
    SENSE COUNTS:             188    133    165    96     34     1.20e-15      1.23e-14             0.000  -0.499 -0.188 -0.970 -2.467
    TAGS:
      d+1      CTTGTGATGG     186    133    152    95     34     1.07e-14      1.93e-13      1372   0.000  -0.484 -0.291 -0.969 -2.452
      d+2      ATGAGTGCTG     2      0      13     1      0      2.60e-06      1.62e-05      739    0.000  0.000  2.700  -1.000 0.000

LOCUS: AT5G54270
DESCRIPTION:  chlorophyll A-B binding protein / LHCII type III (LHCB3), identical to Lhcb3 protein (Arabidopsis thaliana) GI
DATA:                         Control 30min  2hours 2days  1week  p-value       B&H           Pos    Fold change relative to control (log2)
SENSE COUNTS:                 67     12     83     43     19     3.42e-15      3.46e-14             0.000  -2.481 0.309  -0.640 -1.818
  GENES:
  AT5G54270.1
    SENSE COUNTS:             67     12     83     43     19     3.42e-15      3.45e-14             0.000  -2.481 0.309  -0.640 -1.818
    TAGS:
      d+1      TCTCTCACAG     64     12     59     41     14     4.47e-11      5.87e-10      1006   0.000  -2.415 -0.117 -0.642 -2.193
      d+2      CTCAGAGCAT     1      0      1      0      0      6.01e-01      6.48e-01      553    0.000  0.000  0.000  0.000  0.000
      d+2      GGAGATGGGC     2      0      23     2      5      5.25e-10      5.80e-09      394    0.000  0.000  3.524  0.000  1.322

LOCUS: AT4G37930
DESCRIPTION:  glycine hydroxymethyltransferase / serine hydroxymethyltransferase / serine/threonine aldolase (SHM1), identical to serine hydroxymethyl
transferase (Arabidopsis thaliana) GI
DATA:                         Control 30min  2hours 2days  1week  p-value       B&H           Pos    Fold change relative to control (log2)
SENSE COUNTS:                 195    60     131    142    134    4.70e-15      4.70e-14             0.000  -1.700 -0.574 -0.458 -0.541
  GENES:
  AT4G37930.1
    SENSE COUNTS:             195    60     131    142    134    4.70e-15      4.70e-14             0.000  -1.700 -0.574 -0.458 -0.541
    TAGS:
      d+1      TAAAACTCTA     195    60     127    142    134    3.80e-15      7.17e-14      1745   0.000  -1.700 -0.619 -0.458 -0.541
      d+2      AAGTACAAGA     0      0      2      0      0      1.21e-01      1.86e-01      1663   0.000  0.000  1.000  0.000  0.000
      d+2      AAGTCGAGGA     0      0      1      0      0      4.55e-01      5.35e-01      1607   0.000  0.000  0.000  0.000  0.000
      d+2      GCAGAAACCT     0      0      1      0      0      4.55e-01      5.24e-01      484    0.000  0.000  0.000  0.000  0.000

LOCUS: AT3G08940
DESCRIPTION:  chlorophyll A-B binding protein (LHCB4.2), contains Pfam profile
DATA:                         Control 30min  2hours 2days  1week  p-value       B&H           Pos    Fold change relative to control (log2)
SENSE COUNTS:                 112    65     37     44     12     5.63e-15      5.57e-14             0.000  -0.785 -1.598 -1.348 -3.222
  GENES:
  AT3G08940.2
    SENSE COUNTS:             112    65     37     44     12     5.63e-15      5.58e-14             0.000  -0.785 -1.598 -1.348 -3.222
    TAGS:
      d+1      TAATGATGTA     111    65     34     44     12     2.50e-15      4.77e-14      976    0.000  -0.772 -1.707 -1.335 -3.209
      d+2      TAATGATGTG     1      0      1      0      0      6.01e-01      6.50e-01      938    0.000  0.000  0.000  0.000  0.000
      d+2      CTCGTTTAGC     0      0      0      0      0      6.15e-01      6.33e-01      769    0.000  0.000  0.000  0.000  0.000
      d+2      GGTACCCGGG     0      0      2      0      0      1.21e-01      1.86e-01      84     0.000  0.000  1.000  0.000  0.000
  AT3G08940.1
    SENSE COUNTS:             112    65     37     44     12     5.63e-15      5.53e-14             0.000  -0.785 -1.598 -1.348 -3.222
    TAGS:
      d+1      TAATGATGTA     111    65     34     44     12     2.50e-15      4.77e-14      1061   0.000  -0.772 -1.707 -1.335 -3.209
      d+2      TAATGATGTG     1      0      1      0      0      6.01e-01      6.50e-01      1023   0.000  0.000  0.000  0.000  0.000
      d+2      CTCGTTTAGC     0      0      0      0      0      6.15e-01      6.33e-01      854    0.000  0.000  0.000  0.000  0.000
      d+2      GGTACCCGGG     0      0      2      0      0      1.21e-01      1.86e-01      84     0.000  0.000  1.000  0.000  0.000

LOCUS: AT5G24120
DESCRIPTION:  RNA polymerase sigma subunit SigE (sigE) / sigma-like factor (SIG5), identical to RNA polymerase sigma subunit SigE (Arabidopsis
thaliana) GI
DATA:                         Control 30min  2hours 2days  1week  p-value       B&H           Pos    Fold change relative to control (log2)
SENSE COUNTS:                 17     2      36     67     26     8.25e-15      8.07e-14             0.000  -3.087 1.082  1.979  0.613
  GENES:
  AT5G24120.1
```

```
    SENSE COUNTS:            17     2      36     67     26     8.25e-15      8.03e-14             0.000  -3.087  1.082  1.979  0.613
    TAGS:
      d+1     TAAAGTTTGA     16     2      35     62     25     7.71e-14      1.30e-12      1873   0.000  -3.000  1.129  1.954  0.644
      d+2     CTTGGTTGTT     1      0      1      5      1      1.87e-01      2.63e-01      783    0.000  0.000   0.000  2.322  0.000

LOCUS: AT4G21100
DESCRIPTION:  UV-damaged DNA-binding protein, putative, similar to UV-damaged DNA binding protein (GI
DATA:                        Control 30min  2hours 2days  1week  p-value       B&H           Pos    Fold change relative to control (log2)
SENSE COUNTS:                120    74     33     41     124    8.61e-15      8.34e-14             0.000  -0.697  -1.862 -1.549 0.047
  GENES:
  AT4G21100.1
    SENSE COUNTS:            120    74     33     41     124    8.61e-15      8.30e-14             0.000  -0.697  -1.862 -1.549 0.047
    TAGS:
      d+2     AATATAGAGG     120    74     33     41     124    8.61e-15      1.57e-13      1291   0.000  -0.697  -1.862 -1.549 0.047

LOCUS: AT2G26500
DESCRIPTION:  cytochrome b6f complex subunit (petM), putative, nearly identical to cytochrome b6f complex subunit (GI
DATA:                        Control 30min  2hours 2days  1week  p-value       B&H           Pos    Fold change relative to control (log2)
SENSE COUNTS:                134    74     49     45     138    9.72e-15      9.32e-14             0.000  -0.857  -1.451 -1.574 0.042
  GENES:
  AT2G26500.1
    SENSE COUNTS:            134    74     49     45     138    9.72e-15      9.29e-14             0.000  -0.857  -1.451 -1.574 0.042
    TAGS:
      d+1     CCCTCCATTG     1      2      4      1      3      6.51e-01      6.66e-01      682    0.000  1.000   2.000  0.000  1.585
      d+2     TTACATTTGT     133    72     43     43     134    5.07e-16      1.03e-14      588    0.000  -0.885  -1.629 -1.629 0.011
      d+2     AACGCTCTTA     0      0      2      1      1      4.09e-01      5.15e-01      422    0.000  0.000   1.000  0.000  0.000
  AT2G26500.2
    SENSE COUNTS:            134    74     49     45     138    9.72e-15      9.20e-14             0.000  -0.857  -1.451 -1.574 0.042
    TAGS:
      d+1     CCCTCCATTG     1      2      4      1      3      6.51e-01      6.66e-01      650    0.000  1.000   2.000  0.000  1.585
      d+2     TTACATTTGT     133    72     43     43     134    5.07e-16      1.03e-14      556    0.000  -0.885  -1.629 -1.629 0.011
      d+2     AACGCTCTTA     0      0      2      1      1      4.09e-01      5.15e-01      390    0.000  0.000   1.000  0.000  0.000

LOCUS: AT5G66400
DESCRIPTION:  dehydrin (RAB18), nearly identical to SP|P30185 Dehydrin Rab18 {Arabidopsis thaliana}
DATA:                        Control 30min  2hours 2days  1week  p-value       B&H           Pos    Fold change relative to control (log2)
SENSE COUNTS:                8      0      3      5      39     1.20e-14      1.14e-13             0.000  0.000   -1.415 -0.678 2.285
  GENES:
  AT5G66400.1
    SENSE COUNTS:            8      0      3      5      39     1.20e-14      1.13e-13             0.000  0.000   -1.415 -0.678 2.285
    TAGS:
      d+1     AGTGTGTAAT     8      0      3      5      39     1.20e-14      2.14e-13      776    0.000  0.000   -1.415 -0.678 2.285

LOCUS: AT4G32020
DESCRIPTION:  expressed protein, NuLL
DATA:                        Control 30min  2hours 2days  1week  p-value       B&H           Pos    Fold change relative to control (log2)
SENSE COUNTS:                23     25     94     33     58     1.60e-14      1.50e-13             0.000  0.120   2.031  0.521  1.334
  GENES:
  AT4G32020.1
    SENSE COUNTS:            23     25     94     33     58     1.60e-14      1.49e-13             0.000  0.120   2.031  0.521  1.334
    TAGS:
      d+1     AGATTAAATA     0      0      2      2      1      3.02e-01      4.06e-01      1474   0.000  0.000   1.000  1.000  0.000
      d+2     TAACCGTTTG     22     25     88     31     57     6.11e-13      9.59e-12      1362   0.000  0.184   2.000  0.495  1.373
      d+2     ACCTCGCCGC     1      0      4      0      0      2.82e-02      4.80e-02      583    0.000  0.000   2.000  0.000  0.000

LOCUS: AT2G15890
DESCRIPTION:  expressed protein
DATA:                        Control 30min  2hours 2days  1week  p-value       B&H           Pos    Fold change relative to control (log2)
SENSE COUNTS:                6      39     5      3      3      3.30e-14      3.07e-13             0.000  2.700   -0.263 -1.000 -1.000
  GENES:
```

```
  AT2G15890.1
    SENSE COUNTS:              6      39     5      3      3      3.30e-14      3.04e-13                0.000  2.700  -0.263  -1.000  -1.000
    TAGS:
      d+1     TAGTCTATCT       6      38     5      1      3      2.17e-14      3.72e-13      727       0.000  2.663  -0.263  -2.585  -1.000
      d+2     TAGGAGAAGA       0      1      0      2      0      2.43e-01      3.34e-01      443       0.000  0.000  0.000   1.000   0.000

LOCUS: AT1G23290
DESCRIPTION:  60S ribosomal protein L27A (RPL27aB), similar to 60S RIBOSOMAL PROTEIN L27A GB
DATA:                          Control 30min  2hours 2days  1week  p-value       B&H           Pos       Fold change relative to control (log2)
SENSE COUNTS:                  31     13     14     30     80     6.93e-14      6.38e-13                0.000  -1.254 -1.147  -0.047  1.368
  GENES:
  AT1G23290.1
    SENSE COUNTS:              31     13     14     30     80     6.93e-14      6.34e-13                0.000  -1.254 -1.147  -0.047  1.368
    TAGS:
      d+1     TTATTGAACT       31     13     14     30     80     6.93e-14      1.18e-12      505       0.000  -1.254 -1.147  -0.047  1.368

LOCUS: AT4G17090
DESCRIPTION:  beta-amylase (CT-BMY) / 1,4-alpha-D-glucan maltohydrolase, identical to beta-amylase enzyme GI
DATA:                          Control 30min  2hours 2days  1week  p-value       B&H           Pos       Fold change relative to control (log2)
SENSE COUNTS:                  2      3      35     48     40     8.11e-14      7.39e-13                0.000  0.585  4.129   4.585   4.322
  GENES:
  AT4G17090.1
    SENSE COUNTS:              2      3      35     48     40     8.11e-14      7.35e-13                0.000  0.585  4.129   4.585   4.322
    TAGS:
      d+1     GGAGGAGACT       2      1      34     47     37     1.22e-14      2.16e-13      1631      0.000  -1.000 4.087   4.555   4.209
      d+2     GAGATGAAAG       0      2      1      0      3      2.60e-01      3.52e-01      1345      0.000  1.000  0.000   0.000   1.585
      d+2     ACCTTTGCGA       0      0      0      1      0      3.09e-01      4.03e-01      175       0.000  0.000  0.000   0.000   0.000

LOCUS: AT3G16140
DESCRIPTION:  photosystem I reaction center subunit VI, chloroplast, putative / PSI-H, putative (PSAH1), identical to SP|Q9SUI7; similar to PSI-H
precursor (Nicotiana sylvestris) GI
DATA:                          Control 30min  2hours 2days  1week  p-value       B&H           Pos       Fold change relative to control (log2)
SENSE COUNTS:                  119    82     60     33     21     9.05e-14      8.16e-13                0.000  -0.537 -0.988  -1.850  -2.503
  GENES:
  AT3G16140.1
    SENSE COUNTS:              119    82     60     33     21     9.05e-14      8.14e-13                0.000  -0.537 -0.988  -1.850  -2.503
    TAGS:
      d+1     TTACCTTTCT       119    82     54     33     21     1.86e-14      3.25e-13      512       0.000  -0.537 -1.140  -1.850  -2.503
      d+2     GCGTCTCTTG       0      0      6      0      0      2.08e-04      7.87e-04      65        0.000  0.000  2.585   0.000   0.000

LOCUS: AT3G47650
DESCRIPTION:  bundle-sheath defective protein 2 family / bsd2 family, similar to bundle sheath defective protein 2 (Zea mays) GI
DATA:                          Control 30min  2hours 2days  1week  p-value       B&H           Pos       Fold change relative to control (log2)
SENSE COUNTS:                  9      8      7      5      52     1.45e-13      1.30e-12                0.000  -0.170 -0.363  -0.848  2.531
  GENES:
  AT3G47650.1
    SENSE COUNTS:              9      8      7      5      52     1.45e-13      1.29e-12                0.000  -0.170 -0.363  -0.848  2.531
    TAGS:
      d+1     TATTACTATT       9      8      6      5      52     2.10e-14      3.64e-13      572       0.000  -0.170 -0.585  -0.848  2.531
      d+2     TTCTCAATGC       0      0      1      0      0      7.06e-01      7.16e-01      287       0.000  0.000  0.000   0.000   0.000

LOCUS: AT5G54770
DESCRIPTION:  thiazole biosynthetic enzyme, chloroplast (ARA6) (THI1) (THI4), identical to SP|Q38814 Thiazole biosynthetic enzyme, chloroplast
precursor (ARA6) {Arabidopsis thaliana}
DATA:                          Control 30min  2hours 2days  1week  p-value       B&H           Pos       Fold change relative to control (log2)
SENSE COUNTS:                  55     147    95     69     49     1.52e-13      1.34e-12                0.000  1.418  0.788   0.327   -0.167
  GENES:
  AT5G54770.1
    SENSE COUNTS:              55     147    95     69     49     1.52e-13      1.34e-12                0.000  1.418  0.788   0.327   -0.167
    TAGS:
```

```
      d+1      AACACTGCTG     55      147     93      69      48      8.24e-14       1.37e-12       826     0.000   1.418   0.758   0.327   -0.196
      d+2      TTCCTGGGAT     0       0       1       0       1       3.96e-01       5.04e-01       800     0.000   0.000   0.000   0.000   0.000
      d+2      ACGGATATGA     0       0       1       0       0       4.55e-01       5.43e-01       265     0.000   0.000   0.000   0.000   0.000

LOCUS: AT5G20250
DESCRIPTION:  raffinose synthase family protein / seed imbibition protein, putative (din10), similar to seed imbibition protein GB
DATA:                          Control 30min   2hours  2days   1week   p-value        B&H            Pos     Fold change relative to control (log2)
SENSE COUNTS:                  8       37      5       4       0       1.62e-13       1.42e-12               0.000   2.209   -0.678  -1.000  0.000
  GENES:
  AT5G20250.1
    SENSE COUNTS:              8       37      5       4       0       1.62e-13       1.41e-12               0.000   2.209   -0.678  -1.000  0.000
    TAGS:
      d+1      TACAATTCGG     8       36      5       4       0       1.49e-12       2.20e-11       2307    0.000   2.170   -0.678  -1.000  0.000
      d+2      CCTCTGCTAG     0       1       0       0       0       2.54e-01       3.46e-01       1771    0.000   0.000   0.000   0.000   0.000
  AT5G20250.2
    SENSE COUNTS:              8       37      5       4       0       1.62e-13       1.42e-12               0.000   2.209   -0.678  -1.000  0.000
    TAGS:
      d+1      TACAATTCGG     8       36      5       4       0       1.49e-12       2.20e-11       2328    0.000   2.170   -0.678  -1.000  0.000
      d+2      CCTCTGCTAG     0       1       0       0       0       2.54e-01       3.46e-01       1792    0.000   0.000   0.000   0.000   0.000

LOCUS: AT3G62250
DESCRIPTION:  ubiquitin extension protein 5 (UBQ5) / 40S ribosomal protein S27A (RPS27aC), identical to GI
DATA:                          Control 30min   2hours  2days   1week   p-value        B&H            Pos     Fold change relative to control (log2)
SENSE COUNTS:                  47      10      26      41      88      1.85e-13       1.61e-12               0.000   -2.233  -0.854  -0.197  0.905
  GENES:
  AT3G62250.1
    SENSE COUNTS:              47      10      26      41      88      1.85e-13       1.60e-12               0.000   -2.233  -0.854  -0.197  0.905
    TAGS:
      d+1      GATTCTATGT     47      10      26      41      88      1.85e-13       3.05e-12       620     0.000   -2.233  -0.854  -0.197  0.905

LOCUS: AT3G59790
DESCRIPTION:  mitogen-activated protein kinase, putative / MAPK, putative (MPK10), mitogen-activated protein kinase (MAPK)(AtMPK10), PMID
DATA:                          Control 30min   2hours  2days   1week   p-value        B&H            Pos     Fold change relative to control (log2)
SENSE COUNTS:                  75      10      43      47      14      2.52e-13       2.17e-12               0.000   -2.907  -0.803  -0.674  -2.421
  GENES:
  AT3G59790.1
    SENSE COUNTS:              75      10      43      47      14      2.52e-13       2.16e-12               0.000   -2.907  -0.803  -0.674  -2.421
    TAGS:
      v+1      AGCTATTGTC     0       0       1       0       0       4.55e-01       5.16e-01       1771    0.000   0.000   0.000   0.000   0.000
      v+2      AGAATATTGT     75      10      42      47      14      2.36e-13       3.81e-12       700     0.000   -2.907  -0.837  -0.674  -2.421

LOCUS: AT3G09440
DESCRIPTION:  heat shock cognate 70 kDa protein 3 (HSC70-3) (HSP70-3), identical to SP|O65719 Heat shock cognate 70 kDa protein 3 (Hsc70.3)
{Arabidopsis thaliana}
DATA:                          Control 30min   2hours  2days   1week   p-value        B&H            Pos     Fold change relative to control (log2)
SENSE COUNTS:                  2       0       0       23      0       2.74e-13       2.33e-12               0.000   0.000   0.000   3.524   0.000
  GENES:
  AT3G09440.1
    SENSE COUNTS:              2       0       0       23      0       2.74e-13       2.33e-12               0.000   0.000   0.000   3.524   0.000
    TAGS:
      d+1      AGGAACACAA     2       0       0       23      0       2.74e-13       4.38e-12       1756    0.000   0.000   0.000   3.524   0.000

LOCUS: AT4G02770
DESCRIPTION:  photosystem I reaction center subunit II, chloroplast, putative / photosystem I 20 kDa subunit, putative / PSI-D, putative (PSAD1),
similar to SP|P12353 Photosystem I reaction center subunit II, chloroplast precursor (Photosystem I 20 kDa subunit) (PSI-D)
DATA:                          Control 30min   2hours  2days   1week   p-value        B&H            Pos     Fold change relative to control (log2)
SENSE COUNTS:                  241     116     171     177     97      2.82e-13       2.38e-12               0.000   -1.055  -0.495  -0.445  -1.313
  GENES:
  AT4G02770.1
    SENSE COUNTS:              241     116     171     177     97      2.82e-13       2.38e-12               0.000   -1.055  -0.495  -0.445  -1.313
```

```
    TAGS:
      d+1      TTAATTTTTA     236    115    163    177    97     1.28e-12       1.92e-11       764    0.000  -1.037 -0.534 -0.415 -1.283
      d+2      AGATCTATTG     4      1      7      0      0      2.04e-02       3.54e-02       631    0.000  -2.000 0.807  0.000  0.000
      d+2      AGAGAAGGTC     1      0      1      0      0      6.01e-01       6.47e-01       433    0.000  0.000  0.000  0.000  0.000

LOCUS: AT5G48250
DESCRIPTION:  zinc finger (B-box type) family protein, contains similarity to CONSTANS homologs
DATA:                         Control 30min  2hours 2days  1week  p-value        B&H            Pos    Fold change relative to control (log2)
SENSE COUNTS:                 0      1      31     6      5      4.21e-13       3.52e-12              0.000  0.000  4.954  2.585  2.322
  GENES:
  AT5G48250.1
    SENSE COUNTS:             0      1      31     6      5      4.21e-13       3.52e-12              0.000  0.000  4.954  2.585  2.322
    TAGS:
      d+1      TAGAAGTTTG     0      1      30     6      5      1.95e-12       2.85e-11       1658   0.000  0.000  4.907  2.585  2.322
      d+2      ATCCTTAAAT     0      0      1      0      0      4.55e-01       5.44e-01       1569   0.000  0.000  0.000  0.000  0.000

LOCUS: AT4G17615
DESCRIPTION:  calcineurin B-like protein 1 (CBL1), identical to calcineurin B-like protein 1 (GI
DATA:                         Control 30min  2hours 2days  1week  p-value        B&H            Pos    Fold change relative to control (log2)
SENSE COUNTS:                 38     0      41     19     3      6.99e-13       5.79e-12              0.000  0.000  0.110  -1.000 -3.663
  GENES:
  AT4G17615.2
    SENSE COUNTS:             38     0      41     19     3      6.99e-13       5.75e-12              0.000  0.000  0.110  -1.000 -3.663
    TAGS:
      d+1      AAGAAACAAA     38     0      41     19     3      6.99e-13       1.09e-11       1109   0.000  0.000  0.110  -1.000 -3.663
  AT4G17615.1
    SENSE COUNTS:             38     0      41     19     3      6.99e-13       5.80e-12              0.000  0.000  0.110  -1.000 -3.663
    TAGS:
      d+1      AAGAAACAAA     38     0      41     19     3      6.99e-13       1.09e-11       903    0.000  0.000  0.110  -1.000 -3.663

LOCUS: AT2G36880
DESCRIPTION:  S-adenosylmethionine synthetase, putative, similar to S-adenosylmethionine synthetase 3 (Methionine adenosyltransferase 3, AdoMet
synthetase 3) (Lycopersicon esculentum) SWISS-PROT
DATA:                         Control 30min  2hours 2days  1week  p-value        B&H            Pos    Fold change relative to control (log2)
SENSE COUNTS:                 28     7      49     76     42     8.05e-13       6.61e-12              0.000  -2.000 0.807  1.441  0.585
  GENES:
  AT2G36880.1
    SENSE COUNTS:             28     7      49     76     42     8.05e-13       6.58e-12              0.000  -2.000 0.807  1.441  0.585
    TAGS:
      d+1      CAAGTTAGAA     27     7      45     74     37     1.20e-12       1.83e-11       1660   0.000  -1.948 0.737  1.455  0.455
      d+2      GTGGAGGTGC     0      0      4      2      5      1.42e-01       2.16e-01       951    0.000  0.000  2.000  1.000  2.322
      X+4      AGAATACTGT     1      0      0      0      0      4.28e-01       5.28e-01       -75    0.000  0.000  0.000  0.000  0.000

LOCUS: AT5G19510
DESCRIPTION:  elongation factor 1B alpha-subunit 2 (eEF1Balpha2), identical to elongation factor 1B alpha-subunit (Arabidopsis thaliana) GI
DATA:                         Control 30min  2hours 2days  1week  p-value        B&H            Pos    Fold change relative to control (log2)
SENSE COUNTS:                 17     9      6      6      53     1.51e-12       1.23e-11              0.000  -0.918 -1.503 -1.503 1.640
  GENES:
  AT5G19510.1
    SENSE COUNTS:             17     9      6      6      53     1.51e-12       1.21e-11              0.000  -0.918 -1.503 -1.503 1.640
    TAGS:
      d+1      TTACAATAAC     16     9      6      6      53     1.01e-12       1.56e-11       856    0.000  -0.830 -1.415 -1.415 1.728
      d+2      GATCTCTTTG     1      0      0      0      0      4.28e-01       5.24e-01       384    0.000  0.000  0.000  0.000  0.000

LOCUS: AT3G44010
DESCRIPTION:  40S ribosomal protein S29 (RPS29B), ribosomal protein S29, rat, PIR
DATA:                         Control 30min  2hours 2days  1week  p-value        B&H            Pos    Fold change relative to control (log2)
SENSE COUNTS:                 33     11     24     23     80     1.54e-12       1.24e-11              0.000  -1.585 -0.459 -0.521 1.278
  GENES:
  AT3G44010.1
```

```
    SENSE COUNTS:             33      11      24      23      80      1.54e-12        1.23e-11                0.000   -1.585  -0.459  -0.521  1.278
    TAGS:
      d+1      TCTTATTTTG     30      11      21      22      70      2.90e-10        3.43e-09        393     0.000   -1.447  -0.515  -0.447  1.222
      d+2      AAGGCGTCGA     3       0       3       1       10      1.68e-02        2.94e-02        283     0.000   0.000   0.000   -1.585  1.737

LOCUS: AT2G39930
DESCRIPTION:  isoamylase, putative / starch debranching enzyme, putative, similar to isoamylase from (Solanum tuberosum) GI
DATA:                         Control 30min   2hours  2days   1week   p-value         B&H             Pos     Fold change relative to control (log2)
SENSE COUNTS:                 69      8       78      72      66      3.43e-12        2.74e-11                0.000   -3.109  0.177   0.061   -0.064
  GENES:
  AT2G39930.1
    SENSE COUNTS:             69      8       78      72      66      3.43e-12        2.72e-11                0.000   -3.109  0.177   0.061   -0.064
    TAGS:
      d+1      AAACAAAAAA     69      8       78      72      66      1.35e-12        2.01e-11        2949    0.000   -3.109  0.177   0.061   -0.064
      d+2      GTCTTGCTCC     0       0       0       0       0       6.15e-01        6.42e-01        2061    0.000   0.000   0.000   0.000   0.000

LOCUS: AT1G64720
DESCRIPTION:  expressed protein, weak similarity to SP|P53809 Phosphatidylcholine transfer protein (PC-TP) {Rattus norvegicus}
DATA:                         Control 30min   2hours  2days   1week   p-value         B&H             Pos     Fold change relative to control (log2)
SENSE COUNTS:                 116     123     85      59      21      6.22e-12        4.93e-11                0.000   0.085   -0.449  -0.975  -2.466
  GENES:
  AT1G64720.1
    SENSE COUNTS:             116     123     85      59      21      6.22e-12        4.89e-11                0.000   0.085   -0.449  -0.975  -2.466
    TAGS:
      d+1      TAATAGCAAA     113     111     85      59      21      4.96e-10        5.56e-09        1320    0.000   -0.026  -0.411  -0.938  -2.428
      d+2      TAAGTAGACA     1       0       0       0       0       4.28e-01        5.27e-01        1235    0.000   0.000   0.000   0.000   0.000
      d+2      TACATTGGAC     1       12      0       0       0       1.06e-06        7.18e-06        1138    0.000   3.585   0.000   0.000   0.000
      d+2      AATGAGAGGG     1       0       0       0       0       4.28e-01        5.20e-01        1040    0.000   0.000   0.000   0.000   0.000

LOCUS: AT3G12690
DESCRIPTION:  protein kinase, putative, similar to viroid symptom modulation protein (Lycopersicon esculentum) gi|7672777|gb|AAF66637
DATA:                         Control 30min   2hours  2days   1week   p-value         B&H             Pos     Fold change relative to control (log2)
SENSE COUNTS:                 225     229     163     247     86      6.77e-12        5.32e-11                0.000   0.025   -0.465  0.135   -1.388
  GENES:
  AT3G12690.2
    SENSE COUNTS:             225     229     163     247     86      6.77e-12        5.21e-11                0.000   0.025   -0.465  0.135   -1.388
    TAGS:
      d+1      GAGGTGGTGA     224     229     163     247     86      7.47e-12        1.07e-10        1972    0.000   0.032   -0.459  0.141   -1.381
      d+2      CATCCATCCG     1       0       0       0       0       4.28e-01        5.35e-01        1434    0.000   0.000   0.000   0.000   0.000
  AT3G12690.1
    SENSE COUNTS:             225     229     163     247     86      6.77e-12        5.29e-11                0.000   0.025   -0.465  0.135   -1.388
    TAGS:
      d+1      GAGGTGGTGA     224     229     163     247     86      7.47e-12        1.07e-10        1944    0.000   0.032   -0.459  0.141   -1.381
      d+2      CATCCATCCG     1       0       0       0       0       4.28e-01        5.35e-01        1406    0.000   0.000   0.000   0.000   0.000
  AT3G12690.3
    SENSE COUNTS:             225     229     163     247     86      6.77e-12        5.25e-11                0.000   0.025   -0.465  0.135   -1.388
    TAGS:
      d+1      GAGGTGGTGA     224     229     163     247     86      7.47e-12        1.07e-10        1842    0.000   0.032   -0.459  0.141   -1.381
      d+2      CATCCATCCG     1       0       0       0       0       4.28e-01        5.35e-01        1304    0.000   0.000   0.000   0.000   0.000

LOCUS: AT3G08640
DESCRIPTION:  alphavirus core protein family, contains Pfam profile
DATA:                         Control 30min   2hours  2days   1week   p-value         B&H             Pos     Fold change relative to control (log2)
SENSE COUNTS:                 0       0       0       0       19      7.73e-12        6.03e-11                0.000   0.000   0.000   0.000   4.248
  GENES:
  AT3G08640.1
    SENSE COUNTS:             0       0       0       0       19      7.73e-12        5.87e-11                0.000   0.000   0.000   0.000   4.248
    TAGS:
      d+1      AATATAATAA     0       0       0       0       19      4.24e-13        6.72e-12        1299    0.000   0.000   0.000   0.000   4.248
      X+4      CTTAGTTGGT     0       0       0       0       0       6.15e-01        6.55e-01        -151    0.000   0.000   0.000   0.000   0.000
```

```
LOCUS: AT1G66580
DESCRIPTION:  60S ribosomal protein L10 (RPL10C), contains Pfam profile
DATA:                         Control 30min  2hours  2days  1week  p-value       B&H           Pos    Fold change relative to control (log2)
SENSE COUNTS:                 19      5      26      28     68     7.74e-12      5.98e-11             0.000  -1.926  0.453  0.559  1.840
  GENES:
  AT1G66580.1
    SENSE COUNTS:             19      5      26      28     68     7.74e-12      5.83e-11             0.000  -1.926  0.453  0.559  1.840
    TAGS:
      d+1      TGTTTCTTAC     16      5      24      26     68     2.26e-13      3.69e-12      842    0.000  -1.678  0.585  0.700  2.087
      d+2      GTCCATTGGC     3       0      2       2      0      5.37e-01      5.86e-01      654    0.000  0.000  -0.585  -0.585  0.000

LOCUS: AT1G20440
DESCRIPTION:  dehydrin (COR47), identical to dehydrin COR47 (Cold-induced COR47 protein) (Arabidopsis thaliana) SWISS-PROT
DATA:                         Control 30min  2hours  2days  1week  p-value       B&H           Pos    Fold change relative to control (log2)
SENSE COUNTS:                 7       11     35      62     38     9.09e-12      6.97e-11             0.000  0.652  2.322  3.147  2.441
  GENES:
  AT1G20440.1
    SENSE COUNTS:             7       11     35      62     38     9.09e-12      6.80e-11             0.000  0.652  2.322  3.147  2.441
    TAGS:
      d+2      CCAAGACCAC     6       11     31      59     37     2.73e-11      3.67e-10      857    0.000  0.874  2.369  3.298  2.624
      X+4      CATAAGAGGA     0       0      1       1      0      5.21e-01      5.69e-01      780    0.000  0.000  0.000  0.000  0.000
      d+2      ACCATCCCGA     1       0      3       2      1      7.11e-01      7.18e-01      638    0.000  0.000  1.585  1.000  0.000

LOCUS: AT2G05520
DESCRIPTION:  glycine-rich protein (GRP), identical to glycine-rich protein; atGRP (GI
DATA:                         Control 30min  2hours  2days  1week  p-value       B&H           Pos    Fold change relative to control (log2)
SENSE COUNTS:                 149     142    192     247    280    9.98e-12      7.59e-11             0.000  -0.069  0.366  0.729  0.910
  GENES:
  AT2G05520.1
    SENSE COUNTS:             149     142    192     247    280    9.98e-12      7.42e-11             0.000  -0.069  0.366  0.729  0.910
    TAGS:
      d+1      GTGCGTTTGT     148     142    191     247    276    2.00e-11      2.78e-10      563    0.000  -0.060  0.368  0.739  0.899
      d+2      TATTGCATTA     1       0      1       0      3      4.58e-01      5.11e-01      526    0.000  0.000  0.000  0.000  1.585
      i+3      TACGTTTACA     0       0      0       0      1      1.65e-01      2.42e-01      310    0.000  0.000  0.000  0.000  0.000

LOCUS: AT4G27440
DESCRIPTION:  protochlorophyllide reductase B, chloroplast / PCR B / NADPH-protochlorophyllide oxidoreductase B (PORB), identical to SP
DATA:                         Control 30min  2hours  2days  1week  p-value       B&H           Pos    Fold change relative to control (log2)
SENSE COUNTS:                 0       29     4       2      7      1.05e-11      7.92e-11             0.000  4.858  2.000  1.000  2.807
  GENES:
  AT4G27440.1
    SENSE COUNTS:             0       29     4       2      7      1.05e-11      7.75e-11             0.000  4.858  2.000  1.000  2.807
    TAGS:
      d+1      AAGAAACTGG     0       29     4       2      7      1.05e-11      1.48e-10      944    0.000  4.858  2.000  1.000  2.807

LOCUS: AT1G51260
DESCRIPTION:  acyl-CoA
DATA:                         Control 30min  2hours  2days  1week  p-value       B&H           Pos    Fold change relative to control (log2)
SENSE COUNTS:                 1       0      18      0      0      1.25e-11      9.35e-11             0.000  0.000  4.170  0.000  0.000
  GENES:
  AT1G51260.1
    SENSE COUNTS:             1       0      18      0      0      1.25e-11      9.16e-11             0.000  0.000  4.170  0.000  0.000
    TAGS:
      i+3      ATTTTTCAAA     1       0      18      0      0      1.25e-11      1.75e-10      809    0.000  0.000  4.170  0.000  0.000

LOCUS: AT1G77120
DESCRIPTION:  Catalyzes the reduction of acetaldehyde using NADH as reductant. Requires zinc for activity. Dimer. Anaerobic response polypeptide (ANP).
Fermentation.
DATA:                         Control 30min  2hours  2days  1week  p-value       B&H           Pos    Fold change relative to control (log2)
```

```
SENSE COUNTS:                 0       2       5       31      16      2.49e-11        1.85e-10                0.000   1.000   2.322   4.954   4.000
  GENES:
  AT1G77120.1
    SENSE COUNTS:             0       2       5       31      16      2.49e-11        1.81e-10                0.000   1.000   2.322   4.954   4.000
    TAGS:
      d+1      GGTGCTTGAA     0       2       4       31      16      7.89e-12        1.12e-10        1190    0.000   1.000   2.000   4.954   4.000
      d+2      CTGAAGGGAG     0       0       1       0       0       4.55e-01        5.31e-01        1154    0.000   0.000   0.000   0.000   0.000

LOCUS: AT4G39260
DESCRIPTION:  glycine-rich RNA-binding protein 8 (GRP8) (CCR1), SP|Q03251 Glycine-rich RNA-binding protein 8 (CCR1 protein) (GRP8) {Arabidopsis
thaliana} isoform contains a non-consensus CG acceptor splice site at intron 2
DATA:                         Control 30min   2hours  2days   1week   p-value         B&H             Pos     Fold change relative to control (log2)
SENSE COUNTS:                 4       44      11      8       11      2.63e-11        1.94e-10                0.000   3.459   1.459   1.000   1.459
  GENES:
  AT4G39260.4
    SENSE COUNTS:             3       34      6       6       8       1.15e-09        7.07e-09                0.000   3.503   1.000   1.000   1.415
    TAGS:
      d+1      AGGGATGCGA     3       34      6       6       8       1.15e-09        1.20e-08        240     0.000   3.503   1.000   1.000   1.415
  AT4G39260.1
    SENSE COUNTS:             1       10      5       2       3       4.15e-02        4.20e-02                0.000   3.322   2.322   1.000   1.585
    TAGS:
      d+1      TATTTAATCG     1       10      5       2       3       4.15e-02        6.92e-02        808     0.000   3.322   2.322   1.000   1.585
  AT4G39260.3
    SENSE COUNTS:             1       10      5       2       3       4.15e-02        4.21e-02                0.000   3.322   2.322   1.000   1.585
    TAGS:
      d+1      TATTTAATCG     1       10      5       2       3       4.15e-02        6.92e-02        577     0.000   3.322   2.322   1.000   1.585
  AT4G39260.2
    SENSE COUNTS:             1       10      5       2       3       4.15e-02        4.21e-02                0.000   3.322   2.322   1.000   1.585
    TAGS:
      d+1      TATTTAATCG     1       10      5       2       3       4.15e-02        6.92e-02        679     0.000   3.322   2.322   1.000   1.585

LOCUS: AT1G12900
DESCRIPTION:  glyceraldehyde 3-phosphate dehydrogenase, chloroplast, putative / NADP-dependent glyceraldehydephosphate dehydrogenase, putative,
similar to SP|P25856 Glyceraldehyde 3-phosphate dehydrogenase A, chloroplast precursor (EC 1.2.1.13) (NADP-dependent glyceral
DATA:                         Control 30min   2hours  2days   1week   p-value         B&H             Pos     Fold change relative to control (log2)
SENSE COUNTS:                 100     25      85      65      44      3.17e-11        2.31e-10                0.000   -2.000  -0.234  -0.621  -1.184
  GENES:
  AT1G12900.1
    SENSE COUNTS:             100     25      85      65      44      3.17e-11        2.29e-10                0.000   -2.000  -0.234  -0.621  -1.184
    TAGS:
      d+1      ATTCGGAATG     100     25      84      65      44      4.00e-11        5.29e-10        1320    0.000   -2.000  -0.252  -0.621  -1.184
      d+2      AAGATACGAT     0       0       1       0       0       4.55e-01        5.41e-01        689     0.000   0.000   0.000   0.000   0.000

LOCUS: AT2G21210
DESCRIPTION:  auxin-responsive protein, putative, similar to small auxin-up regulated protein SAUR (GI
DATA:                         Control 30min   2hours  2days   1week   p-value         B&H             Pos     Fold change relative to control (log2)
SENSE COUNTS:                 38      8       4       6       3       3.19e-11        2.31e-10                0.000   -2.248  -3.248  -2.663  -3.663
  GENES:
  AT2G21210.1
    SENSE COUNTS:             38      8       4       6       3       3.19e-11        2.29e-10                0.000   -2.248  -3.248  -2.663  -3.663
    TAGS:
      d+1      ATTCCTTCTA     38      8       4       6       3       3.19e-11        4.25e-10        524     0.000   -2.248  -3.248  -2.663  -3.663

LOCUS: AT5G56030
DESCRIPTION:  heat shock protein 81-2 (HSP81-2), nearly identical to SP|P55737 Heat shock protein 81-2 (HSP81-2) {Arabidopsis thaliana}
DATA:                         Control 30min   2hours  2days   1week   p-value         B&H             Pos     Fold change relative to control (log2)
SENSE COUNTS:                 9       5       9       47      21      3.94e-11        2.83e-10                0.000   -0.848  0.000   2.385   1.222
  GENES:
  AT5G56030.1
    SENSE COUNTS:             9       5       9       47      21      3.94e-11        2.81e-10                0.000   -0.848  0.000   2.385   1.222
```

```
    TAGS:
      d+1      GATGAGTTGA     3       2       4       16      8       2.48e-03       6.15e-03       1934    0.000   -0.585  0.415   2.415   1.415
      d+2      TCGAGTAAGA     0       0       1       0       0       4.55e-01       5.44e-01       1889    0.000   0.000   0.000   0.000   0.000
      d+2      GTTGATGCGA     5       3       4       29      12      2.30e-07       1.85e-06       1568    0.000   -0.737  -0.322  2.536   1.263
      d+2      ATTGGTCAGT     1       0       0       2       1       3.00e-01       4.04e-01       443     0.000   0.000   0.000   1.000   0.000

LOCUS: AT5G07440
DESCRIPTION:  glutamate dehydrogenase 2 (GDH2), identical to glutamate dehydrogenase 2 (GDH 2) (Arabidopsis thaliana) SWISS-PROT
DATA:                         Control 30min   2hours  2days   1week   p-value        B&H            Pos     Fold change relative to control (log2)
SENSE COUNTS:                 3       28      7       0       0       5.48e-11       3.91e-10               0.000   3.222   1.222   0.000   0.000
  GENES:
  AT5G07440.1
    SENSE COUNTS:             3       28      7       0       0       5.48e-11       3.88e-10               0.000   3.222   1.222   0.000   0.000
    TAGS:
      d+1      ACTCGAGCCT     3       28      7       0       0       5.48e-11       7.14e-10       1327    0.000   3.222   1.222   0.000   0.000

LOCUS: AT5G02030
DESCRIPTION:  homeodomain protein (BELLRINGER), several homeodomain proteins;
DATA:                         Control 30min   2hours  2days   1week   p-value        B&H            Pos     Fold change relative to control (log2)
SENSE COUNTS:                 6       0       2       18      32      7.04e-11       4.98e-10               0.000   0.000   -1.585  1.585   2.415
  GENES:
  AT5G02030.1
    SENSE COUNTS:             6       0       2       18      32      7.04e-11       4.96e-10               0.000   0.000   -1.585  1.585   2.415
    TAGS:
      d+1      ATTTTGTAGG     5       0       2       18      32      2.64e-11       3.58e-10       1835    0.000   0.000   -1.322  1.848   2.678
      i+3      ATCAATTCTA     1       0       0       0       0       4.28e-01       5.36e-01       856     0.000   0.000   0.000   0.000   0.000

LOCUS: AT2G39460
DESCRIPTION:  60S ribosomal protein L23A (RPL23aA), identical to GB
DATA:                         Control 30min   2hours  2days   1week   p-value        B&H            Pos     Fold change relative to control (log2)
SENSE COUNTS:                 25      23      34      59      91      7.93e-11       5.57e-10               0.000   -0.120  0.444   1.239   1.864
  GENES:
  AT2G39460.1
    SENSE COUNTS:             25      23      34      59      91      7.93e-11       5.55e-10               0.000   -0.120  0.444   1.239   1.864
    TAGS:
      d+1      TTGCAGTTTA     25      22      34      59      91      2.55e-11       3.48e-10       731     0.000   -0.184  0.444   1.239   1.864
      d+2      TCTCCGGCTA     0       1       0       0       0       2.54e-01       3.44e-01       130     0.000   0.000   0.000   0.000   0.000

LOCUS: AT1G20630
DESCRIPTION:  catalase 1, identical to catalase 1 GI
DATA:                         Control 30min   2hours  2days   1week   p-value        B&H            Pos     Fold change relative to control (log2)
SENSE COUNTS:                 169     98      106     118     41      1.12e-10       7.81e-10               0.000   -0.786  -0.673  -0.518  -2.043
  GENES:
  AT1G20630.1
    SENSE COUNTS:             169     98      106     118     41      1.12e-10       7.78e-10               0.000   -0.786  -0.673  -0.518  -2.043
    TAGS:
      d+1      CCGAAAAATA     1       1       0       0       0       4.77e-01       5.29e-01       1290    0.000   0.000   0.000   0.000   0.000
      d+2      TTTTCATTTC     0       0       0       1       0       3.09e-01       4.04e-01       638     0.000   0.000   0.000   0.000   0.000
      d+2      CATTGAAACC     0       0       0       0       0       6.15e-01       6.37e-01       552     0.000   0.000   0.000   0.000   0.000
      d+2      GATCCATACA     168     97      106     117     41      1.11e-10       1.40e-09       86      0.000   -0.792  -0.664  -0.522  -2.035

LOCUS: AT5G17460
DESCRIPTION:  expressed protein
DATA:                         Control 30min   2hours  2days   1week   p-value        B&H            Pos     Fold change relative to control (log2)
SENSE COUNTS:                 0       0       16      22      31      1.22e-10       8.44e-10               0.000   0.000   4.000   4.459   4.954
  GENES:
  AT5G17460.1
    SENSE COUNTS:             0       0       16      22      31      1.22e-10       8.42e-10               0.000   0.000   4.000   4.459   4.954
    TAGS:
      d+2      AAAGATATTC     0       0       13      18      30      6.11e-10       6.62e-09       554     0.000   0.000   3.700   4.170   4.907
```

```
      d+2      CTTTGTATTT     0       0       3       4       1       1.12e-01        1.75e-01        343     0.000   0.000   1.585   2.000   0.000

LOCUS: AT2G20670
DESCRIPTION:  expressed protein, contains Pfam profile PF04720
DATA:                         Control 30min   2hours  2days   1week   p-value         B&H             Pos     Fold change relative to control (log2)
SENSE COUNTS:                 3       26      2       5       0       1.37e-10        9.41e-10                0.000   3.115   -0.585  0.737   0.000
  GENES:
  AT2G20670.1
    SENSE COUNTS:             3       26      2       5       0       1.37e-10        9.40e-10                0.000   3.115   -0.585  0.737   0.000
    TAGS:
      d+1      AGTGTTAATT     3       26      2       5       0       4.30e-10        5.02e-09        1008    0.000   3.115   -0.585  0.737   0.000
      d+2      AGATTTCTCG     0       0       0       0       0       6.15e-01        6.33e-01        93      0.000   0.000   0.000   0.000   0.000

LOCUS: AT2G40000
DESCRIPTION:  expressed protein
DATA:                         Control 30min   2hours  2days   1week   p-value         B&H             Pos     Fold change relative to control (log2)
SENSE COUNTS:                 0       18      1       0       0       1.55e-10        1.06e-09                0.000   4.170   0.000   0.000   0.000
  GENES:
  AT2G40000.1
    SENSE COUNTS:             0       18      1       0       0       1.55e-10        1.06e-09                0.000   4.170   0.000   0.000   0.000
    TAGS:
      d+1      TTCAGCACCT     0       18      1       0       0       1.55e-10        1.94e-09        204     0.000   4.170   0.000   0.000   0.000

LOCUS: AT3G02480
DESCRIPTION:  ABA-responsive protein-related, similar to ABA-inducible protein (Fagus sylvatica) GI
DATA:                         Control 30min   2hours  2days   1week   p-value         B&H             Pos     Fold change relative to control (log2)
SENSE COUNTS:                 1       0       1       0       17      1.91e-10        1.29e-09                0.000   0.000   0.000   0.000   4.087
  GENES:
  AT3G02480.1
    SENSE COUNTS:             1       0       1       0       17      1.91e-10        1.29e-09                0.000   0.000   0.000   0.000   4.087
    TAGS:
      d+1      AACAAAAGCC     1       0       1       0       17      1.91e-10        2.33e-09        263     0.000   0.000   0.000   0.000   4.087

LOCUS: AT1G66020
DESCRIPTION:  terpene synthase/cyclase family protein, contains Pfam profile
DATA:                         Control 30min   2hours  2days   1week   p-value         B&H             Pos     Fold change relative to control (log2)
SENSE COUNTS:                 89      76      40      54      7       2.24e-10        1.50e-09                0.000   -0.228  -1.154  -0.721  -3.668
  GENES:
  AT1G66020.1
    SENSE COUNTS:             89      76      40      54      7       2.24e-10        1.51e-09                0.000   -0.228  -1.154  -0.721  -3.668
    TAGS:
      d+1      ATGTTTTTGA     89      76      40      54      7       2.24e-10        2.71e-09        1994    0.000   -0.228  -1.154  -0.721  -3.668

LOCUS: AT2G37040
DESCRIPTION:  phenylalanine ammonia-lyase 1 (PAL1), nearly identical to SP|P35510
DATA:                         Control 30min   2hours  2days   1week   p-value         B&H             Pos     Fold change relative to control (log2)
SENSE COUNTS:                 8       0       5       32      19      2.40e-10        1.60e-09                0.000   0.000   -0.678  2.000   1.248
  GENES:
  AT2G37040.1
    SENSE COUNTS:             8       0       5       32      19      2.40e-10        1.60e-09                0.000   0.000   -0.678  2.000   1.248
    TAGS:
      d+1      TCTGAATAAT     8       0       5       31      19      9.19e-10        9.83e-09        2357    0.000   0.000   -0.678  1.954   1.248
      d+2      GTGATCAACG     0       0       0       1       0       3.09e-01        4.10e-01        211     0.000   0.000   0.000   0.000   0.000

LOCUS: AT3G04720
DESCRIPTION:  hevein-like protein (HEL), identical to SP|P43082 Hevein-like protein precursor {Arabidopsis thaliana}; similar to SP|P09762 Wound-
induced protein WIN2 precursor {Solanum tuberosum}; contains Pfam profile PF00187
DATA:                         Control 30min   2hours  2days   1week   p-value         B&H             Pos     Fold change relative to control (log2)
SENSE COUNTS:                 4       31      5       4       3       2.69e-10        1.78e-09                0.000   2.954   0.322   0.000   -0.415
  GENES:
```

```
  AT3G04720.1
    SENSE COUNTS:           4      31     5      4      3      2.69e-10      1.79e-09             0.000  2.954  0.322  0.000  -0.415
    TAGS:
      d+1     CTTGTTTCGG    4      31     5      4      3      2.69e-10      3.21e-09      644    0.000  2.954  0.322  0.000  -0.415

LOCUS: AT5G40480
DESCRIPTION:  expressed protein, ; expression supported by MPSS
DATA:                       Control 30min  2hours 2days  1week  p-value       B&H           Pos    Fold change relative to control (log2)
SENSE COUNTS:               61     13     54     43     8      3.02e-10      1.98e-09             0.000  -2.230 -0.176 -0.504 -2.931
  GENES:
  AT5G40480.1
    SENSE COUNTS:           61     13     54     43     8      3.02e-10      1.99e-09             0.000  -2.230 -0.176 -0.504 -2.931
    TAGS:
      v+2     AGGAATCTCC    0      0      2      2      1      5.87e-01      6.37e-01      5995   0.000  0.000  1.000  1.000  0.000
      i+3     TGTGTTTAAA    61     13     52     41     7      1.03e-10      1.31e-09      1134   0.000  -2.230 -0.230 -0.573 -3.123

LOCUS: AT4G14170
DESCRIPTION:  pentatricopeptide (PPR) repeat-containing protein, contains Pfam profile PF01535
DATA:                       Control 30min  2hours 2days  1week  p-value       B&H           Pos    Fold change relative to control (log2)
SENSE COUNTS:               3      0      7      30     19     4.28e-10      2.79e-09             0.000  0.000  1.222  3.322  2.663
  GENES:
  AT4G14170.1
    SENSE COUNTS:           3      0      7      30     19     4.28e-10      2.81e-09             0.000  0.000  1.222  3.322  2.663
    TAGS:
      d+2     TTCAGAGAAA    3      0      7      30     19     4.28e-10      5.03e-09      654    0.000  0.000  1.222  3.322  2.663

LOCUS: AT4G17550
DESCRIPTION:  transporter-related, similar to cAMP inducible 2 protein (Mus musculus) GI
DATA:                       Control 30min  2hours 2days  1week  p-value       B&H           Pos    Fold change relative to control (log2)
SENSE COUNTS:               0      0      0      18     5      4.78e-10      3.10e-09             0.000  0.000  0.000  4.170  2.322
  GENES:
  AT4G17550.1
    SENSE COUNTS:           0      0      0      18     5      4.78e-10      3.12e-09             0.000  0.000  0.000  4.170  2.322
    TAGS:
      d+1     GTGGTGTTTC    0      0      0      18     5      4.78e-10      5.46e-09      1870   0.000  0.000  0.000  4.170  2.322

LOCUS: AT1G62380
DESCRIPTION:  Encodes a protein similar to 1-aminocyclopropane-1-carboxylic oxidase (ACC oxidase). Expression of the AtACO2 transcripts is affected by
ethylene.
DATA:                       Control 30min  2hours 2days  1week  p-value       B&H           Pos    Fold change relative to control (log2)
SENSE COUNTS:               22     42     7      10     0      4.84e-10      3.11e-09             0.000  0.933  -1.652 -1.138 0.000
  GENES:
  AT1G62380.1
    SENSE COUNTS:           22     42     7      10     0      4.84e-10      3.14e-09             0.000  0.933  -1.652 -1.138 0.000
    TAGS:
      d+1     AAGCTTTATG    20     42     6      9      0      6.47e-11      8.36e-10      931    0.000  1.070  -1.737 -1.152 0.000
      d+2     CTCAAGTCCA    0      0      1      0      0      4.55e-01      5.36e-01      292    0.000  0.000  0.000  0.000  0.000
      d+2     ACTTAATGGA    1      0      0      1      0      5.06e-01      5.57e-01      218    0.000  0.000  0.000  0.000  0.000
      X+4     CTCCACGAAC    1      0      0      0      0      4.28e-01      5.21e-01      -185   0.000  0.000  0.000  0.000  0.000

LOCUS: AT2G13360
DESCRIPTION:  AGT1 encodes peroxisomal alanine
DATA:                       Control 30min  2hours 2days  1week  p-value       B&H           Pos    Fold change relative to control (log2)
SENSE COUNTS:               1      0      17     0      1      6.23e-10      3.98e-09             0.000  0.000  4.087  0.000  0.000
  GENES:
  AT2G13360.2
    SENSE COUNTS:           1      0      17     0      1      6.23e-10      4.02e-09             0.000  0.000  4.087  0.000  0.000
    TAGS:
      d+2     GCAGAGGTAC    0      0      12     0      0      3.96e-08      3.52e-07      1072   0.000  0.000  3.585  0.000  0.000
      d+2     GGGGCTGAAA    0      0      4      0      0      5.58e-03      1.18e-02      967    0.000  0.000  2.000  0.000  0.000
```

```
      d+2      CTCGTTTGGG     1       0       1       0       1       6.15e-01       6.34e-01       927     0.000   0.000   0.000   0.000   0.000
  AT2G13360.1
    SENSE COUNTS:             1       0       17      0       1       6.23e-10       3.99e-09               0.000   0.000   4.087   0.000   0.000
    TAGS:
      d+2      GCAGAGGTAC     0       0       12      0       0       3.96e-08       3.52e-07       1094    0.000   0.000   3.585   0.000   0.000
      d+2      GGGGCTGAAA     0       0       4       0       0       5.58e-03       1.18e-02       989     0.000   0.000   2.000   0.000   0.000
      d+2      CTCGTTTGGG     1       0       1       0       1       6.15e-01       6.34e-01       949     0.000   0.000   0.000   0.000   0.000

LOCUS: AT2G05310
DESCRIPTION:  expressed protein
DATA:                         Control 30min   2hours  2days   1week   p-value        B&H            Pos     Fold change relative to control (log2)
SENSE COUNTS:                 28      5       2       0       11      7.08e-10       4.49e-09               0.000   -2.485  -3.807  0.000   -1.348
  GENES:
  AT2G05310.1
    SENSE COUNTS:             28      5       2       0       11      7.08e-10       4.51e-09               0.000   -2.485  -3.807  0.000   -1.348
    TAGS:
      d+1      TTTCTTATAC     28      5       2       0       10      4.89e-10       5.51e-09       465     0.000   -2.485  -3.807  0.000   -1.485
      i+3      TAGACTAGTG     0       0       0       0       1       1.65e-01       2.46e-01       463     0.000   0.000   0.000   0.000   0.000

LOCUS: AT1G12780
DESCRIPTION:  Encodes a UDP-glucose epimerase that catalyzes the interconversion of the sugar nucleotides UDP-glucose UDP-galactose via a UDP-4-keto-
hexose intermediate.
DATA:                         Control 30min   2hours  2days   1week   p-value        B&H            Pos     Fold change relative to control (log2)
SENSE COUNTS:                 6       38      18      5       3       7.12e-10       4.49e-09               0.000   2.663   1.585   -0.263  -1.000
  GENES:
  AT1G12780.1
    SENSE COUNTS:             6       38      18      5       3       7.12e-10       4.51e-09               0.000   2.663   1.585   -0.263  -1.000
    TAGS:
      d+1      TATCGAACTG     6       38      16      5       3       9.56e-10       1.02e-08       1367    0.000   2.663   1.415   -0.263  -1.000
      d+2      GGGTTACCAG     0       0       2       0       0       3.51e-01       4.52e-01       1243    0.000   0.000   1.000   0.000   0.000

LOCUS: AT3G63410
DESCRIPTION:  Encodes a 37kDz polypeptide precursor of the chloroplast inner envelope membrane with partial sequence similarity to S-
adenosylmethionine-dependent methyltransferase.  Mutant plants lack plastoquinone (PQ), suggesting that the APG1 protein is involved in
DATA:                         Control 30min   2hours  2days   1week   p-value        B&H            Pos     Fold change relative to control (log2)
SENSE COUNTS:                 55      14      32      62      78      7.48e-10       4.68e-09               0.000   -1.974  -0.781  0.173   0.504
  GENES:
  AT3G63410.1
    SENSE COUNTS:             55      14      32      62      78      7.48e-10       4.71e-09               0.000   -1.974  -0.781  0.173   0.504
    TAGS:
      d+1      TTGAGATATC     49      13      22      37      57      4.94e-07       3.69e-06       1267    0.000   -1.914  -1.155  -0.405  0.218
      d+2      TATTACTAGA     0       0       0       1       0       6.04e-01       6.49e-01       1249    0.000   0.000   0.000   0.000   0.000
      d+2      CGGAATATCA     3       1       5       22      17      3.22e-06       1.96e-05       1218    0.000   -1.585  0.737   2.874   2.503
      d+2      ATCATTGTAT     3       0       1       2       3       3.22e-01       4.18e-01       1154    0.000   0.000   -1.585  -0.585  0.000
      d+2      GGATGTTCTG     0       0       2       0       0       1.21e-01       1.88e-01       924     0.000   0.000   1.000   0.000   0.000
      d+2      CGAGTGGTCG     0       0       2       0       1       2.22e-01       3.07e-01       453     0.000   0.000   1.000   0.000   0.000

LOCUS: AT3G44450
DESCRIPTION:  expressed protein,
DATA:                         Control 30min   2hours  2days   1week   p-value        B&H            Pos     Fold change relative to control (log2)
SENSE COUNTS:                 1       0       14      15      33      8.13e-10       5.05e-09               0.000   0.000   3.807   3.907   5.044
  GENES:
  AT3G44450.1
    SENSE COUNTS:             1       0       14      15      33      8.13e-10       5.09e-09               0.000   0.000   3.807   3.907   5.044
    TAGS:
      X+4      TGCGTGAGAC     0       0       3       2       3       1.60e-01       2.42e-01       567     0.000   0.000   1.585   1.000   1.585
      d+1      ATTTTGAATG     1       0       11      13      30      7.72e-09       7.54e-08       440     0.000   0.000   3.459   3.700   4.907

LOCUS: AT1G05135
DESCRIPTION:  expressed protein, isoform contains a non-consensus TG acceptor site at intron.
```

```
DATA:                         Control 30min  2hours  2days  1week  p-value        B&H            Pos    Fold change relative to control (log2)
SENSE COUNTS:                 3       28     2       16     0      1.02e-09       6.30e-09              0.000  3.222  -0.585 2.415  0.000
  GENES:
  AT1G05135.1
    SENSE COUNTS:             3       28     2       16     0      1.02e-09       6.35e-09              0.000  3.222  -0.585 2.415  0.000
    TAGS:
      p+1     GAGGAGGCGT      3       28     2       16     0      1.02e-09       1.08e-08       1043   0.000  3.222  -0.585 2.415  0.000

LOCUS: AT1G52300
DESCRIPTION:  60S ribosomal protein L37 (RPL37B), similar to SP
DATA:                         Control 30min  2hours  2days  1week  p-value        B&H            Pos    Fold change relative to control (log2)
SENSE COUNTS:                 38      19     61      83     73     1.05e-09       6.44e-09              0.000  -1.000 0.683  1.127  0.942
  GENES:
  AT1G52300.1
    SENSE COUNTS:             38      19     61      83     73     1.05e-09       6.49e-09              0.000  -1.000 0.683  1.127  0.942
    TAGS:
      d+1     GATGGATTTC      38      19     61      83     73     1.05e-09       1.10e-08       479    0.000  -1.000 0.683  1.127  0.942

LOCUS: AT3G06700
DESCRIPTION:  60S ribosomal protein L29 (RPL29A), similar to ribosomal protein L29 GI
DATA:                         Control 30min  2hours  2days  1week  p-value        B&H            Pos    Fold change relative to control (log2)
SENSE COUNTS:                 24      15     21      38     73     1.20e-09       7.31e-09              0.000  -0.678 -0.193 0.663  1.605
  GENES:
  AT3G06700.1
    SENSE COUNTS:             24      15     21      38     73     1.20e-09       7.34e-09              0.000  -0.678 -0.193 0.663  1.605
    TAGS:
      d+1     TTTACGCAAT      24      15     21      38     73     1.20e-09       1.24e-08       361    0.000  -0.678 -0.193 0.663  1.605

LOCUS: AT1G15825
DESCRIPTION:  hydroxyproline-rich glycoprotein family protein, contains proline-rich extensin domains, INTERPRO
DATA:                         Control 30min  2hours  2days  1week  p-value        B&H            Pos    Fold change relative to control (log2)
SENSE COUNTS:                 0       0      14      0      0      1.21e-09       7.32e-09              0.000  0.000  3.807  0.000  0.000
  GENES:
  AT1G15825.1
    SENSE COUNTS:             0       0      14      0      0      1.21e-09       7.36e-09              0.000  0.000  3.807  0.000  0.000
    TAGS:
      v+1     GCGATGGCGG      0       0      14      0      0      1.21e-09       1.24e-08       1207   0.000  0.000  3.807  0.000  0.000

LOCUS: AT3G47070
DESCRIPTION:  expressed protein
DATA:                         Control 30min  2hours  2days  1week  p-value        B&H            Pos    Fold change relative to control (log2)
SENSE COUNTS:                 64      10     67      64     53     1.22e-09       7.34e-09              0.000  -2.678 0.066  0.000  -0.272
  GENES:
  AT3G47070.1
    SENSE COUNTS:             64      10     67      64     53     1.22e-09       7.38e-09              0.000  -2.678 0.066  0.000  -0.272
    TAGS:
      d+1     ATCTAGTTAT      10      3      11      8      5      2.62e-01       3.54e-01       646    0.000  -1.737 0.138  -0.322 -1.000
      d+2     CCTCTGTTTT      54      7      56      56     48     6.75e-09       6.67e-08       565    0.000  -2.948 0.052  0.052  -0.170

LOCUS: AT5G26742
DESCRIPTION:  DEAD box RNA helicase (RH3), nearly identical to RNA helicase (Arabidopsis thaliana) GI
DATA:                         Control 30min  2hours  2days  1week  p-value        B&H            Pos    Fold change relative to control (log2)
SENSE COUNTS:                 20      10     24      37     68     1.34e-09       8.01e-09              0.000  -1.000 0.263  0.888  1.766
  GENES:
  AT5G26742.1
    SENSE COUNTS:             20      10     24      37     68     1.34e-09       8.06e-09              0.000  -1.000 0.263  0.888  1.766
    TAGS:
      v+1     TGTTTGGACA      20      10     23      37     68     4.37e-10       5.06e-09       3008   0.000  -1.000 0.202  0.888  1.766
      v+2     ATAACAAAGT      0       0      1       0      0      7.06e-01       7.15e-01       2223   0.000  0.000  0.000  0.000  0.000
```

```
LOCUS: AT3G20430
DESCRIPTION:  expressed protein,
DATA:                         Control 30min  2hours 2days  1week  p-value       B&H           Pos    Fold change relative to control (log2)
SENSE COUNTS:                 44      3      8      24     19     1.48e-09      8.78e-09             0.000  -3.874 -2.459 -0.874 -1.212
  GENES:
  AT3G20430.1
    SENSE COUNTS:             44      3      8      24     19     1.48e-09      8.85e-09             0.000  -3.874 -2.459 -0.874 -1.212
    TAGS:
      d+1      AGAGGATCAG     0       0      0      0      0      6.15e-01      6.57e-01      713    0.000  0.000  0.000  0.000  0.000
      d+2      ATTTAGATTC     44      3      8      24     19     5.16e-10      5.74e-09      113    0.000  -3.874 -2.459 -0.874 -1.212

LOCUS: AT1G30680
DESCRIPTION:  toprim domain-containing protein, contains Pfam profile
DATA:                         Control 30min  2hours 2days  1week  p-value       B&H           Pos    Fold change relative to control (log2)
SENSE COUNTS:                 0       0      2      0      14     2.53e-09      1.49e-08             0.000  0.000  1.000  0.000  3.807
  GENES:
  AT1G30680.1
    SENSE COUNTS:             0       0      2      0      14     2.53e-09      1.50e-08             0.000  0.000  1.000  0.000  3.807
    TAGS:
      i+3      ATATGTTAAA     0       0      0      0      1      1.65e-01      2.41e-01      3504   0.000  0.000  0.000  0.000  0.000
      d+1      GTGTTTCTCT     0       0      2      0      12     7.73e-07      5.43e-06      2403   0.000  0.000  1.000  0.000  3.585
      d+2      TCTGGTTTGT     0       0      0      0      1      1.65e-01      2.36e-01      1897   0.000  0.000  0.000  0.000  0.000

LOCUS: AT3G57660
DESCRIPTION:  DNA-directed RNA polymerase family protein, similar to SP|O35134 DNA-directed RNA polymerase I largest subunit (EC 2.7.7.6) (RNA
polymerase I 194 kDa subunit) (RPA194) {Mus musculus}; contains InterPro accession IPR000722
DATA:                         Control 30min  2hours 2days  1week  p-value       B&H           Pos    Fold change relative to control (log2)
SENSE COUNTS:                 3       29     7      1      6      2.89e-09      1.69e-08             0.000  3.273  1.222  -1.585 1.000
  GENES:
  AT3G57660.1
    SENSE COUNTS:             3       29     7      1      6      2.89e-09      1.71e-08             0.000  3.273  1.222  -1.585 1.000
    TAGS:
      v+2      GAGACAATCC     0       0      0      0      1      1.65e-01      2.37e-01      4867   0.000  0.000  0.000  0.000  0.000
      i+3      AGATTAATTT     3       29     7      1      5      1.18e-09      1.22e-08      732    0.000  3.273  1.222  -1.585 0.737

LOCUS: AT1G20450
DESCRIPTION:  dehydrin (ERD10), identical to dehydrin ERD10 (Low-temperature-induced protein LTI45) (Arabidopsis thaliana) SWISS-PROT
DATA:                         Control 30min  2hours 2days  1week  p-value       B&H           Pos    Fold change relative to control (log2)
SENSE COUNTS:                 2       15     20     49     23     3.04e-09      1.77e-08             0.000  2.907  3.322  4.615  3.524
  GENES:
  AT1G20450.2
    SENSE COUNTS:             2       15     19     47     23     1.86e-08      9.80e-08             0.000  2.907  3.248  4.555  3.524
    TAGS:
      d+1      AAAAGTTTTT     1       0      2      6      3      1.18e-01      1.84e-01      1498   0.000  0.000  1.000  2.585  1.585
      d+2      CCAGCACCAC     1       15     17     41     19     2.98e-07      2.33e-06      1010   0.000  3.907  4.087  5.358  4.248
      i+3      AACAGTCGAG     0       0      0      0      1      1.65e-01      2.46e-01      915    0.000  0.000  0.000  0.000  0.000
  AT1G20450.1
    SENSE COUNTS:             2       15     20     49     23     3.04e-09      1.79e-08             0.000  2.907  3.322  4.615  3.524
    TAGS:
      d+1      AAAAGTTTTT     1       0      2      6      3      1.18e-01      1.84e-01      1501   0.000  0.000  1.000  2.585  1.585
      d+2      CCAGCACCAC     1       15     17     41     19     2.98e-07      2.33e-06      1013   0.000  3.907  4.087  5.358  4.248
      i+3      AACAGTCGAG     0       0      0      0      1      1.65e-01      2.46e-01      915    0.000  0.000  0.000  0.000  0.000
      X+4      CAAGAGTTAG     0       0      1      2      0      1.79e-01      2.52e-01      719    0.000  0.000  0.000  1.000  0.000

LOCUS: AT3G60750
DESCRIPTION:  transketolase, putative, strong similarity to transketolase 1 (Capsicum annuum) GI
DATA:                         Control 30min  2hours 2days  1week  p-value       B&H           Pos    Fold change relative to control (log2)
SENSE COUNTS:                 106     31     60     53     58     3.58e-09      2.07e-08             0.000  -1.774 -0.821 -1.000 -0.870
  GENES:
  AT3G60750.1
```

```
    SENSE COUNTS:             106    31     60     53     58     3.58e-09      2.09e-08              0.000  -1.774  -0.821  -1.000  -0.870
    TAGS:
      d+1      ATCATTCGTG     105    28     59     52     57     4.81e-10      5.46e-09      2731    0.000  -1.907  -0.832  -1.014  -0.881
      d+2      ATTCCATTGG     1      3      1      1      0      4.67e-01      5.18e-01      1904    0.000  1.585   0.000   0.000   0.000
      d+2      AGAATCTCAG     0      0      0      0      1      1.65e-01      2.46e-01      1855    0.000  0.000   0.000   0.000   0.000

LOCUS: AT4G31985
DESCRIPTION:  60S ribosomal protein L39 (RPL39C)
DATA:                         Control 30min  2hours 2days  1week  p-value       B&H           Pos     Fold change relative to control (log2)
SENSE COUNTS:                 27     10     20     51     59     4.41e-09      2.54e-08              0.000  -1.433  -0.433  0.918   1.128
  GENES:
  AT4G31985.1
    SENSE COUNTS:             27     10     20     51     59     4.41e-09      2.56e-08              0.000  -1.433  -0.433  0.918   1.128
    TAGS:
      d+1      TTACCAAATT     27     10     20     51     59     4.41e-09      4.44e-08      317     0.000  -1.433  -0.433  0.918   1.128

LOCUS: AT1G71380
DESCRIPTION:  glycosyl hydrolase family 9 protein, similar to beta-glucanase GB
DATA:                         Control 30min  2hours 2days  1week  p-value       B&H           Pos     Fold change relative to control (log2)
SENSE COUNTS:                 1      0      17     1      1      4.70e-09      2.69e-08              0.000  0.000   4.087   0.000   0.000
  GENES:
  AT1G71380.1
    SENSE COUNTS:             1      0      17     1      1      4.70e-09      2.72e-08              0.000  0.000   4.087   0.000   0.000
    TAGS:
      d+1      GTTGGATTCT     1      0      17     1      1      4.70e-09      4.70e-08      1211    0.000  0.000   4.087   0.000   0.000

LOCUS: AT1G59870
DESCRIPTION:  ABC transporter family protein, similar to PDR5-like ABC transporter GI
DATA:                         Control 30min  2hours 2days  1week  p-value       B&H           Pos     Fold change relative to control (log2)
SENSE COUNTS:                 10     35     7      2      10     4.81e-09      2.73e-08              0.000  1.807   -0.515  -2.322  0.000
  GENES:
  AT1G59870.1
    SENSE COUNTS:             10     35     7      2      10     4.81e-09      2.77e-08              0.000  1.807   -0.515  -2.322  0.000
    TAGS:
      d+1      TAATATAATT     6      21     4      2      10     1.31e-04      5.32e-04      4731    0.000  1.807   -0.585  -1.585  0.737
      d+2      GACCGTGTAT     4      14     3      0      0      4.70e-05      2.10e-04      4343    0.000  1.807   -0.415  0.000   0.000

LOCUS: AT1G13930
DESCRIPTION:  expressed protein, weakly similar to drought-induced protein SDi-6 (PIR
DATA:                         Control 30min  2hours 2days  1week  p-value       B&H           Pos     Fold change relative to control (log2)
SENSE COUNTS:                 67     49     80     122    125    6.41e-09      3.62e-08              0.000  -0.451  0.256   0.865   0.900
  GENES:
  AT1G13930.1
    SENSE COUNTS:             67     49     80     122    125    6.41e-09      3.67e-08              0.000  -0.451  0.256   0.865   0.900
    TAGS:
      d+1      AATCTAATCC     66     44     74     120    124    6.06e-10      6.61e-09      652     0.000  -0.585  0.165   0.862   0.910
      d+2      GCAAGTGCCA     1      5      6      2      1      2.93e-01      3.95e-01      173     0.000  2.322   2.585   1.000   0.000

LOCUS: AT1G18080
DESCRIPTION:  WD-40 repeat family protein / auxin-dependent protein (ARCA) / guanine nucleotide-binding protein beta subunit, putative, identical to
SP|O24456 Guanine nucleotide-binding protein beta subunit-like protein (WD-40 repeat auxin-dependent protein ARCA) {Arab
DATA:                         Control 30min  2hours 2days  1week  p-value       B&H           Pos     Fold change relative to control (log2)
SENSE COUNTS:                 45     9      13     24     51     6.73e-09      3.78e-08              0.000  -2.322  -1.791  -0.907  0.181
  GENES:
  AT1G18080.1
    SENSE COUNTS:             45     9      13     24     51     6.73e-09      3.83e-08              0.000  -2.322  -1.791  -0.907  0.181
    TAGS:
      d+1      AAGTACCTTG     45     9      12     24     50     7.20e-09      7.07e-08      1194    0.000  -2.322  -1.907  -0.907  0.152
      d+2      GTATTAAGAT     0      0      1      0      1      3.96e-01      5.04e-01      894     0.000  0.000   0.000   0.000   0.000
```

```
LOCUS: AT4G33070
DESCRIPTION:  pyruvate decarboxylase, putative, strong similarity to SP|P51846 Pyruvate decarboxylase isozyme 2 (EC 4.1.1.1) (PDC) {Nicotiana
tabacum}; contains InterPro entry IPR000399
DATA:                          Control 30min  2hours  2days  1week  p-value       B&H           Pos    Fold change relative to control (log2)
SENSE COUNTS:                  1       0      0       4      17     7.52e-09      4.19e-08             0.000  0.000  0.000  2.000  4.087
  GENES:
  AT4G33070.1
    SENSE COUNTS:              1       0      0       4      17     7.52e-09      4.26e-08             0.000  0.000  0.000  2.000  4.087
    TAGS:
      d+1      GAATAAAGTA      1       0      0       4      16     1.05e-07      8.78e-07      2053   0.000  0.000  0.000  2.000  4.000
      X+4      TTTTGCTAGA      0       0      0       0      1      1.65e-01      2.41e-01      434    0.000  0.000  0.000  0.000  0.000

LOCUS: AT2G27385
DESCRIPTION:  expressed protein
DATA:                          Control 30min  2hours  2days  1week  p-value       B&H           Pos    Fold change relative to control (log2)
SENSE COUNTS:                  45      10     47      37     5      7.61e-09      4.22e-08             0.000  -2.170 0.063  -0.282 -3.170
  GENES:
  AT2G27385.1
    SENSE COUNTS:              45      10     47      37     5      7.61e-09      4.29e-08             0.000  -2.170 0.063  -0.282 -3.170
    TAGS:
      d+1      CTTGAACTGA      44      10     46      37     5      1.60e-08      1.49e-07      619    0.000  -2.138 0.064  -0.250 -3.138
      d+2      TAACTATCTT      1       0      1       0      0      6.01e-01      6.51e-01      587    0.000  0.000  0.000  0.000  0.000

LOCUS: AT4G38130
DESCRIPTION:  histone deacetylase (RPD3A), identical to SP|O22446 Histone deacetylase (HD) {Arabidopsis thaliana}
DATA:                          Control 30min  2hours  2days  1week  p-value       B&H           Pos    Fold change relative to control (log2)
SENSE COUNTS:                  9       2      4       8      34     8.66e-09      4.77e-08             0.000  -2.170 -1.170 -0.170 1.918
  GENES:
  AT4G38130.1
    SENSE COUNTS:              9       2      4       8      34     8.66e-09      4.85e-08             0.000  -2.170 -1.170 -0.170 1.918
    TAGS:
      d+1      TCTGAATGAT      9       2      4       8      34     8.66e-09      8.36e-08      1824   0.000  -2.170 -1.170 -0.170 1.918

LOCUS: AT5G20830
DESCRIPTION:  sucrose synthase / sucrose-UDP glucosyltransferase (SUS1), identical to SP|P49040 Sucrose synthase (EC 2.4.1.13) (Sucrose-UDP
glucosyltransferase) {Arabidopsis thaliana}
DATA:                          Control 30min  2hours  2days  1week  p-value       B&H           Pos    Fold change relative to control (log2)
SENSE COUNTS:                  1       0      0       19     7      9.37e-09      5.13e-08             0.000  0.000  0.000  4.248  2.807
  GENES:
  AT5G20830.1
    SENSE COUNTS:              1       0      0       19     7      9.37e-09      5.22e-08             0.000  0.000  0.000  4.248  2.807
    TAGS:
      d+1      TCTCGAACCT      1       0      0       19     7      9.37e-09      9.00e-08      2411   0.000  0.000  0.000  4.248  2.807

LOCUS: AT3G50970
DESCRIPTION:  dehydrin xero2 (XERO2) / low-temperature-induced protein LTI30 (LTI30), identical to dehydrin Xero 2 (Low-temperature-induced protein
LTI30) (Arabidopsis thaliana) SWISS-PROT
DATA:                          Control 30min  2hours  2days  1week  p-value       B&H           Pos    Fold change relative to control (log2)
SENSE COUNTS:                  1       0      3       13     22     9.59e-09      5.22e-08             0.000  0.000  1.585  3.700  4.459
  GENES:
  AT3G50970.1
    SENSE COUNTS:              1       0      3       13     22     9.59e-09      5.32e-08             0.000  0.000  1.585  3.700  4.459
    TAGS:
      d+1      AAAATAAAAG      1       0      3       13     21     5.13e-08      4.51e-07      575    0.000  0.000  1.585  3.700  4.392
      d+2      GAACTCACAA      0       0      0       0      1      1.65e-01      2.37e-01      518    0.000  0.000  0.000  0.000  0.000

LOCUS: AT1G22530
DESCRIPTION:  SEC14 cytosolic factor family protein / phosphoglyceride transfer family protein, contains Pfam PF00650
DATA:                          Control 30min  2hours  2days  1week  p-value       B&H           Pos    Fold change relative to control (log2)
SENSE COUNTS:                  4       29     5       8      1      1.02e-08      5.52e-08             0.000  2.858  0.322  1.000  -2.000
```

```
  GENES:
  AT1G22530.1
    SENSE COUNTS:            4      29     5      8      1      1.02e-08      5.63e-08             0.000  2.858  0.322  1.000  -2.000
    TAGS:
      d+1      GGAGCTTAGG    4      29     5      8      1      1.02e-08      9.74e-08      1940   0.000  2.858  0.322  1.000  -2.000

LOCUS: AT5G24740
DESCRIPTION:  expressed protein
DATA:                        Control 30min  2hours 2days  1week  p-value       B&H           Pos    Fold change relative to control (log2)
SENSE COUNTS:                2      5      1      3      27     1.03e-08      5.54e-08             0.000  1.322  -1.000 0.585  3.755
  GENES:
  AT5G24740.1
    SENSE COUNTS:            2      5      1      3      27     1.03e-08      5.65e-08             0.000  1.322  -1.000 0.585  3.755
    TAGS:
      d+1      TTTTGGCTAT    1      1      0      0      0      4.77e-01      5.28e-01      10022  0.000  0.000  0.000  0.000  0.000
      d+2      CATTTATTCA    0      4      1      2      26     2.42e-11      3.33e-10      6918   0.000  2.000  0.000  1.000  4.700
      i+3      AAAAGGGTTT    1      0      0      0      1      3.83e-01      4.90e-01      5121   0.000  0.000  0.000  0.000  0.000
      d+2      TGTTATGTCA    0      0      0      1      0      6.04e-01      6.49e-01      3591   0.000  0.000  0.000  0.000  0.000
      d+2      GGATGGATGC    0      0      0      0      0      6.15e-01      6.37e-01      2324   0.000  0.000  0.000  0.000  0.000

LOCUS: AT1G62180
DESCRIPTION:  5'adenylylphosphosulfate reductase (APR2) mRNA, complete
DATA:                        Control 30min  2hours 2days  1week  p-value       B&H           Pos    Fold change relative to control (log2)
SENSE COUNTS:                3      0      0      0      17     1.05e-08      5.62e-08             0.000  0.000  0.000  0.000  2.503
  GENES:
  AT1G62180.1
    SENSE COUNTS:            3      0      0      0      17     1.05e-08      5.73e-08             0.000  0.000  0.000  0.000  2.503
    TAGS:
      d+1      ACTTTGTATA    3      0      0      0      16     1.46e-08      1.38e-07      1631   0.000  0.000  0.000  0.000  2.415
      d+2      TCGTTTGTGA    0      0      0      0      1      4.65e-01      5.17e-01      1479   0.000  0.000  0.000  0.000  0.000

LOCUS: AT5G67030
DESCRIPTION:  Involved in the first step of abscisic acid (ABA) biosynthesis
DATA:                        Control 30min  2hours 2days  1week  p-value       B&H           Pos    Fold change relative to control (log2)
SENSE COUNTS:                13     2      6      0      30     1.20e-08      6.38e-08             0.000  -2.700 -1.115 0.000  1.206
  GENES:
  AT5G67030.2
    SENSE COUNTS:            13     2      6      0      30     1.20e-08      6.52e-08             0.000  -2.700 -1.115 0.000  1.206
    TAGS:
      d+1      ACAATGTATA    11     2      5      0      30     3.86e-09      3.91e-08      2264   0.000  -2.459 -1.138 0.000  1.447
      d+2      CAATGGCGAG    1      0      1      0      0      6.01e-01      6.49e-01      1453   0.000  0.000  0.000  0.000  0.000
      d+2      TGTTTAGTCC    1      0      0      0      0      4.28e-01      5.23e-01      217    0.000  0.000  0.000  0.000  0.000
  AT5G67030.1
    SENSE COUNTS:            13     2      6      0      30     1.20e-08      6.48e-08             0.000  -2.700 -1.115 0.000  1.206
    TAGS:
      d+1      ACAATGTATA    11     2      5      0      30     3.86e-09      3.91e-08      2260   0.000  -2.459 -1.138 0.000  1.447
      d+2      CAATGGCGAG    1      0      1      0      0      6.01e-01      6.49e-01      1453   0.000  0.000  0.000  0.000  0.000
      d+2      TGTTTAGTCC    1      0      0      0      0      4.28e-01      5.23e-01      217    0.000  0.000  0.000  0.000  0.000

LOCUS: AT5G46110
DESCRIPTION:  mutant has Altered acclimation responses; Chloroplast Triose Phosphate Translocator
DATA:                        Control 30min  2hours 2days  1week  p-value       B&H           Pos    Fold change relative to control (log2)
SENSE COUNTS:                18     60     38     27     7      1.36e-08      7.19e-08             0.000  1.737  1.078  0.585  -1.363
  GENES:
  AT5G46110.2
    SENSE COUNTS:            18     60     38     27     7      1.36e-08      7.28e-08             0.000  1.737  1.078  0.585  -1.363
    TAGS:
      d+1      GTTTCGCCGA    18     60     37     27     7      1.32e-08      1.25e-07      1304   0.000  1.737  1.040  0.585  -1.363
      d+2      TCACTAGCAA    0      0      1      0      0      4.55e-01      5.10e-01      932    0.000  0.000  0.000  0.000  0.000
  AT5G46110.1
```

SENSE COUNTS: 18 60 38 27 7 1.36e-08 7.31e-08 0.000 1.737 1.078 0.585 -1.363
TAGS:
d+1 GTTTCGCCGA 18 60 37 27 7 1.32e-08 1.25e-07 1297 0.000 1.737 1.040 0.585 -1.363
d+2 TCACTAGCAA 0 0 1 0 0 4.55e-01 5.10e-01 925 0.000 0.000 0.000 0.000 0.000

LOCUS: AT1G80130
DESCRIPTION: expressed protein
DATA: Control 30min 2hours 2days 1week p-value B&H Pos Fold change relative to control (log2)
SENSE COUNTS: 0 0 3 10 23 1.39e-08 7.31e-08 0.000 0.000 1.585 3.322 4.524
GENES:
AT1G80130.1
SENSE COUNTS: 0 0 3 10 23 1.39e-08 7.40e-08 0.000 0.000 1.585 3.322 4.524
TAGS:
d+1 TCCTATCGAG 0 0 1 8 16 2.41e-06 1.52e-05 1297 0.000 0.000 0.000 3.000 4.000
d+2 TTCCGTGATT 0 0 2 2 7 1.57e-02 2.76e-02 1042 0.000 0.000 1.000 1.000 2.807

LOCUS: AT3G23810
DESCRIPTION: adenosylhomocysteinase, putative / S-adenosyl-L-homocysteine hydrolase, putative / AdoHcyase, putative, strong similarity to SP|P50248|SAHH_TOBAC Adenosylhomocysteinase (EC 3.3.1.1) (S-adenosyl-L-homocysteine hydrolase) (AdoHcyase) {Nicotiana sylvestris};
DATA: Control 30min 2hours 2days 1week p-value B&H Pos Fold change relative to control (log2)
SENSE COUNTS: 1 5 5 29 7 1.56e-08 8.15e-08 0.000 2.322 2.322 4.858 2.807
GENES:
AT3G23810.1
SENSE COUNTS: 1 5 5 29 7 1.56e-08 8.26e-08 0.000 2.322 2.322 4.858 2.807
TAGS:
d+1 CTTGGACTTG 1 5 5 29 7 1.56e-08 1.46e-07 1114 0.000 2.322 2.322 4.858 2.807

LOCUS: AT1G09690
DESCRIPTION: 60S ribosomal protein L21 (RPL21C), Similar to ribosomal protein L21 (gb|L38826). ESTs gb|AA395597,gb|ATTS5197 come from this gene
DATA: Control 30min 2hours 2days 1week p-value B&H Pos Fold change relative to control (log2)
SENSE COUNTS: 26 8 13 22 55 2.00e-08 1.04e-07 0.000 -1.700 -1.000 -0.241 1.081
GENES:
AT1G09690.1
SENSE COUNTS: 26 8 13 22 55 2.00e-08 1.05e-07 0.000 -1.700 -1.000 -0.241 1.081
TAGS:
d+1 TGTCTTAGCT 26 8 13 22 52 2.20e-07 1.79e-06 593 0.000 -1.700 -1.000 -0.241 1.000
d+2 TGCAGCAATC 0 0 0 0 3 1.12e-02 2.02e-02 328 0.000 0.000 0.000 0.000 1.585

LOCUS: AT2G30860
DESCRIPTION: glutathione S-transferase, putative, identical to GB
DATA: Control 30min 2hours 2days 1week p-value B&H Pos Fold change relative to control (log2)
SENSE COUNTS: 16 36 3 6 12 2.30e-08 1.19e-07 0.000 1.170 -2.415 -1.415 -0.415
GENES:
AT2G30860.1
SENSE COUNTS: 16 36 3 6 12 2.30e-08 1.20e-07 0.000 1.170 -2.415 -1.415 -0.415
TAGS:
d+1 ATCAAAGATA 16 36 3 6 12 2.30e-08 2.12e-07 688 0.000 1.170 -2.415 -1.415 -0.415

LOCUS: AT5G09660
DESCRIPTION: encodes a microbody NAD-dependent malate dehydrogenase
DATA: Control 30min 2hours 2days 1week p-value B&H Pos Fold change relative to control (log2)
SENSE COUNTS: 84 33 73 40 26 2.53e-08 1.30e-07 0.000 -1.348 -0.202 -1.070 -1.692
GENES:
AT5G09660.1
SENSE COUNTS: 84 33 73 40 26 2.53e-08 1.31e-07 0.000 -1.348 -0.202 -1.070 -1.692
TAGS:
d+1 CCTATGTCTC 84 33 73 40 26 2.53e-08 2.32e-07 1265 0.000 -1.348 -0.202 -1.070 -1.692

LOCUS: AT3G56240
DESCRIPTION: copper homeostasis factor / copper chaperone (CCH) (ATX1), identical to gi

DATA:		Control	30min	2hours	2days	1week	p-value	B&H	Pos	Fold change relative to control (log2)				
SENSE COUNTS:		76	43	120	98	70	3.03e-08	1.55e-07		0.000	-0.822	0.659	0.367	-0.119
GENES:														
AT3G56240.1														
SENSE COUNTS:		76	43	120	98	70	3.03e-08	1.57e-07		0.000	-0.822	0.659	0.367	-0.119
TAGS:														
d+1	TTGGTCCGAC	75	43	120	98	70	2.73e-08	2.49e-07	613	0.000	-0.803	0.678	0.386	-0.100
d+2	GCTCAGACCG	1	0	0	0	0	4.28e-01	5.29e-01	93	0.000	0.000	0.000	0.000	0.000

LOCUS: AT3G18080
DESCRIPTION: glycosyl hydrolase family 1 protein, contains Pfam PF00232

DATA:		Control	30min	2hours	2days	1week	p-value	B&H	Pos	Fold change relative to control (log2)				
SENSE COUNTS:		1	20	4	0	3	3.11e-08	1.58e-07		0.000	4.322	2.000	0.000	1.585
GENES:														
AT3G18080.1														
SENSE COUNTS:		1	20	4	0	3	3.11e-08	1.60e-07		0.000	4.322	2.000	0.000	1.585
TAGS:														
d+1	ACACCACGAG	1	18	4	0	3	8.65e-07	5.98e-06	1334	0.000	4.170	2.000	0.000	1.585
d+2	GCCTCTCCTC	0	2	0	0	0	9.14e-02	1.46e-01	54	0.000	1.000	0.000	0.000	0.000

LOCUS: AT1G29070
DESCRIPTION: ribosomal protein L34 family protein, similar to plastid ribosomal protein L34 precursor GB

DATA:		Control	30min	2hours	2days	1week	p-value	B&H	Pos	Fold change relative to control (log2)				
SENSE COUNTS:		22	1	2	1	17	3.22e-08	1.63e-07		0.000	-4.459	-3.459	-4.459	-0.372
GENES:														
AT1G29070.1														
SENSE COUNTS:		22	1	2	1	17	3.22e-08	1.65e-07		0.000	-4.459	-3.459	-4.459	-0.372
TAGS:														
d+1	TACTACTATA	22	1	2	1	17	3.22e-08	2.92e-07	730	0.000	-4.459	-3.459	-4.459	-0.372

LOCUS: AT5G64570
DESCRIPTION: glycosyl hydrolase family 3 protein,

DATA:		Control	30min	2hours	2days	1week	p-value	B&H	Pos	Fold change relative to control (log2)				
SENSE COUNTS:		13	38	18	5	1	3.37e-08	1.69e-07		0.000	1.547	0.469	-1.379	-3.700
GENES:														
AT5G64570.1														
SENSE COUNTS:		13	38	18	5	1	3.37e-08	1.72e-07		0.000	1.547	0.469	-1.379	-3.700
TAGS:														
d+1	TCGGAGATTT	13	38	17	4	0	3.01e-09	3.06e-08	2344	0.000	1.547	0.387	-1.700	0.000
d+2	AGACCCGATA	0	0	1	1	1	5.61e-01	6.10e-01	1863	0.000	0.000	0.000	0.000	0.000

LOCUS: AT1G23300
DESCRIPTION: MATE efflux family protein, similar to ripening regulated protein DDTFR18 (Lycopersicon esculentum) GI

DATA:		Control	30min	2hours	2days	1week	p-value	B&H	Pos	Fold change relative to control (log2)				
SENSE COUNTS:		2	7	6	8	33	3.50e-08	1.75e-07		0.000	1.807	1.585	2.000	4.044
GENES:														
AT1G23300.1														
SENSE COUNTS:		2	7	6	8	33	3.50e-08	1.77e-07		0.000	1.807	1.585	2.000	4.044
TAGS:														
v+1	AAATAAATTA	2	7	6	8	32	2.24e-07	1.81e-06	2242	0.000	1.807	1.585	2.000	4.000
v+2	CCTCCGATCT	0	0	0	0	1	1.65e-01	2.38e-01	435	0.000	0.000	0.000	0.000	0.000

LOCUS: AT1G62510
DESCRIPTION: protease inhibitor/seed storage/lipid transfer protein (LTP) family protein, contains Pfam protease inhibitor/seed storage/LTP family domain PF00234

DATA:		Control	30min	2hours	2days	1week	p-value	B&H	Pos	Fold change relative to control (log2)				
SENSE COUNTS:		16	33	10	4	0	3.74e-08	1.86e-07		0.000	1.044	-0.678	-2.000	0.000
GENES:														
AT1G62510.1														
SENSE COUNTS:		16	33	10	4	0	3.74e-08	1.89e-07		0.000	1.044	-0.678	-2.000	0.000

```
    TAGS:
      d+1      TTGCACCCTC     16      33      10      4       0       3.74e-08        3.36e-07        401     0.000   1.044   -0.678  -2.000  0.000

LOCUS: AT1G21310
DESCRIPTION:  proline-rich extensin-like family protein, contains extensin-like region, Pfam
DATA:                         Control 30min   2hours  2days   1week   p-value         B&H             Pos     Fold change relative to control (log2)
SENSE COUNTS:                 6       38      15      49      47      4.46e-08        2.21e-07                0.000   2.663   1.322   3.030   2.970
  GENES:
  AT1G21310.1
    SENSE COUNTS:             6       38      15      49      47      4.46e-08        2.24e-07                0.000   2.663   1.322   3.030   2.970
    TAGS:
      d+1      TGCAAGTTTG     6       31      15      44      44      1.37e-06        9.13e-06        1592    0.000   2.369   1.322   2.874   2.874
      d+2      CAATCAATAG     0       7       0       5       3       1.13e-02        2.01e-02        1522    0.000   2.807   0.000   2.322   1.585

LOCUS: AT5G47930
DESCRIPTION:  40S ribosomal protein S27 (RPS27D)
DATA:                         Control 30min   2hours  2days   1week   p-value         B&H             Pos     Fold change relative to control (log2)
SENSE COUNTS:                 16      3       6       17      40      4.46e-08        2.19e-07                0.000   -2.415  -1.415  0.087   1.322
  GENES:
  AT5G47930.1
    SENSE COUNTS:             16      3       6       17      40      4.46e-08        2.23e-07                0.000   -2.415  -1.415  0.087   1.322
    TAGS:
      d+1      TTTACAATTC     1       0       1       2       14      2.25e-05        1.11e-04        543     0.000   0.000   0.000   1.000   3.807
      d+2      ATAAAACTAC     15      3       5       15      25      4.28e-04        1.45e-03        427     0.000   -2.322  -1.585  0.000   0.737
      i+3      TGAGTTTACT     0       0       0       0       1       1.65e-01        2.37e-01        392     0.000   0.000   0.000   0.000   0.000

LOCUS: AT3G15353
DESCRIPTION:  metallothionein protein, putative
DATA:                         Control 30min   2hours  2days   1week   p-value         B&H             Pos     Fold change relative to control (log2)
SENSE COUNTS:                 62      56      27      12      23      4.78e-08        2.34e-07                0.000   -0.147  -1.199  -2.369  -1.431
  GENES:
  AT3G15353.1
    SENSE COUNTS:             62      56      27      12      23      4.78e-08        2.38e-07                0.000   -0.147  -1.199  -2.369  -1.431
    TAGS:
      d+1      TCTCTCTTAT     62      56      23      12      23      7.90e-09        7.67e-08        352     0.000   -0.147  -1.431  -2.369  -1.431
      d+2      GACGTTGGTG     0       0       4       0       0       5.58e-03        1.18e-02        196     0.000   0.000   2.000   0.000   0.000

LOCUS: AT2G41100
DESCRIPTION:  touch-responsive protein / calmodulin-related protein 3, touch-induced (TCH3), identical to calmodulin-related protein 3, touch-induced
SP
DATA:                         Control 30min   2hours  2days   1week   p-value         B&H             Pos     Fold change relative to control (log2)
SENSE COUNTS:                 22      39      14      1       10      5.78e-08        2.81e-07                0.000   0.826   -0.652  -4.459  -1.138
  GENES:
  AT2G41100.2
    SENSE COUNTS:             22      39      14      1       10      5.78e-08        2.86e-07                0.000   0.826   -0.652  -4.459  -1.138
    TAGS:
      d+1      TGTTAGTGTG     22      37      13      0       10      9.29e-08        7.88e-07        867     0.000   0.750   -0.759  0.000   -1.138
      d+2      AAAGATAGGT     0       2       1       0       0       2.47e-01        3.39e-01        714     0.000   1.000   0.000   0.000   0.000
      d+2      ATCAAGCGCC     0       0       0       1       0       3.09e-01        4.10e-01        286     0.000   0.000   0.000   0.000   0.000
  AT2G41100.1
    SENSE COUNTS:             22      39      14      2       10      1.68e-07        7.68e-07                0.000   0.826   -0.652  -3.459  -1.138
    TAGS:
      d+1      TGTTAGTGTG     22      37      13      0       10      9.29e-08        7.88e-07        1134    0.000   0.750   -0.759  0.000   -1.138
      d+2      AAAGATAGGT     0       2       1       0       0       2.47e-01        3.39e-01        981     0.000   1.000   0.000   0.000   0.000
      d+2      ATCAAGCGCC     0       0       0       1       0       3.09e-01        4.10e-01        286     0.000   0.000   0.000   0.000   0.000

LOCUS: AT4G28670
DESCRIPTION:  protein kinase family protein, contains Pfam domain, PF00069
DATA:                         Control 30min   2hours  2days   1week   p-value         B&H             Pos     Fold change relative to control (log2)
SENSE COUNTS:                 5       0       3       4       25      7.03e-08        3.40e-07                0.000   0.000   -0.737  -0.322  2.322
```

```
  GENES:
  AT4G28670.1
    SENSE COUNTS:             5      0      3      4      25     7.03e-08       3.47e-07              0.000  0.000  -0.737  -0.322  2.322
    TAGS:
      v+2      ATTTTACTAA     5      0      3      4      25     7.03e-08       6.09e-07       75     0.000  0.000  -0.737  -0.322  2.322

LOCUS: AT1G70420
DESCRIPTION:  expressed protein
DATA:                         Control 30min  2hours 2days  1week  p-value        B&H            Pos    Fold change relative to control (log2)
SENSE COUNTS:                 0      3      19     2      1      7.37e-08       3.55e-07              0.000  1.585  4.248  1.000  0.000
  GENES:
  AT1G70420.1
    SENSE COUNTS:             0      3      19     2      1      7.37e-08       3.62e-07              0.000  1.585  4.248  1.000  0.000
    TAGS:
      d+1      TTGGGTTCTT     0      3      18     2      1      3.25e-07       2.53e-06       871    0.000  1.585  4.170  1.000  0.000
      d+2      GTGGAATCAG     0      0      1      0      0      4.55e-01       5.48e-01       251    0.000  0.000  0.000  0.000  0.000

LOCUS: AT3G09260
DESCRIPTION:  glycosyl hydrolase family 1 protein, contains Pfam PF00232
DATA:                         Control 30min  2hours 2days  1week  p-value        B&H            Pos    Fold change relative to control (log2)
SENSE COUNTS:                 57     70     67     39     2      7.46e-08       3.57e-07              0.000  0.296  0.233  -0.547  -4.833
  GENES:
  AT3G09260.1
    SENSE COUNTS:             57     70     67     39     2      7.46e-08       3.64e-07              0.000  0.296  0.233  -0.547  -4.833
    TAGS:
      d+1      ATTTGCCAGA     38     52     44     30     1      1.13e-05       5.97e-05       1742   0.000  0.453  0.212  -0.341  -5.248
      d+2      AGGTCTTGGT     19     18     23     9      1      1.13e-02       2.01e-02       1684   0.000  -0.078  0.276  -1.078  -4.248

LOCUS: AT4G37980
DESCRIPTION:  mannitol dehydrogenase, putative (ELI3-1), identical to GI
DATA:                         Control 30min  2hours 2days  1week  p-value        B&H            Pos    Fold change relative to control (log2)
SENSE COUNTS:                 44     9      15     13     8      7.53e-08       3.59e-07              0.000  -2.290  -1.553  -1.759  -2.459
  GENES:
  AT4G37980.1
    SENSE COUNTS:             44     9      15     13     8      7.53e-08       3.66e-07              0.000  -2.290  -1.553  -1.759  -2.459
    TAGS:
      d+1      TACTTTATCA     44     9      14     13     8      4.94e-08       4.37e-07       1263   0.000  -2.290  -1.652  -1.759  -2.459
      d+2      AAGTATCACG     0      0      1      0      0      4.55e-01       5.24e-01       595    0.000  0.000  0.000  0.000  0.000

LOCUS: AT4G08390
DESCRIPTION:  L-ascorbate peroxidase, stromal (sAPX), identical to stromal ascorbate peroxidase (Arabidopsis thaliana) gi|1419388|emb|CAA67425
DATA:                         Control 30min  2hours 2days  1week  p-value        B&H            Pos    Fold change relative to control (log2)
SENSE COUNTS:                 2      0      2      4      20     7.97e-08       3.78e-07              0.000  0.000  0.000  1.000  3.322
  GENES:
  AT4G08390.1
    SENSE COUNTS:             2      0      2      4      20     7.97e-08       3.86e-07              0.000  0.000  0.000  1.000  3.322
    TAGS:
      d+1      TTTTTTCTTG     2      0      2      4      19     8.49e-07       5.89e-06       1411   0.000  0.000  0.000  1.000  3.248
      d+2      CGAAGCTCAG     0      0      0      0      1      1.65e-01       2.45e-01       1158   0.000  0.000  0.000  0.000  0.000
  AT4G08390.2
    SENSE COUNTS:             2      0      2      4      20     7.97e-08       3.84e-07              0.000  0.000  0.000  1.000  3.322
    TAGS:
      d+1      TTTTTTCTTG     2      0      2      4      19     8.49e-07       5.89e-06       1352   0.000  0.000  0.000  1.000  3.248
      d+2      CGAAGCTCAG     0      0      0      0      1      1.65e-01       2.45e-01       1096   0.000  0.000  0.000  0.000  0.000

LOCUS: AT4G31390
DESCRIPTION:  ABC1 family protein, contains Pfam domain, PF03109
DATA:                         Control 30min  2hours 2days  1week  p-value        B&H            Pos    Fold change relative to control (log2)
SENSE COUNTS:                 0      0      0      1      12     8.09e-08       3.82e-07              0.000  0.000  0.000  0.000  3.585
  GENES:
```

AT4G31390.1
SENSE COUNTS: 0 0 0 1 12 8.09e-08 3.88e-07 0.000 0.000 0.000 0.000 3.585
TAGS:
d+1 TAATTATATA 0 0 0 1 12 8.09e-08 6.93e-07 2256 0.000 0.000 0.000 0.000 3.585

LOCUS: AT1G62600
DESCRIPTION: flavin-containing monooxygenase family protein / FMO family protein, low similarity to flavin-containing monooxygenase 2 from Cavia porcellus (SP|P36366); contains Pfam profile PF00743 Flavin-binding monooxygenase-like
DATA: Control 30min 2hours 2days 1week p-value B&H Pos Fold change relative to control (log2)
SENSE COUNTS: 2 18 3 0 0 8.50e-08 3.99e-07 0.000 3.170 0.585 0.000 0.000
GENES:
AT1G62600.1
SENSE COUNTS: 2 18 3 0 0 8.50e-08 4.06e-07 0.000 3.170 0.585 0.000 0.000
TAGS:
X+4 AGTTTTTGGG 2 18 3 0 0 8.50e-08 7.25e-07 -9 0.000 3.170 0.585 0.000 0.000

LOCUS: AT2G39010
DESCRIPTION: aquaporin, putative, similar to plasma membrane aquaporin 2b GI
DATA: Control 30min 2hours 2days 1week p-value B&H Pos Fold change relative to control (log2)
SENSE COUNTS: 16 39 12 11 3 9.02e-08 4.21e-07 0.000 1.285 -0.415 -0.541 -2.415
GENES:
AT2G39010.1
SENSE COUNTS: 16 39 12 11 3 9.02e-08 4.29e-07 0.000 1.285 -0.415 -0.541 -2.415
TAGS:
d+1 CTTAAATTTG 16 39 11 11 3 1.11e-07 9.23e-07 953 0.000 1.285 -0.541 -0.541 -2.415
i+3 TGAAAACACC 0 0 0 0 0 6.15e-01 6.38e-01 802 0.000 0.000 0.000 0.000 0.000
d+2 CTCTCCGACG 0 0 1 0 0 4.55e-01 5.11e-01 565 0.000 0.000 0.000 0.000 0.000

LOCUS: AT2G34430
DESCRIPTION: chlorophyll A-B binding protein / LHCII type I (LHB1B1), identical to photosystem II type I chlorophyll a/b binding protein (Arabidopsis thaliana) GI
DATA: Control 30min 2hours 2days 1week p-value B&H Pos Fold change relative to control (log2)
SENSE COUNTS: 61 78 45 41 8 9.46e-08 4.40e-07 0.000 0.355 -0.439 -0.573 -2.931
GENES:
AT2G34430.1
SENSE COUNTS: 61 78 45 41 8 9.46e-08 4.48e-07 0.000 0.355 -0.439 -0.573 -2.931
TAGS:
d+1 GGCCTTCGCT 61 78 45 41 8 9.46e-08 7.99e-07 830 0.000 0.355 -0.439 -0.573 -2.931

LOCUS: AT5G01410
DESCRIPTION: stress-responsive protein, putative, similar to ethylene-inducible protein HEVER (Hevea brasiliensis) SWISS-PROT
DATA: Control 30min 2hours 2days 1week p-value B&H Pos Fold change relative to control (log2)
SENSE COUNTS: 5 2 6 26 3 1.05e-07 4.85e-07 0.000 -1.322 0.263 2.379 -0.737
GENES:
AT5G01410.1
SENSE COUNTS: 5 2 6 26 3 1.05e-07 4.95e-07 0.000 -1.322 0.263 2.379 -0.737
TAGS:
d+1 GTTGGGATCA 4 2 5 24 3 5.38e-07 4.00e-06 983 0.000 -1.000 0.322 2.585 -0.415
d+2 ATGCAGCTTG 1 0 1 2 0 3.84e-01 4.91e-01 830 0.000 0.000 0.000 1.000 0.000

LOCUS: AT3G17800
DESCRIPTION: expressed protein
DATA: Control 30min 2hours 2days 1week p-value B&H Pos Fold change relative to control (log2)
SENSE COUNTS: 10 7 42 26 16 1.10e-07 5.06e-07 0.000 -0.515 2.070 1.379 0.678
GENES:
AT3G17800.1
SENSE COUNTS: 10 7 42 26 16 1.10e-07 5.16e-07 0.000 -0.515 2.070 1.379 0.678
TAGS:
d+2 GTCTGTAATG 10 7 41 26 16 2.56e-07 2.03e-06 1552 0.000 -0.515 2.036 1.379 0.678
d+2 TAGATGCGAG 0 0 1 0 0 4.55e-01 5.21e-01 1513 0.000 0.000 0.000 0.000 0.000

```
LOCUS: AT3G23400
DESCRIPTION:  plastid-lipid associated protein PAP / fibrillin family protein, contains Pfam profile PF04755
DATA:                         Control 30min   2hours  2days   1week   p-value         B&H             Pos     Fold change relative to control (log2)
SENSE COUNTS:                 15      2       12      20      41      1.18e-07        5.40e-07                0.000   -2.907  -0.322  0.415   1.451
  GENES:
  AT3G23400.1
    SENSE COUNTS:             15      2       12      20      41      1.18e-07        5.51e-07                0.000   -2.907  -0.322  0.415   1.451
    TAGS:
      d+1      TGAACATATA     15      2       12      20      41      1.18e-07        9.72e-07        1008    0.000   -2.907  -0.322  0.415   1.451

LOCUS: AT1G62750
DESCRIPTION:  elongation factor Tu family protein, similar to elongation factor G SP
DATA:                         Control 30min   2hours  2days   1week   p-value         B&H             Pos     Fold change relative to control (log2)
SENSE COUNTS:                 17      1       11      10      37      1.27e-07        5.78e-07                0.000   -4.087  -0.628  -0.766  1.122
  GENES:
  AT1G62750.1
    SENSE COUNTS:             17      1       11      10      37      1.27e-07        5.91e-07                0.000   -4.087  -0.628  -0.766  1.122
    TAGS:
      d+2      TAAGCTAATC     17      1       11      9       37      7.43e-08        6.40e-07        2542    0.000   -4.087  -0.628  -0.918  1.122
      d+2      AGTGATCCTT     0       0       0       1       0       3.09e-01        4.01e-01        1339    0.000   0.000   0.000   0.000   0.000

LOCUS: AT1G05010
DESCRIPTION:  1-aminocyclopropane-1-carboxylate oxidase / ACC oxidase / ethylene-forming enzyme (ACO) (EAT1), Identical to 1-aminocyclopropane-1-
carboxylate oxidase (ACC oxidase) gb|X66719 (EAT1). ESTs gb|T43073, gb|T5714, gb|R90435, gb|R44023, gb|AA597926, gb|AI099676
DATA:                         Control 30min   2hours  2days   1week   p-value         B&H             Pos     Fold change relative to control (log2)
SENSE COUNTS:                 54      31      18      5       24      1.41e-07        6.39e-07                0.000   -0.801  -1.585  -3.433  -1.170
  GENES:
  AT1G05010.1
    SENSE COUNTS:             54      31      18      5       24      1.41e-07        6.53e-07                0.000   -0.801  -1.585  -3.433  -1.170
    TAGS:
      d+1      TTGTTGTATG     54      29      17      5       23      5.77e-08        5.05e-07        1239    0.000   -0.897  -1.667  -3.433  -1.231
      d+2      AAAGCTATGG     0       2       1       0       1       4.01e-01        5.06e-01        1082    0.000   1.000   0.000   0.000   0.000

LOCUS: AT5G64940
DESCRIPTION:  ABC1 family protein, contains Pfam domain, PF03109
DATA:                         Control 30min   2hours  2days   1week   p-value         B&H             Pos     Fold change relative to control (log2)
SENSE COUNTS:                 35      7       13      2       19      1.51e-07        6.81e-07                0.000   -2.322  -1.429  -4.129  -0.881
  GENES:
  AT5G64940.2
    SENSE COUNTS:             35      7       13      2       19      1.51e-07        6.96e-07                0.000   -2.322  -1.429  -4.129  -0.881
    TAGS:
      d+1      TATTGCTTGT     35      7       13      2       19      1.51e-07        1.24e-06        2620    0.000   -2.322  -1.429  -4.129  -0.881
  AT5G64940.1
    SENSE COUNTS:             35      7       13      2       19      1.51e-07        6.93e-07                0.000   -2.322  -1.429  -4.129  -0.881
    TAGS:
      d+1      TATTGCTTGT     35      7       13      2       19      1.51e-07        1.24e-06        2675    0.000   -2.322  -1.429  -4.129  -0.881

LOCUS: AT3G16080
DESCRIPTION:  60S ribosomal protein L37 (RPL37C), similar to ribosomal protein L37 GB
DATA:                         Control 30min   2hours  2days   1week   p-value         B&H             Pos     Fold change relative to control (log2)
SENSE COUNTS:                 10      6       3       19      35      2.13e-07        9.56e-07                0.000   -0.737  -1.737  0.926   1.807
  GENES:
  AT3G16080.1
    SENSE COUNTS:             10      6       3       19      35      2.13e-07        9.70e-07                0.000   -0.737  -1.737  0.926   1.807
    TAGS:
      d+1      TGGATCTCAT     10      6       3       19      35      2.13e-07        1.74e-06        444     0.000   -0.737  -1.737  0.926   1.807

LOCUS: AT1G73660
DESCRIPTION:  protein kinase family protein, contains Pfam profile
```

```
DATA:                          Control 30min   2hours  2days   1week   p-value         B&H             Pos     Fold change relative to control (log2)
SENSE COUNTS:                  2       24      7       3       4       2.25e-07        1.00e-06                0.000   3.585   1.807   0.585   1.000
  GENES:
  AT1G73660.1
    SENSE COUNTS:              2       24      7       3       4       2.25e-07        1.02e-06                0.000   3.585   1.807   0.585   1.000
    TAGS:
      d+1      CGTAATTTCC      1       1       3       2       3       8.34e-01        8.36e-01        3821    0.000   0.000   1.585   1.000   1.585
      d+2      GAAGATGACG      0       0       0       0       0       6.15e-01        6.43e-01        3741    0.000   0.000   0.000   0.000   0.000
      d+2      GGAGCTGTGA      1       23      4       1       0       2.67e-10        3.20e-09        2921    0.000   4.524   2.000   0.000   0.000
      d+2      TGGATTTAGG      0       0       0       0       1       1.65e-01        2.46e-01        879     0.000   0.000   0.000   0.000   0.000

LOCUS: AT4G26660
DESCRIPTION:  expressed protein, weak similarity to phragmoplast-associated kinesin-related protein 1 (Arabidopsis thaliana) GI
DATA:                          Control 30min   2hours  2days   1week   p-value         B&H             Pos     Fold change relative to control (log2)
SENSE COUNTS:                  31      5       30      15      0       2.44e-07        1.08e-06                0.000   -2.632  -0.047  -1.047  0.000
  GENES:
  AT4G26660.1
    SENSE COUNTS:              31      5       30      15      0       2.44e-07        1.10e-06                0.000   -2.632  -0.047  -1.047  0.000
    TAGS:
      d+1      TATTGATTGA      0       0       0       0       0       6.15e-01        6.29e-01        2643    0.000   0.000   0.000   0.000   0.000
      d+2      TAGAAAGAAA      31      5       30      15      0       1.01e-07        8.48e-07        1330    0.000   -2.632  -0.047  -1.047  0.000

LOCUS: AT1G61800
DESCRIPTION:  glucose-6-phosphate/phosphate translocator, putative, similar to glucose-6-phosphate/phosphate-translocator precursor GI
DATA:                          Control 30min   2hours  2days   1week   p-value         B&H             Pos     Fold change relative to control (log2)
SENSE COUNTS:                  1       0       1       6       16      2.61e-07        1.15e-06                0.000   0.000   0.000   2.585   4.000
  GENES:
  AT1G61800.1
    SENSE COUNTS:              1       0       1       6       16      2.61e-07        1.17e-06                0.000   0.000   0.000   2.585   4.000
    TAGS:
      d+1      GATATGAAGA      1       0       0       5       10      1.65e-04        6.34e-04        1383    0.000   0.000   0.000   2.322   3.322
      d+2      TCATTAGACC      0       0       0       0       3       1.12e-02        2.01e-02        1084    0.000   0.000   0.000   0.000   1.585
      X+4      TGTAATAGTT      0       0       1       1       3       1.91e-01        2.68e-01        346     0.000   0.000   0.000   0.000   1.585

LOCUS: AT4G26970
DESCRIPTION:  aconitate hydratase, cytoplasmic, putative / citrate hydro-lyase/aconitase, putative, strong similarity to SP|P49608 Aconitate hydratase,
cytoplasmic (EC 4.2.1.3) (Citrate hydro-lyase) (Aconitase) {Cucurbita maxima}; contains Pfam profiles PF00330
DATA:                          Control 30min   2hours  2days   1week   p-value         B&H             Pos     Fold change relative to control (log2)
SENSE COUNTS:                  5       23      3       5       0       2.72e-07        1.20e-06                0.000   2.202   -0.737  0.000   0.000
  GENES:
  AT4G26970.1
    SENSE COUNTS:              5       23      3       5       0       2.72e-07        1.22e-06                0.000   2.202   -0.737  0.000   0.000
    TAGS:
      d+2      AACGCTACAC      1       2       1       0       0       4.78e-01        5.28e-01        2963    0.000   1.000   0.000   0.000   0.000
      d+2      TATTTCTGGT      0       0       1       0       0       4.55e-01        5.20e-01        1569    0.000   0.000   0.000   0.000   0.000
      d+2      GTGTTGTCGG      4       21      1       5       0       2.47e-07        1.97e-06        1277    0.000   2.392   -2.000  0.322   0.000

LOCUS: AT1G03600
DESCRIPTION:  photosystem II family protein, similar to SP
DATA:                          Control 30min   2hours  2days   1week   p-value         B&H             Pos     Fold change relative to control (log2)
SENSE COUNTS:                  69      17      53      34      37      2.83e-07        1.24e-06                0.000   -2.021  -0.381  -1.021  -0.899
  GENES:
  AT1G03600.1
    SENSE COUNTS:              69      17      53      34      37      2.83e-07        1.26e-06                0.000   -2.021  -0.381  -1.021  -0.899
    TAGS:
      d+1      ATAATTGCTT      69      17      53      34      37      2.83e-07        2.22e-06        717     0.000   -2.021  -0.381  -1.021  -0.899

LOCUS: AT4G30190
DESCRIPTION:  belongs to the P-type ATPase superfamily of cation-transporting ATPases, pumps protons out of the cell, generating a proton gradient
that drives the active transport of nutrients by proton symport. has two autoinhibitory  regions within the C-terminal dom
```

```
DATA:                         Control 30min  2hours  2days  1week  p-value       B&H           Pos    Fold change relative to control (log2)
SENSE COUNTS:                 11      5      4       9      35     3.13e-07      1.36e-06             0.000  -1.138 -1.459 -0.290 1.670
  GENES:
  AT4G30190.1
    SENSE COUNTS:             11      5      4       9      35     3.13e-07      1.39e-06             0.000  -1.138 -1.459 -0.290 1.670
    TAGS:
      d+1     AGCCTATGTA      10      5      2       8      28     6.12e-06      3.56e-05      3243   0.000  -1.000 -2.322 -0.322 1.485
      d+2     CCTTTGTGTT      1       0      2       1      7      9.17e-02      1.46e-01      3188   0.000  0.000  1.000  0.000  2.807

LOCUS: AT3G18740
DESCRIPTION:  60S ribosomal protein L30 (RPL30C), similar to 60S RIBOSOMAL PROTEIN L30 GB
DATA:                         Control 30min  2hours  2days  1week  p-value       B&H           Pos    Fold change relative to control (log2)
SENSE COUNTS:                 26      14     13      12     53     3.61e-07      1.57e-06             0.000  -0.893 -1.000 -1.115 1.027
  GENES:
  AT3G18740.1
    SENSE COUNTS:             26      14     13      12     53     3.61e-07      1.59e-06             0.000  -0.893 -1.000 -1.115 1.027
    TAGS:
      d+1     TTGTTCTCTA      26      14     13      12     53     3.61e-07      2.77e-06      434    0.000  -0.893 -1.000 -1.115 1.027

LOCUS: AT2G23930
DESCRIPTION:  small nuclear ribonucleoprotein G, putative / snRNP-G, putative / Sm protein G, putative, similar to small nuclear ribonucleoprotein G
(snRNP-G, Sm protein G, Sm-G, SmG) (Homo sapiens) SWISS-PROT
DATA:                         Control 30min  2hours  2days  1week  p-value       B&H           Pos    Fold change relative to control (log2)
SENSE COUNTS:                 2       1      4       4      23     3.61e-07      1.56e-06             0.000  -1.000 1.000  1.000  3.524
  GENES:
  AT2G23930.1
    SENSE COUNTS:             2       1      4       4      23     3.61e-07      1.60e-06             0.000  -1.000 1.000  1.000  3.524
    TAGS:
      d+1     AATACTCTTT      2       1      4       4      23     3.61e-07      2.76e-06      440    0.000  -1.000 1.000  1.000  3.524

LOCUS: AT2G34480
DESCRIPTION:  60S ribosomal protein L18A (RPL18aB)
DATA:                         Control 30min  2hours  2days  1week  p-value       B&H           Pos    Fold change relative to control (log2)
SENSE COUNTS:                 40      21     32      44     79     3.62e-07      1.56e-06             0.000  -0.930 -0.322 0.138  0.982
  GENES:
  AT2G34480.1
    SENSE COUNTS:             40      21     32      44     79     3.62e-07      1.59e-06             0.000  -0.930 -0.322 0.138  0.982
    TAGS:
      d+1     AATGTGAACT      39      21     31      44     71     1.63e-05      8.25e-05      690    0.000  -0.893 -0.331 0.174  0.864
      d+2     TAAAACGTCT      1       0      1       0      8      2.95e-04      1.06e-03      575    0.000  0.000  0.000  0.000  3.000

LOCUS: AT2G41090
DESCRIPTION:  calmodulin-like calcium-binding protein, 22 kDa (CaBP-22), identical to SP|P30187 22 kDa calmodulin-like calcium-binding protein (CABP-
22) (Arabidopsis thaliana)
DATA:                         Control 30min  2hours  2days  1week  p-value       B&H           Pos    Fold change relative to control (log2)
SENSE COUNTS:                 3       17     4       11     35     4.34e-07      1.86e-06             0.000  2.503  0.415  1.874  3.544
  GENES:
  AT2G41090.1
    SENSE COUNTS:             3       17     4       11     35     4.34e-07      1.90e-06             0.000  2.503  0.415  1.874  3.544
    TAGS:
      d+1     CAATTTATTA      3       17     4       11     35     4.34e-07      3.28e-06      744    0.000  2.503  0.415  1.874  3.544

LOCUS: AT4G38770
DESCRIPTION:  proline-rich family protein (PRP4), similar to proline-rich protein (Arabidopsis thaliana) gi|6782442|gb|AAF28388; contains proline-rich
extensin domains, INTERPRO
DATA:                         Control 30min  2hours  2days  1week  p-value       B&H           Pos    Fold change relative to control (log2)
SENSE COUNTS:                 48      18     36      43     76     4.46e-07      1.90e-06             0.000  -1.415 -0.415 -0.159 0.663
  GENES:
  AT4G38770.1
    SENSE COUNTS:             48      18     36      43     76     4.46e-07      1.94e-06             0.000  -1.415 -0.415 -0.159 0.663
```

TAGS:
d+1 TATAATCAAC 44 18 28 40 68 9.09e-06 4.89e-05 1505 0.000 -1.290 -0.652 -0.138 0.628
d+2 TCCGCCACTT 0 0 5 1 3 2.64e-02 4.53e-02 1355 0.000 0.000 2.322 0.000 1.585
d+2 CCCTCCGCCG 0 0 1 1 1 5.61e-01 6.09e-01 1298 0.000 0.000 0.000 0.000 0.000
d+2 TCCGCCTAAG 2 0 2 0 1 3.93e-01 5.01e-01 1013 0.000 0.000 0.000 0.000 -1.000
d+2 TCCACCTAAA 2 0 0 1 3 1.97e-01 2.76e-01 662 0.000 0.000 0.000 -1.000 0.585

LOCUS: AT1G64230
DESCRIPTION: ubiquitin-conjugating enzyme, putative, identical or nearly so to Ubiquitin-conjugating enzymes SP|P35132, SP|P35131, SP|P35133 from {Arabidopsis thaliana}; contains Pfam profile PF00179
DATA: Control 30min 2hours 2days 1week p-value B&H Pos Fold change relative to control (log2)
SENSE COUNTS: 17 47 15 18 10 5.22e-07 2.21e-06 0.000 1.467 -0.181 0.082 -0.766
GENES:
AT1G64230.1
SENSE COUNTS: 17 47 15 18 10 5.22e-07 2.26e-06 0.000 1.467 -0.181 0.082 -0.766
TAGS:
d+1 AAATAAGTTA 16 47 15 18 10 3.31e-07 2.56e-06 673 0.000 1.555 -0.093 0.170 -0.678
X+4 ATTTTGAAGA 1 0 0 0 0 4.28e-01 5.25e-01 537 0.000 0.000 0.000 0.000 0.000

LOCUS: AT1G15670
DESCRIPTION: kelch repeat-containing F-box family protein, similar to SP|Q9ER30 Kelch-related protein 1 (Sarcosin) {Rattus norvegicus}; contains Pfam profiles PF01344
DATA: Control 30min 2hours 2days 1week p-value B&H Pos Fold change relative to control (log2)
SENSE COUNTS: 0 10 0 0 0 5.38e-07 2.27e-06 0.000 3.322 0.000 0.000 0.000
GENES:
AT1G15670.1
SENSE COUNTS: 0 10 0 0 0 5.38e-07 2.32e-06 0.000 3.322 0.000 0.000 0.000
TAGS:
d+1 TACTATTTGG 0 3 0 0 0 3.05e-02 5.17e-02 1499 0.000 1.585 0.000 0.000 0.000
d+2 ATAAATATGA 0 7 0 0 0 7.93e-05 3.36e-04 1201 0.000 2.807 0.000 0.000 0.000

LOCUS: AT1G22410
DESCRIPTION: 2-dehydro-3-deoxyphosphoheptonate aldolase, putative / 3-deoxy-D-arabino-heptulosonate 7-phosphate synthase, putative / DAHP synthetase, putative, similar to 3-deoxy-D-arabino-heptulosonate 7-phosphate GI
DATA: Control 30min 2hours 2days 1week p-value B&H Pos Fold change relative to control (log2)
SENSE COUNTS: 8 3 13 31 5 5.50e-07 2.31e-06 0.000 -1.415 0.700 1.954 -0.678
GENES:
AT1G22410.1
SENSE COUNTS: 8 3 13 31 5 5.50e-07 2.36e-06 0.000 -1.415 0.700 1.954 -0.678
TAGS:
d+1 GATGGTTGGG 8 3 13 31 5 5.50e-07 4.07e-06 1822 0.000 -1.415 0.700 1.954 -0.678

LOCUS: AT5G21090
DESCRIPTION: leucine-rich repeat protein, putative, similar to leucine rich repeat protein (LRP) GI
DATA: Control 30min 2hours 2days 1week p-value B&H Pos Fold change relative to control (log2)
SENSE COUNTS: 1 14 0 2 0 6.13e-07 2.56e-06 0.000 3.807 0.000 1.000 0.000
GENES:
AT5G21090.1
SENSE COUNTS: 1 14 0 2 0 6.13e-07 2.62e-06 0.000 3.807 0.000 1.000 0.000
TAGS:
d+1 TCACCTGTAA 1 14 0 2 0 6.13e-07 4.46e-06 337 0.000 3.807 0.000 1.000 0.000

LOCUS: AT4G00810
DESCRIPTION: 60S acidic ribosomal protein P1 (RPP1B), similar to acidic ribosomal protein p1
DATA: Control 30min 2hours 2days 1week p-value B&H Pos Fold change relative to control (log2)
SENSE COUNTS: 15 6 4 24 36 6.95e-07 2.89e-06 0.000 -1.322 -1.907 0.678 1.263
GENES:
AT4G00810.2
SENSE COUNTS: 15 6 4 24 36 6.95e-07 2.96e-06 0.000 -1.322 -1.907 0.678 1.263
TAGS:

```
      d+1      AGAGTTATCT     10      6       4       18      35      6.45e-07        4.67e-06        519     0.000   -0.737  -1.322  0.848   1.807
      d+2      TACTGGAGTT     5       0       0       6       1       2.56e-02        4.41e-02        475     0.000   0.000   0.000   0.263   -2.322
  AT4G00810.1
    SENSE COUNTS:             15      6       4       24      36      6.95e-07        2.95e-06                0.000   -1.322  -1.907  0.678   1.263
    TAGS:
      d+1      AGAGTTATCT     10      6       4       18      35      6.45e-07        4.67e-06        597     0.000   -0.737  -1.322  0.848   1.807
      d+2      TACTGGAGTT     5       0       0       6       1       2.56e-02        4.41e-02        553     0.000   0.000   0.000   0.263   -2.322

LOCUS: AT1G56220
DESCRIPTION:  dormancy/auxin associated family protein, similar to Auxin-repressed 12.5 kDa protein (Swiss-Prot
DATA:                         Control 30min   2hours  2days   1week   p-value         B&H             Pos     Fold change relative to control (log2)
SENSE COUNTS:                 24      63      32      31      14      7.40e-07        3.07e-06                0.000   1.392   0.415   0.369   -0.778
  GENES:
  AT1G56220.1
    SENSE COUNTS:             24      63      32      31      14      7.40e-07        3.11e-06                0.000   1.392   0.415   0.369   -0.778
    TAGS:
      d+1      AAGTGGTGGT     24      63      32      31      14      7.40e-07        5.31e-06        568     0.000   1.392   0.415   0.369   -0.778
  AT1G56220.3
    SENSE COUNTS:             24      63      32      31      14      7.40e-07        3.13e-06                0.000   1.392   0.415   0.369   -0.778
    TAGS:
      d+1      AAGTGGTGGT     24      63      32      31      14      7.40e-07        5.31e-06        577     0.000   1.392   0.415   0.369   -0.778
  AT1G56220.2
    SENSE COUNTS:             24      63      32      31      14      7.40e-07        3.12e-06                0.000   1.392   0.415   0.369   -0.778
    TAGS:
      d+1      AAGTGGTGGT     24      63      32      31      14      7.40e-07        5.31e-06        582     0.000   1.392   0.415   0.369   -0.778

LOCUS: AT2G23120
DESCRIPTION:  expressed protein
DATA:                         Control 30min   2hours  2days   1week   p-value         B&H             Pos     Fold change relative to control (log2)
SENSE COUNTS:                 8       18      42      48      39      7.63e-07        3.15e-06                0.000   1.170   2.392   2.585   2.285
  GENES:
  AT2G23120.1
    SENSE COUNTS:             8       18      42      48      39      7.63e-07        3.19e-06                0.000   1.170   2.392   2.585   2.285
    TAGS:
      d+1      GCGACGGGTA     8       18      42      48      39      7.63e-07        5.43e-06        159     0.000   1.170   2.392   2.585   2.285

LOCUS: AT5G52650
DESCRIPTION:  40S ribosomal protein S10 (RPS10C), contains similarity to 40S ribosomal protein S10
DATA:                         Control 30min   2hours  2days   1week   p-value         B&H             Pos     Fold change relative to control (log2)
SENSE COUNTS:                 20      10      11      44      37      7.70e-07        3.16e-06                0.000   -1.000  -0.862  1.138   0.888
  GENES:
  AT5G52650.1
    SENSE COUNTS:             20      10      11      44      37      7.70e-07        3.21e-06                0.000   -1.000  -0.862  1.138   0.888
    TAGS:
      i+3      AACATATTTA     0       0       1       0       0       4.55e-01        5.18e-01        1054    0.000   0.000   0.000   0.000   0.000
      d+1      GAGGAGTTTT     20      10      10      44      37      3.94e-07        3.00e-06        647     0.000   -1.000  -1.000  1.138   0.888

LOCUS: AT3G11120
DESCRIPTION:  60S ribosomal protein L41 (RPL41E), identical to ribosomal protein L41 GB
DATA:                         Control 30min   2hours  2days   1week   p-value         B&H             Pos     Fold change relative to control (log2)
SENSE COUNTS:                 14      4       11      26      39      7.88e-07        3.22e-06                0.000   -1.807  -0.348  0.893   1.478
  GENES:
  AT3G11120.1
    SENSE COUNTS:             14      4       11      26      39      7.88e-07        3.27e-06                0.000   -1.807  -0.348  0.893   1.478
    TAGS:
      d+1      AGGGCTAAGT     13      4       11      26      39      5.57e-07        4.09e-06        80      0.000   -1.700  -0.241  1.000   1.585
      i+3      AGGGCTAAGG     1       0       0       0       0       4.28e-01        5.32e-01        80      0.000   0.000   0.000   0.000   0.000

LOCUS: AT1G65930
```

```
DESCRIPTION:  isocitrate dehydrogenase, putative / NADP+ isocitrate dehydrogenase, putative, strong similarity to isocitrate dehydrogenase SP|Q40345
from (Medicago sativa)
DATA:                         Control 30min   2hours  2days   1week   p-value         B&H             Pos     Fold change relative to control (log2)
SENSE COUNTS:                 1       21      2       11      1       7.96e-07        3.24e-06                0.000   4.392   1.000   3.459   0.000
  GENES:
  AT1G65930.1
    SENSE COUNTS:             1       21      2       11      1       7.96e-07        3.29e-06                0.000   4.392   1.000   3.459   0.000
    TAGS:
      d+1      GAACTGTCAC     1       21      2       11      1       7.96e-07        5.57e-06        1093    0.000   4.392   1.000   3.459   0.000

LOCUS: AT3G05590
DESCRIPTION:  60S ribosomal protein L18 (RPL18B), similar to GB
DATA:                         Control 30min   2hours  2days   1week   p-value         B&H             Pos     Fold change relative to control (log2)
SENSE COUNTS:                 16      16      22      38      58      8.18e-07        3.32e-06                0.000   0.000   0.459   1.248   1.858
  GENES:
  AT3G05590.1
    SENSE COUNTS:             16      16      22      38      58      8.18e-07        3.37e-06                0.000   0.000   0.459   1.248   1.858
    TAGS:
      d+1      CTACTAAGTT     16      16      21      38      57      1.54e-06        1.01e-05        708     0.000   0.000   0.392   1.248   1.833
      d+2      AAAGTGACTG     0       0       1       0       1       3.96e-01        5.03e-01        323     0.000   0.000   0.000   0.000   0.000

LOCUS: AT1G09210
DESCRIPTION:  calreticulin 2 (CRT2), identical to SP|Q38858 Calreticulin 2 precursor {Arabidopsis thaliana}
DATA:                         Control 30min   2hours  2days   1week   p-value         B&H             Pos     Fold change relative to control (log2)
SENSE COUNTS:                 19      9       31      34      54      8.37e-07        3.38e-06                0.000   -1.078  0.706   0.840   1.507
  GENES:
  AT1G09210.1
    SENSE COUNTS:             19      9       31      34      54      8.37e-07        3.43e-06                0.000   -1.078  0.706   0.840   1.507
    TAGS:
      i+3      GTCACACCAG     0       0       1       0       0       4.55e-01        5.42e-01        2424    0.000   0.000   0.000   0.000   0.000
      d+1      AAGAAGTTTT     19      9       30      34      53      1.99e-06        1.28e-05        1521    0.000   -1.078  0.659   0.840   1.480
      d+2      TGAAACTGAC     0       0       0       0       1       1.65e-01        2.43e-01        572     0.000   0.000   0.000   0.000   0.000

LOCUS: AT5G24330
DESCRIPTION:  PHD finger family protein / SET domain-containing protein, contains Pfam domain, PF00628
DATA:                         Control 30min   2hours  2days   1week   p-value         B&H             Pos     Fold change relative to control (log2)
SENSE COUNTS:                 17      25      54      13      19      8.68e-07        3.49e-06                0.000   0.556   1.667   -0.387  0.160
  GENES:
  AT5G24330.1
    SENSE COUNTS:             17      25      54      13      19      8.68e-07        3.54e-06                0.000   0.556   1.667   -0.387  0.160
    TAGS:
      v+2      ATTCCGATTC     17      25      54      13      19      8.68e-07        5.98e-06        427     0.000   0.556   1.667   -0.387  0.160

LOCUS: AT1G79600
DESCRIPTION:  ABC1 family protein, contains Pfam domain, PF03109
DATA:                         Control 30min   2hours  2days   1week   p-value         B&H             Pos     Fold change relative to control (log2)
SENSE COUNTS:                 17      1       1       0       10      9.70e-07        3.88e-06                0.000   -4.087  -4.087  0.000   -0.766
  GENES:
  AT1G79600.1
    SENSE COUNTS:             17      1       1       0       10      9.70e-07        3.95e-06                0.000   -4.087  -4.087  0.000   -0.766
    TAGS:
      d+1      TTACAGTAAT     17      1       1       0       10      9.70e-07        6.62e-06        2387    0.000   -4.087  -4.087  0.000   -0.766

LOCUS: AT3G52180
DESCRIPTION:  protein tyrosine phosphatase/kinase interaction sequence protein (PTPKIS1), identical to PTPKIS1 protein (Arabidopsis thaliana) GI
DATA:                         Control 30min   2hours  2days   1week   p-value         B&H             Pos     Fold change relative to control (log2)
SENSE COUNTS:                 1       2       2       1       18      9.81e-07        3.91e-06                0.000   1.000   1.000   0.000   4.170
  GENES:
  AT3G52180.1
    SENSE COUNTS:             1       2       2       1       18      9.81e-07        3.98e-06                0.000   1.000   1.000   0.000   4.170
```

```
    TAGS:
      d+1      AAGTTAGTAG     1       2       2       1       17      3.36e-06        2.03e-05        1392    0.000   1.000   1.000   0.000   4.087
      i+3      AGTTGATTTT     0       0       0       0       0       6.15e-01        6.52e-01        1380    0.000   0.000   0.000   0.000   0.000
      d+2      TTAGTAATAC     0       0       0       0       1       1.65e-01        2.41e-01        1344    0.000   0.000   0.000   0.000   0.000

LOCUS: AT3G62290
DESCRIPTION:  ADP-ribosylation factor, identical to GP
DATA:                         Control 30min   2hours  2days   1week   p-value         B&H             Pos     Fold change relative to control (log2)
SENSE COUNTS:                 18      52      32      30      5       1.02e-06        4.04e-06                0.000   1.531   0.830   0.737   -1.848
  GENES:
  AT3G62290.1
    SENSE COUNTS:             18      52      32      30      5       1.02e-06        4.12e-06                0.000   1.531   0.830   0.737   -1.848
    TAGS:
      d+1      TGCTACCTCC     18      52      30      29      5       7.04e-07        5.08e-06        584     0.000   1.531   0.737   0.688   -1.848
      d+2      GTGGGTCTCG     0       0       2       1       0       2.74e-01        3.70e-01        174     0.000   0.000   1.000   0.000   0.000

LOCUS: AT5G05270
DESCRIPTION:  chalcone-flavanone isomerase family protein, contains very low similarity to chalcone-flavonone isomerase (chalcone isomerase), GI
DATA:                         Control 30min   2hours  2days   1week   p-value         B&H             Pos     Fold change relative to control (log2)
SENSE COUNTS:                 0       0       1       13      10      1.06e-06        4.19e-06                0.000   0.000   0.000   3.700   3.322
  GENES:
  AT5G05270.2
    SENSE COUNTS:             0       0       1       13      10      1.06e-06        4.25e-06                0.000   0.000   0.000   3.700   3.322
    TAGS:
      d+1      GCCCCTTACA     0       0       1       13      10      1.06e-06        7.21e-06        752     0.000   0.000   0.000   3.700   3.322
  AT5G05270.1
    SENSE COUNTS:             0       0       1       13      10      1.06e-06        4.26e-06                0.000   0.000   0.000   3.700   3.322
    TAGS:
      d+1      GCCCCTTACA     0       0       1       13      10      1.06e-06        7.21e-06        811     0.000   0.000   0.000   3.700   3.322

LOCUS: AT4G23950
DESCRIPTION:  expressed protein, ; expression supported by MPSS
DATA:                         Control 30min   2hours  2days   1week   p-value         B&H             Pos     Fold change relative to control (log2)
SENSE COUNTS:                 6       0       0       1       16      1.07e-06        4.21e-06                0.000   0.000   0.000   -2.585  1.415
  GENES:
  AT4G23950.1
    SENSE COUNTS:             6       0       0       1       16      1.07e-06        4.27e-06                0.000   0.000   0.000   -2.585  1.415
    TAGS:
      v+2      ATACTGCTAA     6       0       0       1       16      1.07e-06        7.22e-06        1704    0.000   0.000   0.000   -2.585  1.415

LOCUS: AT5G56320
DESCRIPTION:  expansin, putative (EXP14), similar to alpha-expansin 3 GI
DATA:                         Control 30min   2hours  2days   1week   p-value         B&H             Pos     Fold change relative to control (log2)
SENSE COUNTS:                 0       0       3       1       15      1.15e-06        4.50e-06                0.000   0.000   1.585   0.000   3.907
  GENES:
  AT5G56320.1
    SENSE COUNTS:             0       0       3       1       15      1.15e-06        4.57e-06                0.000   0.000   1.585   0.000   3.907
    TAGS:
      d+1      TCAAGAAACT     0       0       2       0       1       2.22e-01        3.07e-01        702     0.000   0.000   1.000   0.000   0.000
      i+3      GGTAATTTGC     0       0       1       1       14      7.66e-07        5.41e-06        352     0.000   0.000   0.000   0.000   3.807

LOCUS: ATCG00740
DESCRIPTION:  RNA polymerase alpha subunit
DATA:                         Control 30min   2hours  2days   1week   p-value         B&H             Pos     Fold change relative to control (log2)
SENSE COUNTS:                 148     79      93      79      71      1.20e-06        4.68e-06                0.000   -0.906  -0.670  -0.906  -1.060
  GENES:
  ATCG00740.1
    SENSE COUNTS:             148     79      93      79      71      1.20e-06        4.76e-06                0.000   -0.906  -0.670  -0.906  -1.060
    TAGS:
      d+1      TAGAAGAAGA     147     79      91      77      71      1.13e-06        7.59e-06        695     0.000   -0.896  -0.692  -0.933  -1.050
```

```
      d+2      CCTGTTGAAA     1       0       2       2       0       3.64e-01        4.68e-01        532     0.000   0.000   1.000   1.000   0.000

LOCUS: AT5G50920
DESCRIPTION:  ATP-dependent Clp protease ATP-binding subunit / ClpC, almost identical to ClpC GI
DATA:                          Control 30min   2hours  2days   1week   p-value         B&H             Pos     Fold change relative to control (log2)
SENSE COUNTS:                  81      29      83      81      79      1.21e-06        4.70e-06                0.000   -1.482  0.035   0.000   -0.036
  GENES:
  AT5G50920.1
    SENSE COUNTS:              81      29      83      81      79      1.21e-06        4.78e-06                0.000   -1.482  0.035   0.000   -0.036
    TAGS:
      d+1      CAAAGAGGAG     11      0       14      11      10      5.46e-03        1.17e-02        3150    0.000   0.000   0.348   0.000   -0.138
      d+2      CTTTTTAAGG     52      21      44      41      46      8.38e-03        1.57e-02        3120    0.000   -1.308  -0.241  -0.343  -0.177
      d+2      GGGGCTTTCT     17      7       25      27      23      6.38e-03        1.27e-02        3038    0.000   -1.280  0.556   0.667   0.436
      d+2      ACATCAAACG     0       0       0       2       0       4.80e-02        7.92e-02        2424    0.000   0.000   0.000   1.000   0.000
      d+2      CACAGGTCCC     1       1       0       0       0       4.77e-01        5.28e-01        1657    0.000   0.000   0.000   0.000   0.000

LOCUS: AT2G27680
DESCRIPTION:  aldo/keto reductase family protein, contains Pfam profile PF00248
DATA:                          Control 30min   2hours  2days   1week   p-value         B&H             Pos     Fold change relative to control (log2)
SENSE COUNTS:                  12      3       3       4       28      1.28e-06        4.95e-06                0.000   -2.000  -2.000  -1.585  1.222
  GENES:
  AT2G27680.1
    SENSE COUNTS:              12      3       3       4       28      1.28e-06        5.04e-06                0.000   -2.000  -2.000  -1.585  1.222
    TAGS:
      d+1      TTTGGAGAGA     0       0       0       0       0       6.15e-01        6.33e-01        1306    0.000   0.000   0.000   0.000   0.000
      d+2      TAATTACACT     12      3       3       4       28      4.92e-07        3.71e-06        1225    0.000   -2.000  -2.000  -1.585  1.222

LOCUS: AT1G73330
DESCRIPTION:  protease inhibitor, putative (DR4), identical to Dr4 GI
DATA:                          Control 30min   2hours  2days   1week   p-value         B&H             Pos     Fold change relative to control (log2)
SENSE COUNTS:                  5       24      5       12      0       1.31e-06        5.04e-06                0.000   2.263   0.000   1.263   0.000
  GENES:
  AT1G73330.1
    SENSE COUNTS:              5       24      5       12      0       1.31e-06        5.13e-06                0.000   2.263   0.000   1.263   0.000
    TAGS:
      d+1      TGTTTTTTGC     5       24      5       12      0       7.01e-06        3.98e-05        823     0.000   2.263   0.000   1.263   0.000
      d+2      AACGTTGGCA     0       0       0       0       0       6.15e-01        6.43e-01        556     0.000   0.000   0.000   0.000   0.000
      d+2      AAGGCCACCA     0       0       0       0       0       6.15e-01        6.51e-01        70      0.000   0.000   0.000   0.000   0.000

LOCUS: AT1G53240
DESCRIPTION:  malate dehydrogenase (NAD), mitochondrial, identical to mitochondrial NAD-dependent malate dehydrogenase GI
DATA:                          Control 30min   2hours  2days   1week   p-value         B&H             Pos     Fold change relative to control (log2)
SENSE COUNTS:                  29      8       24      17      53      1.41e-06        5.41e-06                0.000   -1.858  -0.273  -0.771  0.870
  GENES:
  AT1G53240.1
    SENSE COUNTS:              29      8       24      17      53      1.41e-06        5.51e-06                0.000   -1.858  -0.273  -0.771  0.870
    TAGS:
      d+1      AACAAGAGAT     24      8       15      12      48      5.94e-07        4.34e-06        1378    0.000   -1.585  -0.678  -1.000  1.000
      d+2      CGACTTTTTT     2       0       3       5       3       4.58e-01        5.11e-01        1362    0.000   0.000   0.585   1.322   0.585
      d+2      TCAAAGTTGT     1       0       6       0       0       7.29e-03        1.41e-02        1325    0.000   0.000   2.585   0.000   0.000
      d+2      TTTGTGCCTC     2       0       0       0       0       1.04e-01        1.64e-01        1252    0.000   0.000   0.000   0.000   0.000
      d+2      CTTGAAAGGA     0       0       0       0       1       1.65e-01        2.47e-01        936     0.000   0.000   0.000   0.000   0.000
      d+2      CGCTTATTAA     0       0       0       0       1       1.65e-01        2.37e-01        554     0.000   0.000   0.000   0.000   0.000

LOCUS: AT3G13790
DESCRIPTION:  beta-fructosidase (BFRUCT1) / beta-fructofuranosidase / cell wall invertase, identical to beta-fructofuranosidase GI
DATA:                          Control 30min   2hours  2days   1week   p-value         B&H             Pos     Fold change relative to control (log2)
SENSE COUNTS:                  0       0       0       0       8       1.50e-06        5.73e-06                0.000   0.000   0.000   0.000   3.000
  GENES:
  AT3G13790.1
```

```
    SENSE COUNTS:             0      0      0      0      8      1.50e-06       5.79e-06              0.000  0.000  0.000  0.000  3.000
    TAGS:
      d+1      TAAACACATT     0      0      0      0      8      1.50e-06       9.84e-06       1804   0.000  0.000  0.000  0.000  3.000

LOCUS: AT4G22880
DESCRIPTION:  leucoanthocyanidin dioxygenase, putative / anthocyanidin synthase, putative, similar to SP|P51091 (Malus domestica); contains PF03171
2OG-Fe(II) oxygenase superfamily
DATA:                         Control 30min  2hours 2days  1week  p-value        B&H            Pos    Fold change relative to control (log2)
SENSE COUNTS:                 0      0      0      0      8      1.50e-06       5.70e-06              0.000  0.000  0.000  0.000  3.000
  GENES:
  AT4G22880.1
    SENSE COUNTS:             0      0      0      0      8      1.50e-06       5.82e-06              0.000  0.000  0.000  0.000  3.000
    TAGS:
      d+1      AATTTGATGT     0      0      0      0      8      1.50e-06       9.88e-06       1195   0.000  0.000  0.000  0.000  3.000

LOCUS: AT4G30890
DESCRIPTION:  ubiquitin-specific protease 24, putative (UBP24), identical to ubiquitin-specific protease 24 (Arabidopsis thaliana) GI
DATA:                         Control 30min  2hours 2days  1week  p-value        B&H            Pos    Fold change relative to control (log2)
SENSE COUNTS:                 0      0      0      0      8      1.50e-06       5.68e-06              0.000  0.000  0.000  0.000  3.000
  GENES:
  AT4G30890.1
    SENSE COUNTS:             0      0      0      0      8      1.50e-06       5.77e-06              0.000  0.000  0.000  0.000  3.000
    TAGS:
      d+1      TCTTCTTATC     0      0      0      0      7      3.14e-05       1.49e-04       2109   0.000  0.000  0.000  0.000  2.807
      d+2      TCTTATGCTA     0      0      0      0      1      1.65e-01       2.36e-01       2095   0.000  0.000  0.000  0.000  0.000
  AT4G30890.2
    SENSE COUNTS:             0      0      0      0      8      1.50e-06       5.84e-06              0.000  0.000  0.000  0.000  3.000
    TAGS:
      d+1      TCTTCTTATC     0      0      0      0      7      3.14e-05       1.49e-04       2334   0.000  0.000  0.000  0.000  2.807
      d+2      TCTTATGCTA     0      0      0      0      1      1.65e-01       2.36e-01       2320   0.000  0.000  0.000  0.000  0.000

LOCUS: AT1G43160
DESCRIPTION:  encodes a member of the ERF (ethylene response factor) subfamily B-4 of ERF/AP2 transcription factor family (RAP2.6). The protein
contains one AP2 domain. There are 7 members in this subfamily.
DATA:                         Control 30min  2hours 2days  1week  p-value        B&H            Pos    Fold change relative to control (log2)
SENSE COUNTS:                 0      0      1      0      10     1.70e-06       6.41e-06              0.000  0.000  0.000  0.000  3.322
  GENES:
  AT1G43160.1
    SENSE COUNTS:             0      0      1      0      10     1.70e-06       6.52e-06              0.000  0.000  0.000  0.000  3.322
    TAGS:
      d+1      GGTGTATATA     0      0      0      0      7      3.14e-05       1.49e-04       879    0.000  0.000  0.000  0.000  2.807
      d+2      GACCGAATAT     0      0      1      0      3      7.32e-02       1.19e-01       716    0.000  0.000  0.000  0.000  1.585

LOCUS: AT4G36040
DESCRIPTION:  DNAJ heat shock N-terminal domain-containing protein (J11), identical to dnaJ heat shock protein J11 (Arabidopsis thaliana) GI
DATA:                         Control 30min  2hours 2days  1week  p-value        B&H            Pos    Fold change relative to control (log2)
SENSE COUNTS:                 37     37     38     8      3      1.73e-06       6.50e-06              0.000  0.000  0.038  -2.209 -3.624
  GENES:
  AT4G36040.1
    SENSE COUNTS:             37     37     38     8      3      1.73e-06       6.61e-06              0.000  0.000  0.038  -2.209 -3.624
    TAGS:
      d+1      TGTTACTACT     2      0      0      0      0      1.04e-01       1.64e-01       1055   0.000  0.000  0.000  0.000  0.000
      d+2      TGCTTACCGT     35     37     37     8      3      3.04e-06       1.86e-05       694    0.000  0.080  0.080  -2.129 -3.544
      d+2      CCGCTTACTG     0      0      1      0      0      4.55e-01       5.18e-01       425    0.000  0.000  0.000  0.000  0.000

LOCUS: AT1G26610
DESCRIPTION:  zinc finger (C2H2 type) family protein, contains Pfam domain, PF00096
DATA:                         Control 30min  2hours 2days  1week  p-value        B&H            Pos    Fold change relative to control (log2)
SENSE COUNTS:                 22     6      23     43     43     1.75e-06       6.54e-06              0.000  -1.874 0.064  0.967  0.967
  GENES:
```

```
  AT1G26610.1
    SENSE COUNTS:              22     6      23     43     43     1.75e-06      6.66e-06                0.000  -1.874  0.064  0.967  0.967
    TAGS:
      d+1      AGATGCATAC      0      0      0      0      0      6.15e-01      6.54e-01      1627      0.000  0.000   0.000  0.000  0.000
      d+2      AATGAGAATT      22     6      23     43     43     7.52e-07      5.38e-06      405       0.000  -1.874  0.064  0.967  0.967

LOCUS: AT5G59180
DESCRIPTION:  DNA-directed RNA polymerase II, identical to Swiss-Prot
DATA:                          Control 30min 2hours 2days  1week  p-value       B&H           Pos       Fold change relative to control (log2)
SENSE COUNTS:                  0      0      0      0      9      1.81e-06      6.74e-06                0.000  0.000   0.000  0.000  3.170
  GENES:
  AT5G59180.1
    SENSE COUNTS:              0      0      0      0      9      1.81e-06      6.82e-06                0.000  0.000   0.000  0.000  3.170
    TAGS:
      d+2      CCTAACTATA      0      0      0      0      1      4.65e-01      5.18e-01      455       0.000  0.000   0.000  0.000  0.000
      X+4      TCTCAATGAA      0      0      0      0      8      1.50e-06      9.92e-06      289       0.000  0.000   0.000  0.000  3.000

LOCUS: AT3G04730
DESCRIPTION:  auxin-responsive protein / indoleacetic acid-induced protein 16 (IAA16), identical to SP|O24407 Auxin-responsive protein IAA16
(Indoleacetic acid-induced protein 16) {Arabidopsis thaliana}
DATA:                          Control 30min 2hours 2days  1week  p-value       B&H           Pos       Fold change relative to control (log2)
SENSE COUNTS:                  10     28     15     1      1      1.89e-06      7.01e-06                0.000  1.485   0.585  -3.322 -3.322
  GENES:
  AT3G04730.1
    SENSE COUNTS:              10     28     15     1      1      1.89e-06      7.10e-06                0.000  1.485   0.585  -3.322 -3.322
    TAGS:
      d+1      CAAACGTATA      10     28     14     1      1      1.61e-06      1.05e-05      817       0.000  1.485   0.485  -3.322 -3.322
      d+2      GACGGTGCAC      0      0      1      0      0      4.55e-01      5.34e-01      563       0.000  0.000   0.000  0.000  0.000

LOCUS: AT3G09735
DESCRIPTION:  DNA-binding S1FA family protein, contains Pfam profile
DATA:                          Control 30min 2hours 2days  1week  p-value       B&H           Pos       Fold change relative to control (log2)
SENSE COUNTS:                  11     4      2      15     30     1.93e-06      7.13e-06                0.000  -1.459  -2.459 0.447  1.447
  GENES:
  AT3G09735.1
    SENSE COUNTS:              11     4      2      15     30     1.93e-06      7.22e-06                0.000  -1.459  -2.459 0.447  1.447
    TAGS:
      d+1      ATATTTTCTT      11     4      2      15     30     1.93e-06      1.24e-05      411       0.000  -1.459  -2.459 0.447  1.447

LOCUS: AT3G56880
DESCRIPTION:  VQ motif-containing protein, contains PF05678
DATA:                          Control 30min 2hours 2days  1week  p-value       B&H           Pos       Fold change relative to control (log2)
SENSE COUNTS:                  0      7      19     4      0      2.01e-06      7.40e-06                0.000  2.807   4.248  2.000  0.000
  GENES:
  AT3G56880.1
    SENSE COUNTS:              0      7      19     4      0      2.01e-06      7.49e-06                0.000  2.807   4.248  2.000  0.000
    TAGS:
      d+1      GAAAGTTATG      0      7      15     4      0      1.58e-04      6.19e-04      855       0.000  2.807   3.907  2.000  0.000
      d+2      GCGTCGTCGG      0      0      4      0      0      5.58e-03      1.18e-02      133       0.000  0.000   2.000  0.000  0.000

LOCUS: AT2G28140
DESCRIPTION:  expressed protein
DATA:                          Control 30min 2hours 2days  1week  p-value       B&H           Pos       Fold change relative to control (log2)
SENSE COUNTS:                  13     26     56     20     39     2.07e-06      7.59e-06                0.000  1.000   2.107  0.621  1.585
  GENES:
  AT2G28140.1
    SENSE COUNTS:              13     26     56     20     39     2.07e-06      7.69e-06                0.000  1.000   2.107  0.621  1.585
    TAGS:
      d+2      TTAAACAAAA      13     26     56     20     39     2.07e-06      1.32e-05      882       0.000  1.000   2.107  0.621  1.585
```

```
LOCUS: AT4G04640
DESCRIPTION:  ATP synthase gamma chain 1, chloroplast (ATPC1), identical to SP|Q01908 ATP synthase gamma chain 1, chloroplast precursor (EC 3.6.3.14)
{Arabidopsis thaliana}
DATA:                         Control 30min  2hours  2days  1week  p-value       B&H           Pos    Fold change relative to control (log2)
SENSE COUNTS:                 83      29     65      66     37     2.11e-06      7.70e-06             0.000  -1.517  -0.353  -0.331  -1.166
  GENES:
  AT4G04640.1
    SENSE COUNTS:             83      29     65      66     37     2.11e-06      7.81e-06             0.000  -1.517  -0.353  -0.331  -1.166
    TAGS:
      d+1      CAATTACAAA     83      29     65      66     37     2.11e-06      1.34e-05      1379   0.000  -1.517  -0.353  -0.331  -1.166

LOCUS: AT5G55620
DESCRIPTION:  expressed protein, similar to unknown protein (gb|AAF04428.1)
DATA:                         Control 30min  2hours  2days  1week  p-value       B&H           Pos    Fold change relative to control (log2)
SENSE COUNTS:                 13      0      5       6      25     2.16e-06      7.85e-06             0.000  0.000   -1.379  -1.115  0.943
  GENES:
  AT5G55620.1
    SENSE COUNTS:             13      0      5       6      25     2.16e-06      7.94e-06             0.000  0.000   -1.379  -1.115  0.943
    TAGS:
      X+4      CTGGATATTT     0       0      0       1      1      3.25e-01      4.20e-01      835    0.000  0.000   0.000   0.000   0.000
      X+4      TATAACACTA     11      0      2       4      12     5.15e-04      1.68e-03      727    0.000  0.000   -2.459  -1.459  0.126
      d+2      TATATCAATC     2       0      3       1      12     1.68e-03      4.45e-03      582    0.000  0.000   0.585   -1.000  2.585

LOCUS: AT1G71500
DESCRIPTION:  Rieske (2Fe-2S) domain-containing protein, contains Pfam profile PF00355
DATA:                         Control 30min  2hours  2days  1week  p-value       B&H           Pos    Fold change relative to control (log2)
SENSE COUNTS:                 10      1      0       2      19     2.16e-06      7.82e-06             0.000  -3.322  0.000   -2.322  0.926
  GENES:
  AT1G71500.1
    SENSE COUNTS:             10      1      0       2      19     2.16e-06      7.97e-06             0.000  -3.322  0.000   -2.322  0.926
    TAGS:
      d+1      CTAGGCCACT     2       0      0       2      0      1.57e-01      2.37e-01      1174   0.000  0.000   0.000   0.000   0.000
      d+2      TAATAGTATA     8       1      0       0      19     1.12e-07      9.27e-07      989    0.000  -3.000  0.000   0.000   1.248

LOCUS: AT5G65720
DESCRIPTION:  cysteine desulfurase, mitochondrial (NIFS), identical to Cysteine desulfurase, mitochondrial precursor  (SP
DATA:                         Control 30min  2hours  2days  1week  p-value       B&H           Pos    Fold change relative to control (log2)
SENSE COUNTS:                 3       0      6       20     5      2.31e-06      8.33e-06             0.000  0.000   1.000   2.737   0.737
  GENES:
  AT5G65720.1
    SENSE COUNTS:             3       0      6       20     5      2.31e-06      8.46e-06             0.000  0.000   1.000   2.737   0.737
    TAGS:
      d+1      TGAGAGTTTC     3       0      6       20     5      2.31e-06      1.46e-05      1634   0.000  0.000   1.000   2.737   0.737

LOCUS: AT4G09010
DESCRIPTION:  L-ascorbate peroxidase, chloroplast, putative, identical to SP|P82281|TL29_ARATH (EC 1.11.1.11) {Arabidopsis thaliana}; ascorbate
peroxidase - Spinacia oleracea, (gi
DATA:                         Control 30min  2hours  2days  1week  p-value       B&H           Pos    Fold change relative to control (log2)
SENSE COUNTS:                 37      19     49      41     3      2.50e-06      8.98e-06             0.000  -0.962  0.405   0.148   -3.624
  GENES:
  AT4G09010.1
    SENSE COUNTS:             37      19     49      41     3      2.50e-06      9.13e-06             0.000  -0.962  0.405   0.148   -3.624
    TAGS:
      d+1      AGAGGAAACT     37      19     49      41     3      2.50e-06      1.57e-05      1160   0.000  -0.962  0.405   0.148   -3.624

LOCUS: AT4G34190
DESCRIPTION:  stress enhanced protein 1 (SEP1), identical to stress enhanced protein 1 (SEP1) GI
DATA:                         Control 30min  2hours  2days  1week  p-value       B&H           Pos    Fold change relative to control (log2)
SENSE COUNTS:                 3       1      4       14     23     2.58e-06      9.24e-06             0.000  -1.585  0.415   2.222   2.939
  GENES:
```

```
  AT4G34190.1
    SENSE COUNTS:              3      1      4      14     23     2.58e-06       9.39e-06              0.000  -1.585  0.415  2.222  2.939
    TAGS:
      d+1     TATTAAGTTT     1      1      1      6      3      1.31e-01       1.99e-01       755    0.000  0.000  0.000  2.585  1.585
      d+2     ATCTATAACA     0      0      0      0      1      1.65e-01       2.36e-01       743    0.000  0.000  0.000  0.000  0.000
      d+2     TCTTTTGGCC     1      0      2      8      19     9.61e-07       6.59e-06       704    0.000  0.000  1.000  3.000  4.248
      d+2     TTCCGCAACT     1      0      1      0      0      6.01e-01       6.48e-01       222    0.000  0.000  0.000  0.000  0.000

LOCUS: AT1G78820
DESCRIPTION:  curculin-like (mannose-binding) lectin family protein / PAN domain-containing protein, similar to S locus glycoprotein (Brassica rapa)
GI
DATA:                          Control 30min  2hours  2days  1week  p-value        B&H            Pos    Fold change relative to control (log2)
SENSE COUNTS:                  0      15     1      2      3      2.79e-06       9.95e-06              0.000  3.907  0.000  1.000  1.585
  GENES:
  AT1G78820.1
    SENSE COUNTS:              0      15     1      2      3      2.79e-06       1.01e-05              0.000  3.907  0.000  1.000  1.585
    TAGS:
      d+1     TACGTTAACA     0      15     1      2      3      2.79e-06       1.72e-05       670    0.000  3.907  0.000  1.000  1.585

LOCUS: AT1G55450
DESCRIPTION:  embryo-abundant protein-related, similar to embryo-abundant protein GI
DATA:                          Control 30min  2hours  2days  1week  p-value        B&H            Pos    Fold change relative to control (log2)
SENSE COUNTS:                  0      2      17     5      1      2.81e-06       9.98e-06              0.000  1.000  4.087  2.322  0.000
  GENES:
  AT1G55450.1
    SENSE COUNTS:              0      2      17     5      1      2.81e-06       1.02e-05              0.000  1.000  4.087  2.322  0.000
    TAGS:
      d+2     ACTCAAGGGG     0      2      17     5      1      2.81e-06       1.73e-05       1162   0.000  1.000  4.087  2.322  0.000

LOCUS: AT3G27690
DESCRIPTION:  chlorophyll A-B binding protein (LHCB2
DATA:                          Control 30min  2hours  2days  1week  p-value        B&H            Pos    Fold change relative to control (log2)
SENSE COUNTS:                  13     35     32     6      5      2.86e-06       1.01e-05              0.000  1.429  1.300  -1.115 -1.379
  GENES:
  AT3G27690.1
    SENSE COUNTS:              13     35     32     6      5      2.86e-06       1.03e-05              0.000  1.429  1.300  -1.115 -1.379
    TAGS:
      d+1     CGTCGTACCG     13     35     32     6      5      2.86e-06       1.75e-05       170    0.000  1.429  1.300  -1.115 -1.379

LOCUS: AT1G20220
DESCRIPTION:  expressed protein
DATA:                          Control 30min  2hours  2days  1week  p-value        B&H            Pos    Fold change relative to control (log2)
SENSE COUNTS:                  3      0      3      17     3      2.89e-06       1.02e-05              0.000  0.000  0.000  2.503  0.000
  GENES:
  AT1G20220.1
    SENSE COUNTS:              3      0      3      17     3      2.89e-06       1.04e-05              0.000  0.000  0.000  2.503  0.000
    TAGS:
      d+1     GATCTTGGAT     3      0      2      13     3      1.59e-04       6.21e-04       1317   0.000  0.000  -0.585 2.115  0.000
      d+2     TCGCTTTAGG     0      0      1      4      0      3.72e-02       6.22e-02       1278   0.000  0.000  0.000  2.000  0.000

LOCUS: AT5G67300
DESCRIPTION:  myb family transcription factor, contains PFAM profile
DATA:                          Control 30min  2hours  2days  1week  p-value        B&H            Pos    Fold change relative to control (log2)
SENSE COUNTS:                  0      22     15     6      1      2.99e-06       1.05e-05              0.000  4.459  3.907  2.585  0.000
  GENES:
  AT5G67300.1
    SENSE COUNTS:              0      22     15     6      1      2.99e-06       1.07e-05              0.000  4.459  3.907  2.585  0.000
    TAGS:
      d+1     ATTCCGATGA     0      22     12     6      1      2.64e-06       1.64e-05       967    0.000  4.459  3.585  2.585  0.000
      d+2     ACGGAGATGC     0      0      1      0      0      4.55e-01       5.21e-01       907    0.000  0.000  0.000  0.000  0.000
```

```
      d+2      AGCCCAGGAA     0       0       2       0       0       1.21e-01        1.86e-01        511     0.000   0.000   1.000   0.000   0.000

LOCUS: ATCG01130
DESCRIPTION:  hypothetical protein
DATA:                         Control 30min   2hours  2days   1week   p-value         B&H             Pos     Fold change relative to control (log2)
SENSE COUNTS:                 286     224     210     182     161     3.26e-06        1.14e-05                0.000   -0.353  -0.446  -0.652  -0.829
  GENES:
  ATCG01130.1
    SENSE COUNTS:             286     224     210     182     161     3.26e-06        1.16e-05                0.000   -0.353  -0.446  -0.652  -0.829
    TAGS:
      d+1      TATACGCGAT     1       0       0       1       0       5.06e-01        5.56e-01        5338    0.000   0.000   0.000   0.000   0.000
      d+2      GGTCATACCA     54      70      32      44      32      6.30e-04        1.95e-03        4305    0.000   0.374   -0.755  -0.295  -0.755
      d+2      TCTATAGGCA     13      10      6       6       7       4.77e-01        5.29e-01        3937    0.000   -0.379  -1.115  -1.115  -0.893
      d+2      GGCTAGATTA     56      47      66      44      35      8.19e-02        1.32e-01        3702    0.000   -0.253  0.237   -0.348  -0.678
      d+2      AACTATCTGT     93      38      36      26      32      6.68e-11        8.57e-10        2978    0.000   -1.291  -1.369  -1.839  -1.539
      d+2      TTAAATGTAC     15      7       6       8       16      1.65e-01        2.42e-01        2465    0.000   -1.100  -1.322  -0.907  0.093
      d+2      CGTTCCCAAA     25      21      46      27      21      1.02e-02        1.85e-02        2065    0.000   -0.252  0.880   0.111   -0.252
      d+2      GCGAGATTTA     1       0       0       0       1       3.83e-01        4.90e-01        1949    0.000   0.000   0.000   0.000   0.000
      d+2      GTCTACTTCT     13      6       3       12      8       1.01e-01        1.60e-01        1574    0.000   -1.115  -2.115  -0.115  -0.700
      d+2      GAATCTCTCG     2       0       1       4       1       5.72e-01        6.21e-01        1472    0.000   0.000   -1.000  1.000   -1.000
      d+2      CCAAAGTGAT     12      6       11      6       7       4.90e-01        5.41e-01        1167    0.000   -1.000  -0.126  -1.000  -0.778
      d+1      AAATGGGTTG     1       19      3       4       1       1.35e-06        9.04e-06        559     0.000   4.248   1.585   2.000   0.000

LOCUS: AT5G40370
DESCRIPTION:  glutaredoxin, putative, similar to glutaredoxin (Ricinus communis) SWISS-PROT
DATA:                         Control 30min   2hours  2days   1week   p-value         B&H             Pos     Fold change relative to control (log2)
SENSE COUNTS:                 4       1       3       2       21      3.34e-06        1.16e-05                0.000   -2.000  -0.415  -1.000  2.392
  GENES:
  AT5G40370.1
    SENSE COUNTS:             4       1       3       2       21      3.34e-06        1.18e-05                0.000   -2.000  -0.415  -1.000  2.392
    TAGS:
      d+1      ATGTTATTAT     4       1       3       2       21      3.34e-06        2.03e-05        588     0.000   -2.000  -0.415  -1.000  2.392

LOCUS: AT5G61820
DESCRIPTION:  expressed protein, MtN19, Medicago truncatula, EMBL
DATA:                         Control 30min   2hours  2days   1week   p-value         B&H             Pos     Fold change relative to control (log2)
SENSE COUNTS:                 11      2       10      14      33      3.41e-06        1.18e-05                0.000   -2.459  -0.138  0.348   1.585
  GENES:
  AT5G61820.1
    SENSE COUNTS:             11      2       10      14      33      3.41e-06        1.20e-05                0.000   -2.459  -0.138  0.348   1.585
    TAGS:
      d+1      TCGACTCTAT     11      2       10      13      30      4.32e-05        1.95e-04        1516    0.000   -2.459  -0.138  0.241   1.447
      d+2      TGGAATACGA     0       0       0       1       3       6.27e-02        1.02e-01        959     0.000   0.000   0.000   0.000   1.585

LOCUS: AT1G31580
DESCRIPTION:  expressed protein, identical to ORF1 (Arabidopsis thaliana) gi|457716|emb|CAA50905
DATA:                         Control 30min   2hours  2days   1week   p-value         B&H             Pos     Fold change relative to control (log2)
SENSE COUNTS:                 15      0       1       1       13      3.55e-06        1.23e-05                0.000   0.000   -3.907  -3.907  -0.206
  GENES:
  AT1G31580.1
    SENSE COUNTS:             15      0       1       1       13      3.55e-06        1.25e-05                0.000   0.000   -3.907  -3.907  -0.206
    TAGS:
      d+2      TATGATGTAT     15      0       1       1       12      7.23e-06        4.06e-05        604     0.000   0.000   -3.907  -3.907  -0.322
      d+2      TATGTGGCTC     0       0       0       0       1       1.65e-01        2.39e-01        541     0.000   0.000   0.000   0.000   0.000

LOCUS: AT4G32480
DESCRIPTION:  expressed protein, contains Pfam profile PF04720
DATA:                         Control 30min   2hours  2days   1week   p-value         B&H             Pos     Fold change relative to control (log2)
SENSE COUNTS:                 0       10      1       0       0       3.66e-06        1.26e-05                0.000   3.322   0.000   0.000   0.000
  GENES:
```

AT4G32480.1														
SENSE COUNTS:		0	10	1	0	0	3.66e-06	1.28e-05		0.000	3.322	0.000	0.000	0.000
TAGS:														
d+1	GTATACAAAG	0	8	0	0	0	2.30e-05	1.12e-04	1092	0.000	3.000	0.000	0.000	0.000
d+2	CAGGCTAAAT	0	2	0	0	0	9.14e-02	1.46e-01	789	0.000	1.000	0.000	0.000	0.000
d+2	AAATTTCTCG	0	0	1	0	0	4.55e-01	5.25e-01	114	0.000	0.000	0.000	0.000	0.000

LOCUS: AT3G07430
DESCRIPTION: YGGT family protein, contains Pfam profile PF02325

DATA:		Control	30min	2hours	2days	1week	p-value	B&H	Pos	Fold change relative to control (log2)				
SENSE COUNTS:		3	5	6	10	29	3.92e-06	1.35e-05		0.000	0.737	1.000	1.737	3.273
GENES:														
AT3G07430.1														
SENSE COUNTS:		3	5	6	10	29	3.92e-06	1.37e-05		0.000	0.737	1.000	1.737	3.273
TAGS:														
d+1	TTCAGACTAC	3	2	2	6	19	8.62e-05	3.60e-04	967	0.000	-0.585	-0.585	1.000	2.663
d+2	GCAGCACTGG	0	3	4	4	10	4.41e-02	7.34e-02	744	0.000	1.585	2.000	2.000	3.322

LOCUS: AT5G19550
DESCRIPTION: Nitrogen metabolism. Major cytosolic isoenzyme controlling aspartate biosynthesis in the light.

DATA:		Control	30min	2hours	2days	1week	p-value	B&H	Pos	Fold change relative to control (log2)				
SENSE COUNTS:		3	2	1	6	21	3.98e-06	1.36e-05		0.000	-0.585	-1.585	1.000	2.807
GENES:														
AT5G19550.1														
SENSE COUNTS:		3	2	1	6	21	3.98e-06	1.39e-05		0.000	-0.585	-1.585	1.000	2.807
TAGS:														
d+1	TCTTTTGACT	1	2	0	4	16	1.49e-05	7.66e-05	1435	0.000	1.000	0.000	2.000	4.000
d+2	CTGCAGTGAC	2	0	1	2	5	1.75e-01	2.47e-01	1291	0.000	0.000	-1.000	0.000	1.322

LOCUS: AT4G37910
DESCRIPTION: heat shock protein 70, mitochondrial, putative / HSP70, mitochondrial, putative, strong similarity to SP|Q01899 Heat shock 70 kDa protein, mitochondrial precursor {Phaseolus vulgaris}

DATA:		Control	30min	2hours	2days	1week	p-value	B&H	Pos	Fold change relative to control (log2)				
SENSE COUNTS:		14	2	5	27	22	4.22e-06	1.44e-05		0.000	-2.807	-1.485	0.948	0.652
GENES:														
AT4G37910.1														
SENSE COUNTS:		14	2	5	27	22	4.22e-06	1.47e-05		0.000	-2.807	-1.485	0.948	0.652
TAGS:														
d+1	AAGTTTACGG	12	2	5	27	21	4.80e-06	2.82e-05	2308	0.000	-2.585	-1.263	1.170	0.807
d+2	CTTATCTCCT	2	0	0	0	1	2.03e-01	2.83e-01	2186	0.000	0.000	0.000	0.000	-1.000

LOCUS: AT2G30870
DESCRIPTION: glutathione S-transferase, putative, supported by cDNA GI

DATA:		Control	30min	2hours	2days	1week	p-value	B&H	Pos	Fold change relative to control (log2)				
SENSE COUNTS:		2	19	4	2	1	4.26e-06	1.45e-05		0.000	3.248	1.000	0.000	-1.000
GENES:														
AT2G30870.1														
SENSE COUNTS:		2	19	4	2	1	4.26e-06	1.48e-05		0.000	3.248	1.000	0.000	-1.000
TAGS:														
d+2	TTAGCGCTTG	2	19	4	2	1	4.26e-06	2.55e-05	612	0.000	3.248	1.000	0.000	-1.000

LOCUS: AT4G22890
DESCRIPTION: expressed protein

DATA:		Control	30min	2hours	2days	1week	p-value	B&H	Pos	Fold change relative to control (log2)				
SENSE COUNTS:		62	21	56	40	21	4.55e-06	1.54e-05		0.000	-1.562	-0.147	-0.632	-1.562
GENES:														
AT4G22890.2														
SENSE COUNTS:		0	0	1	1	0	5.21e-01	5.21e-01		0.000	0.000	0.000	0.000	0.000
TAGS:														
d+1	GAGCTTCCAG	0	0	1	1	0	5.21e-01	5.70e-01	780	0.000	0.000	0.000	0.000	0.000

```
  AT4G22890.3
    SENSE COUNTS:             62     21     55     39     21     5.35e-06      1.82e-05             0.000  -1.562  -0.173  -0.669  -1.562
    TAGS:
      d+1     AAAGACCAAA      6      1      13     5      1      9.61e-03      1.78e-02      1379   0.000  -2.585  1.115   -0.263  -2.585
      d+2     TTTTCTGATT      55     20     42     34     19     1.34e-04      5.41e-04      1259   0.000  -1.459  -0.389  -0.694  -1.533
      d+2     TTAGCTCTCT      1      0      0      0      0      4.28e-01      5.33e-01      1237   0.000  0.000   0.000   0.000   0.000
      X+4     TTTCGAAAAA      0      0      0      0      1      1.65e-01      2.35e-01      -209   0.000  0.000   0.000   0.000   0.000
  AT4G22890.1
    SENSE COUNTS:             62     21     55     39     20     3.22e-06      1.15e-05             0.000  -1.562  -0.173  -0.669  -1.632
    TAGS:
      d+1     AAAGACCAAA      6      1      13     5      1      9.61e-03      1.78e-02      1383   0.000  -2.585  1.115   -0.263  -2.585
      d+2     TTTTCTGATT      55     20     42     34     19     1.34e-04      5.41e-04      1263   0.000  -1.459  -0.389  -0.694  -1.533
      d+2     TTAGCTCTCT      1      0      0      0      0      4.28e-01      5.33e-01      1241   0.000  0.000   0.000   0.000   0.000

LOCUS: AT5G42500
DESCRIPTION:  disease resistance-responsive family protein, similar to disease resistance response protein 206-d (Pisum sativum) gi|508844|gb|AAB18669
DATA:                         Control 30min  2hours 2days  1week  p-value       B&H           Pos    Fold change relative to control (log2)
SENSE COUNTS:                 0      12     2      0      0      4.66e-06      1.57e-05             0.000  3.585   1.000   0.000   0.000
  GENES:
  AT5G42500.1
    SENSE COUNTS:             0      12     2      0      0      4.66e-06      1.61e-05             0.000  3.585   1.000   0.000   0.000
    TAGS:
      d+1     AAGAACATAA      0      12     2      0      0      4.66e-06      2.75e-05      342    0.000  3.585   1.000   0.000   0.000

LOCUS: AT3G05220
DESCRIPTION:  heavy-metal-associated domain-containing protein, similar to farnesylated protein 1 (GI
DATA:                         Control 30min  2hours 2days  1week  p-value       B&H           Pos    Fold change relative to control (log2)
SENSE COUNTS:                 6      23     5      5      3      4.76e-06      1.60e-05             0.000  1.939   -0.263  -0.263  -1.000
  GENES:
  AT3G05220.2
    SENSE COUNTS:             6      23     5      5      3      4.76e-06      1.63e-05             0.000  1.939   -0.263  -0.263  -1.000
    TAGS:
      d+1     TATGCTTGTC      3      7      5      0      3      1.57e-01      2.38e-01      1789   0.000  1.222   0.737   0.000   0.000
      d+2     GTGGGGGAGG      0      0      0      0      0      6.15e-01      6.56e-01      1116   0.000  0.000   0.000   0.000   0.000
      d+2     GTAATGGTGG      3      16     0      5      0      4.50e-06      2.66e-05      762    0.000  2.415   0.000   0.737   0.000
  AT3G05220.1
    SENSE COUNTS:             6      23     5      5      3      4.76e-06      1.64e-05             0.000  1.939   -0.263  -0.263  -1.000
    TAGS:
      d+1     TATGCTTGTC      3      7      5      0      3      1.57e-01      2.38e-01      1976   0.000  1.222   0.737   0.000   0.000
      d+2     GTGGGGGAGG      0      0      0      0      0      6.15e-01      6.56e-01      1303   0.000  0.000   0.000   0.000   0.000
      d+2     GTAATGGTGG      3      16     0      5      0      4.50e-06      2.66e-05      949    0.000  2.415   0.000   0.737   0.000

LOCUS: AT5G03545
DESCRIPTION:  expressed protein, No ATG start, annotated according to PMID
DATA:                         Control 30min  2hours 2days  1week  p-value       B&H           Pos    Fold change relative to control (log2)
SENSE COUNTS:                 1      19     2      5      7      4.83e-06      1.62e-05             0.000  4.248   1.000   2.322   2.807
  GENES:
  AT5G03545.1
    SENSE COUNTS:             1      19     2      5      7      4.83e-06      1.65e-05             0.000  4.248   1.000   2.322   2.807
    TAGS:
      d+1     TTTGTGTTGA      1      19     2      5      7      1.27e-05      6.61e-05      584    0.000  4.248   1.000   2.322   2.807
      d+2     ACCAAAGACA      0      0      0      0      0      6.15e-01      6.44e-01      11     0.000  0.000   0.000   0.000   0.000

LOCUS: AT5G08180
DESCRIPTION:  ribosomal protein L7Ae/L30e/S12e/Gadd45 family protein, Similar to NHP2/L7Ae family proteins, see SWISSPROT
DATA:                         Control 30min  2hours 2days  1week  p-value       B&H           Pos    Fold change relative to control (log2)
SENSE COUNTS:                 2      0      0      6      17     5.11e-06      1.70e-05             0.000  0.000   0.000   1.585   3.087
  GENES:
  AT5G08180.1
    SENSE COUNTS:             2      0      0      6      17     5.11e-06      1.74e-05             0.000  0.000   0.000   1.585   3.087
```

```
    TAGS:
      d+1      AATCCTATGG     2      0      0      5      17     7.64e-07       5.42e-06       685    0.000  0.000  0.000  1.322  3.087
      i+3      GTACTGTACA     0      0      0      0      0      6.15e-01       6.41e-01       590    0.000  0.000  0.000  0.000  0.000
      X+4      TCTTCGTGCT     0      0      0      1      0      3.09e-01       4.12e-01       406    0.000  0.000  0.000  0.000  0.000

LOCUS: AT4G11600
DESCRIPTION:  glutathione peroxidase, putative
DATA:                         Control 30min  2hours 2days  1week  p-value        B&H            Pos    Fold change relative to control (log2)
SENSE COUNTS:                 0      3      9      23     7      5.74e-06       1.91e-05              0.000  1.585  3.170  4.524  2.807
  GENES:
  AT4G11600.1
    SENSE COUNTS:             0      3      9      23     7      5.74e-06       1.94e-05              0.000  1.585  3.170  4.524  2.807
    TAGS:
      d+1      AAACTGTGTC     0      0      4      0      0      3.11e-02       5.25e-02       938    0.000  0.000  2.000  0.000  0.000
      d+2      GTTTCGAGAT     0      3      5      23     7      3.42e-07       2.64e-06       403    0.000  1.585  2.322  4.524  2.807

LOCUS: AT3G55980
DESCRIPTION:  zinc finger (CCCH-type) family protein, contains Pfam domain, PF00642
DATA:                         Control 30min  2hours 2days  1week  p-value        B&H            Pos    Fold change relative to control (log2)
SENSE COUNTS:                 0      2      16     2      1      5.76e-06       1.91e-05              0.000  1.000  4.000  1.000  0.000
  GENES:
  AT3G55980.1
    SENSE COUNTS:             0      2      16     2      1      5.76e-06       1.94e-05              0.000  1.000  4.000  1.000  0.000
    TAGS:
      d+1      AAATGGCTAA     0      0      10     0      0      1.21e-05       6.35e-05       2119   0.000  0.000  3.322  0.000  0.000
      X+4      TCATCAACAC     0      0      0      0      1      1.65e-01       2.45e-01       2010   0.000  0.000  0.000  0.000  0.000
      d+2      GGTTAACTCT     0      2      6      2      0      5.32e-02       8.72e-02       1787   0.000  1.000  2.585  1.000  0.000

LOCUS: AT1G13440
DESCRIPTION:  glyceraldehyde 3-phosphate dehydrogenase, cytosolic, putative / NAD-dependent glyceraldehyde-3-phosphate dehydrogenase, putative, very
strong similarity to SP|P25858 Glyceraldehyde 3-phosphate dehydrogenase, cytosolic (EC 1.2.1.12) {Arabidopsis thaliana};
DATA:                         Control 30min  2hours 2days  1week  p-value        B&H            Pos    Fold change relative to control (log2)
SENSE COUNTS:                 62     128    101    116    68     5.94e-06       1.96e-05              0.000  1.046  0.704  0.904  0.133
  GENES:
  AT1G13440.1
    SENSE COUNTS:             62     128    101    116    68     5.94e-06       2.00e-05              0.000  1.046  0.704  0.904  0.133
    TAGS:
      d+1      AGAGTTTGTA     62     128    96     116    68     6.80e-06       3.89e-05       1206   0.000  1.046  0.631  0.904  0.133
      d+2      ACCACTGTCC     0      0      5      0      0      7.30e-03       1.41e-02       612    0.000  0.000  2.322  0.000  0.000

LOCUS: AT3G17020
DESCRIPTION:  universal stress protein (USP) family protein, similar to early nodulin ENOD18 (Vicia faba) GI
DATA:                         Control 30min  2hours 2days  1week  p-value        B&H            Pos    Fold change relative to control (log2)
SENSE COUNTS:                 3      25     7      4      8      6.11e-06       2.01e-05              0.000  3.059  1.222  0.415  1.415
  GENES:
  AT3G17020.1
    SENSE COUNTS:             3      25     7      4      8      6.11e-06       2.05e-05              0.000  3.059  1.222  0.415  1.415
    TAGS:
      d+1      CCCTGTGACC     3      25     7      4      8      6.11e-06       3.56e-05       523    0.000  3.059  1.222  0.415  1.415

LOCUS: AT1G54960
DESCRIPTION:  member of MEKK subfamily
DATA:                         Control 30min  2hours 2days  1week  p-value        B&H            Pos    Fold change relative to control (log2)
SENSE COUNTS:                 4      2      7      20     25     6.50e-06       2.13e-05              0.000  -1.000 0.807  2.322  2.644
  GENES:
  AT1G54960.1
    SENSE COUNTS:             4      2      7      20     25     6.50e-06       2.17e-05              0.000  -1.000 0.807  2.322  2.644
    TAGS:
      i+3      ATTTCGATTA     4      2      7      20     25     6.50e-06       3.75e-05       1346   0.000  -1.000 0.807  2.322  2.644
```

```
LOCUS: AT4G15210
DESCRIPTION:  beta-amylase (BMY1) / 1,4-alpha-D-glucan maltohydrolase, identical to Beta-amylase (EC 3.2.1.2) (1,4-alpha-D-glucan maltohydrolase) SP
DATA:                         Control 30min   2hours  2days   1week   p-value        B&H            Pos     Fold change relative to control (log2)
SENSE COUNTS:                 0       9       0       0       0       6.62e-06       2.16e-05               0.000   3.170   0.000   0.000   0.000
  GENES:
  AT4G15210.2
    SENSE COUNTS:             0       9       0       0       0       6.62e-06       2.21e-05               0.000   3.170   0.000   0.000   0.000
    TAGS:
      d+1      AGATTGTGCC     0       9       0       0       0       6.62e-06       3.81e-05       1376    0.000   3.170   0.000   0.000   0.000
  AT4G15210.1
    SENSE COUNTS:             0       9       0       0       0       6.62e-06       2.20e-05               0.000   3.170   0.000   0.000   0.000
    TAGS:
      d+1      AGATTGTGCC     0       9       0       0       0       6.62e-06       3.81e-05       1492    0.000   3.170   0.000   0.000   0.000

LOCUS: AT1G61960
DESCRIPTION:  mitochondrial transcription termination factor-related / mTERF-related, contains Pfam profile PF02536
DATA:                         Control 30min   2hours  2days   1week   p-value        B&H            Pos     Fold change relative to control (log2)
SENSE COUNTS:                 1       2       2       5       19      6.67e-06       2.17e-05               0.000   1.000   1.000   2.322   4.248
  GENES:
  AT1G61960.1
    SENSE COUNTS:             1       2       2       5       19      6.67e-06       2.21e-05               0.000   1.000   1.000   2.322   4.248
    TAGS:
      X+4      AAGCACATAT     1       2       1       4       19      8.31e-07       5.79e-06       1769    0.000   1.000   0.000   2.000   4.248
      d+1      AGAAGTGAAA     0       0       1       1       0       5.21e-01       5.71e-01       1580    0.000   0.000   0.000   0.000   0.000

LOCUS: AT4G23670
DESCRIPTION:  major latex protein-related / MLP-related, low similarity to major latex protein {Papaver somniferum}(GI
DATA:                         Control 30min   2hours  2days   1week   p-value        B&H            Pos     Fold change relative to control (log2)
SENSE COUNTS:                 33      57      51      36      5       6.74e-06       2.18e-05               0.000   0.788   0.628   0.126   -2.722
  GENES:
  AT4G23670.1
    SENSE COUNTS:             33      57      51      36      5       6.74e-06       2.23e-05               0.000   0.788   0.628   0.126   -2.722
    TAGS:
      d+1      AAATTCGTCA     33      57      51      36      5       6.74e-06       3.86e-05       551     0.000   0.788   0.628   0.126   -2.722

LOCUS: AT4G02380
DESCRIPTION:  late embryogenesis abundant 3 family protein / LEA3 family protein, similar to several small proteins (~100 aa) that are induced by heat,
auxin, ethylene and wounding such as Phaseolus aureus indole-3-acetic acid induced protein ARG (SW
DATA:                         Control 30min   2hours  2days   1week   p-value        B&H            Pos     Fold change relative to control (log2)
SENSE COUNTS:                 6       26      11      22      44      6.91e-06       2.23e-05               0.000   2.115   0.874   1.874   2.874
  GENES:
  AT4G02380.1
    SENSE COUNTS:             6       26      11      22      44      6.91e-06       2.27e-05               0.000   2.115   0.874   1.874   2.874
    TAGS:
      d+1      TAATTTTGTC     6       26      11      22      44      6.91e-06       3.94e-05       501     0.000   2.115   0.874   1.874   2.874

LOCUS: AT5G62360
DESCRIPTION:  invertase/pectin methylesterase inhibitor family protein, similar to pectinesterase from Arabidosis thaliana SP|Q42534, Lycopersicon
esculentum SP|Q43143; contains Pfam profile PF04043
DATA:                         Control 30min   2hours  2days   1week   p-value        B&H            Pos     Fold change relative to control (log2)
SENSE COUNTS:                 0       3       15      1       1       7.12e-06       2.29e-05               0.000   1.585   3.907   0.000   0.000
  GENES:
  AT5G62360.1
    SENSE COUNTS:             0       3       15      1       1       7.12e-06       2.34e-05               0.000   1.585   3.907   0.000   0.000
    TAGS:
      d+1      AAAATTGATT     0       3       11      1       1       1.49e-03       3.99e-03       787     0.000   1.585   3.459   0.000   0.000
      d+2      GCTAAAGAGA     0       0       2       0       0       1.21e-01       1.86e-01       651     0.000   0.000   1.000   0.000   0.000
      d+2      TACTGACGGA     0       0       1       0       0       4.55e-01       5.27e-01       523     0.000   0.000   0.000   0.000   0.000
      d+2      AGAGACTGCG     0       0       1       0       0       4.55e-01       5.37e-01       374     0.000   0.000   0.000   0.000   0.000
```

LOCUS: AT2G38540
DESCRIPTION: nonspecific lipid transfer protein 1 (LTP1), identical to SP|Q42589

DATA:		Control	30min	2hours	2days	1week	p-value	B&H	Pos	Fold change relative to control (log2)				
SENSE COUNTS:		19	36	3	13	28	7.15e-06	2.29e-05		0.000	0.922	-2.663	-0.547	0.559
GENES:														
AT2G38540.1														
SENSE COUNTS:		19	36	3	13	28	7.15e-06	2.34e-05		0.000	0.922	-2.663	-0.547	0.559
TAGS:														
d+1	TGGAGTCAAT	19	36	3	13	28	7.15e-06	4.03e-05	368	0.000	0.922	-2.663	-0.547	0.559

LOCUS: AT5G21020
DESCRIPTION: expressed protein

DATA:		Control	30min	2hours	2days	1week	p-value	B&H	Pos	Fold change relative to control (log2)				
SENSE COUNTS:		1	13	2	0	0	7.25e-06	2.32e-05		0.000	3.700	1.000	0.000	0.000
GENES:														
AT5G21020.2														
SENSE COUNTS:		1	13	2	0	0	7.25e-06	2.37e-05		0.000	3.700	1.000	0.000	0.000
TAGS:														
d+1	TTTTCGGCTT	1	13	2	0	0	7.25e-06	4.06e-05	265	0.000	3.700	1.000	0.000	0.000

LOCUS: AT2G17360
DESCRIPTION: 40S ribosomal protein S4 (RPS4A), contains ribosomal protein S4 signature from residues 8 to 22

DATA:		Control	30min	2hours	2days	1week	p-value	B&H	Pos	Fold change relative to control (log2)				
SENSE COUNTS:		5	8	8	18	35	7.40e-06	2.36e-05		0.000	0.678	0.678	1.848	2.807
GENES:														
AT2G17360.1														
SENSE COUNTS:		5	8	8	18	35	7.40e-06	2.41e-05		0.000	0.678	0.678	1.848	2.807
TAGS:														
d+1	CTTTAGTTTG	5	8	8	18	35	7.40e-06	4.13e-05	944	0.000	0.678	0.678	1.848	2.807

LOCUS: AT3G19740
DESCRIPTION: AAA-type ATPase family protein, contains Pfam domain, PF00004

DATA:		Control	30min	2hours	2days	1week	p-value	B&H	Pos	Fold change relative to control (log2)				
SENSE COUNTS:		1	0	2	0	12	7.41e-06	2.35e-05		0.000	0.000	1.000	0.000	3.585
GENES:														
AT3G19740.1														
SENSE COUNTS:		1	0	2	0	12	7.41e-06	2.40e-05		0.000	0.000	1.000	0.000	3.585
TAGS:														
d+1	TCGTAACAAT	1	0	2	0	12	7.41e-06	4.12e-05	1386	0.000	0.000	1.000	0.000	3.585

LOCUS: AT4G39800
DESCRIPTION: inositol-3-phosphate synthase isozyme 1 / myo-inositol-1-phosphate synthase 1 / MI-1-P synthase 1 / IPS 1, identical to SP|P42801 Inositol-3-phosphate synthase isozyme 1 (EC 5.5.1.4) (Myo-inositol-1- phosphate synthase 1) (MI-1-P synthase 1) (IPS 1) {Arab

DATA:		Control	30min	2hours	2days	1week	p-value	B&H	Pos	Fold change relative to control (log2)				
SENSE COUNTS:		23	4	22	40	38	7.50e-06	2.37e-05		0.000	-2.524	-0.064	0.798	0.724
GENES:														
AT4G39800.1														
SENSE COUNTS:		23	4	22	40	38	7.50e-06	2.42e-05		0.000	-2.524	-0.064	0.798	0.724
TAGS:														
d+1	GTTTTGGTTC	20	4	20	40	35	1.38e-06	9.16e-06	1904	0.000	-2.322	0.000	1.000	0.807
d+2	AATCTAAGAC	2	0	2	0	3	5.19e-01	5.70e-01	1623	0.000	0.000	0.000	0.000	0.585
d+2	GCTAGAGCCA	1	0	0	0	0	6.89e-01	7.01e-01	534	0.000	0.000	0.000	0.000	0.000

LOCUS: AT5G19120
DESCRIPTION: expressed protein, low similarity to extracellular dermal glycoprotein EDGP precursor (Daucus carota) GI

DATA:		Control	30min	2hours	2days	1week	p-value	B&H	Pos	Fold change relative to control (log2)				
SENSE COUNTS:		3	14	2	0	0	7.75e-06	2.44e-05		0.000	2.222	-0.585	0.000	0.000
GENES:														
AT5G19120.1														
SENSE COUNTS:		3	14	2	0	0	7.75e-06	2.50e-05		0.000	2.222	-0.585	0.000	0.000

```
    TAGS:
      d+1      GGAAGAATCT     3       14      2       0       0       7.75e-06        4.28e-05        997     0.000   2.222   -0.585  0.000   0.000

LOCUS: AT5G51110
DESCRIPTION:  expressed protein
DATA:                         Control 30min   2hours  2days   1week   p-value         B&H             Pos     Fold change relative to control (log2)
SENSE COUNTS:                 23      11      17      32      51      7.87e-06        2.47e-05                0.000   -1.064  -0.436  0.476   1.149
  GENES:
  AT5G51110.1
    SENSE COUNTS:             23      11      17      32      51      7.87e-06        2.53e-05                0.000   -1.064  -0.436  0.476   1.149
    TAGS:
      d+1      TCTTTGAGAT     5       5       7       12      21      1.09e-02        1.97e-02        859     0.000   0.000   0.485   1.263   2.070
      d+2      TCGAGTTATA     18      6       10      20      30      1.07e-03        3.03e-03        805     0.000   -1.585  -0.848  0.152   0.737

LOCUS: AT1G15930
DESCRIPTION:  40S ribosomal protein S12 (RPS12A), similar to 40S ribosomal protein S12 GI
DATA:                         Control 30min   2hours  2days   1week   p-value         B&H             Pos     Fold change relative to control (log2)
SENSE COUNTS:                 34      15      39      63      35      8.17e-06        2.56e-05                0.000   -1.181  0.198   0.890   0.042
  GENES:
  AT1G15930.2
    SENSE COUNTS:             34      15      39      63      35      8.17e-06        2.61e-05                0.000   -1.181  0.198   0.890   0.042
    TAGS:
      d+1      ATCAAGTTTT     34      15      39      63      35      8.17e-06        4.49e-05        639     0.000   -1.181  0.198   0.890   0.042
  AT1G15930.1
    SENSE COUNTS:             34      15      39      63      35      8.17e-06        2.62e-05                0.000   -1.181  0.198   0.890   0.042
    TAGS:
      d+1      ATCAAGTTTT     34      15      39      63      35      8.17e-06        4.49e-05        742     0.000   -1.181  0.198   0.890   0.042

LOCUS: AT3G15630
DESCRIPTION:  expressed protein
DATA:                         Control 30min   2hours  2days   1week   p-value         B&H             Pos     Fold change relative to control (log2)
SENSE COUNTS:                 1       13      0       1       1       8.33e-06        2.60e-05                0.000   3.700   0.000   0.000   0.000
  GENES:
  AT3G15630.1
    SENSE COUNTS:             1       13      0       1       1       8.33e-06        2.65e-05                0.000   3.700   0.000   0.000   0.000
    TAGS:
      d+1      TCATCGGAAC     1       13      0       1       1       8.33e-06        4.56e-05        206     0.000   3.700   0.000   0.000   0.000

LOCUS: AT2G45660
DESCRIPTION:  Controls flowering and is required for CO to promote flowering.Overexpression of (SOC1) AGL20 suppresses not only the late flowering of
plants that have functional FRI and FLC alleles but also the delayed phase transitions during the vegetative stages of

DATA:                         Control 30min   2hours  2days   1week   p-value         B&H             Pos     Fold change relative to control (log2)
SENSE COUNTS:                 0       0       0       8       0       8.74e-06        2.72e-05                0.000   0.000   0.000   3.000   0.000
  GENES:
  AT2G45660.1
    SENSE COUNTS:             0       0       0       8       0       8.74e-06        2.78e-05                0.000   0.000   0.000   3.000   0.000
    TAGS:
      d+1      TATCAATTTA     0       0       0       8       0       8.74e-06        4.74e-05        1271    0.000   0.000   0.000   3.000   0.000

LOCUS: AT1G75380
DESCRIPTION:  wound-responsive protein-related, similar to wound inducive gene GI
DATA:                         Control 30min   2hours  2days   1week   p-value         B&H             Pos     Fold change relative to control (log2)
SENSE COUNTS:                 26      41      20      17      1       9.09e-06        2.82e-05                0.000   0.657   -0.379  -0.613  -4.700
  GENES:
  AT1G75380.2
    SENSE COUNTS:             25      40      19      17      1       1.87e-05        5.53e-05                0.000   0.678   -0.396  -0.556  -4.644
    TAGS:
      d+1      AGAGAGCTCG     24      36      17      15      1       1.08e-04        4.45e-04        1142    0.000   0.585   -0.497  -0.678  -4.585
      d+2      TATCAAGTGG     0       0       0       0       0       6.15e-01        6.56e-01        687     0.000   0.000   0.000   0.000   0.000
```

```
      d+2      TGCTTCACTA     1       4       2       2       0       4.29e-01       5.19e-01       227     0.000   2.000   1.000   1.000   0.000
  AT1G75380.3
    SENSE COUNTS:             25      40      19      17      1       1.87e-05       5.51e-05               0.000   0.678   -0.396  -0.556  -4.644
    TAGS:
      d+1      AGAGAGCTCG     24      36      17      15      1       1.08e-04       4.45e-04       1134    0.000   0.585   -0.497  -0.678  -4.585
      d+2      TATCAAGTGG     0       0       0       0       0       6.15e-01       6.56e-01       679     0.000   0.000   0.000   0.000   0.000
      d+2      TGCTTCACTA     1       4       2       2       0       4.29e-01       5.19e-01       219     0.000   2.000   1.000   1.000   0.000
  AT1G75380.1
    SENSE COUNTS:             2       5       3       2       0       1.79e-01       1.81e-01               0.000   1.322   0.585   0.000   0.000
    TAGS:
      d+1      AGAGAGGTAA     1       1       1       0       0       7.28e-01       7.34e-01       1147    0.000   0.000   0.000   0.000   0.000
      d+2      TATCAAGTGG     0       0       0       0       0       6.15e-01       6.56e-01       692     0.000   0.000   0.000   0.000   0.000
      d+2      TGCTTCACTA     1       4       2       2       0       4.29e-01       5.19e-01       232     0.000   2.000   1.000   1.000   0.000

LOCUS: AT5G49450
DESCRIPTION:  bZIP family transcription factor, similar to bZIP transcription factor GI
DATA:                         Control 30min   2hours  2days   1week   p-value        B&H            Pos     Fold change relative to control (log2)
SENSE COUNTS:                 10      15      1       0       0       9.33e-06       2.88e-05               0.000   0.585   -3.322  0.000   0.000
  GENES:
  AT5G49450.1
    SENSE COUNTS:             10      15      1       0       0       9.33e-06       2.95e-05               0.000   0.585   -3.322  0.000   0.000
    TAGS:
      d+1      AGATTTGTGT     9       15      0       0       0       2.27e-06       1.44e-05       754     0.000   0.737   0.000   0.000   0.000
      d+2      AGATCTCCAG     0       0       1       0       0       4.55e-01       5.14e-01       450     0.000   0.000   0.000   0.000   0.000
      d+2      GCAAACGCAG     1       0       0       0       0       4.28e-01       5.33e-01       320     0.000   0.000   0.000   0.000   0.000

LOCUS: AT1G27760
DESCRIPTION:  interferon-related developmental regulator family protein / IFRD protein family, contains Pfam PF05004
DATA:                         Control 30min   2hours  2days   1week   p-value        B&H            Pos     Fold change relative to control (log2)
SENSE COUNTS:                 0       0       1       13      8       9.40e-06       2.89e-05               0.000   0.000   0.000   3.700   3.000
  GENES:
  AT1G27760.3
    SENSE COUNTS:             0       0       0       13      8       1.77e-06       6.69e-06               0.000   0.000   0.000   3.700   3.000
    TAGS:
      d+1      TTTTTGATTT     0       0       0       13      8       1.77e-06       1.14e-05       1719    0.000   0.000   0.000   3.700   3.000
  AT1G27760.2
    SENSE COUNTS:             0       0       1       13      8       9.40e-06       2.97e-05               0.000   0.000   0.000   3.700   3.000
    TAGS:
      d+1      TTTTTGATTT     0       0       0       13      8       1.77e-06       1.14e-05       1897    0.000   0.000   0.000   3.700   3.000
      X+4      TATTGGTGAG     0       0       1       0       0       4.55e-01       5.28e-01       357     0.000   0.000   0.000   0.000   0.000
  AT1G27760.1
    SENSE COUNTS:             0       0       0       13      8       1.77e-06       6.72e-06               0.000   0.000   0.000   3.700   3.000
    TAGS:
      d+1      TTTTTGATTT     0       0       0       13      8       1.77e-06       1.14e-05       1707    0.000   0.000   0.000   3.700   3.000

LOCUS: AT3G56940
DESCRIPTION:  Encodes a putative ZIP protein with varying mRNA accumulation in leaves, stems and roots.  Has a consensus carboxylate-bridged di-iron
binding site.
DATA:                         Control 30min   2hours  2days   1week   p-value        B&H            Pos     Fold change relative to control (log2)
SENSE COUNTS:                 118     86      106     79      37      9.49e-06       2.91e-05               0.000   -0.456  -0.155  -0.579  -1.673
  GENES:
  AT3G56940.1
    SENSE COUNTS:             118     86      106     79      37      9.49e-06       2.99e-05               0.000   -0.456  -0.155  -0.579  -1.673
    TAGS:
      d+1      CCTCCTGTTG     118     86      105     79      37      1.09e-05       5.79e-05       1220    0.000   -0.456  -0.168  -0.579  -1.673
      d+2      CCGGGTTCTT     0       0       1       0       0       4.55e-01       5.31e-01       585     0.000   0.000   0.000   0.000   0.000

LOCUS: AT1G68010
DESCRIPTION:  glycerate dehydrogenase / NADH-dependent hydroxypyruvate reductase, identical to hydroxypyruvate reductase (HPR) GB
DATA:                         Control 30min   2hours  2days   1week   p-value        B&H            Pos     Fold change relative to control (log2)
```

```
SENSE COUNTS:                 50     11     31     28     20     1.03e-05      3.15e-05             0.000  -2.184  -0.690  -0.837  -1.322
  GENES:
  AT1G68010.1
    SENSE COUNTS:             50     11     31     28     20     1.03e-05      3.21e-05             0.000  -2.184  -0.690  -0.837  -1.322
    TAGS:
      d+1    AATCTACCAT       5      0      3      1      1      1.07e-01      1.68e-01      1471   0.000  0.000   -0.737  -2.322  -2.322
      d+2    TTGGATCATA       45     11     23     27     19     1.00e-04      4.15e-04      1322   0.000  -2.032  -0.968  -0.737  -1.244
      d+2    ACCCGAACCG       0      0      3      0      0      2.70e-02      4.61e-02      1120   0.000  0.000   1.585   0.000   0.000
      d+2    AAACCAGGGC       0      0      1      0      0      4.55e-01      5.40e-01      981    0.000  0.000   0.000   0.000   0.000
      X+4    CAAAACCGAC       0      0      1      0      0      4.55e-01      5.33e-01      -86    0.000  0.000   0.000   0.000   0.000

LOCUS: AT4G03520
DESCRIPTION:  thioredoxin M-type 2, chloroplast (TRX-M2), nearly identical to SP|Q9SEU8 Thioredoxin M-type 2, chloroplast precursor (TRX-M2)
{Arabidopsis thaliana}
DATA:                         Control 30min  2hours 2days  1week  p-value       B&H           Pos    Fold change relative to control (log2)
SENSE COUNTS:                 34     14     17     13     50     1.03e-05      3.14e-05             0.000  -1.280  -1.000  -1.387  0.556
  GENES:
  AT4G03520.1
    SENSE COUNTS:             34     14     17     13     50     1.03e-05      3.22e-05             0.000  -1.280  -1.000  -1.387  0.556
    TAGS:
      d+1    TCTTATGTCA       32     14     16     13     50     1.15e-05      6.06e-05      792    0.000  -1.193  -1.000  -1.300  0.644
      d+2    AAATATCACT       2      0      0      0      0      1.04e-01      1.64e-01      672    0.000  0.000   0.000   0.000   0.000
      d+2    ATCTTTGTCG       0      0      1      0      0      4.55e-01      5.39e-01      583    0.000  0.000   0.000   0.000   0.000

LOCUS: AT3G30775
DESCRIPTION:  proline oxidase, mitochondrial / osmotic stress-responsive proline dehydrogenase (POX) (PRO1) (ERD5), nearly identical to SP|P92983
Proline oxidase, mitochondrial precursor (EC 1.5.3.-) (Osmotic stress- induced proline dehydrogenase) (Arabidopsis thaliana
DATA:                         Control 30min  2hours 2days  1week  p-value       B&H           Pos    Fold change relative to control (log2)
SENSE COUNTS:                 2      18     3      3      4      1.03e-05      3.13e-05             0.000  3.170   0.585   0.585   1.000
  GENES:
  AT3G30775.1
    SENSE COUNTS:             2      18     3      3      4      1.03e-05      3.23e-05             0.000  3.170   0.585   0.585   1.000
    TAGS:
      d+1    AGGGACTATG       2      9      3      2      3      9.47e-02      1.50e-01      1646   0.000  2.170   0.585   0.000   0.585
      d+2    AGGATGGAAC       0      9      0      1      1      1.86e-04      7.07e-04      1459   0.000  3.170   0.000   0.000   0.000
      d+2    TCTAGCGAAG       0      0      0      0      0      6.15e-01      6.35e-01      1054   0.000  0.000   0.000   0.000   0.000

LOCUS: AT3G61440
DESCRIPTION:  cysteine synthase, putative / O-acetylserine (thiol)-lyase, putative / O-acetylserine sulfhydrylase, putative, identical to cysteine
synthase (EC 4.2.99.8) (Arabidopsis thaliana) GI
DATA:                         Control 30min  2hours 2days  1week  p-value       B&H           Pos    Fold change relative to control (log2)
SENSE COUNTS:                 27     57     47     47     7      1.08e-05      3.27e-05             0.000  1.078   0.800   0.800   -1.948
  GENES:
  AT3G61440.1
    SENSE COUNTS:             27     57     47     47     7      1.08e-05      3.35e-05             0.000  1.078   0.800   0.800   -1.948
    TAGS:
      d+1    CGAGCTTCGG       26     57     46     47     7      7.99e-06      4.40e-05      1157   0.000  1.132   0.823   0.854   -1.893
      d+2    CTATCACAGG       0      0      1      0      0      4.55e-01      5.45e-01      935    0.000  0.000   0.000   0.000   0.000
      d+2    GCTGCTATGA       1      0      0      0      0      4.28e-01      5.36e-01      526    0.000  0.000   0.000   0.000   0.000

LOCUS: AT4G33865
DESCRIPTION:  40S ribosomal protein S29 (RPS29C)
DATA:                         Control 30min  2hours 2days  1week  p-value       B&H           Pos    Fold change relative to control (log2)
SENSE COUNTS:                 32     14     24     37     61     1.08e-05      3.26e-05             0.000  -1.193  -0.415  0.209   0.931
  GENES:
  AT4G33865.1
    SENSE COUNTS:             32     14     24     37     61     1.08e-05      3.36e-05             0.000  -1.193  -0.415  0.209   0.931
    TAGS:
      d+1    TCTTTTGAGT       32     14     24     37     61     1.08e-05      5.76e-05      285    0.000  -1.193  -0.415  0.209   0.931
```

```
LOCUS: AT3G08850
DESCRIPTION:  transducin family protein / WD-40 repeat family protein, similar to WD-repeat protein mip1 (SP
DATA:                         Control 30min   2hours  2days   1week   p-value         B&H             Pos     Fold change relative to control (log2)
SENSE COUNTS:                 49      19      52      47      12      1.09e-05        3.28e-05                0.000   -1.367  0.086   -0.060  -2.030
  GENES:
  AT3G08850.1
    SENSE COUNTS:             49      19      52      47      12      1.09e-05        3.37e-05                0.000   -1.367  0.086   -0.060  -2.030
    TAGS:
      d+1      TGTAAAAAAA     49      19      52      47      12      3.92e-06        2.35e-05        4758    0.000   -1.367  0.086   -0.060  -2.030
      d+2      ATTTTCCTGA     0       0       0       0       0       6.15e-01        6.54e-01        3595    0.000   0.000   0.000   0.000   0.000
      d+2      CAGGTAATCT     0       0       0       0       0       6.15e-01        6.32e-01        3097    0.000   0.000   0.000   0.000   0.000

LOCUS: AT4G37020
DESCRIPTION:  expressed protein
DATA:                         Control 30min   2hours  2days   1week   p-value         B&H             Pos     Fold change relative to control (log2)
SENSE COUNTS:                 10      30      6       26      5       1.15e-05        3.45e-05                0.000   1.585   -0.737  1.379   -1.000
  GENES:
  AT4G37020.1
    SENSE COUNTS:             10      30      6       26      5       1.15e-05        3.55e-05                0.000   1.585   -0.737  1.379   -1.000
    TAGS:
      d+2      GATCTCCGAT     9       30      6       26      5       6.39e-06        3.70e-05        730     0.000   1.737   -0.585  1.531   -0.848
      i+3      TTCTTCCCAC     1       0       0       0       0       4.28e-01        5.32e-01        724     0.000   0.000   0.000   0.000   0.000

LOCUS: AT2G34460
DESCRIPTION:  flavin reductase-related, low similarity to SP|P30043 Flavin reductase {Homo sapiens}
DATA:                         Control 30min   2hours  2days   1week   p-value         B&H             Pos     Fold change relative to control (log2)
SENSE COUNTS:                 6       1       18      17      28      1.18e-05        3.52e-05                0.000   -2.585  1.585   1.503   2.222
  GENES:
  AT2G34460.1
    SENSE COUNTS:             6       1       18      17      28      1.18e-05        3.63e-05                0.000   -2.585  1.585   1.503   2.222
    TAGS:
      d+2      CTCAAACCTT     6       1       18      15      28      1.29e-05        6.67e-05        894     0.000   -2.585  1.585   1.322   2.222
      d+2      GGACAGATAC     0       0       0       2       0       4.80e-02        7.91e-02        535     0.000   0.000   0.000   1.000   0.000

LOCUS: AT3G44260
DESCRIPTION:  CCR4-NOT transcription complex protein, putative, similar to SWISS-PROT
DATA:                         Control 30min   2hours  2days   1week   p-value         B&H             Pos     Fold change relative to control (log2)
SENSE COUNTS:                 1       10      0       0       0       1.24e-05        3.69e-05                0.000   3.322   0.000   0.000   0.000
  GENES:
  AT3G44260.1
    SENSE COUNTS:             1       10      0       0       0       1.24e-05        3.80e-05                0.000   3.322   0.000   0.000   0.000
    TAGS:
      d+1      CTGGGGTTTT     1       10      0       0       0       1.24e-05        6.47e-05        841     0.000   3.322   0.000   0.000   0.000

LOCUS: AT5G13650
DESCRIPTION:  elongation factor family protein, contains Pfam profiles
DATA:                         Control 30min   2hours  2days   1week   p-value         B&H             Pos     Fold change relative to control (log2)
SENSE COUNTS:                 8       13      19      19      46      1.25e-05        3.71e-05                0.000   0.700   1.248   1.248   2.524
  GENES:
  AT5G13650.1
    SENSE COUNTS:             8       13      19      19      46      1.25e-05        3.81e-05                0.000   0.700   1.248   1.248   2.524
    TAGS:
      i+3      TTAATTTCTC     1       0       1       0       0       6.01e-01        6.48e-01        3744    0.000   0.000   0.000   0.000   0.000
      i+3      AGCTATCTTA     0       0       1       0       0       4.55e-01        5.29e-01        2675    0.000   0.000   0.000   0.000   0.000
      d+1      CCCTTTGATC     6       13      15      16      43      9.62e-06        5.16e-05        2256    0.000   1.115   1.322   1.415   2.841
      d+2      GGGCCTGTTG     1       0       2       2       3       4.13e-01        5.20e-01        1515    0.000   0.000   1.000   1.000   1.585
      d+2      GGAAGCCTCT     0       0       0       1       0       3.09e-01        4.04e-01        1159    0.000   0.000   0.000   0.000   0.000
  AT5G13650.2
    SENSE COUNTS:             8       13      19      19      46      1.25e-05        3.82e-05                0.000   0.700   1.248   1.248   2.524
    TAGS:
```

```
      i+3      TTAATTTCTC     1       0       1       0       0       6.01e-01       6.48e-01       3770    0.000   0.000   0.000   0.000   0.000
      i+3      AGCTATCTTA     0       0       1       0       0       4.55e-01       5.29e-01       2701    0.000   0.000   0.000   0.000   0.000
      d+1      CCCTTTGATC     6       13      15      16      43      9.62e-06       5.16e-05       2285    0.000   1.115   1.322   1.415   2.841
      d+2      GGGCCTGTTG     1       0       2       2       3       4.13e-01       5.20e-01       1544    0.000   0.000   1.000   1.000   1.585
      d+2      GGAAGCCTCT     0       0       0       1       0       3.09e-01       4.04e-01       1188    0.000   0.000   0.000   0.000   0.000

LOCUS: AT1G73610
DESCRIPTION:  GDSL-motif lipase/hydrolase family protein, similar to family II lipase EXL6 (GI
DATA:                         Control 30min   2hours  2days   1week   p-value        B&H            Pos     Fold change relative to control (log2)
SENSE COUNTS:                 24      28      67      41      51      1.39e-05       4.11e-05               0.000   0.222   1.481   0.773   1.087
  GENES:
  AT1G73610.1
    SENSE COUNTS:             24      28      67      41      51      1.39e-05       4.23e-05               0.000   0.222   1.481   0.773   1.087
    TAGS:
      v+1      TCAAAAAAAA     24      28      67      41      50      1.61e-05       8.17e-05       1794    0.000   0.222   1.481   0.773   1.059
      v+2      ATCAAGTTAT     0       0       0       0       1       1.65e-01       2.44e-01       1385    0.000   0.000   0.000   0.000   0.000

LOCUS: AT5G05440
DESCRIPTION:  expressed protein, low similarity to cytokinin-specific binding protein (Vigna radiata) GI
DATA:                         Control 30min   2hours  2days   1week   p-value        B&H            Pos     Fold change relative to control (log2)
SENSE COUNTS:                 0       11      3       0       0       1.40e-05       4.13e-05               0.000   3.459   1.585   0.000   0.000
  GENES:
  AT5G05440.1
    SENSE COUNTS:             0       11      3       0       0       1.40e-05       4.24e-05               0.000   3.459   1.585   0.000   0.000
    TAGS:
      d+1      GTATCATTCT     0       0       1       0       0       7.06e-01       7.15e-01       1045    0.000   0.000   0.000   0.000   0.000
      d+2      GTGGTCTCTG     0       11      2       0       0       1.52e-05       7.76e-05       455     0.000   3.459   1.000   0.000   0.000

LOCUS: AT3G09922
DESCRIPTION:  hypothetical protein, no ATG start, annotated according to PMID
DATA:                         Control 30min   2hours  2days   1week   p-value        B&H            Pos     Fold change relative to control (log2)
SENSE COUNTS:                 0       10      2       0       0       1.52e-05       4.47e-05               0.000   3.322   1.000   0.000   0.000
  GENES:
  AT3G09922.1
    SENSE COUNTS:             0       10      2       0       0       1.52e-05       4.58e-05               0.000   3.322   1.000   0.000   0.000
    TAGS:
      d+1      TAAATTTGCT     0       2       1       0       0       2.47e-01       3.38e-01       504     0.000   1.000   0.000   0.000   0.000
      d+2      TAAGGAAAGC     0       8       1       0       0       1.41e-04       5.57e-04       151     0.000   3.000   0.000   0.000   0.000

LOCUS: AT5G06860
DESCRIPTION:  polygalacturonase inhibiting protein 1 (PGIP1), identical to polygalacturonase inhibiting protein 1 (PGIP1) (Arabidopsis thaliana)
gi|7800199|gb|AAF69827; contains leucine rich-repeat (LRR) domains Pfam
DATA:                         Control 30min   2hours  2days   1week   p-value        B&H            Pos     Fold change relative to control (log2)
SENSE COUNTS:                 17      16      24      44      52      1.55e-05       4.54e-05               0.000   -0.087  0.497   1.372   1.613
  GENES:
  AT5G06860.1
    SENSE COUNTS:             17      16      24      44      52      1.55e-05       4.66e-05               0.000   -0.087  0.497   1.372   1.613
    TAGS:
      d+1      AACCAAACCT     17      16      23      44      52      1.22e-05       6.39e-05       1109    0.000   -0.087  0.436   1.372   1.613
      d+2      GGACCCTCAA     0       0       1       0       0       4.55e-01       5.28e-01       170     0.000   0.000   0.000   0.000   0.000

LOCUS: AT4G25470
DESCRIPTION:  encodes a member of the DREB subfamily A-1 of ERF/AP2 transcription factor family (CBF2). The protein contains one AP2 domain. There are
six members in this subfamily, including CBF1, CBF2, and CBF3. This gene is involved in response to low temperature an
DATA:                         Control 30min   2hours  2days   1week   p-value        B&H            Pos     Fold change relative to control (log2)
SENSE COUNTS:                 0       4       14      1       0       1.56e-05       4.56e-05               0.000   2.000   3.807   0.000   0.000
  GENES:
  AT4G25470.1
    SENSE COUNTS:             0       4       14      1       0       1.56e-05       4.68e-05               0.000   2.000   3.807   0.000   0.000
    TAGS:
```

```
      d+1      GCCGAAGGGA     0      4      14     1      0      1.56e-05       7.94e-05       693    0.000  2.000  3.807  0.000  0.000

LOCUS: AT5G26717
DESCRIPTION:  hypothetical protein
DATA:                         Control 30min  2hours 2days  1week  p-value        B&H            Pos    Fold change relative to control (log2)
SENSE COUNTS:                 13     5      30     32     33     1.60e-05       4.66e-05              0.000  -1.379 1.206  1.300  1.344
  GENES:
  AT5G26717.1
    SENSE COUNTS:             13     5      30     32     33     1.60e-05       4.78e-05              0.000  -1.379 1.206  1.300  1.344
    TAGS:
      v+1      GAAACTGATC     2      0      3      2      7      2.54e-01       3.44e-01       1158   0.000  0.000  0.585  0.000  1.807
      v+2      TGATCCGAAT     11     5      27     30     26     5.56e-05       2.45e-04       1050   0.000  -1.138 1.295  1.447  1.241

LOCUS: AT2G21330
DESCRIPTION:  fructose-bisphosphate aldolase, putative, strong similarity to plastidic fructose-bisphosphate aldolase (EC 4.1.2.13) from Nicotiana
paniculata (NPALDP1) (GI
DATA:                         Control 30min  2hours 2days  1week  p-value        B&H            Pos    Fold change relative to control (log2)
SENSE COUNTS:                 58     16     43     31     50     1.70e-05       4.93e-05              0.000  -1.858 -0.432 -0.904 -0.214
  GENES:
  AT2G21330.1
    SENSE COUNTS:             58     16     43     31     50     1.70e-05       5.07e-05              0.000  -1.858 -0.432 -0.904 -0.214
    TAGS:
      d+1      GTTAATATCT     42     4      27     18     35     2.78e-07       2.19e-06       1398   0.000  -3.392 -0.637 -1.222 -0.263
      d+2      TGAGACTAGT     3      0      4      4      3      6.13e-01       6.56e-01       1374   0.000  0.000  0.415  0.415  0.000
      d+2      GGGAGGCAAG     13     12     12     9      12     9.65e-01       9.66e-01       1110   0.000  -0.115 -0.115 -0.531 -0.115

LOCUS: AT1G09070
DESCRIPTION:  C2 domain-containing protein / src2-like protein, putative, similar to cold-regulated gene SRC2 (Glycine max) GI
DATA:                         Control 30min  2hours 2days  1week  p-value        B&H            Pos    Fold change relative to control (log2)
SENSE COUNTS:                 5      19     31     8      7      1.91e-05       5.53e-05              0.000  1.926  2.632  0.678  0.485
  GENES:
  AT1G09070.1
    SENSE COUNTS:             5      19     31     8      7      1.91e-05       5.61e-05              0.000  1.926  2.632  0.678  0.485
    TAGS:
      X+4      CCATAGAAAA     0      0      1      0      0      7.06e-01       7.17e-01       1276   0.000  0.000  0.000  0.000  0.000
      d+1      TGAAGAACGT     5      19     25     8      7      1.17e-03       3.23e-03       1107   0.000  1.926  2.322  0.678  0.485
      d+2      GGTGGTTTCG     0      0      3      0      0      2.70e-02       4.62e-02       1022   0.000  0.000  1.585  0.000  0.000
      d+2      GTAAGGCTGG     0      0      1      0      0      4.55e-01       5.26e-01       897    0.000  0.000  0.000  0.000  0.000
      d+2      GAGTGTAGAT     0      0      1      0      0      4.55e-01       5.16e-01       68     0.000  0.000  0.000  0.000  0.000

LOCUS: AT2G37130
DESCRIPTION:  peroxidase 21 (PER21) (P21) (PRXR5), identical to SP|Q42580 Peroxidase 21 precursor (EC 1.11.1.7) (Atperox P21) (PRXR5) (ATP2a/ATP2b)
{Arabidopsis thaliana}
DATA:                         Control 30min  2hours 2days  1week  p-value        B&H            Pos    Fold change relative to control (log2)
SENSE COUNTS:                 2      16     5      2      0      1.92e-05       5.54e-05              0.000  3.000  1.322  0.000  0.000
  GENES:
  AT2G37130.1
    SENSE COUNTS:             2      16     5      2      0      1.92e-05       5.63e-05              0.000  3.000  1.322  0.000  0.000
    TAGS:
      d+1      AGCAGTTCTC     2      16     5      2      0      5.09e-05       2.26e-04       944    0.000  3.000  1.322  0.000  0.000
      i+3      GTGCGTTTAT     0      0      0      0      0      6.15e-01       6.50e-01       751    0.000  0.000  0.000  0.000  0.000

LOCUS: AT4G02520
DESCRIPTION:  glutathione S-transferase, putative
DATA:                         Control 30min  2hours 2days  1week  p-value        B&H            Pos    Fold change relative to control (log2)
SENSE COUNTS:                 2      20     13     4      0      1.94e-05       5.58e-05              0.000  3.322  2.700  1.000  0.000
  GENES:
  AT4G02520.1
    SENSE COUNTS:             2      20     13     4      0      1.94e-05       5.67e-05              0.000  3.322  2.700  1.000  0.000
    TAGS:
```

d+1 GCCATTGGAA 2 20 12 4 0 1.96e-05 9.80e-05 394 0.000 3.322 2.585 1.000 0.000
d+2 TCGAACTCAA 0 0 1 0 0 4.55e-01 5.46e-01 194 0.000 0.000 0.000 0.000 0.000

LOCUS: AT5G07810
DESCRIPTION: SNF2 domain-containing protein / helicase domain-containing protein / HNH endonuclease domain-containing protein, similar to HepA-related protein HARP (Homo sapiens) GI
DATA: Control 30min 2hours 2days 1week p-value B&H Pos Fold change relative to control (log2)
SENSE COUNTS: 1 0 1 0 10 2.03e-05 5.82e-05 0.000 0.000 0.000 0.000 3.322
GENES:
AT5G07810.1
SENSE COUNTS: 1 0 1 0 10 2.03e-05 5.92e-05 0.000 0.000 0.000 0.000 3.322
TAGS:
d+1 ATGCCTCATA 1 0 1 0 10 2.03e-05 1.01e-04 3563 0.000 0.000 0.000 0.000 3.322

LOCUS: AT5G26010
DESCRIPTION: protein phosphatase 2C, putative / PP2C, putative, protein phosphatase-2C, Mesembryanthemum crystallinum, AF075579
DATA: Control 30min 2hours 2days 1week p-value B&H Pos Fold change relative to control (log2)
SENSE COUNTS: 0 0 0 1 9 2.04e-05 5.83e-05 0.000 0.000 0.000 0.000 3.170
GENES:
AT5G26010.1
SENSE COUNTS: 0 0 0 1 9 2.04e-05 5.93e-05 0.000 0.000 0.000 0.000 3.170
TAGS:
v+1 CGGATTTGAT 0 0 0 0 1 1.65e-01 2.39e-01 1552 0.000 0.000 0.000 0.000 0.000
i+3 ACATTTCTCA 0 0 0 1 8 2.96e-04 1.06e-03 451 0.000 0.000 0.000 0.000 3.000

LOCUS: AT3G53490
DESCRIPTION: expressed protein, ADAM 13, Xenopus laevis, EMBL
DATA: Control 30min 2hours 2days 1week p-value B&H Pos Fold change relative to control (log2)
SENSE COUNTS: 8 0 1 2 16 2.05e-05 5.84e-05 0.000 0.000 -3.000 -2.000 1.000
GENES:
AT3G53490.1
SENSE COUNTS: 8 0 1 2 16 2.05e-05 5.94e-05 0.000 0.000 -3.000 -2.000 1.000
TAGS:
v+1 ATTCTTACTA 8 0 1 2 16 2.05e-05 1.02e-04 1362 0.000 0.000 -3.000 -2.000 1.000

LOCUS: AT1G56200
DESCRIPTION: expressed protein
DATA: Control 30min 2hours 2days 1week p-value B&H Pos Fold change relative to control (log2)
SENSE COUNTS: 0 8 0 0 0 2.30e-05 6.53e-05 0.000 3.000 0.000 0.000 0.000
GENES:
AT1G56200.1
SENSE COUNTS: 0 8 0 0 0 2.30e-05 6.65e-05 0.000 3.000 0.000 0.000 0.000
TAGS:
d+1 GCTATGGCGG 0 8 0 0 0 2.30e-05 1.13e-04 41 0.000 3.000 0.000 0.000 0.000

LOCUS: AT1G02280
DESCRIPTION: GTP-binding protein (TOC33), identical to atToc33 protein (GI
DATA: Control 30min 2hours 2days 1week p-value B&H Pos Fold change relative to control (log2)
SENSE COUNTS: 5 2 7 15 26 2.40e-05 6.79e-05 0.000 -1.322 0.485 1.585 2.379
GENES:
AT1G02280.1
SENSE COUNTS: 5 2 7 15 26 2.40e-05 6.92e-05 0.000 -1.322 0.485 1.585 2.379
TAGS:
d+1 CTTTCTGGTT 5 2 7 15 26 2.40e-05 1.17e-04 1124 0.000 -1.322 0.485 1.585 2.379

LOCUS: AT4G24280
DESCRIPTION: heat shock protein 70, putative / HSP70, putative, strong similarity to heat shock protein 70 (Arabidopsis thaliana) GI
DATA: Control 30min 2hours 2days 1week p-value B&H Pos Fold change relative to control (log2)
SENSE COUNTS: 19 4 24 30 37 2.76e-05 7.79e-05 0.000 -2.248 0.337 0.659 0.962
GENES:

```
  AT4G24280.1
    SENSE COUNTS:             19     4      24     30     37     2.76e-05       7.94e-05              0.000  -2.248  0.337  0.659  0.962
    TAGS:
      d+1      GGATGCGATG     19     4      24     30     37     2.76e-05       1.34e-04       2300   0.000  -2.248  0.337  0.659  0.962

LOCUS: AT1G22690
DESCRIPTION:  gibberellin-responsive protein, putative, similar to SP|P46688 Gibberellin-regulated protein 2 precursor {Arabidopsis thaliana};
contains Pfam profile PF02704
DATA:                         Control 30min  2hours 2days  1week  p-value        B&H            Pos    Fold change relative to control (log2)
SENSE COUNTS:                 51     20     34     19     12     2.78e-05       7.82e-05              0.000  -1.350  -0.585  -1.424  -2.087
  GENES:
  AT1G22690.1
    SENSE COUNTS:             51     20     34     19     12     2.78e-05       7.97e-05              0.000  -1.350  -0.585  -1.424  -2.087
    TAGS:
      d+1      TGTGATGTGT     51     20     34     19     12     2.78e-05       1.34e-04       451    0.000  -1.350  -0.585  -1.424  -2.087

LOCUS: AT2G35260
DESCRIPTION:  expressed protein
DATA:                         Control 30min  2hours 2days  1week  p-value        B&H            Pos    Fold change relative to control (log2)
SENSE COUNTS:                 19     5      2      2      3      2.79e-05       7.83e-05              0.000  -1.926  -3.248  -3.248  -2.663
  GENES:
  AT2G35260.1
    SENSE COUNTS:             19     5      2      2      3      2.79e-05       7.98e-05              0.000  -1.926  -3.248  -3.248  -2.663
    TAGS:
      d+1      CTTTATTGAC     0      0      0      1      0      3.09e-01       4.11e-01       1532   0.000  0.000   0.000   0.000   0.000
      d+2      ATACAGTAGA     19     5      1      1      3      2.78e-06       1.72e-05       1377   0.000  -1.926  -4.248  -4.248  -2.663
      d+2      GGCTGTGGAA     0      0      1      0      0      4.55e-01       5.46e-01       1170   0.000  0.000   0.000   0.000   0.000

LOCUS: AT4G29660
DESCRIPTION:  expressed protein, predicted proteins, Arabidopsis thaliana
DATA:                         Control 30min  2hours 2days  1week  p-value        B&H            Pos    Fold change relative to control (log2)
SENSE COUNTS:                 0      0      0      1      8      2.84e-05       7.94e-05              0.000  0.000   0.000   0.000   3.000
  GENES:
  AT4G29660.1
    SENSE COUNTS:             0      0      0      1      8      2.84e-05       8.08e-05              0.000  0.000   0.000   0.000   3.000
    TAGS:
      d+1      TAATGACAGT     0      0      0      1      8      2.84e-05       1.36e-04       625    0.000  0.000   0.000   0.000   3.000

LOCUS: AT5G17220
DESCRIPTION:  glutathione S-transferase, putative
DATA:                         Control 30min  2hours 2days  1week  p-value        B&H            Pos    Fold change relative to control (log2)
SENSE COUNTS:                 0      0      0      1      8      2.84e-05       7.92e-05              0.000  0.000   0.000   0.000   3.000
  GENES:
  AT5G17220.1
    SENSE COUNTS:             0      0      0      1      8      2.84e-05       8.10e-05              0.000  0.000   0.000   0.000   3.000
    TAGS:
      X+4      TCAATGCTAA     0      0      0      1      8      2.84e-05       1.36e-04       564    0.000  0.000   0.000   0.000   3.000

LOCUS: AT4G31850
DESCRIPTION:  pentatricopeptide (PPR) repeat-containing protein, contains Pfam profile PF01535
DATA:                         Control 30min  2hours 2days  1week  p-value        B&H            Pos    Fold change relative to control (log2)
SENSE COUNTS:                 11     0      2      1      0      3.00e-05       8.34e-05              0.000  0.000   -2.459  -3.459  0.000
  GENES:
  AT4G31850.1
    SENSE COUNTS:             11     0      2      1      0      3.00e-05       8.51e-05              0.000  0.000   -2.459  -3.459  0.000
    TAGS:
      v+2      AGACAAAAGT     1      0      0      0      0      4.28e-01       5.20e-01       3874   0.000  0.000   0.000   0.000   0.000
      v+2      ATGATATGTT     9      0      2      1      0      6.61e-04       2.04e-03       3804   0.000  0.000   -2.170  -3.170  0.000
      v+2      TGAAGTGTAC     1      0      0      0      0      4.28e-01       5.25e-01       3747   0.000  0.000   0.000   0.000   0.000
```

```
LOCUS: AT4G15520
DESCRIPTION:  tRNA/rRNA methyltransferase (SpoU) family protein, similar to SP|P19396 tRNA (Guanosine-2'-O-)-methyltransferase (EC 2.1.1.34)
{Escherichia coli O157
DATA:                         Control 30min   2hours  2days   1week   p-value         B&H             Pos     Fold change relative to control (log2)
SENSE COUNTS:                 0       0       0       0       7       3.14e-05        8.70e-05                0.000   0.000   0.000   0.000   2.807
  GENES:
  AT4G15520.1
    SENSE COUNTS:             0       0       0       0       7       3.14e-05        8.89e-05                0.000   0.000   0.000   0.000   2.807
    TAGS:
      X+4      AAGGCTTTAT     0       0       0       0       7       3.14e-05        1.48e-04        554     0.000   0.000   0.000   0.000   2.807

LOCUS: AT2G42750
DESCRIPTION:  DNAJ heat shock N-terminal domain-containing protein, low similarity to GFA2 (Arabidopsis thaliana) GI
DATA:                         Control 30min   2hours  2days   1week   p-value         B&H             Pos     Fold change relative to control (log2)
SENSE COUNTS:                 27      7       31      26      3       3.18e-05        8.79e-05                0.000   -1.948  0.199   -0.054  -3.170
  GENES:
  AT2G42750.1
    SENSE COUNTS:             27      7       31      26      3       3.18e-05        8.98e-05                0.000   -1.948  0.199   -0.054  -3.170
    TAGS:
      d+1      TCGCGTTCTA     27      7       31      26      3       3.18e-05        1.49e-04        1216    0.000   -1.948  0.199   -0.054  -3.170

LOCUS: AT4G15000
DESCRIPTION:  60S ribosomal protein L27 (RPL27C)
DATA:                         Control 30min   2hours  2days   1week   p-value         B&H             Pos     Fold change relative to control (log2)
SENSE COUNTS:                 42      18      33      54      62      3.33e-05        9.17e-05                0.000   -1.222  -0.348  0.363   0.562
  GENES:
  AT4G15000.1
    SENSE COUNTS:             42      18      33      54      62      3.33e-05        9.38e-05                0.000   -1.222  -0.348  0.363   0.562
    TAGS:
      d+1      GTGAAAAATG     42      18      33      54      62      3.33e-05        1.55e-04        552     0.000   -1.222  -0.348  0.363   0.562

LOCUS: AT1G55930
DESCRIPTION:  CBS domain-containing protein / transporter associated domain-containing protein, contains Pfam profiles PF00571
DATA:                         Control 30min   2hours  2days   1week   p-value         B&H             Pos     Fold change relative to control (log2)
SENSE COUNTS:                 0       0       0       0       8       3.35e-05        9.20e-05                0.000   0.000   0.000   0.000   3.000
  GENES:
  AT1G55930.1
    SENSE COUNTS:             0       0       0       0       8       3.35e-05        9.41e-05                0.000   0.000   0.000   0.000   3.000
    TAGS:
      d+1      TATAGAAAGA     0       0       0       0       3       1.12e-02        2.00e-02        2179    0.000   0.000   0.000   0.000   1.585
      X+4      TTGGGAAACA     0       0       0       0       5       7.96e-03        1.51e-02        317     0.000   0.000   0.000   0.000   2.322

LOCUS: AT2G25900
DESCRIPTION:  zinc finger (CCCH-type) family protein, contains Pfam domain, PF00642
DATA:                         Control 30min   2hours  2days   1week   p-value         B&H             Pos     Fold change relative to control (log2)
SENSE COUNTS:                 2       16      2       6       0       3.43e-05        9.39e-05                0.000   3.000   0.000   1.585   0.000
  GENES:
  AT2G25900.1
    SENSE COUNTS:             2       16      2       6       0       3.43e-05        9.61e-05                0.000   3.000   0.000   1.585   0.000
    TAGS:
      d+1      GAGTTTTCGA     2       16      2       6       0       3.43e-05        1.59e-04        563     0.000   3.000   0.000   1.585   0.000

LOCUS: AT5G54940
DESCRIPTION:  eukaryotic translation initiation factor SUI1, putative, similar to SP|P32911 Protein translation factor SUI1 {Saccharomyces
cerevisiae}; contains Pfam profile PF01253
DATA:                         Control 30min   2hours  2days   1week   p-value         B&H             Pos     Fold change relative to control (log2)
SENSE COUNTS:                 2       19      15      2       1       3.52e-05        9.61e-05                0.000   3.248   2.907   0.000   -1.000
  GENES:
  AT5G54940.2
    SENSE COUNTS:             2       19      15      2       1       3.52e-05        9.83e-05                0.000   3.248   2.907   0.000   -1.000
```

```
    TAGS:
      d+1      GTTGAGCTAG     2       19      15      2       1       3.52e-05       1.63e-04       108     0.000   3.248   2.907   0.000   -1.000
  AT5G54940.1
    SENSE COUNTS:             2       19      15      2       1       3.52e-05       9.81e-05               0.000   3.248   2.907   0.000   -1.000
    TAGS:
      d+1      GTTGAGCTAG     2       19      15      2       1       3.52e-05       1.63e-04       174     0.000   3.248   2.907   0.000   -1.000

LOCUS: AT2G07080
DESCRIPTION:  copia-like retrotransposon family, has a 5.0e-74 P-value blast match to gb|AAG52949.1| gag/pol polyprotein (Endovir1-1) (Arabidopsis
thaliana) (Ty1_Copia-family)
DATA:                         Control 30min   2hours  2days   1week   p-value        B&H            Pos     Fold change relative to control (log2)
SENSE COUNTS:                 106     123     85      112     46      3.70e-05       1.01e-04               0.000   0.215   -0.319  0.079   -1.204
  GENES:
  AT2G07080.1
    SENSE COUNTS:             106     123     85      112     46      3.70e-05       1.03e-04               0.000   0.215   -0.319  0.079   -1.204
    TAGS:
      p+1      GATAAGCTCA     106     123     85      112     46      3.70e-05       1.71e-04       2393    0.000   0.215   -0.319  0.079   -1.204

LOCUS: AT3G24190
DESCRIPTION:  ABC1 family protein, contains Pfam domain, PF03109
DATA:                         Control 30min   2hours  2days   1week   p-value        B&H            Pos     Fold change relative to control (log2)
SENSE COUNTS:                 6       0       7       20      16      3.93e-05       1.07e-04               0.000   0.000   0.222   1.737   1.415
  GENES:
  AT3G24190.1
    SENSE COUNTS:             6       0       7       20      16      3.93e-05       1.09e-04               0.000   0.000   0.222   1.737   1.415
    TAGS:
      d+1      GCGAGAATAG     6       0       7       20      16      3.93e-05       1.79e-04       2382    0.000   0.000   0.222   1.737   1.415

LOCUS: AT1G69870
DESCRIPTION:  proton-dependent oligopeptide transport (POT) family protein, contains Pfam profile
DATA:                         Control 30min   2hours  2days   1week   p-value        B&H            Pos     Fold change relative to control (log2)
SENSE COUNTS:                 2       22      4       16      6       3.98e-05       1.08e-04               0.000   3.459   1.000   3.000   1.585
  GENES:
  AT1G69870.1
    SENSE COUNTS:             2       22      4       16      6       3.98e-05       1.10e-04               0.000   3.459   1.000   3.000   1.585
    TAGS:
      d+1      ATCGTCCGGA     0       3       3       8       3       1.07e-01       1.68e-01       1836    0.000   1.585   1.585   3.000   1.585
      i+3      GGTCAGTGCT     2       19      1       8       3       8.73e-06       4.75e-05       1335    0.000   3.248   -1.000  2.000   0.585

LOCUS: AT2G37250
DESCRIPTION:  adenylate kinase family protein, contains Pfam profile
DATA:                         Control 30min   2hours  2days   1week   p-value        B&H            Pos     Fold change relative to control (log2)
SENSE COUNTS:                 5       1       2       4       18      4.08e-05       1.10e-04               0.000   -2.322  -1.322  -0.322  1.848
  GENES:
  AT2G37250.1
    SENSE COUNTS:             5       1       2       4       18      4.08e-05       1.13e-04               0.000   -2.322  -1.322  -0.322  1.848
    TAGS:
      d+1      ATTGTTTGAG     4       0       2       4       14      5.07e-04       1.66e-03       1112    0.000   0.000   -1.000  0.000   1.807
      d+2      AGAGAAATAT     0       0       0       0       1       1.65e-01       2.44e-01       1091    0.000   0.000   0.000   0.000   0.000
      d+2      TGGAATACAT     1       1       0       0       3       2.97e-01       4.00e-01       949     0.000   0.000   0.000   0.000   1.585

LOCUS: AT5G51570
DESCRIPTION:  band 7 family protein, similar to hypersensitive-induced response protein (Zea mays) GI
DATA:                         Control 30min   2hours  2days   1week   p-value        B&H            Pos     Fold change relative to control (log2)
SENSE COUNTS:                 1       3       6       22      12      4.13e-05       1.11e-04               0.000   1.585   2.585   4.459   3.585
  GENES:
  AT5G51570.1
    SENSE COUNTS:             1       3       6       22      12      4.13e-05       1.14e-04               0.000   1.585   2.585   4.459   3.585
    TAGS:
      d+1      AGAAAGTTGA     1       3       6       22      12      1.78e-05       8.93e-05       1168    0.000   1.585   2.585   4.459   3.585
```

```
      d+2      TGAGGGACAT     0      0      0      0      0      6.15e-01       6.57e-01       891    0.000  0.000  0.000  0.000  0.000

LOCUS: AT5G35920
DESCRIPTION:  cytochrome P450, putative, similar to cytochrome P450 (Sinapis alba) gi|3283433|gb|AAD03415
DATA:                         Control 30min  2hours 2days  1week  p-value        B&H            Pos    Fold change relative to control (log2)
SENSE COUNTS:                 1      0      1      2      12     4.19e-05       1.12e-04              0.000  0.000  0.000  1.000  3.585
  GENES:
  AT5G35920.1
    SENSE COUNTS:             1      0      1      2      12     4.19e-05       1.15e-04              0.000  0.000  0.000  1.000  3.585
    TAGS:
      v+2      TCCACTACTG     1      0      1      2      12     4.19e-05       1.91e-04       423    0.000  0.000  0.000  1.000  3.585

LOCUS: AT5G64260
DESCRIPTION:  phosphate-responsive protein, putative, similar to phi-1 (phosphate-induced gene) (Nicotiana tabacum) GI
DATA:                         Control 30min  2hours 2days  1week  p-value        B&H            Pos    Fold change relative to control (log2)
SENSE COUNTS:                 4      17     0      9      1      4.21e-05       1.13e-04              0.000  2.087  0.000  1.170  -2.000
  GENES:
  AT5G64260.1
    SENSE COUNTS:             4      17     0      9      1      4.21e-05       1.15e-04              0.000  2.087  0.000  1.170  -2.000
    TAGS:
      d+1      TGTAGTCAAA     4      17     0      9      1      4.21e-05       1.91e-04       1035   0.000  2.087  0.000  1.170  -2.000

LOCUS: AT5G61600
DESCRIPTION:  encodes a member of the ERF (ethylene response factor) subfamily B-3 of ERF/AP2 transcription factor family. The protein contains one
AP2 domain. There are 18 members in this subfamily including ATERF-1, ATERF-2, AND ATERF-5.
DATA:                         Control 30min  2hours 2days  1week  p-value        B&H            Pos    Fold change relative to control (log2)
SENSE COUNTS:                 0      9      1      0      0      4.22e-05       1.13e-04              0.000  3.170  0.000  0.000  0.000
  GENES:
  AT5G61600.1
    SENSE COUNTS:             0      9      1      0      0      4.22e-05       1.15e-04              0.000  3.170  0.000  0.000  0.000
    TAGS:
      d+1      ATTTCATTTA     0      9      1      0      0      4.22e-05       1.91e-04       841    0.000  3.170  0.000  0.000  0.000

LOCUS: AT2G05540
DESCRIPTION:  glycine-rich protein
DATA:                         Control 30min  2hours 2days  1week  p-value        B&H            Pos    Fold change relative to control (log2)
SENSE COUNTS:                 27     3      18     13     2      4.28e-05       1.14e-04              0.000  -3.170 -0.585 -1.054 -3.755
  GENES:
  AT2G05540.1
    SENSE COUNTS:             27     3      18     13     2      4.28e-05       1.17e-04              0.000  -3.170 -0.585 -1.054 -3.755
    TAGS:
      d+1      AACTTTTAAA     10     0      8      5      1      2.14e-02       3.70e-02       704    0.000  0.000  -0.322 -1.000 -3.322
      X+4      AAGTGTTTGT     17     3      10     8      1      5.28e-03       1.14e-02       540    0.000  -2.503 -0.766 -1.087 -4.087

LOCUS: AT4G13940
DESCRIPTION:  adenosylhomocysteinase / S-adenosyl-L-homocysteine hydrolase / AdoHcyase (SAHH), identical to SP|O23255 Adenosylhomocysteinase (EC
3.3.1.1) (S-adenosyl-L-homocysteine hydrolase) (AdoHcyase) {Arabidopsis thaliana}; strong similarity to SP|P50248 Adenosylho
DATA:                         Control 30min  2hours 2days  1week  p-value        B&H            Pos    Fold change relative to control (log2)
SENSE COUNTS:                 29     34     33     70     63     4.30e-05       1.14e-04              0.000  0.229  0.186  1.271  1.119
  GENES:
  AT4G13940.1
    SENSE COUNTS:             29     34     33     70     63     4.30e-05       1.17e-04              0.000  0.229  0.186  1.271  1.119
    TAGS:
      d+1      GTTCGATTCG     1      0      1      1      1      7.91e-01       7.95e-01       1908   0.000  0.000  0.000  0.000  0.000
      d+2      AAACTTAAAT     28     34     32     69     62     4.27e-05       1.93e-04       1707   0.000  0.280  0.193  1.301  1.147
      i+3      AAAAGTCAAT     0      0      0      0      0      6.15e-01       6.53e-01       970    0.000  0.000  0.000  0.000  0.000

LOCUS: AT3G58730
DESCRIPTION:  vacuolar ATP synthase subunit D (VATD) / V-ATPase D subunit / vacuolar proton pump D subunit (VATPD), identical to Vacuolar ATP synthase
subunit D (EC 3.6.3.14) (V-ATPase D subunit) (Vacuolar proton pump D subunit) (Swiss-Prot
```

```
DATA:                         Control 30min  2hours  2days  1week  p-value       B&H           Pos    Fold change relative to control (log2)
SENSE COUNTS:                 1       12     0       4      0      4.78e-05      1.26e-04             0.000  3.585  0.000  2.000  0.000
  GENES:
  AT3G58730.1
    SENSE COUNTS:             1       12     0       4      0      4.78e-05      1.29e-04             0.000  3.585  0.000  2.000  0.000
    TAGS:
      d+1     TTGTCCTCGA      1       12     0       4      0      4.78e-05      2.13e-04      405    0.000  3.585  0.000  2.000  0.000

LOCUS: AT1G58983
DESCRIPTION:  40S ribosomal protein S2, putative, similar to ribosomal protein S2 GI
DATA:                         Control 30min  2hours  2days  1week  p-value       B&H           Pos    Fold change relative to control (log2)
SENSE COUNTS:                 10      5      11      16     34     5.17e-05      1.36e-04             0.000  -1.000 0.138  0.678  1.766
  GENES:
  AT1G58983.1
    SENSE COUNTS:             10      5      11      16     34     5.17e-05      1.40e-04             0.000  -1.000 0.138  0.678  1.766
    TAGS:
      d+1     TATAAATTGT      1       0      1       0      0      6.01e-01      6.47e-01      1098   0.000  0.000  0.000  0.000  0.000
      d+2     AAATGACATT      9       5      10      16     34     2.20e-05      1.09e-04      1035   0.000  -0.848 0.152  0.830  1.918

LOCUS: AT5G17990
DESCRIPTION:  anthranilate phosphoribosyltransferase, identical to anthranilate phosphoribosyltransferase, chloroplast precursor (EC 2.4.2.18) SP
DATA:                         Control 30min  2hours  2days  1week  p-value       B&H           Pos    Fold change relative to control (log2)
SENSE COUNTS:                 11      0      2       0      10     5.19e-05      1.36e-04             0.000  0.000  -2.459 0.000  -0.138
  GENES:
  AT5G17990.1
    SENSE COUNTS:             11      0      2       0      10     5.19e-05      1.40e-04             0.000  0.000  -2.459 0.000  -0.138
    TAGS:
      d+1     TAAACACTAC      11      0      1       0      10     1.48e-05      7.63e-05      1626   0.000  0.000  -3.459 0.000  -0.138
      d+2     TGGTCTAGAC      0       0      1       0      0      4.55e-01      5.38e-01      1092   0.000  0.000  0.000  0.000  0.000

LOCUS: AT5G58070
DESCRIPTION:  lipocalin, putative, similar to temperature stress-induced lipocalin (Triticum aestivum) GI
DATA:                         Control 30min  2hours  2days  1week  p-value       B&H           Pos    Fold change relative to control (log2)
SENSE COUNTS:                 2       1      8       20     11     5.23e-05      1.37e-04             0.000  -1.000 2.000  3.322  2.459
  GENES:
  AT5G58070.1
    SENSE COUNTS:             2       1      8       20     11     5.23e-05      1.41e-04             0.000  -1.000 2.000  3.322  2.459
    TAGS:
      d+1     AATTTGTTTT      0       0      1       0      0      4.55e-01      5.18e-01      1018   0.000  0.000  0.000  0.000  0.000
      d+2     CAAACTACAG      1       0      0       1      1      5.50e-01      6.00e-01      898    0.000  0.000  0.000  0.000  0.000
      d+2     GGCCGTTGGT      1       1      7       19     10     4.79e-05      2.13e-04      111    0.000  0.000  2.807  4.248  3.322

LOCUS: AT5G22920
DESCRIPTION:  zinc finger (C3HC4-type RING finger) family protein, contains Pfam profiles
DATA:                         Control 30min  2hours  2days  1week  p-value       B&H           Pos    Fold change relative to control (log2)
SENSE COUNTS:                 2       16     3       5      0      5.53e-05      1.45e-04             0.000  3.000  0.585  1.322  0.000
  GENES:
  AT5G22920.1
    SENSE COUNTS:             2       16     3       5      0      5.53e-05      1.48e-04             0.000  3.000  0.585  1.322  0.000
    TAGS:
      d+1     AGGAGATTAT      2       16     3       5      0      5.53e-05      2.44e-04      1069   0.000  3.000  0.585  1.322  0.000

LOCUS: AT1G50920
DESCRIPTION:  GTP-binding protein-related, similar to GTP-binding protein SP
DATA:                         Control 30min  2hours  2days  1week  p-value       B&H           Pos    Fold change relative to control (log2)
SENSE COUNTS:                 5       18     3       1      2      5.60e-05      1.46e-04             0.000  1.848  -0.737 -2.322 -1.322
  GENES:
  AT1G50920.1
    SENSE COUNTS:             5       18     3       1      2      5.60e-05      1.50e-04             0.000  1.848  -0.737 -2.322 -1.322
    TAGS:
```

```
      d+1      AAGTTGTGCC     4      5      1      1      1      3.43e-01       4.43e-01       1947   0.000  0.322  -2.000 -2.000 -2.000
      d+2      TGAGAGGCTG     0      0      1      0      0      4.55e-01       5.43e-01       1156   0.000  0.000  0.000  0.000  0.000
      d+2      TCTGTGAAGA     1      13     1      0      1      8.58e-06       4.68e-05       1136   0.000  3.700  0.000  0.000  0.000

LOCUS: AT1G13260
DESCRIPTION:  DNA-binding protein RAV1 (RAV1), identical to SP|Q9ZWM9 DNA-binding protein RAV1 {Arabidopsis thaliana}, RAV1 GI
DATA:                        Control 30min  2hours 2days  1week  p-value        B&H            Pos    Fold change relative to control (log2)
SENSE COUNTS:                1      3      16     4      1      5.63e-05       1.46e-04              0.000  1.585  4.000  2.000  0.000
  GENES:
  AT1G13260.1
    SENSE COUNTS:            1      3      16     4      1      5.63e-05       1.50e-04              0.000  1.585  4.000  2.000  0.000
    TAGS:
      d+1      AGATGATGAA     1      3      16     4      1      5.63e-05       2.48e-04       1461   0.000  1.585  4.000  2.000  0.000

LOCUS: AT3G57020
DESCRIPTION:  strictosidine synthase family protein, similar to strictosidine synthase (Rauvolfia serpentina)(SP|P15324)
DATA:                        Control 30min  2hours 2days  1week  p-value        B&H            Pos    Fold change relative to control (log2)
SENSE COUNTS:                0      0      5      1      12     5.84e-05       1.51e-04              0.000  0.000  2.322  0.000  3.585
  GENES:
  AT3G57020.1
    SENSE COUNTS:            0      0      5      1      12     5.84e-05       1.55e-04              0.000  0.000  2.322  0.000  3.585
    TAGS:
      d+1      TTGTATCTAC     0      0      2      1      12     7.09e-06       4.01e-05       1170   0.000  0.000  1.000  0.000  3.585
      d+2      CGAGTCCGGC     0      0      2      0      0      1.21e-01       1.87e-01       737    0.000  0.000  1.000  0.000  0.000
      d+2      AAAGTTGGAC     0      0      1      0      0      4.55e-01       5.47e-01       444    0.000  0.000  0.000  0.000  0.000

LOCUS: AT5G65480
DESCRIPTION:  expressed protein
DATA:                        Control 30min  2hours 2days  1week  p-value        B&H            Pos    Fold change relative to control (log2)
SENSE COUNTS:                42     10     30     23     13     5.85e-05       1.51e-04              0.000  -2.070 -0.485 -0.869 -1.692
  GENES:
  AT5G65480.1
    SENSE COUNTS:            42     10     30     23     13     5.85e-05       1.55e-04              0.000  -2.070 -0.485 -0.869 -1.692
    TAGS:
      d+1      TGCTTACTTT     12     3      13     8      3      5.24e-02       8.60e-02       1185   0.000  -2.000 0.115  -0.585 -2.000
      d+2      TACATATGTT     2      0      0      1      3      1.97e-01       2.76e-01       1151   0.000  0.000  0.000  -1.000 0.585
      d+2      TGGGTGTTTT     8      1      4      2      0      8.12e-02       1.31e-01       1004   0.000  -3.000 -1.000 -2.000 0.000
      d+2      GAACAGTGGC     20     6      13     12     7      3.80e-02       6.34e-02       684    0.000  -1.737 -0.621 -0.737 -1.515

LOCUS: AT4G16460
DESCRIPTION:  hypothetical protein
DATA:                        Control 30min  2hours 2days  1week  p-value        B&H            Pos    Fold change relative to control (log2)
SENSE COUNTS:                0      10     1      0      1      6.05e-05       1.56e-04              0.000  3.322  0.000  0.000  0.000
  GENES:
  AT4G16460.1
    SENSE COUNTS:            0      10     1      0      1      6.05e-05       1.60e-04              0.000  3.322  0.000  0.000  0.000
    TAGS:
      i+3      CAGGTGTGGT     0      10     1      0      1      6.05e-05       2.65e-04       358    0.000  3.322  0.000  0.000  0.000

LOCUS: AT5G02110
DESCRIPTION:  cyclin family protein, low similarity to cyclin D3.1 from (Lycopersicon esculentum) GI
DATA:                        Control 30min  2hours 2days  1week  p-value        B&H            Pos    Fold change relative to control (log2)
SENSE COUNTS:                23     1      7      9      7      6.13e-05       1.58e-04              0.000  -4.524 -1.716 -1.354 -1.716
  GENES:
  AT5G02110.1
    SENSE COUNTS:            23     1      7      9      7      6.13e-05       1.62e-04              0.000  -4.524 -1.716 -1.354 -1.716
    TAGS:
      v+1      ATTTTGTTTT     23     1      7      9      7      6.13e-05       2.67e-04       1776   0.000  -4.524 -1.716 -1.354 -1.716

LOCUS: AT1G23310
```

DESCRIPTION: glutamate

DATA:		Control	30min	2hours	2days	1week	p-value	B&H	Pos	Fold change relative to control (log2)				
SENSE COUNTS:		78	49	55	23	42	6.36e-05	1.63e-04		0.000	-0.671	-0.504	-1.762	-0.893
GENES:														
AT1G23310.1														
SENSE COUNTS:		78	49	55	23	42	6.36e-05	1.68e-04		0.000	-0.671	-0.504	-1.762	-0.893
TAGS:														
d+1	ACTCAGTATG	78	49	47	23	41	3.90e-05	1.79e-04	1710	0.000	-0.671	-0.731	-1.762	-0.928
d+2	GATAGCTTCA	0	0	2	0	0	3.51e-01	4.53e-01	1677	0.000	0.000	1.000	0.000	0.000
d+2	ACAGATGGAT	0	0	6	0	0	2.08e-04	7.89e-04	1413	0.000	0.000	2.585	0.000	0.000
X+4	TCCTAGTTTG	0	0	0	0	1	1.65e-01	2.48e-01	443	0.000	0.000	0.000	0.000	0.000

LOCUS: AT4G33010

DESCRIPTION: glycine dehydrogenase (decarboxylating), putative / glycine decarboxylase, putative / glycine cleavage system P-protein, putative, strong similarity to SP|P49361 Glycine dehydrogenase (decarboxylating) A, mitochondrial precursor (EC 1.4.4.2) {Flaveria pri

DATA:		Control	30min	2hours	2days	1week	p-value	B&H	Pos	Fold change relative to control (log2)				
SENSE COUNTS:		32	13	44	33	10	6.39e-05	1.63e-04		0.000	-1.300	0.459	0.044	-1.678
GENES:														
AT4G33010.1														
SENSE COUNTS:		32	13	44	33	10	6.39e-05	1.68e-04		0.000	-1.300	0.459	0.044	-1.678
TAGS:														
d+1	GAAAAAGCCG	31	13	42	33	10	1.02e-04	4.22e-04	3131	0.000	-1.254	0.438	0.090	-1.632
d+2	GACCCACAAT	0	0	2	0	0	1.21e-01	1.87e-01	2926	0.000	0.000	1.000	0.000	0.000
i+3	TATTATTCTG	0	0	0	0	0	6.15e-01	6.48e-01	2117	0.000	0.000	0.000	0.000	0.000
d+2	GCGAAAAACT	1	0	0	0	0	4.28e-01	5.23e-01	101	0.000	0.000	0.000	0.000	0.000

LOCUS: AT2G42870

DESCRIPTION: expressed protein

DATA:		Control	30min	2hours	2days	1week	p-value	B&H	Pos	Fold change relative to control (log2)				
SENSE COUNTS:		4	14	2	1	0	6.58e-05	1.68e-04		0.000	1.807	-1.000	-2.000	0.000
GENES:														
AT2G42870.1														
SENSE COUNTS:		4	14	2	1	0	6.58e-05	1.73e-04		0.000	1.807	-1.000	-2.000	0.000
TAGS:														
d+1	GGATTTTAGC	4	11	2	1	0	2.90e-03	7.00e-03	519	0.000	1.459	-1.000	-2.000	0.000
d+2	AAGTTCGGAG	0	3	0	0	0	3.05e-02	5.15e-02	406	0.000	1.585	0.000	0.000	0.000

LOCUS: AT5G08260

DESCRIPTION: serine carboxypeptidase S10 family protein, similar to Serine carboxypeptidase II chains A and B (SP

DATA:		Control	30min	2hours	2days	1week	p-value	B&H	Pos	Fold change relative to control (log2)				
SENSE COUNTS:		0	2	2	1	14	6.63e-05	1.68e-04		0.000	1.000	1.000	0.000	3.807
GENES:														
AT5G08260.1														
SENSE COUNTS:		0	2	2	1	14	6.63e-05	1.73e-04		0.000	1.000	1.000	0.000	3.807
TAGS:														
d+1	ATGGCAAGTT	0	2	1	1	14	1.74e-05	8.75e-05	1534	0.000	1.000	0.000	0.000	3.807
d+2	CACTGAAAGT	0	0	1	0	0	4.55e-01	5.20e-01	1062	0.000	0.000	0.000	0.000	0.000

LOCUS: AT1G50450

DESCRIPTION: expressed protein

DATA:		Control	30min	2hours	2days	1week	p-value	B&H	Pos	Fold change relative to control (log2)				
SENSE COUNTS:		6	1	11	6	25	6.80e-05	1.72e-04		0.000	-2.585	0.874	0.000	2.059
GENES:														
AT1G50450.1														
SENSE COUNTS:		6	1	11	6	25	6.80e-05	1.77e-04		0.000	-2.585	0.874	0.000	2.059
TAGS:														
d+1	TTCATCAGAA	6	1	11	6	25	6.80e-05	2.92e-04	1405	0.000	-2.585	0.874	0.000	2.059

LOCUS: AT2G17450

DESCRIPTION: zinc finger (C3HC4-type RING finger) family protein, contains Pfam domain, PF00097

```
DATA:                         Control 30min  2hours  2days  1week  p-value    B&H        Pos   Fold change relative to control (log2)
SENSE COUNTS:                 2       11     0       1      0      6.89e-05   1.74e-04         0.000  2.459  0.000  -1.000 0.000
  GENES:
  AT2G17450.1
    SENSE COUNTS:             2       11     0       1      0      6.89e-05   1.79e-04         0.000  2.459  0.000  -1.000 0.000
    TAGS:
      d+1     CTTCTACGGC      2       11     0       1      0      6.89e-05   2.95e-04   576   0.000  2.459  0.000  -1.000 0.000

LOCUS: AT5G26000
DESCRIPTION:  glycosyl hydrolase family 1 protein, contains Pfam PF00232
DATA:                         Control 30min  2hours  2days  1week  p-value    B&H        Pos   Fold change relative to control (log2)
SENSE COUNTS:                 89      48     51      41     39     6.90e-05   1.74e-04         0.000  -0.891 -0.803 -1.118 -1.190
  GENES:
  AT5G26000.1
    SENSE COUNTS:             89      48     51      41     39     6.90e-05   1.79e-04         0.000  -0.891 -0.803 -1.118 -1.190
    TAGS:
      d+1     TTTCCTCTTT      89      48     50      41     39     6.12e-05   2.67e-04   1701  0.000  -0.891 -0.832 -1.118 -1.190
      d+2     AGGGAATATG      0       0      1       0      0      4.55e-01   5.39e-01   985   0.000  0.000  0.000  0.000  0.000
  AT5G26000.2
    SENSE COUNTS:             89      48     51      41     39     6.90e-05   1.79e-04         0.000  -0.891 -0.803 -1.118 -1.190
    TAGS:
      d+1     TTTCCTCTTT      89      48     50      41     39     6.12e-05   2.67e-04   1701  0.000  -0.891 -0.832 -1.118 -1.190
      d+2     AGGGAATATG      0       0      1       0      0      4.55e-01   5.39e-01   985   0.000  0.000  0.000  0.000  0.000

LOCUS: AT3G26060
DESCRIPTION:  peroxiredoxin Q, putative, similar to peroxiredoxin Q (Sedum lineare) GI
DATA:                         Control 30min  2hours  2days  1week  p-value    B&H        Pos   Fold change relative to control (log2)
SENSE COUNTS:                 12      0      1       9      17     7.03e-05   1.77e-04         0.000  0.000  -3.585 -0.415 0.503
  GENES:
  AT3G26060.1
    SENSE COUNTS:             12      0      1       9      17     7.03e-05   1.82e-04         0.000  0.000  -3.585 -0.415 0.503
    TAGS:
      d+1     TTATACCAAT      11      0      1       8      17     8.62e-05   3.61e-04   797   0.000  0.000  -3.459 -0.459 0.628
      d+2     ACCTTCTTAT      1       0      0       1      0      5.06e-01   5.57e-01   749   0.000  0.000  0.000  0.000  0.000

LOCUS: AT1G57660
DESCRIPTION:  60S ribosomal protein L21 (RPL21E), similar to 60S ribosomal protein L21 GB
DATA:                         Control 30min  2hours  2days  1week  p-value    B&H        Pos   Fold change relative to control (log2)
SENSE COUNTS:                 6       4      3       4      23     7.04e-05   1.76e-04         0.000  -0.585 -1.000 -0.585 1.939
  GENES:
  AT1G57660.1
    SENSE COUNTS:             6       4      3       4      23     7.04e-05   1.82e-04         0.000  -0.585 -1.000 -0.585 1.939
    TAGS:
      d+1     GTCGAAGGTA      6       4      3       4      23     7.04e-05   3.01e-04   468   0.000  -0.585 -1.000 -0.585 1.939

LOCUS: AT5G67360
DESCRIPTION:  subtilisin-like protease that has been  located in stem and siliques but not roots.
DATA:                         Control 30min  2hours  2days  1week  p-value    B&H        Pos   Fold change relative to control (log2)
SENSE COUNTS:                 21      7      17      24     44     7.31e-05   1.83e-04         0.000  -1.585 -0.305 0.193  1.067
  GENES:
  AT5G67360.1
    SENSE COUNTS:             21      7      17      24     44     7.31e-05   1.88e-04         0.000  -1.585 -0.305 0.193  1.067
    TAGS:
      d+1     TGACTTGAAA      6       0      5       12     12     1.46e-02   2.58e-02   2617  0.000  0.000  -0.263 1.000  1.000
      d+2     CAGTCTGTTA      15      7      12      11     32     1.14e-03   3.17e-03   2501  0.000  -1.100 -0.322 -0.447 1.093
      d+2     GATCACAACT      0       0      0       1      0      6.04e-01   6.48e-01   1098  0.000  0.000  0.000  0.000  0.000

LOCUS: AT1G07440
DESCRIPTION:  tropinone reductase, putative / tropine dehydrogenase, putative, similar to tropinone reductase SP
DATA:                         Control 30min  2hours  2days  1week  p-value    B&H        Pos   Fold change relative to control (log2)
```

```
SENSE COUNTS:                 0       7       0       0       0       7.93e-05       1.98e-04                 0.000   2.807   0.000   0.000   0.000
  GENES:
  AT1G07440.1
    SENSE COUNTS:             0       7       0       0       0       7.93e-05       2.03e-04                 0.000   2.807   0.000   0.000   0.000
    TAGS:
      d+1      TGAGTGGGCT     0       7       0       0       0       7.93e-05       3.37e-04       563       0.000   2.807   0.000   0.000   0.000

LOCUS: AT5G28237
DESCRIPTION:  tryptophan synthase, beta subunit, putative, similar to SP|P14671 Tryptophan synthase beta chain 1, chloroplast precursor (EC 4.2.1.20)
{Arabidopsis thaliana}; contains Pfam profile PF00291
DATA:                         Control 30min   2hours  2days   1week   p-value        B&H            Pos       Fold change relative to control (log2)
SENSE COUNTS:                 0       7       0       0       0       7.93e-05       1.97e-04                 0.000   2.807   0.000   0.000   0.000
  GENES:
  AT5G28237.1
    SENSE COUNTS:             0       7       0       0       0       7.93e-05       2.03e-04                 0.000   2.807   0.000   0.000   0.000
    TAGS:
      d+2      GGAGCTGCTG     0       7       0       0       0       7.93e-05       3.38e-04       675       0.000   2.807   0.000   0.000   0.000
  AT5G28237.2
    SENSE COUNTS:             0       7       0       0       0       7.93e-05       2.03e-04                 0.000   2.807   0.000   0.000   0.000
    TAGS:
      d+2      GGAGCTGCTG     0       7       0       0       0       7.93e-05       3.38e-04       675       0.000   2.807   0.000   0.000   0.000

LOCUS: AT4G10730
DESCRIPTION:  protein kinase family protein, contains protein kinase domain, Pfam
DATA:                         Control 30min   2hours  2days   1week   p-value        B&H            Pos       Fold change relative to control (log2)
SENSE COUNTS:                 0       0       0       2       10      8.00e-05       1.98e-04                 0.000   0.000   0.000   1.000   3.322
  GENES:
  AT4G10730.1
    SENSE COUNTS:             0       0       0       2       10      8.00e-05       2.04e-04                 0.000   0.000   0.000   1.000   3.322
    TAGS:
      i+3      AAACAAATTT     0       0       0       0       3       8.36e-02       1.34e-01       4954      0.000   0.000   0.000   0.000   1.585
      d+1      GAAAATTGTA     0       0       0       2       7       1.31e-03       3.57e-03       2463      0.000   0.000   0.000   1.000   2.807

LOCUS: AT1G78460
DESCRIPTION:  SOUL heme-binding family protein, weak similarity to SOUL protein (Mus musculus) GI
DATA:                         Control 30min   2hours  2days   1week   p-value        B&H            Pos       Fold change relative to control (log2)
SENSE COUNTS:                 3       13      1       1       0       8.09e-05       2.00e-04                 0.000   2.115   -1.585  -1.585  0.000
  GENES:
  AT1G78460.1
    SENSE COUNTS:             3       13      1       1       0       8.09e-05       2.06e-04                 0.000   2.115   -1.585  -1.585  0.000
    TAGS:
      d+1      TTAATGACGC     3       13      1       1       0       8.09e-05       3.42e-04       686       0.000   2.115   -1.585  -1.585  0.000

LOCUS: AT5G05987
DESCRIPTION:  prenylated rab acceptor (PRA1) family protein, contains Pfam profile PF03208
DATA:                         Control 30min   2hours  2days   1week   p-value        B&H            Pos       Fold change relative to control (log2)
SENSE COUNTS:                 0       0       0       5       0       8.11e-05       2.00e-04                 0.000   0.000   0.000   2.322   0.000
  GENES:
  AT5G05987.1
    SENSE COUNTS:             0       0       0       5       0       8.11e-05       2.06e-04                 0.000   0.000   0.000   2.322   0.000
    TAGS:
      d+1      GTTAAGATGG     0       0       0       2       0       4.80e-02       7.95e-02       789       0.000   0.000   0.000   1.000   0.000
      i+3      TGTATTCATA     0       0       0       2       0       4.80e-02       7.94e-02       755       0.000   0.000   0.000   1.000   0.000
      d+2      CAAGCATAAG     0       0       0       1       0       3.09e-01       4.09e-01       574       0.000   0.000   0.000   0.000   0.000

LOCUS: AT1G06040
DESCRIPTION:  zinc finger (B-box type) family protein / salt-tolerance protein (STO), identical to SP|Q96288 Salt-tolerance protein (Arabidopsis
thaliana); contains Pfam profile PF00643
DATA:                         Control 30min   2hours  2days   1week   p-value        B&H            Pos       Fold change relative to control (log2)
SENSE COUNTS:                 14      2       21      24      30      8.16e-05       2.01e-04                 0.000   -2.807  0.585   0.778   1.100
```

```
  GENES:
  AT1G06040.1
    SENSE COUNTS:             14     2      21     24     30     8.16e-05      2.06e-04                0.000  -2.807 0.585  0.778  1.100
    TAGS:
      d+1     GTCTATGCTT      13     2      21     24     30     6.16e-05      2.67e-04      1158      0.000  -2.700 0.692  0.885  1.206
      d+2     TTCATTCATA      1      0      0      0      0      4.28e-01      5.30e-01      869       0.000  0.000  0.000  0.000  0.000
  AT1G06040.2
    SENSE COUNTS:             14     2      21     24     30     8.16e-05      2.06e-04                0.000  -2.807 0.585  0.778  1.100
    TAGS:
      d+1     GTCTATGCTT      13     2      21     24     30     6.16e-05      2.67e-04      1240      0.000  -2.700 0.692  0.885  1.206
      d+2     TTCATTCATA      1      0      0      0      0      4.28e-01      5.30e-01      951       0.000  0.000  0.000  0.000  0.000

LOCUS: AT5G61030
DESCRIPTION:  RNA-binding protein, putative, similar to RNA-binding protein from (Solanum tuberosum) GI
DATA:                         Control 30min  2hours 2days  1week  p-value       B&H           Pos       Fold change relative to control (log2)
SENSE COUNTS:                 0      0      1      9      1      8.39e-05      2.06e-04                0.000  0.000  0.000  3.170  0.000
  GENES:
  AT5G61030.1
    SENSE COUNTS:             0      0      1      9      1      8.39e-05      2.11e-04                0.000  0.000  0.000  3.170  0.000
    TAGS:
      d+1     GAGAAAAGTG      0      0      1      9      1      8.39e-05      3.53e-04      922       0.000  0.000  0.000  3.170  0.000

LOCUS: AT2G47640
DESCRIPTION:  small nuclear ribonucleoprotein D2, putative / snRNP core protein D2, putative / Sm protein D2, putative, similar to small nuclear
ribonucleoprotein Sm D2 (snRNP core protein D2) (Sm-D2) (Mus musculus) SWISS-PROT
DATA:                         Control 30min  2hours 2days  1week  p-value       B&H           Pos       Fold change relative to control (log2)
SENSE COUNTS:                 5      1      1      1      15     8.42e-05      2.06e-04                0.000  -2.322 -2.322 -2.322 1.585
  GENES:
  AT2G47640.1
    SENSE COUNTS:             5      1      1      1      15     8.42e-05      2.11e-04                0.000  -2.322 -2.322 -2.322 1.585
    TAGS:
      d+1     TTCCTTGTTT      5      1      1      1      14     5.36e-04      1.74e-03      599       0.000  -2.322 -2.322 -2.322 1.485
      X+4     TCCAAGCTCA      0      0      0      0      1      1.65e-01      2.43e-01      295       0.000  0.000  0.000  0.000  0.000
  AT2G47640.2
    SENSE COUNTS:             5      1      1      1      14     5.36e-04      1.04e-03                0.000  -2.322 -2.322 -2.322 1.485
    TAGS:
      d+1     TTCCTTGTTT      5      1      1      1      14     5.36e-04      1.74e-03      566       0.000  -2.322 -2.322 -2.322 1.485
  AT2G47640.3
    SENSE COUNTS:             5      1      1      1      14     5.36e-04      1.03e-03                0.000  -2.322 -2.322 -2.322 1.485
    TAGS:
      d+1     TTCCTTGTTT      5      1      1      1      14     5.36e-04      1.74e-03      574       0.000  -2.322 -2.322 -2.322 1.485

LOCUS: AT1G23740
DESCRIPTION:  oxidoreductase, zinc-binding dehydrogenase family protein, contains Pfam profile PF00107
DATA:                         Control 30min  2hours 2days  1week  p-value       B&H           Pos       Fold change relative to control (log2)
SENSE COUNTS:                 16     1      11     2      0      8.54e-05      2.08e-04                0.000  -4.000 -0.541 -3.000 0.000
  GENES:
  AT1G23740.1
    SENSE COUNTS:             16     1      11     2      0      8.54e-05      2.14e-04                0.000  -4.000 -0.541 -3.000 0.000
    TAGS:
      d+1     CCACAGGGAA      16     1      11     2      0      8.54e-05      3.59e-04      1199      0.000  -4.000 -0.541 -3.000 0.000

LOCUS: AT3G14420
DESCRIPTION:  (S)-2-hydroxy-acid oxidase, peroxisomal, putative / glycolate oxidase, putative / short chain alpha-hydroxy acid oxidase, putative,
similar to (S)-2-hydroxy-acid oxidase, peroxisomal (Glycolate oxidase, GOX) (Short chain alpha-hydroxy acid oxidase) (Spina
DATA:                         Control 30min  2hours 2days  1week  p-value       B&H           Pos       Fold change relative to control (log2)
SENSE COUNTS:                 86     48     72     36     45     9.11e-05      2.22e-04                0.000  -0.841 -0.256 -1.256 -0.934
  GENES:
  AT3G14420.2
    SENSE COUNTS:             86     48     72     36     45     9.11e-05      2.28e-04                0.000  -0.841 -0.256 -1.256 -0.934
```

```
    TAGS:
      d+1      TACTTACATT     64      17      30      18      37      3.26e-08       2.94e-07       1428    0.000   -1.913  -1.093  -1.830  -0.791
      d+2      GCACTGAGTG     22      31      39      18      8       3.32e-03       7.87e-03       1126    0.000   0.495   0.826   -0.290  -1.459
      d+2      GGCTACTTCC     0       0       2       0       0       1.21e-01       1.87e-01       435     0.000   0.000   1.000   0.000   0.000
      d+2      CAAAAGATGG     0       0       1       0       0       4.55e-01       5.19e-01       352     0.000   0.000   0.000   0.000   0.000
  AT3G14420.1
    SENSE COUNTS:             86      48      72      36      45      9.11e-05       2.27e-04               0.000   -0.841  -0.256  -1.256  -0.934
    TAGS:
      d+1      TACTTACATT     64      17      30      18      37      3.26e-08       2.94e-07       1387    0.000   -1.913  -1.093  -1.830  -0.791
      d+2      GCACTGAGTG     22      31      39      18      8       3.32e-03       7.87e-03       1085    0.000   0.495   0.826   -0.290  -1.459
      d+2      GGCTACTTCC     0       0       2       0       0       1.21e-01       1.87e-01       394     0.000   0.000   1.000   0.000   0.000
      d+2      CAAAAGATGG     0       0       1       0       0       4.55e-01       5.19e-01       311     0.000   0.000   0.000   0.000   0.000
  AT3G14420.3
    SENSE COUNTS:             86      48      72      36      45      9.11e-05       2.27e-04               0.000   -0.841  -0.256  -1.256  -0.934
    TAGS:
      d+1      TACTTACATT     64      17      30      18      37      3.26e-08       2.94e-07       1520    0.000   -1.913  -1.093  -1.830  -0.791
      d+2      GCACTGAGTG     22      31      39      18      8       3.32e-03       7.87e-03       1218    0.000   0.495   0.826   -0.290  -1.459
      d+2      GGCTACTTCC     0       0       2       0       0       1.21e-01       1.87e-01       527     0.000   0.000   1.000   0.000   0.000
      d+2      CAAAAGATGG     0       0       1       0       0       4.55e-01       5.19e-01       444     0.000   0.000   0.000   0.000   0.000

LOCUS: AT5G39850
DESCRIPTION:  40S ribosomal protein S9 (RPS9C), 40S ribosomal protein S9 - Chlamydomonas sp.,EMBL
DATA:                         Control 30min   2hours  2days   1week   p-value        B&H            Pos     Fold change relative to control (log2)
SENSE COUNTS:                 1       0       1       9       0       9.62e-05       2.34e-04               0.000   0.000   0.000   3.170   0.000
  GENES:
  AT5G39850.1
    SENSE COUNTS:             1       0       1       9       0       9.62e-05       2.39e-04               0.000   0.000   0.000   3.170   0.000
    TAGS:
      d+1      TCACTTTCGA     1       0       1       9       0       9.62e-05       4.01e-04       827     0.000   0.000   0.000   3.170   0.000

LOCUS: AT5G44650
DESCRIPTION:  expressed protein
DATA:                         Control 30min   2hours  2days   1week   p-value        B&H            Pos     Fold change relative to control (log2)
SENSE COUNTS:                 12      0       6       19      21      9.74e-05       2.36e-04               0.000   0.000   -1.000  0.663   0.807
  GENES:
  AT5G44650.1
    SENSE COUNTS:             12      0       6       19      21      9.74e-05       2.41e-04               0.000   0.000   -1.000  0.663   0.807
    TAGS:
      d+1      AAATGAGTTT     12      0       6       19      21      9.74e-05       4.05e-04       1165    0.000   0.000   -1.000  0.663   0.807

LOCUS: AT2G34860
DESCRIPTION:  chaperone protein dnaJ-related, contains Pfam PF00684
DATA:                         Control 30min   2hours  2days   1week   p-value        B&H            Pos     Fold change relative to control (log2)
SENSE COUNTS:                 5       1       3       1       17      1.02e-04       2.46e-04               0.000   -2.322  -0.737  -2.322  1.766
  GENES:
  AT2G34860.1
    SENSE COUNTS:             5       1       3       1       17      1.02e-04       2.51e-04               0.000   -2.322  -0.737  -2.322  1.766
    TAGS:
      d+1      TTTTTATAAT     5       1       3       0       17      2.63e-05       1.28e-04       698     0.000   -2.322  -0.737  0.000   1.766
      d+2      TGTGTGCGCA     0       0       0       1       0       3.09e-01       4.02e-01       225     0.000   0.000   0.000   0.000   0.000

LOCUS: AT1G11860
DESCRIPTION:  aminomethyltransferase, putative, similar to aminomethyltransferase, mitochondrial precursor SP
DATA:                         Control 30min   2hours  2days   1week   p-value        B&H            Pos     Fold change relative to control (log2)
SENSE COUNTS:                 31      9       8       5       19      1.02e-04       2.46e-04               0.000   -1.784  -1.954  -2.632  -0.706
  GENES:
  AT1G11860.2
    SENSE COUNTS:             31      9       8       5       19      1.02e-04       2.52e-04               0.000   -1.784  -1.954  -2.632  -0.706
    TAGS:
      d+1      GTTTTAATGT     0       0       0       0       0       6.15e-01       6.32e-01       1557    0.000   0.000   0.000   0.000   0.000
```

```
      d+2      TAATAGTATC     9       0       1       0       7       4.12e-04       1.40e-03       1537    0.000   0.000   -3.170  0.000   -0.363
      d+2      ACTTTGTCTC     22      9       5       5       12      6.76e-03       1.33e-02       1409    0.000   -1.290  -2.138  -2.138  -0.874
      d+2      GGATATGTGA     0       0       2       0       0       3.51e-01       4.53e-01       1260    0.000   0.000   1.000   0.000   0.000
  AT1G11860.1
    SENSE COUNTS:             31      9       8       5       19      1.02e-04       2.51e-04               0.000   -1.784  -1.954  -2.632  -0.706
    TAGS:
      d+1      GTTTTAATGT     0       0       0       0       0       6.15e-01       6.32e-01       1556    0.000   0.000   0.000   0.000   0.000
      d+2      TAATAGTATC     9       0       1       0       7       4.12e-04       1.40e-03       1536    0.000   0.000   -3.170  0.000   -0.363
      d+2      ACTTTGTCTC     22      9       5       5       12      6.76e-03       1.33e-02       1408    0.000   -1.290  -2.138  -2.138  -0.874
      d+2      GGATATGTGA     0       0       2       0       0       3.51e-01       4.53e-01       1259    0.000   0.000   1.000   0.000   0.000

LOCUS: AT1G73920
DESCRIPTION:  lipase family protein, similar to lipase GB
DATA:                         Control 30min   2hours  2days   1week   p-value        B&H            Pos     Fold change relative to control (log2)
SENSE COUNTS:                 10      1       0       0       0       1.04e-04       2.50e-04               0.000   -3.322  0.000   0.000   0.000
  GENES:
  AT1G73920.2
    SENSE COUNTS:             10      1       0       0       0       1.04e-04       2.55e-04               0.000   -3.322  0.000   0.000   0.000
    TAGS:
      d+1      TGTGTGTTCT     9       1       0       0       0       1.63e-04       6.28e-04       2523    0.000   -3.170  0.000   0.000   0.000
      d+2      TGAACAAGAA     1       0       0       0       0       6.89e-01       7.02e-01       1465    0.000   0.000   0.000   0.000   0.000
  AT1G73920.1
    SENSE COUNTS:             10      1       0       0       0       1.04e-04       2.55e-04               0.000   -3.322  0.000   0.000   0.000
    TAGS:
      d+1      TGTGTGTTCT     9       1       0       0       0       1.63e-04       6.28e-04       2518    0.000   -3.170  0.000   0.000   0.000
      d+2      TGAACAAGAA     1       0       0       0       0       6.89e-01       7.02e-01       1460    0.000   0.000   0.000   0.000   0.000

LOCUS: AT3G12120
DESCRIPTION:  omega-6 fatty acid desaturase, endoplasmic reticulum (FAD2) / delta-12 desaturase, identical to omega-6 fatty acid desaturase,
endoplasmic reticulum (FAD2) SP
DATA:                         Control 30min   2hours  2days   1week   p-value        B&H            Pos     Fold change relative to control (log2)
SENSE COUNTS:                 8       22      12      1       5       1.05e-04       2.52e-04               0.000   1.459   0.585   -3.000  -0.678
  GENES:
  AT3G12120.1
    SENSE COUNTS:             8       22      12      1       5       1.05e-04       2.57e-04               0.000   1.459   0.585   -3.000  -0.678
    TAGS:
      d+1      ATGTTAACCG     8       22      12      1       5       2.13e-04       8.01e-04       697     0.000   1.459   0.585   -3.000  -0.678
      d+2      CTTCTACTAC     0       0       0       0       0       6.15e-01       6.41e-01       351     0.000   0.000   0.000   0.000   0.000

LOCUS: AT1G10960
DESCRIPTION:  ferredoxin, chloroplast, putative, strong similarity to FERREDOXIN PRECURSOR GB
DATA:                         Control 30min   2hours  2days   1week   p-value        B&H            Pos     Fold change relative to control (log2)
SENSE COUNTS:                 15      14      27      31      0       1.06e-04       2.53e-04               0.000   -0.100  0.848   1.047   0.000
  GENES:
  AT1G10960.1
    SENSE COUNTS:             15      14      27      31      0       1.06e-04       2.58e-04               0.000   -0.100  0.848   1.047   0.000
    TAGS:
      d+1      CCGTGCCGGT     15      14      27      30      0       1.76e-04       6.74e-04       332     0.000   -0.100  0.848   1.000   0.000
      d+2      GCTACCTACA     0       0       0       1       0       3.09e-01       4.13e-01       216     0.000   0.000   0.000   0.000   0.000

LOCUS: AT2G27710
DESCRIPTION:  60S acidic ribosomal protein P2 (RPP2B)
DATA:                         Control 30min   2hours  2days   1week   p-value        B&H            Pos     Fold change relative to control (log2)
SENSE COUNTS:                 18      16      34      45      50      1.08e-04       2.57e-04               0.000   -0.170  0.918   1.322   1.474
  GENES:
  AT2G27710.3
    SENSE COUNTS:             18      16      34      45      50      1.08e-04       2.63e-04               0.000   -0.170  0.918   1.322   1.474
    TAGS:
      d+1      AAAAGTCCGG     18      16      34      45      50      6.63e-05       2.86e-04       502     0.000   -0.170  0.918   1.322   1.474
      d+2      GGTTTCAGTC     0       0       0       0       0       6.15e-01       6.41e-01       466     0.000   0.000   0.000   0.000   0.000
```

AT2G27710.2														
SENSE COUNTS:		18	16	34	45	50	1.08e-04	2.62e-04		0.000	-0.170	0.918	1.322	1.474
TAGS:														
d+1	AAAAGTCCGG	18	16	34	45	50	6.63e-05	2.86e-04	432	0.000	-0.170	0.918	1.322	1.474
d+2	GGTTTCAGTC	0	0	0	0	0	6.15e-01	6.41e-01	396	0.000	0.000	0.000	0.000	0.000
AT2G27710.1														
SENSE COUNTS:		18	16	34	45	50	1.08e-04	2.61e-04		0.000	-0.170	0.918	1.322	1.474
TAGS:														
d+1	AAAAGTCCGG	18	16	34	45	50	6.63e-05	2.86e-04	436	0.000	-0.170	0.918	1.322	1.474
d+2	GGTTTCAGTC	0	0	0	0	0	6.15e-01	6.41e-01	400	0.000	0.000	0.000	0.000	0.000

LOCUS: AT1G17010
DESCRIPTION: oxidoreductase, 2OG-Fe(II) oxygenase family protein, similar to flavonol synthase (Petunia x hybrida)(GI

DATA:		Control	30min	2hours	2days	1week	p-value	B&H	Pos	Fold change relative to control (log2)				
SENSE COUNTS:		21	0	11	21	15	1.09e-04	2.59e-04		0.000	0.000	-0.933	0.000	-0.485
GENES:														
AT1G17010.1														
SENSE COUNTS:		21	0	11	21	15	1.09e-04	2.63e-04		0.000	0.000	-0.933	0.000	-0.485
TAGS:														
i+3	CATAAAAAAA	1	0	0	1	1	5.50e-01	6.00e-01	836	0.000	0.000	0.000	0.000	0.000
d+1	GAAGAAAAAA	20	0	11	20	14	2.54e-04	9.36e-04	432	0.000	0.000	-0.862	0.000	-0.515

LOCUS: AT5G42050
DESCRIPTION: expressed protein, similar to gda-1 (Pisum sativum) GI

DATA:		Control	30min	2hours	2days	1week	p-value	B&H	Pos	Fold change relative to control (log2)				
SENSE COUNTS:		0	14	5	2	1	1.11e-04	2.63e-04		0.000	3.807	2.322	1.000	0.000
GENES:														
AT5G42050.1														
SENSE COUNTS:		0	14	5	2	1	1.11e-04	2.67e-04		0.000	3.807	2.322	1.000	0.000
TAGS:														
d+1	GAATCTACGA	0	12	4	2	1	1.10e-03	3.09e-03	944	0.000	3.585	2.000	1.000	0.000
d+2	GGTTTAGGAT	0	2	1	0	0	2.47e-01	3.38e-01	403	0.000	1.000	0.000	0.000	0.000

LOCUS: AT1G08640
DESCRIPTION: expressed protein

DATA:		Control	30min	2hours	2days	1week	p-value	B&H	Pos	Fold change relative to control (log2)				
SENSE COUNTS:		4	0	15	16	2	1.11e-04	2.63e-04		0.000	0.000	1.907	2.000	-1.000
GENES:														
AT1G08640.1														
SENSE COUNTS:		4	0	15	16	2	1.11e-04	2.67e-04		0.000	0.000	1.907	2.000	-1.000
TAGS:														
i+3	AGTTGGATAT	0	0	0	1	0	3.09e-01	4.11e-01	2352	0.000	0.000	0.000	0.000	0.000
d+1	TGGAAGGTTT	4	0	15	15	1	8.39e-05	3.54e-04	1173	0.000	0.000	1.907	1.907	-2.000
d+2	CTCTATGTTA	0	0	0	0	1	1.65e-01	2.46e-01	925	0.000	0.000	0.000	0.000	0.000

LOCUS: AT2G32060
DESCRIPTION: 40S ribosomal protein S12 (RPS12C)

DATA:		Control	30min	2hours	2days	1week	p-value	B&H	Pos	Fold change relative to control (log2)				
SENSE COUNTS:		9	10	14	26	37	1.13e-04	2.67e-04		0.000	0.152	0.637	1.531	2.040
GENES:														
AT2G32060.3														
SENSE COUNTS:		9	10	14	26	37	1.13e-04	2.70e-04		0.000	0.152	0.637	1.531	2.040
TAGS:														
d+1	AAAGTTTTGA	9	10	14	26	37	1.13e-04	4.64e-04	657	0.000	0.152	0.637	1.531	2.040
AT2G32060.2														
SENSE COUNTS:		9	10	14	26	37	1.13e-04	2.70e-04		0.000	0.152	0.637	1.531	2.040
TAGS:														
d+1	AAAGTTTTGA	9	10	14	26	37	1.13e-04	4.64e-04	659	0.000	0.152	0.637	1.531	2.040
AT2G32060.1														
SENSE COUNTS:		9	10	14	26	37	1.13e-04	2.71e-04		0.000	0.152	0.637	1.531	2.040

TAGS:
d+1 AAAGTTTTGA 9 10 14 26 37 1.13e-04 4.64e-04 695 0.000 0.152 0.637 1.531 2.040

LOCUS: AT3G55610
DESCRIPTION: delta 1-pyrroline-5-carboxylate synthetase B / P5CS B (P5CS2), identical to SP|P54888
DATA: Control 30min 2hours 2days 1week p-value B&H Pos Fold change relative to control (log2)
SENSE COUNTS: 7 4 15 30 18 1.14e-04 2.68e-04 0.000 -0.807 1.100 2.100 1.363
GENES:
AT3G55610.1
SENSE COUNTS: 7 4 15 30 18 1.14e-04 2.72e-04 0.000 -0.807 1.100 2.100 1.363
TAGS:
d+1 CAGAATAAGT 6 2 15 22 16 8.29e-04 2.46e-03 2520 0.000 -1.585 1.322 1.874 1.415
d+2 CCCGTGGTCC 1 2 0 8 1 1.67e-02 2.93e-02 2134 0.000 1.000 0.000 3.000 0.000
d+2 GAAGTGCACA 0 0 0 0 1 1.65e-01 2.47e-01 1966 0.000 0.000 0.000 0.000 0.000

LOCUS: AT1G12660
DESCRIPTION: hypothetical protein
DATA: Control 30min 2hours 2days 1week p-value B&H Pos Fold change relative to control (log2)
SENSE COUNTS: 33 15 45 47 17 1.15e-04 2.70e-04 0.000 -1.138 0.447 0.510 -0.957
GENES:
AT1G12660.1
SENSE COUNTS: 33 15 45 47 17 1.15e-04 2.73e-04 0.000 -1.138 0.447 0.510 -0.957
TAGS:
i+3 ATAACCCAAG 1 0 0 1 1 5.50e-01 5.99e-01 1926 0.000 0.000 0.000 0.000 0.000
i+3 AGAGACTCTA 1 0 0 2 0 4.17e-01 5.25e-01 1284 0.000 0.000 0.000 1.000 0.000
v+1 TGTGAAAAAA 31 15 45 44 16 1.13e-04 4.63e-04 759 0.000 -1.047 0.538 0.505 -0.954

LOCUS: AT2G02390
DESCRIPTION: glutathione S-transferase zeta 1 (GSTZ1) (GST18), identical to SP|Q9ZVQ3|GTZ1_ARATH Glutathione S-transferase zeta-class 1 (EC 2.5.1.18) (AtGSTZ1) (Maleylacetone isomerase) (EC 5.2.1.-) (MAI) {Arabidopsis thaliana}; contains Pfam profiles PF02798
DATA: Control 30min 2hours 2days 1week p-value B&H Pos Fold change relative to control (log2)
SENSE COUNTS: 4 2 0 2 14 1.16e-04 2.72e-04 0.000 -1.000 0.000 -1.000 1.807
GENES:
AT2G02390.3
SENSE COUNTS: 4 2 0 2 13 7.48e-04 1.35e-03 0.000 -1.000 0.000 -1.000 1.700
TAGS:
d+1 TATCATATAA 4 2 0 2 10 1.80e-02 3.15e-02 858 0.000 -1.000 0.000 -1.000 1.322
d+2 GAACCGTACC 0 0 0 0 3 1.12e-02 2.03e-02 657 0.000 0.000 0.000 0.000 1.585
AT2G02390.1
SENSE COUNTS: 4 2 0 2 13 7.48e-04 1.35e-03 0.000 -1.000 0.000 -1.000 1.700
TAGS:
d+1 TATCATATAA 4 2 0 2 10 1.80e-02 3.15e-02 813 0.000 -1.000 0.000 -1.000 1.322
d+2 GAACCGTACC 0 0 0 0 3 1.12e-02 2.03e-02 612 0.000 0.000 0.000 0.000 1.585
AT2G02390.2
SENSE COUNTS: 0 0 0 0 1 1.65e-01 1.67e-01 0.000 0.000 0.000 0.000 0.000
TAGS:
d+1 GTACTTTTCC 0 0 0 0 1 1.65e-01 2.44e-01 636 0.000 0.000 0.000 0.000 0.000

LOCUS: AT2G40510
DESCRIPTION: 40S ribosomal protein S26 (RPS26A)
DATA: Control 30min 2hours 2days 1week p-value B&H Pos Fold change relative to control (log2)
SENSE COUNTS: 23 9 21 16 44 1.17e-04 2.73e-04 0.000 -1.354 -0.131 -0.524 0.936
GENES:
AT2G40510.1
SENSE COUNTS: 23 9 21 16 44 1.17e-04 2.78e-04 0.000 -1.354 -0.131 -0.524 0.936
TAGS:
d+1 TGGATCTTGA 23 9 20 16 41 6.34e-04 1.96e-03 564 0.000 -1.354 -0.202 -0.524 0.834
d+2 TCGTTAGAGT 0 0 1 0 3 7.32e-02 1.19e-01 319 0.000 0.000 0.000 0.000 1.585

LOCUS: AT5G49330

```
DESCRIPTION:  myb family transcription factor, contains Pfam profile
DATA:                        Control 30min  2hours  2days  1week  p-value        B&H            Pos     Fold change relative to control (log2)
SENSE COUNTS:                0       0      0       2      8      1.20e-04       2.79e-04               0.000  0.000  0.000  1.000  3.000
  GENES:
  AT5G49330.1
    SENSE COUNTS:            0       0      0       2      8      1.20e-04       2.84e-04               0.000  0.000  0.000  1.000  3.000
    TAGS:
      X+4      TTATGCCTTT    0       0      0       2      8      1.20e-04       4.89e-04       955     0.000  0.000  0.000  1.000  3.000

LOCUS: AT5G44565
DESCRIPTION:  hypothetical protein
DATA:                        Control 30min  2hours  2days  1week  p-value        B&H            Pos     Fold change relative to control (log2)
SENSE COUNTS:                0       0      0       2      8      1.20e-04       2.79e-04               0.000  0.000  0.000  1.000  3.000
  GENES:
  AT5G44565.1
    SENSE COUNTS:            0       0      0       2      8      1.20e-04       2.83e-04               0.000  0.000  0.000  1.000  3.000
    TAGS:
      d+1      TAAGGATTTT    0       0      0       2      8      1.20e-04       4.91e-04       509     0.000  0.000  0.000  1.000  3.000

LOCUS: AT1G13245
DESCRIPTION:  Expressed protein
DATA:                        Control 30min  2hours  2days  1week  p-value        B&H            Pos     Fold change relative to control (log2)
SENSE COUNTS:                0       9      0       2      0      1.31e-04       3.04e-04               0.000  3.170  0.000  1.000  0.000
  GENES:
  AT1G13245.1
    SENSE COUNTS:            0       9      0       2      0      1.31e-04       3.09e-04               0.000  3.170  0.000  1.000  0.000
    TAGS:
      d+1      CTTCTTTGTT    0       9      0       2      0      1.31e-04       5.33e-04       193     0.000  3.170  0.000  1.000  0.000

LOCUS: AT5G18580
DESCRIPTION:  tonneau 2 (TON2), identical to tonneau 2 protein (TON2) GI
DATA:                        Control 30min  2hours  2days  1week  p-value        B&H            Pos     Fold change relative to control (log2)
SENSE COUNTS:                10      0      1       0      7      1.32e-04       3.05e-04               0.000  0.000  -3.322 0.000  -0.515
  GENES:
  AT5G18580.1
    SENSE COUNTS:            10      0      1       0      7      1.32e-04       3.10e-04               0.000  0.000  -3.322 0.000  -0.515
    TAGS:
      d+2      TGTGTGTATC    10      0      1       0      7      1.32e-04       5.34e-04       1687    0.000  0.000  -3.322 0.000  -0.515

LOCUS: AT2G23370
DESCRIPTION:  expressed protein
DATA:                        Control 30min  2hours  2days  1week  p-value        B&H            Pos     Fold change relative to control (log2)
SENSE COUNTS:                0       8      0       1      0      1.34e-04       3.09e-04               0.000  3.000  0.000  0.000  0.000
  GENES:
  AT2G23370.1
    SENSE COUNTS:            0       8      0       1      0      1.34e-04       3.14e-04               0.000  3.000  0.000  0.000  0.000
    TAGS:
      i+3      TTTCTCTTCT    0       8      0       1      0      1.34e-04       5.39e-04       732     0.000  3.000  0.000  0.000  0.000

LOCUS: AT5G17050
DESCRIPTION:  UDP-glucoronosyl/UDP-glucosyl transferase family protein, similar to UDP glucose
DATA:                        Control 30min  2hours  2days  1week  p-value        B&H            Pos     Fold change relative to control (log2)
SENSE COUNTS:                0       0      0       8      7      1.35e-04       3.10e-04               0.000  0.000  0.000  3.000  2.807
  GENES:
  AT5G17050.1
    SENSE COUNTS:            0       0      0       8      7      1.35e-04       3.16e-04               0.000  0.000  0.000  3.000  2.807
    TAGS:
      d+1      GGCACCGCAA    0       0      0       8      7      1.35e-04       5.40e-04       1114    0.000  0.000  0.000  3.000  2.807

LOCUS: AT2G38210
```

```
DESCRIPTION:  ethylene-responsive protein, putative, very strong similarity to ethylene-inducible protein HEVER SP
DATA:                         Control 30min  2hours  2days  1week  p-value   B&H       Pos   Fold change relative to control (log2)
SENSE COUNTS:                 3       0      1       5      14     1.37e-04  3.14e-04        0.000  0.000  -1.585  0.737  2.222
  GENES:
  AT2G38210.1
    SENSE COUNTS:             3       0      1       5      14     1.37e-04  3.20e-04        0.000  0.000  -1.585  0.737  2.222
    TAGS:
      d+1      GTCATACTTA     3       0      1       5      14     1.37e-04  5.47e-04  435   0.000  0.000  -1.585  0.737  2.222

LOCUS: AT5G53320
DESCRIPTION:  leucine-rich repeat transmembrane protein kinase, putative,
DATA:                         Control 30min  2hours  2days  1week  p-value   B&H       Pos   Fold change relative to control (log2)
SENSE COUNTS:                 53      20     46      51     23     1.39e-04  3.18e-04        0.000  -1.406  -0.204  -0.055  -1.204
  GENES:
  AT5G53320.1
    SENSE COUNTS:             53      20     46      51     23     1.39e-04  3.24e-04        0.000  -1.406  -0.204  -0.055  -1.204
    TAGS:
      d+1      ATTTCAGCTA     53      20     46      51     23     1.39e-04  5.53e-04  2120  0.000  -1.406  -0.204  -0.055  -1.204

LOCUS: AT5G60390
DESCRIPTION:  elongation factor 1-alpha / EF-1-alpha, identical to SWISS-PROT
DATA:                         Control 30min  2hours  2days  1week  p-value   B&H       Pos   Fold change relative to control (log2)
SENSE COUNTS:                 33      36     43      67     76     1.40e-04  3.20e-04        0.000  0.126  0.382  1.022  1.204
  GENES:
  AT5G60390.1
    SENSE COUNTS:             33      36     43      67     76     1.40e-04  3.26e-04        0.000  0.126  0.382  1.022  1.204
    TAGS:
      d+1      AGGCAGACCG     32      36     41      67     71     3.00e-04  1.07e-03  1318  0.000  0.170  0.358  1.066  1.150
      d+2      GTTGTTGAGA     1       0      2       0      5      5.71e-02  9.34e-02  1261  0.000  0.000  1.000  0.000  2.322

LOCUS: AT4G39730
DESCRIPTION:  lipid-associated family protein, contains PLAT/LH2 (Polycystin-1, Lipoxygenase, Alpha-Toxin/Lipoxygenase homology) domain Pfam
DATA:                         Control 30min  2hours  2days  1week  p-value   B&H       Pos   Fold change relative to control (log2)
SENSE COUNTS:                 2       7      3       16     21     1.42e-04  3.23e-04        0.000  1.807  0.585  3.000  3.392
  GENES:
  AT4G39730.1
    SENSE COUNTS:             2       7      3       16     21     1.42e-04  3.30e-04        0.000  1.807  0.585  3.000  3.392
    TAGS:
      d+1      GTTGGTACGT     2       7      3       16     21     1.42e-04  5.60e-04  392   0.000  1.807  0.585  3.000  3.392

LOCUS: AT1G79140
DESCRIPTION:  expressed protein
DATA:                         Control 30min  2hours  2days  1week  p-value   B&H       Pos   Fold change relative to control (log2)
SENSE COUNTS:                 1       0      1       1      10     1.44e-04  3.27e-04        0.000  0.000  0.000  0.000  3.322
  GENES:
  AT1G79140.1
    SENSE COUNTS:             1       0      1       1      10     1.44e-04  3.34e-04        0.000  0.000  0.000  0.000  3.322
    TAGS:
      d+1      GTTAAGCTCA     0       0      0       1      3      6.27e-02  1.02e-01  766   0.000  0.000  0.000  0.000  1.585
      d+2      AACACTTCTC     1       0      1       0      7      3.62e-03  8.42e-03  592   0.000  0.000  0.000  0.000  2.807

LOCUS: AT1G35620
DESCRIPTION:  thioredoxin family protein, similar to SP|Q43116 Protein disulfide isomerase precursor (PDI) (EC 5.3.4.1) {Ricinus communis}; contains
Pfam profile PF00085
DATA:                         Control 30min  2hours  2days  1week  p-value   B&H       Pos   Fold change relative to control (log2)
SENSE COUNTS:                 2       0      0       0      8      1.55e-04  3.51e-04        0.000  0.000  0.000  0.000  2.000
  GENES:
  AT1G35620.1
    SENSE COUNTS:             2       0      0       0      8      1.55e-04  3.57e-04        0.000  0.000  0.000  0.000  2.000
    TAGS:
```

```
      d+1      CTTATTTATC     2      0      0      0      8      1.55e-04       6.10e-04       1447   0.000  0.000  0.000  0.000  2.000

LOCUS: AT1G54610
DESCRIPTION:  protein kinase family protein, contains Pfam domain, PF00069
DATA:                         Control 30min  2hours 2days  1week  p-value        B&H            Pos    Fold change relative to control (log2)
SENSE COUNTS:                 1      0      2      2      11     1.57e-04       3.55e-04              0.000  0.000  1.000  1.000  3.459
  GENES:
  AT1G54610.1
    SENSE COUNTS:             1      0      2      2      11     1.57e-04       3.61e-04              0.000  0.000  1.000  1.000  3.459
    TAGS:
      d+1      TATTTGTAAC     1      0      2      2      10     1.35e-03       3.68e-03       1989   0.000  0.000  1.000  1.000  3.322
      d+2      ATTTGGCTGG     0      0      0      0      1      1.65e-01       2.45e-01       757    0.000  0.000  0.000  0.000  0.000

LOCUS: AT1G16830
DESCRIPTION:  pentatricopeptide (PPR) repeat-containing protein, contains Pfam profile PF01535
DATA:                         Control 30min  2hours 2days  1week  p-value        B&H            Pos    Fold change relative to control (log2)
SENSE COUNTS:                 17     24     10     2      3      1.60e-04       3.61e-04              0.000  0.497  -0.766 -3.087 -2.503
  GENES:
  AT1G16830.1
    SENSE COUNTS:             17     24     10     2      3      1.60e-04       3.67e-04              0.000  0.497  -0.766 -3.087 -2.503
    TAGS:
      v+2      CGTTGCACTA     17     24     10     2      3      1.60e-04       6.21e-04       24     0.000  0.497  -0.766 -3.087 -2.503

LOCUS: AT2G03320
DESCRIPTION:  hypothetical protein
DATA:                         Control 30min  2hours 2days  1week  p-value        B&H            Pos    Fold change relative to control (log2)
SENSE COUNTS:                 4      0      5      12     17     1.61e-04       3.62e-04              0.000  0.000  0.322  1.585  2.087
  GENES:
  AT2G03320.1
    SENSE COUNTS:             4      0      5      12     17     1.61e-04       3.67e-04              0.000  0.000  0.322  1.585  2.087
    TAGS:
      i+3      AATGGTTAAC     4      0      5      12     17     1.61e-04       6.23e-04       208    0.000  0.000  0.322  1.585  2.087

LOCUS: AT1G64350
DESCRIPTION:  transducin family protein / WD-40 repeat family protein, contains 5 WD-40 repeats (PF00400); similar to nuclear pore protein SEH1 (SP
DATA:                         Control 30min  2hours 2days  1week  p-value        B&H            Pos    Fold change relative to control (log2)
SENSE COUNTS:                 1      0      1      0      9      1.61e-04       3.61e-04              0.000  0.000  0.000  0.000  3.170
  GENES:
  AT1G64350.1
    SENSE COUNTS:             1      0      1      0      9      1.61e-04       3.68e-04              0.000  0.000  0.000  0.000  3.170
    TAGS:
      d+1      TAGTCTTTGT     1      0      1      0      8      1.85e-03       4.83e-03       1225   0.000  0.000  0.000  0.000  3.000
      d+2      TTCGTTCCAT     0      0      0      0      1      1.65e-01       2.49e-01       1083   0.000  0.000  0.000  0.000  0.000

LOCUS: AT4G03280
DESCRIPTION:  cytochrome B6-F complex iron-sulfur subunit, chloroplast / Rieske iron-sulfur protein / plastoquinol-plastocyanin reductase (petC),
identical to gi
DATA:                         Control 30min  2hours 2days  1week  p-value        B&H            Pos    Fold change relative to control (log2)
SENSE COUNTS:                 80     59     55     45     21     1.62e-04       3.63e-04              0.000  -0.439 -0.541 -0.830 -1.930
  GENES:
  AT4G03280.2
    SENSE COUNTS:             80     59     54     45     21     1.54e-04       3.56e-04              0.000  -0.439 -0.567 -0.830 -1.930
    TAGS:
      d+1      GTGGTCTTAA     80     59     52     45     21     1.34e-04       5.40e-04       909    0.000  -0.439 -0.621 -0.830 -1.930
      d+2      GTCCCGGTGA     0      0      2      0      0      1.21e-01       1.88e-01       596    0.000  0.000  1.000  0.000  0.000
  AT4G03280.1
    SENSE COUNTS:             80     59     55     45     21     1.62e-04       3.69e-04              0.000  -0.439 -0.541 -0.830 -1.930
    TAGS:
      d+1      GTGGTCTTAA     80     59     52     45     21     1.34e-04       5.40e-04       810    0.000  -0.439 -0.621 -0.830 -1.930
      d+2      GTCCCGGTGA     0      0      2      0      0      1.21e-01       1.88e-01       497    0.000  0.000  1.000  0.000  0.000
```

```
      X+4      TAAGATCAGG     0       0       1       0       0       4.55e-01       5.43e-01       327     0.000   0.000   0.000   0.000   0.000

LOCUS: AT5G17290
DESCRIPTION:  autophagy protein Apg5 family, contains Pfam profile
DATA:                         Control 30min   2hours  2days   1week   p-value        B&H            Pos     Fold change relative to control (log2)
SENSE COUNTS:                 24      4       13      5       23      1.66e-04       3.71e-04               0.000   -2.585  -0.885  -2.263  -0.061
  GENES:
  AT5G17290.1
    SENSE COUNTS:             24      4       13      5       23      1.66e-04       3.77e-04               0.000   -2.585  -0.885  -2.263  -0.061
    TAGS:
      i+3      ATACAAAGTA     24      4       12      5       23      1.35e-04       5.41e-04       2134    0.000   -2.585  -1.000  -2.263  -0.061
      d+1      AACGGTGATC     0       0       1       0       0       4.55e-01       5.36e-01       527     0.000   0.000   0.000   0.000   0.000

LOCUS: AT5G11090
DESCRIPTION:  serine-rich protein-related, contains some similarity to serine-rich proteins
DATA:                         Control 30min   2hours  2days   1week   p-value        B&H            Pos     Fold change relative to control (log2)
SENSE COUNTS:                 1       10      3       0       0       1.81e-04       4.03e-04               0.000   3.322   1.585   0.000   0.000
  GENES:
  AT5G11090.1
    SENSE COUNTS:             1       10      3       0       0       1.81e-04       4.10e-04               0.000   3.322   1.585   0.000   0.000
    TAGS:
      d+1      GCGAAAGCTG     1       10      3       0       0       5.14e-04       1.68e-03       750     0.000   3.322   1.585   0.000   0.000
      X+4      GTTAAATTTG     0       0       0       0       0       6.15e-01       6.45e-01       -133    0.000   0.000   0.000   0.000   0.000

LOCUS: AT1G58360
DESCRIPTION:  neutral amino acid transporter expressed in seeds
DATA:                         Control 30min   2hours  2days   1week   p-value        B&H            Pos     Fold change relative to control (log2)
SENSE COUNTS:                 8       0       6       1       18      1.82e-04       4.04e-04               0.000   0.000   -0.415  -3.000  1.170
  GENES:
  AT1G58360.1
    SENSE COUNTS:             8       0       6       1       18      1.82e-04       4.12e-04               0.000   0.000   -0.415  -3.000  1.170
    TAGS:
      d+1      AATCTCTCTG     5       0       4       1       17      6.37e-05       2.76e-04       1653    0.000   0.000   -0.322  -2.322  1.766
      d+2      AGTGAGTTTG     2       0       2       0       1       7.01e-01       7.12e-01       1541    0.000   0.000   0.000   0.000   -1.000
      d+2      TCCTTTACTT     1       0       0       0       0       4.28e-01       5.31e-01       694     0.000   0.000   0.000   0.000   0.000

LOCUS: AT1G48240
DESCRIPTION:  novel plant SNARE 12 (NPSN12), identical to  Novel plant SNARE 12 (AtNPSN12) (Swiss-Prot
DATA:                         Control 30min   2hours  2days   1week   p-value        B&H            Pos     Fold change relative to control (log2)
SENSE COUNTS:                 0       8       0       1       1       1.86e-04       4.12e-04               0.000   3.000   0.000   0.000   0.000
  GENES:
  AT1G48240.1
    SENSE COUNTS:             0       8       0       1       1       1.86e-04       4.19e-04               0.000   3.000   0.000   0.000   0.000
    TAGS:
      d+1      TTGTATAATT     0       8       0       1       1       5.78e-04       1.85e-03       1199    0.000   3.000   0.000   0.000   0.000
      i+3      AGAACTAAAA     0       0       0       0       0       6.15e-01       6.39e-01       689     0.000   0.000   0.000   0.000   0.000

LOCUS: AT2G04030
DESCRIPTION:  heat shock protein, putative, strong similarity to heat shock protein (Arabidopsis thaliana) GI
DATA:                         Control 30min   2hours  2days   1week   p-value        B&H            Pos     Fold change relative to control (log2)
SENSE COUNTS:                 9       7       15      34      20      1.86e-04       4.11e-04               0.000   -0.363  0.737   1.918   1.152
  GENES:
  AT2G04030.1
    SENSE COUNTS:             9       7       15      34      20      1.86e-04       4.18e-04               0.000   -0.363  0.737   1.918   1.152
    TAGS:
      d+1      GGCCTAATGA     9       5       14      33      19      2.77e-05       1.34e-04       2502    0.000   -0.848  0.637   1.874   1.078
      d+2      GGAGAGAACC     0       1       1       0       1       6.08e-01       6.51e-01       1665    0.000   0.000   0.000   0.000   0.000
      d+2      GCAGGAGAAA     0       1       0       1       0       4.31e-01       5.21e-01       950     0.000   0.000   0.000   0.000   0.000
  AT2G04030.2
    SENSE COUNTS:             9       7       15      34      20      1.86e-04       4.20e-04               0.000   -0.363  0.737   1.918   1.152
```

```
    TAGS:
      d+1      GGCCTAATGA     9       5       14      33      19      2.77e-05       1.34e-04       2493    0.000   -0.848  0.637   1.874   1.078
      d+2      GGAGAGAACC     0       1       1       0       1       6.08e-01       6.51e-01       1656    0.000   0.000   0.000   0.000   0.000
      d+2      GCAGGAGAAA     0       1       0       1       0       4.31e-01       5.21e-01       950     0.000   0.000   0.000   0.000   0.000

LOCUS: AT5G35970
DESCRIPTION:  DNA-binding protein, putative, similar to SWISS-PROT
DATA:                         Control 30min   2hours  2days   1week   p-value        B&H            Pos     Fold change relative to control (log2)
SENSE COUNTS:                 11      0       14      18      24      1.99e-04       4.39e-04               0.000   0.000   0.348   0.710   1.126
  GENES:
  AT5G35970.1
    SENSE COUNTS:             11      0       14      18      24      1.99e-04       4.46e-04               0.000   0.000   0.348   0.710   1.126
    TAGS:
      d+2      CAGACCCTGG     0       0       0       0       1       1.65e-01       2.34e-01       3084    0.000   0.000   0.000   0.000   0.000
      d+2      GCTGGAGCAA     0       0       1       0       0       4.55e-01       5.13e-01       1654    0.000   0.000   0.000   0.000   0.000
      X+4      TTACCACAGA     11      0       13      18      23      3.99e-04       1.37e-03       430     0.000   0.000   0.241   0.710   1.064

LOCUS: AT5G49910
DESCRIPTION:  heat shock protein 70 / HSP70 (HSC70-7), identical to heat shock protein 70 (Arabidopsis thaliana) GI
DATA:                         Control 30min   2hours  2days   1week   p-value        B&H            Pos     Fold change relative to control (log2)
SENSE COUNTS:                 3       0       3       2       14      2.00e-04       4.40e-04               0.000   0.000   0.000   -0.585  2.222
  GENES:
  AT5G49910.1
    SENSE COUNTS:             3       0       3       2       14      2.00e-04       4.48e-04               0.000   0.000   0.000   -0.585  2.222
    TAGS:
      d+2      TACAATAAAC     0       0       1       0       1       3.96e-01       5.03e-01       2395    0.000   0.000   0.000   0.000   0.000
      d+2      ATTGAAGCAA     3       0       2       2       12      3.86e-03       8.87e-03       2336    0.000   0.000   -0.585  -0.585  2.000
      d+2      ACCAAGATCA     0       0       0       0       1       1.65e-01       2.38e-01       1494    0.000   0.000   0.000   0.000   0.000

LOCUS: AT2G21160
DESCRIPTION:  translocon-associated protein alpha (TRAP alpha) family protein, contains Pfam profile
DATA:                         Control 30min   2hours  2days   1week   p-value        B&H            Pos     Fold change relative to control (log2)
SENSE COUNTS:                 10      2       7       24      14      2.10e-04       4.61e-04               0.000   -2.322  -0.515  1.263   0.485
  GENES:
  AT2G21160.1
    SENSE COUNTS:             10      2       7       24      14      2.10e-04       4.69e-04               0.000   -2.322  -0.515  1.263   0.485
    TAGS:
      d+1      AAACAAGTTA     10      2       7       24      14      2.10e-04       7.91e-04       1051    0.000   -2.322  -0.515  1.263   0.485

LOCUS: AT2G40140
DESCRIPTION:  zinc finger (CCCH-type) family protein, contains Pfam domain, PF00642
DATA:                         Control 30min   2hours  2days   1week   p-value        B&H            Pos     Fold change relative to control (log2)
SENSE COUNTS:                 1       5       17      5       1       2.10e-04       4.60e-04               0.000   2.322   4.087   2.322   0.000
  GENES:
  AT2G40140.1
    SENSE COUNTS:             1       5       17      5       1       2.10e-04       4.68e-04               0.000   2.322   4.087   2.322   0.000
    TAGS:
      d+1      AGTTCAAATG     1       5       17      4       1       6.64e-05       2.86e-04       2106    0.000   2.322   4.087   2.000   0.000
      d+2      AGCAGCAGAT     0       0       0       1       0       6.04e-01       6.48e-01       1877    0.000   0.000   0.000   0.000   0.000

LOCUS: AT2G41945
DESCRIPTION:  expressed protein
DATA:                         Control 30min   2hours  2days   1week   p-value        B&H            Pos     Fold change relative to control (log2)
SENSE COUNTS:                 0       0       0       8       3       2.17e-04       4.74e-04               0.000   0.000   0.000   3.000   1.585
  GENES:
  AT2G41945.1
    SENSE COUNTS:             0       0       0       8       3       2.17e-04       4.83e-04               0.000   0.000   0.000   3.000   1.585
    TAGS:
      d+1      GTATTTGATT     0       0       0       4       3       2.21e-02       3.81e-02       722     0.000   0.000   0.000   2.000   1.585
      d+2      CACAGATAAA     0       0       0       4       0       6.18e-03       1.24e-02       533     0.000   0.000   0.000   2.000   0.000
```

```
LOCUS: AT4G37830
DESCRIPTION:  cytochrome c oxidase-related, contains weak similarity to cytochrome c oxidase polypeptide VIa-liver, mitochondrial precursor (EC 1.9.3.1) (Swiss-Prot
DATA:                        Control 30min  2hours  2days  1week  p-value    B&H        Pos   Fold change relative to control (log2)
SENSE COUNTS:                2       10     1       0      0      2.18e-04   4.75e-04         0.000  2.322  -1.000  0.000  0.000
  GENES:
  AT4G37830.1
    SENSE COUNTS:            2       10     1       0      0      2.18e-04   4.84e-04         0.000  2.322  -1.000  0.000  0.000
    TAGS:
      d+1    ACGATGCTTA      2       10     1       0      0      2.18e-04   8.18e-04   172   0.000  2.322  -1.000  0.000  0.000

LOCUS: AT1G66410
DESCRIPTION:  calmodulin-1/4 (CAM4), identical to calmodulin (Arabidopsis thaliana) GI
DATA:                        Control 30min  2hours  2days  1week  p-value    B&H        Pos   Fold change relative to control (log2)
SENSE COUNTS:                8       21     6       17     35     2.23e-04   4.85e-04         0.000  1.392  -0.415  1.087  2.129
  GENES:
  AT1G66410.1
    SENSE COUNTS:            8       21     6       17     35     2.23e-04   4.94e-04         0.000  1.392  -0.415  1.087  2.129
    TAGS:
      d+1    TTTAGATTTC      3       4      2       15     21     3.72e-05   1.71e-04   718   0.000  0.415  -0.585  2.322  2.807
      d+2    TGATGACGAA      5       17     4       2      14     2.37e-03   5.91e-03   394   0.000  1.766  -0.322  -1.322 1.485

LOCUS: AT1G09560
DESCRIPTION:  germin-like protein (GLP4) (GLP5), identical to Arabidopsis germin-like protein subfamily 2 member 1 (SP|P94014); Location of EST 180L10T7, gi|906417
DATA:                        Control 30min  2hours  2days  1week  p-value    B&H        Pos   Fold change relative to control (log2)
SENSE COUNTS:                9       4      2       4      21     2.24e-04   4.86e-04         0.000  -1.170 -2.170  -1.170 1.222
  GENES:
  AT1G09560.1
    SENSE COUNTS:            9       4      2       4      21     2.24e-04   4.95e-04         0.000  -1.170 -2.170  -1.170 1.222
    TAGS:
      d+1    TATTCGTCTT      9       4      2       4      21     2.24e-04   8.36e-04   864   0.000  -1.170 -2.170  -1.170 1.222

LOCUS: AT2G16600
DESCRIPTION:  peptidyl-prolyl cis-trans isomerase, cytosolic / cyclophilin / rotamase (ROC3), identical to cytosolic cyclophilin (Arabidopsis thaliana) GI
DATA:                        Control 30min  2hours  2days  1week  p-value    B&H        Pos   Fold change relative to control (log2)
SENSE COUNTS:                4       19     1       9      17     2.27e-04   4.91e-04         0.000  2.248  -2.000  1.170  2.087
  GENES:
  AT2G16600.1
    SENSE COUNTS:            4       19     1       9      17     2.27e-04   5.01e-04         0.000  2.248  -2.000  1.170  2.087
    TAGS:
      d+1    TTGTGTTTGG      4       19     1       9      17     2.27e-04   8.46e-04   459   0.000  2.248  -2.000  1.170  2.087

LOCUS: AT1G11260
DESCRIPTION:  glucose transporter (STP1), nearly identical to glucose transporter GB
DATA:                        Control 30min  2hours  2days  1week  p-value    B&H        Pos   Fold change relative to control (log2)
SENSE COUNTS:                31      42     32      11     10     2.31e-04   4.99e-04         0.000  0.438  0.046   -1.495 -1.632
  GENES:
  AT1G11260.1
    SENSE COUNTS:            31      42     32      11     10     2.31e-04   5.09e-04         0.000  0.438  0.046   -1.495 -1.632
    TAGS:
      d+1    AAGAAATTAT      16      30     25      9      5      9.08e-04   2.66e-03   1827  0.000  0.907  0.644   -0.830 -1.678
      d+2    TTTGATTTAT      15      12     6       2      5      5.53e-02   9.05e-02   1696  0.000  -0.322 -1.322  -2.907 -1.585
      d+2    TTTGGATGCT      0       0      1       0      0      4.55e-01   5.35e-01   529   0.000  0.000  0.000   0.000  0.000

LOCUS: AT4G10450
DESCRIPTION:  60S ribosomal protein L9 (RPL90D), ribosomal protein L9, cytosolic - garden pea, PIR2
DATA:                        Control 30min  2hours  2days  1week  p-value    B&H        Pos   Fold change relative to control (log2)
```

```
SENSE COUNTS:                1      0      3      0      10     2.32e-04       5.00e-04              0.000   0.000   1.585   0.000   3.322
  GENES:
  AT4G10450.1
    SENSE COUNTS:            1      0      3      0      10     2.32e-04       5.10e-04              0.000   0.000   1.585   0.000   3.322
    TAGS:
      d+1      TTTGGTCTTT    1      0      3      0      10     2.32e-04       8.60e-04       800    0.000   0.000   1.585   0.000   3.322

LOCUS: AT5G12040
DESCRIPTION:  carbon-nitrogen hydrolase family protein, similar to Nit protein 2 (Homo sapiens) GI
DATA:                        Control 30min  2hours 2days  1week  p-value        B&H            Pos    Fold change relative to control (log2)
SENSE COUNTS:                1      0      4      0      11     2.34e-04       5.03e-04              0.000   0.000   2.000   0.000   3.459
  GENES:
  AT5G12040.2
    SENSE COUNTS:            1      0      4      0      11     2.34e-04       5.13e-04              0.000   0.000   2.000   0.000   3.459
    TAGS:
      d+1      TGTTTGTTGT    1      0      3      0      10     1.16e-03       3.21e-03       1524   0.000   0.000   1.585   0.000   3.322
      d+2      ATCAGTCATT    0      0      1      0      1      3.96e-01       5.04e-01       1400   0.000   0.000   0.000   0.000   0.000
  AT5G12040.1
    SENSE COUNTS:            1      0      4      0      11     2.34e-04       5.12e-04              0.000   0.000   2.000   0.000   3.459
    TAGS:
      d+1      TGTTTGTTGT    1      0      3      0      10     1.16e-03       3.21e-03       1316   0.000   0.000   1.585   0.000   3.322
      d+2      ATCAGTCATT    0      0      1      0      1      3.96e-01       5.04e-01       1192   0.000   0.000   0.000   0.000   0.000

LOCUS: AT5G38660
DESCRIPTION:  mutant has Altered acclimation responses;
DATA:                        Control 30min  2hours 2days  1week  p-value        B&H            Pos    Fold change relative to control (log2)
SENSE COUNTS:                8      7      24     5      5      2.46e-04       5.28e-04              0.000   -0.193  1.585   -0.678  -0.678
  GENES:
  AT5G38660.1
    SENSE COUNTS:            8      7      24     5      5      2.46e-04       5.37e-04              0.000   -0.193  1.585   -0.678  -0.678
    TAGS:
      d+1      TATACGGCGA    8      7      24     5      5      2.46e-04       9.10e-04       915    0.000   -0.193  1.585   -0.678  -0.678

LOCUS: AT5G25350
DESCRIPTION:  F-box family protein, contains Pfam PF00646
DATA:                        Control 30min  2hours 2days  1week  p-value        B&H            Pos    Fold change relative to control (log2)
SENSE COUNTS:                2      13     2      0      3      2.47e-04       5.28e-04              0.000   2.700   0.000   0.000   0.585
  GENES:
  AT5G25350.1
    SENSE COUNTS:            2      13     2      0      3      2.47e-04       5.38e-04              0.000   2.700   0.000   0.000   0.585
    TAGS:
      d+1      CATACAAAAA    2      13     2      0      3      2.47e-04       9.12e-04       1885   0.000   2.700   0.000   0.000   0.585

LOCUS: AT4G03020
DESCRIPTION:  transducin family protein / WD-40 repeat family protein, contains 6 WD-40 repeats (PF00400); similar to L. erythrorhizon LEC14B, GenBank
accession number Q40153
DATA:                        Control 30min  2hours 2days  1week  p-value        B&H            Pos    Fold change relative to control (log2)
SENSE COUNTS:                2      0      4      15     12     2.48e-04       5.29e-04              0.000   0.000   1.000   2.907   2.585
  GENES:
  AT4G03020.1
    SENSE COUNTS:            2      0      4      15     12     2.48e-04       5.39e-04              0.000   0.000   1.000   2.907   2.585
    TAGS:
      d+1      GTTTGGTTTC    2      0      4      13     12     6.20e-04       1.96e-03       1902   0.000   0.000   1.000   2.700   2.585
      d+2      AGGTGCTAAG    0      0      0      2      0      2.03e-01       2.84e-01       1240   0.000   0.000   0.000   1.000   0.000

LOCUS: AT5G55920
DESCRIPTION:  nucleolar protein, putative, similar to SP|P46087 Proliferating-cell nucleolar antigen p120 (Proliferation-associated nucleolar protein
p120) {Homo sapiens}, SP|P40991 Nucleolar protein NOP2 {Saccharomyces cerevisiae}; contains Pfam profile PF01189
DATA:                        Control 30min  2hours 2days  1week  p-value        B&H            Pos    Fold change relative to control (log2)
SENSE COUNTS:                0      3      0      10     12     2.51e-04       5.35e-04              0.000   1.585   0.000   3.322   3.585
```

```
  GENES:
  AT5G55920.1
    SENSE COUNTS:             0      3      0      10     12     2.51e-04       5.45e-04              0.000  1.585  0.000  3.322  3.585
    TAGS:
      d+1      CGGGAGAAGG     0      1      0      6      5      1.28e-02       2.28e-02       2087   0.000  0.000  0.000  2.585  2.322
      d+2      GACGGATTTT     0      2      0      4      7      3.25e-02       5.47e-02       1724   0.000  1.000  0.000  2.000  2.807

LOCUS: AT4G33950
DESCRIPTION:  protein kinase, putative, similar to abscisic acid-activated protein kinase (Vicia faba) gi|6739629|gb|AAF27340; contains protein kinase
domain, Pfam
DATA:                         Control 30min  2hours 2days  1week  p-value        B&H            Pos    Fold change relative to control (log2)
SENSE COUNTS:                 1      0      2      1      12     2.66e-04       5.65e-04              0.000  0.000  1.000  0.000  3.585
  GENES:
  AT4G33950.1
    SENSE COUNTS:             1      0      2      1      12     2.66e-04       5.76e-04              0.000  0.000  1.000  0.000  3.585
    TAGS:
      d+1      GTTTCTGCTA     1      0      2      1      12     2.66e-04       9.76e-04       1156   0.000  0.000  1.000  0.000  3.585

LOCUS: AT2G15765
DESCRIPTION:  pseudogene, hypothetical protein
DATA:                         Control 30min  2hours 2days  1week  p-value        B&H            Pos    Fold change relative to control (log2)
SENSE COUNTS:                 35     17     30     45     57     2.67e-04       5.66e-04              0.000  -1.042 -0.222 0.363  0.704
  GENES:
  AT2G15765.1
    SENSE COUNTS:             35     17     30     45     57     2.67e-04       5.76e-04              0.000  -1.042 -0.222 0.363  0.704
    TAGS:
      p+1      TTTGAGAGAG     35     17     30     45     57     2.67e-04       9.77e-04       173    0.000  -1.042 -0.222 0.363  0.704

LOCUS: AT3G62880
DESCRIPTION:  mitochondrial import inner membrane translocase subunit Tim17/Tim22/Tim23 family protein, contains PFam PF02466
DATA:                         Control 30min  2hours 2days  1week  p-value        B&H            Pos    Fold change relative to control (log2)
SENSE COUNTS:                 2      0      14     5      14     2.67e-04       5.65e-04              0.000  0.000  2.807  1.322  2.807
  GENES:
  AT3G62880.1
    SENSE COUNTS:             2      0      14     5      14     2.67e-04       5.77e-04              0.000  0.000  2.807  1.322  2.807
    TAGS:
      d+2      TACACACAAA     2      0      14     5      14     2.67e-04       9.75e-04       610    0.000  0.000  2.807  1.322  2.807

LOCUS: AT2G23810
DESCRIPTION:  senescence-associated family protein, similar to senescence-associated protein 5 (Hemerocallis hybrid cultivar) gi|3551954|gb|AAC34855
DATA:                         Control 30min  2hours 2days  1week  p-value        B&H            Pos    Fold change relative to control (log2)
SENSE COUNTS:                 0      7      0      0      0      2.70e-04       5.70e-04              0.000  2.807  0.000  0.000  0.000
  GENES:
  AT2G23810.1
    SENSE COUNTS:             0      7      0      0      0      2.70e-04       5.79e-04              0.000  2.807  0.000  0.000  0.000
    TAGS:
      d+1      TTCCTTTTGA     0      7      0      0      0      2.70e-04       9.84e-04       13     0.000  2.807  0.000  0.000  0.000

LOCUS: AT3G02550
DESCRIPTION:  LOB domain protein 41 / lateral organ boundaries domain protein 41 (LBD41), identical to LOB DOMAIN 41 (Arabidopsis thaliana) GI
DATA:                         Control 30min  2hours 2days  1week  p-value        B&H            Pos    Fold change relative to control (log2)
SENSE COUNTS:                 0      7      0      0      0      2.70e-04       5.68e-04              0.000  2.807  0.000  0.000  0.000
  GENES:
  AT3G02550.1
    SENSE COUNTS:             0      7      0      0      0      2.70e-04       5.80e-04              0.000  2.807  0.000  0.000  0.000
    TAGS:
      d+1      TGGTACCTGT     0      7      0      0      0      2.70e-04       9.78e-04       1058   0.000  2.807  0.000  0.000  0.000

LOCUS: AT5G16340
DESCRIPTION:  AMP-binding protein, putative, similar to AMP-binding protein GI
```

```
DATA:                         Control 30min  2hours  2days  1week  p-value    B&H        Pos    Fold change relative to control (log2)
SENSE COUNTS:                 0       7      0       0      0      2.70e-04   5.67e-04          0.000  2.807  0.000  0.000  0.000
  GENES:
  AT5G16340.1
    SENSE COUNTS:             0       7      0       0      0      2.70e-04   5.81e-04          0.000  2.807  0.000  0.000  0.000
    TAGS:
      d+1     CTCGGTTACT      0       7      0       0      0      2.70e-04   9.82e-04   1201   0.000  2.807  0.000  0.000  0.000

LOCUS: AT5G11070
DESCRIPTION:  expressed protein
DATA:                         Control 30min  2hours  2days  1week  p-value    B&H        Pos    Fold change relative to control (log2)
SENSE COUNTS:                 0       7      0       0      0      2.70e-04   5.66e-04          0.000  2.807  0.000  0.000  0.000
  GENES:
  AT5G11070.1
    SENSE COUNTS:             0       7      0       0      0      2.70e-04   5.78e-04          0.000  2.807  0.000  0.000  0.000
    TAGS:
      d+1     ATGATCGGAG      0       7      0       0      0      2.70e-04   9.75e-04   218    0.000  2.807  0.000  0.000  0.000

LOCUS: AT5G48300
DESCRIPTION:  encodes ADPG pyrophosphorylase small subunit. The presence of the small subunit is required for large subunit stability.
DATA:                         Control 30min  2hours  2days  1week  p-value    B&H        Pos    Fold change relative to control (log2)
SENSE COUNTS:                 10      3      3       8      23     2.80e-04   5.85e-04          0.000  -1.737 -1.737 -0.322 1.202
  GENES:
  AT5G48300.1
    SENSE COUNTS:             10      3      3       8      23     2.80e-04   5.98e-04          0.000  -1.737 -1.737 -0.322 1.202
    TAGS:
      d+1     TTTTCACAAT      10      3      3       8      23     2.80e-04   1.01e-03   1780   0.000  -1.737 -1.737 -0.322 1.202

LOCUS: AT2G16365
DESCRIPTION:  expressed protein
DATA:                         Control 30min  2hours  2days  1week  p-value    B&H        Pos    Fold change relative to control (log2)
SENSE COUNTS:                 4       0      12      1      0      2.85e-04   5.95e-04          0.000  0.000  1.585  -2.000 0.000
  GENES:
  AT2G16365.1
    SENSE COUNTS:             1       0      0       0      0      4.28e-01   4.31e-01          0.000  0.000  0.000  0.000  0.000
    TAGS:
      i+3     ACAGACGTCT      1       0      0       0      0      4.28e-01   5.33e-01   3231   0.000  0.000  0.000  0.000  0.000
  AT2G16365.2
    SENSE COUNTS:             3       0      12      1      0      1.59e-04   3.65e-04          0.000  0.000  2.000  -1.585 0.000
    TAGS:
      d+1     GTTTGGAATG      3       0      12      1      0      1.59e-04   6.20e-04   1472   0.000  0.000  2.000  -1.585 0.000

LOCUS: AT1G37130
DESCRIPTION:  nitrate reductase 2 (NR2), identical to SP|P11035 Nitrate reductase 2 (formerly EC 1.6.6.1) (NR2) {Arabidopsis thaliana}
DATA:                         Control 30min  2hours  2days  1week  p-value    B&H        Pos    Fold change relative to control (log2)
SENSE COUNTS:                 24      29     38      39     1      2.90e-04   6.04e-04          0.000  0.273  0.663  0.700  -4.585
  GENES:
  AT1G37130.1
    SENSE COUNTS:             24      29     38      39     1      2.90e-04   6.18e-04          0.000  0.273  0.663  0.700  -4.585
    TAGS:
      d+1     GCTTGCGGAC      23      29     37      37     1      4.77e-04   1.58e-03   2752   0.000  0.334  0.686  0.686  -4.524
      d+2     ATGTTCGTAA      0       0      0       2      0      4.80e-02   7.93e-02   2117   0.000  0.000  0.000  1.000  0.000
      d+2     GAACGTATGT      0       0      1       0      0      4.55e-01   5.15e-01   1332   0.000  0.000  0.000  0.000  0.000
      d+2     TCACGAGGAG      1       0      0       0      0      6.89e-01   7.02e-01   1203   0.000  0.000  0.000  0.000  0.000
      d+2     AAATTCACCA      0       0      0       0      0      6.15e-01   6.52e-01   598    0.000  0.000  0.000  0.000  0.000

LOCUS: AT4G01560
DESCRIPTION:  brix domain-containing protein, contains Pfam domain, PF04427
DATA:                         Control 30min  2hours  2days  1week  p-value    B&H        Pos    Fold change relative to control (log2)
SENSE COUNTS:                 1       1      3       13     1      2.98e-04   6.19e-04          0.000  0.000  1.585  3.700  0.000
```

```
  GENES:
  AT4G01560.1
    SENSE COUNTS:             1       1       3       13      1       2.98e-04        6.34e-04                0.000   0.000   1.585   3.700   0.000
    TAGS:
      d+1      AGCCATCGAA     1       1       3       13      1       2.98e-04        1.06e-03        1220    0.000   0.000   1.585   3.700   0.000

LOCUS: AT4G25100
DESCRIPTION:  superoxide dismutase (Fe), chloroplast (SODB) / iron superoxide dismutase (FSD1), identical to Fe-superoxide dismutase (Arabidopsis
thaliana) gi|166700|gb|AAA32791; supported by cDNA, Ceres
DATA:                         Control 30min   2hours  2days   1week   p-value         B&H             Pos     Fold change relative to control (log2)
SENSE COUNTS:                 155     218     249     185     208     2.99e-04        6.20e-04                0.000   0.492   0.684   0.255   0.424
  GENES:
  AT4G25100.1
    SENSE COUNTS:             26      8       24      12      20      2.25e-02        2.29e-02                0.000   -1.700  -0.115  -1.115  -0.379
    TAGS:
      d+1      GCCCATTATT     26      8       20      12      17      2.91e-02        4.94e-02        1093    0.000   -1.700  -0.379  -1.115  -0.613
      d+2      ACCAATCTTG     0       0       4       0       3       7.65e-02        1.24e-01        768     0.000   0.000   2.000   0.000   1.585
  AT4G25100.3
    SENSE COUNTS:             129     210     229     173     191     1.44e-05        4.35e-05                0.000   0.703   0.828   0.423   0.566
    TAGS:
      d+1      CTCTTTTCTG     129     210     225     173     188     3.18e-05        1.49e-04        853     0.000   0.703   0.803   0.423   0.543
      d+2      ACCAATCTTG     0       0       4       0       3       7.65e-02        1.24e-01        632     0.000   0.000   2.000   0.000   1.585
  AT4G25100.2
    SENSE COUNTS:             26      8       24      12      20      2.25e-02        2.29e-02                0.000   -1.700  -0.115  -1.115  -0.379
    TAGS:
      d+1      GCCCATTATT     26      8       20      12      17      2.91e-02        4.94e-02        957     0.000   -1.700  -0.379  -1.115  -0.613
      d+2      ACCAATCTTG     0       0       4       0       3       7.65e-02        1.24e-01        632     0.000   0.000   2.000   0.000   1.585

LOCUS: AT4G16740
DESCRIPTION:  terpene synthase/cyclase family protein, similar to myrcene/ocimene synthase (GI
DATA:                         Control 30min   2hours  2days   1week   p-value         B&H             Pos     Fold change relative to control (log2)
SENSE COUNTS:                 1       0       7       0       0       3.02e-04        6.24e-04                0.000   0.000   2.807   0.000   0.000
  GENES:
  AT4G16740.1
    SENSE COUNTS:             1       0       7       0       0       3.02e-04        6.41e-04                0.000   0.000   2.807   0.000   0.000
    TAGS:
      d+2      GAGAAAAAGA     1       0       2       0       0       3.07e-01        4.11e-01        1619    0.000   0.000   1.000   0.000   0.000
      d+2      GTTTCAGTAT     0       0       5       0       0       1.10e-03        3.10e-03        501     0.000   0.000   2.322   0.000   0.000

LOCUS: AT3G13330
DESCRIPTION:  expressed protein
DATA:                         Control 30min   2hours  2days   1week   p-value         B&H             Pos     Fold change relative to control (log2)
SENSE COUNTS:                 4       5       3       19      19      3.07e-04        6.33e-04                0.000   0.322   -0.415  2.248   2.248
  GENES:
  AT3G13330.1
    SENSE COUNTS:             4       5       3       19      19      3.07e-04        6.50e-04                0.000   0.322   -0.415  2.248   2.248
    TAGS:
      d+1      GTTTCGGTTT     2       5       3       19      19      2.97e-05        1.41e-04        5261    0.000   1.322   0.585   3.248   3.248
      d+2      TGACCTTACT     1       0       0       0       0       4.28e-01        5.31e-01        4958    0.000   0.000   0.000   0.000   0.000
      d+2      GCCTAGAACT     0       0       0       0       0       6.15e-01        6.30e-01        3528    0.000   0.000   0.000   0.000   0.000
      i+3      TATAGATGAC     1       0       0       0       0       4.28e-01        5.34e-01        2377    0.000   0.000   0.000   0.000   0.000

LOCUS: AT3G17390
DESCRIPTION:  S-adenosylmethionine synthetase, putative, similar to S-adenosylmethionine synthetase 2 (Methionine adenosyltransferase 2, AdoMet
synthetase 2) (Catharanthus roseus) SWISS-PROT
DATA:                         Control 30min   2hours  2days   1week   p-value         B&H             Pos     Fold change relative to control (log2)
SENSE COUNTS:                 5       23      11      4       7       3.08e-04        6.34e-04                0.000   2.202   1.138   -0.322  0.485
  GENES:
  AT3G17390.1
    SENSE COUNTS:             5       23      11      4       7       3.08e-04        6.51e-04                0.000   2.202   1.138   -0.322  0.485
```

```
    TAGS:
      d+1      GAGATGCAGG     5       23      11      4       7       3.08e-04       1.09e-03       943     0.000   2.202   1.138   -0.322  0.485

LOCUS: AT3G10860
DESCRIPTION:  ubiquinol-cytochrome C reductase complex ubiquinone-binding protein, putative / ubiquinol-cytochrome C reductase complex 8.2 kDa protein,
putative, similar to ubiquinol--cytochrome c reductase GI
DATA:                        Control 30min   2hours  2days   1week   p-value        B&H            Pos     Fold change relative to control (log2)
SENSE COUNTS:                13      3       6       4       23      3.11e-04       6.39e-04               0.000   -2.115  -1.115  -1.700  0.823
  GENES:
  AT3G10860.1
    SENSE COUNTS:            13      3       6       4       23      3.11e-04       6.56e-04               0.000   -2.115  -1.115  -1.700  0.823
    TAGS:
      d+1      ACAATGACTC     13      3       6       4       23      3.11e-04       1.10e-03       449     0.000   -2.115  -1.115  -1.700  0.823

LOCUS: AT3G02470
DESCRIPTION:  adenosylmethionine decarboxylase family protein, contains Pfam profile
DATA:                        Control 30min   2hours  2days   1week   p-value        B&H            Pos     Fold change relative to control (log2)
SENSE COUNTS:                12      43      23      33      15      3.15e-04       6.45e-04               0.000   1.841   0.939   1.459   0.322
  GENES:
  AT3G02470.1
    SENSE COUNTS:            12      43      23      33      15      3.15e-04       6.63e-04               0.000   1.841   0.939   1.459   0.322
    TAGS:
      d+1      TGACCCCTGA     10      43      22      33      14      4.44e-05       1.99e-04       1354    0.000   2.104   1.138   1.722   0.485
      d+2      ACTGGTCTGG     1       0       0       0       1       3.83e-01       4.91e-01       1170    0.000   0.000   0.000   0.000   0.000
      d+2      CACGATCGTT     1       0       1       0       0       6.01e-01       6.49e-01       764     0.000   0.000   0.000   0.000   0.000

LOCUS: AT1G21110
DESCRIPTION:  O-methyltransferase, putative, similar to GI
DATA:                        Control 30min   2hours  2days   1week   p-value        B&H            Pos     Fold change relative to control (log2)
SENSE COUNTS:                0       0       7       0       0       3.23e-04       6.60e-04               0.000   0.000   2.807   0.000   0.000
  GENES:
  AT1G21110.1
    SENSE COUNTS:            0       0       7       0       0       3.23e-04       6.79e-04               0.000   0.000   2.807   0.000   0.000
    TAGS:
      d+1      CCTCTCTTTG     0       0       7       0       0       3.23e-04       1.14e-03       1340    0.000   0.000   2.807   0.000   0.000

LOCUS: AT2G35910
DESCRIPTION:  zinc finger (C3HC4-type RING finger) family protein, low similarity to RING-H2 zinc finger protein ATL6 (Arabidopsis thaliana) GI
DATA:                        Control 30min   2hours  2days   1week   p-value        B&H            Pos     Fold change relative to control (log2)
SENSE COUNTS:                0       0       8       0       1       3.32e-04       6.77e-04               0.000   0.000   3.000   0.000   0.000
  GENES:
  AT2G35910.1
    SENSE COUNTS:            0       0       8       0       1       3.32e-04       6.97e-04               0.000   0.000   3.000   0.000   0.000
    TAGS:
      d+1      TCATCATCGA     0       0       8       0       1       3.32e-04       1.16e-03       544     0.000   0.000   3.000   0.000   0.000

LOCUS: AT3G50820
DESCRIPTION:  oxygen-evolving enhancer protein, chloroplast, putative / 33 kDa subunit of oxygen evolving system of photosystem II, putative (PSBO2),
identical to SP
DATA:                        Control 30min   2hours  2days   1week   p-value        B&H            Pos     Fold change relative to control (log2)
SENSE COUNTS:                12      28      12      13      1       3.42e-04       6.96e-04               0.000   1.222   0.000   0.115   -3.585
  GENES:
  AT3G50820.1
    SENSE COUNTS:            12      28      12      13      1       3.42e-04       7.16e-04               0.000   1.222   0.000   0.115   -3.585
    TAGS:
      d+1      ACCCGTCTCA     12      28      12      13      1       3.42e-04       1.19e-03       605     0.000   1.222   0.000   0.115   -3.585

LOCUS: AT4G31550
DESCRIPTION:  WRKY family transcription factor, contains Pfam profile
DATA:                        Control 30min   2hours  2days   1week   p-value        B&H            Pos     Fold change relative to control (log2)
```

```
SENSE COUNTS:               0      12     4      2      0      3.42e-04      6.95e-04             0.000  3.585  2.000  1.000  0.000
  GENES:
  AT4G31550.3
    SENSE COUNTS:           0      0      1      0      0      4.55e-01      4.58e-01             0.000  0.000  0.000  0.000  0.000
    TAGS:
      d+2      TCGTCGGCGA   0      0      1      0      0      4.55e-01      5.11e-01      547    0.000  0.000  0.000  0.000  0.000
  AT4G31550.1
    SENSE COUNTS:           0      12     4      2      0      3.42e-04      7.15e-04             0.000  3.585  2.000  1.000  0.000
    TAGS:
      d+1      AGCACTCAGA   0      12     3      2      0      1.77e-04      6.76e-04      683    0.000  3.585  1.585  1.000  0.000
      d+2      TCGTCGGCGA   0      0      1      0      0      4.55e-01      5.11e-01      547    0.000  0.000  0.000  0.000  0.000
  AT4G31550.2
    SENSE COUNTS:           0      12     4      2      0      3.42e-04      7.13e-04             0.000  3.585  2.000  1.000  0.000
    TAGS:
      d+1      AGCACTCAGA   0      12     3      2      0      1.77e-04      6.76e-04      683    0.000  3.585  1.585  1.000  0.000
      d+2      TCGTCGGCGA   0      0      1      0      0      4.55e-01      5.11e-01      547    0.000  0.000  0.000  0.000  0.000

LOCUS: AT3G21890
DESCRIPTION:  zinc finger (B-box type) family protein, contains Pfam profile
DATA:                       Control 30min 2hours 2days 1week  p-value       B&H           Pos    Fold change relative to control (log2)
SENSE COUNTS:               0      0      8      9      1      3.43e-04      6.95e-04             0.000  0.000  3.000  3.170  0.000
  GENES:
  AT3G21890.1
    SENSE COUNTS:           0      0      8      9      1      3.43e-04      7.11e-04             0.000  0.000  3.000  3.170  0.000
    TAGS:
      d+1      AGGTTCCGTT   0      0      8      9      1      3.43e-04      1.20e-03      414    0.000  0.000  3.000  3.170  0.000

LOCUS: AT1G19000
DESCRIPTION:  myb family transcription factor, similar to MybSt1 GI
DATA:                       Control 30min 2hours 2days 1week  p-value       B&H           Pos    Fold change relative to control (log2)
SENSE COUNTS:               29     13     28     9      5      3.43e-04      6.94e-04             0.000  -1.158 -0.051 -1.688 -2.536
  GENES:
  AT1G19000.2
    SENSE COUNTS:           29     13     28     9      5      3.43e-04      7.13e-04             0.000  -1.158 -0.051 -1.688 -2.536
    TAGS:
      d+1      TGGCCCATCT   28     13     26     9      5      7.83e-04      2.36e-03      1334   0.000  -1.107 -0.107 -1.637 -2.485
      d+2      GAGCAAGATC   1      0      2      0      0      5.95e-01      6.45e-01      626    0.000  0.000  1.000  0.000  0.000
  AT1G19000.1
    SENSE COUNTS:           29     13     28     9      5      3.43e-04      7.14e-04             0.000  -1.158 -0.051 -1.688 -2.536
    TAGS:
      d+1      TGGCCCATCT   28     13     26     9      5      7.83e-04      2.36e-03      1161   0.000  -1.107 -0.107 -1.637 -2.485
      d+2      GAGCAAGATC   1      0      2      0      0      5.95e-01      6.45e-01      631    0.000  0.000  1.000  0.000  0.000

LOCUS: AT1G75350
DESCRIPTION:  ribosomal protein L31 family protein, similar to SP
DATA:                       Control 30min 2hours 2days 1week  p-value       B&H           Pos    Fold change relative to control (log2)
SENSE COUNTS:               1      12     2      1      1      3.50e-04      7.06e-04             0.000  3.585  1.000  0.000  0.000
  GENES:
  AT1G75350.1
    SENSE COUNTS:           1      12     2      1      1      3.50e-04      7.24e-04             0.000  3.585  1.000  0.000  0.000
    TAGS:
      d+1      TGTTCCTTCT   1      12     2      1      1      3.50e-04      1.21e-03      127    0.000  3.585  1.000  0.000  0.000

LOCUS: AT2G11290
DESCRIPTION:  gypsy-like retrotransposon family, has a 1.2e-92 P-value blast match to GB
DATA:                       Control 30min 2hours 2days 1week  p-value       B&H           Pos    Fold change relative to control (log2)
SENSE COUNTS:               0      0      8      1      0      3.59e-04      7.23e-04             0.000  0.000  3.000  0.000  0.000
  GENES:
  AT2G11290.1
    SENSE COUNTS:           0      0      8      1      0      3.59e-04      7.42e-04             0.000  0.000  3.000  0.000  0.000
```

```
    TAGS:
      p+1      GCATTTCTCA     0      0      8      1      0      3.59e-04       1.24e-03       1507   0.000  0.000  3.000  0.000  0.000

LOCUS: AT4G18010
DESCRIPTION:  inositol polyphosphate 5-phosphatase II (IP5PII), nearly identical to inositol polyphosphate 5-phosphatase II (Arabidopsis thaliana) GI
DATA:                         Control 30min  2hours 2days  1week  p-value        B&H            Pos    Fold change relative to control (log2)
SENSE COUNTS:                 2      2      17     8      5      3.70e-04       7.43e-04              0.000  0.000  3.087  2.000  1.322
  GENES:
  AT4G18010.2
    SENSE COUNTS:             2      2      17     8      5      3.70e-04       7.63e-04              0.000  0.000  3.087  2.000  1.322
    TAGS:
      d+1      GGATTCGTTT     2      2      17     8      5      3.70e-04       1.28e-03       2024   0.000  0.000  3.087  2.000  1.322
  AT4G18010.1
    SENSE COUNTS:             2      2      17     8      5      3.70e-04       7.61e-04              0.000  0.000  3.087  2.000  1.322
    TAGS:
      d+1      GGATTCGTTT     2      2      17     8      5      3.70e-04       1.28e-03       2332   0.000  0.000  3.087  2.000  1.322

LOCUS: AT1G01620
DESCRIPTION:  plasma membrane intrinsic protein 1C (PIP1C) / aquaporin PIP1.3 (PIP1.3) / transmembrane protein B (TMPB), identical to plasma membrane
intrinsic protein 1c SP
DATA:                         Control 30min  2hours 2days  1week  p-value        B&H            Pos    Fold change relative to control (log2)
SENSE COUNTS:                 32     35     16     13     7      3.73e-04       7.48e-04              0.000  0.129  -1.000 -1.300 -2.193
  GENES:
  AT1G01620.1
    SENSE COUNTS:             32     35     16     13     7      3.73e-04       7.66e-04              0.000  0.129  -1.000 -1.300 -2.193
    TAGS:
      d+1      TGTTTATTTT     32     35     16     13     7      3.73e-04       1.28e-03       1071   0.000  0.129  -1.000 -1.300 -2.193

LOCUS: AT5G56010
DESCRIPTION:  heat shock protein, putative, strong similarity to SP|P55737 Heat shock protein 81-2 (HSP81-2) {Arabidopsis thaliana}; contains Pfam
profiles PF02518
DATA:                         Control 30min  2hours 2days  1week  p-value        B&H            Pos    Fold change relative to control (log2)
SENSE COUNTS:                 4      6      1      18     7      3.83e-04       7.66e-04              0.000  0.585  -2.000 2.170  0.807
  GENES:
  AT5G56010.1
    SENSE COUNTS:             4      6      1      18     7      3.83e-04       7.85e-04              0.000  0.585  -2.000 2.170  0.807
    TAGS:
      d+1      CCTCCACTTG     2      6      1      16     7      9.66e-04       2.79e-03       2149   0.000  1.585  -1.000 3.000  1.807
      X+4      AAGCAGCTCC     2      0      0      2      0      1.57e-01       2.38e-01       2107   0.000  0.000  0.000  0.000  0.000

LOCUS: AT2G36120
DESCRIPTION:  pseudogene, glycine-rich protein,
DATA:                         Control 30min  2hours 2days  1week  p-value        B&H            Pos    Fold change relative to control (log2)
SENSE COUNTS:                 4      14     2      4      0      4.02e-04       8.02e-04              0.000  1.807  -1.000 0.000  0.000
  GENES:
  AT2G36120.1
    SENSE COUNTS:             4      14     2      4      0      4.02e-04       8.22e-04              0.000  1.807  -1.000 0.000  0.000
    TAGS:
      p+1      AAAAATTATC     4      14     2      4      0      4.02e-04       1.38e-03       804    0.000  1.807  -1.000 0.000  0.000

LOCUS: AT3G03780
DESCRIPTION:  5-methyltetrahydropteroyltriglutamate--homocysteine methyltransferase, putative / vitamin-B12-independent methionine synthase, putative
/ cobalamin-independent methionine synthase, putative, very strong similarity to SP|O50008 5-methyltetrahydropteroyltri
DATA:                         Control 30min  2hours 2days  1week  p-value        B&H            Pos    Fold change relative to control (log2)
SENSE COUNTS:                 8      2      18     22     8      4.06e-04       8.08e-04              0.000  -2.000 1.170  1.459  0.000
  GENES:
  AT3G03780.1
    SENSE COUNTS:             8      2      18     22     8      4.06e-04       8.27e-04              0.000  -2.000 1.170  1.459  0.000
    TAGS:
      d+1      GTTGACGCGG     8      2      17     22     8      5.87e-04       1.86e-03       2330   0.000  -2.000 1.087  1.459  0.000
```

d+2 GACGCTGATG 0 0 1 0 0 4.55e-01 5.20e-01 2069 0.000 0.000 0.000 0.000 0.000
AT3G03780.2
SENSE COUNTS: 8 2 18 22 8 4.06e-04 8.29e-04 0.000 -2.000 1.170 1.459 0.000
TAGS:
d+1 GTTGACGCGG 8 2 17 22 8 5.87e-04 1.86e-03 2323 0.000 -2.000 1.087 1.459 0.000
d+2 GACGCTGATG 0 0 1 0 0 4.55e-01 5.20e-01 2062 0.000 0.000 0.000 0.000 0.000

LOCUS: AT1G12890
DESCRIPTION: encodes a member of the ERF (ethylene response factor) subfamily B-1 of ERF/AP2 transcription factor family. The protein contains one AP2 domain. There are 15 members in this subfamily including ATERF-3, ATERF-4, ATERF-7, and leafy petiole.
DATA: Control 30min 2hours 2days 1week p-value B&H Pos Fold change relative to control (log2)
SENSE COUNTS: 19 0 17 8 12 4.07e-04 8.09e-04 0.000 0.000 -0.160 -1.248 -0.663
GENES:
AT1G12890.1
SENSE COUNTS: 19 0 17 8 12 4.07e-04 8.28e-04 0.000 0.000 -0.160 -1.248 -0.663
TAGS:
v+1 TAACAGTAAA 19 0 17 8 12 4.07e-04 1.39e-03 1205 0.000 0.000 -0.160 -1.248 -0.663

LOCUS: AT4G31800
DESCRIPTION: WRKY family transcription factor
DATA: Control 30min 2hours 2days 1week p-value B&H Pos Fold change relative to control (log2)
SENSE COUNTS: 0 1 13 8 3 4.13e-04 8.19e-04 0.000 0.000 3.700 3.000 1.585
GENES:
AT4G31800.1
SENSE COUNTS: 0 1 13 8 3 4.13e-04 8.39e-04 0.000 0.000 3.700 3.000 1.585
TAGS:
d+1 AACGTTTTTA 0 1 1 2 3 5.00e-01 5.51e-01 1026 0.000 0.000 0.000 1.000 1.585
d+2 AGCAGAAAAA 0 0 12 6 0 1.11e-05 5.88e-05 506 0.000 0.000 3.585 2.585 0.000

LOCUS: AT1G64780
DESCRIPTION: ammonium transporter 1, member 2 (AMT1.2), nearly identical to SP|Q9ZPJ8 Ammonium transporter 1, member 2 (AtAMT1;2) {Arabidopsis thaliana}
DATA: Control 30min 2hours 2days 1week p-value B&H Pos Fold change relative to control (log2)
SENSE COUNTS: 12 0 4 2 1 4.20e-04 8.31e-04 0.000 0.000 -1.585 -2.585 -3.585
GENES:
AT1G64780.1
SENSE COUNTS: 12 0 4 2 1 4.20e-04 8.51e-04 0.000 0.000 -1.585 -2.585 -3.585
TAGS:
d+1 ATCAGTATGT 12 0 3 2 1 2.28e-04 8.47e-04 1795 0.000 0.000 -2.000 -2.585 -3.585
d+2 TTGGACGCAT 0 0 1 0 0 4.55e-01 5.23e-01 1690 0.000 0.000 0.000 0.000 0.000

LOCUS: AT1G52220
DESCRIPTION: expressed protein
DATA: Control 30min 2hours 2days 1week p-value B&H Pos Fold change relative to control (log2)
SENSE COUNTS: 39 14 28 29 7 4.31e-04 8.51e-04 0.000 -1.478 -0.478 -0.427 -2.478
GENES:
AT1G52220.1
SENSE COUNTS: 39 14 28 29 7 4.31e-04 8.72e-04 0.000 -1.478 -0.478 -0.427 -2.478
TAGS:
d+1 CATTTGAAAC 39 14 28 29 7 4.31e-04 1.46e-03 668 0.000 -1.478 -0.478 -0.427 -2.478

LOCUS: AT5G04800
DESCRIPTION: 40S ribosomal protein S17 (RPS17D), 40S ribosomal protein S17, Lycopersicon esculentum, EMBL
DATA: Control 30min 2hours 2days 1week p-value B&H Pos Fold change relative to control (log2)
SENSE COUNTS: 8 3 6 13 25 4.34e-04 8.55e-04 0.000 -1.415 -0.415 0.700 1.644
GENES:
AT5G04800.2
SENSE COUNTS: 8 3 6 13 25 4.34e-04 8.75e-04 0.000 -1.415 -0.415 0.700 1.644
TAGS:
d+1 TGTGTTCTTG 8 3 6 13 25 4.34e-04 1.46e-03 623 0.000 -1.415 -0.415 0.700 1.644

```
  AT5G04800.1
    SENSE COUNTS:              8      3      6      13     25     4.34e-04       8.76e-04              0.000  -1.415  -0.415  0.700   1.644
    TAGS:
      d+1      TGTGTTCTTG      8      3      6      13     25     4.34e-04       1.46e-03       617    0.000  -1.415  -0.415  0.700   1.644

LOCUS: AT5G63790
DESCRIPTION:  no apical meristem (NAM) family protein, contains Pfam PF02365
DATA:                          Control 30min  2hours 2days  1week  p-value        B&H            Pos    Fold change relative to control (log2)
SENSE COUNTS:                  2      2      17     6      6      4.39e-04       8.63e-04              0.000  0.000   3.087   1.585   1.585
  GENES:
  AT5G63790.1
    SENSE COUNTS:              2      2      17     6      6      4.39e-04       8.83e-04              0.000  0.000   3.087   1.585   1.585
    TAGS:
      d+1      AGCTTATTTC      2      0      16     4      3      4.45e-06       2.64e-05       1275   0.000  0.000   3.000   1.000   0.585
      d+2      TTTAGTTCCA      0      2      1      2      3      5.00e-01       5.51e-01       802    0.000  1.000   0.000   1.000   1.585

LOCUS: AT4G16980
DESCRIPTION:  arabinogalactan-protein family, similar to arabinogalactan protein (Arabidopsis thaliana) gi|10880495|gb|AAG24277; contains proline-rich
extensin domains, INTERPRO
DATA:                          Control 30min  2hours 2days  1week  p-value        B&H            Pos    Fold change relative to control (log2)
SENSE COUNTS:                  59     30     42     50     16     4.44e-04       8.71e-04              0.000  -0.976  -0.490  -0.239  -1.883
  GENES:
  AT4G16980.1
    SENSE COUNTS:              59     30     42     50     16     4.44e-04       8.91e-04              0.000  -0.976  -0.490  -0.239  -1.883
    TAGS:
      d+1      CGTTGTTGGA      59     30     40     49     16     4.95e-04       1.63e-03       565    0.000  -0.976  -0.561  -0.268  -1.883
      d+2      CCGCCTATGT      0      0      2      0      0      1.21e-01       1.86e-01       224    0.000  0.000   1.000   0.000   0.000
      d+2      GCCTCTTCTT      0      0      0      1      0      3.09e-01       4.08e-01       113    0.000  0.000   0.000   0.000   0.000

LOCUS: AT3G18820
DESCRIPTION:  Ras-related GTP-binding protein, putative, similar to Ras-related protein RAB7 GI
DATA:                          Control 30min  2hours 2days  1week  p-value        B&H            Pos    Fold change relative to control (log2)
SENSE COUNTS:                  2      9      4      3      20     4.56e-04       8.93e-04              0.000  2.170   1.000   0.585   3.322
  GENES:
  AT3G18820.1
    SENSE COUNTS:              2      9      4      3      20     4.56e-04       9.14e-04              0.000  2.170   1.000   0.585   3.322
    TAGS:
      d+1      CTAGCTAAAT      2      4      4      2      19     1.57e-04       6.16e-04       973    0.000  1.000   1.000   0.000   3.248
      d+2      AAATCATTTG      0      5      0      1      1      3.77e-02       6.30e-02       445    0.000  2.322   0.000   0.000   0.000

LOCUS: AT5G66190
DESCRIPTION:  ferredoxin--NADP(+) reductase, putative / adrenodoxin reductase, putative, strong similarity to Ferredoxin--NADP reductase, chloroplast
precursor (EC 1.18.1.2) (FNR) from {Pisum sativum} SP|P10933, {Mesembryanthemum crystallinum} SP|P41343, {Spinacia oler
DATA:                          Control 30min  2hours 2days  1week  p-value        B&H            Pos    Fold change relative to control (log2)
SENSE COUNTS:                  1      9      0      0      3      4.57e-04       8.93e-04              0.000  3.170   0.000   0.000   1.585
  GENES:
  AT5G66190.1
    SENSE COUNTS:              1      9      0      0      3      4.57e-04       9.14e-04              0.000  3.170   0.000   0.000   1.585
    TAGS:
      d+1      TTATATTTCT      1      9      0      0      3      4.57e-04       1.54e-03       1397   0.000  3.170   0.000   0.000   1.585

LOCUS: AT4G27280
DESCRIPTION:  calcium-binding EF hand family protein, similar to EF-hand Ca2+-binding protein CCD1 (Triticum aestivum) GI
DATA:                          Control 30min  2hours 2days  1week  p-value        B&H            Pos    Fold change relative to control (log2)
SENSE COUNTS:                  0      7      1      0      0      4.60e-04       8.97e-04              0.000  2.807   0.000   0.000   0.000
  GENES:
  AT4G27280.1
    SENSE COUNTS:              0      7      1      0      0      4.60e-04       9.18e-04              0.000  2.807   0.000   0.000   0.000
    TAGS:
      d+1      GCCGGAAACC      0      7      1      0      0      4.60e-04       1.54e-03       157    0.000  2.807   0.000   0.000   0.000
```

LOCUS: AT1G76100
DESCRIPTION: plastocyanin, identical to plastocyanin GI

DATA:		Control	30min	2hours	2days	1week	p-value	B&H	Pos	Fold change relative to control (log2)				
SENSE COUNTS:		17	2	4	6	1	4.61e-04	8.97e-04		0.000	-3.087	-2.087	-1.503	-4.087
GENES:														
AT1G76100.1														
SENSE COUNTS:		17	2	4	6	1	4.61e-04	9.19e-04		0.000	-3.087	-2.087	-1.503	-4.087
TAGS:														
d+1	ATTTATAAAT	17	2	4	6	1	4.61e-04	1.54e-03	588	0.000	-3.087	-2.087	-1.503	-4.087

LOCUS: AT1G19960
DESCRIPTION: expressed protein

DATA:		Control	30min	2hours	2days	1week	p-value	B&H	Pos	Fold change relative to control (log2)				
SENSE COUNTS:		0	0	0	1	6	4.62e-04	8.97e-04		0.000	0.000	0.000	0.000	2.585
GENES:														
AT1G19960.1														
SENSE COUNTS:		0	0	0	1	6	4.62e-04	9.17e-04		0.000	0.000	0.000	0.000	2.585
TAGS:														
v+2	TTAACTGTGT	0	0	0	0	5	6.24e-04	1.96e-03	636	0.000	0.000	0.000	0.000	2.322
v+2	TGTAGTGAAA	0	0	0	1	1	3.25e-01	4.21e-01	586	0.000	0.000	0.000	0.000	0.000

LOCUS: AT5G64480
DESCRIPTION: expressed protein,

DATA:		Control	30min	2hours	2days	1week	p-value	B&H	Pos	Fold change relative to control (log2)				
SENSE COUNTS:		0	0	0	1	7	4.62e-04	8.95e-04		0.000	0.000	0.000	0.000	2.807
GENES:														
AT5G64480.1														
SENSE COUNTS:		0	0	0	1	7	4.62e-04	9.19e-04		0.000	0.000	0.000	0.000	2.807
TAGS:														
d+2	TTTCTTTAGC	0	0	0	1	7	4.62e-04	1.54e-03	618	0.000	0.000	0.000	0.000	2.807

LOCUS: AT1G07473
DESCRIPTION: hypothetical protein

DATA:		Control	30min	2hours	2days	1week	p-value	B&H	Pos	Fold change relative to control (log2)				
SENSE COUNTS:		28	9	7	13	7	4.69e-04	9.06e-04		0.000	-1.637	-2.000	-1.107	-2.000
GENES:														
AT1G07473.1														
SENSE COUNTS:		28	9	7	13	7	4.69e-04	9.29e-04		0.000	-1.637	-2.000	-1.107	-2.000
TAGS:														
v+1	TCTCTCACTC	28	9	7	13	7	4.69e-04	1.55e-03	1008	0.000	-1.637	-2.000	-1.107	-2.000

LOCUS: AT5G48490
DESCRIPTION: protease inhibitor/seed storage/lipid transfer protein (LTP) family protein, contains Pfam protease inhibitor/seed storage/LTP family domain PF00234

DATA:		Control	30min	2hours	2days	1week	p-value	B&H	Pos	Fold change relative to control (log2)				
SENSE COUNTS:		11	0	1	1	8	4.78e-04	9.22e-04		0.000	0.000	-3.459	-3.459	-0.459
GENES:														
AT5G48490.1														
SENSE COUNTS:		11	0	1	1	8	4.78e-04	9.45e-04		0.000	0.000	-3.459	-3.459	-0.459
TAGS:														
d+1	TTTAATGGAA	10	0	0	1	8	3.44e-04	1.19e-03	392	0.000	0.000	0.000	-3.322	-0.322
d+2	CTGACTACAC	1	0	1	0	0	6.01e-01	6.51e-01	229	0.000	0.000	0.000	0.000	0.000

LOCUS: AT4G29905
DESCRIPTION: Expressed protein

DATA:		Control	30min	2hours	2days	1week	p-value	B&H	Pos	Fold change relative to control (log2)				
SENSE COUNTS:		2	6	18	5	3	4.81e-04	9.26e-04		0.000	1.585	3.170	1.322	0.585
GENES:														
AT4G29905.1														

```
    SENSE COUNTS:             2      6      18     5      3      4.81e-04       9.50e-04              0.000  1.585  3.170  1.322  0.585
    TAGS:
      d+1      TTCTTCGTGC     2      6      18     5      3      4.81e-04       1.59e-03       168    0.000  1.585  3.170  1.322  0.585

LOCUS: AT3G48990
DESCRIPTION:  AMP-dependent synthetase and ligase family protein, similar to peroxisomal-coenzyme A synthetase (FAT2) (gi
DATA:                         Control 30min  2hours 2days  1week  p-value        B&H            Pos    Fold change relative to control (log2)
SENSE COUNTS:                 34     13     42     38     46     4.87e-04       9.35e-04              0.000  -1.387 0.305  0.160  0.436
  GENES:
  AT3G48990.1
    SENSE COUNTS:             34     13     42     38     46     4.87e-04       9.60e-04              0.000  -1.387 0.305  0.160  0.436
    TAGS:
      d+1      GTGGTAAAGG     34     13     42     38     46     4.87e-04       1.60e-03       1731   0.000  -1.387 0.305  0.160  0.436

LOCUS: AT5G55450
DESCRIPTION:  protease inhibitor/seed storage/lipid transfer protein (LTP) family protein, contains Pfam protease inhibitor/seed storage/LTP family
domain PF00234
DATA:                         Control 30min  2hours 2days  1week  p-value        B&H            Pos    Fold change relative to control (log2)
SENSE COUNTS:                 5      1      1      4      16     4.96e-04       9.51e-04              0.000  -2.322 -2.322 -0.322 1.678
  GENES:
  AT5G55450.1
    SENSE COUNTS:             5      1      1      4      16     4.96e-04       9.76e-04              0.000  -2.322 -2.322 -0.322 1.678
    TAGS:
      d+1      TTCTACTATT     4      1      1      4      16     3.43e-04       1.19e-03       546    0.000  -2.000 -2.000 0.000  2.000
      d+2      AAGAACTGAG     1      0      0      0      0      4.28e-01       5.37e-01       391    0.000  0.000  0.000  0.000  0.000

LOCUS: AT5G03380
DESCRIPTION:  heavy-metal-associated domain-containing protein, similar to farnesylated protein ATFP2 (GI
DATA:                         Control 30min  2hours 2days  1week  p-value        B&H            Pos    Fold change relative to control (log2)
SENSE COUNTS:                 3      9      20     8      0      5.01e-04       9.58e-04              0.000  1.585  2.737  1.415  0.000
  GENES:
  AT5G03380.1
    SENSE COUNTS:             3      9      20     8      0      5.01e-04       9.84e-04              0.000  1.585  2.737  1.415  0.000
    TAGS:
      d+1      CGTGCGCCTG     3      9      20     8      0      5.01e-04       1.64e-03       1232   0.000  1.585  2.737  1.415  0.000

LOCUS: AT2G19870
DESCRIPTION:  tRNA/rRNA methyltransferase (SpoU) family protein, similar to SP|P25270 Ribose methyltransferase PET56 (EC 2.1.1.-) {Saccharomyces
cerevisiae}; contains Pfam profile PF00588
DATA:                         Control 30min  2hours 2days  1week  p-value        B&H            Pos    Fold change relative to control (log2)
SENSE COUNTS:                 1      0      0      0      6      5.04e-04       9.62e-04              0.000  0.000  0.000  0.000  2.585
  GENES:
  AT2G19870.1
    SENSE COUNTS:             1      0      0      0      6      5.04e-04       9.88e-04              0.000  0.000  0.000  0.000  2.585
    TAGS:
      d+1      ACCTGATCTA     0      0      0      0      5      6.24e-04       1.94e-03       2035   0.000  0.000  0.000  0.000  2.322
      d+2      TACCGATTTA     1      0      0      0      1      3.83e-01       4.92e-01       1755   0.000  0.000  0.000  0.000  0.000

LOCUS: AT2G38470
DESCRIPTION:  WRKY family transcription factor, contains Pfam profile
DATA:                         Control 30min  2hours 2days  1week  p-value        B&H            Pos    Fold change relative to control (log2)
SENSE COUNTS:                 1      10     3      0      0      5.14e-04       9.79e-04              0.000  3.322  1.585  0.000  0.000
  GENES:
  AT2G38470.1
    SENSE COUNTS:             1      10     3      0      0      5.14e-04       1.01e-03              0.000  3.322  1.585  0.000  0.000
    TAGS:
      d+1      AACAACAATA     1      10     2      0      0      2.22e-04       8.31e-04       1458   0.000  3.322  1.000  0.000  0.000
      d+2      AGAGCAGTAA     0      0      1      0      0      4.55e-01       5.27e-01       1233   0.000  0.000  0.000  0.000  0.000

LOCUS: AT1G17100
```

```
DESCRIPTION:  SOUL heme-binding family protein, similar to SOUL protein (Mus musculus) GI
DATA:                         Control 30min   2hours  2days   1week   p-value         B&H             Pos     Fold change relative to control (log2)
SENSE COUNTS:                 0       1       0       8       0       5.21e-04        9.90e-04                0.000   0.000   0.000   3.000   0.000
  GENES:
  AT1G17100.1
    SENSE COUNTS:             0       1       0       8       0       5.21e-04        1.02e-03                0.000   0.000   0.000   3.000   0.000
    TAGS:
      d+2      GCCACCGGTT     0       1       0       8       0       5.21e-04        1.69e-03        34      0.000   0.000   0.000   3.000   0.000

LOCUS: AT1G64770
DESCRIPTION:  expressed protein
DATA:                         Control 30min   2hours  2days   1week   p-value         B&H             Pos     Fold change relative to control (log2)
SENSE COUNTS:                 12      1       2       2       14      5.23e-04        9.92e-04                0.000   -3.585  -2.585  -2.585  0.222
  GENES:
  AT1G64770.1
    SENSE COUNTS:             12      1       2       2       14      5.23e-04        1.02e-03                0.000   -3.585  -2.585  -2.585  0.222
    TAGS:
      d+1      TTAATGACTA     11      1       2       2       14      9.71e-04        2.80e-03        1242    0.000   -3.459  -2.459  -2.459  0.348
      d+2      GCTACAGCTC     1       0       0       0       0       4.28e-01        5.28e-01        594     0.000   0.000   0.000   0.000   0.000

LOCUS: AT3G26570
DESCRIPTION:  phosphate transporter family protein, contains Pfam profile
DATA:                         Control 30min   2hours  2days   1week   p-value         B&H             Pos     Fold change relative to control (log2)
SENSE COUNTS:                 26      3       19      12      13      5.27e-04        9.98e-04                0.000   -3.115  -0.453  -1.115  -1.000
  GENES:
  AT3G26570.1
    SENSE COUNTS:             26      3       19      12      13      5.27e-04        1.02e-03                0.000   -3.115  -0.453  -1.115  -1.000
    TAGS:
      d+1      AAATTGATCT     26      3       14      12      12      5.56e-04        1.79e-03        1917    0.000   -3.115  -0.893  -1.115  -1.115
      d+2      TCATTCGCTC     0       0       5       0       0       1.10e-03        3.08e-03        1408    0.000   0.000   2.322   0.000   0.000
      d+2      AAGTCCCTGG     0       0       0       0       1       1.65e-01        2.34e-01        544     0.000   0.000   0.000   0.000   0.000
  AT3G26570.2
    SENSE COUNTS:             26      3       19      12      13      5.27e-04        1.03e-03                0.000   -3.115  -0.453  -1.115  -1.000
    TAGS:
      d+1      AAATTGATCT     26      3       14      12      12      5.56e-04        1.79e-03        2028    0.000   -3.115  -0.893  -1.115  -1.115
      d+2      TCATTCGCTC     0       0       5       0       0       1.10e-03        3.08e-03        1519    0.000   0.000   2.322   0.000   0.000
      d+2      AAGTCCCTGG     0       0       0       0       1       1.65e-01        2.34e-01        655     0.000   0.000   0.000   0.000   0.000

LOCUS: AT1G01910
DESCRIPTION:  anion-transporting ATPase, putative, similar to SP|O43681 Arsenical pump-driving ATPase (EC 3.6.3.16) (Arsenite-translocating ATPase)
(Arsenical resistance ATPase) (Arsenite-transporting ATPase) (ARSA) (ASNA-I) {Homo sapiens}; contains Pfam profile PF0237
DATA:                         Control 30min   2hours  2days   1week   p-value         B&H             Pos     Fold change relative to control (log2)
SENSE COUNTS:                 9       0       0       0       5       5.28e-04        9.97e-04                0.000   0.000   0.000   0.000   -0.848
  GENES:
  AT1G01910.1
    SENSE COUNTS:             7       0       0       0       5       5.57e-03        6.79e-03                0.000   0.000   0.000   0.000   -0.485
    TAGS:
      d+1      ACTACATAGA     6       0       0       0       5       5.74e-03        1.19e-02        1276    0.000   0.000   0.000   0.000   -0.263
      d+2      GAGGATGAGT     1       0       0       0       0       6.89e-01        7.01e-01        624     0.000   0.000   0.000   0.000   0.000
  AT1G01910.2
    SENSE COUNTS:             9       0       0       0       5       5.28e-04        1.02e-03                0.000   0.000   0.000   0.000   -0.848
    TAGS:
      d+1      ACTACATAGA     6       0       0       0       5       5.74e-03        1.19e-02        1424    0.000   0.000   0.000   0.000   -0.263
      d+2      GGGGTCTTCT     2       0       0       0       0       1.04e-01        1.64e-01        1212    0.000   0.000   0.000   0.000   0.000
      d+2      GAGGATGAGT     1       0       0       0       0       6.89e-01        7.01e-01        624     0.000   0.000   0.000   0.000   0.000

LOCUS: AT4G14030
DESCRIPTION:  selenium-binding protein, putative, contains Pfam profile PF05694
DATA:                         Control 30min   2hours  2days   1week   p-value         B&H             Pos     Fold change relative to control (log2)
SENSE COUNTS:                 3       17      16      6       0       5.33e-04        1.00e-03                0.000   2.503   2.415   1.000   0.000
```

GENES:
AT4G14030.1
SENSE COUNTS: 3 17 16 6 0 5.33e-04 1.03e-03 0.000 2.503 2.415 1.000 0.000
TAGS:
d+1 GAGAAAGGCT 3 17 16 6 0 5.33e-04 1.73e-03 1386 0.000 2.503 2.415 1.000 0.000

LOCUS: AT5G19630
DESCRIPTION: expressed protein
DATA: Control 30min 2hours 2days 1week p-value B&H Pos Fold change relative to control (log2)
SENSE COUNTS: 11 0 2 2 10 5.47e-04 1.03e-03 0.000 0.000 -2.459 -2.459 -0.138
GENES:
AT5G19630.1
SENSE COUNTS: 11 0 2 2 10 5.47e-04 1.05e-03 0.000 0.000 -2.459 -2.459 -0.138
TAGS:
d+1 AAAAGATAGA 11 0 2 2 10 5.47e-04 1.77e-03 757 0.000 0.000 -2.459 -2.459 -0.138

LOCUS: AT5G04140
DESCRIPTION: glutamate synthase (GLU1) / ferredoxin-dependent glutamate synthase (Fd-GOGAT 1), identical to ferredoxin-dependent glutamate synthase precursor (Arabidopsis thaliana) GI
DATA: Control 30min 2hours 2days 1week p-value B&H Pos Fold change relative to control (log2)
SENSE COUNTS: 68 25 48 41 44 5.48e-04 1.03e-03 0.000 -1.444 -0.503 -0.730 -0.628
GENES:
AT5G04140.1
SENSE COUNTS: 68 25 48 41 44 5.48e-04 1.05e-03 0.000 -1.444 -0.503 -0.730 -0.628
TAGS:
d+1 GTTGCTATAA 68 23 45 41 43 1.40e-04 5.56e-04 5215 0.000 -1.564 -0.596 -0.730 -0.661
d+2 TAGATCAAAT 0 0 0 0 1 1.65e-01 2.35e-01 5156 0.000 0.000 0.000 0.000 0.000
d+2 TGGAAAAAAC 0 0 1 0 0 4.55e-01 5.23e-01 4885 0.000 0.000 0.000 0.000 0.000
d+2 ACTGGTGGCT 0 0 1 0 0 4.55e-01 5.22e-01 4695 0.000 0.000 0.000 0.000 0.000
d+2 ATCTTTCTTC 0 2 1 0 0 2.47e-01 3.38e-01 723 0.000 1.000 0.000 0.000 0.000
AT5G04140.2
SENSE COUNTS: 68 25 48 41 44 5.48e-04 1.05e-03 0.000 -1.444 -0.503 -0.730 -0.628
TAGS:
d+1 GTTGCTATAA 68 23 45 41 43 1.40e-04 5.56e-04 5114 0.000 -1.564 -0.596 -0.730 -0.661
d+2 TAGATCAAAT 0 0 0 0 1 1.65e-01 2.35e-01 5055 0.000 0.000 0.000 0.000 0.000
d+2 TGGAAAAAAC 0 0 1 0 0 4.55e-01 5.23e-01 4784 0.000 0.000 0.000 0.000 0.000
d+2 ACTGGTGGCT 0 0 1 0 0 4.55e-01 5.22e-01 4594 0.000 0.000 0.000 0.000 0.000
d+2 ATCTTTCTTC 0 2 1 0 0 2.47e-01 3.38e-01 622 0.000 1.000 0.000 0.000 0.000

LOCUS: AT4G14920
DESCRIPTION: PHD finger transcription factor, putative
DATA: Control 30min 2hours 2days 1week p-value B&H Pos Fold change relative to control (log2)
SENSE COUNTS: 24 5 10 18 3 5.54e-04 1.04e-03 0.000 -2.263 -1.263 -0.415 -3.000
GENES:
AT4G14920.1
SENSE COUNTS: 24 5 10 18 3 5.54e-04 1.06e-03 0.000 -2.263 -1.263 -0.415 -3.000
TAGS:
v+1 GCCTTGAGCC 1 0 0 2 0 4.17e-01 5.24e-01 3654 0.000 0.000 0.000 1.000 0.000
v+2 AAGAGTAAGG 1 0 0 0 0 4.28e-01 5.22e-01 1224 0.000 0.000 0.000 0.000 0.000
i+3 CGTTGTTGTT 22 5 10 16 3 1.92e-03 4.97e-03 626 0.000 -2.138 -1.138 -0.459 -2.874

LOCUS: AT3G05560
DESCRIPTION: 60S ribosomal protein L22-2 (RPL22B), identical to 60S ribosomal protein L22-2 SP
DATA: Control 30min 2hours 2days 1week p-value B&H Pos Fold change relative to control (log2)
SENSE COUNTS: 19 9 7 9 32 5.55e-04 1.04e-03 0.000 -1.078 -1.441 -1.078 0.752
GENES:
AT3G05560.1
SENSE COUNTS: 19 9 7 9 32 5.55e-04 1.06e-03 0.000 -1.078 -1.441 -1.078 0.752
TAGS:
d+1 TTTCTGTTTT 19 9 7 9 32 5.55e-04 1.79e-03 508 0.000 -1.078 -1.441 -1.078 0.752

AT3G05560.2
SENSE COUNTS: 19 9 7 9 32 5.55e-04 1.06e-03 0.000 -1.078 -1.441 -1.078 0.752
TAGS:
d+1 TTTCTGTTTT 19 9 7 9 32 5.55e-04 1.79e-03 711 0.000 -1.078 -1.441 -1.078 0.752

LOCUS: AT3G25940
DESCRIPTION: transcription factor S-II (TFIIS) domain-containing protein, similar to SP|O94703 DNA-directed RNA polymerase I 13.1 kDa polypeptide (EC 2.7.7.6) {Schizosaccharomyces pombe}; contains Pfam profile PF01096
DATA: Control 30min 2hours 2days 1week p-value B&H Pos Fold change relative to control (log2)
SENSE COUNTS: 0 0 0 0 7 5.61e-04 1.05e-03 0.000 0.000 0.000 0.000 2.807
GENES:
AT3G25940.1
SENSE COUNTS: 0 0 0 0 7 5.61e-04 1.07e-03 0.000 0.000 0.000 0.000 2.807
TAGS:
d+1 TAGTGTTCTT 0 0 0 0 7 5.61e-04 1.80e-03 578 0.000 0.000 0.000 0.000 2.807

LOCUS: AT2G35110
DESCRIPTION: HEM protein-related, weak similarity to Membrane-associated protein Hem (Dhem-2) (Swiss-Prot
DATA: Control 30min 2hours 2days 1week p-value B&H Pos Fold change relative to control (log2)
SENSE COUNTS: 1 0 2 0 10 5.66e-04 1.05e-03 0.000 0.000 1.000 0.000 3.322
GENES:
AT2G35110.1
SENSE COUNTS: 1 0 2 0 10 5.66e-04 1.08e-03 0.000 0.000 1.000 0.000 3.322
TAGS:
d+1 TCTTTATCTT 1 0 2 0 10 5.66e-04 1.81e-03 4177 0.000 0.000 1.000 0.000 3.322

LOCUS: AT1G38203
DESCRIPTION: gypsy-like retrotransposon family (Athila), has a 3.5e-30 P-value blast match to GB
DATA: Control 30min 2hours 2days 1week p-value B&H Pos Fold change relative to control (log2)
SENSE COUNTS: 3 0 0 8 12 5.79e-04 1.08e-03 0.000 0.000 0.000 1.415 2.000
GENES:
AT1G38203.1
SENSE COUNTS: 3 0 0 8 12 5.79e-04 1.10e-03 0.000 0.000 0.000 1.415 2.000
TAGS:
p+2 AACTGAAAAT 3 0 0 8 12 5.79e-04 1.85e-03 661 0.000 0.000 0.000 1.415 2.000

LOCUS: AT1G66100
DESCRIPTION: thionin, putative, similar to thionin (Arabidopsis thaliana) GI
DATA: Control 30min 2hours 2days 1week p-value B&H Pos Fold change relative to control (log2)
SENSE COUNTS: 12 2 12 1 0 5.79e-04 1.07e-03 0.000 -2.585 0.000 -3.585 0.000
GENES:
AT1G66100.1
SENSE COUNTS: 12 2 12 1 0 5.79e-04 1.10e-03 0.000 -2.585 0.000 -3.585 0.000
TAGS:
d+1 TTAAGATAAT 12 2 12 1 0 5.79e-04 1.85e-03 584 0.000 -2.585 0.000 -3.585 0.000

LOCUS: AT1G72610
DESCRIPTION: germin-like protein (GER1), identical to germin-like protein subfamily 3 member 1 SP|P94040; contains Pfam profile
DATA: Control 30min 2hours 2days 1week p-value B&H Pos Fold change relative to control (log2)
SENSE COUNTS: 24 21 13 24 50 5.89e-04 1.09e-03 0.000 -0.193 -0.885 0.000 1.059
GENES:
AT1G72610.1
SENSE COUNTS: 24 21 13 24 50 5.89e-04 1.11e-03 0.000 -0.193 -0.885 0.000 1.059
TAGS:
d+1 GTTTTCCCAC 23 21 11 24 43 4.10e-03 9.26e-03 531 0.000 -0.131 -1.064 0.061 0.903
d+2 TCAAAGCTAC 1 0 2 0 7 9.07e-03 1.69e-02 247 0.000 0.000 1.000 0.000 2.807

LOCUS: AT5G51040
DESCRIPTION: expressed protein
DATA: Control 30min 2hours 2days 1week p-value B&H Pos Fold change relative to control (log2)

```
SENSE COUNTS:                 5      1      11     20     14     5.93e-04       1.10e-03              0.000  -2.322 1.138  2.000  1.485
  GENES:
  AT5G51040.1
    SENSE COUNTS:             5      1      11     20     14     5.93e-04       1.12e-03              0.000  -2.322 1.138  2.000  1.485
    TAGS:
      d+1      GCGTTGAGAT     5      1      11     20     14     5.93e-04       1.88e-03       737    0.000  -2.322 1.138  2.000  1.485

LOCUS: AT4G00730
DESCRIPTION:  homeodomain protein AHDP mRNA, complete cds
DATA:                         Control 30min 2hours 2days  1week  p-value        B&H            Pos    Fold change relative to control (log2)
SENSE COUNTS:                 0      5      0      8      0      6.05e-04       1.12e-03              0.000  2.322  0.000  3.000  0.000
  GENES:
  AT4G00730.1
    SENSE COUNTS:             0      5      0      8      0      6.05e-04       1.14e-03              0.000  2.322  0.000  3.000  0.000
    TAGS:
      d+1      CACCGTTCAG     0      5      0      6      0      6.76e-03       1.33e-02       2750   0.000  2.322  0.000  2.585  0.000
      d+2      TGGTGATGAA     0      0      0      2      0      2.03e-01       2.82e-01       2527   0.000  0.000  0.000  1.000  0.000
      d+2      CATTGACGCA     0      0      0      0      0      6.15e-01       6.51e-01       2468   0.000  0.000  0.000  0.000  0.000

LOCUS: AT2G22122
DESCRIPTION:  expressed protein
DATA:                         Control 30min 2hours 2days  1week  p-value        B&H            Pos    Fold change relative to control (log2)
SENSE COUNTS:                 0      0      0      0      4      6.24e-04       1.15e-03              0.000  0.000  0.000  0.000  2.000
  GENES:
  AT2G22122.1
    SENSE COUNTS:             0      0      0      0      4      6.24e-04       1.16e-03              0.000  0.000  0.000  0.000  2.000
    TAGS:
      d+1      TAAGATATAT     0      0      0      0      3      1.12e-02       2.00e-02       507    0.000  0.000  0.000  0.000  1.585
      X+4      GAGAAGCATA     0      0      0      0      1      1.65e-01       2.39e-01       -97    0.000  0.000  0.000  0.000  0.000

LOCUS: AT5G17760
DESCRIPTION:  AAA-type ATPase family protein, contains Pfam profile
DATA:                         Control 30min 2hours 2days  1week  p-value        B&H            Pos    Fold change relative to control (log2)
SENSE COUNTS:                 0      0      0      0      4      6.24e-04       1.15e-03              0.000  0.000  0.000  0.000  2.000
  GENES:
  AT5G17760.1
    SENSE COUNTS:             0      0      0      0      4      6.24e-04       1.16e-03              0.000  0.000  0.000  0.000  2.000
    TAGS:
      d+1      GGACATTGCT     0      0      0      0      3      1.12e-02       2.00e-02       1283   0.000  0.000  0.000  0.000  1.585
      d+2      GCTAATTACC     0      0      0      0      1      1.65e-01       2.38e-01       941    0.000  0.000  0.000  0.000  0.000
  AT5G17760.2
    SENSE COUNTS:             0      0      0      0      4      6.24e-04       1.17e-03              0.000  0.000  0.000  0.000  2.000
    TAGS:
      d+1      GGACATTGCT     0      0      0      0      3      1.12e-02       2.00e-02       1476   0.000  0.000  0.000  0.000  1.585
      d+2      GCTAATTACC     0      0      0      0      1      1.65e-01       2.38e-01       941    0.000  0.000  0.000  0.000  0.000

LOCUS: AT2G30270
DESCRIPTION:  expressed protein, contains Pfam profile PF04525
DATA:                         Control 30min 2hours 2days  1week  p-value        B&H            Pos    Fold change relative to control (log2)
SENSE COUNTS:                 0      0      0      0      5      6.24e-04       1.14e-03              0.000  0.000  0.000  0.000  2.322
  GENES:
  AT2G30270.1
    SENSE COUNTS:             0      0      0      0      5      6.24e-04       1.17e-03              0.000  0.000  0.000  0.000  2.322
    TAGS:
      d+1      AATATTGAGA     0      0      0      0      5      6.24e-04       1.95e-03       748    0.000  0.000  0.000  0.000  2.322

LOCUS: AT2G47770
DESCRIPTION:  benzodiazepine receptor-related, contains weak similarity to Peripheral-type benzodiazepine receptor (PBR) (PKBS) (Mitochondrial
benzodiazepine receptor) (Swiss-Prot
DATA:                         Control 30min 2hours 2days  1week  p-value        B&H            Pos    Fold change relative to control (log2)
```

```
SENSE COUNTS:                0      0      0      0      5      6.24e-04      1.14e-03            0.000  0.000  0.000  0.000  2.322
  GENES:
  AT2G47770.1
    SENSE COUNTS:            0      0      0      0      5      6.24e-04      1.17e-03            0.000  0.000  0.000  0.000  2.322
    TAGS:
      d+1      ATATACTTGA    0      0      0      0      5      6.24e-04      1.96e-03      974   0.000  0.000  0.000  0.000  2.322

LOCUS: AT2G28056
DESCRIPTION:  expressed protein
DATA:                        Control 30min  2hours 2days  1week  p-value       B&H           Pos   Fold change relative to control (log2)
SENSE COUNTS:                0      0      0      0      5      6.24e-04      1.14e-03            0.000  0.000  0.000  0.000  2.322
  GENES:
  AT2G28056.1
    SENSE COUNTS:            0      0      0      0      5      6.24e-04      1.16e-03            0.000  0.000  0.000  0.000  2.322
    TAGS:
      d+1      ATGATATCTT    0      0      0      0      5      6.24e-04      1.97e-03      981   0.000  0.000  0.000  0.000  2.322

LOCUS: AT4G27360
DESCRIPTION:  dynein light chain, putative, similar to SP|O02414 Dynein light chain LC6, flagellar outer arm {Anthocidaris crassispina}; contains Pfam
profile PF01221
DATA:                        Control 30min  2hours 2days  1week  p-value       B&H           Pos   Fold change relative to control (log2)
SENSE COUNTS:                0      0      0      0      5      6.24e-04      1.14e-03            0.000  0.000  0.000  0.000  2.322
  GENES:
  AT4G27360.1
    SENSE COUNTS:            0      0      0      0      5      6.24e-04      1.17e-03            0.000  0.000  0.000  0.000  2.322
    TAGS:
      d+1      ATTCGTCGTT    0      0      0      0      5      6.24e-04      1.96e-03      483   0.000  0.000  0.000  0.000  2.322

LOCUS: AT3G45850
DESCRIPTION:  kinesin motor protein-related, kinesin-related protein TKRP125, Nicotiana tabacum, PIR
DATA:                        Control 30min  2hours 2days  1week  p-value       B&H           Pos   Fold change relative to control (log2)
SENSE COUNTS:                0      0      0      0      5      6.24e-04      1.13e-03            0.000  0.000  0.000  0.000  2.322
  GENES:
  AT3G45850.1
    SENSE COUNTS:            0      0      0      0      5      6.24e-04      1.17e-03            0.000  0.000  0.000  0.000  2.322
    TAGS:
      d+2      GATGCCAATG    0      0      0      0      5      6.24e-04      1.94e-03      2674  0.000  0.000  0.000  0.000  2.322

LOCUS: AT4G26190
DESCRIPTION:  expressed protein
DATA:                        Control 30min  2hours 2days  1week  p-value       B&H           Pos   Fold change relative to control (log2)
SENSE COUNTS:                23     11     35     37     16     6.36e-04      1.15e-03            0.000  -1.064 0.606  0.686  -0.524
  GENES:
  AT4G26190.1
    SENSE COUNTS:            23     11     35     37     16     6.36e-04      1.18e-03            0.000  -1.064 0.606  0.686  -0.524
    TAGS:
      d+1      GGAGTTTTAC    22     11     32     34     14     2.47e-03      6.13e-03      3271  0.000  -1.000 0.541  0.628  -0.652
      d+2      CATCAGTTGT    0      0      1      0      0      4.55e-01      5.17e-01      3010  0.000  0.000  0.000  0.000  0.000
      d+2      AAAAGCGGGC    0      0      1      0      1      3.96e-01      5.02e-01      1454  0.000  0.000  0.000  0.000  0.000
      d+2      TGAGAAGCCT    0      0      0      2      0      4.80e-02      7.96e-02      679   0.000  0.000  0.000  1.000  0.000
      d+2      TGAAGATTCT    1      0      1      1      1      7.91e-01      7.95e-01      463   0.000  0.000  0.000  0.000  0.000

LOCUS: AT4G13340
DESCRIPTION:  leucine-rich repeat family protein / extensin family protein, similar to extensin-like protein (Lycopersicon esculentum)
gi|5917664|gb|AAD55979; contains leucine-rich repeats, Pfam
DATA:                        Control 30min  2hours 2days  1week  p-value       B&H           Pos   Fold change relative to control (log2)
SENSE COUNTS:                1      15     4      8      1      6.36e-04      1.15e-03            0.000  3.907  2.000  3.000  0.000
  GENES:
  AT4G13340.1
    SENSE COUNTS:            1      15     4      8      1      6.36e-04      1.18e-03            0.000  3.907  2.000  3.000  0.000
```

TAGS:														
d+1	GTTCACCACA	1	15	3	8	1	3.10e-04	1.09e-03	2155	0.000	3.907	1.585	3.000	0.000
d+2	AACAATGGTC	0	0	1	0	0	4.55e-01	5.40e-01	793	0.000	0.000	0.000	0.000	0.000

LOCUS: AT5G63160
DESCRIPTION: speckle-type POZ protein-related, contains Pfam PF00651

DATA:		Control	30min	2hours	2days	1week	p-value	B&H	Pos	Fold change relative to control (log2)				
SENSE COUNTS:		1	11	3	0	1	6.39e-04	1.15e-03		0.000	3.459	1.585	0.000	0.000
GENES:														
AT5G63160.1														
SENSE COUNTS:		1	11	3	0	1	6.39e-04	1.18e-03		0.000	3.459	1.585	0.000	0.000
TAGS:														
d+1	TCTTCGTTGT	1	11	3	0	0	1.81e-04	6.90e-04	1090	0.000	3.459	1.585	0.000	0.000
d+2	TCAAGCAAAA	0	0	0	0	1	1.65e-01	2.38e-01	804	0.000	0.000	0.000	0.000	0.000

LOCUS: AT1G68050
DESCRIPTION: F-box family protein (FKF1) / adagio 3 (ADO3), E3 ubiquitin ligase SCF complex F-box subunit; identical to FKF1 GI

DATA:		Control	30min	2hours	2days	1week	p-value	B&H	Pos	Fold change relative to control (log2)				
SENSE COUNTS:		0	1	11	1	8	6.53e-04	1.18e-03		0.000	0.000	3.459	0.000	3.000
GENES:														
AT1G68050.1														
SENSE COUNTS:		0	1	11	1	8	6.53e-04	1.20e-03		0.000	0.000	3.459	0.000	3.000
TAGS:														
d+1	TAATGTTAAT	0	1	10	1	8	1.61e-03	4.27e-03	2068	0.000	0.000	3.322	0.000	3.000
d+2	CGATCGGAGA	0	0	1	0	0	4.55e-01	5.09e-01	78	0.000	0.000	0.000	0.000	0.000

LOCUS: AT3G02170
DESCRIPTION: expressed protein

DATA:		Control	30min	2hours	2days	1week	p-value	B&H	Pos	Fold change relative to control (log2)				
SENSE COUNTS:		1	8	1	0	0	6.67e-04	1.20e-03		0.000	3.000	0.000	0.000	0.000
GENES:														
AT3G02170.1														
SENSE COUNTS:		1	8	1	0	0	6.67e-04	1.23e-03		0.000	3.000	0.000	0.000	0.000
TAGS:														
d+1	AACTTGAAGG	1	7	1	0	0	6.05e-03	1.24e-02	3009	0.000	2.807	0.000	0.000	0.000
d+2	ATAAGCTTTC	0	1	0	0	0	2.54e-01	3.45e-01	2613	0.000	0.000	0.000	0.000	0.000

LOCUS: AT5G62280
DESCRIPTION: expressed protein

DATA:		Control	30min	2hours	2days	1week	p-value	B&H	Pos	Fold change relative to control (log2)				
SENSE COUNTS:		1	8	1	0	0	6.67e-04	1.20e-03		0.000	3.000	0.000	0.000	0.000
GENES:														
AT5G62280.1														
SENSE COUNTS:		1	8	1	0	0	6.67e-04	1.22e-03		0.000	3.000	0.000	0.000	0.000
TAGS:														
d+1	TGTTTAGGGT	1	8	1	0	0	6.67e-04	2.06e-03	748	0.000	3.000	0.000	0.000	0.000

LOCUS: AT4G30690
DESCRIPTION: translation initiation factor 3 (IF-3) family protein, similar to SP|P33319 Translation initiation factor IF-3 {Proteus vulgaris}; contains Pfam profiles PF00707

DATA:		Control	30min	2hours	2days	1week	p-value	B&H	Pos	Fold change relative to control (log2)				
SENSE COUNTS:		12	16	4	3	0	6.67e-04	1.20e-03		0.000	0.415	-1.585	-2.000	0.000
GENES:														
AT4G30690.1														
SENSE COUNTS:		12	16	4	3	0	6.67e-04	1.23e-03		0.000	0.415	-1.585	-2.000	0.000
TAGS:														
d+1	TAATTGTAAC	9	9	2	1	0	9.61e-03	1.78e-02	987	0.000	0.000	-2.170	-3.170	0.000
d+2	AAAGGCCGAG	3	7	2	2	0	1.75e-01	2.47e-01	661	0.000	1.222	-0.585	-0.585	0.000
d+2	ATTGGTTTAG	0	0	0	0	0	6.15e-01	6.35e-01	370	0.000	0.000	0.000	0.000	0.000

```
LOCUS: AT1G56580
DESCRIPTION:  expressed protein, contains Pfam profile PF04398
DATA:                         Control 30min   2hours  2days   1week   p-value         B&H             Pos     Fold change relative to control (log2)
SENSE COUNTS:                 10      4       1       19      12      6.78e-04        1.21e-03                0.000   -1.322  -3.322  0.926   0.263
  GENES:
  AT1G56580.1
    SENSE COUNTS:             10      4       1       19      12      6.78e-04        1.24e-03                0.000   -1.322  -3.322  0.926   0.263
    TAGS:
      d+1      AATTACTTTC     10      4       1       19      12      6.78e-04        2.08e-03        593     0.000   -1.322  -3.322  0.926   0.263

LOCUS: AT1G48750
DESCRIPTION:  protease inhibitor/seed storage/lipid transfer protein (LTP) family protein, similar to TED4 (Zinnia elegans) GI
DATA:                         Control 30min   2hours  2days   1week   p-value         B&H             Pos     Fold change relative to control (log2)
SENSE COUNTS:                 10      2       8       5       23      6.80e-04        1.21e-03                0.000   -2.322  -0.322  -1.000  1.202
  GENES:
  AT1G48750.1
    SENSE COUNTS:             10      2       8       5       23      6.80e-04        1.24e-03                0.000   -2.322  -0.322  -1.000  1.202
    TAGS:
      d+1      CTTGAATGTT     10      2       8       5       23      6.80e-04        2.08e-03        481     0.000   -2.322  -0.322  -1.000  1.202

LOCUS: AT3G47520
DESCRIPTION:  malate dehydrogenase (NAD), chloroplast (MDH), identical to chloroplast NAD-malate dehydrogenase (Arabidopsis thaliana) GI
DATA:                         Control 30min   2hours  2days   1week   p-value         B&H             Pos     Fold change relative to control (log2)
SENSE COUNTS:                 2       0       6       13      14      6.85e-04        1.22e-03                0.000   0.000   1.585   2.700   2.807
  GENES:
  AT3G47520.1
    SENSE COUNTS:             2       0       6       13      14      6.85e-04        1.25e-03                0.000   0.000   1.585   2.700   2.807
    TAGS:
      d+1      GCATTGAAGC     2       0       6       11      14      7.03e-04        2.15e-03        1520    0.000   0.000   1.585   2.459   2.807
      d+2      CTGGAATCAC     0       0       0       2       0       2.03e-01        2.83e-01        948     0.000   0.000   0.000   1.000   0.000

LOCUS: AT2G24820
DESCRIPTION:  Rieske (2Fe-2S) domain-containing protein, similar to Rieske iron-sulfur protein Tic55 from Pisum sativum (gi
DATA:                         Control 30min   2hours  2days   1week   p-value         B&H             Pos     Fold change relative to control (log2)
SENSE COUNTS:                 4       2       16      2       5       7.09e-04        1.26e-03                0.000   -1.000  2.000   -1.000  0.322
  GENES:
  AT2G24820.1
    SENSE COUNTS:             4       2       16      2       5       7.09e-04        1.29e-03                0.000   -1.000  2.000   -1.000  0.322
    TAGS:
      d+2      AAATAAATTT     4       2       15      2       5       2.10e-03        5.34e-03        1729    0.000   -1.000  1.907   -1.000  0.322
      d+2      GGTCGCTGAA     0       0       1       0       0       4.55e-01        5.36e-01        1241    0.000   0.000   0.000   0.000   0.000

LOCUS: AT3G21560
DESCRIPTION:  UDP-glucosyltransferase, putative, similar to UDP-glucose
DATA:                         Control 30min   2hours  2days   1week   p-value         B&H             Pos     Fold change relative to control (log2)
SENSE COUNTS:                 0       0       6       11      8       7.16e-04        1.27e-03                0.000   0.000   2.585   3.459   3.000
  GENES:
  AT3G21560.1
    SENSE COUNTS:             0       0       6       11      8       7.16e-04        1.30e-03                0.000   0.000   2.585   3.459   3.000
    TAGS:
      d+1      TCTTGGCTGG     0       0       5       11      7       8.94e-04        2.63e-03        1541    0.000   0.000   2.322   3.459   2.807
      d+2      ATCGATGTTT     0       0       1       0       1       3.96e-01        5.01e-01        1276    0.000   0.000   0.000   0.000   0.000

LOCUS: AT1G54370
DESCRIPTION:  sodium proton exchanger, putative (NHX5), contains similarity to Na+/H+ antiporter GI
DATA:                         Control 30min   2hours  2days   1week   p-value         B&H             Pos     Fold change relative to control (log2)
SENSE COUNTS:                 5       0       0       0       0       7.17e-04        1.27e-03                0.000   0.000   0.000   0.000   0.000
  GENES:
  AT1G54370.1
    SENSE COUNTS:             5       0       0       0       0       7.17e-04        1.30e-03                0.000   0.000   0.000   0.000   0.000
```

TAGS:
d+1 TCAATAATCT 5 0 0 0 0 7.17e-04 2.19e-03 1748 0.000 0.000 0.000 0.000 0.000

LOCUS: AT5G52450
DESCRIPTION: MATE efflux protein-related, strong similarity to unknown protein (pir||T02324); contains Pfam profile PF01554 Uncharacterized membrane protein family
DATA: Control 30min 2hours 2days 1week p-value B&H Pos Fold change relative to control (log2)
SENSE COUNTS: 0 0 0 5 0 7.27e-04 1.29e-03 0.000 0.000 0.000 2.322 0.000
GENES:
AT5G52450.1
SENSE COUNTS: 0 0 0 5 0 7.27e-04 1.32e-03 0.000 0.000 0.000 2.322 0.000
TAGS:
d+1 AAATTTCTAG 0 0 0 5 0 7.27e-04 2.21e-03 1704 0.000 0.000 0.000 2.322 0.000

LOCUS: AT4G26600
DESCRIPTION: nucleolar protein, putative, similar to SP|P46087 Proliferating-cell nucleolar antigen p120 (Proliferation-associated nucleolar protein p120) {Homo sapiens}; contains Pfam profile PF01189
DATA: Control 30min 2hours 2days 1week p-value B&H Pos Fold change relative to control (log2)
SENSE COUNTS: 0 0 0 5 0 7.27e-04 1.28e-03 0.000 0.000 0.000 2.322 0.000
GENES:
AT4G26600.1
SENSE COUNTS: 0 0 0 5 0 7.27e-04 1.32e-03 0.000 0.000 0.000 2.322 0.000
TAGS:
d+1 TCCAAAAGTT 0 0 0 5 0 7.27e-04 2.21e-03 2263 0.000 0.000 0.000 2.322 0.000

LOCUS: AT1G17050
DESCRIPTION: geranyl diphosphate synthase, putative / GPPS, putative / dimethylallyltransferase, putative / prenyl transferase, putative, similar to GI
DATA: Control 30min 2hours 2days 1week p-value B&H Pos Fold change relative to control (log2)
SENSE COUNTS: 0 0 0 5 0 7.27e-04 1.28e-03 0.000 0.000 0.000 2.322 0.000
GENES:
AT1G17050.1
SENSE COUNTS: 0 0 0 5 0 7.27e-04 1.31e-03 0.000 0.000 0.000 2.322 0.000
TAGS:
X+4 CTTATTATTG 0 0 0 5 0 7.27e-04 2.20e-03 666 0.000 0.000 0.000 2.322 0.000

LOCUS: AT3G28740
DESCRIPTION: cytochrome P450 family protein, contains Pfam profile
DATA: Control 30min 2hours 2days 1week p-value B&H Pos Fold change relative to control (log2)
SENSE COUNTS: 4 0 13 13 14 7.34e-04 1.29e-03 0.000 0.000 1.700 1.700 1.807
GENES:
AT3G28740.1
SENSE COUNTS: 4 0 13 13 14 7.34e-04 1.32e-03 0.000 0.000 1.700 1.700 1.807
TAGS:
d+1 AGAGAAAGTG 1 0 12 9 14 1.72e-04 6.60e-04 1427 0.000 0.000 3.585 3.170 3.807
d+2 GGCCATCCAT 3 0 1 4 0 1.19e-01 1.85e-01 1210 0.000 0.000 -1.585 0.415 0.000

LOCUS: AT5G58740
DESCRIPTION: nuclear movement family protein, contains Pfam profile
DATA: Control 30min 2hours 2days 1week p-value B&H Pos Fold change relative to control (log2)
SENSE COUNTS: 0 7 1 0 10 7.96e-04 1.40e-03 0.000 2.807 0.000 0.000 3.322
GENES:
AT5G58740.1
SENSE COUNTS: 0 7 1 0 10 7.96e-04 1.43e-03 0.000 2.807 0.000 0.000 3.322
TAGS:
d+1 TCTCATAAAT 0 7 1 0 10 7.96e-04 2.40e-03 605 0.000 2.807 0.000 0.000 3.322

LOCUS: AT1G31660
DESCRIPTION: bystin family, contains Pfam profile
DATA: Control 30min 2hours 2days 1week p-value B&H Pos Fold change relative to control (log2)

SENSE COUNTS:		1	0	0	5	10	8.06e-04	1.41e-03		0.000	0.000	0.000	2.322	3.322
GENES:														
AT1G31660.1														
SENSE COUNTS:		1	0	0	5	10	8.06e-04	1.44e-03		0.000	0.000	0.000	2.322	3.322
TAGS:														
X+4	GTTTAATCTT	1	0	0	5	7	1.64e-02	2.88e-02	245	0.000	0.000	0.000	2.322	2.807
X+4	ATAGTGTATC	0	0	0	0	3	1.12e-02	2.02e-02	225	0.000	0.000	0.000	0.000	1.585

LOCUS: AT5G15520
DESCRIPTION: 40S ribosomal protein S19 (RPS19B), 40S RIBOSOMAL PROTEIN S19 - Oryza sativa, SWISSPROT

DATA:		Control	30min	2hours	2days	1week	p-value	B&H	Pos	Fold change relative to control (log2)				
SENSE COUNTS:		0	0	1	2	8	8.12e-04	1.42e-03		0.000	0.000	0.000	1.000	3.000
GENES:														
AT5G15520.1														
SENSE COUNTS:		0	0	1	2	8	8.12e-04	1.45e-03		0.000	0.000	0.000	1.000	3.000
TAGS:														
d+1	AAGAGCTGTT	0	0	1	2	8	8.12e-04	2.43e-03	463	0.000	0.000	0.000	1.000	3.000

LOCUS: AT3G27060
DESCRIPTION: ribonucleoside-diphosphate reductase small chain, putative / ribonucleotide reductase, putative, similar to ribonucleotide reductase R2 (Nicotiana tabacum) GI

DATA:		Control	30min	2hours	2days	1week	p-value	B&H	Pos	Fold change relative to control (log2)				
SENSE COUNTS:		9	1	1	0	0	8.15e-04	1.42e-03		0.000	-3.170	-3.170	0.000	0.000
GENES:														
AT3G27060.1														
SENSE COUNTS:		9	1	1	0	0	8.15e-04	1.46e-03		0.000	-3.170	-3.170	0.000	0.000
TAGS:														
d+1	TCTTCTCTCT	9	1	1	0	0	8.15e-04	2.44e-03	1212	0.000	-3.170	-3.170	0.000	0.000

LOCUS: AT2G36830
DESCRIPTION: major intrinsic family protein / MIP family protein, contains Pfam profile

DATA:		Control	30min	2hours	2days	1week	p-value	B&H	Pos	Fold change relative to control (log2)				
SENSE COUNTS:		20	21	6	8	1	8.18e-04	1.43e-03		0.000	0.070	-1.737	-1.322	-4.322
GENES:														
AT2G36830.1														
SENSE COUNTS:		20	21	6	8	1	8.18e-04	1.46e-03		0.000	0.070	-1.737	-1.322	-4.322
TAGS:														
d+1	GACCAACCAC	20	21	6	8	1	8.18e-04	2.43e-03	703	0.000	0.070	-1.737	-1.322	-4.322

LOCUS: AT2G42220
DESCRIPTION: rhodanese-like domain-containing protein, contains rhodanese-like domain PF

DATA:		Control	30min	2hours	2days	1week	p-value	B&H	Pos	Fold change relative to control (log2)				
SENSE COUNTS:		39	19	14	18	10	8.22e-04	1.43e-03		0.000	-1.037	-1.478	-1.115	-1.963
GENES:														
AT2G42220.1														
SENSE COUNTS:		39	19	14	18	10	8.22e-04	1.46e-03		0.000	-1.037	-1.478	-1.115	-1.963
TAGS:														
d+1	TTTTCACATT	39	19	14	18	10	8.22e-04	2.44e-03	755	0.000	-1.037	-1.478	-1.115	-1.963

LOCUS: AT1G70890
DESCRIPTION: major latex protein-related / MLP-related, low similarity to major latex protein {Papaver somniferum}(GI

DATA:		Control	30min	2hours	2days	1week	p-value	B&H	Pos	Fold change relative to control (log2)				
SENSE COUNTS:		2	10	1	1	0	8.30e-04	1.44e-03		0.000	2.322	-1.000	-1.000	0.000
GENES:														
AT1G70890.1														
SENSE COUNTS:		2	10	1	1	0	8.30e-04	1.48e-03		0.000	2.322	-1.000	-1.000	0.000
TAGS:														
d+1	ATGGAAAGTT	1	10	1	1	0	2.84e-04	1.02e-03	255	0.000	3.322	0.000	0.000	0.000
d+2	GCAGAAGCGT	1	0	0	0	0	4.28e-01	5.30e-01	59	0.000	0.000	0.000	0.000	0.000

```
LOCUS: AT3G17210
DESCRIPTION:  stable protein 1-related, similar to stable protein 1 (GI
DATA:                         Control 30min   2hours  2days   1week   p-value        B&H            Pos     Fold change relative to control (log2)
SENSE COUNTS:                 17      8       8       24      32      8.38e-04       1.45e-03               0.000   -1.087  -1.087  0.497   0.913
  GENES:
  AT3G17210.1
    SENSE COUNTS:             17      8       8       24      32      8.38e-04       1.49e-03               0.000   -1.087  -1.087  0.497   0.913
    TAGS:
      d+1      AGTTTATTAA     17      8       8       24      32      8.38e-04       2.48e-03       549     0.000   -1.087  -1.087  0.497   0.913

LOCUS: AT2G02510
DESCRIPTION:  expressed protein
DATA:                         Control 30min   2hours  2days   1week   p-value        B&H            Pos     Fold change relative to control (log2)
SENSE COUNTS:                 4       2       6       4       19      8.39e-04       1.45e-03               0.000   -1.000  0.585   0.000   2.248
  GENES:
  AT2G02510.1
    SENSE COUNTS:             4       2       6       4       19      8.39e-04       1.49e-03               0.000   -1.000  0.585   0.000   2.248
    TAGS:
      X+4      TATGGTTAAA     0       0       1       0       0       4.55e-01       5.17e-01       447     0.000   0.000   0.000   0.000   0.000
      d+1      GTTTCGTTAT     4       2       5       4       19      5.85e-04       1.86e-03       427     0.000   -1.000  0.322   0.000   2.248

LOCUS: AT5G42300
DESCRIPTION:  ubiquitin family protein, contains INTERPRO
DATA:                         Control 30min   2hours  2days   1week   p-value        B&H            Pos     Fold change relative to control (log2)
SENSE COUNTS:                 20      5       4       12      20      8.40e-04       1.45e-03               0.000   -2.000  -2.322  -0.737  0.000
  GENES:
  AT5G42300.1
    SENSE COUNTS:             20      5       4       12      20      8.40e-04       1.49e-03               0.000   -2.000  -2.322  -0.737  0.000
    TAGS:
      d+1      TTACATCTAA     20      5       4       12      19      1.43e-03       3.86e-03       391     0.000   -2.000  -2.322  -0.737  -0.074
      d+2      GGTCTTGAGC     0       0       0       0       1       1.65e-01       2.42e-01       297     0.000   0.000   0.000   0.000   0.000

LOCUS: AT2G46680
DESCRIPTION:  homeobox-leucine zipper protein 7 (HB-7) / HD-ZIP transcription factor 7, identical to homeobox-leucine zipper protein ATHB-7 (HD-ZIP
protein ATHB-7) (SP
DATA:                         Control 30min   2hours  2days   1week   p-value        B&H            Pos     Fold change relative to control (log2)
SENSE COUNTS:                 0       0       0       2       8       8.59e-04       1.48e-03               0.000   0.000   0.000   1.000   3.000
  GENES:
  AT2G46680.1
    SENSE COUNTS:             0       0       0       2       8       8.59e-04       1.52e-03               0.000   0.000   0.000   1.000   3.000
    TAGS:
      d+1      CTTTGTAGCT     0       0       0       2       8       8.59e-04       2.53e-03       1228    0.000   0.000   0.000   1.000   3.000

LOCUS: AT4G34350
DESCRIPTION:  LytB family protein, contains Pfam profile
DATA:                         Control 30min   2hours  2days   1week   p-value        B&H            Pos     Fold change relative to control (log2)
SENSE COUNTS:                 22      7       9       1       8       8.70e-04       1.50e-03               0.000   -1.652  -1.290  -4.459  -1.459
  GENES:
  AT4G34350.1
    SENSE COUNTS:             22      7       9       1       8       8.70e-04       1.53e-03               0.000   -1.652  -1.290  -4.459  -1.459
    TAGS:
      d+1      TCAAGAGTGT     22      7       8       1       8       6.77e-04       2.08e-03       1515    0.000   -1.652  -1.459  -4.459  -1.459
      d+2      GAGAAATTCA     0       0       1       0       0       4.55e-01       5.26e-01       934     0.000   0.000   0.000   0.000   0.000

LOCUS: AT4G05320
DESCRIPTION:  polyubiquitin (UBQ10) (SEN3), senescence-associated protein; identical to GI
DATA:                         Control 30min   2hours  2days   1week   p-value        B&H            Pos     Fold change relative to control (log2)
SENSE COUNTS:                 120     105     153     149     89      8.85e-04       1.52e-03               0.000   -0.193  0.350   0.312   -0.431
  GENES:
  AT4G05320.1
```

```
    SENSE COUNTS:              120    105    153    148    89     1.04e-03       1.79e-03              0.000   -0.193 0.350   0.303   -0.431
    TAGS:
      d+1      TAAAACTTTG      120    105    153    148    89     1.04e-03       2.96e-03       1276   0.000   -0.193 0.350   0.303   -0.431
  AT4G05320.4
    SENSE COUNTS:              120    105    153    148    89     1.04e-03       1.78e-03              0.000   -0.193 0.350   0.303   -0.431
    TAGS:
      d+1      TAAAACTTTG      120    105    153    148    89     1.04e-03       2.96e-03       1469   0.000   -0.193 0.350   0.303   -0.431
  AT4G05320.5
    SENSE COUNTS:              120    105    153    148    89     1.04e-03       1.79e-03              0.000   -0.193 0.350   0.303   -0.431
    TAGS:
      d+1      TAAAACTTTG      120    105    153    148    89     1.04e-03       2.96e-03       1048   0.000   -0.193 0.350   0.303   -0.431
  AT4G05320.2
    SENSE COUNTS:              0      0      0      1      0      3.09e-01       3.12e-01              0.000   0.000  0.000   0.000   0.000
    TAGS:
      d+1      TTAATCTTTT      0      0      0      1      0      3.09e-01       4.13e-01       1793   0.000   0.000  0.000   0.000   0.000
  AT4G05320.3
    SENSE COUNTS:              120    105    153    148    89     1.04e-03       1.79e-03              0.000   -0.193 0.350   0.303   -0.431
    TAGS:
      d+1      TAAAACTTTG      120    105    153    148    89     1.04e-03       2.96e-03       1276   0.000   -0.193 0.350   0.303   -0.431

LOCUS: AT2G35490
DESCRIPTION:  plastid-lipid associated protein PAP, putative, similar to plastid-lipid associated protein PAP3 (Brassica rapa) GI
DATA:                          Control 30min  2hours 2days  1week  p-value        B&H            Pos    Fold change relative to control (log2)
SENSE COUNTS:                  11     2      1      1      12     9.04e-04       1.55e-03              0.000   -2.459 -3.459  -3.459  0.126
  GENES:
  AT2G35490.1
    SENSE COUNTS:              11     2      1      1      12     9.04e-04       1.59e-03              0.000   -2.459 -3.459  -3.459  0.126
    TAGS:
      d+1      TAAAAACCGT      11     2      1      1      12     9.04e-04       2.66e-03       1248   0.000   -2.459 -3.459  -3.459  0.126

LOCUS: AT3G05500
DESCRIPTION:  rubber elongation factor (REF) family protein, contains Pfam profile
DATA:                          Control 30min  2hours 2days  1week  p-value        B&H            Pos    Fold change relative to control (log2)
SENSE COUNTS:                  2      1      6      15     14     9.07e-04       1.55e-03              0.000   -1.000 1.585   2.907   2.807
  GENES:
  AT3G05500.1
    SENSE COUNTS:              2      1      6      15     14     9.07e-04       1.59e-03              0.000   -1.000 1.585   2.907   2.807
    TAGS:
      d+1      AACTTTTGTT      2      1      6      15     14     9.07e-04       2.66e-03       954    0.000   -1.000 1.585   2.907   2.807

LOCUS: AT5G16110
DESCRIPTION:  expressed protein, hypothetical protein T26J14.6 - Arabidopsis thaliana, EMBL
DATA:                          Control 30min  2hours 2days  1week  p-value        B&H            Pos    Fold change relative to control (log2)
SENSE COUNTS:                  0      6      0      0      0      9.09e-04       1.55e-03              0.000   2.585  0.000   0.000   0.000
  GENES:
  AT5G16110.1
    SENSE COUNTS:              0      6      0      0      0      9.09e-04       1.59e-03              0.000   2.585  0.000   0.000   0.000
    TAGS:
      d+1      GCTTAGTAGA      0      6      0      0      0      9.09e-04       2.66e-03       902    0.000   2.585  0.000   0.000   0.000

LOCUS: AT3G23600
DESCRIPTION:  dienelactone hydrolase family protein, similar to SP|Q9ZT66 Endo-1,3;1,4-beta-D-glucanase precursor (EC 3.2.1.-) {Zea mays}; contains
Pfam profile
DATA:                          Control 30min  2hours 2days  1week  p-value        B&H            Pos    Fold change relative to control (log2)
SENSE COUNTS:                  0      6      0      0      0      9.09e-04       1.55e-03              0.000   2.585  0.000   0.000   0.000
  GENES:
  AT3G23600.1
    SENSE COUNTS:              0      6      0      0      0      9.09e-04       1.59e-03              0.000   2.585  0.000   0.000   0.000
    TAGS:
      d+1      GCTGTGATAA      0      6      0      0      0      9.09e-04       2.64e-03       331    0.000   2.585  0.000   0.000   0.000
```

```
LOCUS: AT1G77210
DESCRIPTION:  sugar transporter, putative, similar to monosaccharide transporter PaMst-1 (Picea abies) GI
DATA:                          Control 30min  2hours  2days  1week  p-value     B&H         Pos   Fold change relative to control (log2)
SENSE COUNTS:                  0       6      0       0      0      9.09e-04    1.55e-03          0.000  2.585  0.000  0.000  0.000
  GENES:
  AT1G77210.1
    SENSE COUNTS:              0       6      0       0      0      9.09e-04    1.59e-03          0.000  2.585  0.000  0.000  0.000
    TAGS:
      d+1     GTTGTAGTAG       0       6      0       0      0      9.09e-04    2.65e-03    1205  0.000  2.585  0.000  0.000  0.000

LOCUS: AT4G29350
DESCRIPTION:  profilin 2 (PRO2) (PFN2) (PRF2), identical to profilin 2 SP
DATA:                          Control 30min  2hours  2days  1week  p-value     B&H         Pos   Fold change relative to control (log2)
SENSE COUNTS:                  11      32     11      11     17     9.36e-04    1.59e-03          0.000  1.541  0.000  0.000  0.628
  GENES:
  AT4G29350.1
    SENSE COUNTS:              11      32     11      11     17     9.36e-04    1.63e-03          0.000  1.541  0.000  0.000  0.628
    TAGS:
      d+1     TATTGTGATT       11      32     6       11     17     5.76e-05    2.53e-04    577   0.000  1.541  -0.874 0.000  0.628
      d+2     GTTGTCCAAG       0       0      3       0      0      2.70e-02    4.61e-02    326   0.000  0.000  1.585  0.000  0.000
      d+2     TGCGAGGTCG       0       0      2       0      0      1.21e-01    1.88e-01    143   0.000  0.000  1.000  0.000  0.000

LOCUS: AT1G45760
DESCRIPTION:  hAT-like transposase family (hobo/Ac/Tam3), has a 2.6e-37 P-value blast match to GB
DATA:                          Control 30min  2hours  2days  1week  p-value     B&H         Pos   Fold change relative to control (log2)
SENSE COUNTS:                  9       0      1       4      0      9.64e-04    1.63e-03          0.000  0.000  -3.170 -1.170 0.000
  GENES:
  AT1G45760.1
    SENSE COUNTS:              9       0      1       4      0      9.64e-04    1.68e-03          0.000  0.000  -3.170 -1.170 0.000
    TAGS:
      p+1     GAGGCTTTTG       9       0      1       4      0      9.64e-04    2.79e-03    369   0.000  0.000  -3.170 -1.170 0.000

LOCUS: AT1G60190
DESCRIPTION:  armadillo/beta-catenin repeat family protein / U-box domain-containing protein, contains Pfam domain, PF00514
DATA:                          Control 30min  2hours  2days  1week  p-value     B&H         Pos   Fold change relative to control (log2)
SENSE COUNTS:                  0       0      0       6      5      9.65e-04    1.63e-03          0.000  0.000  0.000  2.585  2.322
  GENES:
  AT1G60190.1
    SENSE COUNTS:              0       0      0       6      5      9.65e-04    1.68e-03          0.000  0.000  0.000  2.585  2.322
    TAGS:
      d+1     GGGTCGCTTT       0       0      0       6      5      9.65e-04    2.79e-03    1912  0.000  0.000  0.000  2.585  2.322

LOCUS: AT1G67740
DESCRIPTION:  photosystem II core complex proteins psbY, chloroplast (PSBY) / L-arginine metabolising enzyme, identical to SP
DATA:                          Control 30min  2hours  2days  1week  p-value     B&H         Pos   Fold change relative to control (log2)
SENSE COUNTS:                  18      36     13      28     10     9.78e-04    1.65e-03          0.000  1.000  -0.469 0.637  -0.848
  GENES:
  AT1G67740.1
    SENSE COUNTS:              18      36     13      28     10     9.78e-04    1.70e-03          0.000  1.000  -0.469 0.637  -0.848
    TAGS:
      d+1     GGTCCTTTAC       9       36     8       20     10     3.81e-06    2.29e-05    447   0.000  2.000  -0.170 1.152  0.152
      d+2     TCTCTAAACC       9       0      5       8      0      1.80e-02    3.14e-02    193   0.000  0.000  -0.848 -0.170 0.000

LOCUS: AT5G53970
DESCRIPTION:  aminotransferase, putative, similar to nicotianamine aminotransferase from Hordeum vulgare (GI
DATA:                          Control 30min  2hours  2days  1week  p-value     B&H         Pos   Fold change relative to control (log2)
SENSE COUNTS:                  5       0      2       8      14     1.01e-03    1.70e-03          0.000  0.000  -1.322 0.678  1.485
  GENES:
  AT5G53970.1
```

SENSE COUNTS: 5 0 2 8 14 1.01e-03 1.75e-03 0.000 0.000 -1.322 0.678 1.485
TAGS:
d+1 TTTAAAAACA 5 0 2 8 14 1.01e-03 2.90e-03 1424 0.000 0.000 -1.322 0.678 1.485

LOCUS: AT1G02820
DESCRIPTION: late embryogenesis abundant 3 family protein / LEA3 family protein, similar to late embryogenis abundant protein 5 GI
DATA: Control 30min 2hours 2days 1week p-value B&H Pos Fold change relative to control (log2)
SENSE COUNTS: 1 0 1 9 3 1.03e-03 1.73e-03 0.000 0.000 0.000 3.170 1.585
GENES:
AT1G02820.1
SENSE COUNTS: 1 0 1 9 3 1.03e-03 1.78e-03 0.000 0.000 0.000 3.170 1.585
TAGS:
d+1 GGTTCCAGAT 1 0 1 9 3 1.03e-03 2.95e-03 347 0.000 0.000 0.000 3.170 1.585

LOCUS: AT3G19680
DESCRIPTION: expressed protein
DATA: Control 30min 2hours 2days 1week p-value B&H Pos Fold change relative to control (log2)
SENSE COUNTS: 4 13 3 0 1 1.03e-03 1.73e-03 0.000 1.700 -0.415 0.000 -2.000
GENES:
AT3G19680.1
SENSE COUNTS: 4 13 3 0 1 1.03e-03 1.78e-03 0.000 1.700 -0.415 0.000 -2.000
TAGS:
d+1 AGTTGTCTTC 4 13 3 0 1 1.03e-03 2.94e-03 1676 0.000 1.700 -0.415 0.000 -2.000

LOCUS: AT4G09130
DESCRIPTION: zinc finger (C3HC4-type RING finger) family protein, contains Pfam profile
DATA: Control 30min 2hours 2days 1week p-value B&H Pos Fold change relative to control (log2)
SENSE COUNTS: 1 0 2 0 8 1.04e-03 1.74e-03 0.000 0.000 1.000 0.000 3.000
GENES:
AT4G09130.1
SENSE COUNTS: 1 0 2 0 8 1.04e-03 1.79e-03 0.000 0.000 1.000 0.000 3.000
TAGS:
v+2 TAATAATAAT 1 0 2 0 8 1.04e-03 2.96e-03 965 0.000 0.000 1.000 0.000 3.000

LOCUS: AT3G48420
DESCRIPTION: haloacid dehalogenase-like hydrolase family protein, low similarity to SP|P95649 CbbY protein {Rhodobacter sphaeroides}; contains InterPro accession IPR005834
DATA: Control 30min 2hours 2days 1week p-value B&H Pos Fold change relative to control (log2)
SENSE COUNTS: 13 3 6 0 14 1.04e-03 1.74e-03 0.000 -2.115 -1.115 0.000 0.107
GENES:
AT3G48420.1
SENSE COUNTS: 13 3 6 0 14 1.04e-03 1.78e-03 0.000 -2.115 -1.115 0.000 0.107
TAGS:
i+3 AATATGCGAG 0 0 2 0 1 2.22e-01 3.07e-01 1214 0.000 0.000 1.000 0.000 0.000
d+1 TAAAACCATA 13 3 3 0 12 8.53e-04 2.52e-03 1133 0.000 -2.115 -2.115 0.000 -0.115
i+3 ATGCTATGAA 0 0 0 0 1 1.65e-01 2.40e-01 941 0.000 0.000 0.000 0.000 0.000
d+2 CTTGCTTGGA 0 0 1 0 0 4.55e-01 5.32e-01 729 0.000 0.000 0.000 0.000 0.000

LOCUS: AT1G04690
DESCRIPTION: potassium channel protein, putative, nearly identical to K+ channel protein (Arabidopsis thaliana) GI
DATA: Control 30min 2hours 2days 1week p-value B&H Pos Fold change relative to control (log2)
SENSE COUNTS: 1 0 2 0 8 1.04e-03 1.74e-03 0.000 0.000 1.000 0.000 3.000
GENES:
AT1G04690.1
SENSE COUNTS: 1 0 2 0 8 1.04e-03 1.78e-03 0.000 0.000 1.000 0.000 3.000
TAGS:
d+1 ACGTCAATAT 0 0 2 0 8 1.62e-04 6.26e-04 1356 0.000 0.000 1.000 0.000 3.000
d+2 GTATAGGTCT 1 0 0 0 0 4.28e-01 5.35e-01 686 0.000 0.000 0.000 0.000 0.000

LOCUS: AT2G38530

DESCRIPTION: nonspecific lipid transfer protein 2 (LTP2), identical to nonspecific lipid-transfer protein 2 from Arabidopsis thaliana (SP|Q9S7I3); contains Pfam protease inhibitor/seed storage/LTP family domain PF00234
DATA: Control 30min 2hours 2days 1week p-value B&H Pos Fold change relative to control (log2)
SENSE COUNTS: 3 13 0 4 3 1.05e-03 1.75e-03 0.000 2.115 0.000 0.415 0.000
GENES:
AT2G38530.1
SENSE COUNTS: 3 13 0 4 3 1.05e-03 1.79e-03 0.000 2.115 0.000 0.415 0.000
TAGS:
d+1 CAAAGTCAAT 3 13 0 4 3 1.05e-03 2.98e-03 403 0.000 2.115 0.000 0.415 0.000

LOCUS: AT1G31812
DESCRIPTION: acyl-CoA binding protein / ACBP, identical to acyl-CoA-binding protein (ACBP) (Arabidopsis thaliana) SWISS-PROT
DATA: Control 30min 2hours 2days 1week p-value B&H Pos Fold change relative to control (log2)
SENSE COUNTS: 82 45 85 65 47 1.06e-03 1.76e-03 0.000 -0.866 0.052 -0.335 -0.803
GENES:
AT1G31812.1
SENSE COUNTS: 82 45 85 65 47 1.06e-03 1.81e-03 0.000 -0.866 0.052 -0.335 -0.803
TAGS:
d+1 ATGCAAACTA 40 30 52 45 32 1.23e-01 1.88e-01 532 0.000 -0.415 0.379 0.170 -0.322
d+2 GTGTGATCTC 38 14 31 19 14 2.02e-03 5.17e-03 484 0.000 -1.441 -0.294 -1.000 -1.441
d+2 AATGACTATA 4 1 2 0 1 3.88e-01 4.95e-01 307 0.000 -2.000 -1.000 0.000 -2.000
d+2 GGTTTGAAGG 0 0 0 1 0 3.09e-01 4.07e-01 94 0.000 0.000 0.000 0.000 0.000

LOCUS: AT5G20520
DESCRIPTION: expressed protein
DATA: Control 30min 2hours 2days 1week p-value B&H Pos Fold change relative to control (log2)
SENSE COUNTS: 6 3 0 0 12 1.06e-03 1.76e-03 0.000 -1.000 0.000 0.000 1.000
GENES:
AT5G20520.1
SENSE COUNTS: 6 3 0 0 12 1.06e-03 1.80e-03 0.000 -1.000 0.000 0.000 1.000
TAGS:
d+1 GTTATATGAT 6 3 0 0 12 1.06e-03 3.00e-03 1412 0.000 -1.000 0.000 0.000 1.000

LOCUS: AT3G01690
DESCRIPTION: expressed protein
DATA: Control 30min 2hours 2days 1week p-value B&H Pos Fold change relative to control (log2)
SENSE COUNTS: 8 21 26 20 1 1.07e-03 1.77e-03 0.000 1.392 1.700 1.322 -3.000
GENES:
AT3G01690.1
SENSE COUNTS: 8 21 26 20 1 1.07e-03 1.82e-03 0.000 1.392 1.700 1.322 -3.000
TAGS:
d+1 TGGATGTCGC 8 21 26 19 1 1.13e-03 3.16e-03 1374 0.000 1.392 1.700 1.248 -3.000
d+2 AAAAGACTCG 0 0 0 1 0 3.09e-01 4.08e-01 1205 0.000 0.000 0.000 0.000 0.000

LOCUS: AT3G05280
DESCRIPTION: integral membrane Yip1 family protein, contains Pfam domain, PF04893
DATA: Control 30min 2hours 2days 1week p-value B&H Pos Fold change relative to control (log2)
SENSE COUNTS: 7 0 1 0 0 1.09e-03 1.80e-03 0.000 0.000 -2.807 0.000 0.000
GENES:
AT3G05280.1
SENSE COUNTS: 7 0 1 0 0 1.09e-03 1.85e-03 0.000 0.000 -2.807 0.000 0.000
TAGS:
d+1 ATTTACTTTG 6 0 1 0 0 9.60e-04 2.79e-03 1079 0.000 0.000 -2.585 0.000 0.000
d+2 TCCGCAACAT 1 0 0 0 0 6.89e-01 7.02e-01 791 0.000 0.000 0.000 0.000 0.000

LOCUS: AT5G39570
DESCRIPTION: expressed protein
DATA: Control 30min 2hours 2days 1week p-value B&H Pos Fold change relative to control (log2)
SENSE COUNTS: 0 0 3 9 10 1.09e-03 1.80e-03 0.000 0.000 1.585 3.170 3.322
GENES:

```
  AT5G39570.1
    SENSE COUNTS:              0      0      3      9      10     1.09e-03       1.85e-03              0.000  0.000  1.585  3.170  3.322
    TAGS:
      d+1      AATTGGAATT      0      0      3      8      10     2.15e-03       5.46e-03       1530   0.000  0.000  1.585  3.000  3.322
      i+3      AATAGAAAGC      0      0      0      1      0      3.09e-01       4.02e-01       1324   0.000  0.000  0.000  0.000  0.000

LOCUS: AT1G74470
DESCRIPTION:  geranylgeranyl reductase, identical to geranylgeranyl reductase GB
DATA:                          Control 30min  2hours 2days  1week  p-value        B&H            Pos    Fold change relative to control (log2)
SENSE COUNTS:                  15     27     10     16     1      1.09e-03       1.80e-03              0.000  0.848  -0.585 0.093  -3.907
  GENES:
  AT1G74470.1
    SENSE COUNTS:              15     27     10     16     1      1.09e-03       1.84e-03              0.000  0.848  -0.585 0.093  -3.907
    TAGS:
      d+1      TAGCTGTTGG      15     27     10     16     1      1.09e-03       3.08e-03       904    0.000  0.848  -0.585 0.093  -3.907

LOCUS: AT5G57630
DESCRIPTION:  CBL-interacting protein kinase 21, putative (CIPK21), identical to CBL-interacting protein kinase 21 (Arabidopsis thaliana)
gi|14334390|gb|AAK59696
DATA:                          Control 30min  2hours 2days  1week  p-value        B&H            Pos    Fold change relative to control (log2)
SENSE COUNTS:                  0      0      5      0      0      1.10e-03       1.81e-03              0.000  0.000  2.322  0.000  0.000
  GENES:
  AT5G57630.1
    SENSE COUNTS:              0      0      5      0      0      1.10e-03       1.85e-03              0.000  0.000  2.322  0.000  0.000
    TAGS:
      i+3      GAGGTAAGTT      0      0      2      0      0      1.21e-01       1.86e-01       1958   0.000  0.000  1.000  0.000  0.000
      d+1      GAGTTTTGCC      0      0      3      0      0      2.70e-02       4.61e-02       1262   0.000  0.000  1.585  0.000  0.000

LOCUS: AT4G23750
DESCRIPTION:  encodes a member of the ERF (ethylene response factor) subfamily B-5 of ERF/AP2 transcription factor family. The protein contains one
AP2 domain. There are 7 members in this subfamily.
DATA:                          Control 30min  2hours 2days  1week  p-value        B&H            Pos    Fold change relative to control (log2)
SENSE COUNTS:                  0      0      5      0      0      1.10e-03       1.81e-03              0.000  0.000  2.322  0.000  0.000
  GENES:
  AT4G23750.2
    SENSE COUNTS:              0      0      5      0      0      1.10e-03       1.85e-03              0.000  0.000  2.322  0.000  0.000
    TAGS:
      d+1      GAGGACCATT      0      0      5      0      0      1.10e-03       3.10e-03       1319   0.000  0.000  2.322  0.000  0.000
  AT4G23750.1
    SENSE COUNTS:              0      0      5      0      0      1.10e-03       1.86e-03              0.000  0.000  2.322  0.000  0.000
    TAGS:
      d+1      GAGGACCATT      0      0      5      0      0      1.10e-03       3.10e-03       1530   0.000  0.000  2.322  0.000  0.000

LOCUS: ATCG00280
DESCRIPTION:  chloroplast gene encoding a CP43 subunit of  the photosystem II reaction center. promoter contains a blue-light responsive element.
DATA:                          Control 30min  2hours 2days  1week  p-value        B&H            Pos    Fold change relative to control (log2)
SENSE COUNTS:                  15     0      6      6      2      1.11e-03       1.82e-03              0.000  0.000  -1.322 -1.322 -2.907
  GENES:
  ATCG00280.1
    SENSE COUNTS:              15     0      6      6      2      1.11e-03       1.86e-03              0.000  0.000  -1.322 -1.322 -2.907
    TAGS:
      X+4      CTAAGAAGTA      13     0      4      2      0      2.95e-05       1.41e-04       1542   0.000  0.000  -1.700 -2.700 0.000
      d+2      GGCTCGCCGC      1      0      0      0      1      3.83e-01       4.91e-01       777    0.000  0.000  0.000  0.000  0.000
      d+2      TATGGTTAGG      1      0      0      0      0      4.28e-01       5.18e-01       713    0.000  0.000  0.000  0.000  0.000
      d+2      CACTTCTGGG      0      0      1      4      0      1.31e-01       2.00e-01       398    0.000  0.000  0.000  2.000  0.000
      d+2      CCGGATTAAT      0      0      1      0      1      3.96e-01       5.01e-01       170    0.000  0.000  0.000  0.000  0.000

LOCUS: AT2G17845
DESCRIPTION:  short-chain dehydrogenase/reductase (SDR) family protein, contains similarity to 3-oxoacyl-(acyl-carrier protein) reductase SP
DATA:                          Control 30min  2hours 2days  1week  p-value        B&H            Pos    Fold change relative to control (log2)
```

```
SENSE COUNTS:                  32      26      61      51      46      1.13e-03       1.85e-03              0.000   -0.300  0.931   0.672   0.524
  GENES:
  AT2G17845.1
    SENSE COUNTS:              32      26      61      51      46      1.13e-03       1.90e-03              0.000   -0.300  0.931   0.672   0.524
    TAGS:
      v+2      ATAAAAAAAA      32      26      61      51      46      1.13e-03       3.15e-03       22     0.000   -0.300  0.931   0.672   0.524

LOCUS: AT2G18030
DESCRIPTION:  peptide methionine sulfoxide reductase family protein, similar to SP|P08761 Ecdysone-induced protein 28/29 kDa {Drosophila
melanogaster}; contains Pfam profile PF01625
DATA:                          Control 30min   2hours  2days   1week   p-value        B&H            Pos    Fold change relative to control (log2)
SENSE COUNTS:                  9       8       0       1       16      1.15e-03       1.88e-03              0.000   -0.170  0.000   -3.170  0.830
  GENES:
  AT2G18030.1
    SENSE COUNTS:              1       1       0       0       0       4.77e-01       4.78e-01              0.000   0.000   0.000   0.000   0.000
    TAGS:
      d+1      GTAGAAGAAG      1       1       0       0       0       4.77e-01       5.27e-01       718    0.000   0.000   0.000   0.000   0.000
  AT2G18030.2
    SENSE COUNTS:              9       8       0       1       16      1.15e-03       1.92e-03              0.000   -0.170  0.000   -3.170  0.830
    TAGS:
      d+1      GTAGAAGAAG      1       1       0       0       0       4.77e-01       5.27e-01       863    0.000   0.000   0.000   0.000   0.000
      X+4      CCTTAATTGT      8       7       0       1       16      1.15e-03       3.18e-03       -87    0.000   -0.193  0.000   -3.000  1.000

LOCUS: AT5G08640
DESCRIPTION:  flavonol synthase 1 (FLS1), identical to SP|Q96330; contains PF03171 2OG-Fe(II) oxygenase superfamily
DATA:                          Control 30min   2hours  2days   1week   p-value        B&H            Pos    Fold change relative to control (log2)
SENSE COUNTS:                  0       0       1       4       8       1.15e-03       1.88e-03              0.000   0.000   0.000   2.000   3.000
  GENES:
  AT5G08640.1
    SENSE COUNTS:              0       0       1       4       8       1.15e-03       1.93e-03              0.000   0.000   0.000   2.000   3.000
    TAGS:
      d+1      TTTTTATTGT      0       0       1       4       8       1.15e-03       3.19e-03       1221   0.000   0.000   0.000   2.000   3.000

LOCUS: AT2G44430
DESCRIPTION:  DNA-binding bromodomain-containing protein, contains Pfam domains, Pfam PF00439
DATA:                          Control 30min   2hours  2days   1week   p-value        B&H            Pos    Fold change relative to control (log2)
SENSE COUNTS:                  15      10      6       28      6       1.18e-03       1.92e-03              0.000   -0.585  -1.322  0.900   -1.322
  GENES:
  AT2G44430.1
    SENSE COUNTS:              15      10      6       28      6       1.18e-03       1.97e-03              0.000   -0.585  -1.322  0.900   -1.322
    TAGS:
      d+1      TTTGCCCCTT      0       0       0       0       1       4.65e-01       5.17e-01       2012   0.000   0.000   0.000   0.000   0.000
      d+2      GGGCACGTGG      15      10      6       27      5       1.13e-03       3.15e-03       206    0.000   -0.585  -1.322  0.848   -1.585
      d+2      AACGTCGACG      0       0       0       1       0       3.09e-01       4.11e-01       99     0.000   0.000   0.000   0.000   0.000

LOCUS: AT5G13850
DESCRIPTION:  nascent polypeptide-associated complex (NAC) domain-containing protein, similar to alpha-NAC, non-muscle form (Mus musculus) GI
DATA:                          Control 30min   2hours  2days   1week   p-value        B&H            Pos    Fold change relative to control (log2)
SENSE COUNTS:                  7       0       0       2       8       1.24e-03       2.02e-03              0.000   0.000   0.000   -1.807  0.193
  GENES:
  AT5G13850.1
    SENSE COUNTS:              7       0       0       2       8       1.24e-03       2.07e-03              0.000   0.000   0.000   -1.807  0.193
    TAGS:
      d+1      AGTTTATACT      6       0       0       2       7       5.97e-03       1.23e-02       805    0.000   0.000   0.000   -1.585  0.222
      d+2      TTGTATCATT      1       0       0       0       1       3.83e-01       4.90e-01       739    0.000   0.000   0.000   0.000   0.000

LOCUS: AT3G53430
DESCRIPTION:  60S ribosomal protein L12 (RPL12B), 60S RIBOSOMAL PROTEIN L12, Prunus armeniaca, SWISSPROT
DATA:                          Control 30min   2hours  2days   1week   p-value        B&H            Pos    Fold change relative to control (log2)
SENSE COUNTS:                  2       15      2       6       5       1.25e-03       2.03e-03              0.000   2.907   0.000   1.585   1.322
```

```
  GENES:
  AT3G53430.1
    SENSE COUNTS:          2      15     2      6      5      1.25e-03      2.08e-03             0.000  2.907  0.000  1.585  1.322
    TAGS:
      d+1     CCGCCGAAGT   2      15     2      6      5      1.25e-03      3.44e-03      78     0.000  2.907  0.000  1.585  1.322

LOCUS: AT3G47460
DESCRIPTION:  SMC2-like condensin, putative, similar to SMC2-like condensin (TITAN3) (Arabidopsis thaliana) GI
DATA:                      Control 30min  2hours 2days  1week  p-value       B&H           Pos    Fold change relative to control (log2)
SENSE COUNTS:              37     39     42     25     6      1.28e-03      2.07e-03             0.000  0.076  0.183  -0.566 -2.624
  GENES:
  AT3G47460.1
    SENSE COUNTS:          37     39     42     25     6      1.28e-03      2.13e-03             0.000  0.076  0.183  -0.566 -2.624
    TAGS:
      d+1     GGTCAGACAA   11     2      21     5      0      1.50e-05      7.68e-05      3995   0.000  -2.459 0.933  -1.138 0.000
      d+2     GAGCTACAAC   23     37     21     19     5      1.13e-03      3.14e-03      3833   0.000  0.686  -0.131 -0.276 -2.202
      d+2     AATAATAGAT   3      0      0      0      0      9.89e-02      1.57e-01      3755   0.000  0.000  0.000  0.000  0.000
      i+3     GTTTAGTTAG   0      0      0      1      1      3.25e-01      4.21e-01      3634   0.000  0.000  0.000  0.000  0.000
      d+2     TCAAAATTAG   0      0      0      0      0      6.15e-01      6.53e-01      3145   0.000  0.000  0.000  0.000  0.000
      d+2     ATCAGTCTCT   0      0      0      0      0      6.15e-01      6.31e-01      2591   0.000  0.000  0.000  0.000  0.000

LOCUS: AT5G53400
DESCRIPTION:  nuclear movement family protein, contains Pfam profile
DATA:                      Control 30min  2hours 2days  1week  p-value       B&H           Pos    Fold change relative to control (log2)
SENSE COUNTS:              1      1      0      0      8      1.30e-03      2.10e-03             0.000  0.000  0.000  0.000  3.000
  GENES:
  AT5G53400.1
    SENSE COUNTS:          1      1      0      0      8      1.30e-03      2.15e-03             0.000  0.000  0.000  0.000  3.000
    TAGS:
      d+1     TCTGAGCATC   1      1      0      0      8      1.30e-03      3.56e-03      996    0.000  0.000  0.000  0.000  3.000

LOCUS: AT5G15850
DESCRIPTION:  Zinc finger protein CONSTANS-LIKE 1 (COL1), identical to Zinc finger protein CONSTANS-LIKE 1 SP
DATA:                      Control 30min  2hours 2days  1week  p-value       B&H           Pos    Fold change relative to control (log2)
SENSE COUNTS:              3      0      3      11     14     1.30e-03      2.10e-03             0.000  0.000  0.000  1.874  2.222
  GENES:
  AT5G15850.1
    SENSE COUNTS:          3      0      3      11     14     1.30e-03      2.16e-03             0.000  0.000  0.000  1.874  2.222
    TAGS:
      d+1     CTTGTAAATT   3      0      3      11     14     1.30e-03      3.57e-03      1521   0.000  0.000  0.000  1.874  2.222

LOCUS: AT2G06850
DESCRIPTION:  xyloglucan
DATA:                      Control 30min  2hours 2days  1week  p-value       B&H           Pos    Fold change relative to control (log2)
SENSE COUNTS:              24     14     5      4      19     1.31e-03      2.11e-03             0.000  -0.778 -2.263 -2.585 -0.337
  GENES:
  AT2G06850.1
    SENSE COUNTS:          24     14     5      4      19     1.31e-03      2.17e-03             0.000  -0.778 -2.263 -2.585 -0.337
    TAGS:
      d+1     TTATAATTCA   24     13     4      4      16     7.68e-04      2.32e-03      1085   0.000  -0.885 -2.585 -2.585 -0.585
      d+2     ATGTTTATGC   0      1      0      0      3      1.27e-01      1.94e-01      1051   0.000  0.000  0.000  0.000  1.585
      d+2     TACCAGATCG   0      0      1      0      0      7.06e-01      7.14e-01      542    0.000  0.000  0.000  0.000  0.000

LOCUS: AT1G41850
DESCRIPTION:  non-LTR retrotransposon family (LINE), has a 1.3e-29 P-value blast match to GB
DATA:                      Control 30min  2hours 2days  1week  p-value       B&H           Pos    Fold change relative to control (log2)
SENSE COUNTS:              0      0      0      2      7      1.31e-03      2.11e-03             0.000  0.000  0.000  1.000  2.807
  GENES:
  AT1G41850.1
    SENSE COUNTS:          0      0      0      2      7      1.31e-03      2.16e-03             0.000  0.000  0.000  1.000  2.807
```

```
    TAGS:
      p+1      GGGATTATCA     0       0       0       2       7       1.31e-03       3.58e-03       3741    0.000   0.000   0.000   1.000   2.807

LOCUS: AT3G61060
DESCRIPTION:  F-box family protein / lectin-related, low similarity to PP2 lectin polypeptide (Cucurbita maxima) GI
DATA:                         Control 30min   2hours  2days   1week   p-value        B&H            Pos     Fold change relative to control (log2)
SENSE COUNTS:                 2       13      7       2       0       1.35e-03       2.17e-03               0.000   2.700   1.807   0.000   0.000
  GENES:
  AT3G61060.1
    SENSE COUNTS:             2       13      7       2       0       1.35e-03       2.22e-03               0.000   2.700   1.807   0.000   0.000
    TAGS:
      d+1      GTTGGGTTGT     2       13      7       2       0       2.82e-03       6.87e-03       1258    0.000   2.700   1.807   0.000   0.000
      d+2      ACTCAAATCG     0       0       0       0       0       6.15e-01       6.56e-01       1022    0.000   0.000   0.000   0.000   0.000
  AT3G61060.2
    SENSE COUNTS:             2       13      7       2       0       1.35e-03       2.22e-03               0.000   2.700   1.807   0.000   0.000
    TAGS:
      d+1      GTTGGGTTGT     2       13      7       2       0       2.82e-03       6.87e-03       1270    0.000   2.700   1.807   0.000   0.000
      d+2      ACTCAAATCG     0       0       0       0       0       6.15e-01       6.56e-01       1034    0.000   0.000   0.000   0.000   0.000

LOCUS: AT4G15540
DESCRIPTION:  nodulin-related, low similarity to MtN21 (Medicago truncatula) GI
DATA:                         Control 30min   2hours  2days   1week   p-value        B&H            Pos     Fold change relative to control (log2)
SENSE COUNTS:                 4       12      1       1       1       1.36e-03       2.18e-03               0.000   1.585   -2.000  -2.000  -2.000
  GENES:
  AT4G15540.1
    SENSE COUNTS:             4       12      1       1       1       1.36e-03       2.23e-03               0.000   1.585   -2.000  -2.000  -2.000
    TAGS:
      v+1      ATACATTGAA     4       12      1       1       1       1.36e-03       3.70e-03       1307    0.000   1.585   -2.000  -2.000  -2.000

LOCUS: AT4G13510
DESCRIPTION:  A.thaliana amt1 mRNA
DATA:                         Control 30min   2hours  2days   1week   p-value        B&H            Pos     Fold change relative to control (log2)
SENSE COUNTS:                 4       2       15      13      19      1.38e-03       2.21e-03               0.000   -1.000  1.907   1.700   2.248
  GENES:
  AT4G13510.1
    SENSE COUNTS:             4       2       15      13      19      1.38e-03       2.26e-03               0.000   -1.000  1.907   1.700   2.248
    TAGS:
      d+1      GGGTTGTGAT     4       2       13      13      19      2.21e-03       5.58e-03       1668    0.000   -1.000  1.700   1.700   2.248
      d+2      GGGACACTTT     0       0       2       0       0       1.21e-01       1.88e-01       1337    0.000   0.000   1.000   0.000   0.000

LOCUS: AT3G52800
DESCRIPTION:  zinc finger (AN1-like) family protein, contains Pfam domain, PF01428
DATA:                         Control 30min   2hours  2days   1week   p-value        B&H            Pos     Fold change relative to control (log2)
SENSE COUNTS:                 1       9       3       0       0       1.41e-03       2.25e-03               0.000   3.170   1.585   0.000   0.000
  GENES:
  AT3G52800.1
    SENSE COUNTS:             1       9       3       0       0       1.41e-03       2.30e-03               0.000   3.170   1.585   0.000   0.000
    TAGS:
      d+1      GATGCAGCTA     1       9       3       0       0       1.41e-03       3.82e-03       677     0.000   3.170   1.585   0.000   0.000

LOCUS: AT2G17230
DESCRIPTION:  phosphate-responsive 1 family protein, similar to phi-1 (phosphate-induced gene) (Nicotiana tabacum) GI
DATA:                         Control 30min   2hours  2days   1week   p-value        B&H            Pos     Fold change relative to control (log2)
SENSE COUNTS:                 0       7       2       0       0       1.41e-03       2.25e-03               0.000   2.807   1.000   0.000   0.000
  GENES:
  AT2G17230.1
    SENSE COUNTS:             0       7       2       0       0       1.41e-03       2.31e-03               0.000   2.807   1.000   0.000   0.000
    TAGS:
      d+1      AGTTAGCTGA     0       7       1       0       0       4.60e-04       1.54e-03       869     0.000   2.807   0.000   0.000   0.000
      d+2      GGACACGGTG     0       0       1       0       0       4.55e-01       5.08e-01       790     0.000   0.000   0.000   0.000   0.000
```

```
LOCUS: AT1G68670
DESCRIPTION:  myb family transcription factor, contains Pfam domain, PF00249
DATA:                         Control 30min  2hours  2days  1week  p-value      B&H          Pos    Fold change relative to control (log2)
SENSE COUNTS:                 0       7      2       0      0      1.41e-03     2.24e-03            0.000  2.807  1.000  0.000  0.000
  GENES:
  AT1G68670.1
    SENSE COUNTS:             0       7      2       0      0      1.41e-03     2.30e-03            0.000  2.807  1.000  0.000  0.000
    TAGS:
      d+1     AAGGTTGATG      0       7      2       0      0      1.41e-03     3.83e-03     1013   0.000  2.807  1.000  0.000  0.000

LOCUS: AT5G05740
DESCRIPTION:  peptidase M50 family protein / sterol-regulatory element binding protein (SREBP) site 2 protease family protein, contains Pfam PF02163
DATA:                         Control 30min  2hours  2days  1week  p-value      B&H          Pos    Fold change relative to control (log2)
SENSE COUNTS:                 0       0      2       2      10     1.42e-03     2.26e-03            0.000  0.000  1.000  1.000  3.322
  GENES:
  AT5G05740.1
    SENSE COUNTS:             0       0      2       2      10     1.42e-03     2.31e-03            0.000  0.000  1.000  1.000  3.322
    TAGS:
      d+1     GAGCAGTTTA      0       0      2       2      10     1.42e-03     3.84e-03     1803   0.000  0.000  1.000  1.000  3.322
  AT5G05740.2
    SENSE COUNTS:             0       0      2       2      10     1.42e-03     2.31e-03            0.000  0.000  1.000  1.000  3.322
    TAGS:
      d+1     GAGCAGTTTA      0       0      2       2      10     1.42e-03     3.84e-03     1716   0.000  0.000  1.000  1.000  3.322

LOCUS: AT5G08330
DESCRIPTION:  TCP family transcription factor, putative, similar to PCF1 (GI
DATA:                         Control 30min  2hours  2days  1week  p-value      B&H          Pos    Fold change relative to control (log2)
SENSE COUNTS:                 1       7      0       0      0      1.46e-03     2.32e-03            0.000  2.807  0.000  0.000  0.000
  GENES:
  AT5G08330.1
    SENSE COUNTS:             1       7      0       0      0      1.46e-03     2.37e-03            0.000  2.807  0.000  0.000  0.000
    TAGS:
      d+1     GCCGACAACG      1       7      0       0      0      1.46e-03     3.92e-03     88     0.000  2.807  0.000  0.000  0.000

LOCUS: AT1G79010
DESCRIPTION:  NADH-ubiquinone oxidoreductase 23 kDa subunit, mitochondrial (TYKY), identical to SP|Q42599 NADH-ubiquinone oxidoreductase 23 kDa
subunit, mitochondrial precursor (EC 1.6.5.3) (EC 1.6.99.3) (Complex I-23KD) (CI-23KD) (Complex I- 28.5KD) (CI-28.5KD) {Arabi
DATA:                         Control 30min  2hours  2days  1week  p-value      B&H          Pos    Fold change relative to control (log2)
SENSE COUNTS:                 12      4      3       13     23     1.46e-03     2.31e-03            0.000  -1.585 -2.000 0.115  0.939
  GENES:
  AT1G79010.1
    SENSE COUNTS:             12      4      3       13     23     1.46e-03     2.36e-03            0.000  -1.585 -2.000 0.115  0.939
    TAGS:
      d+1     AGTTCCGGTT      12      2      3       12     23     1.60e-04     6.22e-04     932    0.000  -2.585 -2.000 0.000  0.939
      d+2     ACAAAATGCA      0       2      0       1      0      2.23e-01     3.08e-01     562    0.000  1.000  0.000  0.000  0.000

LOCUS: AT5G05930
DESCRIPTION:  guanylyl cyclase-related (GC1), similar to guanylyl cyclase (GC1) GI
DATA:                         Control 30min  2hours  2days  1week  p-value      B&H          Pos    Fold change relative to control (log2)
SENSE COUNTS:                 1       5      0       0      0      1.46e-03     2.31e-03            0.000  2.322  0.000  0.000  0.000
  GENES:
  AT5G05930.2
    SENSE COUNTS:             1       5      0       0      0      1.46e-03     2.36e-03            0.000  2.322  0.000  0.000  0.000
    TAGS:
      i+3     TTTTCCAATA      0       0      0       0      0      6.15e-01     6.45e-01     2524   0.000  0.000  0.000  0.000  0.000
      d+1     AAATATCTCA      0       5      0       0      0      3.00e-03     7.18e-03     1242   0.000  2.322  0.000  0.000  0.000
      d+2     AGGTGAAAAG      1       0      0       0      0      4.28e-01     5.27e-01     1159   0.000  0.000  0.000  0.000  0.000
      d+2     AGATTTCTTG      0       0      0       0      0      6.15e-01     6.49e-01     767    0.000  0.000  0.000  0.000  0.000
  AT5G05930.1
```

```
    SENSE COUNTS:              1      5      0      0      0      1.46e-03       2.37e-03              0.000  2.322  0.000  0.000  0.000
    TAGS:
      i+3      TTTTCCAATA     0      0      0      0      0      6.15e-01       6.45e-01       2558   0.000  0.000  0.000  0.000  0.000
      d+1      AAATATCTCA     0      5      0      0      0      3.00e-03       7.18e-03       1341   0.000  2.322  0.000  0.000  0.000
      d+2      AGGTGAAAAG     1      0      0      0      0      4.28e-01       5.27e-01       1258   0.000  0.000  0.000  0.000  0.000
      d+2      AGATTTCTTG     0      0      0      0      0      6.15e-01       6.49e-01       866    0.000  0.000  0.000  0.000  0.000

LOCUS: AT3G27090
DESCRIPTION:  expressed protein, similar to gda-1 (Pisum sativum) GI
DATA:                          Control 30min  2hours 2days  1week  p-value        B&H            Pos    Fold change relative to control (log2)
SENSE COUNTS:                  0      7      1      0      0      1.47e-03       2.32e-03              0.000  2.807  0.000  0.000  0.000
  GENES:
  AT3G27090.1
    SENSE COUNTS:              0      7      1      0      0      1.47e-03       2.37e-03              0.000  2.807  0.000  0.000  0.000
    TAGS:
      d+1      GTATCTTTGA     0      7      1      0      0      1.47e-03       3.95e-03       768    0.000  2.807  0.000  0.000  0.000

LOCUS: AT2G45570
DESCRIPTION:  cytochrome P450 76C2, putative (CYP76C2) (YLS6), identical to SP|O64637 Cytochrome P450 76C2 (EC 1.14.-.-) {Arabidopsis thaliana}, cDNA
YLS6 mRNA for cytochrome P450 (CYP76C2), partial cds GI
DATA:                          Control 30min  2hours 2days  1week  p-value        B&H            Pos    Fold change relative to control (log2)
SENSE COUNTS:                  0      0      0      4      6      1.48e-03       2.33e-03              0.000  0.000  0.000  2.000  2.585
  GENES:
  AT2G45570.1
    SENSE COUNTS:              0      0      0      4      6      1.48e-03       2.38e-03              0.000  0.000  0.000  2.000  2.585
    TAGS:
      d+1      CTCTGATTTT     0      0      0      4      5      7.47e-03       1.43e-02       1648   0.000  0.000  0.000  2.000  2.322
      X+4      AGTTGTTGAT     0      0      0      0      1      1.65e-01       2.49e-01       772    0.000  0.000  0.000  0.000  0.000

LOCUS: AT3G27240
DESCRIPTION:  cytochrome c1, putative, cytochrome c1, heme protein, mitochondrial precursor (Clone PC13III) (Solanum tuberosum) SWISS-PROT
DATA:                          Control 30min  2hours 2days  1week  p-value        B&H            Pos    Fold change relative to control (log2)
SENSE COUNTS:                  13     7      13     22     32     1.49e-03       2.34e-03              0.000  -0.893 0.000  0.759  1.300
  GENES:
  AT3G27240.1
    SENSE COUNTS:              13     7      13     22     32     1.49e-03       2.39e-03              0.000  -0.893 0.000  0.759  1.300
    TAGS:
      d+1      ATTTTGAGAT     13     7      13     22     32     1.49e-03       3.98e-03       1319   0.000  -0.893 0.000  0.759  1.300

LOCUS: AT1G44770
DESCRIPTION:  expressed protein
DATA:                          Control 30min  2hours 2days  1week  p-value        B&H            Pos    Fold change relative to control (log2)
SENSE COUNTS:                  0      0      7      0      1      1.49e-03       2.34e-03              0.000  0.000  2.807  0.000  0.000
  GENES:
  AT1G44770.1
    SENSE COUNTS:              0      0      7      0      1      1.49e-03       2.40e-03              0.000  0.000  2.807  0.000  0.000
    TAGS:
      d+1      CGACCAGCAA     0      0      7      0      1      1.49e-03       3.99e-03       839    0.000  0.000  2.807  0.000  0.000

LOCUS: AT4G26850
DESCRIPTION:  expressed protein
DATA:                          Control 30min  2hours 2days  1week  p-value        B&H            Pos    Fold change relative to control (log2)
SENSE COUNTS:                  12     3      23     18     8      1.52e-03       2.38e-03              0.000  -2.000 0.939  0.585  -0.585
  GENES:
  AT4G26850.1
    SENSE COUNTS:              12     3      23     18     8      1.52e-03       2.44e-03              0.000  -2.000 0.939  0.585  -0.585
    TAGS:
      d+1      GAGGTCCGAT     10     3      21     18     8      3.09e-03       7.38e-03       1783   0.000  -1.737 1.070  0.848  -0.322
      d+2      CCTTTCCCAC     1      0      1      0      0      6.01e-01       6.46e-01       1242   0.000  0.000  0.000  0.000  0.000
      d+2      TGCTGCTGAT     0      0      1      0      0      4.55e-01       5.44e-01       1063   0.000  0.000  0.000  0.000  0.000
```

```
      d+2      TAAGAATCTG     1       0       0       0       0       4.28e-01        5.28e-01        641     0.000   0.000   0.000   0.000   0.000

LOCUS: AT5G13300
DESCRIPTION:  ARF GTPase-activating domain-containing protein, similar to GCN4-complementing protein (GCP1) GI
DATA:                         Control 30min   2hours  2days   1week   p-value         B&H             Pos     Fold change relative to control (log2)
SENSE COUNTS:                 1       0       7       1       0       1.53e-03        2.39e-03                0.000   0.000   2.807   0.000   0.000
  GENES:
  AT5G13300.1
    SENSE COUNTS:             1       0       7       1       0       1.53e-03        2.45e-03                0.000   0.000   2.807   0.000   0.000
    TAGS:
      d+1      TCCAGACGTT     0       0       2       1       0       2.74e-01        3.70e-01        2851    0.000   0.000   1.000   0.000   0.000
      i+3      CTACATTTGT     1       0       5       0       0       6.70e-03        1.32e-02        2730    0.000   0.000   2.322   0.000   0.000

LOCUS: AT5G18800
DESCRIPTION:  NADH-ubiquinone oxidoreductase 19 kDa subunit (NDUFA8) family protein, contains Pfam profile
DATA:                         Control 30min   2hours  2days   1week   p-value         B&H             Pos     Fold change relative to control (log2)
SENSE COUNTS:                 15      4       1       4       3       1.53e-03        2.39e-03                0.000   -1.907  -3.907  -1.907  -2.322
  GENES:
  AT5G18800.2
    SENSE COUNTS:             15      4       1       4       3       1.53e-03        2.44e-03                0.000   -1.907  -3.907  -1.907  -2.322
    TAGS:
      d+1      TTTTGATTTG     15      4       1       4       3       1.53e-03        4.08e-03        434     0.000   -1.907  -3.907  -1.907  -2.322
  AT5G18800.1
    SENSE COUNTS:             15      4       1       4       3       1.53e-03        2.45e-03                0.000   -1.907  -3.907  -1.907  -2.322
    TAGS:
      d+1      TTTTGATTTG     15      4       1       4       3       1.53e-03        4.08e-03        447     0.000   -1.907  -3.907  -1.907  -2.322

LOCUS: AT1G76080
DESCRIPTION:  thioredoxin family protein, low similarity to thioredoxin (TRX) (Fasciola hepatica) GI
DATA:                         Control 30min   2hours  2days   1week   p-value         B&H             Pos     Fold change relative to control (log2)
SENSE COUNTS:                 18      21      6       8       1       1.55e-03        2.42e-03                0.000   0.222   -1.585  -1.170  -4.170
  GENES:
  AT1G76080.1
    SENSE COUNTS:             18      21      6       8       1       1.55e-03        2.47e-03                0.000   0.222   -1.585  -1.170  -4.170
    TAGS:
      d+1      AATGTTATCG     18      21      6       8       1       1.55e-03        4.13e-03        852     0.000   0.222   -1.585  -1.170  -4.170

LOCUS: AT2G40475
DESCRIPTION:  Expressed protein
DATA:                         Control 30min   2hours  2days   1week   p-value         B&H             Pos     Fold change relative to control (log2)
SENSE COUNTS:                 7       0       2       0       11      1.58e-03        2.46e-03                0.000   0.000   -1.807  0.000   0.652
  GENES:
  AT2G40475.1
    SENSE COUNTS:             7       0       2       0       11      1.58e-03        2.52e-03                0.000   0.000   -1.807  0.000   0.652
    TAGS:
      d+1      TTTTGATTAT     6       0       0       0       10      2.56e-04        9.41e-04        1089    0.000   0.000   0.000   0.000   0.737
      d+2      GTGTTCTTAT     1       0       2       0       1       7.50e-01        7.56e-01        921     0.000   0.000   1.000   0.000   0.000

LOCUS: AT3G14230
DESCRIPTION:  encodes a member of the ERF (ethylene response factor) subfamily B-2 of ERF/AP2 transcription factor family (RAP2.2). The protein
contains one AP2 domain. There are 5 members in this subfamily including RAP2.2 AND RAP2.12.
DATA:                         Control 30min   2hours  2days   1week   p-value         B&H             Pos     Fold change relative to control (log2)
SENSE COUNTS:                 3       14      3       1       2       1.60e-03        2.49e-03                0.000   2.222   0.000   -1.585  -0.585
  GENES:
  AT3G14230.1
    SENSE COUNTS:             3       14      3       1       2       1.60e-03        2.54e-03                0.000   2.222   0.000   -1.585  -0.585
    TAGS:
      d+1      AGCCTCTCTT     2       14      3       1       1       3.06e-04        1.08e-03        1379    0.000   2.807   0.585   -1.000  -1.000
      d+2      CTGGAAGGAG     1       0       0       0       1       3.83e-01        4.92e-01        1259    0.000   0.000   0.000   0.000   0.000
  AT3G14230.3
```

```
    SENSE COUNTS:              3      14     3      1      2      1.60e-03       2.54e-03              0.000  2.222  0.000  -1.585  -0.585
    TAGS:
      d+1      AGCCTCTCTT      2      14     3      1      1      3.06e-04       1.08e-03       1364   0.000  2.807  0.585  -1.000  -1.000
      d+2      CTGGAAGGAG      1      0      0      0      1      3.83e-01       4.92e-01       1244   0.000  0.000  0.000  0.000   0.000
  AT3G14230.2
    SENSE COUNTS:              3      14     3      1      2      1.60e-03       2.54e-03              0.000  2.222  0.000  -1.585  -0.585
    TAGS:
      d+1      AGCCTCTCTT      2      14     3      1      1      3.06e-04       1.08e-03       1367   0.000  2.807  0.585  -1.000  -1.000
      d+2      CTGGAAGGAG      1      0      0      0      1      3.83e-01       4.92e-01       1247   0.000  0.000  0.000  0.000   0.000

LOCUS: AT4G08850
DESCRIPTION:  leucine-rich repeat family protein / protein kinase family protein, contains Pfam domains PF00560
DATA:                          Control 30min  2hours 2days  1week  p-value        B&H            Pos    Fold change relative to control (log2)
SENSE COUNTS:                  2      6      19     5      8      1.63e-03       2.53e-03              0.000  1.585  3.248  1.322   2.000
  GENES:
  AT4G08850.2
    SENSE COUNTS:              0      0      1      0      0      4.87e-01       4.88e-01              0.000  0.000  0.000  0.000   0.000
    TAGS:
      d+1      ATCGGTCTCC      0      0      1      0      0      7.06e-01       7.17e-01       2827   0.000  0.000  0.000  0.000   0.000
      d+2      TTCAATCACA      0      0      0      0      0      6.15e-01       6.43e-01       2225   0.000  0.000  0.000  0.000   0.000
  AT4G08850.1
    SENSE COUNTS:              2      6      18     5      8      2.76e-03       4.01e-03              0.000  1.585  3.170  1.322   2.000
    TAGS:
      d+1      GAATTTTAGT      2      6      18     5      8      1.83e-03       4.80e-03       3381   0.000  1.585  3.170  1.322   2.000
      d+2      TTCAATCACA      0      0      0      0      0      6.15e-01       6.43e-01       2225   0.000  0.000  0.000  0.000   0.000

LOCUS: AT5G42980
DESCRIPTION:  thioredoxin H-type 3 (TRX-H-3) (GIF1), identical to SP|Q42403 Thioredoxin H-type 3 (TRX-H-3) {Arabidopsis thaliana}; identical to cDNA
(GIF1) mRNA for thioredoxin GI
DATA:                          Control 30min  2hours 2days  1week  p-value        B&H            Pos    Fold change relative to control (log2)
SENSE COUNTS:                  20     7      15     4      25     1.65e-03       2.56e-03              0.000  -1.515 -0.415 -2.322  0.322
  GENES:
  AT5G42980.1
    SENSE COUNTS:              20     7      15     4      25     1.65e-03       2.61e-03              0.000  -1.515 -0.415 -2.322  0.322
    TAGS:
      d+1      GTTATGTTTA      20     7      14     4      25     1.59e-03       4.23e-03       655    0.000  -1.515 -0.515 -2.322  0.322
      d+2      GTGCCCACCT      0      0      1      0      0      4.55e-01       5.21e-01       249    0.000  0.000  0.000  0.000   0.000

LOCUS: AT5G41670
DESCRIPTION:  6-phosphogluconate dehydrogenase family protein, contains Pfam profiles
DATA:                          Control 30min  2hours 2days  1week  p-value        B&H            Pos    Fold change relative to control (log2)
SENSE COUNTS:                  2      1      1      9      12     1.67e-03       2.58e-03              0.000  -1.000 -1.000 2.170   2.585
  GENES:
  AT5G41670.2
    SENSE COUNTS:              2      1      1      8      7      5.00e-02       5.06e-02              0.000  -1.000 -1.000 2.000   1.807
    TAGS:
      d+1      TTGTTGTGTA      2      1      1      8      7      5.00e-02       8.22e-02       1618   0.000  -1.000 -1.000 2.000   1.807
  AT5G41670.1
    SENSE COUNTS:              0      0      0      1      5      6.31e-03       7.23e-03              0.000  0.000  0.000  0.000   2.322
    TAGS:
      d+1      GACACAGTCC      0      0      0      1      5      6.31e-03       1.26e-02       1532   0.000  0.000  0.000  0.000   2.322

LOCUS: AT3G07110
DESCRIPTION:  60S ribosomal protein L13A (RPL13aA), similar to ribosomal protein L13A GB
DATA:                          Control 30min  2hours 2days  1week  p-value        B&H            Pos    Fold change relative to control (log2)
SENSE COUNTS:                  1      2      0      10     3      1.67e-03       2.58e-03              0.000  1.000  0.000  3.322   1.585
  GENES:
  AT3G07110.1
    SENSE COUNTS:              1      2      0      10     3      1.67e-03       2.64e-03              0.000  1.000  0.000  3.322   1.585
    TAGS:
```

```
      d+1      CGCCTCCTCC     0       0       0       4       0       6.18e-03        1.25e-02        840     0.000   0.000   0.000   2.000   0.000
      d+2      AGGTTTCTCC     1       2       0       6       3       8.00e-02        1.29e-01        245     0.000   1.000   0.000   2.585   1.585

LOCUS: AT4G32260
DESCRIPTION:  ATP synthase family, contains Pfam profile
DATA:                         Control 30min   2hours  2days   1week   p-value         B&H             Pos     Fold change relative to control (log2)
SENSE COUNTS:                 19      27      9       12      3       1.69e-03        2.60e-03                0.000   0.507   -1.078  -0.663  -2.663
  GENES:
  AT4G32260.1
    SENSE COUNTS:             19      27      9       12      3       1.69e-03        2.67e-03                0.000   0.507   -1.078  -0.663  -2.663
    TAGS:
      d+1      GATCAAAGAG     19      27      8       12      3       1.03e-03        2.94e-03        494     0.000   0.507   -1.248  -0.663  -2.663
      X+4      CGAGTCTTAT     0       0       1       0       0       4.55e-01        5.13e-01        402     0.000   0.000   0.000   0.000   0.000

LOCUS: AT5G48240
DESCRIPTION:  hypothetical protein,
DATA:                         Control 30min   2hours  2days   1week   p-value         B&H             Pos     Fold change relative to control (log2)
SENSE COUNTS:                 0       1       1       9       1       1.73e-03        2.66e-03                0.000   0.000   0.000   3.170   0.000
  GENES:
  AT5G48240.1
    SENSE COUNTS:             0       1       1       9       1       1.73e-03        2.73e-03                0.000   0.000   0.000   3.170   0.000
    TAGS:
      v+2      CAAATTTTGT     0       1       1       9       1       1.73e-03        4.58e-03        1425    0.000   0.000   0.000   3.170   0.000

LOCUS: AT2G30620
DESCRIPTION:  histone H1.2, nearly identical to SP|P26569 Histone H1.2 {Arabidopsis thaliana}
DATA:                         Control 30min   2hours  2days   1week   p-value         B&H             Pos     Fold change relative to control (log2)
SENSE COUNTS:                 11      21      9       7       0       1.73e-03        2.66e-03                0.000   0.933   -0.290  -0.652  0.000
  GENES:
  AT2G30620.1
    SENSE COUNTS:             11      21      9       7       0       1.73e-03        2.72e-03                0.000   0.933   -0.290  -0.652  0.000
    TAGS:
      d+1      GTGGTGGTTA     9       21      7       6       0       6.15e-04        1.95e-03        1297    0.000   1.222   -0.363  -0.585  0.000
      X+4      TTTAGTTCTG     2       0       2       1       0       7.53e-01        7.58e-01        914     0.000   0.000   0.000   -1.000  0.000

LOCUS: AT3G58690
DESCRIPTION:  protein kinase family protein, contains protein kinase domain, Pfam
DATA:                         Control 30min   2hours  2days   1week   p-value         B&H             Pos     Fold change relative to control (log2)
SENSE COUNTS:                 1       0       0       1       8       1.74e-03        2.67e-03                0.000   0.000   0.000   0.000   3.000
  GENES:
  AT3G58690.1
    SENSE COUNTS:             1       0       0       1       8       1.74e-03        2.73e-03                0.000   0.000   0.000   0.000   3.000
    TAGS:
      d+1      GAGTCATAGA     1       0       0       1       8       1.74e-03        4.59e-03        1478    0.000   0.000   0.000   0.000   3.000

LOCUS: AT1G29020
DESCRIPTION:  calcium-binding EF hand family protein, contains INTERPRO
DATA:                         Control 30min   2hours  2days   1week   p-value         B&H             Pos     Fold change relative to control (log2)
SENSE COUNTS:                 2       0       8       0       7       1.74e-03        2.66e-03                0.000   0.000   2.000   0.000   1.807
  GENES:
  AT1G29020.1
    SENSE COUNTS:             2       0       8       0       7       1.74e-03        2.73e-03                0.000   0.000   2.000   0.000   1.807
    TAGS:
      i+3      TGTTGAACTA     2       0       8       0       7       1.74e-03        4.60e-03        2952    0.000   0.000   2.000   0.000   1.807

LOCUS: AT1G42970
DESCRIPTION:  glyceraldehyde-3-phosphate dehydrogenase B, chloroplast (GAPB) / NADP-dependent glyceraldehydephosphate dehydrogenase subunit B,
identical to SP|P25857 Glyceraldehyde 3-phosphate dehydrogenase B, chloroplast precursor (EC 1.2.1.13) (NADP-dependent glycera
DATA:                         Control 30min   2hours  2days   1week   p-value         B&H             Pos     Fold change relative to control (log2)
SENSE COUNTS:                 67      43      74      50      87      1.79e-03        2.74e-03                0.000   -0.640  0.143   -0.422  0.377
```

```
  GENES:
  AT1G42970.1
    SENSE COUNTS:              67     43     74     50     87     1.79e-03       2.80e-03              0.000  -0.640 0.143  -0.422 0.377
    TAGS:
      d+2      TTAAAACGGA      15     9      23     12     14     1.49e-01       2.26e-01       1642   0.000  -0.737 0.617  -0.322 -0.100
      d+2      AGTTACTTAT      48     34     44     36     71     9.22e-03       1.72e-02       1518   0.000  -0.497 -0.126 -0.415 0.565
      i+3      TAGAAACCTA      1      0      0      0      0      4.28e-01       5.31e-01       1445   0.000  0.000  0.000  0.000  0.000
      i+3      CTTCAGTTTT      1      0      0      0      0      4.28e-01       5.22e-01       1199   0.000  0.000  0.000  0.000  0.000
      d+2      GGCTGAGCTC      0      0      6      2      1      1.12e-02       2.00e-02       619    0.000  0.000  2.585  1.000  0.000
      d+2      CTTGGAACCT      0      0      1      0      0      4.55e-01       5.14e-01       512    0.000  0.000  0.000  0.000  0.000
      X+4      AGTGTGCAAC      2      0      0      0      1      2.03e-01       2.83e-01       385    0.000  0.000  0.000  0.000  -1.000

LOCUS: AT5G16130
DESCRIPTION:  40S ribosomal protein S7 (RPS7C), 40S ribosomal protein S7 homolog - Brassica oleracea, EMBL
DATA:                          Control 30min  2hours 2days  1week  p-value        B&H            Pos    Fold change relative to control (log2)
SENSE COUNTS:                  1      6      5      9      18     1.80e-03       2.75e-03              0.000  2.585  2.322  3.170  4.170
  GENES:
  AT5G16130.1
    SENSE COUNTS:              1      6      5      9      18     1.80e-03       2.82e-03              0.000  2.585  2.322  3.170  4.170
    TAGS:
      d+1      AAGGTCTTTT      1      6      5      9      17     5.84e-03       1.21e-02       583    0.000  2.585  2.322  3.170  4.087
      d+2      AAGCTATGCT      0      0      0      0      1      1.65e-01       2.34e-01       500    0.000  0.000  0.000  0.000  0.000

LOCUS: AT4G27000
DESCRIPTION:  RNA-binding protein 45 (RBP45), putative, DNA binding protein ACBF - Nicotiana tabacum, PID
DATA:                          Control 30min  2hours 2days  1week  p-value        B&H            Pos    Fold change relative to control (log2)
SENSE COUNTS:                  15     6      4      2      19     1.81e-03       2.76e-03              0.000  -1.322 -1.907 -2.907 0.341
  GENES:
  AT4G27000.1
    SENSE COUNTS:              15     6      4      2      19     1.81e-03       2.83e-03              0.000  -1.322 -1.907 -2.907 0.341
    TAGS:
      d+1      ACTAATATGA      15     6      2      2      19     3.72e-04       1.28e-03       1563   0.000  -1.322 -2.907 -2.907 0.341
      d+2      ATGTGTAAGT      0      0      1      0      0      4.55e-01       5.30e-01       1346   0.000  0.000  0.000  0.000  0.000
      d+2      GGGTCGCAGT      0      0      1      0      0      4.55e-01       5.38e-01       1132   0.000  0.000  0.000  0.000  0.000

LOCUS: AT1G27290
DESCRIPTION:  expressed protein
DATA:                          Control 30min  2hours 2days  1week  p-value        B&H            Pos    Fold change relative to control (log2)
SENSE COUNTS:                  15     6      10     1      0      1.81e-03       2.75e-03              0.000  -1.322 -0.585 -3.907 0.000
  GENES:
  AT1G27290.1
    SENSE COUNTS:              15     6      10     1      0      1.81e-03       2.82e-03              0.000  -1.322 -0.585 -3.907 0.000
    TAGS:
      d+1      ATCATTTCTT      10     4      4      1      0      3.03e-02       5.14e-02       951    0.000  -1.322 -1.322 -3.322 0.000
      d+2      TGGATATCAG      5      2      6      0      0      6.19e-02       1.01e-01       828    0.000  -1.322 0.263  0.000  0.000

LOCUS: AT1G25400
DESCRIPTION:  expressed protein, similar to unknown protein GI
DATA:                          Control 30min  2hours 2days  1week  p-value        B&H            Pos    Fold change relative to control (log2)
SENSE COUNTS:                  0      9      4      0      2      1.81e-03       2.75e-03              0.000  3.170  2.000  0.000  1.000
  GENES:
  AT1G25400.1
    SENSE COUNTS:              0      9      4      0      2      1.81e-03       2.82e-03              0.000  3.170  2.000  0.000  1.000
    TAGS:
      d+1      TTGTAAGTAG      0      2      2      0      1      4.26e-01       5.35e-01       1194   0.000  1.000  1.000  0.000  0.000
      d+2      GTAGGGTCTA      0      7      2      0      1      4.83e-03       1.05e-02       990    0.000  2.807  1.000  0.000  0.000

LOCUS: AT3G62550
DESCRIPTION:  universal stress protein (USP) family protein, similar to ER6 protein (Lycopersicon esculentum) GI
DATA:                          Control 30min  2hours 2days  1week  p-value        B&H            Pos    Fold change relative to control (log2)
```

```
SENSE COUNTS:                1       7       13      1       1       1.82e-03        2.76e-03                0.000   2.807   3.700   0.000   0.000
  GENES:
  AT3G62550.1
    SENSE COUNTS:            1       7       13      1       1       1.82e-03        2.83e-03                0.000   2.807   3.700   0.000   0.000
    TAGS:
      d+1      ACTATGGCTT    1       7       13      1       1       1.82e-03        4.78e-03        593     0.000   2.807   3.700   0.000   0.000

LOCUS: AT4G39680
DESCRIPTION:  SAP domain-containing protein, contains Pfam domain PF02037
DATA:                        Control 30min   2hours  2days   1week   p-value         B&H             Pos     Fold change relative to control (log2)
SENSE COUNTS:                0       0       2       0       7       1.84e-03        2.78e-03                0.000   0.000   1.000   0.000   2.807
  GENES:
  AT4G39680.1
    SENSE COUNTS:            0       0       2       0       7       1.84e-03        2.86e-03                0.000   0.000   1.000   0.000   2.807
    TAGS:
      d+1      GATCTATGAA    0       0       2       0       7       1.84e-03        4.81e-03        2413    0.000   0.000   1.000   0.000   2.807

LOCUS: AT4G29110
DESCRIPTION:  hypothetical protein
DATA:                        Control 30min   2hours  2days   1week   p-value         B&H             Pos     Fold change relative to control (log2)
SENSE COUNTS:                0       0       2       0       6       1.84e-03        2.78e-03                0.000   0.000   1.000   0.000   2.585
  GENES:
  AT4G29110.1
    SENSE COUNTS:            0       0       2       0       6       1.84e-03        2.85e-03                0.000   0.000   1.000   0.000   2.585
    TAGS:
      v+1      GCTATGTATA    0       0       2       0       5       1.57e-02        2.76e-02        1142    0.000   0.000   1.000   0.000   2.322
      v+2      TATAAATAAT    0       0       0       0       1       1.65e-01        2.48e-01        1036    0.000   0.000   0.000   0.000   0.000

LOCUS: AT1G10370
DESCRIPTION:  glutathione S-transferase, putative (ERD9), similar to glutathione S-transferase TSI-1 (Aegilops tauschii) gi
DATA:                        Control 30min   2hours  2days   1week   p-value         B&H             Pos     Fold change relative to control (log2)
SENSE COUNTS:                0       0       6       9       3       1.85e-03        2.79e-03                0.000   0.000   2.585   3.170   1.585
  GENES:
  AT1G10370.1
    SENSE COUNTS:            0       0       6       9       3       1.85e-03        2.86e-03                0.000   0.000   2.585   3.170   1.585
    TAGS:
      d+1      CCCGAGACTG    0       0       6       9       3       1.85e-03        4.83e-03        709     0.000   0.000   2.585   3.170   1.585

LOCUS: AT1G03090
DESCRIPTION:  methylcrotonyl-CoA carboxylase alpha chain, mitochondrial / 3-methylcrotonyl-CoA carboxylase 1 (MCCA), nearly identical to SP|Q42523
Methylcrotonyl-CoA carboxylase alpha chain, mitochondrial precursor (EC 6.4.1.4) (3-Methylcrotonyl-CoA carboxylase 1) (MCC
DATA:                        Control 30min   2hours  2days   1week   p-value         B&H             Pos     Fold change relative to control (log2)
SENSE COUNTS:                0       8       2       1       0       1.85e-03        2.79e-03                0.000   3.000   1.000   0.000   0.000
  GENES:
  AT1G03090.1
    SENSE COUNTS:            0       8       2       1       0       1.85e-03        2.86e-03                0.000   3.000   1.000   0.000   0.000
    TAGS:
      d+1      GCTGGTTTGG    0       8       2       1       0       1.85e-03        4.82e-03        1982    0.000   3.000   1.000   0.000   0.000
  AT1G03090.2
    SENSE COUNTS:            0       8       2       1       0       1.85e-03        2.86e-03                0.000   3.000   1.000   0.000   0.000
    TAGS:
      d+1      GCTGGTTTGG    0       8       2       1       0       1.85e-03        4.82e-03        2042    0.000   3.000   1.000   0.000   0.000

LOCUS: AT2G21170
DESCRIPTION:  triosephosphate isomerase, chloroplast, putative, similar to Triosephosphate isomerase, chloroplast precursor
DATA:                        Control 30min   2hours  2days   1week   p-value         B&H             Pos     Fold change relative to control (log2)
SENSE COUNTS:                52      24      36      24      19      1.86e-03        2.80e-03                0.000   -1.115  -0.531  -1.115  -1.453
  GENES:
  AT2G21170.1
    SENSE COUNTS:            52      24      36      24      19      1.86e-03        2.87e-03                0.000   -1.115  -0.531  -1.115  -1.453
```

```
    TAGS:
      d+1      GAACAGATGA     52      24      34      24      19      2.03e-03        5.19e-03        1104    0.000   -1.115  -0.613  -1.115  -1.453
      d+2      TAGCTGTCCG     0       0       1       0       0       4.55e-01        5.37e-01        854     0.000   0.000   0.000   0.000   0.000
      d+2      TCATCGGAGA     0       0       1       0       0       4.55e-01        5.15e-01        599     0.000   0.000   0.000   0.000   0.000

LOCUS: AT3G14690
DESCRIPTION:  cytochrome P450, putative, similar to GB
DATA:                         Control 30min   2hours  2days   1week   p-value         B&H             Pos     Fold change relative to control (log2)
SENSE COUNTS:                 11      6       7       24      22      1.87e-03        2.81e-03                0.000   -0.874  -0.652  1.126   1.000
  GENES:
  AT3G14690.1
    SENSE COUNTS:             11      6       7       24      22      1.87e-03        2.88e-03                0.000   -0.874  -0.652  1.126   1.000
    TAGS:
      d+1      CACAAGCTCT     11      6       6       24      21      9.89e-04        2.84e-03        1559    0.000   -0.874  -0.874  1.126   0.933
      d+2      TGAGGTAGAC     0       0       0       0       1       4.65e-01        5.16e-01        610     0.000   0.000   0.000   0.000   0.000
      d+2      GTTTGGACCT     0       0       1       0       0       4.55e-01        5.14e-01        328     0.000   0.000   0.000   0.000   0.000

LOCUS: AT1G67870
DESCRIPTION:  glycine-rich protein, contains non-consensus GG donor splice site at exon2; modeled to est match.
DATA:                         Control 30min   2hours  2days   1week   p-value         B&H             Pos     Fold change relative to control (log2)
SENSE COUNTS:                 16      4       11      2       0       1.90e-03        2.85e-03                0.000   -2.000  -0.541  -3.000  0.000
  GENES:
  AT1G67870.1
    SENSE COUNTS:             16      4       11      2       0       1.90e-03        2.92e-03                0.000   -2.000  -0.541  -3.000  0.000
    TAGS:
      d+1      TTTCTGTATC     16      4       11      1       0       4.19e-04        1.42e-03        1003    0.000   -2.000  -0.541  -4.000  0.000
      d+2      GGATGAAGCA     0       0       0       1       0       3.09e-01        4.12e-01        449     0.000   0.000   0.000   0.000   0.000
      d+2      TCTGGGATGA     0       0       0       0       0       6.15e-01        6.45e-01        260     0.000   0.000   0.000   0.000   0.000

LOCUS: AT1G54010
DESCRIPTION:  myrosinase-associated protein, putative, similar to myrosinase-associated protein GI
DATA:                         Control 30min   2hours  2days   1week   p-value         B&H             Pos     Fold change relative to control (log2)
SENSE COUNTS:                 13      7       1       4       0       1.90e-03        2.84e-03                0.000   -0.893  -3.700  -1.700  0.000
  GENES:
  AT1G54010.1
    SENSE COUNTS:             13      7       1       4       0       1.90e-03        2.92e-03                0.000   -0.893  -3.700  -1.700  0.000
    TAGS:
      d+1      TTTCTCTAGT     13      7       1       4       0       1.90e-03        4.93e-03        1341    0.000   -0.893  -3.700  -1.700  0.000

LOCUS: AT2G31380
DESCRIPTION:  zinc finger (B-box type) family protein / salt tolerance-like protein (STH), contains Pfam profile PF00643
DATA:                         Control 30min   2hours  2days   1week   p-value         B&H             Pos     Fold change relative to control (log2)
SENSE COUNTS:                 6       0       3       5       16      1.92e-03        2.87e-03                0.000   0.000   -1.000  -0.263  1.415
  GENES:
  AT2G31380.1
    SENSE COUNTS:             6       0       3       5       16      1.92e-03        2.94e-03                0.000   0.000   -1.000  -0.263  1.415
    TAGS:
      d+1      ATCTGATATG     6       0       3       5       16      1.92e-03        4.96e-03        997     0.000   0.000   -1.000  -0.263  1.415

LOCUS: AT3G28900
DESCRIPTION:  60S ribosomal protein L34 (RPL34C), similar to 60S ribosomal protein L34 GB
DATA:                         Control 30min   2hours  2days   1week   p-value         B&H             Pos     Fold change relative to control (log2)
SENSE COUNTS:                 6       2       5       16      17      1.93e-03        2.88e-03                0.000   -1.585  -0.263  1.415   1.503
  GENES:
  AT3G28900.1
    SENSE COUNTS:             6       2       5       16      17      1.93e-03        2.95e-03                0.000   -1.585  -0.263  1.415   1.503
    TAGS:
      d+1      AGTTTTTGTT     6       2       5       16      17      1.93e-03        4.98e-03        464     0.000   -1.585  -0.263  1.415   1.503

LOCUS: AT2G38310
```

```
DESCRIPTION:  expressed protein, low similarity to early flowering protein 1 (Asparagus officinalis) GI
DATA:                         Control 30min   2hours  2days   1week   p-value         B&H             Pos     Fold change relative to control (log2)
SENSE COUNTS:                 9       2       1       0       0       1.97e-03        2.93e-03                0.000   -2.170  -3.170  0.000   0.000
  GENES:
  AT2G38310.1
    SENSE COUNTS:             9       2       1       0       0       1.97e-03        3.01e-03                0.000   -2.170  -3.170  0.000   0.000
    TAGS:
      d+1      CATATCATAA     9       2       1       0       0       1.97e-03        5.07e-03        944     0.000   -2.170  -3.170  0.000   0.000

LOCUS: AT5G44110
DESCRIPTION:  ABC transporter family protein
DATA:                         Control 30min   2hours  2days   1week   p-value         B&H             Pos     Fold change relative to control (log2)
SENSE COUNTS:                 0       0       1       6       7       1.97e-03        2.93e-03                0.000   0.000   0.000   2.585   2.807
  GENES:
  AT5G44110.1
    SENSE COUNTS:             0       0       1       6       7       1.97e-03        3.01e-03                0.000   0.000   0.000   2.585   2.807
    TAGS:
      d+1      AAAGAATTTC     0       0       1       6       7       1.97e-03        5.06e-03        1029    0.000   0.000   0.000   2.585   2.807

LOCUS: AT1G48300
DESCRIPTION:  expressed protein
DATA:                         Control 30min   2hours  2days   1week   p-value         B&H             Pos     Fold change relative to control (log2)
SENSE COUNTS:                 20      1       16      7       16      1.99e-03        2.95e-03                0.000   -4.322  -0.322  -1.515  -0.322
  GENES:
  AT1G48300.1
    SENSE COUNTS:             20      1       16      7       16      1.99e-03        3.03e-03                0.000   -4.322  -0.322  -1.515  -0.322
    TAGS:
      d+2      TAATTGCGAT     18      0       12      6       16      8.16e-04        2.43e-03        985     0.000   0.000   -0.585  -1.585  -0.170
      d+2      GGGAAGTGTC     2       1       4       1       0       4.49e-01        5.42e-01        708     0.000   -1.000  1.000   -1.000  0.000

LOCUS: AT5G49360
DESCRIPTION:  glycosyl hydrolase family 3 protein
DATA:                         Control 30min   2hours  2days   1week   p-value         B&H             Pos     Fold change relative to control (log2)
SENSE COUNTS:                 6       20      12      6       1       2.03e-03        3.01e-03                0.000   1.737   1.000   0.000   -2.585
  GENES:
  AT5G49360.1
    SENSE COUNTS:             6       20      12      6       1       2.03e-03        3.09e-03                0.000   1.737   1.000   0.000   -2.585
    TAGS:
      d+1      GCAGGGGCAA     6       19      11      5       1       3.77e-03        8.71e-03        2256    0.000   1.663   0.874   -0.263  -2.585
      d+2      AGAGCATCCG     0       0       0       1       0       3.09e-01        4.03e-01        1884    0.000   0.000   0.000   0.000   0.000
      d+2      TGAGAGTTCA     0       1       1       0       0       4.87e-01        5.38e-01        256     0.000   0.000   0.000   0.000   0.000

LOCUS: AT1G18570
DESCRIPTION:  myb family transcription factor (MYB51), contains PFAM profile
DATA:                         Control 30min   2hours  2days   1week   p-value         B&H             Pos     Fold change relative to control (log2)
SENSE COUNTS:                 1       7       1       0       0       2.04e-03        3.02e-03                0.000   2.807   0.000   0.000   0.000
  GENES:
  AT1G18570.1
    SENSE COUNTS:             1       7       1       0       0       2.04e-03        3.10e-03                0.000   2.807   0.000   0.000   0.000
    TAGS:
      d+1      AATGTAGTTC     1       7       1       0       0       6.05e-03        1.24e-02        1541    0.000   2.807   0.000   0.000   0.000
      d+2      TCAATATGAT     0       0       0       0       0       6.15e-01        6.49e-01        1450    0.000   0.000   0.000   0.000   0.000

LOCUS: AT4G03400
DESCRIPTION:  auxin-responsive GH3 family protein, similar to auxin-responsive GH3 product (Glycine max) GI
DATA:                         Control 30min   2hours  2days   1week   p-value         B&H             Pos     Fold change relative to control (log2)
SENSE COUNTS:                 2       2       15      9       14      2.08e-03        3.07e-03                0.000   0.000   2.907   2.170   2.807
  GENES:
  AT4G03400.1
    SENSE COUNTS:             2       2       15      9       14      2.08e-03        3.16e-03                0.000   0.000   2.907   2.170   2.807
```

TAGS:
d+1 GCGGCTGTGT 2 2 15 9 14 2.08e-03 5.31e-03 1963 0.000 0.000 2.907 2.170 2.807

LOCUS: AT1G78510
DESCRIPTION: solanesyl diphosphate synthase (SPS), identical to solanesyl diphosphate synthase (Arabidopsis thaliana) GI
DATA: Control 30min 2hours 2days 1week p-value B&H Pos Fold change relative to control (log2)
SENSE COUNTS: 3 0 8 13 14 2.09e-03 3.08e-03 0.000 0.000 1.415 2.115 2.222
GENES:
AT1G78510.1
SENSE COUNTS: 3 0 8 13 14 2.09e-03 3.17e-03 0.000 0.000 1.415 2.115 2.222
TAGS:
d+1 ATTTCGATTT 3 0 8 13 14 2.09e-03 5.33e-03 1587 0.000 0.000 1.415 2.115 2.222

LOCUS: AT2G47400
DESCRIPTION: CP12 domain-containing protein, contains Pfam profile
DATA: Control 30min 2hours 2days 1week p-value B&H Pos Fold change relative to control (log2)
SENSE COUNTS: 56 64 42 40 20 2.09e-03 3.08e-03 0.000 0.193 -0.415 -0.485 -1.485
GENES:
AT2G47400.1
SENSE COUNTS: 56 64 42 40 20 2.09e-03 3.16e-03 0.000 0.193 -0.415 -0.485 -1.485
TAGS:
d+1 CTAGGGACAA 54 64 28 40 12 4.33e-06 2.58e-05 316 0.000 0.245 -0.948 -0.433 -2.170
d+2 CGCTGATGAT 2 0 14 0 8 5.72e-06 3.35e-05 239 0.000 0.000 2.807 0.000 2.000

LOCUS: AT1G21680
DESCRIPTION: expressed protein, similar to TolB protein precursor (SP
DATA: Control 30min 2hours 2days 1week p-value B&H Pos Fold change relative to control (log2)
SENSE COUNTS: 1 13 4 8 0 2.10e-03 3.09e-03 0.000 3.700 2.000 3.000 0.000
GENES:
AT1G21680.1
SENSE COUNTS: 1 13 4 8 0 2.10e-03 3.17e-03 0.000 3.700 2.000 3.000 0.000
TAGS:
d+1 GGCCCCTCGG 1 13 4 8 0 2.10e-03 5.35e-03 2018 0.000 3.700 2.000 3.000 0.000

LOCUS: AT2G28000
DESCRIPTION: RuBisCO subunit binding-protein alpha subunit, chloroplast / 60 kDa chaperonin alpha subunit / CPN-60 alpha, identical to SWISS-PROT
DATA: Control 30min 2hours 2days 1week p-value B&H Pos Fold change relative to control (log2)
SENSE COUNTS: 13 7 6 11 28 2.19e-03 3.21e-03 0.000 -0.893 -1.115 -0.241 1.107
GENES:
AT2G28000.1
SENSE COUNTS: 13 7 6 11 28 2.19e-03 3.31e-03 0.000 -0.893 -1.115 -0.241 1.107
TAGS:
d+1 GTGTAATCAA 13 7 6 11 28 2.19e-03 5.55e-03 1893 0.000 -0.893 -1.115 -0.241 1.107

LOCUS: AT4G38840
DESCRIPTION: auxin-responsive protein, putative, auxin-inducible SAUR gene, Raphanus sativus,AB000708
DATA: Control 30min 2hours 2days 1week p-value B&H Pos Fold change relative to control (log2)
SENSE COUNTS: 12 4 1 4 0 2.20e-03 3.22e-03 0.000 -1.585 -3.585 -1.585 0.000
GENES:
AT4G38840.1
SENSE COUNTS: 12 4 1 4 0 2.20e-03 3.32e-03 0.000 -1.585 -3.585 -1.585 0.000
TAGS:
d+1 ACTCACTCGA 12 4 1 4 0 2.20e-03 5.57e-03 358 0.000 -1.585 -3.585 -1.585 0.000

LOCUS: AT5G24530
DESCRIPTION: oxidoreductase, 2OG-Fe(II) oxygenase family protein, similar to flavanone 3-hydroxylase (Persea americana)(GI
DATA: Control 30min 2hours 2days 1week p-value B&H Pos Fold change relative to control (log2)
SENSE COUNTS: 0 0 7 9 1 2.22e-03 3.25e-03 0.000 0.000 2.807 3.170 0.000
GENES:
AT5G24530.1

```
    SENSE COUNTS:             0      0      7      9      1      2.22e-03       3.33e-03              0.000  0.000  2.807  3.170  0.000
    TAGS:
      d+1      GGCATATTTG     0      0      7      8      1      5.02e-03       1.09e-02       1316   0.000  0.000  2.807  3.000  0.000
      d+2      AGCCCGGCCA     0      0      0      1      0      3.09e-01       4.07e-01       1014   0.000  0.000  0.000  0.000  0.000

LOCUS: AT5G16300
DESCRIPTION:  expressed protein,
DATA:                         Control 30min  2hours 2days  1week  p-value        B&H            Pos    Fold change relative to control (log2)
SENSE COUNTS:                 4      0      0      0      6      2.22e-03       3.24e-03              0.000  0.000  0.000  0.000  0.585
  GENES:
  AT5G16300.2
    SENSE COUNTS:             4      0      0      0      6      2.22e-03       3.34e-03              0.000  0.000  0.000  0.000  0.585
    TAGS:
      d+2      TAATTAAGTA     3      0      0      0      5      1.39e-02       2.46e-02       3318   0.000  0.000  0.000  0.000  0.737
      d+2      AACCAAAAAC     0      0      0      0      1      1.65e-01       2.35e-01       3203   0.000  0.000  0.000  0.000  0.000
      d+2      CTGTACTCTT     1      0      0      0      0      4.28e-01       5.23e-01       2756   0.000  0.000  0.000  0.000  0.000
  AT5G16300.1
    SENSE COUNTS:             4      0      0      0      6      2.22e-03       3.34e-03              0.000  0.000  0.000  0.000  0.585
    TAGS:
      d+2      TAATTAAGTA     3      0      0      0      5      1.39e-02       2.46e-02       3420   0.000  0.000  0.000  0.000  0.737
      d+2      AACCAAAAAC     0      0      0      0      1      1.65e-01       2.35e-01       3305   0.000  0.000  0.000  0.000  0.000
      d+2      CTGTACTCTT     1      0      0      0      0      4.28e-01       5.23e-01       2756   0.000  0.000  0.000  0.000  0.000

LOCUS: AT3G48360
DESCRIPTION:  speckle-type POZ  protein-related, contains Pfam PF00651
DATA:                         Control 30min  2hours 2days  1week  p-value        B&H            Pos    Fold change relative to control (log2)
SENSE COUNTS:                 4      10     2      0      0      2.23e-03       3.25e-03              0.000  1.322  -1.000 0.000  0.000
  GENES:
  AT3G48360.1
    SENSE COUNTS:             4      10     2      0      0      2.23e-03       3.34e-03              0.000  1.322  -1.000 0.000  0.000
    TAGS:
      d+1      TAATACAAAA     4      10     2      0      0      2.23e-03       5.63e-03       1365   0.000  1.322  -1.000 0.000  0.000

LOCUS: AT5G25610
DESCRIPTION:  dehydration-responsive protein (RD22), identical to SP|Q08298 Dehydration-responsive protein RD22 precursor {Arabidopsis thaliana}
DATA:                         Control 30min  2hours 2days  1week  p-value        B&H            Pos    Fold change relative to control (log2)
SENSE COUNTS:                 24     3      14     12     17     2.23e-03       3.25e-03              0.000  -3.000 -0.778 -1.000 -0.497
  GENES:
  AT5G25610.1
    SENSE COUNTS:             24     3      14     12     17     2.23e-03       3.34e-03              0.000  -3.000 -0.778 -1.000 -0.497
    TAGS:
      d+1      TTGTGTGGTT     24     3      13     12     17     2.01e-03       5.16e-03       1197   0.000  -3.000 -0.885 -1.000 -0.497
      d+2      GTAGTGGCGA     0      0      1      0      0      4.55e-01       5.46e-01       83     0.000  0.000  0.000  0.000  0.000

LOCUS: AT1G77760
DESCRIPTION:  nitrate reductase 1 (NR1), identical to SP|P11832 Nitrate reductase 1 (formerly EC 1.6.6.1) (NR1){Arabidopsis thaliana}
DATA:                         Control 30min  2hours 2days  1week  p-value        B&H            Pos    Fold change relative to control (log2)
SENSE COUNTS:                 12     7      24     27     12     2.24e-03       3.26e-03              0.000  -0.778 1.000  1.170  0.000
  GENES:
  AT1G77760.1
    SENSE COUNTS:             12     7      24     27     12     2.24e-03       3.35e-03              0.000  -0.778 1.000  1.170  0.000
    TAGS:
      d+1      GTTATTTGGG     10     7      24     26     12     1.83e-03       4.80e-03       2905   0.000  -0.515 1.263  1.379  0.263
      d+2      CGGACCACCG     2      0      0      0      0      1.04e-01       1.63e-01       2745   0.000  0.000  0.000  0.000  0.000
      i+3      CGGGGCAATT     0      0      0      1      0      3.09e-01       4.08e-01       1984   0.000  0.000  0.000  0.000  0.000

LOCUS: AT5G28060
DESCRIPTION:  40S ribosomal protein S24 (RPS24B), 40S ribosomal protein S19, Cyanophora paradoxa, EMBL
DATA:                         Control 30min  2hours 2days  1week  p-value        B&H            Pos    Fold change relative to control (log2)
SENSE COUNTS:                 27     14     27     36     48     2.25e-03       3.26e-03              0.000  -0.948 0.000  0.415  0.830
```

```
  GENES:
  AT5G28060.1
    SENSE COUNTS:              27      14      27      36      48      2.25e-03        3.36e-03                0.000   -0.948  0.000   0.415   0.830
    TAGS:
      d+1      TGGTTAAGAT     27      14      27      36      48      2.25e-03        5.67e-03        667     0.000   -0.948  0.000   0.415   0.830

LOCUS: AT1G75690
DESCRIPTION:  chaperone protein dnaJ-related, contains Pfam PF00684
DATA:                         Control 30min   2hours  2days   1week   p-value         B&H             Pos     Fold change relative to control (log2)
SENSE COUNTS:                 22      4       10      8       19      2.26e-03        3.27e-03                0.000   -2.459  -1.138  -1.459  -0.212
  GENES:
  AT1G75690.1
    SENSE COUNTS:             22      4       10      8       19      2.26e-03        3.37e-03                0.000   -2.459  -1.138  -1.459  -0.212
    TAGS:
      d+1      GATATTGTAA     22      4       10      8       19      2.26e-03        5.68e-03        619     0.000   -2.459  -1.138  -1.459  -0.212

LOCUS: AT4G17720
DESCRIPTION:  RNA recognition motif (RRM)-containing protein
DATA:                         Control 30min   2hours  2days   1week   p-value         B&H             Pos     Fold change relative to control (log2)
SENSE COUNTS:                 0       2       11      1       5       2.28e-03        3.30e-03                0.000   1.000   3.459   0.000   2.322
  GENES:
  AT4G17720.1
    SENSE COUNTS:             0       2       11      1       5       2.28e-03        3.39e-03                0.000   1.000   3.459   0.000   2.322
    TAGS:
      d+1      GTCGATTTGG     0       2       11      1       5       2.28e-03        5.73e-03        1245    0.000   1.000   3.459   0.000   2.322

LOCUS: AT1G48210
DESCRIPTION:  serine/threonine protein kinase, putative, similar to Pto kinase interactor 1 (Lycopersicon esculentum) gi|3668069|gb|AAC61805; contains
protein kinase domain, Pfam
DATA:                         Control 30min   2hours  2days   1week   p-value         B&H             Pos     Fold change relative to control (log2)
SENSE COUNTS:                 2       0       1       1       10      2.31e-03        3.34e-03                0.000   0.000   -1.000  -1.000  2.322
  GENES:
  AT1G48210.1
    SENSE COUNTS:             2       0       1       1       10      2.31e-03        3.43e-03                0.000   0.000   -1.000  -1.000  2.322
    TAGS:
      i+3      TCTTCATTCA     0       0       0       0       0       6.15e-01        6.47e-01        2286    0.000   0.000   0.000   0.000   0.000
      d+1      TGTATGTCTC     2       0       1       1       10      5.01e-04        1.64e-03        1390    0.000   0.000   -1.000  -1.000  2.322

LOCUS: AT3G52060
DESCRIPTION:  expressed protein, contains Pfam profile PF03267
DATA:                         Control 30min   2hours  2days   1week   p-value         B&H             Pos     Fold change relative to control (log2)
SENSE COUNTS:                 2       9       1       1       0       2.33e-03        3.36e-03                0.000   2.170   -1.000  -1.000  0.000
  GENES:
  AT3G52060.2
    SENSE COUNTS:             2       9       1       1       0       2.33e-03        3.46e-03                0.000   2.170   -1.000  -1.000  0.000
    TAGS:
      d+1      CCTTGATGTT     2       9       1       1       0       2.33e-03        5.83e-03        1018    0.000   2.170   -1.000  -1.000  0.000
  AT3G52060.1
    SENSE COUNTS:             2       9       1       1       0       2.33e-03        3.45e-03                0.000   2.170   -1.000  -1.000  0.000
    TAGS:
      d+1      CCTTGATGTT     2       9       1       1       0       2.33e-03        5.83e-03        1018    0.000   2.170   -1.000  -1.000  0.000

LOCUS: AT1G08520
DESCRIPTION:  magnesium-chelatase subunit chlD, chloroplast, putative / Mg-protoporphyrin IX chelatase, putative (CHLD), similar to Mg-chelatase
SP|O24133 from Nicotiana tabacum, GB
DATA:                         Control 30min   2hours  2days   1week   p-value         B&H             Pos     Fold change relative to control (log2)
SENSE COUNTS:                 16      9       1       8       21      2.34e-03        3.37e-03                0.000   -0.830  -4.000  -1.000  0.392
  GENES:
  AT1G08520.1
    SENSE COUNTS:             16      9       1       8       21      2.34e-03        3.46e-03                0.000   -0.830  -4.000  -1.000  0.392
```

TAGS:
d+1 AAATTATAAG 16 9 1 8 21 1.89e-03 4.91e-03 2488 0.000 -0.830 -4.000 -1.000 0.392
d+2 AAATCCTCCC 0 0 0 0 0 6.15e-01 6.32e-01 512 0.000 0.000 0.000 0.000 0.000

LOCUS: AT4G09320
DESCRIPTION: nucleoside diphosphate kinase 1 (NDK1), identical to identical to Nucleoside diphosphate kinase I (NDK I) (NDP kinase I) (NDPK I) (SP
DATA: Control 30min 2hours 2days 1week p-value B&H Pos Fold change relative to control (log2)
SENSE COUNTS: 19 11 10 18 35 2.35e-03 3.38e-03 0.000 -0.788 -0.926 -0.078 0.881
GENES:
AT4G09320.1
SENSE COUNTS: 19 11 10 18 35 2.35e-03 3.47e-03 0.000 -0.788 -0.926 -0.078 0.881
TAGS:
d+1 GTAGTGACTC 19 11 10 18 35 2.35e-03 5.87e-03 431 0.000 -0.788 -0.926 -0.078 0.881

LOCUS: AT3G24170
DESCRIPTION: glutathione reductase, putative, identical to GB
DATA: Control 30min 2hours 2days 1week p-value B&H Pos Fold change relative to control (log2)
SENSE COUNTS: 1 2 5 12 14 2.47e-03 3.55e-03 0.000 1.000 2.322 3.585 3.807
GENES:
AT3G24170.1
SENSE COUNTS: 1 2 5 12 14 2.47e-03 3.64e-03 0.000 1.000 2.322 3.585 3.807
TAGS:
d+1 AAAACTCGGT 1 2 5 11 14 4.29e-03 9.65e-03 1750 0.000 1.000 2.322 3.459 3.807
d+2 GCGAGGAAGA 0 0 0 1 0 3.09e-01 4.09e-01 223 0.000 0.000 0.000 0.000 0.000

LOCUS: AT1G05385
DESCRIPTION: photosystem II 11 kDa protein-related, similar to Photosystem II 11 kDa protein precursor (SP|P74367)(strain PCC 6803) {Synechocystis sp.}
DATA: Control 30min 2hours 2days 1week p-value B&H Pos Fold change relative to control (log2)
SENSE COUNTS: 3 0 0 0 7 2.56e-03 3.67e-03 0.000 0.000 0.000 0.000 1.222
GENES:
AT1G05385.1
SENSE COUNTS: 3 0 0 0 7 2.56e-03 3.77e-03 0.000 0.000 0.000 0.000 1.222
TAGS:
d+1 TTATACATAT 3 0 0 0 7 2.56e-03 6.33e-03 700 0.000 0.000 0.000 0.000 1.222

LOCUS: AT2G47940
DESCRIPTION: DegP2 protease (DEGP2), identical to DegP2 protease GI
DATA: Control 30min 2hours 2days 1week p-value B&H Pos Fold change relative to control (log2)
SENSE COUNTS: 16 8 23 22 1 2.57e-03 3.68e-03 0.000 -1.000 0.524 0.459 -4.000
GENES:
AT2G47940.1
SENSE COUNTS: 16 8 23 22 1 2.57e-03 3.78e-03 0.000 -1.000 0.524 0.459 -4.000
TAGS:
d+1 AGAATGCTCG 16 8 22 22 1 3.74e-03 8.67e-03 2079 0.000 -1.000 0.459 0.459 -4.000
d+2 AATAACCAGC 0 0 1 0 0 4.55e-01 5.34e-01 1575 0.000 0.000 0.000 0.000 0.000

LOCUS: AT3G13740
DESCRIPTION: URF 4-related, similar to URF 4 (GI
DATA: Control 30min 2hours 2days 1week p-value B&H Pos Fold change relative to control (log2)
SENSE COUNTS: 1 0 3 4 12 2.59e-03 3.70e-03 0.000 0.000 1.585 2.000 3.585
GENES:
AT3G13740.1
SENSE COUNTS: 1 0 3 4 12 2.59e-03 3.80e-03 0.000 0.000 1.585 2.000 3.585
TAGS:
d+1 GGTTGATTCT 1 0 3 4 12 2.59e-03 6.40e-03 994 0.000 0.000 1.585 2.000 3.585

LOCUS: AT5G49460
DESCRIPTION: One of the two genes encoding subunit B of the cytosolic enzyme ATP Citrate Lyase (ACL)
DATA: Control 30min 2hours 2days 1week p-value B&H Pos Fold change relative to control (log2)

```
SENSE COUNTS:                 11     1      3      0      1      2.62e-03       3.74e-03              0.000   -3.459  -1.874  0.000   -3.459
  GENES:
  AT5G49460.1
    SENSE COUNTS:             11     1      3      0      1      2.62e-03       3.84e-03              0.000   -3.459  -1.874  0.000   -3.459
    TAGS:
      d+1      TCTATATCTT     0      0      0      0      0      6.15e-01       6.38e-01       2199   0.000   0.000   0.000   0.000   0.000
      d+2      TATGTTTGTT     9      1      2      0      1      7.40e-03       1.42e-02       2085   0.000   -3.170  -2.170  0.000   -3.170
      d+2      GGAAGATGTG     2      0      1      0      0      2.87e-01       3.87e-01       1937   0.000   0.000   -1.000  0.000   0.000

LOCUS: AT5G32482
DESCRIPTION:  zinc knuckle (CCHC-type) family protein, contains Pfam domain, PF00098
DATA:                         Control 30min  2hours 2days  1week  p-value        B&H            Pos    Fold change relative to control (log2)
SENSE COUNTS:                 13     0      8      2      12     2.63e-03       3.75e-03              0.000   0.000   -0.700  -2.700  -0.115
  GENES:
  AT5G32482.1
    SENSE COUNTS:             13     0      8      2      12     2.63e-03       3.85e-03              0.000   0.000   -0.700  -2.700  -0.115
    TAGS:
      v+1      TACTCTTACC     13     0      8      2      12     2.63e-03       6.48e-03       1684   0.000   0.000   -0.700  -2.700  -0.115

LOCUS: AT1G48830
DESCRIPTION:  40S ribosomal protein S7 (RPS7A), similar to 40S ribosomal protein S7 homolog GI
DATA:                         Control 30min  2hours 2days  1week  p-value        B&H            Pos    Fold change relative to control (log2)
SENSE COUNTS:                 5      2      0      0      10     2.64e-03       3.75e-03              0.000   -1.322  0.000   0.000   1.000
  GENES:
  AT1G48830.1
    SENSE COUNTS:             5      2      0      0      10     2.64e-03       3.85e-03              0.000   -1.322  0.000   0.000   1.000
    TAGS:
      d+1      AAGGTCTTCT     5      2      0      0      10     2.64e-03       6.49e-03       557    0.000   -1.322  0.000   0.000   1.000
  AT1G48830.2
    SENSE COUNTS:             5      2      0      0      10     2.64e-03       3.84e-03              0.000   -1.322  0.000   0.000   1.000
    TAGS:
      d+1      AAGGTCTTCT     5      2      0      0      10     2.64e-03       6.49e-03       497    0.000   -1.322  0.000   0.000   1.000

LOCUS: AT5G42900
DESCRIPTION:  expressed protein, similar to unknown protein (pir||T05226)
DATA:                         Control 30min  2hours 2days  1week  p-value        B&H            Pos    Fold change relative to control (log2)
SENSE COUNTS:                 0      1      12     6      7      2.64e-03       3.75e-03              0.000   0.000   3.585   2.585   2.807
  GENES:
  AT5G42900.2
    SENSE COUNTS:             0      1      12     6      7      2.64e-03       3.85e-03              0.000   0.000   3.585   2.585   2.807
    TAGS:
      d+1      TGAGTGGATT     0      1      12     6      7      2.64e-03       6.48e-03       963    0.000   0.000   3.585   2.585   2.807
  AT5G42900.1
    SENSE COUNTS:             0      1      12     6      7      2.64e-03       3.86e-03              0.000   0.000   3.585   2.585   2.807
    TAGS:
      d+1      TGAGTGGATT     0      1      12     6      7      2.64e-03       6.48e-03       966    0.000   0.000   3.585   2.585   2.807

LOCUS: AT3G46620
DESCRIPTION:  zinc finger (C3HC4-type RING finger) family protein, contains Pfam domain, PF00097
DATA:                         Control 30min  2hours 2days  1week  p-value        B&H            Pos    Fold change relative to control (log2)
SENSE COUNTS:                 0      9      6      1      0      2.66e-03       3.77e-03              0.000   3.170   2.585   0.000   0.000
  GENES:
  AT3G46620.1
    SENSE COUNTS:             0      9      6      1      0      2.66e-03       3.87e-03              0.000   3.170   2.585   0.000   0.000
    TAGS:
      d+1      AGACACCGGA     0      9      6      1      0      2.66e-03       6.51e-03       1032   0.000   3.170   2.585   0.000   0.000

LOCUS: AT1G78040
DESCRIPTION:  pollen Ole e 1 allergen and extensin family protein, contains Pfam domain, PF01190
DATA:                         Control 30min  2hours 2days  1week  p-value        B&H            Pos    Fold change relative to control (log2)
```

```
SENSE COUNTS:                 19      43      25      20      17      2.78e-03        3.93e-03                0.000   1.178   0.396   0.074   -0.160
  GENES:
  AT1G78040.1
    SENSE COUNTS:             19      43      25      20      17      2.78e-03        4.03e-03                0.000   1.178   0.396   0.074   -0.160
    TAGS:
      d+1      GGATTTGAGA     19      43      25      20      16      1.86e-03        4.84e-03        576     0.000   1.178   0.396   0.074   -0.248
      d+2      CAAAGACAGG     0       0       0       0       1       1.65e-01        2.39e-01        344     0.000   0.000   0.000   0.000   0.000

LOCUS: AT4G38160
DESCRIPTION:  mitochondrial transcription termination factor-related / mTERF-related, contains Pfam profile PF02536
DATA:                         Control 30min   2hours  2days   1week   p-value         B&H             Pos     Fold change relative to control (log2)
SENSE COUNTS:                 3       1       4       15      5       2.78e-03        3.93e-03                0.000   -1.585  0.415   2.322   0.737
  GENES:
  AT4G38160.2
    SENSE COUNTS:             1       0       0       0       0       4.28e-01        4.31e-01                0.000   0.000   0.000   0.000   0.000
    TAGS:
      i+3      GATTCTACAC     1       0       0       0       0       4.28e-01        5.21e-01        1306    0.000   0.000   0.000   0.000   0.000
  AT4G38160.1
    SENSE COUNTS:             2       1       4       15      5       1.31e-03        2.16e-03                0.000   -1.000  1.000   2.907   1.322
    TAGS:
      d+1      AGAAGTTTGG     2       1       4       15      5       1.31e-03        3.58e-03        1032    0.000   -1.000  1.000   2.907   1.322

LOCUS: AT2G21390
DESCRIPTION:  coatomer protein complex, subunit alpha, putative, contains Pfam PF00400
DATA:                         Control 30min   2hours  2days   1week   p-value         B&H             Pos     Fold change relative to control (log2)
SENSE COUNTS:                 1       0       3       9       1       2.79e-03        3.94e-03                0.000   0.000   1.585   3.170   0.000
  GENES:
  AT2G21390.1
    SENSE COUNTS:             1       0       3       9       1       2.79e-03        4.04e-03                0.000   0.000   1.585   3.170   0.000
    TAGS:
      d+1      AGAACTTTAT     1       0       3       9       1       2.79e-03        6.81e-03        4050    0.000   0.000   1.585   3.170   0.000

LOCUS: AT3G17790
DESCRIPTION:  acid phosphatase type 5 (ACP5), contains Pfam profile
DATA:                         Control 30min   2hours  2days   1week   p-value         B&H             Pos     Fold change relative to control (log2)
SENSE COUNTS:                 0       5       10      1       0       2.80e-03        3.94e-03                0.000   2.322   3.322   0.000   0.000
  GENES:
  AT3G17790.1
    SENSE COUNTS:             0       5       10      1       0       2.80e-03        4.05e-03                0.000   2.322   3.322   0.000   0.000
    TAGS:
      d+2      TCTGCTCGGT     0       5       10      1       0       2.80e-03        6.83e-03        1067    0.000   2.322   3.322   0.000   0.000

LOCUS: AT2G45180
DESCRIPTION:  protease inhibitor/seed storage/lipid transfer protein (LTP) family protein, similar to 14 kDa polypeptide (Catharanthus roseus) GI
DATA:                         Control 30min   2hours  2days   1week   p-value         B&H             Pos     Fold change relative to control (log2)
SENSE COUNTS:                 393     353     295     320     303     2.84e-03        4.00e-03                0.000   -0.155  -0.414  -0.296  -0.375
  GENES:
  AT2G45180.1
    SENSE COUNTS:             393     353     295     320     303     2.84e-03        4.10e-03                0.000   -0.155  -0.414  -0.296  -0.375
    TAGS:
      X+4      ATAGAACCTT     389     346     290     315     298     2.39e-03        5.95e-03        585     0.000   -0.169  -0.424  -0.304  -0.384
      d+1      GTTTCCAATG     4       7       5       5       5       9.70e-01        9.70e-01        427     0.000   0.807   0.322   0.322   0.322

LOCUS: AT3G26450
DESCRIPTION:  major latex protein-related / MLP-related, low similarity to major latex protein {Papaver somniferum}(GI
DATA:                         Control 30min   2hours  2days   1week   p-value         B&H             Pos     Fold change relative to control (log2)
SENSE COUNTS:                 18      8       17      5       0       2.85e-03        4.00e-03                0.000   -1.170  -0.082  -1.848  0.000
  GENES:
  AT3G26450.1
    SENSE COUNTS:             18      8       17      5       0       2.85e-03        4.11e-03                0.000   -1.170  -0.082  -1.848  0.000
```

```
    TAGS:
      i+3      TCCTATCTTT     2       0       0       0       0       1.04e-01       1.64e-01       1221    0.000   0.000   0.000   0.000   0.000
      d+1      CAACTCCTCA     16      8       17      5       0       4.54e-03       1.01e-02       475     0.000   -1.000  0.087   -1.678  0.000
      i+3      GGTTGATACC     0       0       0       0       0       6.15e-01       6.51e-01       314     0.000   0.000   0.000   0.000   0.000

LOCUS: AT4G16720
DESCRIPTION:  60S ribosomal protein L15 (RPL15A)
DATA:                         Control 30min   2hours  2days   1week   p-value        B&H            Pos     Fold change relative to control (log2)
SENSE COUNTS:                 20      15      21      23      46      2.86e-03       4.01e-03               0.000   -0.415  0.070   0.202   1.202
  GENES:
  AT4G16720.1
    SENSE COUNTS:             20      15      21      23      46      2.86e-03       4.12e-03               0.000   -0.415  0.070   0.202   1.202
    TAGS:
      d+1      TTGTTGCACT     20      15      21      23      46      2.86e-03       6.95e-03       730     0.000   -0.415  0.070   0.202   1.202

LOCUS: AT5G46860
DESCRIPTION:  syntaxin 22 (SYP22) (VAM3), identical to GP|8809669| syntaxin related protein AtVam3p (Arabidopsis thaliana)
DATA:                         Control 30min   2hours  2days   1week   p-value        B&H            Pos     Fold change relative to control (log2)
SENSE COUNTS:                 1       9       1       1       1       2.87e-03       4.02e-03               0.000   3.170   0.000   0.000   0.000
  GENES:
  AT5G46860.1
    SENSE COUNTS:             1       9       1       1       1       2.87e-03       4.12e-03               0.000   3.170   0.000   0.000   0.000
    TAGS:
      d+1      CTTGCTCTTG     1       9       1       1       1       2.87e-03       6.97e-03       919     0.000   3.170   0.000   0.000   0.000

LOCUS: AT3G10920
DESCRIPTION:  superoxide dismutase (Mn), mitochondrial (SODA) / manganese superoxide dismutase (MSD1), identical to manganese superoxide dismutase
(Arabidopsis thaliana) gi|3273751|gb|AAC24832
DATA:                         Control 30min   2hours  2days   1week   p-value        B&H            Pos     Fold change relative to control (log2)
SENSE COUNTS:                 1       9       1       1       1       2.87e-03       4.01e-03               0.000   3.170   0.000   0.000   0.000
  GENES:
  AT3G10920.1
    SENSE COUNTS:             1       9       1       1       1       2.87e-03       4.13e-03               0.000   3.170   0.000   0.000   0.000
    TAGS:
      d+1      TCAACCATTC     1       9       1       1       1       2.87e-03       6.96e-03       340     0.000   3.170   0.000   0.000   0.000

LOCUS: AT5G57020
DESCRIPTION:  myristoyl-CoA
DATA:                         Control 30min   2hours  2days   1week   p-value        B&H            Pos     Fold change relative to control (log2)
SENSE COUNTS:                 4       12      2       2       0       2.88e-03       4.02e-03               0.000   1.585   -1.000  -1.000  0.000
  GENES:
  AT5G57020.1
    SENSE COUNTS:             4       12      2       2       0       2.88e-03       4.12e-03               0.000   1.585   -1.000  -1.000  0.000
    TAGS:
      d+1      GTTTACTGAA     4       12      2       2       0       2.88e-03       6.97e-03       1659    0.000   1.585   -1.000  -1.000  0.000

LOCUS: AT2G32480
DESCRIPTION:  membrane-associated zinc metalloprotease, putative, similar to Hypothetical zinc metalloprotease All3971 (SP
DATA:                         Control 30min   2hours  2days   1week   p-value        B&H            Pos     Fold change relative to control (log2)
SENSE COUNTS:                 5       2       3       13      16      2.88e-03       4.01e-03               0.000   -1.322  -0.737  1.379   1.678
  GENES:
  AT2G32480.2
    SENSE COUNTS:             5       2       3       13      16      2.88e-03       4.11e-03               0.000   -1.322  -0.737  1.379   1.678
    TAGS:
      d+1      TAATAGTTCT     4       2       3       13      16      1.78e-03       4.69e-03       1400    0.000   -1.000  -0.415  1.700   2.000
      d+2      CTTGTGATAT     1       0       0       0       0       4.28e-01       5.24e-01       1210    0.000   0.000   0.000   0.000   0.000
  AT2G32480.1
    SENSE COUNTS:             5       2       3       13      16      2.88e-03       4.13e-03               0.000   -1.322  -0.737  1.379   1.678
    TAGS:
      d+1      TAATAGTTCT     4       2       3       13      16      1.78e-03       4.69e-03       1511    0.000   -1.000  -0.415  1.700   2.000
```

```
      d+2      CTTGTGATAT     1       0       0       0       0       4.28e-01        5.24e-01        1321    0.000   0.000   0.000   0.000   0.000

LOCUS: AT5G06320
DESCRIPTION:  harpin-induced family protein / HIN1 family protein / harpin-responsive family protein / NDR1/HIN1-like protein 3, similar to harpin-
induced protein hin1 (GI
DATA:                         Control 30min   2hours  2days   1week   p-value         B&H             Pos     Fold change relative to control (log2)
SENSE COUNTS:                 1       13      3       4       10      2.88e-03        4.01e-03                0.000   3.700   1.585   2.000   3.322
  GENES:
  AT5G06320.1
    SENSE COUNTS:             1       13      3       4       10      2.88e-03        4.13e-03                0.000   3.700   1.585   2.000   3.322
    TAGS:
      d+2      AAGCATTGAT     0       0       0       0       0       6.15e-01        6.35e-01        979     0.000   0.000   0.000   0.000   0.000
      d+2      TAAATAAATA     1       13      3       4       10      5.47e-03        1.17e-02        914     0.000   3.700   1.585   2.000   3.322

LOCUS: AT1G80230
DESCRIPTION:  cytochrome c oxidase family protein, contains Pfam domain, PF01215
DATA:                         Control 30min   2hours  2days   1week   p-value         B&H             Pos     Fold change relative to control (log2)
SENSE COUNTS:                 0       0       3       9       1       2.92e-03        4.06e-03                0.000   0.000   1.585   3.170   0.000
  GENES:
  AT1G80230.1
    SENSE COUNTS:             0       0       3       9       1       2.92e-03        4.17e-03                0.000   0.000   1.585   3.170   0.000
    TAGS:
      d+1      TGTTTATACG     0       0       3       0       1       7.97e-02        1.28e-01        843     0.000   0.000   1.585   0.000   0.000
      d+2      GTGATGACCA     0       0       0       9       0       1.28e-05        6.64e-05        597     0.000   0.000   0.000   3.170   0.000

LOCUS: AT5G01600
DESCRIPTION:  ferritin 1 (FER1), identical to ferritin (Arabidopsis thaliana) GI
DATA:                         Control 30min   2hours  2days   1week   p-value         B&H             Pos     Fold change relative to control (log2)
SENSE COUNTS:                 5       25      11      23      12      2.99e-03        4.15e-03                0.000   2.322   1.138   2.202   1.263
  GENES:
  AT5G01600.1
    SENSE COUNTS:             5       25      11      23      12      2.99e-03        4.26e-03                0.000   2.322   1.138   2.202   1.263
    TAGS:
      d+1      CTGAAAAAGG     5       25      10      22      12      2.60e-03        6.41e-03        975     0.000   2.322   1.000   2.138   1.263
      d+2      CCTCTCTCGC     0       0       1       1       0       5.21e-01        5.71e-01        705     0.000   0.000   0.000   0.000   0.000

LOCUS: AT5G27420
DESCRIPTION:  zinc finger (C3HC4-type RING finger) family protein, similar to RING-H2 zinc finger protein ATL6 (Arabidopsis thaliana)
gi|4928403|gb|AAD33584.1|AF132016_1(4928403); contains Pfam domain, PF00097
DATA:                         Control 30min   2hours  2days   1week   p-value         B&H             Pos     Fold change relative to control (log2)
SENSE COUNTS:                 0       5       0       0       0       3.00e-03        4.16e-03                0.000   2.322   0.000   0.000   0.000
  GENES:
  AT5G27420.1
    SENSE COUNTS:             0       5       0       0       0       3.00e-03        4.25e-03                0.000   2.322   0.000   0.000   0.000
    TAGS:
      d+2      GGCTTCTTCA     0       5       0       0       0       3.00e-03        7.19e-03        225     0.000   2.322   0.000   0.000   0.000

LOCUS: AT1G55280
DESCRIPTION:  expressed protein
DATA:                         Control 30min   2hours  2days   1week   p-value         B&H             Pos     Fold change relative to control (log2)
SENSE COUNTS:                 0       5       0       0       0       3.00e-03        4.15e-03                0.000   2.322   0.000   0.000   0.000
  GENES:
  AT1G55280.1
    SENSE COUNTS:             0       5       0       0       0       3.00e-03        4.24e-03                0.000   2.322   0.000   0.000   0.000
    TAGS:
      d+2      GTTTACGAAA     0       5       0       0       0       3.00e-03        7.20e-03        142     0.000   2.322   0.000   0.000   0.000

LOCUS: AT2G31810
DESCRIPTION:  acetolactate synthase small subunit, putative, similar to gi
DATA:                         Control 30min   2hours  2days   1week   p-value         B&H             Pos     Fold change relative to control (log2)
```

SENSE COUNTS:		0	5	0	0	0	3.00e-03	4.14e-03		0.000	2.322	0.000	0.000	0.000
GENES:														
AT2G31810.1														
SENSE COUNTS:		0	5	0	0	0	3.00e-03	4.26e-03		0.000	2.322	0.000	0.000	0.000
TAGS:														
d+1	ATCTTACTCA	0	5	0	0	0	3.00e-03	7.21e-03	1224	0.000	2.322	0.000	0.000	0.000
AT2G31810.2														
SENSE COUNTS:		0	5	0	0	0	3.00e-03	4.23e-03		0.000	2.322	0.000	0.000	0.000
TAGS:														
d+1	ATCTTACTCA	0	5	0	0	0	3.00e-03	7.21e-03	1227	0.000	2.322	0.000	0.000	0.000
AT2G31810.3														
SENSE COUNTS:		0	5	0	0	0	3.00e-03	4.26e-03		0.000	2.322	0.000	0.000	0.000
TAGS:														
d+1	ATCTTACTCA	0	5	0	0	0	3.00e-03	7.21e-03	1224	0.000	2.322	0.000	0.000	0.000

LOCUS: AT1G70850
DESCRIPTION: Bet v I allergen family protein, similar to Csf-2 (Cucumis sativus)(GI

DATA:		Control	30min	2hours	2days	1week	p-value	B&H	Pos	Fold change relative to control (log2)				
SENSE COUNTS:		0	4	0	0	0	3.00e-03	4.14e-03		0.000	2.000	0.000	0.000	0.000
GENES:														
AT1G70850.2														
SENSE COUNTS:		0	4	0	0	0	3.00e-03	4.27e-03		0.000	2.000	0.000	0.000	0.000
TAGS:														
d+1	TTGTATGTTT	0	4	0	0	0	9.72e-03	1.78e-02	1124	0.000	2.000	0.000	0.000	0.000
d+2	CCGATTTTTA	0	0	0	0	0	6.15e-01	6.55e-01	1060	0.000	0.000	0.000	0.000	0.000
AT1G70850.1														
SENSE COUNTS:		0	4	0	0	0	3.00e-03	4.25e-03		0.000	2.000	0.000	0.000	0.000
TAGS:														
d+1	TTGTATGTTT	0	4	0	0	0	9.72e-03	1.78e-02	1120	0.000	2.000	0.000	0.000	0.000
d+2	CCGATTTTTA	0	0	0	0	0	6.15e-01	6.55e-01	1056	0.000	0.000	0.000	0.000	0.000

LOCUS: AT5G65010
DESCRIPTION: asparagine synthetase (ASN2) mRNA, complete cds

DATA:		Control	30min	2hours	2days	1week	p-value	B&H	Pos	Fold change relative to control (log2)				
SENSE COUNTS:		15	2	19	20	11	3.03e-03	4.17e-03		0.000	-2.907	0.341	0.415	-0.447
GENES:														
AT5G65010.1														
SENSE COUNTS:		14	2	19	19	8	2.45e-03	3.62e-03		0.000	-2.807	0.441	0.441	-0.807
TAGS:														
d+1	GATCTCCAGT	12	2	18	19	8	3.40e-03	7.99e-03	1896	0.000	-2.585	0.585	0.663	-0.585
i+3	TGTAAATAAA	2	0	0	0	0	1.04e-01	1.64e-01	472	0.000	0.000	0.000	0.000	0.000
d+2	TTGGATGGAA	0	0	1	0	0	4.55e-01	5.41e-01	383	0.000	0.000	0.000	0.000	0.000
AT5G65010.2														
SENSE COUNTS:		15	2	19	20	11	3.03e-03	4.27e-03		0.000	-2.907	0.341	0.415	-0.447
TAGS:														
d+1	GATCTCCAGT	12	2	18	19	8	3.40e-03	7.99e-03	1899	0.000	-2.585	0.585	0.663	-0.585
X+4	TTCTGGCTTG	1	0	0	1	3	1.87e-01	2.63e-01	531	0.000	0.000	0.000	0.000	1.585
i+3	TGTAAATAAA	2	0	0	0	0	1.04e-01	1.64e-01	472	0.000	0.000	0.000	0.000	0.000
d+2	TTGGATGGAA	0	0	1	0	0	4.55e-01	5.41e-01	383	0.000	0.000	0.000	0.000	0.000

LOCUS: AT5G53300
DESCRIPTION: ubiquitin-conjugating enzyme 10 (UBC10), E2; identical to gi

DATA:		Control	30min	2hours	2days	1week	p-value	B&H	Pos	Fold change relative to control (log2)				
SENSE COUNTS:		48	75	58	67	30	3.10e-03	4.26e-03		0.000	0.644	0.273	0.481	-0.678
GENES:														
AT5G53300.2														
SENSE COUNTS:		48	75	58	67	30	3.10e-03	4.35e-03		0.000	0.644	0.273	0.481	-0.678
TAGS:														
d+1	GGCTAAATGG	48	75	56	67	30	3.56e-03	8.32e-03	544	0.000	0.644	0.222	0.481	-0.678
d+2	TTTCACTGGC	0	0	2	0	0	3.51e-01	4.52e-01	193	0.000	0.000	1.000	0.000	0.000

AT5G53300.1
SENSE COUNTS: 48 75 58 67 30 3.10e-03 4.36e-03 0.000 0.644 0.273 0.481 -0.678
TAGS:
d+1 GGCTAAATGG 48 75 56 67 30 3.56e-03 8.32e-03 563 0.000 0.644 0.222 0.481 -0.678
d+2 TTTCACTGGC 0 0 2 0 0 3.51e-01 4.52e-01 212 0.000 0.000 1.000 0.000 0.000

LOCUS: AT2G26740
DESCRIPTION: epoxide hydrolase, soluble (sEH), identical to ATsEH (Arabidopsis thaliana) GI
DATA: Control 30min 2hours 2days 1week p-value B&H Pos Fold change relative to control (log2)
SENSE COUNTS: 14 12 5 2 0 3.10e-03 4.26e-03 0.000 -0.222 -1.485 -2.807 0.000
GENES:
AT2G26740.1
SENSE COUNTS: 14 12 5 2 0 3.10e-03 4.36e-03 0.000 -0.222 -1.485 -2.807 0.000
TAGS:
d+1 TGATTGTTTA 10 4 3 1 0 2.12e-02 3.67e-02 1160 0.000 -1.322 -1.737 -3.322 0.000
d+2 TATGGTTGTA 4 8 2 1 0 3.18e-02 5.36e-02 1063 0.000 1.000 -1.000 -2.000 0.000

LOCUS: AT1G52230
DESCRIPTION: photosystem I reaction center subunit VI, chloroplast, putative / PSI-H, putative (PSAH2), identical to SP|Q9SUI6; similar to PSI-H precursor (Nicotiana sylvestris) GI
DATA: Control 30min 2hours 2days 1week p-value B&H Pos Fold change relative to control (log2)
SENSE COUNTS: 19 31 21 20 1 3.14e-03 4.31e-03 0.000 0.706 0.144 0.074 -4.248
GENES:
AT1G52230.1
SENSE COUNTS: 19 31 21 20 1 3.14e-03 4.40e-03 0.000 0.706 0.144 0.074 -4.248
TAGS:
d+1 GCGTCTTTTG 19 31 21 20 1 3.14e-03 7.49e-03 70 0.000 0.706 0.144 0.074 -4.248

LOCUS: AT4G19880
DESCRIPTION: glutathione S-transferase-related, contains weak hit to Pfam profile PF00043
DATA: Control 30min 2hours 2days 1week p-value B&H Pos Fold change relative to control (log2)
SENSE COUNTS: 3 7 17 3 3 3.14e-03 4.30e-03 0.000 1.222 2.503 0.000 0.000
GENES:
AT4G19880.1
SENSE COUNTS: 3 7 17 3 3 3.14e-03 4.40e-03 0.000 1.222 2.503 0.000 0.000
TAGS:
d+1 AGTTGAGTTC 2 5 6 2 3 6.14e-01 6.57e-01 1096 0.000 1.322 1.585 0.000 0.585
X+4 TGGCGGATTA 1 2 11 1 0 8.11e-04 2.43e-03 474 0.000 1.000 3.459 0.000 0.000

LOCUS: AT1G04820
DESCRIPTION: tubulin alpha-2/alpha-4 chain (TUA4), nearly identical to SP
DATA: Control 30min 2hours 2days 1week p-value B&H Pos Fold change relative to control (log2)
SENSE COUNTS: 26 9 9 6 21 3.15e-03 4.31e-03 0.000 -1.531 -1.531 -2.115 -0.308
GENES:
AT1G04820.1
SENSE COUNTS: 26 9 9 6 21 3.15e-03 4.41e-03 0.000 -1.531 -1.531 -2.115 -0.308
TAGS:
d+1 TTATCTCTCT 25 9 7 4 21 4.15e-04 1.41e-03 1598 0.000 -1.474 -1.837 -2.644 -0.252
d+2 ATCTCAAACT 1 0 2 2 0 6.66e-01 6.81e-01 1241 0.000 0.000 1.000 1.000 0.000

LOCUS: AT5G10630
DESCRIPTION: elongation factor 1-alpha, putative / EF-1-alpha, putative, contains similarity to SWISS-PROT
DATA: Control 30min 2hours 2days 1week p-value B&H Pos Fold change relative to control (log2)
SENSE COUNTS: 2 0 5 10 0 3.18e-03 4.34e-03 0.000 0.000 1.322 2.322 0.000
GENES:
AT5G10630.1
SENSE COUNTS: 2 0 5 10 0 3.18e-03 4.44e-03 0.000 0.000 1.322 2.322 0.000
TAGS:
d+1 CAAAATTCGA 2 0 5 9 0 1.03e-02 1.87e-02 2202 0.000 0.000 1.322 2.170 0.000
d+2 GAAAACCAAA 0 0 0 1 0 3.09e-01 4.01e-01 1376 0.000 0.000 0.000 0.000 0.000

LOCUS: AT5G37260
DESCRIPTION: myb family transcription factor, contains Pfam profile

DATA:		Control	30min	2hours	2days	1week	p-value	B&H	Pos	Fold change relative to control (log2)				
SENSE COUNTS:		0	0	7	2	1	3.20e-03	4.36e-03		0.000	0.000	2.807	1.000	0.000
GENES:														
AT5G37260.1														
SENSE COUNTS:		0	0	7	2	1	3.20e-03	4.46e-03		0.000	0.000	2.807	1.000	0.000
TAGS:														
v+1	CCTTACAAAA	0	0	7	2	1	3.20e-03	7.61e-03	1146	0.000	0.000	2.807	1.000	0.000

LOCUS: AT1G06200
DESCRIPTION: expressed protein

DATA:		Control	30min	2hours	2days	1week	p-value	B&H	Pos	Fold change relative to control (log2)				
SENSE COUNTS:		1	0	3	9	0	3.22e-03	4.38e-03		0.000	0.000	1.585	3.170	0.000
GENES:														
AT1G06200.1														
SENSE COUNTS:		1	0	3	9	0	3.22e-03	4.49e-03		0.000	0.000	1.585	3.170	0.000
TAGS:														
d+1	GTTTTAAACC	0	0	1	4	0	3.72e-02	6.23e-02	926	0.000	0.000	0.000	2.000	0.000
d+2	AGCTTCATCC	1	0	2	5	0	1.37e-01	2.08e-01	873	0.000	0.000	1.000	2.322	0.000

LOCUS: AT4G25340
DESCRIPTION: immunophilin-related / FKBP-type peptidyl-prolyl cis-trans isomerase-related, immunophilin FKBP46 - Spodoptera frugiperda (fall armyworm),PIR2

DATA:		Control	30min	2hours	2days	1week	p-value	B&H	Pos	Fold change relative to control (log2)				
SENSE COUNTS:		1	0	1	10	4	3.25e-03	4.42e-03		0.000	0.000	0.000	3.322	2.000
GENES:														
AT4G25340.1														
SENSE COUNTS:		1	0	1	10	4	3.25e-03	4.52e-03		0.000	0.000	0.000	3.322	2.000
TAGS:														
d+1	CGCGTTGGTG	0	0	0	5	1	2.33e-02	4.01e-02	1376	0.000	0.000	0.000	2.322	0.000
d+2	GGAAAACCCA	0	0	1	1	0	5.21e-01	5.70e-01	1202	0.000	0.000	0.000	0.000	0.000
d+2	TTCCAGTTTT	1	0	0	4	3	2.03e-01	2.84e-01	678	0.000	0.000	0.000	2.000	1.585

LOCUS: AT5G14520
DESCRIPTION: pescadillo-related, similar to pescadillo (Zebrafish, Danio rerio) SWISS-PROT

DATA:		Control	30min	2hours	2days	1week	p-value	B&H	Pos	Fold change relative to control (log2)				
SENSE COUNTS:		4	2	11	17	17	3.26e-03	4.42e-03		0.000	-1.000	1.459	2.087	2.087
GENES:														
AT5G14520.1														
SENSE COUNTS:		4	2	11	17	17	3.26e-03	4.53e-03		0.000	-1.000	1.459	2.087	2.087
TAGS:														
v+1	AGTACGCCTA	2	2	8	6	12	4.96e-02	8.16e-02	2444	0.000	0.000	2.000	1.585	2.585
v+2	TACTTCCAAA	1	0	2	11	5	2.98e-03	7.17e-03	2216	0.000	0.000	1.000	3.459	2.322
v+2	CACGAGCCTC	1	0	1	0	0	6.01e-01	6.50e-01	564	0.000	0.000	0.000	0.000	0.000

LOCUS: AT2G47730
DESCRIPTION: glutathione S-transferase 6 (GST6), identical to GB

DATA:		Control	30min	2hours	2days	1week	p-value	B&H	Pos	Fold change relative to control (log2)				
SENSE COUNTS:		8	16	19	31	8	3.30e-03	4.47e-03		0.000	1.000	1.248	1.954	0.000
GENES:														
AT2G47730.1														
SENSE COUNTS:		8	16	19	31	8	3.30e-03	4.58e-03		0.000	1.000	1.248	1.954	0.000
TAGS:														
d+1	ACCACTGACC	8	16	16	30	7	3.64e-03	8.46e-03	542	0.000	1.000	1.000	1.907	-0.193
d+2	TCCACCGCCA	0	0	3	1	1	1.97e-01	2.76e-01	191	0.000	0.000	1.585	0.000	0.000

LOCUS: AT2G31360

```
DESCRIPTION:  homologous to delta 9 acyl-lipid desaturases of cyanobacteria and acyl-CoA desaturases of yeast and mammals. expression up-regulated by
cold temperature.
DATA:                        Control 30min  2hours  2days  1week  p-value       B&H           Pos    Fold change relative to control (log2)
SENSE COUNTS:                1       12     14      5      1      3.30e-03      4.46e-03             0.000  3.585  3.807  2.322  0.000
  GENES:
  AT2G31360.1
    SENSE COUNTS:            1       12     14      5      1      3.30e-03      4.57e-03             0.000  3.585  3.807  2.322  0.000
    TAGS:
      d+1      CGTTCGAGTC    1       12     13      5      1      5.39e-03      1.16e-02      833    0.000  3.585  3.700  2.322  0.000
      d+2      TCCTTCGTTA    0       0      1       0      0      4.55e-01      5.30e-01      655    0.000  0.000  0.000  0.000  0.000

LOCUS: AT4G02580
DESCRIPTION:  NADH-ubiquinone oxidoreductase 24 kDa subunit, putative, similar to NADH-ubiquinone oxidoreductase 24 kDa subunit, mitochondrial
precursor (EC 1.6.5.3) (EC 1.6.99.3) (Polypeptide II) (Swiss-Prot
DATA:                        Control 30min  2hours  2days  1week  p-value       B&H           Pos    Fold change relative to control (log2)
SENSE COUNTS:                2       5      5       12     19     3.32e-03      4.49e-03             0.000  1.322  1.322  2.585  3.248
  GENES:
  AT4G02580.1
    SENSE COUNTS:            2       5      5       12     19     3.32e-03      4.60e-03             0.000  1.322  1.322  2.585  3.248
    TAGS:
      d+1      TGAAATTGTA    2       4      5       11     14     3.26e-02      5.48e-02      1054   0.000  1.000  1.322  2.459  2.807
      d+2      CTAAAGCTAA    0       1      0       0      5      2.20e-02      3.80e-02      857    0.000  0.000  0.000  0.000  2.322
      d+2      GGAGTTTTCA    0       0      0       1      0      3.09e-01      4.05e-01      233    0.000  0.000  0.000  0.000  0.000

LOCUS: AT1G71480
DESCRIPTION:  nuclear transport factor 2 (NTF2) family protein, contains Pfam domain, PF02136
DATA:                        Control 30min  2hours  2days  1week  p-value       B&H           Pos    Fold change relative to control (log2)
SENSE COUNTS:                3       0      13      6      3      3.32e-03      4.48e-03             0.000  0.000  2.115  1.000  0.000
  GENES:
  AT1G71480.1
    SENSE COUNTS:            3       0      13      6      3      3.32e-03      4.59e-03             0.000  0.000  2.115  1.000  0.000
    TAGS:
      d+1      TGTATCCAAC    3       0      13      6      3      3.32e-03      7.88e-03      930    0.000  0.000  2.115  1.000  0.000

LOCUS: AT2G32850
DESCRIPTION:  protein kinase family protein, contains protein kinase domain, Pfam
DATA:                        Control 30min  2hours  2days  1week  p-value       B&H           Pos    Fold change relative to control (log2)
SENSE COUNTS:                1       0      0       1      7      3.33e-03      4.49e-03             0.000  0.000  0.000  0.000  2.807
  GENES:
  AT2G32850.2
    SENSE COUNTS:            1       0      0       1      7      3.33e-03      4.57e-03             0.000  0.000  0.000  0.000  2.807
    TAGS:
      d+1      TTGCTGCCAT    1       0      0       1      7      3.33e-03      7.87e-03      2070   0.000  0.000  0.000  0.000  2.807
  AT2G32850.1
    SENSE COUNTS:            1       0      0       1      7      3.33e-03      4.59e-03             0.000  0.000  0.000  0.000  2.807
    TAGS:
      d+1      TTGCTGCCAT    1       0      0       1      7      3.33e-03      7.87e-03      2243   0.000  0.000  0.000  0.000  2.807

LOCUS: AT3G57030
DESCRIPTION:  strictosidine synthase family protein, similar to strictosidine synthase (Rauvolfia serpentina)(SP|P15324)
DATA:                        Control 30min  2hours  2days  1week  p-value       B&H           Pos    Fold change relative to control (log2)
SENSE COUNTS:                1       0      0       1      7      3.33e-03      4.48e-03             0.000  0.000  0.000  0.000  2.807
  GENES:
  AT3G57030.1
    SENSE COUNTS:            1       0      0       1      7      3.33e-03      4.58e-03             0.000  0.000  0.000  0.000  2.807
    TAGS:
      d+1      TGTATACTTT    1       0      0       1      7      3.33e-03      7.85e-03      1333   0.000  0.000  0.000  0.000  2.807

LOCUS: AT3G48830
```

```
DESCRIPTION:  polynucleotide adenylyltransferase family protein / RNA recognition motif (RRM)-containing protein, similar to SP|P13685 Poly(A)
polymerase (EC 2.7.7.19) {Escherichia coli O157
DATA:                         Control 30min   2hours  2days   1week   p-value         B&H             Pos     Fold change relative to control (log2)
SENSE COUNTS:                 1       0       0       1       7       3.33e-03        4.47e-03                0.000   0.000   0.000   0.000   2.807
  GENES:
  AT3G48830.1
    SENSE COUNTS:             1       0       0       1       7       3.33e-03        4.60e-03                0.000   0.000   0.000   0.000   2.807
    TAGS:
      i+3      TATTAAAAAT     1       0       0       1       7       3.33e-03        7.86e-03        4002    0.000   0.000   0.000   0.000   2.807

LOCUS: AT5G49080
DESCRIPTION:  proline-rich extensin-like family protein, contains proline-rich extensin domains, INTERPRO
DATA:                         Control 30min   2hours  2days   1week   p-value         B&H             Pos     Fold change relative to control (log2)
SENSE COUNTS:                 1       0       0       1       7       3.33e-03        4.47e-03                0.000   0.000   0.000   0.000   2.807
  GENES:
  AT5G49080.1
    SENSE COUNTS:             1       0       0       1       7       3.33e-03        4.59e-03                0.000   0.000   0.000   0.000   2.807
    TAGS:
      d+1      AAATTTCTAA     1       0       0       1       7       3.33e-03        7.88e-03        2001    0.000   0.000   0.000   0.000   2.807

LOCUS: AT3G53740
DESCRIPTION:  60S ribosomal protein L36 (RPL36B), 60S RIBOSOMAL PROTEIN L36 - Schizosaccharomyces pombe, swissprot
DATA:                         Control 30min   2hours  2days   1week   p-value         B&H             Pos     Fold change relative to control (log2)
SENSE COUNTS:                 3       10      0       1       3       3.36e-03        4.50e-03                0.000   1.737   0.000   -1.585  0.000
  GENES:
  AT3G53740.1
    SENSE COUNTS:             3       10      0       1       3       3.36e-03        4.61e-03                0.000   1.737   0.000   -1.585  0.000
    TAGS:
      d+1      TTGTTACCAG     3       10      0       1       3       3.36e-03        7.91e-03        122     0.000   1.737   0.000   -1.585  0.000
  AT3G53740.2
    SENSE COUNTS:             3       10      0       1       3       3.36e-03        4.60e-03                0.000   1.737   0.000   -1.585  0.000
    TAGS:
      d+1      TTGTTACCAG     3       10      0       1       3       3.36e-03        7.91e-03        106     0.000   1.737   0.000   -1.585  0.000

LOCUS: AT1G65820
DESCRIPTION:  microsomal glutathione s-transferase, putative, similar to MGST3_HUMAN SP
DATA:                         Control 30min   2hours  2days   1week   p-value         B&H             Pos     Fold change relative to control (log2)
SENSE COUNTS:                 17      9       14      22      35      3.41e-03        4.56e-03                0.000   -0.918  -0.280  0.372   1.042
  GENES:
  AT1G65820.1
    SENSE COUNTS:             17      9       14      22      35      3.41e-03        4.67e-03                0.000   -0.918  -0.280  0.372   1.042
    TAGS:
      d+1      GCTTCATAGA     17      9       14      22      35      3.41e-03        8.00e-03        684     0.000   -0.918  -0.280  0.372   1.042

LOCUS: AT4G13290
DESCRIPTION:  cytochrome P450 71A19, putative (CYP71A19), Identical to Cytochrome P450 (SP
DATA:                         Control 30min   2hours  2days   1week   p-value         B&H             Pos     Fold change relative to control (log2)
SENSE COUNTS:                 10      0       3       1       5       3.44e-03        4.59e-03                0.000   0.000   -1.737  -3.322  -1.000
  GENES:
  AT4G13290.1
    SENSE COUNTS:             10      0       3       1       5       3.44e-03        4.70e-03                0.000   0.000   -1.737  -3.322  -1.000
    TAGS:
      X+4      CAAATCCAAA     10      0       3       1       5       3.44e-03        8.06e-03        513     0.000   0.000   -1.737  -3.322  -1.000

LOCUS: AT2G26560
DESCRIPTION:  patatin, putative, similar to patatin-like latex allergen (Hevea brasiliensis)(PMID
DATA:                         Control 30min   2hours  2days   1week   p-value         B&H             Pos     Fold change relative to control (log2)
SENSE COUNTS:                 0       9       7       1       1       3.53e-03        4.71e-03                0.000   3.170   2.807   0.000   0.000
  GENES:
  AT2G26560.1
```

```
    SENSE COUNTS:              0      9      7      1      1      3.53e-03       4.82e-03              0.000  3.170  2.807  0.000  0.000
    TAGS:
      d+1      CAAAAGCTCC      0      8      7      1      1      1.12e-02       2.01e-02       1297   0.000  3.000  2.807  0.000  0.000
      d+2      CTCTTATAAA      0      1      0      0      0      2.54e-01       3.45e-01       1231   0.000  0.000  0.000  0.000  0.000

LOCUS: AT3G21150
DESCRIPTION:  zinc finger (B-box type) family protein,  contains Pfam profile
DATA:                          Control 30min  2hours 2days  1week  p-value        B&H            Pos    Fold change relative to control (log2)
SENSE COUNTS:                  1      0      1      7      0      3.55e-03       4.73e-03              0.000  0.000  0.000  2.807  0.000
  GENES:
  AT3G21150.1
    SENSE COUNTS:              1      0      1      7      0      3.55e-03       4.84e-03              0.000  0.000  0.000  2.807  0.000
    TAGS:
      d+1      GGTCCCGCAT      0      0      1      6      0      8.17e-04       2.43e-03       845    0.000  0.000  0.000  2.585  0.000
      d+2      AACCATAAAA      1      0      0      1      0      7.78e-01       7.83e-01       19     0.000  0.000  0.000  0.000  0.000

LOCUS: AT2G20260
DESCRIPTION:  photosystem I reaction center subunit IV, chloroplast, putative / PSI-E, putative (PSAE2), identical to SP|Q9S714; similar to SP|P12354
Photosystem I reaction center subunit IV, chloroplast precursor (PSI-E) {Spinacia oleracea}; contains Pfam profile PF02
DATA:                          Control 30min  2hours 2days  1week  p-value        B&H            Pos    Fold change relative to control (log2)
SENSE COUNTS:                  42     49     32     24     16     3.56e-03       4.73e-03              0.000  0.222  -0.392 -0.807 -1.392
  GENES:
  AT2G20260.1
    SENSE COUNTS:              42     49     32     24     16     3.56e-03       4.85e-03              0.000  0.222  -0.392 -0.807 -1.392
    TAGS:
      d+1      GTTTATCTCT      42     49     32     24     16     3.56e-03       8.31e-03       490    0.000  0.222  -0.392 -0.807 -1.392

LOCUS: AT3G06530
DESCRIPTION:  BAP28-related, similar to Protein BAP28 (Swiss-Prot
DATA:                          Control 30min  2hours 2days  1week  p-value        B&H            Pos    Fold change relative to control (log2)
SENSE COUNTS:                  0      0      0      1      7      3.63e-03       4.82e-03              0.000  0.000  0.000  0.000  2.807
  GENES:
  AT3G06530.1
    SENSE COUNTS:              0      0      0      1      7      3.63e-03       4.94e-03              0.000  0.000  0.000  0.000  2.807
    TAGS:
      v+2      TTTGCAGAAA      0      0      0      1      7      4.62e-04       1.53e-03       6005   0.000  0.000  0.000  0.000  2.807
      v+2      CATCGGTCAA      0      0      0      0      0      6.15e-01       6.44e-01       5552   0.000  0.000  0.000  0.000  0.000

LOCUS: AT3G01060
DESCRIPTION:  expressed protein
DATA:                          Control 30min  2hours 2days  1week  p-value        B&H            Pos    Fold change relative to control (log2)
SENSE COUNTS:                  2      0      8      2      12     3.63e-03       4.81e-03              0.000  0.000  2.000  0.000  2.585
  GENES:
  AT3G01060.2
    SENSE COUNTS:              2      0      8      2      12     3.63e-03       4.90e-03              0.000  0.000  2.000  0.000  2.585
    TAGS:
      d+1      TTGGATAAAG      2      0      6      2      12     6.40e-03       1.27e-02       1446   0.000  0.000  1.585  0.000  2.585
      d+2      GGCTGAGGAG      0      0      1      0      0      4.55e-01       5.15e-01       655    0.000  0.000  0.000  0.000  0.000
      d+2      GCCGTTCATC      0      0      1      0      0      4.55e-01       5.32e-01       14     0.000  0.000  0.000  0.000  0.000
  AT3G01060.1
    SENSE COUNTS:              2      0      8      2      12     3.63e-03       4.92e-03              0.000  0.000  2.000  0.000  2.585
    TAGS:
      d+1      TTGGATAAAG      2      0      6      2      12     6.40e-03       1.27e-02       1533   0.000  0.000  1.585  0.000  2.585
      d+2      GGCTGAGGAG      0      0      1      0      0      4.55e-01       5.15e-01       718    0.000  0.000  0.000  0.000  0.000
      d+2      GCCGTTCATC      0      0      1      0      0      4.55e-01       5.32e-01       77     0.000  0.000  0.000  0.000  0.000
  AT3G01060.3
    SENSE COUNTS:              2      0      8      2      12     3.63e-03       4.92e-03              0.000  0.000  2.000  0.000  2.585
    TAGS:
      d+1      TTGGATAAAG      2      0      6      2      12     6.40e-03       1.27e-02       1508   0.000  0.000  1.585  0.000  2.585
      d+2      GGCTGAGGAG      0      0      1      0      0      4.55e-01       5.15e-01       718    0.000  0.000  0.000  0.000  0.000
```

d+2	GCCGTTCATC	0	0	1	0	0	4.55e-01	5.32e-01	77	0.000	0.000	0.000	0.000	0.000

LOCUS: AT3G56580
DESCRIPTION: zinc finger (C3HC4-type RING finger) family protein, contains INTERPRO domain, IPR001841, RING finger

DATA:		Control	30min	2hours	2days	1week	p-value	B&H	Pos	Fold change relative to control (log2)				
SENSE COUNTS:		0	0	0	1	6	3.63e-03	4.81e-03		0.000	0.000	0.000	0.000	2.585
GENES:														
AT3G56580.1														
SENSE COUNTS:		0	0	0	1	6	3.63e-03	4.91e-03		0.000	0.000	0.000	0.000	2.585
TAGS:														
d+1	TTAGCTTGTT	0	0	0	0	3	1.12e-02	2.02e-02	1483	0.000	0.000	0.000	0.000	1.585
d+2	TCTCTCTTTC	0	0	0	1	3	2.09e-01	2.90e-01	1383	0.000	0.000	0.000	0.000	1.585
AT3G56580.2														
SENSE COUNTS:		0	0	0	1	6	3.63e-03	4.93e-03		0.000	0.000	0.000	0.000	2.585
TAGS:														
d+1	TTAGCTTGTT	0	0	0	0	3	1.12e-02	2.02e-02	1357	0.000	0.000	0.000	0.000	1.585
d+2	TCTCTCTTTC	0	0	0	1	3	2.09e-01	2.90e-01	1257	0.000	0.000	0.000	0.000	1.585

LOCUS: AT5G43830
DESCRIPTION: expressed protein, similar to auxin down-regulated protein ARG10 (Vigna radiata) GI

DATA:		Control	30min	2hours	2days	1week	p-value	B&H	Pos	Fold change relative to control (log2)				
SENSE COUNTS:		18	23	13	5	5	3.76e-03	4.97e-03		0.000	0.354	-0.469	-1.848	-1.848
GENES:														
AT5G43830.1														
SENSE COUNTS:		18	23	13	5	5	3.76e-03	5.07e-03		0.000	0.354	-0.469	-1.848	-1.848
TAGS:														
d+1	TTTGAGGTGG	18	23	13	5	5	5.82e-03	1.21e-02	970	0.000	0.354	-0.469	-1.848	-1.848
d+2	TTTGAGTTAC	0	0	0	0	0	6.15e-01	6.47e-01	909	0.000	0.000	0.000	0.000	0.000

LOCUS: AT5G11670
DESCRIPTION: malate oxidoreductase, putative, similar to NADP-dependent malic enzyme (EC 1.1.1.40) (NADP-ME) (SP|P12628) {Phaseolus vulgaris}

DATA:		Control	30min	2hours	2days	1week	p-value	B&H	Pos	Fold change relative to control (log2)				
SENSE COUNTS:		6	20	13	6	1	3.77e-03	4.98e-03		0.000	1.737	1.115	0.000	-2.585
GENES:														
AT5G11670.1														
SENSE COUNTS:		6	20	13	6	1	3.77e-03	5.08e-03		0.000	1.737	1.115	0.000	-2.585
TAGS:														
d+1	TACAGCCCTG	6	20	13	6	1	3.77e-03	8.72e-03	1841	0.000	1.737	1.115	0.000	-2.585

LOCUS: AT2G46330
DESCRIPTION: arabinogalactan protein (AGP16) mRNA, complete cds

DATA:		Control	30min	2hours	2days	1week	p-value	B&H	Pos	Fold change relative to control (log2)				
SENSE COUNTS:		19	30	13	11	8	3.79e-03	5.00e-03		0.000	0.659	-0.547	-0.788	-1.248
GENES:														
AT2G46330.2														
SENSE COUNTS:		19	30	13	11	8	3.79e-03	5.10e-03		0.000	0.659	-0.547	-0.788	-1.248
TAGS:														
d+1	TTCTCGATTA	19	30	13	11	8	3.79e-03	8.75e-03	647	0.000	0.659	-0.547	-0.788	-1.248
AT2G46330.1														
SENSE COUNTS:		19	30	13	11	8	3.79e-03	5.09e-03		0.000	0.659	-0.547	-0.788	-1.248
TAGS:														
d+1	TTCTCGATTA	19	30	13	11	8	3.79e-03	8.75e-03	490	0.000	0.659	-0.547	-0.788	-1.248

LOCUS: AT2G21320
DESCRIPTION: zinc finger (B-box type) family protein

DATA:		Control	30min	2hours	2days	1week	p-value	B&H	Pos	Fold change relative to control (log2)				
SENSE COUNTS:		1	0	1	2	8	3.81e-03	5.01e-03		0.000	0.000	0.000	1.000	3.000
GENES:														
AT2G21320.1														
SENSE COUNTS:		1	0	1	2	8	3.81e-03	5.12e-03		0.000	0.000	0.000	1.000	3.000

```
    TAGS:
      d+1      AATAGTGATC     1      0      1      2      8      3.81e-03       8.77e-03       754    0.000  0.000  0.000  1.000  3.000

LOCUS: AT3G62670
DESCRIPTION:  member of Response Regulator
DATA:                         Control 30min  2hours 2days  1week  p-value        B&H            Pos    Fold change relative to control (log2)
SENSE COUNTS:                 0      0      1      6      1      3.89e-03       5.11e-03              0.000  0.000  0.000  2.585  0.000
  GENES:
  AT3G62670.1
    SENSE COUNTS:             0      0      1      6      1      3.89e-03       5.22e-03              0.000  0.000  0.000  2.585  0.000
    TAGS:
      v+2      AGATCGATCT     0      0      1      6      1      3.89e-03       8.92e-03       601    0.000  0.000  0.000  2.585  0.000

LOCUS: AT4G34550
DESCRIPTION:  expressed protein,
DATA:                         Control 30min  2hours 2days  1week  p-value        B&H            Pos    Fold change relative to control (log2)
SENSE COUNTS:                 0      0      1      6      1      3.89e-03       5.11e-03              0.000  0.000  0.000  2.585  0.000
  GENES:
  AT4G34550.1
    SENSE COUNTS:             0      0      1      6      1      3.89e-03       5.20e-03              0.000  0.000  0.000  2.585  0.000
    TAGS:
      v+2      CCAATCTTGC     0      0      1      6      1      3.89e-03       8.93e-03       1206   0.000  0.000  0.000  2.585  0.000

LOCUS: AT3G04000
DESCRIPTION:  short-chain dehydrogenase/reductase (SDR) family protein, similar to SP|Q08632 Short-chain type dehydrogenase/reductase (EC 1.-.-.-)
{Picea abies}; contains Pfam
DATA:                         Control 30min  2hours 2days  1week  p-value        B&H            Pos    Fold change relative to control (log2)
SENSE COUNTS:                 1      0      0      0      6      3.89e-03       5.10e-03              0.000  0.000  0.000  0.000  2.585
  GENES:
  AT3G04000.1
    SENSE COUNTS:             1      0      0      0      6      3.89e-03       5.21e-03              0.000  0.000  0.000  0.000  2.585
    TAGS:
      d+1      TATCAATAAC     1      0      0      0      5      3.71e-02       6.22e-02       917    0.000  0.000  0.000  0.000  2.322
      d+2      GCTAATGGTG     0      0      0      0      1      1.65e-01       2.47e-01       796    0.000  0.000  0.000  0.000  0.000

LOCUS: AT1G79930
DESCRIPTION:  heat shock protein, putative, contains Pfam profile
DATA:                         Control 30min  2hours 2days  1week  p-value        B&H            Pos    Fold change relative to control (log2)
SENSE COUNTS:                 0      2      1      9      1      3.90e-03       5.10e-03              0.000  1.000  0.000  3.170  0.000
  GENES:
  AT1G79930.1
    SENSE COUNTS:             0      2      1      9      1      3.90e-03       5.21e-03              0.000  1.000  0.000  3.170  0.000
    TAGS:
      d+1      GCTGCGGGGA     0      2      1      9      1      3.90e-03       8.93e-03       2388   0.000  1.000  0.000  3.170  0.000

LOCUS: AT2G43060
DESCRIPTION:  expressed protein, similar to cDNA bHLH transcription factor (bHLH zeta gene) GI
DATA:                         Control 30min  2hours 2days  1week  p-value        B&H            Pos    Fold change relative to control (log2)
SENSE COUNTS:                 2      7      0      0      0      3.91e-03       5.11e-03              0.000  1.807  0.000  0.000  0.000
  GENES:
  AT2G43060.1
    SENSE COUNTS:             2      7      0      0      0      3.91e-03       5.22e-03              0.000  1.807  0.000  0.000  0.000
    TAGS:
      d+1      CAGTGTCTCG     2      7      0      0      0      3.91e-03       8.91e-03       501    0.000  1.807  0.000  0.000  0.000

LOCUS: AT4G29580
DESCRIPTION:  cytidine deaminase, putative / cytidine aminohydrolase, putative, identical to cytidine deaminase homolog DesB (Arabidopsis thaliana) GI
DATA:                         Control 30min  2hours 2days  1week  p-value        B&H            Pos    Fold change relative to control (log2)
SENSE COUNTS:                 2      7      0      0      0      3.91e-03       5.10e-03              0.000  1.807  0.000  0.000  0.000
  GENES:
```

```
  AT4G29580.1
    SENSE COUNTS:             2      7      0      0      0      3.91e-03       5.21e-03              0.000  1.807  0.000  0.000  0.000
    TAGS:
      v+1      GTGTCAGAAT     2      7      0      0      0      3.91e-03       8.93e-03       1690   0.000  1.807  0.000  0.000  0.000

LOCUS: AT4G34135
DESCRIPTION:  UDP-glucoronosyl/UDP-glucosyl transferase family protein, contains Pfam profile
DATA:                         Control 30min  2hours 2days  1week  p-value        B&H            Pos    Fold change relative to control (log2)
SENSE COUNTS:                 0      0      4      8      1      3.92e-03       5.11e-03              0.000  0.000  2.000  3.000  0.000
  GENES:
  AT4G34135.1
    SENSE COUNTS:             0      0      4      8      1      3.92e-03       5.22e-03              0.000  0.000  2.000  3.000  0.000
    TAGS:
      d+1      GAAGAGTTTA     0      0      4      8      1      3.92e-03       8.92e-03       1552   0.000  0.000  2.000  3.000  0.000

LOCUS: AT5G09810
DESCRIPTION:  Member of Actin gene family.Mutants are defective in germination and root growth.
DATA:                         Control 30min  2hours 2days  1week  p-value        B&H            Pos    Fold change relative to control (log2)
SENSE COUNTS:                 8      18     8      5      1      3.94e-03       5.13e-03              0.000  1.170  0.000  -0.678 -3.000
  GENES:
  AT5G09810.1
    SENSE COUNTS:             8      18     8      5      1      3.94e-03       5.24e-03              0.000  1.170  0.000  -0.678 -3.000
    TAGS:
      d+1      AAGATCAAGG     8      16     7      5      1      1.95e-02       3.39e-02       1085   0.000  1.000  -0.193 -0.678 -3.000
      d+2      TATGTTGCCA     0      2      1      0      0      2.47e-01       3.37e-01       506    0.000  1.000  0.000  0.000  0.000

LOCUS: AT3G48530
DESCRIPTION:  CBS domain-containing protein, low similarity to SP|Q9UGI9 5'-AMP-activated protein kinase, gamma-3 subunit (AMPK gamma-3 chain) (AMPK
gamma3) {Homo sapiens}; contains Pfam profile PF00571
DATA:                         Control 30min  2hours 2days  1week  p-value        B&H            Pos    Fold change relative to control (log2)
SENSE COUNTS:                 1      11     5      2      0      3.95e-03       5.13e-03              0.000  3.459  2.322  1.000  0.000
  GENES:
  AT3G48530.1
    SENSE COUNTS:             1      11     5      2      0      3.95e-03       5.24e-03              0.000  3.459  2.322  1.000  0.000
    TAGS:
      d+1      AGCCATCCGG     1      11     5      2      0      3.95e-03       8.98e-03       1335   0.000  3.459  2.322  1.000  0.000

LOCUS: AT1G54440
DESCRIPTION:  3'-5' exonuclease domain-containing protein / helicase and RNase D C-terminal domain-containing protein / HRDC domain-containing protein,
similar to SP|Q01780 Polymyositis/scleroderma autoantigen 2 {Homo sapiens}; contains Pfam profiles PF00570
DATA:                         Control 30min  2hours 2days  1week  p-value        B&H            Pos    Fold change relative to control (log2)
SENSE COUNTS:                 4      0      0      0      0      4.01e-03       5.20e-03              0.000  0.000  0.000  0.000  0.000
  GENES:
  AT1G54440.1
    SENSE COUNTS:             4      0      0      0      0      4.01e-03       5.31e-03              0.000  0.000  0.000  0.000  0.000
    TAGS:
      d+1      AATATGAAAC     4      0      0      0      0      4.01e-03       9.10e-03       2188   0.000  0.000  0.000  0.000  0.000

LOCUS: AT3G02790
DESCRIPTION:  zinc finger (C2H2 type) family protein, contains Pfam profile
DATA:                         Control 30min  2hours 2days  1week  p-value        B&H            Pos    Fold change relative to control (log2)
SENSE COUNTS:                 4      0      0      0      0      4.01e-03       5.20e-03              0.000  0.000  0.000  0.000  0.000
  GENES:
  AT3G02790.1
    SENSE COUNTS:             4      0      0      0      0      4.01e-03       5.30e-03              0.000  0.000  0.000  0.000  0.000
    TAGS:
      d+1      TTAAATCAAC     4      0      0      0      0      4.01e-03       9.09e-03       533    0.000  0.000  0.000  0.000  0.000

LOCUS: AT1G30230
DESCRIPTION:  elongation factor 1-beta / EF-1-beta, identical to SP|P48006 Elongation factor 1-beta (EF-1-beta) {Arabidopsis thaliana}
```

```
DATA:                         Control 30min   2hours  2days   1week   p-value         B&H             Pos     Fold change relative to control (log2)
SENSE COUNTS:                 32      27      56      54      39      4.01e-03        5.19e-03                0.000   -0.245  0.807   0.755   0.285
  GENES:
  AT1G30230.1
    SENSE COUNTS:             32      27      56      54      39      4.01e-03        5.32e-03                0.000   -0.245  0.807   0.755   0.285
    TAGS:
      d+1      TGGTCTGGTT     32      27      56      54      39      4.01e-03        9.08e-03        807     0.000   -0.245  0.807   0.755   0.285

LOCUS: AT1G09750
DESCRIPTION:  chloroplast nucleoid DNA-binding protein-related, contains Pfam profile PF00026
DATA:                         Control 30min   2hours  2days   1week   p-value         B&H             Pos     Fold change relative to control (log2)
SENSE COUNTS:                 15      14      30      30      7       4.06e-03        5.25e-03                0.000   -0.100  1.000   1.000   -1.100
  GENES:
  AT1G09750.1
    SENSE COUNTS:             15      14      30      30      7       4.06e-03        5.36e-03                0.000   -0.100  1.000   1.000   -1.100
    TAGS:
      d+1      GCCTGCAGTT     15      14      27      30      7       6.70e-03        1.33e-02        1532    0.000   -0.100  0.848   1.000   -1.100
      d+2      GCCGGGATAC     0       0       2       0       0       1.21e-01        1.87e-01        1265    0.000   0.000   1.000   0.000   0.000
      d+2      ACGTCACTCG     0       0       1       0       0       4.55e-01        5.19e-01        1187    0.000   0.000   0.000   0.000   0.000
      d+2      GCCTCCTCAG     0       0       0       0       0       6.15e-01        6.30e-01        242     0.000   0.000   0.000   0.000   0.000
      d+2      GCCTCCTCAA     0       0       0       0       0       6.15e-01        6.39e-01        44      0.000   0.000   0.000   0.000   0.000

LOCUS: AT3G55360
DESCRIPTION:  3-oxo-5-alpha-steroid 4-dehydrogenase family protein / steroid 5-alpha-reductase family protein, similar to synaptic glycoprotein SC2
spliced variant from Homo sapiens (EMBL
DATA:                         Control 30min   2hours  2days   1week   p-value         B&H             Pos     Fold change relative to control (log2)
SENSE COUNTS:                 4       2       0       2       12      4.06e-03        5.24e-03                0.000   -1.000  0.000   -1.000  1.585
  GENES:
  AT3G55360.1
    SENSE COUNTS:             4       2       0       2       12      4.06e-03        5.36e-03                0.000   -1.000  0.000   -1.000  1.585
    TAGS:
      d+1      AGTCAATAAT     4       2       0       2       12      4.06e-03        9.18e-03        1289    0.000   -1.000  0.000   -1.000  1.585

LOCUS: AT1G27950
DESCRIPTION:  lipid transfer protein-related, low similarity to lipid transfer protein Picea abies GI
DATA:                         Control 30min   2hours  2days   1week   p-value         B&H             Pos     Fold change relative to control (log2)
SENSE COUNTS:                 15      6       14      14      29      4.13e-03        5.32e-03                0.000   -1.322  -0.100  -0.100  0.951
  GENES:
  AT1G27950.1
    SENSE COUNTS:             15      6       14      14      29      4.13e-03        5.44e-03                0.000   -1.322  -0.100  -0.100  0.951
    TAGS:
      d+1      TCACATTAGG     15      6       12      13      28      6.54e-03        1.30e-02        602     0.000   -1.322  -0.322  -0.206  0.900
      d+2      ACGATCGTTG     0       0       2       1       1       4.09e-01        5.16e-01        76      0.000   0.000   1.000   0.000   0.000

LOCUS: AT4G09040
DESCRIPTION:  RNA recognition motif (RRM)-containing protein, low similarity to enhancer binding protein-1; EBP1 (Entamoeba histolytica) GI
DATA:                         Control 30min   2hours  2days   1week   p-value         B&H             Pos     Fold change relative to control (log2)
SENSE COUNTS:                 1       1       7       5       13      4.13e-03        5.31e-03                0.000   0.000   2.807   2.322   3.700
  GENES:
  AT4G09040.1
    SENSE COUNTS:             1       1       7       5       13      4.13e-03        5.44e-03                0.000   0.000   2.807   2.322   3.700
    TAGS:
      d+1      TGGCTCCTAC     1       1       7       5       12      1.28e-02        2.27e-02        1192    0.000   0.000   2.807   2.322   3.585
      d+2      GACATCTACA     0       0       0       0       1       1.65e-01        2.36e-01        328     0.000   0.000   0.000   0.000   0.000

LOCUS: AT5G19440
DESCRIPTION:  similar to Eucalyptus gunnii alcohol dehydrogenase of unknown physiological function (GI
DATA:                         Control 30min   2hours  2days   1week   p-value         B&H             Pos     Fold change relative to control (log2)
SENSE COUNTS:                 4       3       3       6       17      4.16e-03        5.35e-03                0.000   -0.415  -0.415  0.585   2.087
  GENES:
```

```
  AT5G19440.1
    SENSE COUNTS:             4      3      3      6      17     4.16e-03       5.47e-03              0.000  -0.415  -0.415  0.585  2.087
    TAGS:
      d+1      TCAATGTTCT     4      3      2      6      17     1.93e-03       4.97e-03       1136   0.000  -0.415  -1.000  0.585  2.087
      X+4      GTCGGGAAAG     0      0      1      0      0      4.55e-01       5.33e-01       420    0.000  0.000   0.000   0.000  0.000

LOCUS: AT5G20010
DESCRIPTION:  Ras-related GTP-binding nuclear protein (RAN-1), identical to GTP-binding nuclear protein RAN-1 SP
DATA:                         Control 30min  2hours 2days  1week  p-value        B&H            Pos    Fold change relative to control (log2)
SENSE COUNTS:                 0      9      1      5      1      4.16e-03       5.34e-03              0.000  3.170   0.000   2.322  0.000
  GENES:
  AT5G20010.1
    SENSE COUNTS:             0      9      1      5      1      4.16e-03       5.46e-03              0.000  3.170   0.000   2.322  0.000
    TAGS:
      d+1      GCACCGTGAT     0      9      1      5      1      4.16e-03       9.38e-03       401    0.000  3.170   0.000   2.322  0.000

LOCUS: AT2G27020
DESCRIPTION:  20S proteasome alpha subunit G (PAG1) (PRC8), identical to proteasome subunit alpha type 3 SP
DATA:                         Control 30min  2hours 2days  1week  p-value        B&H            Pos    Fold change relative to control (log2)
SENSE COUNTS:                 13     2      5      4      17     4.20e-03       5.38e-03              0.000  -2.700  -1.379  -1.700 0.387
  GENES:
  AT2G27020.1
    SENSE COUNTS:             13     2      5      4      17     4.20e-03       5.51e-03              0.000  -2.700  -1.379  -1.700 0.387
    TAGS:
      d+1      AACCATTGGA     13     1      5      4      17     8.02e-04       2.41e-03       977    0.000  -3.700  -1.379  -1.700 0.387
      d+2      CAAAGAAGGC     0      1      0      0      0      2.54e-01       3.45e-01       676    0.000  0.000   0.000   0.000  0.000

LOCUS: AT2G30490
DESCRIPTION:  trans-cinnamate 4-monooxygenase / cinnamic acid 4-hydroxylase (C4H) (CA4H) / cytochrome P450 73 (CYP73) (CYP73A5), identical to
SP|P92994| Trans-cinnamate 4-monooxygenase (EC 1.14.13.11) (Cinnamic acid 4-hydroxylase) (CA4H) (C4H) (P450C4H) (Cytochrome P45
DATA:                         Control 30min  2hours 2days  1week  p-value        B&H            Pos    Fold change relative to control (log2)
SENSE COUNTS:                 2      8      5      18     3      4.23e-03       5.41e-03              0.000  2.000   1.322   3.170  0.585
  GENES:
  AT2G30490.1
    SENSE COUNTS:             2      8      5      18     3      4.23e-03       5.54e-03              0.000  2.000   1.322   3.170  0.585
    TAGS:
      d+1      ATGCGAAGCT     2      8      5      18     3      4.23e-03       9.53e-03       1232   0.000  2.000   1.322   3.170  0.585

LOCUS: AT5G66610
DESCRIPTION:  LIM domain-containing protein, contains Pfam profile PF00412
DATA:                         Control 30min  2hours 2days  1week  p-value        B&H            Pos    Fold change relative to control (log2)
SENSE COUNTS:                 0      1      0      1      8      4.29e-03       5.48e-03              0.000  0.000   0.000   0.000  3.000
  GENES:
  AT5G66610.1
    SENSE COUNTS:             0      1      0      1      8      4.29e-03       5.61e-03              0.000  0.000   0.000   0.000  3.000
    TAGS:
      i+3      AGATAAAGAA     0      0      0      1      8      2.96e-04       1.06e-03       1348   0.000  0.000   0.000   0.000  3.000
      d+1      TGGTTGGAGT     0      0      0      0      0      6.15e-01       6.48e-01       1321   0.000  0.000   0.000   0.000  0.000
      X+4      AGCACGTCCA     0      1      0      0      0      2.54e-01       3.46e-01       449    0.000  0.000   0.000   0.000  0.000

LOCUS: AT3G56130
DESCRIPTION:  biotin/lipoyl attachment domain-containing protein, low similarity to SP|Q06881 Biotin carboxyl carrier protein of acetyl-CoA
carboxylase (BCCP) {Anabaena sp.}; contains Pfam profile PF00364
DATA:                         Control 30min  2hours 2days  1week  p-value        B&H            Pos    Fold change relative to control (log2)
SENSE COUNTS:                 0      6      0      1      0      4.32e-03       5.51e-03              0.000  2.585   0.000   0.000  0.000
  GENES:
  AT3G56130.1
    SENSE COUNTS:             0      6      0      1      0      4.32e-03       5.64e-03              0.000  2.585   0.000   0.000  0.000
    TAGS:
      d+1      TGTGCCACGG     0      6      0      1      0      4.32e-03       9.69e-03       308    0.000  2.585   0.000   0.000  0.000
```

```
LOCUS: AT2G17880
DESCRIPTION:  DNAJ heat shock protein, putative, similar to J11 protein (Arabidopsis thaliana) GI
DATA:                          Control 30min   2hours  2days   1week   p-value         B&H             Pos     Fold change relative to control (log2)
SENSE COUNTS:                  0       6       0       1       0       4.32e-03        5.50e-03                0.000   2.585   0.000   0.000   0.000
  GENES:
  AT2G17880.1
    SENSE COUNTS:              0       6       0       1       0       4.32e-03        5.64e-03                0.000   2.585   0.000   0.000   0.000
    TAGS:
      d+1      AAGATCCACG      0       6       0       1       0       4.32e-03        9.70e-03        613     0.000   2.585   0.000   0.000   0.000

LOCUS: AT5G17870
DESCRIPTION:  plastid-specific ribosomal protein-related, contains similarity to plastid-specific ribosomal protein 6 precursor GI
DATA:                          Control 30min   2hours  2days   1week   p-value         B&H             Pos     Fold change relative to control (log2)
SENSE COUNTS:                  5       11      1       1       1       4.42e-03        5.62e-03                0.000   1.138   -2.322  -2.322  -2.322
  GENES:
  AT5G17870.1
    SENSE COUNTS:              5       11      1       1       1       4.42e-03        5.74e-03                0.000   1.138   -2.322  -2.322  -2.322
    TAGS:
      d+1      TCTGTGTCAG      5       11      1       1       1       4.42e-03        9.87e-03        46      0.000   1.138   -2.322  -2.322  -2.322

LOCUS: AT5G16400
DESCRIPTION:  thioredoxin, putative, similar to SP|P29450 Thioredoxin F-type, chloroplast precursor (TRX-F) {Pisum sativum}; contains Pfam profile
DATA:                          Control 30min   2hours  2days   1week   p-value         B&H             Pos     Fold change relative to control (log2)
SENSE COUNTS:                  8       0       1       1       0       4.44e-03        5.64e-03                0.000   0.000   -3.000  -3.000  0.000
  GENES:
  AT5G16400.1
    SENSE COUNTS:              8       0       1       1       0       4.44e-03        5.75e-03                0.000   0.000   -3.000  -3.000  0.000
    TAGS:
      d+1      TAAACTATAA      8       0       1       1       0       4.44e-03        9.89e-03        834     0.000   0.000   -3.000  -3.000  0.000

LOCUS: AT1G35720
DESCRIPTION:  Encodes a member of the annexin gene family, a diverse, multigene family of calcium-dependent, membrane-binding proteins.  Annat1 mRNA
is expressed in flowers, roots,leaves and stems and is most abundant in stems. mRNA levels are increased in response to
DATA:                          Control 30min   2hours  2days   1week   p-value         B&H             Pos     Fold change relative to control (log2)
SENSE COUNTS:                  1       20      13      10      10      4.44e-03        5.63e-03                0.000   4.322   3.700   3.322   3.322
  GENES:
  AT1G35720.1
    SENSE COUNTS:              1       20      13      10      10      4.44e-03        5.76e-03                0.000   4.322   3.700   3.322   3.322
    TAGS:
      d+1      GCGAGGAAAT      1       20      12      9       10      3.29e-03        7.82e-03        673     0.000   4.322   3.585   3.170   3.322
      d+2      AGAAAATCAA      0       0       1       1       0       5.21e-01        5.72e-01        565     0.000   0.000   0.000   0.000   0.000

LOCUS: AT3G02180
DESCRIPTION:  expressed protein
DATA:                          Control 30min   2hours  2days   1week   p-value         B&H             Pos     Fold change relative to control (log2)
SENSE COUNTS:                  5       13      5       1       0       4.50e-03        5.70e-03                0.000   1.379   0.000   -2.322  0.000
  GENES:
  AT3G02180.1
    SENSE COUNTS:              5       13      5       1       0       4.50e-03        5.81e-03                0.000   1.379   0.000   -2.322  0.000
    TAGS:
      d+1      CAGCTCCTGG      5       13      5       1       0       4.50e-03        1.00e-02        401     0.000   1.379   0.000   -2.322  0.000
  AT3G02180.2
    SENSE COUNTS:              5       13      5       1       0       4.50e-03        5.80e-03                0.000   1.379   0.000   -2.322  0.000
    TAGS:
      d+1      CAGCTCCTGG      5       13      5       1       0       4.50e-03        1.00e-02        401     0.000   1.379   0.000   -2.322  0.000

LOCUS: AT5G13130
DESCRIPTION:  hypothetical protein, low similarity to microrchidia (Mus musculus) GI
DATA:                          Control 30min   2hours  2days   1week   p-value         B&H             Pos     Fold change relative to control (log2)
```

```
SENSE COUNTS:                 11      0       8       15      10      4.53e-03        5.73e-03                0.000   0.000   -0.459  0.447   -0.138
  GENES:
  AT5G13130.1
    SENSE COUNTS:             11      0       8       15      10      4.53e-03        5.83e-03                0.000   0.000   -0.459  0.447   -0.138
    TAGS:
      v+2      ATGAAGATTG     11      0       8       15      10      4.53e-03        1.01e-02        1506    0.000   0.000   -0.459  0.447   -0.138

LOCUS: AT4G38470
DESCRIPTION:  protein kinase family protein, similar to protein kinase (gi
DATA:                         Control 30min   2hours  2days   1week   p-value         B&H             Pos     Fold change relative to control (log2)
SENSE COUNTS:                 4       10      3       0       0       4.53e-03        5.72e-03                0.000   1.322   -0.415  0.000   0.000
  GENES:
  AT4G38470.1
    SENSE COUNTS:             4       10      3       0       0       4.53e-03        5.83e-03                0.000   1.322   -0.415  0.000   0.000
    TAGS:
      d+1      ATTCGACGCA     3       10      3       0       0       2.89e-03        6.99e-03        1574    0.000   1.737   0.000   0.000   0.000
      d+2      AGATAACCTT     1       0       0       0       0       4.28e-01        5.21e-01        533     0.000   0.000   0.000   0.000   0.000

LOCUS: AT1G67280
DESCRIPTION:  lactoylglutathione lyase, putative / glyoxalase I, putative, similar to putative lactoylglutathione lyase SP
DATA:                         Control 30min   2hours  2days   1week   p-value         B&H             Pos     Fold change relative to control (log2)
SENSE COUNTS:                 0       7       1       2       0       4.54e-03        5.73e-03                0.000   2.807   0.000   1.000   0.000
  GENES:
  AT1G67280.1
    SENSE COUNTS:             0       7       1       2       0       4.54e-03        5.83e-03                0.000   2.807   0.000   1.000   0.000
    TAGS:
      d+1      ATGGGATATG     0       7       0       2       0       1.17e-03        3.22e-03        925     0.000   2.807   0.000   1.000   0.000
      d+2      TTGTTTATCG     0       0       1       0       0       4.55e-01        5.30e-01        422     0.000   0.000   0.000   0.000   0.000

LOCUS: AT3G56150
DESCRIPTION:  eukaryotic translation initiation factor 3 subunit 8 / eIF3 p110 / eIF3c / p105 (TIF3C1), nearly identical to SP|O49160 Eukaryotic
translation initiation factor 3 subunit 8 (eIF3 p110) (eIF3c) (p105) {Arabidopsis thaliana}
DATA:                         Control 30min   2hours  2days   1week   p-value         B&H             Pos     Fold change relative to control (log2)
SENSE COUNTS:                 1       6       0       0       0       4.55e-03        5.73e-03                0.000   2.585   0.000   0.000   0.000
  GENES:
  AT3G56150.1
    SENSE COUNTS:             1       6       0       0       0       4.55e-03        5.82e-03                0.000   2.585   0.000   0.000   0.000
    TAGS:
      d+1      AAGTGCAACA     1       6       0       0       0       4.55e-03        1.01e-02        2442    0.000   2.585   0.000   0.000   0.000

LOCUS: AT1G73500
DESCRIPTION:  mitogen-activated protein kinase kinase (MAPKK), putative (MKK9), mitogen-activated protein kinase kinase (MAPKK) family, PMID
DATA:                         Control 30min   2hours  2days   1week   p-value         B&H             Pos     Fold change relative to control (log2)
SENSE COUNTS:                 1       6       0       0       0       4.55e-03        5.73e-03                0.000   2.585   0.000   0.000   0.000
  GENES:
  AT1G73500.1
    SENSE COUNTS:             1       6       0       0       0       4.55e-03        5.83e-03                0.000   2.585   0.000   0.000   0.000
    TAGS:
      d+1      GATCCGATTT     1       6       0       0       0       4.55e-03        1.00e-02        425     0.000   2.585   0.000   0.000   0.000

LOCUS: AT5G48150
DESCRIPTION:  phytochrome A signal transduction 1 (PAT1)
DATA:                         Control 30min   2hours  2days   1week   p-value         B&H             Pos     Fold change relative to control (log2)
SENSE COUNTS:                 1       6       0       0       0       4.55e-03        5.72e-03                0.000   2.585   0.000   0.000   0.000
  GENES:
  AT5G48150.2
    SENSE COUNTS:             1       6       0       0       0       4.55e-03        5.84e-03                0.000   2.585   0.000   0.000   0.000
    TAGS:
      d+1      TGAAGGAGCT     1       6       0       0       0       4.55e-03        1.01e-02        1467    0.000   2.585   0.000   0.000   0.000
  AT5G48150.1
```

```
    SENSE COUNTS:            1      6      0      0      0      4.55e-03       5.82e-03              0.000  2.585  0.000  0.000  0.000
    TAGS:
      d+1      TGAAGGAGCT    1      6      0      0      0      4.55e-03       1.01e-02       1392   0.000  2.585  0.000  0.000  0.000

LOCUS: AT4G17230
DESCRIPTION:  scarecrow-like transcription factor 13 (SCL13)
DATA:                        Control 30min  2hours 2days  1week  p-value        B&H            Pos    Fold change relative to control (log2)
SENSE COUNTS:                0      2      7      0      0      4.57e-03       5.74e-03              0.000  1.000  2.807  0.000  0.000
  GENES:
  AT4G17230.1
    SENSE COUNTS:            0      2      7      0      0      4.57e-03       5.84e-03              0.000  1.000  2.807  0.000  0.000
    TAGS:
      d+1      TGGTTGTGGG    0      0      6      0      0      2.08e-04       7.85e-04       1970   0.000  0.000  2.585  0.000  0.000
      d+2      TTCCGTGTGG    0      2      1      0      0      2.47e-01       3.38e-01       1763   0.000  1.000  0.000  0.000  0.000
      d+2      TTTTTGATTC    0      0      0      0      0      6.15e-01       6.36e-01       1033   0.000  0.000  0.000  0.000  0.000

LOCUS: AT2G43970
DESCRIPTION:  La domain-containing protein, contains Pfam profile PF05383
DATA:                        Control 30min  2hours 2days  1week  p-value        B&H            Pos    Fold change relative to control (log2)
SENSE COUNTS:                28     16     41     19     17     4.58e-03       5.74e-03              0.000  -0.807 0.550  -0.559 -0.720
  GENES:
  AT2G43970.1
    SENSE COUNTS:            28     16     41     19     17     4.58e-03       5.84e-03              0.000  -0.807 0.550  -0.559 -0.720
    TAGS:
      d+1      GGGATTGGCT    27     16     41     18     17     3.66e-03       8.49e-03       1932   0.000  -0.755 0.603  -0.585 -0.667
      d+2      GGGAAACAGC    0      0      0      1      0      3.09e-01       4.14e-01       1733   0.000  0.000  0.000  0.000  0.000
      d+2      CTTTTGTTGA    1      0      0      0      0      4.28e-01       5.35e-01       1227   0.000  0.000  0.000  0.000  0.000
  AT2G43970.2
    SENSE COUNTS:            27     16     41     19     17     4.85e-03       6.08e-03              0.000  -0.755 0.603  -0.507 -0.667
    TAGS:
      d+1      GGGATTGGCT    27     16     41     18     17     3.66e-03       8.49e-03       1884   0.000  -0.755 0.603  -0.585 -0.667
      d+2      GGGAAACAGC    0      0      0      1      0      3.09e-01       4.14e-01       1685   0.000  0.000  0.000  0.000  0.000

LOCUS: AT4G13615
DESCRIPTION:  four F5 protein-related / 4F5 protein-related, contains weak hit to Pfam profile PF04419
DATA:                        Control 30min  2hours 2days  1week  p-value        B&H            Pos    Fold change relative to control (log2)
SENSE COUNTS:                5      12     1      2      1      4.58e-03       5.73e-03              0.000  1.263  -2.322 -1.322 -2.322
  GENES:
  AT4G13615.1
    SENSE COUNTS:            5      12     1      2      1      4.58e-03       5.83e-03              0.000  1.263  -2.322 -1.322 -2.322
    TAGS:
      d+1      ACTCGTGGAA    5      12     1      2      1      4.58e-03       1.01e-02       31     0.000  1.263  -2.322 -1.322 -2.322

LOCUS: AT3G46600
DESCRIPTION:  scarecrow transcription factor family protein, scarecrow-like 11 - Arabidopsis thaliana, EMBL
DATA:                        Control 30min  2hours 2days  1week  p-value        B&H            Pos    Fold change relative to control (log2)
SENSE COUNTS:                0      5      1      0      0      4.60e-03       5.75e-03              0.000  2.322  0.000  0.000  0.000
  GENES:
  AT3G46600.1
    SENSE COUNTS:            0      5      1      0      0      4.60e-03       5.85e-03              0.000  2.322  0.000  0.000  0.000
    TAGS:
      d+1      TATGAGACCA    0      5      1      0      0      1.38e-02       2.44e-02       1550   0.000  2.322  0.000  0.000  0.000
      d+2      TTGATGGAAG    0      0      0      0      0      6.15e-01       6.47e-01       332    0.000  0.000  0.000  0.000  0.000
  AT3G46600.2
    SENSE COUNTS:            0      5      1      0      0      4.60e-03       5.85e-03              0.000  2.322  0.000  0.000  0.000
    TAGS:
      d+1      TATGAGACCA    0      5      1      0      0      1.38e-02       2.44e-02       1471   0.000  2.322  0.000  0.000  0.000
      d+2      TTGATGGAAG    0      0      0      0      0      6.15e-01       6.47e-01       312    0.000  0.000  0.000  0.000  0.000

LOCUS: AT2G43150
```

```
DESCRIPTION:  proline-rich extensin-like family protein, similar to CRANTZ hydroxyproline-rich glycoprotein (Manihot esculenta)
gi|7211797|gb|AAF40442; similar to extensin gi|1165322|gb|AAB53156; contains proline-rich extensin domains, INTERPRO
DATA:                        Control 30min   2hours  2days   1week   p-value         B&H             Pos     Fold change relative to control (log2)
SENSE COUNTS:                0       6       1       0       0       4.60e-03        5.74e-03                0.000   2.585   0.000   0.000   0.000
  GENES:
  AT2G43150.1
    SENSE COUNTS:            0       6       1       0       0       4.60e-03        5.83e-03                0.000   2.585   0.000   0.000   0.000
    TAGS:
      d+1      TTGGCGTTAC    0       6       1       0       0       4.60e-03        1.01e-02        52      0.000   2.585   0.000   0.000   0.000

LOCUS: AT2G25490
DESCRIPTION:  F-box family protein (FBL6), contains similarity to grr1 GI
DATA:                        Control 30min   2hours  2days   1week   p-value         B&H             Pos     Fold change relative to control (log2)
SENSE COUNTS:                0       6       1       0       0       4.60e-03        5.73e-03                0.000   2.585   0.000   0.000   0.000
  GENES:
  AT2G25490.1
    SENSE COUNTS:            0       6       1       0       0       4.60e-03        5.84e-03                0.000   2.585   0.000   0.000   0.000
    TAGS:
      d+1      AAAAAGGCGA    0       6       1       0       0       4.60e-03        1.01e-02        1343    0.000   2.585   0.000   0.000   0.000

LOCUS: AT1G12130
DESCRIPTION:  flavin-containing monooxygenase family protein / FMO family protein, contains similarity to flavin-containing monooxygenase 2 (FMO2)
from Homo sapiens (GI
DATA:                        Control 30min   2hours  2days   1week   p-value         B&H             Pos     Fold change relative to control (log2)
SENSE COUNTS:                0       0       0       5       1       4.62e-03        5.75e-03                0.000   0.000   0.000   2.322   0.000
  GENES:
  AT1G12130.1
    SENSE COUNTS:            0       0       0       5       1       4.62e-03        5.85e-03                0.000   0.000   0.000   2.322   0.000
    TAGS:
      v+1      TCATTAAATT    0       0       0       1       0       3.09e-01        4.13e-01        2117    0.000   0.000   0.000   0.000   0.000
      v+2      GCATTGCAAT    0       0       0       4       1       2.76e-02        4.70e-02        1341    0.000   0.000   0.000   2.000   0.000

LOCUS: AT1G17220
DESCRIPTION:  translation initiation factor IF-2, chloroplast, putative, similar to SP|P57997|IF2C_PHAVU Translation initiation factor IF-2,
chloroplast precursor (PvIF2cp) {Phaseolus vulgaris}
DATA:                        Control 30min   2hours  2days   1week   p-value         B&H             Pos     Fold change relative to control (log2)
SENSE COUNTS:                11      2       11      19      19      4.62e-03        5.74e-03                0.000   -2.459  0.000   0.788   0.788
  GENES:
  AT1G17220.1
    SENSE COUNTS:            11      2       11      19      19      4.62e-03        5.84e-03                0.000   -2.459  0.000   0.788   0.788
    TAGS:
      d+1      GCAAATGTTC    10      2       11      19      19      3.90e-03        8.91e-03        3516    0.000   -2.322  0.138   0.926   0.926
      d+2      TCGGTGTTCT    1       0       0       0       0       4.28e-01        5.26e-01        3179    0.000   0.000   0.000   0.000   0.000

LOCUS: AT4G27680
DESCRIPTION:  MSP1 protein, putative / intramitochondrial sorting protein, putative, similar to Swiss-Prot
DATA:                        Control 30min   2hours  2days   1week   p-value         B&H             Pos     Fold change relative to control (log2)
SENSE COUNTS:                0       0       0       5       1       4.62e-03        5.74e-03                0.000   0.000   0.000   2.322   0.000
  GENES:
  AT4G27680.1
    SENSE COUNTS:            0       0       0       5       1       4.62e-03        5.84e-03                0.000   0.000   0.000   2.322   0.000
    TAGS:
      d+2      GCAGCGGGAT    0       0       0       5       1       4.62e-03        1.01e-02        1324    0.000   0.000   0.000   2.322   0.000

LOCUS: AT5G44190
DESCRIPTION:  myb family transcription factor (GLK2), contains Pfam profile
DATA:                        Control 30min   2hours  2days   1week   p-value         B&H             Pos     Fold change relative to control (log2)
SENSE COUNTS:                28      11      23      30      8       4.63e-03        5.74e-03                0.000   -1.348  -0.284  0.100   -1.807
  GENES:
  AT5G44190.1
```

```
    SENSE COUNTS:              28      11      23      30      8       4.63e-03        5.84e-03                0.000   -1.348  -0.284  0.100   -1.807
    TAGS:
      d+1      TATCGAAGTT      28      11      23      30      8       4.63e-03        1.01e-02        1361    0.000   -1.348  -0.284  0.100   -1.807

LOCUS: AT2G32540
DESCRIPTION:  cellulose synthase family protein, similar to cellulose synthase catalytic subunit from Arabidopsis thaliana (gi
DATA:                          Control 30min   2hours  2days   1week   p-value         B&H             Pos     Fold change relative to control (log2)
SENSE COUNTS:                  6       0       3       0       0       4.67e-03        5.78e-03                0.000   0.000   -1.000  0.000   0.000
  GENES:
  AT2G32540.1
    SENSE COUNTS:              6       0       3       0       0       4.67e-03        5.89e-03                0.000   0.000   -1.000  0.000   0.000
    TAGS:
      v+1      ACATTTTCTC      6       0       3       0       0       4.67e-03        1.02e-02        2667    0.000   0.000   -1.000  0.000   0.000

LOCUS: AT5G10560
DESCRIPTION:  glycosyl hydrolase family 3 protein, beta-xylosidase, Aspergllus nidulans, EMBL
DATA:                          Control 30min   2hours  2days   1week   p-value         B&H             Pos     Fold change relative to control (log2)
SENSE COUNTS:                  6       0       0       1       0       4.77e-03        5.90e-03                0.000   0.000   0.000   -2.585  0.000
  GENES:
  AT5G10560.1
    SENSE COUNTS:              6       0       0       1       0       4.77e-03        6.00e-03                0.000   0.000   0.000   -2.585  0.000
    TAGS:
      d+1      TCTTGTTCTT      5       0       0       1       0       2.02e-02        3.51e-02        2348    0.000   0.000   0.000   -2.322  0.000
      d+2      TTTCTTCATT      1       0       0       0       0       4.28e-01        5.20e-01        1310    0.000   0.000   0.000   0.000   0.000

LOCUS: AT5G23010
DESCRIPTION:  2-isopropylmalate synthase 3 (IMS3), identical to 2-isopropylmalate synthase (IMS3) (Arabidopsis thaliana) GI
DATA:                          Control 30min   2hours  2days   1week   p-value         B&H             Pos     Fold change relative to control (log2)
SENSE COUNTS:                  1       13      7       6       0       4.77e-03        5.89e-03                0.000   3.700   2.807   2.585   0.000
  GENES:
  AT5G23010.1
    SENSE COUNTS:              1       13      7       6       0       4.77e-03        6.00e-03                0.000   3.700   2.807   2.585   0.000
    TAGS:
      d+1      AGAGCGGCAT      1       13      7       6       0       4.77e-03        1.04e-02        1244    0.000   3.700   2.807   2.585   0.000

LOCUS: AT3G14620
DESCRIPTION:  cytochrome P450, putative, similar to GB
DATA:                          Control 30min   2hours  2days   1week   p-value         B&H             Pos     Fold change relative to control (log2)
SENSE COUNTS:                  5       0       1       0       0       4.82e-03        5.94e-03                0.000   0.000   -2.322  0.000   0.000
  GENES:
  AT3G14620.1
    SENSE COUNTS:              5       0       1       0       0       4.82e-03        6.05e-03                0.000   0.000   -2.322  0.000   0.000
    TAGS:
      d+1      AACACGTGAT      5       0       1       0       0       4.82e-03        1.05e-02        1780    0.000   0.000   -2.322  0.000   0.000

LOCUS: AT1G02560
DESCRIPTION:  ATP-dependent Clp protease proteolytic subunit (ClpP1), identical to nClpP1 GB
DATA:                          Control 30min   2hours  2days   1week   p-value         B&H             Pos     Fold change relative to control (log2)
SENSE COUNTS:                  59      31      61      63      65      4.83e-03        5.95e-03                0.000   -0.928  0.048   0.095   0.140
  GENES:
  AT1G02560.1
    SENSE COUNTS:              59      31      61      63      65      4.83e-03        6.06e-03                0.000   -0.928  0.048   0.095   0.140
    TAGS:
      d+1      AAGACCAAAG      57      31      61      62      64      7.33e-03        1.41e-02        1152    0.000   -0.879  0.098   0.121   0.167
      d+2      AAATATGTTT      1       0       0       0       1       3.83e-01        4.92e-01        1082    0.000   0.000   0.000   0.000   0.000
      d+2      GCTATATTCG      1       0       0       1       0       5.06e-01        5.56e-01        623     0.000   0.000   0.000   0.000   0.000

LOCUS: AT5G11740
DESCRIPTION:  arabinogalactan protein (AGP15) mRNA, complete cds
DATA:                          Control 30min   2hours  2days   1week   p-value         B&H             Pos     Fold change relative to control (log2)
```

```
SENSE COUNTS:                 5       21      5       12      14      4.87e-03      5.99e-03              0.000   2.070   0.000   1.263   1.485
  GENES:
  AT5G11740.1
    SENSE COUNTS:             5       21      5       12      14      4.87e-03      6.09e-03              0.000   2.070   0.000   1.263   1.485
    TAGS:
      d+1      ATGGTGATTA     5       21      5       12      14      4.87e-03      1.06e-02      104     0.000   2.070   0.000   1.263   1.485

LOCUS: AT1G04250
DESCRIPTION:  auxin-responsive protein / indoleacetic acid-induced protein 17 (IAA17), Identical to SP|P93830 Auxin-responsive protein IAA17
(Indoleacetic acid-induced protein 17) {Arabidopsis thaliana}; ESTs gb|H36782 and gb|F14074 come from this gene
DATA:                         Control 30min   2hours  2days   1week   p-value       B&H           Pos     Fold change relative to control (log2)
SENSE COUNTS:                 1       0       0       6       0       4.90e-03      6.02e-03              0.000   0.000   0.000   2.585   0.000
  GENES:
  AT1G04250.1
    SENSE COUNTS:             1       0       0       6       0       4.90e-03      6.13e-03              0.000   0.000   0.000   2.585   0.000
    TAGS:
      d+1      AAAGGATCGG     1       0       0       6       0       4.90e-03      1.06e-02      752     0.000   0.000   0.000   2.585   0.000

LOCUS: AT3G47800
DESCRIPTION:  aldose 1-epimerase family protein, similar to ALDOSE 1-EPIMERASE PRECURSOR GB
DATA:                         Control 30min   2hours  2days   1week   p-value       B&H           Pos     Fold change relative to control (log2)
SENSE COUNTS:                 0       2       0       0       6       4.98e-03      6.11e-03              0.000   1.000   0.000   0.000   2.585
  GENES:
  AT3G47800.1
    SENSE COUNTS:             0       2       0       0       6       4.98e-03      6.22e-03              0.000   1.000   0.000   0.000   2.585
    TAGS:
      d+2      TTATGCTCTT     0       2       0       0       5       2.94e-02      4.99e-02      1210    0.000   1.000   0.000   0.000   2.322
      i+3      GTACTCAAAA     0       0       0       0       1       1.65e-01      2.44e-01      872     0.000   0.000   0.000   0.000   0.000

LOCUS: AT4G16520
DESCRIPTION:  autophagy 8f (APG8f), identical to autophagy 8f (Arabidopsis thaliana) GI
DATA:                         Control 30min   2hours  2days   1week   p-value       B&H           Pos     Fold change relative to control (log2)
SENSE COUNTS:                 2       7       0       1       0       5.00e-03      6.13e-03              0.000   1.807   0.000   -1.000  0.000
  GENES:
  AT4G16520.1
    SENSE COUNTS:             2       7       0       1       0       5.00e-03      6.23e-03              0.000   1.807   0.000   -1.000  0.000
    TAGS:
      d+1      TCTTCTGTGT     2       7       0       1       0       5.00e-03      1.09e-02      402     0.000   1.807   0.000   -1.000  0.000
  AT4G16520.2
    SENSE COUNTS:             2       7       0       1       0       5.00e-03      6.24e-03              0.000   1.807   0.000   -1.000  0.000
    TAGS:
      d+1      TCTTCTGTGT     2       7       0       1       0       5.00e-03      1.09e-02      606     0.000   1.807   0.000   -1.000  0.000

LOCUS: AT1G72320
DESCRIPTION:  pumilio/Puf RNA-binding domain-containing protein, contains Pfam profile
DATA:                         Control 30min   2hours  2days   1week   p-value       B&H           Pos     Fold change relative to control (log2)
SENSE COUNTS:                 0       0       1       7       5       5.01e-03      6.13e-03              0.000   0.000   0.000   2.807   2.322
  GENES:
  AT1G72320.1
    SENSE COUNTS:             0       0       0       1       0       6.04e-01      6.04e-01              0.000   0.000   0.000   0.000   0.000
    TAGS:
      d+1      CAAAGGATAA     0       0       0       1       0       6.04e-01      6.48e-01      2248    0.000   0.000   0.000   0.000   0.000
  AT1G72320.2
    SENSE COUNTS:             0       0       1       6       5       4.47e-03      5.78e-03              0.000   0.000   0.000   2.585   2.322
    TAGS:
      d+1      AAACTTTTTG     0       0       1       6       5       4.47e-03      9.95e-03      2722    0.000   0.000   0.000   2.585   2.322
  AT1G72320.3
    SENSE COUNTS:             0       0       1       6       5       4.47e-03      5.78e-03              0.000   0.000   0.000   2.585   2.322
    TAGS:
      d+1      AAACTTTTTG     0       0       1       6       5       4.47e-03      9.95e-03      2713    0.000   0.000   0.000   2.585   2.322
```

```
LOCUS: AT3G52870
DESCRIPTION:  calmodulin-binding family protein, contains Pfam profile PF00612
DATA:                         Control 30min   2hours  2days   1week   p-value         B&H             Pos     Fold change relative to control (log2)
SENSE COUNTS:                 0       0       6       4       10      5.04e-03        6.16e-03                0.000   0.000   2.585   2.000   3.322
  GENES:
  AT3G52870.1
    SENSE COUNTS:             0       0       6       4       10      5.04e-03        6.27e-03                0.000   0.000   2.585   2.000   3.322
    TAGS:
      d+1      GATGATTGTG     0       0       5       4       10      5.90e-03        1.22e-02        1609    0.000   0.000   2.322   2.000   3.322
      d+2      GATCGGAAGG     0       0       1       0       0       4.55e-01        5.10e-01        807     0.000   0.000   0.000   0.000   0.000

LOCUS: AT3G11400
DESCRIPTION:  eukaryotic translation initiation factor 3G / eIF3g, nearly identical to eukaryotic translation initiation factor 3g (Arabidopsis
thaliana) GI
DATA:                         Control 30min   2hours  2days   1week   p-value         B&H             Pos     Fold change relative to control (log2)
SENSE COUNTS:                 3       6       15      10      21      5.16e-03        6.30e-03                0.000   1.000   2.322   1.737   2.807
  GENES:
  AT3G11400.1
    SENSE COUNTS:             3       6       15      10      21      5.16e-03        6.41e-03                0.000   1.000   2.322   1.737   2.807
    TAGS:
      d+1      CTTTTTAAAA     3       6       15      9       21      4.43e-03        9.88e-03        1185    0.000   1.000   2.322   1.585   2.807
      i+3      TTAGCCGTGA     0       0       0       1       0       3.09e-01        4.04e-01        797     0.000   0.000   0.000   0.000   0.000

LOCUS: AT3G53890
DESCRIPTION:  40S ribosomal protein S21 (RPS21B), ribosomal protein S21, cytosolic - Oryza sativa, PIR
DATA:                         Control 30min   2hours  2days   1week   p-value         B&H             Pos     Fold change relative to control (log2)
SENSE COUNTS:                 26      7       21      27      26      5.18e-03        6.31e-03                0.000   -1.893  -0.308  0.054   0.000
  GENES:
  AT3G53890.1
    SENSE COUNTS:             26      7       21      27      26      5.18e-03        6.43e-03                0.000   -1.893  -0.308  0.054   0.000
    TAGS:
      d+1      TTTTCGATTT     26      7       21      27      26      5.18e-03        1.12e-02        449     0.000   -1.893  -0.308  0.054   0.000

LOCUS: AT2G27810
DESCRIPTION:  xanthine/uracil permease family protein, contains Pfam profile
DATA:                         Control 30min   2hours  2days   1week   p-value         B&H             Pos     Fold change relative to control (log2)
SENSE COUNTS:                 5       0       0       0       0       5.18e-03        6.30e-03                0.000   0.000   0.000   0.000   0.000
  GENES:
  AT2G27810.2
    SENSE COUNTS:             5       0       0       0       0       5.18e-03        6.42e-03                0.000   0.000   0.000   0.000   0.000
    TAGS:
      d+1      TTGTTGTAAG     5       0       0       0       0       7.17e-04        2.19e-03        2440    0.000   0.000   0.000   0.000   0.000
      d+2      AGGGAGCTGC     0       0       0       0       0       6.15e-01        6.40e-01        1087    0.000   0.000   0.000   0.000   0.000
  AT2G27810.1
    SENSE COUNTS:             5       0       0       0       0       5.18e-03        6.42e-03                0.000   0.000   0.000   0.000   0.000
    TAGS:
      d+1      TTGTTGTAAG     5       0       0       0       0       7.17e-04        2.19e-03        2587    0.000   0.000   0.000   0.000   0.000
      d+2      AGGGAGCTGC     0       0       0       0       0       6.15e-01        6.40e-01        1087    0.000   0.000   0.000   0.000   0.000

LOCUS: AT5G53500
DESCRIPTION:  WD-40 repeat family protein, contains Pfam PF00400
DATA:                         Control 30min   2hours  2days   1week   p-value         B&H             Pos     Fold change relative to control (log2)
SENSE COUNTS:                 13      7       11      1       21      5.24e-03        6.37e-03                0.000   -0.893  -0.241  -3.700  0.692
  GENES:
  AT5G53500.1
    SENSE COUNTS:             13      7       11      1       21      5.24e-03        6.48e-03                0.000   -0.893  -0.241  -3.700  0.692
    TAGS:
      d+1      TAAAGGAGTG     13      7       11      1       21      5.24e-03        1.13e-02        2761    0.000   -0.893  -0.241  -3.700  0.692
```

```
LOCUS: AT2G28800
DESCRIPTION:  member of Chloroplast membrane protein ALBINO3 family
DATA:                        Control 30min  2hours  2days  1week  p-value       B&H           Pos    Fold change relative to control (log2)
SENSE COUNTS:                11      5      16      24     21     5.25e-03      6.37e-03             0.000  -1.138  0.541  1.126  0.933
  GENES:
  AT2G28800.1
    SENSE COUNTS:            11      5      16      24     21     5.25e-03      6.49e-03             0.000  -1.138  0.541  1.126  0.933
    TAGS:
      d+1      CCCGCTTCAG    11      5      16      24     21     5.25e-03      1.13e-02      1726   0.000  -1.138  0.541  1.126  0.933

LOCUS: AT2G35840
DESCRIPTION:  sucrose-phosphatase 1 (SPP1), identical to sucrose-phosphatase (SPP1) (Arabidopsis thaliana) GI
DATA:                        Control 30min  2hours  2days  1week  p-value       B&H           Pos    Fold change relative to control (log2)
SENSE COUNTS:                10      0      7       2      14     5.26e-03      6.38e-03             0.000  0.000  -0.515  -2.322  0.485
  GENES:
  AT2G35840.2
    SENSE COUNTS:            10      0      7       2      14     5.26e-03      6.49e-03             0.000  0.000  -0.515  -2.322  0.485
    TAGS:
      d+1      AGATCCAATA    10      0      7       2      14     5.26e-03      1.13e-02      1471   0.000  0.000  -0.515  -2.322  0.485
  AT2G35840.1
    SENSE COUNTS:            10      0      7       2      14     5.26e-03      6.48e-03             0.000  0.000  -0.515  -2.322  0.485
    TAGS:
      d+1      AGATCCAATA    10      0      7       2      14     5.26e-03      1.13e-02      1410   0.000  0.000  -0.515  -2.322  0.485

LOCUS: AT5G08570
DESCRIPTION:  pyruvate kinase, putative, similar to pyruvate kinase, cytosolic isozyme (Glycine max) SWISS-PROT
DATA:                        Control 30min  2hours  2days  1week  p-value       B&H           Pos    Fold change relative to control (log2)
SENSE COUNTS:                3       0      4       5      14     5.29e-03      6.40e-03             0.000  0.000  0.415  0.737  2.222
  GENES:
  AT5G08570.1
    SENSE COUNTS:            3       0      4       5      14     5.29e-03      6.51e-03             0.000  0.000  0.415  0.737  2.222
    TAGS:
      d+1      AAATAAAGAA    3       0      0       5      14     1.41e-04      5.59e-04      1753   0.000  0.000  0.000  0.737  2.222
      d+2      GCTAAGATCT    0       0      4       0      0      5.58e-03      1.18e-02      1190   0.000  0.000  2.000  0.000  0.000

LOCUS: AT5G30440
DESCRIPTION:  CACTA-like transposase family (Ptta/En/Spm), has a 4.6e-22 P-value blast match to At5g36655.1/81-333 CACTA-like transposase family
(Ptta/En/Spm) (CACTA-element) (Arabidopsis thaliana)
DATA:                        Control 30min  2hours  2days  1week  p-value       B&H           Pos    Fold change relative to control (log2)
SENSE COUNTS:                1       7      0       0      1      5.34e-03      6.46e-03             0.000  2.807  0.000  0.000  0.000
  GENES:
  AT5G30440.1
    SENSE COUNTS:            1       7      0       0      1      5.34e-03      6.57e-03             0.000  2.807  0.000  0.000  0.000
    TAGS:
      p+1      CTCGGGATGA    1       7      0       0      1      5.34e-03      1.15e-02      993    0.000  2.807  0.000  0.000  0.000

LOCUS: AT1G05490
DESCRIPTION:  C protein immunoglobulin-A-binding beta antigen-related, contains weak similarity to C protein immunoglobulin-A-binding beta antigen
(Streptococcus agalactiae) gi|18028989|gb|AAL56250
DATA:                        Control 30min  2hours  2days  1week  p-value       B&H           Pos    Fold change relative to control (log2)
SENSE COUNTS:                12      7      18      15     30     5.44e-03      6.57e-03             0.000  -0.778  0.585  0.322  1.322
  GENES:
  AT1G05490.1
    SENSE COUNTS:            12      7      18      15     30     5.44e-03      6.68e-03             0.000  -0.778  0.585  0.322  1.322
    TAGS:
      d+2      TCTGAAAGAG    12      7      18      15     30     5.44e-03      1.17e-02      503    0.000  -0.778  0.585  0.322  1.322

LOCUS: AT3G51260
DESCRIPTION:  20S proteasome alpha subunit D (PAD1)
DATA:                        Control 30min  2hours  2days  1week  p-value       B&H           Pos    Fold change relative to control (log2)
```

SENSE COUNTS: 1 0 0 1 8 5.47e-03 6.60e-03 0.000 0.000 0.000 0.000 3.000
GENES:
AT3G51260.1
SENSE COUNTS: 1 0 0 1 8 5.47e-03 6.71e-03 0.000 0.000 0.000 0.000 3.000
TAGS:
d+2 TTCAGTATTC 0 0 0 1 8 2.96e-04 1.06e-03 1041 0.000 0.000 0.000 0.000 3.000
d+2 AAATGGTTTG 1 0 0 0 0 6.89e-01 7.03e-01 940 0.000 0.000 0.000 0.000 0.000

LOCUS: AT5G61210
DESCRIPTION: SNAP25 homologous protein SNAP33 (SNAP33) (SNAP33B) / synaptosomal-associated protein SNAP25-like 1 / snap25a, identical to SNAP25 homologous protein SNAP33 (AtSNAP33) (Synaptosomal-associated protein SNAP25-like 1) (SNAP-25 like protein 1) (Snap25a) (Swi
DATA: Control 30min 2hours 2days 1week p-value B&H Pos Fold change relative to control (log2)
SENSE COUNTS: 1 1 0 1 8 5.47e-03 6.59e-03 0.000 0.000 0.000 0.000 3.000
GENES:
AT5G61210.1
SENSE COUNTS: 1 1 0 1 8 5.47e-03 6.70e-03 0.000 0.000 0.000 0.000 3.000
TAGS:
d+1 TTAAATACAT 1 1 0 1 8 5.47e-03 1.17e-02 1278 0.000 0.000 0.000 0.000 3.000

LOCUS: AT4G28240
DESCRIPTION: wound-responsive protein-related, wound-induced protein - tomato (fragment), PIR2
DATA: Control 30min 2hours 2days 1week p-value B&H Pos Fold change relative to control (log2)
SENSE COUNTS: 8 13 1 13 1 5.48e-03 6.59e-03 0.000 0.700 -3.000 0.700 -3.000
GENES:
AT4G28240.1
SENSE COUNTS: 8 13 1 13 1 5.48e-03 6.71e-03 0.000 0.700 -3.000 0.700 -3.000
TAGS:
d+1 AACTCCGCTG 8 13 1 13 1 5.48e-03 1.17e-02 401 0.000 0.700 -3.000 0.700 -3.000

LOCUS: AT2G29530
DESCRIPTION: mitochondrial import inner membrane translocase (TIM10), identical to mitochondrial import inner membrane translocase subunit Tim10 (Arabidopsis thaliana) Swiss-Prot
DATA: Control 30min 2hours 2days 1week p-value B&H Pos Fold change relative to control (log2)
SENSE COUNTS: 3 0 0 8 3 5.49e-03 6.59e-03 0.000 0.000 0.000 1.415 0.000
GENES:
AT2G29530.1
SENSE COUNTS: 3 0 0 8 3 5.49e-03 6.71e-03 0.000 0.000 0.000 1.415 0.000
TAGS:
d+1 TGAACCCATT 3 0 0 8 3 5.49e-03 1.17e-02 456 0.000 0.000 0.000 1.415 0.000

LOCUS: AT3G20310
DESCRIPTION: encodes a member of the ERF (ethylene response factor) subfamily B-1 of ERF/AP2 transcription factor family (ATERF-7). The protein contains one AP2 domain. There are 15 members in this subfamily including ATERF-3, ATERF-4, ATERF-7, and leafy petiole.
DATA: Control 30min 2hours 2days 1week p-value B&H Pos Fold change relative to control (log2)
SENSE COUNTS: 1 9 2 1 2 5.51e-03 6.61e-03 0.000 3.170 1.000 0.000 1.000
GENES:
AT3G20310.1
SENSE COUNTS: 1 9 2 1 2 5.51e-03 6.73e-03 0.000 3.170 1.000 0.000 1.000
TAGS:
d+1 GTCAATGGGC 0 2 1 0 1 4.01e-01 5.07e-01 1580 0.000 1.000 0.000 0.000 0.000
d+2 CGATTAAGAG 1 7 0 1 1 6.47e-03 1.29e-02 629 0.000 2.807 0.000 0.000 0.000
d+2 AGGAAAGGGA 0 0 1 0 0 4.55e-01 5.42e-01 118 0.000 0.000 0.000 0.000 0.000

LOCUS: AT2G20890
DESCRIPTION: Chloroplast-localized Thylakoid formation1 gene product involved in vesicle-mediated formation of thylakoid membranes
DATA: Control 30min 2hours 2days 1week p-value B&H Pos Fold change relative to control (log2)
SENSE COUNTS: 36 16 34 21 45 5.57e-03 6.67e-03 0.000 -1.170 -0.082 -0.778 0.322
GENES:
AT2G20890.1
SENSE COUNTS: 36 16 34 21 45 5.57e-03 6.80e-03 0.000 -1.170 -0.082 -0.778 0.322

```
    TAGS:
      d+1      TAGTATTTTA     4      4      1      4      10     1.30e-01       1.98e-01       1165   0.000  0.000  -2.000 0.000  1.322
      d+2      AGTTTACATA     31     12     31     16     34     6.14e-03       1.25e-02       1142   0.000  -1.369 0.000  -0.954 0.133
      d+2      GAAGGATATC     1      0      2      1      0      8.12e-01       8.14e-01       488    0.000  0.000  1.000  0.000  0.000
      d+2      TCCAATGTTA     0      0      0      0      1      1.65e-01       2.43e-01       287    0.000  0.000  0.000  0.000  0.000

LOCUS: AT4G18480
DESCRIPTION:  magnesium-chelatase subunit chlI, chloroplast / Mg-protoporphyrin IX chelatase (CHLI) (CS) (CH42), identical to SP|P161127 Magnesium-
chelatase subunit chlI, chloroplast precursor (Mg-protoporphyrin IX chelatase) (Protein CS/CH-42) {Arabidopsis thaliana}
DATA:                         Control 30min  2hours  2days  1week  p-value        B&H            Pos    Fold change relative to control (log2)
SENSE COUNTS:                 0      0      4      0      0      5.58e-03       6.68e-03              0.000  0.000  2.000  0.000  0.000
  GENES:
  AT4G18480.1
    SENSE COUNTS:             0      0      4      0      0      5.58e-03       6.74e-03              0.000  0.000  2.000  0.000  0.000
    TAGS:
      d+1      CACAAGTAGG     0      0      3      0      0      2.70e-02       4.63e-02       942    0.000  0.000  1.585  0.000  0.000
      d+2      TTTCGGTTAT     0      0      1      0      0      4.55e-01       5.38e-01       258    0.000  0.000  0.000  0.000  0.000

LOCUS: AT2G17040
DESCRIPTION:  no apical meristem (NAM) family protein, contains Pfam PF02365
DATA:                         Control 30min  2hours  2days  1week  p-value        B&H            Pos    Fold change relative to control (log2)
SENSE COUNTS:                 0      0      4      0      0      5.58e-03       6.67e-03              0.000  0.000  2.000  0.000  0.000
  GENES:
  AT2G17040.1
    SENSE COUNTS:             0      0      4      0      0      5.58e-03       6.77e-03              0.000  0.000  2.000  0.000  0.000
    TAGS:
      d+1      GCTCCAGAAT     0      0      4      0      0      5.58e-03       1.18e-02       790    0.000  0.000  2.000  0.000  0.000

LOCUS: AT4G13970
DESCRIPTION:  expressed protein
DATA:                         Control 30min  2hours  2days  1week  p-value        B&H            Pos    Fold change relative to control (log2)
SENSE COUNTS:                 0      0      4      0      0      5.58e-03       6.66e-03              0.000  0.000  2.000  0.000  0.000
  GENES:
  AT4G13970.1
    SENSE COUNTS:             0      0      4      0      0      5.58e-03       6.76e-03              0.000  0.000  2.000  0.000  0.000
    TAGS:
      v+1      AAAGCCACTA     0      0      4      0      0      5.58e-03       1.17e-02       2689   0.000  0.000  2.000  0.000  0.000

LOCUS: AT4G33040
DESCRIPTION:  glutaredoxin family protein, contains INTERPRO Domain IPR002109, Glutaredoxin (thioltransferase)
DATA:                         Control 30min  2hours  2days  1week  p-value        B&H            Pos    Fold change relative to control (log2)
SENSE COUNTS:                 0      0      4      0      0      5.58e-03       6.65e-03              0.000  0.000  2.000  0.000  0.000
  GENES:
  AT4G33040.1
    SENSE COUNTS:             0      0      4      0      0      5.58e-03       6.79e-03              0.000  0.000  2.000  0.000  0.000
    TAGS:
      d+1      TAGAAAATGA     0      0      4      0      0      5.58e-03       1.17e-02       698    0.000  0.000  2.000  0.000  0.000

LOCUS: AT4G21460
DESCRIPTION:  expressed protein
DATA:                         Control 30min  2hours  2days  1week  p-value        B&H            Pos    Fold change relative to control (log2)
SENSE COUNTS:                 0      0      4      0      0      5.58e-03       6.64e-03              0.000  0.000  2.000  0.000  0.000
  GENES:
  AT4G21460.1
    SENSE COUNTS:             0      0      4      0      0      5.58e-03       6.78e-03              0.000  0.000  2.000  0.000  0.000
    TAGS:
      d+2      CTAACTTCTA     0      0      4      0      0      5.58e-03       1.17e-02       1509   0.000  0.000  2.000  0.000  0.000

LOCUS: AT4G25490
```

```
DESCRIPTION:  Transcriptional activator that binds to the DRE/CRT regulatory element and induces COR (cold-regulated) gene expression increasing plant
freezing tolerance. It encodes a member of the DREB subfamily A-1 of ERF/AP2 transcription factor family (CBF1). The p
DATA:                         Control 30min  2hours  2days  1week  p-value        B&H            Pos    Fold change relative to control (log2)
SENSE COUNTS:                 0       0      4       0      0      5.58e-03       6.63e-03              0.000   0.000   2.000   0.000   0.000
  GENES:
  AT4G25490.1
    SENSE COUNTS:             0       0      4       0      0      5.58e-03       6.75e-03              0.000   0.000   2.000   0.000   0.000
    TAGS:
      d+1      CTTTTACCGC     0       0      4       0      0      5.58e-03       1.18e-02       725    0.000   0.000   2.000   0.000   0.000

LOCUS: AT1G80260
DESCRIPTION:  tubulin family protein
DATA:                         Control 30min  2hours  2days  1week  p-value        B&H            Pos    Fold change relative to control (log2)
SENSE COUNTS:                 0       0      4       0      0      5.58e-03       6.62e-03              0.000   0.000   2.000   0.000   0.000
  GENES:
  AT1G80260.1
    SENSE COUNTS:             0       0      4       0      0      5.58e-03       6.78e-03              0.000   0.000   2.000   0.000   0.000
    TAGS:
      d+1      CTTTTCTTGT     0       0      3       0      0      2.70e-02       4.63e-02       2926   0.000   0.000   1.585   0.000   0.000
      d+2      GCAGATGCTG     0       0      1       0      0      4.55e-01       5.48e-01       1591   0.000   0.000   0.000   0.000   0.000

LOCUS: AT1G38221
DESCRIPTION:  gypsy-like retrotransposon family (Athila), has a 8.2e-114 P-value blast match to GB
DATA:                         Control 30min  2hours  2days  1week  p-value        B&H            Pos    Fold change relative to control (log2)
SENSE COUNTS:                 0       0      4       0      0      5.58e-03       6.62e-03              0.000   0.000   2.000   0.000   0.000
  GENES:
  AT1G38221.1
    SENSE COUNTS:             0       0      4       0      0      5.58e-03       6.75e-03              0.000   0.000   2.000   0.000   0.000
    TAGS:
      p+1      TGACCTTGAT     0       0      4       0      0      5.58e-03       1.19e-02       2493   0.000   0.000   2.000   0.000   0.000

LOCUS: AT4G34390
DESCRIPTION:  extra-large guanine nucleotide binding protein, putative / G-protein, putative, similar to extra-large G-protein (XLG) (Arabidopsis
thaliana) GI
DATA:                         Control 30min  2hours  2days  1week  p-value        B&H            Pos    Fold change relative to control (log2)
SENSE COUNTS:                 2       12     4       4      0      5.59e-03       6.62e-03              0.000   2.585   1.000   1.000   0.000
  GENES:
  AT4G34390.1
    SENSE COUNTS:             2       12     4       4      0      5.59e-03       6.74e-03              0.000   2.585   1.000   1.000   0.000
    TAGS:
      d+1      TGATTGATTT     2       12     4       4      0      5.59e-03       1.17e-02       3093   0.000   2.585   1.000   1.000   0.000

LOCUS: AT1G70640
DESCRIPTION:  octicosapeptide/Phox/Bem1p (PB1) domain-containing protein, contains Pfam profile PF00564
DATA:                         Control 30min  2hours  2days  1week  p-value        B&H            Pos    Fold change relative to control (log2)
SENSE COUNTS:                 0       0      6       1      1      5.61e-03       6.63e-03              0.000   0.000   2.585   0.000   0.000
  GENES:
  AT1G70640.1
    SENSE COUNTS:             0       0      6       1      1      5.61e-03       6.76e-03              0.000   0.000   2.585   0.000   0.000
    TAGS:
      d+1      ATGTTGATGA     0       0      6       1      1      5.61e-03       1.17e-02       656    0.000   0.000   2.585   0.000   0.000

LOCUS: AT5G49890
DESCRIPTION:  chloride channel protein (CLC-c), identical to gi
DATA:                         Control 30min  2hours  2days  1week  p-value        B&H            Pos    Fold change relative to control (log2)
SENSE COUNTS:                 3       1      0       0      8      5.65e-03       6.67e-03              0.000   -1.585  0.000   0.000   1.415
  GENES:
  AT5G49890.1
    SENSE COUNTS:             3       1      0       0      8      5.65e-03       6.80e-03              0.000   -1.585  0.000   0.000   1.415
    TAGS:
```

```
      d+1      TAATTATATT     2      1      0      0      8      3.57e-03       8.32e-03       2802   0.000  -1.000 0.000  0.000  2.000
      i+3      TTTGTCTAGT     1      0      0      0      0      4.28e-01       5.18e-01       2009   0.000  0.000  0.000  0.000  0.000

LOCUS: AT3G08510
DESCRIPTION:  phosphoinositide-specific phospholipase C (PLC2), identical to phosphoinositide specific phospholipase C(AtPLC2) GI
DATA:                          Control 30min  2hours 2days  1week  p-value        B&H            Pos    Fold change relative to control (log2)
SENSE COUNTS:                  1      9      1      1      3      5.67e-03       6.69e-03              0.000  3.170  0.000  0.000  1.585
  GENES:
  AT3G08510.1
    SENSE COUNTS:              1      9      1      1      3      5.67e-03       6.82e-03              0.000  3.170  0.000  0.000  1.585
    TAGS:
      d+1      ATTAAGAAAG     1      9      1      1      3      5.67e-03       1.18e-02       2048   0.000  3.170  0.000  0.000  1.585

LOCUS: AT3G23325
DESCRIPTION:  splicing factor, putative, similar to Splicing factor 3B subunit 10 (SF3b10) (Pre-mRNA splicing factor SF3b 10 kDa subunit) (Swiss-Prot
DATA:                          Control 30min  2hours 2days  1week  p-value        B&H            Pos    Fold change relative to control (log2)
SENSE COUNTS:                  5      0      0      0      3      5.67e-03       6.68e-03              0.000  0.000  0.000  0.000  -0.737
  GENES:
  AT3G23325.1
    SENSE COUNTS:              5      0      0      0      3      5.67e-03       6.81e-03              0.000  0.000  0.000  0.000  -0.737
    TAGS:
      d+1      ATCTAAACAT     5      0      0      0      3      5.67e-03       1.18e-02       441    0.000  0.000  0.000  0.000  -0.737

LOCUS: AT1G21500
DESCRIPTION:  expressed protein
DATA:                          Control 30min  2hours 2days  1week  p-value        B&H            Pos    Fold change relative to control (log2)
SENSE COUNTS:                  0      8      5      1      0      5.70e-03       6.71e-03              0.000  3.000  2.322  0.000  0.000
  GENES:
  AT1G21500.1
    SENSE COUNTS:              0      8      5      1      0      5.70e-03       6.84e-03              0.000  3.000  2.322  0.000  0.000
    TAGS:
      d+1      GTGGCACATT     0      8      5      1      0      5.70e-03       1.19e-02       75     0.000  3.000  2.322  0.000  0.000

LOCUS: AT5G60790
DESCRIPTION:  ABC transporter family protein, similar to ABC transporter homolog PnATH GI
DATA:                          Control 30min  2hours 2days  1week  p-value        B&H            Pos    Fold change relative to control (log2)
SENSE COUNTS:                  1      3      4      11     14     5.70e-03       6.70e-03              0.000  1.585  2.000  3.459  3.807
  GENES:
  AT5G60790.1
    SENSE COUNTS:              1      3      4      11     14     5.70e-03       6.83e-03              0.000  1.585  2.000  3.459  3.807
    TAGS:
      d+1      TGCACCTTCT     1      3      4      11     14     5.70e-03       1.19e-02       2116   0.000  1.585  2.000  3.459  3.807

LOCUS: AT1G67785
DESCRIPTION:  expressed protein
DATA:                          Control 30min  2hours 2days  1week  p-value        B&H            Pos    Fold change relative to control (log2)
SENSE COUNTS:                  10     4      0      3      0      5.79e-03       6.79e-03              0.000  -1.322 0.000  -1.737 0.000
  GENES:
  AT1G67785.1
    SENSE COUNTS:              10     4      0      3      0      5.79e-03       6.93e-03              0.000  -1.322 0.000  -1.737 0.000
    TAGS:
      d+1      CTTTTGGGTG     10     4      0      2      0      3.47e-03       8.12e-03       211    0.000  -1.322 0.000  -2.322 0.000
      d+2      TCTGGATCGC     0      0      0      1      0      3.09e-01       4.09e-01       77     0.000  0.000  0.000  0.000  0.000

LOCUS: AT2G45520
DESCRIPTION:  expressed protein
DATA:                          Control 30min  2hours 2days  1week  p-value        B&H            Pos    Fold change relative to control (log2)
SENSE COUNTS:                  0      6      1      1      0      5.80e-03       6.80e-03              0.000  2.585  0.000  0.000  0.000
  GENES:
  AT2G45520.1
```

```
    SENSE COUNTS:             0      6      1      1      0      5.80e-03       6.93e-03              0.000  2.585  0.000  0.000  0.000
    TAGS:
      d+2      AATGTACTCT     0      3      1      1      0      2.38e-01       3.28e-01       650    0.000  1.585  0.000  0.000  0.000
      d+2      ATTCTACTAA     0      3      0      0      0      3.05e-02       5.15e-02       175    0.000  1.585  0.000  0.000  0.000

LOCUS: AT4G35450
DESCRIPTION:  AFT protein (AFT) mRNA, complete cds
DATA:                         Control 30min  2hours 2days  1week  p-value        B&H            Pos    Fold change relative to control (log2)
SENSE COUNTS:                 0      9      2      1      4      5.81e-03       6.80e-03              0.000  3.170  1.000  0.000  2.000
  GENES:
  AT4G35450.3
    SENSE COUNTS:             0      9      2      1      3      4.41e-03       5.73e-03              0.000  3.170  1.000  0.000  1.585
    TAGS:
      d+1      ATGAAGTACT     0      9      2      1      3      4.41e-03       9.87e-03       624    0.000  3.170  1.000  0.000  1.585
  AT4G35450.2
    SENSE COUNTS:             0      9      2      1      3      4.41e-03       5.74e-03              0.000  3.170  1.000  0.000  1.585
    TAGS:
      d+1      ATGAAGTACT     0      9      2      1      3      4.41e-03       9.87e-03       704    0.000  3.170  1.000  0.000  1.585
  AT4G35450.4
    SENSE COUNTS:             0      9      2      1      3      4.41e-03       5.75e-03              0.000  3.170  1.000  0.000  1.585
    TAGS:
      d+1      ATGAAGTACT     0      9      2      1      3      4.41e-03       9.87e-03       559    0.000  3.170  1.000  0.000  1.585
  AT4G35450.1
    SENSE COUNTS:             0      9      2      1      4      5.81e-03       6.93e-03              0.000  3.170  1.000  0.000  2.000
    TAGS:
      d+1      ATGAAGTACT     0      9      2      1      3      4.41e-03       9.87e-03       695    0.000  3.170  1.000  0.000  1.585
      d+2      AGCGTTTGCC     0      0      0      0      1      1.65e-01       2.45e-01       119    0.000  0.000  0.000  0.000  0.000

LOCUS: AT5G64840
DESCRIPTION:  ABC transporter family protein
DATA:                         Control 30min  2hours 2days  1week  p-value        B&H            Pos    Fold change relative to control (log2)
SENSE COUNTS:                 3      4      15     2      6      5.81e-03       6.79e-03              0.000  0.415  2.322  -0.585 1.000
  GENES:
  AT5G64840.1
    SENSE COUNTS:             3      4      15     2      6      5.81e-03       6.94e-03              0.000  0.415  2.322  -0.585 1.000
    TAGS:
      d+1      GAACCGAACC     0      0      2      0      1      2.22e-01       3.07e-01       2377   0.000  0.000  1.000  0.000  0.000
      d+2      GTGACGCCAT     3      4      12     2      5      6.43e-02       1.04e-01       1792   0.000  0.415  2.000  -0.585 0.737
      d+2      ACAGAGCCTT     0      0      1      0      0      4.55e-01       5.12e-01       1061   0.000  0.000  0.000  0.000  0.000

LOCUS: AT1G80720
DESCRIPTION:  mitochondrial glycoprotein family protein / MAM33 family protein, similar to SUAPRGA1 (Emericella nidulans) GI
DATA:                         Control 30min  2hours 2days  1week  p-value        B&H            Pos    Fold change relative to control (log2)
SENSE COUNTS:                 1      0      0      5      0      5.84e-03       6.82e-03              0.000  0.000  0.000  2.322  0.000
  GENES:
  AT1G80720.1
    SENSE COUNTS:             1      0      0      5      0      5.84e-03       6.96e-03              0.000  0.000  0.000  2.322  0.000
    TAGS:
      d+1      GGTACTCATT     1      0      0      5      0      5.84e-03       1.21e-02       921    0.000  0.000  0.000  2.322  0.000

LOCUS: AT3G05800
DESCRIPTION:  expressed protein
DATA:                         Control 30min  2hours 2days  1week  p-value        B&H            Pos    Fold change relative to control (log2)
SENSE COUNTS:                 0      0      7      1      1      5.87e-03       6.84e-03              0.000  0.000  2.807  0.000  0.000
  GENES:
  AT3G05800.1
    SENSE COUNTS:             0      0      7      1      1      5.87e-03       6.99e-03              0.000  0.000  2.807  0.000  0.000
    TAGS:
      d+1      ACAGCTCTAT     0      0      7      1      1      5.87e-03       1.21e-02       709    0.000  0.000  2.807  0.000  0.000
```

```
LOCUS: AT1G20020
DESCRIPTION:  ferredoxin--NADP(+) reductase, putative / adrenodoxin reductase, putative, strong similarity to Ferredoxin--NADP reductase, chloroplast
precursor (EC 1.18.1.2) (FNR) from {Pisum sativum} SP|P10933, {Mesembryanthemum crystallinum} SP|P41343, {Spinacia oler
DATA:                         Control 30min  2hours  2days  1week  p-value       B&H           Pos    Fold change relative to control (log2)
SENSE COUNTS:                 5       22     10      20     8      5.90e-03      6.87e-03             0.000  2.138  1.000  2.000  0.678
  GENES:
  AT1G20020.1
    SENSE COUNTS:             5       22     10      20     8      5.90e-03      7.01e-03             0.000  2.138  1.000  2.000  0.678
    TAGS:
      d+1     TGTGGACTCA      5       22     10      20     8      5.90e-03      1.22e-02      1025   0.000  2.138  1.000  2.000  0.678

LOCUS: AT4G21790
DESCRIPTION:  transmembrane protein-related (TOM1), contains some similarity to transmembrane protein TOM3 GI
DATA:                         Control 30min  2hours  2days  1week  p-value       B&H           Pos    Fold change relative to control (log2)
SENSE COUNTS:                 0       8      2       1      1      5.90e-03      6.86e-03             0.000  3.000  1.000  0.000  0.000
  GENES:
  AT4G21790.1
    SENSE COUNTS:             0       8      2       1      1      5.90e-03      7.01e-03             0.000  3.000  1.000  0.000  0.000
    TAGS:
      i+3     TATTTGATGT      0       0      1       0      0      4.55e-01      5.12e-01      1466   0.000  0.000  0.000  0.000  0.000
      d+1     CTAAGAAGGT      0       8      1       1      1      2.31e-03      5.79e-03      744    0.000  3.000  0.000  0.000  0.000

LOCUS: AT3G48560
DESCRIPTION:  acetolactate synthase, chloroplast / acetohydroxy-acid synthase (ALS), nearly identical to SP|P17597 Acetolactate synthase, chloroplast
precursor (EC 2.2.1.6, formerly EC 4.1.3.18) (Acetohydroxy-acid synthase) (ALS) {Arabidopsis thaliana}
DATA:                         Control 30min  2hours  2days  1week  p-value       B&H           Pos    Fold change relative to control (log2)
SENSE COUNTS:                 11      25     16      19     1      5.94e-03      6.90e-03             0.000  1.184  0.541  0.788  -3.459
  GENES:
  AT3G48560.1
    SENSE COUNTS:             11      25     16      19     1      5.94e-03      7.05e-03             0.000  1.184  0.541  0.788  -3.459
    TAGS:
      d+1     TGTTGCCGAT      11      25     16      19     1      5.94e-03      1.22e-02      1979   0.000  1.184  0.541  0.788  -3.459

LOCUS: AT1G73650
DESCRIPTION:  expressed protein
DATA:                         Control 30min  2hours  2days  1week  p-value       B&H           Pos    Fold change relative to control (log2)
SENSE COUNTS:                 1       4      0       11     5      5.95e-03      6.90e-03             0.000  2.000  0.000  3.459  2.322
  GENES:
  AT1G73650.3
    SENSE COUNTS:             1       4      0       11     5      5.95e-03      7.03e-03             0.000  2.000  0.000  3.459  2.322
    TAGS:
      d+1     GAAACTCAGG      1       4      0       11     5      5.95e-03      1.22e-02      861    0.000  2.000  0.000  3.459  2.322
  AT1G73650.2
    SENSE COUNTS:             1       4      0       11     5      5.95e-03      7.05e-03             0.000  2.000  0.000  3.459  2.322
    TAGS:
      d+1     GAAACTCAGG      1       4      0       11     5      5.95e-03      1.22e-02      861    0.000  2.000  0.000  3.459  2.322
  AT1G73650.1
    SENSE COUNTS:             1       4      0       11     5      5.95e-03      7.03e-03             0.000  2.000  0.000  3.459  2.322
    TAGS:
      d+1     GAAACTCAGG      1       4      0       11     5      5.95e-03      1.22e-02      861    0.000  2.000  0.000  3.459  2.322

LOCUS: AT1G19450
DESCRIPTION:  integral membrane protein, putative / sugar transporter family protein, similar to GB
DATA:                         Control 30min  2hours  2days  1week  p-value       B&H           Pos    Fold change relative to control (log2)
SENSE COUNTS:                 2       0      2       1      9      5.95e-03      6.89e-03             0.000  0.000  0.000  -1.000 2.170
  GENES:
  AT1G19450.1
    SENSE COUNTS:             2       0      2       1      9      5.95e-03      7.04e-03             0.000  0.000  0.000  -1.000 2.170
    TAGS:
      d+1     CTTTGTTCAA      2       0      2       1      8      3.47e-02      5.82e-02      1814   0.000  0.000  0.000  -1.000 2.000
```

```
      d+2      GAGCAGTGGG     0       0       0       0       1       1.65e-01       2.47e-01       1593    0.000   0.000   0.000   0.000   0.000

LOCUS: AT1G80830
DESCRIPTION:  NRAMP metal ion transporter 1 (NRAMP1), identical to NRAMP1 protein (Arabidopsis thaliana) gi|7108911|gb|AAF36535; member of the natural
resistance-associated macrophage protein (NRAMP) metal transporter family, PMID
DATA:                         Control 30min   2hours  2days   1week   p-value        B&H            Pos     Fold change relative to control (log2)
SENSE COUNTS:                 0       0       0       5       2       5.98e-03       6.92e-03               0.000   0.000   0.000   2.322   1.000
  GENES:
  AT1G80830.1
    SENSE COUNTS:             0       0       0       5       2       5.98e-03       7.05e-03               0.000   0.000   0.000   2.322   1.000
    TAGS:
      d+1      GCATAATAAA     0       0       0       4       1       2.76e-02       4.70e-02       1804    0.000   0.000   0.000   2.000   0.000
      d+2      TAGAGTATCT     0       0       0       0       1       1.65e-01       2.38e-01       1624    0.000   0.000   0.000   0.000   0.000
      d+2      CAGTTACCAT     0       0       0       1       0       3.09e-01       4.03e-01       1613    0.000   0.000   0.000   0.000   0.000

LOCUS: AT1G47710
DESCRIPTION:  serpin, putative / serine protease inhibitor, putative, similar to phloem serpin-1 (Cucurbita maxima) GI
DATA:                         Control 30min   2hours  2days   1week   p-value        B&H            Pos     Fold change relative to control (log2)
SENSE COUNTS:                 0       0       0       5       3       5.98e-03       6.91e-03               0.000   0.000   0.000   2.322   1.585
  GENES:
  AT1G47710.1
    SENSE COUNTS:             0       0       0       5       3       5.98e-03       7.05e-03               0.000   0.000   0.000   2.322   1.585
    TAGS:
      d+1      TGATGCATCT     0       0       0       4       0       6.18e-03       1.25e-02       1423    0.000   0.000   0.000   2.000   0.000
      d+2      TACTGAACTG     0       0       0       1       0       3.09e-01       4.07e-01       1396    0.000   0.000   0.000   0.000   0.000
      X+4      GTTACATACA     0       0       0       0       3       1.12e-02       2.01e-02       1009    0.000   0.000   0.000   0.000   1.585

LOCUS: AT3G13410
DESCRIPTION:  expressed protein
DATA:                         Control 30min   2hours  2days   1week   p-value        B&H            Pos     Fold change relative to control (log2)
SENSE COUNTS:                 1       7       1       0       0       6.05e-03       6.98e-03               0.000   2.807   0.000   0.000   0.000
  GENES:
  AT3G13410.1
    SENSE COUNTS:             1       7       1       0       0       6.05e-03       7.12e-03               0.000   2.807   0.000   0.000   0.000
    TAGS:
      d+1      TCTGCAAAGG     1       7       1       0       0       6.05e-03       1.24e-02       286     0.000   2.807   0.000   0.000   0.000

LOCUS: AT1G27970
DESCRIPTION:  nuclear transport factor 2 (NTF2), putative, similar to Swiss-Prot
DATA:                         Control 30min   2hours  2days   1week   p-value        B&H            Pos     Fold change relative to control (log2)
SENSE COUNTS:                 16      13      6       8       28      6.05e-03       6.97e-03               0.000   -0.300  -1.415  -1.000  0.807
  GENES:
  AT1G27970.1
    SENSE COUNTS:             16      13      6       8       28      6.05e-03       7.11e-03               0.000   -0.300  -1.415  -1.000  0.807
    TAGS:
      d+1      TATTTGGTTT     16      13      6       8       28      6.05e-03       1.24e-02       636     0.000   -0.300  -1.415  -1.000  0.807

LOCUS: AT1G24160
DESCRIPTION:  expressed protein, Location of EST gb|H36355
DATA:                         Control 30min   2hours  2days   1week   p-value        B&H            Pos     Fold change relative to control (log2)
SENSE COUNTS:                 1       6       1       0       0       6.05e-03       6.97e-03               0.000   2.585   0.000   0.000   0.000
  GENES:
  AT1G24160.1
    SENSE COUNTS:             1       6       1       0       0       6.05e-03       7.11e-03               0.000   2.585   0.000   0.000   0.000
    TAGS:
      d+1      GACTCAAAAG     1       6       0       0       0       4.55e-03       1.00e-02       1574    0.000   2.585   0.000   0.000   0.000
      d+2      GAGCGAATGG     0       0       1       0       0       4.55e-01       5.10e-01       1345    0.000   0.000   0.000   0.000   0.000
      d+2      TGCAGGAGAG     0       0       0       0       0       6.15e-01       6.34e-01       800     0.000   0.000   0.000   0.000   0.000

LOCUS: AT3G18490
```

```
DESCRIPTION:  aspartyl protease family protein, contains Pfam domain, PF00026
DATA:                         Control 30min   2hours  2days   1week   p-value         B&H             Pos     Fold change relative to control (log2)
SENSE COUNTS:                 26      6       20      13      14      6.07e-03        6.98e-03                0.000   -2.115  -0.379  -1.000  -0.893
  GENES:
  AT3G18490.1
    SENSE COUNTS:             26      6       20      13      14      6.07e-03        7.11e-03                0.000   -2.115  -0.379  -1.000  -0.893
    TAGS:
      d+1      TCAAAGTAGA     26      6       20      13      14      6.07e-03        1.24e-02        1585    0.000   -2.115  -0.379  -1.000  -0.893

LOCUS: AT3G02730
DESCRIPTION:  thioredoxin, putative, similar to SP|P29450 Thioredoxin F-type, chloroplast precursor (TRX-F) {Pisum sativum}; contains Pfam profile
DATA:                         Control 30min   2hours  2days   1week   p-value         B&H             Pos     Fold change relative to control (log2)
SENSE COUNTS:                 32      13      18      31      12      6.07e-03        6.97e-03                0.000   -1.300  -0.830  -0.046  -1.415
  GENES:
  AT3G02730.1
    SENSE COUNTS:             32      13      18      31      12      6.07e-03        7.12e-03                0.000   -1.300  -0.830  -0.046  -1.415
    TAGS:
      d+1      AAAATGATTA     32      13      18      31      12      6.07e-03        1.24e-02        767     0.000   -1.300  -0.830  -0.046  -1.415

LOCUS: AT4G19580
DESCRIPTION:  DNAJ heat shock N-terminal domain-containing protein, low similarity to SP|Q9QYI4 DnaJ homolog subfamily B member 12 {Mus musculus};
contains Pfam profile PF00226
DATA:                         Control 30min   2hours  2days   1week   p-value         B&H             Pos     Fold change relative to control (log2)
SENSE COUNTS:                 5       0       1       0       8       6.09e-03        6.99e-03                0.000   0.000   -2.322  0.000   0.678
  GENES:
  AT4G19580.1
    SENSE COUNTS:             5       0       1       0       8       6.09e-03        7.13e-03                0.000   0.000   -2.322  0.000   0.678
    TAGS:
      v+1      TTTTGAATAT     5       0       1       0       8       6.09e-03        1.24e-02        1733    0.000   0.000   -2.322  0.000   0.678

LOCUS: AT1G80180
DESCRIPTION:  expressed protein
DATA:                         Control 30min   2hours  2days   1week   p-value         B&H             Pos     Fold change relative to control (log2)
SENSE COUNTS:                 9       9       2       1       0       6.10e-03        6.99e-03                0.000   0.000   -2.170  -3.170  0.000
  GENES:
  AT1G80180.1
    SENSE COUNTS:             9       9       2       1       0       6.10e-03        7.13e-03                0.000   0.000   -2.170  -3.170  0.000
    TAGS:
      X+4      TATATTCTTG     5       4       1       0       0       6.73e-02        1.09e-01        759     0.000   -0.322  -2.322  0.000   0.000
      d+2      AACTAAAGTC     3       5       1       1       0       2.06e-01        2.86e-01        534     0.000   0.737   -1.585  -1.585  0.000
      X+4      ATAATCGCGA     1       0       0       0       0       4.28e-01        5.29e-01        -146    0.000   0.000   0.000   0.000   0.000

LOCUS: AT5G15100
DESCRIPTION:  auxin efflux carrier family protein, contains auxin efflux carrier domain, Pfam
DATA:                         Control 30min   2hours  2days   1week   p-value         B&H             Pos     Fold change relative to control (log2)
SENSE COUNTS:                 12      3       22      13      11      6.13e-03        7.01e-03                0.000   -2.000  0.874   0.115   -0.126
  GENES:
  AT5G15100.1
    SENSE COUNTS:             12      3       22      13      11      6.13e-03        7.16e-03                0.000   -2.000  0.874   0.115   -0.126
    TAGS:
      v+2      TTTTATACGT     0       0       0       0       1       1.65e-01        2.49e-01        1482    0.000   0.000   0.000   0.000   0.000
      v+2      TTCAAAAAAA     12      3       22      13      10      5.16e-03        1.12e-02        1465    0.000   -2.000  0.874   0.115   -0.263

LOCUS: AT1G78930
DESCRIPTION:  mitochondrial transcription termination factor-related / mTERF-related, contains Pfam profile PF02536
DATA:                         Control 30min   2hours  2days   1week   p-value         B&H             Pos     Fold change relative to control (log2)
SENSE COUNTS:                 0       0       0       4       0       6.18e-03        7.06e-03                0.000   0.000   0.000   2.000   0.000
  GENES:
  AT1G78930.1
    SENSE COUNTS:             0       0       0       4       0       6.18e-03        7.17e-03                0.000   0.000   0.000   2.000   0.000
```

```
    TAGS:
      d+1      GAGAAGCCGG     0      0      0      4      0      6.18e-03       1.24e-02       1685   0.000  0.000  0.000  2.000  0.000

LOCUS: AT1G73390
DESCRIPTION:  expressed protein
DATA:                         Control 30min  2hours  2days  1week  p-value        B&H            Pos    Fold change relative to control (log2)
SENSE COUNTS:                 0      0      0      4      0      6.18e-03       7.05e-03              0.000  0.000  0.000  2.000  0.000
  GENES:
  AT1G73390.3
    SENSE COUNTS:             0      0      0      4      0      6.18e-03       7.13e-03              0.000  0.000  0.000  2.000  0.000
    TAGS:
      d+1      GTCTGAAGCC     0      0      0      4      0      6.18e-03       1.24e-02       1467   0.000  0.000  0.000  2.000  0.000
  AT1G73390.1
    SENSE COUNTS:             0      0      0      4      0      6.18e-03       7.15e-03              0.000  0.000  0.000  2.000  0.000
    TAGS:
      d+1      GTCTGAAGCC     0      0      0      4      0      6.18e-03       1.24e-02       1265   0.000  0.000  0.000  2.000  0.000
  AT1G73390.2
    SENSE COUNTS:             0      0      0      4      0      6.18e-03       7.20e-03              0.000  0.000  0.000  2.000  0.000
    TAGS:
      d+1      GTCTGAAGCC     0      0      0      4      0      6.18e-03       1.24e-02       1439   0.000  0.000  0.000  2.000  0.000

LOCUS: AT5G25890
DESCRIPTION:  auxin-responsive protein / indoleacetic acid-induced protein 28 (IAA28), identical to SP|Q9XFM0|AXIS_ARATH Auxin-responsive protein
IAA28 (Indoleacetic acid-induced protein 28) {Arabidopsis thaliana}
DATA:                         Control 30min  2hours  2days  1week  p-value        B&H            Pos    Fold change relative to control (log2)
SENSE COUNTS:                 0      0      0      3      0      6.18e-03       7.05e-03              0.000  0.000  0.000  1.585  0.000
  GENES:
  AT5G25890.1
    SENSE COUNTS:             0      0      0      3      0      6.18e-03       7.21e-03              0.000  0.000  0.000  1.585  0.000
    TAGS:
      X+4      CCTTCGTTTC     0      0      0      2      0      4.80e-02       7.94e-02       272    0.000  0.000  0.000  1.000  0.000
      i+3      AATGATTTGA     0      0      0      1      0      3.09e-01       4.06e-01       216    0.000  0.000  0.000  0.000  0.000

LOCUS: AT2G36500
DESCRIPTION:  CBS domain-containing protein / octicosapeptide/Phox/Bemp1 (PB1) domain-containing protein, contains Pfam profiles
DATA:                         Control 30min  2hours  2days  1week  p-value        B&H            Pos    Fold change relative to control (log2)
SENSE COUNTS:                 0      0      0      4      0      6.18e-03       7.04e-03              0.000  0.000  0.000  2.000  0.000
  GENES:
  AT2G36500.1
    SENSE COUNTS:             0      0      0      4      0      6.18e-03       7.20e-03              0.000  0.000  0.000  2.000  0.000
    TAGS:
      d+2      AGAGTTGTAG     0      0      0      4      0      6.18e-03       1.25e-02       997    0.000  0.000  0.000  2.000  0.000

LOCUS: AT4G03120
DESCRIPTION:  proline-rich family protein, similar to U1 small nuclear ribonucleoprotein C; contains proline rich extensin domains, INTERPRO
DATA:                         Control 30min  2hours  2days  1week  p-value        B&H            Pos    Fold change relative to control (log2)
SENSE COUNTS:                 0      0      0      4      0      6.18e-03       7.03e-03              0.000  0.000  0.000  2.000  0.000
  GENES:
  AT4G03120.1
    SENSE COUNTS:             0      0      0      4      0      6.18e-03       7.14e-03              0.000  0.000  0.000  2.000  0.000
    TAGS:
      d+1      AAAAGGAAAA     0      0      0      4      0      6.18e-03       1.25e-02       874    0.000  0.000  0.000  2.000  0.000

LOCUS: AT5G50800
DESCRIPTION:  nodulin MtN3 family protein, similar to MtN3 GI
DATA:                         Control 30min  2hours  2days  1week  p-value        B&H            Pos    Fold change relative to control (log2)
SENSE COUNTS:                 0      0      0      4      0      6.18e-03       7.02e-03              0.000  0.000  0.000  2.000  0.000
  GENES:
  AT5G50800.1
    SENSE COUNTS:             0      0      0      4      0      6.18e-03       7.18e-03              0.000  0.000  0.000  2.000  0.000
```

```
    TAGS:
      d+1      ACCGATCCGA     0      0      0      4      0      6.18e-03       1.25e-02       996    0.000  0.000  0.000  2.000  0.000

LOCUS: AT4G17650
DESCRIPTION:  aromatic-rich family protein, contains Pfam PF03654
DATA:                         Control 30min  2hours 2days  1week  p-value        B&H            Pos    Fold change relative to control (log2)
SENSE COUNTS:                 0      0      0      3      0      6.18e-03       7.01e-03              0.000  0.000  0.000  1.585  0.000
  GENES:
  AT4G17650.1
    SENSE COUNTS:             0      0      0      3      0      6.18e-03       7.19e-03              0.000  0.000  0.000  1.585  0.000
    TAGS:
      d+1      TTTTGTTTTC     0      0      0      2      0      4.80e-02       7.91e-02       1088   0.000  0.000  0.000  1.000  0.000
      d+2      GAAGTAACAA     0      0      0      1      0      3.09e-01       4.05e-01       1025   0.000  0.000  0.000  0.000  0.000

LOCUS: AT2G01170
DESCRIPTION:  amino acid permease family protein, weak similarity to GABA permease (Emericella nidulans) GI
DATA:                         Control 30min  2hours 2days  1week  p-value        B&H            Pos    Fold change relative to control (log2)
SENSE COUNTS:                 0      0      0      4      0      6.18e-03       7.00e-03              0.000  0.000  0.000  2.000  0.000
  GENES:
  AT2G01170.1
    SENSE COUNTS:             0      0      0      4      0      6.18e-03       7.13e-03              0.000  0.000  0.000  2.000  0.000
    TAGS:
      X+4      GGCCTCGACG     0      0      0      4      0      6.18e-03       1.25e-02       515    0.000  0.000  0.000  2.000  0.000

LOCUS: AT5G45900
DESCRIPTION:  Component of autophagy conjugation pathway. Required for proper senescence.
DATA:                         Control 30min  2hours 2days  1week  p-value        B&H            Pos    Fold change relative to control (log2)
SENSE COUNTS:                 0      0      0      4      0      6.18e-03       6.99e-03              0.000  0.000  0.000  2.000  0.000
  GENES:
  AT5G45900.1
    SENSE COUNTS:             0      0      0      4      0      6.18e-03       7.16e-03              0.000  0.000  0.000  2.000  0.000
    TAGS:
      d+1      GTGCTGGCCC     0      0      0      4      0      6.18e-03       1.24e-02       1614   0.000  0.000  0.000  2.000  0.000

LOCUS: AT5G02490
DESCRIPTION:  heat shock cognate 70 kDa protein 2 (HSC70-2) (HSP70-2), identical to SP|P22954 Heat shock cognate 70 kDa protein 2 (Hsc70.2)
{Arabidopsis thaliana}
DATA:                         Control 30min  2hours 2days  1week  p-value        B&H            Pos    Fold change relative to control (log2)
SENSE COUNTS:                 0      0      0      4      0      6.18e-03       6.98e-03              0.000  0.000  0.000  2.000  0.000
  GENES:
  AT5G02490.1
    SENSE COUNTS:             0      0      0      4      0      6.18e-03       7.16e-03              0.000  0.000  0.000  2.000  0.000
    TAGS:
      d+1      TCAGATTCAC     0      0      0      4      0      6.18e-03       1.26e-02       2228   0.000  0.000  0.000  2.000  0.000

LOCUS: AT5G20730
DESCRIPTION:  auxin-responsive factor (ARF7), identical to auxin response factor 7 GI
DATA:                         Control 30min  2hours 2days  1week  p-value        B&H            Pos    Fold change relative to control (log2)
SENSE COUNTS:                 0      0      5      1      0      6.25e-03       7.06e-03              0.000  0.000  2.322  0.000  0.000
  GENES:
  AT5G20730.3
    SENSE COUNTS:             0      0      1      0      0      4.55e-01       4.57e-01              0.000  0.000  0.000  0.000  0.000
    TAGS:
      i+3      ATGAATGTCA     0      0      1      0      0      4.55e-01       5.28e-01       2603   0.000  0.000  0.000  0.000  0.000
  AT5G20730.1
    SENSE COUNTS:             0      0      1      0      0      4.55e-01       4.57e-01              0.000  0.000  0.000  0.000  0.000
    TAGS:
      i+3      ATGAATGTCA     0      0      1      0      0      4.55e-01       5.28e-01       2603   0.000  0.000  0.000  0.000  0.000
  AT5G20730.2
    SENSE COUNTS:             0      0      5      1      0      6.25e-03       7.19e-03              0.000  0.000  2.322  0.000  0.000
```

```
    TAGS:
      d+1      AAATCAGCAA     0       0       4       1       0       2.60e-02        4.47e-02        4262    0.000   0.000   2.000   0.000   0.000
      i+3      ATGAATGTCA     0       0       1       0       0       4.55e-01        5.28e-01        2532    0.000   0.000   0.000   0.000   0.000

LOCUS: AT1G25275
DESCRIPTION:  Expressed protein
DATA:                         Control 30min   2hours  2days   1week   p-value         B&H             Pos     Fold change relative to control (log2)
SENSE COUNTS:                 12      28      10      13      12      6.25e-03        7.05e-03                0.000   1.222   -0.263  0.115   0.000
  GENES:
  AT1G25275.1
    SENSE COUNTS:             12      28      10      13      12      6.25e-03        7.20e-03                0.000   1.222   -0.263  0.115   0.000
    TAGS:
      d+1      CATTTGGATT     12      28      10      13      12      6.25e-03        1.25e-02        298     0.000   1.222   -0.263  0.115   0.000

LOCUS: AT3G51870
DESCRIPTION:  mitochondrial substrate carrier family protein, peroxisomal Ca-dependent solute carrier - Oryctolagus cuniculus, EMBL
DATA:                         Control 30min   2hours  2days   1week   p-value         B&H             Pos     Fold change relative to control (log2)
SENSE COUNTS:                 0       0       0       1       5       6.31e-03        7.11e-03                0.000   0.000   0.000   0.000   2.322
  GENES:
  AT3G51870.1
    SENSE COUNTS:             0       0       0       1       5       6.31e-03        7.24e-03                0.000   0.000   0.000   0.000   2.322
    TAGS:
      d+1      TTTGGGGAAC     0       0       0       1       5       6.31e-03        1.26e-02        1304    0.000   0.000   0.000   0.000   2.322

LOCUS: AT1G79150
DESCRIPTION:  expressed protein, ; expression supported by MPSS
DATA:                         Control 30min   2hours  2days   1week   p-value         B&H             Pos     Fold change relative to control (log2)
SENSE COUNTS:                 0       0       0       1       5       6.31e-03        7.10e-03                0.000   0.000   0.000   0.000   2.322
  GENES:
  AT1G79150.1
    SENSE COUNTS:             0       0       0       1       5       6.31e-03        7.24e-03                0.000   0.000   0.000   0.000   2.322
    TAGS:
      v+1      TATTTGAACT     0       0       0       1       0       3.09e-01        4.05e-01        2107    0.000   0.000   0.000   0.000   0.000
      v+2      TCCATTCGCT     0       0       0       0       5       6.24e-04        1.95e-03        1497    0.000   0.000   0.000   0.000   2.322

LOCUS: AT3G57800
DESCRIPTION:  basic helix-loop-helix (bHLH) family protein, contains Pfam profile
DATA:                         Control 30min   2hours  2days   1week   p-value         B&H             Pos     Fold change relative to control (log2)
SENSE COUNTS:                 0       0       0       1       5       6.31e-03        7.09e-03                0.000   0.000   0.000   0.000   2.322
  GENES:
  AT3G57800.1
    SENSE COUNTS:             0       0       0       1       5       6.31e-03        7.21e-03                0.000   0.000   0.000   0.000   2.322
    TAGS:
      d+1      AATGTGTATA     0       0       0       1       5       6.31e-03        1.26e-02        1597    0.000   0.000   0.000   0.000   2.322
  AT3G57800.2
    SENSE COUNTS:             0       0       0       1       5       6.31e-03        7.22e-03                0.000   0.000   0.000   0.000   2.322
    TAGS:
      d+1      AATGTGTATA     0       0       0       1       5       6.31e-03        1.26e-02        1565    0.000   0.000   0.000   0.000   2.322

LOCUS: AT5G09930
DESCRIPTION:  ABC transporter family protein,
DATA:                         Control 30min   2hours  2days   1week   p-value         B&H             Pos     Fold change relative to control (log2)
SENSE COUNTS:                 0       0       0       1       5       6.31e-03        7.08e-03                0.000   0.000   0.000   0.000   2.322
  GENES:
  AT5G09930.1
    SENSE COUNTS:             0       0       0       1       5       6.31e-03        7.25e-03                0.000   0.000   0.000   0.000   2.322
    TAGS:
      v+2      AAGGTATCAT     0       0       0       1       5       6.31e-03        1.26e-02        2525    0.000   0.000   0.000   0.000   2.322

LOCUS: AT4G21700
```

DESCRIPTION: hypothetical protein,

DATA:		Control	30min	2hours	2days	1week	p-value	B&H	Pos	Fold change relative to control (log2)				
SENSE COUNTS:		0	0	0	4	0	6.47e-03	7.25e-03		0.000	0.000	0.000	2.000	0.000
GENES:														
AT4G21700.1														
SENSE COUNTS:		0	0	0	4	0	6.47e-03	7.39e-03		0.000	0.000	0.000	2.000	0.000
TAGS:														
v+1	GACTTCTTGT	0	0	0	2	0	2.03e-01	2.83e-01	3660	0.000	0.000	0.000	1.000	0.000
v+2	AGATTTGTCA	0	0	0	1	0	3.09e-01	4.02e-01	1791	0.000	0.000	0.000	0.000	0.000
v+2	TAAAAGCTTC	0	0	0	1	0	3.09e-01	4.10e-01	324	0.000	0.000	0.000	0.000	0.000

LOCUS: AT4G13250
DESCRIPTION: short-chain dehydrogenase/reductase (SDR) family protein, contains INTERPRO family IPR002198 Short-chain dehydrogenase/reductase (SDR) superfamily

DATA:		Control	30min	2hours	2days	1week	p-value	B&H	Pos	Fold change relative to control (log2)				
SENSE COUNTS:		9	1	2	0	8	6.64e-03	7.43e-03		0.000	-3.170	-2.170	0.000	-0.170
GENES:														
AT4G13250.1														
SENSE COUNTS:		9	1	2	0	8	6.64e-03	7.57e-03		0.000	-3.170	-2.170	0.000	-0.170
TAGS:														
d+1	TAAACACTTC	9	1	1	0	8	2.65e-03	6.50e-03	1699	0.000	-3.170	-3.170	0.000	-0.170
d+2	GGTCTATAGT	0	0	1	0	0	4.55e-01	5.08e-01	1141	0.000	0.000	0.000	0.000	0.000

LOCUS: AT5G14120
DESCRIPTION: nodulin family protein, similar to nodulin-like protein (Arabidopsis thaliana) GI

DATA:		Control	30min	2hours	2days	1week	p-value	B&H	Pos	Fold change relative to control (log2)				
SENSE COUNTS:		12	15	5	5	0	6.70e-03	7.49e-03		0.000	0.322	-1.263	-1.263	0.000
GENES:														
AT5G14120.1														
SENSE COUNTS:		12	15	5	5	0	6.70e-03	7.63e-03		0.000	0.322	-1.263	-1.263	0.000
TAGS:														
d+1	AGTAGACTTA	12	12	5	4	0	2.02e-02	3.51e-02	2262	0.000	0.000	-1.263	-1.585	0.000
d+2	TTGAGTATGA	0	3	0	1	0	9.54e-02	1.51e-01	1998	0.000	1.585	0.000	0.000	0.000

LOCUS: AT1G07770
DESCRIPTION: 40S ribosomal protein S15A (RPS15aA), identical to GB

DATA:		Control	30min	2hours	2days	1week	p-value	B&H	Pos	Fold change relative to control (log2)				
SENSE COUNTS:		1	0	5	0	0	6.70e-03	7.48e-03		0.000	0.000	2.322	0.000	0.000
GENES:														
AT1G07770.1														
SENSE COUNTS:		1	0	5	0	0	6.70e-03	7.61e-03		0.000	0.000	2.322	0.000	0.000
TAGS:														
d+2	CAGAAGCACG	1	0	5	0	0	6.70e-03	1.32e-02	218	0.000	0.000	2.322	0.000	0.000
AT1G07770.2														
SENSE COUNTS:		1	0	5	0	0	6.70e-03	7.62e-03		0.000	0.000	2.322	0.000	0.000
TAGS:														
d+2	CAGAAGCACG	1	0	5	0	0	6.70e-03	1.32e-02	178	0.000	0.000	2.322	0.000	0.000

LOCUS: AT5G50730
DESCRIPTION: expressed protein, ; expression supported by MPSS

DATA:		Control	30min	2hours	2days	1week	p-value	B&H	Pos	Fold change relative to control (log2)				
SENSE COUNTS:		1	0	5	0	0	6.70e-03	7.47e-03		0.000	0.000	2.322	0.000	0.000
GENES:														
AT5G50730.1														
SENSE COUNTS:		1	0	5	0	0	6.70e-03	7.63e-03		0.000	0.000	2.322	0.000	0.000
TAGS:														
v+2	CCGCGGTTGT	1	0	5	0	0	6.70e-03	1.33e-02	475	0.000	0.000	2.322	0.000	0.000

LOCUS: AT4G02940

```
DESCRIPTION:  oxidoreductase, 2OG-Fe(II) oxygenase family protein, similar to A. thaliana hypothetical protein T13L16.2, GenBank accession number
2708738; contains Pfam domain PF03171 2OG-Fe(II) oxygenase superfamily
DATA:                        Control 30min  2hours  2days  1week  p-value     B&H         Pos    Fold change relative to control (log2)
SENSE COUNTS:                0       0      4       4      8      6.74e-03    7.51e-03           0.000  0.000  2.000  2.000  3.000
  GENES:
  AT4G02940.1
    SENSE COUNTS:            0       0      4       4      8      6.74e-03    7.65e-03           0.000  0.000  2.000  2.000  3.000
    TAGS:
      d+1     TGTTTTCTAC     0       0      4       4      8      6.74e-03    1.33e-02    2035   0.000  0.000  2.000  2.000  3.000

LOCUS: AT1G07080
DESCRIPTION:  gamma interferon responsive lysosomal thiol reductase family protein / GILT family protein, similar to SP|P13284 Gamma-interferon
inducible lysosomal thiol reductase precursor {Homo sapiens}; contains Pfam profile PF03227
DATA:                        Control 30min  2hours  2days  1week  p-value     B&H         Pos    Fold change relative to control (log2)
SENSE COUNTS:                13      2      12      16     21     6.81e-03    7.58e-03           0.000  -2.700 -0.115 0.300  0.692
  GENES:
  AT1G07080.1
    SENSE COUNTS:            13      2      12      16     21     6.81e-03    7.72e-03           0.000  -2.700 -0.115 0.300  0.692
    TAGS:
      d+1     TTGGGTAAAA     11      0      10      15     21     3.29e-04    1.15e-03    1000   0.000  0.000  -0.138 0.447  0.933
      d+2     TAACAAACCA     2       2      2       1      0      8.09e-01    8.12e-01    883    0.000  0.000  0.000  -1.000 0.000

LOCUS: AT5G10740
DESCRIPTION:  protein phosphatase 2C-related / PP2C-related, protein phosphatase 2C, alfalfa, PIR
DATA:                        Control 30min  2hours  2days  1week  p-value     B&H         Pos    Fold change relative to control (log2)
SENSE COUNTS:                0       0      1       2      7      6.90e-03    7.67e-03           0.000  0.000  0.000  1.000  2.807
  GENES:
  AT5G10740.1
    SENSE COUNTS:            0       0      1       2      7      6.90e-03    7.81e-03           0.000  0.000  0.000  1.000  2.807
    TAGS:
      d+1     ATAGTTTATT     0       0      1       2      7      6.90e-03    1.36e-02    1496   0.000  0.000  0.000  1.000  2.807

LOCUS: AT5G27230
DESCRIPTION:  expressed protein,  ; expression supported by MPSS
DATA:                        Control 30min  2hours  2days  1week  p-value     B&H         Pos    Fold change relative to control (log2)
SENSE COUNTS:                1       0      0       0      5      7.00e-03    7.77e-03           0.000  0.000  0.000  0.000  2.322
  GENES:
  AT5G27230.1
    SENSE COUNTS:            1       0      0       0      5      7.00e-03    7.90e-03           0.000  0.000  0.000  0.000  2.322
    TAGS:
      i+3     TAACACTTTT     1       0      0       0      5      7.00e-03    1.37e-02    1790   0.000  0.000  0.000  0.000  2.322

LOCUS: AT5G41810
DESCRIPTION:  expressed protein
DATA:                        Control 30min  2hours  2days  1week  p-value     B&H         Pos    Fold change relative to control (log2)
SENSE COUNTS:                1       0      0       0      5      7.00e-03    7.76e-03           0.000  0.000  0.000  0.000  2.322
  GENES:
  AT5G41810.1
    SENSE COUNTS:            1       0      0       0      5      7.00e-03    7.92e-03           0.000  0.000  0.000  0.000  2.322
    TAGS:
      d+1     TTATATGACT     1       0      0       0      5      7.00e-03    1.37e-02    1157   0.000  0.000  0.000  0.000  2.322
  AT5G41810.2
    SENSE COUNTS:            1       0      0       0      5      7.00e-03    7.91e-03           0.000  0.000  0.000  0.000  2.322
    TAGS:
      d+1     TTATATGACT     1       0      0       0      5      7.00e-03    1.37e-02    1135   0.000  0.000  0.000  0.000  2.322

LOCUS: AT3G08740
DESCRIPTION:  elongation factor P (EF-P) family protein, similar to SP|P33398 Elongation factor P (EF-P) {Escherichia coli O157
DATA:                        Control 30min  2hours  2days  1week  p-value     B&H         Pos    Fold change relative to control (log2)
SENSE COUNTS:                10      5      18      5      19     7.12e-03    7.88e-03           0.000  -1.000 0.848  -1.000 0.926
```

```
  GENES:
  AT3G08740.1
    SENSE COUNTS:             10     5      18     5      19     7.12e-03       8.03e-03                     0.000  -1.000  0.848  -1.000  0.926
    TAGS:
      d+1      TTTGTTTTTC     10     5      18     5      19     7.12e-03       1.39e-02       889    0.000  -1.000  0.848  -1.000  0.926

LOCUS: AT2G42460
DESCRIPTION:  meprin and TRAF homology domain-containing protein / MATH domain-containing protein, weak similarity to ubiquitin-specific protease 12
(Arabidopsis thaliana) GI
DATA:                         Control 30min  2hours 2days  1week  p-value        B&H            Pos    Fold change relative to control (log2)
SENSE COUNTS:                 17     1      8      8      12     7.14e-03       7.90e-03                     0.000  -4.087  -1.087  -1.087  -0.503
  GENES:
  AT2G42460.1
    SENSE COUNTS:             17     1      8      8      12     7.14e-03       8.04e-03                     0.000  -4.087  -1.087  -1.087  -0.503
    TAGS:
      v+1      TCTATTTCTA     17     1      8      8      12     7.14e-03       1.39e-02       2121   0.000  -4.087  -1.087  -1.087  -0.503

LOCUS: AT5G11010
DESCRIPTION:  pre-mRNA cleavage complex-related, low similarity to SP|Q92989 Pre-mRNA cleavage complex II protein Clp1 {Homo sapiens}
DATA:                         Control 30min  2hours 2days  1week  p-value        B&H            Pos    Fold change relative to control (log2)
SENSE COUNTS:                 0      0      1      0      4      7.15e-03       7.90e-03                     0.000  0.000  0.000  0.000  2.000
  GENES:
  AT5G11010.3
    SENSE COUNTS:             0      0      1      0      4      7.15e-03       8.02e-03                     0.000  0.000  0.000  0.000  2.000
    TAGS:
      d+1      TTTTTTTATA     0      0      0      0      3      1.12e-02       2.01e-02       1232   0.000  0.000  0.000  0.000  1.585
      X+4      AGAAAAGAGT     0      0      1      0      1      3.96e-01       5.01e-01       398    0.000  0.000  0.000  0.000  0.000
  AT5G11010.2
    SENSE COUNTS:             0      0      0      0      3      1.12e-02       1.14e-02                     0.000  0.000  0.000  0.000  1.585
    TAGS:
      d+1      TTTTTTTATA     0      0      0      0      3      1.12e-02       2.01e-02       1359   0.000  0.000  0.000  0.000  1.585
  AT5G11010.1
    SENSE COUNTS:             0      0      0      0      3      1.12e-02       1.14e-02                     0.000  0.000  0.000  0.000  1.585
    TAGS:
      d+1      TTTTTTTATA     0      0      0      0      3      1.12e-02       2.01e-02       1445   0.000  0.000  0.000  0.000  1.585

LOCUS: AT4G21060
DESCRIPTION:  galactosyltransferase family protein, contains Pfam profile
DATA:                         Control 30min  2hours 2days  1week  p-value        B&H            Pos    Fold change relative to control (log2)
SENSE COUNTS:                 0      0      1      0      5      7.15e-03       7.89e-03                     0.000  0.000  0.000  0.000  2.322
  GENES:
  AT4G21060.1
    SENSE COUNTS:             0      0      1      0      5      7.15e-03       7.99e-03                     0.000  0.000  0.000  0.000  2.322
    TAGS:
      v+2      GTTCAAACGG     0      0      1      0      0      4.55e-01       5.45e-01       1508   0.000  0.000  0.000  0.000  0.000
      i+3      TTATACACTT     0      0      0      0      5      6.24e-04       1.94e-03       406    0.000  0.000  0.000  0.000  2.322

LOCUS: AT3G09830
DESCRIPTION:  protein kinase, putative, similar to protein kinase (Lophopyrum elongatum) gi|13022177|gb|AAK11674

DATA:                         Control 30min  2hours 2days  1week  p-value        B&H            Pos    Fold change relative to control (log2)
SENSE COUNTS:                 0      0      1      0      5      7.15e-03       7.88e-03                     0.000  0.000  0.000  0.000  2.322
  GENES:
  AT3G09830.1
    SENSE COUNTS:             0      0      1      0      5      7.15e-03       8.04e-03                     0.000  0.000  0.000  0.000  2.322
    TAGS:
      d+1      GAGATTCCGA     0      0      1      0      5      7.15e-03       1.40e-02       1684   0.000  0.000  0.000  0.000  2.322
  AT3G09830.2
    SENSE COUNTS:             0      0      1      0      5      7.15e-03       8.04e-03                     0.000  0.000  0.000  0.000  2.322
    TAGS:
```

d+1 GAGATTCCGA 0 0 1 0 5 7.15e-03 1.40e-02 1617 0.000 0.000 0.000 0.000 2.322

LOCUS: AT1G69010
DESCRIPTION: basic helix-loop-helix (bHLH) family protein, contains Pfam profile
DATA: Control 30min 2hours 2days 1week p-value B&H Pos Fold change relative to control (log2)
SENSE COUNTS: 0 0 1 0 5 7.15e-03 7.87e-03 0.000 0.000 0.000 0.000 2.322
GENES:
AT1G69010.1
SENSE COUNTS: 0 0 1 0 5 7.15e-03 8.03e-03 0.000 0.000 0.000 0.000 2.322
TAGS:
d+1 TAACCTATCA 0 0 1 0 5 7.15e-03 1.39e-02 1208 0.000 0.000 0.000 0.000 2.322

LOCUS: AT3G17920
DESCRIPTION: leucine-rich repeat family protein, contains leucine rich repeat (LRR) domains, Pfam
DATA: Control 30min 2hours 2days 1week p-value B&H Pos Fold change relative to control (log2)
SENSE COUNTS: 0 0 1 0 4 7.15e-03 7.86e-03 0.000 0.000 0.000 0.000 2.000
GENES:
AT3G17920.1
SENSE COUNTS: 0 0 1 0 4 7.15e-03 8.01e-03 0.000 0.000 0.000 0.000 2.000
TAGS:
v+1 ACTTGAGACT 0 0 0 0 3 1.12e-02 2.01e-02 3696 0.000 0.000 0.000 0.000 1.585
v+2 GCTTCCACGG 0 0 0 0 1 1.65e-01 2.48e-01 2873 0.000 0.000 0.000 0.000 0.000
v+2 AAGAATAGAA 0 0 1 0 0 4.55e-01 5.47e-01 1716 0.000 0.000 0.000 0.000 0.000

LOCUS: AT4G12830
DESCRIPTION: hydrolase, alpha/beta fold family protein, low similarity to haloalkane dehalogenase from (Mycobacterium avium subsp. avium) GI
DATA: Control 30min 2hours 2days 1week p-value B&H Pos Fold change relative to control (log2)
SENSE COUNTS: 0 0 1 0 5 7.15e-03 7.85e-03 0.000 0.000 0.000 0.000 2.322
GENES:
AT4G12830.1
SENSE COUNTS: 0 0 1 0 5 7.15e-03 8.00e-03 0.000 0.000 0.000 0.000 2.322
TAGS:
d+1 TGCTTTCTAT 0 0 1 0 5 7.15e-03 1.39e-02 1481 0.000 0.000 0.000 0.000 2.322

LOCUS: AT3G23980
DESCRIPTION: dentin sialophosphoprotein-related, contains weak similarity to Dentin sialophosphoprotein precursor (Swiss-Prot
DATA: Control 30min 2hours 2days 1week p-value B&H Pos Fold change relative to control (log2)
SENSE COUNTS: 0 0 1 0 5 7.15e-03 7.84e-03 0.000 0.000 0.000 0.000 2.322
GENES:
AT3G23980.1
SENSE COUNTS: 0 0 1 0 5 7.15e-03 7.99e-03 0.000 0.000 0.000 0.000 2.322
TAGS:
d+1 AACTTGAAGA 0 0 1 0 5 7.15e-03 1.39e-02 2675 0.000 0.000 0.000 0.000 2.322

LOCUS: AT4G39940
DESCRIPTION: adenosine-5'-phosphosulfate-kinase (akn2) mRNA, complete
DATA: Control 30min 2hours 2days 1week p-value B&H Pos Fold change relative to control (log2)
SENSE COUNTS: 1 7 1 1 0 7.18e-03 7.86e-03 0.000 2.807 0.000 0.000 0.000
GENES:
AT4G39940.1
SENSE COUNTS: 1 7 1 1 0 7.18e-03 8.01e-03 0.000 2.807 0.000 0.000 0.000
TAGS:
d+1 TGTGCGAGTC 1 7 1 1 0 7.18e-03 1.39e-02 744 0.000 2.807 0.000 0.000 0.000

LOCUS: AT3G24503
DESCRIPTION: Arabidopsis thaliana aldehyde dehydrogenase AtALDH1a mRNA. a sinapaldehyde dehydrogenase
DATA: Control 30min 2hours 2days 1week p-value B&H Pos Fold change relative to control (log2)
SENSE COUNTS: 0 2 7 9 12 7.22e-03 7.90e-03 0.000 1.000 2.807 3.170 3.585
GENES:
AT3G24503.1

SENSE COUNTS: 0 2 7 9 12 7.22e-03 8.05e-03 0.000 1.000 2.807 3.170 3.585
TAGS:
d+1 GACGCTCTCG 0 2 7 9 12 7.22e-03 1.40e-02 1476 0.000 1.000 2.807 3.170 3.585

LOCUS: AT1G15270
DESCRIPTION: expressed protein, ESTs gb|AA650895, gb|AA720043 and gb|R29777 come from this gene
DATA: Control 30min 2hours 2days 1week p-value B&H Pos Fold change relative to control (log2)
SENSE COUNTS: 23 8 16 26 32 7.26e-03 7.93e-03 0.000 -1.524 -0.524 0.177 0.476
GENES:
AT1G15270.1
SENSE COUNTS: 23 8 16 26 32 7.26e-03 8.08e-03 0.000 -1.524 -0.524 0.177 0.476
TAGS:
d+1 AAAAAGAAAA 23 8 16 26 32 7.26e-03 1.41e-02 426 0.000 -1.524 -0.524 0.177 0.476

LOCUS: AT3G57560
DESCRIPTION: aspartate/glutamate/uridylate kinase family protein, similar to acetylglutamate kinase from Porphyra purpurea (SP|P31595), Campylobacter jejuni (GI
DATA: Control 30min 2hours 2days 1week p-value B&H Pos Fold change relative to control (log2)
SENSE COUNTS: 1 0 6 0 0 7.29e-03 7.96e-03 0.000 0.000 2.585 0.000 0.000
GENES:
AT3G57560.1
SENSE COUNTS: 1 0 6 0 0 7.29e-03 8.11e-03 0.000 0.000 2.585 0.000 0.000
TAGS:
d+1 AGATTATGTC 1 0 6 0 0 7.29e-03 1.41e-02 1106 0.000 0.000 2.585 0.000 0.000

LOCUS: AT1G27050
DESCRIPTION: homeobox-leucine zipper family protein, contains Pfam profile
DATA: Control 30min 2hours 2days 1week p-value B&H Pos Fold change relative to control (log2)
SENSE COUNTS: 0 0 5 0 0 7.30e-03 7.96e-03 0.000 0.000 2.322 0.000 0.000
GENES:
AT1G27050.1
SENSE COUNTS: 0 0 5 0 0 7.30e-03 8.11e-03 0.000 0.000 2.322 0.000 0.000
TAGS:
d+1 GGAGAAGCAG 0 0 5 0 0 7.30e-03 1.41e-02 1190 0.000 0.000 2.322 0.000 0.000

LOCUS: AT2G03820
DESCRIPTION: nonsense-mediated mRNA decay NMD3 family protein, contains Pfam profile
DATA: Control 30min 2hours 2days 1week p-value B&H Pos Fold change relative to control (log2)
SENSE COUNTS: 5 1 1 9 12 7.31e-03 7.96e-03 0.000 -2.322 -2.322 0.848 1.263
GENES:
AT2G03820.1
SENSE COUNTS: 5 1 1 9 12 7.31e-03 8.11e-03 0.000 -2.322 -2.322 0.848 1.263
TAGS:
d+1 GCTGCTGAAT 4 1 1 9 12 5.52e-03 1.17e-02 1638 0.000 -2.000 -2.000 1.170 1.585
d+2 TGTGTGAATT 1 0 0 0 0 4.28e-01 5.24e-01 204 0.000 0.000 0.000 0.000 0.000

LOCUS: AT1G25540
DESCRIPTION: phytochrome and flowering time regulatory protein (PFT1), PMID
DATA: Control 30min 2hours 2days 1week p-value B&H Pos Fold change relative to control (log2)
SENSE COUNTS: 0 0 0 2 7 7.34e-03 7.98e-03 0.000 0.000 0.000 1.000 2.807
GENES:
AT1G25540.1
SENSE COUNTS: 0 0 0 2 7 7.34e-03 8.14e-03 0.000 0.000 0.000 1.000 2.807
TAGS:
d+1 TCCTATTCTG 0 0 0 2 7 7.34e-03 1.41e-02 2871 0.000 0.000 0.000 1.000 2.807

LOCUS: AT3G04840
DESCRIPTION: 40S ribosomal protein S3A (RPS3aA), similar to 40S ribosomal protein S3A (S phase specific protein GBIS289) GB
DATA: Control 30min 2hours 2days 1week p-value B&H Pos Fold change relative to control (log2)
SENSE COUNTS: 18 5 20 16 26 7.40e-03 8.04e-03 0.000 -1.848 0.152 -0.170 0.531

```
  GENES:
  AT3G04840.1
    SENSE COUNTS:              18      5       20      16      26      7.40e-03        8.20e-03                0.000   -1.848  0.152   -0.170  0.531
    TAGS:
      d+1      GATGTTCACG      18      5       16      16      25      1.76e-02        3.08e-02        746     0.000   -1.848  -0.170  -0.170  0.474
      i+3      TACGGACTTT      0       0       1       0       0       4.55e-01        5.32e-01        647     0.000   0.000   0.000   0.000   0.000
      d+2      TGACCTTAAG      0       0       3       0       1       7.97e-02        1.29e-01        595     0.000   0.000   1.585   0.000   0.000

LOCUS: AT5G16620
DESCRIPTION:  hydroxyproline-rich glycoprotein family protein, contains proline rich extensin domains, INTERPRO
DATA:                          Control 30min   2hours  2days   1week   p-value         B&H             Pos     Fold change relative to control (log2)
SENSE COUNTS:                  4       0       0       1       8       7.44e-03        8.07e-03                0.000   0.000   0.000   -2.000  1.000
  GENES:
  AT5G16620.1
    SENSE COUNTS:              4       0       0       1       8       7.44e-03        8.22e-03                0.000   0.000   0.000   -2.000  1.000
    TAGS:
      d+1      TTAATCTTAA      4       0       0       1       8       7.44e-03        1.43e-02        1584    0.000   0.000   0.000   -2.000  1.000

LOCUS: AT3G12490
DESCRIPTION:  cysteine protease inhibitor, putative / cystatin, putative, similar to PRLI-interacting factor M (Arabidopsis thaliana) GI
DATA:                          Control 30min   2hours  2days   1week   p-value         B&H             Pos     Fold change relative to control (log2)
SENSE COUNTS:                  3       7       2       9       17      7.44e-03        8.06e-03                0.000   1.222   -0.585  1.585   2.503
  GENES:
  AT3G12490.2
    SENSE COUNTS:              3       7       2       9       17      7.44e-03        8.22e-03                0.000   1.222   -0.585  1.585   2.503
    TAGS:
      d+2      ACTAGTCCCT      2       7       2       8       16      1.07e-02        1.94e-02        690     0.000   1.807   0.000   2.000   3.000
      d+2      CTACTGAAGT      1       0       0       1       1       5.50e-01        5.99e-01        587     0.000   0.000   0.000   0.000   0.000
  AT3G12490.1
    SENSE COUNTS:              3       7       2       9       17      7.44e-03        8.23e-03                0.000   1.222   -0.585  1.585   2.503
    TAGS:
      d+2      ACTAGTCCCT      2       7       2       8       16      1.07e-02        1.94e-02        656     0.000   1.807   0.000   2.000   3.000
      d+2      CTACTGAAGT      1       0       0       1       1       5.50e-01        5.99e-01        553     0.000   0.000   0.000   0.000   0.000

LOCUS: AT5G50360
DESCRIPTION:  expressed protein
DATA:                          Control 30min   2hours  2days   1week   p-value         B&H             Pos     Fold change relative to control (log2)
SENSE COUNTS:                  0       0       0       4       5       7.47e-03        8.09e-03                0.000   0.000   0.000   2.000   2.322
  GENES:
  AT5G50360.1
    SENSE COUNTS:              0       0       0       4       5       7.47e-03        8.24e-03                0.000   0.000   0.000   2.000   2.322
    TAGS:
      d+1      CAAGTTAGAT      0       0       0       4       5       7.47e-03        1.43e-02        972     0.000   0.000   0.000   2.000   2.322

LOCUS: AT3G29240
DESCRIPTION:  expressed protein, similar to At1g33780 (Arabidopsis thaliana); contains Pfam profile PF02622
DATA:                          Control 30min   2hours  2days   1week   p-value         B&H             Pos     Fold change relative to control (log2)
SENSE COUNTS:                  16      4       6       6       0       7.49e-03        8.10e-03                0.000   -2.000  -1.415  -1.415  0.000
  GENES:
  AT3G29240.1
    SENSE COUNTS:              16      4       6       6       0       7.49e-03        8.25e-03                0.000   -2.000  -1.415  -1.415  0.000
    TAGS:
      d+1      GTCTTTGGGA      16      4       5       6       0       3.18e-03        7.58e-03        1060    0.000   -2.000  -1.678  -1.415  0.000
      d+2      GCAGGAACGT      0       0       1       0       0       4.55e-01        5.35e-01        723     0.000   0.000   0.000   0.000   0.000
      d+2      AGCCAGAGAC      0       0       0       0       0       6.15e-01        6.46e-01        550     0.000   0.000   0.000   0.000   0.000
  AT3G29240.2
    SENSE COUNTS:              16      4       6       6       0       7.49e-03        8.25e-03                0.000   -2.000  -1.415  -1.415  0.000
    TAGS:
      d+1      GTCTTTGGGA      16      4       5       6       0       3.18e-03        7.58e-03        1073    0.000   -2.000  -1.678  -1.415  0.000
      d+2      GCAGGAACGT      0       0       1       0       0       4.55e-01        5.35e-01        736     0.000   0.000   0.000   0.000   0.000
```

d+2 AGCCAGAGAC 0 0 0 0 0 6.15e-01 6.46e-01 563 0.000 0.000 0.000 0.000 0.000

LOCUS: AT3G18680
DESCRIPTION: aspartate/glutamate/uridylate kinase family protein, similar to UMP-kinase GB
DATA: Control 30min 2hours 2days 1week p-value B&H Pos Fold change relative to control (log2)
SENSE COUNTS: 4 2 3 15 7 7.50e-03 8.10e-03 0.000 -1.000 -0.415 1.907 0.807
GENES:
AT3G18680.1
SENSE COUNTS: 4 2 3 15 7 7.50e-03 8.25e-03 0.000 -1.000 -0.415 1.907 0.807
TAGS:
d+2 AAGCTCATTT 4 2 3 15 7 7.50e-03 1.44e-02 1291 0.000 -1.000 -0.415 1.907 0.807

LOCUS: AT5G14320
DESCRIPTION: 30S ribosomal protein S13, chloroplast (CS13), ribosomal protein S13 precursor, chloroplast Arabidopsis thaliana, PIR
DATA: Control 30min 2hours 2days 1week p-value B&H Pos Fold change relative to control (log2)
SENSE COUNTS: 8 11 10 4 25 7.51e-03 8.10e-03 0.000 0.459 0.322 -1.000 1.644
GENES:
AT5G14320.1
SENSE COUNTS: 8 11 10 4 25 7.51e-03 8.25e-03 0.000 0.459 0.322 -1.000 1.644
TAGS:
d+1 ATCTCCTTCT 8 11 10 4 25 7.51e-03 1.44e-02 647 0.000 0.459 0.322 -1.000 1.644

LOCUS: AT3G53260
DESCRIPTION: phenylalanine ammonia-lyase 2 (PAL2), nearly identical to SP|P45724
DATA: Control 30min 2hours 2days 1week p-value B&H Pos Fold change relative to control (log2)
SENSE COUNTS: 2 0 0 8 1 7.59e-03 8.18e-03 0.000 0.000 0.000 2.000 -1.000
GENES:
AT3G53260.1
SENSE COUNTS: 2 0 0 8 1 7.59e-03 8.33e-03 0.000 0.000 0.000 2.000 -1.000
TAGS:
d+1 TTCAATCAGC 2 0 0 8 1 7.59e-03 1.45e-02 1603 0.000 0.000 0.000 2.000 -1.000

LOCUS: AT1G19380
DESCRIPTION: expressed protein
DATA: Control 30min 2hours 2days 1week p-value B&H Pos Fold change relative to control (log2)
SENSE COUNTS: 1 11 10 1 3 7.60e-03 8.18e-03 0.000 3.459 3.322 0.000 1.585
GENES:
AT1G19380.1
SENSE COUNTS: 1 11 10 1 3 7.60e-03 8.33e-03 0.000 3.459 3.322 0.000 1.585
TAGS:
d+1 AATTGCTTCG 1 11 10 1 3 7.60e-03 1.45e-02 629 0.000 3.459 3.322 0.000 1.585

LOCUS: AT3G58370
DESCRIPTION: K-Cl Co-transporter type 1 protein-related / KCC1 protein-related, contains weak hit to Pfam profile PF03522
DATA: Control 30min 2hours 2days 1week p-value B&H Pos Fold change relative to control (log2)
SENSE COUNTS: 13 0 4 8 7 7.68e-03 8.25e-03 0.000 0.000 -1.700 -0.700 -0.893
GENES:
AT3G58370.1
SENSE COUNTS: 13 0 4 8 7 7.68e-03 8.41e-03 0.000 0.000 -1.700 -0.700 -0.893
TAGS:
v+1 GATTATACAA 13 0 4 8 7 7.68e-03 1.46e-02 1394 0.000 0.000 -1.700 -0.700 -0.893

LOCUS: AT3G61430
DESCRIPTION: plasma membrane intrinsic protein 1A (PIP1A) / aquaporin PIP1.1 (PIP1.1) (AQ1), identical to plasma membrane intrinsic protein 1A SP
DATA: Control 30min 2hours 2days 1week p-value B&H Pos Fold change relative to control (log2)
SENSE COUNTS: 24 9 13 5 16 7.69e-03 8.26e-03 0.000 -1.415 -0.885 -2.263 -0.585
GENES:
AT3G61430.1
SENSE COUNTS: 24 9 13 5 16 7.69e-03 8.41e-03 0.000 -1.415 -0.885 -2.263 -0.585
TAGS:

```
      d+1      TTTATTACAG     4       0       0       0       5       8.28e-03       1.56e-02       1229    0.000   0.000   0.000   0.000   0.322
      d+2      TATGTGTGCA     20      9       11      5       10      6.19e-02       1.01e-01       1126    0.000   -1.152  -0.862  -2.000  -1.000
      d+2      TGGTTGTCAT     0       0       1       0       0       4.55e-01       5.22e-01       1004    0.000   0.000   0.000   0.000   0.000
      d+2      TCCCTATTCT     0       0       1       0       0       4.55e-01       5.41e-01       803     0.000   0.000   0.000   0.000   0.000
      X+4      GATATTGGTA     0       0       0       0       1       1.65e-01       2.42e-01       309     0.000   0.000   0.000   0.000   0.000

LOCUS: AT3G55440
DESCRIPTION:  triosephosphate isomerase, cytosolic, putative, strong similarity to triosephosphate isomerase, cytosolic from Petunia hybrida
(SP|P48495), from Coptis japonica (SP|P21820)
DATA:                          Control 30min   2hours  2days   1week   p-value        B&H            Pos     Fold change relative to control (log2)
SENSE COUNTS:                  3       21      8       11      10      7.76e-03       8.32e-03               0.000   2.807   1.415   1.874   1.737
  GENES:
  AT3G55440.1
    SENSE COUNTS:              3       21      8       11      10      7.76e-03       8.48e-03               0.000   2.807   1.415   1.874   1.737
    TAGS:
      d+1      CTGTCAACCA     0       0       0       0       1       1.65e-01       2.40e-01       1245    0.000   0.000   0.000   0.000   0.000
      i+3      TCTATGAAAT     1       0       0       0       0       4.28e-01       5.26e-01       601     0.000   0.000   0.000   0.000   0.000
      d+2      GATGTTGTGG     2       21      8       11      8       2.32e-03       5.81e-03       524     0.000   3.392   2.000   2.459   2.000
      d+2      GGTTATCCTT     0       0       0       0       1       1.65e-01       2.41e-01       373     0.000   0.000   0.000   0.000   0.000

LOCUS: AT5G19350
DESCRIPTION:  RNA-binding protein 45 (RBP45), putative
DATA:                          Control 30min   2hours  2days   1week   p-value        B&H            Pos     Fold change relative to control (log2)
SENSE COUNTS:                  12      3       1       4       10      7.78e-03       8.33e-03               0.000   -2.000  -3.585  -1.585  -0.263
  GENES:
  AT5G19350.1
    SENSE COUNTS:              12      3       1       4       10      7.78e-03       8.49e-03               0.000   -2.000  -3.585  -1.585  -0.263
    TAGS:
      d+1      GTCAATGATA     12      3       1       4       10      7.78e-03       1.48e-02       1505    0.000   -2.000  -3.585  -1.585  -0.263

LOCUS: AT4G00130
DESCRIPTION:  hypothetical protein, contains Pfam profile PF04504
DATA:                          Control 30min   2hours  2days   1week   p-value        B&H            Pos     Fold change relative to control (log2)
SENSE COUNTS:                  0       0       1       6       7       7.83e-03       8.38e-03               0.000   0.000   0.000   2.585   2.807
  GENES:
  AT4G00130.1
    SENSE COUNTS:              0       0       1       6       7       7.83e-03       8.54e-03               0.000   0.000   0.000   2.585   2.807
    TAGS:
      v+1      AAAAAAACAA     0       0       1       6       7       7.83e-03       1.49e-02       1573    0.000   0.000   0.000   2.585   2.807

LOCUS: AT2G04842
DESCRIPTION:  threonyl-tRNA synthetase, putative / threonine--tRNA ligase, putative, similar to SP|P18256 Threonyl-tRNA synthetase 2 (EC 6.1.1.3)
(Threonine--tRNA ligase) (ThrRS) {Bacillus subtilis}; contains Pfam profiles PF00587
DATA:                          Control 30min   2hours  2days   1week   p-value        B&H            Pos     Fold change relative to control (log2)
SENSE COUNTS:                  13      0       4       7       8       7.85e-03       8.39e-03               0.000   0.000   -1.700  -0.893  -0.700
  GENES:
  AT2G04842.1
    SENSE COUNTS:              13      0       4       7       8       7.85e-03       8.55e-03               0.000   0.000   -1.700  -0.893  -0.700
    TAGS:
      v+2      ACCATCAAAA     1       0       1       2       1       5.52e-01       6.01e-01       2419    0.000   0.000   0.000   1.000   0.000
      v+2      TAAGTGTAGA     12      0       3       4       7       5.81e-03       1.21e-02       2312    0.000   0.000   -2.000  -1.585  -0.778
      v+2      TCGAGACCAC     0       0       0       1       0       3.09e-01       4.12e-01       937     0.000   0.000   0.000   0.000   0.000

LOCUS: AT2G19110
DESCRIPTION:  ATPase E1-E2 type family protein / haloacid dehalogenase-like hydrolase family protein / heavy-metal-associated domain-containing
protein, similar to cadmium efflux pump protein from Geobacillus stearothermophilus (GI
DATA:                          Control 30min   2hours  2days   1week   p-value        B&H            Pos     Fold change relative to control (log2)
SENSE COUNTS:                  0       0       0       0       3       7.96e-03       8.50e-03               0.000   0.000   0.000   0.000   1.585
  GENES:
  AT2G19110.1
```

```
    SENSE COUNTS:              0      0      0      0      3      7.96e-03       8.65e-03              0.000  0.000  0.000  0.000  1.585
    TAGS:
      d+2      AGAAGCCTGC     0      0      0      0      1      1.65e-01       2.35e-01       3373   0.000  0.000  0.000  0.000  0.000
      d+2      ACCTGAAAAT     0      0      0      0      0      6.15e-01       6.50e-01       2578   0.000  0.000  0.000  0.000  0.000
      d+2      TAAGAAGTCT     0      0      0      0      1      1.65e-01       2.34e-01       2552   0.000  0.000  0.000  0.000  0.000
      i+3      ATAATGATAA     0      0      0      0      1      1.65e-01       2.44e-01       473    0.000  0.000  0.000  0.000  0.000

LOCUS: AT2G17280
DESCRIPTION:  phosphoglycerate/bisphosphoglycerate mutase family protein, contains Pfam profile PF00300
DATA:                          Control 30min  2hours  2days  1week  p-value        B&H            Pos    Fold change relative to control (log2)
SENSE COUNTS:                  0      0      0      0      5      7.96e-03       8.49e-03              0.000  0.000  0.000  0.000  2.322
  GENES:
  AT2G17280.1
    SENSE COUNTS:              0      0      0      0      5      7.96e-03       8.63e-03              0.000  0.000  0.000  0.000  2.322
    TAGS:
      d+1      AATTTATTGT     0      0      0      0      5      7.96e-03       1.50e-02       1038   0.000  0.000  0.000  0.000  2.322

LOCUS: AT4G04800
DESCRIPTION:  methionine sulfoxide reductase domain-containing protein / SeIR domain-containing protein, low similarity to pilin-like transcription
factor (Homo sapiens) GI
DATA:                          Control 30min  2hours  2days  1week  p-value        B&H            Pos    Fold change relative to control (log2)
SENSE COUNTS:                  0      0      0      0      4      7.96e-03       8.48e-03              0.000  0.000  0.000  0.000  2.000
  GENES:
  AT4G04800.1
    SENSE COUNTS:              0      0      0      0      4      7.96e-03       8.64e-03              0.000  0.000  0.000  0.000  2.000
    TAGS:
      d+1      ACTATTTAAA     0      0      0      0      3      8.36e-02       1.34e-01       684    0.000  0.000  0.000  0.000  1.585
      X+4      TAAATTAACA     0      0      0      0      1      1.65e-01       2.41e-01       -62    0.000  0.000  0.000  0.000  0.000

LOCUS: AT4G26230
DESCRIPTION:  60S ribosomal protein L31 (RPL31B), ribosomal protein L31, Nicotiana glutinosa, U23784
DATA:                          Control 30min  2hours  2days  1week  p-value        B&H            Pos    Fold change relative to control (log2)
SENSE COUNTS:                  0      0      0      0      5      7.96e-03       8.47e-03              0.000  0.000  0.000  0.000  2.322
  GENES:
  AT4G26230.1
    SENSE COUNTS:              0      0      0      0      5      7.96e-03       8.66e-03              0.000  0.000  0.000  0.000  2.322
    TAGS:
      d+1      ATAATATCTG     0      0      0      0      5      7.96e-03       1.50e-02       640    0.000  0.000  0.000  0.000  2.322

LOCUS: AT5G66550
DESCRIPTION:  Maf family protein, contains Pfam domain PF02545
DATA:                          Control 30min  2hours  2days  1week  p-value        B&H            Pos    Fold change relative to control (log2)
SENSE COUNTS:                  0      0      0      0      5      7.96e-03       8.46e-03              0.000  0.000  0.000  0.000  2.322
  GENES:
  AT5G66550.1
    SENSE COUNTS:              0      0      0      0      5      7.96e-03       8.62e-03              0.000  0.000  0.000  0.000  2.322
    TAGS:
      d+1      TTTTTAAGTA     0      0      0      0      5      7.96e-03       1.51e-02       737    0.000  0.000  0.000  0.000  2.322

LOCUS: AT1G55150
DESCRIPTION:  DEAD box RNA helicase, putative (RH20), similar to ethylene-responsive RNA helicase GI
DATA:                          Control 30min  2hours  2days  1week  p-value        B&H            Pos    Fold change relative to control (log2)
SENSE COUNTS:                  0      0      0      0      5      7.96e-03       8.45e-03              0.000  0.000  0.000  0.000  2.322
  GENES:
  AT1G55150.1
    SENSE COUNTS:              0      0      0      0      5      7.96e-03       8.66e-03              0.000  0.000  0.000  0.000  2.322
    TAGS:
      d+1      TAAGATAACA     0      0      0      0      5      7.96e-03       1.51e-02       1777   0.000  0.000  0.000  0.000  2.322

LOCUS: AT3G13750
```

```
DESCRIPTION:  beta-galactosidase, putative / lactase, putative, similar to beta-galactosidase precursor SP
DATA:                        Control 30min   2hours  2days   1week   p-value         B&H             Pos     Fold change relative to control (log2)
SENSE COUNTS:                13      23      16      15      0       8.10e-03        8.59e-03                0.000   0.823   0.300   0.206   0.000
  GENES:
  AT3G13750.1
    SENSE COUNTS:            13      23      16      15      0       8.10e-03        8.76e-03                0.000   0.823   0.300   0.206   0.000
    TAGS:
      d+1      TGTAGATTCT    13      23      15      15      0       7.68e-03        1.46e-02        3053    0.000   0.823   0.206   0.206   0.000
      d+2      ATGAAAGCTG    0       0       1       0       0       4.55e-01        5.25e-01        719     0.000   0.000   0.000   0.000   0.000

LOCUS: AT5G23740
DESCRIPTION:  40S ribosomal protein S11 (RPS11C)
DATA:                        Control 30min   2hours  2days   1week   p-value         B&H             Pos     Fold change relative to control (log2)
SENSE COUNTS:                19      7       27      24      21      8.15e-03        8.63e-03                0.000   -1.441  0.507   0.337   0.144
  GENES:
  AT5G23740.1
    SENSE COUNTS:            19      7       27      24      21      8.15e-03        8.81e-03                0.000   -1.441  0.507   0.337   0.144
    TAGS:
      d+1      GGAGATTTTG    19      7       27      24      21      8.15e-03        1.54e-02        604     0.000   -1.441  0.507   0.337   0.144

LOCUS: AT1G14400
DESCRIPTION:  ubiquitin-conjugating enzyme 1 (UBC1), E2; identical to gi
DATA:                        Control 30min   2hours  2days   1week   p-value         B&H             Pos     Fold change relative to control (log2)
SENSE COUNTS:                8       9       1       9       18      8.15e-03        8.62e-03                0.000   0.170   -3.000  0.170   1.170
  GENES:
  AT1G14400.1
    SENSE COUNTS:            8       9       1       9       18      8.15e-03        8.80e-03                0.000   0.170   -3.000  0.170   1.170
    TAGS:
      d+1      CTTTGATGTA    8       9       1       9       17      1.91e-02        3.33e-02        800     0.000   0.170   -3.000  0.170   1.087
      d+2      TCTTGTGTTT    0       0       0       0       1       1.65e-01        2.40e-01        765     0.000   0.000   0.000   0.000   0.000
  AT1G14400.2
    SENSE COUNTS:            8       9       1       9       18      8.15e-03        8.79e-03                0.000   0.170   -3.000  0.170   1.170
    TAGS:
      d+1      CTTTGATGTA    8       9       1       9       17      1.91e-02        3.33e-02        671     0.000   0.170   -3.000  0.170   1.087
      d+2      TCTTGTGTTT    0       0       0       0       1       1.65e-01        2.40e-01        636     0.000   0.000   0.000   0.000   0.000

LOCUS: AT4G20440
DESCRIPTION:  small nuclear ribonucleoprotein associated protein B, putative / snRNP-B, putative / Sm protein B, putative, similar to SP|Q05856 Small
nuclear ribonucleoprotein associated protein B (snRNP-B) (Sm protein B) (Sm-B) (SmB) {Drosophila melanogaster}
DATA:                        Control 30min   2hours  2days   1week   p-value         B&H             Pos     Fold change relative to control (log2)
SENSE COUNTS:                6       0       1       2       9       8.16e-03        8.62e-03                0.000   0.000   -2.585  -1.585  0.585
  GENES:
  AT4G20440.2
    SENSE COUNTS:            6       0       1       2       9       8.16e-03        8.79e-03                0.000   0.000   -2.585  -1.585  0.585
    TAGS:
      d+1      TTGCCTTTCA    6       0       1       0       8       3.81e-03        8.78e-03        934     0.000   0.000   -2.585  0.000   0.415
      d+2      CCACCACATA    0       0       0       2       1       1.24e-01        1.89e-01        883     0.000   0.000   0.000   1.000   0.000
  AT4G20440.1
    SENSE COUNTS:            6       0       1       2       9       8.16e-03        8.78e-03                0.000   0.000   -2.585  -1.585  0.585
    TAGS:
      d+1      TTGCCTTTCA    6       0       1       0       8       3.81e-03        8.78e-03        1042    0.000   0.000   -2.585  0.000   0.415
      d+2      CCACCACATA    0       0       0       2       1       1.24e-01        1.89e-01        887     0.000   0.000   0.000   1.000   0.000

LOCUS: AT2G46820
DESCRIPTION:  expressed protein
DATA:                        Control 30min   2hours  2days   1week   p-value         B&H             Pos     Fold change relative to control (log2)
SENSE COUNTS:                46      25      23      29      16      8.28e-03        8.74e-03                0.000   -0.880  -1.000  -0.666  -1.524
  GENES:
  AT2G46820.1
    SENSE COUNTS:            46      25      23      29      16      8.28e-03        8.90e-03                0.000   -0.880  -1.000  -0.666  -1.524
```

```
    TAGS:
      d+1      AATTGGAATG     46      25      22      29      16      6.60e-03       1.31e-02       711     0.000   -0.880  -1.064  -0.666  -1.524
      X+4      GTTAGCTTAG     0       0       1       0       0       4.55e-01       5.26e-01       350     0.000   0.000   0.000   0.000   0.000

LOCUS: AT2G46830
DESCRIPTION:  myb-related transcription factor (CCA1), identical to myb-related transcription factor (CCA1) GI
DATA:                         Control 30min   2hours  2days   1week   p-value        B&H            Pos     Fold change relative to control (log2)
SENSE COUNTS:                 5       0       3       11      12      8.28e-03       8.73e-03               0.000   0.000   -0.737  1.138   1.263
  GENES:
  AT2G46830.1
    SENSE COUNTS:             5       0       3       11      12      8.28e-03       8.90e-03               0.000   0.000   -0.737  1.138   1.263
    TAGS:
      d+1      AGACTCTATT     5       0       3       11      12      2.77e-03       6.77e-03       2064    0.000   0.000   -0.737  1.138   1.263
      d+2      AGCAGCCTTC     0       0       0       0       0       6.15e-01       6.36e-01       1430    0.000   0.000   0.000   0.000   0.000
  AT2G46830.2
    SENSE COUNTS:             5       0       3       11      12      8.28e-03       8.89e-03               0.000   0.000   -0.737  1.138   1.263
    TAGS:
      d+1      AGACTCTATT     5       0       3       11      12      2.77e-03       6.77e-03       2478    0.000   0.000   -0.737  1.138   1.263
      d+2      AGCAGCCTTC     0       0       0       0       0       6.15e-01       6.36e-01       1844    0.000   0.000   0.000   0.000   0.000

LOCUS: AT5G20180
DESCRIPTION:  ribosomal protein L36 family protein, contains Pfam profile
DATA:                         Control 30min   2hours  2days   1week   p-value        B&H            Pos     Fold change relative to control (log2)
SENSE COUNTS:                 4       0       4       5       14      8.35e-03       8.79e-03               0.000   0.000   0.000   0.322   1.807
  GENES:
  AT5G20180.2
    SENSE COUNTS:             4       0       4       5       14      8.35e-03       8.94e-03               0.000   0.000   0.000   0.322   1.807
    TAGS:
      d+1      ACCTGAACAA     4       0       4       5       14      8.35e-03       1.57e-02       574     0.000   0.000   0.000   0.322   1.807
  AT5G20180.1
    SENSE COUNTS:             4       0       4       5       14      8.35e-03       8.95e-03               0.000   0.000   0.000   0.322   1.807
    TAGS:
      d+1      ACCTGAACAA     4       0       4       5       14      8.35e-03       1.57e-02       499     0.000   0.000   0.000   0.322   1.807

LOCUS: AT1G04420
DESCRIPTION:  aldo/keto reductase family protein, Similar to SP|Q46933 Tas protein {Escherichia coli}, Babesia aldo-keto reductase SP|P40690; contains
Pfam profile PF00248
DATA:                         Control 30min   2hours  2days   1week   p-value        B&H            Pos     Fold change relative to control (log2)
SENSE COUNTS:                 8       0       3       2       12      8.39e-03       8.82e-03               0.000   0.000   -1.415  -2.000  0.585
  GENES:
  AT1G04420.1
    SENSE COUNTS:             8       0       3       2       12      8.39e-03       8.98e-03               0.000   0.000   -1.415  -2.000  0.585
    TAGS:
      d+1      TAATGACGTA     8       0       3       2       12      8.39e-03       1.57e-02       1383    0.000   0.000   -1.415  -2.000  0.585

LOCUS: AT5G59320
DESCRIPTION:  lipid transfer protein 3 (LTP3), identical to lipid transfer protein 3 from Arabidopsis thaliana (gi
DATA:                         Control 30min   2hours  2days   1week   p-value        B&H            Pos     Fold change relative to control (log2)
SENSE COUNTS:                 4       0       3       9       10      8.44e-03       8.86e-03               0.000   0.000   -0.415  1.170   1.322
  GENES:
  AT5G59320.1
    SENSE COUNTS:             4       0       3       9       10      8.44e-03       9.02e-03               0.000   0.000   -0.415  1.170   1.322
    TAGS:
      d+1      TATTTGTTTT     4       0       3       9       10      8.44e-03       1.58e-02       525     0.000   0.000   -0.415  1.170   1.322

LOCUS: AT1G20110
DESCRIPTION:  zinc finger (FYVE type) family protein, contains Pfam profile
DATA:                         Control 30min   2hours  2days   1week   p-value        B&H            Pos     Fold change relative to control (log2)
SENSE COUNTS:                 4       2       12      1       2       8.65e-03       9.07e-03               0.000   -1.000  1.585   -2.000  -1.000
  GENES:
```

```
  AT1G20110.1
    SENSE COUNTS:            4      2      12     1      2      8.65e-03       9.24e-03                 0.000   -1.000  1.585   -2.000  -1.000
    TAGS:
      d+1      TAGTCTGTTG    2      1      4      0      1      4.21e-01       5.29e-01       2060      0.000   -1.000  1.000   0.000   -1.000
      d+2      TGGATGGTGT    2      1      7      1      1      8.52e-02       1.36e-01       1992      0.000   -1.000  1.807   -1.000  -1.000
      d+2      CACTGTGCAC    0      0      1      0      0      4.55e-01       5.29e-01       1782      0.000   0.000   0.000   0.000   0.000

LOCUS: AT3G18050
DESCRIPTION:  expressed protein
DATA:                        Control 30min  2hours 2days  1week  p-value        B&H            Pos       Fold change relative to control (log2)
SENSE COUNTS:                9      7      1      0      1      8.75e-03       9.17e-03                 0.000   -0.363  -3.170  0.000   -3.170
  GENES:
  AT3G18050.1
    SENSE COUNTS:            9      7      1      0      1      8.75e-03       9.33e-03                 0.000   -0.363  -3.170  0.000   -3.170
    TAGS:
      d+1      TTCTTTAACA    9      7      1      0      1      8.75e-03       1.64e-02       1238      0.000   -0.363  -3.170  0.000   -3.170

LOCUS: AT4G12290
DESCRIPTION:  copper amine oxidase, putative, similar to copper amine oxidase (Cicer arietinum) gi|3819099|emb|CAA08855
DATA:                        Control 30min  2hours 2days  1week  p-value        B&H            Pos       Fold change relative to control (log2)
SENSE COUNTS:                1      3      14     5      5      8.81e-03       9.22e-03                 0.000   1.585   3.807   2.322   2.322
  GENES:
  AT4G12290.1
    SENSE COUNTS:            1      3      14     5      5      8.81e-03       9.39e-03                 0.000   1.585   3.807   2.322   2.322
    TAGS:
      d+1      CGGATTATGG    1      2      12     1      5      2.43e-03       6.04e-03       2411      0.000   1.000   3.585   0.000   2.322
      d+2      ATCTCCCGGT    0      1      0      2      0      2.43e-01       3.35e-01       2218      0.000   0.000   0.000   1.000   0.000
      d+2      GACCAAAAAA    0      0      2      2      0      1.68e-01       2.38e-01       32        0.000   0.000   1.000   1.000   0.000

LOCUS: AT3G04210
DESCRIPTION:  disease resistance protein (TIR-NBS class), putative, domain signature TIR-NBS exists, suggestive of a disease resistance protein.
DATA:                        Control 30min  2hours 2days  1week  p-value        B&H            Pos       Fold change relative to control (log2)
SENSE COUNTS:                1      5      10     1      0      8.82e-03       9.22e-03                 0.000   2.322   3.322   0.000   0.000
  GENES:
  AT3G04210.1
    SENSE COUNTS:            1      5      10     1      0      8.82e-03       9.39e-03                 0.000   2.322   3.322   0.000   0.000
    TAGS:
      d+1      TAGAACCAAC    1      5      10     1      0      8.82e-03       1.65e-02       1850      0.000   2.322   3.322   0.000   0.000

LOCUS: AT4G26370
DESCRIPTION:  antitermination NusB domain-containing protein, contains Pfam profile
DATA:                        Control 30min  2hours 2days  1week  p-value        B&H            Pos       Fold change relative to control (log2)
SENSE COUNTS:                4      0      2      3      11     8.83e-03       9.22e-03                 0.000   0.000   -1.000  -0.415  1.459
  GENES:
  AT4G26370.1
    SENSE COUNTS:            4      0      2      3      11     8.83e-03       9.39e-03                 0.000   0.000   -1.000  -0.415  1.459
    TAGS:
      d+1      TTTCTGTCTT    0      0      2      2      10     1.42e-03       3.84e-03       1261      0.000   0.000   1.000   1.000   3.322
      d+2      TCTGTATTAA    1      0      0      0      0      4.28e-01       5.37e-01       1206      0.000   0.000   0.000   0.000   0.000
      d+2      AGCTTTGGAG    3      0      0      1      1      1.73e-01       2.44e-01       519       0.000   0.000   0.000   -1.585  -1.585
  AT4G26370.2
    SENSE COUNTS:            4      0      2      3      11     8.83e-03       9.38e-03                 0.000   0.000   -1.000  -0.415  1.459
    TAGS:
      d+1      TTTCTGTCTT    0      0      2      2      10     1.42e-03       3.84e-03       1444      0.000   0.000   1.000   1.000   3.322
      d+2      TCTGTATTAA    1      0      0      0      0      4.28e-01       5.37e-01       1389      0.000   0.000   0.000   0.000   0.000
      d+2      AGCTTTGGAG    3      0      0      1      1      1.73e-01       2.44e-01       464       0.000   0.000   0.000   -1.585  -1.585

LOCUS: AT3G08520
DESCRIPTION:  60S ribosomal protein L41 (RPL41D)
DATA:                        Control 30min  2hours 2days  1week  p-value        B&H            Pos       Fold change relative to control (log2)
```

```
SENSE COUNTS:                4      3      1      13     7      8.95e-03      9.34e-03             0.000  -0.415  -2.000  1.700   0.807
  GENES:
  AT3G08520.1
    SENSE COUNTS:            4      3      1      13     7      8.95e-03      9.50e-03             0.000  -0.415  -2.000  1.700   0.807
    TAGS:
      v+2      AGAGCCAAGT    4      3      1      13     7      8.95e-03      1.67e-02      354    0.000  -0.415  -2.000  1.700   0.807

LOCUS: AT2G29670
DESCRIPTION:  expressed protein
DATA:                        Control 30min 2hours 2days  1week  p-value       B&H           Pos    Fold change relative to control (log2)
SENSE COUNTS:                11     12     11     0      1      9.04e-03      9.42e-03             0.000  0.126   0.000   0.000   -3.459
  GENES:
  AT2G29670.1
    SENSE COUNTS:            11     12     11     0      1      9.04e-03      9.59e-03             0.000  0.126   0.000   0.000   -3.459
    TAGS:
      d+1      TTTGTCACCA    9      11     11     0      1      1.87e-02      3.26e-02      1909   0.000  0.290   0.290   0.000   -3.170
      d+2      AAGACACCAT    1      1      0      0      0      4.77e-01      5.28e-01      1785   0.000  0.000   0.000   0.000   0.000
      d+2      TAGAGGCTCG    1      0      0      0      0      4.28e-01      5.26e-01      1401   0.000  0.000   0.000   0.000   0.000

LOCUS: AT5G52300
DESCRIPTION:  low-temperature-responsive 65 kD protein (LTI65) / desiccation-responsive protein 29B (RD29B), nearly identical to SP|Q04980 Low-
temperature-induced 65 kDa protein (Desiccation-responsive protein 29B) {Arabidopsis thaliana}
DATA:                        Control 30min 2hours 2days  1week  p-value       B&H           Pos    Fold change relative to control (log2)
SENSE COUNTS:                1      0      2      0      5      9.07e-03      9.44e-03             0.000  0.000   1.000   0.000   2.322
  GENES:
  AT5G52300.1
    SENSE COUNTS:            1      0      2      0      5      9.07e-03      9.61e-03             0.000  0.000   1.000   0.000   2.322
    TAGS:
      d+1      GTGAGGCGGT    1      0      1      0      1      6.15e-01      6.46e-01      1785   0.000  0.000   0.000   0.000   0.000
      d+2      AGACAAAACC    0      0      1      0      0      4.55e-01      5.39e-01      1275   0.000  0.000   0.000   0.000   0.000
      d+2      AGGCACTGGC    0      0      0      0      1      1.65e-01      2.48e-01      579    0.000  0.000   0.000   0.000   0.000
      d+2      AGAAGGGAGC    0      0      0      0      3      1.12e-02      2.02e-02      237    0.000  0.000   0.000   0.000   1.585

LOCUS: AT1G01470
DESCRIPTION:  late embryogenesis abundant protein, putative / LEA protein, putative, similar to SP|P46518 Late embryogenesis abundant protein Lea14-A
{Gossypium hirsutum}; contains Pfam profile PF03168
DATA:                        Control 30min 2hours 2days  1week  p-value       B&H           Pos    Fold change relative to control (log2)
SENSE COUNTS:                0      1      2      9      5      9.16e-03      9.52e-03             0.000  0.000   1.000   3.170   2.322
  GENES:
  AT1G01470.1
    SENSE COUNTS:            0      1      2      9      5      9.16e-03      9.69e-03             0.000  0.000   1.000   3.170   2.322
    TAGS:
      d+1      ACGGCTCTTG    0      1      2      9      5      9.16e-03      1.71e-02      359    0.000  0.000   1.000   3.170   2.322

LOCUS: AT1G76490
DESCRIPTION:  3-hydroxy-3-methylglutaryl-CoA reductase 1 / HMG-CoA reductase 1 (HMG1), identical to HMG-CoA reductase 1 (SP|P14891)
DATA:                        Control 30min 2hours 2days  1week  p-value       B&H           Pos    Fold change relative to control (log2)
SENSE COUNTS:                19     22     34     21     5      9.21e-03      9.56e-03             0.000  0.212   0.840   0.144   -1.926
  GENES:
  AT1G76490.1
    SENSE COUNTS:            19     22     34     21     5      9.21e-03      9.74e-03             0.000  0.212   0.840   0.144   -1.926
    TAGS:
      d+1      GATAATTCGT    15     22     30     19     5      1.66e-02      2.91e-02      2107   0.000  0.553   1.000   0.341   -1.585
      d+2      TGCGTATGCG    4      0      4      2      0      1.11e-01      1.74e-01      1963   0.000  0.000   0.000   -1.000  0.000

LOCUS: AT3G09250
DESCRIPTION:  expressed protein
DATA:                        Control 30min 2hours 2days  1week  p-value       B&H           Pos    Fold change relative to control (log2)
SENSE COUNTS:                22     10     15     6      2      9.21e-03      9.55e-03             0.000  -1.138  -0.553  -1.874  -3.459
  GENES:
```

```
  AT3G09250.1
    SENSE COUNTS:              22     10     15     6      2      9.21e-03       9.73e-03              0.000  -1.138  -0.553  -1.874  -3.459
    TAGS:
      d+1      AATCTTGGTC      6      3      2      0      1      1.40e-01       2.13e-01       1121   0.000  -1.000  -1.585  0.000   -2.585
      d+2      TAGACAATGA      16     7      13     6      1      5.09e-02       8.36e-02       1063   0.000  -1.193  -0.300  -1.415  -4.000

LOCUS: AT4G16260
DESCRIPTION:  glycosyl hydrolase family 17 protein, similar to glucan endo-1,3-beta-glucosidase, basic vacuolar isoform precursor SP
DATA:                          Control 30min  2hours 2days  1week  p-value        B&H            Pos    Fold change relative to control (log2)
SENSE COUNTS:                  1      7      3      0      0      9.27e-03       9.60e-03              0.000  2.807   1.585   0.000   0.000
  GENES:
  AT4G16260.1
    SENSE COUNTS:              1      7      3      0      0      9.27e-03       9.78e-03              0.000  2.807   1.585   0.000   0.000
    TAGS:
      d+2      CGTAACGTTT      0      7      1      0      0      4.60e-04       1.54e-03       478    0.000  2.807   0.000   0.000   0.000
      i+3      ATTTATTCTC      1      0      2      0      0      3.07e-01       4.12e-01       177    0.000  0.000   1.000   0.000   0.000

LOCUS: AT5G07150
DESCRIPTION:  leucine-rich repeat family protein, contains weak similarity to LRR receptor-like protein kinase (Nicotiana tabacum)
gi|7672732|gb|AAF66615; contains Pfam PF00560 domain Leucine Rich Repeat
DATA:                          Control 30min  2hours 2days  1week  p-value        B&H            Pos    Fold change relative to control (log2)
SENSE COUNTS:                  2      0      0      0      7      9.31e-03       9.63e-03              0.000  0.000   0.000   0.000   1.807
  GENES:
  AT5G07150.1
    SENSE COUNTS:              2      0      0      0      7      9.31e-03       9.81e-03              0.000  0.000   0.000   0.000   1.807
    TAGS:
      v+1      TTTATTCCTT      2      0      0      0      7      9.31e-03       1.73e-02       2426   0.000  0.000   0.000   0.000   1.807

LOCUS: AT5G12890
DESCRIPTION:  UDP-glucoronosyl/UDP-glucosyl transferase family protein, contains Pfam profile
DATA:                          Control 30min  2hours 2days  1week  p-value        B&H            Pos    Fold change relative to control (log2)
SENSE COUNTS:                  8      8      16     15     28     9.38e-03       9.70e-03              0.000  0.000   1.000   0.907   1.807
  GENES:
  AT5G12890.1
    SENSE COUNTS:              8      8      16     15     28     9.38e-03       9.88e-03              0.000  0.000   1.000   0.907   1.807
    TAGS:
      d+1      TCCGGTAATT      8      8      16     15     28     9.38e-03       1.74e-02       1637   0.000  0.000   1.000   0.907   1.807

LOCUS: AT5G58470
DESCRIPTION:  zinc finger (Ran-binding) family protein, weak similarity to SP|Q01844 RNA-binding protein EWS (EWS oncogene) (Ewing sarcoma breakpoint
region 1 protein) {Homo sapiens}; contains Pfam profiles PF00076
DATA:                          Control 30min  2hours 2days  1week  p-value        B&H            Pos    Fold change relative to control (log2)
SENSE COUNTS:                  3      0      1      8      1      9.46e-03       9.77e-03              0.000  0.000   -1.585  1.415   -1.585
  GENES:
  AT5G58470.2
    SENSE COUNTS:              3      0      1      8      1      9.46e-03       9.95e-03              0.000  0.000   -1.585  1.415   -1.585
    TAGS:
      d+2      ACCTTTTGGT      0      0      0      1      0      3.09e-01       4.06e-01       2142   0.000  0.000   0.000   0.000   0.000
      d+2      CATCTCCGGT      3      0      1      5      1      1.12e-01       1.75e-01       1541   0.000  0.000   -1.585  0.737   -1.585
      d+2      GGGGAAACCG      0      0      0      2      0      2.03e-01       2.83e-01       1310   0.000  0.000   0.000   1.000   0.000
  AT5G58470.1
    SENSE COUNTS:              0      0      0      3      0      4.12e-02       4.18e-02              0.000  0.000   0.000   1.585   0.000
    TAGS:
      d+2      ACCTTTTGGT      0      0      0      1      0      3.09e-01       4.06e-01       1990   0.000  0.000   0.000   0.000   0.000
      d+2      GGGGAAACCG      0      0      0      2      0      2.03e-01       2.83e-01       1310   0.000  0.000   0.000   1.000   0.000

LOCUS: AT5G48620
DESCRIPTION:  disease resistance protein (CC-NBS-LRR class), putative, domain signature CC-NBS-LRR exists, suggestive of a disease resistance protein.
DATA:                          Control 30min  2hours 2days  1week  p-value        B&H            Pos    Fold change relative to control (log2)
SENSE COUNTS:                  6      7      16     5      0      9.54e-03       9.84e-03              0.000  0.222   1.415   -0.263  0.000
```

```
  GENES:
  AT5G48620.1
    SENSE COUNTS:             6      7      16     5      0      9.54e-03      1.00e-02             0.000  0.222  1.415  -0.263 0.000
    TAGS:
      d+1      GAACTTGCTT     6      7      14     5      0      3.21e-02      5.40e-02      3241   0.000  0.222  1.222  -0.263 0.000
      d+2      TGGACAGTCT     0      0      1      0      0      7.06e-01      7.14e-01      2936   0.000  0.000  0.000  0.000  0.000
      d+2      ATTGCGAAAA     0      0      1      0      0      4.55e-01      5.19e-01      2689   0.000  0.000  0.000  0.000  0.000

LOCUS: AT4G20850
DESCRIPTION:  subtilase family protein, contains similarity to Tripeptidyl-peptidase II (EC 3.4.14.10) (TPP-II) (Tripeptidyl aminopeptidase) (Swiss-
Prot
DATA:                         Control 30min  2hours  2days  1week  p-value       B&H           Pos    Fold change relative to control (log2)
SENSE COUNTS:                 0      8      3      2      1      9.56e-03      9.85e-03             0.000  3.000  1.585  1.000  0.000
  GENES:
  AT4G20850.1
    SENSE COUNTS:             0      8      3      2      1      9.56e-03      1.00e-02             0.000  3.000  1.585  1.000  0.000
    TAGS:
      i+3      AAAGCTGAAT     0      0      0      0      1      1.65e-01      2.39e-01      8015   0.000  0.000  0.000  0.000  0.000
      d+1      GCGAGAATCG     0      0      3      2      0      2.44e-01      3.35e-01      3802   0.000  0.000  1.585  1.000  0.000
      d+2      AAAATGTGGA     0      8      0      0      0      2.30e-05      1.13e-04      3041   0.000  3.000  0.000  0.000  0.000

LOCUS: AT4G03260
DESCRIPTION:  leucine-rich repeat family protein, contains leucine rich repeat (LRR) domains, Pfam
DATA:                         Control 30min  2hours  2days  1week  p-value       B&H           Pos    Fold change relative to control (log2)
SENSE COUNTS:                 6      1      1      1      10     9.71e-03      9.99e-03             0.000  -2.585 -2.585 -2.585 0.737
  GENES:
  AT4G03260.1
    SENSE COUNTS:             6      1      1      1      10     9.71e-03      1.02e-02             0.000  -2.585 -2.585 -2.585 0.737
    TAGS:
      d+1      GTATACATTC     6      1      1      1      10     9.71e-03      1.80e-02      2797   0.000  -2.585 -2.585 -2.585 0.737

LOCUS: AT5G57480
DESCRIPTION:  AAA-type ATPase family protein, contains Pfam profile
DATA:                         Control 30min  2hours  2days  1week  p-value       B&H           Pos    Fold change relative to control (log2)
SENSE COUNTS:                 0      4      0      0      0      9.72e-03      9.99e-03             0.000  2.000  0.000  0.000  0.000
  GENES:
  AT5G57480.1
    SENSE COUNTS:             0      4      0      0      0      9.72e-03      1.01e-02             0.000  2.000  0.000  0.000  0.000
    TAGS:
      v+2      AGTTTTTGTA     0      4      0      0      0      9.72e-03      1.78e-02      1530   0.000  2.000  0.000  0.000  0.000

LOCUS: AT1G26270
DESCRIPTION:  phosphatidylinositol 3- and 4-kinase family protein, similar to phosphatidylinositol 4-kinase type-II beta (Homo sapiens) GI
DATA:                         Control 30min  2hours  2days  1week  p-value       B&H           Pos    Fold change relative to control (log2)
SENSE COUNTS:                 0      4      0      0      0      9.72e-03      9.98e-03             0.000  2.000  0.000  0.000  0.000
  GENES:
  AT1G26270.1
    SENSE COUNTS:             0      4      0      0      0      9.72e-03      1.01e-02             0.000  2.000  0.000  0.000  0.000
    TAGS:
      d+1      GTCTTTGCTT     0      4      0      0      0      9.72e-03      1.77e-02      1442   0.000  2.000  0.000  0.000  0.000

LOCUS: AT5G19220
DESCRIPTION:  Biochemical studies of adg2-1 mutant indicate that mutant plants do not accumulate ADP glucose pyrophosphorylase (ADGase)large subunit
protein and that ADGase small subunits assemble as homotetramers. ADG2 gene may encode the large subunit of ADGase.
DATA:                         Control 30min  2hours  2days  1week  p-value       B&H           Pos    Fold change relative to control (log2)
SENSE COUNTS:                 0      3      0      0      0      9.72e-03      9.97e-03             0.000  1.585  0.000  0.000  0.000
  GENES:
  AT5G19220.1
    SENSE COUNTS:             0      3      0      0      0      9.72e-03      1.00e-02             0.000  1.585  0.000  0.000  0.000
    TAGS:
```

```
      i+3      ATATTTCCTT     0      2      0      0      0      9.14e-02       1.46e-01       2156   0.000  1.000  0.000  0.000  0.000
      i+3      GTAGACGTGC     0      1      0      0      0      2.54e-01       3.46e-01       1326   0.000  0.000  0.000  0.000  0.000

LOCUS: AT5G13140
DESCRIPTION:  expressed protein

DATA:                         Control 30min  2hours  2days  1week  p-value        B&H            Pos    Fold change relative to control (log2)
SENSE COUNTS:                 0      4      0      0      0      9.72e-03       9.96e-03              0.000  2.000  0.000  0.000  0.000
  GENES:
  AT5G13140.1
    SENSE COUNTS:             0      4      0      0      0      9.72e-03       1.01e-02              0.000  2.000  0.000  0.000  0.000
    TAGS:
      d+1      TAGATTCAAA     0      4      0      0      0      9.72e-03       1.79e-02       263    0.000  2.000  0.000  0.000  0.000

LOCUS: AT4G37540
DESCRIPTION:  LOB domain protein 39 / lateral organ boundaries domain protein 39 (LBD39), identical to SP|Q9SZE8 LOB domain protein 39 {Arabidopsis
thaliana}
DATA:                         Control 30min  2hours  2days  1week  p-value        B&H            Pos    Fold change relative to control (log2)
SENSE COUNTS:                 0      4      0      0      0      9.72e-03       9.95e-03              0.000  2.000  0.000  0.000  0.000
  GENES:
  AT4G37540.1
    SENSE COUNTS:             0      4      0      0      0      9.72e-03       1.02e-02              0.000  2.000  0.000  0.000  0.000
    TAGS:
      d+1      TCTTTCATCT     0      4      0      0      0      9.72e-03       1.78e-02       296    0.000  2.000  0.000  0.000  0.000

LOCUS: AT2G36810
DESCRIPTION:  expressed protein,
DATA:                         Control 30min  2hours  2days  1week  p-value        B&H            Pos    Fold change relative to control (log2)
SENSE COUNTS:                 0      3      0      0      0      9.72e-03       9.94e-03              0.000  1.585  0.000  0.000  0.000
  GENES:
  AT2G36810.1
    SENSE COUNTS:             0      3      0      0      0      9.72e-03       1.01e-02              0.000  1.585  0.000  0.000  0.000
    TAGS:
      i+3      GACTTGCAAG     0      3      0      0      0      3.05e-02       5.16e-02       7996   0.000  1.585  0.000  0.000  0.000
      i+3      TTTTGAGCCT     0      0      0      0      0      6.15e-01       6.49e-01       666    0.000  0.000  0.000  0.000  0.000

LOCUS: AT2G45130
DESCRIPTION:  SPX (SYG1/Pho81/XPR1) domain-containing protein, weak similarity to NUC-2 (Neurospora crassa) GI
DATA:                         Control 30min  2hours  2days  1week  p-value        B&H            Pos    Fold change relative to control (log2)
SENSE COUNTS:                 0      4      0      0      0      9.72e-03       9.92e-03              0.000  2.000  0.000  0.000  0.000
  GENES:
  AT2G45130.1
    SENSE COUNTS:             0      4      0      0      0      9.72e-03       1.02e-02              0.000  2.000  0.000  0.000  0.000
    TAGS:
      d+1      ATGTTGTTGT     0      4      0      0      0      9.72e-03       1.79e-02       817    0.000  2.000  0.000  0.000  0.000

LOCUS: AT1G09740
DESCRIPTION:  ethylene-responsive protein, putative, similar to ER6 protein (Lycopersicon esculentum) GI
DATA:                         Control 30min  2hours  2days  1week  p-value        B&H            Pos    Fold change relative to control (log2)
SENSE COUNTS:                 0      4      0      0      0      9.72e-03       9.91e-03              0.000  2.000  0.000  0.000  0.000
  GENES:
  AT1G09740.1
    SENSE COUNTS:             0      4      0      0      0      9.72e-03       1.01e-02              0.000  2.000  0.000  0.000  0.000
    TAGS:
      d+1      GGCTCCCGGG     0      4      0      0      0      9.72e-03       1.79e-02       468    0.000  2.000  0.000  0.000  0.000

LOCUS: AT3G48680
DESCRIPTION:  bacterial transferase hexapeptide repeat-containing protein, contains Pfam profile PF00132
DATA:                         Control 30min  2hours  2days  1week  p-value        B&H            Pos    Fold change relative to control (log2)
SENSE COUNTS:                 0      4      0      0      0      9.72e-03       9.90e-03              0.000  2.000  0.000  0.000  0.000
```

```
  GENES:
  AT3G48680.1
    SENSE COUNTS:             0      4      0      0      0      9.72e-03       1.00e-02              0.000  2.000  0.000  0.000  0.000
    TAGS:
      d+1      CACTATCGAA     0      4      0      0      0      9.72e-03       1.79e-02       462    0.000  2.000  0.000  0.000  0.000

LOCUS: AT5G60710
DESCRIPTION:  zinc finger (C3HC4-type RING finger) family protein, low similarity to SP|P97280 Inter-alpha-trypsin inhibitor heavy chain H3 precursor
{Mesocricetus auratus}; contains Pfam profiles PF00097
DATA:                         Control 30min  2hours 2days  1week  p-value        B&H            Pos    Fold change relative to control (log2)
SENSE COUNTS:                 0      4      0      0      0      9.72e-03       9.89e-03              0.000  2.000  0.000  0.000  0.000
  GENES:
  AT5G60710.1
    SENSE COUNTS:             0      4      0      0      0      9.72e-03       1.01e-02              0.000  2.000  0.000  0.000  0.000
    TAGS:
      d+1      ACTTTCGGAA     0      4      0      0      0      9.72e-03       1.79e-02       2236   0.000  2.000  0.000  0.000  0.000

LOCUS: AT5G46710
DESCRIPTION:  zinc-binding family protein, similar zinc-binding protein (Pisum sativum) GI
DATA:                         Control 30min  2hours 2days  1week  p-value        B&H            Pos    Fold change relative to control (log2)
SENSE COUNTS:                 0      4      0      0      0      9.72e-03       9.88e-03              0.000  2.000  0.000  0.000  0.000
  GENES:
  AT5G46710.1
    SENSE COUNTS:             0      4      0      0      0      9.72e-03       1.01e-02              0.000  2.000  0.000  0.000  0.000
    TAGS:
      d+1      GTCTGTTATC     0      4      0      0      0      9.72e-03       1.77e-02       614    0.000  2.000  0.000  0.000  0.000

LOCUS: AT3G04810
DESCRIPTION:  protein kinase, putative, similar to LSTK-1-like kinase (Lycopersicon esculentum) GI
DATA:                         Control 30min  2hours 2days  1week  p-value        B&H            Pos    Fold change relative to control (log2)
SENSE COUNTS:                 0      4      0      0      0      9.72e-03       9.87e-03              0.000  2.000  0.000  0.000  0.000
  GENES:
  AT3G04810.2
    SENSE COUNTS:             0      4      0      0      0      9.72e-03       1.01e-02              0.000  2.000  0.000  0.000  0.000
    TAGS:
      d+1      CCTAGCTCAA     0      4      0      0      0      9.72e-03       1.80e-02       1126   0.000  2.000  0.000  0.000  0.000
  AT3G04810.1
    SENSE COUNTS:             0      4      0      0      0      9.72e-03       1.02e-02              0.000  2.000  0.000  0.000  0.000
    TAGS:
      d+1      CCTAGCTCAA     0      4      0      0      0      9.72e-03       1.80e-02       1210   0.000  2.000  0.000  0.000  0.000

LOCUS: AT5G13050
DESCRIPTION:  5-formyltetrahydrofolate cyclo-ligase (5-FCL), nearly identical to gi
DATA:                         Control 30min  2hours 2days  1week  p-value        B&H            Pos    Fold change relative to control (log2)
SENSE COUNTS:                 0      4      0      0      0      9.72e-03       9.86e-03              0.000  2.000  0.000  0.000  0.000
  GENES:
  AT5G13050.1
    SENSE COUNTS:             0      4      0      0      0      9.72e-03       1.00e-02              0.000  2.000  0.000  0.000  0.000
    TAGS:
      d+1      TGAGCTCAGG     0      4      0      0      0      9.72e-03       1.80e-02       913    0.000  2.000  0.000  0.000  0.000

LOCUS: AT1G76850
DESCRIPTION:  expressed protein
DATA:                         Control 30min  2hours 2days  1week  p-value        B&H            Pos    Fold change relative to control (log2)
SENSE COUNTS:                 0      4      0      0      0      9.72e-03       9.85e-03              0.000  2.000  0.000  0.000  0.000
  GENES:
  AT1G76850.1
    SENSE COUNTS:             0      4      0      0      0      9.72e-03       1.01e-02              0.000  2.000  0.000  0.000  0.000
    TAGS:
      d+1      CTTTGCAGAG     0      4      0      0      0      9.72e-03       1.77e-02       3185   0.000  2.000  0.000  0.000  0.000
```

```
LOCUS: AT4G01250
DESCRIPTION:  WRKY family transcription factor, contains Pfam profile
DATA:                         Control 30min  2hours  2days  1week  p-value     B&H         Pos   Fold change relative to control (log2)
SENSE COUNTS:                 0       4      0       0      0      9.72e-03    9.84e-03          0.000  2.000  0.000  0.000  0.000
  GENES:
  AT4G01250.1
    SENSE COUNTS:             0       4      0       0      0      9.72e-03    1.01e-02          0.000  2.000  0.000  0.000  0.000
    TAGS:
      d+1     GCGAAAGTAC      0       4      0       0      0      9.72e-03    1.78e-02    486   0.000  2.000  0.000  0.000  0.000

LOCUS: AT3G12570
DESCRIPTION:  expressed protein
DATA:                         Control 30min  2hours  2days  1week  p-value     B&H         Pos   Fold change relative to control (log2)
SENSE COUNTS:                 0       4      0       0      0      9.72e-03    9.83e-03          0.000  2.000  0.000  0.000  0.000
  GENES:
  AT3G12570.1
    SENSE COUNTS:             0       4      0       0      0      9.72e-03    1.00e-02          0.000  2.000  0.000  0.000  0.000
    TAGS:
      d+1     TATCATTGGA      0       4      0       0      0      9.72e-03    1.78e-02    1615  0.000  2.000  0.000  0.000  0.000
  AT3G12570.3
    SENSE COUNTS:             0       4      0       0      0      9.72e-03    1.02e-02          0.000  2.000  0.000  0.000  0.000
    TAGS:
      d+1     TATCATTGGA      0       4      0       0      0      9.72e-03    1.78e-02    1722  0.000  2.000  0.000  0.000  0.000
  AT3G12570.2
    SENSE COUNTS:             0       4      0       0      0      9.72e-03    1.00e-02          0.000  2.000  0.000  0.000  0.000
    TAGS:
      d+1     TATCATTGGA      0       4      0       0      0      9.72e-03    1.78e-02    1894  0.000  2.000  0.000  0.000  0.000

LOCUS: AT3G11780
DESCRIPTION:  MD-2-related lipid recognition domain-containing protein / ML domain-containing protein, weak similarity to
phosphatidylglycerol/phosphatidylinositol transfer protein (Aspergillus oryzae) GI
DATA:                         Control 30min  2hours  2days  1week  p-value     B&H         Pos   Fold change relative to control (log2)
SENSE COUNTS:                 12      4      20      10     4      9.74e-03    9.84e-03          0.000  -1.585 0.737  -0.263 -1.585
  GENES:
  AT3G11780.1
    SENSE COUNTS:             12      4      20      10     4      9.74e-03    1.00e-02          0.000  -1.585 0.737  -0.263 -1.585
    TAGS:
      d+1     AATCCCCCCA      3       2      7       5      1      3.44e-01    4.44e-01    717   0.000  -0.585 1.222  0.737  -1.585
      d+2     GAACCACTCT      9       2      13      5      3      3.79e-02    6.33e-02    620   0.000  -2.170 0.531  -0.848 -1.585

LOCUS: AT3G14180
DESCRIPTION:  expressed protein, similar to 6b-interacting protein 1 (NtSIP1) (Nicotiana tabacum) GI
DATA:                         Control 30min  2hours  2days  1week  p-value     B&H         Pos   Fold change relative to control (log2)
SENSE COUNTS:                 0       0      2       0      6      9.75e-03    9.84e-03          0.000  0.000  1.000  0.000  2.585
  GENES:
  AT3G14180.1
    SENSE COUNTS:             0       0      2       0      6      9.75e-03    1.00e-02          0.000  0.000  1.000  0.000  2.585
    TAGS:
      d+1     AGTGTATTAA      0       0      2       0      5      6.19e-02    1.01e-01    1649  0.000  0.000  1.000  0.000  2.322
      d+2     GGAGGAGAAT      0       0      0       0      1      1.65e-01    2.47e-01    1462  0.000  0.000  0.000  0.000  0.000

LOCUS: AT5G47190
DESCRIPTION:  ribosomal protein L19 family protein, similar to plastid ribosomal protein L19 precursor (Spinacia oleracea) gi|7582403|gb|AAF64312
DATA:                         Control 30min  2hours  2days  1week  p-value     B&H         Pos   Fold change relative to control (log2)
SENSE COUNTS:                 17      14     4       5      21     9.78e-03    9.85e-03          0.000  -0.280 -2.087 -1.766 0.305
  GENES:
  AT5G47190.1
    SENSE COUNTS:             17      14     4       5      21     9.78e-03    1.00e-02          0.000  -0.280 -2.087 -1.766 0.305
    TAGS:
```

```
      d+1      TAATTTACAG     17      14      4       5       21      1.02e-02        1.85e-02        858     0.000   -0.280  -2.087  -1.766  0.305
      i+3      GTGGGTTTGA     0       0       0       0       0       6.15e-01        6.58e-01        821     0.000   0.000   0.000   0.000   0.000

LOCUS: AT5G27350
DESCRIPTION:  sugar-porter family protein 1 (SFP1), identical to sugar-porter family protein 1 (Arabidopsis thaliana) GI
DATA:                         Control 30min   2hours  2days   1week   p-value         B&H             Pos     Fold change relative to control (log2)
SENSE COUNTS:                 6       0       0       1       6       9.84e-03        9.90e-03                0.000   0.000   0.000   -2.585  0.000
  GENES:
  AT5G27350.1
    SENSE COUNTS:             6       0       0       1       6       9.84e-03        1.01e-02                0.000   0.000   0.000   -2.585  0.000
    TAGS:
      d+1      TGTATTTTAT     6       0       0       1       5       1.86e-02        3.24e-02        1896    0.000   0.000   0.000   -2.585  -0.263
      d+2      CTACTTGGGG     0       0       0       0       1       1.65e-01        2.43e-01        1321    0.000   0.000   0.000   0.000   0.000

LOCUS: AT2G27510
DESCRIPTION:  ferredoxin, putative, similar to non-photosynthetic ferredoxin from Citrus sinensis (GI
DATA:                         Control 30min   2hours  2days   1week   p-value         B&H             Pos     Fold change relative to control (log2)
SENSE COUNTS:                 5       1       2       13      10      9.84e-03        9.89e-03                0.000   -2.322  -1.322  1.379   1.000
  GENES:
  AT2G27510.1
    SENSE COUNTS:             5       1       2       13      10      9.84e-03        1.01e-02                0.000   -2.322  -1.322  1.379   1.000
    TAGS:
      d+1      AATCGTCACC     5       1       2       13      10      4.40e-03        9.86e-03        707     0.000   -2.322  -1.322  1.379   1.000
      d+2      ACCAAGGCCG     0       0       0       0       0       6.15e-01        6.31e-01        118     0.000   0.000   0.000   0.000   0.000

LOCUS: AT4G33700
DESCRIPTION:  CBS domain-containing protein, contains Pfam profiles PF00571
DATA:                         Control 30min   2hours  2days   1week   p-value         B&H             Pos     Fold change relative to control (log2)
SENSE COUNTS:                 0       0       2       2       8       9.88e-03        9.92e-03                0.000   0.000   1.000   1.000   3.000
  GENES:
  AT4G33700.1
    SENSE COUNTS:             0       0       2       2       8       9.88e-03        1.01e-02                0.000   0.000   1.000   1.000   3.000
    TAGS:
      d+1      AACAACAACT     0       0       1       1       5       1.07e-01        1.68e-01        1675    0.000   0.000   0.000   0.000   2.322
      i+3      CAAATCAGGA     0       0       1       1       3       1.91e-01        2.68e-01        1313    0.000   0.000   0.000   0.000   1.585

LOCUS: AT2G05060
DESCRIPTION:  protein kinase family protein, contains protein kinase domain, Pfam
DATA:                         Control 30min   2hours  2days   1week   p-value         B&H             Pos     Fold change relative to control (log2)
SENSE COUNTS:                 18      6       16      13      1       9.89e-03        9.92e-03                0.000   -1.585  -0.170  -0.469  -4.170
  GENES:
  AT2G05060.1
    SENSE COUNTS:             18      6       16      13      1       9.89e-03        1.01e-02                0.000   -1.585  -0.170  -0.469  -4.170
    TAGS:
      v+1      GCAAGACGAC     17      6       16      13      1       1.42e-02        2.51e-02        1771    0.000   -1.503  -0.087  -0.387  -4.087
      v+2      TGATTTTGAC     1       0       0       0       0       4.28e-01        5.29e-01        1493    0.000   0.000   0.000   0.000   0.000

LOCUS: AT5G42520
DESCRIPTION:  expressed protein
DATA:                         Control 30min   2hours  2days   1week   p-value         B&H             Pos     Fold change relative to control (log2)
SENSE COUNTS:                 0       0       4       0       5       9.97e-03        9.99e-03                0.000   0.000   2.000   0.000   2.322
  GENES:
  AT5G42520.1
    SENSE COUNTS:             0       0       4       0       5       9.97e-03        1.02e-02                0.000   0.000   2.000   0.000   2.322
    TAGS:
      d+1      TGTACTAAAT     0       0       3       0       5       1.57e-02        2.77e-02        1323    0.000   0.000   1.585   0.000   2.322
      d+2      TACAGGAGTT     0       0       1       0       0       4.55e-01        5.09e-01        1004    0.000   0.000   0.000   0.000   0.000

LOCUS: AT4G33980
DESCRIPTION:  expressed protein
```

DATA:		Control	30min	2hours	2days	1week	p-value	B&H	Pos	Fold change relative to control (log2)				
SENSE COUNTS:		0	2	6	0	0	9.98e-03	9.99e-03		0.000	1.000	2.585	0.000	0.000
GENES:														
AT4G33980.1														
SENSE COUNTS:		0	2	6	0	0	9.98e-03	1.02e-02		0.000	1.000	2.585	0.000	0.000
TAGS:														
d+1	ACGAAACCAA	0	2	4	0	0	7.95e-02	1.28e-01	603	0.000	1.000	2.000	0.000	0.000
X+4	CGACTTTTAA	0	0	2	0	0	1.21e-01	1.88e-01	390	0.000	0.000	1.000	0.000	0.000

LOCUS: AT5G47240
DESCRIPTION: MutT/nudix family protein, similar to SP|P53370 Nucleoside diphosphate-linked moiety X motif 6 {Homo sapiens}; contains Pfam profile PF00293

DATA:		Control	30min	2hours	2days	1week	p-value	B&H	Pos	Fold change relative to control (log2)				
SENSE COUNTS:		0	2	6	0	0	9.98e-03	9.98e-03		0.000	1.000	2.585	0.000	0.000
GENES:														
AT5G47240.1														
SENSE COUNTS:		0	2	6	0	0	9.98e-03	1.02e-02		0.000	1.000	2.585	0.000	0.000
TAGS:														
d+1	TAGCATCGAA	0	2	6	0	0	9.98e-03	1.82e-02	1327	0.000	1.000	2.585	0.000	0.000
