# Supplementary material for: Differential SAGE analysis in Arabidopsis uncovers increased transcriptome complexity in response to low temperature
Source: BMC Genomics. 2008 Sep 22;9:434. doi: 10.1186/1471-2164-9-434 (PMC2568001; doi:10.1186/1471-2164-9-434)
Supplement: Additional file 5 — SAGE tags suggesting evidence of alternative transcript processing events. [file 1471-2164-9-434-S5.pdf]

Additional file 5: SAGE tags suggesting evidence of alternative transcript processing. Non-canonical and/or multiple SAGE tags were uniquely matched to each gene code. The relative base pair position of each possible tag within the annotated gene is indicated (pos column), including tags not found in the experimental data (dashed lines). The tag counts were normalised to 50,000 per library to facilitate comparative expression analysis

```
LOCUS: AT2G34420
DESCRIPTION: chlorophyll A-B binding protein / LHCII type I (LHB1B2), identical to GB:X64460 photosystem II
type I chlorophyll a/b binding protein (Arabidopsis thaliana) GI:16364
DATA:                 Control 30min   2hours  2days   1week   p-value         pos
SENSE COUNTS:         339     1023    247     317     35      4.50e-249
GENES (3 total):
  AT2G34420.2
    SENSE COUNTS:     3       0       26      2       1       7.72e-12
    TAGS: (2 total)
           ----------                                                         371
           ----------                                                         199
      d+2  AGGAAGACTG 1       0       5       1       0       8.03e-02        155
      d+2  GCTTTGTCCT 2       0       21      1       1       1.79e-10        74
  AT2G34420.1
    SENSE COUNTS:     339     1023    247     317     35      4.50e-249
    TAGS: (3 total)
      d+1  GGAGCTGTTG 336     1023    221     315     34      9.00e-259       557
           ----------                                                         371
           ----------                                                         199
      d+2  AGGAAGACTG 1       0       5       1       0       8.03e-02        155
      d+2  GCTTTGTCCT 2       0       21      1       1       1.79e-10        74

LOCUS: AT3G50450
DESCRIPTION: hypersensitive response protein 1 (HR1), identical to HR1 (Arabidopsis thaliana) GI:12958166;
contains Pfam profile PF05659: Arabidopsis broad-spectrum mildew resistance protein RPW8
DATA:                 Control 30min   2hours  2days   1week   p-value         pos
SENSE COUNTS:         55      244     24      48      41      3.48e-75
GENES (2 total):
  AT3G50450.1
    SENSE COUNTS:     55      244     24      48      41      3.48e-75
    TAGS: (1 total)
           ----------                                                         1411
           ----------                                                         1354
           ----------                                                         67
      v+2  ATATTTCTTT 55      244     24      48      41      3.48e-75        37

LOCUS: AT3G16770
DESCRIPTION: encodes a member of the ERF (ethylene response factor) subfamily B-2 of ERF/AP2 transcription
factor family (RAP2.3). The protein contains one AP2 domain. There are 5 members in this subfamily including
RAP2.2 AND RAP2.12.
DATA:                 Control 30min   2hours  2days   1week   p-value         pos
SENSE COUNTS:         27      178     15      15      10      3.02e-74
GENES (1 total):
  AT3G16770.1
    SENSE COUNTS:     27      178     15      15      10      3.02e-74
    TAGS: (2 total)
      d+1  TGTAAATAAG 9       11      2       0       7       1.36e-02        1011
      d+2  GCTTATGATG 18      167     13      15      3       6.54e-76        418
           ----------                                                         330

LOCUS: AT1G30380
DESCRIPTION: photosystem I reaction center subunit psaK, chloroplast, putative / photosystem I subunit X,
putative / PSI-K, putative (PSAK), identical to SP|Q9SUI5; strong similarity to SP|P36886 Photosystem I
reaction center subunit psaK, chloroplast precursor (Photo
DATA:                 Control 30min   2hours  2days   1week   p-value         pos
SENSE COUNTS:         413     123     398     327     49      8.89e-69
GENES (1 total):
  AT1G30380.1
    SENSE COUNTS:     413     123     398     327     49      8.89e-69
    TAGS: (3 total)
      d+1  GAAGAAGCGG 27      4       18      12      3       3.35e-05        622
      d+2  CACCTGAACG 384     118     353     315     46      6.80e-61        521
      d+2  AGACGCAAGG 2       1       27      0       0       6.85e-15        166

LOCUS: AT4G14690
DESCRIPTION: chlorophyll A-B binding family protein / early light-induced protein, putative, strong similarity
to early light-induced protein; ELIP (Arabidopsis thaliana) GI:1872544; contains Pfam profile: PF00504
chlorophyll A-B binding protein
DATA:                 Control 30min   2hours  2days   1week   p-value         pos
SENSE COUNTS:         2       0       4       131     87      5.05e-67
GENES (2 total):
  AT4G14690.1
    SENSE COUNTS:     2       0       4       131     87      5.05e-67
    TAGS: (3 total)
           ----------                                                         796
```

```
        ----------                                                      776
     X+4  TGTCTGCCAT  0       0       0       1       1       3.25e-01       671
     d+2  ACTTCAGACG  2       0       3       130     86      5.29e-67       577
        ----------                                                      430
     d+2  GCTCAGGGCG  0       0       1       0       0       4.55e-01       220
        ----------                                                      115

LOCUS: AT5G66570
DESCRIPTION: oxygen-evolving enhancer protein 1-1, chloroplast / 33 kDa subunit of oxygen evolving system of
photosystem II (PSBO1) (PSBO), identical to SP:P23321 Oxygen-evolving enhancer protein 1-1, chloroplast
precursor (OEE1) (33 kDa subunit of oxygen evolving sys
DATA:                 Control 30min   2hours  2days   1week   p-value        pos
SENSE COUNTS:         476     118     341     239     134     4.80e-63
GENES (2 total):
  AT5G66570.1
    SENSE COUNTS:     476     118     341     239     134     4.80e-63
    TAGS: (4 total)
     d+1  TCCTTCATCG  141     43      95      43      50      2.47e-17       1212
     d+2  CCTAAGATCT  335     75      242     195     84      2.54e-47       1181
        ----------                                                      1091
     d+2  ACCCGTCTTA  0       0       3       1       0       9.46e-02       579
        ----------                                                      396
     d+2  GCAGCCTCTC  0       0       1       0       0       4.55e-01       90

LOCUS: AT1G15820
DESCRIPTION: chlorophyll A-B binding protein, chloroplast (LHCB6), nearly identical to Lhcb6 protein
(Arabidopsis thaliana) GI:4741960; contains Pfam profile PF00504: Chlorophyll A-B binding protein
DATA:                 Control 30min   2hours  2days   1week   p-value        pos
SENSE COUNTS:         205     43      272     160     25      1.90e-55
GENES (2 total):
  AT1G15820.1
    SENSE COUNTS:     205     43      272     160     25      1.90e-55
    TAGS: (4 total)
        ----------                                                      1117
     d+2  AGGCTTGTTC  204     43      254     159     25      1.25e-50       1012
        ----------                                                      895
        ----------                                                      823
     d+2  GTCGAAGACC  1       0       13      1       0       5.56e-07       639
     d+2  GGTTGGGTGG  0       0       1       0       0       4.55e-01       565
        ----------                                                      486
     d+2  GCCGATGGGC  0       0       4       0       0       5.58e-03       422
        ----------                                                      109

LOCUS: AT3G47470
DESCRIPTION: chlorophyll A-B binding protein 4, chloroplast / LHCI type III CAB-4 (CAB4), identical to
SP|P27521 Chlorophyll A-B binding protein 4, chloroplast precursor (LHCI type III CAB-4) (LHCP) {Arabidopsis
thaliana}
DATA:                 Control 30min   2hours  2days   1week   p-value        pos
SENSE COUNTS:         144     49      248     125     43      2.87e-40
GENES (2 total):
  AT3G47470.1
    SENSE COUNTS:     144     49      248     125     43      2.87e-40
    TAGS: (4 total)
     d+1  TGGCAACAGT  136     49      235     124     43      2.73e-37       1226
        ----------                                                      1148
     d+2  TCTGTCTGTG  4       0       11      1       0       9.79e-04       1071
     d+2  GCACAACACT  3       0       1       0       0       2.51e-01       986
     d+2  CCTCGGCCTC  1       0       1       0       0       8.46e-01       283

LOCUS: AT3G16640
DESCRIPTION: translationally controlled tumor family protein, similar to translationally controlled tumor
protein GB:AAD10032 from (Hevea brasiliensis)
DATA:                 Control 30min   2hours  2days   1week   p-value        pos
SENSE COUNTS:         119     277     161     105     38      1.78e-36
GENES (2 total):
  AT3G16640.1
    SENSE COUNTS:     119     277     161     105     38      1.78e-36
    TAGS: (4 total)
     d+1  TTTATAGTAG  1       0       0       0       1       6.74e-01       882
     d+2  GTTTGAAGGA  118     277     150     102     37      5.83e-38       573
     d+2  ATGACAGCAC  0       0       11      2       0       8.97e-06       501
     d+2  TTGGTGTACC  0       0       0       1       0       3.09e-01       92

LOCUS: AT3G54890
DESCRIPTION: chlorophyll A-B binding protein / LHCI type I (CAB), identical to chlorophyll A/B-binding protein
(Arabidopsis thaliana) GI:16207; contains Pfam profile: PF00504 chlorophyll A-B binding protein
DATA:                 Control 30min   2hours  2days   1week   p-value        pos
SENSE COUNTS:         411     257     330     190     80      2.18e-35
GENES (3 total):
  AT3G54890.1
    SENSE COUNTS:     411     257     330     190     80      2.18e-35
```

```
    TAGS: (3 total)
      d+1  CTTCCTACAG 1       1       4       0       0       1.70e-01        969
      d+2  GCACAACAAC 409     256     326     190     80      7.60e-35        755
      i+3  ATGCAACATT 1       0       0       0       0       4.28e-01        344
  AT3G54890.2
    SENSE COUNTS:     411     257     330     190     80      2.18e-35
    TAGS: (3 total)
      d+1  CTTCCTACAG 1       1       4       0       0       1.70e-01        867
      d+2  GCACAACAAC 409     256     326     190     80      7.60e-35        653
      i+3  ATGCAACATT 1       0       0       0       0       4.28e-01        344
  AT3G54890.3
    SENSE COUNTS:     411     257     330     190     80      2.18e-35
    TAGS: (3 total)
      d+1  CTTCCTACAG 1       1       4       0       0       1.70e-01        902
      d+2  GCACAACAAC 409     256     326     190     80      7.60e-35        688
      i+3  ATGCAACATT 1       0       0       0       0       4.28e-01        344

LOCUS: AT2G02100
DESCRIPTION: plant defensin-fusion protein, putative (PDF2.2), plant defensin protein family member, personal
communication, Bart Thomma (Bart.Thomma@agr.kuleuven.ac.be); similar to SWISS-PROT:O65740
DATA:                 Control 30min   2hours  2days   1week   p-value         pos
SENSE COUNTS:         10      14      14      21      109     7.24e-34
GENES (1 total):
  AT2G02100.1
    SENSE COUNTS:     10      14      14      21      109     7.24e-34
    TAGS: (3 total)
      X+4  TCTCACCTTG 0       0       1       0       1       3.96e-01        428
      d+1  AAGTACCGTA 10      14      12      20      107     2.52e-33        367
           ----------                                                         298
      d+2  CGTGAGTGCA 0       0       1       1       1       5.61e-01        179
           ----------                                                         96
           ----------                                                         90

LOCUS: AT4G21960
DESCRIPTION: peroxidase 42 (PER42) (P42) (PRXR1), identical to SP|Q9SB81 Peroxidase 42 precursor (EC 1.11.1.7)
(Atperox P42) (PRXR1) (ATP1a/ATP1b) {Arabidopsis thaliana}
DATA:                 Control 30min   2hours  2days   1week   p-value         pos
SENSE COUNTS:         363     529     388     254     189     3.08e-32
GENES (2 total):
  AT4G21960.1
    SENSE COUNTS:     363     529     388     254     189     3.08e-32
    TAGS: (5 total)
      d+1  GTATACATAA 2       0       0       1       3       4.33e-01        1403
           ----------                                                         1399
      d+2  TCCGAATCTT 316     475     344     234     150     3.29e-31        1369
      d+2  TATGACGATG 3       4       2       0       1       4.52e-01        1341
      d+2  GTAGTGACCA 41      43      42      19      35      7.61e-02        1166
      d+2  GTGCTAGACA 1       7       0       0       0       1.46e-03        806
           ----------                                                         731
           ----------                                                         424
           ----------                                                         283
           ----------                                                         264

LOCUS: AT1G29930
DESCRIPTION: chlorophyll A-B binding protein 2, chloroplast / LHCII type I CAB-2 / CAB-140 (CAB2B), identical
to SP|P04778 Chlorophyll A-B binding protein 2, chloroplast precursor (LHCII type I CAB-2) (CAB-140) (LHCP)
{Arabidopsis thaliana}
DATA:                 Control 30min   2hours  2days   1week   p-value         pos
SENSE COUNTS:         748     866     713     1022    468     1.04e-29
GENES (2 total):
  AT1G29930.1
    SENSE COUNTS:     748     866     713     1022    468     1.04e-29
    TAGS: (2 total)
      X+4  TCAAAGTTAA 0       0       2       1       3       2.08e-01        1094
      d+1  GGCCTTCGCC 748     866     711     1021    465     4.71e-30        837
           ----------                                                         388
           ----------                                                         216

LOCUS: AT4G10340
DESCRIPTION: chlorophyll A-B binding protein CP26, chloroplast / light-harvesting complex II protein 5 /
LHCIIc (LHCB5), identical to SP|Q9XF89 Chlorophyll A/B-binding protein CP26, chloroplast precursor (Light-
harvesting complex II protein 5) (LHCB5) (LHCIIc) {Arabid
DATA:                 Control 30min   2hours  2days   1week   p-value         pos
SENSE COUNTS:         304     352     337     238     52      1.22e-29
GENES (2 total):
  AT4G10340.1
    SENSE COUNTS:     304     352     337     238     52      1.22e-29
    TAGS: (3 total)
      d+1  CTTCTAAGGA 82      16      62      43      23      6.87e-12        1160
      d+2  TTTGCGATGC 221     336     258     195     28      6.78e-34        884
      d+2  CGAGATGGGC 1       0       17      0       1       4.96e-09        537
```

```
LOCUS: AT5G42270
DESCRIPTION: FtsH protease, putative, similar to FtsH protease GI:13183728 from (Medicago sativa)
DATA:                 Control 30min   2hours  2days   1week   p-value         pos
SENSE COUNTS:         45      12      59      107     144     8.30e-28
GENES (2 total):
  AT5G42270.1
    SENSE COUNTS:     45      12      59      107     144     8.30e-28
    TAGS: (3 total)
           ----------                                                         2946
           ----------                                                         2920
           ----------                                                         2888
      d+2  TTCAGAGACT 45      12      54      101     141     1.11e-26        2364
      d+2  AGTCTTTTCA 0       0       2       5       3       4.65e-02        2247
      d+2  CAAGTGTCGC 0       0       3       1       0       9.46e-02        1959
           ----------                                                         1723
           ----------                                                         1711

LOCUS: AT5G40450
DESCRIPTION: expressed protein
DATA:                 Control 30min   2hours  2days   1week   p-value         pos
SENSE COUNTS:         8       67      6       7       4       1.33e-27
GENES (1 total):
  AT5G40450.1
    SENSE COUNTS:     8       74      6       7       4       9.43e-32
    TAGS: (12 total)
           ----------                                                         8549
      d+2  AAGTTTCCGT 1       19      2       2       3       4.94e-07        8057
           ----------                                                         8024
           ----------                                                         7961
      d+2  AAGACCCTGT 0       6       0       0       0       9.09e-04        7454
           ----------                                                         7181
           ----------                                                         7043
      d+2  ATAAAAGAGC 0       6       1       1       1       4.45e-02        6736
           ----------                                                         6626
           ----------                                                         6461
           ----------                                                         6385
           ----------                                                         6107
           ----------                                                         6086
      d+2  TCGAGACTCC 2       14      2       2       0       1.02e-04        5966
           ----------                                                         5942
           ----------                                                         5797
           ----------                                                         5600
           ----------                                                         5389
           ----------                                                         5306
           ----------                                                         5054
           ----------                                                         4817
      d+2  TATTGGAGAG 0       0       0       0       0       6.15e-01        4019
           ----------                                                         4013
      d+2  CTCCAAAGAG 1       2       0       0       0       2.43e-01        3444
           ----------                                                         3347
      d+2  TGACTGTTGC 0       2       0       0       0       9.14e-02        3254
      d+2  AACCAACAAA 2       2       0       2       0       4.35e-01        3029
      d+2  ATGTACTTAA 1       4       1       0       0       1.14e-01        2276
           ----------                                                         2207
           ----------                                                         1979
           ----------                                                         1973
      d+2  AATCTGAAAG 0       7       0       0       0       2.70e-04        3743
      d+2  AATCTGAAAG 0       7       0       0       0       2.70e-04        3743
           ----------                                                         1583
           ----------                                                         1502
           ----------                                                         1169
           ----------                                                         1121
           ----------                                                         893
           ----------                                                         800
      d+2  GAAGTACAAA 1       5       0       0       0       1.37e-02        112
           ----------                                                         12

LOCUS: AT3G22120
DESCRIPTION: protease inhibitor/seed storage/lipid transfer protein (LTP) family protein, similar to
SP|Q00451|PRF1_LYCES 36.4 kDa proline-rich protein Lycopersicon esculentum, proline-rich cell wall protein
(Medicago sativa) GI:3818416; contains Pfam profile PF00234
DATA:                 Control 30min   2hours  2days   1week   p-value         pos
SENSE COUNTS:         79      91      23      55      193     5.39e-27
GENES (2 total):
  AT3G22120.1
    SENSE COUNTS:     79      91      24      55      193     1.30e-26
    TAGS: (5 total)
      X+4  CGCATTGCAC 0       0       1       0       0       4.55e-01        1367
      X+4  CGCATTGCAC 0       0       1       0       0       4.55e-01        1367
      d+1  TTTCATTTGA 77      89      21      52      192     2.97e-28        1155
```

```
      d+2  ATCTATTTGG 2       0       0       1       1       6.87e-01        1102
      d+2  CCCACCACCA 0       2       1       2       0       3.87e-01        512

LOCUS: AT1G61520
DESCRIPTION: chlorophyll A-B binding protein / LHCI type III (LHCA3.1), nearly identical to PSI type III
chlorophyll a/b-binding protein GI:430947; contains Pfam profile: PF00504 chlorophyll A-B binding protein;
similar to PSI type III chlorophyll a/b-binding protein
DATA:                  Control 30min   2hours  2days   1week   p-value         pos
SENSE COUNTS:          387     327     294     197     98      6.12e-27
GENES (2 total):
  AT1G61520.1
    SENSE COUNTS:      387     327     294     197     98      6.12e-27
    TAGS: (3 total)
      d+1  TGTTTTTATG 386     327     281     197     97      3.42e-27        983
      i+3  ATGTTCAAAA 1       0       0       0       0       4.28e-01        487
      d+2  TTGGGTGCAG 0       0       13      0       1       6.58e-08        467

LOCUS: AT1G67090
DESCRIPTION: ribulose bisphosphate carboxylase small chain 1A / RuBisCO small subunit 1A (RBCS-1A) (ATS1A),
identical to SP|P10795 Ribulose bisphosphate carboxylase small chain 1A, chloroplast precursor (EC 4.1.1.39)
(RuBisCO small subunit 1A) {Arabidopsis thaliana}
DATA:                  Control 30min   2hours  2days   1week   p-value         pos
SENSE COUNTS:          372     473     255     476     206     1.34e-26
GENES (2 total):
  AT1G67090.1
    SENSE COUNTS:      372     473     255     476     206     1.34e-26
    TAGS: (2 total)
      d+1  AGTCGCTAAA 1       0       4       9       16      4.51e-05        843
      d+2  CAGGTGTGGC 371     473     251     467     190     1.88e-28        197
  AT1G67090.2
    SENSE COUNTS:      372     473     255     476     206     1.34e-26
    TAGS: (2 total)
      d+1  AGTCGCTAAA 1       0       4       9       16      4.51e-05        836
      d+2  CAGGTGTGGC 371     473     251     467     190     1.88e-28        197

LOCUS: AT5G15950
DESCRIPTION: adenosylmethionine decarboxylase family protein, contains Pfam profile: PF01536
adenosylmethionine decarboxylase
DATA:                  Control 30min   2hours  2days   1week   p-value         pos
SENSE COUNTS:          1       0       6       9       55      2.42e-26
GENES (1 total):
  AT5G15950.1
    SENSE COUNTS:      1       0       6       9       55      2.42e-26
    TAGS: (3 total)
      d+1  ATTTTAGTGT 1       0       1       0       26      9.45e-16        1731
      X+4  ACACCTTTGG 0       0       5       8       26      1.81e-10        1609
      d+2  GACTTGAGCC 0       0       0       1       3       6.27e-02        1268
           ----------                                                         1203
           ----------                                                         954
           ----------                                                         476

LOCUS: AT1G79040
DESCRIPTION: photosystem II 10 kDa polypeptide, identical to photosystem II 10 kDa polypeptide, chloroplast
(precursor) SP:P27202 from (Arabidopsis thaliana); contains Pfam profile: PF04725 photosystem II 10 kDa
polypeptide PsbR
DATA:                  Control 30min   2hours  2days   1week   p-value         pos
SENSE COUNTS:          303     236     124     93      193     2.23e-25
GENES (1 total):
  AT1G79040.1
    SENSE COUNTS:      303     236     124     93      193     2.23e-25
    TAGS: (3 total)
      d+1  TTTCTATAAA 299     235     86      88      183     1.35e-33        523
           ----------                                                         513
      X+4  CACAAGAATG 2       1       5       5       0       2.23e-01        306
      d+2  GACTTGAGGG 2       0       33      0       10      1.28e-15        245
           ----------                                                         9

LOCUS: AT2G30570
DESCRIPTION: photosystem II reaction center W (PsbW) protein-related, similar to photosystem II reaction
center W protein SP:Q41387 from (Spinacia oleracea)
DATA:                  Control 30min   2hours  2days   1week   p-value         pos
SENSE COUNTS:          323     124     235     180     126     2.65e-25
GENES (2 total):
  AT2G30570.1
    SENSE COUNTS:      175     61      129     107     60      1.49e-15
    TAGS: (2 total)
      d+1  TTCATCAAAA 86      35      75      48      39      1.67e-06        748
           ----------                                                         743
           ----------                                                         739
           ----------                                                         661
      d+2  AATCAAAAGT 89      26      54      59      21      4.50e-10        595
```

```
          ----------                                                    585
          ----------                                                    313
  AT2G30570.2
    SENSE COUNTS:      237     89      160     132     87      6.66e-19
    TAGS: (2 total)
      d+1  TGTGTTTACT  148     63      106     73      66      6.84e-10       661
      d+2  AATCAAAAGT  89      26      54      59      21      4.50e-10       595
           ----------                                                     585
           ----------                                                     313

LOCUS: AT2G05380
DESCRIPTION: glycine-rich protein (GRP3S), identical to cDNA glycine-rich protein 3 short isoform (GRP3S)
GI:4206766
DATA:                  Control 30min   2hours  2days   1week   p-value        pos
SENSE COUNTS:          74      48      56      100     199     1.12e-24
GENES (2 total):
  AT2G05380.1
    SENSE COUNTS:      74      48      56      100     199     1.12e-24
    TAGS: (3 total)
      d+1  AGTGTACGAT  73      48      45      98      190     4.87e-24       409
           ----------                                                     324
      d+2  GTGGCCACGG  0       0       11      0       8       9.75e-06       207
      d+2  GTGGTGGATT  1       0       0       2       1       3.00e-01       153

LOCUS: AT3G26520
DESCRIPTION: tonoplast intrinsic protein, putative, similar to tonoplast intrinsic protein GI:5081419 from
(Brassica napus)
DATA:                  Control 30min   2hours  2days   1week   p-value        pos
SENSE COUNTS:          51      136     62      71      3       9.73e-22
GENES (2 total):
  AT3G26520.1
    SENSE COUNTS:      51      136     62      71      3       9.73e-22
    TAGS: (3 total)
           ----------                                                     1187
      d+2  AACCCAGCCG  51      136     49      70      3       6.21e-24       710
      d+2  CTTTCGGTCT  0       0       12      0       0       3.96e-08       312
      d+2  CCGACCAGAA  0       0       1       1       0       5.21e-01       116

LOCUS: AT4G35770
DESCRIPTION: senescence-associated protein (SEN1), identical to senescence-associated protein GI:1046270 from
(Arabidopsis thaliana)
DATA:                  Control 30min   2hours  2days   1week   p-value        pos
SENSE COUNTS:          6       51      5       6       0       1.74e-21
GENES (2 total):
  AT4G35770.1
    SENSE COUNTS:      6       51      5       6       0       1.74e-21
    TAGS: (4 total)
      i+3  TACAGGGCTT  0       2       1       0       0       2.47e-01       964
      d+1  TACAGAGTCG  5       49      2       6       0       6.98e-22       466
      X+4  CCTACACACG  0       0       0       0       0       6.15e-01       261
      d+2  TGGATCTTTC  1       0       2       0       0       3.07e-01       210

LOCUS: AT2G15970
DESCRIPTION: cold-acclimation protein, putative (FL3-5A3), similar to cold acclimation WCOR413-like protein
gamma form (Hordeum vulgare) gi|18449100|gb|AAL69988; similar to stress-regulated protein SAP1 (Xerophyta
viscosa) gi|21360378|gb|AAM47505; identical to cDNA co
DATA:                  Control 30min   2hours  2days   1week   p-value        pos
SENSE COUNTS:          32      23      46      99      122     1.38e-20
GENES (3 total):
  AT2G15970.1
    SENSE COUNTS:      32      23      46      99      122     1.38e-20
    TAGS: (3 total)
      d+1  AGGTGTTAGT  10      3       17      18      41      2.39e-07       811
      d+2  TAAGAGTGAT  22      20      28      81      80      4.39e-15       715
      d+2  ATCGGATCCG  0       0       1       0       1       3.96e-01       124
           ----------                                                     107

LOCUS: AT4G27520
DESCRIPTION: plastocyanin-like domain-containing protein, similar to PIR|JC7196 phytocyanin-related protein
Pn14 {Ipomoea nil}; contains Pfam profile PF02298: Plastocyanin-like domain
DATA:                  Control 30min   2hours  2days   1week   p-value        pos
SENSE COUNTS:          25      7       37      92      33      1.43e-19
GENES (1 total):
  AT4G27520.1
    SENSE COUNTS:      25      7       37      92      33      1.43e-19
    TAGS: (2 total)
           ----------                                                     1358
      d+2  ATAACTCCAC  1       0       4       4       1       1.69e-01       1295
      d+2  TCGGATTCGA  24      7       33      88      32      7.68e-19       1162
           ----------                                                     90
```

```
LOCUS: AT1G09310
DESCRIPTION: expressed protein, contains Pfam profile PF04398: Protein of unknown function, DUF538
DATA:                  Control 30min   2hours  2days   1week   p-value         pos
SENSE COUNTS:          103     23      27      20      60      2.08e-19
GENES (1 total):
  AT1G09310.1
    SENSE COUNTS:      103     23      27      20      60      2.08e-19
    TAGS: (3 total)
      X+4  GGAAACCTCT  0       0       0       0       1       1.65e-01        835
      d+1  TATTATCTAC  103     23      26      20      59      1.20e-19        700
      d+2  GAGAGTTAGT  0       0       1       0       0       4.55e-01        665

LOCUS: AT4G39090
DESCRIPTION: cysteine proteinase RD19a (RD19A) / thiol protease, identical to cysteine proteinase RD19a, thiol
protease SP:P43296, GI:435618 from (Arabidopsis thaliana)
DATA:                  Control 30min   2hours  2days   1week   p-value         pos
SENSE COUNTS:          3       45      4       4       5       7.00e-19
GENES (2 total):
  AT4G39090.1
    SENSE COUNTS:      3       45      4       4       5       7.00e-19
    TAGS: (2 total)
           ----------                                                          2285
           ----------                                                          2060
           ----------                                                          1942
      d+2  CCCTTACATA  3       45      1       4       5       6.89e-21        1032
           ----------                                                          832
      d+2  GCGCCGTTAC  0       0       3       0       0       2.70e-02        590
           ----------                                                          440
           ----------                                                          362

LOCUS: AT1G03130
DESCRIPTION: photosystem I reaction center subunit II, chloroplast, putative / photosystem I 20 kDa subunit,
putative / PSI-D, putative (PSAD2), similar to SP|P12353 Photosystem I reaction center subunit II, chloroplast
precursor (Photosystem I 20 kDa subunit) (PSI-D)
DATA:                  Control 30min   2hours  2days   1week   p-value         pos
SENSE COUNTS:          153     72      166     98      30      1.22e-18
GENES (1 total):
  AT1G03130.1
    SENSE COUNTS:      153     72      166     98      30      1.22e-18
    TAGS: (3 total)
      d+1  TGGTAAGTGA  2       0       3       0       0       1.23e-01        829
      d+2  TAAAATCTTG  150     72      162     97      30      1.41e-17        743
           ----------                                                          603
      d+2  GAACTCACCG  1       0       1       1       0       7.18e-01        350

LOCUS: AT2G36530
DESCRIPTION: enolase, identical to SWISS-PROT:P25696 enolase (EC 4.2.1.11) (2-phosphoglycerate dehydratase)(2-
phospho-D- glycerate hydro-lyase) (Arabidopsis thaliana)
DATA:                  Control 30min   2hours  2days   1week   p-value         pos
SENSE COUNTS:          26      14      38      77      103     3.05e-18
GENES (2 total):
  AT2G36530.1
    SENSE COUNTS:      26      14      38      77      103     3.05e-18
    TAGS: (2 total)
      d+1  TGATTGTTAT  0       0       1       4       10      7.67e-04        1669
      d+2  AGAATGCTCT  26      14      37      73      93      1.49e-15        1592
           ----------                                                          642
           ----------                                                          571
           ----------                                                          367
           ----------                                                          360

LOCUS: AT5G01530
DESCRIPTION: chlorophyll A-B binding protein CP29 (LHCB4), identical to CP29 (Arabidopsis thaliana) GI:298036;
contains Pfam profile: PF00504 chlorophyll A-B binding protein
DATA:                  Control 30min   2hours  2days   1week   p-value         pos
SENSE COUNTS:          259     135     199     196     66      3.29e-18
GENES (1 total):
  AT5G01530.1
    SENSE COUNTS:      259     135     199     196     66      3.29e-18
    TAGS: (4 total)
      d+1  ACATTAAATT  108     43      82      91      25      1.76e-10        1133
           ----------                                                          1122
      d+2  TGAGATTCTA  151     92      109     105     41      1.69e-08        1034
           ----------                                                          884
      d+2  GCAAGACGCT  0       0       7       0       0       3.86e-05        642
      d+2  GGTACTCGGG  0       0       1       0       0       4.55e-01        193

LOCUS: AT4G31500
DESCRIPTION: cytochrome P450 83B1 (CYP83B1), Identical to Cytochrome P450 (SP:O65782 )(Arabidopsis thaliana)
DATA:                  Control 30min   2hours  2days   1week   p-value         pos
SENSE COUNTS:          131     19      71      78      92      1.36e-17
```

```
GENES (3 total):
  AT4G31500.1
    SENSE COUNTS:      131     19      71      78      92      1.36e-17
    TAGS: (3 total)
      d+1  ACTGGACTCG  4       10      22      5       8       2.95e-03       1480
           ----------                                                         1372
           ----------                                                         1288
           ----------                                                         1221
           ----------                                                         976
           ----------                                                         916
      X+4  TCGCTAACAA  0       1       0       0       0       2.54e-01       888
      d+2  TCGTATCAAG  127     8       49      73      84      1.47e-24       358

LOCUS: AT1G34430
DESCRIPTION: dihydrolipoamide S-acetyltransferase, putative, similar to dihydrolipoamide S-acetyltransferase
(LTA2) (Arabidopsis thaliana) GI:5881963; contains Pfam profiles PF00198: 2-oxo acid dehydrogenases
acyltransferase (catalytic domain), PF00364: Biotin-requiri
DATA:                  Control 30min   2hours  2days   1week   p-value        pos
SENSE COUNTS:          56      6       12      4       13      1.33e-16
GENES (1 total):
  AT1G34430.1
    SENSE COUNTS:      56      6       12      4       13      1.33e-16
    TAGS: (5 total)
           ----------                                                         1716
      d+2  ATTAATAACA  51      5       10      4       12      1.51e-16       1704
      d+2  CGATGAATCT  0       0       0       0       0       6.15e-01       1669
      d+2  ATTTTGCTAC  3       0       0       0       0       2.13e-02       1634
           ----------                                                         1616
      d+2  GGAGCTTGTA  2       1       2       0       1       8.10e-01       1507
           ----------                                                         1337
      d+2  GTTGAAGAAG  0       0       0       0       0       6.15e-01       350
           ----------                                                         191

LOCUS: AT3G55700
DESCRIPTION: UDP-glucoronosyl/UDP-glucosyl transferase family protein, glucuronosyl transferase homolog,
Lycopersicon esculentum, PIR:S39507 ;contains Pfam profile: PF00201 UDP-glucoronosyl and UDP-glucosyl
transferase
DATA:                  Control 30min   2hours  2days   1week   p-value        pos
SENSE COUNTS:          3       1       18      49      43      2.32e-16
GENES (1 total):
  AT3G55700.1
    SENSE COUNTS:      3       1       18      49      43      2.32e-16
    TAGS: (1 total)
           ----------                                                         1436
           ----------                                                         1220
           ----------                                                         1203
           ----------                                                         688
           ----------                                                         157
           ----------                                                         121
      X+4  AAACTTTATT  3       1       18      49      43      2.32e-16       -248

LOCUS: AT4G12800
DESCRIPTION: photosystem I reaction center subunit XI, chloroplast (PSI-L) / PSI subunit V, identical to
Photosystem I reaction center subunit XI, chloroplast precursor (PSI-L) (PSI subunit V) (Swiss-Prot:Q9SUI4)
(Arabidopsis thaliana); contains Pfam profile PF02605:
DATA:                  Control 30min   2hours  2days   1week   p-value        pos
SENSE COUNTS:          45      83      24      26      1       3.24e-16
GENES (1 total):
  AT4G12800.1
    SENSE COUNTS:      45      83      24      26      1       3.24e-16
    TAGS: (1 total)
           ----------                                                         985
      d+2  TGCCTCACCA  45      83      24      26      1       3.24e-16       592
           ----------                                                         482

LOCUS: AT2G39730
DESCRIPTION: ribulose bisphosphate carboxylase/oxygenase activase / RuBisCO activase, identical to SWISS-
PROT:P10896 ribulose bisphosphate carboxylase/oxygenase activase, chloroplast precursor (RuBisCO activase,
RA)(Arabidopsis thaliana)
DATA:                  Control 30min   2hours  2days   1week   p-value        pos
SENSE COUNTS:          188     133     165     96      34      1.20e-15
GENES (4 total):
  AT2G39730.2
    SENSE COUNTS:      188     133     165     96      34      1.20e-15
    TAGS: (2 total)
      d+1  CTTGTGATGG  186     133     152     95      34      1.07e-14       1372
           ----------                                                         940
      d+2  ATGAGTGCTG  2       0       13      1       0       2.60e-06       739
           ----------                                                         712
           ----------                                                         589
           ----------                                                         556
```

```
  AT2G39730.3
    SENSE COUNTS:      188     133     165     96      34      1.20e-15
    TAGS: (2 total)
      d+1  CTTGTGATGG  186     133     152     95      34      1.07e-14       1372
           ----------                                                         940
      d+2  ATGAGTGCTG  2       0       13      1       0       2.60e-06       739
           ----------                                                         712
           ----------                                                         589
           ----------                                                         556
  AT2G39730.1
    SENSE COUNTS:      188     133     165     96      34      1.20e-15
    TAGS: (2 total)
      d+1  CTTGTGATGG  186     133     152     95      34      1.07e-14       1401
           ----------                                                         969
      d+2  ATGAGTGCTG  2       0       13      1       0       2.60e-06       768
           ----------                                                         741
           ----------                                                         618
           ----------                                                         585

LOCUS: AT5G54270
DESCRIPTION: chlorophyll A-B binding protein / LHCII type III (LHCB3), identical to Lhcb3 protein (Arabidopsis
thaliana) GI:4741952; contains Pfam profile PF00504: Chlorophyll A-B binding protein
DATA:                  Control 30min   2hours  2days   1week   p-value        pos
SENSE COUNTS:          67      12      83      43      19      3.42e-15
GENES (2 total):
  AT5G54270.1
    SENSE COUNTS:      67      12      83      43      19      3.42e-15
    TAGS: (3 total)
      d+1  TCTCTCACAG  64      12      59      41      14      4.47e-11       1006
           ----------                                                         597
      d+2  CTCAGAGCAT  1       0       1       0       0       6.01e-01       553
      d+2  GGAGATGGGC  2       0       23      2       5       5.25e-10       394

LOCUS: AT5G24120
DESCRIPTION: RNA polymerase sigma subunit SigE (sigE) / sigma-like factor (SIG5), identical to RNA polymerase
sigma subunit SigE (Arabidopsis thaliana) GI:4972299, sigma-like factor (Arabidopsis thaliana) GI:4033838;
contains Pfam profiles PF04545: Sigma-70, region 4,
DATA:                  Control 30min   2hours  2days   1week   p-value        pos
SENSE COUNTS:          17      2       36      67      26      8.25e-15
GENES (2 total):
  AT5G24120.1
    SENSE COUNTS:      17      2       36      67      26      8.25e-15
    TAGS: (2 total)
      d+1  TAAAGTTTGA  16      2       35      62      25      7.71e-14       1873
           ----------                                                         1593
           ----------                                                         1140
           ----------                                                         1008
      d+2  CTTGGTTGTT  1       0       1       5       1       1.87e-01       783

LOCUS: AT4G21100
DESCRIPTION: UV-damaged DNA-binding protein, putative, similar to UV-damaged DNA binding protein (GI:12082087)
(Oryza sativa) and damage-specific DNA binding protein 1, Homo sapiens, PIR2:I38908; contains Pfam PF03178 :
CPSF A subunit region
DATA:                  Control 30min   2hours  2days   1week   p-value        pos
SENSE COUNTS:          120     74      33      41      124     8.61e-15
GENES (2 total):
  AT4G21100.1
    SENSE COUNTS:      120     74      33      41      124     8.61e-15
    TAGS: (1 total)
           ----------                                                         2920
           ----------                                                         2906
           ----------                                                         2659
           ----------                                                         2144
           ----------                                                         1928
           ----------                                                         1577
           ----------                                                         1364
      d+2  AATATAGAGG  120     74      33      41      124     8.61e-15       1291
           ----------                                                         854
           ----------                                                         821
           ----------                                                         621
           ----------                                                         569
           ----------                                                         215

LOCUS: AT2G26500
DESCRIPTION: cytochrome b6f complex subunit (petM), putative, nearly identical to cytochrome b6f complex
subunit (GI:3090403) (Arabidopsis thaliana); alternative splice forms exist
DATA:                  Control 30min   2hours  2days   1week   p-value        pos
SENSE COUNTS:          134     74      49      45      138     9.72e-15
GENES (2 total):
  AT2G26500.1
    SENSE COUNTS:      134     74      49      45      138     9.72e-15
```

```
    TAGS: (3 total)
      d+1  CCCTCCATTG  1       2       4       1       3       6.51e-01        682
      d+2  TTACATTTGT  133     72      43      43      134     5.07e-16        588
      d+2  AACGCTCTTA  0       0       2       1       1       4.09e-01        422
           ----------                                                          163
  AT2G26500.2
    SENSE COUNTS:      134     74      49      45      138     9.72e-15
    TAGS: (3 total)
      d+1  CCCTCCATTG  1       2       4       1       3       6.51e-01        650
      d+2  TTACATTTGT  133     72      43      43      134     5.07e-16        556
      d+2  AACGCTCTTA  0       0       2       1       1       4.09e-01        390
           ----------                                                          131

LOCUS: AT4G32020
DESCRIPTION: expressed protein, NuLL
DATA:                  Control 30min   2hours  2days   1week   p-value         pos
SENSE COUNTS:          23      25      94      33      58      1.60e-14
GENES (1 total):
  AT4G32020.1
    SENSE COUNTS:      23      25      94      33      58      1.60e-14
    TAGS: (3 total)
      d+1  AGATTAAATA  0       0       2       2       1       3.02e-01        1474
      d+2  TAACCGTTTG  22      25      88      31      57      6.11e-13        1362
      d+2  ACCTCGCCGC  1       0       4       0       0       2.82e-02        583
           ----------                                                          216
           ----------                                                          190

LOCUS: AT4G17090
DESCRIPTION: beta-amylase (CT-BMY) / 1,4-alpha-D-glucan maltohydrolase, identical to beta-amylase enzyme
GI:6065749 from (Arabidopsis thaliana)
DATA:                  Control 30min   2hours  2days   1week   p-value         pos
SENSE COUNTS:          2       3       35      48      40      8.11e-14
GENES (2 total):
  AT4G17090.1
    SENSE COUNTS:      2       3       35      48      40      8.11e-14
    TAGS: (3 total)
      d+1  GGAGGAGACT  2       1       34      47      37      1.22e-14        1631
           ----------                                                          1615
      d+2  GAGATGAAAG  0       2       1       0       3       2.60e-01        1345
           ----------                                                          1319
           ----------                                                          1280
           ----------                                                          1133
           ----------                                                          1102
           ----------                                                          1074
           ----------                                                          1013
           ----------                                                          781
           ----------                                                          636
           ----------                                                          412
           ----------                                                          358
      d+2  ACCTTTGCGA  0       0       0       1       0       3.09e-01        175

LOCUS: AT3G59790
DESCRIPTION: mitogen-activated protein kinase, putative / MAPK, putative (MPK10), mitogen-activated protein
kinase (MAPK)(AtMPK10), PMID:12119167
DATA:                  Control 30min   2hours  2days   1week   p-value         pos
SENSE COUNTS:          75      10      43      47      14      2.52e-13
GENES (2 total):
  AT3G59790.1
    SENSE COUNTS:      75      10      43      47      14      2.52e-13
    TAGS: (2 total)
      v+1  AGCTATTGTC  0       0       1       0       0       4.55e-01        1771
           ----------                                                          1722
           ----------                                                          1107
           ----------                                                          852
           ----------                                                          843
           ----------                                                          838
      v+2  AGAATATTGT  75      10      42      47      14      2.36e-13        700
           ----------                                                          469
           ----------                                                          354

LOCUS: AT4G02770
DESCRIPTION: photosystem I reaction center subunit II, chloroplast, putative / photosystem I 20 kDa subunit,
putative / PSI-D, putative (PSAD1), similar to SP|P12353 Photosystem I reaction center subunit II, chloroplast
precursor (Photosystem I 20 kDa subunit) (PSI-D)
DATA:                  Control 30min   2hours  2days   1week   p-value         pos
SENSE COUNTS:          241     116     171     177     97      2.82e-13
GENES (1 total):
  AT4G02770.1
    SENSE COUNTS:      241     116     171     177     97      2.82e-13
    TAGS: (3 total)
      d+1  TTAATTTTTA  236     115     163     177     97      1.28e-12        764
```

```
          ----------                                                     737
      d+2  AGATCTATTG  4       1       7       0       0       2.04e-02       631
      d+2  AGAGAAGGTC  1       0       1       0       0       6.01e-01       433

LOCUS: AT1G64720
DESCRIPTION: expressed protein, weak similarity to SP|P53809 Phosphatidylcholine transfer protein (PC-TP)
{Rattus norvegicus}
DATA:                  Control 30min   2hours  2days   1week   p-value        pos
SENSE COUNTS:          116     123     85      59      21      6.22e-12
GENES (2 total):
  AT1G64720.1
    SENSE COUNTS:      116     123     85      59      21      6.22e-12
    TAGS: (4 total)
      d+1  TAATAGCAAA  113     111     85      59      21      4.96e-10       1320
      d+2  TAAGTAGACA  1       0       0       0       0       4.28e-01       1235
      d+2  TACATTGGAC  1       12      0       0       0       1.06e-06       1138
      d+2  AATGAGAGGG  1       0       0       0       0       4.28e-01       1040
           ----------                                                     858
           ----------                                                     835

LOCUS: AT1G20440
DESCRIPTION: dehydrin (COR47), identical to dehydrin COR47 (Cold-induced COR47 protein) (Arabidopsis thaliana)
SWISS-PROT:P31168
DATA:                  Control 30min   2hours  2days   1week   p-value        pos
SENSE COUNTS:          7       11      35      62      38      9.09e-12
GENES (2 total):
  AT1G20440.1
    SENSE COUNTS:      7       11      35      62      38      9.09e-12
    TAGS: (3 total)
           ----------                                                     1188
      d+2  CCAAGACCAC  6       11      31      59      37      2.73e-11       857
      X+4  CATAAGAGGA  0       0       1       1       0       5.21e-01       780
      d+2  ACCATCCCGA  1       0       3       2       1       7.11e-01       638

LOCUS: AT5G56030
DESCRIPTION: heat shock protein 81-2 (HSP81-2), nearly identical to SP|P55737 Heat shock protein 81-2 (HSP81-
2) {Arabidopsis thaliana}
DATA:                  Control 30min   2hours  2days   1week   p-value        pos
SENSE COUNTS:          9       5       9       47      21      3.94e-11
GENES (2 total):
  AT5G56030.1
    SENSE COUNTS:      9       5       9       47      21      3.94e-11
    TAGS: (4 total)
      d+1  GATGAGTTGA  3       2       4       16      8       2.48e-03       1934
      d+2  TCGAGTAAGA  0       0       1       0       0       4.55e-01       1889
           ----------                                                     1877
           ----------                                                     1847
           ----------                                                     1835
      d+2  GTTGATGCGA  5       3       4       29      12      2.30e-07       1568
           ----------                                                     1356
           ----------                                                     1118
           ----------                                                     861
      d+2  ATTGGTCAGT  1       0       0       2       1       3.00e-01       443
           ----------                                                     410
           ----------                                                     347

LOCUS: AT1G20630
DESCRIPTION: catalase 1, identical to catalase 1 GI:2511725 from (Arabidopsis thaliana)
DATA:                  Control 30min   2hours  2days   1week   p-value        pos
SENSE COUNTS:          169     98      106     118     41      1.12e-10
GENES (2 total):
  AT1G20630.1
    SENSE COUNTS:      169     98      106     118     41      1.12e-10
    TAGS: (4 total)
      d+1  CCGAAAAATA  1       1       0       0       0       4.77e-01       1290
           ----------                                                     1235
           ----------                                                     1215
           ----------                                                     918
           ----------                                                     834
           ----------                                                     716
           ----------                                                     689
      d+2  TTTTCATTTC  0       0       0       1       0       3.09e-01       638
      d+2  CATTGAAACC  0       0       0       0       0       6.15e-01       552
           ----------                                                     545
           ----------                                                     408
           ----------                                                     321
           ----------                                                     279
           ----------                                                     120
      d+2  GATCCATACA  168     97      106     117     41      1.11e-10       86

LOCUS: AT5G17460
```

```
DESCRIPTION: expressed protein
DATA:                 Control 30min   2hours  2days   1week   p-value         pos
SENSE COUNTS:         0       0       16      22      31      1.22e-10
GENES (1 total):
  AT5G17460.1
    SENSE COUNTS:     0       0       16      22      31      1.22e-10
    TAGS: (2 total)
           ----------                                                         1835
           ----------                                                         1636
           ----------                                                         1068
           ----------                                                         748
      d+2  AAAGATATTC 0       0       13      18      30      6.11e-10        554
      d+2  CTTTGTATTT 0       0       3       4       1       1.12e-01        343

LOCUS: AT5G40480
DESCRIPTION: expressed protein, ; expression supported by MPSS
DATA:                 Control 30min   2hours  2days   1week   p-value         pos
SENSE COUNTS:         61      13      54      43      8       3.02e-10
GENES (1 total):
  AT5G40480.1
    SENSE COUNTS:     61      13      54      43      8       3.02e-10
    TAGS: (2 total)
           ----------                                                         6423
           ----------                                                         6327
      v+2  AGGAATCTCC 0       0       2       2       1       5.87e-01        5995
           ----------                                                         5680
           ----------                                                         5443
           ----------                                                         5433
           ----------                                                         5415
           ----------                                                         5342
           ----------                                                         5281
           ----------                                                         5128
           ----------                                                         4879
           ----------                                                         4780
           ----------                                                         4589
           ----------                                                         4280
           ----------                                                         4078
           ----------                                                         3861
           ----------                                                         3619
           ----------                                                         2651
           ----------                                                         2637
           ----------                                                         2614
           ----------                                                         2416
           ----------                                                         2220
           ----------                                                         1793
           ----------                                                         1504
           ----------                                                         1155
      i+3  TGTGTTTAAA 61      13      52      41      7       1.03e-10        1134
           ----------                                                         1086
           ----------                                                         1006
           ----------                                                         877
           ----------                                                         762
           ----------                                                         541
           ----------                                                         530

LOCUS: AT4G14170
DESCRIPTION: pentatricopeptide (PPR) repeat-containing protein, contains Pfam profile PF01535: PPR repeat
DATA:                 Control 30min   2hours  2days   1week   p-value         pos
SENSE COUNTS:         3       0       7       30      19      4.28e-10
GENES (1 total):
  AT4G14170.1
    SENSE COUNTS:     3       0       7       30      19      4.28e-10
    TAGS: (1 total)
           ----------                                                         1691
           ----------                                                         1623
           ----------                                                         1567
           ----------                                                         1462
           ----------                                                         1033
           ----------                                                         763
      d+2  TTCAGAGAAA 3       0       7       30      19      4.28e-10        654
           ----------                                                         550
           ----------                                                         460
           ----------                                                         267

LOCUS: AT2G13360
DESCRIPTION: AGT1 encodes peroxisomal alanine : glyoxylate aminotransferase. It is involved in
photorespiration.
DATA:                 Control 30min   2hours  2days   1week   p-value         pos
SENSE COUNTS:         1       0       17      0       1       6.23e-10
GENES (3 total):
  AT2G13360.1
```

```
    SENSE COUNTS:      1       0       17      0       1       6.23e-10
    TAGS: (3 total)
           ----------                                                  1375
           ----------                                                  1326
      d+2  GCAGAGGTAC  0       0       12      0       0       3.96e-08        1094
      d+2  GGGGCTGAAA  0       0       4       0       0       5.58e-03        989
      d+2  CTCGTTTGGG  1       0       1       0       1       6.15e-01        949
  AT2G13360.2
    SENSE COUNTS:      1       0       17      0       1       6.23e-10
    TAGS: (3 total)
           ----------                                                  1353
           ----------                                                  1304
      d+2  GCAGAGGTAC  0       0       12      0       0       3.96e-08        1072
      d+2  GGGGCTGAAA  0       0       4       0       0       5.58e-03        967
      d+2  CTCGTTTGGG  1       0       1       0       1       6.15e-01        927

LOCUS: AT3G63410
DESCRIPTION: Encodes a 37kDz polypeptide precursor of the chloroplast inner envelope membrane with partial
sequence similarity to S-adenosylmethionine-dependent methyltransferase.  Mutant plants lack plastoquinone
(PQ), suggesting that the APG1 protein is involved in
DATA:                  Control 30min   2hours  2days   1week   p-value         pos
SENSE COUNTS:          55      14      32      62      78      7.48e-10
GENES (3 total):
  AT3G63410.1
    SENSE COUNTS:      55      14      32      62      78      7.48e-10
    TAGS: (6 total)
      d+1  TTGAGATATC  49      13      22      37      57      4.94e-07        1267
      d+2  TATTACTAGA  0       0       0       1       0       6.04e-01        1249
      d+2  CGGAATATCA  3       1       5       22      17      3.22e-06        1218
      d+2  ATCATTGTAT  3       0       1       2       3       3.22e-01        1154
           ----------                                                  1092
      d+2  GGATGTTCTG  0       0       2       0       0       1.21e-01        924
      d+2  CGAGTGGTCG  0       0       2       0       1       2.22e-01        453
           ----------                                                  376
           ----------                                                  190

LOCUS: AT3G44450
DESCRIPTION: expressed protein,
DATA:                  Control 30min   2hours  2days   1week   p-value         pos
SENSE COUNTS:          1       0       14      15      33      8.13e-10
GENES (1 total):
  AT3G44450.1
    SENSE COUNTS:      1       0       14      15      33      8.13e-10
    TAGS: (2 total)
      X+4  TGCGTGAGAC  0       0       3       2       3       1.60e-01        567
      d+1  ATTTTGAATG  1       0       11      13      30      7.72e-09        440
           ----------                                                  135

LOCUS: AT3G47070
DESCRIPTION: expressed protein
DATA:                  Control 30min   2hours  2days   1week   p-value         pos
SENSE COUNTS:          64      10      67      64      53      1.22e-09
GENES (1 total):
  AT3G47070.1
    SENSE COUNTS:      64      10      67      64      53      1.22e-09
    TAGS: (2 total)
      d+1  ATCTAGTTAT  10      3       11      8       5       2.62e-01        646
      d+2  CCTCTGTTTT  54      7       56      56      48      6.75e-09        565

LOCUS: AT3G20430
DESCRIPTION: expressed protein,
DATA:                  Control 30min   2hours  2days   1week   p-value         pos
SENSE COUNTS:          44      3       8       24      19      1.48e-09
GENES (1 total):
  AT3G20430.1
    SENSE COUNTS:      44      3       8       24      19      1.48e-09
    TAGS: (2 total)
      d+1  AGAGGATCAG  0       0       0       0       0       6.15e-01        713
           ----------                                                  328
           ----------                                                  268
      d+2  ATTTAGATTC  44      3       8       24      19      5.16e-10        113

LOCUS: AT1G30680
DESCRIPTION: toprim domain-containing protein, contains Pfam profile: PF01751 toprim domain
DATA:                  Control 30min   2hours  2days   1week   p-value         pos
SENSE COUNTS:          0       0       2       0       14      2.53e-09
GENES (1 total):
  AT1G30680.1
    SENSE COUNTS:      0       0       2       0       14      2.53e-09
    TAGS: (3 total)
      i+3  ATATGTTAAA  0       0       0       0       1       1.65e-01        3504
```

```
      d+1  GTGTTTCTCT 0       0       2       0       12      7.73e-07        2403
      d+2  TCTGGTTTGT 0       0       0       0       1       1.65e-01        1897
           ----------                                                         1561
           ----------                                                         1363
           ----------                                                         554
           ----------                                                         138

LOCUS: AT3G57660
DESCRIPTION: DNA-directed RNA polymerase family protein, similar to SP|O35134 DNA-directed RNA polymerase I
largest subunit (EC 2.7.7.6) (RNA polymerase I 194 kDa subunit) (RPA194) {Mus musculus}; contains InterPro
accession IPR000722: RNA polymerase, alpha subunit
DATA:                 Control 30min   2hours  2days   1week   p-value         pos
SENSE COUNTS:         3       29      7       1       6       2.89e-09
GENES (1 total):
  AT3G57660.1
    SENSE COUNTS:     3       29      7       1       6       2.89e-09
    TAGS: (2 total)
           ----------                                                         5808
      v+2  GAGACAATCC 0       0       0       0       1       1.65e-01        4867
           ----------                                                         4263
           ----------                                                         4080
           ----------                                                         3989
           ----------                                                         3642
           ----------                                                         3390
           ----------                                                         3305
           ----------                                                         3006
           ----------                                                         2776
           ----------                                                         2666
           ----------                                                         2430
           ----------                                                         2388
           ----------                                                         1696
           ----------                                                         1050
      i+3  AGATTAATTT 3       29      7       1       5       1.18e-09        732
           ----------                                                         596
           ----------                                                         456
           ----------                                                         414

LOCUS: AT1G20450
DESCRIPTION: dehydrin (ERD10), identical to dehydrin ERD10 (Low-temperature-induced protein LTI45)
(Arabidopsis thaliana) SWISS-PROT:P42759
DATA:                 Control 30min   2hours  2days   1week   p-value         pos
SENSE COUNTS:         2       15      20      49      23      3.04e-09
GENES (4 total):
  AT1G20450.2
    SENSE COUNTS:     2       15      19      47      23      1.86e-08
    TAGS: (3 total)
      d+1  AAAAGTTTTT 1       0       2       6       3       1.18e-01        1498
      d+2  CCAGCACCAC 1       15      17      41      19      2.98e-07        1010
      i+3  AACAGTCGAG 0       0       0       0       1       1.65e-01        915
           ----------                                                         903
           ----------                                                         77
  AT1G20450.1
    SENSE COUNTS:     2       15      20      49      23      3.04e-09
    TAGS: (4 total)
      d+1  AAAAGTTTTT 1       0       2       6       3       1.18e-01        1501
      d+2  CCAGCACCAC 1       15      17      41      19      2.98e-07        1013
      i+3  AACAGTCGAG 0       0       0       0       1       1.65e-01        915
           ----------                                                         906
      X+4  CAAGAGTTAG 0       0       1       2       0       1.79e-01        719
           ----------                                                         77

LOCUS: AT5G24740
DESCRIPTION: expressed protein
DATA:                 Control 30min   2hours  2days   1week   p-value         pos
SENSE COUNTS:         2       5       1       3       27      1.03e-08
GENES (1 total):
  AT5G24740.1
    SENSE COUNTS:     2       5       1       3       27      1.03e-08
    TAGS: (5 total)
      d+1  TTTTGGCTAT 1       1       0       0       0       4.77e-01        10022
           ----------                                                         9411
           ----------                                                         9241
           ----------                                                         9209
           ----------                                                         9086
           ----------                                                         8875
           ----------                                                         8828
           ----------                                                         8465
           ----------                                                         8117
           ----------                                                         7862
           ----------                                                         7501
           ----------                                                         7463
```

	----------							7457
	----------							7390
	----------							7116
	----------							6961
d+2	CATTTATTCA	0	4	1	2	26	2.42e-11	6918
	----------							6779
	----------							6486
	----------							6296
	----------							6029
	----------							6015
	----------							5728
	----------							5315
i+3	AAAAGGGTTT	1	0	0	0	1	3.83e-01	5121
	----------							4944
	----------							4798
	----------							4742
	----------							4712
	----------							4669
	----------							4421
	----------							4405
	----------							3997
d+2	TGTTATGTCA	0	0	0	1	0	6.04e-01	3591
	----------							2837
	----------							2810
d+2	GGATGGATGC	0	0	0	0	0	6.15e-01	2324
	----------							2318
	----------							2036
	----------							2002
	----------							1834
	----------							1727
	----------							1475
	----------							1358
	----------							1279
	----------							1216
	----------							817
	----------							219

LOCUS: AT1G09690
DESCRIPTION: 60S ribosomal protein L21 (RPL21C), Similar to ribosomal protein L21 (gb|L38826). ESTs gb|AA395597,gb|ATTS5197 come from this gene

DATA:		Control	30min	2hours	2days	1week	p-value	pos
SENSE COUNTS:		26	8	13	22	55	2.00e-08	
GENES (1 total):								
AT1G09690.1								
SENSE COUNTS:		26	8	13	22	55	2.00e-08	
TAGS: (2 total)								
d+1	TGTCTTAGCT	26	8	13	22	52	2.20e-07	593
	----------							462
d+2	TGCAGCAATC	0	0	0	0	3	1.12e-02	328
	----------							313
	----------							58

LOCUS: AT5G47930
DESCRIPTION: 40S ribosomal protein S27 (RPS27D)

DATA:		Control	30min	2hours	2days	1week	p-value	pos
SENSE COUNTS:		16	3	6	17	40	4.46e-08	
GENES (1 total):								
AT5G47930.1								
SENSE COUNTS:		16	3	6	17	40	4.46e-08	
TAGS: (3 total)								
d+1	TTTACAATTC	1	0	1	2	14	2.25e-05	543
d+2	ATAAAACTAC	15	3	5	15	25	4.28e-04	427
i+3	TGAGTTTACT	0	0	0	0	1	1.65e-01	392
	----------							175
	----------							73

LOCUS: AT4G28670
DESCRIPTION: protein kinase family protein, contains Pfam domain, PF00069: Protein kinase domain

DATA:		Control	30min	2hours	2days	1week	p-value	pos
SENSE COUNTS:		5	0	3	4	25	7.03e-08	
GENES (1 total):								
AT4G28670.1								
SENSE COUNTS:		5	0	3	4	25	7.03e-08	
TAGS: (1 total)								
	----------							2702
	----------							2238
	----------							2226
	----------							2218
	----------							1545
	----------							1438
	----------							1134

```
          ----------                                                        568
          ----------                                                        562
          ----------                                                        544
          ----------                                                        423
          ----------                                                        354
      v+2  ATTTTACTAA  5       0       3       4       25      7.03e-08     75

LOCUS: AT1G62600
DESCRIPTION: flavin-containing monooxygenase family protein / FMO family protein, low similarity to flavin-
containing monooxygenase 2 from Cavia porcellus (SP|P36366); contains Pfam profile PF00743 Flavin-binding
monooxygenase-like
DATA:                  Control 30min   2hours  2days   1week   p-value      pos
SENSE COUNTS:          2       18      3       0       0       8.50e-08
GENES (1 total):
  AT1G62600.1
    SENSE COUNTS:      2       18      3       0       0       8.50e-08
    TAGS: (1 total)
          ----------                                                        1833
          ----------                                                        1067
          ----------                                                        868
          ----------                                                        778
          ----------                                                        400
      X+4  AGTTTTTGGG  2       18      3       0       0       8.50e-08     -9

LOCUS: AT3G17800
DESCRIPTION: expressed protein
DATA:                  Control 30min   2hours  2days   1week   p-value      pos
SENSE COUNTS:          10      7       42      26      16      1.10e-07
GENES (1 total):
  AT3G17800.1
    SENSE COUNTS:      10      7       42      26      16      1.10e-07
    TAGS: (2 total)
          ----------                                                        2167
          ----------                                                        2143
          ----------                                                        2075
          ----------                                                        2065
          ----------                                                        1916
      d+2  GTCTGTAATG  10      7       41      26      16      2.56e-07     1552
      d+2  TAGATGCGAG  0       0       1       0       0       4.55e-01     1513
          ----------                                                        816
          ----------                                                        282
          ----------                                                        38

LOCUS: AT1G62750
DESCRIPTION: elongation factor Tu family protein, similar to elongation factor G SP:P34811 (Glycine max
(Soybean))
DATA:                  Control 30min   2hours  2days   1week   p-value      pos
SENSE COUNTS:          17      1       11      10      37      1.27e-07
GENES (1 total):
  AT1G62750.1
    SENSE COUNTS:      17      1       11      10      37      1.27e-07
    TAGS: (2 total)
          ----------                                                        2593
      d+2  TAAGCTAATC  17      1       11      9       37      7.43e-08     2542
          ----------                                                        2203
          ----------                                                        2105
          ----------                                                        2014
      d+2  AGTGATCCTT  0       0       0       1       0       3.09e-01     1339
          ----------                                                        1066
          ----------                                                        835
          ----------                                                        518

LOCUS: AT1G73660
DESCRIPTION: protein kinase family protein, contains Pfam profile: PF00069 eukaryotic protein kinase domain
DATA:                  Control 30min   2hours  2days   1week   p-value      pos
SENSE COUNTS:          2       24      7       3       4       2.25e-07
GENES (1 total):
  AT1G73660.1
    SENSE COUNTS:      2       24      7       3       4       2.25e-07
    TAGS: (4 total)
      d+1  CGTAATTTCC  1       1       3       2       3       8.34e-01     3821
          ----------                                                        3815
      d+2  GAAGATGACG  0       0       0       0       0       6.15e-01     3741
          ----------                                                        3573
      d+2  GGAGCTGTGA  1       23      4       1       0       2.67e-10     2921
          ----------                                                        2885
          ----------                                                        2631
          ----------                                                        2601
          ----------                                                        2429
          ----------                                                        2304
          ----------                                                        2082
```

```
          ----------                                                    1725
          ----------                                                    1436
          ----------                                                    1382
          ----------                                                    1352
          ----------                                                    1195
      d+2 TGGATTTAGG 0      0      0      0      1      1.65e-01        879
          ----------                                                    593
          ----------                                                    42

LOCUS: AT4G26660
DESCRIPTION: expressed protein, weak similarity to phragmoplast-associated kinesin-related protein 1
(Arabidopsis thaliana) GI:8745333
DATA:                  Control 30min  2hours 2days  1week  p-value         pos
SENSE COUNTS:          31     5      30     15     0      2.44e-07
GENES (1 total):
  AT4G26660.1
    SENSE COUNTS:      31     5      30     15     0      2.44e-07
    TAGS: (2 total)
      d+1 TATTGATTGA 0      0      0      0      0      6.15e-01        2643
          ----------                                                    2403
          ----------                                                    2359
          ----------                                                    2019
          ----------                                                    1403
      d+2 TAGAAAGAAA 31     5      30     15     0      1.01e-07        1330
          ----------                                                    1064
          ----------                                                    828
          ----------                                                    757
          ----------                                                    620
          ----------                                                    74

LOCUS: AT1G61800
DESCRIPTION: glucose-6-phosphate/phosphate translocator, putative, similar to glucose-6-phosphate/phosphate-
translocator precursor GI:2997591 from (Pisum sativum)
DATA:                  Control 30min  2hours 2days  1week  p-value         pos
SENSE COUNTS:          1      0      1      6      16     2.61e-07
GENES (1 total):
  AT1G61800.1
    SENSE COUNTS:      1      0      1      6      16     2.61e-07
    TAGS: (3 total)
      d+1 GATATGAAGA 1      0      0      5      10     1.65e-04        1383
      d+2 TCATTAGACC 0      0      0      0      3      1.12e-02        1084
          ----------                                                    709
      X+4 TGTAATAGTT 0      0      1      1      3      1.91e-01        346

LOCUS: AT4G26970
DESCRIPTION: aconitate hydratase, cytoplasmic, putative / citrate hydro-lyase/aconitase, putative, strong
similarity to SP|P49608 Aconitate hydratase, cytoplasmic (EC 4.2.1.3) (Citrate hydro-lyase) (Aconitase)
{Cucurbita maxima}; contains Pfam profiles PF00330: Aconit
DATA:                  Control 30min  2hours 2days  1week  p-value         pos
SENSE COUNTS:          5      23     3      5      0      2.72e-07
GENES (1 total):
  AT4G26970.1
    SENSE COUNTS:      5      23     3      5      0      2.72e-07
    TAGS: (3 total)
          ----------                                                    3461
      d+2 AACGCTACAC 1      2      1      0      0      4.78e-01        2963
          ----------                                                    2396
          ----------                                                    2287
          ----------                                                    1980
          ----------                                                    1860
          ----------                                                    1801
          ----------                                                    1742
          ----------                                                    1638
          ----------                                                    1634
          ----------                                                    1595
      d+2 TATTTCTGGT 0      0      1      0      0      4.55e-01        1569
          ----------                                                    1559
          ----------                                                    1406
      d+2 GTGTTGTCGG 4      21     1      5      0      2.47e-07        1277
          ----------                                                    1174
          ----------                                                    1168
          ----------                                                    1093
          ----------                                                    964
          ----------                                                    454
          ----------                                                    362
          ----------                                                    163

LOCUS: AT4G30190
DESCRIPTION: belongs to the P-type ATPase superfamily of cation-transporting ATPases, pumps protons out of the
cell, generating a proton gradient that drives the active transport of nutrients by proton symport. has two
autoinhibitory  regions within the C-terminal dom
```

```
DATA:                  Control 30min   2hours  2days   1week   p-value         pos
SENSE COUNTS:          11      5       4       9       35      3.13e-07
GENES (2 total):
  AT4G30190.1
    SENSE COUNTS:      11      5       4       9       35      3.13e-07
    TAGS: (2 total)
      d+1  AGCCTATGTA  10      5       2       8       28      6.12e-06        3243
           ----------                                                          3214
      d+2  CCTTTGTGTT  1       0       2       1       7       9.17e-02        3188
           ----------                                                          3019
           ----------                                                          2919
           ----------                                                          2303
           ----------                                                          2198
           ----------                                                          2159
           ----------                                                          2117
           ----------                                                          1727
           ----------                                                          1608
           ----------                                                          1564
           ----------                                                          1268
           ----------                                                          1025
           ----------                                                          920
           ----------                                                          371
           ----------                                                          353
           ----------                                                          346

LOCUS: AT1G15670
DESCRIPTION: kelch repeat-containing F-box family protein, similar to SP|Q9ER30 Kelch-related protein 1
(Sarcosin) {Rattus norvegicus}; contains Pfam profiles PF01344: Kelch motif, PF00646: F-box domain
DATA:                  Control 30min   2hours  2days   1week   p-value         pos
SENSE COUNTS:          0       10      0       0       0       5.38e-07
GENES (1 total):
  AT1G15670.1
    SENSE COUNTS:      0       10      0       0       0       5.38e-07
    TAGS: (2 total)
      d+1  TACTATTTGG  0       3       0       0       0       3.05e-02        1499
      d+2  ATAAATATGA  0       7       0       0       0       7.93e-05        1201
           ----------                                                          902
           ----------                                                          829
           ----------                                                          627
           ----------                                                          560

LOCUS: AT5G52650
DESCRIPTION: 40S ribosomal protein S10 (RPS10C), contains similarity to 40S ribosomal protein S10
DATA:                  Control 30min   2hours  2days   1week   p-value         pos
SENSE COUNTS:          20      10      11      44      37      7.70e-07
GENES (1 total):
  AT5G52650.1
    SENSE COUNTS:      20      10      11      44      37      7.70e-07
    TAGS: (2 total)
      i+3  AACATATTTA  0       0       1       0       0       4.55e-01        1054
      d+1  GAGGAGTTTT  20      10      10      44      37      3.94e-07        647
           ----------                                                          419

LOCUS: AT3G11120
DESCRIPTION: 60S ribosomal protein L41 (RPL41E), identical to ribosomal protein L41 GB:AAA79268 (Pisum
sativum)
DATA:                  Control 30min   2hours  2days   1week   p-value         pos
SENSE COUNTS:          14      4       11      26      39      7.88e-07
GENES (1 total):
  AT3G11120.1
    SENSE COUNTS:      14      4       11      26      39      7.88e-07
    TAGS: (2 total)
      i+3  AGGGCTAAGG  1       0       0       0       0       4.28e-01        80
      d+1  AGGGCTAAGT  13      4       11      26      39      5.57e-07        80

LOCUS: AT1G09210
DESCRIPTION: calreticulin 2 (CRT2), identical to SP|Q38858 Calreticulin 2 precursor {Arabidopsis thaliana}
DATA:                  Control 30min   2hours  2days   1week   p-value         pos
SENSE COUNTS:          19      9       31      34      54      8.37e-07
GENES (1 total):
  AT1G09210.1
    SENSE COUNTS:      19      9       31      34      54      8.37e-07
    TAGS: (3 total)
      i+3  GTCACACCAG  0       0       1       0       0       4.55e-01        2424
      d+1  AAGAAGTTTT  19      9       30      34      53      1.99e-06        1521
           ----------                                                          1339
           ----------                                                          1127
           ----------                                                          903
           ----------                                                          595
      d+2  TGAAACTGAC  0       0       0       0       1       1.65e-01        572
           ----------                                                          520
```

```
          ----------                                                        477
          ----------                                                        414

LOCUS: AT5G24330
DESCRIPTION: PHD finger family protein / SET domain-containing protein, contains Pfam domain, PF00628: PHD-
finger and PF00856: SET domain
DATA:                 Control 30min   2hours  2days   1week   p-value        pos
SENSE COUNTS:         17      25      54      13      19      8.68e-07
GENES (1 total):
  AT5G24330.1
    SENSE COUNTS:     17      25      54      13      19      8.68e-07
    TAGS: (1 total)
          ----------                                                        1516
          ----------                                                        1375
          ----------                                                        1153
          ----------                                                        921
      v+2  ATTCCGATTC 17      25      54      13      19      8.68e-07       427
          ----------                                                        417

LOCUS: AT4G23950
DESCRIPTION: expressed protein, ; expression supported by MPSS
DATA:                 Control 30min   2hours  2days   1week   p-value        pos
SENSE COUNTS:         6       0       0       1       16      1.07e-06
GENES (1 total):
  AT4G23950.1
    SENSE COUNTS:     6       0       0       1       16      1.07e-06
    TAGS: (1 total)
          ----------                                                        2218
          ----------                                                        1728
      v+2  ATACTGCTAA 6       0       0       1       16      1.07e-06       1704
          ----------                                                        865
          ----------                                                        589
          ----------                                                        288
          ----------                                                        57

LOCUS: AT5G56320
DESCRIPTION: expansin, putative (EXP14), similar to alpha-expansin 3 GI:6942322 from (Triphysaria versicolor);
alpha-expansin gene family, PMID:11641069
DATA:                 Control 30min   2hours  2days   1week   p-value        pos
SENSE COUNTS:         0       0       3       1       15      1.15e-06
GENES (2 total):
  AT5G56320.1
    SENSE COUNTS:     0       0       3       1       15      1.15e-06
    TAGS: (2 total)
      d+1  TCAAGAAACT 0       0       2       0       1       2.22e-01       702
      i+3  GGTAATTTGC 0       0       1       1       14      7.66e-07       352
          ----------                                                        243

LOCUS: AT5G50920
DESCRIPTION: ATP-dependent Clp protease ATP-binding subunit / ClpC, almost identical to ClpC GI:2921158 from
(Arabidopsis thaliana); contains Pfam profile PF02861: Clp amino terminal domain; contains Pfam profile
PF00004: ATPase, AAA family; contains Pfam profile PF02
DATA:                 Control 30min   2hours  2days   1week   p-value        pos
SENSE COUNTS:         81      29      83      81      79      1.21e-06
GENES (2 total):
  AT5G50920.1
    SENSE COUNTS:     81      29      83      81      79      1.21e-06
    TAGS: (5 total)
      d+1  CAAAGAGGAG 11      0       14      11      10      5.46e-03       3150
      d+2  CTTTTTAAGG 52      21      44      41      46      8.38e-03       3120
      d+2  GGGGCTTTCT 17      7       25      27      23      6.38e-03       3038
          ----------                                                        2775
      d+2  ACATCAAACG 0       0       0       2       0       4.80e-02       2424
          ----------                                                        2343
          ----------                                                        2190
          ----------                                                        2163
          ----------                                                        1904
      d+2  CACAGGTCCC 1       1       0       0       0       4.77e-01       1657
          ----------                                                        1170
          ----------                                                        858
          ----------                                                        591
          ----------                                                        159

LOCUS: AT2G27680
DESCRIPTION: aldo/keto reductase family protein, contains Pfam profile PF00248: oxidoreductase, aldo/keto
reductase family
DATA:                 Control 30min   2hours  2days   1week   p-value        pos
SENSE COUNTS:         12      3       3       4       28      1.28e-06
GENES (1 total):
  AT2G27680.1
    SENSE COUNTS:     12      3       3       4       28      1.28e-06
```

```
    TAGS: (2 total)
      d+1  TTTGGAGAGA  0       0       0       0       0       6.15e-01        1306
      d+2  TAATTACACT  12      3       3       4       28      4.92e-07        1225
           ----------                                                          949
           ----------                                                          887
           ----------                                                          705
           ----------                                                          27

LOCUS: AT1G53240
DESCRIPTION: malate dehydrogenase (NAD), mitochondrial, identical to mitochondrial NAD-dependent malate
dehydrogenase GI:3929649 SP|Q9ZP06 from (Arabidopsis thaliana); contains InterPro entry IPR001236:
Lactate/malate dehydrogenase
DATA:                  Control 30min   2hours  2days   1week   p-value         pos
SENSE COUNTS:          29      8       24      17      53      1.41e-06
GENES (1 total):
  AT1G53240.1
    SENSE COUNTS:      29      8       24      17      53      1.41e-06
    TAGS: (6 total)
      d+1  AACAAGAGAT  24      8       15      12      48      5.94e-07        1378
      d+2  CGACTTTTTT  2       0       3       5       3       4.58e-01        1362
      d+2  TCAAAGTTGT  1       0       6       0       0       7.29e-03        1325
      d+2  TTTGTGCCTC  2       0       0       0       0       1.04e-01        1252
      d+2  CTTGAAAGGA  0       0       0       0       1       1.65e-01        936
           ----------                                                          904
           ----------                                                          752
      d+2  CGCTTATTAA  0       0       0       0       1       1.65e-01        554
           ----------                                                          394
           ----------                                                          283

LOCUS: AT4G36040
DESCRIPTION: DNAJ heat shock N-terminal domain-containing protein (J11), identical to dnaJ heat shock protein
J11 (Arabidopsis thaliana) GI:9843641; contains Pfam profile PF00226 DnaJ domain
DATA:                  Control 30min   2hours  2days   1week   p-value         pos
SENSE COUNTS:          37      37      38      8       3       1.73e-06
GENES (1 total):
  AT4G36040.1
    SENSE COUNTS:      37      37      38      8       3       1.73e-06
    TAGS: (3 total)
      d+1  TGTTACTACT  2       0       0       0       0       1.04e-01        1055
      d+2  TGCTTACCGT  35      37      37      8       3       3.04e-06        694
           ----------                                                          663
      d+2  CCGCTTACTG  0       0       1       0       0       4.55e-01        425
           ----------                                                          415

LOCUS: AT1G26610
DESCRIPTION: zinc finger (C2H2 type) family protein, contains Pfam domain, PF00096: Zinc finger, C2H2 type
DATA:                  Control 30min   2hours  2days   1week   p-value         pos
SENSE COUNTS:          22      6       23      43      43      1.75e-06
GENES (1 total):
  AT1G26610.1
    SENSE COUNTS:      22      6       23      43      43      1.75e-06
    TAGS: (2 total)
      d+1  AGATGCATAC  0       0       0       0       0       6.15e-01        1627
           ----------                                                          1342
           ----------                                                          1227
           ----------                                                          645
      d+2  AATGAGAATT  22      6       23      43      43      7.52e-07        405

LOCUS: AT5G59180
DESCRIPTION: DNA-directed RNA polymerase II, identical to Swiss-Prot:P38421 DNA-directed RNA polymerase II 19
kDa polypeptide (EC 2.7.7.6) (RNA polymerase II subunit 5) (Arabidopsis thaliana)
DATA:                  Control 30min   2hours  2days   1week   p-value         pos
SENSE COUNTS:          0       0       0       0       9       1.81e-06
GENES (1 total):
  AT5G59180.1
    SENSE COUNTS:      0       0       0       0       9       1.81e-06
    TAGS: (2 total)
           ----------                                                          840
      d+2  CCTAACTATA  0       0       0       0       1       4.65e-01        455
      X+4  TCTCAATGAA  0       0       0       0       8       1.50e-06        289
           ----------                                                          210
           ----------                                                          176
           ----------                                                          116

LOCUS: AT2G28140
DESCRIPTION: expressed protein
DATA:                  Control 30min   2hours  2days   1week   p-value         pos
SENSE COUNTS:          13      26      56      20      39      2.07e-06
GENES (1 total):
  AT2G28140.1
    SENSE COUNTS:      13      26      56      20      39      2.07e-06
```

```
    TAGS: (1 total)
          ----------                                                    932
      d+2  TTAAACAAAA 13      26      56      20      39      2.07e-06        882

LOCUS: AT1G71500
DESCRIPTION: Rieske (2Fe-2S) domain-containing protein, contains Pfam profile PF00355: iron-sulfur cluster-
binding protein, rieske family
DATA:                 Control 30min   2hours  2days   1week   p-value         pos
SENSE COUNTS:         10      1       0       2       19      2.16e-06
GENES (1 total):
  AT1G71500.1
    SENSE COUNTS:     10      1       0       2       19      2.16e-06
    TAGS: (2 total)
      d+1  CTAGGCCACT 2       0       0       2       0       1.57e-01        1174
      d+2  TAATAGTATA 8       1       0       0       19      1.12e-07        989

LOCUS: AT5G55620
DESCRIPTION: expressed protein, similar to unknown protein (gb|AAF04428.1)
DATA:                 Control 30min   2hours  2days   1week   p-value         pos
SENSE COUNTS:         13      0       5       6       25      2.16e-06
GENES (1 total):
  AT5G55620.1
    SENSE COUNTS:     13      0       5       6       25      2.16e-06
    TAGS: (3 total)
      X+4  CTGGATATTT 0       0       0       1       1       3.25e-01        835
           ----------                                                         728
      X+4  TATAACACTA 11      0       2       4       12      5.15e-04        727
      d+2  TATATCAATC 2       0       3       1       12      1.68e-03        582
           ----------                                                         521

LOCUS: AT4G34190
DESCRIPTION: stress enhanced protein 1 (SEP1), identical to stress enhanced protein 1 (SEP1) GI:7384978 from
(Arabidopsis thaliana)
DATA:                 Control 30min   2hours  2days   1week   p-value         pos
SENSE COUNTS:         3       1       4       14      23      2.58e-06
GENES (2 total):
  AT4G34190.1
    SENSE COUNTS:     3       1       4       14      23      2.58e-06
    TAGS: (4 total)
      d+1  TATTAAGTTT 1       1       1       6       3       1.31e-01        755
      d+2  ATCTATAACA 0       0       0       0       1       1.65e-01        743
      d+2  TCTTTTGGCC 1       0       2       8       19      9.61e-07        704
           ----------                                                         693
           ----------                                                         395
      d+2  TTCCGCAACT 1       0       1       0       0       6.01e-01        222
           ----------                                                         211

LOCUS: AT1G55450
DESCRIPTION: embryo-abundant protein-related, similar to embryo-abundant protein GI:1350531 from (Picea
glauca)
DATA:                 Control 30min   2hours  2days   1week   p-value         pos
SENSE COUNTS:         0       2       17      5       1       2.81e-06
GENES (1 total):
  AT1G55450.1
    SENSE COUNTS:     0       2       17      5       1       2.81e-06
    TAGS: (1 total)
           ----------                                                         1347
      d+2  ACTCAAGGGG 0       2       17      5       1       2.81e-06        1162
           ----------                                                         1083
           ----------                                                         1075
           ----------                                                         1067
           ----------                                                         519

LOCUS: ATCG01130
DESCRIPTION: hypothetical protein
DATA:                 Control 30min   2hours  2days   1week   p-value         pos
SENSE COUNTS:         286     224     210     182     161     3.26e-06
GENES (1 total):
  ATCG01130.1
    SENSE COUNTS:     286     224     210     182     161     3.26e-06
    TAGS: (12 total)
      d+1  TATACGCGAT 1       0       0       1       0       5.06e-01        5338
      d+2  GGTCATACCA 54      70      32      44      32      6.30e-04        4305
      d+2  TCTATAGGCA 13      10      6       6       7       4.77e-01        3937
      d+2  GGCTAGATTA 56      47      66      44      35      8.19e-02        3702
      d+2  AACTATCTGT 93      38      36      26      32      6.68e-11        2978
      d+2  TTAAATGTAC 15      7       6       8       16      1.65e-01        2465
      d+2  CGTTCCCAAA 25      21      46      27      21      1.02e-02        2065
      d+2  GCGAGATTTA 1       0       0       0       1       3.83e-01        1949
      d+2  GTCTACTTCT 13      6       3       12      8       1.01e-01        1574
      d+2  GAATCTCTCG 2       0       1       4       1       5.72e-01        1472
```

```
      d+2  CCAAAGTGAT 12      6       11      6       7       4.90e-01        1167
      d+1  AAATGGGTTG 1       19      3       4       1       1.35e-06        559

LOCUS: AT1G31580
DESCRIPTION: expressed protein, identical to ORF1 (Arabidopsis thaliana) gi|457716|emb|CAA50905
DATA:                 Control 30min   2hours  2days   1week   p-value         pos
SENSE COUNTS:         15      0       1       1       13      3.55e-06
GENES (2 total):
  AT1G31580.1
    SENSE COUNTS:     15      0       1       1       13      3.55e-06
    TAGS: (2 total)
           ----------                                                         641
      d+2  TATGATGTAT 15      0       1       1       12      7.23e-06        604
           ----------                                                         570
      d+2  TATGTGGCTC 0       0       0       0       1       1.65e-01        541
           ----------                                                         509
           ----------                                                         291
           ----------                                                         155

LOCUS: AT2G30870
DESCRIPTION: glutathione S-transferase, putative, supported by cDNA GI:443698 GB:D17673
DATA:                 Control 30min   2hours  2days   1week   p-value         pos
SENSE COUNTS:         2       19      4       2       1       4.26e-06
GENES (2 total):
  AT2G30870.1
    SENSE COUNTS:     2       19      4       2       1       4.26e-06
    TAGS: (1 total)
           ----------                                                         1491
           ----------                                                         1032
           ----------                                                         879
      d+2  TTAGCGCTTG 2       19      4       2       1       4.26e-06        612
           ----------                                                         251
           ----------                                                         146

LOCUS: AT4G22890
DESCRIPTION: expressed protein
DATA:                 Control 30min   2hours  2days   1week   p-value         pos
SENSE COUNTS:         62      21      56      40      21      4.55e-06
GENES (3 total):
  AT4G22890.1
    SENSE COUNTS:     62      21      55      39      20      3.22e-06
    TAGS: (3 total)
      d+1  AAAGACCAAA 6       1       13      5       1       9.61e-03        1383
      d+2  TTTTCTGATT 55      20      42      34      19      1.34e-04        1263
      d+2  TTAGCTCTCT 1       0       0       0       0       4.28e-01        1241
           ----------                                                         865
           ----------                                                         616
           ----------                                                         514
  AT4G22890.2
    SENSE COUNTS:     0       0       1       1       0       5.21e-01
    TAGS: (1 total)
      d+1  GAGCTTCCAG 0       0       1       1       0       5.21e-01        780
           ----------                                                         531
           ----------                                                         429
  AT4G22890.3
    SENSE COUNTS:     62      21      55      39      21      5.35e-06
    TAGS: (4 total)
      d+1  AAAGACCAAA 6       1       13      5       1       9.61e-03        1379
      d+2  TTTTCTGATT 55      20      42      34      19      1.34e-04        1259
      d+2  TTAGCTCTCT 1       0       0       0       0       4.28e-01        1237
           ----------                                                         865
           ----------                                                         616
           ----------                                                         514
      X+4  TTTCGAAAAA 0       0       0       0       1       1.65e-01        -209

LOCUS: AT3G05220
DESCRIPTION: heavy-metal-associated domain-containing protein, similar to farnesylated protein 1 (GI:23304411)
{Hordeum vulgare subsp. spontaneum}; contains Pfam profile PF00403: Heavy-metal-associated domain
DATA:                 Control 30min   2hours  2days   1week   p-value         pos
SENSE COUNTS:         6       23      5       5       3       4.76e-06
GENES (2 total):
  AT3G05220.1
    SENSE COUNTS:     6       23      5       5       3       4.76e-06
    TAGS: (3 total)
      d+1  TATGCTTGTC 3       7       5       0       3       1.57e-01        1976
           ----------                                                         1806
           ----------                                                         1774
           ----------                                                         1404
           ----------                                                         1378
      d+2  GTGGGGGAGG 0       0       0       0       0       6.15e-01        1303
           ----------                                                         1279
```

```
      d+2  GTAATGGTGG 3       16      0       5       0       4.50e-06       949
           ----------                                                        891
           ----------                                                        868
           ----------                                                        862
           ----------                                                        856
           ----------                                                        601
           ----------                                                        595
           ----------                                                        507
           ----------                                                        418
  AT3G05220.2
    SENSE COUNTS:     6       23      5       5       3       4.76e-06
    TAGS: (3 total)
      d+1  TATGCTTGTC 3       7       5       0       3       1.57e-01       1789
           ----------                                                        1619
           ----------                                                        1587
           ----------                                                        1217
           ----------                                                        1191
      d+2  GTGGGGGAGG 0       0       0       0       0       6.15e-01       1116
           ----------                                                        1092
      d+2  GTAATGGTGG 3       16      0       5       0       4.50e-06       762
           ----------                                                        704
           ----------                                                        681
           ----------                                                        675
           ----------                                                        669
           ----------                                                        414
           ----------                                                        408
           ----------                                                        320
           ----------                                                        231
           ----------                                                        36

LOCUS: AT4G11600
DESCRIPTION: glutathione peroxidase, putative
DATA:                 Control 30min   2hours  2days   1week   p-value        pos
SENSE COUNTS:         0       3       9       23      7       5.74e-06
GENES (2 total):
  AT4G11600.1
    SENSE COUNTS:     0       3       9       23      7       5.74e-06
    TAGS: (2 total)
      d+1  AAACTGTGTC 0       0       4       0       0       3.11e-02       938
      d+2  GTTTCGAGAT 0       3       5       23      7       3.42e-07       403

LOCUS: AT3G55980
DESCRIPTION: zinc finger (CCCH-type) family protein, contains Pfam domain, PF00642: Zinc finger C-x8-C-x5-C-
x3-H type (and similar) and Pfam domain, PF00023: Ankyrin repeat
DATA:                 Control 30min   2hours  2days   1week   p-value        pos
SENSE COUNTS:         0       2       16      2       1       5.76e-06
GENES (1 total):
  AT3G55980.1
    SENSE COUNTS:     0       2       16      2       1       5.76e-06
    TAGS: (3 total)
      d+1  AAATGGCTAA 0       0       10      0       0       1.21e-05       2119
      X+4  TCATCAACAC 0       0       0       0       1       1.65e-01       2010
      d+2  GGTTAACTCT 0       2       6       2       0       5.32e-02       1787
           ----------                                                        1732
           ----------                                                        1473
           ----------                                                        1271
           ----------                                                        962
           ----------                                                        853
           ----------                                                        833

LOCUS: AT1G13440
DESCRIPTION: glyceraldehyde 3-phosphate dehydrogenase, cytosolic, putative / NAD-dependent glyceraldehyde-3-
phosphate dehydrogenase, putative, very strong similarity to SP|P25858 Glyceraldehyde 3-phosphate
dehydrogenase, cytosolic (EC 1.2.1.12) {Arabidopsis thaliana};
DATA:                 Control 30min   2hours  2days   1week   p-value        pos
SENSE COUNTS:         62      128     101     116     68      5.94e-06
GENES (1 total):
  AT1G13440.1
    SENSE COUNTS:     62      128     101     116     68      5.94e-06
    TAGS: (2 total)
      d+1  AGAGTTTGTA 62      128     96      116     68      6.80e-06       1206
           ----------                                                        1196
           ----------                                                        1080
           ----------                                                        1025
      d+2  ACCACTGTCC 0       0       5       0       0       7.30e-03       612
           ----------                                                        477
           ----------                                                        347
           ----------                                                        259
           ----------                                                        219
           ----------                                                        210
```

```
LOCUS: AT5G51110
DESCRIPTION: expressed protein
DATA:                  Control 30min   2hours  2days   1week   p-value         pos
SENSE COUNTS:          23      11      17      32      51      7.87e-06
GENES (1 total):
  AT5G51110.1
    SENSE COUNTS:      23      11      17      32      51      7.87e-06
    TAGS: (2 total)
      d+1  TCTTTGAGAT  5       5       7       12      21      1.09e-02        859
      d+2  TCGAGTTATA  18      6       10      20      30      1.07e-03        805
           ----------                                                          640

LOCUS: AT1G75380
DESCRIPTION: wound-responsive protein-related, similar to wound inducive gene GI:8096273 from (Nicotiana
tabacum)
DATA:                  Control 30min   2hours  2days   1week   p-value         pos
SENSE COUNTS:          26      41      20      17      1       9.09e-06
GENES (3 total):
  AT1G75380.1
    SENSE COUNTS:      2       5       3       2       0       1.79e-01
    TAGS: (3 total)
      d+1  AGAGAGGTAA  1       1       1       0       0       7.28e-01        1147
      d+2  TATCAAGTGG  0       0       0       0       0       6.15e-01        692
      d+2  TGCTTCACTA  1       4       2       2       0       4.29e-01        232
  AT1G75380.2
    SENSE COUNTS:      25      40      19      17      1       1.87e-05
    TAGS: (3 total)
      d+1  AGAGAGCTCG  24      36      17      15      1       1.08e-04        1142
      d+2  TATCAAGTGG  0       0       0       0       0       6.15e-01        687
      d+2  TGCTTCACTA  1       4       2       2       0       4.29e-01        227
  AT1G75380.3
    SENSE COUNTS:      25      40      19      17      1       1.87e-05
    TAGS: (3 total)
      d+1  AGAGAGCTCG  24      36      17      15      1       1.08e-04        1134
      d+2  TATCAAGTGG  0       0       0       0       0       6.15e-01        679
      d+2  TGCTTCACTA  1       4       2       2       0       4.29e-01        219

LOCUS: AT1G68010
DESCRIPTION: glycerate dehydrogenase / NADH-dependent hydroxypyruvate reductase, identical to hydroxypyruvate
reductase (HPR) GB:D85339 (Arabidopsis thaliana) (Plant Cell Physiol 1997 Apr;38(4):449-55)
DATA:                  Control 30min   2hours  2days   1week   p-value         pos
SENSE COUNTS:          50      11      31      28      20      1.03e-05
GENES (2 total):
  AT1G68010.1
    SENSE COUNTS:      50      11      31      28      20      1.03e-05
    TAGS: (5 total)
      d+1  AATCTACCAT  5       0       3       1       1       1.07e-01        1471
      d+2  TTGGATCATA  45      11      23      27      19      1.00e-04        1322
      d+2  ACCCGAACCG  0       0       3       0       0       2.70e-02        1120
      d+2  AAACCAGGGC  0       0       1       0       0       4.55e-01        981
           ----------                                                          849
           ----------                                                          756
           ----------                                                          737
           ----------                                                          501
           ----------                                                          345
           ----------                                                          69
      X+4  CAAAACCGAC  0       0       1       0       0       4.55e-01        -86

LOCUS: AT4G37020
DESCRIPTION: expressed protein
DATA:                  Control 30min   2hours  2days   1week   p-value         pos
SENSE COUNTS:          10      30      6       26      5       1.15e-05
GENES (1 total):
  AT4G37020.1
    SENSE COUNTS:      10      30      6       26      5       1.15e-05
    TAGS: (2 total)
           ----------                                                          980
      d+2  GATCTCCGAT  9       30      6       26      5       6.39e-06        730
      i+3  TTCTTCCCAC  1       0       0       0       0       4.28e-01        724
           ----------                                                          625
           ----------                                                          228
           ----------                                                          94

LOCUS: AT2G34460
DESCRIPTION: flavin reductase-related, low similarity to SP|P30043 Flavin reductase {Homo sapiens}
DATA:                  Control 30min   2hours  2days   1week   p-value         pos
SENSE COUNTS:          6       1       18      17      28      1.18e-05
GENES (1 total):
  AT2G34460.1
    SENSE COUNTS:      6       1       18      17      28      1.18e-05
    TAGS: (2 total)
```

```
          ----------                                                        1086
          ----------                                                        1081
          ----------                                                        1073
          ----------                                                        1065
     d+2  CTCAAACCTT  6      1      18     15     28     1.29e-05           894
          ----------                                                        697
     d+2  GGACAGATAC  0      0      0      2      0      4.80e-02           535

LOCUS: AT5G13650
DESCRIPTION: elongation factor family protein, contains Pfam profiles: PF00009 elongation factor Tu GTP
binding domain,PF00679 elongation factor G C-terminus,  PF03144 elongation factor Tu domain 2
DATA:                 Control 30min  2hours 2days  1week  p-value            pos
SENSE COUNTS:         8      13     19     19     46     1.25e-05
GENES (2 total):
  AT5G13650.1
    SENSE COUNTS:     8      13     19     19     46     1.25e-05
    TAGS: (5 total)
     i+3  TTAATTTCTC  1      0      1      0      0      6.01e-01           3744
     i+3  AGCTATCTTA  0      0      1      0      0      4.55e-01           2675
     d+1  CCCTTTGATC  6      13     15     16     43     9.62e-06           2256
     d+2  GGGCCTGTTG  1      0      2      2      3      4.13e-01           1515
          ----------                                                        1398
     d+2  GGAAGCCTCT  0      0      0      1      0      3.09e-01           1159
          ----------                                                        643
          ----------                                                        552
          ----------                                                        408
          ----------                                                        393
          ----------                                                        328
          ----------                                                        319
  AT5G13650.2
    SENSE COUNTS:     8      13     19     19     46     1.25e-05
    TAGS: (5 total)
     i+3  TTAATTTCTC  1      0      1      0      0      6.01e-01           3770
     i+3  AGCTATCTTA  0      0      1      0      0      4.55e-01           2701
     d+1  CCCTTTGATC  6      13     15     16     43     9.62e-06           2285
     d+2  GGGCCTGTTG  1      0      2      2      3      4.13e-01           1544
          ----------                                                        1427
     d+2  GGAAGCCTCT  0      0      0      1      0      3.09e-01           1188
          ----------                                                        672
          ----------                                                        581
          ----------                                                        437
          ----------                                                        422
          ----------                                                        357
          ----------                                                        348

LOCUS: AT5G05440
DESCRIPTION: expressed protein, low similarity to cytokinin-specific binding protein (Vigna radiata)
GI:4190976
DATA:                 Control 30min  2hours 2days  1week  p-value            pos
SENSE COUNTS:         0      11     3      0      0      1.40e-05
GENES (1 total):
  AT5G05440.1
    SENSE COUNTS:     0      11     3      0      0      1.40e-05
    TAGS: (2 total)
     d+1  GTATCATTCT  0      0      1      0      0      7.06e-01           1045
     d+2  GTGGTCTCTG  0      11     2      0      0      1.52e-05           455
          ----------                                                        246
          ----------                                                        128

LOCUS: AT3G09922
DESCRIPTION: hypothetical protein, no ATG start, annotated according to PMID:11123795;  IPS1 mRNA, complete
sequence GI:8164007
DATA:                 Control 30min  2hours 2days  1week  p-value            pos
SENSE COUNTS:         0      10     2      0      0      1.52e-05
GENES (1 total):
  AT3G09922.1
    SENSE COUNTS:     0      10     2      0      0      1.52e-05
    TAGS: (2 total)
     d+1  TAAATTTGCT  0      2      1      0      0      2.47e-01           504
     d+2  TAAGGAAAGC  0      8      1      0      0      1.41e-04           151

LOCUS: AT5G26717
DESCRIPTION: hypothetical protein
DATA:                 Control 30min  2hours 2days  1week  p-value            pos
SENSE COUNTS:         13     5      30     32     33     1.60e-05
GENES (1 total):
  AT5G26717.1
    SENSE COUNTS:     13     5      30     32     33     1.60e-05
    TAGS: (2 total)
     v+1  GAAACTGATC  2      0      3      2      7      2.54e-01           1158
     v+2  TGATCCGAAT  11     5      27     30     26     5.56e-05           1050
```

```
          ----------                                                  602
          ----------                                                  587
          ----------                                                  546
          ----------                                                  396
          ----------                                                  253
          ----------                                                  229

LOCUS: AT2G21330
DESCRIPTION: fructose-bisphosphate aldolase, putative, strong similarity to plastidic fructose-bisphosphate
aldolase (EC 4.1.2.13) from Nicotiana paniculata (NPALDP1) (GI:4827251), Oryza sativa, PIR2:T02057 (SP|Q40677)
DATA:                 Control 30min  2hours  2days  1week  p-value       pos
SENSE COUNTS:         58      16     43      31     50     1.70e-05
GENES (1 total):
  AT2G21330.1
    SENSE COUNTS:     58      16     43      31     50     1.70e-05
    TAGS: (3 total)
      d+1  GTTAATATCT 42      4      27      18     35     2.78e-07      1398
      d+2  TGAGACTAGT 3       0      4       4      3      6.13e-01      1374
      d+2  GGGAGGCAAG 13      12     12      9      12     9.65e-01      1110
           ----------                                                  1056
           ----------                                                  982
           ----------                                                  874
           ----------                                                  844
           ----------                                                  549

LOCUS: AT1G09070
DESCRIPTION: C2 domain-containing protein / src2-like protein, putative, similar to cold-regulated gene SRC2
(Glycine max) GI:2055230; contains Pfam profile PF00168: C2 domain; identical to cDNA  src2-like protein
GI:3426059
DATA:                 Control 30min  2hours  2days  1week  p-value       pos
SENSE COUNTS:         5       19     31      8      7      1.91e-05
GENES (1 total):
  AT1G09070.1
    SENSE COUNTS:     5       19     31      8      7      1.91e-05
    TAGS: (5 total)
      X+4  CCATAGAAAA 0       0      1       0      0      7.06e-01      1276
      d+1  TGAAGAACGT 5       19     25      8      7      1.17e-03      1107
      d+2  GGTGGTTTCG 0       0      3       0      0      2.70e-02      1022
           ----------                                                  1013
           ----------                                                  1004
      d+2  GTAAGGCTGG 0       0      1       0      0      4.55e-01      897
           ----------                                                  870
           ----------                                                  642
      d+2  GAGTGTAGAT 0       0      1       0      0      4.55e-01      68

LOCUS: AT5G26010
DESCRIPTION: protein phosphatase 2C, putative / PP2C, putative, protein phosphatase-2C, Mesembryanthemum
crystallinum, AF075579
DATA:                 Control 30min  2hours  2days  1week  p-value       pos
SENSE COUNTS:         0       0      0       1      9      2.04e-05
GENES (1 total):
  AT5G26010.1
    SENSE COUNTS:     0       0      0       1      9      2.04e-05
    TAGS: (2 total)
      v+1  CGGATTTGAT 0       0      0       0      1      1.65e-01      1552
           ----------                                                  1537
           ----------                                                  1283
           ----------                                                  1099
           ----------                                                  1062
           ----------                                                  609
           ----------                                                  529
      i+3  ACATTTCTCA 0       0      0       1      8      2.96e-04      451
           ----------                                                  397

LOCUS: AT2G35260
DESCRIPTION: expressed protein
DATA:                 Control 30min  2hours  2days  1week  p-value       pos
SENSE COUNTS:         19      5      2       2      3      2.79e-05
GENES (1 total):
  AT2G35260.1
    SENSE COUNTS:     19      5      2       2      3      2.79e-05
    TAGS: (3 total)
      d+1  CTTTATTGAC 0       0      0       1      0      3.09e-01      1532
      d+2  ATACAGTAGA 19      5      1       1      3      2.78e-06      1377
      d+2  GGCTGTGGAA 0       0      1       0      0      4.55e-01      1170
           ----------                                                  1140
           ----------                                                  839
           ----------                                                  712
           ----------                                                  648
           ----------                                                  599
           ----------                                                  368
```

```
LOCUS: AT5G17220
DESCRIPTION: glutathione S-transferase, putative
DATA:                  Control 30min   2hours  2days   1week   p-value        pos
SENSE COUNTS:          0       0       0       1       8       2.84e-05
GENES (2 total):
  AT5G17220.1
    SENSE COUNTS:      0       0       0       1       8       2.84e-05
    TAGS: (1 total)
           ----------                                                         579
      X+4  TCAATGCTAA  0       0       0       1       8       2.84e-05       564

LOCUS: AT4G31850
DESCRIPTION: pentatricopeptide (PPR) repeat-containing protein, contains Pfam profile PF01535: PPR repeat
DATA:                  Control 30min   2hours  2days   1week   p-value        pos
SENSE COUNTS:          11      0       2       1       0       3.00e-05
GENES (2 total):
  AT4G31850.1
    SENSE COUNTS:      11      0       2       1       0       3.00e-05
    TAGS: (3 total)
           ----------                                                         4128
           ----------                                                         4122
      v+2  AGACAAAAGT  1       0       0       0       0       4.28e-01       3874
      v+2  ATGATATGTT  9       0       2       1       0       6.61e-04       3804
      v+2  TGAAGTGTAC  1       0       0       0       0       4.28e-01       3747
           ----------                                                         2899
           ----------                                                         2262
           ----------                                                         1875
           ----------                                                         1429
           ----------                                                         723
           ----------                                                         543

LOCUS: AT4G15520
DESCRIPTION: tRNA/rRNA methyltransferase (SpoU) family protein, similar to SP|P19396 tRNA (Guanosine-2'-O-)-
methyltransferase (EC 2.1.1.34) {Escherichia coli O157:H7}; contains Pfam profile PF00588: SpoU rRNA Methylase
(RNA methyltransferase, TrmH) family; contains no
DATA:                  Control 30min   2hours  2days   1week   p-value        pos
SENSE COUNTS:          0       0       0       0       7       3.14e-05
GENES (1 total):
  AT4G15520.1
    SENSE COUNTS:      0       0       0       0       7       3.14e-05
    TAGS: (1 total)
           ----------                                                         898
      X+4  AAGGCTTTAT  0       0       0       0       7       3.14e-05       554
           ----------                                                         160

LOCUS: AT1G55930
DESCRIPTION: CBS domain-containing protein / transporter associated domain-containing protein, contains Pfam
profiles PF00571: CBS domain, PF03471: Transporter associated domain, PF01595: Domain of unknown function
DATA:                  Control 30min   2hours  2days   1week   p-value        pos
SENSE COUNTS:          0       0       0       0       8       3.35e-05
GENES (1 total):
  AT1G55930.1
    SENSE COUNTS:      0       0       0       0       8       3.35e-05
    TAGS: (2 total)
      d+1  TATAGAAAGA  0       0       0       0       3       1.12e-02       2179
           ----------                                                         2018
           ----------                                                         1523
           ----------                                                         1439
           ----------                                                         1224
           ----------                                                         692
           ----------                                                         414
      X+4  TTGGGAAACA  0       0       0       0       5       7.96e-03       317
           ----------                                                         250

LOCUS: AT1G69870
DESCRIPTION: proton-dependent oligopeptide transport (POT) family protein, contains Pfam profile: PF00854 POT
family
DATA:                  Control 30min   2hours  2days   1week   p-value        pos
SENSE COUNTS:          2       22      4       16      6       3.98e-05
GENES (1 total):
  AT1G69870.1
    SENSE COUNTS:      2       22      4       16      6       3.98e-05
    TAGS: (2 total)
      d+1  ATCGTCCGGA  0       3       3       8       3       1.07e-01       1836
           ----------                                                         1736
           ----------                                                         1667
           ----------                                                         1472
      i+3  GGTCAGTGCT  2       19      1       8       3       8.73e-06       1335
           ----------                                                         1316
           ----------                                                         938
```

```
          ----------                                                       839
          ----------                                                       643
          ----------                                                       422
          ----------                                                       81

LOCUS: AT5G35920
DESCRIPTION: cytochrome P450, putative, similar to cytochrome P450 (Sinapis alba) gi|3283433|gb|AAD03415
DATA:                  Control 30min  2hours  2days  1week  p-value        pos
SENSE COUNTS:          1       0      1       2      12     4.19e-05
GENES (2 total):
  AT5G35920.1
    SENSE COUNTS:      1       0      1       2      12     4.19e-05
    TAGS: (1 total)
          ----------                                                       1213
          ----------                                                       1147
          ----------                                                       898
          ----------                                                       666
          ----------                                                       657
          ----------                                                       569
      v+2  TCCACTACTG  1       0      1       2      12     4.19e-05       423
          ----------                                                       397

LOCUS: AT2G05540
DESCRIPTION: glycine-rich protein
DATA:                  Control 30min  2hours  2days  1week  p-value        pos
SENSE COUNTS:          27      3      18      13     2      4.28e-05
GENES (1 total):
  AT2G05540.1
    SENSE COUNTS:      27      3      18      13     2      4.28e-05
    TAGS: (2 total)
      d+1  AACTTTTAAA  10      0      8       5      1      2.14e-02       704
      X+4  AAGTGTTTGT  17      3      10      8      1      5.28e-03       540

LOCUS: AT4G13940
DESCRIPTION: adenosylhomocysteinase / S-adenosyl-L-homocysteine hydrolase / AdoHcyase (SAHH), identical to
SP|O23255 Adenosylhomocysteinase (EC 3.3.1.1) (S-adenosyl-L-homocysteine hydrolase) (AdoHcyase) {Arabidopsis
thaliana}; strong similarity to SP|P50248 Adenosylho
DATA:                  Control 30min  2hours  2days  1week  p-value        pos
SENSE COUNTS:          29      34     33      70     63     4.30e-05
GENES (2 total):
  AT4G13940.1
    SENSE COUNTS:      29      34     33      70     63     4.30e-05
    TAGS: (3 total)
      d+1  GTTCGATTCG  1       0      1       1      1      7.91e-01       1908
      d+2  AAACTTAAAT  28      34     32      69     62     4.27e-05       1707
          ----------                                                       1141
          ----------                                                       1078
          ----------                                                       1066
      i+3  AAAAGTCAAT  0       0      0       0      0      6.15e-01       970
          ----------                                                       913
          ----------                                                       847
          ----------                                                       829
          ----------                                                       515
          ----------                                                       259
          ----------                                                       190
          ----------                                                       127
          ----------                                                       73

LOCUS: AT1G58983
DESCRIPTION: 40S ribosomal protein S2, putative, similar to ribosomal protein S2 GI:939717 from (Urechis
caupo)
DATA:                  Control 30min  2hours  2days  1week  p-value        pos
SENSE COUNTS:          10      5      11      16     34     5.17e-05
GENES (1 total):
  AT1G58983.1
    SENSE COUNTS:      10      5      11      16     34     5.17e-05
    TAGS: (2 total)
      d+1  TATAAATTGT  1       0      1       0      0      6.01e-01       1098
          ----------                                                       1060
      d+2  AAATGACATT  9       5      10      16     34     2.20e-05       1035
          ----------                                                       878
          ----------                                                       401
          ----------                                                       322

LOCUS: AT5G58070
DESCRIPTION: lipocalin, putative, similar to temperature stress-induced lipocalin (Triticum aestivum)
GI:18650668
DATA:                  Control 30min  2hours  2days  1week  p-value        pos
SENSE COUNTS:          2       1      8       20     11     5.23e-05
GENES (1 total):
  AT5G58070.1
```

```
    SENSE COUNTS:      2       1       8       20      11      5.23e-05
    TAGS: (3 total)
      d+1  AATTTGTTTT  0       0       1       0       0       4.55e-01        1018
      d+2  CAAACTACAG  1       0       0       1       1       5.50e-01        898
      d+2  GGCCGTTGGT  1       1       7       19      10      4.79e-05        111

LOCUS: AT1G50920
DESCRIPTION: GTP-binding protein-related, similar to GTP-binding protein SP:Q99ME9 from (Mus musculus)
DATA:                  Control 30min   2hours  2days   1week   p-value         pos
SENSE COUNTS:          5       18      3       1       2       5.60e-05
GENES (1 total):
  AT1G50920.1
    SENSE COUNTS:      5       18      3       1       2       5.60e-05
    TAGS: (3 total)
      d+1  AAGTTGTGCC  4       5       1       1       1       3.43e-01        1947
           ----------                                                          1419
           ----------                                                          1233
      d+2  TGAGAGGCTG  0       0       1       0       0       4.55e-01        1156
      d+2  TCTGTGAAGA  1       13      1       0       1       8.58e-06        1136
           ----------                                                          1007
           ----------                                                          901
           ----------                                                          593
           ----------                                                          372
           ----------                                                          297
           ----------                                                          263

LOCUS: AT5G65480
DESCRIPTION: expressed protein
DATA:                  Control 30min   2hours  2days   1week   p-value         pos
SENSE COUNTS:          42      10      30      23      13      5.85e-05
GENES (1 total):
  AT5G65480.1
    SENSE COUNTS:      42      10      30      23      13      5.85e-05
    TAGS: (4 total)
      d+1  TGCTTACTTT  12      3       13      8       3       5.24e-02        1185
           ----------                                                          1180
      d+2  TACATATGTT  2       0       0       1       3       1.97e-01        1151
      d+2  TGGGTGTTTT  8       1       4       2       0       8.12e-02        1004
           ----------                                                          962
      d+2  GAACAGTGGC  20      6       13      12      7       3.80e-02        684
           ----------                                                          544
           ----------                                                          184

LOCUS: AT1G23310
DESCRIPTION: glutamate:glyoxylate aminotransferase 1 (GGT1), identical to glutamate:glyoxylate
aminotransferase 1 (Arabidopsis thaliana) GI:24461827; similar to alanine aminotransferase GI:4730884 from
(Oryza sativa); contains Pfam profile PF00155: aminotransferase, c
DATA:                  Control 30min   2hours  2days   1week   p-value         pos
SENSE COUNTS:          78      49      55      23      42      6.36e-05
GENES (2 total):
  AT1G23310.1
    SENSE COUNTS:      78      49      55      23      42      6.36e-05
    TAGS: (4 total)
      d+1  ACTCAGTATG  78      49      47      23      41      3.90e-05        1710
      d+2  GATAGCTTCA  0       0       2       0       0       3.51e-01        1677
      d+2  ACAGATGGAT  0       0       6       0       0       2.08e-04        1413
           ----------                                                          451
      X+4  TCCTAGTTTG  0       0       0       0       1       1.65e-01        443
           ----------                                                          107

LOCUS: AT5G67360
DESCRIPTION: subtilisin-like protease that has been  located in stem and siliques but not roots.
DATA:                  Control 30min   2hours  2days   1week   p-value         pos
SENSE COUNTS:          21      7       17      24      44      7.31e-05
GENES (2 total):
  AT5G67360.1
    SENSE COUNTS:      21      7       17      24      44      7.31e-05
    TAGS: (3 total)
      d+1  TGACTTGAAA  6       0       5       12      12      1.46e-02        2617
      d+2  CAGTCTGTTA  15      7       12      11      32      1.14e-03        2501
      d+2  GATCACAACT  0       0       0       1       0       6.04e-01        1098
           ----------                                                          1021
           ----------                                                          382
           ----------                                                          338
           ----------                                                          223
           ----------                                                          118

LOCUS: AT5G28237
DESCRIPTION: tryptophan synthase, beta subunit, putative, similar to SP|P14671 Tryptophan synthase beta chain
1, chloroplast precursor (EC 4.2.1.20) {Arabidopsis thaliana}; contains Pfam profile PF00291: Pyridoxal-
phosphate dependent enzyme
```

```
DATA:                  Control 30min  2hours  2days  1week  p-value     pos
SENSE COUNTS:          0       7      0       0      0      7.93e-05
GENES (2 total):
  AT5G28237.1
    SENSE COUNTS:      0       7      0       0      0      7.93e-05
    TAGS: (1 total)
           ----------                                                   1437
           ----------                                                   1275
           ----------                                                   1212
           ----------                                                   1113
           ----------                                                   1105
           ----------                                                   991
           ----------                                                   720
      d+2  GGAGCTGCTG  0       7      0       0      0      7.93e-05    675
           ----------                                                   641
           ----------                                                   619
           ----------                                                   561
  AT5G28237.2
    SENSE COUNTS:      0       7      0       0      0      7.93e-05
    TAGS: (1 total)
           ----------                                                   1113
           ----------                                                   1105
           ----------                                                   991
           ----------                                                   720
      d+2  GGAGCTGCTG  0       7      0       0      0      7.93e-05    675
           ----------                                                   641
           ----------                                                   619
           ----------                                                   561

LOCUS: AT4G10730
DESCRIPTION: protein kinase family protein, contains protein kinase domain, Pfam:PF00069
DATA:                  Control 30min  2hours  2days  1week  p-value     pos
SENSE COUNTS:          0       0      0       2      10     8.00e-05
GENES (1 total):
  AT4G10730.1
    SENSE COUNTS:      0       0      0       2      10     8.00e-05
    TAGS: (2 total)
      i+3  AAACAAATTT  0       0      0       0      3      8.36e-02    4954
      d+1  GAAAATTGTA  0       0      0       2      7      1.31e-03    2463
           ----------                                                   2323
           ----------                                                   1570
           ----------                                                   1353
           ----------                                                   835
           ----------                                                   734
           ----------                                                   677

LOCUS: AT3G14420
DESCRIPTION: (S)-2-hydroxy-acid oxidase, peroxisomal, putative / glycolate oxidase, putative / short chain
alpha-hydroxy acid oxidase, putative, similar to (S)-2-hydroxy-acid oxidase, peroxisomal (Glycolate oxidase,
GOX) (Short chain alpha-hydroxy acid oxidase) (Spina
DATA:                  Control 30min  2hours  2days  1week  p-value     pos
SENSE COUNTS:          86      48     72      36     45     9.11e-05
GENES (3 total):
  AT3G14420.1
    SENSE COUNTS:      86      48     72      36     45     9.11e-05
    TAGS: (4 total)
      d+1  TACTTACATT  64      17     30      18     37     3.26e-08    1387
      d+2  GCACTGAGTG  22      31     39      18     8      3.32e-03    1085
           ----------                                                   834
      d+2  GGCTACTTCC  0       0      2       0      0      1.21e-01    394
           ----------                                                   380
      d+2  CAAAAGATGG  0       0      1       0      0      4.55e-01    311
           ----------                                                   293
           ----------                                                   251
  AT3G14420.2
    SENSE COUNTS:      86      48     72      36     45     9.11e-05
    TAGS: (4 total)
      d+1  TACTTACATT  64      17     30      18     37     3.26e-08    1428
      d+2  GCACTGAGTG  22      31     39      18     8      3.32e-03    1126
           ----------                                                   875
      d+2  GGCTACTTCC  0       0      2       0      0      1.21e-01    435
           ----------                                                   421
      d+2  CAAAAGATGG  0       0      1       0      0      4.55e-01    352
           ----------                                                   334
           ----------                                                   292
           ----------                                                   8
  AT3G14420.3
    SENSE COUNTS:      86      48     72      36     45     9.11e-05
    TAGS: (4 total)
      d+1  TACTTACATT  64      17     30      18     37     3.26e-08    1520
      d+2  GCACTGAGTG  22      31     39      18     8      3.32e-03    1218
```

```
          ----------                                                          967
      d+2  GGCTACTTCC 0       0       2       0       0       1.21e-01        527
          ----------                                                          513
      d+2  CAAAAGATGG 0       0       1       0       0       4.55e-01        444
          ----------                                                          426
          ----------                                                          384
          ----------                                                          8

LOCUS: AT1G11860
DESCRIPTION: aminomethyltransferase, putative, similar to aminomethyltransferase, mitochondrial precursor
SP:O49849 from (Flaveria anomala)
DATA:                 Control 30min   2hours  2days   1week   p-value         pos
SENSE COUNTS:         31      9       8       5       19      1.02e-04
GENES (2 total):
  AT1G11860.1
    SENSE COUNTS:     31      9       8       5       19      1.02e-04
    TAGS: (4 total)
      d+1  GTTTTAATGT 0       0       0       0       0       6.15e-01        1556
      d+2  TAATAGTATC 9       0       1       0       7       4.12e-04        1536
      d+2  ACTTTGTCTC 22      9       5       5       12      6.76e-03        1408
          ----------                                                          1384
      d+2  GGATATGTGA 0       0       2       0       0       3.51e-01        1259
          ----------                                                          1185
          ----------                                                          1042
          ----------                                                          1004
          ----------                                                          885
          ----------                                                          820
          ----------                                                          647
          ----------                                                          303
          ----------                                                          294
  AT1G11860.2
    SENSE COUNTS:     31      9       8       5       19      1.02e-04
    TAGS: (4 total)
      d+1  GTTTTAATGT 0       0       0       0       0       6.15e-01        1557
      d+2  TAATAGTATC 9       0       1       0       7       4.12e-04        1537
      d+2  ACTTTGTCTC 22      9       5       5       12      6.76e-03        1409
          ----------                                                          1385
      d+2  GGATATGTGA 0       0       2       0       0       3.51e-01        1260
          ----------                                                          1186
          ----------                                                          1043
          ----------                                                          1005
          ----------                                                          886
          ----------                                                          821
          ----------                                                          648
          ----------                                                          304
          ----------                                                          295

LOCUS: AT1G17010
DESCRIPTION: oxidoreductase, 2OG-Fe(II) oxygenase family protein, similar to flavonol synthase (Petunia x
hybrida)(GI:311658), (Solanum tuberosum)(GI:1039356); contains PF03171 2OG-Fe(II) oxygenase superfamily domain
DATA:                 Control 30min   2hours  2days   1week   p-value         pos
SENSE COUNTS:         21      0       11      21      15      1.09e-04
GENES (1 total):
  AT1G17010.1
    SENSE COUNTS:     21      0       11      21      15      1.09e-04
    TAGS: (2 total)
      i+3  CATAAAAAAA 1       0       0       1       1       5.50e-01        836
      d+1  GAAGAAAAAA 20      0       11      20      14      2.54e-04        432
          ----------                                                          367
          ----------                                                          273
          ----------                                                          235
          ----------                                                          99

LOCUS: AT1G08640
DESCRIPTION: expressed protein
DATA:                 Control 30min   2hours  2days   1week   p-value         pos
SENSE COUNTS:         4       0       15      16      2       1.11e-04
GENES (1 total):
  AT1G08640.1
    SENSE COUNTS:     4       0       15      16      2       1.11e-04
    TAGS: (3 total)
      i+3  AGTTGGATAT 0       0       0       1       0       3.09e-01        2352
      d+1  TGGAAGGTTT 4       0       15      15      1       8.39e-05        1173
      d+2  CTCTATGTTA 0       0       0       0       1       1.65e-01        925
          ----------                                                          874
          ----------                                                          856
          ----------                                                          652
          ----------                                                          606
          ----------                                                          531
          ----------                                                          433
          ----------                                                          109
```

```
          ----------                                                    73

LOCUS: AT3G55610
DESCRIPTION: delta 1-pyrroline-5-carboxylate synthetase B / P5CS B (P5CS2), identical to SP|P54888
DATA:                Control 30min   2hours  2days   1week   p-value        pos
SENSE COUNTS:        7       4       15      30      18      1.14e-04
GENES (1 total):
  AT3G55610.1
    SENSE COUNTS:    7       4       15      30      18      1.14e-04
    TAGS: (3 total)
      d+1  CAGAATAAGT 6      2       15      22      16      8.29e-04       2520
           ----------                                                       2474
           ----------                                                       2454
      d+2  CCCGTGGTCC 1      2       0       8       1       1.67e-02       2134
      d+2  GAAGTGCACA 0      0       0       0       1       1.65e-01       1966
           ----------                                                       1686
           ----------                                                       1654
           ----------                                                       1636
           ----------                                                       999
           ----------                                                       417

LOCUS: AT1G12660
DESCRIPTION: hypothetical protein
DATA:                Control 30min   2hours  2days   1week   p-value        pos
SENSE COUNTS:        33      15      45      47      17      1.15e-04
GENES (1 total):
  AT1G12660.1
    SENSE COUNTS:    33      15      45      47      17      1.15e-04
    TAGS: (3 total)
      i+3  ATAACCCAAG 1      0       0       1       1       5.50e-01       1926
      i+3  AGAGACTCTA 1      0       0       2       0       4.17e-01       1284
      v+1  TGTGAAAAAA 31     15      45      44      16      1.13e-04       759
           ----------                                                       705
           ----------                                                       693
           ----------                                                       408
           ----------                                                       375
           ----------                                                       53
           ----------                                                       16

LOCUS: AT5G49330
DESCRIPTION: myb family transcription factor, contains Pfam profile: PF00249 myb-like DNA binding domain;
identical to cDNA putative transcription factor (At5g49330) GI:15420625
DATA:                Control 30min   2hours  2days   1week   p-value        pos
SENSE COUNTS:        0       0       0       2       8       1.20e-04
GENES (1 total):
  AT5G49330.1
    SENSE COUNTS:    0       0       0       2       8       1.20e-04
    TAGS: (1 total)
           ----------                                                       1304
      X+4  TTATGCCTTT 0      0       0       2       8       1.20e-04       955
           ----------                                                       900
           ----------                                                       581

LOCUS: AT5G18580
DESCRIPTION: tonneau 2 (TON2), identical to tonneau 2 protein (TON2) GI:11494362 from (Arabidopsis thaliana);
contains Pfam profile: PF00036 EF hand
DATA:                Control 30min   2hours  2days   1week   p-value        pos
SENSE COUNTS:        10      0       1       0       7       1.32e-04
GENES (2 total):
  AT5G18580.1
    SENSE COUNTS:    10      0       1       0       7       1.32e-04
    TAGS: (1 total)
           ----------                                                       1912
           ----------                                                       1846
           ----------                                                       1834
      d+2  TGTGTGTATC 10     0       1       0       7       1.32e-04       1687
           ----------                                                       1543
           ----------                                                       1512
           ----------                                                       1434
           ----------                                                       1177
           ----------                                                       843
           ----------                                                       648
           ----------                                                       193

LOCUS: AT2G23370
DESCRIPTION: expressed protein
DATA:                Control 30min   2hours  2days   1week   p-value        pos
SENSE COUNTS:        0       8       0       1       0       1.34e-04
GENES (1 total):
  AT2G23370.1
    SENSE COUNTS:    0       8       0       1       0       1.34e-04
```

```
    TAGS: (1 total)
           ----------                                                    1012
      i+3  TTTCTCTTCT 0       8       0       1       0       1.34e-04       732
           ----------                                                    669
           ----------                                                    414
           ----------                                                    183
           ----------                                                    129

LOCUS: AT1G79140
DESCRIPTION: expressed protein
DATA:                 Control 30min   2hours  2days   1week   p-value        pos
SENSE COUNTS:         1       0       1       1       10      1.44e-04
GENES (1 total):
  AT1G79140.1
    SENSE COUNTS:     1       0       1       1       10      1.44e-04
    TAGS: (2 total)
      d+1  GTTAAGCTCA 0       0       0       1       3       6.27e-02       766
      d+2  AACACTTCTC 1       0       1       0       7       3.62e-03       592
           ----------                                                    292
           ----------                                                    139

LOCUS: AT1G16830
DESCRIPTION: pentatricopeptide (PPR) repeat-containing protein, contains Pfam profile PF01535: PPR repeat
DATA:                 Control 30min   2hours  2days   1week   p-value        pos
SENSE COUNTS:         17      24      10      2       3       1.60e-04
GENES (1 total):
  AT1G16830.1
    SENSE COUNTS:     17      24      10      2       3       1.60e-04
    TAGS: (1 total)
           ----------                                                    2971
           ----------                                                    2758
           ----------                                                    2674
           ----------                                                    2286
           ----------                                                    1971
           ----------                                                    1497
           ----------                                                    1179
           ----------                                                    1143
           ----------                                                    975
           ----------                                                    640
      v+2  CGTTGCACTA 17      24      10      2       3       1.60e-04       24

LOCUS: AT2G03320
DESCRIPTION: hypothetical protein
DATA:                 Control 30min   2hours  2days   1week   p-value        pos
SENSE COUNTS:         4       0       5       12      17      1.61e-04
GENES (1 total):
  AT2G03320.1
    SENSE COUNTS:     4       0       5       12      17      1.61e-04
    TAGS: (1 total)
           ----------                                                    414
           ----------                                                    391
           ----------                                                    354
      i+3  AATGGTTAAC 4       0       5       12      17      1.61e-04       208

LOCUS: AT5G35970
DESCRIPTION: DNA-binding protein, putative, similar to SWISS-PROT:Q60560 DNA-binding protein SMUBP-2
(Immunoglobulin MU binding protein 2, SMUBP-2) (Mesocricetus auratus)
DATA:                 Control 30min   2hours  2days   1week   p-value        pos
SENSE COUNTS:         11      0       14      18      24      1.99e-04
GENES (1 total):
  AT5G35970.1
    SENSE COUNTS:     11      0       14      18      24      1.99e-04
    TAGS: (3 total)
           ----------                                                    3313
      d+2  CAGACCCTGG 0       0       0       0       1       1.65e-01       3084
           ----------                                                    2433
           ----------                                                    2347
           ----------                                                    2296
           ----------                                                    1904
      d+2  GCTGGAGCAA 0       0       1       0       0       4.55e-01       1654
           ----------                                                    1521
           ----------                                                    1464
           ----------                                                    1346
           ----------                                                    1200
           ----------                                                    782
      X+4  TTACCACAGA 11      0       13      18      23      3.99e-04       430

LOCUS: AT5G49910
DESCRIPTION: heat shock protein 70 / HSP70 (HSC70-7), identical to heat shock protein 70 (Arabidopsis
thaliana) GI:6746592
DATA:                 Control 30min   2hours  2days   1week   p-value        pos
```

```
SENSE COUNTS:          3       0       3       2       14      2.00e-04
GENES (2 total):
  AT5G49910.1
    SENSE COUNTS:      3       0       3       2       14      2.00e-04
    TAGS: (3 total)
           ----------                                                        2573
           ----------                                                        2462
      d+2  TACAATAAAC  0       0       1       0       1       3.96e-01      2395
      d+2  ATTGAAGCAA  3       0       2       2       12      3.86e-03      2336
           ----------                                                        2259
           ----------                                                        2034
      d+2  ACCAAGATCA  0       0       0       0       1       1.65e-01      1494
           ----------                                                        1113
           ----------                                                        60

LOCUS: AT1G66410
DESCRIPTION: calmodulin-1/4 (CAM4), identical to calmodulin (Arabidopsis thaliana) GI:16223; nearly identical
to SP|P25854 Calmodulin-1/4 {Arabidopsis thaliana}
DATA:                  Control 30min   2hours  2days   1week   p-value       pos
SENSE COUNTS:          8       21      6       17      35      2.23e-04
GENES (2 total):
  AT1G66410.1
    SENSE COUNTS:      8       21      6       17      35      2.23e-04
    TAGS: (2 total)
      d+1  TTTAGATTTC  3       4       2       15      21      3.72e-05      718
      d+2  TGATGACGAA  5       17      4       2       14      2.37e-03      394
           ----------                                                        225

LOCUS: AT3G62880
DESCRIPTION: mitochondrial import inner membrane translocase subunit Tim17/Tim22/Tim23 family protein,
contains PFam PF02466: Mitochondrial import inner membrane translocase subunit Tim17
DATA:                  Control 30min   2hours  2days   1week   p-value       pos
SENSE COUNTS:          2       0       14      5       14      2.67e-04
GENES (1 total):
  AT3G62880.1
    SENSE COUNTS:      2       0       14      5       14      2.67e-04
    TAGS: (1 total)
           ----------                                                        751
      d+2  TACACACAAA  2       0       14      5       14      2.67e-04      610
           ----------                                                        380

LOCUS: AT4G25100
DESCRIPTION: superoxide dismutase (Fe), chloroplast (SODB) / iron superoxide dismutase (FSD1), identical to
Fe-superoxide dismutase (Arabidopsis thaliana) gi|166700|gb|AAA32791; supported by cDNA, Ceres:32935
DATA:                  Control 30min   2hours  2days   1week   p-value       pos
SENSE COUNTS:          155     218     249     185     208     2.99e-04
GENES (4 total):
  AT4G25100.2
    SENSE COUNTS:      26      8       24      12      20      2.25e-02
    TAGS: (2 total)
      d+1  GCCCATTATT  26      8       20      12      17      2.91e-02      957
           ----------                                                        853
      d+2  ACCAATCTTG  0       0       4       0       3       7.65e-02      632
           ----------                                                        579
  AT4G25100.3
    SENSE COUNTS:      129     210     229     173     191     1.44e-05
    TAGS: (2 total)
      d+1  CTCTTTTCTG  129     210     225     173     188     3.18e-05      853
      d+2  ACCAATCTTG  0       0       4       0       3       7.65e-02      632
           ----------                                                        579
  AT4G25100.1
    SENSE COUNTS:      26      8       24      12      20      2.25e-02
    TAGS: (2 total)
      d+1  GCCCATTATT  26      8       20      12      17      2.91e-02      1093
           ----------                                                        989
      d+2  ACCAATCTTG  0       0       4       0       3       7.65e-02      768
           ----------                                                        715
           ----------                                                        100
           ----------                                                        86
           ----------                                                        16

LOCUS: AT4G16740
DESCRIPTION: terpene synthase/cyclase family protein, similar to myrcene/ocimene synthase (GI:9957293);
contains Pfam profile  PF01397: Terpene synthase, N-terminal domain; contains Pfam profile PF03936: Terpene
synthase family, metal binding domain; identical to cDNA
DATA:                  Control 30min   2hours  2days   1week   p-value       pos
SENSE COUNTS:          1       0       7       0       0       3.02e-04
GENES (2 total):
  AT4G16740.1
    SENSE COUNTS:      1       0       7       0       0       3.02e-04
    TAGS: (2 total)
```

```
          ----------                                                  1749
          ----------                                                  1716
     d+2  GAGAAAAAGA  1       0       2       0       0       3.07e-01        1619
          ----------                                                  1542
          ----------                                                  1538
          ----------                                                  1331
          ----------                                                  1151
          ----------                                                  1031
          ----------                                                  809
          ----------                                                  726
     d+2  GTTTCAGTAT  0       0       5       0       0       1.10e-03        501
          ----------                                                  405

LOCUS: AT4G31800
DESCRIPTION: WRKY family transcription factor
DATA:                 Control 30min   2hours  2days   1week   p-value         pos
SENSE COUNTS:         0       1       13      8       3       4.13e-04
GENES (2 total):
  AT4G31800.1
    SENSE COUNTS:     0       1       13      8       3       4.13e-04
    TAGS: (2 total)
     d+1  AACGTTTTTA  0       1       1       2       3       5.00e-01        1026
          ----------                                                  531
     d+2  AGCAGAAAAA  0       0       12      6       0       1.11e-05        506

LOCUS: AT1G19960
DESCRIPTION: expressed protein
DATA:                 Control 30min   2hours  2days   1week   p-value         pos
SENSE COUNTS:         0       0       0       1       6       4.62e-04
GENES (1 total):
  AT1G19960.1
    SENSE COUNTS:     0       0       0       1       6       4.62e-04
    TAGS: (2 total)
          ----------                                                  935
     v+2  TTAACTGTGT  0       0       0       0       5       6.24e-04        636
     v+2  TGTAGTGAAA  0       0       0       1       1       3.25e-01        586
          ----------                                                  175
          ----------                                                  32

LOCUS: AT5G64480
DESCRIPTION: expressed protein,
DATA:                 Control 30min   2hours  2days   1week   p-value         pos
SENSE COUNTS:         0       0       0       1       7       4.62e-04
GENES (1 total):
  AT5G64480.1
    SENSE COUNTS:     0       0       0       1       7       4.62e-04
    TAGS: (1 total)
          ----------                                                  670
     d+2  TTTCTTTAGC  0       0       0       1       7       4.62e-04        618

LOCUS: AT1G17100
DESCRIPTION: SOUL heme-binding family protein, similar to SOUL protein (Mus musculus) GI:4886906; contains
Pfam profile PF04832: SOUL heme-binding protein
DATA:                 Control 30min   2hours  2days   1week   p-value         pos
SENSE COUNTS:         0       1       0       8       0       5.21e-04
GENES (1 total):
  AT1G17100.1
    SENSE COUNTS:     0       1       0       8       0       5.21e-04
    TAGS: (1 total)
          ----------                                                  1102
     d+2  GCCACCGGTT  0       1       0       8       0       5.21e-04        34

LOCUS: AT4G14920
DESCRIPTION: PHD finger transcription factor, putative
DATA:                 Control 30min   2hours  2days   1week   p-value         pos
SENSE COUNTS:         24      5       10      18      3       5.54e-04
GENES (1 total):
  AT4G14920.1
    SENSE COUNTS:     24      5       10      18      3       5.54e-04
    TAGS: (3 total)
     v+1  GCCTTGAGCC  1       0       0       2       0       4.17e-01        3654
          ----------                                                  3417
          ----------                                                  3330
          ----------                                                  3300
          ----------                                                  3101
          ----------                                                  2995
          ----------                                                  2953
          ----------                                                  2724
          ----------                                                  2621
          ----------                                                  2599
          ----------                                                  2482
```

```
           ----------                                                      2237
           ----------                                                      2155
           ----------                                                      2073
           ----------                                                      2049
           ----------                                                      2000
           ----------                                                      1903
      v+2  AAGAGTAAGG  1       0       0       0       0       4.28e-01       1224
           ----------                                                      1212
           ----------                                                      966
      i+3  CGTTGTTGTT  22      5       10      16      3       1.92e-03       626
           ----------                                                      288
           ----------                                                      134

LOCUS: AT1G38203
DESCRIPTION: gypsy-like retrotransposon family (Athila), has a 3.5e-30 P-value blast match to GB:CAA57397
Athila ORF 1 (Arabidopsis thaliana)
DATA:                  Control 30min   2hours  2days   1week   p-value        pos
SENSE COUNTS:          3       0       0       8       12      5.79e-04
GENES (1 total):
  AT1G38203.1
    SENSE COUNTS:      3       0       0       8       12      5.79e-04
    TAGS: (1 total)
           ----------                                                      797
           ----------                                                      787
      p+2  AACTGAAAAT  3       0       0       8       12      5.79e-04       661
           ----------                                                      20

LOCUS: AT3G45850
DESCRIPTION: kinesin motor protein-related, kinesin-related protein TKRP125, Nicotiana tabacum, PIR:T02017
DATA:                  Control 30min   2hours  2days   1week   p-value        pos
SENSE COUNTS:          0       0       0       0       5       6.24e-04
GENES (1 total):
  AT3G45850.1
    SENSE COUNTS:      0       0       0       0       5       6.24e-04
    TAGS: (1 total)
           ----------                                                      3281
           ----------                                                      2943
           ----------                                                      2832
           ----------                                                      2821
           ----------                                                      2802
      d+2  GATGCCAATG  0       0       0       0       5       6.24e-04       2674
           ----------                                                      2479
           ----------                                                      2420
           ----------                                                      2192
           ----------                                                      2038
           ----------                                                      1994
           ----------                                                      1816
           ----------                                                      1804
           ----------                                                      1219
           ----------                                                      655
           ----------                                                      547

LOCUS: AT2G24820
DESCRIPTION: Rieske (2Fe-2S) domain-containing protein, similar to Rieske iron-sulfur protein Tic55 from Pisum
sativum (gi:2764524); contains Pfam PF00355 Rieske (2Fe-2S) domain
DATA:                  Control 30min   2hours  2days   1week   p-value        pos
SENSE COUNTS:          4       2       16      2       5       7.09e-04
GENES (1 total):
  AT2G24820.1
    SENSE COUNTS:      4       2       16      2       5       7.09e-04
    TAGS: (2 total)
           ----------                                                      2128
           ----------                                                      1911
           ----------                                                      1809
      d+2  AAATAAATTT  4       2       15      2       5       2.10e-03       1729
           ----------                                                      1697
           ----------                                                      1417
           ----------                                                      1348
           ----------                                                      1282
      d+2  GGTCGCTGAA  0       0       1       0       0       4.55e-01       1241
           ----------                                                      1152
           ----------                                                      802
           ----------                                                      787
           ----------                                                      733
           ----------                                                      514
           ----------                                                      167
           ----------                                                      63

LOCUS: AT1G17050
```

```
DESCRIPTION: geranyl diphosphate synthase, putative / GPPS, putative / dimethylallyltransferase, putative /
prenyl transferase, putative, similar to GI:11322965; Except for first 55 amino acids, 52% identical to Prenyl
transferase (Cyanophora paradoxa) (gi 99282). Loc
DATA:                 Control 30min  2hours  2days  1week  p-value       pos
SENSE COUNTS:         0       0      0       5      0      7.27e-04
GENES (1 total):
  AT1G17050.1
    SENSE COUNTS:     0       0      0       5      0      7.27e-04
    TAGS: (1 total)
           ----------                                                    1562
           ----------                                                    977
           ----------                                                    847
           ----------                                                    830
      X+4  CTTATTATTG 0       0      0       5      0      7.27e-04      666
           ----------                                                    205

LOCUS: AT1G31660
DESCRIPTION: bystin family, contains Pfam profile: PF05291 Bystin
DATA:                 Control 30min  2hours  2days  1week  p-value       pos
SENSE COUNTS:         1       0      0       5      10     8.06e-04
GENES (1 total):
  AT1G31660.1
    SENSE COUNTS:     1       0      0       5      10     8.06e-04
    TAGS: (2 total)
           ----------                                                    1152
           ----------                                                    1025
           ----------                                                    956
           ----------                                                    249
      X+4  GTTTAATCTT 1       0      0       5      7      1.64e-02      245
      X+4  ATAGTGTATC 0       0      0       0      3      1.12e-02      225

LOCUS: AT2G02510
DESCRIPTION: expressed protein
DATA:                 Control 30min  2hours  2days  1week  p-value       pos
SENSE COUNTS:         4       2      6       4      19     8.39e-04
GENES (1 total):
  AT2G02510.1
    SENSE COUNTS:     4       2      6       4      19     8.39e-04
    TAGS: (2 total)
      X+4  TATGGTTAAA 0       0      1       0      0      4.55e-01      447
      d+1  GTTTCGTTAT 4       2      5       4      19     5.85e-04      427
           ----------                                                    202

LOCUS: AT3G48420
DESCRIPTION: haloacid dehalogenase-like hydrolase family protein, low similarity to SP|P95649 CbbY protein
{Rhodobacter sphaeroides}; contains InterPro accession IPR005834: Haloacid dehalogenase-like hydrolase
DATA:                 Control 30min  2hours  2days  1week  p-value       pos
SENSE COUNTS:         13      3      6       0      14     1.04e-03
GENES (1 total):
  AT3G48420.1
    SENSE COUNTS:     13      3      6       0      14     1.04e-03
    TAGS: (4 total)
      i+3  AATATGCGAG 0       0      2       0      1      2.22e-01      1214
      d+1  TAAAACCATA 13      3      3       0      12     8.53e-04      1133
      i+3  ATGCTATGAA 0       0      0       0      1      1.65e-01      941
      d+2  CTTGCTTGGA 0       0      1       0      0      4.55e-01      729
           ----------                                                    595

LOCUS: AT4G09130
DESCRIPTION: zinc finger (C3HC4-type RING finger) family protein, contains Pfam profile: PF00097: Zinc finger,
C3HC4 type (RING finger)
DATA:                 Control 30min  2hours  2days  1week  p-value       pos
SENSE COUNTS:         1       0      2       0      8      1.04e-03
GENES (1 total):
  AT4G09130.1
    SENSE COUNTS:     1       0      2       0      8      1.04e-03
    TAGS: (1 total)
           ----------                                                    1876
           ----------                                                    1800
           ----------                                                    1738
           ----------                                                    1410
           ----------                                                    1088
           ----------                                                    1030
      v+2  TAATAATAAT 1       0      2       0      8      1.04e-03      965
           ----------                                                    824
           ----------                                                    784
           ----------                                                    595
           ----------                                                    395
           ----------                                                    354
           ----------                                                    305
           ----------                                                    82
```

```
LOCUS: ATCG00280
DESCRIPTION: chloroplast gene encoding a CP43 subunit of  the photosystem II reaction center. promoter
contains a blue-light responsive element.
DATA:                  Control 30min  2hours  2days  1week  p-value       pos
SENSE COUNTS:          15      0      6       6      2      1.11e-03
GENES (1 total):
  ATCG00280.1
    SENSE COUNTS:      15      0      6       6      2      1.11e-03
    TAGS: (5 total)
      X+4  CTAAGAAGTA  13      0      4       2      0      2.95e-05      1542
      d+2  GGCTCGCCGC  1       0      0       0      1      3.83e-01      777
      d+2  TATGGTTAGG  1       0      0       0      0      4.28e-01      713
      d+2  CACTTCTGGG  0       0      1       4      0      1.31e-01      398
      d+2  CCGGATTAAT  0       0      1       0      1      3.96e-01      170

LOCUS: AT2G17845
DESCRIPTION: short-chain dehydrogenase/reductase (SDR) family protein, contains similarity to 3-oxoacyl-(acyl-
carrier protein) reductase SP:P51831 from (Bacillus subtilis)
DATA:                  Control 30min  2hours  2days  1week  p-value       pos
SENSE COUNTS:          32      26     61      51     46     1.13e-03
GENES (1 total):
  AT2G17845.1
    SENSE COUNTS:      32      26     61      51     46     1.13e-03
    TAGS: (1 total)
           ----------                                                     1764
           ----------                                                     1643
           ----------                                                     1528
           ----------                                                     1498
           ----------                                                     1392
           ----------                                                     1005
           ----------                                                     435
           ----------                                                     183
      v+2  ATAAAAAAAA  32      26     61      51     46     1.13e-03      22

LOCUS: AT2G18030
DESCRIPTION: peptide methionine sulfoxide reductase family protein, similar to SP|P08761 Ecdysone-induced
protein 28/29 kDa {Drosophila melanogaster}; contains Pfam profile PF01625: Peptide methionine sulfoxide
reductase
DATA:                  Control 30min  2hours  2days  1week  p-value       pos
SENSE COUNTS:          9       8      0       1      16     1.15e-03
GENES (2 total):
  AT2G18030.1
    SENSE COUNTS:      1       1      0       0      0      4.77e-01
    TAGS: (1 total)
      d+1  GTAGAAGAAG  1       1      0       0      0      4.77e-01      718
           ----------                                                     473
  AT2G18030.2
    SENSE COUNTS:      9       8      0       1      16     1.15e-03
    TAGS: (2 total)
      d+1  GTAGAAGAAG  1       1      0       0      0      4.77e-01      863
           ----------                                                     514
           ----------                                                     40
      X+4  CCTTAATTGT  8       7      0       1      16     1.15e-03      -87

LOCUS: AT2G44430
DESCRIPTION: DNA-binding bromodomain-containing protein, contains Pfam domains, Pfam PF00439: Bromodomain and
PF00249: Myb-like DNA-binding domain
DATA:                  Control 30min  2hours  2days  1week  p-value       pos
SENSE COUNTS:          15      10     6       28     6      1.18e-03
GENES (1 total):
  AT2G44430.1
    SENSE COUNTS:      15      10     6       28     6      1.18e-03
    TAGS: (3 total)
      d+1  TTTGCCCCTT  0       0      0       0      1      4.65e-01      2012
           ----------                                                     1086
           ----------                                                     236
      d+2  GGGCACGTGG  15      10     6       27     5      1.13e-03      206
      d+2  AACGTCGACG  0       0      0       1      0      3.09e-01      99

LOCUS: AT3G47460
DESCRIPTION: SMC2-like condensin, putative, similar to SMC2-like condensin (TITAN3) (Arabidopsis thaliana)
GI:14279543; contains Pfam profiles PF02483: SMC family C-terminal domain, PF02463: RecF/RecN/SMC N terminal
domain
DATA:                  Control 30min  2hours  2days  1week  p-value       pos
SENSE COUNTS:          37      39     42      25     6      1.28e-03
GENES (2 total):
  AT3G47460.1
    SENSE COUNTS:      37      39     42      25     6      1.28e-03
    TAGS: (6 total)
      d+1  GGTCAGACAA  11      2      21      5      0      1.50e-05      3995
```

```
          ----------                                                  3910
     d+2  GAGCTACAAC  23      37      21      19      5       1.13e-03        3833
     d+2  AATAATAGAT  3       0       0       0       0       9.89e-02        3755
     i+3  GTTTAGTTAG  0       0       0       1       1       3.25e-01        3634
     d+2  TCAAAATTAG  0       0       0       0       0       6.15e-01        3145
          ----------                                                  3087
          ----------                                                  2805
          ----------                                                  2704
          ----------                                                  2624
     d+2  ATCAGTCTCT  0       0       0       0       0       6.15e-01        2591
          ----------                                                  2432
          ----------                                                  2336
          ----------                                                  2164
          ----------                                                  2131
          ----------                                                  2024
          ----------                                                  1846
          ----------                                                  755
          ----------                                                  469
          ----------                                                  287

LOCUS: AT5G05930
DESCRIPTION: guanylyl cyclase-related (GC1), similar to guanylyl cyclase (GC1) GI:21436486 from (Arabidopsis
thaliana); identical to cDNA  putative guanylate cyclase GI:21436486
DATA:                  Control 30min   2hours  2days   1week   p-value         pos
SENSE COUNTS:          1       5       0       0       0       1.46e-03
GENES (2 total):
  AT5G05930.1
    SENSE COUNTS:      1       5       0       0       0       1.46e-03
    TAGS: (4 total)
      i+3  TTTTCCAATA  0       0       0       0       0       6.15e-01        2558
      d+1  AAATATCTCA  0       5       0       0       0       3.00e-03        1341
      d+2  AGGTGAAAAG  1       0       0       0       0       4.28e-01        1258
           ----------                                                  1076
      d+2  AGATTTCTTG  0       0       0       0       0       6.15e-01        866
           ----------                                                  824
           ----------                                                  561
           ----------                                                  542
           ----------                                                  529
  AT5G05930.2
    SENSE COUNTS:      1       5       0       0       0       1.46e-03
    TAGS: (4 total)
      i+3  TTTTCCAATA  0       0       0       0       0       6.15e-01        2524
      d+1  AAATATCTCA  0       5       0       0       0       3.00e-03        1242
      d+2  AGGTGAAAAG  1       0       0       0       0       4.28e-01        1159
           ----------                                                  977
      d+2  AGATTTCTTG  0       0       0       0       0       6.15e-01        767
           ----------                                                  725
           ----------                                                  495

LOCUS: AT5G13300
DESCRIPTION: ARF GTPase-activating domain-containing protein, similar to GCN4-complementing protein (GCP1)
GI:6465806 from (Arabidopsis thaliana)
DATA:                  Control 30min   2hours  2days   1week   p-value         pos
SENSE COUNTS:          1       0       7       1       0       1.53e-03
GENES (1 total):
  AT5G13300.1
    SENSE COUNTS:      1       0       7       1       0       1.53e-03
    TAGS: (2 total)
      d+1  TCCAGACGTT  0       0       2       1       0       2.74e-01        2851
      i+3  CTACATTTGT  1       0       5       0       0       6.70e-03        2730
           ----------                                                  1461
           ----------                                                  1228
           ----------                                                  1216
           ----------                                                  1210
           ----------                                                  646
           ----------                                                  354
           ----------                                                  330

LOCUS: AT3G07110
DESCRIPTION: 60S ribosomal protein L13A (RPL13aA), similar to ribosomal protein L13A GB:O49885 (Lupinus
luteus)
DATA:                  Control 30min   2hours  2days   1week   p-value         pos
SENSE COUNTS:          1       2       0       10      3       1.67e-03
GENES (1 total):
  AT3G07110.1
    SENSE COUNTS:      1       2       0       10      3       1.67e-03
    TAGS: (2 total)
      d+1  CGCCTCCTCC  0       0       0       4       0       6.18e-03        840
      d+2  AGGTTTCTCC  1       2       0       6       3       8.00e-02        245
           ----------                                                  125
```

```
LOCUS: AT5G48240
DESCRIPTION: hypothetical protein,
DATA:                 Control 30min   2hours  2days   1week   p-value         pos
SENSE COUNTS:         0       1       1       9       1       1.73e-03
GENES (1 total):
  AT5G48240.1
    SENSE COUNTS:     0       1       1       9       1       1.73e-03
    TAGS: (1 total)
           ----------                                                         1786
           ----------                                                         1721
           ----------                                                         1676
           ----------                                                         1587
      v+2  CAAATTTTGT 0       1       1       9       1       1.73e-03        1425
           ----------                                                         1272
           ----------                                                         1018
           ----------                                                         946
           ----------                                                         916
           ----------                                                         691
           ----------                                                         627
           ----------                                                         378
           ----------                                                         354
           ----------                                                         121

LOCUS: AT1G29020
DESCRIPTION: calcium-binding EF hand family protein, contains INTERPRO:IPR002048 calcium-binding EF-hand
domain
DATA:                 Control 30min   2hours  2days   1week   p-value         pos
SENSE COUNTS:         2       0       8       0       7       1.74e-03
GENES (1 total):
  AT1G29020.1
    SENSE COUNTS:     2       0       8       0       7       1.74e-03
    TAGS: (1 total)
           ----------                                                         3344
           ----------                                                         3146
           ----------                                                         2957
      i+3  TGTTGAACTA 2       0       8       0       7       1.74e-03        2952
           ----------                                                         2884
           ----------                                                         2480
           ----------                                                         1981
           ----------                                                         1962
           ----------                                                         1526
           ----------                                                         1046
           ----------                                                         406
           ----------                                                         387
           ----------                                                         197
           ----------                                                         52

LOCUS: AT1G42970
DESCRIPTION: glyceraldehyde-3-phosphate dehydrogenase B, chloroplast (GAPB) / NADP-dependent
glyceraldehydephosphate dehydrogenase subunit B, identical to SP|P25857 Glyceraldehyde 3-phosphate
dehydrogenase B, chloroplast precursor (EC 1.2.1.13) (NADP-dependent glycera
DATA:                 Control 30min   2hours  2days   1week   p-value         pos
SENSE COUNTS:         67      43      74      50      87      1.79e-03
GENES (2 total):
  AT1G42970.1
    SENSE COUNTS:     67      43      74      50      87      1.79e-03
    TAGS: (7 total)
           ----------                                                         1715
      d+2  TTAAAACGGA 15      9       23      12      14      1.49e-01        1642
      d+2  AGTTACTTAT 48      34      44      36      71      9.22e-03        1518
      i+3  TAGAAACCTA 1       0       0       0       0       4.28e-01        1445
      i+3  CTTCAGTTTT 1       0       0       0       0       4.28e-01        1199
      d+2  GGCTGAGCTC 0       0       6       2       1       1.12e-02        619
      d+2  CTTGGAACCT 0       0       1       0       0       4.55e-01        512
           ----------                                                         423
      X+4  AGTGTGCAAC 2       0       0       0       1       2.03e-01        385
           ----------                                                         132

LOCUS: AT1G25400
DESCRIPTION: expressed protein, similar to unknown protein GI:6714347 from (Arabidopsis thaliana)
DATA:                 Control 30min   2hours  2days   1week   p-value         pos
SENSE COUNTS:         0       9       4       0       2       1.81e-03
GENES (1 total):
  AT1G25400.1
    SENSE COUNTS:     0       9       4       0       2       1.81e-03
    TAGS: (2 total)
      d+1  TTGTAAGTAG 0       2       2       0       1       4.26e-01        1194
      d+2  GTAGGGTCTA 0       7       2       0       1       4.83e-03        990
           ----------                                                         856
           ----------                                                         458
           ----------                                                         112
```

```
LOCUS: AT1G27290
DESCRIPTION: expressed protein
DATA:                  Control 30min   2hours  2days   1week   p-value         pos
SENSE COUNTS:          15      6       10      1       0       1.81e-03
GENES (1 total):
  AT1G27290.1
    SENSE COUNTS:      15      6       10      1       0       1.81e-03
    TAGS: (2 total)
      d+1  ATCATTTCTT  10      4       4       1       0       3.03e-02        951
           ----------                                                          929
      d+2  TGGATATCAG  5       2       6       0       0       6.19e-02        828

LOCUS: AT1G48300
DESCRIPTION: expressed protein
DATA:                  Control 30min   2hours  2days   1week   p-value         pos
SENSE COUNTS:          20      1       16      7       16      1.99e-03
GENES (1 total):
  AT1G48300.1
    SENSE COUNTS:      20      1       16      7       16      1.99e-03
    TAGS: (2 total)
           ----------                                                          1200
           ----------                                                          1155
           ----------                                                          1134
      d+2  TAATTGCGAT  18      0       12      6       16      8.16e-04        985
      d+2  GGGAAGTGTC  2       1       4       1       0       4.49e-01        708
           ----------                                                          369
           ----------                                                          282

LOCUS: AT5G16300
DESCRIPTION: expressed protein,
DATA:                  Control 30min   2hours  2days   1week   p-value         pos
SENSE COUNTS:          4       0       0       0       6       2.22e-03
GENES (2 total):
  AT5G16300.1
    SENSE COUNTS:      4       0       0       0       6       2.22e-03
    TAGS: (3 total)
           ----------                                                          3703
      d+2  TAATTAAGTA  3       0       0       0       5       1.39e-02        3420
      d+2  AACCAAAAAC  0       0       0       0       1       1.65e-01        3305
           ----------                                                          3052
           ----------                                                          2967
           ----------                                                          2850
           ----------                                                          2845
      d+2  CTGTACTCTT  1       0       0       0       0       4.28e-01        2756
           ----------                                                          1874
           ----------                                                          1391
           ----------                                                          1168
           ----------                                                          886
           ----------                                                          626
           ----------                                                          479
           ----------                                                          466
           ----------                                                          238
  AT5G16300.2
    SENSE COUNTS:      4       0       0       0       6       2.22e-03
    TAGS: (3 total)
           ----------                                                          3601
      d+2  TAATTAAGTA  3       0       0       0       5       1.39e-02        3318
      d+2  AACCAAAAAC  0       0       0       0       1       1.65e-01        3203
           ----------                                                          2850
           ----------                                                          2845
      d+2  CTGTACTCTT  1       0       0       0       0       4.28e-01        2756
           ----------                                                          1874
           ----------                                                          1391
           ----------                                                          1168
           ----------                                                          886
           ----------                                                          626
           ----------                                                          479
           ----------                                                          466
           ----------                                                          238

LOCUS: AT1G48210
DESCRIPTION: serine/threonine protein kinase, putative, similar to Pto kinase interactor 1 (Lycopersicon
esculentum) gi|3668069|gb|AAC61805; contains protein kinase domain, Pfam:PF00069
DATA:                  Control 30min   2hours  2days   1week   p-value         pos
SENSE COUNTS:          2       0       1       1       10      2.31e-03
GENES (1 total):
  AT1G48210.1
    SENSE COUNTS:      2       0       1       1       10      2.31e-03
    TAGS: (2 total)
      i+3  TCTTCATTCA  0       0       0       0       0       6.15e-01        2286
```

```
      d+1  TGTATGTCTC 2       0       1       1       10      5.01e-04       1390
           ----------                                                        1285
           ----------                                                        1165
           ----------                                                        1020
           ----------                                                        832
           ----------                                                        716
           ----------                                                        614
           ----------                                                        602
           ----------                                                        515
           ----------                                                        298
           ----------                                                        277

LOCUS: AT5G49460
DESCRIPTION: One of the two genes encoding subunit B of the cytosolic enzyme ATP Citrate Lyase (ACL)
DATA:                 Control 30min   2hours  2days   1week   p-value        pos
SENSE COUNTS:         11      1       3       0       1       2.62e-03
GENES (2 total):
  AT5G49460.1
    SENSE COUNTS:     11      1       3       0       1       2.62e-03
    TAGS: (3 total)
      d+1  TCTATATCTT 0       0       0       0       0       6.15e-01       2199
           ----------                                                        2195
           ----------                                                        2189
      d+2  TATGTTTGTT 9       1       2       0       1       7.40e-03       2085
      d+2  GGAAGATGTG 2       0       1       0       0       2.87e-01       1937
           ----------                                                        1686
           ----------                                                        1382
           ----------                                                        1232
           ----------                                                        955
           ----------                                                        423
           ----------                                                        334

LOCUS: AT3G17790
DESCRIPTION: acid phosphatase type 5 (ACP5), contains Pfam profile: PF00149 calcineurin-like phosphoesterase;
nearly identical to acid phosphatase type 5 (GI:10278031) (Arabidopsis thaliana)
DATA:                 Control 30min   2hours  2days   1week   p-value        pos
SENSE COUNTS:         0       5       10      1       0       2.80e-03
GENES (2 total):
  AT3G17790.1
    SENSE COUNTS:     0       5       10      1       0       2.80e-03
    TAGS: (1 total)
           ----------                                                        2167
           ----------                                                        1470
           ----------                                                        1431
      d+2  TCTGCTCGGT 0       5       10      1       0       2.80e-03       1067
           ----------                                                        918
           ----------                                                        908
           ----------                                                        840
           ----------                                                        816
           ----------                                                        534
           ----------                                                        450

LOCUS: AT3G26450
DESCRIPTION: major latex protein-related / MLP-related, low similarity to major latex protein {Papaver
somniferum}(GI:294060) ; contains Pfam profile PF00407: Pathogenesis-related protein Bet v I family
DATA:                 Control 30min   2hours  2days   1week   p-value        pos
SENSE COUNTS:         18      8       17      5       0       2.85e-03
GENES (1 total):
  AT3G26450.1
    SENSE COUNTS:     18      8       17      5       0       2.85e-03
    TAGS: (3 total)
      i+3  TCCTATCTTT 2       0       0       0       0       1.04e-01       1221
      d+1  CAACTCCTCA 16      8       17      5       0       4.54e-03       475
      i+3  GGTTGATACC 0       0       0       0       0       6.15e-01       314

LOCUS: AT5G06320
DESCRIPTION: harpin-induced family protein / HIN1 family protein / harpin-responsive family protein /
NDR1/HIN1-like protein 3, similar to harpin-induced protein hin1 (GI:1619321)(Nicotiana tabacum)
DATA:                 Control 30min   2hours  2days   1week   p-value        pos
SENSE COUNTS:         1       13      3       4       10      2.88e-03
GENES (2 total):
  AT5G06320.1
    SENSE COUNTS:     1       13      3       4       10      2.88e-03
    TAGS: (2 total)
           ----------                                                        1075
      d+2  AAGCATTGAT 0       0       0       0       0       6.15e-01       979
      d+2  TAAATAAATA 1       13      3       4       10      5.47e-03       914

LOCUS: AT1G80230
DESCRIPTION: cytochrome c oxidase family protein, contains Pfam domain, PF01215: Cytochrome c oxidase subunit
Vb
```

```
DATA:                 Control 30min   2hours  2days   1week   p-value        pos
SENSE COUNTS:         0       0       3       9       1       2.92e-03
GENES (1 total):
  AT1G80230.1
    SENSE COUNTS:     0       0       3       9       1       2.92e-03
    TAGS: (2 total)
      d+1  TGTTTATACG 0       0       3       0       1       7.97e-02       843
      d+2  GTGATGACCA 0       0       0       9       0       1.28e-05       597
           ----------                                                        489
           ----------                                                        446

LOCUS: AT1G55280
DESCRIPTION: expressed protein
DATA:                 Control 30min   2hours  2days   1week   p-value        pos
SENSE COUNTS:         0       5       0       0       0       3.00e-03
GENES (1 total):
  AT1G55280.1
    SENSE COUNTS:     0       5       0       0       0       3.00e-03
    TAGS: (1 total)
           ----------                                                        1365
           ----------                                                        1353
           ----------                                                        1306
           ----------                                                        1136
           ----------                                                        851
      d+2  GTTTACGAAA 0       5       0       0       0       3.00e-03       142

LOCUS: AT5G27420
DESCRIPTION: zinc finger (C3HC4-type RING finger) family protein, similar to RING-H2 zinc finger protein ATL6
(Arabidopsis thaliana) gi|4928403|gb|AAD33584.1|AF132016_1(4928403); contains Pfam domain, PF00097: Zinc
finger, C3HC4 type (RING finger)
DATA:                 Control 30min   2hours  2days   1week   p-value        pos
SENSE COUNTS:         0       5       0       0       0       3.00e-03
GENES (1 total):
  AT5G27420.1
    SENSE COUNTS:     0       5       0       0       0       3.00e-03
    TAGS: (1 total)
           ----------                                                        1458
           ----------                                                        1386
      d+2  GGCTTCTTCA 0       5       0       0       0       3.00e-03       225
           ----------                                                        183
           ----------                                                        145

LOCUS: AT5G65010
DESCRIPTION: asparagine synthetase (ASN2) mRNA, complete cds
DATA:                 Control 30min   2hours  2days   1week   p-value        pos
SENSE COUNTS:         15      2       19      20      11      3.03e-03
GENES (3 total):
  AT5G65010.1
    SENSE COUNTS:     14      2       19      19      8       2.45e-03
    TAGS: (3 total)
      d+1  GATCTCCAGT 12      2       18      19      8       3.40e-03       1896
           ----------                                                        1686
           ----------                                                        1452
           ----------                                                        1440
           ----------                                                        1141
           ----------                                                        1131
      i+3  TGTAAATAAA 2       0       0       0       0       1.04e-01       472
      d+2  TTGGATGGAA 0       0       1       0       0       4.55e-01       383
           ----------                                                        363
           ----------                                                        180
  AT5G65010.2
    SENSE COUNTS:     15      2       19      20      11      3.03e-03
    TAGS: (4 total)
      d+1  GATCTCCAGT 12      2       18      19      8       3.40e-03       1899
           ----------                                                        1689
           ----------                                                        1452
           ----------                                                        1440
           ----------                                                        1141
           ----------                                                        1131
      X+4  TTCTGGCTTG 1       0       0       1       3       1.87e-01       531
      i+3  TGTAAATAAA 2       0       0       0       0       1.04e-01       472
      d+2  TTGGATGGAA 0       0       1       0       0       4.55e-01       383
           ----------                                                        363
           ----------                                                        180

LOCUS: AT2G26740
DESCRIPTION: epoxide hydrolase, soluble (sEH), identical to ATsEH (Arabidopsis thaliana) GI:1109600
DATA:                 Control 30min   2hours  2days   1week   p-value        pos
SENSE COUNTS:         14      12      5       2       0       3.10e-03
GENES (2 total):
  AT2G26740.1
```

```
    SENSE COUNTS:      14      12      5       2       0       3.10e-03
    TAGS: (2 total)
      d+1  TGATTGTTTA  10      4       3       1       0       2.12e-02        1160
      d+2  TATGGTTGTA  4       8       2       1       0       3.18e-02        1063
           ----------                                                          908
           ----------                                                          858
           ----------                                                          766
           ----------                                                          554
           ----------                                                          458
           ----------                                                          330
           ----------                                                          247
           ----------                                                          139
           ----------                                                          114

LOCUS: AT4G19880
DESCRIPTION: glutathione S-transferase-related, contains weak hit to Pfam profile PF00043: Glutathione S-
transferase, C-terminal domain
DATA:                  Control 30min   2hours  2days   1week   p-value         pos
SENSE COUNTS:          3       7       17      3       3       3.14e-03
GENES (1 total):
  AT4G19880.1
    SENSE COUNTS:      3       7       17      3       3       3.14e-03
    TAGS: (2 total)
      d+1  AGTTGAGTTC  2       5       6       2       3       6.14e-01        1096
           ----------                                                          1037
           ----------                                                          1007
           ----------                                                          940
      X+4  TGGCGGATTA  1       2       11      1       0       8.11e-04        474
           ----------                                                          234

LOCUS: AT4G25340
DESCRIPTION: immunophilin-related / FKBP-type peptidyl-prolyl cis-trans isomerase-related, immunophilin FKBP46
- Spodoptera frugiperda (fall armyworm),PIR2:A55320
DATA:                  Control 30min   2hours  2days   1week   p-value         pos
SENSE COUNTS:          1       0       1       10      4       3.25e-03
GENES (1 total):
  AT4G25340.1
    SENSE COUNTS:      1       0       1       10      4       3.25e-03
    TAGS: (3 total)
      d+1  CGCGTTGGTG  0       0       0       5       1       2.33e-02        1376
      d+2  GGAAAACCCA  0       0       1       1       0       5.21e-01        1202
           ----------                                                          1019
      d+2  TTCCAGTTTT  1       0       0       4       3       2.03e-01        678
           ----------                                                          390

LOCUS: AT5G14520
DESCRIPTION: pescadillo-related, similar to pescadillo (Zebrafish, Danio rerio) SWISS-PROT:P79741
DATA:                  Control 30min   2hours  2days   1week   p-value         pos
SENSE COUNTS:          4       2       11      17      17      3.26e-03
GENES (1 total):
  AT5G14520.1
    SENSE COUNTS:      4       2       11      17      17      3.26e-03
    TAGS: (3 total)
      v+1  AGTACGCCTA  2       2       8       6       12      4.96e-02        2444
           ----------                                                          2328
      v+2  TACTTCCAAA  1       0       2       11      5       2.98e-03        2216
           ----------                                                          2123
           ----------                                                          2037
           ----------                                                          1870
           ----------                                                          1173
           ----------                                                          964
           ----------                                                          877
           ----------                                                          847
      v+2  CACGAGCCTC  1       0       1       0       0       6.01e-01        564
           ----------                                                          541
           ----------                                                          354
           ----------                                                          190
           ----------                                                          128

LOCUS: AT2G47730
DESCRIPTION: glutathione S-transferase 6 (GST6), identical to GB:X95295. Based on identical cDNA hits, the
translation is now 40 AAs longer at the N-terminal, and start of exon2 is also corrected.
DATA:                  Control 30min   2hours  2days   1week   p-value         pos
SENSE COUNTS:          8       16      19      31      8       3.30e-03
GENES (2 total):
  AT2G47730.1
    SENSE COUNTS:      8       16      19      31      8       3.30e-03
    TAGS: (2 total)
      d+1  ACCACTGACC  8       16      16      30      7       3.64e-03        542
           ----------                                                          533
      d+2  TCCACCGCCA  0       0       3       1       1       1.97e-01        191
```

```
          ----------                                                    161

LOCUS: AT4G13290
DESCRIPTION: cytochrome P450 71A19, putative (CYP71A19), Identical to Cytochrome P450 (SP:Q9T0K0) (Arabidopsis
thaliana); similar to cytochrome P450LXXIA1, Persea americana, M32885
DATA:                 Control 30min   2hours  2days   1week   p-value        pos
SENSE COUNTS:         10      0       3       1       5       3.44e-03
GENES (2 total):
  AT4G13290.1
    SENSE COUNTS:     10      0       3       1       5       3.44e-03
    TAGS: (1 total)
          ----------                                                    1403
          ----------                                                    1191
          ----------                                                    949
          ----------                                                    776
      X+4  CAAATCCAAA 10      0       3       1       5       3.44e-03  513
          ----------                                                    263
          ----------                                                    205
          ----------                                                    114

LOCUS: AT3G06530
DESCRIPTION: BAP28-related, similar to Protein BAP28 (Swiss-Prot:Q9H583) (Homo sapiens)
DATA:                 Control 30min   2hours  2days   1week   p-value        pos
SENSE COUNTS:         0       0       0       1       7       3.63e-03
GENES (1 total):
  AT3G06530.1
    SENSE COUNTS:     0       0       0       1       7       3.63e-03
    TAGS: (2 total)
          ----------                                                    6313
      v+2  TTTGCAGAAA 0       0       0       1       7       4.62e-04  6005
      v+2  CATCGGTCAA 0       0       0       0       0       6.15e-01  5552
          ----------                                                    5512
          ----------                                                    5401
          ----------                                                    4939
          ----------                                                    4710
          ----------                                                    4532
          ----------                                                    3582
          ----------                                                    3507
          ----------                                                    3428
          ----------                                                    3063
          ----------                                                    2922
          ----------                                                    2324
          ----------                                                    2214
          ----------                                                    2187
          ----------                                                    1906
          ----------                                                    1650
          ----------                                                    1219
          ----------                                                    1140
          ----------                                                    787
          ----------                                                    778
          ----------                                                    558
          ----------                                                    131

LOCUS: AT4G34550
DESCRIPTION: expressed protein,
DATA:                 Control 30min   2hours  2days   1week   p-value        pos
SENSE COUNTS:         0       0       1       6       1       3.89e-03
GENES (1 total):
  AT4G34550.1
    SENSE COUNTS:     0       0       1       6       1       3.89e-03
    TAGS: (1 total)
          ----------                                                    1668
      v+2  CCAATCTTGC 0       0       1       6       1       3.89e-03  1206
          ----------                                                    1021
          ----------                                                    980
          ----------                                                    825
          ----------                                                    591
          ----------                                                    513
          ----------                                                    406
          ----------                                                    225
          ----------                                                    187
          ----------                                                    165

LOCUS: AT3G62670
DESCRIPTION: member of Response Regulator: B- Type
DATA:                 Control 30min   2hours  2days   1week   p-value        pos
SENSE COUNTS:         0       0       1       6       1       3.89e-03
GENES (2 total):
  AT3G62670.1
    SENSE COUNTS:     0       0       1       6       1       3.89e-03
    TAGS: (1 total)
```

```
          ----------                                                  2115
          ----------                                                  1871
          ----------                                                  1545
          ----------                                                  1524
          ----------                                                  1482
          ----------                                                  1383
          ----------                                                  1323
          ----------                                                  1267
          ----------                                                  811
          ----------                                                  709
          ----------                                                  702
     v+2  AGATCGATCT 0      0      1      6      1      3.89e-03      601
          ----------                                                  576
          ----------                                                  525
          ----------                                                  6

LOCUS: AT5G13130
DESCRIPTION: hypothetical protein, low similarity to microrchidia (Mus musculus) GI:5410255
DATA:                Control 30min  2hours 2days  1week  p-value       pos
SENSE COUNTS:        11     0      8      15     10     4.53e-03
GENES (1 total):
  AT5G13130.1
    SENSE COUNTS:    11     0      8      15     10     4.53e-03
    TAGS: (1 total)
          ----------                                                  2659
     v+2  ATGAAGATTG 11     0      8      15     10     4.53e-03      1506
          ----------                                                  1309
          ----------                                                  772
          ----------                                                  676
          ----------                                                  354

LOCUS: AT1G12130
DESCRIPTION: flavin-containing monooxygenase family protein / FMO family protein, contains similarity to
flavin-containing monooxygenase 2 (FMO2) from Homo sapiens (GI:1834493); contains Pfam profile PF00743 Flavin-
binding monooxygenase-like
DATA:                Control 30min  2hours 2days  1week  p-value       pos
SENSE COUNTS:        0      0      0      5      1      4.62e-03
GENES (1 total):
  AT1G12130.1
    SENSE COUNTS:    0      0      0      5      1      4.62e-03
    TAGS: (2 total)
     v+1  TCATTAAATT 0      0      0      1      0      3.09e-01      2117
          ----------                                                  1819
          ----------                                                  1549
     v+2  GCATTGCAAT 0      0      0      4      1      2.76e-02      1341
          ----------                                                  1288
          ----------                                                  917
          ----------                                                  693
          ----------                                                  175
          ----------                                                  139

LOCUS: AT4G27680
DESCRIPTION: MSP1 protein, putative / intramitochondrial sorting protein, putative, similar to Swiss-
Prot:P28737 MSP1 protein (TAT-binding homolog 4) (Saccharomyces cerevisiae); contains Pfam domain, PF00004:
ATPase, AAA family
DATA:                Control 30min  2hours 2days  1week  p-value       pos
SENSE COUNTS:        0      0      0      5      1      4.62e-03
GENES (1 total):
  AT4G27680.1
    SENSE COUNTS:    0      0      0      5      1      4.62e-03
    TAGS: (1 total)
          ----------                                                  1556
     d+2  GCAGCGGGAT 0      0      0      5      1      4.62e-03      1324
          ----------                                                  1108
          ----------                                                  822
          ----------                                                  773
          ----------                                                  756
          ----------                                                  370
          ----------                                                  161

LOCUS: AT3G47800
DESCRIPTION: aldose 1-epimerase family protein, similar to ALDOSE 1-EPIMERASE PRECURSOR GB:P05149 (SP|P05149)
from (Acinetobacter calcoaceticus); contains Pfam profile PF01263 Aldose 1-epimerase
DATA:                Control 30min  2hours 2days  1week  p-value       pos
SENSE COUNTS:        0      2      0      0      6      4.98e-03
GENES (1 total):
  AT3G47800.1
    SENSE COUNTS:    0      2      0      0      6      4.98e-03
    TAGS: (2 total)
          ----------                                                  1792
          ----------                                                  1767
```

```
      d+2  TTATGCTCTT 0      2      0      0      5      2.94e-02       1210
      i+3  GTACTCAAAA 0      0      0      0      1      1.65e-01       872
           ----------                                                 657
           ----------                                                 520
           ----------                                                 346
           ----------                                                 318

LOCUS: AT5G08570
DESCRIPTION: pyruvate kinase, putative, similar to pyruvate kinase, cytosolic isozyme (Glycine max) SWISS-
PROT:Q42806
DATA:                 Control 30min  2hours 2days  1week  p-value        pos
SENSE COUNTS:         3      0      4      5      14     5.29e-03
GENES (1 total):
  AT5G08570.1
    SENSE COUNTS:     3      0      4      5      14     5.29e-03
    TAGS: (2 total)
      d+1  AAATAAAGAA 3      0      0      5      14     1.41e-04       1753
      d+2  GCTAAGATCT 0      0      4      0      0      5.58e-03       1190
           ----------                                                 929
           ----------                                                 860
           ----------                                                 840
           ----------                                                 610
           ----------                                                 524
           ----------                                                 392
           ----------                                                 321
           ----------                                                 312
           ----------                                                 257

LOCUS: AT1G05490
DESCRIPTION: C protein immunoglobulin-A-binding beta antigen-related, contains weak similarity to C protein
immunoglobulin-A-binding beta antigen (Streptococcus agalactiae) gi|18028989|gb|AAL56250
DATA:                 Control 30min  2hours 2days  1week  p-value        pos
SENSE COUNTS:         12     7      18     15     30     5.44e-03
GENES (1 total):
  AT1G05490.1
    SENSE COUNTS:     12     7      18     15     30     5.44e-03
    TAGS: (1 total)
           ----------                                                 641
      d+2  TCTGAAAGAG 12     7      18     15     30     5.44e-03       503
           ----------                                                 381

LOCUS: AT3G51260
DESCRIPTION: 20S proteasome alpha subunit D (PAD1)
DATA:                 Control 30min  2hours 2days  1week  p-value        pos
SENSE COUNTS:         1      0      0      1      8      5.47e-03
GENES (2 total):
  AT3G51260.1
    SENSE COUNTS:     1      0      0      1      8      5.47e-03
    TAGS: (2 total)
           ----------                                                 1122
      d+2  TTCAGTATTC 0      0      0      1      8      2.96e-04       1041
      d+2  AAATGGTTTG 1      0      0      0      0      6.89e-01       940
           ----------                                                 844

LOCUS: AT3G20310
DESCRIPTION: encodes a member of the ERF (ethylene response factor) subfamily B-1 of ERF/AP2 transcription
factor family (ATERF-7). The protein contains one AP2 domain. There are 15 members in this subfamily including
ATERF-3, ATERF-4, ATERF-7, and leafy petiole.
DATA:                 Control 30min  2hours 2days  1week  p-value        pos
SENSE COUNTS:         1      9      2      1      2      5.51e-03
GENES (1 total):
  AT3G20310.1
    SENSE COUNTS:     1      9      2      1      2      5.51e-03
    TAGS: (3 total)
      d+1  GTCAATGGGC 0      2      1      0      1      4.01e-01       1580
      d+2  CGATTAAGAG 1      7      0      1      1      6.47e-03       629
           ----------                                                 547
      d+2  AGGAAAGGGA 0      0      1      0      0      4.55e-01       118

LOCUS: AT2G20890
DESCRIPTION: Chloroplast-localized Thylakoid formation1 gene product involved in vesicle-mediated formation of
thylakoid membranes
DATA:                 Control 30min  2hours 2days  1week  p-value        pos
SENSE COUNTS:         36     16     34     21     45     5.57e-03
GENES (1 total):
  AT2G20890.1
    SENSE COUNTS:     36     16     34     21     45     5.57e-03
    TAGS: (4 total)
      d+1  TAGTATTTTA 4      4      1      4      10     1.30e-01       1165
      d+2  AGTTTACATA 31     12     31     16     34     6.14e-03       1142
      d+2  GAAGGATATC 1      0      2      1      0      8.12e-01       488
```

```
      d+2  TCCAATGTTA 0      0      0      0      1      1.65e-01       287

LOCUS: AT4G21460
DESCRIPTION: expressed protein
DATA:                 Control 30min  2hours 2days  1week  p-value        pos
SENSE COUNTS:         0      0      4      0      0      5.58e-03
GENES (1 total):
  AT4G21460.1
    SENSE COUNTS:     0      0      4      0      0      5.58e-03
    TAGS: (1 total)
           ----------                                                    1571
      d+2  CTAACTTCTA 0      0      4      0      0      5.58e-03       1509
           ----------                                                    1430
           ----------                                                    1422
           ----------                                                    573
           ----------                                                    551
           ----------                                                    527
           ----------                                                    17

LOCUS: AT2G45520
DESCRIPTION: expressed protein
DATA:                 Control 30min  2hours 2days  1week  p-value        pos
SENSE COUNTS:         0      6      1      1      0      5.80e-03
GENES (1 total):
  AT2G45520.1
    SENSE COUNTS:     0      6      1      1      0      5.80e-03
    TAGS: (2 total)
           ----------                                                    954
           ----------                                                    863
           ----------                                                    809
      d+2  AATGTACTCT 0      3      1      1      0      2.38e-01       650
           ----------                                                    454
      d+2  ATTCTACTAA 0      3      0      0      0      3.05e-02       175
           ----------                                                    150

LOCUS: AT5G64840
DESCRIPTION: ABC transporter family protein
DATA:                 Control 30min  2hours 2days  1week  p-value        pos
SENSE COUNTS:         3      4      15     2      6      5.81e-03
GENES (2 total):
  AT5G64840.1
    SENSE COUNTS:     3      4      15     2      6      5.81e-03
    TAGS: (3 total)
      d+1  GAACCGAACC 0      0      2      0      1      2.22e-01       2377
      d+2  GTGACGCCAT 3      4      12     2      5      6.43e-02       1792
           ----------                                                    1726
      d+2  ACAGAGCCTT 0      0      1      0      0      4.55e-01       1061
           ----------                                                    667
           ----------                                                    471
           ----------                                                    121

LOCUS: AT4G21790
DESCRIPTION: transmembrane protein-related (TOM1), contains some similarity to transmembrane protein TOM3
GI:15425641 from (Arabidopsis thaliana); identical to cDNA TOM1 GI:9967414
DATA:                 Control 30min  2hours 2days  1week  p-value        pos
SENSE COUNTS:         0      8      2      1      1      5.90e-03
GENES (2 total):
  AT4G21790.1
    SENSE COUNTS:     0      8      2      1      1      5.90e-03
    TAGS: (2 total)
      i+3  TATTTGATGT 0      0      1      0      0      4.55e-01       1466
      d+1  CTAAGAAGGT 0      8      1      1      1      2.31e-03       744
           ----------                                                    643
           ----------                                                    423

LOCUS: AT1G47710
DESCRIPTION: serpin, putative / serine protease inhibitor, putative, similar to phloem serpin-1 (Cucurbita
maxima) GI:9937311; contains Pfam profile PF00079: Serpin (serine protease inhibitor)
DATA:                 Control 30min  2hours 2days  1week  p-value        pos
SENSE COUNTS:         0      0      0      5      3      5.98e-03
GENES (1 total):
  AT1G47710.1
    SENSE COUNTS:     0      0      0      5      3      5.98e-03
    TAGS: (3 total)
      d+1  TGATGCATCT 0      0      0      4      0      6.18e-03       1423
      d+2  TACTGAACTG 0      0      0      1      0      3.09e-01       1396
      X+4  GTTACATACA 0      0      0      0      3      1.12e-02       1009
           ----------                                                    771
           ----------                                                    675
           ----------                                                    593
           ----------                                                    485
```

```
          ----------                                                  108

LOCUS: AT1G80180
DESCRIPTION: expressed protein
DATA:                 Control 30min  2hours  2days  1week  p-value        pos
SENSE COUNTS:         9       9      2       1      0      6.10e-03
GENES (1 total):
  AT1G80180.1
    SENSE COUNTS:     9       9      2       1      0      6.10e-03
    TAGS: (3 total)
          ----------                                                  760
      X+4 TATATTCTTG  5       4      1       0      0      6.73e-02       759
      d+2 AACTAAAGTC  3       5      1       1      0      2.06e-01       534
          ----------                                                  115
      X+4 ATAATCGCGA  1       0      0       0      0      4.28e-01       -146

LOCUS: AT5G15100
DESCRIPTION: auxin efflux carrier family protein, contains auxin efflux carrier domain, Pfam:PF03547
DATA:                 Control 30min  2hours  2days  1week  p-value        pos
SENSE COUNTS:         12      3      22      13     11     6.13e-03
GENES (1 total):
  AT5G15100.1
    SENSE COUNTS:     12      3      22      13     11     6.13e-03
    TAGS: (2 total)
          ----------                                                  1635
      v+2 TTTTATACGT  0       0      0       0      1      1.65e-01       1482
      v+2 TTCAAAAAAA  12      3      22      13     10     5.16e-03       1465
          ----------                                                  1190
          ----------                                                  1164
          ----------                                                  1143
          ----------                                                  615
          ----------                                                  379
          ----------                                                  354

LOCUS: AT2G36500
DESCRIPTION: CBS domain-containing protein / octicosapeptide/Phox/Bemp1 (PB1) domain-containing protein,
contains Pfam profiles: PF00571 CBS domain, PF00564: PB1 domain
DATA:                 Control 30min  2hours  2days  1week  p-value        pos
SENSE COUNTS:         0       0      0       4      0      6.18e-03
GENES (1 total):
  AT2G36500.1
    SENSE COUNTS:     0       0      0       4      0      6.18e-03
    TAGS: (1 total)
          ----------                                                  1115
      d+2 AGAGTTGTAG  0       0      0       4      0      6.18e-03       997
          ----------                                                  968
          ----------                                                  562

LOCUS: AT2G01170
DESCRIPTION: amino acid permease family protein, weak similarity to GABA permease (Emericella nidulans)
GI:4972245; contains Pfam profile PF00324: Amino acid permease
DATA:                 Control 30min  2hours  2days  1week  p-value        pos
SENSE COUNTS:         0       0      0       4      0      6.18e-03
GENES (2 total):
  AT2G01170.1
    SENSE COUNTS:     0       0      0       4      0      6.18e-03
    TAGS: (1 total)
          ----------                                                  1806
          ----------                                                  1138
          ----------                                                  871
          ----------                                                  829
          ----------                                                  706
          ----------                                                  596
      X+4 GGCCTCGACG  0       0      0       4      0      6.18e-03       515
          ----------                                                  127

LOCUS: AT5G09930
DESCRIPTION: ABC transporter family protein,
DATA:                 Control 30min  2hours  2days  1week  p-value        pos
SENSE COUNTS:         0       0      0       1      5      6.31e-03
GENES (2 total):
  AT5G09930.1
    SENSE COUNTS:     0       0      0       1      5      6.31e-03
    TAGS: (1 total)
          ----------                                                  2723
      v+2 AAGGTATCAT  0       0      0       1      5      6.31e-03       2525
          ----------                                                  1983
          ----------                                                  1917
          ----------                                                  1252
          ----------                                                  1018
          ----------                                                  858
```

```
          ----------                                                    214

LOCUS: AT1G79150
DESCRIPTION: expressed protein, ; expression supported by MPSS
DATA:                 Control 30min  2hours 2days  1week  p-value       pos
SENSE COUNTS:         0       0      0      1      5      6.31e-03
GENES (1 total):
  AT1G79150.1
    SENSE COUNTS:     0       0      0      1      5      6.31e-03
    TAGS: (2 total)
      v+1  TATTTGAACT 0       0      0      1      0      3.09e-01      2107
      v+2  TCCATTCGCT 0       0      0      0      5      6.24e-04      1497
           ----------                                                   1447
           ----------                                                   1402
           ----------                                                   811
           ----------                                                   748
           ----------                                                   182
           ----------                                                   54

LOCUS: AT5G50730
DESCRIPTION: expressed protein, ; expression supported by MPSS
DATA:                 Control 30min  2hours 2days  1week  p-value       pos
SENSE COUNTS:         1       0      5      0      0      6.70e-03
GENES (1 total):
  AT5G50730.1
    SENSE COUNTS:     1       0      5      0      0      6.70e-03
    TAGS: (1 total)
           ----------                                                   1095
           ----------                                                   998
      v+2  CCGCGGTTGT 1       0      5      0      0      6.70e-03      475
           ----------                                                   354

LOCUS: AT1G07770
DESCRIPTION: 40S ribosomal protein S15A (RPS15aA), identical to GB:AAA61608 from (Arabidopsis thaliana) (Plant
Physiol. 106 (1), 401-402 (1994))
DATA:                 Control 30min  2hours 2days  1week  p-value       pos
SENSE COUNTS:         1       0      5      0      0      6.70e-03
GENES (3 total):
  AT1G07770.2
    SENSE COUNTS:     1       0      5      0      0      6.70e-03
    TAGS: (1 total)
           ----------                                                   395
      d+2  CAGAAGCACG 1       0      5      0      0      6.70e-03      178
           ----------                                                   133
           ----------                                                   97
  AT1G07770.1
    SENSE COUNTS:     1       0      5      0      0      6.70e-03
    TAGS: (1 total)
           ----------                                                   435
      d+2  CAGAAGCACG 1       0      5      0      0      6.70e-03      218
           ----------                                                   173
           ----------                                                   137

LOCUS: AT5G27230
DESCRIPTION: expressed protein,  ; expression supported by MPSS
DATA:                 Control 30min  2hours 2days  1week  p-value       pos
SENSE COUNTS:         1       0      0      0      5      7.00e-03
GENES (1 total):
  AT5G27230.1
    SENSE COUNTS:     1       0      0      0      5      7.00e-03
    TAGS: (1 total)
           ----------                                                   3355
           ----------                                                   3317
           ----------                                                   3224
           ----------                                                   3034
           ----------                                                   2970
           ----------                                                   2112
           ----------                                                   2007
           ----------                                                   1807
      i+3  TAACACTTTT 1       0      0      0      5      7.00e-03      1790
           ----------                                                   1605
           ----------                                                   1339
           ----------                                                   1294
           ----------                                                   900

LOCUS: AT4G21060
DESCRIPTION: galactosyltransferase family protein, contains Pfam profile: PF01762 galactosyltransferase
DATA:                 Control 30min  2hours 2days  1week  p-value       pos
SENSE COUNTS:         0       0      1      0      5      7.15e-03
GENES (1 total):
  AT4G21060.1
```

```
    SENSE COUNTS:     0       0       1       0       0       5       7.15e-03
    TAGS: (2 total)
         ----------                                                              2873
         ----------                                                              2394
         ----------                                                              2255
         ----------                                                              1898
         ----------                                                              1881
         ----------                                                              1842
         ----------                                                              1732
         ----------                                                              1642
      v+2  GTTCAAACGG 0       0       1       0       0       4.55e-01       1508
         ----------                                                              1476
         ----------                                                              1352
         ----------                                                              1101
         ----------                                                              1046
         ----------                                                              891
         ----------                                                              750
      i+3  TTATACACTT 0       0       0       0       5       6.24e-04       406

LOCUS: AT3G12490
DESCRIPTION: cysteine protease inhibitor, putative / cystatin, putative, similar to PRLI-interacting factor M
(Arabidopsis thaliana) GI:11139270, cysteine proteinase inhibitor (Brassica rapa) GI:762785; contains Pfam
profile PF00031: Cystatin domain
DATA:                  Control 30min   2hours  2days   1week   p-value        pos
SENSE COUNTS:          3       7       2       9       17      7.44e-03
GENES (2 total):
  AT3G12490.1
    SENSE COUNTS:      3       7       2       9       17      7.44e-03
    TAGS: (2 total)
         ----------                                                    1223
         ----------                                                    788
      d+2  ACTAGTCCCT 2       7       2       8       16      1.07e-02       656
         ----------                                                    617
      d+2  CTACTGAAGT 1       0       0       1       1       5.50e-01       553
         ----------                                                    512
         ----------                                                    434
         ----------                                                    386
  AT3G12490.2
    SENSE COUNTS:      3       7       2       9       17      7.44e-03
    TAGS: (2 total)
         ----------                                                    1257
         ----------                                                    822
      d+2  ACTAGTCCCT 2       7       2       8       16      1.07e-02       690
         ----------                                                    651
      d+2  CTACTGAAGT 1       0       0       1       1       5.50e-01       587
         ----------                                                    546
         ----------                                                    468
         ----------                                                    420

LOCUS: AT3G18680
DESCRIPTION: aspartate/glutamate/uridylate kinase family protein, similar to UMP-kinase GB:CAB38122 gi:4468612
from (Lactococcus lactis) ; contains Pfam profile PF00696: Amino acid kinase family
DATA:                  Control 30min   2hours  2days   1week   p-value        pos
SENSE COUNTS:          4       2       3       15      7       7.50e-03
GENES (2 total):
  AT3G18680.1
    SENSE COUNTS:      4       2       3       15      7       7.50e-03
    TAGS: (1 total)
         ----------                                                    1802
      d+2  AAGCTCATTT 4       2       3       15      7       7.50e-03       1291
         ----------                                                    1146
         ----------                                                    721
         ----------                                                    316

LOCUS: AT3G61430
DESCRIPTION: plasma membrane intrinsic protein 1A (PIP1A) / aquaporin PIP1.1 (PIP1.1) (AQ1), identical to
plasma membrane intrinsic protein 1A SP:P43285 from (Arabidopsis thaliana)
DATA:                  Control 30min   2hours  2days   1week   p-value        pos
SENSE COUNTS:          24      9       13      5       16      7.69e-03
GENES (2 total):
  AT3G61430.1
    SENSE COUNTS:      24      9       13      5       16      7.69e-03
    TAGS: (5 total)
      d+1  TTTATTACAG 4       0       0       0       5       8.28e-03       1229
      d+2  TATGTGTGCA 20      9       11      5       10      6.19e-02       1126
      d+2  TGGTTGTCAT 0       0       1       0       0       4.55e-01       1004
      d+2  TCCCTATTCT 0       0       1       0       0       4.55e-01       803
         ----------                                                    695
         ----------                                                    427
         ----------                                                    327
      X+4  GATATTGGTA 0       0       0       0       1       1.65e-01       309
```

```
LOCUS: AT3G55440
DESCRIPTION: triosephosphate isomerase, cytosolic, putative, strong similarity to triosephosphate isomerase,
cytosolic from Petunia hybrida (SP|P48495), from Coptis japonica (SP|P21820)
DATA:                 Control 30min   2hours  2days   1week   p-value         pos
SENSE COUNTS:         3       21      8       11      10      7.76e-03
GENES (2 total):
  AT3G55440.1
    SENSE COUNTS:     3       21      8       11      10      7.76e-03
    TAGS: (4 total)
      d+1  CTGTCAACCA 0       0       0       0       1       1.65e-01        1245
           ----------                                                         1227
      i+3  TCTATGAAAT 1       0       0       0       0       4.28e-01        601
      d+2  GATGTTGTGG 2       21      8       11      8       2.32e-03        524
      d+2  GGTTATCCTT 0       0       0       0       1       1.65e-01        373

LOCUS: AT2G04842
DESCRIPTION: threonyl-tRNA synthetase, putative / threonine--tRNA ligase, putative, similar to SP|P18256
Threonyl-tRNA synthetase 2 (EC 6.1.1.3) (Threonine--tRNA ligase) (ThrRS) {Bacillus subtilis}; contains Pfam
profiles PF00587: tRNA synthetase class II core domain
DATA:                 Control 30min   2hours  2days   1week   p-value         pos
SENSE COUNTS:         13      0       4       7       8       7.85e-03
GENES (1 total):
  AT2G04842.1
    SENSE COUNTS:     13      0       4       7       8       7.85e-03
    TAGS: (3 total)
           ----------                                                         2652
      v+2  ACCATCAAAA 1       0       1       2       1       5.52e-01        2419
      v+2  TAAGTGTAGA 12      0       3       4       7       5.81e-03        2312
           ----------                                                         2110
           ----------                                                         1279
           ----------                                                         1252
           ----------                                                         1243
      v+2  TCGAGACCAC 0       0       0       1       0       3.09e-01        937
           ----------                                                         828
           ----------                                                         616
           ----------                                                         475

LOCUS: AT1G20110
DESCRIPTION: zinc finger (FYVE type) family protein, contains Pfam profile: PF01363 FYVE zinc finger
DATA:                 Control 30min   2hours  2days   1week   p-value         pos
SENSE COUNTS:         4       2       12      1       2       8.65e-03
GENES (1 total):
  AT1G20110.1
    SENSE COUNTS:     4       2       12      1       2       8.65e-03
    TAGS: (3 total)
      d+1  TAGTCTGTTG 2       1       4       0       1       4.21e-01        2060
      d+2  TGGATGGTGT 2       1       7       1       1       8.52e-02        1992
      d+2  CACTGTGCAC 0       0       1       0       0       4.55e-01        1782
           ----------                                                         1673
           ----------                                                         1603
           ----------                                                         1461
           ----------                                                         311

LOCUS: AT4G26370
DESCRIPTION: antitermination NusB domain-containing protein, contains Pfam profile: PF01029 NusB family
DATA:                 Control 30min   2hours  2days   1week   p-value         pos
SENSE COUNTS:         4       0       2       3       11      8.83e-03
GENES (2 total):
  AT4G26370.1
    SENSE COUNTS:     4       0       2       3       11      8.83e-03
    TAGS: (3 total)
      d+1  TTTCTGTCTT 0       0       2       2       10      1.42e-03        1261
      d+2  TCTGTATTAA 1       0       0       0       0       4.28e-01        1206
           ----------                                                         1177
           ----------                                                         709
           ----------                                                         583
      d+2  AGCTTTGGAG 3       0       0       1       1       1.73e-01        519
           ----------                                                         413
  AT4G26370.2
    SENSE COUNTS:     4       0       2       3       11      8.83e-03
    TAGS: (3 total)
      d+1  TTTCTGTCTT 0       0       2       2       10      1.42e-03        1444
      d+2  TCTGTATTAA 1       0       0       0       0       4.28e-01        1389
           ----------                                                         1360
           ----------                                                         791
           ----------                                                         655
           ----------                                                         632
           ----------                                                         528
      d+2  AGCTTTGGAG 3       0       0       1       1       1.73e-01        464
           ----------                                                         358
```

```
LOCUS: AT3G08520
DESCRIPTION: 60S ribosomal protein L41 (RPL41D)
DATA:                 Control 30min   2hours  2days   1week   p-value         pos
SENSE COUNTS:         4       3       1       13      7       8.95e-03
GENES (1 total):
  AT3G08520.1
    SENSE COUNTS:     4       3       1       13      7       8.95e-03
    TAGS: (1 total)
           ----------                                                         929
           ----------                                                         890
           ----------                                                         847
           ----------                                                         568
      v+2  AGAGCCAAGT 4       3       1       13      7       8.95e-03        354
           ----------                                                         79

LOCUS: AT5G52300
DESCRIPTION: low-temperature-responsive 65 kD protein (LTI65) / desiccation-responsive protein 29B (RD29B),
nearly identical to SP|Q04980 Low-temperature-induced 65 kDa protein (Desiccation-responsive protein 29B)
{Arabidopsis thaliana}
DATA:                 Control 30min   2hours  2days   1week   p-value         pos
SENSE COUNTS:         1       0       2       0       5       9.07e-03
GENES (2 total):
  AT5G52300.1
    SENSE COUNTS:     1       0       2       0       5       9.07e-03
    TAGS: (4 total)
      d+1  GTGAGGCGGT 1       0       1       0       1       6.15e-01        1785
      d+2  AGACAAAACC 0       0       1       0       0       4.55e-01        1275
           ----------                                                         1200
           ----------                                                         1047
      d+2  AGGCACTGGC 0       0       0       0       1       1.65e-01        579
           ----------                                                         450
           ----------                                                         324
           ----------                                                         312
      d+2  AGAAGGGAGC 0       0       0       0       3       1.12e-02        237
           ----------                                                         183

LOCUS: AT3G09250
DESCRIPTION: expressed protein
DATA:                 Control 30min   2hours  2days   1week   p-value         pos
SENSE COUNTS:         22      10      15      6       2       9.21e-03
GENES (1 total):
  AT3G09250.1
    SENSE COUNTS:     22      10      15      6       2       9.21e-03
    TAGS: (2 total)
      d+1  AATCTTGGTC 6       3       2       0       1       1.40e-01        1121
      d+2  TAGACAATGA 16      7       13      6       1       5.09e-02        1063
           ----------                                                         795
           ----------                                                         663
           ----------                                                         203
           ----------                                                         158
           ----------                                                         125
           ----------                                                         93
           ----------                                                         83
           ----------                                                         21

LOCUS: AT4G16260
DESCRIPTION: glycosyl hydrolase family 17 protein, similar to glucan endo-1,3-beta-glucosidase, basic vacuolar
isoform precursor SP:P52407 from (Hevea brasiliensis)
DATA:                 Control 30min   2hours  2days   1week   p-value         pos
SENSE COUNTS:         1       7       3       0       0       9.27e-03
GENES (1 total):
  AT4G16260.1
    SENSE COUNTS:     1       7       3       0       0       9.27e-03
    TAGS: (2 total)
           ----------                                                         1275
      d+2  CGTAACGTTT 0       7       1       0       0       4.60e-04        478
           ----------                                                         369
      i+3  ATTTATTCTC 1       0       2       0       0       3.07e-01        177

LOCUS: AT5G58470
DESCRIPTION: zinc finger (Ran-binding) family protein, weak similarity to SP|Q01844 RNA-binding protein EWS
(EWS oncogene) (Ewing sarcoma breakpoint region 1 protein) {Homo sapiens}; contains Pfam profiles PF00076: RNA
recognition motif. (a.k.a. RRM, RBD, or RNP domai
DATA:                 Control 30min   2hours  2days   1week   p-value         pos
SENSE COUNTS:         3       0       1       8       1       9.46e-03
GENES (2 total):
  AT5G58470.1
    SENSE COUNTS:     0       0       0       3       0       4.12e-02
    TAGS: (2 total)
           ----------                                                         2038
```

```
      d+2  ACCTTTTGGT 0       0       0       1       0       3.09e-01        1990
           ----------                                                         1721
           ----------                                                         1622
      d+2  GGGGAAACCG 0       0       0       2       0       2.03e-01        1310
           ----------                                                         70
  AT5G58470.2
    SENSE COUNTS:     3       0       1       8       1       9.46e-03
    TAGS: (3 total)
           ----------                                                         2190
      d+2  ACCTTTTGGT 0       0       0       1       0       3.09e-01        2142
           ----------                                                         1873
           ----------                                                         1774
      d+2  CATCTCCGGT 3       0       1       5       1       1.12e-01        1541
      d+2  GGGGAAACCG 0       0       0       2       0       2.03e-01        1310
           ----------                                                         70

LOCUS: AT4G20850
DESCRIPTION: subtilase family protein, contains similarity to Tripeptidyl-peptidase II (EC 3.4.14.10) (TPP-II)
(Tripeptidyl aminopeptidase) (Swiss-Prot:P29144) (Homo sapiens)
DATA:                 Control 30min   2hours  2days   1week   p-value         pos
SENSE COUNTS:         0       8       3       2       1       9.56e-03
GENES (1 total):
  AT4G20850.1
    SENSE COUNTS:     0       8       3       2       1       9.56e-03
    TAGS: (3 total)
      i+3  AAAGCTGAAT 0       0       0       0       1       1.65e-01        8015
      d+1  GCGAGAATCG 0       0       3       2       0       2.44e-01        3802
      d+2  AAAATGTGGA 0       8       0       0       0       2.30e-05        3041
           ----------                                                         2621
           ----------                                                         2370
           ----------                                                         2360
           ----------                                                         2021
           ----------                                                         1890
           ----------                                                         1663
           ----------                                                         1657
           ----------                                                         1638
           ----------                                                         1306
           ----------                                                         1127
           ----------                                                         1050
           ----------                                                         911
           ----------                                                         615
           ----------                                                         470
           ----------                                                         352

LOCUS: AT5G57480
DESCRIPTION: AAA-type ATPase family protein, contains Pfam profile: PF00004 ATPase family
DATA:                 Control 30min   2hours  2days   1week   p-value         pos
SENSE COUNTS:         0       4       0       0       0       9.72e-03
GENES (1 total):
  AT5G57480.1
    SENSE COUNTS:     0       4       0       0       0       9.72e-03
    TAGS: (1 total)
           ----------                                                         2304
           ----------                                                         2287
           ----------                                                         2019
           ----------                                                         1861
      v+2  AGTTTTTGTA 0       4       0       0       0       9.72e-03        1530
           ----------                                                         999
           ----------                                                         831

LOCUS: AT3G11780
DESCRIPTION: MD-2-related lipid recognition domain-containing protein / ML domain-containing protein, weak
similarity to phosphatidylglycerol/phosphatidylinositol transfer protein (Aspergillus oryzae) GI:10178615;
contains Pfam profile PF02221: ML domain
DATA:                 Control 30min   2hours  2days   1week   p-value         pos
SENSE COUNTS:         12      4       20      10      4       9.74e-03
GENES (1 total):
  AT3G11780.1
    SENSE COUNTS:     12      4       20      10      4       9.74e-03
    TAGS: (2 total)
      d+1  AATCCCCCCA 3       2       7       5       1       3.44e-01        717
      d+2  GAACCACTCT 9       2       13      5       3       3.79e-02        620
           ----------                                                         573
           ----------                                                         382
           ----------                                                         117

LOCUS: AT4G33700
DESCRIPTION: CBS domain-containing protein, contains Pfam profiles PF00571: CBS domain, PF01595: Domain of
unknown function
DATA:                 Control 30min   2hours  2days   1week   p-value         pos
SENSE COUNTS:         0       0       2       2       8       9.88e-03
```

```
GENES (1 total):
  AT4G33700.1
    SENSE COUNTS:      0       0       2       2       8       9.88e-03
    TAGS: (2 total)
      d+1  AACAACAACT  0       0       1       1       5       1.07e-01        1675
           ----------                                                          1580
      i+3  CAAATCAGGA  0       0       1       1       3       1.91e-01        1313
           ----------                                                          1198
           ----------                                                          860
           ----------                                                          824
           ----------                                                          732
           ----------                                                          585
           ----------                                                          379

LOCUS: AT3G60340
DESCRIPTION: palmitoyl protein thioesterase family protein, palmitoyl-protein thioesterase precursor, Mus
musculus, EMBL:AF071025
DATA:                  Control 30min   2hours  2days   1week   p-value         pos
SENSE COUNTS:          8       0       2       0       1       1.00e-02
GENES (2 total):
  AT3G60340.1
    SENSE COUNTS:      8       0       2       0       1       1.00e-02
    TAGS: (1 total)
           ----------                                                          1443
      d+2  TGTAAATCTG  8       0       2       0       1       1.00e-02        1104
           ----------                                                          935
           ----------                                                          772
           ----------                                                          741
           ----------                                                          629
           ----------                                                          527
           ----------                                                          477
           ----------                                                          401
           ----------                                                          295
           ----------                                                          286
           ----------                                                          180
  AT3G60340.2
    SENSE COUNTS:      8       0       2       0       1       1.00e-02
    TAGS: (1 total)
           ----------                                                          1574
      d+2  TGTAAATCTG  8       0       2       0       1       1.00e-02        1235
           ----------                                                          1066
           ----------                                                          903
           ----------                                                          872
           ----------                                                          760
           ----------                                                          658
           ----------                                                          608
           ----------                                                          532
           ----------                                                          426
           ----------                                                          417
           ----------                                                          311

LOCUS: AT1G25260
DESCRIPTION: acidic ribosomal protein P0-related, contains similarity to 60S acidic ribosomal protein
GI:5815233 from (Homo sapiens)
DATA:                  Control 30min   2hours  2days   1week   p-value         pos
SENSE COUNTS:          2       0       1       7       8       1.01e-02
GENES (1 total):
  AT1G25260.1
    SENSE COUNTS:      2       0       1       7       8       1.01e-02
    TAGS: (2 total)
      d+1  TTTTGTAGAA  1       0       0       1       7       3.33e-03        928
      d+2  GTTTGCATTC  1       0       1       6       1       5.21e-02        882
           ----------                                                          861
           ----------                                                          540
           ----------                                                          239

LOCUS: AT4G18200
DESCRIPTION: purine permease family protein, similar to purine permease (Arabidopsis thaliana) GI:7620007;
contains Pfam profile PF03151: Domain of unknown function, DUF250
DATA:                  Control 30min   2hours  2days   1week   p-value         pos
SENSE COUNTS:          0       2       10      2       1       1.06e-02
GENES (3 total):
  AT4G18200.1
    SENSE COUNTS:      0       2       10      2       1       1.06e-02
    TAGS: (3 total)
           ----------                                                          4108
      v+2  TCAATTTTGT  0       2       10      0       1       7.48e-04        3958
           ----------                                                          3842
      v+2  TAAATAACCT  0       0       0       1       0       3.09e-01        3793
           ----------                                                          3595
           ----------                                                          2671
```

```
      v+2  ATAGAATGGA 0      0      0      1      0      6.04e-01      2470
           ----------                                                1591
           ----------                                                1387
           ----------                                                611
           ----------                                                510
           ----------                                                354

LOCUS: AT5G02710
DESCRIPTION: expressed protein, contains Pfam PF03692: Uncharacterised protein family (UPF0153)
DATA:                  Control 30min  2hours 2days  1week  p-value       pos
SENSE COUNTS:          2      0      2      1      7      1.06e-02
GENES (1 total):
  AT5G02710.1
    SENSE COUNTS:      2      0      2      1      7      1.06e-02
    TAGS: (3 total)
           ----------                                                909
      d+2  TTATTAATAG 1      0      0      0      3      7.14e-02      850
      X+4  ATTCTTGACC 1      0      1      1      1      7.91e-01      333
      d+2  ATCGGAGATG 0      0      1      0      3      7.32e-02      303

LOCUS: AT1G67230
DESCRIPTION: expressed protein
DATA:                  Control 30min  2hours 2days  1week  p-value       pos
SENSE COUNTS:          0      6      0      2      1      1.06e-02
GENES (1 total):
  AT1G67230.1
    SENSE COUNTS:      0      6      0      2      1      1.06e-02
    TAGS: (2 total)
      d+1  AAGCTACGGC 0      0      0      2      1      3.32e-01      3456
           ----------                                                2933
           ----------                                                2620
           ----------                                                2510
           ----------                                                2423
           ----------                                                2243
           ----------                                                2076
           ----------                                                1961
           ----------                                                1412
      d+2  GTGACCCCAG 0      6      0      0      0      9.09e-04      347
           ----------                                                330

LOCUS: AT5G19850
DESCRIPTION: hydrolase, alpha/beta fold family protein, low similarity to hydrolase (Terrabacter sp. DBF63)
GI:14196240; contains Pfam profile PF00561: hydrolase, alpha/beta fold family
DATA:                  Control 30min  2hours 2days  1week  p-value       pos
SENSE COUNTS:          0      0      4      6      7      1.08e-02
GENES (1 total):
  AT5G19850.1
    SENSE COUNTS:      0      0      4      6      7      1.08e-02
    TAGS: (2 total)
           ----------                                                1487
           ----------                                                1346
           ----------                                                1326
      d+2  AACAGTCACA 0      0      3      6      7      8.82e-03      1192
      d+2  GGAGCCCATC 0      0      1      0      0      4.55e-01      947
           ----------                                                929
           ----------                                                772
           ----------                                                470
           ----------                                                316
           ----------                                                245
           ----------                                                188
           ----------                                                15

LOCUS: AT5G12140
DESCRIPTION: cysteine protease inhibitor, putative / cystatin, putative, similar to SP|P31726 Cystatin I
precursor (CORN kernel cysteine proteinase inhibitor) {Zea mays}; contains Pfam profile PF00031: Cystatin
domain
DATA:                  Control 30min  2hours 2days  1week  p-value       pos
SENSE COUNTS:          15     14     18     29     36     1.11e-02
GENES (2 total):
  AT5G12140.1
    SENSE COUNTS:      15     14     18     29     36     1.11e-02
    TAGS: (3 total)
      d+1  AGTAAAAGCT 2      2      2      2      7      4.81e-01      634
      d+2  ATGAAGATAC 2      0      0      0      1      2.03e-01      616
      d+2  TGTTTGAACT 11     12     16     27     28     2.14e-02      513

LOCUS: AT3G26080
DESCRIPTION: plastid-lipid associated protein PAP / fibrillin family protein, low similarity to Plastid-lipid-
Associated Protein (Nicotiana tabacum) GI:2632088; contains Pfam profile PF04755: PAP_fibrillin
DATA:                  Control 30min  2hours 2days  1week  p-value       pos
SENSE COUNTS:          0      0      1      2      8      1.12e-02
```

```
GENES (1 total):
  AT3G26080.1
    SENSE COUNTS:      0      0      1      2      8      1.12e-02
    TAGS: (3 total)
      i+3  TTCCATTGTG  0      0      1      0      0      7.06e-01       1009
      d+1  TATGTAAAAA  0      0      0      2      8      1.20e-04       907
      d+2  AACTTGTCGA  0      0      0      0      0      6.15e-01       332
           ----------                                                  133

LOCUS: AT2G30460
DESCRIPTION: expressed protein, contains 4 predicted transmembrane domains; similar to c_pp004044298r
(GI:14597790) (Physcomitrella patens)
DATA:                  Control 30min  2hours  2days  1week  p-value        pos
SENSE COUNTS:          0      0      0      0      3      1.12e-02
GENES (1 total):
  AT2G30460.1
    SENSE COUNTS:      0      0      0      0      3      1.12e-02
    TAGS: (1 total)
           ----------                                                  1334
      v+2  GTTTATTCTA  0      0      0      0      3      1.12e-02       1223
           ----------                                                  674
           ----------                                                  523
           ----------                                                  270

LOCUS: AT4G10040
DESCRIPTION: cytochrome c, putative, similar to cytochrome c (Pumpkin, Winter squash) SWISS-PROT:P00051
DATA:                  Control 30min  2hours  2days  1week  p-value        pos
SENSE COUNTS:          0      0      0      0      3      1.12e-02
GENES (1 total):
  AT4G10040.1
    SENSE COUNTS:      0      0      0      0      3      1.12e-02
    TAGS: (1 total)
           ----------                                                  638
      d+2  AACTGCTTTT  0      0      0      0      3      1.12e-02       506
           ----------                                                  278

LOCUS: AT3G60870
DESCRIPTION: DNA-binding protein-related, contains Pfam domain PF03479: Domain of unknown function (DUF296),
found in AT-hook motifs Pfam:PF02178
DATA:                  Control 30min  2hours  2days  1week  p-value        pos
SENSE COUNTS:          0      0      0      0      3      1.12e-02
GENES (1 total):
  AT3G60870.1
    SENSE COUNTS:      0      0      0      0      3      1.12e-02
    TAGS: (1 total)
           ----------                                                  1473
      v+2  TTTTTTGATA  0      0      0      0      3      1.12e-02       1413
           ----------                                                  624
           ----------                                                  180
           ----------                                                  63

LOCUS: AT1G69090
DESCRIPTION: F-box family protein, contains F-box domain Pfam:PF00646
DATA:                  Control 30min  2hours  2days  1week  p-value        pos
SENSE COUNTS:          0      0      0      0      3      1.12e-02
GENES (1 total):
  AT1G69090.1
    SENSE COUNTS:      0      0      0      0      3      1.12e-02
    TAGS: (1 total)
           ----------                                                  1992
           ----------                                                  1855
           ----------                                                  1522
           ----------                                                  1374
           ----------                                                  1256
           ----------                                                  1191
      v+2  ATATACAAAG  0      0      0      0      3      1.12e-02       996
           ----------                                                  984
           ----------                                                  974
           ----------                                                  462
           ----------                                                  156
           ----------                                                  130
           ----------                                                  5

LOCUS: AT3G57980
DESCRIPTION: DNA-binding bromodomain-containing protein, contains bromodomain, INTERPRO:IPR001487
DATA:                  Control 30min  2hours  2days  1week  p-value        pos
SENSE COUNTS:          0      0      0      0      3      1.12e-02
GENES (1 total):
  AT3G57980.1
    SENSE COUNTS:      0      0      0      0      3      1.12e-02
    TAGS: (1 total)
```

```
           ----------                                                   2652
           ----------                                                   2456
           ----------                                                   404
      v+2  TTATCAACGA  0      0      0      0      3      1.12e-02      98

LOCUS: AT2G21550
DESCRIPTION: bifunctional dihydrofolate reductase-thymidylate synthase, putative / DHFR-TS, putative, similar
to THY-1 (SP| Q05762) and THY-2 (SP|Q05763) from Arabidopsis thaliana; contains Pfam profiles PF00303
thymidylate synthase and PF00186 dihydrofolate reductase
DATA:                  Control 30min  2hours 2days  1week  p-value       pos
SENSE COUNTS:          0      0      0      0      3      1.12e-02
GENES (1 total):
  AT2G21550.1
    SENSE COUNTS:      0      0      0      0      3      1.12e-02
    TAGS: (1 total)
           ----------                                                   1821
           ----------                                                   1675
           ----------                                                   1642
           ----------                                                   1628
           ----------                                                   1461
           ----------                                                   1447
           ----------                                                   1354
           ----------                                                   1104
      v+2  AGGAGTTTGG  0      0      0      0      3      1.12e-02      1051
           ----------                                                   845
           ----------                                                   693
           ----------                                                   578
           ----------                                                   471
           ----------                                                   453

LOCUS: AT3G13050
DESCRIPTION: transporter-related, low similarity to apical organic cation transporter (Sus scrofa) GI:2062135,
SP|Q02563 Synaptic vesicle protein 2 (SV2) {Rattus norvegicus}; contains Pfam profile PF00083: major
facilitator superfamily protein
DATA:                  Control 30min  2hours 2days  1week  p-value       pos
SENSE COUNTS:          0      0      0      0      3      1.12e-02
GENES (1 total):
  AT3G13050.1
    SENSE COUNTS:      0      0      0      0      3      1.12e-02
    TAGS: (1 total)
           ----------                                                   1231
      i+3  CCTTTCTTTG  0      0      0      0      3      1.12e-02      1185
           ----------                                                   617
           ----------                                                   443
           ----------                                                   363
           ----------                                                   320
           ----------                                                   218

LOCUS: AT3G10790
DESCRIPTION: F-box family protein, contains F-box domain Pfam:PF00646
DATA:                  Control 30min  2hours 2days  1week  p-value       pos
SENSE COUNTS:          0      0      0      0      3      1.12e-02
GENES (1 total):
  AT3G10790.1
    SENSE COUNTS:      0      0      0      0      3      1.12e-02
    TAGS: (1 total)
           ----------                                                   1554
           ----------                                                   1487
           ----------                                                   903
      v+2  TTAAGGATGA  0      0      0      0      3      1.12e-02      874
           ----------                                                   552
           ----------                                                   465
           ----------                                                   354

LOCUS: AT4G02465
DESCRIPTION: hypothetical protein
DATA:                  Control 30min  2hours 2days  1week  p-value       pos
SENSE COUNTS:          0      0      0      0      3      1.12e-02
GENES (1 total):
  AT4G02465.1
    SENSE COUNTS:      0      0      0      0      3      1.12e-02
    TAGS: (1 total)
           ----------                                                   1260
           ----------                                                   1185
      v+2  TATTAGATTA  0      0      0      0      3      1.12e-02      980

LOCUS: AT3G03730
DESCRIPTION: F-box family protein, contains F-box domain Pfam:PF00646
DATA:                  Control 30min  2hours 2days  1week  p-value       pos
SENSE COUNTS:          0      0      0      0      3      1.12e-02
GENES (1 total):
```

```
  AT3G03730.1
    SENSE COUNTS:      0       0       0       0       3       1.12e-02
    TAGS: (1 total)
           ----------                                                  1758
      v+2  AGTTACTTAG  0       0       0       0       3       1.12e-02        1363
           ----------                                                  1026
           ----------                                                  838
           ----------                                                  559
           ----------                                                  533
           ----------                                                  503
           ----------                                                  377

LOCUS: AT3G51360
DESCRIPTION: aspartyl protease family protein, contains Eukaryotic and viral aspartyl proteases active site,
PROSITE:PS00141
DATA:                  Control 30min   2hours  2days   1week   p-value         pos
SENSE COUNTS:          0       0       0       0       3       1.12e-02
GENES (1 total):
  AT3G51360.1
    SENSE COUNTS:      0       0       0       0       3       1.12e-02
    TAGS: (1 total)
           ----------                                                  2299
           ----------                                                  2007
           ----------                                                  1940
           ----------                                                  1593
           ----------                                                  1301
      i+3  ATCTAAACAC  0       0       0       0       3       1.12e-02        1201
           ----------                                                  1071
           ----------                                                  936
           ----------                                                  475

LOCUS: AT2G14880
DESCRIPTION: SWIB complex BAF60b domain-containing protein, contains Pfam profile PF02201: BAF60b domain of
the SWIB complex
DATA:                  Control 30min   2hours  2days   1week   p-value         pos
SENSE COUNTS:          3       0       3       9       8       1.16e-02
GENES (1 total):
  AT2G14880.1
    SENSE COUNTS:      3       0       3       9       8       1.16e-02
    TAGS: (3 total)
      d+1  TACATACTCC  0       0       0       1       0       3.09e-01        830
      d+2  TTAGTTACGT  0       0       0       4       5       7.47e-03        808
      d+2  TTTTTCCGTT  3       0       3       4       3       3.75e-01        772
           ----------                                                  592
           ----------                                                  399
           ----------                                                  296

LOCUS: AT1G70820
DESCRIPTION: phosphoglucomutase, putative / glucose phosphomutase, putative, similar to phosphoglucomutase
GI:534981 from (Spinacia oleracea), phosphomannomutase (Pseudomonas aeruginosa) GI:150994; contains InterPro
accession IPR006352: Phosphoglucosamine mutase
DATA:                  Control 30min   2hours  2days   1week   p-value         pos
SENSE COUNTS:          16      9       4       8       0       1.19e-02
GENES (1 total):
  AT1G70820.1
    SENSE COUNTS:      16      9       4       8       0       1.19e-02
    TAGS: (1 total)
           ----------                                                  2084
      d+2  AATTCTTATG  16      9       4       8       0       1.19e-02        1969
           ----------                                                  1829
           ----------                                                  1700
           ----------                                                  1403
           ----------                                                  1359
           ----------                                                  1340
           ----------                                                  569
           ----------                                                  539
           ----------                                                  278

LOCUS: AT3G48690
DESCRIPTION: expressed protein, similar to PrMC3 (Pinus radiata) GI:5487873
DATA:                  Control 30min   2hours  2days   1week   p-value         pos
SENSE COUNTS:          5       5       16      9       1       1.23e-02
GENES (1 total):
  AT3G48690.1
    SENSE COUNTS:      5       5       16      9       1       1.23e-02
    TAGS: (1 total)
           ----------                                                  1776
      d+2  CATAAATTCT  5       5       16      9       1       1.23e-02        1014
           ----------                                                  967
           ----------                                                  588
           ----------                                                  526
```

```
          ----------                                                    150
          ----------                                                    78

LOCUS: AT5G13640
DESCRIPTION: lecithin:cholesterol acyltransferase family protein / LACT family protein, similar to SP|P40345
Phospholipid:diacylglycerol acyltransferase (EC 2.3.1.158) (PDAT) {Saccharomyces cerevisiae}; contains Pfam
profile PF02450: Lecithin:cholesterol acyltransfera
DATA:                 Control 30min   2hours  2days   1week   p-value         pos
SENSE COUNTS:         5       0       0       2       8       1.23e-02
GENES (2 total):
  AT5G13640.1
    SENSE COUNTS:     5       0       0       2       8       1.23e-02
    TAGS: (3 total)
      i+3  TGACTCTTGC 1       0       0       0       0       4.28e-01        3622
      d+1  TTTGTTTGTC 3       0       0       2       7       5.99e-02        2716
      d+2  ATGCTCAACT 1       0       0       0       1       3.83e-01        2580
           ----------                                                         2492
           ----------                                                         2428
           ----------                                                         2388
           ----------                                                         2358
           ----------                                                         2347
           ----------                                                         2220
           ----------                                                         1809
           ----------                                                         1804
           ----------                                                         1502
           ----------                                                         1480
           ----------                                                         1233
           ----------                                                         1098
           ----------                                                         939
           ----------                                                         200
           ----------                                                         30

LOCUS: AT4G02120
DESCRIPTION: CTP synthase, putative / UTP--ammonia ligase, putative, similar to SP|P17812 CTP synthase (EC
6.3.4.2) (UTP--ammonia ligase) {Homo sapiens}; contains Pfam profile PF00117: glutamine amidotransferase
class-I
DATA:                 Control 30min   2hours  2days   1week   p-value         pos
SENSE COUNTS:         11      2       6       2       0       1.28e-02
GENES (1 total):
  AT4G02120.1
    SENSE COUNTS:     11      2       6       2       0       1.28e-02
    TAGS: (2 total)
      d+1  CTGCTGCTTG 0       0       0       1       0       3.09e-01        1093
      d+2  TTCATTGAAG 11      2       6       1       0       5.81e-03        1013
           ----------                                                         1000
           ----------                                                         925
           ----------                                                         790
           ----------                                                         769
           ----------                                                         739
           ----------                                                         680
           ----------                                                         199

LOCUS: AT1G68680
DESCRIPTION: expressed protein
DATA:                 Control 30min   2hours  2days   1week   p-value         pos
SENSE COUNTS:         0       0       3       0       7       1.28e-02
GENES (1 total):
  AT1G68680.1
    SENSE COUNTS:     0       0       3       0       7       1.28e-02
    TAGS: (1 total)
           ----------                                                         612
           ----------                                                         604
           ----------                                                         544
      d+2  AACTATGGAT 0       0       3       0       7       1.28e-02        453
           ----------                                                         306
           ----------                                                         125

LOCUS: AT5G61020
DESCRIPTION: YT521-B-like family protein, contains Pfam profile PF04146: YT521-B-like family
DATA:                 Control 30min   2hours  2days   1week   p-value         pos
SENSE COUNTS:         1       0       3       3       9       1.33e-02
GENES (2 total):
  AT5G61020.1
    SENSE COUNTS:     1       0       3       3       9       1.33e-02
    TAGS: (2 total)
      d+1  AGCATTCATC 1       0       0       1       8       2.74e-04        1727
      d+2  CATCCTCGAT 0       0       3       2       1       4.21e-01        1297
           ----------                                                         654
           ----------                                                         602
           ----------                                                         592
           ----------                                                         512
```

```
          ----------                                                        408
          ----------                                                        99
          ----------                                                        83
  AT5G61020.2
    SENSE COUNTS:      1       0       3       3       9       1.33e-02
    TAGS: (2 total)
      d+1  AGCATTCATC  1       0       0       1       8       2.74e-04        1741
      d+2  CATCCTCGAT  0       0       3       2       1       4.21e-01        1311
          ----------                                                        668
          ----------                                                        616
          ----------                                                        606
          ----------                                                        526
          ----------                                                        422
          ----------                                                        119
          ----------                                                        103

LOCUS: AT1G68440
DESCRIPTION: expressed protein
DATA:                  Control 30min   2hours  2days   1week   p-value         pos
SENSE COUNTS:          0       7       1       2       1       1.35e-02
GENES (1 total):
  AT1G68440.1
    SENSE COUNTS:      0       7       1       2       1       1.35e-02
    TAGS: (3 total)
      X+4  TGAAGTGAAG  0       2       0       0       0       9.14e-02        1262
      d+1  AGAATGTTGT  0       5       1       2       0       6.72e-02        663
      d+2  GCCTGATCTT  0       0       0       0       1       1.65e-01        586
          ----------                                                        541

LOCUS: AT3G23920
DESCRIPTION: beta-amylase, putative / 1,4-alpha-D-glucan maltohydrolase, putative, similar to beta-amylase
enzyme (Arabidopsis thaliana) GI:6065749, beta-amylase PCT-BMYI from (Solanum tuberosum); contains Pfam
profile PF01373: Glycosyl hydrolase family 14
DATA:                  Control 30min   2hours  2days   1week   p-value         pos
SENSE COUNTS:          6       1       3       6       14      1.38e-02
GENES (1 total):
  AT3G23920.1
    SENSE COUNTS:      6       1       3       6       14      1.38e-02
    TAGS: (3 total)
      d+1  TTCTTTAATG  5       0       1       1       8       1.87e-02        2091
      d+2  CTACTACTTT  0       0       0       0       3       1.12e-02        2056
      d+2  GTTCTATGGT  1       1       2       5       3       4.60e-01        1901
          ----------                                                        1473
          ----------                                                        1309
          ----------                                                        1239
          ----------                                                        1029
          ----------                                                        910
          ----------                                                        833
          ----------                                                        511

LOCUS: AT5G39550
DESCRIPTION: zinc finger (C3HC4-type RING finger) family protein, contains zinc finger, C3HC4 type (RING
finger), signature, PROSITE:PS00518
DATA:                  Control 30min   2hours  2days   1week   p-value         pos
SENSE COUNTS:          4       1       11      5       0       1.40e-02
GENES (1 total):
  AT5G39550.1
    SENSE COUNTS:      4       1       11      5       0       1.40e-02
    TAGS: (1 total)
          ----------                                                        1989
          ----------                                                        1904
          ----------                                                        1694
          ----------                                                        1612
          ----------                                                        1595
          ----------                                                        1383
          ----------                                                        1285
      v+2  TGGCAAAAAA  4       1       11      5       0       1.40e-02        877
          ----------                                                        461

LOCUS: AT2G37620
DESCRIPTION: Member of the actin gene family. Expressed in mature pollen.
DATA:                  Control 30min   2hours  2days   1week   p-value         pos
SENSE COUNTS:          2       0       5       0       0       1.47e-02
GENES (2 total):
  AT2G37620.1
    SENSE COUNTS:      2       0       5       0       0       1.47e-02
    TAGS: (2 total)
          ----------                                                        1443
      d+2  CCAAATACAT  0       0       1       0       0       4.55e-01        1426
          ----------                                                        1146
          ----------                                                        997
```

```
          ----------                                                      691
          ----------                                                      540
     X+4  GATAAACTGA 2       0       4       0       0       4.70e-02       436
          ----------                                                      417
          ----------                                                      391

LOCUS: AT5G23630
DESCRIPTION: ATPase E1-E2 type family protein / haloacid dehalogenase-like hydrolase familiy protein, similar
to SP|O14072 Cation-transporting ATPase 4 (EC 3.6.3.-) {Schizosaccharomyces pombe}; contains InterPro
accession IPR001757: ATPase, E1-E2 type; contains Pfam p
DATA:                  Control 30min   2hours  2days   1week   p-value        pos
SENSE COUNTS:          1       0       2       9       1       1.49e-02
GENES (1 total):
  AT5G23630.1
    SENSE COUNTS:      1       0       2       9       1       1.49e-02
    TAGS: (4 total)
      i+3  CTACTGTTTT 0       0       0       0       0       6.15e-01       5217
      d+1  GGAGCGGTTA 0       0       0       1       0       3.09e-01       3441
           ----------                                                      3241
           ----------                                                      3205
           ----------                                                      3196
           ----------                                                      3130
           ----------                                                      2990
           ----------                                                      2783
      d+2  TTGGAGTTGC 1       0       0       0       0       4.28e-01       2474
           ----------                                                      2273
           ----------                                                      2126
           ----------                                                      2081
           ----------                                                      1969
           ----------                                                      1604
           ----------                                                      1504
      X+4  AGCATTTTTC 0       0       2       8       1       7.84e-03       1194
           ----------                                                      1028
           ----------                                                      694
           ----------                                                      685
           ----------                                                      598
           ----------                                                      497
           ----------                                                      373
           ----------                                                      279

LOCUS: AT3G25920
DESCRIPTION: 50S ribosomal protein L15, chloroplast (CL15), identical to GB:P25873 from (Arabidopsis thaliana)
DATA:                  Control 30min   2hours  2days   1week   p-value        pos
SENSE COUNTS:          2       17      12      8       7       1.52e-02
GENES (2 total):
  AT3G25920.1
    SENSE COUNTS:      2       17      12      8       7       1.52e-02
    TAGS: (1 total)
           ----------                                                      1743
      d+2  AAGCTCACCT 2       17      12      8       7       1.52e-02       688
           ----------                                                      64

LOCUS: AT1G73655
DESCRIPTION: immunophilin / FKBP-type peptidyl-prolyl cis-trans isomerase family protein, similar to
(Peptidyl-prolyl cis-trans isomerase) (PPiase) (Rotamase) (SP:Q26486) (Spodoptera frugiperda); contains Pfam
PF00254: peptidyl-prolyl cis-trans isomerase, FKBP-type
DATA:                  Control 30min   2hours  2days   1week   p-value        pos
SENSE COUNTS:          1       7       2       0       1       1.57e-02
GENES (1 total):
  AT1G73655.1
    SENSE COUNTS:      1       7       2       0       1       1.57e-02
    TAGS: (3 total)
      d+1  GTGTTTAGCT 0       0       0       0       0       6.15e-01       829
      d+2  TTCTCAGGTC 1       7       2       0       0       1.41e-02       569
      X+4  GGAGTATTAG 0       0       0       0       1       1.65e-01       481
           ----------                                                      461

LOCUS: AT1G10590
DESCRIPTION: DNA-binding protein-related, contains weak similarity to G-quartet DNA binding protein 3
(Tetrahymena thermophila) gi|4583503|gb|AAD25098
DATA:                  Control 30min   2hours  2days   1week   p-value        pos
SENSE COUNTS:          0       0       2       0       5       1.57e-02
GENES (3 total):
  AT1G10590.1
    SENSE COUNTS:      0       0       2       0       5       1.57e-02
    TAGS: (1 total)
           ----------                                                      705
      d+2  TACAAGGGTT 0       0       2       0       5       1.57e-02       383
           ----------                                                      239
  AT1G10590.2
    SENSE COUNTS:      0       0       2       0       5       1.57e-02
```

```
    TAGS: (1 total)
           ----------                                                 654
      d+2  TACAAGGGTT 0      0      2      0      5      1.57e-02       332
           ----------                                                 188
  AT1G10590.3
    SENSE COUNTS:     0      0      2      0      5      1.57e-02
    TAGS: (1 total)
           ----------                                                 702
      d+2  TACAAGGGTT 0      0      2      0      5      1.57e-02       380
           ----------                                                 236

LOCUS: AT1G53550
DESCRIPTION: F-box family protein, similar to F-box family protein TIGR_Ath1:At3g23960
DATA:                 Control 30min  2hours 2days  1week  p-value        pos
SENSE COUNTS:         18     6      18     24     23     1.58e-02
GENES (1 total):
  AT1G53550.1
    SENSE COUNTS:     18     6      18     24     23     1.58e-02
    TAGS: (2 total)
           ----------                                                 2077
      v+2  ACACCAAAAA 0      0      0      1      0      3.09e-01       1876
           ----------                                                 1320
           ----------                                                 1179
           ----------                                                 1013
           ----------                                                 941
           ----------                                                 748
           ----------                                                 354
           ----------                                                 92
      v+2  AAGAACCATA 18     6      18     23     23     2.13e-02       29

LOCUS: AT1G31130
DESCRIPTION: expressed protein
DATA:                 Control 30min  2hours 2days  1week  p-value        pos
SENSE COUNTS:         3      3      8      12     0      1.58e-02
GENES (1 total):
  AT1G31130.1
    SENSE COUNTS:     3      3      8      12     0      1.58e-02
    TAGS: (2 total)
           ----------                                                 1475
      d+2  ATTTGAGTTT 3      3      7      12     0      1.92e-02       1422
           ----------                                                 1001
           ----------                                                 812
      d+2  ATTGGACTGT 0      0      1      0      0      4.55e-01       488
           ----------                                                 271

LOCUS: AT5G47020
DESCRIPTION: glycine-rich protein, strong similarity to unknown protein (emb|CAB87688.1)
DATA:                 Control 30min  2hours 2days  1week  p-value        pos
SENSE COUNTS:         12     6      7      10     23     1.59e-02
GENES (1 total):
  AT5G47020.1
    SENSE COUNTS:     12     6      7      10     23     1.59e-02
    TAGS: (3 total)
      d+1  TAAATCTTTT 1      2      0      2      1      5.93e-01       4309
      d+2  ATTATAAAGA 11     4      7      8      21     1.51e-02       4266
           ----------                                                 3638
           ----------                                                 3585
           ----------                                                 3428
           ----------                                                 3404
           ----------                                                 3063
           ----------                                                 2791
           ----------                                                 2771
           ----------                                                 2209
           ----------                                                 1796
           ----------                                                 1586
           ----------                                                 1548
           ----------                                                 1246
      d+2  ACTACAGAGA 0      0      0      0      1      1.65e-01       970
           ----------                                                 875
           ----------                                                 680
           ----------                                                 575
           ----------                                                 494
           ----------                                                 269
           ----------                                                 48

LOCUS: AT2G44350
DESCRIPTION: citrate synthase, mitochondrial, putative, strong similarity to SP|P20115 Citrate synthase,
mitochondrial precursor  {Arabidopsis thaliana}; contains Pfam profile PF00285: Citrate synthase
DATA:                 Control 30min  2hours 2days  1week  p-value        pos
SENSE COUNTS:         12     1      11     10     18     1.63e-02
GENES (3 total):
```

```
  AT2G44350.2
    SENSE COUNTS:      12      1       11      10      18      1.63e-02
    TAGS: (3 total)
      d+1  AGTTTATCAA  1       0       1       0       3       2.09e-01        1865
      d+2  AATTAAAGCT  6       1       3       4       5       4.60e-01        1796
      d+2  CTTGGTGCAA  5       0       7       6       10      9.21e-02        1703
           ----------                                                          1677
           ----------                                                          1516
           ----------                                                          1058
           ----------                                                          950
           ----------                                                          919
           ----------                                                          883
           ----------                                                          323
  AT2G44350.1
    SENSE COUNTS:      12      1       11      10      18      1.63e-02
    TAGS: (3 total)
      d+1  AGTTTATCAA  1       0       1       0       3       2.09e-01        1862
      d+2  AATTAAAGCT  6       1       3       4       5       4.60e-01        1793
      d+2  CTTGGTGCAA  5       0       7       6       10      9.21e-02        1700
           ----------                                                          1674
           ----------                                                          1513
           ----------                                                          1055
           ----------                                                          947
           ----------                                                          916
           ----------                                                          880
           ----------                                                          320

LOCUS: AT5G35630
DESCRIPTION: glutamine synthetase (GS2), identical to glutamine synthetase, chloroplast precursor (glutamate--
ammonia ligase, GS2) (Arabidopsis thaliana) SWISS-PROT:Q43127
DATA:                  Control 30min   2hours  2days   1week   p-value         pos
SENSE COUNTS:          13      1       11      4       5       1.63e-02
GENES (2 total):
  AT5G35630.1
    SENSE COUNTS:      13      1       11      4       5       1.63e-02
    TAGS: (3 total)
      d+1  TCCAATGGTT  13      1       7       4       5       1.57e-02        1607
           ----------                                                          1451
      d+2  GACCCATACA  0       0       4       0       0       5.58e-03        1326
           ----------                                                          1229
           ----------                                                          1083
           ----------                                                          952
           ----------                                                          686
      d+2  TCAGATGAGA  0       0       0       0       0       6.15e-01        170

LOCUS: AT5G08790
DESCRIPTION: no apical meristem (NAM) family protein, contains Pfam PF02365: No apical meristem (NAM) domain;
DATA:                  Control 30min   2hours  2days   1week   p-value         pos
SENSE COUNTS:          3       13      17      6       5       1.66e-02
GENES (2 total):
  AT5G08790.1
    SENSE COUNTS:      3       13      17      6       5       1.66e-02
    TAGS: (2 total)
      d+1  CTCTCCGGAA  0       0       1       0       0       4.55e-01        1241
      d+2  AGAAGTTTAT  3       13      16      6       5       2.76e-02        1136
           ----------                                                          846
           ----------                                                          606
           ----------                                                          499

LOCUS: AT4G22030
DESCRIPTION: F-box family protein, PF0064: F-box domain; similar to F-box protein family, AtFBX7 (GI:20197899)
(Arabidopsis thaliana)
DATA:                  Control 30min   2hours  2days   1week   p-value         pos
SENSE COUNTS:          0       0       6       2       3       1.68e-02
GENES (1 total):
  AT4G22030.1
    SENSE COUNTS:      0       0       6       2       3       1.68e-02
    TAGS: (1 total)
           ----------                                                          2495
           ----------                                                          2231
           ----------                                                          2192
           ----------                                                          1725
      i+3  TAAGTTATGT  0       0       6       2       3       1.68e-02        1480
           ----------                                                          1041
           ----------                                                          618

LOCUS: AT5G17560
DESCRIPTION: BolA-like family protein, contains Pfam profile: PF01722 BolA-like protein
DATA:                  Control 30min   2hours  2days   1week   p-value         pos
SENSE COUNTS:          5       7       1       2       14      1.70e-02
GENES (1 total):
```

```
  AT5G17560.1
    SENSE COUNTS:      5       7       1       2       14      1.70e-02
    TAGS: (2 total)
      d+1  TTAAGTGTTG  2       2       0       1       14      6.46e-05        722
      d+2  CCGTTGATCA  3       5       1       1       0       2.06e-01        514
           ----------                                                          409
           ----------                                                          336

LOCUS: AT1G61380
DESCRIPTION: S-locus protein kinase, putative, similar to KI domain interacting kinase 1 (Zea mays)
gi|2735017|gb|AAB93834; contains S-locus glycoprotein family domain, Pfam:PF00954
DATA:                  Control 30min   2hours  2days   1week   p-value         pos
SENSE COUNTS:          0       0       5       5       9       1.70e-02
GENES (1 total):
  AT1G61380.1
    SENSE COUNTS:      0       0       5       5       9       1.70e-02
    TAGS: (2 total)
      d+1  TGATCTCATA  0       0       1       0       1       6.85e-01        2646
      d+2  AACATTTAAA  0       0       4       5       8       2.06e-02        2596
           ----------                                                          2395
           ----------                                                          2298
           ----------                                                          2238
           ----------                                                          2109
           ----------                                                          1851
           ----------                                                          1813
           ----------                                                          1711
           ----------                                                          1503
           ----------                                                          963
           ----------                                                          627
           ----------                                                          473
           ----------                                                          75

LOCUS: AT1G63750
DESCRIPTION: disease resistance protein (TIR-NBS-LRR class), putative, domain signature TIR-NBS-LRR exists,
suggestive of a disease resistance protein.
DATA:                  Control 30min   2hours  2days   1week   p-value         pos
SENSE COUNTS:          1       0       1       0       7       1.71e-02
GENES (1 total):
  AT1G63750.1
    SENSE COUNTS:      1       0       1       0       7       1.71e-02
    TAGS: (2 total)
           ----------                                                          3149
           ----------                                                          3139
           ----------                                                          2796
      d+2  ATATCACAAA  1       0       0       0       0       6.89e-01        2738
           ----------                                                          2110
           ----------                                                          1709
           ----------                                                          1691
           ----------                                                          1393
      d+2  AAAATGATCA  0       0       1       0       7       5.13e-04        1319
           ----------                                                          1133
           ----------                                                          1058
           ----------                                                          753
           ----------                                                          523
           ----------                                                          307
           ----------                                                          34
           ----------                                                          22

LOCUS: AT4G37300
DESCRIPTION: expressed protein
DATA:                  Control 30min   2hours  2days   1week   p-value         pos
SENSE COUNTS:          5       7       0       0       2       1.73e-02
GENES (1 total):
  AT4G37300.1
    SENSE COUNTS:      5       7       0       0       2       1.73e-02
    TAGS: (2 total)
           ----------                                                          695
      i+3  TTCGGTTATC  0       0       0       0       1       1.65e-01        274
      d+2  GTAAAGTTCT  5       7       0       0       1       9.57e-03        274
           ----------                                                          19

LOCUS: AT3G59140
DESCRIPTION: ABC transporter family protein, putative multi resistance protein mrp - Arabidopsis thaliana,
EMBL:ATMRPPROT
DATA:                  Control 30min   2hours  2days   1week   p-value         pos
SENSE COUNTS:          3       0       1       4       8       1.74e-02
GENES (2 total):
  AT3G59140.1
    SENSE COUNTS:      3       0       1       4       8       1.74e-02
    TAGS: (3 total)
           ----------                                                          5081
```

```
      v+2  AGATCATAAT 3      0      0      4      7      2.08e-02       4866
      v+2  TAATTGTTTG 0      0      0      0      1      1.65e-01       4829
           ----------                                                 4389
           ----------                                                 4186
           ----------                                                 3744
           ----------                                                 3456
           ----------                                                 3216
           ----------                                                 3003
           ----------                                                 2375
           ----------                                                 2318
           ----------                                                 1767
           ----------                                                 1716
      v+2  GTTATTCATT 0      0      1      0      0      4.55e-01       650
           ----------                                                 257

LOCUS: AT4G39160
DESCRIPTION: myb family transcription factor, contains Pfam profile: PF00249 myb-like DNA-binding domain
DATA:                 Control 30min  2hours 2days  1week  p-value        pos
SENSE COUNTS:         4      0      0      0      1      1.74e-02
GENES (1 total):
  AT4G39160.1
    SENSE COUNTS:     4      0      0      0      1      1.74e-02
    TAGS: (1 total)
           ----------                                                 2451
      v+2  TAAACTATAG 4      0      0      0      1      1.74e-02       1986
           ----------                                                 1506
           ----------                                                 1252
           ----------                                                 687
           ----------                                                 388

LOCUS: AT4G09000
DESCRIPTION: 14-3-3-like protein GF14 chi /  general regulatory factor 1 (GRF1), identical to 14-3-3 protein
GF14 chi chain GI:1702986, SP:P42643 from (Arabidopsis thaliana)
DATA:                 Control 30min  2hours 2days  1week  p-value        pos
SENSE COUNTS:         6      15     7      2      3      1.76e-02
GENES (2 total):
  AT4G09000.1
    SENSE COUNTS:     6      15     7      2      3      1.76e-02
    TAGS: (1 total)
           ----------                                                 1248
      d+2  GAGAAAGTCG 6      15     7      2      3      1.76e-02       221
           ----------                                                 170

LOCUS: AT1G07390
DESCRIPTION: leucine-rich repeat family protein, contains leucine rich-repeat (LRR) domains Pfam:PF00560,
INTERPRO:IPR001611; contains similarity to Hcr2-5D (Lycopersicon esculentum) gi|3894393|gb|AAC78596
DATA:                 Control 30min  2hours 2days  1week  p-value        pos
SENSE COUNTS:         2      0      3      0      8      1.77e-02
GENES (1 total):
  AT1G07390.1
    SENSE COUNTS:     2      0      3      0      8      1.77e-02
    TAGS: (3 total)
      i+3  GTGTGGTTTT 0      0      1      0      0      4.55e-01       4600
      v+1  ATACCAGTTT 1      0      1      0      0      6.01e-01       3400
           ----------                                                 3216
           ----------                                                 3172
           ----------                                                 2788
           ----------                                                 2604
      v+2  TTTCAGTAAA 1      0      1      0      8      1.85e-03       2558
           ----------                                                 2169
           ----------                                                 2121
           ----------                                                 1993
           ----------                                                 1858
           ----------                                                 1631
           ----------                                                 1276
           ----------                                                 1256
           ----------                                                 1177
           ----------                                                 1108
           ----------                                                 828
           ----------                                                 792
           ----------                                                 451
           ----------                                                 440
           ----------                                                 12

LOCUS: AT5G35330
DESCRIPTION: methyl-CpG-binding domain-containing protein, similar to methyl-CpG binding protein MBD4 (Mus
musculus) GI:3800807; contains Pfam profile PF01429: Methyl-CpG binding domain
DATA:                 Control 30min  2hours 2days  1week  p-value        pos
SENSE COUNTS:         6      0      1      1      0      1.79e-02
GENES (3 total):
  AT5G35330.2
```

```
    SENSE COUNTS:     6       0       1       1       0       1.79e-02
    TAGS: (2 total)
          ----------                                                      1182
      d+2  TGGTTAGCTT 3       0       1       0       0       8.96e-02    1070
      d+2  GATATCTCCA 3       0       0       1       0       2.31e-01    824
          ----------                                                      366
  AT5G35330.1
    SENSE COUNTS:     6       0       1       1       0       1.79e-02
    TAGS: (2 total)
          ----------                                                      1188
      d+2  TGGTTAGCTT 3       0       1       0       0       8.96e-02    1076
      d+2  GATATCTCCA 3       0       0       1       0       2.31e-01    830
          ----------                                                      372

LOCUS: AT4G38480
DESCRIPTION: transducin family protein / WD-40 repeat family protein, contains contains Pfam PF00400: WD
domain, G-beta repeat (7 copies, 3 weak);similar to gene PC326 protein - mouse, PIR2:S37694
DATA:                 Control 30min   2hours  2days   1week   p-value     pos
SENSE COUNTS:         1       5       0       0       5       1.79e-02
GENES (1 total):
  AT4G38480.1
    SENSE COUNTS:     1       5       0       0       5       1.79e-02
    TAGS: (3 total)
      d+1  TATTTATCTA 0       0       0       0       5       7.96e-03    1474
          ----------                                                      1396
          ----------                                                      1134
      d+2  TTGTCAATTG 0       5       0       0       0       3.00e-03    1102
          ----------                                                      1086
      d+2  TAGGAATAAC 1       0       0       0       0       4.28e-01    796
          ----------                                                      634

LOCUS: AT3G61400
DESCRIPTION: 2-oxoglutarate-dependent dioxygenase, putative, similar to 2A6 (GI:599622) and tomato ethylene
synthesis regulatory protein E8 (SP|P10967); 1-aminocyclopropane-1-carboxylate oxidase homolog - Arabidopsis
thaliana, PIR:S59548
DATA:                 Control 30min   2hours  2days   1week   p-value     pos
SENSE COUNTS:         8       3       4       9       17      1.82e-02
GENES (1 total):
  AT3G61400.1
    SENSE COUNTS:     8       3       4       9       17      1.82e-02
    TAGS: (1 total)
          ----------                                                      1954
          ----------                                                      1594
          ----------                                                      1413
          ----------                                                      1295
      v+2  ATAAACAATA 8       3       4       9       17      1.82e-02    1141
          ----------                                                      993
          ----------                                                      828
          ----------                                                      600
          ----------                                                      214

LOCUS: AT5G05200
DESCRIPTION: ABC1 family protein, contains Pfam domain, PF03109: ABC1 family
DATA:                 Control 30min   2hours  2days   1week   p-value     pos
SENSE COUNTS:         5       0       0       0       1       1.82e-02
GENES (1 total):
  AT5G05200.1
    SENSE COUNTS:     5       0       0       0       1       1.82e-02
    TAGS: (1 total)
          ----------                                                      2210
          ----------                                                      1867
      d+2  ACTATATACA 5       0       0       0       1       1.82e-02    1802
          ----------                                                      1610
          ----------                                                      1286
          ----------                                                      1231
          ----------                                                      1149
          ----------                                                      1137
          ----------                                                      938
          ----------                                                      687
          ----------                                                      464

LOCUS: AT3G46530
DESCRIPTION: disease resistance protein, RPP13-like (CC-NBS class), putative, domain signature CC-NBS exists,
suggestive of a disease resistance protein. Closest homolog in Col-0 to RPP13.
DATA:                 Control 30min   2hours  2days   1week   p-value     pos
SENSE COUNTS:         0       3       8       1       1       1.85e-02
GENES (2 total):
  AT3G46530.1
    SENSE COUNTS:     0       3       8       1       1       1.85e-02
    TAGS: (4 total)
      X+4  TGCTGAATAG 0       0       1       0       0       4.55e-01    2997
```

```
      d+1  ATTTTTTGTG 0      1      4      0      0      6.49e-02       2875
           ----------                                                 2827
           ----------                                                 2802
      d+2  TGAACCAGGC 0      2      3      0      1      3.12e-01       2634
           ----------                                                 2505
           ----------                                                 2280
           ----------                                                 2173
           ----------                                                 2005
      d+2  TGGCCCAACA 0      0      0      1      0      3.09e-01       1630
           ----------                                                 1561
           ----------                                                 1339
           ----------                                                 1294
           ----------                                                 982
           ----------                                                 752
           ----------                                                 220

LOCUS: AT3G26290
DESCRIPTION: cytochrome P450 71B26, putative (CYP71B26), identical to cytochrome P450 71B26 (SP:Q9LTL0)
(Arabidopsis thaliana); contains Pfam profile: PF00067 cytochrome P450
DATA:                 Control 30min  2hours 2days  1week  p-value        pos
SENSE COUNTS:         8      1      10     3      0      1.85e-02
GENES (2 total):
  AT3G26290.1
    SENSE COUNTS:     8      1      10     3      0      1.85e-02
    TAGS: (2 total)
      d+1  GTAGTCGAAG 6      1      7      2      0      6.39e-02       1435
           ----------                                                 1363
           ----------                                                 1242
           ----------                                                 1205
           ----------                                                 1107
           ----------                                                 963
      X+4  AAGCCTCCTC 2      0      3      1      0      5.41e-01       752
           ----------                                                 107

LOCUS: AT1G18950
DESCRIPTION: aminoacyl-tRNA synthetase family, contains aminoacyl-transfer RNA synthetases class-II signature
1, PROSITE:PS00179
DATA:                 Control 30min  2hours 2days  1week  p-value        pos
SENSE COUNTS:         3      10     16     3      7      1.88e-02
GENES (1 total):
  AT1G18950.1
    SENSE COUNTS:     3      10     16     3      7      1.88e-02
    TAGS: (3 total)
      d+1  AAGAAAGAAA 3      9      13     2      7      7.05e-02       2716
           ----------                                                 1408
      X+4  GAGCAAATCT 0      0      3      1      0      2.61e-01       1247
      d+2  GAGTTAATTT 0      1      0      0      0      2.54e-01       994
           ----------                                                 583

LOCUS: AT4G35100
DESCRIPTION: plasma membrane intrinsic protein (SIMIP), nearly identical to plasma membrane intrinsic protein
(Arabidopsis thaliana) GI:2306917
DATA:                 Control 30min  2hours 2days  1week  p-value        pos
SENSE COUNTS:         2      3      10     4      0      1.89e-02
GENES (2 total):
  AT4G35100.1
    SENSE COUNTS:     2      3      10     4      0      1.89e-02
    TAGS: (2 total)
      d+1  TCAGTTCGTA 1      3      7      2      0      7.55e-02       1114
           ----------                                                 1101
           ----------                                                 671
      d+2  AAAACTCCTT 1      0      3      2      0      2.27e-01       470
           ----------                                                 416
           ----------                                                 134
           ----------                                                 90

LOCUS: AT2G46900
DESCRIPTION: expressed protein, contains Pfam profile PF04910: Protein of unknown function, DUF654
DATA:                 Control 30min  2hours 2days  1week  p-value        pos
SENSE COUNTS:         2      10     2      3      9      1.97e-02
GENES (1 total):
  AT2G46900.1
    SENSE COUNTS:     2      10     2      3      9      1.97e-02
    TAGS: (3 total)
      d+1  TTCTGGTGGA 0      0      0      1      1      6.17e-01       2133
      d+2  ATGCTCACCA 1      3      0      0      5      7.85e-02       1954
           ----------                                                 1916
           ----------                                                 1726
           ----------                                                 1469
           ----------                                                 1395
           ----------                                                 1098
```

```
          ----------                                                  1089
          ----------                                                  847
      d+2  TGCAGCCGGT  1      7      2      2      3      2.18e-01     334
          ----------                                                  142

LOCUS: AT2G37710
DESCRIPTION: lectin protein kinase, putative, similar to receptor lectin kinase 3 (Arabidopsis thaliana)
gi|4100060|gb|AAD00733; contains protein kinase domain, Pfam:PF00069; contains legume lectins alpha and beta
domains, Pfam:PF00138 and Pfam:PF00139
DATA:                  Control 30min  2hours  2days  1week  p-value      pos
SENSE COUNTS:          2       0      6       0      3      1.98e-02
GENES (1 total):
  AT2G37710.1
    SENSE COUNTS:      2       0      6       0      3      1.98e-02
    TAGS: (3 total)
      X+4  TGGGCATAAT  0       0      1       0      0      4.55e-01     2338
      d+1  ATAATGACGT  2       0      4       0      3      1.13e-01     2266
           ----------                                                  1971
      d+2  CGGTAGACGT  0       0      1       0      0      4.55e-01     1672
           ----------                                                  1566
           ----------                                                  1428
           ----------                                                  1316
           ----------                                                  1185
           ----------                                                  522
           ----------                                                  459
           ----------                                                  354
           ----------                                                  47

LOCUS: AT1G36030
DESCRIPTION: F-box family protein, similar to hypothetical protein GB:AAC61810
DATA:                  Control 30min  2hours  2days  1week  p-value      pos
SENSE COUNTS:          1       0      4       5      8      1.99e-02
GENES (1 total):
  AT1G36030.1
    SENSE COUNTS:      1       0      4       5      8      1.99e-02
    TAGS: (1 total)
           ----------                                                  894
           ----------                                                  885
           ----------                                                  800
      v+2  TTGAAAAAAA  1       0      4       5      8      1.99e-02     771
           ----------                                                  760
           ----------                                                  721
           ----------                                                  169

LOCUS: AT3G61580
DESCRIPTION: delta-8 sphingolipid desaturase (SLD1), identical to delta-8 sphingolipid desaturase GI:3819710
from (Arabidopsis thaliana); contains Pfam profile PF00487: Fatty acid desaturase; contains Pfam profile
PF00173: Heme/Steroid binding domain
DATA:                  Control 30min  2hours  2days  1week  p-value      pos
SENSE COUNTS:          21      8      29      26     25     2.04e-02
GENES (1 total):
  AT3G61580.1
    SENSE COUNTS:      21      8      29      26     25     2.04e-02
    TAGS: (4 total)
      d+1  GCTCTTTTTT  0       0      2       0      0      1.21e-01     1795
      d+2  GAAGAGATGT  20      8      23      26     25     3.93e-02     1662
      d+2  GCTAAATGAT  1       0      3       0      0      2.79e-01     1552
      d+2  GATTGGTTCT  0       0      1       0      0      4.55e-01     1299
           ----------                                                  1115
           ----------                                                  711
           ----------                                                  579

LOCUS: AT2G43910
DESCRIPTION: thiol methyltransferase, putative, similar to thiol methyltransferase 1 GI:14583119 from
(Brassica oleracea)
DATA:                  Control 30min  2hours  2days  1week  p-value      pos
SENSE COUNTS:          1       9      6       0      4      2.09e-02
GENES (1 total):
  AT2G43910.1
    SENSE COUNTS:      1       9      6       0      4      2.09e-02
    TAGS: (3 total)
      i+3  GCAAATAGGT  0       0      1       0      0      4.55e-01     2262
      d+1  TATCCGATTA  1       9      4       0      1      6.63e-03     588
           ----------                                                  533
      d+2  GGACCAAGGG  0       0      1       0      3      7.32e-02     215
           ----------                                                  78

LOCUS: AT1G68725
DESCRIPTION: arabinogalactan-protein, putative (AGP19), non-consensus splice site at the intron:exon boundary
(AT:exon)
DATA:                  Control 30min  2hours  2days  1week  p-value      pos
```

```
SENSE COUNTS:          3      0      0      0      0      2.13e-02
GENES (1 total):
  AT1G68725.1
    SENSE COUNTS:      3      0      0      0      0      2.13e-02
    TAGS: (1 total)
           ----------                                                1562
           ----------                                                1527
           ----------                                                1459
      v+2  TATACCAATA  3      0      0      0      0      2.13e-02   1158
           ----------                                                910
           ----------                                                10

LOCUS: AT1G70260
DESCRIPTION: nodulin MtN21 family protein, contains similarity to MtN21 (Medicago truncatula) GI:2598575;
contains Pfam profile PF00892: Integral membrane protein
DATA:                  Control 30min  2hours 2days  1week  p-value    pos
SENSE COUNTS:          3      0      0      0      0      2.13e-02
GENES (1 total):
  AT1G70260.1
    SENSE COUNTS:      3      0      0      0      0      2.13e-02
    TAGS: (1 total)
           ----------                                                1423
           ----------                                                865
           ----------                                                777
      X+4  GATCAACTAT  3      0      0      0      0      2.13e-02   424
           ----------                                                178

LOCUS: AT3G01360
DESCRIPTION: expressed protein, contains Pfam profile PF04819: Family of unknown function (DUF716) (Plant
viral-response family)
DATA:                  Control 30min  2hours 2days  1week  p-value    pos
SENSE COUNTS:          3      0      0      0      0      2.13e-02
GENES (1 total):
  AT3G01360.1
    SENSE COUNTS:      3      0      0      0      0      2.13e-02
    TAGS: (1 total)
           ----------                                                1790
           ----------                                                1570
      i+3  ATTAAAGGTA  3      0      0      0      0      2.13e-02   956
           ----------                                                956
           ----------                                                591
           ----------                                                475

LOCUS: AT2G17820
DESCRIPTION: histidine kinase 1, 99% identical to GP:4586626
DATA:                  Control 30min  2hours 2days  1week  p-value    pos
SENSE COUNTS:          2      7      12     2      1      2.23e-02
GENES (2 total):
  AT2G17820.1
    SENSE COUNTS:      2      7      12     2      1      2.23e-02
    TAGS: (3 total)
      d+1  CAATGTCTTC  0      0      1      1      0      5.21e-01   3692
           ----------                                                3586
           ----------                                                2944
           ----------                                                2939
           ----------                                                2894
           ----------                                                2566
           ----------                                                2193
           ----------                                                2156
           ----------                                                1963
           ----------                                                1733
           ----------                                                1406
           ----------                                                1087
      d+2  TGACAGTGAG  0      0      0      0      0      6.15e-01   1067
           ----------                                                931
           ----------                                                735
      d+2  GCATTTCACA  2      7      11     1      1      2.33e-02   507
           ----------                                                26

LOCUS: AT5G07350
DESCRIPTION: tudor domain-containing protein / nuclease family protein, contains Pfam domains PF00567: Tudor
domain and PF00565: Staphylococcal nuclease homologue
DATA:                  Control 30min  2hours 2days  1week  p-value    pos
SENSE COUNTS:          3      0      4      10     8      2.26e-02
GENES (1 total):
  AT5G07350.1
    SENSE COUNTS:      3      0      4      10     8      2.26e-02
    TAGS: (3 total)
      d+1  CTTACAGTTT  1      0      0      4      1      9.54e-02   3351
      d+2  TAGGACTTGA  1      0      3      4      7      1.62e-01   3318
      d+2  TCGAACTTTT  1      0      1      2      0      3.84e-01   3230
```

```
          ----------                                                            3180
          ----------                                                            2148
          ----------                                                            2085
          ----------                                                            1991
          ----------                                                            1848
          ----------                                                            1587
          ----------                                                            1288
          ----------                                                            1173
          ----------                                                            690
          ----------                                                            347
          ----------                                                            162

LOCUS: AT1G72770
DESCRIPTION: protein phosphatase 2C P2C-HA / PP2C P2C-HA (P2C-HA), identical to protein phosphatase 2C (AtP2C-
HA) GB:AJ003119 (Arabidopsis thaliana) (Plant Mol. Biol. 38 (5), 879-883 (1998))
DATA:                 Control 30min  2hours  2days  1week  p-value        pos
SENSE COUNTS:         3       0      7       2      8      2.27e-02
GENES (2 total):
  AT1G72770.1
    SENSE COUNTS:     3       0      7       2      8      2.27e-02
    TAGS: (4 total)
      d+1  TTGTGTTCTT 0       0      1       0      0      4.55e-01       2502
      d+2  GTACTTGAAT 2       0      2       2      1      5.91e-01       2341
      d+2  TTAATTACTC 1       0      3       0      7      1.20e-02       2197
           ----------                                                   1804
           ----------                                                   1738
           ----------                                                   1624
      d+2  GAGGCCATAA 0       0      1       0      0      4.55e-01       1286
           ----------                                                   1223
           ----------                                                   607
           ----------                                                   553
           ----------                                                   430

LOCUS: AT1G16340
DESCRIPTION: 2-dehydro-3-deoxyphosphooctonate aldolase, putative / phospho-2-dehydro-3-deoxyoctonate aldolase,
putative / 3-deoxy-D-manno-octulosonic acid 8-phosphate synthetase, putative, similar to Swiss-Prot:Q9AV97 2-
dehydro-3-deoxyphosphooctonate aldolase (EC 4.1.
DATA:                 Control 30min  2hours  2days  1week  p-value        pos
SENSE COUNTS:         6       0      1       0      3      2.38e-02
GENES (1 total):
  AT1G16340.1
    SENSE COUNTS:     6       0      1       0      3      2.38e-02
    TAGS: (1 total)
           ----------                                                   876
           ----------                                                   866
           ----------                                                   820
           ----------                                                   656
           ----------                                                   587
           ----------                                                   435
           ----------                                                   365
      d+2  TTCTTCGTAT 6       0      1       0      3      2.38e-02       243

LOCUS: AT3G23820
DESCRIPTION: NAD-dependent epimerase/dehydratase family protein, similar to nucleotide sugar epimerase from
Vibrio vulnificus GI:3093975 (PID:g3093975), WbnF (Escherichia coli) GI:5739472, CAPI protein {Staphylococcus
aureus} SP|P39858; contains Pfam profile: PF01370
DATA:                 Control 30min  2hours  2days  1week  p-value        pos
SENSE COUNTS:         22      11     27      15     31     2.47e-02
GENES (2 total):
  AT3G23820.1
    SENSE COUNTS:     22      11     27      15     31     2.47e-02
    TAGS: (4 total)
      X+4  GCATTGGACC 0       0      1       0      0      4.55e-01       2200
      X+4  CCAAGACCGA 0       0      3       2      7      2.09e-02       2077
      d+1  CTTGTTTATT 22      11     22      13     23     1.98e-01       1624
      d+2  CTAATGTGAG 0       0      1       0      1      3.96e-01       1433
           ----------                                                   1108
           ----------                                                   1092

LOCUS: AT5G55220
DESCRIPTION: trigger factor type chaperone family protein, contains Pfam profiles PF05697: Bacterial trigger
factor protein (TF), PF05698: Bacterial trigger factor protein (TF) C-terminus, PF00254: peptidyl-prolyl cis-
trans isomerase, FKBP-type
DATA:                 Control 30min  2hours  2days  1week  p-value        pos
SENSE COUNTS:         5       5      14      13     19     2.49e-02
GENES (1 total):
  AT5G55220.1
    SENSE COUNTS:     5       5      14      13     19     2.49e-02
    TAGS: (1 total)
           ----------                                                   1740
           ----------                                                   1391
```

```
           ----------                                                 1075
      d+2  GAATCAGTGA 5      5      14     13     19     2.49e-02     590
           ----------                                                 455
           ----------                                                 270
           ----------                                                 221

LOCUS: AT2G15610
DESCRIPTION: expressed protein
DATA:                 Control 30min  2hours 2days  1week  p-value      pos
SENSE COUNTS:         0      0      4      1      0      2.60e-02
GENES (1 total):
  AT2G15610.1
    SENSE COUNTS:     0      0      4      1      0      2.60e-02
    TAGS: (1 total)
           ----------                                                 1381
      v+2  ACAACAAAAA 0      0      4      1      0      2.60e-02     1321
           ----------                                                 1309
           ----------                                                 1001
           ----------                                                 811
           ----------                                                 656

LOCUS: AT1G72040
DESCRIPTION: deoxynucleoside kinase family, contains Pfam profile: PF01712 deoxynucleoside kinase
DATA:                 Control 30min  2hours 2days  1week  p-value      pos
SENSE COUNTS:         4      0      10     8      7      2.61e-02
GENES (1 total):
  AT1G72040.1
    SENSE COUNTS:     4      0      10     8      7      2.61e-02
    TAGS: (1 total)
           ----------                                                 2093
      d+2  GTCGTGCCAC 4      0      10     8      7      2.61e-02     1989
           ----------                                                 1776
           ----------                                                 1490
           ----------                                                 1455
           ----------                                                 1212
           ----------                                                 801

LOCUS: AT5G20500
DESCRIPTION: glutaredoxin, putative, similar to glutaredoxin (Populus tremula x Populus tremuloides)
gi|19548658|gb|AAL90750
DATA:                 Control 30min  2hours 2days  1week  p-value      pos
SENSE COUNTS:         10     8      15     20     26     2.64e-02
GENES (1 total):
  AT5G20500.1
    SENSE COUNTS:     10     8      15     20     26     2.64e-02
    TAGS: (1 total)
           ----------                                                 1040
           ----------                                                 946
           ----------                                                 900
           ----------                                                 655
      d+2  ACCTTGTGAC 10     8      15     20     26     2.64e-02     559
           ----------                                                 82
           ----------                                                 58

LOCUS: AT5G57345
DESCRIPTION: Expressed protein
DATA:                 Control 30min  2hours 2days  1week  p-value      pos
SENSE COUNTS:         5      2      2      12     3      2.66e-02
GENES (1 total):
  AT5G57345.1
    SENSE COUNTS:     5      2      2      12     3      2.66e-02
    TAGS: (2 total)
      i+3  TATGGGCTTT 0      0      1      0      0      4.55e-01     594
      d+1  TTTTTTGGAG 5      2      1      12     3      1.18e-02     538

LOCUS: AT1G22930
DESCRIPTION: T-complex protein 11, contains Pfam PF05794: T-complex protein 11
DATA:                 Control 30min  2hours 2days  1week  p-value      pos
SENSE COUNTS:         1      9      2      6      1      2.67e-02
GENES (1 total):
  AT1G22930.1
    SENSE COUNTS:     1      9      2      6      1      2.67e-02
    TAGS: (2 total)
      d+1  CCACAACATT 0      4      2      6      0      5.48e-02     3509
           ----------                                                 3493
           ----------                                                 3486
      d+2  GGTATTTCTC 1      5      0      0      1      3.96e-02     2834
           ----------                                                 2636
           ----------                                                 2234
           ----------                                                 2225
           ----------                                                 2118
```

```
          ----------                                                      1820
          ----------                                                      1725
          ----------                                                      801
          ----------                                                      297

LOCUS: AT4G23900
DESCRIPTION: nucleoside diphosphate kinase 4 (NDK4), contains Pfam PF00334 : Nucleoside diphosphate kinase
domain; identical to nucleoside diphosphate kinase 4 (GI:11990430)  (Arabidopsis thaliana)
DATA:                 Control 30min  2hours  2days  1week  p-value       pos
SENSE COUNTS:         0       0      3       0      0      2.70e-02
GENES (1 total):
  AT4G23900.1
    SENSE COUNTS:     0       0      3       0      0      2.70e-02
    TAGS: (1 total)
          ----------                                                      940
      i+3  CATAATGTTT  0       0      3       0      0      2.70e-02       762
          ----------                                                      655
          ----------                                                      528
          ----------                                                      460
          ----------                                                      423
          ----------                                                      279
          ----------                                                      169

LOCUS: AT1G43170
DESCRIPTION: cytoplasmic ribosomal protein mRNA, complete cds
DATA:                 Control 30min  2hours  2days  1week  p-value       pos
SENSE COUNTS:         0       0      3       0      0      2.70e-02
GENES (3 total):
  AT1G43170.1
    SENSE COUNTS:     0       0      3       0      0      2.70e-02
    TAGS: (1 total)
          ----------                                                      1283
      d+2  GTCGCTTCCA  0       0      3       0      0      2.70e-02       1189
          ----------                                                      972
          ----------                                                      618
          ----------                                                      293
          ----------                                                      216
          ----------                                                      109
  AT1G43170.2
    SENSE COUNTS:     0       0      3       0      0      2.70e-02
    TAGS: (1 total)
          ----------                                                      1249
      d+2  GTCGCTTCCA  0       0      3       0      0      2.70e-02       1155
          ----------                                                      938
          ----------                                                      584
          ----------                                                      259
          ----------                                                      182
          ----------                                                      75

LOCUS: AT5G54570
DESCRIPTION: glycosyl hydrolase family 1 protein, contains Pfam PF00232 : Glycosyl hydrolase family 1 domain;
TIGRFAM TIGR01233: 6-phospho-beta-galactosidase; similar to amygdalin hydrolase isoform AH I precursor
(GI:16757966)  (Prunus serotina)
DATA:                 Control 30min  2hours  2days  1week  p-value       pos
SENSE COUNTS:         0       0      3       0      0      2.70e-02
GENES (1 total):
  AT5G54570.1
    SENSE COUNTS:     0       0      3       0      0      2.70e-02
    TAGS: (1 total)
          ----------                                                      2017
          ----------                                                      1629
      v+2  GATGAGAAAA  0       0      3       0      0      2.70e-02       1599
          ----------                                                      1523
          ----------                                                      1084
          ----------                                                      949
          ----------                                                      340

LOCUS: AT2G39170
DESCRIPTION: expressed protein,
DATA:                 Control 30min  2hours  2days  1week  p-value       pos
SENSE COUNTS:         0       0      3       0      0      2.70e-02
GENES (1 total):
  AT2G39170.1
    SENSE COUNTS:     0       0      3       0      0      2.70e-02
    TAGS: (1 total)
          ----------                                                      802
      d+2  ACGGCTAAGG  0       0      3       0      0      2.70e-02       605
          ----------                                                      383
          ----------                                                      171

LOCUS: AT3G26740
```

```
DESCRIPTION: light responsive protein-related, similar to light regulated protein precursor SP:Q03200 (Oryza
sativa) (Plant Mol. Biol. 22 (1), 165-170 (1993)), ccr protein GB:S52663 (Citrus X paradisi) (Plant Mol. Biol.
26 (1), 165-173 (1994))
DATA:                 Control 30min  2hours  2days  1week  p-value      pos
SENSE COUNTS:         0       0      3       0      0      2.70e-02
GENES (1 total):
  AT3G26740.1
    SENSE COUNTS:     0       0      3       0      0      2.70e-02
    TAGS: (1 total)
           ----------                                                   516
      d+2  CGAAGTAATC 0       0      3       0      0      2.70e-02     285

LOCUS: AT5G19760
DESCRIPTION: dicarboxylate/tricarboxylate carrier (DTC), identical to dicarboxylate/tricarboxylate carrier
(Arabidopsis thaliana) GI:19913113
DATA:                 Control 30min  2hours  2days  1week  p-value      pos
SENSE COUNTS:         0       0      3       0      0      2.70e-02
GENES (1 total):
  AT5G19760.1
    SENSE COUNTS:     0       0      3       0      0      2.70e-02
    TAGS: (1 total)
           ----------                                                   1506
           ----------                                                   995
           ----------                                                   987
      d+2  AGAGATAATC 0       0      3       0      0      2.70e-02     732
           ----------                                                   693
           ----------                                                   604
           ----------                                                   315
           ----------                                                   258

LOCUS: AT3G52130
DESCRIPTION: protease inhibitor/seed storage/lipid transfer protein (LTP) family protein, similar to cysteine-
rich 5B protein - Lycopersicon esculentum, PIR2:S39552 (GI:415833); contains Pfam protease inhibitor/seed
storage/LTP family domain PF00234
DATA:                 Control 30min  2hours  2days  1week  p-value      pos
SENSE COUNTS:         0       0      3       0      0      2.70e-02
GENES (1 total):
  AT3G52130.1
    SENSE COUNTS:     0       0      3       0      0      2.70e-02
    TAGS: (1 total)
           ----------                                                   669
      v+2  TAGAGCAGCA 0       0      3       0      0      2.70e-02     536
           ----------                                                   399
           ----------                                                   354
           ----------                                                   265

LOCUS: AT1G14345
DESCRIPTION: expressed protein, contains one transmembrane domain
DATA:                 Control 30min  2hours  2days  1week  p-value      pos
SENSE COUNTS:         10      1      7       1      3      2.73e-02
GENES (1 total):
  AT1G14345.1
    SENSE COUNTS:     10      1      7       1      3      2.73e-02
    TAGS: (2 total)
      X+4  GATATTAATA 1       0      0       0      0      4.28e-01     848
      d+1  TTCTTTCATT 9       1      7       1      3      5.16e-02     695
           ----------                                                   34

LOCUS: AT1G08190
DESCRIPTION: vacuolar assembly protein, putative (VPS41), 99.8% identical to Vacuolar assembly protein VPS41
homolog (SP:P93043) (Arabidopsis thaliana); similar to vacuolar assembly protein vps41 GI:1835787 from
(Lycopersicon esculentum)
DATA:                 Control 30min  2hours  2days  1week  p-value      pos
SENSE COUNTS:         10      1      15      9      4      2.77e-02
GENES (1 total):
  AT1G08190.1
    SENSE COUNTS:     10      1      15      9      4      2.77e-02
    TAGS: (3 total)
      i+3  AATTTTTTCT 0       0      1       0      0      4.55e-01     3555
      d+1  TGTGCCAGAA 9       1      13      8      1      1.26e-02     3243
      d+2  TGTTTGTGAA 1       0      1       1      3      7.06e-01     3112
           ----------                                                   2902
           ----------                                                   2879
           ----------                                                   2684
           ----------                                                   2389
           ----------                                                   2162
           ----------                                                   2141
           ----------                                                   2120
           ----------                                                   1925
           ----------                                                   1394
           ----------                                                   1253
```

```
          ----------                                                    1170
          ----------                                                    577

LOCUS: AT1G52210
DESCRIPTION: copia-like retrotransposon family, has a 7.7e-186 P-value blast match to GB:CAA72989 open reading
frame 1 (Ty1_Copia-element) (Brassica oleracea)
DATA:                 Control 30min  2hours  2days  1week  p-value       pos
SENSE COUNTS:         1       0      5       0      3      2.77e-02
GENES (1 total):
  AT1G52210.1
    SENSE COUNTS:     1       0      5       0      3      2.77e-02
    TAGS: (1 total)
          ----------                                                    4337
          ----------                                                    4238
          ----------                                                    3763
          ----------                                                    3475
          ----------                                                    3465
          ----------                                                    3190
          ----------                                                    3164
          ----------                                                    2909
          ----------                                                    2818
          ----------                                                    2510
          ----------                                                    2266
          ----------                                                    1990
          ----------                                                    1293
          ----------                                                    1208
          ----------                                                    1030
      p+2  GCAGAAAGCT 1       0      5       0      3      2.77e-02      861
          ----------                                                    618
          ----------                                                    607
          ----------                                                    510
          ----------                                                    302
          ----------                                                    127

LOCUS: AT5G61140
DESCRIPTION: DEAD box RNA helicase, putative, similar to ASC-1 complex subunit P200 (Homo sapiens)
GI:12061185; contains Pfam profiles PF00270: DEAD/DEAH box helicase, PF00271: Helicase conserved C-terminal
domain, PF02889: Sec63 domain
DATA:                 Control 30min  2hours  2days  1week  p-value       pos
SENSE COUNTS:         1       4      0       1      7      2.79e-02
GENES (1 total):
  AT5G61140.1
    SENSE COUNTS:     1       4      0       1      7      2.79e-02
    TAGS: (3 total)
      d+1  GAGTTACCTC 0       4      0       0      0      9.72e-03      6328
          ----------                                                    5953
          ----------                                                    5949
          ----------                                                    5911
          ----------                                                    5902
          ----------                                                    5830
          ----------                                                    5714
          ----------                                                    5530
          ----------                                                    5322
          ----------                                                    5174
          ----------                                                    5150
          ----------                                                    5007
          ----------                                                    4916
          ----------                                                    4678
          ----------                                                    4064
          ----------                                                    3890
          ----------                                                    3866
          ----------                                                    3701
          ----------                                                    3254
          ----------                                                    3188
          ----------                                                    3109
      i+3  AGGTAAGTTT 0       0      0       0      0      6.15e-01      2827
          ----------                                                    2702
          ----------                                                    2632
          ----------                                                    2441
          ----------                                                    1888
          ----------                                                    1640
          ----------                                                    1511
          ----------                                                    1097
          ----------                                                    900
          ----------                                                    596
          ----------                                                    217
      d+2  TAATAAACAT 1       0      0       1      7      1.60e-02      21

LOCUS: AT4G14810
DESCRIPTION: hypothetical protein
DATA:                 Control 30min  2hours  2days  1week  p-value       pos
```

```
SENSE COUNTS:          6      0      0      2      1      2.82e-02
GENES (1 total):
  AT4G14810.1
    SENSE COUNTS:      6      0      0      2      1      2.82e-02
    TAGS: (1 total)
           ----------                                                 1066
           ----------                                                 685
           ----------                                                 655
      v+2  GAGCTGTTGA  6      0      0      2      1      2.82e-02    436
           ----------                                                 313
           ----------                                                 179

LOCUS: AT4G14040
DESCRIPTION: selenium-binding protein, putative, contains Pfam profile PF05694: 56kDa selenium binding protein
(SBP56); similar to Putative selenium-binding protein (Swiss-Prot:O23264) (Arabidopsis thaliana); similar to
selenium binding protein (GI:15485232) (Arabidop
DATA:                  Control 30min  2hours 2days  1week  p-value        pos
SENSE COUNTS:          1      0      8      2      1      2.82e-02
GENES (1 total):
  AT4G14040.1
    SENSE COUNTS:      1      0      8      2      1      2.82e-02
    TAGS: (3 total)
      d+1  GAGACGTATT  1      0      2      1      0      5.07e-01    1816
      d+2  GACCAGATTT  0      0      6      1      1      2.15e-02    1734
           ----------                                                 1347
           ----------                                                 1319
           ----------                                                 1081
           ----------                                                 955
           ----------                                                 865
           ----------                                                 745
           ----------                                                 687
           ----------                                                 637
           ----------                                                 542
           ----------                                                 343
      d+2  GCCGGTCCAC  0      0      0      0      0      6.15e-01    147

LOCUS: AT5G12150
DESCRIPTION: pleckstrin homology (PH) domain-containing protein / RhoGAP domain-containing protein, weak
similarity to glucocorticoid receptor DNA binding factor 1 (Canis familiaris) GI:23266717; contains Pfam
profiles PF00169: PH domain, PF00620: RhoGAP domain
DATA:                  Control 30min  2hours 2days  1week  p-value        pos
SENSE COUNTS:          15     3      5      13     5      2.83e-02
GENES (1 total):
  AT5G12150.1
    SENSE COUNTS:      15     3      5      13     5      2.83e-02
    TAGS: (3 total)
      d+1  CTTGTAAAAC  9      2      4      2      3      2.18e-01    2762
      d+2  GAATTAGGCT  6      1      1      11     1      8.06e-03    2683
           ----------                                                 2558
           ----------                                                 1922
           ----------                                                 1160
           ----------                                                 1156
           ----------                                                 1133
           ----------                                                 927
           ----------                                                 903
           ----------                                                 670
      X+4  TCTTTGTCGT  0      0      0      0      1      1.65e-01    -114

LOCUS: AT2G44610
DESCRIPTION: Ras-related GTP-binding protein, putative, similar to GTP-binding protein GI:623586 from
(Nicotiana tabacum) ; contains an ADP-ribosylation factors family signature for proteins involved in protein
trafficking
DATA:                  Control 30min  2hours 2days  1week  p-value        pos
SENSE COUNTS:          1      2      2      9      1      2.83e-02
GENES (2 total):
  AT2G44610.1
    SENSE COUNTS:      1      2      2      9      1      2.83e-02
    TAGS: (2 total)
      i+3  ATGTGCAAAT  0      0      0      1      0      3.09e-01    1445
      d+1  ATTCGATCAG  1      2      2      8      1      9.29e-02    901
           ----------                                                 281
           ----------                                                 194

LOCUS: AT2G40810
DESCRIPTION: WD-40 repeat protein family, similar to Gsa12p(GI:18307769)(Pichia pastoris); contains 3 Pfam
PF00400: WD domain, G-beta repeats
DATA:                  Control 30min  2hours 2days  1week  p-value        pos
SENSE COUNTS:          0      4      2      0      0      2.85e-02
GENES (2 total):
  AT2G40810.1
    SENSE COUNTS:      0      4      2      0      0      2.85e-02
```

```
    TAGS: (2 total)
      i+3  AATGTAGAAA 0       0       0       0       0       6.15e-01        1420
           ----------                                                         1367
           ----------                                                         1330
           ----------                                                         1216
      d+2  TATCAGAAGT 0       4       2       0       0       6.74e-02        1151
           ----------                                                         734
           ----------                                                         712
           ----------                                                         284
  AT2G40810.2
    SENSE COUNTS:     0       4       2       0       0       2.85e-02
    TAGS: (2 total)
      i+3  AATGTAGAAA 0       0       0       0       0       6.15e-01        1390
           ----------                                                         1347
           ----------                                                         1310
           ----------                                                         1196
      d+2  TATCAGAAGT 0       4       2       0       0       6.74e-02        1131
           ----------                                                         714
           ----------                                                         692
           ----------                                                         264

LOCUS: AT3G47540
DESCRIPTION: chitinase, putative, similar to basic endochitinase CHB4 precursor SP:Q06209 from (Brassica
napus)
DATA:                 Control 30min   2hours  2days   1week   p-value         pos
SENSE COUNTS:         2       0       7       5       1       2.86e-02
GENES (1 total):
  AT3G47540.1
    SENSE COUNTS:     2       0       7       5       1       2.86e-02
    TAGS: (2 total)
      d+1  AATTAACATT 1       0       0       0       0       4.28e-01        759
      X+4  GAGATCGATG 1       0       7       5       1       1.33e-02        647
           ----------                                                         366
           ----------                                                         275
           ----------                                                         265
           ----------                                                         19

LOCUS: AT2G47115
DESCRIPTION: expressed protein
DATA:                 Control 30min   2hours  2days   1week   p-value         pos
SENSE COUNTS:         1       0       0       3       5       2.91e-02
GENES (1 total):
  AT2G47115.1
    SENSE COUNTS:     1       0       0       3       5       2.91e-02
    TAGS: (3 total)
      d+1  AAAGTCCCAG 0       0       0       1       0       3.09e-01        1244
      d+2  CAATAAGATT 1       0       0       1       5       3.10e-02        992
           ----------                                                         862
           ----------                                                         837
           ----------                                                         789
      d+2  CACACGGCAA 0       0       0       1       0       3.09e-01        658
           ----------                                                         538
           ----------                                                         382
           ----------                                                         307
           ----------                                                         61

LOCUS: AT1G72930
DESCRIPTION: Toll-Interleukin-Resistance (TIR) domain-containing protein, domain signature TIR exists,
suggestive of a disease resistance protein.
DATA:                 Control 30min   2hours  2days   1week   p-value         pos
SENSE COUNTS:         3       4       8       6       17      2.93e-02
GENES (2 total):
  AT1G72930.1
    SENSE COUNTS:     3       4       8       6       17      2.93e-02
    TAGS: (2 total)
      d+1  TTGTTTCTAG 3       4       8       5       14      9.42e-02        755
           ----------                                                         611
      d+2  CGAGTAGAGA 0       0       0       1       3       2.09e-01        398
           ----------                                                         353
           ----------                                                         289
           ----------                                                         80

LOCUS: AT5G41190
DESCRIPTION: expressed protein,  ; expression supported by MPSS
DATA:                 Control 30min   2hours  2days   1week   p-value         pos
SENSE COUNTS:         0       0       1       4       3       2.94e-02
GENES (1 total):
  AT5G41190.1
    SENSE COUNTS:     0       0       1       4       3       2.94e-02
    TAGS: (3 total)
           ----------                                                         2475
```

```
      v+2  TAAACCACGA 0      0      1      4      1      9.69e-02       1859
      v+2  CTTGCTACAC 0      0      0      0      1      1.65e-01       1741
      v+2  ATATTGAAGT 0      0      0      0      1      1.65e-01       1462
           ----------                                                 1021
           ----------                                                 957
           ----------                                                 642
           ----------                                                 354

LOCUS: AT4G37920
DESCRIPTION: expressed protein,
DATA:                 Control 30min  2hours 2days  1week  p-value        pos
SENSE COUNTS:         10     0      4      1      2      2.99e-02
GENES (1 total):
  AT4G37920.1
    SENSE COUNTS:     10     0      4      1      2      2.99e-02
    TAGS: (3 total)
      v+1  TAGGGTCCAG 0      0      0      0      1      1.65e-01       2551
           ----------                                                 2390
      i+3  TTCCTTACTC 0      0      0      1      1      6.17e-01       969
      i+3  ACATATGCTA 10     0      4      0      0      6.03e-04       840
           ----------                                                 13

LOCUS: AT1G76650
DESCRIPTION: calcium-binding EF hand family protein, similar to regulator of gene silencing calmodulin-related
protein GI:12963415 from (Nicotiana tabacum); contains INTERPRO:IPR002048 calcium-binding EF-hand domain
DATA:                 Control 30min  2hours 2days  1week  p-value        pos
SENSE COUNTS:         0      3      0      0      0      3.05e-02
GENES (1 total):
  AT1G76650.1
    SENSE COUNTS:     0      3      0      0      0      3.05e-02
    TAGS: (1 total)
           ----------                                                 769
      d+2  ACATTGGGAG 0      3      0      0      0      3.05e-02       283
           ----------                                                 229
           ----------                                                 79

LOCUS: AT1G68840
DESCRIPTION: DNA-binding protein RAV2 (RAV2) / AP2 domain-containing protein RAP2.8, identical to RAV2
GI:3868859 from (Arabidopsis thaliana), AP2 domain containing protein RAP2.8 (Arabidopsis thaliana)
GI:2281641; contains Pfam profile: PF00847 AP2-domain
DATA:                 Control 30min  2hours 2days  1week  p-value        pos
SENSE COUNTS:         5      7      1      2      0      3.08e-02
GENES (2 total):
  AT1G68840.1
    SENSE COUNTS:     5      7      1      2      0      3.08e-02
    TAGS: (2 total)
      X+4  ATAAATCGGA 0      0      0      0      0      6.15e-01       1424
      d+1  CTAGGTTGTA 5      7      1      2      0      5.55e-02       1252
           ----------                                                 539

LOCUS: AT1G22250
DESCRIPTION: expressed protein
DATA:                 Control 30min  2hours 2days  1week  p-value        pos
SENSE COUNTS:         0      0      4      0      0      3.11e-02
GENES (1 total):
  AT1G22250.1
    SENSE COUNTS:     0      0      4      0      0      3.11e-02
    TAGS: (1 total)
           ----------                                                 642
           ----------                                                 635
           ----------                                                 584
           ----------                                                 572
      X+4  GTTGTGCCGC 0      0      4      0      0      3.11e-02       508
           ----------                                                 410
           ----------                                                 275
           ----------                                                 174
           ----------                                                 114

LOCUS: AT1G54470
DESCRIPTION: hypothetical protein
DATA:                 Control 30min  2hours 2days  1week  p-value        pos
SENSE COUNTS:         9      1      6      2      0      3.12e-02
GENES (1 total):
  AT1G54470.1
    SENSE COUNTS:     9      1      6      2      0      3.12e-02
    TAGS: (1 total)
           ----------                                                 1189
           ----------                                                 1131
           ----------                                                 728
           ----------                                                 587
      v+2  TCAAATCCAA 9      1      6      2      0      3.12e-02       44
```

```
          ----------                                                        37

LOCUS: AT1G75900
DESCRIPTION: family II extracellular lipase 3 (EXL3), EXL3 (PMID:11431566); similar to anter-specific proline-
rich protein (APG) SP:P40602 (Arabidopsis thaliana)
DATA:                 Control 30min  2hours  2days  1week  p-value        pos
SENSE COUNTS:         0       0      1       1      5      3.15e-02
GENES (1 total):
  AT1G75900.1
    SENSE COUNTS:     0       0      1       1      5      3.15e-02
    TAGS: (1 total)
          ----------                                                        1400
          ----------                                                        1380
      d+2  TGTTTTGGGA 0       0      1       1      5      3.15e-02       1124
          ----------                                                        121

LOCUS: AT2G41905
DESCRIPTION: expressed protein
DATA:                 Control 30min  2hours  2days  1week  p-value        pos
SENSE COUNTS:         0       0      5       4      1      3.21e-02
GENES (1 total):
  AT2G41905.1
    SENSE COUNTS:     0       0      5       4      1      3.21e-02
    TAGS: (1 total)
          ----------                                                        397
          ----------                                                        141
          ----------                                                        129
      X+4  CCGGAAAATA 0       0      5       4      1      3.21e-02       -238

LOCUS: AT1G21660
DESCRIPTION: expressed protein, low similarity to SP|O14976 Cyclin G-associated kinase (EC 2.7.1.-) {Homo
sapiens}; supporting cDNA gi|20466222|gb|AY099577.1|
DATA:                 Control 30min  2hours  2days  1week  p-value        pos
SENSE COUNTS:         3       0      3       0      7      3.21e-02
GENES (1 total):
  AT1G21660.1
    SENSE COUNTS:     3       0      3       0      7      3.21e-02
    TAGS: (3 total)
      i+3  GAAACGATGT 1       0      1       0      0      6.01e-01       2973
      d+1  GGGGATATTG 2       0      1       0      7      8.76e-03       1952
      d+2  CGTGCGCTGT 0       0      1       0      0      4.55e-01       1555
          ----------                                                        1054

LOCUS: AT5G44720
DESCRIPTION: molybdenum cofactor sulfurase family protein, weak similarity to molybdenum cofactor sulfurase
(LOS5/ABA3) (Arabidopsis thaliana) GI:15407262; contains Pfam profiles PF03476: MOSC N-terminal beta barrel
domain, PF03473: MOSC domain
DATA:                 Control 30min  2hours  2days  1week  p-value        pos
SENSE COUNTS:         21      10     26      15     8      3.35e-02
GENES (2 total):
  AT5G44720.1
    SENSE COUNTS:     21      10     26      15     8      3.35e-02
    TAGS: (3 total)
      d+1  AGGATTTCGT 1       0      1       0      0      6.01e-01       1178
      d+2  TTAATTCCAA 2       1      4       4      3      7.97e-01       1111
          ----------                                                        1033
      i+3  TGTGTTAAAA 18      9      21      11     5      4.60e-02       837
          ----------                                                        569
  AT5G44720.2
    SENSE COUNTS:     18      9      21      11     5      4.60e-02
    TAGS: (1 total)
      i+3  TGTGTTAAAA 18      9      21      11     5      4.60e-02       837
          ----------                                                        569

LOCUS: AT4G06477
DESCRIPTION: hypothetical protein
DATA:                 Control 30min  2hours  2days  1week  p-value        pos
SENSE COUNTS:         0       6      2       1      0      3.40e-02
GENES (2 total):
  AT4G06477.1
    SENSE COUNTS:     0       6      2       1      0      3.40e-02
    TAGS: (1 total)
          ----------                                                        4269
          ----------                                                        4229
          ----------                                                        3977
      p+2  CCAAGTTTGG 0       6      2       1      0      3.40e-02       3960
          ----------                                                        3602
          ----------                                                        3563
          ----------                                                        3458
          ----------                                                        3440
          ----------                                                        3428
```

```
           ----------                                                     3230
           ----------                                                     3221
           ----------                                                     3185
           ----------                                                     3159
           ----------                                                     3144
           ----------                                                     2752
           ----------                                                     2689
           ----------                                                     2192
           ----------                                                     1641
           ----------                                                     1449
           ----------                                                     1391
           ----------                                                     1119
           ----------                                                     844

LOCUS: AT3G42182
DESCRIPTION: copia-like retrotransposon family, has a 0. P-value blast match to GB:BAA22288 pol polyprotein
(Ty1_Copia-element) (Oryza australiensis)GB:BAA22288 polyprotein (Ty1_Copia-element) (Oryza
australiensis)gi|2443320|dbj|BAA22288.1| polyprotein (RIRE1) (Oryza
DATA:                  Control 30min   2hours  2days   1week   p-value         pos
SENSE COUNTS:          1       0       1       0       5       3.41e-02
GENES (1 total):
  AT3G42182.1
    SENSE COUNTS:      1       0       1       0       5       3.41e-02
    TAGS: (1 total)
           ----------                                                     3923
           ----------                                                     3827
           ----------                                                     3406
           ----------                                                     3393
           ----------                                                     3362
           ----------                                                     3306
           ----------                                                     3267
           ----------                                                     3234
           ----------                                                     2830
           ----------                                                     2680
           ----------                                                     2619
           ----------                                                     2530
      p+2  ATCTATTTAT  1       0       1       0       5       3.41e-02        2428
           ----------                                                     2284
           ----------                                                     1513
           ----------                                                     1355
           ----------                                                     1235
           ----------                                                     1054
           ----------                                                     335
           ----------                                                     189
           ----------                                                     78
           ----------                                                     64

LOCUS: AT1G31200
DESCRIPTION: expressed protein
DATA:                  Control 30min   2hours  2days   1week   p-value         pos
SENSE COUNTS:          1       0       1       0       4       3.41e-02
GENES (1 total):
  AT1G31200.1
    SENSE COUNTS:      1       0       1       0       4       3.41e-02
    TAGS: (2 total)
           ----------                                                     1328
           ----------                                                     1201
      v+2  TCTGCTAAAA  1       0       1       0       3       2.09e-01        642
      v+2  GATTTCACAA  0       0       0       0       1       1.65e-01        422
           ----------                                                     269

LOCUS: AT5G18250
DESCRIPTION: expressed protein, similar to unknown protein (dbj|BAA90342.1)
DATA:                  Control 30min   2hours  2days   1week   p-value         pos
SENSE COUNTS:          0       0       0       1       5       3.42e-02
GENES (1 total):
  AT5G18250.1
    SENSE COUNTS:      0       0       0       1       5       3.42e-02
    TAGS: (1 total)
           ----------                                                     357
      d+2  TGGTTACATT  0       0       0       1       5       3.42e-02        304
           ----------                                                     266
           ----------                                                     113

LOCUS: AT5G28056
DESCRIPTION: CACTA-like transposase family (Tnp2/En/Spm), has a 0. P-value blast match to
gb|AAG52024.1|AC022456_5 Tam1-homologous transposon protein TNP2, putative; 12762-16371 (Arabidopsis thaliana)
(CACTA-element)
DATA:                  Control 30min   2hours  2days   1week   p-value         pos
SENSE COUNTS:          0       0       0       1       4       3.42e-02
GENES (1 total):
```

```
  AT5G28056.1
    SENSE COUNTS:      0       0       0       1       4       3.42e-02
    TAGS: (2 total)
           ----------                                                         5166
           ----------                                                         4765
           ----------                                                         4667
           ----------                                                         4429
           ----------                                                         4422
      p+2  AGGTTTGGTT  0       0       0       0       1       1.65e-01       4246
           ----------                                                         4241
           ----------                                                         3979
           ----------                                                         3966
           ----------                                                         3667
           ----------                                                         3268
           ----------                                                         3177
           ----------                                                         3033
           ----------                                                         2963
           ----------                                                         2588
           ----------                                                         2424
           ----------                                                         1590
           ----------                                                         1560
           ----------                                                         1537
           ----------                                                         1476
           ----------                                                         1109
           ----------                                                         1067
      p+2  TGAAAAGATG  0       0       0       1       3       2.09e-01       1015
           ----------                                                         750
           ----------                                                         549
           ----------                                                         387
           ----------                                                         324
           ----------                                                         18

LOCUS: AT4G30825
DESCRIPTION: pentatricopeptide (PPR) repeat-containing protein, contains Pfam profile PF01535: PPR repeat
DATA:                  Control 30min   2hours  2days   1week   p-value        pos
SENSE COUNTS:          1       0       2       0       6       3.42e-02
GENES (1 total):
  AT4G30825.1
    SENSE COUNTS:      1       0       2       0       6       3.42e-02
    TAGS: (3 total)
           ----------                                                         3430
           ----------                                                         3417
      v+2  CGTGTATATT  0       0       2       0       5       6.19e-02       3144
           ----------                                                         2778
      v+2  TCATCAGCGA  1       0       0       0       0       4.28e-01       2607
           ----------                                                         2208
           ----------                                                         1855
      v+2  GGTCTTTACC  0       0       0       0       1       1.65e-01       1107
           ----------                                                         1056
           ----------                                                         1010
           ----------                                                         964
           ----------                                                         28

LOCUS: AT2G28700
DESCRIPTION: MADS-box protein-related, contains INTERPRO: IPR002100 MADS-box domain
DATA:                  Control 30min   2hours  2days   1week   p-value        pos
SENSE COUNTS:          2       3       5       5       14      3.48e-02
GENES (1 total):
  AT2G28700.1
    SENSE COUNTS:      2       3       5       5       14      3.48e-02
    TAGS: (1 total)
           ----------                                                         813
           ----------                                                         596
           ----------                                                         569
           ----------                                                         398
           ----------                                                         374
           ----------                                                         154
      d+2  GCAAGAAAAA  2       3       5       5       14      3.48e-02       26

LOCUS: AT5G18380
DESCRIPTION: 40S ribosomal protein S16 (RPS16C)
DATA:                  Control 30min   2hours  2days   1week   p-value        pos
SENSE COUNTS:          1       0       4       0       5       3.49e-02
GENES (1 total):
  AT5G18380.1
    SENSE COUNTS:      1       0       4       0       5       3.49e-02
    TAGS: (1 total)
           ----------                                                         270
      d+2  GCGACTCAAC  1       0       4       0       5       3.49e-02       69

LOCUS: AT1G07890
```

```
DESCRIPTION: Encodes a cytosolic ascorbate peroxidase. Ascorbate peroxidases scavenge hydrogen peroxide in the
cytosol and chloroplasts of plants
DATA:                  Control 30min   2hours  2days   1week   p-value         pos
SENSE COUNTS:          16      24      20      37      13      3.51e-02
GENES (4 total):
  AT1G07890.1
    SENSE COUNTS:      16      24      20      37      13      3.51e-02
    TAGS: (3 total)
      d+1  GCTGTTTTTG  16      24      16      37      12      1.13e-02        946
           ----------                                                          864
      d+2  GACATCAAAC  0       0       3       0       1       7.97e-02        680
      d+2  GAGCCAACAG  0       0       1       0       0       4.55e-01        349
           ----------                                                          266
           ----------                                                          252
           ----------                                                          16
  AT1G07890.2
    SENSE COUNTS:      16      24      20      37      13      3.51e-02
    TAGS: (3 total)
      d+1  GCTGTTTTTG  16      24      16      37      12      1.13e-02        829
           ----------                                                          747
      d+2  GACATCAAAC  0       0       3       0       1       7.97e-02        563
      d+2  GAGCCAACAG  0       0       1       0       0       4.55e-01        232
           ----------                                                          149
           ----------                                                          135
  AT1G07890.3
    SENSE COUNTS:      16      24      20      37      13      3.51e-02
    TAGS: (3 total)
      d+1  GCTGTTTTTG  16      24      16      37      12      1.13e-02        842
           ----------                                                          760
      d+2  GACATCAAAC  0       0       3       0       1       7.97e-02        576
      d+2  GAGCCAACAG  0       0       1       0       0       4.55e-01        245
           ----------                                                          162
           ----------                                                          148

LOCUS: AT1G06460
DESCRIPTION: ACD32.1 encodes an alpha-crystallin domain  containing protein with homology to small heat shock
proteins.
DATA:                  Control 30min   2hours  2days   1week   p-value         pos
SENSE COUNTS:          20      7       15      5       12      3.62e-02
GENES (2 total):
  AT1G06460.1
    SENSE COUNTS:      20      7       15      5       12      3.62e-02
    TAGS: (1 total)
           ----------                                                          1440
      d+2  TAAACAATCC  20      7       15      5       12      3.62e-02        1155
           ----------                                                          1070
           ----------                                                          484
           ----------                                                          133

LOCUS: AT4G23330
DESCRIPTION: eukaryotic translation initiation factor-related, contains weak similarity to Swiss-Prot:Q9LD55
eukaryotic translation initiation factor 3 subunit 10 (eIF-3 theta) (Eukaryotic translation initiation factor
3 large subunit) (eIF3a) (p114) (Arabidopsis thal
DATA:                  Control 30min   2hours  2days   1week   p-value         pos
SENSE COUNTS:          0       3       0       1       0       3.64e-02
GENES (1 total):
  AT4G23330.1
    SENSE COUNTS:      0       3       0       1       0       3.64e-02
    TAGS: (2 total)
           ----------                                                          1631
           ----------                                                          1480
      v+2  ATTTTCCCAA  0       3       0       0       0       3.05e-02        1458
      i+3  AGAAGTCGTC  0       0       0       1       0       6.04e-01        1248
           ----------                                                          594
           ----------                                                          194

LOCUS: AT5G16030
DESCRIPTION: expressed protein
DATA:                  Control 30min   2hours  2days   1week   p-value         pos
SENSE COUNTS:          7       13      4       5       1       3.69e-02
GENES (1 total):
  AT5G16030.1
    SENSE COUNTS:      7       13      4       5       1       3.69e-02
    TAGS: (2 total)
           ----------                                                          1247
           ----------                                                          1198
      d+2  GGTGAGATCA  5       3       3       5       1       7.49e-01        998
      d+2  TCTGCTCCTC  2       10      1       0       0       2.18e-04        495
           ----------                                                          85
           ----------                                                          63
```

```
LOCUS: AT5G66240
DESCRIPTION: transducin family protein / WD-40 repeat family protein, contains Pfam PF00400: WD domain, G-beta
repeat (4 copies, 1 weak); similar to Will die slowly protein. {Drosophila melanogaster} (SP:Q9V3J8)
{Drosophila melanogaster}
DATA:                 Control 30min  2hours  2days  1week  p-value       pos
SENSE COUNTS:         0       2      0       6      3      3.70e-02
GENES (2 total):
  AT5G66240.1
    SENSE COUNTS:     0       2      0       6      3      3.70e-02
    TAGS: (4 total)
      i+3  CACACACTCA 0       0      0       0      0      6.15e-01      1636
      d+1  AAGATAAACC 0       2      0       4      3      1.71e-01      1175
      d+2  TAAATGAGGC 0       0      0       1      0      6.04e-01      1070
      d+2  TTTGTGACAG 0       0      0       1      0      3.09e-01      968
           ----------                                                   880
           ----------                                                   876
           ----------                                                   741
           ----------                                                   725
           ----------                                                   710
           ----------                                                   411
           ----------                                                   250
           ----------                                                   119
  AT5G66240.2
    SENSE COUNTS:     0       2      0       6      3      3.70e-02
    TAGS: (4 total)
      i+3  CACACACTCA 0       0      0       0      0      6.15e-01      1642
      d+1  AAGATAAACC 0       2      0       4      3      1.71e-01      1270
      d+2  TAAATGAGGC 0       0      0       1      0      6.04e-01      1165
      d+2  TTTGTGACAG 0       0      0       1      0      3.09e-01      1063
           ----------                                                   975
           ----------                                                   971
           ----------                                                   836
           ----------                                                   820
           ----------                                                   805
           ----------                                                   506
           ----------                                                   345
           ----------                                                   214

LOCUS: AT5G14490
DESCRIPTION: no apical meristem (NAM) family protein, similar to CUC2 (GI:1944132) (Arabidopsis thaliana);
DATA:                 Control 30min  2hours  2days  1week  p-value       pos
SENSE COUNTS:         0       0      1       4      0      3.72e-02
GENES (1 total):
  AT5G14490.1
    SENSE COUNTS:     0       0      1       4      0      3.72e-02
    TAGS: (1 total)
           ----------                                                   1309
           ----------                                                   876
      v+2  CATATCAAAA 0       0      1       4      0      3.72e-02      739
           ----------                                                   515
           ----------                                                   392
           ----------                                                   34

LOCUS: AT3G15000
DESCRIPTION: expressed protein, similar to DAG protein (required for chloroplast differentiation and palisade
development) GB:Q38732 (Antirrhinum majus)
DATA:                 Control 30min  2hours  2days  1week  p-value       pos
SENSE COUNTS:         4       1      7       13     14     3.81e-02
GENES (1 total):
  AT3G15000.1
    SENSE COUNTS:     4       1      7       13     14     3.81e-02
    TAGS: (4 total)
      d+1  TCTCCCACAG 1       0      1       2      7      7.87e-02      1544
      d+2  GTTAGAACTT 3       1      4       11     7      6.93e-02      1414
      d+2  CATTCTGGGC 0       0      1       0      0      7.06e-01      1284
      d+2  GGAGGAGCAC 0       0      1       0      0      4.55e-01      1054
           ----------                                                   976
           ----------                                                   925
           ----------                                                   889
           ----------                                                   802
           ----------                                                   769
           ----------                                                   61

LOCUS: AT2G46220
DESCRIPTION: Expressed protein
DATA:                 Control 30min  2hours  2days  1week  p-value       pos
SENSE COUNTS:         9       6      0       4      1      3.81e-02
GENES (1 total):
  AT2G46220.1
    SENSE COUNTS:     9       6      0       4      1      3.81e-02
    TAGS: (3 total)
```

```
          ----------                                                  1124
      d+2  AATACATTAC 8      4      0      2      1      6.18e-02      1038
          ----------                                                  938
          ----------                                                  699
          ----------                                                  609
      X+4  ATACATAAAT 1      1      0      0      0      4.77e-01      601
          ----------                                                  563
      d+2  TGTTTATGAG 0      1      0      2      0      2.43e-01      270
          ----------                                                  135
          ----------                                                  21

LOCUS: AT5G50375
DESCRIPTION: cyclopropyl isomerase (CPI1)
DATA:                 Control 30min  2hours  2days  1week  p-value       pos
SENSE COUNTS:         2      0      7      2      3      3.85e-02
GENES (2 total):
  AT5G50375.1
    SENSE COUNTS:     2      0      7      2      3      3.85e-02
    TAGS: (4 total)
      d+1  TTTCTAAGGC 1      0      0      0      0      4.28e-01      1153
      d+2  GGACTGAGAG 0      0      1      0      0      4.55e-01      1078
          ----------                                                  1020
      i+3  ACATTTTGGC 0      0      3      1      0      9.46e-02      964
          ----------                                                  793
          ----------                                                  702
          ----------                                                  596
      d+2  GAAAATGAAT 1      0      3      1      3      3.10e-01      552
          ----------                                                  397

LOCUS: AT5G03360
DESCRIPTION: DC1 domain-containing protein, contains Pfam profile PF03107: DC1 domain
DATA:                 Control 30min  2hours  2days  1week  p-value       pos
SENSE COUNTS:         1      3      0      0      0      3.89e-02
GENES (1 total):
  AT5G03360.1
    SENSE COUNTS:     1      3      0      0      0      3.89e-02
    TAGS: (2 total)
          ----------                                                  5562
          ----------                                                  5497
          ----------                                                  5110
          ----------                                                  4936
          ----------                                                  4900
          ----------                                                  4674
          ----------                                                  4667
          ----------                                                  4573
          ----------                                                  4279
          ----------                                                  3892
          ----------                                                  3332
          ----------                                                  2496
      v+2  AAACCTTATT 0      0      0      0      0      6.15e-01      2292
          ----------                                                  2178
          ----------                                                  2131
          ----------                                                  2027
          ----------                                                  1948
          ----------                                                  1774
      v+2  TGCGAATCTT 1      3      0      0      0      1.03e-01      1736
          ----------                                                  1729
          ----------                                                  1667
          ----------                                                  1183
          ----------                                                  958
          ----------                                                  534
          ----------                                                  235

LOCUS: AT1G15390
DESCRIPTION: peptide deformylase, mitochondrial / polypeptide deformylase 1A (PDF1A), nearly identical to
SP|Q9FV53 Peptide deformylase, mitochondrial precursor (EC 3.5.1.88) (PDF) (Polypeptide deformylase)
{Arabidopsis thaliana}; contains Pfam profile PF01327: polype
DATA:                 Control 30min  2hours  2days  1week  p-value       pos
SENSE COUNTS:         14     3      9      9      1      3.90e-02
GENES (2 total):
  AT1G15390.1
    SENSE COUNTS:     14     3      9      9      1      3.90e-02
    TAGS: (2 total)
      d+1  AGTGTGATCA 2      0      1      0      0      5.71e-01      716
      i+3  GTAAATAATA 12     3      8      9      1      6.65e-02      606
          ----------                                                  526
          ----------                                                  114
          ----------                                                  58

LOCUS: AT5G44580
DESCRIPTION: expressed protein
```

```
DATA:                 Control 30min  2hours  2days  1week  p-value      pos
SENSE COUNTS:         11      22     13      27     10     3.92e-02
GENES (1 total):
  AT5G44580.1
    SENSE COUNTS:     11      22     13      27     10     3.92e-02
    TAGS: (3 total)
      X+4  TACTAGACAC 2       0      1       1      3      4.10e-01     364
      d+1  GTGGGGGTCG 9       22     11      26     7      5.63e-03     288
      d+2  AACTGTTCTT 0       0      1       0      0      4.55e-01     123

LOCUS: AT3G10430
DESCRIPTION: F-box family protein, contains F-box domain Pfam:PF00646
DATA:                 Control 30min  2hours  2days  1week  p-value      pos
SENSE COUNTS:         9       8      8       4      21     4.04e-02
GENES (1 total):
  AT3G10430.1
    SENSE COUNTS:     9       8      8       4      21     4.04e-02
    TAGS: (2 total)
           ----------                                                   1939
      v+2  AATAATGAAA 9       7      8       4      21     2.73e-02     1522
           ----------                                                   1387
           ----------                                                   1286
           ----------                                                   1138
           ----------                                                   648
           ----------                                                   480
           ----------                                                   354
      v+2  GTTAAAGCTT 0       1      0       0      0      2.54e-01     147

LOCUS: AT3G20230
DESCRIPTION: 50S ribosomal protein L18 family, similar to ribosomal protein L18 (GI:29342325) (Enterococcus
faecalis V583); similar to 50S ribosomal protein L18 (Swiss-Prot:O24704) (Synechococcus sp.)
DATA:                 Control 30min  2hours  2days  1week  p-value      pos
SENSE COUNTS:         11      0      6       4      8      4.07e-02
GENES (1 total):
  AT3G20230.1
    SENSE COUNTS:     11      0      6       4      8      4.07e-02
    TAGS: (2 total)
           ----------                                                   953
      d+2  TATCTAATCA 9       0      3       2      7      4.58e-02     891
           ----------                                                   787
      d+2  GCATTATTTT 2       0      3       2      1      4.79e-01     624
           ----------                                                   344
           ----------                                                   338
           ----------                                                   319
           ----------                                                   178

LOCUS: AT3G55250
DESCRIPTION: expressed protein, predicted pectate-lyase, Arabidopsis thaliana, PIR:T06728
DATA:                 Control 30min  2hours  2days  1week  p-value      pos
SENSE COUNTS:         2       1      2       5      9      4.10e-02
GENES (1 total):
  AT3G55250.1
    SENSE COUNTS:     2       1      2       5      9      4.10e-02
    TAGS: (2 total)
      d+1  AGCAAGTAAT 2       0      0       4      1      1.27e-01     931
      d+2  TTTACTTTTT 0       1      2       1      8      1.30e-02     875
           ----------                                                   17

LOCUS: AT5G53880
DESCRIPTION: expressed protein
DATA:                 Control 30min  2hours  2days  1week  p-value      pos
SENSE COUNTS:         9       12     2       3      11     4.11e-02
GENES (1 total):
  AT5G53880.1
    SENSE COUNTS:     9       12     2       3      11     4.11e-02
    TAGS: (2 total)
      d+1  TTATTTTTAT 8       2      1       2      10     2.77e-02     396
      d+2  AAACCAAAGC 1       10     1       1      1      1.01e-03     157
           ----------                                                   135

LOCUS: AT3G16200
DESCRIPTION: expressed protein
DATA:                 Control 30min  2hours  2days  1week  p-value      pos
SENSE COUNTS:         2       9      3       6      0      4.13e-02
GENES (1 total):
  AT3G16200.1
    SENSE COUNTS:     2       9      3       6      0      4.13e-02
    TAGS: (1 total)
           ----------                                                   1064
      i+3  ATAATGATTT 2       9      3       6      0      4.13e-02     782
           ----------                                                   772
```

```
          ----------                                                    537
          ----------                                                    403

LOCUS: AT5G32450
DESCRIPTION: RNA recognition motif (RRM)-containing protein, various predicted proteins, Arabidopsis thaliana
and others
DATA:                 Control 30min  2hours  2days  1week  p-value       pos
SENSE COUNTS:         3       7      4       0      0      4.13e-02
GENES (1 total):
  AT5G32450.1
    SENSE COUNTS:     3       7      4       0      0      4.13e-02
    TAGS: (2 total)
      d+1  GTCGTTGTTT 0       4      2       0      0      6.74e-02      1105
      d+2  TTTCTCATTA 3       3      2       0      0      3.51e-01      1052
           ----------                                                  423

LOCUS: AT4G08290
DESCRIPTION: nodulin MtN21 family protein, similar to MtN21 GI:2598575 (root nodule development) from
(Medicago truncatula)
DATA:                 Control 30min  2hours  2days  1week  p-value       pos
SENSE COUNTS:         5       5      12      11     0      4.17e-02
GENES (2 total):
  AT4G08290.1
    SENSE COUNTS:     5       5      12      11     0      4.17e-02
    TAGS: (2 total)
      d+1  AAATTATTTT 2       3      7       0      0      3.22e-02      1431
      d+2  AACAAATCCA 3       2      5       11     0      3.06e-02      1096
           ----------                                                  1059
           ----------                                                  513
           ----------                                                  483
           ----------                                                  186
           ----------                                                  68
           ----------                                                  35
  AT4G08290.2
    SENSE COUNTS:     5       5      12      11     0      4.17e-02
    TAGS: (2 total)
      d+1  AAATTATTTT 2       3      7       0      0      3.22e-02      1567
      d+2  AACAAATCCA 3       2      5       11     0      3.06e-02      1149
           ----------                                                  1112
           ----------                                                  492
           ----------                                                  462
           ----------                                                  165
           ----------                                                  47
           ----------                                                  14

LOCUS: AT5G56670
DESCRIPTION: 40S ribosomal protein S30 (RPS30C),
DATA:                 Control 30min  2hours  2days  1week  p-value       pos
SENSE COUNTS:         3       2      6       8      14     4.25e-02
GENES (1 total):
  AT5G56670.1
    SENSE COUNTS:     3       2      6       8      14     4.25e-02
    TAGS: (1 total)
           ----------                                                  1019
           ----------                                                  956
      v+2  GGAAAGGTTC 3       2      6       8      14     4.25e-02      354

LOCUS: AT1G49970
DESCRIPTION: ATP-dependent Clp protease proteolytic subunit (ClpR1) (nClpP5), identical to nClpP5 GB:BAA82069
GI:5360595 from (Arabidopsis thaliana); identical to cDNA nClpP5 (nuclear encoded ClpP5) GI:5360594
DATA:                 Control 30min  2hours  2days  1week  p-value       pos
SENSE COUNTS:         9       3      6       4      16     4.28e-02
GENES (2 total):
  AT1G49970.1
    SENSE COUNTS:     9       3      6       4      16     4.28e-02
    TAGS: (2 total)
           ----------                                                  1677
           ----------                                                  1486
      d+2  TGGTAGTAAC 9       3      5       4      16     3.31e-02      1401
      d+2  TGGATAAAGG 0       0      1       0      0      4.55e-01      1042
           ----------                                                  916
           ----------                                                  706
           ----------                                                  592
           ----------                                                  454
           ----------                                                  268
           ----------                                                  238
           ----------                                                  194

LOCUS: AT3G56310
DESCRIPTION: alpha-galactosidase, putative / melibiase, putative / alpha-D-galactoside galactohydrolase,
putative, similar to alpha-galactosidase SP:Q42656 from (Coffea arabica)
```

```
DATA:                  Control 30min   2hours  2days   1week   p-value         pos
SENSE COUNTS:          1       6       10      2       1       4.36e-02
GENES (2 total):
  AT3G56310.1
    SENSE COUNTS:      1       6       10      2       1       4.36e-02
    TAGS: (2 total)
           ----------                                                          1861
           ----------                                                          1774
      d+2  GGATATGATC  1       6       10      2       1       4.76e-02        1232
           ----------                                                          1032
           ----------                                                          842
           ----------                                                          607
      i+3  TTTCATCTGA  0       0       0       0       0       6.15e-01        605
           ----------                                                          418
  AT3G56310.2
    SENSE COUNTS:      1       6       10      2       1       4.36e-02
    TAGS: (2 total)
           ----------                                                          1765
           ----------                                                          1678
      d+2  GGATATGATC  1       6       10      2       1       4.76e-02        1136
           ----------                                                          936
           ----------                                                          746
      i+3  TTTCATCTGA  0       0       0       0       0       6.15e-01        581
           ----------                                                          511
           ----------                                                          394

LOCUS: AT3G06730
DESCRIPTION: thioredoxin family protein, contains Pfam profile: PF00085 Thioredoxin
DATA:                  Control 30min   2hours  2days   1week   p-value         pos
SENSE COUNTS:          0       5       1       1       0       4.37e-02
GENES (1 total):
  AT3G06730.1
    SENSE COUNTS:      0       5       1       1       0       4.37e-02
    TAGS: (1 total)
           ----------                                                          845
      d+2  CAGGTTCGAG  0       5       1       1       0       4.37e-02        475
           ----------                                                          357
           ----------                                                          116

LOCUS: AT2G23760
DESCRIPTION: BEL1-like homeobox 4 protein (BLH4)
DATA:                  Control 30min   2hours  2days   1week   p-value         pos
SENSE COUNTS:          1       1       1       6       0       4.43e-02
GENES (3 total):
  AT2G23760.2
    SENSE COUNTS:      1       1       1       6       0       4.43e-02
    TAGS: (1 total)
           ----------                                                          2293
      d+2  GAGACCGCAA  1       1       1       6       0       4.43e-02        1452
           ----------                                                          856
           ----------                                                          564
  AT2G23760.1
    SENSE COUNTS:      1       1       1       6       0       4.43e-02
    TAGS: (1 total)
           ----------                                                          2203
      d+2  GAGACCGCAA  1       1       1       6       0       4.43e-02        1362
           ----------                                                          766
           ----------                                                          474

LOCUS: AT4G08685
DESCRIPTION: pollen Ole e 1 allergen and extensin family protein, contains Pfam domain, PF01190: Pollen
proteins Ole e I family
DATA:                  Control 30min   2hours  2days   1week   p-value         pos
SENSE COUNTS:          5       0       3       1       0       4.50e-02
GENES (2 total):
  AT4G08685.1
    SENSE COUNTS:      5       0       3       1       0       4.50e-02
    TAGS: (1 total)
           ----------                                                          903
           ----------                                                          809
      d+2  AAGCTATACC  5       0       3       1       0       4.50e-02        588
           ----------                                                          543
           ----------                                                          475
           ----------                                                          403

LOCUS: AT4G19620
DESCRIPTION: hypothetical protein,
DATA:                  Control 30min   2hours  2days   1week   p-value         pos
SENSE COUNTS:          0       0       2       6       3       4.55e-02
GENES (1 total):
  AT4G19620.1
```

```
    SENSE COUNTS:     0      0      2      6      3      4.55e-02
    TAGS: (1 total)
           ----------                                                 1175
           ----------                                                 1117
           ----------                                                 1052
           ----------                                                 827
           ----------                                                 702
           ----------                                                 265
      v+2  AACAAAAAAT 0      0      2      6      3      4.55e-02       21

LOCUS: AT3G22150
DESCRIPTION: pentatricopeptide (PPR) repeat-containing protein, contains INTERPRO:IPR002885 PPR repeats
DATA:                 Control 30min  2hours 2days  1week  p-value        pos
SENSE COUNTS:         0      1      3      8      2      4.58e-02
GENES (1 total):
  AT3G22150.1
    SENSE COUNTS:     0      1      3      8      2      4.58e-02
    TAGS: (2 total)
      X+4  ACTGGAATCG 0      1      2      4      1      4.60e-01       2732
           ----------                                                 1862
           ----------                                                 1795
      d+2  GTTTCTCTAT 0      0      1      4      1      9.69e-02       1694
           ----------                                                 1447
           ----------                                                 1198
           ----------                                                 1085

LOCUS: AT4G03770
DESCRIPTION: hypothetical protein,
DATA:                 Control 30min  2hours 2days  1week  p-value        pos
SENSE COUNTS:         6      0      3      8      7      4.63e-02
GENES (2 total):
  AT4G03770.2
    SENSE COUNTS:     6      0      3      8      7      4.63e-02
    TAGS: (1 total)
           ----------                                                 5655
           ----------                                                 5495
           ----------                                                 4838
           ----------                                                 4776
           ----------                                                 4631
           ----------                                                 4600
           ----------                                                 4219
           ----------                                                 3860
           ----------                                                 3759
      p+2  ACAAAAAAAG 6      0      3      8      7      4.63e-02       3138
           ----------                                                 3123
           ----------                                                 2952
           ----------                                                 2923
           ----------                                                 2341
           ----------                                                 2293
           ----------                                                 2178
           ----------                                                 2011
           ----------                                                 1307
           ----------                                                 1181
           ----------                                                 667

LOCUS: AT3G12020
DESCRIPTION: kinesin motor protein-related, similar to putative kinesin heavy chain GB:AAD23684 GI:4567271
from (Arabidopsis thaliana)
DATA:                 Control 30min  2hours 2days  1week  p-value        pos
SENSE COUNTS:         10     5      9      1      16     4.70e-02
GENES (1 total):
  AT3G12020.1
    SENSE COUNTS:     10     5      9      1      16     4.70e-02
    TAGS: (3 total)
           ----------                                                 3895
           ----------                                                 3787
      i+3  TCCTTTTAAA 8      3      8      1      16     1.32e-02       3302
      v+2  ACAGCCCATC 2      2      0      0      0      2.42e-01       3172
           ----------                                                 2795
           ----------                                                 2493
           ----------                                                 2467
           ----------                                                 2212
      v+2  AGATTAGAGA 0      0      1      0      0      4.55e-01       1951
           ----------                                                 1902
           ----------                                                 1420
           ----------                                                 1363
           ----------                                                 1102
           ----------                                                 1063
           ----------                                                 936
           ----------                                                 832
           ----------                                                 748
```

```
           ----------                                               626
           ----------                                               139
           ----------                                               96

LOCUS: AT4G29060
DESCRIPTION: elongation factor Ts family protein, similar to SP|P35019 Elongation factor Ts (EF-Ts) {Galdieria
sulphuraria}; contains Pfam profiles PF00627: UBA/TS-N domain, PF00889: Elongation factor TS, PF00575: S1 RNA
binding domain
DATA:                  Control 30min  2hours  2days  1week  p-value    pos
SENSE COUNTS:          39      17     34      34     36     4.77e-02
GENES (1 total):
  AT4G29060.1
    SENSE COUNTS:      39      17     34      34     36     4.77e-02
    TAGS: (3 total)
      d+1  AAAATATGAG  35      11     25      29     34     5.94e-03   3249
      d+2  GAGTTTAAAG  4       0      8       1      1      2.50e-02   3149
      d+2  ATGATGGGTG  0       6      1       4      1      8.00e-02   1099
           ----------                                               728
           ----------                                               422

LOCUS: AT2G43180
DESCRIPTION: expressed protein,
DATA:                  Control 30min  2hours  2days  1week  p-value    pos
SENSE COUNTS:          4       2      3       2      12     4.79e-02
GENES (4 total):
  AT2G43180.1
    SENSE COUNTS:      4       2      3       2      12     4.79e-02
    TAGS: (2 total)
           ----------                                               2208
           ----------                                               2202
           ----------                                               2148
           ----------                                               2082
           ----------                                               2076
           ----------                                               2070
           ----------                                               2064
           ----------                                               1998
      d+2  TTTGGCTTGA  3       2      2       2      12     2.07e-02   1904
      d+2  GCGTCTTTAG  1       0      1       0      0      6.01e-01   1021
           ----------                                               505
           ----------                                               294
  AT2G43180.2
    SENSE COUNTS:      4       2      3       2      12     4.79e-02
    TAGS: (2 total)
           ----------                                               2237
           ----------                                               2231
           ----------                                               2177
           ----------                                               2111
           ----------                                               2105
           ----------                                               2099
           ----------                                               2093
           ----------                                               2027
      d+2  TTTGGCTTGA  3       2      2       2      12     2.07e-02   1933
      d+2  GCGTCTTTAG  1       0      1       0      0      6.01e-01   1021
           ----------                                               505
           ----------                                               294
  AT2G43180.3
    SENSE COUNTS:      4       2      3       2      12     4.79e-02
    TAGS: (2 total)
           ----------                                               2205
           ----------                                               2199
           ----------                                               2145
           ----------                                               2079
           ----------                                               2073
           ----------                                               2067
           ----------                                               2061
           ----------                                               1995
      d+2  TTTGGCTTGA  3       2      2       2      12     2.07e-02   1901
      d+2  GCGTCTTTAG  1       0      1       0      0      6.01e-01   1021
           ----------                                               505
           ----------                                               294
  AT2G43180.4
    SENSE COUNTS:      4       2      3       2      12     4.79e-02
    TAGS: (2 total)
           ----------                                               2282
           ----------                                               2276
           ----------                                               2222
           ----------                                               2156
           ----------                                               2150
           ----------                                               2144
           ----------                                               2138
           ----------                                               2072
```

```
      d+2  TTTGGCTTGA 3       2       2       2       12      2.07e-02        1978
      d+2  GCGTCTTTAG 1       0       1       0       0       6.01e-01        1021
           ----------                                                         505
           ----------                                                         294

LOCUS: AT2G33040
DESCRIPTION: ATP synthase gamma chain, mitochondrial (ATPC), identical to SP|Q96250 ATP synthase gamma chain,
mitochondrial precursor (EC 3.6.3.14) {Arabidopsis thaliana}; contains Pfam profile: PF00231 ATP synthase
DATA:                 Control 30min   2hours  2days   1week   p-value         pos
SENSE COUNTS:         5       11      21      16      15      4.88e-02
GENES (1 total):
  AT2G33040.1
    SENSE COUNTS:     5       11      21      16      15      4.88e-02
    TAGS: (3 total)
      d+1  GACAGCTCAA 0       5       8       4       1       5.64e-02        977
           ----------                                                         946
      X+4  TACTCCGGTT 5       6       12      12      14      2.55e-01        451
      d+2  AAGAGTGTTA 0       0       1       0       0       4.55e-01        290

LOCUS: AT1G27020
DESCRIPTION: expressed protein
DATA:                 Control 30min   2hours  2days   1week   p-value         pos
SENSE COUNTS:         0       4       1       0       3       4.92e-02
GENES (1 total):
  AT1G27020.1
    SENSE COUNTS:     0       4       1       0       3       4.92e-02
    TAGS: (3 total)
      d+1  TAATATCTTT 0       2       0       0       0       9.14e-02        1189
      d+2  GTGGGGTGTG 0       2       1       0       0       2.47e-01        1157
      d+2  GACGCTTGTC 0       0       0       0       3       1.12e-02        599
           ----------                                                         285
           ----------                                                         54

LOCUS: AT5G01160
DESCRIPTION: e-cadherin binding protein-related, contains weak similarity to E-cadherin binding protein E7
(Mus musculus GP|9622093|gb|AAF89617
DATA:                 Control 30min   2hours  2days   1week   p-value         pos
SENSE COUNTS:         1       0       2       2       6       4.93e-02
GENES (1 total):
  AT5G01160.1
    SENSE COUNTS:     1       0       2       2       6       4.93e-02
    TAGS: (2 total)
      d+1  TAATTCGAGT 1       0       1       1       1       7.91e-01        1377
      d+2  GAATCCCGGA 0       0       1       1       5       3.15e-02        1167
           ----------                                                         638
           ----------                                                         629
           ----------                                                         623
           ----------                                                         464

LOCUS: AT1G52590
DESCRIPTION: expressed protein
DATA:                 Control 30min   2hours  2days   1week   p-value         pos
SENSE COUNTS:         6       0       2       6       3       4.94e-02
GENES (1 total):
  AT1G52590.1
    SENSE COUNTS:     6       0       2       6       3       4.94e-02
    TAGS: (2 total)
      d+1  TACTCGTCAA 5       0       0       5       0       5.26e-03        703
      d+2  TGAGCTCTAA 1       0       2       1       3       4.23e-01        552
           ----------                                                         424
           ----------                                                         269
           ----------                                                         211

LOCUS: AT5G14130
DESCRIPTION: peroxidase, putative, identical to peroxidase ATP20a (Arabidopsis thaliana)
gi|1546694|emb|CAA67338
DATA:                 Control 30min   2hours  2days   1week   p-value         pos
SENSE COUNTS:         2       7       4       0       0       5.03e-02
GENES (1 total):
  AT5G14130.1
    SENSE COUNTS:     2       7       4       0       0       5.03e-02
    TAGS: (1 total)
           ----------                                                         1520
      d+2  GTAGATGTTG 2       7       4       0       0       5.03e-02        1256
           ----------                                                         700
           ----------                                                         616
           ----------                                                         420
           ----------                                                         162
           ----------                                                         112

LOCUS: AT3G63490
```

```
DESCRIPTION: ribosomal protein L1 family protein, ribosomal protein L1, S.oleracea, EMBL:SORPL1
DATA:                 Control 30min   2hours  2days   1week   p-value         pos
SENSE COUNTS:         29      10      27      27      18      5.04e-02
GENES (2 total):
  AT3G63490.1
    SENSE COUNTS:     29      10      27      27      18      5.04e-02
    TAGS: (4 total)
           ----------                                                         1907
      d+2  TGATTTACTA 2       0       0       0       0       3.22e-01        1387
      d+2  TCAGGACGCA 27      9       25      26      17      2.54e-02        1291
      d+2  CCAAATCCCA 0       1       2       0       1       5.32e-01        790
      d+2  GAGTTTGACA 0       0       0       1       0       3.09e-01        706
           ----------                                                         88
  AT3G63490.2
    SENSE COUNTS:     29      10      27      27      18      5.04e-02
    TAGS: (4 total)
           ----------                                                         1897
      d+2  TGATTTACTA 2       0       0       0       0       3.22e-01        1377
      d+2  TCAGGACGCA 27      9       25      26      17      2.54e-02        1281
      d+2  CCAAATCCCA 0       1       2       0       1       5.32e-01        790
      d+2  GAGTTTGACA 0       0       0       1       0       3.09e-01        706
           ----------                                                         88

LOCUS: AT5G27630
DESCRIPTION: acyl-CoA binding family protein, similar to RING finger rngB protein, cytosolic - Dictyostelium
discoideum, PIR:S68824; contains Pfam profiles PF01344: Kelch motif, PF00887: Acyl CoA binding protein (ACBP)
DATA:                 Control 30min   2hours  2days   1week   p-value         pos
SENSE COUNTS:         1       0       4       5       6       5.05e-02
GENES (1 total):
  AT5G27630.1
    SENSE COUNTS:     1       0       4       5       6       5.05e-02
    TAGS: (3 total)
           ----------                                                         2590
      d+2  AAGGAACCAA 0       0       4       4       5       5.16e-02        2360
           ----------                                                         2313
           ----------                                                         2035
           ----------                                                         2025
           ----------                                                         1957
           ----------                                                         1412
           ----------                                                         1383
           ----------                                                         1184
           ----------                                                         1130
           ----------                                                         1075
           ----------                                                         927
      i+3  TTTACTAGTT 1       0       0       0       1       3.83e-01        916
           ----------                                                         892
           ----------                                                         837
           ----------                                                         719
           ----------                                                         644
           ----------                                                         437
           ----------                                                         334
      d+2  GTGAGAGCGA 0       0       0       1       0       3.09e-01        91

LOCUS: AT3G09450
DESCRIPTION: hypothetical protein
DATA:                 Control 30min   2hours  2days   1week   p-value         pos
SENSE COUNTS:         3       2       2       3       12      5.06e-02
GENES (1 total):
  AT3G09450.1
    SENSE COUNTS:     3       2       2       3       12      5.06e-02
    TAGS: (4 total)
      v+1  TTGAGTCTGA 0       0       0       0       0       6.15e-01        3163
           ----------                                                         2986
      v+2  TAAATCCAAA 2       2       1       0       0       4.77e-01        2912
      v+2  CTACTGAAGG 1       0       1       2       12      4.19e-05        2523
           ----------                                                         2469
           ----------                                                         2425
           ----------                                                         2308
           ----------                                                         2104
           ----------                                                         1782
           ----------                                                         1683
           ----------                                                         1464
           ----------                                                         1303
           ----------                                                         1200
           ----------                                                         1132
           ----------                                                         1067
           ----------                                                         1054
           ----------                                                         954
           ----------                                                         805
           ----------                                                         792
      i+3  TAAGCCAACT 0       0       0       1       0       3.09e-01        787
```

```
           ----------                                                      784
           ----------                                                      405
           ----------                                                      368
           ----------                                                      34

LOCUS: AT3G06070
DESCRIPTION: expressed protein
DATA:                 Control 30min  2hours  2days  1week  p-value        pos
SENSE COUNTS:         1       0      3       5      0      5.13e-02
GENES (1 total):
  AT3G06070.1
    SENSE COUNTS:     1       0      3       5      0      5.13e-02
    TAGS: (1 total)
           ----------                                                      735
      d+2  GATTTGTGTC 1       0      3       5      0      5.13e-02        559
           ----------                                                      481
           ----------                                                      424
           ----------                                                      418
           ----------                                                      87

LOCUS: AT4G08350
DESCRIPTION: KOW domain-containing transcription factor family protein, chromatin structural protein homolog
Supt5hp - Mus musculus,PID:g2754752
DATA:                 Control 30min  2hours  2days  1week  p-value        pos
SENSE COUNTS:         0       3      0       2      0      5.16e-02
GENES (1 total):
  AT4G08350.1
    SENSE COUNTS:     0       3      0       2      0      5.16e-02
    TAGS: (2 total)
           ----------                                                      3778
           ----------                                                      3720
      v+2  CCCGATATTT 0       3      0       2      0      1.05e-01        3126
           ----------                                                      3119
           ----------                                                      3087
           ----------                                                      3009
           ----------                                                      2950
           ----------                                                      2745
           ----------                                                      2725
           ----------                                                      2425
           ----------                                                      2267
      v+2  CTGGATTTAT 0       0      0       0      0      6.15e-01        2236
           ----------                                                      1945
           ----------                                                      1885
           ----------                                                      1842
           ----------                                                      1825
           ----------                                                      1801
           ----------                                                      1632
           ----------                                                      1458
           ----------                                                      1425
           ----------                                                      1368
           ----------                                                      1083
           ----------                                                      1060
           ----------                                                      832
           ----------                                                      94

LOCUS: AT5G26160
DESCRIPTION: expressed protein,
DATA:                 Control 30min  2hours  2days  1week  p-value        pos
SENSE COUNTS:         0       0      3       0      3      5.23e-02
GENES (1 total):
  AT5G26160.1
    SENSE COUNTS:     0       0      3       0      3      5.23e-02
    TAGS: (1 total)
           ----------                                                      3922
           ----------                                                      3887
           ----------                                                      3520
           ----------                                                      3490
           ----------                                                      3422
      v+2  GTGGAAAATG 0       0      3       0      3      5.23e-02        3275
           ----------                                                      3020
           ----------                                                      2646
           ----------                                                      2604
           ----------                                                      2377
           ----------                                                      2226
           ----------                                                      2178
           ----------                                                      1716
           ----------                                                      1402
           ----------                                                      878
           ----------                                                      808
           ----------                                                      640
           ----------                                                      566
```

```
LOCUS: ATCG00300
DESCRIPTION: encodes PsbZ, which is a subunit of photosystem II. In Chlamydomonas, this protein has been shown
to be essential in the interaction between PS II and the light harvesting complex II.
DATA:                  Control 30min   2hours  2days   1week   p-value         pos
SENSE COUNTS:          0       0       3       0       3       5.23e-02
GENES (1 total):
  ATCG00300.1
    SENSE COUNTS:      0       0       3       0       3       5.23e-02
    TAGS: (2 total)
      X+4  ATAAAATCCC  0       0       1       0       0       4.55e-01        -90
      X+4  TCCGAATAGA  0       0       2       0       3       8.60e-02        -155

LOCUS: AT4G36790
DESCRIPTION: transporter-related, low similarity to spinster membrane proteins from (Drosophila melanogaster)
GI:12003976,  GI:12003972, GI:12003974, GI:12003970; contains Pfam profile PF00083: major facilitator
superfamily protein
DATA:                  Control 30min   2hours  2days   1week   p-value         pos
SENSE COUNTS:          6       5       6       11      19      5.24e-02
GENES (1 total):
  AT4G36790.1
    SENSE COUNTS:      6       5       6       11      19      5.24e-02
    TAGS: (2 total)
      d+1  TTGTGATTCT  0       0       1       0       0       7.06e-01        1681
           ----------                                                          1673
           ----------                                                          1209
           ----------                                                          351
           ----------                                                          282
           ----------                                                          199
           ----------                                                          51
      X+4  TAACGATCCA  6       5       5       11      19      2.38e-02        -114

LOCUS: AT5G54680
DESCRIPTION: basic helix-loop-helix (bHLH) family protein, similar to unknown protein (pir |B71406)
DATA:                  Control 30min   2hours  2days   1week   p-value         pos
SENSE COUNTS:          10      2       7       15      7       5.27e-02
GENES (1 total):
  AT5G54680.1
    SENSE COUNTS:      10      2       7       15      7       5.27e-02
    TAGS: (1 total)
           ----------                                                          1362
           ----------                                                          1249
      d+2  TAAATTTAAG  10      2       7       15      7       5.27e-02        1115
           ----------                                                          893
           ----------                                                          859
           ----------                                                          847
           ----------                                                          724
           ----------                                                          565
           ----------                                                          444

LOCUS: AT4G22970
DESCRIPTION: peptidase C50 family protein, contains Pfam PF03568: Peptidase family C50
DATA:                  Control 30min   2hours  2days   1week   p-value         pos
SENSE COUNTS:          10      11      8       1       1       5.30e-02
GENES (1 total):
  AT4G22970.1
    SENSE COUNTS:      10      11      8       1       1       5.30e-02
    TAGS: (2 total)
      d+1  ATGTATTGTC  1       0       0       0       0       4.28e-01        5525
           ----------                                                          4992
           ----------                                                          4901
           ----------                                                          4877
           ----------                                                          4703
           ----------                                                          4633
           ----------                                                          4535
           ----------                                                          4499
           ----------                                                          4475
           ----------                                                          4077
           ----------                                                          4000
           ----------                                                          3739
           ----------                                                          3095
           ----------                                                          3072
           ----------                                                          3023
           ----------                                                          2598
      d+2  ATTTACATAA  9       11      8       1       1       6.04e-02        2240
           ----------                                                          2169
           ----------                                                          2096
           ----------                                                          1783
           ----------                                                          1748
           ----------                                                          1575
           ----------                                                          1428
```

```
          ----------                                                  1209
          ----------                                                  1105
          ----------                                                  877

LOCUS: AT2G24860
DESCRIPTION: chaperone protein dnaJ-related, similar to Tsi1-interacting protein TSIP1 (GI:4337001) (Nicotiana
tabacum)
DATA:                 Control 30min   2hours  2days   1week   p-value        pos
SENSE COUNTS:         6       0       1       1       3       5.35e-02
GENES (1 total):
  AT2G24860.1
    SENSE COUNTS:     6       0       1       1       3       5.35e-02
    TAGS: (3 total)
      d+1  TGCATCTTCC 0       0       0       0       1       1.65e-01       843
      X+4  TCTAGAGTGT 0       0       0       0       1       1.65e-01       645
      d+2  TGTTAAGTAT 6       0       1       1       1       4.80e-02       642
           ----------                                                  613
           ----------                                                  428
           ----------                                                  396
           ----------                                                  343
           ----------                                                  149
           ----------                                                  105

LOCUS: AT1G47370
DESCRIPTION: Toll-Interleukin-Resistance (TIR) domain-containing protein, domain signature TIR exists,
suggestive of a disease resistance protein.
DATA:                 Control 30min   2hours  2days   1week   p-value        pos
SENSE COUNTS:         0       0       2       1       5       5.35e-02
GENES (1 total):
  AT1G47370.1
    SENSE COUNTS:     0       0       2       1       5       5.35e-02
    TAGS: (1 total)
           ----------                                                  1846
      v+2  TTTTAGGAAA 0       0       2       1       5       5.35e-02       1052
           ----------                                                  480
           ----------                                                  433
           ----------                                                  205

LOCUS: AT3G54050
DESCRIPTION: fructose-1,6-bisphosphatase, putative / D-fructose-1,6-bisphosphate 1-phosphohydrolase, putative
/ FBPase, putative, strong similarity to fructose-1,6-bisphosphatase (Brassica napus) GI:289367; identical to
SP|P25851 Fructose-1,6-bisphosphatase, chloropla
DATA:                 Control 30min   2hours  2days   1week   p-value        pos
SENSE COUNTS:         22      12      22      17      33      5.40e-02
GENES (1 total):
  AT3G54050.1
    SENSE COUNTS:     22      12      22      17      33      5.40e-02
    TAGS: (2 total)
      d+1  TAATACTAGT 0       0       0       1       3       6.27e-02       1503
      d+2  GTATATGCAA 22      12      22      16      30      1.16e-01       1351
           ----------                                                  1299

LOCUS: AT1G06780
DESCRIPTION: glycosyl transferase family 8 protein, contains Pfam profile: PF01501 glycosyl transferase family
8
DATA:                 Control 30min   2hours  2days   1week   p-value        pos
SENSE COUNTS:         2       0       0       4       0       5.40e-02
GENES (1 total):
  AT1G06780.1
    SENSE COUNTS:     2       0       0       4       0       5.40e-02
    TAGS: (2 total)
           ----------                                                  2115
      d+2  AAGCCGTGGT 1       0       0       4       0       3.65e-02       1934
           ----------                                                  1854
      d+2  GGCTTTCGGG 1       0       0       0       0       4.28e-01       1678
           ----------                                                  1642
           ----------                                                  1503
           ----------                                                  1263
           ----------                                                  977
           ----------                                                  905
           ----------                                                  456
           ----------                                                  269

LOCUS: AT3G56260
DESCRIPTION: expressed protein,
DATA:                 Control 30min   2hours  2days   1week   p-value        pos
SENSE COUNTS:         0       0       0       2       3       5.41e-02
GENES (1 total):
  AT3G56260.1
    SENSE COUNTS:     0       0       0       2       3       5.41e-02
    TAGS: (3 total)
```

```
      d+1  TCTAAGTAGA 0       0       0       1       0       3.09e-01        936
      d+2  AATAAAGCTT 0       0       0       0       3       1.12e-02        771
           ----------                                                         736
      i+3  TTCAGTGATC 0       0       0       1       0       3.09e-01        479

LOCUS: AT1G09430
DESCRIPTION: Encodes subunit A of the heteromeric enzyme ATP citrate lyase (ACL).  In animals, ACL is encoded
by a single gene; ACL in Arabidopsis is composed of two polypeptides, ACLA (encoded by 3 genes) and ACLB
(encoded by 2 genes).  The holoenzyme has an A(4)B(4)
DATA:                 Control 30min   2hours  2days   1week   p-value         pos
SENSE COUNTS:         4       9       1       4       1       5.44e-02
GENES (2 total):
  AT1G09430.1
    SENSE COUNTS:     4       9       1       4       1       5.44e-02
    TAGS: (1 total)
           ----------                                                         1664
      d+2  TCCTTCATCT 4       9       1       4       1       5.44e-02        1396
           ----------                                                         1292
           ----------                                                         786
           ----------                                                         372
           ----------                                                         178

LOCUS: AT5G48930
DESCRIPTION: transferase family protein, similar to anthranilate N-hydroxycinnamoyl/benzoyltransferase from
Dianthus caryophyllus (GI:3288180, GI:2239091); contains Pfam profile PF02458 transferase family
DATA:                 Control 30min   2hours  2days   1week   p-value         pos
SENSE COUNTS:         16      8       9       22      10      5.47e-02
GENES (1 total):
  AT5G48930.1
    SENSE COUNTS:     16      8       9       22      10      5.47e-02
    TAGS: (5 total)
      d+1  CAATAACTCT 8       4       4       15      10      7.04e-02        1601
      d+2  CTGTATGATT 1       0       0       1       0       5.06e-01        1578
      d+2  CCGTGATTGT 4       0       3       5       0       8.20e-02        1539
      d+2  AAACTGTTTG 3       4       1       1       0       3.34e-01        1323
      d+2  ATTTCTTGGT 0       0       1       0       0       4.55e-01        1036
           ----------                                                         1013
           ----------                                                         835
           ----------                                                         655
           ----------                                                         557
           ----------                                                         222
           ----------                                                         84

LOCUS: AT1G63660
DESCRIPTION: GMP synthase (glutamine-hydrolyzing), putative / glutamine amidotransferase, putative, similar to
SP|P38625 GMP synthase (glutamine-hydrolyzing) (EC 6.3.5.2) (Glutamine amidotransferase) (GMP synthetase)
{Saccharomyces cerevisiae}; contains Pfam profile P
DATA:                 Control 30min   2hours  2days   1week   p-value         pos
SENSE COUNTS:         5       0       0       1       1       5.52e-02
GENES (2 total):
  AT1G63660.1
    SENSE COUNTS:     5       0       0       1       1       5.52e-02
    TAGS: (2 total)
      i+3  TGGTATTTTA 0       0       0       0       0       6.15e-01        2620
      d+1  TAAAATAGAT 5       0       0       1       1       1.60e-02        1799
           ----------                                                         1572
           ----------                                                         1268
           ----------                                                         1131
           ----------                                                         828
           ----------                                                         567
           ----------                                                         143
  AT1G63660.2
    SENSE COUNTS:     5       0       0       1       1       5.52e-02
    TAGS: (2 total)
      i+3  TGGTATTTTA 0       0       0       0       0       6.15e-01        2676
      d+1  TAAAATAGAT 5       0       0       1       1       1.60e-02        1850
           ----------                                                         1623
           ----------                                                         1324
           ----------                                                         1187
           ----------                                                         884
           ----------                                                         623
           ----------                                                         199

LOCUS: AT5G09370
DESCRIPTION: protease inhibitor/seed storage/lipid transfer protein (LTP) family protein, similar to lipid
transfer protein - Hordeum vulgare, EMBL:AF109195; contains Pfam protease inhibitor/seed storage/LTP family
domain PF00234
DATA:                 Control 30min   2hours  2days   1week   p-value         pos
SENSE COUNTS:         1       4       0       0       5       5.56e-02
GENES (2 total):
  AT5G09370.1
```

```
    SENSE COUNTS:      1       4       0       0       5       5.56e-02
    TAGS: (1 total)
           ----------                                                          599
           ----------                                                          533
      i+3  TTAGATTTCT  1       4       0       0       5       5.56e-02        391
           ----------                                                          232
           ----------                                                          181
           ----------                                                          138
           ----------                                                          71
  AT5G09370.2
    SENSE COUNTS:      1       4       0       0       5       5.56e-02
    TAGS: (1 total)
           ----------                                                          623
           ----------                                                          617
           ----------                                                          555
      i+3  TTAGATTTCT  1       4       0       0       5       5.56e-02        391
           ----------                                                          232
           ----------                                                          181
           ----------                                                          138
           ----------                                                          71

LOCUS: AT1G23390
DESCRIPTION: kelch repeat-containing F-box family protein, similar to hypothetical protein GB:AAF27090
GI:6730669 from (Arabidopsis thaliana); contains Pfam profiles PF01344: Kelch motif, PF00646: F-box domain
DATA:                  Control 30min   2hours  2days   1week   p-value         pos
SENSE COUNTS:          13      17      8       7       3       5.66e-02
GENES (1 total):
  AT1G23390.1
    SENSE COUNTS:      13      17      8       7       3       5.66e-02
    TAGS: (3 total)
      d+1  GACGCAATTT  0       0       1       1       0       7.90e-01        1526
      d+2  GTGCGGCGGA  12      17      7       6       3       4.82e-02        993
      d+2  GCGACGATGG  1       0       0       0       0       4.28e-01        248
           ----------                                                          200
           ----------                                                          157

LOCUS: AT1G56050
DESCRIPTION: GTP-binding protein-related, similar to GTP-binding protein GI:10176676 from (Bacillus
halodurans)
DATA:                  Control 30min   2hours  2days   1week   p-value         pos
SENSE COUNTS:          6       1       4       5       13      5.66e-02
GENES (1 total):
  AT1G56050.1
    SENSE COUNTS:      6       1       4       5       13      5.66e-02
    TAGS: (2 total)
           ----------                                                          1146
           ----------                                                          1127
           ----------                                                          949
           ----------                                                          574
      X+4  TGTATCGACA  3       1       1       1       1       8.23e-01        305
      X+4  GGATTGGCAA  3       0       3       4       12      1.16e-02        271
           ----------                                                          231
           ----------                                                          78

LOCUS: AT1G17710
DESCRIPTION: expressed protein
DATA:                  Control 30min   2hours  2days   1week   p-value         pos
SENSE COUNTS:          0       4       5       1       0       5.67e-02
GENES (1 total):
  AT1G17710.1
    SENSE COUNTS:      0       4       5       1       0       5.67e-02
    TAGS: (2 total)
           ----------                                                          1638
           ----------                                                          1628
           ----------                                                          1516
           ----------                                                          1480
      v+2  CACGTTAAGT  0       4       5       1       0       7.68e-02        1270
      v+2  CAATTATGTA  0       0       0       0       0       6.15e-01        1234
           ----------                                                          1144
           ----------                                                          987
           ----------                                                          936
           ----------                                                          804
           ----------                                                          778
           ----------                                                          757
           ----------                                                          613
           ----------                                                          529
           ----------                                                          523
           ----------                                                          498

LOCUS: AT1G27320
DESCRIPTION: AB046870 Arabidopsis thaliana AHK3 mRNA for histidine kinase, complete cds
```

```
DATA:                 Control 30min   2hours  2days   1week   p-value         pos
SENSE COUNTS:         10      4       3       13      2       5.68e-02
GENES (2 total):
  AT1G27320.1
    SENSE COUNTS:     10      4       3       13      2       5.68e-02
    TAGS: (4 total)
      d+1  AGGAATGTCT 0       0       0       0       1       1.65e-01        2999
           ----------                                                         2821
           ----------                                                         2236
           ----------                                                         2048
      d+2  CAAGTCGGAC 0       4       0       1       0       3.64e-02        2017
           ----------                                                         1382
           ----------                                                         1194
           ----------                                                         1160
           ----------                                                         1084
           ----------                                                         482
           ----------                                                         454
           ----------                                                         443
           ----------                                                         436
      X+4  GTCCAAGATA 8       0       3       12      1       4.37e-03        294
      X+4  ATTTAGGATA 2       0       0       0       0       1.04e-01        234
           ----------                                                         17

LOCUS: AT3G20250
DESCRIPTION: pumilio/Puf RNA-binding domain-containing protein, contains Pfam profile: PF00806 Pumilio-family
RNA binding domains (aka PUM-HD, Pumilio homology domain) (8 copies at C-terminus)
DATA:                 Control 30min   2hours  2days   1week   p-value         pos
SENSE COUNTS:         5       0       3       4       8       5.71e-02
GENES (1 total):
  AT3G20250.1
    SENSE COUNTS:     5       0       3       4       8       5.71e-02
    TAGS: (3 total)
           ----------                                                         3158
      d+2  TCCTGGAGAG 1       0       0       0       0       4.28e-01        2620
           ----------                                                         2590
      d+2  TTATCCAAAA 0       0       1       0       0       4.55e-01        2389
           ----------                                                         2197
           ----------                                                         2056
           ----------                                                         1725
           ----------                                                         1669
           ----------                                                         1435
           ----------                                                         1200
           ----------                                                         1009
           ----------                                                         811
           ----------                                                         333
      X+4  ATCTGTGATT 4       0       2       4       8       4.07e-02        175
           ----------                                                         57

LOCUS: AT2G19220
DESCRIPTION: hypothetical protein
DATA:                 Control 30min   2hours  2days   1week   p-value         pos
SENSE COUNTS:         0       0       3       0       5       5.74e-02
GENES (1 total):
  AT2G19220.1
    SENSE COUNTS:     0       0       3       0       5       5.74e-02
    TAGS: (2 total)
           ----------                                                         2134
           ----------                                                         1735
           ----------                                                         1563
           ----------                                                         1297
           ----------                                                         1186
           ----------                                                         1077
           ----------                                                         862
           ----------                                                         371
      v+2  ATTTGCTTTA 0       0       1       0       5       7.15e-03        213
      v+2  ATGTAAAAAA 0       0       2       0       0       3.51e-01        93
           ----------                                                         40

LOCUS: AT4G13590
DESCRIPTION: expressed protein, contains Pfam profile PF01169: Uncharacterized protein family UPF0016
DATA:                 Control 30min   2hours  2days   1week   p-value         pos
SENSE COUNTS:         4       0       0       1       1       5.81e-02
GENES (1 total):
  AT4G13590.1
    SENSE COUNTS:     4       0       0       1       1       5.81e-02
    TAGS: (1 total)
           ----------                                                         2062
           ----------                                                         1588
      d+2  AGAGCAATAA 4       0       0       1       1       5.81e-02        1315
           ----------                                                         1128
           ----------                                                         854
```

```
          ----------                                              822
          ----------                                              666
          ----------                                              156

LOCUS: AT5G10860
DESCRIPTION: CBS domain-containing protein, contains Pfam profile PF00571: CBS domain
DATA:                 Control 30min  2hours  2days  1week  p-value        pos
SENSE COUNTS:         6       14     10      2      4      5.82e-02
GENES (1 total):
  AT5G10860.1
    SENSE COUNTS:     6       14     10      2      4      5.82e-02
    TAGS: (3 total)
      d+1  AGCATAGAGA 6       13     6       1      0      7.65e-03       691
      d+2  ATTGGAATGG 0       0      0       0      1      1.65e-01       645
      X+4  TGTGTTATGA 0       1      4       1      3      3.00e-01       431
           ----------                                                     387
           ----------                                                     306

LOCUS: AT1G44350
DESCRIPTION: IAA-amino acid hydrolase 6, putative (ILL6) / IAA-Ala hydrolase, putative, virtually identical to
gr1-protein from (Arabidopsis thaliana) GI:3559811; similar to IAA-amino acid hydrolase GI:3421384 from
(Arabidopsis thaliana); contains TIGRfam profile TIGR
DATA:                 Control 30min  2hours  2days  1week  p-value        pos
SENSE COUNTS:         5       0      2       2      0      5.89e-02
GENES (2 total):
  AT1G44350.1
    SENSE COUNTS:     5       0      2       2      0      5.89e-02
    TAGS: (2 total)
           ----------                                                     1757
      d+2  AAATATTTGG 3       0      2       1      0      2.55e-01       1711
      d+2  CCGCCGTGGC 2       0      0       1      0      2.50e-01       1440
           ----------                                                     1400
           ----------                                                     1121
           ----------                                                     792
           ----------                                                     627
           ----------                                                     618
           ----------                                                     610
           ----------                                                     606
           ----------                                                     455
           ----------                                                     378
           ----------                                                     301
           ----------                                                     264

LOCUS: AT2G40840
DESCRIPTION: 4-alpha-glucanotransferase -related, temporary automated functional assignment
DATA:                 Control 30min  2hours  2days  1week  p-value        pos
SENSE COUNTS:         0       5      0       2      1      5.91e-02
GENES (2 total):
  AT2G40840.1
    SENSE COUNTS:     0       5      0       2      1      5.91e-02
    TAGS: (3 total)
      i+3  CTTACAGTTA 0       0      0       1      0      3.09e-01       3723
      d+1  TTGAAGCTCC 0       5      0       0      1      1.16e-02       2794
           ----------                                                     2651
           ----------                                                     2580
           ----------                                                     2495
      d+2  TATGAGTTCA 0       0      0       1      0      3.09e-01       2151
           ----------                                                     1960
           ----------                                                     1758
           ----------                                                     1636
           ----------                                                     1359
           ----------                                                     1059
           ----------                                                     1031
           ----------                                                     1002

LOCUS: AT1G27100
DESCRIPTION: expressed protein,  contains Pfam profile: PF04601 protein of unknown function (DUF569
DATA:                 Control 30min  2hours  2days  1week  p-value        pos
SENSE COUNTS:         0       5      4       1      1      6.02e-02
GENES (1 total):
  AT1G27100.1
    SENSE COUNTS:     0       5      4       1      1      6.02e-02
    TAGS: (4 total)
      d+1  GATTCATAAA 0       0      0       0      0      6.15e-01       1960
      i+3  AATGATTGGA 0       0      1       0      0      4.55e-01       1956
      d+2  AACCTTGCTT 0       5      3       1      1      1.98e-01       1802
      d+2  TCATTTTACT 0       0      0       0      0      6.15e-01       1627
           ----------                                                     1536
           ----------                                                     1423
           ----------                                                     934
           ----------                                                     570
```

```
LOCUS: AT3G26070
DESCRIPTION: plastid-lipid associated protein PAP / fibrillin family protein, contains Pfam profile PF04755:
PAP_fibrillin
DATA:                 Control 30min   2hours  2days   1week   p-value         pos
SENSE COUNTS:         8       8       1       1       5       6.11e-02
GENES (1 total):
  AT3G26070.1
    SENSE COUNTS:     8       8       1       1       5       6.11e-02
    TAGS: (2 total)
      d+1  ACCTTTCTAC 0       1       0       0       0       2.54e-01        1212
           ----------                                                         1200
           ----------                                                         1193
      d+2  TATGTAATCT 8       7       1       1       5       1.12e-01        959
           ----------                                                         646
           ----------                                                         160
           ----------                                                         51

LOCUS: AT3G05350
DESCRIPTION: aminopeptidase P, cytosolic, putative, similar to cytosolic aminopeptidase P from (Homo sapiens)
GI:8489879, (Rattus norvegicus) GI:2760920; contains Pfam profile PF00557: metallopeptidase family M24
DATA:                 Control 30min   2hours  2days   1week   p-value         pos
SENSE COUNTS:         4       0       2       0       6       6.16e-02
GENES (1 total):
  AT3G05350.1
    SENSE COUNTS:     4       0       2       0       6       6.16e-02
    TAGS: (3 total)
      i+3  CAGACAAAAA 0       0       0       0       1       1.65e-01        2809
      d+1  ATTAGTACCA 4       0       1       0       5       9.04e-02        1923
      d+2  ATCAAACCCA 0       0       1       0       0       4.55e-01        1870
           ----------                                                         1635
           ----------                                                         1602
           ----------                                                         1565
           ----------                                                         1544
           ----------                                                         1506
           ----------                                                         1467
           ----------                                                         878
           ----------                                                         511
           ----------                                                         494
           ----------                                                         399
           ----------                                                         309
           ----------                                                         164

LOCUS: AT1G18060
DESCRIPTION: expressed protein
DATA:                 Control 30min   2hours  2days   1week   p-value         pos
SENSE COUNTS:         11      1       9       5       9       6.24e-02
GENES (1 total):
  AT1G18060.1
    SENSE COUNTS:     11      1       9       5       9       6.24e-02
    TAGS: (2 total)
      d+1  AAATGAAAGT 11      1       5       5       8       7.69e-02        893
           ----------                                                         834
      d+2  GAAGAATCGG 0       0       4       0       1       2.25e-02        513
           ----------                                                         360
           ----------                                                         75

LOCUS: AT3G15850
DESCRIPTION: fatty acid desaturase family protein, similar to delta 9 acyl-lipid desaturase (ADS1) GI:2970034
from (Arabidopsis thaliana)
DATA:                 Control 30min   2hours  2days   1week   p-value         pos
SENSE COUNTS:         6       8       12      6       20      6.25e-02
GENES (2 total):
  AT3G15850.1
    SENSE COUNTS:     6       8       12      6       20      6.25e-02
    TAGS: (2 total)
      i+3  CTAAAAGTGA 0       0       0       0       1       1.65e-01        1425
      d+1  GTTATGCCTA 6       8       12      6       19      1.28e-01        1212
           ----------                                                         1043
           ----------                                                         963
           ----------                                                         944
           ----------                                                         709

LOCUS: AT5G01590
DESCRIPTION: expressed protein
DATA:                 Control 30min   2hours  2days   1week   p-value         pos
SENSE COUNTS:         5       0       1       1       2       6.26e-02
GENES (1 total):
  AT5G01590.1
    SENSE COUNTS:     5       0       1       1       2       6.26e-02
    TAGS: (3 total)
```

```
      d+1  GGCTTATGAA 0      0      0      0      1      1.65e-01      1898
      d+2  GTTTGTTTGT 4      0      1      1      1      1.57e-01      1800
           ----------                                                1776
           ----------                                                1412
           ----------                                                1288
           ----------                                                1222
           ----------                                                1108
           ----------                                                931
           ----------                                                907
      i+3  ATGTTTCTGA 1      0      0      0      0      4.28e-01      840
           ----------                                                655
           ----------                                                598
           ----------                                                572

LOCUS: AT1G48600
DESCRIPTION: phosphoethanolamine N-methyltransferase 2, putative (NMT2), very similar to |PEM2_ARATH Putative
phosphoethanolamine N-methyltransferase 2 (EC 2.1.1.103) (SP:Q944H0){Arabidopsis thaliana}; very similar to
Halotolerance protein Hal3b (SP:P94063)(Arabidopsi
DATA:                  Control 30min  2hours 2days  1week  p-value       pos
SENSE COUNTS:          5      0      1      1      2      6.26e-02
GENES (2 total):
  AT1G48600.1
    SENSE COUNTS:      5      0      1      1      2      6.26e-02
    TAGS: (3 total)
           ----------                                                1614
      d+2  GAACTGCAAA 1      0      0      0      0      4.28e-01      1586
           ----------                                                1502
      d+2  ATGTTCAAGC 3      0      1      1      1      3.73e-01      1246
           ----------                                                969
           ----------                                                940
           ----------                                                918
           ----------                                                408
      X+4  GGCTAATTGA 1      0      0      0      1      3.83e-01      364
  AT1G48600.2
    SENSE COUNTS:      4      0      1      1      1      1.57e-01
    TAGS: (2 total)
           ----------                                                1800
      d+2  GAACTGCAAA 1      0      0      0      0      4.28e-01      1772
           ----------                                                1688
      d+2  ATGTTCAAGC 3      0      1      1      1      3.73e-01      1432
           ----------                                                1155
           ----------                                                1126
           ----------                                                1104
           ----------                                                594
           ----------                                                66

LOCUS: AT3G54910
DESCRIPTION: expressed protein, various pedicted proteins, Arabidopsis thaliana
DATA:                  Control 30min  2hours 2days  1week  p-value       pos
SENSE COUNTS:          0      0      0      1      3      6.27e-02
GENES (1 total):
  AT3G54910.1
    SENSE COUNTS:      0      0      0      1      3      6.27e-02
    TAGS: (1 total)
           ----------                                                1515
           ----------                                                1450
           ----------                                                1409
           ----------                                                1381
      d+2  ATATCCTTTA 0      0      0      1      3      6.27e-02      1218
           ----------                                                1209
           ----------                                                803

LOCUS: AT1G73810
DESCRIPTION: expressed protein, contains Pfam profile PF03267: Arabidopsis protein of unknown function, DUF266
DATA:                  Control 30min  2hours 2days  1week  p-value       pos
SENSE COUNTS:          0      0      0      1      3      6.27e-02
GENES (1 total):
  AT1G73810.1
    SENSE COUNTS:      0      0      0      1      3      6.27e-02
    TAGS: (1 total)
           ----------                                                1104
      X+4  ATTTACATCT 0      0      0      1      3      6.27e-02      752
           ----------                                                732
           ----------                                                604
           ----------                                                465
           ----------                                                409

LOCUS: AT1G24580
DESCRIPTION: zinc finger (C3HC4-type RING finger) family protein
DATA:                  Control 30min  2hours 2days  1week  p-value       pos
SENSE COUNTS:          0      0      0      1      3      6.27e-02
```

```
GENES (1 total):
  AT1G24580.1
    SENSE COUNTS:      0       0       0       1       3       6.27e-02
    TAGS: (2 total)
      X+4  ATTAGTGTGA  0       0       0       1       0       3.09e-01       720
      d+1  CCCTCTTTGC  0       0       0       0       3       1.12e-02       370
           ----------                                                        140
           ----------                                                        92

LOCUS: AT2G05610
DESCRIPTION: gypsy-like retrotransposon family, has a 1.2e-179 P-value blast match to GB:AAD27547 polyprotein
(Gypsy_Ty3-element) (Oryza sativa subsp. indica)
DATA:                  Control 30min   2hours  2days   1week   p-value        pos
SENSE COUNTS:          0       0       0       1       3       6.27e-02
GENES (1 total):
  AT2G05610.1
    SENSE COUNTS:      0       0       0       1       3       6.27e-02
    TAGS: (1 total)
           ----------                                                        2601
           ----------                                                        2336
           ----------                                                        2153
           ----------                                                        2149
           ----------                                                        1969
           ----------                                                        1860
           ----------                                                        1683
           ----------                                                        1395
           ----------                                                        1054
           ----------                                                        981
           ----------                                                        908
      p+2  GATCCTCTTG  0       0       0       1       3       6.27e-02       442
           ----------                                                        107

LOCUS: AT4G17560
DESCRIPTION: ribosomal protein L19 family protein, similar to plastid ribosomal protein L19 precursor
(Spinacia oleracea) gi|7582403|gb|AAF64312
DATA:                  Control 30min   2hours  2days   1week   p-value        pos
SENSE COUNTS:          0       7       3       4       1       6.32e-02
GENES (1 total):
  AT4G17560.1
    SENSE COUNTS:      0       7       3       4       1       6.32e-02
    TAGS: (2 total)
      d+1  TTCTCGTCTT  0       0       0       0       1       1.65e-01       1073
      d+2  GGGATACTGA  0       7       3       4       0       2.57e-02       410

LOCUS: AT3G54400
DESCRIPTION: aspartyl protease family protein, contains Pfam profile: PF00026 eukaryotic aspartyl protease
DATA:                  Control 30min   2hours  2days   1week   p-value        pos
SENSE COUNTS:          1       3       6       10      8       6.38e-02
GENES (1 total):
  AT3G54400.1
    SENSE COUNTS:      1       3       6       10      8       6.38e-02
    TAGS: (4 total)
           ----------                                                        1490
      d+2  GCTGCAGCTC  1       3       3       9       5       1.17e-01       1196
      d+2  CTACTCCGGC  0       0       1       1       3       1.91e-01       1075
           ----------                                                        671
      d+2  ACCTACGGTG  0       0       1       0       0       4.55e-01       536
           ----------                                                        523
      d+2  CTCGTGGCTC  0       0       1       0       0       4.55e-01       347
           ----------                                                        190

LOCUS: AT5G49680
DESCRIPTION: cell expansion protein, putative, similar to SABRE (Arabidopsis thaliana) GI:719291
DATA:                  Control 30min   2hours  2days   1week   p-value        pos
SENSE COUNTS:          2       7       8       1       1       6.40e-02
GENES (1 total):
  AT5G49680.1
    SENSE COUNTS:      2       7       8       1       1       6.40e-02
    TAGS: (2 total)
           ----------                                                        4879
           ----------                                                        4674
           ----------                                                        4644
           ----------                                                        4112
           ----------                                                        4042
           ----------                                                        3944
           ----------                                                        3760
           ----------                                                        3632
           ----------                                                        3048
           ----------                                                        2493
           ----------                                                        2371
      v+2  TACAAGCTCA  1       0       1       0       0       6.01e-01       2359
```

```
          ----------                                                2346
          ----------                                                2334
          ----------                                                2324
          ----------                                                2319
          ----------                                                2262
          ----------                                                2248
          ----------                                                1101
     v+2  GTTGATCAGA  1      7      7      1      1      4.79e-02   1079
          ----------                                                1069
          ----------                                                648
          ----------                                                562
          ----------                                                533
          ----------                                                286
          ----------                                                114
          ----------                                                110

LOCUS: AT3G30842
DESCRIPTION: ABC transporter protein, putative, similar to pleiotropic drug resistance like protein (Nicotiana
tabacum) GI:20522008, ABC1 protein (Nicotiana plumbaginifolia) GI:14331118; contains Pfam profile PF00005: ABC
transporter
DATA:                 Control 30min  2hours 2days  1week  p-value    pos
SENSE COUNTS:         2      4      0      0      0      6.45e-02
GENES (1 total):
  AT3G30842.1
    SENSE COUNTS:     2      4      0      0      0      6.45e-02
    TAGS: (1 total)
          ----------                                                4446
          ----------                                                4324
          ----------                                                3852
          ----------                                                3706
          ----------                                                3575
          ----------                                                3351
          ----------                                                3303
          ----------                                                3125
          ----------                                                2423
          ----------                                                2326
          ----------                                                2126
          ----------                                                2007
          ----------                                                1856
          ----------                                                1447
          ----------                                                1374
          ----------                                                994
          ----------                                                985
          ----------                                                622
          ----------                                                549
     v+2  TGTCTATGAG  2      4      0      0      0      6.45e-02   257
          ----------                                                40
          ----------                                                36

LOCUS: AT3G32112
DESCRIPTION: copia-like retrotransposon family, has a 1.8e-135 P-value blast match to gb|AAO73527.1| gag-pol
polyprotein (Glycine max) (SIRE1) (Ty1_Copia-family)
DATA:                 Control 30min  2hours 2days  1week  p-value    pos
SENSE COUNTS:         0      0      4      0      0      6.49e-02
GENES (1 total):
  AT3G32112.1
    SENSE COUNTS:     0      0      4      0      0      6.49e-02
    TAGS: (2 total)
          ----------                                                3460
          ----------                                                3438
          ----------                                                3049
          ----------                                                2836
          ----------                                                2577
          ----------                                                2344
          ----------                                                2230
          ----------                                                1900
          ----------                                                1834
          ----------                                                1710
          ----------                                                1699
     p+2  TGGACAATCA  0      0      0      0      0      6.15e-01   1566
          ----------                                                1480
     p+2  TAAATCTTGC  0      0      4      0      0      3.11e-02   1309
          ----------                                                1294
          ----------                                                1168
          ----------                                                819
          ----------                                                812
          ----------                                                688
          ----------                                                485
          ----------                                                207
          ----------                                                104
```

```
LOCUS: AT5G56980
DESCRIPTION: expressed protein, non-consensus CG donor splice site at exon 1, GA donor splice site at exon 3
DATA:                  Control 30min  2hours  2days  1week  p-value       pos
SENSE COUNTS:          1       6      4       1      0      6.50e-02
GENES (1 total):
  AT5G56980.1
    SENSE COUNTS:      1       6      5       1      0      3.27e-02
    TAGS: (4 total)
      X+4  TGATTGCAAA  0       0      1       0      0      7.06e-01      1739
      X+4  TGATTGCAAA  0       0      1       0      0      7.06e-01      1739
      d+1  ATACAGATTA  1       0      2       0      0      3.07e-01      1577
      d+2  CGGAAGCGCC  0       6      1       1      0      1.64e-02      970
           ----------                                                     859
           ----------                                                     543
           ----------                                                     399
           ----------                                                     325
           ----------                                                     184

LOCUS: AT5G60960
DESCRIPTION: pentatricopeptide (PPR) repeat-containing protein, contains Pfam profile PF01535: PPR repeat
DATA:                  Control 30min  2hours  2days  1week  p-value       pos
SENSE COUNTS:          2       0      3       6      8      6.54e-02
GENES (1 total):
  AT5G60960.1
    SENSE COUNTS:      2       0      3       6      8      6.54e-02
    TAGS: (2 total)
           ----------                                                     1755
      X+4  CTTGGCTCAA  0       0      1       5      1      2.17e-02      1754
      d+2  AGCAAATTGT  2       0      2       1      7      1.50e-01      1695
           ----------                                                     1284
           ----------                                                     1261
           ----------                                                     879

LOCUS: AT3G55530
DESCRIPTION: zinc finger (C3HC4-type RING finger) family protein, contains Pfam profile: PF00097 zinc finger,
C3HC4 type (RING finger)
DATA:                  Control 30min  2hours  2days  1week  p-value       pos
SENSE COUNTS:          1       1      1       0      6      6.56e-02
GENES (1 total):
  AT3G55530.1
    SENSE COUNTS:      1       1      1       0      6      6.56e-02
    TAGS: (3 total)
      d+1  AAATAGTAGT  0       0      0       0      0      6.15e-01      1101
      d+2  TAATGGTCAA  0       1      1       0      1      6.08e-01      1029
           ----------                                                     955
           ----------                                                     882
           ----------                                                     861
           ----------                                                     842
           ----------                                                     771
           ----------                                                     467
           ----------                                                     407
           ----------                                                     230
      d+2  AGCTTTGTTT  1       0      0       0      5      3.71e-02      142

LOCUS: AT2G39670
DESCRIPTION: radical SAM domain-containing protein, similar to hypothetical protein PIR|S76698|S76698 contains
Pfam profile PF04055: radical SAM domain protein
DATA:                  Control 30min  2hours  2days  1week  p-value       pos
SENSE COUNTS:          4       1      0       1      6      6.59e-02
GENES (2 total):
  AT2G39670.1
    SENSE COUNTS:      4       1      0       1      6      6.59e-02
    TAGS: (2 total)
      d+1  GCTCAATCAC  3       1      0       1      3      4.40e-01      1432
           ----------                                                     1068
           ----------                                                     909
           ----------                                                     734
      X+4  GTACATTAAC  1       0      0       0      3      7.14e-02      701
           ----------                                                     666
           ----------                                                     258
  AT2G39670.2
    SENSE COUNTS:      3       1      0       1      3      4.40e-01
    TAGS: (1 total)
      d+1  GCTCAATCAC  3       1      0       1      3      4.40e-01      1441
           ----------                                                     1077
           ----------                                                     918
           ----------                                                     743
           ----------                                                     675
           ----------                                                     258

LOCUS: AT1G20970
```

```
DESCRIPTION: adhesin-related, contains TIGRFAM TIGR01612: reticulocyte binding protein; contains TIGRFAM
TIGR00864: polycystin cation channel protein; similar to fimbriae-associated protein Fap1 (Streptococcus
parasanguinis) (GI:3929312)
DATA:                 Control 30min  2hours  2days  1week  p-value      pos
SENSE COUNTS:         2       6      1       1      3      6.64e-02
GENES (1 total):
  AT1G20970.1
    SENSE COUNTS:     2       6      1       1      3      6.64e-02
    TAGS: (4 total)
           ----------                                                   5298
           ----------                                                   5281
           ----------                                                   5164
           ----------                                                   5102
           ----------                                                   5032
           ----------                                                   4954
           ----------                                                   4678
      v+2  AGGCAGTTCC 1       6      1       1      3      1.49e-01     3760
           ----------                                                   3576
      v+2  CGTGAAATAA 0       0      0       0      0      6.15e-01     3153
           ----------                                                   2970
           ----------                                                   2860
      v+2  TAGCTCCATC 0       0      0       0      0      6.15e-01     2281
           ----------                                                   2235
           ----------                                                   2110
           ----------                                                   1981
           ----------                                                   1903
           ----------                                                   1825
           ----------                                                   1816
           ----------                                                   1146
      v+2  ATTCTGAAGT 1       0      0       0      0      4.28e-01     880
           ----------                                                   481
           ----------                                                   241

LOCUS: AT1G42698
DESCRIPTION: copia-like retrotransposon family, has a 7.4e-54 P-value blast match to GB:BAA11674 ORF(AA 1-
1338) (Ty1_Copia-element) (Nicotiana tabacum)
DATA:                 Control 30min  2hours  2days  1week  p-value      pos
SENSE COUNTS:         2       6      2       0      0      6.65e-02
GENES (1 total):
  AT1G42698.1
    SENSE COUNTS:     2       6      2       0      0      6.65e-02
    TAGS: (1 total)
           ----------                                                   1620
           ----------                                                   1505
           ----------                                                   1401
           ----------                                                   1385
           ----------                                                   1381
           ----------                                                   1359
           ----------                                                   1265
           ----------                                                   1191
           ----------                                                   577
           ----------                                                   429
           ----------                                                   401
      p+2  GAATTGATTA 2       6      2       0      0      6.65e-02     249

LOCUS: AT1G26640
DESCRIPTION: aspartate/glutamate/uridylate kinase family protein, contains Pfam amino acid kinase family
PF00696
DATA:                 Control 30min  2hours  2days  1week  p-value      pos
SENSE COUNTS:         8       0      7       8      10     6.70e-02
GENES (1 total):
  AT1G26640.1
    SENSE COUNTS:     8       0      7       8      10     6.70e-02
    TAGS: (3 total)
      d+1  ACAGACACCA 2       0      0       1      0      2.50e-01     1716
           ----------                                                   1707
      d+2  AACAAACATA 5       0      4       5      10     8.81e-02     1422
      d+2  TAAAGAGTTG 1       0      3       2      0      2.27e-01     1394
           ----------                                                   694
           ----------                                                   602
           ----------                                                   588
           ----------                                                   418
           ----------                                                   318

LOCUS: AT5G35210
DESCRIPTION: peptidase M50 family protein / sterol-regulatory element binding protein (SREBP) site 2 protease
family protein, contains PFam PF02163: sterol-regulatory element binding protein (SREBP) site 2 protease
DATA:                 Control 30min  2hours  2days  1week  p-value      pos
SENSE COUNTS:         4       0      0       0      1      6.71e-02
GENES (2 total):
  AT5G35210.1
```

```
    SENSE COUNTS:      1       0       0       0       1       6.74e-01
    TAGS: (1 total)
      d+1  TAGGTACACT  1       0       0       0       1       6.74e-01        4976
           ----------                                                          4493
           ----------                                                          4426
           ----------                                                          4357
           ----------                                                          4176
           ----------                                                          4092
           ----------                                                          3934
           ----------                                                          3496
           ----------                                                          2965
           ----------                                                          2957
           ----------                                                          2610
           ----------                                                          2448
           ----------                                                          2224
           ----------                                                          2033
           ----------                                                          1884
           ----------                                                          1445
           ----------                                                          1400
           ----------                                                          1350
           ----------                                                          875
           ----------                                                          719
  AT5G35210.2
    SENSE COUNTS:      4       0       0       0       1       6.71e-02
    TAGS: (2 total)
      d+1  TAGGTACACT  1       0       0       0       1       6.74e-01        5397
           ----------                                                          4830
           ----------                                                          4763
           ----------                                                          4694
      d+2  TATTGGTGAT  3       0       0       0       0       2.13e-02        4475
           ----------                                                          4415
           ----------                                                          4176
           ----------                                                          4092
           ----------                                                          3934
           ----------                                                          3496
           ----------                                                          2965
           ----------                                                          2957
           ----------                                                          2610
           ----------                                                          2448
           ----------                                                          2224
           ----------                                                          2033
           ----------                                                          1884
           ----------                                                          1445
           ----------                                                          1400
           ----------                                                          1350
           ----------                                                          875
           ----------                                                          719

LOCUS: AT5G22830
DESCRIPTION: magnesium transporter CorA-like family protein, weak similarity to SP|Q01926 RNA splicing protein
MRS2, mitochondrial precursor {Saccharomyces cerevisiae}; contains Pfam profile PF01544: CorA-like Mg2+
transporter protein; supporting cDNA gi|12007446|gb|A
DATA:                  Control 30min   2hours  2days   1week   p-value         pos
SENSE COUNTS:          3       0       0       0       1       6.72e-02
GENES (2 total):
  AT5G22830.1
    SENSE COUNTS:      3       0       0       0       1       6.72e-02
    TAGS: (1 total)
           ----------                                                          2008
           ----------                                                          1890
      d+2  TATTCCTATC  3       0       0       0       1       6.72e-02        1374
           ----------                                                          1281
           ----------                                                          1120
           ----------                                                          1115
           ----------                                                          981
           ----------                                                          958
           ----------                                                          618
           ----------                                                          577

LOCUS: AT1G31230
DESCRIPTION: bifunctional aspartate kinase/homoserine dehydrogenase / AK-HSDH, nearly identical to gb|X71364
(PIR|S46497) aspartate kinase / homoserine dehydrogenase from Arabidopsis thaliana;  contains ACT domain
DATA:                  Control 30min   2hours  2days   1week   p-value         pos
SENSE COUNTS:          3       0       0       0       1       6.72e-02
GENES (1 total):
  AT1G31230.1
    SENSE COUNTS:      3       0       0       0       1       6.72e-02
    TAGS: (1 total)
           ----------                                                          3084
      d+2  CTAGCATATG  3       0       0       0       1       6.72e-02        2878
           ----------                                                          2551
```

```
          ----------                                                  2114
          ----------                                                  1993
          ----------                                                  707
          ----------                                                  378

LOCUS: AT1G54115
DESCRIPTION: cation exchanger, putative
DATA:                  Control 30min   2hours  2days   1week   p-value        pos
SENSE COUNTS:          3       0       0       0       1       6.72e-02
GENES (1 total):
  AT1G54115.1
    SENSE COUNTS:      3       0       0       0       1       6.72e-02
    TAGS: (2 total)
      v+1  TTACGTAACA  0       0       0       0       1       1.65e-01       2630
      v+2  TTGTTAGAGA  3       0       0       0       0       2.13e-02       2321
           ----------                                                     2262
           ----------                                                     2094
           ----------                                                     1833
           ----------                                                     1812
           ----------                                                     1469
           ----------                                                     1450
           ----------                                                     1375
           ----------                                                     637

LOCUS: AT4G24560
DESCRIPTION: ubiquitin-specific protease 16, putative (UBP16), similar to ubiquitin-specific protease 16
GI:11993477 (Arabidopsis thaliana)
DATA:                  Control 30min   2hours  2days   1week   p-value        pos
SENSE COUNTS:          1       2       1       3       8       6.87e-02
GENES (2 total):
  AT4G24560.1
    SENSE COUNTS:      1       2       1       3       8       6.87e-02
    TAGS: (3 total)
      i+3  AGCTTTATAT  0       0       0       0       1       1.65e-01       3630
      d+1  AAATTGTATC  1       0       0       1       0       5.06e-01       3626
           ----------                                                     3250
           ----------                                                     2771
           ----------                                                     2446
           ----------                                                     2300
      d+2  TACAAAAAAA  0       2       1       2       7       8.83e-02       2148
           ----------                                                     1967
           ----------                                                     1880
           ----------                                                     1664
           ----------                                                     1112
           ----------                                                     554
           ----------                                                     295

LOCUS: AT4G17050
DESCRIPTION: expressed protein
DATA:                  Control 30min   2hours  2days   1week   p-value        pos
SENSE COUNTS:          7       2       3       1       0       6.89e-02
GENES (1 total):
  AT4G17050.1
    SENSE COUNTS:      7       2       3       1       0       6.89e-02
    TAGS: (3 total)
      i+3  AAAAATTCTA  1       0       0       0       0       4.28e-01       2643
      d+1  TCCACTCTAA  6       2       2       1       0       1.21e-01       1050
           ----------                                                     805
           ----------                                                     393
           ----------                                                     313
           ----------                                                     289
      d+2  AATGAAACCT  0       0       1       0       0       4.55e-01       44

LOCUS: AT2G25450
DESCRIPTION: 2-oxoglutarate-dependent dioxygenase, putative, similar to 2A6 (GI:599622) and tomato ethylene
synthesis regulatory protein E8 (SP|P10967)
DATA:                  Control 30min   2hours  2days   1week   p-value        pos
SENSE COUNTS:          1       0       8       3       4       6.89e-02
GENES (1 total):
  AT2G25450.1
    SENSE COUNTS:      1       0       8       3       4       6.89e-02
    TAGS: (4 total)
           ----------                                                     1301
      d+2  TTCAAGACCT  0       0       2       1       3       4.51e-01       1244
      d+2  GAGCATAGAA  0       0       5       0       1       5.51e-03       948
           ----------                                                     606
           ----------                                                     552
      d+2  GGGTTCCACT  1       0       1       1       0       9.46e-01       370
      d+2  TTAACGTAGC  0       0       0       1       0       3.09e-01       214

LOCUS: AT1G54690
```

```
DESCRIPTION: histone H2A, putative, strong similarity to histone H2A GI:3204129 SP|O65759 from Cicer arietinum,
Picea abies SP|P35063; contains Pfam profile PF00125 Core histone H2A/H2B/H3/H4
DATA:                  Control 30min  2hours  2days  1week  p-value       pos
SENSE COUNTS:          1       3      7       6      0      6.99e-02
GENES (1 total):
  AT1G54690.1
    SENSE COUNTS:      1       3      7       6      0      6.99e-02
    TAGS: (1 total)
           ----------                                                 708
      d+2  TTACTCAAAA  1       3      7       6      0      6.99e-02  576
           ----------                                                 541

LOCUS: AT4G11175
DESCRIPTION: translation initiation factor IF-1, chloroplast, putative, similar to Swiss-Prot:P08698
translation initiation factor IF-1, chloroplast (Spinacia oleracea); contains Pfam profile PF00575: S1 RNA
binding domain
DATA:                  Control 30min  2hours  2days  1week  p-value       pos
SENSE COUNTS:          7       0      6       9      10     7.07e-02
GENES (1 total):
  AT4G11175.1
    SENSE COUNTS:      7       0      6       9      10     7.07e-02
    TAGS: (2 total)
      d+1  ATTAGGTTTA  2       0      1       0      0      2.87e-01  689
           ----------                                                 671
           ----------                                                 659
      d+2  TAATCGTACA  5       0      5       9      10     5.80e-02  566

LOCUS: ATCG00270
DESCRIPTION: PSII D2 protein
DATA:                  Control 30min  2hours  2days  1week  p-value       pos
SENSE COUNTS:          1       0      0       0      3      7.14e-02
GENES (1 total):
  ATCG00270.1
    SENSE COUNTS:      1       0      0       0      3      7.14e-02
    TAGS: (2 total)
      d+2  GTGCTACTGT  1       0      0       0      0      4.28e-01  647
      d+2  GATTGGCCAG  0       0      0       0      3      1.12e-02  188

LOCUS: AT1G36580
DESCRIPTION: 2,4-dienoyl-CoA reductase-related, low similarity to peroxisomal 2,4-dienoyl-CoA reductase (Homo
sapiens) GI:9967554
DATA:                  Control 30min  2hours  2days  1week  p-value       pos
SENSE COUNTS:          1       0      0       0      3      7.14e-02
GENES (1 total):
  AT1G36580.1
    SENSE COUNTS:      1       0      0       0      3      7.14e-02
    TAGS: (1 total)
           ----------                                                 1554
           ----------                                                 1410
           ----------                                                 1203
           ----------                                                 999
      v+2  AGTATTACTT  1       0      0       0      3      7.14e-02  945
           ----------                                                 354
           ----------                                                 162
           ----------                                                 151

LOCUS: AT1G13580
DESCRIPTION: longevity-assurance (LAG1) family protein, similar to Alternaria stem canker resistance protein
(ASC1) (Lycopersicon esculentum) GI:7688742; contains Pfam profile PF03798: Longevity-assurance protein (LAG1)
DATA:                  Control 30min  2hours  2days  1week  p-value       pos
SENSE COUNTS:          1       0      0       0      3      7.14e-02
GENES (1 total):
  AT1G13580.1
    SENSE COUNTS:      1       0      0       0      3      7.14e-02
    TAGS: (1 total)
           ----------                                                 1372
           ----------                                                 1281
           ----------                                                 1256
      d+2  TTTAACACTC  1       0      0       0      3      7.14e-02  948
           ----------                                                 132

LOCUS: AT3G16660
DESCRIPTION: expressed protein,
DATA:                  Control 30min  2hours  2days  1week  p-value       pos
SENSE COUNTS:          2       1      0       7      3      7.14e-02
GENES (1 total):
  AT3G16660.1
    SENSE COUNTS:      2       1      0       7      3      7.14e-02
    TAGS: (3 total)
      d+1  AATAAGGTCT  1       1      0       0      0      4.77e-01  718
           ----------                                                 690
```

```
      X+4  CCAAGAAGTA 0       0       0       2       0       2.03e-01        550
           ----------                                                         547
      X+4  TCTAATGCTT 1       0       0       5       3       7.79e-02        459
           ----------                                                         103
           ----------                                                         79

LOCUS: AT3G62410
DESCRIPTION: CP12 domain-containing protein, contains Pfam domain PF02672: CP12 domain
DATA:                 Control 30min   2hours  2days   1week   p-value         pos
SENSE COUNTS:         18      8       20      6       13      7.22e-02
GENES (1 total):
  AT3G62410.1
    SENSE COUNTS:     18      8       20      6       13      7.22e-02
    TAGS: (2 total)
      X+4  AGACACAAGA 0       0       0       0       1       1.65e-01        692
      d+1  TCTTTGAATC 18      8       20      6       12      6.76e-02        546
           ----------                                                         364

LOCUS: AT2G22300
DESCRIPTION: ethylene-responsive calmodulin-binding protein, putative (SR1), identical to partial sequence of
ethylene-induced calmodulin-binding protein GI:11545505 from (Arabidopsis thaliana); contains Pfam profiles
PF03859: CG-1 domain, PF00612: IQ calmodulin-bindi
DATA:                 Control 30min   2hours  2days   1week   p-value         pos
SENSE COUNTS:         1       5       9       4       1       7.26e-02
GENES (1 total):
  AT2G22300.1
    SENSE COUNTS:     1       5       9       4       1       7.26e-02
    TAGS: (3 total)
           ----------                                                         3889
           ----------                                                         3765
           ----------                                                         3609
           ----------                                                         3496
      d+2  ACTCTAATTT 1       5       7       4       1       2.32e-01        3413
           ----------                                                         3103
      d+2  TGAGAGGTTA 0       0       1       0       0       7.06e-01        2864
           ----------                                                         2492
           ----------                                                         2136
      d+2  AATGAAATCT 0       0       1       0       0       4.55e-01        2065
           ----------                                                         1833
           ----------                                                         1815
           ----------                                                         1784
           ----------                                                         1714
           ----------                                                         1552
           ----------                                                         1213
           ----------                                                         1145
           ----------                                                         842
           ----------                                                         713
           ----------                                                         524
           ----------                                                         473
           ----------                                                         242

LOCUS: AT1G11490
DESCRIPTION: zinc finger (C2H2 type) family protein, contains zinc finger, C2H2 type, domain, PROSITE:PS00028
DATA:                 Control 30min   2hours  2days   1week   p-value         pos
SENSE COUNTS:         0       0       1       0       3       7.32e-02
GENES (1 total):
  AT1G11490.1
    SENSE COUNTS:     0       0       1       0       3       7.32e-02
    TAGS: (1 total)
           ----------                                                         1927
           ----------                                                         1705
      v+2  AAACAAGCCC 0       0       1       0       3       7.32e-02        1448
           ----------                                                         1144
           ----------                                                         751
           ----------                                                         354
           ----------                                                         178
           ----------                                                         168

LOCUS: AT5G48470
DESCRIPTION: expressed protein
DATA:                 Control 30min   2hours  2days   1week   p-value         pos
SENSE COUNTS:         0       0       1       0       3       7.32e-02
GENES (1 total):
  AT5G48470.1
    SENSE COUNTS:     0       0       1       0       3       7.32e-02
    TAGS: (2 total)
      i+3  ATGCTGTGTT 0       0       1       0       0       4.55e-01        1680
      d+1  TGACGTAAAA 0       0       0       0       3       1.12e-02        1399
           ----------                                                         851
           ----------                                                         651
           ----------                                                         600
```

```
           ----------                                                     497

LOCUS: AT3G18350
DESCRIPTION: expressed protein, contains Pfam profile: PF04842 plant protein of unknown function (DUF639)
DATA:                  Control 30min   2hours  2days   1week   p-value        pos
SENSE COUNTS:          0       0       1       0       3       7.32e-02
GENES (1 total):
  AT3G18350.1
    SENSE COUNTS:      0       0       1       0       3       7.32e-02
    TAGS: (1 total)
           ----------                                                     2374
           ----------                                                     2019
           ----------                                                     1983
           ----------                                                     1033
           ----------                                                     904
      d+2  ATGGTTGTCT  0       0       1       0       3       7.32e-02       696

LOCUS: AT3G02150
DESCRIPTION: TCP family transcription factor, putative, similar to transcription factor PCF6 (Oryza sativa
(japonica cultivar-group)) GI:20975255; contains Pfam profile PF03634: TCP family transcription factor
DATA:                  Control 30min   2hours  2days   1week   p-value        pos
SENSE COUNTS:          0       0       1       0       3       7.32e-02
GENES (3 total):
  AT3G02150.1
    SENSE COUNTS:      0       0       1       0       3       7.32e-02
    TAGS: (1 total)
           ----------                                                     1317
           ----------                                                     1098
           ----------                                                     780
      X+4  TGTTTTGAAA  0       0       1       0       3       7.32e-02       304

LOCUS: AT5G28535
DESCRIPTION: Mariner-like transposase family, has a 1.2e-97 P-value blast match to GB:AAC28384 mariner
transposase (Mariner_TC1-element) (Glycine max)
DATA:                  Control 30min   2hours  2days   1week   p-value        pos
SENSE COUNTS:          0       0       1       0       3       7.32e-02
GENES (1 total):
  AT5G28535.1
    SENSE COUNTS:      0       0       1       0       3       7.32e-02
    TAGS: (1 total)
           ----------                                                     1191
      p+2  AAAGAAATGA  0       0       1       0       3       7.32e-02       1056
           ----------                                                     982
           ----------                                                     953
           ----------                                                     805
           ----------                                                     739
           ----------                                                     380

LOCUS: AT1G18510
DESCRIPTION: hypothetical protein
DATA:                  Control 30min   2hours  2days   1week   p-value        pos
SENSE COUNTS:          0       0       1       0       3       7.32e-02
GENES (1 total):
  AT1G18510.1
    SENSE COUNTS:      0       0       1       0       3       7.32e-02
    TAGS: (2 total)
      v+1  CAATAATAAA  0       0       1       0       0       4.55e-01       1401
           ----------                                                     1096
      v+2  TTCTCTTATC  0       0       0       0       3       1.12e-02       963
           ----------                                                     865
           ----------                                                     730
           ----------                                                     41

LOCUS: AT1G41810
DESCRIPTION: hypothetical protein
DATA:                  Control 30min   2hours  2days   1week   p-value        pos
SENSE COUNTS:          8       3       10      15      14      7.37e-02
GENES (1 total):
  AT1G41810.1
    SENSE COUNTS:      8       3       10      15      14      7.37e-02
    TAGS: (1 total)
           ----------                                                     1354
           ----------                                                     1287
           ----------                                                     1209
      v+2  TTACCAAAAA  8       3       10      15      14      7.37e-02       1042

LOCUS: AT3G47400
DESCRIPTION: pectinesterase family protein, similar to pectinesterase (EC 3.1.1.11) from Vitis vinifera
GI:15081598, Lycopersicon esculentum SP|Q43143 SP|P14280; contains Pfam profile PF01095 pectinesterase
DATA:                  Control 30min   2hours  2days   1week   p-value        pos
SENSE COUNTS:          4       2       2       11      7       7.43e-02
```

```
GENES (1 total):
  AT3G47400.1
    SENSE COUNTS:      4       2       2       11      7       7.43e-02
    TAGS: (1 total)
           ----------                                                     1839
           ----------                                                     1785
           ----------                                                     1567
           ----------                                                     1397
           ----------                                                     1372
      d+2  GATTCCAAGA  4       2       2       11      7       7.43e-02       1256
           ----------                                                     1162
           ----------                                                     1097
           ----------                                                     1039
           ----------                                                     833
           ----------                                                     776
           ----------                                                     766
           ----------                                                     697
           ----------                                                     330
           ----------                                                     297
           ----------                                                     292
           ----------                                                     113

LOCUS: AT4G34720
DESCRIPTION: vacuolar ATP synthase 16 kDa proteolipid subunit 1 / V-ATPase 16 kDa proteolipid subunit 1
(AVAP1) (AVA-P1), identical to SP|P59227 Vacuolar ATP synthase 16 kDa proteolipid subunit 1/3/5 (EC 3.6.3.14)
(V-ATPase 16 kDa proteolipid subunit 1/3/5) {Arabidops
DATA:                  Control 30min   2hours  2days   1week   p-value        pos
SENSE COUNTS:          0       7       3       2       3       7.45e-02
GENES (1 total):
  AT4G34720.1
    SENSE COUNTS:      0       7       3       2       3       7.45e-02
    TAGS: (2 total)
      d+1  TTGAACGTTT  0       0       1       0       0       4.55e-01       1008
      d+2  TCTACGTTCA  0       7       2       2       3       4.78e-02       134

LOCUS: AT1G08980
DESCRIPTION: amidase family protein, similar to component of chloroplast outer membrane translocon Toc64
(Pisum sativum) GI:7453538; contains Pfam profile PF01425: Amidase; supporting cDNA
gi|11493701|gb|AF202077.1|AF202077
DATA:                  Control 30min   2hours  2days   1week   p-value        pos
SENSE COUNTS:          5       8       1       2       7       7.45e-02
GENES (2 total):
  AT1G08980.1
    SENSE COUNTS:      5       8       1       2       7       7.45e-02
    TAGS: (3 total)
      d+1  TCATAAACTT  1       7       1       1       3       3.33e-02       1317
      d+2  GATCTCGTCG  0       0       0       0       1       1.65e-01       918
           ----------                                                     893
           ----------                                                     856
           ----------                                                     805
           ----------                                                     683
      i+3  TTTTAAGAAA  4       1       0       1       3       2.66e-01       415

LOCUS: AT5G57440
DESCRIPTION: haloacid dehalogenase-like hydrolase family protein, similar to SP|Q08623 GS1 protein {Homo
sapiens}; contains InterPro accession IPR005834: Haloacid dehalogenase-like hydrolase
DATA:                  Control 30min   2hours  2days   1week   p-value        pos
SENSE COUNTS:          1       1       0       0       4       7.47e-02
GENES (2 total):
  AT5G57440.1
    SENSE COUNTS:      1       1       0       0       4       7.47e-02
    TAGS: (2 total)
      d+1  TTTTACACTA  0       0       0       0       1       1.65e-01       1121
           ----------                                                     1098
      d+2  ACGAGAGACC  1       1       0       0       3       2.97e-01       925
           ----------                                                     852
           ----------                                                     840
           ----------                                                     834
           ----------                                                     797
           ----------                                                     364
           ----------                                                     330
           ----------                                                     93

LOCUS: AT3G16290
DESCRIPTION: FtsH protease, putative, contains similarity to cell division protein FtsH GI:1652085 from
(Synechocystis sp. PCC 6803)
DATA:                  Control 30min   2hours  2days   1week   p-value        pos
SENSE COUNTS:          3       0       2       6       1       7.48e-02
GENES (1 total):
  AT3G16290.1
    SENSE COUNTS:      3       0       2       6       1       7.48e-02
```

```
    TAGS: (1 total)
           ----------                                                   3317
           ----------                                                   3307
           ----------                                                   3249
           ----------                                                   3232
      v+2  TTAGATAATT  3      0      2      6      1      7.48e-02      3112
           ----------                                                   2958
           ----------                                                   2793
           ----------                                                   2745
           ----------                                                   2674
           ----------                                                   2502
           ----------                                                   2345
           ----------                                                   2193
           ----------                                                   2181
           ----------                                                   2128
           ----------                                                   1645
           ----------                                                   1500
           ----------                                                   995
           ----------                                                   748
           ----------                                                   481
           ----------                                                   449
           ----------                                                   354
           ----------                                                   315
           ----------                                                   66
           ----------                                                   10

LOCUS: AT5G19610
DESCRIPTION: Sec7 domain-containing protein, similar to SP|Q42510 Pattern formation protein EMB30 (GNOM)
{Arabidopsis thaliana}; contains Pfam profile PF01369: Sec7 domain
DATA:                  Control 30min  2hours  2days  1week  p-value       pos
SENSE COUNTS:          4      1      10     2      3      7.54e-02
GENES (1 total):
  AT5G19610.1
    SENSE COUNTS:      4      1      10     2      3      7.54e-02
    TAGS: (1 total)
           ----------                                                   4549
           ----------                                                   4508
           ----------                                                   4359
           ----------                                                   4347
           ----------                                                   4185
           ----------                                                   4149
           ----------                                                   4093
           ----------                                                   4063
      v+2  AAGATCTTGG  4      1      10     2      3      7.54e-02      4015
           ----------                                                   4008
           ----------                                                   3867
           ----------                                                   3765
           ----------                                                   3485
           ----------                                                   3295
           ----------                                                   3276
           ----------                                                   2925
           ----------                                                   2590
           ----------                                                   1750
           ----------                                                   1374
           ----------                                                   1050
           ----------                                                   952
           ----------                                                   881
           ----------                                                   822
           ----------                                                   405
           ----------                                                   401

LOCUS: AT3G27770
DESCRIPTION: expressed protein
DATA:                  Control 30min  2hours  2days  1week  p-value       pos
SENSE COUNTS:          1      3      7      2      0      7.55e-02
GENES (1 total):
  AT3G27770.1
    SENSE COUNTS:      1      3      7      2      0      7.55e-02
    TAGS: (1 total)
           ----------                                                   1826
           ----------                                                   1573
      d+2  GCTCCAGTGT  1      3      7      2      0      7.55e-02      1035
           ----------                                                   984
           ----------                                                   843
           ----------                                                   672

LOCUS: AT4G18030
DESCRIPTION: dehydration-responsive family protein, similar to early-responsive to dehydration stress ERD3
protein (Arabidopsis thaliana) GI:15320410; contains Pfam profile PF03141: Putative methyltransferase
DATA:                  Control 30min  2hours  2days  1week  p-value       pos
SENSE COUNTS:          3      7      10     2      1      7.59e-02
```

```
GENES (1 total):
  AT4G18030.1
    SENSE COUNTS:      3       7       10      2       1       7.59e-02
    TAGS: (3 total)
      d+1  TGTTTTTGCA  2       2       6       1       1       2.94e-01       2233
      d+2  AAGACGGTCC  1       5       4       0       0       8.40e-02       1888
           ----------                                                         1660
           ----------                                                         1223
           ----------                                                         1079
           ----------                                                         980
           ----------                                                         951
           ----------                                                         468
      X+4  GGTCACCTTT  0       0       0       1       0       3.09e-01       464
           ----------                                                         236
           ----------                                                         135

LOCUS: AT3G22133
DESCRIPTION: pseudogene, hypothetical protein, similar to putative non-LTR retroelement reverse transcriptase
GB:AAC33226.1 from (Arabidopsis thaliana)
DATA:                  Control 30min   2hours  2days   1week   p-value        pos
SENSE COUNTS:          10      7       15      2       7       7.60e-02
GENES (1 total):
  AT3G22133.1
    SENSE COUNTS:      10      7       15      2       7       7.60e-02
    TAGS: (2 total)
           ----------                                                         284
           ----------                                                         150
      p+2  AATTTCAGAA  9       7       14      2       7       1.30e-01       90
           ----------                                                         52
      X+4  GAGATTCATA  1       0       1       0       0       6.01e-01       -171

LOCUS: AT3G08680
DESCRIPTION: leucine-rich repeat transmembrane protein kinase, putative, contains Pfam profile: PF00069
Eukaryotic protein kinase domain, PF00560 leucine Rich Repeat (5 copies)
DATA:                  Control 30min   2hours  2days   1week   p-value        pos
SENSE COUNTS:          6       0       5       5       1       7.72e-02
GENES (2 total):
  AT3G08680.1
    SENSE COUNTS:      2       0       2       1       0       4.40e-01
    TAGS: (1 total)
      d+1  AATGGTTGTA  2       0       2       1       0       4.40e-01       2452
           ----------                                                         2196
           ----------                                                         2144
           ----------                                                         2002
           ----------                                                         1810
           ----------                                                         1765
           ----------                                                         1648
           ----------                                                         1638
           ----------                                                         1040
           ----------                                                         739
  AT3G08680.2
    SENSE COUNTS:      6       0       5       5       1       7.72e-02
    TAGS: (2 total)
      d+1  AATGGTTGTA  2       0       2       1       0       4.40e-01       2527
           ----------                                                         2271
           ----------                                                         2219
           ----------                                                         2077
           ----------                                                         1885
           ----------                                                         1840
           ----------                                                         1723
           ----------                                                         1713
           ----------                                                         1115
      X+4  AGTCCTAATT  4       0       3       4       1       2.61e-01       1027
           ----------                                                         814

LOCUS: AT2G15880
DESCRIPTION: leucine-rich repeat family protein / extensin family protein, similar to extensin-like protein
(Lycopersicon esculentum) gi|5917664|gb|AAD55979; contains leucine-rich repeats, Pfam:PF00560; contains
proline rich extensin domains, INTERPRO:IPR002965
DATA:                  Control 30min   2hours  2days   1week   p-value        pos
SENSE COUNTS:          2       8       4       2       1       7.74e-02
GENES (1 total):
  AT2G15880.1
    SENSE COUNTS:      2       8       4       2       1       7.74e-02
    TAGS: (2 total)
           ----------                                                         3032
      v+2  AGGAAAACTG  2       8       4       2       1       1.43e-01       2574
           ----------                                                         1678
      v+2  TGTTCCAGGT  0       0       0       0       0       6.15e-01       1373
           ----------                                                         1200
           ----------                                                         1098
```

```
          ----------                                                    619
          ----------                                                    610

LOCUS: AT3G14110
DESCRIPTION: tetratricopeptide repeat (TPR)-containing protein, contains Pfam profile PF00515: TPR Domain
DATA:                 Control 30min  2hours  2days  1week  p-value        pos
SENSE COUNTS:         11      6      8       0      4      7.85e-02
GENES (3 total):
  AT3G14110.2
    SENSE COUNTS:     11      6      8       0      4      7.85e-02
    TAGS: (3 total)
          ----------                                                    1503
          ----------                                                    1471
          ----------                                                    1426
     d+2  GGAATACATA  8       4      3       0      3      1.60e-01       1251
     d+2  TAAAATCACA  3       2      4       0      1      4.91e-01       1183
     d+2  GTTCTAGCCA  0       0      1       0      0      4.55e-01       992
          ----------                                                    572
          ----------                                                    563
  AT3G14110.1
    SENSE COUNTS:     11      6      8       0      4      7.85e-02
    TAGS: (3 total)
          ----------                                                    1372
          ----------                                                    1340
          ----------                                                    1295
     d+2  GGAATACATA  8       4      3       0      3      1.60e-01       1120
     d+2  TAAAATCACA  3       2      4       0      1      4.91e-01       1052
     d+2  GTTCTAGCCA  0       0      1       0      0      4.55e-01       861
          ----------                                                    441
          ----------                                                    432

LOCUS: AT5G50320
DESCRIPTION: radical SAM domain-containing protein / GCN5-related N-acetyltransferase (GNAT) family protein,
contains Pfam profiles PF00583: acetyltransferase, GNAT family, PF04055: Radical SAM superfamily
DATA:                 Control 30min  2hours  2days  1week  p-value        pos
SENSE COUNTS:         0       1      0       4      0      7.92e-02
GENES (1 total):
  AT5G50320.1
    SENSE COUNTS:     0       1      0       4      0      7.92e-02
    TAGS: (1 total)
          ----------                                                    2284
          ----------                                                    2078
          ----------                                                    1913
     d+2  GTGAAGCATC  0       1      0       4      0      7.92e-02       1720
          ----------                                                    1550
          ----------                                                    1523
          ----------                                                    1492
          ----------                                                    1170
          ----------                                                    1159
          ----------                                                    988
          ----------                                                    665
          ----------                                                    619
          ----------                                                    499
          ----------                                                    172

LOCUS: AT5G17230
DESCRIPTION: phytoene synthase (PSY) / geranylgeranyl-diphosphate geranylgeranyl transferase, identical to
GB:L25812; synonymous with geranylgeranyl-diphosphate geranylgeranyl transferase
DATA:                 Control 30min  2hours  2days  1week  p-value        pos
SENSE COUNTS:         1       2      3       9      4      7.92e-02
GENES (2 total):
  AT5G17230.1
    SENSE COUNTS:     1       2      3       9      4      7.92e-02
    TAGS: (2 total)
     i+3  AACACCAAGC  0       0      0       0      1      1.65e-01       1613
     d+1  AAAATGCAGC  1       2      3       9      3      7.19e-02       1418
          ----------                                                    1088
          ----------                                                    962

LOCUS: AT1G02170
DESCRIPTION: latex-abundant family protein (AMC1) / caspase family protein, contains similarity to latex-
abundant protein (Hevea brasiliensis) gb:AAD13216; contains Pfam profile PF00656: ICE-like protease (caspase)
p20 domain
DATA:                 Control 30min  2hours  2days  1week  p-value        pos
SENSE COUNTS:         1       3      0       5      0      7.93e-02
GENES (2 total):
  AT1G02170.1
    SENSE COUNTS:     1       3      0       5      0      7.93e-02
    TAGS: (1 total)
          ----------                                                    1382
     d+2  CTTCTGACAG  1       3      0       5      0      7.93e-02       1006
```

```
          ----------                                                  629
          ----------                                                  494
          ----------                                                  421
          ----------                                                  370
          ----------                                                  319
          ----------                                                  233

LOCUS: AT5G54030
DESCRIPTION: DC1 domain-containing protein, contains Pfam profile PF03107: DC1 domain
DATA:                 Control 30min   2hours  2days   1week   p-value         pos
SENSE COUNTS:         0       0       3       0       1       7.97e-02
GENES (1 total):
  AT5G54030.1
    SENSE COUNTS:     0       0       3       0       1       7.97e-02
    TAGS: (1 total)
          ----------                                                  2057
          ----------                                                  1661
          ----------                                                  1540
          ----------                                                  1471
          ----------                                                  1417
          ----------                                                  1286
      v+2  CGATGTCGGT 0       0       3       0       1       7.97e-02        1245
          ----------                                                  1201
          ----------                                                  1192
          ----------                                                  931
          ----------                                                  399
          ----------                                                  79
          ----------                                                  13

LOCUS: AT5G37381
DESCRIPTION: copia-like retrotransposon family, has a 1.5e-121 P-value blast match to GB:BAA78424 polyprotein
(Ty1_Copia-element) (Arabidopsis thaliana)gi|4996363|dbj|BAA78424.1| polyprotein (AtRE2) (Arabidopsis
thaliana) (Ty1_Copia-element)
DATA:                 Control 30min   2hours  2days   1week   p-value         pos
SENSE COUNTS:         2       0       0       0       3       7.98e-02
GENES (1 total):
  AT5G37381.1
    SENSE COUNTS:     2       0       0       0       3       7.98e-02
    TAGS: (2 total)
          ----------                                                  2849
          ----------                                                  2762
          ----------                                                  2705
          ----------                                                  2672
          ----------                                                  2452
          ----------                                                  2415
          ----------                                                  1987
          ----------                                                  1702
          ----------                                                  1676
      p+2  TGGTTATGAT 1       0       0       0       3       7.14e-02        1580
          ----------                                                  1530
      p+2  GAGTATTGTT 1       0       0       0       0       4.28e-01        1416
          ----------                                                  1076
          ----------                                                  1001
          ----------                                                  875
          ----------                                                  685
          ----------                                                  447

LOCUS: AT1G76810
DESCRIPTION: eukaryotic translation initiation factor 2 family protein / eIF-2 family protein, similar to IF2
protein (Drosophila melanogaster) GI:7108770; contains Pfam profile PF03144: Elongation factor Tu domain 2
DATA:                 Control 30min   2hours  2days   1week   p-value         pos
SENSE COUNTS:         3       14      8       7       4       7.98e-02
GENES (1 total):
  AT1G76810.1
    SENSE COUNTS:     3       14      8       7       4       7.98e-02
    TAGS: (2 total)
      d+1  CTGGCAAATT 2       7       5       5       3       6.47e-01        4261
          ----------                                                  4251
          ----------                                                  4076
          ----------                                                  3904
          ----------                                                  3645
          ----------                                                  3209
          ----------                                                  3014
          ----------                                                  2972
          ----------                                                  2806
          ----------                                                  2664
          ----------                                                  2558
          ----------                                                  2288
          ----------                                                  2281
          ----------                                                  2066
          ----------                                                  1385
```

```
      d+2  CGTCTAAGAA 1      7      3      2      1      1.16e-01       593

LOCUS: AT2G44860
DESCRIPTION: 60S ribosomal protein L24, putative
DATA:                 Control 30min  2hours  2days  1week  p-value        pos
SENSE COUNTS:         0      2      1      6      3      8.05e-02
GENES (1 total):
  AT2G44860.1
    SENSE COUNTS:     0      2      1      6      3      8.05e-02
    TAGS: (1 total)
           ----------                                                    1268
           ----------                                                    1192
           ----------                                                    1015
      d+2  CGAATGGTTA 0      2      1      6      3      8.05e-02       810
           ----------                                                    598
           ----------                                                    400
           ----------                                                    316
           ----------                                                    179

LOCUS: AT5G43720
DESCRIPTION: expressed protein
DATA:                 Control 30min  2hours  2days  1week  p-value        pos
SENSE COUNTS:         0      1      5      3      0      8.07e-02
GENES (1 total):
  AT5G43720.1
    SENSE COUNTS:     0      1      5      3      0      8.07e-02
    TAGS: (2 total)
      d+1  GAGCTTTTTT 0      1      1      2      0      4.78e-01       1306
      d+2  GAATTCAGAG 0      0      4      1      0      2.60e-02       1216
           ----------                                                    825
           ----------                                                    466
           ----------                                                    343
           ----------                                                    120

LOCUS: AT2G38950
DESCRIPTION: transcription factor jumonji (jmj) family protein / zinc finger (C5HC2 type) family protein,
contains Pfam domains, PF02375: jmjN domain, PF02373: jmjC domain and PF02928: C5HC2 zinc finger
DATA:                 Control 30min  2hours  2days  1week  p-value        pos
SENSE COUNTS:         3      0      5      6      1      8.11e-02
GENES (1 total):
  AT2G38950.1
    SENSE COUNTS:     3      0      5      6      1      8.11e-02
    TAGS: (3 total)
      d+1  AAGCAACTTG 0      0      2      2      1      3.02e-01       2422
      d+2  CGATAACGAT 0      0      1      0      0      4.55e-01       2259
           ----------                                                    1911
           ----------                                                    1894
           ----------                                                    1860
           ----------                                                    1653
           ----------                                                    1517
           ----------                                                    1288
           ----------                                                    1223
           ----------                                                    856
           ----------                                                    630
           ----------                                                    618
           ----------                                                    404
      X+4  TTGGATAAAA 3      0      2      4      0      1.79e-01       -110

LOCUS: AT1G05790
DESCRIPTION: lipase class 3 family protein, contains Pfam profile PF01764: Lipase
DATA:                 Control 30min  2hours  2days  1week  p-value        pos
SENSE COUNTS:         4      0      1      0      0      8.14e-02
GENES (1 total):
  AT1G05790.1
    SENSE COUNTS:     4      0      1      0      0      8.14e-02
    TAGS: (2 total)
      i+3  GCAGAAAAAT 0      0      0      0      0      6.15e-01       2287
      d+1  AACAGGTTTC 4      0      1      0      0      2.22e-02       2076
           ----------                                                    1855
           ----------                                                    1813
           ----------                                                    1401
           ----------                                                    1374
           ----------                                                    1103
           ----------                                                    1097
           ----------                                                    911
           ----------                                                    872
           ----------                                                    789
           ----------                                                    214
           ----------                                                    184

LOCUS: AT5G13430
```

```
DESCRIPTION: ubiquinol-cytochrome C reductase iron-sulfur subunit, mitochondrial, putative / Rieske iron-
sulfur protein, putative, similar to ubiquinol--cytochrome-c reductase from Solanum tuberosum (SP|P37841),
Nicotiana tabacum (SP|P51132) (SP|P51133); non-consensus
DATA:                 Control 30min   2hours  2days   1week   p-value        pos
SENSE COUNTS:         3       5       4       10      13      8.14e-02
GENES (1 total):
  AT5G13430.1
    SENSE COUNTS:     3       5       4       10      13      8.14e-02
    TAGS: (2 total)
      d+1  CCTATTTGTC 0       0       0       1       1       6.17e-01       1155
      d+2  TTAGAGCAAG 3       5       4       9       12      1.72e-01       1022
           ----------                                                        529
           ----------                                                        410

LOCUS: AT5G60270
DESCRIPTION: lectin protein kinase family protein, contains Pfam domains, PF00069: Protein kinase domain,
PF00139: Legume lectins beta domain, and PF00138: Legume lectins alpha domain
DATA:                 Control 30min   2hours  2days   1week   p-value        pos
SENSE COUNTS:         0       3       0       0       1       8.32e-02
GENES (1 total):
  AT5G60270.1
    SENSE COUNTS:     0       3       0       0       1       8.32e-02
    TAGS: (1 total)
           ----------                                                        2769
           ----------                                                        2711
      v+2  AAGCTTAGCT 0       3       0       0       1       8.32e-02       2473
           ----------                                                        2460
           ----------                                                        2163
           ----------                                                        1884
           ----------                                                        1648
           ----------                                                        1611
           ----------                                                        1585
           ----------                                                        1527
           ----------                                                        1480
           ----------                                                        1128
           ----------                                                        1086
           ----------                                                        808
           ----------                                                        651
           ----------                                                        646
           ----------                                                        544
           ----------                                                        132

LOCUS: AT1G49975
DESCRIPTION: Expressed protein
DATA:                 Control 30min   2hours  2days   1week   p-value        pos
SENSE COUNTS:         0       3       0       0       1       8.32e-02
GENES (1 total):
  AT1G49975.1
    SENSE COUNTS:     0       3       0       0       1       8.32e-02
    TAGS: (2 total)
      X+4  TGGACAGATG 0       0       0       0       1       1.65e-01       540
      d+1  GCTTTAACAG 0       3       0       0       0       3.05e-02       60

LOCUS: AT4G34215
DESCRIPTION: Expressed protein
DATA:                 Control 30min   2hours  2days   1week   p-value        pos
SENSE COUNTS:         0       2       5       1       0       8.33e-02
GENES (2 total):
  AT4G34215.1
    SENSE COUNTS:     0       0       1       0       0       4.55e-01
    TAGS: (1 total)
      d+1  AATCTTTCTG 0       0       1       0       0       4.55e-01       930
           ----------                                                        675
           ----------                                                        621
  AT4G34215.2
    SENSE COUNTS:     0       2       4       1       0       1.88e-01
    TAGS: (1 total)
           ----------                                                        1072
      d+2  GCCGATAACT 0       2       4       1       0       1.88e-01       1039
           ----------                                                        972
           ----------                                                        594
           ----------                                                        540

LOCUS: AT3G48740
DESCRIPTION: nodulin MtN3 family protein, similar to MtN3 GI:1619602 (root nodule development) from (Medicago
truncatula)
DATA:                 Control 30min   2hours  2days   1week   p-value        pos
SENSE COUNTS:         2       7       2       1       1       8.36e-02
GENES (1 total):
  AT3G48740.1
    SENSE COUNTS:     2       7       2       1       1       8.36e-02
```

```
    TAGS: (1 total)
           ----------                                                  1190
      d+2  AATAAAAATA  2      7      2      1      1      8.36e-02       960
           ----------                                                  929
           ----------                                                  589
           ----------                                                  120
           ----------                                                  94

LOCUS: AT4G32000
DESCRIPTION: protein kinase family protein, contains protein kinase domain, Pfam:PF00069
DATA:                 Control 30min  2hours  2days  1week  p-value        pos
SENSE COUNTS:         0      0      0      0      3      8.36e-02
GENES (1 total):
  AT4G32000.1
    SENSE COUNTS:     0      0      0      0      3      8.36e-02
    TAGS: (1 total)
           ----------                                                  1801
      i+3  AAATAAATAA  0      0      0      0      3      8.36e-02       1215
           ----------                                                  1032
           ----------                                                  1025
           ----------                                                  504
           ----------                                                  477
           ----------                                                  117

LOCUS: AT5G38600
DESCRIPTION: proline-rich spliceosome-associated (PSP) family protein / zinc knuckle (CCHC-type) family
protein, contains Pfam domains PF00098: Zinc knuckle, PF04046: PSP
DATA:                 Control 30min  2hours  2days  1week  p-value        pos
SENSE COUNTS:         0      0      0      0      3      8.36e-02
GENES (1 total):
  AT5G38600.1
    SENSE COUNTS:     0      0      0      0      3      8.36e-02
    TAGS: (1 total)
           ----------                                                  2301
      d+2  TTTCTTATTA  0      0      0      0      3      8.36e-02       1975
           ----------                                                  1836
           ----------                                                  1830
           ----------                                                  1416
           ----------                                                  649
           ----------                                                  513

LOCUS: AT1G79780
DESCRIPTION: integral membrane protein, putative, contains 1 transmembrane domain; contains plant integral
membrane protein domain, TIGR01569; contains Pfam PF04535 : Domain of unknown function (DUF588);  similar to
putative ethylene responsive element binding protein
DATA:                 Control 30min  2hours  2days  1week  p-value        pos
SENSE COUNTS:         0      0      0      0      3      8.36e-02
GENES (1 total):
  AT1G79780.1
    SENSE COUNTS:     0      0      0      0      3      8.36e-02
    TAGS: (1 total)
           ----------                                                  1293
           ----------                                                  1179
           ----------                                                  769
           ----------                                                  468
      i+3  ACATTATTGT  0      0      0      0      3      8.36e-02       411
           ----------                                                  24

LOCUS: AT1G25220
DESCRIPTION: Catalyzes the first step of tryptophan biosynthesis: Chorismate L-Glutamine = Anthranilate
Pyruvate L-Glutamate. Functions as a heterocomplex with anthranilate synthase alpha subunit (ASA1 or ASA2).
DATA:                 Control 30min  2hours  2days  1week  p-value        pos
SENSE COUNTS:         0      0      0      0      3      8.36e-02
GENES (2 total):
  AT1G25220.1
    SENSE COUNTS:     0      0      0      0      3      8.36e-02
    TAGS: (1 total)
           ----------                                                  1187
           ----------                                                  1035
           ----------                                                  695
           ----------                                                  550
      d+2  GTCCAATCAT  0      0      0      0      3      8.36e-02       241

LOCUS: AT5G50870
DESCRIPTION: ubiquitin-conjugating enzyme, putative, strong similarity to ubiquitin conjugating enzyme
(Lycopersicon esculentum) GI:886679; contains Pfam profile PF00179: Ubiquitin-conjugating enzyme
DATA:                 Control 30min  2hours  2days  1week  p-value        pos
SENSE COUNTS:         0      0      0      0      3      8.36e-02
GENES (1 total):
  AT5G50870.1
    SENSE COUNTS:     0      0      0      0      3      8.36e-02
```

```
    TAGS: (1 total)
          ----------                                                  472
      X+4  AGATCTTATC  0      0      0      0      3      8.36e-02       253

LOCUS: AT4G38370
DESCRIPTION: phosphoglycerate/bisphosphoglycerate mutase family protein, contains Pfam profile PF00300:
phosphoglycerate mutase family
DATA:                  Control 30min  2hours  2days  1week  p-value        pos
SENSE COUNTS:          0      0      0      0      3      8.36e-02
GENES (1 total):
  AT4G38370.1
    SENSE COUNTS:      0      0      0      0      3      8.36e-02
    TAGS: (1 total)
          ----------                                                  1273
      d+2  CTCATTTATG  0      0      0      0      3      8.36e-02       927
          ----------                                                  898
          ----------                                                  594
          ----------                                                  551
          ----------                                                  542
          ----------                                                  438
          ----------                                                  170

LOCUS: AT5G55630
DESCRIPTION: outward rectifying potassium channel (KCO1), identical to kco1 (Arabidopsis thaliana)
gi|2230761|emb|CAA69158 of the 2 pore, 4 transmembrane (2P/4TM) K+ channel family, PMID:11500563
DATA:                  Control 30min  2hours  2days  1week  p-value        pos
SENSE COUNTS:          0      0      0      0      3      8.36e-02
GENES (3 total):
  AT5G55630.2
    SENSE COUNTS:      0      0      0      0      3      8.36e-02
    TAGS: (2 total)
      d+1  CCTTGTCCTT  0      0      0      0      0      6.15e-01       713
      X+4  GATGATTCTC  0      0      0      0      3      1.12e-02       411
          ----------                                                  354
          ----------                                                  168
  AT5G55630.1
    SENSE COUNTS:      0      0      0      0      0      6.15e-01
    TAGS: (1 total)
      d+1  CCTTGTCCTT  0      0      0      0      0      6.15e-01       921
          ----------                                                  562
          ----------                                                  376
          ----------                                                  267

LOCUS: AT2G07320
DESCRIPTION: SWIM zinc finger family protein, contains Pfam profile PF04434: SWIM zinc finger
DATA:                  Control 30min  2hours  2days  1week  p-value        pos
SENSE COUNTS:          0      0      0      0      3      8.36e-02
GENES (1 total):
  AT2G07320.1
    SENSE COUNTS:      0      0      0      0      3      8.36e-02
    TAGS: (2 total)
      i+3  TCTCGAATTT  0      0      0      0      0      6.15e-01       5277
          ----------                                                  2430
          ----------                                                  1683
      v+2  AGGAAATACA  0      0      0      0      3      1.12e-02       1441
          ----------                                                  1389
          ----------                                                  914
          ----------                                                  846
          ----------                                                  841
          ----------                                                  774
          ----------                                                  293

LOCUS: AT3G51420
DESCRIPTION: strictosidine synthase family protein, similar to hemomucin (Drosophila melanogaster)(GI:1280434),
strictosidine synthase (Rauvolfia serpentina)(SP|P15324); contains strictosidine synthase domain PF03088
DATA:                  Control 30min  2hours  2days  1week  p-value        pos
SENSE COUNTS:          10     4      11     3      1      8.36e-02
GENES (1 total):
  AT3G51420.1
    SENSE COUNTS:      10     4      11     3      1      8.36e-02
    TAGS: (2 total)
          ----------                                                  1563
      d+2  TTTGCTCGTT  0      2      5      2      1      2.52e-01       1421
      d+2  TACGGTTGTT  10     2      6      1      0      1.40e-02       1256
          ----------                                                  1226
          ----------                                                  1036
          ----------                                                  945
          ----------                                                  899
          ----------                                                  759
          ----------                                                  645
```

```
LOCUS: AT3G62030
DESCRIPTION: peptidyl-prolyl cis-trans isomerase, chloroplast / cyclophilin / rotamase / cyclosporin A-binding
protein (ROC4), identical to peptidyl-prolyl cis-trans isomerase, chloroplast precursor, PPIase (cyclophilin,
cyclosporin A-binding protein) (Arabidopsis tha
DATA:                 Control 30min   2hours  2days   1week   p-value         pos
SENSE COUNTS:         37      19      39      36      33      8.47e-02
GENES (2 total):
  AT3G62030.1
    SENSE COUNTS:     37      19      39      36      33      8.47e-02
    TAGS: (4 total)
      d+1  GTTGCAACCG 1       0       0       0       0       6.89e-01        1086
      d+2  AGTTATAAGT 19      6       22      24      19      1.54e-02        1022
      d+2  TCGTGTTTGG 17      13      16      12      14      8.98e-01        751
           ----------                                                         734
      d+2  GCAAACGCTG 0       0       1       0       0       4.55e-01        672
           ----------                                                         555
           ----------                                                         336

LOCUS: AT2G17780
DESCRIPTION: expressed protein, contains Pfam profile PF04749: Protein of unknown function, DUF614
DATA:                 Control 30min   2hours  2days   1week   p-value         pos
SENSE COUNTS:         0       2       0       1       5       8.48e-02
GENES (1 total):
  AT2G17780.1
    SENSE COUNTS:     0       2       0       1       5       8.48e-02
    TAGS: (1 total)
           ----------                                                         1674
           ----------                                                         1201
           ----------                                                         996
           ----------                                                         725
           ----------                                                         175
      i+3  TTTGTTCACT 0       2       0       1       5       8.48e-02        145

LOCUS: AT3G20800
DESCRIPTION: rcd1-like cell differentiation protein, putative, similar to protein involved in sexual
development (Schizosaccharomyces pombe) GI:1620896; contains Pfam profile PF04078: Cell differentiation family,
Rcd1-like
DATA:                 Control 30min   2hours  2days   1week   p-value         pos
SENSE COUNTS:         4       0       4       0       3       8.48e-02
GENES (1 total):
  AT3G20800.1
    SENSE COUNTS:     4       0       4       0       3       8.48e-02
    TAGS: (2 total)
      d+1  TTGAACGGTA 3       0       3       0       0       8.32e-02        1232
           ----------                                                         1064
           ----------                                                         1026
           ----------                                                         993
      i+3  TTCATATATT 1       0       1       0       3       2.09e-01        799
           ----------                                                         609
           ----------                                                         78

LOCUS: AT2G26910
DESCRIPTION: ABC transporter family protein, similar to PDR5-like ABC transporter GI:1514643 from (Spirodela
polyrhiza)
DATA:                 Control 30min   2hours  2days   1week   p-value         pos
SENSE COUNTS:         4       0       4       0       3       8.48e-02
GENES (1 total):
  AT2G26910.1
    SENSE COUNTS:     4       0       4       0       3       8.48e-02
    TAGS: (2 total)
      d+1  AACCAGTACT 3       0       4       0       0       4.29e-02        4493
           ----------                                                         4399
           ----------                                                         4163
           ----------                                                         3979
           ----------                                                         3952
           ----------                                                         3853
           ----------                                                         3252
           ----------                                                         3130
           ----------                                                         2980
           ----------                                                         2840
           ----------                                                         2751
           ----------                                                         2620
           ----------                                                         2428
           ----------                                                         2402
      d+2  GGGTAAGTTT 1       0       0       0       3       7.14e-02        2283
           ----------                                                         1987
           ----------                                                         1806
           ----------                                                         1645
           ----------                                                         1064
           ----------                                                         991
           ----------                                                         882
```

```
          ----------                                                   662
          ----------                                                   419
          ----------                                                   391
          ----------                                                   344

LOCUS: AT3G52230
DESCRIPTION: expressed protein
DATA:                  Control 30min  2hours  2days  1week  p-value        pos
SENSE COUNTS:          1       0      4       1      0      8.52e-02
GENES (1 total):
  AT3G52230.1
    SENSE COUNTS:      1       0      4       1      0      8.52e-02
    TAGS: (2 total)
      i+3  ACTCGAGAAT  0       0      1       0      0      4.55e-01       797
          ----------                                                   753
          ----------                                                   634
      d+2  AAGACTAGTC  1       0      3       1      0      2.40e-01       469

LOCUS: AT5G46250
DESCRIPTION: RNA recognition motif (RRM)-containing protein, contains similarity to RNA-binding protein;
contains InterPro entry IPR000504: RNA-binding region RNP-1 (RNA recognition motif) (RRM)
DATA:                  Control 30min  2hours  2days  1week  p-value        pos
SENSE COUNTS:          1       9      5       5      3      8.55e-02
GENES (2 total):
  AT5G46250.1
    SENSE COUNTS:      1       9      5       5      3      8.55e-02
    TAGS: (3 total)
      i+3  ACTCTTGTTT  0       0      0       1      0      3.09e-01       1360
      d+1  AAGTTTGATC  1       9      5       4      3      1.15e-01       1332
          ----------                                                   1182
          ----------                                                   999
      d+2  CCTTTGTGGA  0       0      0       0      0      6.15e-01       816
          ----------                                                   540
          ----------                                                   195
  AT5G46250.2
    SENSE COUNTS:      1       9      5       5      3      8.55e-02
    TAGS: (3 total)
      i+3  ACTCTTGTTT  0       0      0       1      0      3.09e-01       1360
      d+1  AAGTTTGATC  1       9      5       4      3      1.15e-01       1326
          ----------                                                   1176
          ----------                                                   999
      d+2  CCTTTGTGGA  0       0      0       0      0      6.15e-01       816
          ----------                                                   540
          ----------                                                   195

LOCUS: AT1G53710
DESCRIPTION: calcineurin-like phosphoesterase family protein, contains Pfam profile: PF00149 calcineurin-like
phosphoesterase
DATA:                  Control 30min  2hours  2days  1week  p-value        pos
SENSE COUNTS:          0       0      2       0      3      8.60e-02
GENES (1 total):
  AT1G53710.1
    SENSE COUNTS:      0       0      2       0      3      8.60e-02
    TAGS: (2 total)
          ----------                                                   1957
      v+2  TTTAGTCTAT  0       0      1       0      0      4.55e-01       1654
      v+2  AAATTTTGGA  0       0      1       0      3      7.32e-02       1561
          ----------                                                   1497
          ----------                                                   1455
          ----------                                                   1114
          ----------                                                   1108
          ----------                                                   1052
          ----------                                                   811
          ----------                                                   745
          ----------                                                   615
          ----------                                                   540

LOCUS: AT2G03670
DESCRIPTION: AAA-type ATPase family protein, contains Pfam domain, PF00004: ATPase, AAA family ('A'TPases
'A'ssociated with diverse cellular 'A'ctivities)
DATA:                  Control 30min  2hours  2days  1week  p-value        pos
SENSE COUNTS:          0       0      2       0      3      8.60e-02
GENES (2 total):
  AT2G03670.1
    SENSE COUNTS:      0       0      2       0      3      8.60e-02
    TAGS: (2 total)
      d+1  AGACTTGAAA  0       0      2       0      0      1.21e-01       1883
          ----------                                                   1858
          ----------                                                   1790
          ----------                                                   1494
          ----------                                                   1051
```

```
          ----------                                                    926
          ----------                                                    361
          ----------                                                    75
      X+4 TTAAGACTTA 0       0       0       0       3       1.12e-02   -119

LOCUS: AT5G40770
DESCRIPTION: prohibitin, identical to prohibitin (Arabidopsis thaliana) GI:1946331
DATA:                Control 30min   2hours  2days   1week   p-value    pos
SENSE COUNTS:        1       1       3       0       6       8.64e-02
GENES (2 total):
  AT5G40770.1
    SENSE COUNTS:    1       1       3       0       6       8.64e-02
    TAGS: (2 total)
          ----------                                                    1382
      d+2 CGTGAGAAAA 0       0       1       0       3       7.32e-02   1179
          ----------                                                    1114
      d+2 CTCTTTGCCC 1       1       2       0       3       6.03e-01   883

LOCUS: AT1G16350
DESCRIPTION: inosine-5'-monophosphate dehydrogenase, putative, strong similarity to SP|P47996 gb|L34684
inosine monophosphate dehydrogenase (IMPDH) from Arabidopsis thaliana; member of the PF|00478 IMP
dehydrogenase family
DATA:                Control 30min   2hours  2days   1week   p-value    pos
SENSE COUNTS:        4       1       3       2       10      8.67e-02
GENES (1 total):
  AT1G16350.1
    SENSE COUNTS:    4       1       3       2       10      8.67e-02
    TAGS: (1 total)
          ----------                                                    2165
          ----------                                                    2152
          ----------                                                    2133
      v+2 TAATCCTGTA 4       1       3       2       10      8.67e-02   1937
          ----------                                                    1828
          ----------                                                    1702
          ----------                                                    884
          ----------                                                    849
          ----------                                                    555
          ----------                                                    515

LOCUS: AT4G16620
DESCRIPTION: integral membrane family protein / nodulin MtN21-related, low similarity to MtN21 (Medicago
truncatula) GI:2598575
DATA:                Control 30min   2hours  2days   1week   p-value    pos
SENSE COUNTS:        2       0       2       2       7       8.73e-02
GENES (1 total):
  AT4G16620.1
    SENSE COUNTS:    2       0       2       2       7       8.73e-02
    TAGS: (1 total)
          ----------                                                    1093
          ----------                                                    1005
      i+3 TTTTGATTAA 2       0       2       2       7       8.73e-02   581

LOCUS: AT4G36800
DESCRIPTION: RUB1-conjugating enzyme, putative (RCE1), this gene is frameshifted and may be a pseudogene;
identical over first 79 amino acids to RUB1 conjugating enzyme (Arabidopsis thaliana) GI:6635457
DATA:                Control 30min   2hours  2days   1week   p-value    pos
SENSE COUNTS:        0       3       5       1       0       8.86e-02
GENES (2 total):
  AT4G36800.1
    SENSE COUNTS:    0       3       5       1       0       8.86e-02
    TAGS: (1 total)
          ----------                                                    961
      d+2 ACCGGTGGAT 0       3       5       1       0       8.86e-02   701
          ----------                                                    639
          ----------                                                    462

LOCUS: AT3G05700
DESCRIPTION: drought-responsive family protein, contains similarity to drought-induced mRNA, Di19 (Arabidopsis
thaliana) gi|469110|emb|CAA55321
DATA:                Control 30min   2hours  2days   1week   p-value    pos
SENSE COUNTS:        14      5       14      18      14      8.87e-02
GENES (1 total):
  AT3G05700.1
    SENSE COUNTS:    14      5       14      18      14      8.87e-02
    TAGS: (2 total)
      d+1 GAAACGGCTT 3       0       1       2       0       2.07e-01   1054
      d+2 AGAAAAAAAA 11      5       13      16      14      1.86e-01   949
          ----------                                                    818
          ----------                                                    606
          ----------                                                    484
          ----------                                                    243
```

```
LOCUS: AT2G44950
DESCRIPTION: zinc finger (C3HC4-type RING finger) family protein, contains Pfam profile: PF00097 zinc finger,
C3HC4 type (RING finger)
DATA:                 Control 30min   2hours  2days   1week   p-value         pos
SENSE COUNTS:         3       0       1       0       0       8.96e-02
GENES (1 total):
  AT2G44950.1
    SENSE COUNTS:     3       0       1       0       0       8.96e-02
    TAGS: (1 total)
           ----------                                                         3248
      d+2  AAGAATAATT 3       0       1       0       0       8.96e-02        3112
           ----------                                                         3006
           ----------                                                         2936
           ----------                                                         2891
           ----------                                                         2409
           ----------                                                         2233
           ----------                                                         2224
           ----------                                                         2035
           ----------                                                         1962
           ----------                                                         1936
           ----------                                                         1746
           ----------                                                         1330
           ----------                                                         1263
           ----------                                                         647

LOCUS: AT3G14990
DESCRIPTION: 4-methyl-5(b-hydroxyethyl)-thiazole monophosphate biosynthesis protein, putative, supporting cDNA
gi|11908017|gb|AF326856.1|; contains Pfam profile PF01965:DJ-1/PfpI family; identical to cDNA  EST Athsr7
GI:5281061
DATA:                 Control 30min   2hours  2days   1week   p-value         pos
SENSE COUNTS:         2       6       1       1       0       8.97e-02
GENES (2 total):
  AT3G14990.1
    SENSE COUNTS:     2       6       1       1       0       8.97e-02
    TAGS: (3 total)
      i+3  CTGTGTTGTA 1       0       0       0       0       4.28e-01        1390
      d+1  GTCAAGAAAC 1       6       0       1       0       1.62e-02        373
      d+2  GCATCAAGAT 0       0       1       0       0       4.55e-01        248
  AT3G14990.2
    SENSE COUNTS:     2       6       1       1       0       8.97e-02
    TAGS: (3 total)
      i+3  CTGTGTTGTA 1       0       0       0       0       4.28e-01        1215
      d+1  GTCAAGAAAC 1       6       0       1       0       1.62e-02        302
      d+2  GCATCAAGAT 0       0       1       0       0       4.55e-01        177

LOCUS: AT3G20770
DESCRIPTION: ethylene-insensitive 3 (EIN3), identical to ethylene-insensitive3 GI:2224933 from (Arabidopsis
thaliana)
DATA:                 Control 30min   2hours  2days   1week   p-value         pos
SENSE COUNTS:         2       5       1       1       0       8.97e-02
GENES (2 total):
  AT3G20770.1
    SENSE COUNTS:     2       5       1       1       0       8.97e-02
    TAGS: (2 total)
      X+4  GGTCAATGCT 1       1       0       0       0       4.77e-01        2330
      d+1  TAATATTTTT 1       4       1       1       0       2.51e-01        2211
           ----------                                                         2045
           ----------                                                         1826
           ----------                                                         1751
           ----------                                                         1709
           ----------                                                         1703
           ----------                                                         1581
           ----------                                                         1528
           ----------                                                         1388
           ----------                                                         1341
           ----------                                                         1177
           ----------                                                         981
           ----------                                                         753
           ----------                                                         293

LOCUS: AT1G65960
DESCRIPTION: glutamate decarboxylase 2 (GAD 2), similar to glutamate decarboxylase (gad) GI:294111 from
(Petunia hybrida)
DATA:                 Control 30min   2hours  2days   1week   p-value         pos
SENSE COUNTS:         22      15      14      14      32      9.00e-02
GENES (2 total):
  AT1G65960.1
    SENSE COUNTS:     22      15      14      14      32      9.00e-02
    TAGS: (3 total)
      d+1  CTTGTTGATT 19      5       13      11      25      7.55e-03        1703
```

```
      d+2  AGCTAGATAC 3       10      1       2       7       3.00e-02        1357
           ----------                                                         1083
           ----------                                                         423
      d+2  GACTCTATCA 0       0       0       1       0       3.09e-01        261
           ----------                                                         230
           ----------                                                         81

LOCUS: AT3G15354
DESCRIPTION: WD-40 repeat family protein / phytochrome A-related, contains 7 WD-40 repeats (PF00400);
phytochrome A supressor spa1 (GI:4809171) (Arabidopsis thaliana)
DATA:                 Control 30min   2hours  2days   1week   p-value         pos
SENSE COUNTS:         4       0       1       2       0       9.02e-02
GENES (2 total):
  AT3G15354.1
    SENSE COUNTS:     4       0       1       2       0       9.02e-02
    TAGS: (2 total)
           ----------                                                         3652
      d+2  ACAGTCGCTC 0       0       1       0       0       4.55e-01        3264
      d+2  TTCAACAACA 4       0       0       2       0       2.93e-02        2728
           ----------                                                         749
           ----------                                                         733
           ----------                                                         723
           ----------                                                         476
           ----------                                                         66

LOCUS: AT2G33430
DESCRIPTION: plastid developmental protein DAG, putative, similar to DAG protein, chloroplast precursor
(Garden snapdragon) SWISS-PROT:Q38732
DATA:                 Control 30min   2hours  2days   1week   p-value         pos
SENSE COUNTS:         4       1       4       0       9       9.02e-02
GENES (1 total):
  AT2G33430.1
    SENSE COUNTS:     4       1       4       0       9       9.02e-02
    TAGS: (5 total)
      i+3  CAACTCTAGA 0       0       0       0       0       6.15e-01        1090
      d+1  TGGGAAATGG 3       1       3       0       5       3.74e-01        983
      i+3  AAGGAAATAT 0       0       1       0       0       4.55e-01        920
      X+4  CTAAAAAGAG 1       0       0       0       1       6.74e-01        472
      d+2  TGAGAGGTAT 0       0       0       0       3       8.36e-02        435

LOCUS: AT5G55190
DESCRIPTION: Ras-related GTP-binding protein (RAN3), identical to atran3 (Arabidopsis thaliana) GI:2058280
DATA:                 Control 30min   2hours  2days   1week   p-value         pos
SENSE COUNTS:         5       5       5       13      14      9.05e-02
GENES (2 total):
  AT5G55190.1
    SENSE COUNTS:     5       5       5       13      14      9.05e-02
    TAGS: (1 total)
           ----------                                                         958
      d+2  GTGCTATTTT 5       5       5       13      14      9.05e-02        842
           ----------                                                         711
           ----------                                                         430
           ----------                                                         386
           ----------                                                         366

LOCUS: AT1G51300
DESCRIPTION: acyl-protein thioesterase-related,  contains similarity to acyl-protein thioesterase-1 (Homo
sapiens) gi|9965372|gb|AAG10063
DATA:                 Control 30min   2hours  2days   1week   p-value         pos
SENSE COUNTS:         0       0       4       2       1       9.09e-02
GENES (1 total):
  AT1G51300.1
    SENSE COUNTS:     0       0       4       2       1       9.09e-02
    TAGS: (3 total)
           ----------                                                         1368
      v+2  AATCTTTTCG 0       0       3       0       0       2.70e-02        1169
           ----------                                                         1068
      v+2  TGACTTTTTG 0       0       1       2       0       1.79e-01        1052
           ----------                                                         748
           ----------                                                         702
           ----------                                                         652
           ----------                                                         614
           ----------                                                         484
           ----------                                                         354
      i+3  AGAGAAACAT 0       0       0       0       1       1.65e-01        143

LOCUS: AT1G59820
DESCRIPTION: haloacid dehalogenase-like hydrolase family protein, similar to Potential phospholipid-
transporting ATPase (EC 3.6.3.1) from Mus musculus (SP|P70704), {Bos taurus} SP|Q29449; contains InterPro
accession IPR005834: Haloacid dehalogenase-like hydrolase
DATA:                 Control 30min   2hours  2days   1week   p-value         pos
```

```
SENSE COUNTS:         0      0      3      2      0      9.12e-02
GENES (1 total):
  AT1G59820.1
    SENSE COUNTS:     0      0      3      2      0      9.12e-02
    TAGS: (2 total)
      d+1  TACATAAAAT 0      0      0      1      0      3.09e-01      4139
           ----------                                                 3498
           ----------                                                 3454
           ----------                                                 3361
           ----------                                                 2984
           ----------                                                 2946
           ----------                                                 2844
           ----------                                                 2828
           ----------                                                 2437
           ----------                                                 1926
      d+2  GCTTAAAAGT 0      0      3      1      0      9.46e-02      1623
           ----------                                                 1431
           ----------                                                 1295
           ----------                                                 1115
           ----------                                                 1089
           ----------                                                 886
           ----------                                                 791

LOCUS: AT1G47260
DESCRIPTION: AF249876 Arabidopsis thaliana transcription factor APFI mRNA, complete cds
DATA:                 Control 30min  2hours 2days  1week  p-value       pos
SENSE COUNTS:         0      4      0      1      1      9.15e-02
GENES (2 total):
  AT1G47260.1
    SENSE COUNTS:     0      4      0      1      1      9.15e-02
    TAGS: (2 total)
      d+1  TGACAGATTT 0      0      0      1      0      3.09e-01      1274
      d+2  AGAAAGTTAA 0      4      0      0      1      3.23e-02      705
           ----------                                                 624
           ----------                                                 619
           ----------                                                 544
           ----------                                                 460

LOCUS: AT3G57520
DESCRIPTION: alkaline alpha galactosidase, putative, similar to alkaline alpha galactosidase II (Cucumis melo)
GI:29838631; contains Pfam profile PF05691: Raffinose synthase or seed imbibition protein Sip1
DATA:                 Control 30min  2hours 2days  1week  p-value       pos
SENSE COUNTS:         1      4      2      1      0      9.16e-02
GENES (3 total):
  AT3G57520.1
    SENSE COUNTS:     1      4      2      1      0      9.16e-02
    TAGS: (4 total)
      i+3  AGCTTGGTTT 0      0      0      0      0      6.15e-01      3622
      d+1  TTGAGATTCT 1      4      0      1      0      1.07e-01      2528
           ----------                                                 2233
      d+2  ATACTTCTCC 0      0      1      0      0      4.55e-01      1982
           ----------                                                 1900
           ----------                                                 1703
           ----------                                                 1651
           ----------                                                 1507
           ----------                                                 1353
           ----------                                                 1283
           ----------                                                 1142
           ----------                                                 1137
      d+2  GGACGCTTTC 0      0      1      0      0      7.06e-01      837
           ----------                                                 769
           ----------                                                 710
           ----------                                                 514
           ----------                                                 430
           ----------                                                 392
  AT3G57520.2
    SENSE COUNTS:     1      4      2      1      0      9.16e-02
    TAGS: (4 total)
      i+3  AGCTTGGTTT 0      0      0      0      0      6.15e-01      3622
      d+1  TTGAGATTCT 1      4      0      1      0      1.07e-01      2494
           ----------                                                 2199
      d+2  ATACTTCTCC 0      0      1      0      0      4.55e-01      1982
           ----------                                                 1900
           ----------                                                 1703
           ----------                                                 1651
           ----------                                                 1507
           ----------                                                 1353
           ----------                                                 1283
           ----------                                                 1142
           ----------                                                 1137
      d+2  GGACGCTTTC 0      0      1      0      0      7.06e-01      837
```

```
          ----------                                                    769
          ----------                                                    710
          ----------                                                    514
          ----------                                                    430
          ----------                                                    392
  AT3G57520.3
    SENSE COUNTS:      1       4       2       1       0       9.16e-02
    TAGS: (4 total)
      i+3  AGCTTGGTTT  0       0       0       0       0       6.15e-01       3622
      d+1  TTGAGATTCT  1       4       0       1       0       1.07e-01       2466
          ----------                                                    2171
      d+2  ATACTTCTCC  0       0       1       0       0       4.55e-01       1920
          ----------                                                    1703
          ----------                                                    1651
          ----------                                                    1507
          ----------                                                    1353
          ----------                                                    1283
          ----------                                                    1142
          ----------                                                    1137
      d+2  GGACGCTTTC  0       0       1       0       0       7.06e-01       837
          ----------                                                    769
          ----------                                                    710
          ----------                                                    514
          ----------                                                    430
          ----------                                                    392

LOCUS: AT4G17600
DESCRIPTION: lil3 protein, identical to Lil3 protein (Arabidopsis thaliana) gi|4741966|gb|AAD28780
DATA:                  Control 30min   2hours  2days   1week   p-value        pos
SENSE COUNTS:          7       0       4       6       1       9.23e-02
GENES (2 total):
  AT4G17600.1
    SENSE COUNTS:      7       0       4       6       1       9.23e-02
    TAGS: (2 total)
      d+1  TCTAGTCTCT  4       0       2       0       0       4.03e-02       956
      d+2  GTGGGCTTGG  3       0       2       6       1       1.86e-01       537

LOCUS: AT4G34265
DESCRIPTION: Expressed protein
DATA:                  Control 30min   2hours  2days   1week   p-value        pos
SENSE COUNTS:          4       6       1       9       1       9.29e-02
GENES (2 total):
  AT4G34265.1
    SENSE COUNTS:      4       6       1       9       1       9.29e-02
    TAGS: (2 total)
      i+3  TATGCAATCA  0       0       0       0       0       6.15e-01       818
      d+1  GCGCAGTGCA  4       6       1       9       1       1.05e-01       74
  AT4G34265.2
    SENSE COUNTS:      4       6       1       9       1       9.29e-02
    TAGS: (2 total)
      i+3  TATGCAATCA  0       0       0       0       0       6.15e-01       818
      d+1  GCGCAGTGCA  4       6       1       9       1       1.05e-01       74

LOCUS: AT2G28990
DESCRIPTION: leucine-rich repeat protein kinase, putative, similar to light repressible receptor protein
kinase (Arabidopsis thaliana) gi|1321686|emb|CAA66376; contains leucine rich repeat (LRR) domains,
Pfam:PF00560; contains protein kinase domain, Pfam:PF00069
DATA:                  Control 30min   2hours  2days   1week   p-value        pos
SENSE COUNTS:          1       0       4       1       5       9.32e-02
GENES (1 total):
  AT2G28990.1
    SENSE COUNTS:      1       0       4       1       5       9.32e-02
    TAGS: (2 total)
          ----------                                                    3428
          ----------                                                    3193
          ----------                                                    3113
      v+2  AATATTCAAA  0       0       3       0       0       2.70e-02       3044
          ----------                                                    2940
          ----------                                                    2868
          ----------                                                    2834
          ----------                                                    2350
          ----------                                                    2310
      v+2  GTTTTGTTAA  1       0       1       1       5       9.91e-02       2131
          ----------                                                    1972
          ----------                                                    1707
          ----------                                                    1502
          ----------                                                    1170
          ----------                                                    979
          ----------                                                    378
          ----------                                                    354
          ----------                                                    254
```

```
          ----------                                                    140

LOCUS: AT1G04850
DESCRIPTION: ubiquitin-associated (UBA)/TS-N domain-containing protein, weak similarity to SP|P45974 Ubiquitin
carboxyl-terminal hydrolase 5 (EC 3.1.2.15) {Homo sapiens}; contains Pfam profile PF00627: UBA/TS-N domain
DATA:                 Control 30min  2hours  2days  1week  p-value       pos
SENSE COUNTS:         2       4      9       2      1      9.34e-02
GENES (1 total):
  AT1G04850.1
    SENSE COUNTS:     2       4      9       2      1      9.34e-02
    TAGS: (3 total)
      d+1  CCTCTTCTTA 2       2      3       1      1      9.44e-01      1337
           ----------                                                  1192
           ----------                                                  1084
      i+3  TTAATGTATA 0       0      1       0      0      4.55e-01      1018
      d+2  AGAATGATCC 0       2      5       1      0      8.33e-02      542
           ----------                                                  358
           ----------                                                  91

LOCUS: AT5G35737
DESCRIPTION: hypothetical protein
DATA:                 Control 30min  2hours  2days  1week  p-value       pos
SENSE COUNTS:         13      6      5       16     10     9.38e-02
GENES (1 total):
  AT5G35737.1
    SENSE COUNTS:     13      6      5       16     10     9.38e-02
    TAGS: (1 total)
           ----------                                                  814
           ----------                                                  536
           ----------                                                  427
      v+2  GCGGATGAGT 13      6      5       16     10     9.38e-02      354

LOCUS: AT3G16520
DESCRIPTION: UDP-glucoronosyl/UDP-glucosyl transferase family protein, contains Pfam profile: PF00201 UDP-
glucoronosyl and UDP-glucosyl transferase
DATA:                 Control 30min  2hours  2days  1week  p-value       pos
SENSE COUNTS:         16      8      13      13     1      9.39e-02
GENES (3 total):
  AT3G16520.1
    SENSE COUNTS:     1       0      1       0      0      6.01e-01
    TAGS: (2 total)
      i+3  CGTAGTAAGG 1       0      0       0      0      4.28e-01      1766
      d+1  GAGCCCAAAA 0       0      1       0      0      4.55e-01      1540
           ----------                                                  1510
           ----------                                                  1388
           ----------                                                  1318
           ----------                                                  1044
           ----------                                                  459
           ----------                                                  82
           ----------                                                  25
  AT3G16520.2
    SENSE COUNTS:     1       0      1       0      0      6.01e-01
    TAGS: (2 total)
      i+3  CGTAGTAAGG 1       0      0       0      0      4.28e-01      1785
      d+1  GAGCCCAAAA 0       0      1       0      0      4.55e-01      1584
           ----------                                                  1554
           ----------                                                  1432
           ----------                                                  1337
           ----------                                                  1063
           ----------                                                  478
           ----------                                                  101
           ----------                                                  44
  AT3G16520.3
    SENSE COUNTS:     15      8      12      13     1      1.31e-01
    TAGS: (2 total)
      d+1  CCGATACATC 4       1      2       1      0      4.13e-01      1616
      d+2  TCTTTGGGGG 11      7      10      12     1      2.85e-01      1485
           ----------                                                  1337
           ----------                                                  1063
           ----------                                                  478
           ----------                                                  101
           ----------                                                  44

LOCUS: AT1G03120
DESCRIPTION: seed maturation family protein, similar to embryonic cell protein (Daucus carota) GI:18337;
contains Pfam profile PF04927: Seed maturation protein
DATA:                 Control 30min  2hours  2days  1week  p-value       pos
SENSE COUNTS:         11      4      9       6      0      9.42e-02
GENES (2 total):
  AT1G03120.1
    SENSE COUNTS:     11      4      9       6      0      9.42e-02
```

```
    TAGS: (2 total)
      v+1  TTTAGTCCAA  3       0       4       4       0       3.08e-01        1360
      v+2  TAGTGTGATC  8       4       5       2       0       2.27e-01        1220
           ----------                                                          1134
           ----------                                                          935
           ----------                                                          867
           ----------                                                          412
           ----------                                                          206
           ----------                                                          195
           ----------                                                          14

LOCUS: AT1G21690
DESCRIPTION: replication factor C 37 kDa, putative, Similar to SWISS-PROT:P35249 activator 1 37 kDa subunit
(Replication factor C 37 kDa subunit, A1 37 kDa subunit, RF-C 37 kDa subunit, RFC37) (Homo sapiens); contains
Pfam domain, PF00004: ATPase, AAA family
DATA:                  Control 30min   2hours  2days   1week   p-value         pos
SENSE COUNTS:          1       0       0       4       1       9.54e-02
GENES (2 total):
  AT1G21690.1
    SENSE COUNTS:      1       0       0       4       1       9.54e-02
    TAGS: (2 total)
      d+1  ATCGAATCAA  0       0       0       0       1       1.65e-01        1266
      d+2  CAAAAGGCTA  1       0       0       4       0       3.65e-02        917
           ----------                                                          787
           ----------                                                          569
           ----------                                                          167
           ----------                                                          64
  AT1G21690.2
    SENSE COUNTS:      1       0       0       4       1       9.54e-02
    TAGS: (2 total)
      d+1  ATCGAATCAA  0       0       0       0       1       1.65e-01        1230
      d+2  CAAAAGGCTA  1       0       0       4       0       3.65e-02        881
           ----------                                                          751
           ----------                                                          533
           ----------                                                          131
           ----------                                                          64

LOCUS: AT4G25640
DESCRIPTION: MATE efflux family protein, similar to ripening regulated protein DDTFR18 (Lycopersicon
esculentum) GI:12231296; contains Pfam profile PF01554: Uncharacterized membrane protein family
DATA:                  Control 30min   2hours  2days   1week   p-value         pos
SENSE COUNTS:          0       1       5       6       5       9.56e-02
GENES (1 total):
  AT4G25640.1
    SENSE COUNTS:      0       1       5       6       5       9.56e-02
    TAGS: (3 total)
      i+3  AGTTAAGTCA  0       0       0       1       0       3.09e-01        1807
      d+1  TAACCCAAAT  0       0       4       5       5       3.52e-02        1731
      d+2  GTTCTCAACA  0       1       1       0       0       4.87e-01        1244
           ----------                                                          983

LOCUS: AT2G29630
DESCRIPTION: thiamine biosynthesis family protein / thiC family protein, contains Pfam profile: PF01964 ThiC
family
DATA:                  Control 30min   2hours  2days   1week   p-value         pos
SENSE COUNTS:          6       6       2       2       12      9.61e-02
GENES (2 total):
  AT2G29630.1
    SENSE COUNTS:      6       6       1       2       12      4.93e-02
    TAGS: (2 total)
      i+3  CTAGTATAAA  0       0       0       0       0       6.15e-01        2599
      d+1  TTACTTTTAG  6       6       1       2       12      4.80e-02        2137
           ----------                                                          1809
           ----------                                                          1759
           ----------                                                          1740
           ----------                                                          1667
           ----------                                                          1657
           ----------                                                          1627
           ----------                                                          1372
           ----------                                                          1138
           ----------                                                          1060
           ----------                                                          892
           ----------                                                          864
           ----------                                                          843
           ----------                                                          421
           ----------                                                          406
  AT2G29630.2
    SENSE COUNTS:      0       0       1       0       0       4.87e-01
    TAGS: (3 total)
      i+3  CTAGTATAAA  0       0       0       0       0       6.15e-01        2599
      d+1  TGTGGACCAA  0       0       0       0       0       6.15e-01        1809
```

```
           ----------                                                    1759
           ----------                                                    1740
           ----------                                                    1667
           ----------                                                    1657
           ----------                                                    1627
           ----------                                                    1372
           ----------                                                    1138
           ----------                                                    1060
           ----------                                                    892
           ----------                                                    864
           ----------                                                    843
      X+4  AAACTGATCC 0      0      1      0      0      4.55e-01        484
           ----------                                                    421
           ----------                                                    406

LOCUS: AT4G00430
DESCRIPTION: plasma membrane intrinsic protein, putative, identical to transmembrane protein GI:535780 from
(Arabidopsis thaliana); very strong similarity to SP|Q08733 Plasma membrane intrinsic protein 1C
(Transmembrane protein B) (TMP-B) {Arabidopsis thaliana}; conta
DATA:                 Control 30min  2hours 2days  1week  p-value         pos
SENSE COUNTS:         4      6      9      13     1      9.63e-02
GENES (3 total):
  AT4G00430.2
    SENSE COUNTS:     4      6      8      12     1      1.83e-01
    TAGS: (2 total)
      i+3  TGAGTAAACA 0      0      0      0      0      6.15e-01        1287
      d+1  GCTACACCAA 4      6      8      12     1      1.60e-01        601
           ----------                                                    498
           ----------                                                    381
           ----------                                                    333
           ----------                                                    233
  AT4G00430.1
    SENSE COUNTS:     0      0      1      1      0      7.90e-01
    TAGS: (2 total)
      d+1  ACTTCGTGAA 0      0      1      1      0      5.21e-01        1303
      i+3  TGAGTAAACA 0      0      0      0      0      6.15e-01        1287
           ----------                                                    1285
           ----------                                                    601
           ----------                                                    498
           ----------                                                    381
           ----------                                                    333
           ----------                                                    233

LOCUS: AT3G08610
DESCRIPTION: expressed protein
DATA:                 Control 30min  2hours 2days  1week  p-value         pos
SENSE COUNTS:         24     9      13     10     11     9.70e-02
GENES (1 total):
  AT3G08610.1
    SENSE COUNTS:     24     9      13     10     11     9.70e-02
    TAGS: (3 total)
      X+4  GGGTCCCCTC 0      0      0      1      0      3.09e-01        498
      d+1  ATACACTGAA 24     8      13     9      10     3.24e-02        454
      d+2  ATTCGCTTTA 0      1      0      0      1      3.55e-01        233
           ----------                                                    139
           ----------                                                    102

LOCUS: AT1G67900
DESCRIPTION: phototropic-responsive NPH3 family protein, contains NPH3 family domain, Pfam:PF03000
DATA:                 Control 30min  2hours 2days  1week  p-value         pos
SENSE COUNTS:         2      5      8      1      1      9.71e-02
GENES (2 total):
  AT1G67900.1
    SENSE COUNTS:     2      5      8      1      1      9.71e-02
    TAGS: (3 total)
      i+3  AACTTTTCTT 0      0      0      1      0      3.09e-01        2644
      d+1  AATGGGGACG 2      4      6      0      1      1.69e-01        2114
           ----------                                                    1795
           ----------                                                    1680
           ----------                                                    908
      X+4  AGACACGTGT 0      1      2      0      0      3.64e-01        654
  AT1G67900.2
    SENSE COUNTS:     2      4      6      1      1      3.47e-01
    TAGS: (2 total)
      i+3  AACTTTTCTT 0      0      0      1      0      3.09e-01        2553
      d+1  AATGGGGACG 2      4      6      0      1      1.69e-01        2151
           ----------                                                    1832
           ----------                                                    1717
           ----------                                                    945

LOCUS: AT1G32060
```

```
DESCRIPTION: phosphoribulokinase (PRK) / phosphopentokinase, nearly identical to SP|P25697 Phosphoribulokinase,
chloroplast precursor (EC 2.7.1.19) (Phosphopentokinase) (PRKASE) (PRK) {Arabidopsis thaliana}
DATA:                  Control 30min  2hours  2days  1week  p-value      pos
SENSE COUNTS:          1       0      4       0      0      9.82e-02
GENES (1 total):
  AT1G32060.1
    SENSE COUNTS:      1       0      4       0      0      9.82e-02
    TAGS: (2 total)
           ----------                                                    1414
      d+2  CTGATTTCCC  0       0      4       0      0      3.11e-02     1182
           ----------                                                    1056
           ----------                                                    737
           ----------                                                    570
           ----------                                                    509
      X+4  AGCCAATCAT  1       0      0       0      0      4.28e-01     -205

LOCUS: AT5G13780
DESCRIPTION: GCN5-related N-acetyltransferase, putative, similar to SP|P07347 N-terminal acetyltransferase
complex ARD1 subunit (Arrest-defective protein 1) {Saccharomyces cerevisiae}; contains Pfam profile PF00583:
acetyltransferase, GNAT family
DATA:                  Control 30min  2hours  2days  1week  p-value      pos
SENSE COUNTS:          1       4      0       0      3      9.84e-02
GENES (1 total):
  AT5G13780.1
    SENSE COUNTS:      1       4      0       0      3      9.84e-02
    TAGS: (1 total)
           ----------                                                    1180
           ----------                                                    1168
           ----------                                                    1108
      d+2  GAGGAGGATG  1       4      0       0      3      9.84e-02     540
           ----------                                                    479
           ----------                                                    369
           ----------                                                    332
           ----------                                                    311
           ----------                                                    154
           ----------                                                    101
           ----------                                                    41

LOCUS: AT3G13460
DESCRIPTION: expressed protein, contains Pfam profile PF04146: YT521-B-like family
DATA:                  Control 30min  2hours  2days  1week  p-value      pos
SENSE COUNTS:          9       1      11      11     6      9.85e-02
GENES (2 total):
  AT3G13460.1
    SENSE COUNTS:      9       1      11      11     6      9.85e-02
    TAGS: (2 total)
      d+1  TTTTAAGATT  6       0      7       5      5      2.02e-01     2784
           ----------                                                    2632
      d+2  GAAGGAGCTT  3       1      4       6      1      4.06e-01     2377
           ----------                                                    2290
           ----------                                                    1587
           ----------                                                    1523
           ----------                                                    576
           ----------                                                    400
           ----------                                                    261
  AT3G13460.2
    SENSE COUNTS:      9       1      11      11     6      9.85e-02
    TAGS: (2 total)
      d+1  TTTTAAGATT  6       0      7       5      5      2.02e-01     2775
           ----------                                                    2623
      d+2  GAAGGAGCTT  3       1      4       6      1      4.06e-01     2368
           ----------                                                    2281
           ----------                                                    1578
           ----------                                                    1514
           ----------                                                    567
           ----------                                                    391
           ----------                                                    261

LOCUS: AT5G63190
DESCRIPTION: MA3 domain-containing protein, low similarity to programmed cell death 4 protein (Gallus gallus)
GI:12958564; contains Pfam profile PF02847: MA3 domain
DATA:                  Control 30min  2hours  2days  1week  p-value      pos
SENSE COUNTS:          11      3      6       2      3      9.88e-02
GENES (2 total):
  AT5G63190.1
    SENSE COUNTS:      11      3      6       2      3      9.88e-02
    TAGS: (4 total)
      d+1  GTTAATTTGT  1       0      1       1      0      7.18e-01     2585
      d+2  TGTTTCTGTC  9       3      3       1      3      1.48e-01     2483
      d+2  AGATGTTTAT  1       0      1       0      0      6.01e-01     2464
           ----------                                                    2322
```

```
          ----------                                                     2317
       d+2  GACGCATCAA  0       0       1       0       0       4.55e-01       1797
          ----------                                                     1716
          ----------                                                     1225
          ----------                                                     1066
          ----------                                                     765
          ----------                                                     436
  AT5G63190.2
    SENSE COUNTS:      11      3       6       2       3       9.88e-02
    TAGS: (4 total)
       d+1  GTTAATTTGT  1       0       1       1       0       7.18e-01       2541
       d+2  TGTTTCTGTC  9       3       3       1       3       1.48e-01       2439
       d+2  AGATGTTTAT  1       0       1       0       0       6.01e-01       2420
          ----------                                                     2278
          ----------                                                     2273
       d+2  GACGCATCAA  0       0       1       0       0       4.55e-01       1753
          ----------                                                     1672
          ----------                                                     1181
          ----------                                                     1022
          ----------                                                     721
          ----------                                                     392

LOCUS: AT1G48850
DESCRIPTION: chorismate synthase, putative / 5-enolpyruvylshikimate-3-phosphate phospholyase, putative,
similar to chorismate synthase from Lycopersicon esculentum (SP|Q42884), Corydalis sempervirens (SP|P27793);
contains Pfam chorismate synthase domain PF01264
DATA:                  Control 30min   2hours  2days   1week   p-value        pos
SENSE COUNTS:          1       0       1       1       5       9.91e-02
GENES (1 total):
  AT1G48850.1
    SENSE COUNTS:      1       0       1       1       5       9.91e-02
    TAGS: (1 total)
          ----------                                                     1687
          ----------                                                     1680
       d+2  ATTTTTTATA  1       0       1       1       5       9.91e-02       1561
          ----------                                                     1279
          ----------                                                     1182
          ----------                                                     769
          ----------                                                     621
          ----------                                                     601
          ----------                                                     529
          ----------                                                     340
          ----------                                                     138

LOCUS: AT3G62830
DESCRIPTION: NAD-dependent epimerase/dehydratase family protein, similar to UDP-glucuronic acid decarboxylase
Uxs1p from Filobasidiella neoformans GI:14318327; contains Pfam profile PF01370 NAD dependent
epimerase/dehydratase family; contains non-consensus CA donor sp
DATA:                  Control 30min   2hours  2days   1week   p-value        pos
SENSE COUNTS:          5       2       3       6       12      9.91e-02
GENES (2 total):
  AT3G62830.1
    SENSE COUNTS:      5       2       3       6       12      9.91e-02
    TAGS: (2 total)
       v+1  TTCTCACTGT  1       0       0       0       0       4.28e-01       1993
       v+2  GACCTCACTA  4       2       3       6       12      8.38e-02       1851
          ----------                                                     1602
          ----------                                                     1393
          ----------                                                     1170
          ----------                                                     999
          ----------                                                     465
          ----------                                                     434

LOCUS: AT1G09250
DESCRIPTION: expressed protein
DATA:                  Control 30min   2hours  2days   1week   p-value        pos
SENSE COUNTS:          13      12      18      13      1       9.92e-02
GENES (1 total):
  AT1G09250.1
    SENSE COUNTS:      13      12      18      13      1       9.92e-02
    TAGS: (3 total)
       X+4  TGATTCTCAA  1       0       0       0       0       4.28e-01       1165
       d+1  TATTTTATTT  3       2       1       2       0       6.27e-01       874
          ----------                                                     696
       X+4  AATGACAAAA  9       10      17      11      1       7.49e-02       -158

LOCUS: AT5G17920
DESCRIPTION: 5-methyltetrahydropteroyltriglutamate--homocysteine methyltransferase / vitamin-B12-independent
methionine synthase / cobalamin-independent methionine synthase (CIMS), identical to SP|O50008 5-
methyltetrahydropteroyltriglutamate--homocysteine methyltransf
DATA:                  Control 30min   2hours  2days   1week   p-value        pos
```

```
SENSE COUNTS:         31      49      38      47      26      9.94e-02
GENES (2 total):
  AT5G17920.1
    SENSE COUNTS:     31      49      38      47      26      9.94e-02
    TAGS: (3 total)
      i+3  TAGTGTAATG 0       0       0       0       0       6.15e-01       3641
      d+1  GTTGATGCGG 31      49      37      47      26      1.13e-01       2343
           ----------                                                        2082
           ----------                                                        2037
           ----------                                                        1954
      d+2  ACCTCTCGCC 0       0       1       0       0       4.55e-01       1740
           ----------                                                        1581
           ----------                                                        1142
           ----------                                                        414
           ----------                                                        384
           ----------                                                        315

LOCUS: AT4G23370
DESCRIPTION: hypothetical protein, predicted proteins, Arabidopsis thaliana contains Pfam profile PF03080:
Arabidopsis proteins of unknown function
DATA:                 Control 30min   2hours  2days   1week   p-value        pos
SENSE COUNTS:         1       0       6       2       1       9.96e-02
GENES (1 total):
  AT4G23370.1
    SENSE COUNTS:     1       0       6       2       1       9.96e-02
    TAGS: (1 total)
           ----------                                                        3733
           ----------                                                        3701
           ----------                                                        3631
           ----------                                                        3544
           ----------                                                        3355
           ----------                                                        3324
           ----------                                                        3316
           ----------                                                        3071
           ----------                                                        2933
           ----------                                                        2928
           ----------                                                        2863
           ----------                                                        2854
           ----------                                                        2607
           ----------                                                        2587
           ----------                                                        2154
           ----------                                                        2108
           ----------                                                        2089
           ----------                                                        1986
           ----------                                                        1626
      v+2  GCGATACATT 1       0       6       2       1       9.96e-02       1387
           ----------                                                        1048
           ----------                                                        753
           ----------                                                        354

LOCUS: AT1G22760
DESCRIPTION: polyadenylate-binding protein 3 (PABP3)
DATA:                 Control 30min   2hours  2days   1week   p-value        pos
SENSE COUNTS:         0       3       1       2       6       1.00e-01
GENES (2 total):
  AT1G22760.1
    SENSE COUNTS:     0       3       1       2       6       1.00e-01
    TAGS: (2 total)
           ----------                                                        2231
           ----------                                                        1690
      d+2  TACGTAGGCC 0       3       1       1       1       4.13e-01       1618
      i+3  ATTCTGCTAC 0       0       0       1       5       6.31e-03       832
           ----------                                                        768
           ----------                                                        640
           ----------                                                        488
           ----------                                                        277
           ----------                                                        133

LOCUS: AT2G43010
DESCRIPTION: phytochrome-interacting factor 4 (PIF4) / basic helix-loop-helix protein 9 (bHLH9) / short under
red-light 2 (SRL2), identical to SP|Q8W2F3 Phytochrome-interacting factor 4 (Basic helix-loop-helix protein 9)
(bHLH9) (Short under red-light 2) {Arabidopsis
DATA:                 Control 30min   2hours  2days   1week   p-value        pos
SENSE COUNTS:         5       5       8       1       0       1.00e-01
GENES (3 total):
  AT2G43010.2
    SENSE COUNTS:     5       5       8       1       0       1.00e-01
    TAGS: (3 total)
      d+1  TGATTTTTGT 3       2       3       1       0       6.58e-01       1727
           ----------                                                        1687
      d+2  CAGGCCGCGA 2       3       4       0       0       2.63e-01       1404
```

```
          ----------                                                   778
       d+2  CCTCCTCCAA  0       0       1       0       0       4.55e-01       636
          ----------                                                   594
          ----------                                                   525
          ----------                                                   403
          ----------                                                   237
          ----------                                                   68
  AT2G43010.1
    SENSE COUNTS:      5       5       8       1       0       1.00e-01
    TAGS: (3 total)
      d+1  TGATTTTTGT  3       2       3       1       0       6.58e-01       1733
          ----------                                                   1693
      d+2  CAGGCCGCGA  2       3       4       0       0       2.63e-01       1404
          ----------                                                   778
      d+2  CCTCCTCCAA  0       0       1       0       0       4.55e-01       636
          ----------                                                   594
          ----------                                                   525
          ----------                                                   403
          ----------                                                   237
          ----------                                                   68

LOCUS: AT1G49660
DESCRIPTION: expressed protein
DATA:                  Control 30min   2hours  2days   1week   p-value        pos
SENSE COUNTS:          0       1       2       5       0       1.01e-01
GENES (1 total):
  AT1G49660.1
    SENSE COUNTS:      0       1       2       5       0       1.01e-01
    TAGS: (1 total)
          ----------                                                   1098
      X+4  AATTTCAGAC  0       1       2       5       0       1.01e-01       1097
          ----------                                                   1068
          ----------                                                   1062
          ----------                                                   947
          ----------                                                   580

LOCUS: AT5G62350
DESCRIPTION: invertase/pectin methylesterase inhibitor family protein / DC 1.2 homolog (FL5-2I22), similar to
SP|Q42534 Pectinesterase 2 precursor (EC 3.1.1.11) (Pectin methylesterase 2) (PE 2) {Arabidopsis thaliana};
contains Pfam profile PF04043: Plant invertase/pec
DATA:                  Control 30min   2hours  2days   1week   p-value        pos
SENSE COUNTS:          15      26      18      22      8       1.01e-01
GENES (1 total):
  AT5G62350.1
    SENSE COUNTS:      15      26      18      22      8       1.01e-01
    TAGS: (2 total)
      d+1  AAACCAGCAA  15      26      16      22      5       3.66e-02       638
          ----------                                                   625
      d+2  AGTAATGCTC  0       0       2       0       3       2.43e-01       508

LOCUS: AT1G11390
DESCRIPTION: ABC1 family protein, contains Pfam domain, PF03109: ABC1 family
DATA:                  Control 30min   2hours  2days   1week   p-value        pos
SENSE COUNTS:          4       2       1       3       9       1.02e-01
GENES (1 total):
  AT1G11390.1
    SENSE COUNTS:      4       2       1       3       9       1.02e-01
    TAGS: (5 total)
      d+1  TTGCCTGAGA  1       0       1       0       1       6.15e-01       2342
          ----------                                                   2231
      d+2  TTCTTATGTT  1       0       0       0       0       4.28e-01       2171
          ----------                                                   1556
          ----------                                                   1509
      d+2  AATGGATTAA  2       1       0       2       8       2.38e-02       1470
      d+2  GAGAAAGTGT  0       1       0       0       0       2.54e-01       1428
          ----------                                                   1286
          ----------                                                   805
      X+4  ATGTCTTGTA  0       0       0       1       0       3.09e-01       621
          ----------                                                   607
          ----------                                                   468

LOCUS: AT1G04950
DESCRIPTION: TATA box-binding protein-associated factor (TAF) family protein, contains Pfam profile: PF02969
TATA box binding protein associated factor
DATA:                  Control 30min   2hours  2days   1week   p-value        pos
SENSE COUNTS:          3       1       0       0       4       1.02e-01
GENES (3 total):
  AT1G04950.2
    SENSE COUNTS:      3       1       0       0       4       1.02e-01
    TAGS: (2 total)
      d+1  TATGCTCTCT  0       0       0       0       3       1.12e-02       2033
```

```
      d+2  AAATAATTTC 3       1       0       0       1       2.92e-01        1971
           ----------                                                         1593
           ----------                                                         1398
           ----------                                                         1240
           ----------                                                         863
           ----------                                                         577
           ----------                                                         529
           ----------                                                         216
           ----------                                                         195
  AT1G04950.1
    SENSE COUNTS:     3       1       0       0       4       1.02e-01
    TAGS: (2 total)
      d+1  TATGCTCTCT 0       0       0       0       3       1.12e-02        2129
      d+2  AAATAATTTC 3       1       0       0       1       2.92e-01        2067
           ----------                                                         1689
           ----------                                                         1494
           ----------                                                         1336
           ----------                                                         959
           ----------                                                         673
           ----------                                                         625
           ----------                                                         312
           ----------                                                         291
           ----------                                                         33

LOCUS: AT3G18900
DESCRIPTION: expressed protein, contains Pfam profile PF04784: Protein of unknown function, DUF547; contains
TIGRFAM TIGR01640: F-box protein interaction domain
DATA:                 Control 30min   2hours  2days   1week   p-value         pos
SENSE COUNTS:         1       3       0       0       0       1.03e-01
GENES (1 total):
  AT3G18900.1
    SENSE COUNTS:     1       3       0       0       0       1.03e-01
    TAGS: (1 total)
           ----------                                                         3237
           ----------                                                         3058
           ----------                                                         2914
           ----------                                                         2858
           ----------                                                         2813
           ----------                                                         2652
           ----------                                                         2613
           ----------                                                         2195
           ----------                                                         1933
           ----------                                                         1879
           ----------                                                         1849
           ----------                                                         1785
           ----------                                                         1330
      i+3  TCTTCAGCAA 1       3       0       0       0       1.03e-01        1074
           ----------                                                         952
           ----------                                                         456
           ----------                                                         354

LOCUS: AT4G30760
DESCRIPTION: expressed protein, contains Pfam profile PF04396: Protein of unknown function, DUF537
DATA:                 Control 30min   2hours  2days   1week   p-value         pos
SENSE COUNTS:         1       3       0       0       0       1.03e-01
GENES (2 total):
  AT4G30760.1
    SENSE COUNTS:     0       3       0       0       0       3.05e-02
    TAGS: (1 total)
      d+1  CTTATGCTAA 0       3       0       0       0       3.05e-02        212
           ----------                                                         93
           ----------                                                         34
  AT4G30760.2
    SENSE COUNTS:     1       3       0       0       0       1.03e-01
    TAGS: (2 total)
      X+4  AGTGACTAAG 1       0       0       0       0       4.28e-01        449
      d+1  CTTATGCTAA 0       3       0       0       0       3.05e-02        212
           ----------                                                         93
           ----------                                                         34

LOCUS: AT3G24570
DESCRIPTION: peroxisomal membrane 22 kDa family protein, contains Mpv17 / PMP22 family domain, Pfam:PF04117
DATA:                 Control 30min   2hours  2days   1week   p-value         pos
SENSE COUNTS:         1       3       0       0       0       1.03e-01
GENES (1 total):
  AT3G24570.1
    SENSE COUNTS:     1       3       0       0       0       1.03e-01
    TAGS: (1 total)
           ----------                                                         1003
      d+2  GGTCGAGCAA 1       3       0       0       0       1.03e-01        743
           ----------                                                         644
```

```
          ----------                                                      555
          ----------                                                      381

LOCUS: AT2G23320
DESCRIPTION: WRKY family transcription factor, identical to WRKY DNA-binding protein 15 GI:13506742 from
(Arabidopsis thaliana)
DATA:                 Control 30min   2hours  2days   1week   p-value        pos
SENSE COUNTS:         6       3       4       0       0       1.03e-01
GENES (3 total):
  AT2G23320.2
    SENSE COUNTS:     6       3       3       0       0       9.20e-02
    TAGS: (3 total)
      d+1  TTTACTTGTT 2       0       2       0       0       5.04e-01       1189
      d+2  TTGATTGTTA 3       3       1       0       0       2.52e-01       922
           ----------                                                      893
           ----------                                                      800
      d+2  TTATTTCTCC 1       0       0       0       0       4.28e-01       373
           ----------                                                      84
  AT2G23320.1
    SENSE COUNTS:     6       3       4       0       0       1.03e-01
    TAGS: (4 total)
      d+1  TTTACTTGTT 2       0       2       0       0       5.04e-01       1191
      d+2  TTGATTGTTA 3       3       1       0       0       2.52e-01       924
           ----------                                                      895
           ----------                                                      800
      X+4  TGAAAATGGT 0       0       1       0       0       4.55e-01       468
      d+2  TTATTTCTCC 1       0       0       0       0       4.28e-01       373
           ----------                                                      84

LOCUS: AT5G45820
DESCRIPTION: CBL-interacting protein kinase 20 (CIPK20), identical to CBL-interacting protein kinase 20
(Arabidopsis thaliana) gi|14486384|gb|AAK61493
DATA:                 Control 30min   2hours  2days   1week   p-value        pos
SENSE COUNTS:         2       2       7       2       0       1.04e-01
GENES (2 total):
  AT5G45820.1
    SENSE COUNTS:     2       2       7       2       0       1.04e-01
    TAGS: (2 total)
           ----------                                                      2009
           ----------                                                      1895
      v+2  GAGAATTCCT 2       2       7       1       0       6.08e-02       1134
      v+2  TGGAACACCT 0       0       0       1       0       3.09e-01       866
           ----------                                                      580
           ----------                                                      265

LOCUS: AT3G51710
DESCRIPTION: curculin-like (mannose-binding) lectin family protein / PAN domain-containing protein, contains
Pfam profiles: PF01453 lectin (probable mannose binding), PF00024 PAN domain
DATA:                 Control 30min   2hours  2days   1week   p-value        pos
SENSE COUNTS:         1       0       3       0       0       1.04e-01
GENES (1 total):
  AT3G51710.1
    SENSE COUNTS:     1       0       3       0       0       1.04e-01
    TAGS: (1 total)
           ----------                                                      2245
           ----------                                                      2159
           ----------                                                      1944
           ----------                                                      1834
           ----------                                                      1506
           ----------                                                      1357
           ----------                                                      354
      v+2  AAAGCAGAAA 1       0       3       0       0       1.04e-01       210

LOCUS: AT1G43690
DESCRIPTION: ubiquitin interaction motif-containing protein, contains Pfam profile PF02809: Ubiquitin
interaction motif
DATA:                 Control 30min   2hours  2days   1week   p-value        pos
SENSE COUNTS:         1       0       3       0       0       1.04e-01
GENES (1 total):
  AT1G43690.1
    SENSE COUNTS:     1       0       3       0       0       1.04e-01
    TAGS: (2 total)
           ----------                                                      2209
      d+2  CACCACTGGT 1       0       0       0       0       4.28e-01       1993
           ----------                                                      1928
           ----------                                                      1923
      d+2  TTTCTGAAAG 0       0       3       0       0       2.70e-02       1335
           ----------                                                      1249
           ----------                                                      1140
           ----------                                                      850
           ----------                                                      724
```

```
          ----------                                                    144

LOCUS: AT4G36480
DESCRIPTION: aminotransferase class I and II family protein, similar to Serine palmitoyltransferase 1 (EC
2.3.1.50) from Homo sapiens (SP|O15269), Mus musculus (SP|O35704), Cricetulus griseus (SP|O54695)
DATA:                 Control 30min  2hours  2days  1week  p-value      pos
SENSE COUNTS:         1       0      3       0      0      1.04e-01
GENES (2 total):
  AT4G36480.1
    SENSE COUNTS:     1       0      3       0      0      1.04e-01
    TAGS: (2 total)
          ----------                                                    2056
      d+2  CATTAGCCAC 1       0      0       0      0      4.28e-01     1206
      d+2  ACAAAGTACA 0       0      3       0      0      2.70e-02     965
          ----------                                                    932
          ----------                                                    652
          ----------                                                    531
          ----------                                                    524

LOCUS: AT1G69270
DESCRIPTION: leucine-rich repeat family protein / protein kinase family protein, contains Pfam domains
PF00560: Leucine Rich Repeat and PF00069: Protein kinase domain
DATA:                 Control 30min  2hours  2days  1week  p-value      pos
SENSE COUNTS:         0       3      1       0      0      1.04e-01
GENES (2 total):
  AT1G69270.1
    SENSE COUNTS:     0       3      1       0      0      1.04e-01
    TAGS: (1 total)
          ----------                                                    2348
          ----------                                                    2260
          ----------                                                    2247
          ----------                                                    2211
          ----------                                                    2196
      d+2  AGAACGGGTT 0       3      1       0      0      1.04e-01     1609
          ----------                                                    1511
          ----------                                                    1453
          ----------                                                    1177
          ----------                                                    1114

LOCUS: AT1G21130
DESCRIPTION: O-methyltransferase, putative, similar to GI:2781394
DATA:                 Control 30min  2hours  2days  1week  p-value      pos
SENSE COUNTS:         0       3      1       0      0      1.04e-01
GENES (2 total):
  AT1G21130.2
    SENSE COUNTS:     0       3      1       0      0      1.04e-01
    TAGS: (2 total)
          ----------                                                    1207
          ----------                                                    1201
          ----------                                                    1058
      d+2  GTACTAGAAC 0       0      1       0      0      4.55e-01     1006
      d+2  ATCTTGAAAG 0       3      0       0      0      3.05e-02     948
          ----------                                                    904
          ----------                                                    640

LOCUS: AT2G43080
DESCRIPTION: oxidoreductase, 2OG-Fe(II) oxygenase family protein, similar to prolyl 4-hydroxylase, alpha
subunit, from Homo sapiens (GI:18073925); contains PF03171 2OG-Fe(II) oxygenase superfamily domain
DATA:                 Control 30min  2hours  2days  1week  p-value      pos
SENSE COUNTS:         0       3      1       0      0      1.04e-01
GENES (1 total):
  AT2G43080.1
    SENSE COUNTS:     0       3      1       0      0      1.04e-01
    TAGS: (2 total)
      i+3  TATCGTTTTT 0       0      1       0      0      4.55e-01     1216
      d+1  GAGGATGTGA 0       3      0       0      0      3.05e-02     972
          ----------                                                    935
          ----------                                                    881
          ----------                                                    603
          ----------                                                    462
          ----------                                                    206
          ----------                                                    194

LOCUS: AT3G12830
DESCRIPTION: auxin-responsive family protein, similar to auxin-induced protein (SP:P33082) (Glycine max)
DATA:                 Control 30min  2hours  2days  1week  p-value      pos
SENSE COUNTS:         0       3      1       0      0      1.04e-01
GENES (1 total):
  AT3G12830.1
    SENSE COUNTS:     0       3      1       0      0      1.04e-01
    TAGS: (1 total)
```

```
          ----------                                                    807
      d+2  GAATCGCTTC  0       3       1       0       0       1.04e-01       424
          ----------                                                    401

LOCUS: AT3G30380
DESCRIPTION: expressed protein, ; expression supported by MPSS
DATA:                  Control 30min   2hours  2days   1week   p-value        pos
SENSE COUNTS:          0       3       1       0       0       1.04e-01
GENES (1 total):
  AT3G30380.1
    SENSE COUNTS:      0       3       1       0       0       1.04e-01
    TAGS: (2 total)
      v+1  GTGGTATATA  0       0       1       0       0       4.55e-01       1932
          ----------                                                    1867
          ----------                                                    1816
      v+2  GATCCAAGAC  0       3       0       0       0       3.05e-02       1596
          ----------                                                    1467
          ----------                                                    1452
          ----------                                                    1006
          ----------                                                    885
          ----------                                                    574

LOCUS: AT3G23460
DESCRIPTION: cyclopropane fatty acid synthase-related, similar to cyclopropane synthase (Sterculia foetida)
GI:21069167
DATA:                  Control 30min   2hours  2days   1week   p-value        pos
SENSE COUNTS:          9       9       21      18      10      1.06e-01
GENES (1 total):
  AT3G23460.1
    SENSE COUNTS:      9       9       21      18      10      1.06e-01
    TAGS: (2 total)
          ----------                                                    1533
          ----------                                                    1510
          ----------                                                    1450
          ----------                                                    1431
          ----------                                                    1420
          ----------                                                    1100
          ----------                                                    870
          ----------                                                    660
          ----------                                                    486
          ----------                                                    390
          ----------                                                    384
          ----------                                                    292
          ----------                                                    267
      v+2  AAATGCAAAA  3       2       7       6       5       4.60e-01       147
      v+2  TAAAACGCTA  6       7       14      12      5       3.02e-01       62

LOCUS: AT1G51700
DESCRIPTION: adof1 mRNA for dof zinc finger protein, complete cds
DATA:                  Control 30min   2hours  2days   1week   p-value        pos
SENSE COUNTS:          0       0       1       1       5       1.07e-01
GENES (2 total):
  AT1G51700.1
    SENSE COUNTS:      0       0       1       1       5       1.07e-01
    TAGS: (1 total)
          ----------                                                    1119
      d+2  GGTCCGGGTC  0       0       1       1       5       1.07e-01       525

LOCUS: AT3G51800
DESCRIPTION: metallopeptidase M24 family protein, similar to SP|P50580 Proliferation-associated protein 2G4
{Mus musculus}; contains Pfam profile PF00557: metallopeptidase family M24
DATA:                  Control 30min   2hours  2days   1week   p-value        pos
SENSE COUNTS:          0       0       1       1       5       1.07e-01
GENES (3 total):
  AT3G51800.2
    SENSE COUNTS:      0       0       0       0       0       6.15e-01
    TAGS: (1 total)
      i+3  TTGATTAAAA  0       0       0       0       0       6.15e-01       2300
          ----------                                                    1037
          ----------                                                    991
          ----------                                                    679
          ----------                                                    484
          ----------                                                    423
          ----------                                                    347
          ----------                                                    297
  AT3G51800.1
    SENSE COUNTS:      0       0       1       1       5       1.07e-01
    TAGS: (2 total)
      i+3  TTGATTAAAA  0       0       0       0       0       6.15e-01       2300
      d+1  GTCATTTGCA  0       0       1       1       5       3.15e-02       991
          ----------                                                    679
```

```
           ----------                                                    484
           ----------                                                    423
           ----------                                                    347
           ----------                                                    297

LOCUS: AT1G46912
DESCRIPTION: F-box family protein-related, contains weak hit to TIGRFAM TIGR01640 : F-box protein interaction
domain; similar to hypothetical protein GI:4589954 from (Arabidopsis thaliana) contains weak hit to TIGRFAM
TIGR01640 : F-box protein interaction domain;
DATA:                  Control 30min  2hours  2days  1week  p-value       pos
SENSE COUNTS:          0       4      1       1      0      1.08e-01
GENES (1 total):
  AT1G46912.1
    SENSE COUNTS:      0       4      1       1      0      1.08e-01
    TAGS: (1 total)
           ----------                                                    766
           ----------                                                    205
      v+2  CCAAGTAAAG  0       4      1       1      0      1.08e-01      184
           ----------                                                    135

LOCUS: AT3G57230
DESCRIPTION: MADS-box protein (AGL16), MADS-box transcription factor DEFH125 - Antirrhinum majus, PIR:T17029;
contains Pfam domain PF00319: SRF-type transcription factor (DNA-binding and dimerisation domain); contains
Pfam domain PF01486: K-box region
DATA:                  Control 30min  2hours  2days  1week  p-value       pos
SENSE COUNTS:          4       0      3       0      3      1.08e-01
GENES (1 total):
  AT3G57230.1
    SENSE COUNTS:      4       0      3       0      3      1.08e-01
    TAGS: (2 total)
      d+1  TAGTTTGATG  1       0      0       0      0      4.28e-01      922
      d+2  ATCAGTTTGC  3       0      3       0      3      1.69e-01      829
           ----------                                                    674
           ----------                                                    668
           ----------                                                    638
           ----------                                                    616
           ----------                                                    545
           ----------                                                    535
           ----------                                                    187

LOCUS: AT1G52890
DESCRIPTION: no apical meristem (NAM) family protein, contains Pfam PF02365: No apical meristem (NAM) domain;
similar to NAM (no apical meristem) GB:CAA63101 from (Petunia x hybrida)
DATA:                  Control 30min  2hours  2days  1week  p-value       pos
SENSE COUNTS:          0       1      5       1      4      1.08e-01
GENES (1 total):
  AT1G52890.1
    SENSE COUNTS:      0       1      5       1      4      1.08e-01
    TAGS: (2 total)
      d+1  TAAGTTTAAT  0       0      1       1      1      5.61e-01      1247
      d+2  CATAGAAAAT  0       1      4       0      3      1.42e-01      1118
           ----------                                                    825
           ----------                                                    435
           ----------                                                    431
           ----------                                                    199

LOCUS: AT2G33300
DESCRIPTION: hypothetical protein
DATA:                  Control 30min  2hours  2days  1week  p-value       pos
SENSE COUNTS:          1       0      4       5      0      1.09e-01
GENES (1 total):
  AT2G33300.1
    SENSE COUNTS:      1       0      4       5      0      1.09e-01
    TAGS: (1 total)
           ----------                                                    1020
           ----------                                                    805
           ----------                                                    673
           ----------                                                    549
      v+2  TTCCAGAAAA  1       0      4       5      0      1.09e-01      529
           ----------                                                    490

LOCUS: AT3G52840
DESCRIPTION: beta-galactosidase, putative / lactase, putative, similar to beta-galactosidase precursor
GI:3869280 from (Carica papaya)
DATA:                  Control 30min  2hours  2days  1week  p-value       pos
SENSE COUNTS:          5       4      1       2      10     1.09e-01
GENES (1 total):
  AT3G52840.1
    SENSE COUNTS:      5       4      1       2      10     1.09e-01
    TAGS: (2 total)
      i+3  ATTGATTTAT  1       1      0       2      10     1.59e-03      4812
```

```
      d+1  TACAGATATA 4      3      1      0      0      1.56e-01       2322
           ----------                                                  2308
           ----------                                                  2184
           ----------                                                  2171
           ----------                                                  2005
           ----------                                                  1822
           ----------                                                  1815
           ----------                                                  1601
           ----------                                                  1592
           ----------                                                  1477
           ----------                                                  1184
           ----------                                                  789
           ----------                                                  741
           ----------                                                  344
           ----------                                                  120

LOCUS: AT4G22990
DESCRIPTION: SPX (SYG1/Pho81/XPR1) domain-containing protein, low similarity to SP|P51564 Tetracycline
resistance protein, class H {Pasteurella multocida}, SP|P39843 Multidrug resistance protein 2 (Multidrug-
efflux transporter 2) {Bacillus subtilis}; contains Pfam pro
DATA:                  Control 30min  2hours 2days  1week  p-value        pos
SENSE COUNTS:          7      3      6      0      1      1.11e-01
GENES (1 total):
  AT4G22990.1
    SENSE COUNTS:      7      3      6      0      1      1.11e-01
    TAGS: (4 total)
      d+1  TCGTCGAGGC 2      0      0      0      0      3.22e-01       2171
           ----------                                                  2114
           ----------                                                  2064
      d+2  TTCGAAGACC 0      0      1      0      0      4.55e-01       1994
           ----------                                                  1838
      d+2  TCTCTCTTGT 1      0      0      0      0      4.28e-01       1028
           ----------                                                  941
           ----------                                                  807
           ----------                                                  762
      i+3  AATATTGATA 4      3      5      0      1      3.34e-01       601
           ----------                                                  566
           ----------                                                  476
           ----------                                                  417

LOCUS: AT1G25350
DESCRIPTION: glutamine-tRNA ligase, putative / glutaminyl-tRNA synthetase, putative / GlnRS, putative, similar
to tRNA-glutamine synthetase GI:2995454 from (Lupinus luteus)
DATA:                  Control 30min  2hours 2days  1week  p-value        pos
SENSE COUNTS:          0      0      3      4      1      1.12e-01
GENES (1 total):
  AT1G25350.1
    SENSE COUNTS:      0      0      3      4      1      1.12e-01
    TAGS: (2 total)
           ----------                                                  2583
           ----------                                                  2089
      d+2  GTGGTGCTTA 0      0      1      0      0      4.55e-01       1800
           ----------                                                  1734
           ----------                                                  1524
           ----------                                                  1138
           ----------                                                  916
      d+2  GGCCGATCCT 0      0      2      4      1      1.34e-01       545
           ----------                                                  526
           ----------                                                  401
           ----------                                                  57

LOCUS: AT5G08420
DESCRIPTION: expressed protein
DATA:                  Control 30min  2hours 2days  1week  p-value        pos
SENSE COUNTS:          1      6      3      0      1      1.12e-01
GENES (1 total):
  AT5G08420.1
    SENSE COUNTS:      1      6      3      0      1      1.12e-01
    TAGS: (2 total)
      i+3  AGGAAAATTA 1      0      0      0      0      4.28e-01       1020
      d+1  AGTGATAAAA 0      6      3      0      1      4.34e-02       1004
           ----------                                                  812
           ----------                                                  286
           ----------                                                  232

LOCUS: AT2G35800
DESCRIPTION: mitochondrial substrate carrier family protein, contains INTERPRO:IPR001993 Mitochondrial
substrate carrier family, INTERPRO:IPR002048 calcium-binding EF-hand domain
DATA:                  Control 30min  2hours 2days  1week  p-value        pos
SENSE COUNTS:          6      7      2      1      1      1.12e-01
GENES (1 total):
```

```
  AT2G35800.1
    SENSE COUNTS:      6       7       2       1       1       1.12e-01
    TAGS: (2 total)
      d+1  GGAGACAATA  0       3       0       0       0       3.05e-02        2558
           ----------                                                          2496
           ----------                                                          2379
           ----------                                                          2345
           ----------                                                          2212
           ----------                                                          1908
           ----------                                                          1845
           ----------                                                          859
      X+4  AGAAACTCAA  6       4       2       1       1       3.02e-01        759
           ----------                                                          26

LOCUS: AT1G36310
DESCRIPTION: expressed protein
DATA:                  Control 30min   2hours  2days   1week   p-value         pos
SENSE COUNTS:          3       1       7       6       10      1.12e-01
GENES (1 total):
  AT1G36310.1
    SENSE COUNTS:      3       1       7       6       10      1.12e-01
    TAGS: (3 total)
      d+1  GTTGTAGTTT  2       0       3       4       5       2.44e-01        1374
      d+2  TCTTTAGCGA  0       0       1       0       0       4.55e-01        1165
           ----------                                                          1049
      X+4  ATAAACAAAA  1       1       3       2       5       5.87e-01        291
           ----------                                                          78
           ----------                                                          63

LOCUS: AT1G65020
DESCRIPTION: expressed protein
DATA:                  Control 30min   2hours  2days   1week   p-value         pos
SENSE COUNTS:          2       0       4       0       2       1.13e-01
GENES (1 total):
  AT1G65020.1
    SENSE COUNTS:      2       0       4       0       2       1.13e-01
    TAGS: (2 total)
           ----------                                                          1170
      d+2  ACTGAAGTAA  2       0       3       0       1       2.44e-01        1098
           ----------                                                          1050
           ----------                                                          834
           ----------                                                          532
      X+4  AAAACGTTTC  0       0       1       0       1       3.96e-01        343

LOCUS: AT2G46450
DESCRIPTION: cyclic nucleotide-regulated ion channel, putative (CNGC12), similar to cyclic nucleotide and
calmodulin-regulated ion channel (cngc3) GI:4581201 from (Arabidopsis thaliana)
DATA:                  Control 30min   2hours  2days   1week   p-value         pos
SENSE COUNTS:          1       0       1       0       5       1.14e-01
GENES (2 total):
  AT2G46450.1
    SENSE COUNTS:      1       0       1       0       5       1.14e-01
    TAGS: (1 total)
           ----------                                                          1852
           ----------                                                          1825
           ----------                                                          1590
           ----------                                                          1442
           ----------                                                          1429
           ----------                                                          919
           ----------                                                          752
      X+4  CCCTACAACG  1       0       1       0       5       1.14e-01        347
           ----------                                                          133

LOCUS: AT2G27650
DESCRIPTION: hypothetical protein
DATA:                  Control 30min   2hours  2days   1week   p-value         pos
SENSE COUNTS:          1       0       1       0       4       1.14e-01
GENES (2 total):
  AT2G27650.1
    SENSE COUNTS:      1       0       1       0       4       1.14e-01
    TAGS: (2 total)
           ----------                                                          3979
           ----------                                                          3722
           ----------                                                          3000
           ----------                                                          2793
           ----------                                                          2712
      i+3  TCTAGTAAAC  1       0       1       0       3       4.58e-01        2609
           ----------                                                          2592
           ----------                                                          2229
           ----------                                                          1949
           ----------                                                          1847
```

```
          ----------                                                  1822
          ----------                                                  1177
          ----------                                                  987
          ----------                                                  675
          ----------                                                  637
      v+2  TTGGGTTCTG 0       0       0       0       1       1.65e-01       579
          ----------                                                  45

LOCUS: AT5G47040
DESCRIPTION: Lon protease homolog 1, mitochondrial (LON), identical to Lon protease homolog 1  mitochondrial
precursor SP:O64948 from (Arabidopsis thaliana)
DATA:                  Control 30min   2hours  2days   1week   p-value        pos
SENSE COUNTS:          4       1       3       6       9       1.14e-01
GENES (1 total):
  AT5G47040.1
    SENSE COUNTS:      4       1       3       6       9       1.14e-01
    TAGS: (3 total)
      i+3  AGTTTTAAAT 1       0       1       1       1       7.91e-01       3424
      d+1  GCGGAACAAC 2       0       1       1       1       6.51e-01       2680
          ----------                                                  2459
          ----------                                                  2269
      d+2  CTTGAAAAAA 1       1       1       4       7       1.44e-01       2057
          ----------                                                  2004
          ----------                                                  1884
          ----------                                                  1740
          ----------                                                  1400
          ----------                                                  1122
          ----------                                                  1002

LOCUS: AT5G42310
DESCRIPTION: pentatricopeptide (PPR) repeat-containing protein, contains Pfam profile PF01535: PPR repeat
DATA:                  Control 30min   2hours  2days   1week   p-value        pos
SENSE COUNTS:          7       0       4       5       1       1.14e-01
GENES (1 total):
  AT5G42310.1
    SENSE COUNTS:      7       0       4       5       1       1.14e-01
    TAGS: (2 total)
          ----------                                                  2765
          ----------                                                  2684
          ----------                                                  2639
          ----------                                                  2625
          ----------                                                  2524
      d+2  TAGGAAGTTA 5       0       4       5       1       2.92e-01       2450
      d+2  AAGCAGACGT 2       0       0       0       0       1.04e-01       2184
          ----------                                                  2160
          ----------                                                  2124
          ----------                                                  2061
          ----------                                                  2013
          ----------                                                  1836
          ----------                                                  1555
          ----------                                                  1470
          ----------                                                  1465
          ----------                                                  545
          ----------                                                  260

LOCUS: AT2G32770
DESCRIPTION: purple acid phosphatase (PAP13), identical to purple acid phosphatase (PAP13) (Arabidopsis
thaliana) GI:20257489; contains Pfam profile PF00149: Ser/Thr protein phosphatase
DATA:                  Control 30min   2hours  2days   1week   p-value        pos
SENSE COUNTS:          3       0       2       0       0       1.15e-01
GENES (3 total):
  AT2G32770.2
    SENSE COUNTS:      3       0       2       0       0       1.15e-01
    TAGS: (1 total)
          ----------                                                  2007
      d+2  GATCAAACTC 3       0       2       0       0       1.15e-01       1895
          ----------                                                  1653
          ----------                                                  1542
          ----------                                                  1411
          ----------                                                  1166
          ----------                                                  1136
          ----------                                                  753
          ----------                                                  725
          ----------                                                  539
          ----------                                                  74

LOCUS: AT2G19600
DESCRIPTION: K+ efflux antiporter, putative (KEA4), similar to glutathione-regulated potassium-efflux system
protein KEFB, Escherichia coli, SWISSPROT:P45522; Monovalent cation:proton antiporter family 2 (CPA2 family)
member, PMID:11500563; Note: non-consensus splice
DATA:                  Control 30min   2hours  2days   1week   p-value        pos
```

```
SENSE COUNTS:          3       0       2       0       0       1.15e-01
GENES (2 total):
  AT2G19600.1
    SENSE COUNTS:      3       0       2       0       0       1.15e-01
    TAGS: (3 total)
      i+3  AATCACCAGG  0       0       1       0       0       4.55e-01       2489
      d+1  TGCTCTGAAA  0       0       1       0       0       4.55e-01       2124
           ----------                                                         1895
           ----------                                                         1456
      d+2  TTGATACATA  3       0       0       0       0       2.13e-02       1425
           ----------                                                         1116
           ----------                                                         1003
           ----------                                                         864

LOCUS: AT4G17080
DESCRIPTION: MORN (Membrane Occupation and Recognition Nexus) repeat-containing protein /phosphatidylinositol-
4-phosphate 5-kinase-related, low similarity to phosphatidylinositol-4-phosphate 5-kinase AtPIP5K1
(Arabidopsis thaliana) GI:3702691; contains Pfam profile PF
DATA:                  Control 30min   2hours  2days   1week   p-value        pos
SENSE COUNTS:          3       0       2       0       0       1.15e-01
GENES (1 total):
  AT4G17080.1
    SENSE COUNTS:      3       0       2       0       0       1.15e-01
    TAGS: (1 total)
           ----------                                                         2194
      v+2  TAAGAGAAGT  3       0       2       0       0       1.15e-01       2095
           ----------                                                         1805
           ----------                                                         1441
           ----------                                                         1387
           ----------                                                         1357
           ----------                                                         1288
           ----------                                                         1219
           ----------                                                         1019
           ----------                                                         961
           ----------                                                         780

LOCUS: AT4G28750
DESCRIPTION: photosystem I reaction center subunit IV, chloroplast, putative / PSI-E, putative (PSAE1),
identical to SP|Q9S831; similar to SP|P12354 Photosystem I reaction center subunit IV, chloroplast precursor
(PSI-E) {Spinacia oleracea}; contains Pfam profile PF02
DATA:                  Control 30min   2hours  2days   1week   p-value        pos
SENSE COUNTS:          8       0       4       4       1       1.15e-01
GENES (2 total):
  AT4G28750.1
    SENSE COUNTS:      8       0       4       4       1       1.15e-01
    TAGS: (2 total)
      i+3  CCGGTTTTGT  0       0       0       0       0       6.15e-01       855
      X+4  GTTAGCTCTC  8       0       4       4       1       4.48e-02       289

LOCUS: AT1G27300
DESCRIPTION: expressed protein
DATA:                  Control 30min   2hours  2days   1week   p-value        pos
SENSE COUNTS:          7       2       1       1       3       1.15e-01
GENES (1 total):
  AT1G27300.1
    SENSE COUNTS:      7       2       1       1       3       1.15e-01
    TAGS: (3 total)
      d+1  GTTCAGTGCC  2       2       1       0       0       4.77e-01       874
      d+2  AAAAAGGGTT  4       0       0       0       3       6.43e-02       810
      d+2  GGAATGGATC  1       0       0       1       0       5.06e-01       588
           ----------                                                         458
           ----------                                                         449
           ----------                                                         390
           ----------                                                         120

LOCUS: AT2G44640
DESCRIPTION: expressed protein
DATA:                  Control 30min   2hours  2days   1week   p-value        pos
SENSE COUNTS:          5       1       1       5       0       1.15e-01
GENES (1 total):
  AT2G44640.1
    SENSE COUNTS:      5       1       1       5       0       1.15e-01
    TAGS: (2 total)
           ----------                                                         1676
      d+2  ACTATAAAAA  4       1       0       4       0       1.34e-01       1451
      d+2  ATCGAGCTCC  1       0       1       1       0       7.18e-01       1373
           ----------                                                         1280
           ----------                                                         1155
           ----------                                                         989
           ----------                                                         930
           ----------                                                         579
```

```
LOCUS: AT1G16680
DESCRIPTION: DNAJ heat shock N-terminal domain-containing protein / S-locus protein, putative, similar to S-
locus protein 5 GI:6069485 from (Brassica rapa); contains Pfam profile PF00226 DnaJ domain
DATA:                 Control 30min  2hours 2days  1week  p-value        pos
SENSE COUNTS:         0       0      3      0      0      1.17e-01
GENES (1 total):
  AT1G16680.1
    SENSE COUNTS:     0       0      3      0      0      1.17e-01
    TAGS: (2 total)
      i+3  ATGATATATT 0       0      0      0      0      6.15e-01       2428
      d+1  GGAGCTTGAA 0       0      3      0      0      2.70e-02       1641
           ----------                                                  1282
           ----------                                                  1274
           ----------                                                  619
           ----------                                                  566

LOCUS: AT2G44980
DESCRIPTION: transcription regulatory protein SNF2, putative, similar to SNF2P (Oryza sativa (japonica
cultivar-group)) GI:23193483; contains Pfam profiles PF00271: Helicase conserved C-terminal domain, PF00176:
SNF2 family N-terminal domain; CG donor site annotated i
DATA:                 Control 30min  2hours 2days  1week  p-value        pos
SENSE COUNTS:         4       0      3      0      0      1.17e-01
GENES (2 total):
  AT2G44980.1
    SENSE COUNTS:     4       0      3      0      0      1.17e-01
    TAGS: (3 total)
      v+1  TAACGGTGTC 1       0      2      0      0      5.95e-01       3063
           ----------                                                  2802
           ----------                                                  2458
           ----------                                                  2183
           ----------                                                  1914
           ----------                                                  1810
      v+2  ATCTCTACAA 3       0      0      0      0      2.13e-02       1674
           ----------                                                  1495
           ----------                                                  1379
           ----------                                                  1044
           ----------                                                  799
      v+2  TCGGAAATCC 0       0      1      0      0      4.55e-01       78
  AT2G44980.2
    SENSE COUNTS:     4       0      3      0      0      1.17e-01
    TAGS: (3 total)
      v+1  TAACGGTGTC 1       0      2      0      0      5.95e-01       3120
           ----------                                                  2859
           ----------                                                  2515
           ----------                                                  2240
           ----------                                                  1971
           ----------                                                  1867
      v+2  ATCTCTACAA 3       0      0      0      0      2.13e-02       1731
           ----------                                                  1522
           ----------                                                  1406
           ----------                                                  1062
           ----------                                                  799
      v+2  TCGGAAATCC 0       0      1      0      0      4.55e-01       78

LOCUS: AT4G04900
DESCRIPTION: p21-rho-binding domain-containing protein, contains Pfam PF00786: P21-Rho-binding domain
DATA:                 Control 30min  2hours 2days  1week  p-value        pos
SENSE COUNTS:         6       0      7      4      8      1.19e-01
GENES (2 total):
  AT4G04900.1
    SENSE COUNTS:     6       0      7      4      8      1.19e-01
    TAGS: (2 total)
           ----------                                                  1265
           ----------                                                  1138
      v+2  TGGCTCATAT 0       0      0      0      0      6.15e-01       466
      v+2  TCAATGAAAA 6       0      7      4      8      4.83e-02       354
           ----------                                                  84

LOCUS: AT1G67130
DESCRIPTION: F-box family protein, contains Pfam PF00646: F-box domain
DATA:                 Control 30min  2hours 2days  1week  p-value        pos
SENSE COUNTS:         3       0      1      4      0      1.19e-01
GENES (1 total):
  AT1G67130.1
    SENSE COUNTS:     3       0      1      4      0      1.19e-01
    TAGS: (1 total)
           ----------                                                  1749
           ----------                                                  1626
           ----------                                                  1348
           ----------                                                  1243
```

```
           ----------                                                 1115
           ----------                                                 979
           ----------                                                 471
           ----------                                                 354
      v+2  GCTTCTTGGG 3      0      1      4      0      1.19e-01     214

LOCUS: AT2G37680
DESCRIPTION: phytochrome A specific signal transduction component (PAT3) / far-red elongated hypocotyl protein
1 (FHY1), identical to phytochrome A specific signal transduction component PAT3 (Arabidopsis thaliana)
gi|19421998|gb|AAL87850; identical to far-red elongat
DATA:                 Control 30min  2hours 2days  1week  p-value      pos
SENSE COUNTS:         2      2      2      1      8      1.19e-01
GENES (2 total):
  AT2G37680.1
    SENSE COUNTS:     2      2      2      1      8      1.19e-01
    TAGS: (2 total)
      i+3  CGATGCACGT 1      2      0      0      1      3.96e-01     2071
      v+1  AGACAAAGAA 1      0      2      1      7      3.06e-02     1363
           ----------                                                 1338
           ----------                                                 1067
           ----------                                                 804
           ----------                                                 522
           ----------                                                 501
           ----------                                                 481

LOCUS: AT4G04310
DESCRIPTION: pseudogene, zinc knuckle (CCHC type) protein family, contains Pfam domain PF00098: Zinc knuckle
DATA:                 Control 30min  2hours 2days  1week  p-value      pos
SENSE COUNTS:         1      0      1      4      0      1.20e-01
GENES (1 total):
  AT4G04310.1
    SENSE COUNTS:     1      0      1      4      0      1.20e-01
    TAGS: (1 total)
           ----------                                                 3637
           ----------                                                 3519
           ----------                                                 3502
           ----------                                                 3354
           ----------                                                 3336
           ----------                                                 3111
           ----------                                                 3024
           ----------                                                 2999
           ----------                                                 2959
      p+2  TTTCTATAAT 1      0      1      4      0      1.20e-01     2845
           ----------                                                 2772
           ----------                                                 2601
           ----------                                                 2340
           ----------                                                 2241
           ----------                                                 2131
           ----------                                                 1443
           ----------                                                 806
           ----------                                                 618
           ----------                                                 170
           ----------                                                 22

LOCUS: AT5G34850
DESCRIPTION: calcineurin-like phosphoesterase family protein, contains Pfam profile: PF00149 calcineurin-like
phosphoesterase
DATA:                 Control 30min  2hours 2days  1week  p-value      pos
SENSE COUNTS:         3      0      7      4      1      1.20e-01
GENES (1 total):
  AT5G34850.1
    SENSE COUNTS:     3      0      7      4      1      1.20e-01
    TAGS: (1 total)
           ----------                                                 1775
      d+2  GGTGTGAATT 3      0      7      4      1      1.20e-01     1738
           ----------                                                 1522
           ----------                                                 1128
           ----------                                                 1122
           ----------                                                 1061
           ----------                                                 1046
           ----------                                                 782
           ----------                                                 773
           ----------                                                 759
           ----------                                                 605
           ----------                                                 519
           ----------                                                 447
           ----------                                                 204

LOCUS: AT4G02420
```

```
DESCRIPTION: lectin protein kinase, putative, similar to receptor lectin kinase 3 (Arabidopsis thaliana)
gi|4100060|gb|AAD00733; contains protein kinase domain, Pfam:PF00069; contains legume lectins alpha and beta
domains, Pfam:PF00138 and Pfam:PF00139
DATA:                 Control 30min   2hours  2days   1week   p-value        pos
SENSE COUNTS:         1       0       3       5       1       1.20e-01
GENES (1 total):
  AT4G02420.1
    SENSE COUNTS:     1       0       3       5       1       1.20e-01
    TAGS: (2 total)
          ----------                                                         2547
      v+2  GCTTTTCGAT 1       0       2       4       0       1.59e-01       2514
      v+2  AACTCAAACG 0       0       1       1       1       5.61e-01       2419
          ----------                                                         2310
          ----------                                                         2274
          ----------                                                         1632
          ----------                                                         1458
          ----------                                                         1299
          ----------                                                         984
          ----------                                                         826
          ----------                                                         763
          ----------                                                         663
          ----------                                                         534
          ----------                                                         354
          ----------                                                         212

LOCUS: AT5G47570
DESCRIPTION: expressed protein
DATA:                 Control 30min   2hours  2days   1week   p-value        pos
SENSE COUNTS:         3       8       2       7       1       1.20e-01
GENES (1 total):
  AT5G47570.1
    SENSE COUNTS:     3       8       2       7       1       1.20e-01
    TAGS: (2 total)
      X+4  TGTCGAACCA 0       0       0       1       0       3.09e-01       321
      d+1  GTTGAGTGGC 3       8       2       6       1       1.54e-01       313
          ----------                                                         110

LOCUS: AT3G16850
DESCRIPTION: glycoside hydrolase family 28 protein / polygalacturonase (pectinase) family protein, weak
similarity to SP|P05117 Polygalacturonase 2A precursor (EC 3.2.1.15)  (Pectinase) {Lycopersicon esculentum};
contains PF00295: Glycosyl hydrolases family 28
DATA:                 Control 30min   2hours  2days   1week   p-value        pos
SENSE COUNTS:         9       10      3       6       15      1.20e-01
GENES (1 total):
  AT3G16850.1
    SENSE COUNTS:     9       10      3       6       15      1.20e-01
    TAGS: (3 total)
      d+1  GTGTAACAAA 4       2       2       5       8       3.15e-01       1727
      d+2  AGGTTACGAT 3       7       1       1       7       1.33e-01       1548
      d+2  AGTTCTCCTT 2       1       0       0       0       3.42e-01       1409
          ----------                                                         604
          ----------                                                         122
          ----------                                                         14

LOCUS: AT3G51720
DESCRIPTION: expressed protein, contains Pfam PF05701: Plant protein of unknown function (DUF827)
DATA:                 Control 30min   2hours  2days   1week   p-value        pos
SENSE COUNTS:         0       4       0       2       1       1.21e-01
GENES (1 total):
  AT3G51720.1
    SENSE COUNTS:     0       4       0       2       1       1.21e-01
    TAGS: (1 total)
          ----------                                                         944
          ----------                                                         549
          ----------                                                         375
      X+4  AGAAAAAGAC 0       4       0       2       1       1.21e-01       -52

LOCUS: AT1G09130
DESCRIPTION: ATP-dependent Clp protease proteolytic subunit, putative, similar to nClpP5 GI:5360595 from
(Arabidopsis thaliana)
DATA:                 Control 30min   2hours  2days   1week   p-value        pos
SENSE COUNTS:         20      7       15      21      21      1.21e-01
GENES (1 total):
  AT1G09130.1
    SENSE COUNTS:     20      7       15      21      21      1.21e-01
    TAGS: (3 total)
      i+3  GAAATCTCTG 5       0       1       1       5       1.15e-01       2287
      d+1  CGATTAGGAG 15      7       13      20      16      2.20e-01       1154
          ----------                                                         1075
          ----------                                                         903
      d+2  CCAAAGCGAT 0       0       1       0       0       4.55e-01       724
```

```
          ----------                                                    333
          ----------                                                    66
          ----------                                                    42

LOCUS: AT5G56350
DESCRIPTION: pyruvate kinase, putative, similar to pyruvate kinase, cytosolic isozyme (Nicotiana tabacum)
SWISS-PROT:Q42954
DATA:                 Control 30min   2hours  2days   1week   p-value         pos
SENSE COUNTS:         8       1       9       13      13      1.22e-01
GENES (1 total):
  AT5G56350.1
    SENSE COUNTS:     8       1       9       13      13      1.22e-01
    TAGS: (4 total)
      d+1  ATTTCTTTTT 3       1       4       12      12      8.01e-03        1966
      d+2  GTCCTAACCA 0       0       1       1       1       8.43e-01        1399
           ----------                                                         1309
      d+2  CTCAGTGGTG 0       0       1       0       0       4.55e-01        1192
           ----------                                                         1114
           ----------                                                         1009
           ----------                                                         985
           ----------                                                         916
           ----------                                                         829
           ----------                                                         799
      i+3  ATGTTTTACC 5       0       3       0       0       4.96e-02        794
           ----------                                                         580
           ----------                                                         448
           ----------                                                         377
           ----------                                                         368

LOCUS: AT2G37340
DESCRIPTION: splicing factor RSZ33 (RSZ33), nearly identical to splicing factor RSZ33 (Arabidopsis thaliana)
GI:9843663; contains Pfam profiles PF00076: RNA recognition motif. (a.k.a. RRM, RBD, or RNP domain), PF00098:
Zinc knuckle
DATA:                 Control 30min   2hours  2days   1week   p-value         pos
SENSE COUNTS:         5       4       2       0       0       1.22e-01
GENES (4 total):
  AT2G37340.2
    SENSE COUNTS:     5       4       2       0       0       1.22e-01
    TAGS: (1 total)
           ----------                                                         1302
      d+2  TTACCAAGAG 5       4       2       0       0       1.22e-01        1192
           ----------                                                         1124
           ----------                                                         1097
           ----------                                                         323
           ----------                                                         319
           ----------                                                         268
  AT2G37340.3
    SENSE COUNTS:     5       4       2       0       0       1.22e-01
    TAGS: (1 total)
           ----------                                                         1365
      d+2  TTACCAAGAG 5       4       2       0       0       1.22e-01        1255
           ----------                                                         1187
           ----------                                                         1160
  AT2G37340.1
    SENSE COUNTS:     5       4       2       0       0       1.22e-01
    TAGS: (1 total)
           ----------                                                         1158
      d+2  TTACCAAGAG 5       4       2       0       0       1.22e-01        1048
           ----------                                                         980
           ----------                                                         953

LOCUS: AT4G10300
DESCRIPTION: expressed protein,
DATA:                 Control 30min   2hours  2days   1week   p-value         pos
SENSE COUNTS:         0       5       1       1       3       1.23e-01
GENES (1 total):
  AT4G10300.1
    SENSE COUNTS:     0       5       1       1       3       1.23e-01
    TAGS: (1 total)
           ----------                                                         695
      d+2  GACTTACAGT 0       5       1       1       3       1.23e-01        333
           ----------                                                         199

LOCUS: AT3G09970
DESCRIPTION: calcineurin-like phosphoesterase family protein, contains Pfam profile: PF00149 Calcineurin-like
phosphoesterase
DATA:                 Control 30min   2hours  2days   1week   p-value         pos
SENSE COUNTS:         2       0       3       0       0       1.23e-01
GENES (1 total):
  AT3G09970.1
    SENSE COUNTS:     2       0       3       0       0       1.23e-01
```

```
    TAGS: (2 total)
      d+1  CTATAATCCA 2      0      0      0      0      1.04e-01      1173
      d+2  GAAAACTTCA 0      0      3      0      0      2.70e-02      842
           ----------                                                 680
           ----------                                                 614
           ----------                                                 542
           ----------                                                 284
           ----------                                                 263
           ----------                                                 86

LOCUS: AT4G32250
DESCRIPTION: protein kinase family protein, contains protein kinase domain, Pfam:PF00069
DATA:                 Control 30min  2hours 2days  1week  p-value       pos
SENSE COUNTS:         2      0      3      0      0      1.23e-01
GENES (2 total):
  AT4G32250.1
    SENSE COUNTS:     2      0      3      0      0      1.23e-01
    TAGS: (1 total)
           ----------                                                 2112
      d+2  TACATAATGA 2      0      3      0      0      1.23e-01      1985
           ----------                                                 1687
           ----------                                                 1635
           ----------                                                 1254
           ----------                                                 1245
           ----------                                                 1166
           ----------                                                 1158
           ----------                                                 758
           ----------                                                 443
           ----------                                                 288
  AT4G32250.2
    SENSE COUNTS:     2      0      3      0      0      1.23e-01
    TAGS: (1 total)
           ----------                                                 2252
      d+2  TACATAATGA 2      0      3      0      0      1.23e-01      2125
           ----------                                                 1827
           ----------                                                 1775
           ----------                                                 1394
           ----------                                                 1385
           ----------                                                 1306
           ----------                                                 1298
           ----------                                                 898
           ----------                                                 583
           ----------                                                 428

LOCUS: AT1G72020
DESCRIPTION: expressed protein
DATA:                 Control 30min  2hours 2days  1week  p-value       pos
SENSE COUNTS:         4      9      2      2      3      1.24e-01
GENES (1 total):
  AT1G72020.1
    SENSE COUNTS:     4      9      2      2      3      1.24e-01
    TAGS: (2 total)
      i+3  TTCGTGTTTT 1      0      0      0      0      4.28e-01      521
      d+1  AAGTACCAAG 3      9      2      2      3      9.30e-02      288
           ----------                                                 270
           ----------                                                 232
           ----------                                                 108

LOCUS: AT1G26630
DESCRIPTION: eukaryotic translation initiation factor 5A, putative / eIF-5A, putative, strong similariy to
SP|Q9AXQ6 Eukaryotic translation initiation factor 5A-1 (eIF-5A 1) {Lycopersicon esculentum}
DATA:                 Control 30min  2hours 2days  1week  p-value       pos
SENSE COUNTS:         27     37     43     53     46     1.24e-01
GENES (1 total):
  AT1G26630.1
    SENSE COUNTS:     27     37     43     53     46     1.24e-01
    TAGS: (4 total)
      d+1  ATATTCATTG 6      0      2      6      19     1.30e-05      878
      d+2  AAGCTTGTTT 2      1      3      4      8      1.96e-01      850
      d+2  GGAGAGGAGC 19     36     35     43     19     3.32e-02      565
           ----------                                                 556
      d+2  TGAACCGTGT 0      0      3      0      0      2.70e-02      386
           ----------                                                 136

LOCUS: AT1G80210
DESCRIPTION: expressed protein
DATA:                 Control 30min  2hours 2days  1week  p-value       pos
SENSE COUNTS:         2      0      4      1      0      1.24e-01
GENES (1 total):
  AT1G80210.1
    SENSE COUNTS:     2      0      4      1      0      1.24e-01
```

```
    TAGS: (2 total)
          ----------                                                    1849
          ----------                                                    1653
      v+2 GAAGATTAAC 2      0      3      1      0      2.70e-01        1559
          ----------                                                    1441
      v+2 TTTTGCCGAC 0      0      1      0      0      4.55e-01        1117
          ----------                                                    1032
          ----------                                                    417
          ----------                                                    344

LOCUS: AT2G30560
DESCRIPTION: glycine-rich protein,
DATA:                Control 30min  2hours 2days  1week  p-value         pos
SENSE COUNTS:        9      4      12     6      1      1.24e-01
GENES (1 total):
  AT2G30560.1
    SENSE COUNTS:    9      4      12     6      1      1.24e-01
    TAGS: (1 total)
          ----------                                                    1298
          ----------                                                    1288
      v+2 AAAATAGTTT 9      4      12     6      1      1.24e-01        1222
          ----------                                                    1144
          ----------                                                    1140
          ----------                                                    1135
          ----------                                                    977
          ----------                                                    856
          ----------                                                    771
          ----------                                                    510
          ----------                                                    107

LOCUS: AT4G24680
DESCRIPTION: expressed protein
DATA:                Control 30min  2hours 2days  1week  p-value         pos
SENSE COUNTS:        3      1      9      8      1      1.24e-01
GENES (1 total):
  AT4G24680.1
    SENSE COUNTS:    3      1      9      8      1      1.24e-01
    TAGS: (4 total)
          ----------                                                    5251
      v+2 AGTATTTTTG 3      0      6      8      0      3.34e-02        5057
      v+2 CTGAGGATAC 0      1      2      0      0      3.64e-01        4836
          ----------                                                    4771
          ----------                                                    4246
          ----------                                                    4186
          ----------                                                    4087
          ----------                                                    4071
          ----------                                                    3962
          ----------                                                    3821
          ----------                                                    3814
          ----------                                                    3802
          ----------                                                    3778
          ----------                                                    3376
      v+2 CCAGCGACGT 0      0      1      0      0      7.06e-01        3066
          ----------                                                    2754
          ----------                                                    2721
          ----------                                                    2239
      v+2 CTTATAACAA 0      0      0      0      1      1.65e-01        1765
          ----------                                                    1704
          ----------                                                    1558
          ----------                                                    1509
          ----------                                                    1194
          ----------                                                    984
          ----------                                                    847
          ----------                                                    836
          ----------                                                    683
          ----------                                                    661
          ----------                                                    408
          ----------                                                    161

LOCUS: AT5G22035
DESCRIPTION: ubiquitin-specific protease-related, contains weak similarity to ubiquitin-specific protease;
UBP5 (Arabidopsis thaliana) gi|6648604|gb|AAF21246
DATA:                Control 30min  2hours 2days  1week  p-value         pos
SENSE COUNTS:        6      0      2      1      7      1.24e-01
GENES (1 total):
  AT5G22035.1
    SENSE COUNTS:    6      0      2      1      7      1.24e-01
    TAGS: (2 total)
      i+3 ATTTATTCAT 1      0      0      0      7      3.89e-03        2349
          ----------                                                    1468
          ----------                                                    1348
```

```
          ----------                                                   962
      v+2  ATGATAAAAA  5      0      2      1      0      1.09e-01       796

LOCUS: AT4G36420
DESCRIPTION: ribosomal protein L12 family protein
DATA:                 Control 30min  2hours 2days  1week  p-value        pos
SENSE COUNTS:         2      2      3      9      7      1.25e-01
GENES (1 total):
  AT4G36420.1
    SENSE COUNTS:     2      2      3      9      7      1.25e-01
    TAGS: (1 total)
          ----------                                                   867
          ----------                                                   627
      X+4  TTATTTCCAA  2      2      3      9      7      1.25e-01       261
          ----------                                                   12

LOCUS: AT1G48840
DESCRIPTION: expressed protein, contains Pfam profile: PF04842 plant protein of unknown function (DUF639)
DATA:                 Control 30min  2hours 2days  1week  p-value        pos
SENSE COUNTS:         3      0      1      1      5      1.26e-01
GENES (1 total):
  AT1G48840.1
    SENSE COUNTS:     3      0      1      1      5      1.26e-01
    TAGS: (2 total)
      d+1  TTCTTTGTTT  1      0      0      0      0      4.28e-01       2471
      d+2  TATGTTGTAG  2      0      1      1      5      1.41e-01       2352
          ----------                                                   2345
          ----------                                                   1874
          ----------                                                   1817
          ----------                                                   892

LOCUS: AT2G41220
DESCRIPTION: glutamate synthase, chloroplast (GLU2) / ferredoxin-dependent glutamate synthase (Fd-GOGAT 2),
identical to SP|Q9T0P4 Ferredoxin-dependent glutamate synthase 2, chloroplast precursor (EC 1.4.7.1) (Fd-GOGAT
2) {Arabidopsis thaliana}
DATA:                 Control 30min  2hours 2days  1week  p-value        pos
SENSE COUNTS:         0      0      0      0      3      1.27e-01
GENES (2 total):
  AT2G41220.1
    SENSE COUNTS:     0      0      0      0      3      1.27e-01
    TAGS: (2 total)
      i+3  TCAAAGGTTC  0      0      0      0      0      6.15e-01       6154
      d+1  CTTGTACTTT  0      0      0      0      3      8.36e-02       5093
          ----------                                                   4709
          ----------                                                   4248
          ----------                                                   4117
          ----------                                                   3724
          ----------                                                   3533
          ----------                                                   3329
          ----------                                                   2966
          ----------                                                   2726
          ----------                                                   2392
          ----------                                                   1882
          ----------                                                   1759
          ----------                                                   1477
          ----------                                                   1097
          ----------                                                   1000
          ----------                                                   324

LOCUS: AT3G57790
DESCRIPTION: glycoside hydrolase family 28 protein / polygalacturonase (pectinase) family protein, weak
similarity to SP|P15922 Exo-poly-alpha-D-galacturonosidase precursor (EC 3.2.1.82) (Exo-PG) {Erwinia
chrysanthemi}; contains PF00295: Glycosyl hydrolases family 28
DATA:                 Control 30min  2hours 2days  1week  p-value        pos
SENSE COUNTS:         0      1      0      0      3      1.27e-01
GENES (1 total):
  AT3G57790.1
    SENSE COUNTS:     0      1      0      0      3      1.27e-01
    TAGS: (1 total)
          ----------                                                   2228
          ----------                                                   2169
      v+2  TGAACGGGTT  0      1      0      0      3      1.27e-01       1624
          ----------                                                   1512
          ----------                                                   1260
          ----------                                                   982

LOCUS: AT1G08280
DESCRIPTION: glycosyl transferase family 29 protein / sialyltransferase family protein, contains Pfam profile:
PF00777 sialyltransferase (Glycosyltransferase family 29)
DATA:                 Control 30min  2hours 2days  1week  p-value        pos
SENSE COUNTS:         0      1      0      0      3      1.27e-01
```

```
GENES (1 total):
  AT1G08280.1
    SENSE COUNTS:      0       1       0       0       3       1.27e-01
    TAGS: (1 total)
           ----------                                                         1231
           ----------                                                         1091
           ----------                                                         941
           ----------                                                         853
           ----------                                                         779
           ----------                                                         596
           ----------                                                         448
           ----------                                                         283
           ----------                                                         142
      X+4  AGTTTTCTAA  0       1       0       0       3       1.27e-01       -169

LOCUS: AT1G74870
DESCRIPTION: expressed protein, contains similarity to hypothetical proteins
DATA:                  Control 30min   2hours  2days   1week   p-value        pos
SENSE COUNTS:          2       0       0       4       1       1.27e-01
GENES (1 total):
  AT1G74870.1
    SENSE COUNTS:      2       0       0       4       1       1.27e-01
    TAGS: (1 total)
           ----------                                                         1494
           ----------                                                         1454
      v+2  TTTGTGAATC  2       0       0       4       1       1.27e-01       1355
           ----------                                                         1253
           ----------                                                         1037
           ----------                                                         385
           ----------                                                         354

LOCUS: AT2G39705
DESCRIPTION: Expressed protein
DATA:                  Control 30min   2hours  2days   1week   p-value        pos
SENSE COUNTS:          8       5       3       1       1       1.28e-01
GENES (1 total):
  AT2G39705.1
    SENSE COUNTS:      8       5       3       1       1       1.28e-01
    TAGS: (2 total)
      d+1  TACATAAAGG  5       5       0       0       1       4.55e-02       535
      d+2  CTTATTTGCT  3       0       3       1       0       1.97e-01       411
           ----------                                                         393

LOCUS: AT3G46180
DESCRIPTION: UDP-galactose/UDP-glucose transporter-related, contains weak similarity to UDP-galactose/UDP-
glucose transporter (GI:22651763) (Arabidopsis thaliana)
DATA:                  Control 30min   2hours  2days   1week   p-value        pos
SENSE COUNTS:          1       0       0       4       0       1.29e-01
GENES (1 total):
  AT3G46180.1
    SENSE COUNTS:      1       0       0       4       0       1.29e-01
    TAGS: (2 total)
      i+3  CTTTTTTGCT  0       0       0       0       0       6.15e-01       1489
      d+1  ACGTAGGACG  1       0       0       4       0       3.65e-02       1193
           ----------                                                         904
           ----------                                                         345
           ----------                                                         160
           ----------                                                         118

LOCUS: AT4G34620
DESCRIPTION: ribosomal protein S16 family protein, ribosomal protein S16, Neurospora crassa, PIR2:A29927
DATA:                  Control 30min   2hours  2days   1week   p-value        pos
SENSE COUNTS:          4       2       7       1       9       1.31e-01
GENES (2 total):
  AT4G34620.1
    SENSE COUNTS:      4       2       7       1       9       1.31e-01
    TAGS: (2 total)
      d+1  CCTTTTTACA  4       2       2       1       8       1.65e-01       634
           ----------                                                         390
      d+2  CTTTTCAGGG  0       0       5       0       1       2.43e-02       308

LOCUS: AT1G23760
DESCRIPTION: BURP domain-containing protein / polygalacturonase, putative, similar to polygalacturonase
isoenzyme 1 beta subunit (Lycopersicon esculentum) GI:170480; contains Pfam profile PF03181: BURP domain
DATA:                  Control 30min   2hours  2days   1week   p-value        pos
SENSE COUNTS:          0       0       1       4       0       1.31e-01
GENES (2 total):
  AT1G23760.1
    SENSE COUNTS:      0       0       1       4       0       1.31e-01
    TAGS: (1 total)
           ----------                                                         1971
```

```
          ----------                                                  1885
          ----------                                                  1874
          ----------                                                  1846
          ----------                                                  1826
          ----------                                                  1663
     d+2  TAGTTATAGC  0      0      1      4      0      1.31e-01     149

LOCUS: AT3G04890
DESCRIPTION: expressed protein
DATA:                 Control 30min  2hours 2days  1week  p-value      pos
SENSE COUNTS:         0      0      1      4      0      1.31e-01
GENES (1 total):
  AT3G04890.1
    SENSE COUNTS:     0      0      1      4      0      1.31e-01
    TAGS: (1 total)
          ----------                                                  847
     d+2  GCCCAAACAA  0      0      1      4      0      1.31e-01     750
          ----------                                                  637
          ----------                                                  566

LOCUS: AT1G11475
DESCRIPTION: DNA-directed RNA polymerase II, putative, nearly identical to DNA-directed RNA polymerase II 8.2
kDa polypeptide    SP:Q39290 from (Brassica napus)
DATA:                 Control 30min  2hours 2days  1week  p-value      pos
SENSE COUNTS:         3      0      3      4      8      1.31e-01
GENES (1 total):
  AT1G11475.1
    SENSE COUNTS:     3      0      3      4      8      1.31e-01
    TAGS: (4 total)
          ----------                                                  559
     d+2  TTTCATTAGT  1      0      0      0      0      4.28e-01     528
     d+2  ATTTCCTGAC  2      0      2      2      7      2.14e-01     491
     d+2  TTGATCTGAT  0      0      1      1      1      5.61e-01     279
     X+4  GCCATAACTG  0      0      0      1      0      3.09e-01     247

LOCUS: AT3G06483
DESCRIPTION: pyruvate dehydrogenase (lipoamide) kinase (pdhk), nearly identical to pyruvate dehydrogenase
kinase (Arabidopsis thaliana) GI:3641834
DATA:                 Control 30min  2hours 2days  1week  p-value      pos
SENSE COUNTS:         3      0      0      1      4      1.32e-01
GENES (2 total):
  AT3G06483.1
    SENSE COUNTS:     3      0      0      1      4      1.32e-01
    TAGS: (2 total)
     d+1  GAAGGATATG  0      0      0      0      1      1.65e-01     1042
     X+4  TATTTATGGT  3      0      0      1      3      3.02e-01     452
          ----------                                                  382
          ----------                                                  286
          ----------                                                  103
          ----------                                                  73

LOCUS: AT5G49580
DESCRIPTION: DNAJ heat shock N-terminal domain-containing protein, contains similarity to S-locus protein 5
GI:6069485 from (Brassica rapa); contains Pfam profile PF00226 DnaJ domain
DATA:                 Control 30min  2hours 2days  1week  p-value      pos
SENSE COUNTS:         2      0      1      5      0      1.33e-01
GENES (1 total):
  AT5G49580.1
    SENSE COUNTS:     2      0      1      5      0      1.33e-01
    TAGS: (2 total)
          ----------                                                  2885
          ----------                                                  2718
          ----------                                                  2633
     v+2  GTGAAAGTTT  1      0      1      5      0      8.72e-02     2472
          ----------                                                  2202
          ----------                                                  2194
          ----------                                                  1945
          ----------                                                  1257
     v+2  ACCAAATTTA  1      0      0      0      0      4.28e-01     1092
          ----------                                                  1053

LOCUS: AT1G62040
DESCRIPTION: autophagy 8c (APG8c), identical to autophagy 8c (Arabidopsis thaliana) GI:19912155; contains Pfam
profile PF02991: Microtubule associated protein 1A/1B, light chain 3
DATA:                 Control 30min  2hours 2days  1week  p-value      pos
SENSE COUNTS:         0      0      2      4      1      1.34e-01
GENES (1 total):
  AT1G62040.1
    SENSE COUNTS:     0      0      2      4      1      1.34e-01
    TAGS: (1 total)
          ----------                                                  524
```

```
      d+2  GCTAATAGCT 0       0       2       4       1       1.34e-01        203

LOCUS: AT2G27170
DESCRIPTION: structural maintenance of chromosomes (SMC) family protein, similar to basement membrane-
associated chondroitin proteoglycan Bamacan (Rattus norvegicus) GI:1785540; contains Pfam profile PF02463:
RecF/RecN/SMC N terminal domain. No suitalble start codon w
DATA:                 Control 30min   2hours  2days   1week   p-value         pos
SENSE COUNTS:         3       0       1       0       2       1.34e-01
GENES (2 total):
  AT2G27170.1
    SENSE COUNTS:     3       0       1       0       2       1.34e-01
    TAGS: (2 total)
      v+1  TCAACAAGAA 0       0       1       0       1       3.96e-01        3274
           ----------                                                         2652
           ----------                                                         2572
           ----------                                                         2361
           ----------                                                         1849
      v+2  AACTATTCAA 3       0       0       0       1       6.72e-02        1708
           ----------                                                         1663
           ----------                                                         550
           ----------                                                         538
           ----------                                                         481

LOCUS: AT1G68730
DESCRIPTION: zinc finger (DNL type) family protein, contains Pfam profile PF05180: DNL zinc finger
DATA:                 Control 30min   2hours  2days   1week   p-value         pos
SENSE COUNTS:         6       7       4       2       0       1.34e-01
GENES (1 total):
  AT1G68730.1
    SENSE COUNTS:     6       7       4       2       0       1.34e-01
    TAGS: (3 total)
           ----------                                                         1332
           ----------                                                         1195
      v+2  GGAAAAAATG 4       0       3       1       0       2.57e-01        995
           ----------                                                         730
      v+2  TGGAGAAAGA 2       0       0       1       0       5.24e-01        695
           ----------                                                         354
      v+2  TTAAATAAAA 0       7       1       0       0       1.47e-03        97

LOCUS: AT5G63530
DESCRIPTION: copper chaperone (CCH)-related, low similarity to copper homeostasis factor (GI:3168840); nearly
identical to farnesylated protein ATFP3 (GI:4097547); contains Pfam profile PF00403: Heavy-metal-associated
domain
DATA:                 Control 30min   2hours  2days   1week   p-value         pos
SENSE COUNTS:         1       0       6       2       6       1.35e-01
GENES (2 total):
  AT5G63530.1
    SENSE COUNTS:     1       0       6       2       6       1.35e-01
    TAGS: (5 total)
      d+1  TGGTTTCTTT 0       0       2       1       1       7.15e-01        1202
      d+2  GCATTTGCTG 0       0       1       0       0       4.55e-01        1178
      d+2  TACTGTAATG 1       0       1       1       5       2.53e-01        1092
      X+4  CAGTGTTTGT 0       0       1       0       0       4.55e-01        940
           ----------                                                         745
      d+2  CTGCGATCAT 0       0       1       0       0       4.55e-01        734
           ----------                                                         616
           ----------                                                         574
           ----------                                                         280

LOCUS: AT1G79830
DESCRIPTION: expressed protein, weak similarity to TATA element modulatory factor (TMF) (Swiss-Prot:P82094)
(Homo sapiens)
DATA:                 Control 30min   2hours  2days   1week   p-value         pos
SENSE COUNTS:         4       3       2       0       0       1.35e-01
GENES (1 total):
  AT1G79830.1
    SENSE COUNTS:     4       3       2       0       0       1.35e-01
    TAGS: (3 total)
      d+1  TATTGTCTGT 3       0       1       0       0       2.51e-01        3062
      d+2  CTGGTGAACA 0       0       0       0       0       6.15e-01        2764
           ----------                                                         2518
           ----------                                                         2449
           ----------                                                         2419
           ----------                                                         2067
           ----------                                                         1771
           ----------                                                         1127
           ----------                                                         794
      d+2  GGTAACACCT 1       3       1       0       0       2.52e-01        220

LOCUS: AT2G29290
```

```
DESCRIPTION: tropinone reductase, putative / tropine dehydrogenase, putative, similar to tropinone reductase
SP:P50165 from (Datura stramonium)
DATA:                 Control 30min  2hours  2days  1week  p-value       pos
SENSE COUNTS:         2       3      0       0      0      1.35e-01
GENES (1 total):
  AT2G29290.1
    SENSE COUNTS:     2       3      0       0      0      1.35e-01
    TAGS: (1 total)
           ----------                                                    785
           ----------                                                    649
      d+2  GTTAATCACA 2       3      0       0      0      1.35e-01      570
           ----------                                                    499
           ----------                                                    458
           ----------                                                    433
           ----------                                                    232
           ----------                                                    117

LOCUS: AT5G52920
DESCRIPTION: pyruvate kinase, putative, similar to pyruvate kinase isozyme G, chloroplast precursor (Nicotiana
tabacum) SWISS-PROT:Q40546
DATA:                 Control 30min  2hours  2days  1week  p-value       pos
SENSE COUNTS:         2       3      0       0      0      1.35e-01
GENES (1 total):
  AT5G52920.1
    SENSE COUNTS:     2       3      0       0      0      1.35e-01
    TAGS: (1 total)
           ----------                                                    1946
      d+2  GCCATATTGT 2       3      0       0      0      1.35e-01      1605
           ----------                                                    1551
           ----------                                                    1543
           ----------                                                    1293
           ----------                                                    822
           ----------                                                    657
           ----------                                                    583
           ----------                                                    574

LOCUS: AT1G15740
DESCRIPTION: leucine-rich repeat family protein
DATA:                 Control 30min  2hours  2days  1week  p-value       pos
SENSE COUNTS:         2       5      2       0      0      1.36e-01
GENES (1 total):
  AT1G15740.1
    SENSE COUNTS:     2       5      2       0      0      1.36e-01
    TAGS: (1 total)
           ----------                                                    2599
      d+2  GCTCTTCTAC 2       5      2       0      0      1.36e-01      2075
           ----------                                                    1461
           ----------                                                    1186
           ----------                                                    1166
           ----------                                                    1042
           ----------                                                    1029
           ----------                                                    899
           ----------                                                    752
           ----------                                                    161

LOCUS: AT5G28020
DESCRIPTION: cysteine synthase, putative / O-acetylserine (thiol)-lyase, putative / O-acetylserine
sulfhydrylase, putative, similar to O-acetylserine(thiol) lyase (EC 4.2.99.8) (Brassica juncea) GI:2245144;
contains Pfam profile PF00291: Pyridoxal-phosphate dependent
DATA:                 Control 30min  2hours  2days  1week  p-value       pos
SENSE COUNTS:         0       2      2       5      0      1.36e-01
GENES (3 total):
  AT5G28020.2
    SENSE COUNTS:     0       2      2       5      0      1.36e-01
    TAGS: (2 total)
      d+1  AACGTTTCAT 0       1      2       1      0      5.89e-01      1212
      d+2  AATTTTAATA 0       1      0       4      0      7.92e-02      1183
           ----------                                                    305
           ----------                                                    47
  AT5G28020.1
    SENSE COUNTS:     0       2      2       5      0      1.36e-01
    TAGS: (2 total)
      d+1  AACGTTTCAT 0       1      2       1      0      5.89e-01      1517
      d+2  AATTTTAATA 0       1      0       4      0      7.92e-02      1488
           ----------                                                    610
           ----------                                                    352

LOCUS: AT4G34730
DESCRIPTION: ribosome-binding factor A family protein, contains PFam PF02033: Ribosome-binding factor A
DATA:                 Control 30min  2hours  2days  1week  p-value       pos
SENSE COUNTS:         4       0      6       5      2      1.37e-01
```

```
GENES (1 total):
  AT4G34730.1
    SENSE COUNTS:      4       0       6       5       2       1.37e-01
    TAGS: (4 total)
      i+3  TCAGTCATAT  0       0       0       1       0       3.09e-01       3787
      d+1  TATGCAGTAA  0       0       3       0       1       7.97e-02       1476
      d+2  AGCTCAAGTT  2       0       3       4       1       3.51e-01       1320
      d+2  GAAAGAGGAA  2       0       0       0       0       1.04e-01       958
           ----------                                                         682

LOCUS: AT2G30960
DESCRIPTION: expressed protein
DATA:                  Control 30min   2hours  2days   1week   p-value        pos
SENSE COUNTS:          1       0       2       5       0       1.37e-01
GENES (1 total):
  AT2G30960.1
    SENSE COUNTS:      1       0       2       5       0       1.37e-01
    TAGS: (2 total)
           ----------                                                         1509
           ----------                                                         1469
           ----------                                                         1445
      d+2  GCTTCACGAT  0       0       2       4       0       1.71e-01       1343
      d+2  ATTCCATAAC  1       0       0       1       0       5.06e-01       958

LOCUS: AT5G37360
DESCRIPTION: expressed protein
DATA:                  Control 30min   2hours  2days   1week   p-value        pos
SENSE COUNTS:          8       4       4       1       0       1.37e-01
GENES (1 total):
  AT5G37360.1
    SENSE COUNTS:      8       4       4       1       0       1.37e-01
    TAGS: (3 total)
      i+3  TGTGTGGTTA  0       1       0       0       0       2.54e-01       1232
      d+1  TACAGTGCCA  8       3       3       1       0       9.52e-02       1081
      d+2  GCAAATCAGT  0       0       1       0       0       4.55e-01       852
           ----------                                                         640

LOCUS: AT5G28935
DESCRIPTION: Mutator-like transposase family, has a 1.0e-48 P-value blast match to Q9SHN7 /450-633 Pfam
PF03108 MuDR family transposase (MuDr-element domain)
DATA:                  Control 30min   2hours  2days   1week   p-value        pos
SENSE COUNTS:          3       2       4       6       12      1.38e-01
GENES (1 total):
  AT5G28935.1
    SENSE COUNTS:      3       2       4       6       12      1.38e-01
    TAGS: (3 total)
           ----------                                                         5080
           ----------                                                         5072
           ----------                                                         5036
           ----------                                                         4851
           ----------                                                         4750
           ----------                                                         4692
           ----------                                                         4517
           ----------                                                         4496
           ----------                                                         4490
           ----------                                                         4459
           ----------                                                         4361
           ----------                                                         4274
           ----------                                                         4267
           ----------                                                         3951
           ----------                                                         3681
           ----------                                                         3596
           ----------                                                         3448
           ----------                                                         3410
      p+2  TGTTTTTCTG  0       0       0       1       0       3.09e-01       3354
           ----------                                                         3308
           ----------                                                         2771
           ----------                                                         2450
           ----------                                                         2296
           ----------                                                         2284
      p+2  TTGAGGATAC  0       0       0       0       0       6.15e-01       2053
           ----------                                                         1703
           ----------                                                         1696
           ----------                                                         1686
           ----------                                                         1578
           ----------                                                         1558
           ----------                                                         1436
           ----------                                                         1280
           ----------                                                         1206
           ----------                                                         1041
           ----------                                                         886
```

```
      p+2  ACATTGTTTT 3       2       4       5       12      8.75e-02        751
           ----------                                                         479
           ----------                                                         187

LOCUS: AT2G25670
DESCRIPTION: expressed protein
DATA:                 Control 30min   2hours  2days   1week   p-value         pos
SENSE COUNTS:         1       3       4       3       10      1.38e-01
GENES (2 total):
  AT2G25670.1
    SENSE COUNTS:     1       3       4       3       10      1.38e-01
    TAGS: (2 total)
      d+1  CCTTGTCACA 0       0       1       1       3       4.30e-01        1614
      d+2  TATTTCGCCG 1       3       3       2       7       4.39e-01        1330
           ----------                                                         1143
           ----------                                                         637
           ----------                                                         153
  AT2G25670.2
    SENSE COUNTS:     1       3       4       3       10      1.38e-01
    TAGS: (2 total)
      d+1  CCTTGTCACA 0       0       1       1       3       4.30e-01        1732
      d+2  TATTTCGCCG 1       3       3       2       7       4.39e-01        1448
           ----------                                                         1261
           ----------                                                         755
           ----------                                                         163

LOCUS: AT4G28200
DESCRIPTION: expressed protein
DATA:                 Control 30min   2hours  2days   1week   p-value         pos
SENSE COUNTS:         2       0       2       6       0       1.38e-01
GENES (1 total):
  AT4G28200.1
    SENSE COUNTS:     2       0       2       6       0       1.38e-01
    TAGS: (3 total)
           ----------                                                         2178
      d+2  TTGAAGTTCA 1       0       1       4       0       2.99e-01        2162
           ----------                                                         1632
           ----------                                                         1620
           ----------                                                         1335
           ----------                                                         1026
      d+2  GAAGAATGAA 0       0       1       1       0       7.90e-01        408
      X+4  CCTCTACGTC 1       0       0       1       0       5.06e-01        283
           ----------                                                         32

LOCUS: AT4G32620
DESCRIPTION: expressed protein, predicted protein T10M13.8, Arabidopsis thaliana
DATA:                 Control 30min   2hours  2days   1week   p-value         pos
SENSE COUNTS:         2       7       2       9       3       1.39e-01
GENES (1 total):
  AT4G32620.1
    SENSE COUNTS:     2       7       2       9       3       1.39e-01
    TAGS: (2 total)
           ----------                                                         4561
           ----------                                                         4473
           ----------                                                         4234
           ----------                                                         3694
           ----------                                                         3673
           ----------                                                         3586
           ----------                                                         3463
           ----------                                                         3395
           ----------                                                         3314
           ----------                                                         2921
           ----------                                                         2726
           ----------                                                         2480
           ----------                                                         2461
           ----------                                                         2312
           ----------                                                         2272
           ----------                                                         2171
           ----------                                                         1901
           ----------                                                         1548
           ----------                                                         1404
           ----------                                                         1381
           ----------                                                         1175
           ----------                                                         1133
      X+4  TACCCATCGG 2       7       1       9       3       7.09e-02        750
      X+4  TCCTGTTCAA 0       0       1       0       0       4.55e-01        599
           ----------                                                         261
           ----------                                                         19

LOCUS: AT2G41710
```

```
DESCRIPTION: ovule development protein, putative, similar to ovule development protein AINTEGUMENTA
(GI:1209099) (Arabidopsis thaliana);Pfam domain (PF00847)
DATA:                 Control 30min  2hours  2days  1week  p-value       pos
SENSE COUNTS:         3       0      1       0      4      1.40e-01
GENES (2 total):
  AT2G41710.1
    SENSE COUNTS:     3       0      1       0      4      1.40e-01
    TAGS: (2 total)
      d+1  ATATTTTGTG 2       0      1       0      1      7.29e-01      1540
           ----------                                                  1176
           ----------                                                  1129
      d+2  CAACAGAACC 1       0      0       0      3      7.14e-02      874
           ----------                                                  84
  AT2G41710.2
    SENSE COUNTS:     3       0      1       0      4      1.40e-01
    TAGS: (2 total)
      d+1  ATATTTTGTG 2       0      1       0      1      7.29e-01      1555
           ----------                                                  1191
           ----------                                                  1144
      d+2  CAACAGAACC 1       0      0       0      3      7.14e-02      889
           ----------                                                  84

LOCUS: AT4G21650
DESCRIPTION: subtilase family protein, contains Pfam domain, PF00082: Subtilase family; contains Pfam domain,
PF02225: protease associated (PA) domain
DATA:                 Control 30min  2hours  2days  1week  p-value       pos
SENSE COUNTS:         1       0      5       0      1      1.40e-01
GENES (1 total):
  AT4G21650.1
    SENSE COUNTS:     1       0      5       0      1      1.40e-01
    TAGS: (2 total)
      d+1  ATGTTATAAT 0       0      2       0      0      3.51e-01      2280
           ----------                                                  2263
           ----------                                                  1537
           ----------                                                  1402
           ----------                                                  951
      d+2  ACACCAACTT 1       0      3       0      1      4.56e-01      468
           ----------                                                  354
           ----------                                                  279

LOCUS: AT4G25260
DESCRIPTION: invertase/pectin methylesterase inhibitor family protein, low similarity to pectinesterase from
Phaseolus vulgaris SP|Q43111, Lycopersicon esculentum SP|Q43143, Arabidopsis thaliana SP|Q42534; contains Pfam
profile PF04043: Plant invertase/pectin methyles
DATA:                 Control 30min  2hours  2days  1week  p-value       pos
SENSE COUNTS:         1       5      5       1      0      1.40e-01
GENES (1 total):
  AT4G25260.1
    SENSE COUNTS:     1       5      5       1      0      1.40e-01
    TAGS: (1 total)
           ----------                                                  871
      d+2  GCTGGGGAGC 1       5      5       1      0      1.40e-01      553
           ----------                                                  118

LOCUS: AT4G15080
DESCRIPTION: zinc finger (DHHC type) family protein, contains Pfam profile PF01529: DHHC zinc finger domain
DATA:                 Control 30min  2hours  2days  1week  p-value       pos
SENSE COUNTS:         12      6      6       2      3      1.41e-01
GENES (1 total):
  AT4G15080.1
    SENSE COUNTS:     12      6      6       2      3      1.41e-01
    TAGS: (2 total)
      d+1  TCTGCTATTT 1       0      0       1      3      4.25e-01      2828
           ----------                                                  2540
           ----------                                                  2195
      i+3  CTCTTTGTGA 11      6      6       1      0      2.80e-02      2033
           ----------                                                  2025
           ----------                                                  1979
           ----------                                                  1866
           ----------                                                  1860
           ----------                                                  1653
           ----------                                                  1399
           ----------                                                  1217
           ----------                                                  1175
           ----------                                                  1088
           ----------                                                  560
           ----------                                                  351
           ----------                                                  95

LOCUS: AT3G07250
```

```
DESCRIPTION: nuclear transport factor 2 (NTF2) family protein / RNA recognition motif (RRM)-containing protein,
contains Pfam profiles PF00076: RNA recognition motif. (a.k.a. RRM, RBD, or RNP domain), PF02136: Nuclear
transport factor 2 (NTF2) domain
DATA:                  Control 30min  2hours  2days  1week  p-value       pos
SENSE COUNTS:          5       0      1       1      1      1.44e-01
GENES (1 total):
  AT3G07250.1
    SENSE COUNTS:      5       0      1       1      1      1.44e-01
    TAGS: (3 total)
           ----------                                                 4241
           ----------                                                 4146
      v+2  CAGCTGCTGC  0       0      1       0      0      4.55e-01  4087
           ----------                                                 3522
           ----------                                                 3400
           ----------                                                 3384
      v+2  GTTTTGATAA  0       0      0       0      0      6.15e-01  1642
           ----------                                                 1524
           ----------                                                 1114
           ----------                                                 669
      i+3  TAGATGATTT  5       0      0       1      1      1.60e-02  559
           ----------                                                 189
           ----------                                                 182
           ----------                                                 160

LOCUS: AT5G07180
DESCRIPTION: leucine-rich repeat family protein / protein kinase family protein, contains Pfam domains
PF00560: Leucine Rich Repeat and PF00069: Protein kinase domain
DATA:                  Control 30min  2hours  2days  1week  p-value       pos
SENSE COUNTS:          4       0      3       2      0      1.45e-01
GENES (2 total):
  AT5G07180.1
    SENSE COUNTS:      4       0      3       2      0      1.45e-01
    TAGS: (2 total)
      d+1  GGATAGGTGA  1       0      0       0      0      4.28e-01  2946
           ----------                                                 2626
           ----------                                                 2548
           ----------                                                 2195
           ----------                                                 2108
           ----------                                                 2077
           ----------                                                 1831
           ----------                                                 1804
           ----------                                                 1798
           ----------                                                 1699
           ----------                                                 1448
           ----------                                                 1073
      d+2  GCAACAAGCT  3       0      3       2      0      2.33e-01  857

LOCUS: AT3G51150
DESCRIPTION: kinesin motor family protein, contains Pfam domain, PF00225: Kinesin motor domain
DATA:                  Control 30min  2hours  2days  1week  p-value       pos
SENSE COUNTS:          4       1      1       0      0      1.45e-01
GENES (1 total):
  AT3G51150.1
    SENSE COUNTS:      4       1      1       0      0      1.45e-01
    TAGS: (2 total)
           ----------                                                 3334
           ----------                                                 3319
           ----------                                                 3284
           ----------                                                 3016
           ----------                                                 2939
           ----------                                                 2878
           ----------                                                 2696
      d+2  CCAGCAACTT  1       0      0       0      0      4.28e-01  2575
           ----------                                                 2484
      d+2  TATCCAGAGC  3       1      1       0      0      3.45e-01  2020
           ----------                                                 1934
           ----------                                                 1811
           ----------                                                 1731
           ----------                                                 1414
           ----------                                                 796
           ----------                                                 656
           ----------                                                 544
           ----------                                                 254
           ----------                                                 214

LOCUS: AT1G01140
DESCRIPTION: CBL-interacting protein kinase 9 (CIPK9), identical to CBL-interacting protein kinase 9
(Arabidopsis thaliana) gi|13249117|gb|AAK16684; contains Pfam profiles PF00069: Protein kinase domain and
PF03822: NAF domain; identical to cDNA CBL-interacting protei
DATA:                  Control 30min  2hours  2days  1week  p-value       pos
SENSE COUNTS:          2       3      0       0      1      1.46e-01
```

```
GENES (4 total):
  AT1G01140.2
    SENSE COUNTS:      2       3       0       0       1       1.46e-01
    TAGS: (2 total)
      i+3  AAGTAATAGT  0       0       0       0       1       4.65e-01       2082
      d+1  TATAATGAAT  2       3       0       0       0       1.35e-01       1512
           ----------                                                         1339
           ----------                                                         1071
           ----------                                                         845
           ----------                                                         792
  AT1G01140.3
    SENSE COUNTS:      2       3       0       0       1       1.46e-01
    TAGS: (2 total)
      i+3  AAGTAATAGT  0       0       0       0       1       4.65e-01       2082
      d+1  TATAATGAAT  2       3       0       0       0       1.35e-01       1518
           ----------                                                         1333
           ----------                                                         1065
           ----------                                                         839
           ----------                                                         792
  AT1G01140.1
    SENSE COUNTS:      2       3       0       0       1       1.46e-01
    TAGS: (2 total)
      i+3  AAGTAATAGT  0       0       0       0       1       4.65e-01       2082
      d+1  TATAATGAAT  2       3       0       0       0       1.35e-01       1506
           ----------                                                         1333
           ----------                                                         1065
           ----------                                                         839
           ----------                                                         792

LOCUS: AT4G28480
DESCRIPTION: DNAJ heat shock family protein, contains Pfam profile PF00226: DnaJ domain; ; similar to DnaJ
homolog subfamily B member 1 (Heat shock 40 kDa protein 1) (Heat shock protein 40) (HSP40) (DnaJ protein
homolog 1) (HDJ-1) (Swiss-Prot:P25685) (Homo sapiens) an
DATA:                  Control 30min   2hours  2days   1week   p-value        pos
SENSE COUNTS:          3       0       0       2       3       1.46e-01
GENES (1 total):
  AT4G28480.1
    SENSE COUNTS:      3       0       0       2       3       1.46e-01
    TAGS: (2 total)
           ----------                                                         680
      X+4  TCCTCCATAA  1       0       0       2       3       1.58e-01       587
      X+4  TTCTTTTATG  2       0       0       0       0       1.04e-01       -128

LOCUS: AT3G21110
DESCRIPTION: phosphoribosylamidoimidazole-succinocarboxamide synthase / SAICAR synthetase (PUR7), identical to
phosphoribosylamidoimidazole-succinocarboxamide synthase, chloroplast (precursor) SP:P38025 from (Arabidopsis
thaliana)
DATA:                  Control 30min   2hours  2days   1week   p-value        pos
SENSE COUNTS:          0       0       2       4       5       1.48e-01
GENES (2 total):
  AT3G21110.1
    SENSE COUNTS:      0       0       2       4       5       1.48e-01
    TAGS: (1 total)
           ----------                                                         2007
           ----------                                                         1701
      d+2  AAAAAAACTA  0       0       2       4       5       1.48e-01       1614
           ----------                                                         1559
           ----------                                                         1420
           ----------                                                         1090
           ----------                                                         1011
           ----------                                                         967

LOCUS: AT3G11450
DESCRIPTION: DNAJ heat shock N-terminal domain-containing protein / cell division protein-related, similar to
GlsA (Volvox carteri f. nagariensis) GI:4633129; contains Pfam profiles PF00226 DnaJ domain, PF00249 Myb-like
DNA-binding domain
DATA:                  Control 30min   2hours  2days   1week   p-value        pos
SENSE COUNTS:          3       9       9       11      3       1.49e-01
GENES (1 total):
  AT3G11450.1
    SENSE COUNTS:      3       9       9       11      3       1.49e-01
    TAGS: (3 total)
      d+1  AGAATTTTGG  1       1       2       2       0       7.80e-01       2018
           ----------                                                         1446
           ----------                                                         1234
      d+2  CTCGAATCAG  2       8       7       9       3       2.73e-01       893
      d+2  ATCTTGAACA  0       0       0       0       0       6.15e-01       803
           ----------                                                         239
           ----------                                                         209
           ----------                                                         79
```

```
LOCUS: AT2G24020
DESCRIPTION: expressed protein, contains Pfam domain PF02575: Uncharacterized BCR, YbaB family COG0718
DATA:                  Control 30min  2hours  2days  1week  p-value       pos
SENSE COUNTS:          5       0      2       4      0      1.49e-01
GENES (1 total):
  AT2G24020.1
    SENSE COUNTS:      5       0      2       4      0      1.49e-01
    TAGS: (2 total)
      i+3  TTGTAAAGAT  3       0      0       0      0      2.13e-02      516
      d+1  GCCAAAGCAG  2       0      2       4      0      4.45e-01      114
           ----------                                                   74

LOCUS: AT5G47455
DESCRIPTION: Expressed protein
DATA:                  Control 30min  2hours  2days  1week  p-value       pos
SENSE COUNTS:          2       0      2       1      6      1.50e-01
GENES (6 total):
  AT5G47455.1
    SENSE COUNTS:      1       0      1       0      0      6.01e-01
    TAGS: (2 total)
      i+3  ATCCGTGTAT  0       0      1       0      0      4.55e-01      1153
      i+3  TGAGGACACT  1       0      0       0      0      4.28e-01      667
           ----------                                                   593
           ----------                                                   402
           ----------                                                   337
           ----------                                                   101
  AT5G47455.2
    SENSE COUNTS:      1       0      1       0      1      6.15e-01
    TAGS: (3 total)
      i+3  ATCCGTGTAT  0       0      1       0      0      4.55e-01      1155
      i+3  TGAGGACACT  1       0      0       0      0      4.28e-01      669
      d+1  ACTAGTTTGA  0       0      0       0      1      1.65e-01      643
           ----------                                                   631
           ----------                                                   566
           ----------                                                   466
           ----------                                                   339
           ----------                                                   103
  AT5G47455.3
    SENSE COUNTS:      2       0      2       1      5      4.37e-01
    TAGS: (3 total)
      i+3  ATCCGTGTAT  0       0      1       0      0      4.55e-01      1153
      i+3  TGAGGACACT  1       0      0       0      0      4.28e-01      667
      d+1  AAGGGAAAAA  1       0      1       1      5      2.53e-01      519
           ----------                                                   419
           ----------                                                   337
           ----------                                                   101
  AT5G47455.4
    SENSE COUNTS:      2       0      2       1      5      4.37e-01
    TAGS: (3 total)
      i+3  ATCCGTGTAT  0       0      1       0      0      4.55e-01      1153
      i+3  TGAGGACACT  1       0      0       0      0      4.28e-01      667
      d+1  AAGGGAAAAA  1       0      1       1      5      2.53e-01      517
           ----------                                                   417
           ----------                                                   337
           ----------                                                   101
  AT5G47455.5
    SENSE COUNTS:      2       0      2       1      5      4.37e-01
    TAGS: (3 total)
      i+3  ATCCGTGTAT  0       0      1       0      0      4.55e-01      1153
      i+3  TGAGGACACT  1       0      0       0      0      4.28e-01      667
      d+1  AAGGGAAAAA  1       0      1       1      5      2.53e-01      621
           ----------                                                   521
           ----------                                                   457
           ----------                                                   337
           ----------                                                   101
  AT5G47455.6
    SENSE COUNTS:      2       0      2       1      5      4.37e-01
    TAGS: (3 total)
      i+3  ATCCGTGTAT  0       0      1       0      0      4.55e-01      1153
      i+3  TGAGGACACT  1       0      0       0      0      4.28e-01      667
      d+1  AAGGGAAAAA  1       0      1       1      5      2.53e-01      611
           ----------                                                   511
           ----------                                                   402
           ----------                                                   337
           ----------                                                   101

LOCUS: AT1G11300
DESCRIPTION: S-locus lectin protein kinase family protein, contains protein kinase domain, Pfam:PF00069;
contains S-locus glycoprotein family domain, Pfam:PF00954
DATA:                  Control 30min  2hours  2days  1week  p-value       pos
SENSE COUNTS:          1       0      3       0      4      1.50e-01
```

```
GENES (1 total):
  AT1G11300.1
    SENSE COUNTS:      1       0       3       0       4       1.50e-01
    TAGS: (2 total)
           ----------                                                         5699
           ----------                                                         5609
           ----------                                                         5600
           ----------                                                         5528
      v+2  TATCCATATA  0       0       0       0       1       1.65e-01       5330
           ----------                                                         4951
           ----------                                                         4551
           ----------                                                         4182
           ----------                                                         3999
           ----------                                                         3979
           ----------                                                         3956
           ----------                                                         3552
           ----------                                                         2878
           ----------                                                         2833
           ----------                                                         2809
      i+3  TCAACTAACA  1       0       3       0       3       3.44e-01       2093
           ----------                                                         2061
           ----------                                                         1692
           ----------                                                         1554
           ----------                                                         1534
           ----------                                                         1511
           ----------                                                         1146
           ----------                                                         1107
           ----------                                                         364

LOCUS: AT1G34220
DESCRIPTION: expressed protein,  contains Pfam profile: PF03398 eukaryotic protein of unknown function, DUF292
DATA:                  Control 30min   2hours  2days   1week   p-value        pos
SENSE COUNTS:          0       3       2       0       1       1.51e-01
GENES (2 total):
  AT1G34220.1
    SENSE COUNTS:      0       3       2       0       1       1.51e-01
    TAGS: (3 total)
      d+1  AAGATTATCA  0       0       1       0       0       4.55e-01       2419
      d+2  ATTCGTGATT  0       3       1       0       1       2.14e-01       2032
           ----------                                                         1981
           ----------                                                         1411
           ----------                                                         769
           ----------                                                         718
      d+2  TTTTTGTATT  0       0       0       0       0       6.15e-01       609
           ----------                                                         575
           ----------                                                         93

LOCUS: AT3G44690
DESCRIPTION: expressed protein,
DATA:                  Control 30min   2hours  2days   1week   p-value        pos
SENSE COUNTS:          4       5       1       7       1       1.56e-01
GENES (1 total):
  AT3G44690.1
    SENSE COUNTS:      4       5       1       7       1       1.56e-01
    TAGS: (3 total)
           ----------                                                         4369
           ----------                                                         3811
           ----------                                                         3622
           ----------                                                         3532
           ----------                                                         3253
           ----------                                                         2977
           ----------                                                         2914
      v+2  GATTATAAAA  0       0       0       0       0       6.15e-01       2853
           ----------                                                         2748
           ----------                                                         2728
           ----------                                                         2632
           ----------                                                         2589
           ----------                                                         2473
           ----------                                                         2430
           ----------                                                         2410
           ----------                                                         2314
           ----------                                                         2284
           ----------                                                         2271
           ----------                                                         2217
           ----------                                                         1327
           ----------                                                         1258
      v+2  ATTCACCAGT  0       4       0       5       0       2.16e-02       1150
           ----------                                                         1033
           ----------                                                         787
           ----------                                                         763
      v+2  ATCATCCATC  4       1       1       2       1       6.01e-01       706
```

```
          ----------                                                    444
          ----------                                                    191

LOCUS: AT4G27600
DESCRIPTION: pfkB-type carbohydrate kinase family protein, contains Pfam profile: PF00294 pfkB family
carbohydrate kinase
DATA:                 Control 30min   2hours  2days   1week   p-value        pos
SENSE COUNTS:         10      1       5       6       5       1.56e-01
GENES (1 total):
  AT4G27600.1
    SENSE COUNTS:     10      1       5       6       5       1.56e-01
    TAGS: (2 total)
          ----------                                                    1756
      d+2  TAGTAGTGCC 10      1       4       6       5       1.28e-01       1579
      d+2  CCTTCCGTCT 0       0       1       0       0       4.55e-01       1477
          ----------                                                    1310
          ----------                                                    1292
          ----------                                                    1197
          ----------                                                    498
          ----------                                                    436
          ----------                                                    306
          ----------                                                    111

LOCUS: AT4G26450
DESCRIPTION: expressed protein
DATA:                 Control 30min   2hours  2days   1week   p-value        pos
SENSE COUNTS:         2       0       5       2       1       1.56e-01
GENES (1 total):
  AT4G26450.1
    SENSE COUNTS:     2       0       5       2       1       1.56e-01
    TAGS: (4 total)
      i+3  ACTTTTGATT 1       0       1       1       0       7.18e-01       4829
          ----------                                                    4596
          ----------                                                    4565
          ----------                                                    4050
          ----------                                                    3706
          ----------                                                    3489
          ----------                                                    3468
          ----------                                                    3391
          ----------                                                    2800
      i+3  AGGGAAAAAA 0       0       0       1       0       3.09e-01       2726
      v+2  TCACTCGGAG 1       0       3       0       1       2.11e-01       2418
          ----------                                                    2250
      v+2  AAAGTCTGGT 0       0       1       0       0       4.55e-01       1879
          ----------                                                    1776
          ----------                                                    606
          ----------                                                    457
          ----------                                                    358

LOCUS: AT3G46120
DESCRIPTION: calcineurin-like phosphoesterase family protein, contains Pfam profile: PF00149 calcineurin-like
phosphoesterase
DATA:                 Control 30min   2hours  2days   1week   p-value        pos
SENSE COUNTS:         2       0       2       6       1       1.56e-01
GENES (1 total):
  AT3G46120.1
    SENSE COUNTS:     2       0       2       6       1       1.56e-01
    TAGS: (1 total)
          ----------                                                    1890
          ----------                                                    1405
          ----------                                                    1183
          ----------                                                    1177
      v+2  GTTCGTTGAA 2       0       2       6       1       1.56e-01       1136
          ----------                                                    1116
          ----------                                                    747
          ----------                                                    709
          ----------                                                    442

LOCUS: AT3G21690
DESCRIPTION: MATE efflux family protein, similar to ripening regulated protein DDTFR18 (Lycopersicon
esculentum) GI:12231296; contains Pfam profile: PF01554 MatE uncharacterized membrane protein family
DATA:                 Control 30min   2hours  2days   1week   p-value        pos
SENSE COUNTS:         0       1       5       6       3       1.58e-01
GENES (1 total):
  AT3G21690.1
    SENSE COUNTS:     0       1       5       6       3       1.58e-01
    TAGS: (3 total)
          ----------                                                    1851
      d+2  TGAGCTGGTA 0       1       5       5       3       1.64e-01       1686
      i+3  AATTTTTCTA 0       0       0       0       0       6.15e-01       1465
          ----------                                                    1067
```

```
          ----------                                                        1043
      d+2  GGGAGTGCAG  0      0      0      1      0      3.09e-01       416
          ----------                                                        407
          ----------                                                        311
          ----------                                                        305
          ----------                                                        290
          ----------                                                        89

LOCUS: AT4G33580
DESCRIPTION: expressed protein,
DATA:                 Control 30min  2hours  2days  1week  p-value        pos
SENSE COUNTS:         1       1      1       5      0      1.58e-01
GENES (2 total):
  AT4G33580.1
    SENSE COUNTS:     1       1      1       5      0      1.58e-01
    TAGS: (2 total)
          ----------                                                        1347
      d+2  TGTTTGTTCC  1      1      1      4      0      4.20e-01       1172
          ----------                                                        967
          ----------                                                        477
      X+4  CATTACAGAG  0      0      0      1      0      3.09e-01       465
          ----------                                                        438
          ----------                                                        220
          ----------                                                        186

LOCUS: AT5G21040
DESCRIPTION: F-box family protein / WD-40 repeat family protein, contains G-protein beta WD-40 repeats
DATA:                 Control 30min  2hours  2days  1week  p-value        pos
SENSE COUNTS:         1       0      1       5      0      1.58e-01
GENES (1 total):
  AT5G21040.1
    SENSE COUNTS:     1       0      1       5      0      1.58e-01
    TAGS: (2 total)
      X+4  AGTCTTGGTT  0      0      0      0      0      6.15e-01       2100
      d+1  TTGGCTTTAA  1      0      1      5      0      8.72e-02       1899
          ----------                                                        1679
          ----------                                                        1223
          ----------                                                        1133
          ----------                                                        1041
          ----------                                                        1018
          ----------                                                        986
          ----------                                                        790
          ----------                                                        621
          ----------                                                        220

LOCUS: AT1G44830
DESCRIPTION: encodes a member of the DREB subfamily A-5 of ERF/AP2 transcription factor family. The protein
contains one AP2 domain. There are 15 members in this subfamily including RAP2.1, RAP2.9 and RAP2.10.
DATA:                 Control 30min  2hours  2days  1week  p-value        pos
SENSE COUNTS:         1       0      0       2      3      1.58e-01
GENES (1 total):
  AT1G44830.1
    SENSE COUNTS:     1       0      0       2      3      1.58e-01
    TAGS: (1 total)
          ----------                                                        1420
      v+2  TTAAGAGTTC  1      0      0      2      3      1.58e-01       1163
          ----------                                                        894
          ----------                                                        860
          ----------                                                        816
          ----------                                                        684
          ----------                                                        488
          ----------                                                        217

LOCUS: AT3G28715
DESCRIPTION: H+-transporting two-sector ATPase, putative, similar to SP|P54641 Vacuolar ATP synthase subunit d
(EC 3.6.3.14) (Vacuolar proton pump d subunit) (V-ATPase 41 KDa accessory protein) {Dictyostelium discoideum};
contains Pfam profile PF01992: ATP synthase (C
DATA:                 Control 30min  2hours  2days  1week  p-value        pos
SENSE COUNTS:         1       0      2       4      0      1.59e-01
GENES (1 total):
  AT3G28715.1
    SENSE COUNTS:     1       0      2       4      0      1.59e-01
    TAGS: (1 total)
          ----------                                                        1871
      v+2  TTCTGAGTCT  1      0      2      4      0      1.59e-01       1401
          ----------                                                        879
          ----------                                                        792
          ----------                                                        753
          ----------                                                        388

LOCUS: AT3G08580
```

```
DESCRIPTION: mitochondrial ADP/ATP carrier
DATA:                  Control 30min   2hours  2days   1week   p-value        pos
SENSE COUNTS:          3       0       4       4       8       1.59e-01
GENES (3 total):
  AT3G08580.1
    SENSE COUNTS:      3       0       4       4       8       1.59e-01
    TAGS: (2 total)
      d+1  TTATTTATAG  0       0       0       0       3       1.12e-02       1444
           ----------                                                         1361
      d+2  TGCCCTAAAC  3       0       4       4       5       5.19e-01       1298
           ----------                                                         815
           ----------                                                         169
           ----------                                                         105
  AT3G08580.2
    SENSE COUNTS:      3       0       4       4       8       1.59e-01
    TAGS: (2 total)
      d+1  TTATTTATAG  0       0       0       0       3       1.12e-02       1559
           ----------                                                         1476
      d+2  TGCCCTAAAC  3       0       4       4       5       5.19e-01       1413
           ----------                                                         930
           ----------                                                         284
           ----------                                                         220

LOCUS: AT1G23130
DESCRIPTION: Bet v I allergen family protein, similar to Csf-2 (Cucumis sativus)(GI:5762258)(J Am Soc Hortic
Sci 124, 136-139 (1999)); location of ESTs gb|T45139 and gb|T43456 ; contains Pfam profile PF00407:
Pathogenesis-related protein Bet v I family
DATA:                  Control 30min   2hours  2days   1week   p-value        pos
SENSE COUNTS:          11      13      4       5       8       1.59e-01
GENES (1 total):
  AT1G23130.1
    SENSE COUNTS:      11      13      4       5       8       1.59e-01
    TAGS: (2 total)
      d+1  TTATGTTTTA  10      1       3       1       7       2.40e-02       741
      d+2  AGGGAGAGTT  1       12      1       4       1       6.53e-04       206
           ----------                                                         142

LOCUS: AT1G70770
DESCRIPTION: expressed protein
DATA:                  Control 30min   2hours  2days   1week   p-value        pos
SENSE COUNTS:          5       2       4       0       0       1.62e-01
GENES (1 total):
  AT1G70770.1
    SENSE COUNTS:      5       2       4       0       0       1.62e-01
    TAGS: (3 total)
      d+1  TGAATGCAGA  1       0       0       0       0       4.28e-01       2287
      d+2  TTTGATTTGG  1       1       4       0       0       1.70e-01       2228
      d+2  AATACATCAA  3       1       0       0       0       1.56e-01       1876
           ----------                                                         1073
           ----------                                                         665
           ----------                                                         157

LOCUS: AT3G13110
DESCRIPTION: serine O-acetyltransferase (SAT-1), identical to serine acetyltransferase (Sat-1) GI:1184048
(Arabidopsis thaliana)
DATA:                  Control 30min   2hours  2days   1week   p-value        pos
SENSE COUNTS:          3       5       10      7       14      1.64e-01
GENES (3 total):
  AT3G13110.1
    SENSE COUNTS:      3       5       10      7       14      1.64e-01
    TAGS: (2 total)
      d+1  ATTTCTGTGA  0       0       6       2       7       2.46e-02       1642
      d+2  ACAAGATTCC  3       5       4       5       7       9.04e-01       1297
           ----------                                                         1012
           ----------                                                         892
           ----------                                                         453

LOCUS: AT5G59960
DESCRIPTION: expressed protein
DATA:                  Control 30min   2hours  2days   1week   p-value        pos
SENSE COUNTS:          0       2       3       0       0       1.66e-01
GENES (1 total):
  AT5G59960.1
    SENSE COUNTS:      0       2       3       0       0       1.66e-01
    TAGS: (1 total)
           ----------                                                         1604
           ----------                                                         1597
      d+2  GATAAGCTTT  0       2       3       0       0       1.66e-01       1425
           ----------                                                         1183
           ----------                                                         1077
           ----------                                                         161
```

```
LOCUS: AT1G19140
DESCRIPTION: expressed protein
DATA:                 Control 30min  2hours  2days  1week  p-value       pos
SENSE COUNTS:         5       0      2       3      3      1.66e-01
GENES (2 total):
  AT1G19140.1
    SENSE COUNTS:     5       0      2       3      3      1.66e-01
    TAGS: (2 total)
      d+1  ATTCTGCTTC 1       0      1       2      0      3.84e-01      1061
      i+3  TTAAGCTCAA 4       0      1       1      3      1.50e-01      896
           ----------                                                   870
           ----------                                                   835
           ----------                                                   749
           ----------                                                   652
           ----------                                                   529
           ----------                                                   341
  AT1G19140.2
    SENSE COUNTS:     5       0      2       3      3      1.66e-01
    TAGS: (2 total)
      d+1  ATTCTGCTTC 1       0      1       2      0      3.84e-01      1015
      i+3  TTAAGCTCAA 4       0      1       1      3      1.50e-01      847
           ----------                                                   824
           ----------                                                   786
           ----------                                                   700
           ----------                                                   603
           ----------                                                   480
           ----------                                                   292

LOCUS: AT4G33240
DESCRIPTION: phosphatidylinositol-4-phosphate 5-kinase family protein, similar to SP|Q9Z1T6 FYVE finger-
containing phosphoinositide kinase (EC 2.7.1.68) (1- phosphatidylinositol-4-phosphate kinase) (PIP5K)
(PtdIns(4)P-5-kinase) {Mus musculus}; contains Pfam profiles P
DATA:                 Control 30min  2hours  2days  1week  p-value       pos
SENSE COUNTS:         3       1      4       8      4      1.66e-01
GENES (1 total):
  AT4G33240.1
    SENSE COUNTS:     3       1      4       8      4      1.66e-01
    TAGS: (4 total)
      d+1  AGGCAATACA 1       0      1       1      0      7.18e-01      5014
           ----------                                                   4325
           ----------                                                   4249
           ----------                                                   3967
           ----------                                                   3735
           ----------                                                   3473
           ----------                                                   2870
           ----------                                                   2625
      d+2  TGATTCAGTA 1       0      0       0      3      7.14e-02      1868
           ----------                                                   1841
           ----------                                                   1739
      d+2  TGGAAAAGTT 1       1      3       6      0      1.01e-01      1715
           ----------                                                   740
           ----------                                                   731
           ----------                                                   557
      d+2  GATTCTGAAA 0       0      0       1      1      3.25e-01      493
           ----------                                                   257

LOCUS: AT1G71810
DESCRIPTION: ABC1 family protein, contains Pfam domain, PF03109: ABC1 family
DATA:                 Control 30min  2hours  2days  1week  p-value       pos
SENSE COUNTS:         5       0      2       2      7      1.67e-01
GENES (1 total):
  AT1G71810.1
    SENSE COUNTS:     5       0      2       2      7      1.67e-01
    TAGS: (2 total)
      d+1  TATCAGCTTC 1       0      0       0      0      6.89e-01      2133
           ----------                                                   2004
           ----------                                                   1842
           ----------                                                   1352
           ----------                                                   1344
           ----------                                                   1237
      d+2  ATATTCAAAA 4       0      2       2      7      9.38e-02      138

LOCUS: AT3G17220
DESCRIPTION: invertase/pectin methylesterase inhibitor family protein, similar to SP|P83326 Pectinesterase
inhibitor (Pectin methylesterase inhibitor) (PMEI) {Actinidia chinensis}; contains Pfam profile PF04043: Plant
invertase/pectin methylesterase inhibitor
DATA:                 Control 30min  2hours  2days  1week  p-value       pos
SENSE COUNTS:         7       1      5       2      1      1.67e-01
GENES (1 total):
  AT3G17220.1
```

```
    SENSE COUNTS:      7       1       5       2       1       1.67e-01
    TAGS: (2 total)
      v+1  AGAACAATGC  1       0       0       0       0       4.28e-01       1205
      v+2  ATCTTTTTAC  6       1       5       2       1       2.88e-01       1124
           ----------                                                         867
           ----------                                                         780
           ----------                                                         767
           ----------                                                         647
           ----------                                                         492
           ----------                                                         266
           ----------                                                         55

LOCUS: AT1G47720
DESCRIPTION: expressed protein, contains Pfam PF05329: Protein of unknown function (DUF731)
DATA:                  Control 30min   2hours  2days   1week   p-value        pos
SENSE COUNTS:          0       3       1       4       0       1.69e-01
GENES (1 total):
  AT1G47720.1
    SENSE COUNTS:      0       3       1       4       0       1.69e-01
    TAGS: (1 total)
           ----------                                                         858
      X+4  CACCCAAAAG  0       3       1       4       0       1.69e-01       519
           ----------                                                         238
           ----------                                                         140

LOCUS: AT4G32540
DESCRIPTION: flavin-containing monooxygenase / FMO (YUCCA), identical to gi:16555352
DATA:                  Control 30min   2hours  2days   1week   p-value        pos
SENSE COUNTS:          0       0       2       4       0       1.71e-01
GENES (2 total):
  AT4G32540.1
    SENSE COUNTS:      0       0       2       4       0       1.71e-01
    TAGS: (1 total)
           ----------                                                         2058
           ----------                                                         1239
           ----------                                                         1003
           ----------                                                         936
      v+2  ATAACACAGA  0       0       2       4       0       1.71e-01       748
           ----------                                                         458

LOCUS: AT1G45904
DESCRIPTION: pseudogene, putative NADH-plastoquinone oxidoreductase subunit, blastp match of 52% identity and
1.3e-28 P-value to GP|7636113|emb|CAB88733.1||AJ400848 NADH dehydrogenase 32kDa subunit {Spinacia oleracea}
DATA:                  Control 30min   2hours  2days   1week   p-value        pos
SENSE COUNTS:          0       0       2       4       0       1.71e-01
GENES (1 total):
  AT1G45904.1
    SENSE COUNTS:      0       0       2       4       0       1.71e-01
    TAGS: (1 total)
           ----------                                                         537
      p+2  GTGAAAAGGA  0       0       2       4       0       1.71e-01       194

LOCUS: AT2G30050
DESCRIPTION: transducin family protein / WD-40 repeat family protein, similar to SEC13-related protein
(SP:P55735) (Homo sapiens)
DATA:                  Control 30min   2hours  2days   1week   p-value        pos
SENSE COUNTS:          1       4       2       0       0       1.71e-01
GENES (1 total):
  AT2G30050.1
    SENSE COUNTS:      1       4       2       0       0       1.71e-01
    TAGS: (2 total)
      d+1  ATATATAGTT  0       0       1       0       0       4.55e-01       1245
           ----------                                                         730
           ----------                                                         610
           ----------                                                         480
           ----------                                                         469
      d+2  TTTTCACAGA  1       4       1       0       0       1.14e-01       429
           ----------                                                         364
           ----------                                                         183
           ----------                                                         168
           ----------                                                         140

LOCUS: AT5G16730
DESCRIPTION: expressed protein, weak similarity to microtubule binding protein D-CLIP-190 (Drosophila
melanogaster) GI:2773363, SMC2-like condensin (Arabidopsis thaliana) GI:14279543
DATA:                  Control 30min   2hours  2days   1week   p-value        pos
SENSE COUNTS:          1       4       3       0       0       1.72e-01
GENES (1 total):
  AT5G16730.1
    SENSE COUNTS:      1       4       3       0       0       1.72e-01
    TAGS: (2 total)
```

```
      d+1  TAAAACGTAT 0      0      2      0      0      1.21e-01      2608
           ----------                                                1861
           ----------                                                1568
      d+2  ACACGGAAAC 1      4      1      0      0      1.14e-01      1088
           ----------                                                704
           ----------                                                569
           ----------                                                455

LOCUS: AT2G21660
DESCRIPTION: glycine-rich RNA-binding protein (GRP7), SP|Q03250 Glycine-rich RNA-binding protein 7
{Arabidopsis thaliana}
DATA:                 Control 30min  2hours 2days  1week  p-value       pos
SENSE COUNTS:         0      0      0      2      3      1.73e-01
GENES (3 total):
  AT2G21660.2
    SENSE COUNTS:     0      0      0      2      3      1.73e-01
    TAGS: (1 total)
           ----------                                                244
      d+2  GGCCACTGAT 0      0      0      2      3      1.73e-01      108
  AT2G21660.1
    SENSE COUNTS:     0      0      0      2      3      1.73e-01
    TAGS: (1 total)
           ----------                                                244
      d+2  GGCCACTGAT 0      0      0      2      3      1.73e-01      108

LOCUS: AT1G56500
DESCRIPTION: haloacid dehalogenase-like hydrolase family protein, low similarity to SP|P95649 CbbY protein
{Rhodobacter sphaeroides}; contains InterPro accession IPR005834: Haloacid dehalogenase-like hydrolase
DATA:                 Control 30min  2hours 2days  1week  p-value       pos
SENSE COUNTS:         7      2      5      4      0      1.73e-01
GENES (1 total):
  AT1G56500.1
    SENSE COUNTS:     7      2      5      4      0      1.73e-01
    TAGS: (4 total)
      i+3  CTTTGAGGTA 0      0      1      0      0      4.55e-01      5107
      d+1  ACCGTGGTAT 1      0      0      0      0      4.28e-01      3208
      i+3  ACCTTTTCTT 6      2      3      4      0      2.79e-01      2158
      d+2  CTTTGAGAGA 0      0      1      0      0      4.55e-01      1968
           ----------                                                1511
           ----------                                                1505
           ----------                                                1341
           ----------                                                797
           ----------                                                641

LOCUS: AT1G10760
DESCRIPTION: starch excess protein (SEX1), identical to SEX1 (Arabidopsis thaliana) GI:12044358; supporting
cDNA gi|12044357|gb|AF312027.1|AF312027
DATA:                 Control 30min  2hours 2days  1week  p-value       pos
SENSE COUNTS:         8      12     12     22     9      1.73e-01
GENES (2 total):
  AT1G10760.1
    SENSE COUNTS:     8      12     12     22     9      1.73e-01
    TAGS: (5 total)
      d+1  GCTGTTTTGG 0      3      0      0      1      8.32e-02      3691
           ----------                                                3677
           ----------                                                3558
           ----------                                                3553
           ----------                                                3057
           ----------                                                3014
           ----------                                                2995
           ----------                                                2566
           ----------                                                2467
           ----------                                                2357
           ----------                                                2273
           ----------                                                2197
           ----------                                                2167
           ----------                                                2107
           ----------                                                1540
           ----------                                                1448
      d+2  CCGTTTGTAC 0      0      0      0      0      6.15e-01      1363
           ----------                                                1151
           ----------                                                962
      i+3  TGTGCTAAAA 0      0      1      0      0      4.55e-01      422
      i+3  TCCAAAACTT 8      5      11     22     8      1.16e-02      343
      d+2  GATCCTCAGG 0      4      0      0      0      9.72e-03      238

LOCUS: AT1G76050
DESCRIPTION: pseudouridine synthase family protein, contains Pfam profiles: PF00849 RNA pseudouridylate
synthase, PF01479: S4 domain
DATA:                 Control 30min  2hours 2days  1week  p-value       pos
SENSE COUNTS:         3      1      3      0      7      1.75e-01
```

```
GENES (2 total):
  AT1G76050.1
    SENSE COUNTS:      3       1       3       0       7       1.75e-01
    TAGS: (1 total)
           ----------                                                  1723
      d+2  AATGATAATT  3       1       3       0       7       1.75e-01        1536
           ----------                                                  1370
           ----------                                                  1255
           ----------                                                  1200
           ----------                                                  1109
           ----------                                                  1050
           ----------                                                  929
           ----------                                                  752
           ----------                                                  499
           ----------                                                  470
           ----------                                                  261
  AT1G76050.2
    SENSE COUNTS:      3       1       3       0       7       1.75e-01
    TAGS: (1 total)
           ----------                                                  1739
      d+2  AATGATAATT  3       1       3       0       7       1.75e-01        1552
           ----------                                                  1386
           ----------                                                  1271
           ----------                                                  1216
           ----------                                                  1125
           ----------                                                  1066
           ----------                                                  964
           ----------                                                  787
           ----------                                                  534
           ----------                                                  505
           ----------                                                  296

LOCUS: AT5G61780
DESCRIPTION: tudor domain-containing protein / nuclease family protein, contains Pfam domains PF00567: Tudor
domain and PF00565: Staphylococcal nuclease homologue
DATA:                  Control 30min   2hours  2days   1week   p-value         pos
SENSE COUNTS:          2       1       1       5       6       1.75e-01
GENES (1 total):
  AT5G61780.1
    SENSE COUNTS:      2       1       1       5       6       1.75e-01
    TAGS: (2 total)
      i+3  ATTTCTTCTT  0       0       0       0       1       1.65e-01        4671
      d+1  TTTGAGCTTT  2       1       1       5       5       3.34e-01        3502
           ----------                                                  3268
           ----------                                                  2178
           ----------                                                  1707
           ----------                                                  1408
           ----------                                                  1293
           ----------                                                  837
           ----------                                                  804
           ----------                                                  461
           ----------                                                  270

LOCUS: AT3G58340
DESCRIPTION: meprin and TRAF homology domain-containing protein / MATH domain-containing protein, similar to
ubiquitin-specific protease 12 (Arabidopsis thaliana) GI:11993471; contains Pfam profile PF00917: MATH domain
DATA:                  Control 30min   2hours  2days   1week   p-value         pos
SENSE COUNTS:          2       0       1       2       5       1.75e-01
GENES (1 total):
  AT3G58340.1
    SENSE COUNTS:      2       0       1       2       5       1.75e-01
    TAGS: (1 total)
           ----------                                                  1249
      v+2  TGAAAGAAAA  2       0       1       2       5       1.75e-01        1180
           ----------                                                  1035
           ----------                                                  729
           ----------                                                  669
           ----------                                                  575
           ----------                                                  280
           ----------                                                  163
           ----------                                                  100

LOCUS: AT1G06670
DESCRIPTION: DEIH-box RNA/DNA helicase, identical to DEIH-box RNA/DNA helicase GB:BAA84364 GI:5881579
(Arabidopsis thaliana)
DATA:                  Control 30min   2hours  2days   1week   p-value         pos
SENSE COUNTS:          4       0       1       3       0       1.76e-01
GENES (2 total):
  AT1G06670.1
    SENSE COUNTS:      4       0       1       3       0       1.76e-01
    TAGS: (2 total)
```

```
      d+1  TAACTGAAAG 0       0       0       1       0       3.09e-01        5114
      d+2  AAACCAAGTT 4       0       1       2       0       2.30e-01        4974
           ----------                                                         4566
           ----------                                                         4419
           ----------                                                         4141
           ----------                                                         4011
           ----------                                                         3996
           ----------                                                         3927
           ----------                                                         3918
           ----------                                                         3843
           ----------                                                         3601
           ----------                                                         3030
           ----------                                                         2878
           ----------                                                         2833
           ----------                                                         2781
           ----------                                                         2647
           ----------                                                         2601
           ----------                                                         2570
           ----------                                                         1726
           ----------                                                         1528
           ----------                                                         1472
           ----------                                                         1200
           ----------                                                         1174
           ----------                                                         932

LOCUS: AT1G54730
DESCRIPTION: sugar transporter, putative, similar to ERD6 protein (Arabidopsis thaliana) GI:3123712, sugar-
porter family proteins 1 and 2 (Arabidopsis thaliana) GI:14585699, GI:14585701; contains Pfam profile PF00083:
major facilitator superfamily protein
DATA:                 Control 30min   2hours  2days   1week   p-value         pos
SENSE COUNTS:         7       1       4       2       1       1.77e-01
GENES (3 total):
  AT1G54730.2
    SENSE COUNTS:     7       1       3       2       1       1.57e-01
    TAGS: (2 total)
      i+3  AAAAAAATAG 1       0       2       0       0       3.07e-01        4522
      i+3  CACCTTTAAT 6       1       1       2       1       1.53e-01        2748
           ----------                                                         1242
           ----------                                                         1020
           ----------                                                         582
  AT1G54730.3
    SENSE COUNTS:     7       1       4       2       1       1.77e-01
    TAGS: (3 total)
      i+3  AAAAAAATAG 1       0       2       0       0       3.07e-01        4583
      i+3  CACCTTTAAT 6       1       1       2       1       1.53e-01        2809
           ----------                                                         1584
           ----------                                                         1077
      X+4  GATGAGTGCA 0       0       1       0       0       4.55e-01        1012
           ----------                                                         639

LOCUS: AT3G43590
DESCRIPTION: zinc knuckle (CCHC-type) family protein, contains Pfam domain, PF00098: Zinc knuckle
DATA:                 Control 30min   2hours  2days   1week   p-value         pos
SENSE COUNTS:         0       0       3       0       0       1.77e-01
GENES (1 total):
  AT3G43590.1
    SENSE COUNTS:     0       0       3       0       0       1.77e-01
    TAGS: (2 total)
      d+1  AAATCTATTA 0       0       0       0       0       6.15e-01        1844
           ----------                                                         1739
      d+2  AGTCCAATGG 0       0       3       0       0       1.17e-01        1352
           ----------                                                         1184
           ----------                                                         1032
           ----------                                                         981
           ----------                                                         871
           ----------                                                         866
           ----------                                                         758
           ----------                                                         731
           ----------                                                         443
           ----------                                                         136

LOCUS: AT3G63310
DESCRIPTION: expressed protein, low similarity to N-methyl-D-aspartate receptor-associated protein (Drosophila
melanogaster) GI:567104; contains Pfam profile PF01027: Uncharacterized protein family UPF0005
DATA:                 Control 30min   2hours  2days   1week   p-value         pos
SENSE COUNTS:         4       5       13      11      7       1.77e-01
GENES (1 total):
  AT3G63310.1
    SENSE COUNTS:     4       5       13      11      7       1.77e-01
    TAGS: (1 total)
           ----------                                                         1408
```

```
      v+2  ATAGGAGAAT 4       5       13      11      7       1.77e-01       1198
           ----------                                                        846
           ----------                                                        796
           ----------                                                        370

LOCUS: AT5G65600
DESCRIPTION: legume lectin family protein / protein kinase family protein, contains Pfam domains PF00138:
Legume lectins alpha domain, PF00139: Legume lectins beta domain and PF00069: Protein kinase domain
DATA:                 Control 30min   2hours  2days   1week   p-value        pos
SENSE COUNTS:         3       0       2       3       0       1.79e-01
GENES (1 total):
  AT5G65600.1
    SENSE COUNTS:     3       0       2       3       0       1.79e-01
    TAGS: (2 total)
           ----------                                                        2715
           ----------                                                        2605
           ----------                                                        2454
           ----------                                                        2235
           ----------                                                        2108
           ----------                                                        1905
      v+2  AACTTGGTTC 3       0       2       2       0       2.68e-01       1858
           ----------                                                        1744
           ----------                                                        1317
           ----------                                                        1151
           ----------                                                        955
      v+2  TTGGGATCAA 0       0       0       1       0       3.09e-01       874
           ----------                                                        817
           ----------                                                        700

LOCUS: AT2G34920
DESCRIPTION: ubiquitin-protein ligase-related, contains weak similarity to Ubiquitin-protein ligase E3 Mdm2
(EC 6.3.2.-) (p53-binding protein Mdm2) (Oncoprotein Mdm2) (Double minute 2 protein) (Swiss-Prot:P23804) (Mus
musculus)
DATA:                 Control 30min   2hours  2days   1week   p-value        pos
SENSE COUNTS:         3       0       3       4       0       1.79e-01
GENES (1 total):
  AT2G34920.1
    SENSE COUNTS:     3       0       3       4       0       1.79e-01
    TAGS: (2 total)
      i+3  AACTCATTTT 0       0       1       0       0       4.55e-01       6338
      i+3  AACAAATTGA 3       0       2       4       0       1.79e-01       3831
           ----------                                                        2926
           ----------                                                        2766
           ----------                                                        2662
           ----------                                                        2606
           ----------                                                        2598
           ----------                                                        2381
           ----------                                                        2046
           ----------                                                        1683
           ----------                                                        771
           ----------                                                        761
           ----------                                                        732
           ----------                                                        672
           ----------                                                        477
           ----------                                                        466
           ----------                                                        331
           ----------                                                        130

LOCUS: AT4G19420
DESCRIPTION: pectinacetylesterase family protein, contains Pfam profile: PF03283 pectinacetylesterase
DATA:                 Control 30min   2hours  2days   1week   p-value        pos
SENSE COUNTS:         2       6       3       2       0       1.79e-01
GENES (2 total):
  AT4G19420.1
    SENSE COUNTS:     2       6       3       2       0       1.79e-01
    TAGS: (2 total)
      i+3  ATTCATTATT 0       0       0       1       0       6.04e-01       2892
      d+1  GTTCTGGCAA 2       6       3       1       0       2.00e-01       1293
           ----------                                                        972
           ----------                                                        944
           ----------                                                        742
  AT4G19420.2
    SENSE COUNTS:     2       6       3       2       0       1.79e-01
    TAGS: (2 total)
      i+3  ATTCATTATT 0       0       0       1       0       6.04e-01       2892
      d+1  GTTCTGGCAA 2       6       3       1       0       2.00e-01       1283
           ----------                                                        972
           ----------                                                        944
           ----------                                                        742

LOCUS: AT1G31800
```

```
DESCRIPTION: cytochrome P450 family protein, similar to Cytochrome P450 97B2 (SP:048921) (Glycine max);
contains Pfam profile: PF00067: Cytochrome P450
DATA:                 Control 30min   2hours  2days   1week   p-value        pos
SENSE COUNTS:         9       1       8       8       5       1.80e-01
GENES (1 total):
  AT1G31800.1
    SENSE COUNTS:     9       1       8       8       5       1.80e-01
    TAGS: (1 total)
           ----------                                                        2283
      d+2  ACCAATGCTT 9       1       8       8       5       1.80e-01       1920
           ----------                                                        1599
           ----------                                                        1332
           ----------                                                        1202
           ----------                                                        1177
           ----------                                                        1022
           ----------                                                        339

LOCUS: AT5G57887
DESCRIPTION: expressed protein
DATA:                 Control 30min   2hours  2days   1week   p-value        pos
SENSE COUNTS:         13      11      15      6       2       1.81e-01
GENES (1 total):
  AT5G57887.1
    SENSE COUNTS:     13      11      15      6       2       1.81e-01
    TAGS: (2 total)
           ----------                                                        525
      X+4  TTGTCGTTTT 11      6       14      5       1       5.41e-02       524
      d+2  TATTTGTACA 2       5       1       1       1       3.55e-01       482
           ----------                                                        196

LOCUS: AT1G04160
DESCRIPTION: myosin family protein, contains Pfam profiles: PF02736 myosin N-terminal SH3-like domain, PF00063
myosin head (motor domain), PF00612 IQ calmodulin-binding motif, PF01843: DIL domain
DATA:                 Control 30min   2hours  2days   1week   p-value        pos
SENSE COUNTS:         0       0       4       1       3       1.82e-01
GENES (2 total):
  AT1G04160.1
    SENSE COUNTS:     0       0       4       1       3       1.82e-01
    TAGS: (3 total)
      v+1  ACAGAAGAAT 0       0       0       0       0       6.15e-01       4686
           ----------                                                        4674
           ----------                                                        4567
           ----------                                                        3465
           ----------                                                        3355
           ----------                                                        3059
           ----------                                                        2945
           ----------                                                        2694
      v+2  TTCATTTGTT 0       0       4       0       0       5.58e-03       2099
           ----------                                                        1890
           ----------                                                        1566
           ----------                                                        1485
           ----------                                                        1440
      v+2  CAACAAAAAA 0       0       0       1       3       6.27e-02       1255
           ----------                                                        1131
           ----------                                                        879
           ----------                                                        574
           ----------                                                        416
           ----------                                                        388

LOCUS: AT2G37730
DESCRIPTION: fringe-related protein, similarity to predicted proteins + similar to hypothetical protein
GB:AAC23643 (Arabidopsis thaliana) + weak similarity to Fringe (Schistocerca gregaria)(GI:6573138);Fringe
encodes an extracellular protein that regulates Notch sign
DATA:                 Control 30min   2hours  2days   1week   p-value        pos
SENSE COUNTS:         0       1       1       1       5       1.82e-01
GENES (1 total):
  AT2G37730.1
    SENSE COUNTS:     0       1       1       1       5       1.82e-01
    TAGS: (1 total)
           ----------                                                        2437
      v+2  TTTTTTTAAA 0       1       1       1       5       1.82e-01       2223
           ----------                                                        2090
           ----------                                                        1675
           ----------                                                        1457
           ----------                                                        1423
           ----------                                                        1065
           ----------                                                        782
           ----------                                                        430

LOCUS: AT2G19310
DESCRIPTION: expressed protein
```

```
DATA:                 Control 30min  2hours  2days  1week  p-value     pos
SENSE COUNTS:         9       6      10      2      1      1.83e-01
GENES (1 total):
  AT2G19310.1
    SENSE COUNTS:     9       6      10      2      1      1.83e-01
    TAGS: (2 total)
      X+4  TAACCTTCTC 1       0      0       0      0      4.28e-01    761
      d+1  TTGTCGAGAT 8       6      10      2      1      2.22e-01    530
           ----------                                                  342
           ----------                                                  102

LOCUS: AT3G01040
DESCRIPTION: glycosyl transferase family 8 protein, contains Pfam profile: PF01501 glycosyl transferase family
8
DATA:                 Control 30min  2hours  2days  1week  p-value     pos
SENSE COUNTS:         1       0      4       1      1      1.83e-01
GENES (1 total):
  AT3G01040.1
    SENSE COUNTS:     1       0      4       1      1      1.83e-01
    TAGS: (3 total)
      d+1  ATCACAACAG 1       0      1       0      1      6.15e-01    2308
      d+2  AAAGACCAGT 0       0      0       1      0      3.09e-01    2123
           ----------                                                  1829
           ----------                                                  1331
           ----------                                                  1157
      i+3  TGATGATGAG 0       0      3       0      0      2.70e-02    1105
           ----------                                                  1001
           ----------                                                  913
           ----------                                                  463

LOCUS: AT1G70160
DESCRIPTION: expressed protein, similar to hypothetical protein GI:4455225 from (Arabidopsis thaliana)
DATA:                 Control 30min  2hours  2days  1week  p-value     pos
SENSE COUNTS:         9       1      4       3      6      1.84e-01
GENES (1 total):
  AT1G70160.1
    SENSE COUNTS:     9       1      4       3      6      1.84e-01
    TAGS: (2 total)
      d+1  TATGTCTATC 6       1      4       2      5      4.30e-01    1800
           ----------                                                  1729
      d+2  TGTTGCTTTC 3       0      0       1      1      3.94e-01    1488
           ----------                                                  1291
           ----------                                                  1216
           ----------                                                  1022
           ----------                                                  680
           ----------                                                  552
           ----------                                                  342

LOCUS: AT1G26920
DESCRIPTION: expressed protein, Location of EST 228A16T7A, gb|N65686
DATA:                 Control 30min  2hours  2days  1week  p-value     pos
SENSE COUNTS:         2       5      1       1      0      1.86e-01
GENES (1 total):
  AT1G26920.1
    SENSE COUNTS:     2       5      1       1      0      1.86e-01
    TAGS: (1 total)
           ----------                                                  875
      d+2  TAGAAATTAT 2       5      1       1      0      1.86e-01    592
           ----------                                                  348
           ----------                                                  76

LOCUS: AT2G24060
DESCRIPTION: translation initiation factor 3 (IF-3) family protein, similar to SP|P33319 Translation
initiation factor IF-3 {Proteus vulgaris}; contains Pfam profiles PF00707: Translation initiation factor IF-3,
C-terminal domain, PF05198: Translation initiation facto
DATA:                 Control 30min  2hours  2days  1week  p-value     pos
SENSE COUNTS:         5       1      2       4      8      1.86e-01
GENES (1 total):
  AT2G24060.1
    SENSE COUNTS:     5       1      2       4      8      1.86e-01
    TAGS: (2 total)
           ----------                                                  1075
      d+2  AAAGGACGAG 5       1      2       4      7      3.54e-01    687
           ----------                                                  613
           ----------                                                  570
      d+2  CTTGGTTTAG 0       0      0       0      1      1.65e-01    396
           ----------                                                  72

LOCUS: AT5G55070
```

```
DESCRIPTION: 2-oxoacid dehydrogenase family protein, similar to SP|Q01205 Dihydrolipoamide succinyltransferase
component of 2-oxoglutarate dehydrogenase complex, mitochondrial precursor (EC 2.3.1.61) {Rattus norvegicus};
contains Pfam profiles PF00198: 2-oxo acid dehy
DATA:                 Control 30min   2hours  2days   1week   p-value         pos
SENSE COUNTS:         1       0       1       5       1       1.87e-01
GENES (1 total):
  AT5G55070.1
    SENSE COUNTS:     1       0       1       5       1       1.87e-01
    TAGS: (2 total)
      d+1  GAGTTGCATT 0       0       1       1       0       5.21e-01        1833
      d+2  TGGCACCCTC 1       0       0       4       1       2.52e-01        672
           ----------                                                         446
           ----------                                                         297

LOCUS: AT5G24420
DESCRIPTION: glucosamine/galactosamine-6-phosphate isomerase-related, contains weak similarity to Swiss-
Prot:O95336 6-phosphogluconolactonase (EC 3.1.1.31) (6PGL) (Homo sapiens)
DATA:                 Control 30min   2hours  2days   1week   p-value         pos
SENSE COUNTS:         1       0       0       1       3       1.87e-01
GENES (1 total):
  AT5G24420.1
    SENSE COUNTS:     1       0       0       1       3       1.87e-01
    TAGS: (2 total)
           ----------                                                         1119
           ----------                                                         1068
      d+2  AAGCACAACA 1       0       0       0       0       4.28e-01        756
      d+2  GCGTCTCTCT 0       0       0       1       3       6.27e-02        573
           ----------                                                         360
           ----------                                                         326

LOCUS: AT3G06230
DESCRIPTION: mitogen-activated protein kinase kinase (MAPKK), putative (MKK8), mitogen-activated protein
kinase kinase (MAPKK) family, PMID:12119167
DATA:                 Control 30min   2hours  2days   1week   p-value         pos
SENSE COUNTS:         5       0       1       4       5       1.88e-01
GENES (2 total):
  AT3G06230.1
    SENSE COUNTS:     5       0       1       4       5       1.88e-01
    TAGS: (1 total)
           ----------                                                         1580
           ----------                                                         1197
           ----------                                                         978
           ----------                                                         735
           ----------                                                         654
      v+2  AAAATAAAGA 5       0       1       4       5       1.88e-01        221
           ----------                                                         207

LOCUS: AT1G60780
DESCRIPTION: clathrin adaptor complexes medium subunit family protein, contains Pfam profile: PF00928 adaptor
complexes medium subunit family
DATA:                 Control 30min   2hours  2days   1week   p-value         pos
SENSE COUNTS:         9       4       3       2       2       1.89e-01
GENES (1 total):
  AT1G60780.1
    SENSE COUNTS:     9       4       3       2       2       1.89e-01
    TAGS: (3 total)
      i+3  ATTTGTTTCT 0       0       0       0       1       1.65e-01        2095
      d+1  GTTCATTATT 6       3       3       1       1       3.79e-01        1800
      d+2  AGGCTCTCTG 3       1       0       1       0       3.23e-01        1773
           ----------                                                         1532
           ----------                                                         1513
           ----------                                                         1349
           ----------                                                         278

LOCUS: AT4G04330
DESCRIPTION: expressed protein
DATA:                 Control 30min   2hours  2days   1week   p-value         pos
SENSE COUNTS:         1       7       3       2       5       1.89e-01
GENES (1 total):
  AT4G04330.1
    SENSE COUNTS:     1       7       3       2       5       1.89e-01
    TAGS: (2 total)
      i+3  GCCAATGCAA 0       0       0       1       0       3.09e-01        1527
      d+1  TCGAAACCGA 1       7       3       1       5       1.10e-01        534
           ----------                                                         506
           ----------                                                         389
           ----------                                                         368
           ----------                                                         231

LOCUS: AT3G21250
```

```
DESCRIPTION: ABC transporter family protein, similar to MRP-like ABC transporter GB:AAC49791 from (Arabidopsis
thaliana)
DATA:                 Control 30min   2hours  2days   1week   p-value         pos
SENSE COUNTS:         4       0       8       5       8       1.89e-01
GENES (2 total):
  AT3G21250.1
    SENSE COUNTS:     4       0       8       5       8       1.89e-01
    TAGS: (4 total)
      d+1  AACTTTGAGA 0       0       6       5       1       9.74e-03        4267
      d+2  GATAATGATA 4       0       2       0       7       2.05e-02        4230
      d+2  GTTCTCTCTT 0       0       0       0       0       6.15e-01        3994
           ----------                                                         3988
           ----------                                                         3360
           ----------                                                         3085
           ----------                                                         2835
           ----------                                                         2821
      d+2  ACGGTTGTCG 0       0       0       0       0       6.15e-01        2290
           ----------                                                         1898
           ----------                                                         1882
           ----------                                                         1863
           ----------                                                         1828
           ----------                                                         1700
           ----------                                                         1643
           ----------                                                         1251
           ----------                                                         1197
           ----------                                                         1123
           ----------                                                         528
           ----------                                                         273
           ----------                                                         26

LOCUS: AT4G16960
DESCRIPTION: disease resistance protein (TIR-NBS-LRR class), putative, domain signature TIR-NBS-LRR exists,
suggestive of a disease resistance protein.
DATA:                 Control 30min   2hours  2days   1week   p-value         pos
SENSE COUNTS:         4       1       8       5       8       1.89e-01
GENES (1 total):
  AT4G16960.1
    SENSE COUNTS:     4       1       8       5       8       1.89e-01
    TAGS: (1 total)
           ----------                                                         3858
           ----------                                                         3801
      v+2  GTAACTGAAA 4       1       8       5       8       1.89e-01        3473
           ----------                                                         3408
           ----------                                                         3374
           ----------                                                         2891
           ----------                                                         2532
           ----------                                                         2244
           ----------                                                         1982
           ----------                                                         1972
           ----------                                                         1944
           ----------                                                         1667
           ----------                                                         1351
           ----------                                                         1119
           ----------                                                         846
           ----------                                                         740
           ----------                                                         694
           ----------                                                         486
           ----------                                                         354

LOCUS: AT5G57120
DESCRIPTION: expressed protein, weak similarity to SP|Q14978 Nucleolar phosphoprotein p130 {Homo sapiens}
DATA:                 Control 30min   2hours  2days   1week   p-value         pos
SENSE COUNTS:         1       3       4       8       1       1.90e-01
GENES (1 total):
  AT5G57120.1
    SENSE COUNTS:     1       3       4       8       1       1.90e-01
    TAGS: (1 total)
           ----------                                                         1249
           ----------                                                         1012
      d+2  AAGCTGCAGC 1       3       4       8       1       1.90e-01        322

LOCUS: AT3G20910
DESCRIPTION: CCAAT-binding transcription factor (CBF-B/NF-YA) family protein, contains Pfam profile: PF02045
CCAAT-binding transcription factor (CBF-B/NF-YA) subunit B
DATA:                 Control 30min   2hours  2days   1week   p-value         pos
SENSE COUNTS:         3       5       3       0       0       1.90e-01
GENES (1 total):
  AT3G20910.1
    SENSE COUNTS:     3       5       3       0       0       1.90e-01
    TAGS: (3 total)
      d+1  AAAGAGTATT 1       0       0       0       0       4.28e-01        1075
```

```
            ----------                                                  825
            ----------                                                  729
            ----------                                                  711
      d+2  GCATCACACA  1      5      3      0      0      9.61e-02      411
           ----------                                                   168
      d+2  CATTCAAAAT  1      0      0      0      0      4.28e-01      119

LOCUS: AT1G72030
DESCRIPTION: GCN5-related N-acetyltransferase (GNAT) family protein, contains Pfam profile PF00583:
acetyltransferase, GNAT family
DATA:                  Control 30min  2hours  2days  1week  p-value       pos
SENSE COUNTS:          2      2      2      2      8      1.90e-01
GENES (1 total):
  AT1G72030.1
    SENSE COUNTS:      2      2      2      2      8      1.90e-01
    TAGS: (2 total)
           ----------                                                   980
      d+2  AACTGAACTG  0      0      1      1      1      5.61e-01      876
      d+2  TACATTTTGG  2      2      1      1      7      2.29e-01      808
           ----------                                                   613
           ----------                                                   415
           ----------                                                   344
           ----------                                                   26

LOCUS: AT2G29990
DESCRIPTION: pyridine nucleotide-disulphide oxidoreductase family protein, similar to SP|P32340 Rotenone-
insensitive NADH-ubiquinone oxidoreductase, mitochondrial precursor (EC 1.6.5.3) (Internal NADH dehydrogenase)
{Saccharomyces cerevisiae} ; contains Pfam profile P
DATA:                  Control 30min  2hours  2days  1week  p-value       pos
SENSE COUNTS:          0      0      1      1      3      1.91e-01
GENES (1 total):
  AT2G29990.1
    SENSE COUNTS:      0      0      1      1      3      1.91e-01
    TAGS: (3 total)
           ----------                                                   2180
           ----------                                                   2173
      v+2  ATGACCGATT  0      0      0      0      3      1.12e-02      2104
      v+2  GCTACTATCG  0      0      0      1      0      3.09e-01      1677
           ----------                                                   1186
           ----------                                                   1168
           ----------                                                   985
           ----------                                                   943
           ----------                                                   872
      v+2  AGGTTCATTG  0      0      1      0      0      4.55e-01      823
           ----------                                                   360
           ----------                                                   118

LOCUS: AT2G23130
DESCRIPTION: arabinogalactan protein AGP17 mRNA, complete cds
DATA:                  Control 30min  2hours  2days  1week  p-value       pos
SENSE COUNTS:          6      6      11     1      10     1.91e-01
GENES (3 total):
  AT2G23130.1
    SENSE COUNTS:      6      6      11     1      10     1.91e-01
    TAGS: (2 total)
      d+1  CTATTTTCTG  3      4      10     1      3      1.16e-01      793
      d+2  TATTACAGCT  3      2      1      0      7      1.21e-01      713
           ----------                                                   595

LOCUS: AT2G36020
DESCRIPTION: abscisic acid-responsive HVA22 family protein, weak similarity to SP|Q00765 Polyposis locus
protein 1 (TB2 protein) {Homo sapiens}; contains Pfam profile PF03134: TB2/DP1, HVA22 family
DATA:                  Control 30min  2hours  2days  1week  p-value       pos
SENSE COUNTS:          1      0      1      5      3      1.92e-01
GENES (1 total):
  AT2G36020.1
    SENSE COUNTS:      1      0      1      5      3      1.92e-01
    TAGS: (1 total)
           ----------                                                   1175
           ----------                                                   766
           ----------                                                   601
           ----------                                                   565
      d+2  TGTATGAAAC  1      0      1      5      3      1.92e-01      536
           ----------                                                   459

LOCUS: AT5G10760
DESCRIPTION: aspartyl protease family protein, contains Pfam domain, PF00026: eukaryotic aspartyl protease
DATA:                  Control 30min  2hours  2days  1week  p-value       pos
SENSE COUNTS:          1      0      1      3      3      1.92e-01
GENES (1 total):
  AT5G10760.1
```

```
    SENSE COUNTS:     1       0       1       3       3       1.92e-01
    TAGS: (3 total)
      d+1  CTTCAACATT 0       0       0       2       0       4.80e-02        1664
      d+2  TAGATTTGCT 1       0       1       0       3       2.09e-01        1598
      d+2  TTACGATTTC 0       0       0       1       0       3.09e-01        1190
           ----------                                                         527
           ----------                                                         509
           ----------                                                         295
           ----------                                                         253
           ----------                                                         249

LOCUS: AT1G50930
DESCRIPTION: hypothetical protein
DATA:                 Control 30min   2hours  2days   1week   p-value         pos
SENSE COUNTS:         3       7       6       1       1       1.94e-01
GENES (1 total):
  AT1G50930.1
    SENSE COUNTS:     3       7       6       1       1       1.94e-01
    TAGS: (1 total)
           ----------                                                         921
           ----------                                                         795
           ----------                                                         763
      v+2  ATGATGAAAT 3       7       6       1       1       1.94e-01        478
           ----------                                                         436
           ----------                                                         224
           ----------                                                         10

LOCUS: AT1G27000
DESCRIPTION: bZIP family transcription factor
DATA:                 Control 30min   2hours  2days   1week   p-value         pos
SENSE COUNTS:         6       1       3       2       9       1.95e-01
GENES (1 total):
  AT1G27000.1
    SENSE COUNTS:     6       1       3       2       9       1.95e-01
    TAGS: (5 total)
           ----------                                                         1348
           ----------                                                         1339
      d+2  TGGGGAATAA 3       1       2       2       8       1.62e-01        1309
      d+2  TGTAGTGTCC 2       0       1       0       0       2.87e-01        1261
      d+2  CTAAACTTGT 0       0       0       0       0       6.15e-01        806
           ----------                                                         530
      X+4  ACGGGCAAGT 0       0       0       0       1       1.65e-01        446
           ----------                                                         398
      X+4  TTTGCAATAA 1       0       0       0       0       6.89e-01        312
           ----------                                                         218
           ----------                                                         55

LOCUS: AT3G09860
DESCRIPTION: expressed protein,
DATA:                 Control 30min   2hours  2days   1week   p-value         pos
SENSE COUNTS:         1       9       6       8       5       1.95e-01
GENES (1 total):
  AT3G09860.1
    SENSE COUNTS:     1       9       6       8       5       1.95e-01
    TAGS: (2 total)
      X+4  ACCACCTTCC 0       0       3       8       5       5.86e-03        306
      d+1  ACAAGTGGAT 1       9       3       0       0       1.41e-03        181
           ----------                                                         150
           ----------                                                         73

LOCUS: AT2G13600
DESCRIPTION: pentatricopeptide (PPR) repeat-containing protein, contains INTERPRO:IPR002885 PPR repeats
DATA:                 Control 30min   2hours  2days   1week   p-value         pos
SENSE COUNTS:         2       4       3       6       10      1.96e-01
GENES (1 total):
  AT2G13600.1
    SENSE COUNTS:     2       4       3       6       10      1.96e-01
    TAGS: (2 total)
           ----------                                                         2779
           ----------                                                         2631
      v+2  TAATGTAAGT 0       0       0       1       0       3.09e-01        2442
           ----------                                                         2286
           ----------                                                         2278
           ----------                                                         2272
           ----------                                                         2163
           ----------                                                         2004
           ----------                                                         1956
           ----------                                                         1867
           ----------                                                         1859
           ----------                                                         1839
           ----------                                                         1597
```

```
          ----------                                                1585
          ----------                                                1538
          ----------                                                1412
     v+2  TTTACCAAAA  2      4      3      5      10     2.15e-01     1377
          ----------                                                1329
          ----------                                                1296
          ----------                                                1180
          ----------                                                1031
          ----------                                                939
          ----------                                                742
          ----------                                                481
          ----------                                                437
          ----------                                                319

LOCUS: AT1G06180
DESCRIPTION: myb family transcription factor, identical to  GB:CAA90748 GI:1263093 from (Arabidopsis
thaliana);contains PFAM profile:PF00249
DATA:                 Control 30min  2hours 2days  1week  p-value      pos
SENSE COUNTS:         2      0      2      6      2      1.96e-01
GENES (2 total):
  AT1G06180.1
    SENSE COUNTS:     2      0      2      6      2      1.96e-01
    TAGS: (2 total)
     i+3  TGACATTTGT  0      0      0      0      1      1.65e-01     1186
     d+1  AATAAAAGTC  2      0      2      6      1      1.56e-01     890
          ----------                                                772
          ----------                                                767
          ----------                                                323
          ----------                                                200
          ----------                                                153
          ----------                                                120

LOCUS: AT4G27290
DESCRIPTION: S-locus protein kinase, putative, similar to S-receptor kinase gi|392557|gb|AAA62232; contains
Pfam domains PF00954: S-locus glycoprotein family, PF00069: Protein kinase domain and PF01453: Lectin
(probable mannose binding)
DATA:                 Control 30min  2hours 2days  1week  p-value      pos
SENSE COUNTS:         0      1      4      2      0      1.96e-01
GENES (1 total):
  AT4G27290.1
    SENSE COUNTS:     0      1      4      2      0      1.96e-01
    TAGS: (1 total)
          ----------                                                2979
          ----------                                                2932
          ----------                                                2649
          ----------                                                2508
          ----------                                                2426
          ----------                                                2056
     i+3  AAAAAAAAGA  0      1      4      2      0      1.96e-01     1493
          ----------                                                1469
          ----------                                                1425
          ----------                                                968
          ----------                                                354
          ----------                                                231
          ----------                                                195

LOCUS: AT3G54150
DESCRIPTION: embryo-abundant protein-related, similar to embryo-abundant protein (Picea glauca) GI:1350531
DATA:                 Control 30min  2hours 2days  1week  p-value      pos
SENSE COUNTS:         2      0      0      1      3      1.97e-01
GENES (1 total):
  AT3G54150.1
    SENSE COUNTS:     2      0      0      1      3      1.97e-01
    TAGS: (2 total)
          ----------                                                1124
          ----------                                                966
     i+3  CACATATAGA  1      0      0      1      3      1.87e-01     953
     d+2  ATTGTTGGAA  1      0      0      0      0      4.28e-01     799
          ----------                                                476

LOCUS: AT2G33255
DESCRIPTION: haloacid dehalogenase-like hydrolase family protein, contains InterPro accession IPR005834:
Haloacid dehalogenase-like hydrolase
DATA:                 Control 30min  2hours 2days  1week  p-value      pos
SENSE COUNTS:         4      0      1      2      2      1.97e-01
GENES (1 total):
  AT2G33255.1
    SENSE COUNTS:     4      0      1      2      2      1.97e-01
    TAGS: (3 total)
     d+1  TGGGAAACGA  0      0      0      0      1      1.65e-01     580
          ----------                                                545
```

```
          ----------                                                    520
          ----------                                                    329
     X+4  TGTGTGAGGG 4      0      1      2      0      9.02e-02        327
     X+4  GTCGTCTAGC 0      0      0      0      1      1.65e-01        310
          ----------                                                    65

LOCUS: AT2G36320
DESCRIPTION: zinc finger (AN1-like) family protein, contains Pfam domain, PF01428: AN1-like Zinc finger
DATA:                Control 30min  2hours 2days  1week  p-value         pos
SENSE COUNTS:        3      5      1      4      0      1.99e-01
GENES (1 total):
  AT2G36320.1
    SENSE COUNTS:    3      5      1      4      0      1.99e-01
    TAGS: (2 total)
          ----------                                                    1314
     d+2  TCCTTATTGA 0      0      0      0      0      6.15e-01        1067
     d+2  GTTGTACGTT 3      5      1      4      0      3.00e-01        626
          ----------                                                    376

LOCUS: AT1G47770
DESCRIPTION: hypothetical protein
DATA:                Control 30min  2hours 2days  1week  p-value         pos
SENSE COUNTS:        2      6      3      1      0      2.00e-01
GENES (1 total):
  AT1G47770.1
    SENSE COUNTS:    2      6      3      1      0      2.00e-01
    TAGS: (1 total)
          ----------                                                    1168
          ----------                                                    1150
          ----------                                                    997
          ----------                                                    915
          ----------                                                    733
     v+2  TCAAGTTTTT 2      6      3      1      0      2.00e-01        594
          ----------                                                    354
          ----------                                                    50

LOCUS: AT2G44360
DESCRIPTION: expressed protein,
DATA:                Control 30min  2hours 2days  1week  p-value         pos
SENSE COUNTS:        3      1      6      1      6      2.01e-01
GENES (1 total):
  AT2G44360.1
    SENSE COUNTS:    3      1      6      1      6      2.01e-01
    TAGS: (2 total)
          ----------                                                    891
     d+2  AACAAATGTT 1      0      1      0      1      6.15e-01        730
          ----------                                                    704
     d+2  AGTGATATTG 2      1      5      1      5      3.79e-01        611
          ----------                                                    542

LOCUS: AT2G36080
DESCRIPTION: DNA-binding protein, putative, strong similarity to DNA-binding proteins from (Arabidopsis
thaliana) RAV1 GI:3868857, RAV2 GI:3868859; contains Pfam profile PF02362: B3 DNA binding domain
DATA:                Control 30min  2hours 2days  1week  p-value         pos
SENSE COUNTS:        0      1      4      4      0      2.02e-01
GENES (2 total):
  AT2G36080.1
    SENSE COUNTS:    0      1      3      4      0      2.76e-01
    TAGS: (4 total)
     i+3  TATTAGCTAG 0      0      0      0      0      6.15e-01        1991
     i+3  AAATGTATGA 0      0      1      0      0      4.55e-01        1115
     d+1  GACATAAGTT 0      1      1      4      0      2.06e-01        861
     d+2  GAGTGCCAGC 0      0      1      0      0      4.55e-01        735
          ----------                                                    667
          ----------                                                    631
          ----------                                                    434
          ----------                                                    213
          ----------                                                    63
  AT2G36080.2
    SENSE COUNTS:    0      0      1      0      0      4.55e-01
    TAGS: (1 total)
     d+1  CGATGTTCTT 0      0      1      0      0      4.55e-01        1012
          ----------                                                    969
          ----------                                                    667
          ----------                                                    631
          ----------                                                    434
          ----------                                                    213
          ----------                                                    63

LOCUS: AT4G29510
```

```
DESCRIPTION: protein arginine N-methyltransferase, putative, similar to protein arginine N-methyltransferase
1-variant 2 (Homo sapiens) GI:7453575
DATA:                 Control 30min  2hours  2days  1week  p-value        pos
SENSE COUNTS:         1       0      0       4      3      2.03e-01
GENES (1 total):
  AT4G29510.1
    SENSE COUNTS:     1       0      0       4      3      2.03e-01
    TAGS: (2 total)
      i+3  TTATCGAATA 0       0      0       0      0      6.15e-01       1831
      d+1  TGCCACAAGC 1       0      0       4      3      7.49e-02       1054
           ----------                                                     849
           ----------                                                     844
           ----------                                                     547
           ----------                                                     512
           ----------                                                     362
           ----------                                                     265

LOCUS: AT5G64270
DESCRIPTION: splicing factor, putative, similar to splicing factor 3B subunit 1 (Spliceosome associated
protein 155) (SAP 155) (SF3b155) (Pre-mRNA splicing factor SF3b 155 kDa subunit) (146 kDa nuclear protein)
SP:O57683 from (Xenopus laevis)
DATA:                 Control 30min  2hours  2days  1week  p-value        pos
SENSE COUNTS:         0       0      1       3      3      2.05e-01
GENES (1 total):
  AT5G64270.1
    SENSE COUNTS:     0       0      1       3      3      2.05e-01
    TAGS: (2 total)
           ----------                                                     4119
      X+4  AAATAACGTA 0       0      0       1      0      3.09e-01       4065
      d+2  GACAGGGATT 0       0      1       2      3      3.73e-01       3559
           ----------                                                     3393
           ----------                                                     3110
           ----------                                                     2332
           ----------                                                     2099
           ----------                                                     1995
           ----------                                                     1642
           ----------                                                     1336
           ----------                                                     1240

LOCUS: AT3G10160
DESCRIPTION: dihydrofolate synthetase/folylpolyglutamate synthetase (DHFS/FPGS3), nearly identical to
gi:17976757
DATA:                 Control 30min  2hours  2days  1week  p-value        pos
SENSE COUNTS:         4       0      0       2      3      2.05e-01
GENES (2 total):
  AT3G10160.1
    SENSE COUNTS:     4       0      0       2      3      2.05e-01
    TAGS: (2 total)
      d+1  AGAAGTGAGA 4       0      0       2      0      9.54e-02       1527
           ----------                                                     1196
           ----------                                                     1021
           ----------                                                     925
           ----------                                                     866
           ----------                                                     859
           ----------                                                     784
           ----------                                                     735
      d+2  ATGTAGTTTC 0       0      0       0      3      1.12e-02       686
           ----------                                                     665
           ----------                                                     142

LOCUS: AT4G34670
DESCRIPTION: 40S ribosomal protein S3A (RPS3aB)
DATA:                 Control 30min  2hours  2days  1week  p-value        pos
SENSE COUNTS:         5       6      5       4      14     2.06e-01
GENES (1 total):
  AT4G34670.1
    SENSE COUNTS:     5       6      5       4      14     2.06e-01
    TAGS: (1 total)
           ----------                                                     989
      d+2  GAGATTACAC 5       6      5       4      14     2.06e-01       776
           ----------                                                     766
           ----------                                                     452
           ----------                                                     82

LOCUS: AT4G18240
DESCRIPTION: starch synthase-related protein, contains similarity to starch synthase GI:4582783 from (Vigna
unguiculata)
DATA:                 Control 30min  2hours  2days  1week  p-value        pos
SENSE COUNTS:         1       1      5       2      0      2.07e-01
GENES (1 total):
  AT4G18240.1
```

```
    SENSE COUNTS:      1      1      5      2      0      2.07e-01
    TAGS: (5 total)
      d+1  TCCGGTTGTT  0      1      0      0      0      2.54e-01       2771
           ----------                                                   2765
           ----------                                                   2654
           ----------                                                   2472
      d+2  TCATAATTTT  0      0      0      1      0      3.09e-01       2187
           ----------                                                   2102
           ----------                                                   1607
           ----------                                                   1579
           ----------                                                   860
           ----------                                                   821
      d+2  ATGATGGGAA  0      0      1      0      0      4.55e-01       527
           ----------                                                   506
      d+2  CTGATGAGAA  0      0      1      0      0      4.55e-01       410
           ----------                                                   389
      d+2  GAAGTTCTCG  1      0      3      1      0      2.40e-01       143
           ----------                                                   110
           ----------                                                   73

LOCUS: AT3G55520
DESCRIPTION: immunophilin, putative / FKBP-type peptidyl-prolyl cis-trans isomerase, putative, POSSIBLE
PEPTIDYL-PROLYL CIS-TRANS ISOMERASE) (EC 5.2.1.8) (PPIASE) (ROTAMASE) SP:P30416(Mouse);P59 PROTEIN (HSP
BINDING IMMUNOPHILIN), rabbit, SWISSPROT:P27124:FKB4_RABBIT
DATA:                  Control 30min  2hours  2days  1week  p-value        pos
SENSE COUNTS:          1      2      0      1      4      2.07e-01
GENES (1 total):
  AT3G55520.1
    SENSE COUNTS:      1      2      0      1      4      2.07e-01
    TAGS: (3 total)
      d+1  TCGGAAGCAT  0      0      0      0      1      1.65e-01       931
           ----------                                                   857
      d+2  ATGAATCTAA  1      0      0      1      3      1.87e-01       785
      d+2  TAAGCCAGAA  0      2      0      0      0      9.14e-02       446
           ----------                                                   417

LOCUS: AT3G51520
DESCRIPTION: diacylglycerol acyltransferase family, contains Pfam PF03982: Diacylglycerol acyltransferase
DATA:                  Control 30min  2hours  2days  1week  p-value        pos
SENSE COUNTS:          4      1      3      0      0      2.08e-01
GENES (1 total):
  AT3G51520.1
    SENSE COUNTS:      4      1      3      0      0      2.08e-01
    TAGS: (1 total)
           ----------                                                   1209
      d+2  TCTTTAGCAT  4      1      3      0      0      2.08e-01       1151
           ----------                                                   1142
           ----------                                                   1131
           ----------                                                   953
           ----------                                                   881
           ----------                                                   709
           ----------                                                   656
           ----------                                                   546
           ----------                                                   241
           ----------                                                   157

LOCUS: AT1G79000
DESCRIPTION: p300/CBP acetyltransferase-related protein 2 (PCAT2), contains Pfam domains PF02135: TAZ zinc
finger and PF00569: Zinc finger, ZZ type; identical to cDNA p300/CBP acetyltransferase-related protein 2
GI:12597460
DATA:                  Control 30min  2hours  2days  1week  p-value        pos
SENSE COUNTS:          4      0      3      0      0      2.08e-01
GENES (2 total):
  AT1G79000.1
    SENSE COUNTS:      4      0      3      0      0      2.08e-01
    TAGS: (4 total)
      d+1  CGAGCTTTAA  0      0      1      0      0      7.06e-01       5245
      d+2  TACCTCGTTG  0      0      0      0      0      6.15e-01       4949
           ----------                                                   4919
      d+2  GTATCAACTG  1      0      0      0      0      4.28e-01       4844
           ----------                                                   4775
           ----------                                                   4501
           ----------                                                   4340
           ----------                                                   4174
           ----------                                                   3898
      d+2  TTATAATGAA  3      0      2      0      0      1.15e-01       2967
           ----------                                                   1999
           ----------                                                   1922
           ----------                                                   1475
           ----------                                                   1367
           ----------                                                   845
```

```
          ----------                                                 769
          ----------                                                 628
          ----------                                                 619
          ----------                                                 547

LOCUS: AT4G05526
DESCRIPTION: pseudogene, similar to OSJNBb0043H09.1, similar to En/Spm-like transposon protein, putative;
blastp match of 48% identity and 8.1e-37 P-value to GP|21740634|emb|CAD40195.1||AL606611 OSJNBb0043H09.1
{Oryza sativa (japonica cultivar-group)}
DATA:                 Control 30min  2hours  2days  1week  p-value       pos
SENSE COUNTS:         2       1      8       5      3      2.08e-01
GENES (1 total):
  AT4G05526.1
    SENSE COUNTS:     2       1      8       5      3      2.08e-01
    TAGS: (2 total)
          ----------                                                 2927
          ----------                                                 2813
          ----------                                                 2706
          ----------                                                 2647
          ----------                                                 2452
          ----------                                                 1024
          ----------                                                 927
          ----------                                                 505
          ----------                                                 482
      p+2  ATGTTTCTTC 2       1      8       5      3      1.23e-01      398
      p+2  AATTGGTAAT 0       0      0       0      0      6.15e-01      230
          ----------                                                 196
          ----------                                                 82
          ----------                                                 52

LOCUS: AT5G24800
DESCRIPTION: bZIP transcription factor family protein, contains Pfam profile: PF00170 bZIP transcription
factor; identical to cDNA bZIP protein BZO2H2 GI:10954096
DATA:                 Control 30min  2hours  2days  1week  p-value       pos
SENSE COUNTS:         2       0      5       7      2      2.08e-01
GENES (2 total):
  AT5G24800.1
    SENSE COUNTS:     2       0      5       7      2      2.08e-01
    TAGS: (3 total)
      d+1  AGCAGACAAA 0       0      0       1      0      3.09e-01      1220
          ----------                                                 1210
      d+2  TATAGGCCAA 0       0      1       0      1      6.85e-01      1063
      d+2  TTCGTTAAAT 2       0      4       6      1      1.84e-01      981
          ----------                                                 910
          ----------                                                 889
          ----------                                                 348
          ----------                                                 164

LOCUS: AT2G34250
DESCRIPTION: protein transport protein sec61, putative, similar to PfSec61 (Plasmodium falciparum) GI:3057044;
contains Pfam profile PF00344: eubacterial secY protein
DATA:                 Control 30min  2hours  2days  1week  p-value       pos
SENSE COUNTS:         10      12     3       11     6      2.09e-01
GENES (1 total):
  AT2G34250.1
    SENSE COUNTS:     10      12     3       11     6      2.09e-01
    TAGS: (3 total)
      i+3  CATCAACTGA 0       0      0       0      0      6.15e-01      1981
      d+1  GGAGCCATCG 10      12     3       11     5      1.99e-01      1482
          ----------                                                 1304
          ----------                                                 1272
          ----------                                                 1249
          ----------                                                 1230
      d+2  CCCATCATCC 0       0      0       0      1      1.65e-01      1041
          ----------                                                 402

LOCUS: AT3G44600
DESCRIPTION: peptidyl-prolyl cis-trans isomerase cyclophilin-type family protein, similar to SP|P87051
Peptidyl-prolyl cis-trans isomerase cyp2 (EC 5.2.1.8) (Cyclophilin cyp2) {Schizosaccharomyces pombe}; contains
Pfam profiles PF00160: peptidyl-prolyl cis-trans isome
DATA:                 Control 30min  2hours  2days  1week  p-value       pos
SENSE COUNTS:         1       0      1       0      3      2.09e-01
GENES (1 total):
  AT3G44600.1
    SENSE COUNTS:     1       0      1       0      3      2.09e-01
    TAGS: (2 total)
          ----------                                                 2270
      d+2  ACAGGCCGTT 0       0      1       0      3      7.32e-02      1731
          ----------                                                 1529
          ----------                                                 689
          ----------                                                 512
```

```
          ----------                                                      468
      d+2  TTGCTGTTTC  1      0      0      0      0      4.28e-01       288

LOCUS: AT2G39570
DESCRIPTION: ACT domain-containing protein, contains Pfam ACT domain PF01842
DATA:                  Control 30min  2hours 2days  1week  p-value        pos
SENSE COUNTS:          4      7      3      1      1      2.09e-01
GENES (1 total):
  AT2G39570.1
    SENSE COUNTS:      4      7      3      1      1      2.09e-01
    TAGS: (2 total)
      d+1  TAGTGGCCTT  2      1      3      1      1      9.23e-01       1609
           ----------                                                     1594
      d+2  AACGTTATGC  2      6      0      0      0      1.07e-02       1328
           ----------                                                     618
           ----------                                                     497
           ----------                                                     441

LOCUS: AT4G27430
DESCRIPTION: COP1-interacting protein 7 (CIP7), identical to COP1-Interacting Protein 7 (CIP7) GI:3327868 from
(Arabidopsis thaliana)
DATA:                  Control 30min  2hours 2days  1week  p-value        pos
SENSE COUNTS:          4      2      9      4      1      2.09e-01
GENES (2 total):
  AT4G27430.1
    SENSE COUNTS:      4      2      9      4      1      2.09e-01
    TAGS: (3 total)
      d+1  TTGAGTTCAG  1      0      3      2      1      7.11e-01       3378
           ----------                                                     3151
           ----------                                                     2178
           ----------                                                     2134
           ----------                                                     2114
           ----------                                                     2053
           ----------                                                     1996
           ----------                                                     1835
      d+2  GGAGATGTGG  3      2      5      2      0      4.60e-01       1129
           ----------                                                     1103
           ----------                                                     1046
           ----------                                                     1027
           ----------                                                     926
      d+2  GGTGACTCTG  0      0      1      0      0      4.55e-01       859
           ----------                                                     700
           ----------                                                     519

LOCUS: AT5G54600
DESCRIPTION: 50S ribosomal protein L24, chloroplast  (CL24), identical to SP|P92959 50S ribosomal protein L24,
chloroplast precursor {Arabidopsis thaliana}
DATA:                  Control 30min  2hours 2days  1week  p-value        pos
SENSE COUNTS:          14     21     9      15     12     2.09e-01
GENES (2 total):
  AT5G54600.1
    SENSE COUNTS:      14     21     9      15     12     2.09e-01
    TAGS: (2 total)
      d+1  ATTTAACATA  3      0      1      0      5      1.40e-01       792
           ----------                                                     776
      d+2  AAGAGCCGAG  11     21     8      15     7      5.70e-02       414
           ----------                                                     286
           ----------                                                     206
           ----------                                                     81
  AT5G54600.2
    SENSE COUNTS:      3      0      1      0      5      1.40e-01
    TAGS: (1 total)
      d+1  ATTTAACATA  3      0      1      0      5      1.40e-01       735
           ----------                                                     719
           ----------                                                     286
           ----------                                                     206
           ----------                                                     81

LOCUS: AT5G52430
DESCRIPTION: hydroxyproline-rich glycoprotein family protein, Common family member At4g25620 (Arabidopsis
thaliana)
DATA:                  Control 30min  2hours 2days  1week  p-value        pos
SENSE COUNTS:          0      0      0      1      3      2.09e-01
GENES (1 total):
  AT5G52430.1
    SENSE COUNTS:      0      0      0      1      3      2.09e-01
    TAGS: (1 total)
           ----------                                                     2290
      d+2  TAGTTGGAAA  0      0      0      1      3      2.09e-01       1662
           ----------                                                     1564
           ----------                                                     1154
```

```
           ----------                                                       1140
           ----------                                                       1087
           ----------                                                       1005
           ----------                                                       896
           ----------                                                       295

LOCUS: AT5G17260
DESCRIPTION: no apical meristem (NAM) family protein, contains Pfam PF02365: No apical meristem (NAM) domain;
DATA:                 Control 30min   2hours  2days   1week   p-value       pos
SENSE COUNTS:         0       0       0       1       3       2.09e-01
GENES (1 total):
  AT5G17260.1
    SENSE COUNTS:     0       0       0       1       3       2.09e-01
    TAGS: (2 total)
      i+3  TATCTTAAAA 0       0       0       1       0       6.04e-01      1684
           ----------                                                       1639
           ----------                                                       1047
           ----------                                                       1015
           ----------                                                       786
      i+3  GACCACCACA 0       0       0       0       3       1.12e-02      746
           ----------                                                       739
           ----------                                                       735
           ----------                                                       712
           ----------                                                       497
           ----------                                                       354

LOCUS: AT1G77010
DESCRIPTION: pentatricopeptide (PPR) repeat-containing protein, contains INTERPRO:IPR002885 PPR repeats
DATA:                 Control 30min   2hours  2days   1week   p-value       pos
SENSE COUNTS:         1       0       3       0       1       2.11e-01
GENES (1 total):
  AT1G77010.1
    SENSE COUNTS:     1       0       3       0       1       2.11e-01
    TAGS: (1 total)
           ----------                                                       2658
           ----------                                                       2262
           ----------                                                       2214
           ----------                                                       2126
           ----------                                                       2095
           ----------                                                       2033
           ----------                                                       1907
           ----------                                                       1861
           ----------                                                       1437
           ----------                                                       1381
           ----------                                                       982
           ----------                                                       841
           ----------                                                       801
           ----------                                                       663
           ----------                                                       458
      v+2  TGAGACTAAA 1       0       3       0       1       2.11e-01      307

LOCUS: AT5G64540
DESCRIPTION: hypothetical protein,
DATA:                 Control 30min   2hours  2days   1week   p-value       pos
SENSE COUNTS:         1       0       3       0       1       2.11e-01
GENES (1 total):
  AT5G64540.1
    SENSE COUNTS:     1       0       3       0       1       2.11e-01
    TAGS: (1 total)
           ----------                                                       1785
           ----------                                                       1578
           ----------                                                       1506
      v+2  GGCCTGACCC 1       0       3       0       1       2.11e-01      1117
           ----------                                                       1016
           ----------                                                       596

LOCUS: AT1G32530
DESCRIPTION: zinc finger (C3HC4-type RING finger) family protein, contains Pfam profile: PF00097 zinc finger,
C3HC4 type (RING finger); weak similarity to interaptin (GI:3549261) (Dictyostelium discoideum) weak
similarity to Axoneme-associated protein mst101(2) (Swiss
DATA:                 Control 30min   2hours  2days   1week   p-value       pos
SENSE COUNTS:         1       0       3       0       1       2.11e-01
GENES (1 total):
  AT1G32530.1
    SENSE COUNTS:     1       0       3       0       1       2.11e-01
    TAGS: (1 total)
           ----------                                                       2423
           ----------                                                       1593
           ----------                                                       1546
           ----------                                                       937
           ----------                                                       809
```

```
      X+4  CCTCTCTAGT 1       0       3       0       1       2.11e-01        721
           ----------                                                         668
           ----------                                                         85

LOCUS: AT1G05850
DESCRIPTION: chitinase-like protein 1 (CTL1), similar to class I chitinase GI:7798656 from (Halimolobos
perplexa var. perplexa); contains Pfam profile PF00182: Chitinase class I; identical to cDNA chitinase-like
protein 1 (CTL1) CTL1-ELP1 allele GI:17226328
DATA:                  Control 30min   2hours  2days   1week   p-value         pos
SENSE COUNTS:          19      21      27      10      17      2.12e-01
GENES (2 total):
  AT1G05850.1
    SENSE COUNTS:      19      21      27      10      17      2.12e-01
    TAGS: (4 total)
      d+1  GTTTTTAGTC 2       0       1       1       1       6.51e-01        1391
      d+2  GGTATTGGAA 17      21      23      8       16      1.82e-01        1018
      d+2  TGGTCAAGGT 0       0       1       0       0       4.55e-01        951
           ----------                                                         925
      d+2  TAGCCAGCAA 0       0       2       1       0       2.74e-01        521
           ----------                                                         487
           ----------                                                         398

LOCUS: AT5G06360
DESCRIPTION: ribosomal protein S8e family protein, contains Pfam profile PF01201: Ribosomal protein S8e
DATA:                  Control 30min   2hours  2days   1week   p-value         pos
SENSE COUNTS:          4       5       4       0       8       2.13e-01
GENES (1 total):
  AT5G06360.1
    SENSE COUNTS:      4       5       4       0       8       2.13e-01
    TAGS: (2 total)
      d+1  TTGGCTAGTT 4       5       4       0       5       4.52e-01        908
           ----------                                                         686
      d+2  AAGAGTCATC 0       0       0       0       3       1.12e-02        270
           ----------                                                         248
           ----------                                                         179
           ----------                                                         68

LOCUS: AT4G03415
DESCRIPTION: protein phosphatase 2C family protein / PP2C family protein, similar to protein phosphatase-2C;
PP2C (GI:3643088) (Mesembryanthemum crystallinum); contains Pfam PF00481 : Protein phosphatase 2C domain;
DATA:                  Control 30min   2hours  2days   1week   p-value         pos
SENSE COUNTS:          4       2       4       0       0       2.14e-01
GENES (1 total):
  AT4G03415.1
    SENSE COUNTS:      4       2       4       0       0       2.14e-01
    TAGS: (1 total)
           ----------                                                         2005
      v+2  AATCAAATGT 4       2       4       0       0       2.14e-01        1888
           ----------                                                         1520
           ----------                                                         1146
           ----------                                                         981
           ----------                                                         888
           ----------                                                         664
           ----------                                                         603
           ----------                                                         65

LOCUS: AT1G09280
DESCRIPTION: expressed protein, contains Pfam profile: PF03959 domain of unknown function (DUF341)
DATA:                  Control 30min   2hours  2days   1week   p-value         pos
SENSE COUNTS:          0       3       1       0       1       2.14e-01
GENES (2 total):
  AT1G09280.1
    SENSE COUNTS:      0       3       1       0       1       2.14e-01
    TAGS: (2 total)
           ----------                                                         2331
           ----------                                                         2118
      d+2  GATTTAGGCA 0       3       0       0       1       8.32e-02        1123
           ----------                                                         684
      i+3  AATCGAAAAT 0       0       1       0       0       4.55e-01        346
  AT1G09280.2
    SENSE COUNTS:      0       3       1       0       1       2.14e-01
    TAGS: (2 total)
           ----------                                                         2313
           ----------                                                         2100
      d+2  GATTTAGGCA 0       3       0       0       1       8.32e-02        1105
           ----------                                                         666
      i+3  AATCGAAAAT 0       0       1       0       0       4.55e-01        346

LOCUS: AT2G40750
DESCRIPTION: WRKY family transcription factor, contains Pfam profile: PF03106 WRKY DNA -binding domain
DATA:                  Control 30min   2hours  2days   1week   p-value         pos
```

```
SENSE COUNTS:         3      0      1      1      0      2.14e-01
GENES (2 total):
  AT2G40750.1
    SENSE COUNTS:     3      0      1      1      0      2.14e-01
    TAGS: (1 total)
           ----------                                           1321
           ----------                                           1284
           ----------                                           913
           ----------                                           848
           ----------                                           747
           ----------                                           420
           ----------                                           408
      d+2  TCTAAGATCT 3      0      1      1      0      2.14e-01      313

LOCUS: AT1G43850
DESCRIPTION: SEUSS transcriptional co-regulator, identical to SEUSS transcriptional co-regulator (Arabidopsis
thaliana) gi|18033922|gb|AAL57277
DATA:                 Control 30min  2hours 2days  1week  p-value       pos
SENSE COUNTS:         3      2      0      6      3      2.15e-01
GENES (2 total):
  AT1G43850.1
    SENSE COUNTS:     3      2      0      6      3      2.15e-01
    TAGS: (3 total)
      d+1  ATTTTTTGTT 2      1      0      2      0      4.73e-01      3653
      d+2  TGTTAACCAA 0      0      0      0      3      1.12e-02      3576
      d+2  AACGGAAGAG 1      1      0      4      0      2.03e-01      3128
           ----------                                           3083
           ----------                                           2943
           ----------                                           2933
           ----------                                           2801
           ----------                                           2690
           ----------                                           1986
           ----------                                           1517
           ----------                                           1229
           ----------                                           1149
           ----------                                           1038

LOCUS: AT4G12890
DESCRIPTION: gamma interferon responsive lysosomal thiol reductase family protein / GILT family protein,
similar to SP|P13284 Gamma-interferon inducible lysosomal thiol reductase precursor {Homo sapiens}; contains
Pfam profile PF03227: Gamma interferon inducible lysos
DATA:                 Control 30min  2hours 2days  1week  p-value       pos
SENSE COUNTS:         2      0      4      4      5      2.15e-01
GENES (1 total):
  AT4G12890.1
    SENSE COUNTS:     2      0      4      4      5      2.15e-01
    TAGS: (1 total)
           ----------                                           565
           ----------                                           552
      d+2  TGTTAAAAAA 2      0      4      4      5      2.15e-01      439
           ----------                                           318
           ----------                                           79

LOCUS: AT1G17290
DESCRIPTION: alanine aminotransferase (ALAAT1) mRNA, complete cds
DATA:                 Control 30min  2hours 2days  1week  p-value       pos
SENSE COUNTS:         5      7      4      13     10     2.16e-01
GENES (2 total):
  AT1G17290.1
    SENSE COUNTS:     5      7      4      13     10     2.16e-01
    TAGS: (1 total)
           ----------                                           2013
      d+2  CCGTTTCTTT 5      7      4      13     10     2.16e-01      1811
           ----------                                           1731
           ----------                                           1646
           ----------                                           1469
           ----------                                           1341
           ----------                                           1236
           ----------                                           732

LOCUS: AT1G62880
DESCRIPTION: cornichon family protein, contains Pfam profile: PF03311 cornichon protein
DATA:                 Control 30min  2hours 2days  1week  p-value       pos
SENSE COUNTS:         6      1      3      1      1      2.16e-01
GENES (1 total):
  AT1G62880.1
    SENSE COUNTS:     6      1      3      1      1      2.16e-01
    TAGS: (2 total)
           ----------                                           839
      d+2  AGAAGGAAGG 1      0      0      0      0      4.28e-01      708
           ----------                                           410
```

```
      d+2  GATTATTTCA 5       1       3       1       1       4.19e-01        217

LOCUS: AT1G54880
DESCRIPTION: hypothetical protein
DATA:                 Control 30min   2hours  2days   1week   p-value         pos
SENSE COUNTS:         1       7       2       2       3       2.18e-01
GENES (1 total):
  AT1G54880.1
    SENSE COUNTS:     1       7       2       2       3       2.18e-01
    TAGS: (1 total)
           ----------                                                         760
      v+2  AATATAAATA 1       7       2       2       3       2.18e-01        589
           ----------                                                         382
           ----------                                                         354

LOCUS: AT2G47450
DESCRIPTION: chloroplast signal recognition particle component (CAO), nearly identical to CAO (Arabidopsis
thaliana) GI:4102582
DATA:                 Control 30min   2hours  2days   1week   p-value         pos
SENSE COUNTS:         5       4       2       0       4       2.18e-01
GENES (2 total):
  AT2G47450.1
    SENSE COUNTS:     5       4       2       0       4       2.18e-01
    TAGS: (4 total)
      d+1  TAAACCCAAT 3       0       0       0       1       2.03e-01        1547
      d+2  ATGCTGGCTG 1       0       1       0       3       4.58e-01        1422
           ----------                                                         1386
      d+2  GCGGCTGGTT 0       4       1       0       0       3.94e-02        701
           ----------                                                         674
      d+2  GAGTACCTTA 1       0       0       0       0       4.28e-01        401
           ----------                                                         125

LOCUS: AT3G59820
DESCRIPTION: calcium-binding mitochondrial protein-related, contains weak similarity to Calcium-binding
mitochondrial protein Anon-60Da (Swiss-Prot:P91927) (Drosophila melanogaster)
DATA:                 Control 30min   2hours  2days   1week   p-value         pos
SENSE COUNTS:         0       1       1       4       5       2.20e-01
GENES (1 total):
  AT3G59820.1
    SENSE COUNTS:     0       1       1       4       5       2.20e-01
    TAGS: (3 total)
      i+3  AGATTTGTTT 0       0       0       0       0       6.15e-01        2963
      d+1  TTTTGCTGCT 0       1       0       4       5       6.65e-02        2354
           ----------                                                         1150
           ----------                                                         620
      i+3  AACAGGTTTG 0       0       1       0       0       4.55e-01        577
           ----------                                                         577
           ----------                                                         528
           ----------                                                         316
           ----------                                                         248
           ----------                                                         122

LOCUS: AT3G10620
DESCRIPTION: diadenosine 5',5'''-P1,P4-tetraphosphate hydrolase, putative, similar to diadenosine 5',5'''-
P1,P4-tetraphosphate hydrolase from (Lupinus angustifolius) GI:1888557, (Hordeum vulgare subsp. vulgare)
GI:2564253; contains Pfam profile PF00293: NUDIX domain
DATA:                 Control 30min   2hours  2days   1week   p-value         pos
SENSE COUNTS:         3       0       3       0       0       2.20e-01
GENES (1 total):
  AT3G10620.1
    SENSE COUNTS:     3       0       3       0       0       2.20e-01
    TAGS: (1 total)
           ----------                                                         896
           ----------                                                         813
      d+2  TCAGCTTTCG 3       0       3       0       0       2.20e-01        625
           ----------                                                         325
           ----------                                                         217
           ----------                                                         169

LOCUS: AT4G20830
DESCRIPTION: FAD-binding domain-containing protein, similar to SP|P30986 reticuline oxidase precursor
(Berberine-bridge-forming enzyme) (BBE) (Tetrahydroprotoberberine synthase) (Eschscholzia californica);
contains PF01565 FAD binding domain
DATA:                 Control 30min   2hours  2days   1week   p-value         pos
SENSE COUNTS:         2       7       7       12      7       2.21e-01
GENES (2 total):
  AT4G20830.1
    SENSE COUNTS:     0       0       1       4       0       3.72e-02
    TAGS: (1 total)
           ----------                                                         1739
      d+2  GGGCGAATAG 0       0       1       4       0       3.72e-02        1508
```

```
          ----------                                                    1432
          ----------                                                    631
          ----------                                                    560
          ----------                                                    495
          ----------                                                    448
          ----------                                                    395
  AT4G20830.2
    SENSE COUNTS:     2       7       7       12      7       2.21e-01
    TAGS: (2 total)
      d+1  GAACTCTACA 2       7       6       8       7       5.82e-01      1764
      d+2  GGGCGAATAG 0       0       1       4       0       3.72e-02      1501
          ----------                                                    1425
          ----------                                                    624
          ----------                                                    553
          ----------                                                    488
          ----------                                                    441
          ----------                                                    388

LOCUS: AT3G13070
DESCRIPTION: CBS domain-containing protein / transporter associated domain-containing protein, similar to
SP|Q54318 Hemolysin C (Serpulina hyodysenteriae) {Treponema hyodysenteriae}; contains Pfam profiles PF00571:
CBS domain, PF03471: Transporter associated domain, P
DATA:                 Control 30min   2hours  2days   1week   p-value       pos
SENSE COUNTS:         4       1       5       0       4       2.22e-01
GENES (1 total):
  AT3G13070.1
    SENSE COUNTS:     4       1       5       0       4       2.22e-01
    TAGS: (2 total)
      i+3  TTCTTTAAAA 2       0       2       0       1       3.93e-01      3072
          ----------                                                    2778
      v+2  TACTGGCTGA 2       1       3       0       3       5.97e-01      2305
          ----------                                                    2208
          ----------                                                    1851
          ----------                                                    1713
          ----------                                                    1629
          ----------                                                    1414

LOCUS: AT2G44745
DESCRIPTION: WRKY family transcription factor, contains Pfam profile: PF03106 WRKY DNA -binding domain
DATA:                 Control 30min   2hours  2days   1week   p-value       pos
SENSE COUNTS:         4       3       5       5       11      2.23e-01
GENES (1 total):
  AT2G44745.1
    SENSE COUNTS:     4       3       5       5       11      2.23e-01
    TAGS: (2 total)
      i+3  TCCTTAATTA 0       0       0       0       1       1.65e-01      935
      d+1  TGTTTTTCAA 4       3       5       5       10      4.41e-01      745
          ----------                                                    643
          ----------                                                    211

LOCUS: AT1G72370
DESCRIPTION: 40S ribosomal protein SA (RPSaA), identical to laminin receptor-like protein GB:U01955
(Arabidopsis thaliana); identical to cDNA laminin receptor homologue GI:16379
DATA:                 Control 30min   2hours  2days   1week   p-value       pos
SENSE COUNTS:         1       0       2       0       3       2.23e-01
GENES (2 total):
  AT1G72370.1
    SENSE COUNTS:     1       0       2       0       3       2.23e-01
    TAGS: (1 total)
          ----------                                                    1025
      d+2  GCCAGGAGAA 1       0       2       0       3       2.23e-01      847
          ----------                                                    692
          ----------                                                    641
          ----------                                                    262

LOCUS: AT5G53030
DESCRIPTION: expressed protein
DATA:                 Control 30min   2hours  2days   1week   p-value       pos
SENSE COUNTS:         3       1       1       5       7       2.24e-01
GENES (2 total):
  AT5G53030.1
    SENSE COUNTS:     3       1       1       4       7       2.79e-01
    TAGS: (1 total)
          ----------                                                    935
      d+2  TCTTCTCTAA 3       1       1       4       7       2.79e-01      60
  AT5G53030.2
    SENSE COUNTS:     3       1       1       5       7       2.24e-01
    TAGS: (2 total)
          ----------                                                    1429
      d+2  GTTGCACAAA 0       0       0       1       0       3.09e-01      1391
          ----------                                                    1208
```

```
      d+2  TCTTCTCTAA 3       1       1       4       7       2.79e-01       60

LOCUS: AT5G48810
DESCRIPTION: cytochrome b5, identical to cytochrome b5 (Arabidopsis thaliana) GI:4240122; strong similarity to
Cytochrome B5 SP:P49098 from (Nicotiana tabacum)
DATA:                 Control 30min   2hours  2days   1week   p-value        pos
SENSE COUNTS:         3       7       2       1       1       2.24e-01
GENES (2 total):
  AT5G48810.1
    SENSE COUNTS:     3       7       2       1       1       2.24e-01
    TAGS: (1 total)
           ----------                                                        1101
           ----------                                                        917
      d+2  CTAGATGAGT 3       7       2       1       1       2.24e-01       320

LOCUS: AT5G14540
DESCRIPTION: proline-rich family protein, contains proline rich extensin domains, INTERPRO:IPR002965
DATA:                 Control 30min   2hours  2days   1week   p-value        pos
SENSE COUNTS:         1       0       4       1       0       2.24e-01
GENES (1 total):
  AT5G14540.1
    SENSE COUNTS:     1       0       4       1       0       2.24e-01
    TAGS: (2 total)
           ----------                                                        1830
      d+2  TCAGATTTCC 1       0       4       1       0       8.52e-02       1751
      d+2  GGGTTCCCAA 0       0       0       0       0       6.15e-01       1530
           ----------                                                        748
           ----------                                                        624
           ----------                                                        583
           ----------                                                        481
           ----------                                                        463
           ----------                                                        114

LOCUS: AT1G08630
DESCRIPTION: L-allo-threonine aldolase-related, similar to L-allo-threonine aldolase (EC 4.1.2.-) (L-allo-TA)
(L-allo-threonine acetaldehyde-lyase) (SP:O07051) {Aeromonas jandaei}; similar to ESTs gb|R30517, gb|T42772,
gb|R90493, and gb|R90493
DATA:                 Control 30min   2hours  2days   1week   p-value        pos
SENSE COUNTS:         0       4       3       2       0       2.25e-01
GENES (3 total):
  AT1G08630.1
    SENSE COUNTS:     0       4       3       2       0       2.25e-01
    TAGS: (2 total)
      d+1  GATATGGAGG 0       4       1       0       0       3.94e-02       1182
           ----------                                                        1173
           ----------                                                        1087
           ----------                                                        811
           ----------                                                        742
           ----------                                                        651
           ----------                                                        574
      i+3  TCGAAGGAAC 0       0       2       2       0       1.68e-01       358
           ----------                                                        74
  AT1G08630.2
    SENSE COUNTS:     0       4       3       2       0       2.25e-01
    TAGS: (2 total)
      i+3  TCGAAGGAAC 0       0       2       2       0       1.68e-01       1382
      d+1  GATATGGAGG 0       4       1       0       0       3.94e-02       1045
           ----------                                                        1036
           ----------                                                        950
           ----------                                                        674
           ----------                                                        605
           ----------                                                        514
           ----------                                                        437
  AT1G08630.3
    SENSE COUNTS:     0       4       3       2       0       2.25e-01
    TAGS: (2 total)
      d+1  GATATGGAGG 0       4       1       0       0       3.94e-02       1037
           ----------                                                        1028
           ----------                                                        942
           ----------                                                        666
           ----------                                                        597
           ----------                                                        506
           ----------                                                        429
      i+3  TCGAAGGAAC 0       0       2       2       0       1.68e-01       114

LOCUS: AT1G66840
DESCRIPTION: expressed protein, contains Pfam profile PF05701: Plant protein of unknown function (DUF827);
expression supported by MPSS
DATA:                 Control 30min   2hours  2days   1week   p-value        pos
SENSE COUNTS:         4       0       2       1       1       2.25e-01
GENES (1 total):
```

```
  AT1G66840.1
    SENSE COUNTS:     4      0      2      1      1      2.25e-01
    TAGS: (2 total)
          ----------                                                2444
      v+2 GTCAAATTTT  1      0      1      1      0      7.18e-01   2127
          ----------                                                1747
          ----------                                                1285
      v+2 TATTGGTTGA  3      0      1      0      1      1.89e-01   862

LOCUS: AT1G10950
DESCRIPTION: endomembrane protein 70, putative
DATA:                 Control 30min  2hours 2days  1week  p-value   pos
SENSE COUNTS:         6      9      7      1      2      2.26e-01
GENES (1 total):
  AT1G10950.1
    SENSE COUNTS:     6      9      7      1      2      2.26e-01
    TAGS: (3 total)
          ----------                                                2232
      d+2 TGTTGGGCCT  0      0      1      0      1      3.96e-01   2197
      d+2 ATGTTCTGTC  6      9      5      1      1      1.28e-01   1778
          ----------                                                1559
          ----------                                                1390
      d+2 GTCCTCACGG  0      0      1      0      0      4.55e-01   1184
          ----------                                                942
          ----------                                                797
          ----------                                                621
          ----------                                                564
          ----------                                                389

LOCUS: AT3G20630
DESCRIPTION: ubiquitin-specific protease 14, putative (UBP14), similar to ubiquitin-specific protease 14
GI:11993473 (Arabidopsis thaliana)
DATA:                 Control 30min  2hours 2days  1week  p-value   pos
SENSE COUNTS:         4      0      0      4      4      2.27e-01
GENES (2 total):
  AT3G20630.1
    SENSE COUNTS:     4      0      0      4      4      2.27e-01
    TAGS: (2 total)
      v+1 TGGATTAGTA  4      0      0      4      1      1.56e-01   2878
          ----------                                                2848
          ----------                                                2712
          ----------                                                2601
          ----------                                                2505
          ----------                                                2316
      v+2 CGTAGCAAAG  0      0      0      0      3      8.36e-02   2109
          ----------                                                2022
          ----------                                                1777
          ----------                                                1539
          ----------                                                991
          ----------                                                832
          ----------                                                421

LOCUS: AT4G16130
DESCRIPTION: Similar to galactokinase.
DATA:                 Control 30min  2hours 2days  1week  p-value   pos
SENSE COUNTS:         0      2      4      2      0      2.28e-01
GENES (2 total):
  AT4G16130.1
    SENSE COUNTS:     0      2      4      2      0      2.28e-01
    TAGS: (2 total)
      d+1 CGGACTAGGA  0      0      1      0      0      7.06e-01   2837
          ----------                                                2653
          ----------                                                2616
      d+2 GGGAGAAAGA  0      2      3      2      0      3.72e-01   2421
          ----------                                                2338
          ----------                                                2289
          ----------                                                2231
          ----------                                                2158
          ----------                                                2136
          ----------                                                1905
          ----------                                                1804
          ----------                                                1756
          ----------                                                1576
          ----------                                                551
          ----------                                                304
          ----------                                                295
          ----------                                                235

LOCUS: AT4G10050
DESCRIPTION: hydrolase, alpha/beta fold family protein, similar to protein phosphatase methylesterase-1 (Homo
sapiens) GI:5533003; contains Pfam profile PF00561: hydrolase, alpha/beta fold family
```

```
DATA:                  Control 30min   2hours  2days   1week   p-value         pos
SENSE COUNTS:          1       0       0       0       3       2.28e-01
GENES (1 total):
  AT4G10050.1
    SENSE COUNTS:      1       0       0       0       3       2.28e-01
    TAGS: (2 total)
      i+3  GTGTTGAGGT  0       0       0       0       0       6.15e-01        2537
      d+1  GTGTTGAGAT  1       0       0       0       3       7.14e-02        1108
           ----------                                                          1030
           ----------                                                          687
           ----------                                                          576
           ----------                                                          361
           ----------                                                          316

LOCUS: AT3G59330
DESCRIPTION: anthocyanin-related membrane protein family, similar to anthocyanin-related membrane protein 1
(GI:16416383),  anthocyanin-related membrane protein 2 (GI:16416385), anthocyanin-related membrane protein 3
(GI:16416387) (Arabidopsis thaliana)
DATA:                  Control 30min   2hours  2days   1week   p-value         pos
SENSE COUNTS:          1       0       0       0       3       2.28e-01
GENES (1 total):
  AT3G59330.1
    SENSE COUNTS:      1       0       0       0       3       2.28e-01
    TAGS: (1 total)
           ----------                                                          720
           ----------                                                          589
           ----------                                                          567
      d+2  AACTTACAAA  1       0       0       0       3       2.28e-01        277

LOCUS: AT3G01050
DESCRIPTION: expressed protein
DATA:                  Control 30min   2hours  2days   1week   p-value         pos
SENSE COUNTS:          1       0       0       0       3       2.28e-01
GENES (1 total):
  AT3G01050.1
    SENSE COUNTS:      1       0       0       0       3       2.28e-01
    TAGS: (1 total)
           ----------                                                          577
      X+4  TAAGAGAGTA  1       0       0       0       3       2.28e-01        348

LOCUS: AT5G22410
DESCRIPTION: peroxidase, putative, identical to peroxidase ATP14a (Arabidopsis thaliana)
gi|1546690|emb|CAA67335
DATA:                  Control 30min   2hours  2days   1week   p-value         pos
SENSE COUNTS:          1       0       0       0       3       2.28e-01
GENES (1 total):
  AT5G22410.1
    SENSE COUNTS:      1       0       0       0       3       2.28e-01
    TAGS: (1 total)
           ----------                                                          985
      d+2  GTTGTTATGG  1       0       0       0       3       2.28e-01        90

LOCUS: AT4G36240
DESCRIPTION: zinc finger (GATA type) family protein, NTL1 protein, curled-leaved tobacco, PIR2:S46419
DATA:                  Control 30min   2hours  2days   1week   p-value         pos
SENSE COUNTS:          1       0       0       0       3       2.28e-01
GENES (1 total):
  AT4G36240.1
    SENSE COUNTS:      1       0       0       0       3       2.28e-01
    TAGS: (2 total)
      d+1  ATCATTAAAA  1       0       0       0       0       4.28e-01        890
           ----------                                                          877
      d+2  AAGTTAAGCT  0       0       0       0       3       8.36e-02        830
           ----------                                                          766
           ----------                                                          701

LOCUS: AT2G05550
DESCRIPTION: non-LTR retrotransposon family (LINE), has a 6.6e-37 P-value blast match to GB:NP_038605 L1
repeat, Tf subfamily, member 30 (LINE-element) (Mus musculus)
DATA:                  Control 30min   2hours  2days   1week   p-value         pos
SENSE COUNTS:          1       0       0       0       3       2.28e-01
GENES (1 total):
  AT2G05550.1
    SENSE COUNTS:      1       0       0       0       3       2.28e-01
    TAGS: (1 total)
           ----------                                                          3928
           ----------                                                          3848
           ----------                                                          3739
           ----------                                                          3648
           ----------                                                          3191
           ----------                                                          3080
```

```
          ----------                                                  2988
          ----------                                                  2847
          ----------                                                  2814
          ----------                                                  2118
          ----------                                                  1812
          ----------                                                  1525
          ----------                                                  1515
          ----------                                                  1499
          ----------                                                  1385
          ----------                                                  647
          ----------                                                  527
     p+2  TTTATCTATT 1      0      0      0      3      2.28e-01      408

LOCUS: AT1G48170
DESCRIPTION: Expressed protein
DATA:                 Control 30min  2hours 2days  1week  p-value       pos
SENSE COUNTS:         1      0      0      0      3      2.28e-01
GENES (1 total):
  AT1G48170.1
    SENSE COUNTS:     1      0      0      0      3      2.28e-01
    TAGS: (1 total)
          ----------                                                  863
          ----------                                                  766
     d+2  AAATGATCAC 1      0      0      0      3      2.28e-01      593

LOCUS: AT4G38350
DESCRIPTION: patched family protein, similar to SP|O15118 Niemann-Pick C1 protein precursor from Homo sapiens
(PID:g2276463); contains Pfam profile PF02460 Patched family
DATA:                 Control 30min  2hours 2days  1week  p-value       pos
SENSE COUNTS:         1      0      4      1      5      2.29e-01
GENES (1 total):
  AT4G38350.1
    SENSE COUNTS:     1      0      4      1      5      2.29e-01
    TAGS: (3 total)
          ----------                                                  3747
     v+2  TTGGTACACT 0      0      1      0      5      7.15e-03      3700
     v+2  GCCCAAAGTA 1      0      1      1      0      7.18e-01      3683
     v+2  TTAACTCAAC 0      0      2      0      0      3.51e-01      3571
          ----------                                                  3439
          ----------                                                  3303
          ----------                                                  3244
          ----------                                                  2632
          ----------                                                  2619
          ----------                                                  2433
          ----------                                                  2417
          ----------                                                  2275
          ----------                                                  1886
          ----------                                                  1693
          ----------                                                  1290
          ----------                                                  1256
          ----------                                                  1198
          ----------                                                  961
          ----------                                                  926
          ----------                                                  563
          ----------                                                  169

LOCUS: AT1G18180
DESCRIPTION: expressed protein
DATA:                 Control 30min  2hours 2days  1week  p-value       pos
SENSE COUNTS:         0      0      3      0      1      2.30e-01
GENES (1 total):
  AT1G18180.1
    SENSE COUNTS:     0      0      3      0      1      2.30e-01
    TAGS: (1 total)
          ----------                                                  642
          ----------                                                  527
     i+3  TGACTAAAAA 0      0      3      0      1      2.30e-01      338

LOCUS: AT1G18790
DESCRIPTION: RWP-RK domain-containing protein, contains Pfam profile: PF02042 RWP-RK domain
DATA:                 Control 30min  2hours 2days  1week  p-value       pos
SENSE COUNTS:         2      2      6      1      7      2.30e-01
GENES (1 total):
  AT1G18790.1
    SENSE COUNTS:     2      2      6      1      7      2.30e-01
    TAGS: (1 total)
          ----------                                                  1080
     v+2  AGCCTACAAA 2      2      6      1      7      2.30e-01      870
          ----------                                                  768
          ----------                                                  617
          ----------                                                  580
```

```
           ----------                                                     287
           ----------                                                     283

LOCUS: AT4G36670
DESCRIPTION: mannitol transporter, putative, similar to mannitol transporter (Apium graveolens var. dulce)
GI:12004316; contains Pfam profile PF00083: major facilitator superfamily protein
DATA:                 Control 30min   2hours  2days   1week   p-value        pos
SENSE COUNTS:         2       7       5       1       6       2.31e-01
GENES (1 total):
  AT4G36670.1
    SENSE COUNTS:     2       7       5       1       6       2.31e-01
    TAGS: (2 total)
      d+1  ATGAATATAA 0       6       0       0       1       3.93e-03       1594
           ----------                                                     1077
           ----------                                                     877
           ----------                                                     642
      i+3  AGAATTGATA 2       1       5       1       5       3.79e-01       478
           ----------                                                     393
           ----------                                                     111

LOCUS: AT2G33570
DESCRIPTION: expressed protein
DATA:                 Control 30min   2hours  2days   1week   p-value        pos
SENSE COUNTS:         2       1       1       5       0       2.31e-01
GENES (1 total):
  AT2G33570.1
    SENSE COUNTS:     2       1       1       5       0       2.31e-01
    TAGS: (2 total)
      d+1  TATCATTTTT 0       1       0       0       0       2.54e-01       1700
      d+2  ACCATAATAT 2       0       1       5       0       4.63e-02       1629
           ----------                                                     1558
           ----------                                                     1469
           ----------                                                     1160
           ----------                                                     564
           ----------                                                     238
           ----------                                                     142

LOCUS: AT5G12120
DESCRIPTION: ubiquitin-associated (UBA)/TS-N domain-containing protein, contains Pfam profile PF00627: UBA/TS-
N domain
DATA:                 Control 30min   2hours  2days   1week   p-value        pos
SENSE COUNTS:         1       4       1       0       1       2.32e-01
GENES (1 total):
  AT5G12120.1
    SENSE COUNTS:     1       4       1       0       1       2.32e-01
    TAGS: (3 total)
           ----------                                                     2147
      d+2  AATGGAAGGA 0       4       0       0       0       9.72e-03       1815
      d+2  AATGCATCCT 0       0       1       0       1       3.96e-01       1374
           ----------                                                     1131
      d+2  AGCGGGCAAC 1       0       0       0       0       4.28e-01       619
           ----------                                                     586
           ----------                                                     564
           ----------                                                     541
           ----------                                                     201
           ----------                                                     159

LOCUS: AT1G60060
DESCRIPTION: expressed protein
DATA:                 Control 30min   2hours  2days   1week   p-value        pos
SENSE COUNTS:         0       0       1       0       3       2.32e-01
GENES (1 total):
  AT1G60060.1
    SENSE COUNTS:     0       0       1       0       3       2.32e-01
    TAGS: (1 total)
           ----------                                                     593
           ----------                                                     451
           ----------                                                     172
      X+4  CGAAAGAAAA 0       0       1       0       3       2.32e-01       -216

LOCUS: AT5G65390
DESCRIPTION: arabinogalactan-protein (AGP7)
DATA:                 Control 30min   2hours  2days   1week   p-value        pos
SENSE COUNTS:         0       0       1       0       3       2.32e-01
GENES (1 total):
  AT5G65390.1
    SENSE COUNTS:     0       0       1       0       3       2.32e-01
    TAGS: (1 total)
           ----------                                                     677
           ----------                                                     599
      d+2  CCTCGCTCAA 0       0       1       0       3       2.32e-01       120
```

LOCUS: AT3G22670
DESCRIPTION: pentatricopeptide (PPR) repeat-containing protein, contains Pfam profile PF01535: PPR repeat

DATA:		Control	30min	2hours	2days	1week	p-value	pos
SENSE COUNTS:		0	0	1	0	3	2.32e-01	
GENES (1 total):								
AT3G22670.1								
SENSE COUNTS:		0	0	1	0	3	2.32e-01	
TAGS: (2 total)								
	----------							2483
v+2	TCTTAGATCC	0	0	1	0	0	4.55e-01	2174
v+2	TGACCTTTAC	0	0	0	0	3	8.36e-02	2127
	----------							1782
	----------							1691
	----------							1608
	----------							1563
	----------							1129
	----------							1123
	----------							700
	----------							27

LOCUS: AT1G78570
DESCRIPTION: NAD-dependent epimerase/dehydratase family protein, similar to dTDP-glucose 4,6-dehydratase from Aneurinibacillus thermoaerophilus GI:16357461, RmlB from Leptospira borgpetersenii GI:4234803; contains Pfam profile PF01370 NAD dependent epimerase/dehydrata

DATA:		Control	30min	2hours	2days	1week	p-value	pos
SENSE COUNTS:		3	0	3	6	1	2.33e-01	
GENES (1 total):								
AT1G78570.1								
SENSE COUNTS:		3	0	3	6	1	2.33e-01	
TAGS: (3 total)								
	----------							2619
	----------							2447
d+2	ACTGTGTTGG	0	0	1	6	1	1.67e-02	1883
d+2	GTCGAGGAGC	0	0	2	0	0	1.21e-01	1742
	----------							1482
	----------							989
d+2	TTTACAATAT	3	0	0	0	0	2.13e-02	915
	----------							825
	----------							621
	----------							564
	----------							513
	----------							431
	----------							240
	----------							179

LOCUS: AT2G04760
DESCRIPTION: non-LTR retrotransposon family (LINE), has a 1.8e-38 P-value blast match to GB:NP_038605 L1 repeat, Tf subfamily, member 30 (LINE-element) (Mus musculus)

DATA:		Control	30min	2hours	2days	1week	p-value	pos
SENSE COUNTS:		4	0	2	4	1	2.34e-01	
GENES (1 total):								
AT2G04760.1								
SENSE COUNTS:		4	0	2	4	1	2.34e-01	
TAGS: (2 total)								
	----------							2610
	----------							2168
p+2	GGGATTCCGT	3	0	1	4	1	2.41e-01	1870
	----------							1517
	----------							1111
	----------							963
	----------							945
	----------							720
	----------							533
	----------							333
	----------							277
	----------							217
X+4	AAACAAAAAT	1	0	1	0	0	6.01e-01	3

LOCUS: AT4G14880
DESCRIPTION: cysteine synthase / O-acetylserine (thiol)-lyase / O-acetylserine sulfhydrylase (OAS1), nearly identical to SP|P47998 Cysteine synthase (EC 4.2.99.8) (O-acetylserine sulfhydrylase) (O-acetylserine (Thiol)-lyase) {Arabidopsis thaliana}; identical to cDNA O

DATA:		Control	30min	2hours	2days	1week	p-value	pos
SENSE COUNTS:		6	14	14	9	5	2.34e-01	
GENES (4 total):								
AT4G14880.2								
SENSE COUNTS:		6	14	14	9	5	2.34e-01	
TAGS: (3 total)								
d+1	ATCAAAACTA	1	0	1	0	0	6.01e-01	1296
d+2	ACCTTCGAGG	4	14	12	9	5	1.44e-01	1051

```
      d+2  GCAAGGCAGC 1      0      1      0      0      6.01e-01      859
           ----------                                                529
           ----------                                                472
           ----------                                                256
           ----------                                                100
  AT4G14880.1
    SENSE COUNTS:     6      14     14     9      5      2.34e-01
    TAGS: (3 total)
      d+1  ATCAAAACTA 1      0      1      0      0      6.01e-01      1351
      d+2  ACCTTCGAGG 4      14     12     9      5      1.44e-01      1106
      d+2  GCAAGGCAGC 1      0      1      0      0      6.01e-01      914
           ----------                                                584
           ----------                                                527
           ----------                                                311
           ----------                                                155
           ----------                                                23

LOCUS: AT3G48190
DESCRIPTION: ataxia-telangiectasia mutated protein (Atm), identical to ataxia-telangiectasia mutated protein
(Atm) (Arabidopsis thaliana) GI:7529272; contains Pfam profile PF00855: PWWP domain; contains GA donor splice
site at exon 73
DATA:                 Control 30min  2hours 2days  1week  p-value       pos
SENSE COUNTS:         0      0      5      1      2      2.34e-01
GENES (2 total):
  AT3G48190.1
    SENSE COUNTS:     0      0      5      1      2      2.34e-01
    TAGS: (6 total)
      i+3  GTTTTCTTGA 0      0      0      0      0      6.15e-01      26010
      i+3  TCTAAATAAG 0      0      0      0      1      1.65e-01      22516
      i+3  ATTCCAGCAA 0      0      0      0      0      6.15e-01      15541
      i+3  AAGCAAAAAT 0      0      5      1      0      2.69e-02      12787
           ----------                                                11897
      d+2  GCCAGGCACA 0      0      0      0      0      6.15e-01      11603
           ----------                                                11488
           ----------                                                11411
           ----------                                                11170
           ----------                                                11165
           ----------                                                11037
           ----------                                                10393
           ----------                                                10098
           ----------                                                10056
           ----------                                                9504
           ----------                                                9489
           ----------                                                9402
           ----------                                                9384
           ----------                                                9329
           ----------                                                9257
           ----------                                                9169
           ----------                                                9144
           ----------                                                8922
           ----------                                                8901
           ----------                                                8836
           ----------                                                8745
           ----------                                                8535
           ----------                                                8430
           ----------                                                8412
           ----------                                                8342
           ----------                                                8019
           ----------                                                7731
           ----------                                                7705
           ----------                                                7494
           ----------                                                7490
           ----------                                                6892
      d+2  CCATCATCGG 0      0      0      0      1      1.65e-01      6634
           ----------                                                6596
           ----------                                                6433
           ----------                                                6186
           ----------                                                5733
           ----------                                                5409
           ----------                                                5351
           ----------                                                5299
           ----------                                                5215
           ----------                                                5185
           ----------                                                5168
           ----------                                                4987
           ----------                                                4743
           ----------                                                4714
           ----------                                                4659
           ----------                                                4562
           ----------                                                4557
           ----------                                                4168
```

```
           ----------                                                4022
           ----------                                                3619
           ----------                                                3603
           ----------                                                3526
           ----------                                                3375
           ----------                                                3056
           ----------                                                2928
           ----------                                                2922
           ----------                                                2647
           ----------                                                2463
           ----------                                                2040
           ----------                                                1274
           ----------                                                1239

LOCUS: AT3G57680
DESCRIPTION: peptidase S41 family protein, similar to PSII D1 protein processing enzyme (GI::7268527)
(Arabidopsis thaliana); similar to SP|Q55669 Carboxyl-terminal processing protease precursor (Photosystem II
D1 protein processing peptidase) (EC 3.4.21.102) (strain
DATA:                  Control 30min   2hours  2days   1week   p-value         pos
SENSE COUNTS:          1       3       0       1       0       2.35e-01
GENES (1 total):
  AT3G57680.1
    SENSE COUNTS:      1       3       0       1       0       2.35e-01
    TAGS: (1 total)
           ----------                                                2277
      v+2  TAACAAGAAA  1       3       0       1       0       2.35e-01        2229
           ----------                                                2223
           ----------                                                2181
           ----------                                                1973
           ----------                                                1844
           ----------                                                1525
           ----------                                                1500
           ----------                                                1033
           ----------                                                541
           ----------                                                379
           ----------                                                78

LOCUS: AT3G63500
DESCRIPTION: expressed protein
DATA:                  Control 30min   2hours  2days   1week   p-value         pos
SENSE COUNTS:          4       7       2       1       4       2.35e-01
GENES (2 total):
  AT3G63500.1
    SENSE COUNTS:      4       7       2       1       4       2.35e-01
    TAGS: (4 total)
      d+1  TACAGCCCCT  1       0       0       0       0       4.28e-01        3197
      d+2  TAATGTACAC  3       5       0       0       3       1.03e-01        3101
      d+2  AGAGGGGAAG  0       0       1       0       0       4.55e-01        2581
           ----------                                                2100
           ----------                                                2004
           ----------                                                1998
           ----------                                                1812
           ----------                                                1750
           ----------                                                1681
           ----------                                                1565
           ----------                                                931
           ----------                                                649
      d+2  TTTCTCAAAA  0       2       1       1       1       6.46e-01        499
           ----------                                                495
           ----------                                                481
           ----------                                                435
           ----------                                                397
           ----------                                                81
  AT3G63500.2
    SENSE COUNTS:      4       7       2       1       4       2.35e-01
    TAGS: (4 total)
      d+1  TACAGCCCCT  1       0       0       0       0       4.28e-01        3696
      d+2  TAATGTACAC  3       5       0       0       3       1.03e-01        3600
      d+2  AGAGGGGAAG  0       0       1       0       0       4.55e-01        3080
           ----------                                                2599
           ----------                                                2503
           ----------                                                2497
           ----------                                                2311
           ----------                                                2249
           ----------                                                2180
           ----------                                                2064
           ----------                                                1430
           ----------                                                1148
      d+2  TTTCTCAAAA  0       2       1       1       1       6.46e-01        998
           ----------                                                994
           ----------                                                980
```

```
           ----------                                                      934
           ----------                                                      896
           ----------                                                      770
           ----------                                                      597
           ----------                                                      57

LOCUS: AT1G20693
DESCRIPTION: high mobility group protein beta1 (HMGbeta1) / HMG protein beta1, nearly identical to HMG protein
(HMGbeta1) (Arabidopsis thaliana) GI:2832359
DATA:                 Control 30min   2hours  2days   1week   p-value         pos
SENSE COUNTS:         1       6       3       8       4       2.35e-01
GENES (2 total):
  AT1G20693.1
    SENSE COUNTS:     1       6       3       8       4       2.35e-01
    TAGS: (3 total)
      i+3  ATACGGTTGT 0       0       0       0       0       6.15e-01        1498
      X+4  CTCACAGACT 0       0       2       0       1       2.22e-01        377
      d+1  GAAGATTTCC 1       6       1       8       3       8.57e-02        334

LOCUS: AT2G06290
DESCRIPTION: non-LTR retrotransposon family (LINE), has a 1.2e-30 P-value blast match to GB:AAA39398 ORF2 (Mus
musculus) (LINE-element)
DATA:                 Control 30min   2hours  2days   1week   p-value         pos
SENSE COUNTS:         0       3       2       5       1       2.36e-01
GENES (3 total):
  AT2G06290.1
    SENSE COUNTS:     0       3       2       5       1       2.36e-01
    TAGS: (3 total)
           ----------                                                      8662
           ----------                                                      8529
           ----------                                                      8512
           ----------                                                      8437
      p+2  GCTGGAGAAG 0       0       1       0       0       4.55e-01        7492
           ----------                                                      7172
           ----------                                                      6435
           ----------                                                      6212
           ----------                                                      6096
           ----------                                                      6075
           ----------                                                      5792
           ----------                                                      5617
           ----------                                                      5337
           ----------                                                      4923
           ----------                                                      4700
           ----------                                                      4584
           ----------                                                      4563
           ----------                                                      4280
           ----------                                                      4105
           ----------                                                      3825
           ----------                                                      3411
           ----------                                                      3188
           ----------                                                      3073
           ----------                                                      3052
           ----------                                                      2782
           ----------                                                      2607
           ----------                                                      2327
      p+2  GTTTTTGTCT 0       3       1       5       1       1.74e-01        2154
           ----------                                                      1985
           ----------                                                      1952
           ----------                                                      1864
           ----------                                                      1257
           ----------                                                      843
      X+4  AGCCTTCGCT 0       0       0       0       0       6.15e-01        -164

LOCUS: AT1G76680
DESCRIPTION: 12-oxophytodienoate reductase (OPR1), identical to 12-oxophytodienoate reductase OPR1 GB:AAC78440
(Arabidopsis thaliana)
DATA:                 Control 30min   2hours  2days   1week   p-value         pos
SENSE COUNTS:         2       4       5       1       0       2.36e-01
GENES (3 total):
  AT1G76680.2
    SENSE COUNTS:     2       4       5       1       0       2.36e-01
    TAGS: (2 total)
           ----------                                                      1460
      d+2  CTTGTCCTCA 2       4       4       1       0       4.14e-01        996
      d+2  TGATTGAAGC 0       0       1       0       0       7.06e-01        957
           ----------                                                      875
           ----------                                                      728
           ----------                                                      696
           ----------                                                      399
           ----------                                                      363
           ----------                                                      337
```

```
          ----------                                                     327
  AT1G76680.1
    SENSE COUNTS:     2      4      5      1      0      2.36e-01
    TAGS: (2 total)
          ----------                                                     1451
      d+2  CTTGTCCTCA 2      4      4      1      0      4.14e-01       987
      d+2  TGATTGAAGC 0      0      1      0      0      7.06e-01       948
          ----------                                                     866
          ----------                                                     719
          ----------                                                     687
          ----------                                                     465
          ----------                                                     429
          ----------                                                     403
          ----------                                                     393

LOCUS: AT2G28900
DESCRIPTION: mitochondrial import inner membrane translocase subunit Tim17/Tim22/Tim23 family protein,
contains Pfam PF02466: Mitochondrial import inner membrane translocase subunit Tim17
DATA:                 Control 30min  2hours  2days  1week  p-value        pos
SENSE COUNTS:         1      5      3      4      8      2.36e-01
GENES (1 total):
  AT2G28900.1
    SENSE COUNTS:     1      5      3      4      8      2.36e-01
    TAGS: (2 total)
      i+3  TGTAATCTTT 0      0      0      0      0      6.15e-01       704
      d+1  CGCTTAAGAA 1      5      3      4      8      2.63e-01       316
          ----------                                                     183

LOCUS: AT5G59370
DESCRIPTION: Encodes one of eight Arabidopsis actins.  ACT4 belongs to the reproductive actin subclass which
is predominantly expressed in developing and reproductive tissues, such as pollen, pollen tubes, ovules, and
developing seeds.  Expression of the ACT4/GUS fusi
DATA:                 Control 30min  2hours  2days  1week  p-value        pos
SENSE COUNTS:         5      1      1      2      0      2.37e-01
GENES (2 total):
  AT5G59370.1
    SENSE COUNTS:     5      1      1      2      0      2.37e-01
    TAGS: (2 total)
          ----------                                                     931
      d+2  AAACCACATA 5      1      1      2      0      1.74e-01       908
          ----------                                                     718
          ----------                                                     652
          ----------                                                     451
          ----------                                                     328
          ----------                                                     302
      X+4  ACGACCAAAA 0      0      0      0      0      6.15e-01       -168

LOCUS: AT1G64200
DESCRIPTION: vacuolar ATP synthase subunit E, putative / V-ATPase E subunit, putative / vacuolar proton pump E
subunit, putative, similar to SP|Q39258 Vacuolar ATP synthase subunit E (EC 3.6.3.14) (V-ATPase E subunit)
(Vacuolar proton pump E subunit) {Arabidopsis thal
DATA:                 Control 30min  2hours  2days  1week  p-value        pos
SENSE COUNTS:         0      3      1      1      0      2.38e-01
GENES (1 total):
  AT1G64200.1
    SENSE COUNTS:     0      3      1      1      0      2.38e-01
    TAGS: (1 total)
          ----------                                                     1327
      v+2  CTCTTTCCTG 0      3      1      1      0      2.38e-01       916
          ----------                                                     850
          ----------                                                     670
          ----------                                                     59

LOCUS: AT2G36690
DESCRIPTION: oxidoreductase, 2OG-Fe(II) oxygenase family protein, similar to IDS3 (Hordeum
vulgare)(GI:4514655), leucoanthocyanidin dioxygenase (SP|P51091)(Malus domestica); contains PF03171 2OG-Fe(II)
oxygenase superfamily domain
DATA:                 Control 30min  2hours  2days  1week  p-value        pos
SENSE COUNTS:         0      3      1      1      0      2.38e-01
GENES (1 total):
  AT2G36690.1
    SENSE COUNTS:     0      3      1      1      0      2.38e-01
    TAGS: (1 total)
          ----------                                                     1547
      v+2  TATGATAAAT 0      3      1      1      0      2.38e-01       1447
          ----------                                                     738
          ----------                                                     726
          ----------                                                     640
          ----------                                                     580

LOCUS: AT2G25100
```

```
DESCRIPTION: ribonuclease HII family protein, contains Pfam profile: PF01351 ribonuclease HII
DATA:                 Control 30min  2hours  2days  1week  p-value        pos
SENSE COUNTS:         1       0      1       4      1      2.38e-01
GENES (1 total):
  AT2G25100.1
    SENSE COUNTS:     1       0      1       4      1      2.38e-01
    TAGS: (2 total)
      d+1  CGAGAAAAGA 1       0      1       0      0      6.01e-01       857
           ----------                                                     773
      d+2  GGAGTTCTTC 0       0      0       4      1      2.76e-02       408

LOCUS: AT3G11200
DESCRIPTION: PHD finger family protein, contains Pfam domain, PF00628: PHD-finger
DATA:                 Control 30min  2hours  2days  1week  p-value        pos
SENSE COUNTS:         2       3      0       0      3      2.39e-01
GENES (2 total):
  AT3G11200.1
    SENSE COUNTS:     2       3      0       0      3      2.39e-01
    TAGS: (2 total)
      d+1  GGGTTTGTAT 1       0      0       0      0      4.28e-01       1184
      d+2  TTTTAATCAA 1       3      0       0      3      1.74e-01       971
           ----------                                                     823
           ----------                                                     736
           ----------                                                     597
           ----------                                                     347
           ----------                                                     78
  AT3G11200.2
    SENSE COUNTS:     2       3      0       0      3      2.39e-01
    TAGS: (2 total)
      d+1  GGGTTTGTAT 1       0      0       0      0      4.28e-01       1332
      d+2  TTTTAATCAA 1       3      0       0      3      1.74e-01       1119
           ----------                                                     971
           ----------                                                     884
           ----------                                                     745
           ----------                                                     495
           ----------                                                     42

LOCUS: AT3G56800
DESCRIPTION: calmodulin-2/3/5 (CAM3), identical to calmodulin GI:474183 from (Arabidopsis thaliana); almost
identical to calmodulin-2/3/5 SP:P25069 (Arabidopsis thaliana)
DATA:                 Control 30min  2hours  2days  1week  p-value        pos
SENSE COUNTS:         19      12     23      25     16     2.39e-01
GENES (2 total):
  AT3G56800.1
    SENSE COUNTS:     19      12     23      25     16     2.39e-01
    TAGS: (3 total)
      d+1  GTTTTGTGAA 3       2      4       6      0      3.17e-01       880
      d+2  GGACTCGAGG 16      10     18      19     16     5.30e-01       697
           ----------                                                     593
      d+2  TGATGACAAA 0       0      1       0      0      4.55e-01       480
           ----------                                                     311

LOCUS: AT4G02390
DESCRIPTION: Encodes a DNA dependent nuclear poly (ADP-ribose) polymerase (E.C.2.4.2.30), thought to be
involved in post-translational modification .
DATA:                 Control 30min  2hours  2days  1week  p-value        pos
SENSE COUNTS:         3       1      1       0      5      2.40e-01
GENES (2 total):
  AT4G02390.1
    SENSE COUNTS:     3       1      1       0      5      2.40e-01
    TAGS: (2 total)
      d+1  CTCCAAGGGG 1       0      0       0      0      4.28e-01       1962
           ----------                                                     1807
           ----------                                                     1690
           ----------                                                     1487
           ----------                                                     1474
      d+2  ATTTTGGTTT 2       1      1       0      5      2.60e-01       1244
           ----------                                                     1063
           ----------                                                     918
           ----------                                                     816
           ----------                                                     748
           ----------                                                     629

LOCUS: AT3G66654
DESCRIPTION: peptidyl-prolyl cis-trans isomerase cyclophilin-type family protein, contains Pfam domain,
PF00160: peptidyl-prolyl cis-trans isomerase, cyclophilin-type
DATA:                 Control 30min  2hours  2days  1week  p-value        pos
SENSE COUNTS:         3       1      1       0      5      2.40e-01
GENES (3 total):
  AT3G66654.2
    SENSE COUNTS:     3       1      1       0      5      2.40e-01
```

```
    TAGS: (1 total)
          ----------                                               1140
          ----------                                               925
          ----------                                               914
          ----------                                               605
          ----------                                               321
      X+4 AACTGATTTT 3      1      1      0      5      2.40e-01   294
          ----------                                               186

LOCUS: AT4G28080
DESCRIPTION: expressed protein
DATA:                Control 30min  2hours 2days  1week  p-value    pos
SENSE COUNTS:        4      3      9      7      1      2.41e-01
GENES (1 total):
  AT4G28080.1
    SENSE COUNTS:    4      3      9      7      1      2.41e-01
    TAGS: (6 total)
      d+1 GCCACCAAAA 1      0      4      6      1      3.95e-02   5736
          ----------                                               5546
      d+2 AGACTTGTGA 1      0      3      0      0      1.04e-01   5329
      i+3 GAAGCTAAAT 0      0      0      0      0      6.15e-01   5241
      d+2 TCCGTTACAC 1      0      0      1      0      5.06e-01   5130
          ----------                                               4984
          ----------                                               4801
          ----------                                               4637
          ----------                                               4451
          ----------                                               4240
          ----------                                               3789
      d+2 AGCAGACCAC 0      3      0      0      0      3.05e-02   3211
          ----------                                               3157
          ----------                                               3091
          ----------                                               3070
          ----------                                               2993
          ----------                                               2624
          ----------                                               2467
          ----------                                               2351
          ----------                                               2260
          ----------                                               2239
          ----------                                               1279
          ----------                                               1108
          ----------                                               860
          ----------                                               444
          ----------                                               398
      i+3 GCTAAAAAAA 1      0      2      0      0      3.07e-01   127
          ----------                                               56
          ----------                                               46

LOCUS: AT5G41260
DESCRIPTION: protein kinase family protein, contains protein kinase domain, Pfam:PF00069
DATA:                Control 30min  2hours 2days  1week  p-value    pos
SENSE COUNTS:        11     4      13     11     3      2.42e-01
GENES (1 total):
  AT5G41260.1
    SENSE COUNTS:    11     4      13     11     3      2.42e-01
    TAGS: (4 total)
      d+1 CAAGACAGAG 0      1      0      0      0      2.54e-01   1347
      d+2 AGATCATTGA 1      0      0      0      0      4.28e-01   1143
      d+2 CCTAAGATCA 10     3      12     11     3      1.07e-01   1114
          ----------                                               852
          ----------                                               682
      d+2 ACCTAAATGC 0      0      1      0      0      7.06e-01   627
          ----------                                               282

LOCUS: AT4G16240
DESCRIPTION: hypothetical protein
DATA:                Control 30min  2hours 2days  1week  p-value    pos
SENSE COUNTS:        14     7      7      6      4      2.42e-01
GENES (1 total):
  AT4G16240.1
    SENSE COUNTS:    14     7      7      6      4      2.42e-01
    TAGS: (3 total)
          ----------                                               783
      v+2 ACTTCATTTC 1      0      0      0      0      4.28e-01   605
          ----------                                               466
          ----------                                               412
      v+2 ATTTATTTAT 11     7      7      6      3      5.49e-01   104
      v+2 ACATTGTAAA 2      0      0      0      1      2.03e-01   78

LOCUS: AT5G15680
DESCRIPTION: expressed protein,
DATA:                Control 30min  2hours 2days  1week  p-value    pos
```

```
SENSE COUNTS:          4      3      2      0      0      2.42e-01
GENES (1 total):
  AT5G15680.1
    SENSE COUNTS:      4      3      2      0      0      2.42e-01
    TAGS: (1 total)
           ----------                                                    7207
      v+2  TAAGAAGGAA  4      3      2      0      0      2.42e-01       6958
           ----------                                                    6804
           ----------                                                    6408
           ----------                                                    6384
           ----------                                                    6236
           ----------                                                    6119
           ----------                                                    6078
           ----------                                                    5981
           ----------                                                    5695
           ----------                                                    5673
           ----------                                                    5619
           ----------                                                    5607
           ----------                                                    5510
           ----------                                                    5456
           ----------                                                    5280
           ----------                                                    5008
           ----------                                                    4864
           ----------                                                    4229
           ----------                                                    4148
           ----------                                                    3804
           ----------                                                    3150
           ----------                                                    3133
           ----------                                                    3004
           ----------                                                    2922
           ----------                                                    2888
           ----------                                                    2848
           ----------                                                    2796
           ----------                                                    2422
           ----------                                                    2400
           ----------                                                    2368
           ----------                                                    2346
           ----------                                                    2091
           ----------                                                    2004
           ----------                                                    1912
           ----------                                                    1684
           ----------                                                    1560
           ----------                                                    1341
           ----------                                                    1296
           ----------                                                    1051
           ----------                                                    864

LOCUS: AT5G40440
DESCRIPTION: mitogen-activated protein kinase kinase (MAPKK), putative (MKK3), similar to NPK2 (Nicotiana
tabacum) gi|862342|dbj|BAA06731; mitogen-activated protein kinase kinase (MAPKK) family, PMID:12119167
DATA:                  Control 30min  2hours 2days  1week  p-value        pos
SENSE COUNTS:          3      3      7      1      1      2.42e-01
GENES (2 total):
  AT5G40440.1
    SENSE COUNTS:      3      3      7      1      1      2.42e-01
    TAGS: (1 total)
           ----------                                                    1691
           ----------                                                    1415
           ----------                                                    862
           ----------                                                    710
      X+4  TGAAAAACCC  3      3      7      1      1      2.42e-01       629
           ----------                                                    530
           ----------                                                    311
           ----------                                                    118

LOCUS: AT1G10910
DESCRIPTION: pentatricopeptide (PPR) repeat-containing protein, contains Pfam profile PF01535: PPR repeat
DATA:                  Control 30min  2hours 2days  1week  p-value        pos
SENSE COUNTS:          0      2      0      1      3      2.42e-01
GENES (1 total):
  AT1G10910.1
    SENSE COUNTS:      0      2      0      1      3      2.42e-01
    TAGS: (1 total)
           ----------                                                    2529
      v+2  AAATAGATGA  0      2      0      1      3      2.42e-01       2387
           ----------                                                    2254
           ----------                                                    2044
           ----------                                                    1876
           ----------                                                    1770
           ----------                                                    1500
           ----------                                                    733
```

```
          ----------                                                     296

LOCUS: AT1G21380
DESCRIPTION: VHS domain-containing protein / GAT domain-containing protein, weak similarity to Hrs (Rattus
norvegicus) GI:8547026; contains Pfam profiles PF00790: VHS domain, PF03127: GAT domain
DATA:                Control 30min  2hours 2days  1week  p-value       pos
SENSE COUNTS:        0       2      6      5      2      2.43e-01
GENES (1 total):
  AT1G21380.1
    SENSE COUNTS:    0       2      6      5      2      2.43e-01
    TAGS: (3 total)
          ----------                                                     2125
      d+2 AAAGCAGAAT 0       2      0      0      1      1.90e-01      1977
      d+2 GATTTTTTTA 0       0      6      5      1      3.13e-02      1853
          ----------                                                     1453
          ----------                                                     1398
          ----------                                                     1341
          ----------                                                     1239
          ----------                                                     1101
      d+2 ATGATAAGGC 0       0      0      0      0      6.15e-01      1029
          ----------                                                     845
          ----------                                                     797
          ----------                                                     574
          ----------                                                     341

LOCUS: AT1G60950
DESCRIPTION: ferredoxin, chloroplast (PETF), identical to FERREDOXIN PRECURSOR GB:P16972 (SP|P16972) from
(Arabidopsis thaliana)
DATA:                Control 30min  2hours 2days  1week  p-value       pos
SENSE COUNTS:        2       0      3      0      1      2.44e-01
GENES (2 total):
  AT1G60950.1
    SENSE COUNTS:    2       0      3      0      1      2.44e-01
    TAGS: (1 total)
          ----------                                                     534
      d+2 GCTACATACA 2       0      3      0      1      2.44e-01      202

LOCUS: AT5G57660
DESCRIPTION: zinc finger (B-box type) family protein, contains Pfam domain, PF00643: B-box zinc finger
DATA:                Control 30min  2hours 2days  1week  p-value       pos
SENSE COUNTS:        2       0      3      0      1      2.44e-01
GENES (1 total):
  AT5G57660.1
    SENSE COUNTS:    2       0      3      0      1      2.44e-01
    TAGS: (2 total)
          ----------                                                     1128
      d+2 AAAGGTTCTT 0       0      0      0      1      1.65e-01      614
          ----------                                                     244
      X+4 TTTACACGTG 2       0      3      0      0      1.23e-01      -69

LOCUS: AT3G61230
DESCRIPTION: LIM domain-containing protein, similar to SP|P29675 Pollen specific protein SF3 {Helianthus
annuus}; contains Pfam profile PF00412: LIM domain
DATA:                Control 30min  2hours 2days  1week  p-value       pos
SENSE COUNTS:        0       1      4      4      5      2.49e-01
GENES (1 total):
  AT3G61230.1
    SENSE COUNTS:    0       1      4      4      5      2.49e-01
    TAGS: (1 total)
          ----------                                                     729
          ----------                                                     609
      d+2 GATGGAGTTT 0       1      4      4      5      2.49e-01      254
          ----------                                                     199

LOCUS: AT3G14595
DESCRIPTION: Expressed protein
DATA:                Control 30min  2hours 2days  1week  p-value       pos
SENSE COUNTS:        2       8      5      5      1      2.49e-01
GENES (1 total):
  AT3G14595.1
    SENSE COUNTS:    2       8      5      5      1      2.49e-01
    TAGS: (2 total)
      i+3 AAGAAATGAA 0       0      1      0      0      4.55e-01      531
      d+1 AAGGTGAAGA 2       8      4      5      1      2.22e-01      486

LOCUS: AT4G34120
DESCRIPTION: CBS domain-containing protein, contains Pfam profile PF00571: CBS domain
DATA:                Control 30min  2hours 2days  1week  p-value       pos
SENSE COUNTS:        1       3      0      2      0      2.50e-01
GENES (1 total):
  AT4G34120.1
```

```
    SENSE COUNTS:      1       3       0       2       0       2.50e-01
    TAGS: (1 total)
           ----------                                                  1474
      d+2  ACTCCGAGAC  1       3       0       2       0       2.50e-01       354

LOCUS: AT3G10720
DESCRIPTION: pectinesterase, putative, contains similarity to pectinesterase from Vitis vinifera GI:15081598,
Prunus persica SP|Q43062; contains Pfam profile PF01095 pectinesterase
DATA:                  Control 30min   2hours  2days   1week   p-value        pos
SENSE COUNTS:          2       4       6       2       0       2.50e-01
GENES (2 total):
  AT3G10720.1
    SENSE COUNTS:      2       4       6       2       0       2.50e-01
    TAGS: (2 total)
      d+1  TAAATCTAGA  0       2       1       0       0       2.47e-01       1218
      d+2  GTTGCCTCAA  2       2       5       2       0       4.07e-01       1041
           ----------                                                  1030
           ----------                                                  1003
           ----------                                                  832
           ----------                                                  784
           ----------                                                  689
           ----------                                                  415
           ----------                                                  51
  AT3G10720.2
    SENSE COUNTS:      2       4       6       2       0       2.50e-01
    TAGS: (2 total)
      d+1  TAAATCTAGA  0       2       1       0       0       2.47e-01       2019
      d+2  GTTGCCTCAA  2       2       5       2       0       4.07e-01       1842
           ----------                                                  1831
           ----------                                                  1804
           ----------                                                  1633
           ----------                                                  1585
           ----------                                                  1490
           ----------                                                  1216
           ----------                                                  852
           ----------                                                  587
           ----------                                                  99

LOCUS: AT1G42540
DESCRIPTION: glutamate receptor family protein (GLR3.3), plant glutamate receptor family, PMID:11379626
DATA:                  Control 30min   2hours  2days   1week   p-value        pos
SENSE COUNTS:          1       3       1       0       0       2.52e-01
GENES (2 total):
  AT1G42540.1
    SENSE COUNTS:      1       3       1       0       0       2.52e-01
    TAGS: (1 total)
           ----------                                                  3583
      v+2  GATGAGAAAG  1       3       1       0       0       2.52e-01       3018
           ----------                                                  2982
           ----------                                                  2968
           ----------                                                  2786
           ----------                                                  2253
           ----------                                                  1818
           ----------                                                  1681
           ----------                                                  1033
           ----------                                                  1002
           ----------                                                  987
           ----------                                                  981
           ----------                                                  600
           ----------                                                  208
           ----------                                                  85

LOCUS: AT1G60160
DESCRIPTION: potassium transporter family protein, similar to potassium transporter HAK2p (Mesembryanthemum
crystallinum) gi|14091471|gb|AAK53759; KUP/HAK/KT Transporter family member, PMID:11500563; contains Pfam
profile PF02705: K+ potassium transporter
DATA:                  Control 30min   2hours  2days   1week   p-value        pos
SENSE COUNTS:          3       3       1       0       0       2.52e-01
GENES (1 total):
  AT1G60160.1
    SENSE COUNTS:      3       3       1       0       0       2.52e-01
    TAGS: (1 total)
           ----------                                                  3132
      v+2  AATTAGGGTT  3       3       1       0       0       2.52e-01       2892
           ----------                                                  2680
           ----------                                                  2508
           ----------                                                  2079
           ----------                                                  1752
           ----------                                                  1653
           ----------                                                  1637
           ----------                                                  1617
```

```
           ----------                                                    1485
           ----------                                                    1466
           ----------                                                    1035
           ----------                                                    580
           ----------                                                    189

LOCUS: AT4G28040
DESCRIPTION: nodulin MtN21 family protein, similar to MtN21 GI:2598575 (root nodule development) from
(Medicago truncatula)
DATA:                 Control 30min   2hours  2days   1week   p-value        pos
SENSE COUNTS:         0       4       5       2       1       2.52e-01
GENES (2 total):
  AT4G28040.1
    SENSE COUNTS:     0       4       5       2       1       2.52e-01
    TAGS: (2 total)
           ----------                                                    1612
      d+2  GTGTAAGTGG 0       4       4       2       1       3.54e-01       1166
      d+2  TGAGTTTTAT 0       0       1       0       0       4.55e-01       1138
           ----------                                                    687
           ----------                                                    505
           ----------                                                    379
           ----------                                                    367
           ----------                                                    154
  AT4G28040.2
    SENSE COUNTS:     0       4       5       2       1       2.52e-01
    TAGS: (2 total)
           ----------                                                    1690
      d+2  GTGTAAGTGG 0       4       4       2       1       3.54e-01       1244
      d+2  TGAGTTTTAT 0       0       1       0       0       4.55e-01       1216
           ----------                                                    765
           ----------                                                    583
           ----------                                                    457
           ----------                                                    445
           ----------                                                    232

LOCUS: AT5G35650
DESCRIPTION: hypothetical protein
DATA:                 Control 30min   2hours  2days   1week   p-value        pos
SENSE COUNTS:         3       0       0       4       1       2.52e-01
GENES (1 total):
  AT5G35650.1
    SENSE COUNTS:     3       0       0       4       1       2.52e-01
    TAGS: (1 total)
           ----------                                                    963
      v+2  TTCTTTGAAA 3       0       0       4       1       2.52e-01       915
           ----------                                                    906
           ----------                                                    760
           ----------                                                    501
           ----------                                                    354
           ----------                                                    124

LOCUS: AT4G28810
DESCRIPTION: expressed protein, PMID: 12679534, putative bHLH127 transcription factor
DATA:                 Control 30min   2hours  2days   1week   p-value        pos
SENSE COUNTS:         1       0       0       4       1       2.52e-01
GENES (1 total):
  AT4G28810.1
    SENSE COUNTS:     1       0       0       4       1       2.52e-01
    TAGS: (2 total)
           ----------                                                    3453
           ----------                                                    3447
           ----------                                                    3438
           ----------                                                    3346
           ----------                                                    3300
           ----------                                                    2784
      v+2  GTTTTAAACT 0       0       0       0       1       1.65e-01       2383
           ----------                                                    2312
      i+3  TACTCAAAAA 1       0       0       4       0       1.29e-01       1938
           ----------                                                    1927
           ----------                                                    1773
           ----------                                                    1767
           ----------                                                    1758
           ----------                                                    1735
           ----------                                                    1711
           ----------                                                    1639
           ----------                                                    1610
           ----------                                                    1435
           ----------                                                    107
           ----------                                                    44

LOCUS: AT3G52220
```

```
DESCRIPTION: expressed protein
DATA:                 Control 30min   2hours  2days   1week   p-value         pos
SENSE COUNTS:         14      9       12      14      3       2.54e-01
GENES (1 total):
  AT3G52220.1
    SENSE COUNTS:     14      9       12      14      3       2.54e-01
    TAGS: (3 total)
      d+1  AAAAATGTGG 10      4       6       6       3       4.63e-01        960
           ----------                                                         773
      d+2  AGAAGCGAAG 4       5       5       8       0       3.49e-01        698
           ----------                                                         671
      d+2  AGGGAGGCTC 0       0       1       0       0       4.55e-01        316

LOCUS: AT2G21970
DESCRIPTION: stress enhanced protein 2 (SEP2), nearly identical to stress enhanced protein 2; SEP2
(GI:7384980) (Arabidopsis thaliana)
DATA:                 Control 30min   2hours  2days   1week   p-value         pos
SENSE COUNTS:         5       0       3       6       3       2.54e-01
GENES (2 total):
  AT2G21970.1
    SENSE COUNTS:     5       0       3       6       3       2.54e-01
    TAGS: (4 total)
      d+1  TGAAACAATT 1       0       1       1       0       9.46e-01        815
      d+2  GTGCTTGAAG 4       0       1       4       3       1.70e-01        740
      d+2  ATTGTATTTG 0       0       0       1       0       3.09e-01        382
      X+4  TAGATTTCTC 0       0       1       0       0       4.55e-01        -27

LOCUS: AT4G38260
DESCRIPTION: expressed protein, contains Pfam PF05742: Protein of unknown function (DUF833)
DATA:                 Control 30min   2hours  2days   1week   p-value         pos
SENSE COUNTS:         0       0       1       4       1       2.55e-01
GENES (1 total):
  AT4G38260.1
    SENSE COUNTS:     0       0       1       4       1       2.55e-01
    TAGS: (2 total)
           ----------                                                         1611
           ----------                                                         1542
      d+2  TAAACACAAA 0       0       0       4       1       1.05e-01        970
      d+2  GAAGGAACAC 0       0       1       0       0       4.55e-01        842
           ----------                                                         778
           ----------                                                         717
           ----------                                                         612
           ----------                                                         499
           ----------                                                         432
           ----------                                                         415
           ----------                                                         241

LOCUS: AT4G17940
DESCRIPTION: expressed protein
DATA:                 Control 30min   2hours  2days   1week   p-value         pos
SENSE COUNTS:         0       2       0       2       3       2.56e-01
GENES (1 total):
  AT4G17940.1
    SENSE COUNTS:     0       2       0       2       3       2.56e-01
    TAGS: (1 total)
           ----------                                                         1005
           ----------                                                         856
      d+2  TGGGAGGCAG 0       2       0       2       3       2.56e-01        781
           ----------                                                         661

LOCUS: AT1G21370
DESCRIPTION: expressed protein
DATA:                 Control 30min   2hours  2days   1week   p-value         pos
SENSE COUNTS:         0       1       0       2       3       2.57e-01
GENES (2 total):
  AT1G21370.1
    SENSE COUNTS:     0       1       0       2       3       2.57e-01
    TAGS: (2 total)
           ----------                                                         2175
           ----------                                                         2118
           ----------                                                         2063
      d+2  TTCAGTCAAA 0       0       0       1       3       6.27e-02        1663
      d+2  TGTTGTCGAA 0       1       0       1       0       4.31e-01        1539
           ----------                                                         1391
           ----------                                                         1000
           ----------                                                         528
           ----------                                                         409
           ----------                                                         372
           ----------                                                         174
  AT1G21370.2
    SENSE COUNTS:     0       1       0       2       3       2.57e-01
```

```
    TAGS: (2 total)
           ----------                                                 2094
           ----------                                                 2037
           ----------                                                 1982
      d+2  TTCAGTCAAA  0      0      0      1      3      6.27e-02    1582
      d+2  TGTTGTCGAA  0      1      0      1      0      4.31e-01    1458
           ----------                                                 1310
           ----------                                                 919
           ----------                                                 447
           ----------                                                 328
           ----------                                                 291
           ----------                                                 12

LOCUS: AT1G57680
DESCRIPTION: expressed protein
DATA:                  Control 30min  2hours 2days  1week  p-value     pos
SENSE COUNTS:          2      4      1      2      0      2.58e-01
GENES (2 total):
  AT1G57680.1
    SENSE COUNTS:      1      4      1      2      0      3.06e-01
    TAGS: (1 total)
      d+1  GCCCTGAGAG  1      4      1      2      0      3.06e-01    1316
           ----------                                                 773
           ----------                                                 397
           ----------                                                 382
  AT1G57680.2
    SENSE COUNTS:      3      4      1      2      0      1.77e-01
    TAGS: (3 total)
      X+4  ACTTTAAAAG  1      0      0      0      0      6.89e-01    1564
      X+4  ACTTTAAAAG  1      0      0      0      0      6.89e-01    1564
      d+1  GCCCTGAGAG  1      4      1      2      0      3.06e-01    1269
           ----------                                                 726
           ----------                                                 350
           ----------                                                 335

LOCUS: AT4G28060
DESCRIPTION: cytochrome c oxidase subunit 6b, putative, similar to subunit 6b of cytochrome c oxidase
(Arabidopsis thaliana) gi|6518353|dbj|BAA87883
DATA:                  Control 30min  2hours 2days  1week  p-value     pos
SENSE COUNTS:          7      5      2      9      7      2.60e-01
GENES (1 total):
  AT4G28060.1
    SENSE COUNTS:      7      5      2      9      7      2.60e-01
    TAGS: (4 total)
      v+1  TCAGAATTTT  0      0      2      1      1      4.09e-01    1001
      v+2  AAATCTATAA  2      5      0      1      5      1.51e-01    912
           ----------                                                 571
           ----------                                                 328
      v+2  GTTAAACTTT  0      0      0      1      0      3.09e-01    275
      v+2  GCTCACCAAA  5      0      0      6      1      2.56e-02    44
           ----------                                                 10

LOCUS: AT4G26530
DESCRIPTION: fructose-bisphosphate aldolase, putative, strong similarity to SP|P22197 Fructose-bisphosphate
aldolase, cytoplasmic isozyme (EC 4.1.2.13) {Arabidopsis thaliana}
DATA:                  Control 30min  2hours 2days  1week  p-value     pos
SENSE COUNTS:          2      1      1      5      6      2.61e-01
GENES (1 total):
  AT4G26530.1
    SENSE COUNTS:      2      1      1      5      6      2.61e-01
    TAGS: (2 total)
           ----------                                                 1306
           ----------                                                 932
           ----------                                                 747
           ----------                                                 712
           ----------                                                 640
      X+4  ACATAACGGT  2      0      1      5      5      1.93e-01    532
           ----------                                                 345
      i+3  CACTCTTCTT  0      1      0      0      1      3.55e-01    113
           ----------                                                 66

LOCUS: AT5G06390
DESCRIPTION: beta-Ig-H3 domain-containing protein / fasciclin domain-containing protein, contains Pfam profile
PF02469: Fasciclin domain
DATA:                  Control 30min  2hours 2days  1week  p-value     pos
SENSE COUNTS:          0      0      3      1      0      2.61e-01
GENES (1 total):
  AT5G06390.1
    SENSE COUNTS:      0      0      3      1      0      2.61e-01
    TAGS: (1 total)
           ----------                                                 2001
```

```
      d+2  GTAAGGATTT 0       0       3       1       0       2.61e-01        1791
           ----------                                                         1680
           ----------                                                         1594
           ----------                                                         440
           ----------                                                         92

LOCUS: AT5G58020
DESCRIPTION: expressed protein, contains PF04641: Protein of unknown function, DUF602
DATA:                 Control 30min   2hours  2days   1week   p-value         pos
SENSE COUNTS:         0       0       3       1       0       2.61e-01
GENES (1 total):
  AT5G58020.1
    SENSE COUNTS:     0       0       3       1       0       2.61e-01
    TAGS: (1 total)
           ----------                                                         1303
      d+2  CGGATGTCAA 0       0       3       1       0       2.61e-01        795
           ----------                                                         738
           ----------                                                         308
           ----------                                                         89

LOCUS: AT2G01180
DESCRIPTION: phosphatidic acid phosphatase family protein / PAP2 family protein, similar to phosphatidic acid
phosphatase 2a2 (Cavia porcellus) GI:3641336; contains Pfam profile PF01569 PAP2 superfamily
DATA:                 Control 30min   2hours  2days   1week   p-value         pos
SENSE COUNTS:         2       2       0       5       1       2.62e-01
GENES (3 total):
  AT2G01180.2
    SENSE COUNTS:     2       2       0       5       1       2.62e-01
    TAGS: (2 total)
           ----------                                                         1673
      d+2  AAATTGACGT 2       2       0       4       1       4.86e-01        1578
           ----------                                                         1401
           ----------                                                         1102
      d+2  TGTGTACGAT 0       0       0       1       0       3.09e-01        797
           ----------                                                         681
           ----------                                                         565
  AT2G01180.1
    SENSE COUNTS:     2       2       0       5       1       2.62e-01
    TAGS: (2 total)
           ----------                                                         1458
      d+2  AAATTGACGT 2       2       0       4       1       4.86e-01        1363
           ----------                                                         1186
           ----------                                                         887
      d+2  TGTGTACGAT 0       0       0       1       0       3.09e-01        582
           ----------                                                         466
           ----------                                                         350
           ----------                                                         17

LOCUS: AT3G24160
DESCRIPTION: expressed protein, identical to cDNA putative type 1 membrane protein (PMP)GI:4206764
DATA:                 Control 30min   2hours  2days   1week   p-value         pos
SENSE COUNTS:         5       2       8       1       4       2.65e-01
GENES (2 total):
  AT3G24160.1
    SENSE COUNTS:     5       2       8       1       4       2.65e-01
    TAGS: (3 total)
      d+1  AATCTGAATA 1       1       2       0       1       8.08e-01        1528
      d+2  CCTTTGACGA 4       1       5       1       3       4.86e-01        1158
           ----------                                                         1097
      d+2  GTATTGACAG 0       0       1       0       0       4.55e-01        775
           ----------                                                         252

LOCUS: AT1G76600
DESCRIPTION: expressed protein
DATA:                 Control 30min   2hours  2days   1week   p-value         pos
SENSE COUNTS:         1       4       4       0       1       2.65e-01
GENES (1 total):
  AT1G76600.1
    SENSE COUNTS:     1       4       4       0       1       2.65e-01
    TAGS: (1 total)
           ----------                                                         946
      d+2  TGTACTTATA 1       4       4       0       1       2.65e-01        880

LOCUS: AT1G75500
DESCRIPTION: nodulin MtN21 family protein, similar to MtN21 GB:CAA75575 GI:2598575 from (Medicago truncatula)
(Mol. Plant Microbe Interact. 9 (4), 233-242 (1996)); contains Pfam profile PF00892: Integral membrane protein
DATA:                 Control 30min   2hours  2days   1week   p-value         pos
SENSE COUNTS:         2       0       1       5       1       2.67e-01
GENES (1 total):
  AT1G75500.1
    SENSE COUNTS:     2       0       1       5       1       2.67e-01
```

```
    TAGS: (2 total)
          ----------                                                    1453
      d+2  GTATTGAACG 2       0       0       5       1       1.16e-01      1154
      d+2  GCTTCTATTG 0       0       1       0       0       4.55e-01      1000
          ----------                                                    451
          ----------                                                    418
          ----------                                                    202

LOCUS: AT4G31590
DESCRIPTION: glycosyl transferase family 2 protein, similar to cellulose synthase from Agrobacterium
tumeficiens (gi:710492) and Agrobacterium radiobacter (gi:710493); contains Pfam glycosyl transferase, group 2
family protein domain PF00535
DATA:                 Control 30min   2hours  2days   1week   p-value       pos
SENSE COUNTS:         6       2       3       2       8       2.67e-01
GENES (2 total):
  AT4G31590.1
    SENSE COUNTS:     6       2       3       2       8       2.67e-01
    TAGS: (2 total)
      d+1  TCAAATCACT 1       0       0       0       0       4.28e-01      2622
      d+2  TTGTTGGCAG 5       2       3       2       8       3.32e-01      2572
          ----------                                                    2544
          ----------                                                    1858
          ----------                                                    1801
          ----------                                                    1544

LOCUS: AT1G74450
DESCRIPTION: expressed protein
DATA:                 Control 30min   2hours  2days   1week   p-value       pos
SENSE COUNTS:         5       1       3       7       10      2.69e-01
GENES (1 total):
  AT1G74450.1
    SENSE COUNTS:     5       1       3       7       10      2.69e-01
    TAGS: (4 total)
      X+4  ATATCGAGGC 1       0       0       0       0       4.28e-01      1977
      X+4  AATTAAAAAA 4       0       1       1       7       8.32e-02      1809
      d+1  GAGACAAATT 0       0       1       2       0       4.23e-01      1531
          ----------                                                    1412
          ----------                                                    1164
          ----------                                                    888
      X+4  AAATTAAAAA 0       1       1       4       3       3.21e-01      -95

LOCUS: AT5G05080
DESCRIPTION: ubiquitin-conjugating enzyme, putative, similar to SP|Q16763 Ubiquitin-conjugating enzyme E2-24
kDa (EC 6.3.2.19) (Ubiquitin- protein ligase) (Ubiquitin carrier protein) {Homo sapiens}; contains Pfam
profile PF00179: Ubiquitin-conjugating enzyme
DATA:                 Control 30min   2hours  2days   1week   p-value       pos
SENSE COUNTS:         1       3       3       0       0       2.70e-01
GENES (1 total):
  AT5G05080.1
    SENSE COUNTS:     1       3       3       0       0       2.70e-01
    TAGS: (2 total)
      d+1  GTTTTGCTGA 1       0       2       0       0       3.07e-01      1211
      d+2  CTAAACCAAA 0       3       1       0       0       1.04e-01      758
          ----------                                                    734
          ----------                                                    626
          ----------                                                    532
          ----------                                                    497
          ----------                                                    298

LOCUS: AT4G00460
DESCRIPTION: expressed protein, contains Pfam profile PF03759: Domain of unknown function (DUF315)
DATA:                 Control 30min   2hours  2days   1week   p-value       pos
SENSE COUNTS:         1       3       3       0       0       2.70e-01
GENES (1 total):
  AT4G00460.1
    SENSE COUNTS:     1       3       3       0       0       2.70e-01
    TAGS: (1 total)
          ----------                                                    2220
          ----------                                                    2139
          ----------                                                    1758
          ----------                                                    1506
          ----------                                                    1499
          ----------                                                    1460
          ----------                                                    1414
      i+3  CAGTGTGGTA 1       3       3       0       0       2.70e-01      1395
          ----------                                                    1350
          ----------                                                    1293

LOCUS: AT4G22520
DESCRIPTION: protease inhibitor/seed storage/lipid transfer protein (LTP) family protein, contains Pfam
profile: PF00234 protease inhibitor/seed storage/LTP family
```

```
DATA:                 Control 30min   2hours  2days   1week   p-value         pos
SENSE COUNTS:         2       0       3       1       0       2.70e-01
GENES (1 total):
  AT4G22520.1
    SENSE COUNTS:     2       0       3       1       0       2.70e-01
    TAGS: (1 total)
           ----------                                                         1192
           ----------                                                         916
      v+2  TTCGTTTTGA 2       0       3       1       0       2.70e-01        848
           ----------                                                         548
           ----------                                                         369
           ----------                                                         354

LOCUS: AT1G13350
DESCRIPTION: protein kinase family protein, contains protein kinase domain, Pfam:PF00069 (likely that this
cDNA contains a single unspliced intron. Putative intron removed in this gene model.)
DATA:                 Control 30min   2hours  2days   1week   p-value         pos
SENSE COUNTS:         0       0       3       4       1       2.72e-01
GENES (1 total):
  AT1G13350.1
    SENSE COUNTS:     0       0       3       4       1       2.72e-01
    TAGS: (4 total)
      d+1  TACATTGAAT 0       0       0       0       1       1.65e-01        2373
           ----------                                                         1794
           ----------                                                         1402
           ----------                                                         1392
           ----------                                                         1215
           ----------                                                         955
           ----------                                                         748
           ----------                                                         697
      i+3  CATTTGTAAG 0       0       1       0       0       4.55e-01        576
      X+4  TGATTCAGTC 0       0       1       4       0       3.72e-02        537
      d+2  GCCACCGTCA 0       0       1       0       0       7.06e-01        178
           ----------                                                         76

LOCUS: AT4G16155
DESCRIPTION: dihydrolipoamide dehydrogenase 2, plastidic / lipoamide dehydrogenase 2 (PTLPD2), identical to
plastidic lipoamide dehydrogenase from Arabidopsis thaliana (gi:7159284)
DATA:                 Control 30min   2hours  2days   1week   p-value         pos
SENSE COUNTS:         2       2       6       5       0       2.73e-01
GENES (1 total):
  AT4G16155.1
    SENSE COUNTS:     2       2       6       5       0       2.73e-01
    TAGS: (3 total)
      i+3  TGAACAGATG 0       0       0       0       0       6.15e-01        3266
      d+1  GTTTTGACAA 2       2       5       5       0       3.23e-01        1956
           ----------                                                         1839
           ----------                                                         1733
      d+2  AAGCATCAAA 0       0       1       0       0       4.55e-01        1673
           ----------                                                         1655
           ----------                                                         1412
           ----------                                                         1358
           ----------                                                         860
           ----------                                                         577
           ----------                                                         422

LOCUS: AT3G21790
DESCRIPTION: UDP-glucoronosyl/UDP-glucosyl transferase family protein, contains Pfam profile: PF00201 UDP-
glucoronosyl and UDP-glucosyl transferase
DATA:                 Control 30min   2hours  2days   1week   p-value         pos
SENSE COUNTS:         2       0       4       0       1       2.75e-01
GENES (1 total):
  AT3G21790.1
    SENSE COUNTS:     2       0       4       0       1       2.75e-01
    TAGS: (1 total)
           ----------                                                         2326
           ----------                                                         2094
           ----------                                                         2026
      v+2  TAGCTCTTAA 2       0       4       0       1       2.75e-01        1825
           ----------                                                         1726
           ----------                                                         1662
           ----------                                                         1511
           ----------                                                         1206
           ----------                                                         811
           ----------                                                         714
           ----------                                                         354

LOCUS: AT4G01050
DESCRIPTION: hydroxyproline-rich glycoprotein family protein
DATA:                 Control 30min   2hours  2days   1week   p-value         pos
SENSE COUNTS:         2       4       6       2       0       2.76e-01
```

```
GENES (1 total):
  AT4G01050.1
    SENSE COUNTS:      2      4      6      2      0      2.76e-01
    TAGS: (2 total)
      i+3  GGTTTCTGTT  0      0      1      0      0      4.55e-01       2311
      d+1  GGGAGTTGAG  2      4      5      2      0      4.20e-01       496
           ----------                                                  110

LOCUS: AT3G07950
DESCRIPTION: rhomboid protein-related, contains 6 transmembrane domains; similar to phosphatidyl inositol
glycan class T (GI:14456615) (Homo sapiens)
DATA:                  Control 30min  2hours 2days  1week  p-value        pos
SENSE COUNTS:          0      3      2      0      1      2.76e-01
GENES (1 total):
  AT3G07950.1
    SENSE COUNTS:      0      3      2      0      1      2.76e-01
    TAGS: (1 total)
           ----------                                                  1271
      d+2  TGGACAGTTT  0      3      2      0      1      2.76e-01       1104
           ----------                                                  882
           ----------                                                  522
           ----------                                                  134

LOCUS: AT2G20360
DESCRIPTION: expressed protein
DATA:                  Control 30min  2hours 2days  1week  p-value        pos
SENSE COUNTS:          10     7      3      4      10     2.76e-01
GENES (1 total):
  AT2G20360.1
    SENSE COUNTS:      10     7      3      4      10     2.76e-01
    TAGS: (2 total)
           ----------                                                  1626
      d+2  TACTACTTCC  10     7      3      4      5      3.64e-01       1525
      d+2  CAAGTGTCAC  0      0      0      0      5      7.96e-03       1312
           ----------                                                  1031
           ----------                                                  947
           ----------                                                  884
           ----------                                                  722
           ----------                                                  707
           ----------                                                  461

LOCUS: AT1G63055
DESCRIPTION: expressed protein
DATA:                  Control 30min  2hours 2days  1week  p-value        pos
SENSE COUNTS:          4      1      2      6      7      2.78e-01
GENES (1 total):
  AT1G63055.1
    SENSE COUNTS:      4      1      2      6      7      2.78e-01
    TAGS: (2 total)
           ----------                                                  571
      d+2  AAAAAATGTT  4      1      0      5      7      1.00e-01       335
      d+2  GAAAAAGCTC  0      0      2      1      0      2.74e-01       294
           ----------                                                  260

LOCUS: AT5G55850
DESCRIPTION: nitrate-responsive NOI protein, putative, similar to nitrate-induced NOI protein (Zea mays)
GI:2642213
DATA:                  Control 30min  2hours 2days  1week  p-value        pos
SENSE COUNTS:          8      13     6      6      4      2.81e-01
GENES (2 total):
  AT5G55850.1
    SENSE COUNTS:      8      13     6      6      4      2.81e-01
    TAGS: (2 total)
           ----------                                                  1083
      v+2  CAAGCTCCAG  8      13     4      6      3      1.49e-01       567
      v+2  TTAAATCTGG  0      0      2      0      1      4.86e-01       508
           ----------                                                  354

LOCUS: AT4G10220
DESCRIPTION: hypothetical protein, IB1C3-1 protein, Arabidopsis thaliana, AJ011845
DATA:                  Control 30min  2hours 2days  1week  p-value        pos
SENSE COUNTS:          2      0      3      2      0      2.81e-01
GENES (1 total):
  AT4G10220.1
    SENSE COUNTS:      2      0      3      2      0      2.81e-01
    TAGS: (1 total)
           ----------                                                  1966
           ----------                                                  1606
           ----------                                                  1513
           ----------                                                  1042
           ----------                                                  612
```

```
          ----------                                                    445
          ----------                                                    404
          ----------                                                    393
          ----------                                                    50
      v+2  GTCAAAAATA 2      0      3      2      0      2.81e-01       13

LOCUS: AT1G14320
DESCRIPTION: 60S ribosomal protein L10 (RPL10A) / Wilm's tumor suppressor protein-related, similar to tumor
suppressor GI:575354 from (Oryza sativa)
DATA:                 Control 30min  2hours  2days  1week  p-value       pos
SENSE COUNTS:         3      5      10     5      10     2.82e-01
GENES (1 total):
  AT1G14320.1
    SENSE COUNTS:     3      5      10     5      10     2.82e-01
    TAGS: (3 total)
      d+1  GCTTTAGCTT 0      0      1      1      3      4.30e-01       936
      d+2  GACCTTTGGC 3      5      7      4      7      7.29e-01       667
           ----------                                                   662
      d+2  CTCAAGAGGC 0      0      2      0      0      1.21e-01       499
           ----------                                                   490
           ----------                                                   343
           ----------                                                   285
           ----------                                                   221
           ----------                                                   60

LOCUS: AT1G79750
DESCRIPTION: malate oxidoreductase, putative, similar to malate oxidoreductase (NADP-dependent malic enzyme)
GB:P34105 (Populus balsamifera subsp. trichocarpa)
DATA:                 Control 30min  2hours  2days  1week  p-value       pos
SENSE COUNTS:         9      6      3      4      2      2.84e-01
GENES (1 total):
  AT1G79750.1
    SENSE COUNTS:     9      6      3      4      2      2.84e-01
    TAGS: (2 total)
      d+1  TGATTGCATA 6      1      3      4      1      3.59e-01       2395
      d+2  TACAGCCCTT 3      5      0      0      1      8.08e-02       2119
           ----------                                                   1918
           ----------                                                   1910
           ----------                                                   1672
           ----------                                                   1565
           ----------                                                   1559
           ----------                                                   1171
           ----------                                                   1163
           ----------                                                   887
           ----------                                                   493
           ----------                                                   211

LOCUS: AT3G46430
DESCRIPTION: expressed protein
DATA:                 Control 30min  2hours  2days  1week  p-value       pos
SENSE COUNTS:         8      6      11     12     17     2.85e-01
GENES (1 total):
  AT3G46430.1
    SENSE COUNTS:     8      6      11     12     17     2.85e-01
    TAGS: (2 total)
           ----------                                                   987
      v+2  CGAACGCACA 8      4      11     12     17     8.73e-02       754
      v+2  TTCAATAACA 0      2      0      0      0      9.14e-02       596
           ----------                                                   371
           ----------                                                   33

LOCUS: AT3G50830
DESCRIPTION: stress-responsive protein, putative, similar to cold acclimation WCOR413-like protein gamma form
(Hordeum vulgare) gi|18449100|gb|AAL69988; similar to stress-regulated protein SAP1 (Xerophyta viscosa)
gi|21360378|gb|AAM47505; identical to cDNA cold acclim
DATA:                 Control 30min  2hours  2days  1week  p-value       pos
SENSE COUNTS:         4      1      3      3      8      2.89e-01
GENES (2 total):
  AT3G50830.1
    SENSE COUNTS:     4      1      3      3      8      2.89e-01
    TAGS: (2 total)
           ----------                                                   1006
           ----------                                                   998
      d+2  TAATCCACAT 0      1      1      1      1      8.22e-01       750
      i+3  TTGAATTCGA 4      0      2      2      7      9.38e-02       434

LOCUS: AT5G42250
DESCRIPTION: alcohol dehydrogenase, putative, similar to alcohol dehydrogenase ADH GI:7705214 from
(Lycopersicon esculentum); contains Pfam zinc-binding dehydrogenase domain PF00107
DATA:                 Control 30min  2hours  2days  1week  p-value       pos
SENSE COUNTS:         4      2      1      1      6      2.90e-01
```

```
GENES (1 total):
  AT5G42250.1
    SENSE COUNTS:      4       2       1       1       6       2.90e-01
    TAGS: (5 total)
      d+1  TTTAACTATT  0       0       0       0       3       1.12e-02       1307
      i+3  TTTCGACAGG  0       0       0       0       0       6.15e-01       1196
      d+2  AGATGAAATT  1       0       0       0       0       4.28e-01       1169
           ----------                                                         1055
           ----------                                                         855
           ----------                                                         394
      d+2  GTGGCGCCGC  0       0       1       0       0       7.06e-01       190
      X+4  AATAACAATA  3       2       0       1       3       4.80e-01       -172

LOCUS: AT3G61750
DESCRIPTION: auxin-responsive protein -related, similar to auxin-induced protein AIR12  GI:11357190
(Arabidopsis thaliana);
DATA:                  Control 30min   2hours  2days   1week   p-value        pos
SENSE COUNTS:          3       0       2       0       0       2.90e-01
GENES (1 total):
  AT3G61750.1
    SENSE COUNTS:      3       0       2       0       0       2.90e-01
    TAGS: (1 total)
           ----------                                                         1962
      v+2  TAAGTTGAGT  3       0       2       0       0       2.90e-01       1780
           ----------                                                         1725
           ----------                                                         1012
           ----------                                                         922
           ----------                                                         522
           ----------                                                         354

LOCUS: AT5G38830
DESCRIPTION: tRNA synthetase class I (C) family protein, similar to SP|Q06752 Cysteinyl-tRNA synthetase (EC
6.1.1.16) (Cysteine--tRNA ligase) (CysRS) {Bacillus subtilis}; contains Pfam profile PF01406: tRNA synthetases
class I (C)
DATA:                  Control 30min   2hours  2days   1week   p-value        pos
SENSE COUNTS:          0       2       2       4       0       2.90e-01
GENES (1 total):
  AT5G38830.1
    SENSE COUNTS:      0       2       2       4       0       2.90e-01
    TAGS: (1 total)
           ----------                                                         1564
           ----------                                                         852
           ----------                                                         811
           ----------                                                         802
           ----------                                                         780
           ----------                                                         747
      X+4  GAGATAGTTA  0       2       2       4       0       2.90e-01       357
           ----------                                                         198

LOCUS: AT4G04350
DESCRIPTION: leucyl-tRNA synthetase, putative / leucine--tRNA ligase, putative, similar to SP|P36430 Leucyl-
tRNA synthetase (EC 6.1.1.4) (Leucine--tRNA ligase) (LeuRS) {Bacillus subtilis}; contains Pfam profile
PF00133: tRNA synthetases class I (I, L, M and V)
DATA:                  Control 30min   2hours  2days   1week   p-value        pos
SENSE COUNTS:          2       0       1       4       1       2.92e-01
GENES (1 total):
  AT4G04350.1
    SENSE COUNTS:      2       0       1       4       1       2.92e-01
    TAGS: (2 total)
           ----------                                                         2656
           ----------                                                         2320
           ----------                                                         1940
      d+2  GACCCGAAAA  2       0       0       4       1       1.27e-01       1852
      d+2  GGTGAACACT  0       0       1       0       0       4.55e-01       1752
           ----------                                                         1331
           ----------                                                         1315
           ----------                                                         1078
           ----------                                                         437
           ----------                                                         406
           ----------                                                         230
           ----------                                                         165

LOCUS: AT4G04850
DESCRIPTION: K+ efflux antiporter, putative (KEA3), similar to A. thaliana K+ antiporter KEA1, GenBank
accession number AF003382; Monovalent cation:proton antiporter family 2 (CPA2 family) member, PMID:11500563
DATA:                  Control 30min   2hours  2days   1week   p-value        pos
SENSE COUNTS:          2       0       1       4       1       2.92e-01
GENES (3 total):
  AT4G04850.1
    SENSE COUNTS:      2       0       1       4       1       2.92e-01
    TAGS: (1 total)
```

```
        ----------                                            2298
    d+2  AAGCCAATGC 2     0     1     4     1     2.92e-01    1939
         ----------                                            1474
         ----------                                            889
         ----------                                            229

LOCUS: AT3G16250
DESCRIPTION: ferredoxin-related, contains Pfam profile: PF00111 2Fe-2S iron-sulfur cluster binding domains
DATA:                Control 30min 2hours 2days 1week p-value    pos
SENSE COUNTS:        5     4     9     2     3     2.93e-01
GENES (1 total):
  AT3G16250.1
    SENSE COUNTS:    5     4     9     2     3     2.93e-01
    TAGS: (4 total)
      d+1  GATCCACCGG 0     0     0     1     0     3.09e-01    1181
      d+2  AAGACATTTC 2     0     1     0     0     2.87e-01    1058
      d+2  TTCGGAAGAA 0     0     1     0     0     4.55e-01    1042
           ----------                                          988
      d+2  AGTGGAACAT 3     4     7     1     3     3.83e-01    654
           ----------                                          485
           ----------                                          7

LOCUS: AT4G13430
DESCRIPTION: aconitase family protein / aconitate hydratase family protein, contains Pfam profile PF00330:
Aconitase family (aconitate hydratase
DATA:                Control 30min 2hours 2days 1week p-value    pos
SENSE COUNTS:        3     9     7     5     2     2.93e-01
GENES (1 total):
  AT4G13430.1
    SENSE COUNTS:    3     9     7     5     2     2.93e-01
    TAGS: (3 total)
      d+1  ACCCCATTAG 0     0     0     0     0     6.15e-01    1835
      d+2  TGCGCAGATC 3     9     7     4     1     1.99e-01    1364
      d+2  GTTGTGGAAG 0     0     0     1     1     3.25e-01    960
           ----------                                          622
           ----------                                          525
           ----------                                          379

LOCUS: AT5G60540
DESCRIPTION: SNO glutamine amidotransferase family protein, similar to pyridoxine synthesis protein PDX2
(Cercospora nicotianae) GI:9954418; contains Pfam profile PF01174: SNO glutamine amidotransferase family
DATA:                Control 30min 2hours 2days 1week p-value    pos
SENSE COUNTS:        4     1     3     7     2     2.96e-01
GENES (1 total):
  AT5G60540.1
    SENSE COUNTS:    4     1     3     7     2     2.96e-01
    TAGS: (2 total)
      i+3  AATCAATGGG 3     0     0     1     1     1.73e-01    1209
      d+1  TACTTGTTTC 1     1     3     6     1     2.14e-01    1073
           ----------                                          389
           ----------                                          309

LOCUS: AT2G21370
DESCRIPTION: xylulose kinase, putative, similar to xylulose kinase (Xylulokinase) (Bacillus subtilis) Swiss-
Prot:P39211
DATA:                Control 30min 2hours 2days 1week p-value    pos
SENSE COUNTS:        1     0     5     1     1     2.96e-01
GENES (2 total):
  AT2G21370.1
    SENSE COUNTS:    1     0     2     0     0     5.95e-01
    TAGS: (1 total)
      d+1  TGAACACATT 1     0     2     0     0     5.95e-01    1483
           ----------                                          1217
           ----------                                          714
           ----------                                          561
           ----------                                          315
           ----------                                          298
           ----------                                          283
  AT2G21370.2
    SENSE COUNTS:    1     0     5     1     1     2.96e-01
    TAGS: (2 total)
      d+1  TGAACACATT 1     0     2     0     0     5.95e-01    1477
           ----------                                          1211
           ----------                                          708
           ----------                                          555
      X+4  CGTGTCGATT 0     0     3     1     1     4.33e-01    323
           ----------                                          309
           ----------                                          292
           ----------                                          273

LOCUS: AT5G23140
```

```
DESCRIPTION: ATP-dependent Clp protease proteolytic subunit, putative, nClpP2/nClpP7; similar to SP:Q9X6W8
ATP-dependent Clp protease proteolytic subunit (EC 3.4.21.92) (Endopeptidase Clp) from (Azospirillum
brasilense)
DATA:                 Control 30min   2hours  2days   1week   p-value         pos
SENSE COUNTS:         4       6       2       5       10      2.97e-01
GENES (2 total):
  AT5G23140.1
    SENSE COUNTS:     4       6       2       5       10      2.97e-01
    TAGS: (3 total)
           ----------                                                         1216
      d+2  TTTAGTGTTT 0       0       0       0       0       6.15e-01        961
      d+2  ACTATATTTC 2       6       2       4       10      1.36e-01        931
      d+2  ATCTCTCAAA 2       0       0       1       0       2.50e-01        891
           ----------                                                         666
           ----------                                                         648
           ----------                                                         352

LOCUS: AT5G48790
DESCRIPTION: expressed protein
DATA:                 Control 30min   2hours  2days   1week   p-value         pos
SENSE COUNTS:         6       2       1       5       4       2.99e-01
GENES (1 total):
  AT5G48790.1
    SENSE COUNTS:     6       2       1       5       4       2.99e-01
    TAGS: (3 total)
      i+3  ATTAGATTCA 1       0       0       0       0       4.28e-01        1145
      d+1  CTTGTCAAGC 1       2       0       4       1       3.81e-01        1124
      d+2  TCAAAATTAA 4       0       1       1       3       1.50e-01        1061
           ----------                                                         1041
           ----------                                                         970
           ----------                                                         912

LOCUS: AT3G51540
DESCRIPTION: expressed protein, mucin 5AC, Homo sapiens, PIR:S53363
DATA:                 Control 30min   2hours  2days   1week   p-value         pos
SENSE COUNTS:         3       6       2       7       8       3.00e-01
GENES (1 total):
  AT3G51540.1
    SENSE COUNTS:     3       6       2       7       8       3.00e-01
    TAGS: (2 total)
      v+1  TTTAATGTTT 3       5       2       6       5       6.63e-01        2112
           ----------                                                         1468
           ----------                                                         1117
           ----------                                                         541
           ----------                                                         508
      v+2  AAAACCAAAA 0       1       0       1       3       2.78e-01        202
           ----------                                                         173
           ----------                                                         63

LOCUS: AT5G41020
DESCRIPTION: myb family transcription factor, contains Pfam profile: PF00249 Myb DNA binding domain
DATA:                 Control 30min   2hours  2days   1week   p-value         pos
SENSE COUNTS:         1       3       3       1       6       3.00e-01
GENES (1 total):
  AT5G41020.1
    SENSE COUNTS:     1       3       3       1       6       3.00e-01
    TAGS: (2 total)
      X+4  TTTTTCGGTA 1       1       1       0       1       8.56e-01        1937
           ----------                                                         1527
           ----------                                                         1389
           ----------                                                         1131
      d+2  ATGGTGTGAA 0       2       2       1       5       2.99e-01        546

LOCUS: AT3G06670
DESCRIPTION: expressed protein
DATA:                 Control 30min   2hours  2days   1week   p-value         pos
SENSE COUNTS:         3       0       3       2       6       3.01e-01
GENES (1 total):
  AT3G06670.1
    SENSE COUNTS:     3       0       3       2       6       3.01e-01
    TAGS: (3 total)
      d+1  AGATATAAGG 0       0       1       0       1       3.96e-01        3312
      d+2  GTCTATCAGC 3       0       2       1       5       4.17e-01        3152
           ----------                                                         2971
           ----------                                                         2933
           ----------                                                         2789
           ----------                                                         2630
           ----------                                                         1925
           ----------                                                         1686
           ----------                                                         1630
           ----------                                                         1370
```

```
      d+2  CGAACAATGC 0       0       0       1       0       3.09e-01       1160
           ----------                                                        1013
           ----------                                                        856
           ----------                                                        785
           ----------                                                        522

LOCUS: AT1G12820
DESCRIPTION: transport inhibitor response protein, putative, E3 ubiquitin ligase SCF complex F-box subunit;
similar to transport inhibitor response 1 GI:2352492 from (Arabidopsis thaliana)
DATA:                 Control 30min   2hours  2days   1week   p-value        pos
SENSE COUNTS:         1       0       2       4       1       3.01e-01
GENES (2 total):
  AT1G12820.1
    SENSE COUNTS:     1       0       2       4       1       3.01e-01
    TAGS: (1 total)
           ----------                                                        2316
      d+2  TGTTTTCAAA 1       0       2       4       1       3.01e-01       2129
           ----------                                                        1419
           ----------                                                        1375
           ----------                                                        1136
           ----------                                                        624
           ----------                                                        562
           ----------                                                        472

LOCUS: AT4G08050
DESCRIPTION: gypsy-like retrotransposon family (Athila), has a 0. P-value blast match to GB:CAA57397 Athila
ORF 1 (Arabidopsis thaliana)
DATA:                 Control 30min   2hours  2days   1week   p-value        pos
SENSE COUNTS:         1       0       2       4       1       3.01e-01
GENES (1 total):
  AT4G08050.1
    SENSE COUNTS:     1       0       2       4       1       3.01e-01
    TAGS: (1 total)
           ----------                                                        5848
           ----------                                                        5612
           ----------                                                        5606
           ----------                                                        5533
           ----------                                                        5083
           ----------                                                        4789
           ----------                                                        4504
           ----------                                                        4402
      p+2  GCGAAAGGAG 1       0       2       4       1       3.01e-01       4221
           ----------                                                        4073
           ----------                                                        3985
           ----------                                                        3134
           ----------                                                        2800
           ----------                                                        2159
           ----------                                                        2138
           ----------                                                        1975
           ----------                                                        1518
           ----------                                                        1494
           ----------                                                        1215
           ----------                                                        896
           ----------                                                        579
           ----------                                                        446
           ----------                                                        287

LOCUS: AT1G19740
DESCRIPTION: ATP-dependent protease La (LON) domain-containing protein, weak similarity to SP|P36774 ATP-
dependent protease La 2 (EC 3.4.21.53) {Myxococcus xanthus}; contains Pfam profile PF02190: ATP-dependent
protease La (LON) domain
DATA:                 Control 30min   2hours  2days   1week   p-value        pos
SENSE COUNTS:         2       5       3       2       0       3.01e-01
GENES (1 total):
  AT1G19740.1
    SENSE COUNTS:     2       5       3       2       0       3.01e-01
    TAGS: (2 total)
      X+4  TTCAATGAAA 0       0       2       0       0       3.51e-01       1329
      d+1  ATGCAAACGC 2       5       1       2       0       2.58e-01       372

LOCUS: AT5G61240
DESCRIPTION: leucine-rich repeat family protein, contains leucine rich-repeat (LRR) domains Pfam:PF00560,
INTERPRO:IPR001611; contains similarity to Hcr2-0B (Lycopersicon esculentum) gi|3894387|gb|AAC78593
DATA:                 Control 30min   2hours  2days   1week   p-value        pos
SENSE COUNTS:         0       1       1       0       3       3.01e-01
GENES (1 total):
  AT5G61240.1
    SENSE COUNTS:     0       1       1       0       3       3.01e-01
    TAGS: (1 total)
           ----------                                                        1916
      v+2  TTCAAGTCAG 0       1       1       0       3       3.01e-01       1422
```

```
          ----------                                                        840
          ----------                                                        521
          ----------                                                        354
          ----------                                                        51

LOCUS: AT4G34260
DESCRIPTION: expressed protein,
DATA:                 Control 30min  2hours  2days  1week  p-value       pos
SENSE COUNTS:         3       0      0       1      3      3.02e-01
GENES (1 total):
  AT4G34260.1
    SENSE COUNTS:     3       0      0       1      3      3.02e-01
    TAGS: (1 total)
          ----------                                                        2742
      d+2  TATCTTGGTA 3       0      0       1      3      3.02e-01       2680
          ----------                                                        2206
          ----------                                                        1955
          ----------                                                        1762
          ----------                                                        1560
          ----------                                                        1556
          ----------                                                        1531
          ----------                                                        1293
          ----------                                                        87

LOCUS: AT3G12630
DESCRIPTION: zinc finger (AN1-like) family protein, contains Pfam domain, PF01428: AN1-like Zinc finger
DATA:                 Control 30min  2hours  2days  1week  p-value       pos
SENSE COUNTS:         4       6      8       1      4      3.05e-01
GENES (1 total):
  AT3G12630.1
    SENSE COUNTS:     4       6      8       1      4      3.05e-01
    TAGS: (3 total)
      X+4  AGCTTACTCA 0       0      1       0      1      6.85e-01       1165
      d+1  TCTTTCTGGT 4       1      7       1      3      1.82e-01       906
          ----------                                                        834
          ----------                                                        669
      d+2  ATTGTAGCTA 0       5      0       0      0      3.00e-03       494
          ----------                                                        223

LOCUS: AT2G20760
DESCRIPTION: expressed protein
DATA:                 Control 30min  2hours  2days  1week  p-value       pos
SENSE COUNTS:         0       5      4       2      4      3.07e-01
GENES (1 total):
  AT2G20760.1
    SENSE COUNTS:     0       5      4       2      4      3.07e-01
    TAGS: (3 total)
      d+1  TAAGAAAGTG 0       0      1       0      1      3.96e-01       1618
          ----------                                                        1497
          ----------                                                        1492
          ----------                                                        1471
      d+2  ATGCCGCCTC 0       5      3       1      3      2.42e-01       854
      d+2  ACTTTTCCTC 0       0      0       1      0      3.09e-01       312
          ----------                                                        119

LOCUS: AT3G45780
DESCRIPTION: protein kinase / nonphototropic hypocotyl protein 1 (NPH1) / phototropin, identical to SP|O48963
Nonphototropic hypocotyl protein 1 (EC 2.7.1.37) (Phototropin) {Arabidopsis thaliana}, cDNA nonphototropic
hypocotyl 1 (NPH1) GI:2832240; contains  Pfam profi
DATA:                 Control 30min  2hours  2days  1week  p-value       pos
SENSE COUNTS:         2       6      6       2      1      3.08e-01
GENES (3 total):
  AT3G45780.1
    SENSE COUNTS:     2       6      6       2      1      3.08e-01
    TAGS: (3 total)
      i+3  GTTTTAGGTT 0       0      1       0      0      4.55e-01       4410
      d+1  GCTGAACCAA 1       5      5       2      1      4.16e-01       2590
      d+2  CTCCTAGATC 1       1      0       0      0      4.77e-01       2314
          ----------                                                        1998
          ----------                                                        1924
          ----------                                                        1306
          ----------                                                        1141
          ----------                                                        318

LOCUS: AT5G64250
DESCRIPTION: 2-nitropropane dioxygenase family / NPD family, contains Pfam profile PF03060: oxidoreductase, 2-
nitropropane dioxygenase (NPD) family
DATA:                 Control 30min  2hours  2days  1week  p-value       pos
SENSE COUNTS:         1       0      3       1      3      3.10e-01
GENES (2 total):
  AT5G64250.1
```

```
    SENSE COUNTS:      1       0       3       1       3       3.10e-01
    TAGS: (1 total)
           ----------                                                          1421
      d+2  TGTAATCCAG  1       0       3       1       3       3.10e-01        1248
           ----------                                                          1125
           ----------                                                          1101
           ----------                                                          946
           ----------                                                          899
           ----------                                                          854
           ----------                                                          701
           ----------                                                          527
           ----------                                                          323
           ----------                                                          30
  AT5G64250.2
    SENSE COUNTS:      1       0       3       1       3       3.10e-01
    TAGS: (1 total)
           ----------                                                          1589
      d+2  TGTAATCCAG  1       0       3       1       3       3.10e-01        1416
           ----------                                                          1293
           ----------                                                          1269
           ----------                                                          1114
           ----------                                                          1067
           ----------                                                          1022
           ----------                                                          869
           ----------                                                          695
           ----------                                                          491
           ----------                                                          271

LOCUS: AT3G19670
DESCRIPTION: FF domain-containing protein / WW domain-containing protein, weak similarity to huntingtin-
interacting protein HYPA/FBP11 (Homo sapiens) GI:3341980; contains Pfam profiles PF01846: FF domain, PF00397:
WW domain
DATA:                  Control 30min   2hours  2days   1week   p-value         pos
SENSE COUNTS:          0       1       3       2       0       3.14e-01
GENES (1 total):
  AT3G19670.1
    SENSE COUNTS:      0       1       3       2       0       3.14e-01
    TAGS: (3 total)
      i+3  TCAACTAAAA  0       0       0       0       0       6.15e-01        3458
      d+1  AGCTCCAGCT  0       1       3       2       0       3.56e-01        3011
           ----------                                                          2630
           ----------                                                          2612
           ----------                                                          2513
           ----------                                                          1907
      d+2  TCAGTGAGCT  0       0       0       0       0       6.15e-01        1613
           ----------                                                          1333
           ----------                                                          1320
           ----------                                                          884
           ----------                                                          816
           ----------                                                          556
           ----------                                                          299
           ----------                                                          260
           ----------                                                          247
           ----------                                                          94

LOCUS: AT3G15810
DESCRIPTION: expressed protein, contains Pfam profile PF04525: Protein of unknown function (DUF567)
DATA:                  Control 30min   2hours  2days   1week   p-value         pos
SENSE COUNTS:          0       3       3       2       0       3.14e-01
GENES (1 total):
  AT3G15810.1
    SENSE COUNTS:      0       3       3       2       0       3.14e-01
    TAGS: (2 total)
      X+4  TTAGAAATTT  0       0       0       2       0       4.80e-02        642
      d+1  GACGCGACCG  0       3       3       0       0       1.18e-01        339

LOCUS: AT3G05545
DESCRIPTION: transcription factor, putative / zinc finger (C3HC4 type RING finger) family protein, similar to
VIP2 protein (Avena fatua) gi|6996144|emb|CAB75506; contains Pfam domain PF00097: Zinc finger, C3HC4 type
(RING finger)
DATA:                  Control 30min   2hours  2days   1week   p-value         pos
SENSE COUNTS:          6       1       8       7       3       3.14e-01
GENES (1 total):
  AT3G05545.1
    SENSE COUNTS:      6       1       8       7       3       3.14e-01
    TAGS: (2 total)
      d+1  CTCAAGTTAT  2       1       3       1       0       6.97e-01        1404
      d+2  ATGGACAAAA  4       0       5       6       3       2.98e-01        1330
           ----------                                                          854
           ----------                                                          743
           ----------                                                          513
```

```
           ----------                                                   156

LOCUS: AT1G42150
DESCRIPTION: CACTA-like transposase family (Tnp1/En/Spm), has a 5.3e-161 P-value blast match to
ref|NP_189784.1| TNP1-related protein (Arabidopsis thaliana) (CACTA-element)
DATA:                 Control 30min  2hours  2days  1week  p-value        pos
SENSE COUNTS:         1       0      3       5      3      3.15e-01
GENES (1 total):
  AT1G42150.1
    SENSE COUNTS:     1       0      3       5      3      3.15e-01
    TAGS: (2 total)
           ----------                                                   4021
           ----------                                                   3992
           ----------                                                   3767
      p+2  TGAACAAATT 1       0      3       5      3      1.39e-01       3444
           ----------                                                   3439
           ----------                                                   3177
           ----------                                                   3019
           ----------                                                   2764
           ----------                                                   2513
      p+2  TGAATCTGTA 0       0      0       0      0      6.15e-01       2342
           ----------                                                   2112
           ----------                                                   1479
           ----------                                                   1442
           ----------                                                   1395
           ----------                                                   1311
           ----------                                                   993
           ----------                                                   865
           ----------                                                   812
           ----------                                                   734
           ----------                                                   642
           ----------                                                   380
           ----------                                                   164

LOCUS: AT4G21560
DESCRIPTION: vacuolar protein sorting-associated protein 28 family protein / VPS28 family protein, contains
similarity to Swiss-Prot:Q02767 vacuolar protein sorting-associated protein VPS28 (Saccharomyces cerevisiae)
DATA:                 Control 30min  2hours  2days  1week  p-value        pos
SENSE COUNTS:         1       3      2       0      0      3.16e-01
GENES (3 total):
  AT4G21560.1
    SENSE COUNTS:     1       3      2       0      0      3.16e-01
    TAGS: (1 total)
           ----------                                                   1312
           ----------                                                   1253
      d+2  ACAATTTGCT 1       3      2       0      0      3.16e-01       1149
           ----------                                                   1020
           ----------                                                   813
           ----------                                                   792
  AT4G21560.2
    SENSE COUNTS:     1       3      2       0      0      3.16e-01
    TAGS: (1 total)
           ----------                                                   1295
           ----------                                                   1236
      d+2  ACAATTTGCT 1       3      2       0      0      3.16e-01       1132
           ----------                                                   1003
           ----------                                                   796
           ----------                                                   775
  AT4G21560.3
    SENSE COUNTS:     1       3      2       0      0      3.16e-01
    TAGS: (1 total)
           ----------                                                   1593
           ----------                                                   1534
      d+2  ACAATTTGCT 1       3      2       0      0      3.16e-01       1430
           ----------                                                   1301
           ----------                                                   1094
           ----------                                                   1073
           ----------                                                   394

LOCUS: AT1G78630
DESCRIPTION: ribosomal protein L13 family protein, similar to ribosomal protein L13 GI:170132 from (Spinacia
oleracea)
DATA:                 Control 30min  2hours  2days  1week  p-value        pos
SENSE COUNTS:         11      8      4       9      3      3.18e-01
GENES (1 total):
  AT1G78630.1
    SENSE COUNTS:     11      8      4       9      3      3.18e-01
    TAGS: (3 total)
      d+1  TAATGAATAC 0       0      0       1      0      6.04e-01       981
           ----------                                                   967
      d+2  CTGTTCGTGG 11      8      3       8      3      2.40e-01       622
```

```
         ----------                                                          462
      d+2  TGAACACGGA  0       0       1       0       0       4.55e-01      337
           ----------                                                          314

LOCUS: AT5G18230
DESCRIPTION: transcription regulator NOT2/NOT3/NOT5 family protein, contains Pfam domain PF04153: NOT2 / NOT3
/ NOT5 family
DATA:                 Control 30min   2hours  2days   1week   p-value       pos
SENSE COUNTS:         3       0       1       0       3       3.19e-01
GENES (1 total):
  AT5G18230.1
    SENSE COUNTS:     3       0       1       0       3       3.19e-01
    TAGS: (3 total)
      i+3  TTTTCCGATT  2       0       0       0       0       3.22e-01      5466
      d+1  TTTGTATATA  1       0       0       0       3       7.14e-02      2733
           ----------                                                      2513
           ----------                                                      2239
           ----------                                                      2085
           ----------                                                      1954
           ----------                                                      1908
           ----------                                                      1872
           ----------                                                      1828
           ----------                                                      1723
      d+2  GGGAGTTTGT  0       0       1       0       0       4.55e-01      1227
           ----------                                                      957
           ----------                                                      377

LOCUS: AT3G47370
DESCRIPTION: 40S ribosomal protein S20 (RPS20B), 40S RIBOSOMAL PROTEIN S20 - ARABIDOPSIS THALIANA,PID:g1350956
DATA:                 Control 30min   2hours  2days   1week   p-value       pos
SENSE COUNTS:         2       4       1       0       1       3.20e-01
GENES (2 total):
  AT3G47370.1
    SENSE COUNTS:     2       4       1       0       1       3.20e-01
    TAGS: (2 total)
      i+3  CAAAACAGAA  2       0       0       0       0       1.04e-01      818
      d+1  GCGTATGAAC  0       4       1       0       1       9.77e-02      91
  AT3G47370.2
    SENSE COUNTS:     2       4       1       0       1       3.20e-01
    TAGS: (2 total)
      i+3  CAAAACAGAA  2       0       0       0       0       1.04e-01      799
      d+1  GCGTATGAAC  0       4       1       0       1       9.77e-02      62

LOCUS: AT2G20040
DESCRIPTION: protein kinase, putative, similar to protein kinase (Homo sapiens) gi|1052737|emb|CAA59733
DATA:                 Control 30min   2hours  2days   1week   p-value       pos
SENSE COUNTS:         0       1       1       4       3       3.21e-01
GENES (1 total):
  AT2G20040.1
    SENSE COUNTS:     0       1       1       4       3       3.21e-01
    TAGS: (1 total)
           ----------                                                      1402
           ----------                                                      1390
      d+2  AGAAAGTTTA  0       1       1       4       3       3.21e-01      1217
           ----------                                                      748
           ----------                                                      672
           ----------                                                      166

LOCUS: AT1G32140
DESCRIPTION: F-box family protein, contains F-box domain Pfam:PF00646
DATA:                 Control 30min   2hours  2days   1week   p-value       pos
SENSE COUNTS:         0       1       3       0       1       3.22e-01
GENES (1 total):
  AT1G32140.1
    SENSE COUNTS:     0       1       3       0       1       3.22e-01
    TAGS: (1 total)
           ----------                                                      2517
           ----------                                                      2408
           ----------                                                      2332
           ----------                                                      1787
           ----------                                                      1700
           ----------                                                      1495
      i+3  TGATAAAAAA  0       1       3       0       1       3.22e-01      1329
           ----------                                                      1190
           ----------                                                      1063
           ----------                                                      881
           ----------                                                      864
           ----------                                                      816
           ----------                                                      562
           ----------                                                      546
```

```
LOCUS: AT3G01120
DESCRIPTION: cystathionine gamma-synthase, chloroplast / O-succinylhomoserine (Thiol)-lyase (CGS), identical
to SP|P55217 Cystathionine gamma-synthase, chloroplast precursor (EC 4.2.99.9) (CGS) (O-succinylhomoserine
(Thiol)-lyase) {Arabidopsis thaliana}
DATA:                  Control 30min   2hours  2days   1week   p-value         pos
SENSE COUNTS:          10      12      10      5       17      3.22e-01
GENES (2 total):
  AT3G01120.1
    SENSE COUNTS:      10      12      10      5       17      3.22e-01
    TAGS: (2 total)
           ----------                                                          2206
           ----------                                                          2065
           ----------                                                          1981
      d+2  TCCTACTGGG  10      12      10      5       16      4.58e-01        1628
      d+2  AACTCGCCAA  0       0       0       0       1       1.65e-01        1467
           ----------                                                          1431
           ----------                                                          1343
           ----------                                                          1290
           ----------                                                          932
           ----------                                                          612
           ----------                                                          195

LOCUS: AT1G14380
DESCRIPTION: calmodulin-binding family protein, contains Pfam profile PF00612: IQ calmodulin-binding motif
DATA:                  Control 30min   2hours  2days   1week   p-value         pos
SENSE COUNTS:          3       1       0       1       0       3.23e-01
GENES (2 total):
  AT1G14380.1
    SENSE COUNTS:      3       1       0       1       0       3.23e-01
    TAGS: (2 total)
           ----------                                                          2565
           ----------                                                          2545
      d+2  TCTAGTCTAT  3       0       0       1       0       7.99e-02        2291
           ----------                                                          2209
           ----------                                                          1907
      d+2  TATATGGGGA  0       1       0       0       0       2.54e-01        595
  AT1G14380.2
    SENSE COUNTS:      3       1       0       1       0       3.23e-01
    TAGS: (2 total)
           ----------                                                          2379
           ----------                                                          2359
      d+2  TCTAGTCTAT  3       0       0       1       0       7.99e-02        2105
           ----------                                                          2023
           ----------                                                          1721
      d+2  TATATGGGGA  0       1       0       0       0       2.54e-01        595

LOCUS: AT1G33600
DESCRIPTION: leucine-rich repeat family protein, contains leucine rich-repeat (LRR) domains Pfam:PF00560,
INTERPRO:IPR001611; contains similarity to gi|9294355|dbj|BAB02252 (Arabidopsis thaliana)
DATA:                  Control 30min   2hours  2days   1week   p-value         pos
SENSE COUNTS:          2       2       5       5       0       3.23e-01
GENES (1 total):
  AT1G33600.1
    SENSE COUNTS:      2       2       5       5       0       3.23e-01
    TAGS: (1 total)
           ----------                                                          1542
           ----------                                                          1536
      d+2  GGGAAGCTGA  2       2       5       5       0       3.23e-01        1225
           ----------                                                          1158
           ----------                                                          1036
           ----------                                                          943
           ----------                                                          664
           ----------                                                          189

LOCUS: AT1G21520
DESCRIPTION: expressed protein
DATA:                  Control 30min   2hours  2days   1week   p-value         pos
SENSE COUNTS:          3       1       7       3       3       3.23e-01
GENES (1 total):
  AT1G21520.1
    SENSE COUNTS:      3       1       7       3       3       3.23e-01
    TAGS: (2 total)
      v+1  TGATTGTAGT  2       1       3       2       3       9.47e-01        888
           ----------                                                          873
      v+2  ATGCGTTGTG  1       0       4       1       0       8.52e-02        799
           ----------                                                          520
           ----------                                                          190
           ----------                                                          152

LOCUS: AT5G15980
DESCRIPTION: pentatricopeptide (PPR) repeat-containing protein, contains Pfam profile PF01535: PPR repeat
```

```
DATA:                 Control 30min  2hours  2days  1week  p-value       pos
SENSE COUNTS:         2       1      0       4      4      3.26e-01
GENES (1 total):
  AT5G15980.1
    SENSE COUNTS:     2       1      0       4      4      3.26e-01
    TAGS: (2 total)
      d+1  GATTGTTCCG 2       0      0       0      1      4.58e-01      2443
      d+2  TATCACTCAC 0       1      0       4      3      1.45e-01      2319
           ----------                                                  2169
           ----------                                                  1959
           ----------                                                  1521
           ----------                                                  1066
           ----------                                                  754
           ----------                                                  675

LOCUS: AT3G62420
DESCRIPTION: bZIP transcription factor family protein, similar to common plant regulatory factor 6 GI:9650826
from (Petroselinum crispum)
DATA:                 Control 30min  2hours  2days  1week  p-value       pos
SENSE COUNTS:         8       8      5       1      8      3.27e-01
GENES (1 total):
  AT3G62420.1
    SENSE COUNTS:     8       8      5       1      8      3.27e-01
    TAGS: (1 total)
           ----------                                                  1151
      v+2  TAATTGGTGT 8       8      5       1      8      3.27e-01      1004
           ----------                                                  168

LOCUS: AT1G71170
DESCRIPTION: 6-phosphogluconate dehydrogenase NAD-binding domain-containing protein, contains Pfam profile:
PF03446 NAD binding domain of 6-phosphogluconate
DATA:                 Control 30min  2hours  2days  1week  p-value       pos
SENSE COUNTS:         2       0      1       1      5      3.28e-01
GENES (1 total):
  AT1G71170.1
    SENSE COUNTS:     2       0      1       1      5      3.28e-01
    TAGS: (1 total)
           ----------                                                  1387
           ----------                                                  1214
           ----------                                                  1208
      d+2  GAGTCTCACC 2       0      1       1      5      3.28e-01      969
           ----------                                                  584
           ----------                                                  323

LOCUS: AT2G22870
DESCRIPTION: expressed protein
DATA:                 Control 30min  2hours  2days  1week  p-value       pos
SENSE COUNTS:         2       0      1       1      5      3.28e-01
GENES (1 total):
  AT2G22870.1
    SENSE COUNTS:     2       0      1       1      5      3.28e-01
    TAGS: (2 total)
           ----------                                                  1466
           ----------                                                  1446
           ----------                                                  1392
      d+2  AATTGCCGAA 0       0      0       0      0      6.15e-01      1083
      d+2  TCGCAGCTGA 2       0      1       1      5      1.41e-01      893

LOCUS: AT2G29100
DESCRIPTION: glutamate receptor family protein (GLR2.9), plant glutamate receptor family, PMID:11379626
DATA:                 Control 30min  2hours  2days  1week  p-value       pos
SENSE COUNTS:         0       0      3       1      3      3.28e-01
GENES (2 total):
  AT2G29100.1
    SENSE COUNTS:     0       0      3       1      3      3.28e-01
    TAGS: (1 total)
           ----------                                                  3251
           ----------                                                  3133
           ----------                                                  3035
      v+2  AATTCGCATA 0       0      3       1      3      3.28e-01      2940
           ----------                                                  2574
           ----------                                                  2431
           ----------                                                  2193
           ----------                                                  2027
           ----------                                                  1898
           ----------                                                  1587
           ----------                                                  1189
           ----------                                                  1023
           ----------                                                  990
           ----------                                                  888
           ----------                                                  579
```

```
LOCUS: AT3G06790
DESCRIPTION: plastid developmental protein DAG, putative, similar to DAG protein, chloroplast precursor
(Garden snapdragon) SWISS-PROT:Q38732
DATA:                 Control 30min   2hours  2days   1week   p-value         pos
SENSE COUNTS:         4       0       4       1       5       3.30e-01
GENES (2 total):
  AT3G06790.1
    SENSE COUNTS:     4       0       4       1       5       3.30e-01
    TAGS: (2 total)
           ----------                                                         1700
      d+2  TCTCTAATGA 0       0       0       1       0       3.09e-01        906
      d+2  TGAGAGACTT 4       0       4       0       5       1.59e-01        851
           ----------                                                         832
           ----------                                                         780
           ----------                                                         718
           ----------                                                         337
           ----------                                                         151
  AT3G06790.2
    SENSE COUNTS:     4       0       4       1       5       3.30e-01
    TAGS: (2 total)
           ----------                                                         1700
      d+2  TCTCTAATGA 0       0       0       1       0       3.09e-01        906
      d+2  TGAGAGACTT 4       0       4       0       5       1.59e-01        851
           ----------                                                         832
           ----------                                                         780
           ----------                                                         718
           ----------                                                         337
           ----------                                                         151

LOCUS: AT5G04290
DESCRIPTION: KOW domain-containing transcription factor family protein
DATA:                 Control 30min   2hours  2days   1week   p-value         pos
SENSE COUNTS:         3       0       1       3       4       3.31e-01
GENES (1 total):
  AT5G04290.1
    SENSE COUNTS:     3       0       1       3       4       3.31e-01
    TAGS: (4 total)
           ----------                                                         5209
           ----------                                                         4969
           ----------                                                         4918
           ----------                                                         4751
           ----------                                                         3719
           ----------                                                         3359
           ----------                                                         3182
           ----------                                                         3047
           ----------                                                         2815
           ----------                                                         2609
           ----------                                                         2546
           ----------                                                         2517
           ----------                                                         2491
           ----------                                                         2391
           ----------                                                         2003
      v+2  GGCTCGTGTT 0       0       0       0       0       6.15e-01        1085
      v+2  CAAGAGTTTA 0       0       0       1       0       3.09e-01        974
           ----------                                                         958
           ----------                                                         919
      v+2  CACAAATTTG 2       0       1       2       3       4.02e-01        849
      v+2  TTATTCTAAA 1       0       0       0       1       3.83e-01        6

LOCUS: AT4G06566
DESCRIPTION: gypsy-like retrotransposon family, has a 9.9e-101 P-value blast match to GB:AAD19359 polyprotein
(gypsy_Ty3-element) (Sorghum bicolor)
DATA:                 Control 30min   2hours  2days   1week   p-value         pos
SENSE COUNTS:         3       4       1       1       0       3.34e-01
GENES (1 total):
  AT4G06566.1
    SENSE COUNTS:     3       4       1       1       0       3.34e-01
    TAGS: (1 total)
           ----------                                                         4600
           ----------                                                         4433
      p+2  CCGCGGTGAA 3       4       1       1       0       3.34e-01        4393
           ----------                                                         4353
           ----------                                                         4140
           ----------                                                         3999
           ----------                                                         3482
           ----------                                                         3428
           ----------                                                         3327
           ----------                                                         2897
           ----------                                                         2793
           ----------                                                         2666
```

```
          ----------                                                    2565
          ----------                                                    1924
          ----------                                                    1787
          ----------                                                    1716
          ----------                                                    1699
          ----------                                                    1689
          ----------                                                    1315
          ----------                                                    1098
          ----------                                                    997

LOCUS: AT1G65970
DESCRIPTION: peroxiredoxin type 2, putative, strong similarity to type 2 peroxiredoxin (Brassica rapa subsp.
pekinensis) GI:4928472; contains Pfam profile: PF00578 AhpC/TSA (alkyl hydroperoxide reductase and thiol-
specific antioxidant) family
DATA:                 Control 30min   2hours  2days   1week   p-value       pos
SENSE COUNTS:         4       3       5       0       1       3.34e-01
GENES (2 total):
  AT1G65970.1
    SENSE COUNTS:     4       3       5       0       1       3.34e-01
    TAGS: (1 total)
          ----------                                                    988
      d+2  TGTTTGACTT 4       3       5       0       1       3.34e-01      642
          ----------                                                    353
          ----------                                                    324
          ----------                                                    227
          ----------                                                    213
          ----------                                                    64

LOCUS: AT3G19100
DESCRIPTION: calcium-dependent protein kinase, putative / CDPK, putative, similar to calcium/calmodulin-
dependent protein kinase CaMK3 (Nicotiana tabacum) gi|16904226|gb|AAL30820; contains protein kinase domain,
Pfam:PF00069; contains serine/threonine protein kinase d
DATA:                 Control 30min   2hours  2days   1week   p-value       pos
SENSE COUNTS:         3       0       4       2       3       3.35e-01
GENES (1 total):
  AT3G19100.1
    SENSE COUNTS:     3       0       4       2       3       3.35e-01
    TAGS: (2 total)
      i+3  CTTAACAATT 2       0       0       0       0       1.04e-01      3209
      d+1  GAAGACAGAT 1       0       4       2       3       2.21e-01      2144
          ----------                                                    1704
          ----------                                                    1668

LOCUS: AT5G13620
DESCRIPTION: expressed protein, ; expression supported by MPSS
DATA:                 Control 30min   2hours  2days   1week   p-value       pos
SENSE COUNTS:         3       3       0       1       3       3.36e-01
GENES (1 total):
  AT5G13620.1
    SENSE COUNTS:     3       3       0       1       3       3.36e-01
    TAGS: (3 total)
      v+1  GACTTTTTTT 1       0       0       0       0       4.28e-01      1388
          ----------                                                    1183
          ----------                                                    1122
          ----------                                                    1059
          ----------                                                    1012
          ----------                                                    738
          ----------                                                    483
      v+2  AATAATTGGG 2       3       0       1       3       4.55e-01      194
      v+2  AAAATAGCTA 0       0       0       0       0       6.15e-01      112

LOCUS: AT4G35470
DESCRIPTION: leucine-rich repeat family protein, similar to Leucine-rich repeat protein SHOC-2 (Ras-binding
protein Sur-8) (SP:Q9UQ13 ){Homo sapiens},PIR:T12704; contains Pfam PF00560: Leucine Rich Repeat domains
DATA:                 Control 30min   2hours  2days   1week   p-value       pos
SENSE COUNTS:         5       6       2       2       1       3.42e-01
GENES (1 total):
  AT4G35470.1
    SENSE COUNTS:     5       6       2       2       1       3.42e-01
    TAGS: (6 total)
          ----------                                                    2472
          ----------                                                    2460
          ----------                                                    2436
      d+2  GTCTTTCTTT 1       0       0       0       1       3.83e-01      2422
      d+2  AGTTGGCTTT 1       3       1       0       0       2.52e-01      2304
      d+2  TTTGTCTATC 1       0       0       0       0       4.28e-01      2181
          ----------                                                    2142
      d+2  TCTCTCCACA 1       0       0       2       0       1.75e-01      2095
          ----------                                                    2064
      d+2  GAGATTGTCT 0       0       1       0       0       4.55e-01      1869
          ----------                                                    1758
```

```
          ----------                                                      1563
     X+4  TGAACCAATG 1      3      0      0      0      1.03e-01          559
          ----------                                                      447
          ----------                                                      294

LOCUS: AT4G32940
DESCRIPTION: vacuolar processing enzyme gamma / gamma-VPE, nearly identical to SP|Q39119 Vacuolar processing
enzyme, gamma-isozyme precursor (EC 3.4.22.-) (Gamma-VPE) {Arabidopsis thaliana}
DATA:                 Control 30min  2hours 2days  1week  p-value          pos
SENSE COUNTS:         11      12     9      6      15     3.43e-01
GENES (2 total):
  AT4G32940.1
    SENSE COUNTS:     11      12     9      6      15     3.43e-01
    TAGS: (6 total)
      i+3  GGATAACATC 2       0      0      0      0      3.22e-01         2327
      d+1  GATGGTTTAC 1       5      2      4      7      3.45e-01         1822
      d+2  TACAGAAGCT 8       7      7      2      8      6.56e-01         1741
      d+2  AGGTCTTTTG 0       0      0      0      0      6.15e-01         1467
           ----------                                                      1269
           ----------                                                      1044
           ----------                                                      1039
           ----------                                                      721
           ----------                                                      640
      i+3  TAGAGTTTAG 0       0      0      0      0      6.15e-01         504
           ----------                                                      472
      d+2  TATGATGATA 0       0      0      0      0      6.15e-01         408
           ----------                                                      352

LOCUS: AT1G49140
DESCRIPTION: NADH-ubiquinone oxidoreductase-related, similar to NADH-ubiquinone oxidoreductase 12 kDa subunit,
mitochondrial precursor (EC 1.6.5.3) (EC 1.6.99.3) (Complex I-12KD) (CI-12KD). (Swiss-Prot:Q03015) (Neurospora
crassa)
DATA:                 Control 30min  2hours 2days  1week  p-value          pos
SENSE COUNTS:         5       2      3      1      0      3.44e-01
GENES (1 total):
  AT1G49140.1
    SENSE COUNTS:     5       2      3      1      0      3.44e-01
    TAGS: (2 total)
      i+3  GACTTTCTTC 0       0      0      0      0      6.15e-01         901
      d+1  AATGGAGTCT 5       2      3      1      0      3.08e-01         403

LOCUS: AT3G14100
DESCRIPTION: oligouridylate-binding protein, putative, similar to GB:CAB75429 (GI:6996560) from (Nicotiana
plumbaginifolia), contains Pfam profiles: PF00076 RNA recognition motif (3 copies)
DATA:                 Control 30min  2hours 2days  1week  p-value          pos
SENSE COUNTS:         15      7      7      7      9      3.44e-01
GENES (1 total):
  AT3G14100.1
    SENSE COUNTS:     15      7      7      7      9      3.44e-01
    TAGS: (3 total)
           ----------                                                      1815
      d+2  AACATAAACA 3       1      3      5      1      6.61e-01         1624
      d+2  GATTGTAAGA 12      6      3      2      8      7.92e-02         1556
           ----------                                                      1381
           ----------                                                      1336
      d+2  GAATAAATGC 0       0      1      0      0      4.55e-01         1304
           ----------                                                      1119
           ----------                                                      968

LOCUS: AT2G21380
DESCRIPTION: kinesin motor protein-related
DATA:                 Control 30min  2hours 2days  1week  p-value          pos
SENSE COUNTS:         1       0      3      2      2      3.44e-01
GENES (1 total):
  AT2G21380.1
    SENSE COUNTS:     1       0      3      2      2      3.44e-01
    TAGS: (2 total)
      i+3  TGTGAGCTTC 0       0      0      0      1      1.65e-01         6808
           ----------                                                      3586
      d+2  CTAACAGAAA 1       0      3      2      1      3.98e-01         3496
           ----------                                                      3004
           ----------                                                      2844
           ----------                                                      2675
           ----------                                                      2613
           ----------                                                      2392
           ----------                                                      1974
           ----------                                                      1294
           ----------                                                      1288
           ----------                                                      1231
           ----------                                                      1054
           ----------                                                      976
```

```
          ----------                                                  937
          ----------                                                  706

LOCUS: AT3G21500
DESCRIPTION: 1-deoxy-D-xylulose 5-phosphate synthase, putative / 1-deoxyxylulose-5-phosphate synthase,
putative / DXP-synthase, putative, strong similarity to 1-D-deoxyxylulose 5-phosphate synthase (Lycopersicon
esculentum) GI:5059160, DEF (deficient in photosynthesis
DATA:                 Control 30min  2hours  2days  1week  p-value       pos
SENSE COUNTS:         7       0      3       2      3      3.44e-01
GENES (2 total):
  AT3G21500.1
    SENSE COUNTS:     7       0      3       2      3      3.44e-01
    TAGS: (3 total)
      d+1  AGAGTGCTCT 0       0      0       0      0      6.15e-01      1998
           ----------                                                  1878
           ----------                                                  1817
           ----------                                                  1471
      d+2  ATAGTAATGG 6       0      3       2      3      2.40e-01      1429
           ----------                                                  1416
           ----------                                                  1307
           ----------                                                  1279
           ----------                                                  1208
           ----------                                                  1144
           ----------                                                  1129
           ----------                                                  1121
           ----------                                                  992
           ----------                                                  932
           ----------                                                  670
      d+2  ACTCTTTTGG 1       0      0       0      0      6.89e-01      494
           ----------                                                  362
           ----------                                                  208
  AT3G21500.2
    SENSE COUNTS:     7       0      3       2      3      3.44e-01
    TAGS: (3 total)
      d+1  AGAGTGCTCT 0       0      0       0      0      6.15e-01      2001
           ----------                                                  1881
           ----------                                                  1820
           ----------                                                  1474
      d+2  ATAGTAATGG 6       0      3       2      3      2.40e-01      1432
           ----------                                                  1419
           ----------                                                  1310
           ----------                                                  1282
           ----------                                                  1211
           ----------                                                  1147
           ----------                                                  1132
           ----------                                                  1124
           ----------                                                  992
           ----------                                                  932
           ----------                                                  670
      d+2  ACTCTTTTGG 1       0      0       0      0      6.89e-01      494
           ----------                                                  362
           ----------                                                  208

LOCUS: AT1G55520
DESCRIPTION: transcription initiation factor IID-2 (TFIID-2) / TATA-box factor 2 / TATA sequence-binding
protein 2 (TBP2), identical to Swiss-Prot:P28148|TF22_ARATH Transcription initiation factor TFIID-2 (TATA-box
factor 2) (TATA sequence-binding protein 2) (TBP-2) (
DATA:                 Control 30min  2hours  2days  1week  p-value       pos
SENSE COUNTS:         3       0      4       2      0      3.44e-01
GENES (3 total):
  AT1G55520.2
    SENSE COUNTS:     3       0      4       2      0      3.44e-01
    TAGS: (1 total)
           ----------                                                  1096
           ----------                                                  928
           ----------                                                  908
      d+2  TGATGTCAAA 3       0      4       2      0      3.44e-01      632
           ----------                                                  453
  AT1G55520.1
    SENSE COUNTS:     3       0      4       2      0      3.44e-01
    TAGS: (1 total)
           ----------                                                  1093
           ----------                                                  925
           ----------                                                  905
      d+2  TGATGTCAAA 3       0      4       2      0      3.44e-01      632
           ----------                                                  453

LOCUS: AT1G60010
DESCRIPTION: expressed protein
DATA:                 Control 30min  2hours  2days  1week  p-value       pos
SENSE COUNTS:         3       1      1       0      0      3.45e-01
```

```
GENES (1 total):
  AT1G60010.1
    SENSE COUNTS:      3       1       1       0       0       3.45e-01
    TAGS: (1 total)
           ----------                                                          905
      d+2  GAGGCCATCA  3       1       1       0       0       3.45e-01        626
           ----------                                                          406
           ----------                                                          246

LOCUS: AT2G18160
DESCRIPTION: bZIP transcription factor family protein, contains a bZIP transcription factor basic domain
signature (PDOC00036)
DATA:                  Control 30min   2hours  2days   1week   p-value         pos
SENSE COUNTS:          9       5       12      4       4       3.45e-01
GENES (2 total):
  AT2G18160.1
    SENSE COUNTS:      9       5       12      4       4       3.45e-01
    TAGS: (5 total)
      d+1  ATTTTGTCTT  1       0       1       1       0       7.18e-01        1200
      d+2  TATTGGTGTG  2       2       4       0       3       5.16e-01        1146
      d+2  CATCTTTGAT  3       0       1       1       0       2.14e-01        1066
      d+2  AAGATCCAAG  2       3       2       0       0       3.95e-01        689
      d+2  AATTGCTACA  1       0       4       2       1       4.59e-01        326

LOCUS: AT1G21400
DESCRIPTION: 2-oxoisovalerate dehydrogenase, putative / 3-methyl-2-oxobutanoate dehydrogenase, putative /
branched-chain alpha-keto acid dehydrogenase E1 alpha subunit, putative, similar to branched-chain alpha-keto
acid dehydrogenase E1-alpha subunit (Gallus gallus)
DATA:                  Control 30min   2hours  2days   1week   p-value         pos
SENSE COUNTS:          3       4       1       0       3       3.49e-01
GENES (1 total):
  AT1G21400.1
    SENSE COUNTS:      3       4       1       0       3       3.49e-01
    TAGS: (1 total)
           ----------                                                          1881
           ----------                                                          1639
      d+2  AAATGGCCTT  3       4       1       0       3       3.49e-01        1611
           ----------                                                          1517
           ----------                                                          1468
           ----------                                                          371
           ----------                                                          223

LOCUS: AT5G02740
DESCRIPTION: expressed protein
DATA:                  Control 30min   2hours  2days   1week   p-value         pos
SENSE COUNTS:          5       0       3       2       1       3.50e-01
GENES (2 total):
  AT5G02740.1
    SENSE COUNTS:      5       0       3       2       1       3.50e-01
    TAGS: (2 total)
      d+1  TGGTGAAGTG  3       0       0       1       1       3.94e-01        1067
      d+2  TGTCTTAGTA  2       0       3       1       0       2.70e-01        963
           ----------                                                          541
           ----------                                                          292
  AT5G02740.2
    SENSE COUNTS:      5       0       3       2       1       3.50e-01
    TAGS: (2 total)
      d+1  TGGTGAAGTG  3       0       0       1       1       3.94e-01        1433
      d+2  TGTCTTAGTA  2       0       3       1       0       2.70e-01        1329
           ----------                                                          992
           ----------                                                          963
           ----------                                                          857
           ----------                                                          490
           ----------                                                          241

LOCUS: AT5G19450
DESCRIPTION: calcium-dependent protein kinase 19 (CDPK19), identical to calcium-dependent protein kinase
(Arabidopsis thaliana) gi|836942|gb|AAA67655
DATA:                  Control 30min   2hours  2days   1week   p-value         pos
SENSE COUNTS:          2       4       2       0       5       3.50e-01
GENES (3 total):
  AT5G19450.2
    SENSE COUNTS:      2       4       2       0       5       3.50e-01
    TAGS: (3 total)
      d+1  TTGTCTGGAT  2       0       1       0       5       5.50e-02        2006
      d+2  TACGATATGA  0       0       1       0       0       4.55e-01        1957
      d+2  GCCGAGAGTT  0       4       0       0       0       9.72e-03        1051
           ----------                                                          884
           ----------                                                          735
           ----------                                                          363
  AT5G19450.1
```

```
    SENSE COUNTS:      2       4       2       0       5       3.50e-01
    TAGS: (3 total)
      d+1  TTGTCTGGAT  2       0       1       0       5       5.50e-02        1861
      d+2  TACGATATGA  0       0       1       0       0       4.55e-01        1812
      d+2  GCCGAGAGTT  0       4       0       0       0       9.72e-03        906
           ----------                                                          739
           ----------                                                          590
           ----------                                                          218

LOCUS: AT5G08540
DESCRIPTION: expressed protein, similar to unknown protein (pir||T27191)
DATA:                  Control 30min   2hours  2days   1week   p-value         pos
SENSE COUNTS:          3       4       0       5       3       3.51e-01
GENES (1 total):
  AT5G08540.1
    SENSE COUNTS:      3       4       0       5       3       3.51e-01
    TAGS: (1 total)
           ----------                                                          1742
           ----------                                                          1736
      d+2  ACACAGCTCG  3       4       0       5       3       3.51e-01        1280
           ----------                                                          656

LOCUS: AT5G06110
DESCRIPTION: DNAJ heat shock N-terminal domain-containing protein / cell division protein-related, similar to
GlsA (Volvox carteri f. nagariensis) GI:4633129; contains Pfam profiles PF00226 DnaJ domain, PF00249 Myb-like
DNA-binding domain
DATA:                  Control 30min   2hours  2days   1week   p-value         pos
SENSE COUNTS:          3       7       4       9       10      3.52e-01
GENES (1 total):
  AT5G06110.1
    SENSE COUNTS:      3       7       4       9       10      3.52e-01
    TAGS: (2 total)
      d+1  CTGCTCTTAT  3       2       2       5       10      1.14e-01        2103
           ----------                                                          1703
           ----------                                                          1453
           ----------                                                          1412
           ----------                                                          1382
           ----------                                                          1364
           ----------                                                          1186
           ----------                                                          1169
      d+2  ATATCGAGCA  0       5       2       4       0       1.14e-01        755
           ----------                                                          299
           ----------                                                          134

LOCUS: AT4G12600
DESCRIPTION: ribosomal protein L7Ae/L30e/S12e/Gadd45 family protein, Similar to NHP2/L7Ae family proteins, see
SWISSPROT:P32495 and PMID:2063628.
DATA:                  Control 30min   2hours  2days   1week   p-value         pos
SENSE COUNTS:          3       3       3       0       0       3.53e-01
GENES (1 total):
  AT4G12600.1
    SENSE COUNTS:      3       3       3       0       0       3.53e-01
    TAGS: (2 total)
      X+4  TAAGTCTTCG  0       0       1       0       0       4.55e-01        646
      d+1  TGATGTAACC  3       3       2       0       0       3.51e-01        373

LOCUS: AT4G01940
DESCRIPTION: nitrogen fixation NifU-like family protein, similar to apricot NifU homolog partial CDS, GenBank
accession number U95179; contains Pfam profile: PF01106 NifU-like domain
DATA:                  Control 30min   2hours  2days   1week   p-value         pos
SENSE COUNTS:          1       2       5       4       7       3.55e-01
GENES (2 total):
  AT4G01940.1
    SENSE COUNTS:      1       2       5       4       7       3.55e-01
    TAGS: (1 total)
           ----------                                                          975
      d+2  AAGAAAGCCA  1       2       5       4       7       3.55e-01        776
           ----------                                                          449

LOCUS: AT1G27150
DESCRIPTION: expressed protein
DATA:                  Control 30min   2hours  2days   1week   p-value         pos
SENSE COUNTS:          0       3       1       1       3       3.55e-01
GENES (1 total):
  AT1G27150.1
    SENSE COUNTS:      0       3       1       1       3       3.55e-01
    TAGS: (2 total)
      d+1  TTTTTAAATA  0       3       0       0       0       3.05e-02        1569
           ----------                                                          1557
           ----------                                                          1459
           ----------                                                          1429
```

```
          ----------                                                  1124
          ----------                                                  830
          ----------                                                  808
          ----------                                                  766
          ----------                                                  734
          ----------                                                  722
          ----------                                                  605
          ----------                                                  532
     X+4  AATTTTAAAA  0       0       1       1       3       1.91e-01       274
          ----------                                                  29

LOCUS: AT4G25500
DESCRIPTION: arginine/serine-rich splicing factor RSP40 (RSP40), identical to SP|P92965 Arginine/serine-rich
splicing factor RSP40 {Arabidopsis thaliana}
DATA:                 Control 30min   2hours  2days   1week   p-value        pos
SENSE COUNTS:         0       0       3       1       0       3.56e-01
GENES (3 total):
  AT4G25500.2
    SENSE COUNTS:     0       0       3       1       0       3.56e-01
    TAGS: (2 total)
      i+3  AATCGTAAAC 0       0       0       0       0       6.15e-01       1163
      d+1  AGACCTTCCA 0       0       3       1       0       2.61e-01       212
           ----------                                                        59
  AT4G25500.1
    SENSE COUNTS:     0       0       3       1       0       3.56e-01
    TAGS: (2 total)
      i+3  AATCGTAAAC 0       0       0       0       0       6.15e-01       2583
      d+1  AGACCTTCCA 0       0       3       1       0       2.61e-01       365
           ----------                                                        212
           ----------                                                        89

LOCUS: AT3G23540
DESCRIPTION: expressed protein, ; expression supported by MPSS
DATA:                 Control 30min   2hours  2days   1week   p-value        pos
SENSE COUNTS:         0       1       3       1       0       3.56e-01
GENES (1 total):
  AT3G23540.1
    SENSE COUNTS:     0       1       3       1       0       3.56e-01
    TAGS: (2 total)
      i+3  GTGATCAGCT 0       1       0       0       0       2.54e-01       3807
           ----------                                                        1536
           ----------                                                        1412
           ----------                                                        1205
           ----------                                                        876
      X+4  ATAGGTTAAT 0       0       3       1       0       9.46e-02       519
           ----------                                                        501
           ----------                                                        240

LOCUS: AT4G39170
DESCRIPTION: SEC14 cytosolic factor, putative / phosphoglyceride transfer protein, putative, similar to
phosphatidylinositol transfer-like protein IV (GI:14486707) (Lotus japonicus) and phosphatidylinositol-
phosphatidylcholine transfer protein SEC14, Yarrowia lipolyti
DATA:                 Control 30min   2hours  2days   1week   p-value        pos
SENSE COUNTS:         0       0       3       2       0       3.56e-01
GENES (1 total):
  AT4G39170.1
    SENSE COUNTS:     0       0       3       2       0       3.56e-01
    TAGS: (2 total)
      d+1  GCTTTTGTGA 0       0       0       0       0       6.15e-01       1667
           ----------                                                        1632
           ----------                                                        1342
           ----------                                                        1257
           ----------                                                        1095
           ----------                                                        810
           ----------                                                        593
      d+2  TTTTGAGGGA 0       0       3       2       0       2.44e-01       250

LOCUS: AT5G58120
DESCRIPTION: disease resistance protein (TIR-NBS-LRR class), putative, domain signature TIR-NBS-LRR exists,
suggestive of a disease resistance protein.
DATA:                 Control 30min   2hours  2days   1week   p-value        pos
SENSE COUNTS:         4       0       1       1       1       3.57e-01
GENES (1 total):
  AT5G58120.1
    SENSE COUNTS:     4       0       1       1       1       3.57e-01
    TAGS: (2 total)
           ----------                                                        3980
           ----------                                                        3853
      d+2  CTTGTTGTTG 3       0       1       1       1       6.79e-01       3237
           ----------                                                        3229
           ----------                                                        3188
```

```
          ----------                                                  2287
          ----------                                                  2266
          ----------                                                  2204
          ----------                                                  2080
          ----------                                                  1661
          ----------                                                  1358
          ----------                                                  1177
          ----------                                                  1140
          ----------                                                  1034
          ----------                                                  1013
          ----------                                                  827
      d+2  GTGGGTATTG 1       0       0       0       0       4.28e-01       565
          ----------                                                  68

LOCUS: AT3G53630
DESCRIPTION: expressed protein, predicted proteins, Arabidopsis thaliana
DATA:                  Control 30min   2hours  2days   1week   p-value        pos
SENSE COUNTS:          2       1       1       2       6       3.57e-01
GENES (1 total):
  AT3G53630.1
    SENSE COUNTS:      2       1       1       2       6       3.57e-01
    TAGS: (3 total)
      i+3  GTCGTTAAAT 0       0       0       0       0       6.15e-01       1092
      d+1  TTTGTTTATT 2       1       1       2       5       5.68e-01       963
           ----------                                                     928
      d+2  TTAATGAAGA 0       0       0       0       1       4.65e-01       897
           ----------                                                     833
           ----------                                                     462
           ----------                                                     451
           ----------                                                     412

LOCUS: AT4G11150
DESCRIPTION: vacuolar ATP synthase subunit E / V-ATPase E subunit / vacuolar proton pump E subunit (VATE),
identical to SP|Q39258 Vacuolar ATP synthase subunit E (EC 3.6.3.14) (V-ATPase E subunit) (Vacuolar proton
pump E subunit) {Arabidopsis thaliana}
DATA:                  Control 30min   2hours  2days   1week   p-value        pos
SENSE COUNTS:          32      25      20      23      35      3.58e-01
GENES (1 total):
  AT4G11150.1
    SENSE COUNTS:      32      25      20      23      35      3.58e-01
    TAGS: (2 total)
      d+1  GCTATACAAA 27      10      15      18      32      9.87e-03       1014
      d+2  GTCTCCACTG 5       15      5       5       3       2.10e-02       677
           ----------                                                     611
           ----------                                                     406

LOCUS: AT2G24750
DESCRIPTION: pseudogene, similar to Avr9/Cf-9 rapidly elicited protein 141, similar to GB:AAB61068; blastp
match of 43% identity and 5.0e-152 P-value to GP|30013669|gb|AAP03877.1||AY220478 Avr9/Cf-9 rapidly elicited
protein 141 {Nicotiana tabacum}
DATA:                  Control 30min   2hours  2days   1week   p-value        pos
SENSE COUNTS:          4       1       7       6       5       3.60e-01
GENES (1 total):
  AT2G24750.1
    SENSE COUNTS:      4       1       7       6       5       3.60e-01
    TAGS: (2 total)
           ----------                                                     6153
           ----------                                                     5976
           ----------                                                     5882
           ----------                                                     5733
           ----------                                                     5591
           ----------                                                     5347
           ----------                                                     5160
           ----------                                                     4895
           ----------                                                     4740
           ----------                                                     4431
           ----------                                                     4404
           ----------                                                     4065
           ----------                                                     3927
           ----------                                                     2873
           ----------                                                     2848
      p+2  ATATAAAAAG 1       0       0       0       0       4.28e-01       2483
           ----------                                                     2455
           ----------                                                     2435
           ----------                                                     2415
           ----------                                                     2007
      p+2  CCAAAAAAAA 3       1       7       6       5       2.95e-01       1556
           ----------                                                     1512
           ----------                                                     777
           ----------                                                     719
           ----------                                                     363
```

```
LOCUS: AT2G39770
DESCRIPTION: GDP-mannose  pyrophosphorylase (GMP1), identical to GDP-mannose pyrophosphorylase from
Arabidopsis thaliana (GI:3598958); updated per Conklin PL et al, PNAS 1999, 96(7):4198-203
DATA:                 Control 30min   2hours  2days   1week   p-value        pos
SENSE COUNTS:         1       6       4       2       7       3.61e-01
GENES (2 total):
  AT2G39770.1
    SENSE COUNTS:     1       6       4       2       7       3.61e-01
    TAGS: (2 total)
      i+3  TATTGGTTAT 0       0       0       1       0       3.09e-01       1352
      d+1  TGAGCGATGA 1       6       4       1       7       2.28e-01       1156
           ----------                                                        1131
           ----------                                                        1063
           ----------                                                        1038
           ----------                                                        258

LOCUS: AT5G22640
DESCRIPTION: MORN (Membrane Occupation and Recognition Nexus) repeat-containing protein, contains Pfam profile
PF02493: MORN repeat
DATA:                 Control 30min   2hours  2days   1week   p-value        pos
SENSE COUNTS:         14      10      9       5       6       3.63e-01
GENES (1 total):
  AT5G22640.1
    SENSE COUNTS:     14      10      9       5       6       3.63e-01
    TAGS: (4 total)
      i+3  GTTGAATGCA 1       0       0       0       0       4.28e-01       2962
      d+1  AGCGTGGTTT 8       2       1       4       1       1.21e-01       2600
           ----------                                                        2299
      i+3  AAATATATTG 0       0       0       0       0       6.15e-01       1751
      d+2  GCATTCGACT 5       8       8       1       5       3.07e-01       1644
           ----------                                                        1176
           ----------                                                        1098
           ----------                                                        923
           ----------                                                        822
           ----------                                                        812

LOCUS: AT1G45207
DESCRIPTION: conserved hypothetical protein, contains similarity to hypothetical proteins from (Arabidopsis
thaliana)
DATA:                 Control 30min   2hours  2days   1week   p-value        pos
SENSE COUNTS:         2       2       3       0       0       3.64e-01
GENES (2 total):
  AT1G45207.2
    SENSE COUNTS:     2       2       3       0       0       3.64e-01
    TAGS: (3 total)
      d+1  TATTCTAGTT 1       0       0       0       0       4.28e-01       1661
           ----------                                                        1589
           ----------                                                        1368
           ----------                                                        1355
           ----------                                                        1337
           ----------                                                        1304
           ----------                                                        1052
      d+2  GCTGCTCTGA 0       0       0       0       0       6.15e-01       1007
           ----------                                                        985
      i+3  ATAACAAAAG 1       2       3       0       0       3.56e-01       955
           ----------                                                        877
           ----------                                                        866
           ----------                                                        776

LOCUS: AT4G02930
DESCRIPTION: elongation factor Tu, putative / EF-Tu, putative, similar to mitochondrial elongation factor Tu
(Arabidopsis thaliana) gi|1149571|emb|CAA61511
DATA:                 Control 30min   2hours  2days   1week   p-value        pos
SENSE COUNTS:         2       0       3       5       4       3.65e-01
GENES (1 total):
  AT4G02930.1
    SENSE COUNTS:     2       0       3       5       4       3.65e-01
    TAGS: (4 total)
           ----------                                                        1745
      d+2  AATTTTCGAT 1       0       1       4       3       1.92e-01       1694
      d+2  CCTGTCCCAC 1       0       1       0       0       6.01e-01       1319
           ----------                                                        1120
           ----------                                                        548
           ----------                                                        447
           ----------                                                        411
      d+2  GCAAGACCAC 0       0       0       1       0       3.09e-01       279
           ----------                                                        270
      d+2  TAAATGTTGG 0       0       1       0       1       6.85e-01       246
           ----------                                                        218
```

```
LOCUS: AT1G21830
DESCRIPTION: expressed protein, EST gb|T21171 comes from this gene
DATA:                  Control 30min   2hours  2days   1week   p-value         pos
SENSE COUNTS:          3       2       8       2       3       3.65e-01
GENES (1 total):
  AT1G21830.1
    SENSE COUNTS:      3       2       8       2       3       3.65e-01
    TAGS: (3 total)
      d+1  GATTTAAAAG  1       1       3       2       0       6.32e-01        861
      X+4  TGGTCTGAAT  1       0       5       0       3       8.43e-02        637
      d+2  TGGCACCATC  1       1       0       0       0       4.77e-01        612
           ----------                                                          466

LOCUS: AT1G06550
DESCRIPTION: enoyl-CoA hydratase/isomerase family protein, similar to CHY1 (gi:8572760); contains Pfam profile
PF00388 enoyl-CoA hydratase/isomerase family protein
DATA:                  Control 30min   2hours  2days   1week   p-value         pos
SENSE COUNTS:          1       0       0       4       1       3.66e-01
GENES (1 total):
  AT1G06550.1
    SENSE COUNTS:      1       0       0       4       1       3.66e-01
    TAGS: (2 total)
      d+1  ATATAATTAG  1       0       0       0       0       6.89e-01        1322
           ----------                                                          1312
      d+2  CAGCGATGAT  0       0       0       4       1       1.05e-01        1276

LOCUS: AT5G04750
DESCRIPTION: F1F0-ATPase inhibitor protein, putative, similar to F1F0-ATPase inhibitor protein (Oryza sativa
(japonica cultivar-group)) gi|5106371|dbj|BAA81661
DATA:                  Control 30min   2hours  2days   1week   p-value         pos
SENSE COUNTS:          5       7       1       4       3       3.68e-01
GENES (1 total):
  AT5G04750.1
    SENSE COUNTS:      5       7       1       4       3       3.68e-01
    TAGS: (2 total)
           ----------                                                          899
           ----------                                                          852
      d+2  AGTTTATCAC  4       7       1       4       3       3.84e-01        340
      d+2  TATATCCAGA  1       0       0       0       0       4.28e-01        227

LOCUS: AT2G29060
DESCRIPTION: scarecrow transcription factor family protein,
DATA:                  Control 30min   2hours  2days   1week   p-value         pos
SENSE COUNTS:          1       1       5       3       1       3.68e-01
GENES (1 total):
  AT2G29060.1
    SENSE COUNTS:      1       1       5       3       1       3.68e-01
    TAGS: (2 total)
           ----------                                                          4045
           ----------                                                          3688
           ----------                                                          3580
           ----------                                                          2651
      i+3  ATCTAAAAAA  1       1       3       2       0       6.32e-01        2217
           ----------                                                          2122
      X+4  TAATTTTAGG  0       0       2       1       1       4.09e-01        1963
           ----------                                                          1837
           ----------                                                          1762
           ----------                                                          1174
           ----------                                                          245
           ----------                                                          220

LOCUS: AT3G59010
DESCRIPTION: pectinesterase family protein, contains Pfam profile: PF01095 pectinesterase
DATA:                  Control 30min   2hours  2days   1week   p-value         pos
SENSE COUNTS:          8       3       4       2       1       3.70e-01
GENES (1 total):
  AT3G59010.1
    SENSE COUNTS:      8       3       4       2       1       3.70e-01
    TAGS: (1 total)
           ----------                                                          1891
      d+2  TGGTAAAAAA  8       3       4       2       1       3.70e-01        1790
           ----------                                                          1746
           ----------                                                          1424
           ----------                                                          1050
           ----------                                                          837
           ----------                                                          730
           ----------                                                          657
           ----------                                                          554
           ----------                                                          462
           ----------                                                          411
```

```
LOCUS: AT2G14300
DESCRIPTION: pseudogene, similar to putative helicase,  blastp match of 40% identity and 2.5e-288 P-value to
GP|14140296|gb|AAK54302.1|AC034258_20|AC034258 putative helicase {Oryza sativa (japonica cultivar-group)}
DATA:                 Control 30min  2hours  2days  1week  p-value      pos
SENSE COUNTS:         3       0      1       2      1      3.71e-01
GENES (1 total):
  AT2G14300.1
    SENSE COUNTS:     3       0      1       2      1      3.71e-01
    TAGS: (1 total)
           ----------                                                   4918
           ----------                                                   4654
           ----------                                                   4072
           ----------                                                   3974
           ----------                                                   3853
           ----------                                                   3846
           ----------                                                   3833
      p+2  ATGAAATTAT 3       0      1       2      1      3.71e-01     3614
           ----------                                                   3566
           ----------                                                   3452
           ----------                                                   3115
           ----------                                                   3071
           ----------                                                   2947
           ----------                                                   2807
           ----------                                                   2713
           ----------                                                   2639
           ----------                                                   2190
           ----------                                                   2150
           ----------                                                   2146
           ----------                                                   2119
           ----------                                                   2112
           ----------                                                   1974
           ----------                                                   1883
           ----------                                                   1854
           ----------                                                   1689
           ----------                                                   1431
           ----------                                                   1225
           ----------                                                   1030
           ----------                                                   1006
           ----------                                                   763
           ----------                                                   604
           ----------                                                   400
           ----------                                                   375
           ----------                                                   344
           ----------                                                   208
           ----------                                                   75

LOCUS: AT4G04910
DESCRIPTION: AAA-type ATPase family protein, similar to SP|P18708 Vesicular-fusion protein NSF (N-
ethylmaleimide-sensitive fusion protein) (NEM-sensitive fusion protein) {Cricetulus griseus}; contains Pfam
profiles PF00004: ATPase AAA family, PF02359: Cell division pr
DATA:                 Control 30min  2hours  2days  1week  p-value      pos
SENSE COUNTS:         3       0      1       1      1      3.73e-01
GENES (2 total):
  AT4G04910.1
    SENSE COUNTS:     3       0      1       1      1      3.73e-01
    TAGS: (3 total)
      i+3  TACCTAAATA 0       0      1       0      0      4.55e-01     2570
           ----------                                                   2551
      d+2  TTATTTTGCT 3       0      0       1      0      7.99e-02     2434
           ----------                                                   2340
           ----------                                                   2192
           ----------                                                   2171
           ----------                                                   1457
           ----------                                                   1122
           ----------                                                   1050
           ----------                                                   837
           ----------                                                   810
      d+2  GAACTAATTA 0       0      0       0      1      1.65e-01     534
           ----------                                                   273
           ----------                                                   119
           ----------                                                   92

LOCUS: AT2G37020
DESCRIPTION: translin family protein, similar to SP|Q62348 Translin {Mus musculus}; contains Pfam profile
PF01997: Translin family
DATA:                 Control 30min  2hours  2days  1week  p-value      pos
SENSE COUNTS:         3       0      1       1      1      3.73e-01
GENES (1 total):
  AT2G37020.1
    SENSE COUNTS:     3       0      1       1      1      3.73e-01
    TAGS: (1 total)
```

```
          ----------                                                            1157
     d+2  AAAACGTCAA  3      0      1      1      1      3.73e-01               964
          ----------                                                            759
          ----------                                                            745
          ----------                                                            651
          ----------                                                            484

LOCUS: AT2G32810
DESCRIPTION: beta-galactosidase, putative / lactase, putative, similar to beta-galactosidase GI:7939617 from
(Lycopersicon esculentum)
DATA:                 Control 30min  2hours 2days  1week  p-value               pos
SENSE COUNTS:         3      0      1      1      1      3.73e-01
GENES (2 total):
  AT2G32810.1
    SENSE COUNTS:     3      0      1      1      1      3.73e-01
    TAGS: (2 total)
          ----------                                                            3468
          ----------                                                            3444
     d+2  AACATTCCTA  0      0      0      0      1      1.65e-01               3018
     d+2  AGTGACCTCA  3      0      1      1      0      2.14e-01               2653
          ----------                                                            2595
          ----------                                                            2501
          ----------                                                            2423
          ----------                                                            2189
          ----------                                                            1963
          ----------                                                            1851
          ----------                                                            1612
          ----------                                                            1419
          ----------                                                            1283
          ----------                                                            1214
          ----------                                                            1142
          ----------                                                            1049
          ----------                                                            732
          ----------                                                            655
          ----------                                                            571
          ----------                                                            166

LOCUS: AT5G27640
DESCRIPTION: eukaryotic translation initiation factor 3 subunit 9 / eIF-3 eta / eIF3b (TIF3B1), nearly
identical to SP|Q9C5Z1 Eukaryotic translation initiation factor 3 subunit 9 (eIF-3 eta) (eIF3 p110) (eIF3b)
{Arabidopsis thaliana}
DATA:                 Control 30min  2hours 2days  1week  p-value               pos
SENSE COUNTS:         3      0      1      1      1      3.73e-01
GENES (2 total):
  AT5G27640.1
    SENSE COUNTS:     3      0      1      1      1      3.73e-01
    TAGS: (1 total)
          ----------                                                            2760
          ----------                                                            2692
          ----------                                                            2682
     d+2  TAATGGTACT  3      0      1      1      1      3.73e-01               2404
          ----------                                                            2357
          ----------                                                            2127
          ----------                                                            1848
          ----------                                                            1784
          ----------                                                            1560
          ----------                                                            1525
          ----------                                                            1187
          ----------                                                            857
          ----------                                                            542
          ----------                                                            501
          ----------                                                            125

LOCUS: AT5G59610
DESCRIPTION: DNAJ heat shock N-terminal domain-containing protein, similar to SP|Q9UXR9 Chaperone protein dnaJ
(Heat shock protein 40 Methanosarcina thermophila, SP|Q9QYI6 DnaJ homolog subfamily B member 9 Mus musculus;
contains Pfam profile PF00226 DnaJ domain
DATA:                 Control 30min  2hours 2days  1week  p-value               pos
SENSE COUNTS:         3      0      1      1      1      3.73e-01
GENES (1 total):
  AT5G59610.1
    SENSE COUNTS:     3      0      1      1      1      3.73e-01
    TAGS: (1 total)
          ----------                                                            1444
     v+2  TGGTGAGAAA  3      0      1      1      1      3.73e-01               1227
          ----------                                                            1207
          ----------                                                            709
          ----------                                                            107

LOCUS: AT1G28200
```

```
DESCRIPTION: GRAM domain-containing protein / ABA-responsive protein-related, similar to ABA-responsive
protein (Hordeum vulgare) GI:4103635; contains Pfam profile PF02893: GRAM domain
DATA:                 Control 30min   2hours  2days   1week   p-value         pos
SENSE COUNTS:         0       0       1       2       3       3.73e-01
GENES (2 total):
  AT1G28200.1
    SENSE COUNTS:     0       0       1       2       3       3.73e-01
    TAGS: (2 total)
      d+1  ATTTGTATTC 0       0       0       1       0       3.09e-01        1056
      d+2  GTCCGTGAAG 0       0       1       1       3       4.30e-01        839
           ----------                                                         247
           ----------                                                         95
           ----------                                                         89
           ----------                                                         70

LOCUS: AT5G03160
DESCRIPTION: DNAJ heat shock N-terminal domain-containing protein, similar to P58 protein, Bos primigenius
taurus, PIR:A56534; similar to p58 (GI:1353270) {Homo sapiens}; contains Pfam PF00226: DnaJ domain; contains
Pfam PF00515: TPR Domain
DATA:                 Control 30min   2hours  2days   1week   p-value         pos
SENSE COUNTS:         0       0       1       2       3       3.73e-01
GENES (1 total):
  AT5G03160.1
    SENSE COUNTS:     0       0       1       2       3       3.73e-01
    TAGS: (1 total)
           ----------                                                         2260
           ----------                                                         2237
           ----------                                                         2033
      d+2  TAACTTAAGA 0       0       1       2       3       3.73e-01        1638
           ----------                                                         1627
           ----------                                                         1525
           ----------                                                         1225
           ----------                                                         1215
           ----------                                                         811

LOCUS: AT4G17280
DESCRIPTION: auxin-responsive family protein, similar to auxin-induced protein AIR12 (GI:11357190)
(Arabidopsis thaliana)
DATA:                 Control 30min   2hours  2days   1week   p-value         pos
SENSE COUNTS:         0       0       1       2       3       3.73e-01
GENES (1 total):
  AT4G17280.1
    SENSE COUNTS:     0       0       1       2       3       3.73e-01
    TAGS: (2 total)
      d+1  CTCAAGCAAG 0       0       1       0       0       4.55e-01        1638
           ----------                                                         1616
      d+2  TGAATTCATA 0       0       0       2       3       1.73e-01        1480
           ----------                                                         1426
           ----------                                                         1286
           ----------                                                         803
           ----------                                                         771
           ----------                                                         214
           ----------                                                         188
           ----------                                                         116

LOCUS: AT3G17100
DESCRIPTION: expressed protein
DATA:                 Control 30min   2hours  2days   1week   p-value         pos
SENSE COUNTS:         4       7       4       1       3       3.74e-01
GENES (2 total):
  AT3G17100.1
    SENSE COUNTS:     4       7       4       1       3       3.74e-01
    TAGS: (2 total)
      d+1  TATATCCTTT 0       1       2       1       3       5.80e-01        1136
      d+2  CTTGGTTAGT 4       6       2       0       0       8.40e-02        984
           ----------                                                         496
  AT3G17100.2
    SENSE COUNTS:     4       7       4       1       3       3.74e-01
    TAGS: (2 total)
      d+1  TATATCCTTT 0       1       2       1       3       5.80e-01        1034
      d+2  CTTGGTTAGT 4       6       2       0       0       8.40e-02        882
           ----------                                                         394

LOCUS: AT2G06910
DESCRIPTION: CACTA-like transposase family (En/Spm), has a 4.3e-100 P-value blast match to GB:AAD55677
putative transposase protein (CACTA-element) transposon="Shooter" (Zea mays)
DATA:                 Control 30min   2hours  2days   1week   p-value         pos
SENSE COUNTS:         1       1       3       0       0       3.76e-01
GENES (1 total):
  AT2G06910.1
    SENSE COUNTS:     1       1       3       0       0       3.76e-01
```

```
    TAGS: (1 total)
           ----------                                                   3029
           ----------                                                   2830
           ----------                                                   2696
           ----------                                                   1845
           ----------                                                   1836
           ----------                                                   1803
           ----------                                                   1564
           ----------                                                   1560
           ----------                                                   1542
           ----------                                                   1180
           ----------                                                   1119
           ----------                                                   1115
           ----------                                                   1096
           ----------                                                   1071
           ----------                                                   956
           ----------                                                   952
           ----------                                                   891
           ----------                                                   742
           ----------                                                   656
           ----------                                                   599
           ----------                                                   166
           ----------                                                   115
           ----------                                                   111
           ----------                                                   47
      p+2  TCGGAATCTT 1      1      3      0      0      3.76e-01       24
           ----------                                                   15

LOCUS: AT2G39660
DESCRIPTION: protein kinase, putative, similar to protein kinase gi|166809|gb|AAA18853
DATA:                 Control 30min  2hours 2days  1week  p-value       pos
SENSE COUNTS:         1      1      3      0      0      3.76e-01
GENES (1 total):
  AT2G39660.1
    SENSE COUNTS:     1      1      3      0      0      3.76e-01
    TAGS: (1 total)
           ----------                                                   1245
           ----------                                                   972
           ----------                                                   939
           ----------                                                   740
      X+4  TGTAGCCTTT 1      1      3      0      0      3.76e-01       604

LOCUS: AT2G47390
DESCRIPTION: expressed protein
DATA:                 Control 30min  2hours 2days  1week  p-value       pos
SENSE COUNTS:         1      4      1      2      3      3.76e-01
GENES (1 total):
  AT2G47390.1
    SENSE COUNTS:     1      4      1      2      3      3.76e-01
    TAGS: (2 total)
           ----------                                                   3562
      d+2  CGAGTGCACT 1      3      1      2      3      7.10e-01       3035
           ----------                                                   3028
           ----------                                                   2964
           ----------                                                   2667
           ----------                                                   2627
           ----------                                                   2493
      d+2  CTGAGAAGGA 0      1      0      0      0      2.54e-01       1697
           ----------                                                   1579
           ----------                                                   745
           ----------                                                   299

LOCUS: AT5G35160
DESCRIPTION: endomembrane protein 70, putative, p76, Homo sapiens, EMBL:HSU81006
DATA:                 Control 30min  2hours 2days  1week  p-value       pos
SENSE COUNTS:         1      2      5      1      1      3.76e-01
GENES (1 total):
  AT5G35160.1
    SENSE COUNTS:     1      2      5      1      1      3.76e-01
    TAGS: (2 total)
      v+1  TTGGTGACCG 0      0      1      0      1      3.96e-01       2560
      v+2  CTCGCAACAG 1      2      4      1      0      3.90e-01       2157
           ----------                                                   1977
           ----------                                                   1869
           ----------                                                   1550
           ----------                                                   1461
           ----------                                                   1410
           ----------                                                   1371
           ----------                                                   1341
           ----------                                                   121
           ----------                                                   74
```

```
          ----------                                                    40

LOCUS: AT1G17130
DESCRIPTION: cell cycle control protein-related, contains similarity to Swiss-Prot:Q9P7C5 cell cycle control
protein cwf16 (Schizosaccharomyces pombe)
DATA:                  Control 30min   2hours  2days   1week   p-value         pos
SENSE COUNTS:          0       2       4       2       5       3.77e-01
GENES (1 total):
  AT1G17130.1
    SENSE COUNTS:      0       2       4       2       5       3.77e-01
    TAGS: (2 total)
          ----------                                                    1495
      d+2  AAATTGTTTT  0       2       4       2       5       3.41e-01 1233
      d+2  CTACTGTGAG  0       0       0       0       0       6.15e-01 610
          ----------                                                    120

LOCUS: AT2G06950
DESCRIPTION: copia-like retrotransposon family, has a 2.7e-243 P-value blast match to dbj|BAA78426.1|
polyprotein (AtRE2-1) (Arabidopsis thaliana) (Ty1_Copia-element)
DATA:                  Control 30min   2hours  2days   1week   p-value         pos
SENSE COUNTS:          2       0       3       6       5       3.77e-01
GENES (1 total):
  AT2G06950.1
    SENSE COUNTS:      2       0       3       6       5       3.77e-01
    TAGS: (2 total)
          ----------                                                    4199
          ----------                                                    4121
          ----------                                                    3927
      p+2  GTGATTACAG  0       0       0       0       0       6.15e-01 3367
          ----------                                                    3191
          ----------                                                    3148
          ----------                                                    3122
          ----------                                                    2894
          ----------                                                    2890
          ----------                                                    2862
          ----------                                                    2824
          ----------                                                    2641
          ----------                                                    2316
          ----------                                                    2231
          ----------                                                    1826
      p+2  TCAGCTTGGG  2       0       3       6       5       2.14e-01 1503
          ----------                                                    1239
          ----------                                                    1087
          ----------                                                    1070
          ----------                                                    1025
          ----------                                                    986
          ----------                                                    671
          ----------                                                    568
          ----------                                                    317

LOCUS: AT5G08450
DESCRIPTION: expressed protein, KED, Nicotiana tabacum, EMBL:AB009883
DATA:                  Control 30min   2hours  2days   1week   p-value         pos
SENSE COUNTS:          4       6       3       9       3       3.78e-01
GENES (2 total):
  AT5G08450.1
    SENSE COUNTS:      4       6       3       9       3       3.78e-01
    TAGS: (3 total)
      d+1  CTTTGCTTCT  0       0       1       1       0       7.90e-01 2954
          ----------                                                    2652
          ----------                                                    2578
          ----------                                                    2236
          ----------                                                    2102
          ----------                                                    2013
      d+2  AGTGAACCCA  4       5       1       8       3       3.05e-01 1346
      d+2  CTCACTTGGC  0       1       1       0       0       4.87e-01 1020
  AT5G08450.2
    SENSE COUNTS:      4       6       3       9       3       3.78e-01
    TAGS: (3 total)
      d+1  CTTTGCTTCT  0       0       1       1       0       7.90e-01 2996
          ----------                                                    2694
          ----------                                                    2620
          ----------                                                    2278
          ----------                                                    2144
          ----------                                                    2055
      d+2  AGTGAACCCA  4       5       1       8       3       3.05e-01 1388
      d+2  CTCACTTGGC  0       1       1       0       0       4.87e-01 1062

LOCUS: AT3G16857
DESCRIPTION: ARR1 protein
DATA:                  Control 30min   2hours  2days   1week   p-value         pos
```

```
SENSE COUNTS:          3      4      7      1      2      3.83e-01
GENES (4 total):
  AT3G16857.1
    SENSE COUNTS:      2      4      6      1      1      3.47e-01
    TAGS: (2 total)
           ----------                                                 2322
      d+2  TTTGCGTTCA  2      4      5      1      1      5.26e-01    2262
      d+2  GCCGAAACCA  0      0      1      0      0      4.55e-01    2018
           ----------                                                 1981
           ----------                                                 1864
           ----------                                                 644
           ----------                                                 532
           ----------                                                 500
           ----------                                                 475
           ----------                                                 434
           ----------                                                 340
  AT3G16857.2
    SENSE COUNTS:      1      0      2      0      1      4.42e-01
    TAGS: (2 total)
           ----------                                                 2409
      d+2  AAATTTTAAA  1      0      1      0      1      6.15e-01    2368
      d+2  GCCGAAACCA  0      0      1      0      0      4.55e-01    2018
           ----------                                                 1981
           ----------                                                 1864
           ----------                                                 644
           ----------                                                 532
           ----------                                                 500
           ----------                                                 475
           ----------                                                 434
           ----------                                                 340

LOCUS: AT4G17030
DESCRIPTION: expansin-related, identical to SWISS-PROT:O23547 expansin-related protein 1 precursor (At-
EXPR1)(Arabidopsis thaliana); related to expansins, http://www.bio.psu.edu/expansins/
DATA:                  Control 30min  2hours 2days  1week  p-value     pos
SENSE COUNTS:          1      3      1      1      4      3.86e-01
GENES (2 total):
  AT4G17030.1
    SENSE COUNTS:      1      3      1      1      4      3.86e-01
    TAGS: (2 total)
      d+1  ATTTGCAGAA  1      0      0      0      1      3.83e-01    717
      d+2  AGAAAAGCTA  0      3      1      1      3      3.55e-01    579
           ----------                                                 310

LOCUS: AT2G03140
DESCRIPTION: CAAX amino terminal protease family protein, very low similarity to SP|Q40863 Late embryogenesis
abundant protein EMB8 from Picea glauca; contains Pfam profile PF02517 CAAX amino terminal protease family
protein
DATA:                  Control 30min  2hours 2days  1week  p-value     pos
SENSE COUNTS:          4      0      2      0      1      3.88e-01
GENES (1 total):
  AT2G03140.1
    SENSE COUNTS:      4      0      2      0      1      3.88e-01
    TAGS: (5 total)
           ----------                                                 5806
      d+2  ATATATGGAA  4      0      0      0      0      2.42e-02    5673
           ----------                                                 5574
      d+2  CCCTTTCTAT  0      0      0      0      1      1.65e-01    5462
           ----------                                                 5451
           ----------                                                 4939
           ----------                                                 4591
           ----------                                                 4546
           ----------                                                 4267
           ----------                                                 3736
      d+2  ATGCAGCAGA  0      0      0      0      0      6.15e-01    3602
      d+2  AACAAGAGTT  0      0      1      0      0      4.55e-01    3572
           ----------                                                 3331
           ----------                                                 3254
           ----------                                                 3099
           ----------                                                 2908
           ----------                                                 1801
           ----------                                                 1786
           ----------                                                 1600
      d+2  AAAAGACGGT  0      0      1      0      0      4.55e-01    1538
           ----------                                                 1378
           ----------                                                 1114
           ----------                                                 816

LOCUS: AT4G00040
```

```
DESCRIPTION: chalcone and stilbene synthase family protein, similar to chalcone synthase homolog PrChS1, Pinus
radiata, gb:U90341; similar to anther-specific protein (Nicotiana sylvestris)(GI:2326774), YY2 protein (Oryza
sativa)(GI:2645170)
DATA:                 Control 30min  2hours  2days  1week  p-value     pos
SENSE COUNTS:         3       0      0       2      1      3.89e-01
GENES (1 total):
  AT4G00040.1
    SENSE COUNTS:     3       0      0       2      1      3.89e-01
    TAGS: (2 total)
          ----------                                                   1362
      d+2 TAATGGTTGC  3       0      0       1      1      3.94e-01    1255
      d+2 AGGAACCTCT  0       0      0       1      0      3.09e-01    1173
          ----------                                                   738
          ----------                                                   246

LOCUS: AT5G40340
DESCRIPTION: PWWP domain-containing protein, KED, Nicotiana tabacum, EMBL:AB009883
DATA:                 Control 30min  2hours  2days  1week  p-value     pos
SENSE COUNTS:         4       8      7       3      10     3.89e-01
GENES (1 total):
  AT5G40340.1
    SENSE COUNTS:     4       8      7       3      10     3.89e-01
    TAGS: (4 total)
      v+1 TGGCTTTCTT  0       1      3       0      3      2.52e-01    3073
      v+2 GGAAAATGAA  4       7      4       2      7      6.00e-01    2527
      v+2 ATGGAGCCAA  0       0      0       1      0      3.09e-01    2344
          ----------                                                   2316
          ----------                                                   2301
          ----------                                                   2103
      v+2 ATGAAGTTAA  0       0      0       0      0      6.15e-01    1615
          ----------                                                   1414
          ----------                                                   1279
          ----------                                                   1144
          ----------                                                   1042
          ----------                                                   770
          ----------                                                   721
          ----------                                                   62

LOCUS: AT2G32260
DESCRIPTION: cholinephosphate cytidylyltransferase, putative / phosphorylcholine transferase, putative /
CTP:phosphocholine cytidylyltransferase, putative, strong similarity to CTP:phosphocholine
cytidylyltransferase (Brassica napus) GI:1418125; contains Pfam profile
DATA:                 Control 30min  2hours  2days  1week  p-value     pos
SENSE COUNTS:         5       2      8       5      2      3.91e-01
GENES (1 total):
  AT2G32260.1
    SENSE COUNTS:     5       2      8       5      2      3.91e-01
    TAGS: (2 total)
          ----------                                                   1480
      d+2 ACGGGCAAAG  4       2      7       4      1      3.90e-01    1379
      d+2 TATTTTTTTC  1       0      1       1      1      7.91e-01    1357
          ----------                                                   1027
          ----------                                                   1022
          ----------                                                   667
          ----------                                                   459
          ----------                                                   281

LOCUS: AT4G30600
DESCRIPTION: signal recognition particle receptor alpha subunit family protein, similar to Signal recognition
particle receptor alpha subunit (SR-alpha) (Docking protein alpha) (DP-alpha) (SP:P08240) (Homo sapiens};
similar to Signal recognition particle receptor alph
DATA:                 Control 30min  2hours  2days  1week  p-value     pos
SENSE COUNTS:         4       3      2       2      7      3.93e-01
GENES (1 total):
  AT4G30600.1
    SENSE COUNTS:     4       3      2       2      7      3.93e-01
    TAGS: (3 total)
      d+1 TGAAATAGTG  0       0      2       2      1      5.87e-01    2237
      d+2 ACTTTATATG  3       0      0       0      5      5.20e-02    2184
      d+2 CTGTTGTTTT  1       3      0       0      1      2.11e-01    2083
          ----------                                                   1538
          ----------                                                   1364
          ----------                                                   1183
          ----------                                                   956
          ----------                                                   156

LOCUS: AT1G17060
DESCRIPTION: cytochrome P450, putative, 41% identical to Cytochrome P450 (Catharanthus roseus) (gi|404690)
DATA:                 Control 30min  2hours  2days  1week  p-value     pos
SENSE COUNTS:         3       1      0       3      1      3.93e-01
GENES (2 total):
```

```
  AT1G17060.1
    SENSE COUNTS:      3       1       0       3       1       3.93e-01
    TAGS: (2 total)
      d+1  GAGCTCATTT  0       0       0       1       0       3.09e-01       1604
           ----------                                                         1568
           ----------                                                         1352
           ----------                                                         1263
           ----------                                                         1226
           ----------                                                         1000
           ----------                                                         829
           ----------                                                         812
           ----------                                                         798
           ----------                                                         611
           ----------                                                         504
           ----------                                                         485
      d+2  ATGCCTTTTC  3       1       0       2       1       5.39e-01       451

LOCUS: AT4G32100
DESCRIPTION: hypothetical protein,
DATA:                  Control 30min   2hours  2days   1week   p-value        pos
SENSE COUNTS:          1       0       2       2       5       3.94e-01
GENES (1 total):
  AT4G32100.1
    SENSE COUNTS:      1       0       2       2       5       3.94e-01
    TAGS: (1 total)
           ----------                                                         1218
           ----------                                                         1122
           ----------                                                         959
           ----------                                                         938
           ----------                                                         887
           ----------                                                         755
           ----------                                                         649
           ----------                                                         557
           ----------                                                         480
      i+3  ACAAAGAAAA  1       0       2       2       5       3.94e-01       425
           ----------                                                         416
           ----------                                                         336
           ----------                                                         187
           ----------                                                         72

LOCUS: AT5G25920
DESCRIPTION: hypothetical protein
DATA:                  Control 30min   2hours  2days   1week   p-value        pos
SENSE COUNTS:          1       0       2       2       5       3.94e-01
GENES (1 total):
  AT5G25920.1
    SENSE COUNTS:      1       0       2       2       5       3.94e-01
    TAGS: (1 total)
           ----------                                                         2202
           ----------                                                         2079
      v+2  GACAAGAAAA  1       0       2       2       5       3.94e-01       1806
           ----------                                                         1710
           ----------                                                         1563
           ----------                                                         1128
           ----------                                                         1076
           ----------                                                         911
           ----------                                                         606
           ----------                                                         553
           ----------                                                         504
           ----------                                                         433
           ----------                                                         142

LOCUS: AT5G08580
DESCRIPTION: calcium-binding EF hand family protein, contains INTERPRO:IPR002048 calcium-binding EF-hand
domain
DATA:                  Control 30min   2hours  2days   1week   p-value        pos
SENSE COUNTS:          1       0       2       2       5       3.94e-01
GENES (1 total):
  AT5G08580.1
    SENSE COUNTS:      1       0       2       2       5       3.94e-01
    TAGS: (2 total)
           ----------                                                         1567
      d+2  ACTTGCCTGA  1       0       1       1       0       9.46e-01       998
           ----------                                                         647
           ----------                                                         460
           ----------                                                         419
           ----------                                                         413
           ----------                                                         310
      X+4  CAAAGGTCAG  0       0       1       1       5       3.15e-02       213
           ----------                                                         209
```

```
LOCUS: AT3G27490
DESCRIPTION: DC1 domain-containing protein, contains Pfam profile PF03107: DC1 domain
DATA:                 Control 30min  2hours  2days  1week  p-value     pos
SENSE COUNTS:         3       0      0       1      1      3.94e-01
GENES (1 total):
  AT3G27490.1
    SENSE COUNTS:     3       0      0       1      1      3.94e-01
    TAGS: (2 total)
           ----------                                                  2610
           ----------                                                  2539
           ----------                                                  2399
           ----------                                                  2372
           ----------                                                  2361
           ----------                                                  2326
           ----------                                                  2281
           ----------                                                  1813
           ----------                                                  1621
           ----------                                                  1553
           ----------                                                  1509
           ----------                                                  1495
           ----------                                                  1343
           ----------                                                  1246
           ----------                                                  1234
           ----------                                                  1191
           ----------                                                  1107
           ----------                                                  1087
           ----------                                                  904
           ----------                                                  860
           ----------                                                  749
           ----------                                                  565
      v+2  ATCTATTCAT 3       0      0       1      1      1.73e-01    400
           ----------                                                  394
           ----------                                                  223
           ----------                                                  144
           ----------                                                  85
      v+2  ACTTTTGTTG 0       0      0       0      0      6.15e-01    17

LOCUS: AT2G43100
DESCRIPTION: aconitase C-terminal domain-containing protein, contains Pfam profile PF00694: Aconitase C-
terminal domain
DATA:                 Control 30min  2hours  2days  1week  p-value     pos
SENSE COUNTS:         5       0      3       4      5      3.95e-01
GENES (1 total):
  AT2G43100.1
    SENSE COUNTS:     5       0      3       4      5      3.95e-01
    TAGS: (3 total)
      X+4  TAAACCCAGA 0       0      1       0      0      4.55e-01    1147
      d+1  TTCCAAGATA 5       0      1       4      5      1.88e-01    958
           ----------                                                  815
      d+2  CTCCGGTCTG 0       0      1       0      0      4.55e-01    489

LOCUS: AT5G54430
DESCRIPTION: universal stress protein (USP) family protein, low similarity to early nodulin ENOD18 (Vicia
faba) GI:11602747, ER6 protein (Lycopersicon esculentum) GI:5669654; contains Pfam profile PF00582: universal
stress protein family
DATA:                 Control 30min  2hours  2days  1week  p-value     pos
SENSE COUNTS:         6       3      10      3      7      3.95e-01
GENES (1 total):
  AT5G54430.1
    SENSE COUNTS:     6       3      10      3      7      3.95e-01
    TAGS: (2 total)
      i+3  GGACAAAGAA 0       0      0       1      0      3.09e-01    1262
      d+1  AAGGAGTTGT 6       3      10      2      7      2.75e-01    981
           ----------                                                  775
           ----------                                                  508
           ----------                                                  69

LOCUS: AT3G46920
DESCRIPTION: protein kinase family protein, similar to MAP3K delta-1 protein kinase (Arabidopsis thaliana)
GI:2253010; contains Pfam profile: PF00069 Eukaryotic protein kinase domain
DATA:                 Control 30min  2hours  2days  1week  p-value     pos
SENSE COUNTS:         1       0      1       1      3      3.98e-01
GENES (1 total):
  AT3G46920.1
    SENSE COUNTS:     1       0      1       1      3      3.98e-01
    TAGS: (1 total)
           ----------                                                  4059
      v+2  CAAATAGGTA 1       0      1       1      3      3.98e-01    3929
           ----------                                                  3897
           ----------                                                  3813
           ----------                                                  3723
```

```
          ----------                                                    3533
          ----------                                                    3379
          ----------                                                    3064
          ----------                                                    2830
          ----------                                                    2630
          ----------                                                    2366
          ----------                                                    2344
          ----------                                                    2257
          ----------                                                    2140
          ----------                                                    1881
          ----------                                                    1681
          ----------                                                    1561
          ----------                                                    1467
          ----------                                                    630
          ----------                                                    361
          ----------                                                    90
          ----------                                                    11

LOCUS: AT2G29680
DESCRIPTION: cell division control protein CDC6, putative, almost identical to DNA replication protein CDC6
GI:18056480 from (Arabidopsis thaliana);  identical to cDNA CDC6 protein (2g29680 gene) GI:18056479
DATA:                  Control 30min  2hours  2days  1week  p-value        pos
SENSE COUNTS:          1       0      1       1      3      3.98e-01
GENES (3 total):
  AT2G29680.2
    SENSE COUNTS:      0       0      1       1      3      1.91e-01
    TAGS: (2 total)
      d+1  TAAATGCAGG  0       0      1       0      0      4.55e-01       1680
           ----------                                                    1088
      d+2  AGCTTTTTAT  0       0      0       1      3      6.27e-02       828
           ----------                                                    545
           ----------                                                    402
           ----------                                                    81
  AT2G29680.1
    SENSE COUNTS:      1       0      0       1      3      1.87e-01
    TAGS: (2 total)
      d+1  AGAAAGGCTC  1       0      0       0      0      4.28e-01       1149
      d+2  AGCTTTTTAT  0       0      0       1      3      6.27e-02       796
           ----------                                                    513
           ----------                                                    370
           ----------                                                    49

LOCUS: AT5G59920
DESCRIPTION: DC1 domain-containing protein, contains Pfam profile PF03107: DC1 domain
DATA:                  Control 30min  2hours  2days  1week  p-value        pos
SENSE COUNTS:          1       0      1       1      3      3.98e-01
GENES (2 total):
  AT5G59920.1
    SENSE COUNTS:      1       0      1       1      3      3.98e-01
    TAGS: (1 total)
           ----------                                                    2952
           ----------                                                    2884
           ----------                                                    2880
      v+2  TTTATCGAGT  1       0      1       1      3      3.98e-01       2453
           ----------                                                    2089
           ----------                                                    2063
           ----------                                                    1795
           ----------                                                    1741
           ----------                                                    1603
           ----------                                                    1535
           ----------                                                    1510
           ----------                                                    1468
           ----------                                                    1352
           ----------                                                    1219
           ----------                                                    1100
           ----------                                                    806
           ----------                                                    724
           ----------                                                    718
           ----------                                                    647
           ----------                                                    596
           ----------                                                    456
           ----------                                                    442
           ----------                                                    385
           ----------                                                    106
           ----------                                                    32

LOCUS: AT1G69070
DESCRIPTION: expressed protein
DATA:                  Control 30min  2hours  2days  1week  p-value        pos
SENSE COUNTS:          1       0      3       2      1      3.98e-01
GENES (1 total):
```

```
  AT1G69070.1
    SENSE COUNTS:      1      0      3      2      1      3.98e-01
    TAGS: (1 total)
           ----------                                                 2654
           ----------                                                 2618
           ----------                                                 2594
           ----------                                                 2560
           ----------                                                 2452
           ----------                                                 2245
           ----------                                                 2089
      d+2  CGCTGTCCCA 1      0      3      2      1      3.98e-01     1855
           ----------                                                 1811
           ----------                                                 1794
           ----------                                                 1642
           ----------                                                 1396
           ----------                                                 604
           ----------                                                 589
           ----------                                                 79

LOCUS: AT5G20380
DESCRIPTION: transporter-related, low similarity to vesicular glutamate transporter 3 (Rattus norvegicus)
GI:21685382
DATA:                  Control 30min  2hours 2days  1week  p-value      pos
SENSE COUNTS:          2      0      5      4      3      4.01e-01
GENES (1 total):
  AT5G20380.1
    SENSE COUNTS:      2      0      5      4      3      4.01e-01
    TAGS: (3 total)
      i+3  CTATTTTAGT 0      0      0      0      0      6.15e-01     1960
      d+1  GAGAAACTTT 2      0      4      4      3      2.88e-01     1632
           ----------                                                 1304
           ----------                                                 1269
      d+2  GAAGTCATTT 0      0      1      0      0      4.55e-01     980
           ----------                                                 575
           ----------                                                 326

LOCUS: AT1G51940
DESCRIPTION: protein kinase family protein / peptidoglycan-binding LysM domain-containing protein, contains
protein kinases ATP-binding region signature, PROSITE:PS00107
DATA:                  Control 30min  2hours 2days  1week  p-value      pos
SENSE COUNTS:          4      2      5      2      8      4.02e-01
GENES (1 total):
  AT1G51940.1
    SENSE COUNTS:      4      2      5      2      8      4.02e-01
    TAGS: (2 total)
      d+1  AAGCAAGTGG 1      0      1      0      0      6.01e-01     2026
           ----------                                                 1955
           ----------                                                 1936
           ----------                                                 1903
           ----------                                                 1538
           ----------                                                 1448
           ----------                                                 1226
      i+3  AAACTTAAGT 3      2      4      2      8      3.69e-01     984
           ----------                                                 878
           ----------                                                 331

LOCUS: AT4G11970
DESCRIPTION: YT521-B-like family protein, contains Pfam profile PF04146: YT521-B-like family
DATA:                  Control 30min  2hours 2days  1week  p-value      pos
SENSE COUNTS:          2      0      1      2      3      4.02e-01
GENES (2 total):
  AT4G11970.2
    SENSE COUNTS:      2      0      1      2      3      4.02e-01
    TAGS: (2 total)
      d+1  GTACCAAGTT 0      0      0      1      0      3.09e-01     1567
      d+2  TAAACTTTTG 2      0      1      1      3      4.10e-01     1490
           ----------                                                 1134
           ----------                                                 1123
           ----------                                                 1034
           ----------                                                 483
           ----------                                                 120

LOCUS: AT3G55130
DESCRIPTION: ABC transporter family protein, breast cancer resistance protein 1 BCRP1, Mus musculus,
EMBL:NP_036050
DATA:                  Control 30min  2hours 2days  1week  p-value      pos
SENSE COUNTS:          1      4      2      4      2      4.03e-01
GENES (1 total):
  AT3G55130.1
    SENSE COUNTS:      1      4      2      4      2      4.03e-01
    TAGS: (4 total)
```

```
      d+1  CTTGAGGACG 1       1       1       0       0       7.28e-01        2130
      d+2  GTCTCCATTA 0       0       0       0       1       1.65e-01        1849
      d+2  TTATGTTACA 0       0       0       0       0       6.15e-01        1837
           ----------                                                         1390
           ----------                                                         1324
      d+2  ACACGTGTTT 0       3       1       4       1       3.23e-01        139

LOCUS: AT5G60340
DESCRIPTION: maoC-like dehydratase domain-containing protein, contains similarity to (R)-specific enoyl-CoA
hydratase PhaJ1 (Pseudomonas oleovorans) gi|22506675|gb|AAM97601; contains Pfam domain PF01575: MaoC like
domain
DATA:                 Control 30min   2hours  2days   1week   p-value         pos
SENSE COUNTS:         2       7       5       4       8       4.03e-01
GENES (1 total):
  AT5G60340.1
    SENSE COUNTS:     2       7       5       4       8       4.03e-01
    TAGS: (2 total)
           ----------                                                         1837
           ----------                                                         1341
           ----------                                                         1107
           ----------                                                         1018
           ----------                                                         819
      i+3  GCAACCCTGA 2       7       5       4       7       5.08e-01        785
      v+2  GCTGTGACTT 0       0       0       0       1       1.65e-01        619
           ----------                                                         517
           ----------                                                         354
           ----------                                                         272

LOCUS: AT1G07140
DESCRIPTION: Ran-binding protein 1a (RanBP1a), identical to Ran-binding protein (atranbp1a) GI:2058282 from
(Arabidopsis thaliana)
DATA:                 Control 30min   2hours  2days   1week   p-value         pos
SENSE COUNTS:         3       8       10      7       5       4.03e-01
GENES (2 total):
  AT1G07140.1
    SENSE COUNTS:     3       8       10      7       5       4.03e-01
    TAGS: (4 total)
           ----------                                                         1389
      d+2  TGATTTTTTA 1       0       5       6       3       1.16e-01        1174
           ----------                                                         1125
           ----------                                                         1075
      d+2  TTGGTTATCT 2       0       3       1       1       7.77e-01        1025
      d+2  TTTTGTCGAG 0       0       1       0       0       4.55e-01        887
           ----------                                                         850
      d+2  TGTGTGGCAC 0       8       1       0       1       6.03e-04        449
           ----------                                                         414

LOCUS: AT5G42990
DESCRIPTION: ubiquitin-conjugating enzyme 18 (UBC18), E2; identical to gi:2801448
DATA:                 Control 30min   2hours  2days   1week   p-value         pos
SENSE COUNTS:         5       1       6       8       6       4.03e-01
GENES (1 total):
  AT5G42990.1
    SENSE COUNTS:     5       1       6       8       6       4.03e-01
    TAGS: (2 total)
      d+1  GAGAGTTTAG 1       1       4       2       5       4.95e-01        740
      d+2  GCCTCCCACT 4       0       2       6       1       1.72e-01        628
           ----------                                                         523
           ----------                                                         371
           ----------                                                         54

LOCUS: AT3G11420
DESCRIPTION: fringe-related protein, similar to hypothetical protein GB:AAC23643 (Arabidopsis thaliana)  +
weak similarity to Fringe (Schistocerca gregaria)(GI:6573138);Fringe encodes an extracellular protein that
regulates Notch signalling.
DATA:                 Control 30min   2hours  2days   1week   p-value         pos
SENSE COUNTS:         1       0       4       2       0       4.04e-01
GENES (1 total):
  AT3G11420.1
    SENSE COUNTS:     1       0       4       2       0       4.04e-01
    TAGS: (3 total)
      d+1  AACGTAACTA 0       0       1       1       0       7.90e-01        2073
      d+2  TAAACATTAC 1       0       0       1       0       5.06e-01        2022
      d+2  GATGGTGCCG 0       0       3       0       0       1.17e-01        1611
           ----------                                                         1414
           ----------                                                         924
           ----------                                                         680
           ----------                                                         432

LOCUS: AT2G25170
```

```
DESCRIPTION: chromatin remodeling factor CHD3 (PICKLE), identical to chromatin remodeling factor CHD3
(Arabidopsis thaliana) GI:6478518
DATA:                  Control 30min  2hours  2days  1week  p-value        pos
SENSE COUNTS:          3       1      2       1      6      4.06e-01
GENES (2 total):
  AT2G25170.1
    SENSE COUNTS:      3       1      2       1      6      4.06e-01
    TAGS: (5 total)
      d+1  AAATTATAGT  2       1      1       0      3      5.89e-01       4347
           ----------                                                    4323
      d+2  TATGAGACTT  0       0      0       1      0      3.09e-01       4235
           ----------                                                    4201
           ----------                                                    4149
      d+2  AAGACGACAC  1       0      0       0      0      6.89e-01       3964
           ----------                                                    3510
           ----------                                                    3259
           ----------                                                    3226
           ----------                                                    2095
      d+2  CCCCCCAAAA  0       0      1       0      0      4.55e-01       1551
           ----------                                                    1401
           ----------                                                    1395
           ----------                                                    1263
      d+2  AGTTTTACTT  0       0      0       0      3      1.12e-02       1099
           ----------                                                    1031
           ----------                                                    886
           ----------                                                    223
           ----------                                                    206
           ----------                                                    161

LOCUS: AT5G19780
DESCRIPTION: tubulin alpha-3/alpha-5 chain (TUA5), nearly identical to SP|P20363 Tubulin alpha-3/alpha-5 chain
{Arabidopsis thaliana}
DATA:                  Control 30min  2hours  2days  1week  p-value        pos
SENSE COUNTS:          4       7      3       1      7      4.06e-01
GENES (2 total):
  AT5G19780.1
    SENSE COUNTS:      4       7      3       1      7      4.06e-01
    TAGS: (1 total)
           ----------                                                    2381
           ----------                                                    2275
           ----------                                                    2013
           ----------                                                    1897
           ----------                                                    1785
           ----------                                                    1761
      d+2  TTTGTTTTAA  4       7      3       1      7      4.06e-01       1546
           ----------                                                    1309
           ----------                                                    1246
           ----------                                                    1059
           ----------                                                    1054
           ----------                                                    1018
           ----------                                                    919
           ----------                                                    299
           ----------                                                    200

LOCUS: AT4G23910
DESCRIPTION: expressed protein, various predicted proteins, Arabidopsis thaliana
DATA:                  Control 30min  2hours  2days  1week  p-value        pos
SENSE COUNTS:          1       4      1       2      4      4.07e-01
GENES (1 total):
  AT4G23910.1
    SENSE COUNTS:      1       4      1       2      4      4.07e-01
    TAGS: (2 total)
           ----------                                                    592
           ----------                                                    527
           ----------                                                    459
      X+4  AAAGAATTTT  1       4      1       1      3      4.48e-01       289
      i+3  CTTGGATTAA  0       0      0       1      1      3.25e-01       147
           ----------                                                    86
           ----------                                                    26

LOCUS: AT5G53480
DESCRIPTION: importin beta-2, putative, similar to importin-beta2 (Oryza sativa (japonica cultivar-group))
GI:3983665; contains Pfam profile PF03810: Importin-beta N-terminal domain
DATA:                  Control 30min  2hours  2days  1week  p-value        pos
SENSE COUNTS:          2       0      5       2      0      4.07e-01
GENES (2 total):
  AT5G53480.1
    SENSE COUNTS:      2       0      5       2      0      4.07e-01
    TAGS: (4 total)
           ----------                                                    3769
      d+2  TTAATAGGAG  0       0      1       0      0      7.06e-01       3585
```

```
          ----------                                                    3475
          ----------                                                    3355
          ----------                                                    3102
     d+2  GTCGTTTTCT  2       0       4       0       0       1.40e-01  3046
          ----------                                                    2799
     d+2  CCATAACCCG  0       0       0       1       0       3.09e-01  2713
          ----------                                                    2608
          ----------                                                    2541
          ----------                                                    2529
          ----------                                                    2061
          ----------                                                    2007
          ----------                                                    1996
          ----------                                                    1929
          ----------                                                    1758
          ----------                                                    1527
          ----------                                                    1453
          ----------                                                    1421
          ----------                                                    1402
     X+4  TTTCTGACGT  0       0       0       1       0       6.04e-01  1309
          ----------                                                    1233
          ----------                                                    1228
          ----------                                                    1181
          ----------                                                    1055
          ----------                                                    900
          ----------                                                    789
          ----------                                                    771
          ----------                                                    732
          ----------                                                    640
          ----------                                                    565
          ----------                                                    387

LOCUS: AT1G64710
DESCRIPTION: alcohol dehydrogenase, putative, similar to alcohol dehydrogenase GI:551257 from (Nicotiana
tabacum)
DATA:                 Control 30min   2hours  2days   1week   p-value   pos
SENSE COUNTS:         4       2       4       2       8       4.08e-01
GENES (1 total):
  AT1G64710.1
    SENSE COUNTS:     4       2       4       2       8       4.08e-01
    TAGS: (2 total)
     d+1  AATATGAAAG  0       0       0       1       0       3.09e-01  1462
     d+2  CAGCTGCTTC  4       2       4       1       8       2.62e-01  1212
          ----------                                                    1053
          ----------                                                    1008
          ----------                                                    889
          ----------                                                    391
          ----------                                                    328
          ----------                                                    278
          ----------                                                    164

LOCUS: AT1G64550
DESCRIPTION: ABC transporter family protein, similar to ABC transporter protein GB:AAF31030 GI:6899653 from
(Leishmania major)
DATA:                 Control 30min   2hours  2days   1week   p-value   pos
SENSE COUNTS:         6       2       7       10      8       4.09e-01
GENES (2 total):
  AT1G64550.1
    SENSE COUNTS:     6       2       7       10      8       4.09e-01
    TAGS: (3 total)
     i+3  GTACTCACTC  1       0       0       0       0       4.28e-01  4319
          ----------                                                    2428
     d+2  TTGCGTTTTT  2       0       2       5       0       2.05e-01  2226
          ----------                                                    2159
          ----------                                                    2147
          ----------                                                    2065
          ----------                                                    1903
          ----------                                                    1772
          ----------                                                    1478
          ----------                                                    1226
          ----------                                                    1163
          ----------                                                    752
          ----------                                                    713
          ----------                                                    536
          ----------                                                    482
     X+4  TTTCGCAGAA  3       2       5       5       8       4.54e-01  363
          ----------                                                    284

LOCUS: AT5G51960
DESCRIPTION: expressed protein,
DATA:                 Control 30min   2hours  2days   1week   p-value   pos
SENSE COUNTS:         1       3       0       1       1       4.09e-01
```

```
GENES (1 total):
  AT5G51960.1
    SENSE COUNTS:      1      3      0      1      1      4.09e-01
    TAGS: (1 total)
           ----------                                                   555
           ----------                                                   543
      d+2  CATTCATTCC 1      3      0      1      1      4.09e-01       331
           ----------                                                   81

LOCUS: AT1G75580
DESCRIPTION: auxin-responsive protein, putative, similar to auxin-induced protein TGSAUR22 (GI:10185820)
(Tulipa gesneriana)
DATA:                  Control 30min  2hours 2days  1week  p-value       pos
SENSE COUNTS:          2      0      1      1      3      4.10e-01
GENES (1 total):
  AT1G75580.1
    SENSE COUNTS:      2      0      1      1      3      4.10e-01
    TAGS: (2 total)
      X+4  TGATAATTGA 1      0      0      0      0      4.28e-01       777
      d+1  GTGGTGTGTG 1      0      1      1      3      3.98e-01       645
           ----------                                                   388
           ----------                                                   337
           ----------                                                   332

LOCUS: AT4G36160
DESCRIPTION: no apical meristem (NAM) family protein, similar to NAC2 (GI:6456751)  (Arabidopsis thaliana);
contains Pfam PF02365 : No apical meristem (NAM) protein
DATA:                  Control 30min  2hours 2days  1week  p-value       pos
SENSE COUNTS:          2      0      1      1      3      4.10e-01
GENES (1 total):
  AT4G36160.1
    SENSE COUNTS:      2      0      1      1      3      4.10e-01
    TAGS: (2 total)
      i+3  TAGACCAAAG 2      0      0      0      0      1.04e-01       2081
      d+1  TTTGATAAAA 0      0      1      1      3      1.91e-01       1331
           ----------                                                   1163
           ----------                                                   605
           ----------                                                   601
           ----------                                                   472
           ----------                                                   360
           ----------                                                   225

LOCUS: AT2G42880
DESCRIPTION: mitogen-activated protein kinase, putative / MAPK, putative (MPK20), mitogen-activated protein
kinase (MAPK)(AtMPK20), PMID:12119167
DATA:                  Control 30min  2hours 2days  1week  p-value       pos
SENSE COUNTS:          4      7      4      5      0      4.10e-01
GENES (2 total):
  AT2G42880.1
    SENSE COUNTS:      4      7      4      5      0      4.10e-01
    TAGS: (2 total)
      i+3  GCAAGGATAA 0      0      0      1      0      3.09e-01       3019
      d+1  TCATAAAAGA 4      7      4      4      0      3.93e-01       2294
           ----------                                                   2060
           ----------                                                   1940
           ----------                                                   1823
           ----------                                                   1767
           ----------                                                   1644
           ----------                                                   1399
           ----------                                                   1226
           ----------                                                   743
           ----------                                                   588

LOCUS: AT4G24580
DESCRIPTION: pleckstrin homology (PH) domain-containing protein-related / RhoGAP domain-containing protein,
contains Pfam domain, PF00620: RhoGAP domain
DATA:                  Control 30min  2hours 2days  1week  p-value       pos
SENSE COUNTS:          1      0      1      4      3      4.18e-01
GENES (1 total):
  AT4G24580.1
    SENSE COUNTS:      1      0      1      4      3      4.18e-01
    TAGS: (1 total)
           ----------                                                   3560
           ----------                                                   2838
           ----------                                                   2449
           ----------                                                   2310
           ----------                                                   1846
           ----------                                                   1540
           ----------                                                   1057
           ----------                                                   910
           ----------                                                   874
```

```
      i+3  ATACAACTCT 1       0       1       4       3       4.18e-01        755
           ----------                                                         587
           ----------                                                         463
           ----------                                                         397
           ----------                                                         354
           ----------                                                         230

LOCUS: AT2G30100
DESCRIPTION: ubiquitin family protein, low similarity to SP|Q9UQ13 Leucine-rich repeat protein SHOC-2 (Ras-
binding protein Sur-8) {Homo sapiens}; contains Pfam profiles PF00240: Ubiquitin family, PF01535: PPR repeat,
PF00560: Leucine Rich Repeat
DATA:                 Control 30min   2hours  2days   1week   p-value         pos
SENSE COUNTS:         1       0       1       4       2       4.18e-01
GENES (1 total):
  AT2G30100.1
    SENSE COUNTS:     1       0       1       4       2       4.18e-01
    TAGS: (2 total)
      d+1  CCTATATATT 0       0       0       0       1       1.65e-01        2776
           ----------                                                         2392
           ----------                                                         1807
      d+2  GTTGCTCAGA 1       0       1       4       1       4.93e-01        1251
           ----------                                                         1219
           ----------                                                         1037
           ----------                                                         375
           ----------                                                         17

LOCUS: AT5G41220
DESCRIPTION: glutathione S-transferase, putative, similar to emb|CAA10662
DATA:                 Control 30min   2hours  2days   1week   p-value         pos
SENSE COUNTS:         3       2       2       0       0       4.20e-01
GENES (2 total):
  AT5G41220.1
    SENSE COUNTS:     3       2       2       0       0       4.20e-01
    TAGS: (1 total)
           ----------                                                         1980
           ----------                                                         1410
           ----------                                                         1241
           ----------                                                         1237
      X+4  AAACGTTAGG 3       2       2       0       0       4.20e-01        1159
           ----------                                                         992
           ----------                                                         739
           ----------                                                         561

LOCUS: AT2G20610
DESCRIPTION: aminotransferase, putative, similar to nicotianamine aminotransferase from Hordeum vulgare
(GI:6498122, GI:6469087); contains Pfam profile PF00155 aminotransferase, classes I and II
DATA:                 Control 30min   2hours  2days   1week   p-value         pos
SENSE COUNTS:         5       1       6       2       5       4.22e-01
GENES (3 total):
  AT2G20610.2
    SENSE COUNTS:     5       1       6       2       5       4.22e-01
    TAGS: (2 total)
      d+1  AAAGAGAATT 1       0       1       1       5       9.91e-02        1725
      d+2  CCAAGAAGAC 4       1       5       1       0       2.10e-01        1486
           ----------                                                         1327
           ----------                                                         253
  AT2G20610.1
    SENSE COUNTS:     5       1       6       2       5       4.22e-01
    TAGS: (2 total)
      d+1  AAAGAGAATT 1       0       1       1       5       9.91e-02        1645
      d+2  CCAAGAAGAC 4       1       5       1       0       2.10e-01        1406
           ----------                                                         253

LOCUS: AT2G14170
DESCRIPTION: Arabidopsis thaliana methylmalonate-semialdehyde dehydrogenase
DATA:                 Control 30min   2hours  2days   1week   p-value         pos
SENSE COUNTS:         2       2       2       5       0       4.22e-01
GENES (2 total):
  AT2G14170.1
    SENSE COUNTS:     2       2       2       5       0       4.22e-01
    TAGS: (3 total)
      i+3  TGTAAAAATT 0       0       1       0       0       4.55e-01        2947
      d+1  GGAATAAGAA 2       0       1       5       0       4.63e-02        1979
           ----------                                                         1925
      d+2  CAGGCAAACA 0       2       0       0       0       9.14e-02        1501
           ----------                                                         1456
           ----------                                                         1263
           ----------                                                         1218
           ----------                                                         1177
           ----------                                                         1091
           ----------                                                         828
```

```
          ----------                                                  793
          ----------                                                  665
          ----------                                                  629

LOCUS: AT4G03180
DESCRIPTION: expressed protein
DATA:                 Control 30min  2hours  2days  1week  p-value    pos
SENSE COUNTS:         1       0      2       1      3      4.23e-01
GENES (1 total):
  AT4G03180.1
    SENSE COUNTS:     1       0      2       1      3      4.23e-01
    TAGS: (2 total)
          ----------                                                  1502
          ----------                                                  1341
      d+2  ATTTTAGATG 1       0      1       1      3      3.98e-01   853
      d+2  GTCAGCCTGT 0       0      1       0      0      4.55e-01   567
          ----------                                                  215
          ----------                                                  167
          ----------                                                  120

LOCUS: AT1G02270
DESCRIPTION: endonuclease/exonuclease/phosphatase family protein / calcium-binding EF hand family protein,
contains Pfam profiles: PF03372 endonuclease/exonuclease/phosphatase family, PF00036 EF hand
DATA:                 Control 30min  2hours  2days  1week  p-value    pos
SENSE COUNTS:         1       0      2       1      3      4.23e-01
GENES (1 total):
  AT1G02270.1
    SENSE COUNTS:     1       0      2       1      3      4.23e-01
    TAGS: (3 total)
          ----------                                                  1619
      d+2  CTTGTCTCAC 0       0      1       1      3      1.91e-01   1439
      d+2  ACGATGGTAA 0       0      1       0      0      4.55e-01   1276
          ----------                                                  1178
          ----------                                                  836
      d+2  CCCATTATTC 1       0      0       0      0      4.28e-01   790
          ----------                                                  608
          ----------                                                  439

LOCUS: AT1G02500
DESCRIPTION: S-adenosylmethionine synthetase 1 (SAM1), identical to S-adenosylmethionine synthetase 1
(Methionine adenosyltransferase 1, AdoMet synthetase 1) (Arabidopsis thaliana) SWISS-PROT:P23686
DATA:                 Control 30min  2hours  2days  1week  p-value    pos
SENSE COUNTS:         6       12     7       9      3      4.25e-01
GENES (3 total):
  AT1G02500.2
    SENSE COUNTS:     6       12     7       9      3      4.25e-01
    TAGS: (4 total)
          ----------                                                  1505
      d+2  TTGTTCTTAT 2       0      0       0      0      1.04e-01   1480
      d+2  GAGACGCTGG 4       12     4       9      3      1.25e-01   863
      d+2  TCATCAAGCC 0       0      2       0      0      1.21e-01   770
      d+2  GTCCCAATCC 0       0      1       0      0      4.55e-01   679
          ----------                                                  557
          ----------                                                  508
          ----------                                                  286
          ----------                                                  280
          ----------                                                  264
  AT1G02500.1
    SENSE COUNTS:     6       12     7       9      3      4.25e-01
    TAGS: (4 total)
          ----------                                                  1505
      d+2  TTGTTCTTAT 2       0      0       0      0      1.04e-01   1480
      d+2  GAGACGCTGG 4       12     4       9      3      1.25e-01   863
      d+2  TCATCAAGCC 0       0      2       0      0      1.21e-01   770
      d+2  GTCCCAATCC 0       0      1       0      0      4.55e-01   679
          ----------                                                  557
          ----------                                                  508
          ----------                                                  286
          ----------                                                  280
          ----------                                                  264

LOCUS: AT3G26840
DESCRIPTION: esterase/lipase/thioesterase family protein, contains Pfam profile PF03096: Ndr family
DATA:                 Control 30min  2hours  2days  1week  p-value    pos
SENSE COUNTS:         1       0      0       1      3      4.25e-01
GENES (1 total):
  AT3G26840.1
    SENSE COUNTS:     1       0      0       1      3      4.25e-01
    TAGS: (2 total)
      d+1  AACCTACCTA 1       0      0       0      0      4.28e-01   2249
      d+2  ATGAATAGAG 0       0      0       1      3      2.09e-01   2152
```

```
          ----------                                                        2086
          ----------                                                        1576
          ----------                                                        1455
          ----------                                                        1413
          ----------                                                        1371
          ----------                                                        687
          ----------                                                        673
          ----------                                                        599

LOCUS: AT1G07170
DESCRIPTION: expressed protein, contains Pfam domain PF03660: Uncharacterised protein family (UPF0123)
DATA:                 Control 30min  2hours  2days  1week  p-value        pos
SENSE COUNTS:         1       0      0       1      3      4.25e-01
GENES (1 total):
  AT1G07170.1
    SENSE COUNTS:     1       0      0       1      3      4.25e-01
    TAGS: (1 total)
          ----------                                                        581
      X+4 GGTTCAAACT 1       0      0       1      3      4.25e-01        494
          ----------                                                        165
          ----------                                                        138

LOCUS: AT2G38860
DESCRIPTION: proteaseI (pfpI)-like protein (YLS5), contains Pfam profile PF01965: DJ-1/PfpI family; supporting
cDNA gi|13122287|dbj|AB047808.1|; identical to proteaseI (pfpI)-like protein (Arabidopsis thaliana)
GI:13122288, cDNA proteaseI (pfpI)-like protein GI:131222
DATA:                 Control 30min  2hours  2days  1week  p-value        pos
SENSE COUNTS:         1       0      0       1      3      4.25e-01
GENES (3 total):
  AT2G38860.2
    SENSE COUNTS:     1       0      0       1      3      4.25e-01
    TAGS: (2 total)
      d+1 TAGTTTGGTG 1       0      0       0      0      6.89e-01        1397
          ----------                                                        1348
          ----------                                                        1340
      d+2 TTCTAAGATT 0       0      0       1      3      6.27e-02        1216
          ----------                                                        562
          ----------                                                        523
          ----------                                                        76
  AT2G38860.1
    SENSE COUNTS:     1       0      0       1      3      4.25e-01
    TAGS: (2 total)
      d+1 TAGTTTGGTG 1       0      0       0      0      6.89e-01        1374
          ----------                                                        1325
          ----------                                                        1317
      d+2 TTCTAAGATT 0       0      0       1      3      6.27e-02        1193
          ----------                                                        539
          ----------                                                        500
          ----------                                                        80

LOCUS: AT5G05700
DESCRIPTION: arginine-tRNA-protein transferase 1 / arginyltransferase 1 / arginyl-tRNA-protein transferase 1
(ATE1), identical to SP|Q9ZT48 Arginine-tRNA-protein transferase 1 (EC 2.3.2.8) (R-transferase 1)
(Arginyltransferase 1) (Arginyl-tRNA--protein transferase 1)
DATA:                 Control 30min  2hours  2days  1week  p-value        pos
SENSE COUNTS:         3       1      4       1      0      4.27e-01
GENES (2 total):
  AT5G05700.1
    SENSE COUNTS:     3       1      4       1      0      4.27e-01
    TAGS: (1 total)
          ----------                                                        2167
      d+2 GTGTCTTTCT 3       1      4       1      0      4.27e-01        2079
          ----------                                                        2038
          ----------                                                        1636
          ----------                                                        1628
          ----------                                                        1052
          ----------                                                        284
          ----------                                                        134

LOCUS: AT2G41410
DESCRIPTION: calmodulin, putative, identical to SP|P30188 Calmodulin-like protein {Arabidopsis thaliana}
DATA:                 Control 30min  2hours  2days  1week  p-value        pos
SENSE COUNTS:         7       9      6       3      3      4.29e-01
GENES (1 total):
  AT2G41410.1
    SENSE COUNTS:     7       9      6       3      3      4.29e-01
    TAGS: (3 total)
      d+1 TTATTATCTT 1       0      0       0      0      4.28e-01        1086
      d+2 TTTCTAGTTT 6       8      4       2      3      4.25e-01        982
      d+2 TTTGATTGAT 0       1      2       1      0      5.89e-01        808
          ----------                                                        768
```

```
          ----------                                                    451
          ----------                                                    26

LOCUS: AT2G07783
DESCRIPTION: pseudogene, similar to Ccl1, blastp match of 86% identity and 6.4e-177 P-value to
GP|1694629|dbj|BAA12329.1||D84373 Ccl1 {Brassica napus}
DATA:                 Control 30min   2hours  2days   1week   p-value         pos
SENSE COUNTS:         3       4       1       2       0       4.30e-01
GENES (1 total):
  AT2G07783.1
    SENSE COUNTS:     3       4       1       2       0       4.30e-01
    TAGS: (1 total)
          ----------                                                    2076
          ----------                                                    2065
          ----------                                                    2023
          ----------                                                    1678
      p+2  GTTCGACAAA 3       4       1       2       0       4.30e-01        1353
          ----------                                                    1069
          ----------                                                    1013
          ----------                                                    233
          ----------                                                    222

LOCUS: AT5G38690
DESCRIPTION: expressed protein
DATA:                 Control 30min   2hours  2days   1week   p-value         pos
SENSE COUNTS:         0       0       1       1       3       4.30e-01
GENES (1 total):
  AT5G38690.1
    SENSE COUNTS:     0       0       1       1       3       4.30e-01
    TAGS: (2 total)
      i+3  GAACTGTACA 0       0       0       0       0       6.15e-01        3499
          ----------                                                    1829
          ----------                                                    1676
          ----------                                                    1519
          ----------                                                    1448
      d+2  CGACAACAGA 0       0       1       1       3       1.91e-01        680
          ----------                                                    551
          ----------                                                    358

LOCUS: AT3G10450
DESCRIPTION: serine carboxypeptidase S10 family protein, similar to glucose acyltransferase GB:AAD01263
(Solanum berthaultii); also similar to serine carboxypeptidase I GB:P37890 (Oryza sativa)
DATA:                 Control 30min   2hours  2days   1week   p-value         pos
SENSE COUNTS:         0       0       3       1       1       4.33e-01
GENES (1 total):
  AT3G10450.1
    SENSE COUNTS:     0       0       3       1       1       4.33e-01
    TAGS: (2 total)
      i+3  GAAATGCTAG 0       0       0       1       0       3.09e-01        1810
      d+1  GCCTTTAAAA 0       0       3       0       1       2.30e-01        1499
          ----------                                                    1295
          ----------                                                    1103
          ----------                                                    1049
          ----------                                                    1020
          ----------                                                    960
          ----------                                                    708
          ----------                                                    367

LOCUS: AT3G58680
DESCRIPTION: ethylene-responsive transcriptional coactivator, putative, similar to ethylene-responsive
transcriptional coactivator (Lycopersicon esculentum) gi|5669634|gb|AAD46402
DATA:                 Control 30min   2hours  2days   1week   p-value         pos
SENSE COUNTS:         13      12      9       20      17      4.33e-01
GENES (1 total):
  AT3G58680.1
    SENSE COUNTS:     13      12      9       20      17      4.33e-01
    TAGS: (2 total)
      d+1  TCTCGTAAAC 2       1       2       4       3       8.69e-01        738
      d+2  ATGTAAGAAA 11      11      7       16      14      5.70e-01        666
          ----------                                                    587
          ----------                                                    356
          ----------                                                    321
          ----------                                                    95

LOCUS: AT1G31970
DESCRIPTION: DEAD/DEAH box helicase, putative, similar to p68 RNA helicase (Schizosaccharomyces pombe)
GI:173419
DATA:                 Control 30min   2hours  2days   1week   p-value         pos
SENSE COUNTS:         2       4       2       6       1       4.34e-01
GENES (1 total):
  AT1G31970.1
```

```
    SENSE COUNTS:      2       4       2       6       1       4.34e-01
    TAGS: (1 total)
           ----------                                                         1563
           ----------                                                         1010
           ----------                                                         582
      d+2  AAGTCGAAGA  2       4       2       6       1       4.34e-01       174

LOCUS: AT1G13780
DESCRIPTION: F-box family protein, contains F-box domain Pfam:PF00646
DATA:                  Control 30min   2hours  2days   1week   p-value        pos
SENSE COUNTS:          6       0       4       2       3       4.36e-01
GENES (1 total):
  AT1G13780.1
    SENSE COUNTS:      6       0       4       2       3       4.36e-01
    TAGS: (2 total)
      d+1  AAACACTGAT  1       0       0       0       0       6.89e-01       1474
           ----------                                                         1451
           ----------                                                         1442
           ----------                                                         1335
      d+2  TTGAGATAAA  5       0       4       2       3       4.03e-01       1231
           ----------                                                         867
           ----------                                                         673
           ----------                                                         576
           ----------                                                         474
           ----------                                                         78

LOCUS: AT2G27860
DESCRIPTION: expressed protein
DATA:                  Control 30min   2hours  2days   1week   p-value        pos
SENSE COUNTS:          20      25      24      19      12      4.36e-01
GENES (2 total):
  AT2G27860.1
    SENSE COUNTS:      20      25      24      19      12      4.36e-01
    TAGS: (2 total)
           ----------                                                         1514
      d+2  TATGTCGGAG  20      24      23      18      12      5.33e-01       1423
      d+2  ACCATCATTA  0       1       1       1       0       6.84e-01       1091
           ----------                                                         294
           ----------                                                         122

LOCUS: AT1G27595
DESCRIPTION: expressed protein, similar to Symplekin (SP:Q92797) {Homo sapiens}
DATA:                  Control 30min   2hours  2days   1week   p-value        pos
SENSE COUNTS:          3       0       3       1       4       4.38e-01
GENES (1 total):
  AT1G27595.1
    SENSE COUNTS:      3       0       3       1       4       4.38e-01
    TAGS: (4 total)
      i+3  GAAGGCTTCT  0       0       0       1       0       3.09e-01       5027
      d+1  TAATGGATGT  1       0       2       0       1       7.50e-01       3525
           ----------                                                         3418
      d+2  TCTTTGGGAT  0       0       1       0       0       4.55e-01       3398
      i+3  TTTTATTCCT  2       0       0       0       3       7.98e-02       3380
           ----------                                                         3260
           ----------                                                         3100
           ----------                                                         2902
           ----------                                                         2802
           ----------                                                         2171
           ----------                                                         2061
           ----------                                                         1961
           ----------                                                         1748
           ----------                                                         1591
           ----------                                                         1559
           ----------                                                         985
           ----------                                                         763
           ----------                                                         535
           ----------                                                         215
           ----------                                                         26

LOCUS: AT3G16840
DESCRIPTION: DEAD/DEAH box helicase, putative (RH13), similar to RNA helicase GB:CAA09204 from (Arabidopsis
thaliana); identical to cDNA DEAD box RNA helicase, RH13 GI:3776002
DATA:                  Control 30min   2hours  2days   1week   p-value        pos
SENSE COUNTS:          0       3       1       2       1       4.40e-01
GENES (1 total):
  AT3G16840.1
    SENSE COUNTS:      0       3       1       2       1       4.40e-01
    TAGS: (2 total)
      d+1  CTGAATCAAT  0       0       1       0       1       3.96e-01       2110
           ----------                                                         1603
           ----------                                                         1206
```

```
          ----------                                                  708
          ----------                                                  677
      d+2  GTTACAGGCG  0       3       0       2       0       1.05e-01        105

LOCUS: AT1G15280
DESCRIPTION: glycine-rich protein
DATA:                  Control 30min   2hours  2days   1week   p-value         pos
SENSE COUNTS:          6       5       2       3       1       4.41e-01
GENES (2 total):
  AT1G15280.1
    SENSE COUNTS:      6       5       2       3       1       4.41e-01
    TAGS: (3 total)
      d+1  AATAGGAGCT  0       0       1       2       1       3.05e-01        2289
      d+2  TAAATATTTG  5       4       0       1       0       6.46e-02        2236
      d+2  GAAAGTCCTG  1       1       1       0       0       7.28e-01        1753
           ----------                                                          1596
           ----------                                                          1517
           ----------                                                          989
           ----------                                                          905
           ----------                                                          626
           ----------                                                          554
           ----------                                                          458
           ----------                                                          100
  AT1G15280.2
    SENSE COUNTS:      6       5       2       3       1       4.41e-01
    TAGS: (3 total)
      d+1  AATAGGAGCT  0       0       1       2       1       3.05e-01        2270
      d+2  TAAATATTTG  5       4       0       1       0       6.46e-02        2217
      d+2  GAAAGTCCTG  1       1       1       0       0       7.28e-01        1734
           ----------                                                          1577
           ----------                                                          1498
           ----------                                                          970
           ----------                                                          886
           ----------                                                          607
           ----------                                                          532
           ----------                                                          436
           ----------                                                          78

LOCUS: AT3G50500
DESCRIPTION: protein kinase, putative, similar to abscisic acid-activated protein kinase (Vicia faba)
gi|6739629|gb|AAF27340
DATA:                  Control 30min   2hours  2days   1week   p-value         pos
SENSE COUNTS:          4       6       1       5       7       4.44e-01
GENES (2 total):
  AT3G50500.1
    SENSE COUNTS:      4       6       1       5       7       4.44e-01
    TAGS: (3 total)
      i+3  TGTTTCCTAC  1       0       0       0       0       4.28e-01        1309
      d+1  GATGACTTTG  3       6       1       4       7       3.76e-01        1193
      d+2  GCGGATAATC  0       0       0       1       0       3.09e-01        1163
           ----------                                                          573
           ----------                                                          290

LOCUS: AT2G39080
DESCRIPTION: expressed protein,
DATA:                  Control 30min   2hours  2days   1week   p-value         pos
SENSE COUNTS:          1       4       1       1       1       4.45e-01
GENES (2 total):
  AT2G39080.1
    SENSE COUNTS:      1       4       1       1       1       4.45e-01
    TAGS: (1 total)
           ----------                                                          1239
      d+2  TATCCTCTTG  1       4       1       1       1       4.45e-01        1182
           ----------                                                          860
           ----------                                                          414
           ----------                                                          285

LOCUS: AT1G10900
DESCRIPTION: phosphatidylinositol-4-phosphate 5-kinase family protein, similar to phosphatidylinositol-4-
phosphate 5-kinase AtPIP5K1 (Arabidopsis thaliana) GI:3702691; contains Pfam profiles PF01504:
Phosphatidylinositol-4-phosphate 5-Kinase, PF02493: MORN repeat
DATA:                  Control 30min   2hours  2days   1week   p-value         pos
SENSE COUNTS:          2       0       3       2       3       4.46e-01
GENES (1 total):
  AT1G10900.1
    SENSE COUNTS:      4       0       3       3       6       1.46e-01
    TAGS: (4 total)
      d+1  GGAATCATAG  0       0       2       1       0       2.74e-01        2321
           ----------                                                          2231
           ----------                                                          2094
           ----------                                                          1712
```

```
      d+2  AAAACACACT 0       0       1       0       0       4.55e-01        1632
           ----------                                                         1620
           ----------                                                         1539
           ----------                                                         1460
           ----------                                                         1409
           ----------                                                         1088
           ----------                                                         999
           ----------                                                         994
           ----------                                                         711
           ----------                                                         504
           ----------                                                         449
           ----------                                                         435
      X+4  ATGAAATGCT 2       0       0       1       3       1.97e-01        420
      X+4  ATGAAATGCT 2       0       0       1       3       1.97e-01        420
           ----------                                                         316
           ----------                                                         143

LOCUS: AT4G24220
DESCRIPTION: expressed protein, protein induced upon wounding - Arabidopsis thaliana, PID:e257749
DATA:                 Control 30min   2hours  2days   1week   p-value         pos
SENSE COUNTS:         4       7       2       2       7       4.48e-01
GENES (2 total):
  AT4G24220.1
    SENSE COUNTS:     4       7       2       2       7       4.48e-01
    TAGS: (1 total)
           ----------                                                         1779
      d+2  TTTATGTTTC 4       7       2       2       7       4.48e-01        1483
           ----------                                                         1016
           ----------                                                         963
           ----------                                                         887
           ----------                                                         693
           ----------                                                         576
           ----------                                                         442

LOCUS: AT2G13440
DESCRIPTION: glucose-inhibited division family A protein, similar to GidA from Pseudomonas syringae
(GI:10764670); contains Pfam profile PF01134 Glucose inhibited division protein A
DATA:                 Control 30min   2hours  2days   1week   p-value         pos
SENSE COUNTS:         3       1       2       6       1       4.51e-01
GENES (1 total):
  AT2G13440.1
    SENSE COUNTS:     3       1       2       6       1       4.51e-01
    TAGS: (2 total)
      i+3  ATCAATCCTC 0       0       0       0       0       6.15e-01        3607
           ----------                                                         2230
           ----------                                                         2159
           ----------                                                         2114
           ----------                                                         1955
           ----------                                                         1784
           ----------                                                         1486
           ----------                                                         1385
           ----------                                                         1210
           ----------                                                         983
           ----------                                                         860
      X+4  GCTTTCGTTT 3       1       2       6       1       3.27e-01        433
           ----------                                                         383

LOCUS: AT2G26460
DESCRIPTION: RED family protein, similar to Red protein (RER protein) (Swiss-Prot:Q9Z1M8) (Mus musculus)
DATA:                 Control 30min   2hours  2days   1week   p-value         pos
SENSE COUNTS:         1       1       2       5       3       4.53e-01
GENES (1 total):
  AT2G26460.1
    SENSE COUNTS:     1       1       2       5       3       4.53e-01
    TAGS: (3 total)
      i+3  TTCCTTGTTA 0       0       0       0       1       1.65e-01        3579
      d+1  GGTGGAAAGG 0       0       0       1       1       6.17e-01        1528
           ----------                                                         1481
           ----------                                                         1324
           ----------                                                         1012
      d+2  TCATATCTTC 1       1       2       4       1       7.61e-01        679
           ----------                                                         572
           ----------                                                         218

LOCUS: AT2G04880
DESCRIPTION: WRKY family transcription factor (ZAP1), identical to ZAP1 GI:1064883 from (Arabidopsis
thaliana); contains Pfam profile: PF03106 WRKY DNA -binding domain
DATA:                 Control 30min   2hours  2days   1week   p-value         pos
SENSE COUNTS:         1       0       3       0       1       4.56e-01
GENES (3 total):
  AT2G04880.2
```

```
    SENSE COUNTS:      1      0      3      0      1      4.56e-01
    TAGS: (1 total)
           ----------                                                2256
           ----------                                                2163
           ----------                                                2011
      d+2  GGGATGTATA  1      0      3      0      1      4.56e-01      1895
           ----------                                                1397
           ----------                                                1309
           ----------                                                1226
           ----------                                                896
           ----------                                                884
           ----------                                                746
           ----------                                                23
  AT2G04880.1
    SENSE COUNTS:      1      0      3      0      1      4.56e-01
    TAGS: (1 total)
           ----------                                                2328
           ----------                                                2235
           ----------                                                2083
      d+2  GGGATGTATA  1      0      3      0      1      4.56e-01      1967
           ----------                                                1469
           ----------                                                1381
           ----------                                                1298
           ----------                                                968
           ----------                                                956
           ----------                                                889
           ----------                                                746
           ----------                                                23

LOCUS: AT3G57340
DESCRIPTION: DNAJ heat shock N-terminal domain-containing protein, similar to SP|Q9QYI4 DnaJ homolog subfamily
B member 12 Mus musculus; contains Pfam profile PF00226 DnaJ domain
DATA:                  Control 30min  2hours  2days  1week  p-value       pos
SENSE COUNTS:          1      5      3      2      3      4.56e-01
GENES (2 total):
  AT3G57340.1
    SENSE COUNTS:      0      4      2      2      3      4.25e-01
    TAGS: (1 total)
      d+1  ATGAGGAGAT  0      4      2      2      3      4.25e-01      1171
           ----------                                                919
           ----------                                                100
  AT3G57340.2
    SENSE COUNTS:      1      5      3      2      3      4.56e-01
    TAGS: (3 total)
      X+4  CCTGTTTGAG  0      1      1      0      0      4.87e-01      1481
      d+1  ATGAGGAGAT  0      4      2      2      3      4.25e-01      1242
           ----------                                                990
           ----------                                                171
      d+2  ATTGAGATTT  1      0      0      0      0      4.28e-01      64

LOCUS: AT2G04350
DESCRIPTION: long-chain-fatty-acid--CoA ligase family protein / long-chain acyl-CoA synthetase family protein
(LACS8), similar to LACS 4 (SP|O35547) from Rattus norvegicus, LACS 4 (SP|O60488) from Homo sapiens; contains
Pfam HMM hit: AMP-binding enzymes PF00501
DATA:                  Control 30min  2hours  2days  1week  p-value       pos
SENSE COUNTS:          1      0      1      0      3      4.58e-01
GENES (2 total):
  AT2G04350.1
    SENSE COUNTS:      1      0      1      0      3      4.58e-01
    TAGS: (2 total)
      i+3  CTCTTTCATT  0      0      1      0      0      4.55e-01      2887
      d+1  GTCCACGCAG  1      0      0      0      3      2.28e-01      1978
           ----------                                                1910
           ----------                                                1653
           ----------                                                1546
           ----------                                                1124
           ----------                                                341
  AT2G04350.2
    SENSE COUNTS:      1      0      1      0      3      4.58e-01
    TAGS: (2 total)
      i+3  CTCTTTCATT  0      0      1      0      0      4.55e-01      2842
      d+1  GTCCACGCAG  1      0      0      0      3      2.28e-01      1929
           ----------                                                1861
           ----------                                                1604
           ----------                                                1497
           ----------                                                1075
           ----------                                                292

LOCUS: AT5G62650
DESCRIPTION: expressed protein
DATA:                  Control 30min  2hours  2days  1week  p-value       pos
```

```
SENSE COUNTS:          1       0       1       0       3       4.58e-01
GENES (1 total):
  AT5G62650.1
    SENSE COUNTS:      1       0       1       0       3       4.58e-01
    TAGS: (2 total)
      d+1  CTCTCAATAG  1       0       0       0       0       4.28e-01       1824
      d+2  TTGAAAAAAT  0       0       1       0       3       2.32e-01       1746
           ----------                                                         1727
           ----------                                                         1516
           ----------                                                         963
           ----------                                                         933
           ----------                                                         862
           ----------                                                         501

LOCUS: AT2G17050
DESCRIPTION: disease resistance protein (TIR-NBS-LRR class), putative, domain signature TIR-NBS-LRR exists,
suggestive of a disease resistance protein.
DATA:                  Control 30min   2hours  2days   1week   p-value        pos
SENSE COUNTS:          1       0       1       0       3       4.58e-01
GENES (1 total):
  AT2G17050.1
    SENSE COUNTS:      1       0       1       0       3       4.58e-01
    TAGS: (1 total)
           ----------                                                         4730
           ----------                                                         4339
           ----------                                                         4275
           ----------                                                         3831
      v+2  TGTTGTTGAT  1       0       1       0       3       4.58e-01       3374
           ----------                                                         3342
           ----------                                                         2992
           ----------                                                         2766
           ----------                                                         2653
           ----------                                                         2612
           ----------                                                         2599
           ----------                                                         2569
           ----------                                                         2100
           ----------                                                         2064
           ----------                                                         1680
           ----------                                                         1458
           ----------                                                         1345
           ----------                                                         1216
           ----------                                                         1131
           ----------                                                         1020
           ----------                                                         272
           ----------                                                         190

LOCUS: AT5G46990
DESCRIPTION: invertase/pectin methylesterase inhibitor family protein, contains Pfam profile PF04043: Plant
invertase/pectin methylesterase inhibitor
DATA:                  Control 30min   2hours  2days   1week   p-value        pos
SENSE COUNTS:          1       0       1       0       3       4.58e-01
GENES (1 total):
  AT5G46990.1
    SENSE COUNTS:      1       0       1       0       3       4.58e-01
    TAGS: (1 total)
           ----------                                                         1356
           ----------                                                         1222
           ----------                                                         1209
           ----------                                                         1054
           ----------                                                         763
           ----------                                                         582
      v+2  TTGGAAAATT  1       0       1       0       3       4.58e-01       258
           ----------                                                         239
           ----------                                                         219

LOCUS: AT1G14920
DESCRIPTION: gibberellin response modulator (GAI) (RGA2) / gibberellin-responsive modulator, identical to GAI
GB:CAA75492 GI:2569938 (Arabidopsis thaliana) (Genes Dev. In press)
DATA:                  Control 30min   2hours  2days   1week   p-value        pos
SENSE COUNTS:          3       3       2       5       0       4.61e-01
GENES (2 total):
  AT1G14920.1
    SENSE COUNTS:      3       3       2       5       0       4.61e-01
    TAGS: (2 total)
           ----------                                                         2130
      d+2  TGACATTGGA  3       2       1       4       0       5.03e-01       1964
      d+2  TTGGGTTGGC  0       1       1       1       0       6.84e-01       1731
           ----------                                                         1579
           ----------                                                         1506
           ----------                                                         1144
           ----------                                                         1006
```

```
          ----------                                                    658
          ----------                                                    270
          ----------                                                    192

LOCUS: AT5G12370
DESCRIPTION: exocyst complex component Sec10-related, low similarity to SP|O00471 Exocyst complex component
Sec10 (hSec10) {Homo sapiens}
DATA:                  Control 30min  2hours  2days  1week  p-value     pos
SENSE COUNTS:          4       3      2       1      7      4.63e-01
GENES (1 total):
  AT5G12370.1
    SENSE COUNTS:      4       3      2       1      7      4.63e-01
    TAGS: (3 total)
      i+3  AGATTCTAGA  0       0      0       0      0      6.15e-01    3189
      d+1  AAATCTTGCC  3       3      2       1      7      4.59e-01    2659
      i+3  CAAACTTTCG  1       0      0       0      0      4.28e-01    2531
           ----------                                                   2004
           ----------                                                   1994
           ----------                                                   1926
           ----------                                                   1876
           ----------                                                   1448
           ----------                                                   1067
           ----------                                                   1033
           ----------                                                   883
           ----------                                                   832
           ----------                                                   179

LOCUS: AT5G18590
DESCRIPTION: kelch repeat-containing protein, identical to RanGAP1 interacting protein (GI:21950739)
(Arabidopsis thaliana); similar to Tip elongation aberrant protein 1 (Cell polarity protein tea1) (SP:P87061)
(Schizosaccharomyces pombe); contains Pfam PF01344: Kelch
DATA:                  Control 30min  2hours  2days  1week  p-value     pos
SENSE COUNTS:          4       1      7       2      4      4.65e-01
GENES (2 total):
  AT5G18590.1
    SENSE COUNTS:      4       1      7       2      4      4.65e-01
    TAGS: (3 total)
           ----------                                                   2958
           ----------                                                   2880
           ----------                                                   2849
      d+2  AGATGAGACT  0       0      0       0      1      1.65e-01    2705
      d+2  TGTGTTGTGG  4       1      7       2      3      2.92e-01    2558
           ----------                                                   2546
           ----------                                                   2480
           ----------                                                   2303
           ----------                                                   2272
           ----------                                                   2194
           ----------                                                   2045
      d+2  GTGGCTCTGG  0       0      0       0      0      6.15e-01    1676
           ----------                                                   1301
           ----------                                                   1085
           ----------                                                   1035
           ----------                                                   757
           ----------                                                   705
           ----------                                                   699
           ----------                                                   605
           ----------                                                   385
  AT5G18590.2
    SENSE COUNTS:      4       1      7       2      4      4.65e-01
    TAGS: (3 total)
           ----------                                                   3013
           ----------                                                   2935
           ----------                                                   2904
      d+2  AGATGAGACT  0       0      0       0      1      1.65e-01    2760
      d+2  TGTGTTGTGG  4       1      7       2      3      2.92e-01    2613
           ----------                                                   2601
           ----------                                                   2535
           ----------                                                   2358
           ----------                                                   2327
           ----------                                                   2249
           ----------                                                   2100
      d+2  GTGGCTCTGG  0       0      0       0      0      6.15e-01    1731
           ----------                                                   1356
           ----------                                                   1140
           ----------                                                   1090
           ----------                                                   812
           ----------                                                   760
           ----------                                                   754
           ----------                                                   660
           ----------                                                   440
```

```
LOCUS: AT1G22570
DESCRIPTION: proton-dependent oligopeptide transport (POT) family protein, contains Pfam profile: PF00854 POT
family
DATA:                  Control 30min   2hours  2days   1week   p-value         pos
SENSE COUNTS:          2       4       4       0       3       4.66e-01
GENES (1 total):
  AT1G22570.1
    SENSE COUNTS:      2       4       4       0       3       4.66e-01
    TAGS: (3 total)
      v+1  AAAGTTAATC  0       0       2       0       0       1.21e-01        2418
      v+2  AAATTTTATT  2       4       1       0       0       1.67e-01        2141
      v+2  TGGATTACTT  0       0       1       0       3       7.32e-02        1945
           ----------                                                          1298
           ----------                                                          1099

LOCUS: AT2G18710
DESCRIPTION: preprotein translocase secY subunit, chloroplast (CpSecY), Identical to SP|Q38885 Preprotein
translocase secY subunit, chloroplast precursor (CpSecY) {Arabidopsis thaliana}
DATA:                  Control 30min   2hours  2days   1week   p-value         pos
SENSE COUNTS:          13      6       7       6       6       4.67e-01
GENES (1 total):
  AT2G18710.1
    SENSE COUNTS:      13      6       7       6       6       4.67e-01
    TAGS: (2 total)
      d+1  AGCTTGCCAA  5       2       5       4       1       6.61e-01        1797
      d+2  AACTGGACCT  8       4       2       2       5       3.97e-01        1681
           ----------                                                          199

LOCUS: AT3G62350
DESCRIPTION: hypothetical protein
DATA:                  Control 30min   2hours  2days   1week   p-value         pos
SENSE COUNTS:          2       0       2       1       3       4.72e-01
GENES (1 total):
  AT3G62350.1
    SENSE COUNTS:      2       0       2       1       3       4.72e-01
    TAGS: (1 total)
           ----------                                                          1412
           ----------                                                          1211
      v+2  GATATTTTGA  2       0       2       1       3       4.72e-01        478
           ----------                                                          152

LOCUS: AT5G01450
DESCRIPTION: expressed protein
DATA:                  Control 30min   2hours  2days   1week   p-value         pos
SENSE COUNTS:          2       2       1       0       3       4.74e-01
GENES (1 total):
  AT5G01450.1
    SENSE COUNTS:      2       2       1       0       3       4.74e-01
    TAGS: (2 total)
      i+3  ACACAAGAAT  2       2       1       0       0       4.77e-01        2625
      d+1  TATAGAACTT  0       0       0       0       3       8.36e-02        1840
           ----------                                                          1349
           ----------                                                          1156
           ----------                                                          359
           ----------                                                          205

LOCUS: AT5G05730
DESCRIPTION: branchpoint enzyme in aromatic amino acid biosynthesis
DATA:                  Control 30min   2hours  2days   1week   p-value         pos
SENSE COUNTS:          1       0       2       0       3       4.74e-01
GENES (2 total):
  AT5G05730.1
    SENSE COUNTS:      1       0       2       0       3       4.74e-01
    TAGS: (2 total)
           ----------                                                          2039
      d+2  TGTATAAAAC  1       0       1       0       3       4.58e-01        1928
           ----------                                                          1694
           ----------                                                          1650
           ----------                                                          1450
           ----------                                                          1356
           ----------                                                          1010
           ----------                                                          922
           ----------                                                          768
           ----------                                                          561
      i+3  TTTTTGGACT  0       0       1       0       0       4.55e-01        470

LOCUS: AT1G45332
DESCRIPTION: mitochondrial elongation factor, putative, similar to mitochondrial elongation factor GI:3917
from (Saccharomyces cerevisiae)
DATA:                  Control 30min   2hours  2days   1week   p-value         pos
SENSE COUNTS:          1       0       2       0       3       4.74e-01
```

```
GENES (1 total):
  AT1G45332.1
    SENSE COUNTS:      1       0       2       0       3       4.74e-01
    TAGS: (2 total)
      d+1  ACACAAGGCA  0       0       1       0       0       7.06e-01        2293
           ----------                                                          2263
           ----------                                                          1891
           ----------                                                          1699
           ----------                                                          1418
      i+3  AAACATATGA  1       0       1       0       3       2.09e-01        1334
           ----------                                                          875
           ----------                                                          854
           ----------                                                          747
           ----------                                                          569
           ----------                                                          472
           ----------                                                          419
           ----------                                                          325
           ----------                                                          309

LOCUS: AT4G21970
DESCRIPTION: expressed protein, contains Pfam profile PF04520: Protein of unknown function, DUF584; expression
supported by MPSS
DATA:                  Control 30min   2hours  2days   1week   p-value         pos
SENSE COUNTS:          1       0       3       2       0       4.77e-01
GENES (1 total):
  AT4G21970.1
    SENSE COUNTS:      1       0       3       2       0       4.77e-01
    TAGS: (1 total)
           ----------                                                          1131
           ----------                                                          859
           ----------                                                          837
           ----------                                                          833
           ----------                                                          652
           ----------                                                          608
      i+3  AAAAAAAAAC  1       0       3       2       0       4.77e-01        561
           ----------                                                          289

LOCUS: AT5G57300
DESCRIPTION: UbiE/COQ5 methyltransferase family protein, similar to ubiquinone biosynthesis methyltransferase
COQ5 (Saccharomyces cerevisiae)(SP|P49017), ubiquinone/menaquinone biosynthesis methyltransferase ubiE
(Escherichia coli)(SP|P27851); contains Pfam profile PF
DATA:                  Control 30min   2hours  2days   1week   p-value         pos
SENSE COUNTS:          0       0       1       1       3       4.78e-01
GENES (1 total):
  AT5G57300.1
    SENSE COUNTS:      0       0       1       1       3       4.78e-01
    TAGS: (4 total)
      i+3  GCCGAATCTT  0       0       0       1       0       6.04e-01        2009
      i+3  TTATTATTGA  0       0       0       0       3       8.36e-02        1042
      d+1  TAGAAATTCC  0       0       1       0       0       4.55e-01        759
           ----------                                                          533
      d+2  CCACAAGTTT  0       0       0       0       0       6.15e-01        210

LOCUS: AT3G60600
DESCRIPTION: vesicle-associated membrane protein, putative / VAMP, putative, similar to VAP27 GI:6688926
(Nicotiana plumbaginifolia)
DATA:                  Control 30min   2hours  2days   1week   p-value         pos
SENSE COUNTS:          1       3       6       4       3       4.80e-01
GENES (2 total):
  AT3G60600.1
    SENSE COUNTS:      1       3       6       4       3       4.80e-01
    TAGS: (3 total)
      i+3  GTTTTGAGAA  0       0       0       0       0       6.15e-01        1697
      d+1  TACGTTCTTT  1       3       4       2       3       7.90e-01        851
      d+2  GTTCTGAATT  0       0       2       2       0       1.68e-01        651
           ----------                                                          401

LOCUS: AT5G64960
DESCRIPTION: cyclin-dependent kinase, putative / CDK, putative, similar to cyclin dependent kinase C
(Lycopersicon esculentum) gi|15215944|emb|CAC51391
DATA:                  Control 30min   2hours  2days   1week   p-value         pos
SENSE COUNTS:          2       3       1       3       0       4.80e-01
GENES (1 total):
  AT5G64960.1
    SENSE COUNTS:      2       3       1       3       0       4.80e-01
    TAGS: (2 total)
           ----------                                                          2066
      d+2  CCGCTCCACT  2       3       1       2       0       5.98e-01        1274
           ----------                                                          1142
           ----------                                                          1104
           ----------                                                          1001
```

```
      d+2  TGGTCAGTTG 0       0       0       1       0       3.09e-01       781
           ----------                                                       683
           ----------                                                       578
           ----------                                                       544
           ----------                                                       479
           ----------                                                       472
           ----------                                                       457
           ----------                                                       356
           ----------                                                       235

LOCUS: AT1G53200
DESCRIPTION: expressed protein
DATA:                 Control 30min   2hours  2days   1week   p-value        pos
SENSE COUNTS:         2       1       2       2       6       4.81e-01
GENES (2 total):
  AT1G53200.1
    SENSE COUNTS:     2       1       2       2       6       4.81e-01
    TAGS: (3 total)
      i+3  TTAGTCTTAA 0       0       0       0       1       1.65e-01       2369
      d+1  GACTCTTCGG 0       1       0       0       0       2.54e-01       1821
           ----------                                                       1637
           ----------                                                       1507
           ----------                                                       1445
           ----------                                                       1397
           ----------                                                       1343
           ----------                                                       1227
           ----------                                                       1219
      d+2  AAAAATAAAA 2       0       2       2       5       5.19e-01       1027
           ----------                                                       743
  AT1G53200.2
    SENSE COUNTS:     2       1       2       2       6       4.81e-01
    TAGS: (3 total)
      i+3  TTAGTCTTAA 0       0       0       0       1       1.65e-01       2369
      d+1  GACTCTTCGG 0       1       0       0       0       2.54e-01       1804
           ----------                                                       1620
           ----------                                                       1490
           ----------                                                       1428
           ----------                                                       1380
           ----------                                                       1326
           ----------                                                       1210
           ----------                                                       1202
      d+2  AAAAATAAAA 2       0       2       2       5       5.19e-01       1010
           ----------                                                       726

LOCUS: AT3G26780
DESCRIPTION: phosphoglycerate/bisphosphoglycerate mutase family protein, similar to X4 protein GI:21386798, Y4
protein GI:21386800 from  (Silene dioica); contains Pfam profiles PF00300: phosphoglycerate mutase family,
PF01535: PPR repeat
DATA:                 Control 30min   2hours  2days   1week   p-value        pos
SENSE COUNTS:         6       2       3       6       7       4.81e-01
GENES (1 total):
  AT3G26780.1
    SENSE COUNTS:     6       2       3       6       7       4.81e-01
    TAGS: (2 total)
           ----------                                                       2989
           ----------                                                       2915
           ----------                                                       2795
           ----------                                                       2731
           ----------                                                       2665
           ----------                                                       2396
           ----------                                                       2384
      d+2  ATTGCTGGAT 0       0       1       0       0       4.55e-01       2260
           ----------                                                       2114
      i+3  AGGAAAAAAG 6       2       2       6       7       3.38e-01       1669
           ----------                                                       1440
           ----------                                                       1314
           ----------                                                       1143
           ----------                                                       815
           ----------                                                       566
           ----------                                                       523
           ----------                                                       404
           ----------                                                       353
           ----------                                                       165

LOCUS: AT2G10940
DESCRIPTION: protease inhibitor/seed storage/lipid transfer protein (LTP) family protein, similar to proline-
rich cell wall protein (Medicago sativa) GI:3818416; contains Pfam profile PF00234 Protease inhibitor/seed
storage/LTP family
DATA:                 Control 30min   2hours  2days   1week   p-value        pos
SENSE COUNTS:         32      27      24      32      19      4.85e-01
GENES (2 total):
```

```
  AT2G10940.1
    SENSE COUNTS:      30      27      24      32      19      5.86e-01
    TAGS: (3 total)
      d+1  ATACTGTCAA  24      13      20      30      16      1.03e-01       1210
      d+2  TCCAATAGAC  5       14      2       1       3       2.05e-03       692
      d+2  CGGCTCTTGC  1       0       2       1       0       5.07e-01       152
           ----------                                                         72
  AT2G10940.2
    SENSE COUNTS:      32      27      24      32      19      4.85e-01
    TAGS: (4 total)
      d+1  ATACTGTCAA  24      13      20      30      16      1.03e-01       1564
      d+2  CAAGTGTCCT  2       0       0       0       0       1.04e-01       1205
           ----------                                                         1123
      d+2  TCCAATAGAC  5       14      2       1       3       2.05e-03       692
      d+2  CGGCTCTTGC  1       0       2       1       0       5.07e-01       152
           ----------                                                         72

LOCUS: AT5G13770
DESCRIPTION: pentatricopeptide (PPR) repeat-containing protein, contains Pfam profile PF01535: PPR repeat
DATA:                  Control 30min   2hours  2days   1week   p-value        pos
SENSE COUNTS:          1       2       5       4       1       4.86e-01
GENES (1 total):
  AT5G13770.1
    SENSE COUNTS:      1       2       5       4       1       4.86e-01
    TAGS: (3 total)
      d+1  TCATAAGCAT  0       0       0       0       1       1.65e-01       2330
           ----------                                                         2269
      d+2  AAAGTTGAAG  1       2       4       4       0       4.13e-01       1701
      d+2  GTCGGTGTAT  0       0       1       0       0       4.55e-01       1641
           ----------                                                         1629
           ----------                                                         1444
           ----------                                                         1377
           ----------                                                         1020
           ----------                                                         697
           ----------                                                         157
           ----------                                                         85
           ----------                                                         62
           ----------                                                         42

LOCUS: AT1G79280
DESCRIPTION: expressed protein, weak similarity to Nucleoprotein TPR (Swiss-Prot:P12270) (Homo sapiens)
DATA:                  Control 30min   2hours  2days   1week   p-value        pos
SENSE COUNTS:          1       1       1       2       4       4.86e-01
GENES (1 total):
  AT1G79280.1
    SENSE COUNTS:      1       1       1       2       4       4.86e-01
    TAGS: (6 total)
           ----------                                                         7042
           ----------                                                         6686
      v+2  ATGAGGAGCC  0       0       0       0       0       6.15e-01       6129
           ----------                                                         5617
           ----------                                                         5367
      v+2  CTGAAGATTG  1       0       0       1       3       1.87e-01       4543
      v+2  CAACTCAGGG  0       0       0       0       1       1.65e-01       4251
      i+3  CATAGTTTCT  0       0       0       0       0       6.15e-01       4048
           ----------                                                         3880
      v+2  AGAAATGGCG  0       0       1       1       0       5.21e-01       3646
           ----------                                                         3379
           ----------                                                         3315
      v+2  AAAGAATCTG  0       1       0       0       0       2.54e-01       2547
           ----------                                                         1639
           ----------                                                         1632
           ----------                                                         1497
           ----------                                                         931

LOCUS: AT1G45201
DESCRIPTION: conserved hypothetical protein, contains Pfam profile: PF01764 lipase; contains similarity to
hypothetical proteins from (Arabidopsis thaliana)
DATA:                  Control 30min   2hours  2days   1week   p-value        pos
SENSE COUNTS:          2       2       4       0       1       4.88e-01
GENES (2 total):
  AT1G45201.2
    SENSE COUNTS:      2       2       4       0       1       4.88e-01
    TAGS: (3 total)
      i+3  GTCCGGTAAA  1       0       0       0       0       4.28e-01       2137
      d+1  GAATAGAGTA  1       2       3       0       1       5.74e-01       1096
      X+4  GTCTTCTTGA  0       0       1       0       0       4.55e-01       528

LOCUS: AT3G45980
DESCRIPTION: histone H2B, identical to histone H2B Arabidopsis thaliana GI:2407802; contains Pfam profile
PF00125 Core histone H2A/H2B/H3/H4
```

```
DATA:                  Control 30min   2hours  2days   1week   p-value         pos
SENSE COUNTS:          1       3       1       2       0       4.90e-01
GENES (1 total):
  AT3G45980.1
    SENSE COUNTS:      1       3       1       2       0       4.90e-01
    TAGS: (1 total)
           ----------                                                          1180
           ----------                                                          1048
      d+2  AGAATCATTA  1       3       1       2       0       4.90e-01        721

LOCUS: AT4G30310
DESCRIPTION: ribitol kinase, putative, similar to ribitol kinase (Klebsiella pneumoniae)
gi|2905643|gb|AAC26495
DATA:                  Control 30min   2hours  2days   1week   p-value         pos
SENSE COUNTS:          4       6       5       4       0       4.93e-01
GENES (3 total):
  AT4G30310.1
    SENSE COUNTS:      0       0       1       0       0       4.55e-01
    TAGS: (1 total)
           ----------                                                          1598
           ----------                                                          1590
           ----------                                                          1486
           ----------                                                          1417
           ----------                                                          1239
           ----------                                                          1108
      d+2  GAAAAGTCAG  0       0       1       0       0       4.55e-01        903
           ----------                                                          883
           ----------                                                          757
           ----------                                                          654
           ----------                                                          646
           ----------                                                          226
  AT4G30310.2
    SENSE COUNTS:      4       6       4       4       0       5.06e-01
    TAGS: (1 total)
           ----------                                                          1633
           ----------                                                          1625
           ----------                                                          1517
           ----------                                                          1448
           ----------                                                          1270
           ----------                                                          1139
           ----------                                                          904
           ----------                                                          884
           ----------                                                          758
           ----------                                                          655
           ----------                                                          647
      X+4  AAACTTGGGA  4       6       4       4       0       5.06e-01        261
           ----------                                                          227
  AT4G30310.3
    SENSE COUNTS:      0       0       1       0       0       4.55e-01
    TAGS: (1 total)
           ----------                                                          1438
           ----------                                                          1430
           ----------                                                          1322
           ----------                                                          1253
           ----------                                                          1075
           ----------                                                          944
      d+2  GAAAAGTCAG  0       0       1       0       0       4.55e-01        739
           ----------                                                          719
           ----------                                                          593
           ----------                                                          490
           ----------                                                          482
           ----------                                                          62

LOCUS: AT3G27040
DESCRIPTION: meprin and TRAF homology domain-containing protein / MATH domain-containing protein, similar to
ubiquitin-specific protease 12 (Arabidopsis thaliana) GI:11993471; contains Pfam profile PF00917: MATH domain
DATA:                  Control 30min   2hours  2days   1week   p-value         pos
SENSE COUNTS:          1       0       1       4       1       4.93e-01
GENES (1 total):
  AT3G27040.1
    SENSE COUNTS:      1       0       1       4       1       4.93e-01
    TAGS: (2 total)
           ----------                                                          1910
           ----------                                                          1818
           ----------                                                          1401
           ----------                                                          922
      i+3  TCAATCAAAT  0       0       1       0       0       4.55e-01        907
           ----------                                                          248
      v+2  TGAAGCCATT  1       0       0       4       1       2.52e-01        138
           ----------                                                          55
```

```
LOCUS: AT4G20090
DESCRIPTION: pentatricopeptide (PPR) repeat-containing protein, contains Pfam profile PF01535: PPR repeat
DATA:                  Control 30min   2hours  2days   1week   p-value         pos
SENSE COUNTS:          2       0       2       2       3       4.94e-01
GENES (1 total):
  AT4G20090.1
    SENSE COUNTS:      2       0       2       2       3       4.94e-01
    TAGS: (2 total)
      v+1  TTTGATTTGA  0       0       0       1       0       3.09e-01        2605
      v+2  TTACTTGATT  2       0       2       1       3       4.72e-01        2490
           ----------                                                          2387
           ----------                                                          1930
           ----------                                                          1252
           ----------                                                          894
           ----------                                                          888
           ----------                                                          354

LOCUS: AT1G72570
DESCRIPTION: ovule development protein, putative, similar to ovule development protein AINTEGUMENTA
(GI:1209099) (Arabidopsis thaliana);contains Pfam profile: PF00847 AP2 domain (2 copies); contains non-
consensus TA acceptor splice site at exon 4
DATA:                  Control 30min   2hours  2days   1week   p-value         pos
SENSE COUNTS:          3       0       1       4       1       4.94e-01
GENES (1 total):
  AT1G72570.1
    SENSE COUNTS:      3       0       1       4       1       4.94e-01
    TAGS: (1 total)
           ----------                                                          2107
           ----------                                                          2102
      i+3  CAACATAACA  3       0       1       4       1       4.94e-01        1517
           ----------                                                          1387
           ----------                                                          1314
           ----------                                                          1290
           ----------                                                          892
           ----------                                                          508
           ----------                                                          427
           ----------                                                          154
           ----------                                                          10

LOCUS: AT1G47750
DESCRIPTION: peroxisomal biogenesis factor 11 family protein / PEX11 family protein, contains Pfam PF05648:
Peroxisomal biogenesis factor 11 (PEX11)
DATA:                  Control 30min   2hours  2days   1week   p-value         pos
SENSE COUNTS:          3       0       1       4       1       4.94e-01
GENES (1 total):
  AT1G47750.1
    SENSE COUNTS:      3       0       1       4       1       4.94e-01
    TAGS: (1 total)
           ----------                                                          1246
      d+2  ATTTTGAAAA  3       0       1       4       1       4.94e-01        914
           ----------                                                          781

LOCUS: AT3G05040
DESCRIPTION: expressed protein, weak similarity to exportin 5 (Homo sapiens) GI:10444427
DATA:                  Control 30min   2hours  2days   1week   p-value         pos
SENSE COUNTS:          1       1       0       2       3       4.95e-01
GENES (2 total):
  AT3G05040.1
    SENSE COUNTS:      1       1       0       2       3       4.95e-01
    TAGS: (1 total)
           ----------                                                          2376
      v+2  TCATAAGCAA  1       1       0       2       3       4.95e-01        2175
           ----------                                                          2082
           ----------                                                          1732
           ----------                                                          1669
           ----------                                                          1647
           ----------                                                          1630
           ----------                                                          1506
           ----------                                                          1376
           ----------                                                          1275
           ----------                                                          735
           ----------                                                          526
           ----------                                                          343
           ----------                                                          323
           ----------                                                          261

LOCUS: AT2G18590
DESCRIPTION: transporter-related, low similarity to spinster membrane proteins from (Drosophila melanogaster)
GI:12003976,  GI:12003972, GI:12003974, GI:12003970; contains Pfam profile PF00083: major facilitator
superfamily protein
DATA:                  Control 30min   2hours  2days   1week   p-value         pos
```

```
SENSE COUNTS:          1      1      2      1      5      4.96e-01
GENES (1 total):
  AT2G18590.1
    SENSE COUNTS:      1      1      2      1      5      4.96e-01
    TAGS: (1 total)
           ----------                                                  2184
           ----------                                                  1916
           ----------                                                  1706
           ----------                                                  1587
      v+2  TTAACGACTC  1      1      2      1      5      4.96e-01      1540
           ----------                                                  1497
           ----------                                                  1428
           ----------                                                  1260
           ----------                                                  922
           ----------                                                  775
           ----------                                                  765
           ----------                                                  631
           ----------                                                  418
           ----------                                                  179

LOCUS: AT4G28510
DESCRIPTION: prohibitin, putative, similar to SP|P24142 Prohibitin (B-cell receptor associated protein 32)
(BAP 32) {Rattus norvegicus}; contains Pfam profile PF01145: SPFH domain / Band 7 family
DATA:                  Control 30min  2hours 2days  1week  p-value       pos
SENSE COUNTS:          1      2      0      2      3      4.96e-01
GENES (2 total):
  AT4G28510.1
    SENSE COUNTS:      1      2      0      2      3      4.96e-01
    TAGS: (2 total)
      d+1  TGGGTCATTT  1      2      0      2      0      3.83e-01      983
      i+3  TATTGGGATT  0      0      0      0      3      1.12e-02      440
           ----------                                                  414
           ----------                                                  231

LOCUS: AT3G48100
DESCRIPTION: Transcription repressor that mediates a negative feedback loop in cytokinin signalling.
DATA:                  Control 30min  2hours 2days  1week  p-value       pos
SENSE COUNTS:          1      0      3      1      0      4.99e-01
GENES (2 total):
  AT3G48100.1
    SENSE COUNTS:      1      0      3      1      0      4.99e-01
    TAGS: (1 total)
           ----------                                                  1328
           ----------                                                  1140
           ----------                                                  1082
      d+2  AGAGATATGT  1      0      3      1      0      4.99e-01      918
           ----------                                                  751
           ----------                                                  747
           ----------                                                  214

LOCUS: AT1G36630
DESCRIPTION: CACTA-like transposase family (Tnp1/En/Spm), has a 2.5e-213 P-value blast match to
ref|NP_189784.1| TNP1-related protein (Arabidopsis thaliana) (CACTA-element)
DATA:                  Control 30min  2hours 2days  1week  p-value       pos
SENSE COUNTS:          1      0      3      1      0      4.99e-01
GENES (1 total):
  AT1G36630.1
    SENSE COUNTS:      1      0      3      1      0      4.99e-01
    TAGS: (1 total)
           ----------                                                  7454
           ----------                                                  7330
           ----------                                                  7199
           ----------                                                  6412
           ----------                                                  6279
           ----------                                                  6208
           ----------                                                  6191
           ----------                                                  6181
           ----------                                                  5782
           ----------                                                  5565
           ----------                                                  5465
           ----------                                                  5456
      p+2  AACTCGTCGT  1      0      3      1      0      4.99e-01      5251
           ----------                                                  5005
           ----------                                                  4579
           ----------                                                  3797
           ----------                                                  3171
           ----------                                                  2473
           ----------                                                  2445
           ----------                                                  2050
           ----------                                                  1396
           ----------                                                  887
```

```
          ----------                                                  883
          ----------                                                  333

LOCUS: AT2G36490
DESCRIPTION: HhH-GPD base excision DNA repair family protein (ROS1), similar to DEMETER protein (Arabidopsis
thaliana) GI:21743571; contains Pfam profile PF00730: HhH-GPD superfamily base excision DNA repair protein
DATA:                 Control 30min  2hours  2days  1week  p-value        pos
SENSE COUNTS:         5       1      2       5      8      4.99e-01
GENES (2 total):
  AT2G36490.1
    SENSE COUNTS:     5       1      2       5      8      4.99e-01
    TAGS: (5 total)
      d+1  CATCCAGCCT 0       0      0       1      0      6.04e-01       3932
      d+2  TTCTTATTTG 5       0      1       4      8      4.76e-02       3681
           ----------                                                     3523
           ----------                                                     3457
           ----------                                                     3333
           ----------                                                     3144
           ----------                                                     2774
      d+2  TCAACATTCG 0       1      0       0      0      2.54e-01       2232
           ----------                                                     2096
      d+2  TCGTTGGCTT 0       0      1       0      0      4.55e-01       1858
           ----------                                                     1169
           ----------                                                     859
           ----------                                                     67
           ----------                                                     48
      X+4  TCGCCACTCA 0       0      0       0      0      6.15e-01       -231

LOCUS: AT1G77080
DESCRIPTION: MADS-box protein AGL27-II (AGL27) mRNA, complete cds
DATA:                 Control 30min  2hours  2days  1week  p-value        pos
SENSE COUNTS:         0       0      1       2      3      5.00e-01
GENES (8 total):
  AT1G77080.2
    SENSE COUNTS:     0       0      0       1      0      6.04e-01
    TAGS: (2 total)
      i+3  AACTTTTGTA 0       0      0       1      0      3.09e-01       1248
      d+1  GGAAGAAGAA 0       0      0       0      0      6.15e-01       25
  AT1G77080.3
    SENSE COUNTS:     0       0      0       1      0      3.09e-01
    TAGS: (1 total)
      i+3  AACTTTTGTA 0       0      0       1      0      3.09e-01       1227
           ----------                                                     224
  AT1G77080.4
    SENSE COUNTS:     0       0      0       1      0      3.09e-01
    TAGS: (1 total)
      i+3  AACTTTTGTA 0       0      0       1      0      3.09e-01       1269
           ----------                                                     266
           ----------                                                     46
  AT1G77080.5
    SENSE COUNTS:     0       0      1       2      3      3.73e-01
    TAGS: (2 total)
      i+3  AACTTTTGTA 0       0      0       1      0      3.09e-01       1227
      d+1  AAAACTAAAA 0       0      1       1      3      4.30e-01       581
           ----------                                                     224
  AT1G77080.6
    SENSE COUNTS:     0       0      1       1      3      4.30e-01
    TAGS: (1 total)
      v+1  AAAACTAAAA 0       0      1       1      3      4.30e-01       1185
           ----------                                                     993
           ----------                                                     412

LOCUS: AT3G05760
DESCRIPTION: expressed protein
DATA:                 Control 30min  2hours  2days  1week  p-value        pos
SENSE COUNTS:         0       0      1       2      3      5.00e-01
GENES (1 total):
  AT3G05760.1
    SENSE COUNTS:     0       0      1       2      3      5.00e-01
    TAGS: (2 total)
           ----------                                                     1202
           ----------                                                     846
      d+2  ATGATTATAT 0       0      0       1      3      2.09e-01       687
      d+2  TAGACCTAGA 0       0      1       1      0      7.90e-01       254

LOCUS: AT1G34210
DESCRIPTION: somatic embryogenesis receptor-like kinase 2 (SERK2), nearly identical to somatic embryogenesis
receptor-like kinase 2 (Arabidopsis thaliana) GI:14573457; contains Pfam domains PF00560: Leucine Rich Repeat
and PF00069: Protein kinase domain; identical to
DATA:                 Control 30min  2hours  2days  1week  p-value        pos
SENSE COUNTS:         0       2      1       2      3      5.00e-01
```

```
GENES (2 total):
  AT1G34210.1
    SENSE COUNTS:      0       2       1       2       3       5.00e-01
    TAGS: (2 total)
           ----------                                                         3278
           ----------                                                         2708
           ----------                                                         2657
      d+2  CTATGGAGTT  0       2       1       1       0       4.49e-01       2270
           ----------                                                         1900
           ----------                                                         1799
           ----------                                                         1673
           ----------                                                         1555
           ----------                                                         1477
           ----------                                                         913
           ----------                                                         895
      i+3  AGAATTGAAA  0       0       0       1       3       6.27e-02       576
           ----------                                                         502
           ----------                                                         329

LOCUS: AT4G33630
DESCRIPTION: expressed protein
DATA:                  Control 30min   2hours  2days   1week   p-value        pos
SENSE COUNTS:          2       1       1       4       0       5.00e-01
GENES (2 total):
  AT4G33630.1
    SENSE COUNTS:      2       1       1       4       0       5.00e-01
    TAGS: (1 total)
           ----------                                                         2577
           ----------                                                         2539
      d+2  AGATCGGTTG  2       1       1       4       0       5.00e-01       2173
           ----------                                                         1703
           ----------                                                         719
           ----------                                                         565

LOCUS: AT5G62670
DESCRIPTION: ATPase, plasma membrane-type, putative / proton pump, putative, strong similarity to P-type H(+)-
transporting ATPase from Nicotiana plumbaginifolia (SP|Q08435, SP|Q08436), Lycopersicon esculentum (GI:5901757,
SP|P22180), Solanum tuberosum (GI:435003); con
DATA:                  Control 30min   2hours  2days   1week   p-value        pos
SENSE COUNTS:          4       0       4       4       5       5.01e-01
GENES (1 total):
  AT5G62670.1
    SENSE COUNTS:      4       0       4       4       5       5.01e-01
    TAGS: (2 total)
           ----------                                                         4081
           ----------                                                         3799
           ----------                                                         3637
           ----------                                                         3488
      d+2  TAAGTATCAG  4       0       3       4       5       5.04e-01       3109
           ----------                                                         2955
      d+2  CACAGAGAAC  0       0       1       0       0       4.55e-01       2805
           ----------                                                         2415
           ----------                                                         2321
           ----------                                                         2186
           ----------                                                         2144
           ----------                                                         1754
           ----------                                                         1635
           ----------                                                         1610
           ----------                                                         1494
           ----------                                                         1130
           ----------                                                         1052
           ----------                                                         1028
           ----------                                                         536
           ----------                                                         398
           ----------                                                         356

LOCUS: AT3G12300
DESCRIPTION: expressed protein
DATA:                  Control 30min   2hours  2days   1week   p-value        pos
SENSE COUNTS:          2       0       3       0       1       5.02e-01
GENES (1 total):
  AT3G12300.1
    SENSE COUNTS:      2       0       3       0       1       5.02e-01
    TAGS: (2 total)
      d+1  ATCTTGGGTC  2       0       0       0       0       1.04e-01       866
      d+2  CTTTAACTTT  0       0       3       0       1       2.30e-01       788
           ----------                                                         758
           ----------                                                         636
           ----------                                                         536
           ----------                                                         246
           ----------                                                         179
```

```
          ----------                                                  164

LOCUS: AT4G04470
DESCRIPTION: peroxisomal membrane protein 22 kDa (PMP22), identical to peroxisomal membrane protein
(Arabidopsis thaliana) gi|3980254|emb|CAA06834
DATA:                 Control 30min   2hours  2days   1week   p-value       pos
SENSE COUNTS:         2       0       3       0       1       5.02e-01
GENES (2 total):
  AT4G04470.1
    SENSE COUNTS:     2       0       3       0       1       5.02e-01
    TAGS: (2 total)
      i+3  CTCTTTTAAG 0       0       0       0       1       1.65e-01      986
           ----------                                                       889
      d+2  GATCGTGACA 2       0       3       0       0       3.07e-01      620
           ----------                                                       455
           ----------                                                       363

LOCUS: AT1G24340
DESCRIPTION: monooxygenase family protein, similar to polyketide hydroxylases from several bacterial species;
contains Pfam:PF01360 (Monooxygenase)
DATA:                 Control 30min   2hours  2days   1week   p-value       pos
SENSE COUNTS:         0       3       2       1       1       5.05e-01
GENES (1 total):
  AT1G24340.1
    SENSE COUNTS:     0       3       2       1       1       5.05e-01
    TAGS: (1 total)
           ----------                                                       2697
      v+2  AGAGATGTCT 0       3       2       1       1       5.05e-01      2439
           ----------                                                       2273
           ----------                                                       2164
           ----------                                                       1610
           ----------                                                       1503
           ----------                                                       1480
           ----------                                                       1476
           ----------                                                       1469
           ----------                                                       1432
           ----------                                                       1366
           ----------                                                       1272
           ----------                                                       1072
           ----------                                                       1000
           ----------                                                       891
           ----------                                                       161

LOCUS: AT4G23850
DESCRIPTION: long-chain-fatty-acid--CoA ligase / long-chain acyl-CoA synthetase, nearly identical to acyl-CoA
synthetase (MF7P) from Brassica napus (gi:1617270)
DATA:                 Control 30min   2hours  2days   1week   p-value       pos
SENSE COUNTS:         16      18      24      20      12      5.06e-01
GENES (1 total):
  AT4G23850.1
    SENSE COUNTS:     16      18      24      20      12      5.06e-01
    TAGS: (5 total)
      d+1  ACGAAGACTT 4       9       3       5       7       4.27e-01      2114
      d+2  GAACGAGATC 0       0       2       0       0       3.51e-01      1952
           ----------                                                       1607
           ----------                                                       1311
           ----------                                                       1303
           ----------                                                       1167
           ----------                                                       951
           ----------                                                       599
           ----------                                                       577
      i+3  AATTTGGTTG 2       0       1       0       0       2.87e-01      561
           ----------                                                       453
      d+2  GAGGCTTGTA 10      9       16      15      5       2.76e-01      434
           ----------                                                       341
      X+4  GCGCTGTCTC 0       0       2       0       0       3.51e-01      315
           ----------                                                       230
           ----------                                                       74

LOCUS: AT1G75410
DESCRIPTION: BEL1-like homeodomain 3 protein (BLH3), identical to BEL1-like homeodomain 3 (GI:13877515)
(Arabidopsis thaliana)
DATA:                 Control 30min   2hours  2days   1week   p-value       pos
SENSE COUNTS:         3       0       4       1       3       5.06e-01
GENES (2 total):
  AT1G75410.1
    SENSE COUNTS:     3       0       4       1       3       5.06e-01
    TAGS: (3 total)
      d+1  ATGGTATAAA 0       0       1       1       3       1.91e-01      2075
      d+2  GGTTTGGTAT 3       0       2       0       0       1.15e-01      1707
      d+2  CTTTCAAAGC 0       0       1       0       0       7.06e-01      1373
```

```
          ----------                                                  263

LOCUS: AT1G53320
DESCRIPTION: F-box family protein / tubby family protein (TULP7), similar to Tubby related protein 2 (Tubby-
like protein 2) (P4-6 protein) (Fragment) (SP:P46686) (Mus musculus); similar to phosphodiesterase (GI:467578)
(Mus musculus); similar to Tubby protein homolog
DATA:                 Control 30min  2hours  2days  1week  p-value      pos
SENSE COUNTS:         1       4      4       2      1      5.11e-01
GENES (1 total):
  AT1G53320.1
    SENSE COUNTS:     1       4      4       2      1      5.11e-01
    TAGS: (2 total)
      d+1  TGGTTTTCTT 0       0      3       0      0      1.17e-01     1386
      d+2  TGTAAGTGTA 1       4      1       2      1      5.27e-01     1276
           ----------                                                 988
           ----------                                                 872
           ----------                                                 751
           ----------                                                 194

LOCUS: AT4G12250
DESCRIPTION: NAD-dependent epimerase/dehydratase family protein, similar to nucleotide sugar epimerase from
Vibrio vulnificus GI:3093975 (PID:g3093975), WbnF (Escherichia coli) GI:5739472, CAPI protein {Staphylococcus
aureus} SP|P39858; contains Pfam profile PF01370 N
DATA:                 Control 30min  2hours  2days  1week  p-value      pos
SENSE COUNTS:         0       2      3       4      3      5.13e-01
GENES (2 total):
  AT4G12250.1
    SENSE COUNTS:     0       2      3       4      3      5.13e-01
    TAGS: (1 total)
           ----------                                                 1872
      d+2  TCGGAGGAAT 0       2      3       4      3      5.13e-01     1603
           ----------                                                 1391
           ----------                                                 1238
           ----------                                                 1201
           ----------                                                 575

LOCUS: AT4G13010
DESCRIPTION: oxidoreductase, zinc-binding dehydrogenase family protein, low similarity to probable NADP-
dependent oxidoreductase (zeta-crystallin homolog) P1 (SP|Q39172)(gi:886428) and P2 (SP|Q39173)(gi:886430);
contains Pfam profile PF00107: oxidoreductase, zinc-bind
DATA:                 Control 30min  2hours  2days  1week  p-value      pos
SENSE COUNTS:         3       2      3       5      0      5.18e-01
GENES (1 total):
  AT4G13010.1
    SENSE COUNTS:     3       2      3       5      0      5.18e-01
    TAGS: (2 total)
           ----------                                                 1245
      d+2  CTACTGGGAA 3       2      2       5      0      4.54e-01     1093
      d+2  TCAAAGAAGC 0       0      1       0      0      4.55e-01     933
           ----------                                                 692
           ----------                                                 202
           ----------                                                 150

LOCUS: AT3G10730
DESCRIPTION: sad1/unc-84-like 2 family protein, contains 1 transmembrane domain; similar to Sad1 unc-84 domain
protein 2 (GI:6538749) (Homo sapiens); similar to Sad1/unc-84-like protein 2 (Fragment) (Swiss-Prot:Q9UH99)
(Homo sapiens)
DATA:                 Control 30min  2hours  2days  1week  p-value      pos
SENSE COUNTS:         2       0      2       0      3      5.19e-01
GENES (1 total):
  AT3G10730.1
    SENSE COUNTS:     2       0      2       0      3      5.19e-01
    TAGS: (2 total)
      d+1  CTTGATCTGA 1       0      1       0      0      8.46e-01     1524
           ----------                                                 1491
           ----------                                                 1449
      d+2  TCTCTAAGGC 1       0      1       0      3      2.09e-01     1233
           ----------                                                 972
           ----------                                                 273
           ----------                                                 164

LOCUS: AT5G55530
DESCRIPTION: C2 domain-containing protein, low similarity to cold-regulated gene SRC2 (Glycine max)
GI:2055230; contains Pfam profile PF00168: C2 domain
DATA:                 Control 30min  2hours  2days  1week  p-value      pos
SENSE COUNTS:         2       0      2       0      3      5.19e-01
GENES (3 total):
  AT5G55530.1
    SENSE COUNTS:     2       0      2       0      3      2.55e-01
    TAGS: (2 total)
           ----------                                                 1667
```

```
      d+2  TTGTATCTCC 2       0       1       0       3       2.14e-01        1547
      d+2  CAACAGTTTA 0       0       1       0       0       4.55e-01        1285
           ----------                                                         1276
           ----------                                                         1270
           ----------                                                         1118
           ----------                                                         1001
           ----------                                                         592
           ----------                                                         52
  AT5G55530.2
    SENSE COUNTS:     2       0       2       0       3       5.19e-01
    TAGS: (3 total)
      X+4  TGGTAATAAA 0       0       0       0       0       6.15e-01        1764
           ----------                                                         1589
      d+2  TTGTATCTCC 2       0       1       0       3       2.14e-01        1469
      d+2  CAACAGTTTA 0       0       1       0       0       4.55e-01        1207
           ----------                                                         1198
           ----------                                                         1192
           ----------                                                         1040
           ----------                                                         923
           ----------                                                         514
  AT5G55530.3
    SENSE COUNTS:     2       0       2       0       3       2.55e-01
    TAGS: (2 total)
           ----------                                                         1561
      d+2  TTGTATCTCC 2       0       1       0       3       2.14e-01        1441
      d+2  CAACAGTTTA 0       0       1       0       0       4.55e-01        1179
           ----------                                                         1170
           ----------                                                         1164
           ----------                                                         1012
           ----------                                                         895
           ----------                                                         486

LOCUS: AT4G11420
DESCRIPTION: eukaryotic translation initiation factor 3 subunit 10 / eIF-3 theta / eIF3a (TIF3A1), identical
to eukaryotic translation initiation factor 3 subunit 10 (eIF-3 theta) (Eukaryotic translation initiation
factor 3 large subunit) (eIF3a) (p114). (Arabidopsis
DATA:                 Control 30min   2hours  2days   1week   p-value         pos
SENSE COUNTS:         11      5       13      8       15      5.20e-01
GENES (2 total):
  AT4G11420.1
    SENSE COUNTS:     11      5       13      8       15      5.20e-01
    TAGS: (4 total)
      d+1  TTAGACTCGT 8       5       5       2       14      1.20e-01        3253
           ----------                                                         3125
      d+2  AGAGTGACTT 0       0       1       0       0       7.06e-01        2328
           ----------                                                         2057
      d+2  AAGGGTGTTG 0       0       0       0       0       6.15e-01        1634
           ----------                                                         1619
           ----------                                                         1338
           ----------                                                         1326
           ----------                                                         1018
           ----------                                                         1008
      d+2  GTCAAAAAAA 3       0       7       6       1       8.62e-02        917
           ----------                                                         577
           ----------                                                         278
           ----------                                                         222
           ----------                                                         131

LOCUS: AT3G48810
DESCRIPTION: pentatricopeptide (PPR) repeat-containing protein, contains Pfam profile PF01535: PPR repeat
DATA:                 Control 30min   2hours  2days   1week   p-value         pos
SENSE COUNTS:         3       0       2       2       4       5.20e-01
GENES (1 total):
  AT3G48810.1
    SENSE COUNTS:     3       0       2       2       4       5.20e-01
    TAGS: (3 total)
           ----------                                                         2584
           ----------                                                         2314
           ----------                                                         2264
           ----------                                                         2123
           ----------                                                         1967
           ----------                                                         1960
           ----------                                                         1914
           ----------                                                         1635
           ----------                                                         1485
           ----------                                                         1447
      v+2  ATTACAAAGG 3       0       1       2       3       6.14e-01        1132
      v+2  TTTTGGATAC 0       0       0       0       1       1.65e-01        805
      v+2  TGTTCCATTA 0       0       1       0       0       4.55e-01        515
           ----------                                                         466
           ----------                                                         354
```

```
LOCUS: AT3G06780
DESCRIPTION: glycine-rich protein
DATA:                  Control 30min   2hours  2days   1week   p-value         pos
SENSE COUNTS:          1       1       0       1       3       5.22e-01
GENES (1 total):
  AT3G06780.1
    SENSE COUNTS:      1       1       0       1       3       5.22e-01
    TAGS: (2 total)
      d+1  TAAATACCAT  0       0       0       1       0       3.09e-01        1018
           ----------                                                          999
           ----------                                                          944
      d+2  GAGTTCTCTT  1       1       0       0       3       2.97e-01        150

LOCUS: AT3G02870
DESCRIPTION: inositol-1(or 4)-monophosphatase, putative / inositol monophosphatase, putative / IMPase,
putative, similar to SP|P54928 Inositol-1(or 4)-monophosphatase 3 (EC 3.1.3.25) (IMPase 3) (IMP 3) (Inositol
monophosphatase 3) {Lycopersicon esculentum}; contains P
DATA:                  Control 30min   2hours  2days   1week   p-value         pos
SENSE COUNTS:          1       1       0       1       3       5.22e-01
GENES (1 total):
  AT3G02870.1
    SENSE COUNTS:      1       1       0       1       3       5.22e-01
    TAGS: (1 total)
           ----------                                                          1196
      d+2  AAGTCATCTG  1       1       0       1       3       5.22e-01        979
           ----------                                                          823
           ----------                                                          267

LOCUS: AT4G14020
DESCRIPTION: rapid alkalinization factor (RALF) family protein
DATA:                  Control 30min   2hours  2days   1week   p-value         pos
SENSE COUNTS:          4       4       3       0       2       5.22e-01
GENES (1 total):
  AT4G14020.1
    SENSE COUNTS:      4       4       3       0       2       5.22e-01
    TAGS: (2 total)
      X+4  ATGATCATCG  0       0       0       0       1       1.65e-01        550
      d+1  CAACGTGTGT  4       4       3       0       1       4.32e-01        462
           ----------                                                          264
           ----------                                                          35

LOCUS: AT4G03490
DESCRIPTION: ankyrin repeat family protein, contains ankyrin repeats, Pfam:PF00023
DATA:                  Control 30min   2hours  2days   1week   p-value         pos
SENSE COUNTS:          0       0       1       1       3       5.27e-01
GENES (1 total):
  AT4G03490.1
    SENSE COUNTS:      0       0       1       1       3       5.27e-01
    TAGS: (2 total)
           ----------                                                          2443
      i+3  AGTTGCAAAG  0       0       0       0       0       6.15e-01        2189
           ----------                                                          1782
           ----------                                                          1689
           ----------                                                          1635
           ----------                                                          1543
           ----------                                                          1147
           ----------                                                          790
           ----------                                                          613
           ----------                                                          466
      v+2  AACAGCCGAC  0       0       1       1       3       4.30e-01        101
           ----------                                                          60

LOCUS: AT5G18440
DESCRIPTION: hypothetical protein
DATA:                  Control 30min   2hours  2days   1week   p-value         pos
SENSE COUNTS:          2       2       1       1       4       5.27e-01
GENES (1 total):
  AT5G18440.1
    SENSE COUNTS:      2       2       1       1       4       5.27e-01
    TAGS: (4 total)
           ----------                                                          2072
      v+2  CAAGGAGAAA  0       2       0       0       1       1.90e-01        1951
      v+2  AGAGTAAAAA  0       0       0       0       0       6.15e-01        1834
      v+2  AGCAGTATAA  1       0       0       0       0       4.28e-01        1735
           ----------                                                          1691
           ----------                                                          1527
           ----------                                                          1366
      v+2  CTTTCTCTTC  1       0       1       1       3       3.98e-01        697
           ----------                                                          504
```

```
LOCUS: AT1G52380
DESCRIPTION: Ran-binding protein 1 domain-containing protein / RanBP1 domain-containing protein, weak
similarity to SP|Q09717 Ran-specific GTPase-activating protein 1 (Ran binding protein 1) (RANBP1) (Spi1-
binding protein) {Schizosaccharomyces pombe}; contains Pfam pr
DATA:                 Control 30min  2hours  2days  1week  p-value      pos
SENSE COUNTS:         2       4      7       6      3      5.29e-01
GENES (1 total):
  AT1G52380.1
    SENSE COUNTS:     2       4      7       6      3      5.29e-01
    TAGS: (4 total)
      d+1  TTTCTGTTTG 0       0      3       1      3      3.28e-01     1671
      d+2  TAGAAACACT 1       0      0       0      0      4.28e-01     1519
      d+2  GATAAAAAGG 0       2      2       5      0      1.34e-01     1267
           ----------                                                 1105
      d+2  TGACACAAAC 1       2      2       0      0      4.91e-01     663

LOCUS: AT1G16560
DESCRIPTION: Per1-like family protein, contains Pfam profile PF04080: Per1-like
DATA:                 Control 30min  2hours  2days  1week  p-value      pos
SENSE COUNTS:         5       5      8       2      2      5.32e-01
GENES (3 total):
  AT1G16560.1
    SENSE COUNTS:     5       5      8       2      2      5.32e-01
    TAGS: (2 total)
      d+1  CGAAACCATT 1       0      1       0      1      8.84e-01     1364
           ----------                                                 914
           ----------                                                 794
           ----------                                                 713
           ----------                                                 560
           ----------                                                 473
           ----------                                                 450
           ----------                                                 363
           ----------                                                 235
      d+2  TGTTTCAGAA 4       5      7       2      1      4.92e-01     154
           ----------                                                 97
  AT1G16560.2
    SENSE COUNTS:     5       5      8       2      2      5.32e-01
    TAGS: (2 total)
      d+1  CGAAACCATT 1       0      1       0      1      8.84e-01     1395
           ----------                                                 945
           ----------                                                 825
           ----------                                                 744
           ----------                                                 591
           ----------                                                 504
           ----------                                                 481
           ----------                                                 394
           ----------                                                 266
      d+2  TGTTTCAGAA 4       5      7       2      1      4.92e-01     185
           ----------                                                 128
  AT1G16560.3
    SENSE COUNTS:     5       5      8       2      2      5.32e-01
    TAGS: (2 total)
      d+1  CGAAACCATT 1       0      1       0      1      8.84e-01     1532
           ----------                                                 1082
           ----------                                                 962
           ----------                                                 881
           ----------                                                 728
           ----------                                                 641
           ----------                                                 618
           ----------                                                 531
           ----------                                                 403
      d+2  TGTTTCAGAA 4       5      7       2      1      4.92e-01     322
           ----------                                                 265

LOCUS: AT3G48800
DESCRIPTION: sterile alpha motif (SAM) domain-containing protein, contains Pfam profile PF00536: SAM domain
(Sterile alpha motif)
DATA:                 Control 30min  2hours  2days  1week  p-value      pos
SENSE COUNTS:         1       0      2       3      3      5.34e-01
GENES (1 total):
  AT3G48800.1
    SENSE COUNTS:     1       0      2       3      3      5.34e-01
    TAGS: (2 total)
           ----------                                                 1685
      v+2  AAAGCCTTTT 1       0      2       2      3      7.29e-01     1318
      v+2  GCAGAGTGTA 0       0      0       1      0      3.09e-01     1285
           ----------                                                 1187
           ----------                                                 1063

LOCUS: AT3G07880
```

```
DESCRIPTION: Rho GDP-dissociation inhibitor family protein, similar to SP|P52565 Rho GDP-dissociation
inhibitor 1 (Rho GDI 1) (Rho-GDI alpha) {Homo sapiens}; contains Pfam profile PF02115: RHO protein GDP
dissociation inhibitor
DATA:                 Control 30min  2hours  2days  1week  p-value      pos
SENSE COUNTS:         5       1      6       1      4      5.36e-01
GENES (1 total):
  AT3G07880.1
    SENSE COUNTS:     5       1      6       1      4      5.36e-01
    TAGS: (5 total)
      d+1  TGTTTGATGA 3       1      1       1      0      5.90e-01     1174
      d+2  TTTGCTCGAG 0       0      4       0      1      8.22e-02     817
      d+2  TTAACAACAA 0       0      1       0      0      4.55e-01     662
           ----------                                                 610
      X+4  ATCACCATTC 1       0      0       0      0      4.28e-01     586
      X+4  TATAATGATT 1       0      0       0      3      2.28e-01     359
           ----------                                                 235
           ----------                                                 87

LOCUS: AT1G48090
DESCRIPTION: C2 domain-containing protein, contains Pfam profile: PF00168 C2 domain
DATA:                 Control 30min  2hours  2days  1week  p-value      pos
SENSE COUNTS:         6       1      2       2      4      5.36e-01
GENES (2 total):
  AT1G48090.1
    SENSE COUNTS:     6       1      2       2      4      5.36e-01
    TAGS: (6 total)
      i+3  CATTTTCAAT 0       0      1       0      0      4.55e-01     18643
      d+1  TATACGGGTA 4       1      0       1      3      2.66e-01     12674
      d+2  ACCCGAAGGA 0       0      0       0      0      6.15e-01     12182
           ----------                                                 12103
           ----------                                                 11728
           ----------                                                 11308
           ----------                                                 11294
           ----------                                                 11266
           ----------                                                 11182
           ----------                                                 11060
           ----------                                                 11036
           ----------                                                 10765
           ----------                                                 10742
           ----------                                                 10697
           ----------                                                 10300
           ----------                                                 9922
           ----------                                                 9901
           ----------                                                 9700
      d+2  GCGGTTGGGC 0       0      1       0      0      4.55e-01     9317
           ----------                                                 9266
           ----------                                                 9236
           ----------                                                 9174
           ----------                                                 8874
           ----------                                                 8796
           ----------                                                 8553
           ----------                                                 8417
           ----------                                                 8306
           ----------                                                 7981
           ----------                                                 7459
           ----------                                                 7442
           ----------                                                 7385
           ----------                                                 7236
           ----------                                                 7106
           ----------                                                 6788
           ----------                                                 6171
           ----------                                                 6076
           ----------                                                 5564
           ----------                                                 5220
           ----------                                                 5169
           ----------                                                 4913
           ----------                                                 4708
           ----------                                                 4658
           ----------                                                 4493
           ----------                                                 4445
           ----------                                                 4222
           ----------                                                 3868
      i+3  TAGGATTTCA 1       0      0       1      0      5.06e-01     3748
           ----------                                                 3652
           ----------                                                 3497
           ----------                                                 3464
           ----------                                                 3442
           ----------                                                 3347
           ----------                                                 3312
           ----------                                                 3002
           ----------                                                 2755
```

```
          ----------                                                      2575
          ----------                                                      2512
          ----------                                                      2311
          ----------                                                      2191
          ----------                                                      1901
          ----------                                                      1594
          ----------                                                      1358
     d+2  ATTGGATAAA  1       0       0       0       1       6.74e-01    1082
          ----------                                                      803
          ----------                                                      510
          ----------                                                      434
          ----------                                                      65
  AT1G48090.2
    SENSE COUNTS:     2       0       2       1       1       9.32e-01
    TAGS: (4 total)
     i+3  CATTTTCAAT  0       0       1       0       0       4.55e-01    18643
          ----------                                                      10378
          ----------                                                      9922
          ----------                                                      9901
          ----------                                                      9700
     d+2  GCGGTTGGGC  0       0       1       0       0       4.55e-01    9317
          ----------                                                      9266
          ----------                                                      9236
          ----------                                                      9174
          ----------                                                      8874
          ----------                                                      8796
          ----------                                                      8553
          ----------                                                      8417
          ----------                                                      8306
          ----------                                                      7981
          ----------                                                      7459
          ----------                                                      7442
          ----------                                                      7385
          ----------                                                      7236
          ----------                                                      7106
          ----------                                                      6788
          ----------                                                      6171
          ----------                                                      6076
          ----------                                                      5564
          ----------                                                      5220
          ----------                                                      5169
          ----------                                                      4913
          ----------                                                      4708
          ----------                                                      4658
          ----------                                                      4493
          ----------                                                      4445
          ----------                                                      4222
          ----------                                                      3868
     i+3  TAGGATTTCA  1       0       0       1       0       5.06e-01    3748
          ----------                                                      3652
          ----------                                                      3497
          ----------                                                      3464
          ----------                                                      3442
          ----------                                                      3347
          ----------                                                      3312
          ----------                                                      3002
          ----------                                                      2755
          ----------                                                      2575
          ----------                                                      2512
          ----------                                                      2311
          ----------                                                      2191
          ----------                                                      1901
          ----------                                                      1594
          ----------                                                      1358
     d+2  ATTGGATAAA  1       0       0       0       1       6.74e-01    1082
          ----------                                                      803
          ----------                                                      510
          ----------                                                      434
          ----------                                                      65

LOCUS: AT2G46020
DESCRIPTION: hypothetical protein,  and genefinder
DATA:                 Control 30min   2hours  2days   1week   p-value     pos
SENSE COUNTS:         3       2       4       2       8       5.36e-01
GENES (3 total):
  AT2G46020.1
    SENSE COUNTS:     3       2       4       2       8       5.36e-01
    TAGS: (3 total)
          ----------                                                      7408
     v+2  TATAAAAAAA  3       2       3       2       8       3.34e-01    7154
     v+2  AGGTGAGATC  0       0       1       0       0       7.06e-01    6304
```

```
           ----------                                                        5990
           ----------                                                        5911
           ----------                                                        5647
           ----------                                                        5118
           ----------                                                        5032
           ----------                                                        4936
           ----------                                                        4918
           ----------                                                        4888
           ----------                                                        4852
           ----------                                                        4803
           ----------                                                        4728
           ----------                                                        4268
           ----------                                                        4185
           ----------                                                        3972
           ----------                                                        3850
           ----------                                                        3524
           ----------                                                        3241
      v+2  AAAAGATGTT  0       0       0       0       0       6.15e-01      2830
           ----------                                                        2628
           ----------                                                        1762
           ----------                                                        1384
           ----------                                                        1330
           ----------                                                        1142
           ----------                                                        1092
           ----------                                                        399
           ----------                                                        354
  AT2G46020.2
    SENSE COUNTS:      3       2       4       2       8       5.36e-01
    TAGS: (3 total)
           ----------                                                        7411
      v+2  TATAAAAAAA  3       2       3       2       8       3.34e-01      7157
      v+2  AGGTGAGATC  0       0       1       0       0       7.06e-01      6307
           ----------                                                        5993
           ----------                                                        5914
           ----------                                                        5650
           ----------                                                        5121
           ----------                                                        5035
           ----------                                                        4939
           ----------                                                        4921
           ----------                                                        4891
           ----------                                                        4855
           ----------                                                        4806
           ----------                                                        4731
           ----------                                                        4271
           ----------                                                        4188
           ----------                                                        3975
           ----------                                                        3853
           ----------                                                        3588
           ----------                                                        3524
           ----------                                                        3241
      v+2  AAAAGATGTT  0       0       0       0       0       6.15e-01      2830
           ----------                                                        2628
           ----------                                                        1762
           ----------                                                        1384
           ----------                                                        1330
           ----------                                                        1142
           ----------                                                        1092
           ----------                                                        399
           ----------                                                        354

LOCUS: AT2G29080
DESCRIPTION: FtsH protease, putative, similar to AAA-metalloprotease FtsH (Pisum sativum) GI:15021761;
contains Pfam profiles PF01434: Peptidase family M41, PF00004: ATPase AAA family
DATA:                  Control 30min   2hours  2days   1week   p-value       pos
SENSE COUNTS:          3       0       2       2       0       5.37e-01
GENES (1 total):
  AT2G29080.1
    SENSE COUNTS:      3       0       2       2       0       5.37e-01
    TAGS: (2 total)
           ----------                                                        2996
           ----------                                                        2977
      d+2  ACTCTCGGAG  3       0       2       1       0       5.19e-01      2207
           ----------                                                        2198
           ----------                                                        2174
           ----------                                                        2085
           ----------                                                        2046
           ----------                                                        1824
           ----------                                                        1463
      d+2  AGGTTTGCAC  0       0       0       1       0       3.09e-01      1079
           ----------                                                        1026
           ----------                                                        897
```

```
          ----------                                                674

LOCUS: AT3G32980
DESCRIPTION: peroxidase 32 (PER32) (P32) (PRXR3), identical to SP|Q9LHB9 Peroxidase 32 precursor (EC 1.11.1.7)
(Atperox P32) (PRXR3) (ATP16a) {Arabidopsis thaliana}
DATA:                 Control 30min  2hours  2days  1week  p-value       pos
SENSE COUNTS:         3       0      2       2      0      5.37e-01
GENES (1 total):
  AT3G32980.1
    SENSE COUNTS:     3       0      2       2      0      5.37e-01
    TAGS: (1 total)
          ----------                                                1289
          ----------                                                1215
      d+2  ATGTGGTCGA 3       0      2       2      0      5.37e-01  1067
          ----------                                                423
          ----------                                                405
          ----------                                                165

LOCUS: AT5G19930
DESCRIPTION: integral membrane family protein, contains Pfam domain PF01940: Integral membrane protein
DATA:                 Control 30min  2hours  2days  1week  p-value       pos
SENSE COUNTS:         3       0      2       2      0      5.37e-01
GENES (1 total):
  AT5G19930.1
    SENSE COUNTS:     3       0      2       2      0      5.37e-01
    TAGS: (1 total)
          ----------                                                1179
      d+2  CCAAATTTGG 3       0      2       2      0      5.37e-01  994

LOCUS: AT4G13640
DESCRIPTION: myb family transcription factor, contains Pfam profile: PF00249 myb-like DNA-binding domain
DATA:                 Control 30min  2hours  2days  1week  p-value       pos
SENSE COUNTS:         0       3      3       2      1      5.37e-01
GENES (1 total):
  AT4G13640.1
    SENSE COUNTS:     0       3      3       2      1      5.37e-01
    TAGS: (1 total)
          ----------                                                1283
      d+2  ATGCTGGATG 0       3      3       2      1      5.37e-01  975
          ----------                                                276

LOCUS: AT5G02760
DESCRIPTION: protein phosphatase 2C family protein / PP2C family protein, similar to Ser/Thr protein
phosphatase 2C (PP2C6) (GI:15020818) (Arabidopsis thaliana); similar to protein phosphatase 2C (GI:3608412)
(Mesembryanthemum crystallinum); contains Pfam PF00481 : Pr
DATA:                 Control 30min  2hours  2days  1week  p-value       pos
SENSE COUNTS:         4       4      1       1      2      5.37e-01
GENES (1 total):
  AT5G02760.1
    SENSE COUNTS:     4       4      1       1      2      5.37e-01
    TAGS: (2 total)
          ----------                                                2072
          ----------                                                2026
      d+2  CAATATTCGC 0       0      0       0      1      1.65e-01  1858
          ----------                                                1836
          ----------                                                1769
      d+2  CAATATCATC 4       4      1       1      1      4.64e-01  1422
          ----------                                                1382
          ----------                                                1372
          ----------                                                1236
          ----------                                                549
          ----------                                                362
          ----------                                                247
          ----------                                                204

LOCUS: AT4G29020
DESCRIPTION: glycine-rich protein, supporting cDNA gi|20465684|gb|AY096677.1|
DATA:                 Control 30min  2hours  2days  1week  p-value       pos
SENSE COUNTS:         9       7      5       6      1      5.38e-01
GENES (1 total):
  AT4G29020.1
    SENSE COUNTS:     9       7      5       6      1      5.38e-01
    TAGS: (3 total)
      X+4  CAGCAATGTA 0       0      0       1      0      3.09e-01  850
      d+1  GAGTAGGTGG 9       7      1       5      1      1.11e-01  440
      d+2  GTGTCTCAAG 0       0      4       0      0      5.58e-03  154

LOCUS: AT5G52530
DESCRIPTION: dentin sialophosphoprotein-related, contains weak similarity to dentin sialophosphoprotein
precursor (Dentin matrix protein-3) (DMP- 3) (Swiss-Prot:P97399) (Mus musculus)
DATA:                 Control 30min  2hours  2days  1week  p-value       pos
```

```
SENSE COUNTS:          5       5       6       10      11      5.40e-01
GENES (2 total):
  AT5G52530.1
    SENSE COUNTS:      5       5       6       10      11      5.40e-01
    TAGS: (4 total)
      d+1  GCGTCATCTG  3       3       4       9       8       2.69e-01        3026
      d+2  ATAACAGATA  0       0       0       0       0       6.15e-01        2900
           ----------                                                          2673
           ----------                                                          2093
      d+2  ATGCAATCCA  1       0       0       0       0       4.28e-01        1894
           ----------                                                          1812
           ----------                                                          1729
           ----------                                                          973
           ----------                                                          835
           ----------                                                          760
           ----------                                                          646
      i+3  GAAGATCCCT  1       2       2       1       3       8.69e-01        514
           ----------                                                          417
  AT5G52530.2
    SENSE COUNTS:      5       5       6       10      11      5.40e-01
    TAGS: (4 total)
      d+1  GCGTCATCTG  3       3       4       9       8       2.69e-01        3020
      d+2  ATAACAGATA  0       0       0       0       0       6.15e-01        2894
           ----------                                                          2667
           ----------                                                          2087
      d+2  ATGCAATCCA  1       0       0       0       0       4.28e-01        1888
           ----------                                                          1806
           ----------                                                          1723
           ----------                                                          967
           ----------                                                          829
           ----------                                                          754
           ----------                                                          640
      i+3  GAAGATCCCT  1       2       2       1       3       8.69e-01        492
           ----------                                                          411

LOCUS: AT5G46210
DESCRIPTION: cullin, putative, similar to SP|Q13619 Cullin homolog 4A (CUL-4A) {Homo sapiens}; contains Pfam
profile PF00888: Cullin family
DATA:                  Control 30min   2hours  2days   1week   p-value         pos
SENSE COUNTS:          1       2       5       3       1       5.40e-01
GENES (1 total):
  AT5G46210.1
    SENSE COUNTS:      1       2       5       3       1       5.40e-01
    TAGS: (3 total)
      d+1  AACTAAATGT  0       1       1       2       1       6.75e-01        1893
      d+2  ATCTCTAAGC  0       0       1       0       0       4.55e-01        1679
           ----------                                                          1062
           ----------                                                          1044
           ----------                                                          1010
           ----------                                                          701
      d+2  GGAGAAGTTG  1       1       3       1       0       6.27e-01        445
           ----------                                                          161

LOCUS: AT5G65770
DESCRIPTION: nuclear matrix constituent protein-related, low similarity to nuclear matrix constituent protein
1 (NMCP1) (Daucus carota) GI:2190187
DATA:                  Control 30min   2hours  2days   1week   p-value         pos
SENSE COUNTS:          2       0       3       1       0       5.41e-01
GENES (1 total):
  AT5G65770.1
    SENSE COUNTS:      2       0       3       1       0       5.41e-01
    TAGS: (2 total)
           ----------                                                          3411
      v+2  AACTACCATC  1       0       0       0       0       6.89e-01        3214
           ----------                                                          3133
           ----------                                                          2885
           ----------                                                          2691
           ----------                                                          2632
           ----------                                                          2455
           ----------                                                          2220
      v+2  ACTTGGAAGT  1       0       3       1       0       2.40e-01        1654
           ----------                                                          1111
           ----------                                                          859
           ----------                                                          796
           ----------                                                          591
           ----------                                                          471

LOCUS: AT3G28730
DESCRIPTION: structure-specific recognition protein 1 / high mobility group protein / HMG protein, nearly
identical to SP|Q05153 Structure-specific recognition protein 1 homolog (HMG protein) {Arabidopsis thaliana};
contains Pfam profile PF00505: HMG (high mobility gr
```

```
DATA:                  Control 30min   2hours  2days   1week   p-value         pos
SENSE COUNTS:          2       1       5       2       1       5.43e-01
GENES (2 total):
  AT3G28730.1
    SENSE COUNTS:      2       1       5       2       1       5.43e-01
    TAGS: (3 total)
      d+1  CATTCACCAA  1       0       0       0       0       4.28e-01        2598
      d+2  TCTAGATACT  0       0       0       0       1       1.65e-01        2381
      d+2  TAGTAAACGT  1       1       5       2       0       2.07e-01        2070
           ----------                                                          1776
           ----------                                                          1303
           ----------                                                          1263
           ----------                                                          1243
           ----------                                                          1030
           ----------                                                          523
           ----------                                                          69

LOCUS: AT1G06060
DESCRIPTION: RanBPM-related, similar to RANBPM {GI:13194576)(Homo sapiens)
DATA:                  Control 30min   2hours  2days   1week   p-value         pos
SENSE COUNTS:          2       3       1       1       0       5.47e-01
GENES (1 total):
  AT1G06060.1
    SENSE COUNTS:      2       3       1       1       0       5.47e-01
    TAGS: (1 total)
           ----------                                                          1525
           ----------                                                          1388
      d+2  GCCACAGGCG  2       3       1       1       0       5.47e-01        881
           ----------                                                          827
           ----------                                                          285

LOCUS: AT1G74840
DESCRIPTION: myb family transcription factor, similar to myb-related transcription activator GI:9279717 from
(Arabidopsis thaliana)
DATA:                  Control 30min   2hours  2days   1week   p-value         pos
SENSE COUNTS:          1       5       2       2       3       5.49e-01
GENES (1 total):
  AT1G74840.1
    SENSE COUNTS:      1       5       2       2       3       5.49e-01
    TAGS: (1 total)
           ----------                                                          1186
      d+2  TTTGGAAGTT  1       5       2       2       3       5.49e-01        1095
           ----------                                                          593
           ----------                                                          569
           ----------                                                          501
           ----------                                                          382
           ----------                                                          338
           ----------                                                          92

LOCUS: AT2G26975
DESCRIPTION: copper transporter, putative, similar to SP|Q39065 Copper transporter 1 (COPT1) {Arabidopsis
thaliana}; contains Pfam profile PF04145: Ctr copper transporter family
DATA:                  Control 30min   2hours  2days   1week   p-value         pos
SENSE COUNTS:          2       2       5       3       6       5.49e-01
GENES (1 total):
  AT2G26975.1
    SENSE COUNTS:      2       2       5       3       6       5.49e-01
    TAGS: (2 total)
      X+4  TAGATGATAA  0       0       1       1       1       5.61e-01        715
      d+1  CTCTTCGGAA  2       2       4       2       5       8.20e-01        507
           ----------                                                          279
           ----------                                                          204
           ----------                                                          198
           ----------                                                          150
           ----------                                                          142
           ----------                                                          50

LOCUS: AT3G09210
DESCRIPTION: KOW domain-containing transcription factor family protein, ; est match
DATA:                  Control 30min   2hours  2days   1week   p-value         pos
SENSE COUNTS:          4       0       2       2       1       5.51e-01
GENES (1 total):
  AT3G09210.1
    SENSE COUNTS:      4       0       2       2       1       5.51e-01
    TAGS: (2 total)
      d+1  TTTTGGAAGT  0       0       0       0       0       6.15e-01        1097
      d+2  ATCACTTCAC  4       0       2       2       1       2.79e-01        1084
           ----------                                                          539
           ----------                                                          359

LOCUS: AT1G44100
```

```
DESCRIPTION: amino acid permease 5
DATA:                 Control 30min   2hours  2days   1week   p-value         pos
SENSE COUNTS:         2       2       1       1       5       5.52e-01
GENES (2 total):
  AT1G44100.1
    SENSE COUNTS:     2       2       1       1       5       5.52e-01
    TAGS: (2 total)
      d+1  TATAAGGATA 1       0       0       0       0       4.28e-01        1919
      d+2  TTTCTACCGA 1       2       1       1       5       4.24e-01        1865
           ----------                                                         1663
           ----------                                                         1251
           ----------                                                         1187
           ----------                                                         890
           ----------                                                         803
           ----------                                                         783
           ----------                                                         611
           ----------                                                         469
           ----------                                                         20

LOCUS: AT1G15810
DESCRIPTION: ribosomal protein S15 family protein, contains similarity to ribosomal protein S15
DATA:                 Control 30min   2hours  2days   1week   p-value         pos
SENSE COUNTS:         6       7       8       7       1       5.53e-01
GENES (1 total):
  AT1G15810.1
    SENSE COUNTS:     6       7       8       7       1       5.53e-01
    TAGS: (5 total)
           ----------                                                         2217
           ----------                                                         2153
           ----------                                                         2074
           ----------                                                         2000
      d+2  GCAACCATTG 1       0       2       1       0       5.07e-01        1816
      d+2  CCGTTTTCTC 5       6       4       6       1       7.40e-01        1744
           ----------                                                         1627
      d+2  CATTTGTGGC 0       0       1       0       0       7.06e-01        1522
      d+2  TCCTCTGCGG 0       1       0       0       0       2.54e-01        1051
      d+2  GGGAAGAGAG 0       0       1       0       0       4.55e-01        595
           ----------                                                         82

LOCUS: AT4G18710
DESCRIPTION: shaggy-related protein kinase eta / ASK-eta (ASK7), identical to shaggy-related protein kinase
eta (ASK-eta) (Arabidopsis thaliana) SWISS-PROT:Q39011
DATA:                 Control 30min   2hours  2days   1week   p-value         pos
SENSE COUNTS:         1       4       3       4       1       5.53e-01
GENES (2 total):
  AT4G18710.1
    SENSE COUNTS:     1       4       3       4       1       5.53e-01
    TAGS: (2 total)
      d+1  GTTTTGCTTT 0       1       2       4       1       4.60e-01        1763
           ----------                                                         1751
      d+2  TATCAGATCT 1       3       1       0       0       2.52e-01        637
           ----------                                                         325
           ----------                                                         248
           ----------                                                         205
           ----------                                                         35

LOCUS: AT5G20420
DESCRIPTION: SNF2 domain-containing protein / helicase domain-containing protein, low similarity to SP|Q9U7E0
Transcriptional regulator ATRX homolog {Caenorhabditis elegans}; contains PFam profiles PF00271: Helicase
conserved C-terminal domain, PF00176: SNF2 family N-
DATA:                 Control 30min   2hours  2days   1week   p-value         pos
SENSE COUNTS:         1       0       1       0       3       5.53e-01
GENES (1 total):
  AT5G20420.1
    SENSE COUNTS:     1       0       1       0       3       5.53e-01
    TAGS: (3 total)
      v+1  GGACATCAAA 0       0       1       0       0       4.55e-01        4565
           ----------                                                         4551
      v+2  AACCTTTTGC 1       0       0       0       3       2.28e-01        4446
           ----------                                                         4256
           ----------                                                         4224
           ----------                                                         4110
           ----------                                                         3971
           ----------                                                         3831
           ----------                                                         3549
           ----------                                                         3073
           ----------                                                         2874
           ----------                                                         2788
           ----------                                                         2752
           ----------                                                         2615
           ----------                                                         2392
```

```
          ----------                                                 2170
          ----------                                                 1951
          ----------                                                 1851
          ----------                                                 1641
          ----------                                                 1468
     v+2  AGTCTGAATG  0      0      0      0      0      6.15e-01      679
          ----------                                                 447
          ----------                                                 428
          ----------                                                 398
          ----------                                                 123

LOCUS: AT4G19500
DESCRIPTION: disease resistance protein (TIR-NBS-LRR class), putative, domain signature TIR-NBS-LRR exists,
suggestive of a disease resistance protein. A false intron was added between exons 2 and 3 to circumvent a
frameshift caused by a sequencing error, as per Blake
DATA:                   Control 30min  2hours 2days  1week  p-value       pos
SENSE COUNTS:           1       1      1      0      3      5.53e-01
GENES (1 total):
  AT4G19500.1
    SENSE COUNTS:       1       1      1      0      3      5.53e-01
    TAGS: (2 total)
          ----------                                                 4749
          ----------                                                 4737
          ----------                                                 4636
     v+2  TCTATTCCTT  1      0      1      0      3      2.09e-01      3975
          ----------                                                 3819
          ----------                                                 3804
          ----------                                                 3488
          ----------                                                 3469
          ----------                                                 3450
          ----------                                                 3299
          ----------                                                 2953
          ----------                                                 2850
          ----------                                                 2701
          ----------                                                 2494
          ----------                                                 2250
     v+2  AAAGGAAAGC  0      1      0      0      0      2.54e-01      1506
          ----------                                                 1333
          ----------                                                 1101
          ----------                                                 834
          ----------                                                 728
          ----------                                                 487
          ----------                                                 354

LOCUS: AT4G27010
DESCRIPTION: expressed protein,  ; expression supported by MPSS
DATA:                   Control 30min  2hours 2days  1week  p-value       pos
SENSE COUNTS:           2       0      3      2      0      5.56e-01
GENES (1 total):
  AT4G27010.1
    SENSE COUNTS:       2       0      3      2      0      5.56e-01
    TAGS: (3 total)
     v+1  TCTATTTAAA  2      0      0      0      0      1.04e-01      8170
     v+2  AGTTGTTGGG  0      0      0      1      0      3.09e-01      8025
          ----------                                                 7924
          ----------                                                 7917
          ----------                                                 7825
          ----------                                                 7641
          ----------                                                 7602
          ----------                                                 7533
          ----------                                                 7330
          ----------                                                 7236
          ----------                                                 7086
          ----------                                                 6809
          ----------                                                 6553
          ----------                                                 6407
          ----------                                                 6352
          ----------                                                 6261
          ----------                                                 5887
          ----------                                                 5397
          ----------                                                 5001
          ----------                                                 4852
          ----------                                                 4689
          ----------                                                 4373
          ----------                                                 4368
          ----------                                                 4164
          ----------                                                 4111
          ----------                                                 3654
          ----------                                                 3462
          ----------                                                 2852
          ----------                                                 2577
```

```
           ----------                                                 2525
      v+2  GTTCTTGTTT 0       0       3       1       0       2.61e-01        2507
           ----------                                                 2235
           ----------                                                 1788
           ----------                                                 1663
           ----------                                                 1584
           ----------                                                 1317
           ----------                                                 1219
           ----------                                                 604
           ----------                                                 590
           ----------                                                 466
           ----------                                                 72

LOCUS: AT4G25880
DESCRIPTION: pumilio/Puf RNA-binding domain-containing protein, contains Pfam profile:PF00806 Pumilio-family
RNA binding domains
DATA:                  Control 30min   2hours  2days   1week   p-value         pos
SENSE COUNTS:          4       2       1       1       1       5.56e-01
GENES (2 total):
  AT4G25880.1
    SENSE COUNTS:      4       2       1       1       1       5.56e-01
    TAGS: (3 total)
      i+3  TGCATATAGA 0       0       0       0       1       4.65e-01        4215
      d+1  GTATTCGAAA 3       2       1       1       0       5.78e-01        3148
           ----------                                                 3078
           ----------                                                 2974
           ----------                                                 2816
           ----------                                                 2769
           ----------                                                 2522
           ----------                                                 2432
           ----------                                                 2341
           ----------                                                 2291
           ----------                                                 2260
           ----------                                                 2140
           ----------                                                 2053
           ----------                                                 2039
           ----------                                                 1958
           ----------                                                 1790
           ----------                                                 1481
           ----------                                                 1406
           ----------                                                 1372
           ----------                                                 1318
      d+2  TCTATAACAT 1       0       0       0       0       4.28e-01        1307
           ----------                                                 1151
           ----------                                                 427
  AT4G25880.2
    SENSE COUNTS:      4       2       1       1       1       5.56e-01
    TAGS: (3 total)
      i+3  TGCATATAGA 0       0       0       0       1       4.65e-01        4215
      d+1  GTATTCGAAA 3       2       1       1       0       5.78e-01        3121
           ----------                                                 3051
           ----------                                                 2947
           ----------                                                 2789
           ----------                                                 2742
           ----------                                                 2495
           ----------                                                 2405
           ----------                                                 2314
           ----------                                                 2264
           ----------                                                 2233
           ----------                                                 2113
           ----------                                                 2026
           ----------                                                 2012
           ----------                                                 1790
           ----------                                                 1481
           ----------                                                 1406
           ----------                                                 1372
           ----------                                                 1318
      d+2  TCTATAACAT 1       0       0       0       0       4.28e-01        1307
           ----------                                                 1151
           ----------                                                 427

LOCUS: AT3G30212
DESCRIPTION: copia-like retrotransposon family, has a 4.6e-19 P-value blast match to gb|AAG52949.1| gag/pol
polyprotein (Endovir1-1) (Arabidopsis thaliana) (Ty1_Copia-family)
DATA:                  Control 30min   2hours  2days   1week   p-value         pos
SENSE COUNTS:          5       8       8       4       3       5.59e-01
GENES (1 total):
  AT3G30212.1
    SENSE COUNTS:      5       8       8       4       3       5.59e-01
    TAGS: (1 total)
           ----------                                                 1315
```

```
          ----------                                                  1221
          ----------                                                  1056
          ----------                                                  950
          ----------                                                  939
     p+2  AATTTTTGTT  5       8       8       4       3       5.59e-01       531
          ----------                                                  527

LOCUS: AT3G57890
DESCRIPTION: tubulin-specific chaperone C-related, contains weak similarity to Tubulin-specific chaperone C
(Tubulin-folding cofactor C) (CFC) (Swiss-Prot:Q15814) (Homo sapiens)
DATA:                  Control 30min   2hours  2days   1week   p-value        pos
SENSE COUNTS:          5       8       11      13      10      5.60e-01
GENES (1 total):
  AT3G57890.1
    SENSE COUNTS:      5       8       11      13      10      5.60e-01
    TAGS: (3 total)
      i+3  AAGCATCTAA  0       0       0       0       0       6.15e-01       3009
      d+1  ATGACTTGAA  2       0       4       0       0       4.70e-02       2100
      d+2  TAGAGCTCCT  3       8       7       13      10      2.55e-01       1829
           ----------                                                  1723
           ----------                                                  1456
           ----------                                                  1383
           ----------                                                  1231
           ----------                                                  1108
           ----------                                                  871
           ----------                                                  334
           ----------                                                  72

LOCUS: AT2G45790
DESCRIPTION: eukaryotic phosphomannomutase family protein, contains Pfam profile: PF03332 eukaryotic
phosphomannomutase
DATA:                  Control 30min   2hours  2days   1week   p-value        pos
SENSE COUNTS:          4       4       3       7       7       5.61e-01
GENES (1 total):
  AT2G45790.1
    SENSE COUNTS:      4       4       3       7       7       5.61e-01
    TAGS: (3 total)
      i+3  TTTGCAAAGT  2       3       0       1       3       4.55e-01       1183
      d+1  GTCCTCAAAG  1       1       3       2       3       8.34e-01       1016
      d+2  TCTTGAAACA  1       0       0       4       1       9.54e-02       833
           ----------                                                  39

LOCUS: AT5G11700
DESCRIPTION: glycine-rich protein, predicted protein, Arabidopsis thaliana
DATA:                  Control 30min   2hours  2days   1week   p-value        pos
SENSE COUNTS:          3       1       0       2       2       5.62e-01
GENES (1 total):
  AT5G11700.1
    SENSE COUNTS:      3       1       0       2       2       5.62e-01
    TAGS: (4 total)
      i+3  TGAGTTTTAC  0       0       0       1       0       3.09e-01       7919
      d+1  TAATCTAAAG  3       1       0       1       1       5.22e-01       4452
      i+3  ATCGTTAATG  0       0       0       0       1       1.65e-01       4422
           ----------                                                  4169
           ----------                                                  4090
           ----------                                                  3985
           ----------                                                  3932
           ----------                                                  3624
           ----------                                                  3500
           ----------                                                  3476
           ----------                                                  3304
           ----------                                                  3051
           ----------                                                  3045
           ----------                                                  2843
           ----------                                                  2756
           ----------                                                  2587
           ----------                                                  2553
           ----------                                                  2324
           ----------                                                  1862
      i+3  TAAACTGATC  0       0       0       0       0       6.15e-01       1534
           ----------                                                  1463
           ----------                                                  1390
           ----------                                                  1309
           ----------                                                  959
           ----------                                                  935
           ----------                                                  870
           ----------                                                  557
           ----------                                                  197

LOCUS: AT1G29900
```

```
DESCRIPTION: carbamoyl-phosphate synthase family protein, similar to carbamoylphosphate synthetase GI:6552726
from (Medicago sativa); contains Pfam profiles PF02786: Carbamoyl-phosphate synthase L chain ATP binding
domain, PF00289: Carbamoyl-phosphate synthase L chain
DATA:                 Control 30min   2hours  2days   1week   p-value         pos
SENSE COUNTS:         1       4       3       2       4       5.64e-01
GENES (2 total):
  AT1G29900.1
    SENSE COUNTS:     1       4       3       2       4       5.64e-01
    TAGS: (2 total)
      d+1  TAACGCTTGG 1       1       0       1       1       8.16e-01        3859
      d+2  CTGCTGATAT 0       3       3       1       3       4.57e-01        3427
           ----------                                                         3412
           ----------                                                         3088
           ----------                                                         3033
           ----------                                                         2858
           ----------                                                         2373
           ----------                                                         2367
           ----------                                                         2013
           ----------                                                         1979
           ----------                                                         1755
           ----------                                                         1713
           ----------                                                         1696
           ----------                                                         654
           ----------                                                         549
           ----------                                                         325
           ----------                                                         296

LOCUS: AT4G16490
DESCRIPTION: armadillo/beta-catenin repeat family protein, contains Pfam profile: PF00514 armadillo/beta-
catenin-like repeat
DATA:                 Control 30min   2hours  2days   1week   p-value         pos
SENSE COUNTS:         2       2       3       2       6       5.64e-01
GENES (1 total):
  AT4G16490.1
    SENSE COUNTS:     2       2       3       2       6       5.64e-01
    TAGS: (3 total)
      d+1  TATTGATGGT 2       2       0       2       5       3.66e-01        1662
      d+2  TGGAGGTTTC 0       0       3       0       0       2.70e-02        1528
           ----------                                                         1515
           ----------                                                         960
           ----------                                                         815
      d+2  ATCCAGAGTT 0       0       0       0       1       1.65e-01        547
           ----------                                                         336
           ----------                                                         40

LOCUS: AT1G65490
DESCRIPTION: expressed protein
DATA:                 Control 30min   2hours  2days   1week   p-value         pos
SENSE COUNTS:         1       2       5       2       2       5.66e-01
GENES (1 total):
  AT1G65490.1
    SENSE COUNTS:     1       2       5       2       2       5.66e-01
    TAGS: (3 total)
      X+4  CATTAGAGCC 0       0       0       0       1       1.65e-01        330
      d+1  CAGACGTAGT 1       2       4       2       0       4.71e-01        234
      d+2  TGGAGGTTCT 0       0       1       0       1       3.96e-01        177

LOCUS: AT3G61870
DESCRIPTION: expressed protein, hypothetical protein - Synechocystis sp. (strain PCC 6803), PIR:S75899
DATA:                 Control 30min   2hours  2days   1week   p-value         pos
SENSE COUNTS:         15      8       8       9       6       5.71e-01
GENES (2 total):
  AT3G61870.1
    SENSE COUNTS:     15      8       8       9       6       5.71e-01
    TAGS: (2 total)
      d+1  CAATTAGAGT 15      5       8       9       5       1.83e-01        936
      d+2  TAAAACCCCA 0       3       0       0       1       8.32e-02        121
  AT3G61870.2
    SENSE COUNTS:     15      8       8       9       6       5.71e-01
    TAGS: (2 total)
      d+1  CAATTAGAGT 15      5       8       9       5       1.83e-01        1253
           ----------                                                         955
      d+2  TAAAACCCCA 0       3       0       0       1       8.32e-02        158

LOCUS: AT4G15475
DESCRIPTION: F-box family protein (FBL4), 99.7% identical to F-box protein family, AtFBL4 (GP:21536497)
(Arabidopsis thaliana); similar to grr1 GI:2407790 from (Glycine max)
DATA:                 Control 30min   2hours  2days   1week   p-value         pos
SENSE COUNTS:         2       0       1       4       1       5.72e-01
GENES (1 total):
  AT4G15475.1
```

```
    SENSE COUNTS:      2       0       1       4       1       5.72e-01
    TAGS: (2 total)
      d+1  TGCCGAAGCC  2       0       0       0       0       1.04e-01       2042
      d+2  GATGAAAACT  0       0       1       4       1       2.55e-01       2013
           ----------                                                         1975
           ----------                                                         1876
           ----------                                                         1732
           ----------                                                         1603
           ----------                                                         1082
           ----------                                                         988
           ----------                                                         824
           ----------                                                         281
           ----------                                                         147
           ----------                                                         55

LOCUS: AT1G09570
DESCRIPTION: phytochrome A (PHYA), identical to SP|P14712 Phytochrome A {Arabidopsis thaliana}
DATA:                  Control 30min   2hours  2days   1week   p-value        pos
SENSE COUNTS:          2       3       2       2       0       5.75e-01
GENES (3 total):
  AT1G09570.1
    SENSE COUNTS:      2       3       2       2       0       5.75e-01
    TAGS: (3 total)
      d+1  ATGTATCATT  1       0       1       2       0       6.87e-01       3759
      d+2  CTGATGGCTG  0       3       1       0       0       1.04e-01       3247
           ----------                                                         2932
           ----------                                                         2840
           ----------                                                         2819
           ----------                                                         2613
           ----------                                                         2387
           ----------                                                         2354
           ----------                                                         2334
           ----------                                                         1799
           ----------                                                         1741
           ----------                                                         1661
           ----------                                                         1627
      d+2  GTTGTGTGAA  1       0       0       0       0       4.28e-01       1608
           ----------                                                         1180
           ----------                                                         1171
           ----------                                                         1079
           ----------                                                         923
           ----------                                                         763
           ----------                                                         524
           ----------                                                         287
           ----------                                                         140

LOCUS: AT2G22880
DESCRIPTION: VQ motif-containing protein, contains PF05678: VQ motif
DATA:                  Control 30min   2hours  2days   1week   p-value        pos
SENSE COUNTS:          1       3       3       1       5       5.76e-01
GENES (1 total):
  AT2G22880.1
    SENSE COUNTS:      1       3       3       1       5       5.76e-01
    TAGS: (2 total)
      v+1  TGAAGAAGAA  1       0       2       1       5       1.45e-01       927
      v+2  AATTTGTTTC  0       3       1       0       0       1.04e-01       737
           ----------                                                         428
           ----------                                                         374
           ----------                                                         354
           ----------                                                         132

LOCUS: AT2G28740
DESCRIPTION: histone H4, identical to histone H4 from Lycopersicon esculentum GI:297150, Lolium temulentum
SP|P02308, Acropora formosa GI:455652, Citrus jambhiri GI:16797797
DATA:                  Control 30min   2hours  2days   1week   p-value        pos
SENSE COUNTS:          1       1       5       3       3       5.78e-01
GENES (2 total):
  AT2G28740.1
    SENSE COUNTS:      1       1       5       3       3       5.78e-01
    TAGS: (2 total)
      X+4  GTAAAGGATT  0       0       1       1       0       7.90e-01       531
      d+1  CGAGGAGGAA  1       1       4       2       3       6.67e-01       271

LOCUS: AT5G09320
DESCRIPTION: vacuolar sorting protein 9 domain-containing protein / VPS9 domain-containing protein, contains
similarity to Rab5 GDP/GTP exchange factor, Rabex5 (Bos taurus) gi|2558516|emb|CAA04545; contains Pfam profile
PF02204: Vacuolar sorting protein 9 (VPS9) domai
DATA:                  Control 30min   2hours  2days   1week   p-value        pos
SENSE COUNTS:          2       4       1       2       5       5.78e-01
GENES (1 total):
  AT5G09320.1
```

```
    SENSE COUNTS:      2       4       1       2       5       5.78e-01
    TAGS: (2 total)
      d+1  TCTATGGAGA  0       0       1       0       5       7.15e-03       2371
           ----------                                                        2284
           ----------                                                        2183
           ----------                                                        2066
           ----------                                                        1939
      i+3  TAATTTTACA  2       4       0       2       0       2.05e-01       1935
           ----------                                                        1155
           ----------                                                        1030
           ----------                                                        862
           ----------                                                        773
           ----------                                                        733
           ----------                                                        253

LOCUS: AT1G50730
DESCRIPTION: expressed protein
DATA:                  Control 30min   2hours  2days   1week   p-value        pos
SENSE COUNTS:          1       0       2       4       1       5.84e-01
GENES (1 total):
  AT1G50730.1
    SENSE COUNTS:      1       0       2       4       1       5.84e-01
    TAGS: (3 total)
           ----------                                                        3857
           ----------                                                        3761
           ----------                                                        3735
      v+2  GGTTAATATC  1       0       0       4       1       9.54e-02       3429
           ----------                                                        3306
      v+2  GCGCTTGAAG  0       0       1       0       0       7.06e-01       2961
           ----------                                                        2726
           ----------                                                        2544
           ----------                                                        2326
           ----------                                                        2100
           ----------                                                        2085
           ----------                                                        1597
      v+2  CAGAAGTTTG  0       0       1       0       0       4.55e-01       1329
           ----------                                                        1263
           ----------                                                        1239
           ----------                                                        1103
           ----------                                                        933
           ----------                                                        790
           ----------                                                        769
           ----------                                                        354
           ----------                                                        110

LOCUS: AT4G02290
DESCRIPTION: glycosyl hydrolase family 9 protein, similar to endo-1,4-beta glucanase; ATCEL2 GI:3132891 from
(Arabidopsis thaliana)
DATA:                  Control 30min   2hours  2days   1week   p-value        pos
SENSE COUNTS:          1       1       3       0       1       5.85e-01
GENES (1 total):
  AT4G02290.1
    SENSE COUNTS:      1       1       3       0       1       5.85e-01
    TAGS: (2 total)
      d+1  ATCGCTTCCC  1       0       0       0       0       4.28e-01       1516
           ----------                                                        1452
           ----------                                                        1341
           ----------                                                        1167
           ----------                                                        1069
           ----------                                                        1054
           ----------                                                        615
           ----------                                                        471
      d+2  GAGTGTAATT  0       1       3       0       1       3.22e-01       443
           ----------                                                        358

LOCUS: AT5G67130
DESCRIPTION: expressed protein
DATA:                  Control 30min   2hours  2days   1week   p-value        pos
SENSE COUNTS:          1       1       3       0       1       5.85e-01
GENES (1 total):
  AT5G67130.1
    SENSE COUNTS:      1       1       3       0       1       5.85e-01
    TAGS: (1 total)
           ----------                                                        1886
      d+2  AATTGCCTTA  1       1       3       0       1       5.85e-01       1604
           ----------                                                        1374
           ----------                                                        1337
           ----------                                                        1227
           ----------                                                        990
           ----------                                                        903
           ----------                                                        494
```

```
          ----------                                                    353

LOCUS: AT5G05570
DESCRIPTION: transducin family protein / WD-40 repeat family protein, similar to unknown protein
(pir||T04661); contains Pfam PF00400: WD domain, G-beta repeat (4 copies, 2
weak)|8683726|gb|AV524198.1|AV524198
DATA:                 Control 30min  2hours  2days  1week  p-value      pos
SENSE COUNTS:         2       0      1       0      3      5.89e-01
GENES (1 total):
  AT5G05570.1
    SENSE COUNTS:     2       0      1       0      3      5.89e-01
    TAGS: (5 total)
          ----------                                                    3658
          ----------                                                    3532
          ----------                                                    3525
          ----------                                                    3448
      d+2  ATGGCGCTCC 1       0      1       0      0      6.01e-01     3058
          ----------                                                    2992
          ----------                                                    2899
          ----------                                                    2865
          ----------                                                    2616
      d+2  AGAACAAATC 0       0      0       0      0      6.15e-01     2500
      i+3  TGTCTCTAAA 1       0      0       0      0      4.28e-01     2496
      d+2  CTGTTGGTTC 0       0      0       0      0      6.15e-01     1993
          ----------                                                    1736
      d+2  GCAGGATATC 0       0      0       0      3      1.12e-02     1686
          ----------                                                    937
          ----------                                                    862
          ----------                                                    624
          ----------                                                    610
          ----------                                                    483

LOCUS: AT1G79810
DESCRIPTION: Pex2/Pex12 N-terminal domain-containing protein / zinc finger (C3HC4-type RING finger) family
protein, contains Pfam profiles PF00097: zinc finger C3HC4 type (RING finger), PF04757: Pex2/Pex12 amino
terminal region
DATA:                 Control 30min  2hours  2days  1week  p-value      pos
SENSE COUNTS:         0       2      3       2      3      5.90e-01
GENES (3 total):
  AT1G79810.2
    SENSE COUNTS:     0       2      3       2      3      5.90e-01
    TAGS: (2 total)
      d+1  TTGTTTACCT 0       0      1       0      0      4.55e-01     1513
      d+2  AGTTGAGAAC 0       2      2       2      3      6.19e-01     1408
          ----------                                                    1363
          ----------                                                    1234
          ----------                                                    895
          ----------                                                    517
          ----------                                                    370
          ----------                                                    40
  AT1G79810.1
    SENSE COUNTS:     0       2      3       2      3      5.90e-01
    TAGS: (2 total)
      d+1  TTGTTTACCT 0       0      1       0      0      4.55e-01     1509
      d+2  AGTTGAGAAC 0       2      2       2      3      6.19e-01     1404
          ----------                                                    1359
          ----------                                                    1230
          ----------                                                    891
          ----------                                                    513
          ----------                                                    366
          ----------                                                    40

LOCUS: AT1G05500
DESCRIPTION: C2 domain-containing protein, similar to Ca2+-dependent lipid-binding protein (CLB1) GI:2789434
from (Lycopersicon esculentum)
DATA:                 Control 30min  2hours  2days  1week  p-value      pos
SENSE COUNTS:         2       1      3       4      0      5.91e-01
GENES (1 total):
  AT1G05500.1
    SENSE COUNTS:     2       1      3       4      0      5.91e-01
    TAGS: (1 total)
          ----------                                                    2400
          ----------                                                    2386
      v+2  TAGCCCAGAA 2       1      3       4      0      5.91e-01     2064
          ----------                                                    2010
          ----------                                                    1747

LOCUS: AT1G70900
DESCRIPTION: expressed protein
DATA:                 Control 30min  2hours  2days  1week  p-value      pos
SENSE COUNTS:         5       3      3       1      1      5.96e-01
```

```
GENES (1 total):
  AT1G70900.1
    SENSE COUNTS:      5       3       3       1       1       5.96e-01
    TAGS: (2 total)
      d+1  AAGAAATTGC  1       0       3       0       1       2.11e-01        1126
      d+2  ATGTACACAA  4       3       0       1       0       1.49e-01        766
           ----------                                                          477
           ----------                                                          382
           ----------                                                          353
           ----------                                                          232
           ----------                                                          223
           ----------                                                          174

LOCUS: AT2G29170
DESCRIPTION: short-chain dehydrogenase/reductase (SDR) family protein / tropinone reductase, putative, similar
to tropinone reductase SP:P50165 from (Datura stramonium)
DATA:                  Control 30min   2hours  2days   1week   p-value         pos
SENSE COUNTS:          1       0       3       1       3       5.96e-01
GENES (1 total):
  AT2G29170.1
    SENSE COUNTS:      1       0       3       1       3       5.96e-01
    TAGS: (2 total)
           ----------                                                          1063
           ----------                                                          917
           ----------                                                          853
      v+2  GAAAACGTTG  0       0       1       1       0       7.90e-01        609
           ----------                                                          494
      v+2  TTAGGAGCAA  1       0       2       0       3       2.23e-01        471
           ----------                                                          408
           ----------                                                          24
           ----------                                                          11

LOCUS: AT1G04940
DESCRIPTION: tic20 family protein, similar to Tic20 (GI:3769673) (Pisum sativum); contains TIGRFAM IGR00994:
chloroplast protein import component, Tic20 family
DATA:                  Control 30min   2hours  2days   1week   p-value         pos
SENSE COUNTS:          5       6       5       6       11      5.96e-01
GENES (1 total):
  AT1G04940.1
    SENSE COUNTS:      5       6       5       6       11      5.96e-01
    TAGS: (5 total)
      i+3  TCTAACAAAA  0       0       0       1       0       3.09e-01        2780
      d+1  CCTGAAGCTC  0       0       0       1       0       3.09e-01        1665
      i+3  GATTGGCTAT  0       0       1       0       1       6.85e-01        1524
      i+3  AACGCAGCCG  5       5       4       4       7       9.50e-01        1489
           ----------                                                          1402
           ----------                                                          1196
           ----------                                                          1106
      d+2  TAGTGATGGG  0       1       0       0       3       1.27e-01        653
           ----------                                                          583
           ----------                                                          467
           ----------                                                          283
           ----------                                                          125

LOCUS: AT5G16470
DESCRIPTION: zinc finger (C2H2 type) family protein, contains Pfam profile: PF00096 zinc finger, C2H2 type
DATA:                  Control 30min   2hours  2days   1week   p-value         pos
SENSE COUNTS:          5       7       7       10      3       5.97e-01
GENES (1 total):
  AT5G16470.1
    SENSE COUNTS:      5       7       7       10      3       5.97e-01
    TAGS: (3 total)
      X+4  TTAGCTCATT  0       0       0       1       0       3.09e-01        691
      X+4  TCAAGCTGCT  1       0       0       0       0       4.28e-01        646
      d+1  AGGCTTTAGC  4       7       7       9       3       6.26e-01        348
           ----------                                                          300

LOCUS: AT5G48480
DESCRIPTION: expressed protein
DATA:                  Control 30min   2hours  2days   1week   p-value         pos
SENSE COUNTS:          2       3       1       2       0       5.98e-01
GENES (1 total):
  AT5G48480.1
    SENSE COUNTS:      2       3       1       2       0       5.98e-01
    TAGS: (2 total)
      i+3  TTTAGAAGTT  0       0       1       0       0       4.55e-01        573
      d+1  TTCTCTCTTC  2       3       0       2       0       3.25e-01        348

LOCUS: AT3G30390
DESCRIPTION: amino acid transporter family protein, low similarity to neuronal glutamine transporter (Rattus
norvegicus) GI:6978016; belongs to INTERPRO:IPR002422 amino acid/polyamine transporter, family II
```

```
DATA:                 Control 30min   2hours  2days   1week   p-value         pos
SENSE COUNTS:         24      20      24      13      20      5.98e-01
GENES (1 total):
  AT3G30390.1
    SENSE COUNTS:     24      20      24      13      20      5.98e-01
    TAGS: (2 total)
      d+1  ACTCTTAGCT 2       0       2       0       3       2.55e-01        1891
      d+2  AGTGTTGTTT 22      20      22      13      17      7.56e-01        1826
           ----------                                                         1579
           ----------                                                         1538
           ----------                                                         694
           ----------                                                         466
           ----------                                                         392
           ----------                                                         374

LOCUS: AT1G28580
DESCRIPTION: GDSL-motif lipase, putative, similar to lipase (Arabidopsis thaliana) GI:1145627; contains
InterPro Entry IPR001087 Lipolytic enzyme, G-D-S-L family
DATA:                 Control 30min   2hours  2days   1week   p-value         pos
SENSE COUNTS:         4       3       1       1       3       6.06e-01
GENES (2 total):
  AT1G28580.1
    SENSE COUNTS:     4       3       1       0       0       1.56e-01
    TAGS: (2 total)
           ----------                                                         1370
           ----------                                                         1364
      d+2  TTGTTATGTG 4       3       0       0       0       6.07e-02        1331
           ----------                                                         1319
           ----------                                                         1313
           ----------                                                         1284
           ----------                                                         965
           ----------                                                         891
           ----------                                                         840
           ----------                                                         779
      d+2  GGGGAAATTG 0       0       1       0       0       4.55e-01        569
           ----------                                                         233
  AT1G28580.2
    SENSE COUNTS:     4       3       1       1       3       6.06e-01
    TAGS: (3 total)
           ----------                                                         1637
           ----------                                                         1631
      d+2  TTGTTATGTG 4       3       0       0       0       6.07e-02        1598
           ----------                                                         1586
           ----------                                                         1580
           ----------                                                         1551
           ----------                                                         1232
           ----------                                                         1158
           ----------                                                         1107
           ----------                                                         1046
      d+2  GGGGAAATTG 0       0       1       0       0       4.55e-01        836
      d+2  TTCTTTTCTT 0       0       0       1       3       6.27e-02        557
           ----------                                                         238

LOCUS: AT5G03455
DESCRIPTION: rhodanese-like domain-containing protein, contains Rhodanese-like domain PF:00581
DATA:                 Control 30min   2hours  2days   1week   p-value         pos
SENSE COUNTS:         1       2       3       4       4       6.08e-01
GENES (1 total):
  AT5G03455.1
    SENSE COUNTS:     1       2       3       4       4       6.08e-01
    TAGS: (4 total)
      i+3  AAGTGATTGC 0       0       0       0       1       1.65e-01        1182
      d+1  GAGCACAAAC 0       0       1       2       0       1.79e-01        583
      d+2  TACTAAAGTT 1       2       1       1       3       7.74e-01        523
      d+2  ATCTTGGAAC 0       0       1       1       0       7.90e-01        385

LOCUS: AT2G38650
DESCRIPTION: glycosyl transferase family 8 protein, contains Pfam profile: PF01501 glycosyl transferase family
8
DATA:                 Control 30min   2hours  2days   1week   p-value         pos
SENSE COUNTS:         3       2       1       5       3       6.11e-01
GENES (1 total):
  AT2G38650.1
    SENSE COUNTS:     3       2       1       5       3       6.11e-01
    TAGS: (3 total)
      i+3  GATGTTGTTT 0       0       0       1       0       3.09e-01        2436
      d+1  AAGCCGTGGC 1       0       0       0       0       4.28e-01        1962
           ----------                                                         1279
           ----------                                                         1249
           ----------                                                         1035
      X+4  CAGAAGCCAT 2       2       1       4       3       7.99e-01        417
```

```
LOCUS: AT4G04840
DESCRIPTION: methionine sulfoxide reductase domain-containing protein / SeIR domain-containing protein, low
similarity to pilin-like transcription factor (Homo sapiens) GI:5059062; contains Pfam profile PF01641: SelR
domain
DATA:                  Control 30min   2hours  2days   1week   p-value         pos
SENSE COUNTS:          3       1       5       2       4       6.12e-01
GENES (1 total):
  AT4G04840.1
    SENSE COUNTS:      3       1       5       2       4       6.12e-01
    TAGS: (2 total)
      i+3  CTTGCATTTA  0       0       1       0       3       7.32e-02        730
      d+1  TCTTAAAAAA  3       1       4       2       1       8.06e-01        467

LOCUS: AT1G50570
DESCRIPTION: C2 domain-containing protein, low similarity to cold-regulated gene SRC2 (Glycine max)
GI:2055230; contains Pfam profile PF00168: C2 domain
DATA:                  Control 30min   2hours  2days   1week   p-value         pos
SENSE COUNTS:          1       1       4       3       3       6.13e-01
GENES (1 total):
  AT1G50570.1
    SENSE COUNTS:      1       1       4       3       3       6.13e-01
    TAGS: (2 total)
           ----------                                                          1904
           ----------                                                          1716
      d+2  TAGTGAAAGT  1       1       4       2       3       6.67e-01        1402
      d+2  TACACAAAAA  0       0       0       1       0       3.09e-01        1198
           ----------                                                          1016
           ----------                                                          280

LOCUS: AT1G11750
DESCRIPTION: ATP-dependent Clp protease proteolytic subunit (ClpP), identical to ATP-dependent Clp protease
proteolytic subunit GI:2827888 from (Arabidopsis thaliana); contains Pfam profile PF00574: Clp protease;
contains TIGRfam profile TIGR00493: ATP-dependent Clp p
DATA:                  Control 30min   2hours  2days   1week   p-value         pos
SENSE COUNTS:          1       1       1       3       3       6.15e-01
GENES (2 total):
  AT1G11750.1
    SENSE COUNTS:      1       1       1       3       3       6.15e-01
    TAGS: (4 total)
      i+3  GCTCACTGAT  0       0       0       0       0       6.15e-01        1880
      i+3  CTTCTTGCTT  0       0       0       0       0       6.15e-01        1644
      d+1  TAGAGGACGT  1       1       1       2       3       7.96e-01        760
      d+2  ATACATCAAC  0       0       0       1       0       3.09e-01        726

LOCUS: AT3G24320
DESCRIPTION: DNA mismatch repair MutS family (MSH1), low similarity to SP|Q56239 DNA mismatch repair protein
mutS {Thermus aquaticus}; contains Pfam profiles PF05190: MutS family domain IV, PF01624: MutS domain I,
PF01541: Endo/excinuclease amino terminal domain
DATA:                  Control 30min   2hours  2days   1week   p-value         pos
SENSE COUNTS:          3       3       2       1       0       6.15e-01
GENES (2 total):
  AT3G24320.1
    SENSE COUNTS:      3       3       2       1       0       6.15e-01
    TAGS: (2 total)
      d+1  CAGTTCGGGT  0       0       1       0       0       4.55e-01        3610
           ----------                                                          3575
           ----------                                                          3383
           ----------                                                          3343
           ----------                                                          3201
      d+2  GAATCTTCAG  3       3       1       1       0       4.74e-01        2774
           ----------                                                          2557
           ----------                                                          2548
           ----------                                                          2404
           ----------                                                          2237
           ----------                                                          2105
           ----------                                                          1937
           ----------                                                          1761
           ----------                                                          1550
           ----------                                                          1419
           ----------                                                          1405
           ----------                                                          1013
           ----------                                                          839
           ----------                                                          791
           ----------                                                          393

LOCUS: AT2G01110
DESCRIPTION: mutant is Albino and pale green; Chloroplast Protein Translocation (tatC)
DATA:                  Control 30min   2hours  2days   1week   p-value         pos
SENSE COUNTS:          7       2       4       3       6       6.16e-01
GENES (2 total):
```

```
  AT2G01110.1
    SENSE COUNTS:      7       2       4       3       6       6.16e-01
    TAGS: (2 total)
           ----------                                                        1422
      d+2  ATAATTCAGA  1       2       2       2       5       6.66e-01      1352
      d+2  TATCTTGTAG  6       0       2       1       1       8.88e-02      1200
           ----------                                                        1140

LOCUS: AT1G35760
DESCRIPTION: Mutator-like transposase family, has a 8.7e-50 P-value blast match to Q9SHN7 /450-633 Pfam
PF03108 MuDR family transposase (MuDr-element domain)
DATA:                  Control 30min   2hours  2days   1week   p-value       pos
SENSE COUNTS:          3       1       2       1       5       6.18e-01
GENES (1 total):
  AT1G35760.1
    SENSE COUNTS:      3       1       2       1       5       6.18e-01
    TAGS: (1 total)
           ----------                                                        3261
           ----------                                                        2930
           ----------                                                        2847
           ----------                                                        2706
           ----------                                                        2696
      p+2  TTTTAAAAAA  3       1       2       1       5       6.18e-01      2646
           ----------                                                        2595
           ----------                                                        2501
           ----------                                                        2348
           ----------                                                        2189
           ----------                                                        2165
           ----------                                                        2144
           ----------                                                        1979
           ----------                                                        1907
           ----------                                                        1901
           ----------                                                        1635
           ----------                                                        1398
           ----------                                                        679
           ----------                                                        661
           ----------                                                        77

LOCUS: AT1G76200
DESCRIPTION: expressed protein
DATA:                  Control 30min   2hours  2days   1week   p-value       pos
SENSE COUNTS:          19      17      26      14      22      6.19e-01
GENES (1 total):
  AT1G76200.1
    SENSE COUNTS:      19      17      26      14      22      6.19e-01
    TAGS: (3 total)
      d+1  TTGTTATTTT  10      8       10      6       14      7.45e-01      499
      d+2  ACGAAAAAAG  8       6       16      6       5       8.11e-02      338
           ----------                                                        283
           ----------                                                        121
      X+4  TATCCAAACT  1       3       0       2       3       4.10e-01      -141

LOCUS: AT4G27720
DESCRIPTION: expressed protein, contains Pfam PF05631: Protein of unknown function (DUF791)
DATA:                  Control 30min   2hours  2days   1week   p-value       pos
SENSE COUNTS:          10      6       8       13      6       6.19e-01
GENES (1 total):
  AT4G27720.1
    SENSE COUNTS:      10      6       8       13      6       6.19e-01
    TAGS: (2 total)
      d+1  ATGATTGTTG  0       1       3       2       1       5.74e-01      1386
           ----------                                                        1329
           ----------                                                        1032
      d+2  GTTTCATTTT  10      5       5       11      5       4.34e-01      934
           ----------                                                        740
           ----------                                                        701
           ----------                                                        527
           ----------                                                        398
           ----------                                                        231

LOCUS: AT4G26690
DESCRIPTION: glycerophosphoryl diester phosphodiesterase family protein, weak similarity to
glycerophosphodiester phosphodiesterase (Borrelia hermsii) GI:1399038; contains Pfam profile PF03009:
Glycerophosphoryl diester phosphodiesterase family
DATA:                  Control 30min   2hours  2days   1week   p-value       pos
SENSE COUNTS:          2       3       4       2       0       6.20e-01
GENES (1 total):
  AT4G26690.1
    SENSE COUNTS:      2       3       4       2       0       6.20e-01
    TAGS: (2 total)
           ----------                                                        3037
```

```
          ----------                                                      2995
       d+2  TCGTCTGCTT  2       3       3       2       0       7.34e-01       2576
       d+2  GCCCCTGCCC  0       0       1       0       0       4.55e-01       2155
          ----------                                                      1735
          ----------                                                      1464
          ----------                                                      697
          ----------                                                      237
          ----------                                                      209

LOCUS: AT1G34360
DESCRIPTION: translation initiation factor 3 (IF-3) family protein, low similarity to Translation initiation
factor IF-3 from (subsp. Schizaphis graminum) {Buchnera aphidicola} SP|P46243, {Salmonella typhimurium}
SP|P33321; contains Pfam profiles PF05198: Translation
DATA:                  Control 30min   2hours  2days   1week   p-value        pos
SENSE COUNTS:          2       1       2       4       3       6.25e-01
GENES (1 total):
  AT1G34360.1
    SENSE COUNTS:      2       1       2       4       3       6.25e-01
    TAGS: (3 total)
      i+3  CGTCTCCTTT  0       0       0       1       0       3.09e-01       1273
      i+3  GTACCAAATT  0       0       0       1       0       3.09e-01       1244
      d+1  TAAACCCACA  2       1       2       2       3       9.65e-01       1128
           ----------                                                      201

LOCUS: AT1G64680
DESCRIPTION: expressed protein
DATA:                  Control 30min   2hours  2days   1week   p-value        pos
SENSE COUNTS:          6       4       5       8       1       6.25e-01
GENES (1 total):
  AT1G64680.1
    SENSE COUNTS:      6       4       5       8       1       6.25e-01
    TAGS: (2 total)
      d+1  TTGGTTATAA  2       3       3       0       0       3.64e-01       1021
      d+2  AGTGGAGTGG  4       1       2       8       1       1.48e-01       803
           ----------                                                      672
           ----------                                                      651
           ----------                                                      198
           ----------                                                      54

LOCUS: AT3G19400
DESCRIPTION: cysteine proteinase, putative, non-consensus AT acceptor site at exon 3; contains similarity to
cysteine protease CYP1 GI:2828252, TDI-65 GI:5726641 from (Lycopersicon esculentum)
DATA:                  Control 30min   2hours  2days   1week   p-value        pos
SENSE COUNTS:          2       2       3       1       0       6.32e-01
GENES (2 total):
  AT3G19400.1
    SENSE COUNTS:      2       2       3       1       0       6.32e-01
    TAGS: (2 total)
      d+1  TTCACATTAA  0       1       0       1       0       4.31e-01       1222
      d+2  GGGGTTGAAT  2       1       3       0       0       4.27e-01       987
           ----------                                                      923
           ----------                                                      903
           ----------                                                      640

LOCUS: AT2G17340
DESCRIPTION: pantothenate kinase-related, contains Pfam domain, PF01937: Protein of unknown function;
supported by tandem duplication of pantothenate kinase -related protein (TIGR_Ath1:At2g17320) (Arabidopsis
thaliana)
DATA:                  Control 30min   2hours  2days   1week   p-value        pos
SENSE COUNTS:          2       0       3       1       5       6.33e-01
GENES (1 total):
  AT2G17340.1
    SENSE COUNTS:      2       0       3       1       5       6.33e-01
    TAGS: (2 total)
      i+3  GTAAGTCCCA  0       0       0       0       0       6.15e-01       2140
      d+1  GGTCGTGGAA  2       0       3       1       5       4.30e-01       986
           ----------                                                      802
           ----------                                                      610
           ----------                                                      309

LOCUS: AT1G48490
DESCRIPTION: protein kinase, putative, similar to incomplete root hair elongation (IRE) (Arabidopsis thaliana)
gi|6729346|dbj|BAA89783
DATA:                  Control 30min   2hours  2days   1week   p-value        pos
SENSE COUNTS:          4       3       3       3       0       6.37e-01
GENES (1 total):
  AT1G48490.1
    SENSE COUNTS:      4       3       3       3       0       6.37e-01
    TAGS: (3 total)
      d+1  GTCTCTTAGG  0       3       2       2       0       3.35e-01       3040
      d+2  AAGAAAAACA  4       0       1       1       0       7.05e-02       2910
```

```
         ----------                                                      2398
         ----------                                                      2245
         ----------                                                      2053
         ----------                                                      2044
         ----------                                                      1684
         ----------                                                      1653
         ----------                                                      1306
         ----------                                                      1230
         ----------                                                      1206
         ----------                                                      1169
         ----------                                                      1137
         ----------                                                      1098
         ----------                                                      850
      d+2  TATTTCTGAG 0      0      0      0      0      6.15e-01       788

LOCUS: AT5G35980
DESCRIPTION: protein kinase family protein, contains protein kinase domain, Pfam:PF00069
DATA:                 Control 30min  2hours 2days  1week  p-value        pos
SENSE COUNTS:         7      9      4      10     8      6.38e-01
GENES (2 total):
  AT5G35980.1
    SENSE COUNTS:     7      9      4      10     8      6.38e-01
    TAGS: (4 total)
      i+3  TTTTACCCCA 0      0      0      1      0      3.09e-01       6796
      d+1  GCTTGTGGGC 6      9      4      8      8      7.32e-01       3549
         ----------                                                      3410
         ----------                                                      3383
         ----------                                                      3274
         ----------                                                      3233
      d+2  TCTCCCAGAA 1      0      0      0      0      4.28e-01       3176
         ----------                                                      3131
         ----------                                                      2645
         ----------                                                      2531
         ----------                                                      2498
         ----------                                                      2366
         ----------                                                      2293
         ----------                                                      2156
         ----------                                                      2090
         ----------                                                      2018
      d+2  TGGTCTTTTG 0      0      0      1      0      3.09e-01       1489
         ----------                                                      1396
         ----------                                                      1189
         ----------                                                      959
         ----------                                                      713
         ----------                                                      571

LOCUS: AT3G57380
DESCRIPTION: expressed protein, contains Pfam domain, PF04577: Protein of unknown function (DUF563)
DATA:                 Control 30min  2hours 2days  1week  p-value        pos
SENSE COUNTS:         4      4      1      5      3      6.39e-01
GENES (1 total):
  AT3G57380.1
    SENSE COUNTS:     4      4      1      5      3      6.39e-01
    TAGS: (1 total)
         ----------                                                      1258
      d+2  GATTGATTCA 4      4      1      5      3      6.39e-01       1102
         ----------                                                      528
         ----------                                                      362

LOCUS: AT3G19040
DESCRIPTION: ubiquitin family protein / DNA-binding bromodomain-containing protein, low similarity to
SP|P51123 Transcription initiation factor TFIID 230 kDa subunit {Drosophila melanogaster}; contains Pfam
profiles: PF00439 bromodomain, PF00240: Ubiquitin family
DATA:                 Control 30min  2hours 2days  1week  p-value        pos
SENSE COUNTS:         5      1      5      1      4      6.41e-01
GENES (1 total):
  AT3G19040.1
    SENSE COUNTS:     5      1      5      1      4      6.41e-01
    TAGS: (3 total)
      i+3  AAAACCAATA 0      0      0      0      1      4.65e-01       6352
         ----------                                                      5808
         ----------                                                      4606
      v+2  GGAAAAAAAA 5      1      5      1      3      3.81e-01       4026
         ----------                                                      3415
         ----------                                                      3201
         ----------                                                      2972
         ----------                                                      2697
         ----------                                                      2572
      v+2  TACAAAGCAC 0      0      0      0      0      6.15e-01       2409
         ----------                                                      2341
         ----------                                                      2284
```

```
           ----------                                                  2176
           ----------                                                  2087
           ----------                                                  1636
           ----------                                                  1508
           ----------                                                  1263
           ----------                                                  984
           ----------                                                  977
           ----------                                                  844
           ----------                                                  826
           ----------                                                  526
           ----------                                                  451
           ----------                                                  120

LOCUS: AT3G21410
DESCRIPTION: F-box family protein (FBW1), contains similarity to F-box domain IPR:001810;
DATA:                 Control 30min  2hours  2days  1week  p-value        pos
SENSE COUNTS:         1       1      1       4      1      6.49e-01
GENES (1 total):
  AT3G21410.1
    SENSE COUNTS:     1       1      1       4      1      6.49e-01
    TAGS: (1 total)
           ----------                                                  1841
           ----------                                                  1696
           ----------                                                  1635
           ----------                                                  1623
           ----------                                                  1053
      v+2  TGGTTTTGCG 1       1      1       4      1      6.49e-01     1012
           ----------                                                  779
           ----------                                                  441
           ----------                                                  435
           ----------                                                  392
           ----------                                                  370

LOCUS: AT3G23050
DESCRIPTION: auxin-responsive protein / indoleacetic acid-induced protein 7 (IAA7), identical to
SP|Q38825|AXI7_ARATH Auxin-responsive protein IAA7 (Indoleacetic acid-induced protein 7)
DATA:                 Control 30min  2hours  2days  1week  p-value        pos
SENSE COUNTS:         6       6      7       7      1      6.50e-01
GENES (3 total):
  AT3G23050.2
    SENSE COUNTS:     1       0      0       0      1      3.83e-01
    TAGS: (2 total)
      i+3  AACCTTACCG 1       0      0       0      0      4.28e-01     1203
           ----------                                                  822
           ----------                                                  799
           ----------                                                  684
           ----------                                                  586
           ----------                                                  451
           ----------                                                  346
           ----------                                                  193
      d+2  ATCGGCCAAC 0       0      0       0      1      1.65e-01     61
  AT3G23050.1
    SENSE COUNTS:     6       6      7       7      1      6.50e-01
    TAGS: (4 total)
      i+3  AACCTTACCG 1       0      0       0      0      4.28e-01     1203
      d+1  TGTTTTGATC 1       2      4       6      0      1.48e-01     1109
           ----------                                                  1103
      d+2  TGTCTGTCGG 4       4      3       1      0      4.51e-01     1026
           ----------                                                  684
           ----------                                                  586
           ----------                                                  451
           ----------                                                  346
           ----------                                                  193
      d+2  ATCGGCCAAC 0       0      0       0      1      1.65e-01     61

LOCUS: AT1G45050
DESCRIPTION: ubiquitin-conjugating enzyme 15 (UBC15), E2; identical to ubiquitin-conjugating enzyme 15
GI:2801442 from (Arabidopsis thaliana)
DATA:                 Control 30min  2hours  2days  1week  p-value        pos
SENSE COUNTS:         1       1      1       4      3      6.51e-01
GENES (2 total):
  AT1G45050.1
    SENSE COUNTS:     1       1      1       4      3      6.51e-01
    TAGS: (2 total)
      i+3  GTTATCAAAC 0       0      0       0      0      6.15e-01     1356
      d+1  TCCACAAAAA 1       1      1       4      3      5.90e-01     709
           ----------                                                  529
           ----------                                                  377
           ----------                                                  60

LOCUS: AT4G31900
```

```
DESCRIPTION: chromatin remodeling factor, putative, strong similarity to chromatin remodeling factor CHD3
(PICKLE) (Arabidopsis thaliana) GI:6478518; contains Pfam profiles PF00271: Helicase conserved C-terminal
domain, PF00176: SNF2 family N-terminal domain
DATA:                 Control 30min  2hours  2days  1week  p-value       pos
SENSE COUNTS:         1       2      1       4      2      6.51e-01
GENES (1 total):
  AT4G31900.1
    SENSE COUNTS:     1       2      1       4      2      6.51e-01
    TAGS: (2 total)
           ----------                                                    4434
           ----------                                                    4409
           ----------                                                    4348
      v+2  ATTGTGTTGA 0       0      0       0      1      1.65e-01      3989
           ----------                                                    3916
           ----------                                                    3559
      i+3  ATTTTTTCAC 1       2      1       4      1      6.72e-01      3548
           ----------                                                    3433
           ----------                                                    3400
           ----------                                                    3273
           ----------                                                    3111
           ----------                                                    3006
           ----------                                                    2521
           ----------                                                    2227
           ----------                                                    1410
           ----------                                                    1300
           ----------                                                    1070
           ----------                                                    748
           ----------                                                    344

LOCUS: AT1G10360
DESCRIPTION: glutathione S-transferase, putative, similar to glutathione S-transferase (sp|Q03666|GTX4_TOBAC);
similar to EST gb|H36275 gb:AB039930.
DATA:                 Control 30min  2hours  2days  1week  p-value       pos
SENSE COUNTS:         3       2      2       4      0      6.64e-01
GENES (2 total):
  AT1G10360.1
    SENSE COUNTS:     3       2      2       4      0      6.64e-01
    TAGS: (1 total)
           ----------                                                    900
      d+2  AGTGTACCAA 3       2      2       4      0      6.64e-01      861
           ----------                                                    98
           ----------                                                    79

LOCUS: AT1G75460
DESCRIPTION: ATP-dependent protease La (LON) domain-containing protein, weak similarity to SP|P36774 ATP-
dependent protease La 2 (EC 3.4.21.53) {Myxococcus xanthus}; contains Pfam profile PF02190: ATP-dependent
protease La (LON) domain
DATA:                 Control 30min  2hours  2days  1week  p-value       pos
SENSE COUNTS:         2       3      2       1      0      6.65e-01
GENES (1 total):
  AT1G75460.1
    SENSE COUNTS:     2       3      2       1      0      6.65e-01
    TAGS: (1 total)
           ----------                                                    1760
           ----------                                                    1578
           ----------                                                    1564
      d+2  ATGCACACGC 2       3      2       1      0      6.65e-01      398

LOCUS: AT5G63780
DESCRIPTION: zinc finger (C3HC4-type RING finger) family protein, contains Pfam profile: PF00097 zinc finger,
C3HC4 type (RING finger)
DATA:                 Control 30min  2hours  2days  1week  p-value       pos
SENSE COUNTS:         6       2      5       5      7      6.66e-01
GENES (1 total):
  AT5G63780.1
    SENSE COUNTS:     6       2      5       5      7      6.66e-01
    TAGS: (2 total)
      d+1  GACGTGTCAT 0       0      0       1      0      3.09e-01      2127
      d+2  CTTCATTGAA 6       2      5       4      7      6.25e-01      1654
           ----------                                                    1572
           ----------                                                    1142
           ----------                                                    922
           ----------                                                    33

LOCUS: AT1G20925
DESCRIPTION: auxin efflux carrier family protein, contains auxin efflux carrier domain, Pfam:PF03547
DATA:                 Control 30min  2hours  2days  1week  p-value       pos
SENSE COUNTS:         3       0      3       1      1      6.67e-01
GENES (1 total):
  AT1G20925.1
    SENSE COUNTS:     3       0      3       1      1      6.67e-01
```

```
    TAGS: (2 total)
          ----------                                                  1497
          ----------                                                  582
      v+2 GTGAAAATGT 0      0      2      1      0      2.74e-01      564
      v+2 CAAAGCAAAG 3      0      1      0      1      4.20e-01      61

LOCUS: AT4G28260
DESCRIPTION: expressed protein,
DATA:                Control 30min  2hours 2days  1week  p-value       pos
SENSE COUNTS:        3      2      6      3      1      6.67e-01
GENES (1 total):
  AT4G28260.1
    SENSE COUNTS:    3      2      6      3      1      6.67e-01
    TAGS: (3 total)
      d+1 GGTTAAAAAT 0      0      0      1      0      6.04e-01      1973
          ----------                                                  1934
      d+2 TGTCGTGTGT 0      0      2      0      0      1.21e-01      1690
      d+2 AAGACGGGAA 3      2      4      2      1      9.13e-01      1629
          ----------                                                  1494
          ----------                                                  1231
          ----------                                                  794
          ----------                                                  219
          ----------                                                  165

LOCUS: AT5G17770
DESCRIPTION: NADH-cytochrome b5 reductase, identical to NADH-cytochrome b5 reductase (Arabidopsis thaliana)
GI:4240116
DATA:                Control 30min  2hours 2days  1week  p-value       pos
SENSE COUNTS:        4      4      7      2      3      6.69e-01
GENES (2 total):
  AT5G17770.1
    SENSE COUNTS:    4      4      7      2      3      6.69e-01
    TAGS: (2 total)
      d+1 ACCTACCCTA 1      0      2      0      0      5.95e-01      1235
      d+2 GCTGCAAACC 3      4      5      2      3      9.20e-01      946
          ----------                                                  658
          ----------                                                  157

LOCUS: AT4G26630
DESCRIPTION: expressed protein
DATA:                Control 30min  2hours 2days  1week  p-value       pos
SENSE COUNTS:        11     12     15     7      8      6.71e-01
GENES (1 total):
  AT4G26630.1
    SENSE COUNTS:    11     12     15     7      8      6.71e-01
    TAGS: (2 total)
      d+1 TGACAGGTGA 8      5      12     2      5      1.80e-01      1537
          ----------                                                  1372
      d+2 AAGCTGACAA 3      7      3      5      3      5.48e-01      664
          ----------                                                  480

LOCUS: AT5G59060
DESCRIPTION: expressed protein, ; expression supported by MPSS
DATA:                Control 30min  2hours 2days  1week  p-value       pos
SENSE COUNTS:        2      1      2      0      3      6.75e-01
GENES (1 total):
  AT5G59060.1
    SENSE COUNTS:    2      1      2      0      3      6.75e-01
    TAGS: (1 total)
          ----------                                                  982
      v+2 AAAAAAGAAA 2      1      2      0      3      6.75e-01      741
          ----------                                                  354
          ----------                                                  11
          ----------                                                  5

LOCUS: AT3G07670
DESCRIPTION: SET domain-containing protein, similar to ribulose-1,5-bisphosphate carboxylase/oxygenase small
subunit N-methyltransferase I (Spinacia oleracea) GI:3403236; contains Pfam profile PF00856: SET domain
DATA:                Control 30min  2hours 2days  1week  p-value       pos
SENSE COUNTS:        3      0      1      1      1      6.79e-01
GENES (1 total):
  AT3G07670.1
    SENSE COUNTS:    3      0      1      1      1      6.79e-01
    TAGS: (2 total)
          ----------                                                  1787
      d+2 GTTGGATCTG 3      0      0      1      1      3.94e-01      1674
      d+2 CGAAACAAGC 0      0      1      0      0      4.55e-01      1312
          ----------                                                  832
          ----------                                                  235
          ----------                                                  212
```

```
LOCUS: AT5G11500
DESCRIPTION: expressed protein, contains Pfam profile PF05670: Domain of unknown function (DUF814)
DATA:                 Control 30min   2hours  2days   1week   p-value         pos
SENSE COUNTS:         6       3       3       2       3       6.82e-01
GENES (1 total):
  AT5G11500.1
    SENSE COUNTS:     6       3       3       2       3       6.82e-01
    TAGS: (1 total)
           ----------                                                         1112
      d+2  TAAAAGCTCT 6       3       3       2       3       6.82e-01        753
           ----------                                                         417
           ----------                                                         253
           ----------                                                         162

LOCUS: AT1G52420
DESCRIPTION: glycosyl transferase family 1 protein, contains Pfam profile: PF00534 Glycosyl transferases group
1
DATA:                 Control 30min   2hours  2days   1week   p-value         pos
SENSE COUNTS:         3       0       2       1       3       6.82e-01
GENES (1 total):
  AT1G52420.1
    SENSE COUNTS:     3       0       2       1       3       6.82e-01
    TAGS: (3 total)
           ----------                                                         2521
      d+2  AGACCTTAAA 1       0       1       0       3       4.58e-01        2344
      d+2  AAGCAACATA 2       0       1       0       0       2.87e-01        2296
           ----------                                                         2161
           ----------                                                         1449
           ----------                                                         1303
           ----------                                                         1287
           ----------                                                         1243
      i+3  CTAATCTTTG 0       0       0       1       0       3.09e-01        646
           ----------                                                         572
           ----------                                                         461

LOCUS: AT2G03440
DESCRIPTION: nodulin-related, similar to Early nodulin 12B precursor (N-12B) (Swiss-Prot:Q40339) (Medicago
sativa)
DATA:                 Control 30min   2hours  2days   1week   p-value         pos
SENSE COUNTS:         11      10      6       8       7       6.84e-01
GENES (1 total):
  AT2G03440.1
    SENSE COUNTS:     11      10      6       8       7       6.84e-01
    TAGS: (2 total)
      d+1  TTGTTGACCA 6       6       4       4       7       9.06e-01        866
           ----------                                                         859
      d+2  GGTTTGGAGA 5       4       2       4       0       4.20e-01        667
           ----------                                                         354
           ----------                                                         150

LOCUS: AT4G35090
DESCRIPTION: catalase 2, identical to catalase 2 SP:P25819, GI:17865693 from (Arabidopsis thaliana)
DATA:                 Control 30min   2hours  2days   1week   p-value         pos
SENSE COUNTS:         3       0       3       4       2       6.86e-01
GENES (2 total):
  AT4G35090.1
    SENSE COUNTS:     3       0       3       4       2       6.86e-01
    TAGS: (3 total)
           ----------                                                         1654
      d+2  AAATCCGCAG 2       0       3       4       1       6.53e-01        1457
      d+2  CTGAGAAGTA 0       0       0       0       1       1.65e-01        1280
           ----------                                                         1225
           ----------                                                         1216
           ----------                                                         1205
           ----------                                                         824
           ----------                                                         706
           ----------                                                         679
           ----------                                                         628
      d+2  GTCCACGCTC 1       0       0       0       0       4.28e-01        535
           ----------                                                         398
           ----------                                                         311
           ----------                                                         269
           ----------                                                         169
           ----------                                                         76

LOCUS: AT2G04900
DESCRIPTION: expressed protein
DATA:                 Control 30min   2hours  2days   1week   p-value         pos
SENSE COUNTS:         3       6       2       5       3       6.89e-01
GENES (1 total):
  AT2G04900.1
```

```
    SENSE COUNTS:     3       6       2       5       3       6.89e-01
    TAGS: (2 total)
      i+3  TGTGTTGAAA 1       0       2       0       0       3.07e-01        1006
      d+1  GGCAACTTTA 2       6       0       5       3       1.68e-01        388
           ----------                                                         150

LOCUS: AT5G41940
DESCRIPTION: expressed protein
DATA:                 Control 30min   2hours  2days   1week   p-value         pos
SENSE COUNTS:         4       1       4       1       1       6.91e-01
GENES (2 total):
  AT5G41940.1
    SENSE COUNTS:     4       1       4       1       1       6.91e-01
    TAGS: (3 total)
      d+1  AAAATGTCTT 2       0       0       0       0       3.22e-01        1799
      d+2  ACTTAGTCGT 2       1       3       1       1       9.23e-01        1662
           ----------                                                         1619
      d+2  GGGGAGAATC 0       0       1       0       0       4.55e-01        1480
           ----------                                                         1220
           ----------                                                         908
           ----------                                                         627
           ----------                                                         546
           ----------                                                         227

LOCUS: AT4G39050
DESCRIPTION: kinesin-related protein (MKRP2), kinesin motor protein - Ustilago maydis, PID:g2062750; identical
to cDNA   MKRP2 mRNA for kinesin-related protein GI:16902293, kinesin-related protein (Arabidopsis thaliana)
GI:16902294
DATA:                 Control 30min   2hours  2days   1week   p-value         pos
SENSE COUNTS:         3       3       1       2       1       6.96e-01
GENES (1 total):
  AT4G39050.1
    SENSE COUNTS:     3       3       1       2       1       6.96e-01
    TAGS: (4 total)
      i+3  TAATTGAAAA 3       0       0       0       0       2.13e-02        4432
      d+1  CGGTTCTGAA 0       3       1       2       0       2.52e-01        3170
      d+2  TGGGTACTTG 0       0       0       0       1       1.65e-01        3073
           ----------                                                         2904
           ----------                                                         2809
           ----------                                                         2444
           ----------                                                         2071
           ----------                                                         1703
      d+2  TGTCGCTCAT 0       0       0       0       0       6.15e-01        1484
           ----------                                                         1478
           ----------                                                         1166
           ----------                                                         1127
           ----------                                                         896
           ----------                                                         220

LOCUS: AT1G71190
DESCRIPTION: expressed protein,
DATA:                 Control 30min   2hours  2days   1week   p-value         pos
SENSE COUNTS:         19      22      15      23      16      6.96e-01
GENES (2 total):
  AT1G71190.1
    SENSE COUNTS:     19      22      15      23      16      6.96e-01
    TAGS: (2 total)
      d+1  GAAGAAAGGA 1       0       0       1       0       5.06e-01        822
           ----------                                                         772
           ----------                                                         424
      d+2  ACTATTGCTT 18      22      15      22      16      7.38e-01        400

LOCUS: AT3G11110
DESCRIPTION: zinc finger (C3HC4-type RING finger) family protein, contains Pfam profile: PF00097 Zinc finger,
C3HC4 type (RING finger)
DATA:                 Control 30min   2hours  2days   1week   p-value         pos
SENSE COUNTS:         2       0       3       1       0       6.97e-01
GENES (1 total):
  AT3G11110.1
    SENSE COUNTS:     2       0       3       1       0       6.97e-01
    TAGS: (2 total)
           ----------                                                         1277
      v+2  CTATTTTTTT 0       0       0       0       0       6.15e-01        1205
           ----------                                                         1169
      v+2  GATGGAGGAT 2       0       3       1       0       5.41e-01        931
           ----------                                                         925
           ----------                                                         439
           ----------                                                         43

LOCUS: AT3G17810
```

```
DESCRIPTION: dihydroorotate dehydrogenase family protein / dihydroorotate oxidase family protein, low
similarity to SP|Q12882 Dihydropyrimidine dehydrogenase (NADP+) precursor (EC 1.3.1.2) (DPD) (DHPDHase)
(Dihydrouracil dehydrogenase) (Dihydrothymine dehydrogenase) {
DATA:                 Control 30min  2hours  2days  1week  p-value        pos
SENSE COUNTS:         2       2      5       5      3      6.97e-01
GENES (1 total):
  AT3G17810.1
    SENSE COUNTS:     2       2      5       5      3      6.97e-01
    TAGS: (2 total)
          ----------                                                      1687
      d+2  CAAATTACGA 1       2      5       5      3      4.84e-01       1485
          ----------                                                      1215
          ----------                                                      1180
          ----------                                                      950
          ----------                                                      906
          ----------                                                      709
          ----------                                                      615
          ----------                                                      552
          ----------                                                      366
          ----------                                                      141
      X+4  AAGTTGAAGA 1       0      0       0      0      4.28e-01       -88

LOCUS: AT4G21150
DESCRIPTION: ribophorin II (RPN2) family protein, contains Pfam domain PF05817: Ribophorin II (RPN2)
DATA:                 Control 30min  2hours  2days  1week  p-value        pos
SENSE COUNTS:         1       4      3       4      5      6.98e-01
GENES (1 total):
  AT4G21150.1
    SENSE COUNTS:     1       4      3       4      5      6.98e-01
    TAGS: (2 total)
      d+1  AGTTTCTTAA 1       0      1       4      5      8.64e-02       2409
          ----------                                                      2396
      d+2  AGTCACAGGT 0       4      2       0      0      6.74e-02       1581
          ----------                                                      1323

LOCUS: AT3G19030
DESCRIPTION: expressed protein, contains similarity to phosphoserine aminotransferase GB:P19689 from (Yersinia
enterocolitica)
DATA:                 Control 30min  2hours  2days  1week  p-value        pos
SENSE COUNTS:         3       7      5       5      3      6.98e-01
GENES (1 total):
  AT3G19030.1
    SENSE COUNTS:     3       7      5       5      3      6.98e-01
    TAGS: (2 total)
      d+1  TGATGAGCTT 3       7      3       5      0      1.98e-01       306
      d+2  GTGTCTCAAA 0       0      2       0      3      8.60e-02       129

LOCUS: AT5G30269
DESCRIPTION: copia-like retrotransposon family, has a 2.3e-141 P-value blast match to gb|AAO73529.1| gag-pol
polyprotein (Glycine max) (SIRE1) (Ty1_Copia-family)
DATA:                 Control 30min  2hours  2days  1week  p-value        pos
SENSE COUNTS:         2       0      3       1      2      7.01e-01
GENES (1 total):
  AT5G30269.1
    SENSE COUNTS:     3       0      3       2      3      5.60e-01
    TAGS: (4 total)
          ----------                                                      14483
          ----------                                                      14409
          ----------                                                      14305
          ----------                                                      14301
          ----------                                                      14055
          ----------                                                      13242
          ----------                                                      12689
          ----------                                                      12579
          ----------                                                      12313
          ----------                                                      12298
          ----------                                                      11690
          ----------                                                      11552
          ----------                                                      11528
          ----------                                                      11466
          ----------                                                      11318
      p+2  TACGTTGACG 1       0      3       0      0      2.79e-01       10921
          ----------                                                      10812
          ----------                                                      10799
          ----------                                                      10691
          ----------                                                      10175
          ----------                                                      10063
          ----------                                                      9845
          ----------                                                      8600
          ----------                                                      8544
          ----------                                                      8404
```

```
          ----------                                                        7892
          ----------                                                        7093
          ----------                                                        6832
          ----------                                                        6802
          ----------                                                        6620
          ----------                                                        6132
          ----------                                                        5673
          ----------                                                        5403
          ----------                                                        5082
          ----------                                                        5070
          ----------                                                        4710
          ----------                                                        3102
          ----------                                                        2589
          ----------                                                        2535
          ----------                                                        2509
          ----------                                                        2466
          ----------                                                        2444
          ----------                                                        2277
          ----------                                                        2235
     p+2  TGTGGACACT  0      0      0      0      1      1.65e-01            2104
          ----------                                                        2030
          ----------                                                        1926
          ----------                                                        1922
          ----------                                                        1707
     p+2  ATAATAGAAA  1      0      0      1      1      5.50e-01            13534
     p+2  ATAATAGAAA  1      0      0      1      1      5.50e-01            13534
          ----------                                                        877
          ----------                                                        11

LOCUS: AT5G63060
DESCRIPTION: SEC14 cytosolic factor, putative
DATA:                 Control 30min  2hours 2days  1week  p-value            pos
SENSE COUNTS:         4      1      5      4      3      7.06e-01
GENES (1 total):
  AT5G63060.1
    SENSE COUNTS:     4      1      5      4      3      7.06e-01
    TAGS: (1 total)
          ----------                                                        1430
     v+2  AAAAGCCTTC  4      1      5      4      3      7.06e-01            1329
          ----------                                                        742
          ----------                                                        649
          ----------                                                        376
          ----------                                                        66
          ----------                                                        18

LOCUS: AT1G67430
DESCRIPTION: 60S ribosomal protein L17 (RPL17B), similar to ribosomal protein GI:19101 from (Hordeum vulgare)
DATA:                 Control 30min  2hours 2days  1week  p-value            pos
SENSE COUNTS:         1      0      1      1      3      7.06e-01
GENES (1 total):
  AT1G67430.1
    SENSE COUNTS:     1      0      1      1      3      7.06e-01
    TAGS: (1 total)
          ----------                                                        705
          ----------                                                        583
          ----------                                                        467
          ----------                                                        456
     X+4  GTTCTGCAAG  1      0      1      1      3      7.06e-01            189
          ----------                                                        36

LOCUS: AT5G19860
DESCRIPTION: expressed protein, contains Pfam profile PF04398: Protein of unknown function, DUF538
DATA:                 Control 30min  2hours 2days  1week  p-value            pos
SENSE COUNTS:         1      0      1      1      3      7.06e-01
GENES (1 total):
  AT5G19860.1
    SENSE COUNTS:     1      0      1      1      3      7.06e-01
    TAGS: (2 total)
          ----------                                                        853
          ----------                                                        769
     d+2  TTTGTACATA  0      0      0      0      3      8.36e-02            648
          ----------                                                        521
     X+4  TATGGCATAA  1      0      1      1      0      7.18e-01            316

LOCUS: AT1G15690
DESCRIPTION: pyrophosphate-energized vacuolar membrane proton pump / pyrophosphate-energized inorganic
pyrophosphatase (AVP-3), identical to pyrophosphate-energized vacuolar membrane proton pump (pyrophosphate-
energized inorganic pyrophosphatase) SP:P31414 from (Arabi
DATA:                 Control 30min  2hours 2days  1week  p-value            pos
SENSE COUNTS:         23     20     15     23     25     7.07e-01
GENES (2 total):
```

```
  AT1G15690.1
    SENSE COUNTS:      23      20      15      23      25      7.07e-01
    TAGS: (1 total)
           ----------                                                        2832
           ----------                                                        2813
      d+2  GCTGTTGAGT  23      20      15      23      25      7.07e-01      2370
           ----------                                                        2088
           ----------                                                        2027
           ----------                                                        1914
           ----------                                                        1890
           ----------                                                        1457
           ----------                                                        1280
           ----------                                                        1110
           ----------                                                        1049
           ----------                                                        849
           ----------                                                        438

LOCUS: AT2G47580
DESCRIPTION: small nuclear ribonucleoprotein U1A / spliceosomal protein U1A / U1snRNP-specific protein,
identical to GB:Z49991 U1snRNP-specific protein (Arabidopsis thaliana)
DATA:                  Control 30min   2hours  2days   1week   p-value       pos
SENSE COUNTS:          4       3       8       5       5       7.11e-01
GENES (2 total):
  AT2G47580.1
    SENSE COUNTS:      4       3       8       5       5       7.11e-01
    TAGS: (2 total)
      d+1  GTTTGCGCAA  3       1       3       1       5       6.53e-01      861
           ----------                                                        466
           ----------                                                        460
           ----------                                                        440
      d+2  AGGAGAAAGG  1       2       5       4       0       2.68e-01      398

LOCUS: AT3G54490
DESCRIPTION: eukaryotic rpb5 RNA polymerase subunit family protein, similar to SP|P19388 DNA-directed RNA
polymerase II 23 kDa polypeptide (EC 2.7.7.6) {Homo sapiens}; contains Pfam profiles PF03871: RNA polymerase
Rpb5 N-terminal domain, PF01191: RNA polymerase Rpb5
DATA:                  Control 30min   2hours  2days   1week   p-value       pos
SENSE COUNTS:          5       2       2       2       3       7.12e-01
GENES (1 total):
  AT3G54490.1
    SENSE COUNTS:      5       2       2       2       3       7.12e-01
    TAGS: (1 total)
           ----------                                                        1158
      d+2  GAGATACGTA  5       2       2       2       3       7.12e-01      905
           ----------                                                        568
           ----------                                                        385
           ----------                                                        312

LOCUS: AT4G11330
DESCRIPTION: mitogen-activated protein kinase, putative / MAPK, putative (MPK5), similar to mitogen-activated
protein kinase homolog 5 (AtMPK5)(Arabidopsis thaliana) SWISS-PROT:Q39025; PMID:12119167; possible internal
deletion at position 161, missing one A residue; r
DATA:                  Control 30min   2hours  2days   1week   p-value       pos
SENSE COUNTS:          2       2       3       1       0       7.14e-01
GENES (2 total):
  AT4G11330.1
    SENSE COUNTS:      2       2       3       1       0       7.14e-01
    TAGS: (1 total)
           ----------                                                        1475
      d+2  TTGATCGTTG  2       2       3       1       0       7.14e-01      1313
           ----------                                                        156

LOCUS: AT1G35110
DESCRIPTION: Ulp1 protease family protein, similar to At5g28170, At1g44880, At3g42530, At4g19320, At5g36020,
At4g03970, At3g43010, At2g10350 ; contains Pfam profile PF02902: Ulp1 protease family, C-terminal catalytic
domain
DATA:                  Control 30min   2hours  2days   1week   p-value       pos
SENSE COUNTS:          5       2       4       5       7       7.25e-01
GENES (1 total):
  AT1G35110.1
    SENSE COUNTS:      5       2       4       5       7       7.25e-01
    TAGS: (2 total)
           ----------                                                        4727
      i+3  TCCAAAAAAA  4       2       4       5       7       7.49e-01      4682
           ----------                                                        4233
           ----------                                                        4137
           ----------                                                        3951
           ----------                                                        3885
           ----------                                                        3803
           ----------                                                        3726
           ----------                                                        3237
```

```
          ----------                                                  3171
          ----------                                                  3064
          ----------                                                  2746
          ----------                                                  2431
          ----------                                                  2409
          ----------                                                  2285
          ----------                                                  1785
          ----------                                                  1534
          ----------                                                  1215
          ----------                                                  976
      v+2 TTGATTTATT  1      0      0      0      0      4.28e-01     179
          ----------                                                  71

LOCUS: AT5G65940
DESCRIPTION: 3-hydroxyisobutyryl-coenzyme A hydrolase / CoA-thioester hydrolase (CHY1), identical to
gi:8572760; contains Pfam profile PF00388 enoyl-CoA hydratase/isomerase family protein
DATA:                 Control 30min  2hours 2days  1week  p-value      pos
SENSE COUNTS:         1      4      2      2      3      7.30e-01
GENES (2 total):
  AT5G65940.1
    SENSE COUNTS:     1      4      2      2      3      7.30e-01
    TAGS: (2 total)
      d+1 GCAAACGGAC  0      0      1      0      0      4.55e-01     1356
      d+2 GTGGAGCAGT  1      4      1      2      3      5.52e-01     1073
          ----------                                                  1061
          ----------                                                  957
          ----------                                                  449
          ----------                                                  408
          ----------                                                  380
          ----------                                                  317
          ----------                                                  216
          ----------                                                  140

LOCUS: AT1G23110
DESCRIPTION: hypothetical protein,
DATA:                 Control 30min  2hours 2days  1week  p-value      pos
SENSE COUNTS:         3      3      1      1      1      7.32e-01
GENES (1 total):
  AT1G23110.1
    SENSE COUNTS:     3      3      1      1      1      7.32e-01
    TAGS: (3 total)
          ----------                                                  1430
      v+2 CAATAAACTT  0      3      1      0      0      1.04e-01     1118
          ----------                                                  915
          ----------                                                  763
          ----------                                                  540
          ----------                                                  412
      v+2 AGTCTGCAAA  2      0      0      1      0      2.50e-01     354
      v+2 TATGACCAAT  1      0      0      0      1      3.83e-01     210
          ----------                                                  152

LOCUS: AT3G06510
DESCRIPTION: glycosyl hydrolase family 1 protein, similar to Beta-galactosidase (SP:P22498) (Sulfolobus
solfataricus}; almost identical to beta-glucosidase GB:AAF23823 GI:6685165 from (Arabidopsis thaliana)
DATA:                 Control 30min  2hours 2days  1week  p-value      pos
SENSE COUNTS:         10     5      6      8      8      7.37e-01
GENES (2 total):
  AT3G06510.1
    SENSE COUNTS:     10     5      6      8      8      7.37e-01
    TAGS: (1 total)
          ----------                                                  2016
          ----------                                                  1796
          ----------                                                  1582
          ----------                                                  1491
          ----------                                                  975
          ----------                                                  845
          ----------                                                  833
          ----------                                                  779
          ----------                                                  697
          ----------                                                  560
          ----------                                                  381
      X+4 ATGTTCGGAT  10     5      6      8      8      7.37e-01     351
          ----------                                                  280
          ----------                                                  228

LOCUS: AT2G37330
DESCRIPTION: expressed protein,  and genefinder
DATA:                 Control 30min  2hours 2days  1week  p-value      pos
SENSE COUNTS:         1      0      2      1      3      7.38e-01
GENES (1 total):
  AT2G37330.1
```

```
    SENSE COUNTS:      1      0      2      1      3      7.38e-01
    TAGS: (2 total)
           ----------                                                   1063
           ----------                                                   1036
      d+2  AAACTTAAAA  0      0      1      1      3      1.91e-01      968
      d+2  TCTTCTCCTC  1      0      1      0      0      8.46e-01      858
           ----------                                                   767
           ----------                                                   761
           ----------                                                   521
           ----------                                                   500
           ----------                                                   314
           ----------                                                   56

LOCUS: AT1G07210
DESCRIPTION: 30S ribosomal protein S18 family, contains Pfam profile: PF01084 ribosomal protein S18; similar
to 30S ribosomal protein S18 (SP:P80382) {Thermus thermophilus}
DATA:                  Control 30min  2hours  2days  1week  p-value       pos
SENSE COUNTS:          2      3      2      2      0      7.39e-01
GENES (1 total):
  AT1G07210.1
    SENSE COUNTS:      2      3      2      2      0      7.39e-01
    TAGS: (2 total)
           ----------                                                   1576
           ----------                                                   1394
           ----------                                                   1312
           ----------                                                   1259
      i+3  ATGAATCCCT  0      0      0      1      0      3.09e-01      1209
      d+2  GCTCTAGTTT  2      3      2      1      0      6.65e-01      1059
           ----------                                                   799
           ----------                                                   517

LOCUS: AT4G38680
DESCRIPTION: cold-shock DNA-binding family protein, contains Pfam domains PF00313: 'Cold-shock' DNA-binding
domain and PF00098: Zinc knuckle
DATA:                  Control 30min  2hours  2days  1week  p-value       pos
SENSE COUNTS:          2      3      2      2      0      7.39e-01
GENES (2 total):
  AT4G38680.1
    SENSE COUNTS:      2      3      2      2      0      7.39e-01
    TAGS: (2 total)
      X+4  GACTGGAAGG  1      0      0      0      0      4.28e-01      1382
      d+1  GCGAGAGACT  1      3      2      2      0      6.09e-01      752

LOCUS: AT5G23040
DESCRIPTION: expressed protein, similar to unknown protein (emb|CAB62636.1)
DATA:                  Control 30min  2hours  2days  1week  p-value       pos
SENSE COUNTS:          5      9      6      11     6      7.40e-01
GENES (2 total):
  AT5G23040.1
    SENSE COUNTS:      5      9      6      11     5      6.49e-01
    TAGS: (1 total)
      d+1  GACACTCGAG  5      9      6      11     5      6.49e-01      760
           ----------                                                   731
           ----------                                                   572
           ----------                                                   554
           ----------                                                   515
           ----------                                                   395
           ----------                                                   348
           ----------                                                   56
  AT5G23040.2
    SENSE COUNTS:      5      9      6      11     6      7.40e-01
    TAGS: (2 total)
      i+3  AATTTGTATT  0      0      0      0      1      1.65e-01      2310
      d+1  GACACTCGAG  5      9      6      11     5      6.49e-01      760
           ----------                                                   731
           ----------                                                   572
           ----------                                                   554
           ----------                                                   515
           ----------                                                   395
           ----------                                                   348
           ----------                                                   56

LOCUS: AT5G04550
DESCRIPTION: expressed protein, contains Pfam domain PF05003: protein of unknown function (DUF668)
DATA:                  Control 30min  2hours  2days  1week  p-value       pos
SENSE COUNTS:          3      3      1      1      3      7.40e-01
GENES (1 total):
  AT5G04550.1
    SENSE COUNTS:      3      3      1      1      3      7.40e-01
    TAGS: (1 total)
           ----------                                                   2535
```

```
      d+2  AGAGGTATTA 3       3       1       1       3       7.40e-01       2169
           ----------                                                        1770
           ----------                                                        1614
           ----------                                                        880
           ----------                                                        764
           ----------                                                        256
           ----------                                                        74

LOCUS: AT1G11650
DESCRIPTION: RNA-binding protein 45 (RBP45), putative, similar to gb|U90212 DNA binding protein ACBF from
Nicotiana tabacum and contains 3 PF|00076 RNA recognition motif domains. ESTs gb|T44278, gb|R65195, gb|N65904,
gb|H37499, gb|R90487, gb|N95952, gb|T44278, gb|Z201
DATA:                 Control 30min   2hours  2days   1week   p-value        pos
SENSE COUNTS:         9       12      7       12      7       7.44e-01
GENES (2 total):
  AT1G11650.1
    SENSE COUNTS:     9       12      7       12      7       7.44e-01
    TAGS: (2 total)
      i+3  TGTTTTGTGA 1       0       0       0       0       4.28e-01       2615
      d+1  GGGCCGAAGT 8       12      7       12      7       6.89e-01       1112
           ----------                                                        990
           ----------                                                        462
           ----------                                                        125
  AT1G11650.2
    SENSE COUNTS:     9       12      7       12      7       7.44e-01
    TAGS: (2 total)
      i+3  TGTTTTGTGA 1       0       0       0       0       4.28e-01       2615
      d+1  GGGCCGAAGT 8       12      7       12      7       6.89e-01       1111
           ----------                                                        990
           ----------                                                        462
           ----------                                                        125

LOCUS: AT3G59020
DESCRIPTION: importin beta-2 subunit family protein, similar to D-Importin 7/RanBP7 (Drosophila melanogaster)
GI:7542336; contains Pfam profile PF03810: Importin-beta N-terminal domain
DATA:                 Control 30min   2hours  2days   1week   p-value        pos
SENSE COUNTS:         3       4       2       1       1       7.48e-01
GENES (1 total):
  AT3G59020.1
    SENSE COUNTS:     3       4       2       1       1       7.48e-01
    TAGS: (3 total)
      d+1  TCTTAAGAGA 0       0       1       0       0       4.55e-01       3481
           ----------                                                        3242
           ----------                                                        3163
      d+2  GATGAATTCC 2       4       1       1       1       5.74e-01       2956
           ----------                                                        2849
           ----------                                                        2768
      d+2  CAAGGGCCAG 1       0       0       0       0       4.28e-01       2517
           ----------                                                        2182
           ----------                                                        2162
           ----------                                                        2113
           ----------                                                        1508
           ----------                                                        1226
           ----------                                                        1120
           ----------                                                        735
           ----------                                                        671
           ----------                                                        533
           ----------                                                        505

LOCUS: AT1G17850
DESCRIPTION: expressed protein
DATA:                 Control 30min   2hours  2days   1week   p-value        pos
SENSE COUNTS:         3       0       2       1       1       7.56e-01
GENES (1 total):
  AT1G17850.1
    SENSE COUNTS:     3       0       2       1       1       7.56e-01
    TAGS: (1 total)
           ----------                                                        2008
           ----------                                                        1949
      v+2  TACAAACAAA 3       0       2       1       1       7.56e-01       1749
           ----------                                                        1580
           ----------                                                        1534
           ----------                                                        838

LOCUS: AT1G68140
DESCRIPTION: expressed protein
DATA:                 Control 30min   2hours  2days   1week   p-value        pos
SENSE COUNTS:         2       3       3       1       5       7.60e-01
GENES (1 total):
  AT1G68140.1
    SENSE COUNTS:     2       3       3       1       5       7.60e-01
```

```
    TAGS: (1 total)
          ----------                                                       1581
      d+2  ATCAGTGATG  2      3      3      1      5      7.60e-01         1508
          ----------                                                       1435
          ----------                                                       976
          ----------                                                       799
          ----------                                                       717
          ----------                                                       581
          ----------                                                       465
          ----------                                                       442
          ----------                                                       399
          ----------                                                       330

LOCUS: AT5G64330
DESCRIPTION: non-phototropic hypocotyl 3 (NPH3), identical to non-phototropic hypocotyl 3 (Arabidopsis
thaliana) gi|6224712|gb|AAF05914, PMID:10542152
DATA:                  Control 30min  2hours 2days  1week  p-value         pos
SENSE COUNTS:          1      2      3      1      3      7.66e-01
GENES (2 total):
  AT5G64330.1
    SENSE COUNTS:      1      2      3      1      3      7.66e-01
    TAGS: (3 total)
          ----------                                                       3175
          ----------                                                       2952
          ----------                                                       2778
          ----------                                                       2753
          ----------                                                       2747
      d+2  TGGGTTGAAG  0      0      0      1      0      6.04e-01         2684
      d+2  AAGTGATAGT  1      2      2      0      3      5.90e-01         2472
          ----------                                                       2390
          ----------                                                       2107
          ----------                                                       1974
      d+2  AGAGGAAACG  0      0      1      0      0      4.55e-01         1919
          ----------                                                       1447
          ----------                                                       717
          ----------                                                       421
          ----------                                                       326
          ----------                                                       50

LOCUS: AT3G20362
DESCRIPTION: hypothetical protein, loosely supported by Brassica genome sequence alignments.
DATA:                  Control 30min  2hours 2days  1week  p-value         pos
SENSE COUNTS:          5      3      3      2      1      7.67e-01
GENES (1 total):
  AT3G20362.1
    SENSE COUNTS:      5      3      3      2      1      7.67e-01
    TAGS: (2 total)
      v+1  GATTATCCAT  0      1      0      0      0      2.54e-01         521
      v+2  TGATGAAGAG  5      2      3      2      1      7.02e-01         181

LOCUS: AT4G34100
DESCRIPTION: zinc finger (C3HC4-type RING finger) family protein, contains Pfam profile: PF00097 zinc finger,
C3HC4 type (RING finger)
DATA:                  Control 30min  2hours 2days  1week  p-value         pos
SENSE COUNTS:          4      3      3      1      4      7.78e-01
GENES (1 total):
  AT4G34100.1
    SENSE COUNTS:      4      3      3      1      4      7.78e-01
    TAGS: (3 total)
      v+1  TAAGCCTTAA  0      0      0      0      3      1.12e-02         3891
      v+2  TCTGGTTTAG  4      3      3      1      1      7.88e-01         3427
          ----------                                                       3270
          ----------                                                       2902
          ----------                                                       2812
          ----------                                                       2714
          ----------                                                       2562
      v+2  CTTCTGTTTC  0      0      0      0      0      6.15e-01         2334
          ----------                                                       2182
          ----------                                                       2049
          ----------                                                       2043
          ----------                                                       1869
          ----------                                                       1749
          ----------                                                       1457
          ----------                                                       964
          ----------                                                       793
          ----------                                                       486

LOCUS: AT1G18720
DESCRIPTION: expressed protein, similar to YGL010w-like protein GI:2982301 from (Picea mariana)
DATA:                  Control 30min  2hours 2days  1week  p-value         pos
SENSE COUNTS:          3      1      3      1      4      7.81e-01
```

```
GENES (1 total):
  AT1G18720.1
    SENSE COUNTS:      3       1       3       1       4       7.81e-01
    TAGS: (2 total)
      d+1  TATTACAATC  0       0       0       0       3       8.36e-02        861
           ----------                                                          829
      d+2  GATTGATGTA  3       1       3       1       1       8.54e-01        763
           ----------                                                          756
           ----------                                                          532
           ----------                                                          473
           ----------                                                          61

LOCUS: AT5G04410
DESCRIPTION: no apical meristem (NAM) family protein, contains Pfam PF02365: No apical meristem (NAM) protein;
supporting cDNA gi|6456750|gb|AF201456.1|AF201456
DATA:                  Control 30min   2hours  2days   1week   p-value         pos
SENSE COUNTS:          2       2       4       1       1       7.84e-01
GENES (2 total):
  AT5G04410.1
    SENSE COUNTS:      2       2       4       1       1       7.84e-01
    TAGS: (2 total)
      d+1  TTTCCCCCAC  1       0       0       0       0       4.28e-01        2276
           ----------                                                          2169
      d+2  GTGTCTGCTC  1       2       4       1       1       6.34e-01        1912
           ----------                                                          1864
           ----------                                                          1708
           ----------                                                          1579
           ----------                                                          1231
           ----------                                                          1179
           ----------                                                          917
           ----------                                                          620

LOCUS: AT5G41790
DESCRIPTION: COP1-interactive protein 1 / CIP1, almost identical to CIP1 (GI:836950) (Arabidopsis thaliana)
DATA:                  Control 30min   2hours  2days   1week   p-value         pos
SENSE COUNTS:          7       12      9       9       9       7.91e-01
GENES (2 total):
  AT5G41790.1
    SENSE COUNTS:      7       12      9       9       9       7.91e-01
    TAGS: (6 total)
      i+3  AATGCAAACA  0       1       0       1       0       4.31e-01        4073
      d+1  GTTTTGGACT  1       1       3       0       1       5.85e-01        4030
      d+2  TCAGAAAAAC  4       6       1       5       7       4.44e-01        3655
      d+2  AGACTTATCG  0       0       0       1       0       3.09e-01        3584
           ----------                                                          3577
           ----------                                                          3424
           ----------                                                          3359
           ----------                                                          2230
           ----------                                                          2054
           ----------                                                          1453
      d+2  GATGAATTGG  2       4       4       2       1       8.37e-01        1252
      d+2  TGAAAGAATT  0       0       1       0       0       4.55e-01        827
           ----------                                                          724
           ----------                                                          533
           ----------                                                          460
           ----------                                                          448
           ----------                                                          278

LOCUS: AT1G34575
DESCRIPTION: FAD-binding domain-containing protein, similar to SP|P30986 reticuline oxidase precursor
(Berberine-bridge-forming enzyme) (BBE) (Tetrahydroprotoberberine synthase) (Eschscholzia californica);
contains PF01565 FAD binding domain
DATA:                  Control 30min   2hours  2days   1week   p-value         pos
SENSE COUNTS:          4       1       4       2       3       7.91e-01
GENES (1 total):
  AT1G34575.1
    SENSE COUNTS:      4       1       4       2       3       7.91e-01
    TAGS: (1 total)
           ----------                                                          2334
      v+2  TTTAAGATTC  4       1       4       2       3       7.91e-01        1626
           ----------                                                          1422
           ----------                                                          1263
           ----------                                                          1011
           ----------                                                          924
           ----------                                                          738
           ----------                                                          685

LOCUS: AT1G01320
DESCRIPTION: tetratricopeptide repeat (TPR)-containing protein, low similarity to SP|P46825 Kinesin light
chain (KLC) {Loligo pealeii}; contains Pfam profile PF00515: TPR Domain
DATA:                  Control 30min   2hours  2days   1week   p-value         pos
```

```
SENSE COUNTS:          11      10      8       10      15      7.92e-01
GENES (2 total):
  AT1G01320.1
    SENSE COUNTS:      11      10      8       10      15      7.92e-01
    TAGS: (4 total)
      d+1  TTTAAACATT  9       5       2       4       14      4.44e-02       5712
      d+2  GGAAACTGAG  2       0       5       6       0       2.26e-02       5505
      d+2  TGAACAACGC  0       0       1       0       0       4.55e-01       5177
           ----------                                                         5035
           ----------                                                         4956
           ----------                                                         4795
           ----------                                                         4765
           ----------                                                         3212
           ----------                                                         3158
           ----------                                                         3071
           ----------                                                         3043
           ----------                                                         2994
           ----------                                                         2625
           ----------                                                         2533
           ----------                                                         2323
           ----------                                                         2219
           ----------                                                         2198
      d+2  AGATGGGAGC  0       5       0       0       1       1.16e-02       1651
           ----------                                                         1337
           ----------                                                         978
           ----------                                                         855
           ----------                                                         215

LOCUS: AT3G51660
DESCRIPTION: macrophage migration inhibitory factor family protein / MIF family protein, contains Pfam
profile: PF01187 Macrophage migration inhibitory factor family(MIF)
DATA:                  Control 30min   2hours  2days   1week   p-value        pos
SENSE COUNTS:          2       0       2       1       3       7.94e-01
GENES (1 total):
  AT3G51660.1
    SENSE COUNTS:      2       0       2       1       3       7.94e-01
    TAGS: (1 total)
           ----------                                                         560
      d+2  TTATTCTTTT  2       0       2       1       3       7.94e-01       489

LOCUS: AT4G37460
DESCRIPTION: tetratricopeptide repeat (TPR)-containing protein, contains Pfam profile PF00515: TPR Domain
DATA:                  Control 30min   2hours  2days   1week   p-value        pos
SENSE COUNTS:          4       1       5       2       5       7.95e-01
GENES (1 total):
  AT4G37460.1
    SENSE COUNTS:      4       1       5       2       5       7.95e-01
    TAGS: (4 total)
      d+1  AGCAGTAAGA  1       1       4       1       5       3.79e-01       3670
      d+2  ACATATTACT  0       0       1       0       0       4.55e-01       3271
           ----------                                                         3132
           ----------                                                         3032
      d+2  GATTAGGCGA  0       0       0       0       0       6.15e-01       1780
           ----------                                                         1652
      d+2  ATAATTTAGC  3       0       0       1       0       7.99e-02       955
           ----------                                                         926
           ----------                                                         757
           ----------                                                         724
           ----------                                                         646
           ----------                                                         415

LOCUS: AT3G24630
DESCRIPTION: hypothetical protein
DATA:                  Control 30min   2hours  2days   1week   p-value        pos
SENSE COUNTS:          2       3       1       1       1       7.97e-01
GENES (1 total):
  AT3G24630.1
    SENSE COUNTS:      2       3       1       1       1       7.97e-01
    TAGS: (1 total)
           ----------                                                         2951
      v+2  AGAAAAAAAT  2       3       1       1       1       7.97e-01       2730
           ----------                                                         2017
           ----------                                                         1991
           ----------                                                         1903
           ----------                                                         1883
           ----------                                                         222

LOCUS: AT1G79730
DESCRIPTION: hydroxyproline-rich glycoprotein family protein, contains proline-rich extensin domains,
INTERPRO:IPR002965
DATA:                  Control 30min   2hours  2days   1week   p-value        pos
```

```
SENSE COUNTS:          2       1       2       3       5       7.97e-01
GENES (2 total):
  AT1G79730.1
    SENSE COUNTS:      2       1       2       3       5       7.97e-01
    TAGS: (3 total)
      d+1  AGGTTGAACT  1       0       0       2       0       1.75e-01       1531
           ----------                                                         1400
      d+2  GTTCCTTCCT  0       0       1       1       0       7.90e-01       1363
           ----------                                                         1286
           ----------                                                         1130
           ----------                                                         1110
           ----------                                                         934
      X+4  TACACGTCCA  1       1       1       0       5       1.92e-01       312
           ----------                                                         200

LOCUS: AT5G51720
DESCRIPTION: expressed protein
DATA:                  Control 30min   2hours  2days   1week   p-value        pos
SENSE COUNTS:          2       3       5       5       2       7.99e-01
GENES (1 total):
  AT5G51720.1
    SENSE COUNTS:      2       3       5       5       2       7.99e-01
    TAGS: (2 total)
      i+3  CATAGTAGTG  0       0       0       0       1       1.65e-01       352
      d+1  TGAAGCACAA  2       3       5       5       1       6.61e-01       318

LOCUS: AT1G35160
DESCRIPTION: 14-3-3 protein GF14 phi (grf4), identical to GF14 protein phi chain GI:1493805, SP:P46077 from
(Arabidopsis thaliana)
DATA:                  Control 30min   2hours  2days   1week   p-value        pos
SENSE COUNTS:          4       3       4       1       5       8.01e-01
GENES (2 total):
  AT1G35160.1
    SENSE COUNTS:      4       3       4       1       5       8.01e-01
    TAGS: (3 total)
      i+3  AGCTTCTTGG  0       0       0       0       0       6.15e-01       1212
      d+1  CAGGACGAAA  4       3       3       1       5       7.80e-01       816
           ----------                                                         774
      d+2  TGACCACGAT  0       0       1       0       0       4.55e-01       346
           ----------                                                         186

LOCUS: AT1G11720
DESCRIPTION: starch synthase, putative, strong similarity to soluble-starch-synthase (Solanum tuberosum)
GI:1911166
DATA:                  Control 30min   2hours  2days   1week   p-value        pos
SENSE COUNTS:          3       3       5       2       1       8.01e-01
GENES (1 total):
  AT1G11720.1
    SENSE COUNTS:      3       3       5       2       1       8.01e-01
    TAGS: (2 total)
      d+1  GAACCGTCCT  2       0       2       2       0       6.88e-01       3027
           ----------                                                         2788
      i+3  GTCTCACCAT  1       3       3       0       1       4.71e-01       2781
           ----------                                                         2763
           ----------                                                         2663
           ----------                                                         2142
           ----------                                                         2117
           ----------                                                         2060
           ----------                                                         1946
           ----------                                                         1624
           ----------                                                         1586
           ----------                                                         1004
           ----------                                                         558
           ----------                                                         542
           ----------                                                         478

LOCUS: AT4G21450
DESCRIPTION: vesicle-associated membrane family protein / VAMP family protein, similar to VAP27 GI:6688926
(Nicotiana plumbaginifolia)
DATA:                  Control 30min   2hours  2days   1week   p-value        pos
SENSE COUNTS:          11      6       7       6       6       8.02e-01
GENES (2 total):
  AT4G21450.1
    SENSE COUNTS:      11      6       7       6       6       8.02e-01
    TAGS: (2 total)
           ----------                                                         1211
      d+2  TAAAAAAGAA  2       2       1       2       1       9.46e-01       1164
           ----------                                                         1124
           ----------                                                         1103
           ----------                                                         702
           ----------                                                         532
```

```
      X+4  TTAAGGACTA 9      4      6      4      5      6.36e-01      269
           ----------                                                  93
  AT4G21450.2
    SENSE COUNTS:     2      2      1      2      1      9.46e-01
    TAGS: (1 total)
           ----------                                                  1472
      d+2  TAAAAAAGAA 2      2      1      2      1      9.46e-01      1425
           ----------                                                  1385
           ----------                                                  1364
           ----------                                                  790
           ----------                                                  702
           ----------                                                  532
           ----------                                                  93

LOCUS: AT4G16170
DESCRIPTION: expressed protein
DATA:                 Control 30min  2hours 2days  1week  p-value       pos
SENSE COUNTS:         1      0      1      2      3      8.03e-01
GENES (1 total):
  AT4G16170.1
    SENSE COUNTS:     1      0      1      2      3      8.03e-01
    TAGS: (3 total)
      d+1  AGATGCTGTA 1      0      0      1      0      7.78e-01      1156
      d+2  TACTGGAAAC 0      0      1      1      3      4.30e-01      1130
           ----------                                                  1105
      d+2  AAAGACCTAT 0      0      0      0      0      6.15e-01      671
           ----------                                                  602
           ----------                                                  575
           ----------                                                  466
           ----------                                                  140
           ----------                                                  62
           ----------                                                  14

LOCUS: AT5G42190
DESCRIPTION: Similar to SKP1 in yeast and humans which are involved in mitotic cell cycle control and
ubiquitin mediated proteolysis.
DATA:                 Control 30min  2hours 2days  1week  p-value       pos
SENSE COUNTS:         8      5      4      4      3      8.04e-01
GENES (2 total):
  AT5G42190.1
    SENSE COUNTS:     8      5      4      4      3      8.04e-01
    TAGS: (3 total)
      d+1  TTCGATCCAG 0      0      0      1      0      3.09e-01      1277
           ----------                                                  838
      d+2  TCCTCGACCC 8      5      4      2      3      6.16e-01      596
      d+2  TCGAAGCTGC 0      0      0      1      0      3.09e-01      240

LOCUS: AT4G25740
DESCRIPTION: 40S ribosomal protein S10 (RPS10A), 40S ribosomal protein S10 - Lumbricus rubellus, PID:e1329701
DATA:                 Control 30min  2hours 2days  1week  p-value       pos
SENSE COUNTS:         3      1      4      2      1      8.06e-01
GENES (1 total):
  AT4G25740.1
    SENSE COUNTS:     3      1      4      2      1      8.06e-01
    TAGS: (1 total)
           ----------                                                  741
      d+2  CAGAGTTTCA 3      1      4      2      1      8.06e-01      186

LOCUS: AT1G07110
DESCRIPTION: fructose-6-phosphate 2-kinase / fructose-2,6-bisphosphatase (F2KP), identical to fructose-6-
phosphate 2-kinase/fructose-2,6-bisphosphatase (F2KP) (Arabidopsis thaliana) GI:13096098
DATA:                 Control 30min  2hours 2days  1week  p-value       pos
SENSE COUNTS:         5      3      2      5      5      8.07e-01
GENES (2 total):
  AT1G07110.1
    SENSE COUNTS:     5      3      2      5      5      8.07e-01
    TAGS: (2 total)
      i+3  CAAAATCAGC 0      0      0      0      0      6.15e-01      3645
      d+1  GACTGAATTC 5      3      2      5      5      7.51e-01      2386
           ----------                                                  2101
           ----------                                                  1843
           ----------                                                  1832
           ----------                                                  1408
           ----------                                                  1327
           ----------                                                  1316
           ----------                                                  1271
           ----------                                                  281

LOCUS: AT5G65890
DESCRIPTION: Member of ACT domain containing protein family. ACT domains are amino acid binding domains. Shows
strongest expression in flowers and siliques.
```

```
DATA:                 Control 30min   2hours  2days   1week   p-value         pos
SENSE COUNTS:         1       1       1       1       3       8.15e-01
GENES (2 total):
  AT5G65890.1
    SENSE COUNTS:     1       1       1       1       3       8.15e-01
    TAGS: (1 total)
           ----------                                                         1038
           ----------                                                         851
           ----------                                                         817
           ----------                                                         702
           ----------                                                         436
           ----------                                                         364
      X+4  TGTATCAGTG 1       1       1       1       3       8.15e-01        -80

LOCUS: AT2G05710
DESCRIPTION: aconitate hydratase, cytoplasmic, putative / citrate hydro-lyase/aconitase, putative, nearly
identical to SP|P49608 Aconitate hydratase, cytoplasmic (EC 4.2.1.3) (Citrate hydro-lyase) (Aconitase)
{Cucurbita maxima}; contains Pfam profiles PF00330: Aconita
DATA:                 Control 30min   2hours  2days   1week   p-value         pos
SENSE COUNTS:         9       11      10      10      5       8.16e-01
GENES (1 total):
  AT2G05710.1
    SENSE COUNTS:     9       11      10      10      5       8.16e-01
    TAGS: (2 total)
           ----------                                                         3488
      d+2  TTTCGCAACT 9       11      10      9       5       8.35e-01        3337
      d+2  CAATATATGA 0       0       0       1       0       3.09e-01        3289
           ----------                                                         3122
           ----------                                                         2848
           ----------                                                         2755
           ----------                                                         2683
           ----------                                                         2572
           ----------                                                         2443
           ----------                                                         2437
           ----------                                                         2388
           ----------                                                         2347
           ----------                                                         2305
           ----------                                                         1998
           ----------                                                         1819
           ----------                                                         1760
           ----------                                                         1656
           ----------                                                         1613
           ----------                                                         1424
           ----------                                                         1402
           ----------                                                         1336
           ----------                                                         1295
           ----------                                                         1186
           ----------                                                         982
           ----------                                                         910
           ----------                                                         472
           ----------                                                         196
           ----------                                                         103

LOCUS: AT1G09760
DESCRIPTION: U2 small nuclear ribonucleoprotein A, putative, identical to U2 small nuclear ribonucleoprotein
A' (U2 snRNP-A') (Arabidopsis thaliana) SWISS-PROT:P43333; supported by cDNA:gi_16649064_gb_AY059902.1_
DATA:                 Control 30min   2hours  2days   1week   p-value         pos
SENSE COUNTS:         6       3       6       6       4       8.21e-01
GENES (1 total):
  AT1G09760.1
    SENSE COUNTS:     6       3       6       6       4       8.21e-01
    TAGS: (4 total)
      i+3  ATGTTCCAGG 0       0       0       0       1       1.65e-01        1854
           ----------                                                         1535
           ----------                                                         1457
      d+2  TTATGCGTAG 1       0       1       0       0       6.01e-01        1190
      d+2  GAGAGCGAGG 5       3       4       6       3       8.35e-01        1033
      d+2  CGCAACAGTT 0       0       1       0       0       4.55e-01        924
           ----------                                                         803

LOCUS: AT4G01800
DESCRIPTION: preprotein translocase secA subunit, putative, similar to preprotein translocase secA subunit,
chloroplast (precursor) SP:Q9SYI0 from (Arabidopsis thaliana); non-consensus GA donor splice site at exon 4
DATA:                 Control 30min   2hours  2days   1week   p-value         pos
SENSE COUNTS:         3       1       1       1       1       8.23e-01
GENES (1 total):
  AT4G01800.1
    SENSE COUNTS:     3       1       1       1       1       8.23e-01
    TAGS: (2 total)
      v+1  AATCACGTCG 0       1       1       1       1       8.22e-01        2479
           ----------                                                         2213
```

```
          ----------                                                  2186
          ----------                                                  2088
          ----------                                                  1945
          ----------                                                  1770
      v+2  TGGTTACAGT  3       0       0       0       0       2.13e-02       955
          ----------                                                  753
          ----------                                                  81

LOCUS: AT3G61150
DESCRIPTION: homeobox-leucine zipper family protein / homeodomain GLABRA2 like protein 1 (HD-GL2-1), similar
to Anthocyaninless2 (ANL2) (GP:5702094) Arabidopsis thaliana, EMBL:AF077335
DATA:                 Control 30min   2hours  2days   1week   p-value        pos
SENSE COUNTS:         3       5       5       4       2       8.24e-01
GENES (1 total):
  AT3G61150.1
    SENSE COUNTS:     3       5       5       4       2       8.24e-01
    TAGS: (3 total)
      d+1  GAGGAAGGAG  0       2       2       2       1       6.83e-01       2431
          ----------                                                  2156
          ----------                                                  1852
          ----------                                                  1789
          ----------                                                  1653
          ----------                                                  1627
          ----------                                                  1417
      d+2  AGAATGCTTT  0       0       1       0       0       7.06e-01       677
      X+4  TCGATGAAAT  3       3       2       2       1       9.60e-01       554
          ----------                                                  262
          ----------                                                  163
          ----------                                                  111
          ----------                                                  71
          ----------                                                  65

LOCUS: AT4G27040
DESCRIPTION: expressed protein
DATA:                 Control 30min   2hours  2days   1week   p-value        pos
SENSE COUNTS:         3       1       1       1       1       8.26e-01
GENES (1 total):
  AT4G27040.1
    SENSE COUNTS:     3       1       1       1       1       8.26e-01
    TAGS: (2 total)
          ----------                                                  1735
          ----------                                                  1723
          ----------                                                  1676
          ----------                                                  1648
          ----------                                                  1593
          ----------                                                  1509
      d+2  TATCACTCTG  3       1       1       1       1       8.23e-01       1425
      d+2  CTCAGTTGTT  0       0       0       0       0       6.15e-01       1319
          ----------                                                  1222
          ----------                                                  1164
          ----------                                                  880
          ----------                                                  761

LOCUS: AT1G01930
DESCRIPTION: zinc finger protein-related, contains Pfam PF00023: Ankyrin repeat; contains Pfam PF00096: Zinc
finger, C2H2 type domain and Prosite PS00028: Zinc finger, C2H2 type, domain
DATA:                 Control 30min   2hours  2days   1week   p-value        pos
SENSE COUNTS:         1       0       3       2       3       8.34e-01
GENES (1 total):
  AT1G01930.1
    SENSE COUNTS:     1       0       3       2       3       8.34e-01
    TAGS: (3 total)
      i+3  TCAACATTTG  0       0       1       0       0       4.55e-01       2674
      d+1  AAGCCTTGTT  1       0       2       2       3       7.29e-01       1982
      d+2  ATGCTAAAGT  0       0       0       0       0       6.15e-01       1508
          ----------                                                  1432
          ----------                                                  1334
          ----------                                                  1133
          ----------                                                  980
          ----------                                                  580

LOCUS: AT4G13770
DESCRIPTION: cytochrome P450 family protein
DATA:                 Control 30min   2hours  2days   1week   p-value        pos
SENSE COUNTS:         5       6       7       3       3       8.35e-01
GENES (2 total):
  AT4G13770.1
    SENSE COUNTS:     5       6       7       3       3       8.35e-01
    TAGS: (3 total)
      d+1  ATGTCTTTTG  0       0       0       2       0       4.80e-02       1688
      d+2  ACTGGTCTTG  5       6       6       1       3       5.57e-01       1471
```

```
          ----------                                                  832
      d+2  TACGAGAGGA  0      0      1      0      0      4.55e-01       464
          ----------                                                  370
          ----------                                                  341

LOCUS: AT2G22430
DESCRIPTION: homeobox-leucine zipper protein 6 (HB-6) / HD-ZIP transcription factor 6, identical to homeobox-
leucine zipper protein ATHB-6 (HD-ZIP protein ATHB-6) (SP:P46668) (Arabidopsis thaliana)
DATA:                  Control 30min  2hours  2days  1week  p-value        pos
SENSE COUNTS:          5       6      6       7      3      8.38e-01
GENES (2 total):
  AT2G22430.1
    SENSE COUNTS:      5       6      6       7      3      8.38e-01
    TAGS: (3 total)
      d+1  GGCTCTTTAA  0       2      4       4      3      4.34e-01       1432
      d+2  TCAAAGGCAA  4       2      2       1      0      4.80e-01       1410
      d+2  AGGACTTTCT  1       2      0       2      0      3.83e-01       1060

LOCUS: AT1G79550
DESCRIPTION: phosphoglycerate kinase, putative, similar to SP|P41758 Phosphoglycerate kinase, chloroplast
precursor (EC 2.7.2.3) {Chlamydomonas reinhardtii}; contains Pfam profile PF00162: phosphoglycerate kinase
DATA:                  Control 30min  2hours  2days  1week  p-value        pos
SENSE COUNTS:          5       7      3       6      5      8.41e-01
GENES (3 total):
  AT1G79550.2
    SENSE COUNTS:      5       7      3       6      5      8.41e-01
    TAGS: (1 total)
           ----------                                                  1633
      d+2  GGTGTGTTTG  5       7      3       6      5      8.41e-01       1081
           ----------                                                  874
           ----------                                                  637
           ----------                                                  581
  AT1G79550.1
    SENSE COUNTS:      5       7      3       6      5      8.41e-01
    TAGS: (1 total)
           ----------                                                  1672
      d+2  GGTGTGTTTG  5       7      3       6      5      8.41e-01       1120
           ----------                                                  913
           ----------                                                  676
           ----------                                                  620

LOCUS: AT2G42490
DESCRIPTION: copper amine oxidase, putative, similar to copper methylamine oxidase precursor (MAOXII)
(Arthrobacter sp.) SWISS-PROT:Q07123
DATA:                  Control 30min  2hours  2days  1week  p-value        pos
SENSE COUNTS:          4       3      7       4      7      8.42e-01
GENES (1 total):
  AT2G42490.1
    SENSE COUNTS:      4       3      7       4      7      8.42e-01
    TAGS: (3 total)
      d+1  CGAATTGGAG  3       3      3       2      0      7.46e-01       2572
           ----------                                                  2526
           ----------                                                  2460
           ----------                                                  2392
           ----------                                                  1985
           ----------                                                  1713
           ----------                                                  1701
      d+2  GTGGTGATTG  1       0      4       1      7      9.22e-02       1208
           ----------                                                  1199
      d+2  GATCTAGTGA  0       0      0       1      0      3.09e-01       1034
           ----------                                                  944
           ----------                                                  870
           ----------                                                  653
           ----------                                                  355

LOCUS: AT5G52820
DESCRIPTION: WD-40 repeat family protein / notchless protein, putative, similar to notchless (Xenopus laevis)
GI:3687833; contains Pfam PF00400: WD domain, G-beta repeat (8 copies)
DATA:                  Control 30min  2hours  2days  1week  p-value        pos
SENSE COUNTS:          2       3      5       4      3      8.44e-01
GENES (1 total):
  AT5G52820.1
    SENSE COUNTS:      2       3      5       4      3      8.44e-01
    TAGS: (3 total)
      d+1  CAGATGAGGT  0       1      2       1      1      7.79e-01       1398
           ----------                                                  1272
           ----------                                                  1164
           ----------                                                  912
           ----------                                                  700
      i+3  ACGCCTCTGT  0       0      0       1      1      6.17e-01       307
      X+4  CACAAAAAAA  2       2      3       2      1      9.86e-01       305
```

```
          ----------                                                    152
          ----------                                                    77

LOCUS: AT2G17840
DESCRIPTION: senescence/dehydration-associated protein-related (ERD7), similar to senescence-associated
protein 12 (Hemerocallis hybrid cultivar) gi|3551958|gb|AAC34857; strong similarity to early-responsive to
dehydration stress ERD7 protein (Arabidopsis thaliana) gi
DATA:                 Control 30min  2hours  2days  1week  p-value      pos
SENSE COUNTS:         2       5      5       4      5      8.46e-01
GENES (2 total):
  AT2G17840.1
    SENSE COUNTS:     2       5      5       4      5      8.46e-01
    TAGS: (1 total)
          ----------                                                    1680
          ----------                                                    1637
      d+2 TAATGTCTCT  2       5      5       4      5      8.46e-01     1558
          ----------                                                    916
          ----------                                                    500

LOCUS: AT3G17040
DESCRIPTION: tetratricopeptide repeat (TPR)-containing protein, low similarity to SP|Q9FNS4 PsbB mRNA
maturation factor Mbb1, chloroplast precursor {Chlamydomonas reinhardtii}; contains Pfam profile: PF00515: TPR
Domain
DATA:                 Control 30min  2hours  2days  1week  p-value      pos
SENSE COUNTS:         5       5      7       9      5      8.53e-01
GENES (2 total):
  AT3G17040.1
    SENSE COUNTS:     5       5      7       9      5      8.53e-01
    TAGS: (3 total)
      d+1 AATAGAACCA  2       3      1       1      0      5.47e-01     1837
          ----------                                                    1626
          ----------                                                    1617
          ----------                                                    1274
          ----------                                                    1107
      i+3 AGTTTTGAAG  3       2      5       8      5      4.86e-01     1070
          ----------                                                    899
          ----------                                                    884
      d+2 GAATCTCCGG  0       0      1       0      0      4.55e-01     215
          ----------                                                    29

LOCUS: AT3G07790
DESCRIPTION: DGCR14-related, similar to DGCR14 protein (DiGeorge syndrome critical region 14) (ES2 protein)
(Swiss-Prot:Q96DF8) (Homo sapiens)
DATA:                 Control 30min  2hours  2days  1week  p-value      pos
SENSE COUNTS:         1       0      3       1      1      8.55e-01
GENES (1 total):
  AT3G07790.1
    SENSE COUNTS:     1       0      3       1      1      8.55e-01
    TAGS: (3 total)
      X+4 CTTTTTAAGA  1       0      0       0      0      6.89e-01     1930
      d+1 GGGTGAGATT  0       0      3       0      1      2.30e-01     1217
          ----------                                                    967
      d+2 GTGTTGAAGT  0       0      0       1      0      3.09e-01     571
          ----------                                                    448

LOCUS: AT1G51690
DESCRIPTION: serine/threonine protein phosphatase 2A (PP2A) 55 kDa regulatory subunit B, identical to 55 kDa B
regulatory subunit of phosphatase 2A (GI:710330) (Arabidopsis thaliana); similar to type 2A protein
serine/threonine phosphatase 55 kDa B regulatory GI:14084
DATA:                 Control 30min  2hours  2days  1week  p-value      pos
SENSE COUNTS:         2       0      3       3      1      8.57e-01
GENES (3 total):
  AT1G51690.2
    SENSE COUNTS:     2       0      3       3      1      8.57e-01
    TAGS: (4 total)
      i+3 AATAGAATAA  0       0      0       0      1      1.65e-01     3833
      i+3 TAATACTCAA  0       0      0       0      0      6.15e-01     2840
      d+1 GGGAGAAGGC  2       0      3       2      0      5.56e-01     1957
          ----------                                                    1917
          ----------                                                    1900
      d+2 TACTATGCTT  0       0      0       1      0      3.09e-01     1771
          ----------                                                    1586
          ----------                                                    1391
          ----------                                                    1357
          ----------                                                    941
          ----------                                                    935
          ----------                                                    893
          ----------                                                    786
          ----------                                                    548
  AT1G51690.1
    SENSE COUNTS:     2       0      3       3      1      8.57e-01
```

```
    TAGS: (4 total)
      i+3  AATAGAATAA 0      0      0      0      1      1.65e-01       3833
      i+3  TAATACTCAA 0      0      0      0      0      6.15e-01       2840
      d+1  GGGAGAAGGC 2      0      3      2      0      5.56e-01       1960
           ----------                                                 1920
           ----------                                                 1903
      d+2  TACTATGCTT 0      0      0      1      0      3.09e-01       1774
           ----------                                                 1589
           ----------                                                 1394
           ----------                                                 1360
           ----------                                                 944
           ----------                                                 938
           ----------                                                 896
           ----------                                                 786
           ----------                                                 548

LOCUS: AT1G24330
DESCRIPTION: armadillo/beta-catenin repeat family protein / U-box domain-containing family protein, contains
Pfam domain, PF00514: Armadillo/beta-catenin-like repeats and Pfam, PF04564: U-box domain
DATA:                 Control 30min  2hours  2days  1week  p-value        pos
SENSE COUNTS:         2      2      1      1      3      8.59e-01
GENES (1 total):
  AT1G24330.1
    SENSE COUNTS:     2      2      1      1      3      8.59e-01
    TAGS: (3 total)
      v+1  TAGATAGTCT 1      0      0      0      0      4.28e-01       2811
           ----------                                                 1792
           ----------                                                 1737
      v+2  TCTGACTCTG 0      0      1      0      0      4.55e-01       1449
      v+2  AGAAAATACT 1      2      0      1      3      4.74e-01       984
           ----------                                                 409
           ----------                                                 354
           ----------                                                 291

LOCUS: AT2G04080
DESCRIPTION: MATE efflux family protein, similar to hypothetical protein GB:AAC27412; contains Pfam profile
PF01554: Uncharacterized membrane protein family
DATA:                 Control 30min  2hours  2days  1week  p-value        pos
SENSE COUNTS:         2      2      1      1      3      8.59e-01
GENES (1 total):
  AT2G04080.1
    SENSE COUNTS:     2      2      1      1      3      8.59e-01
    TAGS: (2 total)
      d+1  TAATGTTATT 0      0      1      0      0      4.55e-01       1552
      d+2  TGCAAAAAAA 2      2      0      1      3      5.54e-01       1538
           ----------                                                 1426
           ----------                                                 743
           ----------                                                 738
           ----------                                                 616
           ----------                                                 545
           ----------                                                 223
           ----------                                                 117

LOCUS: AT5G61970
DESCRIPTION: signal recognition particle-related / SRP-related, low similarity to Signal recognition particle
68 kDa protein (SRP68) from Homo sapiens SP|Q9UHB9, Canis familiaris SP|Q00004
DATA:                 Control 30min  2hours  2days  1week  p-value        pos
SENSE COUNTS:         3      1      3      4      1      8.59e-01
GENES (1 total):
  AT5G61970.1
    SENSE COUNTS:     3      1      3      4      1      8.59e-01
    TAGS: (1 total)
           ----------                                                 2258
      d+2  AGATTTCTGT 3      1      3      4      1      8.59e-01       2138
           ----------                                                 1700
           ----------                                                 1160
           ----------                                                 738
           ----------                                                 518
           ----------                                                 510
           ----------                                                 482
           ----------                                                 416
           ----------                                                 341
           ----------                                                 329

LOCUS: AT2G43240
DESCRIPTION: nucleotide-sugar transporter family protein, weak similarity to SP|P78382 CMP-sialic acid
transporter {Homo sapiens}; contains Pfam profile PF04142: Nucleotide-sugar transporter
DATA:                 Control 30min  2hours  2days  1week  p-value        pos
SENSE COUNTS:         2      0      1      1      3      8.59e-01
GENES (1 total):
  AT2G43240.1
```

```
    SENSE COUNTS:      2       0       1       1       3       8.59e-01
    TAGS: (4 total)
      i+3  CAAGCTTTTG  0       0       0       0       0       6.15e-01       5271
      d+1  GTGTTATAAA  1       0       1       1       3       3.98e-01       2541
           ----------                                                         2260
           ----------                                                         2202
           ----------                                                         1321
           ----------                                                         1267
      d+2  ATCCTCTCCT  0       0       0       0       0       6.15e-01       803
      d+2  CAGGTTACTG  1       0       0       0       0       4.28e-01       616
           ----------                                                         268
           ----------                                                         181
           ----------                                                         148

LOCUS: AT4G38040
DESCRIPTION: exostosin family protein, contains Pfam profile: PF03016 Exostosin family
DATA:                  Control 30min   2hours  2days   1week   p-value        pos
SENSE COUNTS:          2       1       1       1       3       8.59e-01
GENES (1 total):
  AT4G38040.1
    SENSE COUNTS:      2       1       1       1       3       8.59e-01
    TAGS: (2 total)
      d+1  TACACCTCTT  1       1       1       0       0       7.28e-01       1419
           ----------                                                         1328
           ----------                                                         1303
           ----------                                                         1297
           ----------                                                         911
      d+2  ATGTTGGTGT  1       0       0       1       3       1.87e-01       680
           ----------                                                         675
           ----------                                                         245

LOCUS: AT3G11950
DESCRIPTION: UbiA prenyltransferase family protein, contains Pfam profile PF01040: UbiA prenyltransferase
family
DATA:                  Control 30min   2hours  2days   1week   p-value        pos
SENSE COUNTS:          2       0       1       1       3       8.59e-01
GENES (1 total):
  AT3G11950.1
    SENSE COUNTS:      2       0       1       1       3       8.59e-01
    TAGS: (3 total)
      i+3  CCTTTGATTG  0       0       1       0       0       4.55e-01       4339
      d+1  TACATACATC  2       0       0       0       3       2.30e-01       2956
           ----------                                                         2880
           ----------                                                         2763
           ----------                                                         2729
           ----------                                                         2689
           ----------                                                         2447
      i+3  GAGTGTATGT  0       0       0       1       0       6.04e-01       1868
           ----------                                                         1594
           ----------                                                         1522
           ----------                                                         1366
           ----------                                                         697
           ----------                                                         559
           ----------                                                         293

LOCUS: AT1G50020
DESCRIPTION: expressed protein
DATA:                  Control 30min   2hours  2days   1week   p-value        pos
SENSE COUNTS:          4       5       3       4       1       8.62e-01
GENES (1 total):
  AT1G50020.1
    SENSE COUNTS:      4       5       3       4       1       8.62e-01
    TAGS: (1 total)
           ----------                                                         1693
           ----------                                                         1647
           ----------                                                         1594
           ----------                                                         1558
      d+2  ATGGAAGTAG  4       5       3       4       1       8.62e-01       628
           ----------                                                         64

LOCUS: AT2G16740
DESCRIPTION: ubiquitin-conjugating enzyme, putative, strong similarity to SP|P35133 Ubiquitin-conjugating
enzyme E2-17 kDa 10 (EC 6.3.2.19) (Ubiquitin- protein ligase 10) (Ubiquitin carrier protein 10) {Arabidopsis
thaliana}; contains Pfam profile PF00179: Ubiquitin-c
DATA:                  Control 30min   2hours  2days   1week   p-value        pos
SENSE COUNTS:          1       3       2       2       1       8.64e-01
GENES (1 total):
  AT2G16740.1
    SENSE COUNTS:      1       3       2       2       1       8.64e-01
    TAGS: (1 total)
           ----------                                                         768
```

```
      d+2  GCTCGAAGCT 1       3       2       2       1       8.64e-01       570
           ----------                                                        221

LOCUS: AT3G55830
DESCRIPTION: glycosyltransferase family protein 47, similar to exostose-related protein 2, Homo sapiens,
PIR:JC5935 (SP|Q93063), EXTL2, Mus musculus (GI:10443633)
DATA:                 Control 30min   2hours  2days   1week   p-value        pos
SENSE COUNTS:         1       3       2       2       1       8.64e-01
GENES (1 total):
  AT3G55830.1
    SENSE COUNTS:     1       3       2       2       1       8.64e-01
    TAGS: (2 total)
      i+3  TATTGCCTTG 0       0       0       1       0       3.09e-01       1803
      d+1  GCAGTACATT 1       3       2       1       1       8.08e-01       1022
           ----------                                                        970
           ----------                                                        739
           ----------                                                        679
           ----------                                                        401
           ----------                                                        291
           ----------                                                        243

LOCUS: AT2G42100
DESCRIPTION: actin, putative, very strong similarity to SP|P53496 Actin 11 {Arabidopsis thaliana}, SP|P53493
Actin 3 {Arabidopsis thaliana}; contains Pfam profile PF00022: Actin
DATA:                 Control 30min   2hours  2days   1week   p-value        pos
SENSE COUNTS:         9       9       7       12      7       8.65e-01
GENES (1 total):
  AT2G42100.1
    SENSE COUNTS:     9       9       7       12      7       8.65e-01
    TAGS: (1 total)
           ----------                                                        1865
           ----------                                                        1528
           ----------                                                        1516
           ----------                                                        1209
           ----------                                                        1186
      v+2  GAAACTTCTG 9       9       7       12      7       8.65e-01       1167
           ----------                                                        939
           ----------                                                        880
           ----------                                                        729
           ----------                                                        606
           ----------                                                        333
           ----------                                                        329
           ----------                                                        220

LOCUS: AT3G19490
DESCRIPTION: sodium hydrogen antiporter, putative, similar to NhaD (Vibrio parahaemolyticus)
gi|3123728|dbj|BAA25994; Na+/H+ aniporter (NhaD) family member, PMID:11500563
DATA:                 Control 30min   2hours  2days   1week   p-value        pos
SENSE COUNTS:         3       1       2       1       3       8.67e-01
GENES (2 total):
  AT3G19490.1
    SENSE COUNTS:     3       1       2       1       3       8.67e-01
    TAGS: (1 total)
           ----------                                                        2607
           ----------                                                        2226
           ----------                                                        2078
      d+2  TACACTCGAT 3       1       2       1       3       8.67e-01       1994
           ----------                                                        1956
           ----------                                                        1707
           ----------                                                        1296
           ----------                                                        1137
           ----------                                                        1057
           ----------                                                        1013
           ----------                                                        921
           ----------                                                        721
           ----------                                                        301

LOCUS: AT5G47480
DESCRIPTION: expressed protein
DATA:                 Control 30min   2hours  2days   1week   p-value        pos
SENSE COUNTS:         7       5       5       6       3       8.72e-01
GENES (1 total):
  AT5G47480.1
    SENSE COUNTS:     7       5       5       6       3       8.72e-01
    TAGS: (3 total)
      d+1  AGTGGTAATG 2       0       0       1       0       2.50e-01       4323
      d+2  AAGCACTTTC 2       3       3       0       3       6.21e-01       4167
           ----------                                                        4147
      d+2  GAGCGGAAGC 3       2       2       5       0       4.54e-01       3933
           ----------                                                        3610
           ----------                                                        3419
```

```
           ----------                                                    3041
           ----------                                                    2443
           ----------                                                    2353
           ----------                                                    1867
           ----------                                                    1493
           ----------                                                    1094
           ----------                                                    1070
           ----------                                                    958
           ----------                                                    915

LOCUS: AT1G20100
DESCRIPTION: expressed protein
DATA:                 Control 30min  2hours  2days  1week  p-value       pos
SENSE COUNTS:         1       1      3       2      1      8.73e-01
GENES (1 total):
  AT1G20100.1
    SENSE COUNTS:     1       1      3       2      1      8.73e-01
    TAGS: (1 total)
           ----------                                                    1276
           ----------                                                    1246
           ----------                                                    1020
           ----------                                                    772
      i+3  TATGTTAGTT 1       1      3       2      1      8.73e-01      589

LOCUS: AT3G58670
DESCRIPTION: expressed protein
DATA:                 Control 30min  2hours  2days  1week  p-value       pos
SENSE COUNTS:         1       1      3       2      1      8.73e-01
GENES (1 total):
  AT3G58670.1
    SENSE COUNTS:     1       1      3       2      1      8.73e-01
    TAGS: (2 total)
      i+3  GTTTAAACCA 1       0      0       0      0      4.28e-01      2092
      d+1  GCTTGAAGAG 0       1      3       2      1      5.74e-01      864
           ----------                                                    770
           ----------                                                    520
           ----------                                                    478
           ----------                                                    443
           ----------                                                    214

LOCUS: AT5G53310
DESCRIPTION: myosin heavy chain-related, contains weak similarity to Myosin IB heavy chain (Swiss-Prot:P34092)
(Dictyostelium discoideum)
DATA:                 Control 30min  2hours  2days  1week  p-value       pos
SENSE COUNTS:         2       2      3       2      5      8.74e-01
GENES (1 total):
  AT5G53310.1
    SENSE COUNTS:     2       2      3       2      5      8.74e-01
    TAGS: (1 total)
           ----------                                                    1175
      d+2  TAACACAAGA 2       2      3       2      5      8.74e-01      855
           ----------                                                    792
           ----------                                                    537
           ----------                                                    501

LOCUS: AT5G13710
DESCRIPTION: sterol 24-C-methyltransferase, putative, similar to SP:P25087 Sterol 24-C-methyltransferase,
Delta(24)-sterol C- methyltransferase, Saccharomyces cerevisiae
DATA:                 Control 30min  2hours  2days  1week  p-value       pos
SENSE COUNTS:         8       5      5       4      4      8.75e-01
GENES (2 total):
  AT5G13710.1
    SENSE COUNTS:     8       5      5       4      4      8.75e-01
    TAGS: (2 total)
      i+3  AGTTTTGAAA 0       0      0       0      1      1.65e-01      1617
      d+1  CCCAAAATAT 8       5      5       4      3      7.94e-01      1300
           ----------                                                    1292
           ----------                                                    1265
           ----------                                                    1249
           ----------                                                    960
           ----------                                                    878
           ----------                                                    622
           ----------                                                    561
           ----------                                                    536
           ----------                                                    231
           ----------                                                    190
           ----------                                                    181

LOCUS: AT3G14067
DESCRIPTION: subtilase family protein, contains similarity to cucumisin-like serine protease GI:3176874 from
(Arabidopsis thaliana)
```

```
DATA:                 Control 30min   2hours  2days   1week   p-value         pos
SENSE COUNTS:         2       5       3       4       2       8.75e-01
GENES (1 total):
  AT3G14067.1
    SENSE COUNTS:     2       5       3       4       2       8.75e-01
    TAGS: (3 total)
      v+1  GTTATCAAAT 1       0       0       0       1       3.83e-01        2963
           ----------                                                         2831
           ----------                                                         2765
      v+2  AATTCGGGTC 1       5       3       4       0       3.12e-01        2593
      v+2  TGAAACGAGC 0       0       0       0       1       1.65e-01        2312
           ----------                                                         1355
           ----------                                                         1167
           ----------                                                         1003
           ----------                                                         961
           ----------                                                         586
           ----------                                                         460
           ----------                                                         354
           ----------                                                         169
           ----------                                                         46
           ----------                                                         41

LOCUS: AT5G51540
DESCRIPTION: peptidase M3 family protein / thimet oligopeptidase family protein, low similarity to SP|Q99797
Mitochondrial intermediate peptidase, mitochondrial precursor (EC 3.4.24.59) {Homo sapiens}; contains Pfam
profile PF01432: Peptidase family M3
DATA:                 Control 30min   2hours  2days   1week   p-value         pos
SENSE COUNTS:         2       0       1       2       3       8.77e-01
GENES (1 total):
  AT5G51540.1
    SENSE COUNTS:     2       0       1       2       3       8.77e-01
    TAGS: (4 total)
           ----------                                                         3379
      v+2  GGCTGACCTG 0       0       0       1       0       3.09e-01        3180
           ----------                                                         3157
           ----------                                                         3132
      v+2  GATATTTGAG 1       0       1       1       3       7.06e-01        2949
           ----------                                                         2670
      v+2  GTGAAGGCAT 1       0       0       0       0       4.28e-01        2419
           ----------                                                         2353
           ----------                                                         2179
           ----------                                                         2040
           ----------                                                         1943
           ----------                                                         1837
           ----------                                                         1406
           ----------                                                         1207
           ----------                                                         1165
           ----------                                                         934
           ----------                                                         579
      i+3  AATTATGATG 0       0       0       0       0       6.15e-01        262
           ----------                                                         237
           ----------                                                         83

LOCUS: AT5G62440
DESCRIPTION: expressed protein
DATA:                 Control 30min   2hours  2days   1week   p-value         pos
SENSE COUNTS:         2       3       4       5       4       8.77e-01
GENES (1 total):
  AT5G62440.1
    SENSE COUNTS:     2       3       4       5       4       8.77e-01
    TAGS: (3 total)
      d+1  GTGGAGGTCG 1       1       1       1       1       9.93e-01        608
           ----------                                                         577
      d+2  GTGCTGCTAG 0       0       1       0       0       4.55e-01        382
      d+2  GAAGTCGAAA 1       2       2       4       3       8.08e-01        142

LOCUS: AT1G45000
DESCRIPTION: 26S proteasome regulatory complex subunit p42D, putative, similar to 26S proteasome regulatory
complex subunit p42D (Drosophila melanogaster) gi|6434958|gb|AAF08391
DATA:                 Control 30min   2hours  2days   1week   p-value         pos
SENSE COUNTS:         2       3       3       4       1       8.81e-01
GENES (1 total):
  AT1G45000.1
    SENSE COUNTS:     2       3       3       4       1       8.81e-01
    TAGS: (4 total)
      i+3  TTTCAAGTGG 1       0       0       0       0       4.28e-01        1823
      d+1  CATCCTGTAT 0       0       1       0       0       4.55e-01        1485
      d+2  AAGGCTGTGA 1       3       2       4       1       7.56e-01        1199
           ----------                                                         1188
      d+2  GAGAAATAGA 0       0       0       0       0       6.15e-01        1065
           ----------                                                         791
```

```
          ----------                                                     557
          ----------                                                     468
          ----------                                                     461

LOCUS: AT3G07460
DESCRIPTION: expressed protein, contains Pfam profile PF04398: Protein of unknown function, DUF538
DATA:                 Control 30min  2hours  2days  1week  p-value       pos
SENSE COUNTS:         8       7      9       5      7      8.82e-01
GENES (2 total):
  AT3G07460.1
    SENSE COUNTS:     8       7      9       5      7      8.82e-01
    TAGS: (2 total)
          ----------                                                     1049
      d+2  TAAGCACAGA 8       7      8       5      7      9.52e-01      986
      d+2  GTCAGATGCT 0       0      1       0      0      4.55e-01      752
          ----------                                                     745
          ----------                                                     300
  AT3G07460.2
    SENSE COUNTS:     8       7      9       5      7      8.82e-01
    TAGS: (2 total)
          ----------                                                     1038
      d+2  TAAGCACAGA 8       7      8       5      7      9.52e-01      975
      d+2  GTCAGATGCT 0       0      1       0      0      4.55e-01      741
          ----------                                                     734
          ----------                                                     300

LOCUS: AT2G31200
DESCRIPTION: actin depolymerizing factor 6 (ADF6) mRNA, complete cds
DATA:                 Control 30min  2hours  2days  1week  p-value       pos
SENSE COUNTS:         6       5      4       6      3      8.85e-01
GENES (2 total):
  AT2G31200.1
    SENSE COUNTS:     6       5      4       6      3      8.85e-01
    TAGS: (3 total)
      d+1  ATGAGAGAGT 0       0      1       0      0      4.55e-01      816
      d+2  TTCTATGTCA 5       5      3       5      3      9.27e-01      656
      d+2  TCTTTCAGAG 1       0      0       1      0      5.06e-01      122

LOCUS: AT2G26650
DESCRIPTION: Shaker-like inward rectifying  potassium channel predominantly expressed in predominantly in root
hairs and root endodermis.
DATA:                 Control 30min  2hours  2days  1week  p-value       pos
SENSE COUNTS:         1       0      2       2      3      8.87e-01
GENES (2 total):
  AT2G26650.1
    SENSE COUNTS:     1       0      2       2      3      8.87e-01
    TAGS: (6 total)
      d+1  GGTTCTTCTC 0       0      0       0      0      6.15e-01      3112
          ----------                                                     3095
      d+2  AGAGGAGAGT 0       0      2       0      0      1.21e-01      2254
          ----------                                                     2218
      d+2  GTTGGACGCC 0       0      0       1      0      3.09e-01      2179
          ----------                                                     2050
          ----------                                                     1957
          ----------                                                     1927
          ----------                                                     1668
          ----------                                                     1593
          ----------                                                     1239
      d+2  GAACAAGTCG 0       0      0       1      0      3.09e-01      1021
          ----------                                                     1002
          ----------                                                     870
      d+2  TTTCTATTAC 1       0      0       0      3      7.14e-02      758
          ----------                                                     624
      d+2  ACATTCTTTG 0       0      0       0      0      6.15e-01      456

LOCUS: AT4G04313
DESCRIPTION: copia-like retrotransposon family, has a 8.8e-52 P-value blast match to GB:CAA32025 ORF
(Ty1_Copia-element) (Nicotiana tabacum)GB:CAA32025 ORF (Ty1_Copia-element) (Nicotiana tabacum)
DATA:                 Control 30min  2hours  2days  1week  p-value       pos
SENSE COUNTS:         3       2      4       4      1      9.02e-01
GENES (1 total):
  AT4G04313.1
    SENSE COUNTS:     3       2      4       4      1      9.02e-01
    TAGS: (1 total)
          ----------                                                     4354
          ----------                                                     4295
          ----------                                                     4169
          ----------                                                     4126
          ----------                                                     3829
          ----------                                                     3661
          ----------                                                     3415
```

```
          ----------                                                     3239
          ----------                                                     3233
          ----------                                                     3188
          ----------                                                     2979
          ----------                                                     2892
          ----------                                                     2741
          ----------                                                     2723
          ----------                                                     2696
          ----------                                                     2651
          ----------                                                     2277
      p+2 CAAAATCACT 3       2       4       4       1       9.02e-01    2033
          ----------                                                     1987
          ----------                                                     1916
          ----------                                                     1907
          ----------                                                     1879
          ----------                                                     1805
          ----------                                                     1727
          ----------                                                     1693
          ----------                                                     1671
          ----------                                                     1551
          ----------                                                     1511
          ----------                                                     1435
          ----------                                                     1417
          ----------                                                     1311
          ----------                                                     913
          ----------                                                     900
          ----------                                                     880
          ----------                                                     867
          ----------                                                     789
          ----------                                                     523
          ----------                                                     492
          ----------                                                     350
          ----------                                                     225
          ----------                                                     145

LOCUS: AT5G01350
DESCRIPTION: expressed protein
DATA:                Control 30min   2hours  2days   1week   p-value     pos
SENSE COUNTS:        4       4       7       5       7       9.08e-01
GENES (1 total):
  AT5G01350.1
    SENSE COUNTS:    4       4       7       5       7       9.08e-01
    TAGS: (2 total)
      i+3 ACGTTAAACG 1       0       0       0       0       6.89e-01    867
      d+1 GGTCAGCTCA 3       4       7       5       7       7.28e-01    360
          ----------                                                     185

LOCUS: AT2G20230
DESCRIPTION: expressed protein
DATA:                Control 30min   2hours  2days   1week   p-value     pos
SENSE COUNTS:        10      9       6       8       7       9.10e-01
GENES (1 total):
  AT2G20230.1
    SENSE COUNTS:    10      9       6       8       7       9.10e-01
    TAGS: (1 total)
          ----------                                                     1593
          ----------                                                     1583
      d+2 TGTCCATATG 10      9       6       8       7       9.10e-01    1069
          ----------                                                     1053
          ----------                                                     828
          ----------                                                     537
          ----------                                                     518
          ----------                                                     481
          ----------                                                     334
          ----------                                                     244

LOCUS: AT5G41520
DESCRIPTION: 40S ribosomal protein S10 (RPS10B), contains similarity to 40S ribosomal protein S10
DATA:                Control 30min   2hours  2days   1week   p-value     pos
SENSE COUNTS:        3       4       4       2       4       9.20e-01
GENES (1 total):
  AT5G41520.1
    SENSE COUNTS:    3       4       4       2       4       9.20e-01
    TAGS: (2 total)
      d+1 AATCTTTTTA 3       0       2       2       3       7.54e-01    786
      d+2 ATCATATCAG 0       4       2       0       1       1.51e-01    82

LOCUS: AT5G60430
DESCRIPTION: expressed protein
DATA:                Control 30min   2hours  2days   1week   p-value     pos
SENSE COUNTS:        2       1       3       1       1       9.23e-01
```

```
GENES (1 total):
  AT5G60430.1
    SENSE COUNTS:      2      1      3      1      1      9.23e-01
    TAGS: (1 total)
           ----------                                                  292
      d+2  TGAGTCAGAT  2      1      3      1      1      9.23e-01     242
           ----------                                                  67

LOCUS: AT2G46540
DESCRIPTION: expressed protein
DATA:                  Control 30min  2hours 2days  1week  p-value      pos
SENSE COUNTS:          3      2      2      1      2      9.23e-01
GENES (1 total):
  AT2G46540.1
    SENSE COUNTS:      3      2      2      1      2      9.23e-01
    TAGS: (2 total)
      i+3  TCTTCCATTA  0      0      0      0      1      1.65e-01     420
      d+1  TGTGTACACT  3      2      2      1      1      9.32e-01     417
           ----------                                                  101

LOCUS: AT1G06150
DESCRIPTION: pentatricopeptide (PPR) repeat-containing protein, contains Pfam profile PF01535: PPR repeat
DATA:                  Control 30min  2hours 2days  1week  p-value      pos
SENSE COUNTS:          3      2      2      3      1      9.27e-01
GENES (1 total):
  AT1G06150.1
    SENSE COUNTS:      3      2      2      3      1      9.27e-01
    TAGS: (4 total)
           ----------                                                  4236
      i+3  ATCTGAATAG  0      0      0      0      1      1.65e-01     4202
      d+2  GTTCATCTAT  0      0      0      0      0      6.15e-01     4029
           ----------                                                  3981
           ----------                                                  3838
           ----------                                                  3503
           ----------                                                  3214
           ----------                                                  2988
           ----------                                                  2892
           ----------                                                  2742
           ----------                                                  2606
           ----------                                                  2460
           ----------                                                  2326
           ----------                                                  2298
           ----------                                                  2093
      X+4  AAAGTAGGAA  0      0      0      1      0      3.09e-01     1813
           ----------                                                  1432
           ----------                                                  1257
           ----------                                                  1206
           ----------                                                  1075
           ----------                                                  1003
      d+2  TACACTCTCT  3      2      2      2      0      7.66e-01     724
           ----------                                                  685
           ----------                                                  679
           ----------                                                  667
           ----------                                                  637
           ----------                                                  145

LOCUS: AT1G34030
DESCRIPTION: 40S ribosomal protein S18 (RPS18B), similar to ribosomal protein S18 GI:38422 from (Homo sapiens)
DATA:                  Control 30min  2hours 2days  1week  p-value      pos
SENSE COUNTS:          3      6      4      4      5      9.29e-01
GENES (1 total):
  AT1G34030.1
    SENSE COUNTS:      3      6      4      4      5      9.29e-01
    TAGS: (2 total)
      i+3  TTGAACCTTG  0      0      1      0      0      4.55e-01     690
      d+1  AAGCTCAGGG  3      6      3      4      5      8.69e-01     401
           ----------                                                  290
           ----------                                                  242

LOCUS: AT1G66550
DESCRIPTION: WRKY family transcription factor, similar to DNA-binding protein 3 (Nicotiana tabacum) GI:7406995
DATA:                  Control 30min  2hours 2days  1week  p-value      pos
SENSE COUNTS:          2      1      2      1      3      9.35e-01
GENES (2 total):
  AT1G66550.1
    SENSE COUNTS:      2      1      2      1      3      9.35e-01
    TAGS: (2 total)
           ----------                                                  1482
           ----------                                                  1275
      v+2  CATTAAATTT  0      1      1      1      3      5.27e-01     1231
           ----------                                                  1169
```

```
          ----------                                                        996
          ----------                                                        871
          ----------                                                        847
     i+3  TAAATAGAGT  2      0      1      0      0      2.87e-01           607
          ----------                                                        564
          ----------                                                        544

LOCUS: AT2G39030
DESCRIPTION: GCN5-related N-acetyltransferase (GNAT) family protein, similar to SP|Q9SMB8 Tyramine N-
feruloyltransferase 4/11 (EC 2.3.1.110) (Hydroxycinnamoyl- CoA: tyramine N-hydroxycinnamoyltransferase)
{Nicotiana tabacum}; contains Pfam profile PF00583: acetyltrans
DATA:                 Control 30min  2hours 2days  1week  p-value           pos
SENSE COUNTS:         3      0      2      2      3      9.36e-01
GENES (1 total):
  AT2G39030.1
    SENSE COUNTS:     3      0      2      2      3      9.36e-01
    TAGS: (3 total)
      X+4  ATAATCAAAT 0      0      0      0      0      6.15e-01           1188
           ----------                                                       915
      d+2  AGACCCATCG 0      0      1      2      0      4.23e-01           740
           ----------                                                       728
      d+2  AGAGAACCTT 3      0      1      0      3      1.34e-01           499
           ----------                                                       308

LOCUS: AT1G56660
DESCRIPTION: expressed protein
DATA:                 Control 30min  2hours 2days  1week  p-value           pos
SENSE COUNTS:         4      2      4      3      4      9.40e-01
GENES (1 total):
  AT1G56660.1
    SENSE COUNTS:     4      2      4      3      4      9.40e-01
    TAGS: (5 total)
      d+1  TCTCTTGGTA 2      0      0      0      1      2.03e-01           1748
      d+2  ATGCAACAGA 0      1      3      0      1      3.22e-01           994
      d+2  TGCGGAGGAA 1      0      0      2      0      4.17e-01           848
           ----------                                                       778
      d+2  AGGATGTATC 0      1      1      0      1      6.08e-01           550
      d+2  AGAAGGAACA 1      0      0      1      1      5.50e-01           406
           ----------                                                       361
           ----------                                                       349
           ----------                                                       115

LOCUS: AT3G51000
DESCRIPTION: epoxide hydrolase, putative, similar to epoxide hydrolase (Glycine max) GI:2764806; contains Pfam
profile PF00561: hydrolase, alpha/beta fold family
DATA:                 Control 30min  2hours 2days  1week  p-value           pos
SENSE COUNTS:         3      1      3      2      3      9.42e-01
GENES (1 total):
  AT3G51000.1
    SENSE COUNTS:     3      1      3      2      3      9.42e-01
    TAGS: (1 total)
           ----------                                                       1234
      d+2  ATGCTCTAGT 3      1      3      2      3      9.42e-01           1042
           ----------                                                       740
           ----------                                                       540
           ----------                                                       342
           ----------                                                       192
           ----------                                                       151
           ----------                                                       135
           ----------                                                       35

LOCUS: AT4G02510
DESCRIPTION: chloroplast outer membrane protein, putative, similar to chloroplast protein import component
Toc159 (Pisum sativum) GI:8489806, chloroplast outer envelope protein 86 (Pisum sativum) GI:599958, GTP-
binding protein (Pisum sativum) GI:576509
DATA:                 Control 30min  2hours 2days  1week  p-value           pos
SENSE COUNTS:         15     11     12     12     15     9.46e-01
GENES (2 total):
  AT4G02510.1
    SENSE COUNTS:     15     11     12     12     15     9.46e-01
    TAGS: (5 total)
      d+1  TATCAGTAAT 6      1      5      4      10     1.63e-01           4664
      d+2  TTTTTCCCCC 9      5      4      6      5      7.31e-01           4571
           ----------                                                       4507
           ----------                                                       4456
      d+2  GCAGGGTTCG 0      0      0      1      0      3.09e-01           3967
      d+2  ACTGTGGATA 0      0      1      0      0      4.55e-01           3809
           ----------                                                       3797
      d+2  CGGCTTCTGC 0      5      2      1      0      7.84e-02           2948
           ----------                                                       716
```

```
LOCUS: AT4G29950
DESCRIPTION: microtubule-associated protein, identical to microtubule-associated protein GI:5032258 from
(Arabidopsis thaliana); similar to TBC1 domain family member 5 (Swiss-Prot:Q92609) (Homo sapiens); contains
Pfam profile PF00566: TBC domain
DATA:                  Control 30min   2hours  2days   1week   p-value         pos
SENSE COUNTS:          3       4       5       5       2       9.47e-01
GENES (2 total):
  AT4G29950.1
    SENSE COUNTS:      3       4       5       5       2       9.47e-01
    TAGS: (3 total)
      d+1  TGTAAGATGT  0       0       1       1       1       5.61e-01        3069
      d+2  ACTGCTCTGA  3       3       4       4       1       9.44e-01        2853
           ----------                                                          2367
           ----------                                                          1225
           ----------                                                          1197
           ----------                                                          1183
           ----------                                                          1176
           ----------                                                          895
           ----------                                                          862
           ----------                                                          795
           ----------                                                          686
      d+2  AGAAAGCAAA  0       1       0       0       0       2.54e-01        96
  AT4G29950.2
    SENSE COUNTS:      3       4       5       5       2       9.47e-01
    TAGS: (3 total)
      d+1  TGTAAGATGT  0       0       1       1       1       5.61e-01        3065
      d+2  ACTGCTCTGA  3       3       4       4       1       9.44e-01        2849
           ----------                                                          2363
           ----------                                                          1221
           ----------                                                          1193
           ----------                                                          1179
           ----------                                                          1172
           ----------                                                          891
           ----------                                                          858
           ----------                                                          791
           ----------                                                          682
      d+2  AGAAAGCAAA  0       1       0       0       0       2.54e-01        96

LOCUS: AT5G14440
DESCRIPTION: surfeit locus 2 protein-related / SURF2 protein-related, contains weak hit to Pfam profile
PF05477: Surfeit locus protein 2 (SURF2)
DATA:                  Control 30min   2hours  2days   1week   p-value         pos
SENSE COUNTS:          3       5       5       5       3       9.47e-01
GENES (1 total):
  AT5G14440.1
    SENSE COUNTS:      3       5       5       5       3       9.47e-01
    TAGS: (1 total)
           ----------                                                          1496
           ----------                                                          1055
      d+2  AAGAACTCGG  3       5       5       5       3       9.47e-01        809
           ----------                                                          788
           ----------                                                          551

LOCUS: AT3G56190
DESCRIPTION: Encodes one of two alpha-SNAPs (soluble NSF attachment protein) in Arabidopsis
DATA:                  Control 30min   2hours  2days   1week   p-value         pos
SENSE COUNTS:          5       1       4       3       2       9.53e-01
GENES (2 total):
  AT3G56190.1
    SENSE COUNTS:      5       1       4       3       2       9.53e-01
    TAGS: (4 total)
      d+1  TTTTCTTGCT  0       0       2       1       1       7.15e-01        1429
      d+2  TCTCGATTAT  4       1       2       2       0       4.95e-01        1335
           ----------                                                          1111
      i+3  CGTCCTAATA  0       0       0       0       1       4.65e-01        1052
           ----------                                                          981
           ----------                                                          968
           ----------                                                          951
           ----------                                                          774
           ----------                                                          501
           ----------                                                          382
           ----------                                                          317
      X+4  TAAGCTTTTC  1       0       0       0       0       4.28e-01        -195

LOCUS: AT3G52880
DESCRIPTION: monodehydroascorbate reductase, putative, monodehydroascorbate reductase (NADH), Lycoperison
esculentum, PIR:T06407
DATA:                  Control 30min   2hours  2days   1week   p-value         pos
SENSE COUNTS:          4       1       3       2       3       9.54e-01
GENES (1 total):
  AT3G52880.1
```

```
    SENSE COUNTS:      4       1       3       2       3       9.54e-01
    TAGS: (2 total)
           ----------                                                 1554
      d+2  AGAAGAGTCT  3       1       3       2       3       9.42e-01      1501
      d+2  GAGGGAGGTA  1       0       0       0       0       6.89e-01      1276
           ----------                                                 1155
           ----------                                                 1031
           ----------                                                 685

LOCUS: AT3G44100
DESCRIPTION: MD-2-related lipid recognition domain-containing protein / ML domain-containing protein, contains
Pfam profile PF02221: ML domain
DATA:                  Control 30min   2hours  2days   1week   p-value       pos
SENSE COUNTS:          3       4       5       3       2       9.69e-01
GENES (1 total):
  AT3G44100.1
    SENSE COUNTS:      3       4       5       3       2       9.69e-01
    TAGS: (2 total)
      d+1  AAACAAAGAG  1       0       3       1       1       4.06e-01      591
      d+2  TCCGGTTGCA  2       4       2       2       1       8.51e-01      319
           ----------                                                 297

LOCUS: AT3G49260
DESCRIPTION: calmodulin-binding family protein, low similarity to SF16 protein (Helianthus annuus) GI:560150;
contains Pfam profile PF00612: IQ calmodulin-binding motif
DATA:                  Control 30min   2hours  2days   1week   p-value       pos
SENSE COUNTS:          3       2       2       3       3       9.73e-01
GENES (2 total):
  AT3G49260.1
    SENSE COUNTS:      3       2       2       3       3       9.73e-01
    TAGS: (2 total)
           ----------                                                 1689
      d+2  TGTGGTTGGT  3       1       1       2       3       8.07e-01      1612
           ----------                                                 1567
      d+2  AAGTGAATCG  0       1       1       1       0       6.84e-01      832
           ----------                                                 721
           ----------                                                 352
           ----------                                                 289
  AT3G49260.2
    SENSE COUNTS:      3       2       2       3       3       9.73e-01
    TAGS: (2 total)
           ----------                                                 1775
      d+2  TGTGGTTGGT  3       1       1       2       3       8.07e-01      1698
           ----------                                                 1653
      d+2  AAGTGAATCG  0       1       1       1       0       6.84e-01      918
           ----------                                                 807
           ----------                                                 438
           ----------                                                 375
           ----------                                                 93
           ----------                                                 52

LOCUS: AT5G54280
DESCRIPTION: myosin heavy chain, putative, similar to myosin (Arabidopsis thaliana) gi|499045|emb|CAA84065
DATA:                  Control 30min   2hours  2days   1week   p-value       pos
SENSE COUNTS:          3       1       2       2       1       9.79e-01
GENES (3 total):
  AT5G54280.1
    SENSE COUNTS:      3       1       2       2       1       9.79e-01
    TAGS: (3 total)
           ----------                                                 4037
           ----------                                                 3878
      d+2  AGGATGACTT  0       0       1       0       0       4.55e-01      3834
      d+2  TATTTTTGAG  3       1       1       2       1       8.45e-01      3425
           ----------                                                 3208
           ----------                                                 2806
           ----------                                                 2442
           ----------                                                 1810
      i+3  TTTTGCATAA  0       0       0       0       0       6.15e-01      1756
           ----------                                                 1262
           ----------                                                 1110
           ----------                                                 489

LOCUS: AT4G22740
DESCRIPTION: glycine-rich protein
DATA:                  Control 30min   2hours  2days   1week   p-value       pos
SENSE COUNTS:          3       2       3       2       1       9.80e-01
GENES (2 total):
  AT4G22740.1
    SENSE COUNTS:      3       2       3       2       1       9.80e-01
    TAGS: (1 total)
           ----------                                                 2239
```

```
          ----------                                                    1939
          ----------                                                    1673
     d+2  ATTTGAGGGT  3       2       3       2       1       9.80e-01  1222
          ----------                                                    1107
          ----------                                                    1053
          ----------                                                    608
          ----------                                                    566
          ----------                                                    524
          ----------                                                    450
          ----------                                                    287
          ----------                                                    257
          ----------                                                    188
  AT4G22740.2
    SENSE COUNTS:     3       2       3       2       1       9.80e-01
    TAGS: (1 total)
          ----------                                                    2274
          ----------                                                    1974
          ----------                                                    1708
     d+2  ATTTGAGGGT  3       2       3       2       1       9.80e-01  1257
          ----------                                                    1142
          ----------                                                    1088
          ----------                                                    643
          ----------                                                    601
          ----------                                                    559
          ----------                                                    485
          ----------                                                    322
          ----------                                                    292
          ----------                                                    223

LOCUS: AT4G20360
DESCRIPTION: elongation factor Tu / EF-Tu (TUFA), identical to SWISS-PROT:P17745 elongation factor Tu,
chloroplast precursor (EF-Tu) (Arabidopsis thaliana)
DATA:                 Control 30min   2hours  2days   1week   p-value       pos
SENSE COUNTS:         30      28      31      31      28      9.82e-01
GENES (1 total):
  AT4G20360.1
    SENSE COUNTS:     30      28      31      31      28      9.82e-01
    TAGS: (2 total)
     d+1  AACGACAAAG  30      28      27      31      28      9.89e-01  1354
          ----------                                                    1315
     d+2  GCTCTCGCTT  0       0       4       0       0       5.58e-03  388
          ----------                                                    356
          ----------                                                    347
          ----------                                                    323
          ----------                                                    88

LOCUS: AT3G60240
DESCRIPTION: MIF4G domain-containing protein / MA3 domain-containing protein, similar to eukaryotic protein
synthesis initiation factor (Homo sapiens) GI:3941724; contains Pfam profiles PF02854: MIF4G domain, PF02847:
MA3 domain
DATA:                 Control 30min   2hours  2days   1week   p-value       pos
SENSE COUNTS:         5       4       5       4       4       9.85e-01
GENES (2 total):
  AT3G60240.1
    SENSE COUNTS:     5       4       5       4       4       9.85e-01
    TAGS: (4 total)
     d+1  CACCTAACCG  2       4       3       4       3       9.55e-01  4598
          ----------                                                    4144
          ----------                                                    3617
     d+2  TTGATCAATG  0       0       1       0       0       4.55e-01  3376
          ----------                                                    3342
          ----------                                                    3227
          ----------                                                    3189
          ----------                                                    3181
          ----------                                                    2336
          ----------                                                    2330
          ----------                                                    2003
          ----------                                                    1799
     d+2  CCTCATCGGA  0       0       1       0       0       4.55e-01  1562
          ----------                                                    1335
     d+2  TTCGTTGAGC  3       0       0       0       1       6.72e-02  1110
          ----------                                                    1067
          ----------                                                    763
          ----------                                                    503
          ----------                                                    455
          ----------                                                    226

LOCUS: AT2G36060
DESCRIPTION: ubiquitin-conjugating enzyme family protein, similar to DNA-binding protein CROC-1B (Homo
sapiens) GI:1066082; contains Pfam profile PF00179: Ubiquitin-conjugating enzyme
DATA:                 Control 30min   2hours  2days   1week   p-value       pos
```

```
SENSE COUNTS:          2      2      3      2      3      9.89e-01
GENES (2 total):
  AT2G36060.1
    SENSE COUNTS:      2      2      3      1      0      7.14e-01
    TAGS: (1 total)
      d+1  ATACCGGCGT  2      2      3      1      0      7.14e-01      471
           ----------                                                 455
           ----------                                                 366
           ----------                                                 188
  AT2G36060.2
    SENSE COUNTS:      2      2      3      2      3      9.89e-01
    TAGS: (2 total)
      d+1  ATACCGGCGT  2      2      3      1      0      7.14e-01      474
           ----------                                                 458
      X+4  TTAGCGAATC  0      0      0      1      3      2.09e-01      386
           ----------                                                 369
           ----------                                                 188

LOCUS: AT3G57410
DESCRIPTION: villin 3 (VLN3), nearly identical to villin 3 (VLN3) (Arabidopsis thaliana) GI:3415117
DATA:                  Control 30min  2hours 2days  1week  p-value       pos
SENSE COUNTS:          5      3      4      4      4      9.91e-01
GENES (2 total):
  AT3G57410.1
    SENSE COUNTS:      5      3      4      4      4      9.91e-01
    TAGS: (5 total)
      i+3  CCATTGTGTT  0      2      0      0      0      9.14e-02      7579
      i+3  TTTTAACATA  0      0      0      0      1      1.65e-01      5350
      d+1  AAATTTCAAT  3      1      3      2      3      9.42e-01      3420
           ----------                                                 3410
      d+2  CGTTCATTTT  2      0      1      2      0      6.49e-01      3308
           ----------                                                 3227
      d+2  GGATTCTACT  0      0      0      0      0      6.15e-01      2351
           ----------                                                 2173
           ----------                                                 1903
           ----------                                                 1872
           ----------                                                 1429
           ----------                                                 808
           ----------                                                 688
           ----------                                                 511
           ----------                                                 313

LOCUS: AT5G38520
DESCRIPTION: hydrolase, alpha/beta fold family protein, low similarity to hydrolase (Terrabacter sp. DBF63)
GI:14196240; contains Pfam profile PF00561: hydrolase, alpha/beta fold family
DATA:                  Control 30min  2hours 2days  1week  p-value       pos
SENSE COUNTS:          4      3      3      4      3      9.92e-01
GENES (1 total):
  AT5G38520.1
    SENSE COUNTS:      4      3      3      4      3      9.92e-01
    TAGS: (1 total)
           ----------                                                 1307
           ----------                                                 1254
      d+2  GCTGGCTCAA  4      3      3      4      3      9.92e-01      1096
           ----------                                                 1080
           ----------                                                 460
           ----------                                                 452
           ----------                                                 324
           ----------                                                 49

LOCUS: AT3G56460
DESCRIPTION: oxidoreductase, zinc-binding dehydrogenase family protein, low similarity to probable NADP-
dependent oxidoreductase (zeta-crystallin homolog) P1 (SP|Q39172)(gi:886428) and P2 (SP|Q39173)(gi:886430),
zeta-crystallin / quinone reductase (NADPH) - Mus muscul
DATA:                  Control 30min  2hours 2days  1week  p-value       pos
SENSE COUNTS:          4      5      4      5      5      9.97e-01
GENES (1 total):
  AT3G56460.1
    SENSE COUNTS:      4      5      4      5      5      9.97e-01
    TAGS: (1 total)
           ----------                                                 1416
      d+2  TAAGCTGAAC  4      5      4      5      5      9.97e-01      1176
           ----------                                                 959
           ----------                                                 886
           ----------                                                 780
           ----------                                                 658
           ----------                                                 481
           ----------                                                 438

LOCUS: AT5G43450
```

DESCRIPTION: 2-oxoglutarate-dependent dioxygenase, putative, similar to 2A6 (GI:599622) and tomato ethylene synthesis regulatory protein E8 (SP|P10967)

DATA:		Control	30min	2hours	2days	1week	p-value	pos
SENSE COUNTS:		4	4	4	5	4	9.97e-01	
GENES (1 total):								
AT5G43450.1								
SENSE COUNTS:		4	4	4	5	4	9.97e-01	
TAGS: (2 total)								
d+1	CTTAAAACAG	0	0	0	4	1	1.05e-01	1302
d+2	ATATGAAGAA	4	4	4	1	3	8.33e-01	1167
	----------							918
	----------							853
	----------							628
	----------							427
	----------							373
	----------							205
	----------							198
	----------							87

LOCUS: AT1G17840
DESCRIPTION: ABC transporter family protein, similar to ABC transporter GI:10280532 from (Homo sapiens)

DATA:		Control	30min	2hours	2days	1week	p-value	pos
SENSE COUNTS:		4	3	4	4	3	9.99e-01	
GENES (1 total):								
AT1G17840.1								
SENSE COUNTS:		4	3	4	4	3	9.99e-01	
TAGS: (2 total)								
	----------							2993
	----------							2907
	----------							2837
	----------							2668
d+2	TAATGTGACA	2	2	3	4	3	9.66e-01	2468
d+2	AAAATTGATC	2	1	1	0	0	6.03e-01	2409
	----------							2202
	----------							2169
	----------							1926
	----------							1887
	----------							1767
	----------							1731
	----------							1638
	----------							1602
	----------							1568
	----------							1446
	----------							1133
	----------							519
	----------							74
	----------							61
	----------							28

TOTALS

DATASET SIZE: (normalized to 50000)

Control: 43125
30 min: 56902
2 hour: 44862
2 day: 36031
Week: 27819

LOCI MATCHED: (1275 total)

Control: 1047
30 min: 1065
2 hour: 1109
2 day: 1027
Week: 1032

UNIQUE TAGS: 2815
