# Supplementary material for: Differential SAGE analysis in Arabidopsis uncovers increased transcriptome complexity in response to low temperature
Source: BMC Genomics. 2008 Sep 22;9:434. doi: 10.1186/1471-2164-9-434 (PMC2568001; doi:10.1186/1471-2164-9-434)
Supplement: Additional file 6 — Differentially expressed SAGE tags uniquely matching Arabidopsis loci in the antisense orientation. [file 1471-2164-9-434-S6.pdf]

**Additional file 6: Differentially expressed SAGE tags uniquely matching Arabidopsis loci in the anti-sense orientation. The total tags were normalised to 50,000 per library to facilitate comparative expression analysis.**

LOCUS: AT3G09390
DESCRIPTION: metallothionein protein, putative (MT2A), identical to Swiss-Prot

| DATA: | | Control | 30min | 2hours | 2days | 1week | p-value | B&H | Pos | Fold change relative to control (log2) | | | | |
|---|---|---|---|---|---|---|---|---|---|---|---|---|---|---|
| ANTISENSE COUNTS: | | 9 | 12 | 20 | 37 | 61 | 1.74e-12 | 8.70e-11 | | 0.000 | 0.415 | 1.152 | 2.040 | 2.761 |
| GENES: | | | | | | | | | | | | | | |
| AT3G09390.1 | | | | | | | | | | | | | | |
| ANTISENSE COUNTS: | | 9 | 12 | 20 | 37 | 61 | 1.74e-12 | 9.05e-11 | | 0.000 | 0.415 | 1.152 | 2.040 | 2.761 |
| TAGS: | | | | | | | | | | | | | | |
| X-4 | GCAGAGTTGC | 9 | 12 | 20 | 37 | 61 | 1.74e-12 | 1.17e-10 | 371 | 0.000 | 0.415 | 1.152 | 2.040 | 2.761 |

LOCUS: AT3G53460
DESCRIPTION: 29 kDa ribonucleoprotein, chloroplast / RNA-binding protein cp 29, nearly identical to SP|Q43349 29 kDa ribonucleoprotein, chloroplast precursor (RNA-binding protein cp29) {Arabidopsis thaliana}

| DATA: | | Control | 30min | 2hours | 2days | 1week | p-value | B&H | Pos | Fold change relative to control (log2) | | | | |
|---|---|---|---|---|---|---|---|---|---|---|---|---|---|---|
| ANTISENSE COUNTS: | | 5 | 0 | 6 | 8 | 28 | 5.44e-10 | 1.36e-08 | | 0.000 | 0.000 | 0.263 | 0.678 | 2.485 |
| GENES: | | | | | | | | | | | | | | |
| AT3G53460.1 | | | | | | | | | | | | | | |
| ANTISENSE COUNTS: | | 5 | 0 | 6 | 8 | 28 | 5.44e-10 | 1.41e-08 | | 0.000 | 0.000 | 0.263 | 0.678 | 2.485 |
| TAGS: | | | | | | | | | | | | | | |
| X-4 | GAATTCCACA | 1 | 0 | 0 | 0 | 0 | 4.25e-01 | 4.83e-01 | 225 | 0.000 | 0.000 | 0.000 | 0.000 | 0.000 |
| X-4 | GGTTCTCTGC | 4 | 0 | 6 | 6 | 24 | 2.21e-08 | 7.40e-07 | 160 | 0.000 | 0.000 | 0.585 | 0.585 | 2.585 |
| X-4 | CATCAAAGGC | 0 | 0 | 0 | 2 | 4 | 1.12e-02 | 1.60e-02 | 95 | 0.000 | 0.000 | 0.000 | 1.000 | 2.000 |

LOCUS: AT3G22840
DESCRIPTION: chlorophyll A-B binding family protein / early light-induced protein (ELIP), identical to early light-induced protein; ELIP (Arabidopsis thaliana) GI

| DATA: | | Control | 30min | 2hours | 2days | 1week | p-value | B&H | Pos | Fold change relative to control (log2) | | | | |
|---|---|---|---|---|---|---|---|---|---|---|---|---|---|---|
| ANTISENSE COUNTS: | | 0 | 0 | 0 | 14 | 7 | 9.86e-09 | 1.64e-07 | | 0.000 | 0.000 | 0.000 | 3.807 | 2.807 |
| GENES: | | | | | | | | | | | | | | |
| AT3G22840.1 | | | | | | | | | | | | | | |
| ANTISENSE COUNTS: | | 0 | 0 | 0 | 14 | 7 | 9.86e-09 | 1.71e-07 | | 0.000 | 0.000 | 0.000 | 3.807 | 2.807 |
| TAGS: | | | | | | | | | | | | | | |
| X-4 | GGCAAAATCG | 0 | 0 | 0 | 13 | 7 | 5.46e-08 | 7.32e-07 | 391 | 0.000 | 0.000 | 0.000 | 3.700 | 2.807 |
| X-4 | CATCTCACTC | 0 | 0 | 0 | 1 | 0 | 3.08e-01 | 3.62e-01 | 248 | 0.000 | 0.000 | 0.000 | 0.000 | 0.000 |

LOCUS: AT2G37220
DESCRIPTION: 29 kDa ribonucleoprotein, chloroplast, putative / RNA-binding protein cp29, putative, similar to SP|Q43349 29 kDa ribonucleoprotein, chloroplast precursor (RNA-binding protein cp29) {Arabidopsis thaliana}

| DATA: | | Control | 30min | 2hours | 2days | 1week | p-value | B&H | Pos | Fold change relative to control (log2) | | | | |
|---|---|---|---|---|---|---|---|---|---|---|---|---|---|---|
| ANTISENSE COUNTS: | | 46 | 34 | 81 | 75 | 97 | 2.61e-08 | 3.26e-07 | | 0.000 | -0.436 | 0.816 | 0.705 | 1.076 |
| GENES: | | | | | | | | | | | | | | |
| AT2G37220.1 | | | | | | | | | | | | | | |
| ANTISENSE COUNTS: | | 46 | 34 | 81 | 75 | 97 | 2.61e-08 | 3.39e-07 | | 0.000 | -0.436 | 0.816 | 0.705 | 1.076 |
| TAGS: | | | | | | | | | | | | | | |
| X-4 | GTCTCTCCAG | 46 | 34 | 81 | 75 | 97 | 2.61e-08 | 5.83e-07 | 158 | 0.000 | -0.436 | 0.816 | 0.705 | 1.076 |

LOCUS: AT1G16870
DESCRIPTION: mitochondrial 28S ribosomal protein S29-related, contains weak similarity to Swiss-Prot

| DATA: | | Control | 30min | 2hours | 2days | 1week | p-value | B&H | Pos | Fold change relative to control (log2) | | | | |
|---|---|---|---|---|---|---|---|---|---|---|---|---|---|---|
| ANTISENSE COUNTS: | | 0 | 13 | 0 | 1 | 1 | 3.30e-08 | 3.30e-07 | | 0.000 | 3.700 | 0.000 | 0.000 | 0.000 |
| GENES: | | | | | | | | | | | | | | |
| AT1G16870.1 | | | | | | | | | | | | | | |
| ANTISENSE COUNTS: | | 0 | 13 | 0 | 1 | 1 | 3.30e-08 | 3.43e-07 | | 0.000 | 3.700 | 0.000 | 0.000 | 0.000 |
| TAGS: | | | | | | | | | | | | | | |
| X-4 | CTTCTGAAAG | 0 | 13 | 0 | 1 | 1 | 3.30e-08 | 5.53e-07 | 16 | 0.000 | 3.700 | 0.000 | 0.000 | 0.000 |

LOCUS: AT2G44120

DESCRIPTION: 60S ribosomal protein L7 (RPL7C),

| DATA: | | Control | 30min | 2hours | 2days | 1week | p-value | B&H | Pos | Fold change relative to control (log2) | | | | |
|---|---|---|---|---|---|---|---|---|---|---|---|---|---|---|
| ANTISENSE COUNTS: | | 4 | 1 | 0 | 2 | 18 | 6.24e-08 | 5.20e-07 | | 0.000 | -2.000 | 0.000 | -1.000 | 2.170 |
| GENES: | | | | | | | | | | | | | | |
| AT2G44120.2 | | | | | | | | | | | | | | |
| ANTISENSE COUNTS: | | 4 | 1 | 0 | 2 | 18 | 6.24e-08 | 5.41e-07 | | 0.000 | -2.000 | 0.000 | -1.000 | 2.170 |
| TAGS: | | | | | | | | | | | | | | |
| X-4 | ATTAGTCATA | 4 | 1 | 0 | 2 | 18 | 6.24e-08 | 6.97e-07 | 438 | 0.000 | -2.000 | 0.000 | -1.000 | 2.170 |

LOCUS: AT4G17245
DESCRIPTION: zinc finger (C3HC4-type RING finger) family protein, contains Pfam profile

| DATA: | | Control | 30min | 2hours | 2days | 1week | p-value | B&H | Pos | Fold change relative to control (log2) | | | | |
|---|---|---|---|---|---|---|---|---|---|---|---|---|---|---|
| ANTISENSE COUNTS: | | 25 | 1 | 4 | 9 | 15 | 3.43e-07 | 2.45e-06 | | 0.000 | -4.644 | -2.644 | -1.474 | -0.737 |
| GENES: | | | | | | | | | | | | | | |
| AT4G17245.1 | | | | | | | | | | | | | | |
| ANTISENSE COUNTS: | | 25 | 1 | 4 | 9 | 15 | 3.43e-07 | 2.55e-06 | | 0.000 | -4.644 | -2.644 | -1.474 | -0.737 |
| TAGS: | | | | | | | | | | | | | | |
| d-2 | ATAGTGGTGT | 25 | 1 | 4 | 9 | 15 | 3.43e-07 | 3.28e-06 | 593 | 0.000 | -4.644 | -2.644 | -1.474 | -0.737 |

LOCUS: AT1G32060
DESCRIPTION: phosphoribulokinase (PRK) / phosphopentokinase, nearly identical to SP|P25697 Phosphoribulokinase, chloroplast precursor (EC 2.7.1.19) (Phosphopentokinase) (PRKASE) (PRK) {Arabidopsis thaliana}

| DATA: | | Control | 30min | 2hours | 2days | 1week | p-value | B&H | Pos | Fold change relative to control (log2) | | | | |
|---|---|---|---|---|---|---|---|---|---|---|---|---|---|---|
| ANTISENSE COUNTS: | | 24 | 3 | 31 | 18 | 9 | 5.53e-07 | 3.46e-06 | | 0.000 | -3.000 | 0.369 | -0.415 | -1.415 |
| GENES: | | | | | | | | | | | | | | |
| AT1G32060.1 | | | | | | | | | | | | | | |
| ANTISENSE COUNTS: | | 24 | 3 | 31 | 18 | 9 | 5.53e-07 | 3.59e-06 | | 0.000 | -3.000 | 0.369 | -0.415 | -1.415 |
| TAGS: | | | | | | | | | | | | | | |
| d-2 | AAAATCCGCG | 23 | 3 | 31 | 18 | 9 | 1.25e-06 | 1.05e-05 | 1409 | 0.000 | -2.939 | 0.431 | -0.354 | -1.354 |
| d-2 | TTTGAGCATT | 1 | 0 | 0 | 0 | 0 | 6.03e-01 | 6.62e-01 | 1177 | 0.000 | 0.000 | 0.000 | 0.000 | 0.000 |

LOCUS: AT1G03600
DESCRIPTION: photosystem II family protein, similar to SP

| DATA: | | Control | 30min | 2hours | 2days | 1week | p-value | B&H | Pos | Fold change relative to control (log2) | | | | |
|---|---|---|---|---|---|---|---|---|---|---|---|---|---|---|
| ANTISENSE COUNTS: | | 47 | 15 | 40 | 26 | 15 | 9.72e-06 | 5.40e-05 | | 0.000 | -1.648 | -0.233 | -0.854 | -1.648 |
| GENES: | | | | | | | | | | | | | | |
| AT1G03600.1 | | | | | | | | | | | | | | |
| ANTISENSE COUNTS: | | 47 | 15 | 40 | 26 | 15 | 9.72e-06 | 5.05e-05 | | 0.000 | -1.648 | -0.233 | -0.854 | -1.648 |
| TAGS: | | | | | | | | | | | | | | |
| d-1 | CAGAACATTA | 47 | 15 | 40 | 26 | 15 | 9.72e-06 | 7.24e-05 | 712 | 0.000 | -1.648 | -0.233 | -0.854 | -1.648 |

LOCUS: AT1G77230
DESCRIPTION: tetratricopeptide repeat (TPR)-containing protein, contains Pfam profile PF00515 TPR Domain

| DATA: | | Control | 30min | 2hours | 2days | 1week | p-value | B&H | Pos | Fold change relative to control (log2) | | | | |
|---|---|---|---|---|---|---|---|---|---|---|---|---|---|---|
| ANTISENSE COUNTS: | | 53 | 16 | 48 | 45 | 29 | 1.65e-05 | 8.25e-05 | | 0.000 | -1.728 | -0.143 | -0.236 | -0.870 |
| GENES: | | | | | | | | | | | | | | |
| AT1G77230.1 | | | | | | | | | | | | | | |
| ANTISENSE COUNTS: | | 53 | 16 | 48 | 45 | 29 | 1.65e-05 | 7.80e-05 | | 0.000 | -1.728 | -0.143 | -0.236 | -0.870 |
| TAGS: | | | | | | | | | | | | | | |
| i-3 | CCTAAGAAAA | 53 | 16 | 48 | 45 | 29 | 1.65e-05 | 1.11e-04 | 1421 | 0.000 | -1.728 | -0.143 | -0.236 | -0.870 |

LOCUS: AT4G13940
DESCRIPTION: adenosylhomocysteinase / S-adenosyl-L-homocysteine hydrolase / AdoHcyase (SAHH), identical to SP|O23255 Adenosylhomocysteinase (EC 3.3.1.1) (S-adenosyl-L-homocysteine hydrolase) (AdoHcyase) {Arabidopsis thaliana}; strong similarity to SP|P50248 Adenosylho

| DATA: | | Control | 30min | 2hours | 2days | 1week | p-value | B&H | Pos | Fold change relative to control (log2) | | | | |
|---|---|---|---|---|---|---|---|---|---|---|---|---|---|---|
| ANTISENSE COUNTS: | | 18 | 3 | 13 | 12 | 30 | 2.83e-05 | 1.29e-04 | | 0.000 | -2.585 | -0.469 | -0.585 | 0.737 |
| GENES: | | | | | | | | | | | | | | |
| AT4G13940.1 | | | | | | | | | | | | | | |
| ANTISENSE COUNTS: | | 18 | 3 | 13 | 12 | 30 | 2.83e-05 | 1.23e-04 | | 0.000 | -2.585 | -0.469 | -0.585 | 0.737 |
| TAGS: | | | | | | | | | | | | | | |

```
      d-2      AAAAATGGAG     18      3       13      12      30      2.83e-05       1.72e-04       1702    0.000   -2.585  -0.469  -0.585  0.737

LOCUS: AT1G27080
DESCRIPTION:  proton-dependent oligopeptide transport (POT) family protein, similar to nitrate transporter NRT1-5 (Glycine max) GI
DATA:                          Control 30min   2hours  2days   1week   p-value        B&H            Pos     Fold change relative to control (log2)
ANTISENSE COUNTS:              8       0       0       0       0       3.05e-05       1.27e-04               0.000   0.000   0.000   0.000   0.000
  GENES:
  AT1G27080.1
    ANTISENSE COUNTS:          8       0       0       0       0       3.05e-05       1.22e-04               0.000   0.000   0.000   0.000   0.000
    TAGS:
      v-2      AAAGCGATAG     8       0       0       0       0       3.05e-05       1.70e-04       1990    0.000   0.000   0.000   0.000   0.000

LOCUS: AT5G56420
DESCRIPTION:  F-box family protein, contains F-box domain Pfam
DATA:                          Control 30min   2hours  2days   1week   p-value        B&H            Pos     Fold change relative to control (log2)
ANTISENSE COUNTS:              0       0       0       0       7       3.91e-05       1.50e-04               0.000   0.000   0.000   0.000   2.807
  GENES:
  AT5G56420.1
    ANTISENSE COUNTS:          0       0       0       0       7       3.91e-05       1.45e-04               0.000   0.000   0.000   0.000   2.807
    TAGS:
      X-4      TTCTCAATGT     0       0       0       0       7       3.91e-05       2.02e-04       779     0.000   0.000   0.000   0.000   2.807

LOCUS: AT4G02970
DESCRIPTION:  ubiquitin family protein, contains INTERPRO
DATA:                          Control 30min   2hours  2days   1week   p-value        B&H            Pos     Fold change relative to control (log2)
ANTISENSE COUNTS:              1       10      0       2       0       7.68e-05       2.74e-04               0.000   3.322   0.000   1.000   0.000
  GENES:
  AT4G02970.1
    ANTISENSE COUNTS:          1       10      0       2       0       7.68e-05       2.66e-04               0.000   3.322   0.000   1.000   0.000
    TAGS:
      i-3      CAGGTTCTAG     1       10      0       2       0       7.68e-05       3.68e-04       1527    0.000   3.322   0.000   1.000   0.000

LOCUS: AT4G21960
DESCRIPTION:  peroxidase 42 (PER42) (P42) (PRXR1), identical to SP|Q9SB81 Peroxidase 42 precursor (EC 1.11.1.7) (Atperox P42) (PRXR1) (ATP1a/ATP1b)
{Arabidopsis thaliana}
DATA:                          Control 30min   2hours  2days   1week   p-value        B&H            Pos     Fold change relative to control (log2)
ANTISENSE COUNTS:              5       15      3       2       1       1.12e-04       3.73e-04               0.000   1.585   -0.737  -1.322  -2.322
  GENES:
  AT4G21960.1
    ANTISENSE COUNTS:          5       15      3       2       1       1.12e-04       3.64e-04               0.000   1.585   -0.737  -1.322  -2.322
    TAGS:
      X-4      ATACCCAACA     5       15      3       2       1       2.78e-04       8.87e-04       703     0.000   1.585   -0.737  -1.322  -2.322
      X-4      TGAGGGACGT     0       0       0       0       0       6.10e-01       6.39e-01       93      0.000   0.000   0.000   0.000   0.000

LOCUS: AT2G30760
DESCRIPTION:  hypothetical protein
DATA:                          Control 30min   2hours  2days   1week   p-value        B&H            Pos     Fold change relative to control (log2)
ANTISENSE COUNTS:              8       0       16      6       4       1.14e-04       3.56e-04               0.000   0.000   1.000   -0.415  -1.000
  GENES:
  AT2G30760.1
    ANTISENSE COUNTS:          8       0       16      6       4       1.14e-04       3.49e-04               0.000   0.000   1.000   -0.415  -1.000
    TAGS:
      X-4      TCGCAAACAG     8       0       16      6       4       1.14e-04       4.49e-04       -126    0.000   0.000   1.000   -0.415  -1.000

LOCUS: AT2G23130
DESCRIPTION:  arabinogalactan protein AGP17 mRNA, complete cds
DATA:                          Control 30min   2hours  2days   1week   p-value        B&H            Pos     Fold change relative to control (log2)
ANTISENSE COUNTS:              17      5       31      24      23      1.14e-04       3.35e-04               0.000   -1.766  0.867   0.497   0.436
  GENES:
  AT2G23130.1
```

```
    ANTISENSE COUNTS:         17     5      31     24     23     1.14e-04       3.29e-04              0.000  -1.766  0.867   0.497   0.436
    TAGS:
      d-2     AGACAAATGG      17     5      31     24     23     1.14e-04       4.24e-04       708    0.000  -1.766  0.867   0.497   0.436

LOCUS: AT5G02960
DESCRIPTION:  40S ribosomal protein S23 (RPS23B), ribosomal protein S23, Fragaria x ananassa, PIR
DATA:                         Control 30min  2hours 2days  1week  p-value        B&H            Pos    Fold change relative to control (log2)
ANTISENSE COUNTS:             10     1      5      10     21     1.81e-04       5.03e-04              0.000  -3.322  -1.000  0.000   1.070
  GENES:
  AT5G02960.1
    ANTISENSE COUNTS:         10     1      5      10     21     1.81e-04       4.95e-04              0.000  -3.322  -1.000  0.000   1.070
    TAGS:
      X-4     TCTTAATTCA      10     1      5      10     21     1.81e-04       6.06e-04       268    0.000  -3.322  -1.000  0.000   1.070

LOCUS: AT5G27970
DESCRIPTION:  expressed protein
DATA:                         Control 30min  2hours 2days  1week  p-value        B&H            Pos    Fold change relative to control (log2)
ANTISENSE COUNTS:             17     1      12     16     4      4.04e-04       1.06e-03              0.000  -4.087  -0.503  -0.087  -2.087
  GENES:
  AT5G27970.1
    ANTISENSE COUNTS:         17     1      12     16     4      4.04e-04       1.05e-03              0.000  -4.087  -0.503  -0.087  -2.087
    TAGS:
      i-3     CACCTGAAAA      17     1      12     16     4      4.04e-04       1.23e-03       9251   0.000  -4.087  -0.503  -0.087  -2.087

LOCUS: AT2G36810
DESCRIPTION:  expressed protein,
DATA:                         Control 30min  2hours 2days  1week  p-value        B&H            Pos    Fold change relative to control (log2)
ANTISENSE COUNTS:             12     0      0      4      4      4.29e-04       1.07e-03              0.000  0.000   0.000   -1.585  -1.585
  GENES:
  AT2G36810.1
    ANTISENSE COUNTS:         12     0      0      4      4      4.29e-04       1.06e-03              0.000  0.000   0.000   -1.585  -1.585
    TAGS:
      X-4     GCCTTCGCCA      11     0      0      4      1      9.10e-05       4.06e-04       119    0.000  0.000   0.000   -1.459  -3.459
      X-4     ATGGGACCAC      1      0      0      0      3      2.15e-01       2.62e-01       -23    0.000  0.000   0.000   0.000   1.585

LOCUS: AT3G48190
DESCRIPTION:  ataxia-telangiectasia mutated protein (Atm), identical to ataxia-telangiectasia mutated protein (Atm) (Arabidopsis thaliana) GI
DATA:                         Control 30min  2hours 2days  1week  p-value        B&H            Pos    Fold change relative to control (log2)
ANTISENSE COUNTS:             0      0      0      0      4      6.97e-04       1.66e-03              0.000  0.000   0.000   0.000   2.000
  GENES:
  AT3G48190.1
    ANTISENSE COUNTS:         0      0      0      0      6      1.82e-06       1.05e-05              0.000  0.000   0.000   0.000   2.585
    TAGS:
      i-3     TGCAGAATGT      0      0      0      0      1      1.70e-01       2.15e-01       19466  0.000  0.000   0.000   0.000   0.000
      d-2     CCATCATAAT      0      0      0      0      3      1.21e-02       1.65e-02       11483  0.000  0.000   0.000   0.000   1.585

LOCUS: AT3G03776
DESCRIPTION:  hydroxyproline-rich glycoprotein family protein, contains proline-rich extensin domains, INTERPRO
DATA:                         Control 30min  2hours 2days  1week  p-value        B&H            Pos    Fold change relative to control (log2)
ANTISENSE COUNTS:             0      0      0      0      4      6.97e-04       1.58e-03              0.000  0.000   0.000   0.000   2.000
  GENES:
  AT3G03776.1
    ANTISENSE COUNTS:         0      0      0      0      4      6.97e-04       1.65e-03              0.000  0.000   0.000   0.000   2.000
    TAGS:
      X-4     TTTATAGTAC      0      0      0      0      4      6.97e-04       2.03e-03       -2     0.000  0.000   0.000   0.000   2.000

LOCUS: AT3G26730
DESCRIPTION:  zinc finger (C3HC4-type RING finger) family protein, contains Pfam profile
DATA:                         Control 30min  2hours 2days  1week  p-value        B&H            Pos    Fold change relative to control (log2)
ANTISENSE COUNTS:             1      0      1      9      4      7.93e-04       1.72e-03              0.000  0.000   0.000   3.170   2.000
```

```
  GENES:
  AT3G26730.1
    ANTISENSE COUNTS:         1      0      1      9      4      7.93e-04       1.79e-03              0.000   0.000   0.000   3.170   2.000
    TAGS:
      d-2      GGTTGCAAAA     1      0      1      9      4      7.93e-04       2.21e-03       2532   0.000   0.000   0.000   3.170   2.000

LOCUS: AT5G24160
DESCRIPTION:  squalene monooxygenase 1,2 / squalene epoxidase 1,2 (SQP1,2), identical to SP|O65402
DATA:                         Control 30min  2hours 2days  1week  p-value        B&H            Pos    Fold change relative to control (log2)
ANTISENSE COUNTS:             0      0      0      3      9      8.61e-04       1.79e-03              0.000   0.000   0.000   1.585   3.170
  GENES:
  AT5G24160.1
    ANTISENSE COUNTS:         0      0      0      3      9      8.61e-04       1.87e-03              0.000   0.000   0.000   1.585   3.170
    TAGS:
      d-2      GCCAAACCAA     0      0      0      0      0      6.10e-01       6.59e-01       1484   0.000   0.000   0.000   0.000   0.000
      X-4      TAATTTTCTG     0      0      0      3      9      1.66e-04       5.85e-04       486    0.000   0.000   0.000   1.585   3.170

LOCUS: AT4G11150
DESCRIPTION:  vacuolar ATP synthase subunit E / V-ATPase E subunit / vacuolar proton pump E subunit (VATE), identical to SP|Q39258 Vacuolar ATP
synthase subunit E (EC 3.6.3.14) (V-ATPase E subunit) (Vacuolar proton pump E subunit) {Arabidopsis thaliana}
DATA:                         Control 30min  2hours 2days  1week  p-value        B&H            Pos    Fold change relative to control (log2)
ANTISENSE COUNTS:             16     4      3      3      15     9.48e-04       1.90e-03              0.000   -2.000  -2.415  -2.415  -0.093
  GENES:
  AT4G11150.1
    ANTISENSE COUNTS:         16     4      3      3      15     9.48e-04       1.90e-03              0.000   -2.000  -2.415  -2.415  -0.093
    TAGS:
      d-2      ATAACACACA     16     4      3      3      15     9.48e-04       2.44e-03       1009   0.000   -2.000  -2.415  -2.415  -0.093

LOCUS: AT5G28920
DESCRIPTION:  expressed protein
DATA:                         Control 30min  2hours 2days  1week  p-value        B&H            Pos    Fold change relative to control (log2)
ANTISENSE COUNTS:             4      1      0      5      15     1.04e-03       2.00e-03              0.000   -2.000  0.000   0.322   1.907
  GENES:
  AT5G28920.1
    ANTISENSE COUNTS:         4      1      0      5      15     1.04e-03       2.00e-03              0.000   -2.000  0.000   0.322   1.907
    TAGS:
      X-4      ATCAAAAGCT     0      0      0      1      0      7.90e-01       7.90e-01       68     0.000   0.000   0.000   0.000   0.000
      X-4      AAGCCCCTAT     4      1      0      4      15     1.05e-04       4.40e-04       -47    0.000   -2.000  0.000   0.000   1.907

LOCUS: AT4G26530
DESCRIPTION:  fructose-bisphosphate aldolase, putative, strong similarity to SP|P22197 Fructose-bisphosphate aldolase, cytoplasmic isozyme (EC
4.1.2.13) {Arabidopsis thaliana}
DATA:                         Control 30min  2hours 2days  1week  p-value        B&H            Pos    Fold change relative to control (log2)
ANTISENSE COUNTS:             17     3      21     14     6      1.20e-03       2.22e-03              0.000   -2.503  0.305   -0.280  -1.503
  GENES:
  AT4G26530.1
    ANTISENSE COUNTS:         17     3      21     14     6      1.20e-03       2.15e-03              0.000   -2.503  0.305   -0.280  -1.503
    TAGS:
      d-2      GCGCGAAAAT     17     3      21     14     6      1.20e-03       2.98e-03       1301   0.000   -2.503  0.305   -0.280  -1.503

LOCUS: AT5G15840
DESCRIPTION:  zinc finger protein CONSTANS (CO), identical to Zinc finger protein CONSTANS SP
DATA:                         Control 30min  2hours 2days  1week  p-value        B&H            Pos    Fold change relative to control (log2)
ANTISENSE COUNTS:             0      0      4      0      0      1.20e-03       2.14e-03              0.000   0.000   2.000   0.000   0.000
  GENES:
  AT5G15840.1
    ANTISENSE COUNTS:         0      0      4      0      0      1.20e-03       2.23e-03              0.000   0.000   2.000   0.000   0.000
    TAGS:
      X-4      ACTCGCAGAC     0      0      4      0      0      1.20e-03       2.87e-03       253    0.000   0.000   2.000   0.000   0.000
```

```
LOCUS: AT2G36620
DESCRIPTION:  60S ribosomal protein L24 (RPL24A)
DATA:                         Control 30min   2hours  2days   1week   p-value       B&H           Pos     Fold change relative to control (log2)
ANTISENSE COUNTS:             0       0       0       3       6       1.59e-03      2.74e-03              0.000   0.000   0.000   1.585   2.585
  GENES:
  AT2G36620.1
    ANTISENSE COUNTS:         0       0       0       3       6       1.59e-03      2.76e-03              0.000   0.000   0.000   1.585   2.585
    TAGS:
      X-4      GCTCCGCCGC     0       0       0       3       6       1.59e-03      3.67e-03      44      0.000   0.000   0.000   1.585   2.585

LOCUS: AT2G32270
DESCRIPTION:  zinc transporter (ZIP3), identical to zinc transporter (Arabidopsis thaliana) gi|3252870|gb|AAC24199; member of the Zinc (Zn2+)-Iron
(Fe2+) permease (ZIP) family, PMID
DATA:                         Control 30min   2hours  2days   1week   p-value       B&H           Pos     Fold change relative to control (log2)
ANTISENSE COUNTS:             33      47      40      15      26      1.73e-03      2.88e-03              0.000   0.510   0.278   -1.138  -0.344
  GENES:
  AT2G32270.1
    ANTISENSE COUNTS:         33      47      40      15      26      1.73e-03      2.90e-03              0.000   0.510   0.278   -1.138  -0.344
    TAGS:
      i-3      TCCGAATCTA     33      47      40      15      26      1.73e-03      3.86e-03      1349    0.000   0.510   0.278   -1.138  -0.344

LOCUS: AT2G10940
DESCRIPTION:  protease inhibitor/seed storage/lipid transfer protein (LTP) family protein, similar to proline-rich cell wall protein (Medicago sativa)
GI
DATA:                         Control 30min   2hours  2days   1week   p-value       B&H           Pos     Fold change relative to control (log2)
ANTISENSE COUNTS:             24      7       25      22      7       2.80e-03      4.52e-03              0.000   -1.778  0.059   -0.126  -1.778
  GENES:
  AT2G10940.2
    ANTISENSE COUNTS:         24      7       25      22      7       9.08e-04      1.89e-03              0.000   -1.778  0.059   -0.126  -1.778
    TAGS:
      X-4      AACGCAGTTC     24      7       25      22      7       9.08e-04      2.43e-03      1558    0.000   -1.778  0.059   -0.126  -1.778
  AT2G10940.1
    ANTISENSE COUNTS:         0       0       0       0       0       2.49e-01      2.49e-01              0.000   0.000   0.000   0.000   0.000
    TAGS:
      X-4      TCGCCTTCCC     0       0       0       0       0       6.10e-01      6.29e-01      686     0.000   0.000   0.000   0.000   0.000
      X-4      CGAGAGAATG     0       0       0       0       0       6.10e-01      6.19e-01      146     0.000   0.000   0.000   0.000   0.000

LOCUS: AT3G11400
DESCRIPTION:  eukaryotic translation initiation factor 3G / eIF3g, nearly identical to eukaryotic translation initiation factor 3g (Arabidopsis
thaliana) GI
DATA:                         Control 30min   2hours  2days   1week   p-value       B&H           Pos     Fold change relative to control (log2)
ANTISENSE COUNTS:             0       2       0       0       7       3.26e-03      5.09e-03              0.000   1.000   0.000   0.000   2.807
  GENES:
  AT3G11400.1
    ANTISENSE COUNTS:         0       2       0       0       7       3.26e-03      5.30e-03              0.000   1.000   0.000   0.000   2.807
    TAGS:
      d-2      CCTTATGTAT     0       2       0       0       7       3.26e-03      7.05e-03      1180    0.000   1.000   0.000   0.000   2.807

LOCUS: AT3G06700
DESCRIPTION:  60S ribosomal protein L29 (RPL29A), similar to ribosomal protein L29 GI
DATA:                         Control 30min   2hours  2days   1week   p-value       B&H           Pos     Fold change relative to control (log2)
ANTISENSE COUNTS:             7       4       11      10      23      3.28e-03      4.97e-03              0.000   -0.807  0.652   0.515   1.716
  GENES:
  AT3G06700.1
    ANTISENSE COUNTS:         7       4       11      10      23      3.28e-03      5.17e-03              0.000   -0.807  0.652   0.515   1.716
    TAGS:
      X-4      ATCAAAAGCG     7       4       11      10      23      3.28e-03      6.87e-03      177     0.000   -0.807  0.652   0.515   1.716

LOCUS: AT4G16760
DESCRIPTION:  Member of a family that includes acyl-CoA oxidases specific for shorter-chain acyl-CoAs. Protein contains peroxisome targeting motif.
```

DATA: Control 30min 2hours 2days 1week p-value B&H Pos Fold change relative to control (log2)
ANTISENSE COUNTS: 1 1 4 9 0 3.51e-03 5.16e-03 0.000 0.000 2.000 3.170 0.000
GENES:
AT4G16760.1
ANTISENSE COUNTS: 1 1 4 9 0 3.51e-03 5.37e-03 0.000 0.000 2.000 3.170 0.000
TAGS:
X-4 TCCACCACAT 0 0 0 1 0 3.08e-01 3.68e-01 35 0.000 0.000 0.000 0.000 0.000
X-4 CCCAAAAAAA 1 1 4 8 0 1.15e-02 1.61e-02 -8 0.000 0.000 2.000 3.000 0.000

LOCUS: AT3G16770
DESCRIPTION: encodes a member of the ERF (ethylene response factor) subfamily B-2 of ERF/AP2 transcription factor family (RAP2.3). The protein contains one AP2 domain. There are 5 members in this subfamily including RAP2.2 AND RAP2.12.
DATA: Control 30min 2hours 2days 1week p-value B&H Pos Fold change relative to control (log2)
ANTISENSE COUNTS: 1 8 2 1 0 4.25e-03 6.07e-03 0.000 3.000 1.000 0.000 0.000
GENES:
AT3G16770.1
ANTISENSE COUNTS: 1 8 2 1 0 4.25e-03 6.31e-03 0.000 3.000 1.000 0.000 0.000
TAGS:
d-1 GACGCTTACG 1 8 2 1 0 4.25e-03 8.63e-03 325 0.000 3.000 1.000 0.000 0.000

LOCUS: AT5G32481
DESCRIPTION: gypsy-like retrotransposon family (Athila), has a 6.5e-294 P-value blast match to GB
DATA: Control 30min 2hours 2days 1week p-value B&H Pos Fold change relative to control (log2)
ANTISENSE COUNTS: 1 0 0 0 6 4.27e-03 5.93e-03 0.000 0.000 0.000 0.000 2.585
GENES:
AT5G32481.1
ANTISENSE COUNTS: 1 0 0 0 6 4.27e-03 6.17e-03 0.000 0.000 0.000 0.000 2.585
TAGS:
X-4 GTGGTTGATA 1 0 0 0 6 4.27e-03 8.17e-03 1842 0.000 0.000 0.000 0.000 2.585

LOCUS: AT4G14690
DESCRIPTION: chlorophyll A-B binding family protein / early light-induced protein, putative, strong similarity to early light-induced protein; ELIP (Arabidopsis thaliana) GI
DATA: Control 30min 2hours 2days 1week p-value B&H Pos Fold change relative to control (log2)
ANTISENSE COUNTS: 0 0 0 4 1 4.57e-03 6.18e-03 0.000 0.000 0.000 2.000 0.000
GENES:
AT4G14690.1
ANTISENSE COUNTS: 0 0 0 4 1 4.57e-03 6.42e-03 0.000 0.000 0.000 2.000 0.000
TAGS:
d-2 GCGATGGCCG 0 0 0 4 1 4.57e-03 8.51e-03 425 0.000 0.000 0.000 2.000 0.000

LOCUS: AT4G01850
DESCRIPTION: S-adenosylmethionine synthetase 2 (SAM2), identical to S-adenosylmethionine synthetase 2 (Methionine adenosyltransferase 2, AdoMet synthetase 2) (Arabidopsis thaliana) SWISS-PROT
DATA: Control 30min 2hours 2days 1week p-value B&H Pos Fold change relative to control (log2)
ANTISENSE COUNTS: 7 0 0 4 6 4.72e-03 6.21e-03 0.000 0.000 0.000 -0.807 -0.222
GENES:
AT4G01850.1
ANTISENSE COUNTS: 7 0 0 4 6 4.72e-03 6.46e-03 0.000 0.000 0.000 -0.807 -0.222
TAGS:
X-4 GTAGATGTTT 7 0 0 3 6 4.25e-03 8.38e-03 1234 0.000 0.000 0.000 -1.222 -0.222
X-4 AGCTCCCCAT 0 0 0 1 0 3.08e-01 3.56e-01 800 0.000 0.000 0.000 0.000 0.000

LOCUS: AT5G10830
DESCRIPTION: embryo-abundant protein-related, similar to embryo-abundant protein (Picea glauca) GI
DATA: Control 30min 2hours 2days 1week p-value B&H Pos Fold change relative to control (log2)
ANTISENSE COUNTS: 5 0 0 1 6 5.59e-03 7.17e-03 0.000 0.000 0.000 -2.322 0.263
GENES:
AT5G10830.1
ANTISENSE COUNTS: 5 0 0 1 6 5.59e-03 7.45e-03 0.000 0.000 0.000 -2.322 0.263

TAGS:
d-2 GCGATTAAGG 5 0 0 1 6 5.59e-03 1.01e-02 1071 0.000 0.000 0.000 -2.322 0.263

LOCUS: AT1G66730
DESCRIPTION: ATP dependent DNA ligase family protein, contains Pfam profile
DATA: Control 30min 2hours 2days 1week p-value B&H Pos Fold change relative to control (log2)
ANTISENSE COUNTS: 0 0 3 0 0 6.01e-03 7.51e-03 0.000 0.000 1.585 0.000 0.000
GENES:
AT1G66730.1
ANTISENSE COUNTS: 0 0 3 0 0 6.01e-03 7.62e-03 0.000 0.000 1.585 0.000 0.000
TAGS:
X-4 GGCCACTAAA 0 0 3 0 0 6.01e-03 1.03e-02 -135 0.000 0.000 1.585 0.000 0.000

LOCUS: AT1G03730
DESCRIPTION: expressed protein, similar to ESTs gb|AA605440 and gb|H37232
DATA: Control 30min 2hours 2days 1week p-value B&H Pos Fold change relative to control (log2)
ANTISENSE COUNTS: 0 0 3 0 0 6.01e-03 7.33e-03 0.000 0.000 1.585 0.000 0.000
GENES:
AT1G03730.1
ANTISENSE COUNTS: 0 0 3 0 0 6.01e-03 7.81e-03 0.000 0.000 1.585 0.000 0.000
TAGS:
X-4 GAAACTGCGT 0 0 3 0 0 6.01e-03 1.06e-02 42 0.000 0.000 1.585 0.000 0.000

LOCUS: AT3G20810
DESCRIPTION: transcription factor jumonji (jmjC) domain-containing protein, contains Pfam domain PF02373
DATA: Control 30min 2hours 2days 1week p-value B&H Pos Fold change relative to control (log2)
ANTISENSE COUNTS: 0 0 0 3 0 6.07e-03 7.23e-03 0.000 0.000 0.000 1.585 0.000
GENES:
AT3G20810.1
ANTISENSE COUNTS: 0 0 0 3 0 6.07e-03 7.52e-03 0.000 0.000 0.000 1.585 0.000
TAGS:
d-2 CTCGGAGCAG 0 0 0 3 0 6.07e-03 9.92e-03 1463 0.000 0.000 0.000 1.585 0.000
AT3G20810.2
ANTISENSE COUNTS: 0 0 0 3 0 6.07e-03 7.17e-03 0.000 0.000 0.000 1.585 0.000
TAGS:
d-2 CTCGGAGCAG 0 0 0 3 0 6.07e-03 9.92e-03 1528 0.000 0.000 0.000 1.585 0.000

LOCUS: AT2G41410
DESCRIPTION: calmodulin, putative, identical to SP|P30188 Calmodulin-like protein {Arabidopsis thaliana}
DATA: Control 30min 2hours 2days 1week p-value B&H Pos Fold change relative to control (log2)
ANTISENSE COUNTS: 0 0 0 3 0 6.07e-03 7.06e-03 0.000 0.000 0.000 1.585 0.000
GENES:
AT2G41410.1
ANTISENSE COUNTS: 0 0 0 3 0 6.07e-03 7.34e-03 0.000 0.000 0.000 1.585 0.000
TAGS:
X-4 CGGCAAAAGT 0 0 0 3 0 6.07e-03 1.02e-02 762 0.000 0.000 0.000 1.585 0.000

LOCUS: AT2G35060
DESCRIPTION: potassium transporter family protein, similar to HAK2 (Hordeum vulgare) GI
DATA: Control 30min 2hours 2days 1week p-value B&H Pos Fold change relative to control (log2)
ANTISENSE COUNTS: 0 0 0 1 4 6.83e-03 7.76e-03 0.000 0.000 0.000 0.000 2.000
GENES:
AT2G35060.1
ANTISENSE COUNTS: 0 0 0 1 4 6.83e-03 7.89e-03 0.000 0.000 0.000 0.000 2.000
TAGS:
X-4 GCAAGAAAAC 0 0 0 1 4 6.83e-03 1.09e-02 -21 0.000 0.000 0.000 0.000 2.000

LOCUS: AT5G24390
DESCRIPTION: RabGAP/TBC domain-containing protein, similar to GTPase activating protein (Yarrowia lipolytica) GI
DATA: Control 30min 2hours 2days 1week p-value B&H Pos Fold change relative to control (log2)

```
ANTISENSE COUNTS:              0       0       0       1       4       6.83e-03       7.59e-03                0.000   0.000   0.000   0.000   2.000
  GENES:
  AT5G24390.1
    ANTISENSE COUNTS:          0       0       0       1       4       6.83e-03       7.72e-03                0.000   0.000   0.000   0.000   2.000
    TAGS:
      X-4      TCTTATGTAA      0       0       0       1       3       6.54e-02       8.59e-02       1574     0.000   0.000   0.000   0.000   1.585
      X-4      TAAGGCATAG      0       0       0       0       1       1.70e-01       2.11e-01       603      0.000   0.000   0.000   0.000   0.000

LOCUS: AT5G54640
DESCRIPTION:  histone H2A, identical to histone H2A Arabidopsis thaliana GI
DATA:                          Control 30min   2hours  2days   1week   p-value        B&H            Pos      Fold change relative to control (log2)
ANTISENSE COUNTS:              7       0       10      8       3       6.92e-03       7.52e-03                0.000   0.000   0.515   0.193   -1.222
  GENES:
  AT5G54640.1
    ANTISENSE COUNTS:          7       0       10      8       3       6.92e-03       7.66e-03                0.000   0.000   0.515   0.193   -1.222
    TAGS:
      d-1      TCGAAACAGA      7       0       10      8       3       6.92e-03       1.08e-02       599      0.000   0.000   0.515   0.193   -1.222

LOCUS: AT1G26880
DESCRIPTION:  60S ribosomal protein L34 (RPL34A), identical to GB
DATA:                          Control 30min   2hours  2days   1week   p-value        B&H            Pos      Fold change relative to control (log2)
ANTISENSE COUNTS:              1       0       1       7       1       7.37e-03       7.84e-03                0.000   0.000   0.000   2.807   0.000
  GENES:
  AT1G26880.1
    ANTISENSE COUNTS:          1       0       1       7       1       7.37e-03       7.98e-03                0.000   0.000   0.000   2.807   0.000
    TAGS:
      X-4      ACTGCACCAA      1       0       1       7       1       7.37e-03       1.12e-02       161      0.000   0.000   0.000   2.807   0.000

LOCUS: AT1G64370
DESCRIPTION:  expressed protein
DATA:                          Control 30min   2hours  2days   1week   p-value        B&H            Pos      Fold change relative to control (log2)
ANTISENSE COUNTS:              1       6       2       0       0       8.66e-03       9.02e-03                0.000   2.585   1.000   0.000   0.000
  GENES:
  AT1G64370.1
    ANTISENSE COUNTS:          1       6       2       0       0       8.66e-03       9.19e-03                0.000   2.585   1.000   0.000   0.000
    TAGS:
      d-2      TAGTAGCGAG      1       6       2       0       0       2.07e-02       2.77e-02       711      0.000   2.585   1.000   0.000   0.000
      d-2      CCCATCCTTG      0       0       0       0       0       6.10e-01       6.49e-01       533      0.000   0.000   0.000   0.000   0.000

LOCUS: AT4G38770
DESCRIPTION:  proline-rich family protein (PRP4), similar to proline-rich protein (Arabidopsis thaliana) gi|6782442|gb|AAF28388; contains proline-rich
extensin domains, INTERPRO
DATA:                          Control 30min   2hours  2days   1week   p-value        B&H            Pos      Fold change relative to control (log2)
ANTISENSE COUNTS:              7       3       7       7       17      8.88e-03       9.06e-03                0.000   -1.222  0.000   0.000   1.280
  GENES:
  AT4G38770.1
    ANTISENSE COUNTS:          7       3       7       7       17      8.88e-03       9.24e-03                0.000   -1.222  0.000   0.000   1.280
    TAGS:
      X-4      ATAAAAGAGA      5       3       7       7       13      8.49e-02       1.09e-01       1396     0.000   -0.737  0.485   0.485   1.379
      X-4      AGTATACGTA      1       0       0       0       4       7.57e-03       1.13e-02       1235     0.000   0.000   0.000   0.000   2.000
      X-4      GGGGGTTTTG      1       0       0       0       0       4.25e-01       4.75e-01       250      0.000   0.000   0.000   0.000   0.000

LOCUS: AT3G54890
DESCRIPTION:  chlorophyll A-B binding protein / LHCI type I (CAB), identical to chlorophyll A/B-binding protein (Arabidopsis thaliana) GI
DATA:                          Control 30min   2hours  2days   1week   p-value        B&H            Pos      Fold change relative to control (log2)
ANTISENSE COUNTS:              5       5       15      8       1       9.87e-03       9.87e-03                0.000   0.000   1.585   0.678   -2.322
  GENES:
  AT3G54890.1
    ANTISENSE COUNTS:          5       5       15      8       1       9.87e-03       1.01e-02                0.000   0.000   1.585   0.678   -2.322
    TAGS:
```

| | | | | | | | | | | | | | | |
|---|---|---|---|---|---|---|---|---|---|---|---|---|---|---|
| X-4 | GATCCGCCAA | 5 | 5 | 15 | 8 | 1 | 9.87e-03 | 1.44e-02 | 98 | 0.000 | 0.000 | 1.585 | 0.678 | -2.322 |
